# Implications of changes in WHO haemoglobin elevation adjustment guidelines on global, regional, and national anaemia burden, 1990–2023: a population-based modelling study

**DOI:** 10.1016/S2352-3026(26)00013-X

**Published:** 2026-04

**Authors:** Corey J Teply, William M Gardner, Taylor S Noyes, Theresa A McHugh, Heidi A Tandiono, Ni Gusti Ayu Nanditha, Ashley A Harris, Nandita Perumal, Jackie Markt-Maloney, Brian C Howatt, Heather J Taylor, Kyle Humphrey, Prof Zulfiqar A Bhutta, Prof Domenico Girelli, Prof Simon I Hay, Prof Christopher JL Murray, Prof Khaled M Musallam, Prof Obiageli Nnodu, Prof Parminder S Suchdev, Prof Nicholas J Kassebaum

**Affiliations:** 1 Institute for Health Metrics and Evaluation, University of Washington, Seattle, WA, USA; 2 Vagelos College of Physicians and Surgeons, Columbia University, New York, NY, USA; 3 Department of Epidemiology and Biostatistics, University of South Carolina, Columbia, SC, USA; 4 Center for Global Child Health, Hospital for Sick Children, Toronto, Canada; 5 Center of Excellence in Women and Child Health, Aga Khan University, Karachi, Pakistan; 6 Section of Internal Medicine, Department of Medicine, University of Verona, Policlinico Giambattista Rossi, Verona, Italy; 7 Department of Health Metrics Sciences, University of Washington, Seattle, WA, USA; 8 Center for Research on Rare Blood Disorders (CR-RBD) and Thalassemia & Sickle Cell Center, Burjeel Cancer Institute, Burjeel Medical City, Abu Dhabi, United Arab Emirates; 9 Department of Public Health & Epidemiology, Khalifa University, Abu Dhabi, United Arab Emirates; 10 Division of Hematology/Oncology, Department of Pediatrics, Weill Cornell Medicine, New York, NY, USA; 11 Department of Haematology and Blood Transfusion, College of Health Sciences and Centre of Excellence for Sickle Cell Disease Research and Training, University of Abuja, Abuja, Nigeria; 12 Emory Global Health Institute, Emory University, Atlanta, GA, USA; 13 Center for Global Health, US Centers for Disease Control and Prevention, Atlanta, Georgia, USA; 14 Department of Anesthesiology and Pain Medicine, University of Washington, Seattle, WA, USA; 15 Department of Global Health, University of Washington, Seattle, WA, USA

## Abstract

**Background:**

Adjustment of haemoglobin concentrations for elevation is essential for anaemia assessments of both individuals and populations. Whereas the previous World Health Organization anaemia definitions released in 2001 (WHO-2001) used an elevation adjustment derived from small sample sizes with limited representativeness, the recently updated global anaemia definitions (WHO-2024) reflect empirical assessments across multiple geographies and settings derived from population-representative surveys. We aimed to assess the impact of these changes on estimates of global anaemia burden.

**Methods:**

Using an identical set of input data from population-based surveys, we created two datasets in parallel, where each input haemoglobin concentration was adjusted using the WHO-2001 and WHO-2024 elevation adjustment methods. We estimated mild, moderate, and severe anaemia prevalence by age and sex for 204 countries and territories from 1990 to 2023 for each dataset using spatiotemporal Gaussian process regression and ensemble distribution modelling. We then analysed the overall differences in anaemia prevalence and years lived with disability to assess the impact that the WHO-2024 elevation adjustment method will have on the global landscape of anaemia.

**Findings:**

Global anaemia prevalence for all ages and both sexes in 2023 was estimated to be 26.4% (95% uncertainty interval [UI] 22·6–31·9) using the WHO-2024 elevation adjustment method, which was 2·4 percentage points (2·2–2·7) higher compared to the total anaemia prevalence estimated using the WHO-2001 method. This increase equates to 198 million (196–199) newly enumerated cases—increasing the rank of anaemia from the third to the second largest cause of disability globally. The largest absolute increases in anaemia prevalence were in locations between 500 and 2000 metres in elevation, which include countries in eastern sub-Saharan Africa and Central America.

**Interpretation:**

Understanding the impact of anaemia requires unbiased and comparable estimates of anaemia burden. To our knowledge, we produced the first set of global estimates of anaemia burden by location, year, age, and sex using the WHO-2024 elevation adjustment method and compared them to estimates using the previous method. Policymakers should consider this modification when designing interventions to manage and prevent anaemia, particularly in regions most affected by changes in elevation adjustment.

**Funding:**

Gates Foundation.

## Introduction

Anaemia affected nearly 2 billion people and was the third leading cause of morbidity worldwide in 2021.^1^ Despite global efforts, its prevalence has remained high for decades, prompting renewed calls for action.^2^ Accurate anaemia prevalence estimates are vital for monitoring progress toward international reduction targets, especially in light of the 2024 WHO guideline updates.^3^

Anaemia is present when an individual has insufficient red blood cell (RBC) oxygen delivery to meet the body’s needs.^4^ Anaemia is diagnosed by proxy as having a haemoglobin concentration below a defined age-, sex-, and pregnancy-specific threshold.^3^ Anaemia is linked to adverse health outcomes, particularly among children, adolescent girls, women, and older adults.^5,6^ There are several factors—most notably elevation and chronic smoke exposure—that cause haemoglobin increases without increased oxygen delivery via hypoxia-induced stimulation of erythropoietin and RBC production.^7,8^

In 2001, WHO published anaemia definitions that included adjusting haemoglobin for elevation.^4^ This adjustment equation was derived from the US Centers for Disease Control and Prevention in 1989 using data from a small, demographically limited subset of the global population.^9-11^ However, recent findings from the Biomarkers Reflecting Inflammation and Nutritional Determinants of Anemia (BRINDA) project indicate that the 2001 adjustment method likely under-adjusts haemoglobin concentrations at lower elevations and over-adjusts them at higher elevations.^12^ Using a recent, more diverse data set, BRINDA proposed a new adjustment equation, now adopted in the 2024 WHO anaemia assessment guidelines.^3^

In this study, we simultaneously generated updated estimates of anaemia burden and quantified the impact of changes between 2001 and 2024 WHO definitions, including changes to elevation adjustment and age-specific thresholds for young children. Understanding the implications of these adjustments will better inform research and policy guidelines for producing high-quality and consistent estimates of anaemia burden.

## Methods

We generated parallel estimates using WHO-2001 and WHO-2024 anaemia definitions using the GBD approach as previously published and detailed in the appendix (pp 5–18).^1^ GBD 2021 anaemia sources^1^ were supplemented with new years of data from the WHO Vitamin and Mineral Nutrition Information System (VMNIS)^13^ through December 2022. We completed parallel data processing and estimation of two unique, continuous distributions of haemoglobin concentrations (g/L), anaemia prevalence, and unadjusted years lived with disability (YLDs) from 1990 to 2023 for 204 countries and territories, males and females, and 25 age groups (0–6 days, 7–27 days, 1–5 months, 6–11 months, 12–23 months, 2–4 years, 5–94 years, in five-year age bins, and ≥95 years). This study complies with the Guidelines for Accurate and Transparent Health Estimates Reporting^14^ (appendix pp 3–4). Ethics approval was not required, as all analyses used publicly available, aggregated data. Analyses were performed in R (version 4.4.2) and Python (version 3.10).

### Data extraction and processing

Input sources were included only if they had either quantitative, individual-level haemoglobin measurements (ie, microdata) or reported a measure of anaemia prevalence with a specified adjustment method in a representative population. All input sources are listed in the appendix (pp 23–99) and at the Global Health Data Exchange (ghdx.healthdata.org). The source-location-years available for both individual-level data and tabulated data can be seen in figure S1.

**Figure 1 –:**
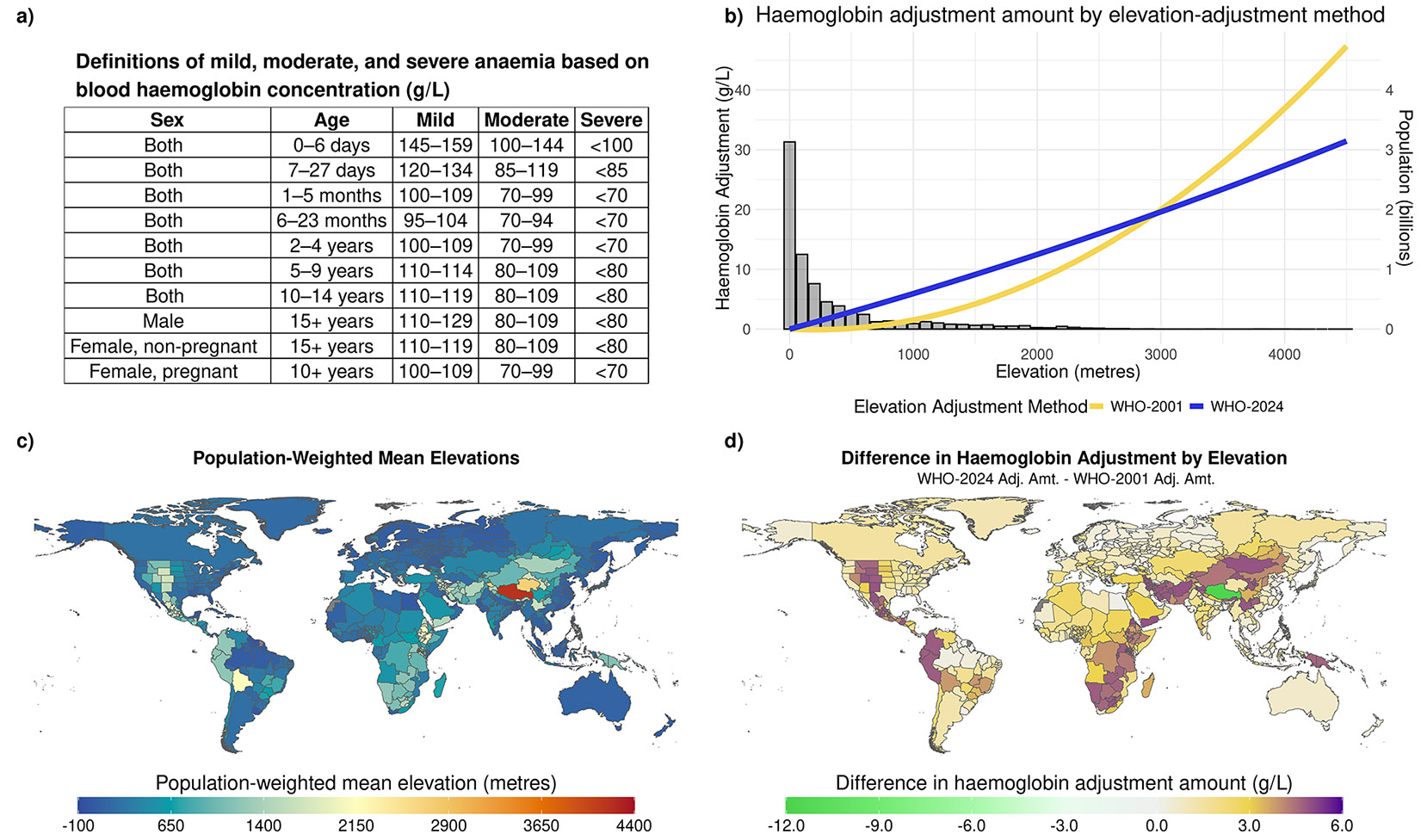
Definitions of anaemia cutoffs and population-weighted mean elevations a) Definitions of mild, moderate, and severe anaemia based on blood haemoglobin concentration. b) Haemoglobin adjustment for elevation. The yellow curve is the WHO elevation adjustment method published in 2001, and the blue curve is the updated elevation adjustment method as proposed by WHO in 2024. Population distribution is added to illustrate the number of people most impacted by the differences in elevation-adjustment methods. c) Population-weighted mean elevations by modelled locations. d) Difference in haemoglobin adjustment between the WHO-2001 and WHO-2024 elevation-adjustment methods. The values represent the WHO-2001 adjustment amount subtracted from the WHO-2024 adjustment amount.

All individuals with microdata were first assigned to a specific location, age, sex, and pregnancy status according to the GBD 2023 demographics hierarchy. Survey-provided elevations were utilised if available. If not available, we used the most granular GBD location to assign each individual a population-weighted mean elevation (figure 1C) derived from combining 5x5 km resolution elevation^15^ and population raster files from 1980 to 2021.^16^ Elevation adjustments from each of the 2001 and 2024 WHO definitions (figures 1B, 1D) were subtracted from raw haemoglobin values according to elevation at each person’s reported residence. No additional adjustments were made for smoking status, method of haemoglobin sampling (eg, whole blood versus capillary), or analysis method (eg, Coulter counter versus point-of-care testing). Severity levels (mild, moderate, severe) were then assigned using specific thresholds that vary by age, sex, and pregnancy status (figure 1A).

Studies containing only tabulated data were first flagged for the elevation-adjustment method used to report the mean haemoglobin concentration and anaemia prevalence. Within each tabulated input data source, if any of the mean haemoglobin concentrations or total, moderate plus severe, or severe anaemia prevalence values were missing for a given age, sex, location, or year combination, those metrics were imputed using meta-regression—Bayesian, regularised, trimmed models (MR-BRT).^17^ The model used either 1) the reported mean haemoglobin to estimate the missing total, moderate plus severe, and/or severe anaemia prevalence; or 2) the reported total or moderate plus severe anaemia prevalence to estimate the missing mean haemoglobin concentration. This was done to ensure internal consistency across both the WHO-2001 and WHO-2024 elevation-adjusted datasets, such that every study had a datapoint for all measures estimated in our models. More detailed processing information can be found in the appendix (pp 8–15; 100–1168).The final output of this process was two parallel datasets—one each for WHO-2001 and WHO-2024 elevation adjustment methods—containing adjusted mean haemoglobin and severity-specific anaemia prevalence observations from each identified data source.

### Estimation of mean haemoglobin and severity-specific anaemia prevalence

We estimated log-transformed mean haemoglobin concentration and logit-transformed prevalence of severe, moderate plus severe, and total anaemia using spatiotemporal Gaussian process regression (ST-GPR) models.^18^ We first estimated a linear prior using covariates known to be biologically or epidemiologically associated with anaemia (appendix p 16). Next, we calculated the residuals between our model and our input data and smoothed these residuals over space, age, and time, producing a revised estimate for every location, year, age, and sex. The final step was a Gaussian process regression that further smoothed the residuals between our data and step two estimates, from which we quantified uncertainty in our final model estimates by taking 1000 draws from the posterior Gaussian process.

### Generation of a continuous distribution of haemoglobin concentration

To derive the form of the final haemoglobin distributions, rather than assume a specific distribution shape *a priori*, we estimated an ensemble distribution consisting of a combination of two-parameter distribution families for each modelled age, sex, location, and year. The ensemble distribution was generated by using both single candidate two-parameter distributions (gamma, mirrored gamma, Weibull, mirrored lognormal, and mirrored Gumbel) and weighted ensembles of those same candidate distributions that best fit individual-level haemoglobin concentrations in our input data using out-of-sample fit metrics (appendix pp 16–17).

### Calculation of anaemia prevalence and years lived with disability

By combining the mean haemoglobin concentrations obtained from the ST-GPR modelling phase and the final ensemble distribution weights, we calculated a variance for each combination of age, sex, year, and location. This variance was chosen to minimise the differences between the ST-GPR-estimated total, moderate plus severe, and severe anaemia prevalence estimates and the corresponding severity-specific anaemia prevalences derived from the probability density curve of the haemoglobin ensemble distribution. Next, using the ST-GPR estimated mean haemoglobin value, the ensemble distribution weights, and the optimised variance, we re-generated the haemoglobin distribution and calculated the area under the probability density curve between the haemoglobin thresholds to derive mild, moderate, and severe anaemia prevalence. Anaemia prevalence estimates by pregnancy status were aggregated to create estimates for all females aged 10–54 years. We assessed for statistically significant differences between elevation adjustment methods using unpaired t-tests to determine those results which are statistically meaningfully different when accounting for the overall uncertainty in the estimates and the difference attributable to changes in the elevation adjustment method.

Unadjusted YLDs were calculated by multiplying the prevalence of mild, moderate, and severe anaemia by their respective GBD disability weights (where a disability weight of 0 is no health loss and a disability weight of 1 is death). The disability weights were 0·004 for mild anaemia, 0·052 for moderate anaemia, and 0·149 for severe anaemia.^1^ The YLD metric allows for standardised comparisons of non-fatal disease burden between severities of anaemia.

### Paediatric threshold analysis

The WHO-2024 guidelines included updated haemoglobin thresholds for children aged 6–23 months.^3^ To assess the combined impact of both the elevation adjustment and threshold changes, we performed a secondary analysis by re-calculating anaemia prevalence for all individual-level data sources using three different combinations: 1) WHO-2001 elevation adjustment with WHO-2001 thresholds, 2) WHO-2024 elevation adjustment with WHO-2001 thresholds, and 3) WHO-2024 elevation adjustment with WHO-2024 thresholds for children aged 6–23 months. We calculated average differences in anaemia prevalence across all location-years to isolate the effects of threshold changes from elevation adjustment changes (appendix pp 20–21).

### Epidemiological transition analysis

To evaluate if the elevation adjustment method led to changes in the historical relationship between anaemia burden and sociodemographic development, we performed an epidemiological transition analysis comparing the results for the two adjustment methods. Briefly, we performed an ecological analysis between Socio-demographic Index (SDI)^19^ and age-specific and sex-specific total, moderate plus severe, and severe anaemia prevalence across all GBD locations and years using MR-BRT models.

The input data for the model were created by matching post-ensemble age- and sex-specific anaemia prevalences with location-year-specific SDI estimates. Prevalence data were logit transformed, and all locations were given an equal weight in the meta-regression (appendix pp 18–19).

### Uncertainty

Uncertainty was propagated in each step of our modelling process, including sample variance from the input data and uncertainty generated in each of the MR-BRT, ST-GPR, and post-ensemble models. We estimated uncertainty by sampling draws from the posterior distribution of each estimated quantity. Reported upper and lower uncertainty intervals (UIs) correspond to the 2·5th and 97·5th percentile of the draws, respectively. Aggregations by geography, age, and sex were made at the draw level, assuming uncorrelated uncertainty.

### Role of the funding source

The funder had no role in study design, data collection, analysis, interpretation, or manuscript preparation. Core research team members had full access to underlying data used to generate the estimates in this paper. All other authors had access to, and reviewed, estimates as part of the research evaluation process.

## Results

Global anaemia prevalence in 2023 aggregated across all ages and both sexes was 24·0% (95% UI 20·6–29·3) for the WHO-2001 elevation adjustment method and 26.4% (22·6–31·9) for the WHO-2024 elevation adjustment method, making anaemia the second largest cause of disability in 2023 (table). This corresponds to an estimated 198 million (196–199) greater number of anaemia cases globally using the WHO-2024 method. The WHO-2024 method resulted in an increase in moderate plus severe anaemia prevalence of 1.5 percentage points (1.4–1.5) and an increase in severe anaemia prevalence of 0.2 percentage points (0.2–0.3). This equates to an estimated 120 million (119–121) more cases of moderate plus severe anaemia and 19.2 million (19.1–19.3) more cases of severe anaemia globally in all ages and both sexes in 2023 when using the WHO-2024 method. Comparisons of all annual age-sex-specific trends from 1990 to 2023 are in the appendix (pp 1169–1990). Selected years from 1990 to 2023 of severity-specific prevalence and YLDs for all-ages, males and females combined are in the appendix (pp 1993–2413).

**Figure 2 –:**
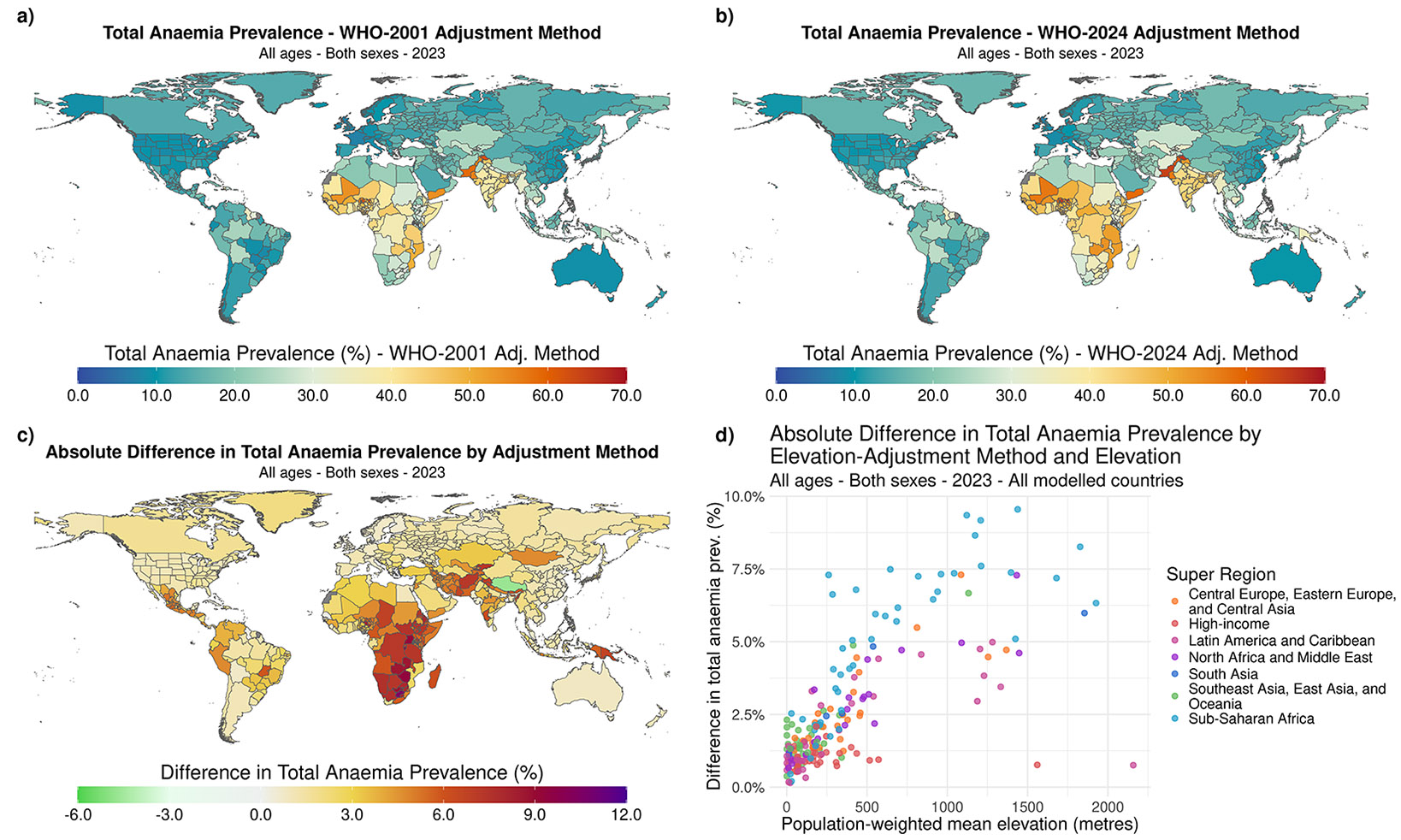
Comparison of total anaemia prevalence between the WHO-2001 and WHO-2024 elevation adjustment methods a) All ages, both sexes total anaemia prevalence, WHO-2001 elevation adjustment method, b) all ages, both sexes total anaemia prevalence, WHO-2024 elevation adjustment method, c) absolute difference in total anaemia prevalence between the WHO-2024 and WHO-2001 elevation adjustment methods, and d) absolute difference in total anaemia prevalence between the WHO-2024 and WHO-2001 elevation adjustment methods plotted against country population-weighted mean elevation.

There were disparities in total anaemia prevalence by geographical region, irrespective of the elevation-adjustment method used. Total anaemia prevalence for all ages and both sexes in 2023, based on the WHO-2024 method, ranged from 7·6% (95% UI 5·6–10·3) in Scotland to 58·7% (53·2–64·6) in Yemen (figure 2B). Overall, the WHO-2024 elevation-adjustment method resulted in a higher anaemia prevalence across all severities for all ages, sexes, and locations with a population-weighted mean elevation below 3000 metres (figure 2C, 2D). The countries that experienced the highest total anaemia prevalence for all ages and both sexes in 2023 were predominantly in sub-Saharan Africa (figure 2A, 2B). For example, Mali had an all-ages, both sexes total anaemia prevalence of 57·1% (44·6–71·6) using the WHO-2024 elevation adjustment method. Subnational units within Nigeria experienced the highest overall total anaemia prevalence for all ages and both sexes in 2023 when using the WHO-2024 elevation adjustment method, where the state of Jigawa, Nigeria, had an estimated total anaemia prevalence of 67·3% (49·2–80·2). Western European high-income countries and their respective subnational units were among the locations that experienced the lowest total anaemia prevalence in 2023 for the same demographics. Within the western European high-income countries, Greece had the highest total anaemia prevalence of 13·5% (10·1–18·4).

Countries with population-weighted mean elevations between 500 and 2000 metres displayed the largest differences in total anaemia prevalence by elevation adjustment method for all ages and both sexes in 2023 (figure 2C, 2D). This included mountainous areas in eastern sub-Saharan Africa, central Asia, and countries in Central and South America. Burundi had the largest difference in total anaemia prevalence between the elevation adjustment methods, where the WHO-2024 method measured a total anaemia prevalence for all ages and both sexes that was 9·6 percentage points (95% UI 9·4–9·7) higher than the total anaemia prevalence using the WHO-2001 method. Tibet (province of China), with a population-weighted mean elevation above 3000 metres, had an estimated total anaemia prevalence of 4·7 percentage points (4·7–4·7) lower using the WHO-2024 method as compared to the WHO-2001 method.

In 2023, female all-age total anaemia prevalence when using the WHO-2024 elevation adjustment method was 31·0% (95% UI 26·9–37·7), while male all-age total anaemia prevalence was 21·9% (18·2–26·7). This corresponds to an unadjusted YLD rate per 100,000 for total anaemia of 1020 (817–1250) for females and 566 (442–766) for males for all ages in 2023 globally, which ranks anaemia as second and fifth overall for causes of disability, respectively.

Among all demographics, females aged 10–54 years (ie, women of reproductive age) and children younger than 5 years experienced the largest burden of anaemia when using the WHO-2024 elevation-adjustment method, with a global total anaemia prevalence in 2023 of 31·9% (95% UI 28·2–38·3) and 39·2% (35·0–45·4), respectively. When comparing global total anaemia prevalence estimates in 2023, we estimated increases of 3·0 percentage points (3·0–3·0) for females aged 10–54 years and 3·9 percentage points (3·9–3·9) for children younger than 5 years when using the WHO-2024 versus WHO-2001 elevation adjustment methods. This corresponded to 75·0 million (74·6–75·5) and 23·4 million (23·2–23·6) more cases globally in 2023 for each of these groups, respectively.

**Figure 3 –:**
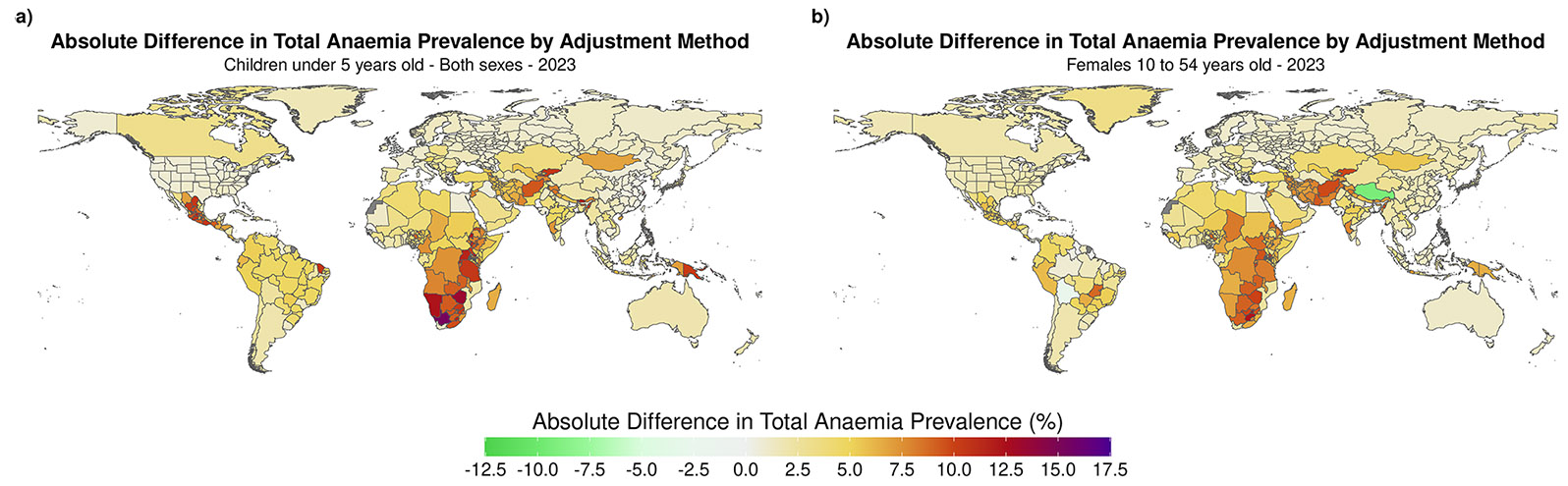
Absolute differences in anaemia prevalence between elevation adjustment methods in non-pregnant females (aged 10–54 years) and children younger than 5 years Absolute differences in total anaemia prevalence between the WHO-2024 and WHO-2001 elevation-adjustment methods in 2023 for a) children younger than 5 years, and b) women of reproductive age.

Geographical trends for differences in total anaemia prevalence between the two elevation adjustment methods for females aged 10–54 years and children younger than 5 years followed that of the total anaemia prevalence for all ages and both sexes (figure 3). The locations that experienced the largest differences in total anaemia prevalence when comparing the WHO-2024 and WHO-2001 elevation adjustment methods were Northern Cape, South Africa (2156 metres), with an increase of 15·4 percentage points (95% UI 15·2–15·5) for children younger than 5 years, and Gauteng, South Africa (1913 metres), with an increase of 12·5 percentage points (12·4–12·5) for females aged 10–54 years.

Moderate plus severe and severe anaemia prevalence estimates for 2023 also showed larger increases in all demographics when using the WHO-2024 elevation-adjustment method compared to the WHO-2001 method for locations with a population-weighted mean between 500 and 2000 metres. For example, in Somali (region of Ethiopia), we observed increases of up to 2·0 percentage points (95% UI 2·0–2·0) in our estimates of severe anaemia prevalence for females aged 10–54 years, which correlates to an increase of 294 (292–296) in unadjusted YLD rate per 100,000. In Yemen, estimates of severe anaemia prevalence for children younger than 5 years showed increases up to 4·4 percentage points (4·4–4·4), resulting in an increase of 651 (649–654) in unadjusted YLD rate per 100,000 in 2023.

In our supplemental analysis of threshold changes in children aged 6–23 months, the updated WHO-2024 elevation adjustment method resulted in an average increase of 5·8 percentage points (95% UI 0·8–14·3) in total anaemia prevalence in this group across all data sources (table S9). However, within the same WHO-2024 elevation adjustment method, the updated paediatric haemoglobin thresholds led to an average decrease in anaemia prevalence of 11·6 percentage points (4·3–17·7). The combined effect of updated elevation adjustment and thresholds resulted in a 5·8 percentage point decrease (0·6–13·3) in anaemia prevalence. Thus, while the WHO-2024 elevation adjustment method increased anaemia prevalence globally, revised thresholds resulted in a net reduction in the estimated number of anaemia cases in the 6–23 months age group (figure S8).

**Figure 4 –:**
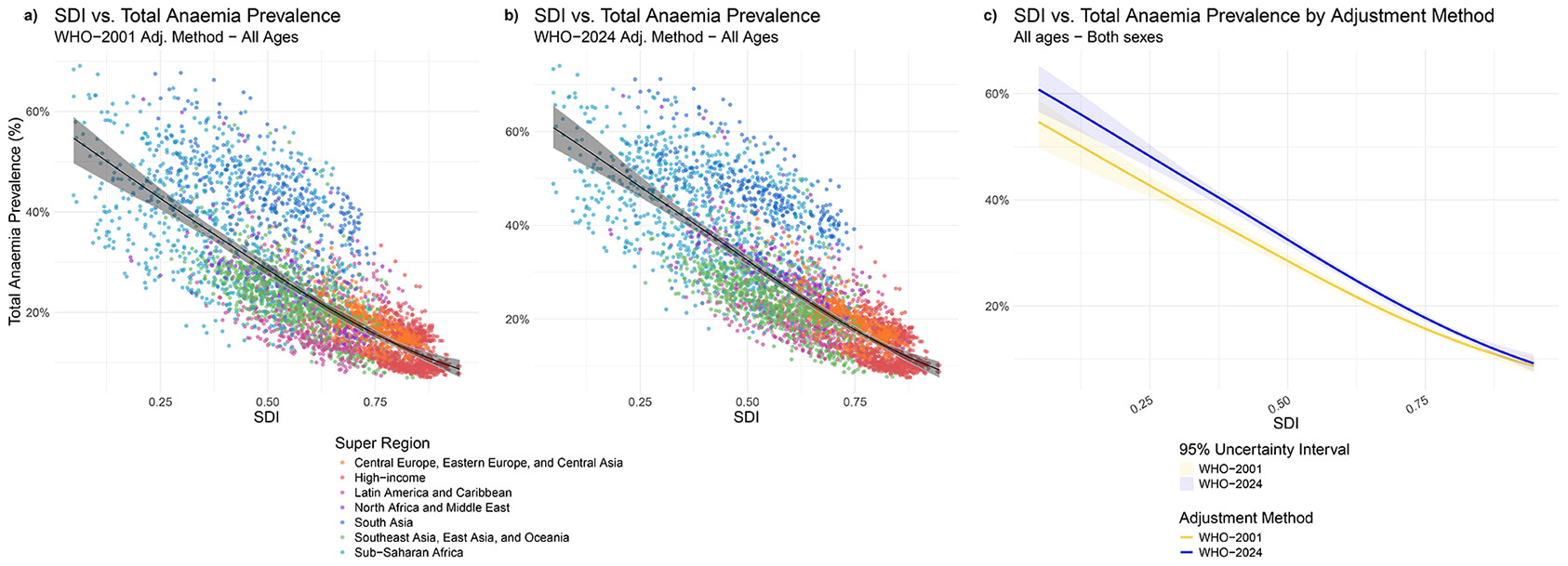
Modelled relationships of Socio-demographic Index and anaemia prevalence Global mean prevalence of total anaemia by Socio-demographic Index (SDI) comparing the WHO-2001 versus WHO-2024 elevation adjustment methods.

Accounting for elevation with the WHO-2024 adjustment method compared to the WHO-2001 method most significantly impacted countries with low to middle SDI (figure 4A-C). We observed a negative correlation between SDI and elevation adjustment amount, meaning that countries with lower SDIs are those where we would expect to see the largest changes in haemoglobin adjustment amounts, and therefore higher anaemia prevalence. Among countries with SDI at or above the 75th percentile, both adjustment methods resulted in similar anaemia prevalences, regardless of severity. However, as SDI decreases, the difference in anaemia prevalence estimated by the WHO-2001 versus the WHO-2024 adjustment method increases, with the WHO-2024 method resulting in a significantly greater anaemia prevalence, particularly in countries with an SDI between the 25th and 75th percentiles. This suggests that the anaemia burden among countries with SDI under the 75th percentile is greater than previously thought. Results for select demographics comparing SDI and anaemia prevalence can be found in the appendix (pp 2414-2420).

## Discussion

There were more than 2.1 billion people with anaemia globally in 2023 according to WHO-2024 definitions. This was a nearly 200 million case increase compared to WHO-2001 definitions and would have made anaemia rank second, compared to third, amongst GBD disability causes (after low back pain). Most newly-enumerated cases were in children younger than 5 years and females 10–54 years, and were concentrated in countries of eastern sub-Saharan Africa, central Asia, and Central and South America that are simultaneously at relatively high elevation, on the lower end of the SDI spectrum, and have comparatively high rates of many anaemia-causing diseases.^1^ These changes translated to an even stronger gradient between SDI and anaemia, especially in lower SDI settings, supporting a notion that sociodemographic factors of income, education, and women’s empowerment are universally key anaemia drivers.

There is little debate about the importance of adjusting haemoglobin for elevation,^20^ given that haemoglobin concentration is only a proxy for blood oxygen supply (the clinical definition of anaemia). Updates to WHO-2024 anaemia definitions facilitate improved monitoring of progress towards international targets and thereby better inform resource allocation for anaemia control.^21-23^ Examining the process and assumptions used to derive both elevation adjustment methods underscores the appropriateness of the WHO-2024 update. The WHO-2001 elevation adjustment method was based on a comparatively small dataset with limited geographical and economic diversity (children and adult males from Peru, Chile, the UK, and the USA), consisting of data collected before the mid-1980s.^9,10^ According to the statistical methods used to calculate the WHO-2001 elevation adjustment equation,^9,10^ it appears that a) there was not any attempt to account for demographics (eg, age and sex), and b) a log-linear functional form was assumed (figure 1B). In contrast, the WHO-2024 elevation adjustment, which leveraged the work of BRINDA,^12,24^ utilised a more comprehensive, multinational set of surveys reflecting broader representation by age, sex, and location. BRINDA used biomarkers to identify a “healthier” subset of respondents, completed multiple sensitivity analyses to look for heterogeneity in associations, and evaluated multiple statistical models to deriving a nearly-linear quadratic functional form (figure 1B).^12,24^

While there have been publications reporting on broader expected changes in anaemia burden assessment as a consequence of WHO-2024 definitional changes,^20,25,26^ our study is the first to our knowledge to comprehensively assess the implications of definitional changes across locations, age, sex, and over time, including reprocessing of all 818 individual-level and tabulated datasets of haemoglobin concentration and anaemia prevalence. For the 6–23 month age group in particular, our secondary analysis (appendix pp 20-21) of the impact of threshold changes found that elevation adjustment changes partially, but did not completely, offset the decreases that accompanied threshold changes and that differences were most pronounced in mild and moderate anaemia (severe anaemia thresholds were unchanged and therefore unaffected). We were unable to assess the impact of WHO-2024 changes to trimester-specific thresholds for anaemia in pregnancy because our models currently evaluate pregnant persons as a single group, but we expect the population-level impact will likely be modest as there were only changes to 2^nd^ trimester thresholds.

While our study provides critical insights, it is not without limitations. First, as is true for all global analyses of anaemia burden, data availability varied by age and sex, with the greatest number of surveys of haemoglobin concentrations available in children younger than 5 years and females aged 10–54 years. More data need to be collected for adult males and individuals (both male and female) over 60 years of age, along with potentially considering different adjustment approaches for populations residing at high elevations, given that these groups may possess unique physiological adaptations to hypoxic environments.^25,27^ Second, we estimated anaemia cutoffs for age groups younger than 6 months. While these cutoffs are generated systematically, additional surveys focusing on infants in this age range are needed to enable WHO to establish more precise anaemia thresholds. Third, in cases where elevation data were not provided in the original survey, our analysis utilised population-weighted mean elevations. While this approach provides a general estimate, it may not fully capture the variation in elevation within certain regions we modelled. Although we assigned population-weighted mean elevations to the most granular locations available in the survey, further efforts could focus on isolating specific locations and elevations to improve accuracy. Fourth, we analysed haemoglobin concentrations collected using both venous and capillary methods, which are known to exhibit differing levels of variability in the amount of haemoglobin detected in the blood sample.^28^ Relatedly, the method of analysis (eg, laboratory vs. point-of-care testing) has also been shown to affect estimates of anaemia prevalence.^29^ Due to limited data availability to formally adjust for these differences, we pooled data using different blood sources and methods of analysis. Further investigation is needed to ensure that haemoglobin measurements from different methods can be reliably used and appropriately adjusted to account for these differences. Potential approaches include developing a crosswalk adjustment factor between measurement site and analysis method or systematically increasing the uncertainty associated with haemoglobin concentrations obtained through non-gold standard methods. Fifth, because the elevation adjustment equation does not incorporate uncertainty, the absolute differences calculated between the WHO-2024 and WHO-2001 envelopes primarily reflect a shift of the distribution rather than additional variability, resulting in narrow uncertainty intervals when comparing differences between adjustment methods. Finally, our analysis does not currently account for smoking, which has a compounding effect that can be added to the elevation adjustment for haemoglobin concentrations.^12^ We intend to produce an updated set of results that incorporates smoking data and re-estimates the global burden of anaemia using haemoglobin concentrations adjusted for both elevation and smoking, in accordance with the WHO-2024 recommendations.

This study highlights the significant increases in estimated anaemia burden with the adoption of the new empirical haemoglobin elevation adjustment method by WHO in 2024. By addressing biases in previous adjustment methods, the WHO-2024 elevation adjustment method offers a more reliable and evidence-based framework for estimating anaemia burden. All future assessments of anaemia burden should adopt these new guidelines for anaemia monitoring and surveillance, including updating of historical estimates. These findings—especially that the newly enumerated cases are concentrated in low SDI locations, young children, and women of reproductive age—underscore the clear and present need to focus efforts on prevention and treatment of anaemia in the most vulnerable populations.

## Supplementary Material

Supplementary Material

## Figures and Tables

**Table:** Mild, moderate, and severe anaemia prevalence and cases using WHO-2001 versus WHO-2024 elevation adjustment definitions for 2023

Location	Sex	Elevation Adjustment Method	Mean Haemoglobin (g/L)	Total Prevalence (per 100k pop)	Moderate+Severe Prevalence (per 100k pop)	Severe Prevalence (per 100k pop)	Total YLDs (per 100k pop)	Total Prevalence (#, thousands)	Moderate+Severe Prevalence (#, thousands)	Severe Prevalence (#, thousands)	Total YLDs (#, in thousands)
Global	Both	WHO-2024	134 (130 to 138)	26,400 (22,600 to 31,900)	11,800 (9,440 to 14,500)	1,260 (740 to 2,110)	794 (630 to 1,010)	2,130,000 (1,820,000 to 2,580,000)	951,000 (762,000 to 1,170,000)	101,000 (59,700 to 170,000)	64,000 (50,800 to 81,100)
		WHO-2001	136 (132 to 140)	24,000 (20,600 to 29,300)	10,300 (8,210 to 12,900)	1,020 (592 to 1,860)	689 (556 to 884)	1,930,000 (1,660,000 to 2,370,000)	831,000 (662,000 to 1,040,000)	82,300 (47,700 to 150,000)	55,600 (44,800 to 71,300)
*Central Asia*	Both	WHO-2024	133 (126 to 139)	28,200 (21,800 to 38,700)	12,000 (8,490 to 16,300)	1,090 (351 to 2,420)	793 (556 to 1,140)	28,200 (21,700 to 38,600)	11,900 (8,480 to 16,300)	1,090 (351 to 2,420)	792 (555 to 1,130)
		WHO-2001	135 (129 to 142)	24,000 (18,600 to 32,700)	10,100 (7,010 to 14,300)	920 (302 to 2,150)	669 (463 to 1,010)	24,000 (18,600 to 32,600)	10,100 (7,010 to 14,300)	919 (302 to 2,150)	668 (462 to 1,010)
*Central Europe*	Both	WHO-2024	141 (132 to 150)	15,400 (9,870 to 23,200)	4,630 (2,000 to 8,840)	357 (45.6 to 1,160)	319 (147 to 608)	17,900 (11,500 to 26,900)	5,380 (2,330 to 10,300)	414 (52.9 to 1,350)	370 (170 to 706)
		WHO-2001	141 (133 to 150)	13,400 (8,550 to 20,600)	3,880 (1,640 to 7,480)	287 (40.3 to 897)	268 (128 to 474)	15,600 (9,920 to 23,900)	4,500 (1,910 to 8,680)	333 (46.8 to 1,040)	311 (149 to 550)
*Eastern Europe*	Both	WHO-2024	142 (135 to 150)	15,300 (10,700 to 21,600)	5,700 (3,310 to 9,460)	552 (127 to 1,490)	389 (219 to 680)	31,700 (22,200 to 44,800)	11,800 (6,860 to 19,600)	1,140 (264 to 3,090)	807 (453 to 1,410)
		WHO-2001	143 (135 to 150)	13,900 (9,780 to 20,100)	5,020 (2,850 to 8,360)	465 (111 to 1,280)	343 (202 to 563)	28,900 (20,300 to 41,600)	10,400 (5,900 to 17,300)	963 (229 to 2,660)	710 (418 to 1,170)
*Australasia*	Both	WHO-2024	143 (139 to 147)	10,600 (6,760 to 16,600)	3,110 (1,480 to 6,330)	247 (62.7 to 722)	216 (108 to 440)	3,390 (2,170 to 5,340)	998 (475 to 2,030)	79.3 (20.1 to 232)	69.2 (34.7 to 141)
		WHO-2001	143 (139 to 148)	9,670 (6,180 to 15,400)	2,760 (1,360 to 5,580)	218 (58.6 to 624)	192 (98.2 to 376)	3,100 (1,980 to 4,920)	886 (436 to 1,790)	70.1 (18.8 to 200)	61.7 (31.5 to 121)
*High-income Asia Pacific*	Both	WHO-2024	143 (139 to 146)	14,300 (12,200 to 17,200)	4,660 (3,550 to 6,280)	418 (204 to 797)	321 (248 to 446)	26,200 (22,300 to 31,500)	8,500 (6,490 to 11,500)	763 (372 to 1,460)	587 (452 to 815)
		WHO-2001	143 (140 to 146)	13,500 (11,200 to 16,300)	4,120 (2,920 to 5,770)	344 (152 to 696)	285 (205 to 394)	24,600 (20,400 to 29,700)	7,510 (5,320 to 10,500)	627 (277 to 1,270)	520 (375 to 720)
*High-income North America*	Both	WHO-2024	140 (138 to 142)	11,300 (9,800 to 13,200)	3,030 (2,480 to 3,810)	186 (106 to 319)	209 (173 to 264)	42,200 (36,600 to 49,500)	11,300 (9,260 to 14,200)	697 (397 to 1,190)	781 (648 to 989)
		WHO-2001	141 (140 to 143)	10,000 (8,630 to 11,600)	2,700 (2,220 to 3,310)	171 (107 to 269)	186 (158 to 231)	37,500 (32,300 to 43,500)	10,100 (8,290 to 12,400)	641 (399 to 1,000)	697 (590 to 862)
*Southern Latin America*	Both	WHO-2024	140 (134 to 146)	12,500 (7,630 to 18,900)	3,560 (1,380 to 7,210)	292 (51.6 to 882)	249 (103 to 506)	8,770 (5,340 to 13,200)	2,490 (967 to 5,050)	205 (36.1 to 618)	174 (72.2 to 354)
		WHO-2001	141 (135 to 148)	11,300 (6,850 to 17,400)	3,050 (1,150 to 6,200)	244 (38.1 to 751)	215 (91.0 to 403)	7,940 (4,800 to 12,200)	2,130 (807 to 4,340)	171 (26.7 to 526)	151 (63.8 to 282)
*Western Europe*	Both	WHO-2024	144 (140 to 149)	9,930 (7,450 to 13,500)	2,920 (1,910 to 4,860)	254 (111 to 564)	204 (135 to 336)	44,200 (33,200 to 60,300)	13,000 (8,480 to 21,600)	1,130 (493 to 2,510)	910 (599 to 1,490)
		WHO-2001	144 (140 to 149)	9,020 (6,690 to 12,400)	2,530 (1,600 to 4,180)	209 (91.8 to 488)	178 (115 to 277)	40,200 (29,800 to 55,100)	11,200 (7,100 to 18,600)	928 (409 to 2,170)	790 (512 to 1,230)
*Andean Latin America*	Both	WHO-2024	135 (131 to 140)	20,100 (15,900 to 26,100)	7,230 (5,170 to 10,000)	563 (242 to 1,140)	482 (335 to 674)	13,300 (10,500 to 17,200)	4,770 (3,410 to 6,630)	372 (159 to 753)	318 (221 to 445)
		WHO-2001	139 (135 to 142)	16,600 (13,100 to 20,800)	6,190 (4,370 to 8,920)	515 (233 to 1,110)	413 (298 to 601)	10,900 (8,650 to 13,700)	4,080 (2,880 to 5,890)	340 (154 to 730)	272 (196 to 396)
*Caribbean*	Both	WHO-2024	132 (126 to 139)	28,800 (22,200 to 37,700)	12,400 (7,930 to 18,200)	1,270 (480 to 2,980)	832 (516 to 1,310)	13,900 (10,800 to 18,300)	5,990 (3,840 to 8,840)	617 (232 to 1,440)	403 (250 to 634)
		WHO-2001	133 (127 to 139)	27,300 (21,200 to 36,700)	11,200 (7,260 to 16,600)	1,060 (407 to 2,570)	751 (510 to 1,140)	13,200 (10,300 to 17,800)	5,440 (3,520 to 8,030)	512 (197 to 1,250)	364 (247 to 552)
*Central Latin America*	Both	WHO-2024	140 (135 to 145)	17,300 (13,500 to 22,200)	6,830 (4,810 to 9,810)	695 (285 to 1,520)	465 (320 to 693)	44,900 (35,100 to 57,600)	17,700 (12,500 to 25,400)	1,800 (739 to 3,940)	1,210 (830 to 1,800)
		WHO-2001	143 (138 to 148)	13,700 (10,800 to 17,600)	5,180 (3,650 to 7,570)	524 (238 to 1,080)	354 (245 to 506)	35,500 (27,900 to 45,600)	13,400 (9,470 to 19,600)	1,360 (616 to 2,800)	918 (635 to 1,310)
*Tropical Latin America*	Both	WHO-2024	137 (133 to 141)	16,200 (13,400 to 21,900)	5,760 (4,470 to 7,190)	528 (253 to 1,010)	392 (293 to 512)	35,300 (29,400 to 47,800)	12,600 (9,770 to 15,700)	1,150 (553 to 2,200)	857 (639 to 1,120)
		WHO-2001	139 (135 to 143)	13,300 (10,900 to 17,000)	4,620 (3,440 to 5,890)	402 (188 to 738)	314 (234 to 400)	29,000 (23,900 to 37,100)	10,100 (7,500 to 12,900)	879 (410 to 1,610)	685 (510 to 875)
*North Africa and Middle East (region)*	Both	WHO-2024	135 (130 to 139)	24,700 (21,100 to 30,000)	10,600 (8,760 to 13,300)	1,260 (894 to 2,070)	731 (600 to 966)	158,000 (135,000 to 192,000)	67,700 (55,900 to 84,700)	8,070 (5,710 to 13,200)	4,660 (3,830 to 6,160)
		WHO-2001	136 (132 to 141)	21,500 (18,200 to 26,500)	8,940 (7,240 to 11,500)	1,000 (698 to 1,720)	612 (500 to 766)	137,000 (116,000 to 169,000)	57,000 (46,200 to 73,400)	6,380 (4,450 to 11,000)	3,910 (3,190 to 4,890)
*South Asia (region)*	Both	WHO-2024	126 (121 to 131)	41,800 (33,600 to 52,900)	20,400 (15,400 to 26,000)	2,270 (1,060 to 4,030)	1,370 (1,030 to 1,780)	791,000 (637,000 to 1,000,000)	387,000 (291,000 to 493,000)	43,000 (20,100 to 76,300)	25,900 (19,500 to 33,700)
		WHO-2001	128 (123 to 132)	39,400 (32,200 to 50,000)	18,500 (13,900 to 23,500)	1,860 (855 to 3,570)	1,230 (922 to 1,630)	746,000 (610,000 to 946,000)	350,000 (263,000 to 446,000)	35,300 (16,200 to 67,600)	23,200 (17,500 to 30,800)
*East Asia*	Both	WHO-2024	144 (137 to 151)	11,600 (9,570 to 14,300)	3,700 (2,730 to 4,740)	305 (112 to 564)	253 (188 to 321)	172,000 (142,000 to 212,000)	54,700 (40,500 to 70,200)	4,520 (1,650 to 8,360)	3,750 (2,790 to 4,760)
		WHO-2001	146 (139 to 153)	10,600 (8,850 to 13,000)	3,390 (2,610 to 4,310)	298 (127 to 537)	234 (183 to 304)	156,000 (131,000 to 193,000)	50,100 (38,600 to 63,800)	4,410 (1,880 to 7,940)	3,460 (2,700 to 4,490)
*Oceania*	Both	WHO-2024	129 (126 to 132)	33,200 (28,500 to 40,700)	14,500 (12,000 to 17,200)	1,510 (743 to 2,520)	979 (778 to 1,210)	4,900 (4,210 to 6,010)	2,140 (1,770 to 2,540)	223 (110 to 372)	145 (115 to 178)
		WHO-2001	132 (129 to 135)	27,700 (23,800 to 32,800)	11,900 (9,090 to 14,500)	1,210 (573 to 2,130)	799 (593 to 1,010)	4,080 (3,510 to 4,840)	1,750 (1,340 to 2,140)	178 (84.6 to 314)	118 (87.5 to 148)
*Southeast Asia*	Both	WHO-2024	135 (132 to 137)	24,400 (21,800 to 28,600)	9,580 (8,140 to 11,300)	848 (519 to 1,340)	640 (536 to 757)	174,000 (156,000 to 204,000)	68,400 (58,100 to 80,400)	6,050 (3,710 to 9,560)	4,570 (3,830 to 5,410)
		WHO-2001	136 (133 to 138)	22,600 (20,000 to 26,100)	8,430 (6,890 to 10,300)	694 (420 to 1,160)	562 (451 to 696)	161,000 (143,000 to 187,000)	60,200 (49,200 to 73,600)	4,950 (3,000 to 8,290)	4,010 (3,220 to 4,970)
*Central Sub-Saharan Africa*	Both	WHO-2024	124 (121 to 127)	42,400 (36,000 to 51,500)	20,800 (17,000 to 24,600)	2,260 (1,270 to 3,660)	1,380 (1,140 to 1,680)	62,200 (52,800 to 75,400)	30,400 (24,900 to 36,100)	3,310 (1,850 to 5,370)	2,030 (1,680 to 2,460)
		WHO-2001	127 (124 to 130)	35,800 (30,700 to 44,900)	16,700 (13,600 to 20,300)	1,630 (842 to 2,910)	1,100 (915 to 1,360)	52,500 (44,900 to 65,700)	24,400 (19,900 to 29,800)	2,390 (1,230 to 4,260)	1,620 (1,340 to 1,990)
*Eastern Sub-Saharan Africa*	Both	WHO-2024	125 (121 to 128)	40,300 (34,500 to 47,500)	20,500 (16,600 to 24,900)	2,510 (1,630 to 3,970)	1,390 (1,120 to 1,740)	182,000 (156,000 to 215,000)	92,900 (75,200 to 113,000)	11,400 (7,360 to 17,900)	6,290 (5,050 to 7,870)
		WHO-2001	128 (125 to 132)	33,700 (28,900 to 40,500)	16,400 (13,200 to 20,000)	1,840 (1,120 to 3,130)	1,100 (896 to 1,400)	152,000 (131,000 to 183,000)	74,000 (59,800 to 90,300)	8,310 (5,060 to 14,200)	4,970 (4,050 to 6,330)
*Southern Sub-Saharan Africa*	Both	WHO-2024	130 (127 to 133)	33,100 (28,600 to 39,800)	14,200 (10,500 to 18,000)	1,380 (648 to 2,530)	948 (701 to 1,270)	29,300 (25,300 to 35,200)	12,500 (9,340 to 15,900)	1,220 (574 to 2,240)	840 (621 to 1,120)
		WHO-2001	133 (131 to 136)	25,900 (22,000 to 30,700)	10,800 (8,070 to 14,200)	1,040 (520 to 1,960)	725 (534 to 981)	22,900 (19,500 to 27,200)	9,570 (7,150 to 12,600)	920 (460 to 1,740)	642 (473 to 869)
*Western Sub-Saharan Africa*	Both	WHO-2024	121 (117 to 126)	47,800 (38,800 to 60,000)	24,700 (18,600 to 31,100)	2,780 (1,420 to 4,880)	1,650 (1,240 to 2,090)	248,000 (202,000 to 312,000)	129,000 (96,600 to 162,000)	14,500 (7,380 to 25,300)	8,570 (6,450 to 10,900)
		WHO-2001	123 (118 to 127)	44,300 (36,100 to 56,800)	21,900 (16,200 to 28,400)	2,250 (1,140 to 4,400)	1,450 (1,070 to 1,880)	230,000 (188,000 to 295,000)	114,000 (84,200 to 147,000)	11,700 (5,940 to 22,900)	7,520 (5,580 to 9,760)

## Data Availability

This study follows the Guidelines for Accurate and Transparent Health Estimates Reporting (GATHER). To download the data used in these analyses and corresponding results, please visit the Global Health Data Exchange at http://ghdx.healthdata.org.

## Research in context

### Evidence before this study

Recent evidence from the Biomarkers Reflecting Inflammation and Nutritional Determinants of Anemia (BRINDA) project found that the World Health Organization (WHO) 2001 guidelines, based on data lacking geographical and demographic diversity, under-adjust haemoglobin levels at lower elevations and over-adjust them at higher elevations, leading to systematic bias in estimates of global anaemia burden. Recognising these limitations, WHO updated its haemoglobin elevation adjustment recommendations in 2024 to align with evidence from BRINDA’s more diverse cohorts. The Global Burden of Diseases, Injuries, and Risk Factors Study (GBD) comprehensively quantifies health loss across space and time—including routine estimation of anaemia burden—but up until this point has not reflected the updated elevation adjustment method for haemoglobin concentration. We conducted a systematic literature search in PubMed using the search terms: (elevation OR altitude) AND (anemia OR anaemia) AND (prevalence OR DALYs OR YLDs), examining all studies published since 2024 (the year of publication of the updated WHO guidelines). We identified several assessments of the implications of the new WHO method, mostly limited to specific age ranges (eg, children aged 6 months to 5 years) or specific countries. One recent analysis by Luo and colleagues (2025) found that anaemia prevalence for children aged 6–59 months increased 4·7 percentage points (ranging from –0·4 to 18·1) using updated haemoglobin cutoffs and the new elevation adjustment method, while anaemia prevalence for non-pregnant people aged 15–49 years increased from 13·5% to 21·3%. We identified no study assessing the global impact of changes to elevation adjustment across all demographics.

### Added value of this study

To our knowledge, this study represents the first comprehensive comparison of the WHO-2001 and WHO-2024 haemoglobin elevation adjustment methods, assessing their impact on both anaemia prevalence and years lived with disability (YLDs) globally. Utilising data from GBD 2023, we evaluated levels and trends in anaemia prevalence and YLDs using both the WHO-2001 and WHO-2024 elevation adjustment methods for 204 countries and territories, 25 age groups, and male and female sexes from 1990 to 2023. Our results provide new insights into how updated elevation adjustment methods reshape global and regional understanding of anaemia burden, and highlight important age, sex, and geographical variations in impact.

### Implications of all the available evidence

The WHO-2024 elevation adjustment method results in higher estimates of anaemia prevalence globally, emphasising the need for updated adjustment strategies based on diverse geographical contexts. Accurate estimates of anaemia prevalence are crucial for monitoring progress towards international reduction targets, as highlighted by extensive research efforts such as BRINDA. Assessing the impact of different elevation adjustment methods is critical to improving the accuracy of anaemia diagnosis and treatment worldwide. With improved methods for elevation adjustments now in place, future efforts can more effectively focus on evaluating the utility of adjustment recommendations specific to regional and demographic differences, enhancing methodologies for data collection and analysis, and revisiting assessments of anaemia prevention and treatment, especially in areas where anaemia has been historically underestimated or overestimated.
