# Supplementary material for: Implications of changes in WHO haemoglobin elevation adjustment guidelines on global, regional, and national anaemia burden, 1990–2023: a population-based modelling study
Source: Lancet Haematol. Author manuscript; Available in PMC 2026 May 11. (PMC13160282; doi:10.1016/S2352-3026(26)00013-X)
Supplement: Supplementary Material [file NIHMS2166597-supplement-Supplementary_Material.pdf]

Appendix to “Implications of changes in WHO haemoglobin elevation adjustment guidelines on global, regional, and national anaemia burden: Comparison of levels and trends from 1990 to 2023”

Table of Contents

Section 1: GATHER Checklist **3**

Section 2: Anaemia Estimation Methods **5**

Figure S0: GBD 2023 estimation flowchart for anaemia........ 5

Input Data and Methodological Summary for Anaemia **6**

Case definition........ 6

Table S1: WHO definitions of mild, moderate, and severe anaemia based on blood haemoglobin concentration (g/L) 6

Data seeking and data extraction........ 6

Figure S1: Maps of all unique source-location-year counts of data used in the anaemia envelope, where a) individual-level data at the country level, b) individual-level data at the most-detailed subnational level, c) tabulated data at the country level, and d) tabulated data at the most-detailed subnational level. 7

Elevation adjustments and changes to WHO guidelines in 2024 7

Figure S2: Haemoglobin adjustment for elevation by adjustment method with populations living within each 100 metre elevation bin. 8

Table S2. Haemoglobin adjustment equations associated with the WHO-2001 and WHO-2024 elevation adjustment methods. 8

Table S3. Example of the application of haemoglobin (Hb) adjustment amounts for both the WHO-2001 and WHO-2024 elevation adjustment methods for an individual residing at 1000 metres with an unadjusted haemoglobin measurement of 120 g/L. 8

Data processing **8**

Summary 8

Step 1: Extracting, elevation adjusting and assigning anaemia status to input data 8

Step 2: Age-sex splitting literature and VMNIS data 9

Table S4: Age/sex weight MR-BRT settings and spline parameters used in each model for S3a) males and females 0-2 years old, S3b) Males and females 2 – 125 years old, S3c) pregnant females 10 – 54 years old. 9

Table S4a. Model parameters for 0 – 2-year-olds, both males and females. 9

Table S4b. Model parameters for 2 – 125-year-olds, both males and females. 10

Table S4c. Model parameters for pregnant females (10 – 54 years old). 10

Table S5: Cascade *thetas* (weights) applied to each of the location hierarchies. 10

Figure S3: Example of cascade model outputs for females in Hidalgo (left; high data coverage) and b) males in Rwanda (right; low data coverage). 10

Step 3: Crosswalking by pregnancy status........ 10

Table S6: MR-BRT crosswalk values for adjusting mean haemoglobin by pregnancy status. 11

Step 4: Measure-to-measure crosswalks to “square the modelling datasets” 11

Table S7: Reference measures used to impute alternative measures, and the transformation used on the alternative measures. 11

Figure S4: Example of using the betas estimated in the women of reproductive age MR-BRT model to predict moderate plus severe anaemia using a reported mean haemoglobin value. 12

Step 5: Creating and assigning elevation to surveys based on modelled locations 12

Figure S5: Elevation raster file and the corresponding population weighted mean elevation output for all GBD modelled locations 13
Step 6: Adjust input data for elevation........ 13
Figure S6. Example of applying elevation crosswalk to estimate the new WHO elevation-adjusted total anaemia prevalence using an old WHO elevation-adjusted survey reported total anaemia prevalence of 25% for non-pregnant WRA living at 1,000 meters........ 15
Step 7: Preventing impossible prevalence combinations........ 15

Modelling strategy........ **16**
Step 1: ST-GPR models of mean haemoglobin and anaemia prevalence........ 16
Step 2: Ensemble distribution modelling........ 16
Generation of ensemble weights 16
Step 3: Haemoglobin variance optimisation 17
Fitting ensemble distributions with method of moments........ 17
Finding the area under the curve to calculate anaemia prevalence 18
Years lived with disability (YLD) calculation 18
Table S8. Anaemia disability weights and health states. 18

Secondary analysis: Epidemiological transition **18**
Figure S7. Differences in predicted total anaemia prevalence between the WHO-2024 and WHO-2001 elevation adjustment envelopes from the epi-trans analysis. The magenta boxes highlight the relevant elevation range, SDI range, and super-region that show the largest differences in predicted total anaemia prevalence. 20

Evaluating the differences in anemia prevalence by threshold used in 6-month-olds to 2-year-olds........ **20**
Table S9. Average differences in anaemia prevalence, by severity (columns), when using different combinations of elevation adjustment and haemoglobin cutoff methods (rows). Blue shaded boxes represent the full change between anaemia assessments using WHO-2024 and WHO-2021 definitions. Note the WHO did not update the severe anaemia haemoglobin cutoffs among children aged 6 months to 2 years. 20
Figure S8. Scatter plots illustrating anaemia prevalence estimates by severity (shown in columns) for children aged 6 months to 2 years, based on various combinations of elevation adjustment and haemoglobin cutoff methods (displayed in rows). Note that the layout of the scatter plots is the same as the layout in table S8........ 21

References........ **22**

Section 3: Anaemia envelope input data sources ***23***

Section 4: MR-BRT age and sex modelled trends........ ***100***

Section 5: MR-BRT measure-to-measure modelled estimates ***1160***

Section 6: MR-BRT modelled elevation adjustment crosswalk betas........ ***1165***

Section 7: Post-ensemble modelled estimates for the WHO-2001 and WHO-2024 envelopes ***1169***

Section 8: Global temporal trends of anaemia prevalence........ ***1991***

Section 9: Anaemia prevalence and YLD estimates........ ***1993***

Section 10: Epidemiological transition analysis ***2414***

Section 1: GATHER Checklist

#	GATHER checklist item	Description of compliance	Reference
**Objectives and funding**			
1	Define the indicator(s), populations (including age, sex, and geographic entities), and time period(s) for which estimates were made.	Narrative provided in paper and appendix describing indicators, definitions, populations, and time periods	Main text (Methods) and Appendix (Section 2)
2	List the funding sources for the work.	Funding sources listed in paper	Summary (Funding)
**Data Inputs**			
For all data inputs from multiple sources that are synthesised as part of the study:			
3	Describe how the data were identified and how the data were accessed.	Narrative description of data seeking methods provided	Main text (Methods) and Appendix (Section 2)
4	Specify the inclusion and exclusion criteria. Identify all ad-hoc exclusions.	Narrative about inclusion and exclusion criteria provided; ad hoc exclusions in appendix supplementary methods	Main text (Methods) and Appendix (Section 2)
5	Provide information on all included data sources and their main characteristics. For each data source used, report reference information or contact name/institution, population represented, data collection method, year(s) of data collection, sex and age range, diagnostic criteria or measurement method, and sample size, as relevant.	An interactive, online data source tool that provides metadata for data sources by component, geography, cause, risk, or impairment has been developed	Appendix (Sections 2-3) and Global Health Data Exchange (ghdx.healthdata.org)
6	Identify and describe any categories of input data that have potentially important biases (e.g., based on characteristics listed in item 5).	Summary of known biases included in appendix supplementary methods	Appendix (Section 2)
For data inputs that contribute to the analysis but were not synthesised as part of the study:			
7	Describe and give sources for any other data inputs.	Included in online data source tool	Global Health Data Exchange (ghdx.healthdata.org)
For all data inputs:			
8	Provide all data inputs in a file format from which data can be efficiently extracted (e.g., a spreadsheet rather than a PDF), including all relevant meta-data listed in item 5. For any data inputs that cannot be shared because of ethical or legal reasons, such as third-party ownership, provide a contact name or the name of the institution that retains the right to the data.	Downloads of input data available through online data tools, including data visualisation tools and data query tools; input data not available in tools will be made available upon request	Online data visualisation tools, data query tools, and the Global Health Data Exchange (ghdx.healthdata.org)
**Data analysis**			
9	Provide a conceptual overview of the data analysis method. A diagram may be helpful.	Flow diagram of methodological process provided, as well as narrative descriptions of modelling process	Main text (Methods) and Appendix (Section 2)
10	Provide a detailed description of all steps of the analysis, including mathematical formulae. This description should cover, as relevant, data cleaning, data pre-processing, data adjustments and weighting of data sources, and mathematical or statistical model(s).	Flow diagram and detailed methods write-up covering all data extraction, processing, and modelling processes provided	Main text (Methods) and Appendix (Section 2)
11	Describe how candidate models were evaluated and how the final model(s) were selected.	Provided in methodological write-up	Appendix (Section 2)

**12**	Provide the results of an evaluation of model performance, if done, as well as the results of any relevant sensitivity analysis.	Provided in methodological write-up	Appendix (Section 2)
**13**	Describe methods for calculating uncertainty of the estimates. State which sources of uncertainty were, and were not, accounted for in the uncertainty analysis.	Provided in main text methods narrative description and appendix methodological write-up	Main text (Methods) and Appendix (Section 2)
**14**	State how analytic or statistical source code used to generate estimates can be accessed.	Remote code repository for access to analytic code provided	Remote code repository (https://github.com/ihmeuw)
**Results and Discussion**			
**15**	Provide published estimates in a file format from which data can be efficiently extracted.	GBD 2023 results available through online data and visualisation tools, the Global Health Data Exchange, and the online data query tool	Main text, appendix, online data repository (ghdx.healthdata.org)
**16**	Report a quantitative measure of the uncertainty of the estimates (e.g. uncertainty intervals).	Uncertainty provided with all results	Main text, appendix, online data and visualisation tools
**17**	Interpret results in light of existing evidence. If updating a previous set of estimates, describe the reasons for changes in estimates.	Discussion of results and methodological changes between GBD rounds and comparisons to prior work provided in manuscript narrative and appendix	Main text (Methods and Discussion) and Appendix (Section 2)
**18**	Discuss limitations of the estimates. Include a discussion of any modelling assumptions or data limitations that affect interpretation of the estimates.	Discussion of limitations, including modelling assumptions and data limitations, included in manuscript narrative and appendix	Main text (Methods and Discussion) and Appendix (Section 2)

Section 2: Anaemia Estimation Methods

Figure S0: GBD 2023 estimation flowchart for anaemia

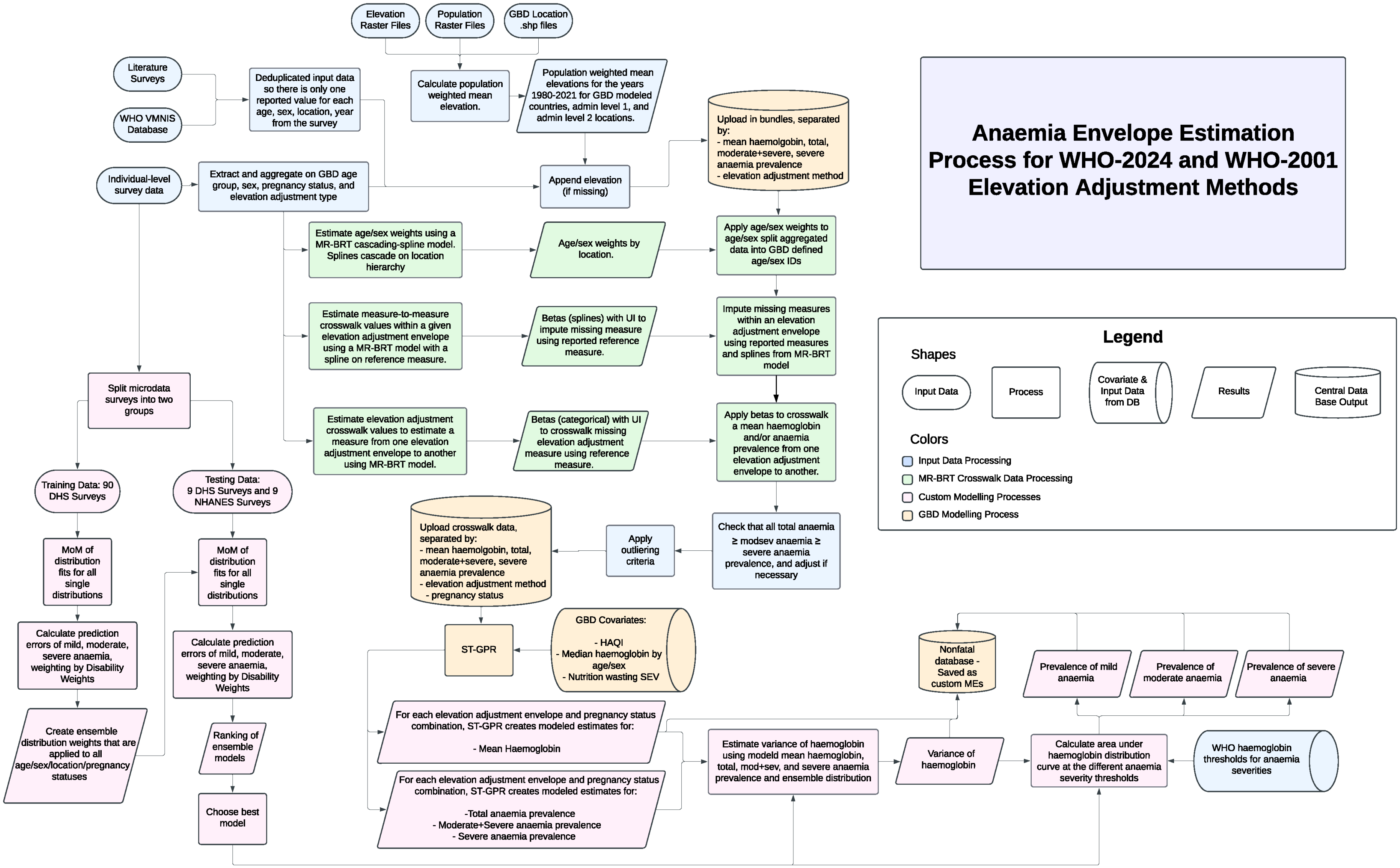

Input Data and Methodological Summary for Anaemia

Case definition

Anaemia is defined as decreased blood concentration of haemoglobin, irrespective of underlying cause, red blood cell morphology, or red blood cell function. Thresholds for defining individuals as being anaemic, as well as thresholds for anaemia severity, are based on the World Health Organization (WHO) thresholds for haemoglobin in g/L as shown below in Table S1.[1]

Table S1: WHO definitions of mild, moderate, and severe anaemia based on blood haemoglobin concentration (g/L)

Sex	Age	Mild	Moderate	Severe
Both	0–6 days	145–159	100–144	<100
Both	7–27 days	120–134	85–119	<85
Both	1–5 months	100–109	70–99	<70
Both	6–23 months	95–104	70–94	<70
Both	2–4 years	100–109	70–99	<70
Both	5–9 years	110–114	80–109	<80
Both	10–14 years	110–119	80–109	<80
Male	15+ years	110–129	80–109	<80
Female, non-pregnant	15+ years	110–119	80–109	<80
Female, pregnant	10+ years	100–109	70–99	<70

There are not any international guidelines on appropriate thresholds for diagnosing anaemia in neonates. To estimate thresholds for the neonatal period that reflect the higher haemoglobin levels typically seen in newborns, we calculated the ratio of "normal" haemoglobin (defined as the 50th percentile of the global haemoglobin distribution) for the 0–6 days, 7–27 days, and 1–5 month periods over the 6–11-month age group by severity, and then multiplied the WHO 6–59-months thresholds by these ratios.

Data seeking and data extraction

We searched the Global Health Data Exchange (GHDx) using the keywords "anemia" and "hemoglobin" to identify potential data sources for inclusion. Population-based individual-level surveys (microdata) – including the Demographic and Health Survey (DHS), Multiple Indicator Cluster Survey (MICS) series, national micronutrient surveys, and other national and subnational nutrition surveys – comprised the bulk of input data. We supplemented these data with additional sources of mean haemoglobin and anaemia prevalence from the WHO Vitamin and Mineral Nutrition Information Systems (VMNIS)[2] and other literature sources where available.

An overview of the source-location-years identified for both individual level data and tabulated data can be seen in Figure S1 along with a full list of all input data used in section 3 of this appendix.

Figure S1: Maps of all unique source-location-year counts of data used in the anaemia envelope, where a) individual-level data at the country level, b) individual-level data at the most-detailed subnational level, c) tabulated data at the country level, and d) tabulated data at the most-detailed subnational level.

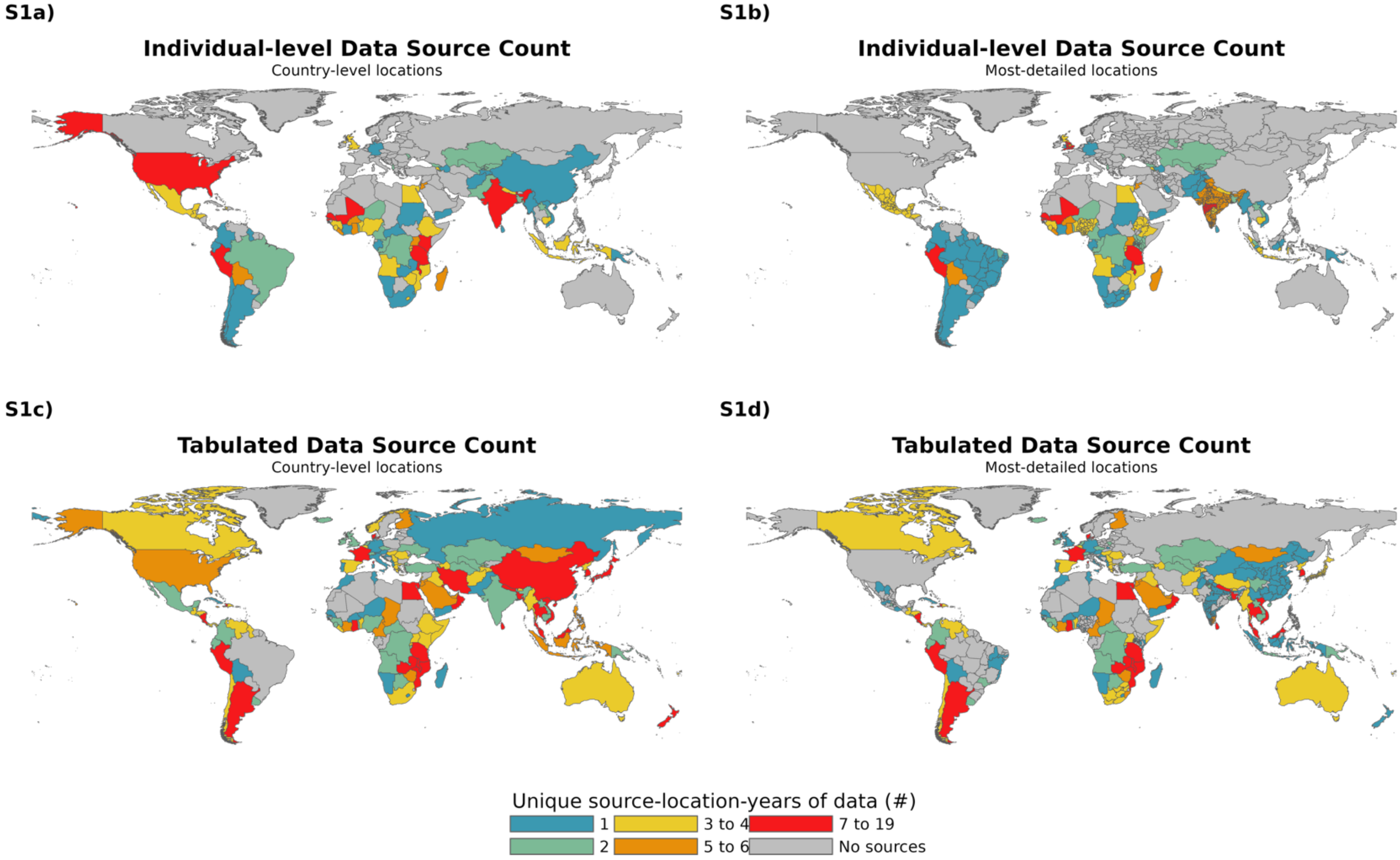

Elevation adjustments and changes to WHO guidelines in 2024

Haemoglobin concentration increases with increasing elevation, a physiological response to lower ambient oxygen levels that aims to maintain oxygen delivery throughout the body.[3] Due to this physiological response, the WHO recommends adjusting haemoglobin for elevation to ensure all haemoglobin concentrations are assigned anaemia statuses using the same haemoglobin thresholds shown in Table S1.[1] The amount that haemoglobin is adjusted for elevation varies based on both the elevation at which the haemoglobin measurement was taken and the adjustment method used.

This study used the newest elevation-adjustment equation developed by Biomarkers Reflecting Inflammation and Nutritional Determinants of Anaemia (BRINDA) working group, which used more input data from a greater diversity of locations than previous elevation adjustment formulas.[4] The previous WHO elevation adjustment method (WHO-2001) was under-adjusting haemoglobin for elevations below 3,000 metres and over-adjusting haemoglobin for elevations above 3,000 metres, seen in Figure S2, with corresponding equations for each adjustment method in Table S2. Given this, the 2024 WHO guidelines for anaemia assessment incorporated BRINDA's revised elevation adjustment equation.[1]

Figure S2: Haemoglobin adjustment for elevation by adjustment method with populations living within each 100 metre elevation bin.

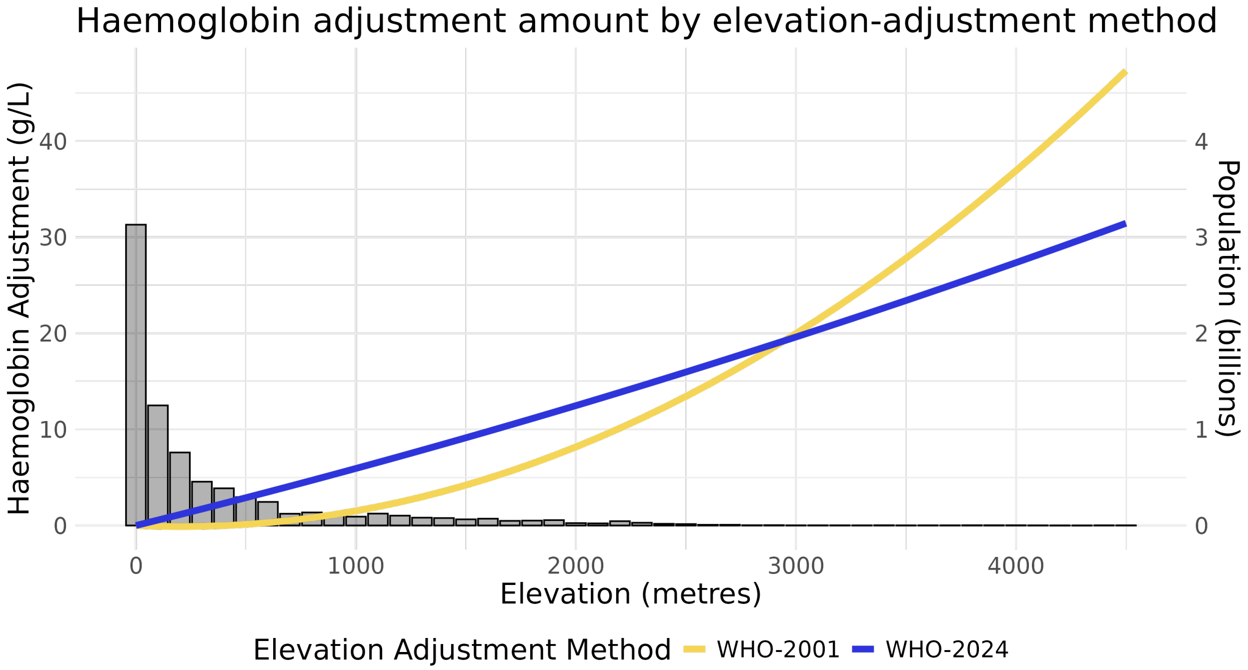

Table S2. Haemoglobin adjustment equations associated with the WHO-2001 and WHO-2024 elevation adjustment methods.

Adjustment Method	Adjustment Equation
WHO-2001	$\Delta Hb = -0.32 \times (elevation + 0.0033) + 0.22 \times (elevation + 0.0033)^2$
WHO-2024	$\Delta Hb = (0.0056384 \times elevation) + (0.0000003 \times elevation^2)$

** ΔHb denotes amount to subtract off of the raw/unadjusted haemoglobin concentration (Hb) in grams per litre (g/L)*
*** elevation is in metres (m)*

Table S3. Example of the application of haemoglobin (Hb) adjustment amounts for both the WHO-2001 and WHO-2024 elevation adjustment methods for an individual residing at 1000 metres with an unadjusted haemoglobin measurement of 120 g/L.

Adjustment Method	Elevation (m)	Unadjusted Hb (g/L)	Hb Adjustment Amount (g/L)	Elevation Adjusted Hb Measurement (g/L)
WHO-2001	1000 m	120 g/L	1.5 g/L	118.5 g/L
WHO-2024	1000 m	120 g/L	5.9 g/L	114.1 g/L

Data processing

Summary

We created two “anaemia envelopes” in parallel to inform the final new WHO elevation-adjusted data set. The two envelopes consisted of mean haemoglobin concentrations and anaemia prevalence values that were: 1) elevation-adjusted using the WHO-2001 method, and 2) elevation-adjusted using the WHO-2024 elevation adjustment method.

Step 1: Extracting, elevation adjusting and assigning anaemia status to input data

We extracted haemoglobin concentrations, elevation in metres (if present), elevation adjustment method, age, sex, location, year, and pregnancy status from survey data. Elevation information was either extracted directly from the

survey or, if the survey provided the latitude and longitude of the haemoglobin measurement site, we used those coordinates to extract elevation values from an elevation raster file.[5]

During the extraction of individual-level data, if the survey contained elevation (in metres) and a raw/unadjusted haemoglobin concentration, we adjusted each individual's haemoglobin concentration using both the WHO-2001 and WHO-2024 elevation adjustment methods, using the equations in Table S2, in parallel. All person-level haemoglobin concentrations were then assigned an anaemia status using the thresholds defined in Table S1.[1] Next, the individual-level data was organized into the WHO-2001 and WHO-2024 elevation-adjustment envelopes and aggregated into GBD age groups by sex and pregnancy status in six different formats: mean haemoglobin concentration, severe anaemia prevalence, moderate anaemia prevalence, moderate plus severe anaemia prevalence, mild anaemia prevalence, and total anaemia prevalence. We used the R *survey* package[6] to calculate the aggregated means and standard errors.

Tabulated data (such as VMNIS data and literature data) were also flagged for elevation-adjustment method used and appended to the appropriate anaemia envelope data set. All remaining aggregated individual-level data and all tabulated data were assigned an elevation in Step 5 (if missing) and adjusted for elevation using the WHO-2001 and WHO-2024 elevation adjustment methods in Step 6.

Step 2: Age-sex splitting literature and VMNIS data

Our modelling framework required that all input data be categorized according to the age and sex groups described above. However, some tabulated data sources reported mean haemoglobin levels and/or anaemia prevalence values that covered multiple age groups or combined both sexes (i.e., reported values for males and females together). As a result, we had to disaggregate all of the aggregated data points so they aligned with our specific age and sex categories to be included in the modelling process. To achieve this, we used collapsed individual-level survey data that had already been aggregated into age-sex-pregnancy groups and derived modelled age-sex patterns for mean haemoglobin, total anaemia, moderate plus severe anaemia, and severe anaemia prevalence using the meta-regression—Bayesian, regularised, trimmed (MR-BRT)[7] meta-analytic tool's cascading spline framework. Males, non-pregnant females, and pregnant females were all modelled separately. Within those subgroups, we modelled 2+ year-olds and 0–2-year-olds separately, which allows us to isolate the varying age trends in the younger age groups. The MR-BRT model uses a quadratic spline on age and is cascaded on location hierarchy, where splines are fit to the input data based on the inverse of the input point's standard error value. Spline parameters used can be seen in Table S4. The cascading framework is shown in Table S5.

We subsequently utilized the age-sex weight patterns derived from the cascading splines to disaggregate any data covering multiple age-sex groups into the specific groups they encompassed. In doing so, we accounted for uncertainty from both the original, unsplit data point and the fixed effects from the MR-BRT model. The location associated with the age-sex weights to be used from the MR-BRT cascade model is assigned by the nearest location level hierarchy modelled in our MR-BRT cascading framework (i.e., either using the same location, if that model is available, or using the model for a location hierarchy above). A full summary of the age-sex patterns that were estimated can be found in section 4 of this appendix.

Table S4: Age/sex weight MR-BRT settings and spline parameters used in each model for S3a) males and females 0-2 years old, S3b) Males and females 2 – 125 years old, S3c) pregnant females 10 – 54 years old.

Table S4a. Model parameters for 0 – 2-year-olds, both males and females.

Measure	Measure Transform	Knot Locations (age in years)	Left Tail Linearity Enforcement	Right Tail Linearity Enforcement	Concavity or Convexity Enforcement
Haemoglobin (g/L)	Log	0.5, 1	False	False	Convex
Anaemia Prevalence	Logit	0.5, 1	False	False	None

Table S4b. Model parameters for 2 – 125-year-olds, both males and females.

Measure	Measure Transform	Knot Locations (age in years)	Left Tail Linearity Enforcement	Right Tail Linearity Enforcement	Concavity or Convexity Enforcement
Haemoglobin (g/L)	Log	10, 15, 55, 70	False	True	Concave
Anaemia Prevalence	Logit	10, 15, 55, 70	False	True	Convex

Table S4c. Model parameters for pregnant females (10 – 54 years old).

Measure	Measure Transform	Knot Locations (age in years)	Left Tail Linearity Enforcement	Right Tail Linearity Enforcement	Concavity or Convexity Enforcement
Haemoglobin (g/L)	Log	25, 35	False	True	None
Anaemia Prevalence	Logit	25, 35	False	True	None

Table S5: Cascade *thetas* (weights) applied to each of the location hierarchies.

Cascade Location Level	Theta Weights
Global	---
Super Region	10
Region	8
Country	2
Admin Level 1	1
Admin Level 2	0.1

**The smaller the theta, the more the cascade model is influenced by the beta weights from its parent model.*

Figure S3: Example of cascade model outputs for females in Hidalgo (left; high data coverage) and b) males in Rwanda (right; low data coverage).

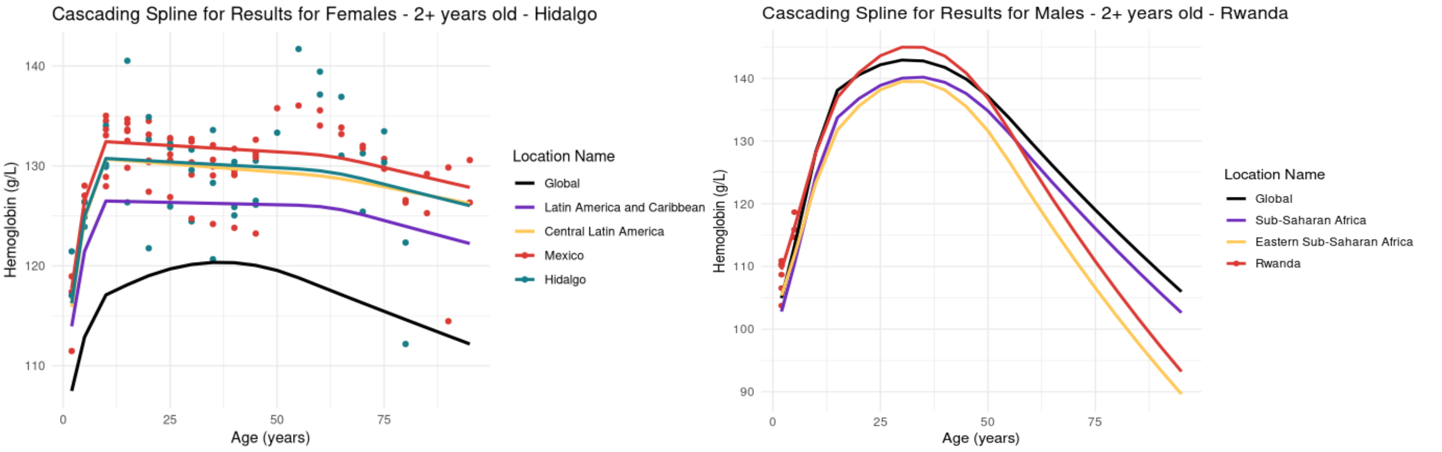

Step 3: Crosswalking by pregnancy status

Pregnancy causes physiologic and metabolic changes which means that haemoglobin levels are fundamentally different than in non-pregnant people. WHO definitions recognize these differences and have different threshold definitions for each of mild, moderate, and severe anaemia in pregnancy. Our models likewise generate estimates of haemoglobin distribution – and anaemia – separately for pregnant and non-pregnant females. The primary mechanism for this on the data processing side was to evaluate the systematic differences between mean haemoglobin level based on pregnancy status and then develop a crosswalk model that allows us to convert from one to the other (including quantification of the uncertainty in that relationship).

To evaluate the relationship, we matched data sources containing age-specific estimates of mean haemoglobin or anaemia prevalence separately by pregnancy status and calculated the ratio of these values among pregnant females versus non-pregnant females within a single study. We log-transformed these ratios and calculated standard errors of the ratios using the delta method. We then meta-analysed these ratios using MR-BRT with a 10% trim setting. Age was tested, but not included, as a fixed effect in the final model because we did not observe a significant age dependence of the ratios. The coefficients from the crosswalk model are illustrated in Table S6 below.

Table S6: MR-BRT crosswalk values for adjusting mean haemoglobin by pregnancy status.

Model	Data input	Reference or alternative case definition	Gamma	Beta coefficient, log (mean [Hb]) (95% UI)*	Adjustment factor**
**Mean haemoglobin**	Non-pregnant females	Ref	0.033	---	---
	Pregnant females	Alt		-0.08 (-0.15 to -0.02)	0.92 (0.86–0.98)

**MR-BRT crosswalk adjustments can be interpreted as the factor the alternative case definition is adjusted by to reflect what it would have been had it been measured using the reference case definition. If the log/logit beta coefficient is negative, then the alternative is adjusted up to the reference. If the log/logit beta coefficient is positive, then the alternative is adjusted down to the reference.*
***The adjustment factor column is the exponentiated beta coefficient. For log beta coefficients, this is the relative rate between the two case definitions. For logit beta coefficients, this is the relative odds between the two case definitions.*

Step 4: Measure-to-measure crosswalks to “square the modelling datasets”

To ensure internal consistency within all the reported mean haemoglobin and total anaemia prevalence, moderate plus severe anaemia prevalence, and severe anaemia prevalence values, we implemented the measure-to-measure crosswalk – an approach we term “squaring the dataset.” The squaring process involved using MR-BRT to impute an input data point for any missing measures (mean haemoglobin, total anaemia prevalence, moderate plus severe anaemia prevalence, severe anaemia prevalence) for all the age, sex, location, year, survey combinations present in the input dataset. For example, if a given source only reports total anaemia prevalence for a specific survey, location, year, age group, and sex combination, we generated an imputed datum for each of mean haemoglobin, moderate plus severe anaemia prevalence, and severe anaemia prevalence using our MR-BRT crosswalk framework.

To perform this operation, we created MR-BRT models to estimate crosswalk values (betas) which use a reported reference measure to estimate a missing alternate measure. Using aggregated individual-level survey data, we generated separate datasets for each of the demographic groups where the threshold definitions for mild, moderate, and severe anaemia are different (see Table S1). Within each group, we then created a dataset with one measure as the reference and a different measure as the alterative. The reference and alternative subsets are then matched by survey, age, sex, location, and year. This was repeated for all demographics and combinations of the varying reference measures that can be used to impute a missing alternative measure, shown in Table S7.

Table S7: Reference measures used to impute alternative measures, and the transformation used on the alternative measures.

Reference Measure	Alternative Measure	Alternative Measure Transform
Mean Haemoglobin	Total Anaemia Prevalence	Logit
Mean Haemoglobin	Moderate plus Severe Anaemia Prevalence	Logit
Mean Haemoglobin	Severe Anaemia Prevalence	Logit
Total Anaemia Prevalence	Mean Haemoglobin	Log
Moderate plus Severe Anaemia Prevalence	Mean Haemoglobin	Log

We modelled the alternative measure as a function of the reference measure. To account for the potential for non-linear relationships, we placed a continuous quadratic spline on the reference measure value with four internal frequency knots at the 10th, 30th, 70th, and 90th quantiles of the input data present along the exposure range. The spline was enforced to be monotonically decreasing. Linearity at the tails was not enforced, nor was convexity. No random effects were included in the model and each of the models were run using a 5% trim setting. Figure S4 provides an example as to how the estimated betas were applied and section 5 in this appendix shows the model fits for each of the two sets of five models (one set for each elevation adjustment method), where uncertainty is derived from fixed effects from the MR-BRT model. Uncertainty from fixed effects was incorporated when crosswalking an input data point from the reference measure to the alternate measure.

Figure S4: Example of using the betas estimated in the women of reproductive age MR-BRT model to predict moderate plus severe anaemia using a reported mean haemoglobin value.

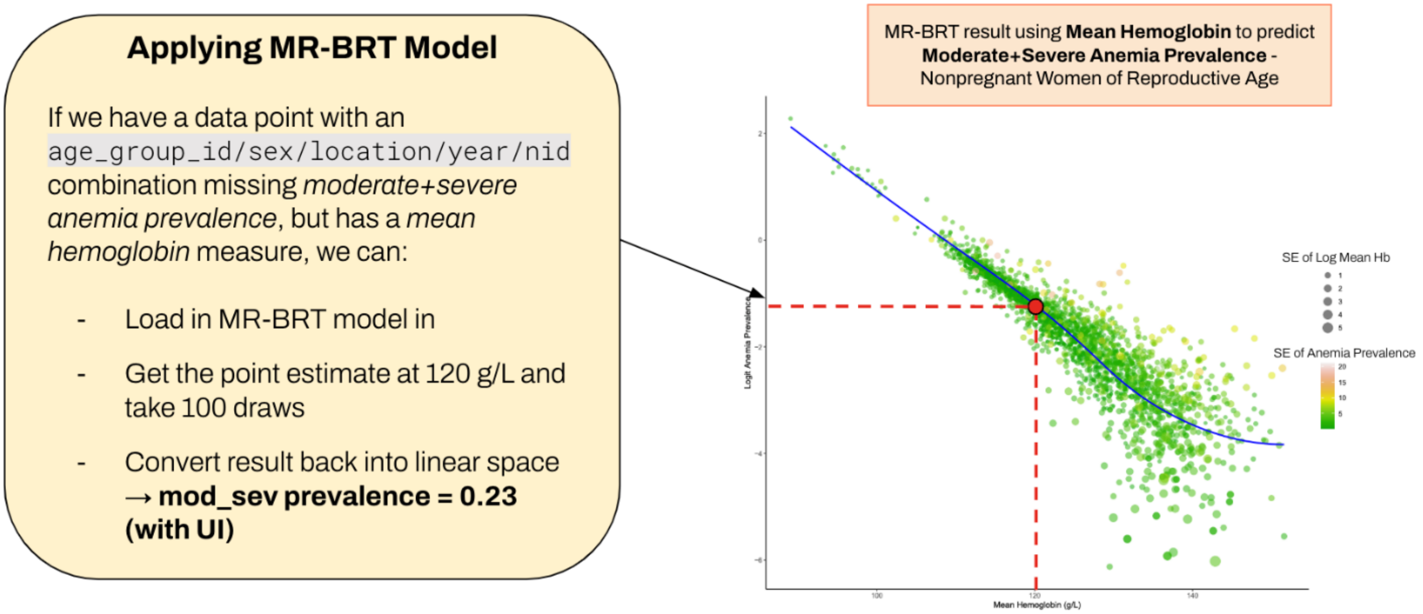

Step 5: Creating and assigning elevation to surveys based on modelled locations

Where individual-level elevation was not ascertainable, elevation was assigned based on the most granular modelled location from which the survey originated. We derived a data set of “population-weighted mean elevations” for each modelled location, using an elevation raster file[5] and population raster files from 1980 to 2021[8] by taking the mean elevation weighted by the population for each 5x5 kilometre grid within the polygon. If a survey was published before 1980 or after 2021 and did not report elevation, it was assigned a population weighted mean elevation from 1980 and 2021, respectively, seen in Figure S3.

Figure S5: Elevation raster file and the corresponding population weighted mean elevation output for all GBD modelled locations

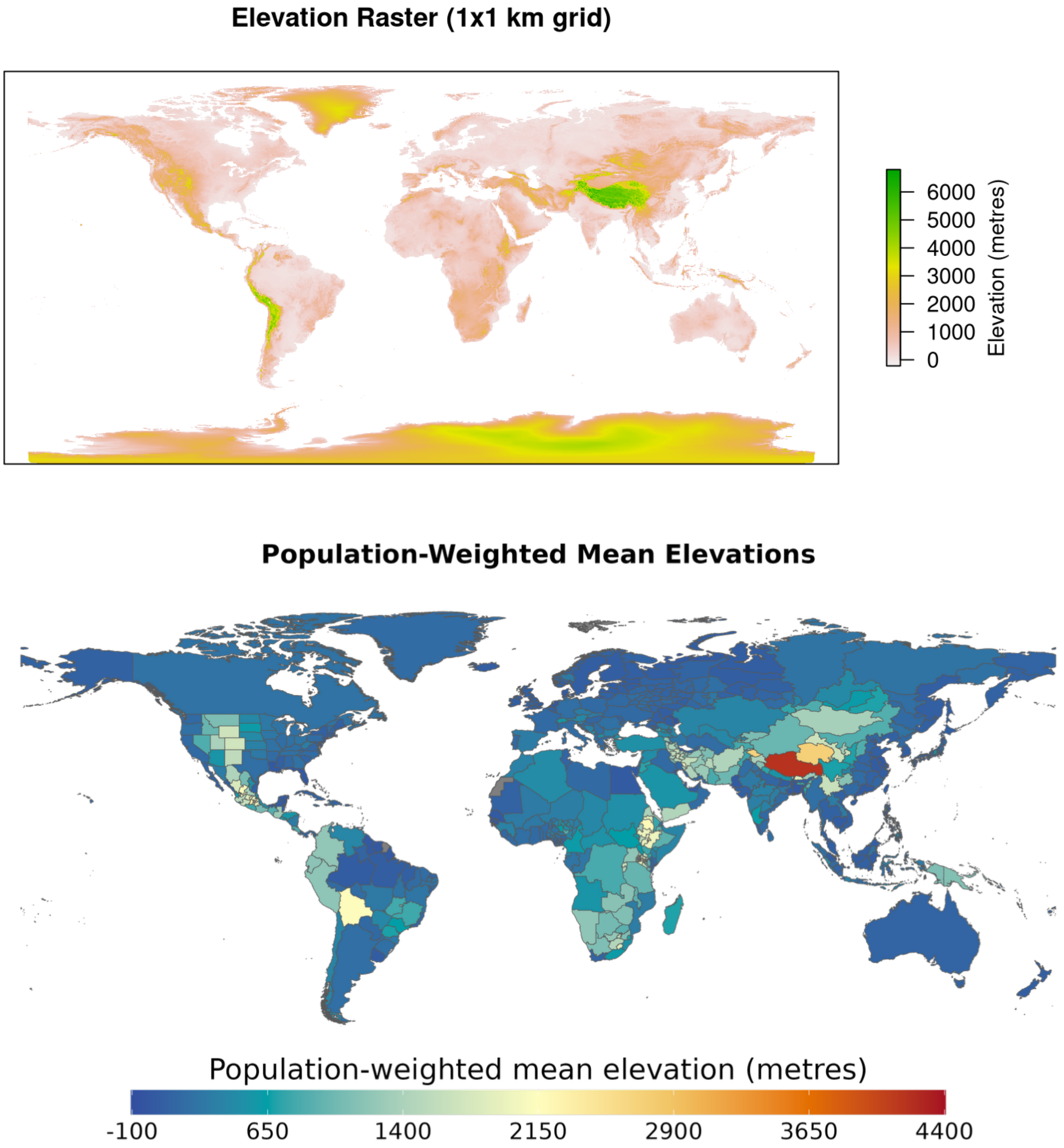

Step 6: Adjust input data for elevation

The final crosswalk step is to ensure all input data is adjusted using both the WHO-2001 and WHO-2024 elevation-adjustment methods. This includes the input data that could not be elevation adjusted in Step 1, where those data points now have both an elevation and mean haemoglobin concentration. This also includes tabulated data that only reported haemoglobin and/or anaemia prevalence values as either: 1) unadjusted for elevation or 2) adjusted using the WHO-2001 elevation adjustment method.

To incorporate mean haemoglobin concentrations into each of the WHO-2001 and WHO-2024 anaemia envelopes, we applied the respective elevation adjustment equations from each guideline. For unadjusted mean haemoglobin values, we applied the corresponding mean haemoglobin and elevation data to the WHO-2001 and WHO-2024 elevation adjustment equations to produce values consistent with each elevation-adjustment method. To adjust mean haemoglobin values from the WHO-2001 elevation-adjustment envelope to the WHO-2024 elevation adjustment envelope, we used the elevation and WHO-2001 elevation adjusted mean haemoglobin to un-adjust the mean haemoglobin concentration, and then subsequently readjusted that value using the same method for unadjusted mean haemoglobin concentrations to WHO-2024 elevation-adjusted values. The resulting mean haemoglobin concentration from each of these methods has the same standard error as it did prior to being adjusted, given there is no uncertainty component present in either of the elevation adjustment equations.

To crosswalk total anaemia prevalence, moderate plus severe anaemia prevalence, severe anaemia prevalence values between elevation adjustment methods, we created a suite of MR-BRT models to estimate betas to perform the crosswalk between elevation adjustment envelopes. Using collapsed microdata where input data from all elevation-adjustment methods are present, we subset the data by total anaemia prevalence, moderate plus severe anaemia prevalence, and severe anaemia prevalence values. Using those subsets, we then create two datasets: 1) reported prevalence value from the unadjusted envelope as the reference and the reported WHO-2024 elevation-adjusted reported value as the alternate, and 2) reported prevalence value from the WHO-2001 elevation-adjusted envelope as the reference and the WHO-2024 elevation-adjusted reported value as the alternate. The reference and alternative data sets are then matched on modelled age, sex, location, year, and study. The reference and alternative prevalence values were then logit transformed and subtracted from each other, and we subsequently delta transformed their standard errors. We then modelled the logit differences in anaemia prevalence using a MR-BRT model, with categorical covariates on elevation (in 400-meter bins), the non-transformed reference prevalence (in 20% increments), and created demographic categories based on the rows from Table S1. An example of the betas predicted for a given demographic can be seen in Figure S6 (note that we incorporated uncertainty from fixed effects when crosswalking from one elevation adjustment envelope to another, which is not shown in the example in Figure S6). All estimated anaemia prevalence crosswalk betas, with uncertainty, can be seen in section 5 of this appendix.

Figure S6. Example of applying elevation crosswalk to estimate the new WHO elevation-adjusted total anaemia prevalence using an old WHO elevation-adjusted survey reported total anaemia prevalence of 25% for non-pregnant WRA living at 1,000 meters.

1. Load in the MR-BRT estimated betas for crosswalking total anaemia prevalence using the old WHO elevation-adjustment method to the new WHO elevation-adjustment method, shown in the table.

2. Identify the categories the data point to be crosswalked correlates to (highlighted in red).

3. Convert the reference total anaemia prevalence to logit space:

$$logit(0.250) = -1.099$$

4. Add the estimated betas to the value calculated in step 3.

$$logit(prev) + \beta_0 + \beta_{elevation} + \beta_{anemia} =$$

$$-1.099 + 0.156 + 0.337 - 0.108 = \mathbf{-0.713}$$

5. Convert back to linear space

$$inv\_logit(-0.713) = \mathbf{0.329}$$

Variable Type	Category	Beta
Intercept	---	0.156
Elevation Category	0 m - 400 m	REFERENCE
	400 m - 800 m	0.132
	800 m - 1200 m	0.337
	1200 m - 1600 m	0.516
	1600 m - 2000 m	0.510
	2000 m - 2400 m	0.461
	2400 m - 2800 m	0.413
	2800 m - 3200 m	0.209
	3200 m - 3600 m	-0.270
	3600 m - 4000 m	-0.259
	4000 m - 4400 m	-0.678
	4400 m - 4800 m	-0.877
	4800 m - 5200 m	-1.959
	5200 m - 5600 m	-2.624
Reference Anaemia Prevalence Category	0% to 20%	REFERENCE
	20% to 40%	-0.108
	40% to 60%	-0.164
	60% to 80%	-0.197
	80% to 100%	-0.128

The result of this process provided us with a square dataset, where all input data is present in each of the WHO-2001 and WHO-2024 elevation-adjustment envelopes and contains a mean haemoglobin concentration and total anaemia prevalence, moderate plus severe anaemia prevalence, and severe anaemia prevalence value.

Step 7: Preventing impossible prevalence combinations

We enforced total anaemia prevalence to be greater than or equal to moderate plus severe anaemia prevalence and, in turn, to be greater or equal to severe anaemia prevalence for each unique age, sex, location, year, study combination in our datasets. This situation occurs when a survey only reports an anaemia prevalence for only one or two of the severity levels along with a mean haemoglobin, where we are then left to impute the remaining one or two missing anaemia prevalence values in Step 5. In some surveys, the reported anaemia prevalence value(s) was (were) far lower than what was estimated when using our measure-to-measure imputation models in Step 5 with the given reported mean haemoglobin concentration. In these cases, the measure-to-measure imputation step can potentially estimate a moderate plus severe anaemia prevalence value that is larger than the reported total anaemia prevalence or estimate a severe anaemia prevalence that is larger than the reported moderate plus severe anaemia prevalence. All observations in violation of this constraint were re-imputed as follows:

To re-estimate anaemia prevalence values that violate the severity constraints, we utilized the MR-BRT models created in the Step 5. To implement this approach, it also required that the mean haemoglobin measure be reported in the survey along with at least one of: 1) total anaemia, 2) moderate plus severe anaemia, or 3) severe anaemia prevalence values also being reported. If a mean haemoglobin and at least one of the anaemia prevalence values were present, then we first use the reported mean haemoglobin to estimate total anaemia, moderate plus severe anaemia, and severe anaemia prevalence values for the given age, sex, location, year, study combination using the MR-BRT measure-to-measure models from Step 5. These estimates are then used to create ratios of the imputed severe to moderate plus severe anaemia prevalence estimates and imputed moderate plus severe to total anaemia prevalence estimates. We

then apply the ratios to the reported anaemia prevalence value to reimpute the anaemia prevalence value(s) that violated the inequality constraints.

In instances where anaemia prevalence values violated the constraints and there is not enough information to implement this alternate imputation approach, the given age, sex, year, location, study combination was outliered from all the mean haemoglobin, total anaemia, moderate plus severe anaemia, and severe anaemia data sets in both the WHO-2001 and WHO-2024 elevation adjustment envelopes.

Modelling strategy

Estimation of overall anaemia prevalence occurred in four steps: 1) spatiotemporal Gaussian process regression (ST-GPR) models of mean haemoglobin and prevalence of total, moderate plus severe, and severe anaemia, 2) calculation of ensemble weights, 3) generation of ensemble distributions, and 4) calculation of anaemia prevalence based on the ensemble distributions. Each of these steps were performed in parallel for each of the WHO-2001 and WHO-2024 datasets constructed above.

Step 1: ST-GPR models of mean haemoglobin and anaemia prevalence

We modelled 1) mean haemoglobin, 2) total anaemia prevalence, 3) moderate plus severe anaemia prevalence, and 4) severe anaemia prevalence using spatiotemporal Gaussian process regression (ST-GPR),[9] a three-step modelling process for generating estimates for every location globally, year, age, and sex. The first step of the ST-GPR process is to estimate a linear prior using predictive covariates to help guide the model's directionality and intercept. The following covariates were used in the stage-one linear prior step: Summary Exposure Value (SEV) for child wasting, Healthcare Access and Quality (HAQ) Index, and 50th percentile of haemoglobin (pooled across all microdata sources).

The second, spatiotemporal smoothing step of ST-GPR calculates the residual between our stage 1 regression estimate and each of our observed datapoints and then smooths this residual, drawing strength over space, age, and time and producing a revised stage 2 estimate for every modelled location, year, age, and sex. The third step of ST-GPR is a Gaussian process regression, using the stage 2 estimates as a prior and the observed datapoints and their variance to 1) further smooth the residual between the stage 2 predictions and observed data and produce a final mean estimate for each location, year, age, and sex, and 2) estimate uncertainty around this mean estimate, quantified by taking 1,000 draws from the posterior Gaussian process.

Step 2: Ensemble distribution modelling

We modelled the full distribution of haemoglobin for each population (location/age/year/sex), from which we applied the WHO thresholds to calculate prevalence of anaemia by severity. This process involves combining multiple two-parameter distributions to create a precise and unbiased ensemble distribution.

Generation of ensemble weights

First, we created a training and testing set of individual-level haemoglobin measurements. The training set consisted of 90 DHS surveys, providing 290 group-specific samples of person-level data from children <5, males 15–45, pregnant females 15–45, and non-pregnant females 15–45 (not all groups were sampled in each DHS). A set of two-parameter distributions (gamma, mirrored gamma, Weibull, mirror lognormal, and mirrored Gumbel) were fit to the sample's haemoglobin mean and variance. These distributions were combined using weights optimised by a loss function of severity-specific prediction error weighted by the ratio of the severity's disability weight (DW) to mild anaemia DW. Weights were constrained to be positive and sum to 1, so that the resultant ensemble distribution is a proper probability density function. All permutations of the five distributions were tested (i.e., we optimised weights for both a mix of all five distributions as well as a gamma-Weibull two-way combination).
The loss function is:

$$\sum_{i=i}^{n_{surveys}} \sum_{j=1}^{n_{demographics}} \sum_{k=1}^{n_{severities}} r_k \left| p_{ijk} - \widehat{p_{ijk}} \right|$$

such that:

$$\widehat{p_{ijk}} = \sum_{z=1}^{n_{distributions}} w_z \int_{t1_{jk}}^{t2_{jk}} PDF_{ijz}\, \mathrm{dt}\,,\ 0 \leq \widehat{p_{ijk}} \leq 1$$

where:

$i \in surveys : 1 \leq i \leq n_{surveys}$, the list of surveys (in either the training or testing set)

$j \in demographics : 1 \leq j \leq n_{demographics}$, the demographics (rows) found in Table 1

$k \in severities : 1 \leq k \leq n_{severities}$, the list of severities (mild, moderate, severe)

r is the ratio of the severity *k* disability weight to that of mild anaemia, r_{mild} = 1, $r_{moderate}$ = 13 and r_{severe} = 40

PDF is a probability density function fit to the sample mean and variance

t1 and t2 are the lower and upper bounds to the WHO anaemia definition for the group found in Table 1

w is the set of distribution weights s.t. $0 \leq w_z \leq 1$ and $\sum_{z=1}^{n_{distributions\ used}} w_z = 1$

$z \in distributions : 1 \leq z \leq n_{distributions}$, the list of distributions (gamma, mirror gamma, Weibull, mirror lognormal, and mirror gumbel)

The testing set consisted of nine National Health and Nutrition Examination Surveys (NHANES) and nine DHS surveys not included in the training data. Inclusion of NHANES as half the testing set ensured out-of-sample predictive validity by challenging the global weights, as it provided the ensemble distribution with high-income data (DHS is from LMIC countries) and data from adults >45 (DHS did not take blood tests from older populations). We selected the combination of distributions (including all individual component distributions) that minimised the loss function. For this analysis, this resulted in an ensemble distribution that was 40% gamma and 60% mirrored Gumbel.

Step 3: Haemoglobin variance optimisation

In order to generate an ensemble distribution of haemoglobin concentration, we first required an estimate of the variance of the haemoglobin distribution. To generate an estimate of distribution variance, we used our ST-GPR estimated draws of mean haemoglobin and prevalence of total, moderate plus severe, and severe anaemia prevalence in a variance-optimisation algorithm. For every location, year, age, and sex, we anchored the distributions at the estimated mean haemoglobin value and found the variance value that minimised the error between our estimates of severe, moderate plus severe, and total anaemia and the corresponding values implied by a given mean haemoglobin and variance combination using the ensemble distribution of 40% gamma and 60% mirrored Gumbel and implementing the loss function shown above. We weighted the errors in the variance optimisation algorithm based on the severity-specific disability weights, such that the severe anaemia error was weighted the most and total anaemia was weighted the least.

Fitting ensemble distributions with method of moments

Using our global model weights and our estimates of mean haemoglobin and standard deviation of haemoglobin, we modelled the full distribution of haemoglobin for each modelled location, year, age, and sex by fitting each component distribution on the modelled mean and standard deviation and weighting each individual distribution to create the ensemble distribution:

$$\sum_{z=1}^{n_{final\ distributions}} w_z * PDF_z$$

where:

$z \in \text{final } distributions : 1 \leq z \leq n_{final\ distributions}$, the list of chosen distributions (40% gamma and 60% mirrored Gumbel)

Because anaemia thresholds depend on pregnancy, we separately modelled the distribution of haemoglobin among pregnant and non-pregnant women. Our modelling process directly provided estimates of mean haemoglobin and variance of haemoglobin concentration among non-pregnant women; to estimate the mean haemoglobin among pregnant women, we shifted the non-pregnant mean haemoglobin estimate for each location, year, age, and sex by the crosswalk adjustment factor calculated, as described in the data processing section. We assumed that the distribution variance was the same among pregnant and non-pregnant women.

We used these estimates of mean haemoglobin and SD of haemoglobin concentration by pregnancy status to produce separate weighted ensemble distributions. To combine these distributions to represent the entire population of females in a given modelled location, year, and age, we used the proportion of pregnant women and weighted each of the haemoglobin distributions among pregnant and non-pregnant women accordingly to produce a final general population distribution.

Finding the area under the curve to calculate anaemia prevalence

Using the WHO anaemia thresholds shown in Table S1, we calculated the prevalence of mild, moderate, and severe anaemia for each location, year, age group, and sex as the area under the mean haemoglobin concentration curve between the thresholds for each severity. Uncertainty about each estimated mean was derived by sampling draws from the posterior distributions estimated in the ST-GPR models.

Years lived with disability (YLD) calculation

Total years lived with disability (YLDs) for a given disease were calculated as the prevalence of the disease multiplied by the unadjusted disability weight (DW) associated with that disease. The disability weight captures the level of health loss due to that illness and ranges between zero (no health loss) and one (death). Table S8 details the disability weights and health state descriptions associated with each severity of anaemia.[10] We calculated YLDs by anaemia severity for every location, year, age, and sex in the analysis.

Table S8. Anaemia disability weights and health states.

Anemia Severity	Health State Description	Disability Weight
Mild Anaemia	Feels slightly tired and weak at times, but this does not interfere with normal daily activities.	0.004 (0.001 – 0.008)
Moderate Anaemia	Feels moderate fatigue, weakness, and shortness of breath after exercise, making daily activities more difficult.	0.052 (0.034 – 0.076)
Severe Anaemia	Feels very weak, tired and short of breath, and has problems with activities that require physical effort or deep concentration.	0.149 (0.101 – 0.209)

Secondary analysis: Epidemiological transition

The epidemiological transition theory is based on the idea that changes in health, mortality, survival, and fertility over time and place are associated with the available health care and technology, in addition to socioeconomic, environmental, and demographic factors.[11] This theory assumes increasing socioeconomic development will lead to increased access to health care and disease prevention, suggesting that non-communicable disease prevalence will increase as the sociodemographic index (SDI)[12] increases. To evaluate if the elevation-adjustment method led to changes in the historical relationship between anaemia burden and sociodemographic development, we performed an epidemiological transition analysis comparing the results for the two adjustment methods. Briefly, this took the form of

ecological analyses between SDI and age-specific and sex-specific total, moderate plus severe, and severe anaemia prevalence across all GBD locations and years using MR-BRT models.[7]

The input data for the model were created by matching post-ensemble age-specific and sex-specific anaemia prevalences with location-year specific SDI estimates. Prevalence data were logit transformed, and all locations were given an equal weight in the meta-regression. We then ran a series of sex-specific and age-specific MR-BRT models, each with a quadratic, continuous spline on SDI with domain knots at the 33.33rd and 66.66th percentiles. Linearity was enforced on the left tail of the spline for each model, but not on the right tail. Monotonicity was not enforced in any of the models. We then re-aggregated results by age and sex to find the total mean relationship between SDI and anaemia. Additionally, we examined the correlation between SDI and adjusted haemoglobin concentration according to the method of elevation adjustment.

As shown in the main text, there is a strong relationship between SDI and the estimated anaemia prevalence in both elevation adjustment envelopes. It is also apparent that the WHO-2024 elevation adjustment envelope estimates a higher anaemia prevalence, as SDI decreases across all anaemia severities when compared to the WHO-2001 elevation adjusted anaemia prevalence estimates (seen in main text Figure 4c, 4f, and 4i). Focusing on the SDI range below 0.25, which is where the predicted anaemia prevalence differs the most between the elevation adjustment methods—as shown in Figure S7 for differences in predicted total anaemia prevalence—the locations that are present in this SDI range are all from sub-Saharan Africa (SSA). These same locations from SSA also have elevations that range from 500 to 2000 metres, which is the elevation range that is expected to have the largest difference in estimated anaemia prevalence between the WHO-2024 and WHO-2001 elevation adjustment methods, as show in main text Figure 1b, 1c, and 1d. While elevations within this range do contribute to differences in predicted anaemia prevalence across the elevation adjustment methods for locations in the SDI range below 0.25, we also observed that total anemia prevalence estimated with the WHO-2024 elevation adjustment shows a more linear relationship with SDI compared to estimates using the WHO-2001 method. This stronger relationship suggests that the societal factors that determine SDI level may be even more relevant for anaemia burden than previously thought and could be relevant across the entire domain of SDI.

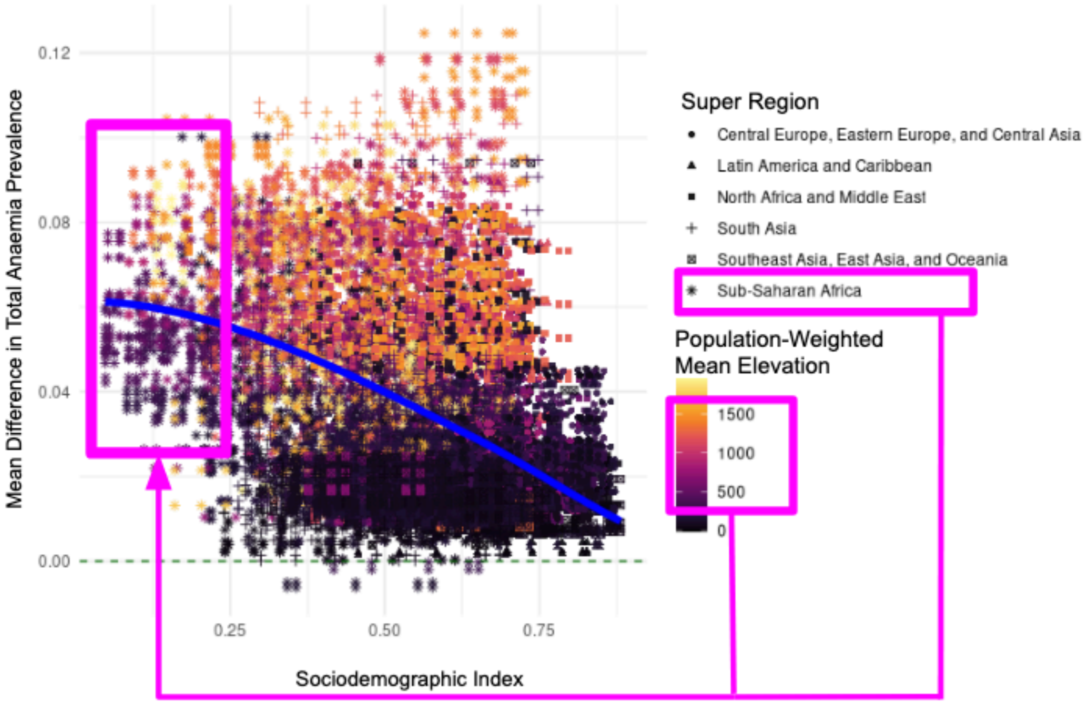

Figure S7. Differences in predicted total anaemia prevalence between the WHO-2024 and WHO-2001 elevation adjustment envelopes from the epi-trans analysis. The magenta boxes highlight the relevant elevation range, SDI range, and super-region that show the largest differences in predicted total anaemia prevalence.

Evaluating the differences in anemia prevalence by threshold used in 6-month-olds to 2-year-olds

In its latest guidelines on haemoglobin thresholds for diagnosing anaemia, the WHO lowered the cutoff values for children aged 6 months to 2 years and for pregnant women in their second trimester. In both groups, the thresholds for mild and moderate anaemia were reduced by 5 g/L—from 110 g/L to 105 g/L for mild anemia, and from 100 g/L to 95 g/L for moderate anaemia, which can impact anaemia prevalence assessments, as shown previously.[13–15]

In our anaemia envelope modeling pipeline, we applied the new WHO haemoglobin cutoffs to define anemia among children aged 6 months to 2 years in all models. We did not incorporate the updated cutoffs for pregnant women in their second trimester, as we did not generate anaemia prevalence estimates by trimester. Instead, we estimated anaemia prevalence for all pregnant women using the haemoglobin thresholds provided in Table S1.

As a parallel secondary analysis, however, we re-calculated anemia prevalence for all individual level data sources using the WHO-2001 versus WHO-2024 anaemia thresholds for 6 months to 2 years. This age range in our analysis is represented by two distinct age groups of 6 to 11 months and 12 to 23 months. After this additional calculation, we had three anaemia prevalence datasets for comparison: 1) WHO-2001 elevation + WHO-2001 thresholds, 2) WHO-2024 elevation + WHO-2001 thresholds, and 3) WHO-2024 elevation + WHO-2024 thresholds.

Simple average differences in anaemia prevalence across all location-years for children aged 6 months to 2 years are shown in Table S9. Scatters of the estimated anaemia prevalence for each survey, location, and year combination, by severity, are shown in Figure S8. When assessing the difference in estimated anaemia prevalence within the same elevation adjustment method envelope, the original versus updated haemoglobin cutoffs using the WHO-2024 elevation adjustment method resulted in an average decrease of 11.6% (17.7 decrease to 4.3 decrease) in total anaemia. To assess the overall impact of applying the new WHO elevation adjustment methods and haemoglobin thresholds, we observed an average decrease in total anaemia prevalence of 5.8% (13.3 decrease to 0.6 decrease) compared to the total anaemia prevalence found using the original WHO recommendations. Although we estimated an overall increase in total anaemia prevalence across all ages and sexes globally using the WHO-2024 elevation adjustment method, the revised haemoglobin thresholds for mild and moderate anaemia for children aged 6 months to 2 years offset these increases, ultimately leading to a reduction in the estimated number of anaemia cases for this age group.

Table S9. Average differences in anaemia prevalence, by severity (columns), when using different combinations of elevation adjustment and haemoglobin cutoff methods (rows). Blue shaded boxes represent the full change between anaemia assessments using WHO-2024 and WHO-2021 definitions. Note the WHO did not update the severe anaemia haemoglobin cutoffs among children aged 6 months to 2 years.

Adjustment/Threshold Comparison	Average Absolute Difference in Anaemia Prevalence (%, range)		
	Total Anaemia	Moderate plus Severe Anaemia	Severe Anaemia
WHO-2024 Adj. (original thresholds) – WHO-2001 Adj. (original thresholds)	5.8% (0.8 to 14.3)	6.7% (-0.2 to 13.3)	1.3% (-0.6 to 4.5)
WHO-2024 Adj. (updated thresholds) – WHO-2024 Adj. (original thresholds)	-11.6% (-17.7 to -4.3)	-13.0% (-16.8 to -7.3)	--
WHO-2024 Adj. (updated thresholds) – WHO-2001 Adj. (original thresholds)	-5.8% (-13.3 to -0.6)	-6.3% (-12.9 to -0.5)	1.3% (-0.6 to 4.5)

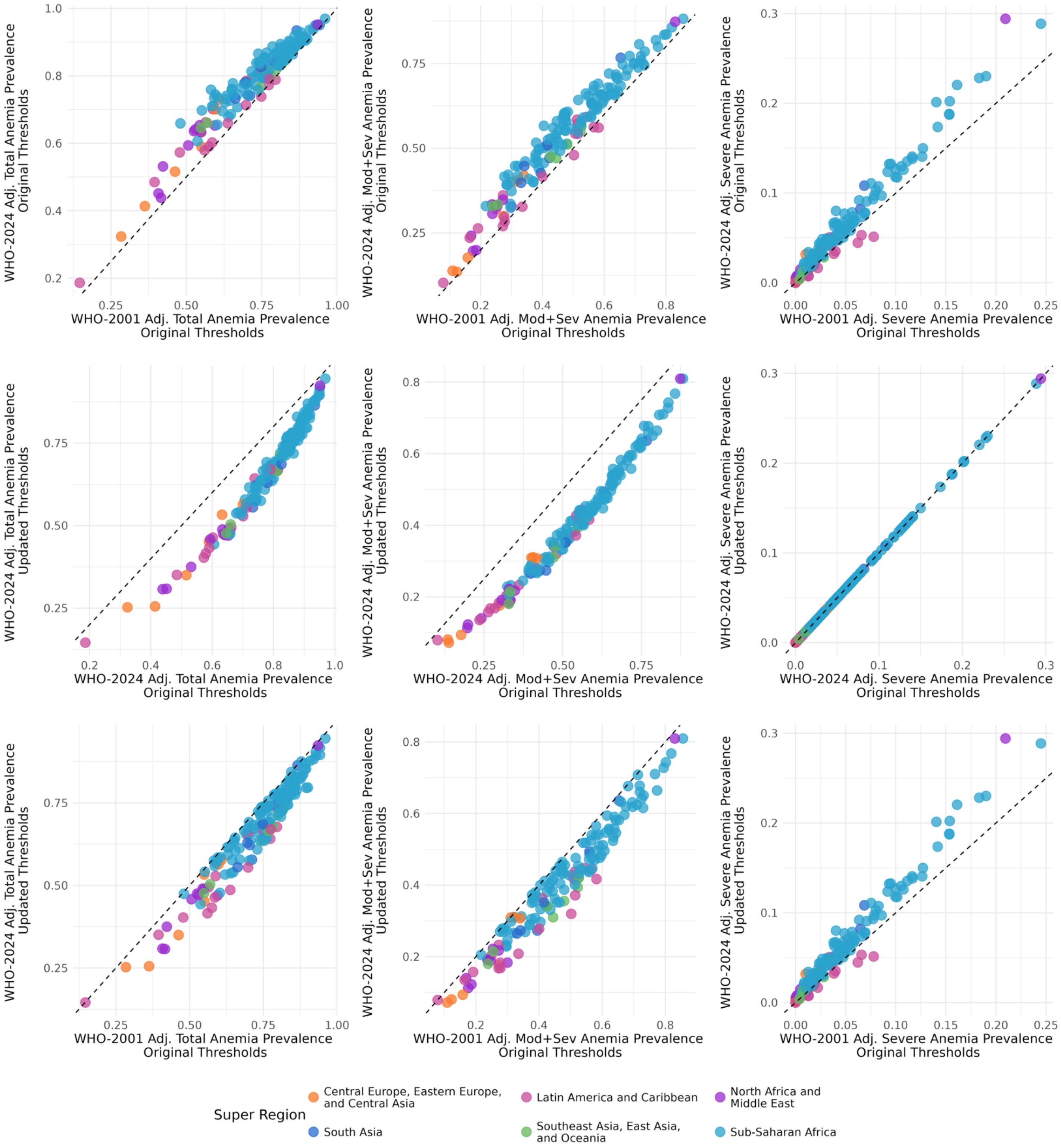

Figure S8. Scatter plots illustrating anaemia prevalence estimates by severity (shown in columns) for children aged 6 months to 2 years, based on various combinations of elevation adjustment and haemoglobin cutoff methods (displayed in rows). Note that the layout of the scatter plots is the same as the layout in table S8.

References

1 WHO. Guideline on haemoglobin cutoffs to define anaemia in individuals and populations. Geneva: World Health Organization, 2024 https://iris.who.int/bitstream/handle/10665/376196/9789240088542-eng.pdf?sequence=1 (accessed Feb 3, 2025).

2 Micronutrients Database [online database]. In: Vitamin and Mineral Nutrition Information System (VMNIS). Geneva: World Health Organization. 2021. https://www.who.int/teams/nutrition-and-food-safety/databases/vitamin-and-mineral-nutrition-information-system/data (accessed Dec 1, 2022).

3 Haase VH. Hypoxic regulation of erythropoiesis and iron metabolism. *Am J Physiol - Ren Physiol* 2010; **299**: F1–13.

4 Sharma AJ, Addo OY, Mei Z, Suchdev PS. Reexamination of hemoglobin adjustments to define anemia: altitude and smoking. *Ann N Y Acad Sci* 2019; **1450**: 190–203.

5 Hastings D, Dunbar. Global Land One-kilometer Base Elevation (GLOBE) v.1. *Natl Geophys Data Cent NOAA* 1999. DOI:10.7289/V52R3PMS.

6 Lumley T. survey: analysis of complex survey samples. 2024.

7 Zheng P, Barber ,Ryan, Sorensen ,Reed J. D., Murray ,Christopher J. L., and Aravkin AY. Trimmed Constrained Mixed Effects Models: Formulations and Algorithms. *J Comput Graph Stat* 2021; **30**: 544–56.

8 Geography and Environmental Science, University of Southampton, WorldPop. Age and Sex Structures, Global Per Country 2000-2020. *Southampt U K WorldPop* 2018.

9 Ferrari AJ, Santomauro DF, Aali A, *et al.* Global incidence, prevalence, years lived with disability (YLDs), disability-adjusted life-years (DALYs), and healthy life expectancy (HALE) for 371 diseases and injuries in 204 countries and territories and 811 subnational locations, 1990–2021: a systematic analysis for the Global Burden of Disease Study 2021. *The Lancet* 2024; **403**: 2133–61.

10 Gardner WM, Razo C, McHugh TA, *et al.* Prevalence, years lived with disability, and trends in anaemia burden by severity and cause, 1990–2021: findings from the Global Burden of Disease Study 2021. *Lancet Haematol* 2023; **10**: e713–34.

11 Omran AR. The epidemiologic transition theory revisited thirty years later. *World Health Stat Q WHO* 1998; **53**: 99–119.

12 Schumacher AE, Kyu HH, Aali A, *et al.* Global age-sex-specific mortality, life expectancy, and population estimates in 204 countries and territories and 811 subnational locations, 1950–2021, and the impact of the COVID-19 pandemic: a comprehensive demographic analysis for the Global Burden of Disease Study 2021. *The Lancet* 2024; **403**: 1989–2056.

13 Gonzales GF, Tapia V, Vásquez-Velásquez C. Changes in hemoglobin levels with age and altitude in preschool-aged children in Peru: the assessment of two individual-based national databases. *Ann N Y Acad Sci* 2021; **1488**: 67–82.

14 Silubonde T, Baumgarter J. Adjusting Haemoglobin Values for Altitude Maximizes Combined Sensitivity and Specificity to Detect Iron Deficiency among Women of Reproductive Age in Johannesburg, South Africa. *Nutrients* 2020; **12**: 633.

15 Luo H, Liu L, Young MF, Suchdev PS. The effects of revised haemoglobin cutoffs on the global burden of anaemia. *Lancet Haematol* 2025; **12**: e325–6.

Section 3: Anaemia envelope input data sources

Overview of input data used in our modelling process, displaying the location, citation, and NID associated with each input data source. The NID is a unique identification number associated with an input data source and can be used to look up and access more information about a given source on the Global Health Data Exchange, a catalog of health data maintained by IHME.

Anaemia Envelope Input Data Sources

NID	Location	Citation
56830	Afghanistan	Central Statistics Organization (Afghanistan), United Nations Children's Fund (UNICEF). Afghanistan Multiple Indicator Cluster Survey 2010-2011. New York, United States of America: United Nations Children's Fund (UNICEF), 2013.
135163	Afghanistan	Centers for Disease Control and Prevention (CDC), Ministry of Public Health (Afghanistan), National Institute for Research on Food and Nutrition (INRAN) (Italy), Tufts University, United Nations Children's Fund (UNICEF). Afghanistan National Nutrition Survey 2004.
139286	Afghanistan	HOPE worldwide, Ministry of Public Health (Afghanistan), World Health Organization (WHO). Afghanistan National Malaria Prevalence Survey 2002.
257841	Afghanistan	Aga Khan University, Central Statistics Organization (Afghanistan), Ministry of Public Health (Afghanistan), Silk Route Training and Research Organization (SRTRO), United Nations Children's Fund (UNICEF). Afghanistan National Nutrition Survey 2013.
18834	Albania	ICF Macro, Institute of Public Health (Albania), Institute of Statistics (Albania). Albania Demographic and Health Survey 2008-2009. Fairfax, United States of America: ICF International, 2009.
143687	Albania	Buonomo E, Cenko F, Altan AMD, Godo A, Marazzi MC, Palombi L. Iron deficiency anemia and feeding practices in Albanian children. Ann Ig. 2005; 17(1): 27-33.
393876	Albania	Albania Institute of Public Health (IPH), Albania Institute of Statistics (INSTAT), ICF International. Albania Demographic and Health Survey 2017-2018. Fairfax, United States of America: ICF International, 2019.
142784	American Samoa	Republic of Kiribati. Pacific Helminths Initiative 2001-2002.
490160	American Samoa	Kemmer TM, Novotny R, Gerber AS, Ah Ping I. Anaemia, its correlation with overweight and growth patterns in children aged 5-10 years living in American Samoa. Public Health Nutr. 2009; 12(5): 660-6.
672	Angola	COSEP-Consulting Ltd., Consaúde Ltd., Macro International, Inc, Ministry of Health (Angola). Angola Malaria Indicator Survey 2006-2007. Fairfax, United States of America: ICF International.
56169	Angola	COSEP-Consulting Ltd., Consaúde Ltd., ICF International, National Malaria Control (Angola), President's Malaria Initiative (PMI). Angola Malaria Indicator Survey 2011. Fairfax, United States of America: ICF International.
136611	Angola	Angola Ministry of Health, United Nations Children's Fund (UNICEF). Assessing Vitamin A and Iron Deficiency

NID	Location	Citation
		Anaemia, Nutritional Anaemia among Children Aged 0-60 Months in the Republic of Angola. 2000.
218555	Angola	ICF International, Ministry of Health (Angola), National Institute of Statistics (Angola), United Nations Children's Fund (UNICEF). Angola Demographic and Health Survey 2015-2016. Fairfax, United States of America: ICF International, 2017.
257552	Angola	Angola Ministry of Health, United Nations Children's Fund (UNICEF). Assessing Vitamin A and Iron Deficiency Anaemia, Nutritional Anaemia among Children Aged 0-60 Months in the Republic of Angola. 2000.
143689	Antigua and Barbuda	Micronutrient Working Group. Iron and vitamin A status in five Caribbean countries. Cajanus. 2002; 4-34.
56715	Argentina	Ministry of Health and Environment (Argentina), United Nations Development Programme (UNDP). Argentina National Survey of Nutrition and Health 2004-2005. Argentina: Ministry of Health and Environment (Argentina).
137334	Argentina	Center for Studies on Infant Nutrition (CESNI) (Argentina), Jorge Macri Foundation (Argentina). Argentina - Tierra del Fuego Project Baseline Health and Nutrition Survey 1995. Buenos Aires, Argentina: Jorge Macri Foundation (Argentina), 1995.
140949	Argentina	Morasso M del C, Molero J, Vinocur P, Acosta L, Paccussi N, Raselli S, Falivene G, Viteri FE. [Iron and vitamin A deficiencies and prevalence of anemia in boys and girls between 6 to 24 months of age in Chaco, Argentina]. Arch Latinoam Nutr. 2003; 53(1): 21-7.
143690	Argentina	Calvo EB, Gnazzo N. Prevalence of iron deficiency in children aged 9-24 mo from a large urban area of Argentina. Am J Clin Nutr. 1990; 52(3): 534-40.
143691	Argentina	Calvo E, Islam J, Gnazzo N, Ibáñez M, de Martínez C, de Vacaliuc R, Quintana E, Carmuega E. Encuesta nutricional en niños de 2 años de la provincia de misiones, II: indicadores dietéticos y hematológicos. Arch Argent Pediatr. 1987; 260-9.
143692	Argentina	Morasso M del C, Molero J, Vinocur P, Acosta L, Paccussi N, Raselli S, Falivene G, Viteri FE. [Iron deficiency and anemia in pregnant women from Chaco, Argentina]. Arch Latinoam Nutr. 2002; 52(4): 336-43.
143693	Argentina	Calvo EB, Sosa EM. Iron status in non-pregnant women of child-bearing age living at Greater Buenos Aires. Eur J Clin Nutr. 1991; 45(4): 215-20.
143694	Argentina	Marín GH, Fazio P, Rubbo S, Baistrocchi A, Sager G, Gelemur A. [Prevalence of anaemia in pregnancy and analysis of the underlying factors]. Aten Primaria. 2002; 29(3): 158-63.
18843	Armenia	Ministry of Health (Armenia), National Statistical Service (NSS), ORC Macro. Armenia Demographic and Health

NID	Location	Citation
		Survey 2000. Fairfax, United States of America: ICF International.
18854	Armenia	MOH Center for Health Information and Statistics, Macro International, Inc, National Statistical Service (NSS). Armenia Demographic and Health Survey 2005. Fairfax, United States of America: ICF International.
139415	Armenia	Branca F, Napoletano A, Coclite D, Rossi L. Armenia Health and Nutritional Status of Children and Women 1998. Rome, Italy: National Institute for Research on Food and Nutrition (INRAN) (Italy), 1998.
144314	Armenia	World Health Organization (WHO). WHO Global Database on Anemia, Nutrition Landscape Information System. Geneva, Switzerland: World Health Organization (WHO).
218563	Armenia	ICF International, Ministry of Health (Armenia), National Statistical Service of the Republic of Armenia. Armenia Demographic and Health Survey 2015-2016. Fairfax, United States of America: ICF International, 2017.
113243	Australia	Australian Bureau of Statistics. Australia National Health Survey 2011-2013.
143697	Australia	Rangan AM, Aitkin I, Blight GD, Binns CW. Factors affecting iron status in 15-30 year old female students. Asia Pac J Clin Nutr. 1997; 6(4): 291-5.
143699	Australia	Fleming AF, Martin JD, Stenhouse NS. Pregnancy anaemia, iron and folate deficiency in Western Australia. Med J Aust. 1974; 2(13): 479-84.
130538	Austria	Federal Ministry of Health (Austria), University of Vienna. Austria Study on Nutritional Status 2010-2012.
18865	Azerbaijan	Macro International, Inc, State Statistical Committee of Azerbaijan. Azerbaijan Demographic and Health Survey 2006. Fairfax, United States of America: ICF International.
27338	Azerbaijan	Adventist Development and Relief Agency (ADRA), Azerbaijan Ministry of Health, State Statistical Committee of Azerbaijan, and Centers for Disease Control and Prevention (CDC). (2003) Azerbaijan Reproductive Health Survey 2001. Atlanta, United States: Centers for Disease Control and Prevention (CDC).
139416	Azerbaijan	Centers for Disease Control and Prevention (CDC). Azerbaijan Health and Nutrition Survey of Internally Displaced and Resident Population 1996.
144314	Azerbaijan	World Health Organization (WHO). WHO Global Database on Anemia, Nutrition Landscape Information System. Geneva, Switzerland: World Health Organization (WHO).
230882	Azerbaijan	Azerbaijan Ministry of Health, GroundWork LLC, State Statistical Committee of Azerbaijan, United Nations Children's Fund (UNICEF). Azerbaijan Nutrition Survey 2013.

NID	Location	Citation
111628	Bahrain	Ministry of Health (Bahrain). Bahrain National Nutrition Survey 1998-1999.
139420	Bahrain	Ministry of Health (Bahrain). Bahrain National Flour Fortification Program First Monitoring Study 2002.
144314	Bahrain	World Health Organization (WHO). WHO Global Database on Anemia, Nutrition Landscape Information System. Geneva, Switzerland: World Health Organization (WHO).
55956	Bangladesh	ICF Macro, Mitra and Associates, National Institute of Population Research and Training (NIPORT). Bangladesh Demographic and Health Survey 2011-2012. Calverton, United States of America: ICF Macro.
139421	Bangladesh	Helen Keller International. Bangladesh Nutritional Surveillance Project Bulletin No. 10, March 2002. New York City, United States of America: Helen Keller International, 2002.
139422	Bangladesh	Helen Keller International. High Anemia Prevalence Among Bangladeshi Children in Urban Slums: An Ethical and Economic Rationale for Multi-micronutrient Supplementation. New York City, United States: Helen Keller International, 2000. (HKI-Nutrition Surveillance Project Bulletin, no. 1).
139424	Bangladesh	Institute of Public Health Nutrition (Bangladesh), International Centre for Diarrhoeal Disease Research, Bangladesh (ICDDR,B), National Institute of Population Research and Training (NIPORT). Bangladesh National Nutrition Programme Baseline Survey 2004-2005.
139426	Bangladesh	Helen Keller International. Bangladesh Nutritional Surveillance Project Bulletin No. 16, April 2006. New York City, United States of America: Helen Keller International, 2006.
143701	Bangladesh	Stoltzfus R, Chakraborty J, Rice A, Briere B, Francisco A. Plausible evidence of effectiveness of an iron supplementation pr ogramme for pregnant and post-partum women in rural Bangladesh. Food Nutr Bull. 1998; 197-204.
143702	Bangladesh	Ahmed F, Khan MR, Karim R, Taj S, Hyderi T, Faruque MO, Margetts BM, Jackson AA. Serum retinol and biochemical measures of iron status in adolescent schoolgirls in urban Bangladesh. Eur J Clin Nutr. 1996; 50(6): 346-51.
143703	Bangladesh	Ahmed F, Mahmuda I, Sattar A, Akhtaruzzaman M. Anaemia and vitamin A deficiency in poor urban pregnant women of Bangladesh. Asia Pac J Clin Nutr. 2003; 12(4): 460-6.
143704	Bangladesh	Bhargava A, Bouis HE, Scrimshaw NS. Dietary intakes and socioeconomic factors are associated with the hemoglobin concentration of Bangladeshi women. J Nutr. 2001; 131(3): 758-64.

NID	Location	Citation
143706	Bangladesh	Kolsteren P, Rahman SR, Hilderbrand K, Diniz A. Treatment for iron deficiency anaemia with a combined supplementation of iron, vitamin A and zinc in women of Dinajpur, Bangladesh. Eur J Clin Nutr. 1999; 53(2): 102-6.
143707	Bangladesh	Hasin A, Begum R, Khan MR, Ahmed F. Relationship between birth weight and biochemical measures of maternal nutritional status at delivery in Bangladeshi urban poors. Int J Food Sci Nutr. 1996; 47(3): 273-9.
143708	Bangladesh	Rahman S, Hilderbrand K, Kolsteren P, Diniz A. A nutritional profile of non-pregnant women from the slums of Dinajpur, Bangladesh. Trop Doct. 1999; 29(4): 221-4.
143710	Bangladesh	Ziauddin Hyder S, Persson Lk, Chowdhury A, Ekström EC. Anaemia among non-pregnant women in rural Bangladesh. Public Health Nutr. 2001; 4(1): 79-83.
143711	Bangladesh	Monawarul Islam AI, Siddiqua R, Rahman A, Rahman A, Ara I. Iron-deficiency anaemia in pregnancy: (biochemical investigations of iron-deficiency and nutritional status). Bangladesh Med Res Counc Bull. 1977; 3(1): 1-8.
143712	Bangladesh	Hussain MA, Khan AK, Abedin Z, Ferdous Z, Ahmad K. Studies on the nutritional status of expectant mothers and newborn babies. Bangladesh Med Res Counc Bull. 1976; 2(2): 120-6.
143713	Bangladesh	Ahmed F, Khan MR, Islam M, Kabir I, Fuchs GJ. Anaemia and iron deficiency among adolescent schoolgirls in peri-urban Bangladesh. Eur J Clin Nutr. 2000; 54(9): 678-83.
261683	Bangladesh	Fogarty International Center, National Institutes of Health (NIH), Foundation for the National Institutes of Health (FNIH), International Centre for Diarrhoeal Disease Research, Bangladesh (ICDDR,B). Bangladesh - Dhaka Malnutrition and Enteric Disease Study 2009-2014.
262810	Bangladesh	Global Alliance for Improved Nutrition (GAIN), Institute of Public Health Nutrition (Bangladesh), International Centre for Diarrhoeal Disease Research, Bangladesh (ICDDR,B), United Nations Children's Fund (UNICEF). Bangladesh National Micronutrients Status Survey 2011.
490191	Bangladesh	Bangladesh Bureau of Statistics (BBS). Anemia Prevalence Survey of Urban Bangladesh and Rural Chittagong Hill Tracts 2003. 2004.
490700	Bangladesh	Helen Keller International. Nutritional Surveillance Project in Bangladesh in 1999: Towards the goals of the 1990 World Summit for Children. 2004.
139427	Barbados	Caribbean Food and Nutrition Institute, Pan American Health Organization (PAHO), Ministry of Health (Barbados). Barbados National Health and Nutrition Survey 1981.
143715	Belgium	Dirren H, Decarli B, Lesourd B, Schlienger JL, Deslypere JP, Kiepurski A. Nutritional status: haematology and albumin. Eur J Clin Nutr. 1991; 45 (Suppl 3): 43-52.

NID	Location	Citation
143716	Belgium	Massot C, Vanderpas J. A survey of iron deficiency anaemia during pregnancy in Belgium: analysis of routine hospital laboratory data in Mons. Acta Clin Belg. 2003; 58(3): 169-77.
143717	Belgium	Elwood PC, Hughes J, Abernethy M, Davies R, Gough R, Johnson AP, Dubourg AY. An international haematological survey. Bull World Health Organ. 1976; 54(1): 87-95.
139429	Belize	Institute of Nutrition of Central America and Panama, Ministry of Health (Belize), Pan American Health Organization (PAHO), United Nations Children's Fund (UNICEF). Belize Iron Deficiency Anemia Survey 1994-1995.
489965	Belize	Rosenthal J, Largaespada N, Bailey LB, Cannon M, Alverson CJ, Ortiz D, Kauwell GP, Sniezek J, Figueroa R, Daly R, Allen P. Folate Deficiency Is Prevalent in Women of Childbearing Age in Belize and Is Negatively Affected by Coexisting Vitamin B-12 Deficiency: Belize National Micronutrient Survey 2011. J Nutr. 2017; 147(6): 1183-1193.
18950	Benin	National Institute of Statistics and Economic Analysis (INSAE) (Benin), ORC Macro. Benin Demographic and Health Survey 2001. Fairfax, United States of America: ICF International.
18959	Benin	Macro International, Inc, National Institute of Statistics and Economic Analysis (INSAE) (Benin), National Program Against AIDS (PNLS) (Benin). Benin Demographic and Health Survey 2006. Fairfax, United States of America: ICF International.
79839	Benin	ICF International, National Institute of Statistics and Economic Analysis (INSAE) (Benin), National Program Against AIDS (PNLS) (Benin). Benin Demographic and Health Survey 2011-2012. Fairfax, United States of America: ICF International, 2014.
143719	Benin	Hercberg S, Chauliac M, Galán P, Devanlay M, Zohoun I, Agboton Y, Soustre Y, Bories C, Christides JP, Potier de Courcy G. Relationship between anaemia, iron and folacin deficiency, haemoglobinopathies and parasitic infection. Hum Nutr Clin Nutr. 1986; 40(5): 371-9.
143720	Benin	Hercberg S, Chauliac M, Galan P, Devanlay M, Zohoun I, Agboton Y, Soustre Y, Auvert B, Masse-Raimbault AM, Dupin H. Prevalence of iron deficiency and iron-deficiency anaemia in Benin. Public Health. 1988; 102(1): 73-83.
143721	Benin	Hercberg S, Chauliac M, Devanlay M, Galán P, Pureur J, Soustre Y, Houdegbe A, Agboton Y, Zohoun I, Masse-Raimbault AM, Dupin H. Evaluation of the iron status of a rural population in south Benin. Nutr Res. 1986; 627-34.
143722	Benin	Ogbeide O, Wagbatsoma V, Orhue A. Anaemia in pregnancy. East Afr Med J. 1994; 672-3.
218565	Benin	Hubert Koutoukou Maga National University Hospital Center (CNHU-HKM)(Benin), ICF International, National Institute of Statistics and Economic Analysis (INSAE) (Benin), National

NID	Location	Citation
		Malaria Control Program, Ministry of Health (Benin), Permanent Secretariat of the Food Council and Nutrition (SP-CAN)(Benin). Benin Demographic and Health Survey 2017-2018. Fairfax, United States of America: ICF International, 2018.
139431	Bhutan	Ministry of Health and Education (Bhutan). Anemia Among Men, Women, and Children. How Big is the Problem? Thimphu, Bhutan: Ministry of Health and Education (Bhutan), 2003.
139433	Bhutan	Sood SK, Sharma S. Occurrence and Causes of Nutritional Anaemia in Bhutan. New Delhi, India: University College of Medical Sciences (India), 1985.
144314	Bhutan	World Health Organization (WHO). WHO Global Database on Anemia, Nutrition Landscape Information System. Geneva, Switzerland: World Health Organization (WHO).
425208	Bhutan	Khesar Gyalpo University of Medical Sciences of Bhutan (KGUMSB), Ministry of Health (Bhutan), National Statistics Bureau (Bhutan), United Nations Children's Fund (UNICEF). Bhutan National Nutrition Survey 2015.
18971	Bolivia (Plurinational State of)	Macro International, Inc, National Institute of Statistics (Bolivia). Bolivia Demographic and Health Survey 1998. Fairfax, United States of America: ICF International.
19001	Bolivia (Plurinational State of)	Macro International, Inc, Ministry of Health and Sports (Bolivia), National Institute of Statistics (Bolivia). Bolivia Demographic and Health Survey 2003-2004. Fairfax, United States of America: ICF International.
19016	Bolivia (Plurinational State of)	Macro International, Inc, Ministry of Health and Sports (Bolivia), National Institute of Statistics (Bolivia). Bolivia Demographic and Health Survey 2008. Fairfax, United States of America: ICF International.
285880	Bolivia (Plurinational State of)	Ministry of Health (Bolivia), Unit for Analysis of Social and Economic Policies (UDAPE) (Bolivia). Bolivia Health and Nutrition Assessment Survey 2012. La Paz, Bolivia: Unit for Analysis of Social and Economic Policies (UDAPE) (Bolivia).
323944	Bolivia (Plurinational State of)	Ministry of Health (Bolivia), National Institute of Statistics (Bolivia). Bolivia Demographic and Health Survey 2016. La Paz, Bolivia: National Institute of Statistics (Bolivia), 2017.
490193	Bolivia (Plurinational State of)	Ministry of Health and Sports (Bolivia). Encuesta nacional de nutrición según niveles de vulnerabilidad a la inseguridad alimentaria (línea de base) [Bolivia National Nutrition Survey According to Levels of Vulnerability to Food Insecurity (Baseline)]. La Paz: Ministry of Health and Sports; 2007.
137418	Botswana	Ministry of Health (Botswana), United Nations Children's Fund (UNICEF). Botswana Micronutrient Malnutrition Survey 1994.
144314	Botswana	World Health Organization (WHO). WHO Global Database on Anemia, Nutrition Landscape Information System.

NID	Location	Citation
		Geneva, Switzerland: World Health Organization (WHO).
139457	Brazil	Piaui State Government, United Nations Children's Fund (UNICEF). Brazil - Piauí Children and Adolescents: Health, Education, and Work 1991. Teresina, Brazil: Piaui State Government, 1992.
139466	Brazil	Almeida APC, Zandonade E, Arantes MM, Lamounier JA. Deficiência de ferro e anemia ferropriva na população de 6 meses a 6 anos em Vitória, Espírito Santo, Sudeste do Brasil [Iron Deficiency and Iron Deficiency Anemia in the Population of 6 Months to 6 Years in Vitória, Espírito Santo, Southeastern Brazil] [master's thesis]. Presented at: UFMG, Belo Horizonte, Brazil; 2000.
139467	Brazil	Stefanini MLR. Merenda escolar: história, evolução e contribuição no atendimento das necessidades nutricionais da criança [School Lunch: History, Evolution and Contribution in Addressing the Nutritional Needs of the Child] [dissertation]. Presented at: University of São Paulo; 1998; São Paulo, Brazil.
140951	Brazil	Martins MC, Santos LMP, Assis AMO. [Prevalence of hypovitaminosis A among preschool children from northeastern Brazil, 1998]. Rev Saude Publica. 2004; 38(4): 537-42.
141948	Brazil	Brazilian Center for Analysis and Planning (CEBRAP), Brazilian Institute of Public Opinion and Statistics (IBOPE), Ministry of Health (Brazil). Brazil National Demographic and Health Survey of Children and Women 2006-2007. Rio de Janeiro, Brazil: Ministry of Health (Brazil).
143730	Brazil	Muniz-Junqueira MI, Queiroz EFO. Relationship between protein-energy malnutrition, vitamin A, and parasitoses in living in Brasília. Rev Soc Bras Med Trop. 2002; 35(2): 133-41.
143731	Brazil	Osório MM, Lira PI, Batista-Filho M, Ashworth A. Prevalence of anemia in children 6-59 months old in the state of Pernambuco, Brazil. Rev Panam Salud Publica. 2001; 10(2): 101-7.
143742	Brazil	Rondo PH, Abbott R, Rodrigues LC, Tomkins AM. Vitamin A, folate, and iron concentrations in cord and maternal blood of intra-uterine growth retarded and appropriate birth weight babies. Eur J Clin Nutr. 1995; 49(6): 391-9.
143753	Brazil	Assis AMO, Gaudenzi EN, Gomes G, Ribeiro R de C, Szarfarc SC, Souza SB de. [Hemoglobin concentration, breastfeeding and complementary feeding in the first year of life]. Rev Saude Publica. 2004; 38(4): 543-51.
195010	Brazil	Brazilian Institute of Geography and Statistics (IBGE), Ministry of Health (Brazil), Ministry of Planning, Budget, and Management (Brazil). Brazil National Survey of Health 2013. Rio de Janeiro, Brazil: Brazilian Institute of Geography and Statistics (IBGE).

NID	Location	Citation
261873	Brazil	Fogarty International Center, National Institutes of Health (NIH), Foundation for the National Institutes of Health (FNIH), National Institute of Science, Technology, and Biomedicine of Semi-Arid Brazil (INCT-IBISAB). Brazil - Fortaleza Malnutrition and Enteric Disease Study 2009-2014.
139609	Brunei Darussalam	Ministry of Health (Brunei Darussalam), Ministry of Health (Laos), Ministry of Health (Malaysia). Nutritional Status of Children Under Five Years Old and Pregnant Women in Brunei 1995-1996.
144314	Brunei Darussalam	World Health Organization (WHO). WHO Global Database on Anemia, Nutrition Landscape Information System. Geneva, Switzerland: World Health Organization (WHO).
149400	Brunei Darussalam	Ministry of Health (Brunei Darussalam). Brunei National Nutritional Status Survey (NNSS) 1997.
149401	Brunei Darussalam	Ministry of Health (Brunei Darussalam). Brunei National Health and Nutritional Status Survey (NHANSS) 2009-2011.
19088	Burkina Faso	Macro International, Inc, National Institute of Statistics and Demography (Burkina Faso). Burkina Faso Demographic and Health Survey 2003. Fairfax, United States of America: ICF International.
19133	Burkina Faso	ICF Macro, Ministry of Health (Burkina Faso), National Institute of Statistics and Demography (Burkina Faso). Burkina Faso Demographic and Health Survey 2010-2011. Fairfax, United States of America: ICF International.
143771	Burkina Faso	Meda N, Cousens S, Kanki B. Anaemia among women of reproductive age in Burkina Faso. World Health Forum. 1996; 17(4): 369-72.
188785	Burkina Faso	ICF International, National Center for Research and Training on Malaria (CNRFP) (Burkina Faso), National Institute of Statistics and Demography (Burkina Faso), National Program for the Fight Against Malaria (PNLP) (Burkina Faso). Burkina Faso Malaria Indicator Survey 2014. Fairfax, United States of America: ICF International, 2015.
283273	Burkina Faso	Institut de Recherche pour le Développement (IRD); Institut de Recherche en Sciences de la Santé (IRSS); and HarvestPlus, International Food Policy Research Institute (IFPRI). 2016. Food consumption and iron status survey in two provinces of rural Burkina Faso. Washington, DC: International Food Policy Research Institute (IFPRI). http://dx.doi.org/10.7910/DVN/5CXCLX
413556	Burkina Faso	ICF International, National Center for Research and Training on Malaria (CNRFP) (Burkina Faso), National Institute of Statistics and Demography (Burkina Faso), National Program for the Fight Against Malaria (PNLP) (Burkina Faso). Burkina Faso Malaria Indicator Survey 2017-2018. Fairfax, United States of America: ICF International, 2019.
30431	Burundi	Burundi Institute of Statistics and Economic Studies, ICF International, Ministry of Public Health and the Fight

NID	Location	Citation
		Against AIDS (Burundi). Burundi Demographic and Health Survey 2010-2011. Fairfax, United States of America: ICF International, 2012.
108080	Burundi	Burundi Institute of Statistics and Economic Studies, ICF Macro, Ministry of Public Health and the Fight Against AIDS (Burundi), National Institute of Public Health (Burundi). Burundi Malaria Indicator Survey 2012-2013. Fairfax, United States of America: ICF International, 2013.
139611	Burundi	Ministry of Public Health (Burundi). Burundi National Anemia Survey 2003.
144314	Burundi	World Health Organization (WHO). WHO Global Database on Anemia, Nutrition Landscape Information System. Geneva, Switzerland: World Health Organization (WHO).
286766	Burundi	Burundi Institute of Statistics and Economic Studies, ICF International, Ministry of Public Health and the Fight Against AIDS (Burundi). Burundi Demographic and Health Survey 2016-2017. Fairfax, United States of America: ICF International, 2018.
140501	Cabo Verde	Ministry of Health (Cape Verde), United Nations Children's Fund (UNICEF). Caracterização deficiência de vitamina "A" e da anemia em crianças pré-escolares de Cabo Verde [Cape Verde Characterization of Vitamin A Deficiency and Anemia in Preschool Children 1996]. Praia, Cape Verde: Ministry of Health (Cape Verde), 1997.
489978	Cabo Verde	Semedo RM, Santos MM, Baião MR, Luiz RR, da Veiga GV. Prevalence of anaemia and associated factors among children below five years of age in Cape Verde, West Africa. J Health Popul Nutr. 2014; 32(4): 646-57.
19156	Cambodia	Macro International, Inc, Ministry of Health (Cambodia), National Institute of Statistics (Cambodia). Cambodia Demographic and Health Survey 2000. Fairfax, United States of America: ICF International.
19167	Cambodia	Macro International, Inc, National Institute of Public Health (Cambodia), National Institute of Statistics (Cambodia). Cambodia Demographic and Health Survey 2005-2006. Fairfax, United States of America: ICF International.
30379	Cambodia	ICF Macro, Ministry of Health (Cambodia), National Institute of Statistics (Cambodia). Cambodia Demographic and Health Survey 2010-2011. Fairfax, United States of America: ICF International.
139612	Cambodia	Helen Keller International. Iron deficiency in Cambodia: The Need for Iron Supplementation Among Preschool-aged Children. HKI Cambodia Nutrition Bulletin. 2001; 2: 1-4.
139614	Cambodia	United Nations Children's Fund (UNICEF). Cambodia Seth Koma Follow-up Survey 2003. 2003.
157024	Cambodia	ICF International, Ministry of Health (Cambodia), National Institute of Statistics (Cambodia). Cambodia Demographic

NID	Location	Citation
		and Health Survey 2014. Fairfax, United States of America: ICF International, 2017.
19211	Cameroon	Macro International, Inc, National Institute of Statistics (Cameroon). Cameroon Demographic and Health Survey 2004. Fairfax, United States of America: ICF International.
19274	Cameroon	ICF International, Ministry of Economy, Planning and Regional Development (Cameroon), Ministry of Public Health (Cameroon), National Institute of Statistics (Cameroon), Pasteur Center of Cameroon. Cameroon Demographic and Health Survey 2011. Fairfax, United States of America: ICF International.
139616	Cameroon	Kurz K, Ngo Som J. Study of the Factors that Influence the Nutritional Status of Adolescent Girls in Cameroon. Washington, DC: International Center for Research on Women, 1994.
139625	Cameroon	Helen Keller International, Ministry of Public Health (Cameroon). Cameroon National Survey on Vitamin A Deficiency and Anemia 2000.
143774	Cameroon	Coulibaly M, Costagliola D, Zittoun J, Mary JY. Modifications of hemato-biological parameters in pregnant women in a migrating population in northern Cameroon: prevalence of anemia, iron and folates deficiencies. Int J Vitam Nutr Res. 1987; 57(2): 173-8.
144314	Cameroon	World Health Organization (WHO). WHO Global Database on Anemia, Nutrition Landscape Information System. Geneva, Switzerland: World Health Organization (WHO).
294308	Cameroon	Engle-Stone R, Nankap M, Ndjebayi AO, Erhardt JG, Brown KH. Plasma ferritin and soluble transferrin receptor concentrations and body iron stores identify similar risk factors for iron deficiency but result in different estimates of the national prevalence of iron deficiency and iron-deficiency anemia. J Nutr. 2013; 143(3): 369-77.
294482	Cameroon	Engle-Stone RC. A National Survey of Micronutrient Status and Consumption of Potentially Fortifiable Foods among Women and Young Children in Cameroon [dissertation]. Ann Arbor, MI: UMI Dissertation Publishing; 2012.
104955	Canada	Health Canada, Public Health Agency of Canada, Statistics Canada (StatCan). Canada Health Measures Survey 2009-2011. Ottawa, Canada: Statistics Canada (StatCan).
143776	Canada	Hodgins S, Dewailly E, Chatwood S, Bruneau S, Bernier F. Iron-deficiency anemia in Nunavik: pregnancy and infancy. Int J Circumpolar Health. 1998; 57 (Suppl 1): 135-40.
143780	Canada	Yeung GS, Zlotkin SH. Efficacy of meat and iron-fortified commercial cereal to prevent iron depletion in cow milk-fed infants 6 to 12 months of age: a randomized controlled trial. Can J Public Health. 2000; 91(4): 263-7.
2223	Central African Republic	United Nations Children's Fund (UNICEF). Central African Republic Multiple Indicator Cluster Survey 2006. New York,

NID	Location	Citation
		United States: United Nations Children's Fund (UNICEF).
139755	Central African Republic	Ministry of Economy, Planning and International Cooperation (Central African Republic), Ministry of Public Health and Population (Central African Republic), United Nations Children's Fund (UNICEF). Central African Republic National Survey on Vitamin A Deficiency, Iron Deficiency and the Consumption of Iodized Salt 1998-1999.
144314	Central African Republic	World Health Organization (WHO). WHO Global Database on Anemia, Nutrition Landscape Information System. Geneva, Switzerland: World Health Organization (WHO).
143786	Chad	Beasley M, Brooker S, Ndinaromtan M, Madjiouroum EM, Baboguel M, Djenguinabe E, Bundy DAP. First nationwide survey of the health of schoolchildren in Chad. Trop Med Int Health. 2002; 7(7): 625-30.
165235	Chad	Ministry of Public Health (Chad), National Institute for Statistics, Economic and Demographic Studies (INSEED) (Chad), National Malaria Control Program (Chad), World Health Organization (WHO). Chad National Malaria Indicators Survey 2010-2011.
341692	Chad	Gallup. Chad World Poll 2017.
425937	Chad	Central Statistics Agency (Ethiopia), Ethiopian Public Health Institute (EPHI), Ministry of Health (Ethiopia). Ethiopia National Malaria Indicator Survey 2017.
490694	Chad	Ministry of Public Health (Chad), National Centre for Nutrition and Food Technology (CNNTA) (Chad), United Nations Children's Fund (UNICEF), World Food Programme (WFP). Chad National Nutrition Survey Using SMART Methodology 2019.
490695	Chad	Bureau for Global Health, United States Agency for International Development (USAID), European Commission for Humanitarian Aid and Civil Protection (ECHO), Ministry of Public Health (Chad), National Centre for Nutrition and Food Technology (CNNTA) (Chad), United Nations Children's Fund (UNICEF), World Food Programme (WFP). Chad National Nutrition Survey Using SMART Methodology 2018.
111907	Chile	Department of Epidemiology, Ministry of Health (Chile), Department of Public Health, Pontifical Catholic University of Chile, Institute of Nutrition and Food Technology (INTA), University of Chile, Pan American Health Organization (PAHO). Chile National Health Survey 2003.
143789	Chile	Mardones F, Rioseco A, Ocqueteau M, Urrutia MT, Javet L, Rojas I, Villarroel del L. [Anemia in pregnant women from the community of Puente Alto, Chile]. Rev Med Chil. 2003; 131(5): 520-5.
143794	Chile	Hertrampf E, Dinamarca M, Llaguno TM, Stekel A. [Iron nutrition and breast feeding in Chilean infants]. Rev Chil Pediatr. 1987; 58(3): 193-7.
143797	Chile	Díaz A, Guerra H, Campos S, Letelier C, Olivares G. [Prevalence of iron deficiency in preschool children from la

NID	Location	Citation
		Pintana county]. Rev Chil Nutr. 2002; 10-3.
119643	China	Ministry of Public Health (China). China National Nutrition Survey 1992 - China CDC.
124479	China	Ministry of Health (China), National Bureau of Statistics of China. China National Nutrition Survey 2002 - China CDC.
143045	China	Dang S, Yan H, Yamamoto S, Wang X, Zeng L. Poor nutritional status of younger Tibetan children living at high altitudes. Eur J Clin Nutr. 2004; 58(6): 938-46.
143801	China	Cai MQ, Yan WY. Study on iron nutritional status in adolescence. Biomed Environ Sci. 1990; 3(1): 113-9.
143804	China	China's National Group On Students' Constitution And Health Survey, Ji C. Dynamic analysis on the detecting rate of iron-deficient anemia of Chinese students. Chin J Prev Med. 2002; 36(2): 81-3.
143808	China	Wang W, Chen X, Liu D. Hematologic response to iron and ascorbic acid administration in preschool children with anemia. Nutr Res. 1986; 241-8.
143814	China	Gao J, Zeng S, Sun BL, Fan HM, Han LH. Menstrual blood loss and hematologic indices in healthy Chinese women. J Reprod Med. 1987; 32(11): 822-6.
143815	China	Dang S, Yan H, Wang X. [Study on the hemoglobin levels of children under the age of three years and the prevalence of anemia at high altitude in Tibet of China]. Chin J Epidemiol. 2003; 24(12): 1108-11.
144314	China	World Health Organization (WHO). WHO Global Database on Anemia, Nutrition Landscape Information System. Geneva, Switzerland: World Health Organization (WHO).
414197	China	Carolina Population Center, University of North Carolina at Chapel Hill, Chinese Center for Disease Control and Prevention (CCDC). China Health and Nutrition Survey 1989-2015. Chapel Hill, United States of America: Carolina Population Center, University of North Carolina at Chapel Hill.
489964	China	Hu YC, Chen J, Li M, Wang R, Li WD, Yang YH, Yang C, Yun CF, Yang LC, Yang XG. [Study on anemia and vitamin A and vitamin D nutritional status of Chinese urban pregnant women in 2010-2012]. Chin J Prev Med. 2017; 51(2): 125-31.
490162	China	Song Y, Wang HJ, Dong B, Wang Z, Ma J, Agardh A. National Trends in Hemoglobin Concentration and Prevalence of Anemia among Chinese School-Aged Children, 1995-2010. J Pediatr. 2017; 183: 164-169e2.
490164	China	Ma Q, Zhang S, Liu J, Wang Q, Shen H, Zhang Y, Liu M. Study on the Prevalence of Severe Anemia among Non-Pregnant Women of Reproductive Age in Rural China: A Large Population-Based Cross-Sectional Study. Nutrients. 2017; 9(12).

NID	Location	Citation
490166	China	Luo D, Xu R, Ma J, Yan X, Hu P, Song Y, Jan C, Raat H, Patton GC. The associations of economic growth and anaemia for school-aged children in China. Matern Child Nutr. 2020; 16(2): e12936.
490213	China	
517021	China	Li M, Hu Y, Mao D, Wang R, Chen J, Li W, Yang X, Piao J, Yang L. Prevalence of Anemia among Chinese Rural Residents. Nutrients. 2017; 9(3).
124467	Colombia	Colombian Family Welfare Institute, National Institute of Health (Colombia), Pan American Health Organization (PAHO), Profamilia (Colombia), University of Antioquia. Colombia National Survey of the Nutritional Situation 2004-2005. Bogotá, Colombia: Colombian Family Welfare Institute.
126331	Colombia	Colombian Family Welfare Institute, Ministry of Social Protection (Colombia), National Institute of Health (Colombia), Profamilia (Colombia). Colombia National Survey of the Nutritional Situation 2010. Bogotá, Colombia: Colombian Family Welfare Institute.
143818	Colombia	Agudelo GM, Cardona OL, Posada M, Montoya MN, Ocampo NE, Marín CM, Correa MC, López C. [Prevalence of iron-deficiency anemia in schoolchildren and adolescents, Medellín, Colombia, 1999]. Rev Panam Salud Publica. 2003; 13(6): 376-86.
19391	Congo	Macro International, Inc, National Center for Statistics and Economic Studies (Congo, Rep.). Congo Demographic and Health Survey 2005. Fairfax, United States of America: ICF International.
56151	Congo	ICF International, Ministry of Health (Congo, Rep.), National Center for Statistics and Economic Studies (Congo, Rep.). Congo Demographic and Health Survey 2011-2012. Fairfax, United States of America: ICF International.
141231	Costa Rica	Costa Rican Institute for Research and Education on Nutrition and Health, Costa Rican Social Security Fund (CCCS), Institute of Nutrition of Central America and Panama, Ministry of Health (Costa Rica), National Institute of Statistics and Censuses (Costa Rica). Costa Rica National Nutrition Survey 2008-2009.
143819	Costa Rica	Rodríguez S, Blanco A, Cunningham L, Ascencio M, Chávez M, Muñoz L. [Prevalence of nutritional anemia in women of reproductive age. Costa Rica. National nutrition survey, 1996]. Arch Latinoam Nutr. 2001; 51(1): 19-24.
143820	Costa Rica	Villarejos VM, Bickers J, Alvaro Riveraaa S, Peña Chavarria A, Hunter GW 3rd, Kotcher E. Pathogenesis of anemia in Costa Rica. Epidemiologic study of hemoglobin and serum protein levels and hookworm infection in children. Am J Trop Med Hyg. 1970; 19(4): 603-9.
143821	Costa Rica	Jiménez E, Brittenham GM, Jiménez R, Lozoff B, Mora L, Gómez I, Carrillo JM. [Iron deficiency anemia in unweaned

NID	Location	Citation
		infants: a prospective study]. Bol Med Hosp Infant Mex. 1987; 44(6): 309-15.
143822	Costa Rica	Cunningham L, Blanco A, Rodríguez S, Ascencio M. [Prevalence of anemia, iron and folate deficiency in children 7 years smaller. Costa Rica, 1996]. Arch Latinoam Nutr. 2001; 51(1): 37-43.
143825	Costa Rica	Blanco A, Rodríguez S, Cunningham L. [Nutritional anemia in nursing women in Costa Rica]. Arch Latinoam Nutr. 2003; 53(1): 28-34.
144314	Costa Rica	World Health Organization (WHO). WHO Global Database on Anemia, Nutrition Landscape Information System. Geneva, Switzerland: World Health Organization (WHO).
144314	Croatia	World Health Organization (WHO). WHO Global Database on Anemia, Nutrition Landscape Information System. Geneva, Switzerland: World Health Organization (WHO).
490170	Cuba	Reboso Pérez J, Cabrera Núñez E, Rodríguez GP, Jiménez Acosta S. [Iron deficiency anemia in children 6 to 24 months and 6 to 12 years old]. Rev Cub Salud Publica. 2005; 31(4).
143717	Czechia	Elwood PC, Hughes J, Abernethy M, Davies R, Gough R, Johnson AP, Dubourg AY. An international haematological survey. Bull World Health Organ. 1976; 54(1): 87-95.
143837	Czechia	Osancová K, Hejda S. Nutrition and its reflection on the health status of the population. Rev Czech Med. 1968; 14(2): 101-17.
18533	Côte d'Ivoire	ICF International, Ministry of the Fight Against AIDS (Côte d'Ivoire), National Institute of Statistics (Côte d'Ivoire). Côte d'Ivoire Demographic and Health Survey 2011-2012. Fairfax, United States of America: ICF International.
143826	Côte d'Ivoire	Asobayire FS, Adou P, Davidsson L, Cook JD, Hurrell RF. Prevalence of iron deficiency with and without concurrent anemia in population groups with high prevalences of malaria and other infections: a study in Côte d'Ivoire. Am J Clin Nutr. 2001; 74(6): 776-82.
143828	Côte d'Ivoire	Reinhardt MC. Maternal anaemia in Abidjan--Its influence on placenta and newborns. Helv Paediatr Acta Suppl. 1978; 43-63.
143829	Côte d'Ivoire	Reinhardt MC, Marti HR. Haematological data of African newborns and their mothers in Abidjan. Helv Paediatr Acta Suppl. 1978; 85-99.
261506	Côte d'Ivoire	Cote D'Ivoire National Survey of Anemia, Vitamin A deficiency, and Iron.
294293	Côte d'Ivoire	Rohner F, Northrop-Clewes C, Tschannen AB, Bosso PE, Kouassi-Gohou V, Erhardt JG, Bui M, Zimmermann MB, Mascie-Taylor CG. Prevalence and public health relevance of micronutrient deficiencies and undernutrition in pre-school children and women of reproductive age in Côte

NID	Location	Citation
		d'Ivoire, west Africa. Public Health Nutr. 2013; 17(9): 2016-28.
7465	Democratic People's Republic of Korea	European Union (EU), Government of North Korea, United Nations Children's Fund (UNICEF), World Food Programme (WFP). North Korea Multiple Indicator Cluster Survey 1998.
58954	Democratic People's Republic of Korea	Central Bureau of Statistics (North Korea), United Nations Children's Fund (UNICEF), World Food Programme (WFP). North Korea Nutrition Assessment 2004.
68833	Democratic People's Republic of Korea	Central Bureau of Statistics (North Korea), United Nations Children's Fund (UNICEF), World Food Programme (WFP). North Korea Nutrition Assessment 2012.
19381	Democratic Republic of the Congo	Macro International, Inc, Ministry of Planning (Congo, DR). Democratic Republic of the Congo Demographic and Health Survey 2007. Fairfax, United States of America: ICF International.
76878	Democratic Republic of the Congo	ICF International, Ministry of Planning and Monitoring Implementation of the Revolution of Modernity (Congo, DR), Ministry of Public Health (Congo, DR), National Institute of Statistics (Congo, DR). Democratic Republic of the Congo Demographic and Health Survey 2013-2014. Fairfax, United States of America: ICF International, 2014.
143839	Democratic Republic of the Congo	Jackson DJ, Klee EB, Green SD, Mokili JL, Elton RA, Cutting WA. Severe anaemia in pregnancy: a problem of primigravidae in rural Zaire. Trans R Soc Trop Med Hyg. 1991; 85(6): 829-32.
144314	Democratic Republic of the Congo	World Health Organization (WHO). WHO Global Database on Anemia, Nutrition Landscape Information System. Geneva, Switzerland: World Health Organization (WHO).
143715	Denmark	Dirren H, Decarli B, Lesourd B, Schlienger JL, Deslypere JP, Kiepurski A. Nutritional status: haematology and albumin. Eur J Clin Nutr. 1991; 45 (Suppl 3): 43-52.
143840	Denmark	Milman N, Cohn J, Pedersen NS. Serum ferritin and iron status of children in the Faroe Islands. Eur J Pediatr. 1984; 142(2): 89-92.
143841	Denmark	Hagerup L, Eriksen M, Schroll M, Hollnagel H, Agner E, Larsen S. The Glostrup population studies. Collection of epidemiologic tables. Reference values for use in cardiovascular population studies. Scand J Soc Med Suppl. 1981; 1-112.
143842	Denmark	Milman N, Heitman B, Lyhne N, Rosdahl N, Jensen K, Graudal N. Iron status in 1113 Danish men and women aged 35-65 years: relation to dietary and supplemental iron intake. Scand J Nutr. 1993; 98-103.
143846	Denmark	Milman N, Schultz-Larsen K. Iron stores in 70-year-old Danish men and women. Evaluation in 469 individuals by serum ferritin and hemoglobin. Aging (Milano). 1994; 6(2): 97-103.

NID	Location	Citation
143848	Denmark	Milman N, Clausen JO, Jordal R. Iron status in young Danish men and women: a population survey comprising 548 individuals. Ann Hematol. 1995; 70(4): 215-21.
143849	Denmark	Milman N, Thomsen H, Mathiassen B. Serum ferritin, iron status and plasma ascorbic acid in 40- to 49-year-old males in the Faroe Islands. Scand J Clin Lab Invest. 1990; 50(5): 559-64.
143850	Denmark	Marner T. Haemoglobin, erythrocyres and serum iron values in normal children 3-6 years of age. Acta Paediatr Scand. 1969; 58(4): 363-8.
143689	Dominica	Micronutrient Working Group. Iron and vitamin A status in five Caribbean countries. Cajanus. 2002; 4-34.
262907	Dominican Republic	Center for Social and Demographic Studies (Dominican Republic) (CESDEM), Centers for Disease Control and Prevention (CDC), Institute of Nutrition of Central America and Panama, Ministry of Public Health and Social Assistance (Dominican Republic), UNICEF International Child Development Center (ICDC), World Bank. Dominican Republic National Micronutrient Survey 2009.
409542	Dominican Republic	Dominican Republic Micronutrient Deficiency in Children Survey 1993.
490208	Dominican Republic	Ministry of Education (Dominican Republic), National Institute for Student Welfare (INABIE) (Dominican Republic). Dominican Republic National Micronutrient Survey in the School Population 2012. 2013.
143852	Ecuador	Quizhpe E, San Sebastián M, Hurtig AK, Llamas A. [Prevalence of anaemia in schoolchildren in the Amazon area of Ecuador]. Rev Panam Salud Publica. 2003; 13(6): 355-61.
153674	Ecuador	Ministry of Public Health (Ecuador), National Institute of Statistics and Censuses (Ecuador). Ecuador National Health and Nutrition Survey 2012.
489967	Ecuador	Rodriguez A, Guamán G, Nelson DP. Vitamin A status of children in five Ecuadorian provinces. Bull Pan Am Health Organ. 1996; 30(3): 234-41.
19511	Egypt	Macro International, Inc, Population Council (Egypt). Egypt Demographic and Health Survey 2000. Fairfax, United States of America: ICF International.
19521	Egypt	El-Zanaty and Associates, Macro International, Inc, Ministry of Health and Population (Egypt), Population Council (Egypt). Egypt Demographic and Health Survey 2005. Fairfax, United States of America: ICF International.
110741	Egypt	Curtale F, Nabil M, el Wakeel A, Shamy MY. Anaemia and intestinal parasitic infections among school age children in Behera Governorate, Egypt Behera Survey Team. J Trop Pediatr. 1998; 44(6): 323-8.

NID	Location	Citation
137578	Egypt	Shaheen FM, Hassan H. Egypt Nutritional Deficiencies Among Primary School Children 1998. Cairo, Egypt: Ministry of Health and Population (Egypt), 1998.
139723	Egypt	Centers for Disease Control and Prevention (CDC), Ministry of Health and Population (Egypt), National Nutrition Institute (Egypt), United States Agency for International Development (USAID). Egypt National Nutrition Survey 1978.
143717	Egypt	Elwood PC, Hughes J, Abernethy M, Davies R, Gough R, Johnson AP, Dubourg AY. An international haematological survey. Bull World Health Organ. 1976; 54(1): 87-95.
143854	Egypt	el-Sahn F, Sallam S, Mandil A, Galal O. Anaemia among Egyptian adolescents: prevalence and determinants. East Mediterr Health J. 2000; 6(5-6): 1017-25.
143858	Egypt	Abdou IA, Ali HE, Said AK, Mousa WA, Demian HG, Soliman AM, el-Hawary LH. Incidence of nutritional deficiencies, goitre and dental caries among school children in Cairo. J Egypt Public Health Assoc. 1967; 42(4): 175-84.
143859	Egypt	Shukry AS, Labib FM, Kamel LM. Assessment of the health and nutritional standards of infants and preschool children in high density Cairo community. Gaz Egypt Paediatr Assoc. 1973; 21(3): 47-56.
154897	Egypt	El-Zanaty and Associates, ICF International, Ministry of Health and Population (Egypt). Egypt Demographic and Health Survey 2014. Fairfax, United States of America: ICF International.
27590	El Salvador	El Salvador Demographic Association (ADS), Division of Reproductive Health-Centers for Disease Control and Prevention (CDC). El Salvador Reproductive Health Survey 1998. Atlanta, United States: Centers for Disease Control and Prevention (CDC).
27599	El Salvador	Asociación Demográfica Salvadoreña (ADS), Division of Reproductive Health-Centers for Disease Control and Prevention (CDC). (2004) El Salvador Reproductive Health Survey 2002-2003. San Salvador, El Salvador: ADS.
27606	El Salvador	Asociación Demográfica Salvadoreña (ADS), Division of Reproductive Health-Centers for Disease Control and Prevention (CDC). (2009) El Salvador Reproductive Health Survey 2008. San Salvador, El Salvador: ADS.
139894	El Salvador	El Salvador Demographic Association (ADS), Institute of Nutrition of Central America and Panama, Ministry of Public Health and Social Assistance (El Salvador). El Salvador Assessment of Nutritional Food Situation 1988. Guatemala City, Guatemala: Institute of Nutrition of Central America and Panama, 1990.
200636	El Salvador	General Administration of Statistics and Censuses (El Salvador), Ministry of Health (El Salvador), United Nations Children's Fund (UNICEF). El Salvador Multiple Indicator

NID	Location	Citation
		Cluster Survey 2014. New York, United States of America: United Nations Children's Fund (UNICEF), 2017.
490693	El Salvador	Ministry of Health (El Salvador). El Salvador - National Study of Ioduria, Evaluation of Nutritional Status and Fortified Foods in First and Second Grade Children 2012. 2012.
76884	Equatorial Guinea	ICF International, Ministry of Health and Social Welfare (Equatorial Guinea), Ministry of Planning, Economic Development and Public Investment (Equatorial Guinea). Equatorial Guinea Demographic and Health Survey 2011. Fairfax, United States of America: ICF International, 2012.
143082	Equatorial Guinea	Custodio E, Descalzo MA, Roche J, Sánchez I, Molina L, Lwanga M, Bernis C, Villamor E, Baylin A. Nutritional status and its correlates in Equatorial Guinean preschool children: results from a nationally representative survey. Food Nutr Bull. 2008; 29(1): 49-58.
20829	Eswatini	Central Statistical Office (Swaziland), Macro International, Inc. Swaziland Demographic and Health Survey 2006-2007. Fairfax, United States of America: ICF International.
19557	Ethiopia	Macro International, Inc, Population and Housing Census Commissions Office (PHCCO). Ethiopia Demographic and Health Survey 2005. Fairfax, United States of America: ICF International.
21301	Ethiopia	Central Statistics Agency (Ethiopia), ICF Macro, Ministry of Health (Ethiopia). Ethiopia Demographic and Health Survey 2010-2011. Fairfax, United States of America: ICF International.
143869	Ethiopia	Ross SM. Haemoglobin and haematocrit values in pregnant women on a high iron intake and living at a high altitude. J Obstet Gynaecol Br Commonw. 1972; 79(12): 1103-7.
143870	Ethiopia	Haidar J, Nekatibeb H, Urga K. Iron deficiency anemia in pregnant and lactating mothers in rural Ethiopia. East Afr Med J. 1999; 76(11): 618-22.
143871	Ethiopia	Desalegn S. Prevalence of anaemia in pregnancy in Jima town, southwestern Ethiopia. Ethiop Med J. 1993; 31(4): 251-8.
218568	Ethiopia	Central Statistics Agency (Ethiopia), ICF International. Ethiopia Demographic and Health Survey 2016. Fairfax, United States of America: ICF International, 2017.
408685	Ethiopia	Ethiopian Public Health Institute (EPHI), Ministry of Health (Ethiopia). Ethiopia National Micronutrient Survey 2015.
490171	Ethiopia	Haidar J, Melaku U, Pobocik Rs. Folate deficiency in women of reproductive age in nine administrative regions of Ethiopia: an emerging public health problem. South Afr J Clin Nutr. 2010; 23(3): 132-7.
124447	Fiji	National Food and Nutrition Centre (Fiji). Fiji National Nutrition Survey 2004.

NID	Location	Citation
124448	Fiji	National Food and Nutrition Committee (Fiji). Fiji National Nutrition Survey 1993.
135526	Fiji	Hill Laboratories (New Zealand), National Food and Nutrition Centre (Fiji), TropicHealth Laboratories, World Health Organization (WHO). Fiji Impact Study of Iron Fortified Flour 2010.
142784	Fiji	Republic of Kiribati. Pacific Helminths Initiative 2001-2002.
143717	Finland	Elwood PC, Hughes J, Abernethy M, Davies R, Gough R, Johnson AP, Dubourg AY. An international haematological survey. Bull World Health Organ. 1976; 54(1): 87-95.
143875	Finland	Lahti-Koski M, Valsta LM, Alfthan G, Tapanainen H, Aro A. Iron status of adults in the capital area of Finland. Eur J Nutr. 2003; 42(5): 287-92.
143876	Finland	Takkunen H. Iron deficiency in the Finnish adult population: an epidemiological survey from 1967 to 1972 inclusive. Scand J Haematol Suppl. 1976; 1-91.
143877	Finland	Siimes MA, Saarinen UM, Dallman PR. Relationship between hemoglobin concentration and transferrin saturation in iron-sufficient infants. Am J Clin Nutr. 1979; 32(11): 2295-300.
143879	Finland	Knuiman JT, Westenbrink S, van der Heyden L, West CE, Burema J, de Boer J, Hautvast JG, Räsänen L, Virkkunen L, Viikari J. Determinants of total and high density lipoprotein cholesterol in boys from Finland, The Netherlands, Italy, the Philippines and Ghana with special reference to diet. Hum Nutr Clin Nutr. 1983; 37(4): 237-54.
143880	Finland	Widholm O, Vartiainen E, Tenhunen T. On iron requirement in menstruating teen-age girls. Iron study among teen-agers. I. Acta Obstet Gynecol Scand. 1967; 46(Suppl 1): 29-46.
143715	France	Dirren H, Decarli B, Lesourd B, Schlienger JL, Deslypere JP, Kiepurski A. Nutritional status: haematology and albumin. Eur J Clin Nutr. 1991; 45 (Suppl 3): 43-52.
143717	France	Elwood PC, Hughes J, Abernethy M, Davies R, Gough R, Johnson AP, Dubourg AY. An international haematological survey. Bull World Health Organ. 1976; 54(1): 87-95.
143883	France	Galan P, Yoon HC, Preziosi P, Viteri F, Valeix P, Fieux B, Briançon S, Malvy D, Roussel AM, Favier A, Hercberg S. Determining factors in the iron status of adult women in the SU.VI.MAX study. SUpplementation en VItamines et Minéraux AntioXydants. Eur J Clin Nutr. 1998; 52(6): 383-8.
143887	France	Galán P, Hercberg S, Soustre Y, Dop MC, Dupin H. Factors affecting iron stores in French female students. Hum Nutr Clin Nutr. 1985; 39(4): 279-87.
143888	France	Blot I, Papiernik E, Kaltwasser JP, Werner E, Tchernia G. Influence of routine administration of folic acid and iron

NID	Location	Citation
		during pregnancy. Gynecol Obstet Invest. 1981; 12(6): 294-304.
143892	France	Blot I, Rey A, Kaltwasser JP, Francoual J, Papiernik E, Tchernia G. Folate and iron deficiencies in mothers and their newborn children. Blut. 1982; 44(5): 297-303.
143893	France	Hercberg S, Galán P, Soustre Y, Dop MC, Devanlay M, Dupin H. Effects of iron supplementation on serum ferritin and other hematological indices of iron status in menstruating women. Ann Nutr Metab. 1985; 29(4): 232-8.
143894	France	Zittoun J, Blot I, Hill C, Zittoun R, Papiernik E, Tchernia G. Iron supplements versus placebo during pregnancy: its effects on iron and folate status on mothers and newborns. Ann Nutr Metab. 1983; 27(4): 320-7.
249660	France	Castetbon K, Vernay M, Malon A, Salanave B, Deschamps V, Roudier C, Oleko A, Szego E, Hercberg S. Dietary intake, physical activity and nutritional status in adults: the French nutrition and health survey (ENNS, 2006-2007). Br J Nutr. 2009; 102(5): 733–43.
489974	France	Valeix P, Zarebska M, Preziosi P, Galan P, Pelletier B, Hercberg S. Iodine deficiency in France. Lancet. 1999; 353(9166): 1766-7.
76706	Gabon	General Directorate of Statistics (Gabon), ICF International, Ministry of Economy, Employment and Sustainable Development (Gabon), Ministry of Health and Social Affairs (Gabon). Gabon Demographic and Health Survey 2012. Fairfax, United States of America: ICF International, 2013.
77384	Gambia	Gambia Bureau of Statistics (GBOS), ICF International, Ministry of Health and Social Welfare (The Gambia). Gambia Demographic and Health Survey 2013. Fairfax, United States of America: ICF International, 2015.
139025	Gambia	National Nutrition Agency (The Gambia). Gambia Nationwide Survey on the Prevalence of Vitamin A and Iron Deficiency in Women and Children 2001.
144314	Gambia	World Health Organization (WHO). WHO Global Database on Anemia, Nutrition Landscape Information System. Geneva, Switzerland: World Health Organization (WHO).
438750	Gambia	Petry N, Jallow B, Sawo Y, Darboe MK, Barrow S, Sarr A, Ceesay PO, Fofana MN, Prentice AM, Wegmüller R, Rohner F, Phall MC, Wirth JP. Micronutrient Deficiencies, Nutritional Status and the Determinants of Anemia in Children 0-59 Months of Age and Non-Pregnant Women of Reproductive Age in The Gambia. Nutrients. 2019; 11(10).
459854	Gambia	Gambia Bureau of Statistics (GBOS), ICF International, Ministry of Health and Social Welfare (The Gambia). Gambia Demographic and Health Survey 2019-2020. Fairfax, United States of America: ICF International, 2021.
3970	Georgia	National Center for Disease Control (Georgia), State Department of Statistics of Georgia, United Nations Children's Fund (UNICEF). Georgia Multiple Indicator Cluster

NID	Location	Citation
		Survey 2005. New York, United States of America: United Nations Children's Fund (UNICEF).
141238	Georgia	National Center for Disease Control and Public Health (Georgia), United Nations Children's Fund (UNICEF). Georgia National Nutrition Survey 2009.
143895	Germany	Bergmann RL, Gravens-Müller L, Hertwig K, Hinkel J, Andres B, Bergmann KE, Dudenhausen JW. Iron deficiency is prevalent in a sample of pregnant women at delivery in Germany. Eur J Obstet Gynecol Reprod Biol. 2002; 102(2): 155-60.
236076	Germany	Robert Koch Institute. Germany Health Interview and Examination Survey for Children and Adolescents 2003-2006, Baseline. Berlin, Germany: Robert Koch Institute, 2008.
19627	Ghana	Ghana Statistical Service, Macro International, Inc. Ghana Demographic and Health Survey 2003. Fairfax, United States of America: ICF International.
21188	Ghana	Ghana Statistical Service, Macro International, Inc, Ministry of Health (Ghana). Ghana Demographic and Health Survey 2008. Fairfax, United States of America: ICF International.
63993	Ghana	Centers for Disease Control and Prevention (CDC), Ghana Statistical Service, Government of Japan, ICF Macro, Ministry of Health (Ghana), Navrongo Health Research Centre, United Nations Children's Fund (UNICEF), United Nations Population Fund (UNFPA), United States Agency for International Development (USAID). Ghana Multiple Indicator Cluster Survey 2011. New York, United States of America: United Nations Children's Fund (UNICEF), 2013.
142049	Ghana	Mockenhaupt FP, Rong B, Günther M, Beck S, Till H, Kohne E, Thompson WN, Bienzle U. Anaemia in pregnant Ghanaian women: importance of malaria, iron deficiency, and haemoglobinopathies. Trans R Soc Trop Med Hyg. 2000; 94(5): 477-83.
143879	Ghana	Knuiman JT, Westenbrink S, van der Heyden L, West CE, Burema J, de Boer J, Hautvast JG, Räsänen L, Virkkunen L, Viikari J. Determinants of total and high density lipoprotein cholesterol in boys from Finland, The Netherlands, Italy, the Philippines and Ghana with special reference to diet. Hum Nutr Clin Nutr. 1983; 37(4): 237-54.
143897	Ghana	Agyepong IA, Wellington EK, Abbey MA. A comparative study of clinical and sociocultural aspects of anaemia among adolescent girls in rural Ghana. Addict Behav Rep. 1997; 65(3): 123-38.
143898	Ghana	Lassey AT, Klufio CA, Annan BD, Wilson JB. Antenatal haemoglobin profile at Korle-Bu Teaching Hospital. East Afr Med J. 1999; 76(4): 228-32.
144314	Ghana	World Health Organization (WHO). WHO Global Database on Anemia, Nutrition Landscape Information System. Geneva, Switzerland: World Health Organization (WHO).

NID	Location	Citation
157027	Ghana	Ghana Health Service, Ghana Statistical Service, ICF International. Ghana Demographic and Health Survey 2014. Fairfax, United States of America: ICF International, 2016.
255115	Ghana	Koram KA, Owusu-Agyei S, Utz G, Binka FN, Baird JK, Hoffman SL, Nkrumah FK. Severe anemia in young children after high and low malaria transmission seasons in the Kassena-Nankana district of northern Ghana. Am J Trop Med Hyg. 2000; 62(6): 670-4.
286788	Ghana	Ghana Health Service, Ghana Statistical Service, ICF International, National Malaria Control Programme (Ghana), National Public Health and Reference Laboratory (NHPRL) (Ghana). Ghana Malaria Indicator Survey 2016. Fairfax, United States of America: ICF International.
409169	Ghana	GroundWork LLC, KEMRI Wellcome Trust Research Programme (KWTRP), University of Ghana, University of Wisconsin - Madison (UW - Madison). Ghana Micronutrient Survey 2017 .
439649	Ghana	Ghana Health Service, Ghana Statistical Service, ICF International, National Malaria Control Programme (Ghana), National Public Health and Reference Laboratory (NHPRL) (Ghana). Ghana Malaria Indicator Survey 2019. Fairfax, United States of America: ICF International, 2020.
143715	Greece	Dirren H, Decarli B, Lesourd B, Schlienger JL, Deslypere JP, Kiepurski A. Nutritional status: haematology and albumin. Eur J Clin Nutr. 1991; 45 (Suppl 3): 43-52.
143899	Greece	Kafatos A, Diacatou A, Labadarios D, Kounali D, Apostolaki J, Vlachonikolis J, Mamalakis G, Megremis S. Nutrition status of the elderly in Anogia, Crete, Greece. J Am Coll Nutr. 1993; 12(6): 685-92.
143900	Greece	Kattamis C, Metaxotou-Mavromnati A, Konidaris C, Touliatos N, Constantsas N, Matsaniotis N. Iron deficiency in Greece. Epidemiologic and hematologic studies. J Pediatr. 1974; 84(5): 666-71.
489996	Greece	Kattamis C. Prospects, inabilities and achievements in epidemiologic studies on iron deficiency and iron deficiency anemia. Paediatr. 1982; 11(3-4): 121-35.
144314	Grenada	World Health Organization (WHO). WHO Global Database on Anemia, Nutrition Landscape Information System. Geneva, Switzerland: World Health Organization (WHO).
4779	Guatemala	Guatemala Ministry of Health and Social Assistance, University of Valle and Division of Reproductive Health-Centers for Disease Control and Prevention (CDC). Guatemala Reproductive Health Survey 2008-2009. Atlanta, United States: Centers for Disease Control and Prevention (CDC).
19656	Guatemala	Guatemala Ministry of Health and Social Assistance, Macro International, Inc, National Statistics Institute (Guatemala).

NID	Location	Citation
		Guatemala Interim Demographic and Health Survey 1998-1999. Fairfax, United States of America: ICF International.
27563	Guatemala	Guatemala Ministry of Health and Social Assistance, University of Valle, Division of Reproductive Health-Centers for Disease Control and Prevention (CDC). (2003) Guatemala Reproductive Health Survey 2002. Atlanta, United States: Centers for Disease Control and Prevention (CDC).
139976	Guatemala	Ministry of Public Health and Social Assistance (Guatemala). Guatemala National Micronutrient Survey 1995.
144314	Guatemala	World Health Organization (WHO). WHO Global Database on Anemia, Nutrition Landscape Information System. Geneva, Switzerland: World Health Organization (WHO).
157031	Guatemala	ICF International, Institute of Nutrition of Central America and Panama, Ministry of Public Health and Social Assistance (Guatemala), National Statistics Institute (Guatemala), Secretary of Planning and Programming of the Presidency (Segeplán) (Guatemala). Guatemala Demographic and Health Survey 2014-2015. Fairfax, United States of America: ICF International, 2017.
409549	Guatemala	Guatemala Ministry of Health and Social Assistance, National Statistics Institute (Guatemala). Guatemala National Micronutrient Survey 2009-2010.
490699	Guatemala	Centers for Disease Control and Prevention (CDC), Institute of Nutrition of Central America and Panama, United States Agency for International Development (USAID). Guatemala Health and Nutrition Epidemiological Surveillance System 2013. Guatemala City, Guatemala: Institute of Nutrition of Central America and Panama, 2021.
19683	Guinea	Macro International, Inc, National Statistics Directorate (Guinea). Guinea Demographic and Health Survey 2005. Fairfax, United States of America: ICF International.
69761	Guinea	ICF Macro, Ministry of Health and Public Hygiene (Guinea), National Institute of Statistics (Guinea). Guinea Demographic and Health Survey 2012. Fairfax, United States of America: ICF International.
141953	Guinea	Ministry of Health and Public Hygiene (Guinea). Guinea National Survey of Iron Deficiency Anemia 2000.
144314	Guinea	World Health Organization (WHO). WHO Global Database on Anemia, Nutrition Landscape Information System. Geneva, Switzerland: World Health Organization (WHO).
396957	Guinea	Ministry of Health (Guinea), Ministry of Planning and Economic Development (Guinea), National Institute of Statistics (Guinea). Guinea Demographic and Health Survey 2018. Fairfax, United States of America: ICF International, 2019.
21348	Guyana	Bureau of Statistics (Guyana), ICF Macro, Ministry of Health (Guyana). Guyana Demographic and Health Survey 2009.

NID	Location	Citation
		Fairfax, United States of America: ICF International, 2011.
143689	Guyana	Micronutrient Working Group. Iron and vitamin A status in five Caribbean countries. Cajanus. 2002; 4-34.
143903	Guyana	Johnson AA, Latham MC, Roe DA. The prevalence and the etiology of the nutritional anemias in Guyana. Am J Clin Nutr. 1982; 35(2): 309-18.
144314	Guyana	World Health Organization (WHO). WHO Global Database on Anemia, Nutrition Landscape Information System. Geneva, Switzerland: World Health Organization (WHO).
19708	Haiti	Haitian Institute of Childhood (IHE), Macro International, Inc. Haiti Demographic and Health Survey 2000. Fairfax, United States of America: ICF International.
19720	Haiti	Haitian Institute of Childhood (IHE), Haitian Institute of Statistics and Informatics, Macro International, Inc. Haiti Demographic and Health Survey 2005-2006. Fairfax, United States of America: ICF International.
65118	Haiti	Centers for Disease Control and Prevention (CDC), Haitian Institute of Childhood (IHE), Haitian Institute of Statistics and Informatics, Macro International, Inc. Haiti Demographic and Health Survey 2012. Fairfax, United States of America: ICF International.
143904	Haiti	Nicklas TA, Kuvibidila S, Gatewood LC, Metzinger AB, Frempong KO. Prevalence of anaemia and iron deficiency in urban Haïtian children two to five years of age. J Trop Pediatr. 1998; 44(3): 133-8.
144314	Haiti	World Health Organization (WHO). WHO Global Database on Anemia, Nutrition Landscape Information System. Geneva, Switzerland: World Health Organization (WHO).
218574	Haiti	Haitian Institute of Childhood (IHE), Haitian Institute of Statistics and Informatics, ICF International, Ministry of Public Health and Population (Haiti). Haiti Demographic and Health Survey 2016-2017. Fairfax, United States of America: ICF International.
19728	Honduras	Macro International, Inc, National Institute of Statistics (Honduras), Secretary of Health (Honduras). Honduras Demographic and Health Survey 2005-2006. Fairfax, United States of America: ICF International.
27551	Honduras	Honduras Family Planning Association (ASHONPLAFA), Ministry of Health (Honduras), and Division of Reproductive Health-Centers for Disease Control and Prevention (CDC). Honduras Reproductive Health Survey 2001. Tegucigalpa, Honduras: Honduras Family Planning Association (ASHONPLAFA).
95440	Honduras	ICF Macro, National Institute of Statistics (Honduras). Honduras Demographic and Health Survey 2011-2012. Fairfax, United States of America: ICF International.
140092	Honduras	International Eye Foundation, John Snow, Inc., Ministry of Health (Honduras), United States Agency for International

NID	Location	Citation
		Development (USAID). Honduras National Micronutrients Survey 1996.
143905	Honduras	Domellöf M, Cohen RJ, Dewey KG, Hernell O, Rivera LL, Lönnerdal B. Iron supplementation of breast-fed Honduran and Swedish infants from 4 to 9 months of age. J Pediatr. 2001; 138(5): 679-87.
144314	Honduras	World Health Organization (WHO). WHO Global Database on Anemia, Nutrition Landscape Information System. Geneva, Switzerland: World Health Organization (WHO).
143715	Hungary	Dirren H, Decarli B, Lesourd B, Schlienger JL, Deslypere JP, Kiepurski A. Nutritional status: haematology and albumin. Eur J Clin Nutr. 1991; 45 (Suppl 3): 43-52.
143717	Hungary	Elwood PC, Hughes J, Abernethy M, Davies R, Gough R, Johnson AP, Dubourg AY. An international haematological survey. Bull World Health Organ. 1976; 54(1): 87-95.
143906	Hungary	Antal M, Regöly-Mérei A, Biró L, Greiner E, Sági K, Agfalvy R, Nagy K, Biró G. Iron status in a healthy population of Hungarian secondary school boys and girls. Ann Nutr Metab. 1998; 42(1): 33-9.
143907	Iceland	Jonsson JJ, Johannesson GM, Sigfusson N, Magnusson B, Thjodleifsson B, Magnusson S. Prevalence of iron deficiency and iron overload in the adult Icelandic population. J Clin Epidemiol. 1991; 44(12): 1289-97.
19950	India	International Institute for Population Sciences (India), Macro International, Inc. India Demographic and Health Survey 1998-1999. Calverton, United States of America: Macro International, Inc.
19963	India	International Institute for Population Sciences (India), Macro International, Inc. India Demographic and Health Survey 2005-2006. Fairfax, United States of America: ICF International.
23219	India	International Institute for Population Sciences (India). India District Level Household Survey 2002-2005. Mumbai, India: International Institute for Population Sciences (India).
129905	India	National Nutrition Monitoring Bureau (India). India Rural Survey of Diet and Nutritional Status 2004-2006.
133397	India	Nangia V, Jonas JB, Sinha A, Matin A, Kulkarni M, Panda-Jonas S. Ocular axial length and its associations in an adult population of Central Rural India. The Central India Eye and Medical Study [Unpublished data]. 2010;117(7):1360-6.
133801	India	National Nutrition Monitoring Bureau (India). India Micronutrient Deficiency Survey 2003-2006.
139573	India	Kapil U, Pathak P, Tandon M, Singh C, Pradhan R, Dwivedi SN. Micronutrient deficiency disorders amongst pregnant women in three urban slum communities of Delhi. Indian Pediatr. 1999; 36(10): 983-9.

NID	Location	Citation
139585	India	Pathak P, Singh P, Kapil U, Raghuvanshi RS. Prevalence of iron, vitamin A, and iodine deficiencies amongst adolescent pregnant mothers. Indian J Pediatr. 2003; 70(4): 299-301.
140228	India	Government of Orissa, Micronutritent Initiative, United Nations Children's Fund (UNICEF), World Health Organization (WHO). India - Orissa Impact of Vitamin A Supplementation Delivered with Oral Polio Vaccine as Part of Immunization Campaign [draft final report].
143912	India	Pathak P, Tandon M, Kapil U, Singh C. Prevalence of iron deficiency anemia amongst pregnant women in urban slum communities of Delhi. Indian Pediatr. 1999; 36(3): 322-3.
143916	India	Brabin L, Nicholas S, Gogate A, Gogate S, Karande A. High prevalence of anaemia among women in Mumbai, India. Food Nutr Bull. 1998; 205-9.
143917	India	Verma M, Chhatwal J, Kaur G. Prevalence of anemia among urban school children of Punjab. Indian Pediatr. 1998; 35(12): 1181-6.
143919	India	Jain S, Chopra H, Garg SK, Bhatnagar M, Singh JV. Anemia in children: early iron supplementation. Indian J Pediatr. 2000; 67(1): 19-21.
143921	India	Abel R, Rajaratnam J, Kalaimani A, Kirubakaran S. Can iron status be improved in each of the three trimesters? A community-based study. Eur J Clin Nutr. 2000; 54(6): 490-3.
143923	India	Agarwal KN, Gomber S, Bisht H, Som M. Anemia prophylaxis in adolescent school girls by weekly or daily iron-folate supplementation. Indian Pediatr. 2003; 40(4): 296-301.
143925	India	Sethi V, Goindi G, Kapil U. Prevalence of anemia amongst primary school age children (6-11 years) in National Capital Territory of Delhi. Indian J Pediatr. 2003; 70(6): 519-20.
143926	India	Agarwal DK, Bhardwaj B, Singla PN, Tripathi AM, Agarwal KN. Etiology of maternal and early childhood deficiency anemia. Indian J Pediatr. 1986; 53(3): 389-95.
143927	India	Agarwal DK, Agarwal KN, Tripathi AM. Nutritional status in rural pregnant women of Bihar and Uttar Pradesh. Indian Pediatr. 1987; 24(2): 119-25.
143931	India	Sharma JB, Soni D, Murthy NS, Malhotra M. Effect of dietary habits on prevalence of anemia in pregnant women of Delhi. J Obstet Gynaecol Res. 2003; 29(2): 73-8.
143932	India	Agarwal AK, Sen AK, Kalra NK, Gupta N. Prevalence of anaemia during pregnancy in district Burdwan, West Bengal. Indian J Public Health. 1999; 43(1): 26-31.
143933	India	Rajaratnam J, Abel R, Asokan JS, Jonathan P. Prevalence of anemia among adolescent girls of rural Tamilnadu. Indian Pediatr. 2000; 37(5): 532-6.
143934	India	Rajaratnam J, Abel R, Ganesan C, Jayaseelan SA. Maternal anaemia: a persistent problem in rural Tamil Nadu. Natl

NID	Location	Citation
		Med J India. 2000; 13(5): 242-5.
143937	India	Chiplonkar S, Agte V, Mengale S. Relative importance of micronutrient deficiencies in iron deficiency anemia. Nutr Res. 2003; 1355-67.
143939	India	Reddaiah VP, Raj PP, Ramachandran K, Nath LM, Sood SK, Madan N, Rusia U. Supplementary iron dose in pregnancy anemia prophylaxis. Indian J Pediatr. 1989; 56(1): 109-14.
143940	India	Brahmam G, Madhavan NK, Laxmaiah A, Reddy C, Ranganathan S, Vishnuvardhana Rao M, Nadamuni Naidu A, Vijayaraghavan K, Sivakumar B, Kamala Krishnaswamy, Gowarinth Sastry J, Mohan Ram M, Praldad Rao N, Vinodini R. Community Trials with Iron and Iodine Fortified Salt (Double Fortified Salt). In: Geertman RM, ed. Proceedings of the 8th World Salt Symposium. Amsterdam, The Netherlands: Elsevier, 2000. 955-60.
143941	India	Abel R, Rajaratnam J, Gnanasekaran VJ, Jayaraman P. Prevalence of anaemia and iron deficiency in three trimesters in Rural Vellore district, South India. Trop Doct. 2001; 31(2): 86-9.
143942	India	Thilothammal N, Sujaritha R, Banu K, Ratnam S, Ezhlarasi. The nutritional status of south Indian women in child bearing age and its influence on birth weight and maturity of offspring. J Obstet Gynaecol India. 1993; 871-7.
143946	India	Bhargava M, Iyer PU, Kumar R, Ramji S, Kapani V, Bhargava SK. Relationship of maternal serum ferritin with foetal serum ferritin, birth weight and gestation. J Trop Pediatr. 1991; 37(4): 149-52.
143947	India	Sarin A. Severe anemia of pregnancy, recent experience. Int J Gynaecol Obstet. 1995; 50(Suppl 2): 45-49.
143948	India	Kumar CSV, Anand Kumar H, Sunita V, Kapur I. Prevalence of anemia and worm infestation in school going girls at Gulbargha, Karnataka. Indian Pediatr. 2003; 40(1): 70-2.
143955	India	Bains K, Mann S. Physical fitness in relation to energy and iron status of female college students. Food Nutr Bull. 2000; 305-10.
143957	India	Thangaleela T, Vijayalakshmi P. Prevalence of anaemia in pregnancy. Indian J Nutr Diet. 1994; 26-9.
143963	India	Koen M, Lemson M, Sampathkumar V, Abel R. Prevalence of anaemia among pregnant mothers in a rural South Indian population. J Obstet Gynaecol India. 1992; 283-7.
143967	India	Bharati P, Ghosh R, Gupta R. Socioeconomic Condition and Anaemia among the Mahishya Population of Southern West Bengal, India. Malays J Nutr. 2004; 10(1): 23-30.
143969	India	Swain S, Singh S, Bhatia BD, Pandey S, Krishna M. Maternal hemoglobin and serum albumin and fetal growth. Indian Pediatr. 1994; 31(7): 777-82.

NID	Location	Citation
143970	India	Ray SK, Mallick S, Kumar S, Biswas B. A rapid assessment of anaemia in pregnancy in West Bengal with special reference to care seeking behaviour of mothers. Indian J Public Health. 2000; 44(2): 58-64.
157050	India	ICF International, International Institute for Population Sciences (India), Ministry of Health and Family Welfare (India). India Demographic and Health Survey 2015-2016. Fairfax, United States of America: ICF International, 2018.
165390	India	International Institute for Population Sciences (India). India District Level Household Survey 2012-2014. New Delhi, India: Ministry of Health and Family Welfare (India).
233917	India	Indian Council of Medical Research (ICMR), National Institute of Health and Family Welfare (India), Nutrition Foundation of India, Office of the Registrar General and Census Commissioner (India). India Clinical, Anthropometric and Bio-chemical Survey 2014.
261875	India	Fogarty International Center, National Institutes of Health (NIH), Foundation for the National Institutes of Health (FNIH). India - Vellore Malnutrition and Enteric Disease Study 2009-2014.
467681	India	ICF International, International Institute for Population Sciences (India), Ministry of Health and Family Welfare (India), United States Agency for International Development (USAID). India Demographic and Health Survey 2019-2020. Fairfax, United States of America: ICF International, 2022.
490175	India	Agarwal KN, Agarwal DK, Sharma A. Anemia in pregnancy - interstate differences. Scientific Report 16. New Delhi: Nutrition Foundation of India; 2005.
5827	Indonesia	Macro International, Inc, RAND Corporation, University of California, Los Angeles (UCLA), University of Indonesia. Indonesia Family Life Survey 1997.
6111	Indonesia	Center for Population and Policy Studies, Gadjah Mada University (Indonesia), RAND Corporation. Indonesia Family Life Survey 2000. Santa Monica, United States of America: RAND Corporation.
6464	Indonesia	Center for Population and Policy Studies, Gadjah Mada University (Indonesia), RAND Corporation, SurveyMETER. Indonesia Family Life Survey 2007-2008. Santa Monica, United States of America: RAND Corporation.
22680	Indonesia	Agency of Health Research and Development (Indonesia). Indonesia Basic Health Research 2007-2008.
140221	Indonesia	Dillon, DHS, Verhoef H, West CE, Hautvast JGAJ, Burema J, Meester HJ, Bos MB. Concentration of Hemoglobin and Plasma Ferritin are not Related to Riboflavin Status but to Vitamin A Status in Indonesian Adolescent Schoolgirls. Wageningen, In: Dillon DHS, ed., Nutrition Health of Indonesian Adolescent Girls: The Role of Riboflavin and

NID	Location	Citation
		Vitamin A on Iron Status. Wageningen, The Netherlands: Wageningen University, 2005. 61-75.
143972	Indonesia	Sari M, de Pee S, Martini E, Herman S, Sugiatmi, Bloem MW, Yip R. Estimating the prevalence of anaemia: a comparison of three methods. Bull World Health Organ. 2001; 79(6): 506-11.
143974	Indonesia	Pegelow K, Gross R, Pietrzik K, Lukito W, Richards AL, Fryauff DJ. Parasitological and nutritional situation of school children in the Sukaraja district, West Java, Indonesia. Southeast Asian J Trop Med Public Health. 1997; 28(1): 173-90.
143975	Indonesia	De Pee S, Bloem MW, Sari M, Kiess L, Yip R, Kosen S. The high prevalence of low hemoglobin concentration among Indonesian infants aged 3-5 months is related to maternal anemia. J Nutr. 2002; 132(8): 2215-21.
143977	Indonesia	Soekarjo DD, de Pee S, Bloem MW, Tjiong R, Yip R, Schreurs WH, Muhilal. Socio-economic status and puberty are the main factors determining anaemia in adolescent girls and boys in East Java, Indonesia. Eur J Clin Nutr. 2001; 55(11): 932-9.
143978	Indonesia	Muslimatun S, Schmidt MK, Schultink W, West CE, Hautvast JA, Gross R, Muhilal. Weekly supplementation with iron and vitamin A during pregnancy increases hemoglobin concentration but decreases serum ferritin concentration in Indonesian pregnant women. J Nutr. 2001; 131(1): 85-90.
143979	Indonesia	Suega K, Dharmayuda TG, Sutarga IM, Bakta IM. Iron-deficiency anemia in pregnant women in Bali, Indonesia: a profile of risk factors and epidemiology. Southeast Asian J Trop Med Public Health. 2002; 33(3): 604-7.
143982	Indonesia	Thorand B, Schultink W, Gross R, Sastroamidjojo S, Wentzel S. Efficiency of the iron supplementation programme for pregnant women in Jeneponto, Sulawesi, Indonesia. Asia Pac J Clin Nutr. 1994; 3(4): 211-5.
143983	Indonesia	Andrade Juguan J, Lukito W, Schultink W. Thiamine deficiency is prevalent in a selected group of urban Indonesian elderly people. J Nutr. 1999; 129(2): 366-71.
143985	Indonesia	Palupi L, Schultink W, Achadi E, Gross R. Effective community intervention to improve hemoglobin status in preschoolers receiving once-weekly iron supplementation. Am J Clin Nutr. 1997; 65(4): 1057-61.
143986	Indonesia	Nurdia DS, Sumarni S, Suyoko, Hakim M, Winkvist A. Impact of intestinal helminth infection on anemia and iron status during pregnancy: a community based study in Indonesia. Southeast Asian J Trop Med Public Health. 2001; 32(1): 14-22.
143987	Indonesia	Dijkhuizen MA, Wieringa FT, West CE, Muherdiyantiningsih, Muhilal. Concurrent micronutrient deficiencies in lactating mothers and their infants in Indonesia. Am J Clin Nutr. 2001; 73(4): 786-91.

NID	Location	Citation
143988	Indonesia	Ridwan E, Schultink W, Dillon D, Gross R. Effects of weekly iron supplementation on pregnant Indonesian women are similar to those of daily supplementation. Am J Clin Nutr. 1996; 63(6): 884-90.
143989	Indonesia	Suharno D, West CE, Muhilal, Logman MH, de Waart FG, Karyadi D, Hautvast JG. Cross-sectional study on the iron and vitamin A status of pregnant women in West Java, Indonesia. Am J Clin Nutr. 1992; 56(6): 988-93.
264956	Indonesia	RAND Corporation, SurveyMETER. Indonesia Family Life Survey 2014-2015. Santa Monica, United States of America: RAND Corporation, 2016.
409577	Indonesia	Sandjaja S, Budiman B, Harahap H, Ernawati F, Soekatri M, Widodo Y, Sumedi E, Rustan E, Sofia G, Syarief SN, Khouw I. Food consumption and nutritional and biochemical status of 05-12-year-old Indonesian children: the SEANUTS study. Br J Nutr. 2013; 110 Suppl 3: S11-20.
489986	Indonesia	Barkley JS, Kendrick KL, Codling K, Muslimatun S, Pachón H. Anaemia prevalence over time in Indonesia: estimates from the 1997, 2000, and 2008 Indonesia Family Life Surveys. Asia Pac J Clin Nutr. 2015; 24(3): 452-5.
140090	Iran (Islamic Republic of)	Institute of Nutrition and Food Technology, Shaheed Beheshti Medical University (Iran), Pasteur Institute of Iran, United Nations Children's Fund (UNICEF). Iran National Integrated Micronutrient Survey 2001. Tehran, Iran (Islamic Republic of): Ministry of Health and Medical Education (Iran).
140096	Iran (Islamic Republic of)	Ministry of Health and Medical Education (Iran), United Nations Children's Fund (UNICEF). Iran Multi-center Study on Iron Deficiency Anemia Among 15 to 49 Year Old Women. Tehran, Iran (Islamic Republic of): Ministry of Health and Medical Education (Iran), 1995.
143990	Iran (Islamic Republic of)	Moshirfatemi M, Faghihi M, Salimi M, Banan A, Khatibi A. Hemoglobin levels in an obstetric population in Isfahan, Iran. Iran J Public Health. 1976; 113-20.
143992	Iran (Islamic Republic of)	Froozani MD, Vahdani F, Montazami K, Maiekafzali H. Maternal and newborn iron status in a public and a private maternity hospital at delivery in Tehran. J Trop Pediatr Environ Child Health. 1978; 24(4): 182-6.
143993	Iran (Islamic Republic of)	Karimi M, Kadivar R, Yarmohammadi H. Assessment of the prevalence of iron deficiency anemia, by serum ferritin, in pregnant women of Southern Iran. Med Sci Monit. 2002; 8(7): 488-492.
144314	Iran (Islamic Republic of)	World Health Organization (WHO). WHO Global Database on Anemia, Nutrition Landscape Information System. Geneva, Switzerland: World Health Organization (WHO).
262117	Iran (Islamic Republic of)	Ministry of Health and Medical Education (Iran), Tehran University of Medical Sciences, United Nations Children's Fund (UNICEF). Iran National Integrated Micronutrient Survey 2012.

NID	Location	Citation
305739	Iran (Islamic Republic of)	Digestive Diseases Research Institute (DDRI) (Iran), Shiraz University of Medical Sciences. Iran - Pars Cohort Study.
23429	Iraq	Ministry of Health (Iraq), Central Organization for Statistics and Information Technology (Iraq), Kurdistan Regional Statistics Office, World Health Organization (WHO), Ministry of Health (Kurdistan). Iraq Family Health Survey 2006-2007.
143996	Iraq	Al-Sharbatti SS, Al-Ward NJ, Al-Timimi DJ. Anemia among adolescents. Saudi Med J. 2003; 24(2): 189-94.
294319	Iraq	United Nations Children's Fund (UNICEF). Iraq National Micronutrient Deficiencies Assessment and Response Survey 2011-2012.
143717	Ireland	Elwood PC, Hughes J, Abernethy M, Davies R, Gough R, Johnson AP, Dubourg AY. An international haematological survey. Bull World Health Organ. 1976; 54(1): 87-95.
143997	Ireland	Armstrong PL. Iron deficiency in adolescents. BMJ. 1989; 298(6672): 499.
143715	Italy	Dirren H, Decarli B, Lesourd B, Schlienger JL, Deslypere JP, Kiepurski A. Nutritional status: haematology and albumin. Eur J Clin Nutr. 1991; 45 (Suppl 3): 43-52.
143717	Italy	Elwood PC, Hughes J, Abernethy M, Davies R, Gough R, Johnson AP, Dubourg AY. An international haematological survey. Bull World Health Organ. 1976; 54(1): 87-95.
143879	Italy	Knuiman JT, Westenbrink S, van der Heyden L, West CE, Burema J, de Boer J, Hautvast JG, Räsänen L, Virkkunen L, Viikari J. Determinants of total and high density lipoprotein cholesterol in boys from Finland, The Netherlands, Italy, the Philippines and Ghana with special reference to diet. Hum Nutr Clin Nutr. 1983; 37(4): 237-54.
143999	Italy	Inelmen EM, D'Alessio M, Gatto MR, Baggio MB, Jimenez G, Bizzotto MG, Enzi G. Descriptive analysis of the prevalence of anemia in a randomly selected sample of elderly people living at home: some results of an Italian multicentric study. Aging (Milano). 1994; 6(2): 81-9.
144000	Italy	Fidanza F, Losito G. Nutritional status of the elderly. Anthropometry, diet and clinical data from old pensioners in Perugia, Italy. Bibl Nutr Dieta. 1981; 70-80.
96462	Jamaica	Miall WE, Milner PF, Lovell HG, Standard KL. Haematological investigations of population samples in Jamaica. Br J Prev Soc Med. 1967; 21(2): 45-55.
140112	Jamaica	Caribbean Food and Nutrition Institute, Pan American Health Organization (PAHO), Ministry of Education (Jamaica), Ministry of Health (Jamaica). Micronutrient Study Report: An Assessment of the Vitamin A, E, Betacarotene, and Iron Status in Jamaica. Kingston, Jamaica: Caribbean Food and Nutrition Institute, Pan American Health Organization (PAHO), 1998.

NID	Location	Citation
144002	Jamaica	Grantham-McGregor SM, Desai P, Milner PF. Haematological levels in Jamaican infants. Arch Dis Child. 1974; 49(7): 525-30.
144003	Jamaica	Simmons WK, Jutsum PJ, Fox K, Spence M, Gueri M, Paradis R, Gurney JM. A survey of the anemia status of preschool age children and pregnant and lactating women in Jamaica. Am J Clin Nutr. 1982; 35(2): 319-26.
144314	Jamaica	World Health Organization (WHO). WHO Global Database on Anemia, Nutrition Landscape Information System. Geneva, Switzerland: World Health Organization (WHO).
81689	Japan	Ministry of Health and Welfare (Japan). Japan National Nutrition Survey 2000.
81905	Japan	Ministry of Health and Welfare (Japan). Japan National Nutrition Survey 1995.
81944	Japan	Ministry of Health and Welfare (Japan). Japan National Nutrition Survey 1994.
81945	Japan	Ministry of Health and Welfare (Japan). Japan National Nutrition Survey 1993.
81946	Japan	Ministry of Health and Welfare (Japan). Japan National Nutrition Survey 1992.
144008	Japan	Fujii M, Miyoshi T, Tada T, Goto H, Ishimoto H. Incidence of anemia in women in fishing villages. Tokushima J Exp Med. 1993; 40(3-4): 183-9.
144314	Japan	World Health Organization (WHO). WHO Global Database on Anemia, Nutrition Landscape Information System. Geneva, Switzerland: World Health Organization (WHO).
20073	Jordan	Department of Statistics (Jordan), Macro International, Inc. Jordan Demographic and Health Survey 2002. Fairfax, United States of America: ICF International.
20083	Jordan	Department of Statistics (Jordan), Macro International, Inc. Jordan Demographic and Health Survey 2007. Fairfax, United States of America: ICF International.
21206	Jordan	Department of Statistics (Jordan), ICF Macro. Jordan Interim Demographic and Health Survey 2009. Fairfax, United States of America: ICF International, 2010.
77517	Jordan	Department of Statistics (Jordan), ICF International. Jordan Demographic and Health Survey 2012. Fairfax, United States of America: ICF International.
144011	Jordan	Albsoul-Younes AM, Al-Ramahi RJ, Al-Safi SA. Frequency of anemia in pregnancy in Northern Jordan. Saudi Med J. 2004; 25(10): 1525-7.
144012	Jordan	Mawajdeh SM, Al-Qutob R, Schmidt A. Measuring reproductive morbidity: a community-based approach, Jordan. Health Care Women Int. 2003; 24(7): 635-49.

NID	Location	Citation
144314	Jordan	World Health Organization (WHO). WHO Global Database on Anemia, Nutrition Landscape Information System. Geneva, Switzerland: World Health Organization (WHO).
149068	Jordan	Centers for Disease Control and Prevention (CDC), Global Alliance for Improved Nutrition (GAIN), Ministry of Health (Jordan), United Nations Children's Fund (UNICEF). Jordan National Micronutrient Survey 2010.
270825	Jordan	Serdula MK, Nichols EK, Aburto NJ, Masa'd H, Obaid B, Wirth J, Tarawneh M, Barham R, Hijawi B, Sullivan KM, the Jordan Fortification Working Group. Micronutrient status in Jordan: 2002 and 2010. Eur J Clin Nutr. 2014; 68(10): 1124–8.
356955	Jordan	Department of Statistics (Jordan), ICF International. Jordan Demographic and Health Survey 2017-2018. Fairfax, United States of America: ICF International, 2019.
20092	Kazakhstan	Macro International, Inc, National Institute of Nutrition (Kazakhstan). Kazakhstan Demographic and Health Survey 1995. Fairfax, United States of America: ICF International.
20103	Kazakhstan	Academy of Preventive Medicine (Kazakhstan), Macro International, Inc. Kazakhstan Demographic and Health Survey 1999. Fairfax, United States of America: ICF International.
143098	Kazakhstan	Dangour AD, Hill HL, Ismail SJ. Height, weight and haemoglobin status of 6 to 59-month-old Kazakh children living in Kzyl-Orda region, Kazakhstan. Eur J Clin Nutr. 2002; 56(10): 1030-8.
144013	Kazakhstan	Dangour AD, Hill HL, Ismail SJ. Haemoglobin status of adult non-pregnant Kazakh women living in Kzyl-Orda region, Kazakhstan. Eur J Clin Nutr. 2001; 55(12): 1068-75.
19557	Kenya	Macro International, Inc, Population and Housing Census Commissions Office (PHCCO). Ethiopia Demographic and Health Survey 2005. Fairfax, United States of America: ICF International.
21301	Kenya	Central Statistics Agency (Ethiopia), ICF Macro, Ministry of Health (Ethiopia). Ethiopia Demographic and Health Survey 2010-2011. Fairfax, United States of America: ICF International.
57990	Kenya	Centers for Disease Control and Prevention (CDC), KEMRI Wellcome Trust Research Programme (KWTRP), Kenya National Bureau of Statistics, Ministry of Public Health and Sanitation (Kenya), National Coordinating Agency for Population and Development (Kenya), Population Services International (PSI). Kenya Malaria Indicator Survey 2007.
58006	Kenya	Centers for Disease Control and Prevention (CDC), ICF Macro, Kenya Medical Research Institute (KEMRI), Kenya National Bureau of Statistics, Measure DHS, Ministry of Public Health and Sanitation (Kenya), Population Services International (PSI), President's Malaria Initiative (PMI), United Nations Children's Fund (UNICEF), Walter Reed

NID	Location	Citation
		Project, World Health Organization (WHO). Kenya Malaria Indicator Survey 2010. Nairobi, Kenya: Kenya National Bureau of Statistics.
140093	Kenya	Kenya Medical Research Institute (KEMRI), Ministry of Health (Kenya), Social Sciences and Medicine Africa Network (SOMA-NET), United Nations Children's Fund (UNICEF), University of Nairobi. Kenya National Micronutrient Survey 1999.
144015	Kenya	Newton CR, Warn PA, Winstanley PA, Peshu N, Snow RW, Pasvol G, Marsh K. Severe anaemia in children living in a malaria endemic area of Kenya. Trop Med Int Health. 1997; 2(2): 165-78.
144017	Kenya	Olsen A, Magnussen P, Ouma JH, Andreassen J, Friis H. The contribution of hookworm and other parasitic infections to haemoglobin and iron status among children and adults in western Kenya. Trans R Soc Trop Med Hyg. 1998; 92(6): 643-9.
144019	Kenya	Akhwale WS, Lum JK, Kaneko A, Eto H, Obonyo C, Björkman A, Kobayakawa T. Anemia and malaria at different altitudes in the western highlands of Kenya. Addict Behav Rep. 2004; 91(2): 167-75.
144020	Kenya	Leenstra T, Kariuki SK, Kurtis JD, Oloo AJ, Kager PA, ter Kuile FO. Prevalence and severity of anemia and iron deficiency: cross-sectional studies in adolescent schoolgirls in western Kenya. Eur J Clin Nutr. 2004; 58(4): 681-91.
144021	Kenya	Friis H, Mwaniki D, Omondi B, Muniu E, Thiong'o F, Ouma J, Magnussen P, Geissler PW, Michaelsen KF. Effects on haemoglobin of multi-micronutrient supplementation and multi-helminth chemotherapy: a randomized, controlled trial in Kenyan school children. Eur J Clin Nutr. 2003; 57(4): 573-9.
144024	Kenya	Ter Kuile FO, Terlouw DJ, Phillips-Howard PA, Hawley WA, Friedman JF, Kolczak MS, Kariuki SK, Shi YP, Kwena AM, Vulule JM, Nahlen BL. Impact of permethrin-treated bed nets on malaria and all-cause morbidity in young children in an area of intense perennial malaria transmission in western Kenya: cross-sectional survey. Am J Trop Med Hyg. 2003; 68(4 Suppl): 100-7.
153981	Kenya	Kenya Medical Research Institute (KEMRI), Kenya National Bureau of Statistics, Ministry of Public Health and Sanitation (Kenya). Kenya National Micronutrient Survey 2011 - IHSN. Nairobi, Kenya: Kenya National Bureau of Statistics.
218568	Kenya	Central Statistics Agency (Ethiopia), ICF International. Ethiopia Demographic and Health Survey 2016. Fairfax, United States of America: ICF International, 2017.
218579	Kenya	ICF International, Kenya National Bureau of Statistics, National Malaria Control Program (NMCP) (Kenya). Kenya Malaria Indicator Survey 2015. Fairfax, United States of America: ICF International, 2015.

NID	Location	Citation
283723	Kenya	Mwaniki DL, Omwega AM, Muniu EM, Mutunga JN, Akelola R, Shako BR, Gotink MH, Pertet AM. Anaemia and status of iron, vitamin A and zinc in Kenya: The 1999 National Survey Report.
294288	Kenya	Petry N, Olofin I, Hurrell RF, Boy E, Wirth JP, Moursi M, Angel MD, Rohner F. The Proportion of Anemia Associated with Iron Deficiency in Low, Medium, and High Human Development Index Countries: A Systematic Analysis of National Surveys. Nutrients. 2016; 8(11).
475926	Kenya	ICF International, Kenya National Bureau of Statistics, Ministry of Health (Kenya). Kenya Malaria Indicator Survey 2020. Fairfax, United States of America: ICF International, 2021.
142784	Kiribati	Republic of Kiribati. Pacific Helminths Initiative 2001-2002.
140203	Kuwait	Al-Mousa Z, Jackson RT, Al-Raqua M, Prakash P. The Epidemiology of Anaemia in Kuwait. In: Musaiger AO, ed. Micronutrient Deficiencies and Diet-related Chronic Diseases in the Arab Gulf Countries. Manama, Bahrain: Bahrain Centre for Studies and Research, 2000. 13-24.
141985	Kuwait	Ministry of Health (Kuwait). Kuwait Nutrition Surveillance System Report: Trends 2001-2005. Kuwait City, Kuwait: Ministry of Health (Kuwait), 2006.
143103	Kuwait	Amine EK, Al-Awadi F. Nutritional status survey of preschool children in Kuwait. East Mediterr Health J. 1996; 2(3): 386-95.
144029	Kuwait	Jackson R, Al Hamad N, Al-Somaie M, Al Guoad N, Prakash P. Gender and age differences in anemia prevalence during the lifecycle in Kuwait. Ecol Food Nutr. 2004; 61-75.
144030	Kuwait	Al-Haifi A, Huffman F. Feeding practices influence weight and hemoglobin status in Kuwaiti infants. Ecol Food Nutr. 2004; 41-59.
144031	Kuwait	Jackson RT, Al-Mousa Z. Iron deficiency is a more important cause of anemia than hemoglobinopathies in Kuwaiti adolescent girls. J Nutr. 2000; 130(5): 1212-6.
144032	Kuwait	Eid N, Al-Hooti S, Bourisly N, Khalafawi M. Anaemia in school children: a preliminary study. J Kuwait Med Assoc. 1986; 39-43.
144033	Kuwait	Dawood J, Prakash P, Shubber K. Iron deficiency anaemia among pregnant Arab women in Kuwait. J Kuwait Med Assoc. 1990; 167-72.
144034	Kuwait	Al Mousa Z, Prakash P, Jackson R, Al Raqua M. A comparison of selected nutrient intakes in anemic and nonanemic adolescent girls in Kuwait. Nutr Res. 2003; 425-433.
144314	Kuwait	World Health Organization (WHO). WHO Global Database on Anemia, Nutrition Landscape Information System. Geneva, Switzerland: World Health Organization (WHO).

NID	Location	Citation
20154	Kyrgyzstan	Macro International, Inc, Ministry of Health (Kyrgyzstan), Research Institute of Obstetrics and Pediatrics (Kyrgyzstan). Kyrgyzstan Demographic and Health Survey 1997. Fairfax, United States of America: ICF International.
77518	Kyrgyzstan	ICF International, Ministry of Health (Kyrgyzstan), National Statistical Committee of the Kyrgyz Republic. Kyrgyzstan Demographic and Health Survey 2012. Fairfax, United States of America: ICF International.
7629	Lao People's Democratic Republic	United Nations Children's Fund (UNICEF), Department of Statistics (Laos), Ministry of Health (Laos). Laos Multiple Indicator Cluster Survey 2006. New York, United States: United Nations Children's Fund (UNICEF).
162427	Lao People's Democratic Republic	Ministry of Health (Laos), National Institute of Public Health (NIOPH), National Statistical Center (Laos). Laos National Health Survey 2000.
294314	Lao People's Democratic Republic	Knowles J, Thurnham DI, Phengdy B, Houamboun K, Philavong K, Keomoungkhone I, Keovilay K. Impact of inflammation on the biomarkers of iron status in a cross-sectional survey of Lao women and children. Br J Nutr. 2013; 110(12): 2285-97.
375362	Lao People's Democratic Republic	Lao Statistics Bureau, Ministry of Education and Sports (Laos), Ministry of Health (Laos), United Nations Children's Fund (UNICEF). Laos Multiple Indicator Cluster Survey 2017. New York, United States of America: United Nations Children's Fund (UNICEF), 2018.
140122	Lebanon	Hwalla N, Adra N. Prevalence and Selected Determinant of Iron Deficiency Anemia in Women and Under Five Children in Lebanon. 1998.
144036	Lebanon	Al Khatib L, Obeid O, Sibai A-M, Batal M, Adra N, Hwalla N. Folate deficiency is associated with nutritional anaemia in Lebanese women of childbearing age. Public Health Nutr. 2006; 9(7): 921-7.
144314	Lebanon	World Health Organization (WHO). WHO Global Database on Anemia, Nutrition Landscape Information System. Geneva, Switzerland: World Health Organization (WHO).
295863	Lebanon	Hwalla N, Adra N, Jackson RT. Iron Deficiency Is an Important Contributor to Anemia Among Reproductive Age Women in Lebanon. Ecol Food Nutr. 2004; 43(1–2): 77–92.
20167	Lesotho	Bureau of Statistics (Lesotho), Macro International, Inc, Ministry of Health and Social Welfare (Lesotho). Lesotho Demographic and Health Survey 2004-2005. Fairfax, United States of America: ICF International.
21382	Lesotho	ICF Macro, Ministry of Health and Social Welfare (Lesotho). Lesotho Demographic and Health Survey 2009-2010. Fairfax, United States of America: ICF International.
124465	Lesotho	Ministry of Health and Social Welfare (Lesotho), United Nations Children's Fund (UNICEF). Lesotho National Micronutrient Survey 1993.

NID	Location	Citation
157058	Lesotho	ICF International, Ministry of Health and Social Welfare (Lesotho). Lesotho Demographic and Health Survey 2014. Fairfax, United States of America: ICF International.
34279	Liberia	ICF Macro, Liberia Institute for Statistics and Geo-information Services (LISGIS), National Malaria Control Program (Liberia). Liberia Malaria Indicator Survey 2008-2009. Fairfax, United States of America: ICF International.
56828	Liberia	ICF International, Liberia Institute for Statistics and Geo-information Services (LISGIS), National Malaria Control Program (Liberia). Liberia Malaria Indicator Survey 2011. Fairfax, United States of America: ICF International, 2012.
140123	Liberia	Ministry of Health and Social Welfare (Liberia), United Nations Children's Fund (UNICEF). Liberia National Micronutrients Survey 1999.
144037	Liberia	Jackson RT. Hemoglobin levels and anemia-associated symptoms in pregnant Liberian women. Nutrition. 1992; 8(6): 430-3.
144314	Liberia	World Health Organization (WHO). WHO Global Database on Anemia, Nutrition Landscape Information System. Geneva, Switzerland: World Health Organization (WHO).
286768	Liberia	ICF International, Liberia Institute for Statistics and Geo-information Services (LISGIS), National Malaria Control Program (Liberia). Liberia Malaria Indicator Survey 2016. Fairfax, United States of America: ICF International, 2017.
294288	Liberia	Petry N, Olofin I, Hurrell RF, Boy E, Wirth JP, Moursi M, Angel MD, Rohner F. The Proportion of Anemia Associated with Iron Deficiency in Low, Medium, and High Human Development Index Countries: A Systematic Analysis of National Surveys. Nutrients. 2016; 8(11).
400126	Liberia	Centers for Disease Control and Prevention (CDC), Emory University (United States), Eunice Shriver Kennedy National Institute of Child Health and Human Development (NICHD), Global Alliance for Improved Nutrition (GAIN), GroundWork LLC, HarvestPlus, International Micronutrient Malnutrition Prevention and Control (IMMPaCt) Program, International Zinc Consultative Group (IZiNCG), Strengthening Partnerships, Results, and Innovations in Nutrition Globally (SPRING). Biomarkers Reflecting Inflammation and Nutrition Determinants of Anemia (BRINDA).
459845	Liberia	ICF International, Liberia Institute for Statistics and Geo-information Services (LISGIS), Ministry of Health and Social Welfare (Liberia). Liberia Demographic and Health Survey 2019-2020. 2021.
20212	Madagascar	Macro International, Inc, National Institute of Statistics (Madagascar). Madagascar Demographic and Health Survey 1997. Fairfax, United States of America: ICF International.
20223	Madagascar	Macro International, Inc, National Institute of Statistics (Madagascar). Madagascar Demographic and Health

NID	Location	Citation
		Survey 2003-2004. Fairfax, United States of America: ICF International.
21409	Madagascar	ICF Macro, National Institute of Statistics (Madagascar). Madagascar Demographic and Health Survey 2008-2009. Fairfax, United States of America: ICF International.
69806	Madagascar	ICF International, National Institute of Statistics (Madagascar), National Program for the Fight Against Malaria (PNLP) (Madagascar), Pasteur Institute of Madagascar (IPM). Madagascar Malaria Indicator Survey 2011. Fairfax, United States of America: ICF International.
111438	Madagascar	ICF International, National Institute of Statistics (Madagascar), National Program for the Fight Against Malaria (PNLP) (Madagascar), Pasteur Institute of Madagascar (IPM). Madagascar Malaria Indicator Survey 2013. Fairfax, United States of America: ICF International, 2013.
140945	Madagascar	Ministry of Agriculture (Madagascar), Ministry of Public Health (Madagascar), Monitoring Education, Schools, and Communities on Food and Expanded Nutrition (SEECALINE), National Institute of Statistics (Madagascar), National Research Center for the Environment (CNRE) (Madagascar), Swiss Vitamin Institute. Madagascar Survey on Vitamin A Deficiency in Women and Children and Survey of Anemia in Schoolchildren from 6-14 Years 2000.
218580	Madagascar	ICF International, Ministry of Public Health (Madagascar), National Institute of Statistics (Madagascar), National Program for the Fight Against Malaria (PNLP) (Madagascar), Pasteur Institute of Madagascar (IPM). Madagascar Malaria Indicator Survey 2016. Fairfax, United States of America: ICF International, 2017.
20263	Malawi	Macro International, Inc, National Statistical Office of Malawi. Malawi Demographic and Health Survey 2004-2005. Fairfax, United States of America: ICF International.
21393	Malawi	ICF Macro, National Statistical Office of Malawi. Malawi Demographic and Health Survey 2010. Fairfax, United States of America: ICF International.
77387	Malawi	ICF International, National Malaria Control Program (Malawi). Malawi Malaria Indicator Survey 2012. Fairfax, United States of America: ICF International.
140495	Malawi	Centers for Disease Control and Prevention (CDC), Ministry of Health (Malawi), National Statistical Office of Malawi, United Nations Children's Fund (UNICEF). Malawi National Micronutrient Survey 2001.
141343	Malawi	Department of Nutrition, HIV and AIDS in the Office of President and Cabinet (DHNA-OPC), Ministry of Health (MOH), National Statistics Office (NSO), UNICEF and CDC. The national micronutrient survey 2009. Lilongwe, Malawi: Ministry of Health, UNICEF, Irish Aid and CDC, 2011.

NID	Location	Citation
144040	Malawi	Verhoeff FH, Brabin BJ, Chimsuku L, Kazembe P, Broadhead RL. Malaria in pregnancy and its consequences for the infant in rural Malawi. Ann Trop Med Parasitol. 1999; 25-33.
144041	Malawi	Van den Broek NR, Rogerson SJ, Mhango CG, Kambala B, White SA, Molyneux ME. Anaemia in pregnancy in southern Malawi: prevalence and risk factors. BJOG. 2000; 107(4): 445-51.
144042	Malawi	Mukiibi JM, Mtimavalye LA, Broadhead R, Mzula E, Dzinyemba WE, Merrick R, Khoromana CO, Ching'ani GW. Some haematological parameters in Malawian neonates. East Afr Med J. 1995; 72(1): 10-4.
144043	Malawi	Semba RD, Kumwenda N, Taha TE, Mtimavalye L, Broadhead R, Garrett E, Miotti PG, Chiphangwi JD. Impact of vitamin A supplementation on anaemia and plasma erythropoietin concentrations in pregnant women: a controlled clinical trial. Eur J Haematol. 2001; 66(6): 389-95.
144045	Malawi	Fazio-Tirrozzo G, Brabin L, Brabin B, Agbaje O, Harper G, Broadhead R. A community based study of vitamin A and vitamin E status of adolescent girls living in the Shire Valley, Southern Malawi. Eur J Clin Nutr. 1998; 52(9): 637-42.
144046	Malawi	Van den Broek NR, Ntonya C, Mhango E, White SA. Diagnosing anaemia in pregnancy in rural clinics: assessing the potential of the Haemoglobin Colour Scale. Bull World Health Organ. 1999; 77(1): 15-21.
157059	Malawi	ICF International, Ministry of Health (Malawi), National Malaria Control Program (Malawi), National Statistical Office of Malawi. Malawi Malaria Indicator Survey 2014. Fairfax, United States of America: ICF International, 2015.
218581	Malawi	Emory University and Centers for Disease Control & Prevention Collaboration, ICF International, Ministry of Health (Malawi), National Statistical Office of Malawi. Malawi Demographic and Health Survey 2015-2016. Fairfax, United States of America: ICF International, 2017.
261359	Malawi	Centers for Disease Control and Prevention (CDC), Malaria Control and Elimination Partnership in Africa (MACEPA), National Malaria Control Program (Malawi), National Statistical Office of Malawi, President's Malaria Initiative (PMI), United Nations Children's Fund (UNICEF), University of Malawi College of Medicine, World Health Organization (WHO). Malawi National Malaria Indicator Survey 2010.
286769	Malawi	ICF International, National Malaria Control Program (Malawi). Malawi Malaria Indicator Survey 2017. Fairfax, United States of America: ICF International, 2018.
317509	Malawi	Centers for Disease Control and Prevention (CDC), ICF International, Ministry of Health (Malawi), National Statistical Office of Malawi, United Nations Children's Fund (UNICEF). Malawi Micronutrient Survey 2015-2016.

NID	Location	Citation
400126	Malawi	Centers for Disease Control and Prevention (CDC), Emory University (United States), Eunice Shriver Kennedy National Institute of Child Health and Human Development (NICHD), Global Alliance for Improved Nutrition (GAIN), GroundWork LLC, HarvestPlus, International Micronutrient Malnutrition Prevention and Control (IMMPaCt) Program, International Zinc Consultative Group (IZiNCG), Strengthening Partnerships, Results, and Innovations in Nutrition Globally (SPRING). Biomarkers Reflecting Inflammation and Nutrition Determinants of Anemia (BRINDA).
144047	Malaysia	Sagin DD, Ismail G, Mohamad M, Pang EKH, Sya OT. Anemia in remote interior communities in Sarawak, Malaysia. Southeast Asian J Trop Med Public Health. 2002; 33(2): 373-7.
144048	Malaysia	Tee ES Jr, Khor G, Tony N, Yassin Z, Chee H, Mdyusof S. Nutritional assessment of rural villages and estates in Peninsular Malaysia*. III. Prevalence of anaemia. Malays J Nutr. 1998; 4(1): 1-29.
144049	Malaysia	Ahmad Z Jr, Jaafar R, Mohd Hassan M, Othman M, Hashim A. Anaemia during pregnancy in rural Kelantan. Malays J Nutr. 1997; 3(1): 83-90.
144050	Malaysia	Foo LH, Khor GL, Tee E-S, Prabakaran D. Iron status and dietary iron intake of adolescents from a rural community in Sabah, Malaysia. Asia Pac J Clin Nutr. 2004; 13(1): 48-55.
144053	Malaysia	Shahar S, Earland J, Powers HJ, Rahman SA. Nutritional status of rural elderly Malays: dietary and biochemical findings. Int J Vitam Nutr Res. 1999; 69(4): 277-84.
144314	Malaysia	World Health Organization (WHO). WHO Global Database on Anemia, Nutrition Landscape Information System. Geneva, Switzerland: World Health Organization (WHO).
289826	Malaysia	Poh BK, Ng BK, Siti Haslinda MD, Nik Shanita S, Wong JE, Budin SB, Ruzita AT, Ng LO, Khouw I, Norimah AK. Nutritional status and dietary intakes of children aged 6 months to 12 years: findings of the Nutrition Survey of Malaysian Children (SEANUTS Malaysia). Br J Nutr. 2013; 110 Suppl 3: S21-35.
489980	Malaysia	Haniff J, Das A, Onn LT, Sun CW, Nordin NM, Rampal S, Bahrin S, Ganeslingam M, Kularatnam KI, Zaher ZM. Anemia in pregnancy in Malaysia: a cross-sectional survey. Asia Pac J Clin Nutr. 2007; 16(3): 527-36.
8033	Maldives	Ministry of Health (Maldives), Republic of Maldives, United Nations Children's Fund (UNICEF). Maldives Multiple Indicator Cluster Survey 2001.
140120	Maldives	Ministry of Health (Maldives). Maldives National Nutrition Survey 1994.
144314	Maldives	World Health Organization (WHO). WHO Global Database on Anemia, Nutrition Landscape Information System. Geneva, Switzerland: World Health Organization (WHO).

NID	Location	Citation
218582	Maldives	ICF International, Ministry of Health (Maldives). Maldives Demographic and Health Survey 2016-2017. Fairfax, United States of America: ICF International, 2019.
236130	Maldives	Aga Khan University, Ministry of Health (Maldives), United Nations Children's Fund (UNICEF). Maldives National Micronutrient Survey 2007-2008.
20274	Mali	Macro International, Inc, Ministry of Health (Mali), National Directorate of Statistics and Informatics (DNSI) (Mali). Mali Demographic and Health Survey 2006. Fairfax, United States of America: ICF International.
20315	Mali	Macro International, Inc, National Directorate of Statistics and Informatics (DNSI) (Mali), Planning and Statistics Unit, Ministry of Health (Mali). Mali Demographic and Health Survey 2001. Fairfax, United States of America: ICF International.
30777	Mali	ICF Macro, INFO-STAT (Mali), National Program for the Fight Against Malaria (Mali). Mali Special Demographic and Health Survey 2010. Fairfax, United States of America: ICF International, 2011.
77388	Mali	ICF International, INFO-STAT (Mali), Ministry of Health (Mali), National Institute of Statistics (INSTAT) (Mali), Planning and Statistics Unit, Ministry of Health (Mali). Mali Demographic and Health Survey 2012-2013. Fairfax, United States of America: ICF International, 2014.
218587	Mali	ICF International, INFO-STAT (Mali), Ministry of Health and Public Hygiene (Mali), National Institute of Public Health Research (INRSP) (Mali), National Institute of Statistics (INSTAT) (Mali), National Program for the Fight Against Malaria (Mali). Mali Malaria Indicator Survey 2015. Fairfax, United States of America: ICF International, 2016.
270627	Mali	Ministry of Health (Mali), National Institute of Statistics (INSTAT) (Mali), United Nations Children's Fund (UNICEF). Mali Multiple Indicator Cluster Survey 2009-2010. New York, United States of America: United Nations Children's Fund (UNICEF), 2017.
398033	Mali	ICF International, National Institute of Statistics (INSTAT) (Mali), Sector Planning and Statistics Unit Health-Social Development and Family Promotion (CPS / SS-DS-PF) (Mali). Mali Demographic and Health Survey 2018. Fairfax, United States of America: ICF International, 2019.
140118	Marshall Islands	Ministry of Health (Marshall Islands), United Nations Children's Fund (UNICEF). Marshall Islands National Nutrition Survey 1990-1991.
142784	Marshall Islands	Republic of Kiribati. Pacific Helminths Initiative 2001-2002.
144057	Marshall Islands	Palafox NA, Gamble MV, Dancheck B, Ricks MO, Briand K, Semba RD. Vitamin A deficiency, iron deficiency, and anemia among preschool children in the Republic of the Marshall Islands. Nutrition. 2003; 19(5): 405-8.

NID	Location	Citation
137516	Mauritius	Ministry of Health and Quality of Life (Mauritius). Mauritius and Rodrigues Survey of Nutrition 1995.
140116	Mauritius	Ministry of Health and Quality of Life (Mauritius), United Nations Children's Fund (UNICEF). Mauritius Survey on the Nutritional Status of Preschool Children 1985.
8618	Mexico	National Institute of Public Health (Mexico). Mexico National Survey of Health and Nutrition 2005-2006. Cuernavaca, Mexico: National Institute of Public Health (Mexico).
81748	Mexico	National Institute of Public Health (Mexico). Mexico National Survey of Health and Nutrition 2011-2012. Cuernavaca, Mexico: National Institute of Public Health (Mexico).
105806	Mexico	National Institute of Public Health (Mexico). Mexico National Nutrition Survey 1988. Cuernavaca, Mexico: National Institute of Public Health (Mexico).
135527	Mexico	Secretariat of Health (Mexico), Secretary of Public Education (Mexico). Mexico National School Health Survey 2008. Cuernavaca, Mexico: National Institute of Public Health (Mexico), 2010.
144061	Mexico	Vásquez-Garibay EM, Romero-Velarde E, Nápoles-Rodríguez F, Nuño-Cosío ME, Trujillo-Contreras F, Sánchez-Mercado O. [Prevalence of iron and iodine deficiency, and parasitosis among children from Arandas, Jalisco, Mexico]. Salud Publica Mex. 2002; 44(3): 195-200.
144062	Mexico	Kordas K, Lopez P, Rosado JL, García Vargas G, Alatorre Rico J, Ronquillo D, Cebrián ME, Stoltzfus RJ. Blood lead, anemia, and short stature are independently associated with cognitive performance in Mexican school children. J Nutr. 2004; 134(2): 363-71.
144063	Mexico	Martínez H, González-Cossío T, Flores M, Rivera-Dommarco J, Lezana MA, Sepúlveda-Amor J. [Anemia in women of reproductive age. The results of a national probability survey]. Salud Publica Mex. 1995; 37(2): 108-19.
144064	Mexico	Tejas AR, Wyatt CJ, Ramírez MJ. Prevalence of undernutrition and iron deficiency in pre-school children from different socioeconomic regions in the city of Oaxaca, Oaxaca, Mexico. J Nutr Sci Vitaminol. 2001; 47(1): 47-51.
144065	Mexico	Ramakrishnan U, González-Cossio T, Neufeld L, Rivera J, Martorell R. Multiple micronutrient supplementation during pregnancy does not lead to greater infant birth size than does iron-only supplementation: a randomized controlled trial in a semirural community in Mexico. Am J Clin Nutr. 2003; 720-5.
144069	Mexico	Maulen-Radovan I, Villagómez S, Soler E, Villicaña R, Hernández-Ronquillo L, Rosado JL. [Nutritional impact of whole milk supplemented with vitamins and minerals in children]]. Salud Publica Mex. 1999; 41(5): 389-96.
144070	Mexico	Vásquez-Molina ME, Corral-Terrazas M, Apezteguia MA, Carmona-Sawasky J, Levario-Carrillo M. [Relationship

NID	Location	Citation
		between maternal and neonatal iron stores]. Salud Publica Mex. 2001; 43(5): 402-7.
144071	Mexico	Black AK, Allen LH, Pelto GH, de Mata MP, Chávez A. Iron, vitamin B-12 and folate status in Mexico: associated factors in men and women and during pregnancy and lactation. J Nutr. 1994; 124(8): 1179-88.
144072	Mexico	Ramírez-Mateos C, Loría A, Nieto-Gómez M, Malacara JM, Piedras J. [Anemia and iron deficiency in 490 Mexican pregnant women]. Rev Invest Clin. 1998; 50(2): 119-26.
144076	Mexico	Allen LH, Rosado JL, Casterline JE, López P, Muñoz E, Garcia OP, Martinez H. Lack of hemoglobin response to iron supplementation in anemic mexican preschoolers with multiple micronutrient deficiencies. Am J Clin Nutr. 2000; 71(6): 1485-94.
316736	Mexico	Ministry of Health (Mexico), National Institute of Public Health (Mexico). Mexico National Survey of Health and Nutrition Mid-way 2016.
139971	Micronesia (Federated States of)	Socorro P, Gonzaga C. Results of vitamin A, Anemia and Blood Lead Survey Among 2-4 Year Old Children and Reproductive-aged Women in Yap Proper and Kosrae State, Federated States of Micronesia. Atlanta, United States: Centers for Disease Control and Prevention (CDC), 2000.
139972	Micronesia (Federated States of)	Auerbach SB. Maternal-Child Health Survey: Pohnpei, Federated States of Micronesia, 1993 [summary table]. Palikir, Pohnpei, US Public Health Service/Department of Health Services [Federated States of Micronesia], 1993.
142784	Micronesia (Federated States of)	Republic of Kiribati. Pacific Helminths Initiative 2001-2002.
143873	Micronesia (Federated States of)	Lloyd-Puryear M, Mahoney J, Humphrey J, Mahoney F, Siren N, Moorman C, West KJ. Vitamin A deficiency in Micronesia: a statewide survey in Chuuk. Nutr Res. 1991; 1101-1110.
137528	Mongolia	Public Health Institute, Ministry of Health (Mongolia), United Nations Children's Fund (UNICEF). Mongolia National Nutrition Survey 2004.
139870	Mongolia	Kachondham Y. Report on the 2nd National Child and Nutrition Survey, Mongolia 1999. Thailand, Institute of Nutrition, Faculty of Medicine, Ramathibodi Hospital Mahidol University, 2000.
139872	Mongolia	Centers for Disease Control and Prevention (CDC), Ministry of Health (Mongolia), United Nations Children's Fund (UNICEF), World Health Organization (WHO). Mongolia Survey Assessing the Nutritional Consequences of the Dzud 2001.
144314	Mongolia	World Health Organization (WHO). WHO Global Database on Anemia, Nutrition Landscape Information System. Geneva, Switzerland: World Health Organization (WHO).
275252	Mongolia	Public Health Institute, Ministry of Health (Mongolia), United Nations Children's Fund (UNICEF). Mongolia National

NID	Location	Citation
		Nutrition Survey 2010. Ulaanbaatar, Mongolia: National Statistical Office of Mongolia.
294288	Mongolia	Petry N, Olofin I, Hurrell RF, Boy E, Wirth JP, Moursi M, Angel MD, Rohner F. The Proportion of Anemia Associated with Iron Deficiency in Low, Medium, and High Human Development Index Countries: A Systematic Analysis of National Surveys. Nutrients. 2016; 8(11).
27024	Montenegro	Institute for Public Health (Montenegro), Institute of Public Health of Serbia, United Nations Children's Fund (UNICEF), World Health Organization (WHO). Yugoslavia, Federal Republic Multiple Indicator Cluster Survey 2000.
139868	Morocco	Ministry of Health (Morocco). Morocco National Survey of Iron Deficiency, Use of Iodized Salt, and Vitamin A Supplementation 2000.
55975	Mozambique	ICF Macro, Manhica Health Research Center (CISM), Ministry of Health (Mozambique), National Institute of Statistics (INE) (Mozambique). Mozambique Demographic and Health Survey 2011. Fairfax, United States of America: ICF International.
137426	Mozambique	Ministry of Health (Mozambique). Mozambique Micronutrient Deficiency Survey 1998.
140214	Mozambique	Helen Keller International, Micronutrient Initiative. Mozambique - Manica School-based Iron/Folate Supplementation for Girls Pilot Project Baseline Survey 2001.
140218	Mozambique	Helen Keller International, Ministry of Health (Mozambique), World Health Organization (WHO). Mozambique Survey on Vitamin A Deficiency and Prevalence of Anemia and Malaria 2001-2002.
144314	Mozambique	World Health Organization (WHO). WHO Global Database on Anemia, Nutrition Landscape Information System. Geneva, Switzerland: World Health Organization (WHO).
157060	Mozambique	Centers for Disease Control and Prevention (CDC), ICF International, Ministry of Health (Mozambique), National Institute of Health (Mozambique), National Institute of Statistics (INE) (Mozambique). Mozambique AIDS Indicator Survey 2015. Fairfax, United States of America: ICF International, 2018.
261361	Mozambique	Centers for Disease Control and Prevention (CDC), Malaria Consortium, National Institute of Health (Mozambique), National Institute of Statistics (INE) (Mozambique), World Health Organization (WHO). Mozambique National Malaria Indicator Survey 2007.
294288	Mozambique	Petry N, Olofin I, Hurrell RF, Boy E, Wirth JP, Moursi M, Angel MD, Rohner F. The Proportion of Anemia Associated with Iron Deficiency in Low, Medium, and High Human Development Index Countries: A Systematic Analysis of National Surveys. Nutrients. 2016; 8(11).

NID	Location	Citation
413934	Mozambique	ICF International, National Institute of Health (Mozambique), National Institute of Statistics (INE) (Mozambique), National Malaria Control Program (Mozambique). Mozambique Malaria Indicator Survey 2018. Fairfax, United States of America: ICF International, 2019.
139863	Myanmar	Ministry of Health (Myanmar). A Study on Hemoglobin Status and Food Practices of Myanmar Women. Naypyidaw, Myanmar: Ministry of Health (Myanmar), 2001.
144314	Myanmar	World Health Organization (WHO). WHO Global Database on Anemia, Nutrition Landscape Information System. Geneva, Switzerland: World Health Organization (WHO).
157061	Myanmar	ICF International, Ministry of Health and Sports (Myanmar). Myanmar Demographic and Health Survey 2015-2016. Fairfax, United States of America: ICF International, 2017.
144082	Namibia	Thomson J. Anaemia in pregnant women in eastern Caprivi, Namibia. S Afr Med J. 1997; 87(11): 1544-7.
150382	Namibia	ICF International, Ministry of Health and Social Services (Namibia), Namibia Institute of Pathology, Namibia Statistics Agency. Namibia Demographic and Health Survey 2013. Fairfax, United States of America: ICF International.
20462	Nepal	Macro International, Inc, Ministry of Health and Population (Nepal), New ERA. Nepal Demographic and Health Survey 2006. Fairfax, United States of America: ICF International.
21240	Nepal	ICF Macro, Ministry of Health and Population (Nepal), New ERA. Nepal Demographic and Health Survey 2011. Fairfax, United States of America: ICF International.
137462	Nepal	Micronutrient Initiative, Ministry of Health and Population (Nepal), National Planning Commission (Nepal), New ERA, United Nations Children's Fund (UNICEF), World Health Organization (WHO). Nepal Micronutrient Status Survey 1997-1998.
144083	Nepal	Christian P, Shrestha J, LeClerq SC, Khatry SK, Jiang T, Wagner T, Katz J, West KP Jr. Supplementation with micronutrients in addition to iron and folic acid does not further improve the hematologic status of pregnant women in rural Nepal. J Nutr. 2003; 133(11): 3492-8.
144084	Nepal	Shrestha PN. Anemia in children of rural Kathmandu. Indian J Pediatr. 1986; 53(5): 647-50.
144088	Nepal	Curtale F, Tilden R, Muhilal, Vaidya Y, Pokhrel RP, Guerra R. Intestinal helminths and risk of anaemia among Nepalese children. Panminerva Med. 1993; 35(3): 159-66.
144089	Nepal	Jiang T, Christian P, Khatry SK, Wu L, West KP Jr. Micronutrient deficiencies in early pregnancy are common, concurrent, and vary by season among rural Nepali pregnant women. J Nutr. 2005; 135(5): 1106-12.
144090	Nepal	Dreyfuss ML, Stoltzfus RJ, Shrestha JB, Pradhan EK, LeClerq SC, Khatry SK, Shrestha SR, Katz J, Albonico M, West KP Jr.

NID	Location	Citation
		Hookworms, malaria and vitamin A deficiency contribute to anemia and iron deficiency among pregnant women in the plains of Nepal. J Nutr. 2000; 130(10): 2527-36.
144196	Nepal	Stoltzfus RJ, Edward-Raj A, Dreyfuss ML, Albonico M, Montresor A, Dhoj Thapa M, West KP Jr, Chwaya HM, Savioli L, Tielsch J. Clinical pallor is useful to detect severe anemia in populations where anemia is prevalent and severe. J Nutr. 1999; 129(9): 1675-81.
261880	Nepal	Fogarty International Center, National Institutes of Health (NIH), Foundation for the National Institutes of Health (FNIH), Institute of Medicine, Tribhuvan University, University of Bergen, Walter Reed/AFRIMS Research Unit Nepal (WARUN). Nepal - Bhaktapur Malnutrition and Enteric Disease Study 2009-2014.
286782	Nepal	ICF International, Ministry of Health (Nepal), New ERA. Nepal Demographic and Health Survey 2016-2017. Fairfax, United States of America: ICF International, 2017.
330624	Nepal	Centers for Disease Control and Prevention (CDC), Department of Health Services, Ministry of Health and Population (Nepal), New ERA, United Nations Children's Fund (UNICEF), United States Agency for International Development (USAID). Nepal National Micronutrient Status Survey 2016.
143715	Netherlands	Dirren H, Decarli B, Lesourd B, Schlienger JL, Deslypere JP, Kiepurski A. Nutritional status: haematology and albumin. Eur J Clin Nutr. 1991; 45 (Suppl 3): 43-52.
143879	Netherlands	Knuiman JT, Westenbrink S, van der Heyden L, West CE, Burema J, de Boer J, Hautvast JG, Räsänen L, Virkkunen L, Viikari J. Determinants of total and high density lipoprotein cholesterol in boys from Finland, The Netherlands, Italy, the Philippines and Ghana with special reference to diet. Hum Nutr Clin Nutr. 1983; 37(4): 237-54.
120232	New Zealand	CBG Health Research Ltd., Ministry of Health (New Zealand), University of Otago (New Zealand). New Zealand Adult Nutrition Survey 2008-2009.
124477	New Zealand	Ministry of Health (New Zealand), University of Otago (New Zealand). New Zealand National Nutrition Survey 1996-1997.
130582	New Zealand	Massey University (New Zealand), University of Auckland (New Zealand), University of Otago (New Zealand). New Zealand National Children's Nutrition Survey 2002.
144091	New Zealand	Fawcett JP, Brooke M, Beresford CH. Iron deficiency and anaemia in a longitudinal study of New Zealanders at ages 11 and 21 years. N Z Med J. 1998; 111(1076): 400-2.
144093	New Zealand	Soh P, Ferguson EL, McKenzie JE, Homs MYV, Gibson RS. Iron deficiency and risk factors for lower iron stores in 6-24-month-old New Zealanders. Eur J Clin Nutr. 2004; 58(1): 71-9.

NID	Location	Citation
144098	New Zealand	Heath AL, Skeaff CM, Williams S, Gibson RS. The role of blood loss and diet in the aetiology of mild iron deficiency in premenopausal adult New Zealand women. Public Health Nutr. 2001; 4(2): 197-206.
264325	New Zealand	Ministry of Health (New Zealand). New Zealand Health Survey 2014-2015. Wellington, New Zealand: Ministry of Health (New Zealand).
139860	Nicaragua	Ministry of Health (Nicaragua). Nicaragua National Micronutrient Survey 2000.
139861	Nicaragua	Ministry of Health (Nicaragua). Nicaragua National Survey of Micronutrient Deficiencies 1993.
144314	Nicaragua	World Health Organization (WHO). WHO Global Database on Anemia, Nutrition Landscape Information System. Geneva, Switzerland: World Health Organization (WHO).
275811	Nicaragua	Centers for Disease Control and Prevention (CDC), Ministry of Health (Nicaragua). Nicaragua Integrated Surveillance System of Nutrition Interventions Progress Report 2003-2005. Managua, Nicaragua: Ministry of Health (Nicaragua), 2008.
294378	Nicaragua	Sullivan KM, Venugopalan B, Jefferds ME, Boy E, Bonilla J, Sandino I, Halleslevens P. Association of elevated α_1-acid glycoprotein (AGP) and the prevalence of anemia in Nicaraguan preschool children. Food Nutr Bull. 2012; 33(2): 137-41.
490177	Nicaragua	Ministry of Health (Nicaragua). [The trend analysis of the nutritional deficiency of less than 5 years and women of childbearing age, 2003-2007]. Managua, Nicaragua: Ministry of Health (Nicaragua), 2009.
20499	Niger	Department of Statistics and National Accounts (Niger), Macro International, Inc. Niger Demographic and Health Survey 2006. Fairfax, United States of America: ICF International.
74393	Niger	ICF International, Ministry of Public Health (Niger), National Institute of Statistics (Niger). Niger Demographic and Health Survey 2012. Fairfax, United States of America: ICF International, 2019.
144101	Niger	Preziosi P, Prual A, Galan P, Daouda H, Boureima H, Hercberg S. Effect of iron supplementation on the iron status of pregnant women: consequences for newborns. Am J Clin Nutr. 1997; 66(5): 1178-82.
30991	Nigeria	ICF Macro, National Malaria Control Programme (Nigeria), National Population Commission of Nigeria. Nigeria Malaria Indicator Survey 2010. Fairfax, United States of America: ICF International.
137400	Nigeria	Federal Ministry of Health (Nigeria), John Snow, Inc., United States Agency for International Development (USAID). Nigeria National Micronutrient Survey 1993.

NID	Location	Citation
144103	Nigeria	Lyko Z, Lyko J, Gaertner H. Anemia in pregnancy in Nigeria: hemoglobin level in pregnant patients of the Ife region hospital. Hamdard Medicus. 1993; 116-24.
144104	Nigeria	Reid H. Physiological changes in packed-cell volume and haemoglobin levels in Nigerian Igbo women during pregnancy. East Afr Med J. 1996; 201-7.
144106	Nigeria	Brabin L, Ikimalo J, Dollimore N, Kemp J, Ikokwu-Wonodi C, Babatunde S, Obunge O, Briggs N. How do they grow? A study of south-eastern Nigerian adolescent girls. Acta Paediatr. 1997; 86(10): 1114-20.
144107	Nigeria	Onadeko MO, Avokey F, Lawoyin TO. Observations of stillbirths, birthweight and maternal haemoglobin in teenage pregnancy in Ibadan, Nigeria. Afr J Med Med Sci. 1996; 25(1): 81-6.
144108	Nigeria	Barr F, Brabin L, Agbaje S, Buseri F, Ikimalo J, Briggs N. Reducing iron deficiency anaemia due to heavy menstrual blood loss in Nigerian rural adolescents. Public Health Nutr. 1998; 1(4): 249-57.
144109	Nigeria	Gwarzo MY, Sen KK, Atiku MK. Diet and serum iron status in pregnant and lactating Hausa women in Kano State, Nigeria. Ann Trop Med Parasitol. 1994; 88(6): 673-6.
218590	Nigeria	ICF International, National Bureau of Statistics (Nigeria), National Malaria Control Programme (Nigeria), National Population Commission of Nigeria. Nigeria Malaria Indicator Survey 2015. Fairfax, United States of America: ICF International, 2016.
283272	Nigeria	International Institute of Tropical Agriculture (IITA), and HarvestPlus of International Food Policy Research Institute (IFPRI). 2015. Dietary intakes, vitamin A, and iron status of women of childbearing age and children 6-59 months of age from Akwa Ibom state in Nigeria. Washington, DC: International Food Policy Research Institute (IFPRI) [datasets]. http://dx.doi.org/10.7910/DVN/29604
408484	Nigeria	Federal Ministry of Health (Nigeria), ICF International, National Population Commission (NPC). Nigeria Demographic and Health Survey 2018. Fairfax, United States of America: ICF International, 2020.
490182	Nigeria	Ekpo A, Jimmy E. Dietary and Haematological Evaluation of Adolescent Females in Nigeria. Pak J Nutr. 2006; 5(3): 278-81.
490178	Niue	Government of Niue, South Pacific Commission. The 1987 National Nutrition and Dietary Survey of Niue. 1993.
124337	North Macedonia	Clinic for Children's Diseases, Clinic Center Skopje (Macedonia), Institute for Mother and Child Health, Health Home Skopje (Macedonia), Ministry of Health (Macedonia), National Nutrition Institute (Italy), Republic Institute for Health Protection (Macedonia), United Nations Children's Fund (UNICEF). Macedonia Multiple Indicator Cluster Survey 1999.

NID	Location	Citation
144314	North Macedonia	World Health Organization (WHO). WHO Global Database on Anemia, Nutrition Landscape Information System. Geneva, Switzerland: World Health Organization (WHO).
261352	North Macedonia	Institute of Public Health (Republic of North Macedonia), UNICEF Macedonia. Macedonia National Nutrition Survey 2011.
143715	Norway	Dirren H, Decarli B, Lesourd B, Schlienger JL, Deslypere JP, Kiepurski A. Nutritional status: haematology and albumin. Eur J Clin Nutr. 1991; 45 (Suppl 3): 43-52.
143717	Norway	Elwood PC, Hughes J, Abernethy M, Davies R, Gough R, Johnson AP, Dubourg AY. An international haematological survey. Bull World Health Organ. 1976; 54(1): 87-95.
144111	Norway	Borch-Iohnsen B, Sandstad B, Asberg A. Iron status among 3005 women aged 20-55 years in Central Norway: the Nord-Trøndelag Health Study (the HUNT study). Scand J Clin Lab Invest. 2005; 65(1): 45-54.
144112	Norway	Eskeland B, Hunskaar S. Anaemia and iron deficiency screening in adolescence: a pilot study of iron stores and haemoglobin response to iron treatment in a population of 14-15-year-olds in Norway. Acta Paediatr. 1999; 88(8): 815-21.
30030	Oman	Centers for Disease Control and Prevention (CDC), Ministry of Health (Oman), United Nations Children's Fund (UNICEF), World Health Organization Regional Office for the Eastern Mediterranean (EMRO-WHO). Oman Food Fortification Study 2004. Muscat, Oman: Ministry of Health (Oman).
30033	Oman	Ministry of Health (Oman). Oman National Health Survey 2000. Muscat, Oman: Ministry of Health (Oman).
110030	Oman	Al-Riyami A, Ebrahim GJ. Genetic Blood Disorders Survey in the Sultanate of Oman. J Trop Pediatr. 2003; 49(Suppl 1): 1-20.
139852	Oman	Alasfoor DH. Nutritional Anemia and Vitamin A Deficiency in Oman: A Multivariate Analysis [thesis]. Ithaca (NY), United States: Cornell University; 1996.
139853	Oman	Musaiger A, United Nations's Children Fund (UNICEF). Health and Nutritional Status of Omani Families. New York, United States: United Nations Children's Fund (UNICEF), 1992.
139854	Oman	Ministry of Health (Oman), United Nations Children's Fund (UNICEF). Oman Nutrition Status Survey 1979.
144314	Oman	World Health Organization (WHO). WHO Global Database on Anemia, Nutrition Landscape Information System. Geneva, Switzerland: World Health Organization (WHO).
137460	Pakistan	Aga Khan University Hospital, Karachi, Pakistan Bureau of Statistics, Pakistan Institute of Development Economics. Pakistan National Nutrition Survey 2001-2002.

NID	Location	Citation
141521	Pakistan	Aga Khan University, Ministry of National Health Services, Regulations & Coordination (Pakistan), Ministry of Planning and Development (Pakistan), Pakistan Medical Research Council, United Nations Children's Fund (UNICEF). Pakistan National Nutrition Survey 2011.
144115	Pakistan	Paracha PI, Hameed A, Simon J, Jamil A, Nawab G. Prevalence of anaemia in semi-urban areas of Peshawar, Pakistan: a challenge for health professionals and policy makers. J Pak Med Assoc. 1997; 47(2): 49-53.
144116	Pakistan	Molla A, Khurshid M, Molla AM. Prevalence of iron deficiency anaemia in children of the urban slums of Karachi. J Pak Med Assoc. 1992; 42(5): 118-21.
144119	Pakistan	Agha F, Hasan TJ, Khan RA, Jafarey S. Iron stores in maternal and cord blood. Asia Oceania J Obstet Gynaecol. 1988; 14(4): 405-9.
144120	Pakistan	Hassan K, Hayat A, Khan U, Ikram N, Dodhy M, Afghan R, Tahir M. Reference values of hematological parameters in full term normal neonates in Rawalpindi and Islamabad. Pak J Obstet Gynecol. 1994; 29-31.
144121	Pakistan	Baig L, Thaver IH, Iqbal R. Risk factors associated with anaemia in children less than 5 years of age residing in a squatter settlement of Karachi. Pak J Med Res. 1996; 35(4): 176-178.
261883	Pakistan	Aga Khan University, Fogarty International Center, National Institutes of Health (NIH), Foundation for the National Institutes of Health (FNIH). Pakistan - Naushahro Feroze Malnutrition and Enteric Disease Study 2009-2014.
9989	Palestine	Birzeit University, Palestinian Central Bureau of Statistics. Palestine Nutrition Survey 2002. Ramallah, Palestine: Palestinian Central Bureau of Statistics.
125591	Palestine	Ministry of Health (Palestine), Palestinian Central Bureau of Statistics, United Nations Children's Fund (UNICEF), United Nations Population Fund (UNFPA). Palestine Multiple Indicator Cluster Survey 2010. New York, United States of America: United Nations Children's Fund (UNICEF), 2014.
261366	Palestine	IBM Global Services, Maram Project (Palestine). Palestine - West Bank and Gaza Strip Prevalence of Vitamin A Deficiency among Children, Aged 12-59 Months. 2004.
285322	Palestine	Ministry of Health (Palestine), United Nations Children's Fund (UNICEF), University of Vienna. Palestine Micronutrient Survey 2013.
489997	Palestine	El Kishawi RR, Soo KL, Abed YA, Wan Muda WA. Anemia among children aged 2-5 years in the Gaza Strip-Palestinian: a cross sectional study. BMC Public Health. 2015; 15: 319.
490168	Palestine	Halileh S, Gordon NH. Determinants of anemia in pre-school children in the occupied Palestinian territory. J Trop Pediatr. 2006; 52(1): 12-8.

NID	Location	Citation
490184	Palestine	Jalambo M, Hamed A, Abed Y. Anemia and risk factors among female secondary students in the Gaza Strip. J Public Health. 2013; 21(3): 271-8.
139825	Panama	Ministry of Health (Panama). Panama National Survey on Vitamin A 1992.
139851	Panama	Ministry of Health (Panama). Panama National Survey on Vitamin A and Iron Deficiency Anemia 1999.
144314	Panama	World Health Organization (WHO). WHO Global Database on Anemia, Nutrition Landscape Information System. Geneva, Switzerland: World Health Organization (WHO).
135757	Papua New Guinea	Centers for Disease Control and Prevention (CDC), National Department of Health (Papua New Guinea), United Nations Children's Fund (UNICEF), University of Papua New Guinea. Papua New Guinea National Nutrition Survey 2005.
144129	Papua New Guinea	Amoa AB, Klufio CA, Kariwiga G, Heywood S. Antenatal haemoglobin profile at the Port Moresby General Hospital. P N G Med J. 1998; 41(3-4): 119-25.
400126	Papua New Guinea	Centers for Disease Control and Prevention (CDC), Emory University (United States), Eunice Shriver Kennedy National Institute of Child Health and Human Development (NICHD), Global Alliance for Improved Nutrition (GAIN), GroundWork LLC, HarvestPlus, International Micronutrient Malnutrition Prevention and Control (IMMPaCt) Program, International Zinc Consultative Group (IZiNCG), Strengthening Partnerships, Results, and Innovations in Nutrition Globally (SPRING). Biomarkers Reflecting Inflammation and Nutrition Determinants of Anemia (BRINDA).
20638	Peru	Macro International, Inc, National Institute of Statistics (Peru). Peru Demographic and Health Survey 1996. Fairfax, United States of America: ICF International.
20649	Peru	Macro International, Inc, National Institute of Statistics (Peru). Peru Demographic and Health Survey 2000. Fairfax, United States of America: ICF International.
139818	Peru	National Institute of Health (Peru). Behavior of Nutrition Indicators in Peru: Monitoring National Nutritional Indicators 2002-2004. Lima, Peru: National Institute of Health (Peru), 2007.
139819	Peru	National Center for Food and Nutrition, National Institute of Health (Peru). National Report of Hemoglobin Levels and Prevalence of Anemia in Children 12 to 36 Months and Women of Childbearing Age 2003. Lima, Peru: Ministry of Health (Peru), 2003.
139823	Peru	Ministry of Health (Peru), National Center for Food and Nutrition, National Institute of Health (Peru), National Institute of Health (Peru). Peru National Survey on Nutritional, Biochemical, Socioeconomic Indicators Related to Chronic Degenerative Diseases 2004-2005.

NID	Location	Citation
140481	Peru	National Center for Food and Nutrition, National Institute of Health (Peru). Peru National Report on Vitamin A Deficiency in Children Under 5 and Women of Childbearing Age 1997-2001. Lima, Peru: Ministry of Health (Peru), 2001.
144130	Peru	Zavaleta N, Caulfield LE, Garcia T. Changes in iron status during pregnancy in peruvian women receiving prenatal iron and folic acid supplements with or without zinc. Am J Clin Nutr. 2000; 71(4): 956-61.
144131	Peru	López de Romaña G, Cusirramos S, López de Romaña D, Gross R. Efficacy of multiple micronutrient supplementation for improving anemia, micronutrient status, growth, and morbidity of Peruvian infants. J Nutr. 2005; 135(3): 646-652.
144133	Peru	Becerra C, Gonzales GF, Villena A, de la Cruz D, Florián A. [Prevalence of anemia in pregnancy, Pucallpa Regional Hospital, Perú]. Rev Panam Salud Publica. 1998; 3(5): 285-92.
144136	Peru	Alarcon K, Kolsteren PW, Prada AM, Chian AM, Velarde RE, Pecho IL, Hoeree TF. Effects of separate delivery of zinc or zinc and vitamin A on hemoglobin response, growth, and diarrhea in young Peruvian children receiving iron therapy for anemia. Am J Clin Nutr. 2004; 80(5): 1276-82.
210182	Peru	ICF International, Ministry of Health (Peru), National Institute of Statistics and Informatics (Peru), National Police of Peru (PNP). Peru Continuous Demographic and Health Survey 2014 - INEI. Lima, Peru: National Institute of Statistics and Informatics (Peru), 2015.
210231	Peru	ICF International, National Institute of Statistics and Informatics (Peru). Peru Continuous Demographic and Health Survey 2013 - INEI. Lima, Peru: National Institute of Statistics and Informatics (Peru), 2014.
261879	Peru	Fogarty International Center, National Institutes of Health (NIH), Foundation for the National Institutes of Health (FNIH), Johns Hopkins Bloomberg School of Public Health. Peru - Loreto Malnutrition and Enteric Disease Study 2009-2014.
270404	Peru	National Institute of Statistics and Informatics (Peru), ORC Macro. Peru Continuous Demographic and Health Survey 2009. Fairfax, United States of America: ICF International.
270469	Peru	National Institute of Statistics and Informatics (Peru). Peru Continuous Demographic and Health Survey 2010. Fairfax, United States of America: ICF International.
270470	Peru	Macro International, Inc, National Institute of Statistics and Informatics (Peru). Peru Continuous Demographic and Health Survey 2011. Fairfax, United States of America: ICF International.
270471	Peru	Macro International, Inc, National Institute of Statistics and Informatics (Peru). Peru Continuous Demographic and

NID	Location	Citation
		Health Survey 2012. Fairfax, United States of America: ICF International.
275090	Peru	Ministry of Economy and Finance (Peru), National Institute of Statistics and Informatics (Peru), ORC Macro. Peru Continuous Demographic and Health Survey 2003-2008. Fairfax, United States of America: ICF International.
303663	Peru	National Institute of Statistics and Informatics (Peru). Peru Demographic and Family Health Survey 2015. Lima, Peru: National Institute of Statistics and Informatics (Peru), 2017.
303664	Peru	National Institute of Statistics and Informatics (Peru). Peru Demographic and Family Health Survey 2016. Lima, Peru: National Institute of Statistics and Informatics (Peru), 2017.
358824	Peru	National Center for Food and Nutrition, National Institute of Health (Peru), National Institute of Statistics and Informatics (Peru), National Police of Peru (PNP). Peru Demographic and Family Health Survey 2017. Lima, Peru: National Institute of Statistics and Informatics (Peru).
359146	Peru	Executive Directorate of Food and Nutritional Surveillance (DEVAN) (Peru), Ministry of Health (Peru), National Center for Food and Nutrition, National Institute of Health (Peru), National Institute of Health (Peru), National Institute of Statistics and Informatics (Peru). Peru Monitoring of Nutritional Indicators in the National Household Survey 2009-2011.
359163	Peru	Executive Directorate of Food and Nutritional Surveillance (DEVAN) (Peru), Ministry of Health (Peru), National Center for Food and Nutrition, National Institute of Health (Peru), National Institute of Health (Peru), National Institute of Statistics and Informatics (Peru). Peru Monitoring of Nutritional Indicators in the National Household Survey 2007-2008.
359165	Peru	Executive Directorate of Food and Nutritional Surveillance (DEVAN) (Peru), Ministry of Health (Peru), National Center for Food and Nutrition, National Institute of Health (Peru), National Institute of Health (Peru), National Institute of Statistics and Informatics (Peru). Peru Monitoring of Nutritional Indicators in the National Household Survey 2011-2012.
407869	Peru	National Institute of Statistics and Informatics (Peru). Peru Demographic and Family Health Survey 2018. Lima, Peru: National Institute of Statistics and Informatics (Peru), 2019.
10950	Philippines	Food and Nutrition Research Institute, Department of Science and Technology (Philippines). Philippines National Nutrition Survey 1998.
124455	Philippines	Food and Nutrition Research Institute, Department of Science and Technology (Philippines). Philippines National Nutrition Survey 2003.
126395	Philippines	Food and Nutrition Research Institute, Department of Science and Technology (Philippines). Philippines National

NID	Location	Citation
		Nutrition Survey 2008-2009.
143879	Philippines	Knuiman JT, Westenbrink S, van der Heyden L, West CE, Burema J, de Boer J, Hautvast JG, Räsänen L, Virkkunen L, Viikari J. Determinants of total and high density lipoprotein cholesterol in boys from Finland, The Netherlands, Italy, the Philippines and Ghana with special reference to diet. Hum Nutr Clin Nutr. 1983; 37(4): 237-54.
144140	Philippines	De Jong N, Romano ABA, Gibson RS. Zinc and iron status during pregnancy of Filipino women. Asia Pac J Clin Nutr. 2002; 11(3): 186-93.
249262	Philippines	Food and Nutrition Research Institute, Department of Science and Technology (Philippines). Philippines National Nutrition Survey 2013-2014.
254226	Philippines	Kuizon MD, Cheong RL, Ancheta LP, Desnacido JA, Macapinlac MP, Baens JS. Effect of anaemia and other maternal characteristics on birthweight. Hum Nutr Clin Nutr. 1985; 39(6): 419-26.
294316	Philippines	Rohner F, Woodruff BA, Aaron GJ, Yakes EA, Lebanan MA, Rayco-Solon P, Saniel OP. Infant and young child feeding practices in urban Philippines and their associations with stunting, anemia, and deficiencies of iron and vitamin A. Food Nutr Bull. 2013; 34(2 Suppl): S17-34.
143715	Poland	Dirren H, Decarli B, Lesourd B, Schlienger JL, Deslypere JP, Kiepurski A. Nutritional status: haematology and albumin. Eur J Clin Nutr. 1991; 45 (Suppl 3): 43-52.
143717	Poland	Elwood PC, Hughes J, Abernethy M, Davies R, Gough R, Johnson AP, Dubourg AY. An international haematological survey. Bull World Health Organ. 1976; 54(1): 87-95.
144142	Poland	Spodaryk K. Disparity between dietary iron intake and iron status of children aged 10-12 years. Arch Physiol Biochem. 1999; 107(5): 361-6.
143715	Portugal	Dirren H, Decarli B, Lesourd B, Schlienger JL, Deslypere JP, Kiepurski A. Nutritional status: haematology and albumin. Eur J Clin Nutr. 1991; 45 (Suppl 3): 43-52.
139137	Qatar	Amine EK. Qatar Nutritional Assessment 1995; Oct 20-Nov 3 [assignment report]. WHO Regional Office for the Eastern Mediterranean, 1995.
144314	Qatar	World Health Organization (WHO). WHO Global Database on Anemia, Nutrition Landscape Information System. Geneva, Switzerland: World Health Organization (WHO).
489999	Qatar	Zainel AJAL, Osman SRO, Al-Kohji SMS, Selim NA. Iron deficiency, its epidemiological features and feeding practices among infants aged 12 months in Qatar: a cross-sectional study. BMJ Open. 2018; 8(5): e020271.
144177	Republic of Korea	Choi CW, Lee J, Park KH, Yoon SY, Choi IK, Oh SC, Seo JH, Kim BS, Shin SW, Kim YH, Kim JS. Prevalence and characteristics of anemia in the elderly: cross-sectional

NID	Location	Citation
		study of three urban Korean population samples. Am J Hematol. 2004; 77(1): 26-30.
144178	Republic of Korea	Choi JW, Kim CS, Pai SH. Erythropoietic activity and soluble transferrin receptor level in neonates and maternal blood. Acta Paediatr. 2000; 89(6): 675-9.
144314	Republic of Korea	World Health Organization (WHO). WHO Global Database on Anemia, Nutrition Landscape Information System. Geneva, Switzerland: World Health Organization (WHO).
20339	Republic of Moldova	Macro International, Inc, National Scientific and Applied Center for Preventive Medicine (Moldova). Moldova Demographic and Health Survey 2005. Fairfax, United States of America: ICF International.
140200	Republic of Moldova	Ministry of Health (Moldova), National Office of Statistics (Moldova), United Nations Children's Fund (UNICEF). Moldova Multiple Indicator Cluster Survey 2012. New York, United States of America: United Nations Children's Fund (UNICEF), 2015.
137566	Romania	Alfred Rusescu Institute for Mother and Child Care. Nutritional Status of Pregnant Women, Children Under 5 Years and School Children Aged 6-7 Years. Bucharest, Romania: Alfred Rusescu Institute for Mother and Child Care, 2005.
139132	Romania	Centers for Disease Control and Prevention (CDC), Ministry of Health (Romania), United Nations Children's Fund (UNICEF). Romania National Nutrition Survey 1991.
144314	Romania	World Health Organization (WHO). WHO Global Database on Anemia, Nutrition Landscape Information System. Geneva, Switzerland: World Health Organization (WHO).
489984	Romania	Stativa E, Rus AV, Stanescu A, Pennings JS, Parris SR, Wenyika R. Prevalence and predictors of anaemia in Romanian infants 6-23 months old. J Public Health (Oxf). 2016; 38(3): e272-e281.
122634	Russian Federation	Rubin CH, Esteban E, Reissman DB, Daley WR, Noonan GP, Karpati A, Gurvitch E, Kuzmin SV, Privalova LI, Zukov A, Zlepko A. Lead poisoning among young children in Russia: concurrent evaluation of childhood lead exposure in Ekaterinburg, Krasnouralsk, and Volgograd. Environ Health Perspect. 2002; 110(6): 559-62.
304148	Russian Federation	Ufa Eye Research Institute. Russia - Ural Eye and Medical Study 2015-2017.
20740	Rwanda	Macro International, Inc, National Institute of Statistics of Rwanda. Rwanda Demographic and Health Survey 2005. Fairfax, United States of America: ICF International.
21222	Rwanda	Macro International, Inc, Ministry of Health (Rwanda), National Institute of Statistics of Rwanda. Rwanda Interim Demographic and Health Survey 2007-2008. Fairfax, United States of America: ICF International.

NID	Location	Citation
56040	Rwanda	ICF Macro, Ministry of Health (Rwanda), National Institute of Statistics of Rwanda. Rwanda Demographic and Health Survey 2010-2011. Fairfax, United States of America: ICF International.
137425	Rwanda	Ministry of Health (Rwanda), United Nations Children's Fund (UNICEF), World Health Organization (WHO). Rwanda National Nutrition Survey of Women and Children 1996.
144314	Rwanda	World Health Organization (WHO). WHO Global Database on Anemia, Nutrition Landscape Information System. Geneva, Switzerland: World Health Organization (WHO).
157063	Rwanda	ICF International, Ministry of Health (Rwanda), National Institute of Statistics of Rwanda. Rwanda Demographic and Health Survey 2014-2015. Fairfax, United States of America: ICF International, 2016.
274703	Rwanda	ICF Macro, MEASURE Evaluation Project, Carolina Population Center, University of North Carolina, Ministry of Health (Rwanda), National Institute of Statistics of Rwanda, School of Public Health, University of Rwanda. Rwanda Child Verbal Autopsy Study 2008.
144314	Samoa	World Health Organization (WHO). WHO Global Database on Anemia, Nutrition Landscape Information System. Geneva, Switzerland: World Health Organization (WHO).
267785	Samoa	Ministry of Finance (Samoa), Ministry of Health (Samoa), Ministry of Women, Community and Social Development (Samoa). Samoa Demographic and Health Survey 2014.
26866	Sao Tome and Principe	ICF Macro, Ministry of Health (Sao Tome and Principe), National Institute of Statistics (Sao Tome and Principe). Sao Tome and Principe Demographic and Health Survey 2008-2009. Fairfax, United States of America: ICF International.
214640	Sao Tome and Principe	Global Fund to Fight Aids Tuberculosis and Malaria (GFATM), ICF International, National Center for Endemic Diseases (CNE) (Sao Tome and Principe), National Institute of Statistics (Sao Tome and Principe), United Nations Children's Fund (UNICEF), United Nations Development Programme (UNDP). Sao Tome and Principe Multiple Indicator Cluster Survey 2014. New York, United States of America: United Nations Children's Fund (UNICEF), 2016.
144146	Saudi Arabia	Al Fawaz I. Surveillance for iron deficiency anemia at a well baby clinic in Riyadh, Saudi Arabia. Saudi Med J. 1993; 27-31.
144147	Saudi Arabia	Ghafouri H, Al Fares A, Islam S, Ahmed A, Jan M. Haematological reference values assessed from birth to adolescence in Saudi subjects in the area of Jeddah. Saudi Med J. 1987; 575-82.
144148	Saudi Arabia	Hawa L, Rowland A, Ahmed G, Dery J. Haemoglobin and iron status in mothers and their babies at delivery. Saudi Med J. 1993; 110-5.

NID	Location	Citation
144149	Saudi Arabia	Ahmed MM. Haematological values and parasitic infections in school children in Riyadh, Saudi Arabia. J Egypt Soc Parasitol. 1991; 21(3): 831-8.
144158	Saudi Arabia	al-Momen AK, al-Meshari A, al-Nuaim L, Saddique A, Abotalib Z, Khashogji T, Abbas M. Intravenous iron sucrose complex in the treatment of iron deficiency anemia during pregnancy. Eur J Obstet Gynecol Reprod Biol. 1996; 69(2): 121-4.
144308	Saudi Arabia	Mahfouz AA, el-Said MM, al-Erian RA, Hamid AM. Teenage pregnancy: are teenagers a high risk group?. Eur J Obstet Gynecol Reprod Biol. 1995; 59(1): 17-20.
11540	Senegal	Macro International, Inc, Research Center for Human Development (Senegal). Senegal Malaria Indicator Survey 2008-2009. Fairfax, United States of America: ICF International.
26855	Senegal	Ministry of Health and Prevention (Senegal), Research Center for Human Development (Senegal). Senegal Demographic and Health Survey 2005. Fairfax, United States of America: ICF International.
56063	Senegal	Center for Research in Human Development (CRDH), Cheikh Anta Diop University, Hospital Aristide Le Dantec, ICF Macro, National Agency of Statistics and Demography (Senegal). Senegal Demographic and Health Survey 2010-2011. Fairfax, United States of America: ICF International.
111432	Senegal	ICF International, Ministry of Health and Social Action (Senegal), National Agency of Statistics and Demography (Senegal). Senegal Continuous Demographic and Health Survey 2012-2013. Fairfax, United States of America: ICF International.
191270	Senegal	Cheikh Anta Diop University, ICF International, National Agency of Statistics and Demography (Senegal). Senegal Continuous Demographic and Health Survey 2014. Fairfax, United States of America: ICF International.
218592	Senegal	Cheikh Anta Diop University, ICF International, National Agency of Statistics and Demography (Senegal). Senegal Continuous Demographic and Health Survey 2015. Fairfax, United States of America: ICF International, 2016.
286772	Senegal	ICF International, Ministry of Health and Social Action (Senegal), National Agency of Statistics and Demography (Senegal). Senegal Continuous Demographic and Health Survey 2016. Fairfax, United States of America: ICF International, 2017.
353526	Senegal	ICF International, Ministry of Health and Social Action (Senegal), National Agency of Statistics and Demography (Senegal), Unit for the Fight Against Malnutrition (Senegal). Senegal Continuous Demographic and Health Survey 2017. Fairfax, United States of America: ICF International, 2018.
490179	Senegal	Laboratoire de Nutrition - UCAD, Comite Senegalais pour la Fortification des Aliments en Micronutriments (COSFAM),

NID	Location	Citation
		Micronutrient Initiative. Rapport Situation de base du statut en vitamine A et en fer chez les enfants de 12-59 mois et chez les femmes en âge de procréer (15-49 ans) dans le cadre du programme de fortification des aliments en micronutriments au Sénégal. [Baseline Vitamin A and Iron Status Report in children 12-59 months and in women of childbearing age (15-49 years) as part of the micronutrient fortification program in Senegal]. Dakar: COFSAM; 2011.
27024	Serbia	Institute for Public Health (Montenegro), Institute of Public Health of Serbia, United Nations Children's Fund (UNICEF), World Health Organization (WHO). Yugoslavia, Federal Republic Multiple Indicator Cluster Survey 2000.
137568	Serbia	National Institute for Research on Food and Nutrition (INRAN) (Italy), National Institute of Public Health (Kosovo), United Nations Children's Fund (UNICEF). Yugoslavia, Federal Republic - Serbia Micronutrient Status Survey in Kosovo 2001.
489982	Serbia	Rakic L, Djokic D, Drakulovic M, Pejic A, Radojicic Z, Marinkovic M. Risk factors associated with anemia among Serbian non-pregnant women 20 to 49 years old. A cross-sectional study. Hippokratia. 2013; 17(1): 47-54.
21258	Sierra Leone	Macro International, Inc, Statistics Sierra Leone. Sierra Leone Demographic and Health Survey 2008. Fairfax, United States of America: ICF International.
77393	Sierra Leone	Catholic Relief Services (CRS), College of Medicine and Allied Health Sciences, University of Sierra Leone (COMAHS), ICF International, National Malaria Control Programme (Sierra Leone), Statistics Sierra Leone. Sierra Leone Malaria Indicator Survey 2013. Fairfax, United States of America: ICF International.
131467	Sierra Leone	ICF International, Ministry of Health and Sanitation (Sierra Leone), Statistics Sierra Leone. Sierra Leone Demographic and Health Survey 2013. Fairfax, United States of America: ICF International, 2014.
262982	Sierra Leone	GroundWork LLC, Helen Keller International, Ministry of Health and Sanitation (Sierra Leone), Sierra Leone Standards Bureau (SLSB), United Nations Children's Fund (UNICEF), World Health Organization (WHO). Sierra Leone Micronutrient Survey 2013.
286773	Sierra Leone	Catholic Relief Services (CRS), College of Medicine and Allied Health Sciences, University of Sierra Leone (COMAHS), ICF International, National Malaria Control Programme (Sierra Leone), Roll Back Malaria Partnership, Statistics Sierra Leone. Sierra Leone Malaria Indicator Survey 2016. Fairfax, United States of America: ICF International, 2017.
294311	Sierra Leone	Wirth JP, Rohner F, Woodruff BA, Chiwile F, Yankson H, Koroma AS, Russel F, Sesay F, Dominguez E, Petry N, Shahab-Ferdows S, de Onis M, Hodges MH. Anemia, Micronutrient Deficiencies, and Malaria in Children and Women in Sierra Leone Prior to the Ebola Outbreak -

NID	Location	Citation
		Findings of a Cross-Sectional Study. PLoS One. 2016; 11(5): e0155031.
144162	Singapore	Singh K, Fong YF, Arulkumaran S. Anaemia in pregnancy--a cross-sectional study in Singapore. Eur J Clin Nutr. 1998; 52(1): 65-70.
21446	Solomon Islands	Macro International, Inc, Ministry of Health (Solomon Islands), Secretariat of the Pacific Community (SPC), Solomon Islands National Statistics Office (SINSO). Solomon Islands Demographic and Health Survey 2006-2007.
142784	Solomon Islands	Republic of Kiribati. Pacific Helminths Initiative 2001-2002.
139113	Somalia	Ministry of Health and Labor (Somalia), United Nations Children's Fund (UNICEF). Somalia - Somaliland Anemia Survey 2001.
152968	Somalia	Centre for International Health and Development, Institute of Child Health (CIHD), Food and Nutrition Security Analysis Unit (Somalia), Ministry of Health (Somalia), Ministry of Health and Labor (Somaliland), Puntland Ministry of Health (Somalia), United Nations Children's Fund (UNICEF), World Food Programme (WFP), World Health Organization (WHO). Somalia National Micronutrient and Anthropometric Survey 2009.
254295	Somalia	Omar MM, Högberg U, Bergström B. Maternal health and child survival in relation to socioeconomic factors. Gynecol Obstet Invest. 1994; 38(2): 107-12.
139810	South Africa	South African Vitamin A Consultative Group (SAVACG). South Africa Anthropometric, Vitamin A, Iron and Immunisation Coverage Status in Children Aged 6 to 71 Months 1994.
144167	South Africa	Oelofse A, Faber M, Benadé JG, Benadé AJ, Kenoyer DG. The nutritional status of a rural community in KwaZulu-Natal, South Africa: the Ndunakazi project. Cent Afr J Med. 1999; 45(1): 14-9.
144174	South Africa	Faber M, Jogessar VB, Benadé AJ. Nutritional status and dietary intakes of children aged 2-5 years and their caregivers in a rural South African community. Int J Food Sci Nutr. 2001; 52(5): 401-11.
155628	South Africa	Human Sciences Research Council, South African Medical Research Council. South Africa National Health and Nutrition Examination Survey 2012.
157064	South Africa	Department of Health (South Africa), ICF International, South African Medical Research Council, Statistics South Africa. South Africa Demographic and Health Survey 2016. Fairfax, United States of America: ICF International, 2019.
255133	South Africa	Van der Westhuyzen J, Van Tonder SV, Gilbertson I, Metz J. Iron, folate and vitamin B12 nutrition and anaemia in black preschool children in the northern Transvaal. S Afr Med J. 1986; 70(3): 143-6.

NID	Location	Citation
261367	South Africa	Department of Health (South Africa), North-West University, South African Medical Research Council, Stellenbosch University, University of Cape Town, University of Kwazulu-Natal, University of Limpopo, University of Pretoria, University of the Free State, University of the Western Cape. South Africa National Food Consumption Survey - Fortification Baseline 2005.
261887	South Africa	Fogarty International Center, National Institutes of Health (NIH), Foundation for the National Institutes of Health (FNIH), University of Venda. South Africa - Venda Malnutrition and Enteric Disease Study 2009-2014.
143715	Spain	Dirren H, Decarli B, Lesourd B, Schlienger JL, Deslypere JP, Kiepurski A. Nutritional status: haematology and albumin. Eur J Clin Nutr. 1991; 45 (Suppl 3): 43-52.
143717	Spain	Elwood PC, Hughes J, Abernethy M, Davies R, Gough R, Johnson AP, Dubourg AY. An international haematological survey. Bull World Health Organ. 1976; 54(1): 87-95.
144179	Spain	Henríquez Sánchez P, Díaz Romero C, Rodríguez Rodríguez E, López Blanco F, Alvárez León E, Díaz Cremádes J, Pastor Ferrer MC, Serra Majem L. [Biochemical assessment of nutritional status in the Canary Islands population (1998)]. Arch Latinoam Nutr. 2000; 50(1 Suppl 1): 43-54.
304111	Spain	Planells E, Sánchez C, Montellano MA, Mataix J, Llopis J. Vitamins B6 and B12 and folate status in an adult Mediterranean population. Eur J Clin Nutr. 2003; 57(6): 777–85.
18815	Sri Lanka	Department of Census and Statistics (Sri Lanka), Macro International, Inc. Sri Lanka Demographic and Health Survey 2006-2007. 2009.
139076	Sri Lanka	Ministry of Health, Nutrition and Welfare (Sri Lanka). Sri Lanka Assessment of Anemia Status 2001.
141592	Sri Lanka	Medical Research Institute (Sri Lanka), United Nations Children's Fund (UNICEF), World Food Programme (WFP). Sri Lanka Nutrition and Food Security Assessment 2009.
144180	Sri Lanka	Mudalige R, Nestel P. Prevalence of anaemia in Sri Lanka. Ceylon J Med Sci. 1996; 9-16.
144181	Sri Lanka	Pathmeswaran A, Jayatissa R, Samarasinghe S, Fernando A, de Silva RP, Thattil RO, de Silva NR. Health status of primary schoolchildren in Sri Lanka. Ceylon Med J. 2005; 50(2): 46-50.
144182	Sri Lanka	Fernando SD, Paranavitane SR, Rajakaruna J, Weerasinghe S, Silva D, Wickremasinghe AR. The health and nutritional status of school children in two rural communities in Sri Lanka. Trop Med Int Health. 2000; 5(6): 450-2.
144183	Sri Lanka	Goonewardene M, Seekkuge J, Liyanage C. Iron stores and its correlation to haemoglobin levels in pregnant women attending an antenatal clinic. Ceylon Med J. 1995; 40(2): 67-9.

NID	Location	Citation
144184	Sri Lanka	Atukorala TM, Lanerolle P. Soil-transmitted helminthic infection and its effect on nutritional status of adolescent schoolgirls of low socioeconomic status in Sri Lanka. J Trop Pediatr. 1999; 45(1): 18-22.
144314	Sri Lanka	World Health Organization (WHO). WHO Global Database on Anemia, Nutrition Landscape Information System. Geneva, Switzerland: World Health Organization (WHO).
153000	Sri Lanka	Medical Research Institute (Sri Lanka), United Nations Children's Fund (UNICEF). Sri Lanka National Nutrition and Micronutrient Survey 2012.
255138	Sri Lanka	Atukorala TM, de Silva LDR. Iron status of adolescent females in three schools in an urban area of Sri Lanka. J Trop Pediatr. 1990; 36(6): 316-21.
490683	Sri Lanka	Department of Nutrition, Medical Research Institute (Sri Lanka). Nutritional Problems Among Sri Lankan Primary School Children Aged 5-9 Years. 2002.
490684	Sri Lanka	Department of Nutrition, Medical Research Institute (Sri Lanka). Challenges with Nutrition of School Children.
490691	Sri Lanka	Department of Nutrition, Medical Research Institute (Sri Lanka). Nutritional Status, Dietary Practices and Pattern of Physical Activity Among School Children Aged 6-12 Years 2016. 2017.
200617	Sudan	Central Bureau of Statistics (Sudan), Federal Ministry of Health (Sudan), United Nations Children's Fund (UNICEF). Sudan Multiple Indicator Cluster Survey 2014. New York, United States of America: United Nations Children's Fund (UNICEF), 2016.
143905	Sweden	Domellöf M, Cohen RJ, Dewey KG, Hernell O, Rivera LL, Lönnerdal B. Iron supplementation of breast-fed Honduran and Swedish infants from 4 to 9 months of age. J Pediatr. 2001; 138(5): 679-87.
144187	Sweden	Hallberg L, Hultén L, Lindstedt G, Lundberg PA, Mark A, Purens J, Svanberg B, Swolin B. Prevalence of iron deficiency in Swedish adolescents. Pediatr Res. 1993; 34(5): 680-7.
144188	Sweden	Samuelson G, Bratteby LE, Berggren K, Elverby JE, Kempe B. Dietary iron intake and iron status in adolescents. Acta Paediatr. 1996; 85(9): 1033-8.
143715	Switzerland	Dirren H, Decarli B, Lesourd B, Schlienger JL, Deslypere JP, Kiepurski A. Nutritional status: haematology and albumin. Eur J Clin Nutr. 1991; 45 (Suppl 3): 43-52.
144189	Switzerland	Hess SY, Zimmermann MB, Brogli S, Hurrell RF. A national survey of iron and folate status in pregnant women in Switzerland. Int J Vitam Nutr Res. 2001; 71(5): 268-73.
155403	Taiwan	Department of Health (Taiwan), Institute of Biomedical Sciences, Academia Sinica. Taiwan Nutrition Survey of the Elderly 1999-2000.

NID	Location	Citation
124414	Tajikistan	Kazakh Academy of Nutrition, Ministry of Health (Tajikistan), National Institute for Research on Food and Nutrition (INRAN) (Italy), United Nations Children's Fund (UNICEF). Tajikistan Micronutrient Status Survey 2003.
126399	Tajikistan	Ministry of Health (Tajikistan), United Nations Children's Fund (UNICEF). Tajikistan Micronutrient Status Survey 2009.
144314	Tajikistan	World Health Organization (WHO). WHO Global Database on Anemia, Nutrition Landscape Information System. Geneva, Switzerland: World Health Organization (WHO).
341838	Tajikistan	ICF International, Statistical Agency under the President of the Republic of Tajikistan. Tajikistan Demographic and Health Survey 2017. Fairfax, United States of America: ICF International, 2018.
409186	Tajikistan	Ministry of Health and Social Protection (Tajikistan), Swiss Tropical and Public Health Institute (TPH), United Nations Children's Fund (UNICEF). Tajikistan Micronutrient Status Survey 2016.
95428	Thailand	Ministry of Public Health (Thailand). Thailand National Health Examination Survey 1991-1992.
108046	Thailand	National Health Examination Survey Office (Thailand). Thailand National Health and Examination Survey 2008-2009.
124416	Thailand	Ministry of Public Health (Thailand). Thailand National Nutrition Survey 1995.
124417	Thailand	Ministry of Public Health (Thailand). Thailand National Nutrition Survey 2003.
144199	Thailand	Bloem MW, Wedel M, Egger RJ, Speek AJ, Schrijver J, Saowakontha S, Schreurs WH. Iron metabolism and vitamin A deficiency in children in northeast Thailand. Am J Clin Nutr. 1989; 50(2): 332-8.
144202	Thailand	Saowakontha S, Pongpaew P, Schelp FP, Rojsathaporn K, Sriboonlue P, Vudhivai N, Intarakhao C, Pipitgool V, Mahaweerawat U, Supawan V. Quetelet index, hemoglobin and parasitic infection of rural women in northeast Thailand. Southeast Asian J Trop Med Public Health. 1994; 25(3): 474-8.
144203	Thailand	Channgam P, Thumpenjit S, Koowatanasiri P. Anemia in Sakon Nakhon Province. J Med Assoc Thai. 1999; 82(6): 545-51.
144207	Thailand	Linpisarn S, Thanangkul O, Suwanraj C, Kaewvichit R, Kricka LJ, Whitehead TP. Iron deficiency in a Northern Thai population: the effects of iron supplements studied by means of plasma ferritin estimations. Ann Clin Biochem. 1984; 21 (Pt 4): 268-74.
144209	Thailand	Sanchaisuriya P, Pongpaew P, Saowakontha S, Supawan V, Migasena P, Schelp FP. Nutritional health and parasitic

NID	Location	Citation
		infection of rural Thai women of the child bearing age. J Med Assoc Thai. 1993; 76(3): 138-45.
144213	Thailand	Linpisarn S, Tienboon P, Promtet N, Putsyainunt P, Santawanpat S, Fuchs GJ. Iron deficiency and anaemia in children with a high prevalence of haemoglobinopathies: implications for screening. Int J Epidemiol. 1996; 25(6): 1262-6.
144214	Thailand	Tungtrongchitr R, Pongpaew P, Schelp FP, Phonrat B, Mahaweerawat U, Paksanont S, Sanchaisuriya P, Jotking P, Intarakhao C, Saowakhontha S. Vitamin B12, folic acid, ferritin and haemoglobin status of rural women in child-bearing age in northeast Thailand. J Med Assoc Thai. 1997; 80(12): 785-90.
283324	Thailand	Health Systems Research Institute (Thailand), Ministry of Public Health (Thailand), Thai Health Promotion Foundation. Thailand National Health and Examination Survey 2013-2015.
409190	Thailand	Bloem MW, Wedel M, Egger RJ, Speek AJ, Chusilp K, Saowakontha S, Schreurs WH. A prevalence study of vitamin A deficiency and xerophthalmia in northeastern Thailand. Am J Epidemiol. 1989; 129(6): 1095-103.
409550	Thailand	Rojroongwasinkul N, Kijboonchoo K, Wimonpeerapattana W, Purttiponthanee S, Yamborisut U, Boonpraderm A, Kunapan P, Thasanasuwan W, Khouw I. SEANUTS: the nutritional status and dietary intakes of 0.5-12-year-old Thai children. Br J Nutr. 2013; 110 Suppl 3: S36-44.
489990	Thailand	Krittaphol W, Bailey KB, Pongcharoen T, Winichagoon P, Thomson C, Gibson RS. Primary school children from northeast Thailand are not at risk of selenium deficiency. Asia Pac J Clin Nutr. 2006; 15(4): 474-81.
20888	Timor-Leste	ACIL Australia Pty Ltd., Australian National University, Ministry of Health (Timor-Leste), National Statistics Directorate (Timor-Leste), University of Newcastle (Australia). Timor-Leste Demographic and Health Survey 2003. Newcastle, Australia: University of Newcastle (Australia).
21274	Timor-Leste	ICF Macro, Ministry of Finance (Timor-Leste), National Statistics Directorate (Timor-Leste). Timor-Leste Demographic and Health Survey 2009-2010. Fairfax, United States of America: ICF International.
286211	Timor-Leste	Ministry of Health (Timor-Leste). Timor-Leste Food and Nutrition Survey 2013.
286785	Timor-Leste	ICF International, National Statistics Directorate (Timor-Leste). Timor-Leste Demographic and Health Survey 2016. Fairfax, United States of America: ICF International, 2018.
77515	Togo	Directorate General of Statistics and National Accounts (Togo), ICF International, Ministry of Health (Togo), Ministry of Planning, Development and Zoning (Togo). Togo

NID	Location	Citation
		Demographic and Health Survey 2013-2014. Fairfax, United States of America: ICF International, 2015.
139019	Togo	National Institute of Hygiene, Ministry of Health (Togo), United Nations Children's Fund (UNICEF), World Health Organization (WHO). Togo Survey on Anemia and Associated Factors in Households 1999-2000.
144216	Togo	Berger J, Dyck JL, Galan P, Aplogan A, Schneider D, Traissac P, Hercberg S. Effect of daily iron supplementation on iron status, cell-mediated immunity, and incidence of infections in 6-36 month old Togolese children. Eur J Clin Nutr. 2000; 54(1): 29-35.
359318	Togo	ICF International, National Institute of Hygiene, Ministry of Health (Togo), National Institute of Statistics and Economic and Demographic Studies (INSEED) (Togo), National Program to Fight Malaria, Ministry of Health (Togo). Togo Malaria Indicator Survey 2017. Fairfax, United States of America: ICF International, 2018.
139017	Tonga	The 1986 National Nutrition Survey of the Kingdom of Tonga.
142784	Tonga	Republic of Kiribati. Pacific Helminths Initiative 2001-2002.
144314	Tunisia	World Health Organization (WHO). WHO Global Database on Anemia, Nutrition Landscape Information System. Geneva, Switzerland: World Health Organization (WHO).
20956	Turkmenistan	Gurbansoltan Eje Clinical Research Center for Maternal and Child Health (GECRCMCH), Macro International, Inc, Ministry of Health and Medical Industry (Turkmenistan). Turkmenistan Demographic and Health Survey 2000.
144314	Turkmenistan	World Health Organization (WHO). WHO Global Database on Anemia, Nutrition Landscape Information System. Geneva, Switzerland: World Health Organization (WHO).
18818	Tuvalu	Macro International, Inc, Secretariat of the Pacific Community (SPC), Tuvalu Central Statistics Division. Tuvalu Demographic and Health Survey 2007.
144220	Türkiye	Kilinç M, Yüregir GT, Ekerbiçer H. Anaemia and iron-deficiency anaemia in south-east Anatolia. Eur J Haematol. 2002; 69(5-6): 280-3.
489976	Türkiye	Gür E, Yildiz I, Celkan T, Can G, Akkus S, Arvas A, Güzelöz S, Cifçili S. Prevalence of anemia and the risk factors among schoolchildren in Istanbul. J Trop Pediatr. 2005; 51(6): 346-50.
13109	Uganda	ICF Macro, Ministry of Health (Uganda), Mulago Hospital, Uganda Bureau of Statistics, United Nations Children's Fund (UNICEF), World Health Organization (WHO). Uganda Malaria Indicator Survey 2009-2010. Fairfax, United States of America: ICF International.
20993	Uganda	Macro International, Inc, Uganda Bureau of Statistics. Uganda Demographic and Health Survey 2000-2001.

NID	Location	Citation
		Fairfax, United States of America: ICF International.
21014	Uganda	Macro International, Inc, Uganda Bureau of Statistics. Uganda Demographic and Health Survey 2006. Fairfax, United States of America: ICF International.
23289	Uganda	MEASURE Evaluation Project, Carolina Population Center, University of North Carolina, Macro International, Inc, Ministry of Health (Uganda), Uganda Bureau of Statistics. Uganda Child Verbal Autopsy Study 2007. Calverton, United States of America: Macro International, Inc.
56021	Uganda	ICF Macro, Uganda Bureau of Statistics. Uganda Demographic and Health Survey 2011. Fairfax, United States of America: ICF International.
110760	Uganda	Koukounari A, Fenwick A, Whawell S, Kabatereine NB, Kazibwe F, Tukahebwa EM, Stothard JR, Donnelly CA, Webster JP. Morbidity indicators of Schistosoma mansoni: relationship between infection and anemia in Ugandan schoolchildren before and after praziquantel and albendazole chemotherapy. Am J Trop Med Hyg. 2006; 75(2): 278-86.
144221	Uganda	Kiwanuka GN, Isharaza WK, Mahmoud S. Iron status of pregnant women at first antenatal booking in Mbarara University Teaching Hospital. Trop Doct. 1999; 29(4): 228-30.
157065	Uganda	ICF International, National Malaria Control Program, Ministry of Health (Uganda), Uganda Bureau of Statistics. Uganda Malaria Indicator Survey 2014-2015. Fairfax, United States of America: ICF International.
281545	Uganda	Save the Children Federation, Inc. (STCFI), Service for Generations (SFG) International (Uganda). Uganda - Luwero and Nakaseke School Health and Nutrition Baseline Survey 2005.
286780	Uganda	ICF International, Uganda Bureau of Statistics. Uganda Demographic and Health Survey 2016. Fairfax, United States of America: ICF International, 2018.
124424	Ukraine	Centers for Disease Control and Prevention (CDC), Ministry of Health (Ukraine), National Academy of Medical Science of Ukraine, State Statistical Committee (Ukraine), United Nations Children's Fund (UNICEF). Ukraine National Micronutrient Survey 2002.
144314	Ukraine	World Health Organization (WHO). WHO Global Database on Anemia, Nutrition Landscape Information System. Geneva, Switzerland: World Health Organization (WHO).
144222	United Arab Emirates	Musaiger AO, Abdulghafoor A, Radwan H. Anaemia among 6 year old children in the United Arab Emirates. Eur J Clin Nutr. 1996; 50(9): 636-7.
144223	United Arab Emirates	Hossain MM, Bakir M, Pugh RN, Sheekh-Hussen M, Bin Ishaq SA, Berg DB, Lindblad BS. The prevalence and correlates of anaemia among young children and women of

NID	Location	Citation
		childbearing age in Al Ain, United Arab Emirates. Ann Trop Paediatr. 1995; 15(3): 227-35.
144307	United Arab Emirates	Hughes PF, Morrison J. Pregnancy outcome data in a United Arab Emirates population: what can they tell us?. Asia Oceania J Obstet Gynaecol. 1994; 20(2): 183-90.
13265	United Kingdom	Joint Health Surveys Unit of Social and Community Planning Research and University College London, [computer file]. Colchester, Essex: UK Data Archive [distributor], July 2001. SN: 4379 , http://dx.doi.org/10.5255/UKDA-SN-4379-1.
13290	United Kingdom	Joint Health Surveys Unit of Social and Community Planning Research and University College London, Scottish Health Survey, 1995 [computer file]. 3rd ed. Colchester, Essex: UK Data Archive [distributor], 11 February 1999. SN: 3807.
22286	United Kingdom	Office of Population Censuses and Surveys. Social Survey Division, Health Survey for England, 1991-1992: Combined Data File. Colchester, Essex: UK Data Archive [distributor], October 1997. SN: 3238, http://dx.doi.org/10.5255/UKDA-SN-3238-1
22293	United Kingdom	Office of Population Censuses and Surveys. Social Survey Division, Health Survey for England, 1993 [Computer file]. Colchester, Essex: UK Data Archive [distributor], April 1995. SN: 3316, http://dx.doi.org/10.5255/UKDA-SN-3316-1
22306	United Kingdom	Joint Health Surveys Unit of Social and Community Planning Research and University College London, Health Survey for England, 1994 [computer file]. 4th ed. Colchester, Essex: UK Data Archive [distributor], 26 March 2001. SN: 3640.
22317	United Kingdom	Joint Health Surveys Unit of Social and Community Planning Research and University College London, Health Survey for England, 1995 [computer file]. 3rd ed. Colchester, Essex: UK Data Archive [distributor], 26 March 2001. SN: 3796.
22329	United Kingdom	
22341	United Kingdom	Joint Health Surveys Unit of Social and Community Planning Research and University College London, Health Survey for England, 1997 [computer file]. 2nd ed. Colchester, Essex: UK Data Archive [distributor], 4 December 2000. SN: 3979.
22352	United Kingdom	National Centre for Social Research, University College London Department of Epidemiology and Public Health, Health Survey for England, 1998 [computer file]. 4th ed. Colchester, Essex: UK Data Archive [distributor], 30 November 2002. SN: 4150.
22364	United Kingdom	National Centre for Social Research and University College London. Department of Epidemiology and Public Health, Health Survey for England, 1999 [computer file]. 3rd Edition. Colchester, Essex: UK Data Archive [distributor], February 2002. SN: 4365.
22374	United Kingdom	National Centre for Social Research, University College London Department of Epidemiology and Public Health, Health Survey for England, 2000 [computer file].

NID	Location	Citation
		Colchester, Essex: UK Data Archive [distributor], 23 April 2002. SN: 4487.
22388	United Kingdom	National Centre for Social Research and University College London. Department of Epidemiology and Public Health, Colchester, Essex: UK Data Archive [distributor], June 2004. SN: 4628.
22403	United Kingdom	National Centre for Social Research and University College London. Department of Epidemiology and Public Health, [computer file]. Colchester, Essex: UK Data Archive [distributor], May 2004. SN: 4912.
22449	United Kingdom	National Centre for Social Research and University College London. Department of Epidemiology and Public Health, [computer file]. Colchester, Essex: UK Data Archive [distributor], July 2006. SN: 5439.
22463	United Kingdom	National Centre for Social Research and University College London. Department of Epidemiology and Public Health, Health Survey for England, 2005 [computer file]. Colchester, Essex: UK Data Archive [distributor], July 2007. SN: 5675.
22476	United Kingdom	National Centre for Social Research and University College London. Department of Epidemiology and Public Health, Colchester, Essex: UK Data Archive [distributor], July 2011. SN: 5809, http://dx.doi.org/10.5255/UKDA-SN-5809-1
95630	United Kingdom	National Centre for Social Research and University College London. Department of Epidemiology and Public Health, Colchester, Essex: UK Data Archive [distributor], July 2011. SN: 6732, http://dx.doi.org/10.5255/UKDA-SN-6732-1
120995	United Kingdom	Human Nutrition Research, Medical Research Council, National Centre for Social Research (NatCen), Northern Ireland Statistics and Research Agency (NISRA), University College London Medical School. United Kingdom National Diet and Nutrition Survey 2008-2011 - Public Health England.
124433	United Kingdom	Office for National Statistics. Social and Vital Statistics Division and Food Standards Agency, National Diet and Nutrition Survey : Adults Aged 19 to 64 Years, 2000-2001 [computer file]. Colchester, Essex: UK Data Archive [distributor], May 2005. SN: 5140 , http://dx.doi.org/10.5255/UKDA-SN-5140-1.
124460	United Kingdom	Office of Population Censuses and Surveys. Social Survey Division, Dietary and Nutritional Survey of British Adults, 1986-1987 [computer file]. Colchester, Essex: UK Data Archive [distributor], September 1991. SN: 2836, http://dx.doi.org/10.5255/UKDA-SN-2836-1.
129281	United Kingdom	NatCen Social Research et al. , Diet and Nutrition Survey of Infants and Young Children, 2011 [computer file]. 2nd Edition. Colchester, Essex: UK Data Archive [distributor], October 2013. SN: 7263 , http://dx.doi.org/10.5255/UKDA-SN-7263-2.

NID	Location	Citation
129421	United Kingdom	Office for National Statistics. Social Survey Division et al. , National Diet and Nutrition Survey : Young People Aged 4 to 18 Years, 1997 [computer file]. Colchester, Essex: UK Data Archive [distributor], January 2001. SN: 4243, http://dx.doi.org/10.5255/UKDA-SN-4243-1.
129449	United Kingdom	Finch, S.A. et al. , National Diet and Nutrition Survey : People Aged 65 Years and Over, 1994-1995 [computer file]. 2nd Edition. Colchester, Essex: UK Data Archive [distributor], November 2001. SN: 4036, http://dx.doi.org/10.5255/UKDA-SN-4036-1.
134368	United Kingdom	Marmot, M. et al. , Colchester, Essex: UK Data Archive [distributor], October 2013. SN: 5050, http://dx.doi.org/10.5255/UKDA-SN-5050-6
143717	United Kingdom	Elwood PC, Hughes J, Abernethy M, Davies R, Gough R, Johnson AP, Dubourg AY. An international haematological survey. Bull World Health Organ. 1976; 54(1): 87-95.
144224	United Kingdom	Fischbacher C, Bhopal R, Patel S, White M, Unwin N, Alberti KG. Anaemia in Chinese, South Asian, and European populations in Newcastle upon Tyne: cross sectional study. BMJ. 2001; 322(7292): 958-9.
144229	United Kingdom	Nelson M, White J, Rhodes C. Haemoglobin, ferritin, and iron intakes in British children aged 12-14 years: a preliminary investigation. Br J Nutr. 1993; 70(1): 147-55.
144230	United Kingdom	Clarke R, Refsum H, Birks J, Evans JG, Johnston C, Sherliker P, Ueland PM, Schneede J, McPartlin J, Nexo E, Scott JM. Screening for vitamin B-12 and folate deficiency in older persons. Am J Clin Nutr. 2003; 77(5): 1241-7.
144231	United Kingdom	Sherriff A, Emond A, Hawkins N, Golding J. Haemoglobin and ferritin concentrations in children aged 12 and 18 months. ALSPAC Children in Focus Study Team. Arch Dis Child. 1999; 80(2): 153-7.
144232	United Kingdom	Emond AM, Hawkins N, Pennock C, Golding J. Haemoglobin and ferritin concentrations in infants at 8 months of age. Arch Dis Child. 1996; 74(1): 36-9.
144234	United Kingdom	Robinson S, Godfrey K, Denne J, Cox V. The determinants of iron status in early pregnancy. Br J Nutr. 1998; 79(3): 249-55.
144237	United Kingdom	Nelson M, Bakaliou F, Trivedi A. Iron-deficiency anaemia and physical performance in adolescent girls from different ethnic backgrounds. Br J Nutr. 1994; 72(3): 427-33.
255142	United Kingdom	Strain JJ, Thompson KA, Barker ME, Carville DGM. Iron sufficiency in the population of Northern Ireland: estimates from blood measurements. Br J Nutr. 1990; 64(1): 219-24.
12644	United Republic of Tanzania	Macro International, Inc, National Bureau of Statistics (Tanzania), Office of the Chief Government Statistician (OCGS) (Zanzibar), Tanzania Commission for AIDS (TACAIDS), Zanzibar AIDS Commission (ZAC). Tanzania

NID	Location	Citation
		HIV/AIDS and Malaria Indicator Survey 2007-2008. Fairfax, United States of America: ICF International.
20875	United Republic of Tanzania	Macro International, Inc, National Bureau of Statistics (Tanzania). Tanzania Demographic and Health Survey 2004-2005. Fairfax, United States of America: ICF International.
21331	United Republic of Tanzania	ICF Macro, National Bureau of Statistics (Tanzania). Tanzania Demographic and Health Survey 2009-2010. Fairfax, United States of America: ICF International.
77395	United Republic of Tanzania	ICF International, National Bureau of Statistics (Tanzania), Office of the Chief Government Statistician (OCGS) (Zanzibar), Tanzania Commission for AIDS (TACAIDS), Zanzibar AIDS Commission (ZAC). Tanzania AIDS Indicator Survey 2011-2012. Fairfax, United States of America: ICF International, 2013.
110765	United Republic of Tanzania	Lwambo NJ, Brooker S, Siza JE, Bundy DA, Guyatt H. Age patterns in stunting and anaemia in African schoolchildren: a cross-sectional study in Tanzania. Eur J Clin Nutr. 2000; 54(1): 36-40.
139599	United Republic of Tanzania	The Partnership for Child Development. The Health and Nutritional Status of Schoolchildren in Africa: Evidence from School-based Health Programmes in Ghana and Tanzania. Trans R Soc Trop Med Hyg. 1998; 92(3): 254-61.
144192	United Republic of Tanzania	Massawe SN, Urassa EN, Nyström L, Lindmark G. Effectiveness of primary level antenatal care in decreasing anemia at term in Tanzania. Acta Obstet Gynecol Scand. 1999; 78(7): 573-9.
144194	United Republic of Tanzania	Stoltzfus RJ, Chwaya HM, Tielsch JM, Schulze KJ, Albonico M, Savioli L. Epidemiology of iron deficiency anemia in Zanzibari schoolchildren: the importance of hookworms. Am J Clin Nutr. 1997; 65(1): 153-9.
144195	United Republic of Tanzania	Bergsjo P, Seha AM, Ole-King'ori N. Hemoglobin concentration in pregnant women. Experience from Moshi, Tanzania. Acta Obstet Gynecol Scand. 1996; 75(3): 241-4.
144196	United Republic of Tanzania	Stoltzfus RJ, Edward-Raj A, Dreyfuss ML, Albonico M, Montresor A, Dhoj Thapa M, West KP Jr, Chwaya HM, Savioli L, Tielsch J. Clinical pallor is useful to detect severe anemia in populations where anemia is prevalent and severe. J Nutr. 1999; 129(9): 1675-81.
218593	United Republic of Tanzania	ICF International, Ministry of Health (Zanzibar), Ministry of Health, Community Development, Gender, Elderly and Children (MoHCDEC) (Tanzania), National Bureau of Statistics (Tanzania), Office of the Chief Government Statistician (OCGS) (Zanzibar). Tanzania Demographic and Health Survey 2015-2016. Fairfax, United States of America: ICF International, 2016.
261889	United Republic of Tanzania	Fogarty International Center, National Institutes of Health (NIH), Foundation for the National Institutes of Health (FNIH), Haydom Lutheran Hospital. Tanzania - Haydom Malnutrition and Enteric Disease Study 2009-2014.

NID	Location	Citation
350798	United Republic of Tanzania	ICF International, Ministry of Health (Zanzibar), Ministry of Health, Community Development, Gender, Elderly and Children (MoHCDEC) (Tanzania), National Bureau of Statistics (Tanzania), Office of the Chief Government Statistician (OCGS) (Zanzibar). Tanzania Malaria Indicator Survey 2017. Fairfax, United States of America: ICF International, 2018.
25914	United States of America	National Center for Health Statistics (NCHS), Centers for Disease Control and Prevention (CDC). United States National Health and Nutrition Examination Survey 2007-2008. Hyattsville, United States: National Center for Health Statistics (NCHS), Centers for Disease Control and Prevention (CDC), 2009.
47478	United States of America	National Center for Health Statistics (NCHS), Centers for Disease Control and Prevention (CDC). United States National Health and Nutrition Examination Survey 2005-2006. Hyattsville, United States: National Center for Health Statistics (NCHS), Centers for Disease Control and Prevention (CDC), 2007.
47962	United States of America	National Center for Health Statistics (NCHS), Centers for Disease Control and Prevention (CDC). United States National Health and Nutrition Examination Survey 2003-2004. Hyattsville, United States: National Center for Health Statistics (NCHS), Centers for Disease Control and Prevention (CDC).
48332	United States of America	National Center for Health Statistics (NCHS), Centers for Disease Control and Prevention (CDC). United States National Health and Nutrition Examination Survey 2009-2010. Hyattsville, United States: National Center for Health Statistics (NCHS), Centers for Disease Control and Prevention (CDC), 2011.
48604	United States of America	National Center for Health Statistics (NCHS), Centers for Disease Control and Prevention (CDC). United States National Health and Nutrition Examination Survey 1988-1994. Hyattsville, United States: National Center for Health Statistics (NCHS), Centers for Disease Control and Prevention (CDC).
49205	United States of America	National Center for Health Statistics (NCHS), Centers for Disease Control and Prevention (CDC). United States National Health and Nutrition Examination Survey 2001-2002. Hyattsville, United States: National Center for Health Statistics (NCHS), Centers for Disease Control and Prevention (CDC).
52110	United States of America	National Center for Health Statistics (NCHS), Centers for Disease Control and Prevention (CDC). United States National Health and Nutrition Examination Survey 1999-2000. Hyattsville, United States: National Center for Health Statistics (NCHS), Centers for Disease Control and Prevention (CDC).
110300	United States of America	National Center for Health Statistics, Centers for Disease Control and Prevention. United States National Health and Nutrition Examination Survey 2011-2012. Hyattsville

NID	Location	Citation
		United States of America: National Center for Health Statistics, Centers for Disease Control and Prevention, 2013.
144254	United States of America	Bodnar LM, Scanlon KS, Freedman DS, Siega-Riz AM, Cogswell ME. High prevalence of postpartum anemia among low-income women in the United States. Am J Obstet Gynecol. 2001; 185(2): 438-43.
144264	United States of America	Salive ME, Cornoni-Huntley J, Guralnik JM, Phillips CL, Wallace RB, Ostfeld AM, Cohen HJ. Anemia and hemoglobin levels in older persons: relationship with age, gender, and health status. J Am Geriatr Soc. 1992; 40(5): 489-96.
144283	United States of America	Mitchell MC, Lerner E. Maternal hematologic measures and pregnancy outcome. J Am Diet Assoc. 1992; 92(4): 484-6.
144284	United States of America	Sarnak MJ, Tighiouart H, Manjunath G, MacLeod B, Griffith J, Salem D, Levey AS. Anemia as a risk factor for cardiovascular disease in The Atherosclerosis Risk in Communities (ARIC) study. J Am Coll Cardiol. 2002; 40(1): 27-33.
165892	United States of America	National Center for Health Statistics, Centers for Disease Control and Prevention. United States National Health and Nutrition Examination Survey 2013-2014. Hyattsville, United States of America: National Center for Health Statistics, Centers for Disease Control and Prevention.
489992	United States of America	Scanlon KS, Yip R, Schieve LA, Cogswell ME. High and low hemoglobin levels during pregnancy: differential risks for preterm birth and small for gestational age. Obstet Gynecol. 2000; 96(5 Pt 1): 741-8.
274952	Uruguay	Ministry of Public Health (Uruguay), Ministry of Social Development (Uruguay), UNICEF Uruguay, Uruguayan Network on Infant Feeding, Nutrition and Development (RUANDI). Uruguay National Survey on Nutritional Status, Feeding Practices and Anemia 2010-2011.
490696	Uruguay	Ministry of Public Health (Uruguay), National Institute of Statistics (Uruguay), UNICEF Uruguay, Uruguayan Network on Infant Feeding, Nutrition and Development (RUANDI). Uruguay - National Survey of Lactation, Feeding Practices and Anemia in children under 24 months 2020. 2020.
21033	Uzbekistan	Institute of Obstetrics and Gynecology, Ministry of Health (Uzbekistan), Macro International, Inc, Ministry of Health (Uzbekistan). Uzbekistan Demographic and Health Survey 1996. Fairfax, United States of America: ICF International.
21039	Uzbekistan	Analytical and Information Center of the Ministry of Health of Uzbekistan, Macro International, Inc, Ministry of Macroeconomics and Statistics (Uzbekistan). Uzbekistan Special Demographic and Health Survey 2002. Fairfax, United States of America: ICF International.
144314	Uzbekistan	World Health Organization (WHO). WHO Global Database on Anemia, Nutrition Landscape Information System. Geneva, Switzerland: World Health Organization (WHO).

NID	Location	Citation
517501	Uzbekistan	Centers for Disease Control and Prevention (CDC), Ministry of Health (Uzbekistan). Uzbekistan National Flour Fortification Program 2008. 2013.
13465	Vanuatu	Ministry of Health (Vanuatu), United Nations Children's Fund (UNICEF). Vanuatu Multiple Indicator Cluster Survey 2007-2008. New York, United States of America: United Nations Children's Fund (UNICEF).
124466	Vanuatu	Australian Agency for International Development (AusAID), Ministry of Health (Vanuatu), Save the Children International, Vanuatu National Statistics Office. Vanuatu National Nutrition Survey 1996.
142784	Vanuatu	Republic of Kiribati. Pacific Helminths Initiative 2001-2002.
144314	Vanuatu	World Health Organization (WHO). WHO Global Database on Anemia, Nutrition Landscape Information System. Geneva, Switzerland: World Health Organization (WHO).
229477	Vanuatu	Ministry of Health (Vanuatu), Secretariat of the Pacific Community (SPC), Vanuatu National Statistics Office. Vanuatu Demographic and Health Survey 2013.
144289	Venezuela (Bolivarian Republic of)	Layrisse M, Chaves JF, Mendez-Castellano, Bosch V, Tropper E, Bastardo B, González E. Early response to the effect of iron fortification in the Venezuelan population. Am J Clin Nutr. 1996; 64(6): 903-7.
144290	Venezuela (Bolivarian Republic of)	Taylor PG, Martínez-Torres C, Méndez-Castellano H, Bosch V, Leets I, Tropper E, Layrisse M. The relationship between iron deficiency and anemia in Venezuelan children. Am J Clin Nutr. 1993; 58(2): 215-8.
144296	Venezuela (Bolivarian Republic of)	Peña E, Sánchez A, Solano L. [Profile of nutritional risk in pregnant adolescents]. Arch Latinoam Nutr. 2003; 53(2): 141-9.
138610	Viet Nam	National Institute of Nutrition (Vietnam). Vietnam National Anemia Survey 2000.
138612	Viet Nam	Centers for Disease Control and Prevention (CDC), National Institute of Nutrition (Vietnam), United Nations Children's Fund (UNICEF). Vietnam National Nutrition Anemia and Intestinal Helminth Survey 1995.
143269	Viet Nam	National Institute of Nutrition (Vietnam). Report on Anemia and Sub-clinical Vitamin A Deficiency in Some Provinces in Viet Nam, March 2006. Hanoi, Vietnam: National Institute of Nutrition (Vietnam), 2006.
144297	Viet Nam	Thu BD, Schultink W, Dillon D, Gross R, Leswara ND, Khoi HH. Effect of daily and weekly micronutrient supplementation on micronutrient deficiencies and growth in young Vietnamese children. Am J Clin Nutr. 1999; 69(1): 80-6.
144314	Viet Nam	World Health Organization (WHO). WHO Global Database on Anemia, Nutrition Landscape Information System. Geneva, Switzerland: World Health Organization (WHO).

NID	Location	Citation
261370	Viet Nam	Laillou A, Pham TV, Tran NT, Le HT, Wieringa F, Rohner F, Fortin S, Le MB, Tran do T, Moench-Pfanner R, Berger J. Micronutrient Deficits Are Still Public Health Issues among Women and Young Children in Vietnam. PLoS One. 2012; 7(4): e34906.
400126	Viet Nam	Centers for Disease Control and Prevention (CDC), Emory University (United States), Eunice Shriver Kennedy National Institute of Child Health and Human Development (NICHD), Global Alliance for Improved Nutrition (GAIN), GroundWork LLC, HarvestPlus, International Micronutrient Malnutrition Prevention and Control (IMMPaCt) Program, International Zinc Consultative Group (IZiNCG), Strengthening Partnerships, Results, and Innovations in Nutrition Globally (SPRING). Biomarkers Reflecting Inflammation and Nutrition Determinants of Anemia (BRINDA).
409579	Viet Nam	Le Nguyen BK, Le Thi H, Nguyen Do VA, Tran Thuy N, Nguyen Huu C, Thanh Do T, Deurenberg P, Khouw I. Double burden of undernutrition and overnutrition in Vietnam in 2011: results of the SEANUTS study in 05-11-year-old children. Br J Nutr. 2013; 110 Suppl 3: S45-56.
466463	Viet Nam	Khan NC, Ninh NX, Van Nhien N, Khoi HH, West CE, Hautvast JG. Sub clinical vitamin A deficiency and anemia among Vietnamese children less than five years of age. Asia Pac J Clin Nutr. 2007; 16(1): 152-7.
112500	Yemen	Central Statistical Organization (Yemen), ICF International, Ministry of Public Health and Population (Yemen). Yemen Demographic and Health Survey 2013. Fairfax, United States of America: ICF International.
144299	Yemen	Raja'a YA, Sulaiman SM, Elkarib SA, Mubarak JS. Nutritional status of Yemeni schoolchildren in Al-Mahweet Governorate. East Mediterr Health J. 2001; 7(1-2): 204-10.
140430	Zambia	Centers for Disease Control and Prevention (CDC), Micronutrient Operational Strategies and Technologies, National Food and Nutrition Commission (Zambia), United Nations Children's Fund (UNICEF), United States Agency for International Development (USAID), University of Zambia. Zambia National Vitamin A Survey 2003.
144314	Zambia	World Health Organization (WHO). WHO Global Database on Anemia, Nutrition Landscape Information System. Geneva, Switzerland: World Health Organization (WHO).
162031	Zambia	Government of Zambia, National Malaria Control Centre (Zambia), World Bank. Zambia Access to ACT Initiative Survey 2009. Washington DC, United States of America: World Bank.
255147	Zambia	Hautvast JL, Tolboom JJ, Kafwembe EM, Musonda RM, Mwanakasale V, van Staveren WA, van 't Hof MA, Sauerwein RW, Willems JL, Monnens LA. Severe linear growth retardation in rural Zambian children: the influence of biological variables. Am J Clin Nutr. 2000; 71(2): 550-9.

NID	Location	Citation
255159	Zambia	Ezeilo GC. Haemotological values in pregnant Zambian women. Trop Geogr Med. 1972; 24(2): 152-6.
262998	Zambia	Mel and Enid Zuckerman College of Public Health, University of Arizona, National Food and Nutrition Commission (Zambia), Tropical Diseases Research Centre, UNICEF International Child Development Center (ICDC). Zambia Food Consumption and Micronutrient Status Survey 2008.
411301	Zambia	Central Statistical Office (Zambia), ICF International, Ministry of Health (Zambia), University Teaching Hospital (Zambia), University of Zambia. Zambia Demographic and Health Survey 2018-2019. Fairfax, United States of America: ICF International, 2020.
490180	Zambia	Luo C, Mwela CM, Campbell J. National baseline survey on prevalence and aetiology of anaemia in Zambia: a random cluster community survey involving children, women and men. Lusaka: National Food and Nutrition Commission (Zambia), 1999.
21163	Zimbabwe	Central Statistical Office (Zimbabwe), Macro International, Inc. Zimbabwe Demographic and Health Survey 2005-2006. Fairfax, United States of America: ICF International.
55992	Zimbabwe	ICF Macro, Zimbabwe National Statistics Agency. Zimbabwe Demographic and Health Survey 2010-2011. Calverton, United States of America: ICF Macro, 2012.
137416	Zimbabwe	Ministry of Health and Child Welfare (Zimbabwe). Zimbabwe National Micronutrient Survey 1999.
144300	Zimbabwe	Sikosana PL, Bhebhe S, Katuli S. A prevalence survey of iron deficiency and iron deficiency anaemia in pregnant and lactating women, adult males and pre-school children in Zimbabwe. Cent Afr J Med. 1998; 44(12): 297-305.
144301	Zimbabwe	Friis H, Gomo E, Koestel P, Ndhlovu P, Nyazema N, Krarup H, Michaelsen KF. HIV and other predictors of serum folate, serum ferritin, and hemoglobin in pregnancy: a cross-sectional study in Zimbabwe. Am J Clin Nutr. 2001; 73(6): 1066-73.
144302	Zimbabwe	Nemapare P. Maternal health and nutrition survey for Masvingo Province, Zimbabwe: an appraisal of pregnant women. Nutr Report Int. 1989; 265-79.
144303	Zimbabwe	Ncube TN, Malaba L, Greiner T, Gebre-Medhin M. Evidence of grave vitamin A deficiency among lactating women in the semi-arid rural area of Makhaza in Zimbabwe. A population-based study. Eur J Clin Nutr. 2001; 55(4): 229-34.
144314	Zimbabwe	World Health Organization (WHO). WHO Global Database on Anemia, Nutrition Landscape Information System. Geneva, Switzerland: World Health Organization (WHO).
157066	Zimbabwe	ICF International, National Microbiology Reference Laboratory, Harare Central Hospital (NMRL) (Zimbabwe),

NID	Location	Citation
		Zimbabwe National Statistics Agency. Zimbabwe Demographic and Health Survey 2015. Fairfax, United States of America: ICF International, 2016.

Section 4: MR-BRT age and sex modelled trends

MR-BRT modelled trends for each age, sex, location, and survey available in our input data set that contained personal level information. Plots are broken up by age range (2 years old and over or under 2 years old), sex, and location hierarchy modelled. Points plotted are the input data used to estimate the given age, sex, location trends. The solid line indicates the location level that was estimated in the cascading-spline framework, where the 95% uncertainty interval is also shown as the bands surrounding the solid line. The darker band represents the uncertainty due to fixed effects, and the lighter band represents the uncertainty due to random effects. The dashed lines represent the MR-BRT modelled estimates of the location hierarchies above the modelled location, all of which informed the shape and direction of the spline for current location being modelled. The location hierarchies modelled are represented by the following line colors: global (black), super region (orange), region (green), country (blue), administrative level one (purple), and administrative level two (red).

The modelled estimates from each age range, sex, pregnancy status, and location are used as inputs to our age and sex splitting pipeline, where we disaggregate tabulated data into the age and sex bins we defined for modelling purposes.

MR−BRT Age/Sex Fits − Males 2+ Years Old − Total Anaemia Prevalence − Global
WHO−2024 Adjustment Method

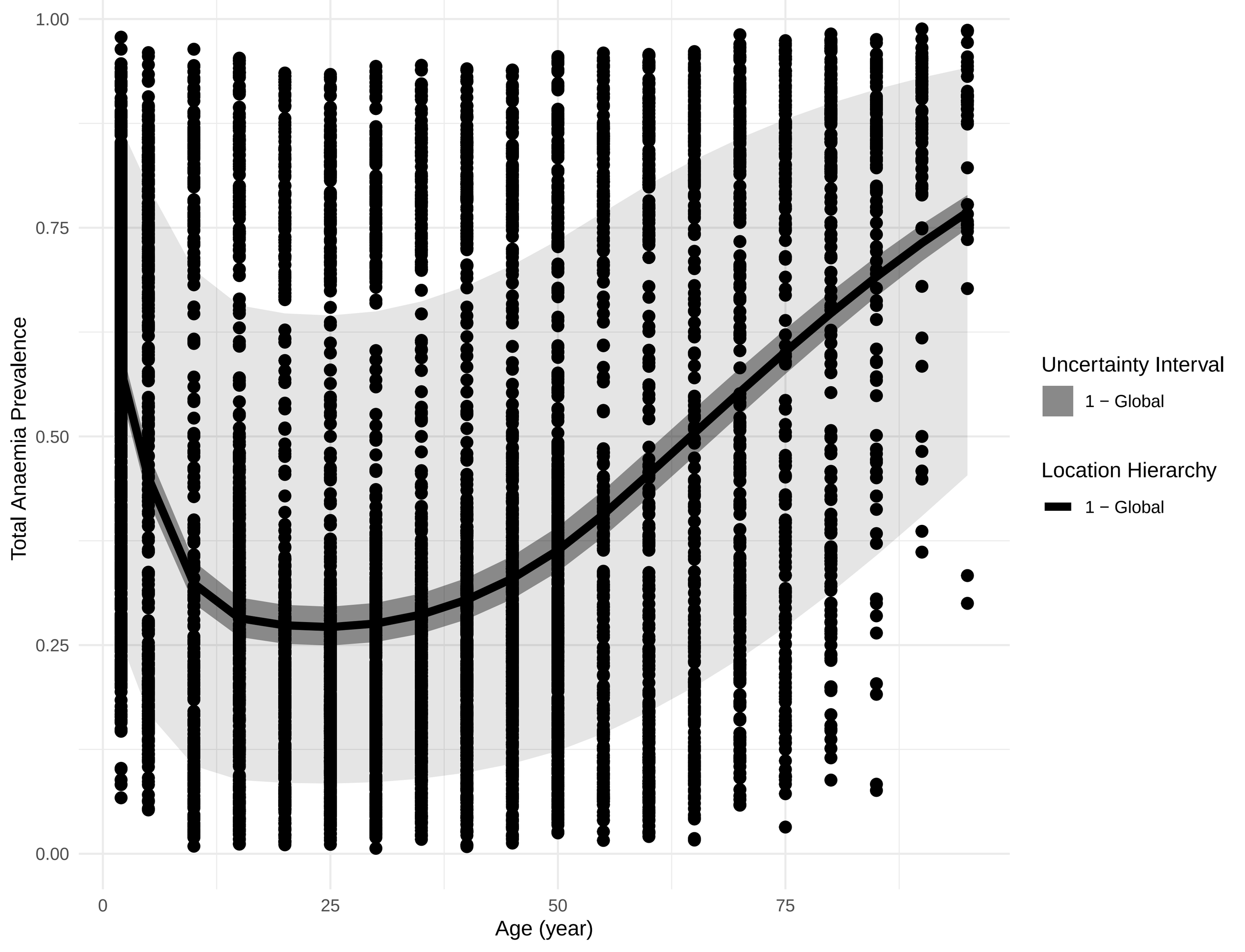

MR−BRT Age/Sex Fits − Males 2+ Years Old − Total Anaemia Prevalence − Central Europe, Eastern Europe, and Central Asia
WHO−2024 Adjustment Method

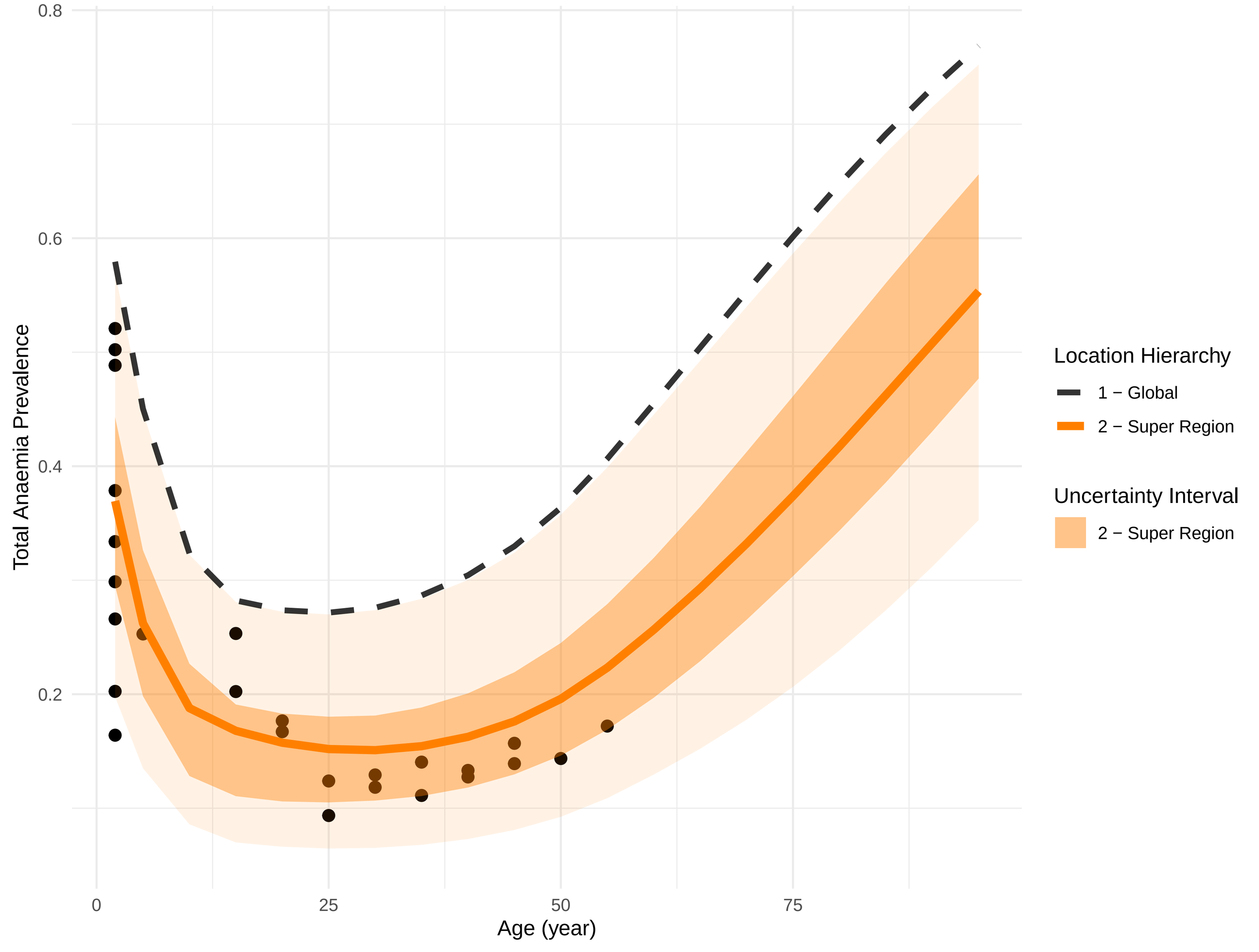

MR−BRT Age/Sex Fits − Males 2+ Years Old − Total Anaemia Prevalence − Central Asia
WHO−2024 Adjustment Method

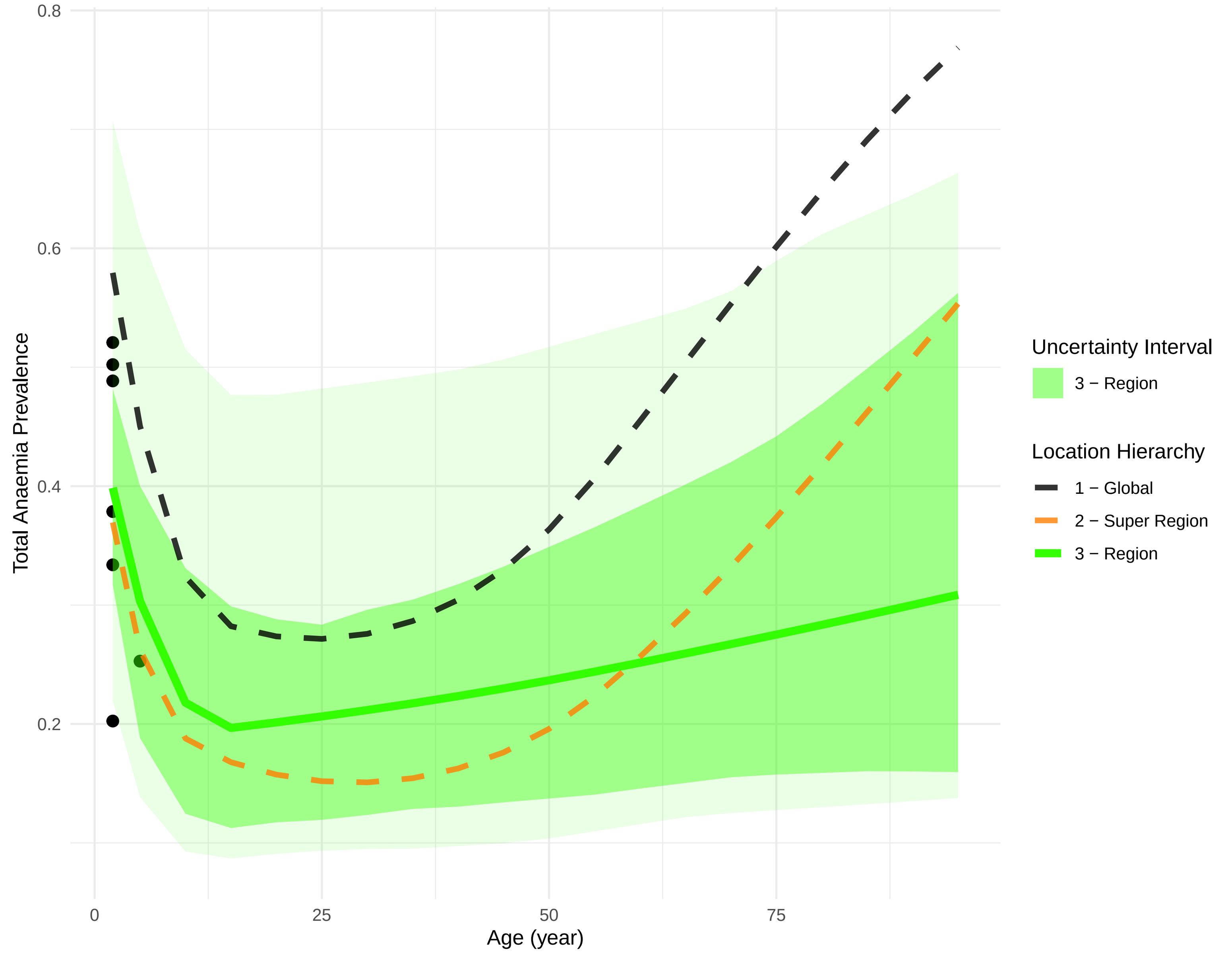

MR−BRT Age/Sex Fits − Males 2+ Years Old − Total Anaemia Prevalence − Armenia
WHO−2024 Adjustment Method

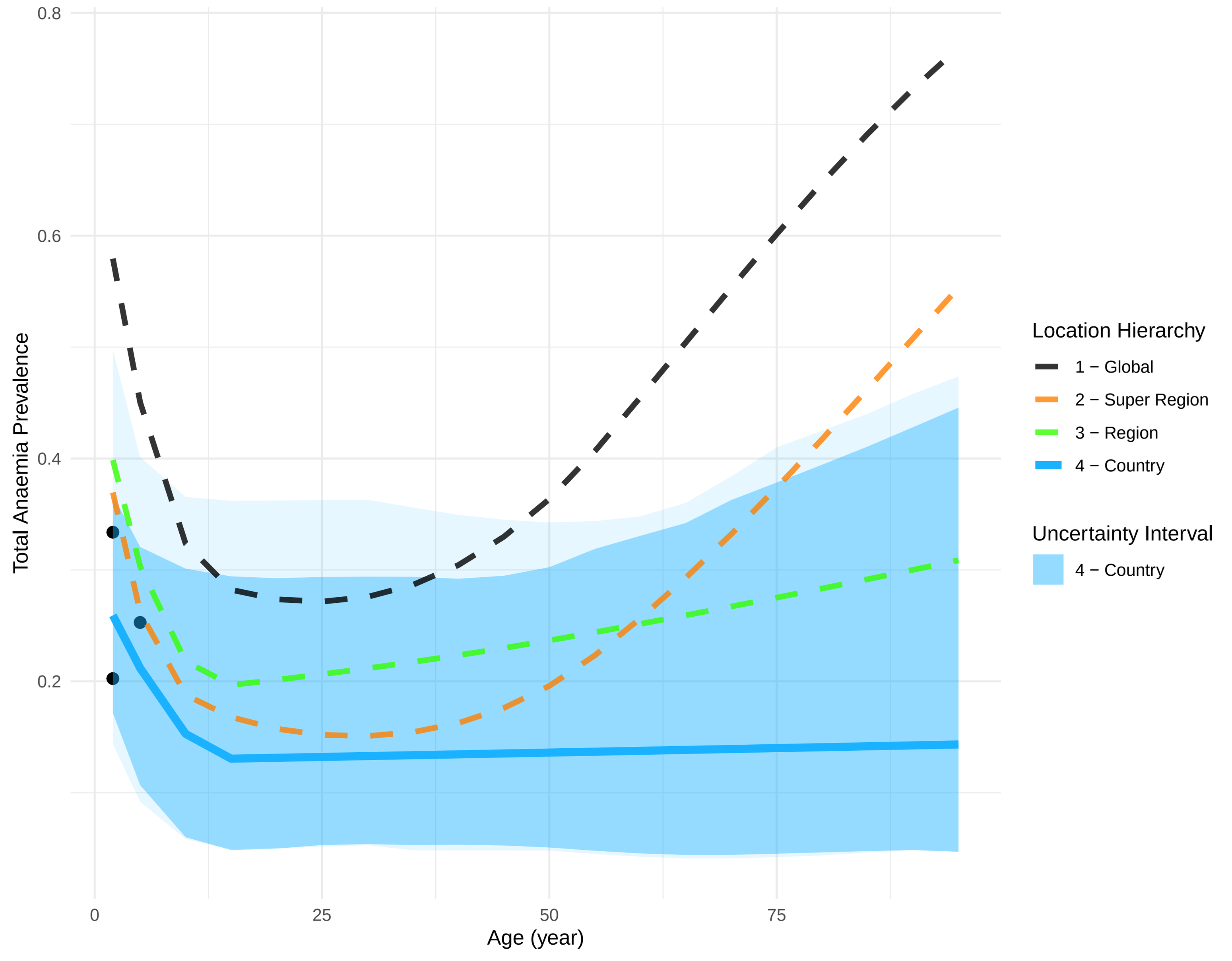

MR−BRT Age/Sex Fits − Males 2+ Years Old − Total Anaemia Prevalence − Azerbaijan
WHO−2024 Adjustment Method

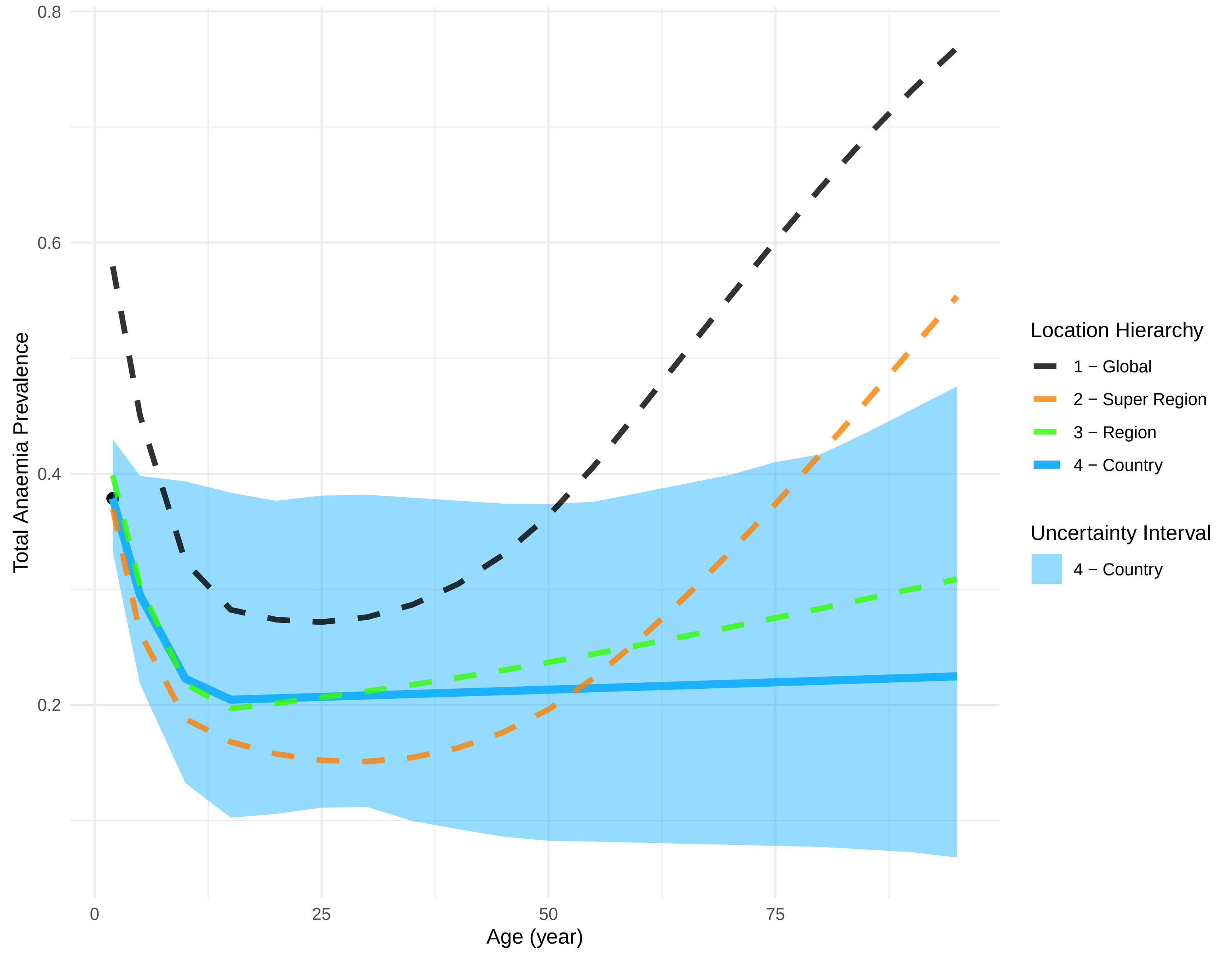

MR−BRT Age/Sex Fits − Males 2+ Years Old − Total Anaemia Prevalence − Kyrgyzstan
WHO−2024 Adjustment Method

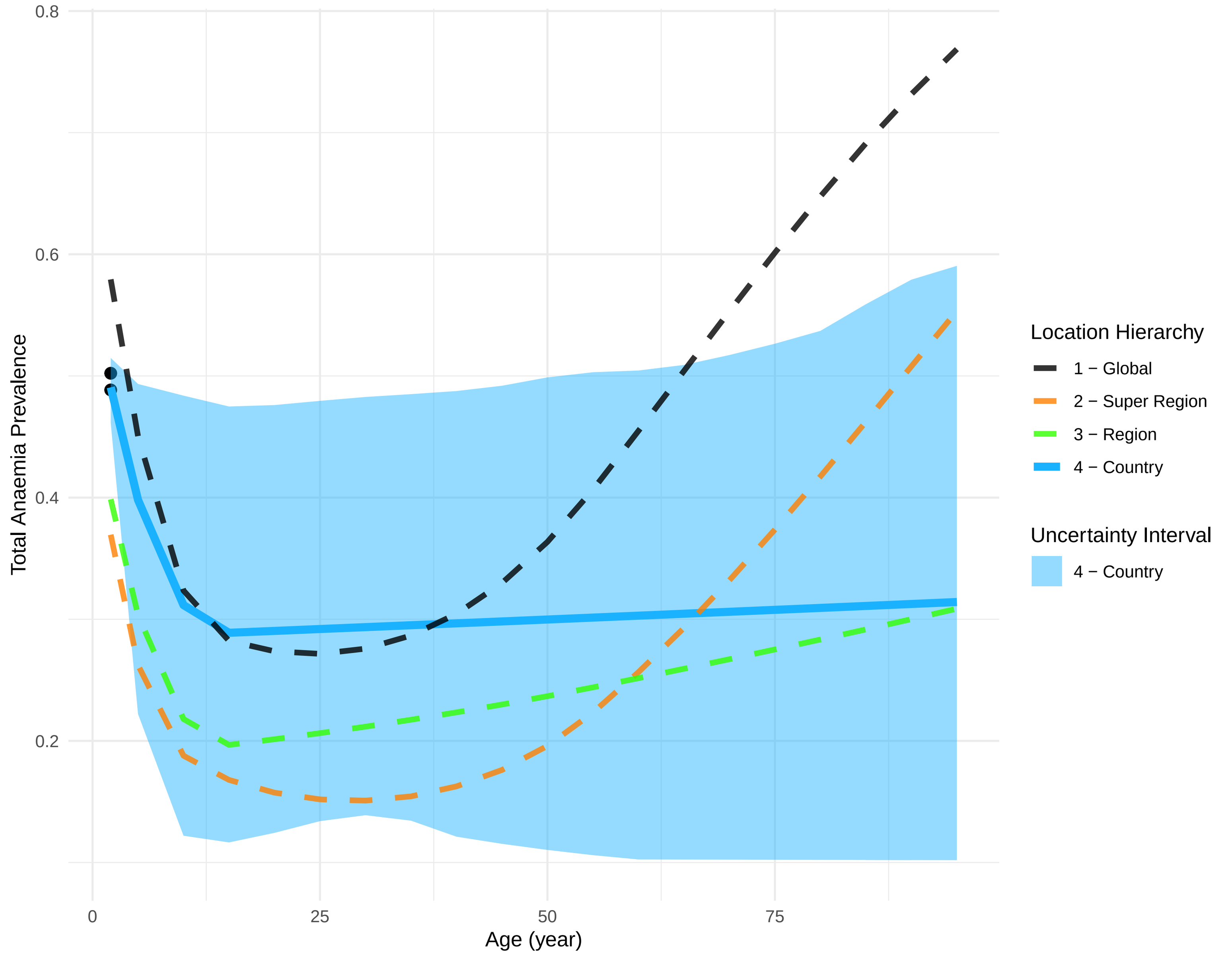

MR−BRT Age/Sex Fits − Males 2+ Years Old − Total Anaemia Prevalence − Uzbekistan
WHO−2024 Adjustment Method

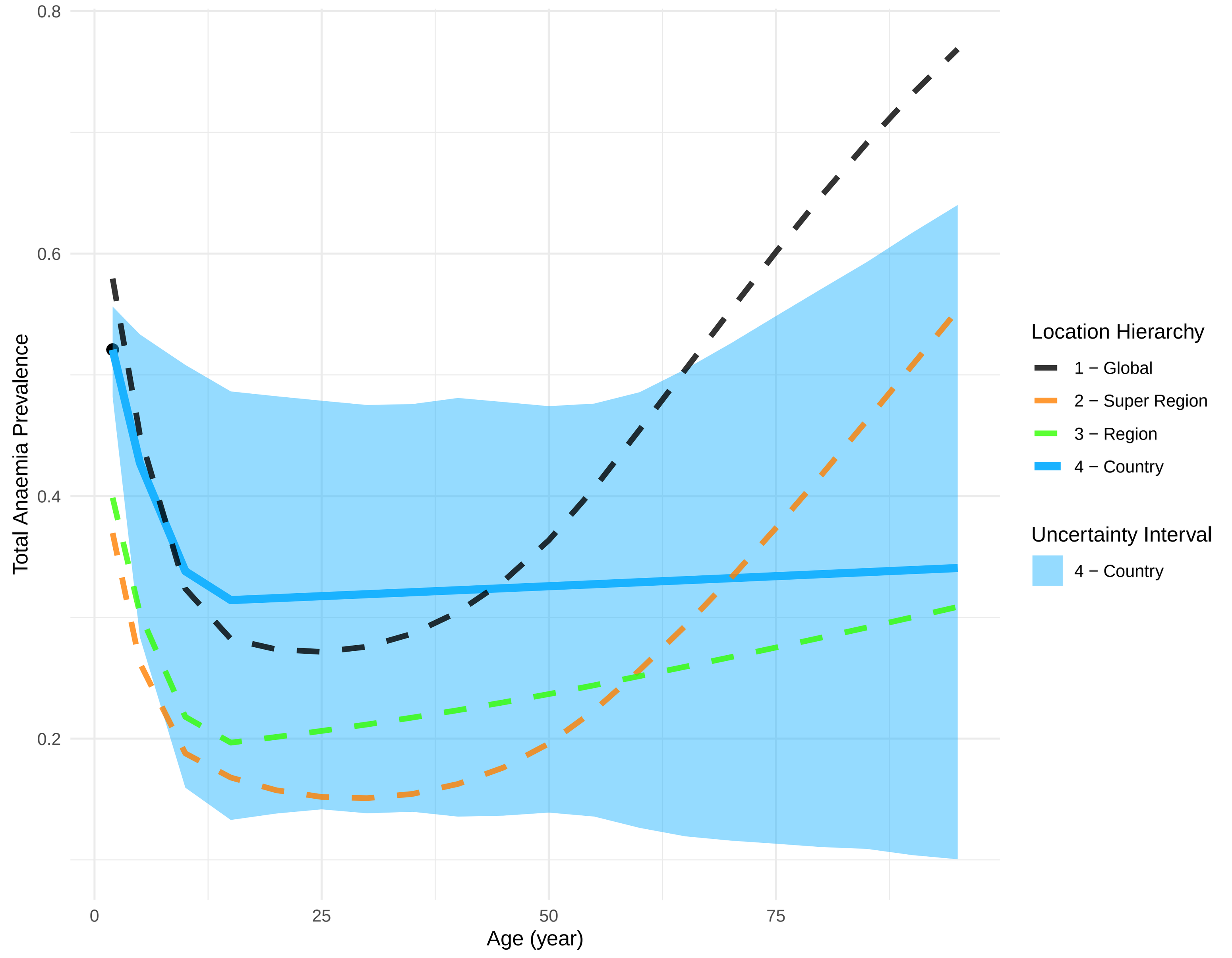

MR−BRT Age/Sex Fits − Males 2+ Years Old − Total Anaemia Prevalence − Central Europe
WHO−2024 Adjustment Method

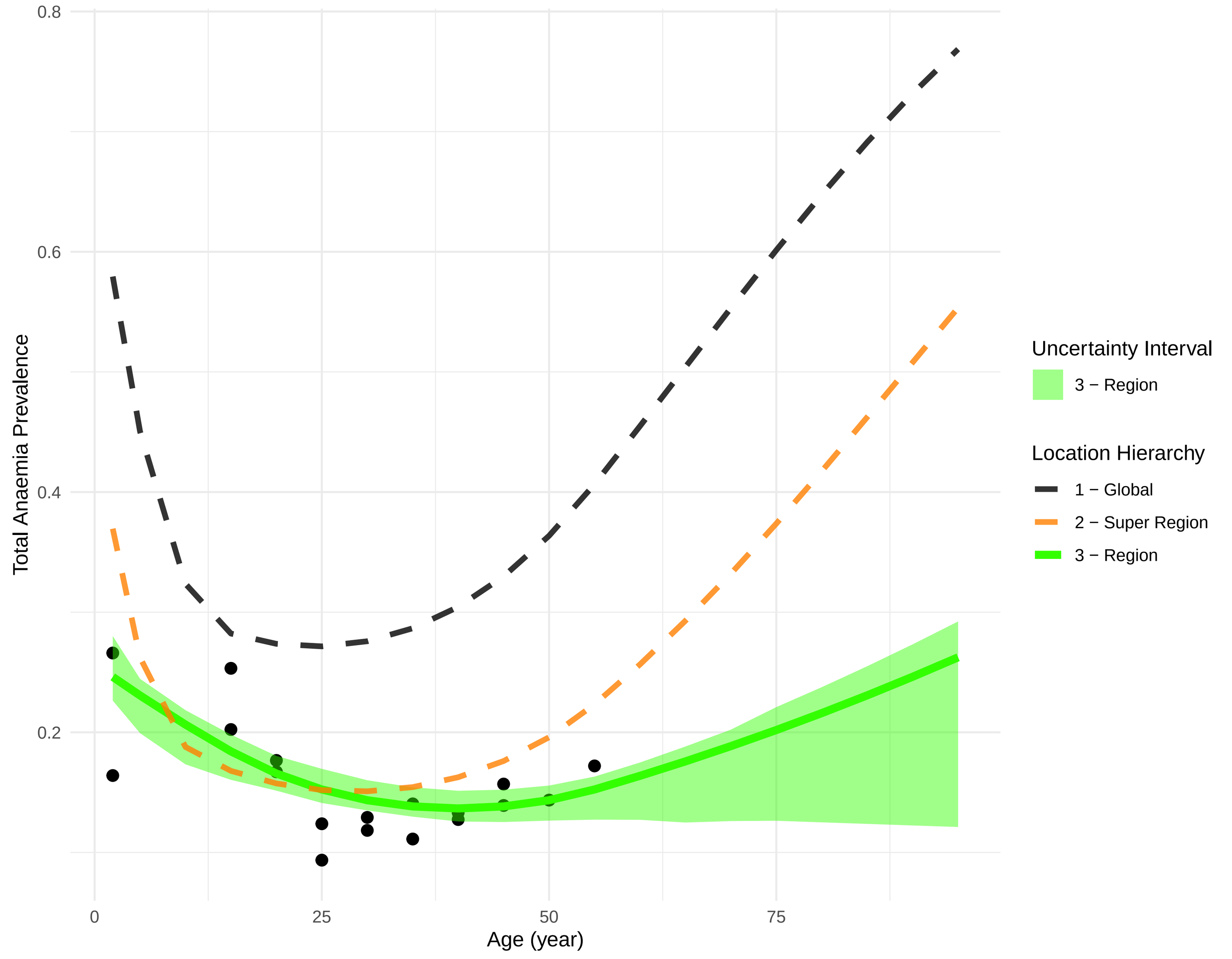

MR−BRT Age/Sex Fits − Males 2+ Years Old − Total Anaemia Prevalence − Albania
WHO−2024 Adjustment Method

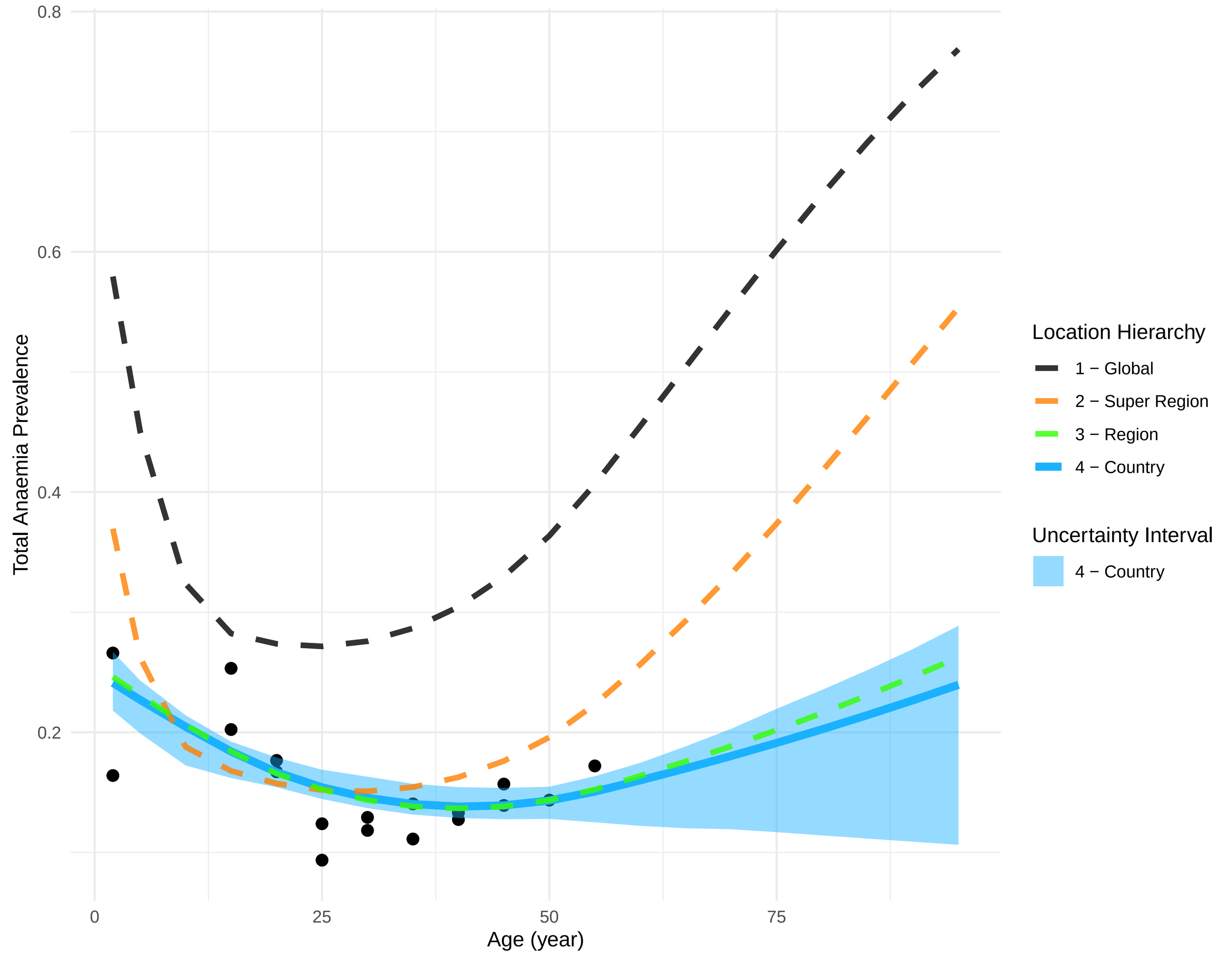

MR−BRT Age/Sex Fits − Males 2+ Years Old − Total Anaemia Prevalence − Eastern Europe
WHO−2024 Adjustment Method

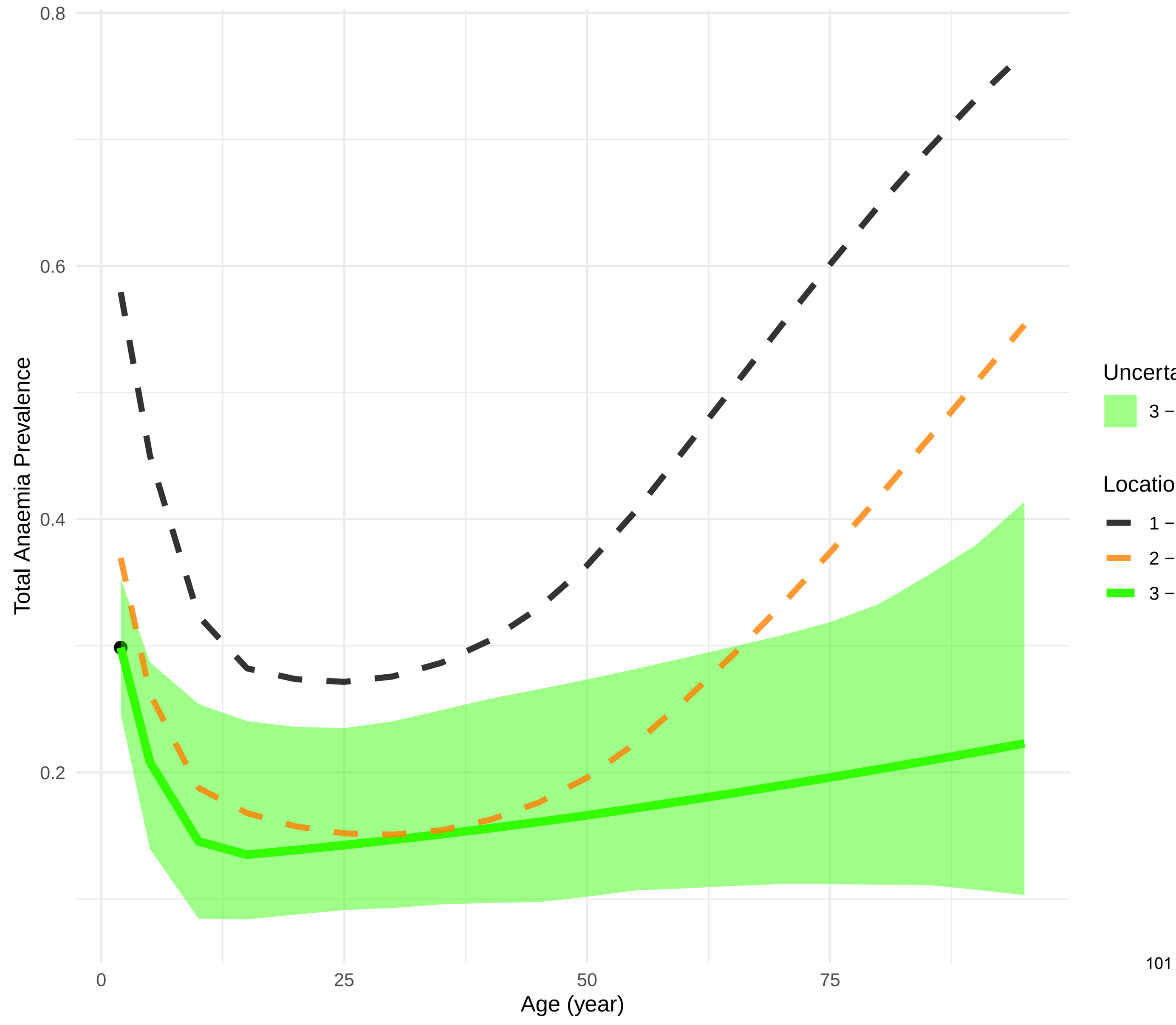

MR−BRT Age/Sex Fits − Males 2+ Years Old − Total Anaemia Prevalence − Republic of moldova
WHO−2024 Adjustment Method

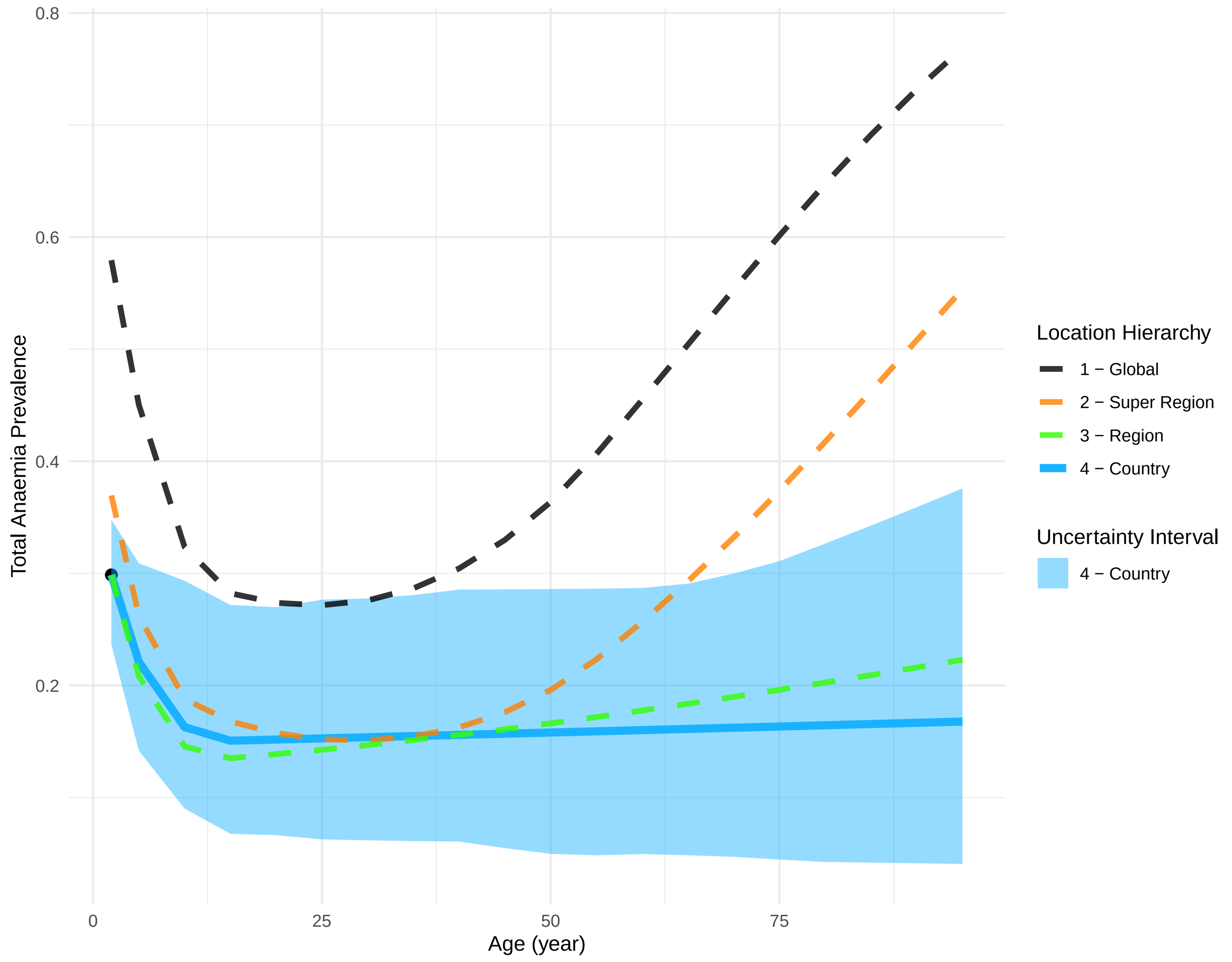

MR−BRT Age/Sex Fits − Males 2+ Years Old − Total Anaemia Prevalence − High−income
WHO−2024 Adjustment Method

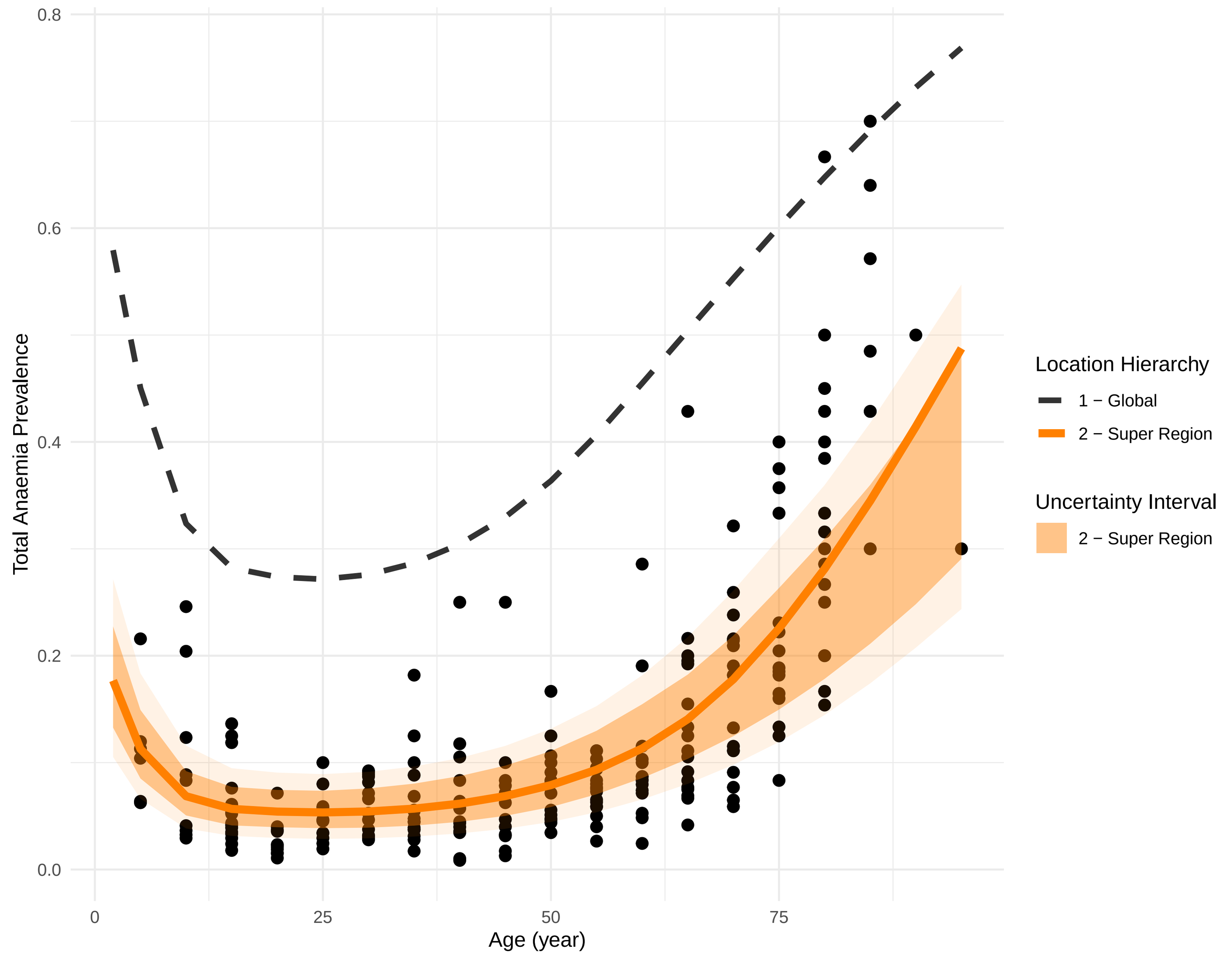

MR−BRT Age/Sex Fits − Males 2+ Years Old − Total Anaemia Prevalence − Western Europe
WHO−2024 Adjustment Method

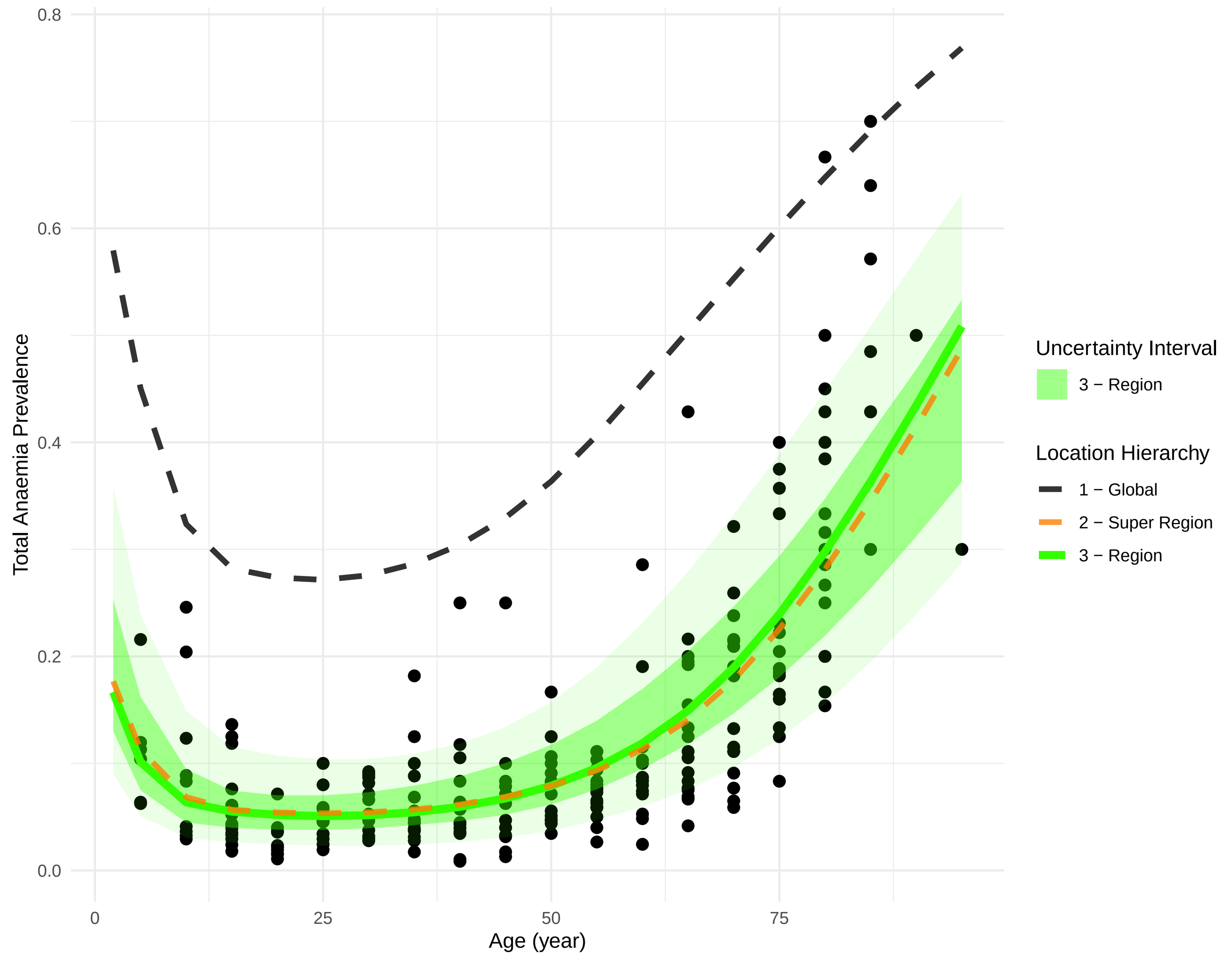

MR−BRT Age/Sex Fits − Males 2+ Years Old − Total Anaemia Prevalence − United Kingdom
WHO−2024 Adjustment Method

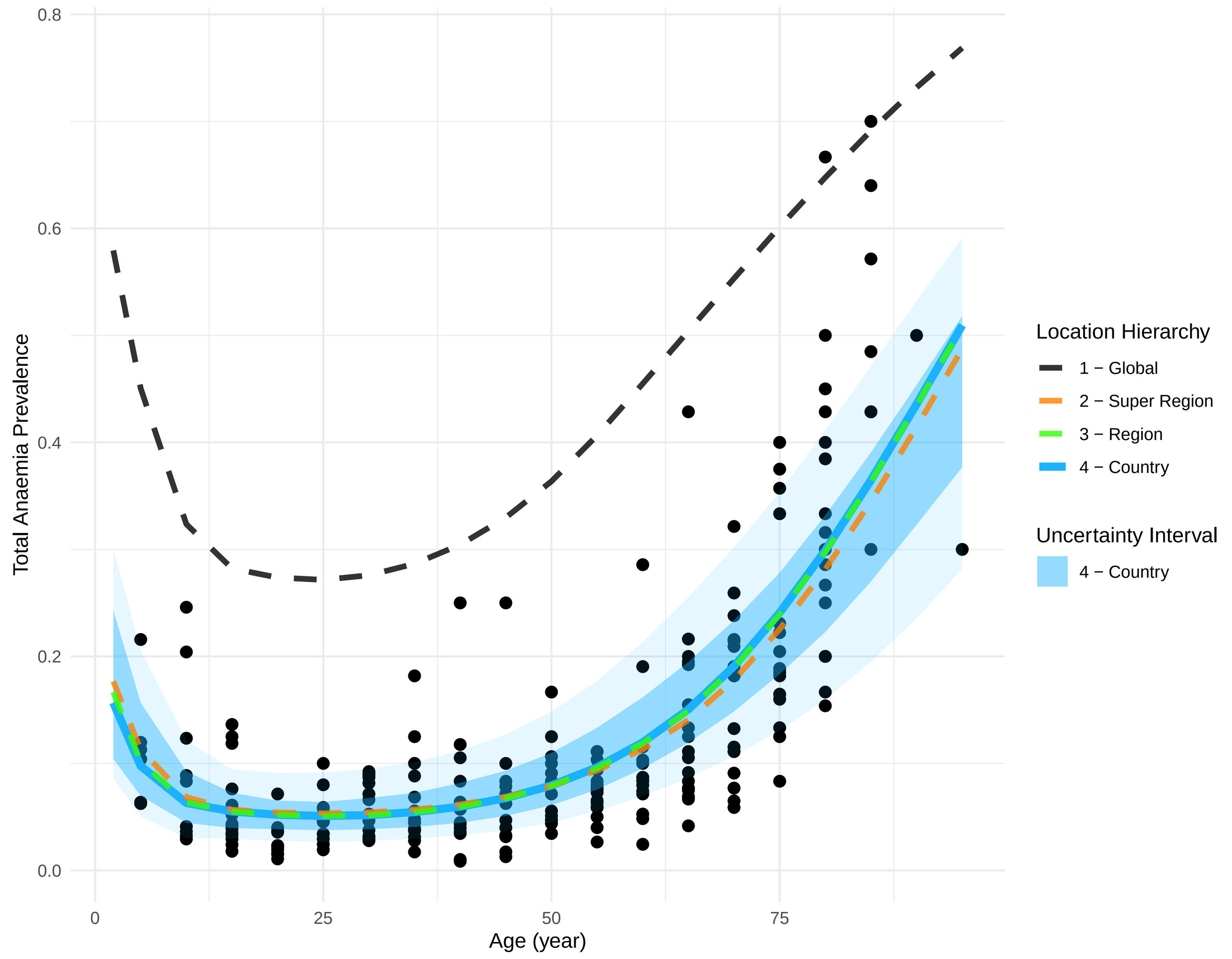

MR−BRT Age/Sex Fits − Males 2+ Years Old − Total Anaemia Prevalence − England
WHO−2024 Adjustment Method

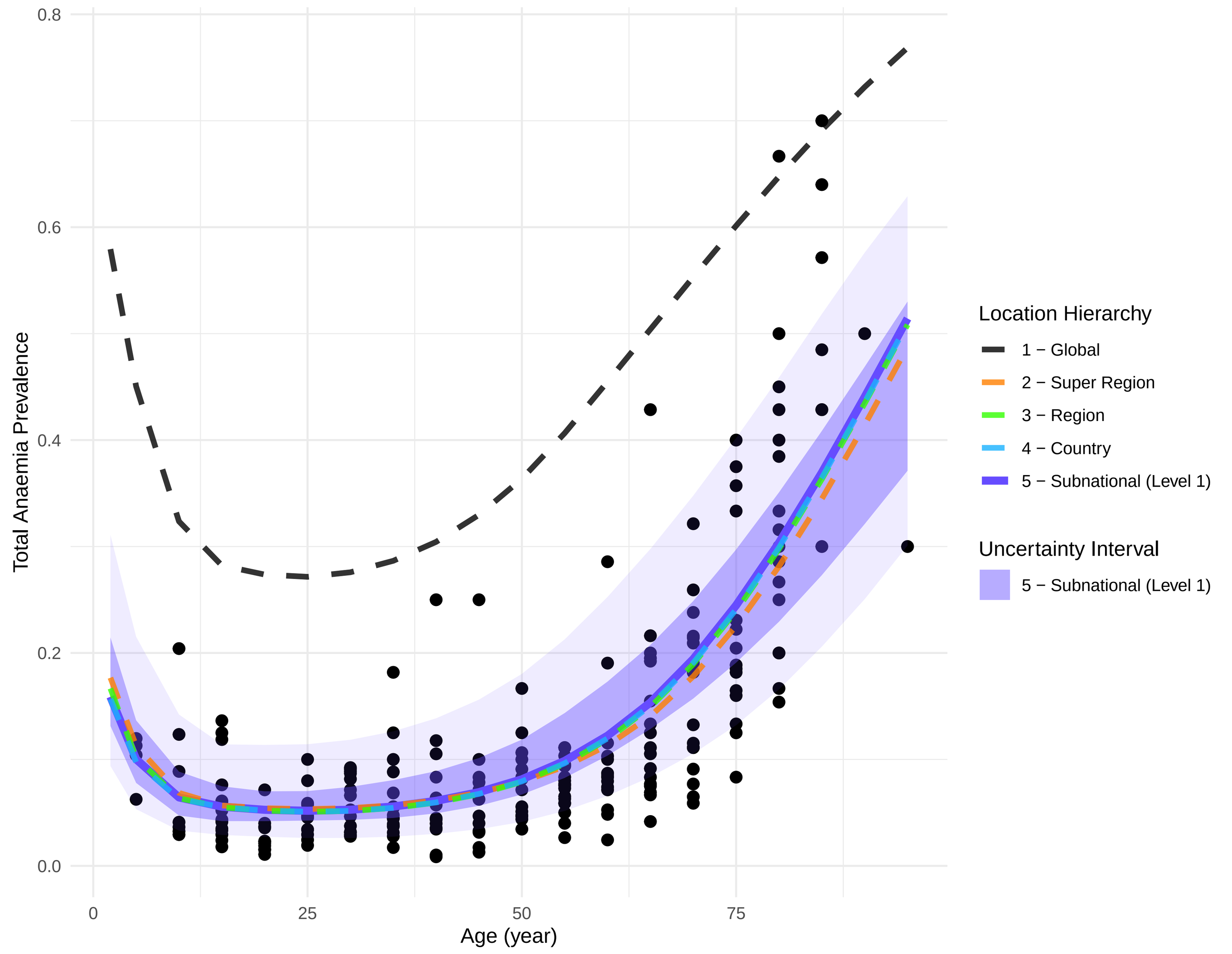

MR−BRT Age/Sex Fits − Males 2+ Years Old − Total Anaemia Prevalence − East Midlands
WHO−2024 Adjustment Method

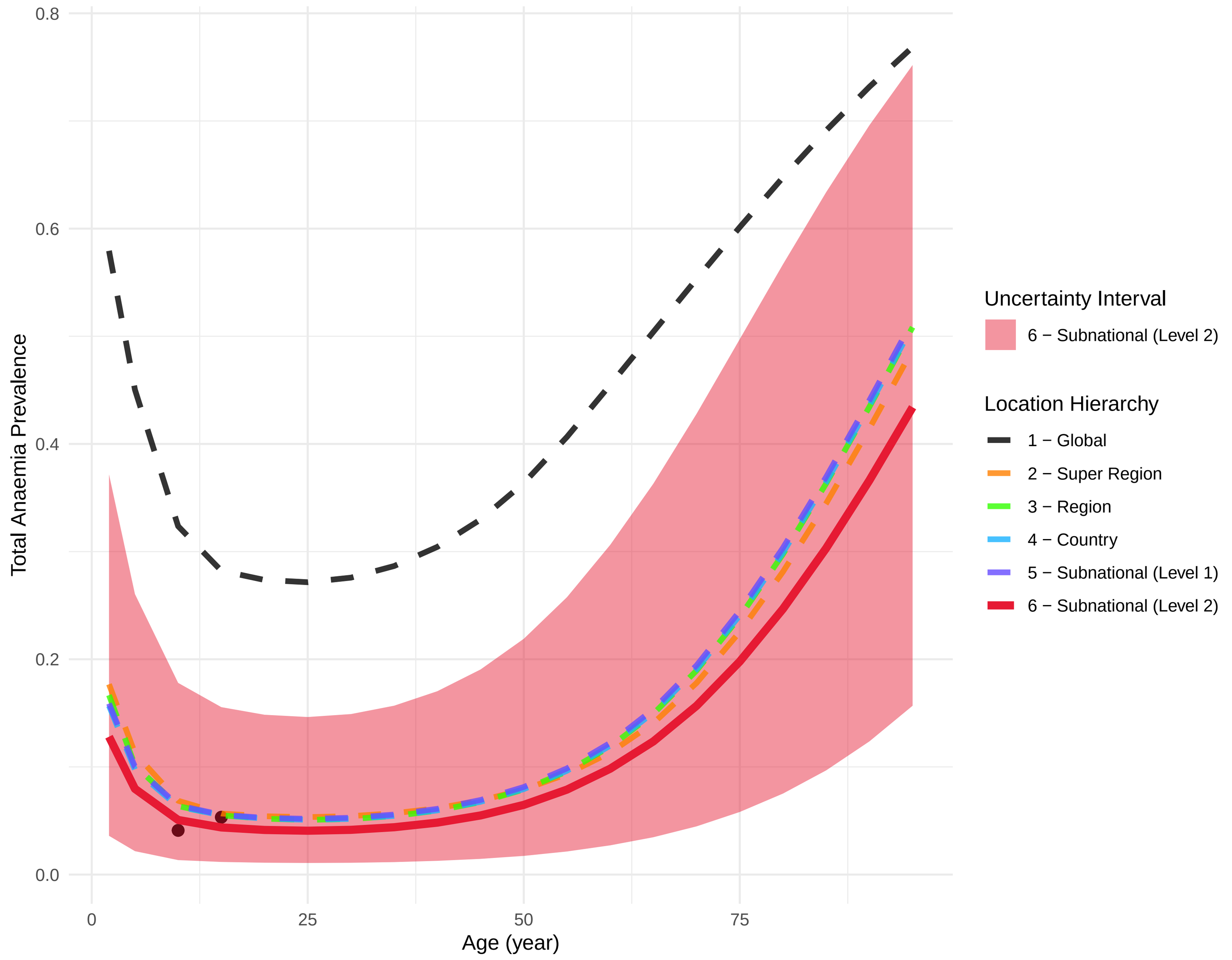

MR−BRT Age/Sex Fits − Males 2+ Years Old − Total Anaemia Prevalence − East of England
WHO−2024 Adjustment Method

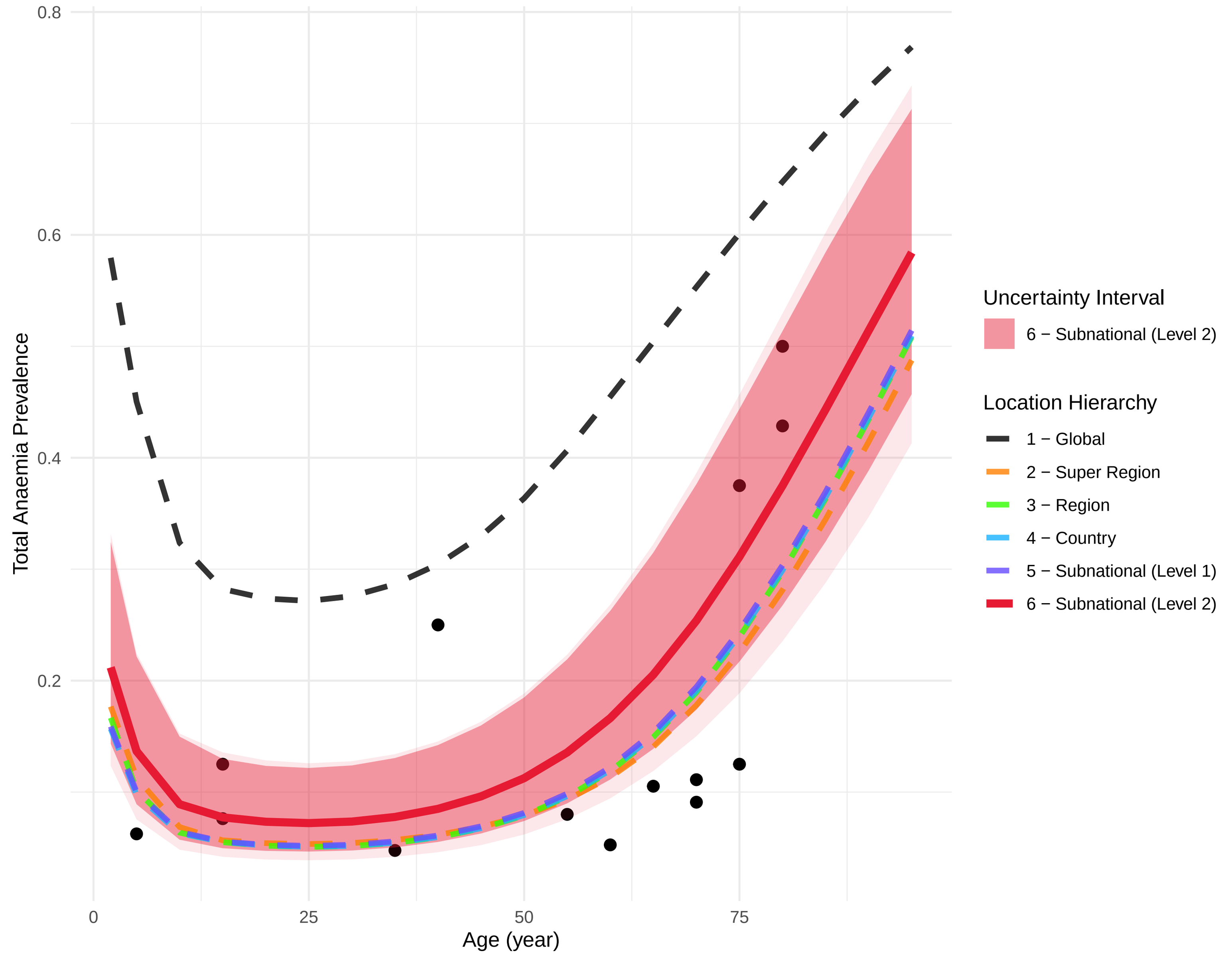

MR−BRT Age/Sex Fits − Males 2+ Years Old − Total Anaemia Prevalence − Greater London
WHO−2024 Adjustment Method

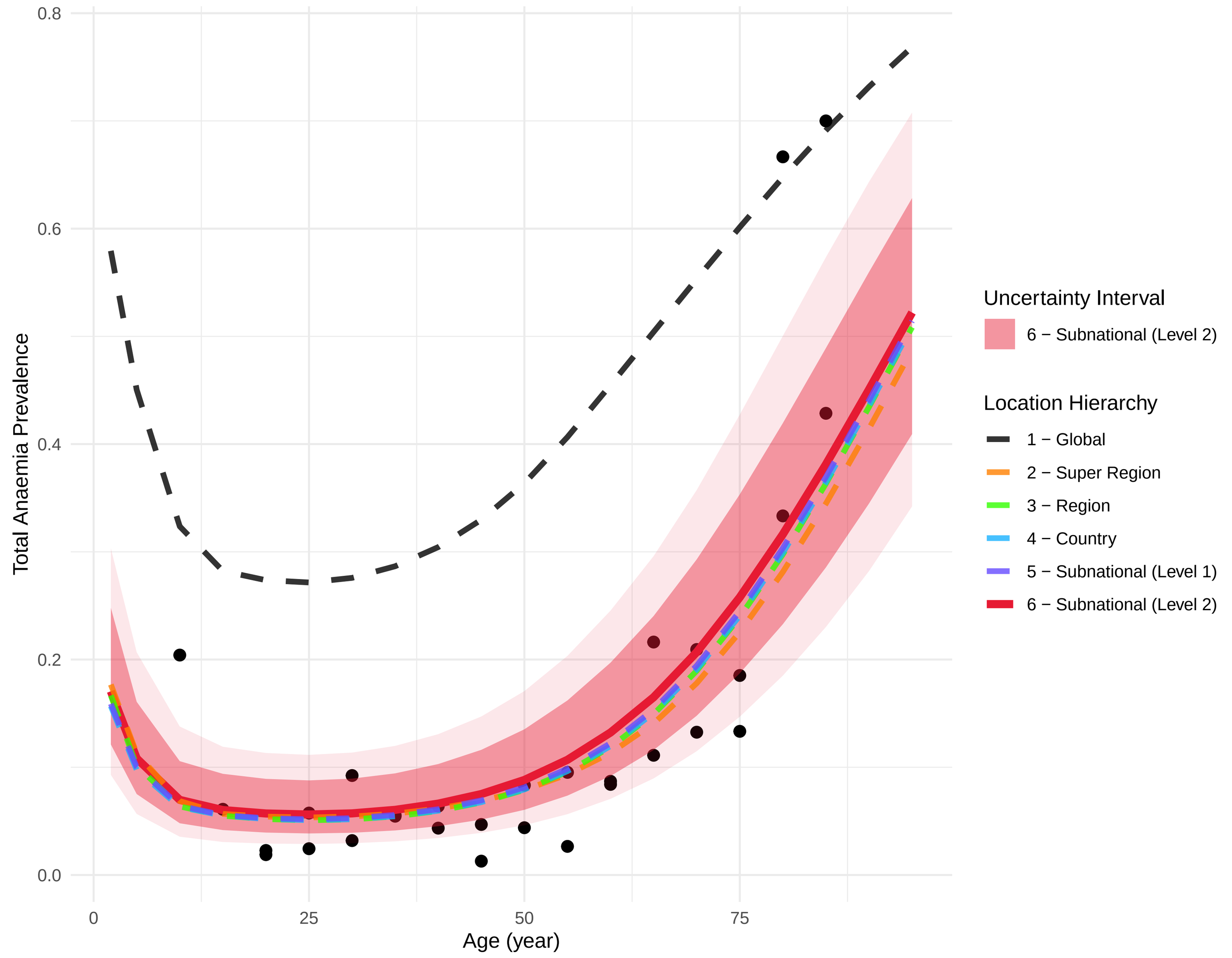

MR−BRT Age/Sex Fits − Males 2+ Years Old − Total Anaemia Prevalence − North West England
WHO−2024 Adjustment Method

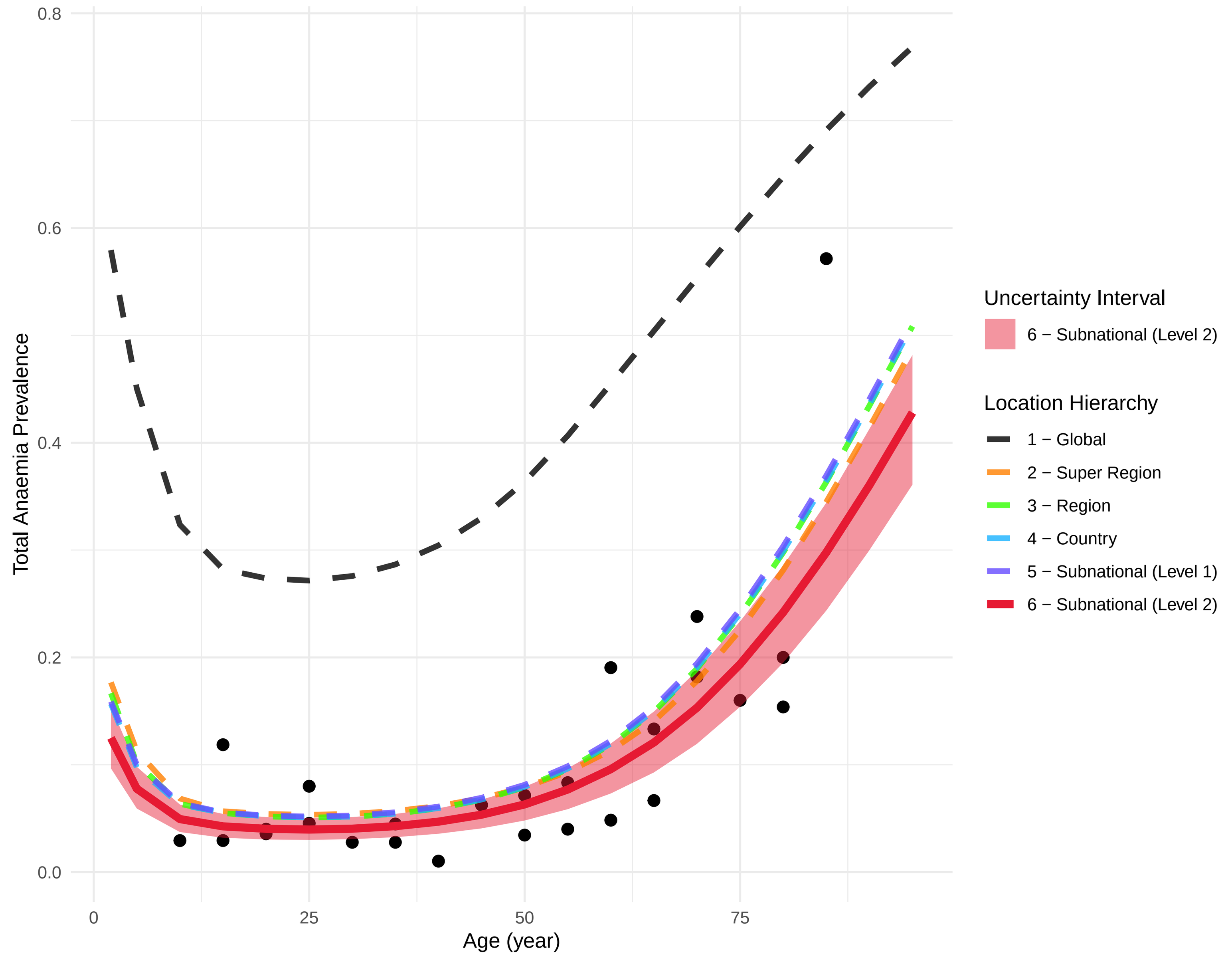

MR−BRT Age/Sex Fits − Males 2+ Years Old − Total Anaemia Prevalence − South East England
WHO−2024 Adjustment Method

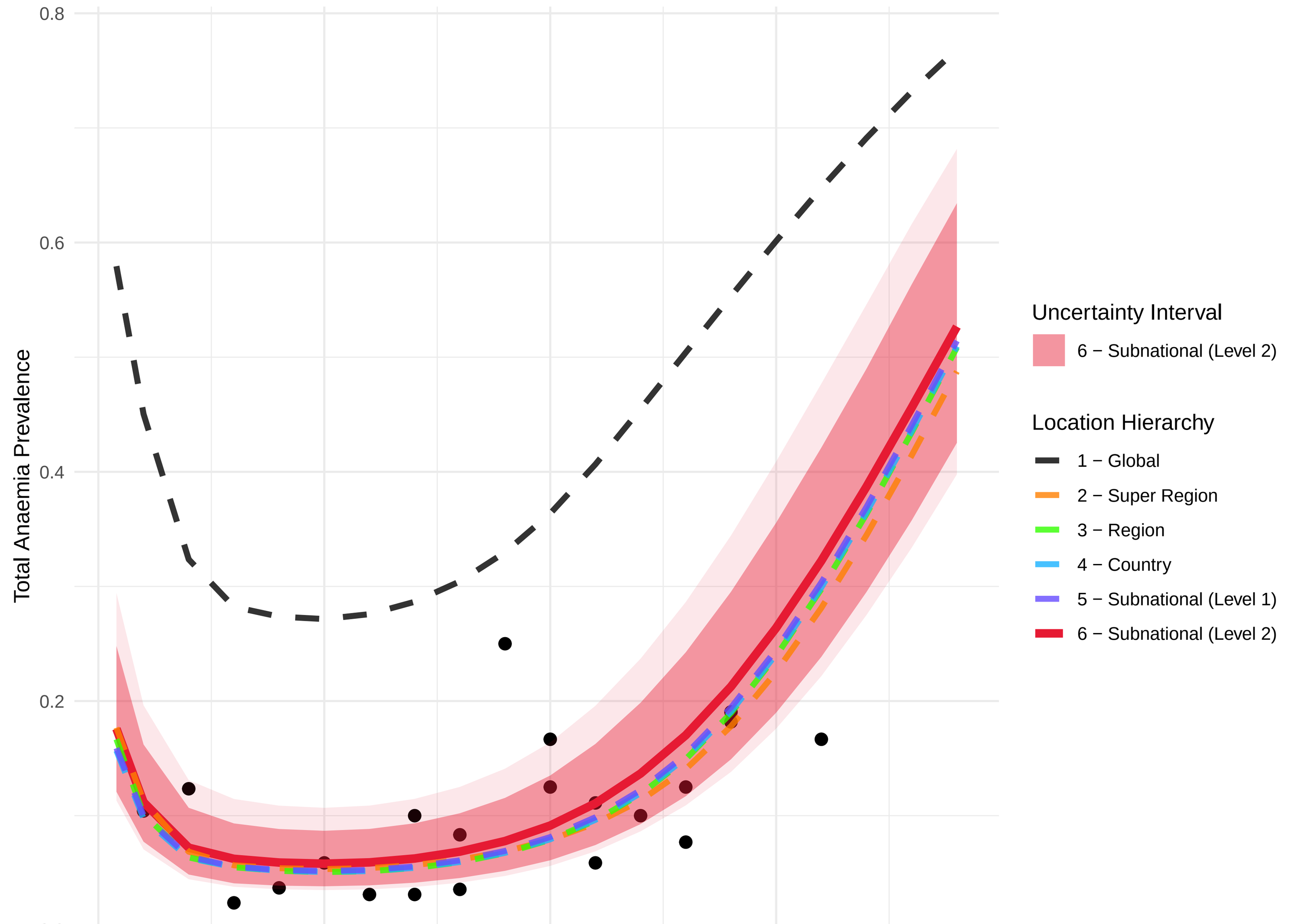

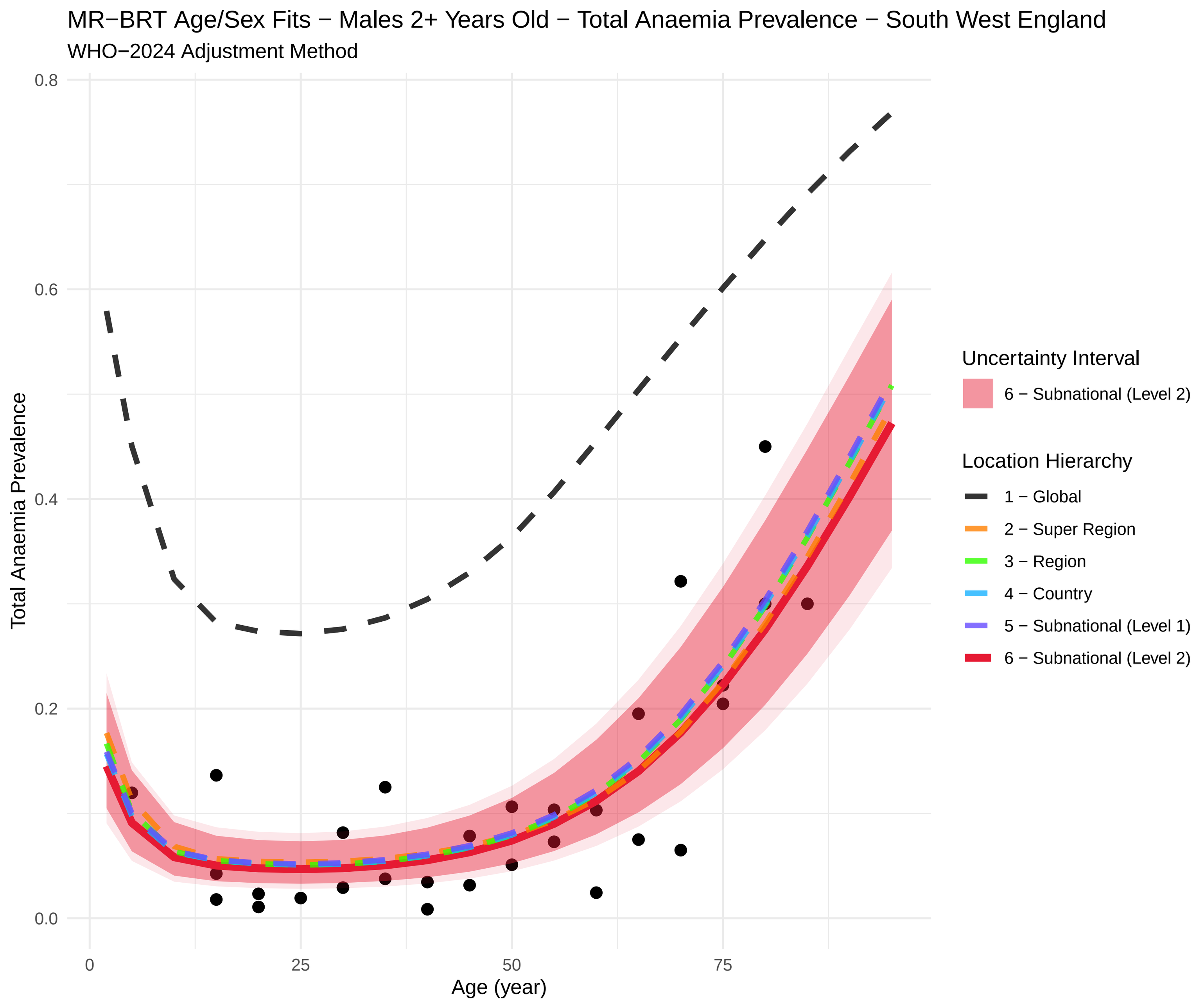
MR−BRT Age/Sex Fits − Males 2+ Years Old − Total Anaemia Prevalence − South West England
WHO−2024 Adjustment Method
Total Anaemia Prevalence
Age (year)
Uncertainty Interval
6 − Subnational (Level 2)
Location Hierarchy
1 − Global
2 − Super Region
3 − Region
4 − Country
5 − Subnational (Level 1)
6 − Subnational (Level 2)

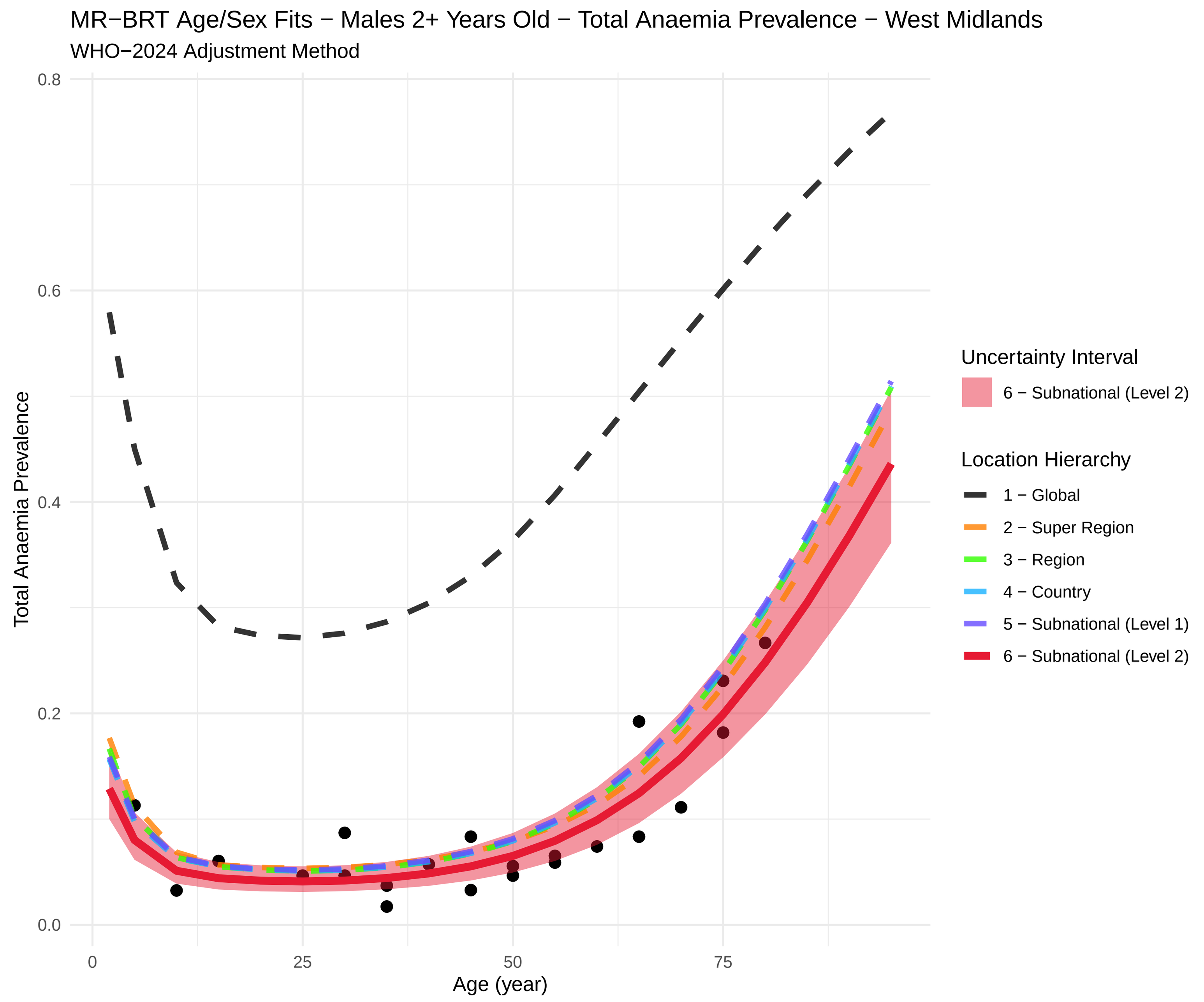
MR−BRT Age/Sex Fits − Males 2+ Years Old − Total Anaemia Prevalence − West Midlands
WHO−2024 Adjustment Method
Total Anaemia Prevalence
Age (year)
Uncertainty Interval
6 − Subnational (Level 2)
Location Hierarchy
1 − Global
2 − Super Region
3 − Region
4 − Country
5 − Subnational (Level 1)
6 − Subnational (Level 2)

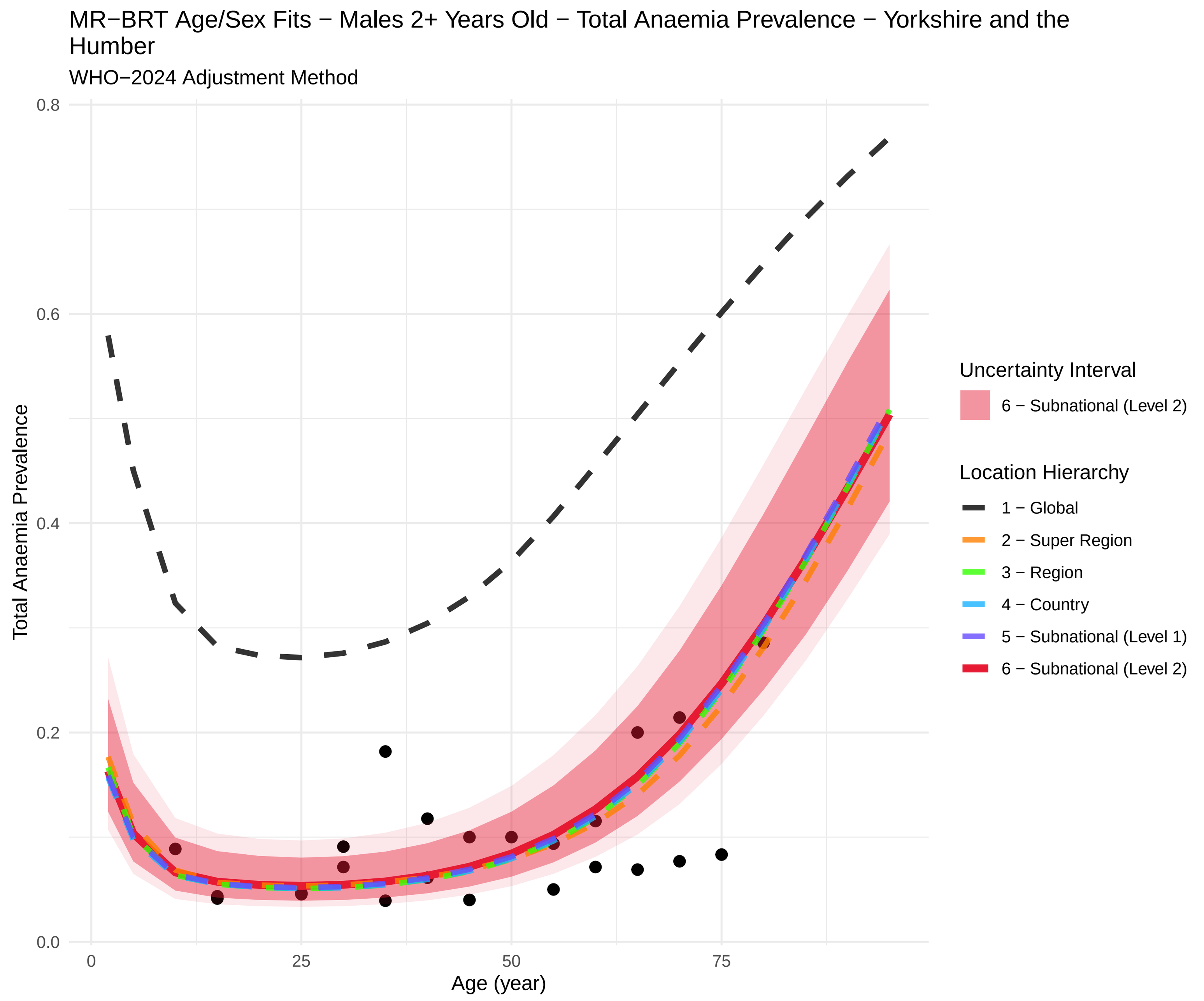
MR−BRT Age/Sex Fits − Males 2+ Years Old − Total Anaemia Prevalence − Yorkshire and the Humber
WHO−2024 Adjustment Method
Total Anaemia Prevalence
Age (year)
Uncertainty Interval
6 − Subnational (Level 2)
Location Hierarchy
1 − Global
2 − Super Region
3 − Region
4 − Country
5 − Subnational (Level 1)
6 − Subnational (Level 2)

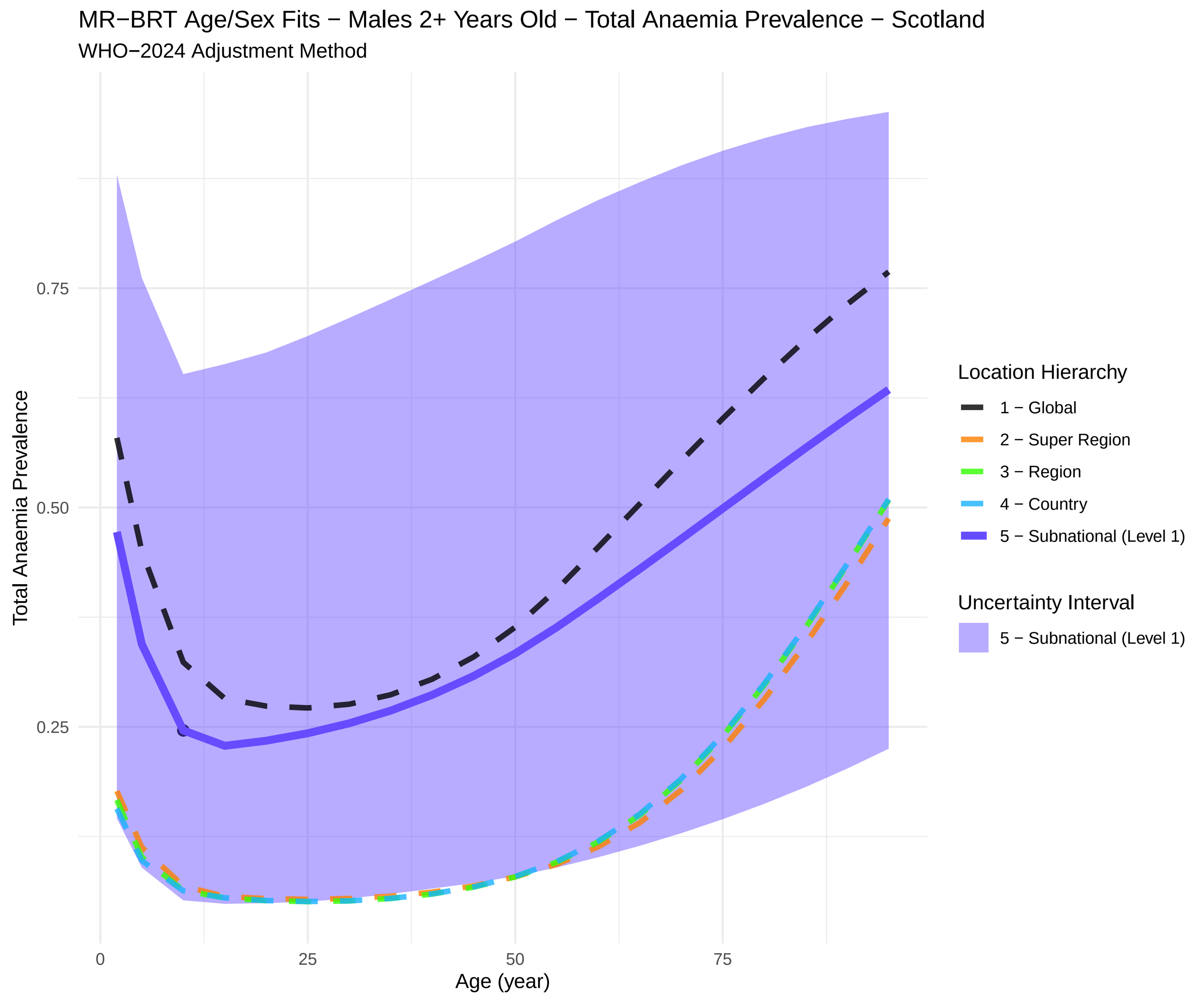
MR−BRT Age/Sex Fits − Males 2+ Years Old − Total Anaemia Prevalence − Scotland
WHO−2024 Adjustment Method
Total Anaemia Prevalence
Age (year)
Location Hierarchy
1 − Global
2 − Super Region
3 − Region
4 − Country
5 − Subnational (Level 1)
Uncertainty Interval
5 − Subnational (Level 1)

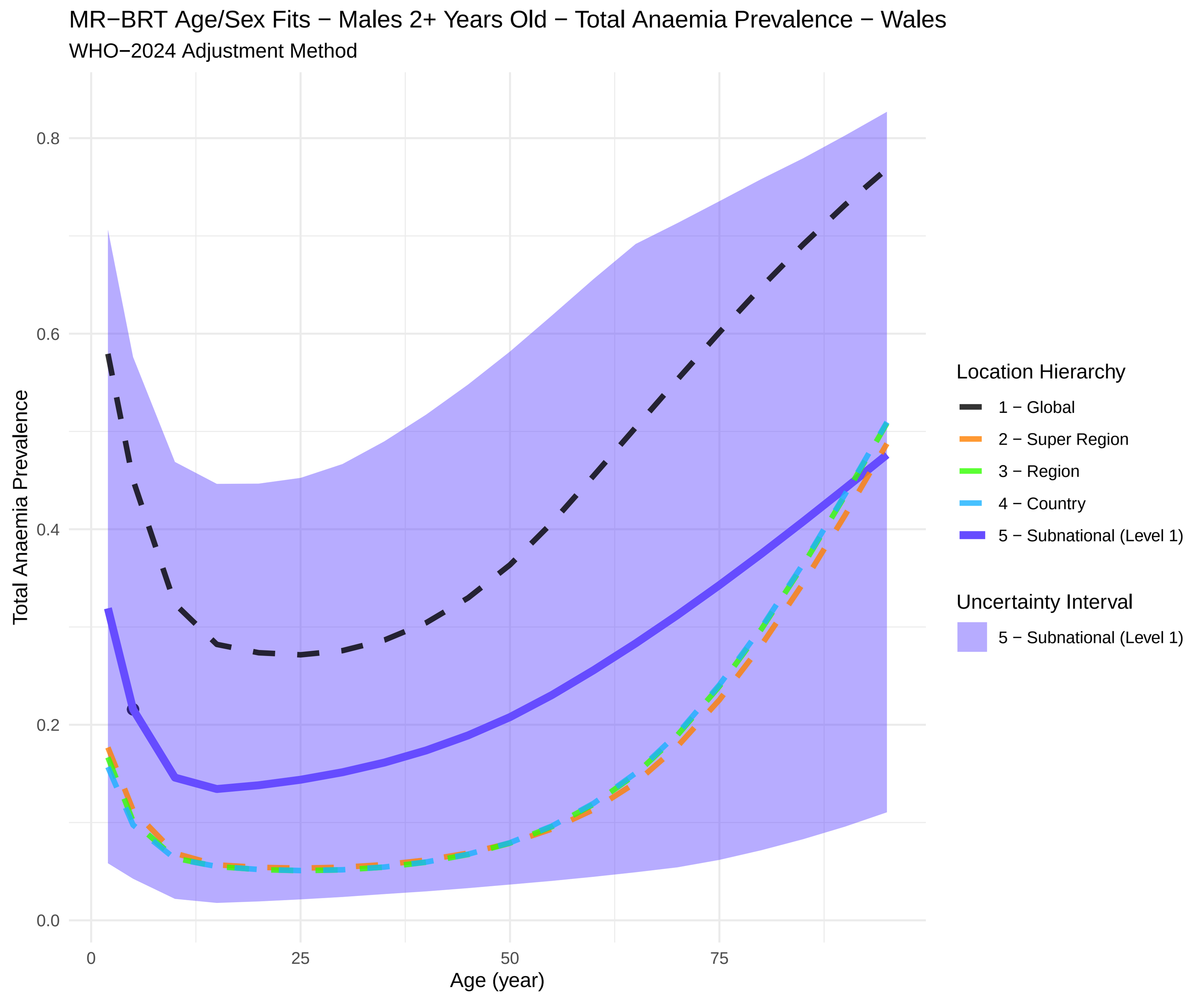
MR−BRT Age/Sex Fits − Males 2+ Years Old − Total Anaemia Prevalence − Wales
WHO−2024 Adjustment Method
Total Anaemia Prevalence
Age (year)
Location Hierarchy
1 − Global
2 − Super Region
3 − Region
4 − Country
5 − Subnational (Level 1)
Uncertainty Interval
5 − Subnational (Level 1)

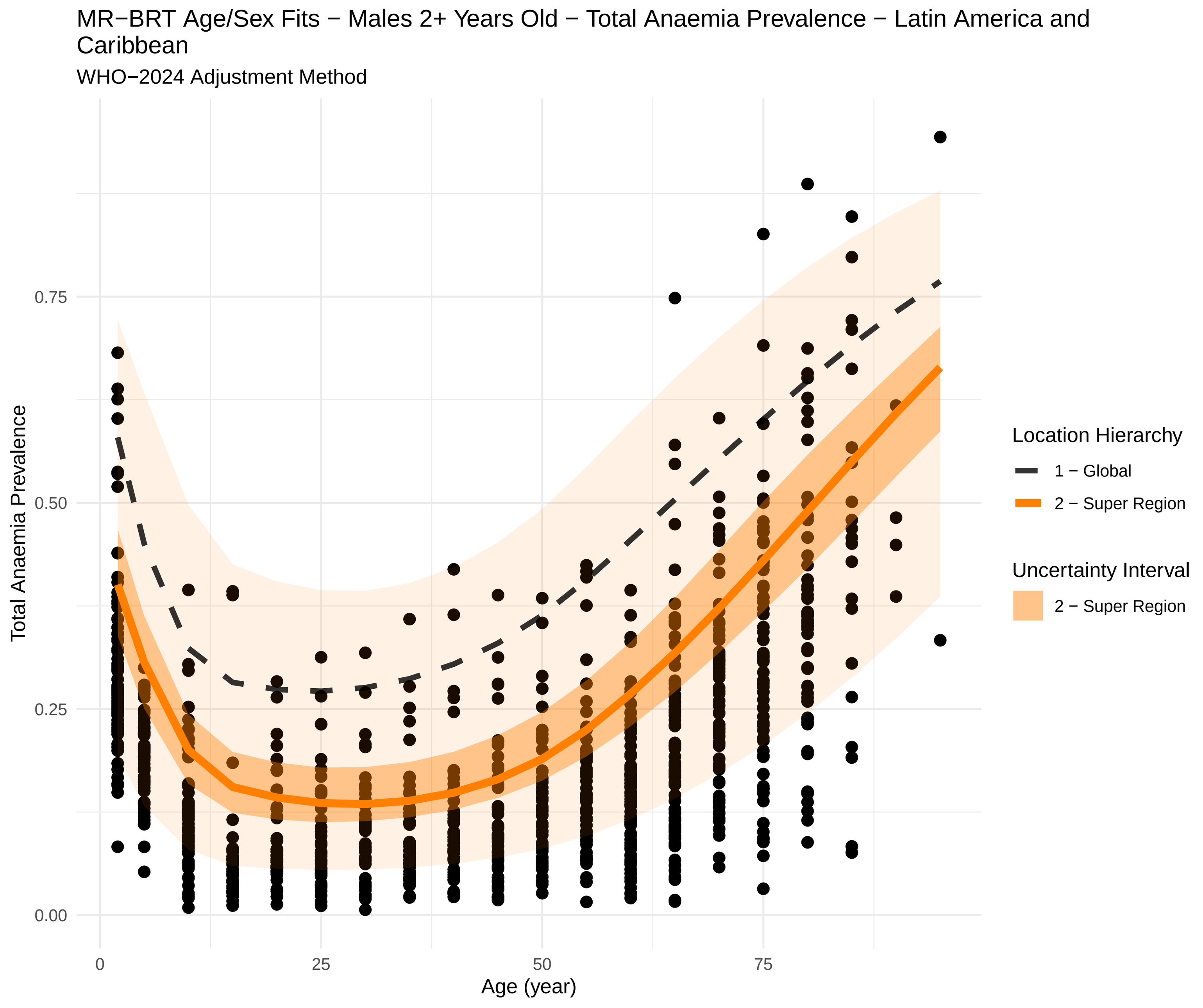
MR−BRT Age/Sex Fits − Males 2+ Years Old − Total Anaemia Prevalence − Latin America and Caribbean
WHO−2024 Adjustment Method
Total Anaemia Prevalence
Age (year)
Location Hierarchy
1 − Global
2 − Super Region
Uncertainty Interval
2 − Super Region

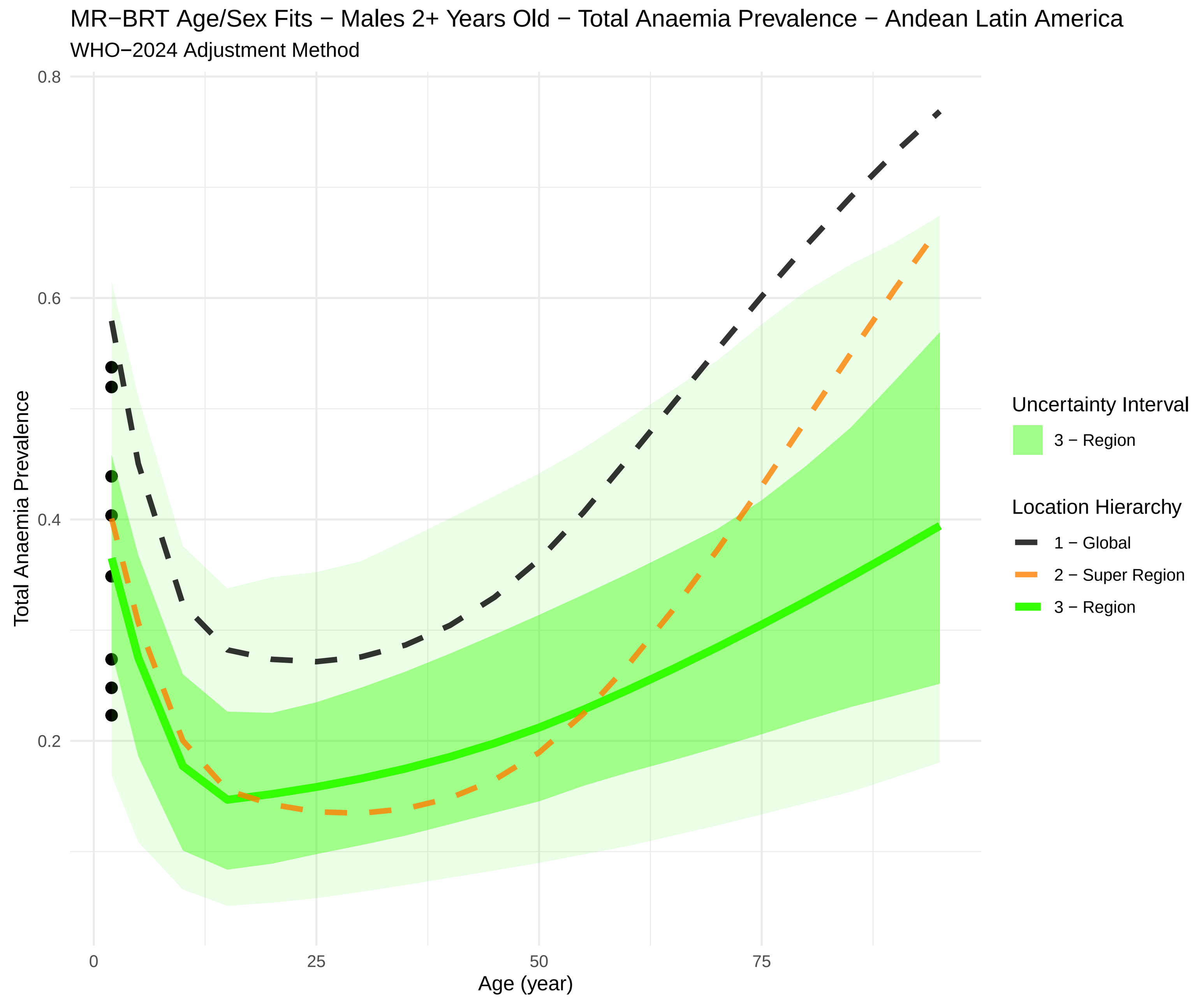
MR−BRT Age/Sex Fits − Males 2+ Years Old − Total Anaemia Prevalence − Andean Latin America
WHO−2024 Adjustment Method
Total Anaemia Prevalence
Age (year)
Uncertainty Interval
3 − Region
Location Hierarchy
1 − Global
2 − Super Region
3 − Region

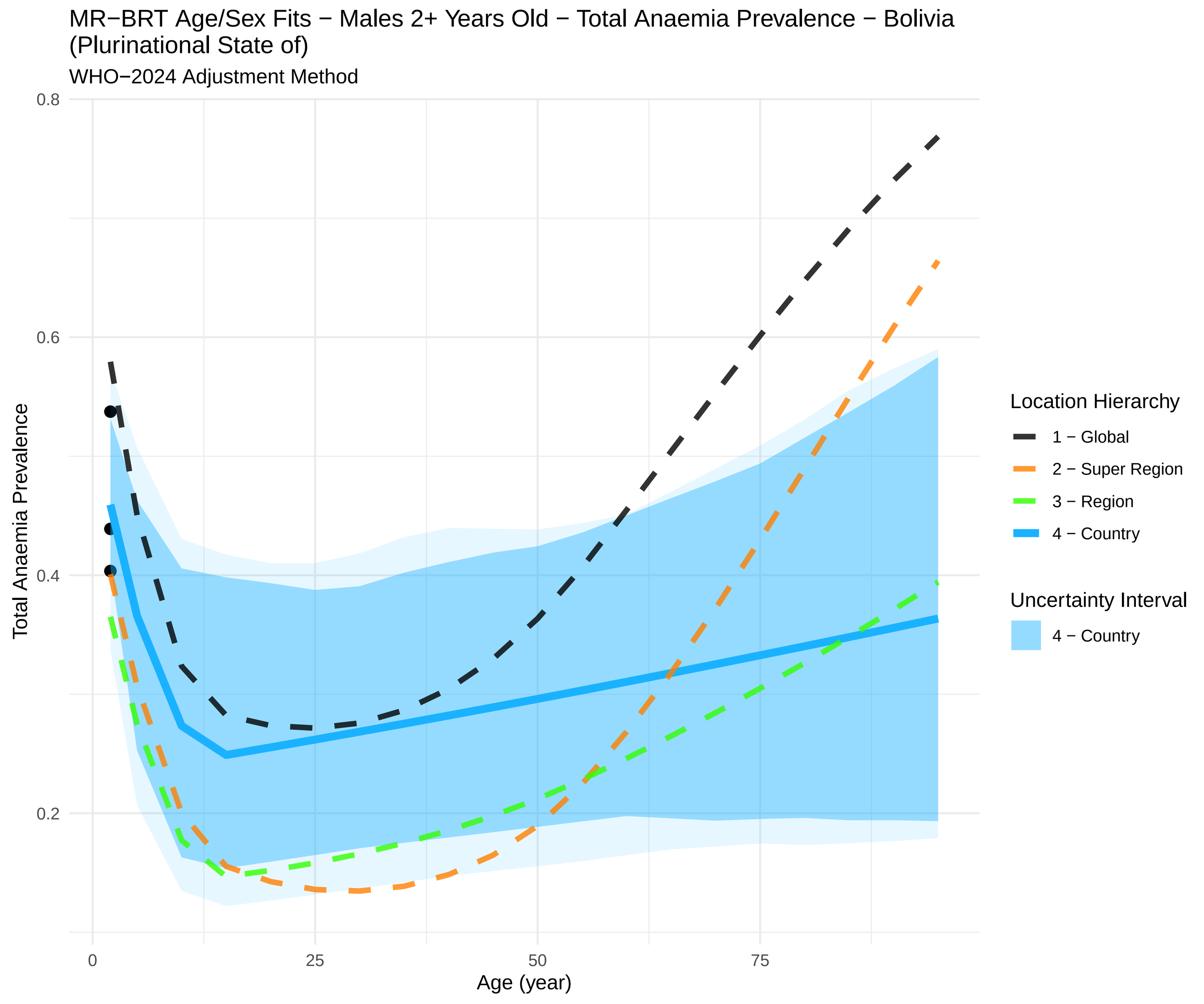
MR−BRT Age/Sex Fits − Males 2+ Years Old − Total Anaemia Prevalence − Bolivia (Plurinational State of)
WHO−2024 Adjustment Method
Total Anaemia Prevalence
Age (year)
Location Hierarchy
1 − Global
2 − Super Region
3 − Region
4 − Country
Uncertainty Interval
4 − Country

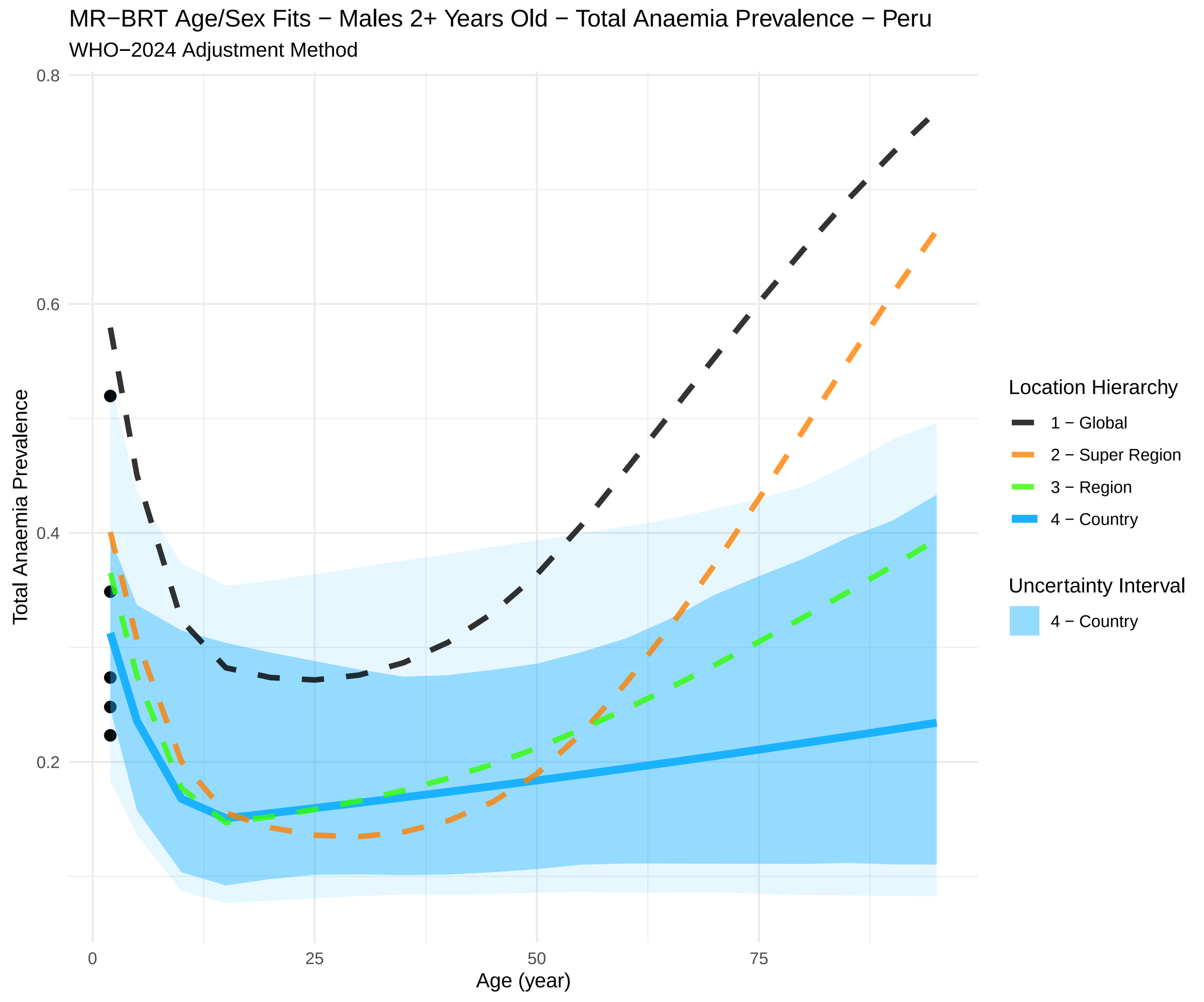
MR−BRT Age/Sex Fits − Males 2+ Years Old − Total Anaemia Prevalence − Peru
WHO−2024 Adjustment Method
Total Anaemia Prevalence
Age (year)
Location Hierarchy
1 − Global
2 − Super Region
3 − Region
4 − Country
Uncertainty Interval
4 − Country

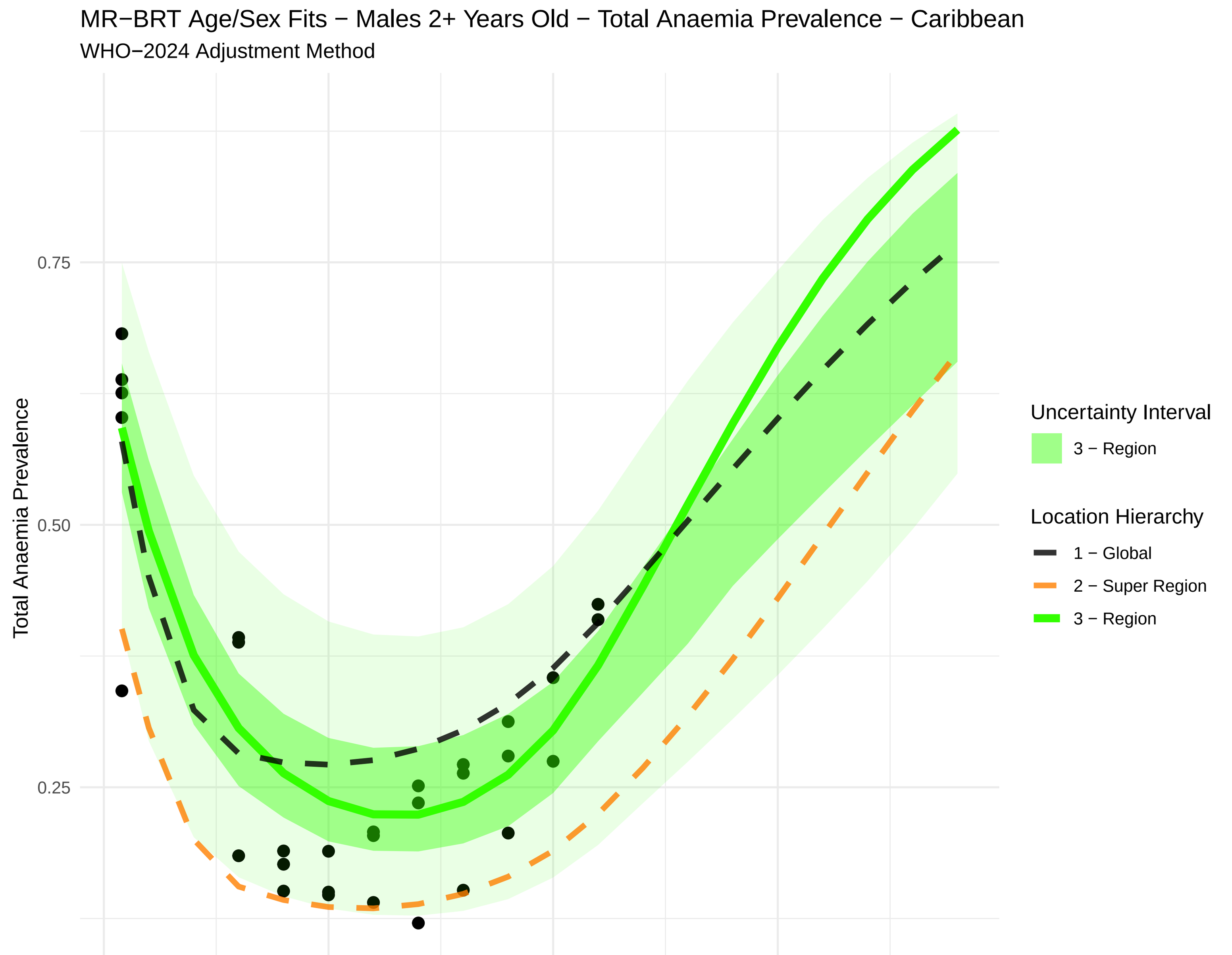
MR−BRT Age/Sex Fits − Males 2+ Years Old − Total Anaemia Prevalence − Caribbean
WHO−2024 Adjustment Method
Total Anaemia Prevalence
Age (year)
Uncertainty Interval
3 − Region
Location Hierarchy
1 − Global
2 − Super Region
3 − Region

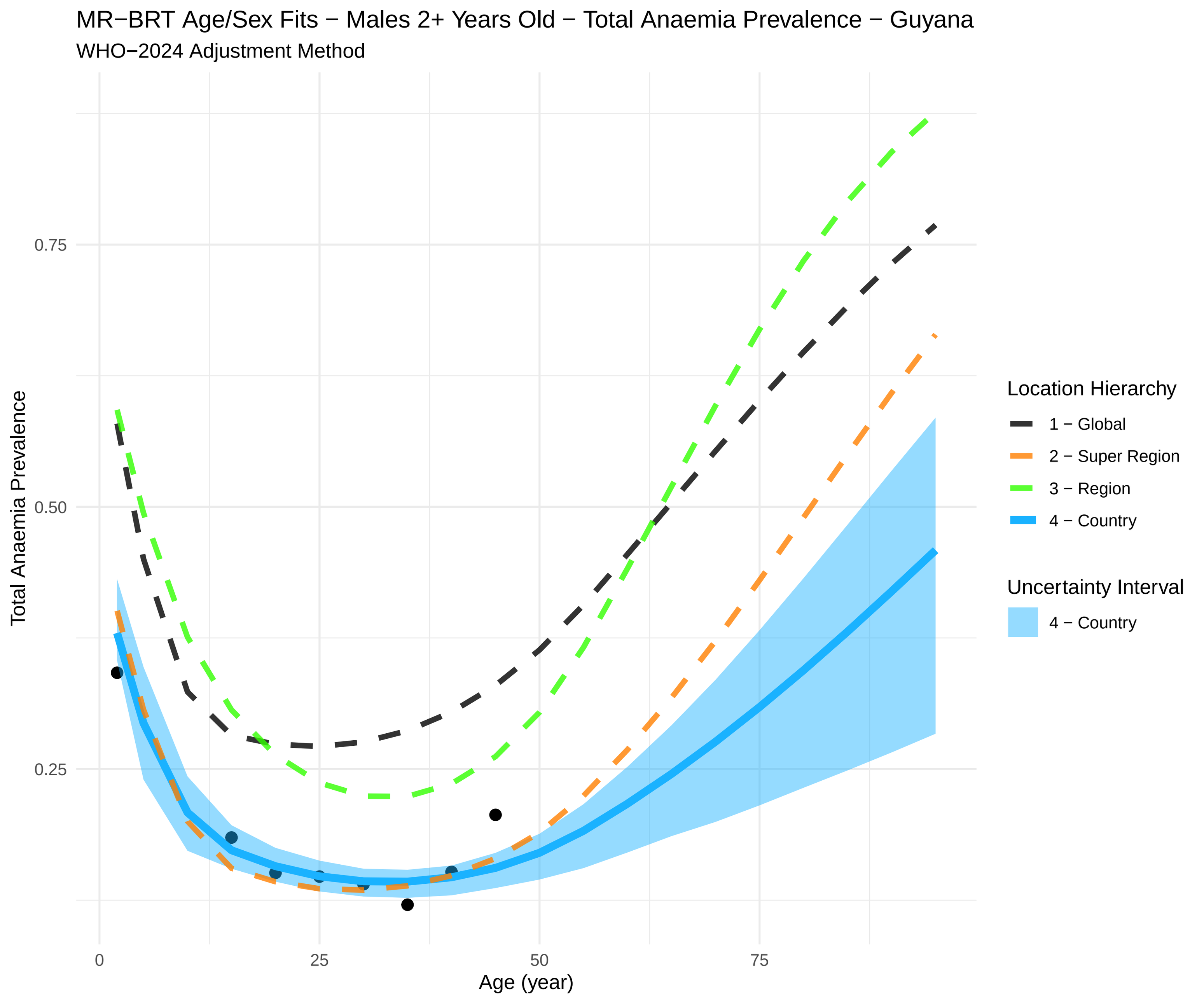
MR−BRT Age/Sex Fits − Males 2+ Years Old − Total Anaemia Prevalence − Guyana
WHO−2024 Adjustment Method
Total Anaemia Prevalence
Age (year)
Location Hierarchy
1 − Global
2 − Super Region
3 − Region
4 − Country
Uncertainty Interval
4 − Country

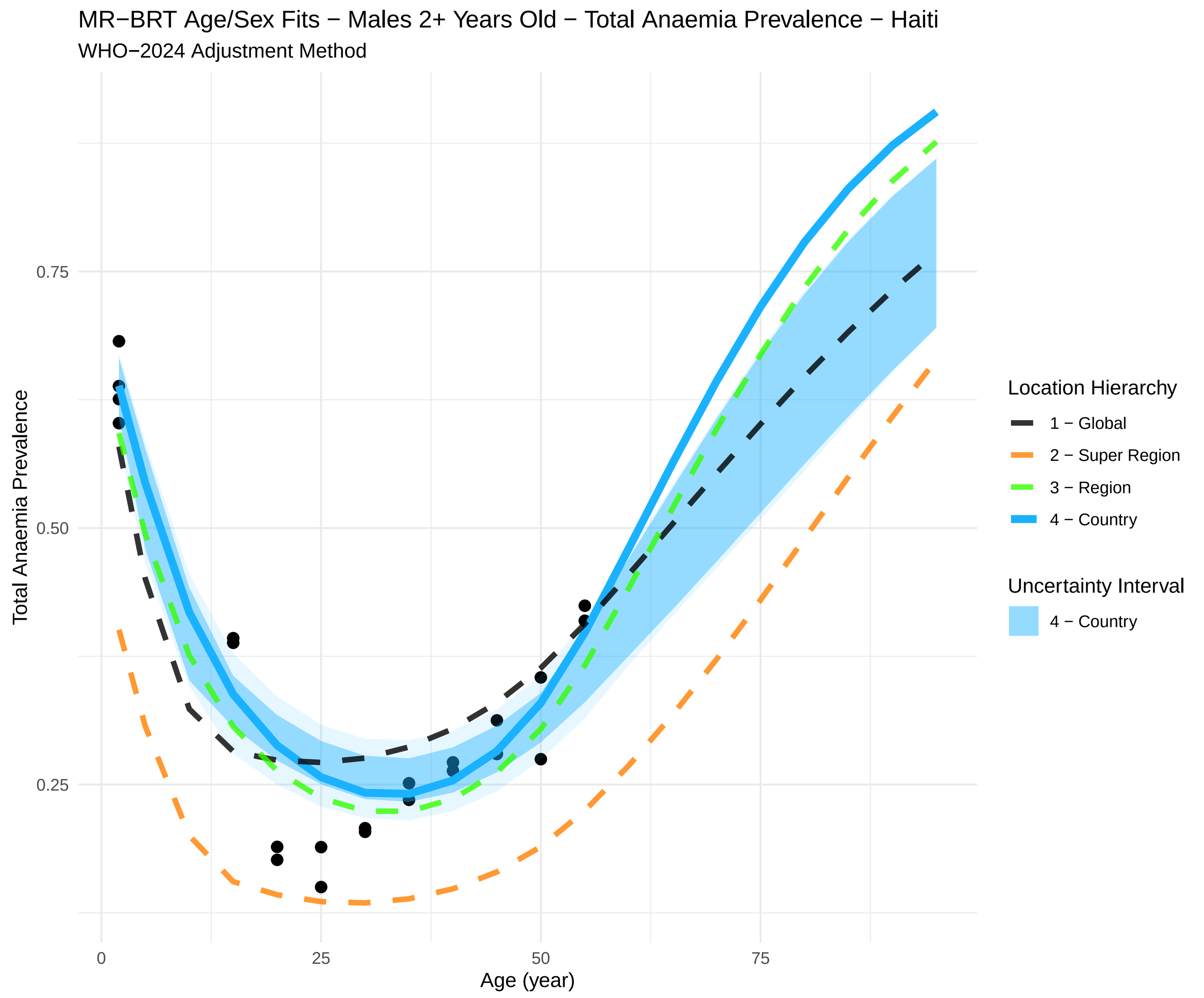
MR−BRT Age/Sex Fits − Males 2+ Years Old − Total Anaemia Prevalence − Haiti
WHO−2024 Adjustment Method
Total Anaemia Prevalence
Age (year)
Location Hierarchy
1 − Global
2 − Super Region
3 − Region
4 − Country
Uncertainty Interval
4 − Country

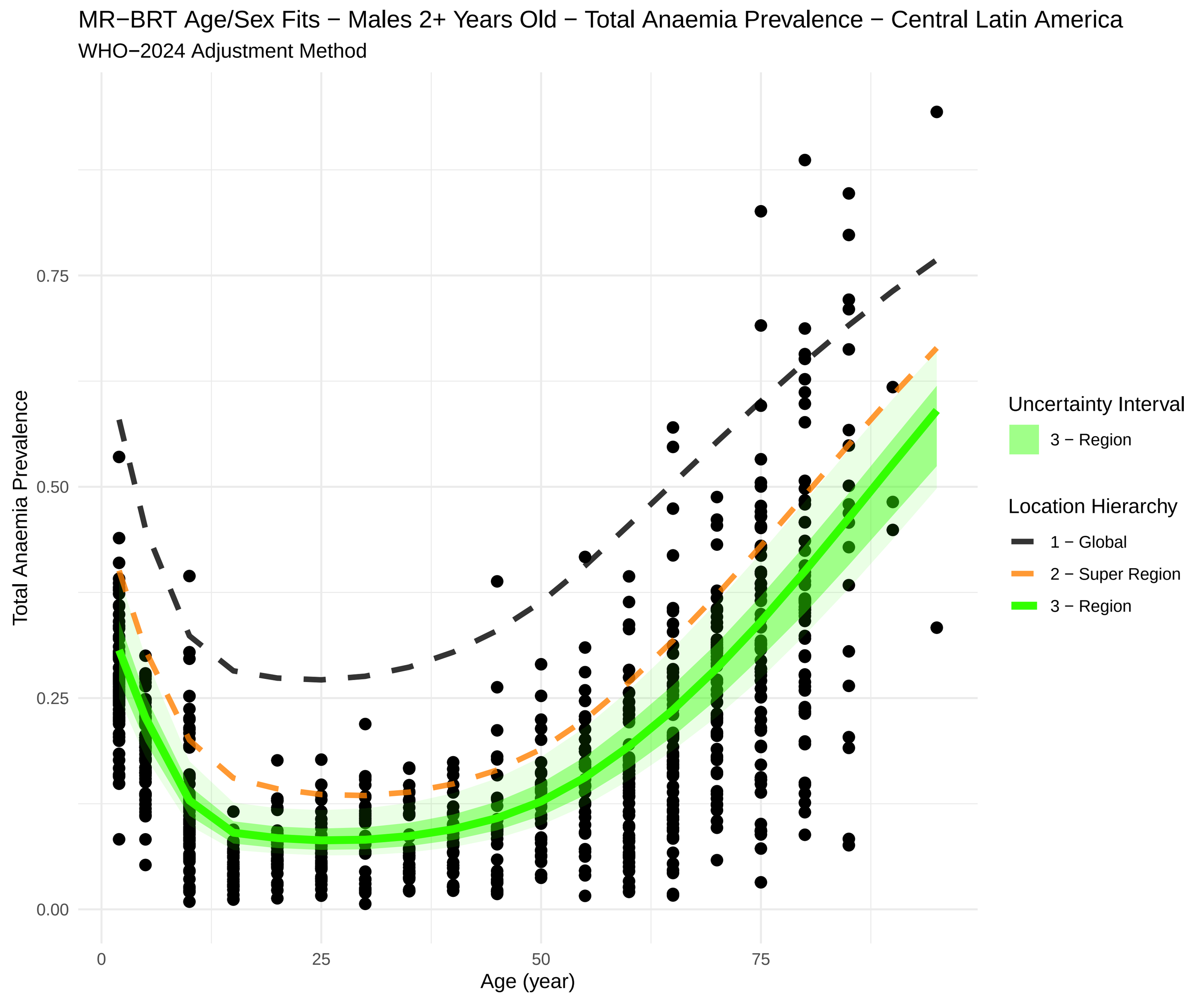
MR−BRT Age/Sex Fits − Males 2+ Years Old − Total Anaemia Prevalence − Central Latin America
WHO−2024 Adjustment Method
Total Anaemia Prevalence
Age (year)
Uncertainty Interval
3 − Region
Location Hierarchy
1 − Global
2 − Super Region
3 − Region

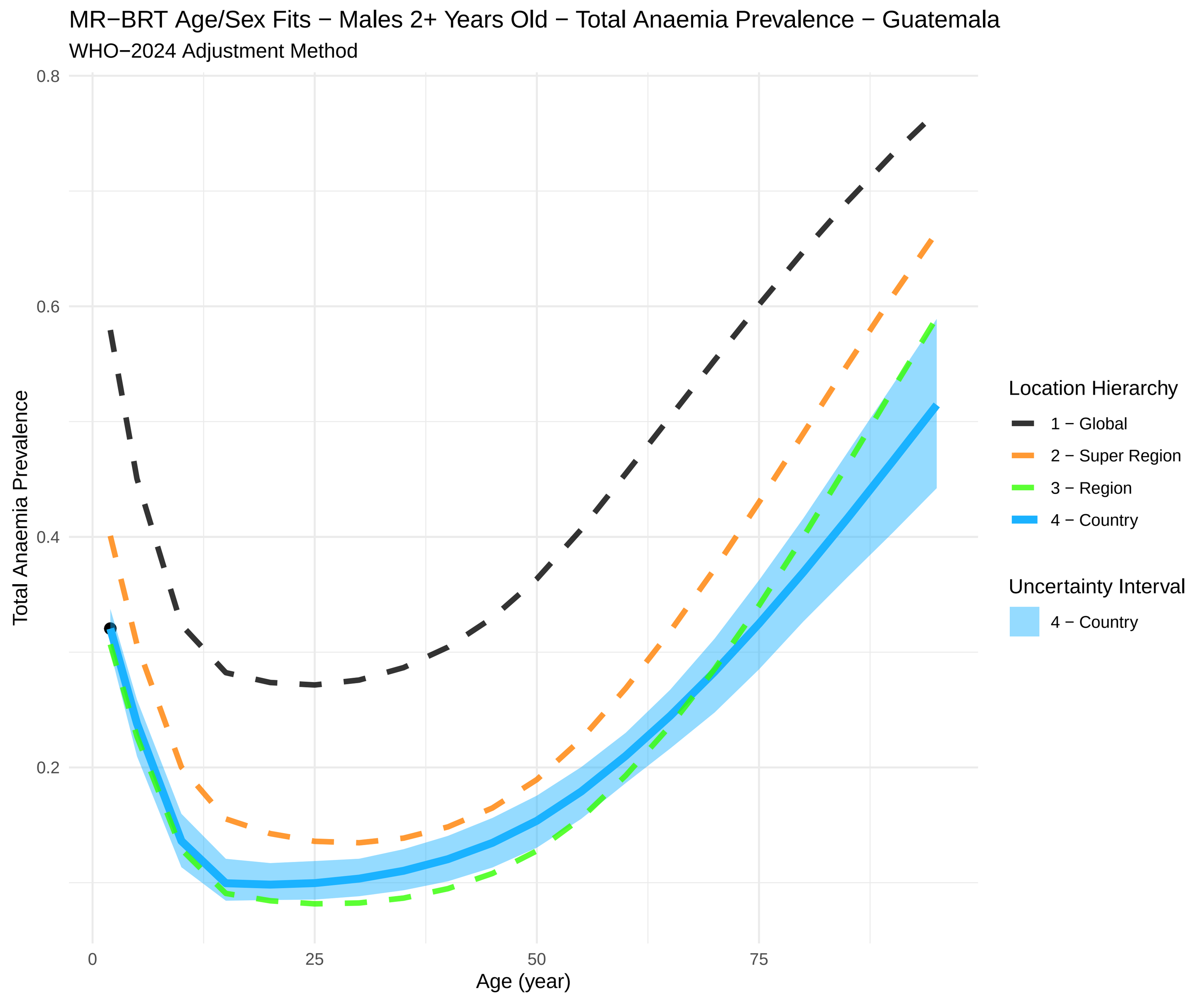
MR−BRT Age/Sex Fits − Males 2+ Years Old − Total Anaemia Prevalence − Guatemala
WHO−2024 Adjustment Method
Total Anaemia Prevalence
Age (year)
Location Hierarchy
1 − Global
2 − Super Region
3 − Region
4 − Country
Uncertainty Interval
4 − Country

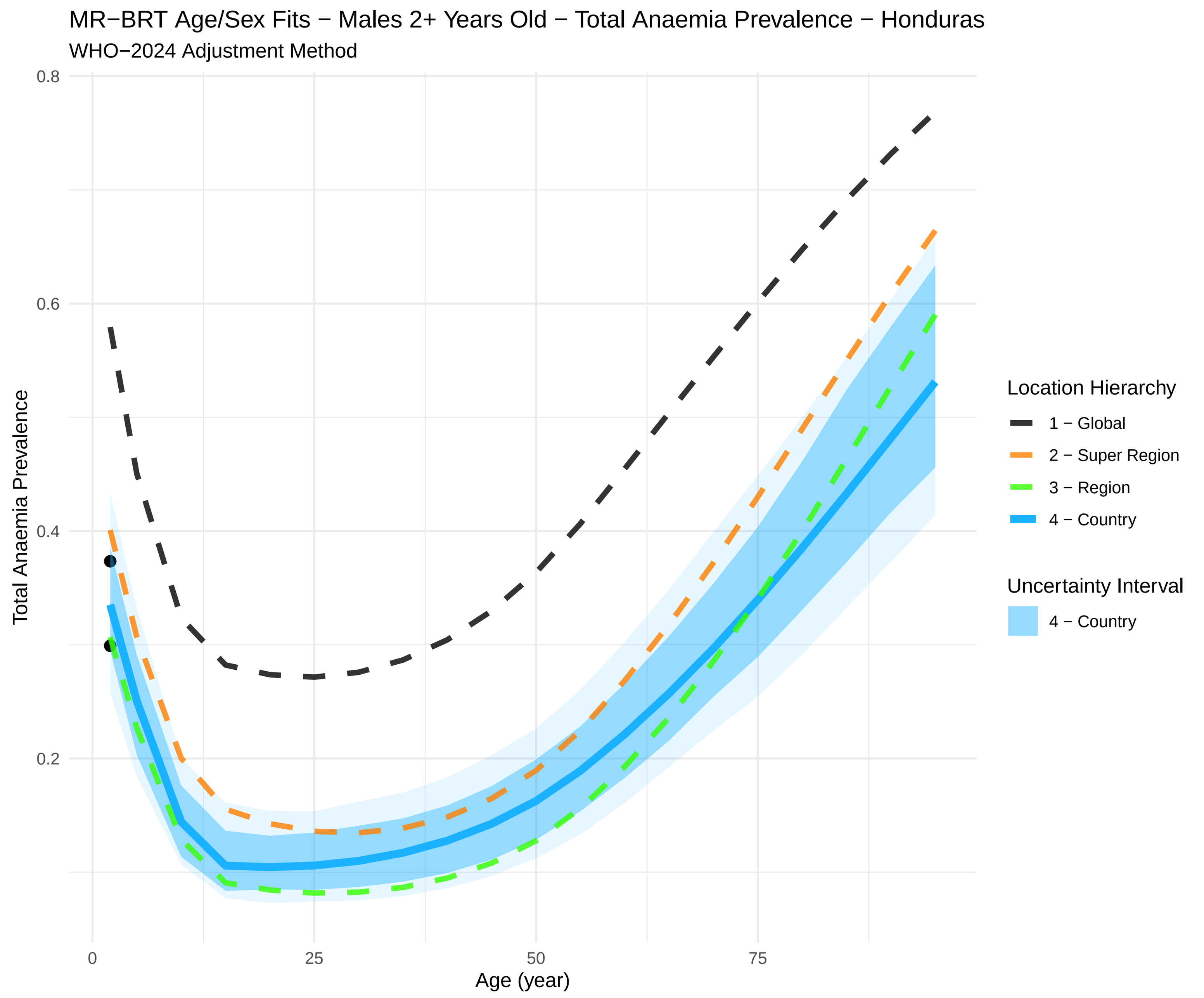
MR−BRT Age/Sex Fits − Males 2+ Years Old − Total Anaemia Prevalence − Honduras
WHO−2024 Adjustment Method
Total Anaemia Prevalence
Age (year)
Location Hierarchy
1 − Global
2 − Super Region
3 − Region
4 − Country
Uncertainty Interval
4 − Country

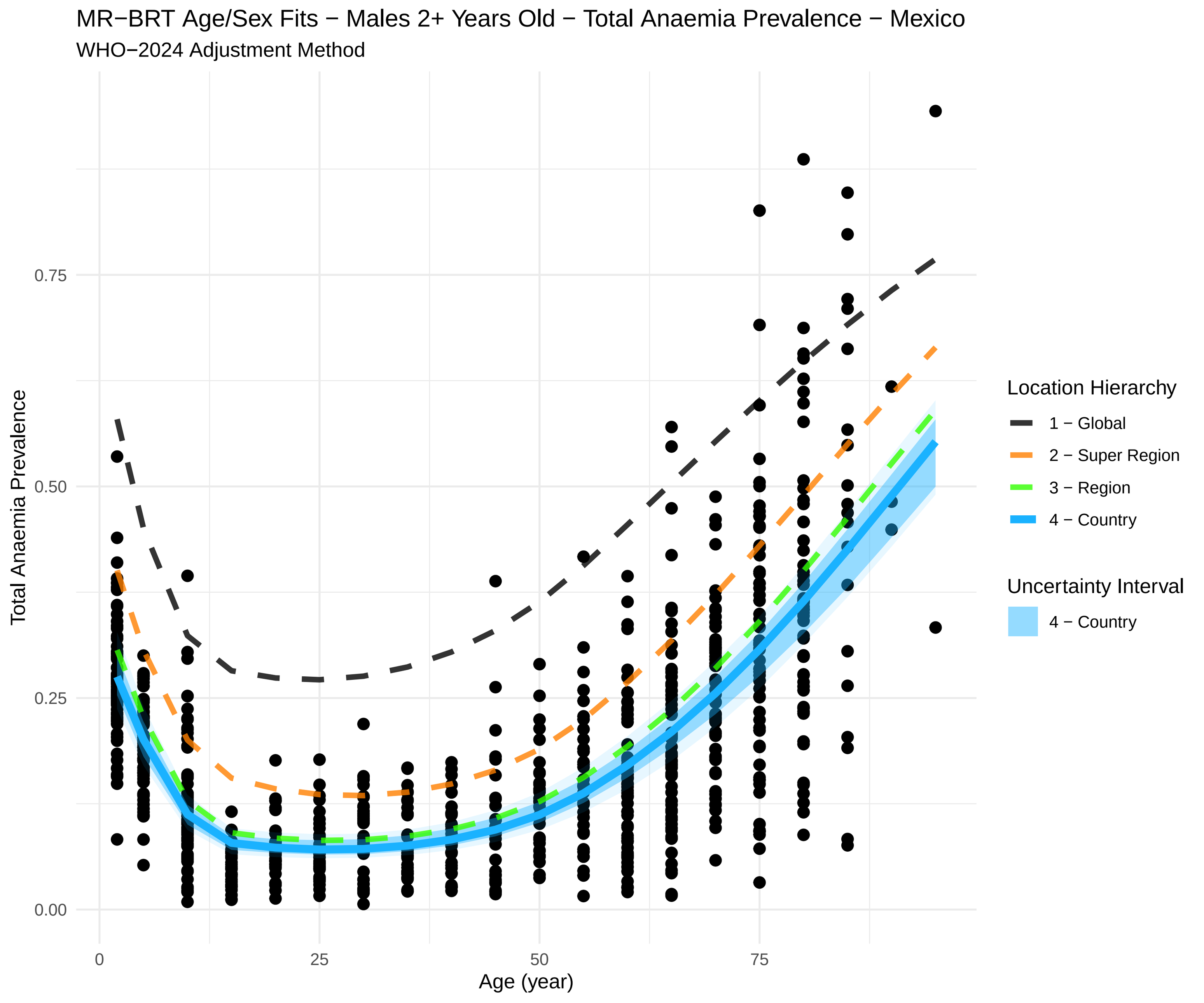
MR−BRT Age/Sex Fits − Males 2+ Years Old − Total Anaemia Prevalence − Mexico
WHO−2024 Adjustment Method
Total Anaemia Prevalence
Age (year)
Location Hierarchy
1 − Global
2 − Super Region
3 − Region
4 − Country
Uncertainty Interval
4 − Country

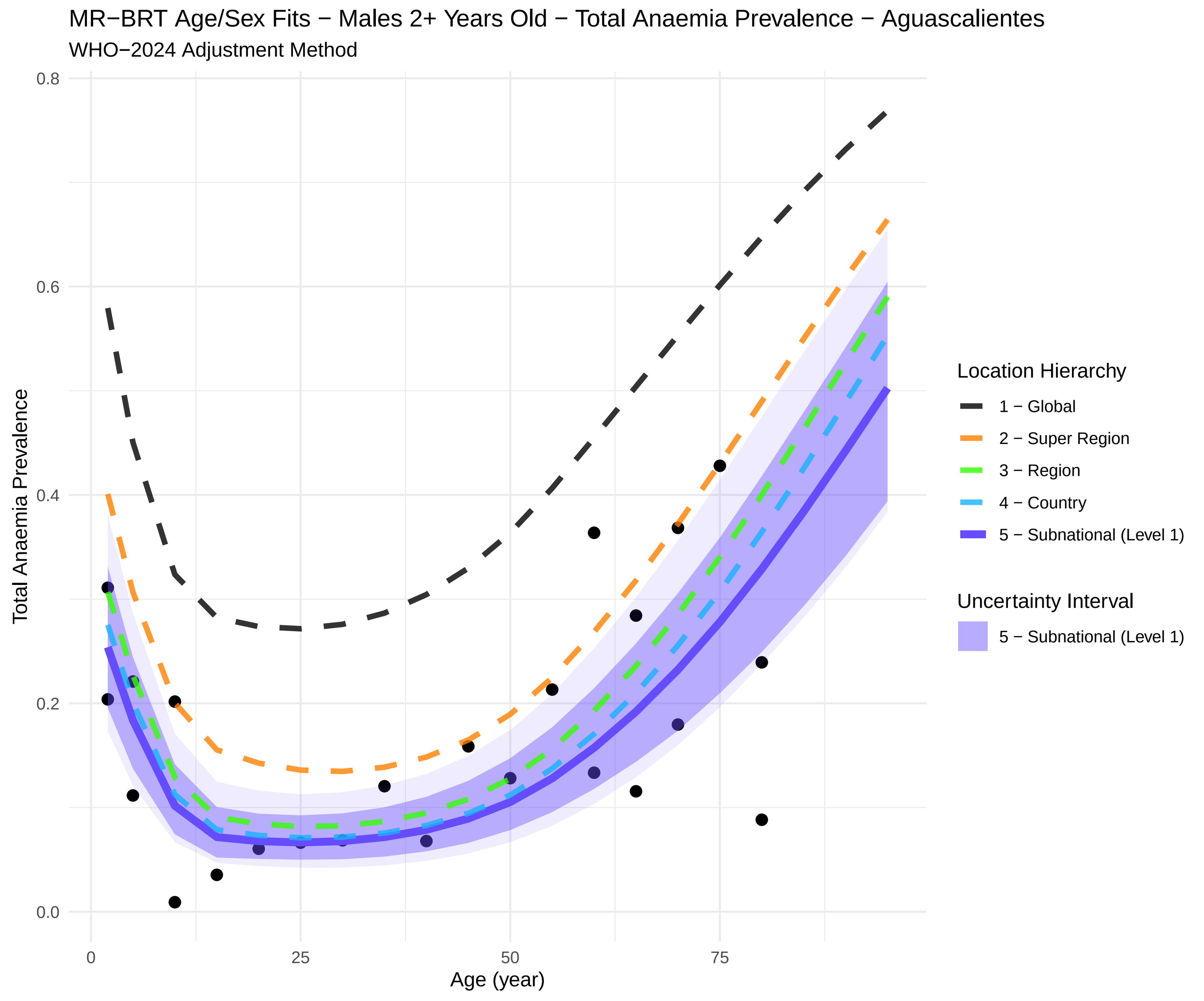
MR−BRT Age/Sex Fits − Males 2+ Years Old − Total Anaemia Prevalence − Aguascalientes
WHO−2024 Adjustment Method
Total Anaemia Prevalence
Age (year)
Location Hierarchy
1 − Global
2 − Super Region
3 − Region
4 − Country
5 − Subnational (Level 1)
Uncertainty Interval
5 − Subnational (Level 1)

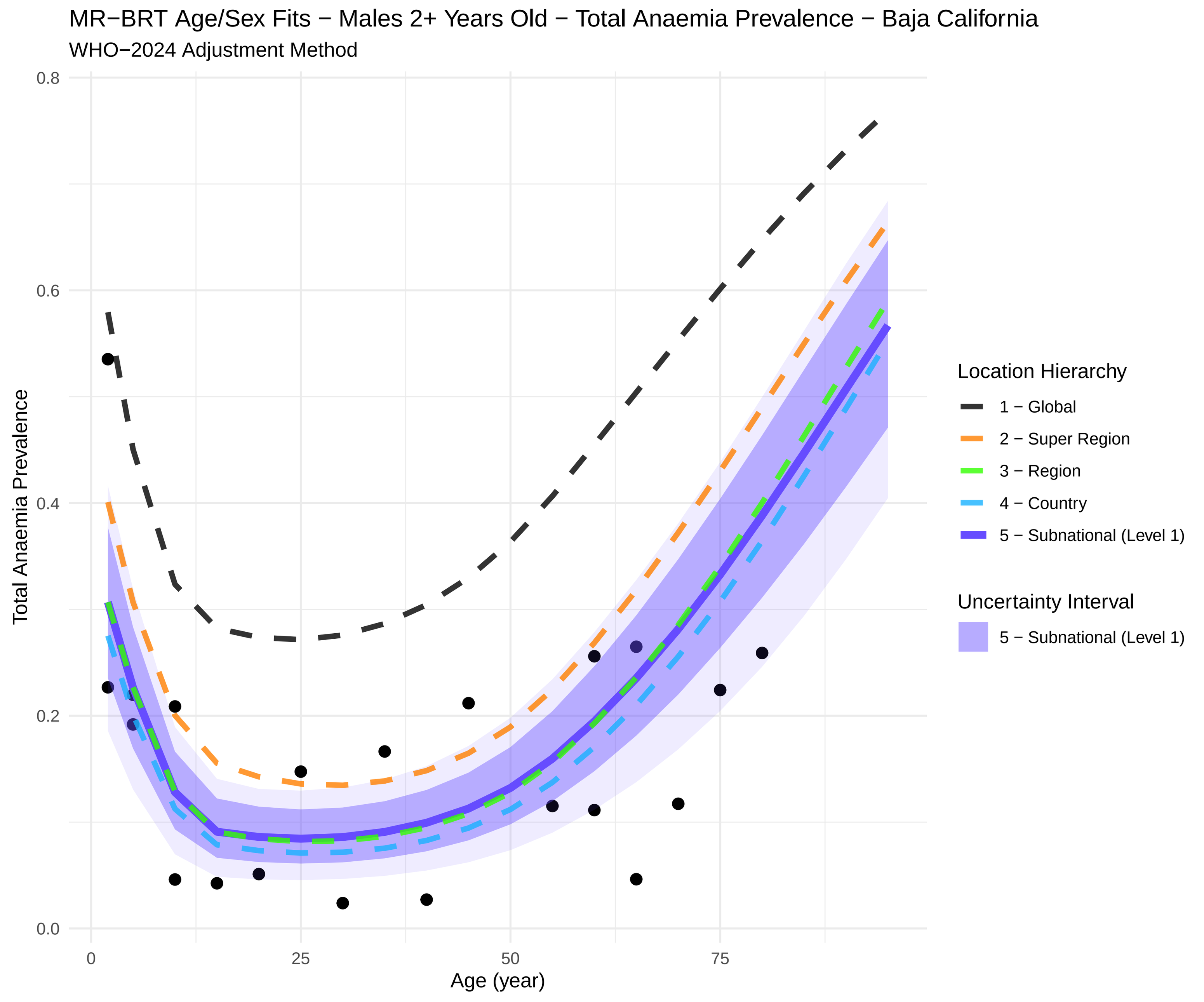
MR−BRT Age/Sex Fits − Males 2+ Years Old − Total Anaemia Prevalence − Baja California
WHO−2024 Adjustment Method
Total Anaemia Prevalence
Age (year)
Location Hierarchy
1 − Global
2 − Super Region
3 − Region
4 − Country
5 − Subnational (Level 1)
Uncertainty Interval
5 − Subnational (Level 1)

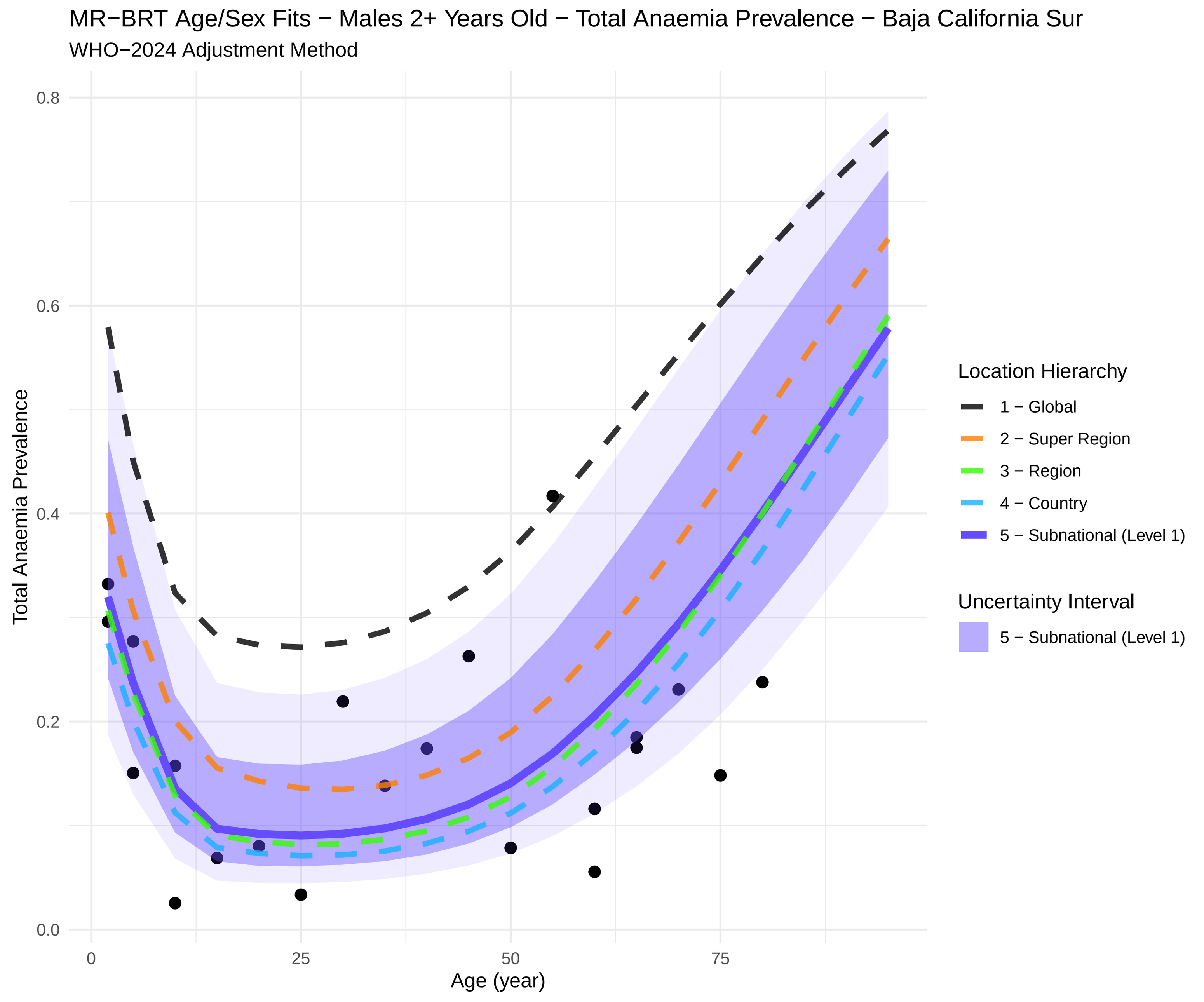
MR−BRT Age/Sex Fits − Males 2+ Years Old − Total Anaemia Prevalence − Baja California Sur
WHO−2024 Adjustment Method
Total Anaemia Prevalence
Age (year)
Location Hierarchy
1 − Global
2 − Super Region
3 − Region
4 − Country
5 − Subnational (Level 1)
Uncertainty Interval
5 − Subnational (Level 1)

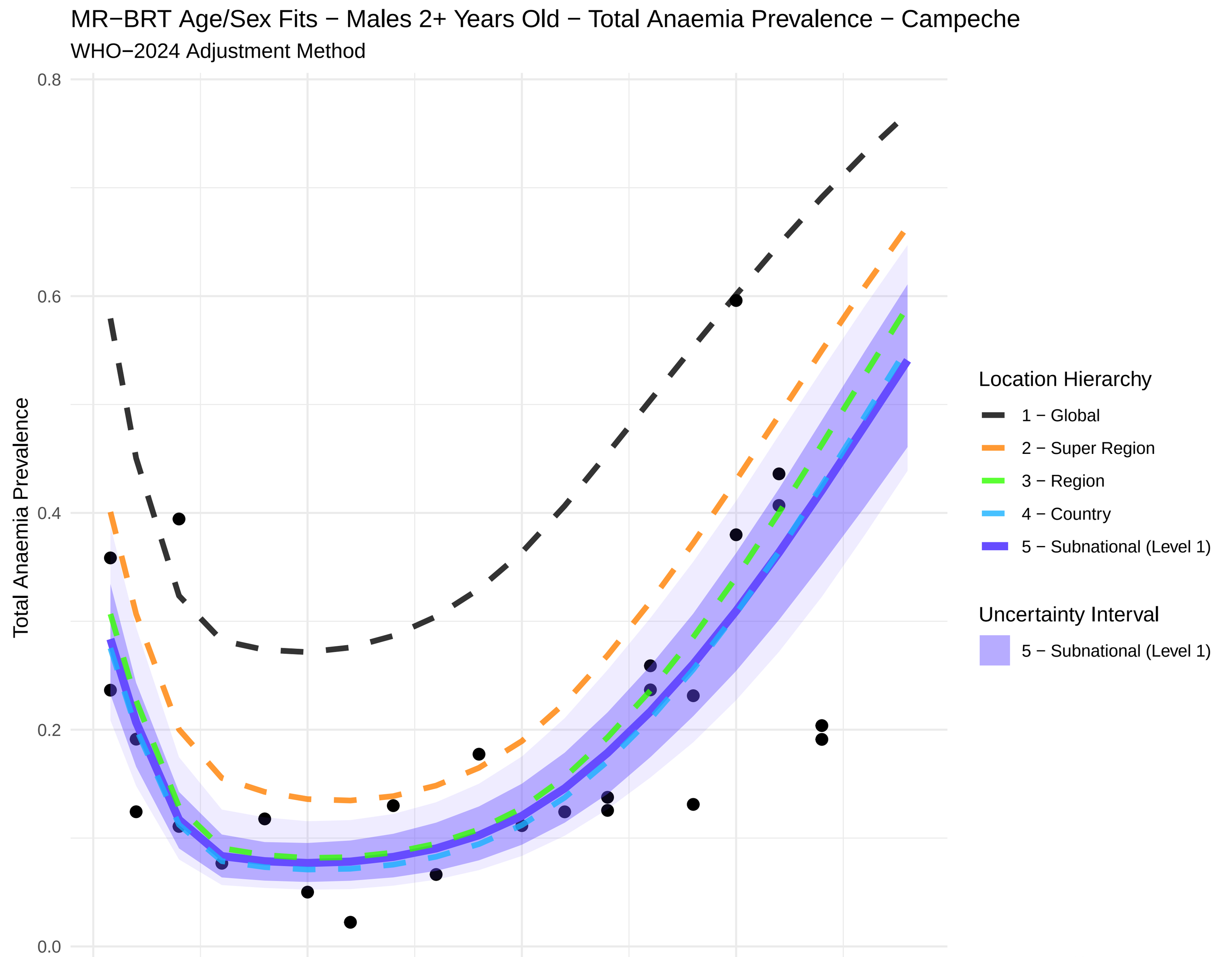
MR−BRT Age/Sex Fits − Males 2+ Years Old − Total Anaemia Prevalence − Campeche
WHO−2024 Adjustment Method
Total Anaemia Prevalence
Age (year)
Location Hierarchy
1 − Global
2 − Super Region
3 − Region
4 − Country
5 − Subnational (Level 1)
Uncertainty Interval
5 − Subnational (Level 1)

MR−BRT Age/Sex Fits − Males 2+ Years Old − Total Anaemia Prevalence − Chiapas
WHO−2024 Adjustment Method

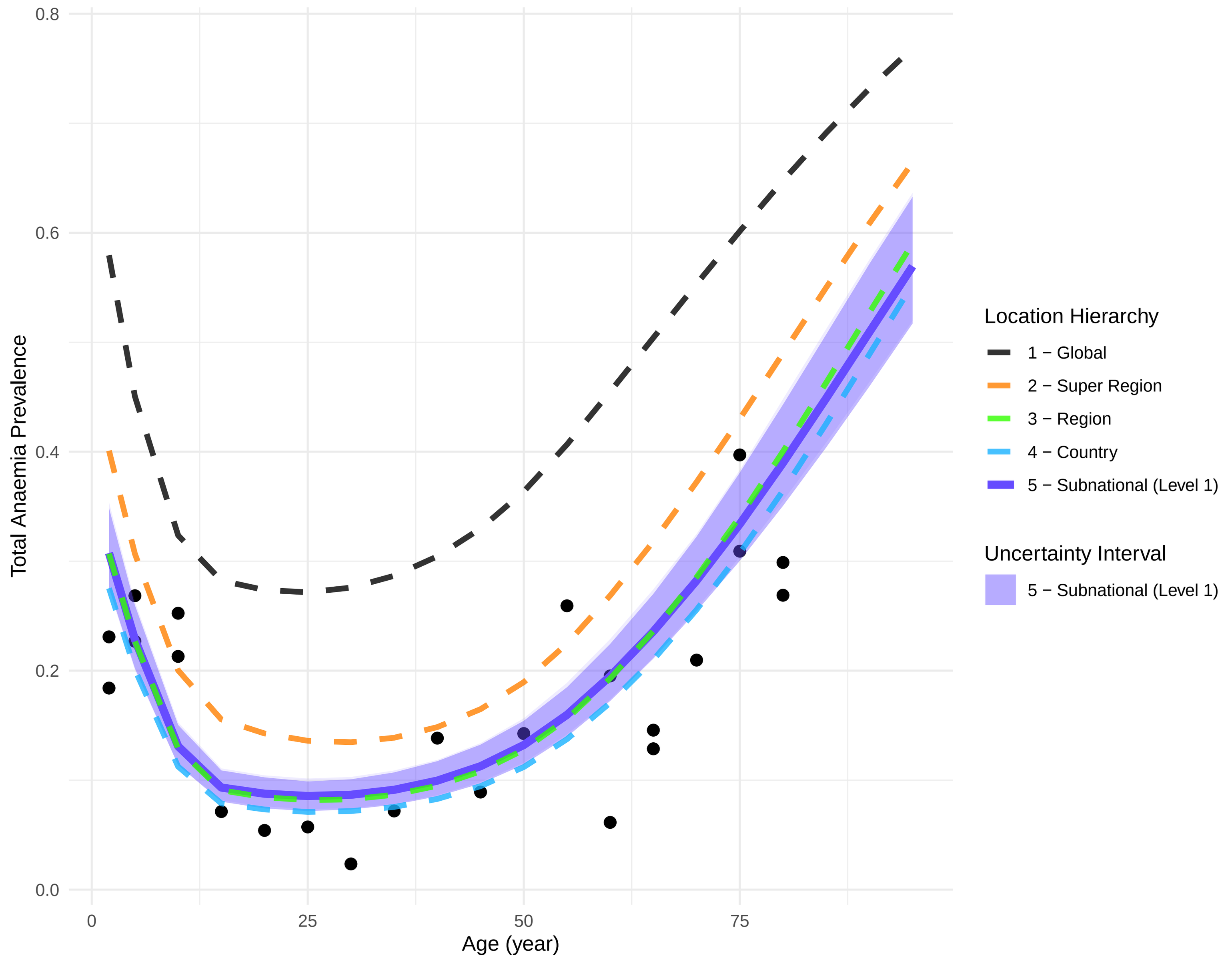

MR−BRT Age/Sex Fits − Males 2+ Years Old − Total Anaemia Prevalence − Chihuahua
WHO−2024 Adjustment Method

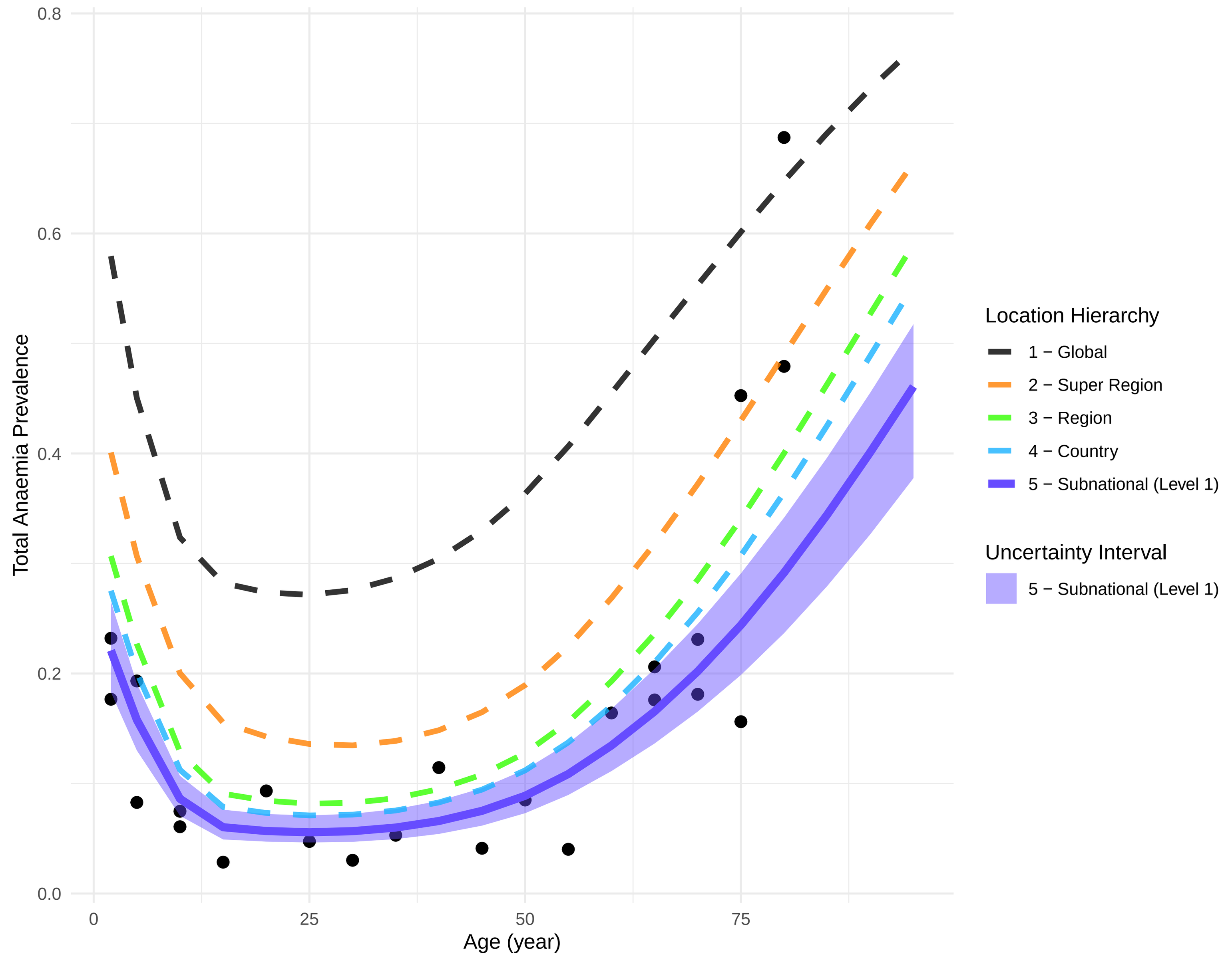

MR−BRT Age/Sex Fits − Males 2+ Years Old − Total Anaemia Prevalence − Coahuila
WHO−2024 Adjustment Method

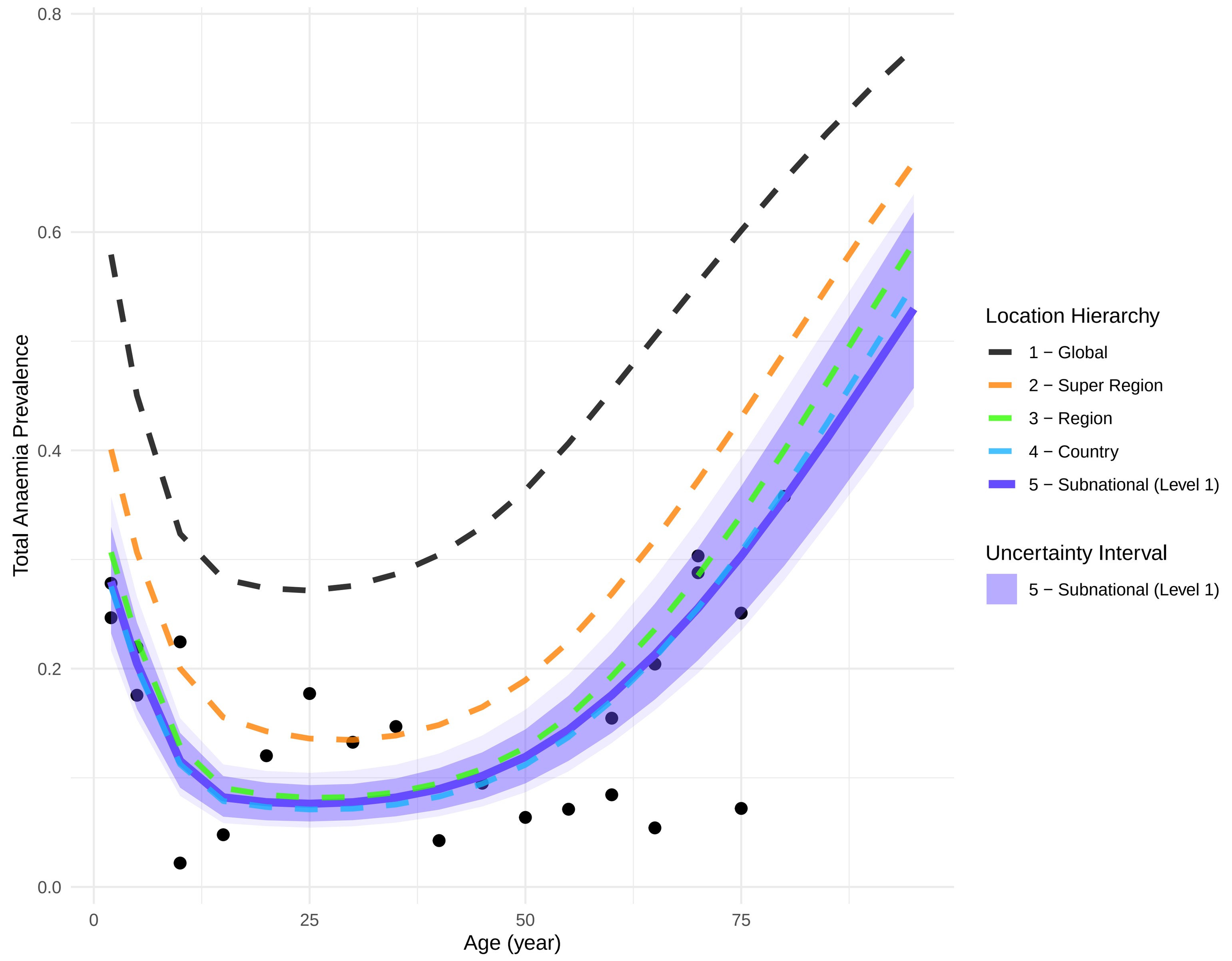

MR−BRT Age/Sex Fits − Males 2+ Years Old − Total Anaemia Prevalence − Colima
WHO−2024 Adjustment Method

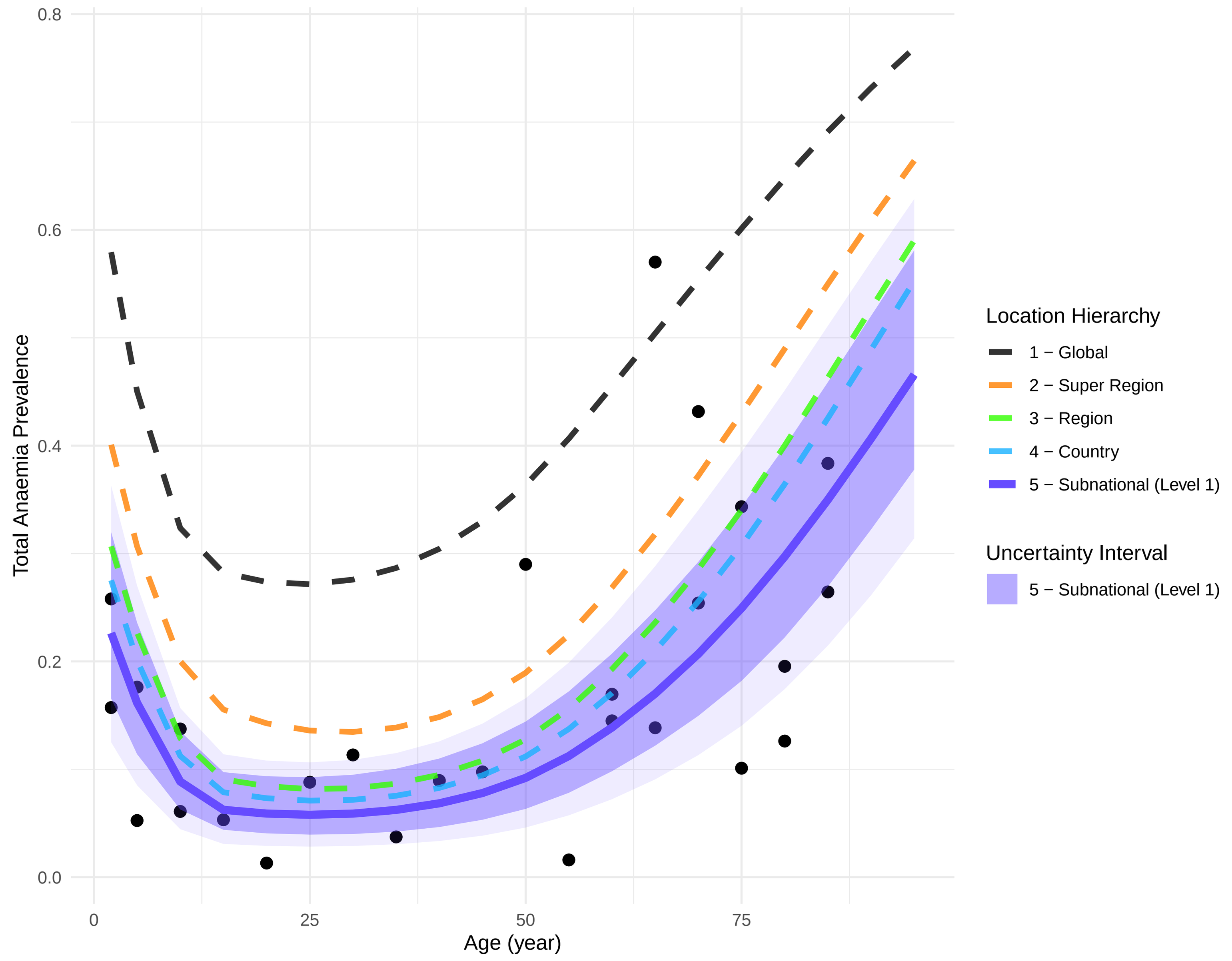

MR−BRT Age/Sex Fits − Males 2+ Years Old − Total Anaemia Prevalence − Durango
WHO−2024 Adjustment Method

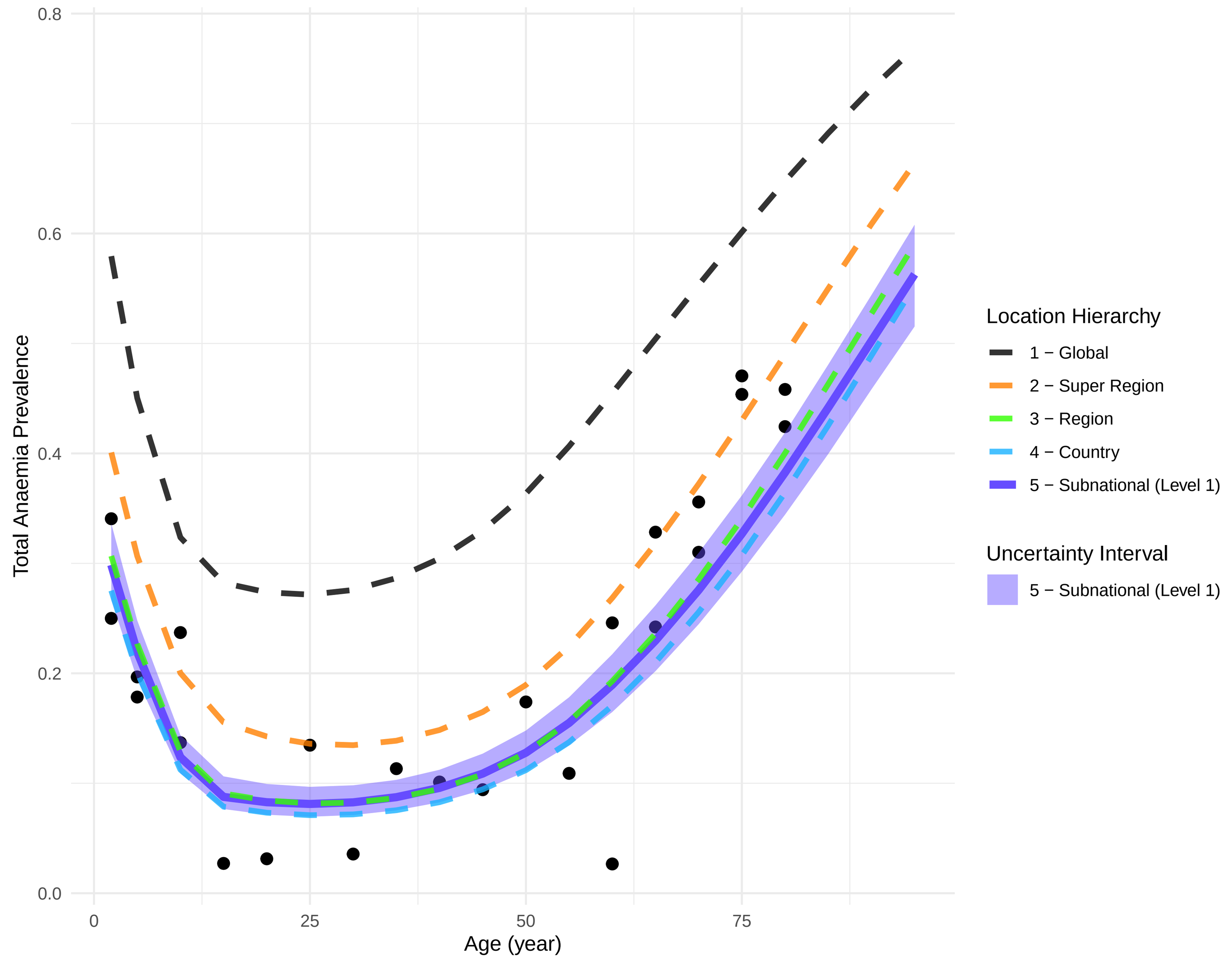

MR−BRT Age/Sex Fits − Males 2+ Years Old − Total Anaemia Prevalence − Guanajuato
WHO−2024 Adjustment Method

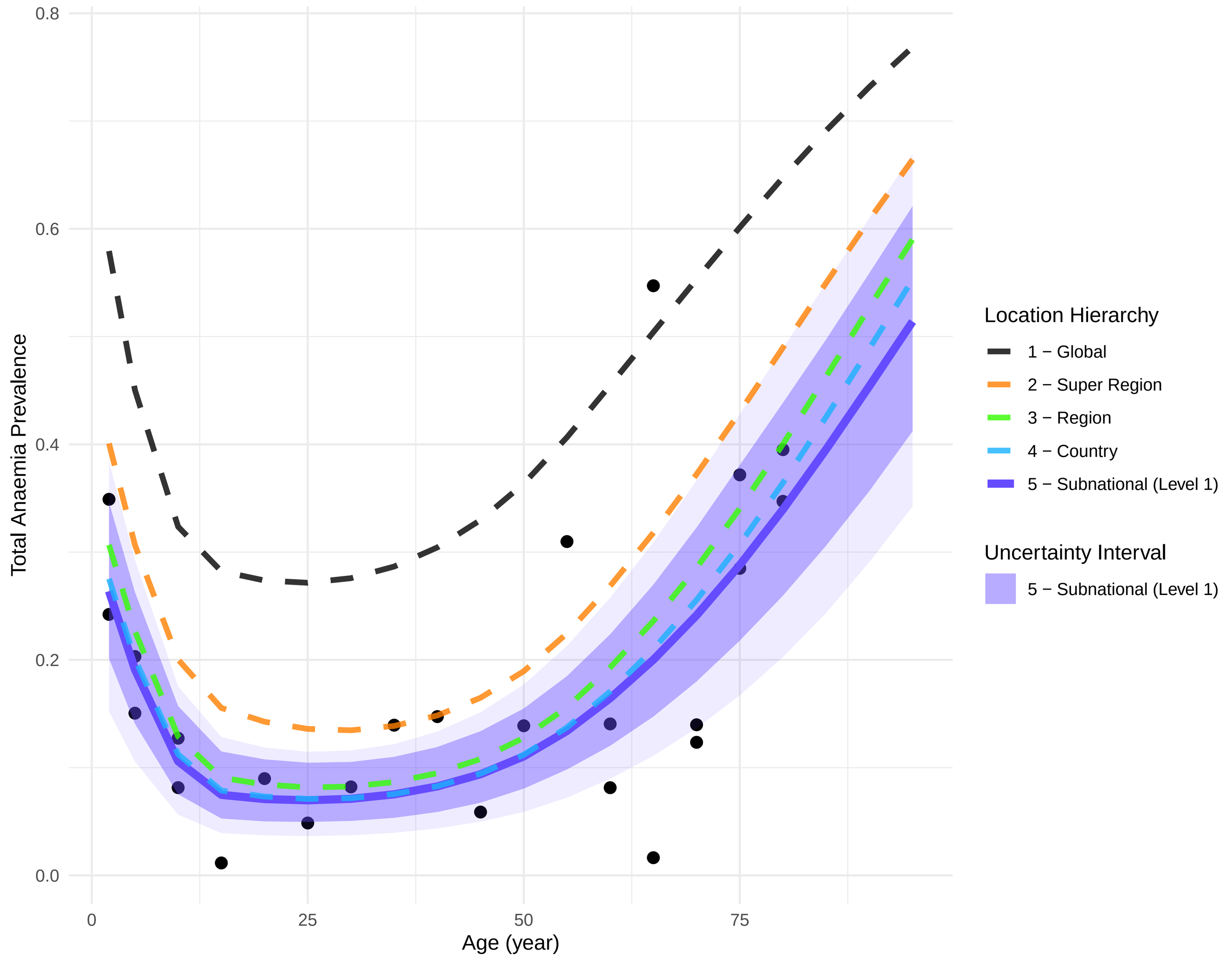

MR−BRT Age/Sex Fits − Males 2+ Years Old − Total Anaemia Prevalence − Guerrero
WHO−2024 Adjustment Method

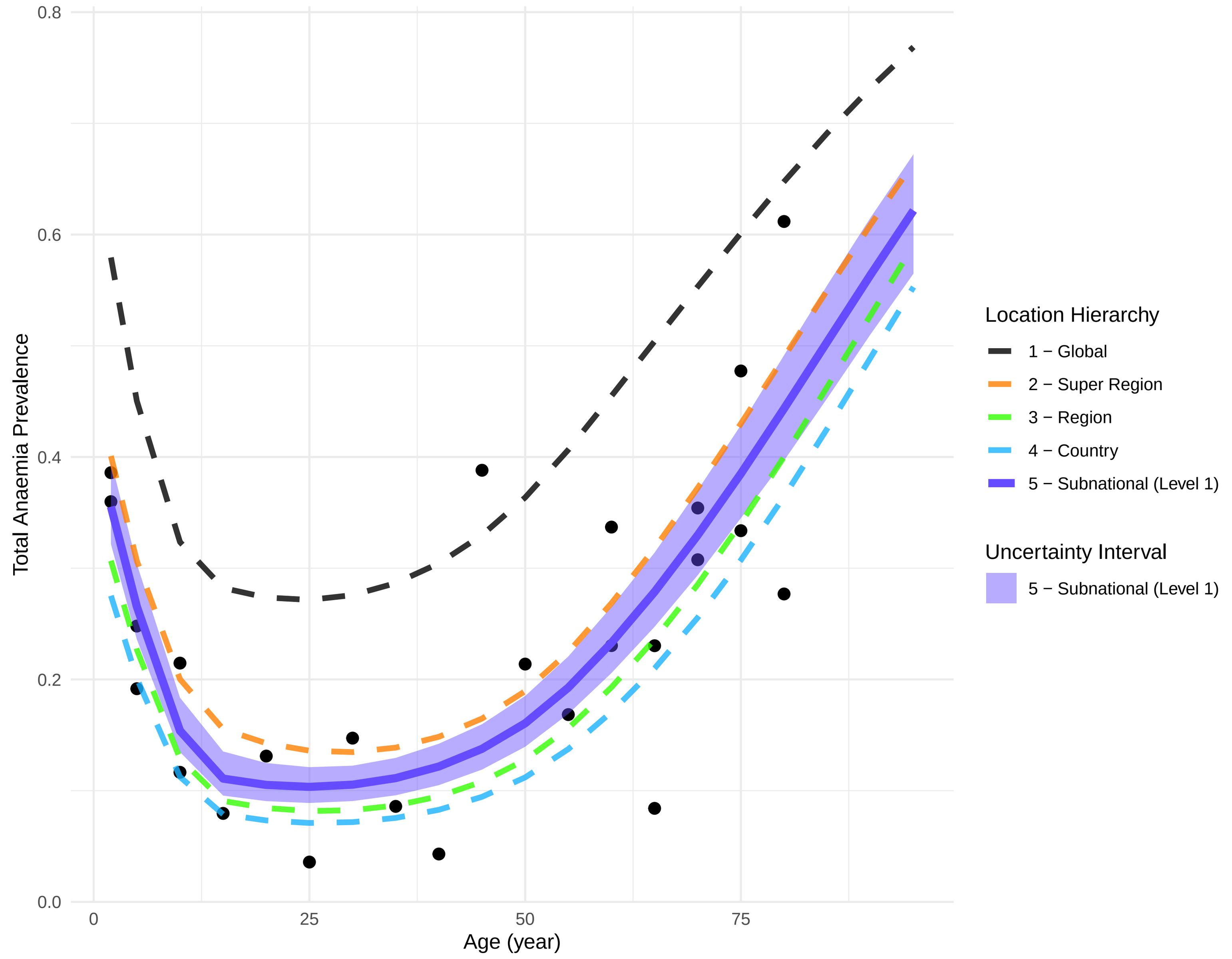

MR−BRT Age/Sex Fits − Males 2+ Years Old − Total Anaemia Prevalence − Hidalgo
WHO−2024 Adjustment Method

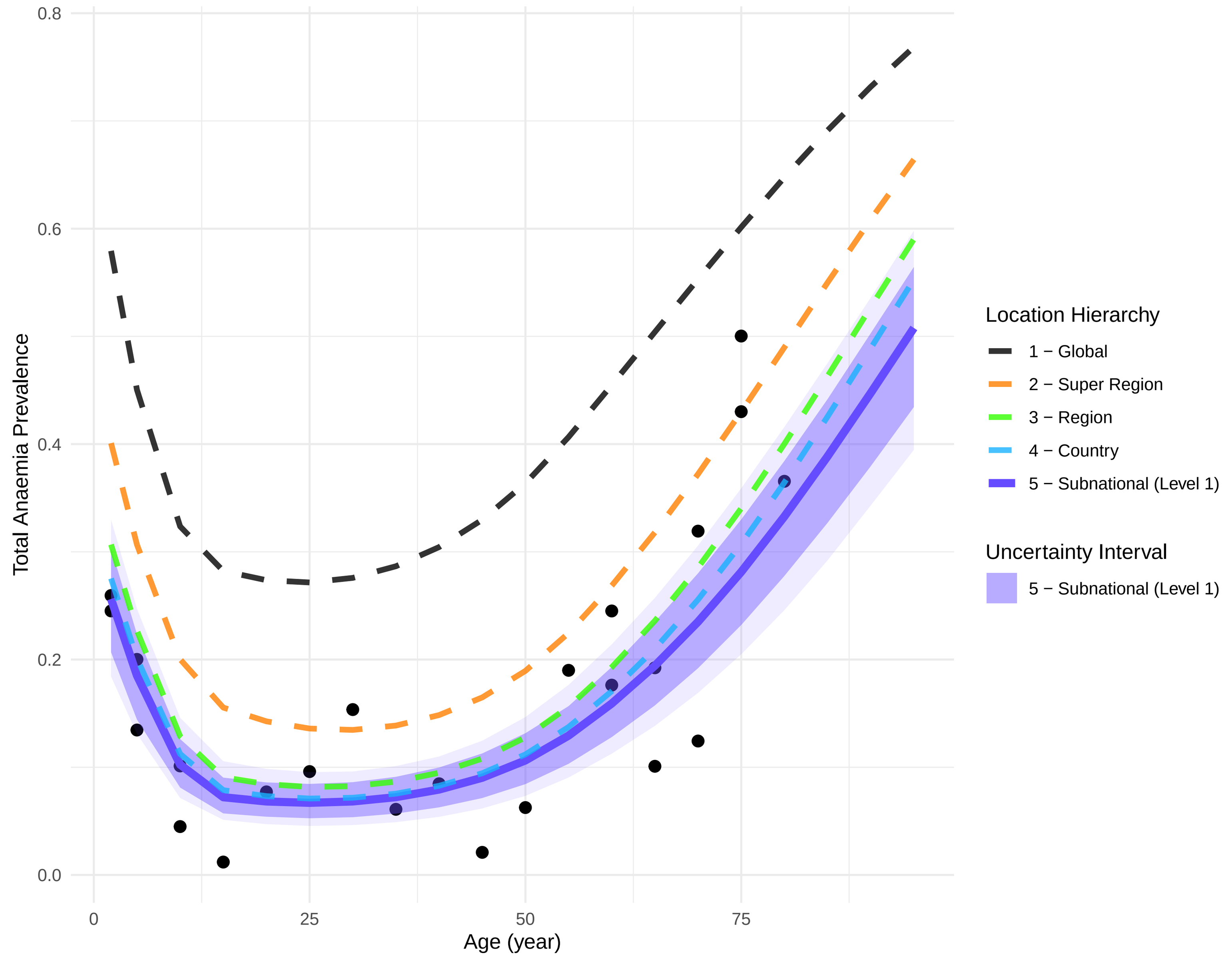

MR−BRT Age/Sex Fits − Males 2+ Years Old − Total Anaemia Prevalence − Jalisco
WHO−2024 Adjustment Method

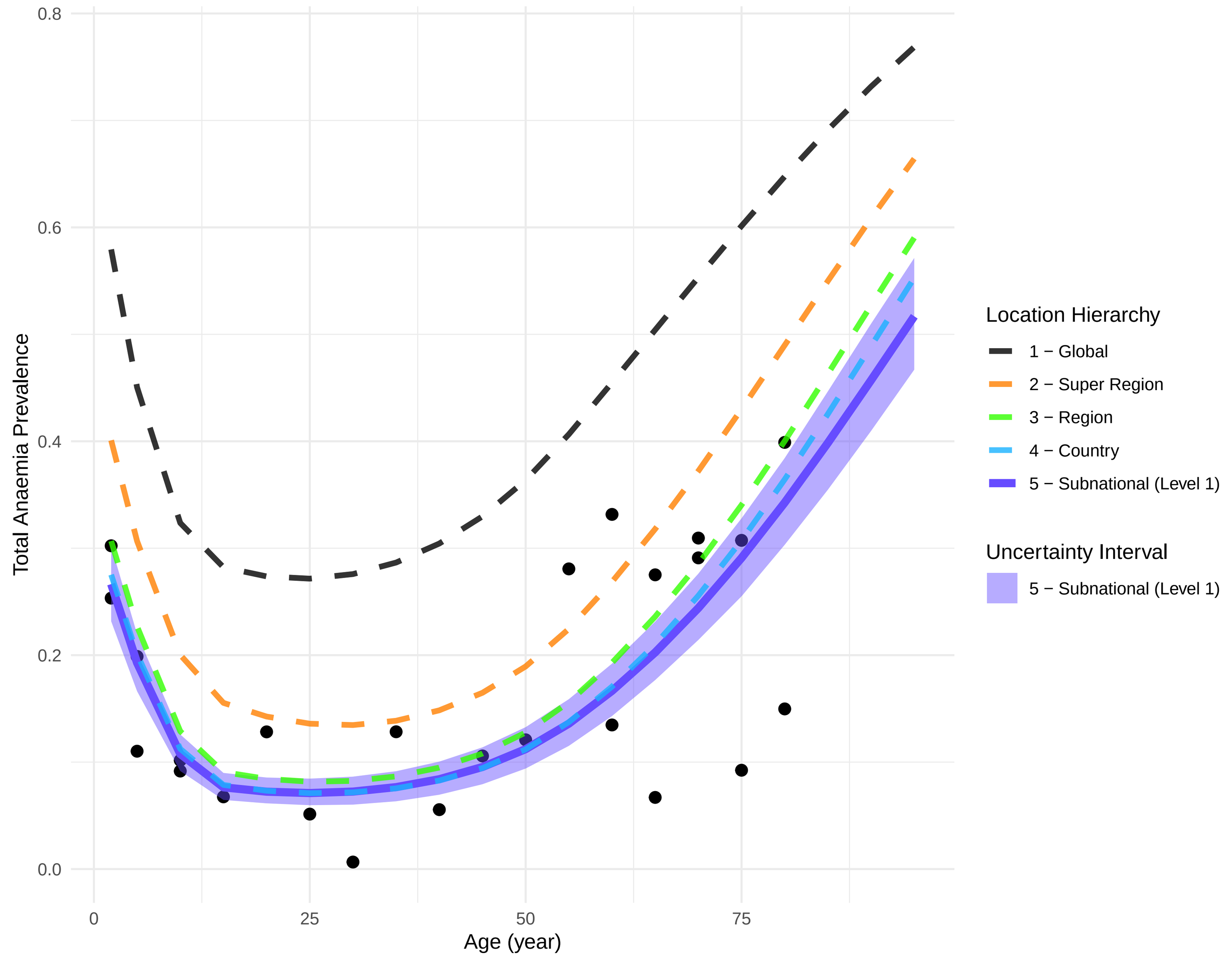

MR−BRT Age/Sex Fits − Males 2+ Years Old − Total Anaemia Prevalence − México
WHO−2024 Adjustment Method

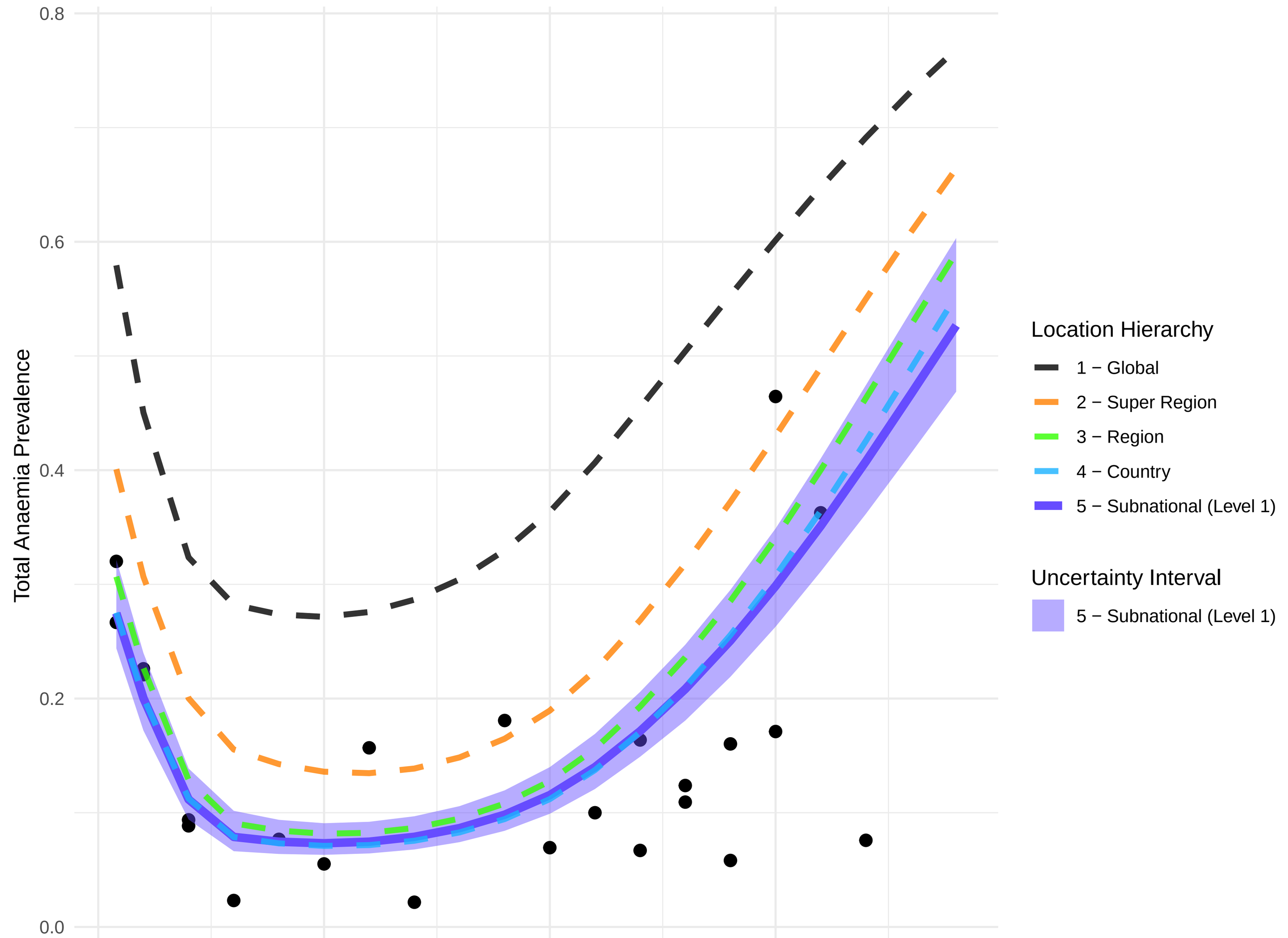

MR−BRT Age/Sex Fits − Males 2+ Years Old − Total Anaemia Prevalence − Mexico City
WHO−2024 Adjustment Method

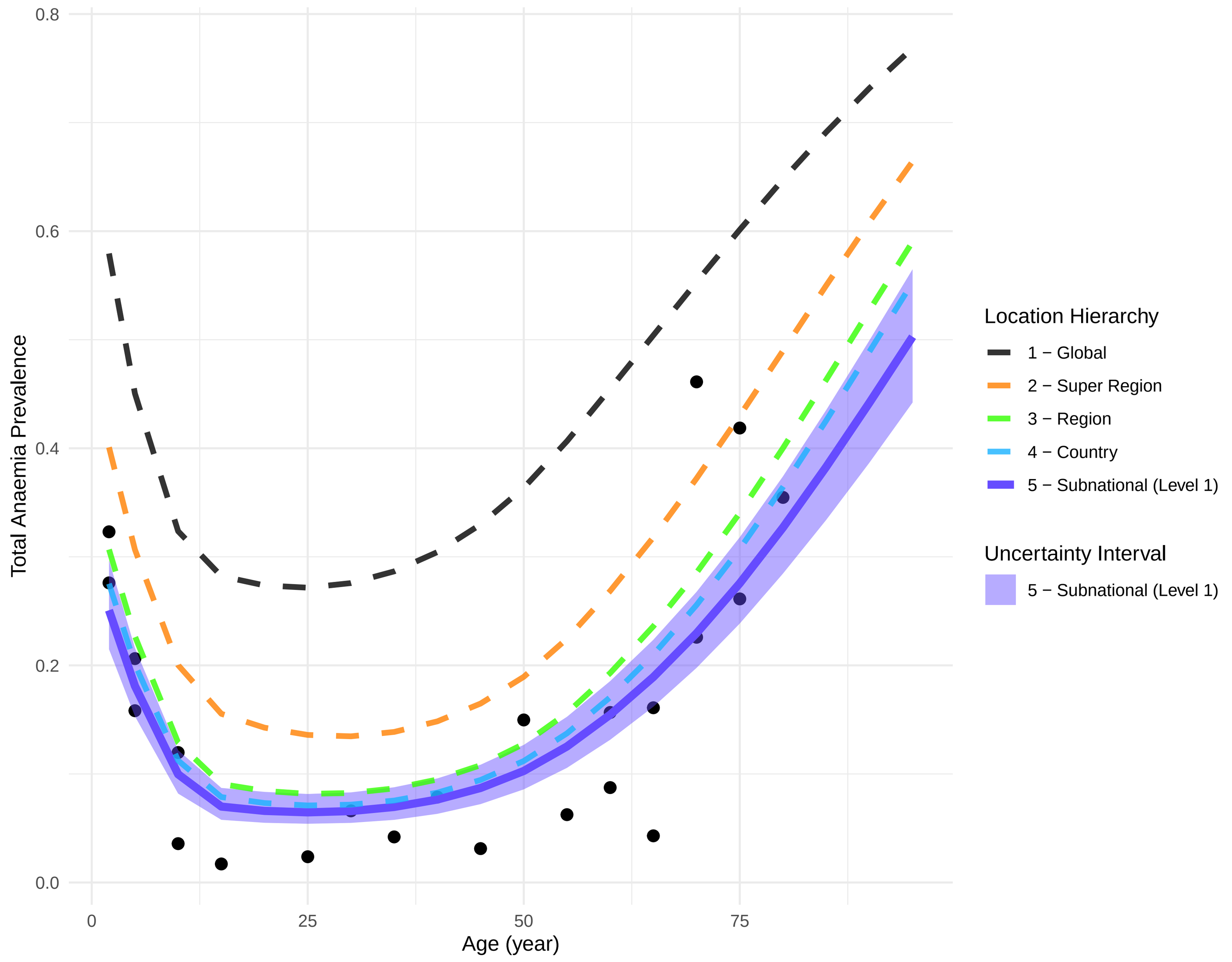

MR−BRT Age/Sex Fits − Males 2+ Years Old − Total Anaemia Prevalence − Michoacán de Ocampo
WHO−2024 Adjustment Method

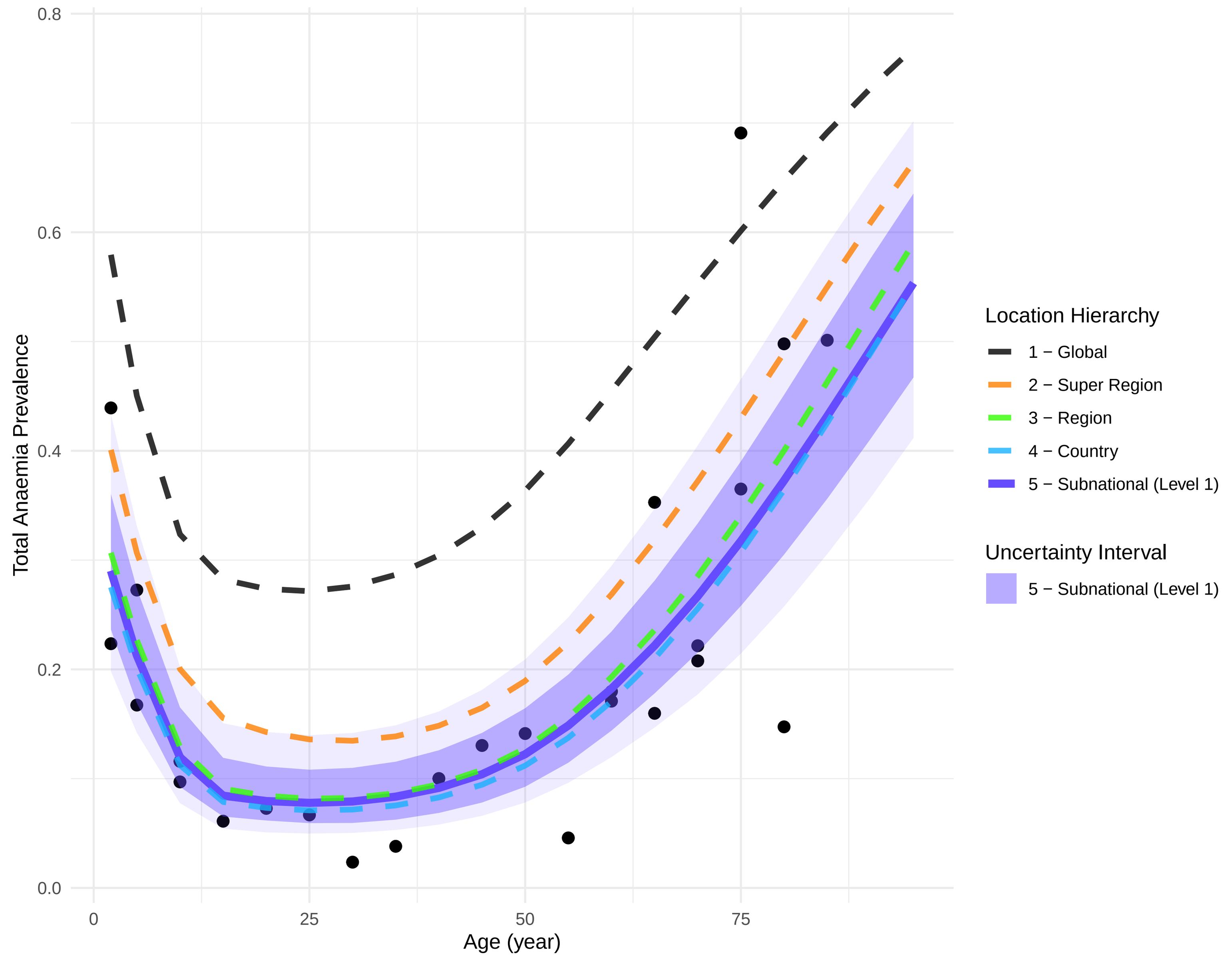

MR−BRT Age/Sex Fits − Males 2+ Years Old − Total Anaemia Prevalence − Morelos
WHO−2024 Adjustment Method

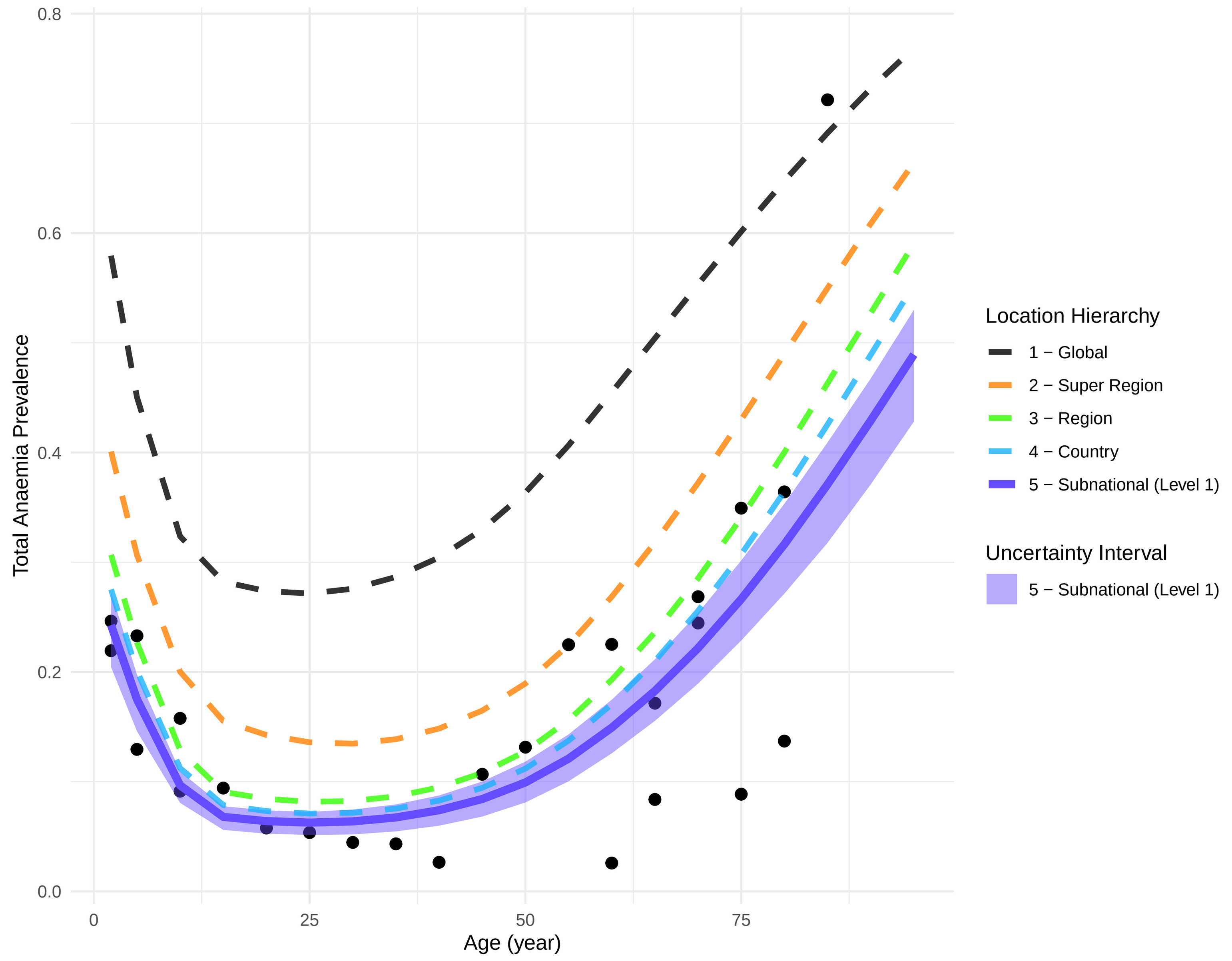

MR−BRT Age/Sex Fits − Males 2+ Years Old − Total Anaemia Prevalence − Nayarit
WHO−2024 Adjustment Method

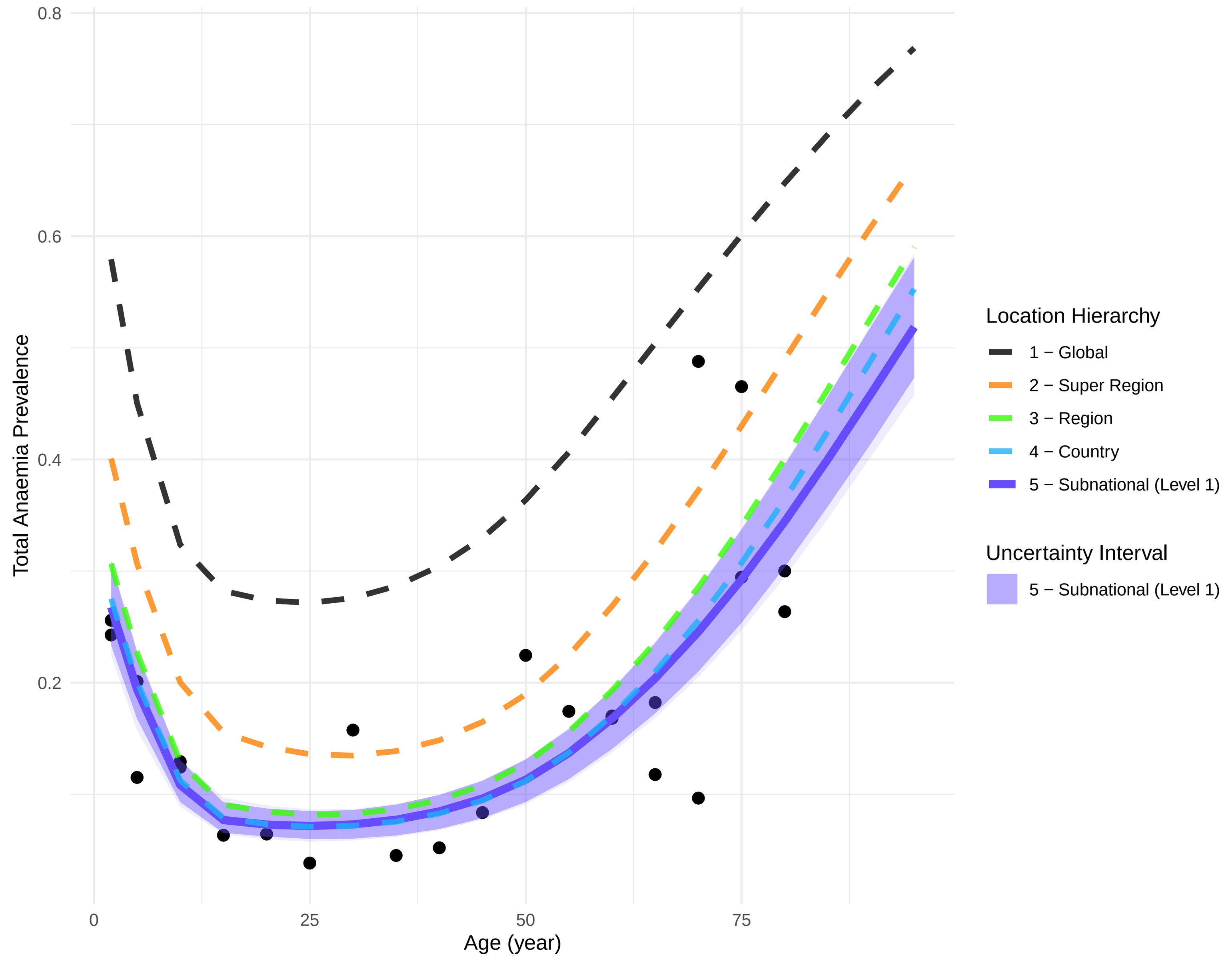

MR−BRT Age/Sex Fits − Males 2+ Years Old − Total Anaemia Prevalence − Nuevo León
WHO−2024 Adjustment Method

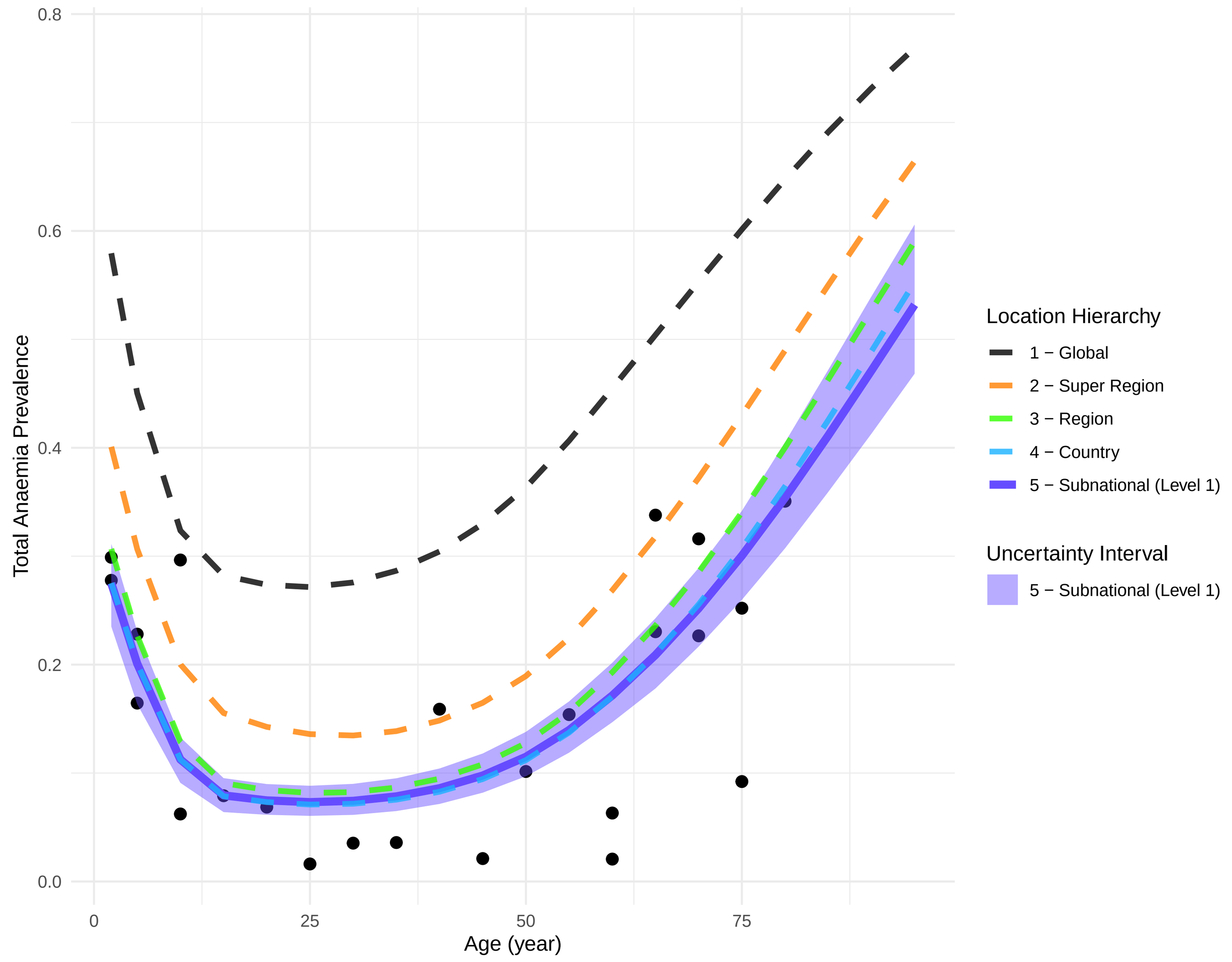

MR−BRT Age/Sex Fits − Males 2+ Years Old − Total Anaemia Prevalence − Oaxaca
WHO−2024 Adjustment Method

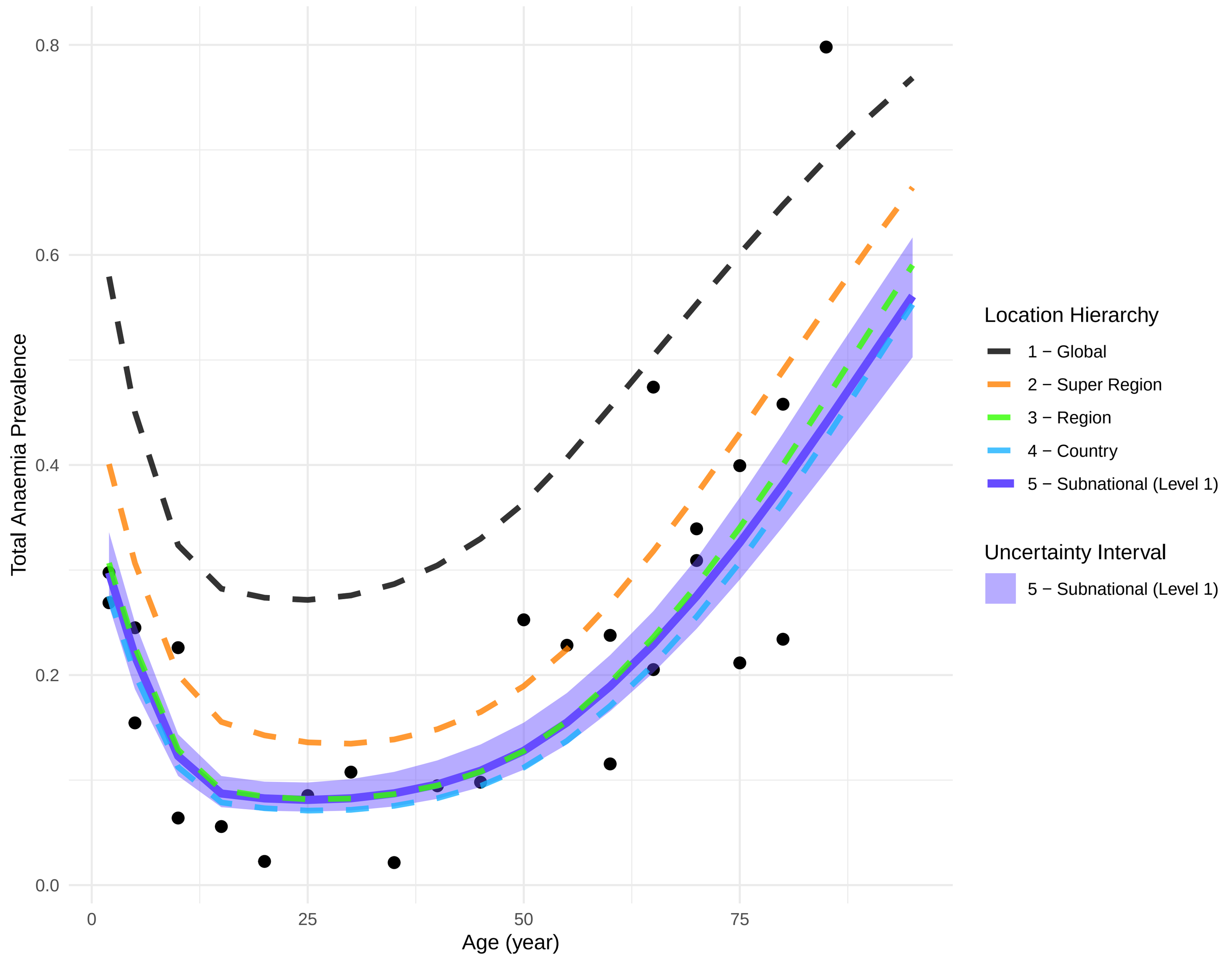

MR−BRT Age/Sex Fits − Males 2+ Years Old − Total Anaemia Prevalence − Puebla
WHO−2024 Adjustment Method

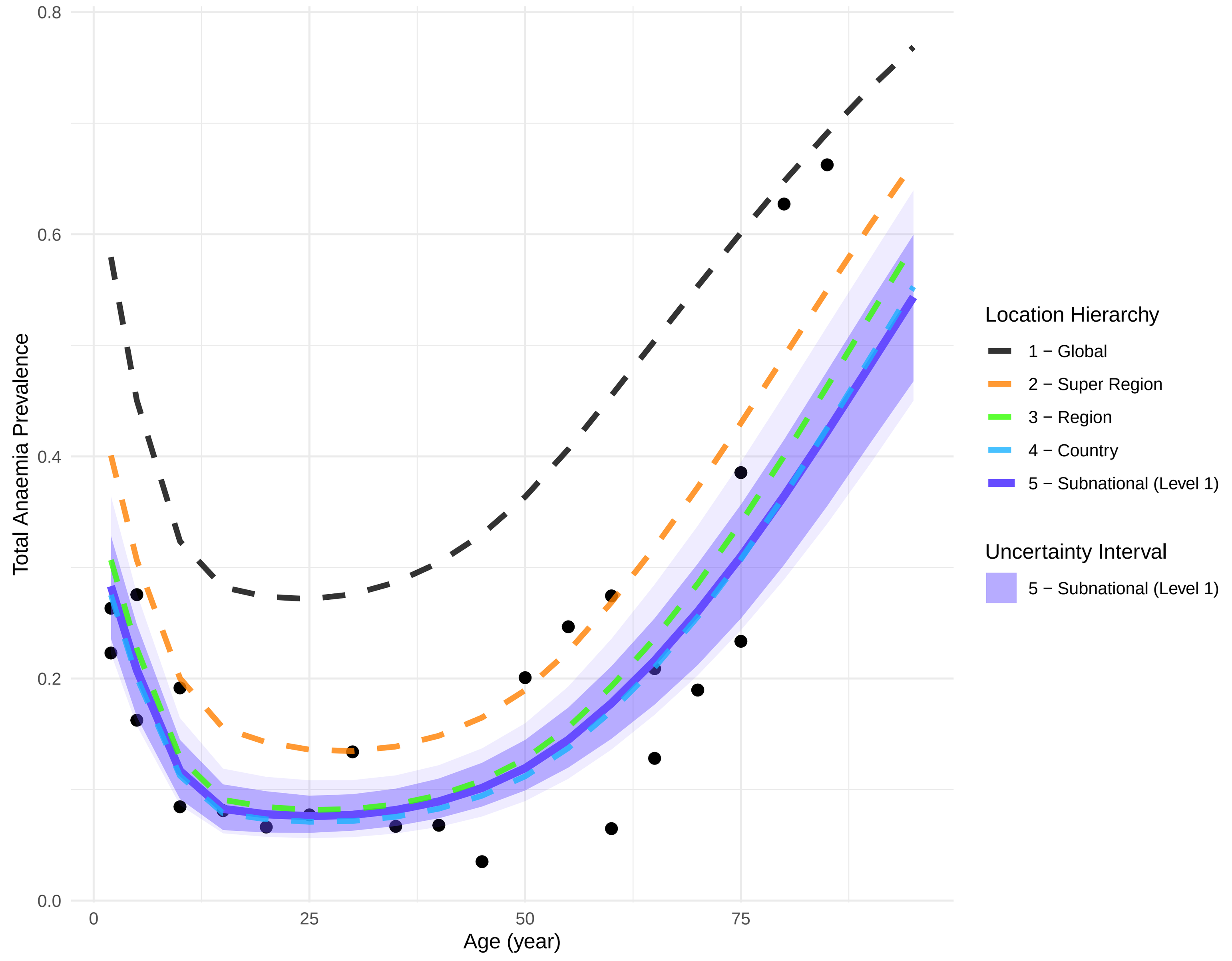

MR−BRT Age/Sex Fits − Males 2+ Years Old − Total Anaemia Prevalence − Querétaro
WHO−2024 Adjustment Method

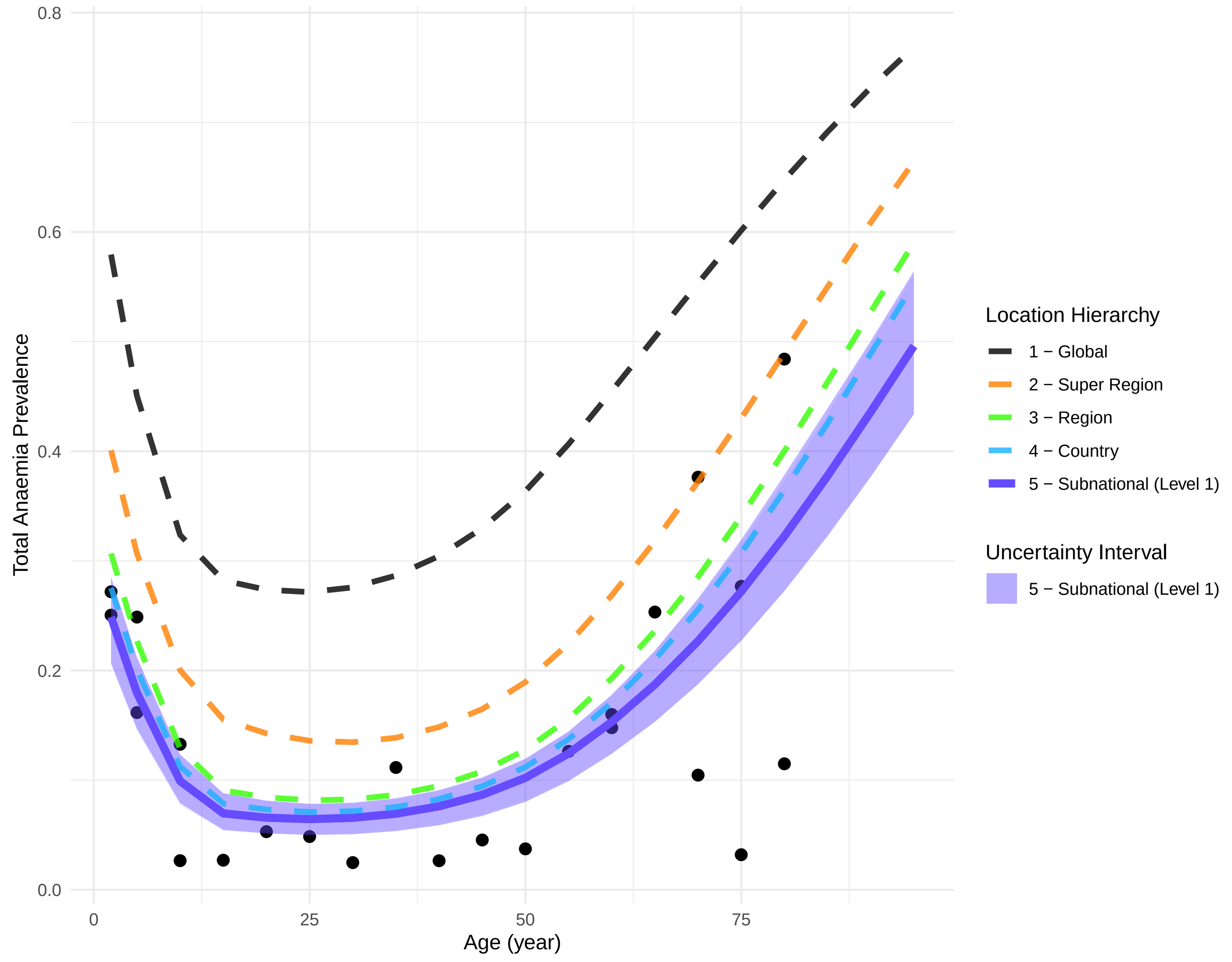

MR−BRT Age/Sex Fits − Males 2+ Years Old − Total Anaemia Prevalence − Quintana Roo
WHO−2024 Adjustment Method

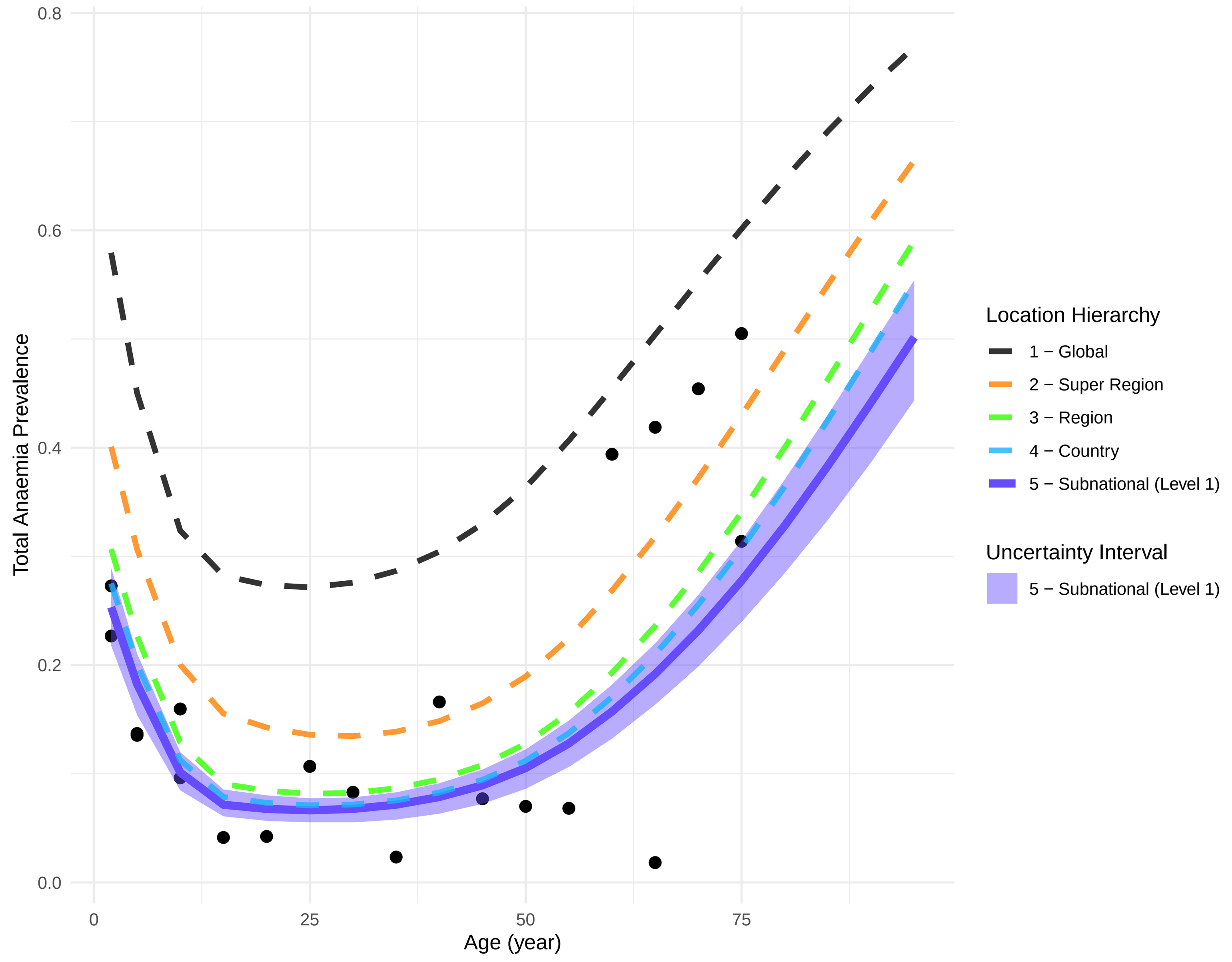

MR−BRT Age/Sex Fits − Males 2+ Years Old − Total Anaemia Prevalence − San Luis Potosí
WHO−2024 Adjustment Method

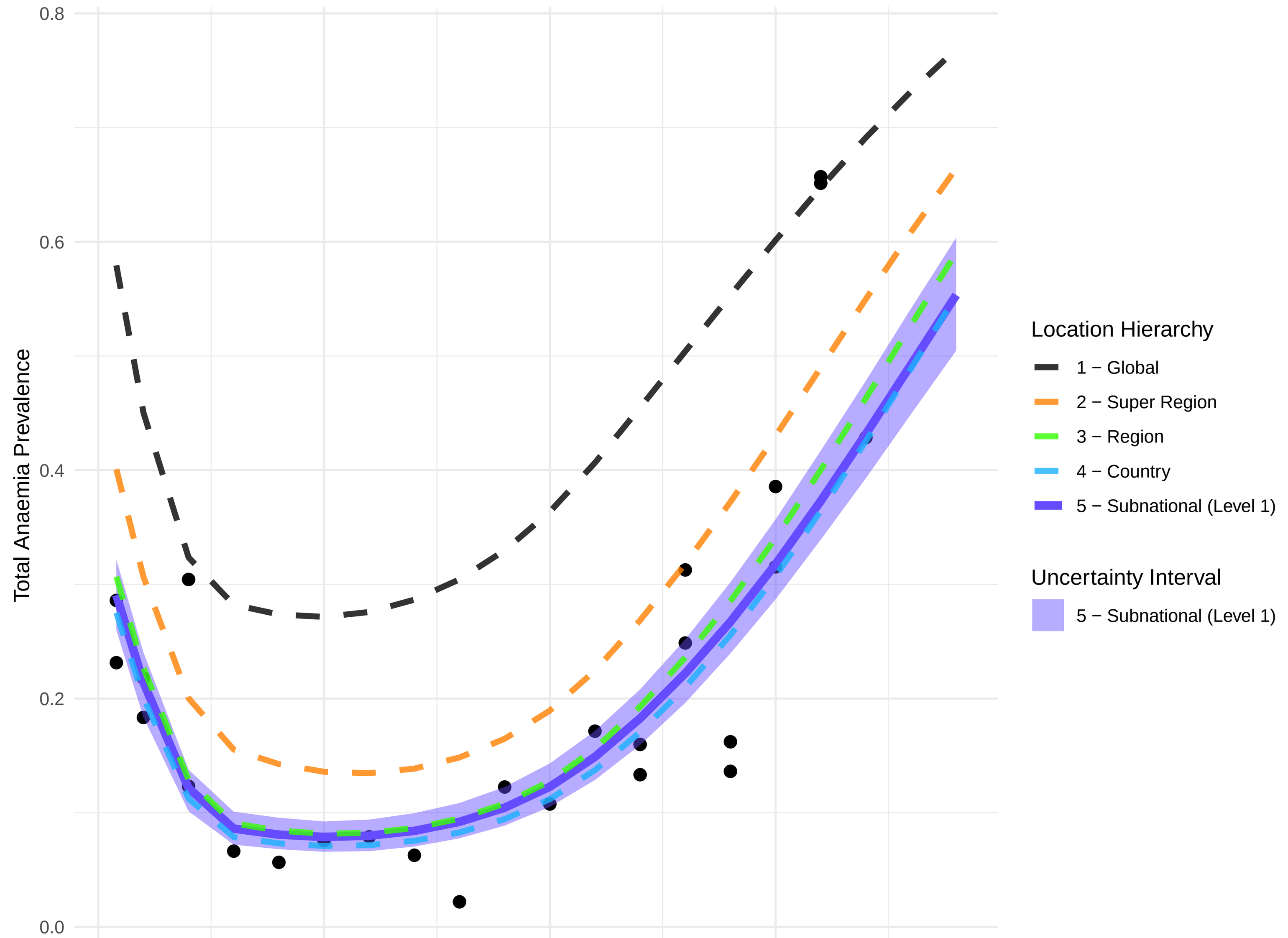

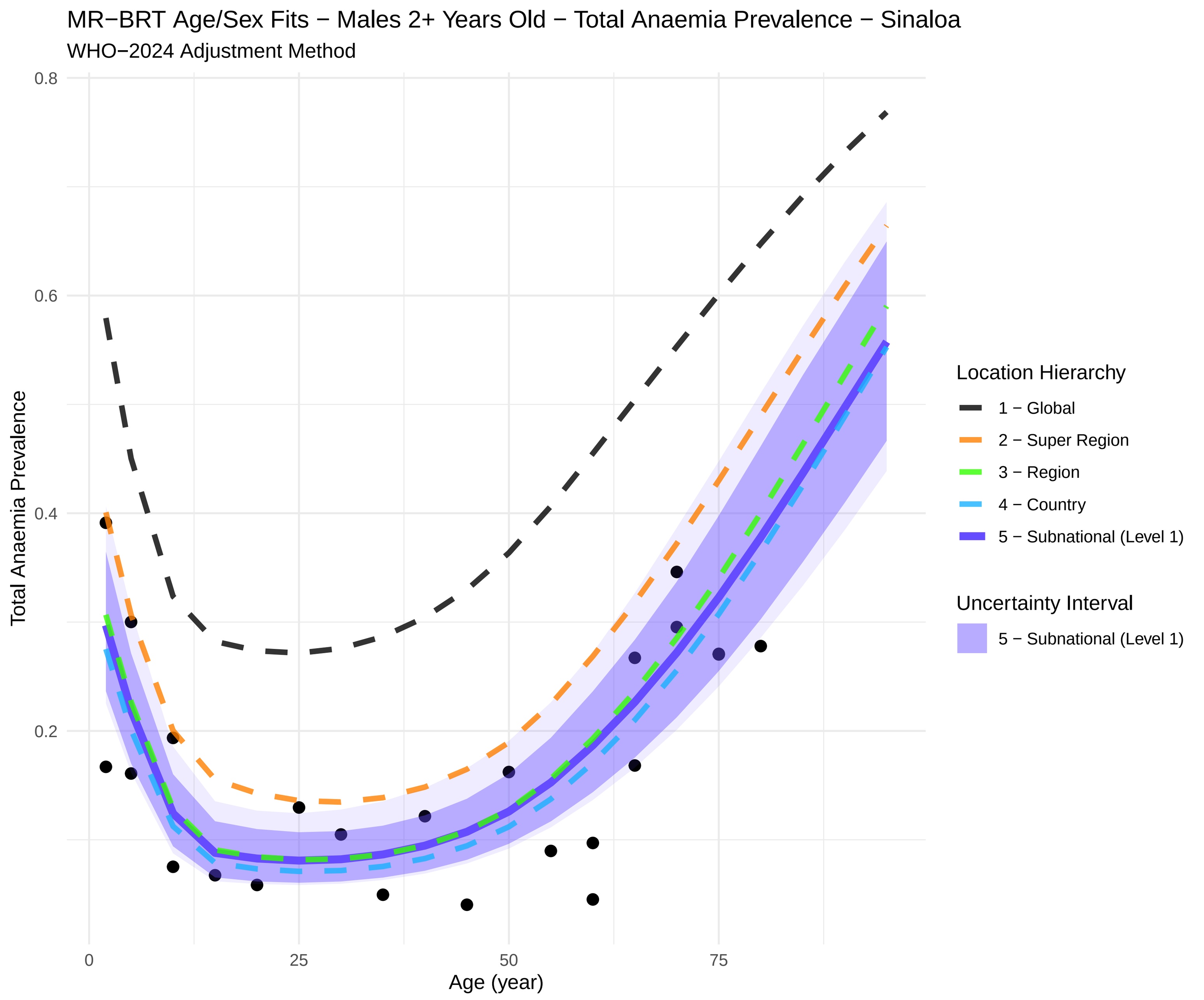
MR−BRT Age/Sex Fits − Males 2+ Years Old − Total Anaemia Prevalence − Sinaloa
WHO−2024 Adjustment Method
Total Anaemia Prevalence
Age (year)
Location Hierarchy
1 − Global
2 − Super Region
3 − Region
4 − Country
5 − Subnational (Level 1)
Uncertainty Interval
5 − Subnational (Level 1)

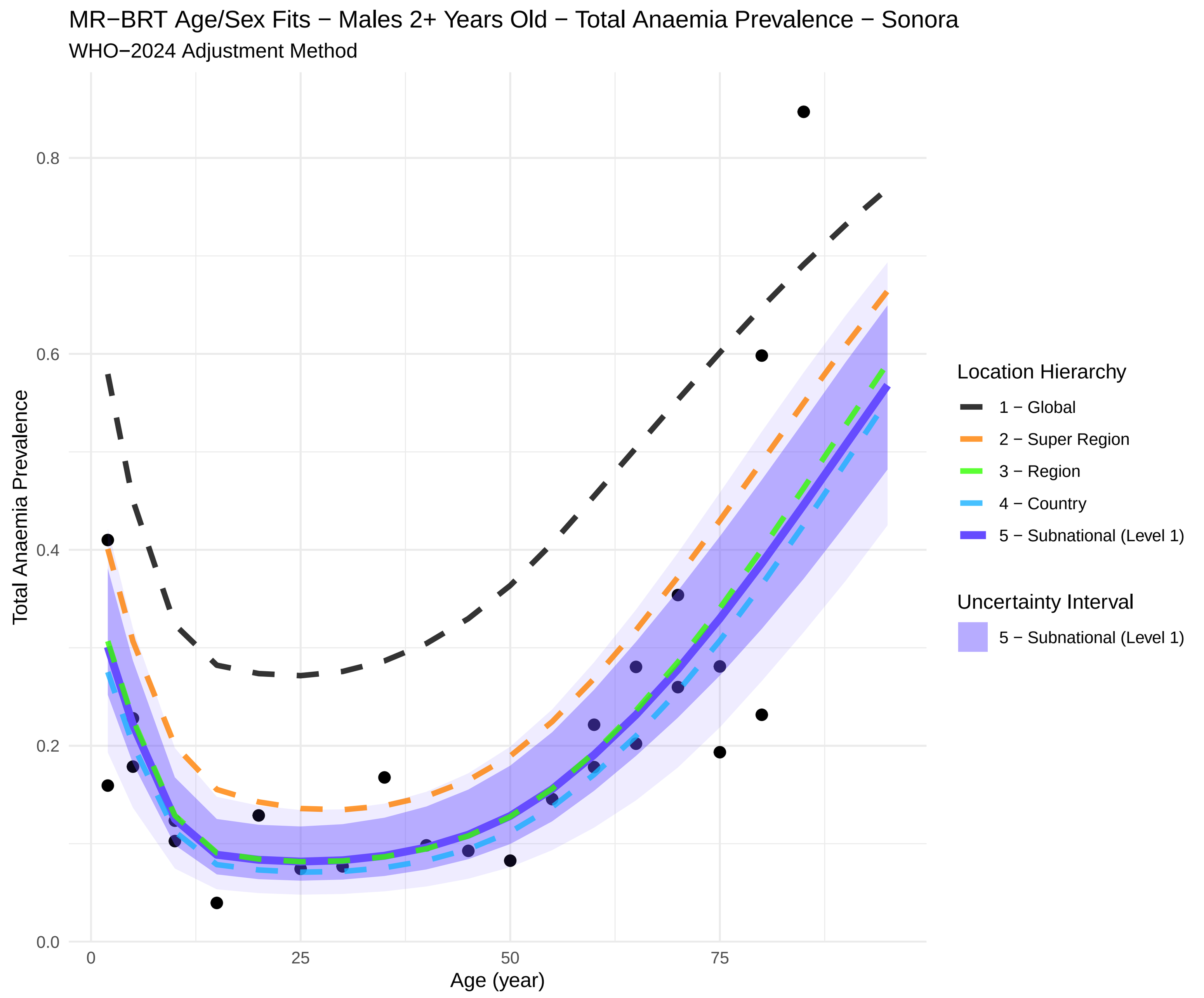
MR−BRT Age/Sex Fits − Males 2+ Years Old − Total Anaemia Prevalence − Sonora
WHO−2024 Adjustment Method
Total Anaemia Prevalence
Age (year)
Location Hierarchy
1 − Global
2 − Super Region
3 − Region
4 − Country
5 − Subnational (Level 1)
Uncertainty Interval
5 − Subnational (Level 1)

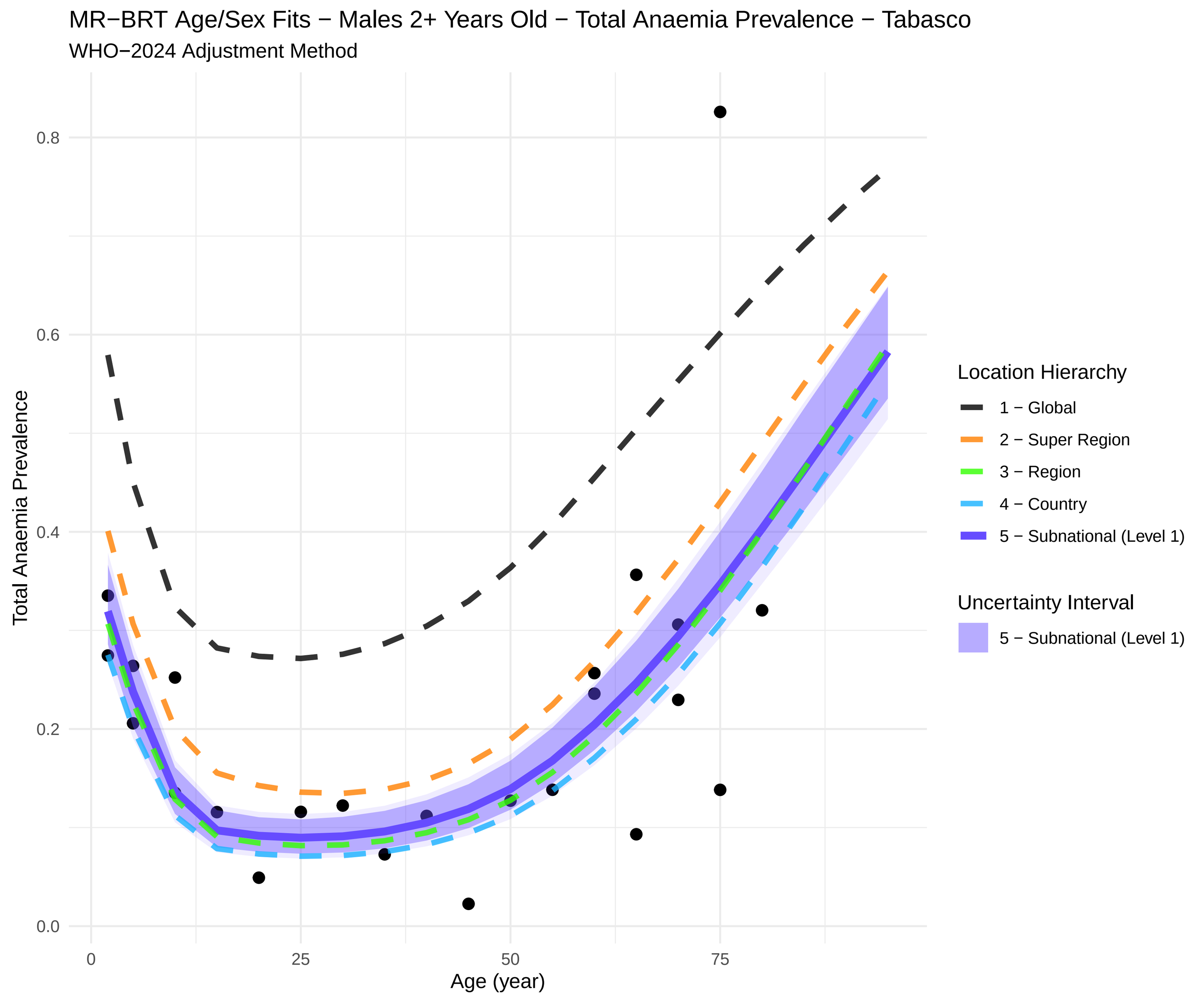
MR−BRT Age/Sex Fits − Males 2+ Years Old − Total Anaemia Prevalence − Tabasco
WHO−2024 Adjustment Method
Total Anaemia Prevalence
Age (year)
Location Hierarchy
1 − Global
2 − Super Region
3 − Region
4 − Country
5 − Subnational (Level 1)
Uncertainty Interval
5 − Subnational (Level 1)

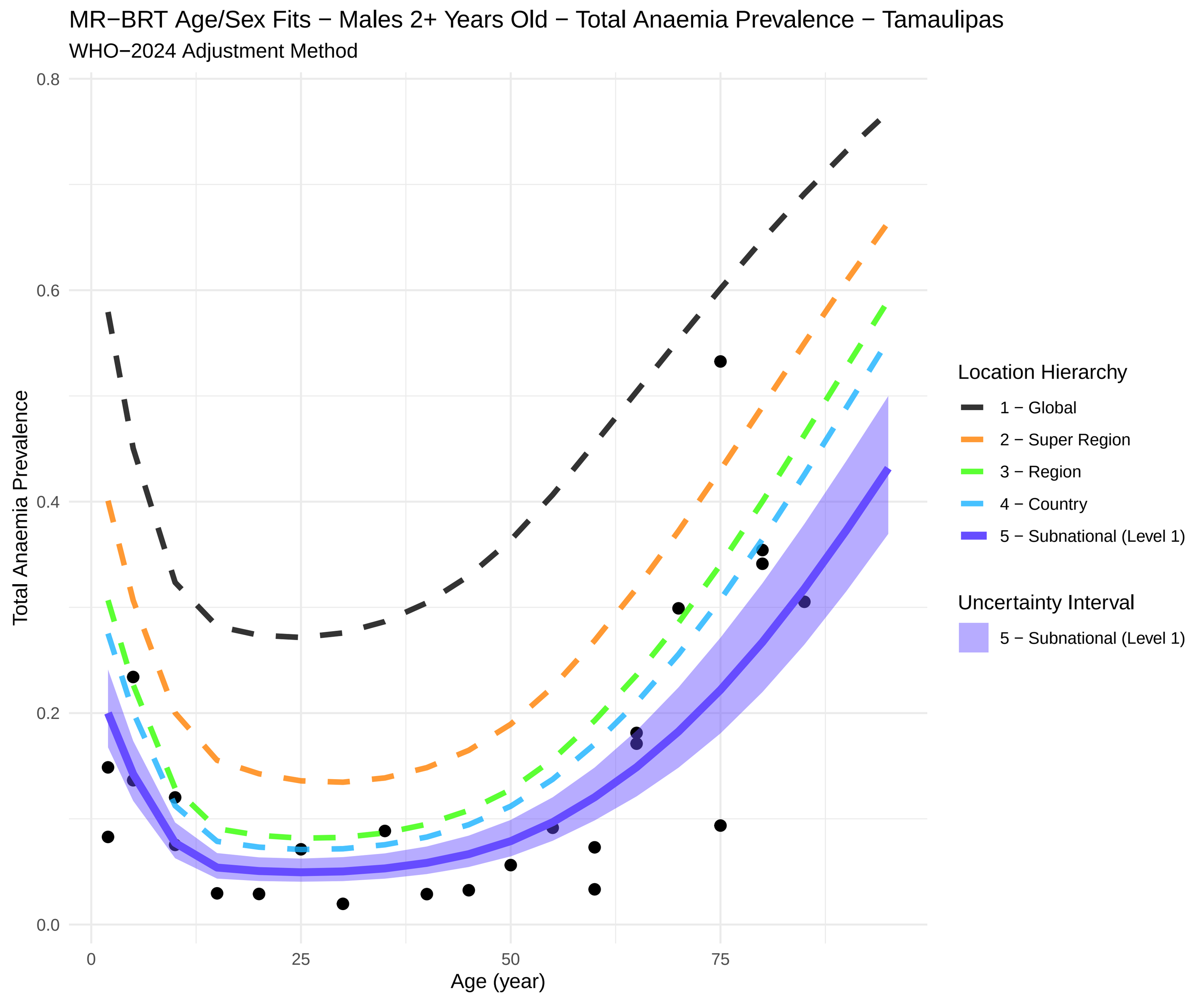
MR−BRT Age/Sex Fits − Males 2+ Years Old − Total Anaemia Prevalence − Tamaulipas
WHO−2024 Adjustment Method
Total Anaemia Prevalence
Age (year)
Location Hierarchy
1 − Global
2 − Super Region
3 − Region
4 − Country
5 − Subnational (Level 1)
Uncertainty Interval
5 − Subnational (Level 1)

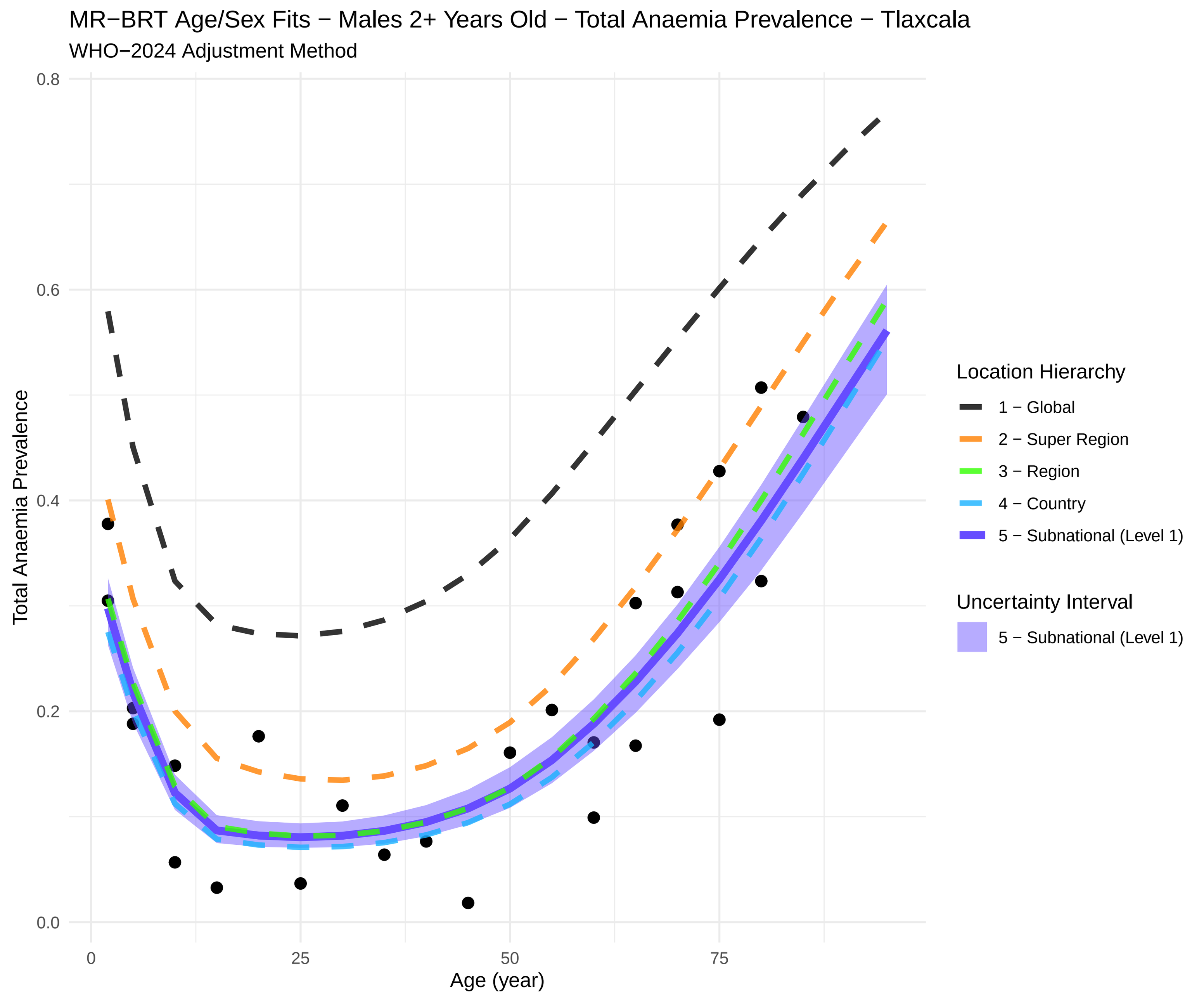
MR−BRT Age/Sex Fits − Males 2+ Years Old − Total Anaemia Prevalence − Tlaxcala
WHO−2024 Adjustment Method
Total Anaemia Prevalence
Age (year)
Location Hierarchy
1 − Global
2 − Super Region
3 − Region
4 − Country
5 − Subnational (Level 1)
Uncertainty Interval
5 − Subnational (Level 1)

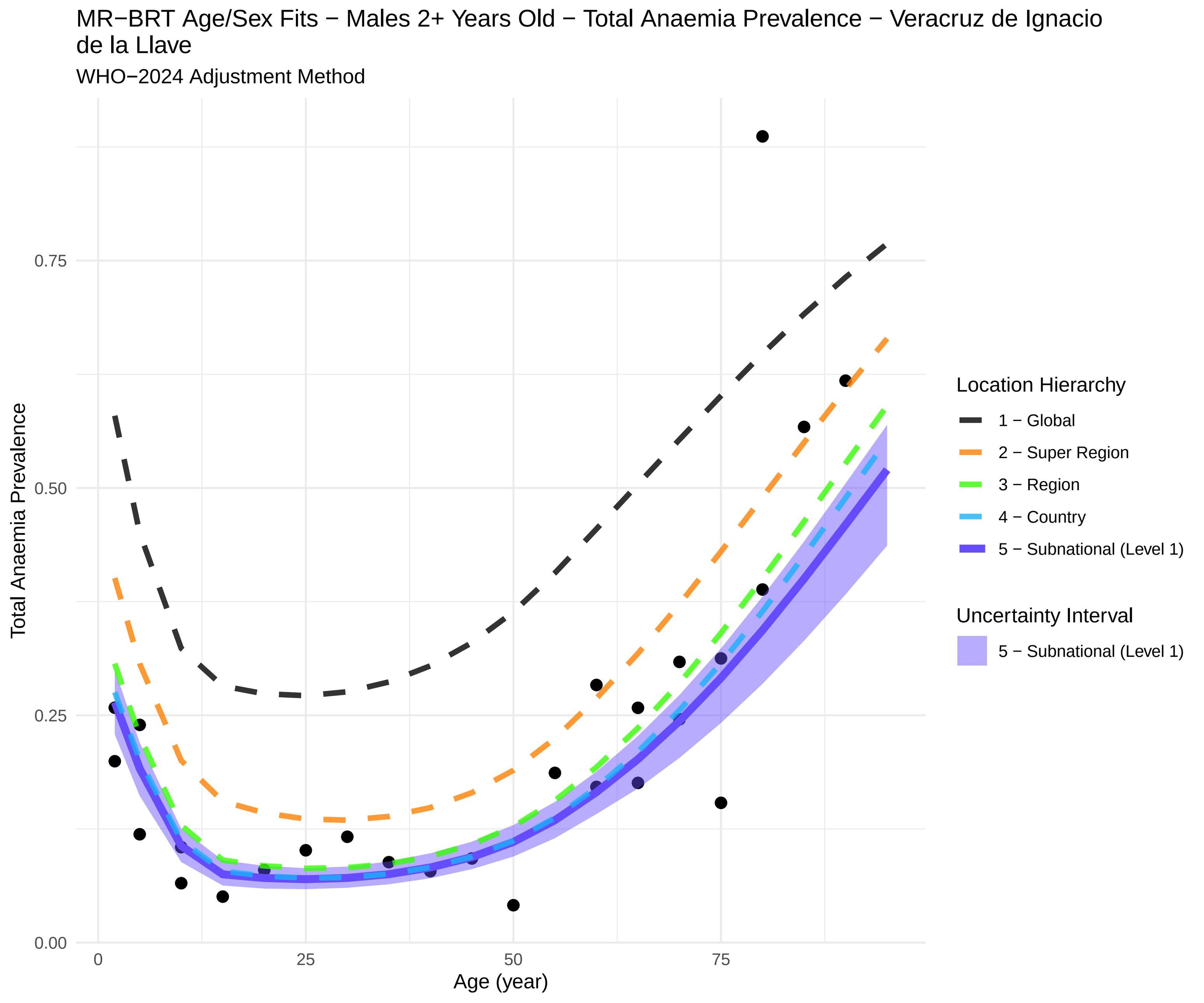
MR−BRT Age/Sex Fits − Males 2+ Years Old − Total Anaemia Prevalence − Veracruz de Ignacio de la Llave
WHO−2024 Adjustment Method
Total Anaemia Prevalence
Age (year)
Location Hierarchy
1 − Global
2 − Super Region
3 − Region
4 − Country
5 − Subnational (Level 1)
Uncertainty Interval
5 − Subnational (Level 1)

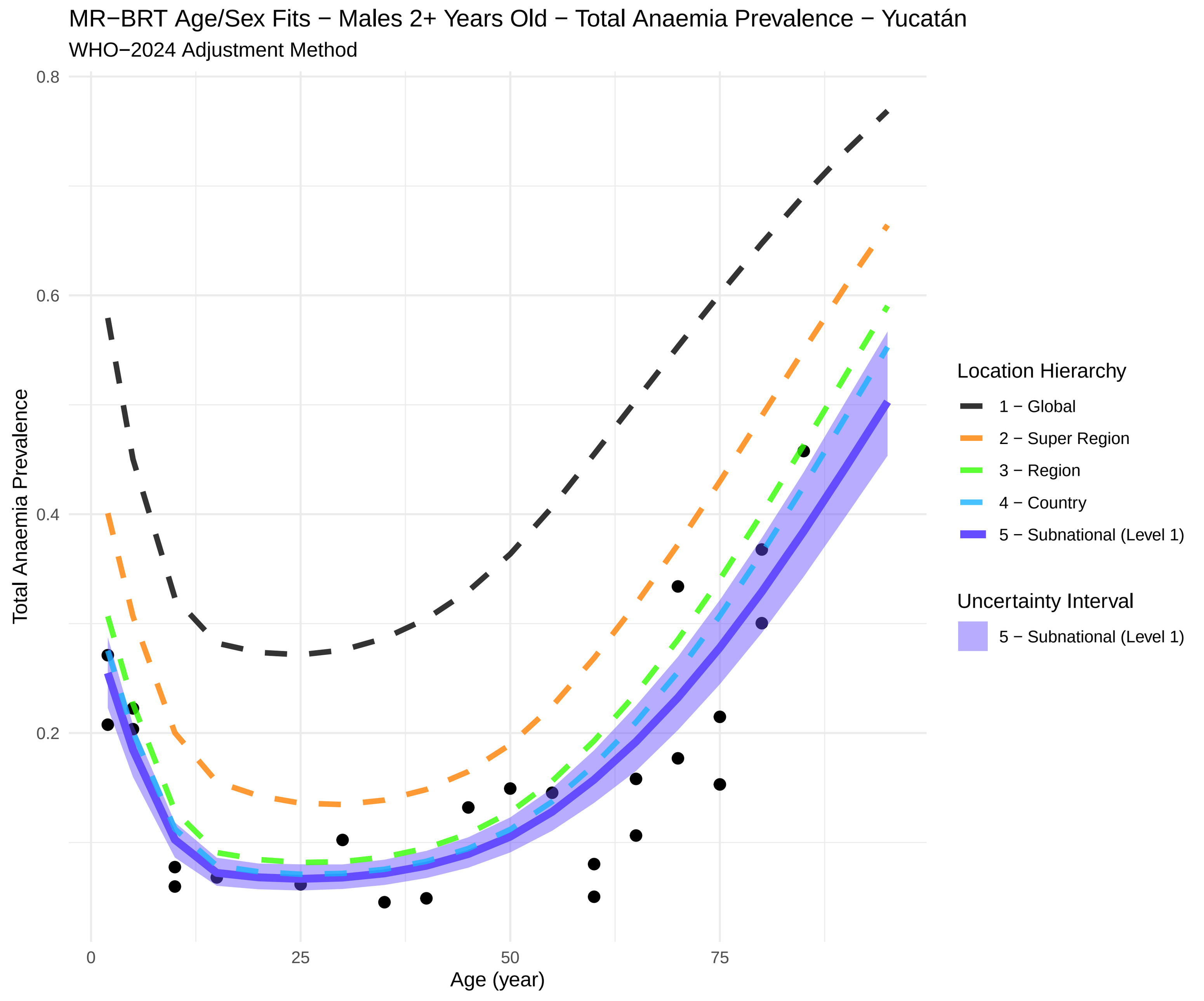
MR−BRT Age/Sex Fits − Males 2+ Years Old − Total Anaemia Prevalence − Yucatán
WHO−2024 Adjustment Method
Total Anaemia Prevalence
Age (year)
Location Hierarchy
1 − Global
2 − Super Region
3 − Region
4 − Country
5 − Subnational (Level 1)
Uncertainty Interval
5 − Subnational (Level 1)

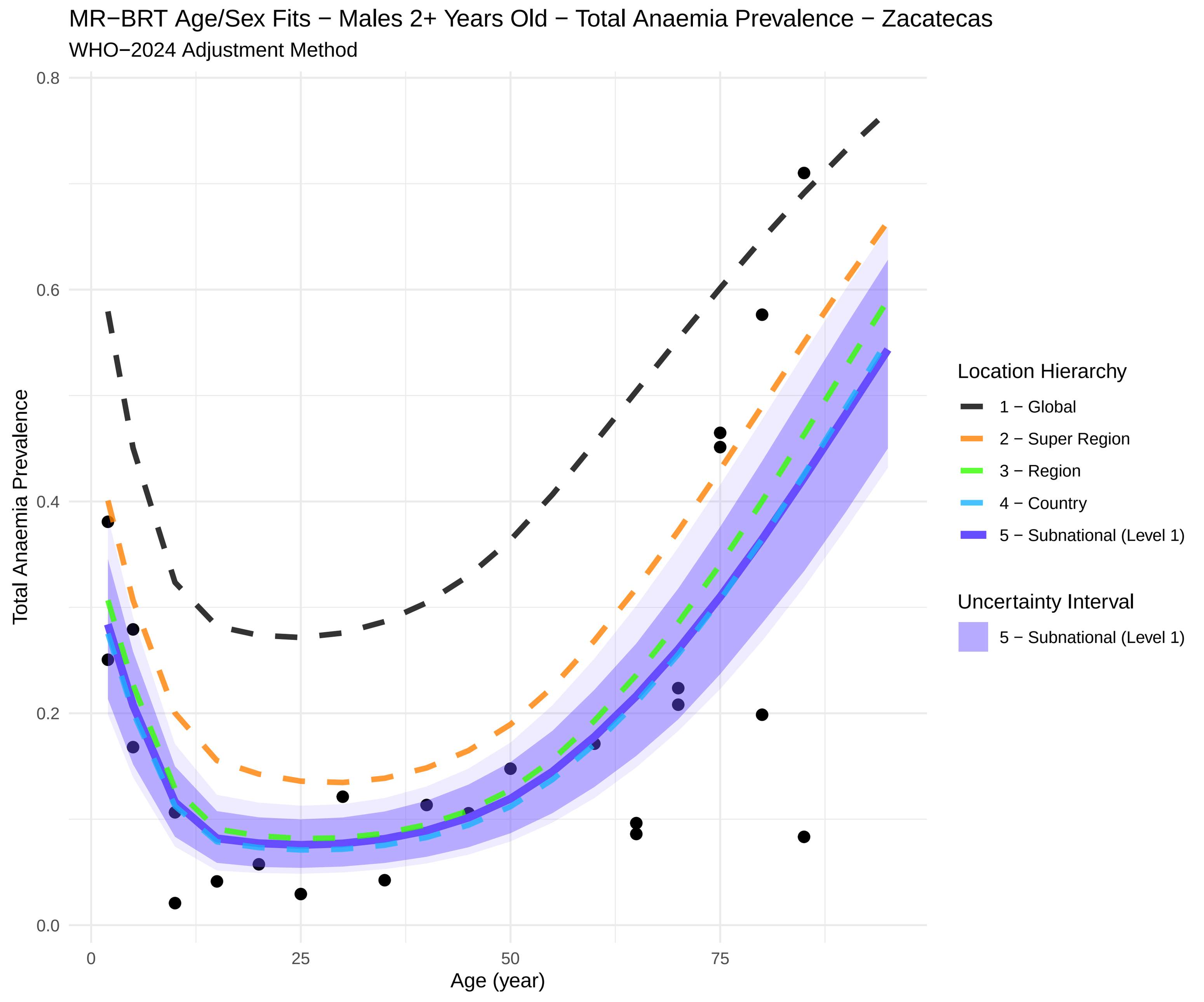
MR−BRT Age/Sex Fits − Males 2+ Years Old − Total Anaemia Prevalence − Zacatecas
WHO−2024 Adjustment Method
Total Anaemia Prevalence
Age (year)
Location Hierarchy
1 − Global
2 − Super Region
3 − Region
4 − Country
5 − Subnational (Level 1)
Uncertainty Interval
5 − Subnational (Level 1)

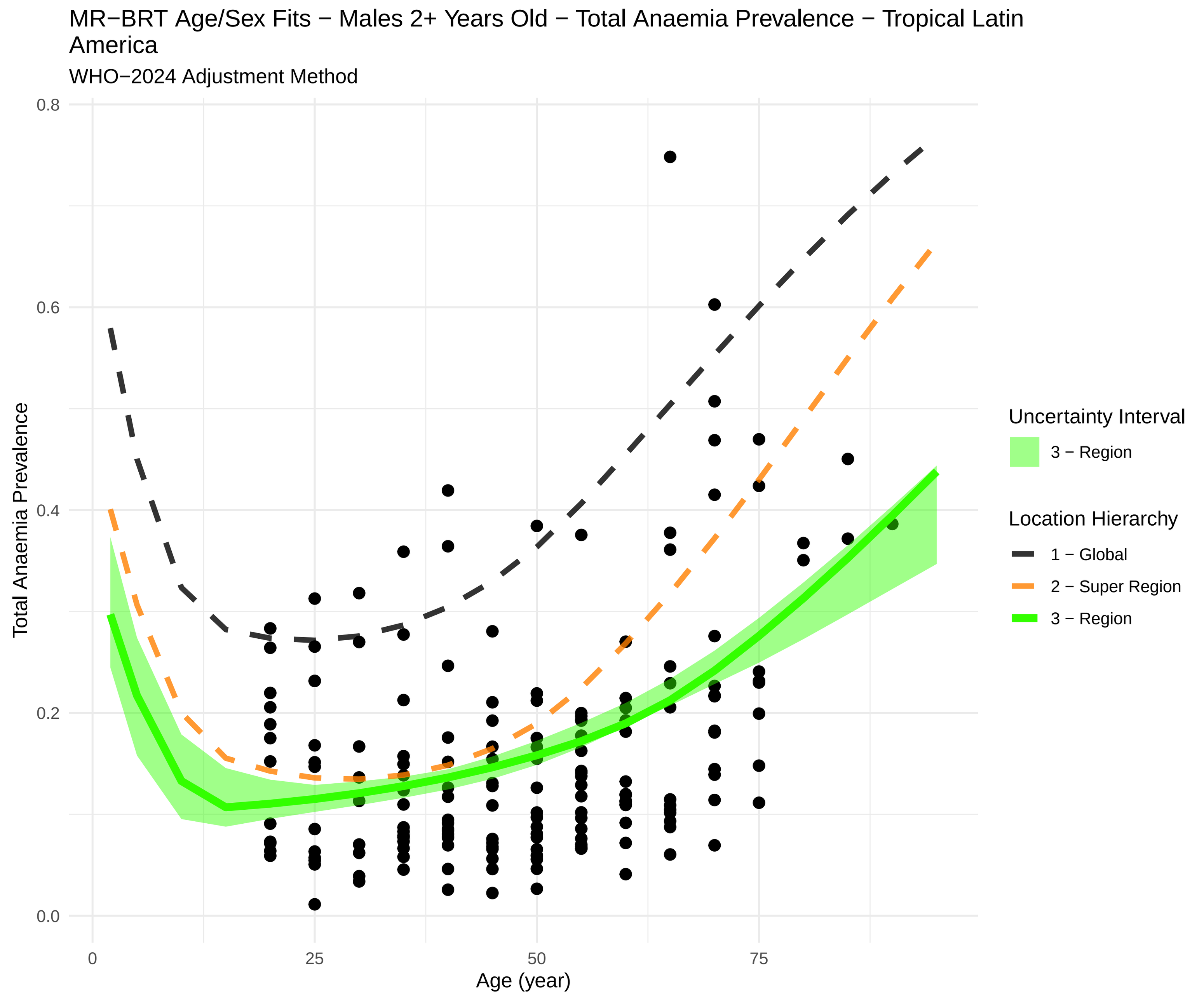
MR−BRT Age/Sex Fits − Males 2+ Years Old − Total Anaemia Prevalence − Tropical Latin America
WHO−2024 Adjustment Method
Total Anaemia Prevalence
Age (year)
Uncertainty Interval
3 − Region
Location Hierarchy
1 − Global
2 − Super Region
3 − Region

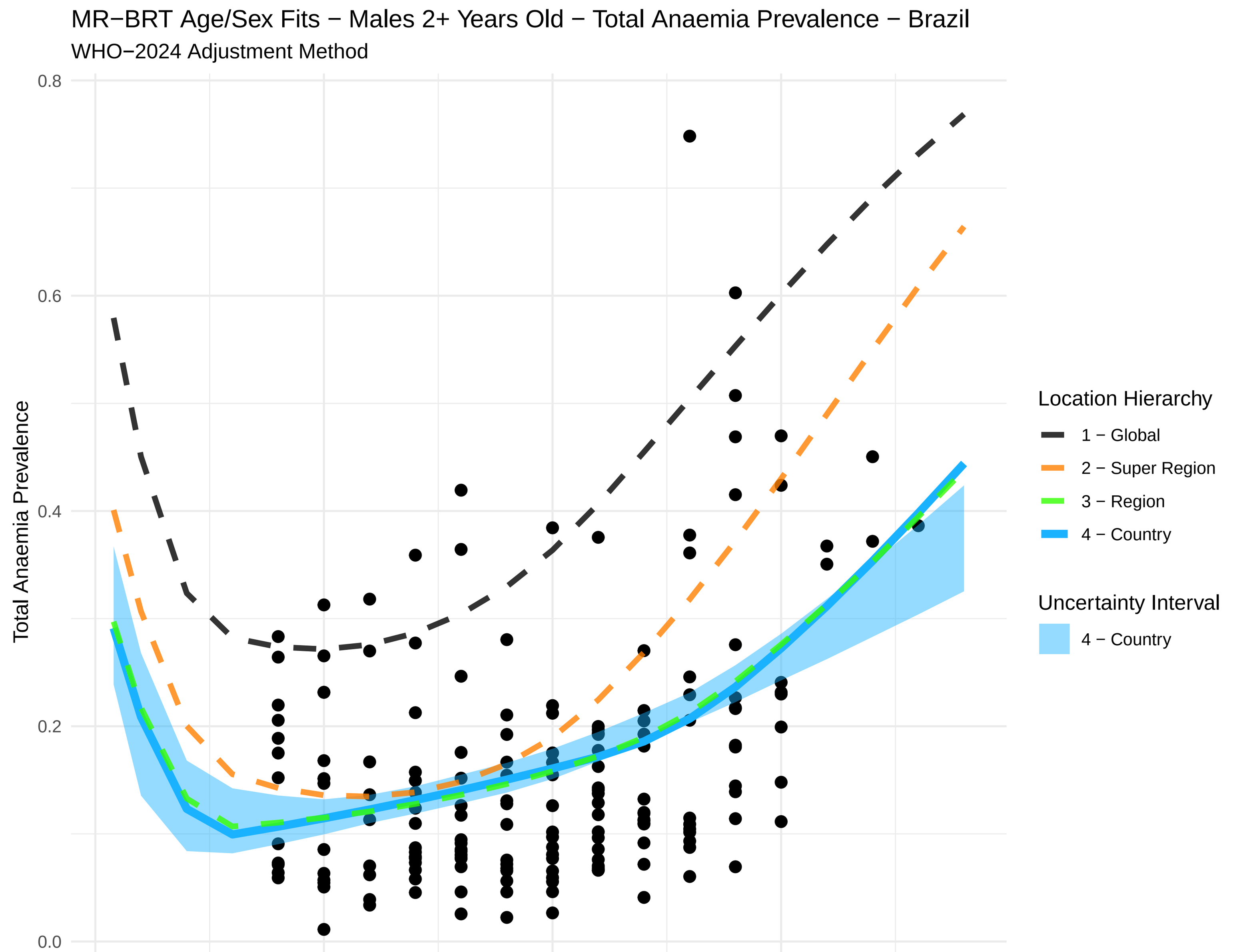
MR−BRT Age/Sex Fits − Males 2+ Years Old − Total Anaemia Prevalence − Brazil
WHO−2024 Adjustment Method
Total Anaemia Prevalence
Age (year)
Location Hierarchy
1 − Global
2 − Super Region
3 − Region
4 − Country
Uncertainty Interval
4 − Country

MR−BRT Age/Sex Fits − Males 2+ Years Old − Total Anaemia Prevalence − Acre
WHO−2024 Adjustment Method

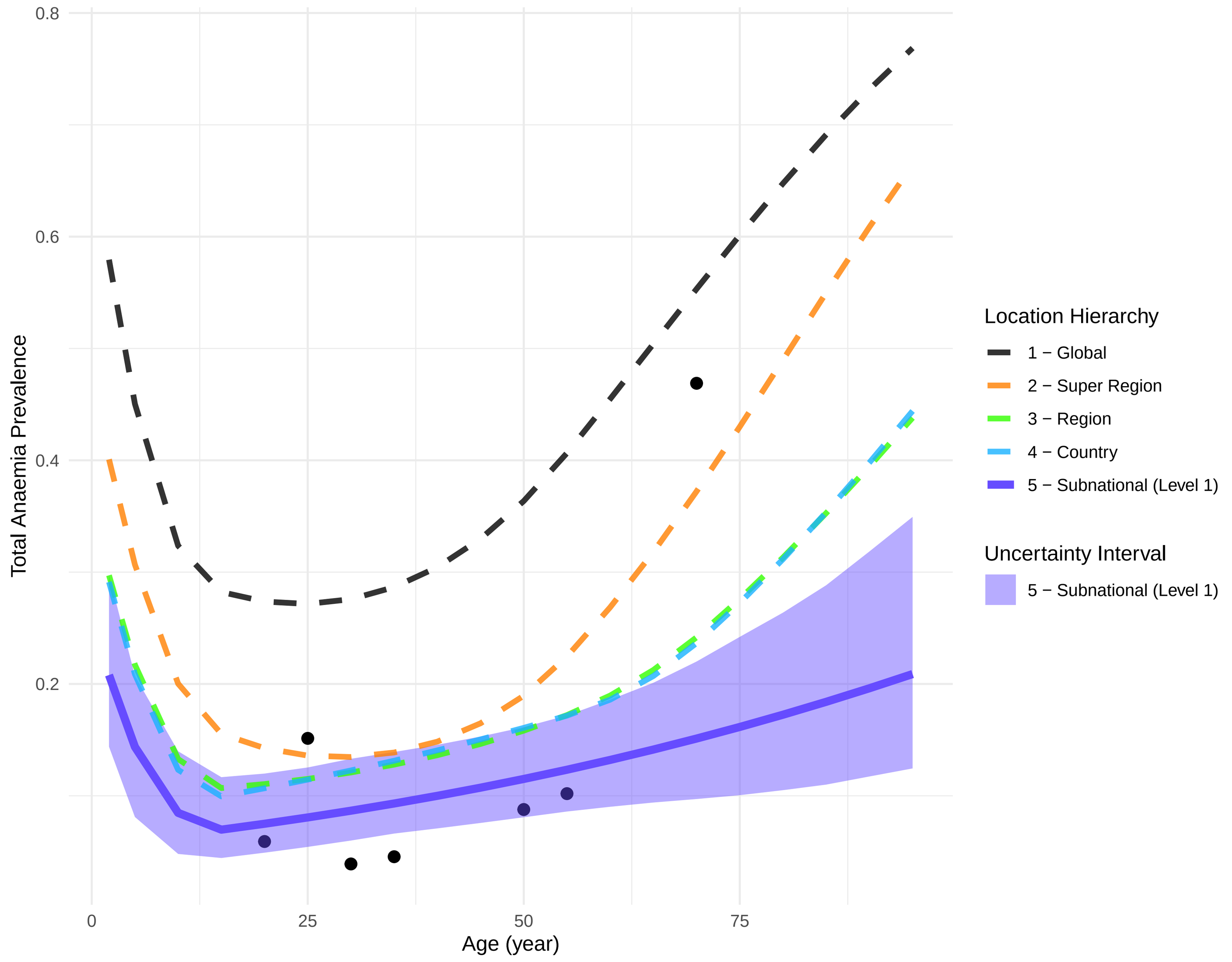

MR−BRT Age/Sex Fits − Males 2+ Years Old − Total Anaemia Prevalence − Alagoas
WHO−2024 Adjustment Method

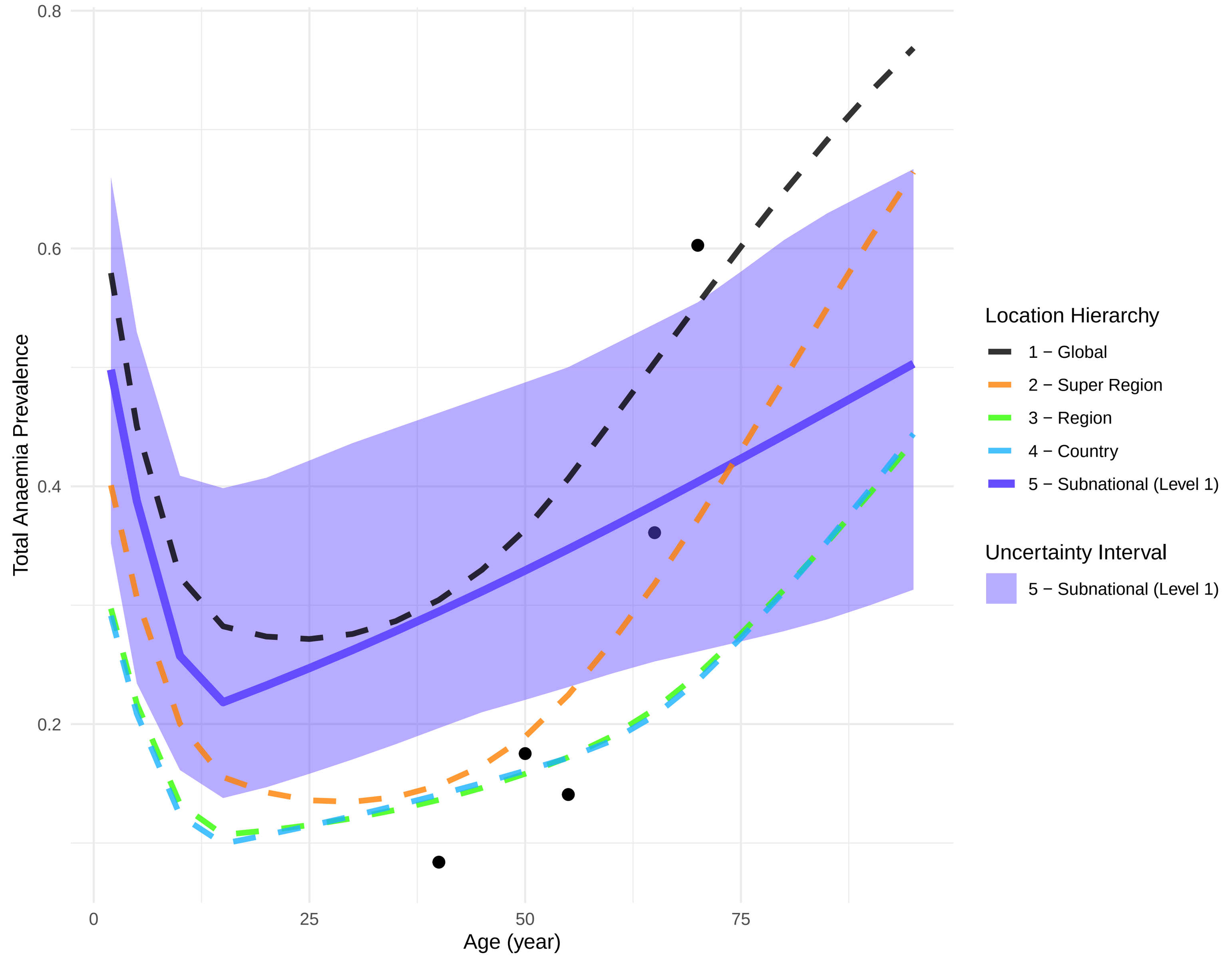

MR−BRT Age/Sex Fits − Males 2+ Years Old − Total Anaemia Prevalence − Amapá
WHO−2024 Adjustment Method

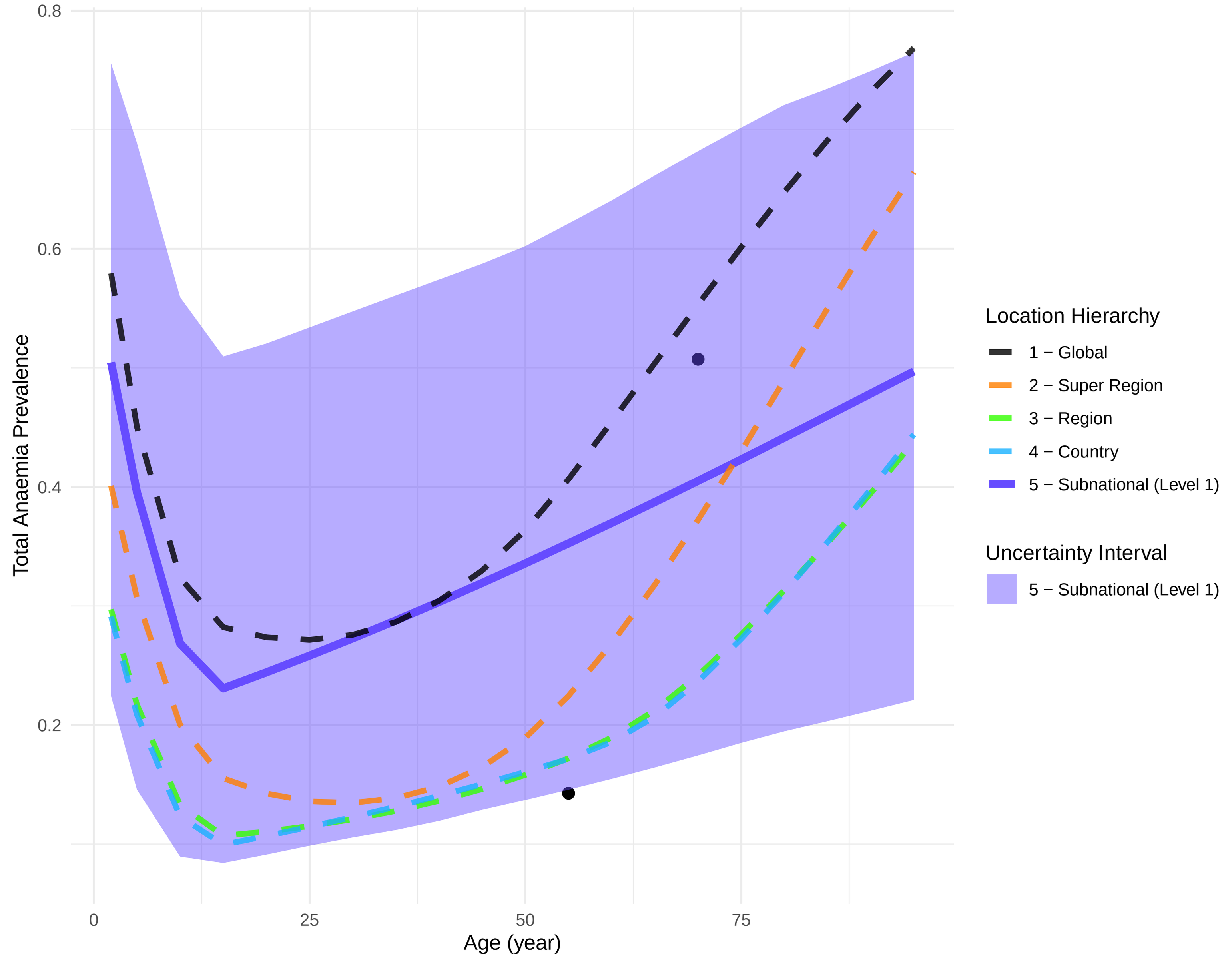

MR−BRT Age/Sex Fits − Males 2+ Years Old − Total Anaemia Prevalence − Amazonas
WHO−2024 Adjustment Method

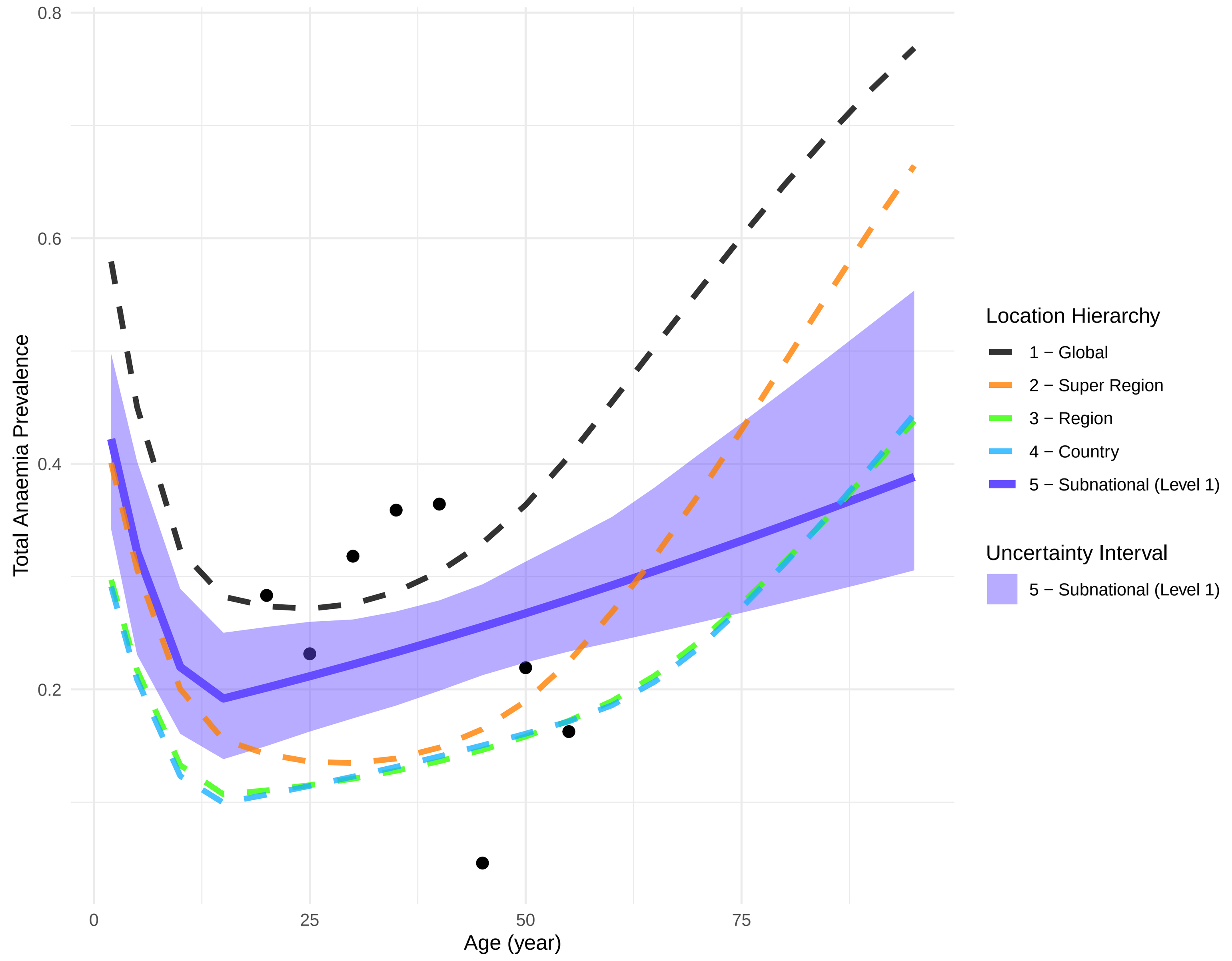

MR−BRT Age/Sex Fits − Males 2+ Years Old − Total Anaemia Prevalence − Bahia
WHO−2024 Adjustment Method

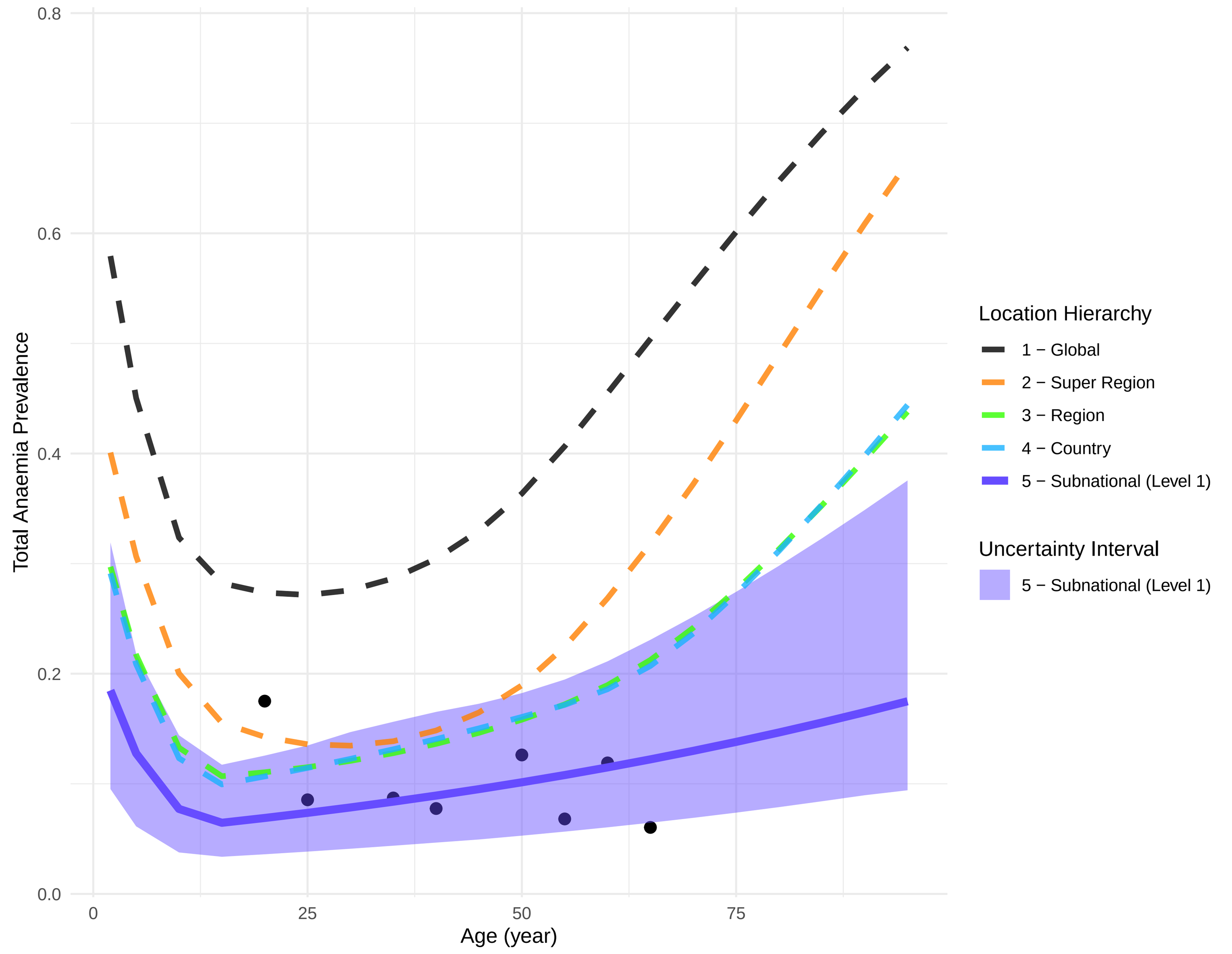

MR−BRT Age/Sex Fits − Males 2+ Years Old − Total Anaemia Prevalence − Ceará
WHO−2024 Adjustment Method

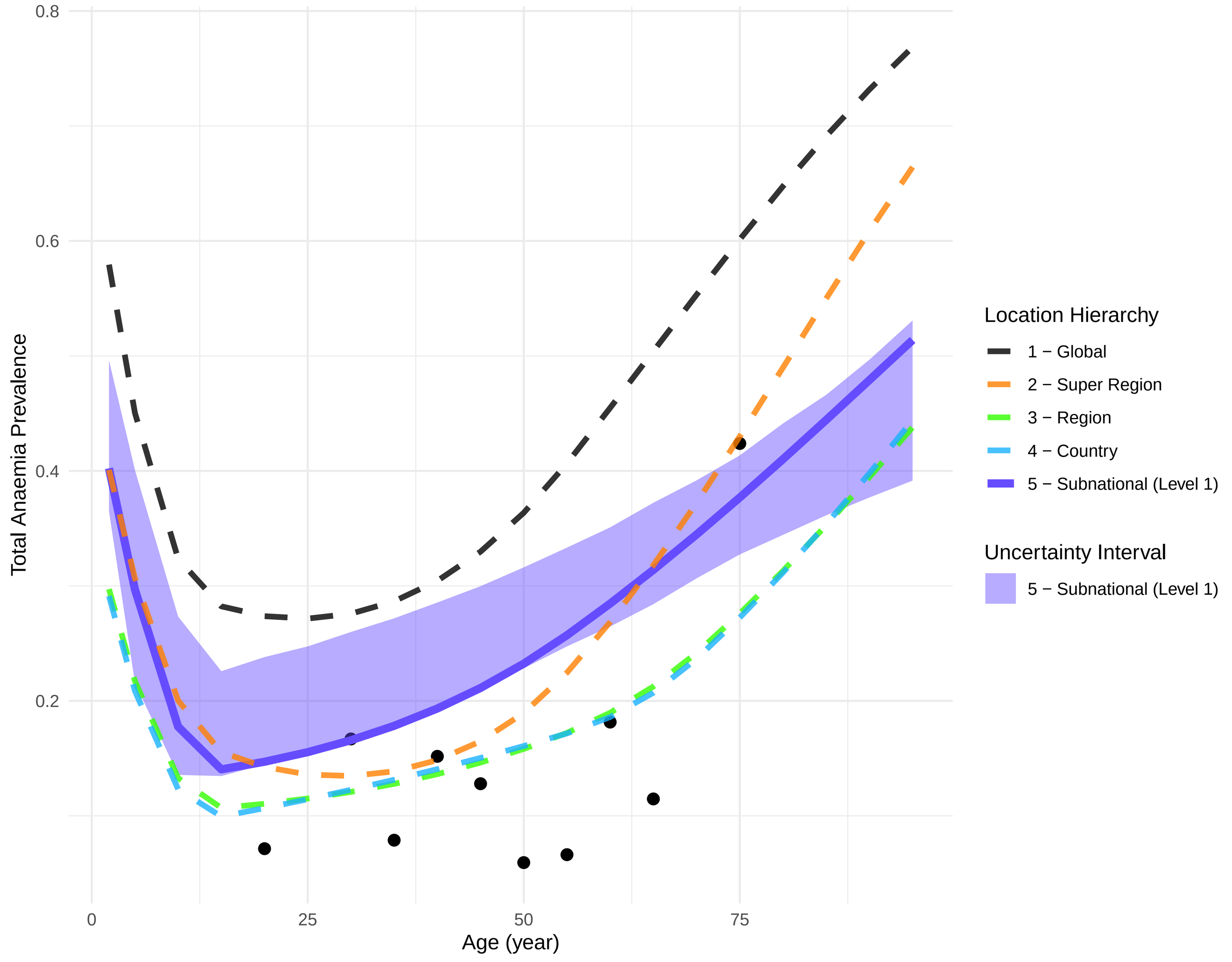

MR−BRT Age/Sex Fits − Males 2+ Years Old − Total Anaemia Prevalence − Distrito Federal
WHO−2024 Adjustment Method

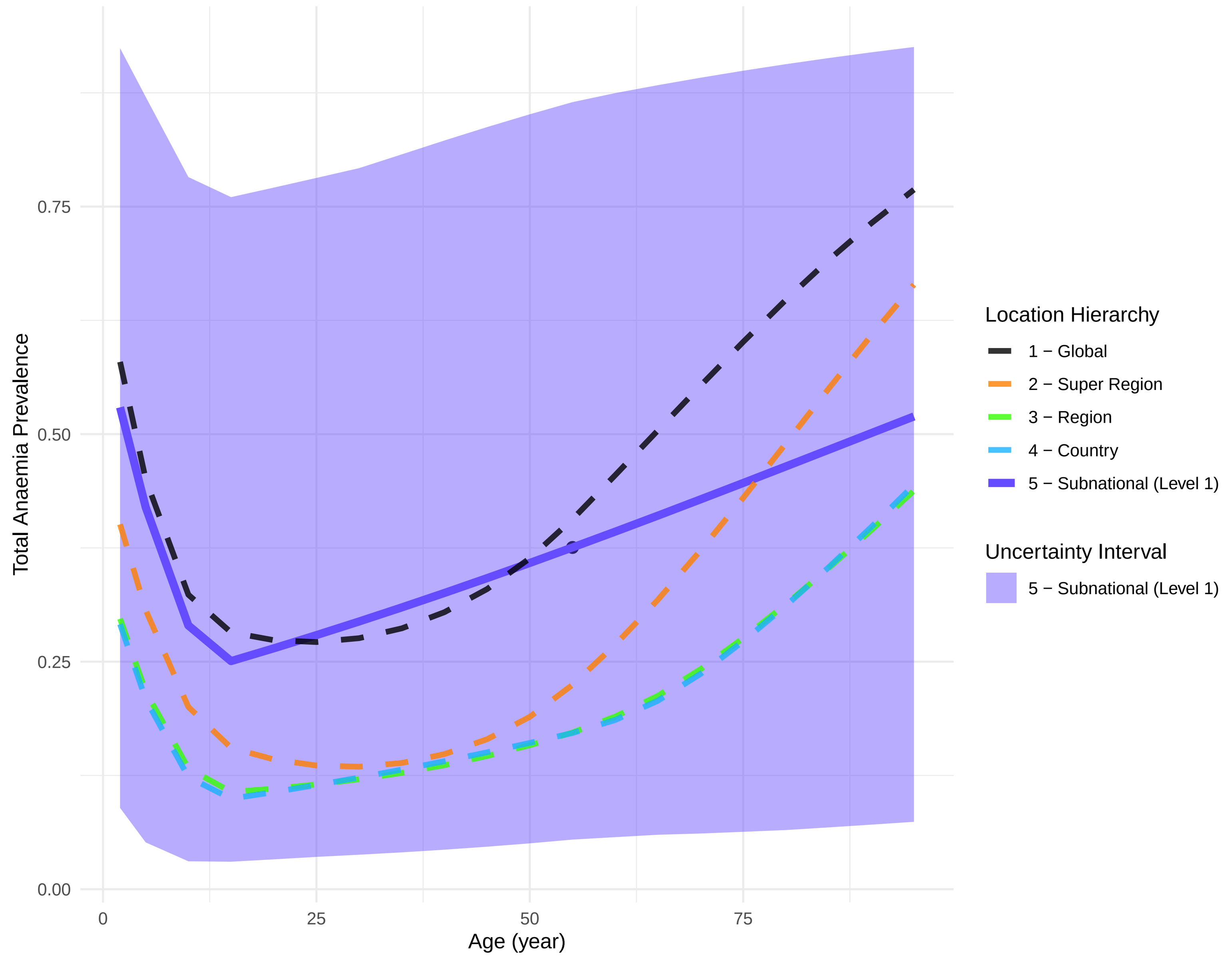

MR−BRT Age/Sex Fits − Males 2+ Years Old − Total Anaemia Prevalence − Espírito Santo
WHO−2024 Adjustment Method

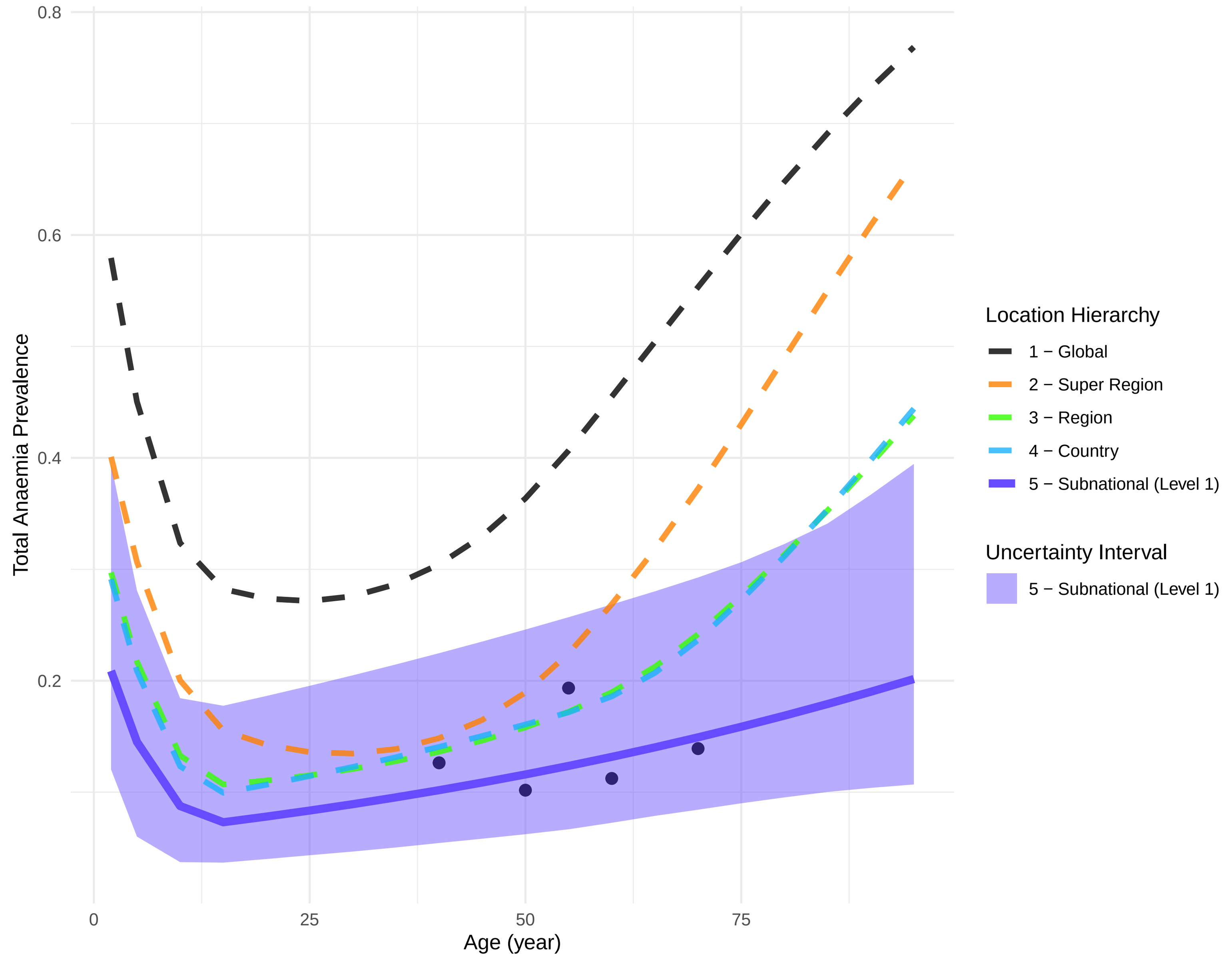

MR−BRT Age/Sex Fits − Males 2+ Years Old − Total Anaemia Prevalence − Goiás
WHO−2024 Adjustment Method

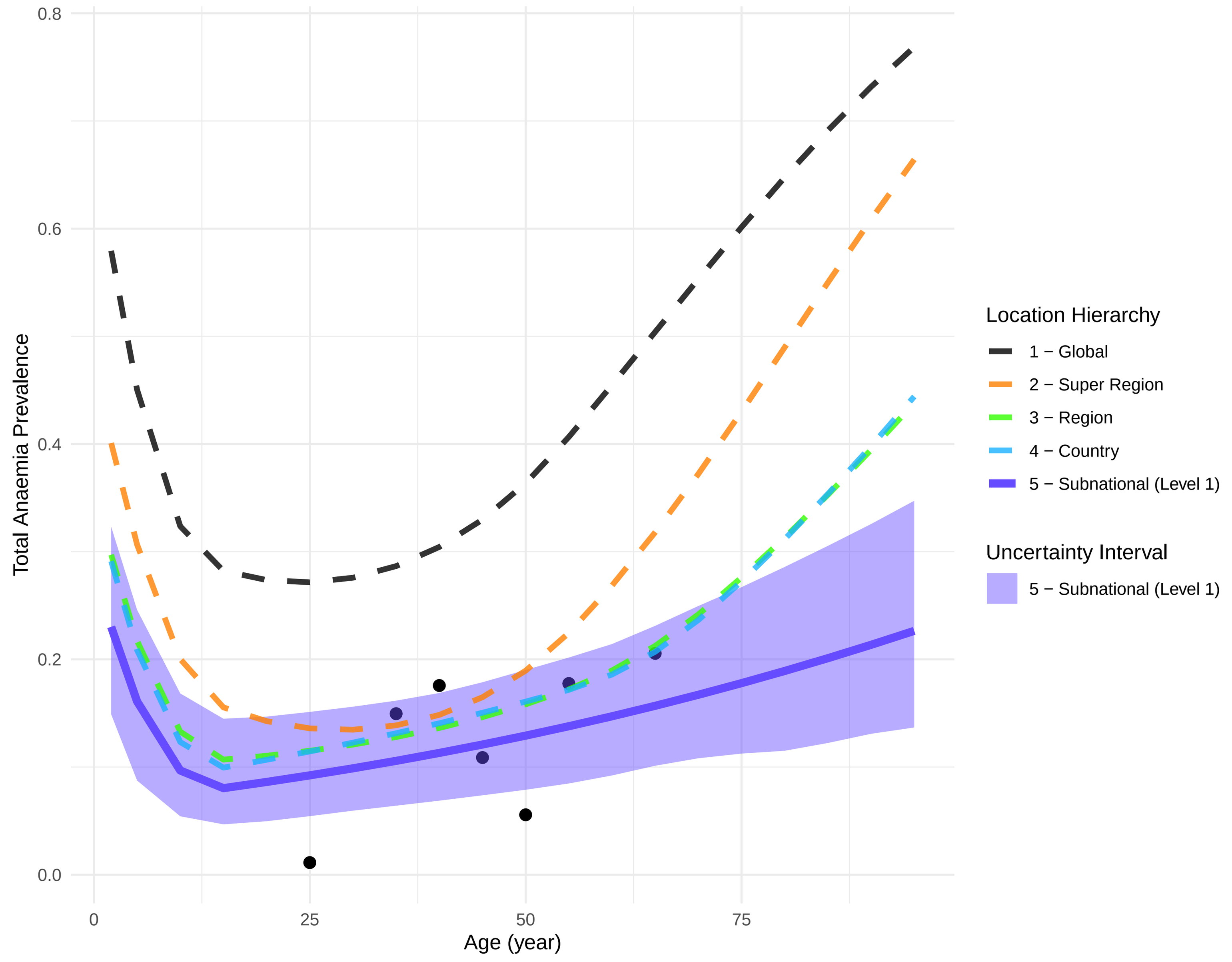

MR−BRT Age/Sex Fits − Males 2+ Years Old − Total Anaemia Prevalence − Maranhão
WHO−2024 Adjustment Method

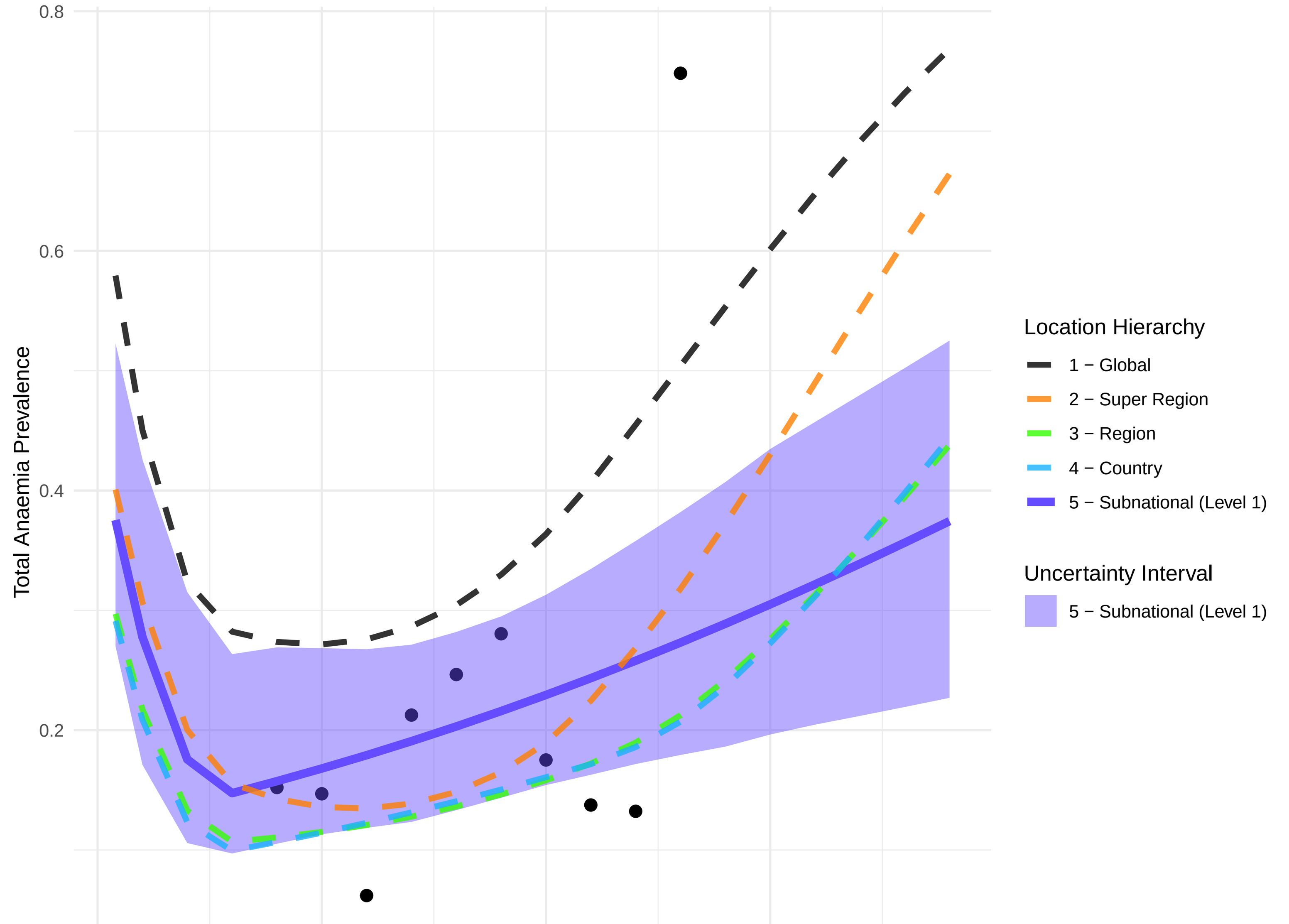

MR−BRT Age/Sex Fits − Males 2+ Years Old − Total Anaemia Prevalence − Mato Grosso
WHO−2024 Adjustment Method

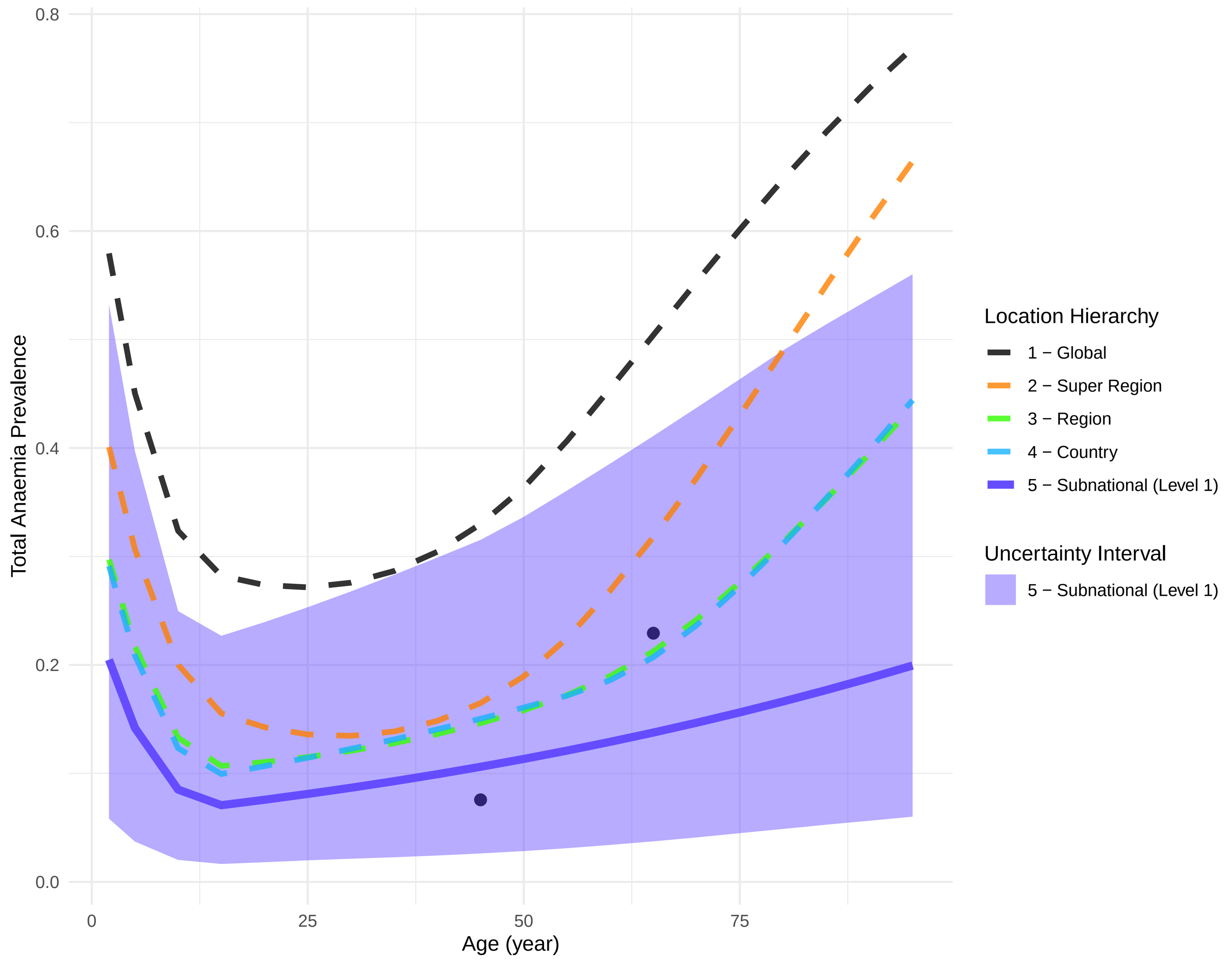

MR−BRT Age/Sex Fits − Males 2+ Years Old − Total Anaemia Prevalence − Mato Grosso do Sul
WHO−2024 Adjustment Method

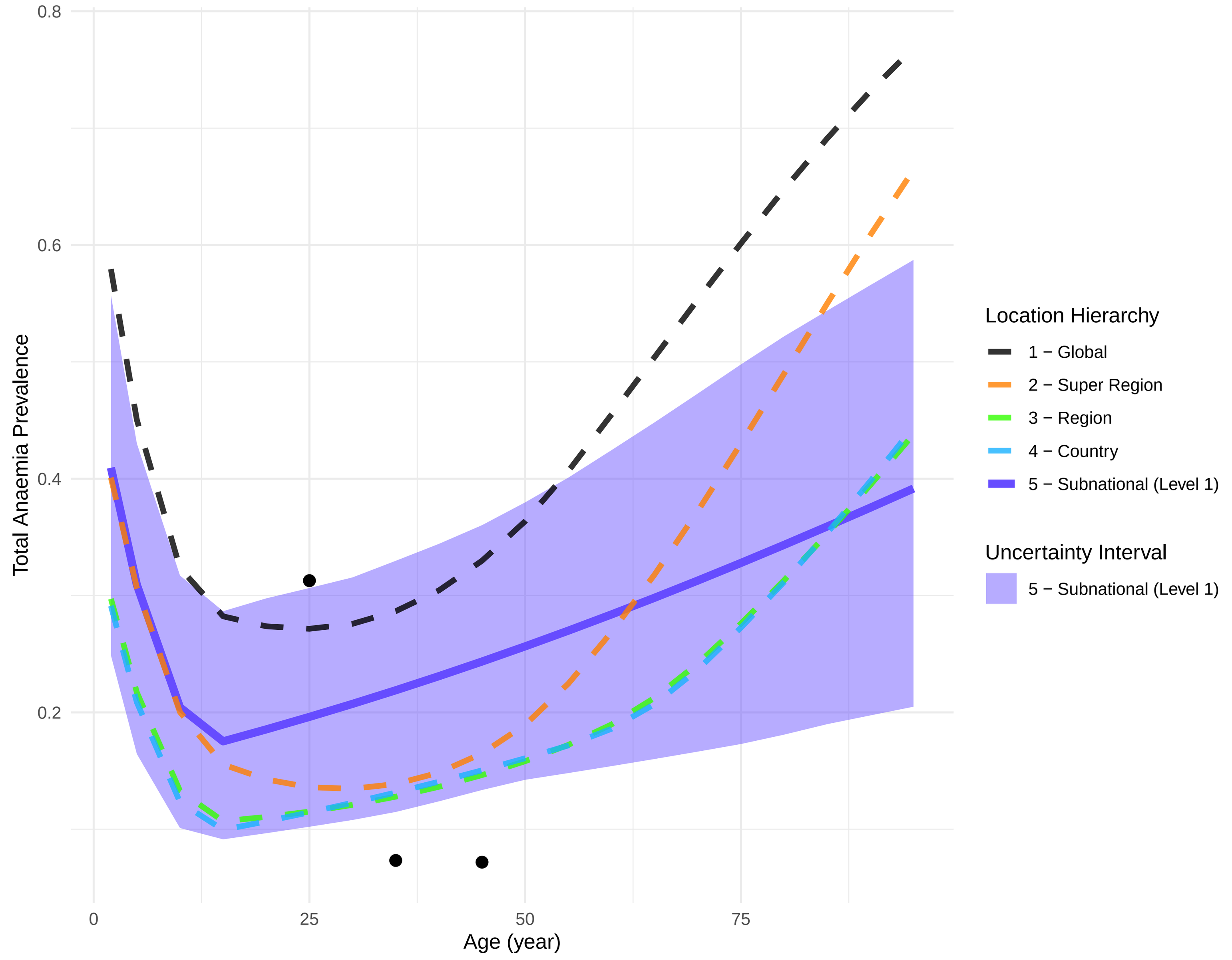

MR−BRT Age/Sex Fits − Males 2+ Years Old − Total Anaemia Prevalence − Minas Gerais
WHO−2024 Adjustment Method

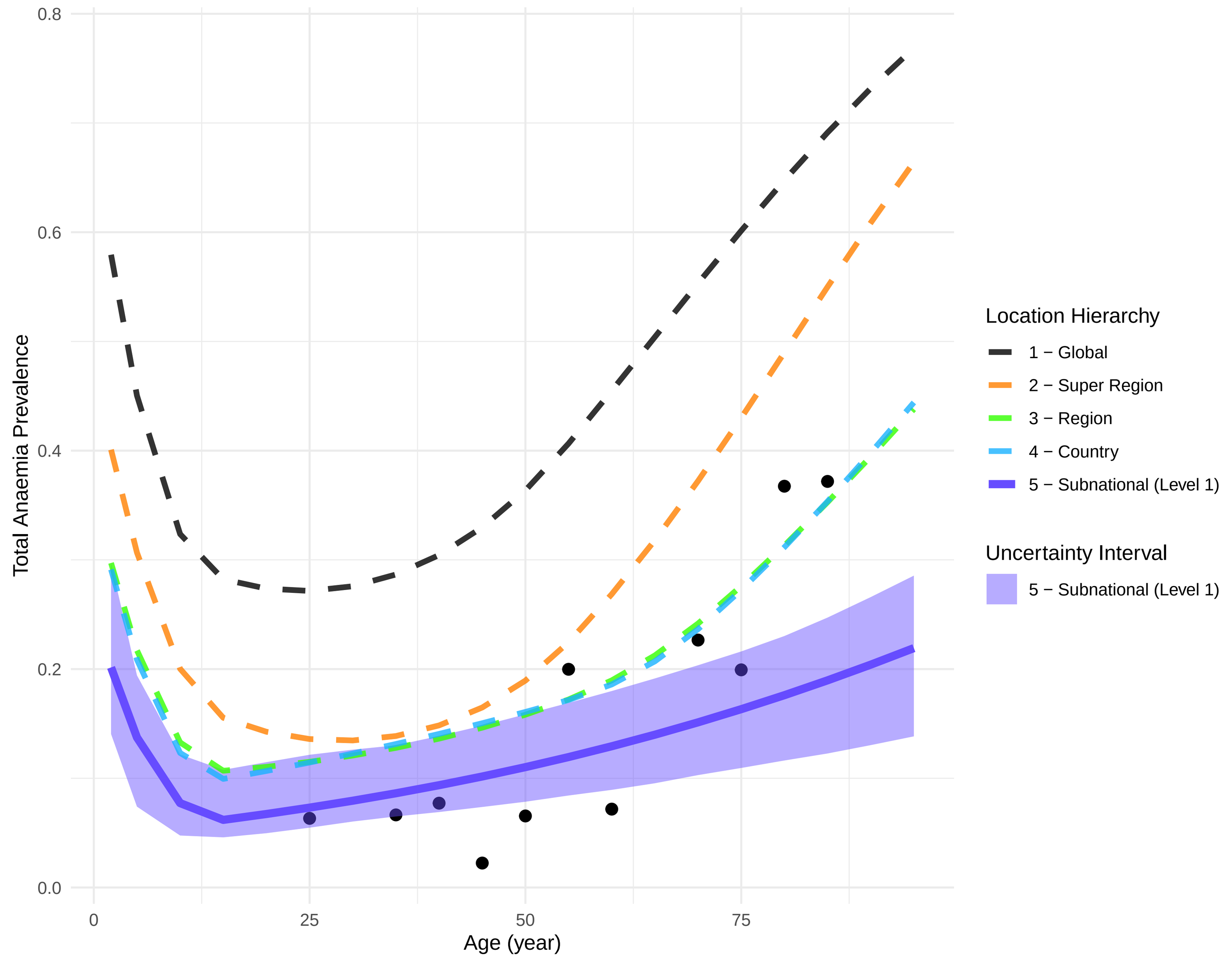

MR−BRT Age/Sex Fits − Males 2+ Years Old − Total Anaemia Prevalence − Pará
WHO−2024 Adjustment Method

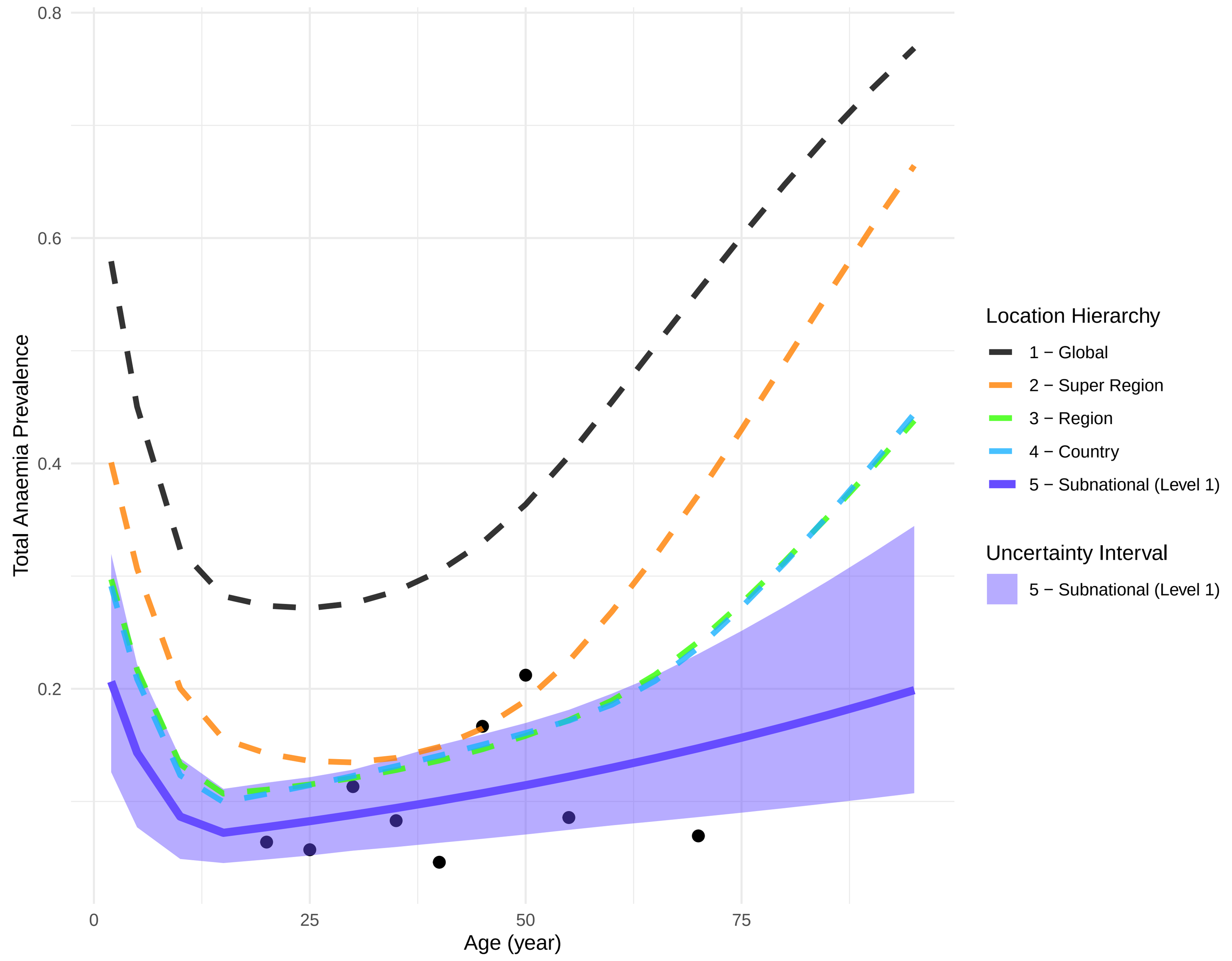

MR−BRT Age/Sex Fits − Males 2+ Years Old − Total Anaemia Prevalence − Paraíba
WHO−2024 Adjustment Method

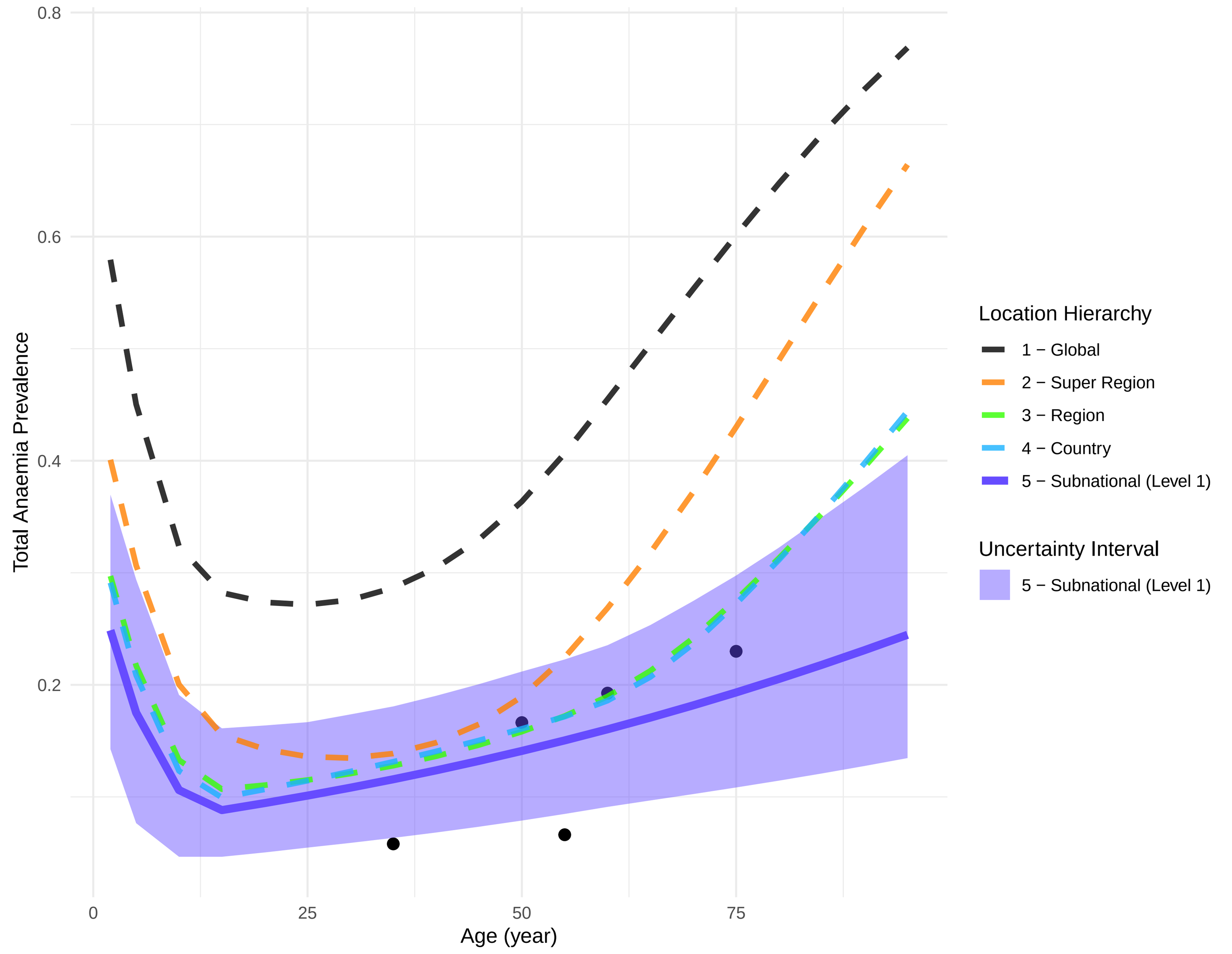

MR−BRT Age/Sex Fits − Males 2+ Years Old − Total Anaemia Prevalence − Paraná
WHO−2024 Adjustment Method

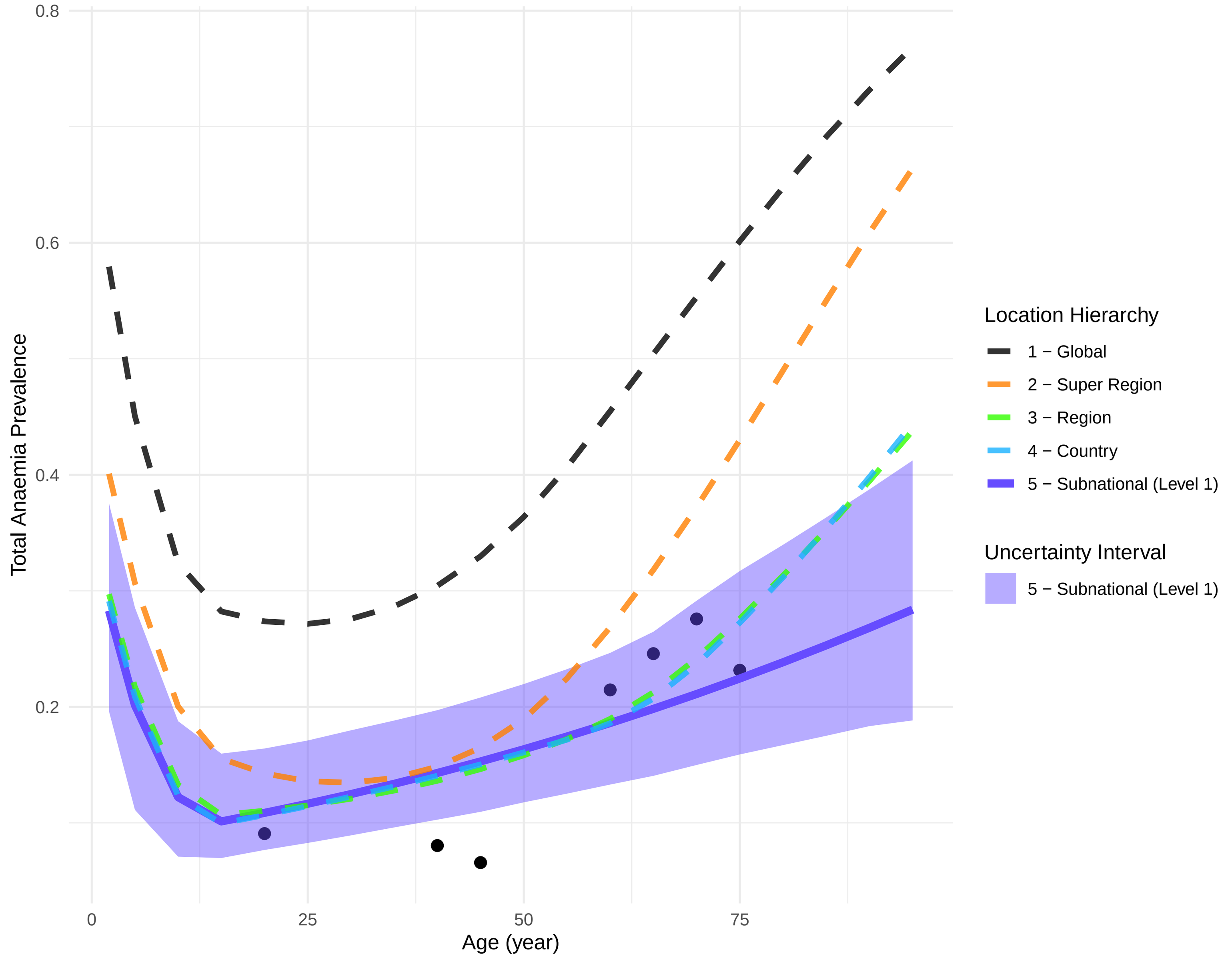

MR−BRT Age/Sex Fits − Males 2+ Years Old − Total Anaemia Prevalence − Pernambuco
WHO−2024 Adjustment Method

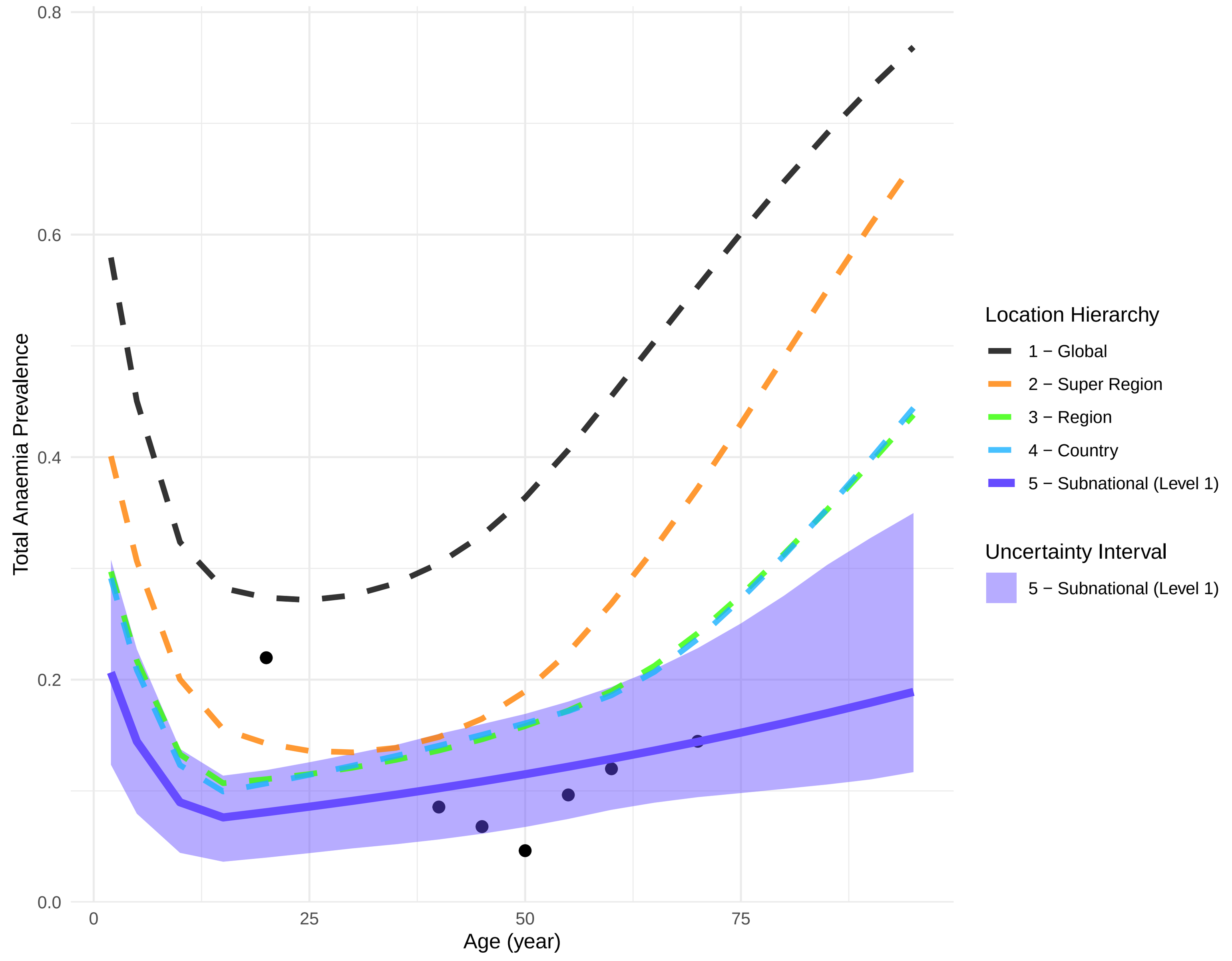

MR−BRT Age/Sex Fits − Males 2+ Years Old − Total Anaemia Prevalence − Piauí
WHO−2024 Adjustment Method

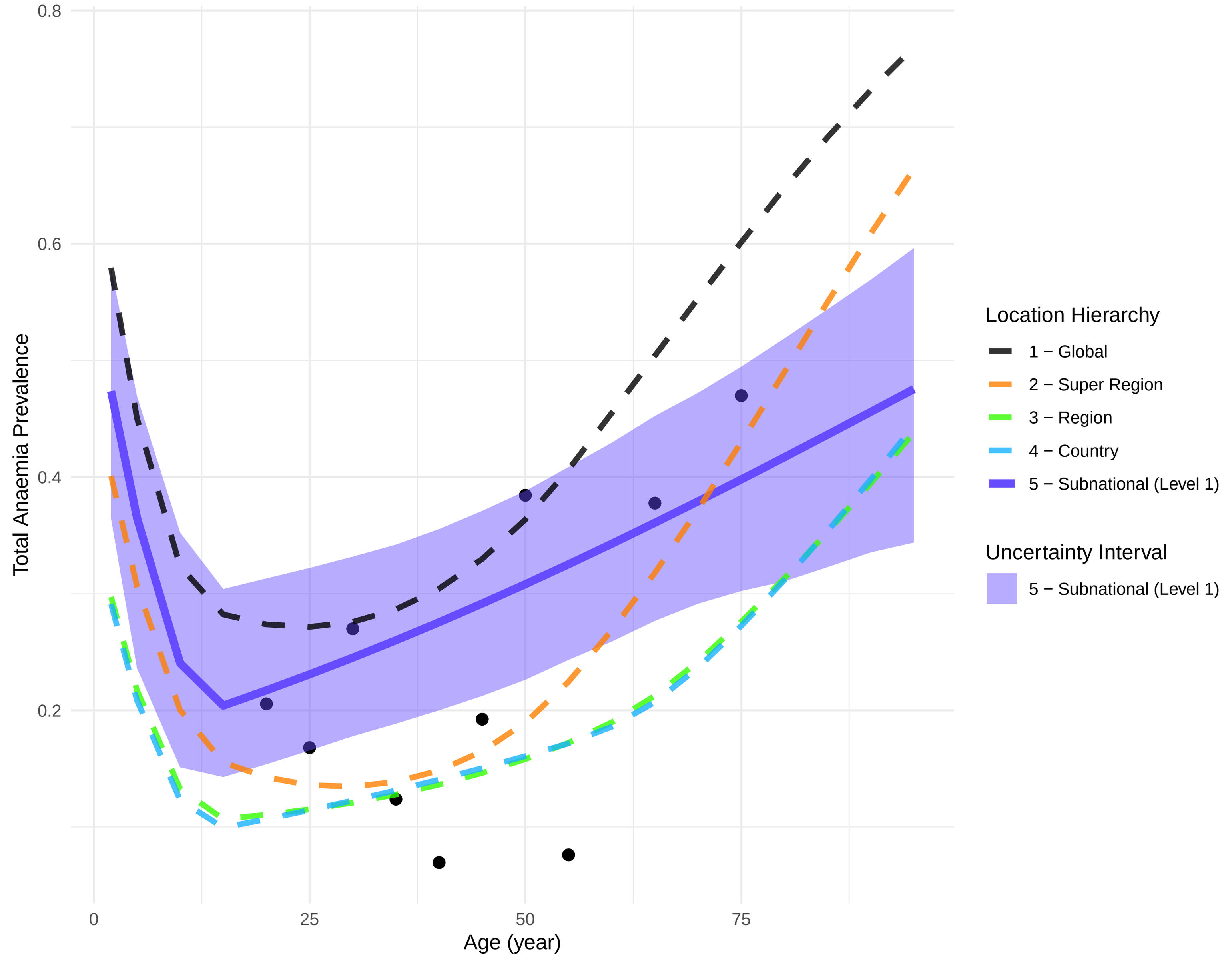

MR−BRT Age/Sex Fits − Males 2+ Years Old − Total Anaemia Prevalence − Rio de Janeiro
WHO−2024 Adjustment Method

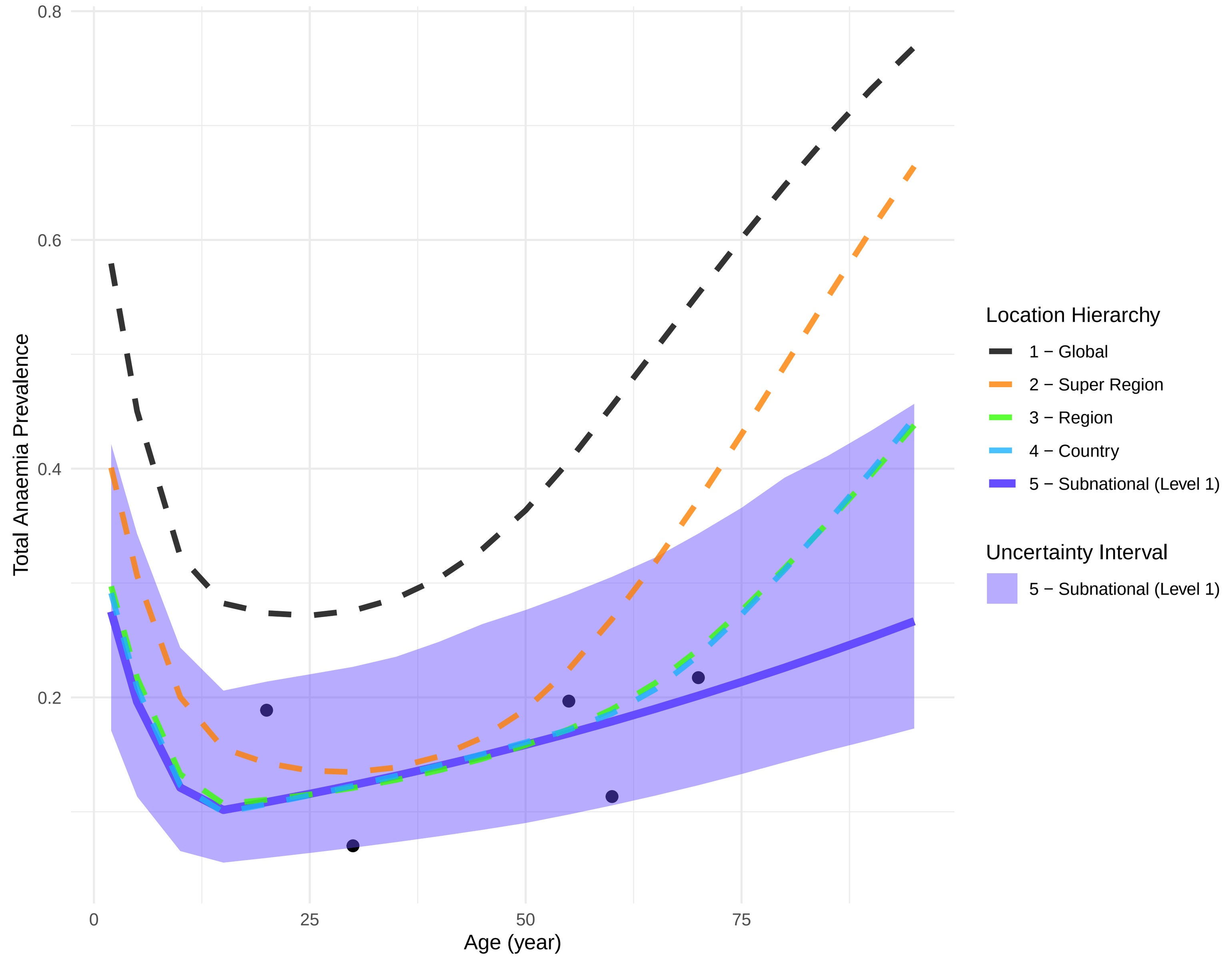

MR−BRT Age/Sex Fits − Males 2+ Years Old − Total Anaemia Prevalence − Rio Grande do Norte
WHO−2024 Adjustment Method

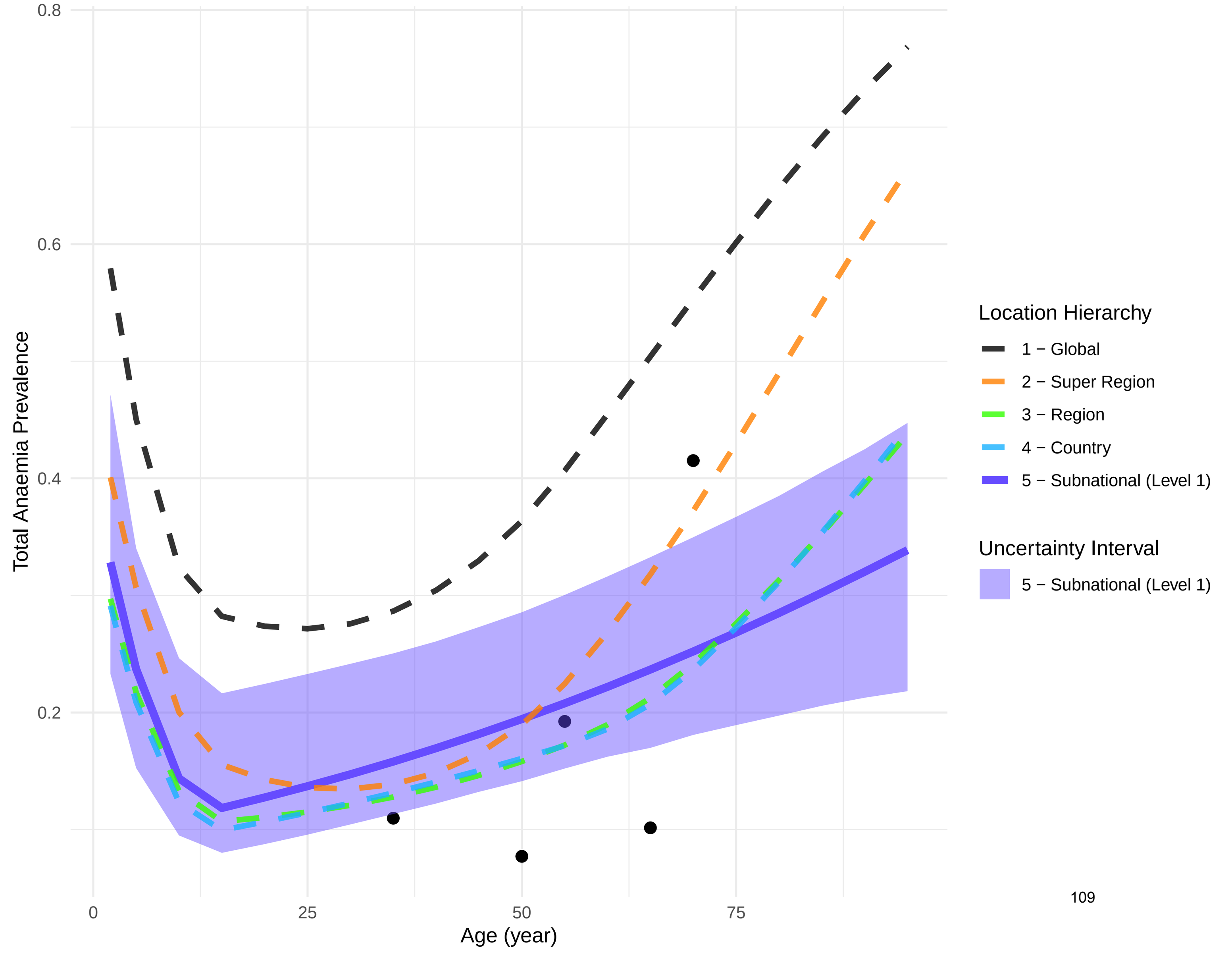

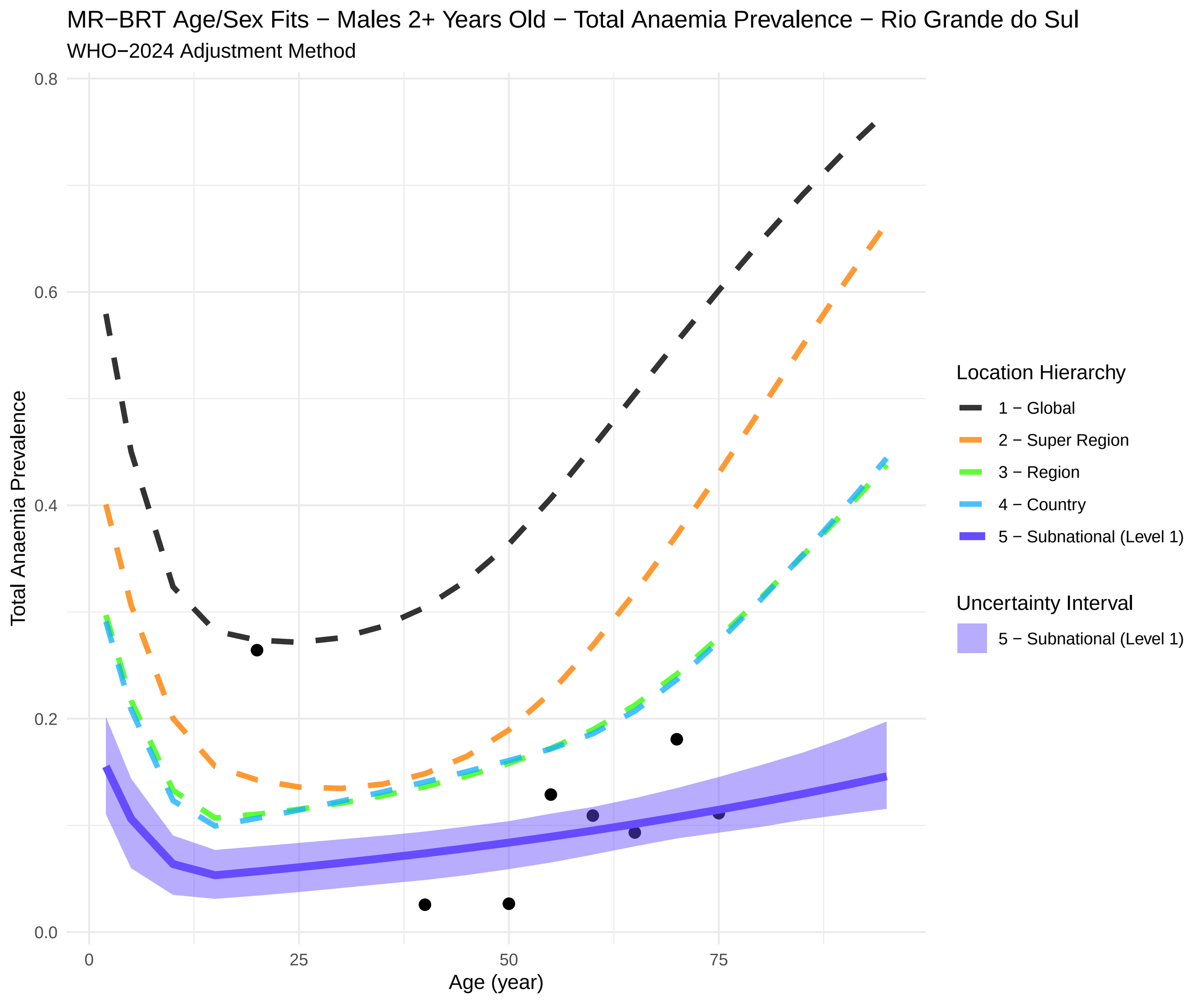
MR−BRT Age/Sex Fits − Males 2+ Years Old − Total Anaemia Prevalence − Rio Grande do Sul
WHO−2024 Adjustment Method
Total Anaemia Prevalence
Age (year)
Location Hierarchy
1 − Global
2 − Super Region
3 − Region
4 − Country
5 − Subnational (Level 1)
Uncertainty Interval
5 − Subnational (Level 1)

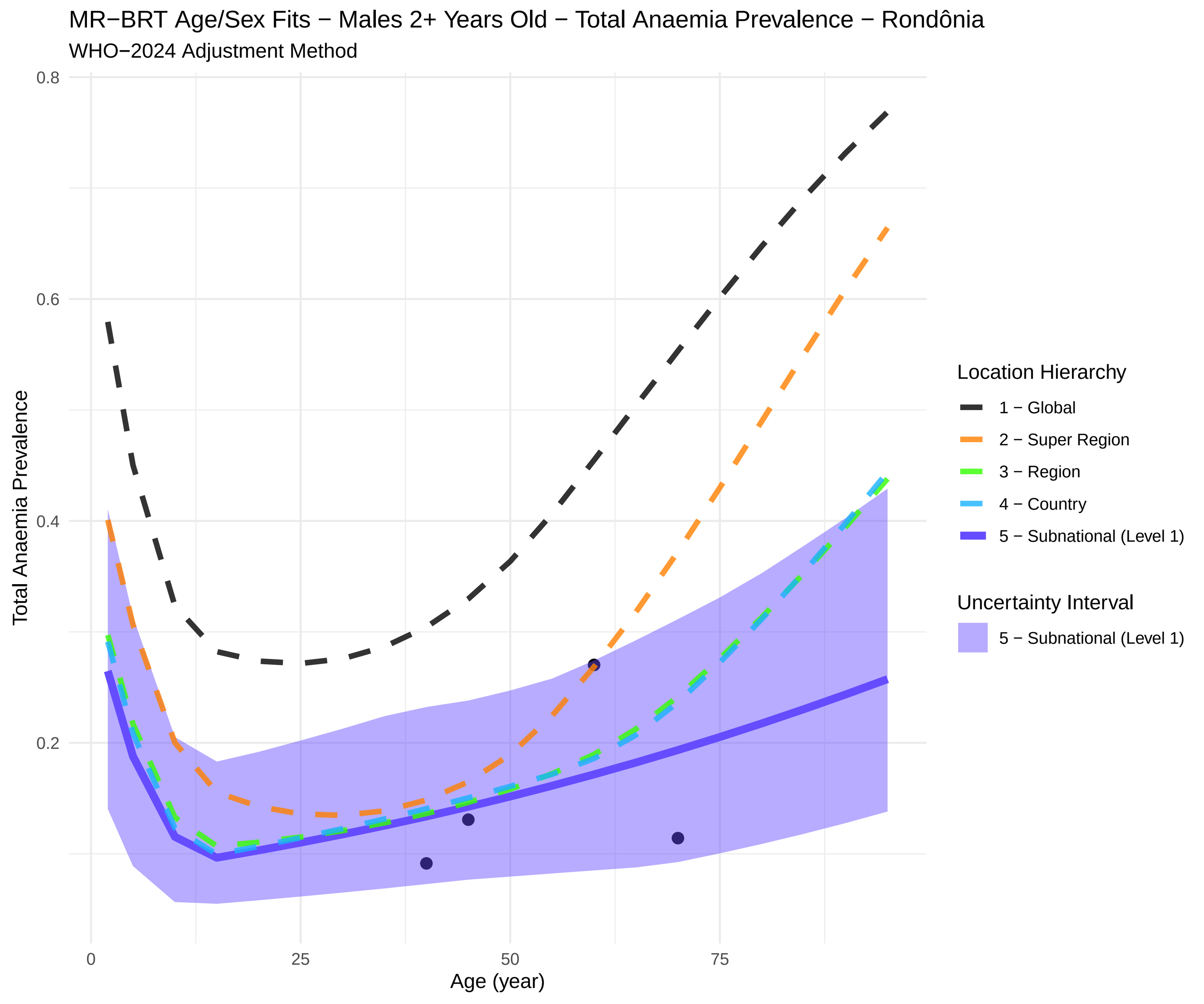
MR−BRT Age/Sex Fits − Males 2+ Years Old − Total Anaemia Prevalence − Rondônia
WHO−2024 Adjustment Method
Total Anaemia Prevalence
Age (year)
Location Hierarchy
1 − Global
2 − Super Region
3 − Region
4 − Country
5 − Subnational (Level 1)
Uncertainty Interval
5 − Subnational (Level 1)

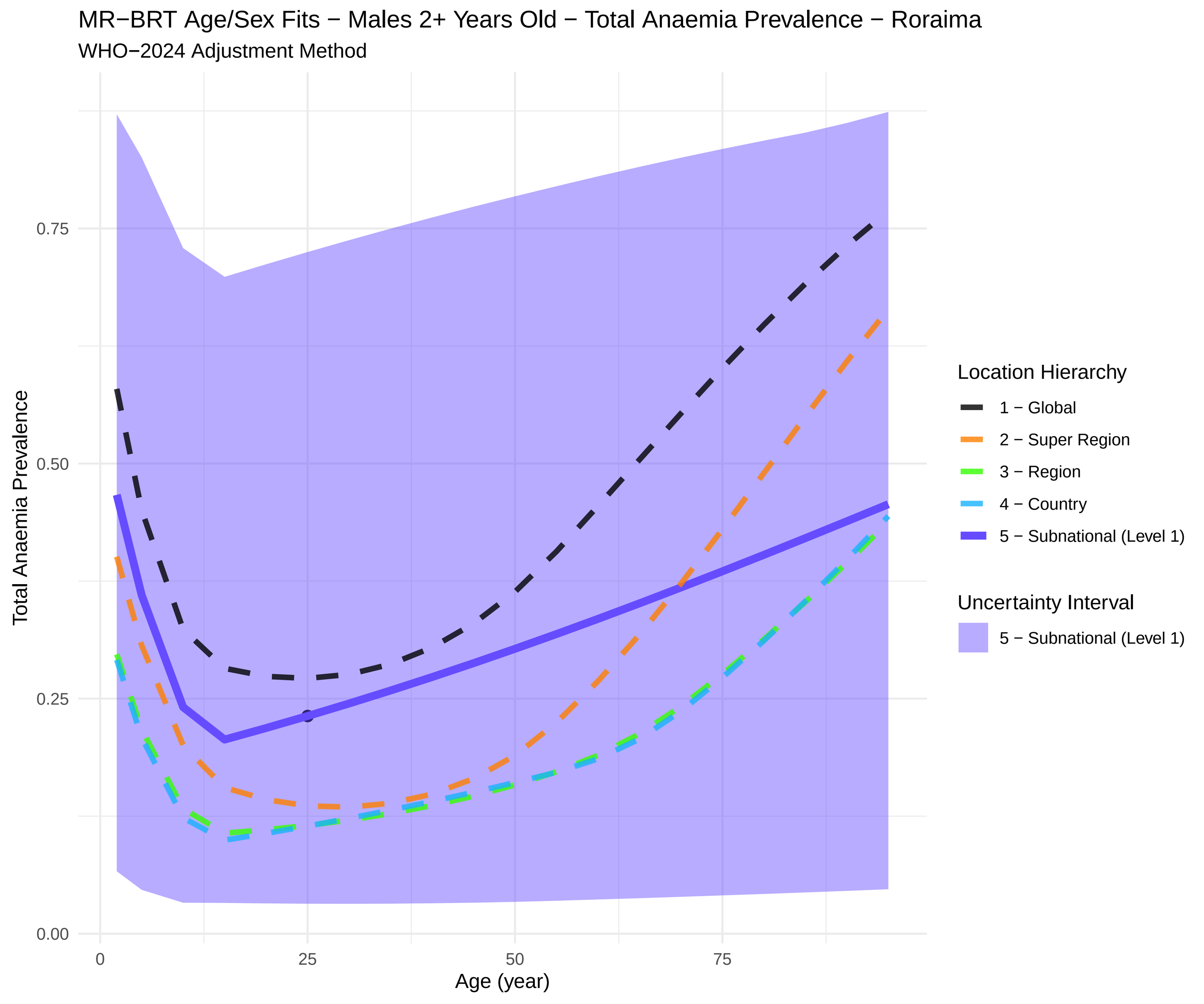
MR−BRT Age/Sex Fits − Males 2+ Years Old − Total Anaemia Prevalence − Roraima
WHO−2024 Adjustment Method
Total Anaemia Prevalence
Age (year)
Location Hierarchy
1 − Global
2 − Super Region
3 − Region
4 − Country
5 − Subnational (Level 1)
Uncertainty Interval
5 − Subnational (Level 1)

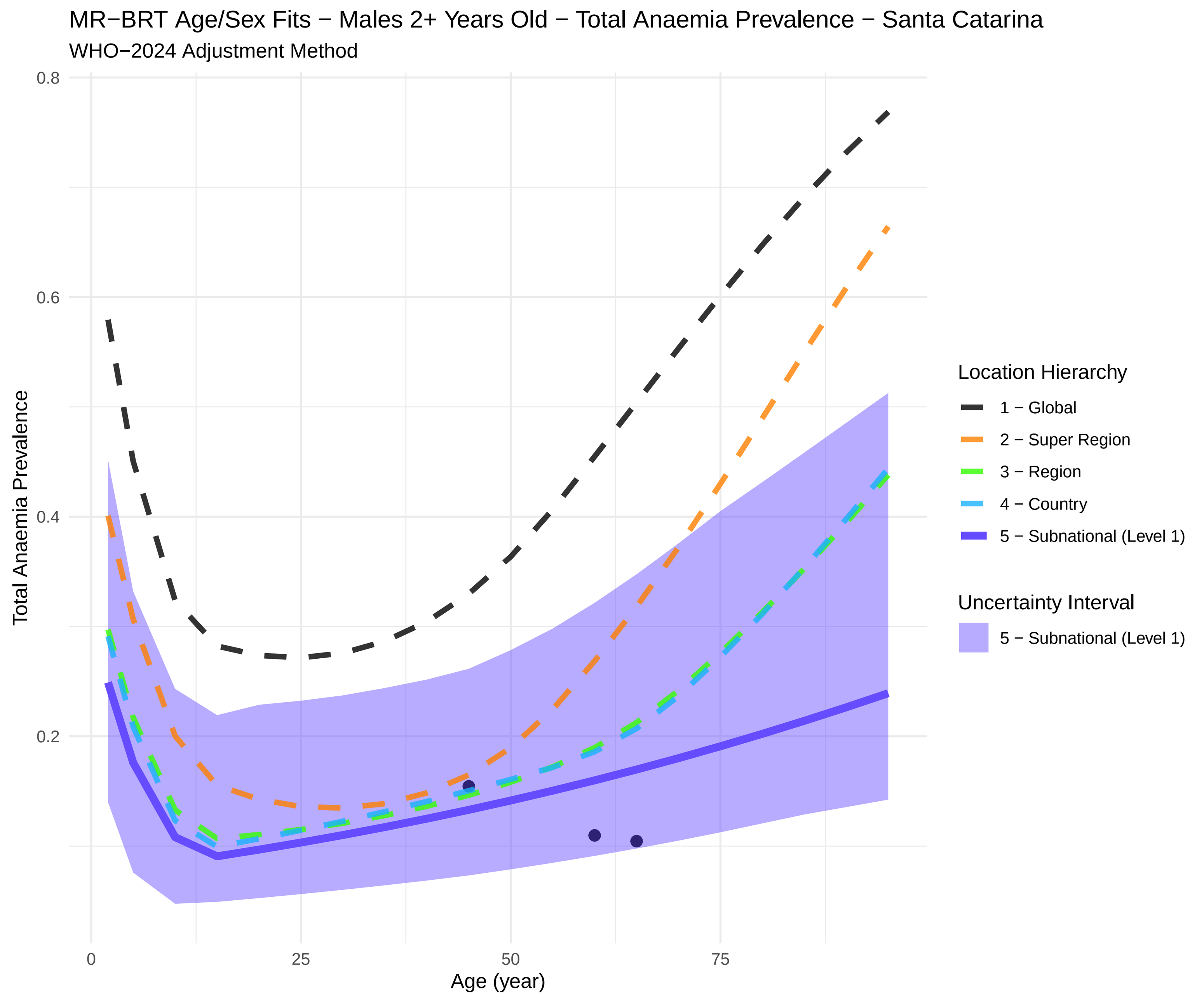
MR−BRT Age/Sex Fits − Males 2+ Years Old − Total Anaemia Prevalence − Santa Catarina
WHO−2024 Adjustment Method
Total Anaemia Prevalence
Age (year)
Location Hierarchy
1 − Global
2 − Super Region
3 − Region
4 − Country
5 − Subnational (Level 1)
Uncertainty Interval
5 − Subnational (Level 1)

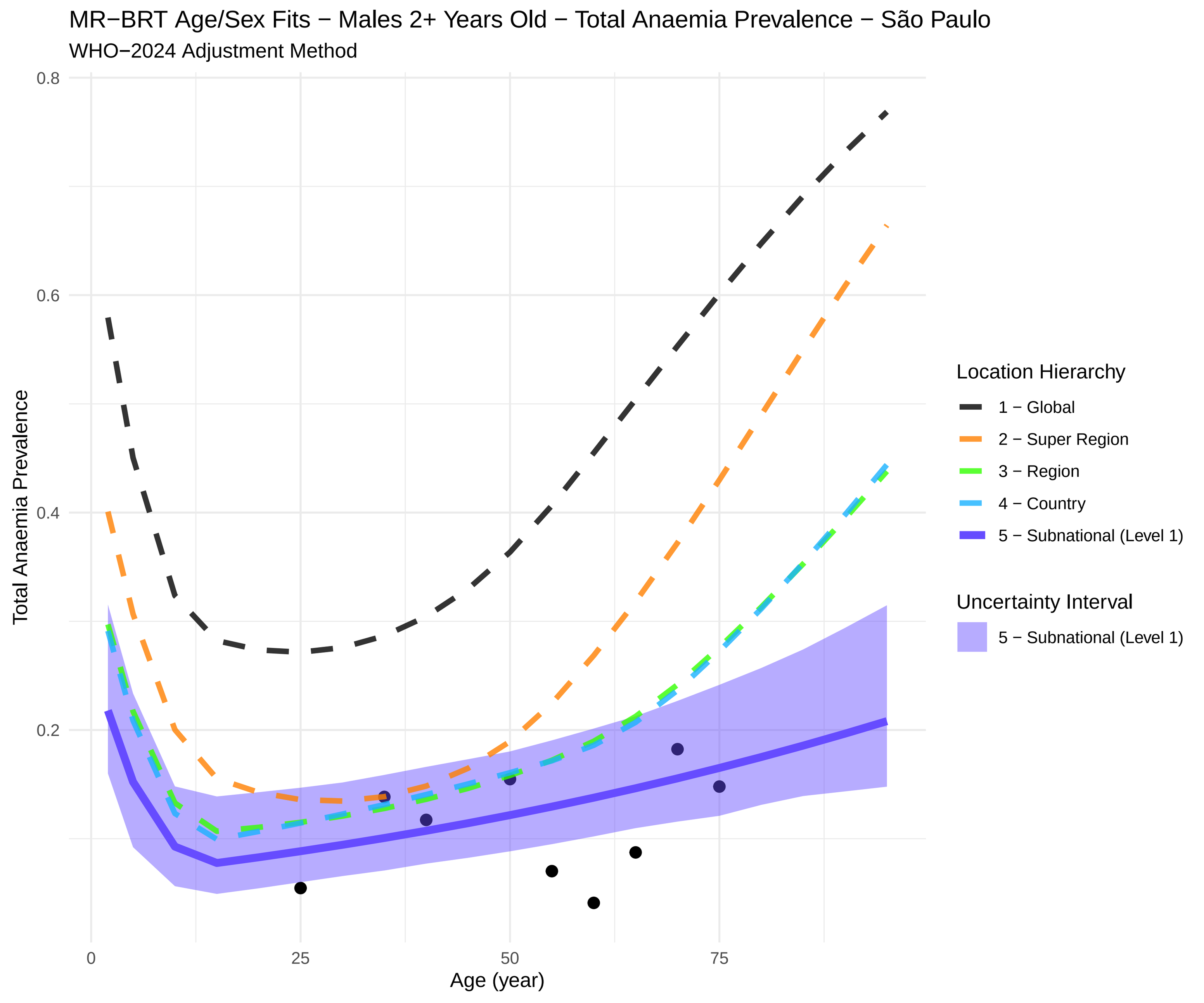
MR−BRT Age/Sex Fits − Males 2+ Years Old − Total Anaemia Prevalence − São Paulo
WHO−2024 Adjustment Method
Total Anaemia Prevalence
Age (year)
Location Hierarchy
1 − Global
2 − Super Region
3 − Region
4 − Country
5 − Subnational (Level 1)
Uncertainty Interval
5 − Subnational (Level 1)

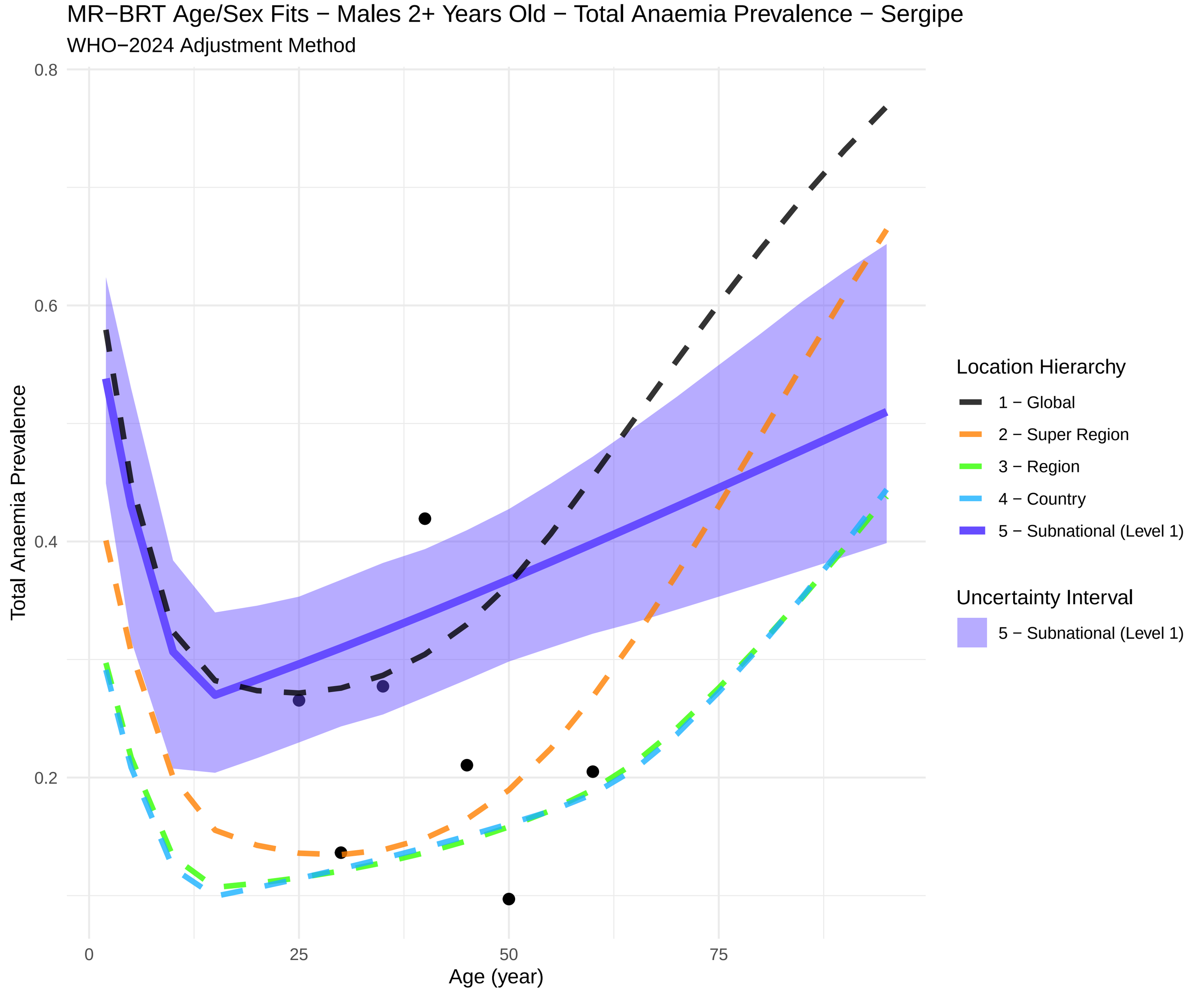
MR−BRT Age/Sex Fits − Males 2+ Years Old − Total Anaemia Prevalence − Sergipe
WHO−2024 Adjustment Method
Total Anaemia Prevalence
Age (year)
Location Hierarchy
1 − Global
2 − Super Region
3 − Region
4 − Country
5 − Subnational (Level 1)
Uncertainty Interval
5 − Subnational (Level 1)

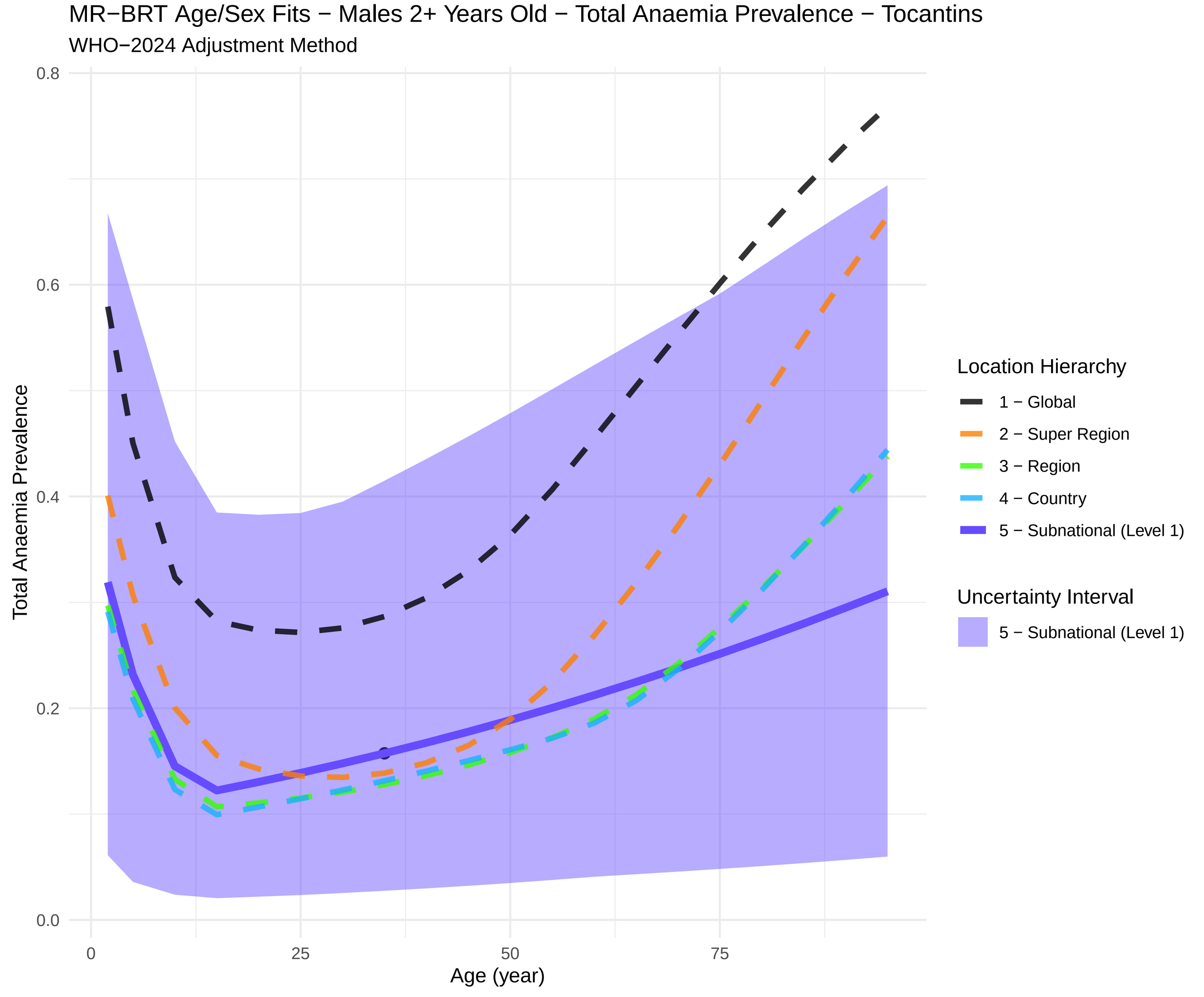
MR−BRT Age/Sex Fits − Males 2+ Years Old − Total Anaemia Prevalence − Tocantins
WHO−2024 Adjustment Method
Total Anaemia Prevalence
Age (year)
Location Hierarchy
1 − Global
2 − Super Region
3 − Region
4 − Country
5 − Subnational (Level 1)
Uncertainty Interval
5 − Subnational (Level 1)

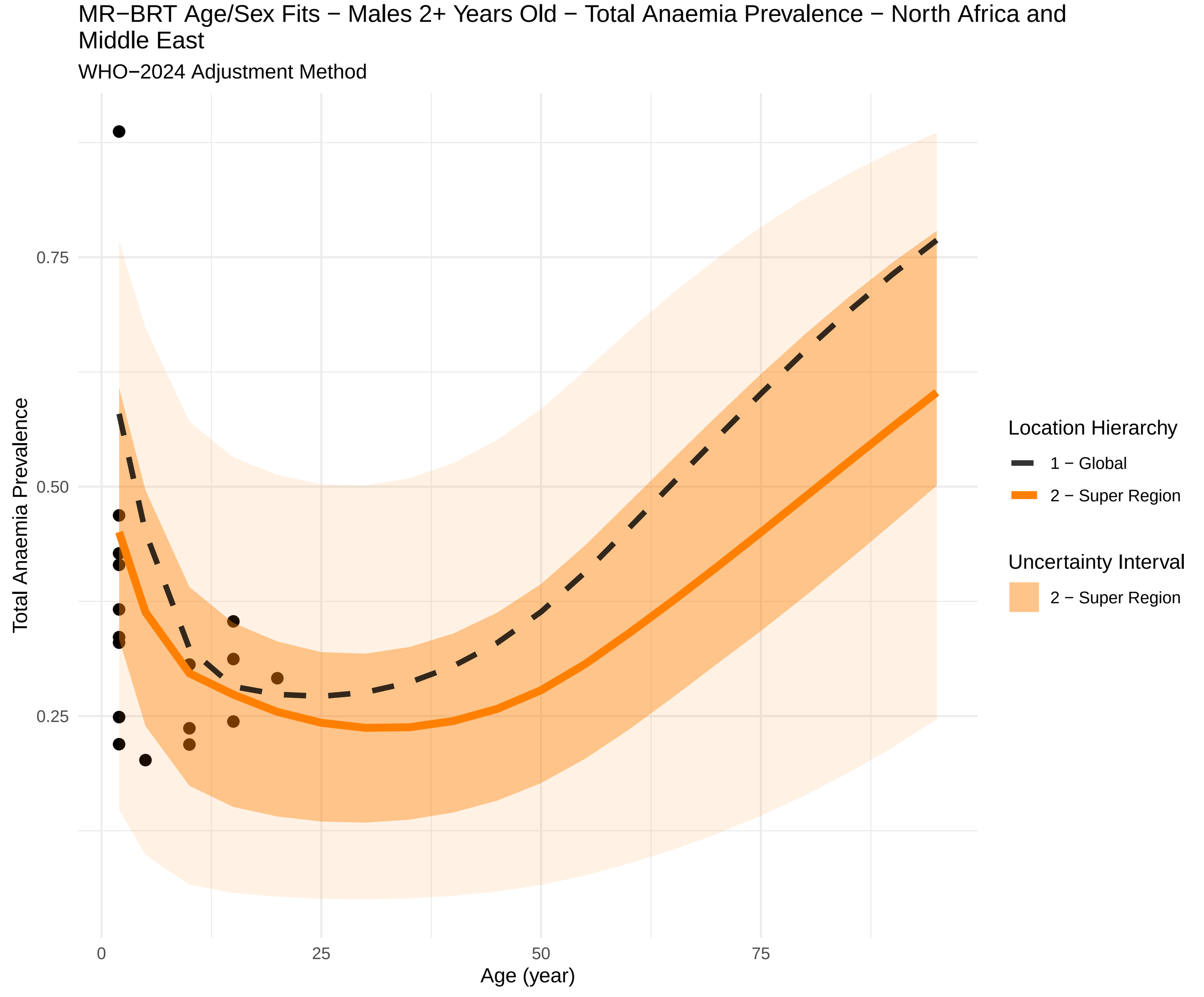
MR−BRT Age/Sex Fits − Males 2+ Years Old − Total Anaemia Prevalence − North Africa and Middle East
WHO−2024 Adjustment Method
Total Anaemia Prevalence
Age (year)
Location Hierarchy
1 − Global
2 − Super Region
Uncertainty Interval
2 − Super Region

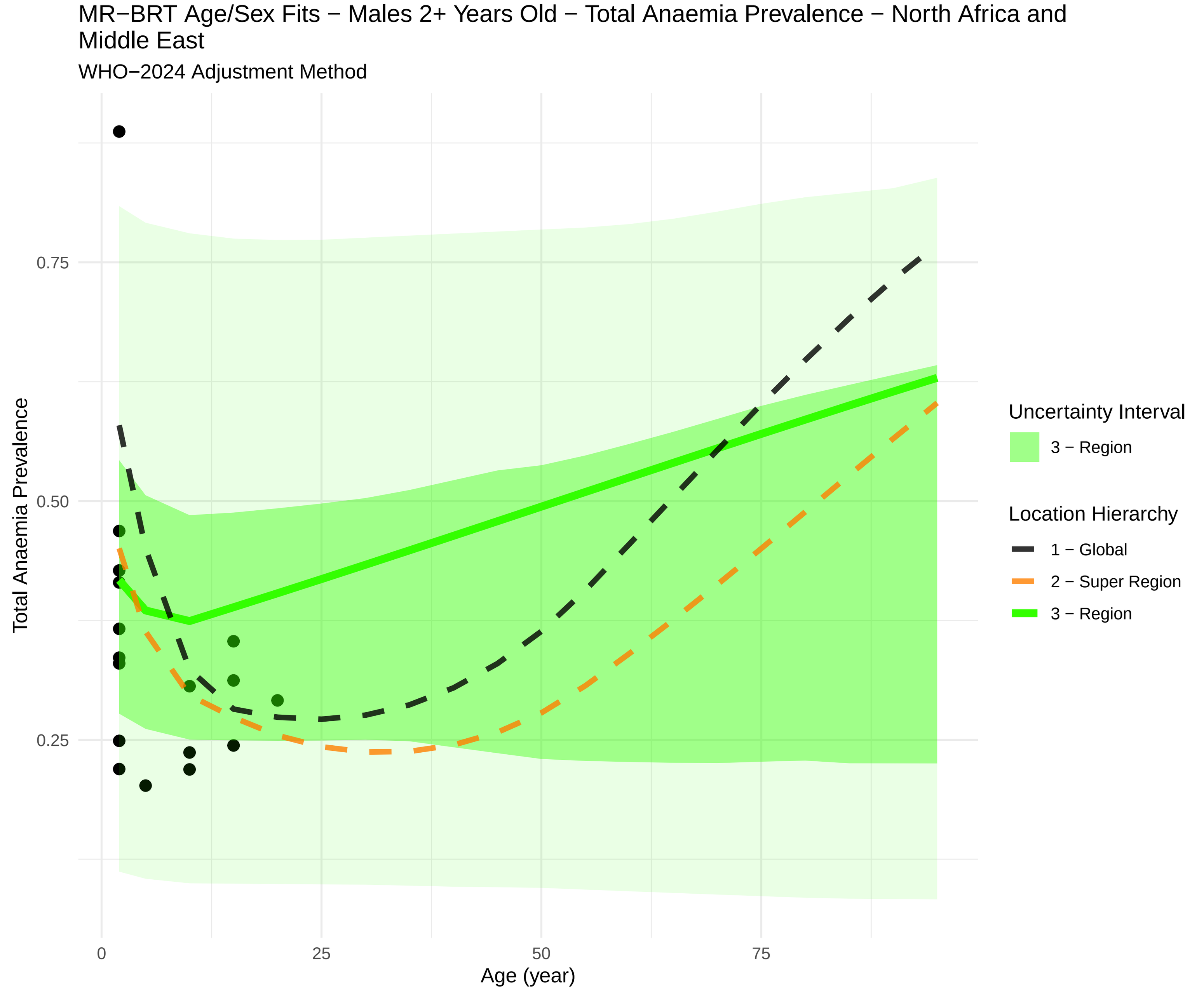
MR−BRT Age/Sex Fits − Males 2+ Years Old − Total Anaemia Prevalence − North Africa and Middle East
WHO−2024 Adjustment Method
Total Anaemia Prevalence
Age (year)
Uncertainty Interval
3 − Region
Location Hierarchy
1 − Global
2 − Super Region
3 − Region

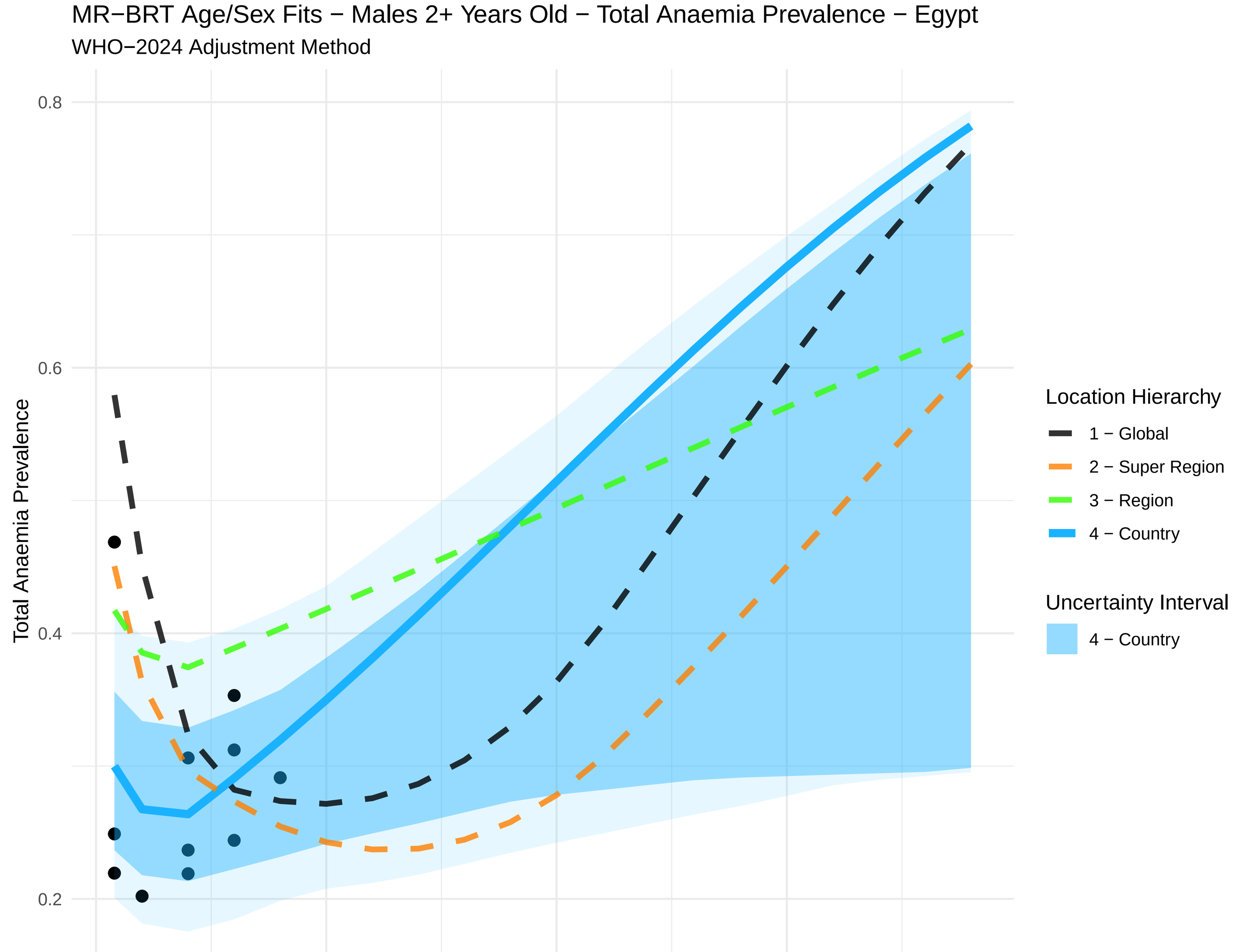
MR−BRT Age/Sex Fits − Males 2+ Years Old − Total Anaemia Prevalence − Egypt
WHO−2024 Adjustment Method
Total Anaemia Prevalence
Age (year)
Location Hierarchy
1 − Global
2 − Super Region
3 − Region
4 − Country
Uncertainty Interval
4 − Country

MR−BRT Age/Sex Fits − Males 2+ Years Old − Total Anaemia Prevalence − Jordan
WHO−2024 Adjustment Method

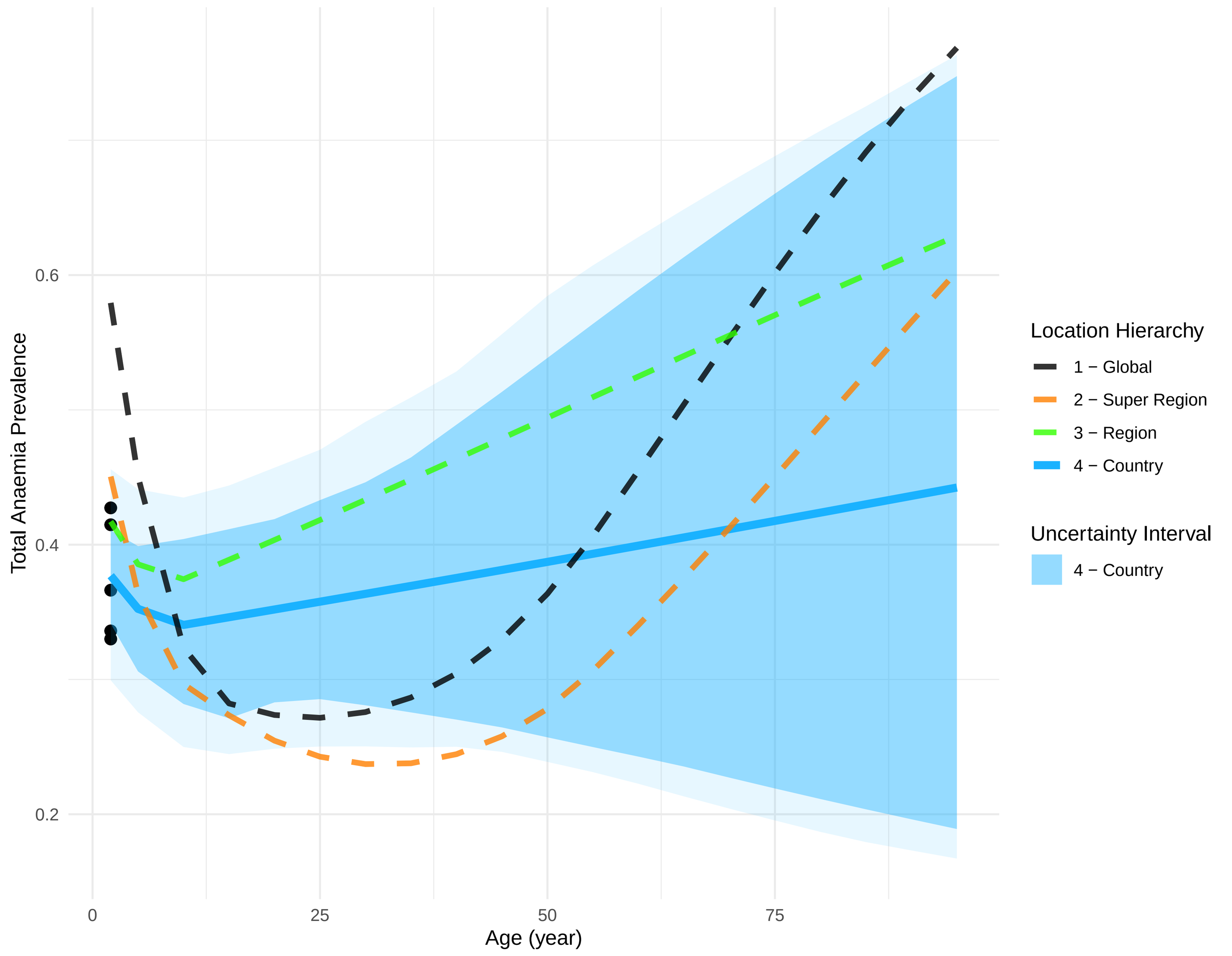

MR−BRT Age/Sex Fits − Males 2+ Years Old − Total Anaemia Prevalence − Yemen
WHO−2024 Adjustment Method

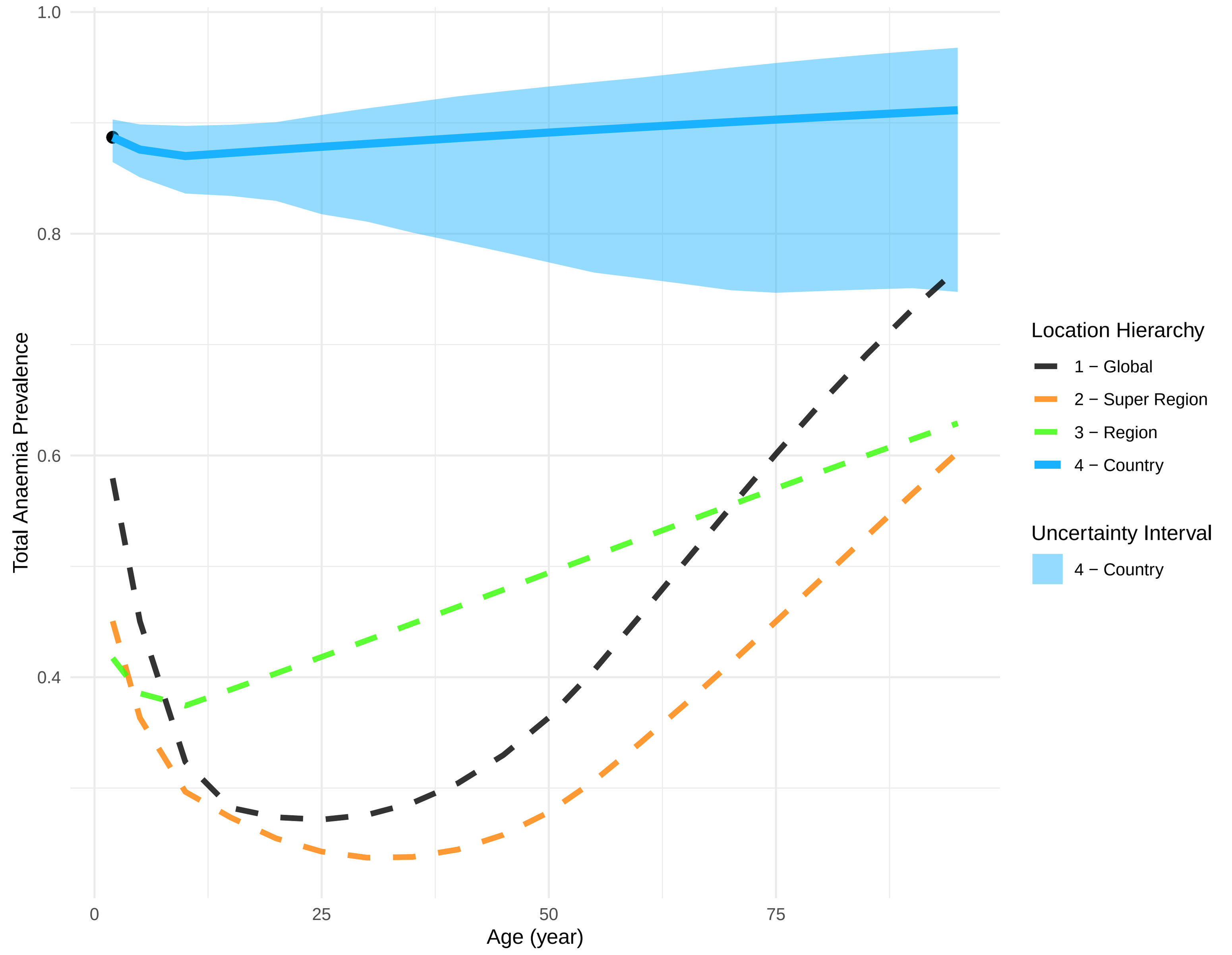

MR−BRT Age/Sex Fits − Males 2+ Years Old − Total Anaemia Prevalence − South Asia
WHO−2024 Adjustment Method

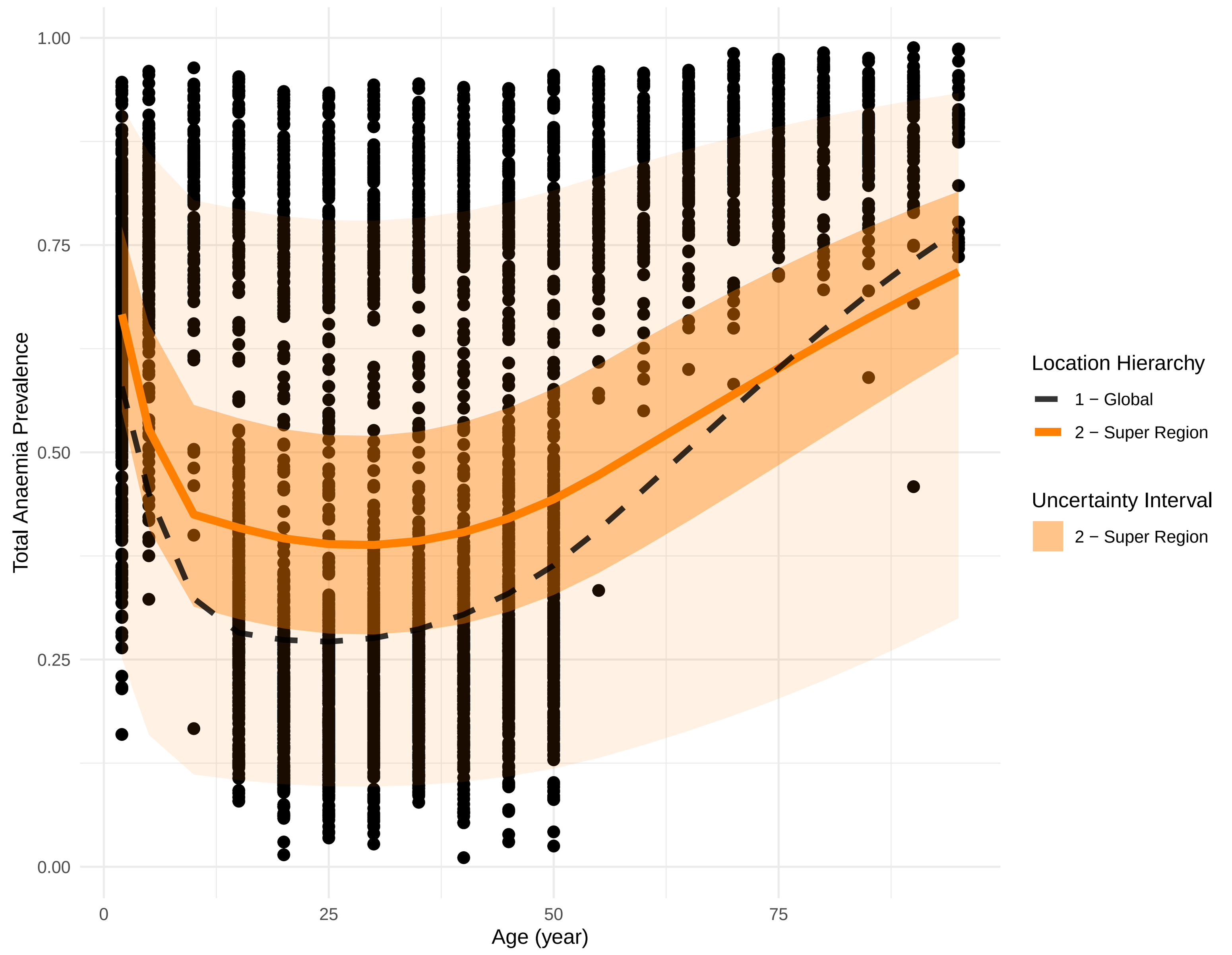

MR−BRT Age/Sex Fits − Males 2+ Years Old − Total Anaemia Prevalence − South Asia
WHO−2024 Adjustment Method

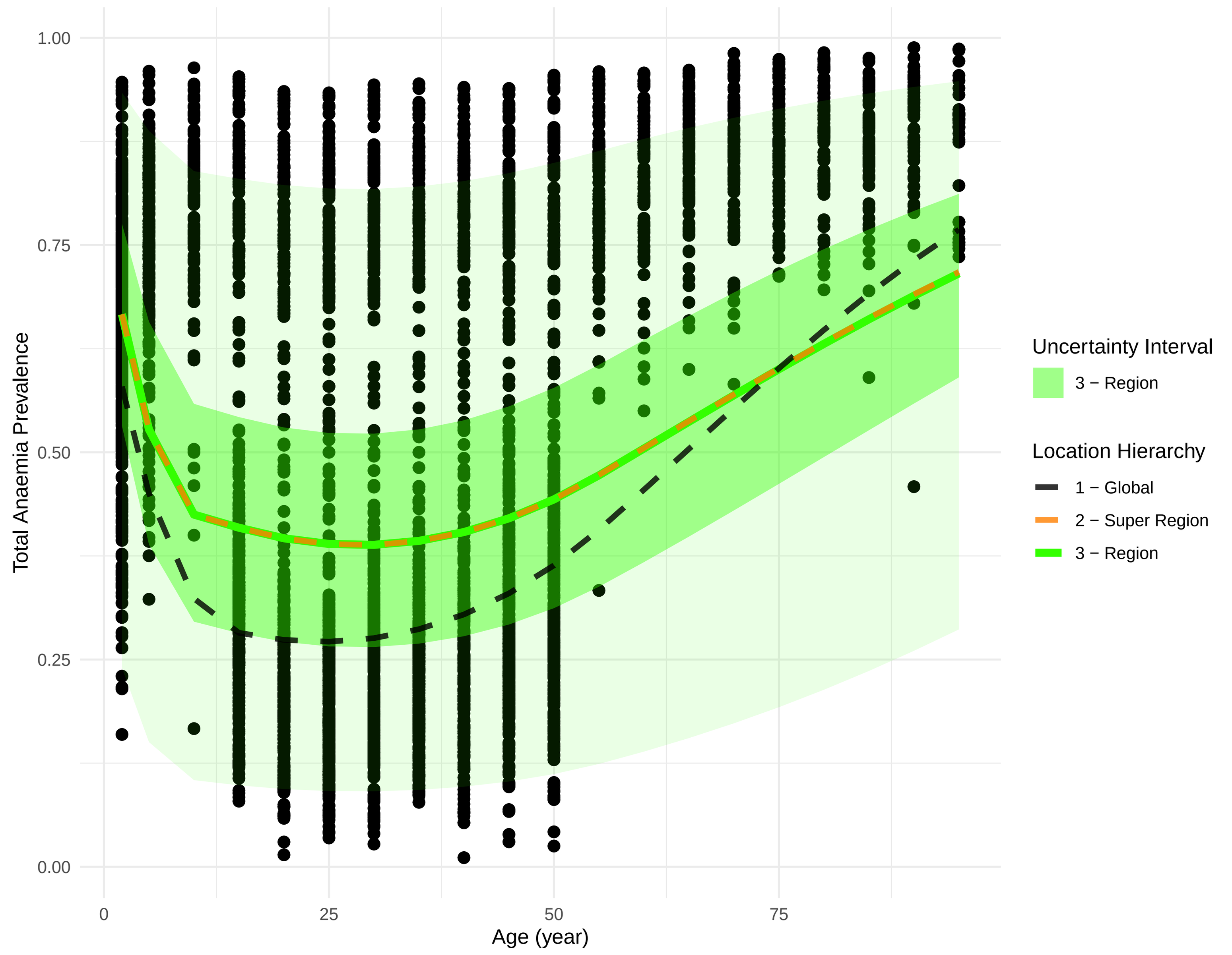

MR−BRT Age/Sex Fits − Males 2+ Years Old − Total Anaemia Prevalence − Bangladesh
WHO−2024 Adjustment Method

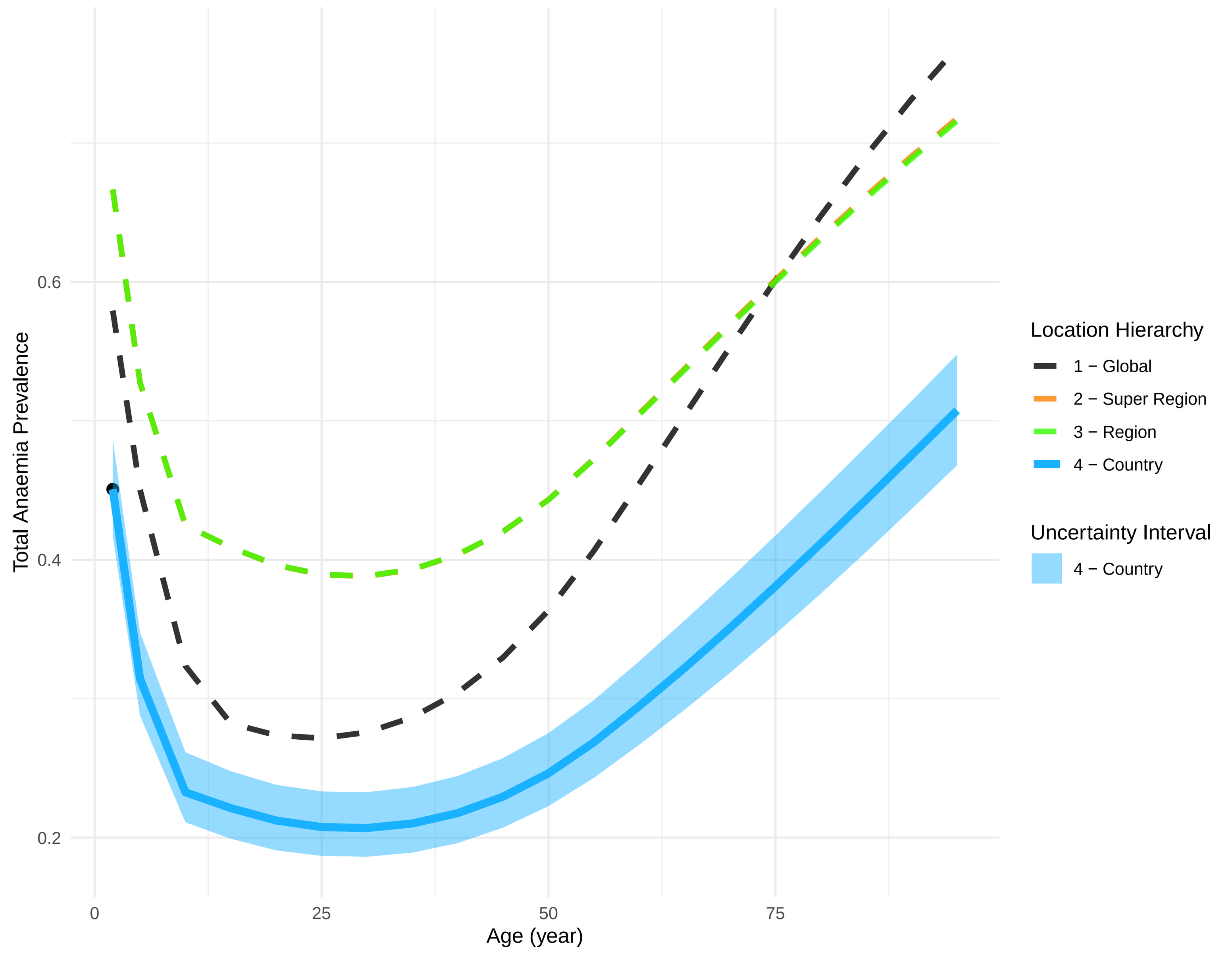

MR−BRT Age/Sex Fits − Males 2+ Years Old − Total Anaemia Prevalence − India
WHO−2024 Adjustment Method

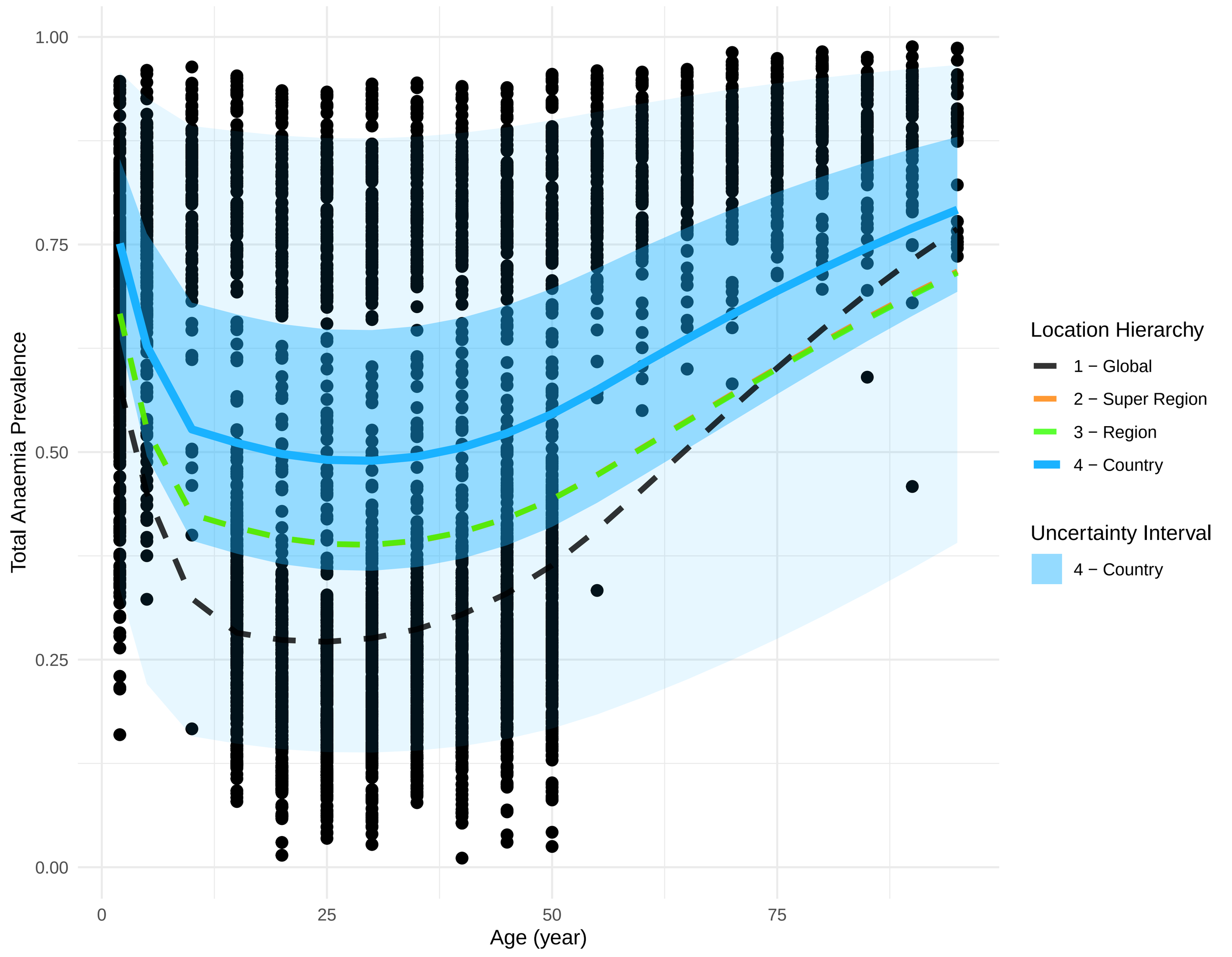

MR−BRT Age/Sex Fits − Males 2+ Years Old − Total Anaemia Prevalence − Andhra Pradesh
WHO−2024 Adjustment Method

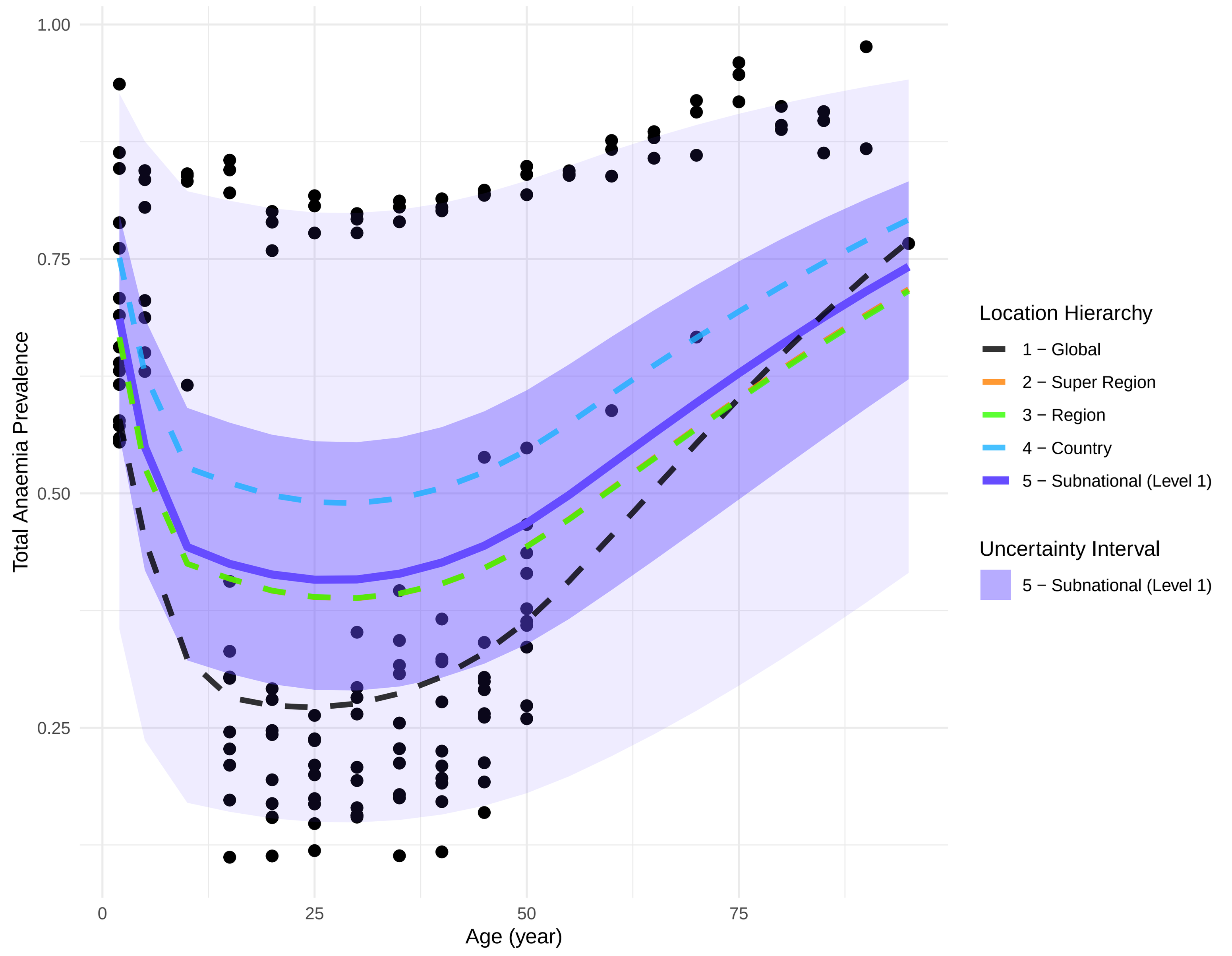

MR−BRT Age/Sex Fits − Males 2+ Years Old − Total Anaemia Prevalence − Andhra Pradesh, Rural
WHO−2024 Adjustment Method

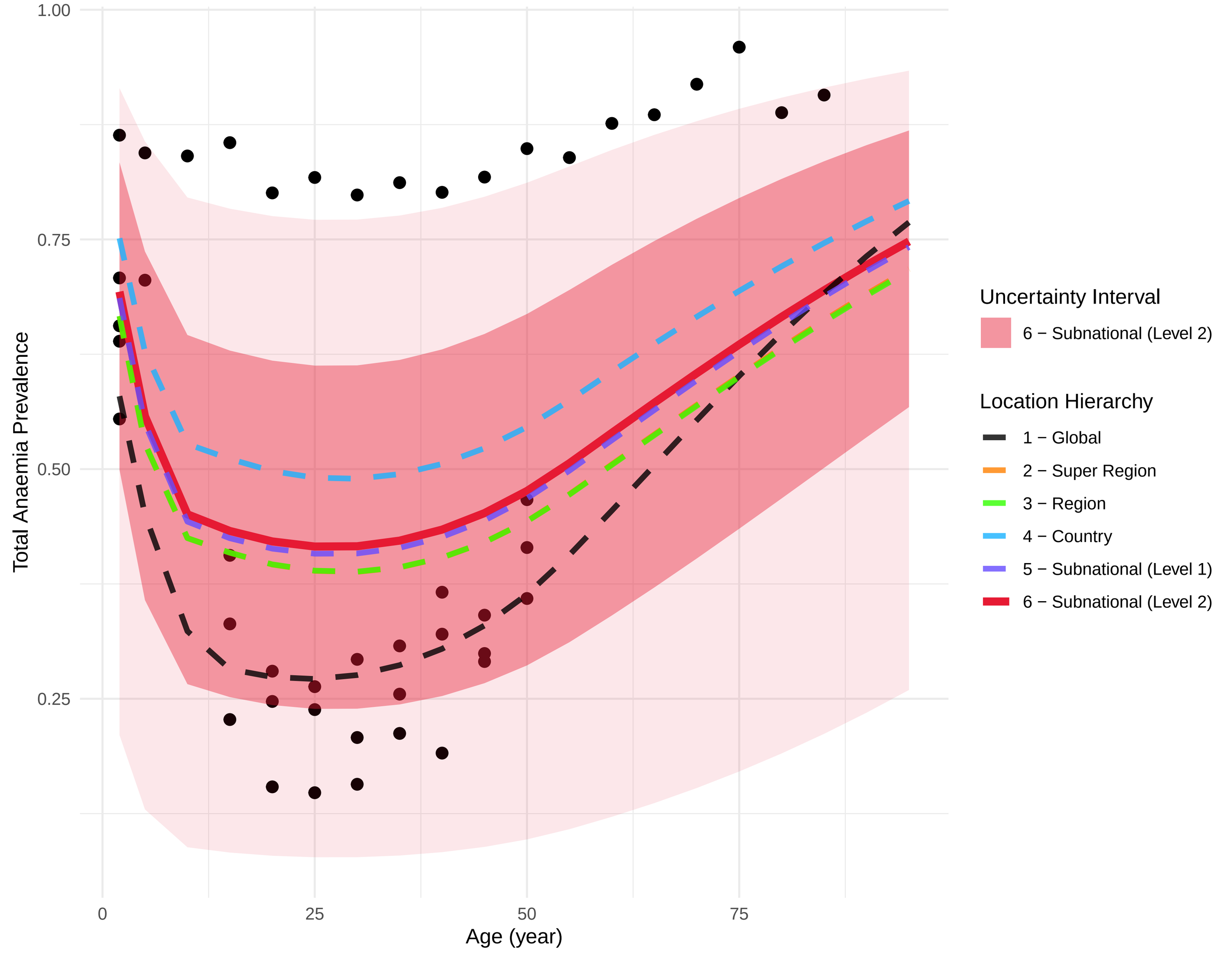

MR−BRT Age/Sex Fits − Males 2+ Years Old − Total Anaemia Prevalence − Andhra Pradesh, Urban
WHO−2024 Adjustment Method

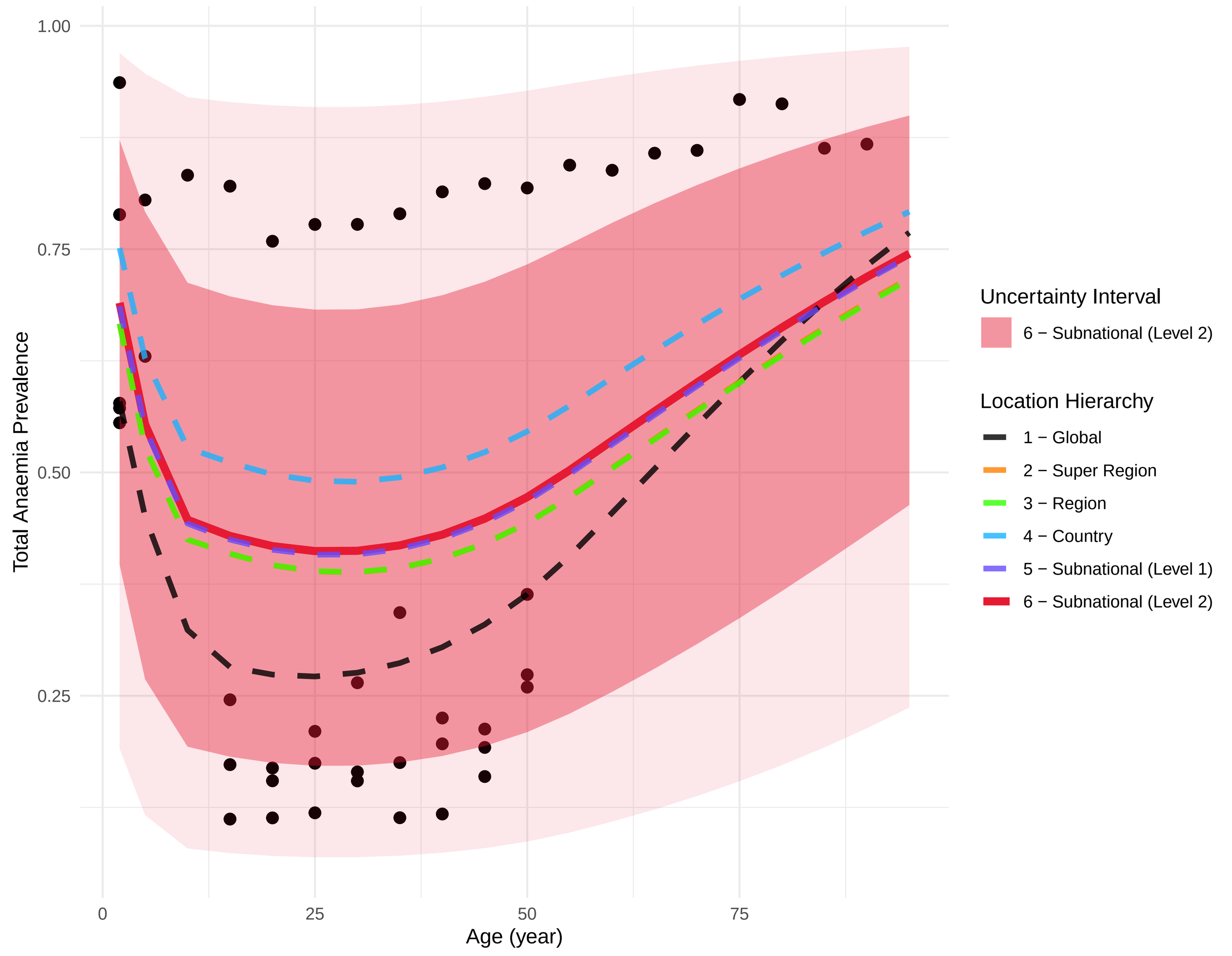

MR−BRT Age/Sex Fits − Males 2+ Years Old − Total Anaemia Prevalence − Arunachal Pradesh
WHO−2024 Adjustment Method

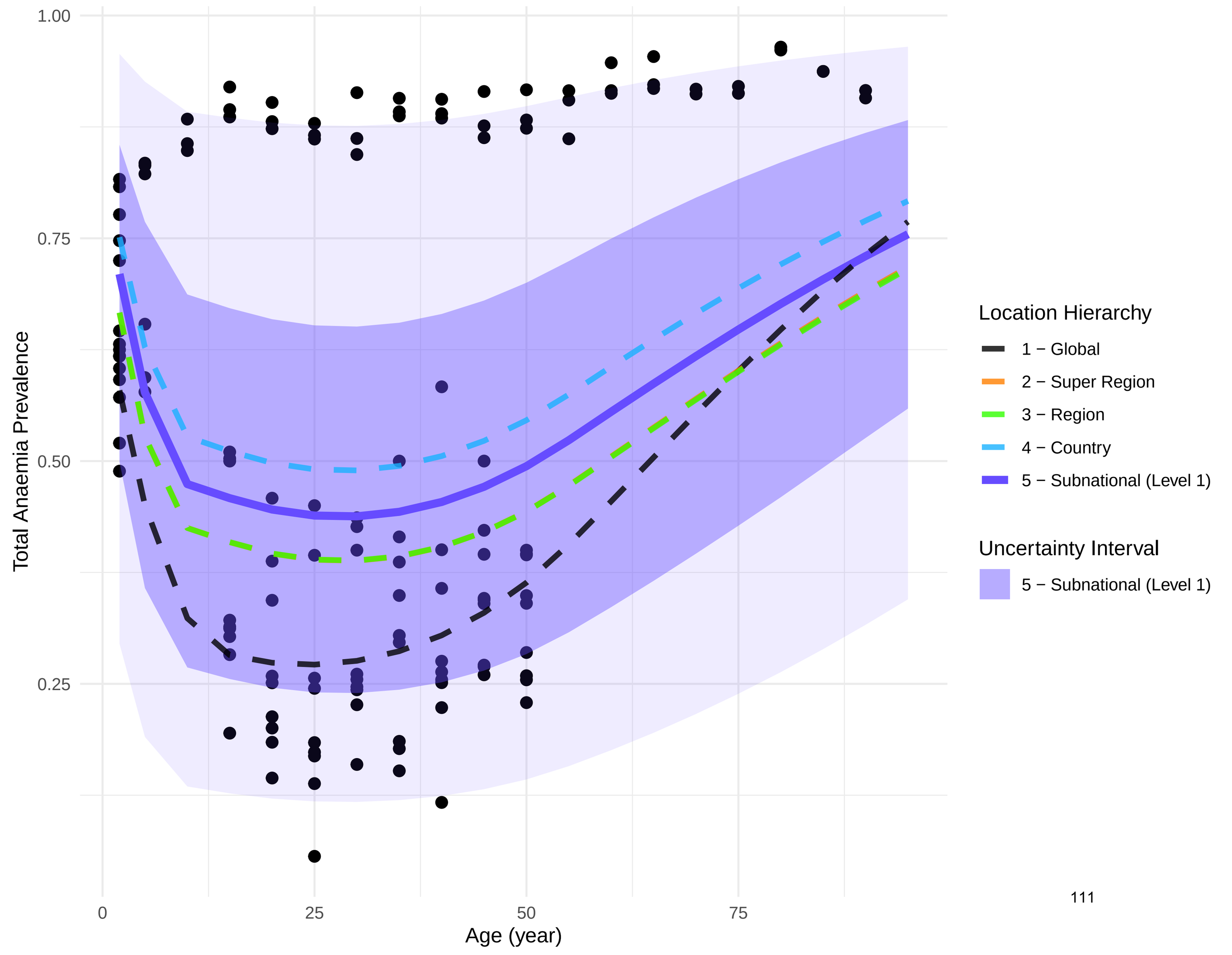

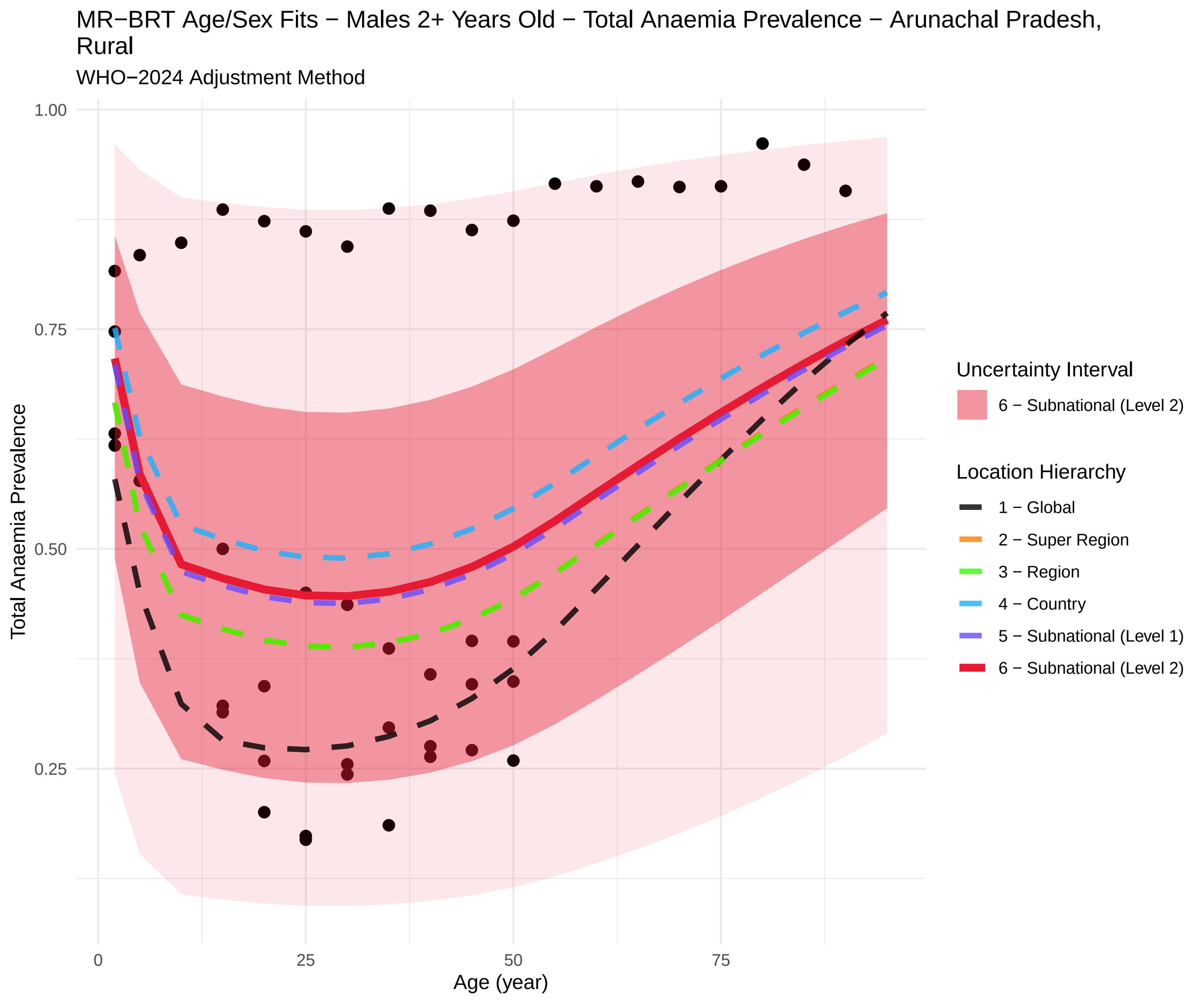
MR−BRT Age/Sex Fits − Males 2+ Years Old − Total Anaemia Prevalence − Arunachal Pradesh, Rural
WHO−2024 Adjustment Method
Total Anaemia Prevalence
Age (year)
Uncertainty Interval
6 − Subnational (Level 2)
Location Hierarchy
1 − Global
2 − Super Region
3 − Region
4 − Country
5 − Subnational (Level 1)
6 − Subnational (Level 2)

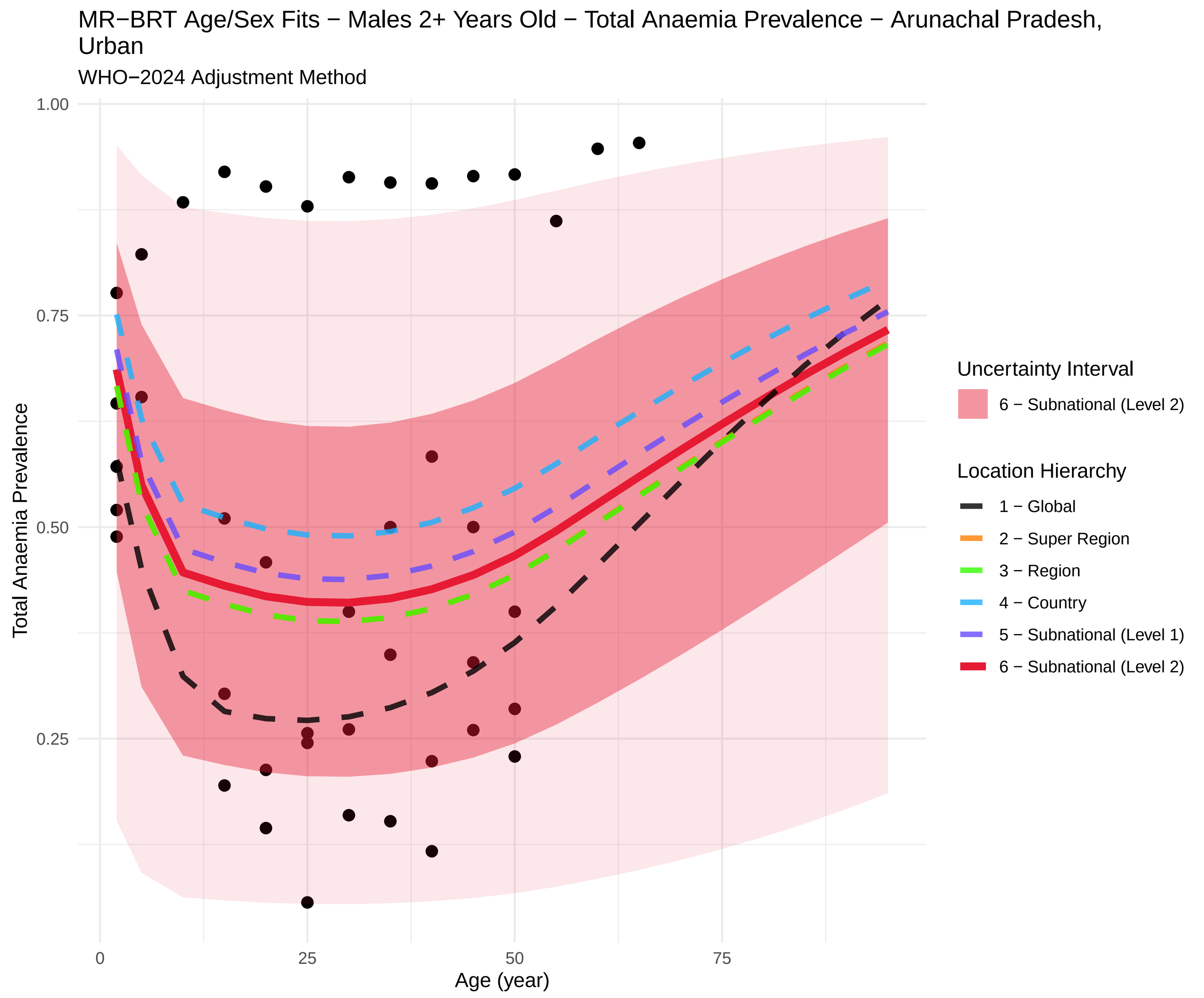
MR−BRT Age/Sex Fits − Males 2+ Years Old − Total Anaemia Prevalence − Arunachal Pradesh, Urban
WHO−2024 Adjustment Method
Total Anaemia Prevalence
Age (year)
Uncertainty Interval
6 − Subnational (Level 2)
Location Hierarchy
1 − Global
2 − Super Region
3 − Region
4 − Country
5 − Subnational (Level 1)
6 − Subnational (Level 2)

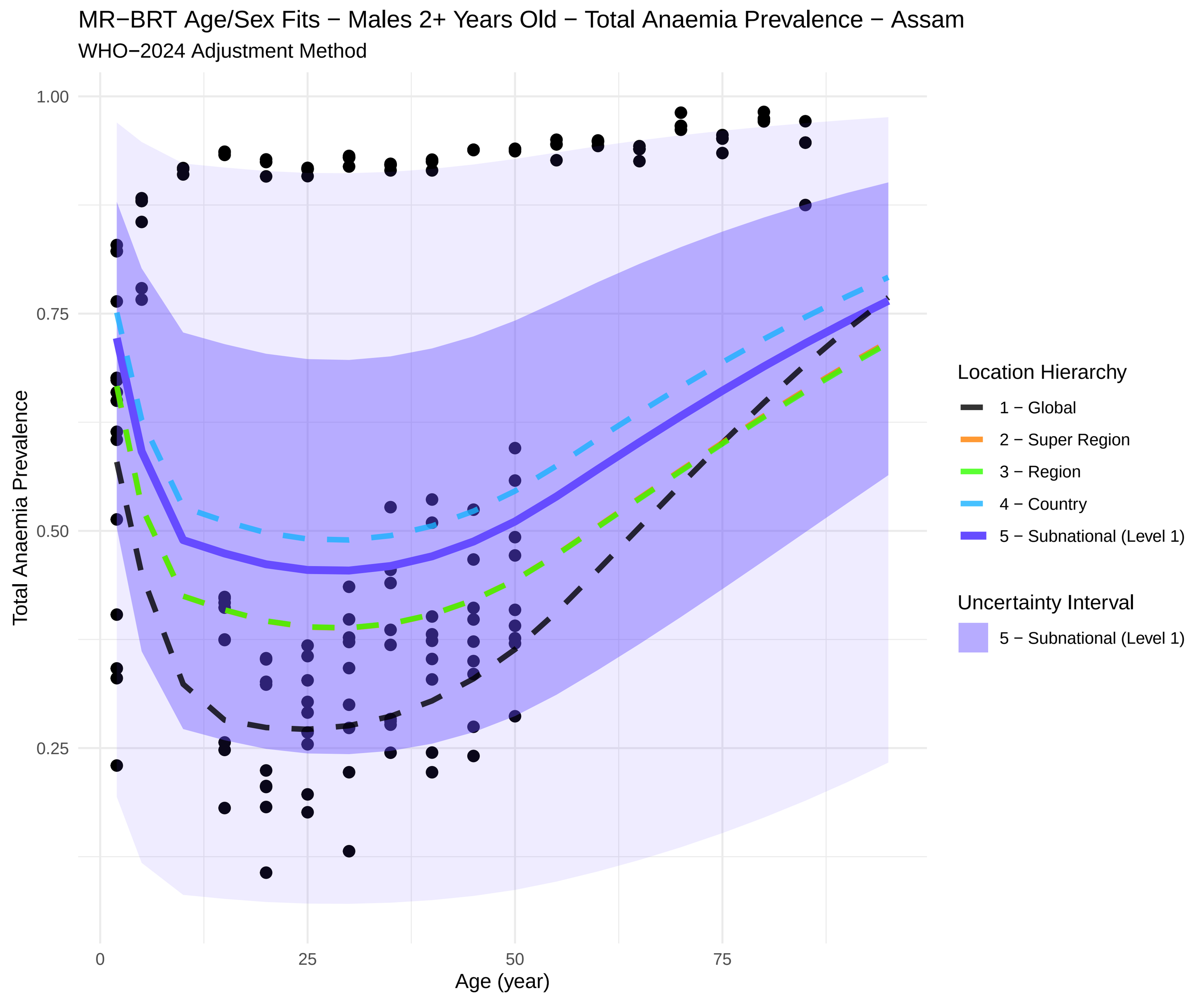
MR−BRT Age/Sex Fits − Males 2+ Years Old − Total Anaemia Prevalence − Assam
WHO−2024 Adjustment Method
Total Anaemia Prevalence
Age (year)
Location Hierarchy
1 − Global
2 − Super Region
3 − Region
4 − Country
5 − Subnational (Level 1)
Uncertainty Interval
5 − Subnational (Level 1)

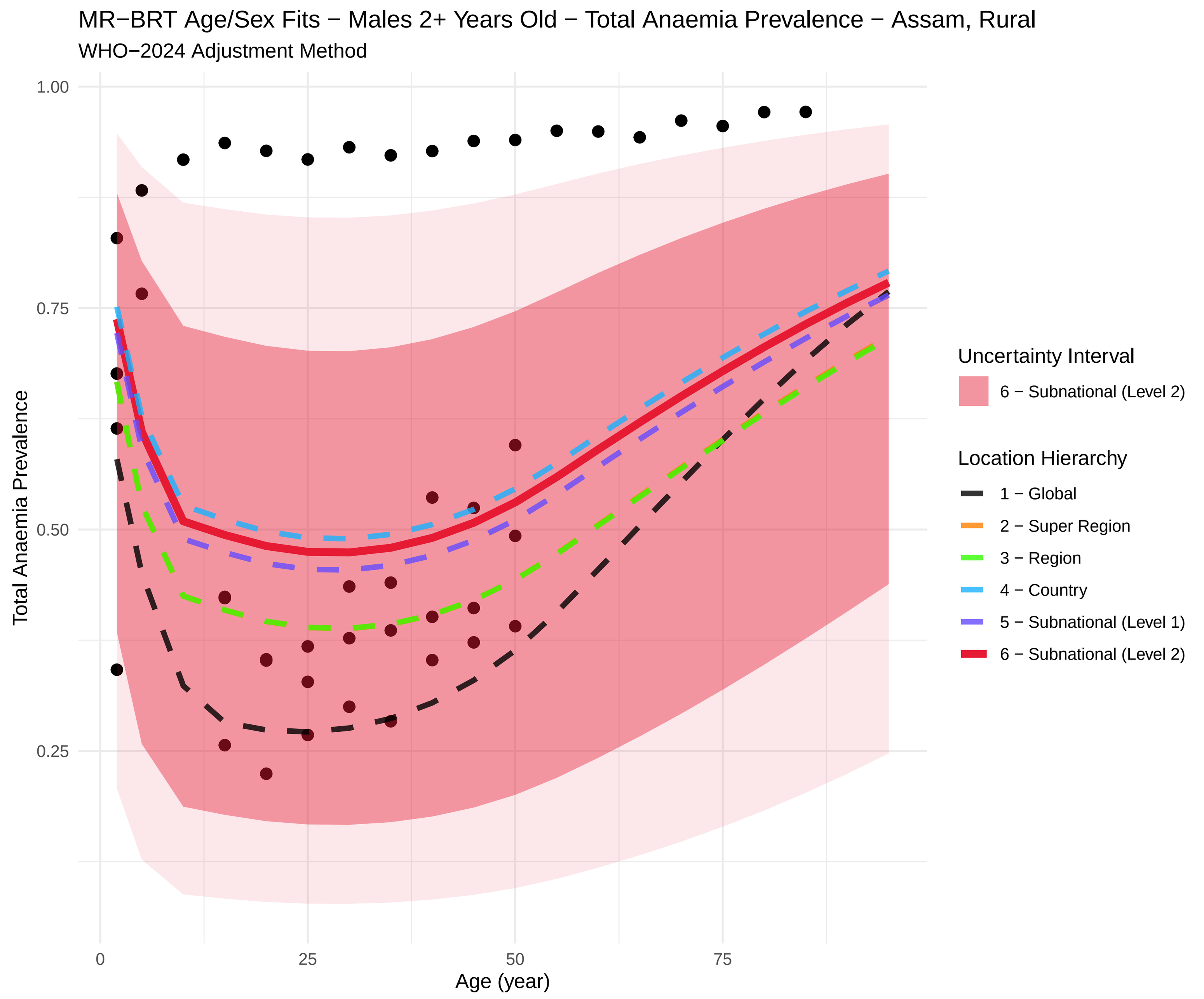
MR−BRT Age/Sex Fits − Males 2+ Years Old − Total Anaemia Prevalence − Assam, Rural
WHO−2024 Adjustment Method
Total Anaemia Prevalence
Age (year)
Uncertainty Interval
6 − Subnational (Level 2)
Location Hierarchy
1 − Global
2 − Super Region
3 − Region
4 − Country
5 − Subnational (Level 1)
6 − Subnational (Level 2)

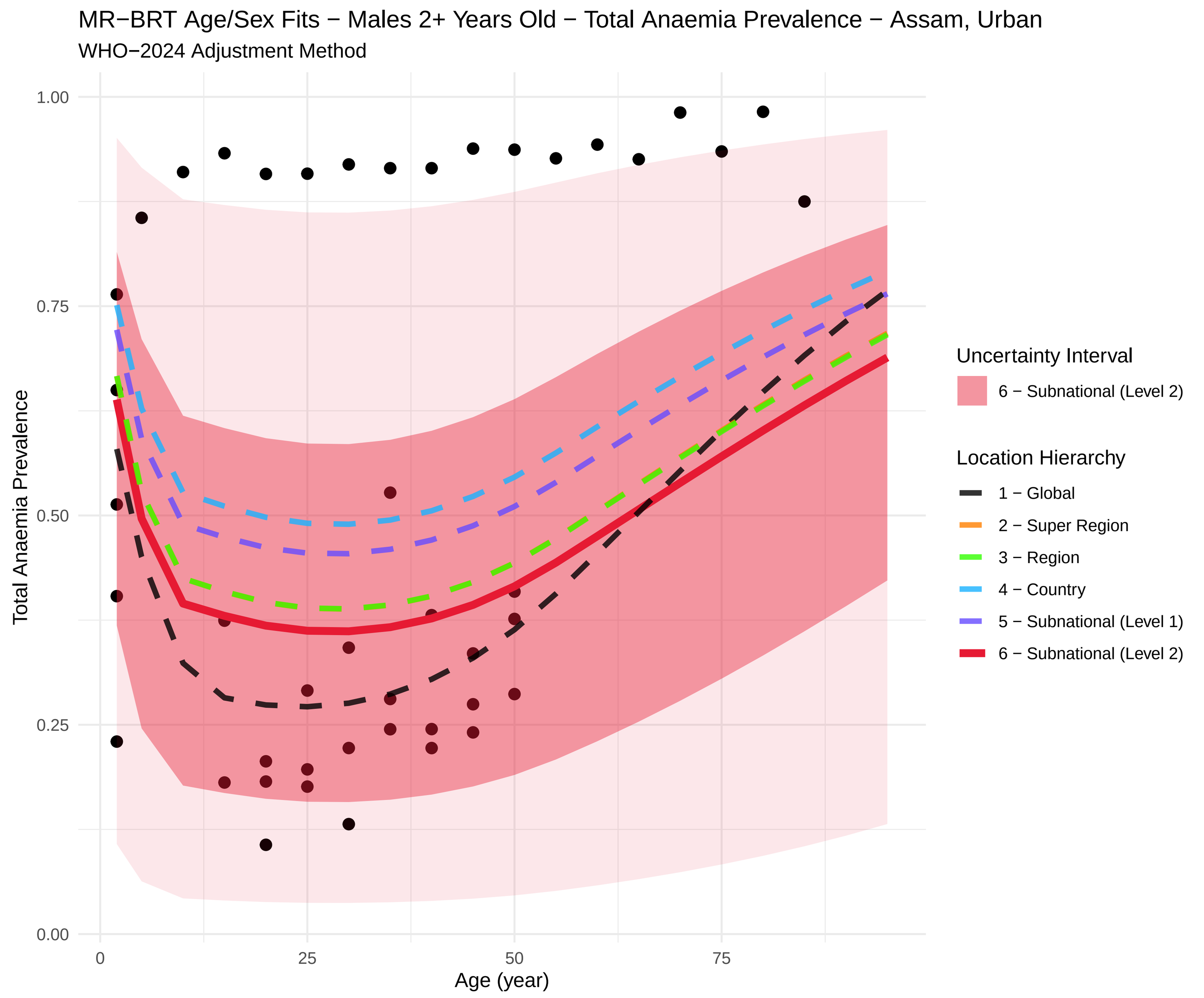
MR−BRT Age/Sex Fits − Males 2+ Years Old − Total Anaemia Prevalence − Assam, Urban
WHO−2024 Adjustment Method
Total Anaemia Prevalence
Age (year)
Uncertainty Interval
6 − Subnational (Level 2)
Location Hierarchy
1 − Global
2 − Super Region
3 − Region
4 − Country
5 − Subnational (Level 1)
6 − Subnational (Level 2)

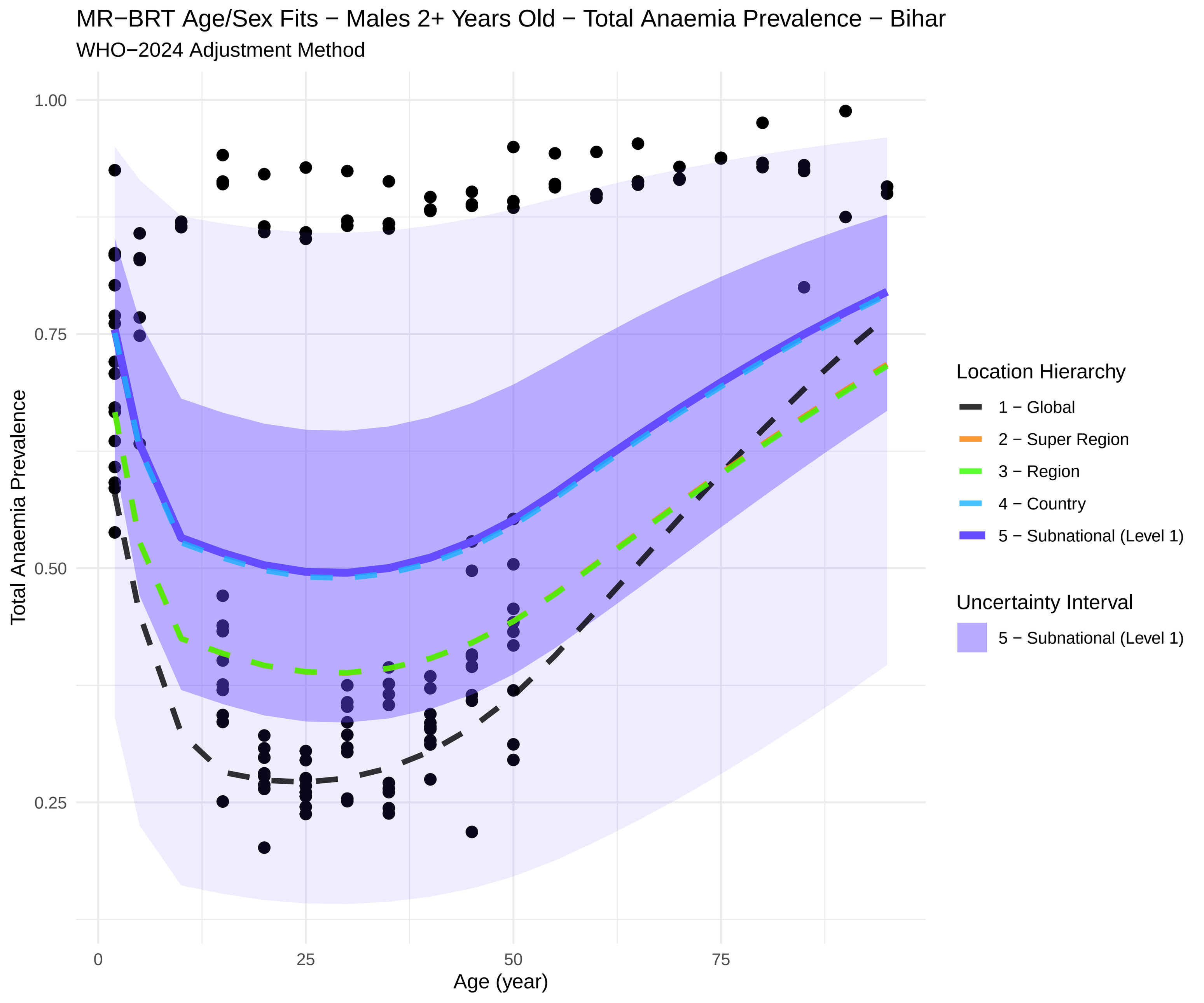
MR−BRT Age/Sex Fits − Males 2+ Years Old − Total Anaemia Prevalence − Bihar
WHO−2024 Adjustment Method
Total Anaemia Prevalence
Age (year)
Location Hierarchy
1 − Global
2 − Super Region
3 − Region
4 − Country
5 − Subnational (Level 1)
Uncertainty Interval
5 − Subnational (Level 1)

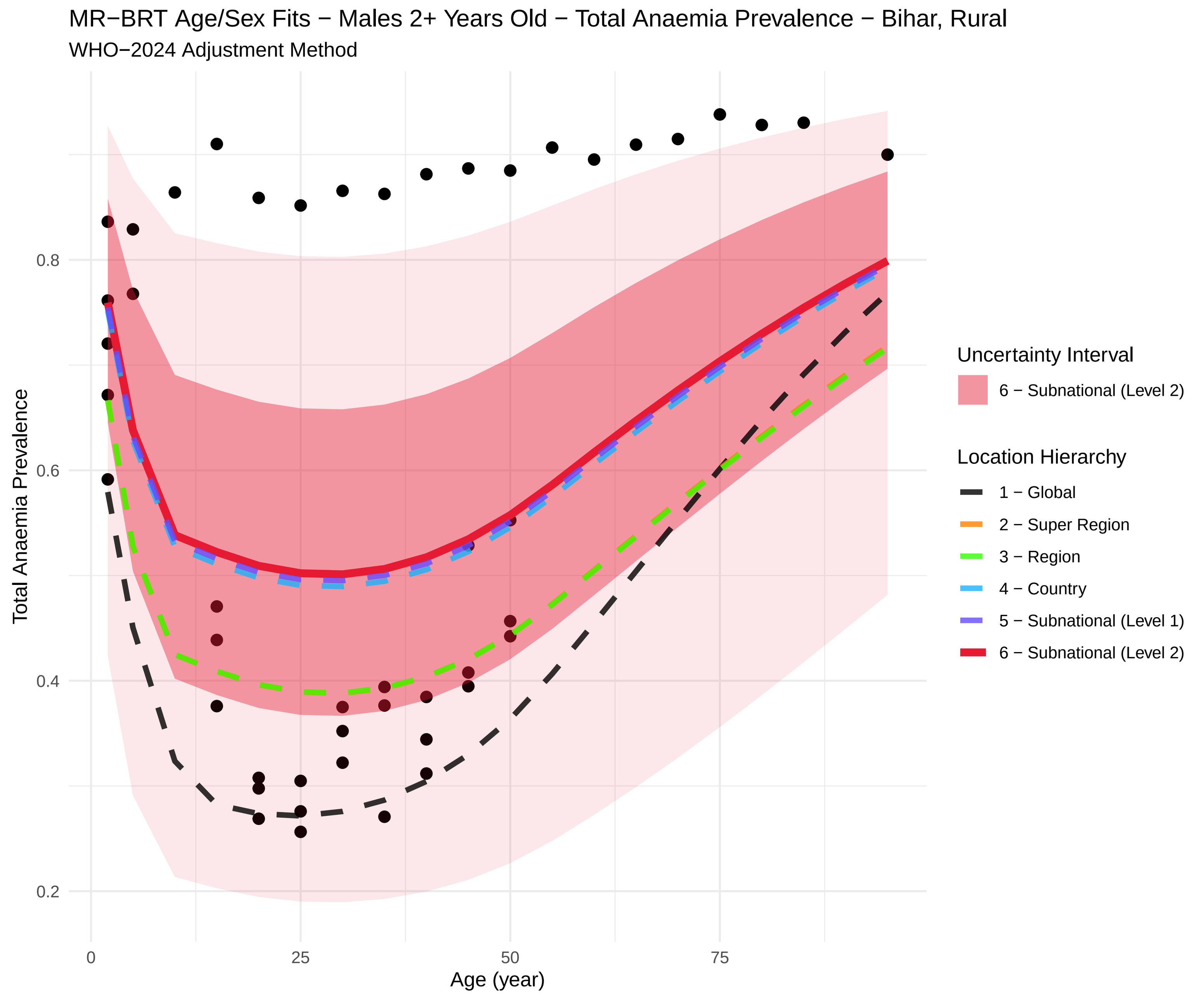
MR−BRT Age/Sex Fits − Males 2+ Years Old − Total Anaemia Prevalence − Bihar, Rural
WHO−2024 Adjustment Method
Total Anaemia Prevalence
Age (year)
Uncertainty Interval
6 − Subnational (Level 2)
Location Hierarchy
1 − Global
2 − Super Region
3 − Region
4 − Country
5 − Subnational (Level 1)
6 − Subnational (Level 2)

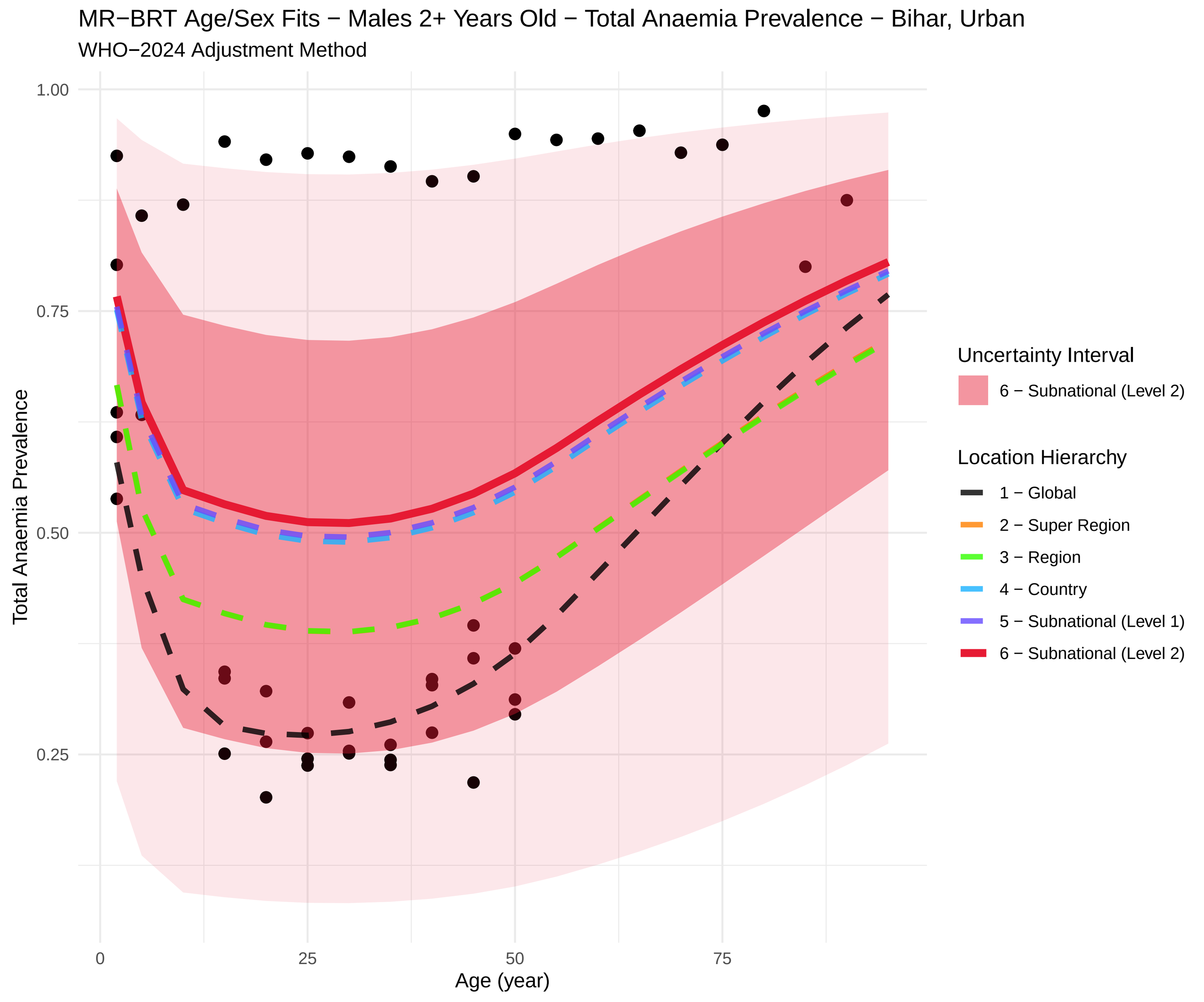
MR−BRT Age/Sex Fits − Males 2+ Years Old − Total Anaemia Prevalence − Bihar, Urban
WHO−2024 Adjustment Method
Total Anaemia Prevalence
Age (year)
Uncertainty Interval
6 − Subnational (Level 2)
Location Hierarchy
1 − Global
2 − Super Region
3 − Region
4 − Country
5 − Subnational (Level 1)
6 − Subnational (Level 2)

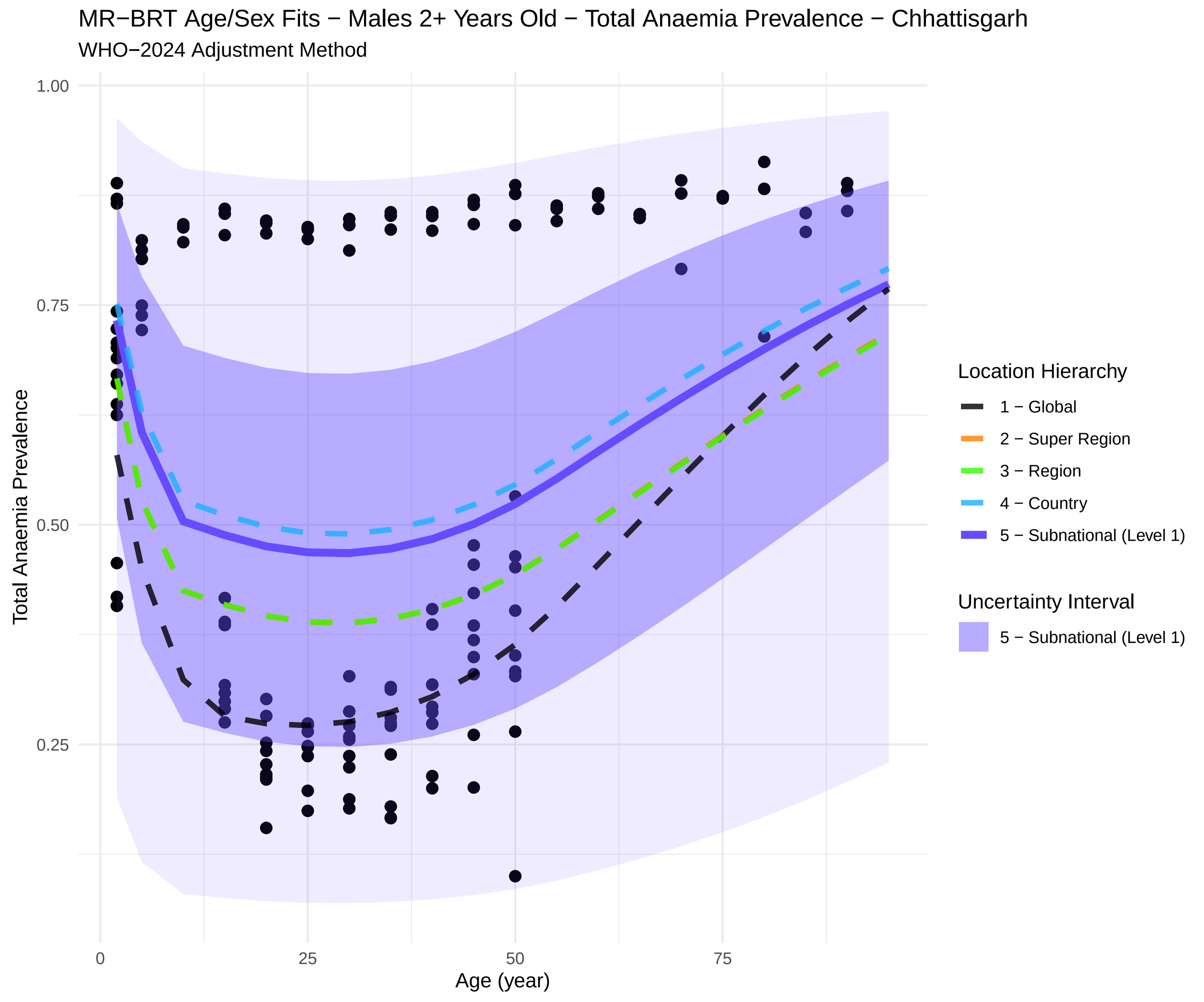
MR−BRT Age/Sex Fits − Males 2+ Years Old − Total Anaemia Prevalence − Chhattisgarh
WHO−2024 Adjustment Method
Total Anaemia Prevalence
Age (year)
Location Hierarchy
1 − Global
2 − Super Region
3 − Region
4 − Country
5 − Subnational (Level 1)
Uncertainty Interval
5 − Subnational (Level 1)

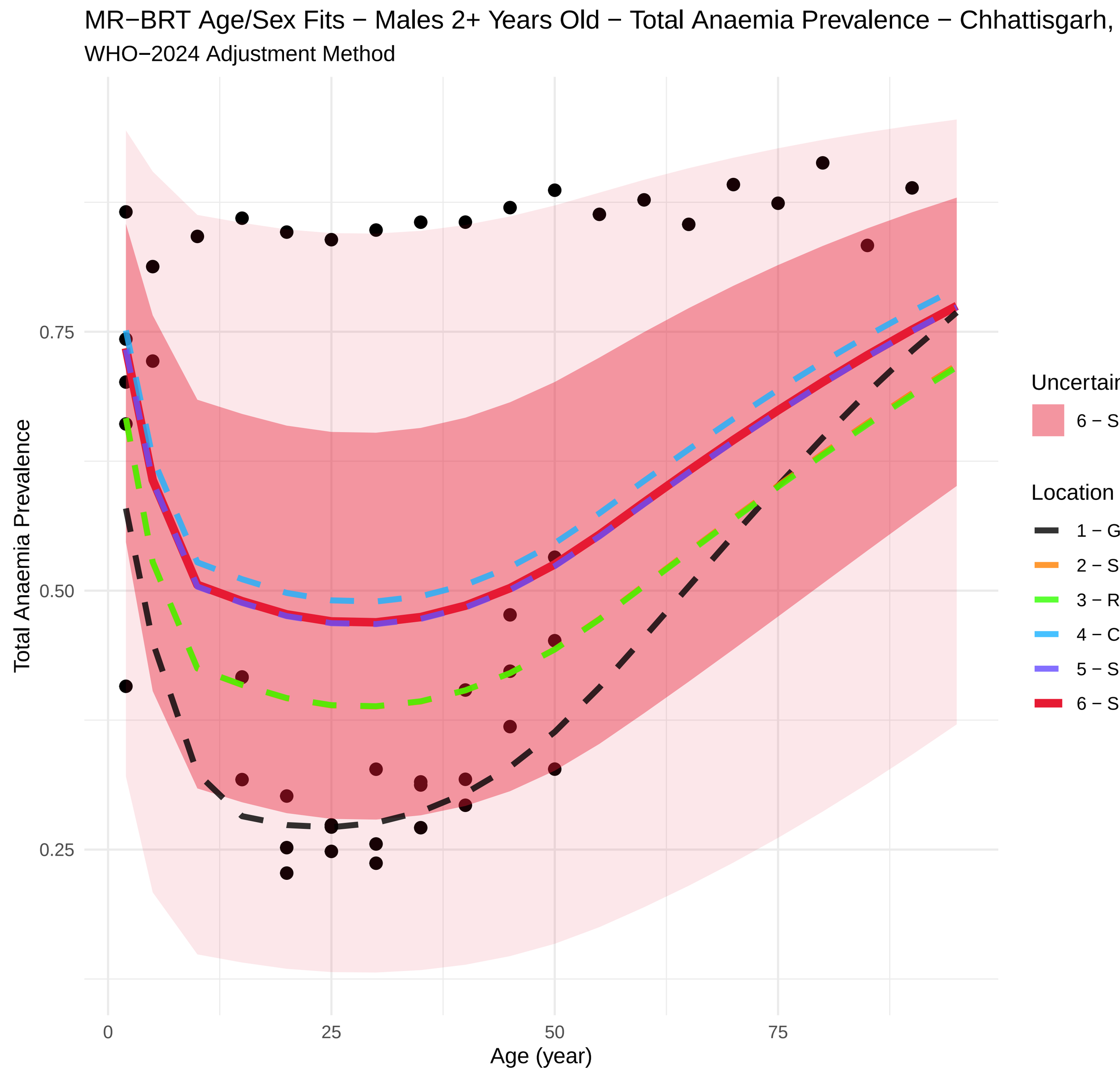
MR−BRT Age/Sex Fits − Males 2+ Years Old − Total Anaemia Prevalence − Chhattisgarh, Rural
WHO−2024 Adjustment Method
Total Anaemia Prevalence
Age (year)
Uncertainty Interval
6 − Subnational (Level 2)
Location Hierarchy
1 − Global
2 − Super Region
3 − Region
4 − Country
5 − Subnational (Level 1)
6 − Subnational (Level 2)

MR–BRT Age/Sex Fits – Males 2+ Years Old – Total Anaemia Prevalence – Chhattisgarh, Urban
WHO–2024 Adjustment Method

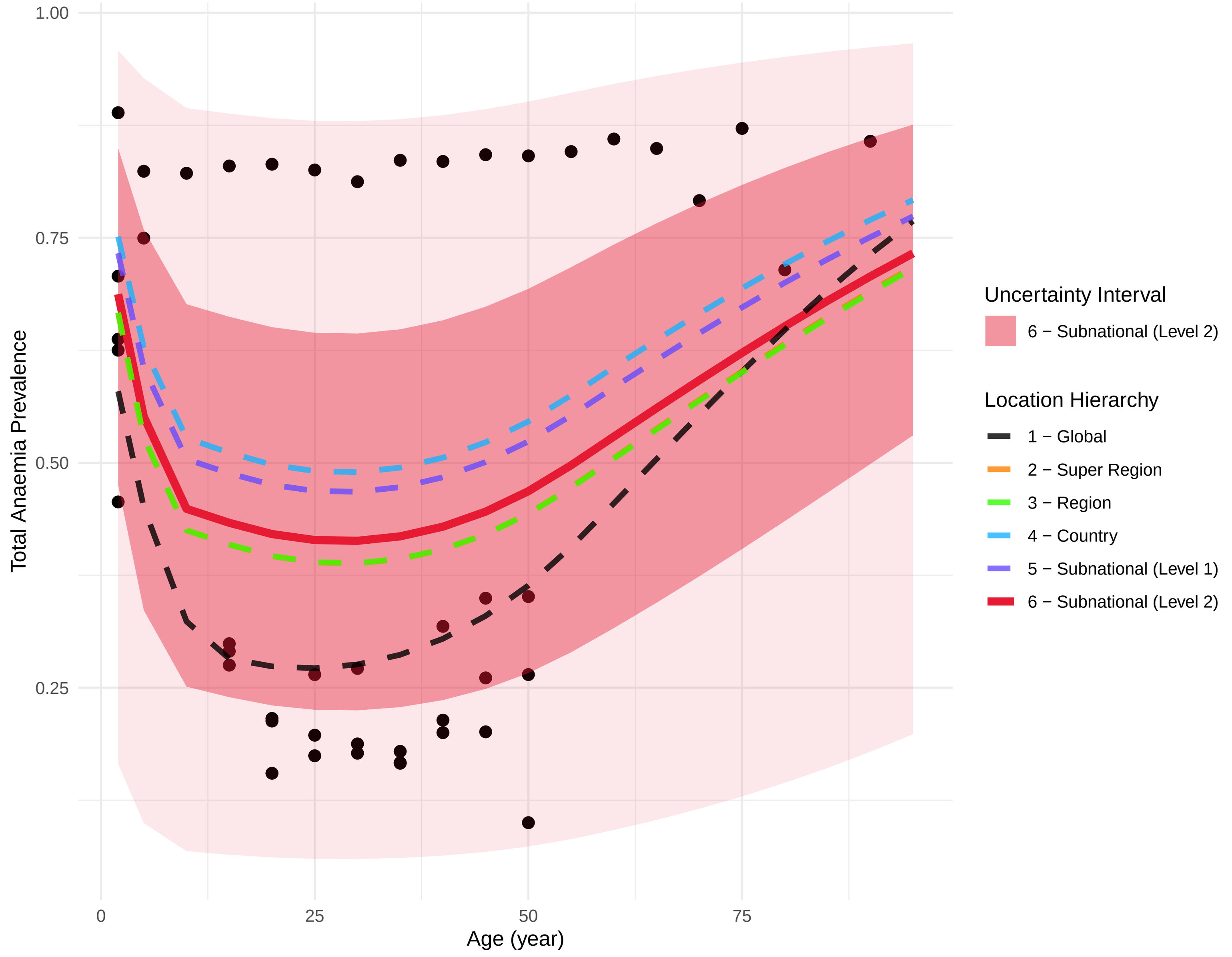

MR–BRT Age/Sex Fits – Males 2+ Years Old – Total Anaemia Prevalence – Delhi
WHO–2024 Adjustment Method

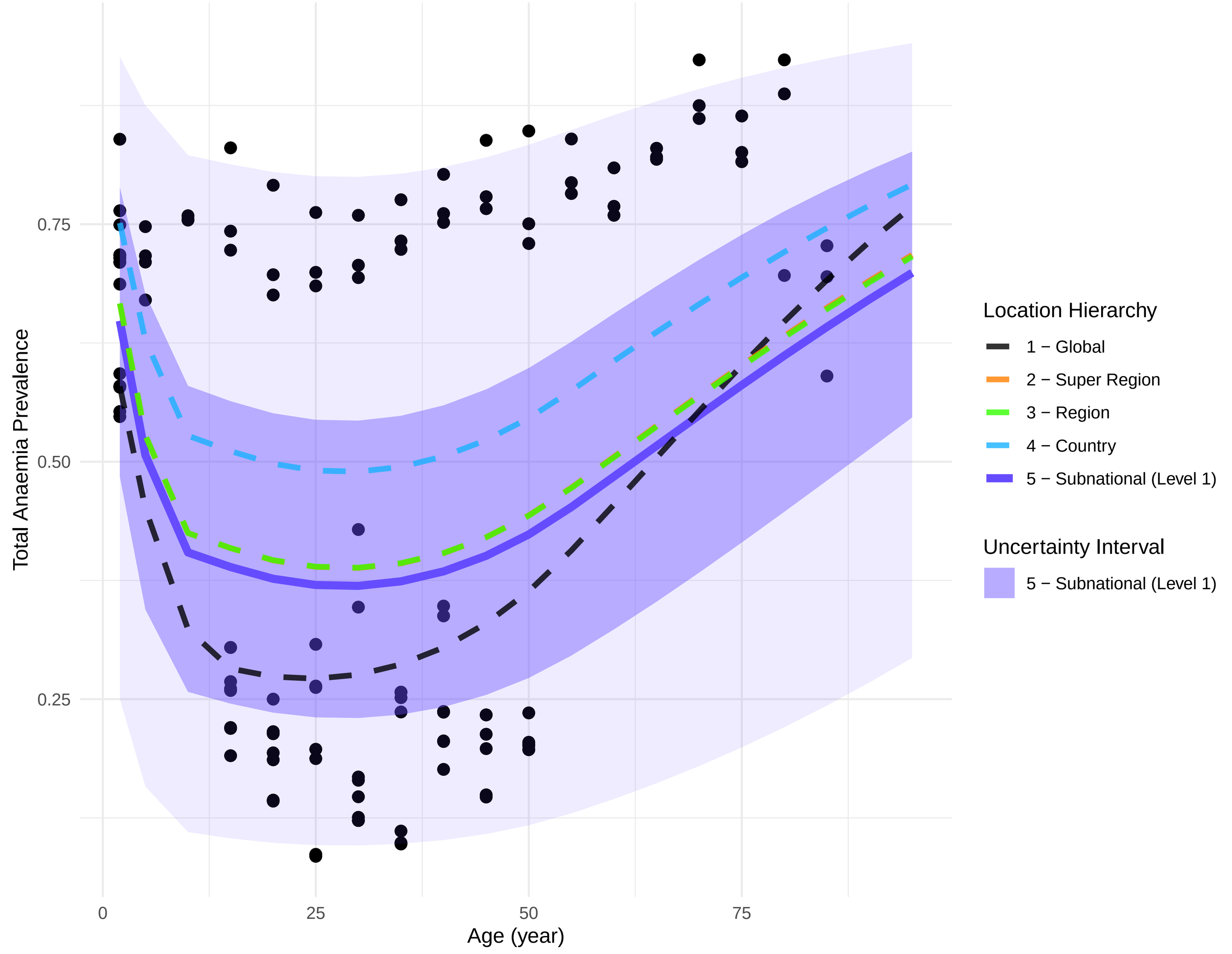

MR–BRT Age/Sex Fits – Males 2+ Years Old – Total Anaemia Prevalence – Delhi, Rural
WHO–2024 Adjustment Method

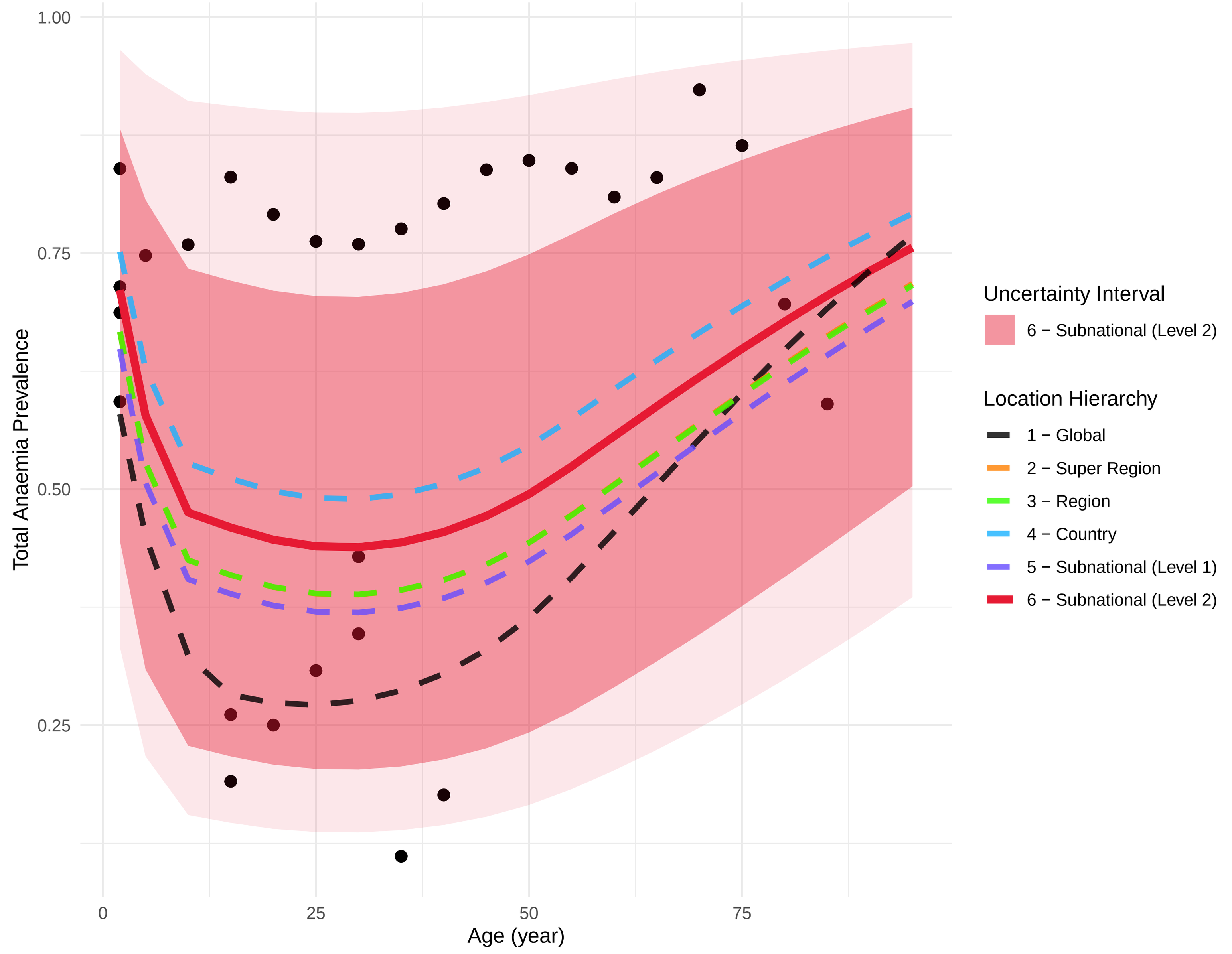

MR–BRT Age/Sex Fits – Males 2+ Years Old – Total Anaemia Prevalence – Delhi, Urban
WHO–2024 Adjustment Method

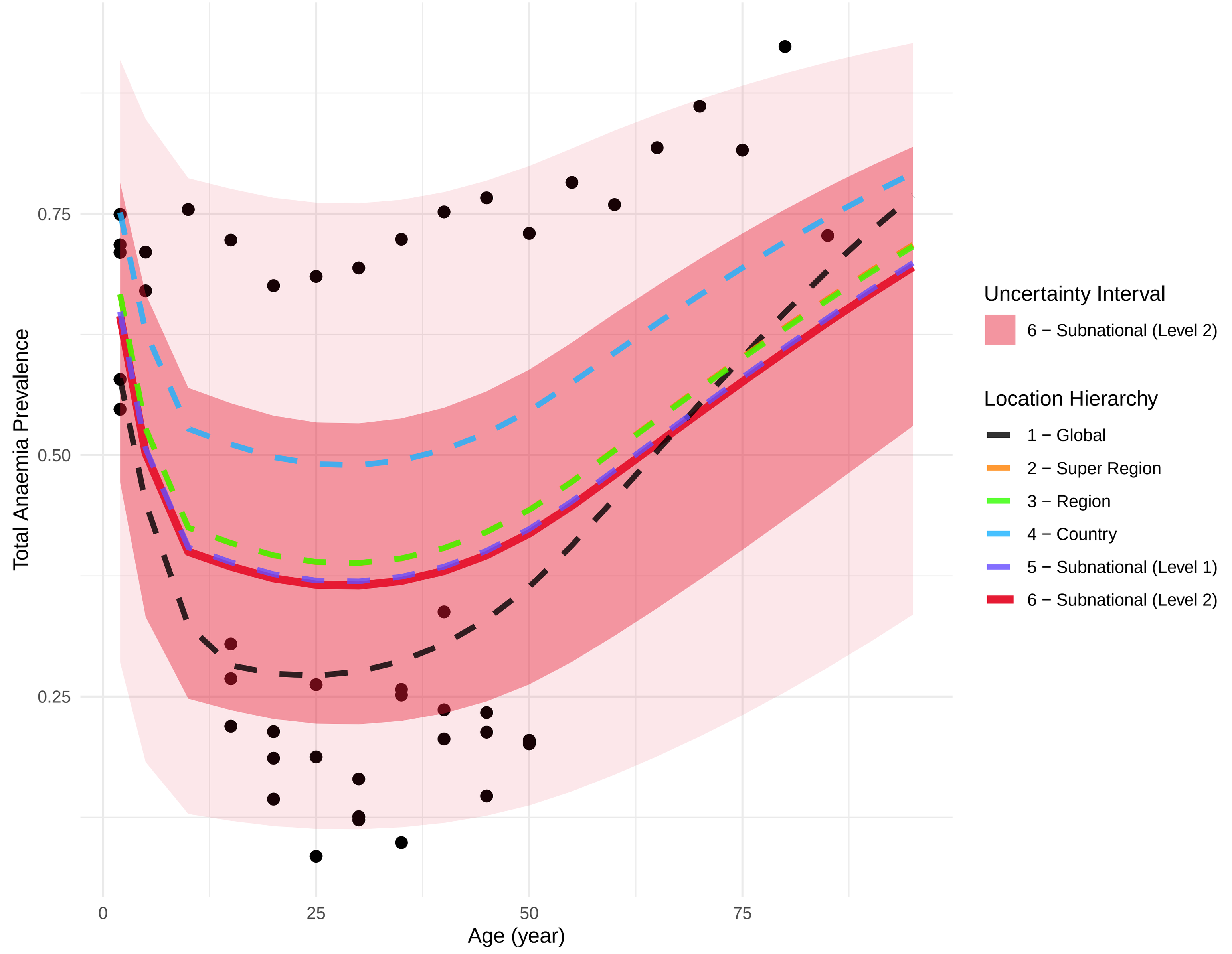

MR–BRT Age/Sex Fits – Males 2+ Years Old – Total Anaemia Prevalence – Goa
WHO–2024 Adjustment Method

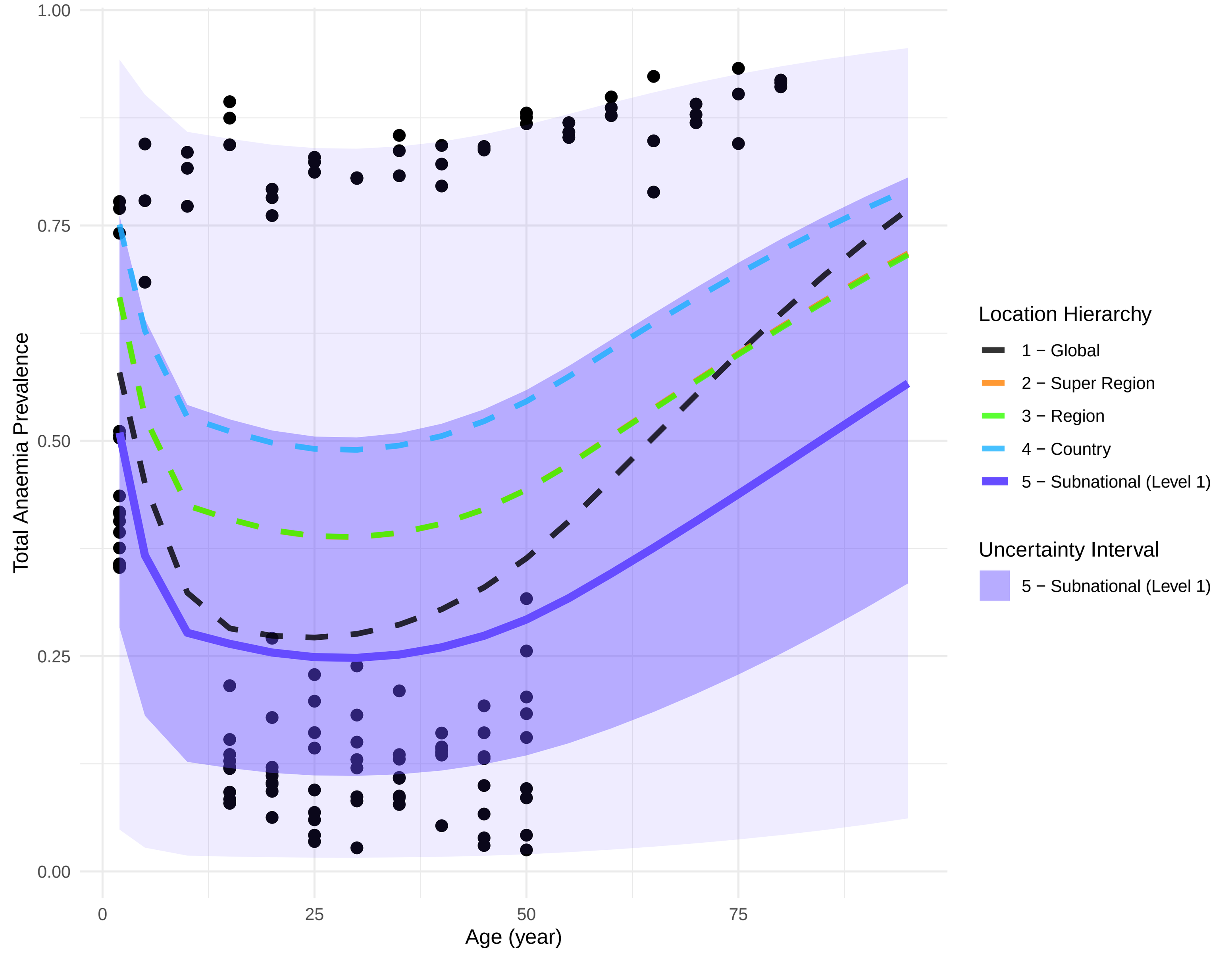

MR–BRT Age/Sex Fits – Males 2+ Years Old – Total Anaemia Prevalence – Goa, Rural
WHO–2024 Adjustment Method

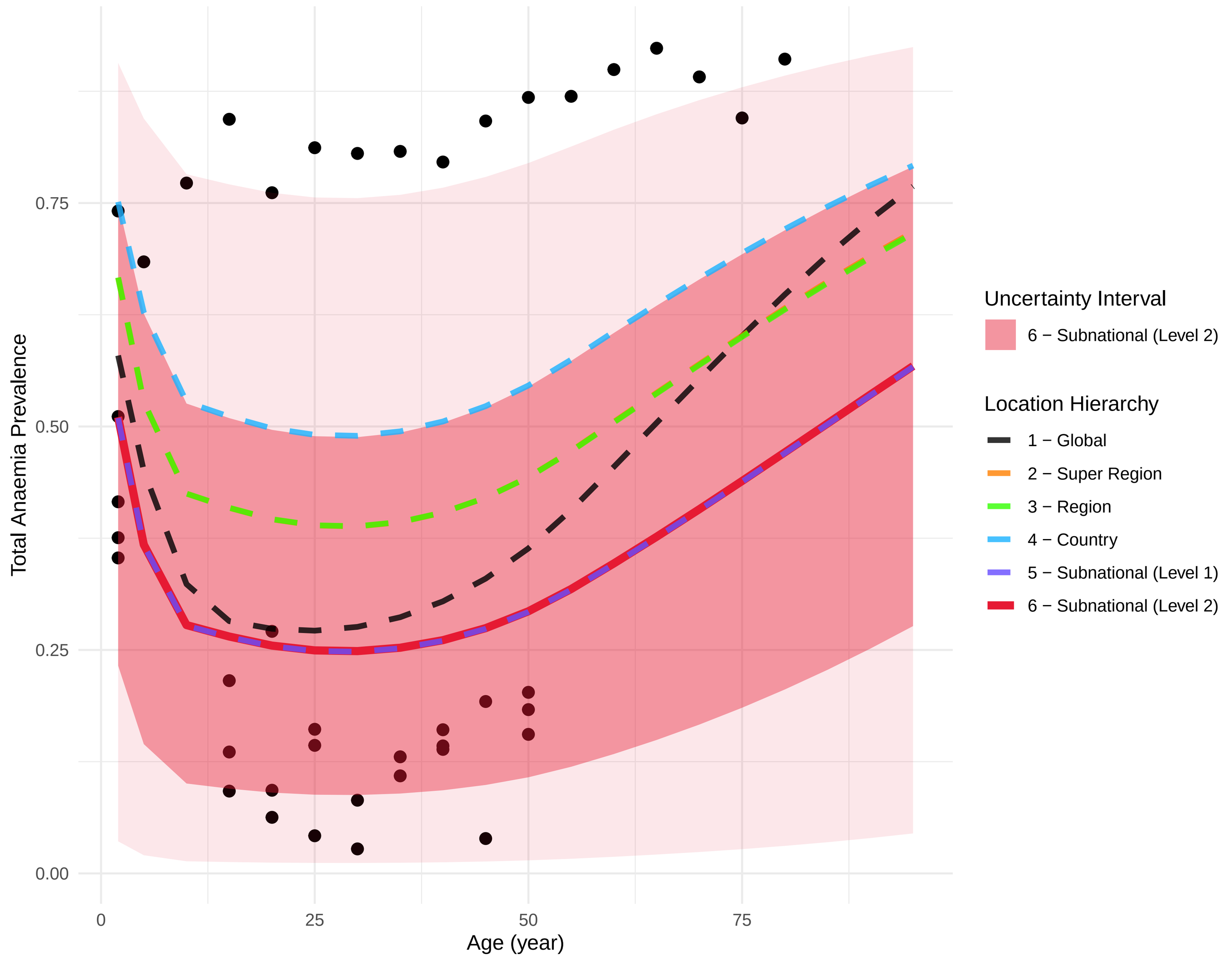

MR–BRT Age/Sex Fits – Males 2+ Years Old – Total Anaemia Prevalence – Goa, Urban
WHO–2024 Adjustment Method

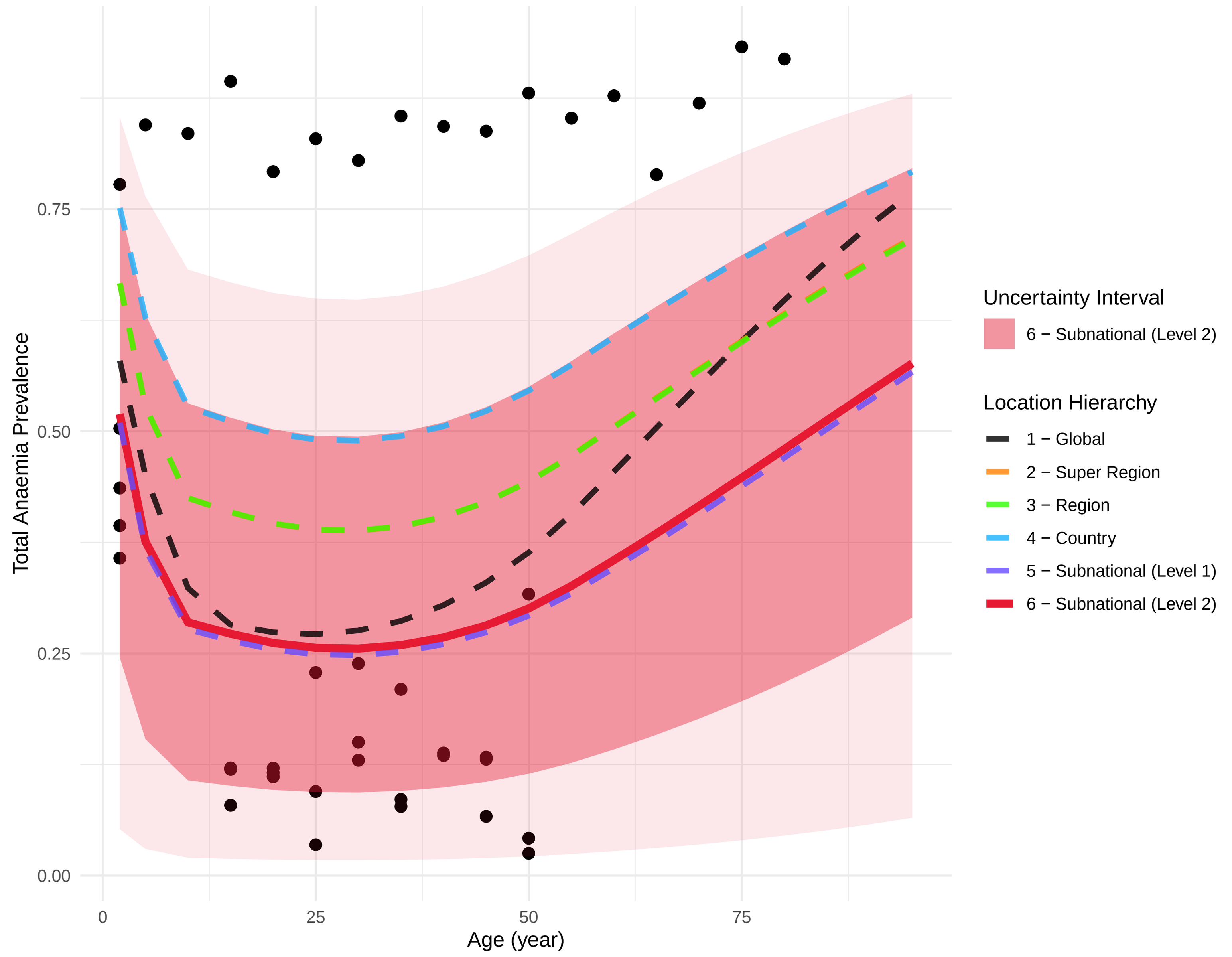

MR–BRT Age/Sex Fits – Males 2+ Years Old – Total Anaemia Prevalence – Gujarat
WHO–2024 Adjustment Method

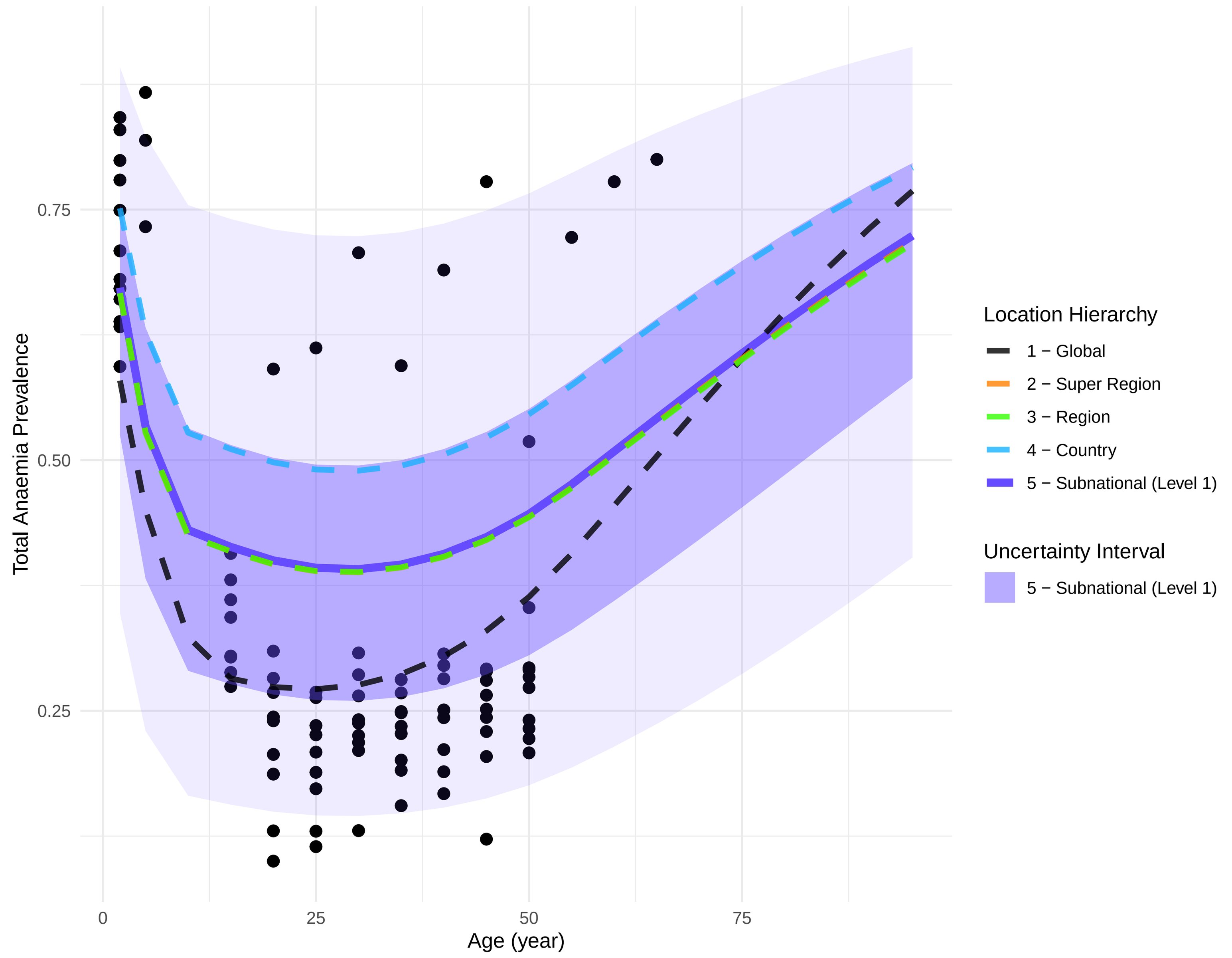

MR–BRT Age/Sex Fits – Males 2+ Years Old – Total Anaemia Prevalence – Gujarat, Rural
WHO–2024 Adjustment Method

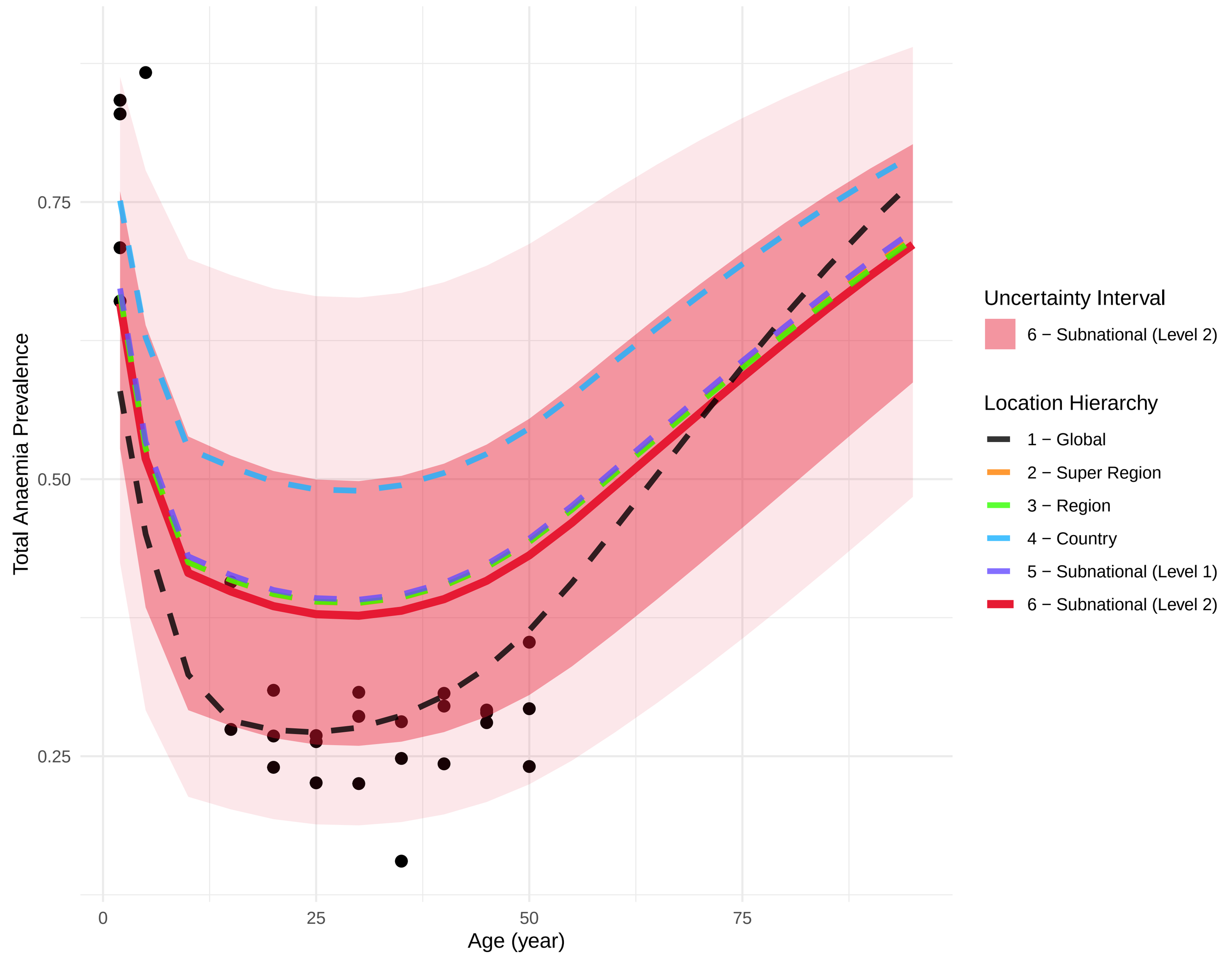

MR–BRT Age/Sex Fits – Males 2+ Years Old – Total Anaemia Prevalence – Gujarat, Urban
WHO–2024 Adjustment Method

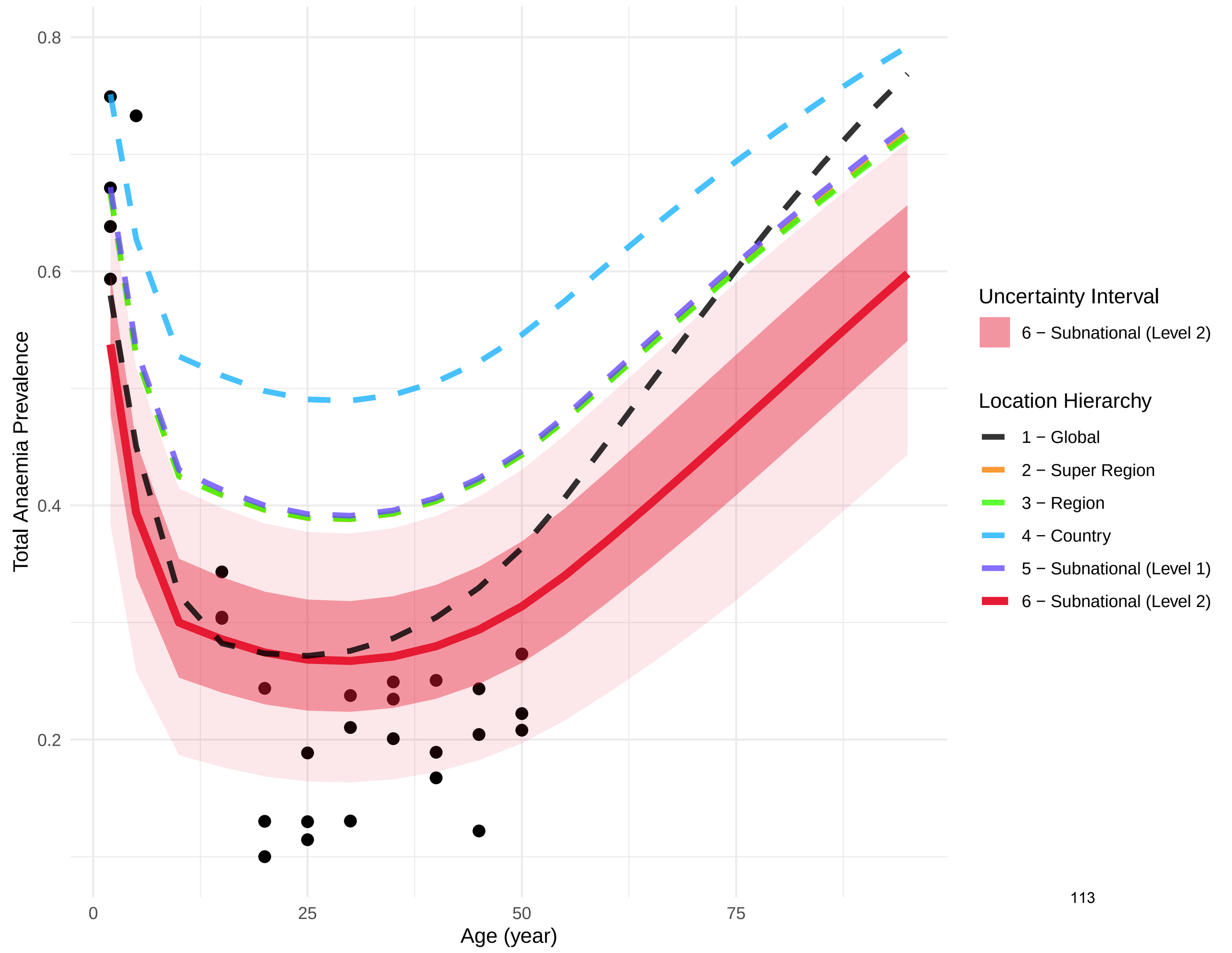

MR−BRT Age/Sex Fits − Males 2+ Years Old − Total Anaemia Prevalence − Haryana
WHO−2024 Adjustment Method

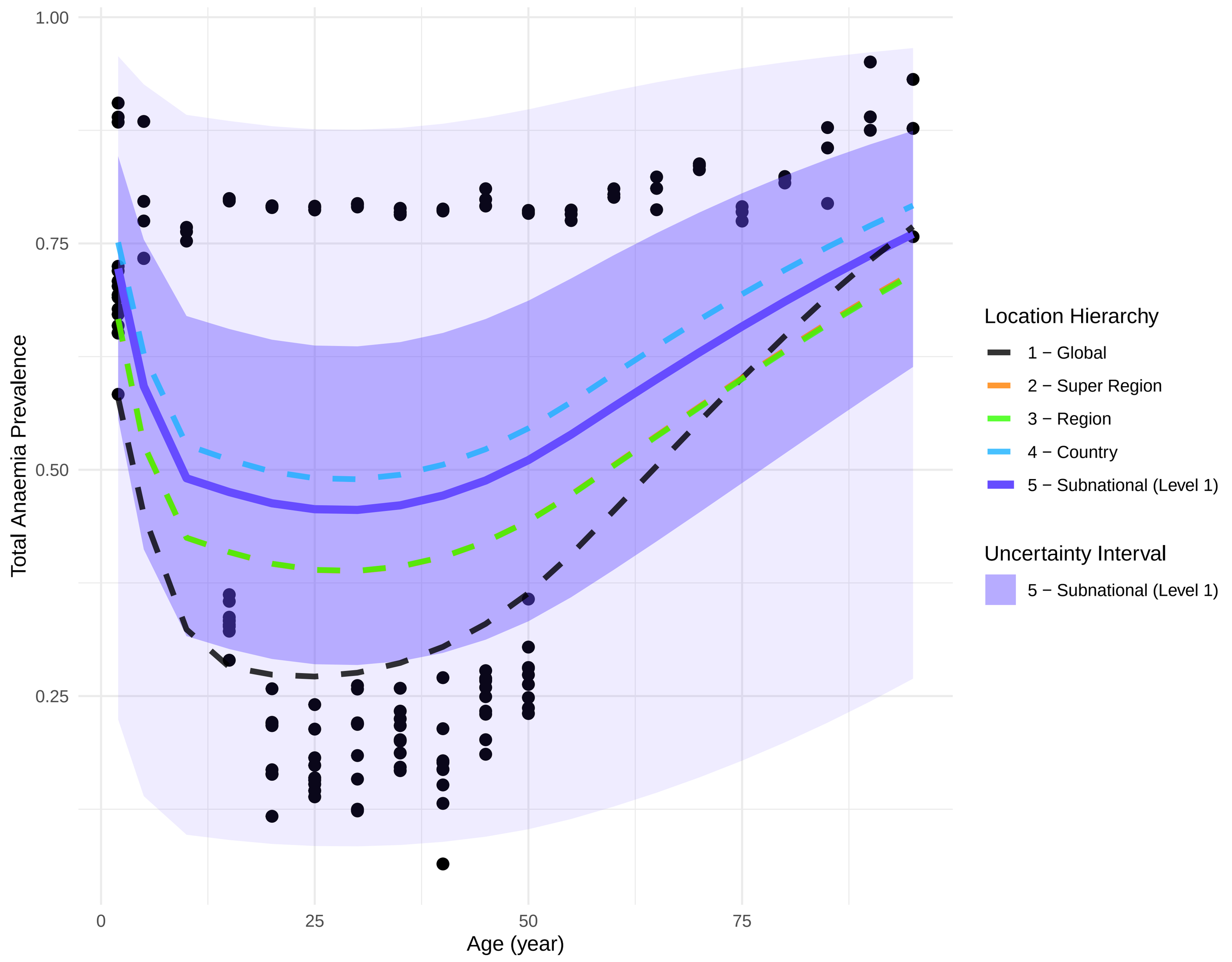

MR−BRT Age/Sex Fits − Males 2+ Years Old − Total Anaemia Prevalence − Haryana, Rural
WHO−2024 Adjustment Method

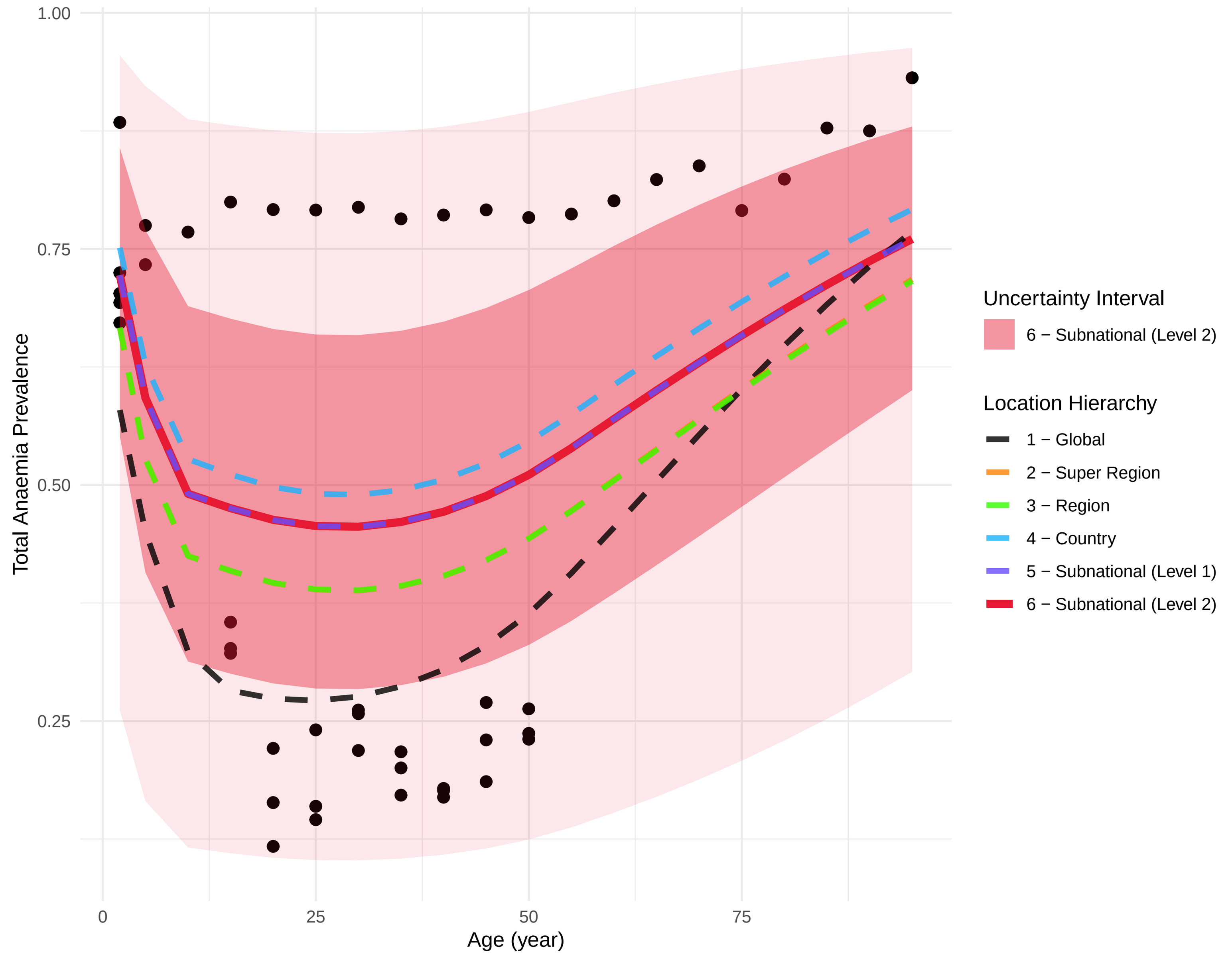

MR−BRT Age/Sex Fits − Males 2+ Years Old − Total Anaemia Prevalence − Haryana, Urban
WHO−2024 Adjustment Method

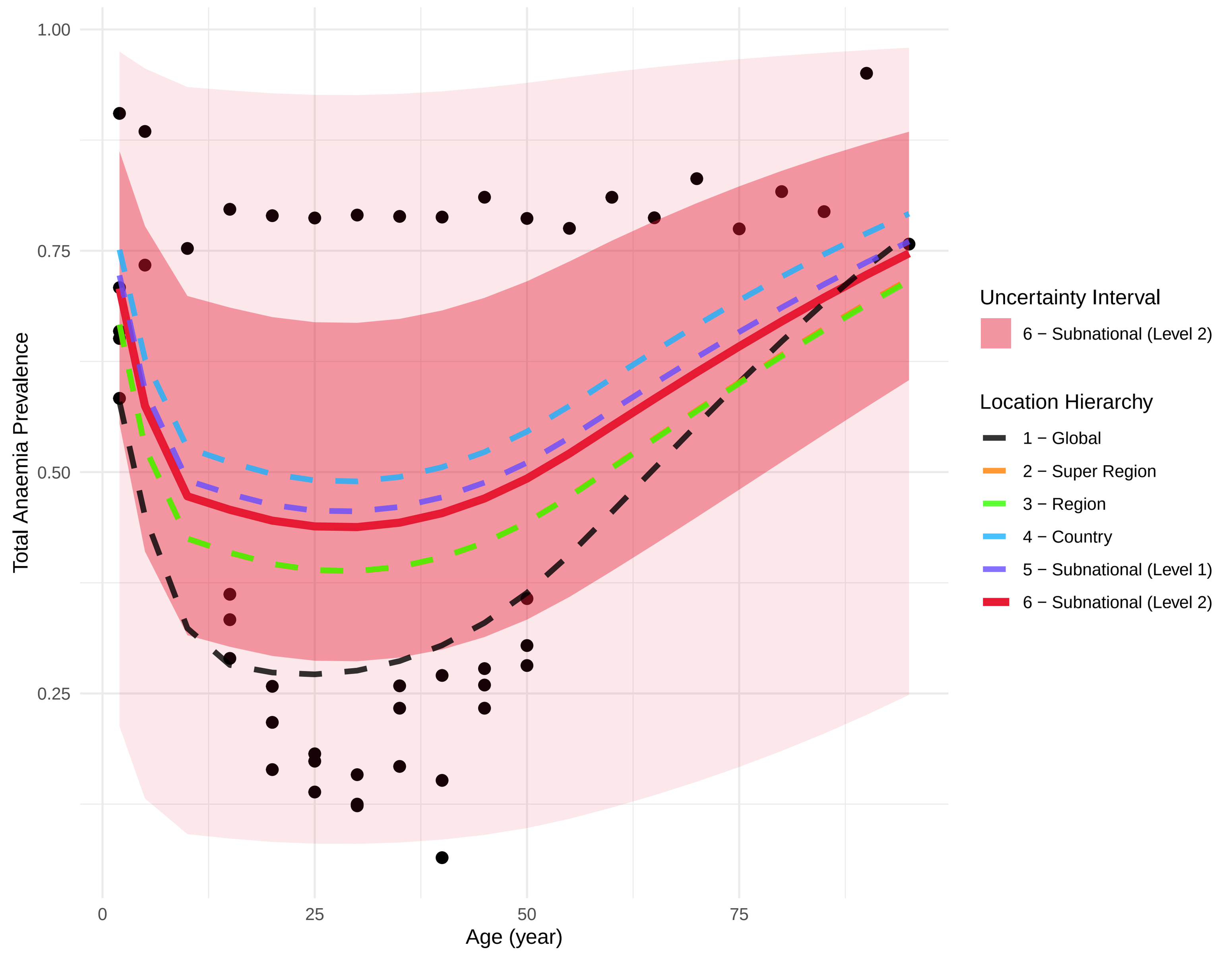

MR−BRT Age/Sex Fits − Males 2+ Years Old − Total Anaemia Prevalence − Himachal Pradesh
WHO−2024 Adjustment Method

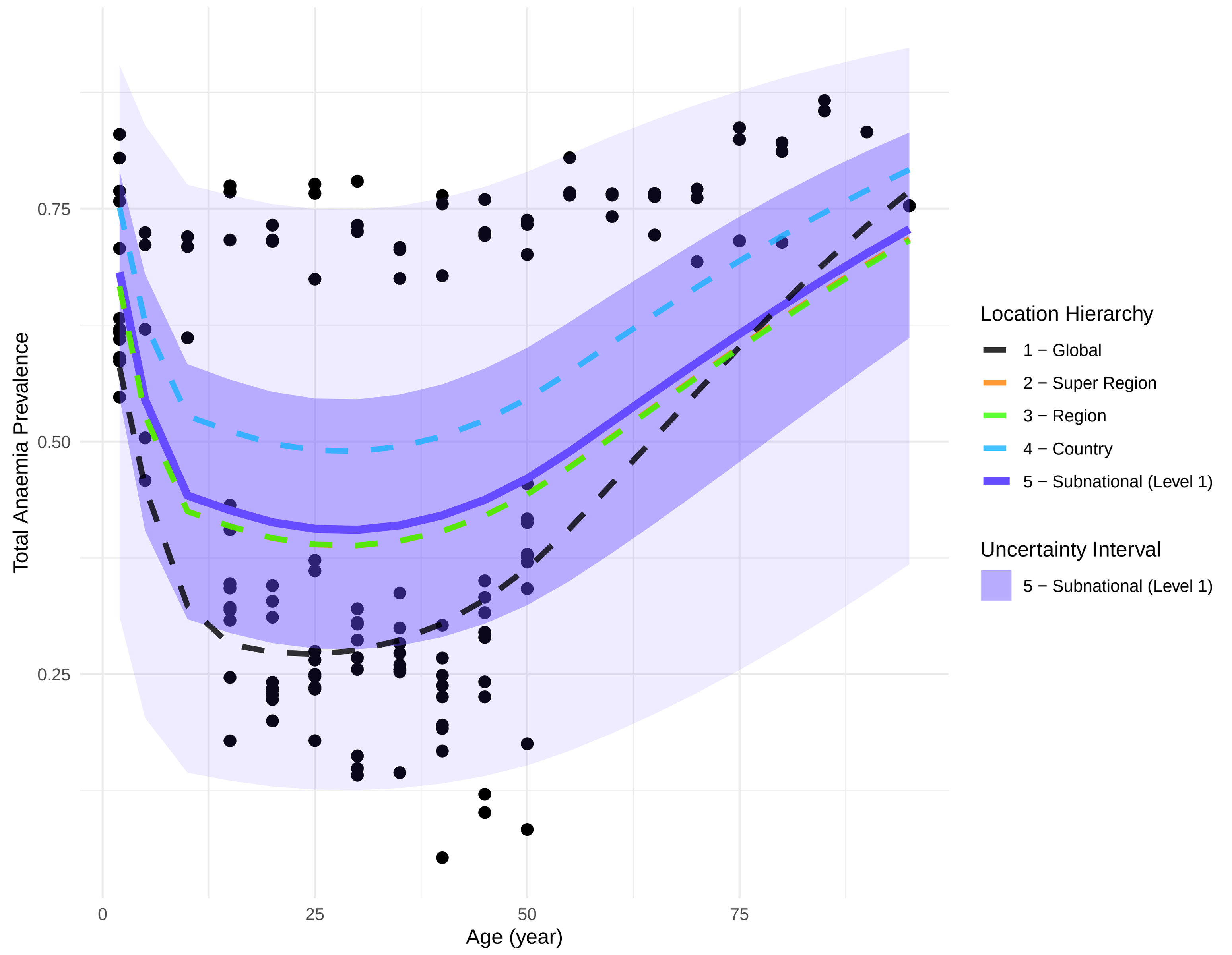

MR−BRT Age/Sex Fits − Males 2+ Years Old − Total Anaemia Prevalence − Himachal Pradesh, Rural
WHO−2024 Adjustment Method

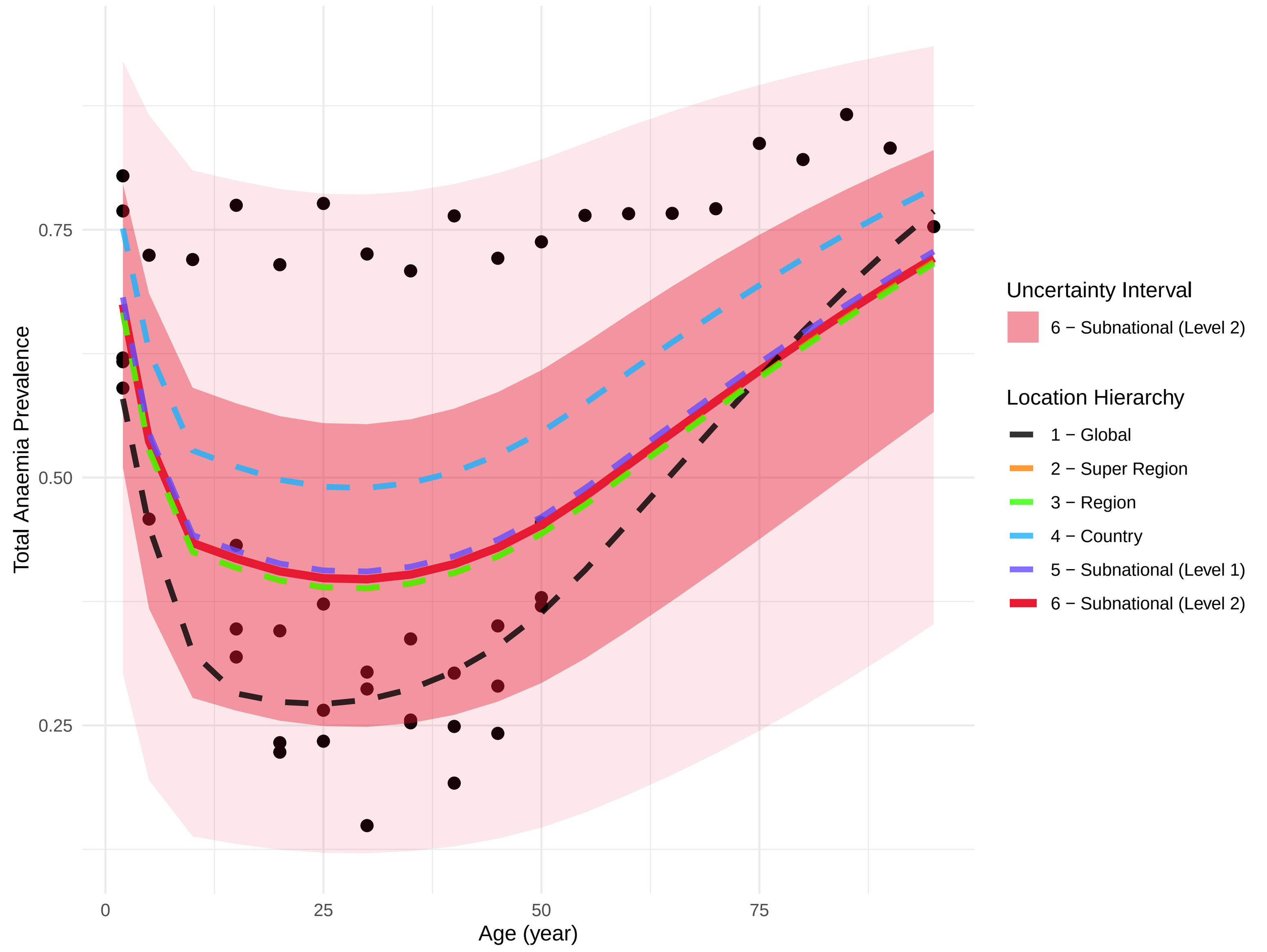

MR−BRT Age/Sex Fits − Males 2+ Years Old − Total Anaemia Prevalence − Himachal Pradesh, Urban
WHO−2024 Adjustment Method

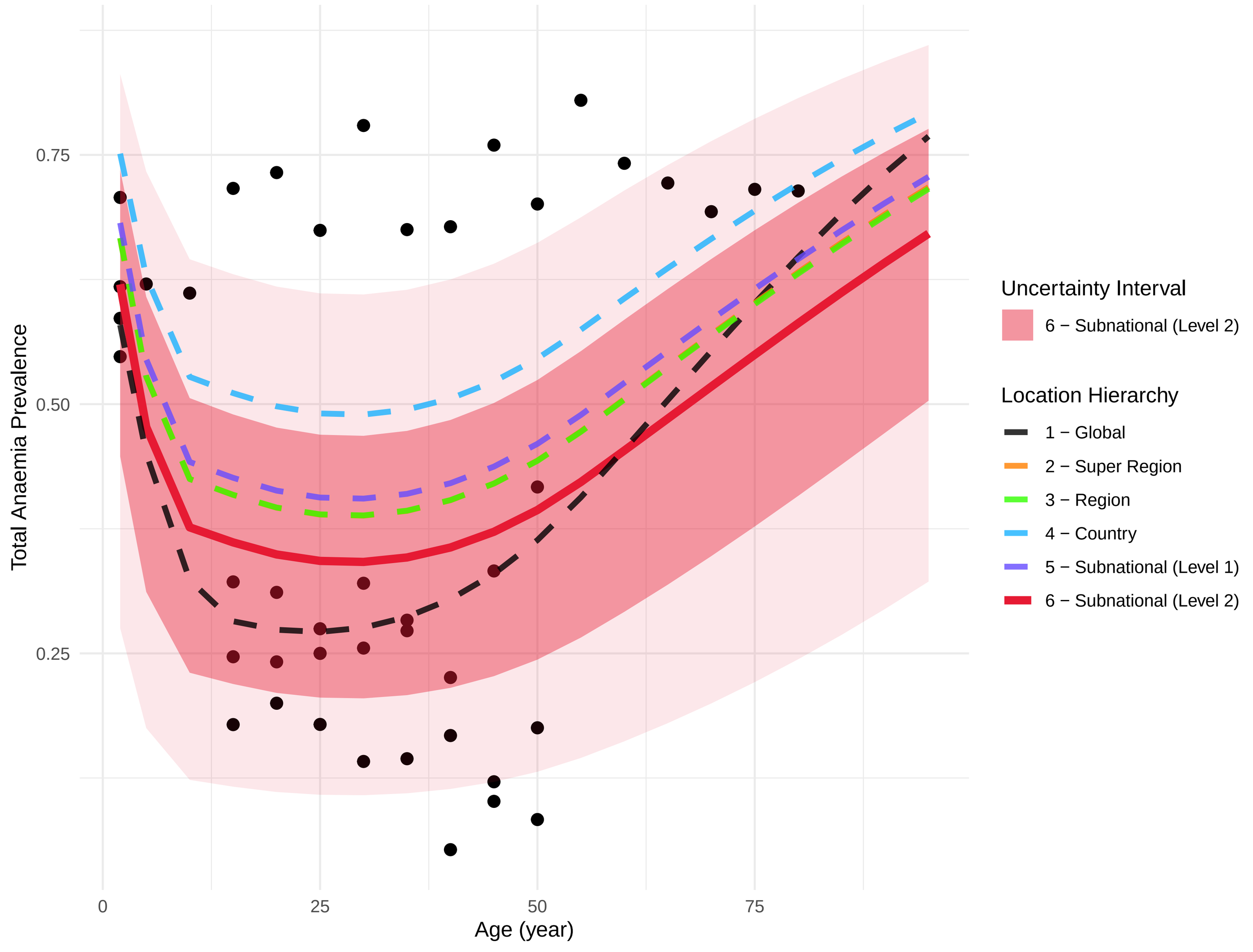

MR−BRT Age/Sex Fits − Males 2+ Years Old − Total Anaemia Prevalence − Jammu & Kashmir and Ladakh
WHO−2024 Adjustment Method

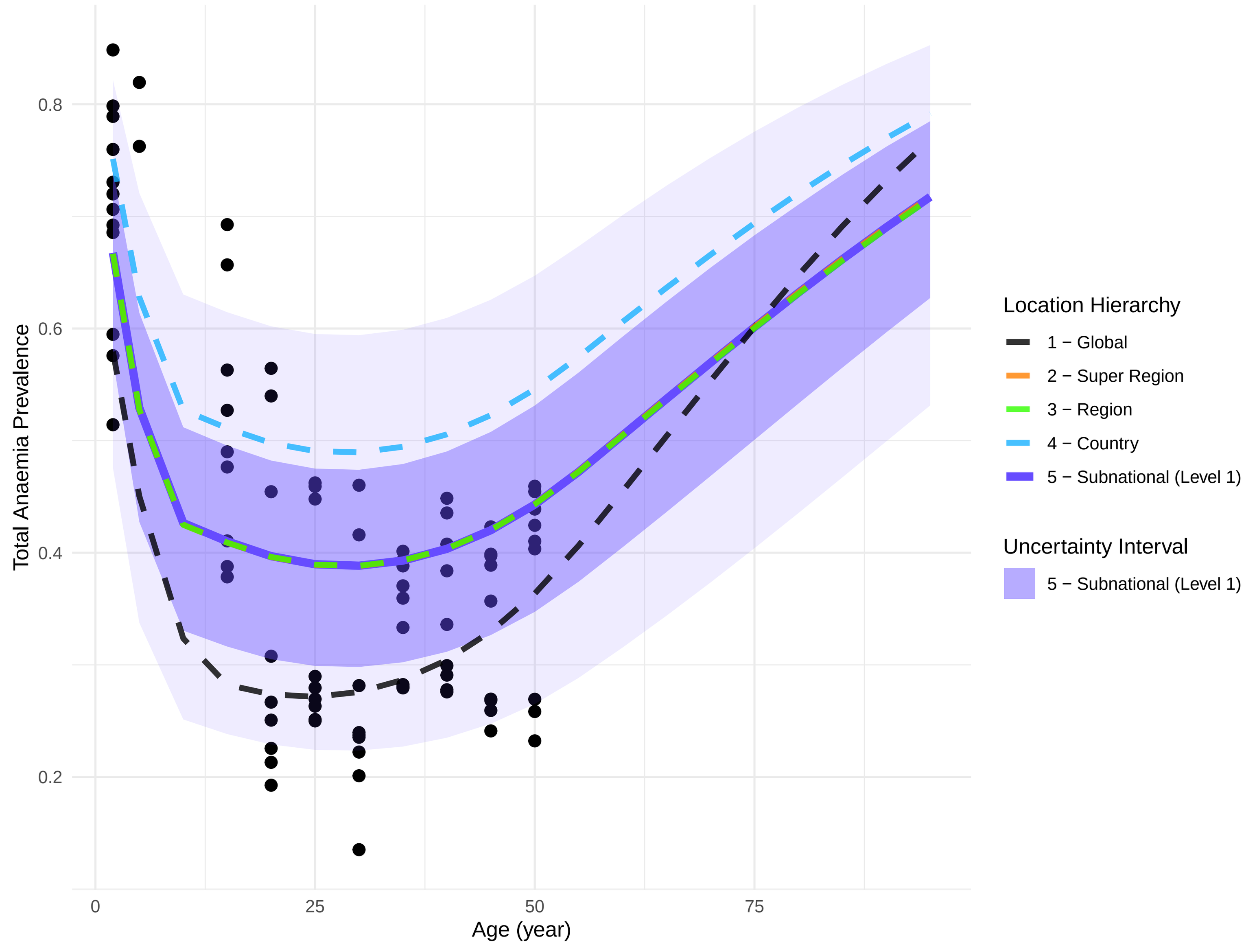

MR−BRT Age/Sex Fits − Males 2+ Years Old − Total Anaemia Prevalence − Jammu & Kashmir and Ladakh, Rural
WHO−2024 Adjustment Method

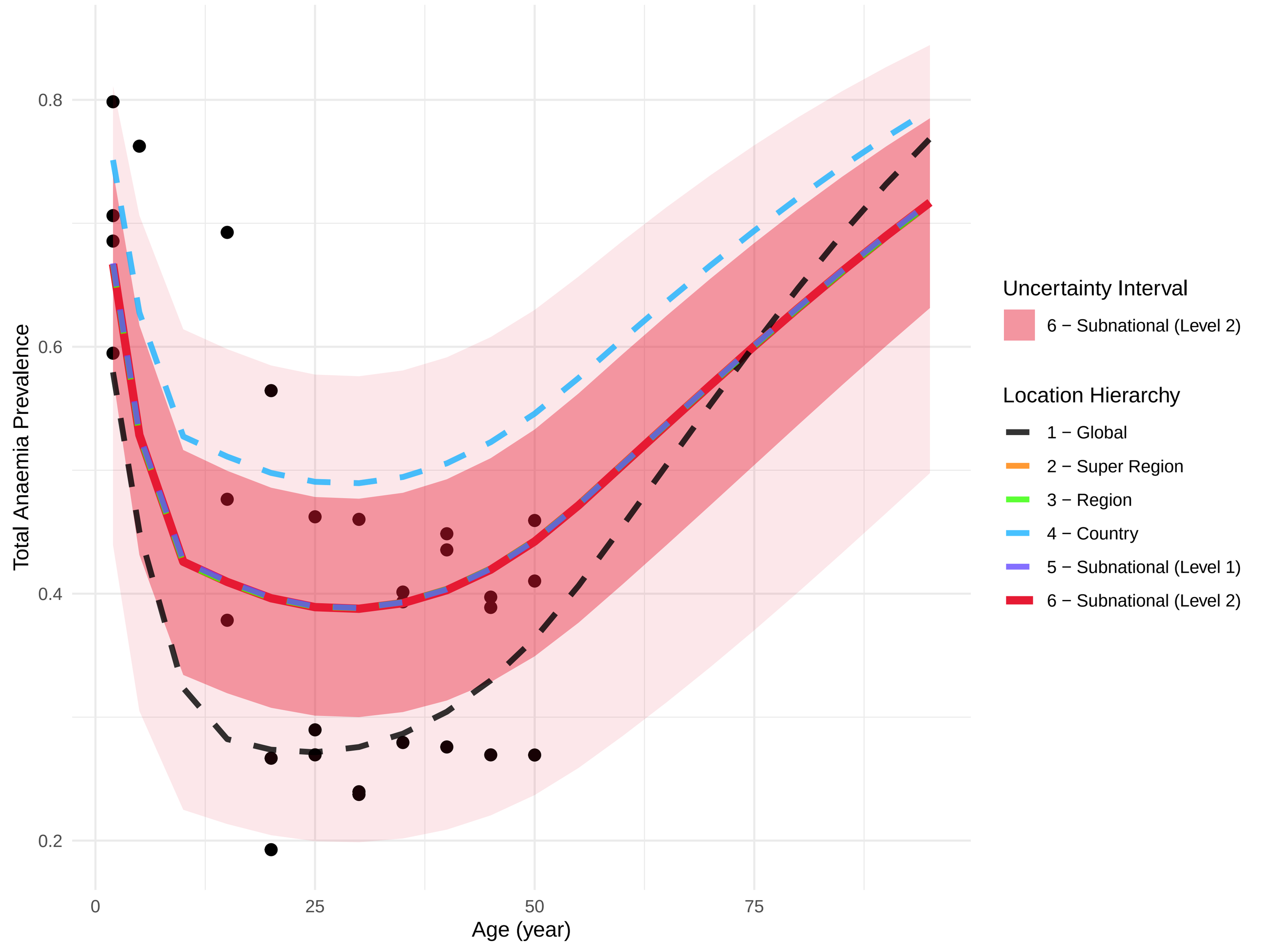

MR−BRT Age/Sex Fits − Males 2+ Years Old − Total Anaemia Prevalence − Jammu & Kashmir and Ladakh, Urban
WHO−2024 Adjustment Method

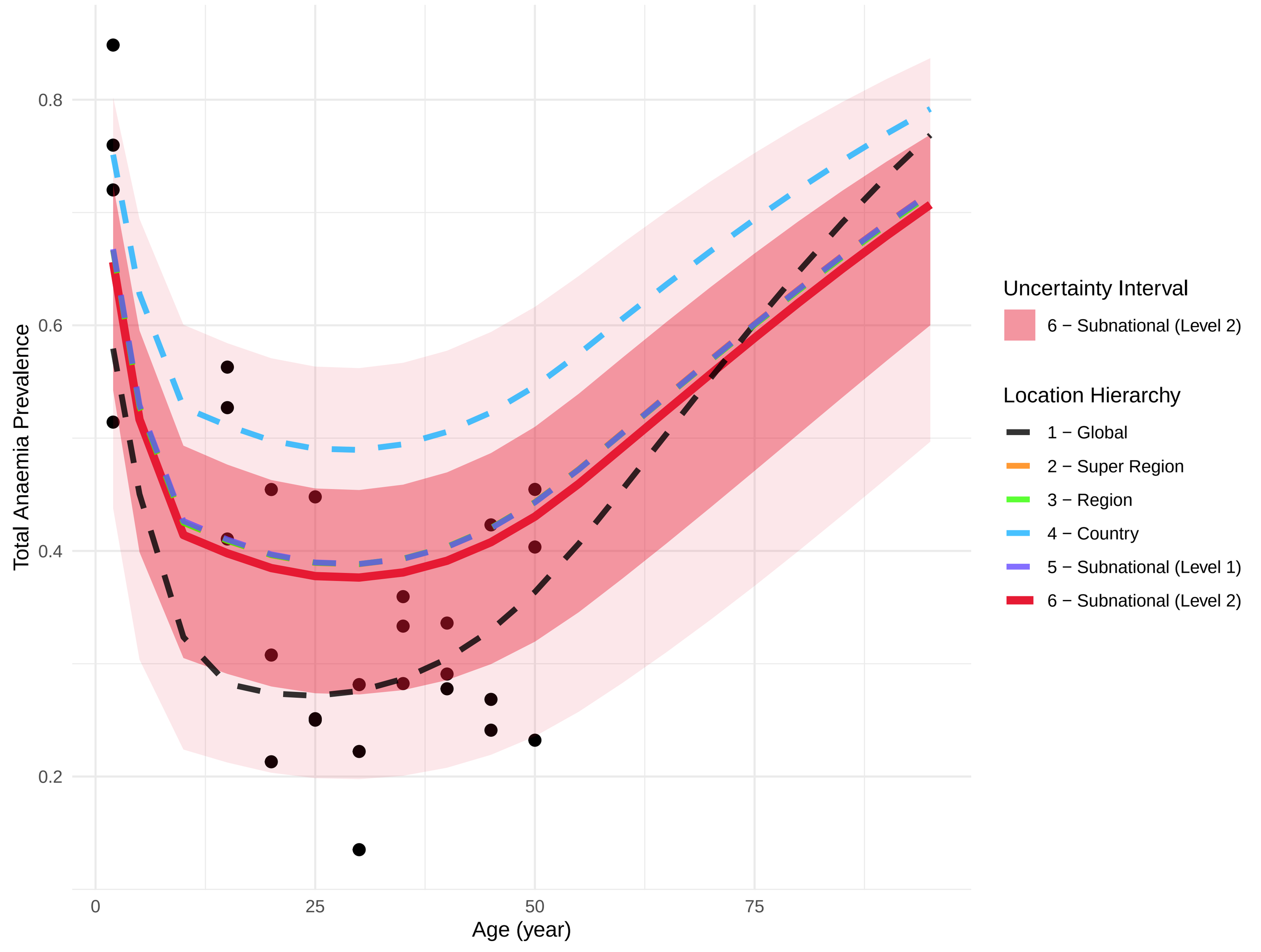

MR−BRT Age/Sex Fits − Males 2+ Years Old − Total Anaemia Prevalence − Jharkhand
WHO−2024 Adjustment Method

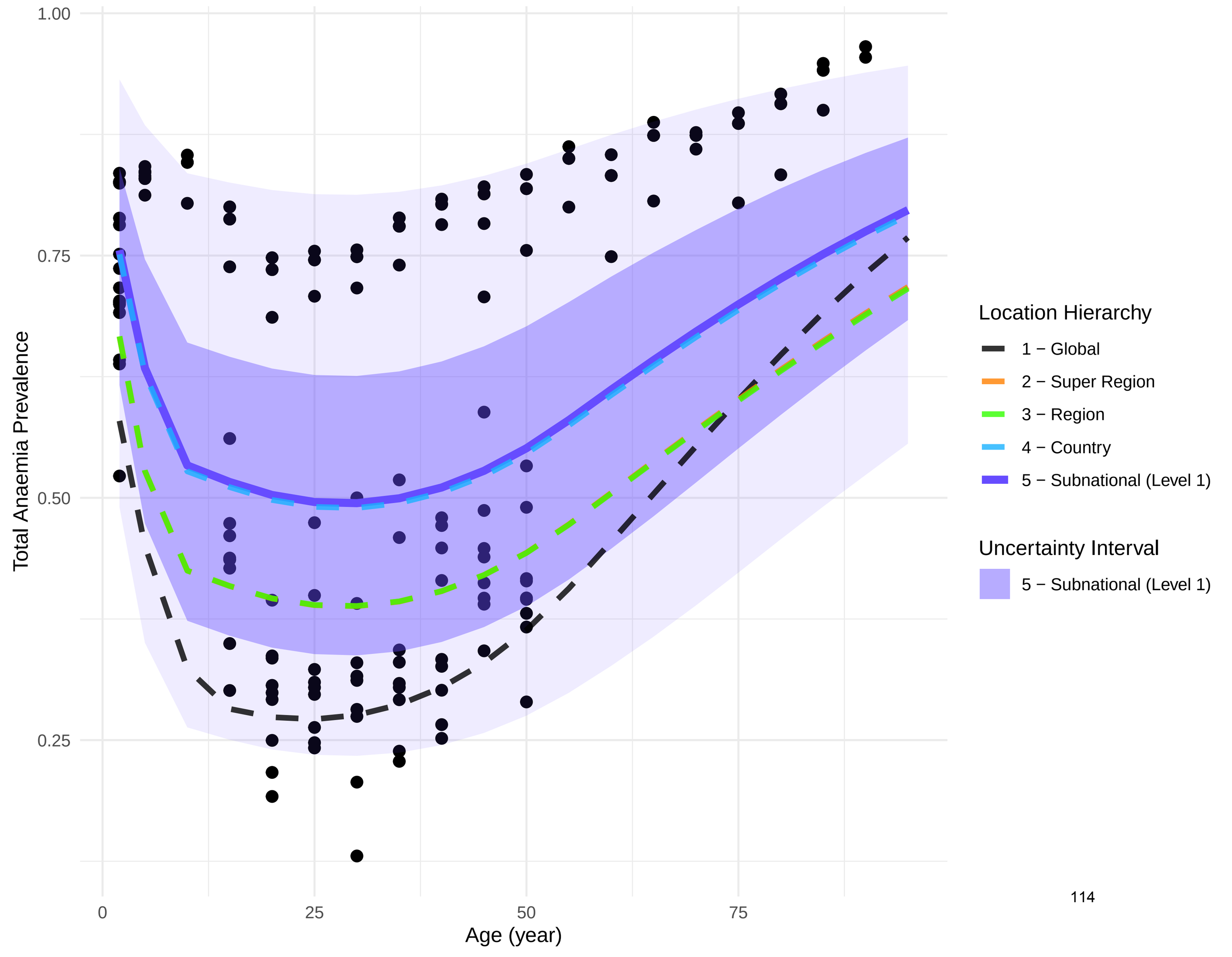

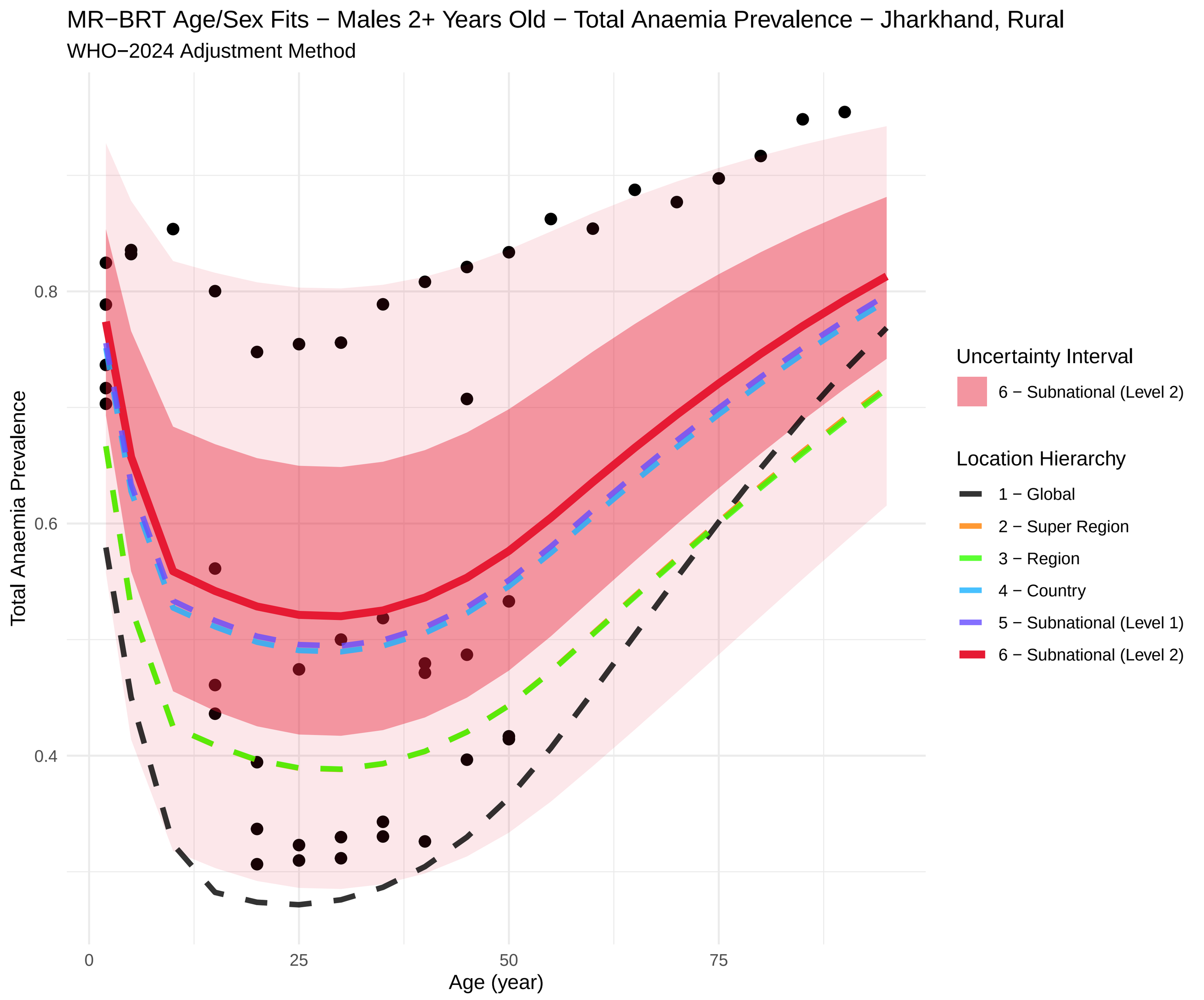
MR−BRT Age/Sex Fits − Males 2+ Years Old − Total Anaemia Prevalence − Jharkhand, Rural
WHO−2024 Adjustment Method
Total Anaemia Prevalence
Age (year)
Uncertainty Interval
6 − Subnational (Level 2)
Location Hierarchy
1 − Global
2 − Super Region
3 − Region
4 − Country
5 − Subnational (Level 1)
6 − Subnational (Level 2)

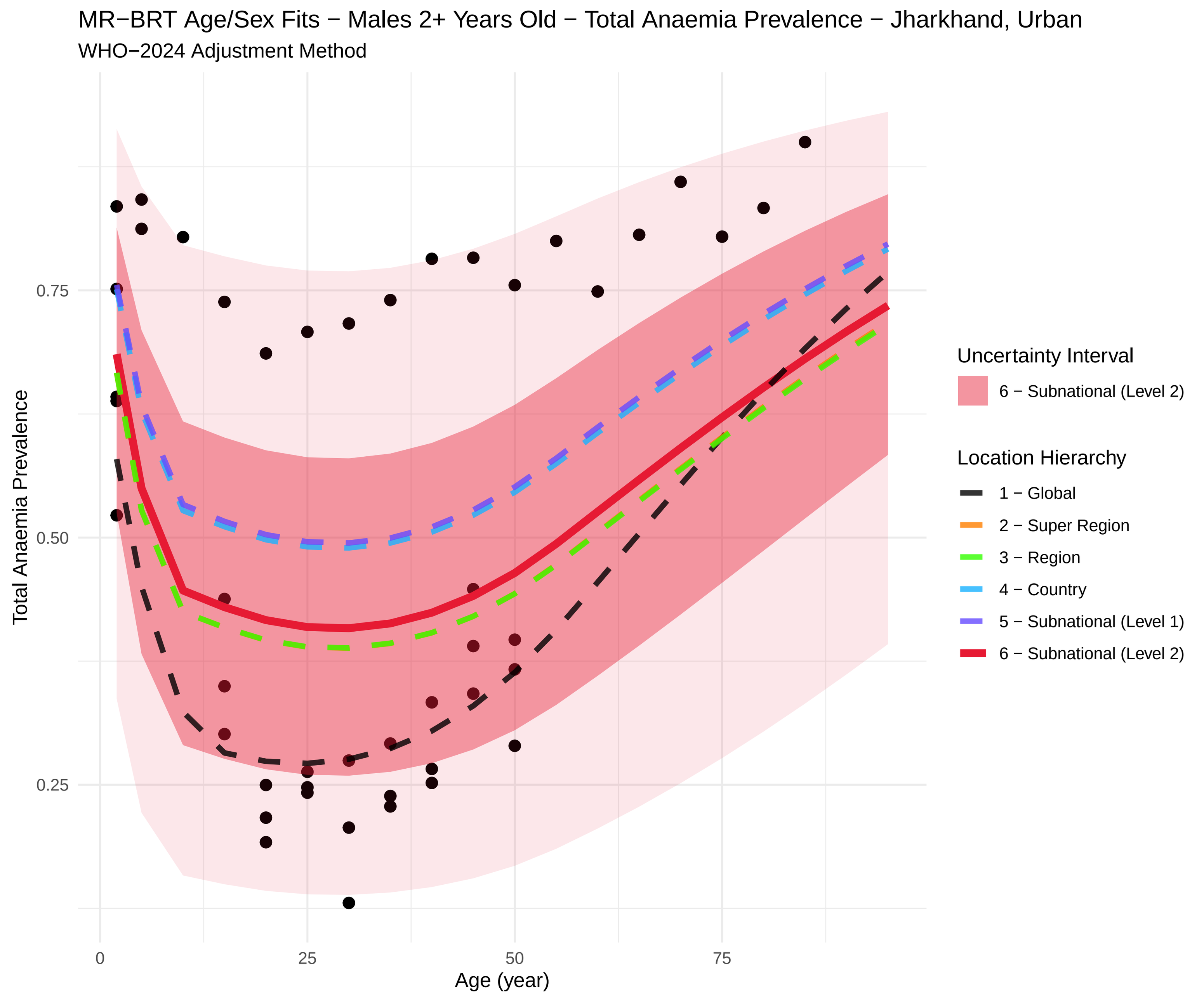
MR−BRT Age/Sex Fits − Males 2+ Years Old − Total Anaemia Prevalence − Jharkhand, Urban
WHO−2024 Adjustment Method
Total Anaemia Prevalence
Age (year)
Uncertainty Interval
6 − Subnational (Level 2)
Location Hierarchy
1 − Global
2 − Super Region
3 − Region
4 − Country
5 − Subnational (Level 1)
6 − Subnational (Level 2)

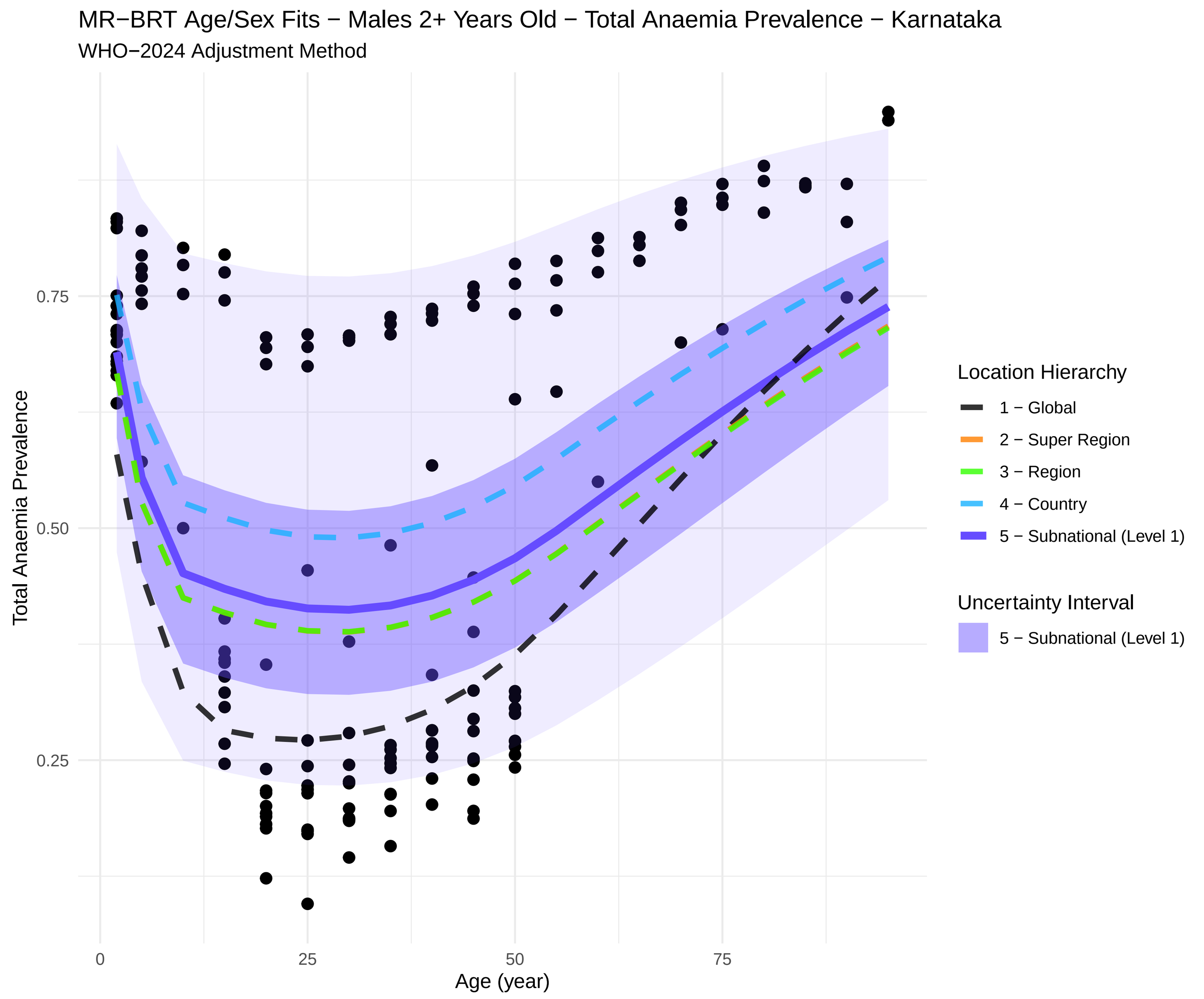
MR−BRT Age/Sex Fits − Males 2+ Years Old − Total Anaemia Prevalence − Karnataka
WHO−2024 Adjustment Method
Total Anaemia Prevalence
Age (year)
Location Hierarchy
1 − Global
2 − Super Region
3 − Region
4 − Country
5 − Subnational (Level 1)
Uncertainty Interval
5 − Subnational (Level 1)

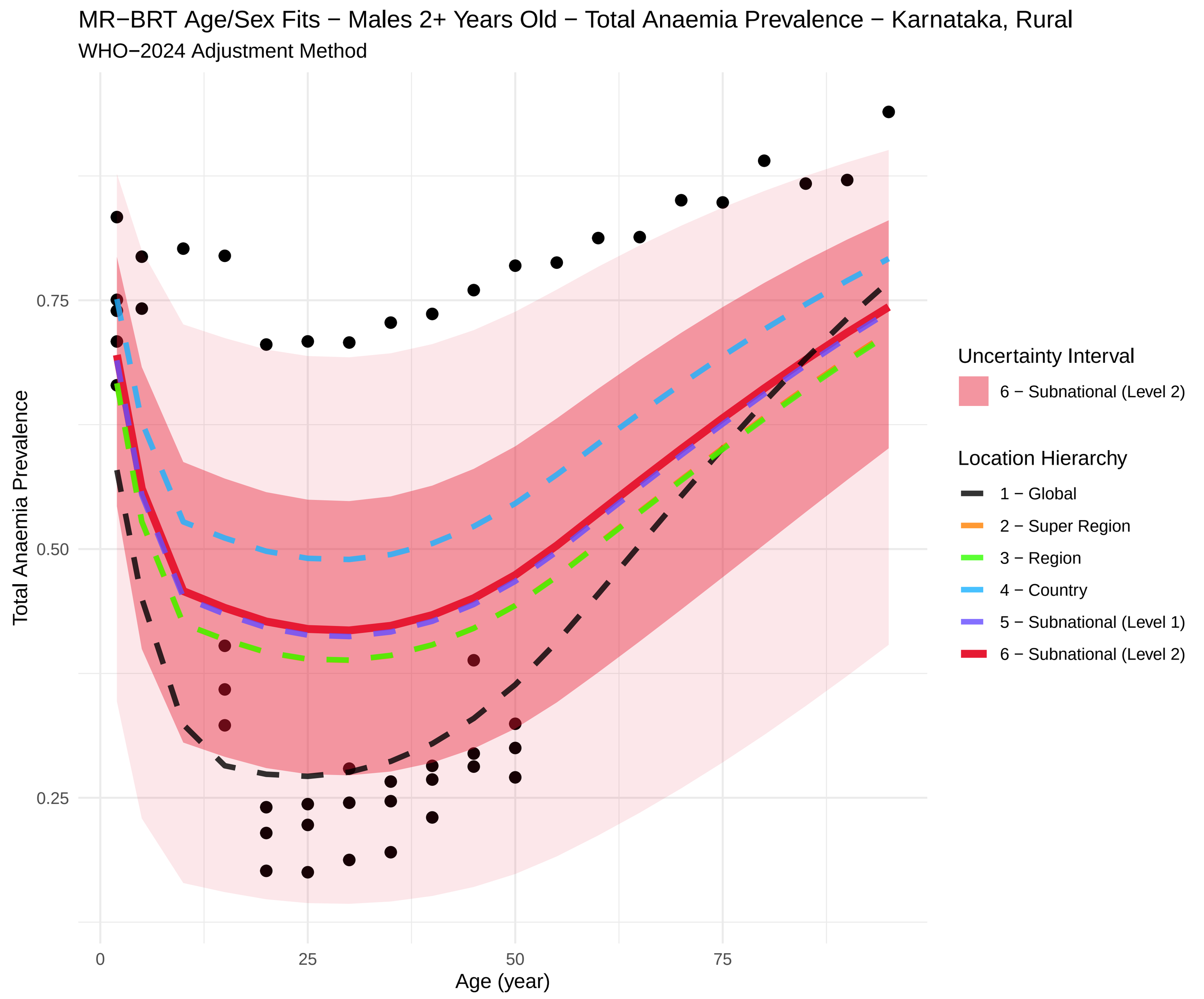
MR−BRT Age/Sex Fits − Males 2+ Years Old − Total Anaemia Prevalence − Karnataka, Rural
WHO−2024 Adjustment Method
Total Anaemia Prevalence
Age (year)
Uncertainty Interval
6 − Subnational (Level 2)
Location Hierarchy
1 − Global
2 − Super Region
3 − Region
4 − Country
5 − Subnational (Level 1)
6 − Subnational (Level 2)

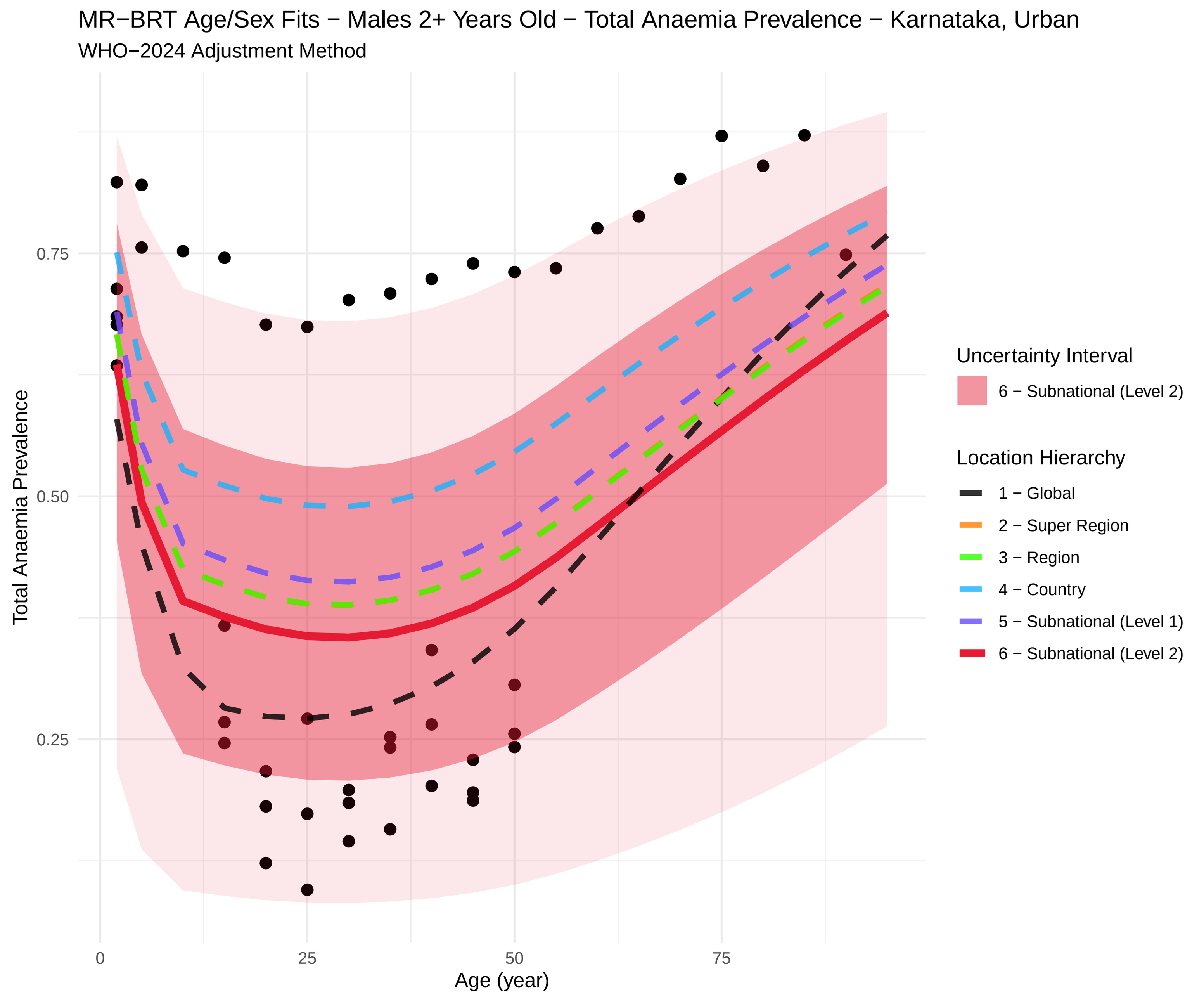
MR−BRT Age/Sex Fits − Males 2+ Years Old − Total Anaemia Prevalence − Karnataka, Urban
WHO−2024 Adjustment Method
Total Anaemia Prevalence
Age (year)
Uncertainty Interval
6 − Subnational (Level 2)
Location Hierarchy
1 − Global
2 − Super Region
3 − Region
4 − Country
5 − Subnational (Level 1)
6 − Subnational (Level 2)

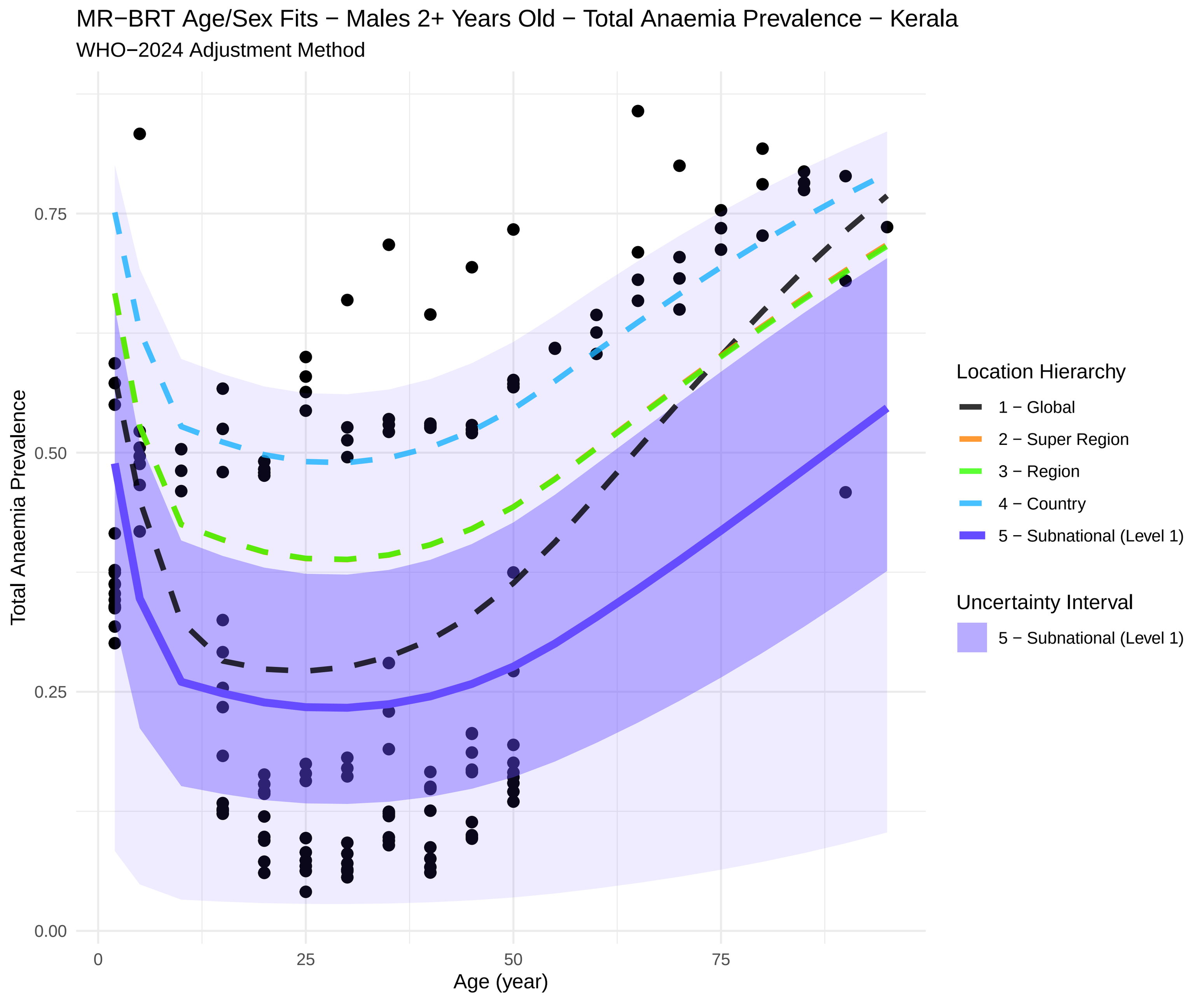
MR−BRT Age/Sex Fits − Males 2+ Years Old − Total Anaemia Prevalence − Kerala
WHO−2024 Adjustment Method
Total Anaemia Prevalence
Age (year)
Location Hierarchy
1 − Global
2 − Super Region
3 − Region
4 − Country
5 − Subnational (Level 1)
Uncertainty Interval
5 − Subnational (Level 1)

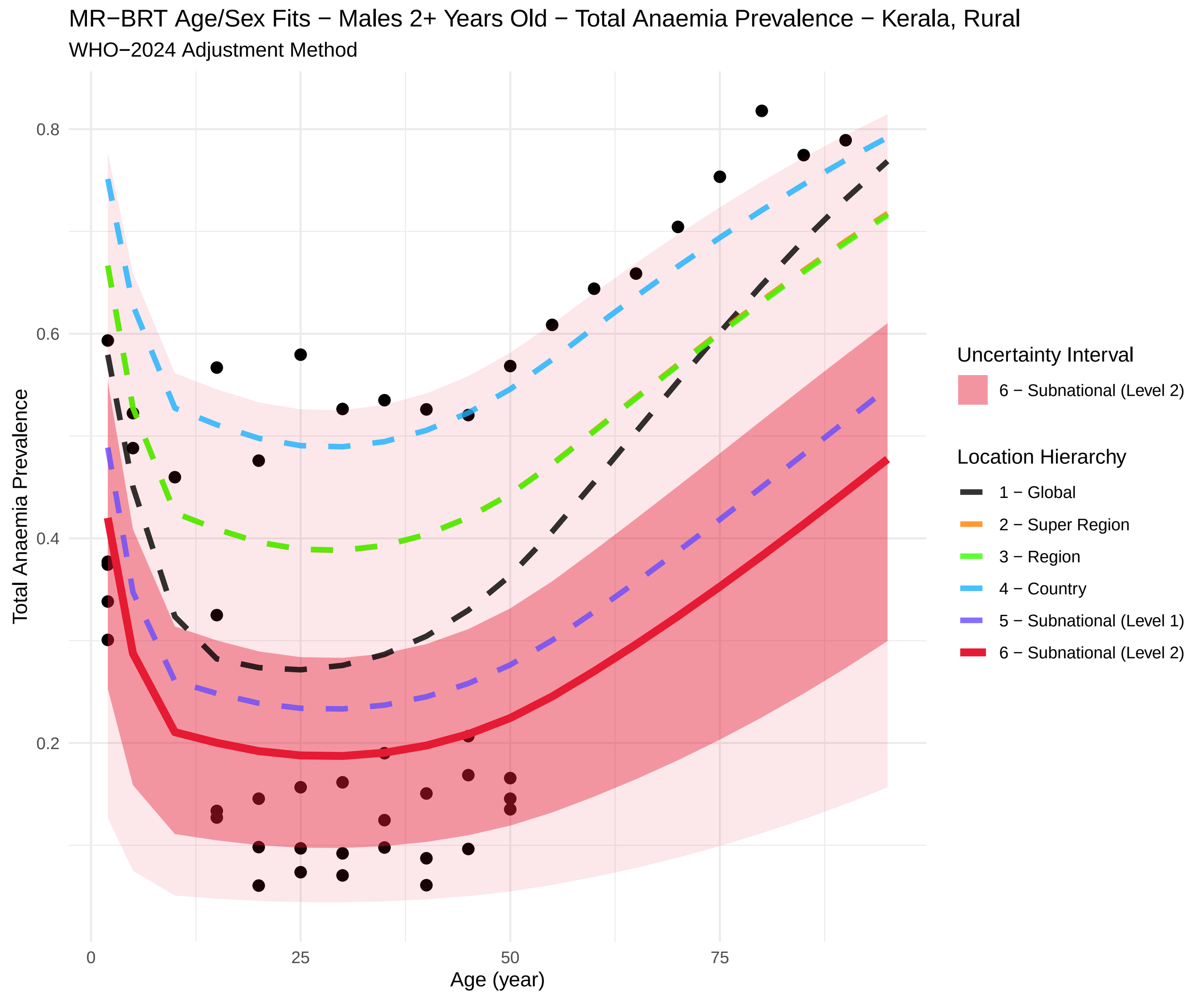
MR−BRT Age/Sex Fits − Males 2+ Years Old − Total Anaemia Prevalence − Kerala, Rural
WHO−2024 Adjustment Method
Total Anaemia Prevalence
Age (year)
Uncertainty Interval
6 − Subnational (Level 2)
Location Hierarchy
1 − Global
2 − Super Region
3 − Region
4 − Country
5 − Subnational (Level 1)
6 − Subnational (Level 2)

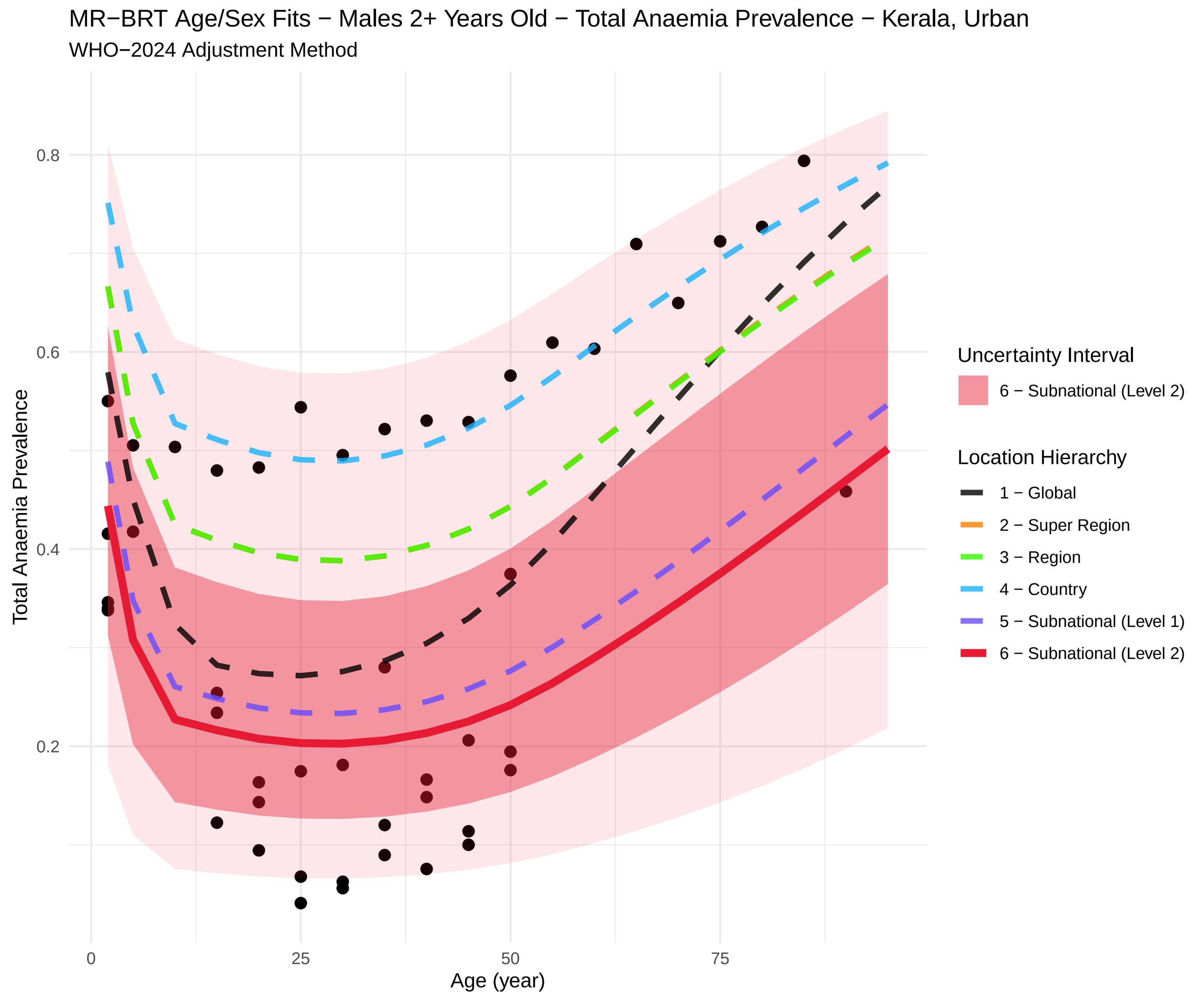
MR−BRT Age/Sex Fits − Males 2+ Years Old − Total Anaemia Prevalence − Kerala, Urban
WHO−2024 Adjustment Method
Total Anaemia Prevalence
Age (year)
Uncertainty Interval
6 − Subnational (Level 2)
Location Hierarchy
1 − Global
2 − Super Region
3 − Region
4 − Country
5 − Subnational (Level 1)
6 − Subnational (Level 2)

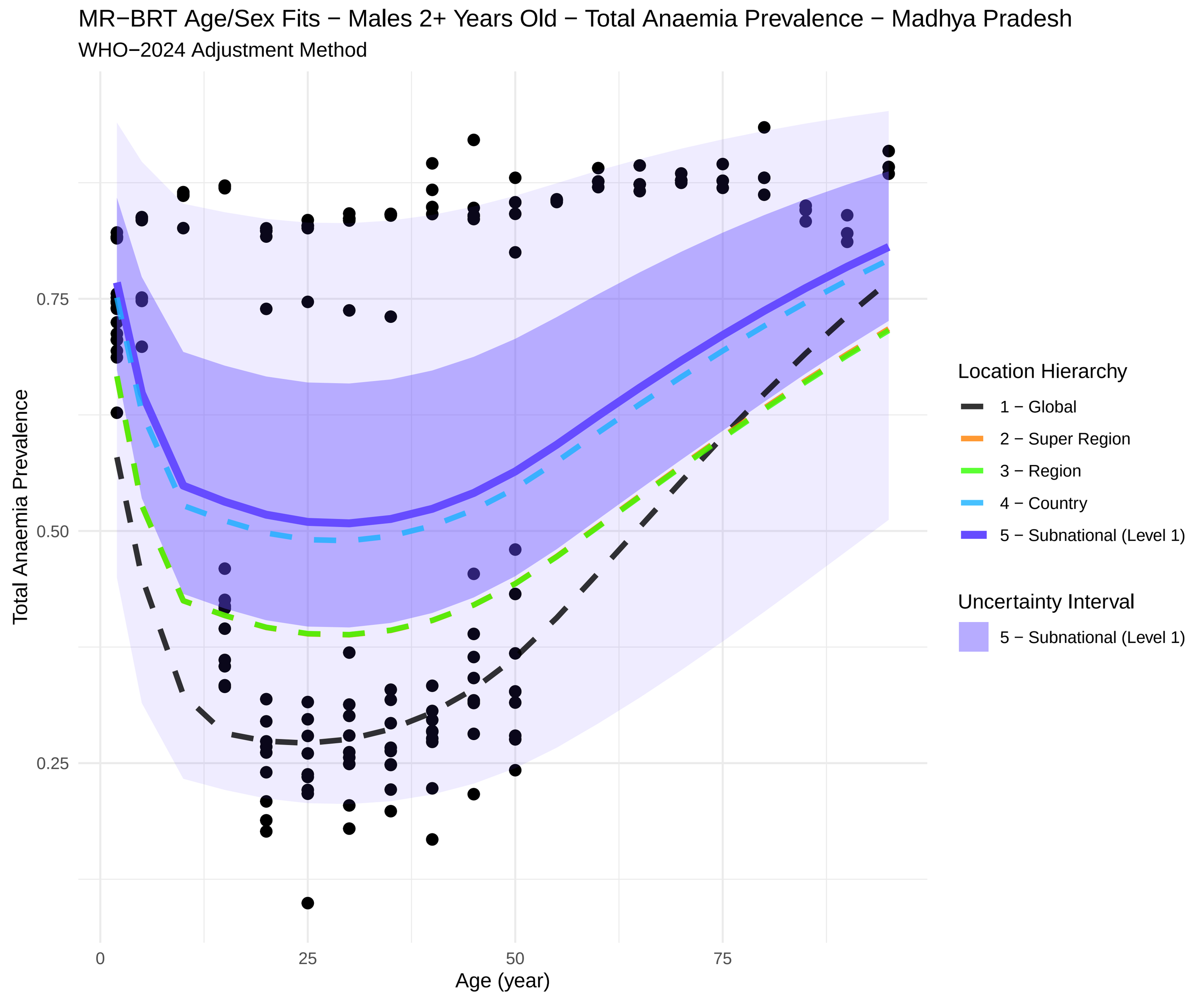
MR−BRT Age/Sex Fits − Males 2+ Years Old − Total Anaemia Prevalence − Madhya Pradesh
WHO−2024 Adjustment Method
Total Anaemia Prevalence
Age (year)
Location Hierarchy
1 − Global
2 − Super Region
3 − Region
4 − Country
5 − Subnational (Level 1)
Uncertainty Interval
5 − Subnational (Level 1)

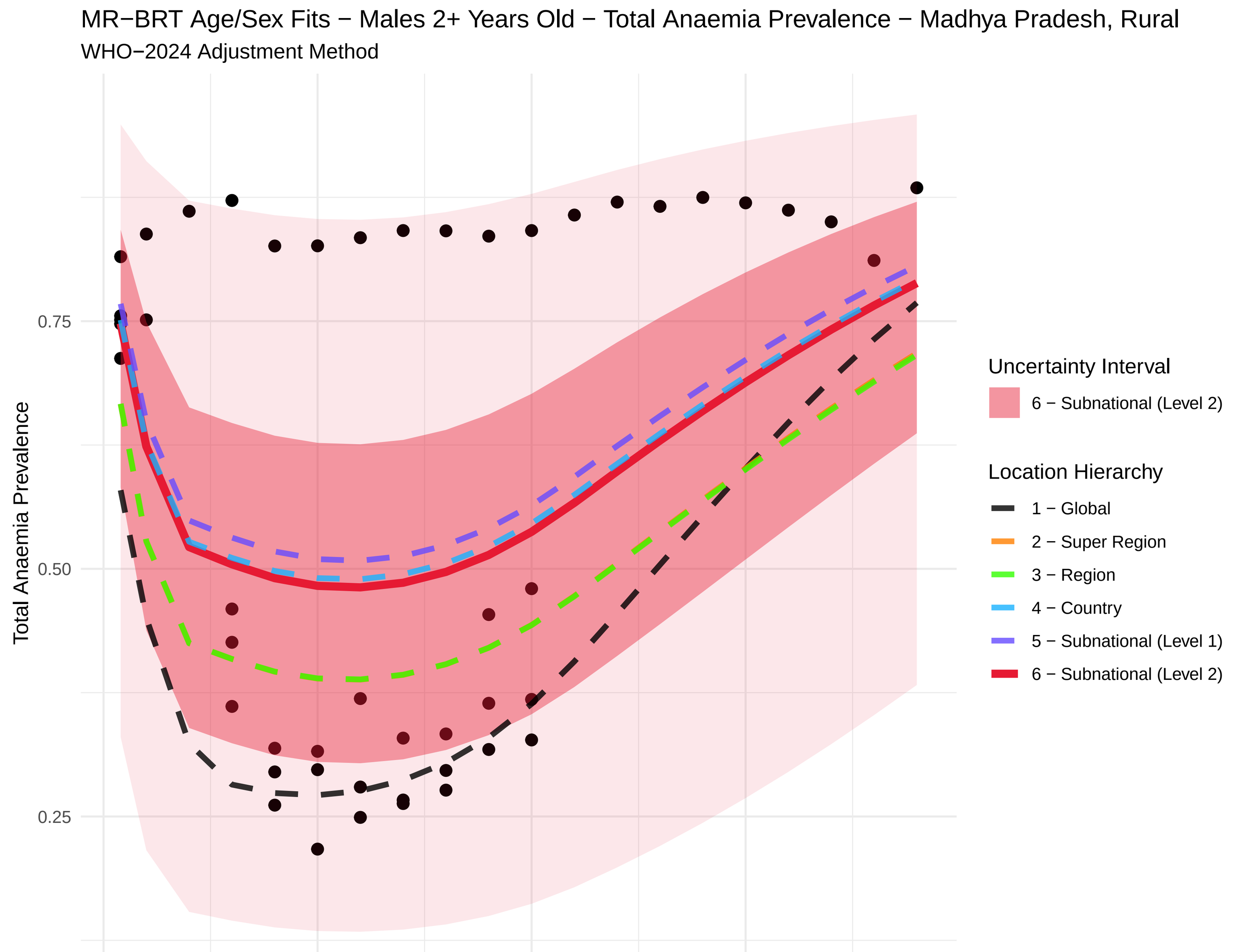
MR−BRT Age/Sex Fits − Males 2+ Years Old − Total Anaemia Prevalence − Madhya Pradesh, Rural
WHO−2024 Adjustment Method
Total Anaemia Prevalence
Age (year)
Uncertainty Interval
6 − Subnational (Level 2)
Location Hierarchy
1 − Global
2 − Super Region
3 − Region
4 − Country
5 − Subnational (Level 1)
6 − Subnational (Level 2)

MR−BRT Age/Sex Fits − Males 2+ Years Old − Total Anaemia Prevalence − Madhya Pradesh, Urban
WHO−2024 Adjustment Method

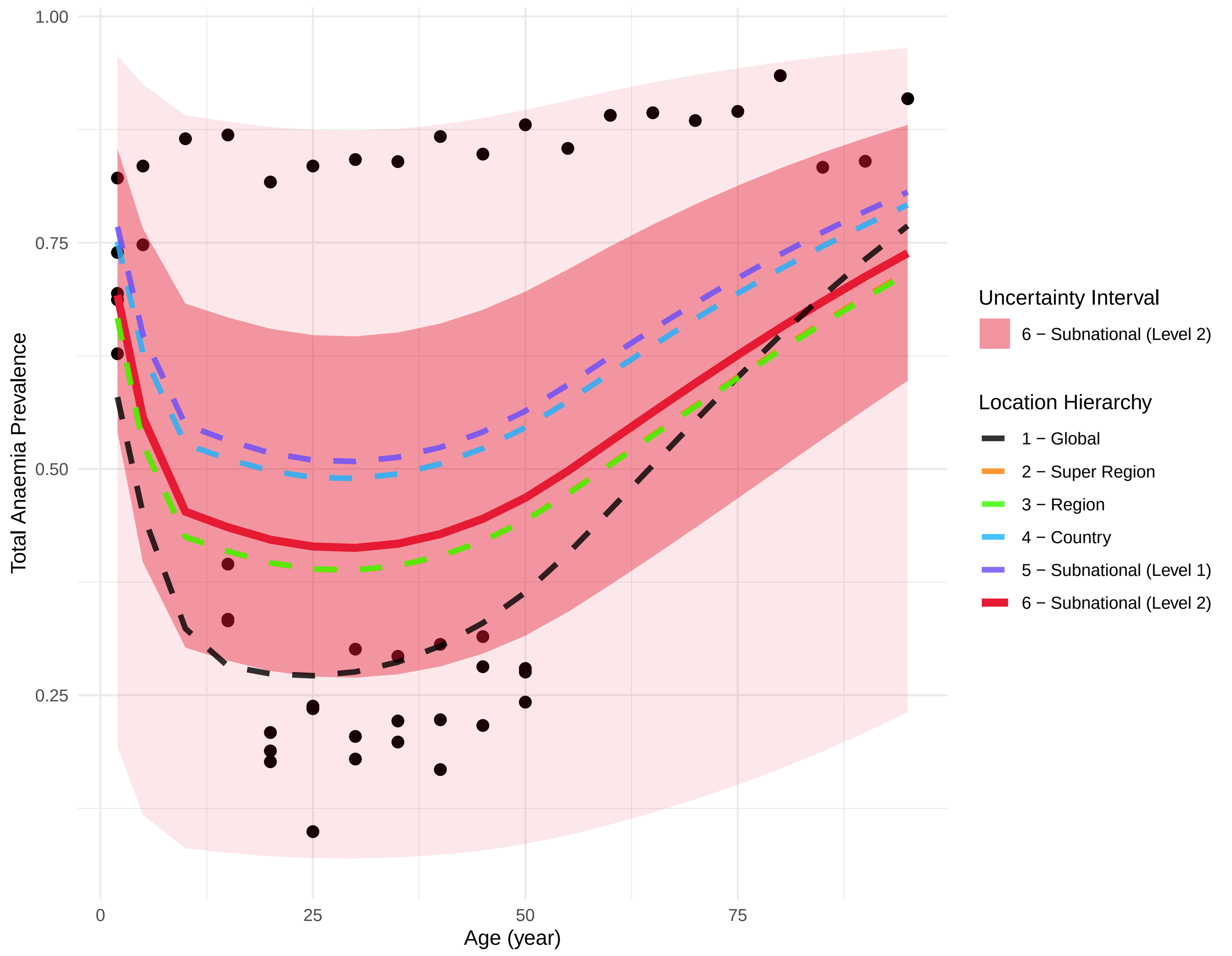

MR−BRT Age/Sex Fits − Males 2+ Years Old − Total Anaemia Prevalence − Maharashtra
WHO−2024 Adjustment Method

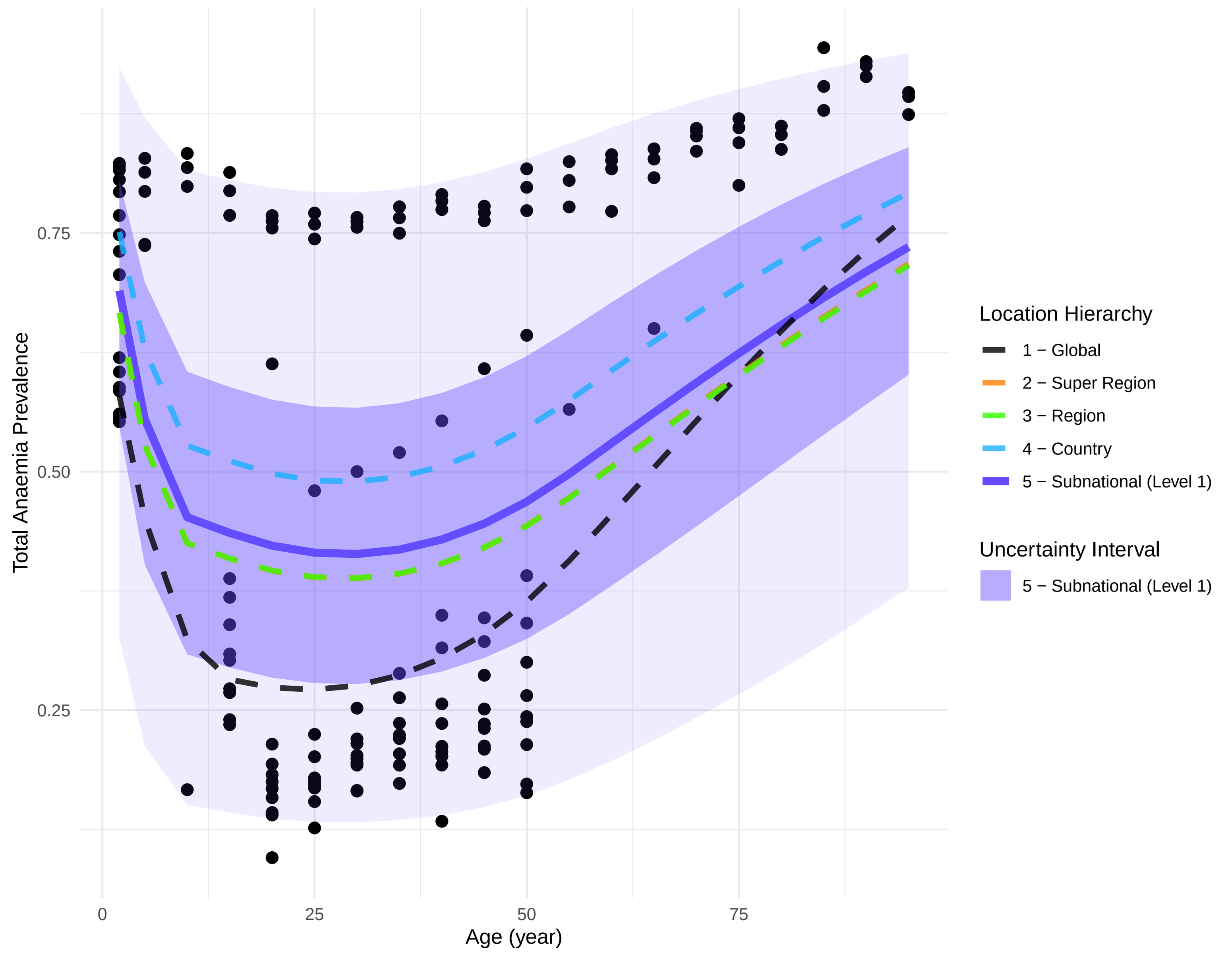

MR−BRT Age/Sex Fits − Males 2+ Years Old − Total Anaemia Prevalence − Maharashtra, Rural
WHO−2024 Adjustment Method

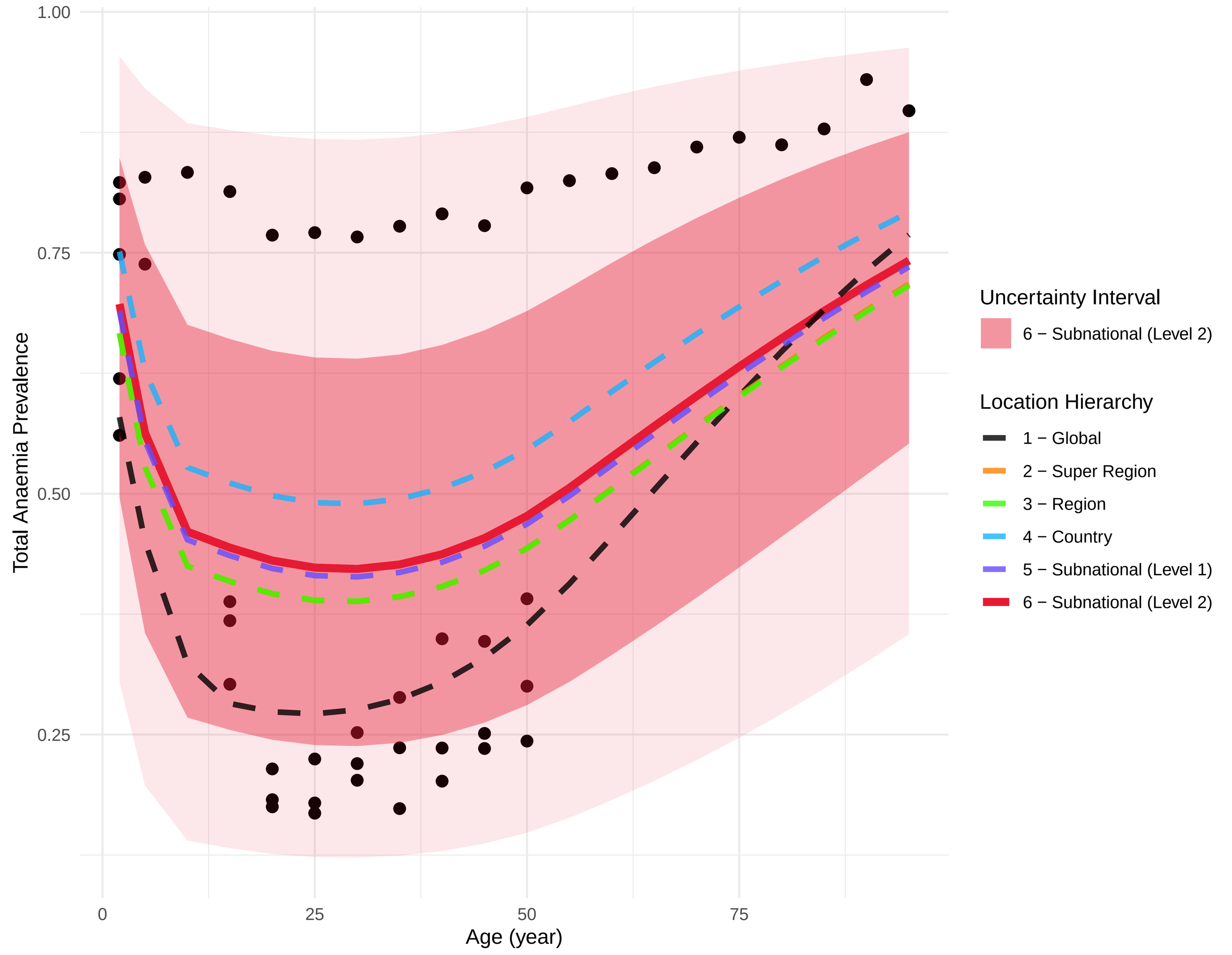

MR−BRT Age/Sex Fits − Males 2+ Years Old − Total Anaemia Prevalence − Maharashtra, Urban
WHO−2024 Adjustment Method

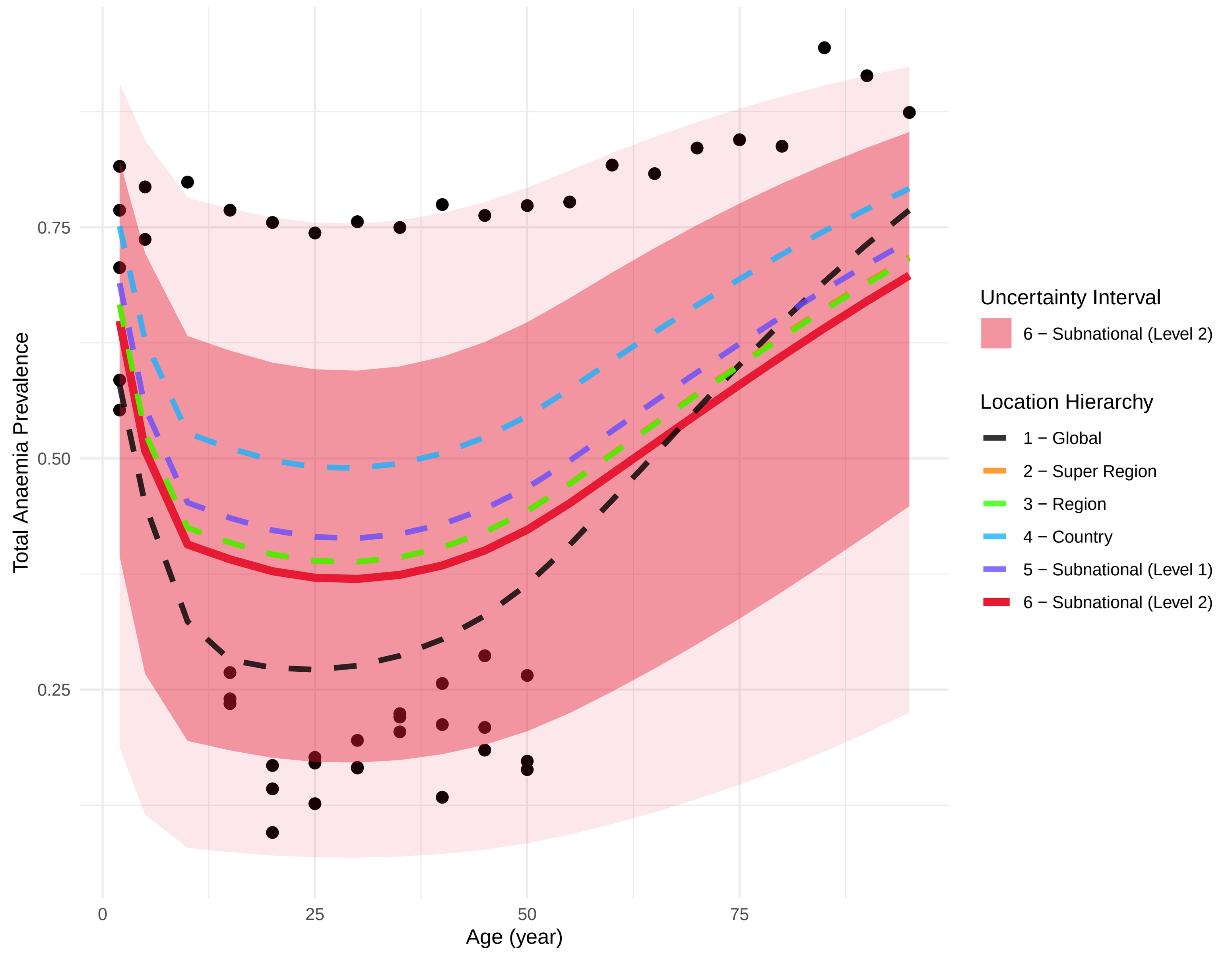

MR−BRT Age/Sex Fits − Males 2+ Years Old − Total Anaemia Prevalence − Manipur
WHO−2024 Adjustment Method

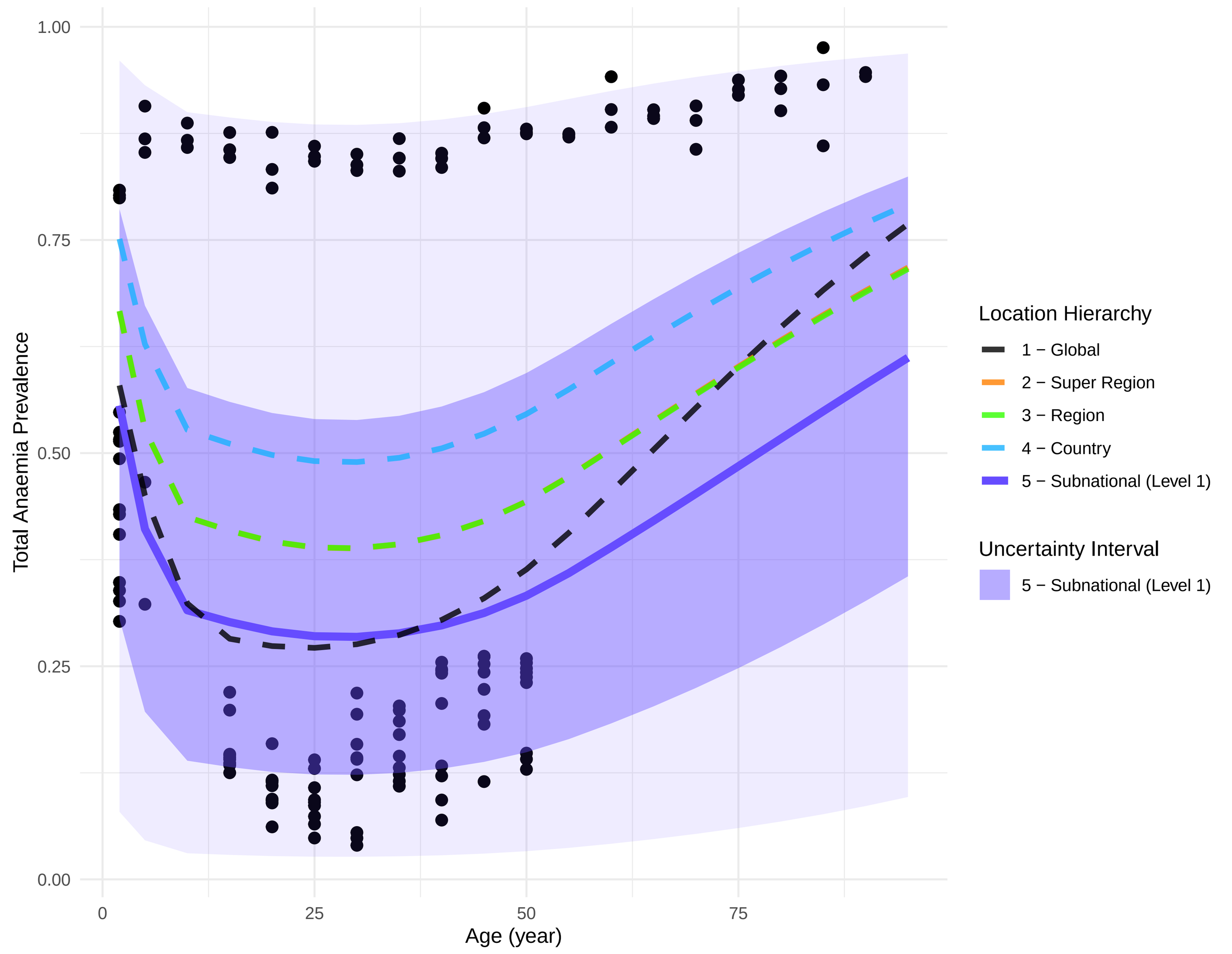

MR−BRT Age/Sex Fits − Males 2+ Years Old − Total Anaemia Prevalence − Manipur, Rural
WHO−2024 Adjustment Method

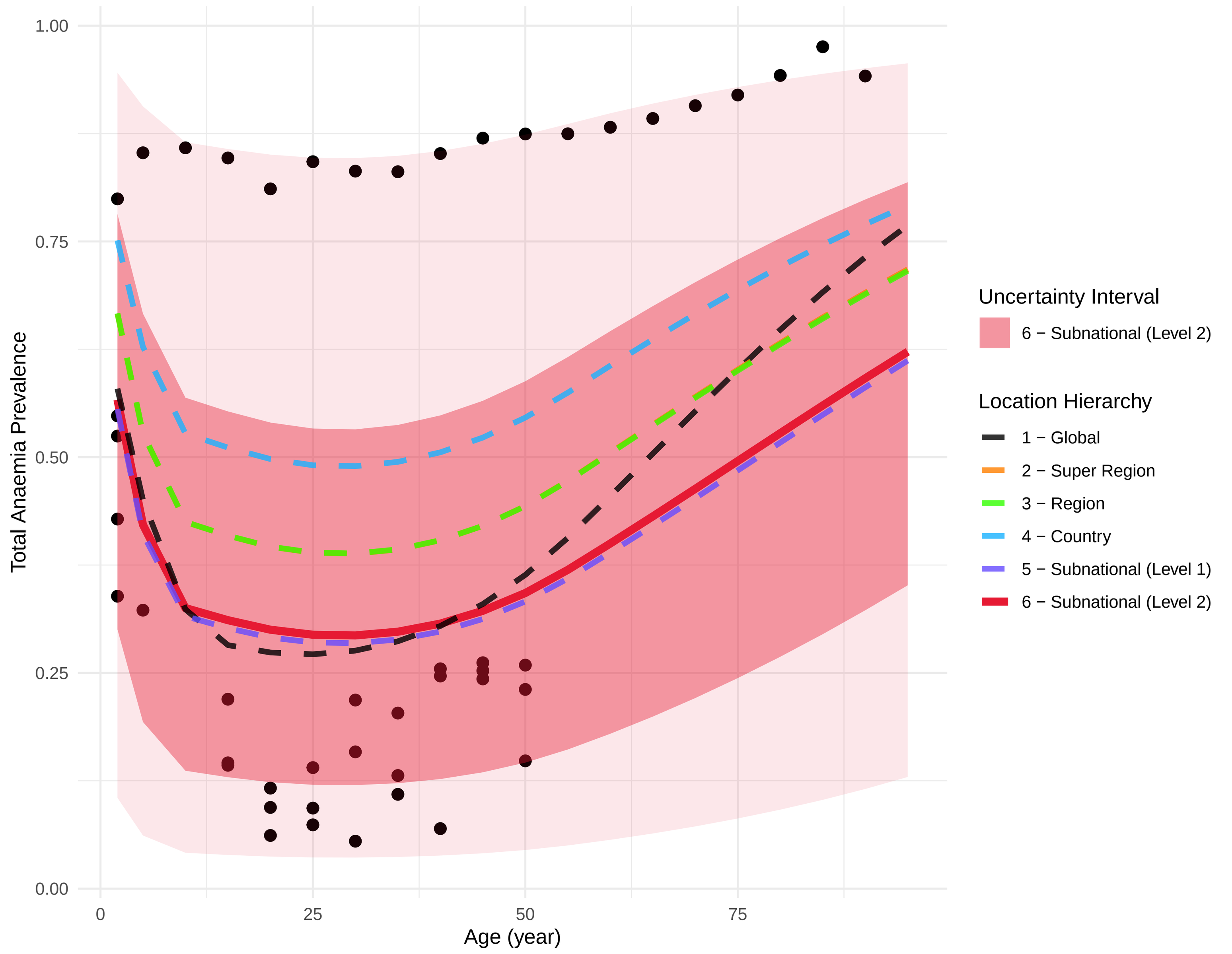

MR−BRT Age/Sex Fits − Males 2+ Years Old − Total Anaemia Prevalence − Manipur, Urban
WHO−2024 Adjustment Method

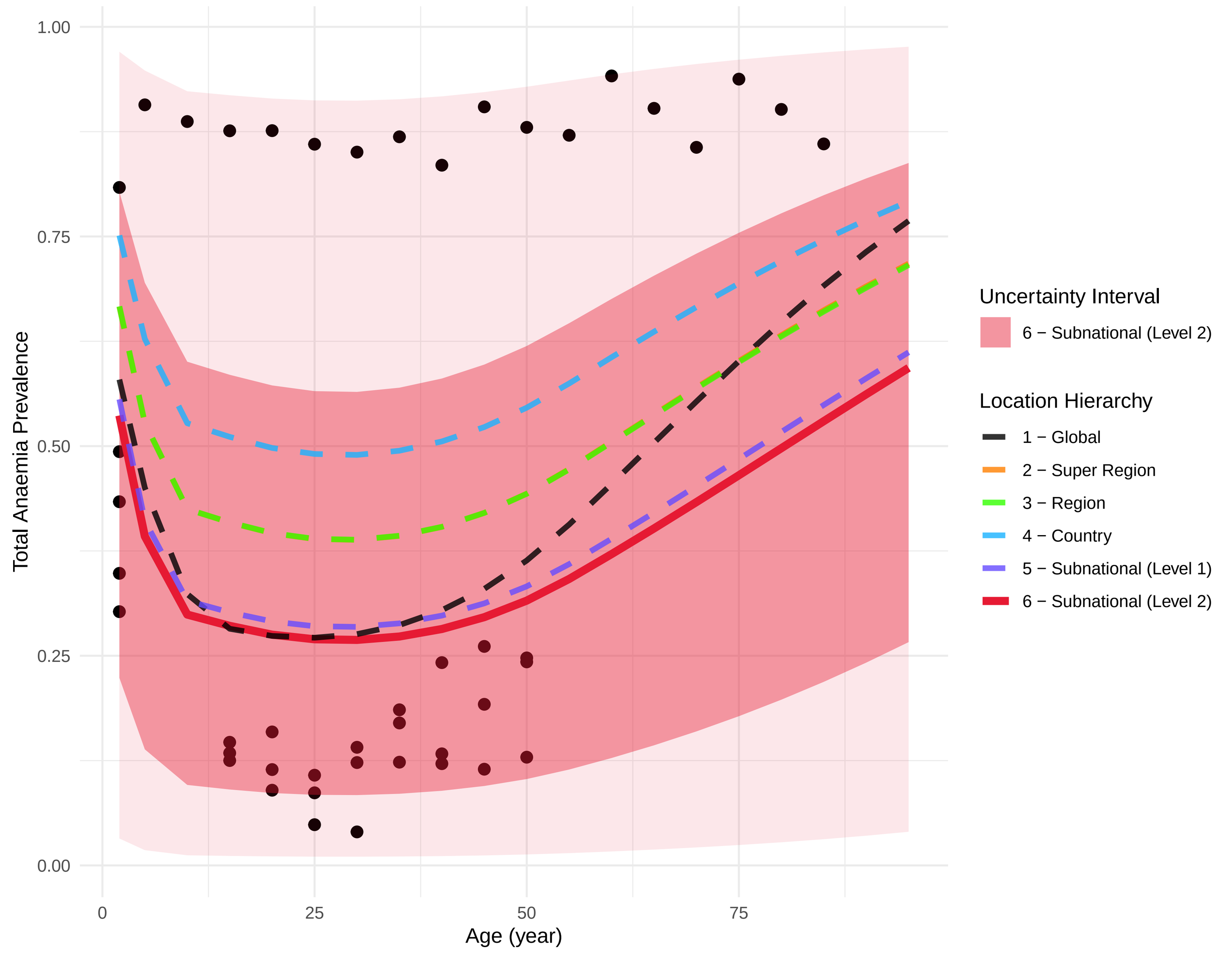

MR−BRT Age/Sex Fits − Males 2+ Years Old − Total Anaemia Prevalence − Meghalaya
WHO−2024 Adjustment Method

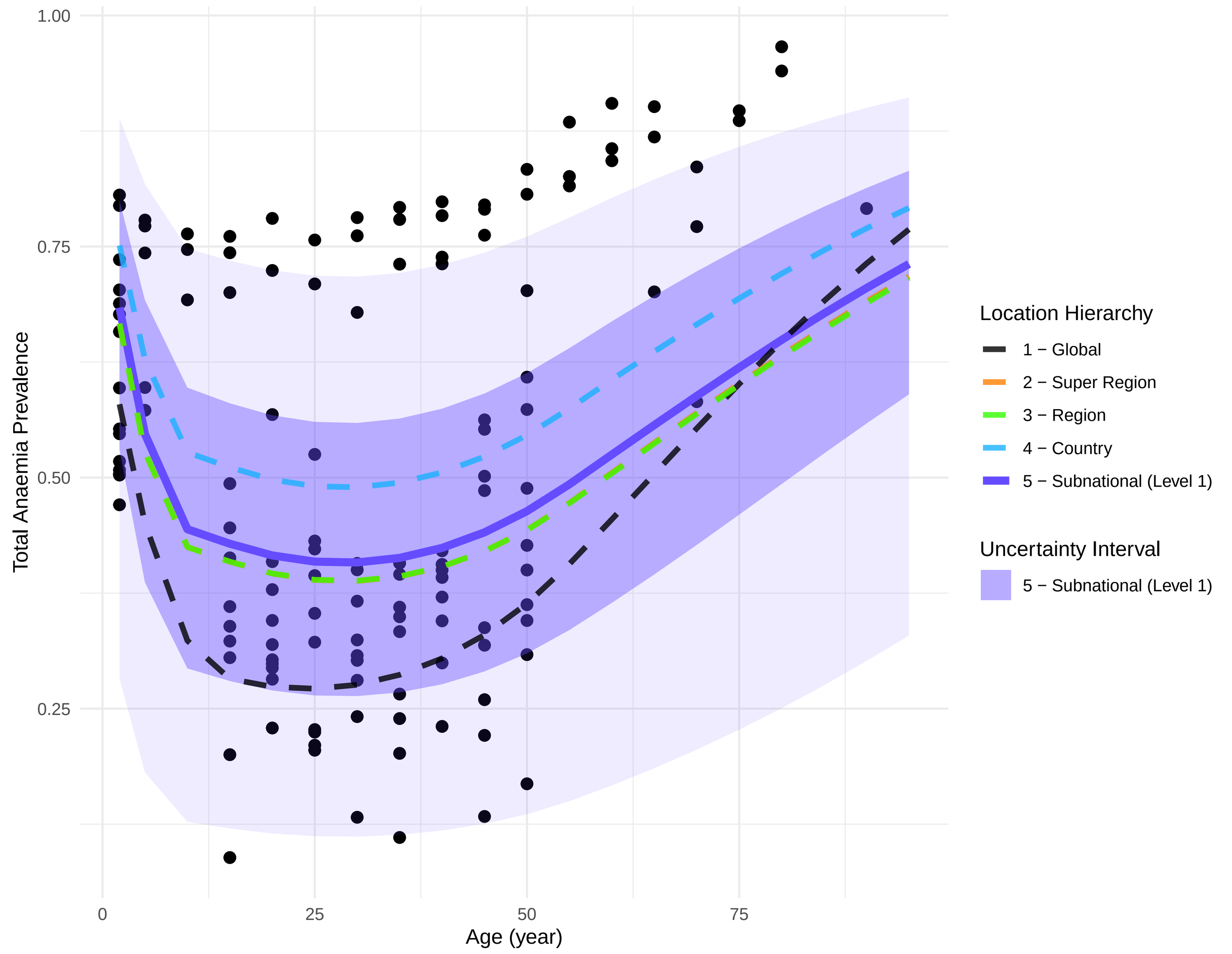

MR−BRT Age/Sex Fits − Males 2+ Years Old − Total Anaemia Prevalence − Meghalaya, Rural
WHO−2024 Adjustment Method

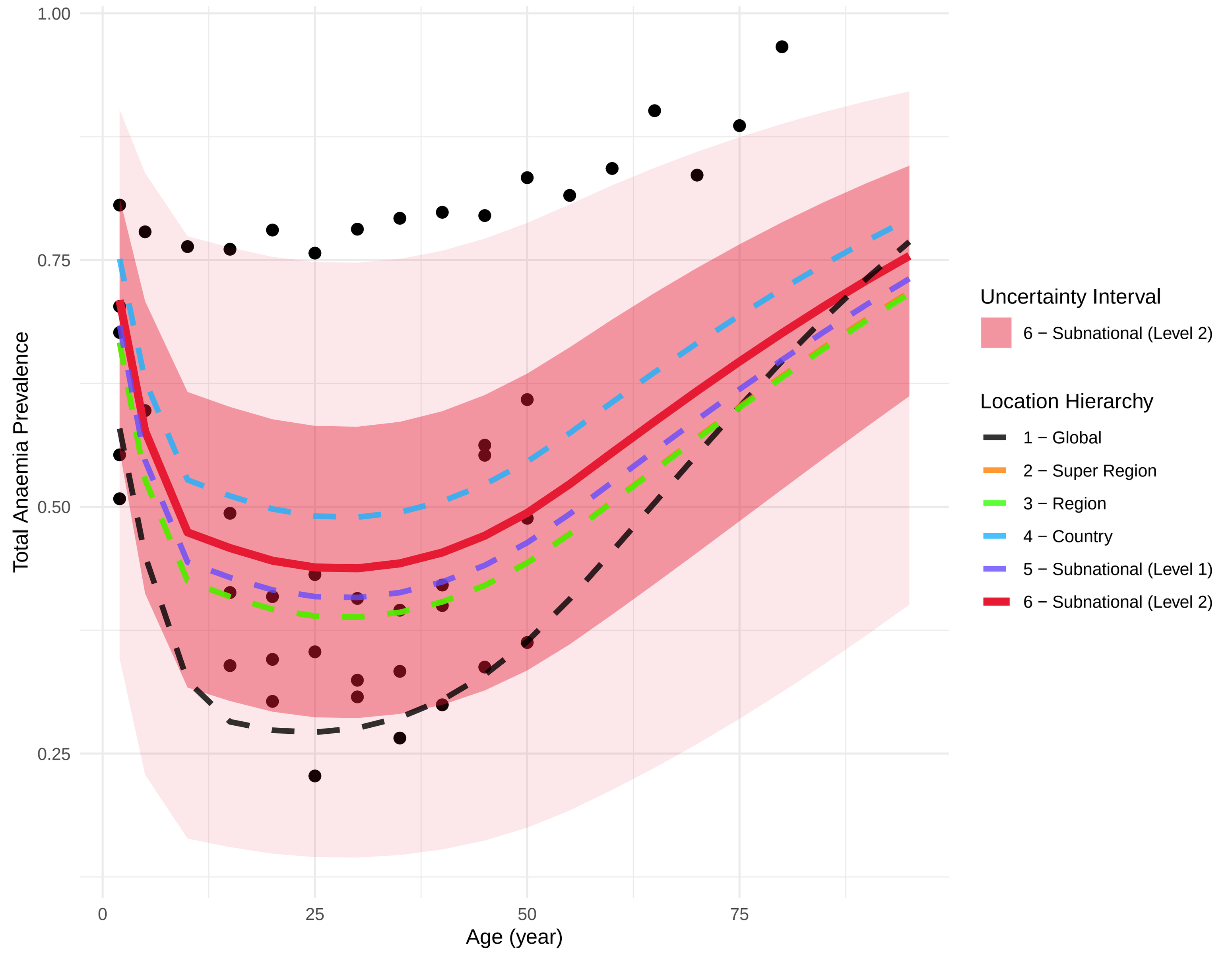

MR−BRT Age/Sex Fits − Males 2+ Years Old − Total Anaemia Prevalence − Meghalaya, Urban
WHO−2024 Adjustment Method

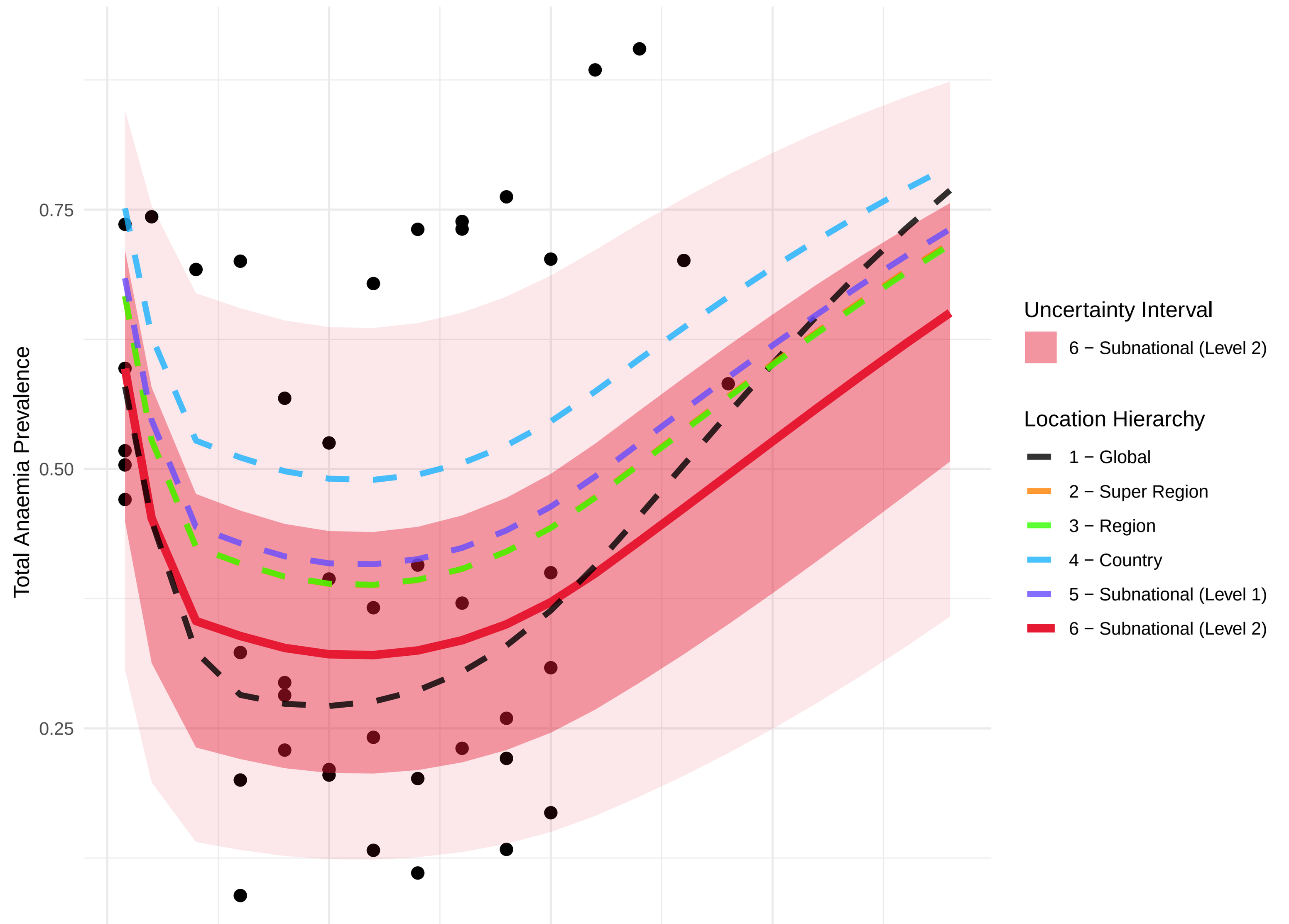

MR−BRT Age/Sex Fits − Males 2+ Years Old − Total Anaemia Prevalence − Mizoram
WHO−2024 Adjustment Method

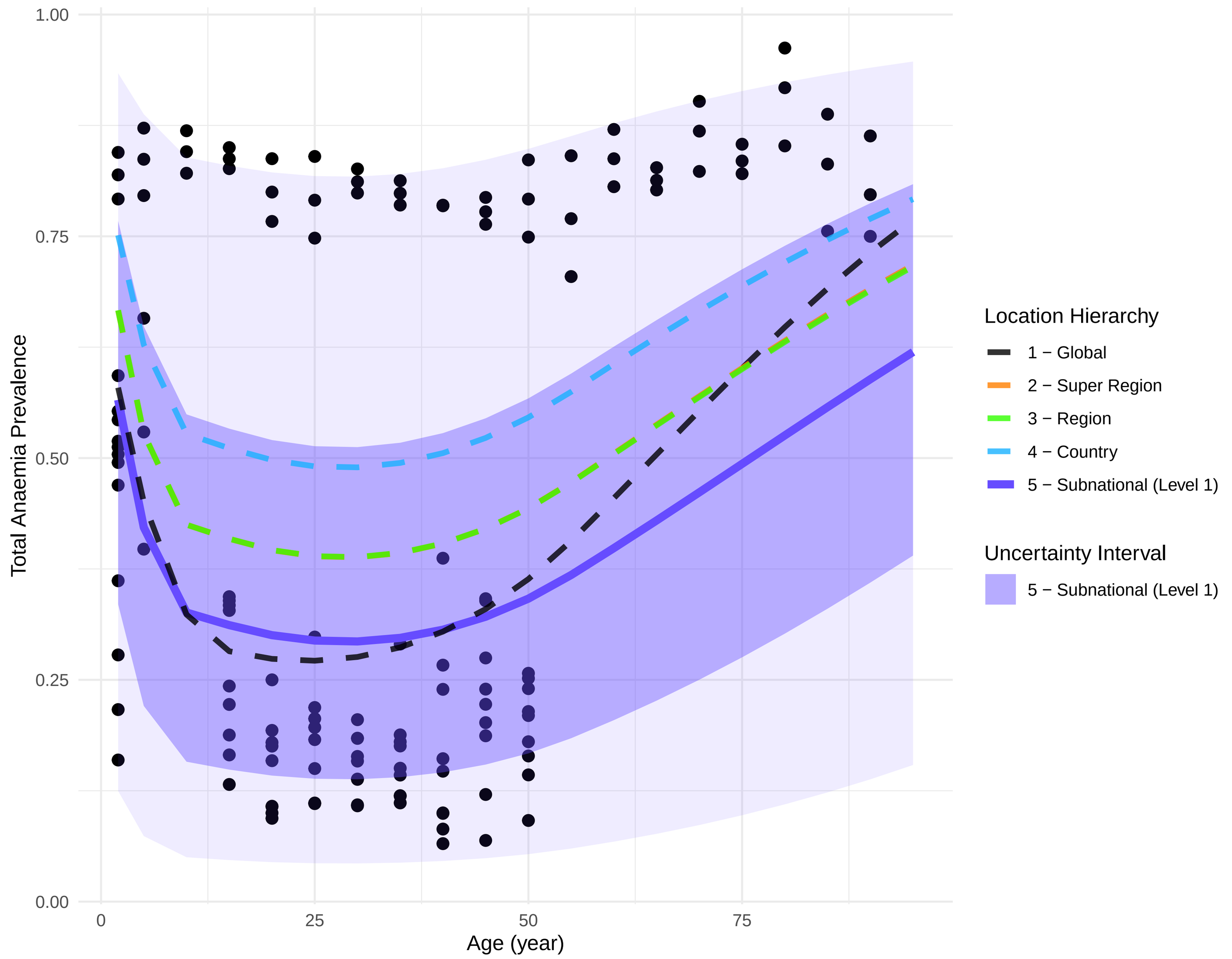

MR−BRT Age/Sex Fits − Males 2+ Years Old − Total Anaemia Prevalence − Mizoram, Rural
WHO−2024 Adjustment Method

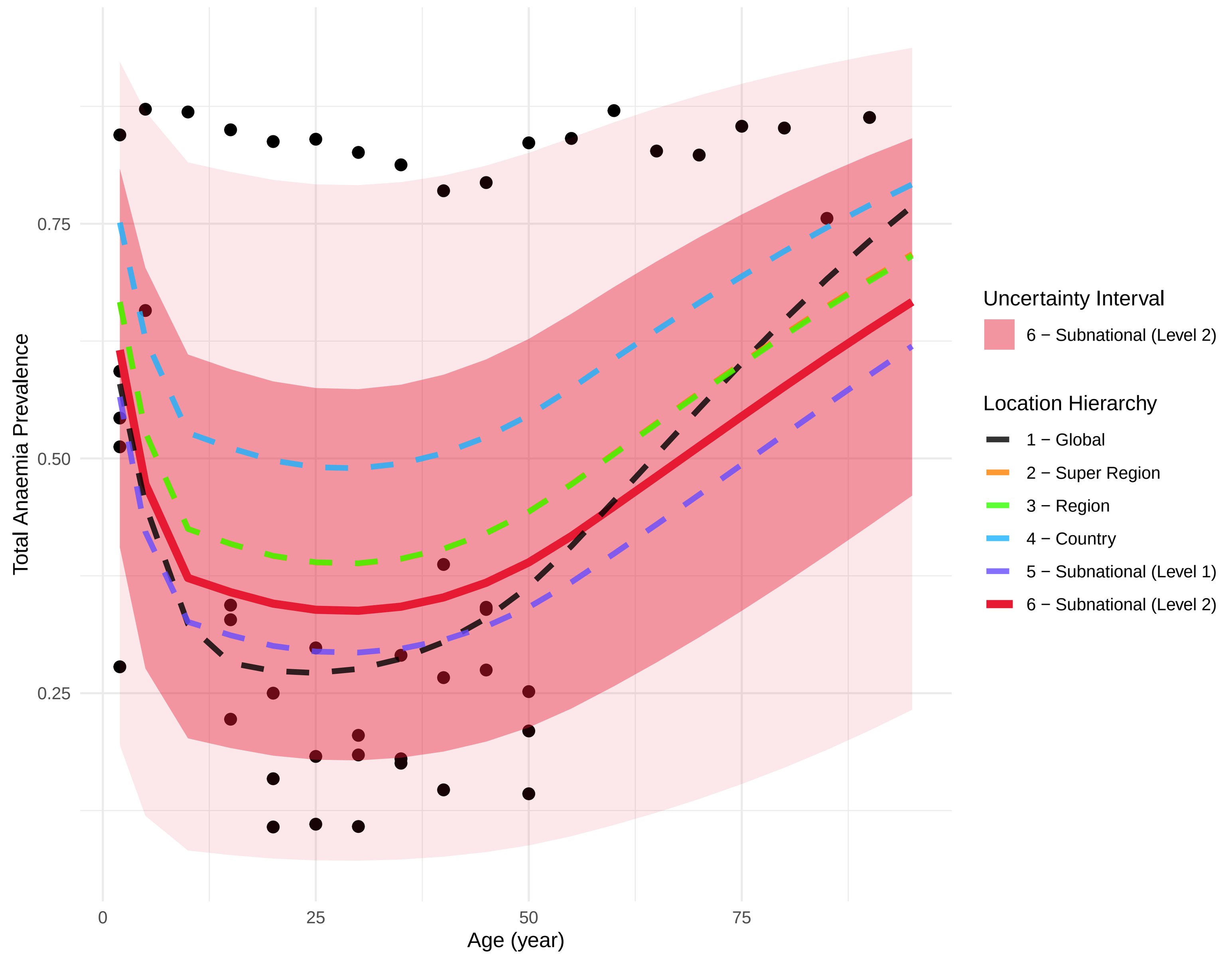

MR−BRT Age/Sex Fits − Males 2+ Years Old − Total Anaemia Prevalence − Mizoram, Urban
WHO−2024 Adjustment Method

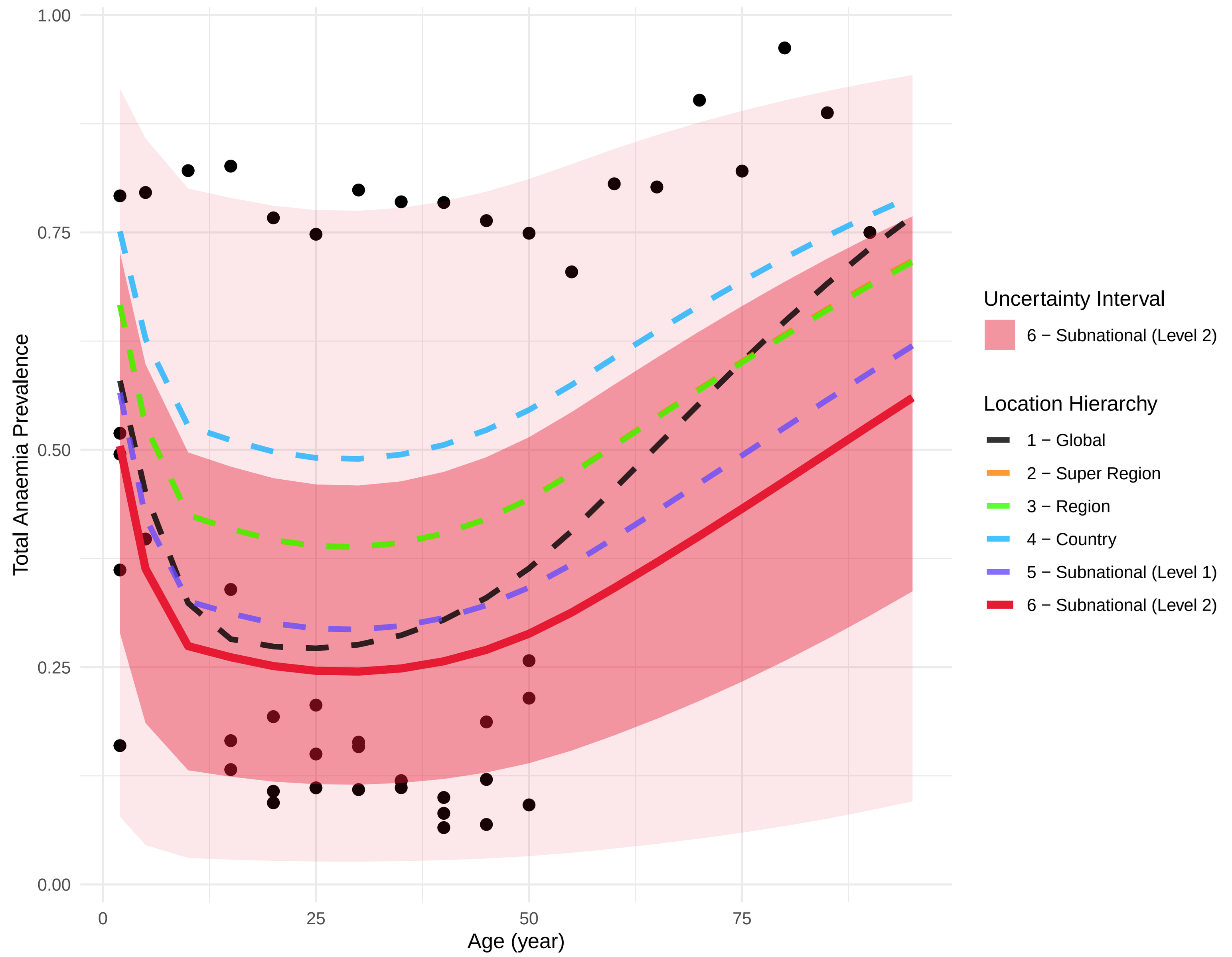

MR−BRT Age/Sex Fits − Males 2+ Years Old − Total Anaemia Prevalence − Nagaland
WHO−2024 Adjustment Method

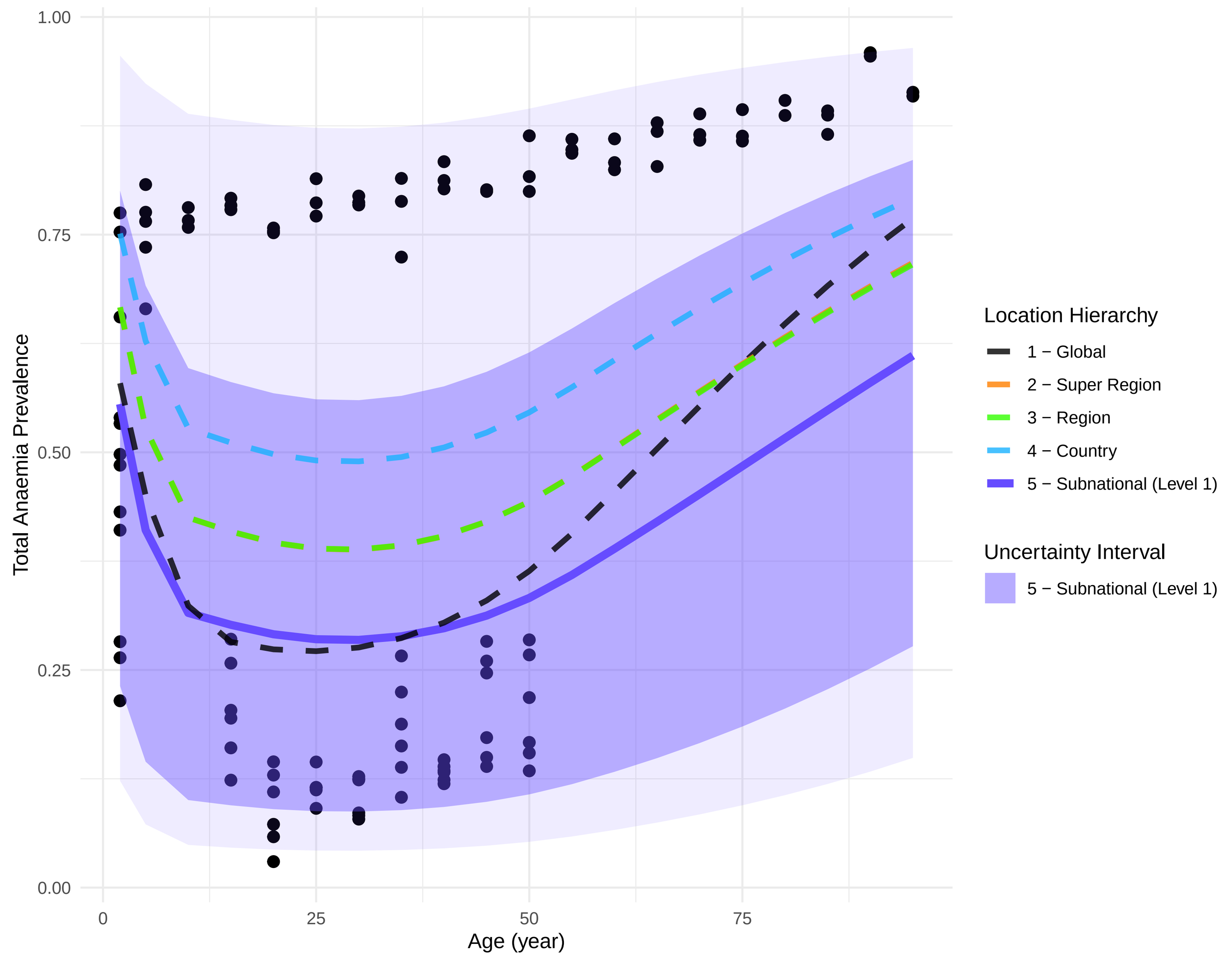

MR−BRT Age/Sex Fits − Males 2+ Years Old − Total Anaemia Prevalence − Nagaland, Rural
WHO−2024 Adjustment Method

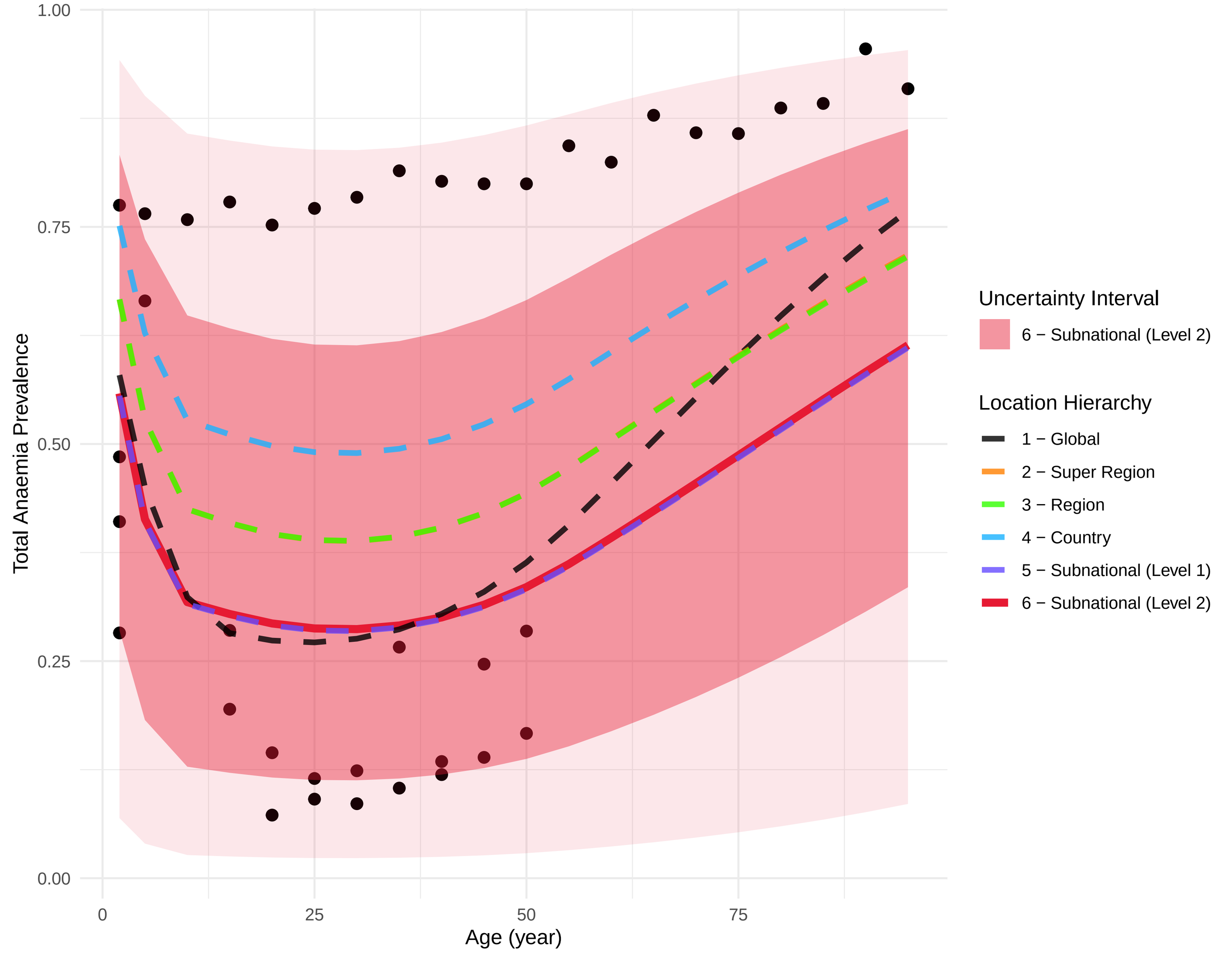

MR−BRT Age/Sex Fits − Males 2+ Years Old − Total Anaemia Prevalence − Nagaland, Urban
WHO−2024 Adjustment Method

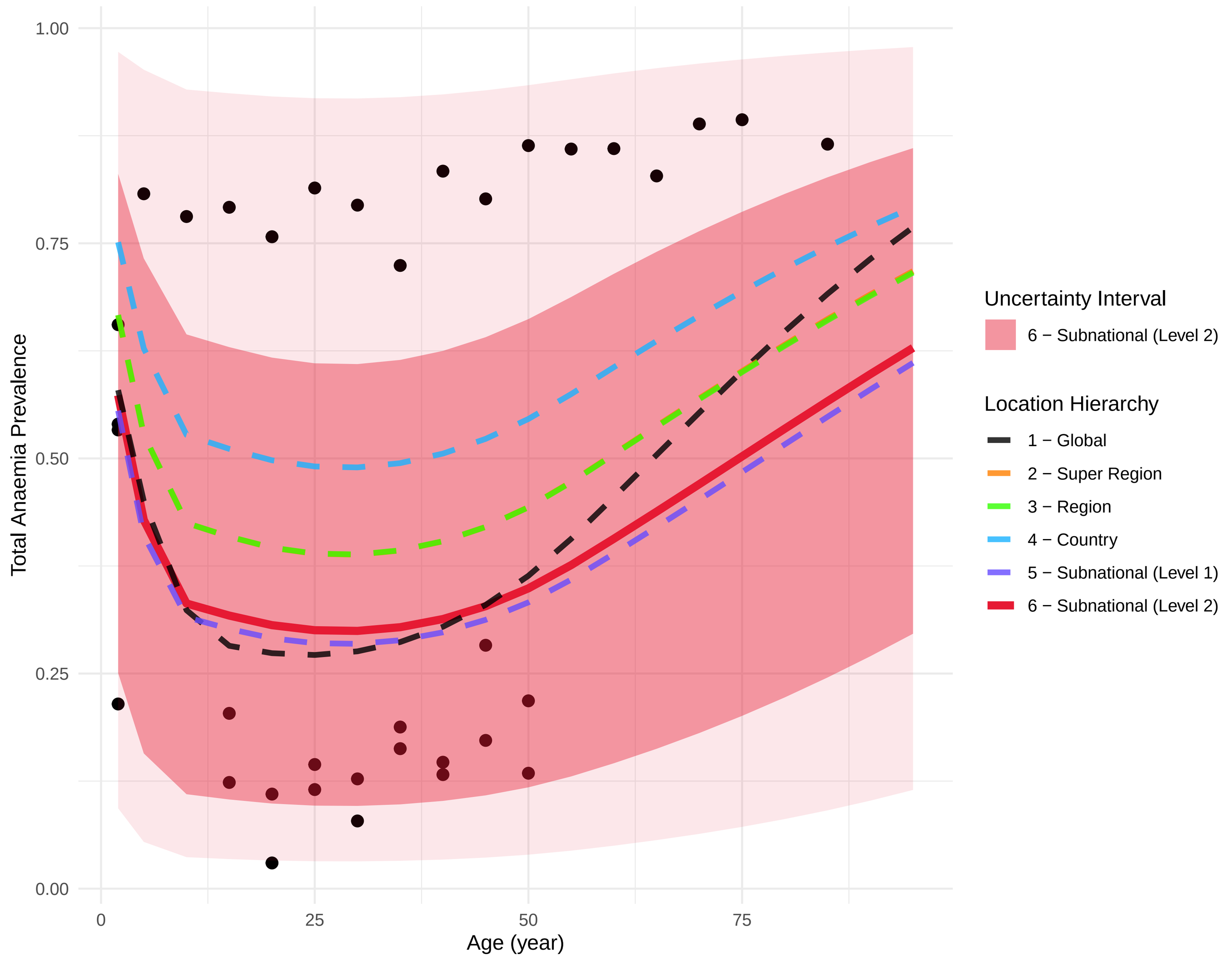

MR−BRT Age/Sex Fits − Males 2+ Years Old − Total Anaemia Prevalence − Odisha
WHO−2024 Adjustment Method

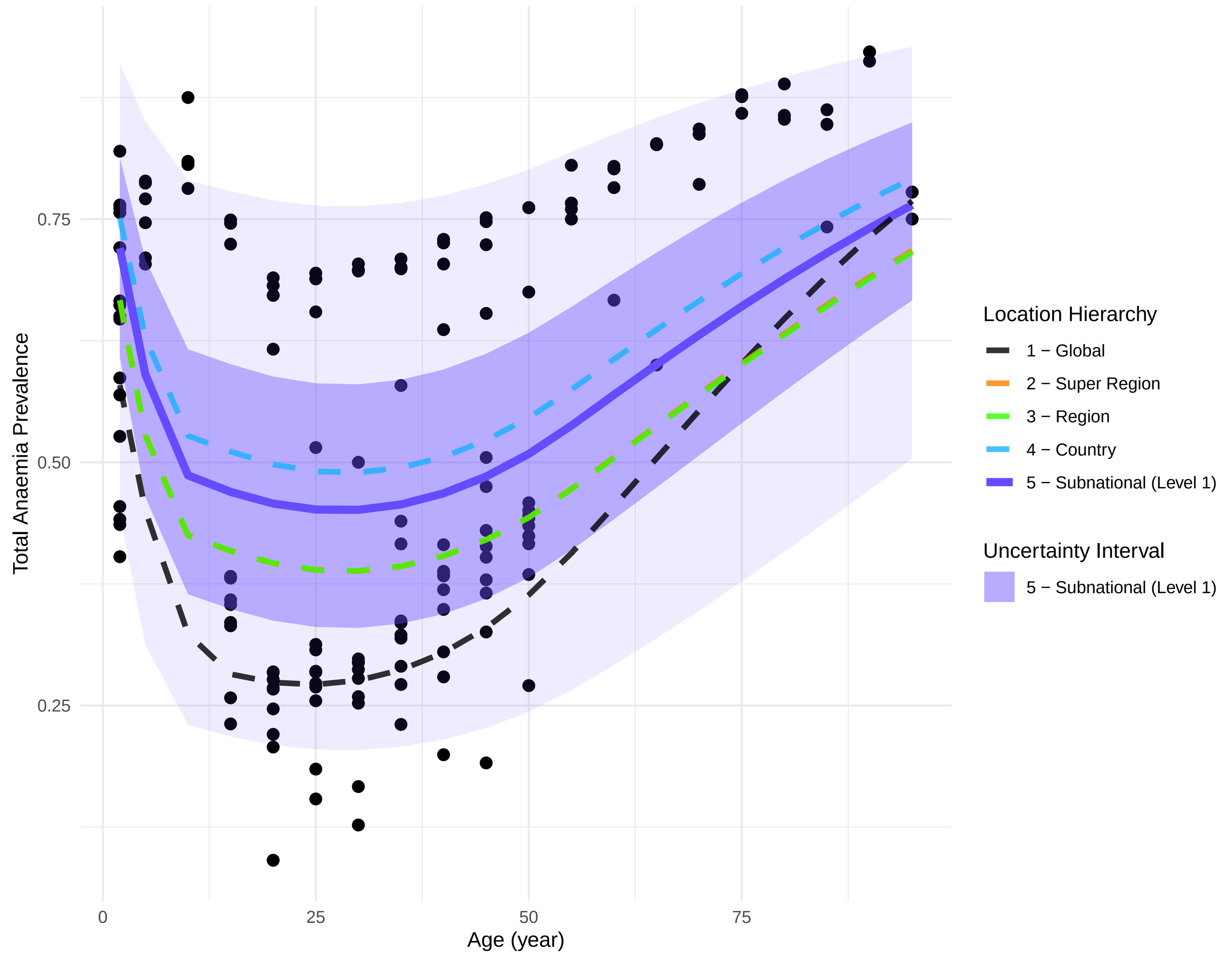

MR−BRT Age/Sex Fits − Males 2+ Years Old − Total Anaemia Prevalence − Odisha, Rural
WHO−2024 Adjustment Method

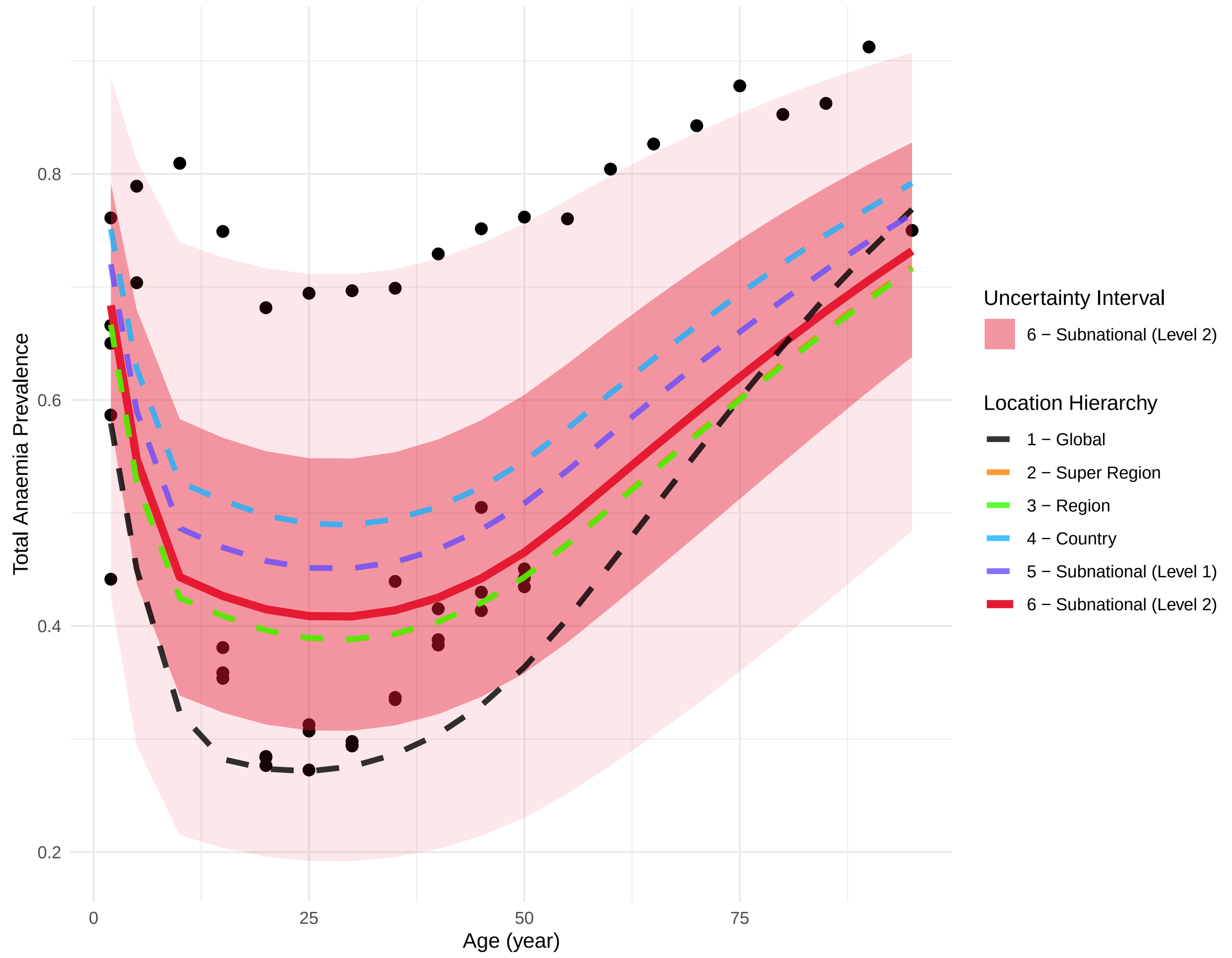

MR−BRT Age/Sex Fits − Males 2+ Years Old − Total Anaemia Prevalence − Odisha, Urban
WHO−2024 Adjustment Method

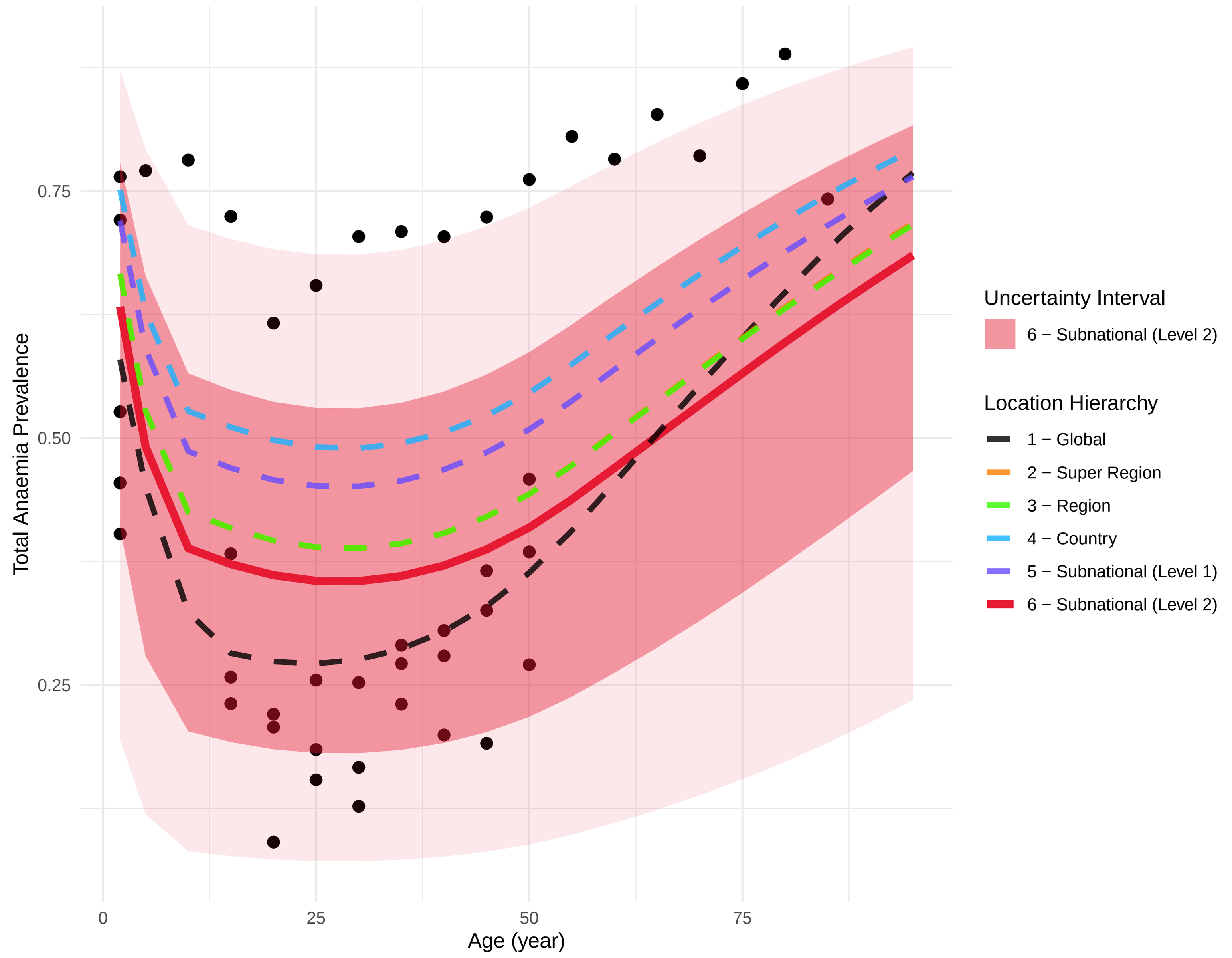

MR−BRT Age/Sex Fits − Males 2+ Years Old − Total Anaemia Prevalence − Other Union Territories
WHO−2024 Adjustment Method

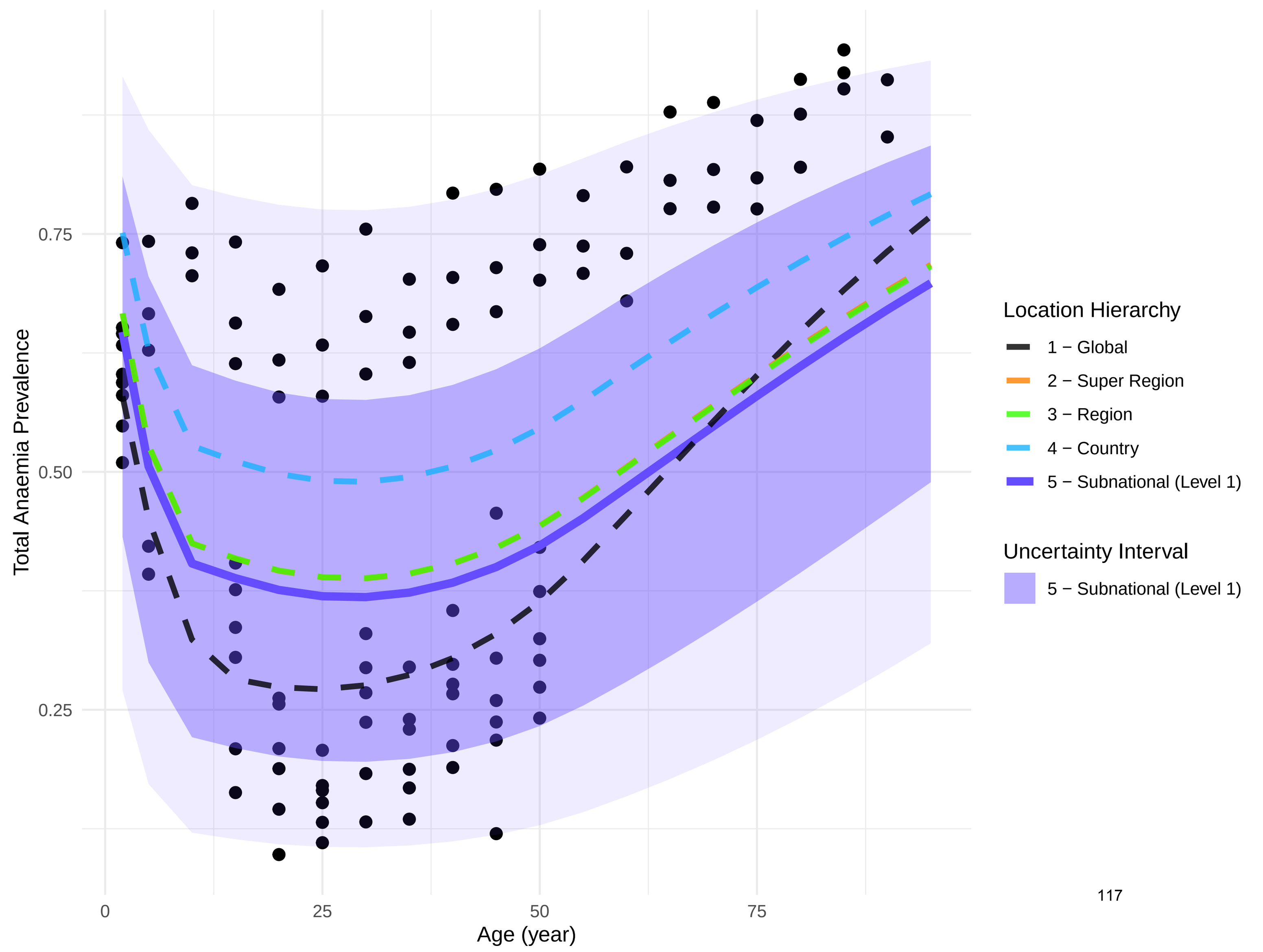

MR−BRT Age/Sex Fits − Males 2+ Years Old − Total Anaemia Prevalence − Other Union Territories, Rural
WHO−2024 Adjustment Method

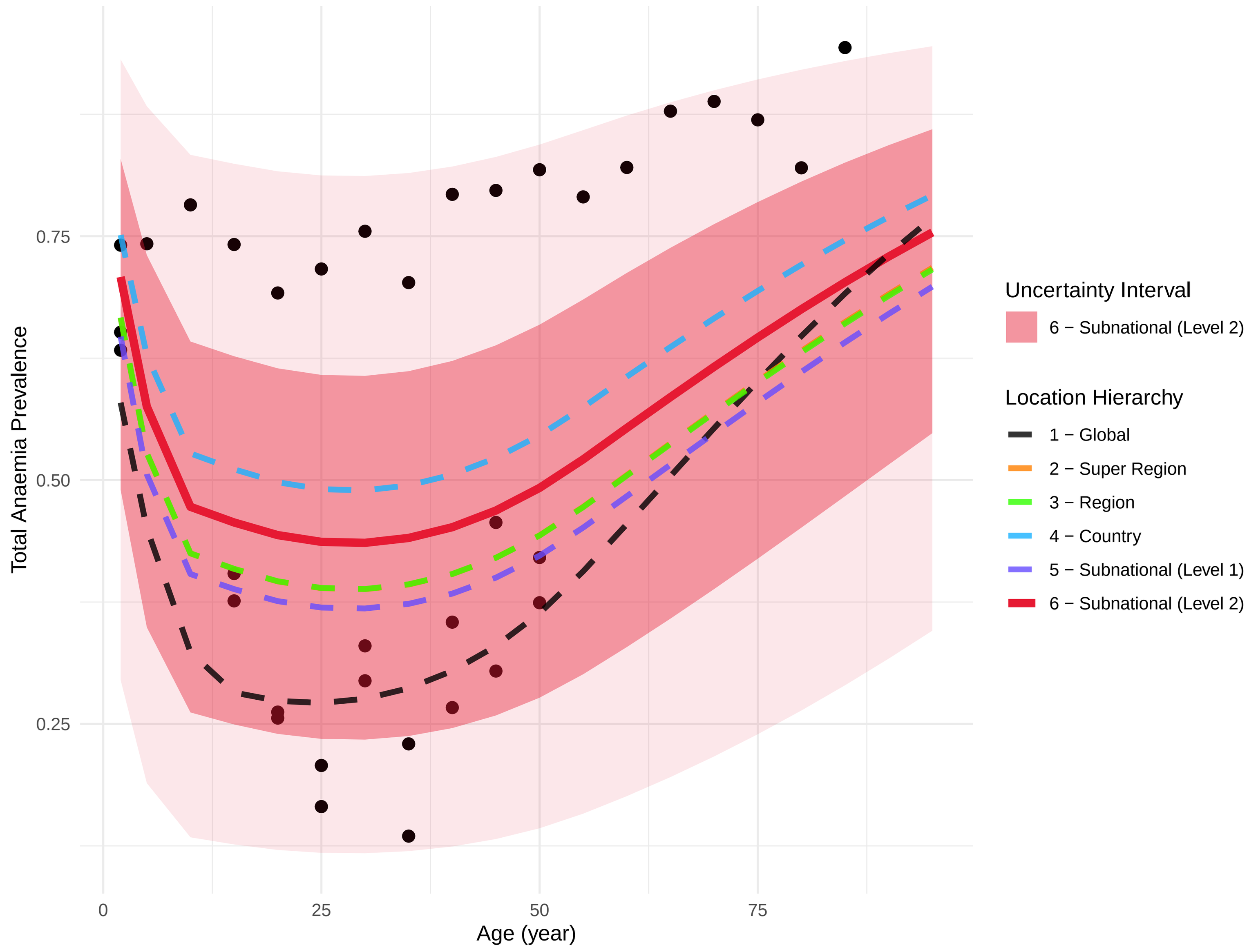

MR−BRT Age/Sex Fits − Males 2+ Years Old − Total Anaemia Prevalence − Other Union Territories, Urban
WHO−2024 Adjustment Method

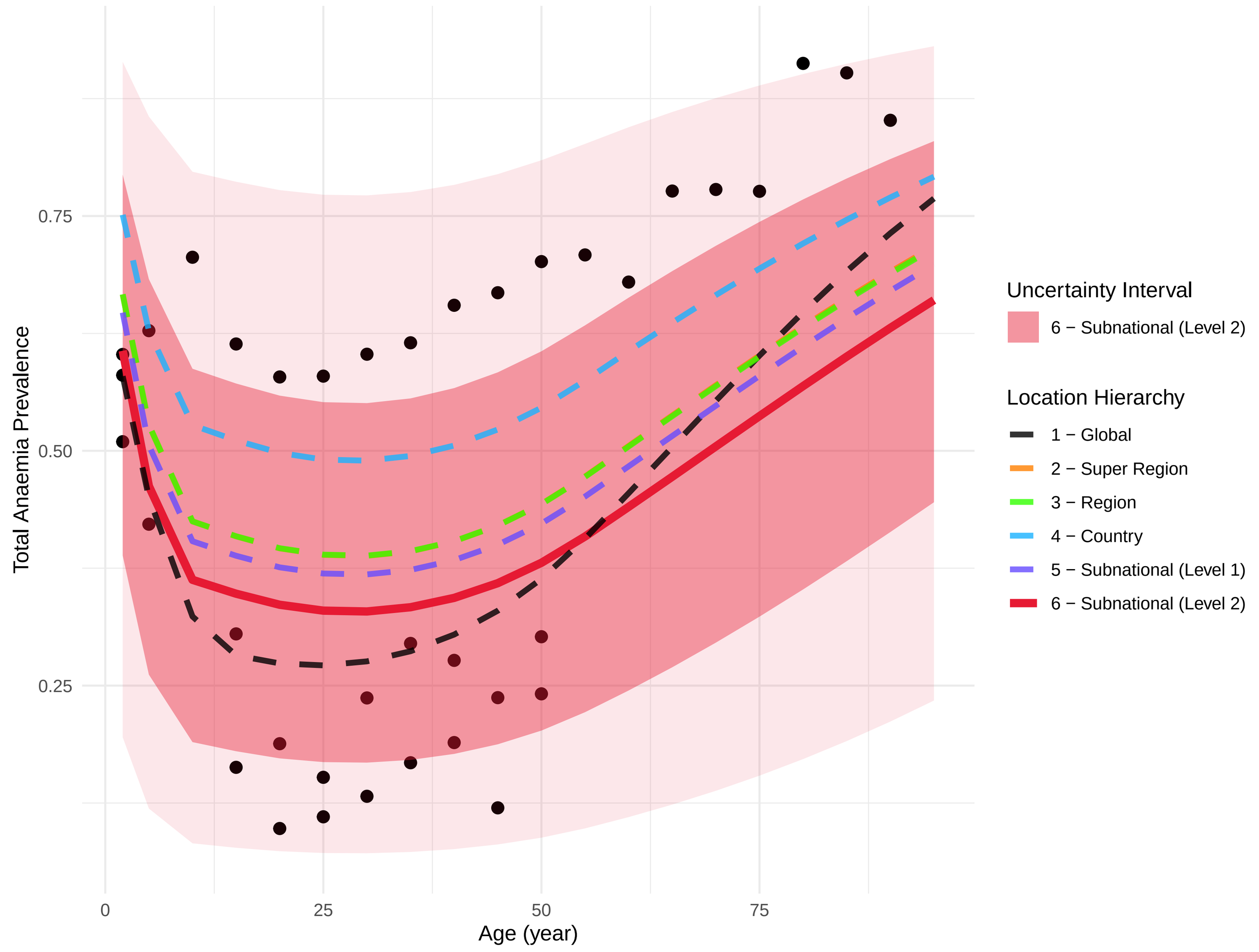

MR−BRT Age/Sex Fits − Males 2+ Years Old − Total Anaemia Prevalence − Punjab
WHO−2024 Adjustment Method

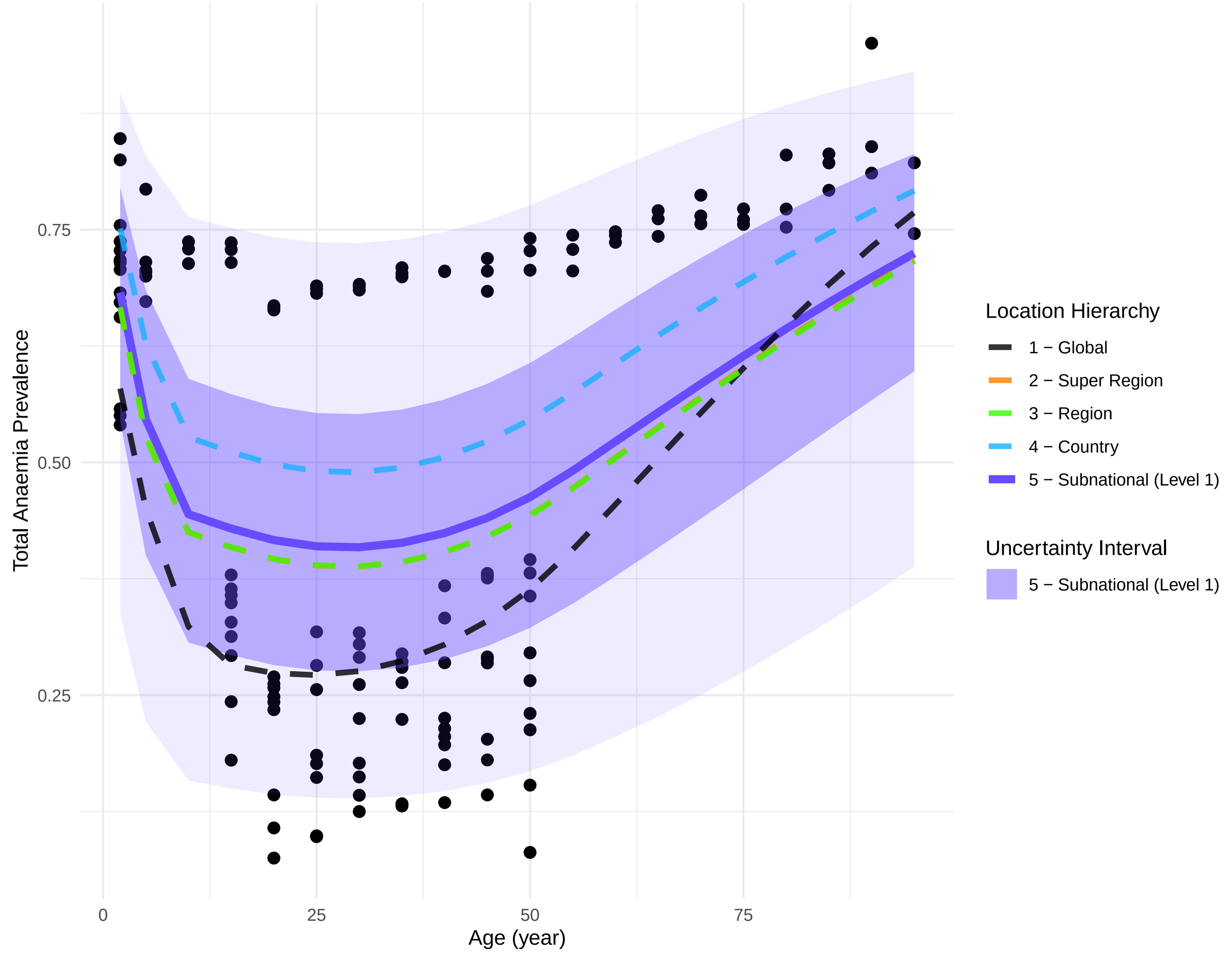

MR−BRT Age/Sex Fits − Males 2+ Years Old − Total Anaemia Prevalence − Punjab, Rural
WHO−2024 Adjustment Method

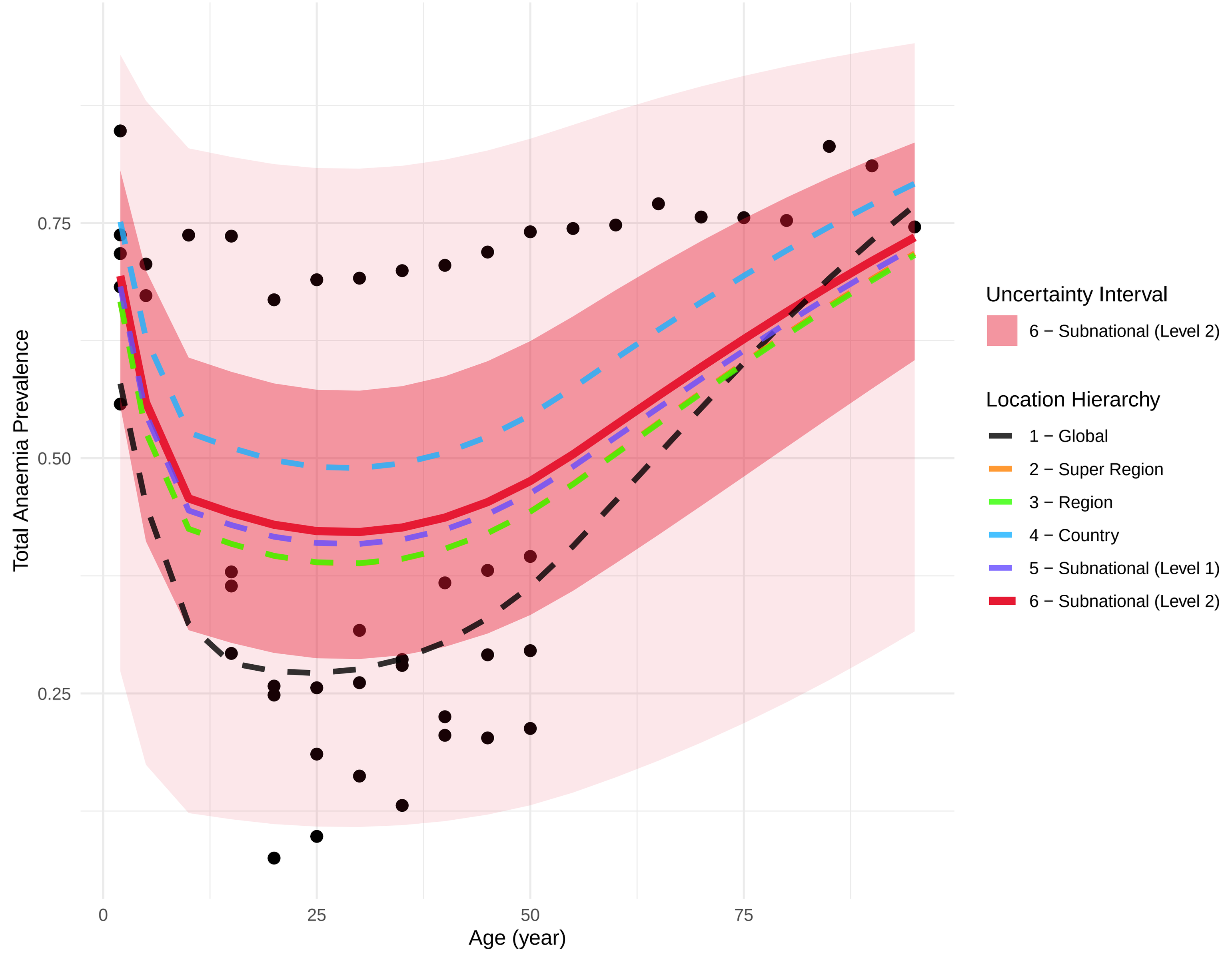

MR−BRT Age/Sex Fits − Males 2+ Years Old − Total Anaemia Prevalence − Punjab, Urban
WHO−2024 Adjustment Method

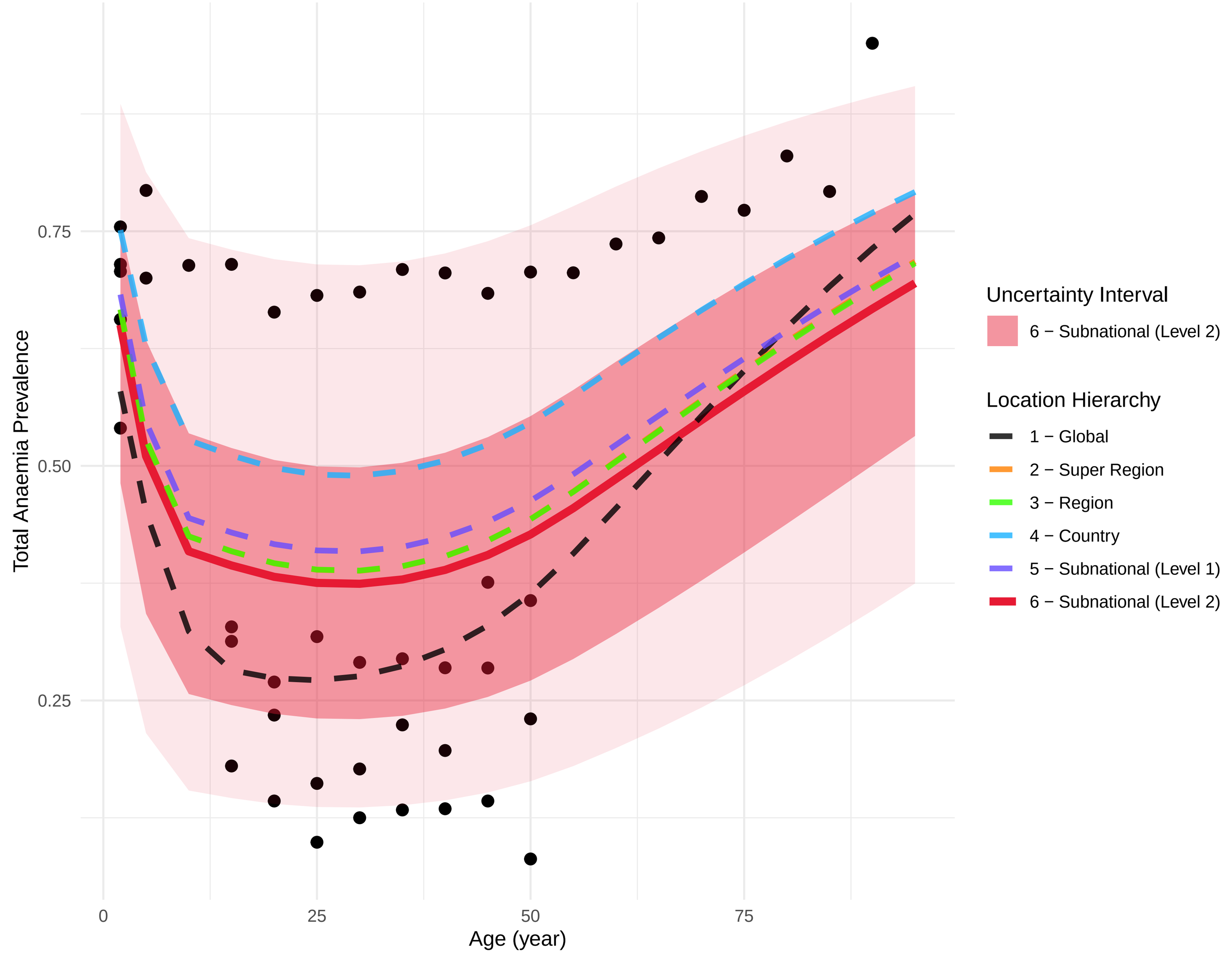

MR−BRT Age/Sex Fits − Males 2+ Years Old − Total Anaemia Prevalence − Rajasthan
WHO−2024 Adjustment Method

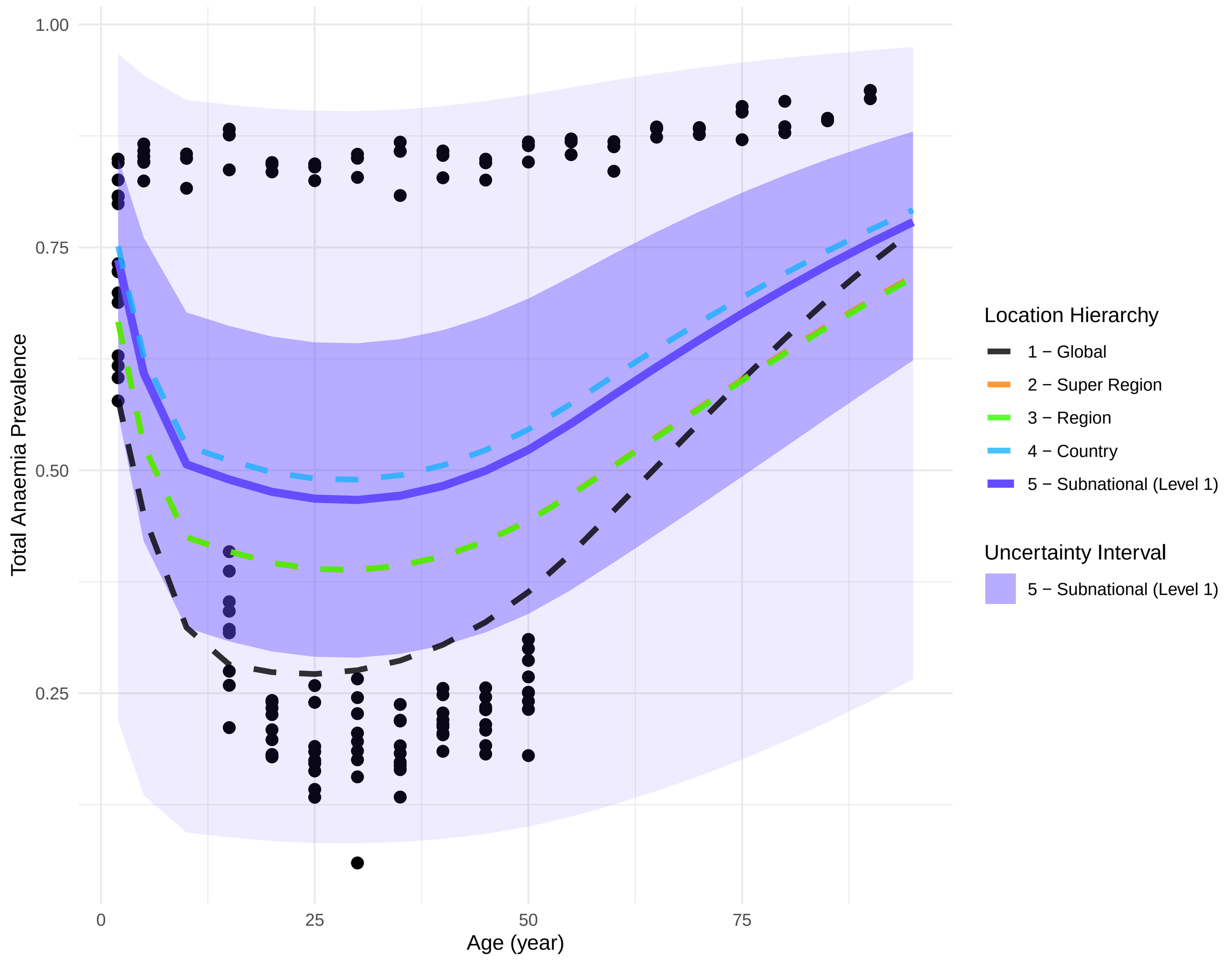

MR−BRT Age/Sex Fits − Males 2+ Years Old − Total Anaemia Prevalence − Rajasthan, Rural
WHO−2024 Adjustment Method

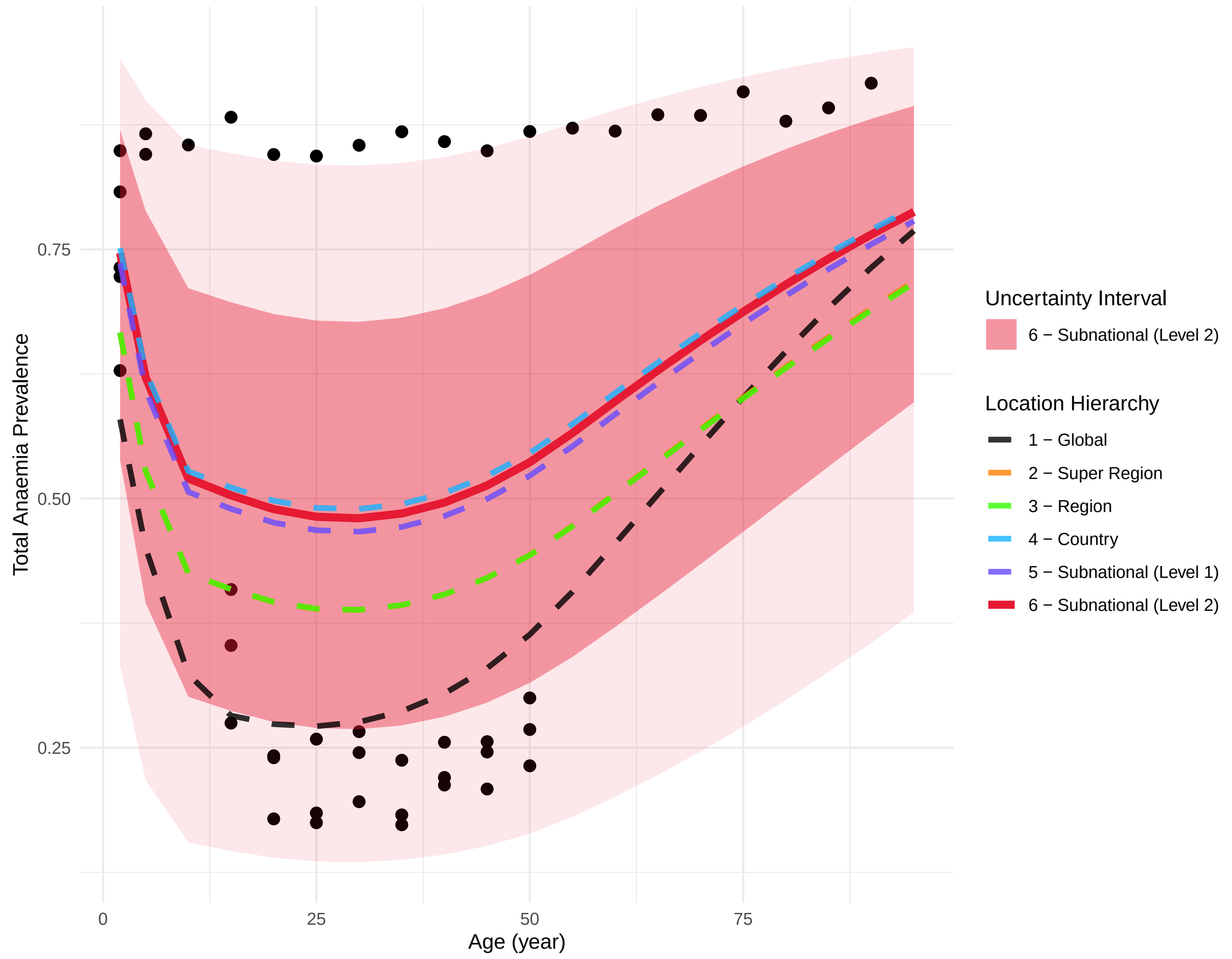

MR−BRT Age/Sex Fits − Males 2+ Years Old − Total Anaemia Prevalence − Rajasthan, Urban
WHO−2024 Adjustment Method

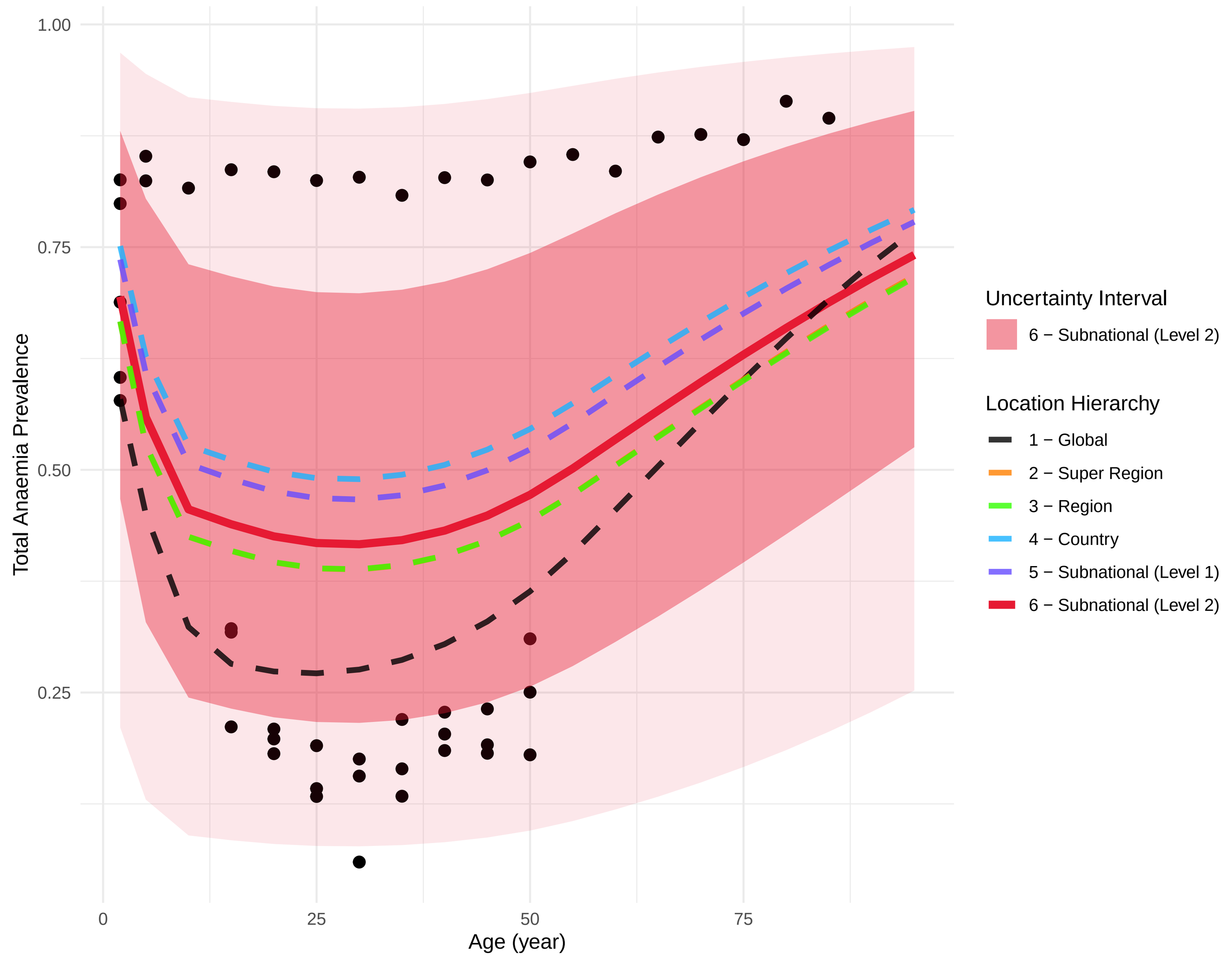

MR−BRT Age/Sex Fits − Males 2+ Years Old − Total Anaemia Prevalence − Sikkim
WHO−2024 Adjustment Method

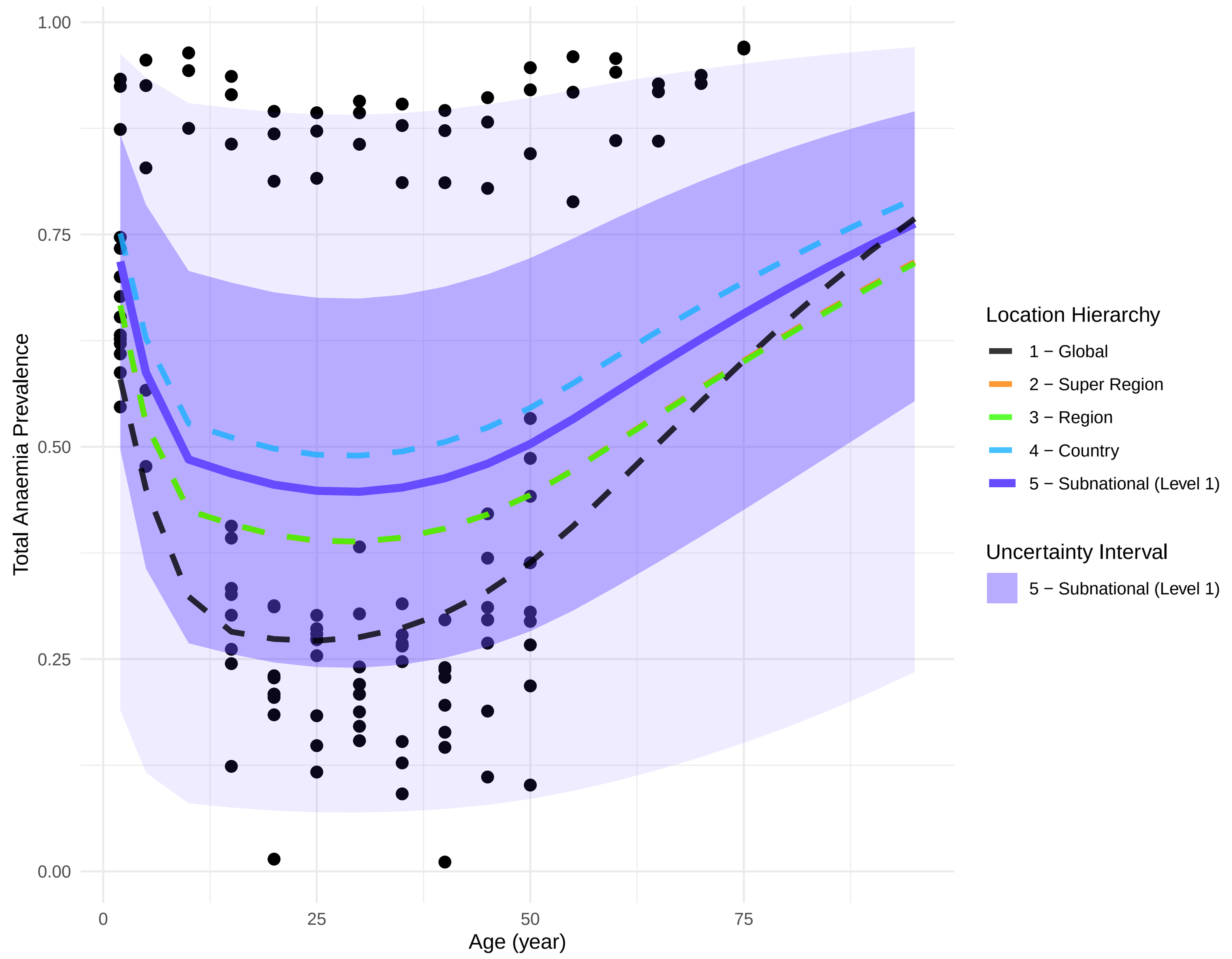

MR−BRT Age/Sex Fits − Males 2+ Years Old − Total Anaemia Prevalence − Sikkim, Rural
WHO−2024 Adjustment Method

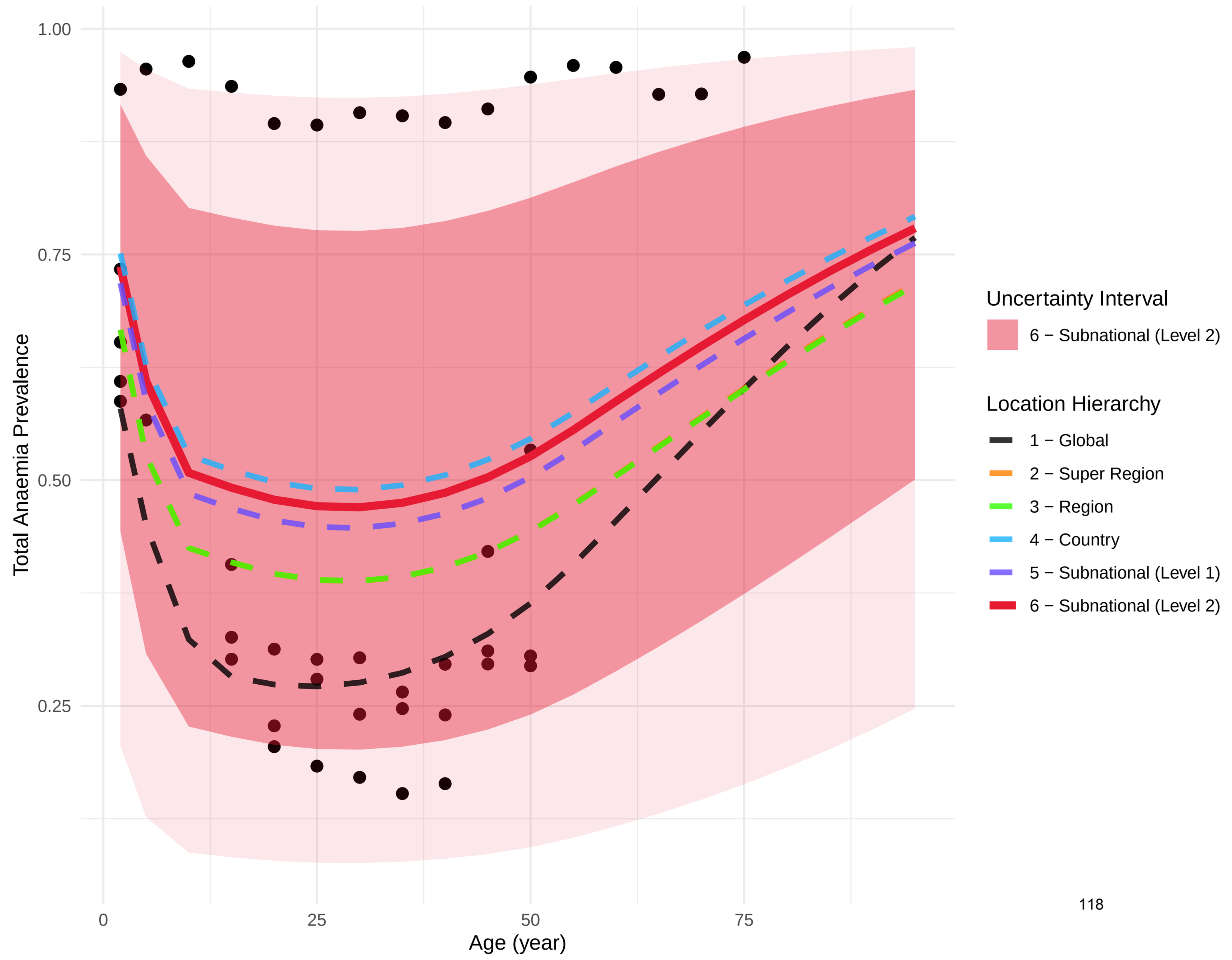

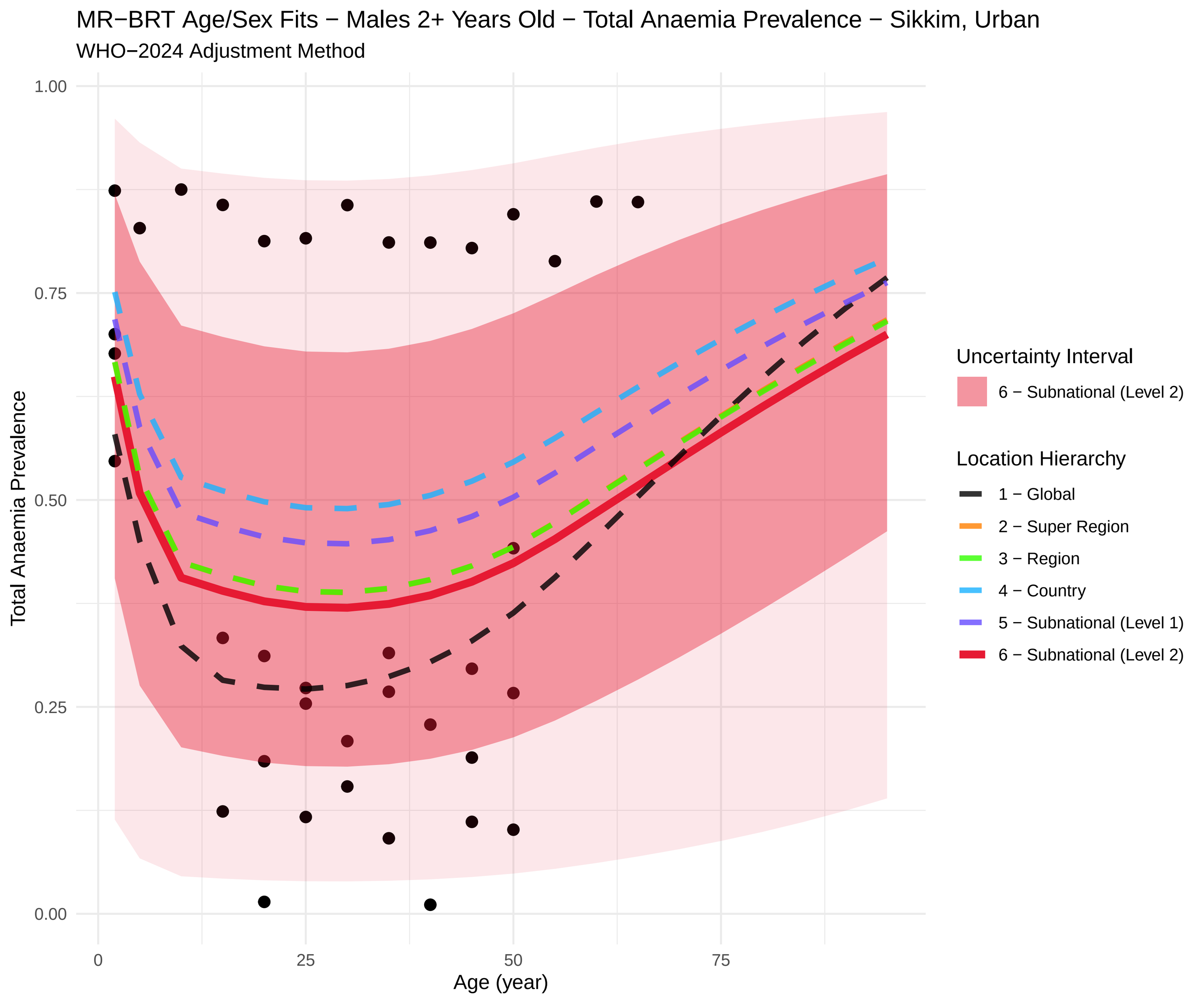
MR−BRT Age/Sex Fits − Males 2+ Years Old − Total Anaemia Prevalence − Sikkim, Urban
WHO−2024 Adjustment Method
Total Anaemia Prevalence
1.00
0.75
0.50
0.25
0.00
0
25
50
75
Age (year)
Uncertainty Interval
6 − Subnational (Level 2)
Location Hierarchy
1 − Global
2 − Super Region
3 − Region
4 − Country
5 − Subnational (Level 1)
6 − Subnational (Level 2)

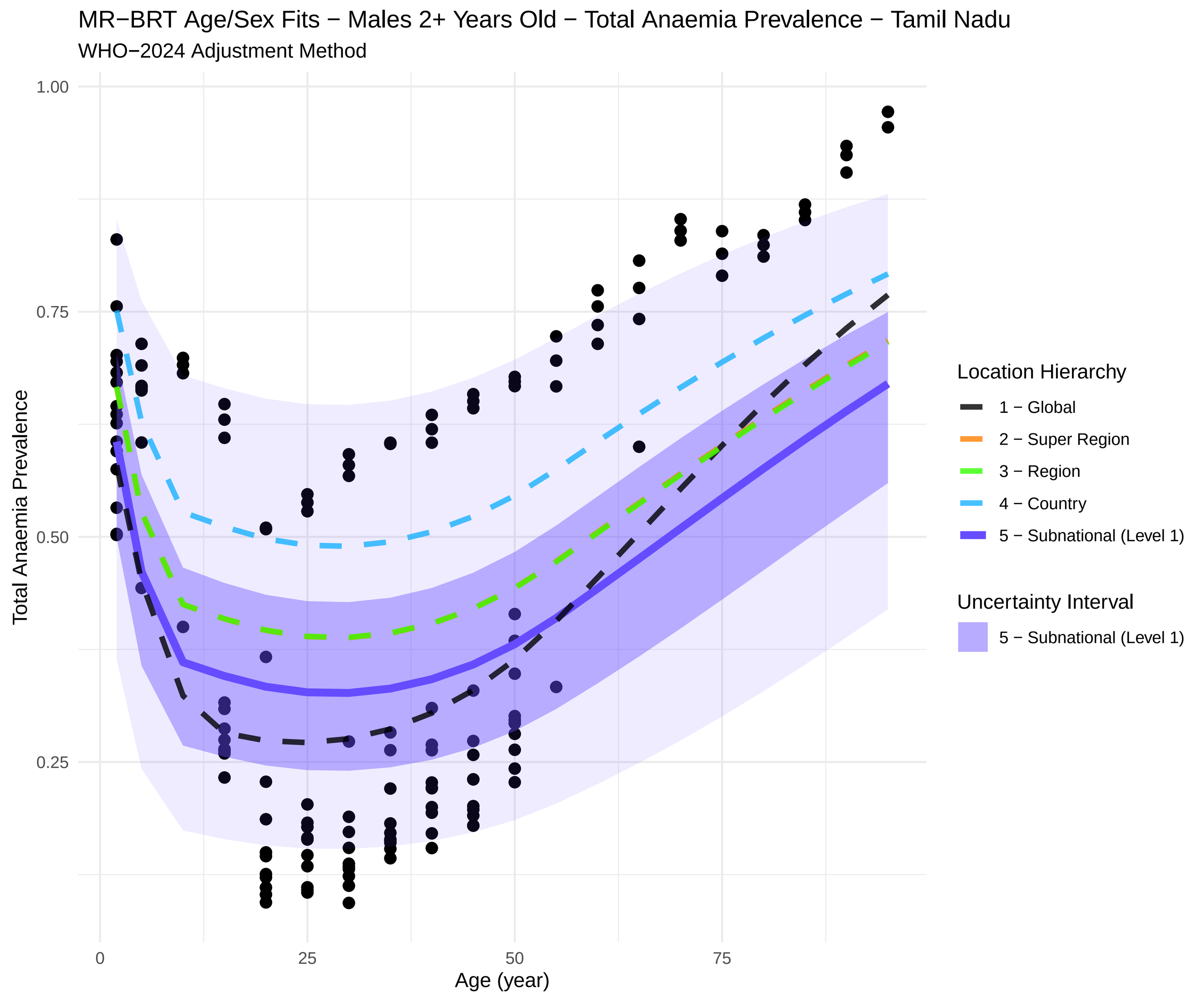
MR−BRT Age/Sex Fits − Males 2+ Years Old − Total Anaemia Prevalence − Tamil Nadu
WHO−2024 Adjustment Method
Total Anaemia Prevalence
1.00
0.75
0.50
0.25
0
25
50
75
Age (year)
Location Hierarchy
1 − Global
2 − Super Region
3 − Region
4 − Country
5 − Subnational (Level 1)
Uncertainty Interval
5 − Subnational (Level 1)

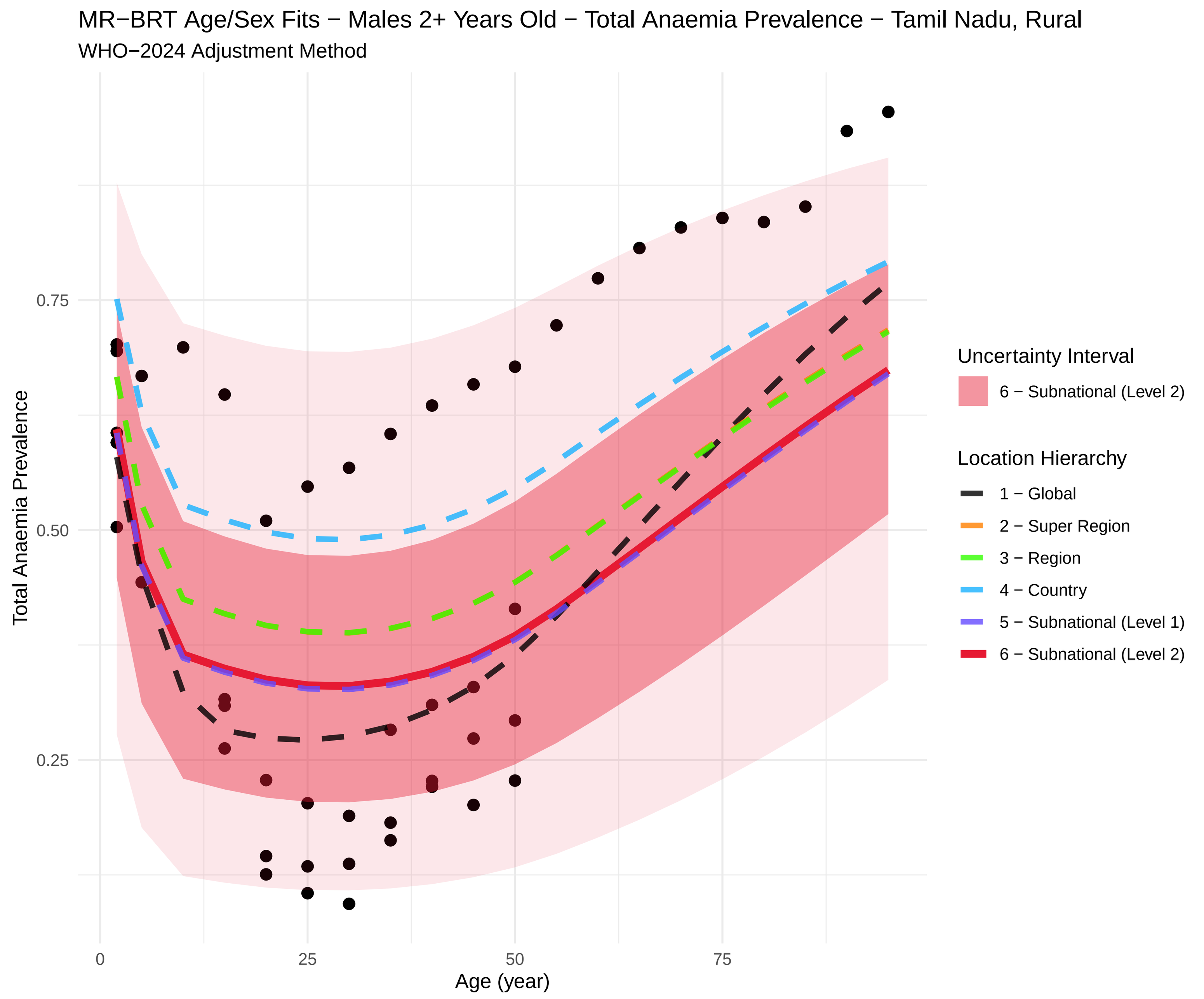
MR−BRT Age/Sex Fits − Males 2+ Years Old − Total Anaemia Prevalence − Tamil Nadu, Rural
WHO−2024 Adjustment Method
Total Anaemia Prevalence
0.75
0.50
0.25
0
25
50
75
Age (year)
Uncertainty Interval
6 − Subnational (Level 2)
Location Hierarchy
1 − Global
2 − Super Region
3 − Region
4 − Country
5 − Subnational (Level 1)
6 − Subnational (Level 2)

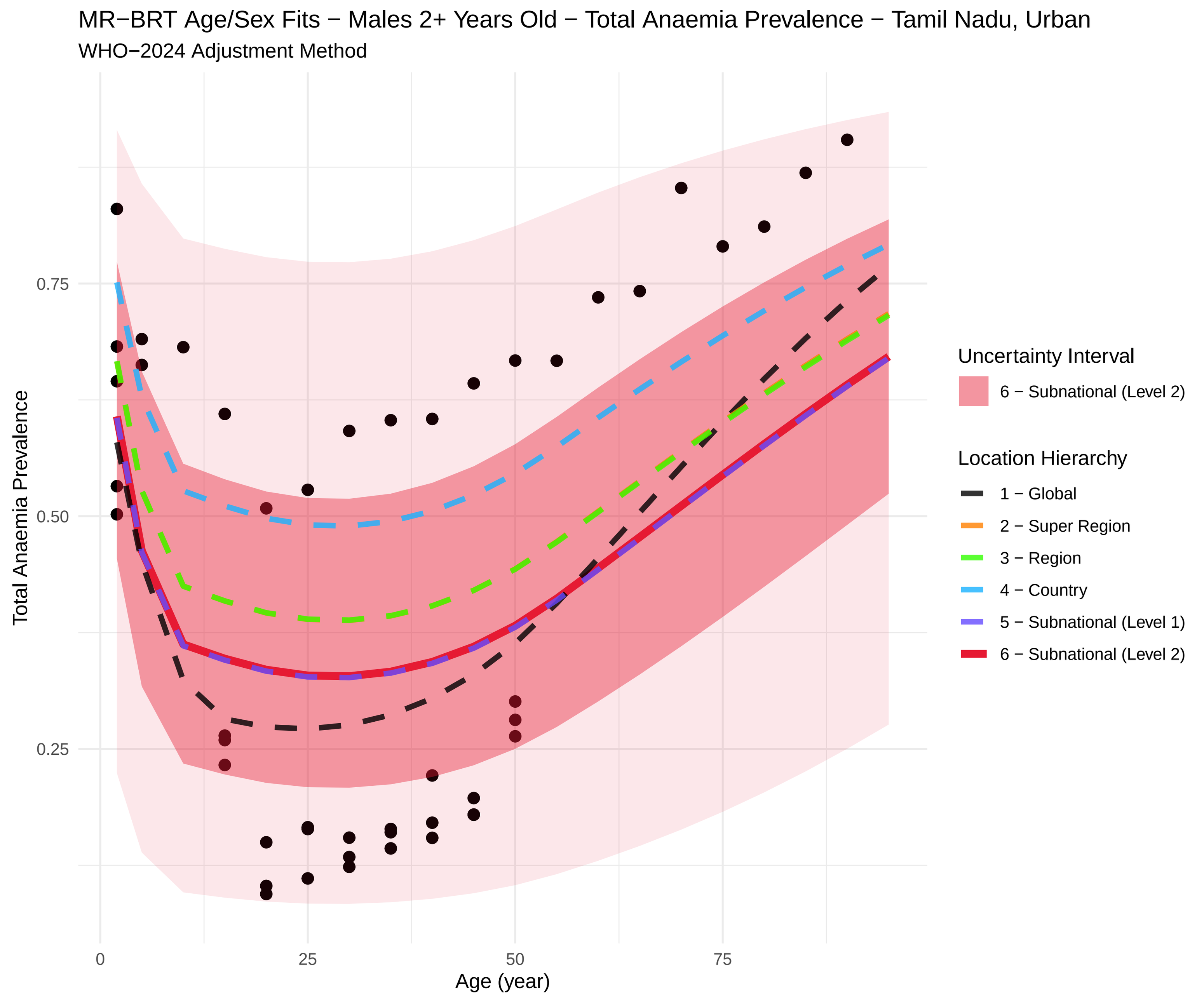
MR−BRT Age/Sex Fits − Males 2+ Years Old − Total Anaemia Prevalence − Tamil Nadu, Urban
WHO−2024 Adjustment Method
Total Anaemia Prevalence
0.75
0.50
0.25
0
25
50
75
Age (year)
Uncertainty Interval
6 − Subnational (Level 2)
Location Hierarchy
1 − Global
2 − Super Region
3 − Region
4 − Country
5 − Subnational (Level 1)
6 − Subnational (Level 2)

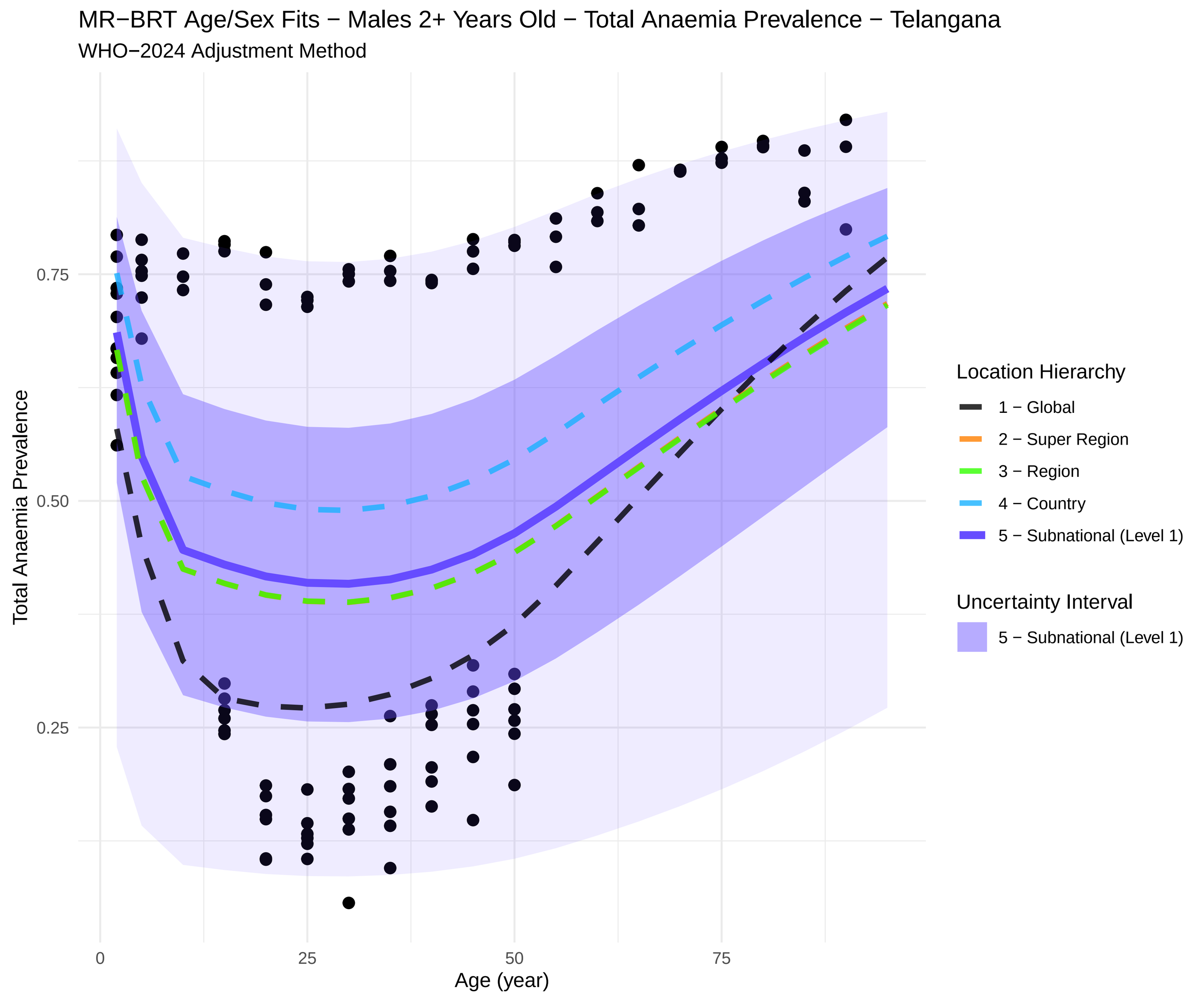
MR−BRT Age/Sex Fits − Males 2+ Years Old − Total Anaemia Prevalence − Telangana
WHO−2024 Adjustment Method
Total Anaemia Prevalence
0.75
0.50
0.25
0
25
50
75
Age (year)
Location Hierarchy
1 − Global
2 − Super Region
3 − Region
4 − Country
5 − Subnational (Level 1)
Uncertainty Interval
5 − Subnational (Level 1)

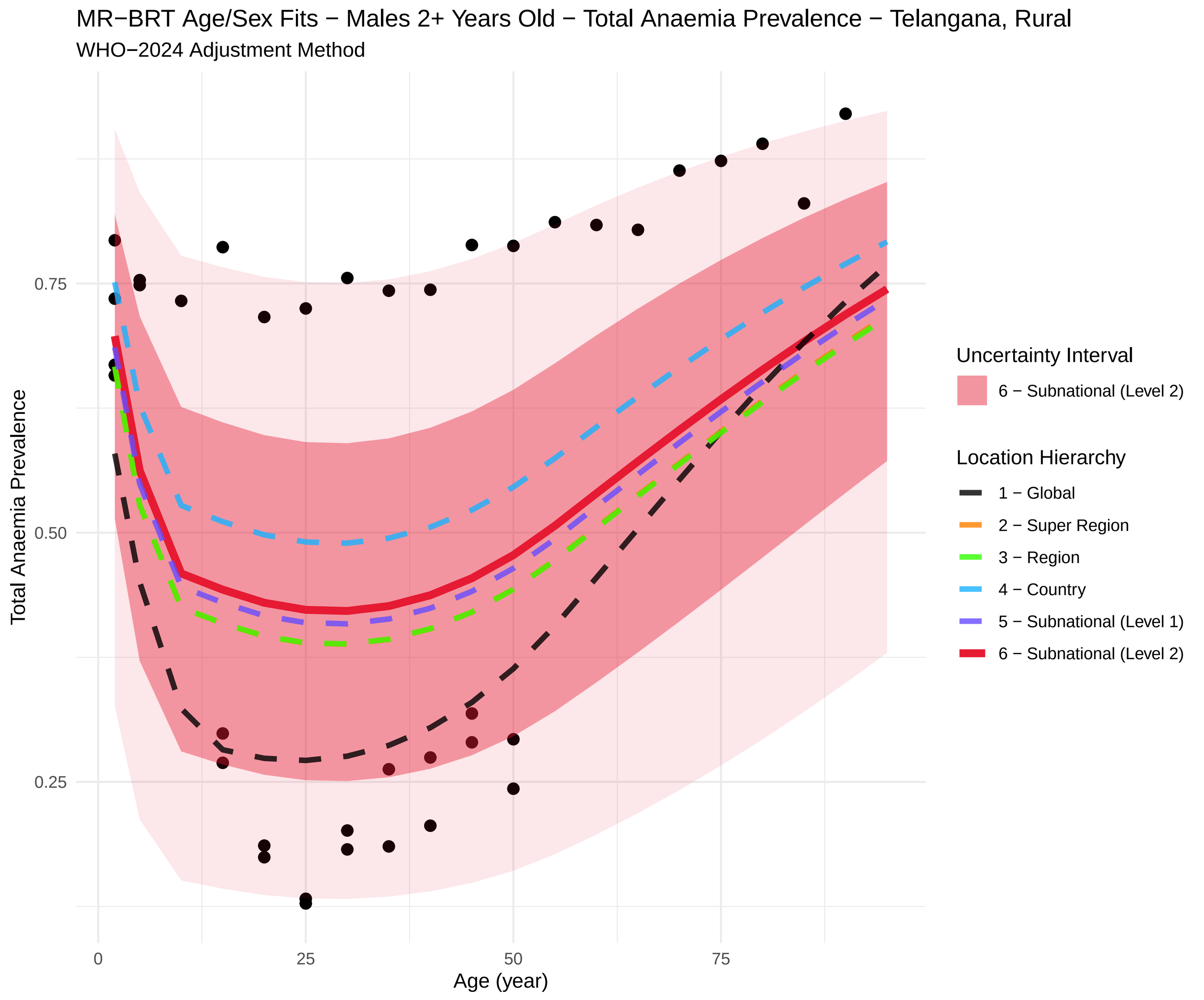
MR−BRT Age/Sex Fits − Males 2+ Years Old − Total Anaemia Prevalence − Telangana, Rural
WHO−2024 Adjustment Method
Total Anaemia Prevalence
0.75
0.50
0.25
0
25
50
75
Age (year)
Uncertainty Interval
6 − Subnational (Level 2)
Location Hierarchy
1 − Global
2 − Super Region
3 − Region
4 − Country
5 − Subnational (Level 1)
6 − Subnational (Level 2)

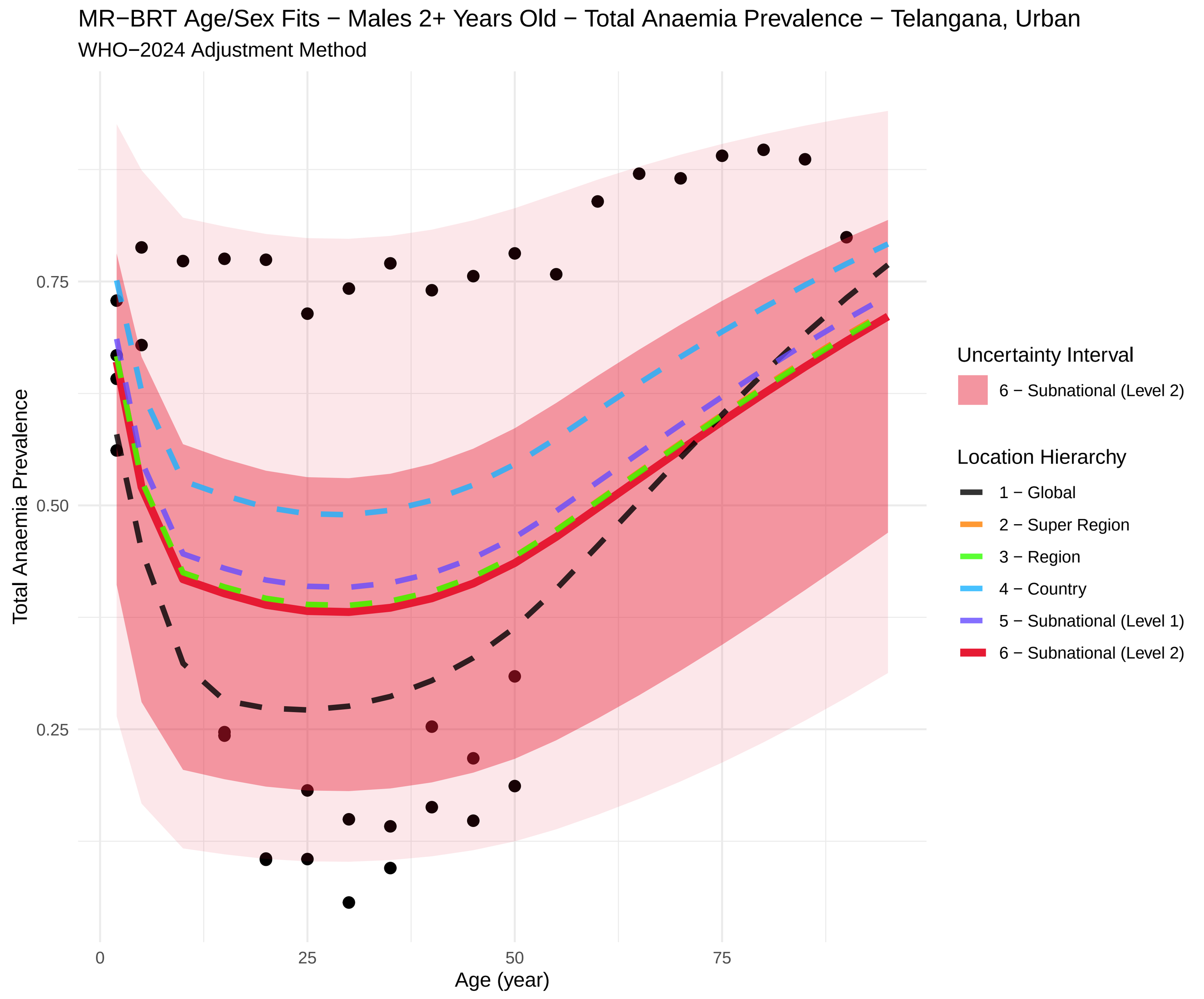
MR−BRT Age/Sex Fits − Males 2+ Years Old − Total Anaemia Prevalence − Telangana, Urban
WHO−2024 Adjustment Method
Total Anaemia Prevalence
0.75
0.50
0.25
0
25
50
75
Age (year)
Uncertainty Interval
6 − Subnational (Level 2)
Location Hierarchy
1 − Global
2 − Super Region
3 − Region
4 − Country
5 − Subnational (Level 1)
6 − Subnational (Level 2)

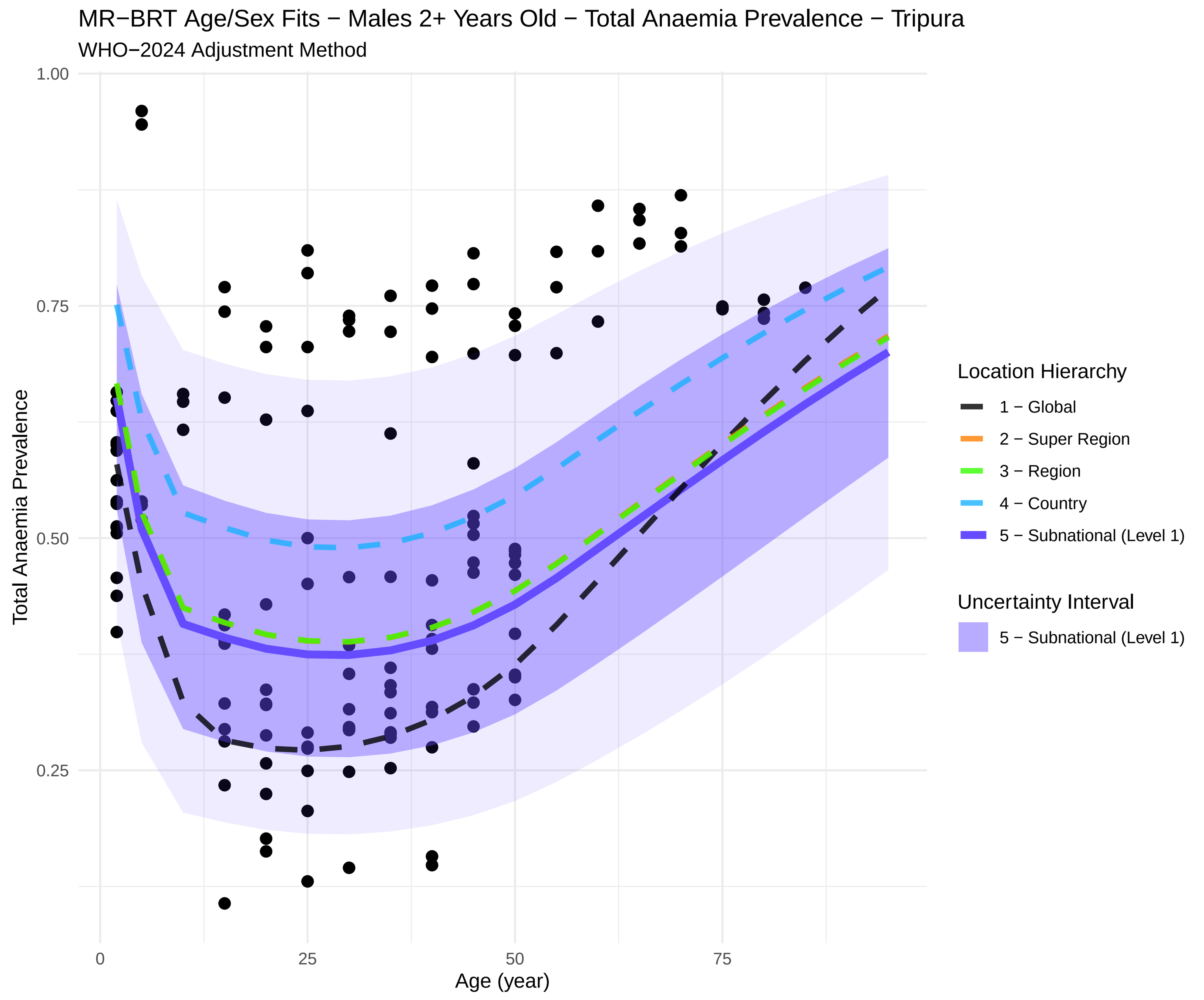
MR−BRT Age/Sex Fits − Males 2+ Years Old − Total Anaemia Prevalence − Tripura
WHO−2024 Adjustment Method
Total Anaemia Prevalence
1.00
0.75
0.50
0.25
0
25
50
75
Age (year)
Location Hierarchy
1 − Global
2 − Super Region
3 − Region
4 − Country
5 − Subnational (Level 1)
Uncertainty Interval
5 − Subnational (Level 1)

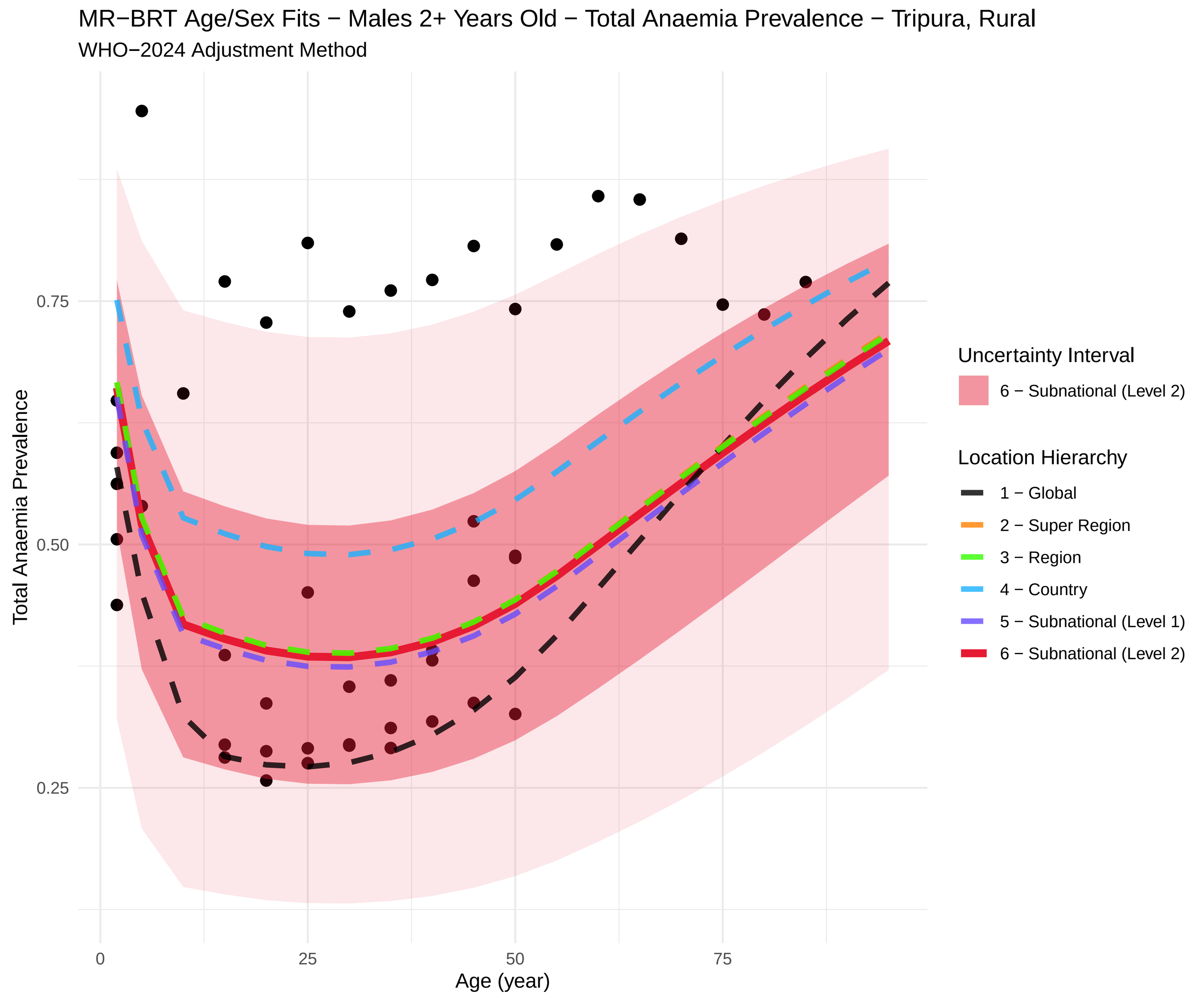
MR−BRT Age/Sex Fits − Males 2+ Years Old − Total Anaemia Prevalence − Tripura, Rural
WHO−2024 Adjustment Method
Total Anaemia Prevalence
0.75
0.50
0.25
0
25
50
75
Age (year)
Uncertainty Interval
6 − Subnational (Level 2)
Location Hierarchy
1 − Global
2 − Super Region
3 − Region
4 − Country
5 − Subnational (Level 1)
6 − Subnational (Level 2)

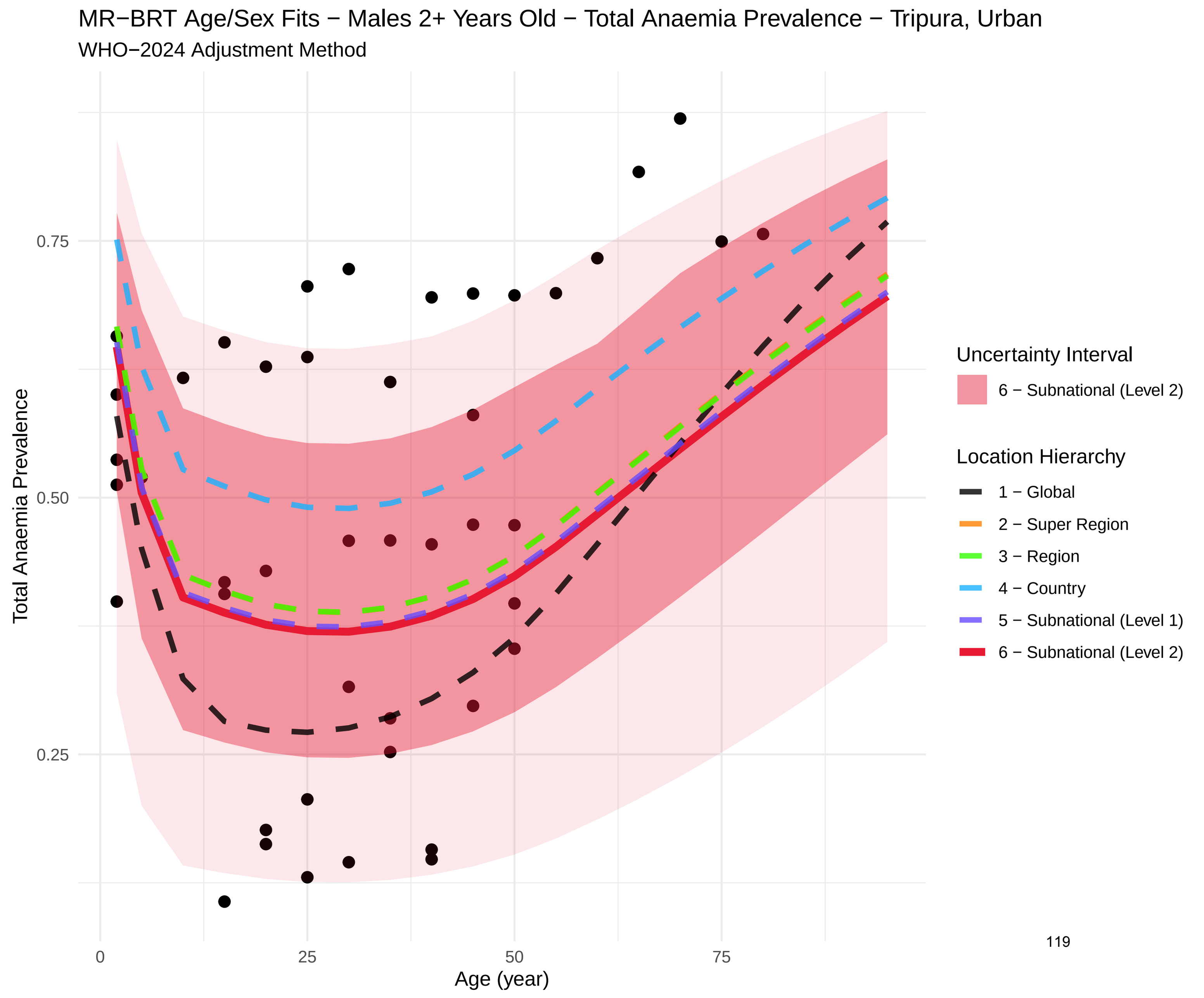
MR−BRT Age/Sex Fits − Males 2+ Years Old − Total Anaemia Prevalence − Tripura, Urban
WHO−2024 Adjustment Method
Total Anaemia Prevalence
0.75
0.50
0.25
0
25
50
75
Age (year)
Uncertainty Interval
6 − Subnational (Level 2)
Location Hierarchy
1 − Global
2 − Super Region
3 − Region
4 − Country
5 − Subnational (Level 1)
6 − Subnational (Level 2)

MR−BRT Age/Sex Fits − Males 2+ Years Old − Total Anaemia Prevalence − Uttar Pradesh
WHO−2024 Adjustment Method

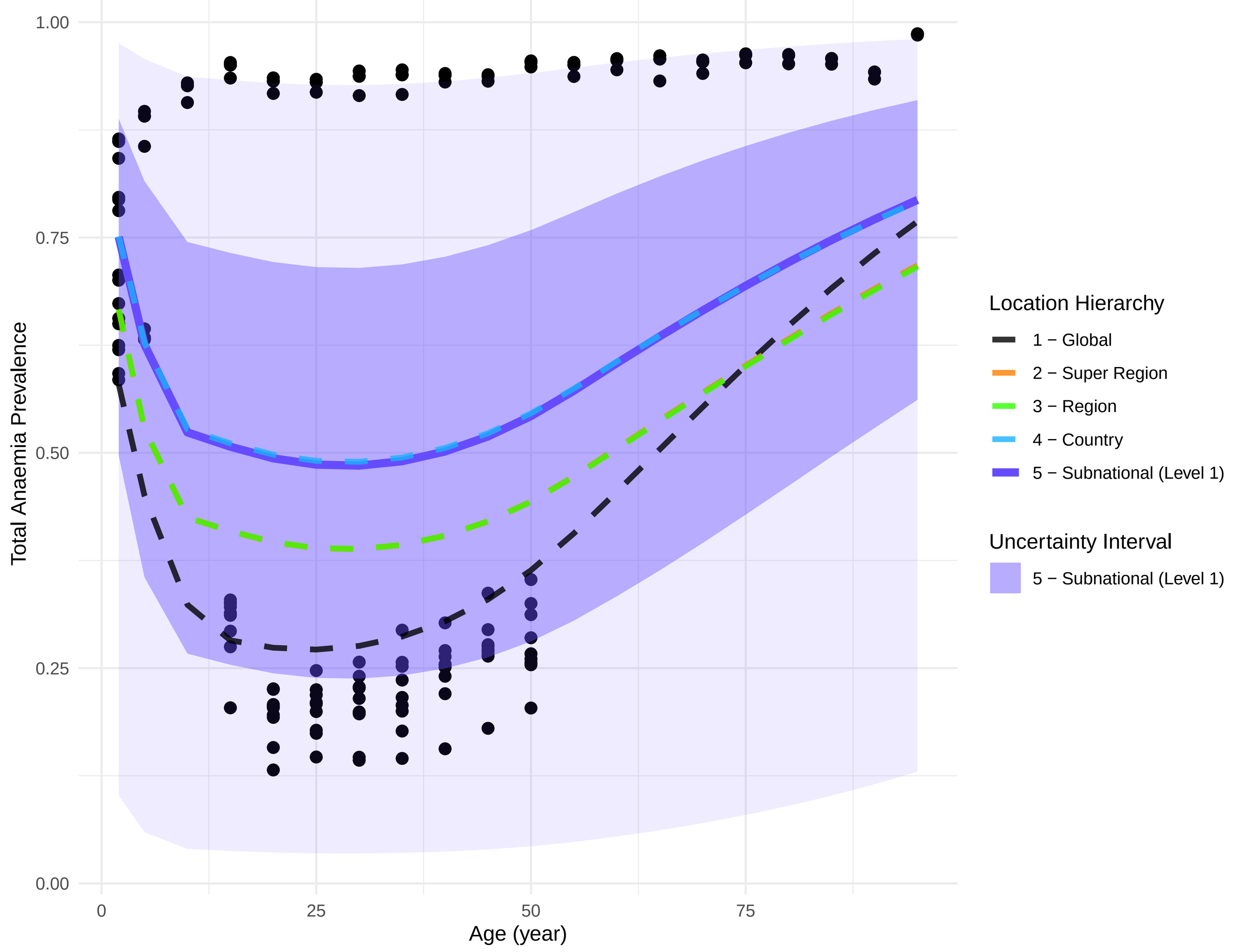

MR−BRT Age/Sex Fits − Males 2+ Years Old − Total Anaemia Prevalence − Uttar Pradesh, Rural
WHO−2024 Adjustment Method

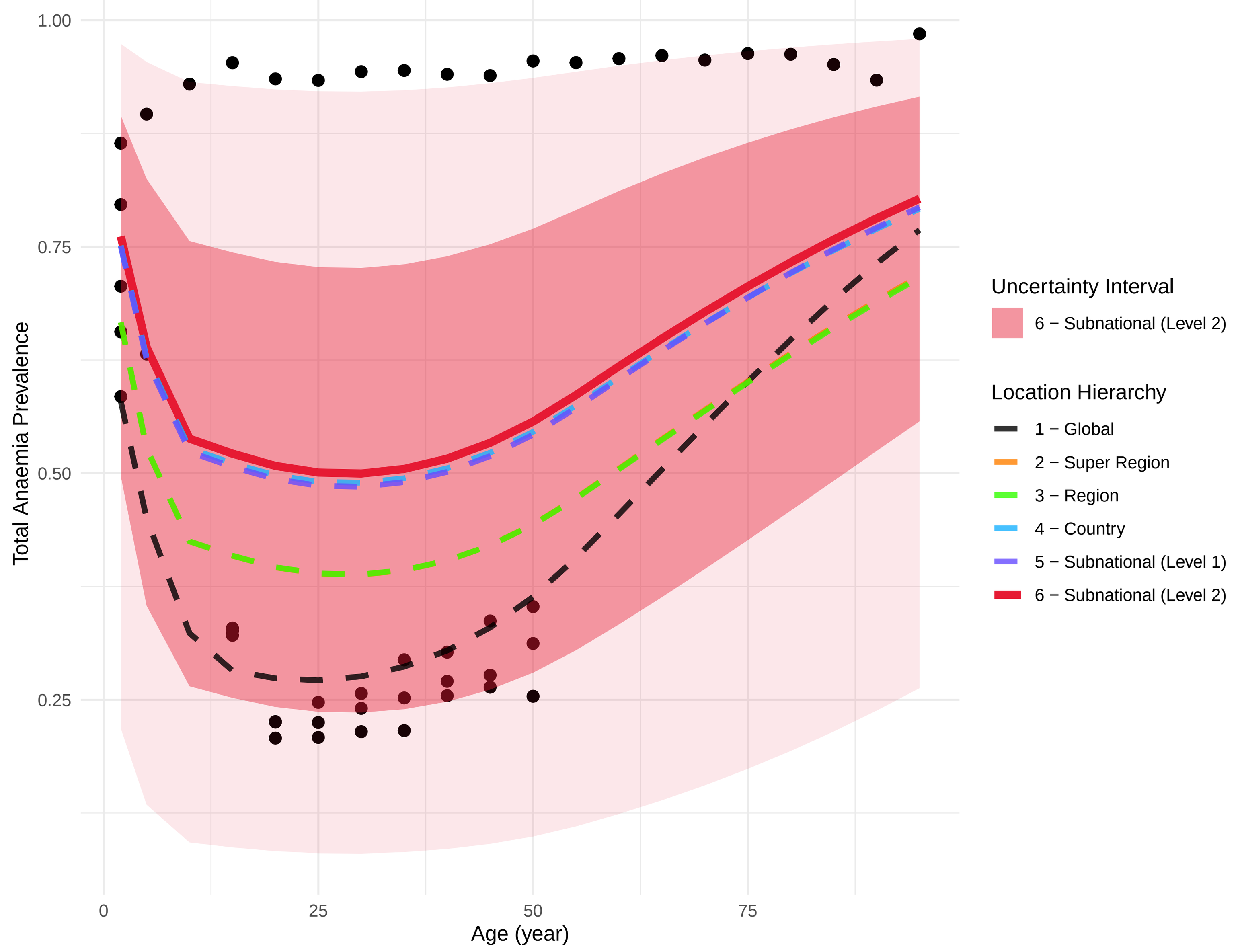

MR−BRT Age/Sex Fits − Males 2+ Years Old − Total Anaemia Prevalence − Uttar Pradesh, Urban
WHO−2024 Adjustment Method

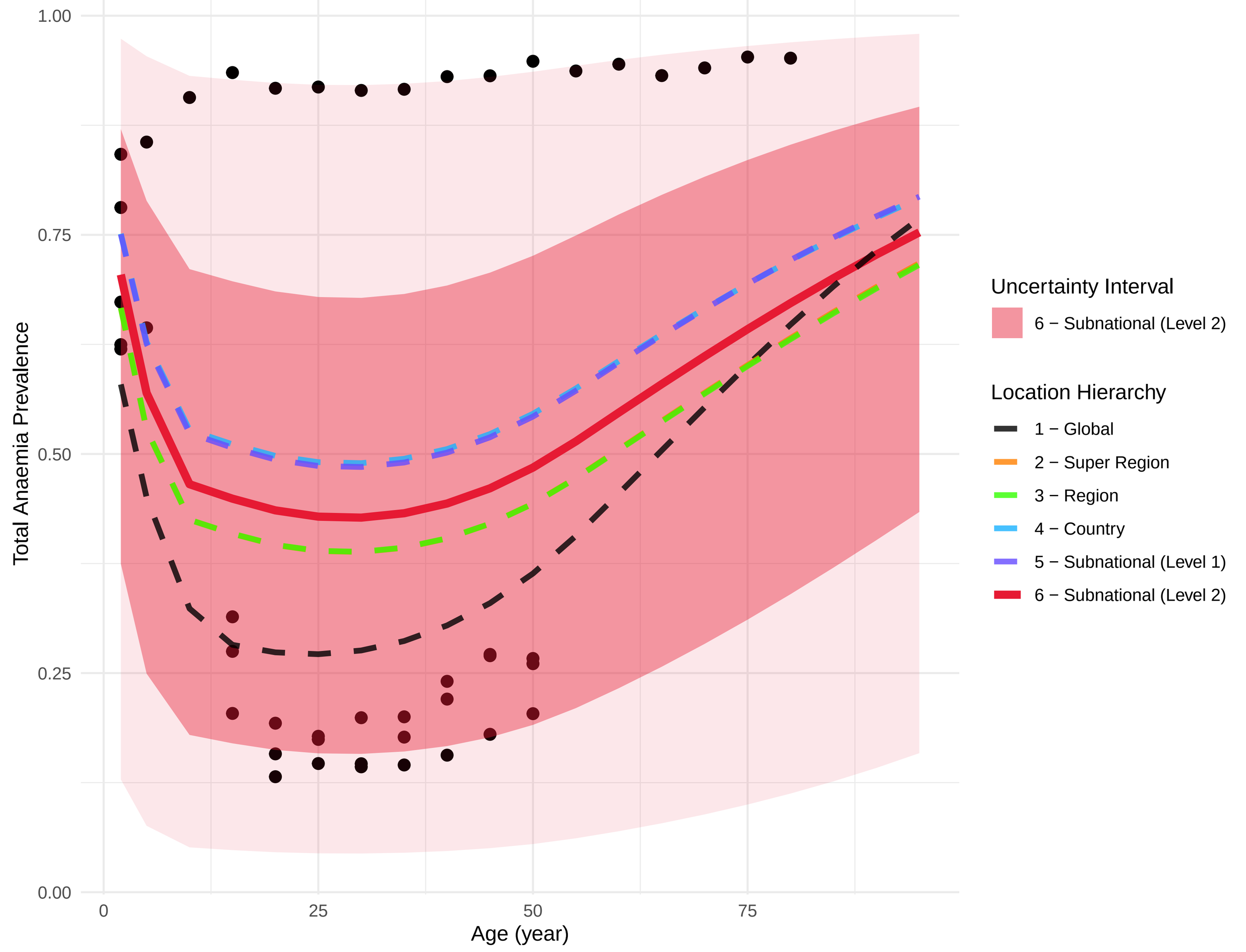

MR−BRT Age/Sex Fits − Males 2+ Years Old − Total Anaemia Prevalence − Uttarakhand
WHO−2024 Adjustment Method

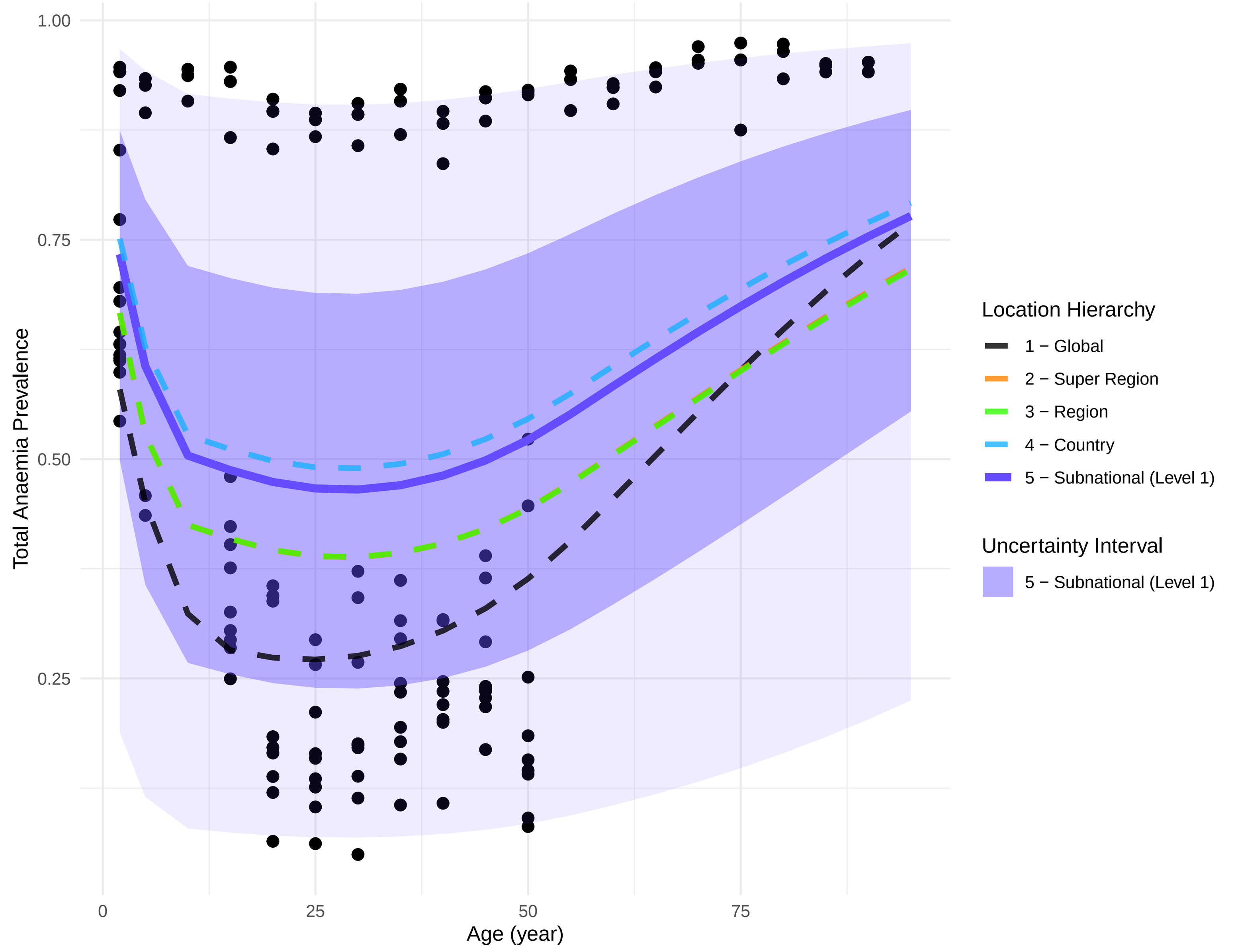

MR−BRT Age/Sex Fits − Males 2+ Years Old − Total Anaemia Prevalence − Uttarakhand, Rural
WHO−2024 Adjustment Method

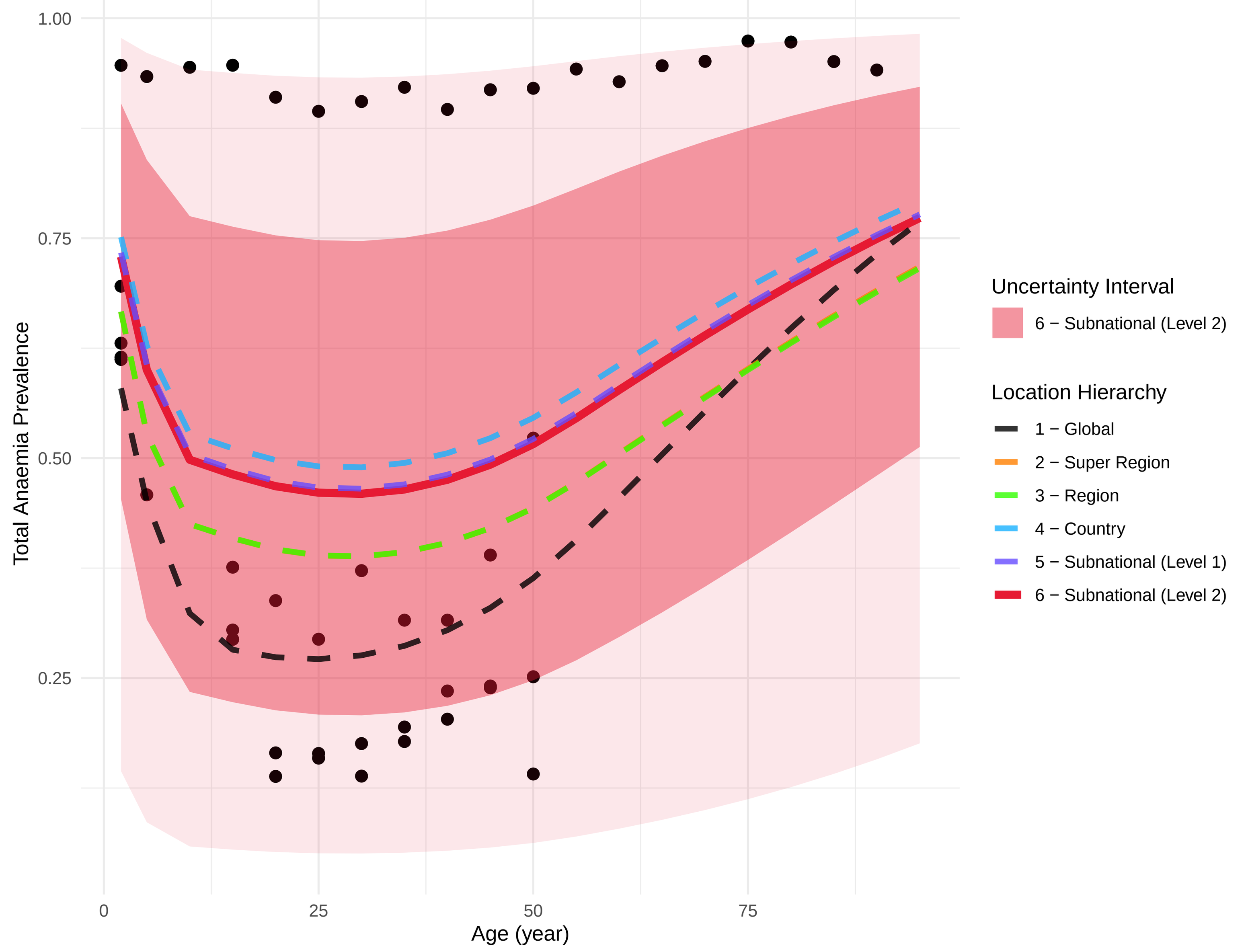

MR−BRT Age/Sex Fits − Males 2+ Years Old − Total Anaemia Prevalence − Uttarakhand, Urban
WHO−2024 Adjustment Method

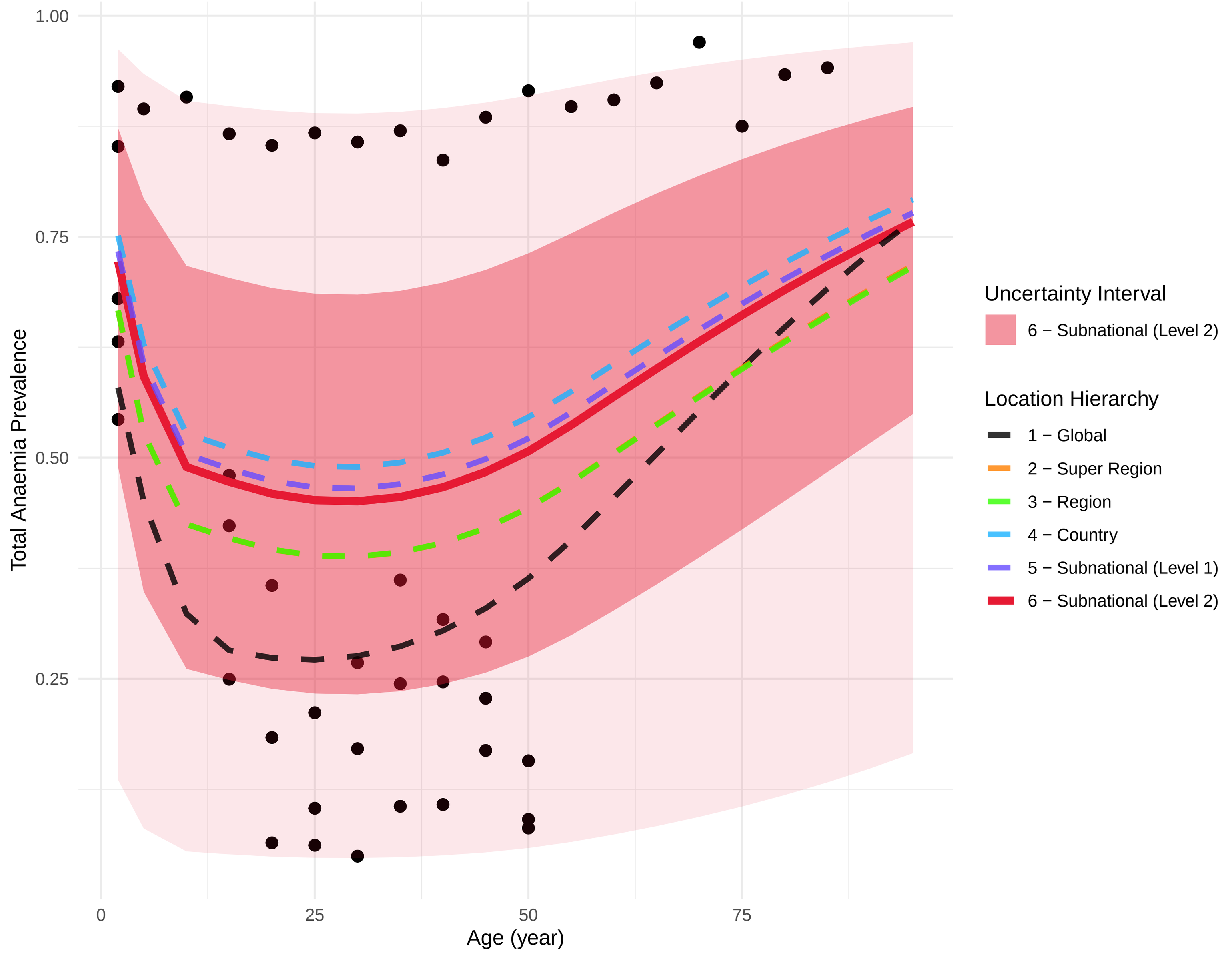

MR−BRT Age/Sex Fits − Males 2+ Years Old − Total Anaemia Prevalence − West Bengal
WHO−2024 Adjustment Method

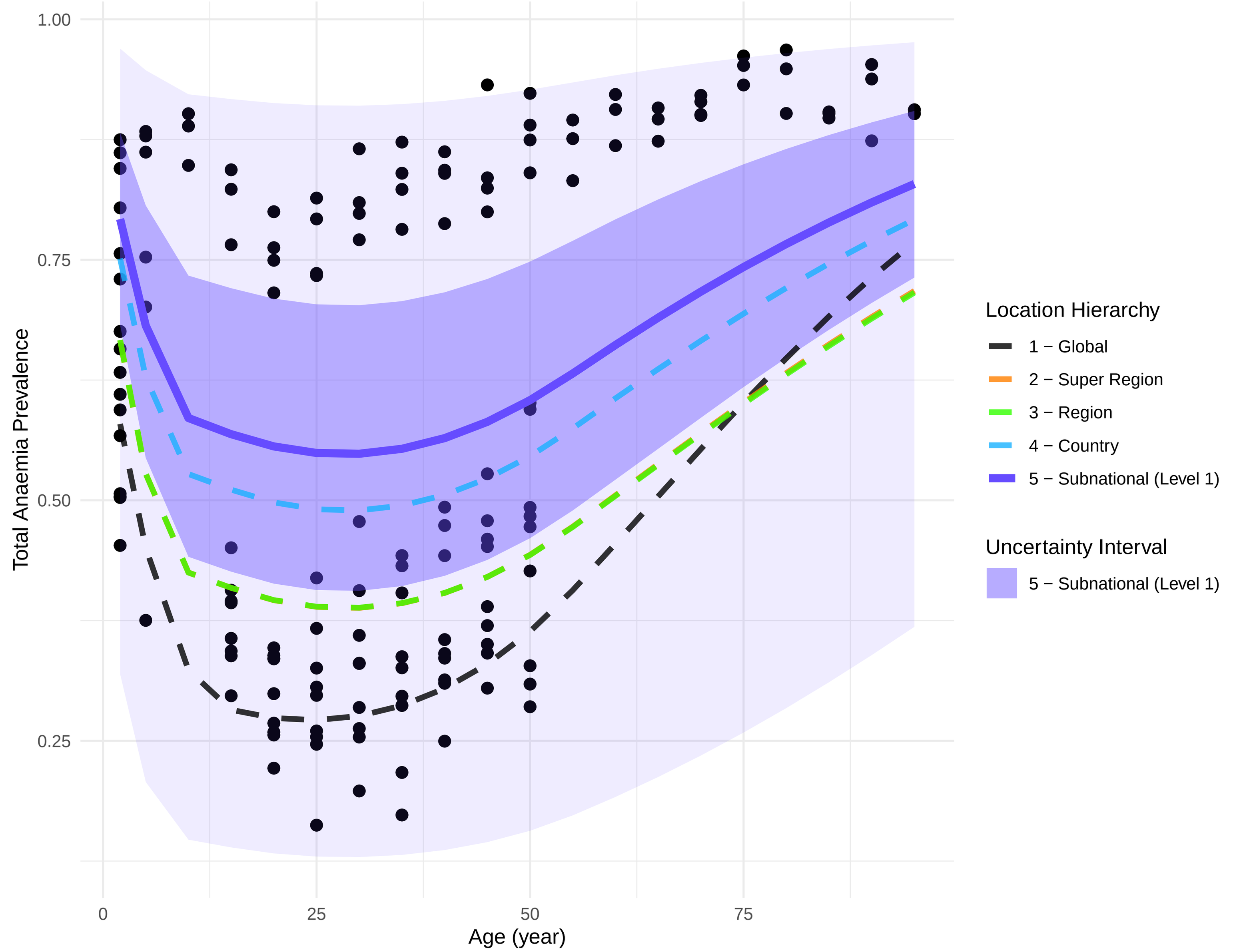

MR−BRT Age/Sex Fits − Males 2+ Years Old − Total Anaemia Prevalence − West Bengal, Rural
WHO−2024 Adjustment Method

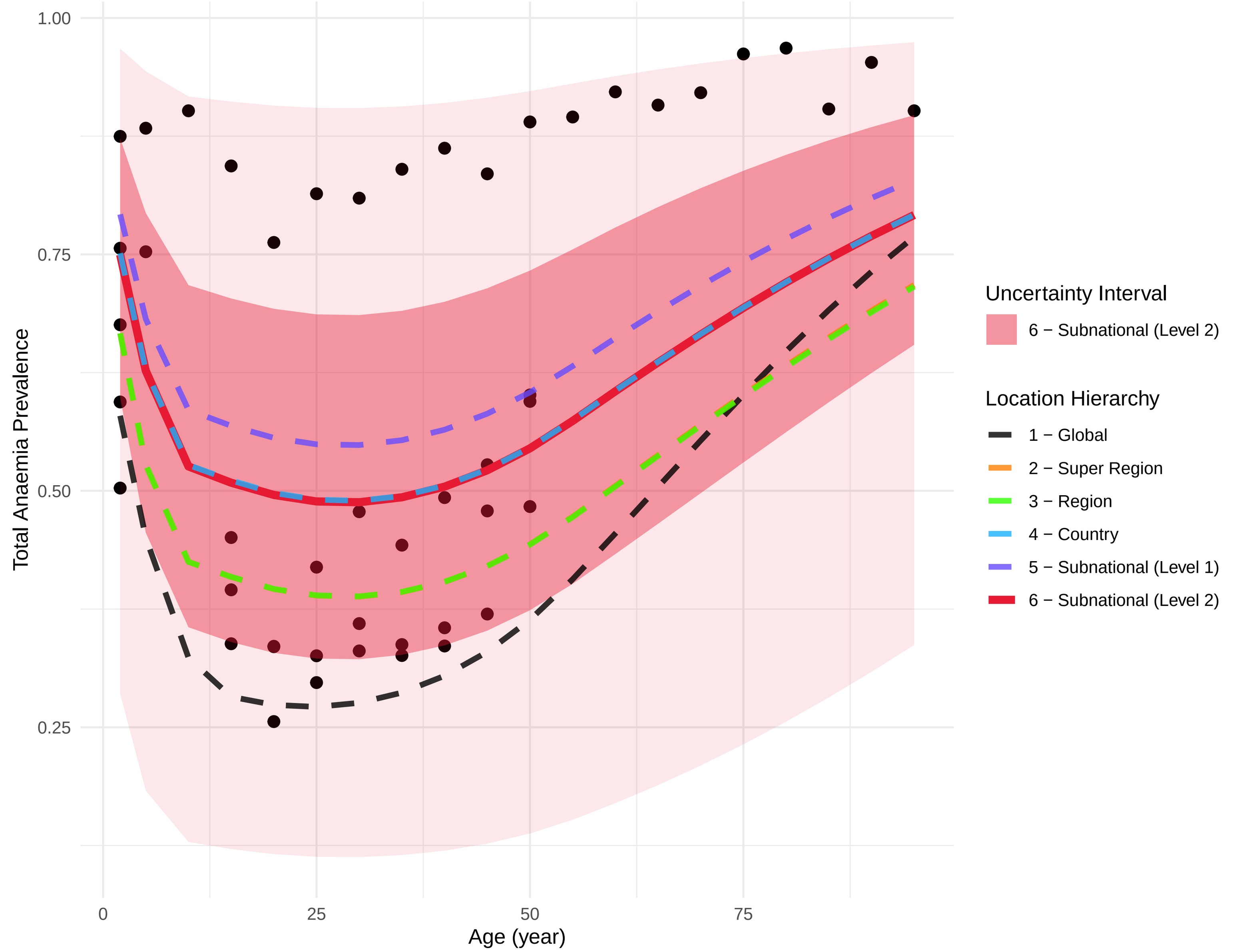

MR−BRT Age/Sex Fits − Males 2+ Years Old − Total Anaemia Prevalence − West Bengal, Urban
WHO−2024 Adjustment Method

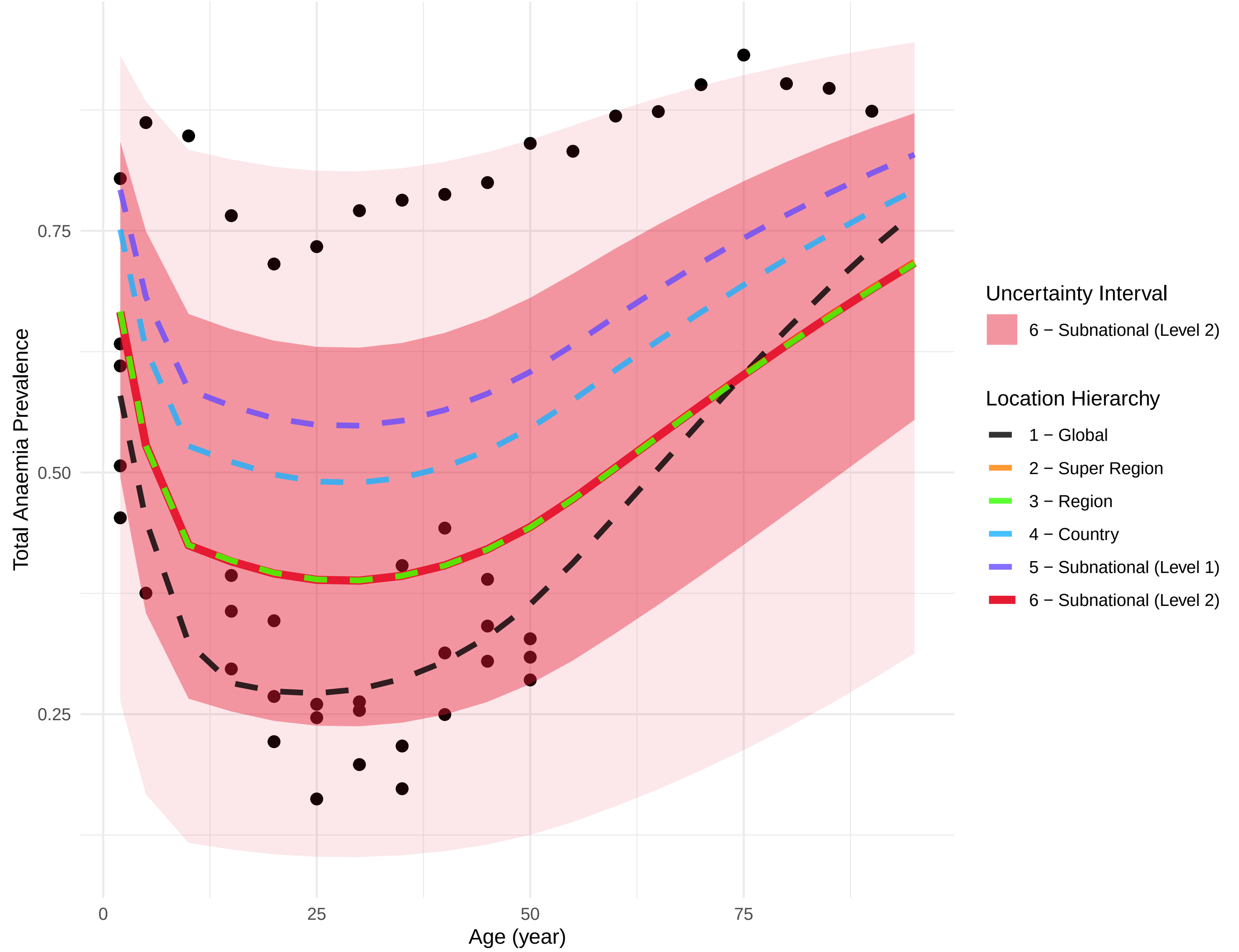

MR−BRT Age/Sex Fits − Males 2+ Years Old − Total Anaemia Prevalence − Nepal
WHO−2024 Adjustment Method

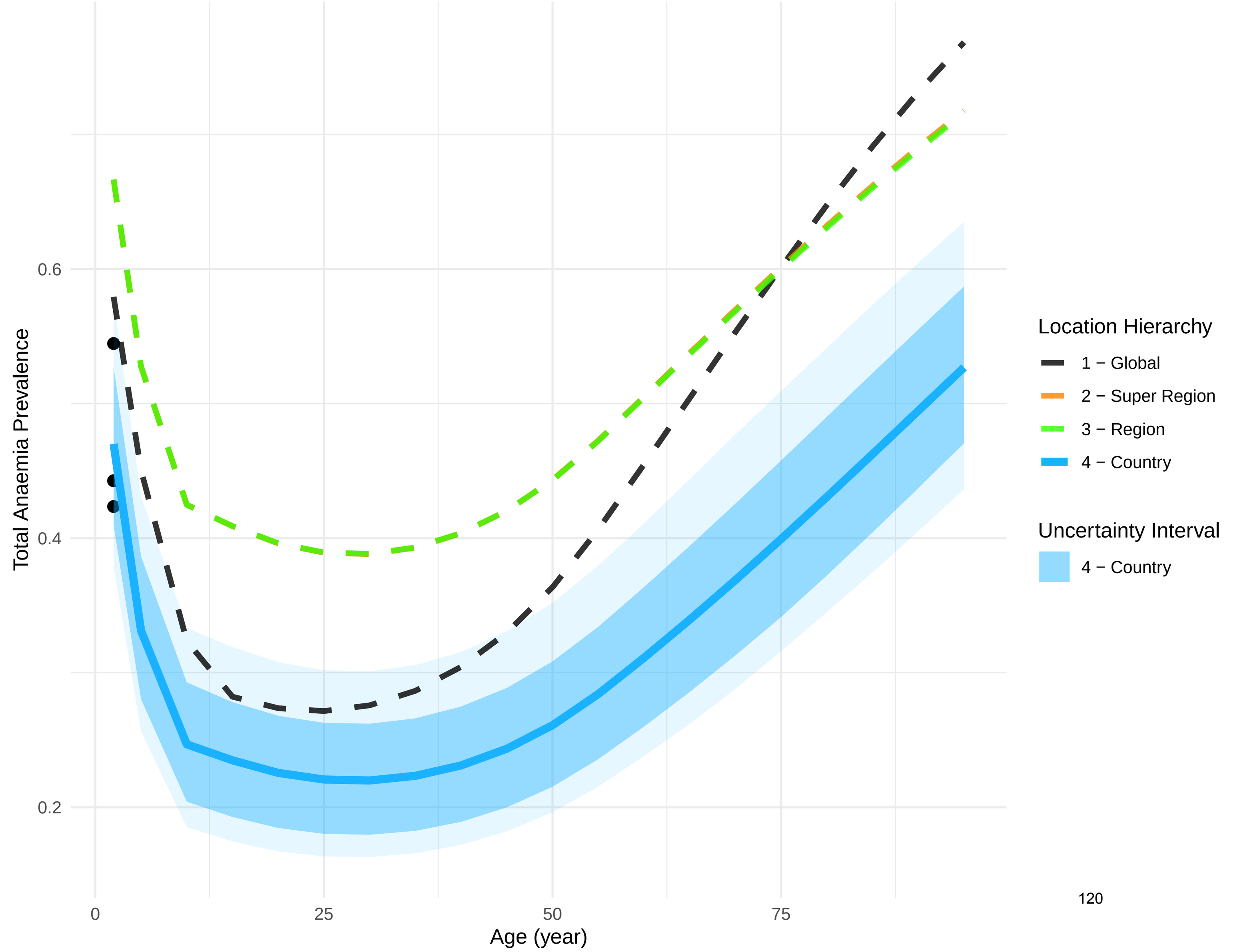

MR−BRT Age/Sex Fits − Males 2+ Years Old − Total Anaemia Prevalence − Southeast Asia, East Asia, and Oceania
WHO−2024 Adjustment Method

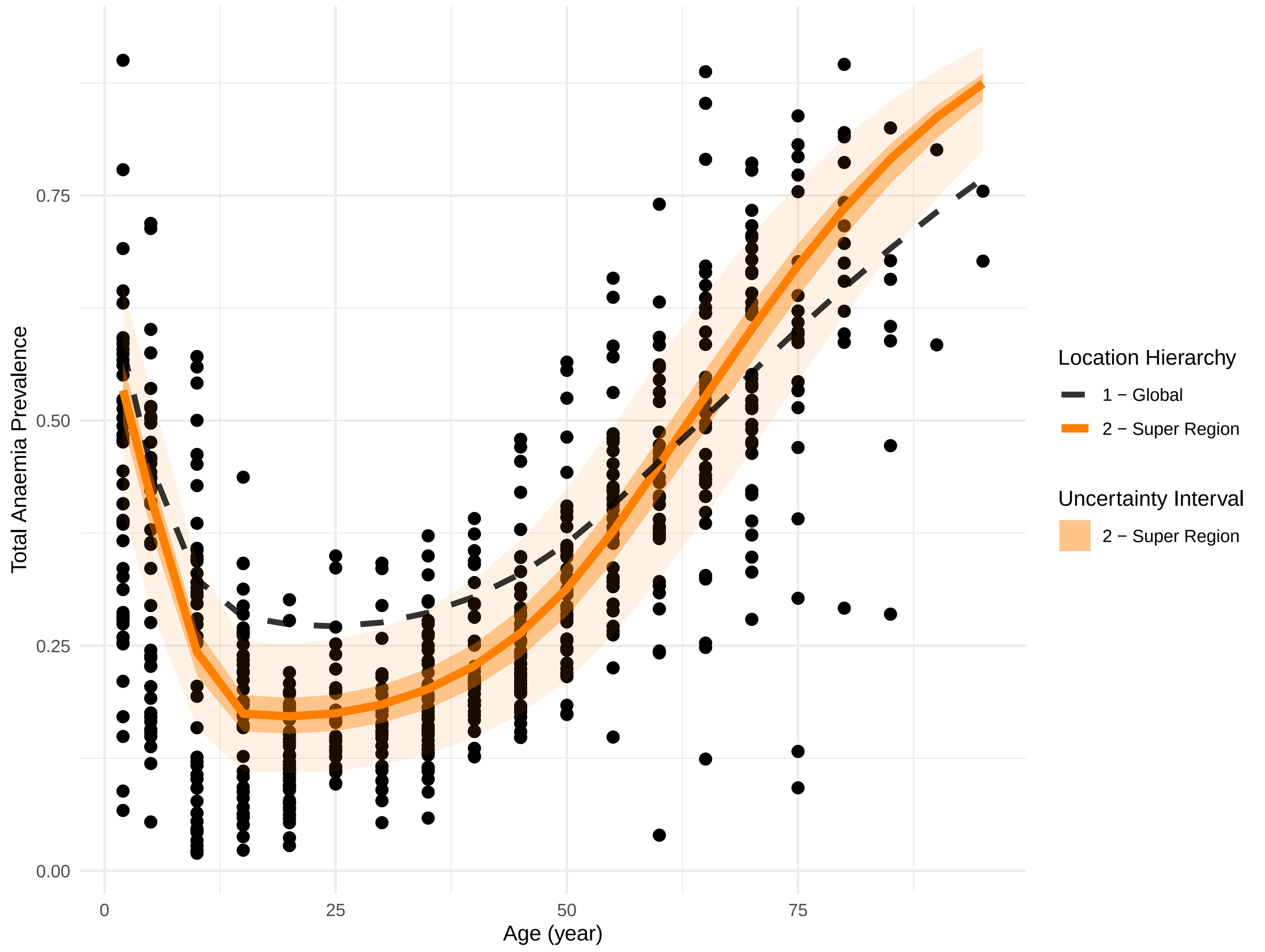

MR−BRT Age/Sex Fits − Males 2+ Years Old − Total Anaemia Prevalence − Southeast Asia
WHO−2024 Adjustment Method

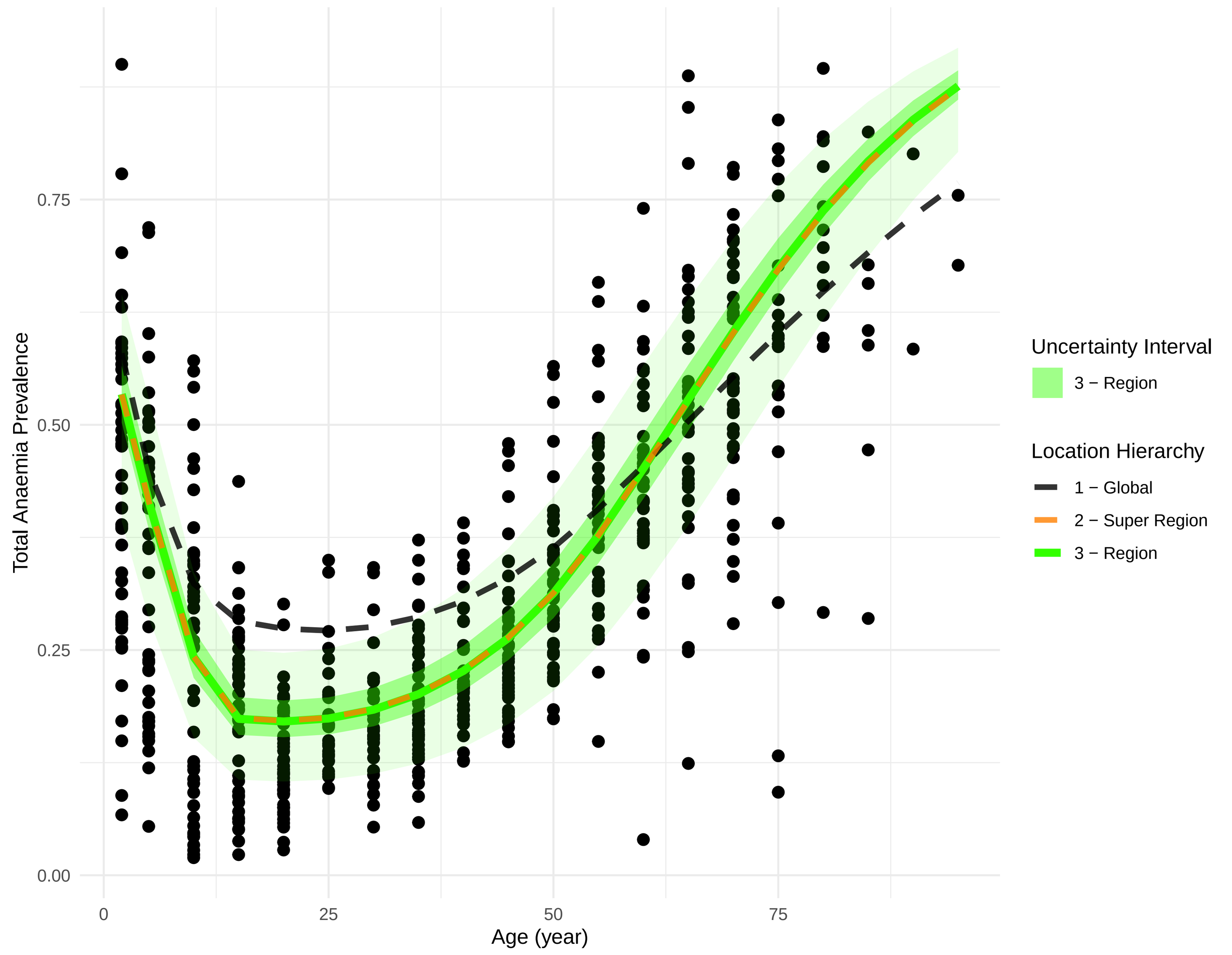

MR−BRT Age/Sex Fits − Males 2+ Years Old − Total Anaemia Prevalence − Cambodia
WHO−2024 Adjustment Method

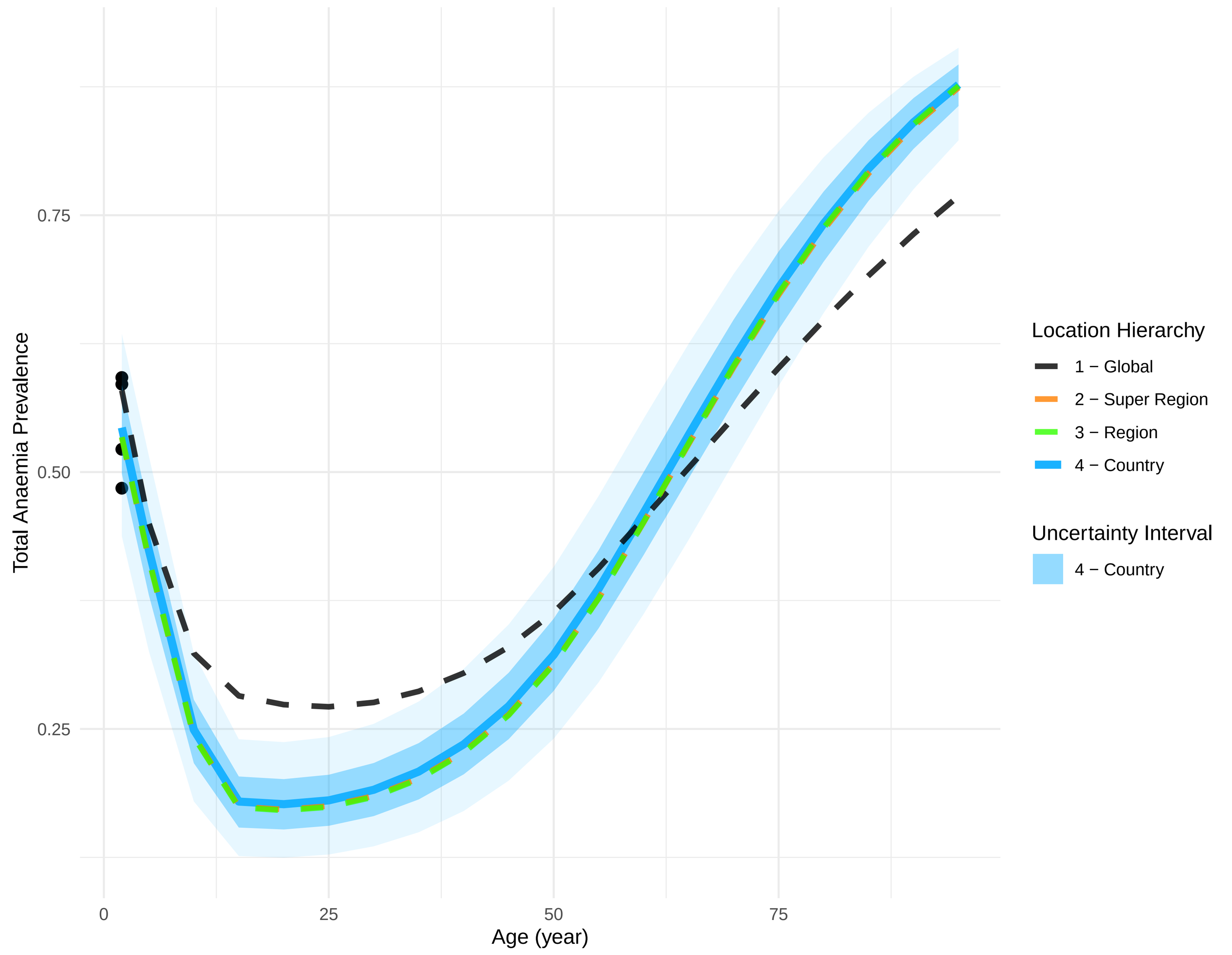

MR−BRT Age/Sex Fits − Males 2+ Years Old − Total Anaemia Prevalence − Indonesia
WHO−2024 Adjustment Method

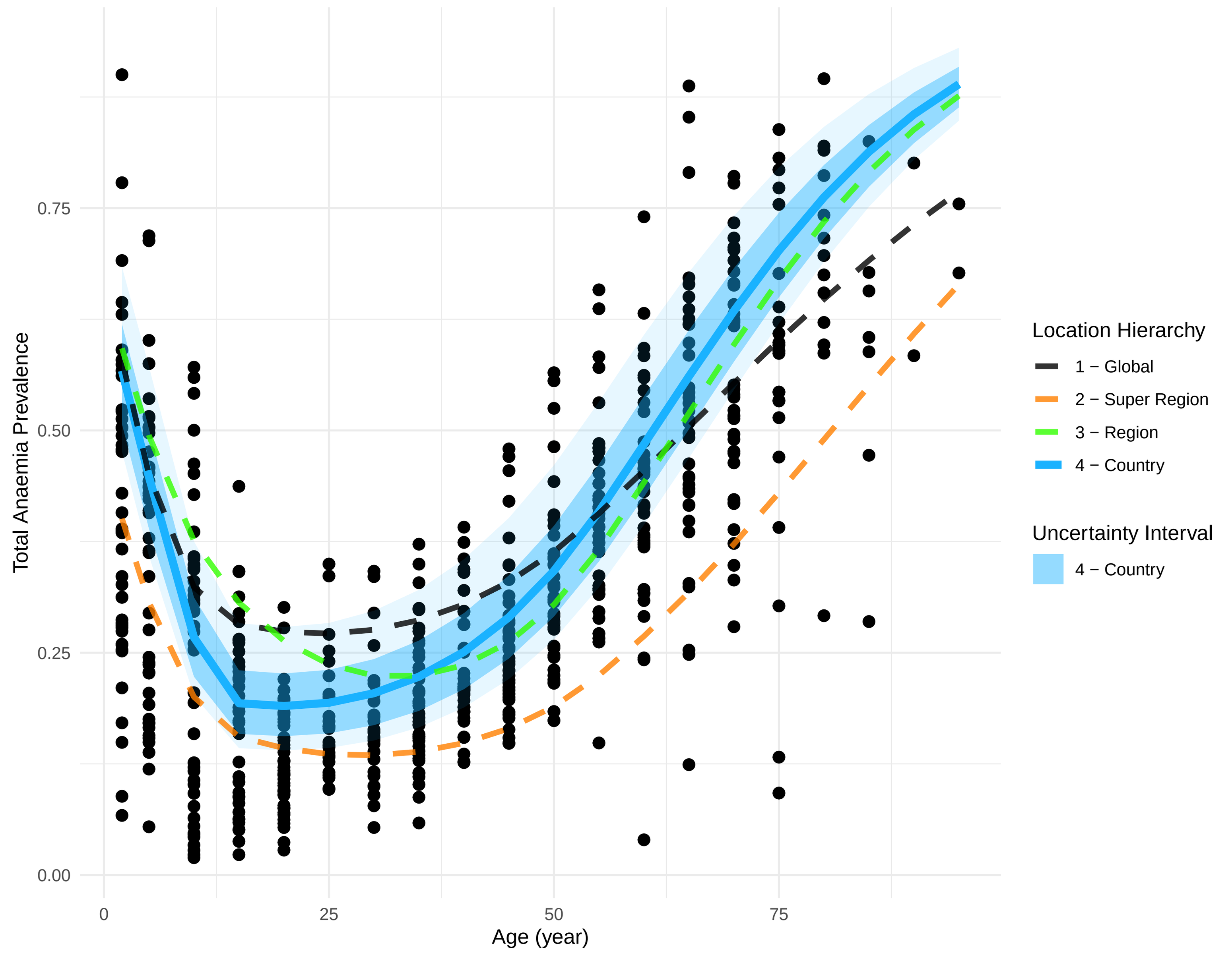

MR−BRT Age/Sex Fits − Males 2+ Years Old − Total Anaemia Prevalence − Bali
WHO−2024 Adjustment Method

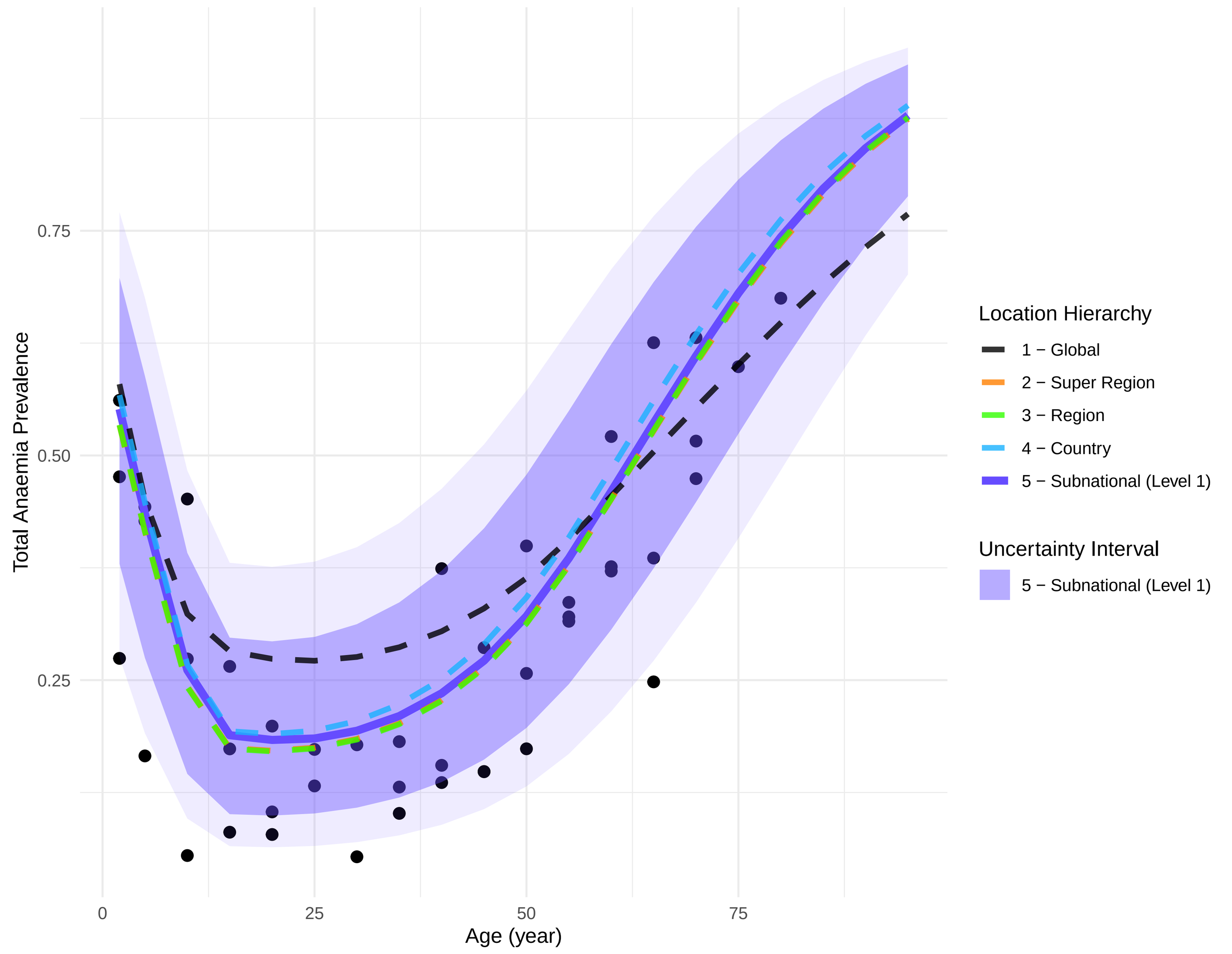

MR−BRT Age/Sex Fits − Males 2+ Years Old − Total Anaemia Prevalence − Bangka−Belitung Islands
WHO−2024 Adjustment Method

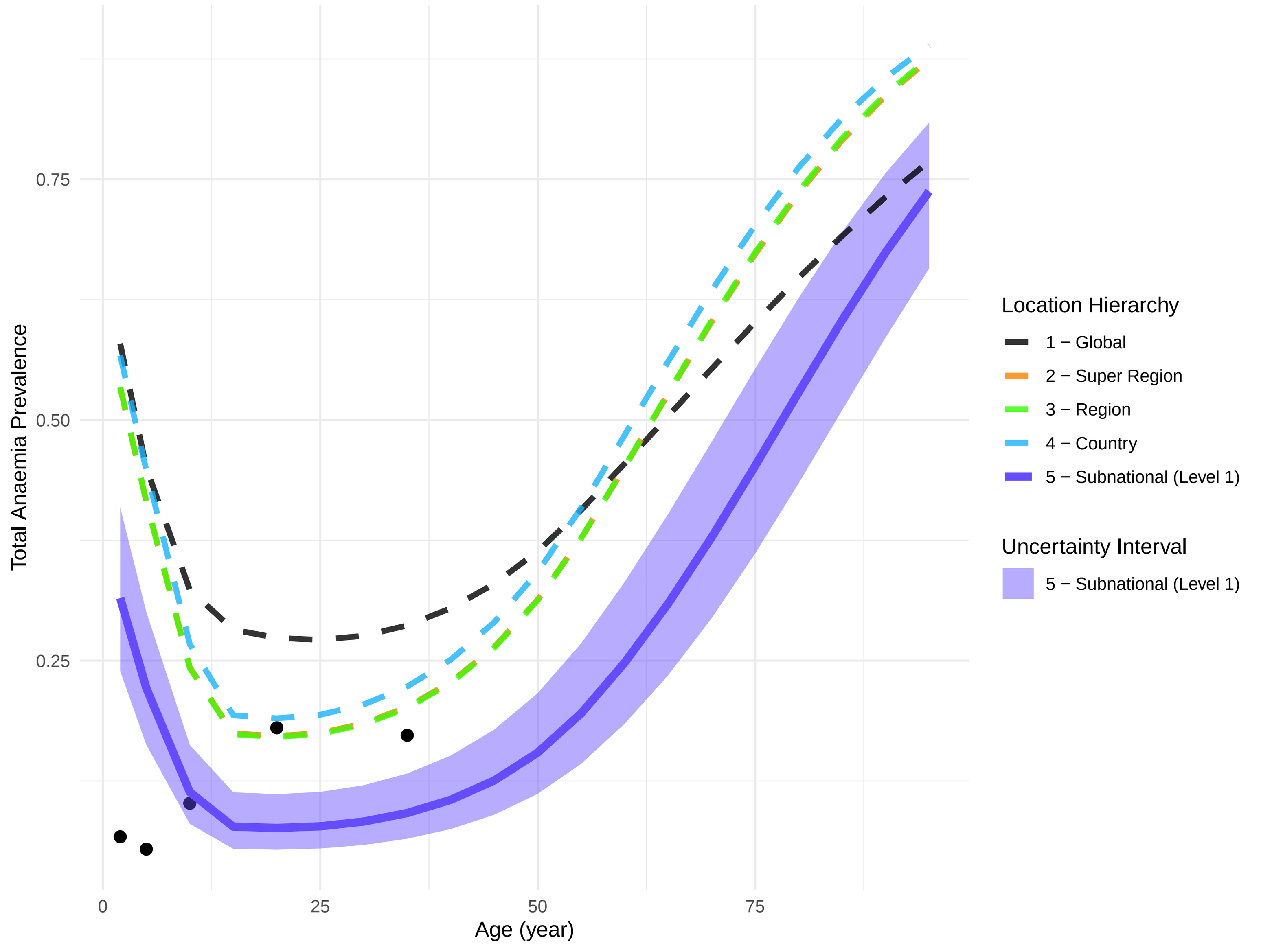

MR−BRT Age/Sex Fits − Males 2+ Years Old − Total Anaemia Prevalence − Banten
WHO−2024 Adjustment Method

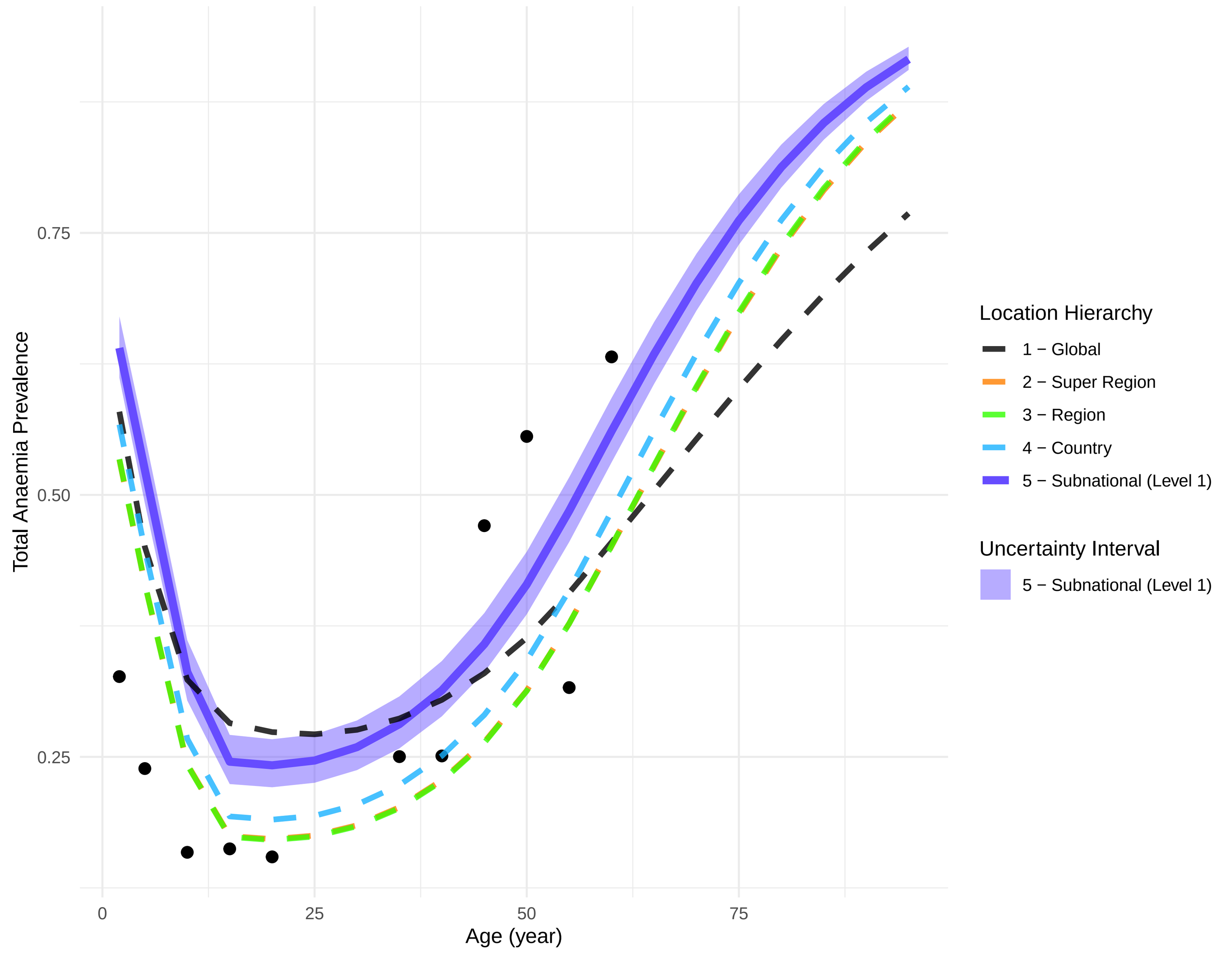

MR−BRT Age/Sex Fits − Males 2+ Years Old − Total Anaemia Prevalence − Jakarta
WHO−2024 Adjustment Method

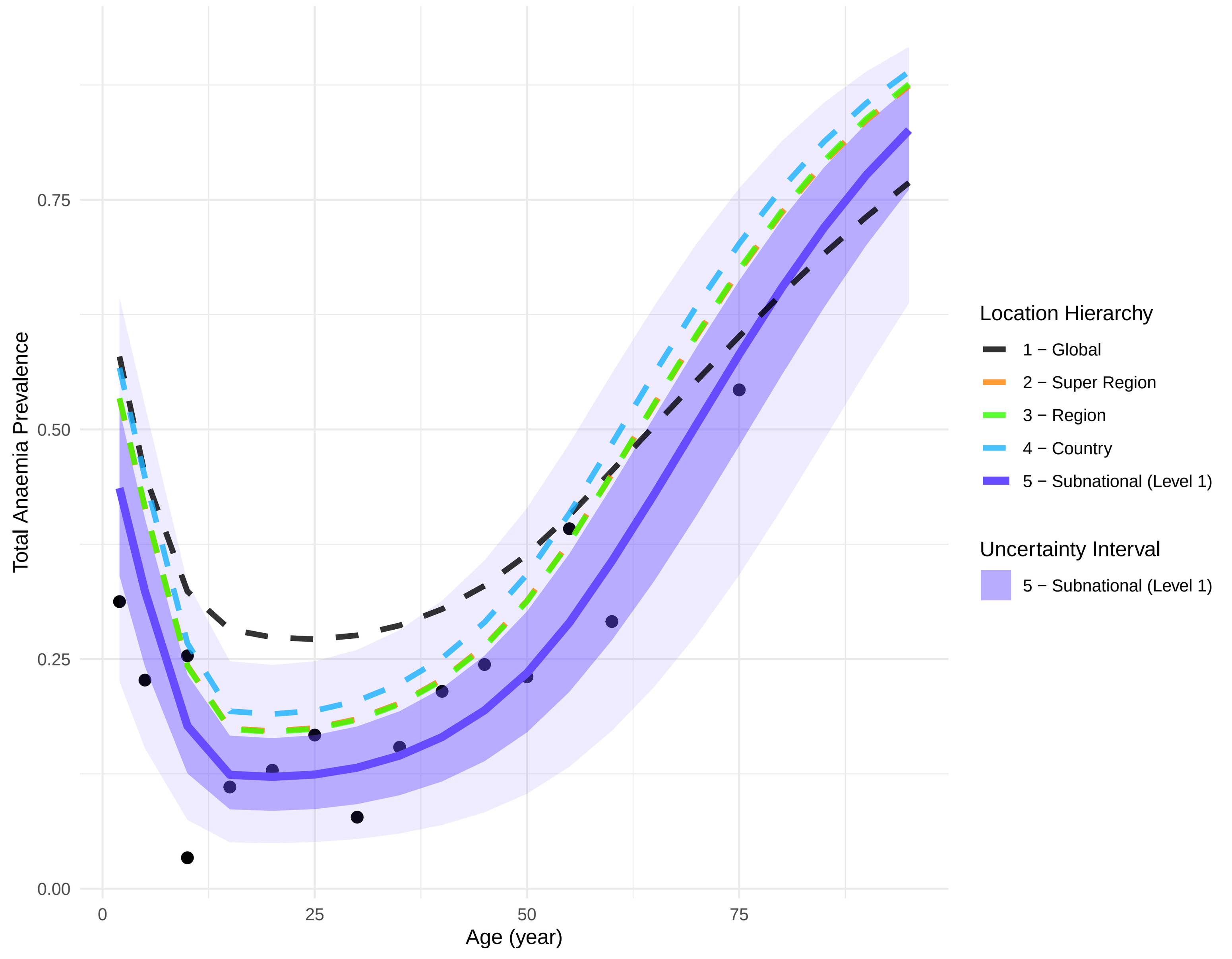

MR−BRT Age/Sex Fits − Males 2+ Years Old − Total Anaemia Prevalence − West Java
WHO−2024 Adjustment Method

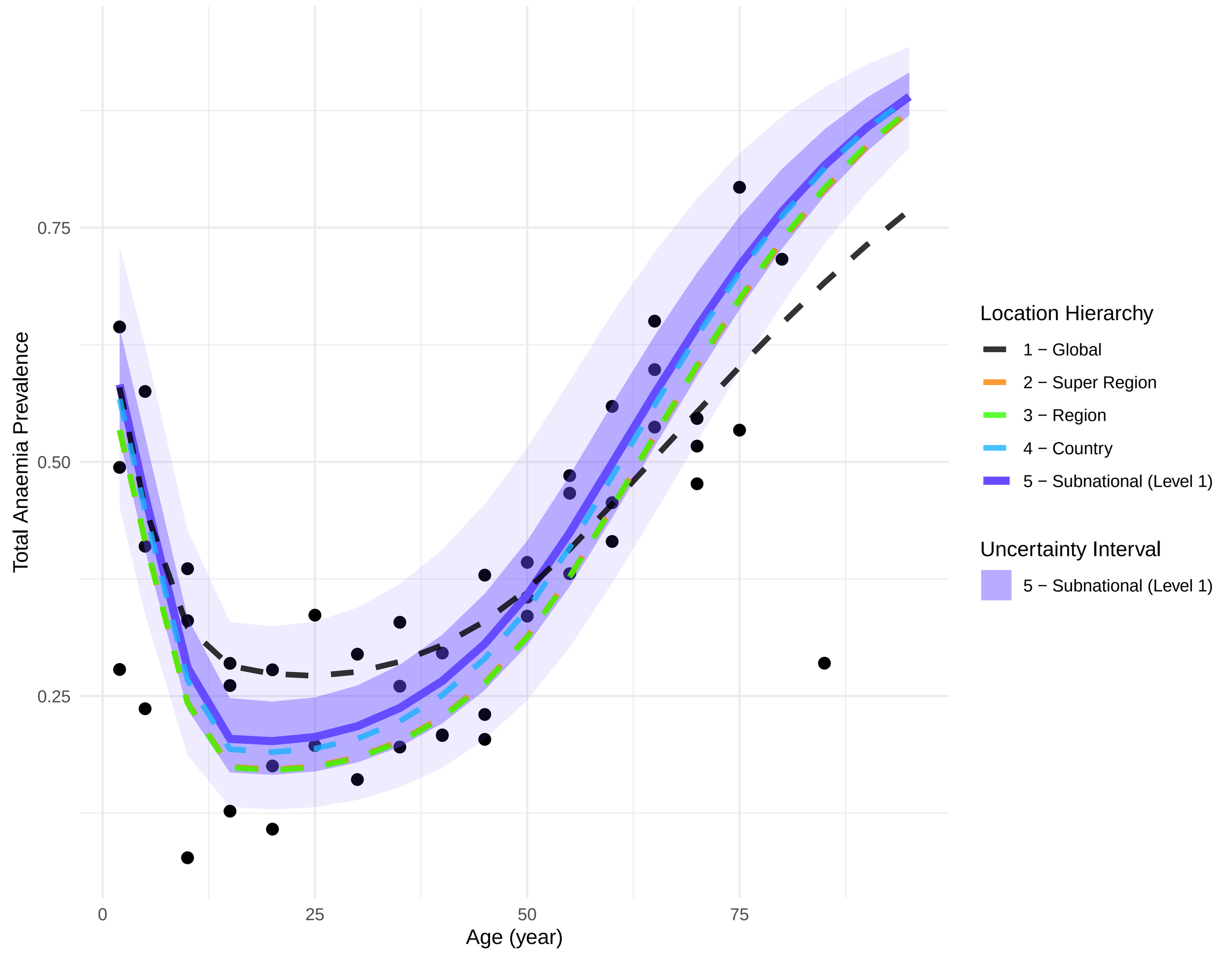

MR−BRT Age/Sex Fits − Males 2+ Years Old − Total Anaemia Prevalence − Central Java
WHO−2024 Adjustment Method

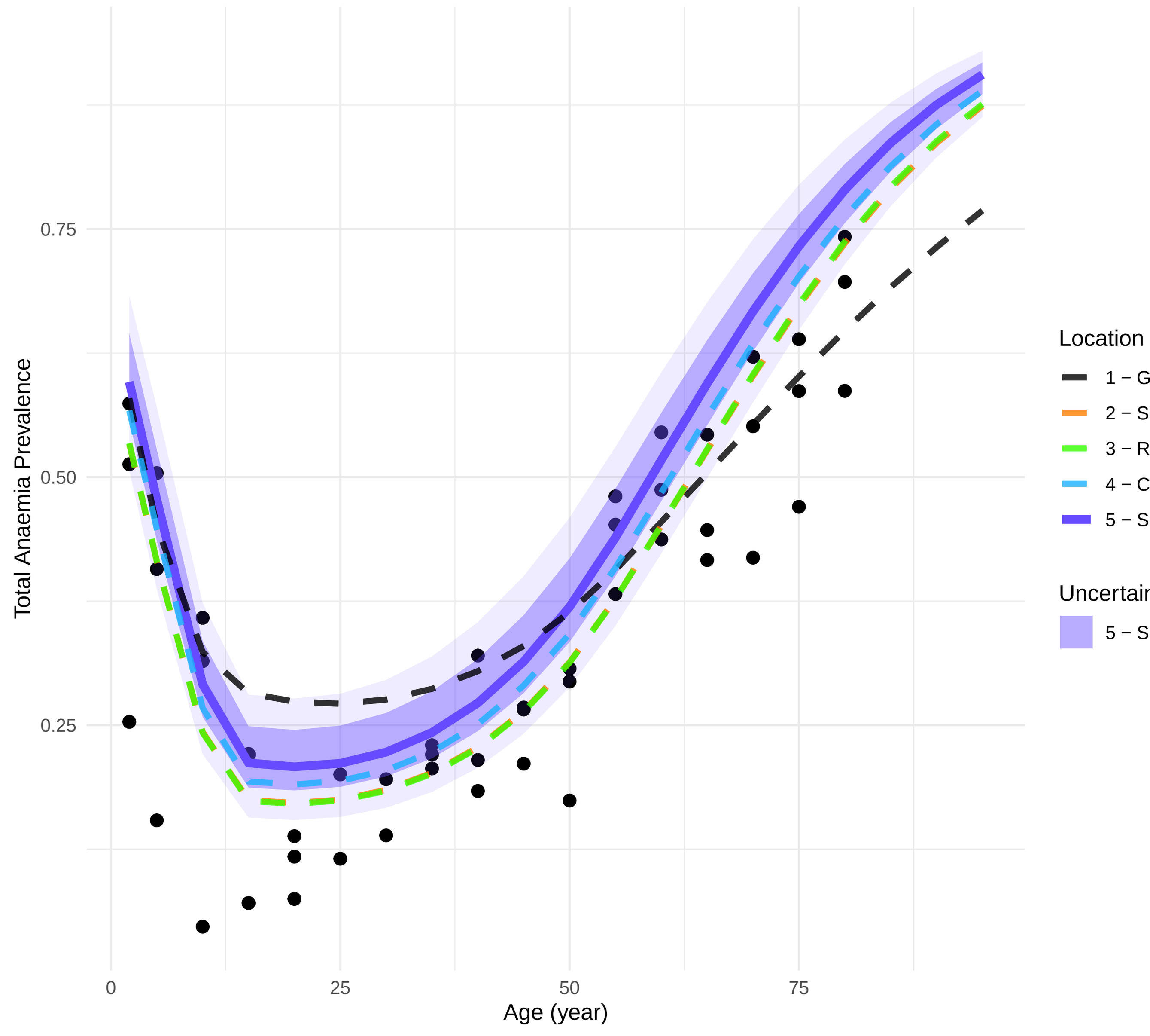

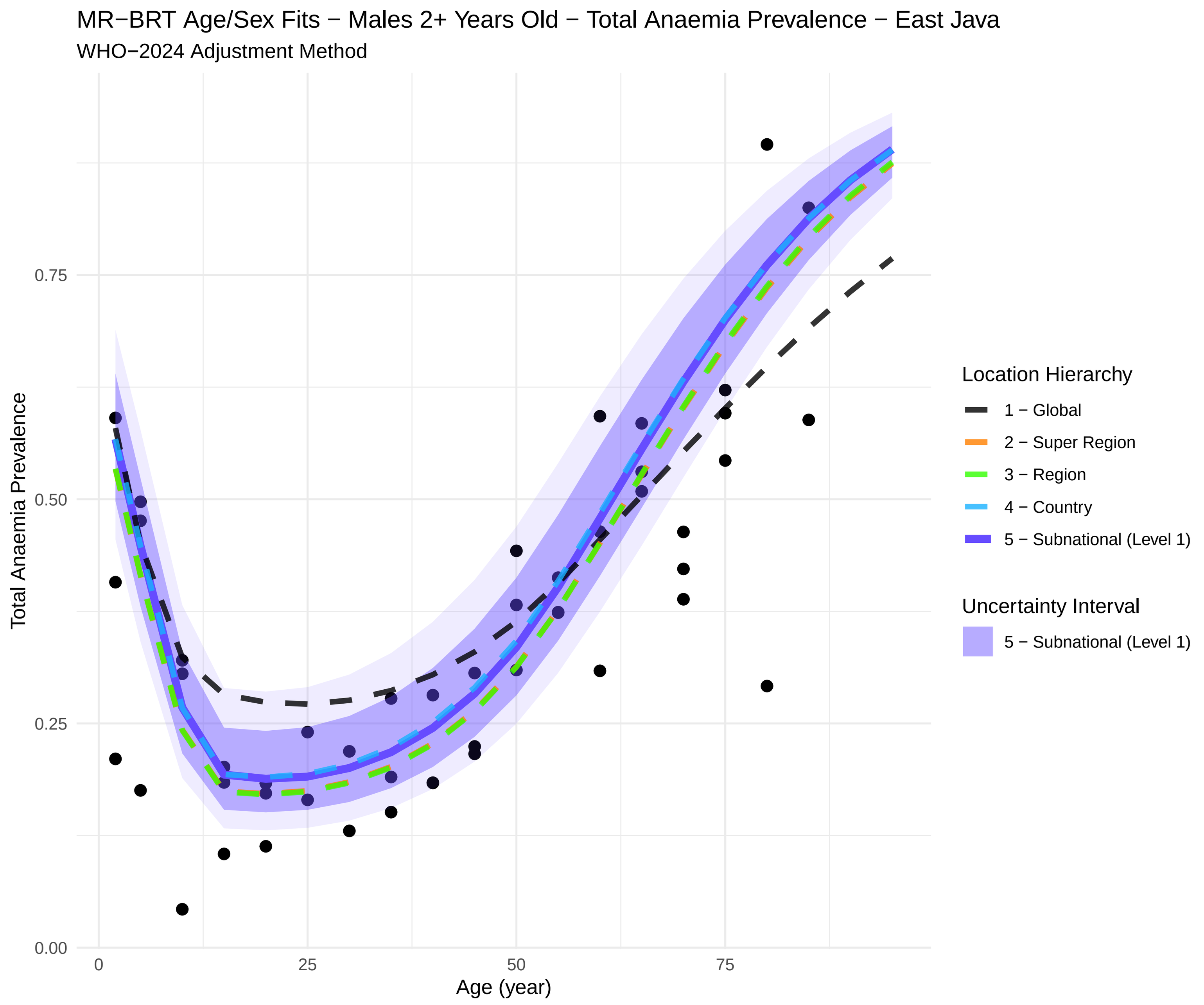
MR−BRT Age/Sex Fits − Males 2+ Years Old − Total Anaemia Prevalence − East Java
WHO−2024 Adjustment Method
Total Anaemia Prevalence
Age (year)
Location Hierarchy
1 − Global
2 − Super Region
3 − Region
4 − Country
5 − Subnational (Level 1)
Uncertainty Interval
5 − Subnational (Level 1)

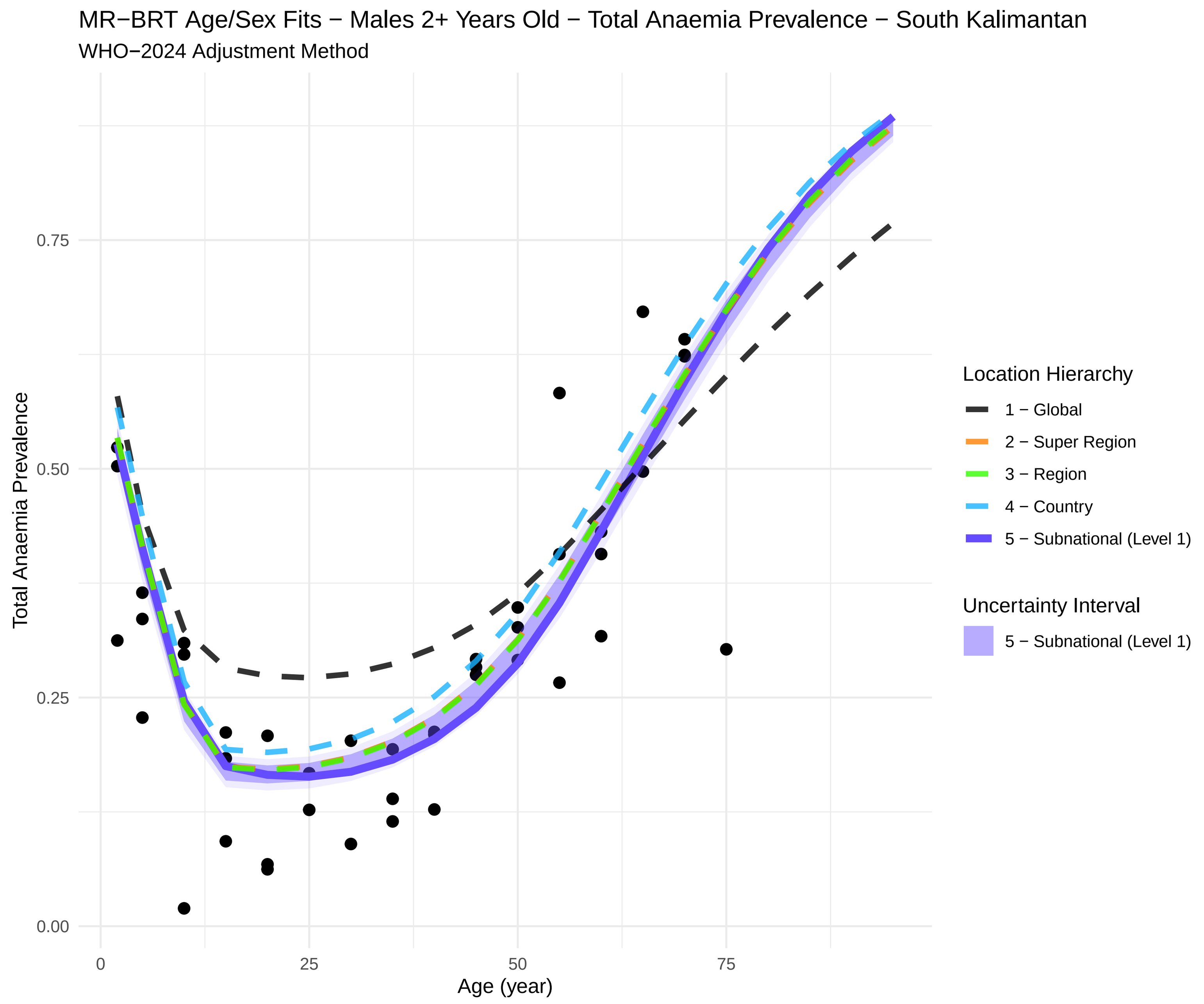
MR−BRT Age/Sex Fits − Males 2+ Years Old − Total Anaemia Prevalence − South Kalimantan
WHO−2024 Adjustment Method
Total Anaemia Prevalence
Age (year)
Location Hierarchy
1 − Global
2 − Super Region
3 − Region
4 − Country
5 − Subnational (Level 1)
Uncertainty Interval
5 − Subnational (Level 1)

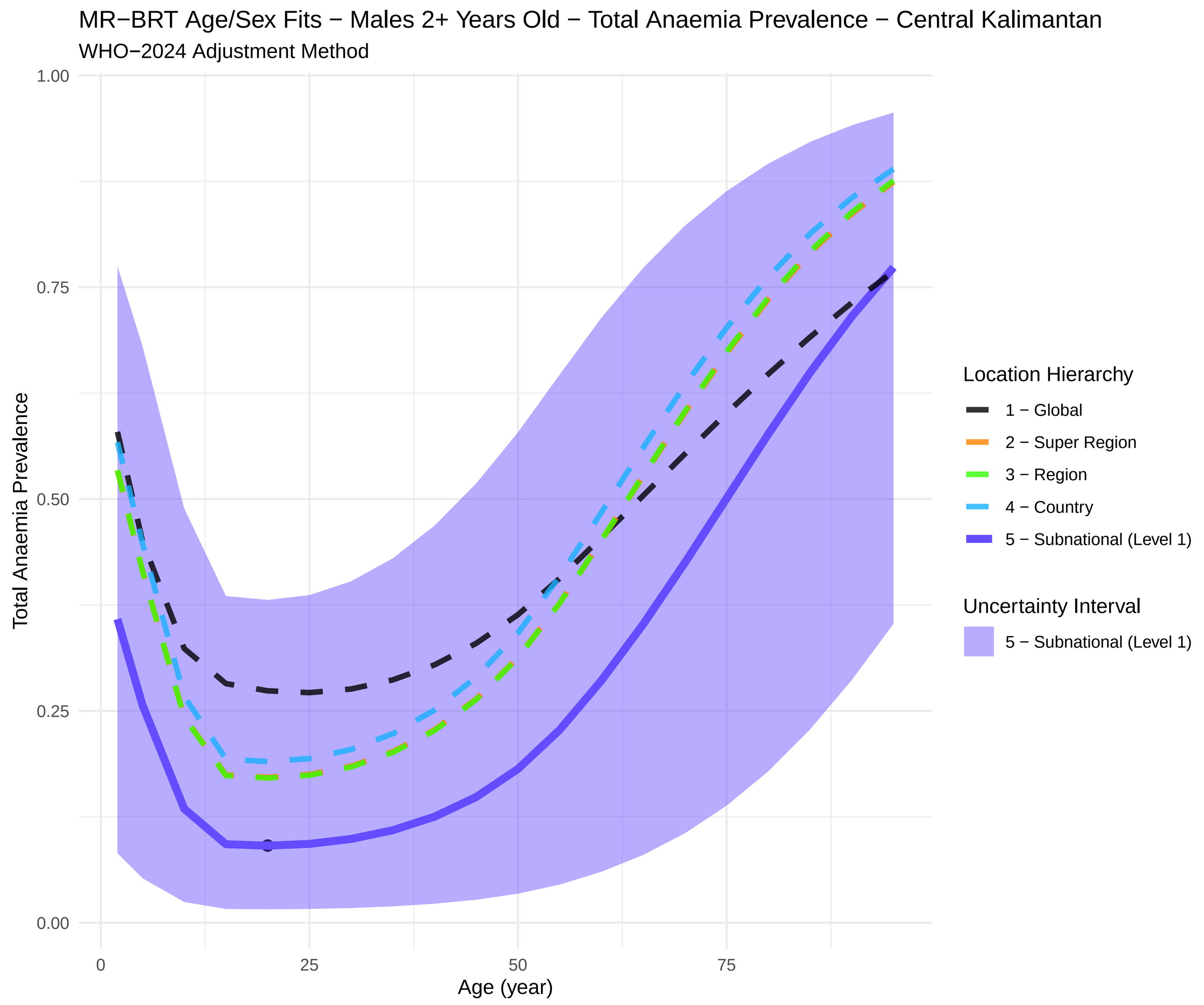
MR−BRT Age/Sex Fits − Males 2+ Years Old − Total Anaemia Prevalence − Central Kalimantan
WHO−2024 Adjustment Method
Total Anaemia Prevalence
Age (year)
Location Hierarchy
1 − Global
2 − Super Region
3 − Region
4 − Country
5 − Subnational (Level 1)
Uncertainty Interval
5 − Subnational (Level 1)

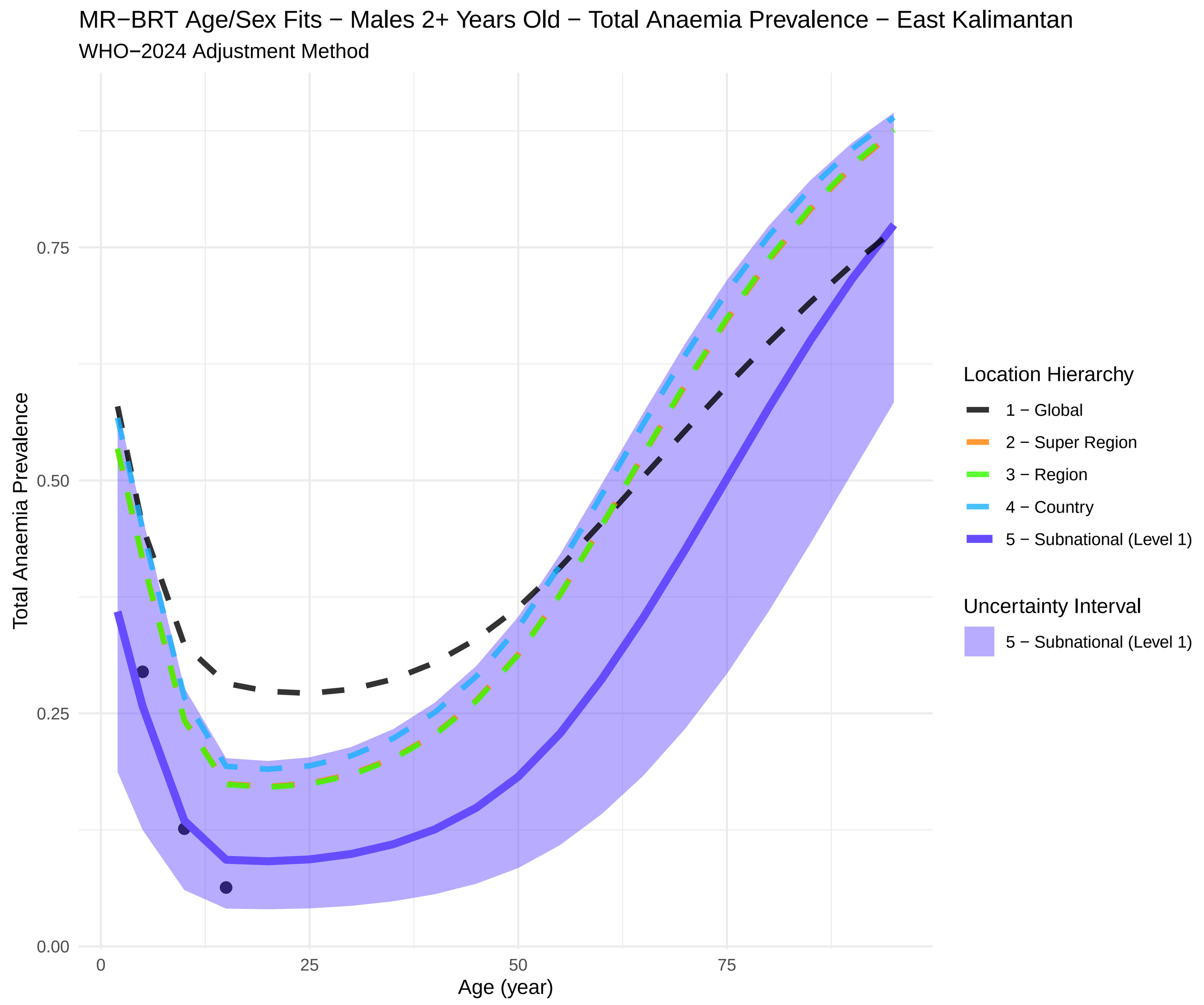
MR−BRT Age/Sex Fits − Males 2+ Years Old − Total Anaemia Prevalence − East Kalimantan
WHO−2024 Adjustment Method
Total Anaemia Prevalence
Age (year)
Location Hierarchy
1 − Global
2 − Super Region
3 − Region
4 − Country
5 − Subnational (Level 1)
Uncertainty Interval
5 − Subnational (Level 1)

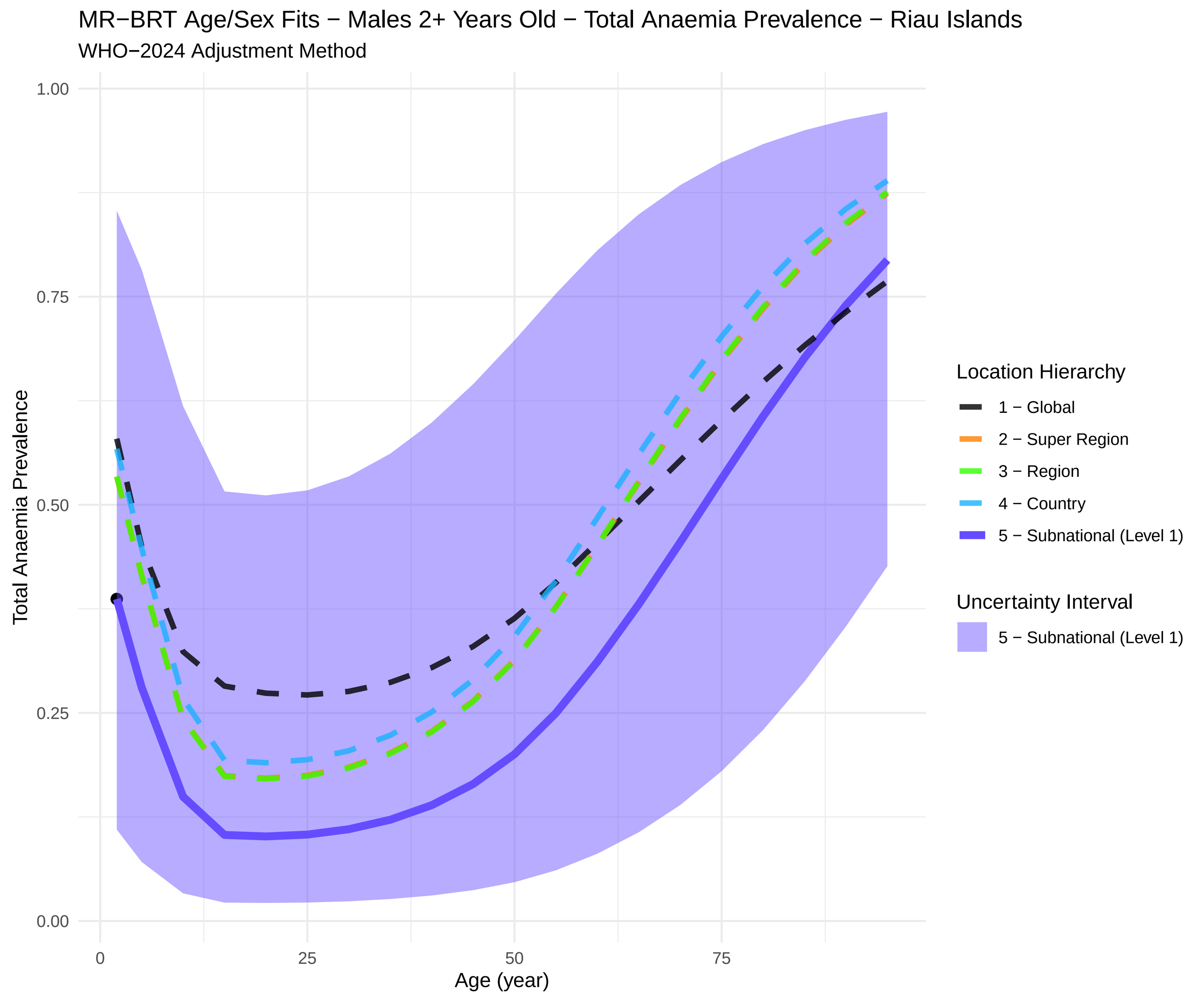
MR−BRT Age/Sex Fits − Males 2+ Years Old − Total Anaemia Prevalence − Riau Islands
WHO−2024 Adjustment Method
Total Anaemia Prevalence
Age (year)
Location Hierarchy
1 − Global
2 − Super Region
3 − Region
4 − Country
5 − Subnational (Level 1)
Uncertainty Interval
5 − Subnational (Level 1)

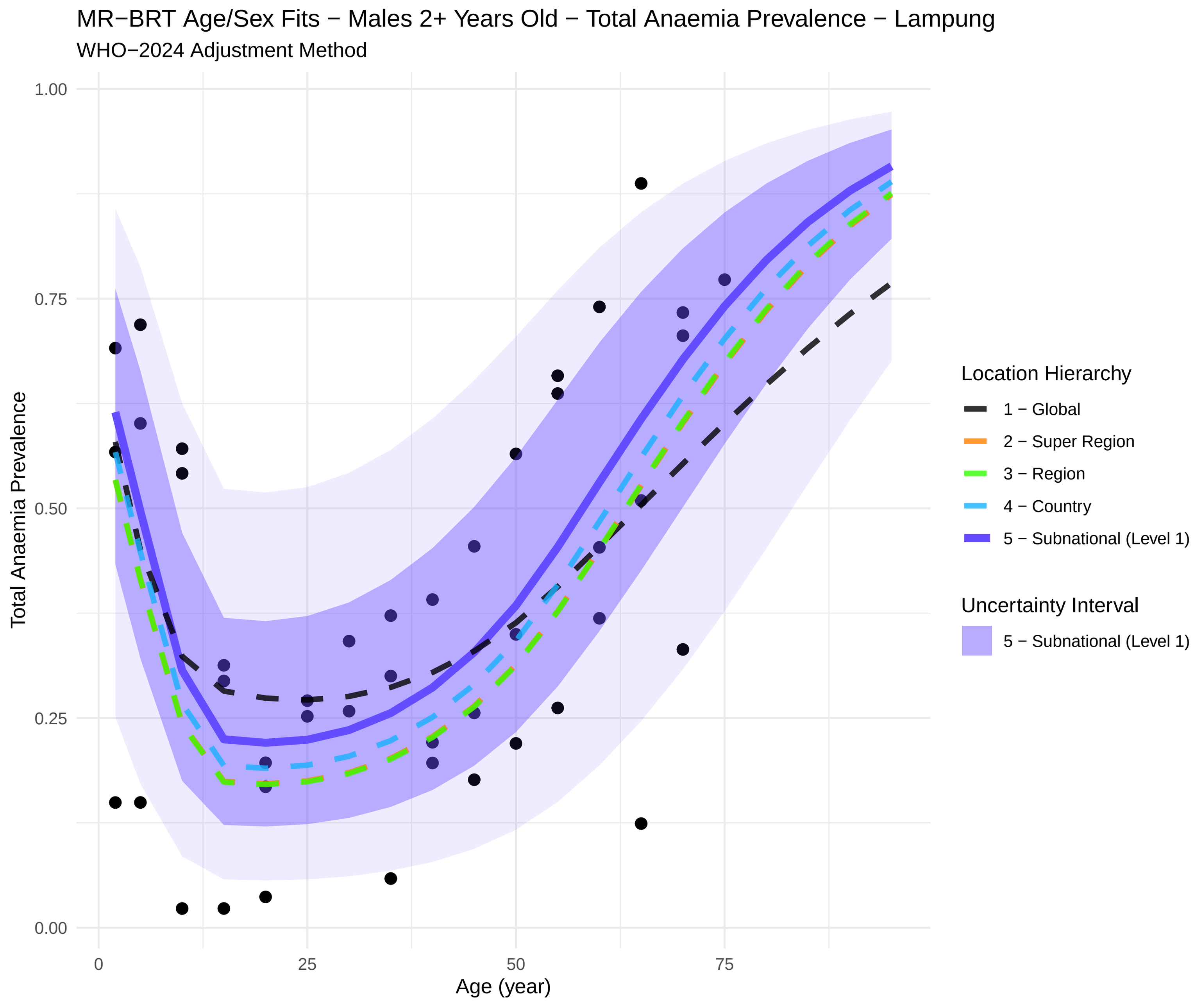
MR−BRT Age/Sex Fits − Males 2+ Years Old − Total Anaemia Prevalence − Lampung
WHO−2024 Adjustment Method
Total Anaemia Prevalence
Age (year)
Location Hierarchy
1 − Global
2 − Super Region
3 − Region
4 − Country
5 − Subnational (Level 1)
Uncertainty Interval
5 − Subnational (Level 1)

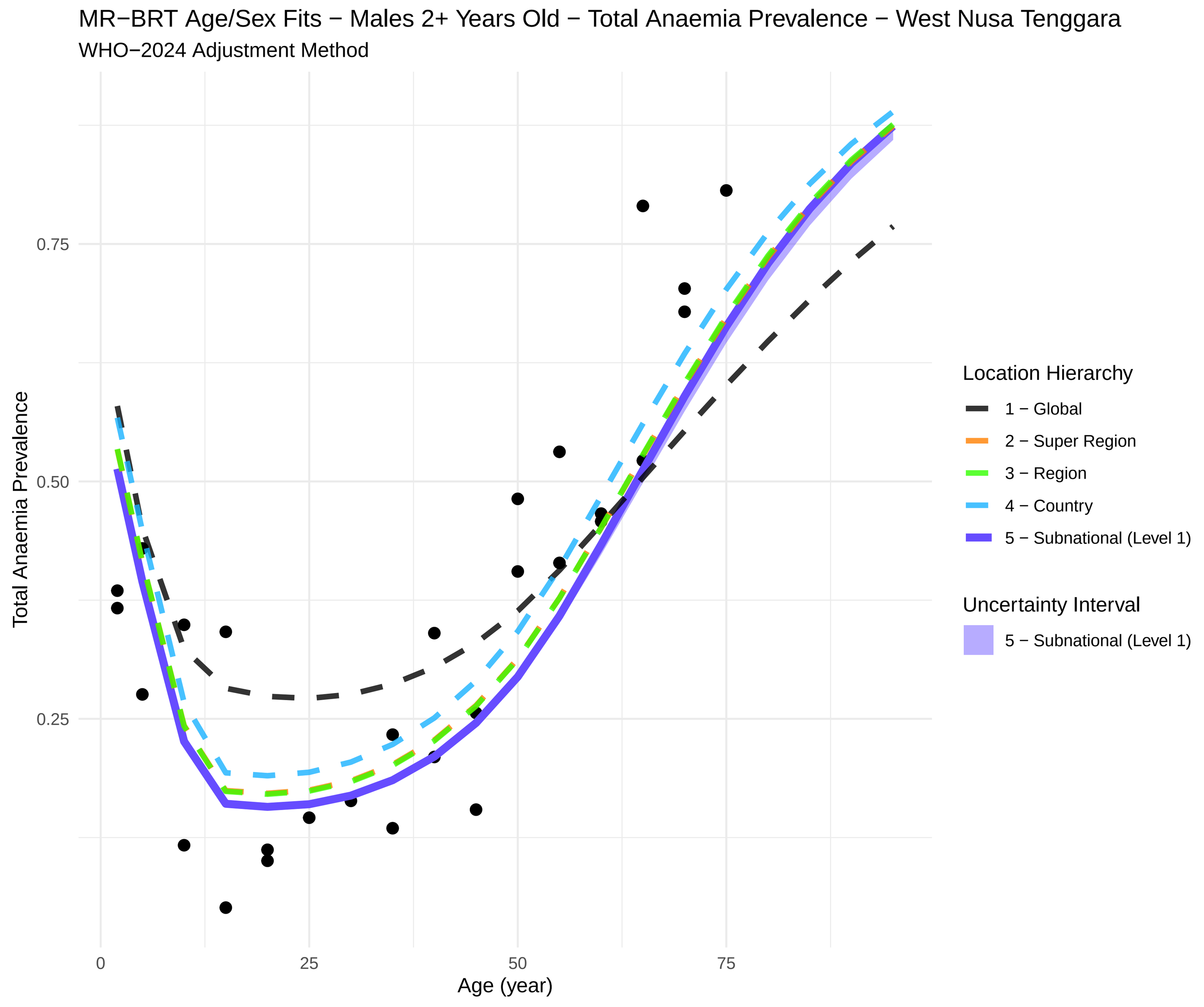
MR−BRT Age/Sex Fits − Males 2+ Years Old − Total Anaemia Prevalence − West Nusa Tenggara
WHO−2024 Adjustment Method
Total Anaemia Prevalence
Age (year)
Location Hierarchy
1 − Global
2 − Super Region
3 − Region
4 − Country
5 − Subnational (Level 1)
Uncertainty Interval
5 − Subnational (Level 1)

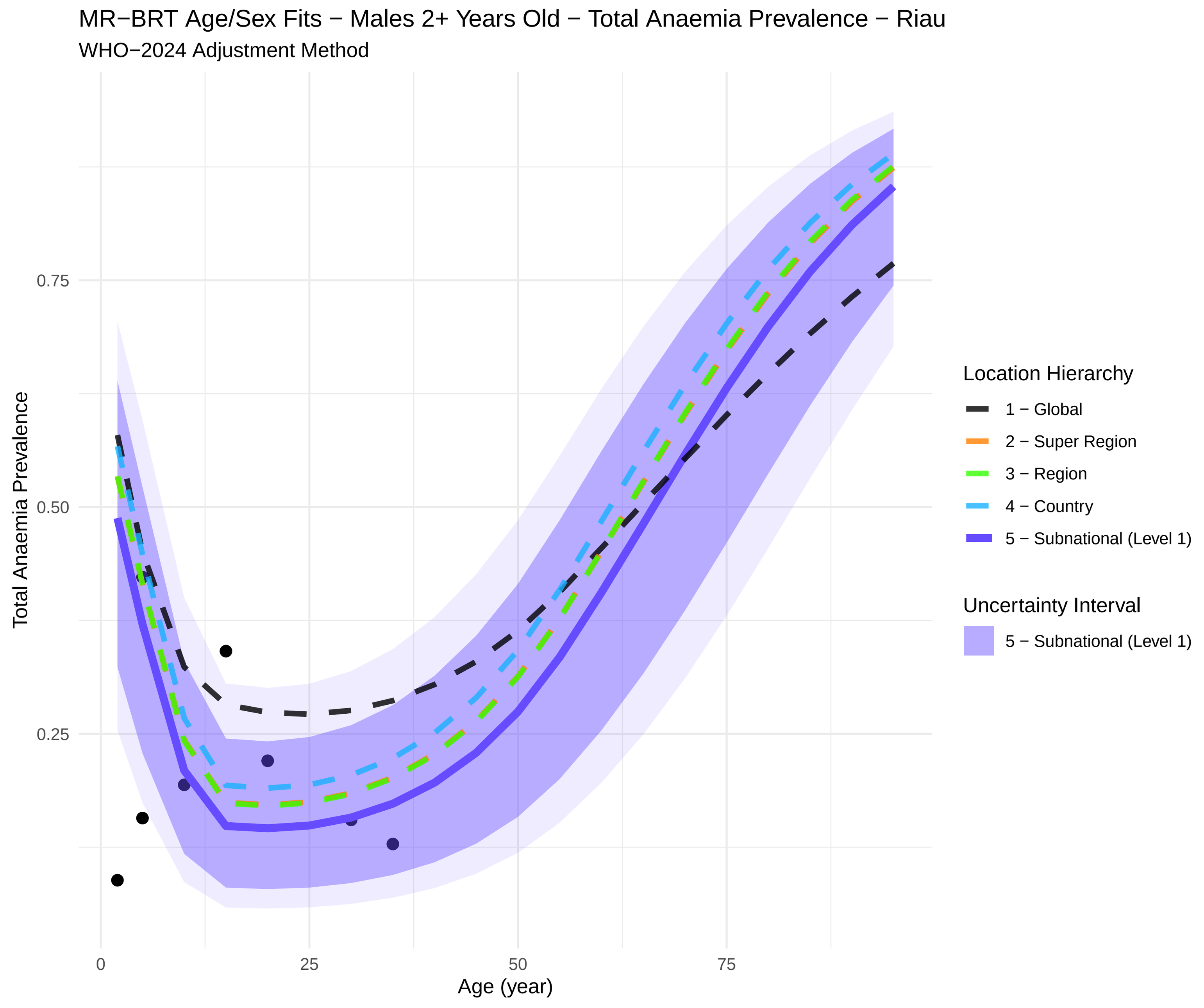
MR−BRT Age/Sex Fits − Males 2+ Years Old − Total Anaemia Prevalence − Riau
WHO−2024 Adjustment Method
Total Anaemia Prevalence
Age (year)
Location Hierarchy
1 − Global
2 − Super Region
3 − Region
4 − Country
5 − Subnational (Level 1)
Uncertainty Interval
5 − Subnational (Level 1)

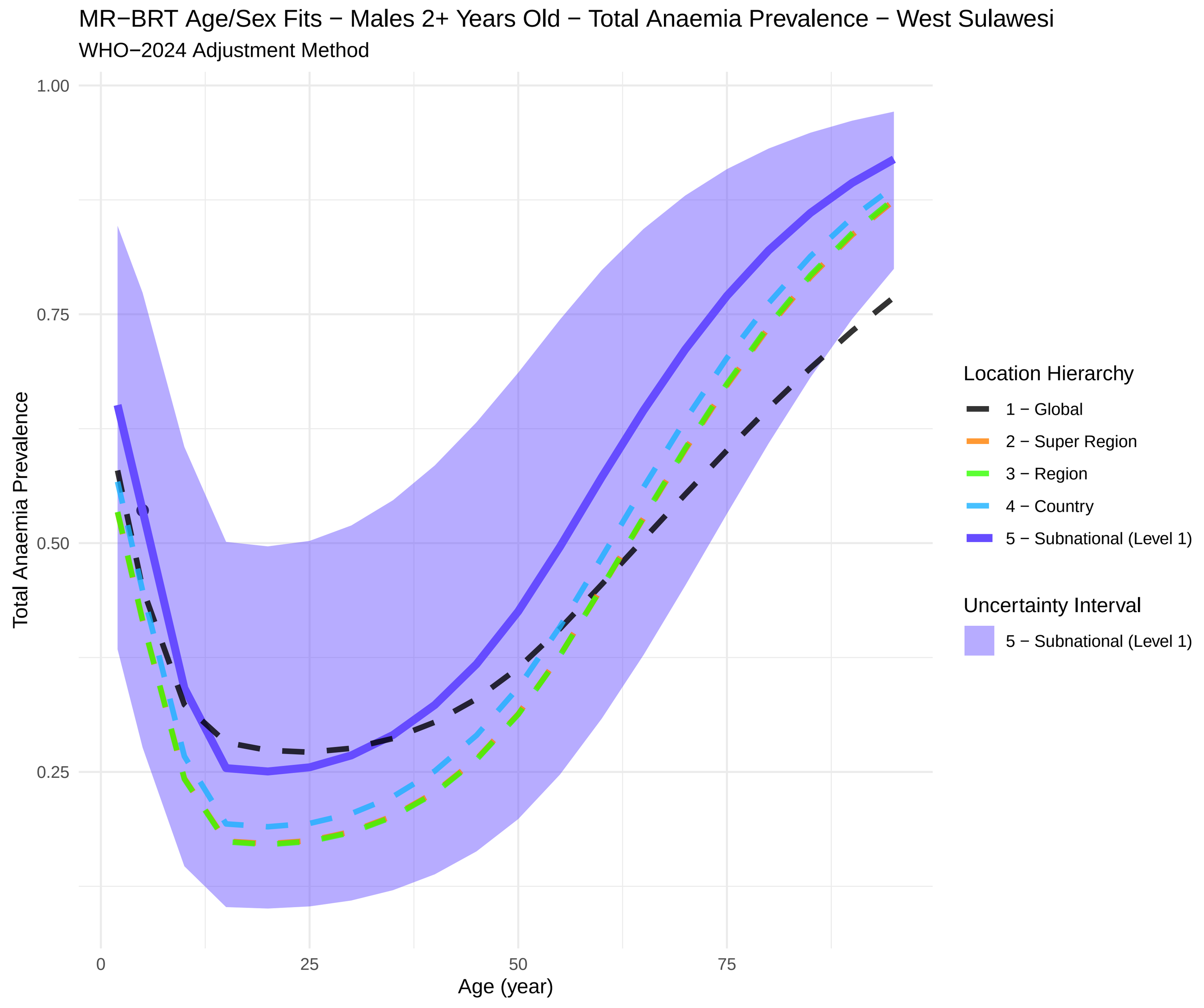
MR−BRT Age/Sex Fits − Males 2+ Years Old − Total Anaemia Prevalence − West Sulawesi
WHO−2024 Adjustment Method
Total Anaemia Prevalence
Age (year)
Location Hierarchy
1 − Global
2 − Super Region
3 − Region
4 − Country
5 − Subnational (Level 1)
Uncertainty Interval
5 − Subnational (Level 1)

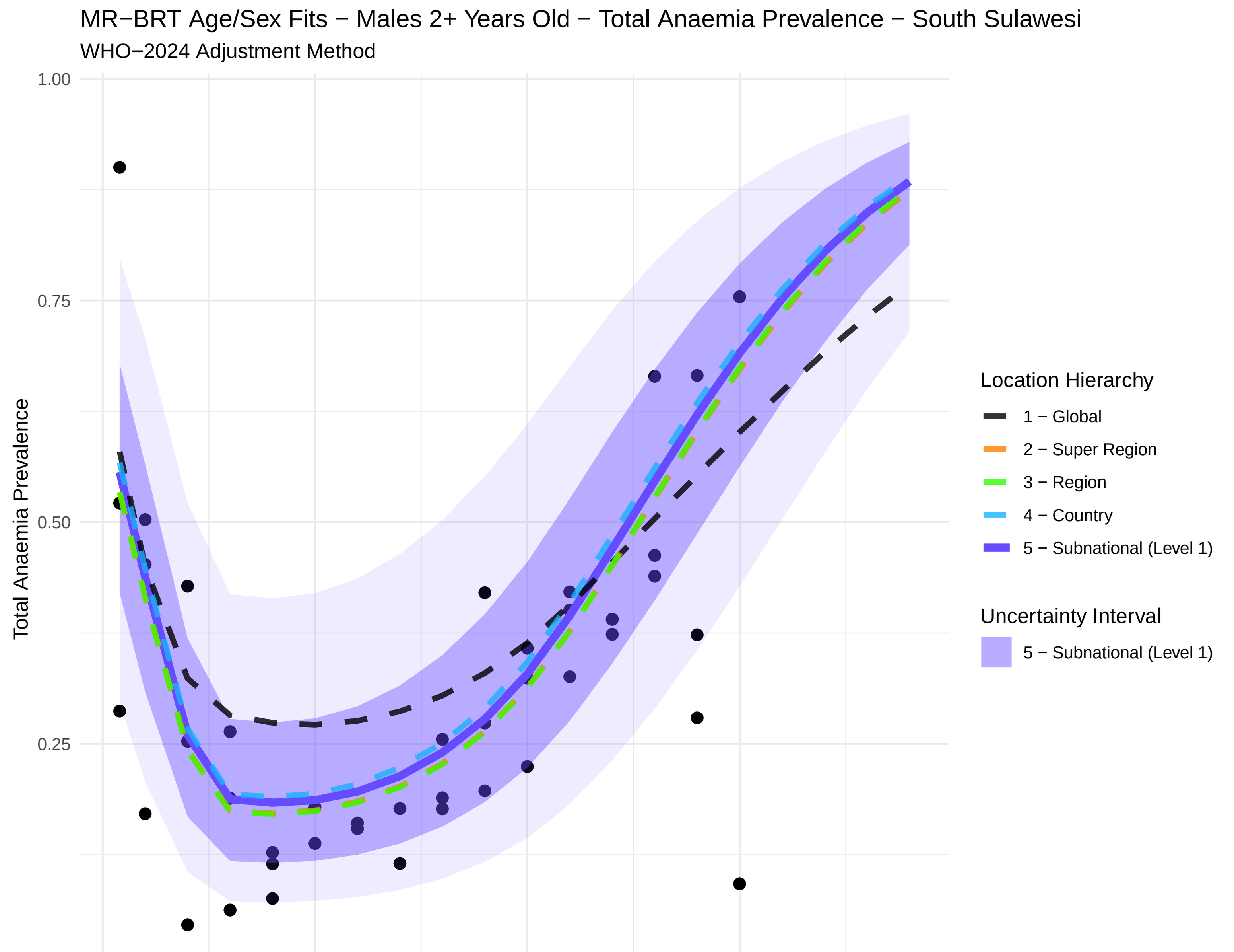
MR−BRT Age/Sex Fits − Males 2+ Years Old − Total Anaemia Prevalence − South Sulawesi
WHO−2024 Adjustment Method
Total Anaemia Prevalence
Age (year)
Location Hierarchy
1 − Global
2 − Super Region
3 − Region
4 − Country
5 − Subnational (Level 1)
Uncertainty Interval
5 − Subnational (Level 1)

MR−BRT Age/Sex Fits − Males 2+ Years Old − Total Anaemia Prevalence − West Sumatra
WHO−2024 Adjustment Method

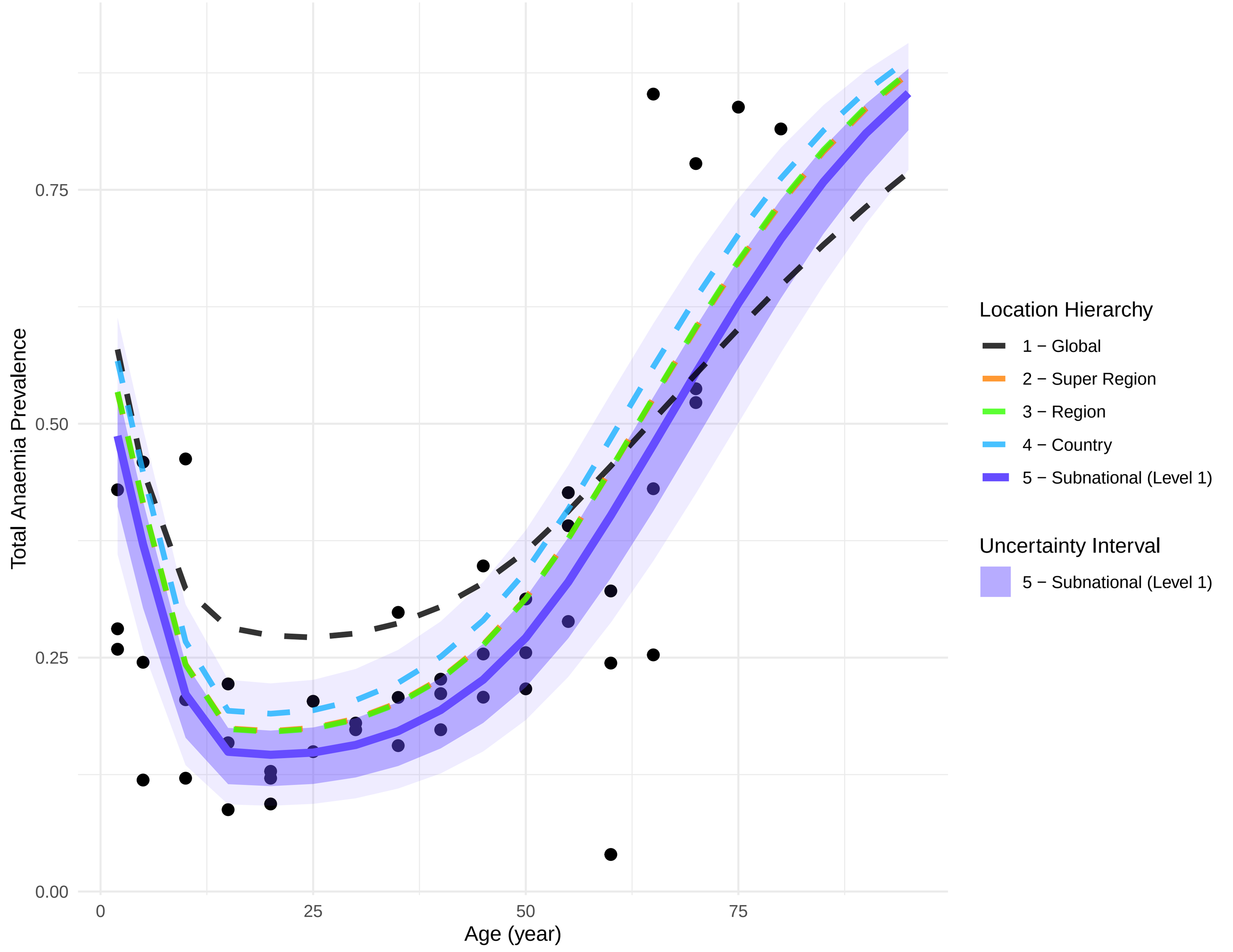

MR−BRT Age/Sex Fits − Males 2+ Years Old − Total Anaemia Prevalence − South Sumatra
WHO−2024 Adjustment Method

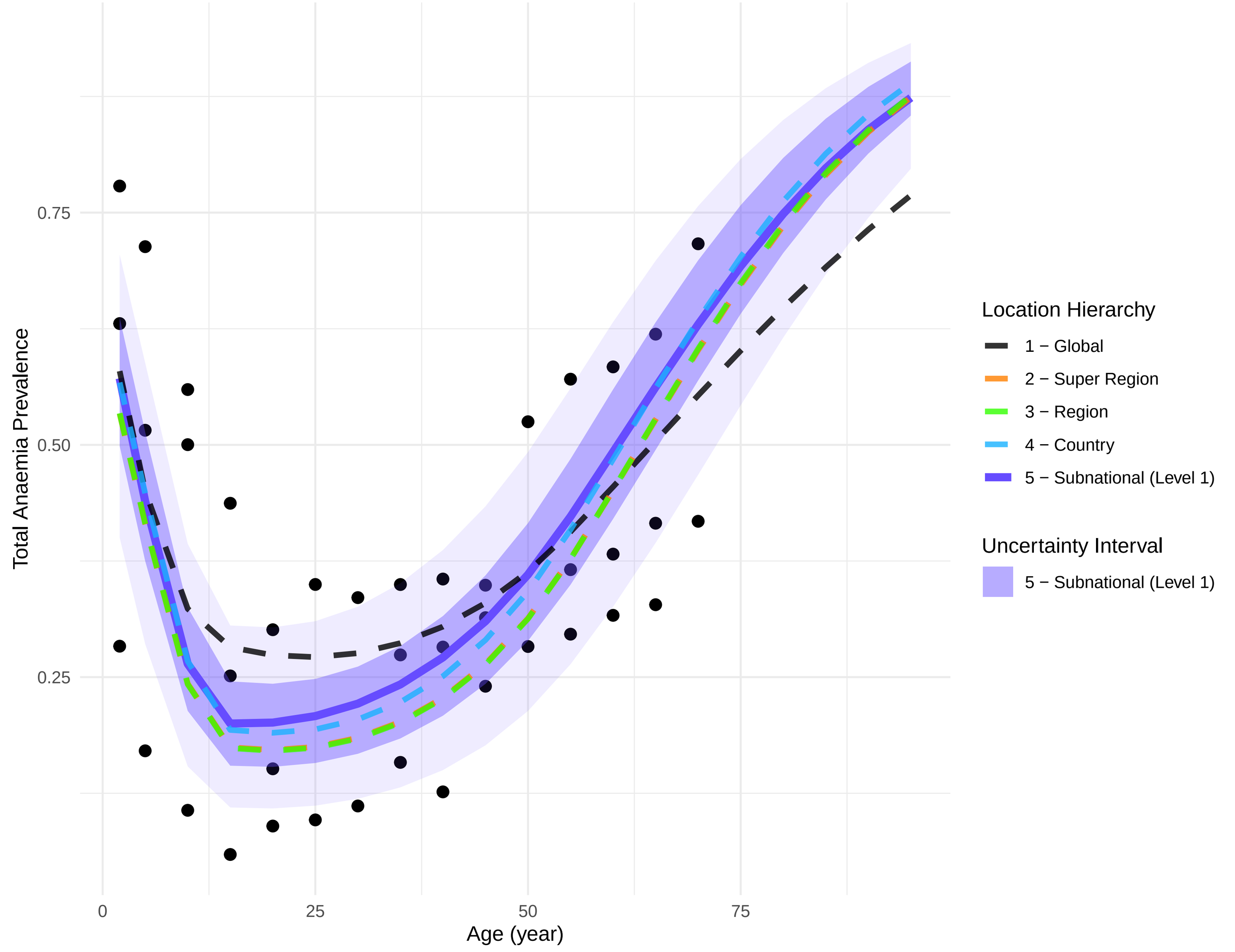

MR−BRT Age/Sex Fits − Males 2+ Years Old − Total Anaemia Prevalence − North Sumatra
WHO−2024 Adjustment Method

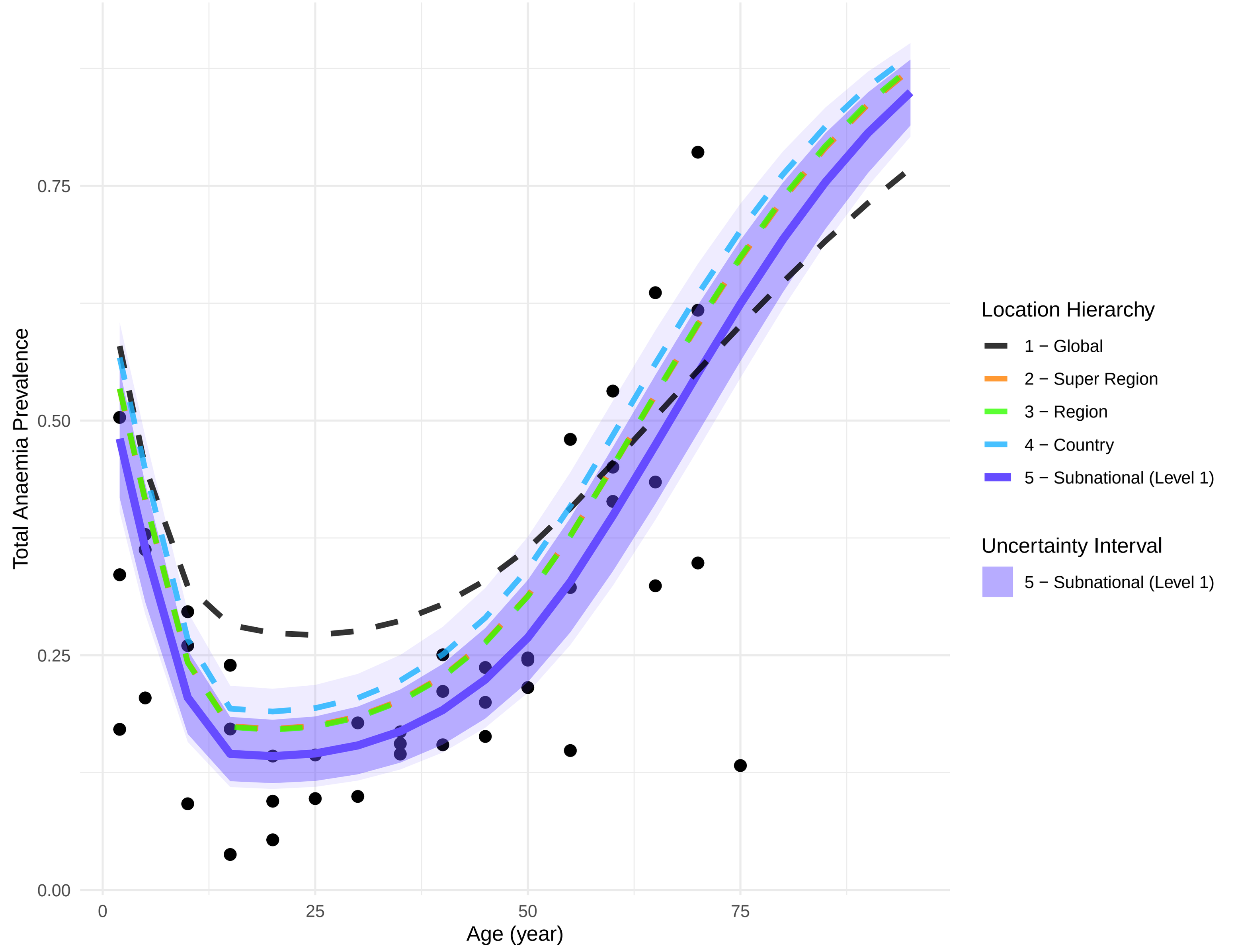

MR−BRT Age/Sex Fits − Males 2+ Years Old − Total Anaemia Prevalence − Yogyakarta
WHO−2024 Adjustment Method

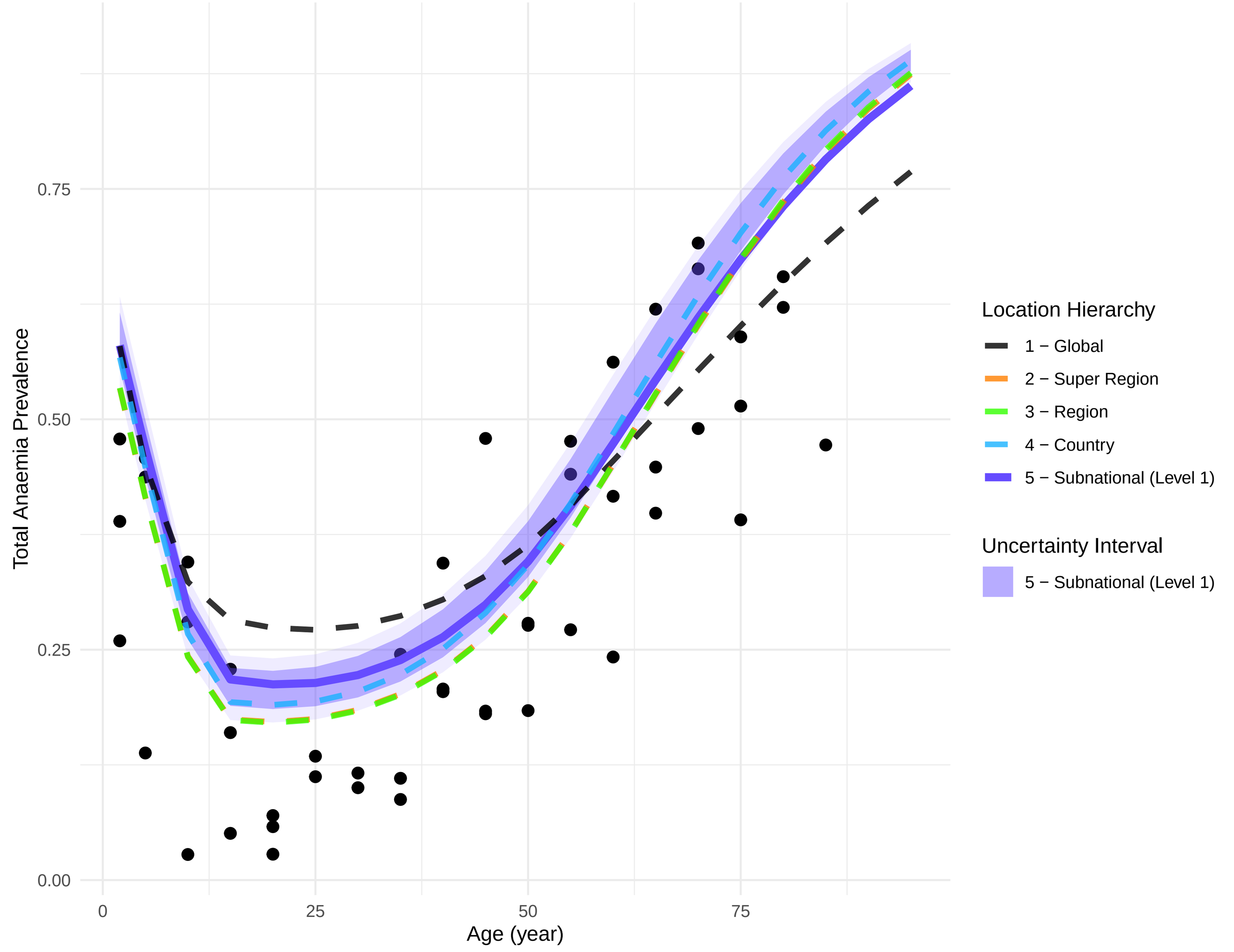

MR−BRT Age/Sex Fits − Males 2+ Years Old − Total Anaemia Prevalence − Myanmar
WHO−2024 Adjustment Method

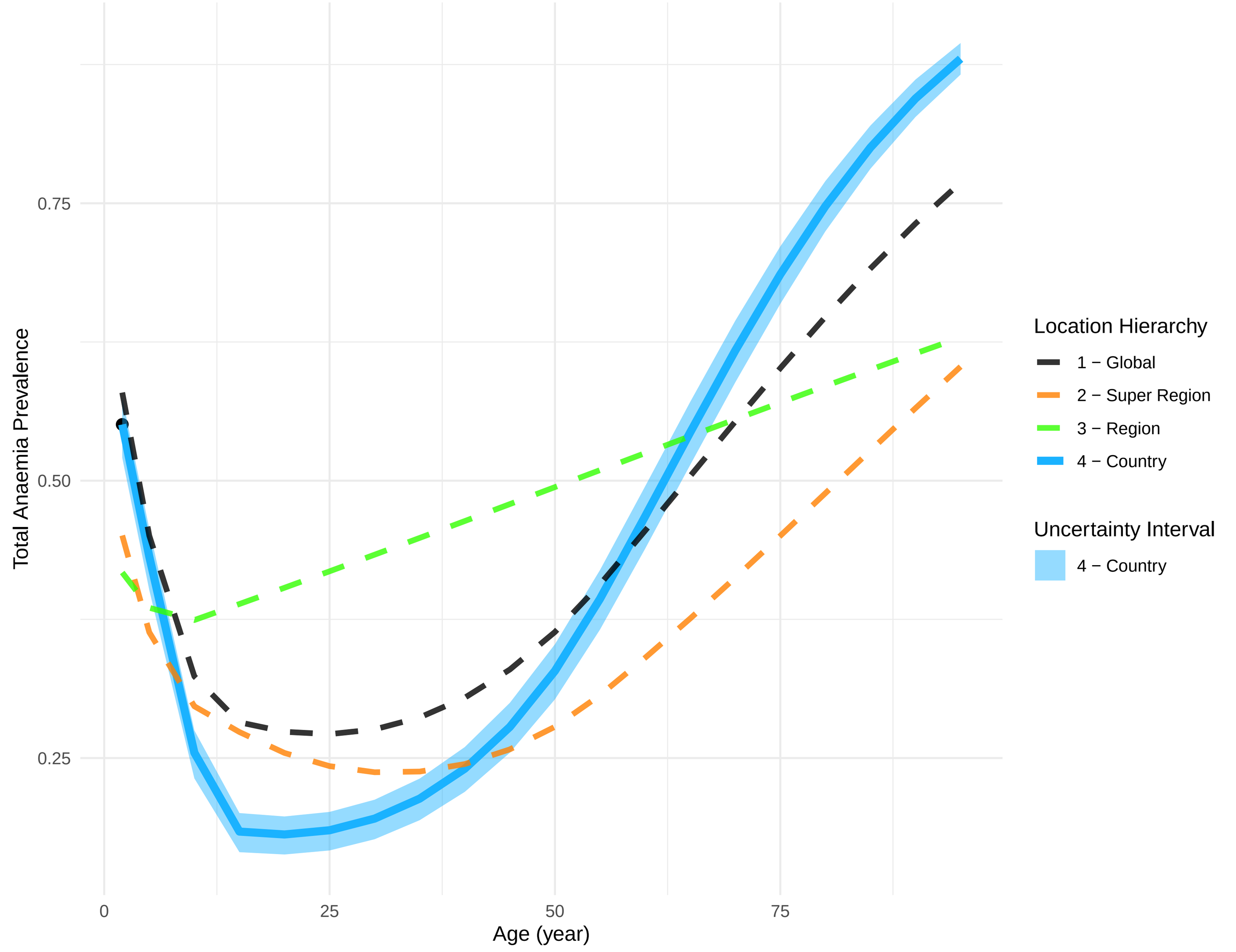

MR−BRT Age/Sex Fits − Males 2+ Years Old − Total Anaemia Prevalence − Timor−Leste
WHO−2024 Adjustment Method

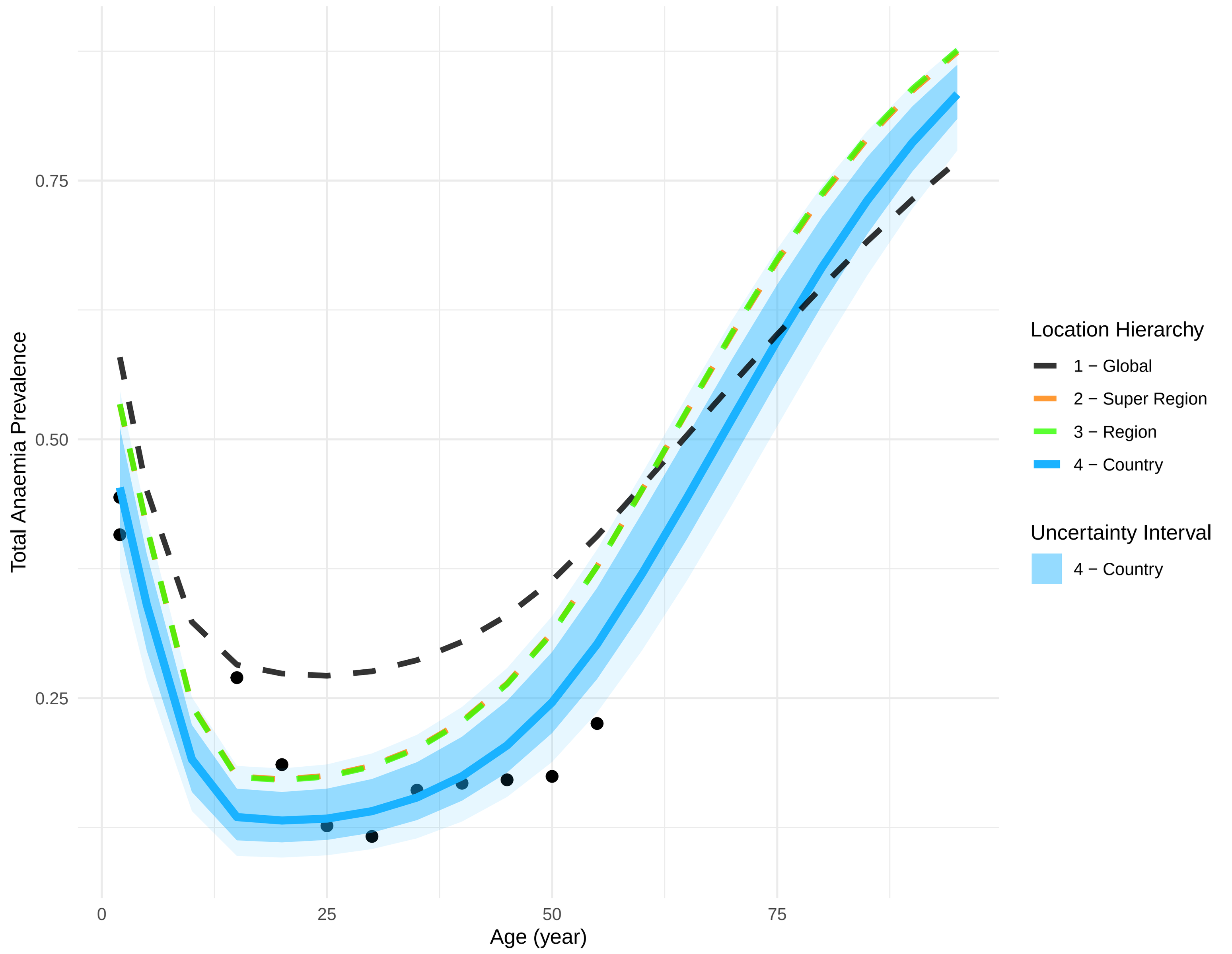

MR−BRT Age/Sex Fits − Males 2+ Years Old − Total Anaemia Prevalence − Sub−Saharan Africa
WHO−2024 Adjustment Method

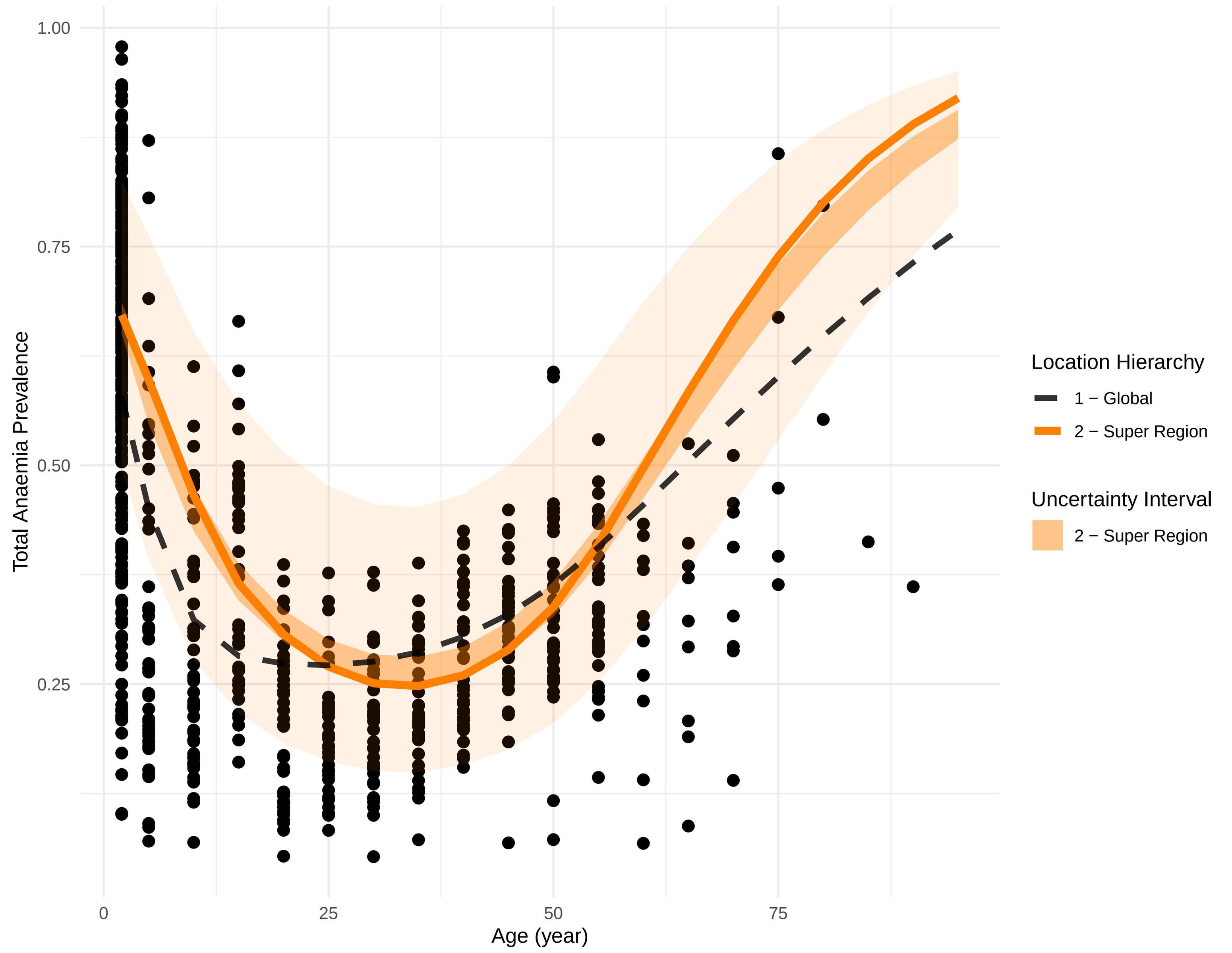

MR−BRT Age/Sex Fits − Males 2+ Years Old − Total Anaemia Prevalence − Central Sub−Saharan Africa
WHO−2024 Adjustment Method

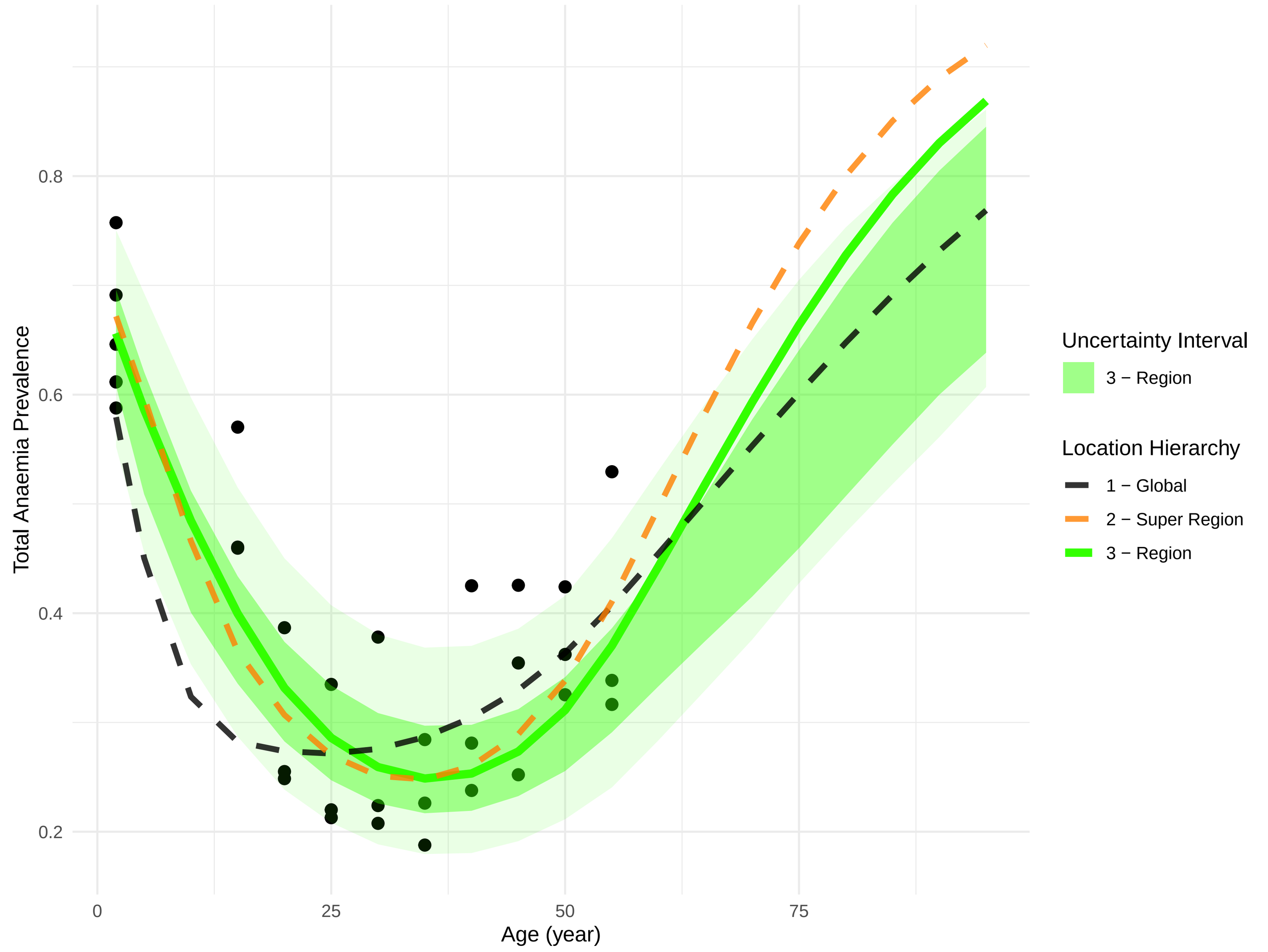

MR−BRT Age/Sex Fits − Males 2+ Years Old − Total Anaemia Prevalence − Angola
WHO−2024 Adjustment Method

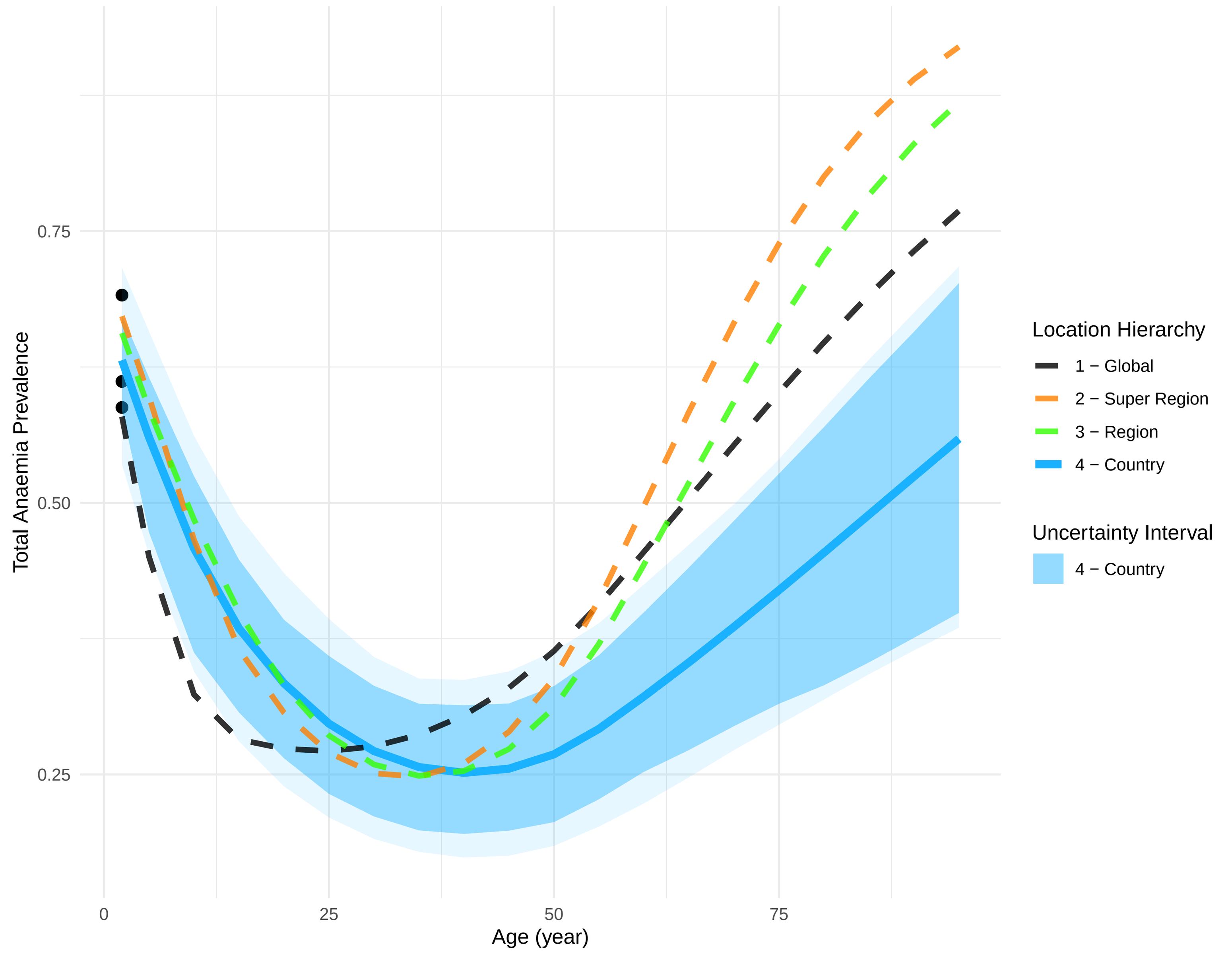

MR−BRT Age/Sex Fits − Males 2+ Years Old − Total Anaemia Prevalence − Democratic Republic of the Congo
WHO−2024 Adjustment Method

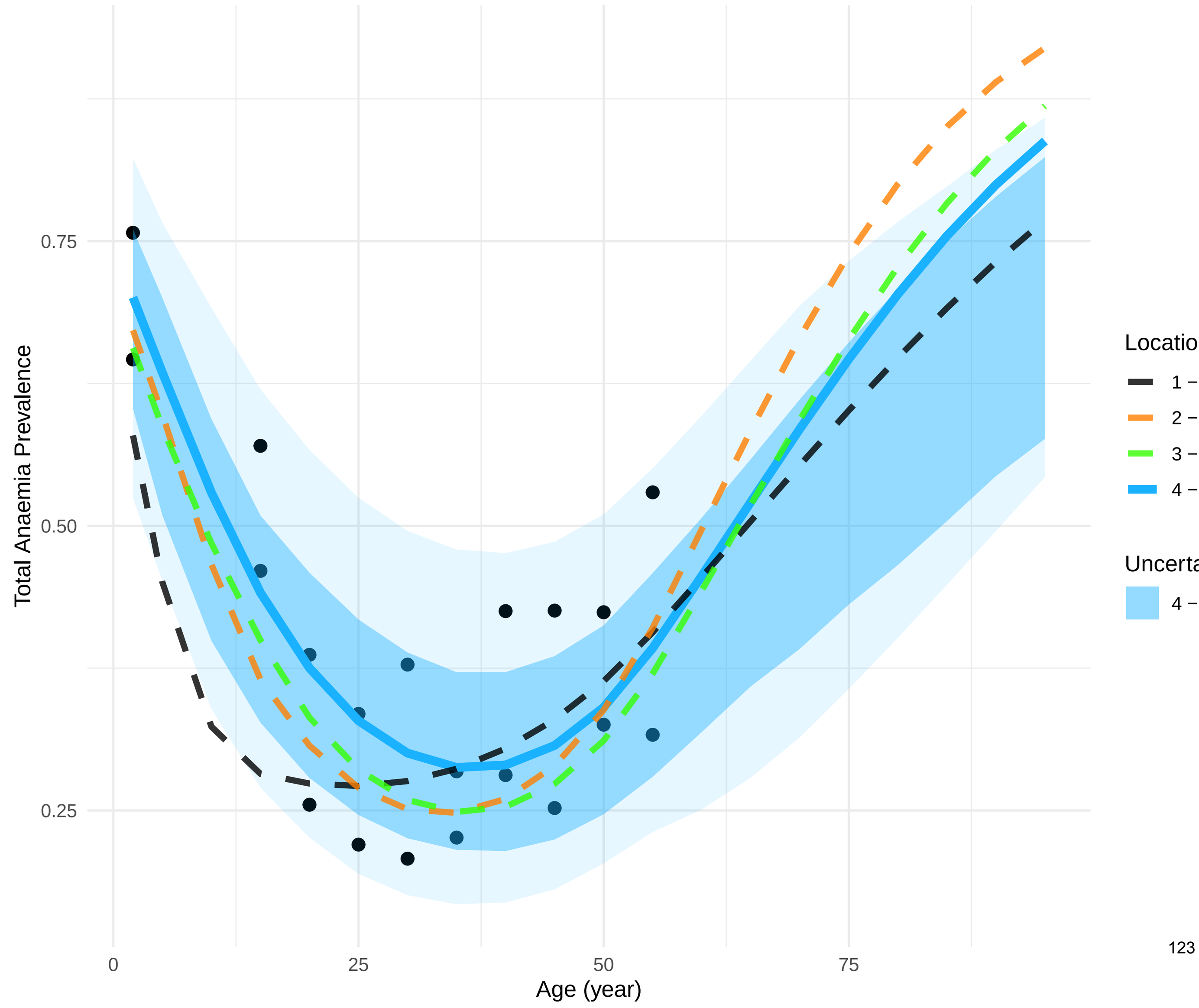

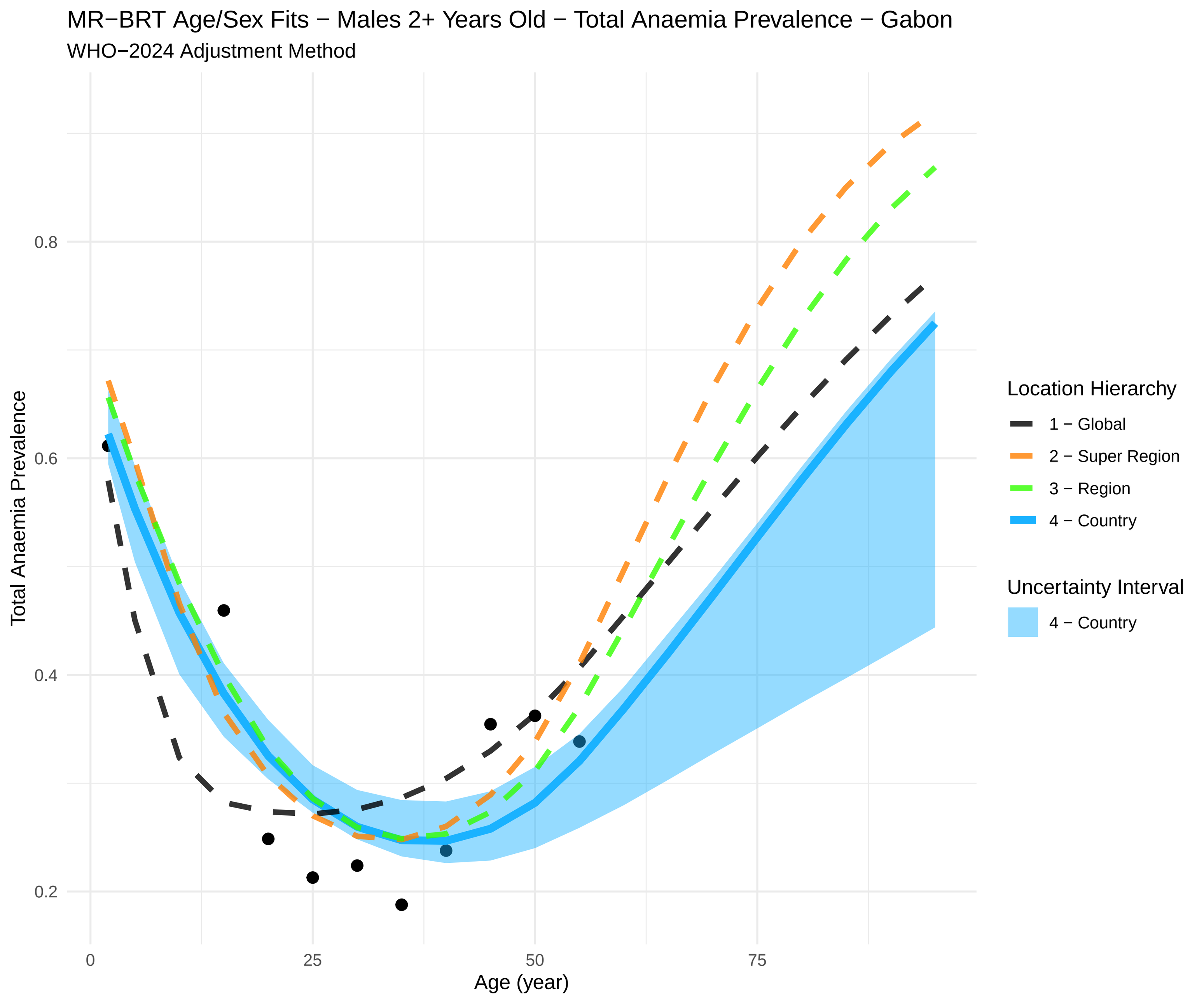
MR−BRT Age/Sex Fits − Males 2+ Years Old − Total Anaemia Prevalence − Gabon
WHO−2024 Adjustment Method
Total Anaemia Prevalence
Age (year)
Location Hierarchy
1 − Global
2 − Super Region
3 − Region
4 − Country
Uncertainty Interval
4 − Country

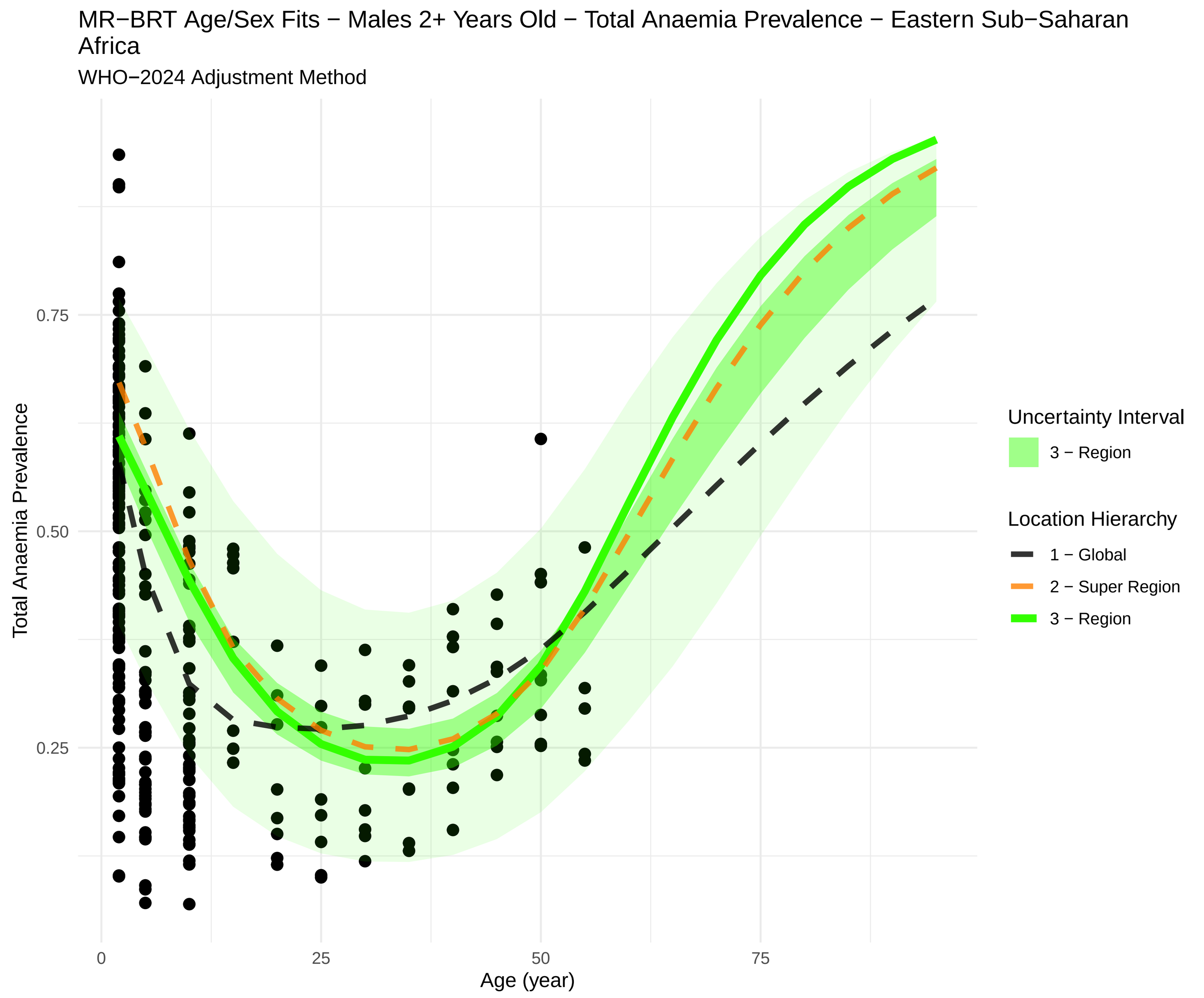
MR−BRT Age/Sex Fits − Males 2+ Years Old − Total Anaemia Prevalence − Eastern Sub−Saharan Africa
WHO−2024 Adjustment Method
Total Anaemia Prevalence
Age (year)
Uncertainty Interval
3 − Region
Location Hierarchy
1 − Global
2 − Super Region
3 − Region

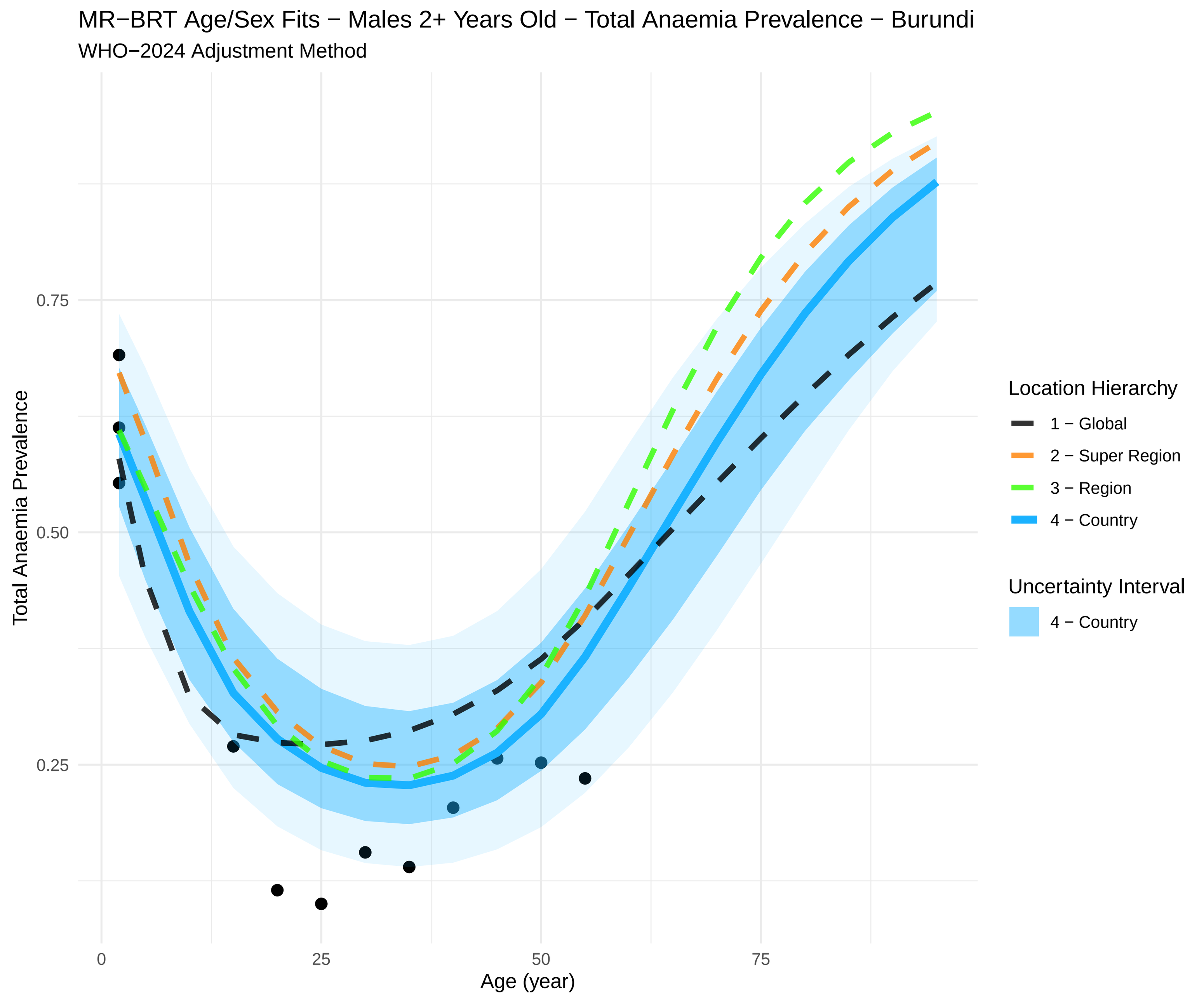
MR−BRT Age/Sex Fits − Males 2+ Years Old − Total Anaemia Prevalence − Burundi
WHO−2024 Adjustment Method
Total Anaemia Prevalence
Age (year)
Location Hierarchy
1 − Global
2 − Super Region
3 − Region
4 − Country
Uncertainty Interval
4 − Country

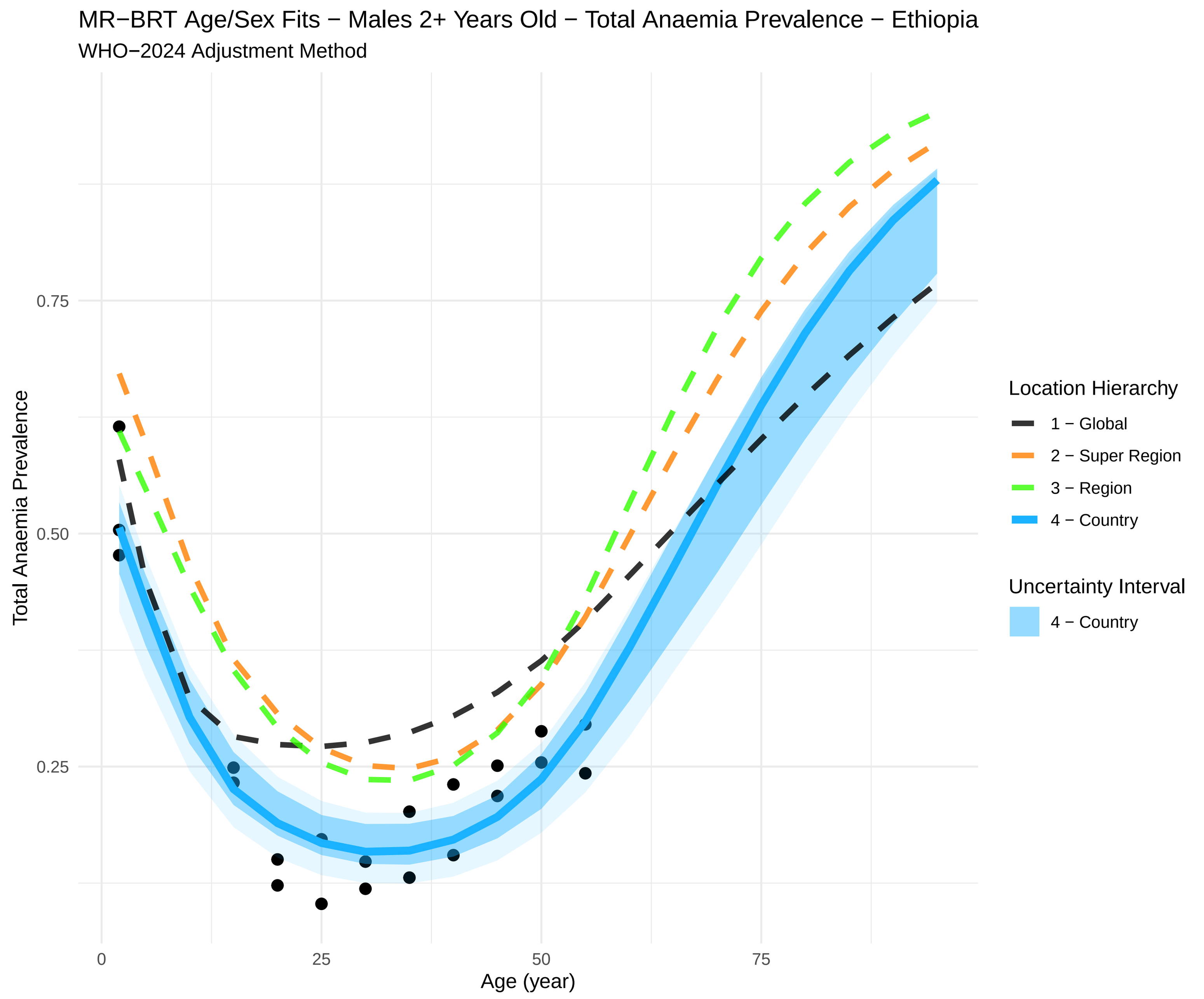
MR−BRT Age/Sex Fits − Males 2+ Years Old − Total Anaemia Prevalence − Ethiopia
WHO−2024 Adjustment Method
Total Anaemia Prevalence
Age (year)
Location Hierarchy
1 − Global
2 − Super Region
3 − Region
4 − Country
Uncertainty Interval
4 − Country

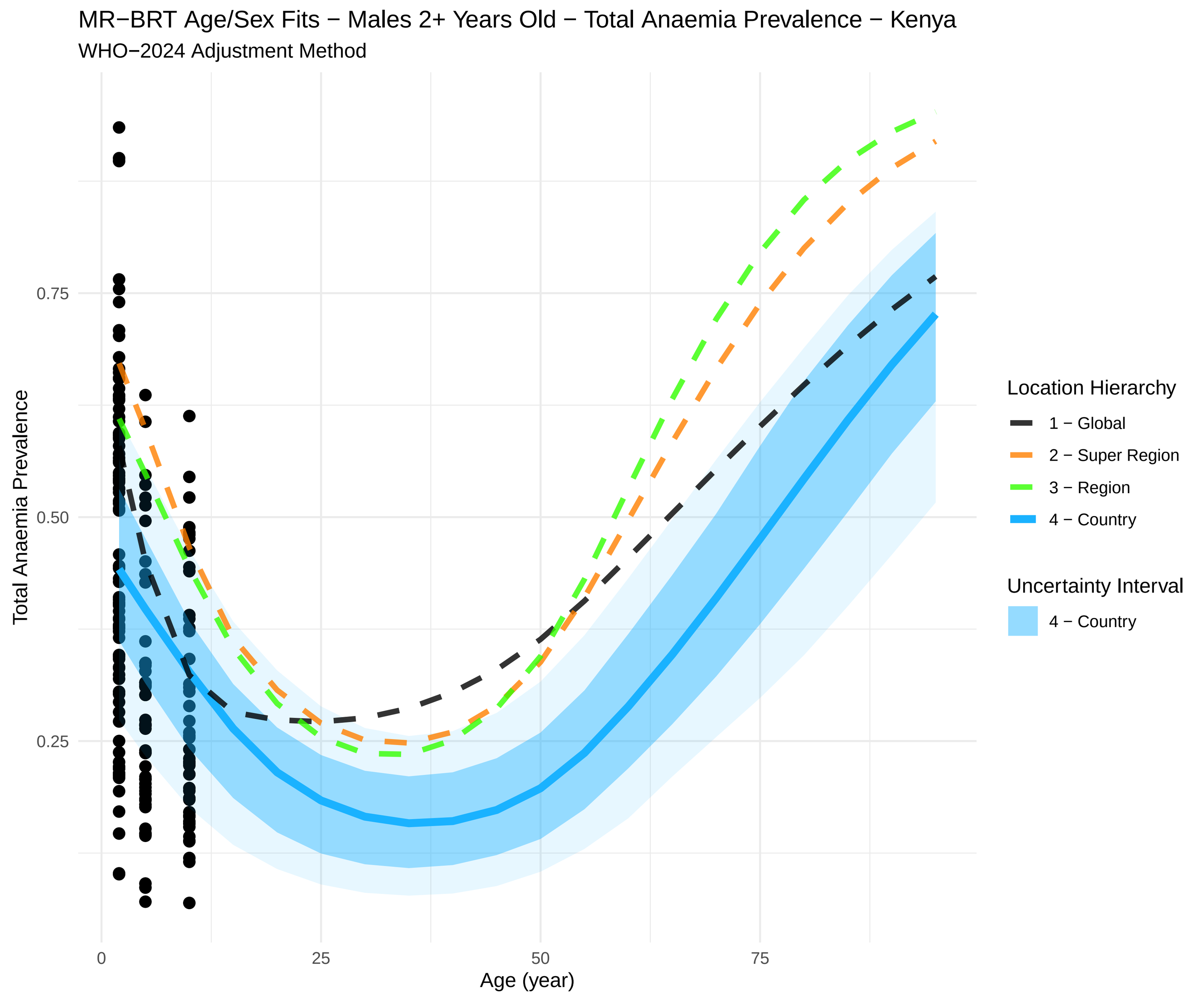
MR−BRT Age/Sex Fits − Males 2+ Years Old − Total Anaemia Prevalence − Kenya
WHO−2024 Adjustment Method
Total Anaemia Prevalence
Age (year)
Location Hierarchy
1 − Global
2 − Super Region
3 − Region
4 − Country
Uncertainty Interval
4 − Country

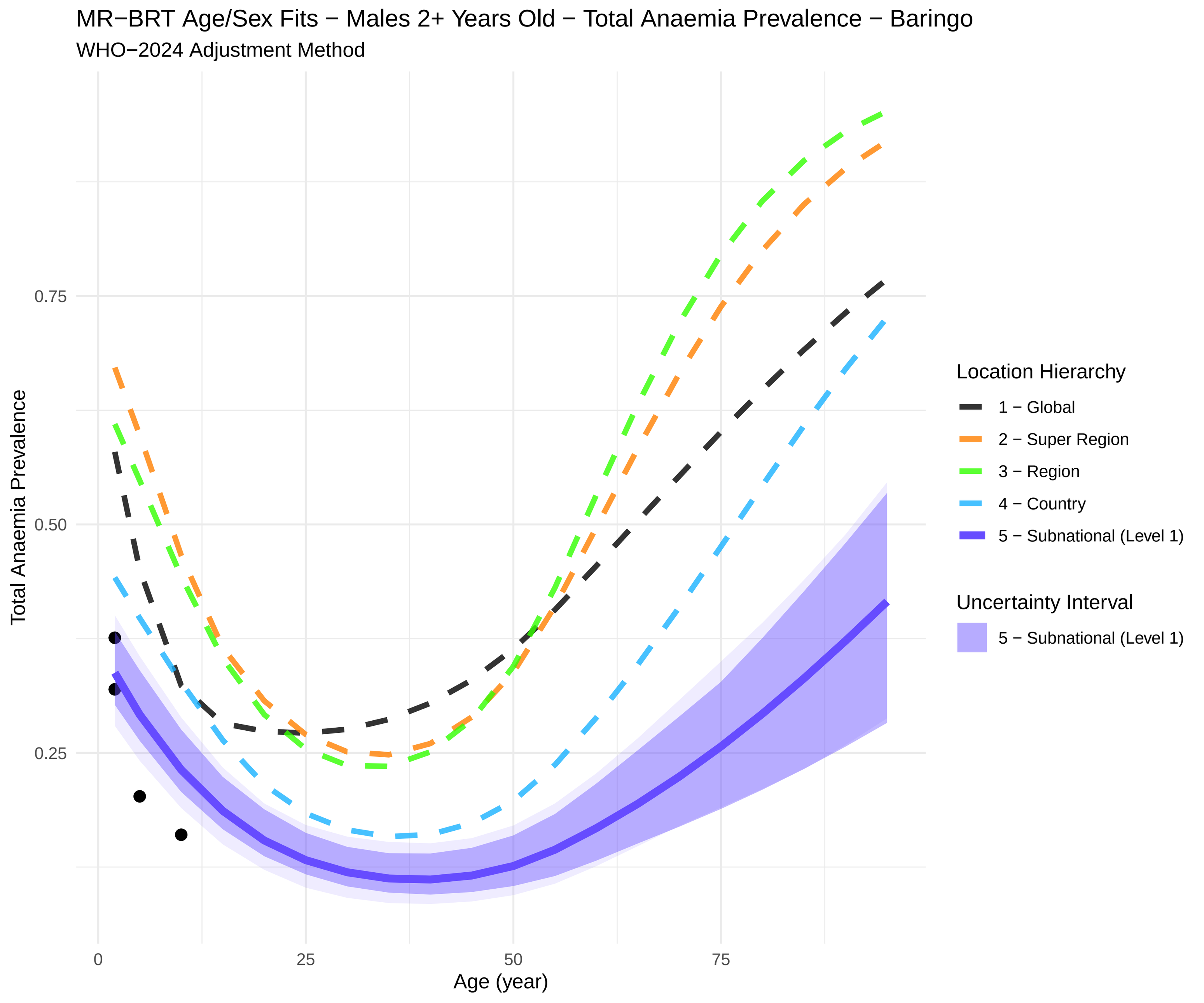
MR−BRT Age/Sex Fits − Males 2+ Years Old − Total Anaemia Prevalence − Baringo
WHO−2024 Adjustment Method
Total Anaemia Prevalence
Age (year)
Location Hierarchy
1 − Global
2 − Super Region
3 − Region
4 − Country
5 − Subnational (Level 1)
Uncertainty Interval
5 − Subnational (Level 1)

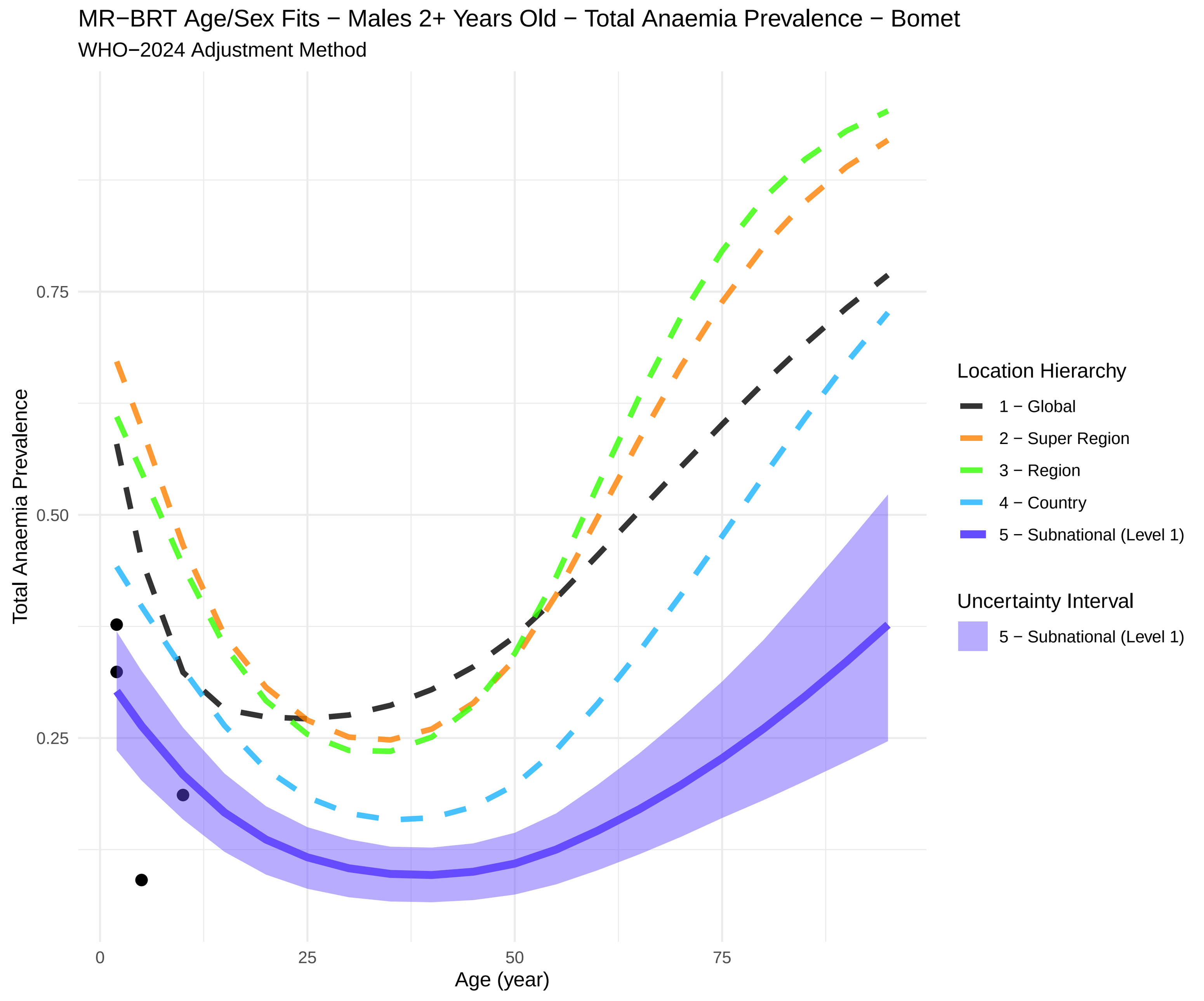
MR−BRT Age/Sex Fits − Males 2+ Years Old − Total Anaemia Prevalence − Bomet
WHO−2024 Adjustment Method
Total Anaemia Prevalence
Age (year)
Location Hierarchy
1 − Global
2 − Super Region
3 − Region
4 − Country
5 − Subnational (Level 1)
Uncertainty Interval
5 − Subnational (Level 1)

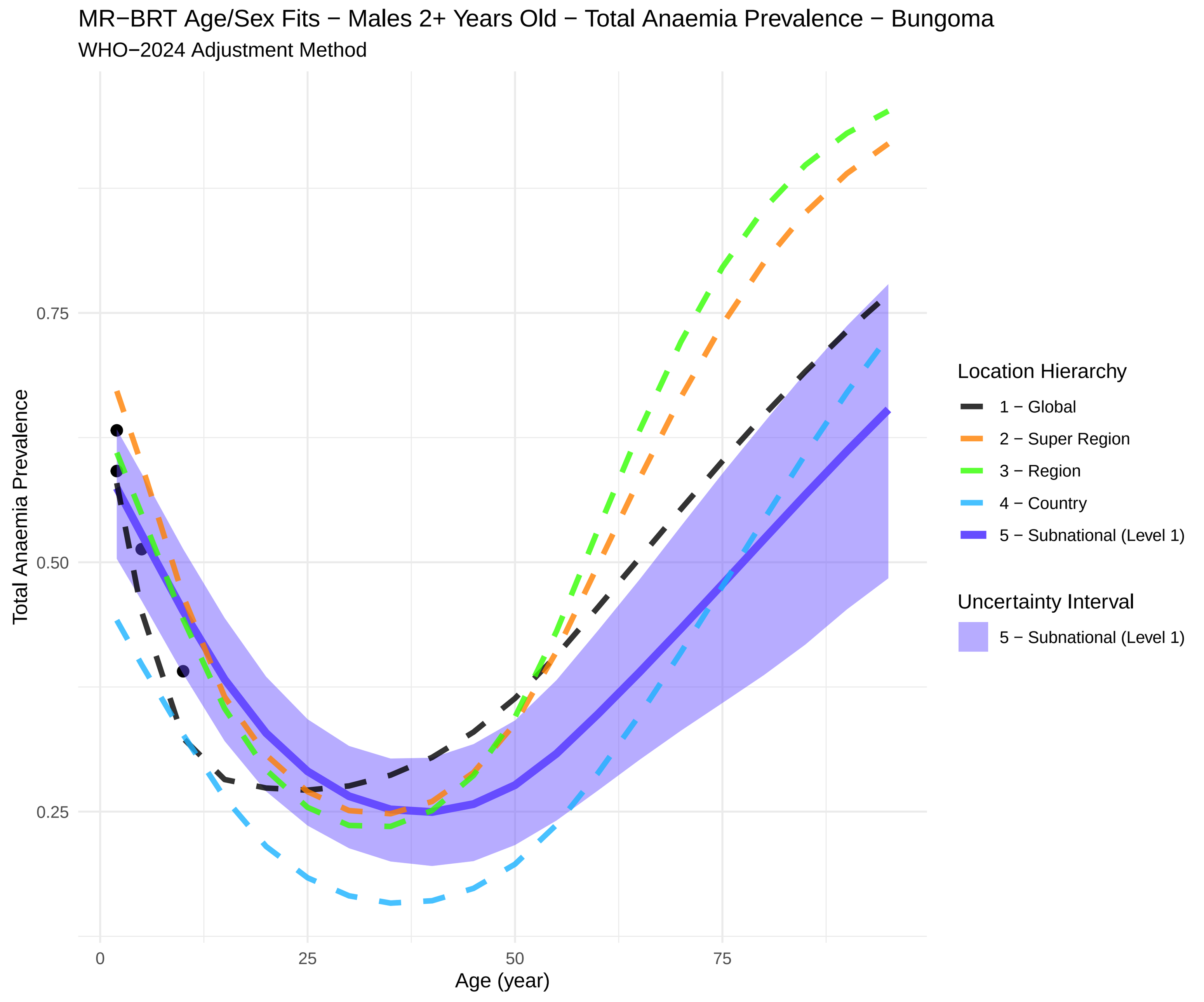
MR−BRT Age/Sex Fits − Males 2+ Years Old − Total Anaemia Prevalence − Bungoma
WHO−2024 Adjustment Method
Total Anaemia Prevalence
Age (year)
Location Hierarchy
1 − Global
2 − Super Region
3 − Region
4 − Country
5 − Subnational (Level 1)
Uncertainty Interval
5 − Subnational (Level 1)

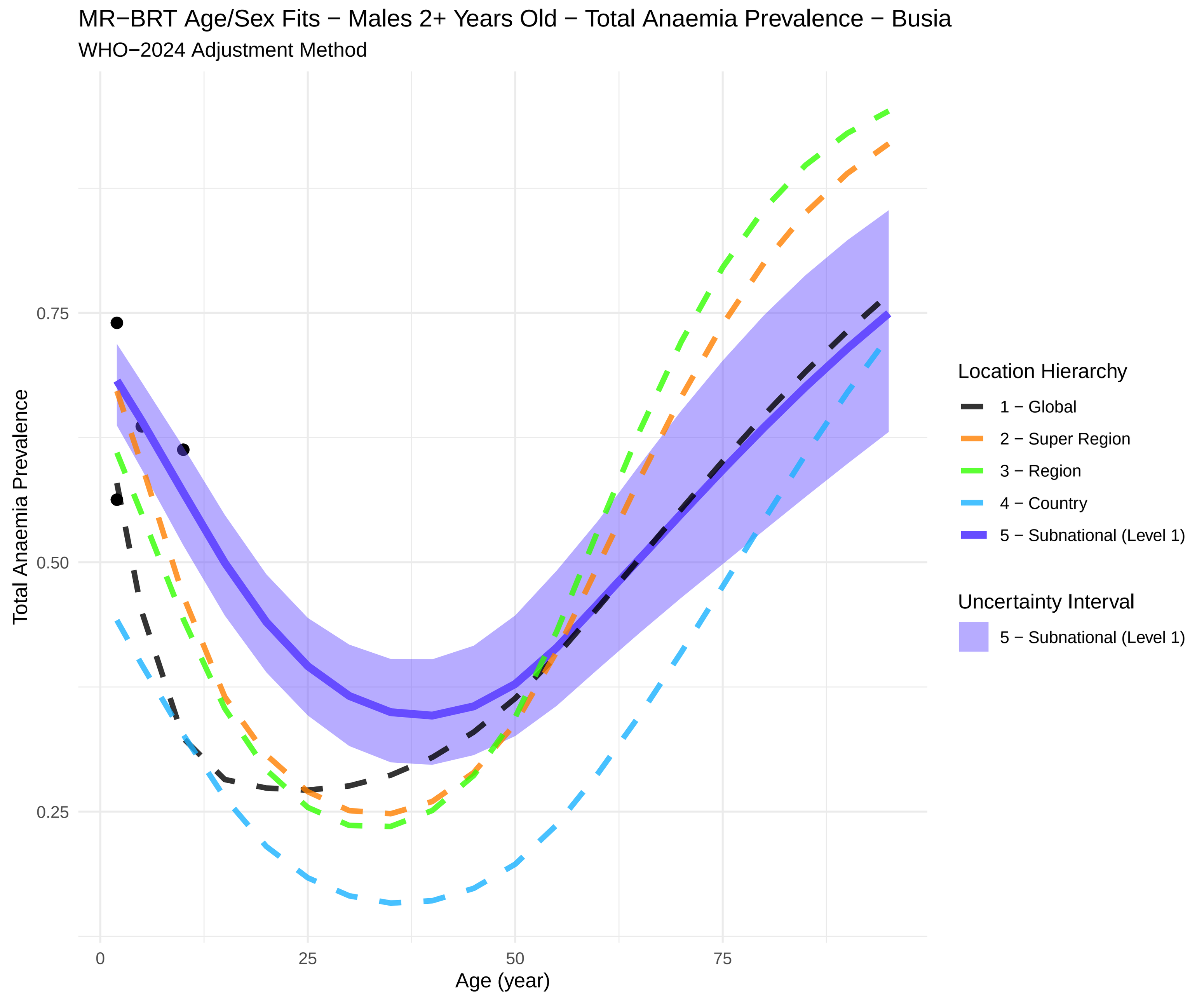
MR−BRT Age/Sex Fits − Males 2+ Years Old − Total Anaemia Prevalence − Busia
WHO−2024 Adjustment Method
Total Anaemia Prevalence
Age (year)
Location Hierarchy
1 − Global
2 − Super Region
3 − Region
4 − Country
5 − Subnational (Level 1)
Uncertainty Interval
5 − Subnational (Level 1)

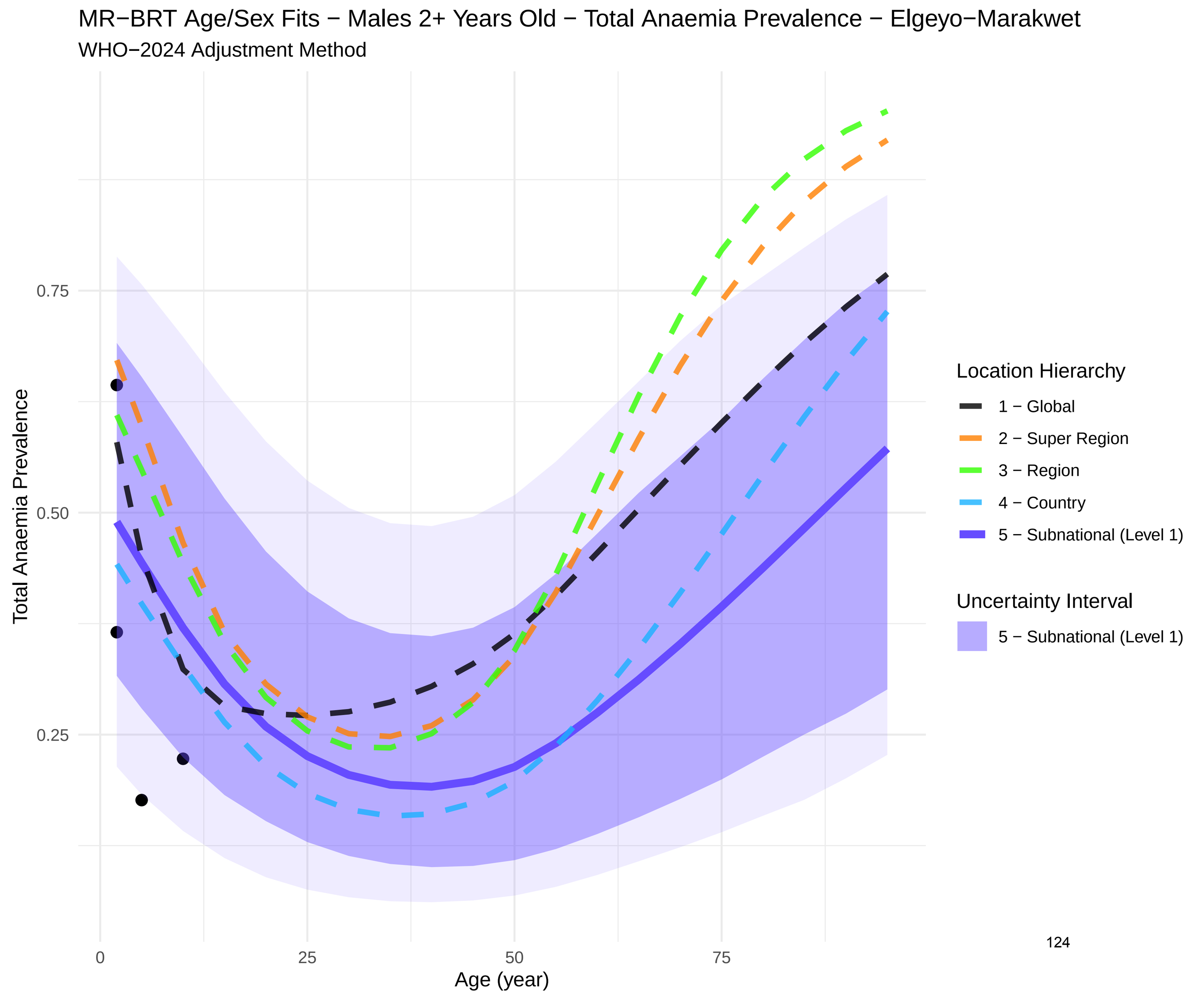
MR−BRT Age/Sex Fits − Males 2+ Years Old − Total Anaemia Prevalence − Elgeyo−Marakwet
WHO−2024 Adjustment Method
Total Anaemia Prevalence
Age (year)
Location Hierarchy
1 − Global
2 − Super Region
3 − Region
4 − Country
5 − Subnational (Level 1)
Uncertainty Interval
5 − Subnational (Level 1)

MR−BRT Age/Sex Fits − Males 2+ Years Old − Total Anaemia Prevalence − Embu
WHO−2024 Adjustment Method

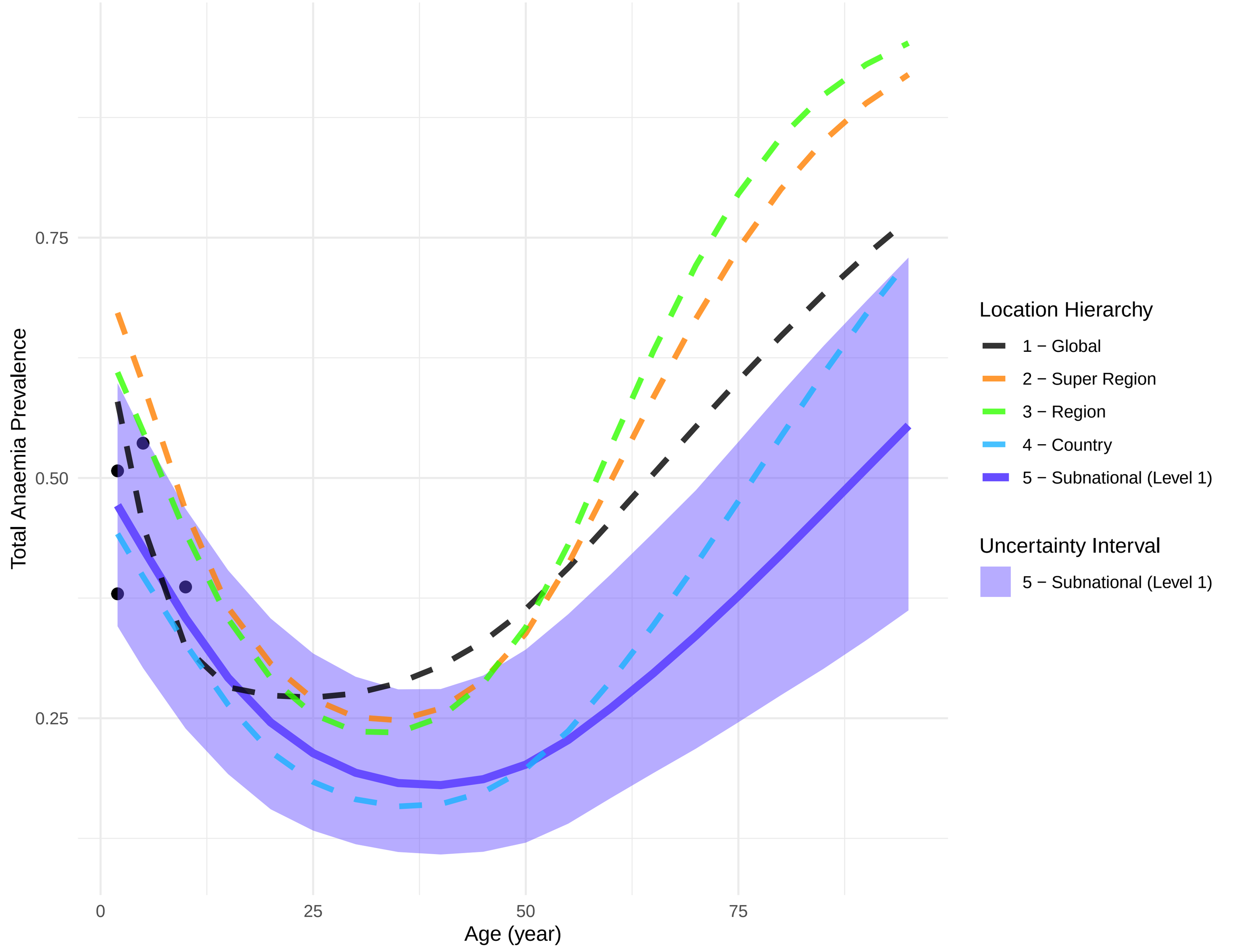

MR−BRT Age/Sex Fits − Males 2+ Years Old − Total Anaemia Prevalence − Garissa
WHO−2024 Adjustment Method

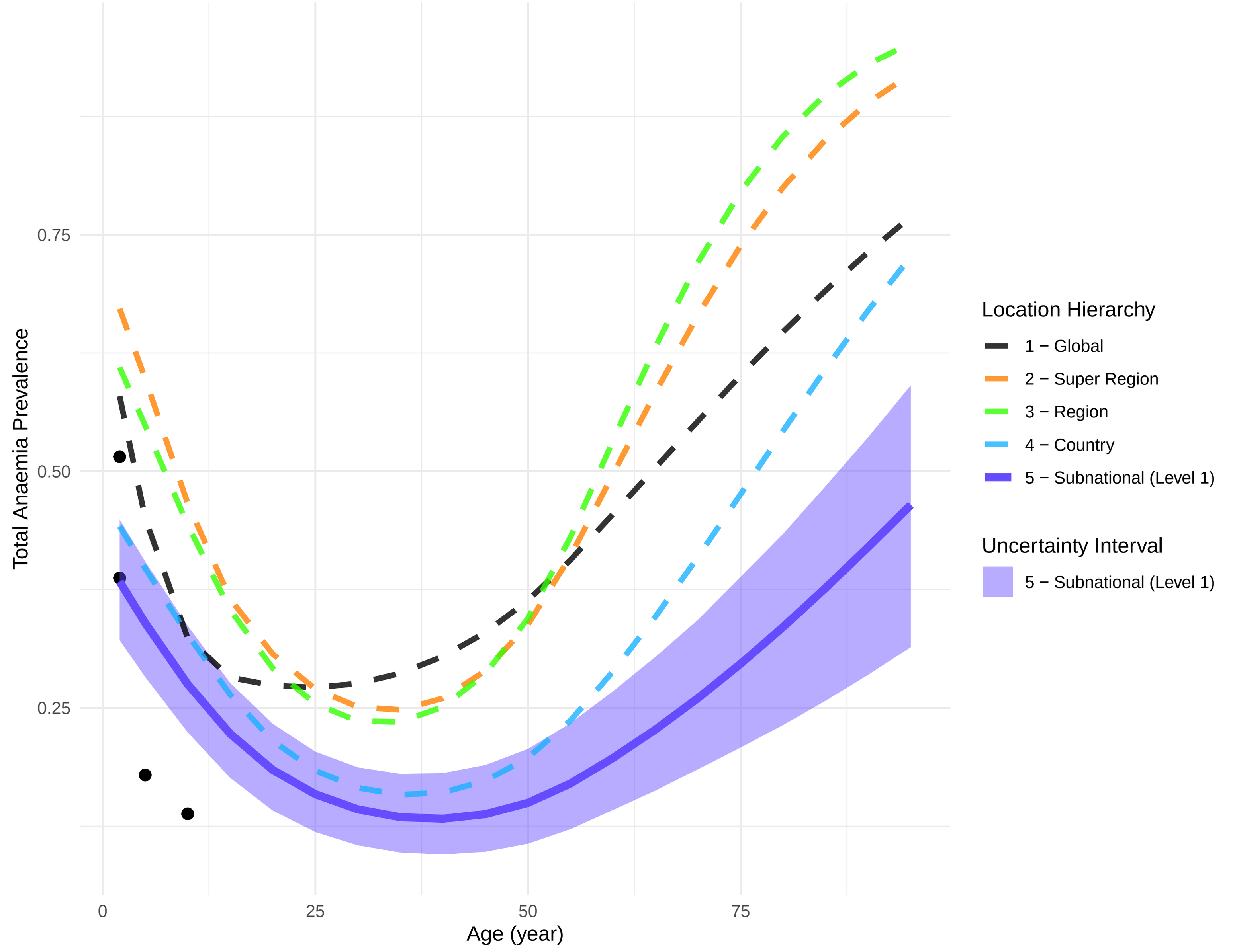

MR−BRT Age/Sex Fits − Males 2+ Years Old − Total Anaemia Prevalence − Homa Bay
WHO−2024 Adjustment Method

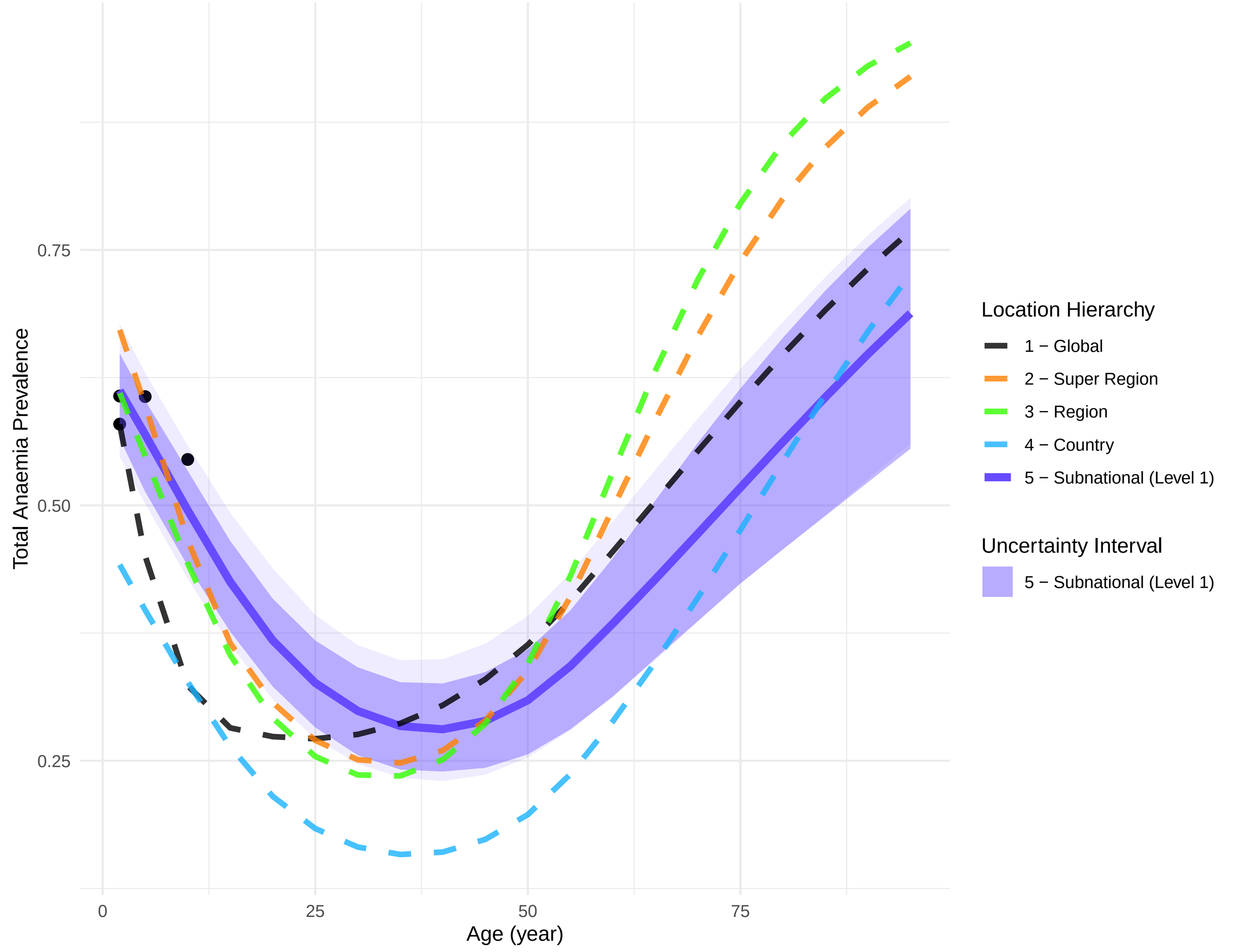

MR−BRT Age/Sex Fits − Males 2+ Years Old − Total Anaemia Prevalence − Isiolo
WHO−2024 Adjustment Method

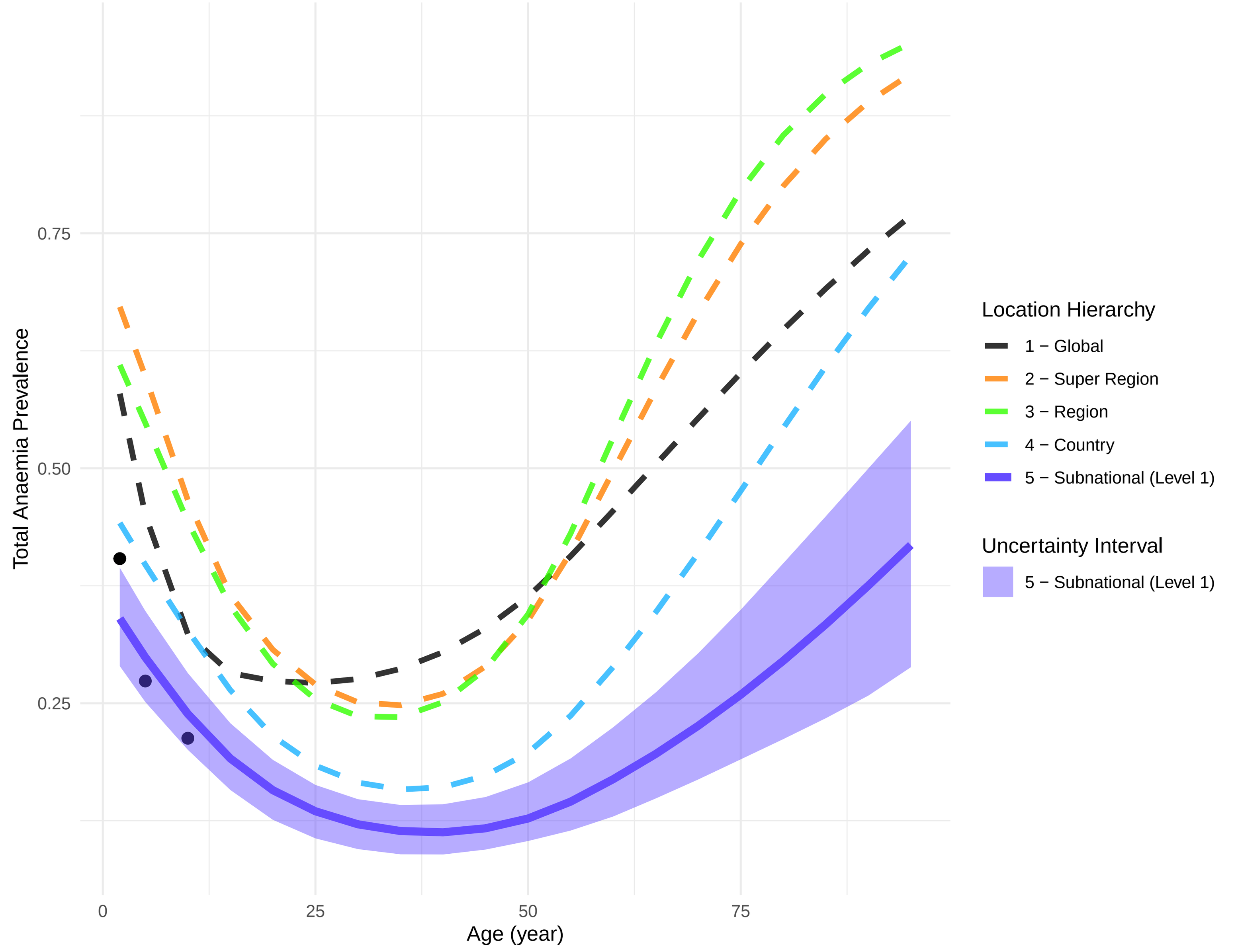

MR−BRT Age/Sex Fits − Males 2+ Years Old − Total Anaemia Prevalence − Kajiado
WHO−2024 Adjustment Method

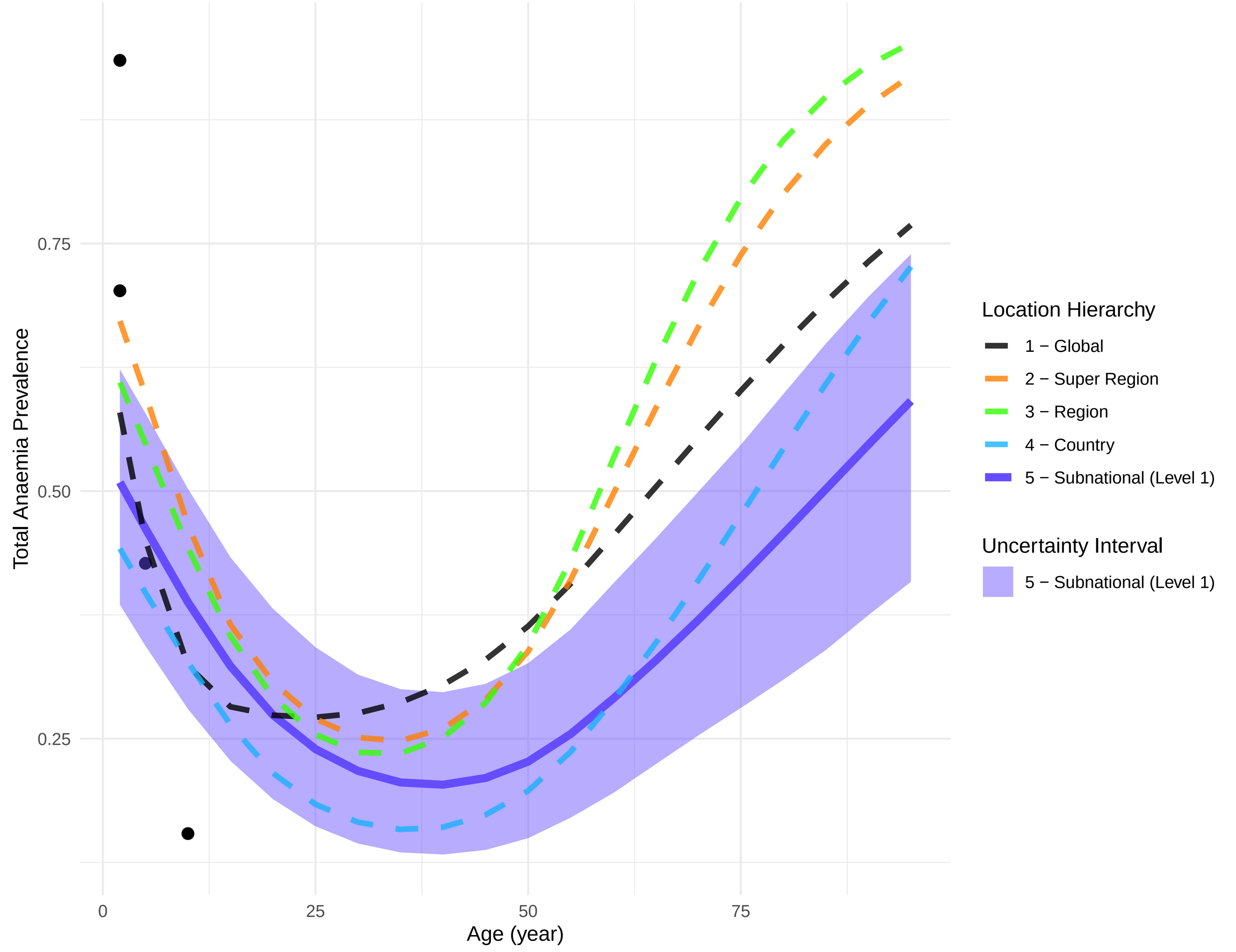

MR−BRT Age/Sex Fits − Males 2+ Years Old − Total Anaemia Prevalence − Kakamega
WHO−2024 Adjustment Method

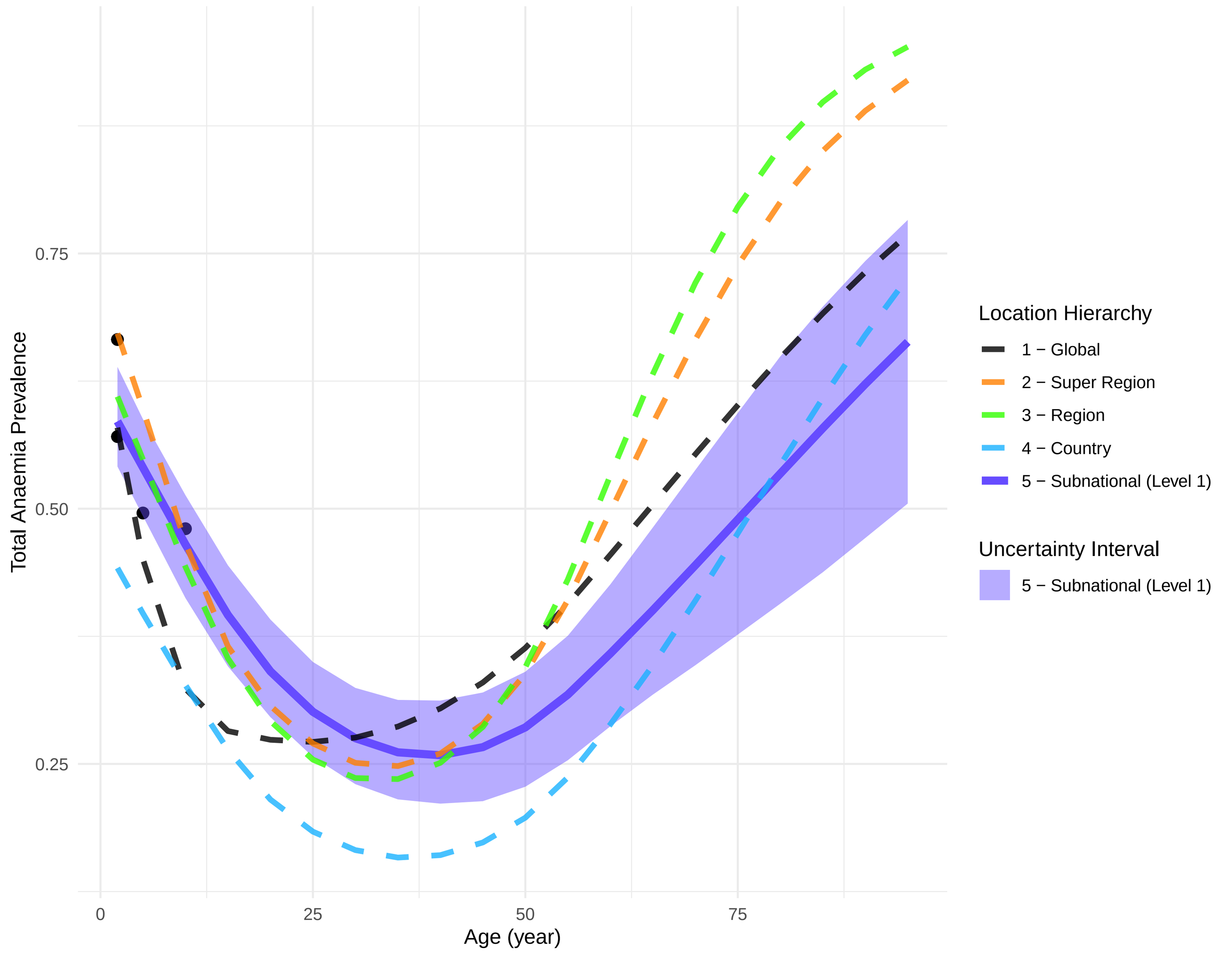

MR−BRT Age/Sex Fits − Males 2+ Years Old − Total Anaemia Prevalence − Kericho
WHO−2024 Adjustment Method

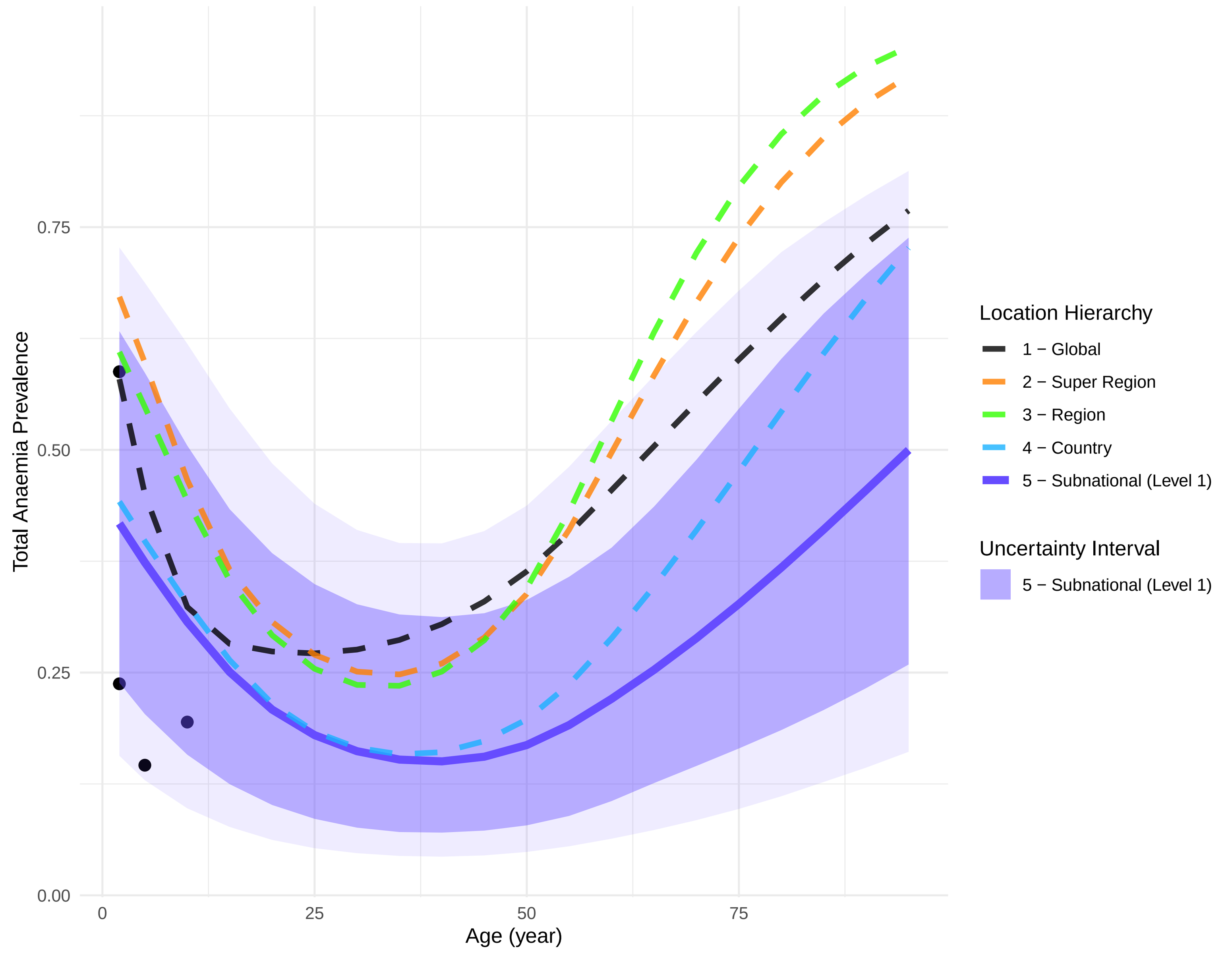

MR−BRT Age/Sex Fits − Males 2+ Years Old − Total Anaemia Prevalence − Kiambu
WHO−2024 Adjustment Method

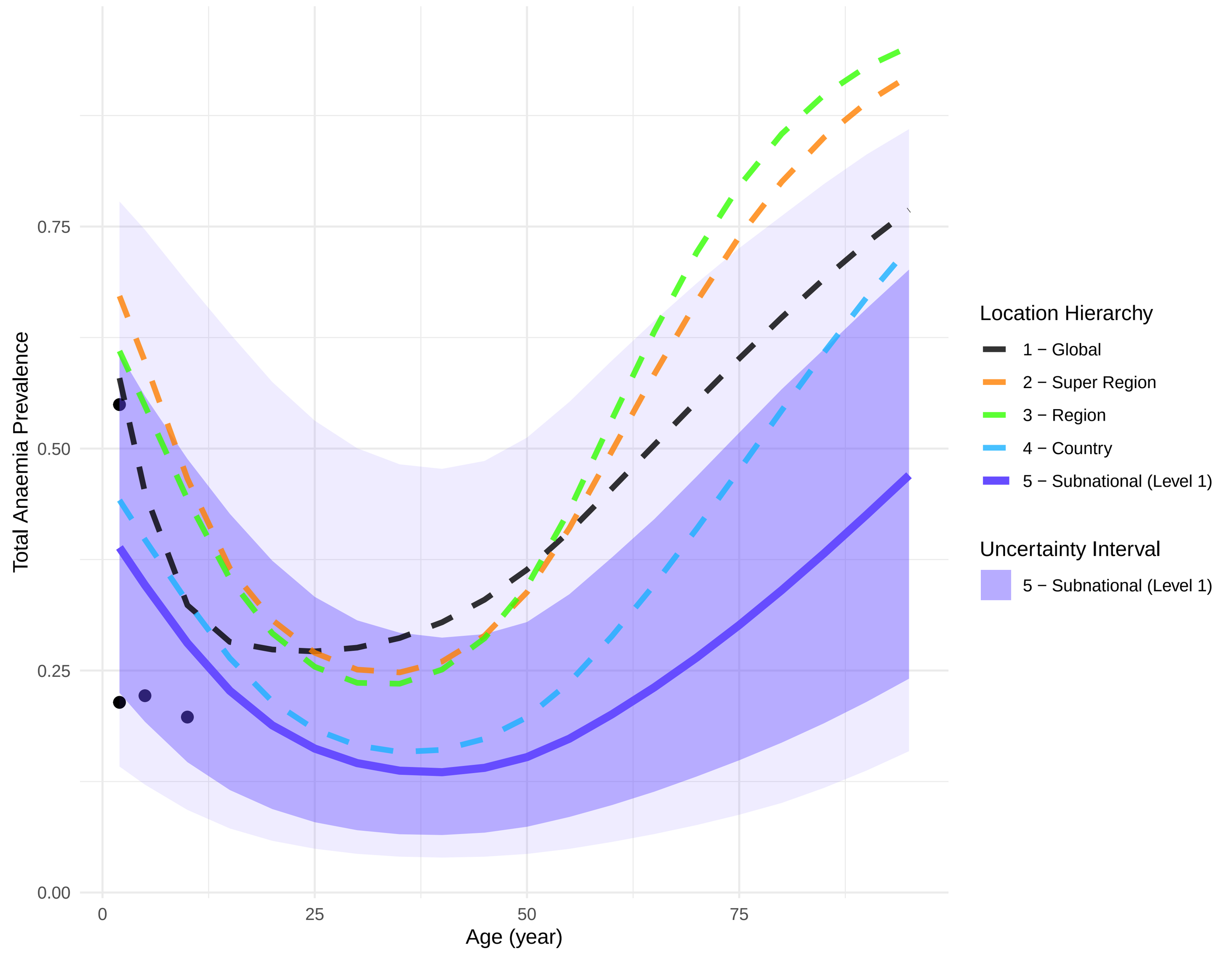

MR−BRT Age/Sex Fits − Males 2+ Years Old − Total Anaemia Prevalence − Kilifi
WHO−2024 Adjustment Method

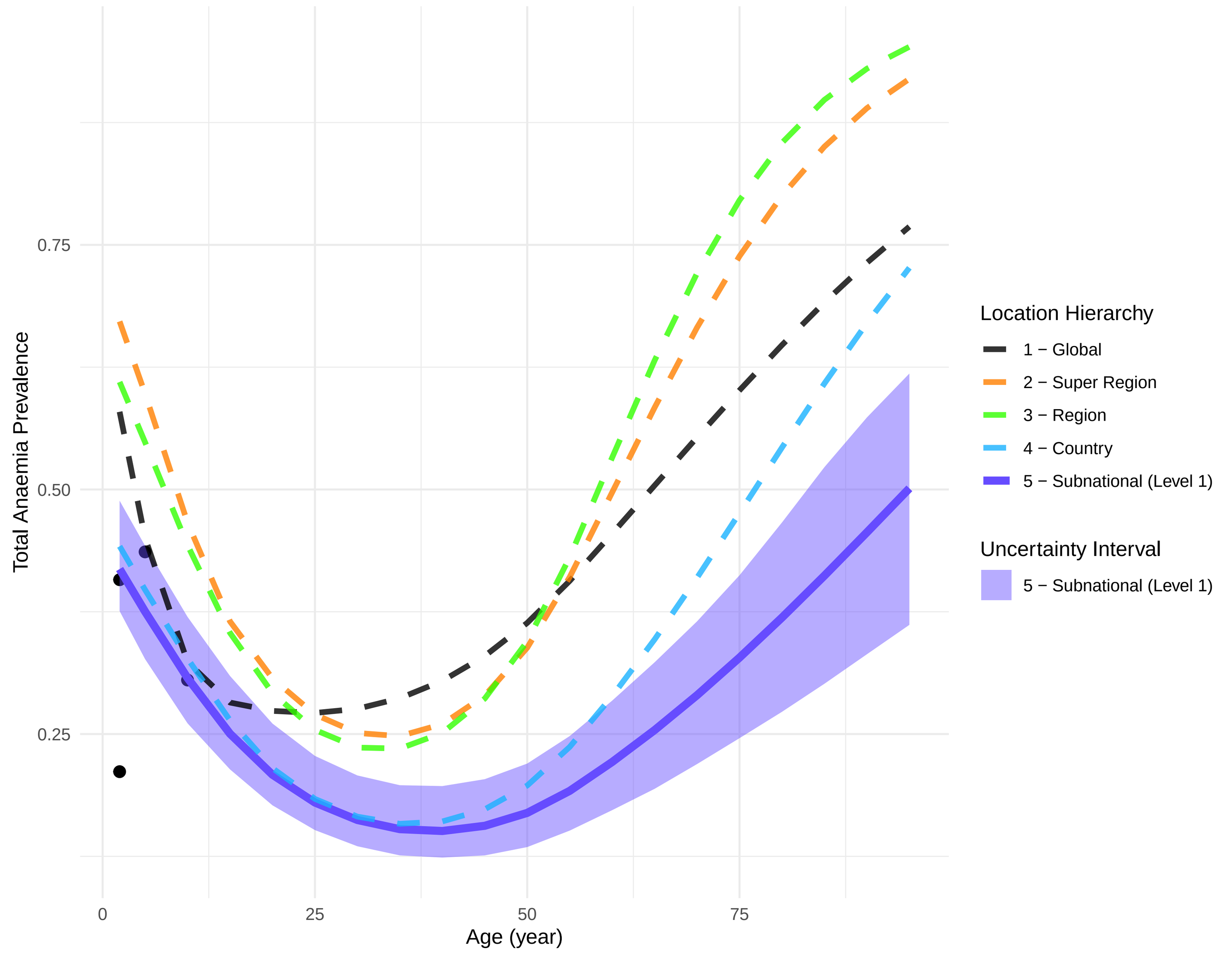

MR−BRT Age/Sex Fits − Males 2+ Years Old − Total Anaemia Prevalence − Kirinyaga
WHO−2024 Adjustment Method

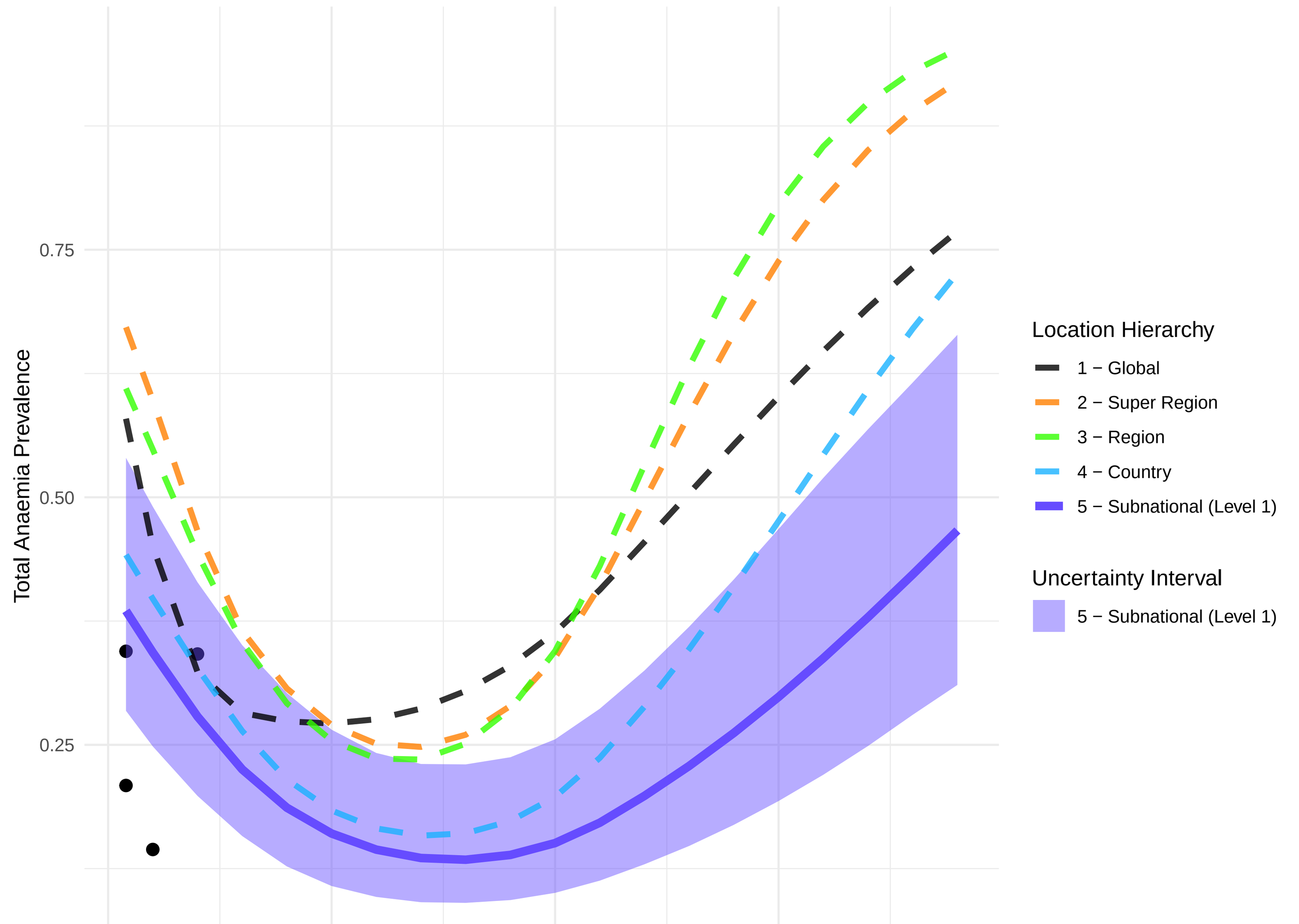

MR−BRT Age/Sex Fits − Males 2+ Years Old − Total Anaemia Prevalence − Kisii
WHO−2024 Adjustment Method

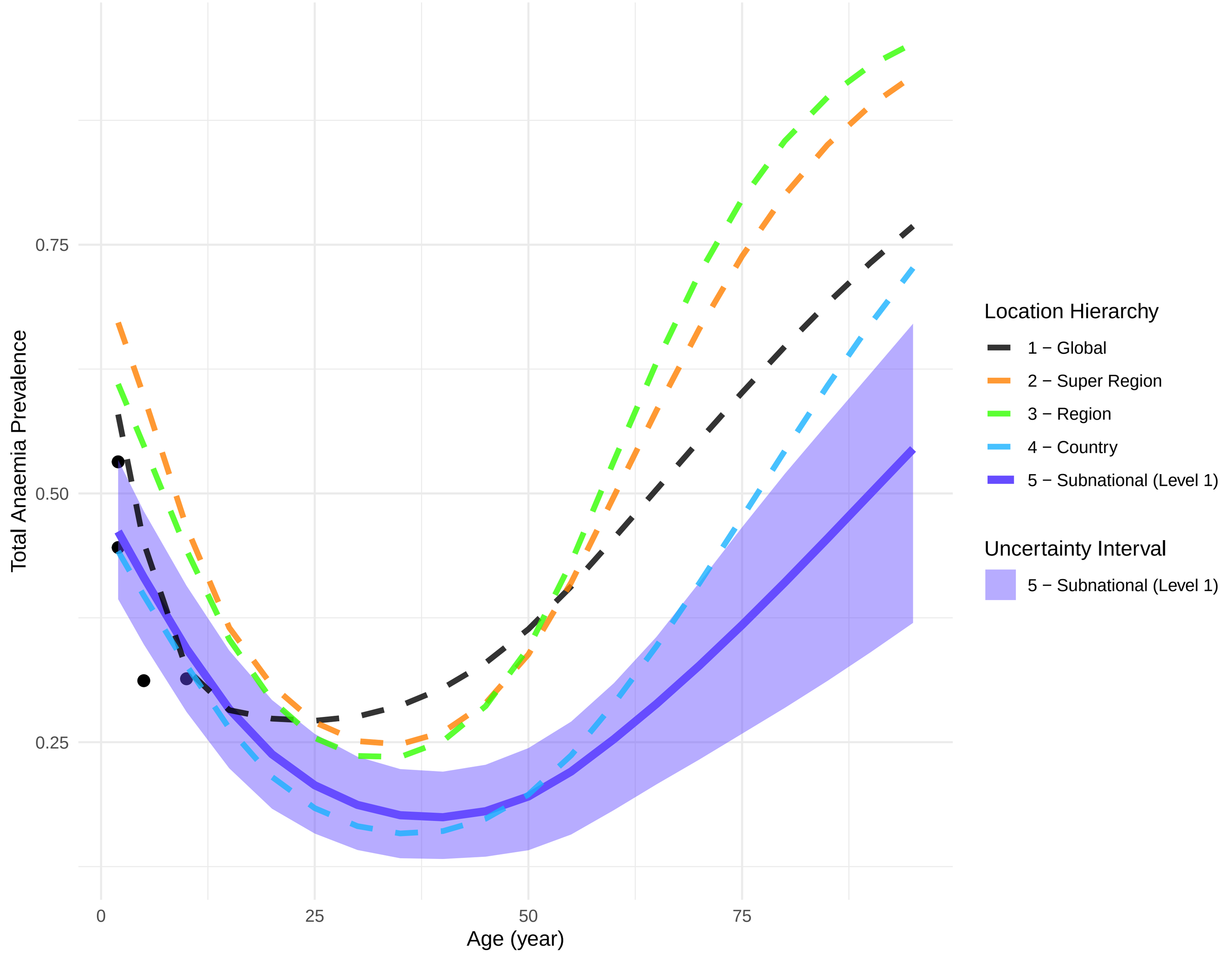

MR−BRT Age/Sex Fits − Males 2+ Years Old − Total Anaemia Prevalence − Kisumu
WHO−2024 Adjustment Method

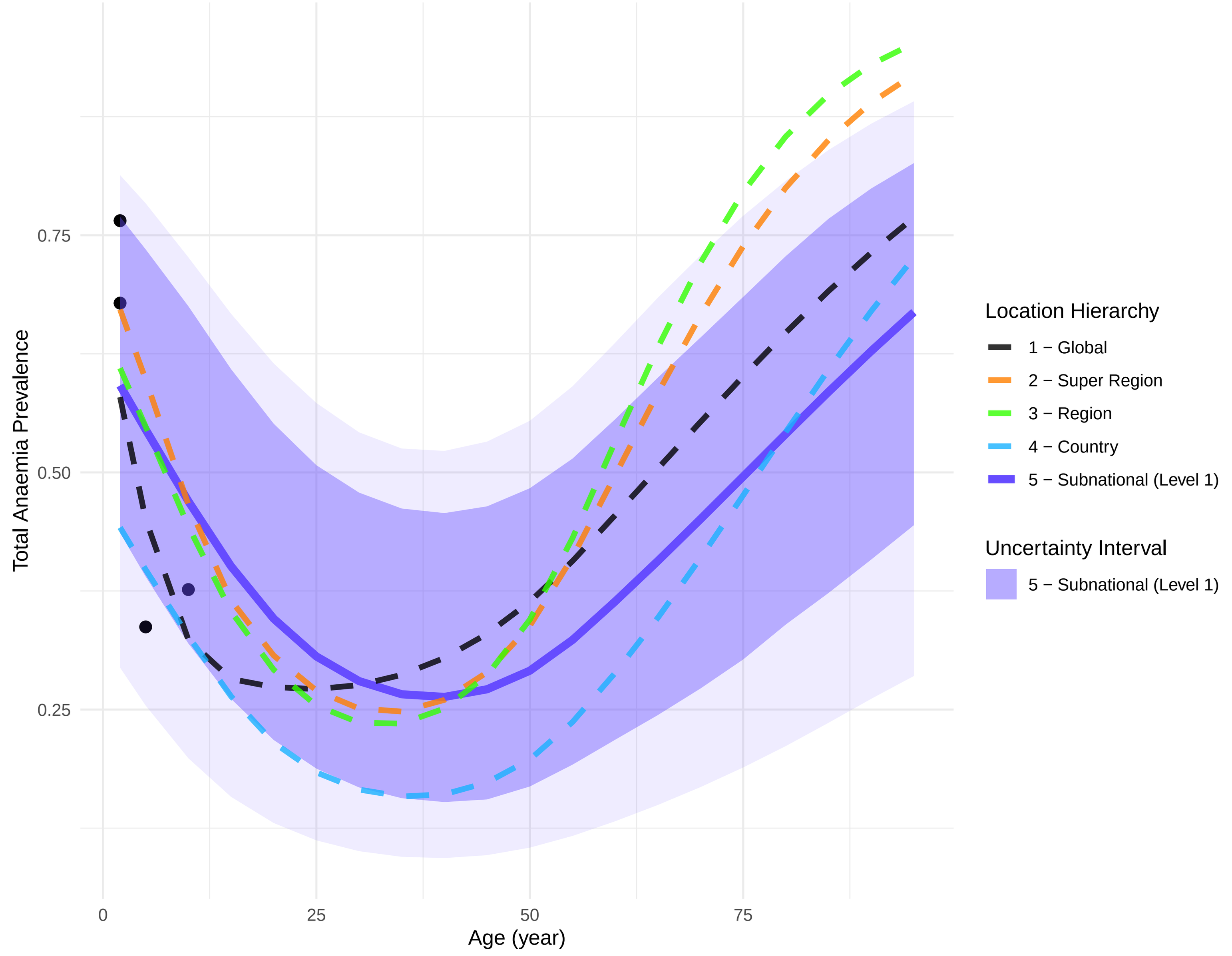

MR−BRT Age/Sex Fits − Males 2+ Years Old − Total Anaemia Prevalence − Kitui
WHO−2024 Adjustment Method

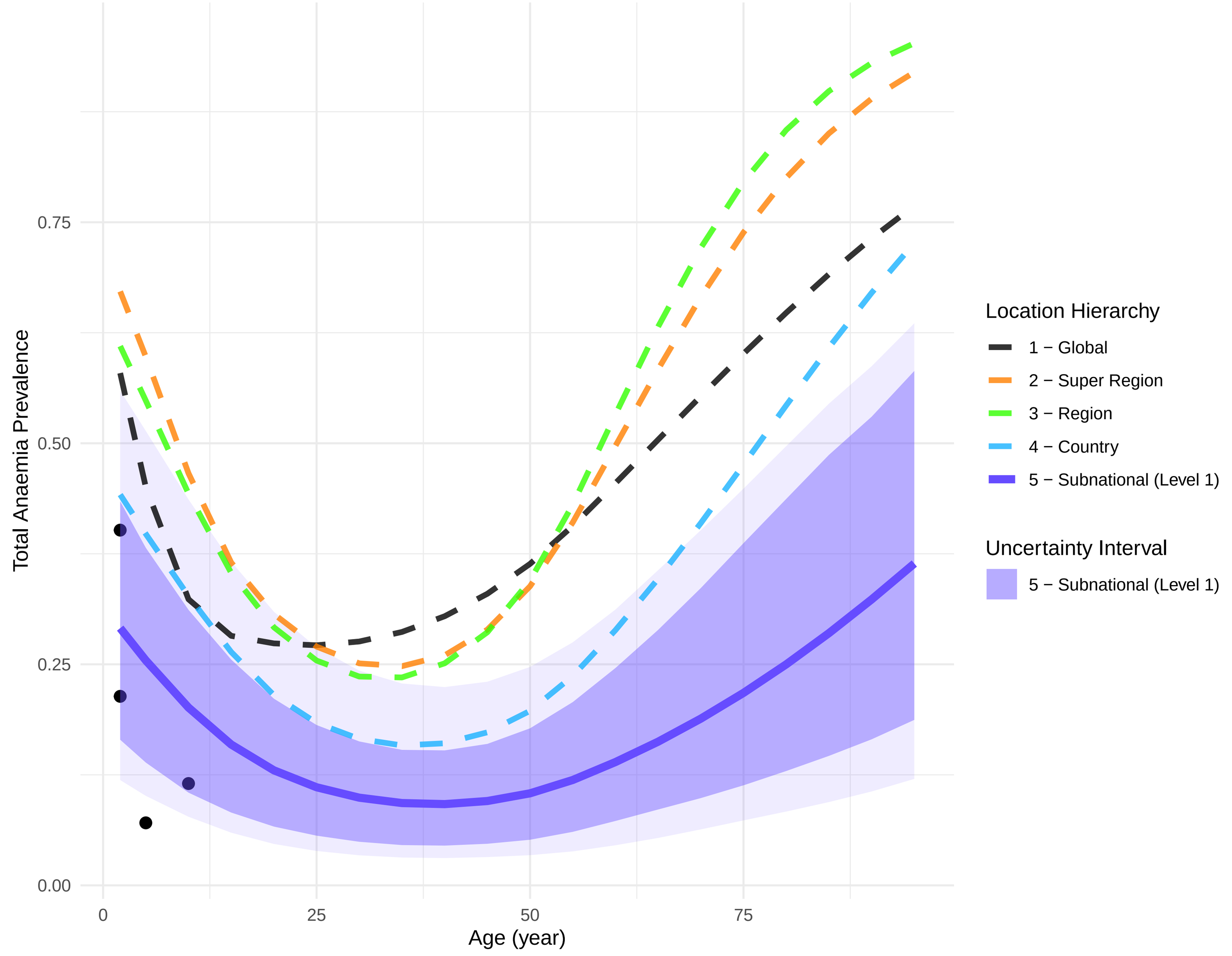

MR−BRT Age/Sex Fits − Males 2+ Years Old − Total Anaemia Prevalence − Kwale
WHO−2024 Adjustment Method

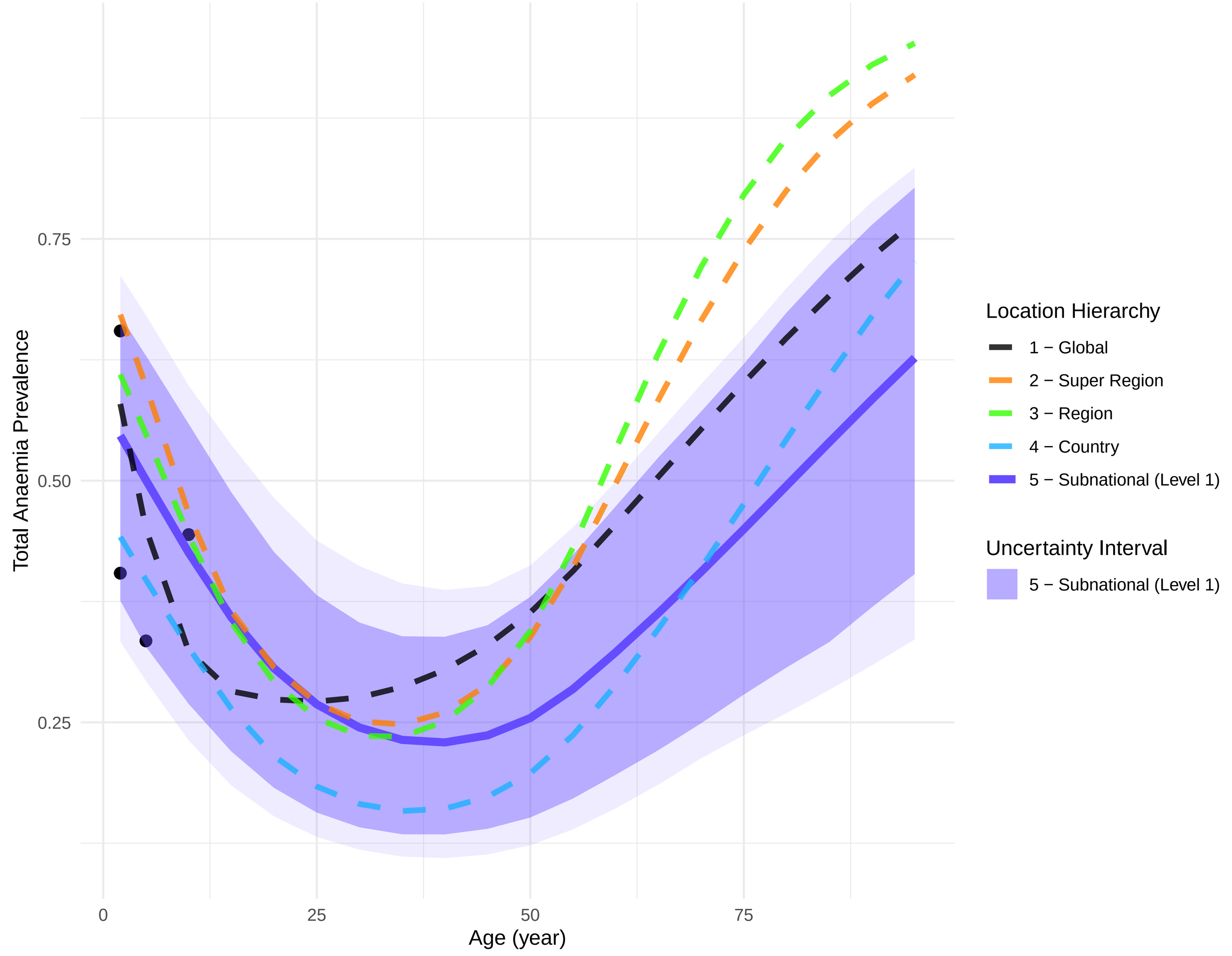

MR−BRT Age/Sex Fits − Males 2+ Years Old − Total Anaemia Prevalence − Laikipia
WHO−2024 Adjustment Method

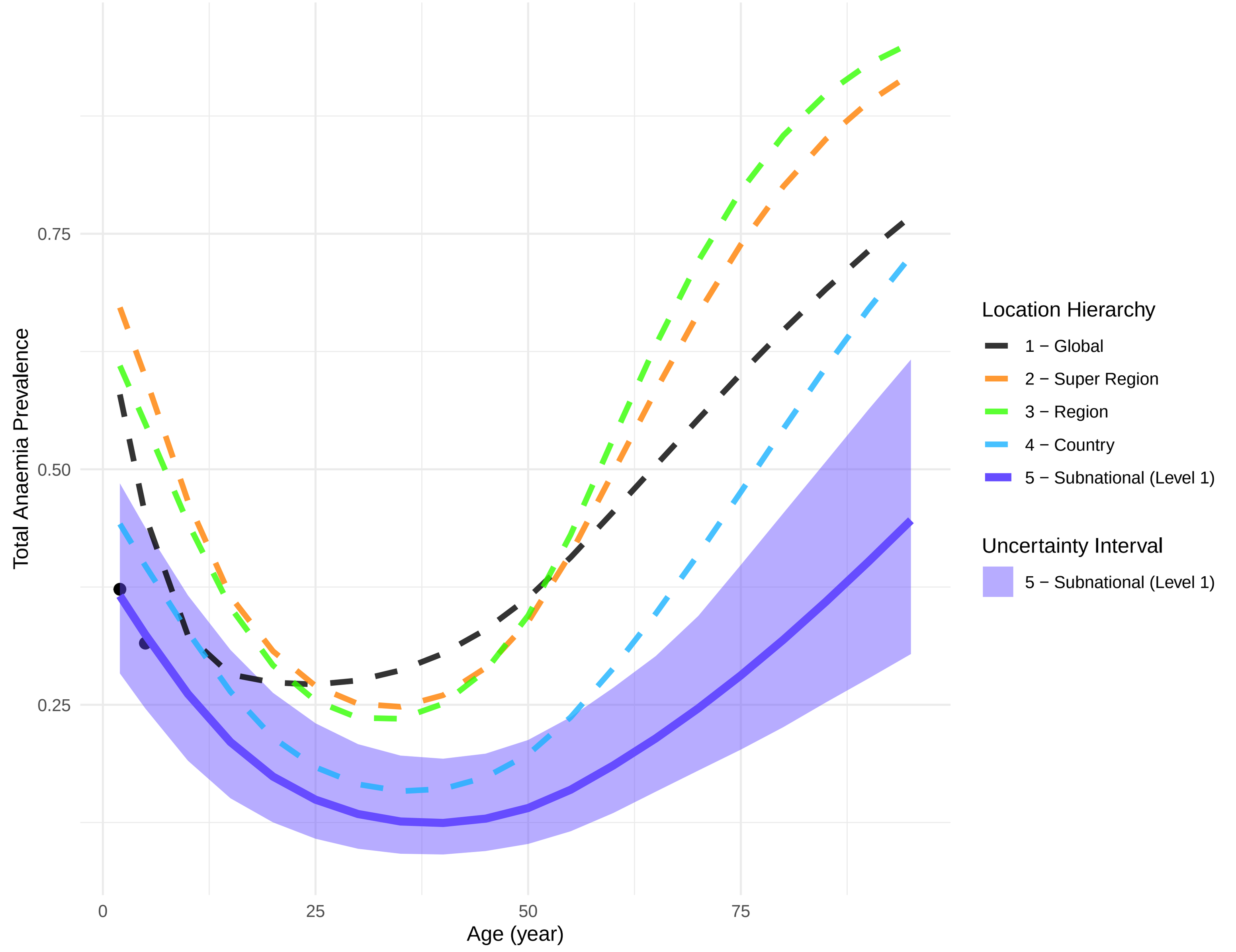

MR−BRT Age/Sex Fits − Males 2+ Years Old − Total Anaemia Prevalence − Lamu
WHO−2024 Adjustment Method

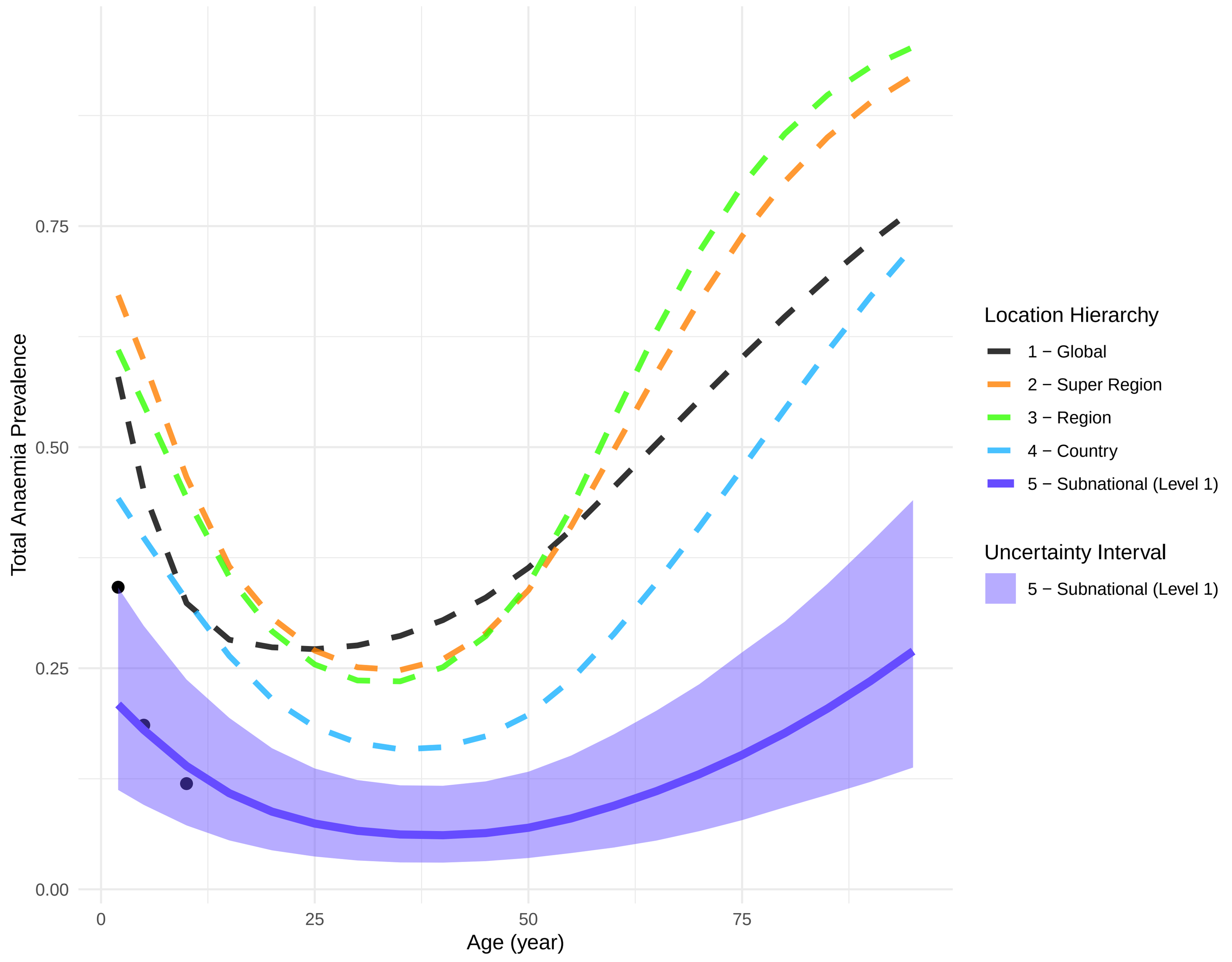

MR−BRT Age/Sex Fits − Males 2+ Years Old − Total Anaemia Prevalence − Machakos
WHO−2024 Adjustment Method

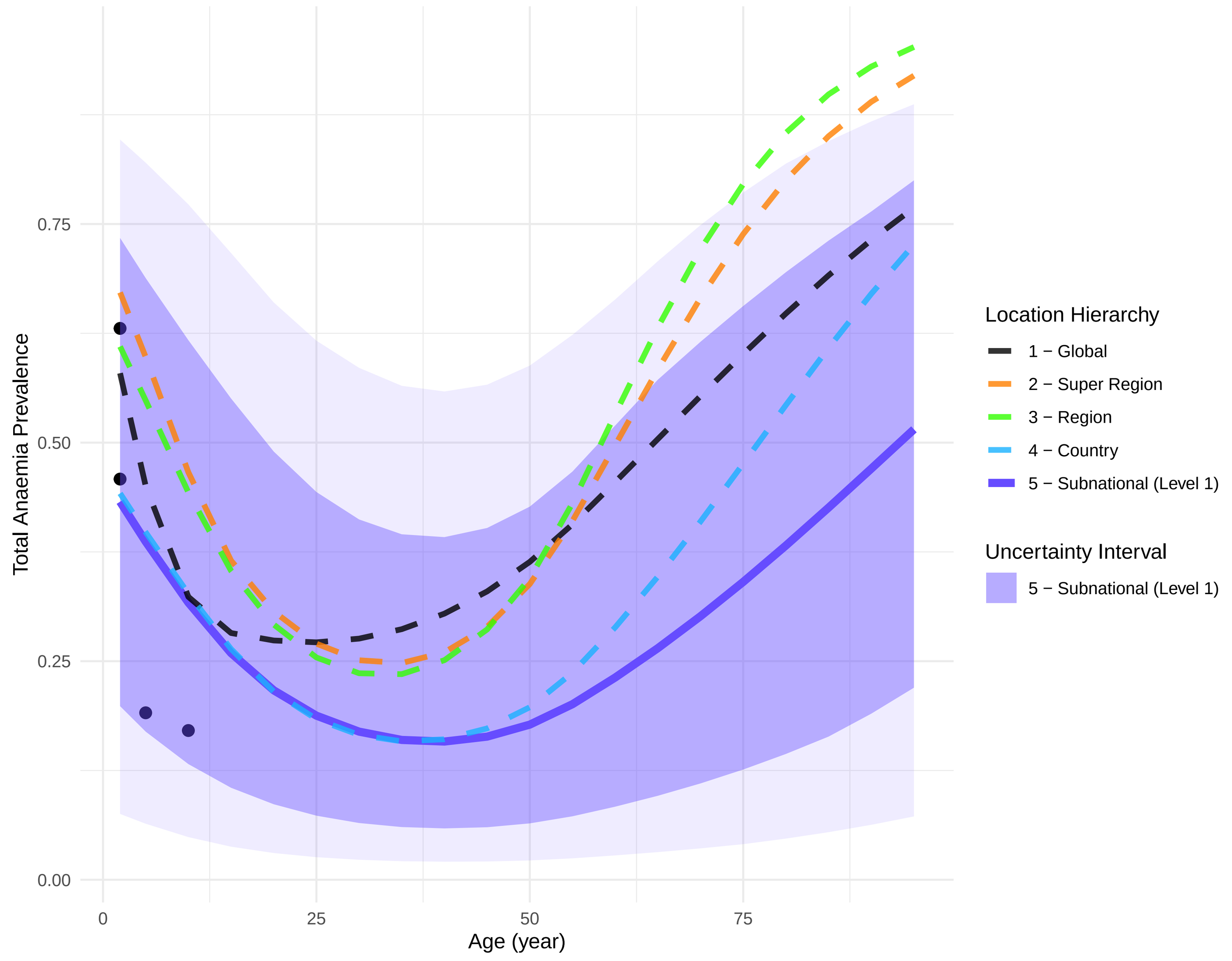

MR−BRT Age/Sex Fits − Males 2+ Years Old − Total Anaemia Prevalence − Makueni
WHO−2024 Adjustment Method

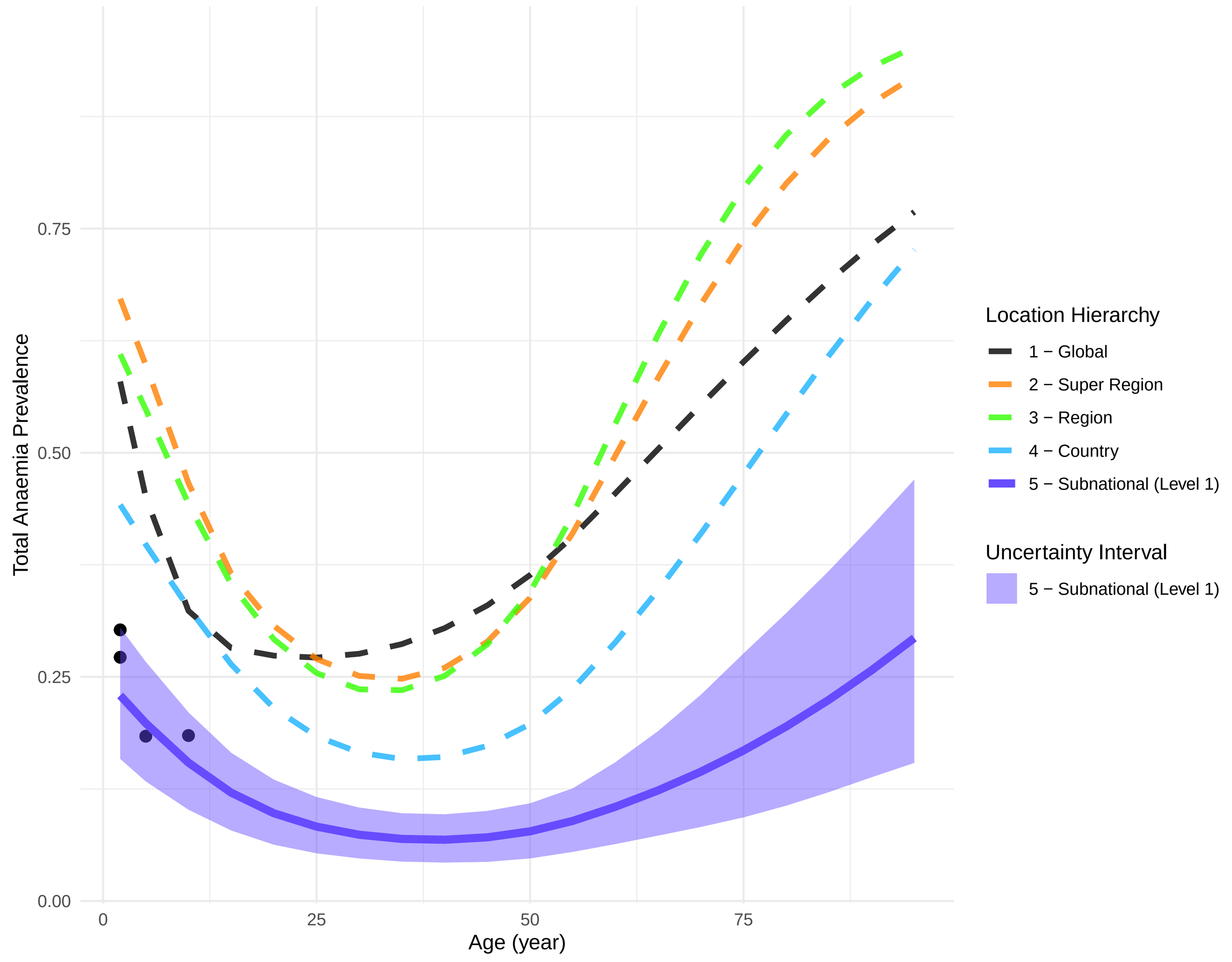

MR−BRT Age/Sex Fits − Males 2+ Years Old − Total Anaemia Prevalence − Mandera
WHO−2024 Adjustment Method

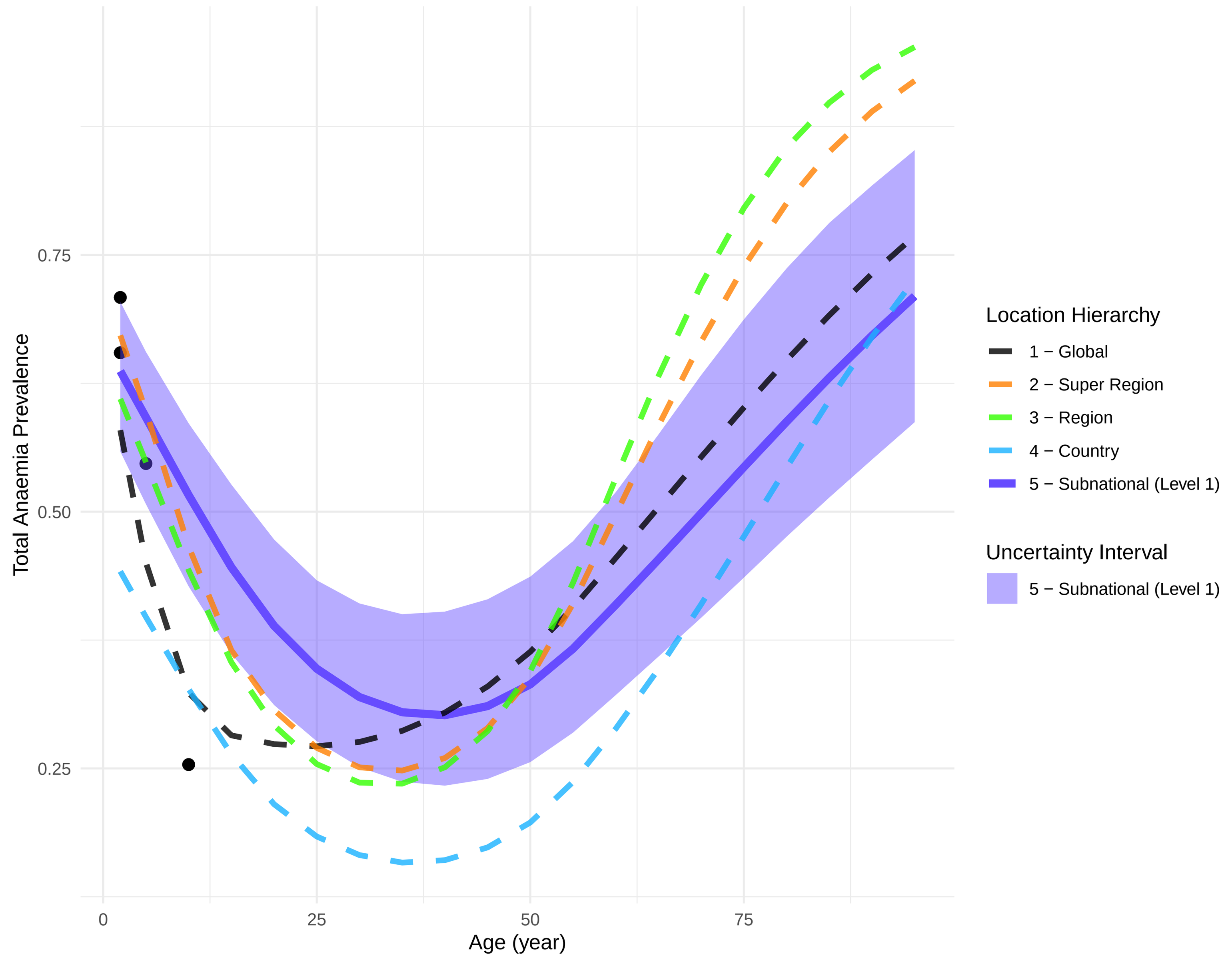

MR−BRT Age/Sex Fits − Males 2+ Years Old − Total Anaemia Prevalence − Marsabit
WHO−2024 Adjustment Method

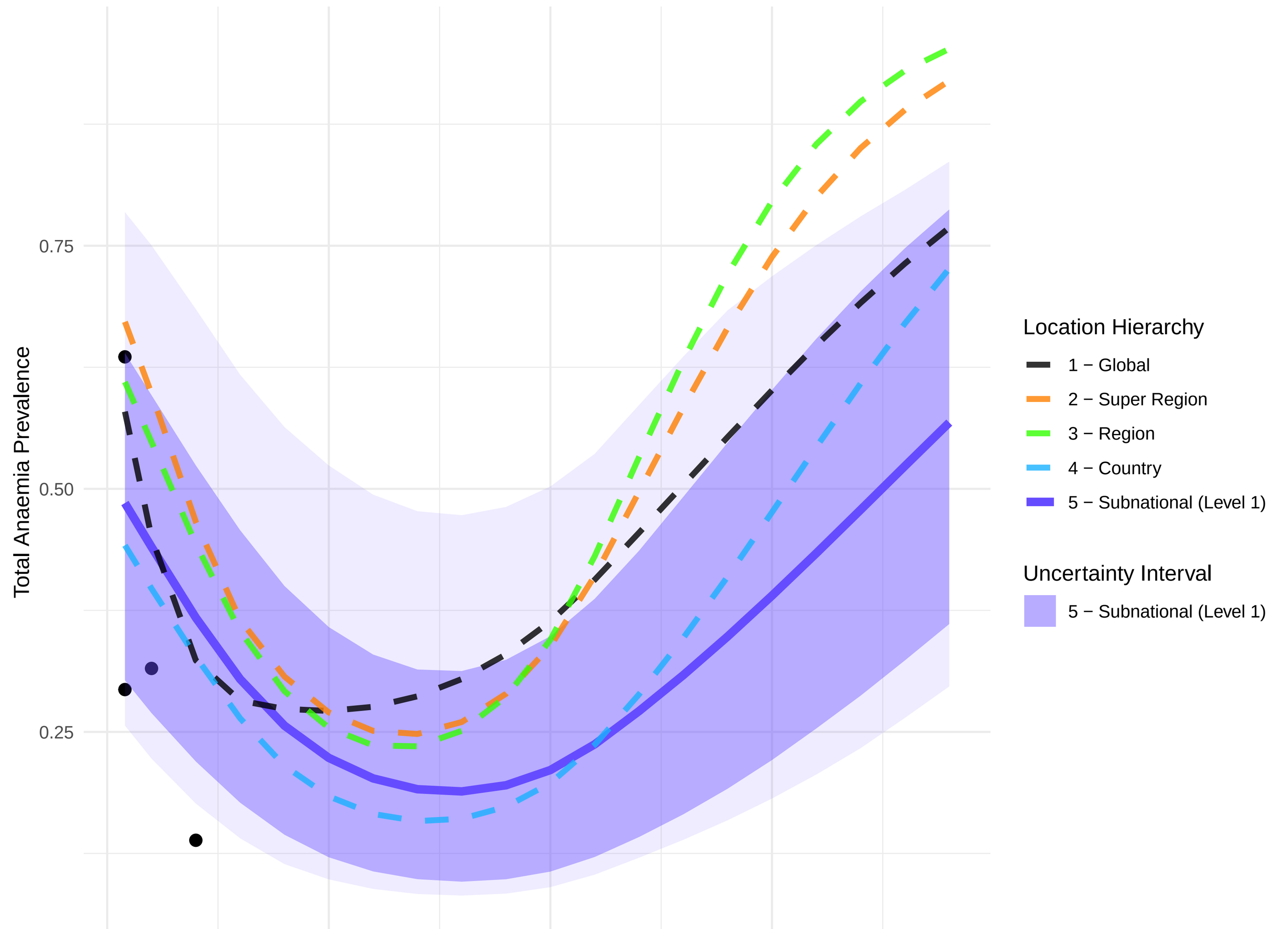

MR−BRT Age/Sex Fits − Males 2+ Years Old − Total Anaemia Prevalence − Meru
WHO−2024 Adjustment Method

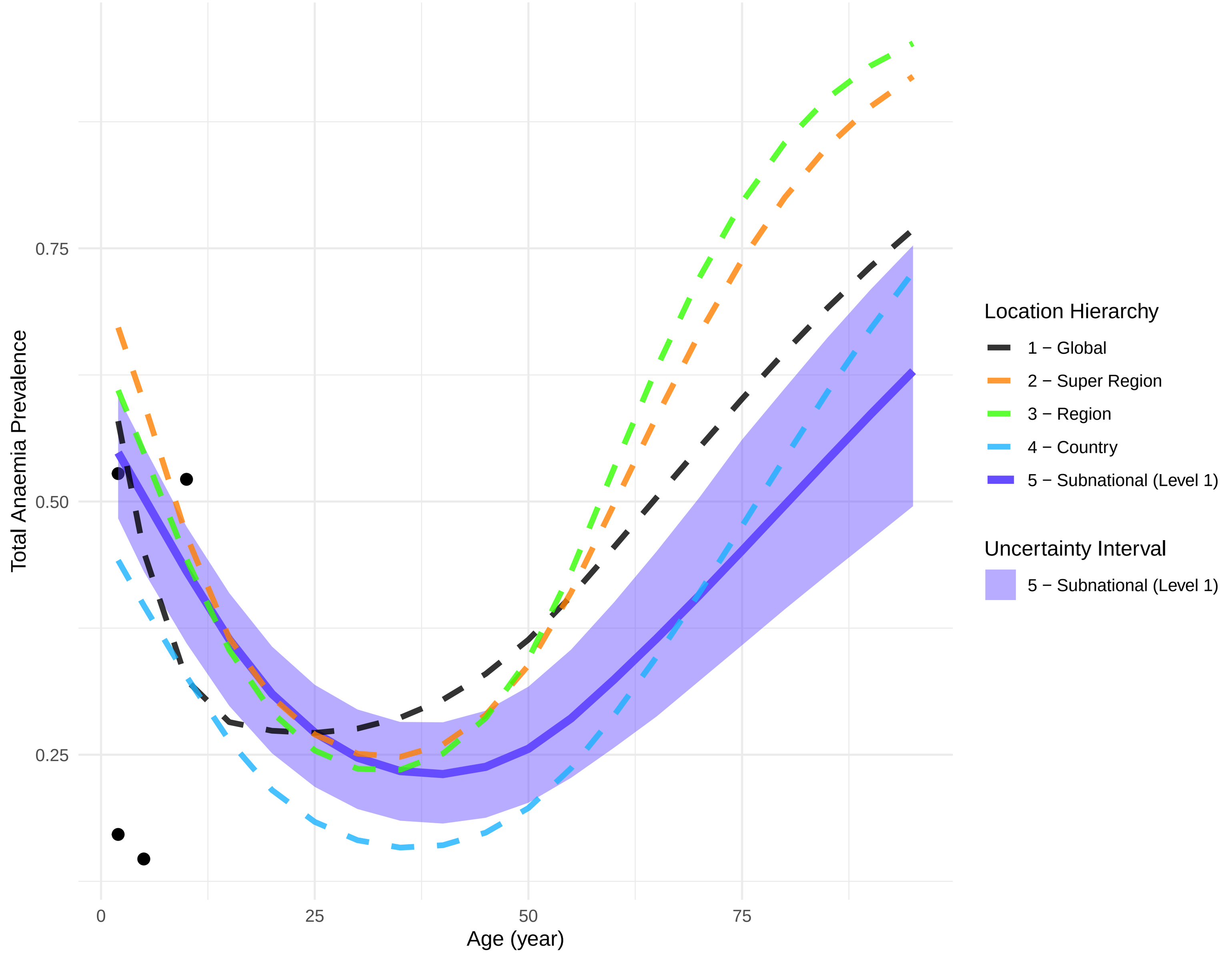

MR−BRT Age/Sex Fits − Males 2+ Years Old − Total Anaemia Prevalence − Migori
WHO−2024 Adjustment Method

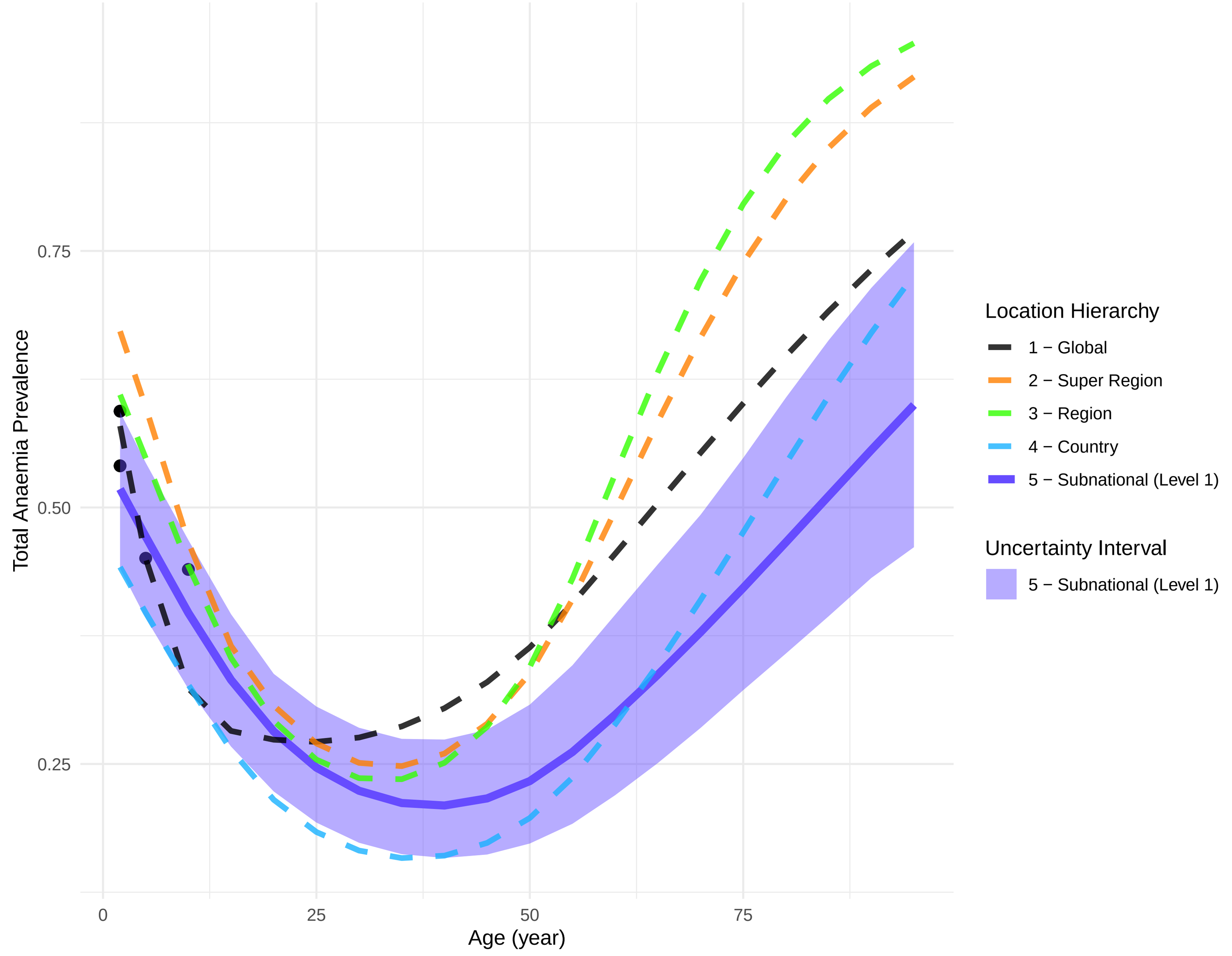

MR−BRT Age/Sex Fits − Males 2+ Years Old − Total Anaemia Prevalence − Mombasa
WHO−2024 Adjustment Method

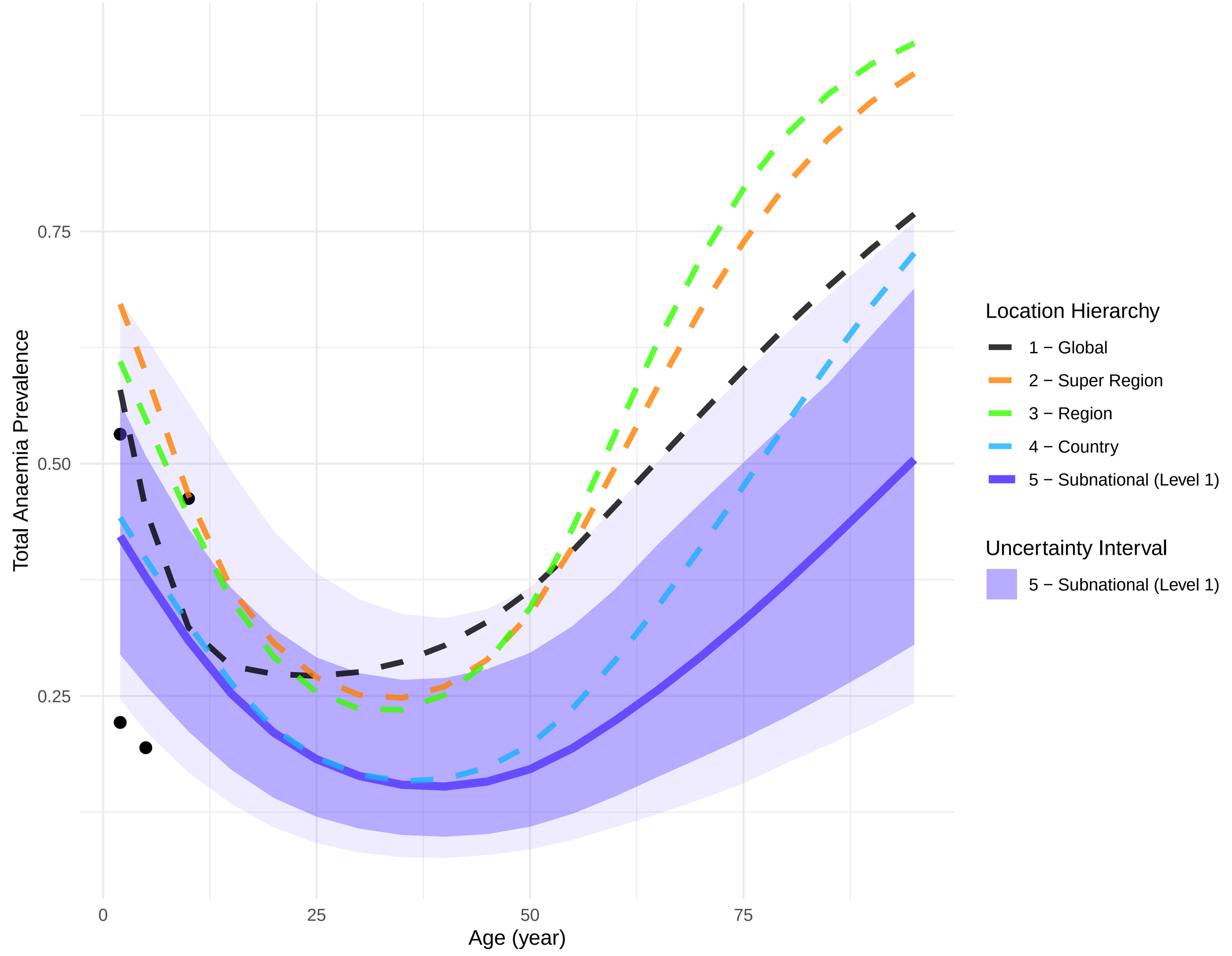

MR−BRT Age/Sex Fits − Males 2+ Years Old − Total Anaemia Prevalence − Murang'a
WHO−2024 Adjustment Method

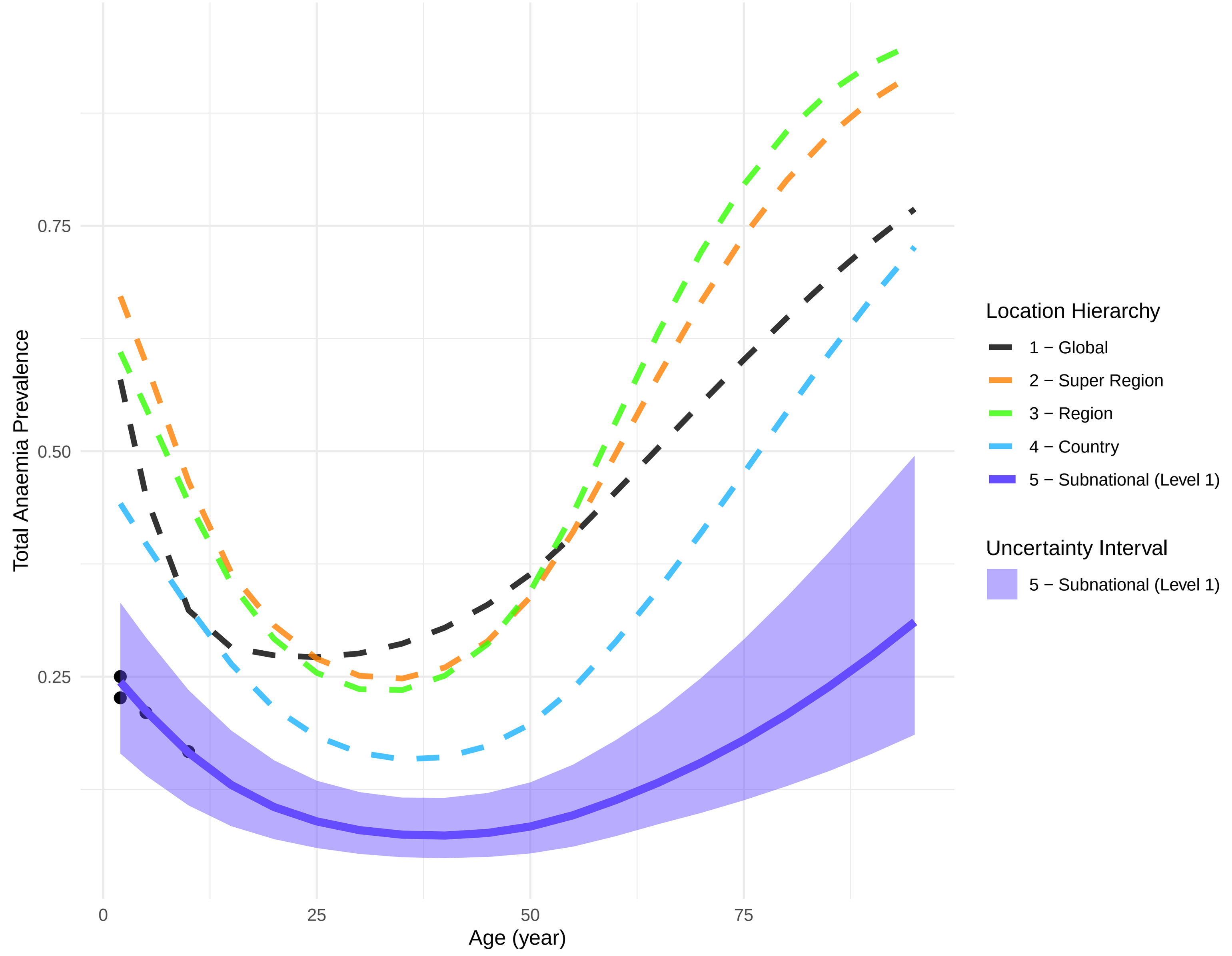

MR−BRT Age/Sex Fits − Males 2+ Years Old − Total Anaemia Prevalence − Nairobi
WHO−2024 Adjustment Method

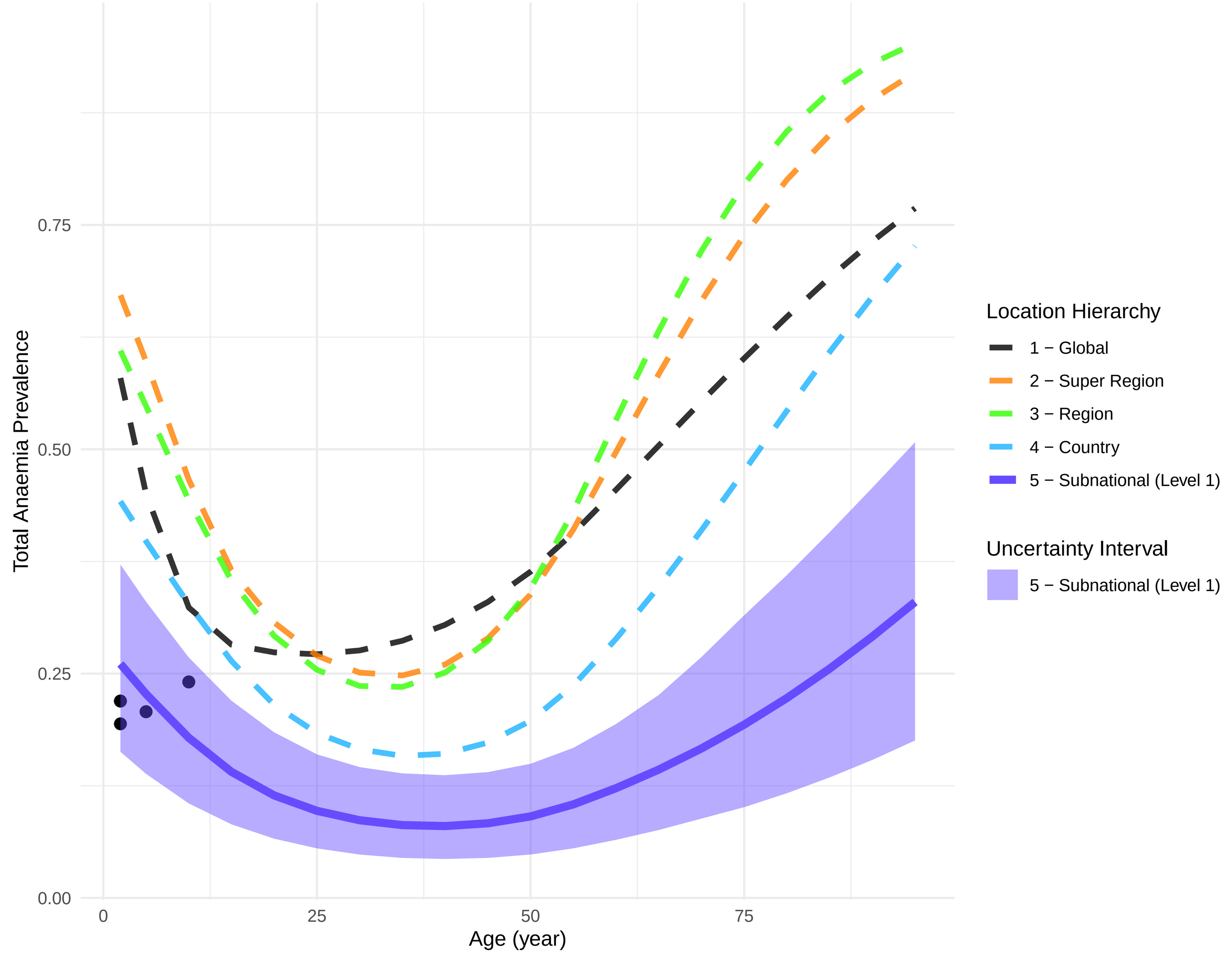

MR−BRT Age/Sex Fits − Males 2+ Years Old − Total Anaemia Prevalence − Nakuru
WHO−2024 Adjustment Method

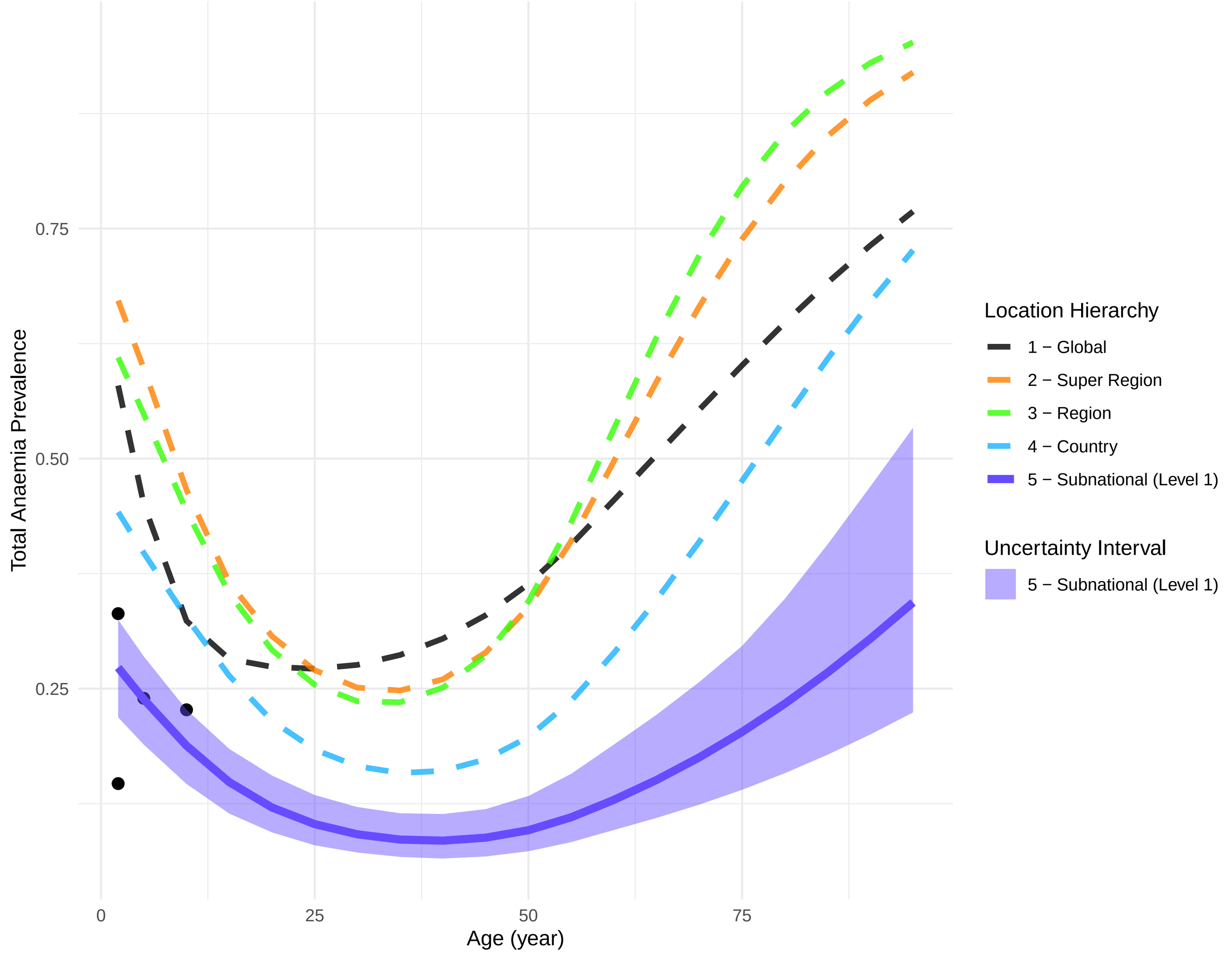

MR−BRT Age/Sex Fits − Males 2+ Years Old − Total Anaemia Prevalence − Nandi
WHO−2024 Adjustment Method

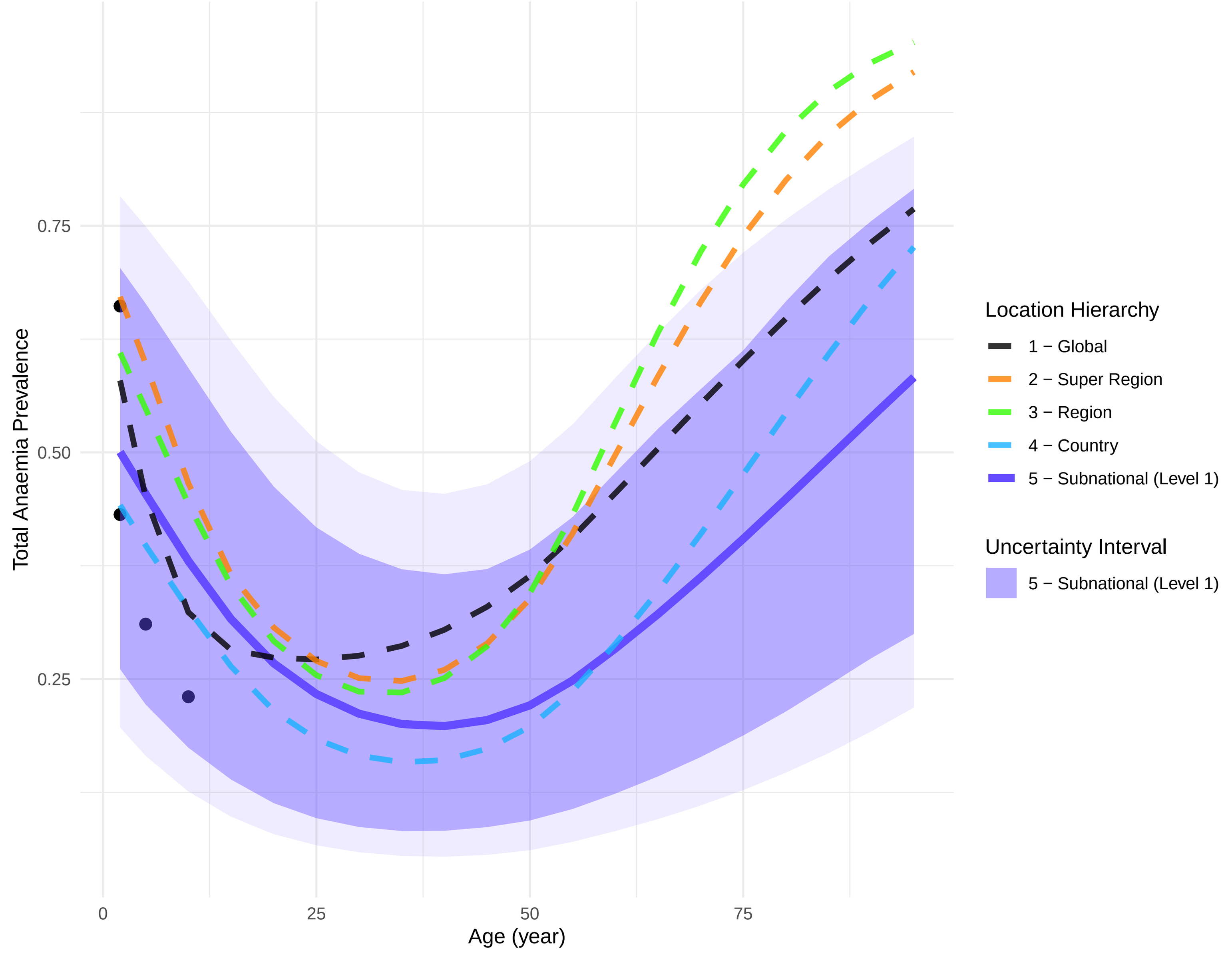

MR−BRT Age/Sex Fits − Males 2+ Years Old − Total Anaemia Prevalence − Narok
WHO−2024 Adjustment Method

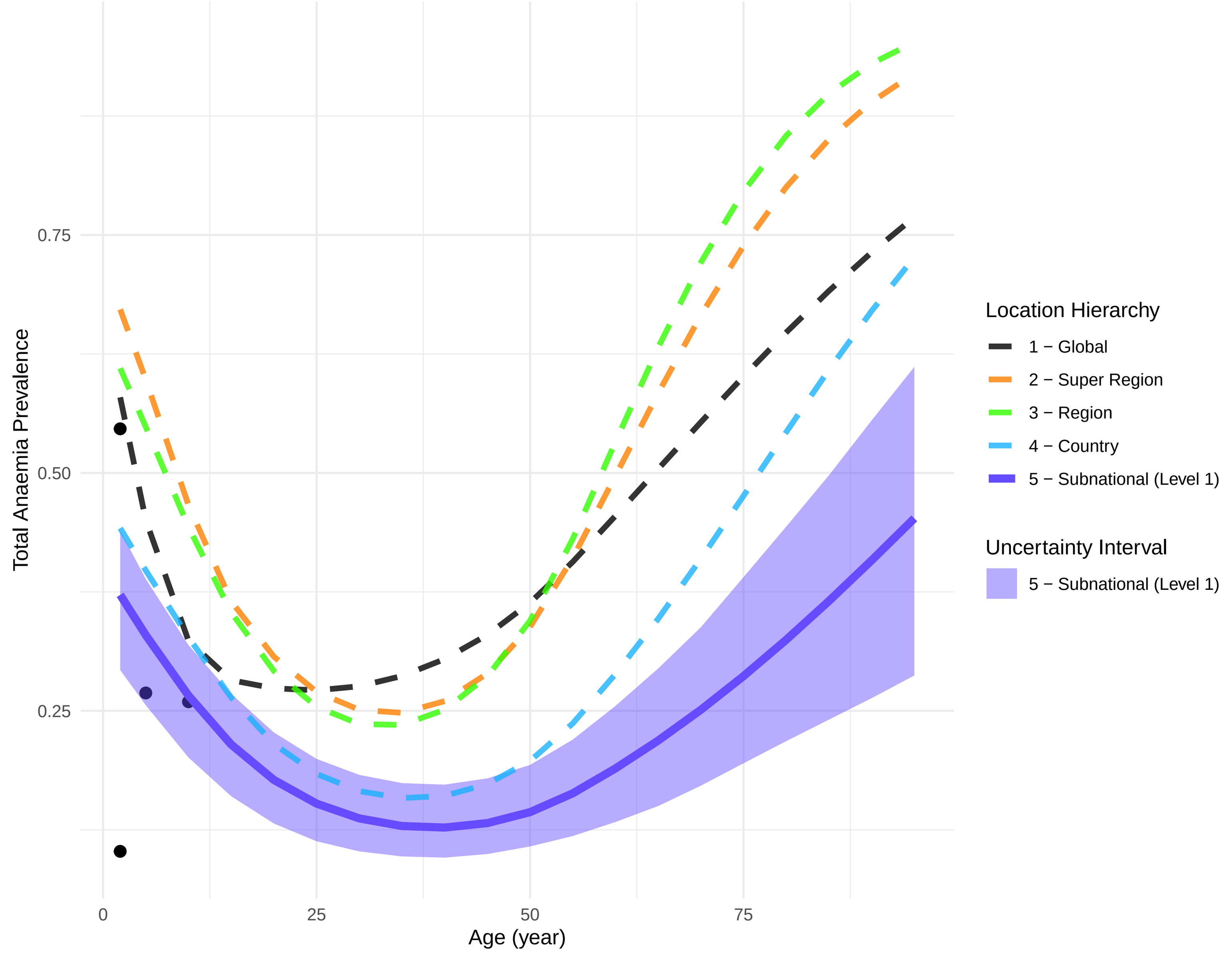

MR−BRT Age/Sex Fits − Males 2+ Years Old − Total Anaemia Prevalence − Nyamira
WHO−2024 Adjustment Method

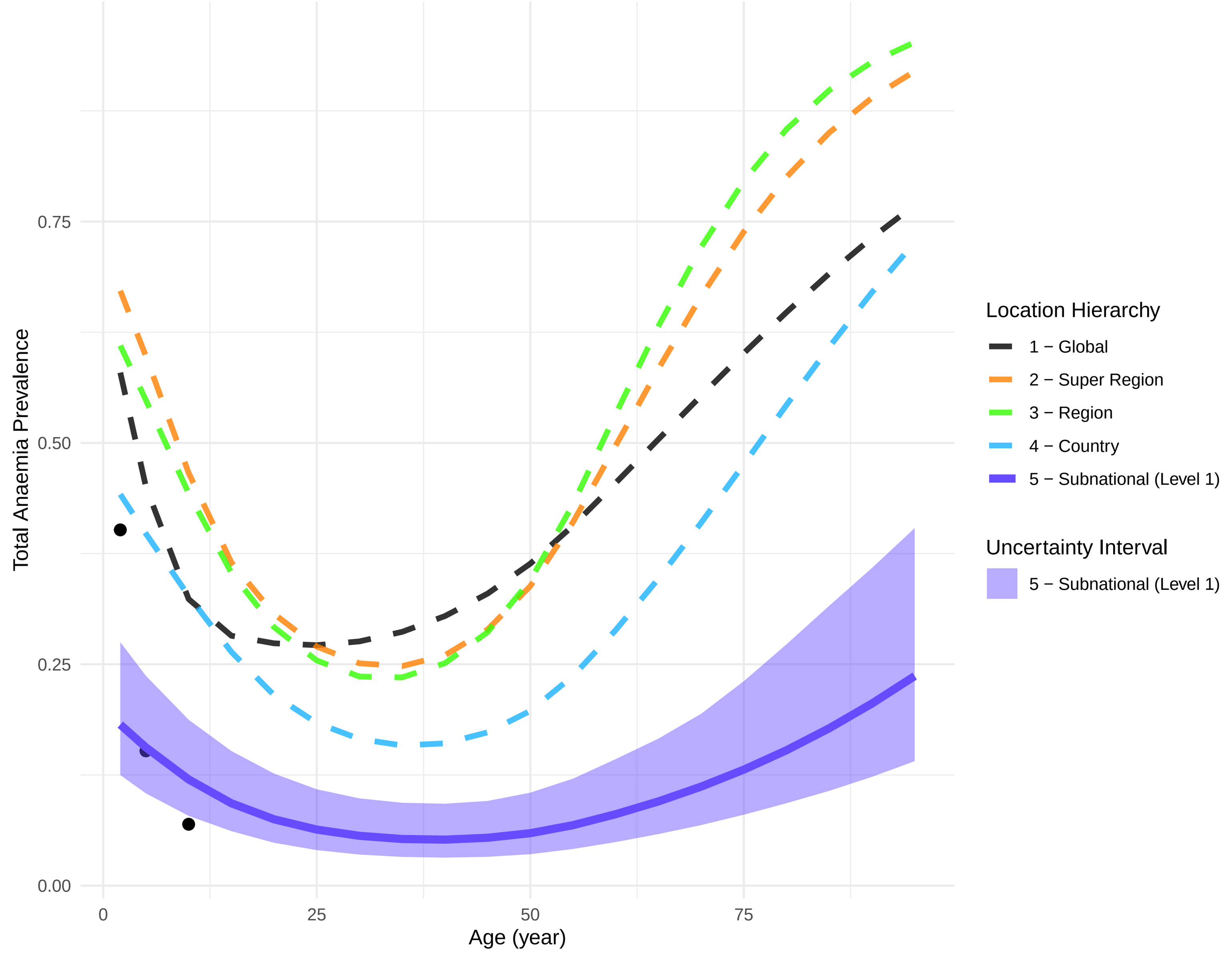

MR−BRT Age/Sex Fits − Males 2+ Years Old − Total Anaemia Prevalence − Nyandarua
WHO−2024 Adjustment Method

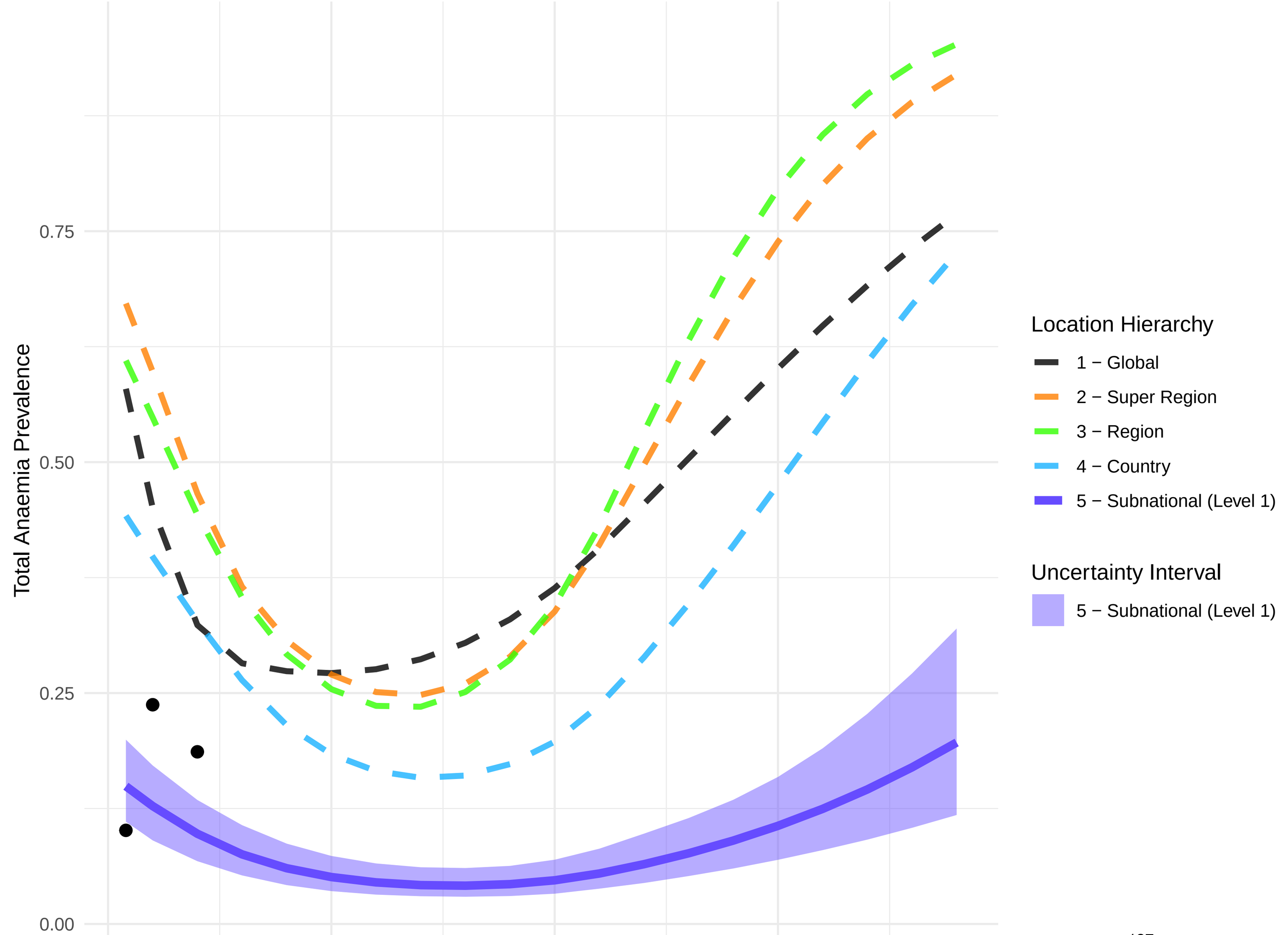

MR−BRT Age/Sex Fits − Males 2+ Years Old − Total Anaemia Prevalence − Nyeri
WHO−2024 Adjustment Method

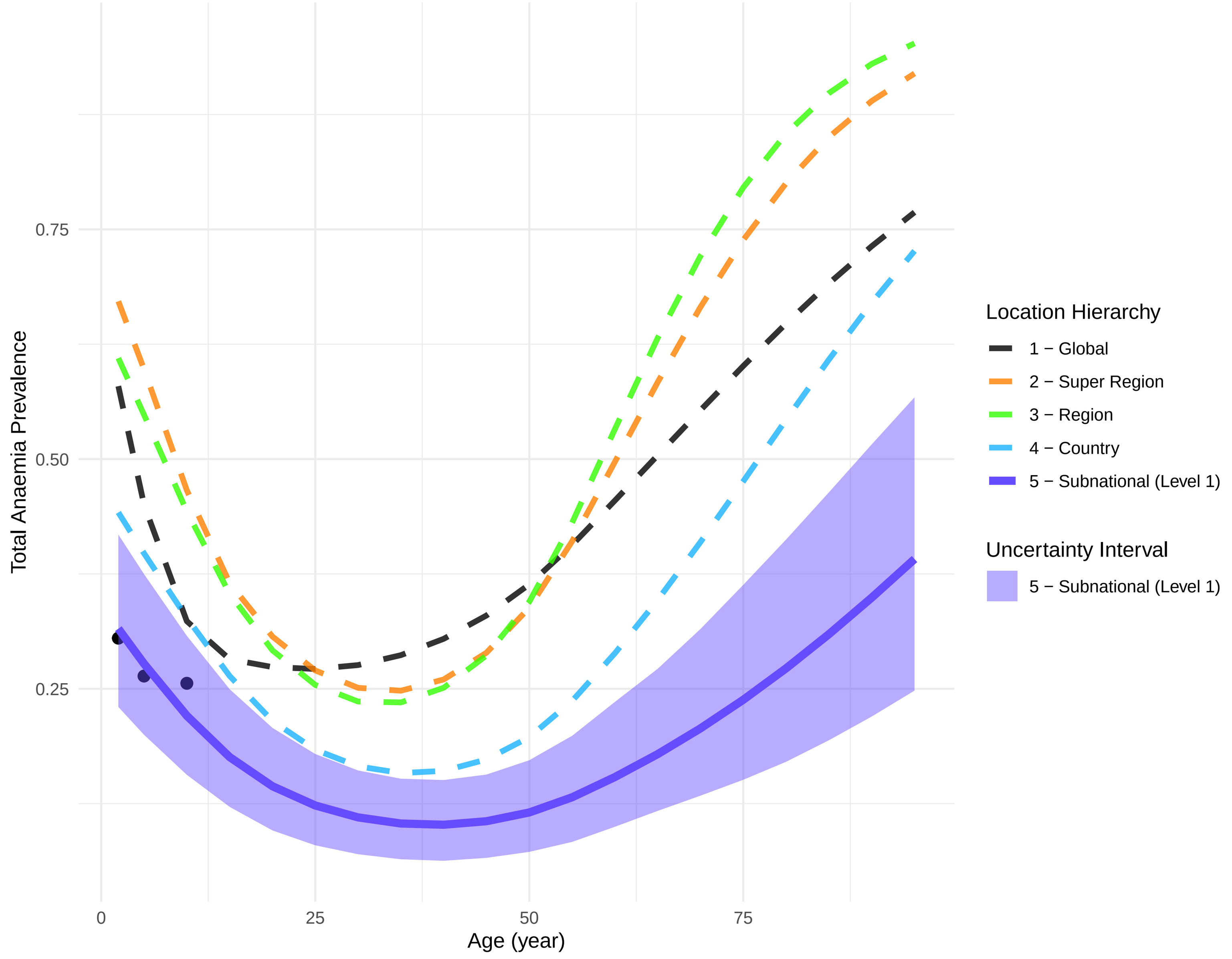

MR−BRT Age/Sex Fits − Males 2+ Years Old − Total Anaemia Prevalence − Samburu
WHO−2024 Adjustment Method

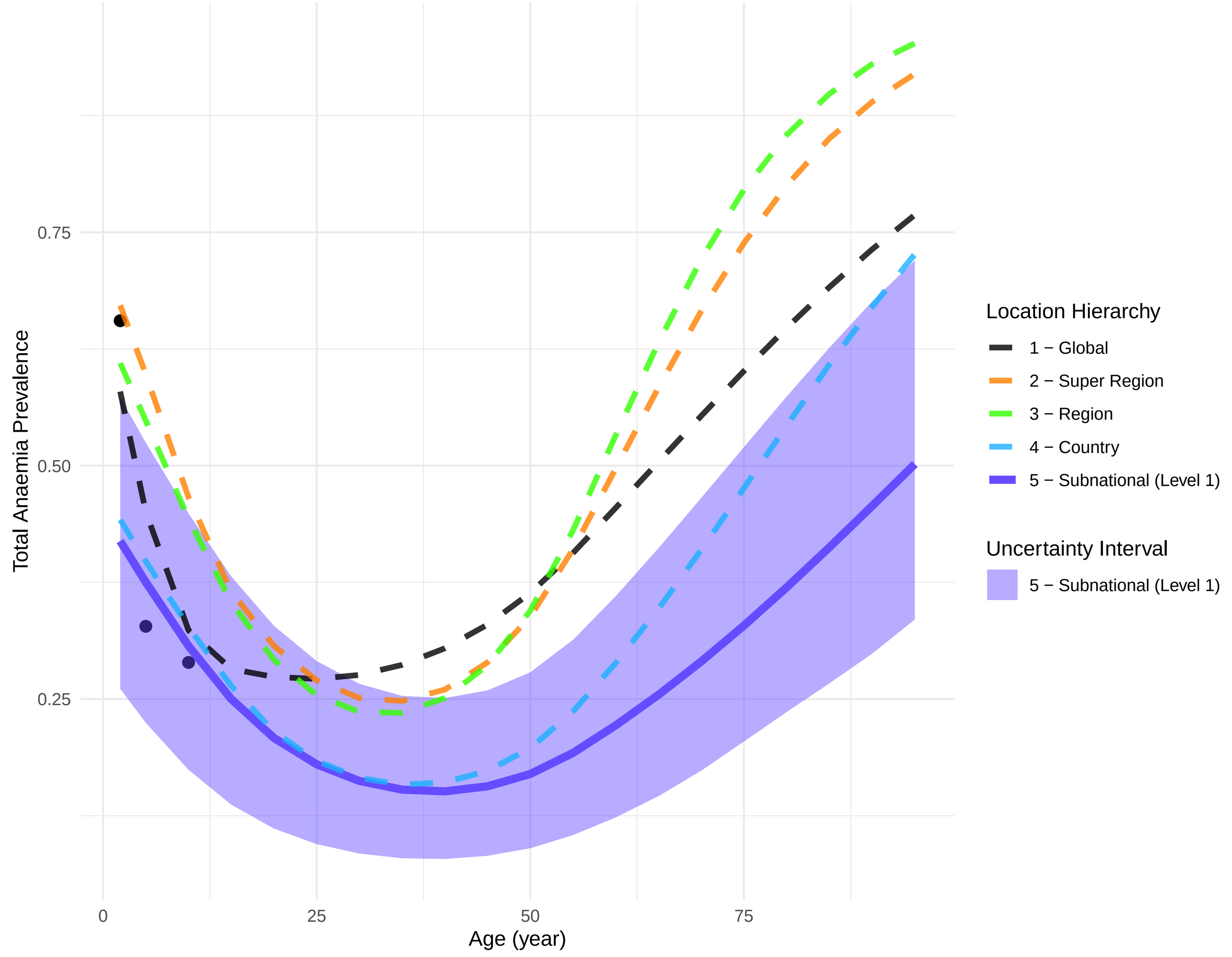

MR−BRT Age/Sex Fits − Males 2+ Years Old − Total Anaemia Prevalence − Siaya
WHO−2024 Adjustment Method

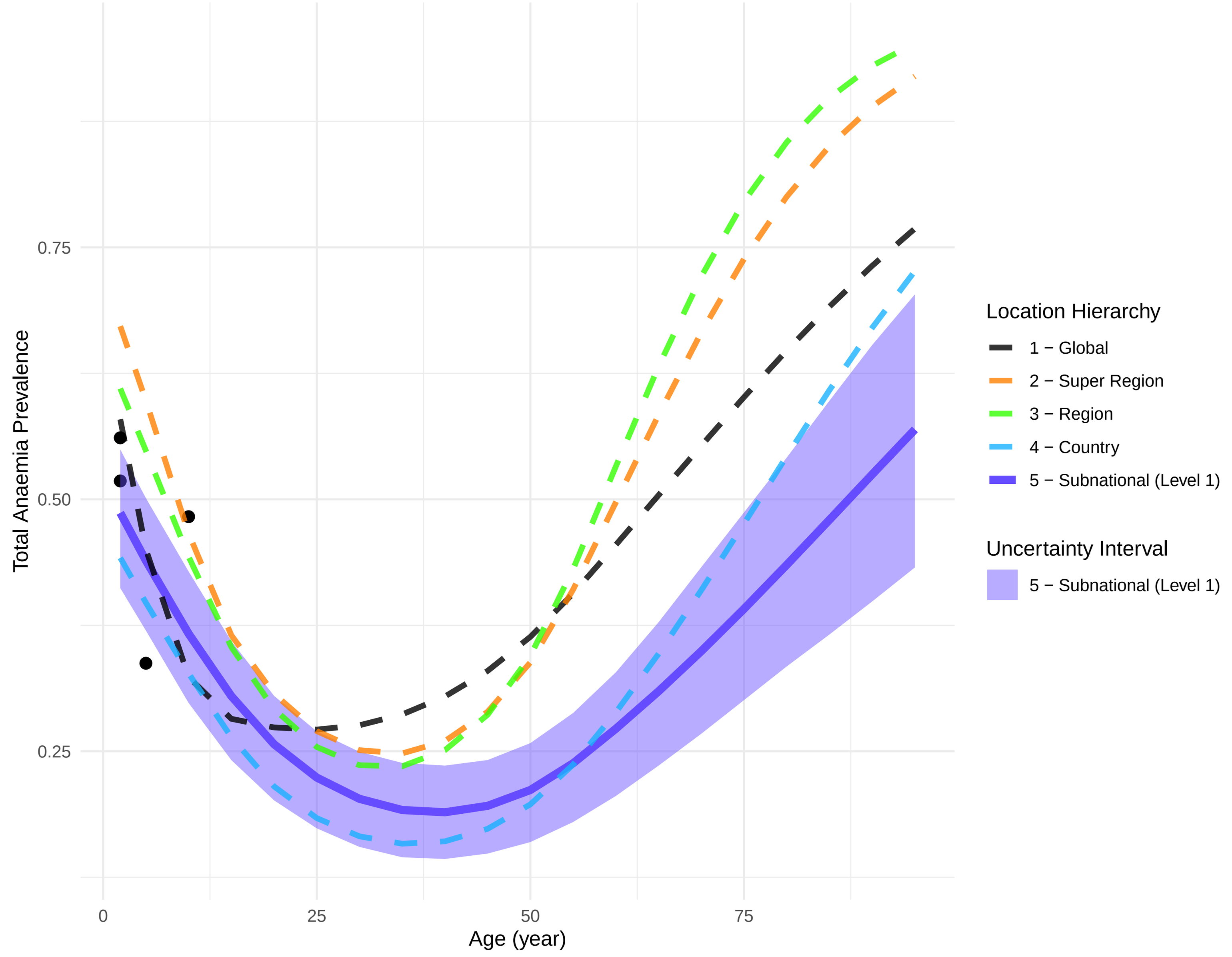

MR−BRT Age/Sex Fits − Males 2+ Years Old − Total Anaemia Prevalence − Taita Taveta
WHO−2024 Adjustment Method

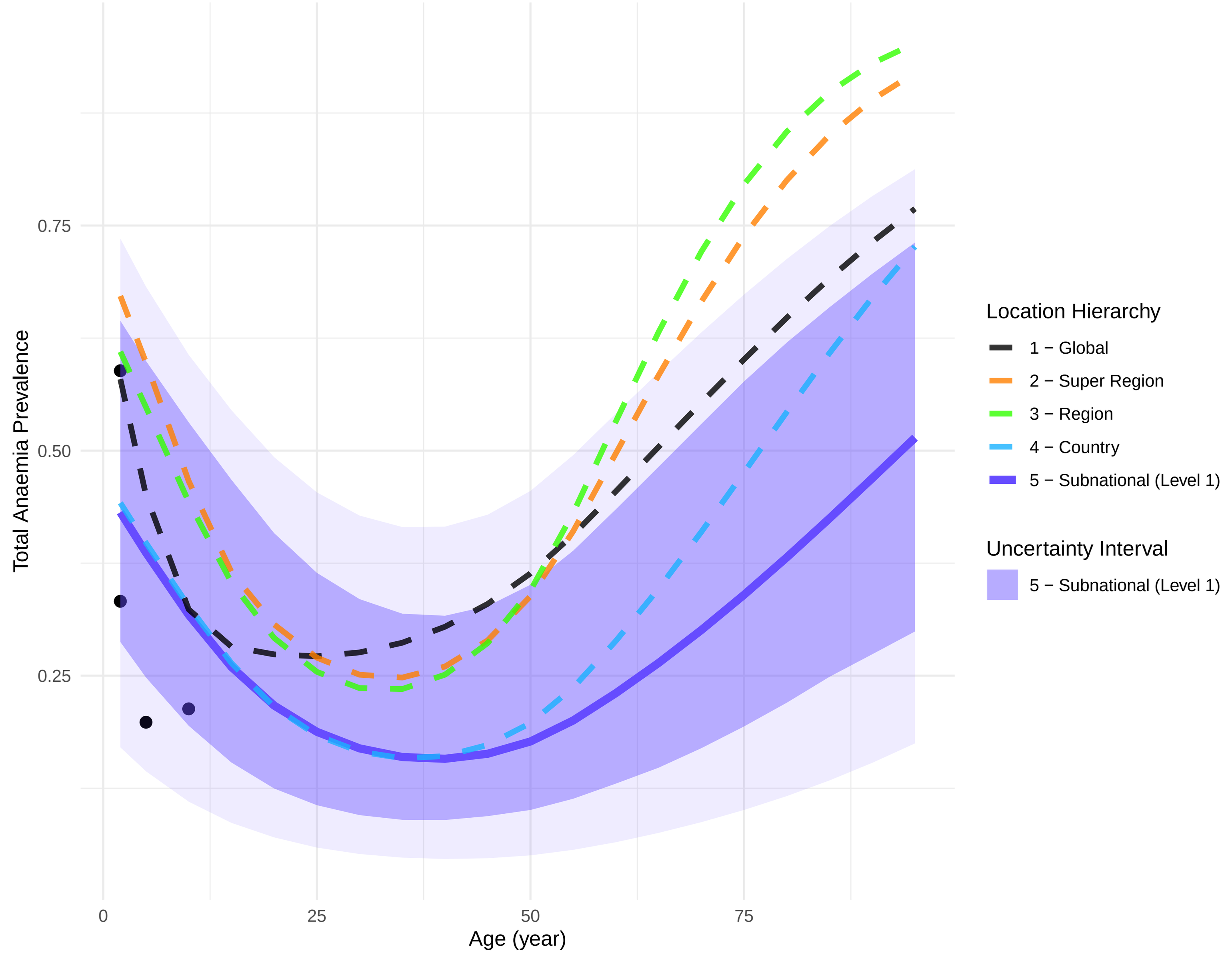

MR−BRT Age/Sex Fits − Males 2+ Years Old − Total Anaemia Prevalence − Tana River
WHO−2024 Adjustment Method

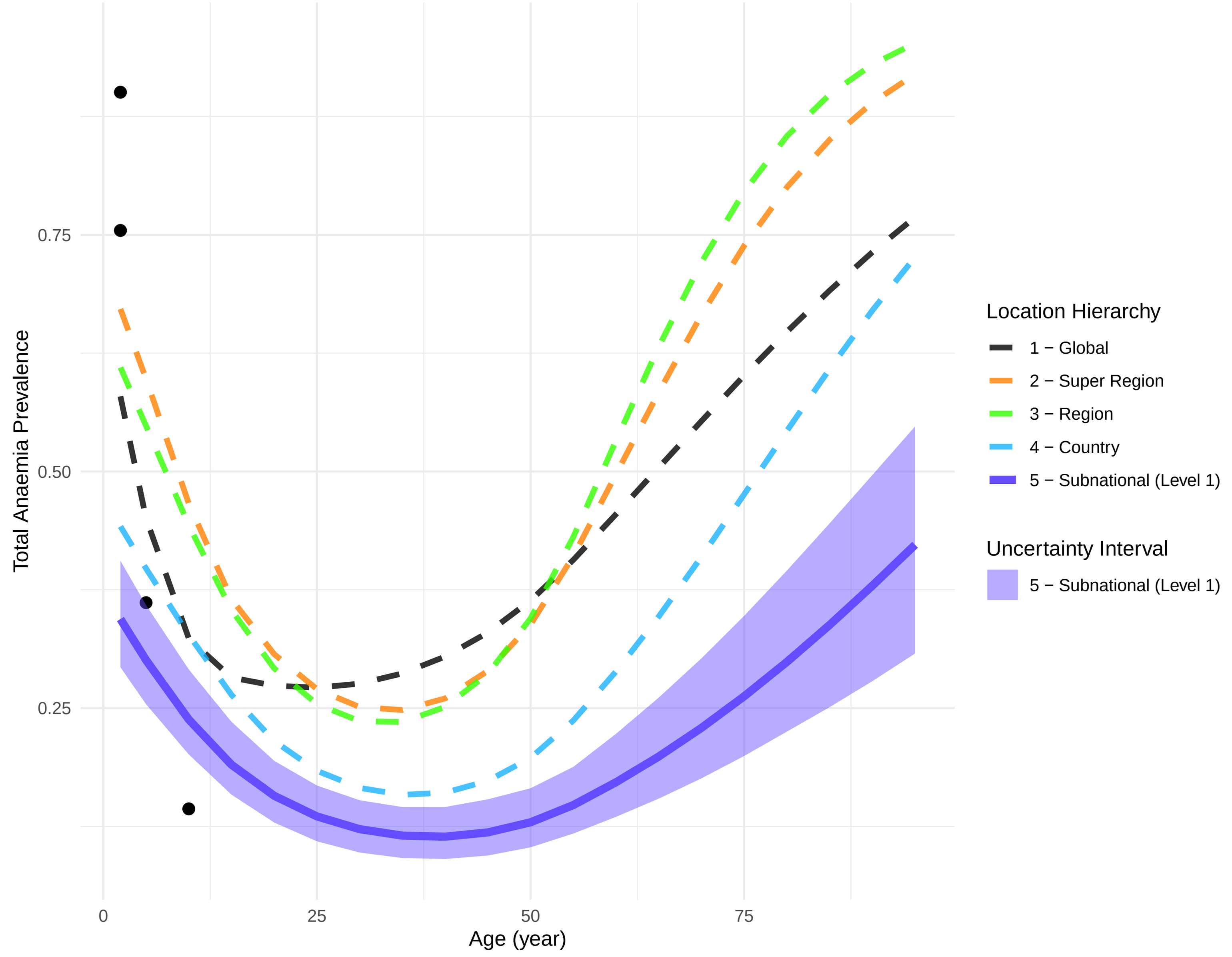

MR−BRT Age/Sex Fits − Males 2+ Years Old − Total Anaemia Prevalence − Tharaka Nithi
WHO−2024 Adjustment Method

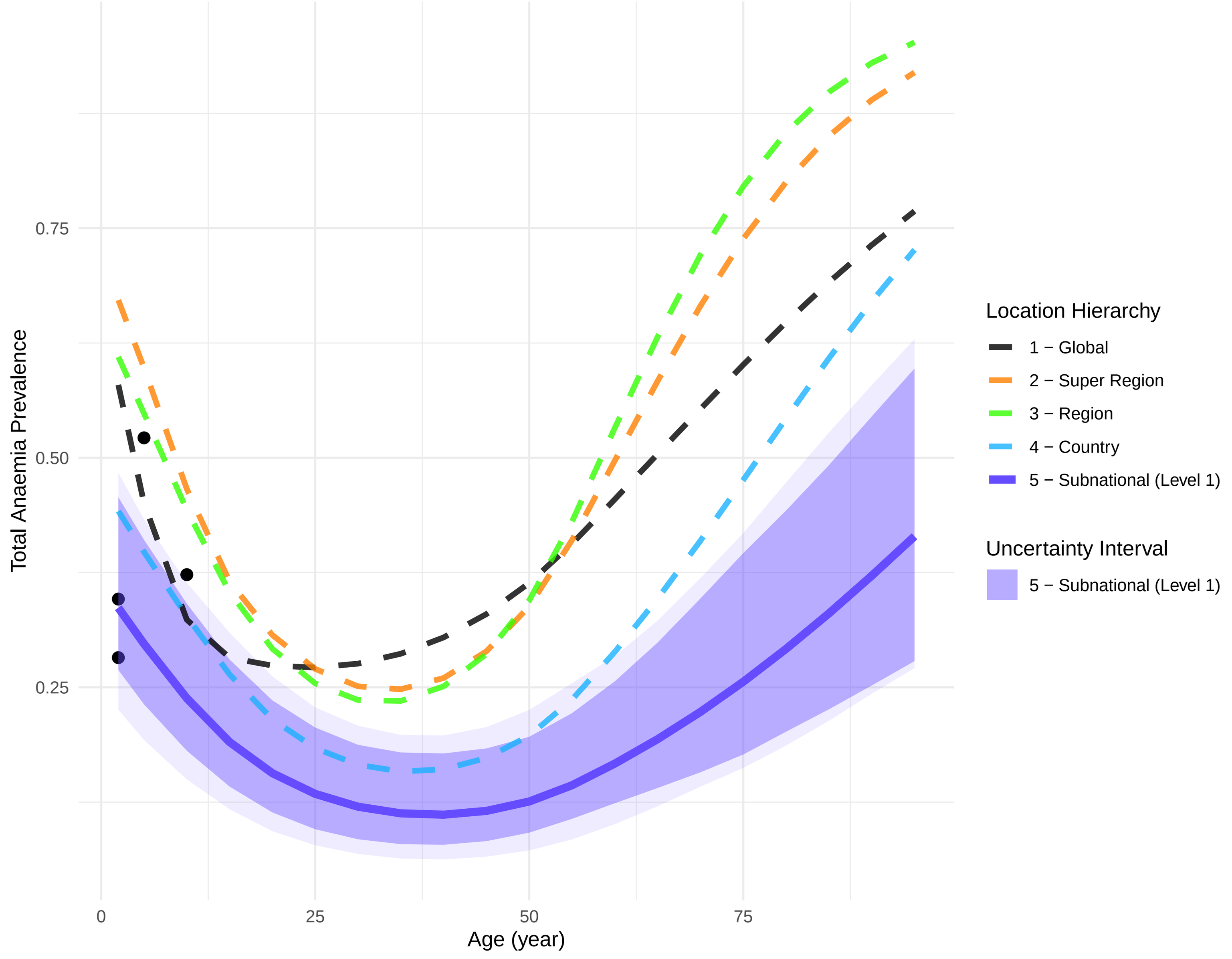

MR−BRT Age/Sex Fits − Males 2+ Years Old − Total Anaemia Prevalence − Trans Nzoia
WHO−2024 Adjustment Method

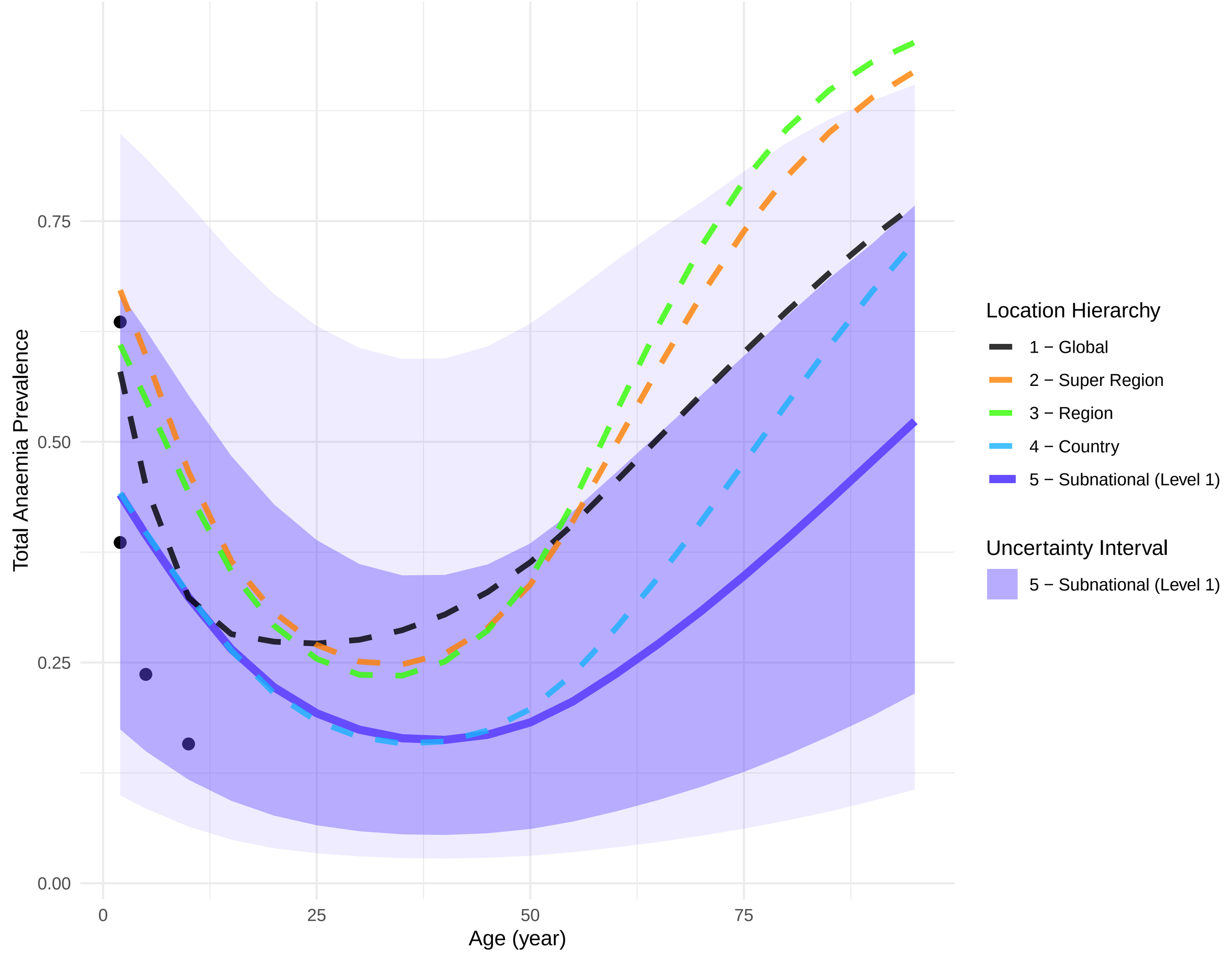

MR−BRT Age/Sex Fits − Males 2+ Years Old − Total Anaemia Prevalence − Turkana
WHO−2024 Adjustment Method

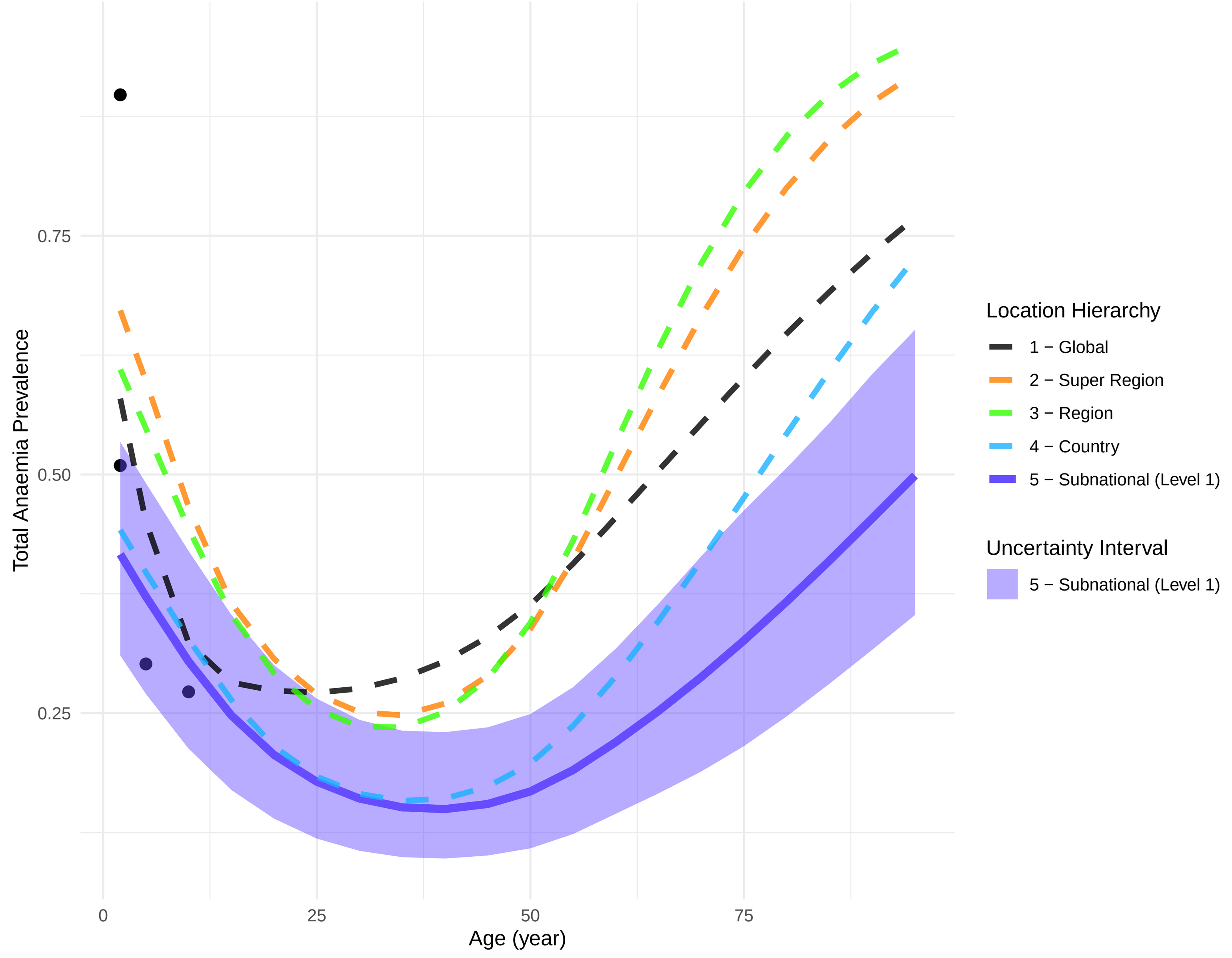

MR−BRT Age/Sex Fits − Males 2+ Years Old − Total Anaemia Prevalence − Uasin Gishu
WHO−2024 Adjustment Method

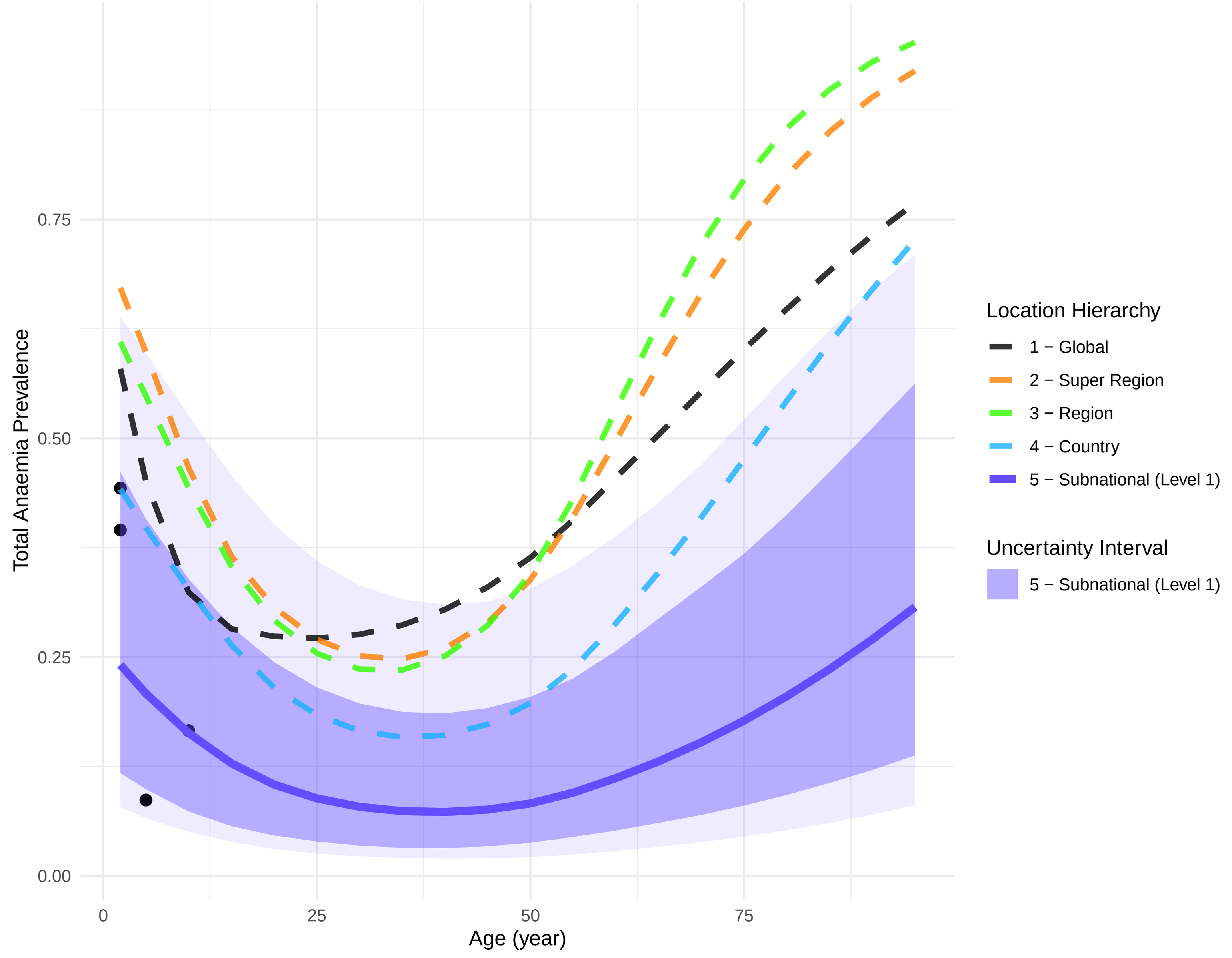

MR−BRT Age/Sex Fits − Males 2+ Years Old − Total Anaemia Prevalence − Vihiga
WHO−2024 Adjustment Method

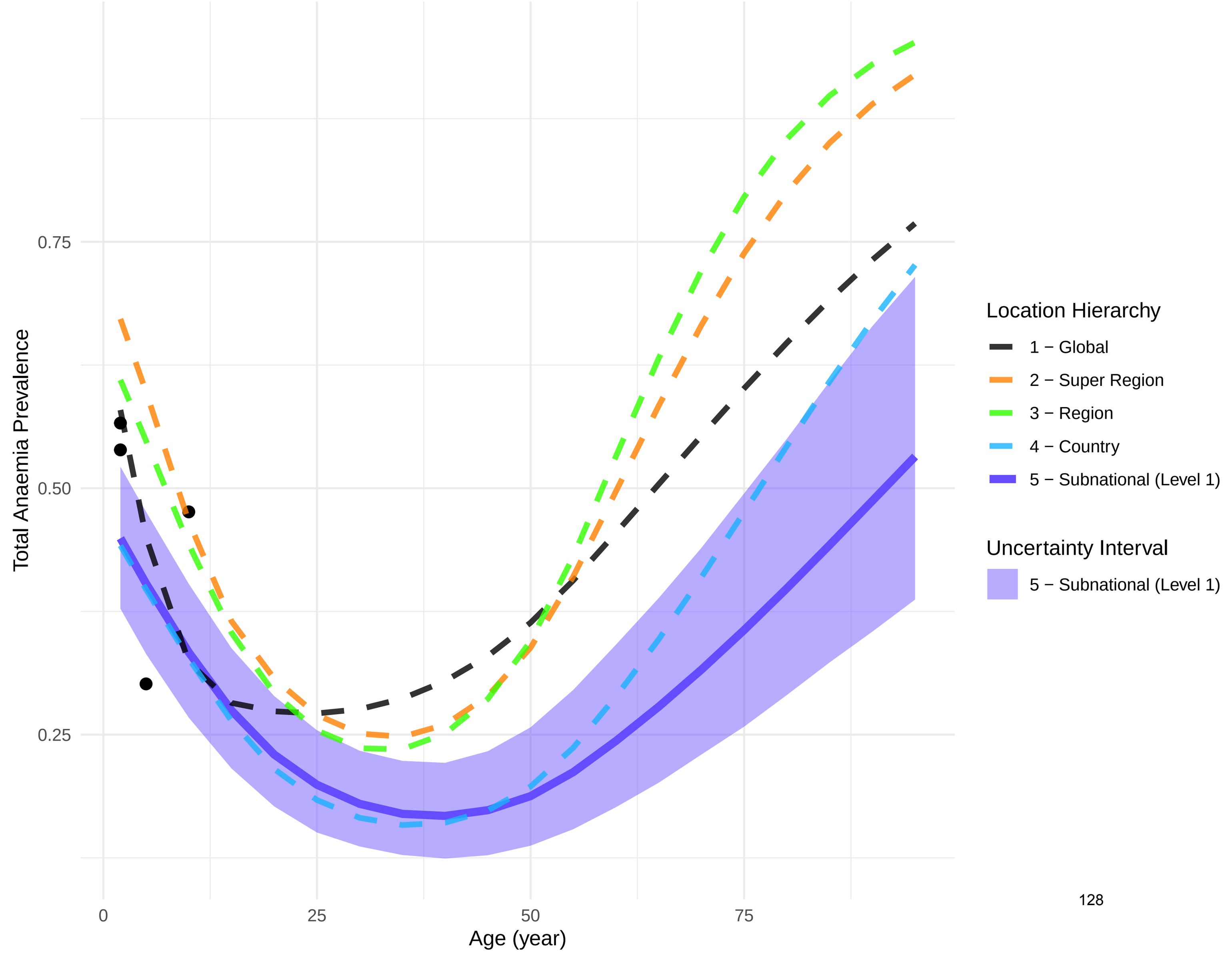

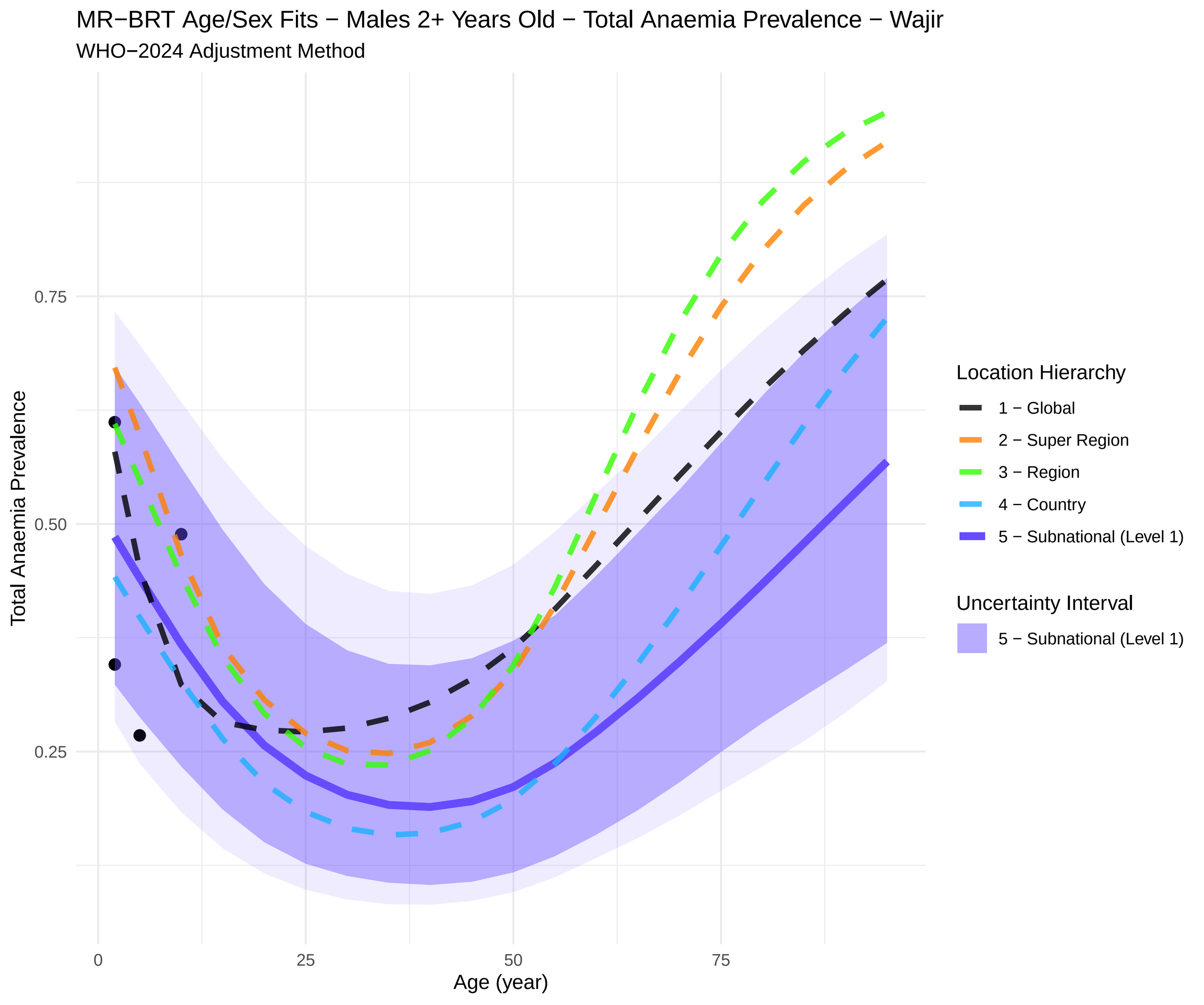
MR−BRT Age/Sex Fits − Males 2+ Years Old − Total Anaemia Prevalence − Wajir
WHO−2024 Adjustment Method
Total Anaemia Prevalence
Age (year)
Location Hierarchy
1 − Global
2 − Super Region
3 − Region
4 − Country
5 − Subnational (Level 1)
Uncertainty Interval
5 − Subnational (Level 1)

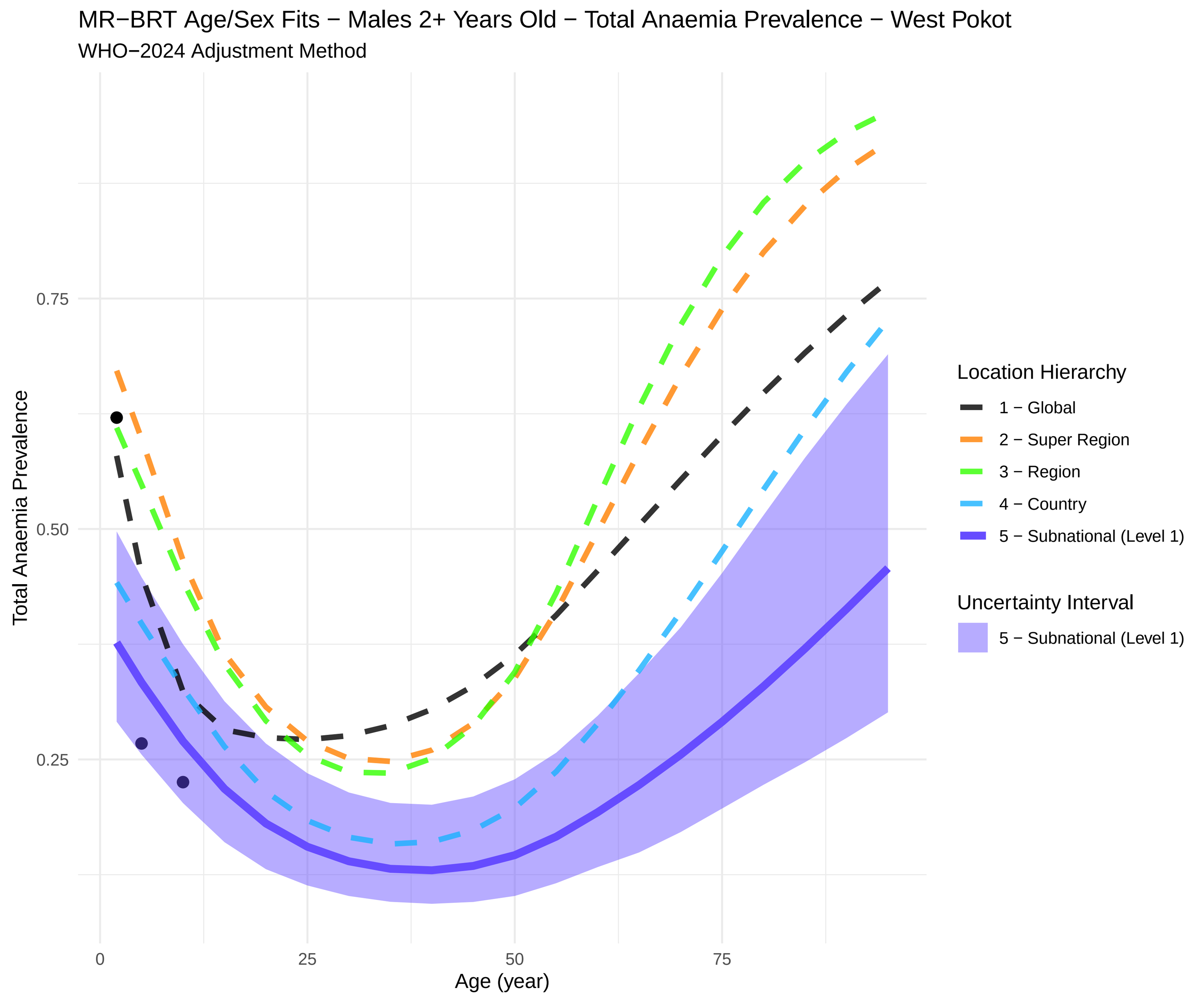
MR−BRT Age/Sex Fits − Males 2+ Years Old − Total Anaemia Prevalence − West Pokot
WHO−2024 Adjustment Method
Total Anaemia Prevalence
Age (year)
Location Hierarchy
1 − Global
2 − Super Region
3 − Region
4 − Country
5 − Subnational (Level 1)
Uncertainty Interval
5 − Subnational (Level 1)

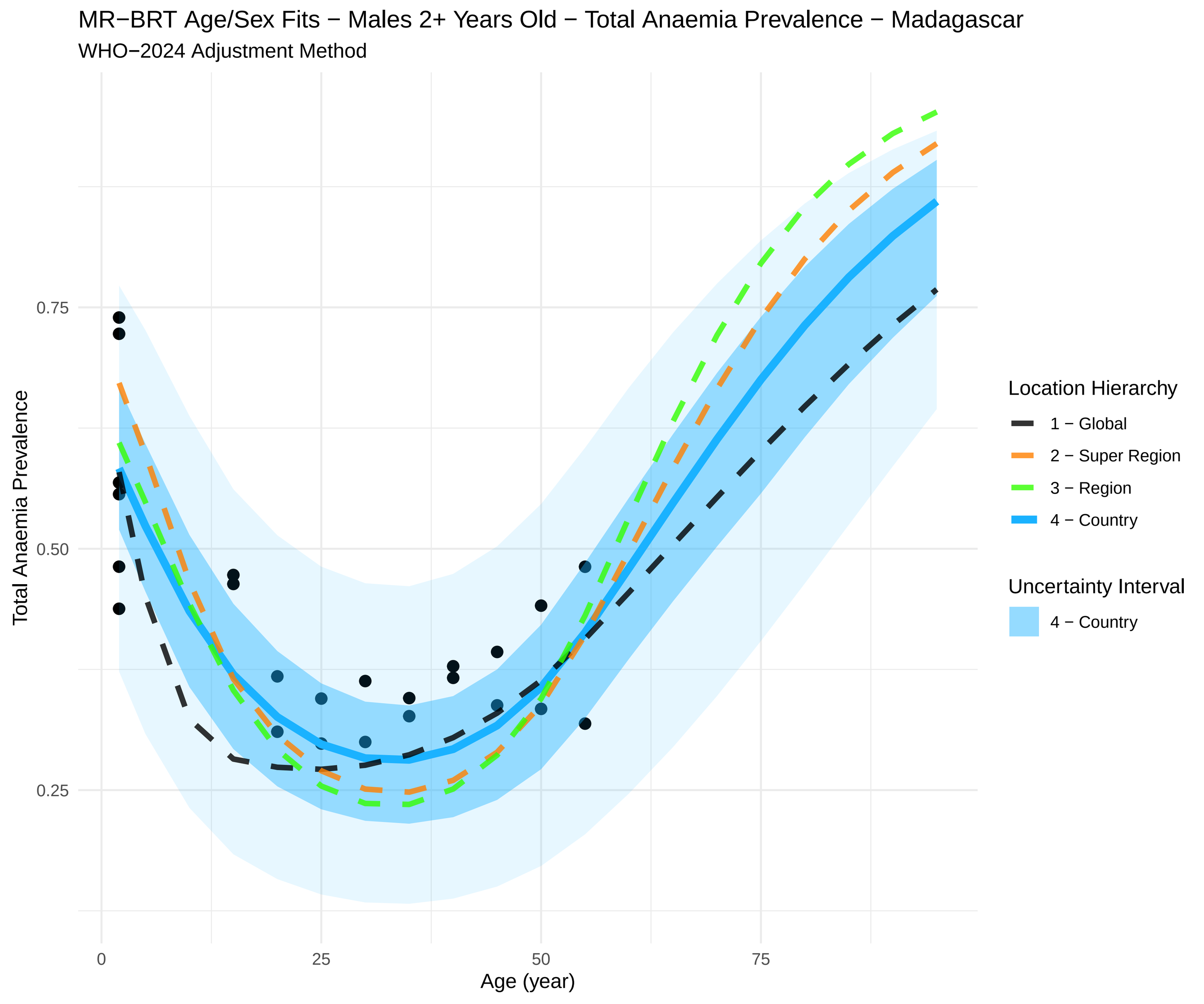
MR−BRT Age/Sex Fits − Males 2+ Years Old − Total Anaemia Prevalence − Madagascar
WHO−2024 Adjustment Method
Total Anaemia Prevalence
Age (year)
Location Hierarchy
1 − Global
2 − Super Region
3 − Region
4 − Country
Uncertainty Interval
4 − Country

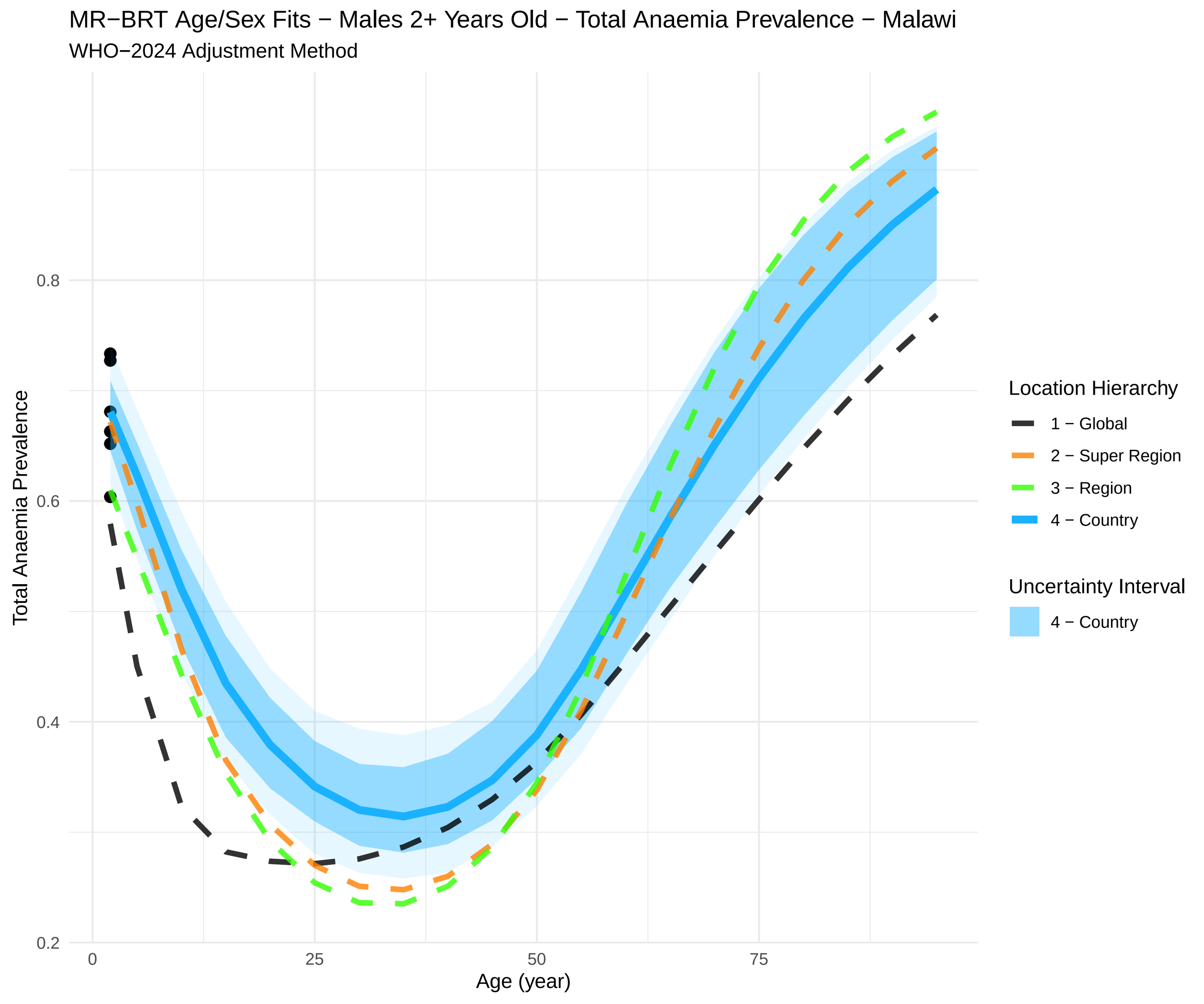
MR−BRT Age/Sex Fits − Males 2+ Years Old − Total Anaemia Prevalence − Malawi
WHO−2024 Adjustment Method
Total Anaemia Prevalence
Age (year)
Location Hierarchy
1 − Global
2 − Super Region
3 − Region
4 − Country
Uncertainty Interval
4 − Country

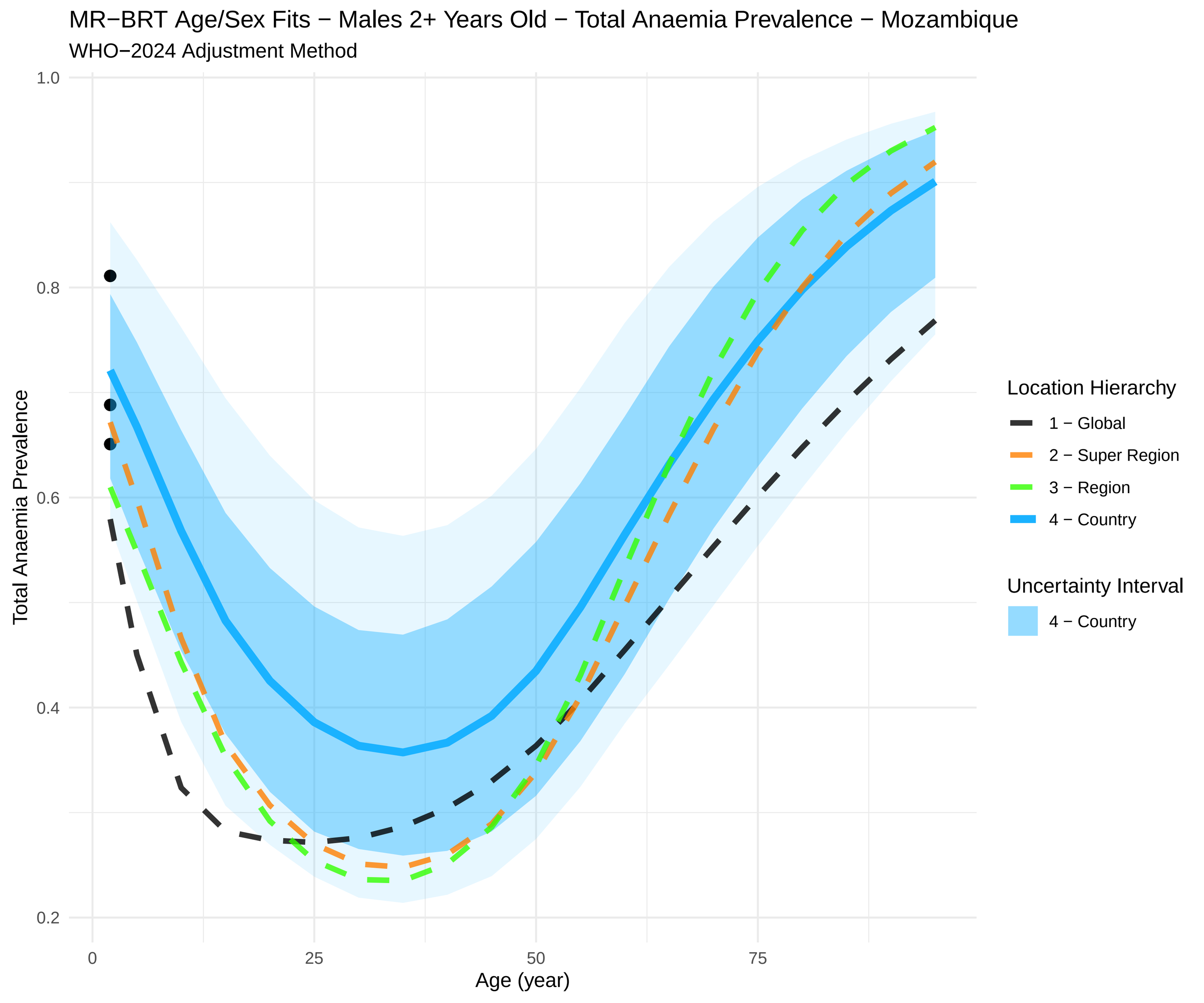
MR−BRT Age/Sex Fits − Males 2+ Years Old − Total Anaemia Prevalence − Mozambique
WHO−2024 Adjustment Method
Total Anaemia Prevalence
Age (year)
Location Hierarchy
1 − Global
2 − Super Region
3 − Region
4 − Country
Uncertainty Interval
4 − Country

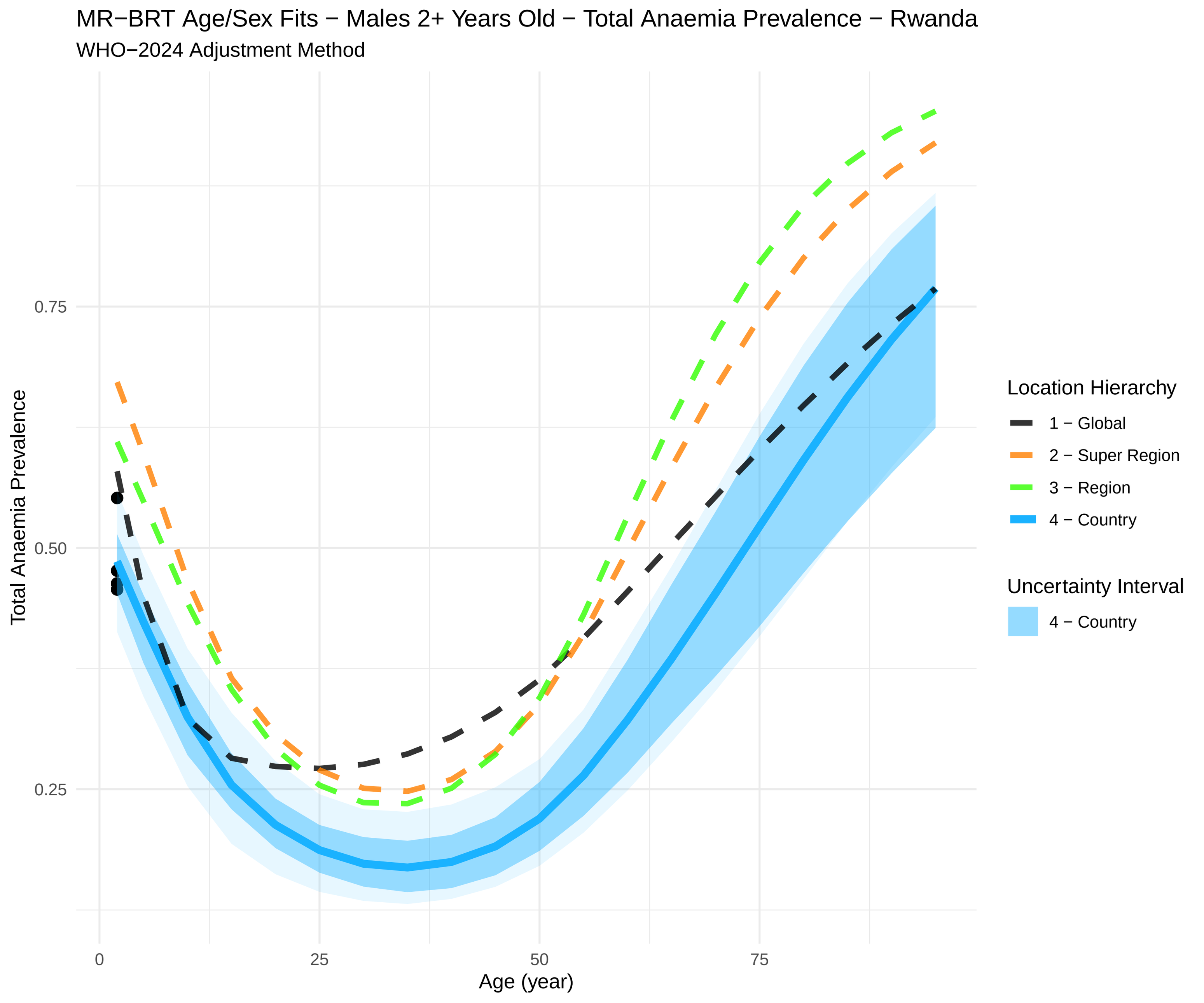
MR−BRT Age/Sex Fits − Males 2+ Years Old − Total Anaemia Prevalence − Rwanda
WHO−2024 Adjustment Method
Total Anaemia Prevalence
Age (year)
Location Hierarchy
1 − Global
2 − Super Region
3 − Region
4 − Country
Uncertainty Interval
4 − Country

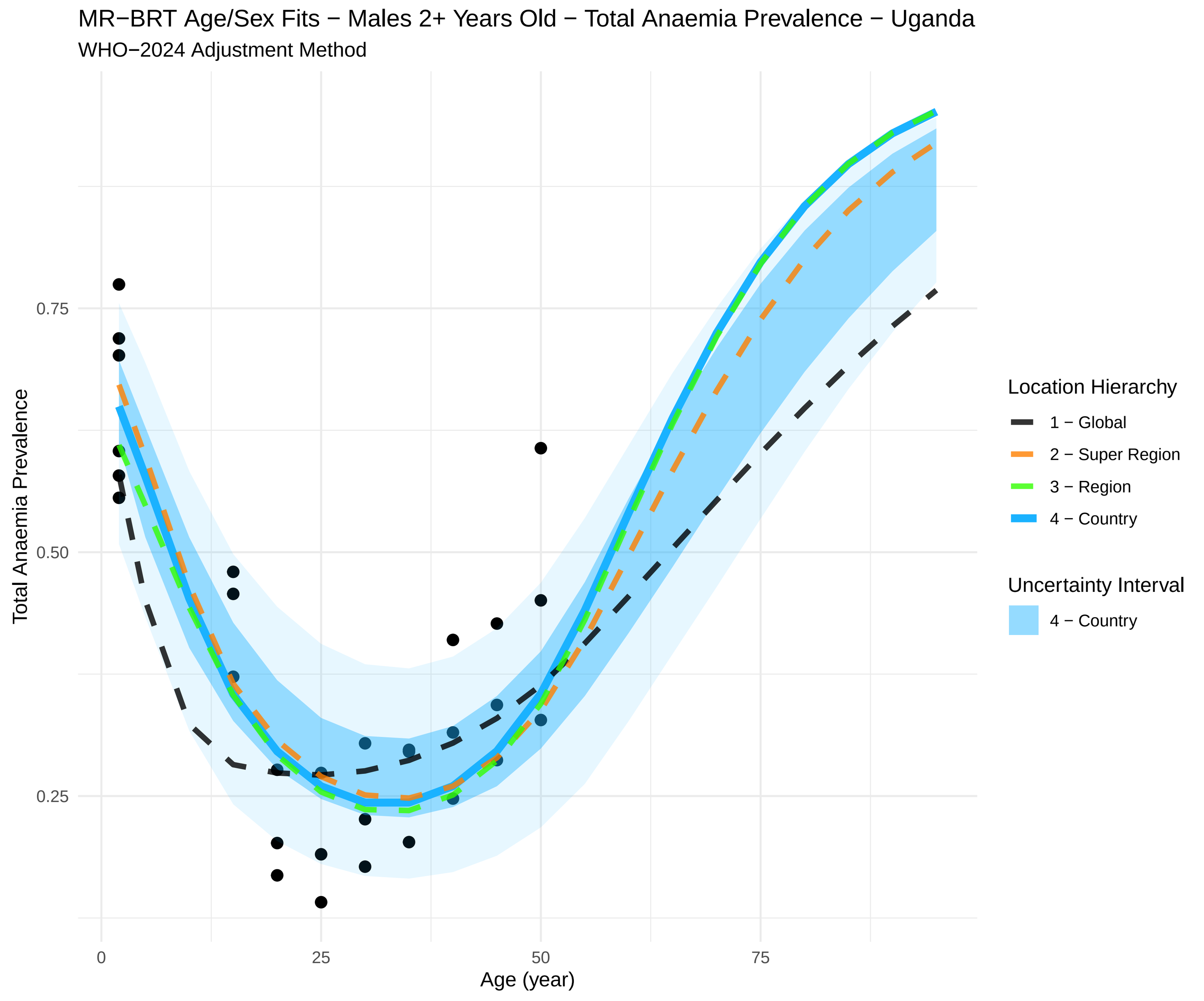
MR−BRT Age/Sex Fits − Males 2+ Years Old − Total Anaemia Prevalence − Uganda
WHO−2024 Adjustment Method
Total Anaemia Prevalence
Age (year)
Location Hierarchy
1 − Global
2 − Super Region
3 − Region
4 − Country
Uncertainty Interval
4 − Country

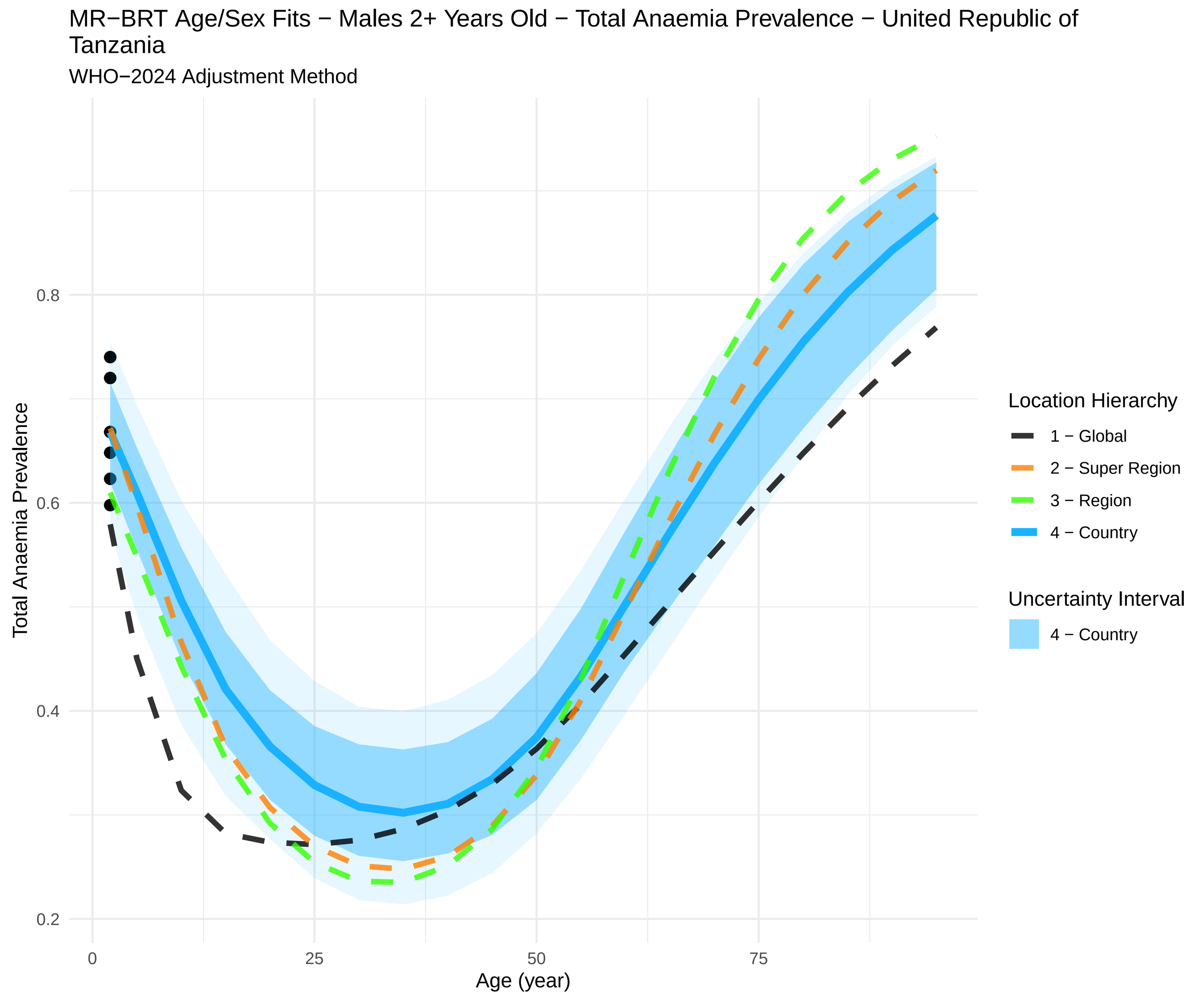
MR−BRT Age/Sex Fits − Males 2+ Years Old − Total Anaemia Prevalence − United Republic of Tanzania
WHO−2024 Adjustment Method
Total Anaemia Prevalence
Age (year)
Location Hierarchy
1 − Global
2 − Super Region
3 − Region
4 − Country
Uncertainty Interval
4 − Country

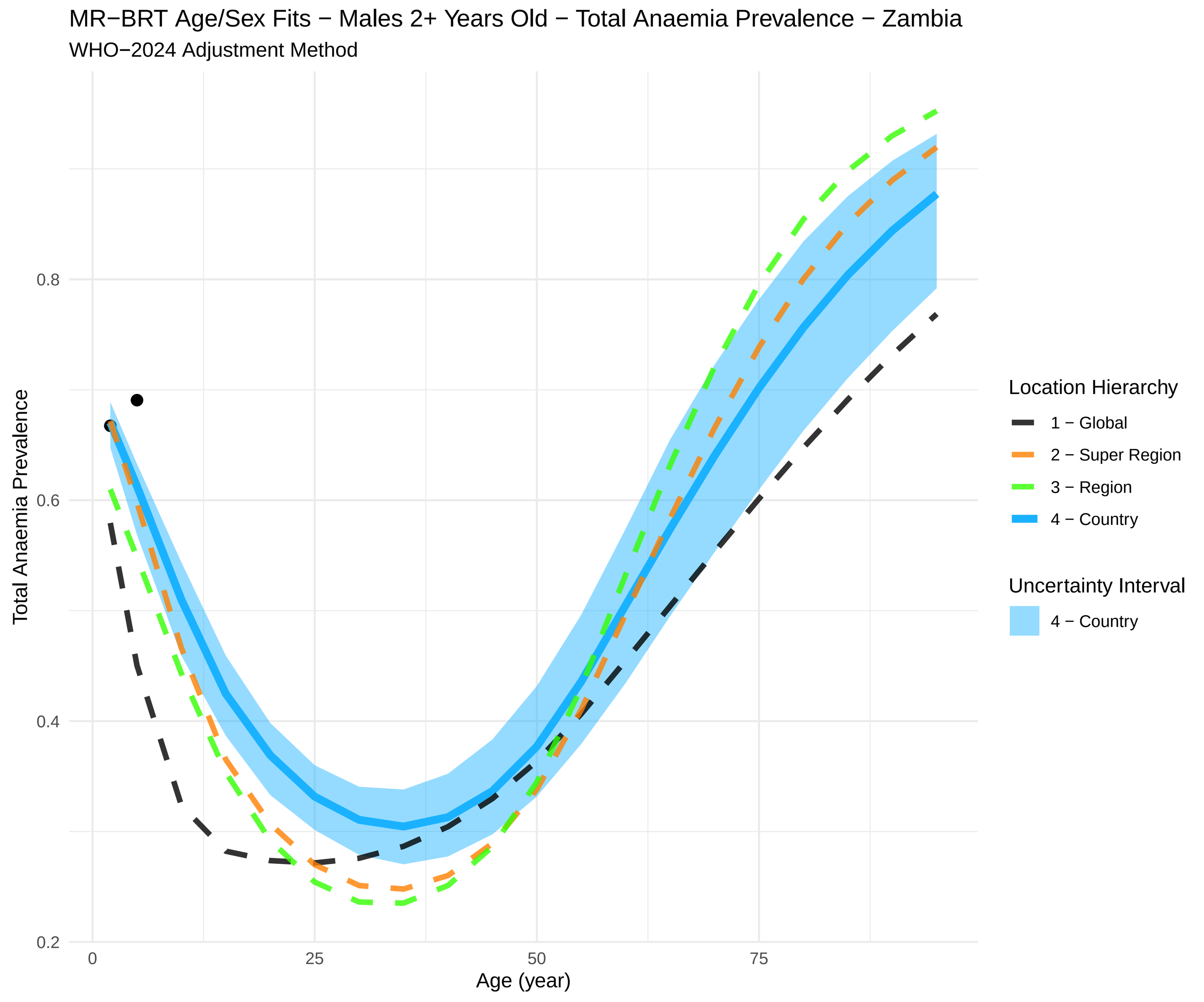
MR−BRT Age/Sex Fits − Males 2+ Years Old − Total Anaemia Prevalence − Zambia
WHO−2024 Adjustment Method
Total Anaemia Prevalence
Age (year)
Location Hierarchy
1 − Global
2 − Super Region
3 − Region
4 − Country
Uncertainty Interval
4 − Country

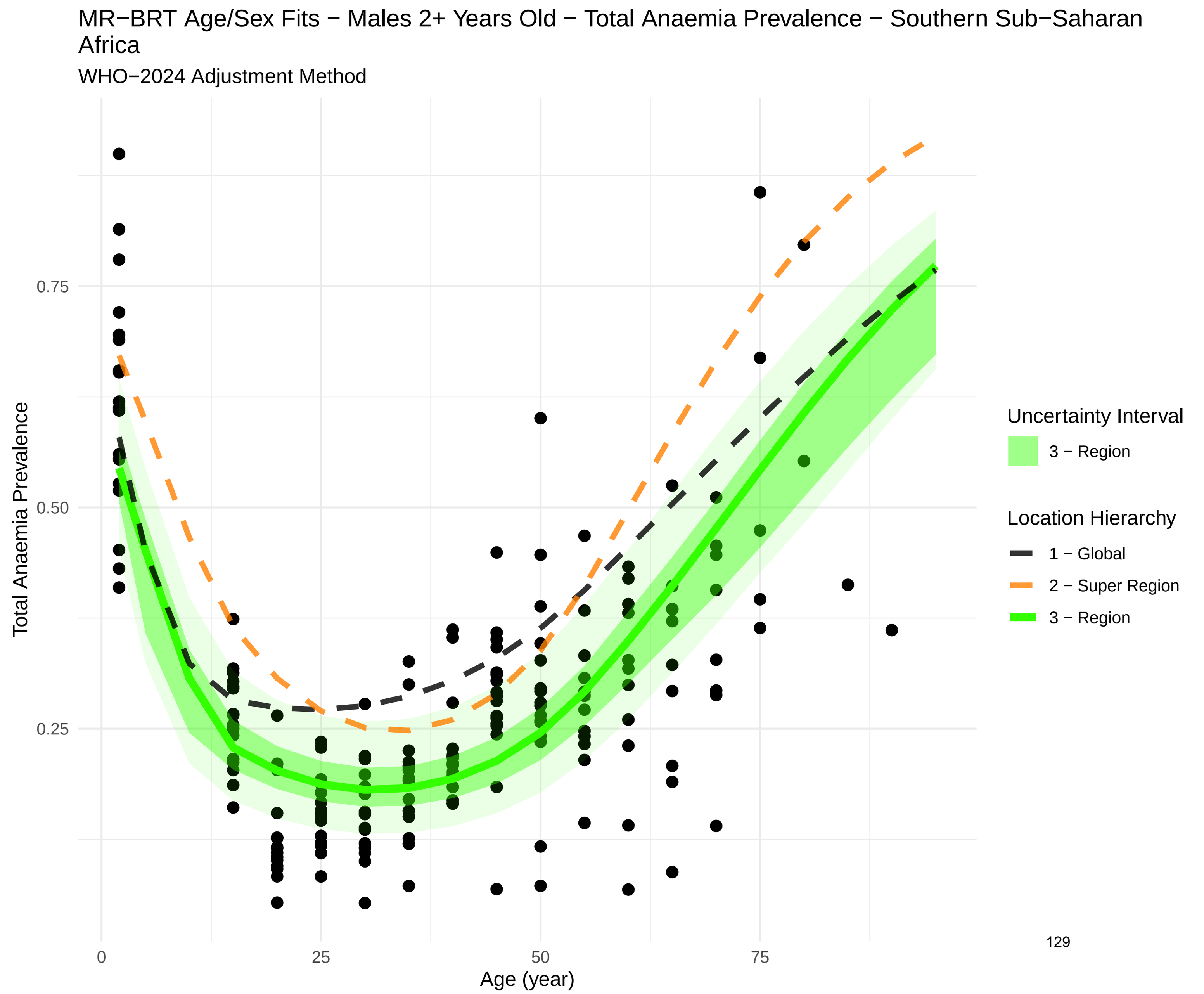
MR−BRT Age/Sex Fits − Males 2+ Years Old − Total Anaemia Prevalence − Southern Sub−Saharan Africa
WHO−2024 Adjustment Method
Total Anaemia Prevalence
Age (year)
Uncertainty Interval
3 − Region
Location Hierarchy
1 − Global
2 − Super Region
3 − Region

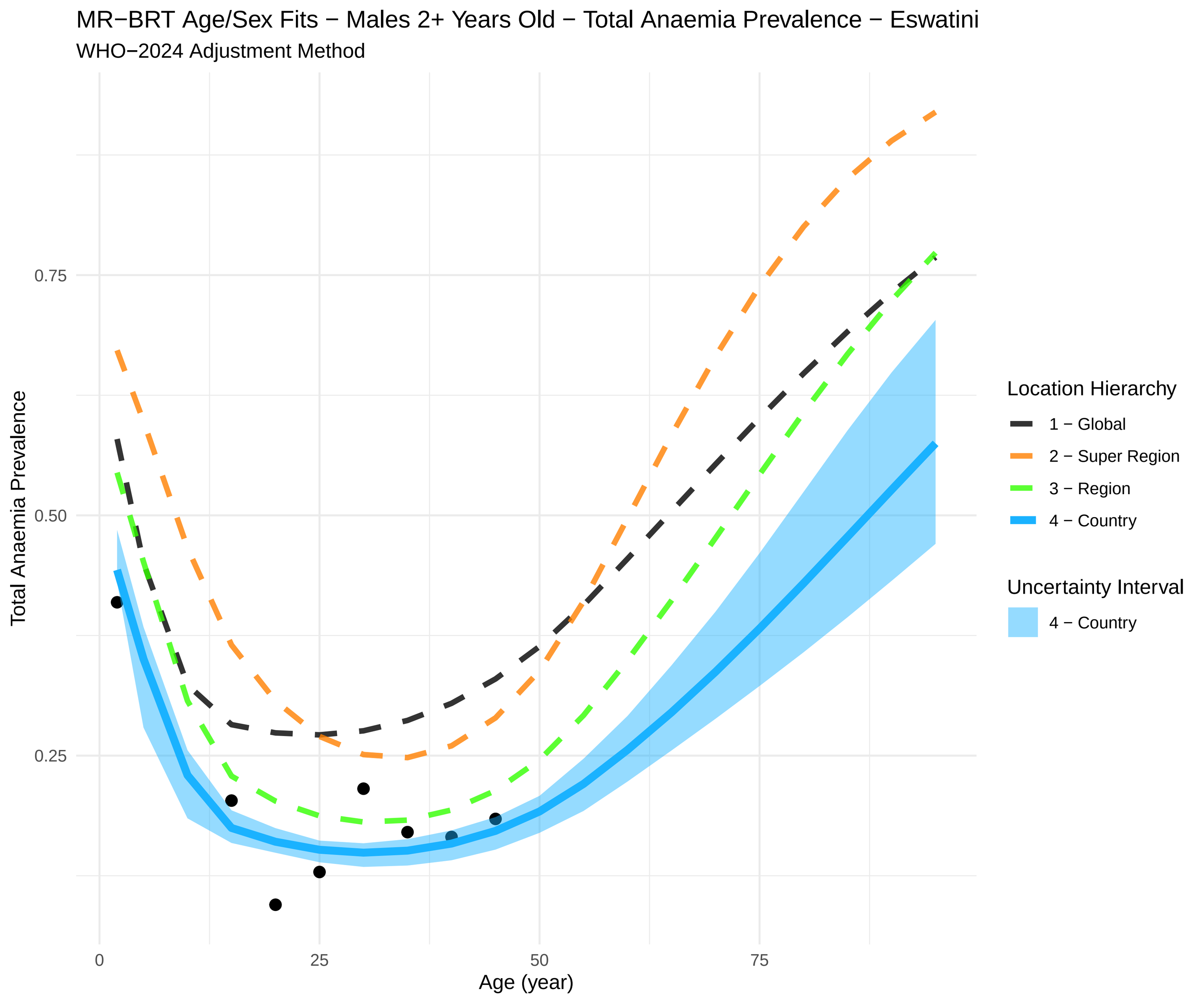
MR−BRT Age/Sex Fits − Males 2+ Years Old − Total Anaemia Prevalence − Eswatini
WHO−2024 Adjustment Method
Total Anaemia Prevalence
Age (year)
Location Hierarchy
1 − Global
2 − Super Region
3 − Region
4 − Country
Uncertainty Interval
4 − Country

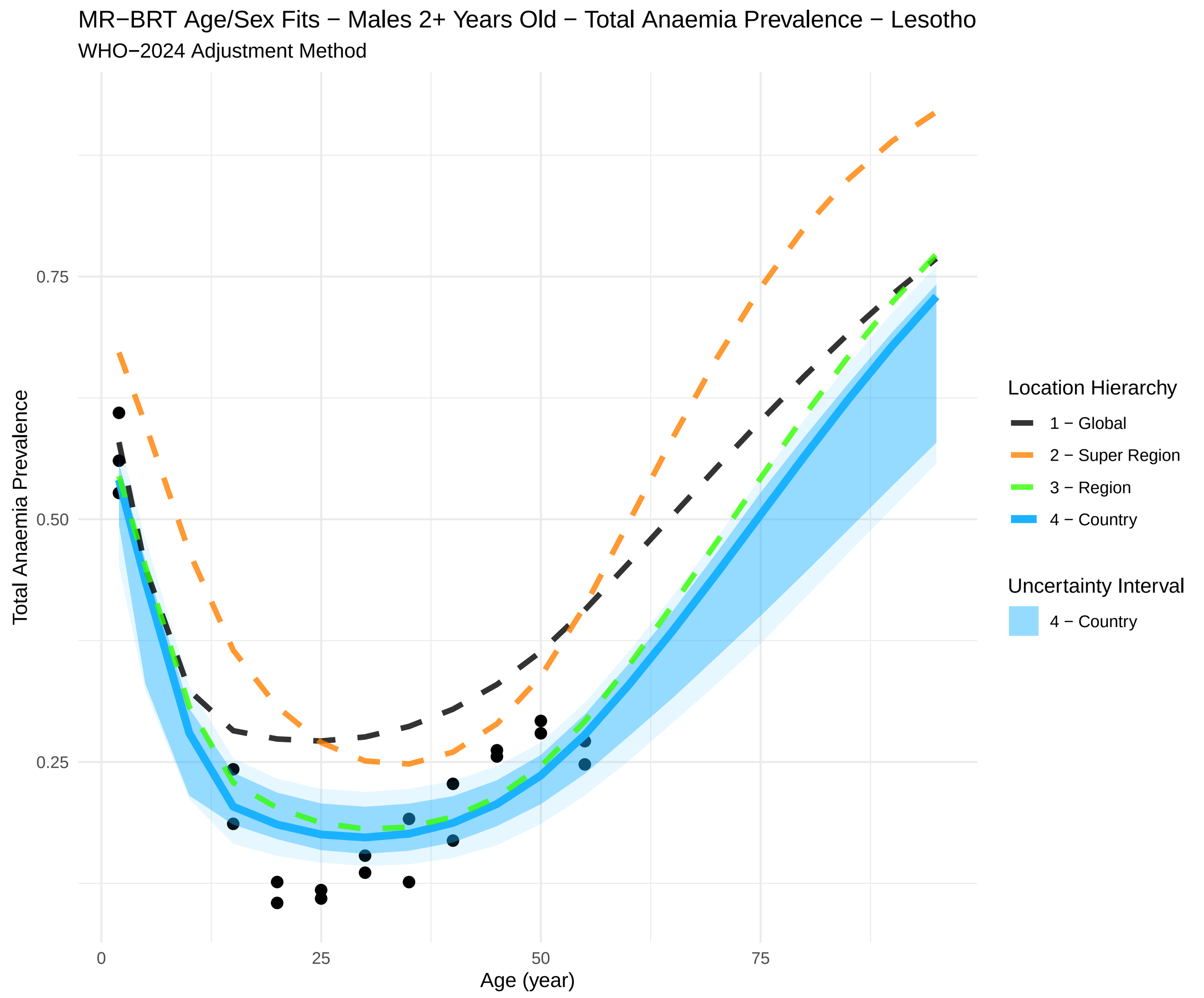
MR−BRT Age/Sex Fits − Males 2+ Years Old − Total Anaemia Prevalence − Lesotho
WHO−2024 Adjustment Method
Total Anaemia Prevalence
Age (year)
Location Hierarchy
1 − Global
2 − Super Region
3 − Region
4 − Country
Uncertainty Interval
4 − Country

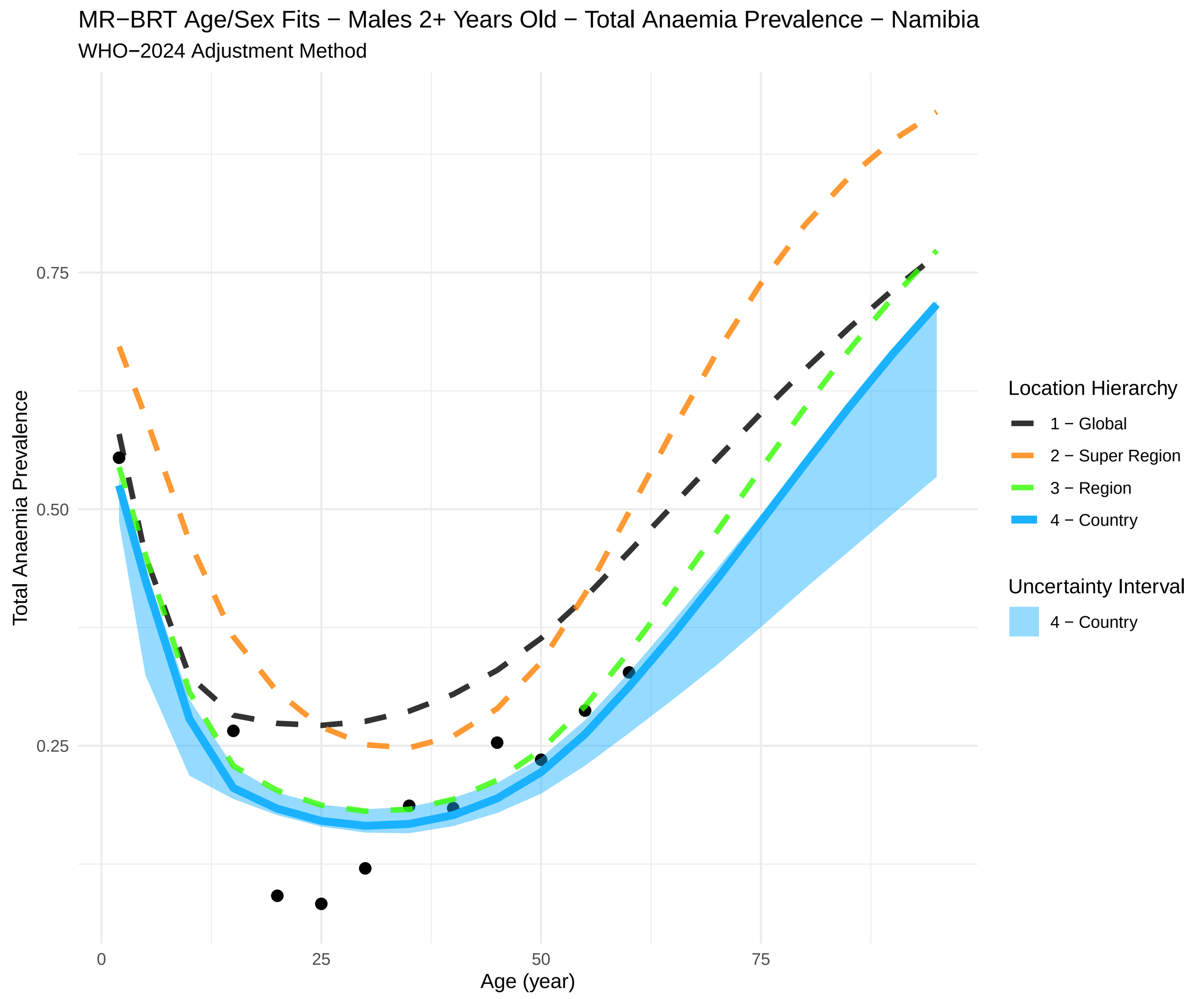
MR−BRT Age/Sex Fits − Males 2+ Years Old − Total Anaemia Prevalence − Namibia
WHO−2024 Adjustment Method
Total Anaemia Prevalence
Age (year)
Location Hierarchy
1 − Global
2 − Super Region
3 − Region
4 − Country
Uncertainty Interval
4 − Country

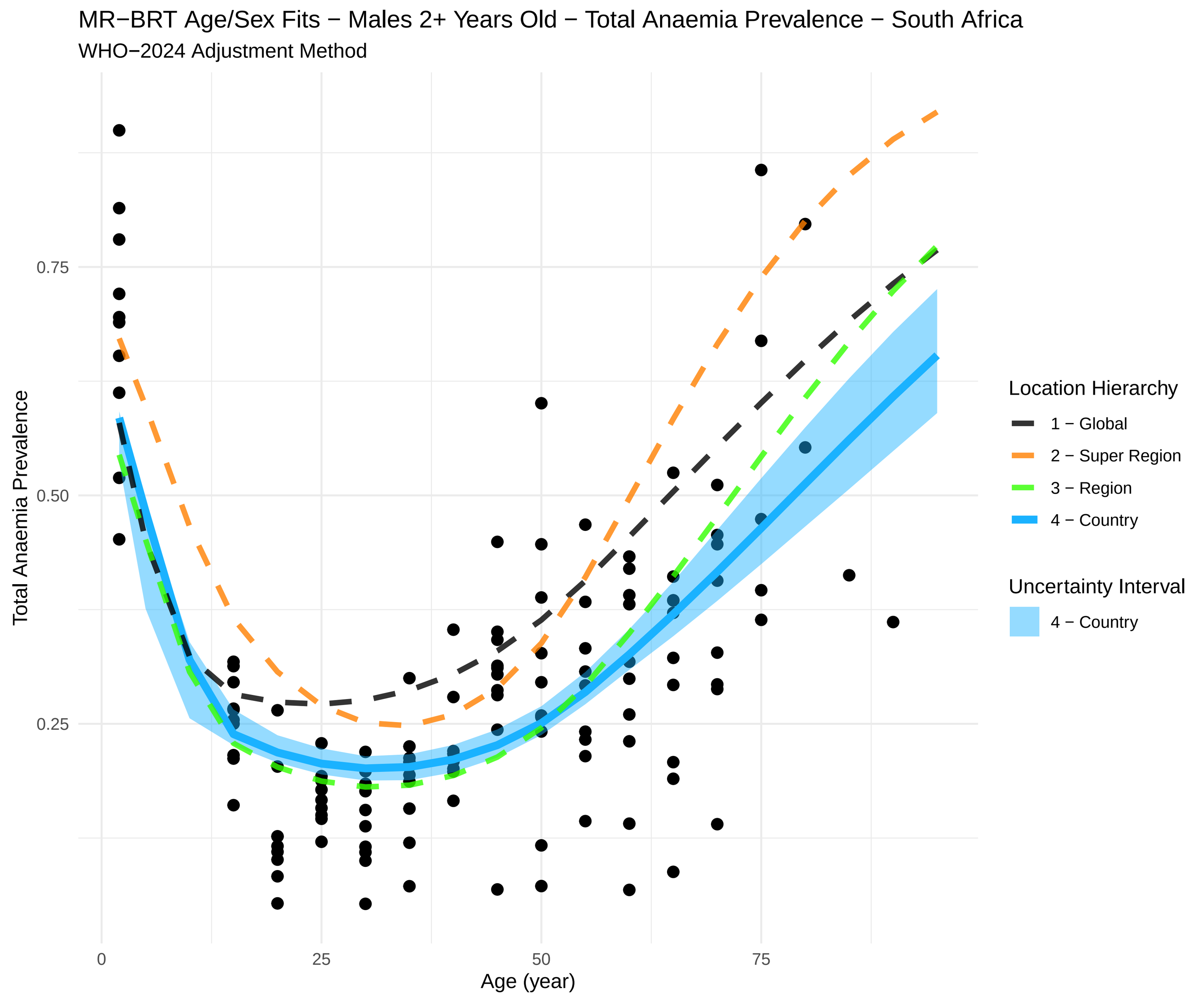
MR−BRT Age/Sex Fits − Males 2+ Years Old − Total Anaemia Prevalence − South Africa
WHO−2024 Adjustment Method
Total Anaemia Prevalence
Age (year)
Location Hierarchy
1 − Global
2 − Super Region
3 − Region
4 − Country
Uncertainty Interval
4 − Country

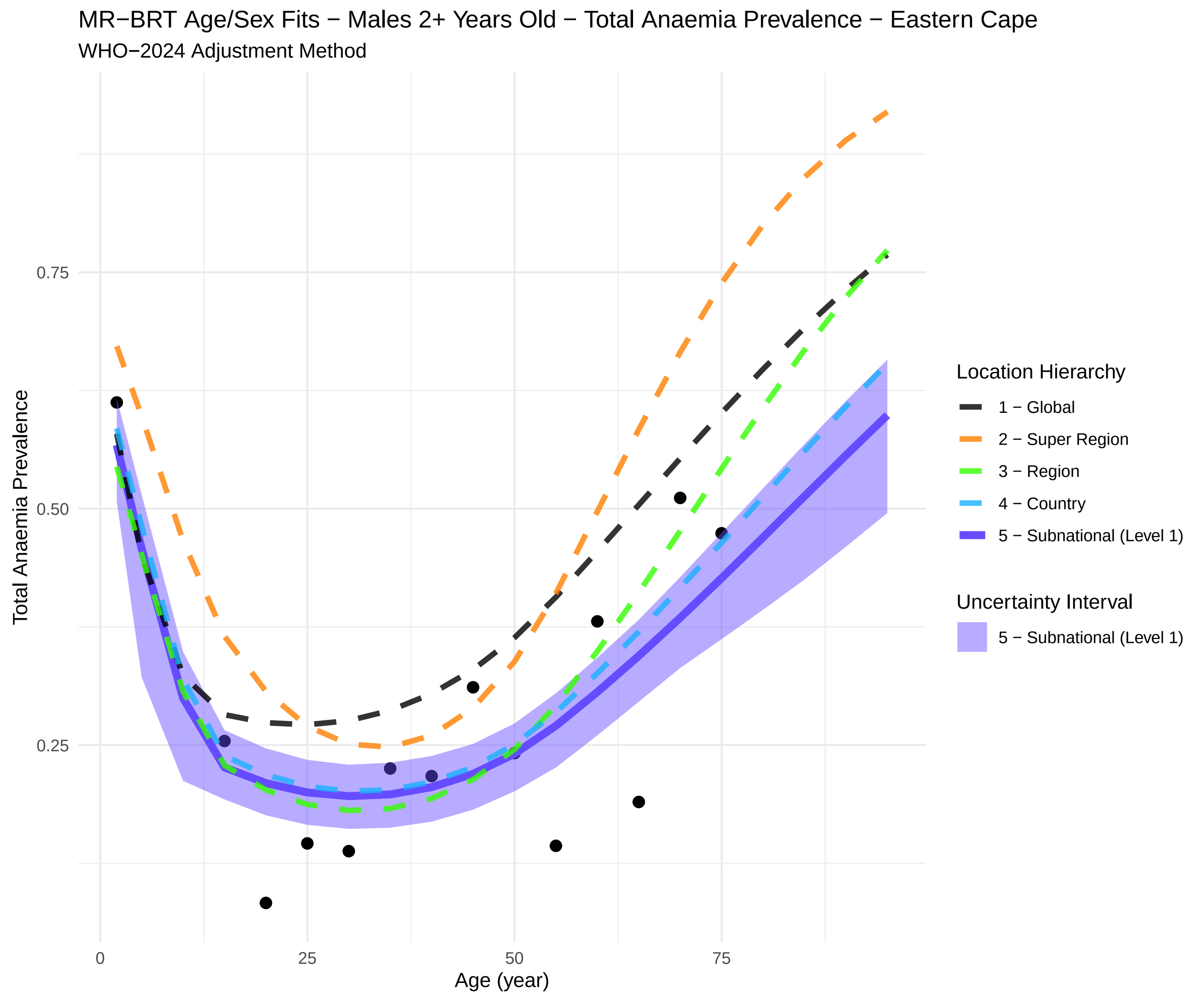
MR−BRT Age/Sex Fits − Males 2+ Years Old − Total Anaemia Prevalence − Eastern Cape
WHO−2024 Adjustment Method
Total Anaemia Prevalence
Age (year)
Location Hierarchy
1 − Global
2 − Super Region
3 − Region
4 − Country
5 − Subnational (Level 1)
Uncertainty Interval
5 − Subnational (Level 1)

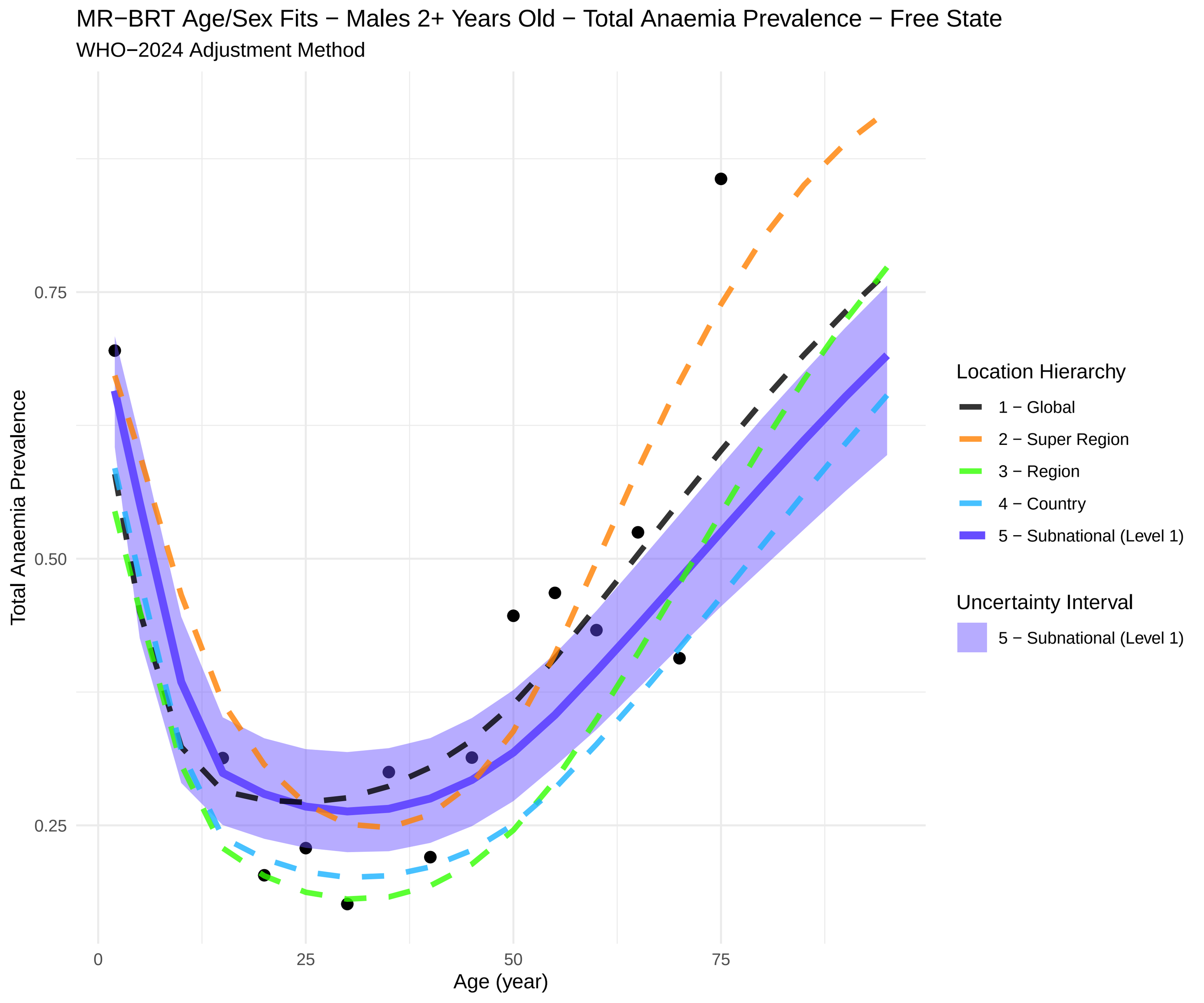
MR−BRT Age/Sex Fits − Males 2+ Years Old − Total Anaemia Prevalence − Free State
WHO−2024 Adjustment Method
Total Anaemia Prevalence
Age (year)
Location Hierarchy
1 − Global
2 − Super Region
3 − Region
4 − Country
5 − Subnational (Level 1)
Uncertainty Interval
5 − Subnational (Level 1)

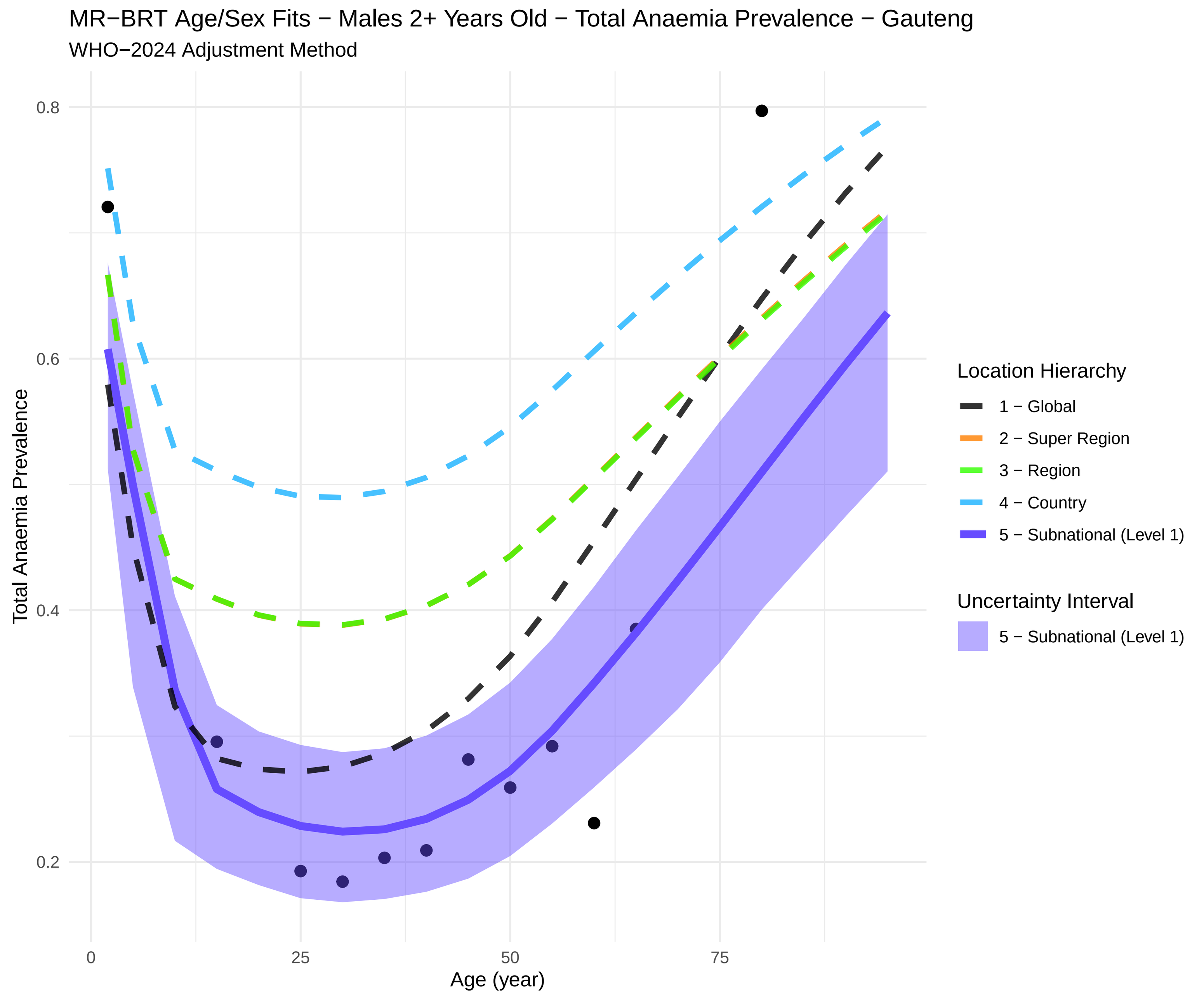
MR−BRT Age/Sex Fits − Males 2+ Years Old − Total Anaemia Prevalence − Gauteng
WHO−2024 Adjustment Method
Total Anaemia Prevalence
Age (year)
Location Hierarchy
1 − Global
2 − Super Region
3 − Region
4 − Country
5 − Subnational (Level 1)
Uncertainty Interval
5 − Subnational (Level 1)

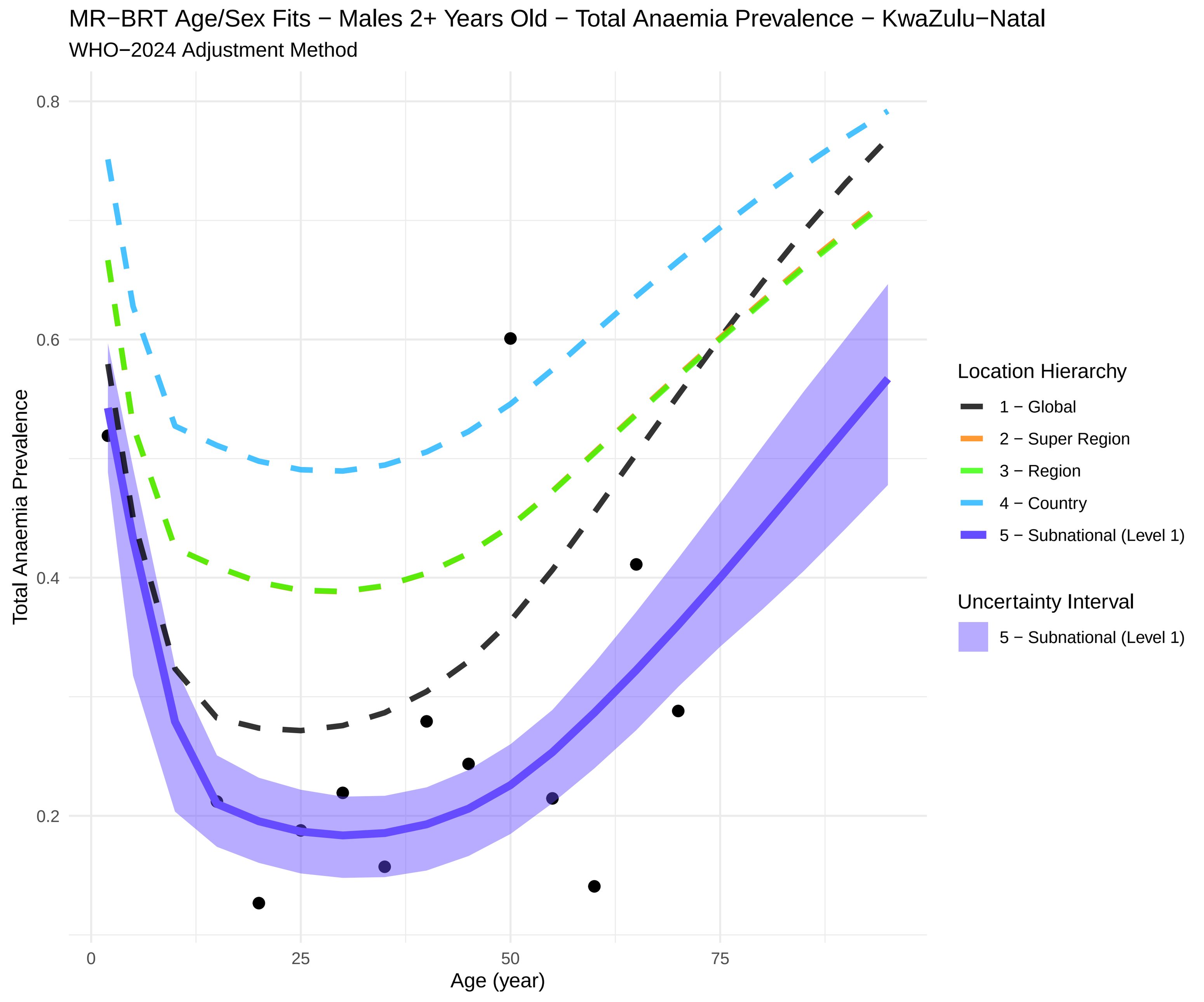
MR−BRT Age/Sex Fits − Males 2+ Years Old − Total Anaemia Prevalence − KwaZulu−Natal
WHO−2024 Adjustment Method
Total Anaemia Prevalence
Age (year)
Location Hierarchy
1 − Global
2 − Super Region
3 − Region
4 − Country
5 − Subnational (Level 1)
Uncertainty Interval
5 − Subnational (Level 1)

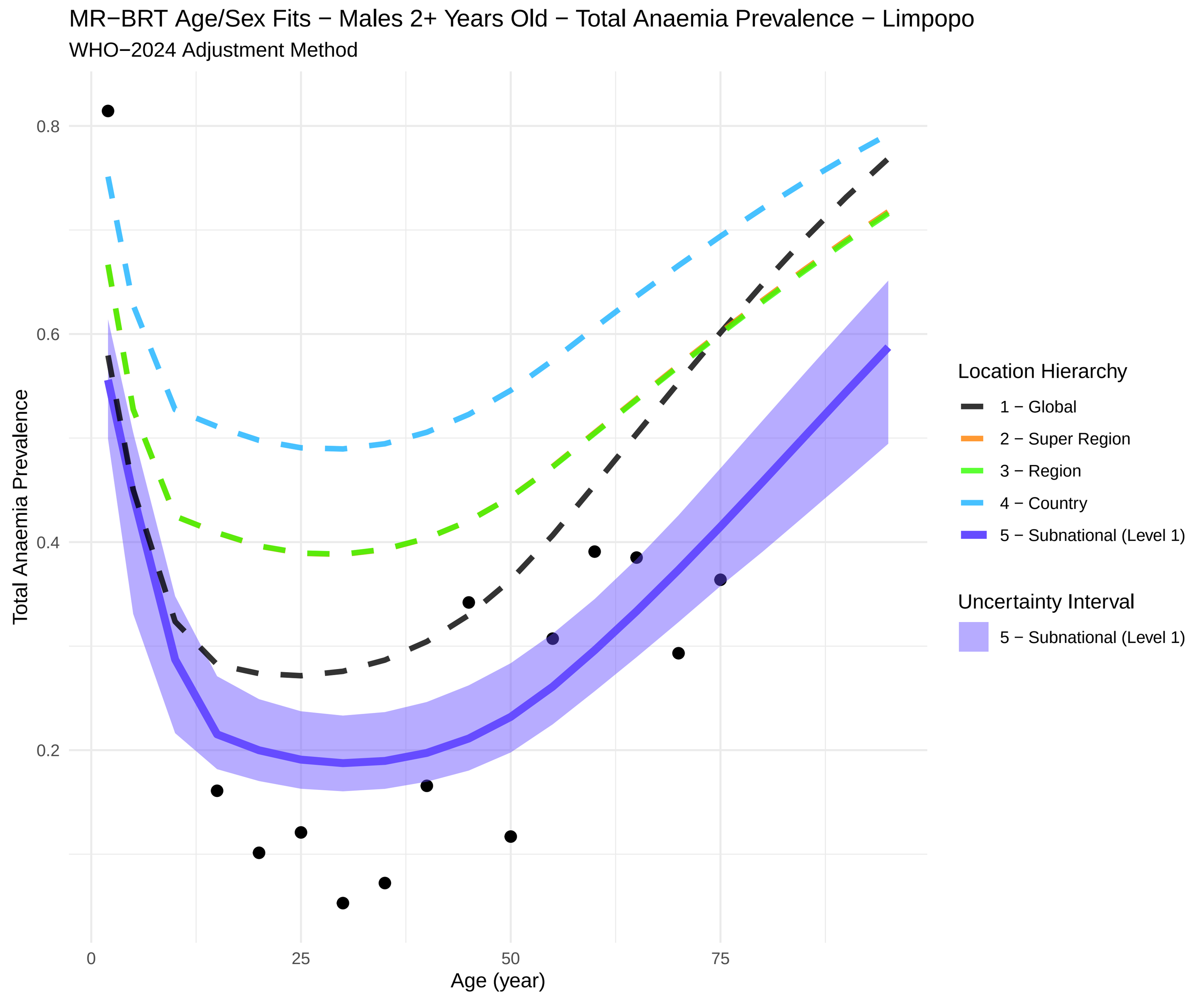
MR−BRT Age/Sex Fits − Males 2+ Years Old − Total Anaemia Prevalence − Limpopo
WHO−2024 Adjustment Method
Total Anaemia Prevalence
Age (year)
Location Hierarchy
1 − Global
2 − Super Region
3 − Region
4 − Country
5 − Subnational (Level 1)
Uncertainty Interval
5 − Subnational (Level 1)

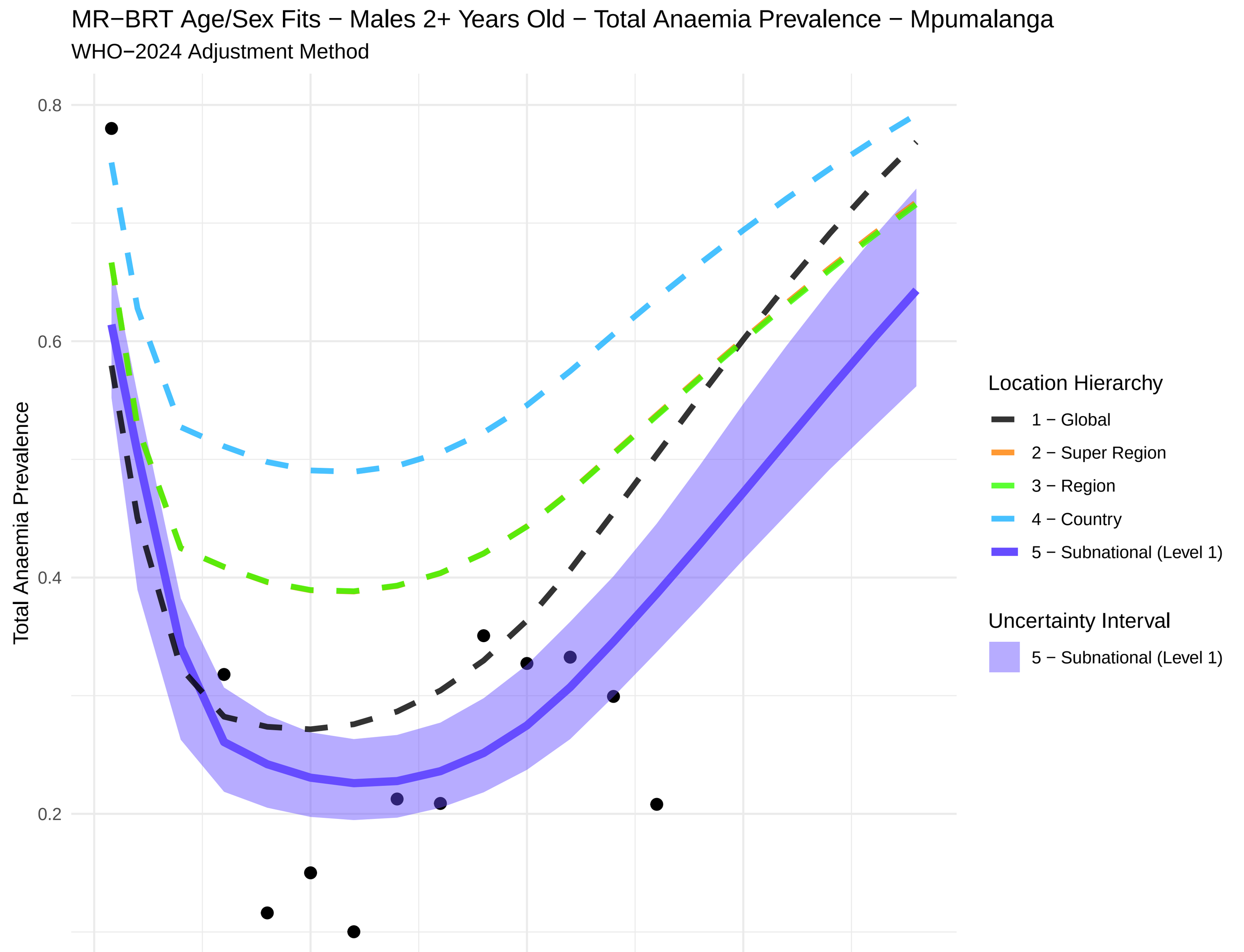
MR−BRT Age/Sex Fits − Males 2+ Years Old − Total Anaemia Prevalence − Mpumalanga
WHO−2024 Adjustment Method
Total Anaemia Prevalence
Age (year)
Location Hierarchy
1 − Global
2 − Super Region
3 − Region
4 − Country
5 − Subnational (Level 1)
Uncertainty Interval
5 − Subnational (Level 1)

MR−BRT Age/Sex Fits − Males 2+ Years Old − Total Anaemia Prevalence − North−West
WHO−2024 Adjustment Method

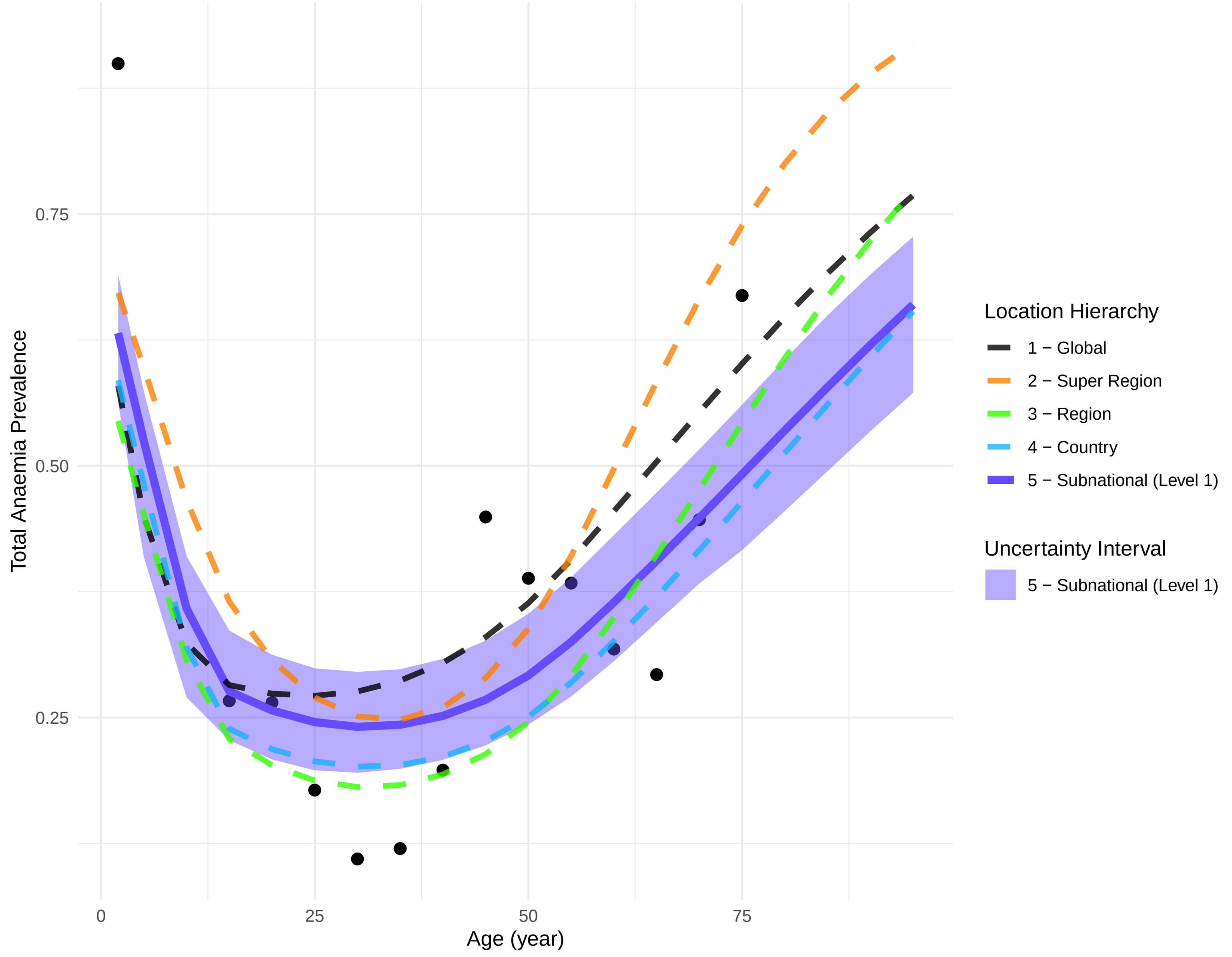

MR−BRT Age/Sex Fits − Males 2+ Years Old − Total Anaemia Prevalence − Northern Cape
WHO−2024 Adjustment Method

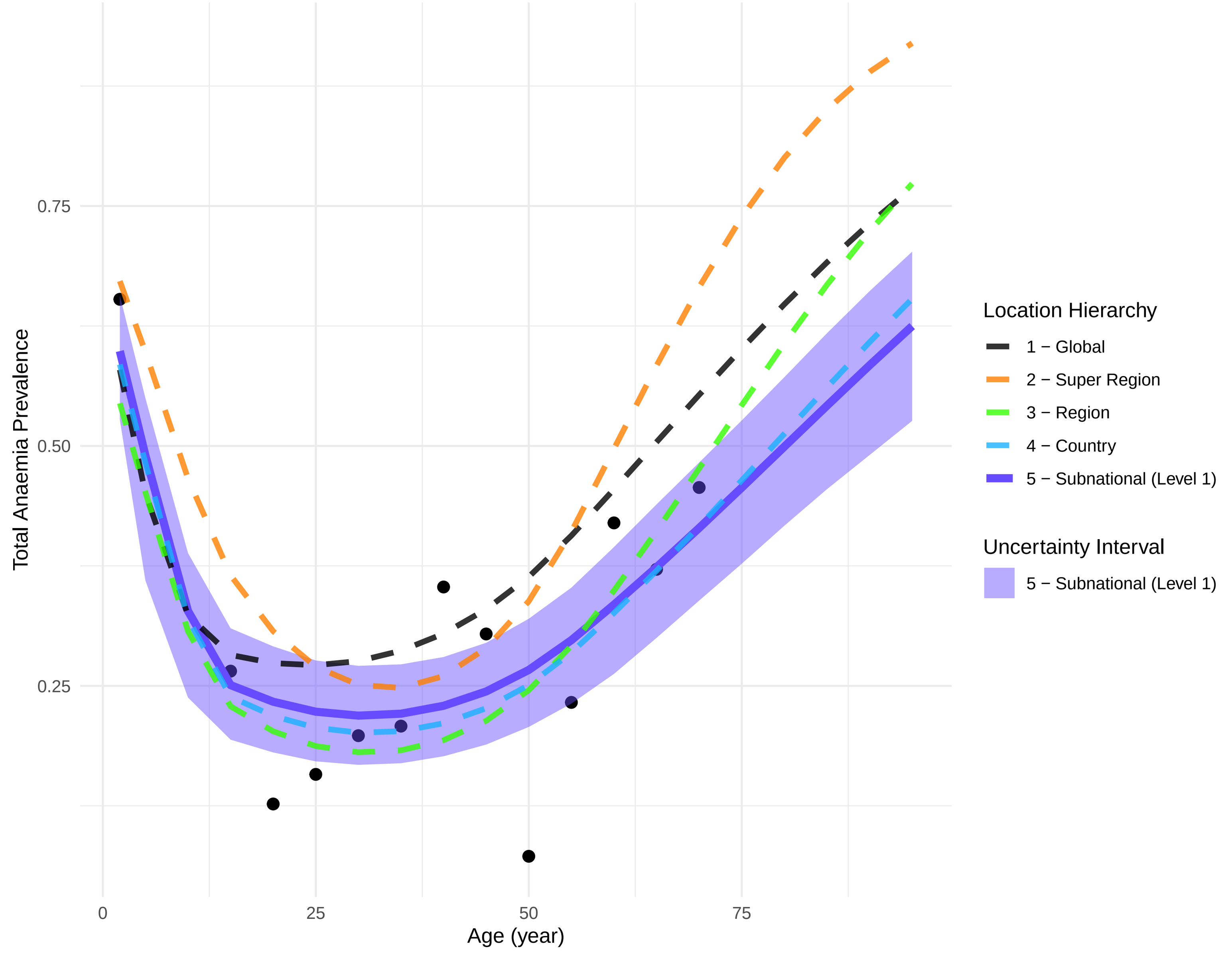

MR−BRT Age/Sex Fits − Males 2+ Years Old − Total Anaemia Prevalence − Western Cape
WHO−2024 Adjustment Method

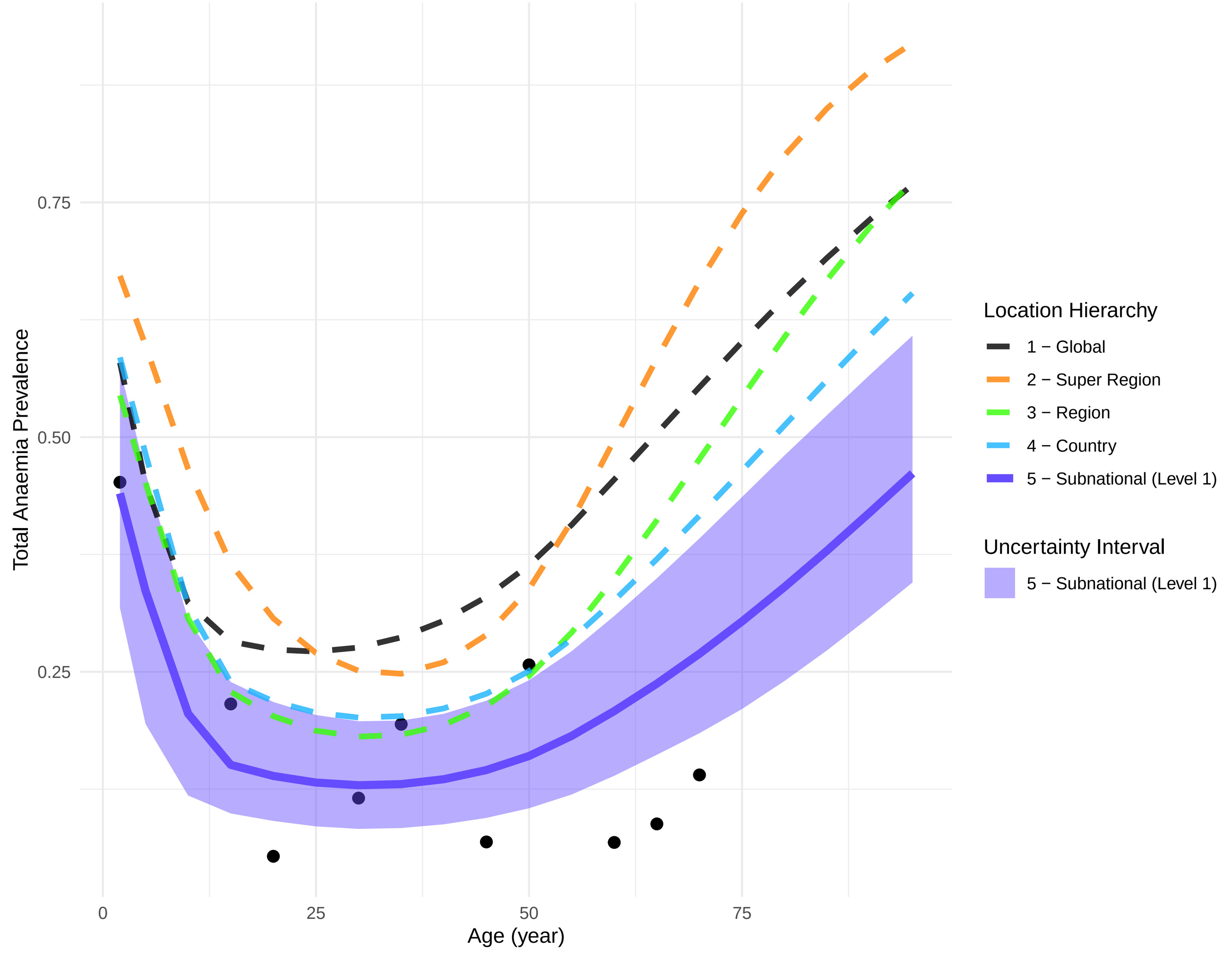

MR−BRT Age/Sex Fits − Males 2+ Years Old − Total Anaemia Prevalence − Zimbabwe
WHO−2024 Adjustment Method

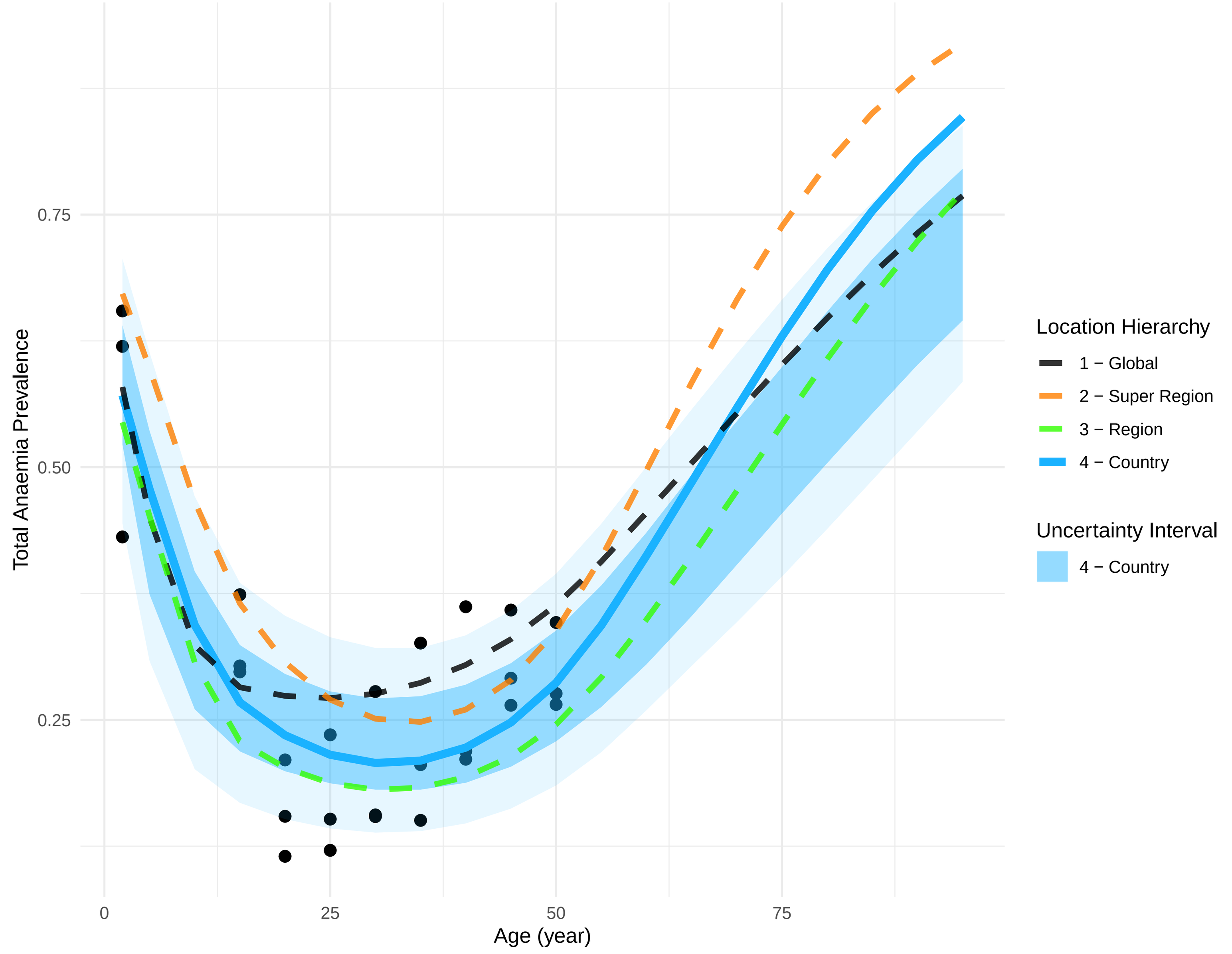

MR−BRT Age/Sex Fits − Males 2+ Years Old − Total Anaemia Prevalence − Western Sub−Saharan Africa
WHO−2024 Adjustment Method

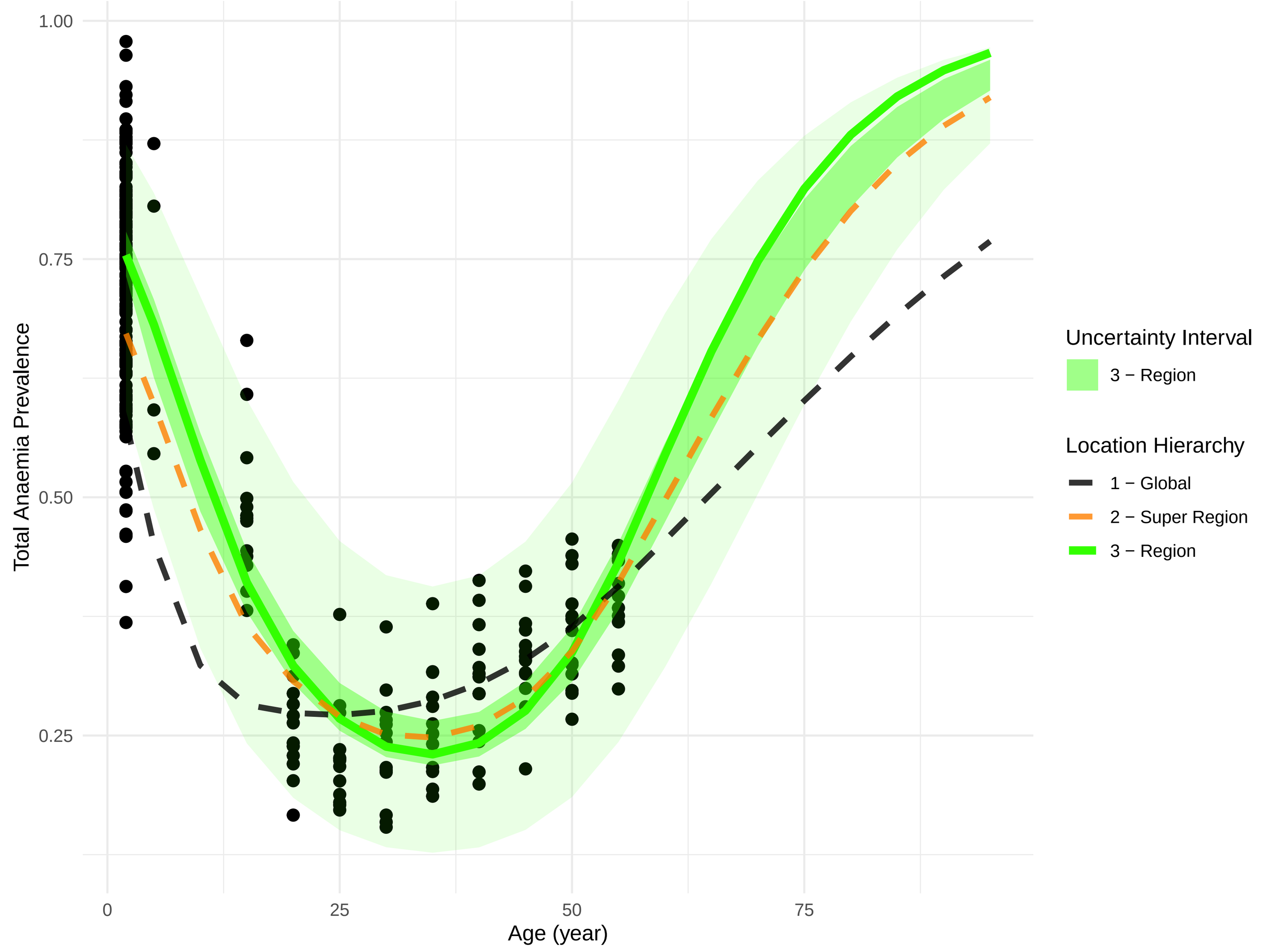

MR−BRT Age/Sex Fits − Males 2+ Years Old − Total Anaemia Prevalence − Benin
WHO−2024 Adjustment Method

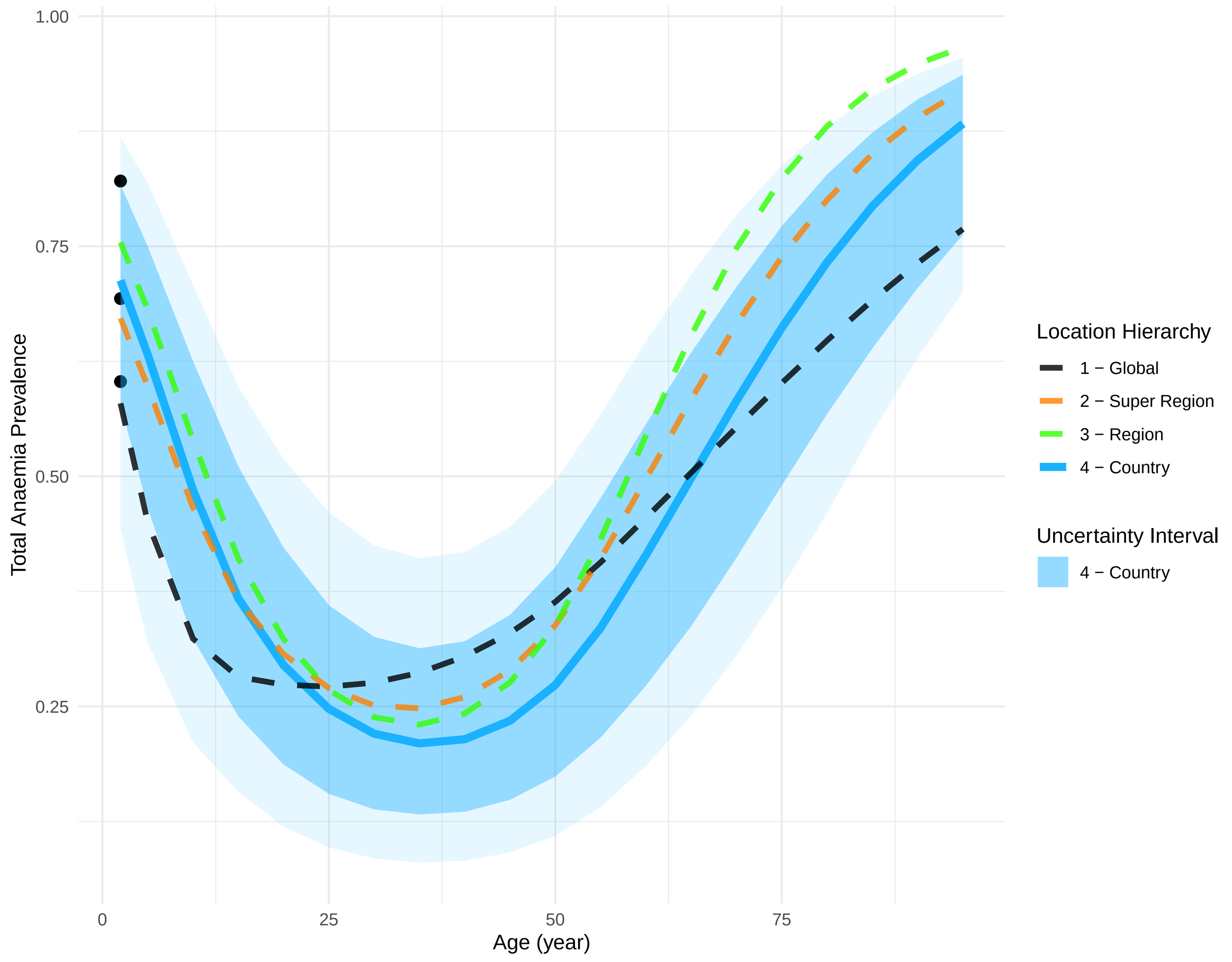

MR−BRT Age/Sex Fits − Males 2+ Years Old − Total Anaemia Prevalence − Burkina Faso
WHO−2024 Adjustment Method

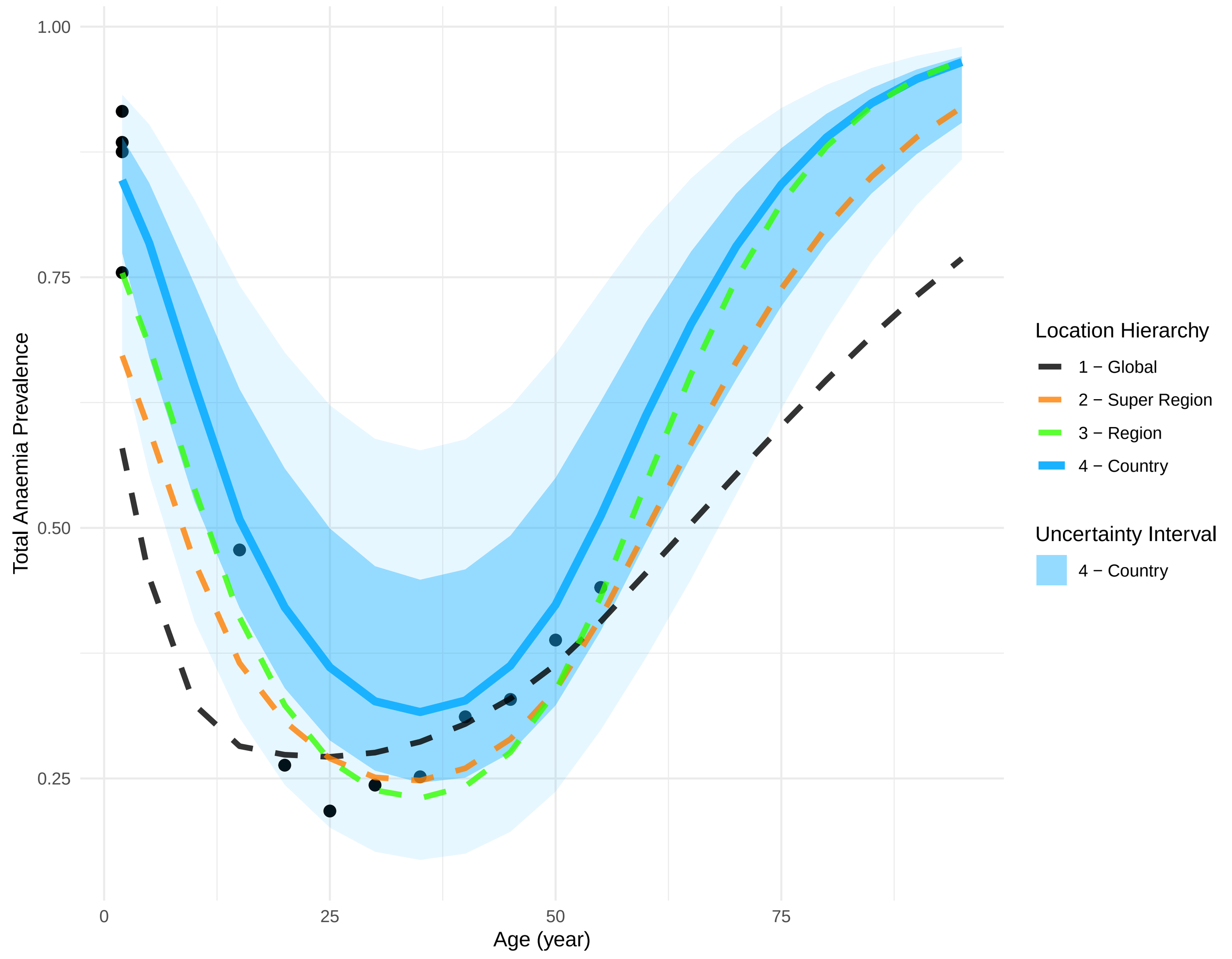

MR−BRT Age/Sex Fits − Males 2+ Years Old − Total Anaemia Prevalence − Cameroon
WHO−2024 Adjustment Method

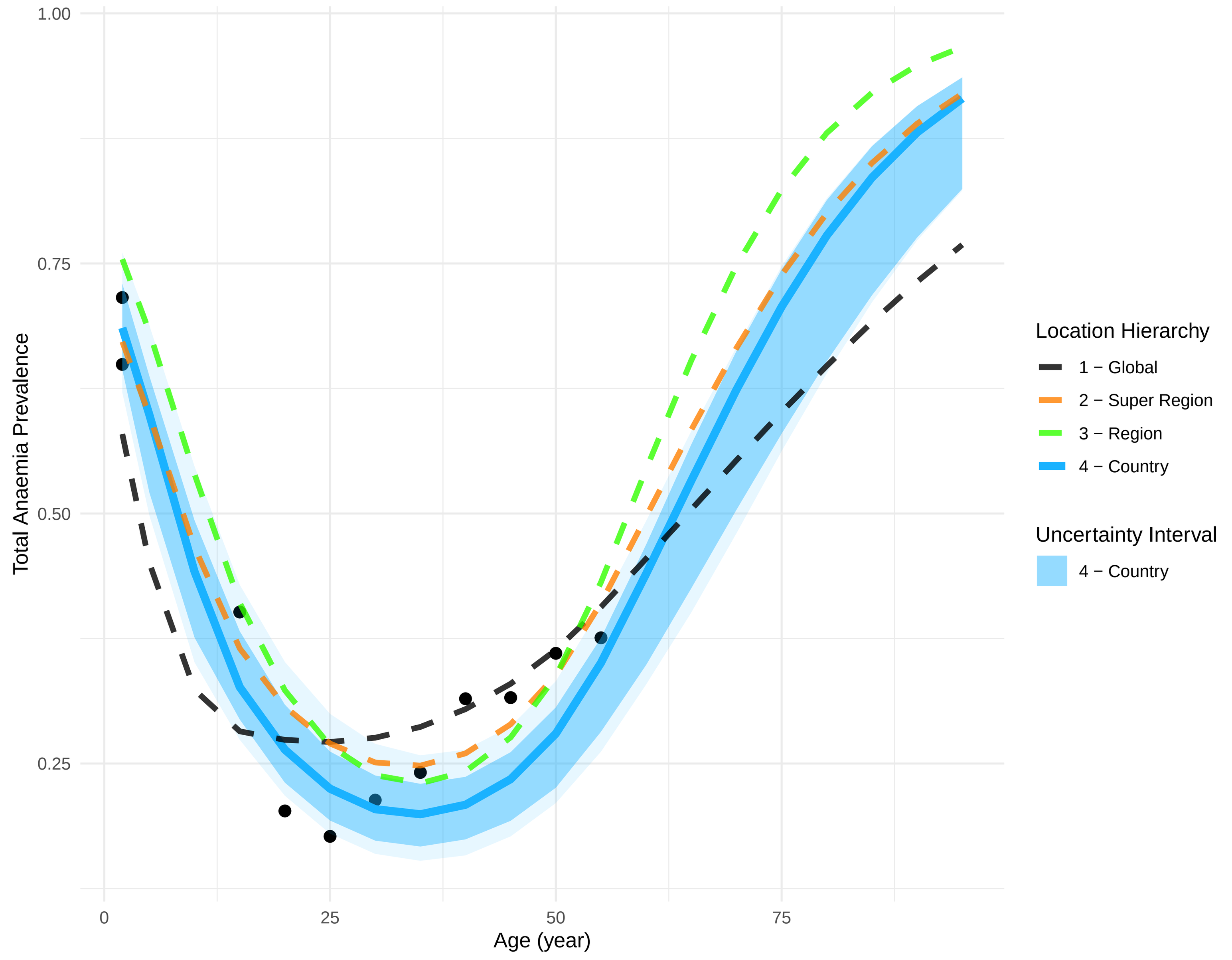

MR−BRT Age/Sex Fits − Males 2+ Years Old − Total Anaemia Prevalence − Côte d'Ivoire
WHO−2024 Adjustment Method

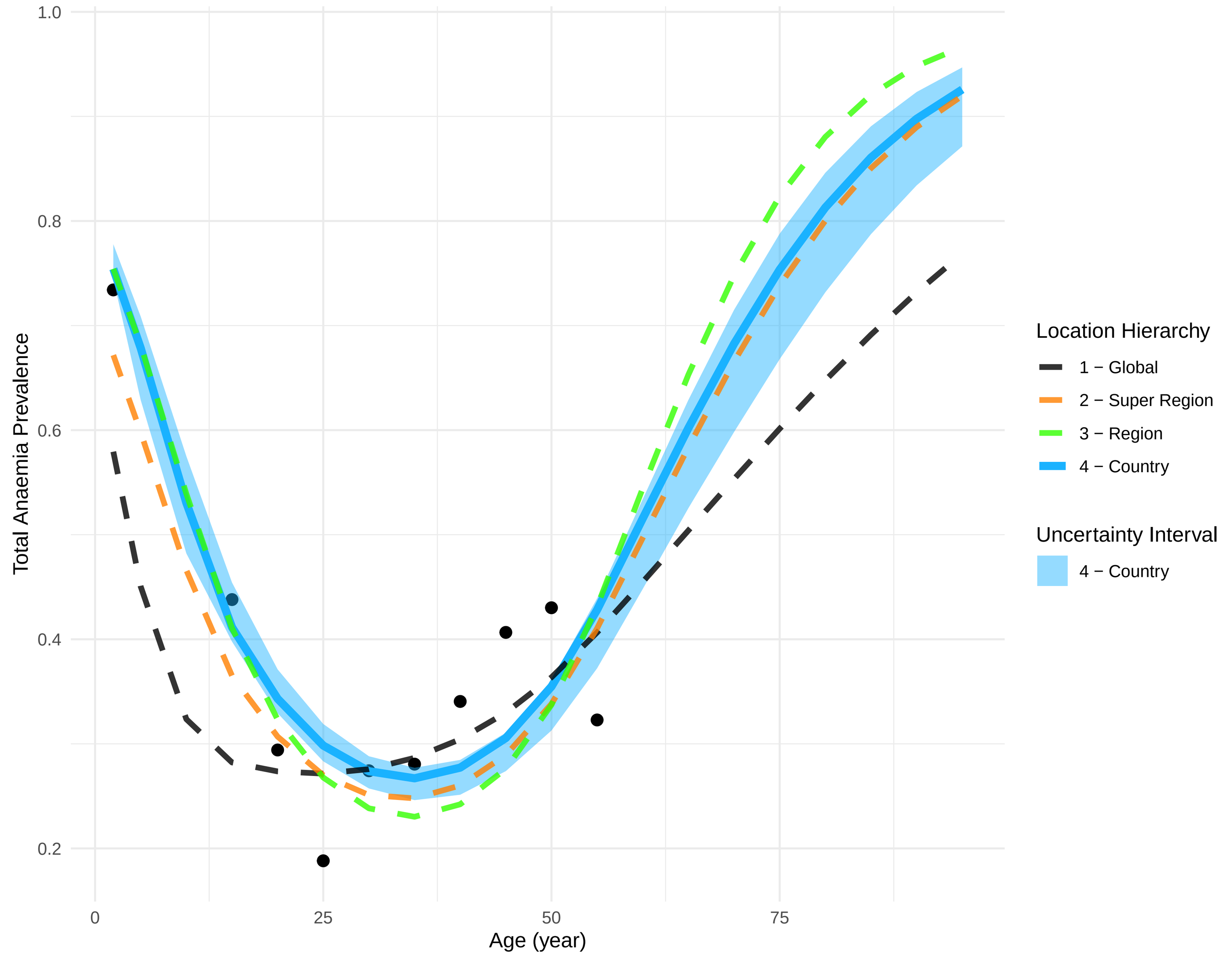

MR−BRT Age/Sex Fits − Males 2+ Years Old − Total Anaemia Prevalence − Gambia
WHO−2024 Adjustment Method

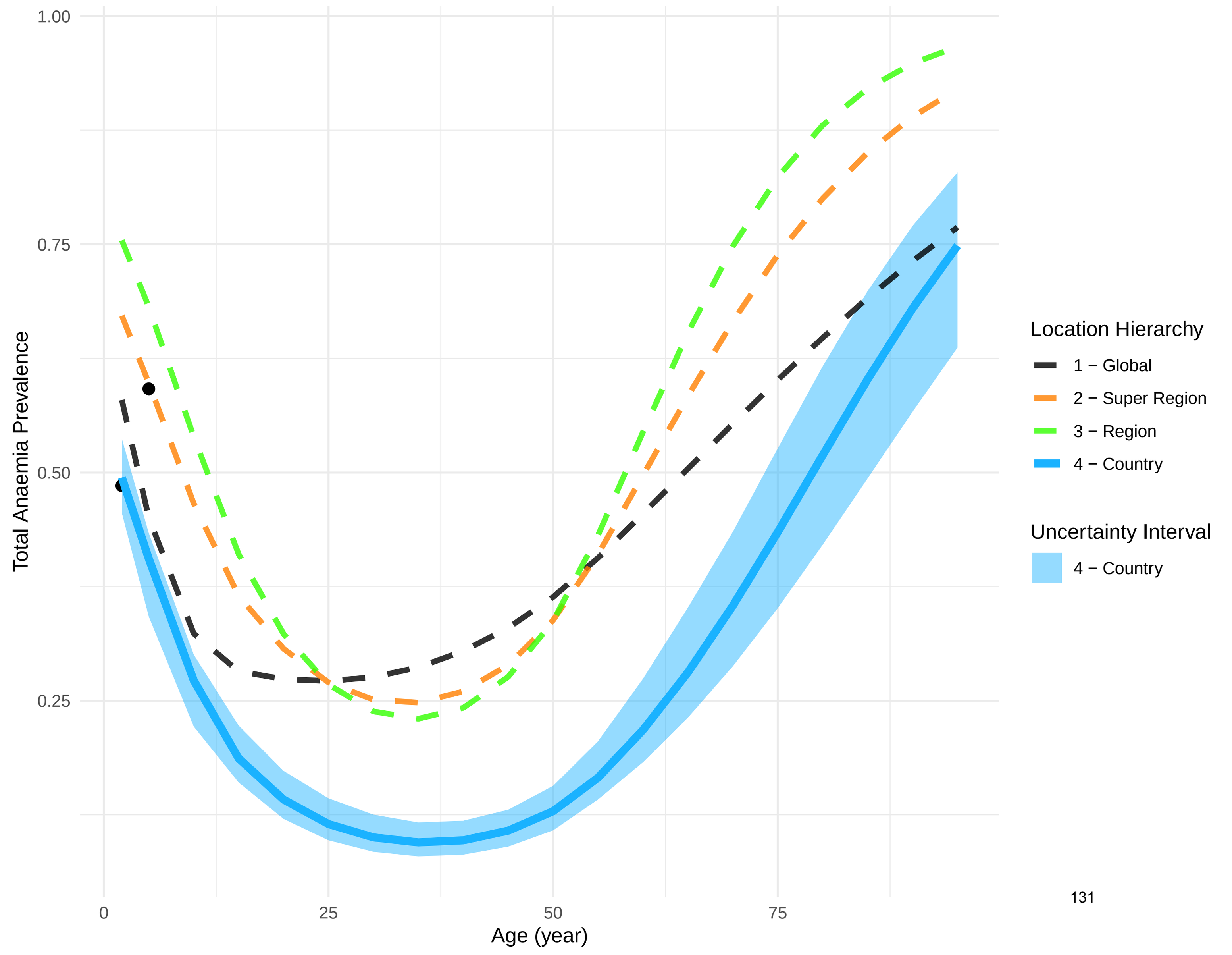

MR−BRT Age/Sex Fits − Males 2+ Years Old − Total Anaemia Prevalence − Ghana
WHO−2024 Adjustment Method

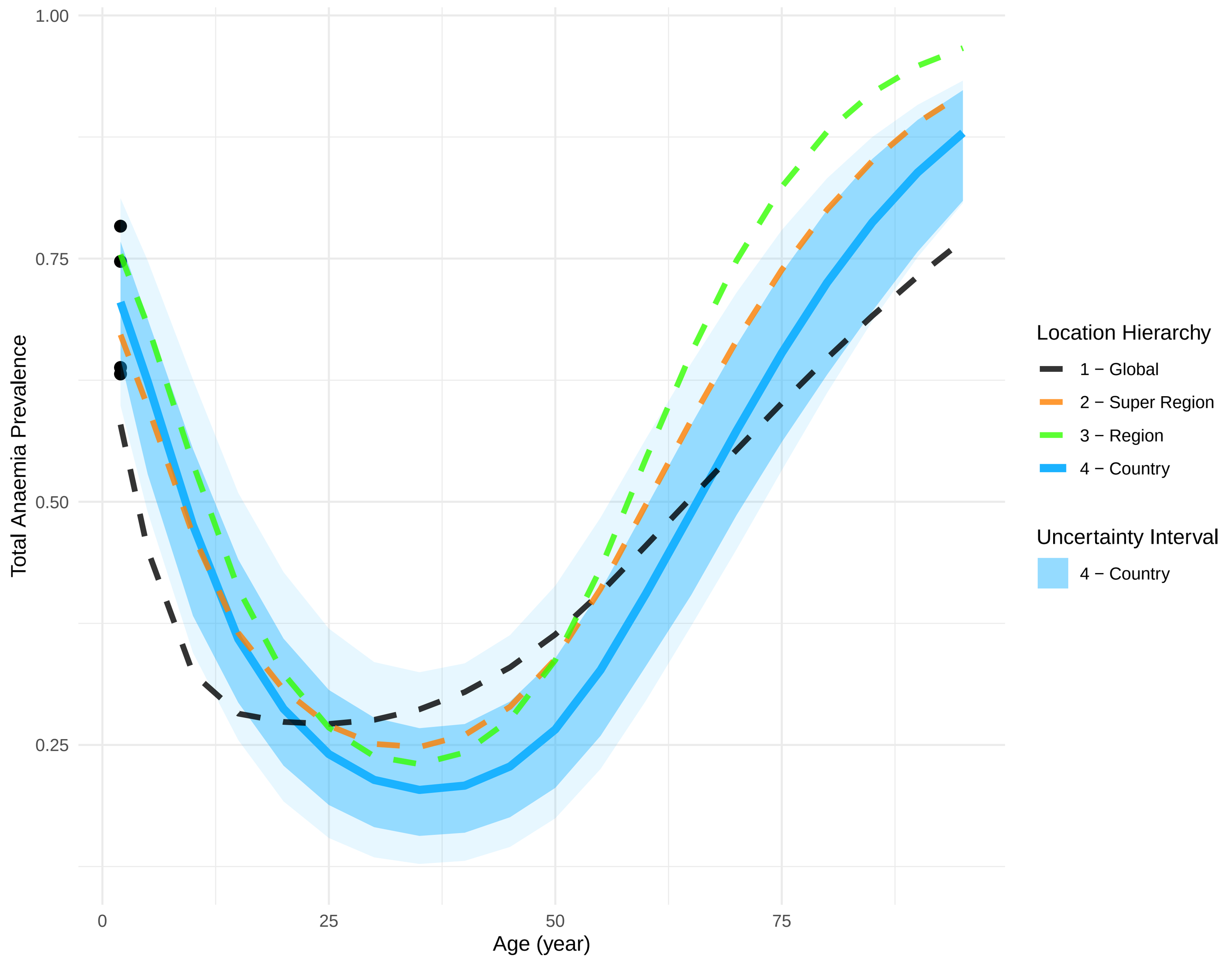

MR−BRT Age/Sex Fits − Males 2+ Years Old − Total Anaemia Prevalence − Guinea
WHO−2024 Adjustment Method

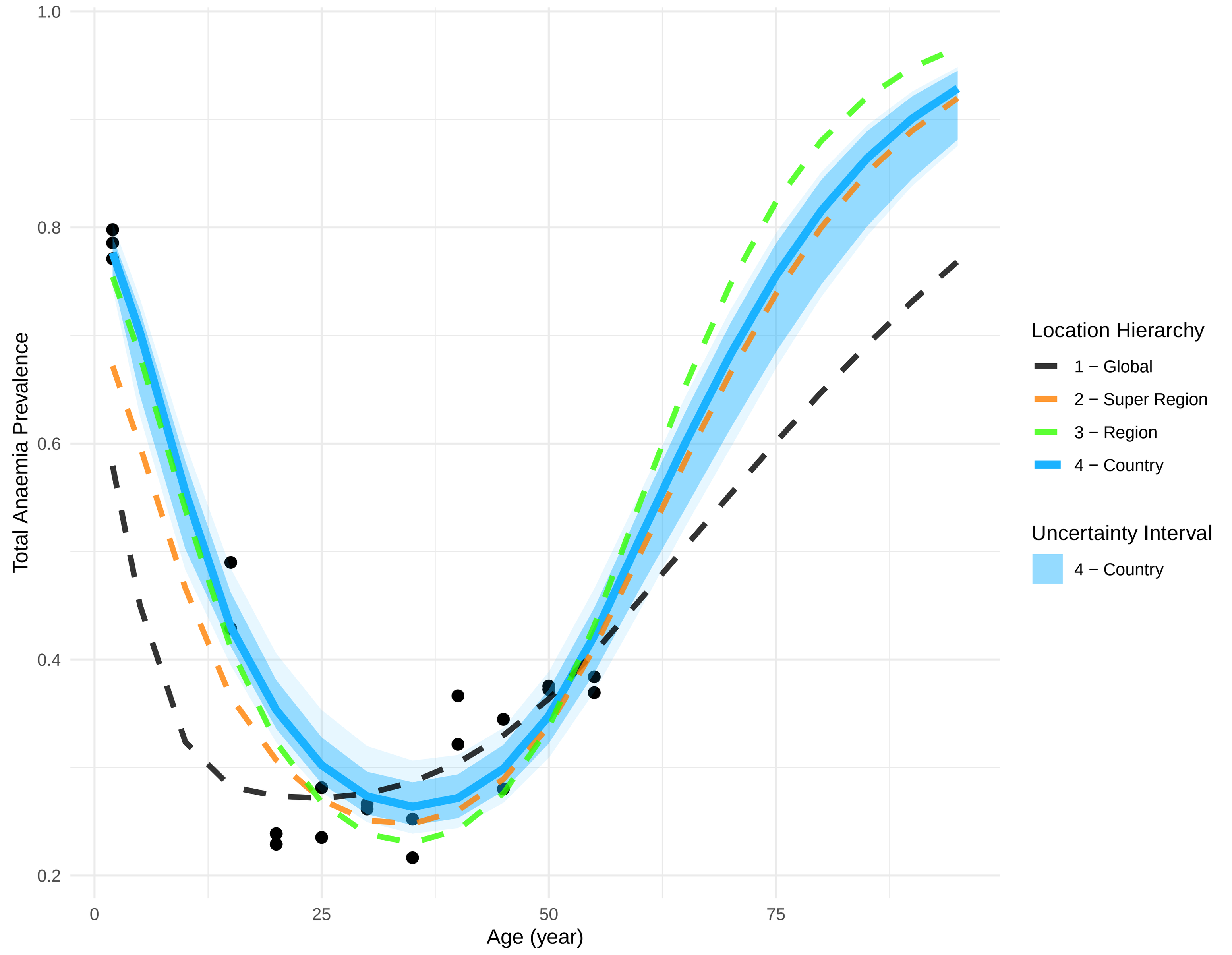

MR−BRT Age/Sex Fits − Males 2+ Years Old − Total Anaemia Prevalence − Liberia
WHO−2024 Adjustment Method

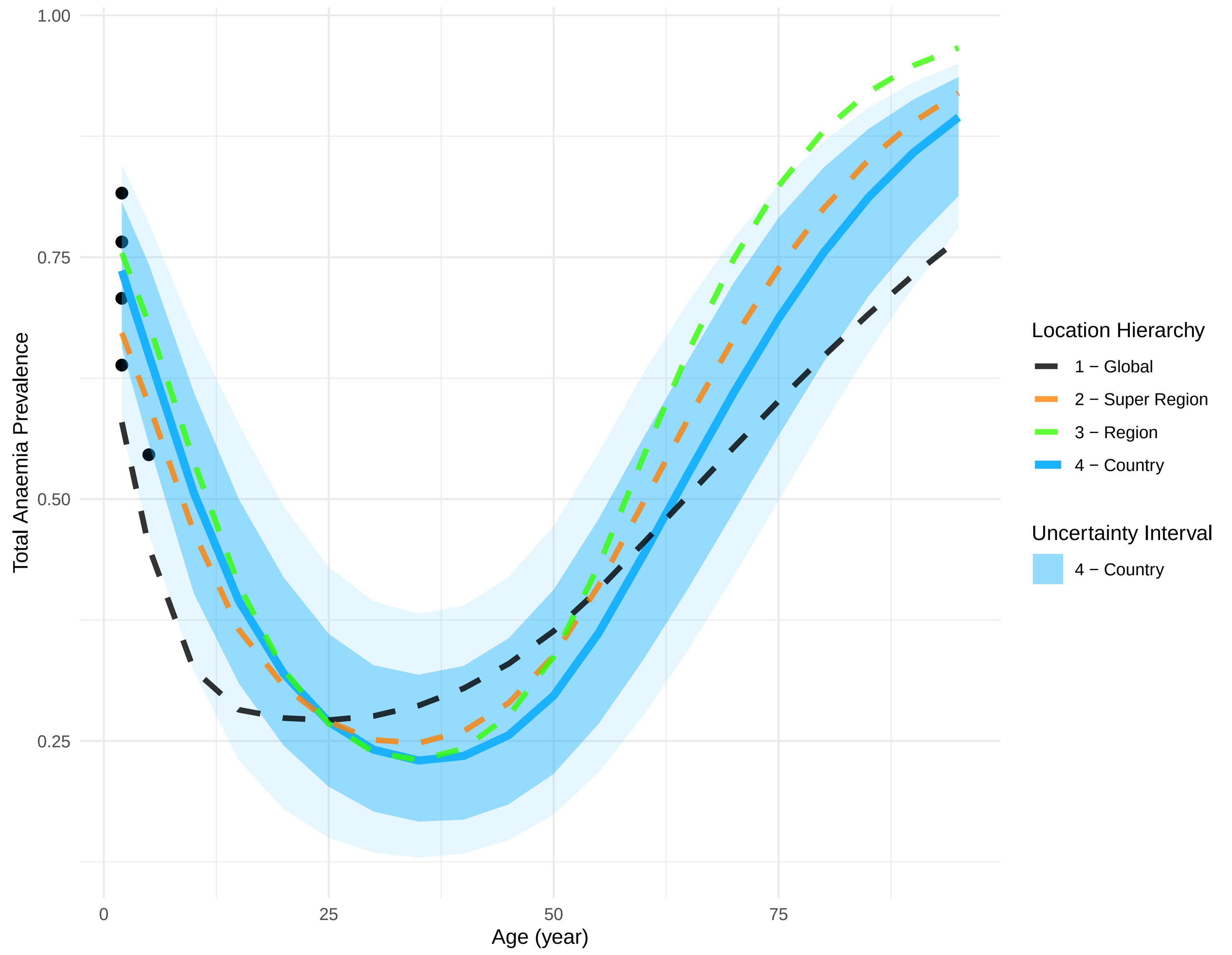

MR−BRT Age/Sex Fits − Males 2+ Years Old − Total Anaemia Prevalence − Mali
WHO−2024 Adjustment Method

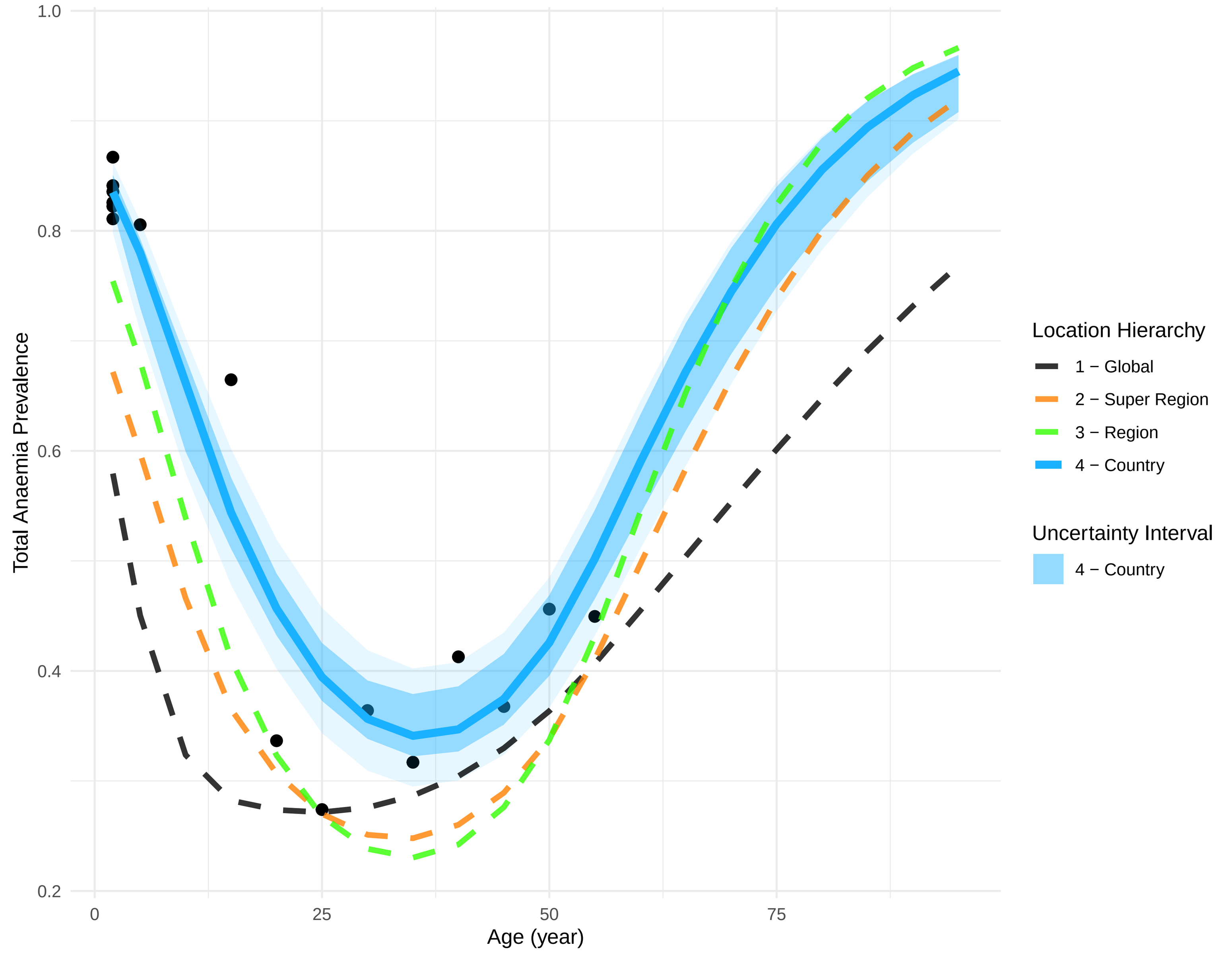

MR−BRT Age/Sex Fits − Males 2+ Years Old − Total Anaemia Prevalence − Niger
WHO−2024 Adjustment Method

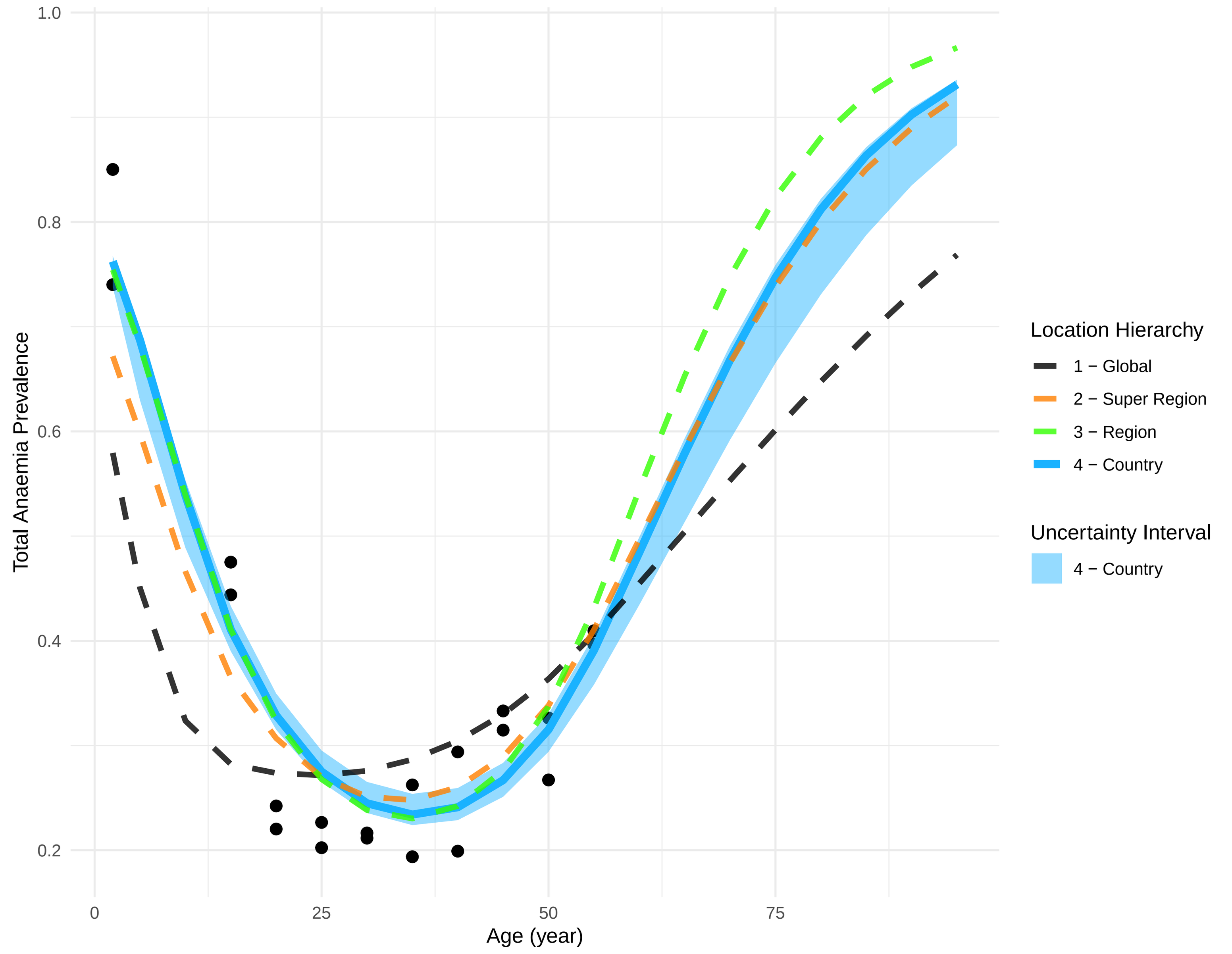

MR−BRT Age/Sex Fits − Males 2+ Years Old − Total Anaemia Prevalence − Nigeria
WHO−2024 Adjustment Method

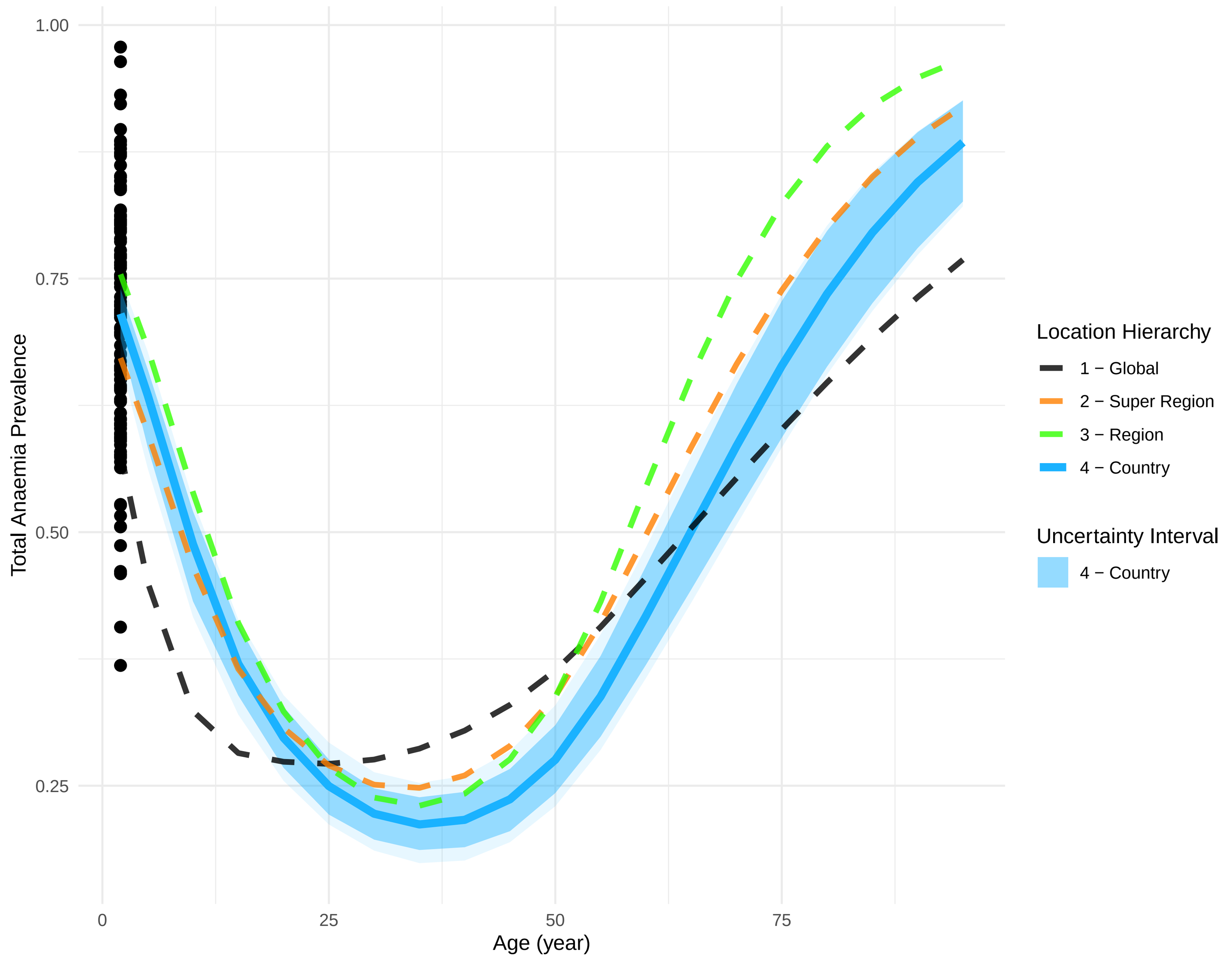

MR−BRT Age/Sex Fits − Males 2+ Years Old − Total Anaemia Prevalence − Abia
WHO−2024 Adjustment Method

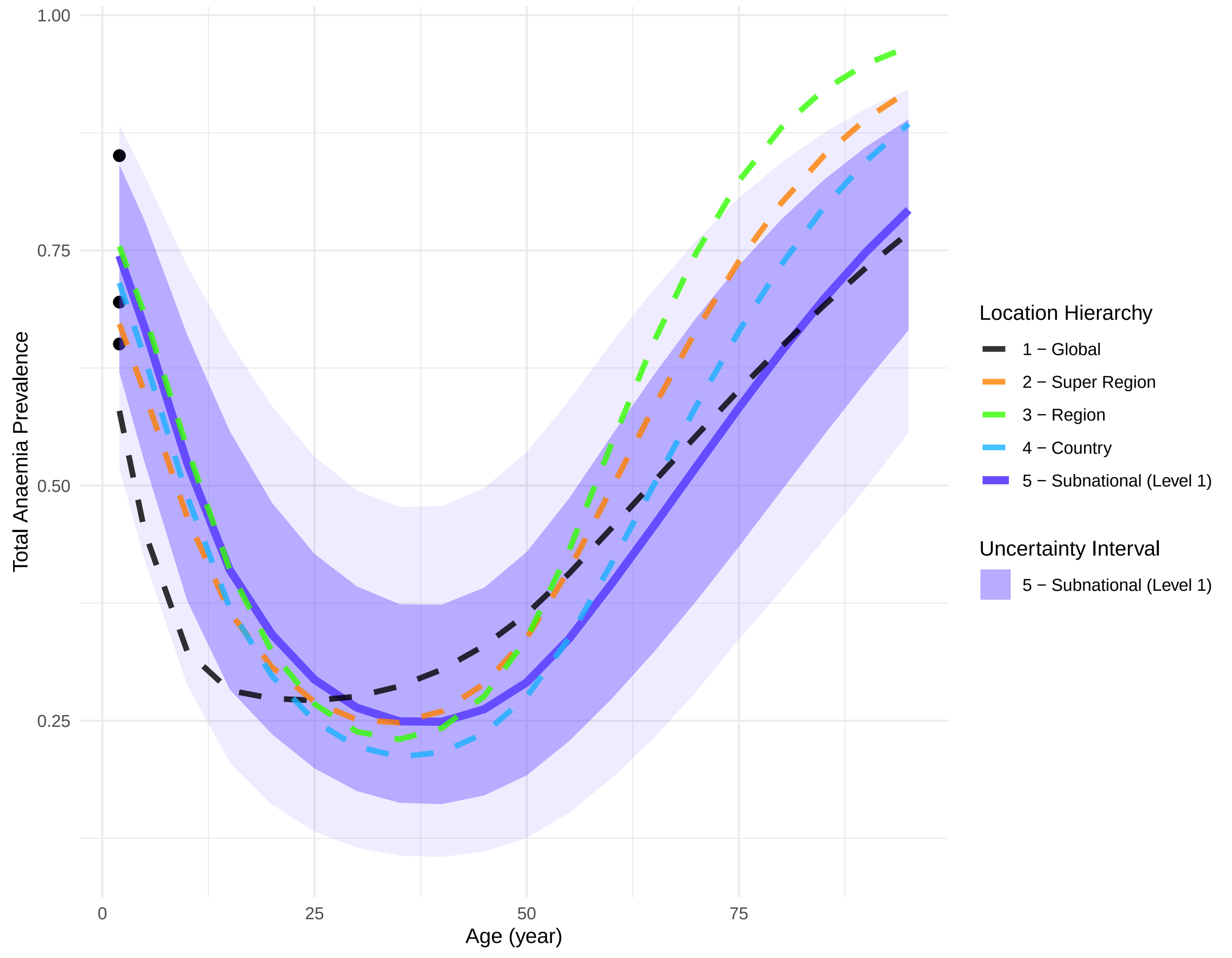

MR−BRT Age/Sex Fits − Males 2+ Years Old − Total Anaemia Prevalence − Adamawa
WHO−2024 Adjustment Method

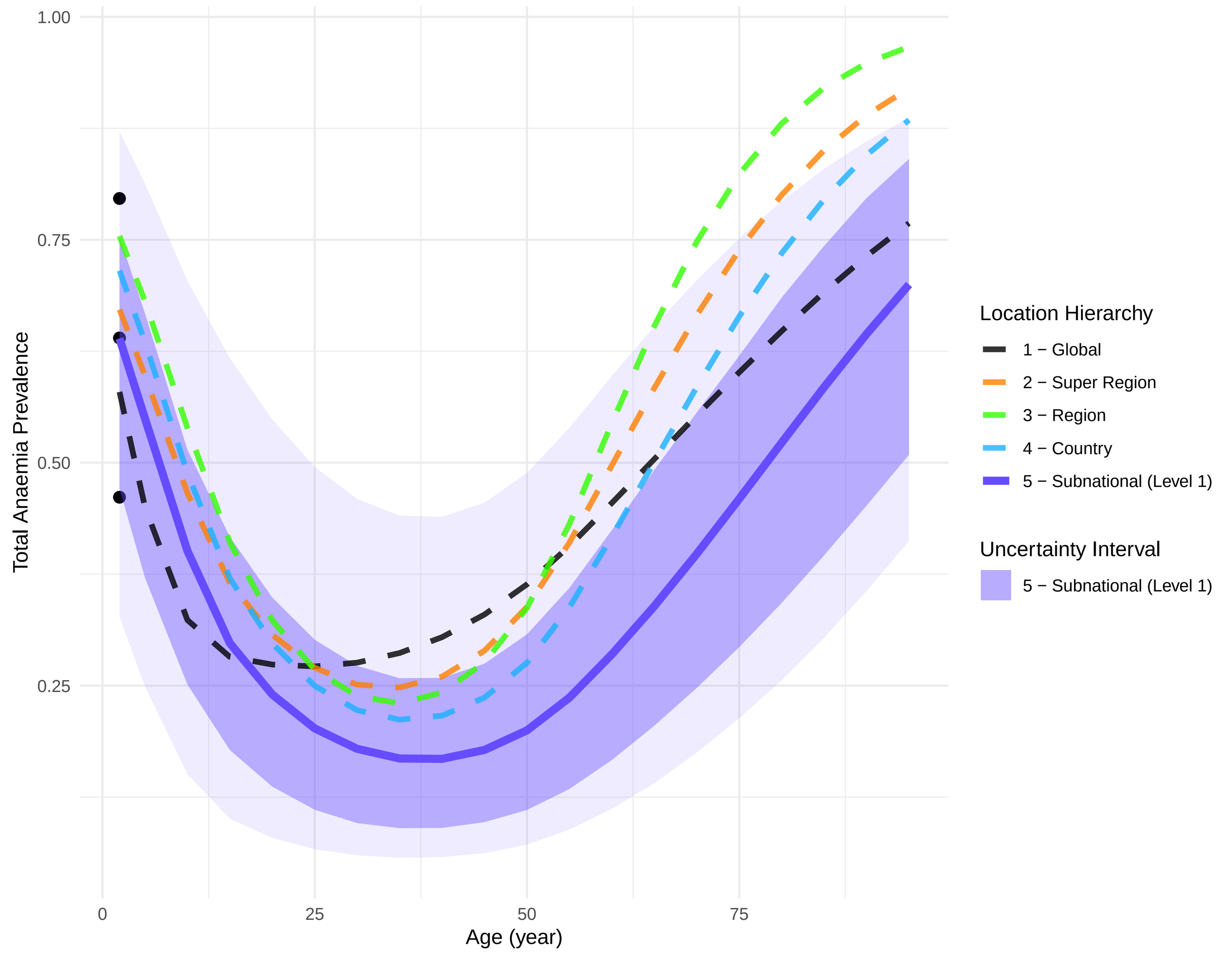

MR−BRT Age/Sex Fits − Males 2+ Years Old − Total Anaemia Prevalence − Akwa Ibom
WHO−2024 Adjustment Method

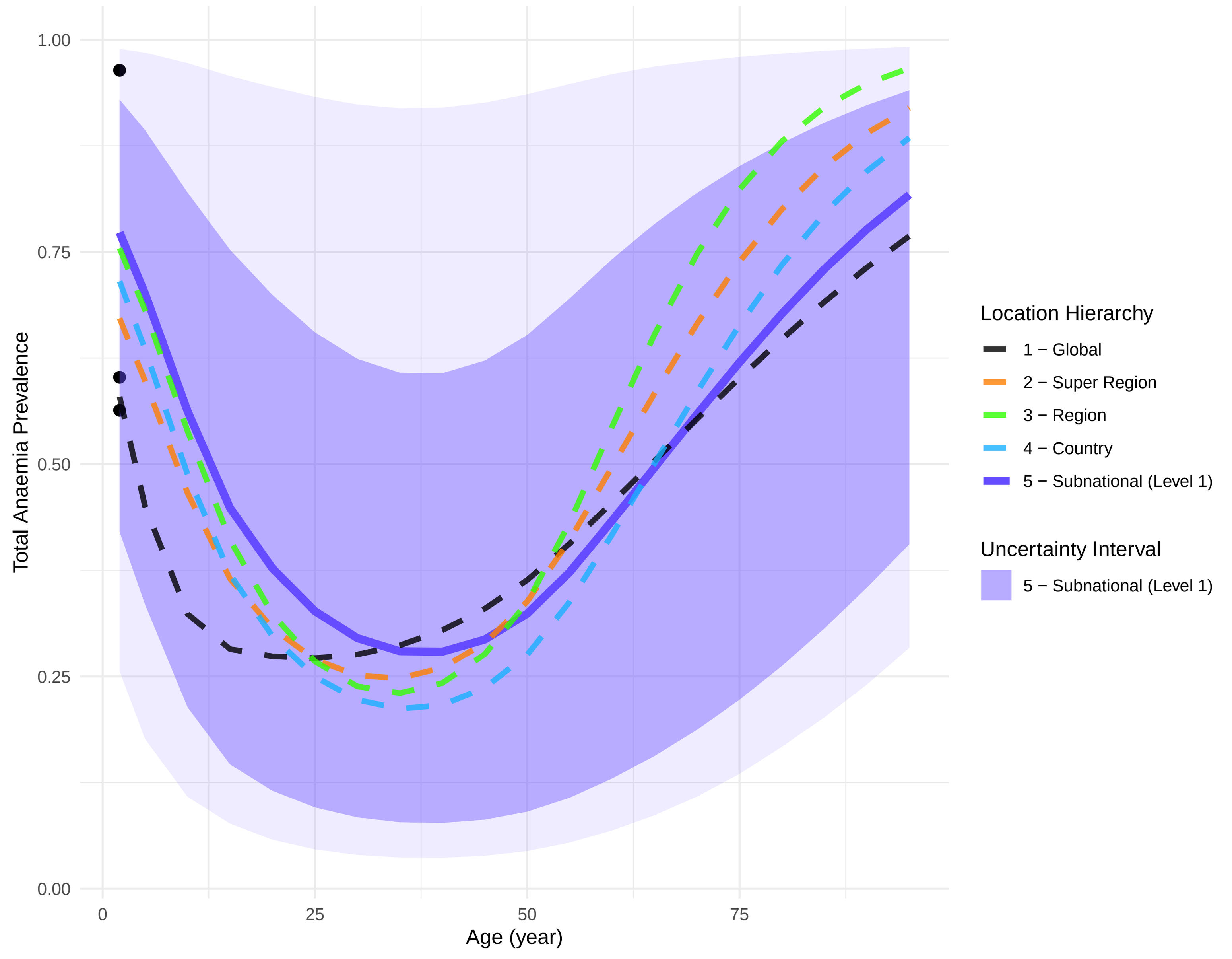

MR−BRT Age/Sex Fits − Males 2+ Years Old − Total Anaemia Prevalence − Anambra
WHO−2024 Adjustment Method

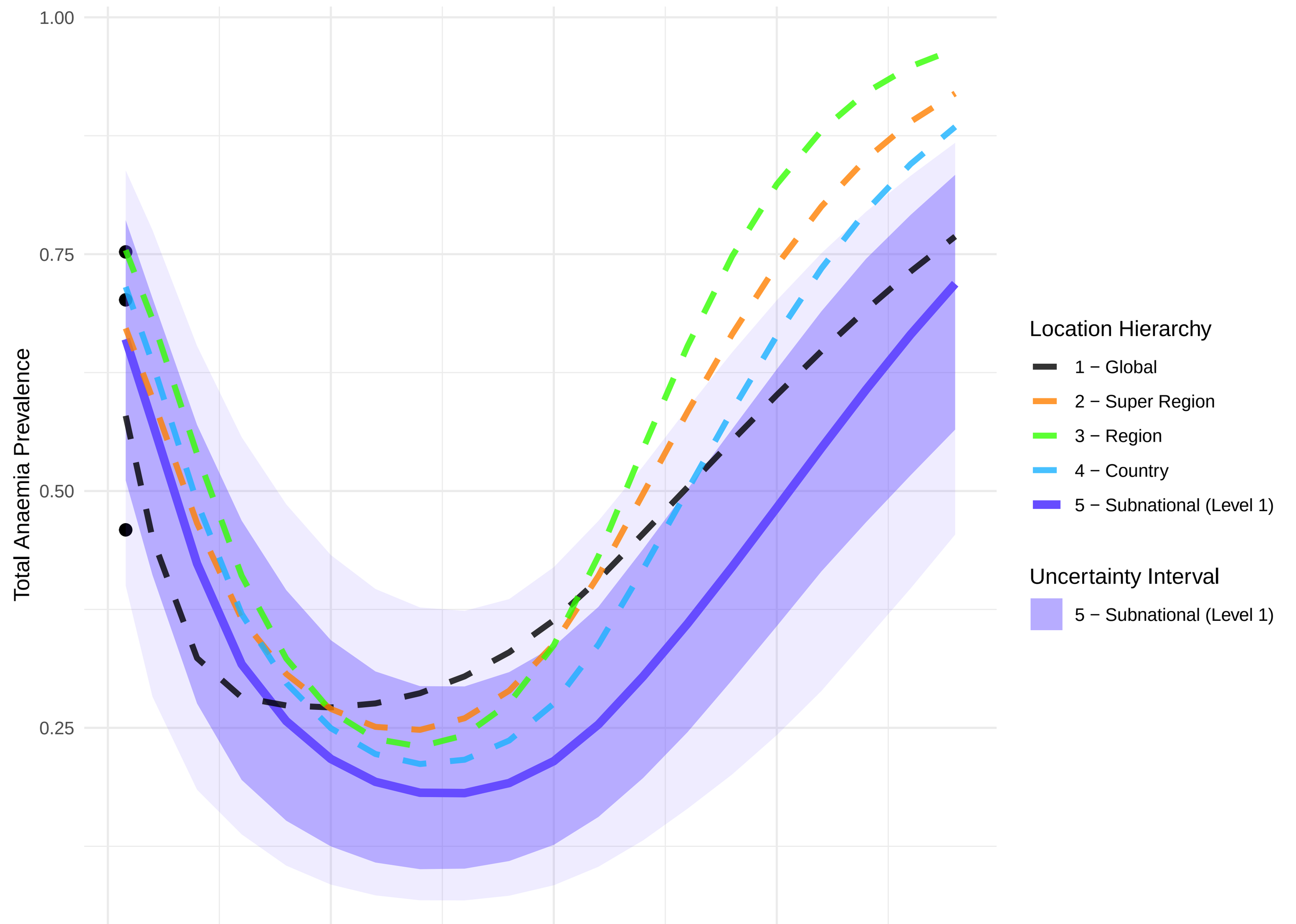

MR−BRT Age/Sex Fits − Males 2+ Years Old − Total Anaemia Prevalence − Bauchi
WHO−2024 Adjustment Method

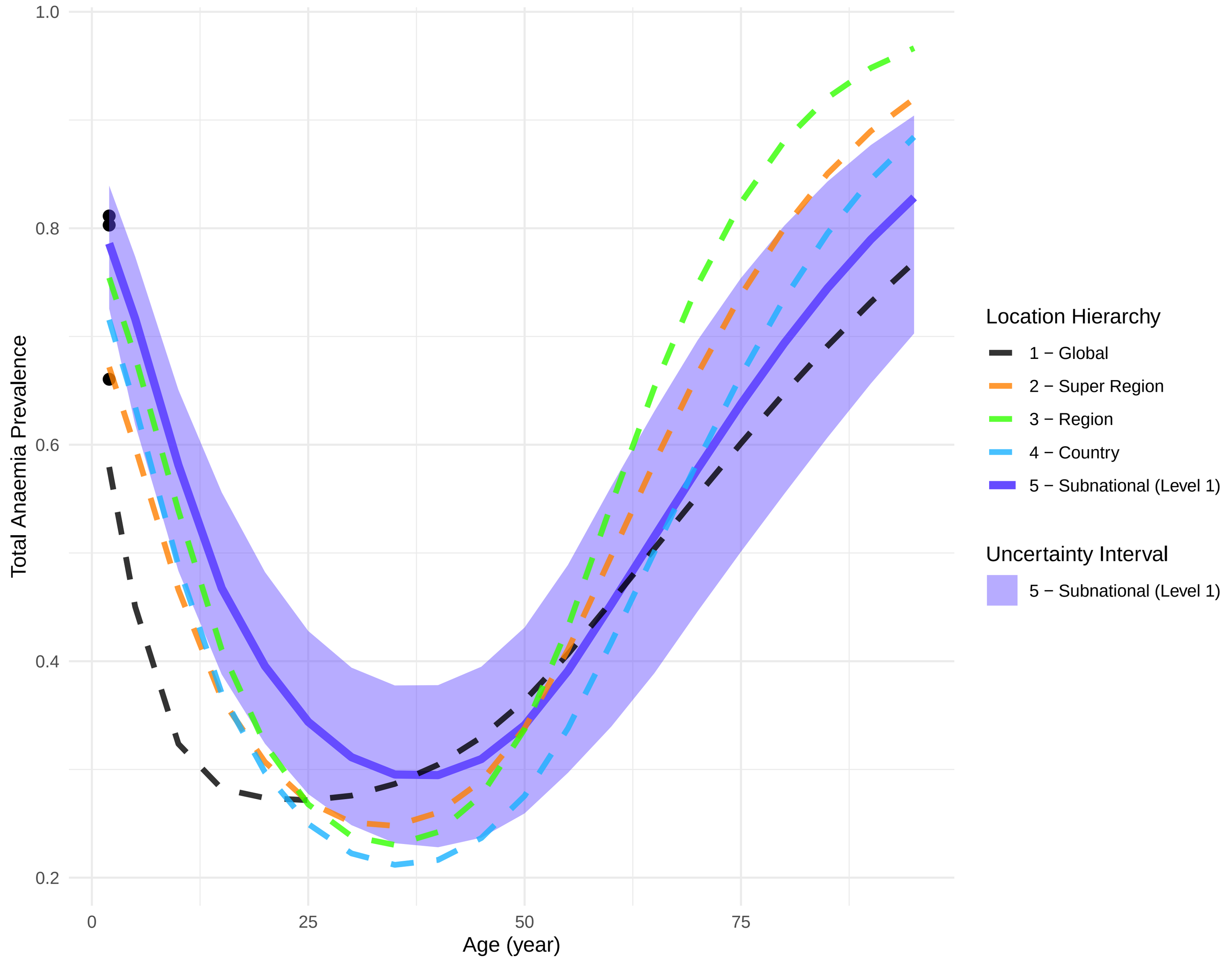

MR−BRT Age/Sex Fits − Males 2+ Years Old − Total Anaemia Prevalence − Bayelsa
WHO−2024 Adjustment Method

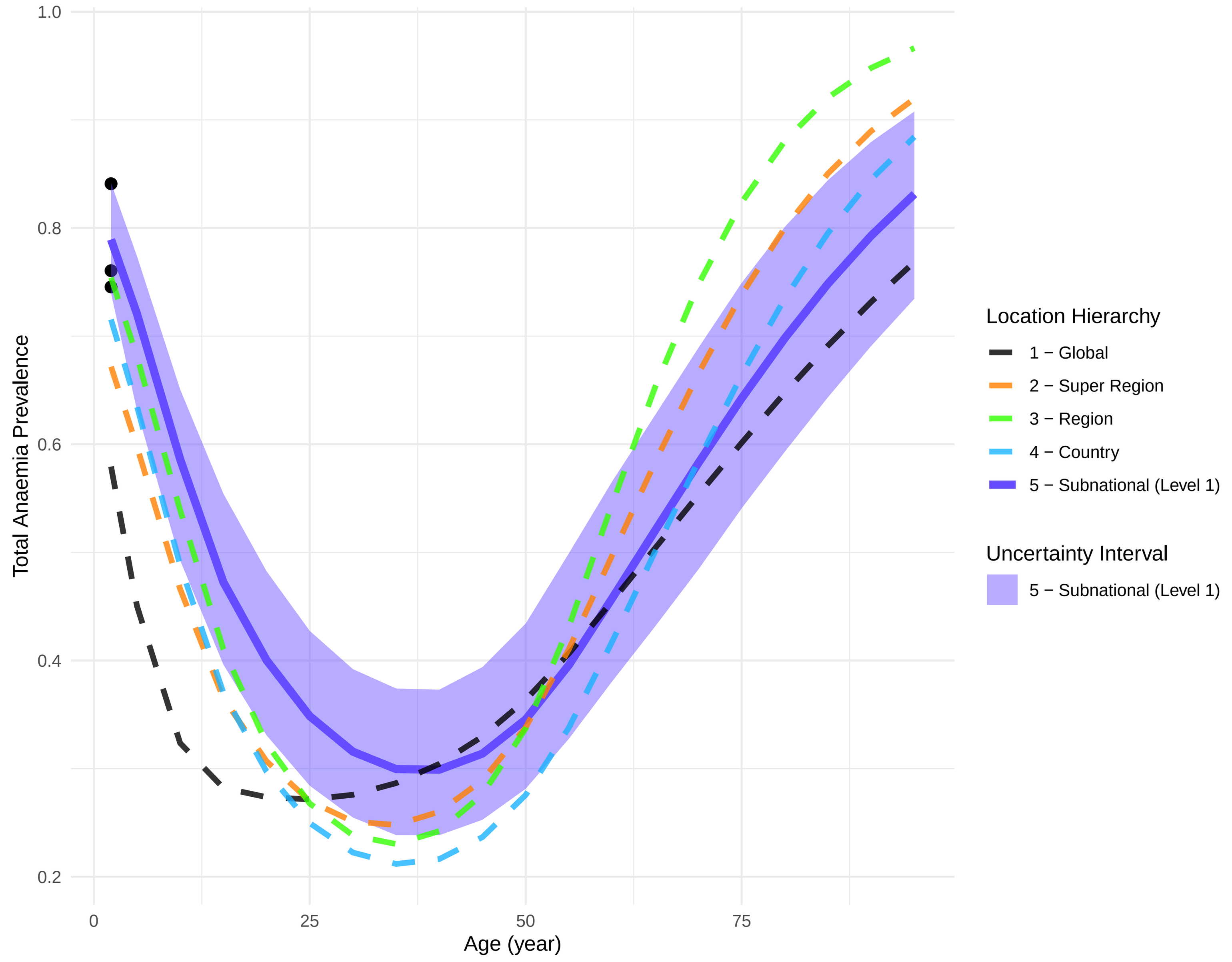

MR−BRT Age/Sex Fits − Males 2+ Years Old − Total Anaemia Prevalence − Benue
WHO−2024 Adjustment Method

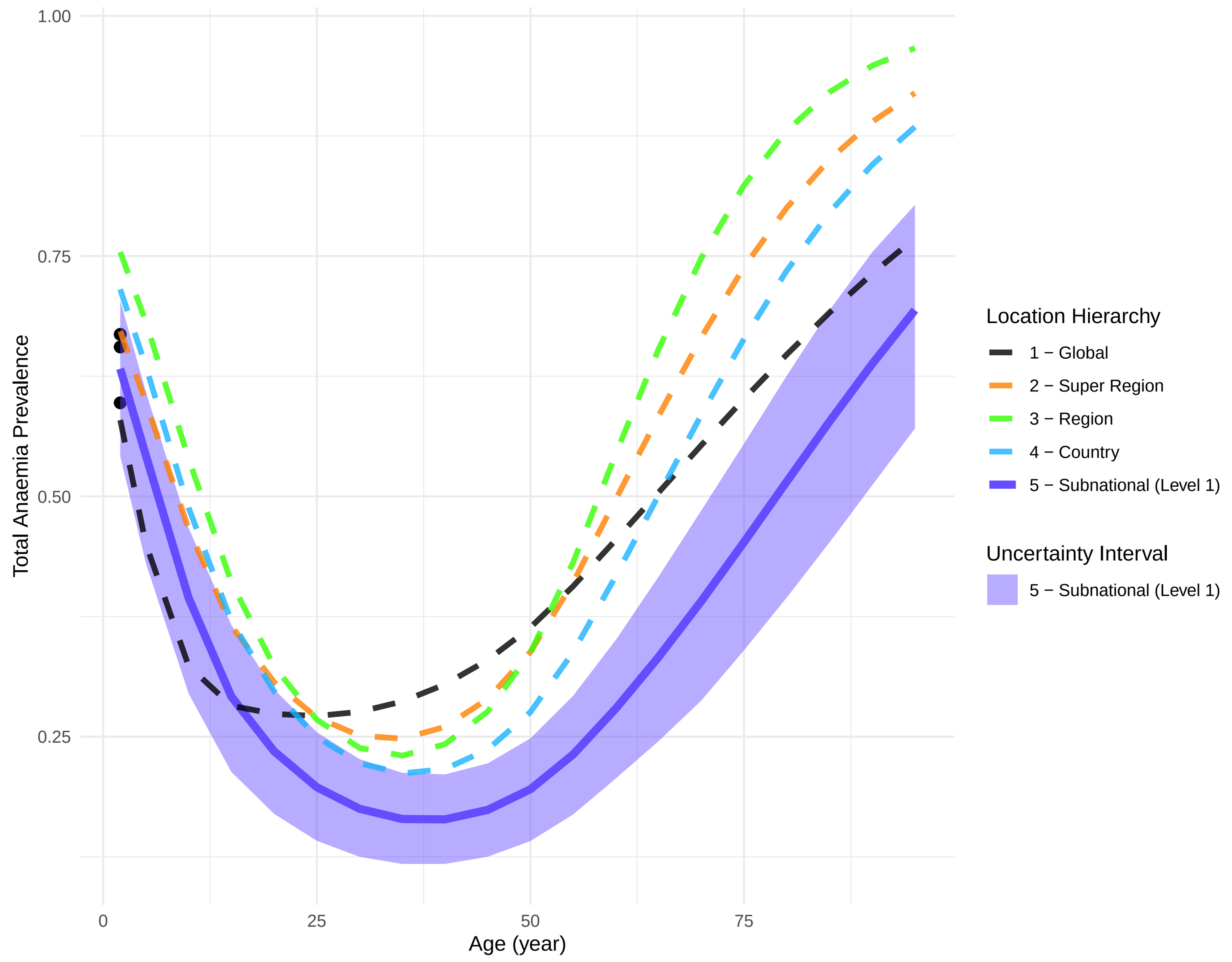

MR−BRT Age/Sex Fits − Males 2+ Years Old − Total Anaemia Prevalence − Borno
WHO−2024 Adjustment Method

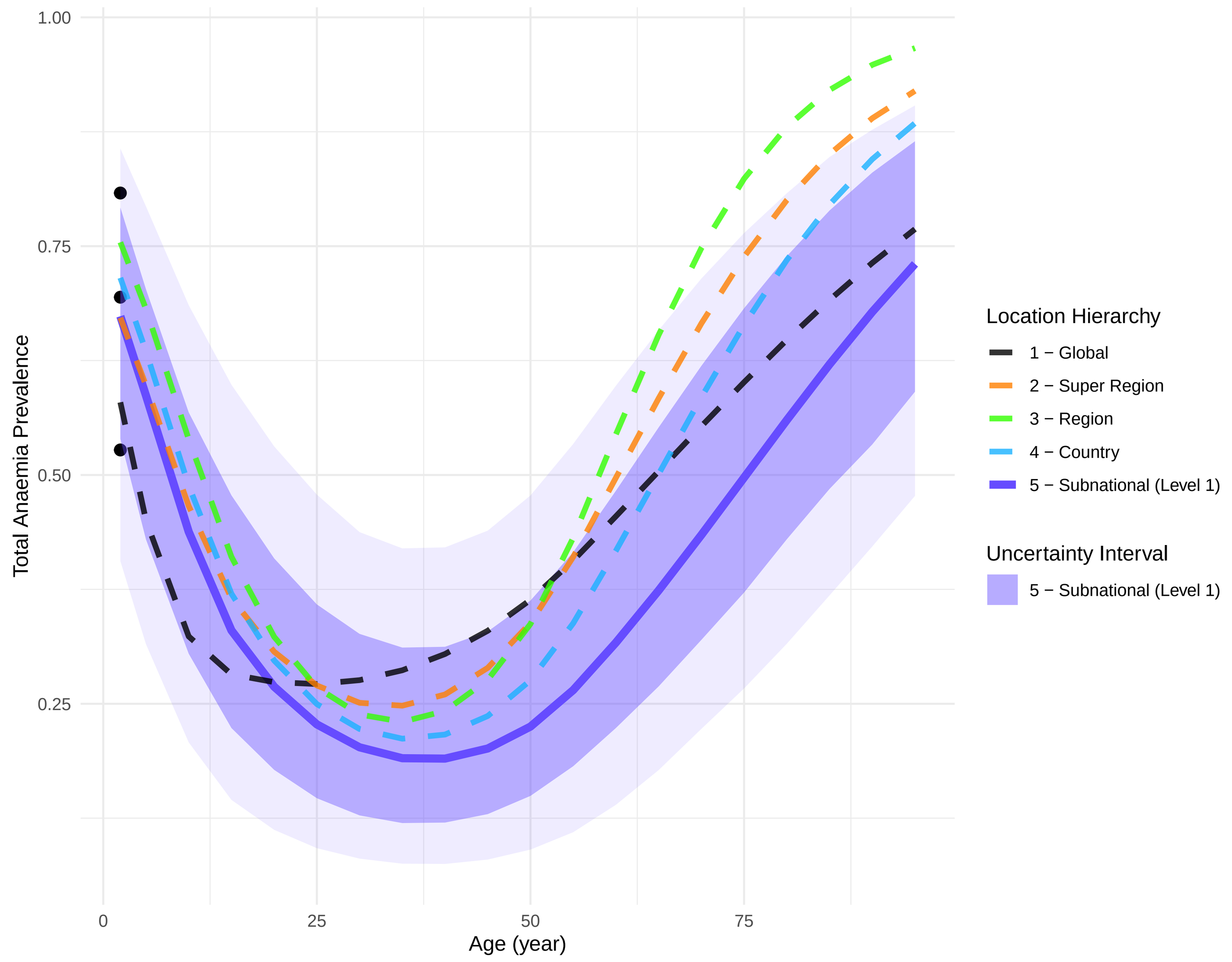

MR−BRT Age/Sex Fits − Males 2+ Years Old − Total Anaemia Prevalence − Cross River
WHO−2024 Adjustment Method

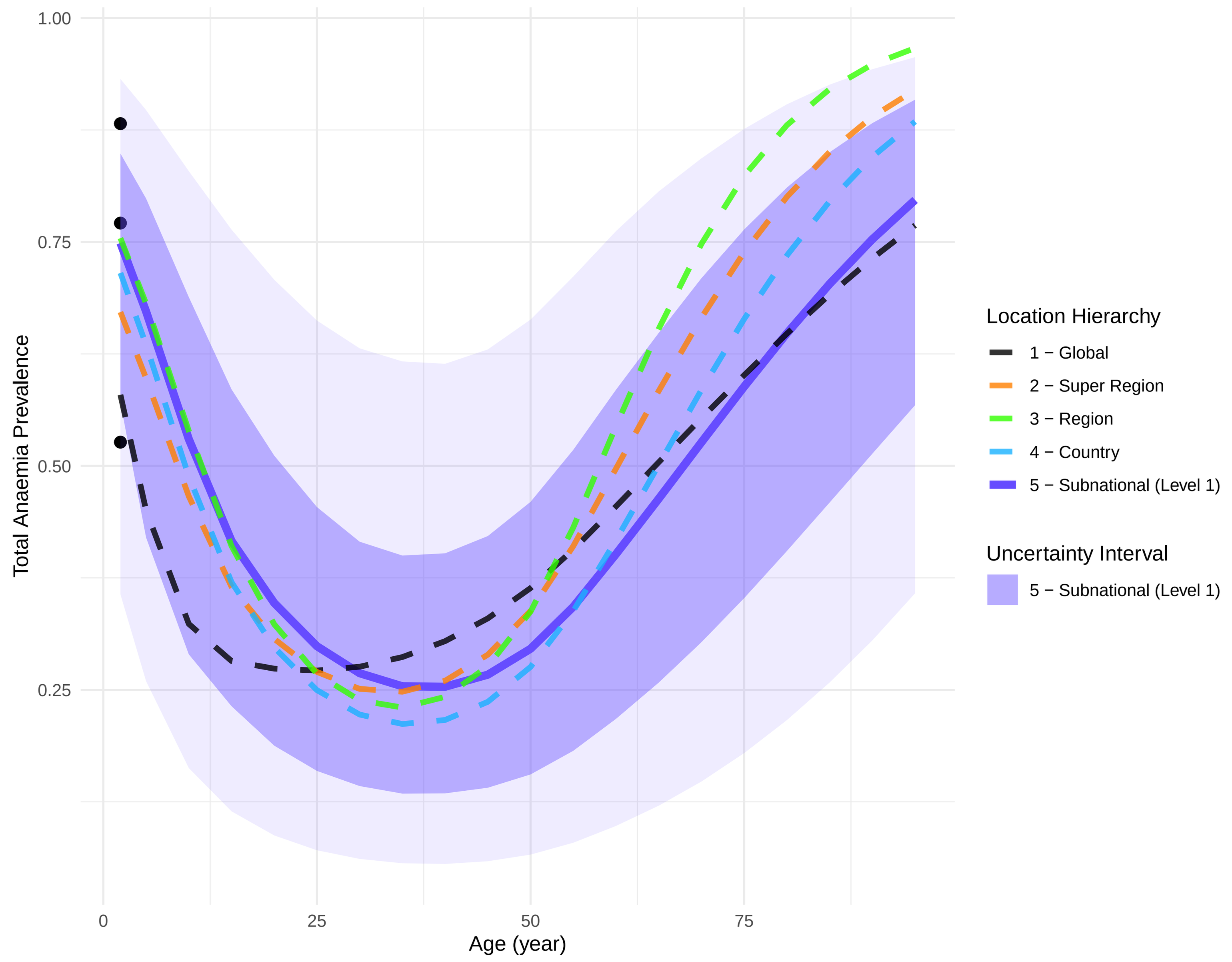

MR−BRT Age/Sex Fits − Males 2+ Years Old − Total Anaemia Prevalence − Delta
WHO−2024 Adjustment Method

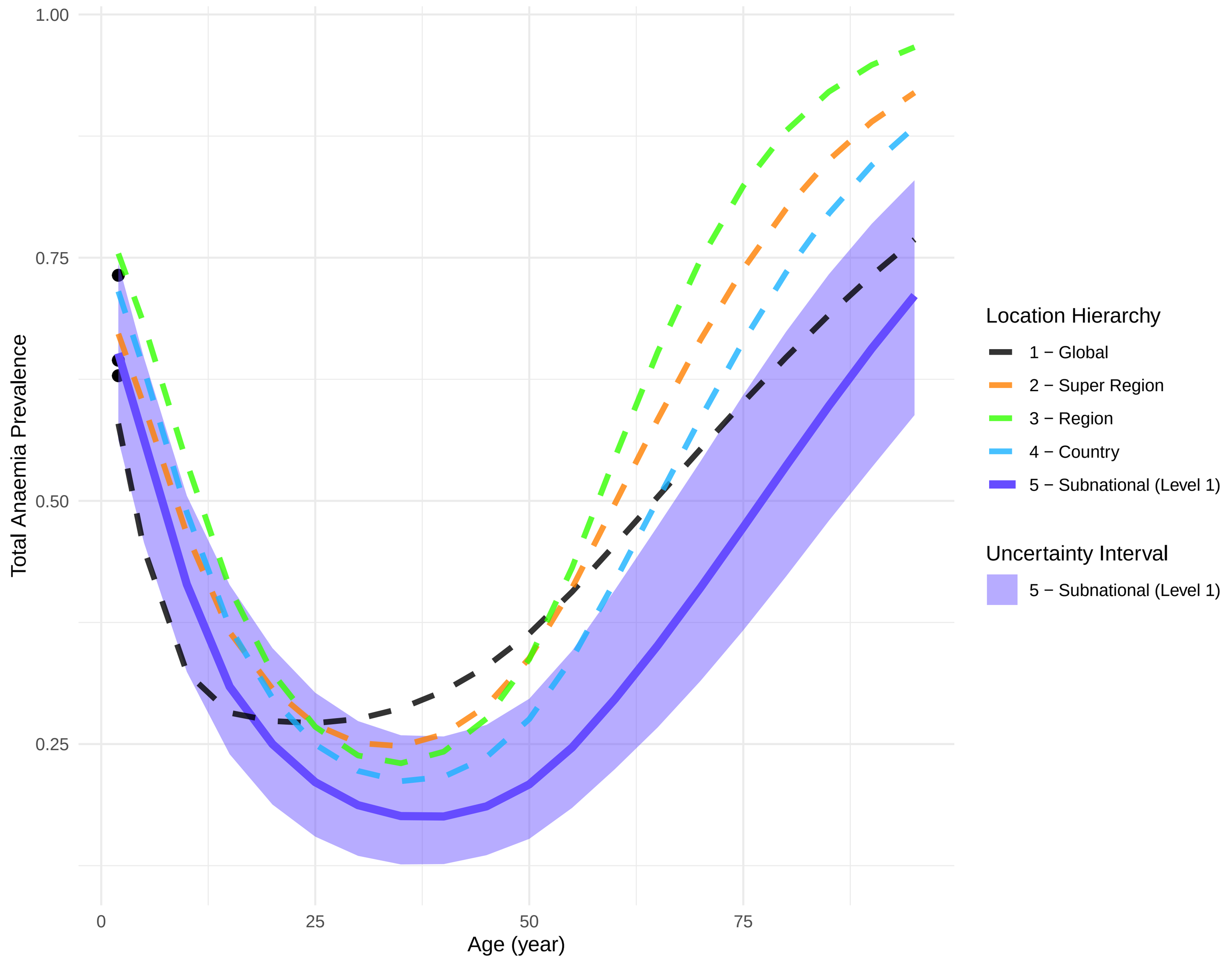

MR−BRT Age/Sex Fits − Males 2+ Years Old − Total Anaemia Prevalence − Ebonyi
WHO−2024 Adjustment Method

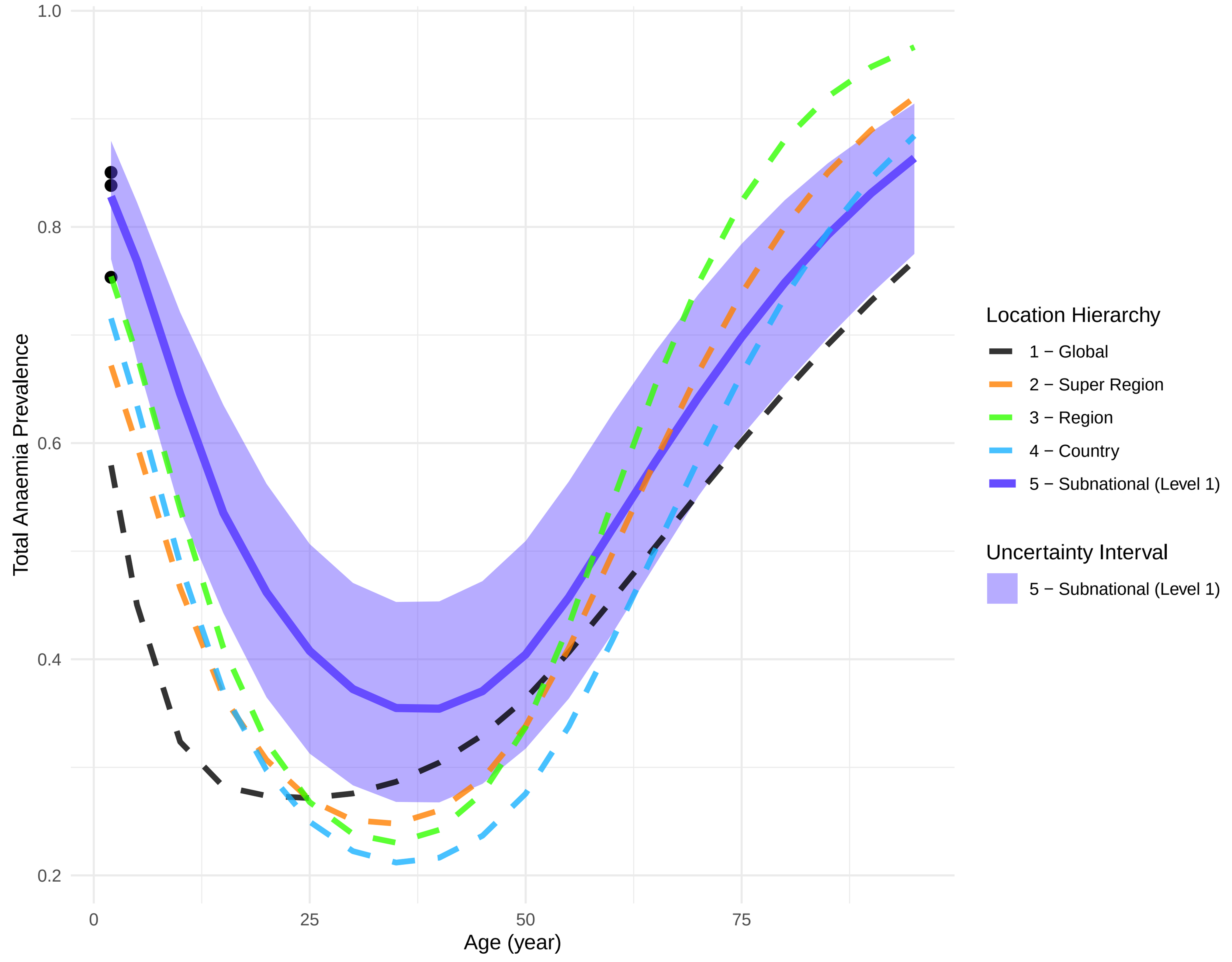

MR−BRT Age/Sex Fits − Males 2+ Years Old − Total Anaemia Prevalence − Edo
WHO−2024 Adjustment Method

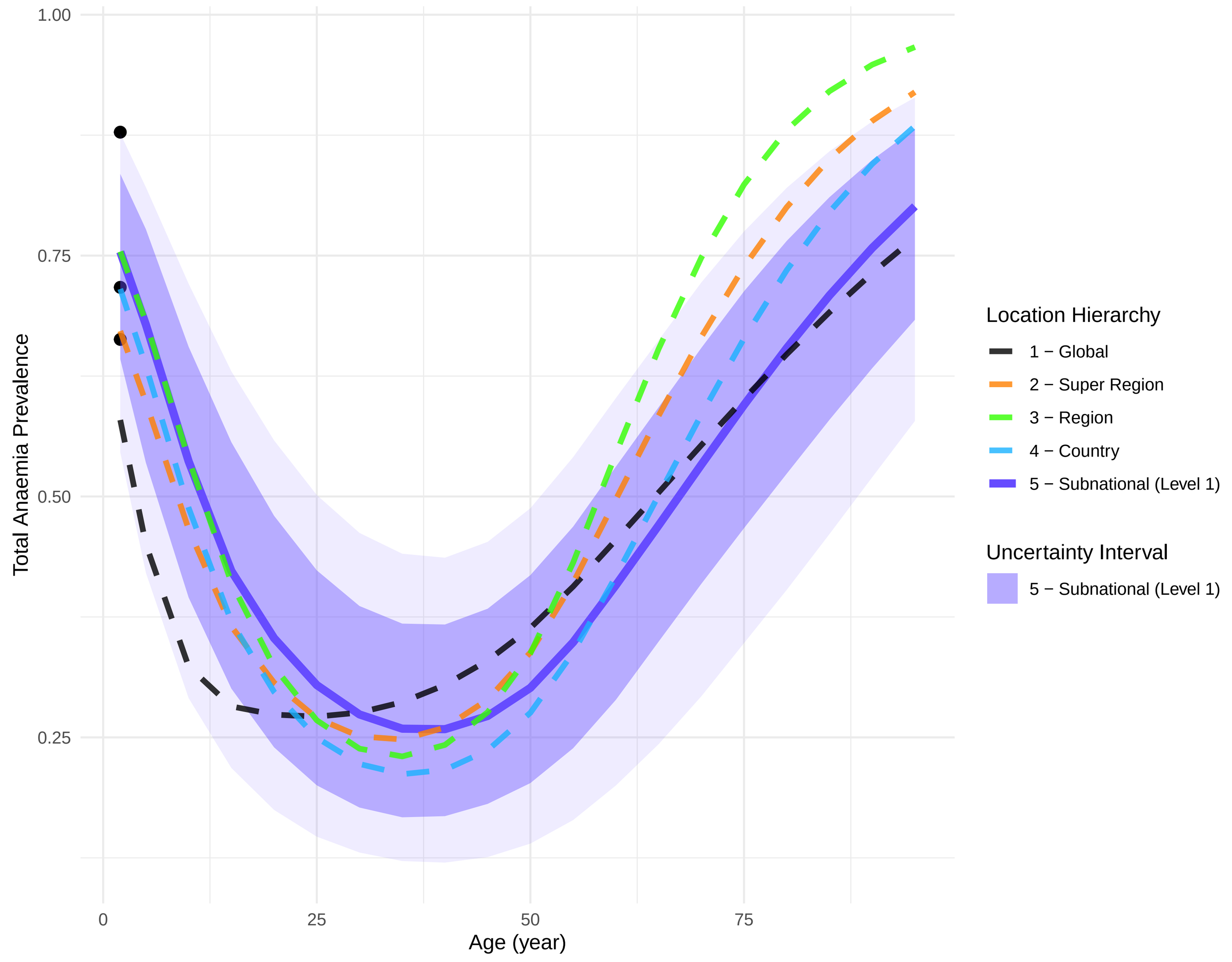

MR−BRT Age/Sex Fits − Males 2+ Years Old − Total Anaemia Prevalence − Ekiti
WHO−2024 Adjustment Method

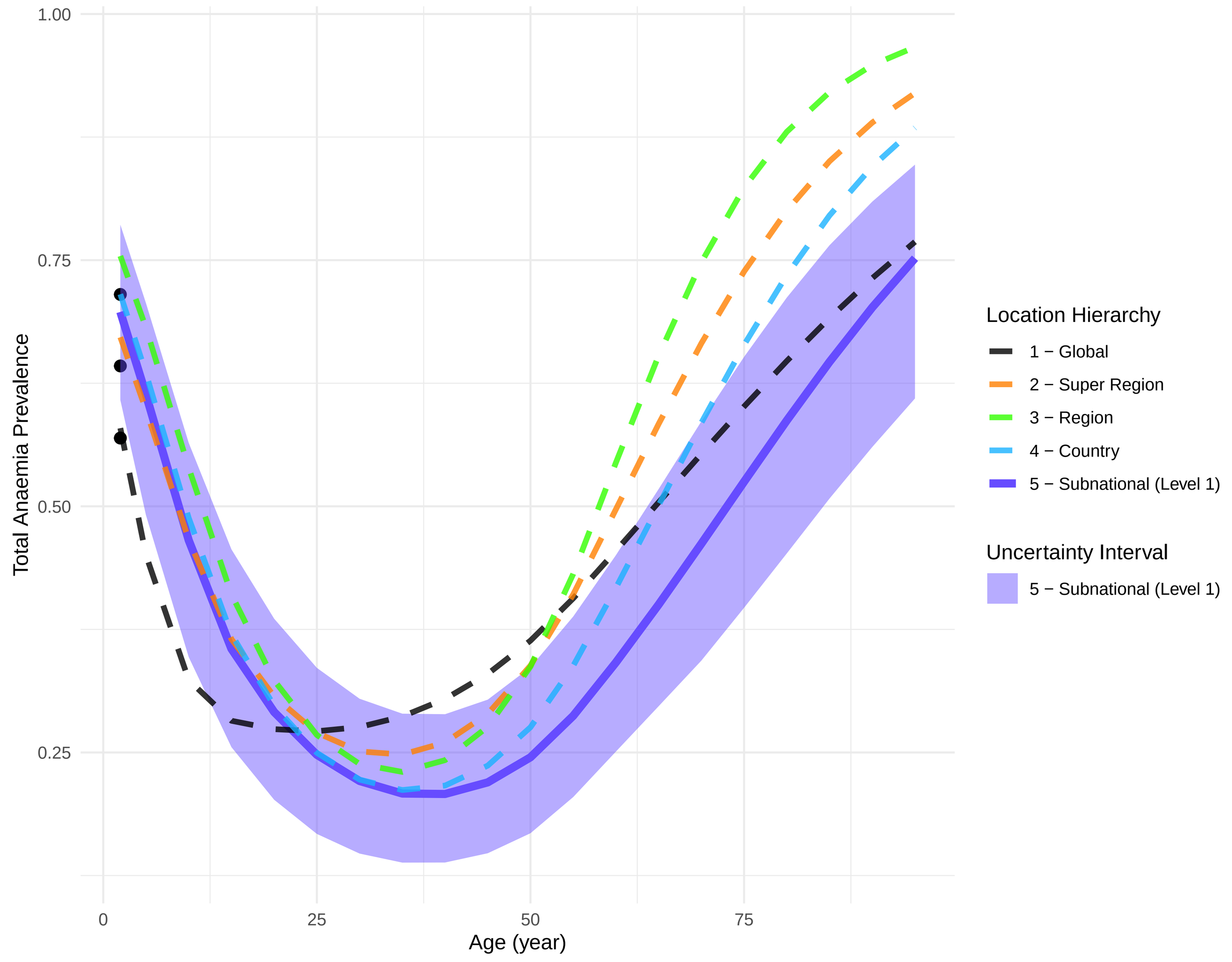

MR−BRT Age/Sex Fits − Males 2+ Years Old − Total Anaemia Prevalence − Enugu
WHO−2024 Adjustment Method

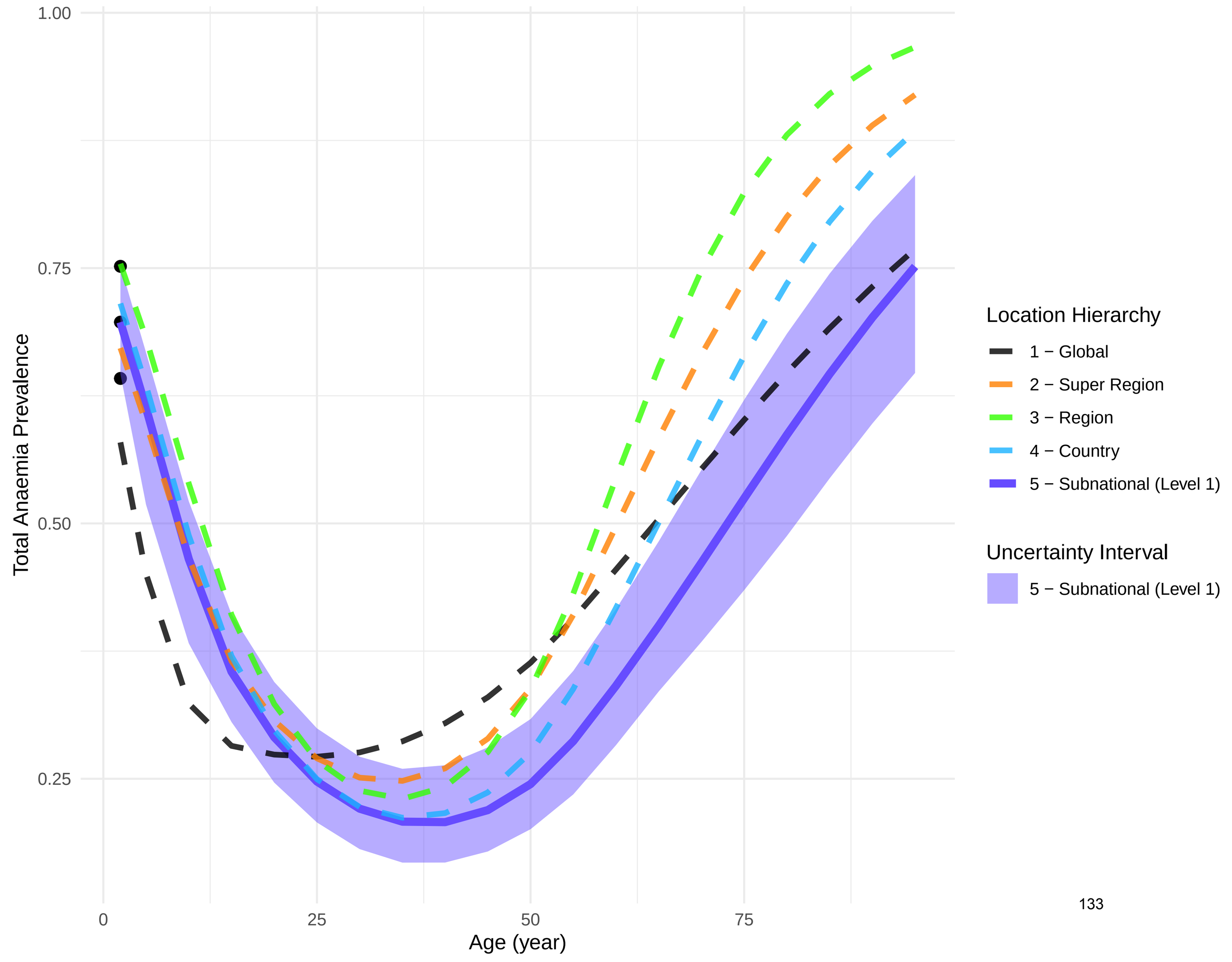

MR−BRT Age/Sex Fits − Males 2+ Years Old − Total Anaemia Prevalence − FCT (Abuja)
WHO−2024 Adjustment Method

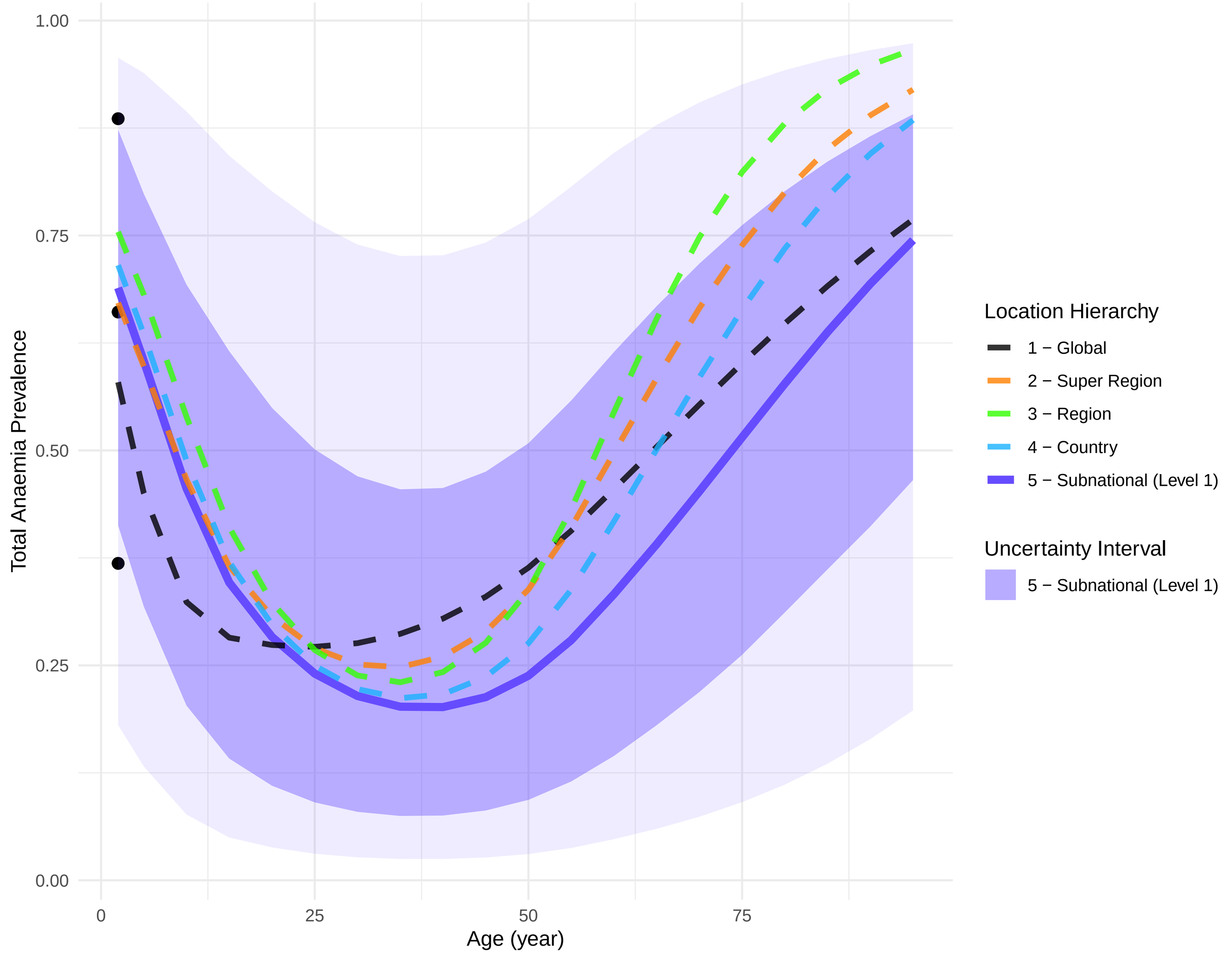

MR−BRT Age/Sex Fits − Males 2+ Years Old − Total Anaemia Prevalence − Gombe
WHO−2024 Adjustment Method

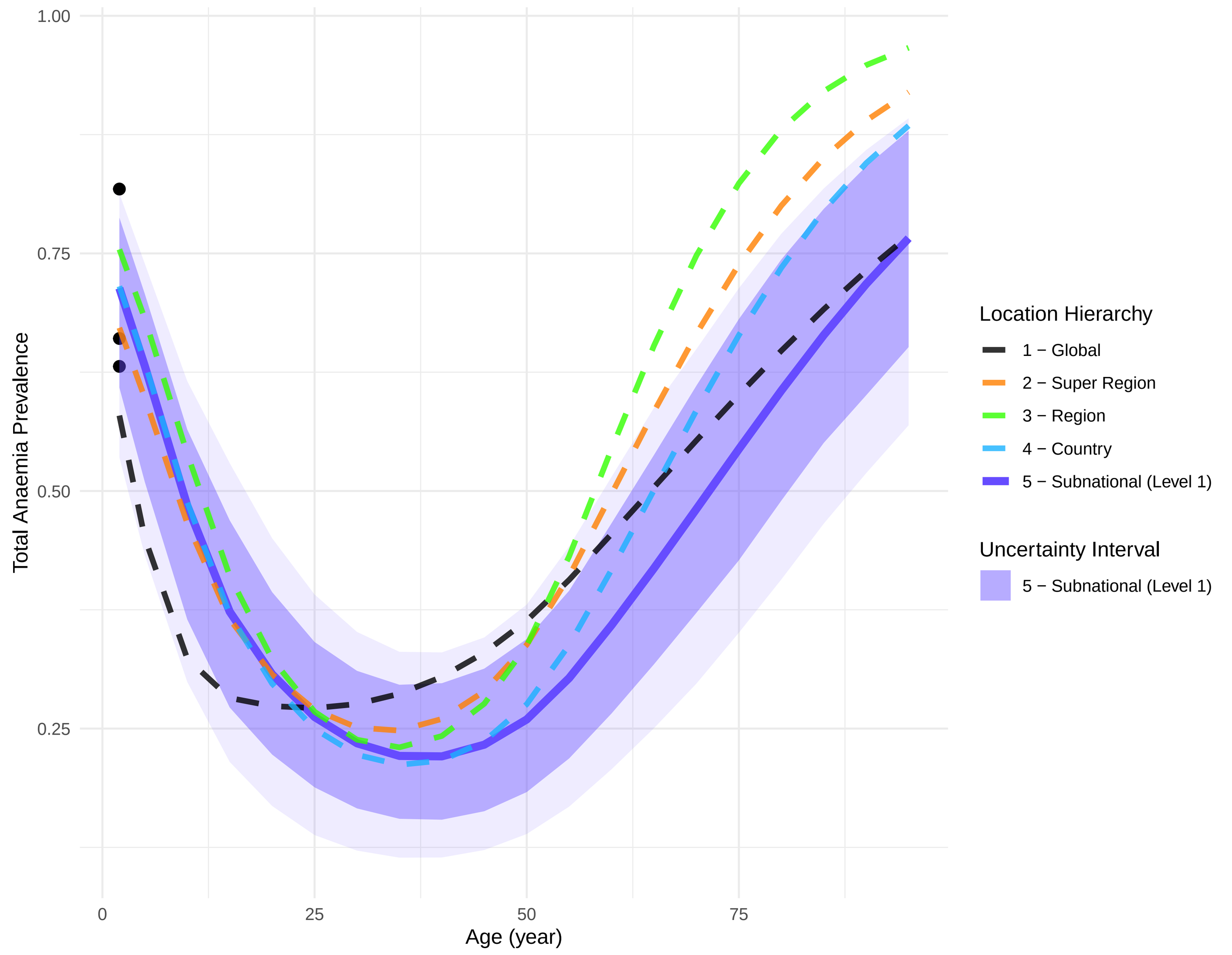

MR−BRT Age/Sex Fits − Males 2+ Years Old − Total Anaemia Prevalence − Imo
WHO−2024 Adjustment Method

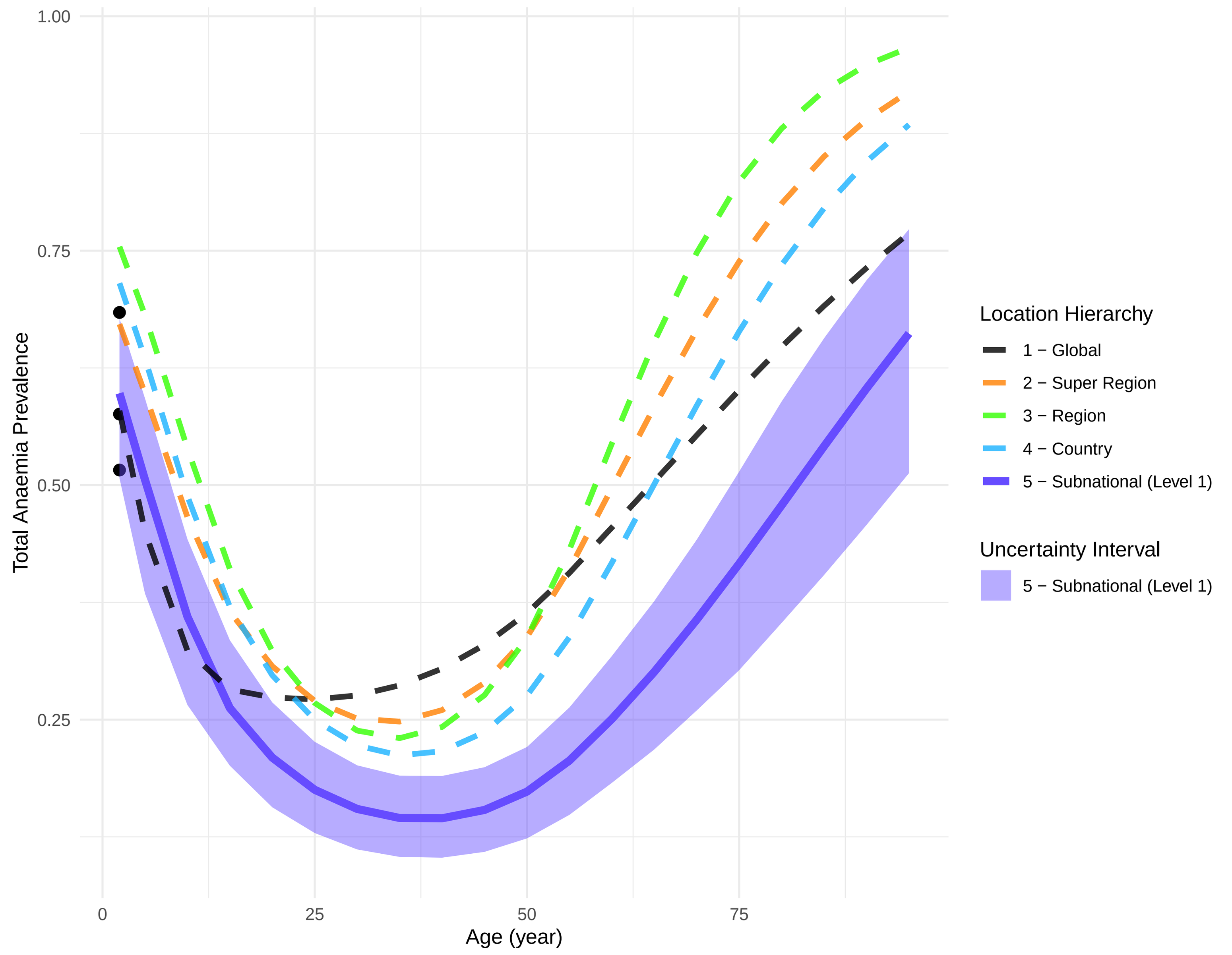

MR−BRT Age/Sex Fits − Males 2+ Years Old − Total Anaemia Prevalence − Jigawa
WHO−2024 Adjustment Method

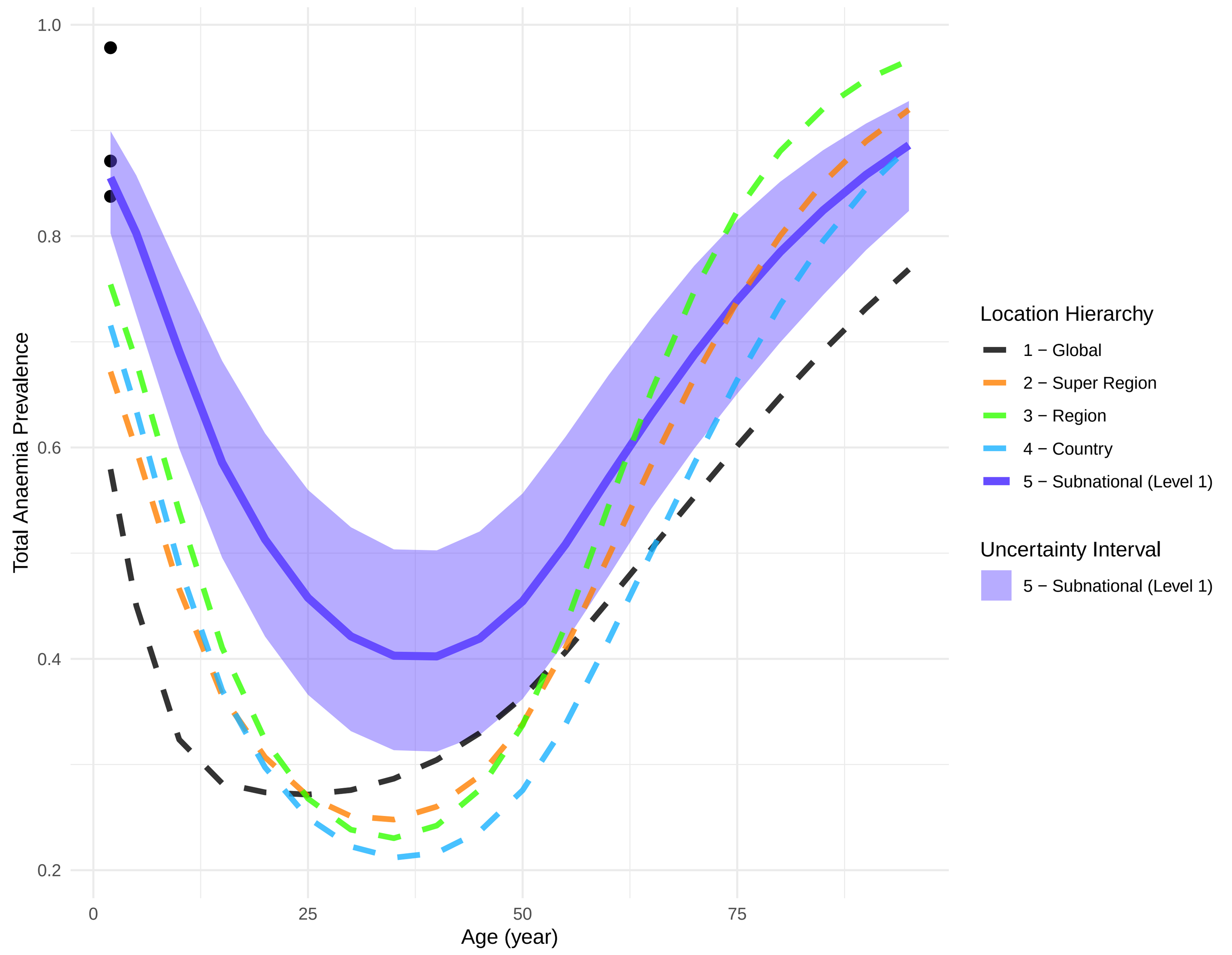

MR−BRT Age/Sex Fits − Males 2+ Years Old − Total Anaemia Prevalence − Kaduna
WHO−2024 Adjustment Method

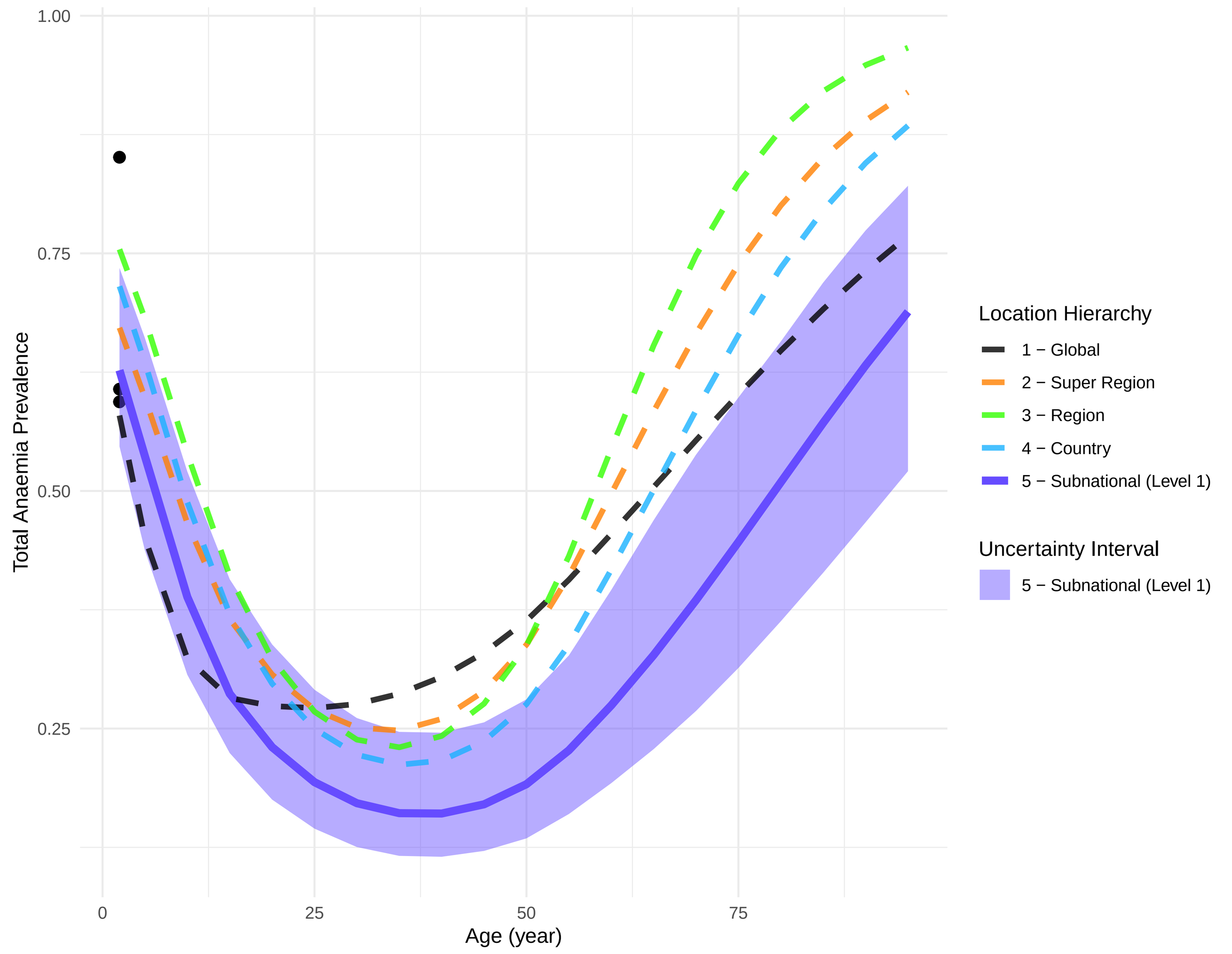

MR−BRT Age/Sex Fits − Males 2+ Years Old − Total Anaemia Prevalence − Kano
WHO−2024 Adjustment Method

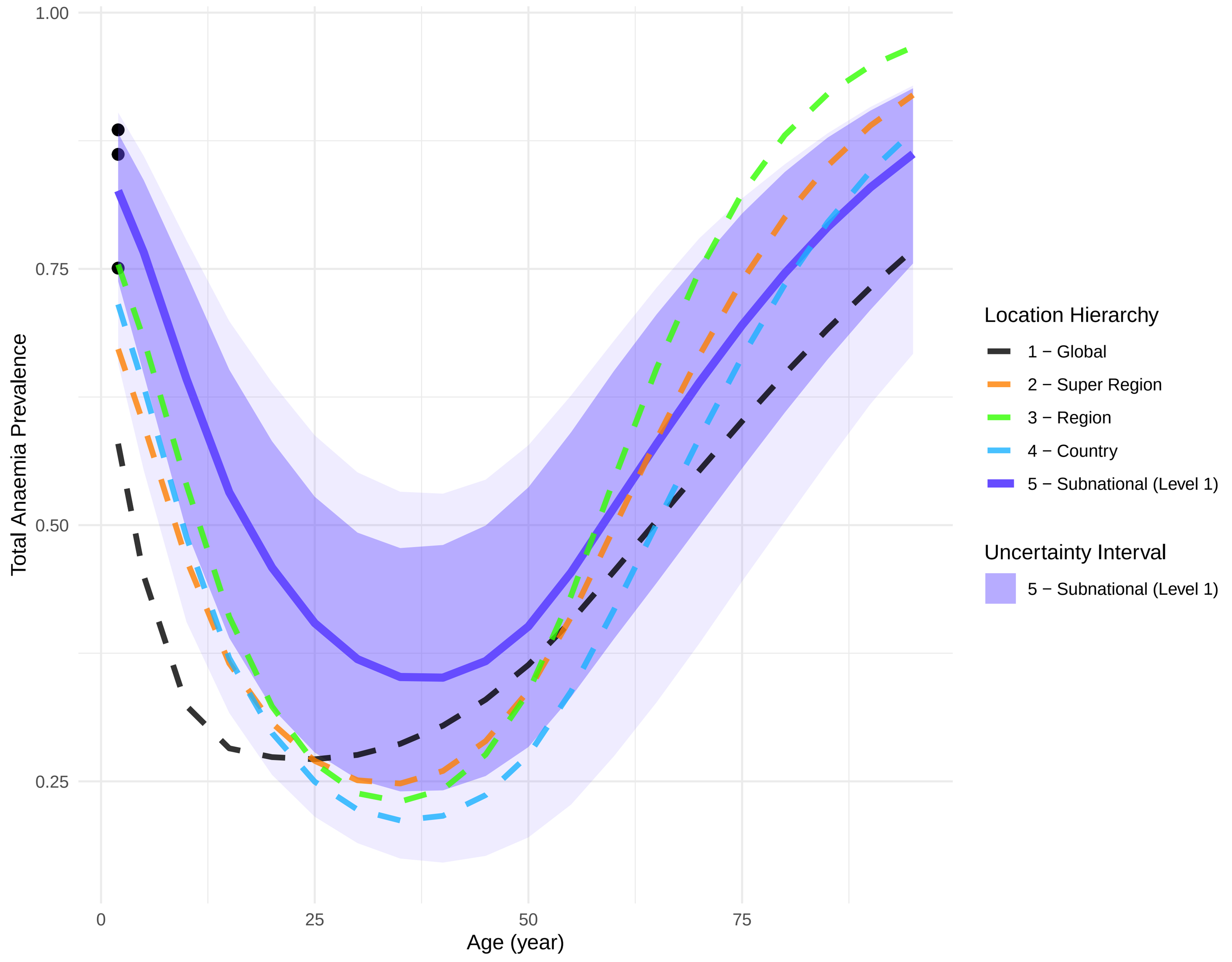

MR−BRT Age/Sex Fits − Males 2+ Years Old − Total Anaemia Prevalence − Katsina
WHO−2024 Adjustment Method

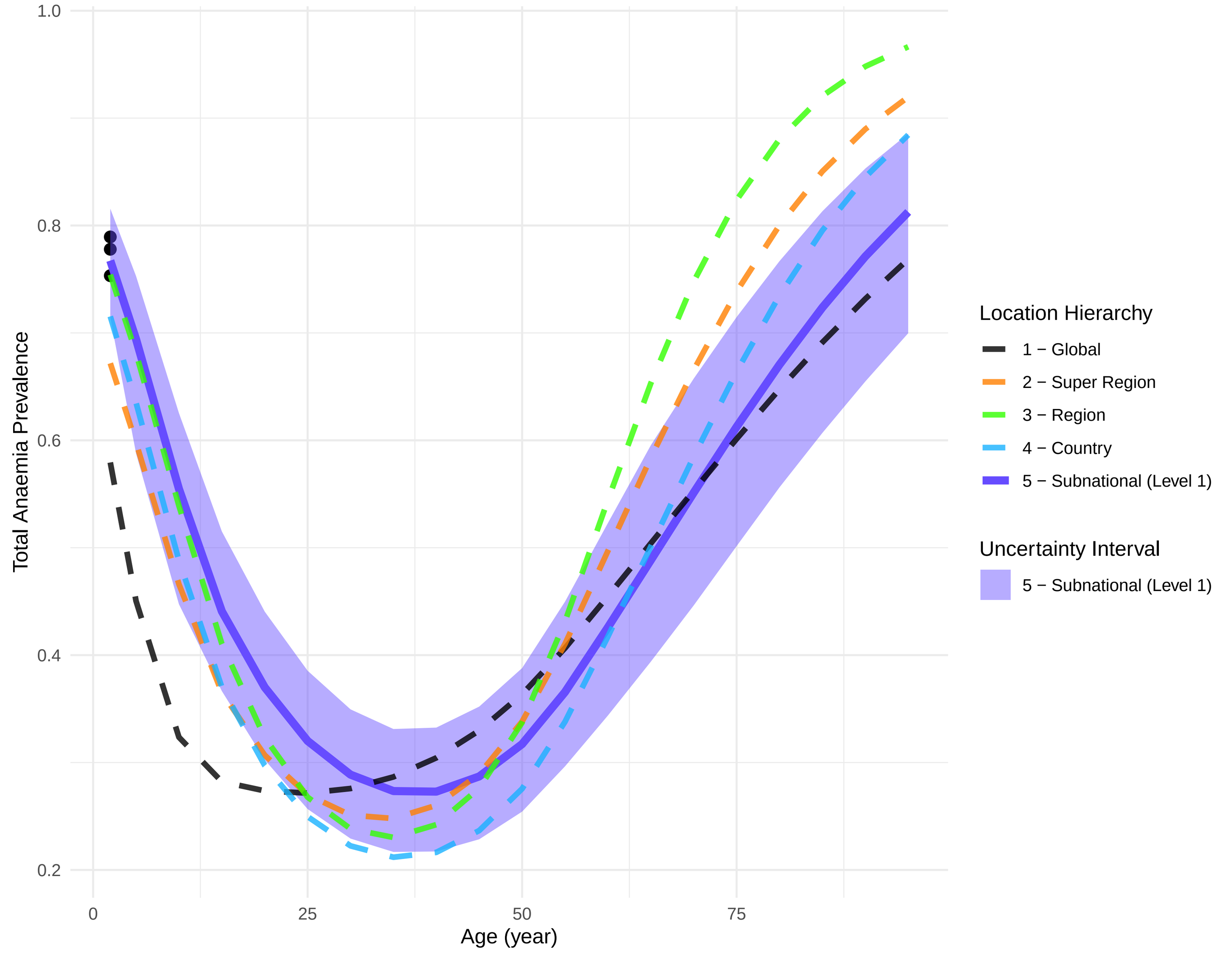

MR−BRT Age/Sex Fits − Males 2+ Years Old − Total Anaemia Prevalence − Kebbi
WHO−2024 Adjustment Method

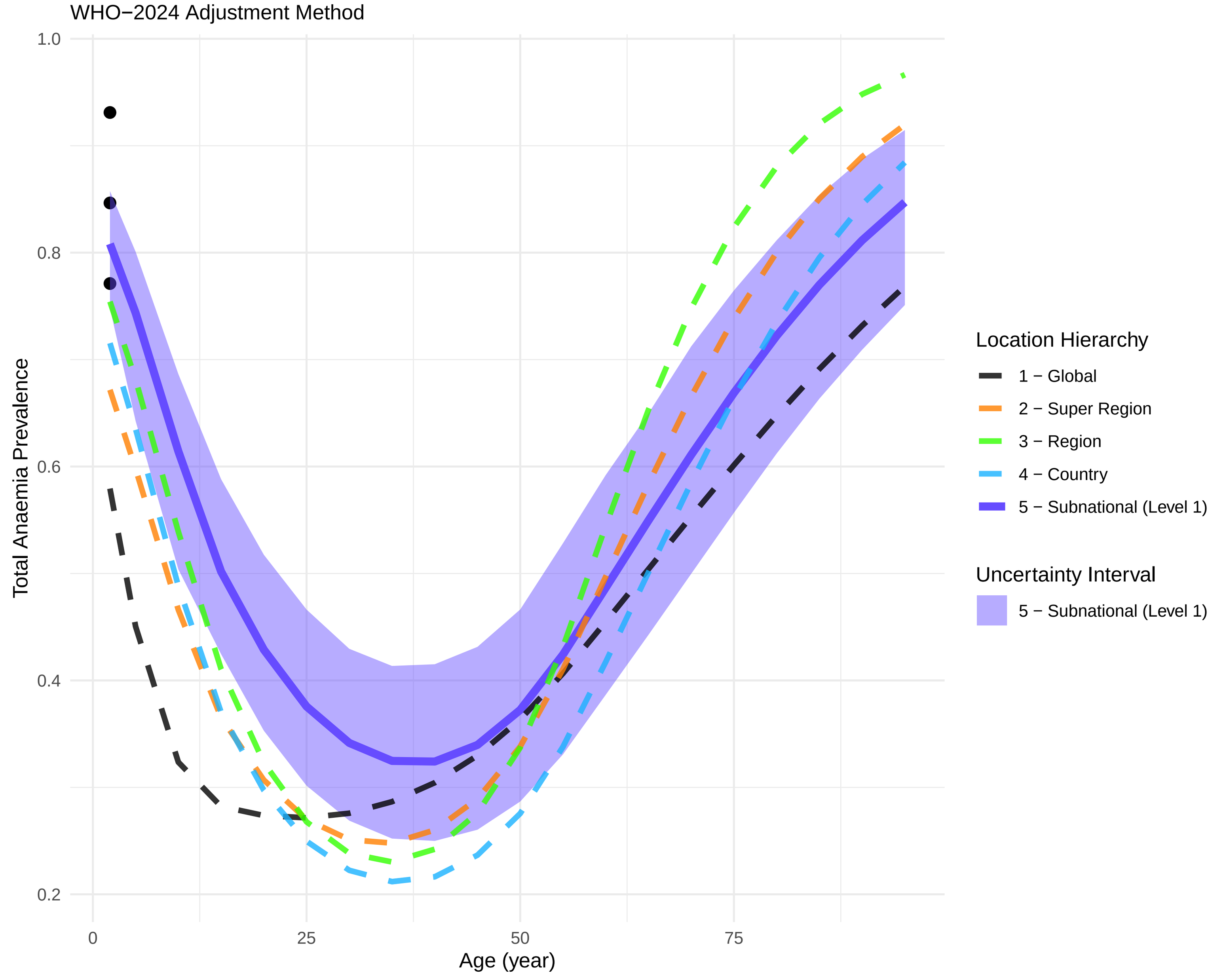

MR−BRT Age/Sex Fits − Males 2+ Years Old − Total Anaemia Prevalence − Kogi
WHO−2024 Adjustment Method

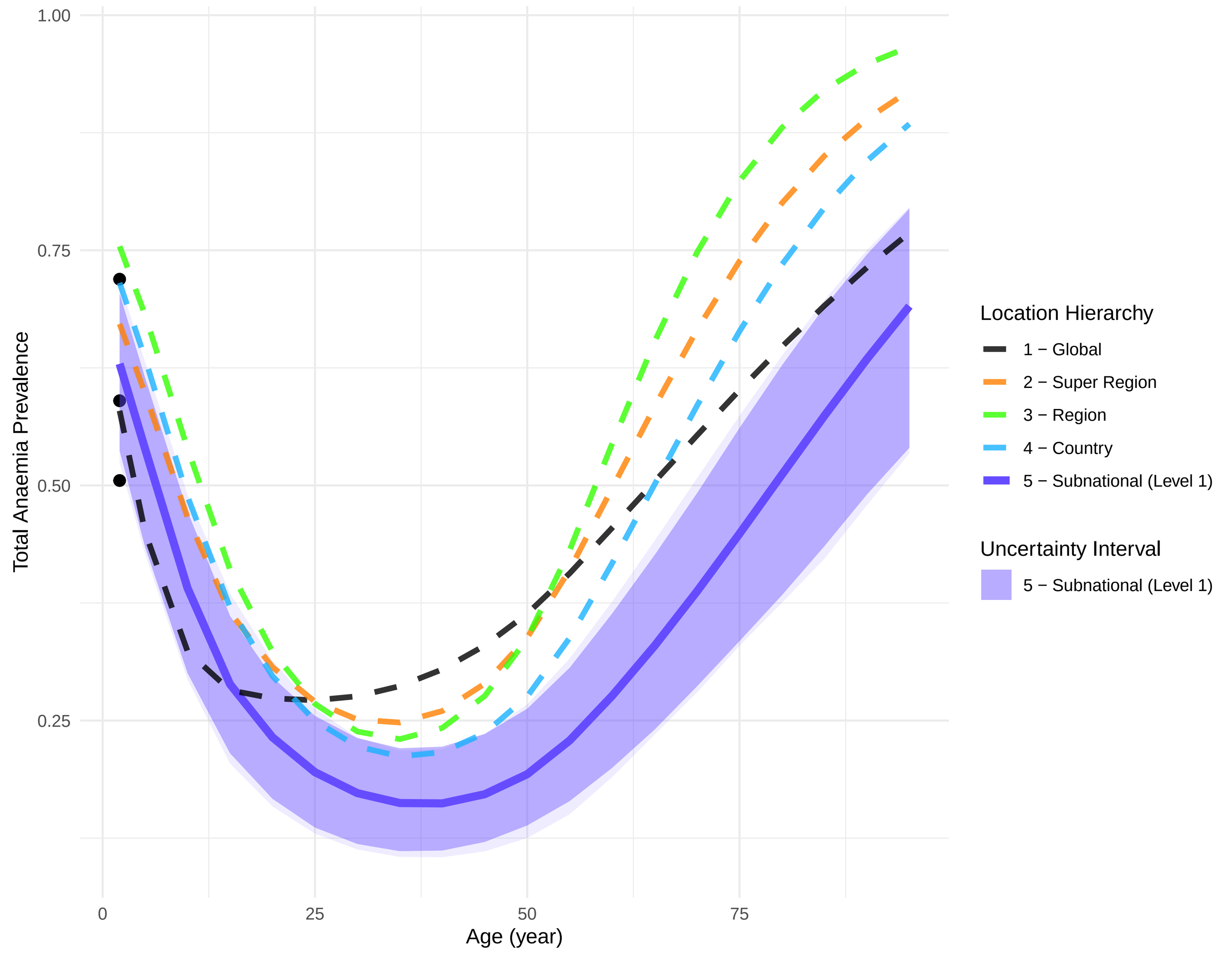

MR−BRT Age/Sex Fits − Males 2+ Years Old − Total Anaemia Prevalence − Kwara
WHO−2024 Adjustment Method

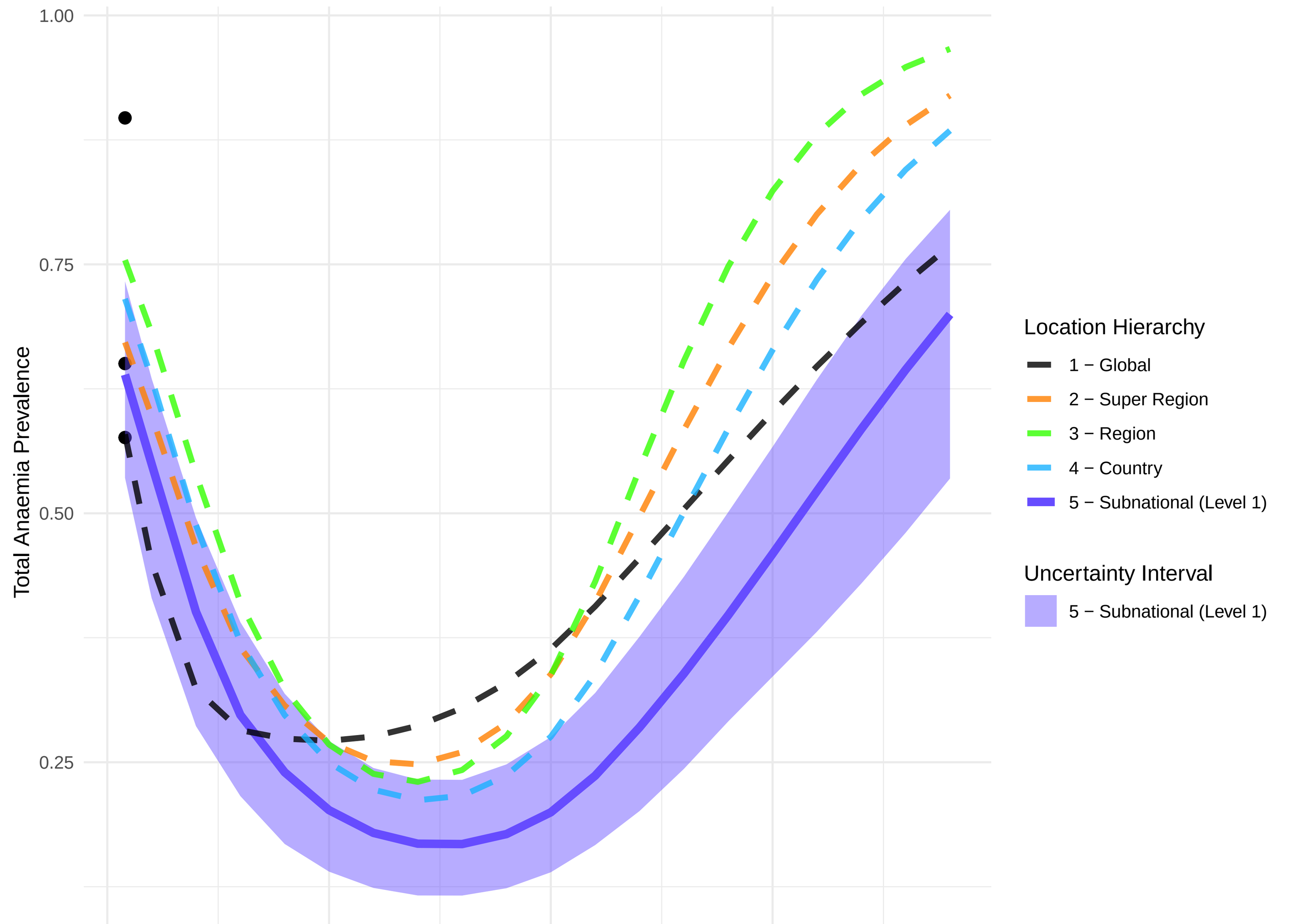

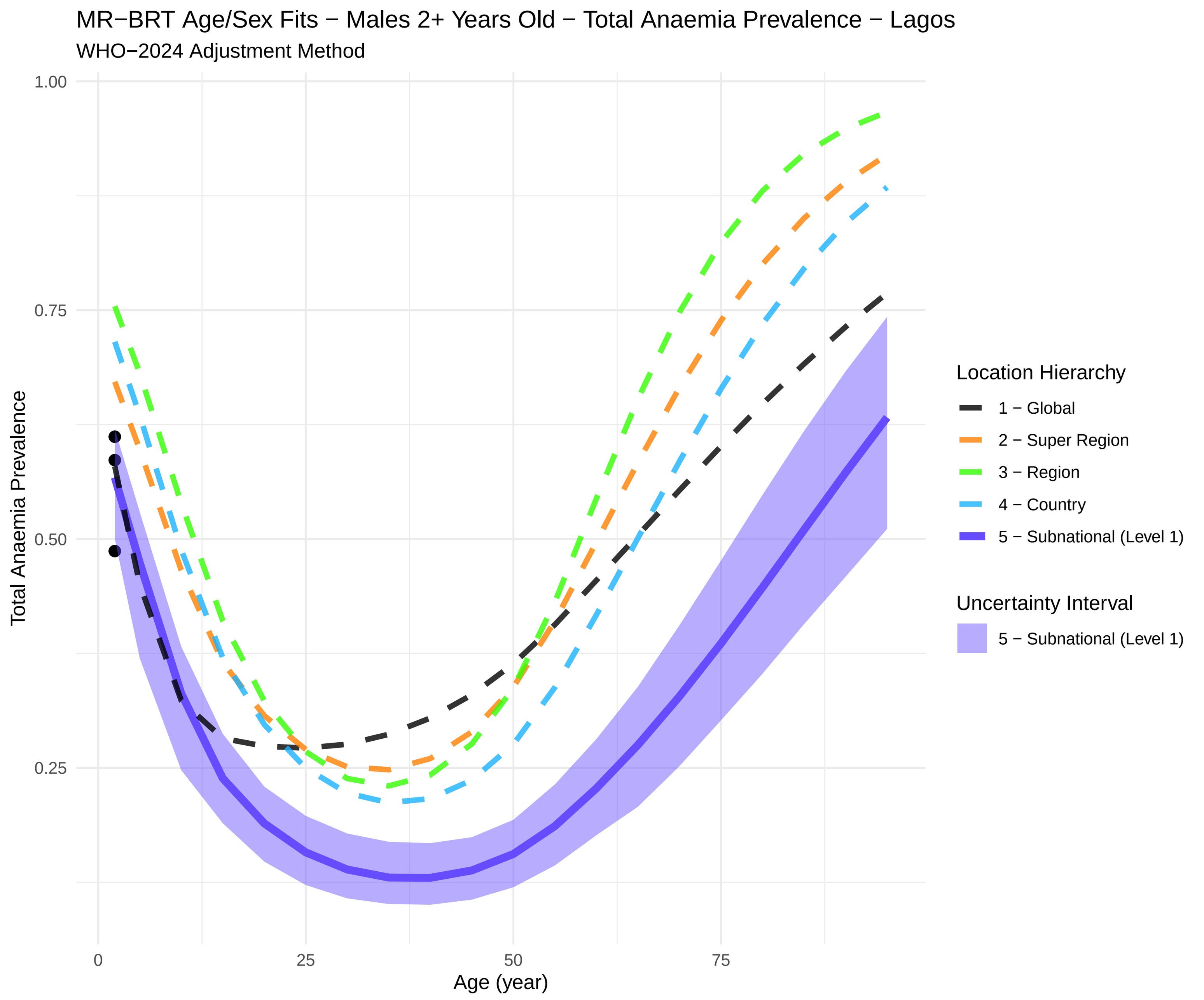
MR−BRT Age/Sex Fits − Males 2+ Years Old − Total Anaemia Prevalence − Lagos
WHO−2024 Adjustment Method
Total Anaemia Prevalence
Age (year)
Location Hierarchy
1 − Global
2 − Super Region
3 − Region
4 − Country
5 − Subnational (Level 1)
Uncertainty Interval
5 − Subnational (Level 1)
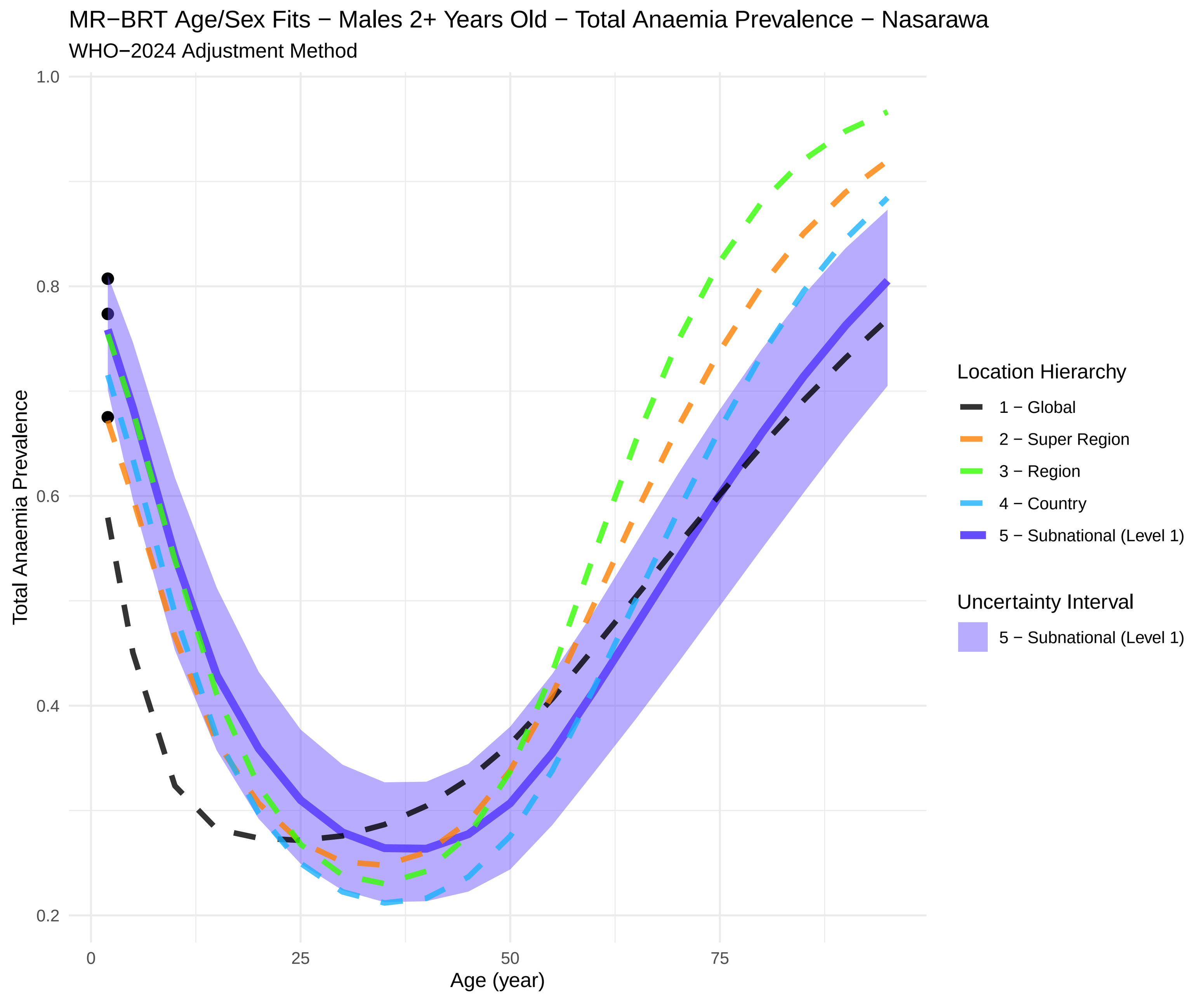
MR−BRT Age/Sex Fits − Males 2+ Years Old − Total Anaemia Prevalence − Nasarawa
WHO−2024 Adjustment Method
Total Anaemia Prevalence
Age (year)
Location Hierarchy
1 − Global
2 − Super Region
3 − Region
4 − Country
5 − Subnational (Level 1)
Uncertainty Interval
5 − Subnational (Level 1)
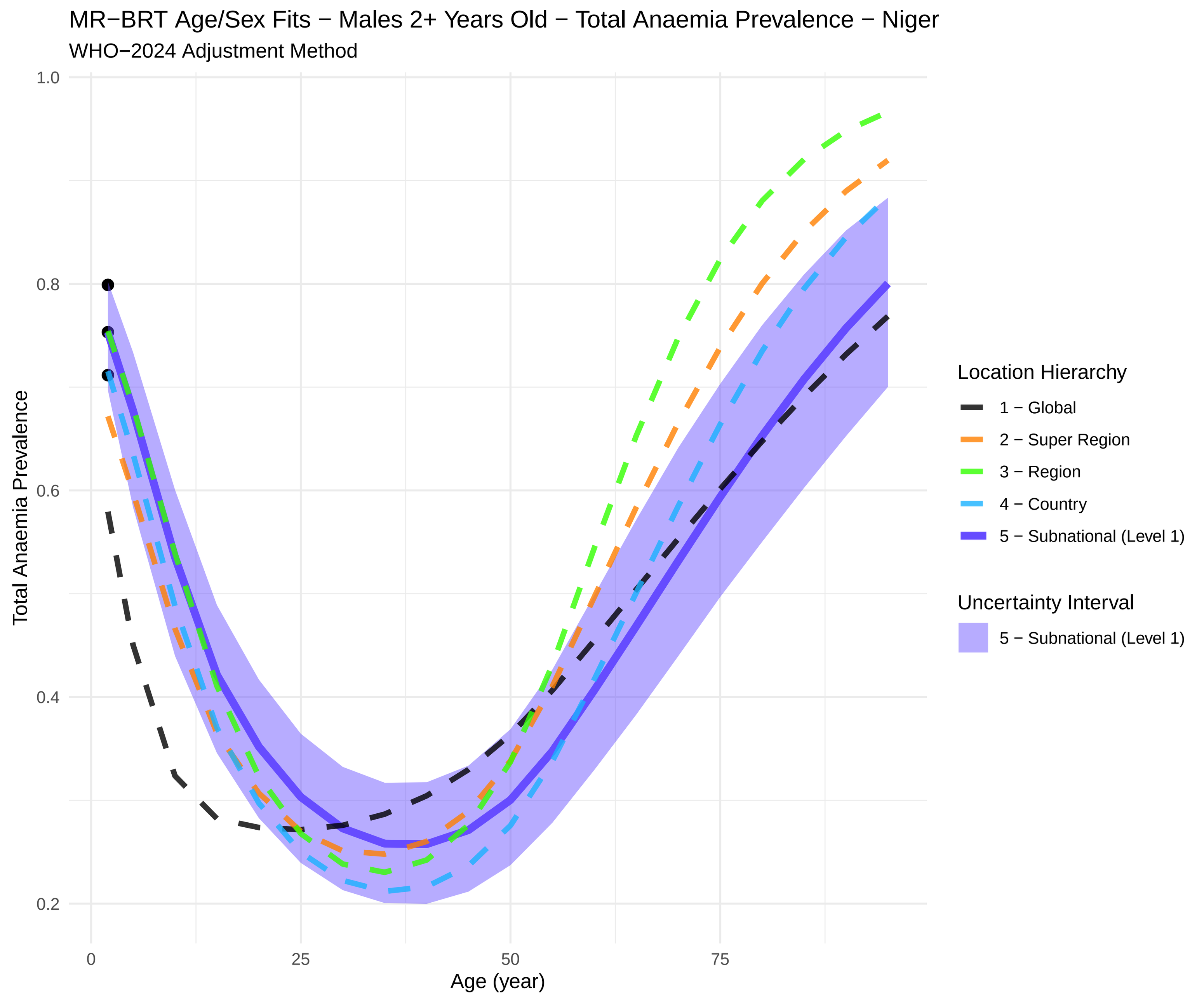
MR−BRT Age/Sex Fits − Males 2+ Years Old − Total Anaemia Prevalence − Niger
WHO−2024 Adjustment Method
Total Anaemia Prevalence
Age (year)
Location Hierarchy
1 − Global
2 − Super Region
3 − Region
4 − Country
5 − Subnational (Level 1)
Uncertainty Interval
5 − Subnational (Level 1)
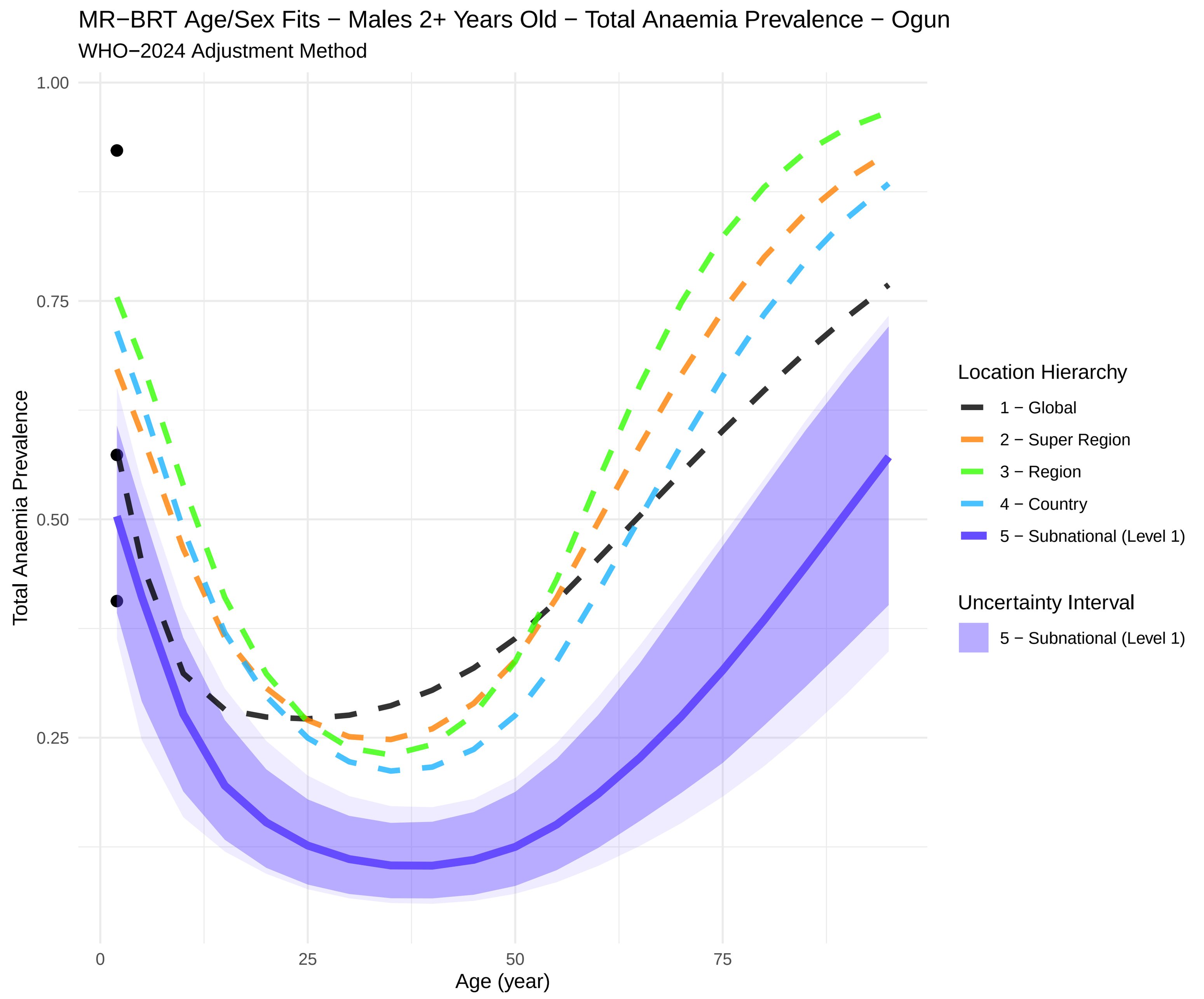
MR−BRT Age/Sex Fits − Males 2+ Years Old − Total Anaemia Prevalence − Ogun
WHO−2024 Adjustment Method
Total Anaemia Prevalence
Age (year)
Location Hierarchy
1 − Global
2 − Super Region
3 − Region
4 − Country
5 − Subnational (Level 1)
Uncertainty Interval
5 − Subnational (Level 1)
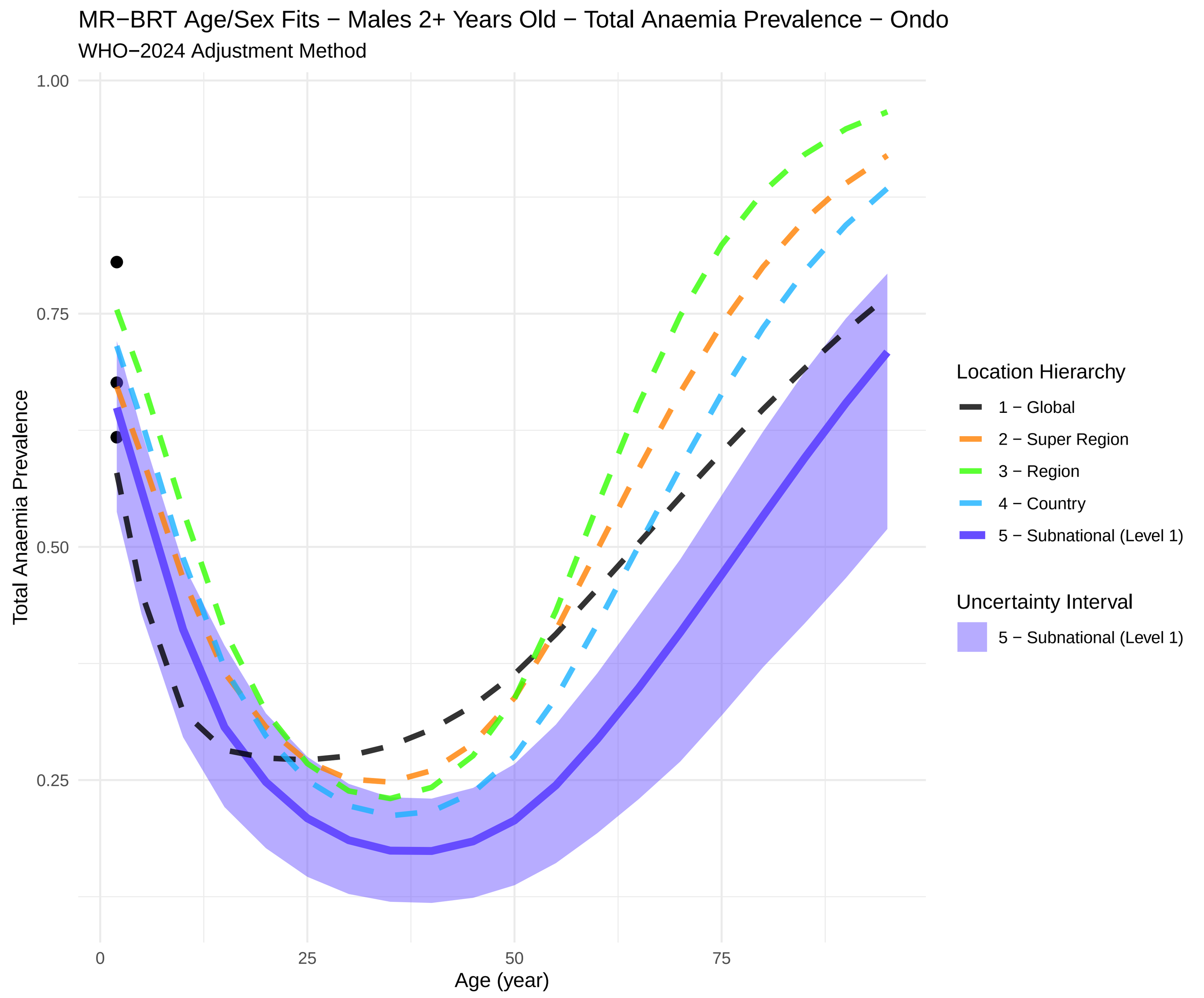
MR−BRT Age/Sex Fits − Males 2+ Years Old − Total Anaemia Prevalence − Ondo
WHO−2024 Adjustment Method
Total Anaemia Prevalence
Age (year)
Location Hierarchy
1 − Global
2 − Super Region
3 − Region
4 − Country
5 − Subnational (Level 1)
Uncertainty Interval
5 − Subnational (Level 1)
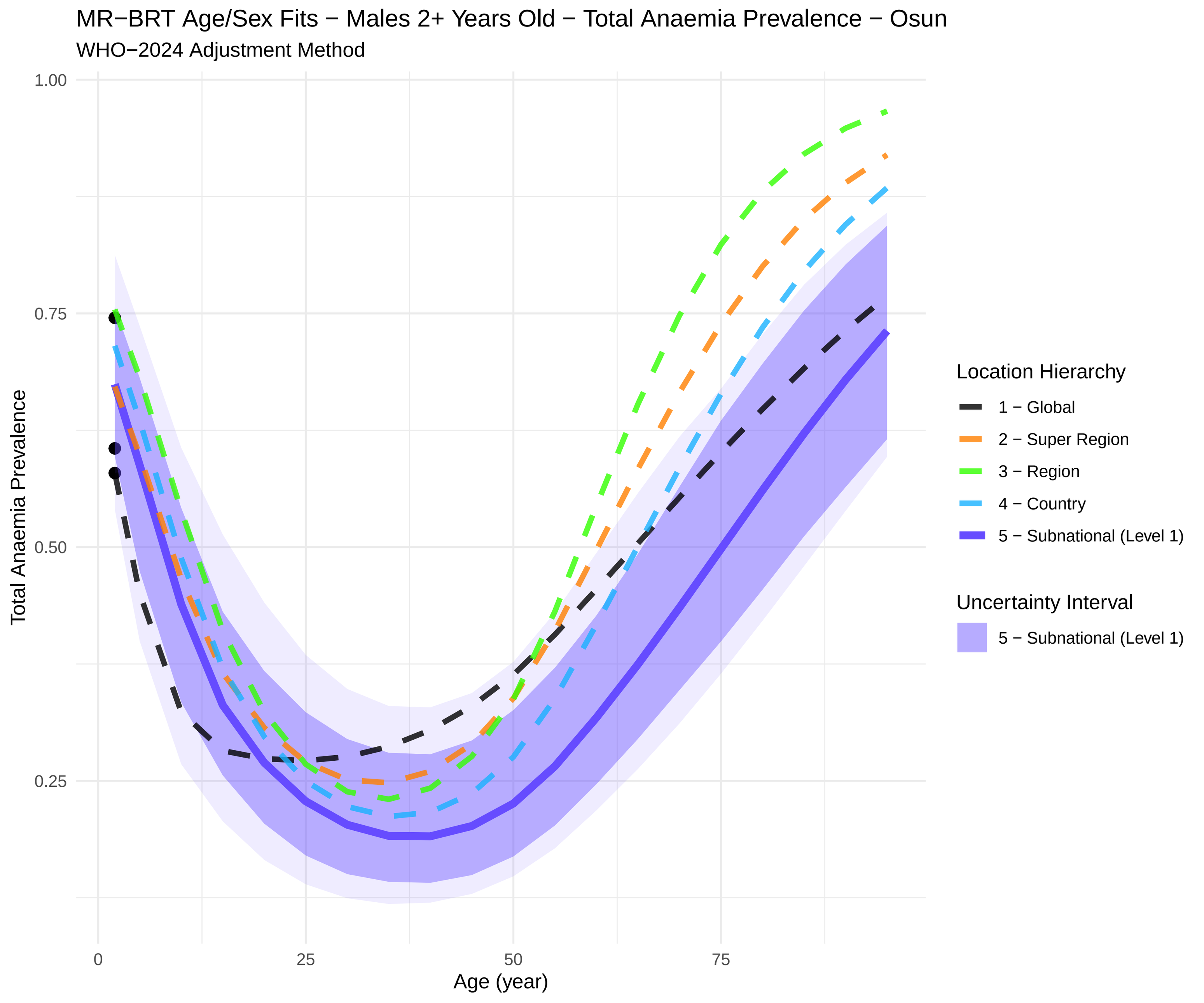
MR−BRT Age/Sex Fits − Males 2+ Years Old − Total Anaemia Prevalence − Osun
WHO−2024 Adjustment Method
Total Anaemia Prevalence
Age (year)
Location Hierarchy
1 − Global
2 − Super Region
3 − Region
4 − Country
5 − Subnational (Level 1)
Uncertainty Interval
5 − Subnational (Level 1)
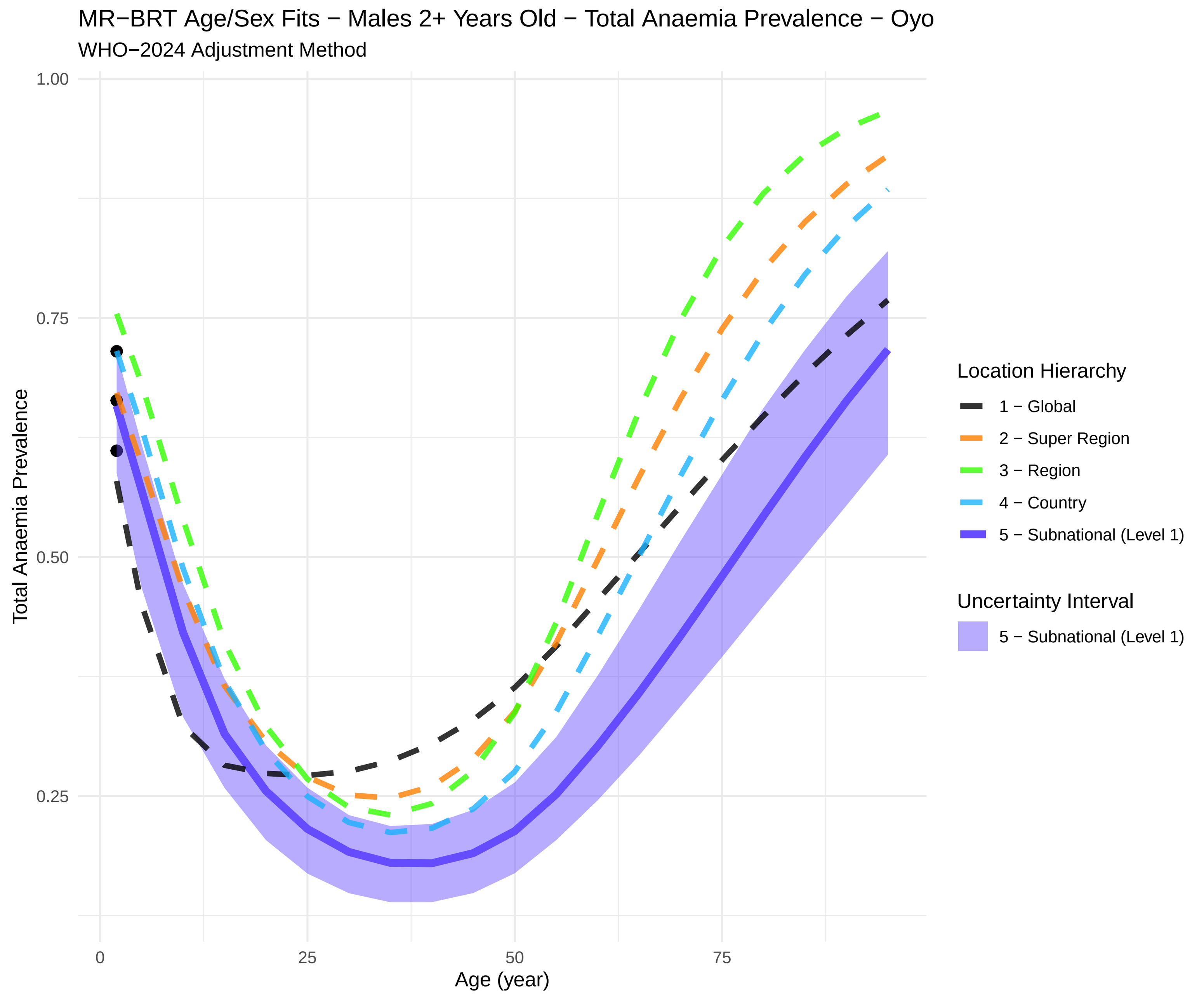
MR−BRT Age/Sex Fits − Males 2+ Years Old − Total Anaemia Prevalence − Oyo
WHO−2024 Adjustment Method
Total Anaemia Prevalence
Age (year)
Location Hierarchy
1 − Global
2 − Super Region
3 − Region
4 − Country
5 − Subnational (Level 1)
Uncertainty Interval
5 − Subnational (Level 1)
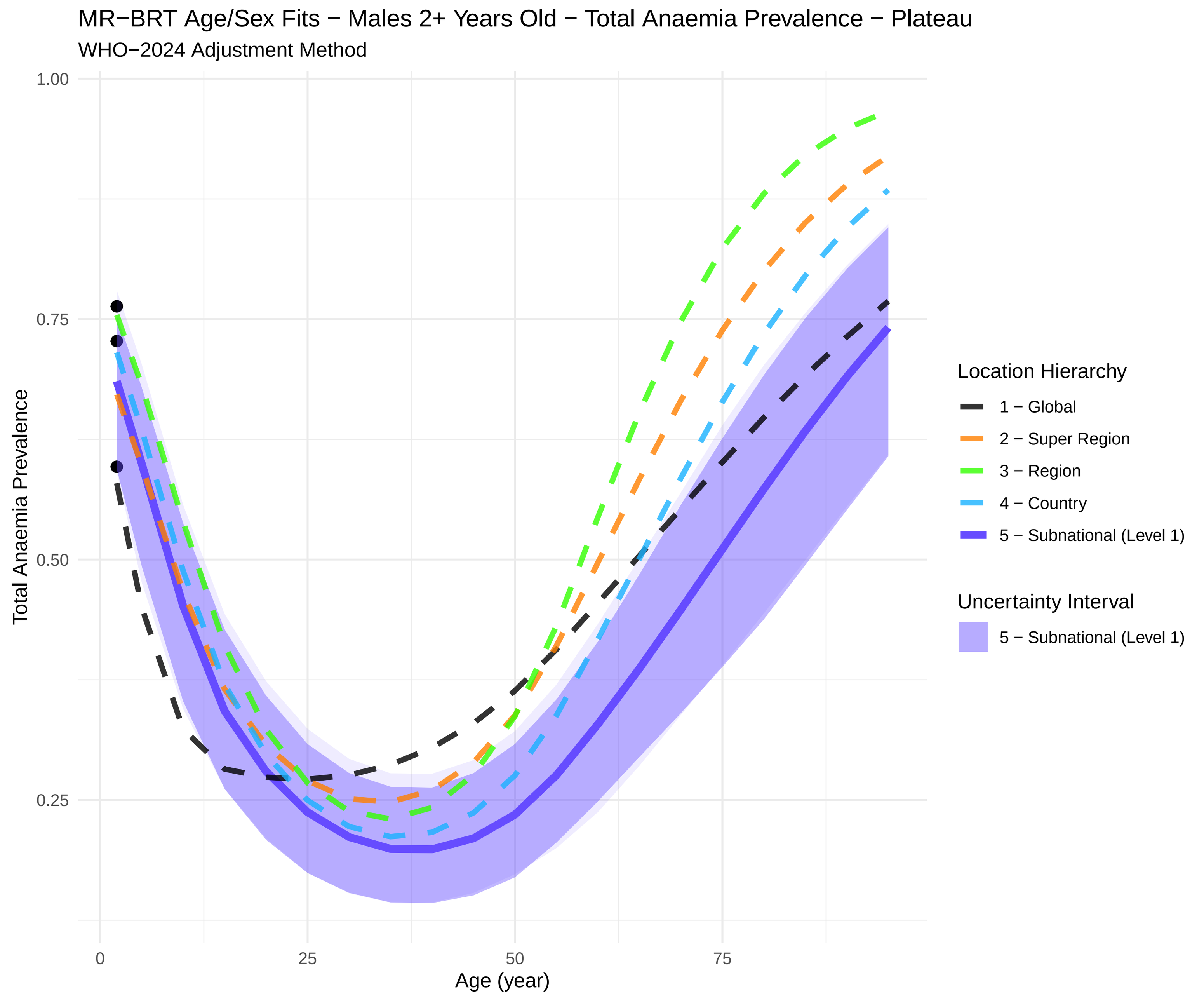
MR−BRT Age/Sex Fits − Males 2+ Years Old − Total Anaemia Prevalence − Plateau
WHO−2024 Adjustment Method
Total Anaemia Prevalence
Age (year)
Location Hierarchy
1 − Global
2 − Super Region
3 − Region
4 − Country
5 − Subnational (Level 1)
Uncertainty Interval
5 − Subnational (Level 1)
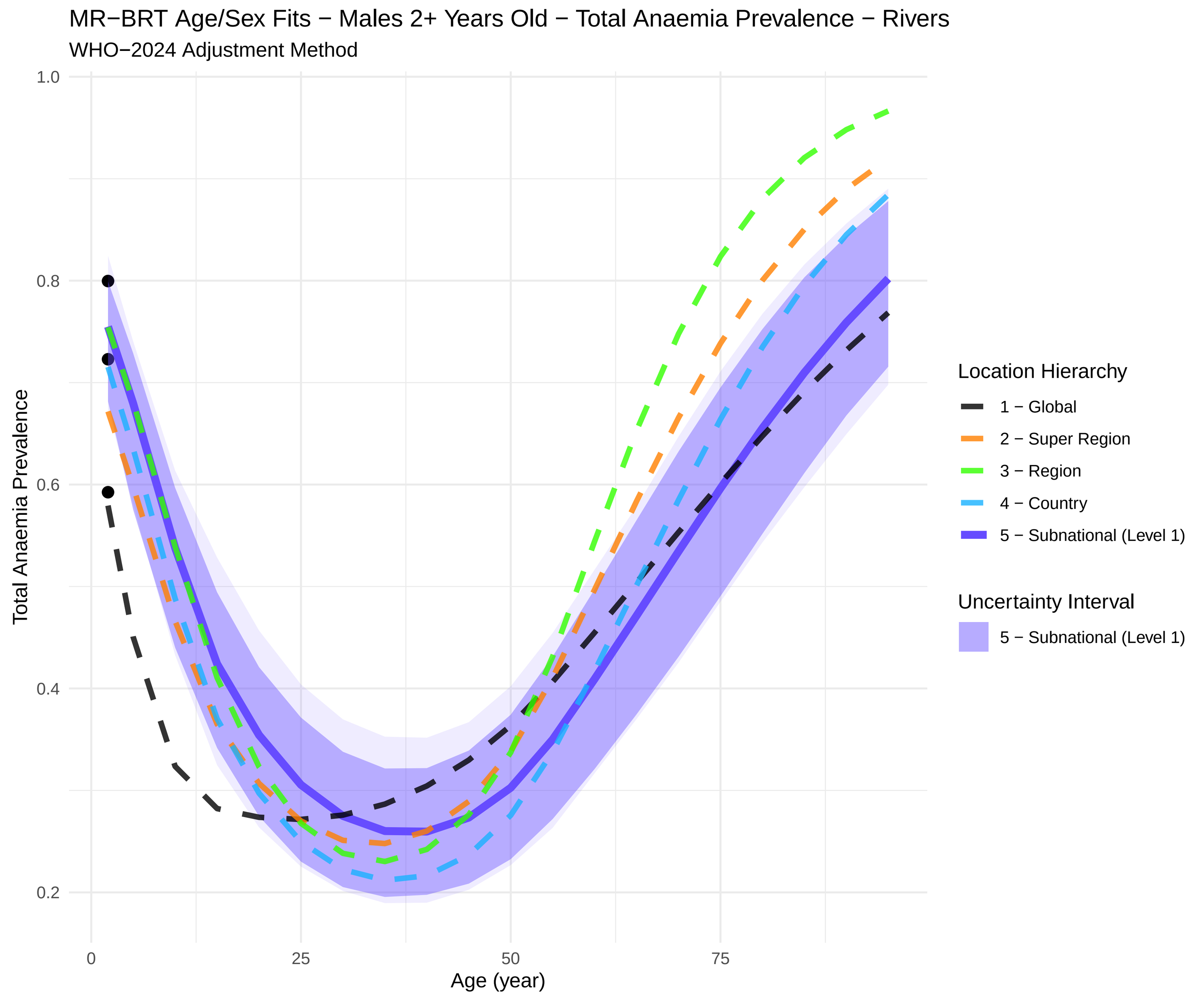
MR−BRT Age/Sex Fits − Males 2+ Years Old − Total Anaemia Prevalence − Rivers
WHO−2024 Adjustment Method
Total Anaemia Prevalence
Age (year)
Location Hierarchy
1 − Global
2 − Super Region
3 − Region
4 − Country
5 − Subnational (Level 1)
Uncertainty Interval
5 − Subnational (Level 1)
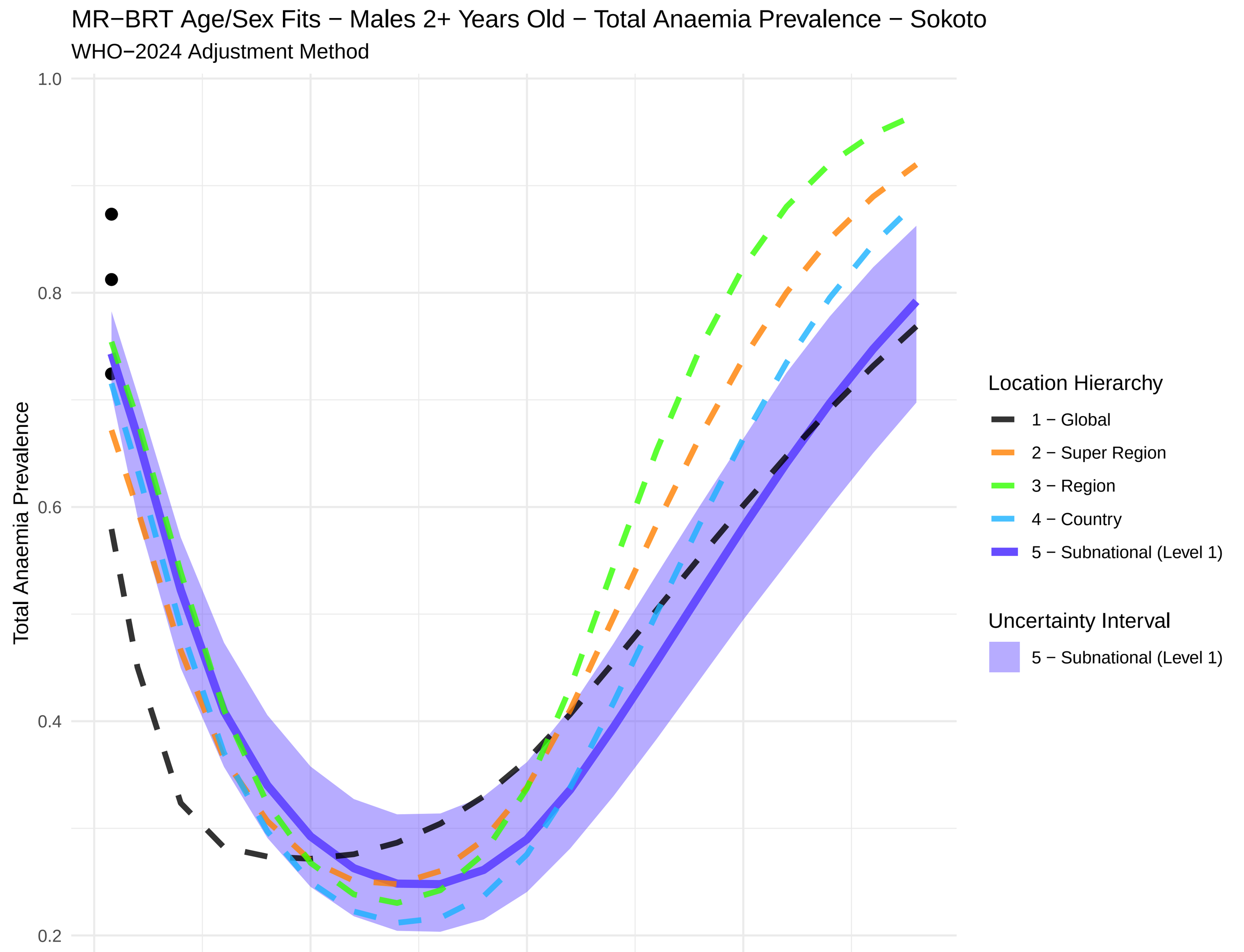
MR−BRT Age/Sex Fits − Males 2+ Years Old − Total Anaemia Prevalence − Sokoto
WHO−2024 Adjustment Method
Total Anaemia Prevalence
Age (year)
Location Hierarchy
1 − Global
2 − Super Region
3 − Region
4 − Country
5 − Subnational (Level 1)
Uncertainty Interval
5 − Subnational (Level 1)

MR−BRT Age/Sex Fits − Males 2+ Years Old − Total Anaemia Prevalence − Taraba

WHO−2024 Adjustment Method

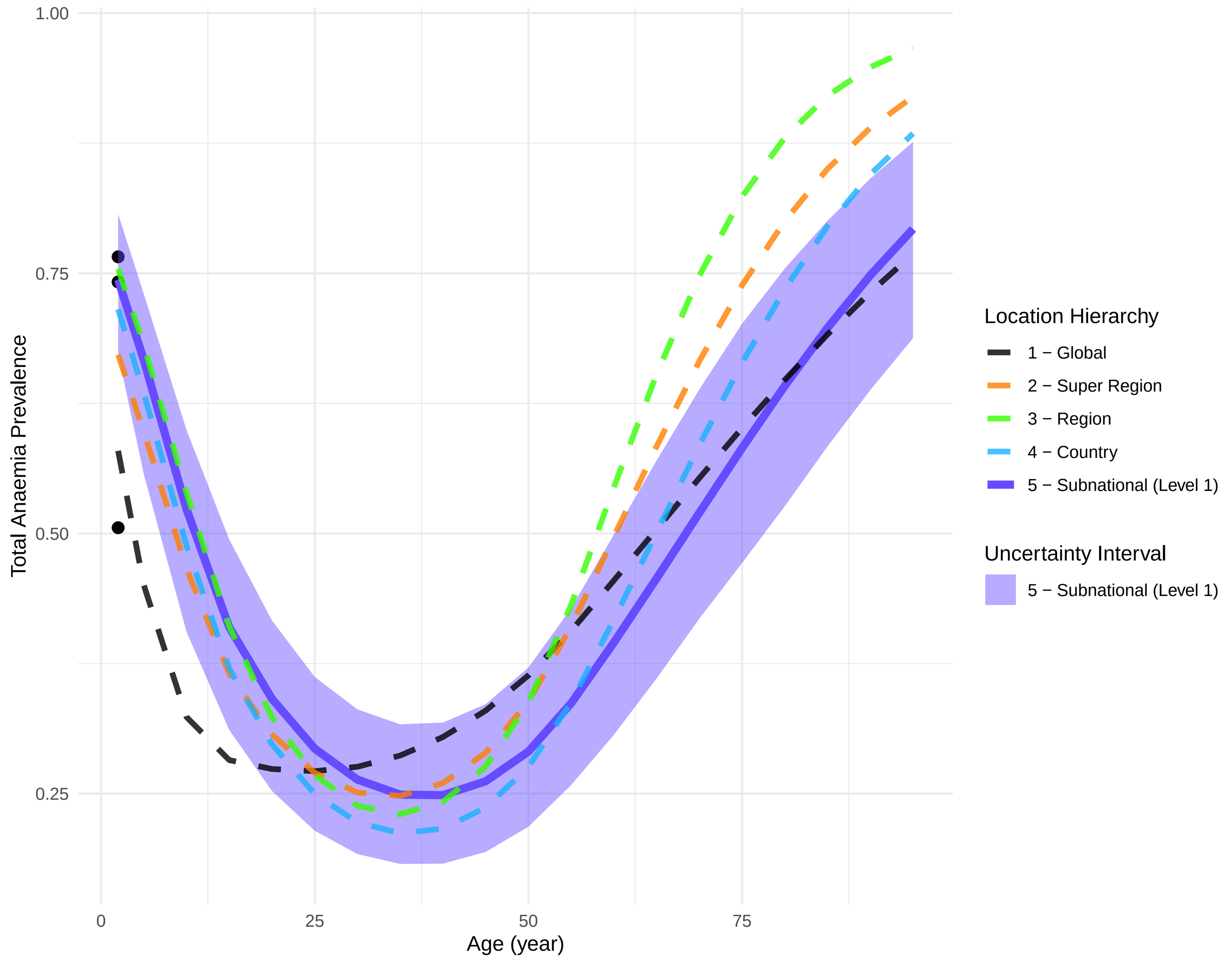

MR−BRT Age/Sex Fits − Males 2+ Years Old − Total Anaemia Prevalence − Yobe

WHO−2024 Adjustment Method

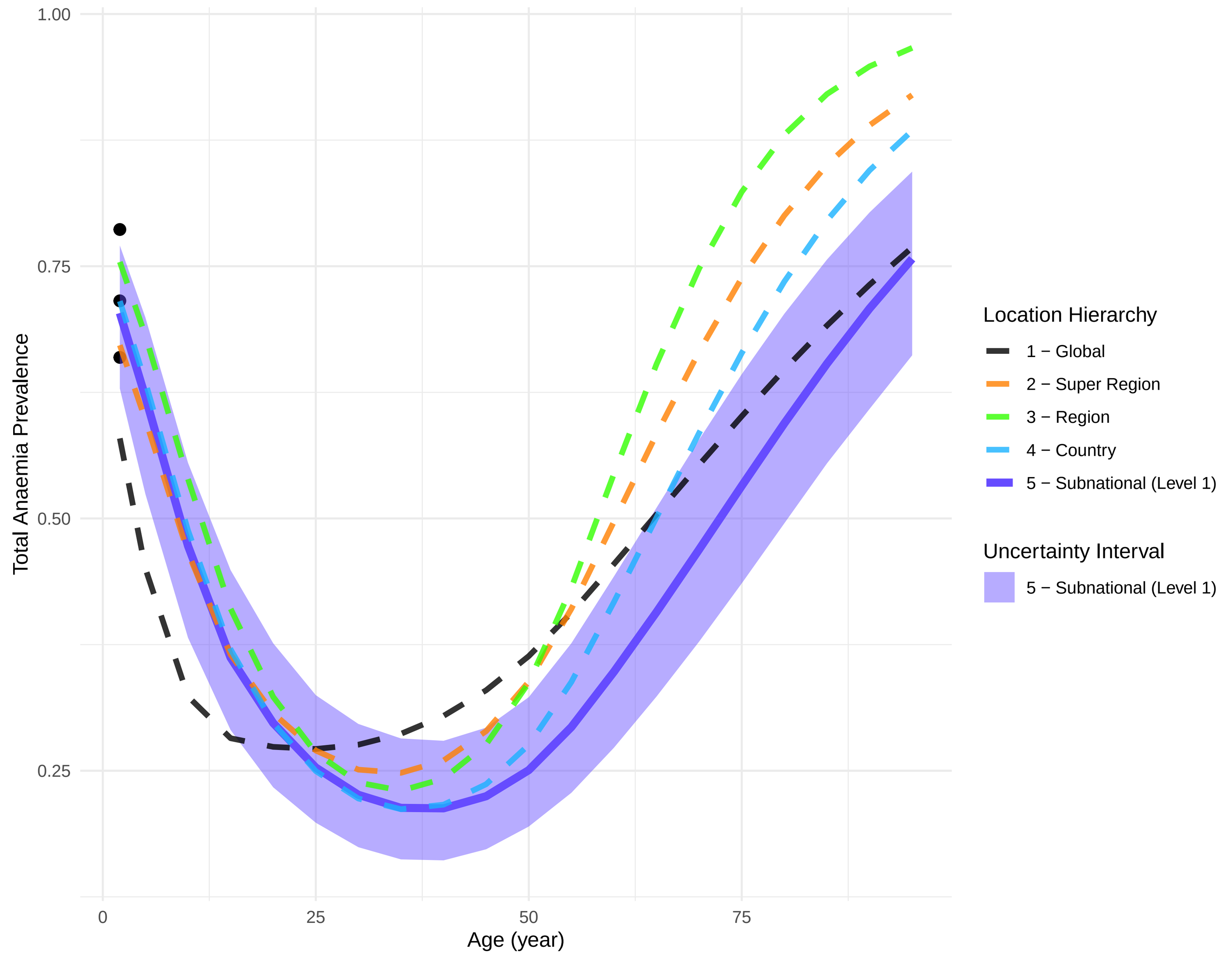

MR−BRT Age/Sex Fits − Males 2+ Years Old − Total Anaemia Prevalence − Zamfara

WHO−2024 Adjustment Method

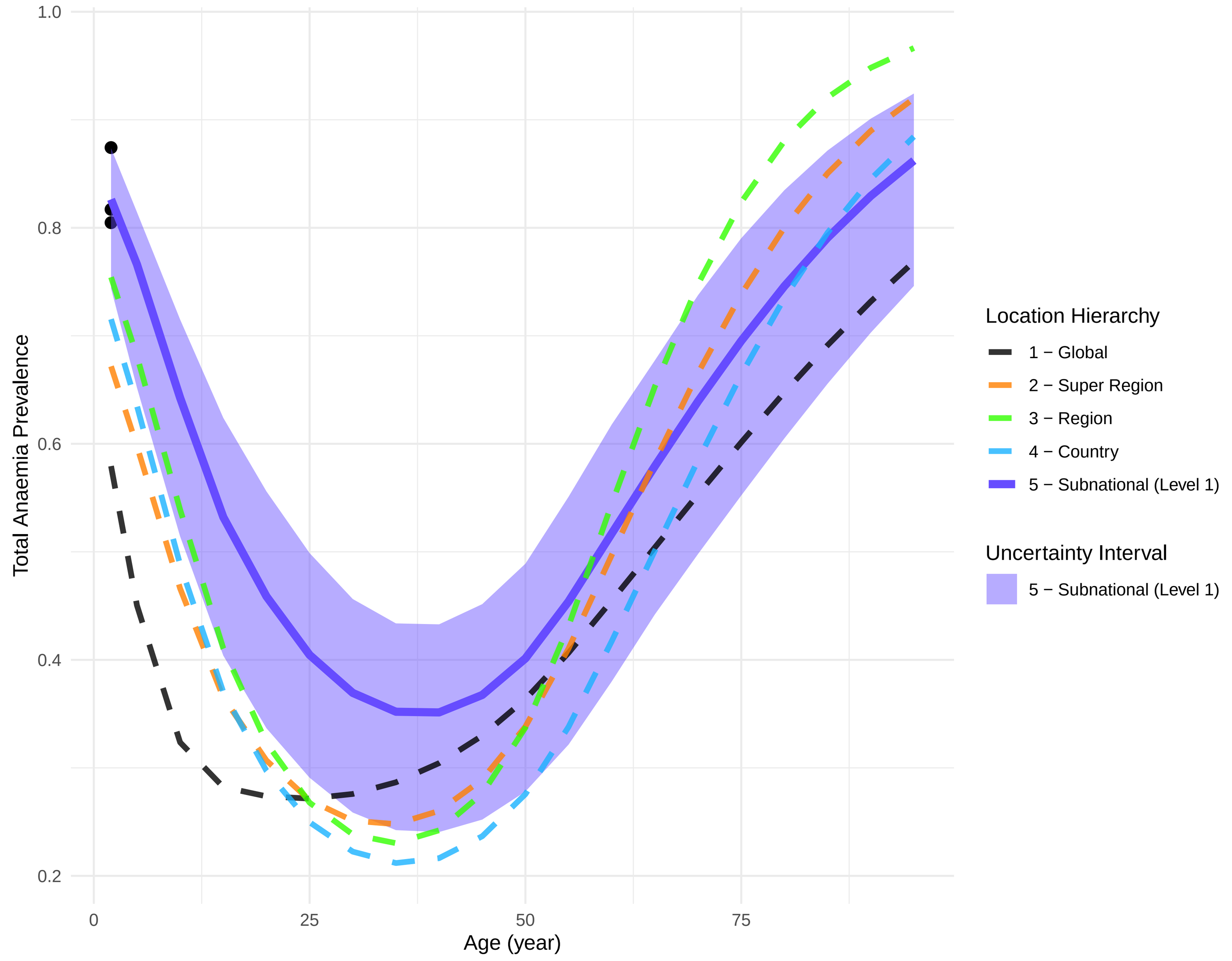

MR−BRT Age/Sex Fits − Males 2+ Years Old − Total Anaemia Prevalence − Senegal

WHO−2024 Adjustment Method

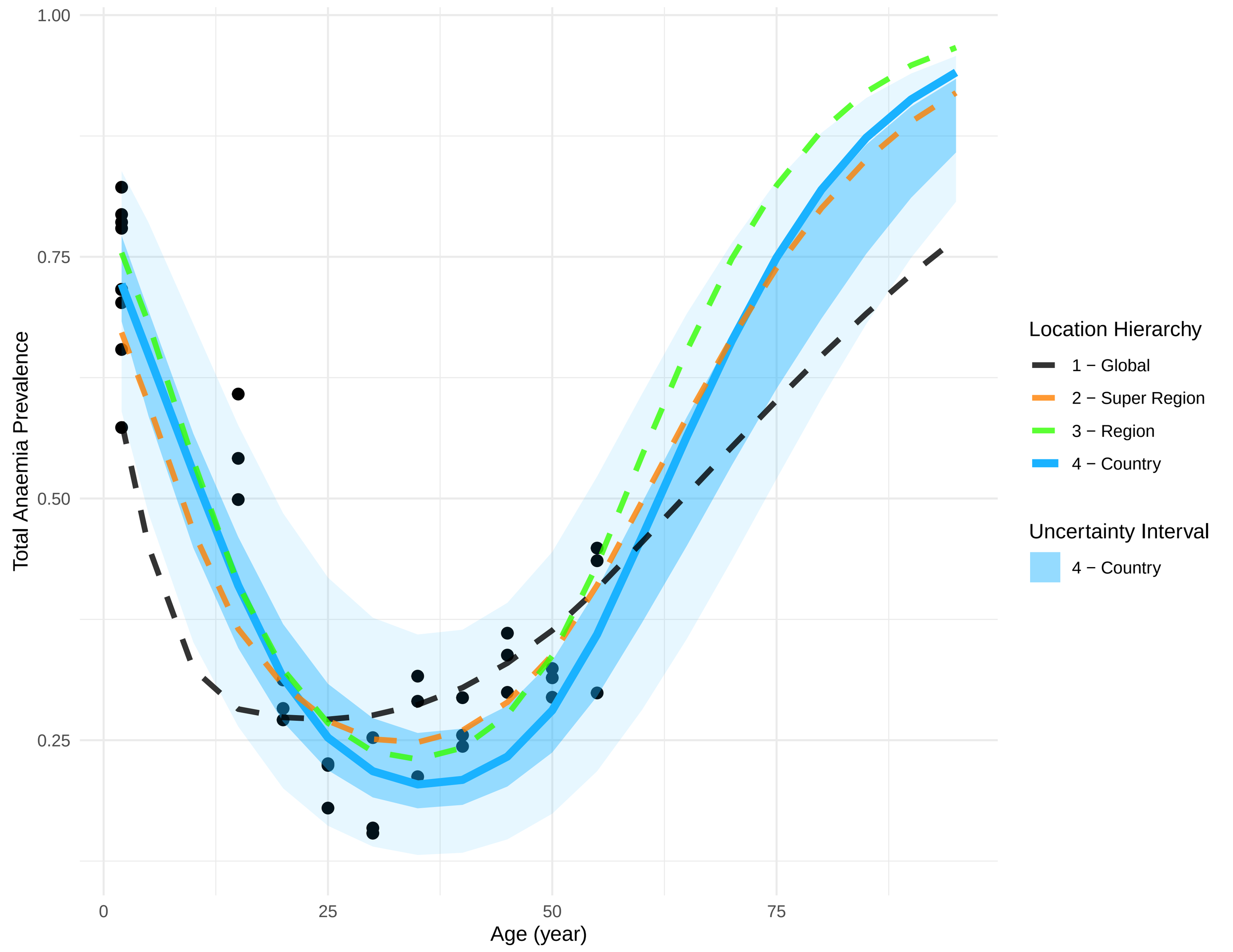

MR−BRT Age/Sex Fits − Males 2+ Years Old − Total Anaemia Prevalence − Sierra Leone

WHO−2024 Adjustment Method

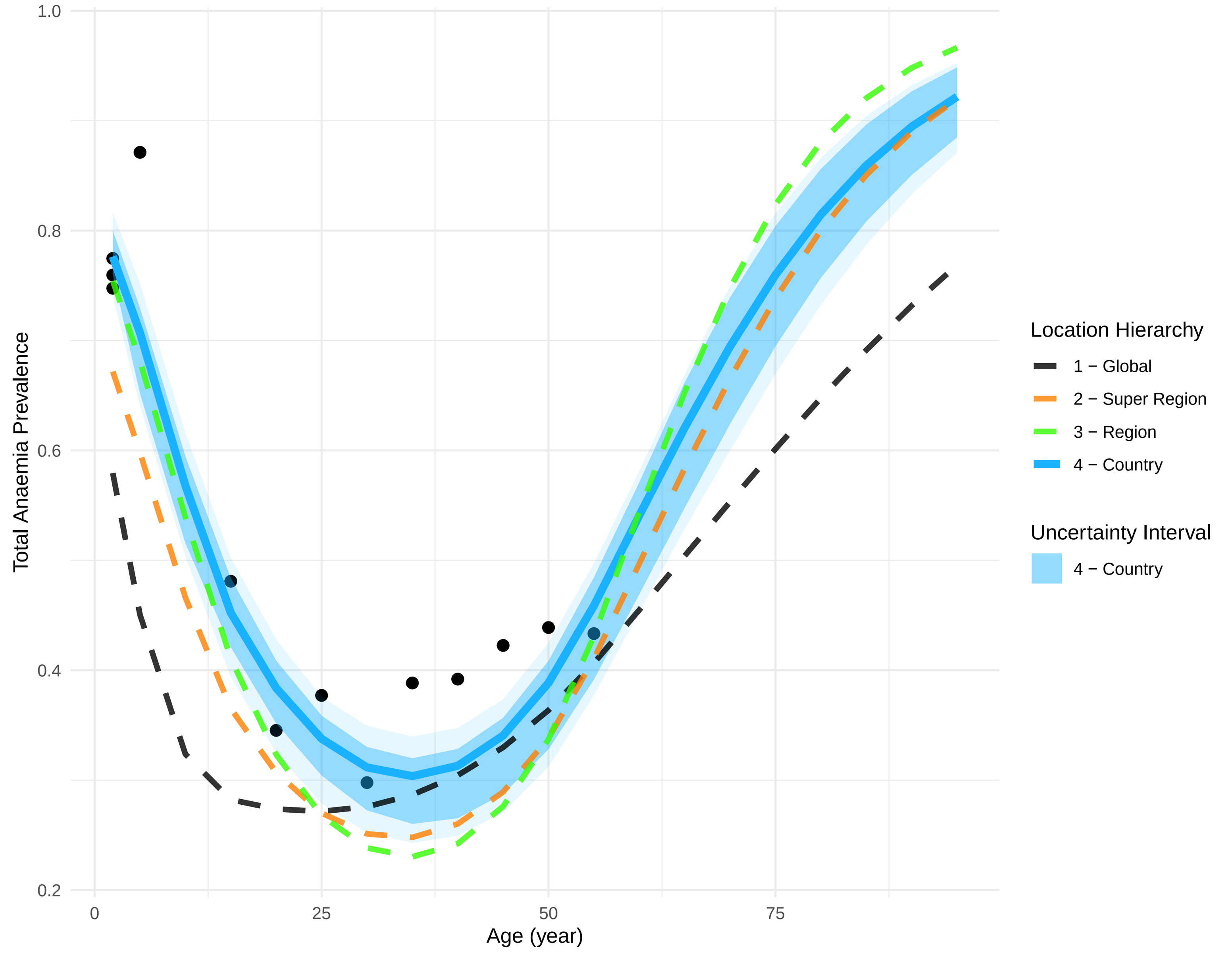

MR−BRT Age/Sex Fits − Males 2+ Years Old − Total Anaemia Prevalence − Togo

WHO−2024 Adjustment Method

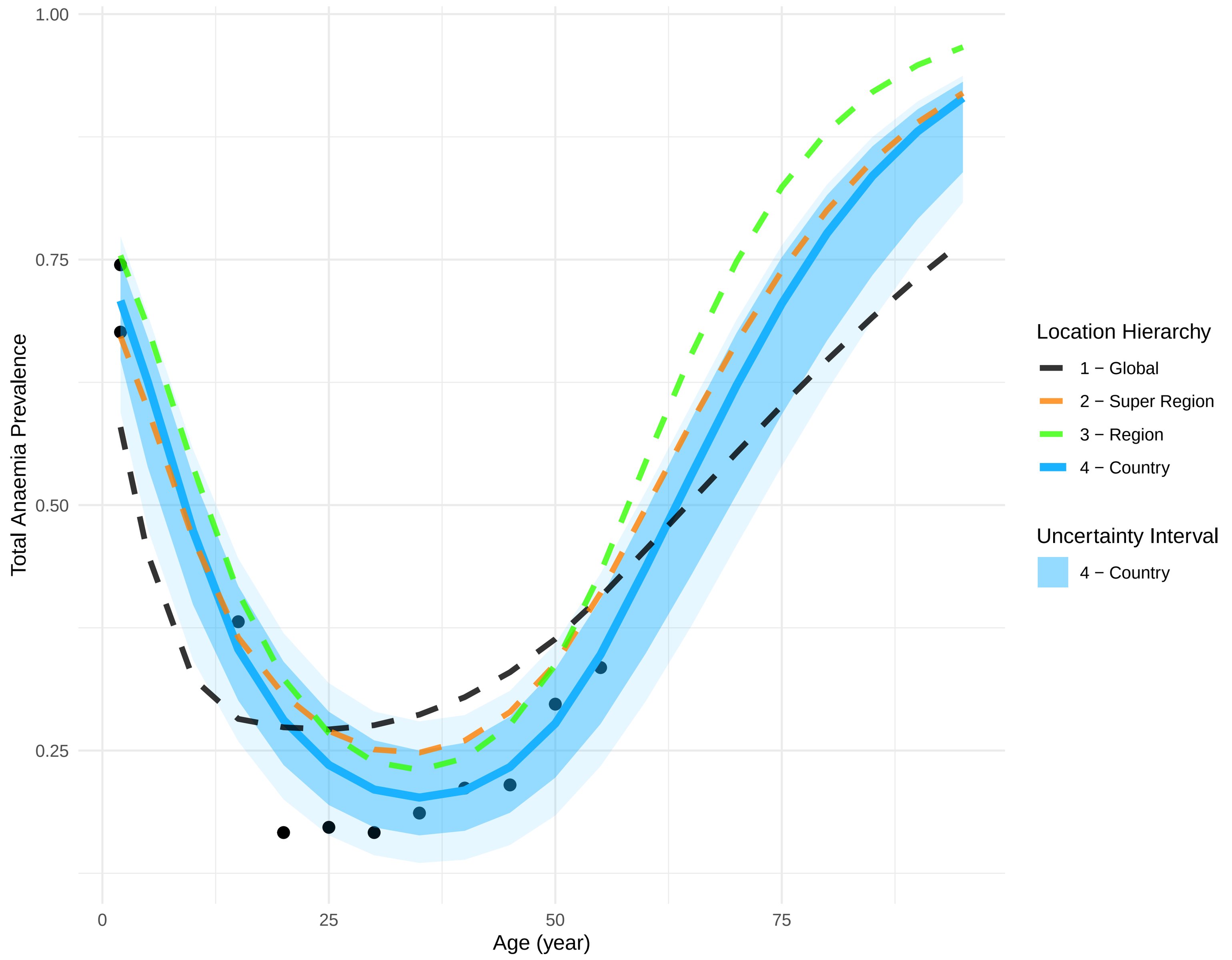

MR−BRT Age/Sex Fits − Non−pregnant Females 2+ Years Old − Total Anaemia Prevalence − Global
WHO−2024 Adjustment Method

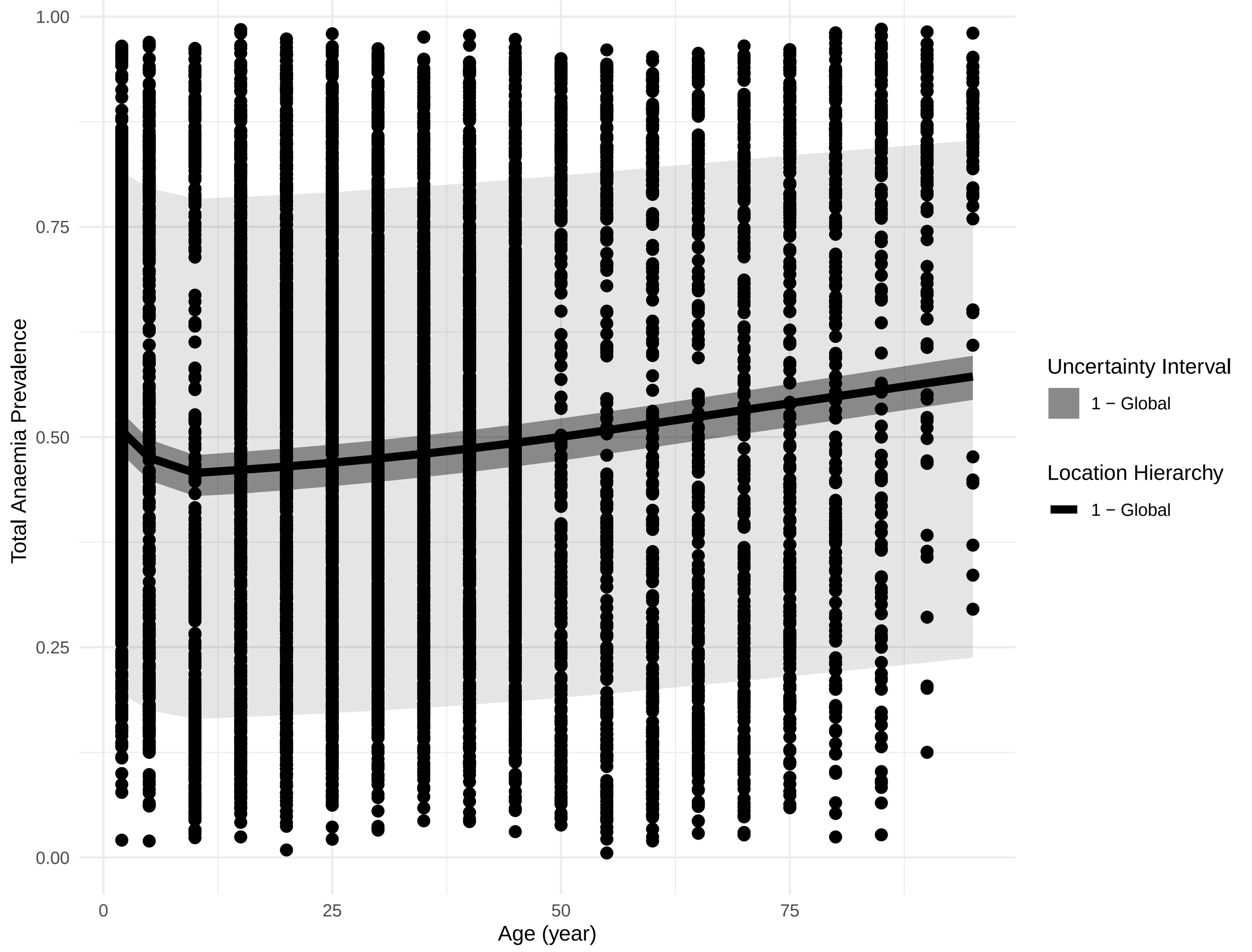

MR−BRT Age/Sex Fits − Non−pregnant Females 2+ Years Old − Total Anaemia Prevalence − Central Europe, Eastern Europe, and Central Asia
WHO−2024 Adjustment Method

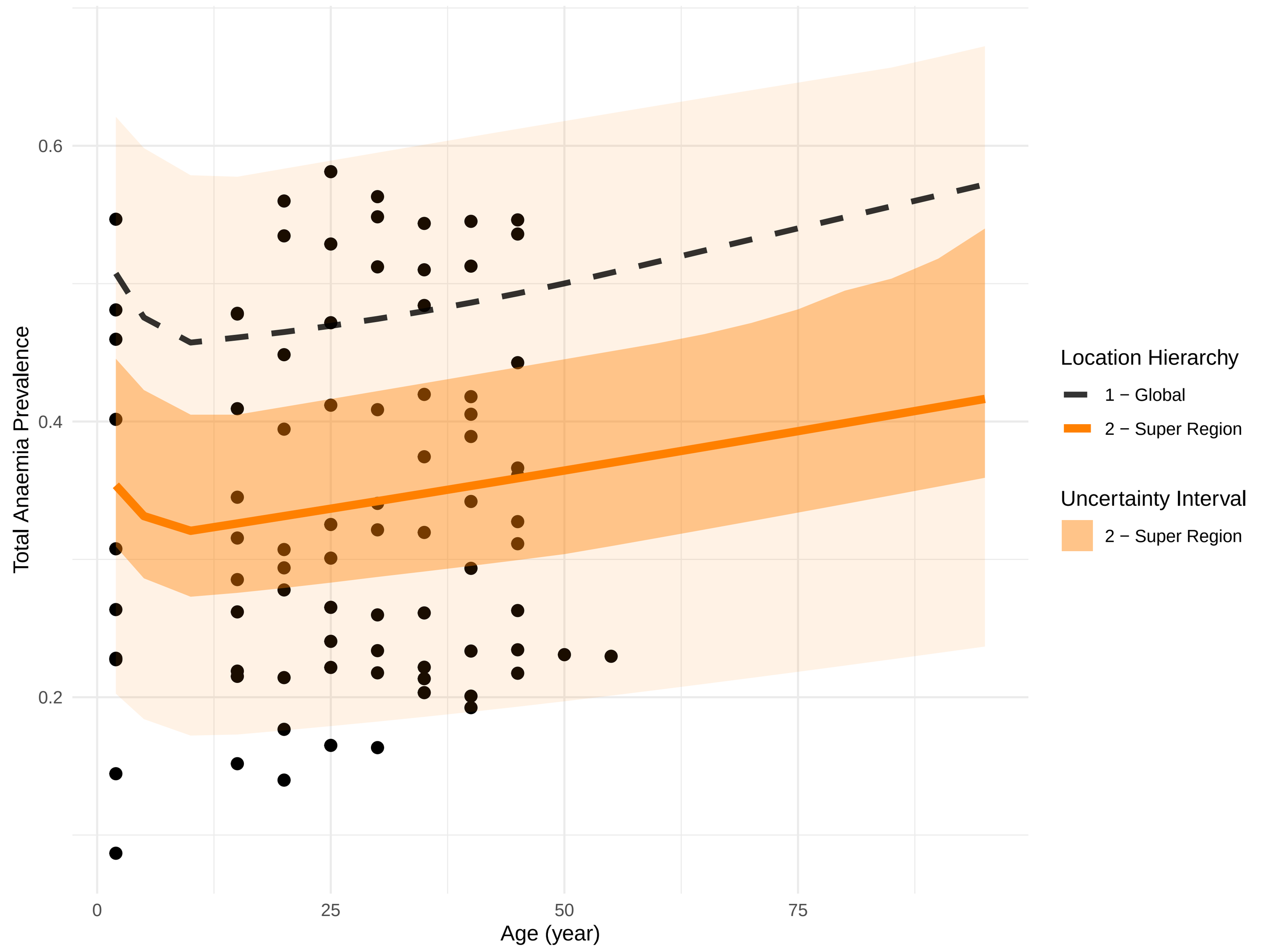

MR−BRT Age/Sex Fits − Non−pregnant Females 2+ Years Old − Total Anaemia Prevalence − Central Asia
WHO−2024 Adjustment Method

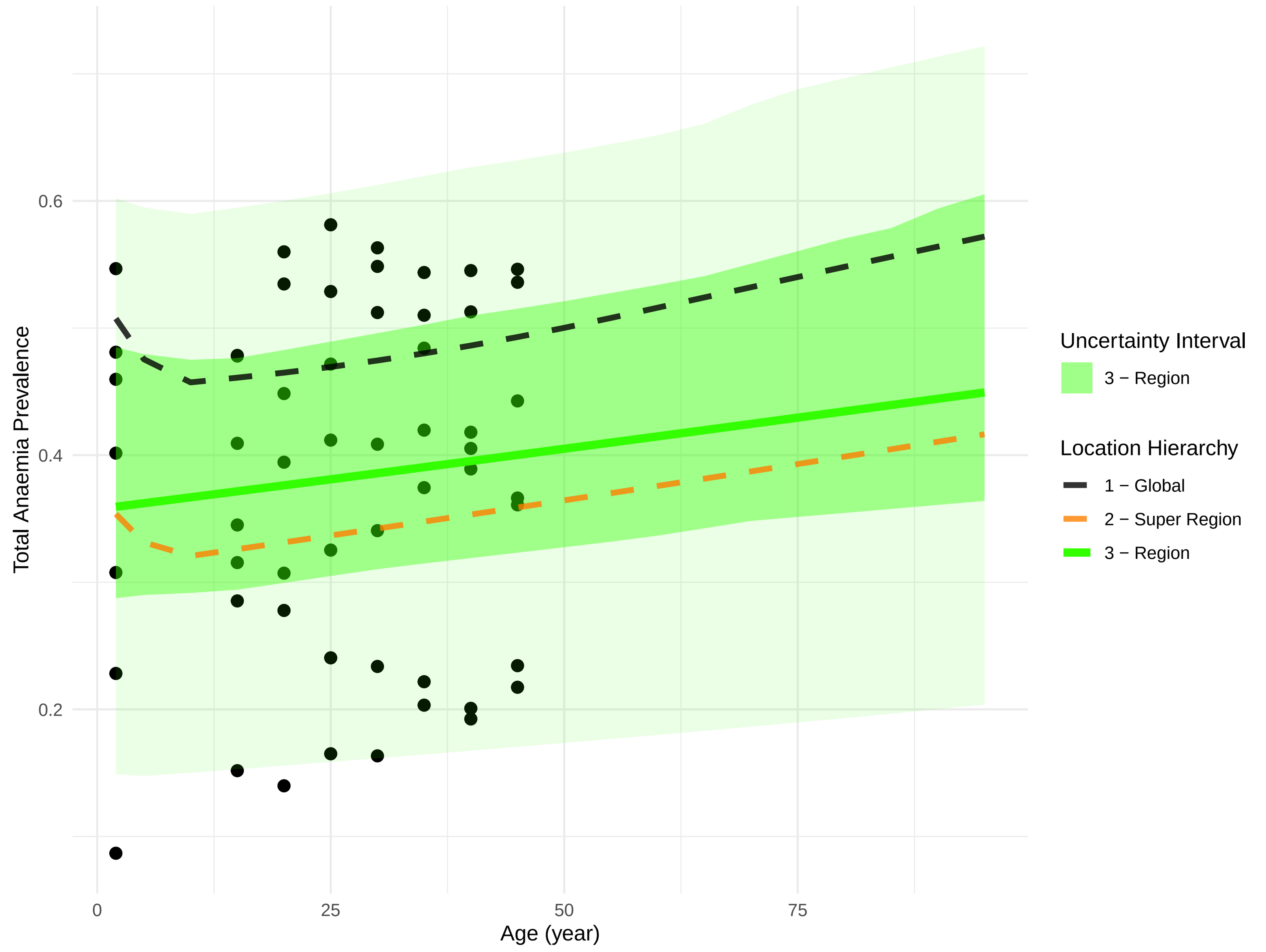

MR−BRT Age/Sex Fits − Non−pregnant Females 2+ Years Old − Total Anaemia Prevalence − Armenia
WHO−2024 Adjustment Method

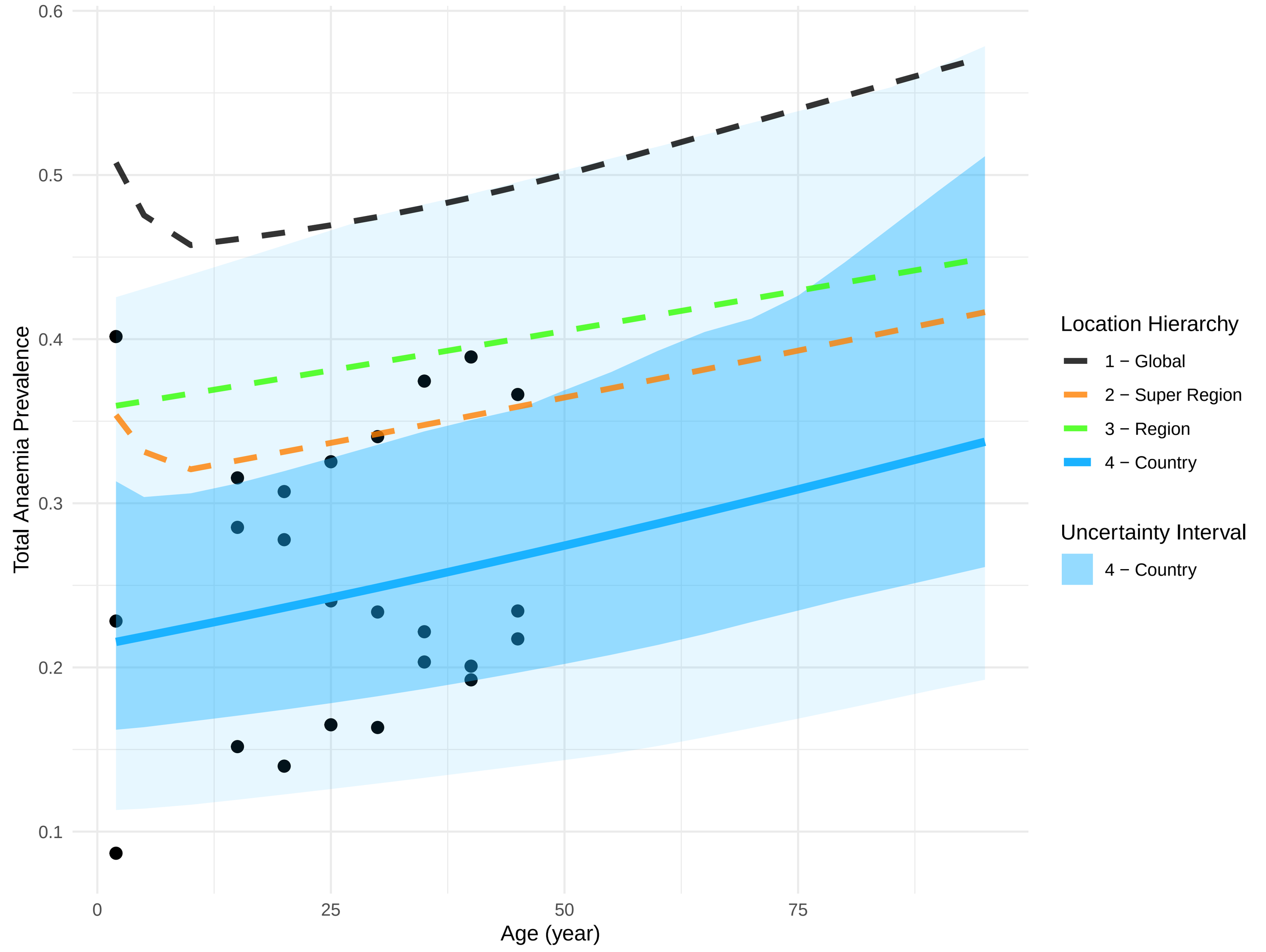

MR−BRT Age/Sex Fits − Non−pregnant Females 2+ Years Old − Total Anaemia Prevalence − Azerbaijan
WHO−2024 Adjustment Method

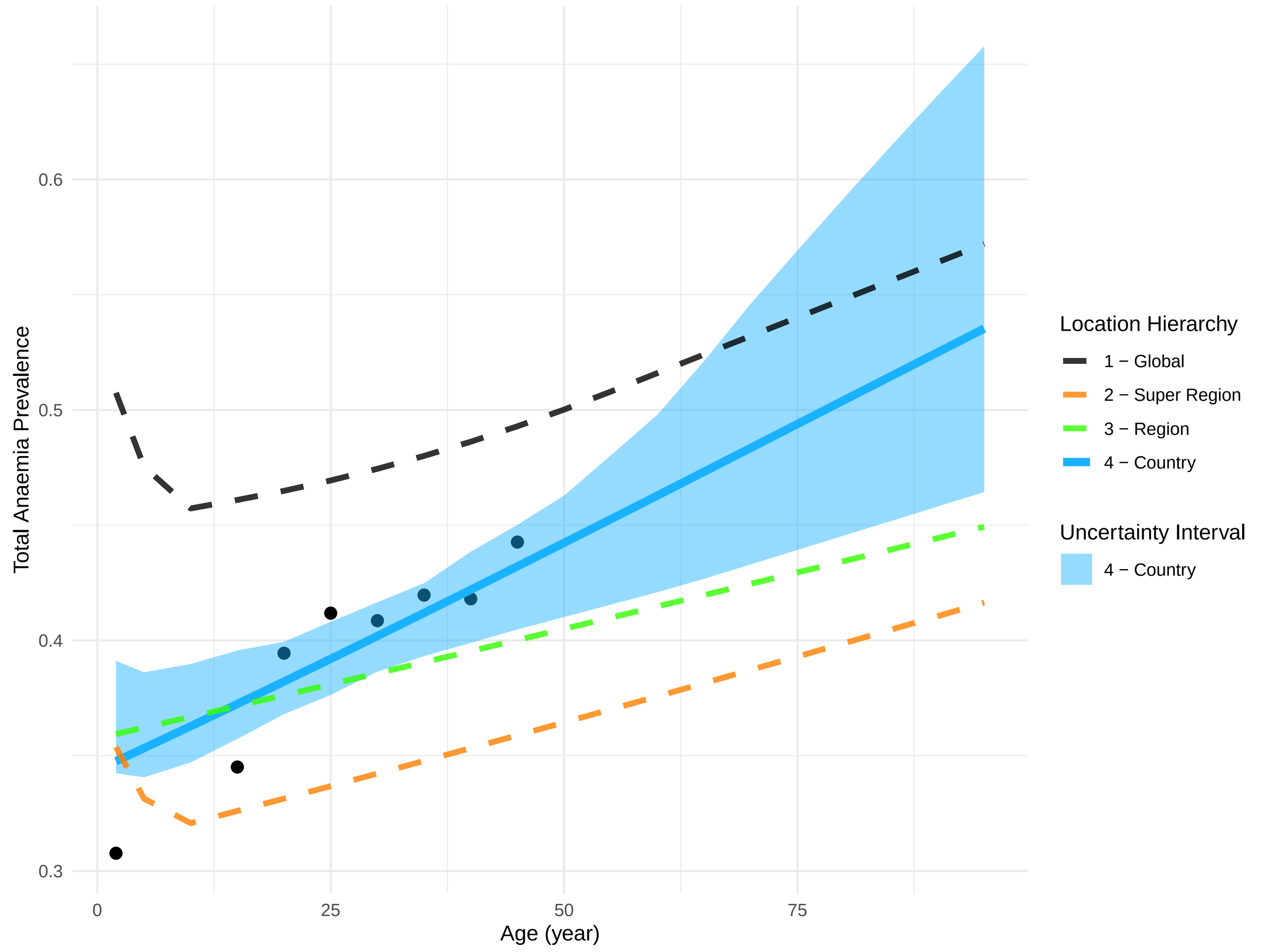

MR−BRT Age/Sex Fits − Non−pregnant Females 2+ Years Old − Total Anaemia Prevalence − Kyrgyzstan
WHO−2024 Adjustment Method

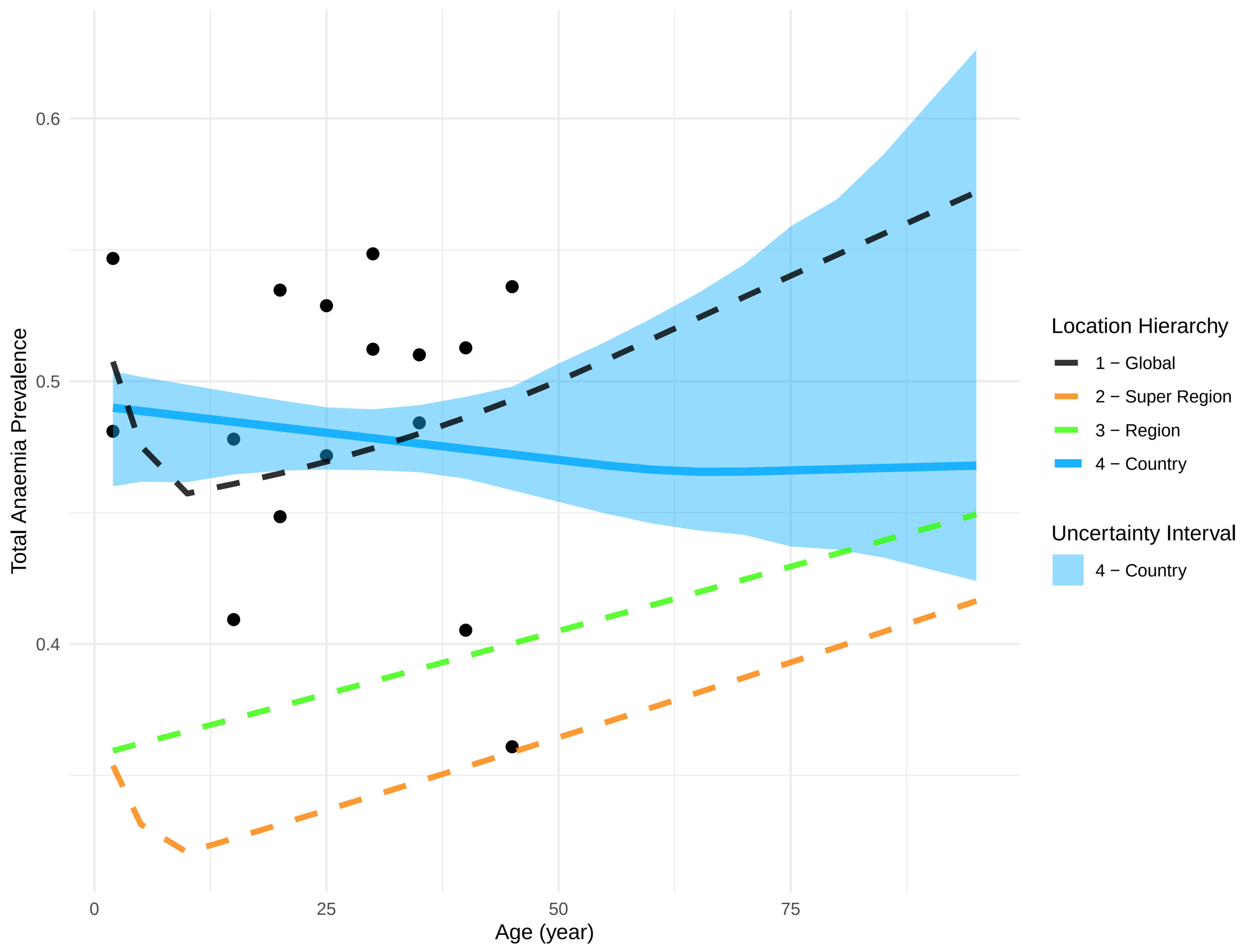

MR−BRT Age/Sex Fits − Non−pregnant Females 2+ Years Old − Total Anaemia Prevalence − Tajikistan
WHO−2024 Adjustment Method

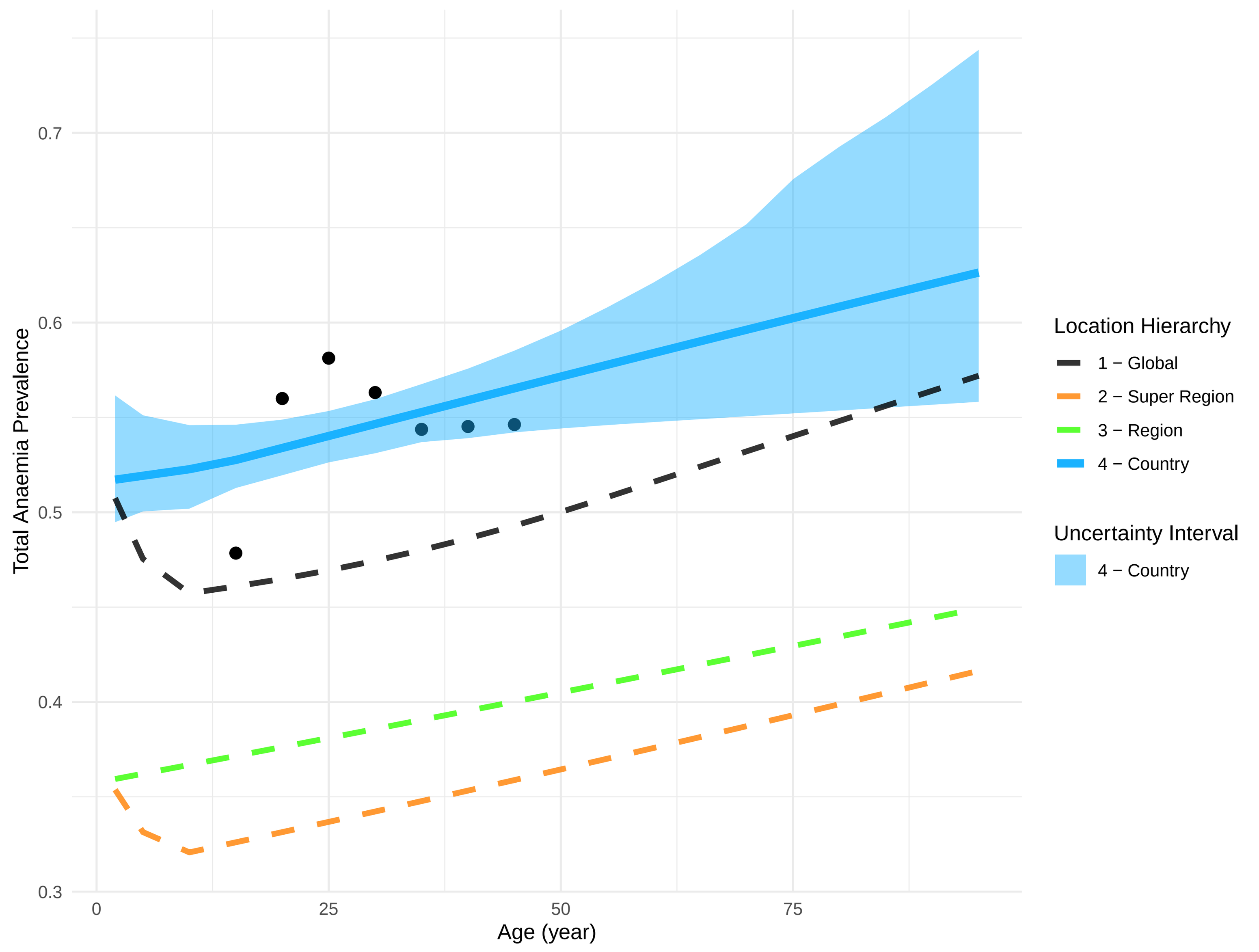

MR−BRT Age/Sex Fits − Non−pregnant Females 2+ Years Old − Total Anaemia Prevalence − Uzbekistan
WHO−2024 Adjustment Method

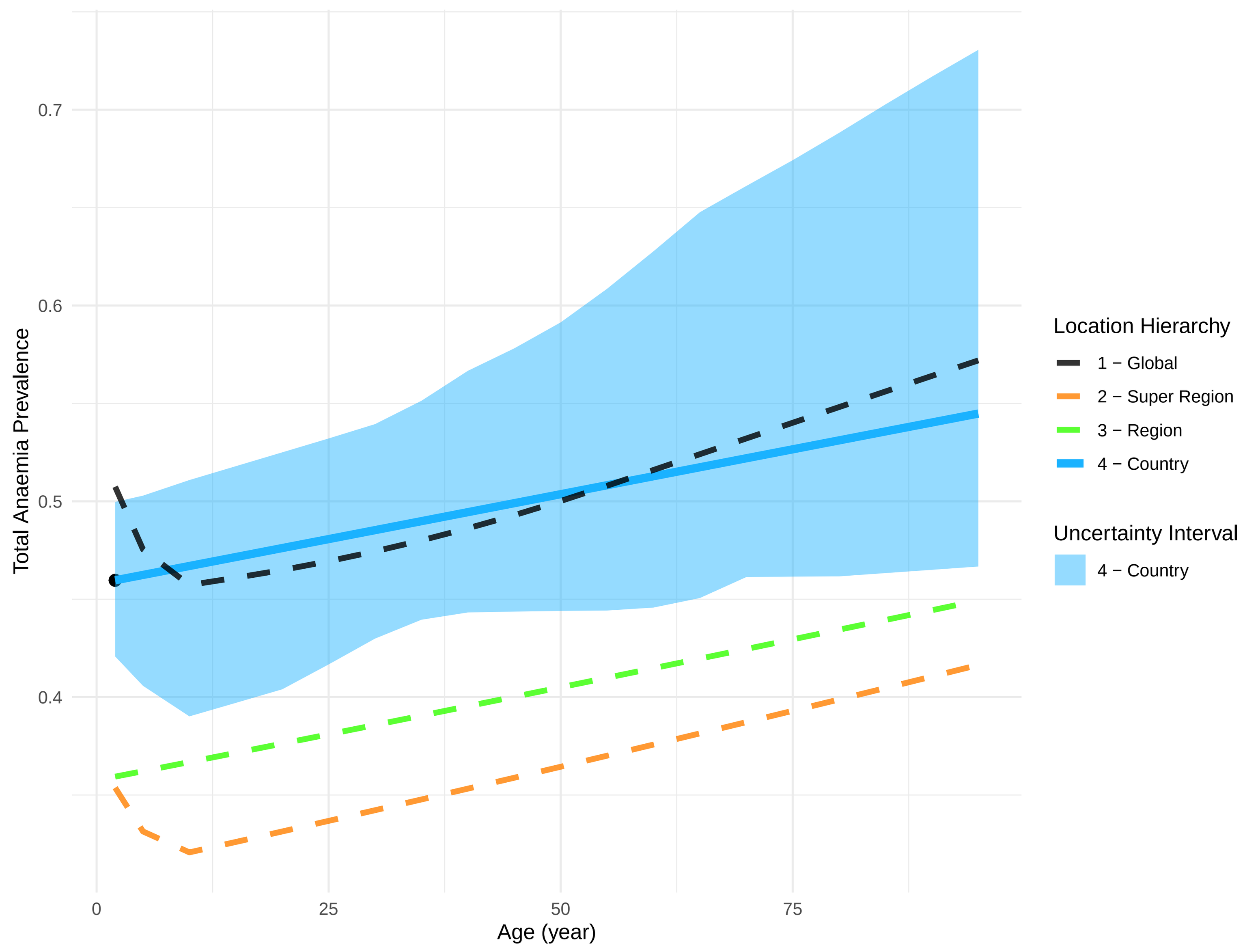

MR−BRT Age/Sex Fits − Non−pregnant Females 2+ Years Old − Total Anaemia Prevalence − Central Europe
WHO−2024 Adjustment Method

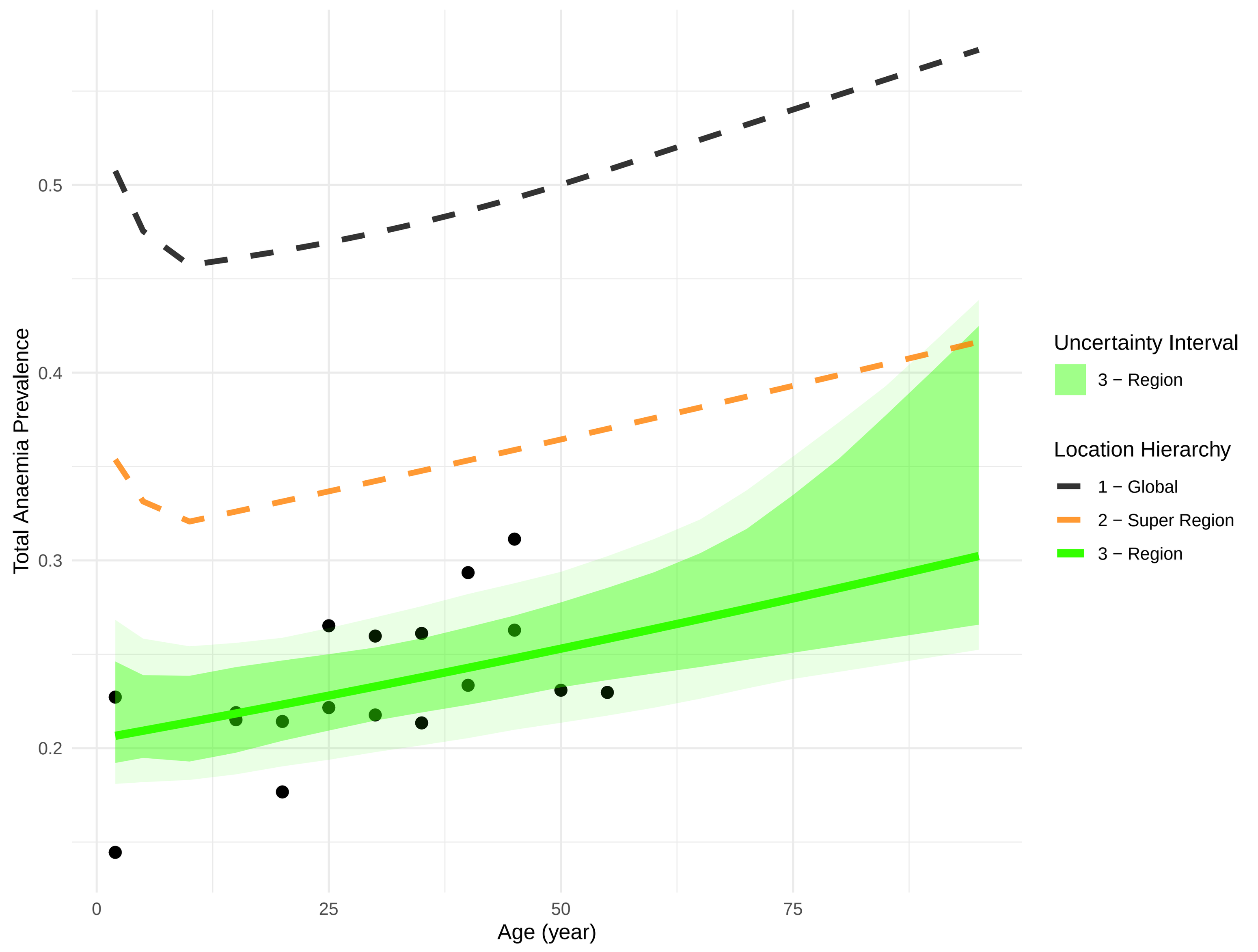

MR−BRT Age/Sex Fits − Non−pregnant Females 2+ Years Old − Total Anaemia Prevalence − Albania
WHO−2024 Adjustment Method

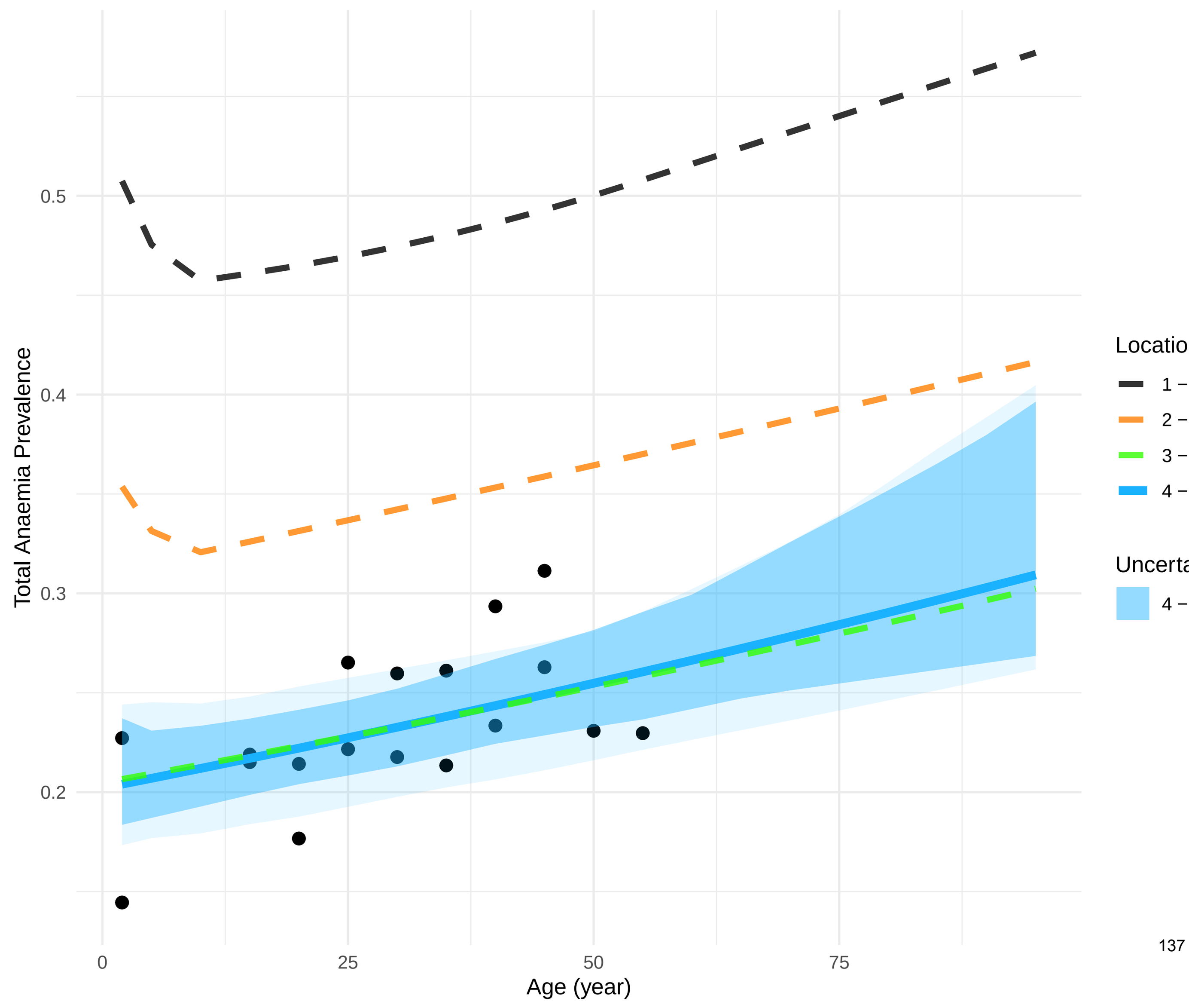

MR−BRT Age/Sex Fits − Non−pregnant Females 2+ Years Old − Total Anaemia Prevalence − Eastern Europe
WHO−2024 Adjustment Method

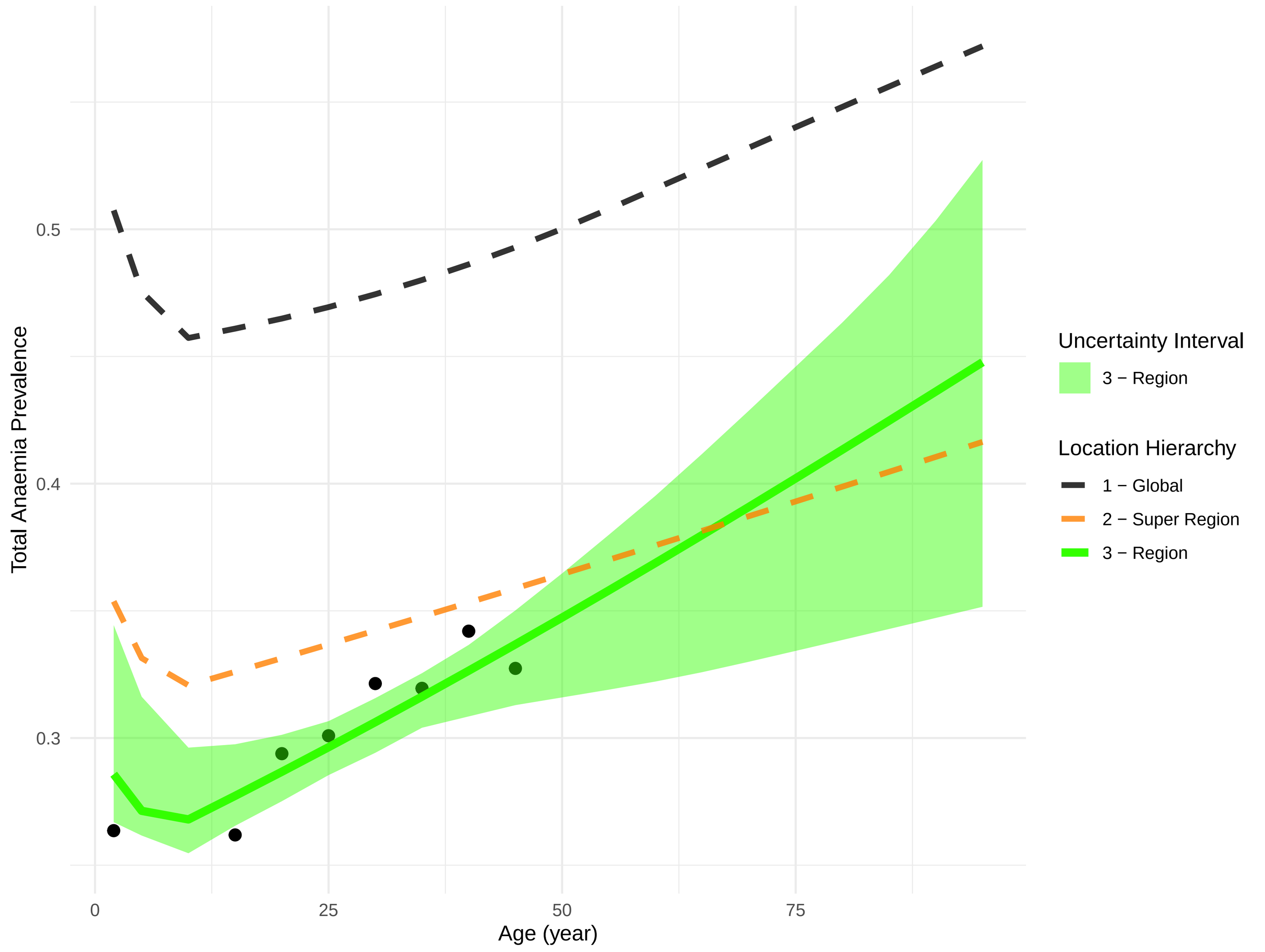

MR−BRT Age/Sex Fits − Non−pregnant Females 2+ Years Old − Total Anaemia Prevalence − Republic of Moldova
WHO−2024 Adjustment Method

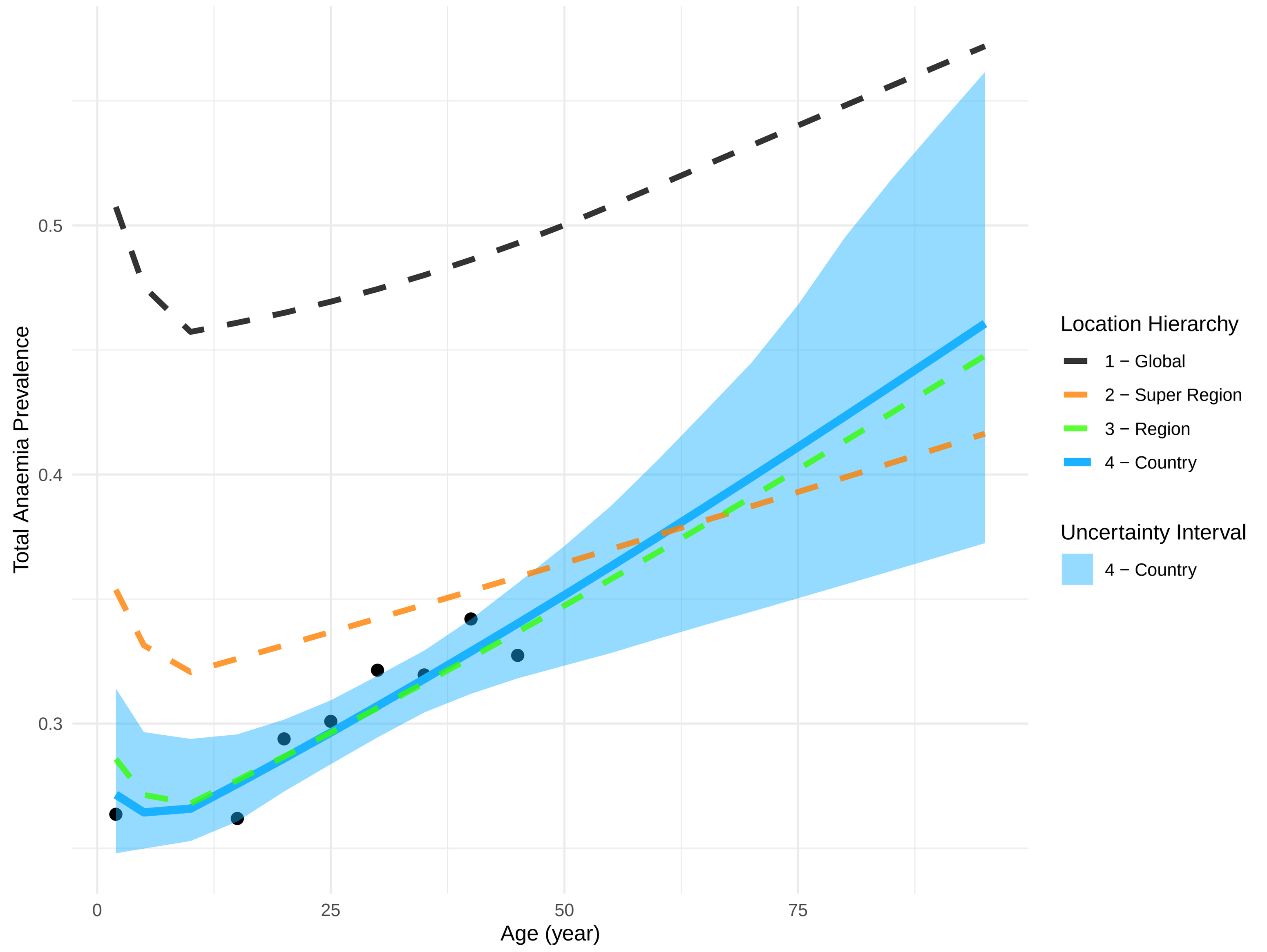

MR−BRT Age/Sex Fits − Non−pregnant Females 2+ Years Old − Total Anaemia Prevalence − High−income
WHO−2024 Adjustment Method

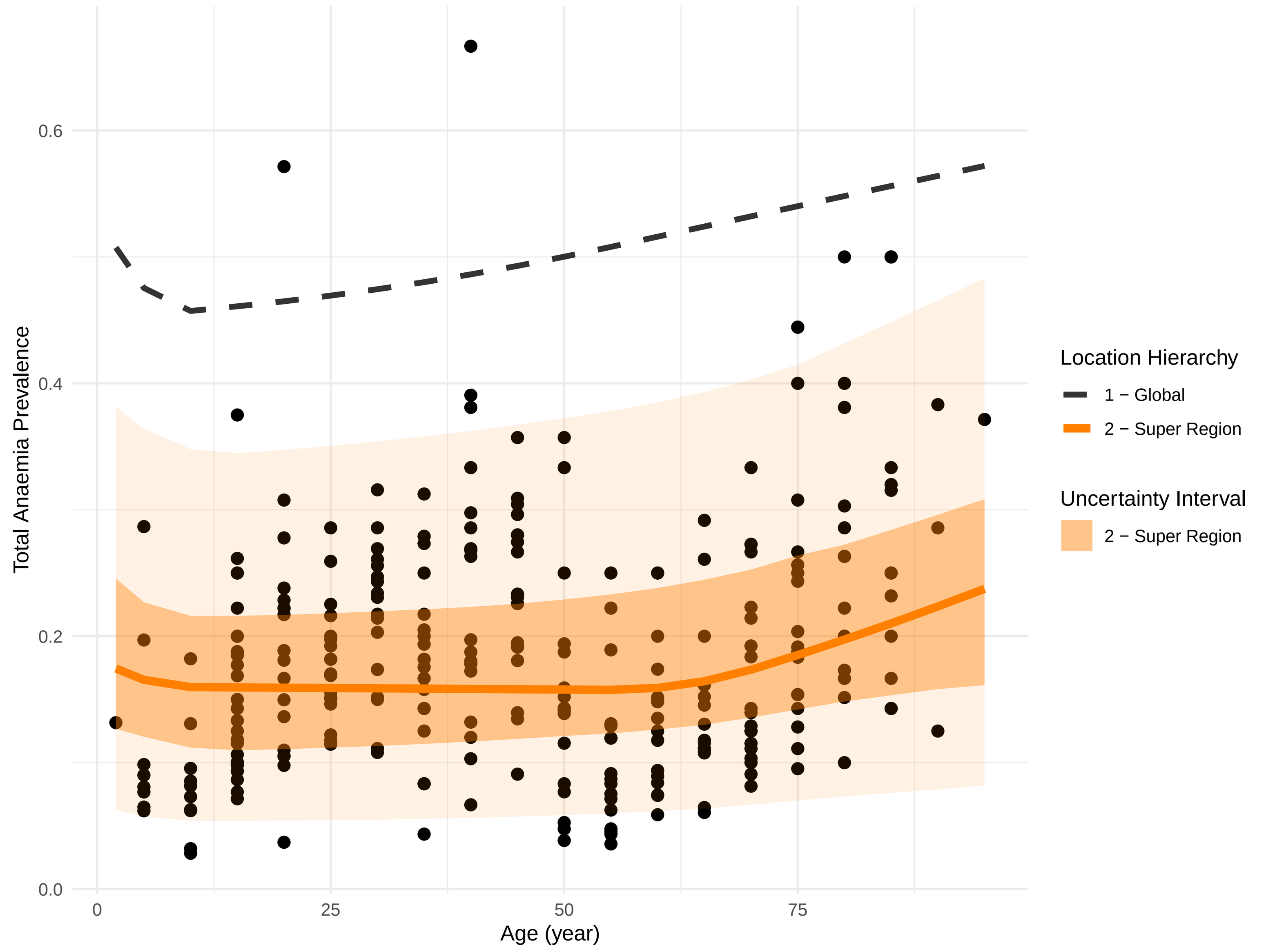

MR−BRT Age/Sex Fits − Non−pregnant Females 2+ Years Old − Total Anaemia Prevalence − Western Europe
WHO−2024 Adjustment Method

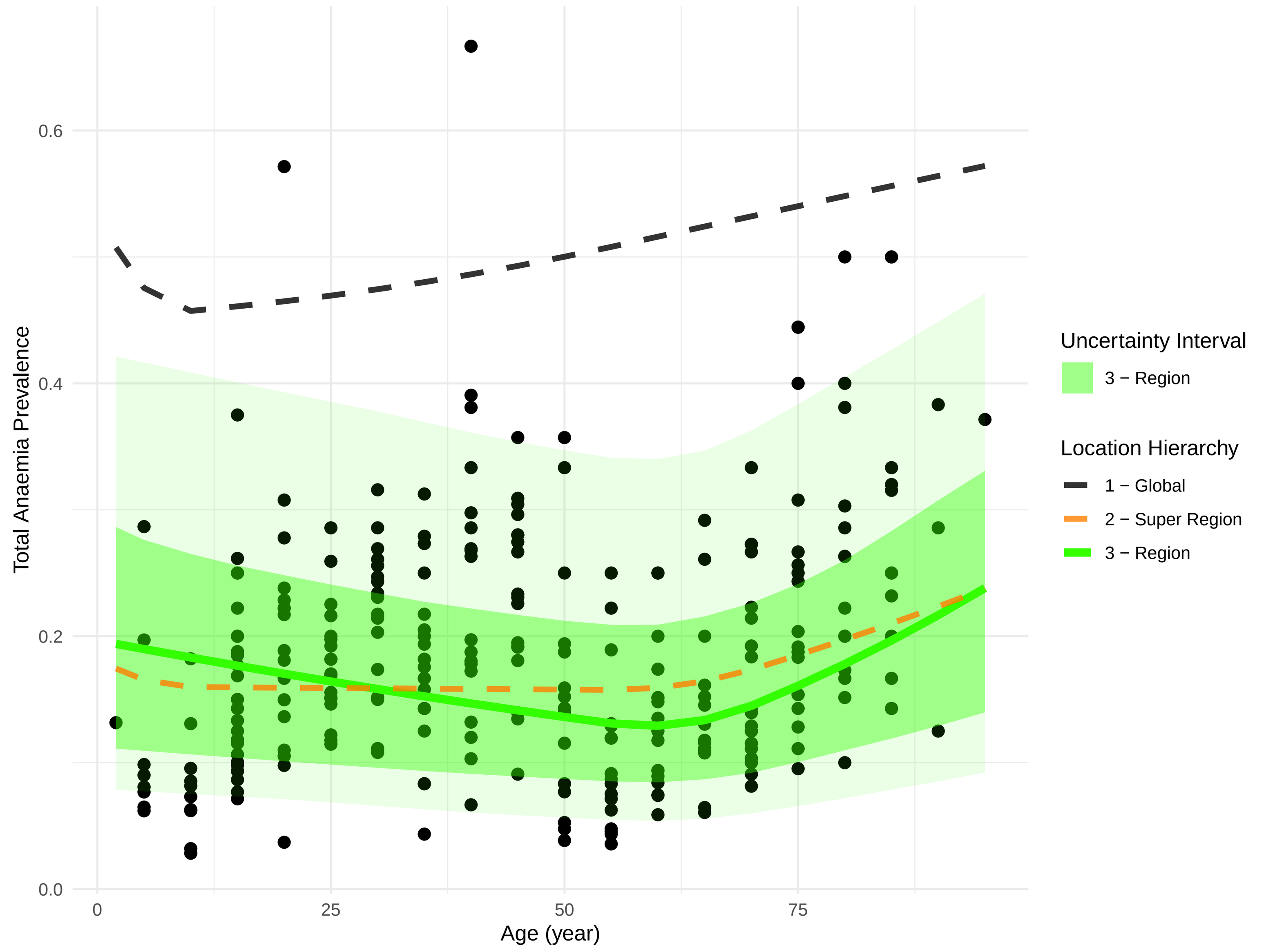

MR−BRT Age/Sex Fits − Non−pregnant Females 2+ Years Old − Total Anaemia Prevalence − United Kingdom
WHO−2024 Adjustment Method

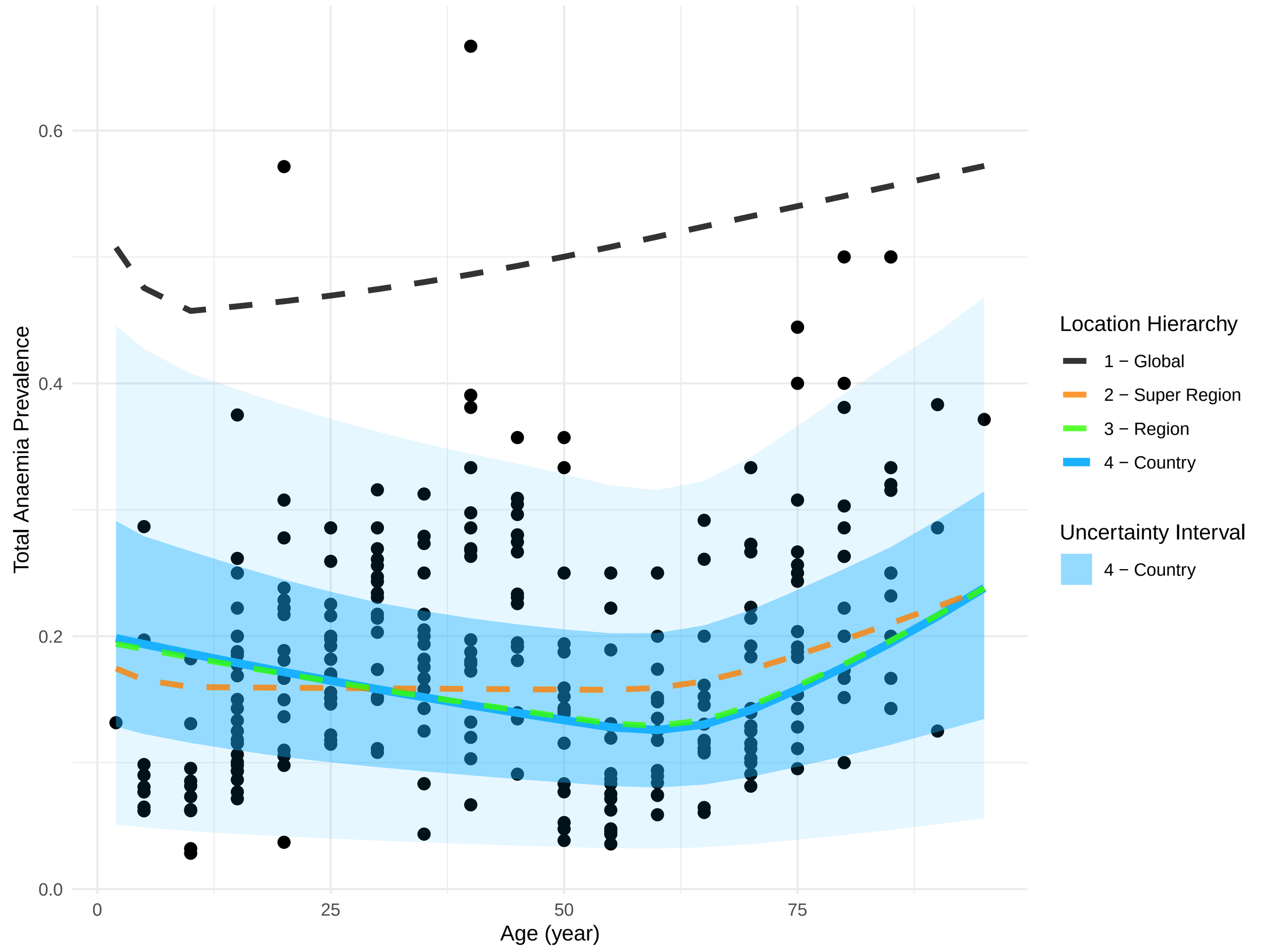

MR−BRT Age/Sex Fits − Non−pregnant Females 2+ Years Old − Total Anaemia Prevalence − England
WHO−2024 Adjustment Method

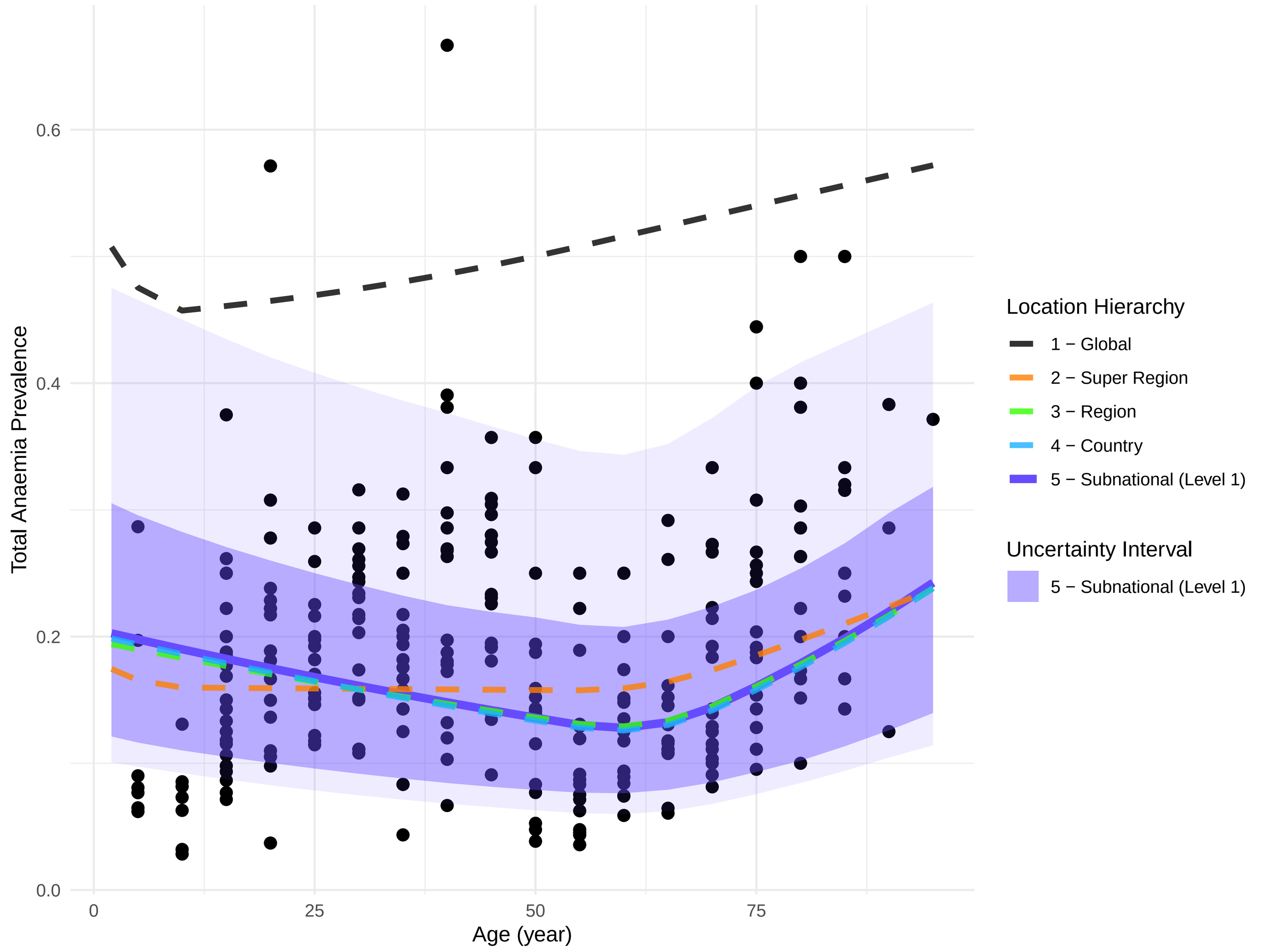

MR−BRT Age/Sex Fits − Non−pregnant Females 2+ Years Old − Total Anaemia Prevalence − East Midlands
WHO−2024 Adjustment Method

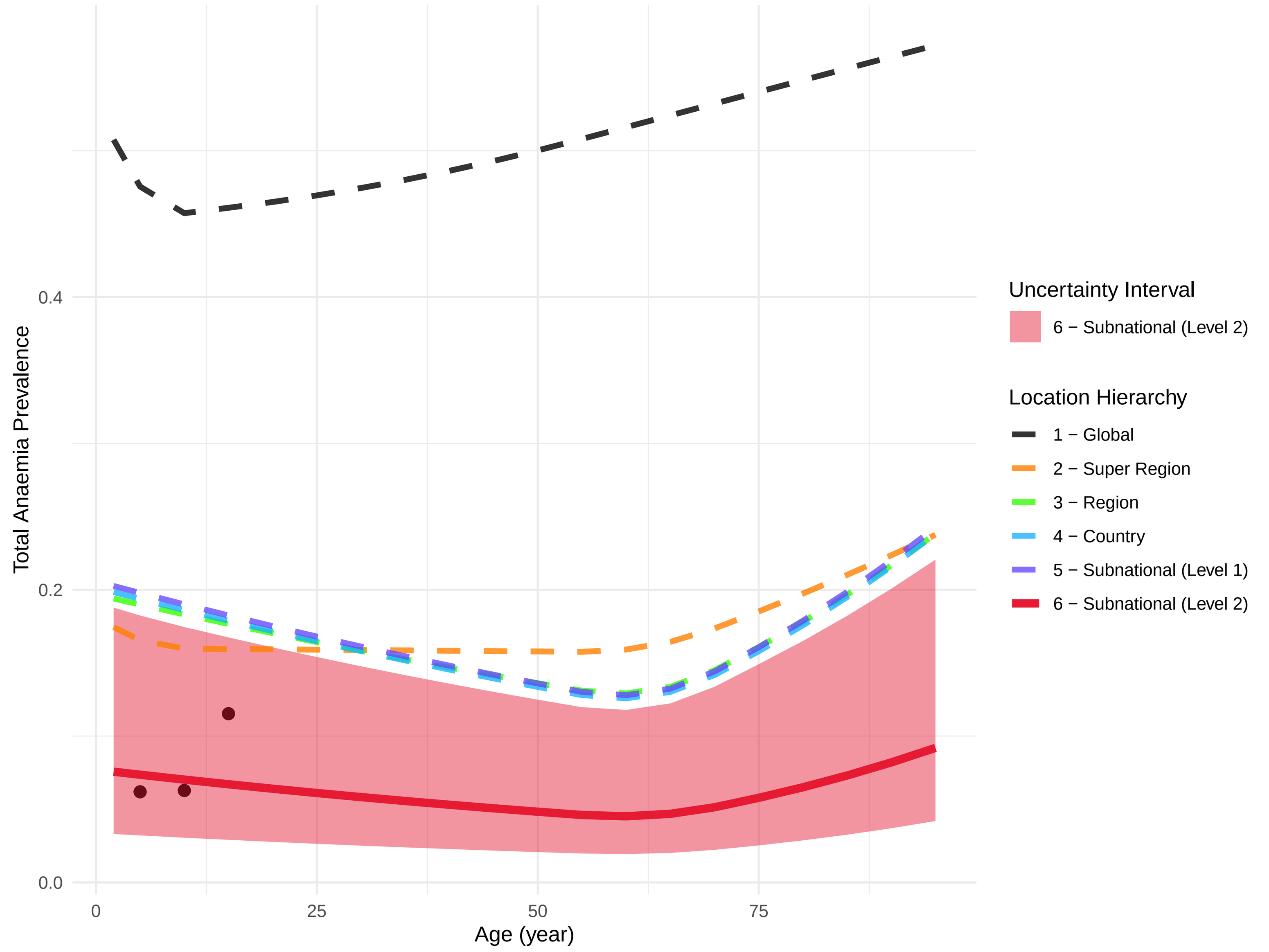

MR−BRT Age/Sex Fits − Non−pregnant Females 2+ Years Old − Total Anaemia Prevalence − East of England
WHO−2024 Adjustment Method

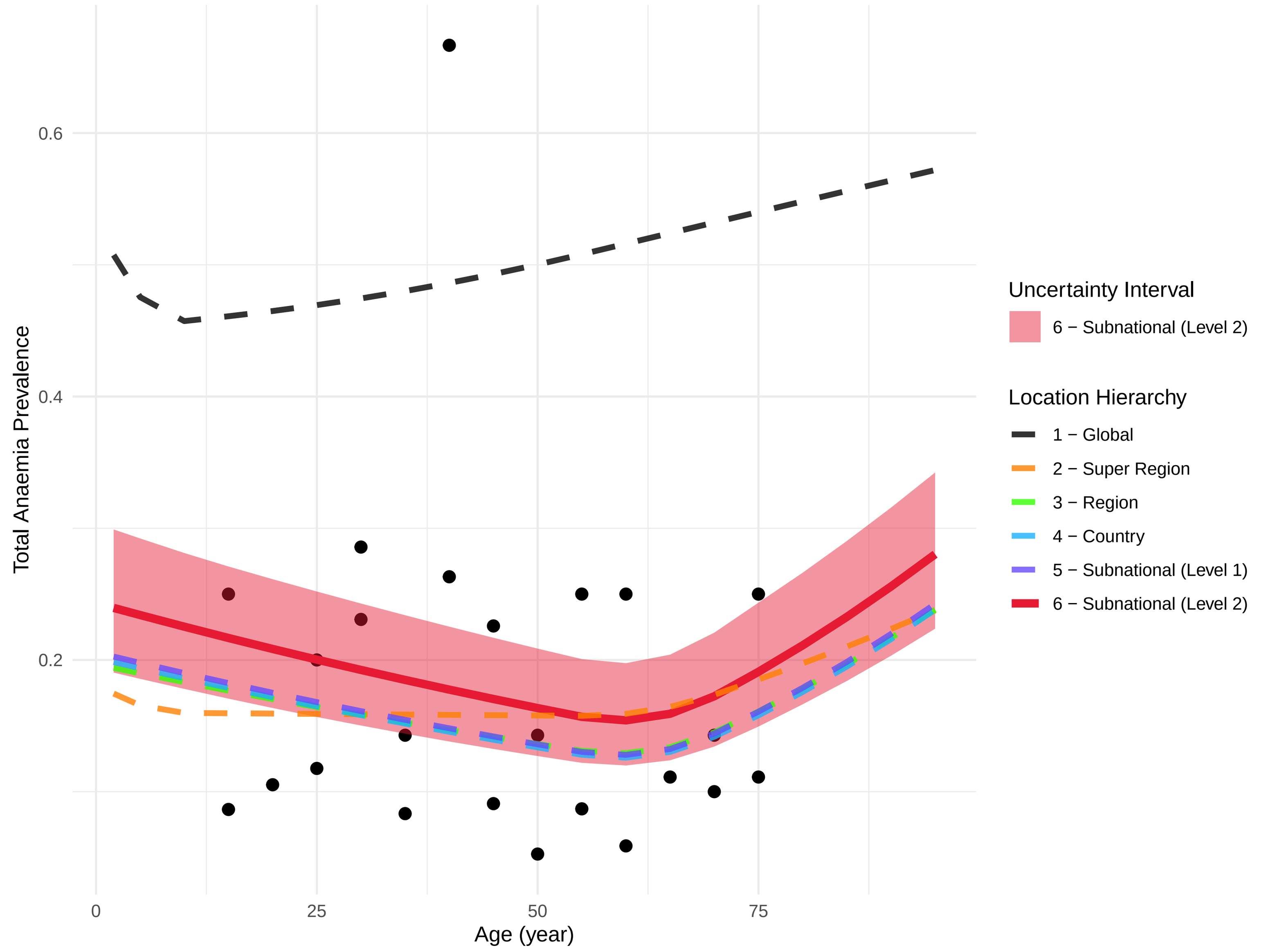

MR−BRT Age/Sex Fits − Non−pregnant Females 2+ Years Old − Total Anaemia Prevalence − Greater London
WHO−2024 Adjustment Method

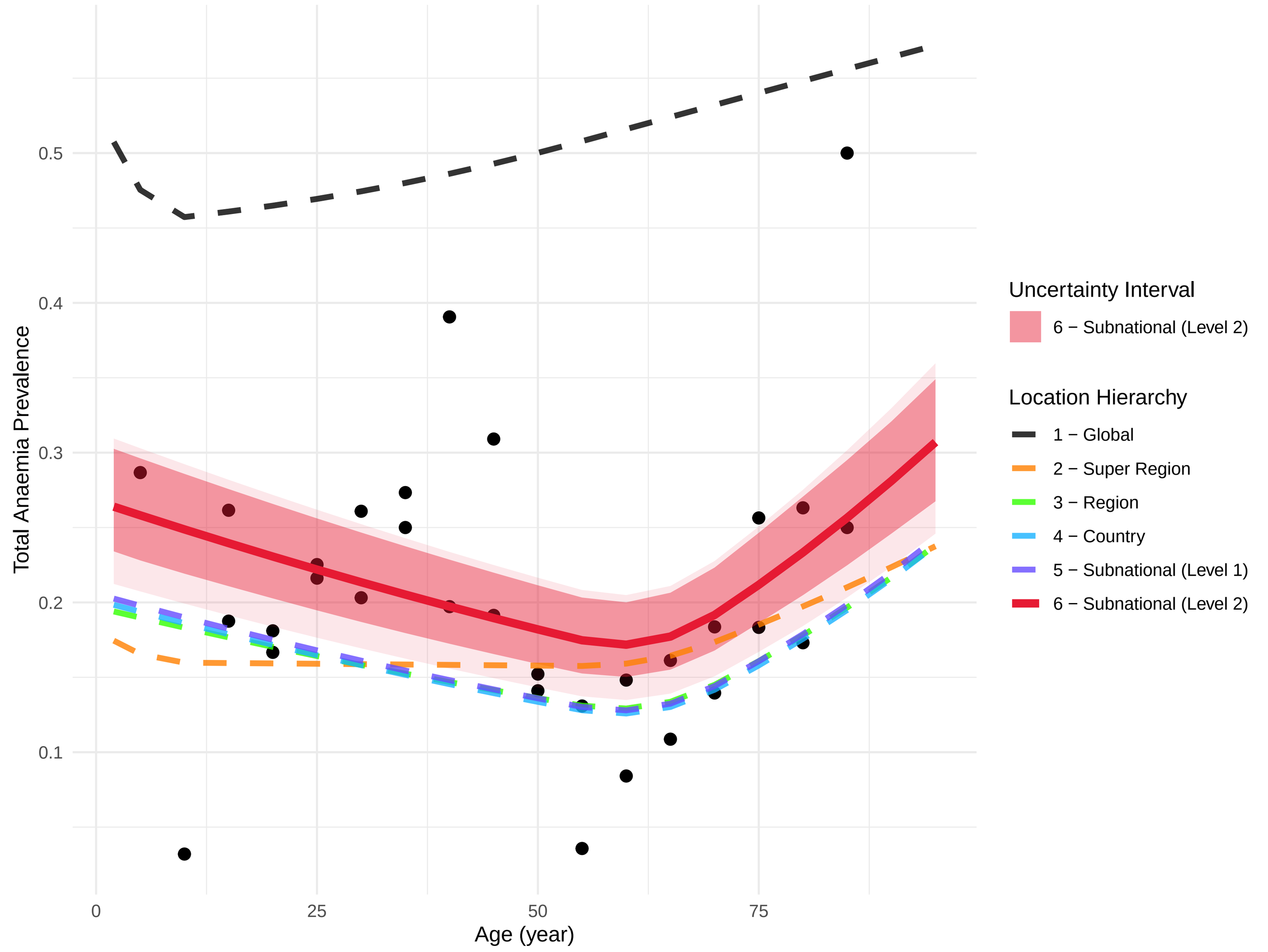

MR−BRT Age/Sex Fits − Non−pregnant Females 2+ Years Old − Total Anaemia Prevalence − North East England
WHO−2024 Adjustment Method

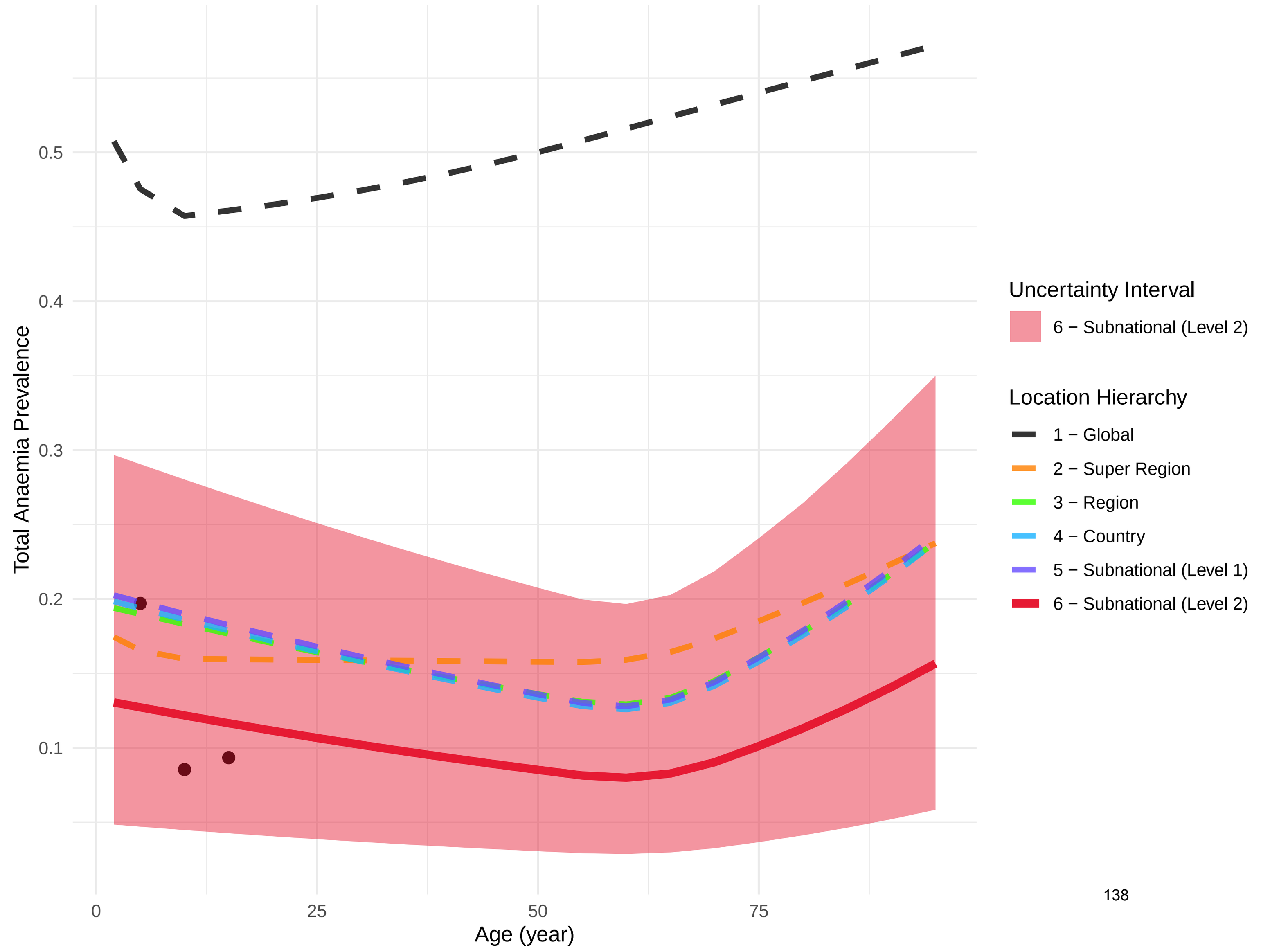

MR−BRT Age/Sex Fits − Non−pregnant Females 2+ Years Old − Total Anaemia Prevalence − North West England
WHO−2024 Adjustment Method

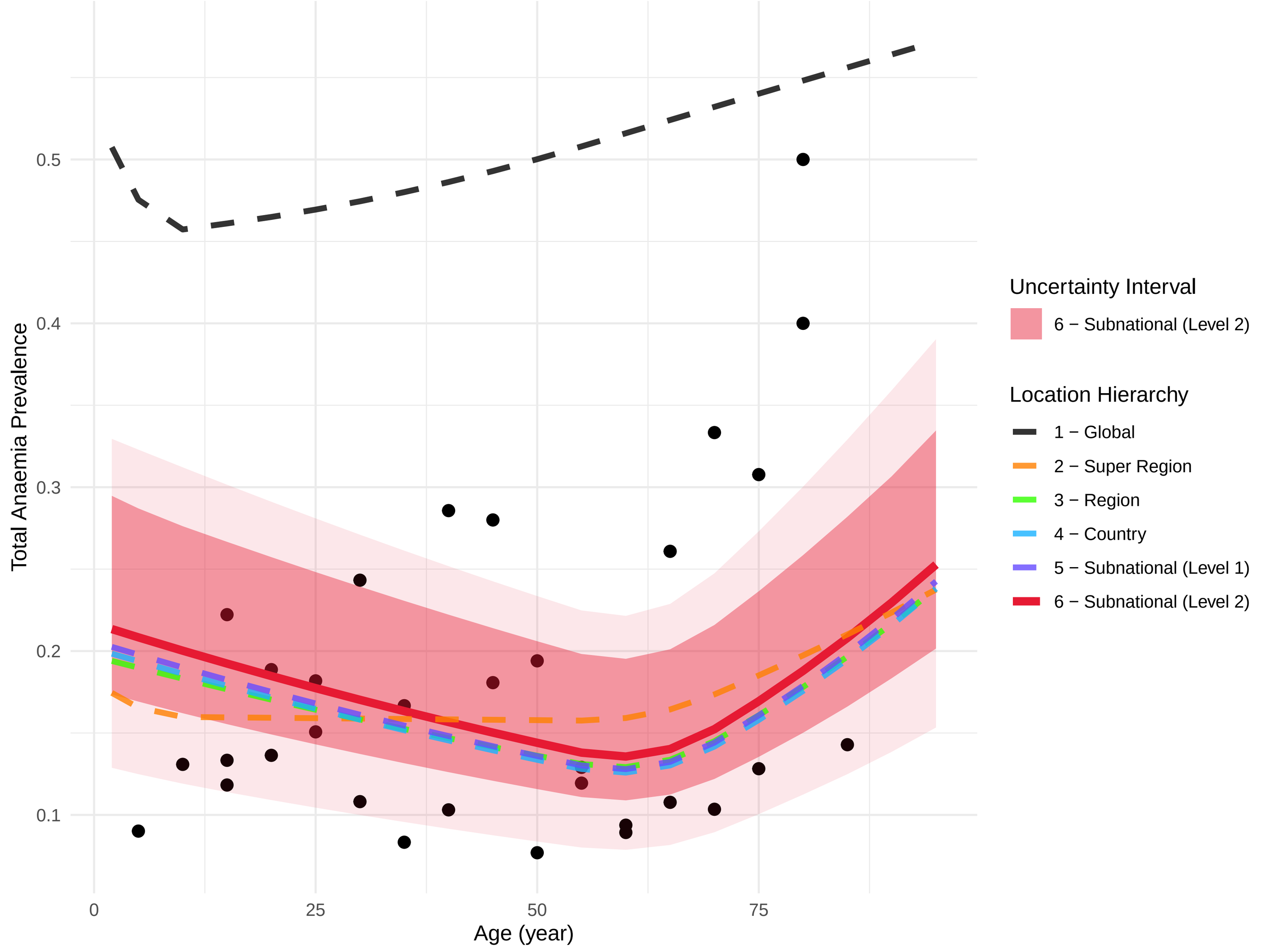

MR−BRT Age/Sex Fits − Non−pregnant Females 2+ Years Old − Total Anaemia Prevalence − South East England
WHO−2024 Adjustment Method

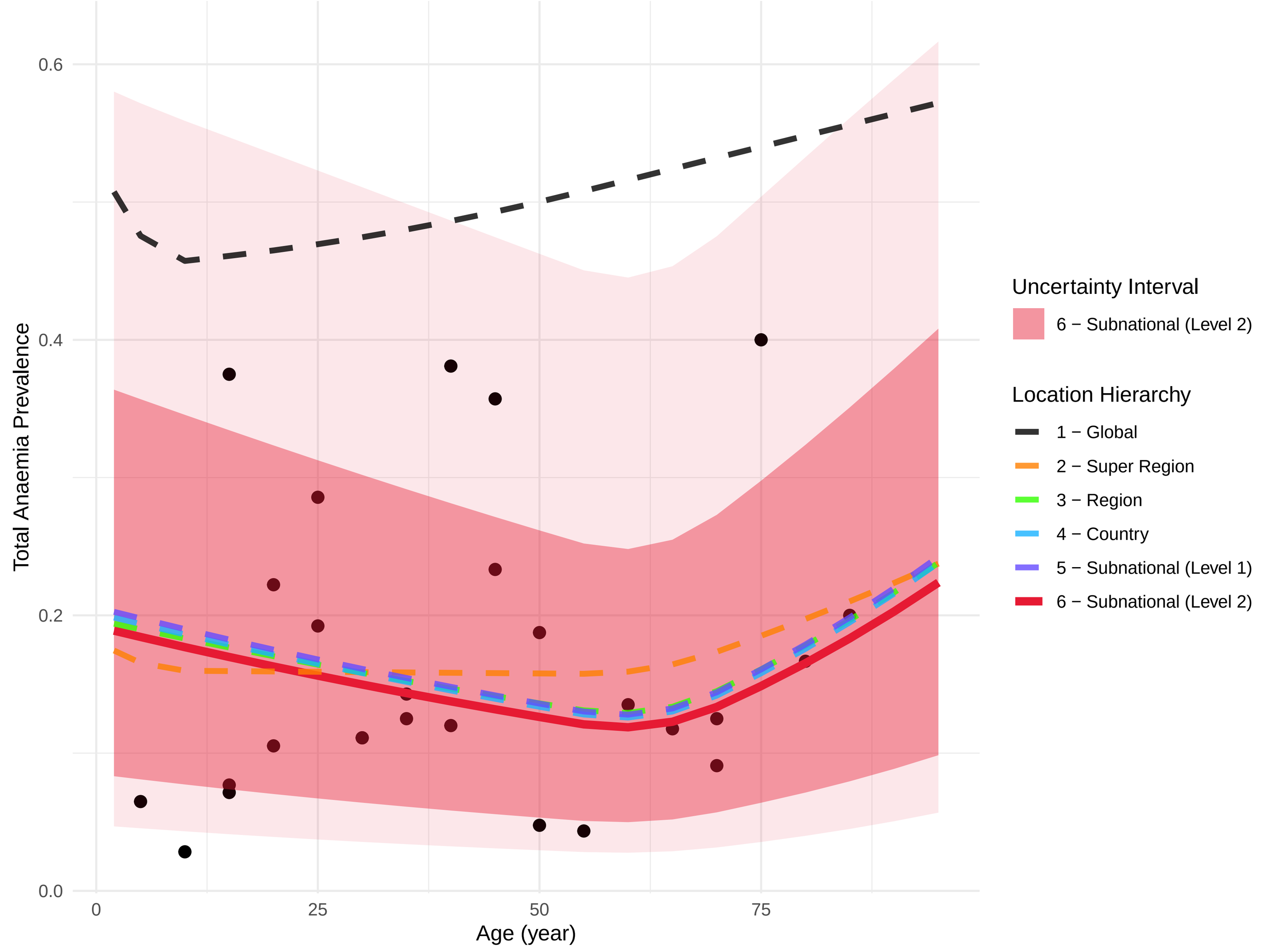

MR−BRT Age/Sex Fits − Non−pregnant Females 2+ Years Old − Total Anaemia Prevalence − South West England
WHO−2024 Adjustment Method

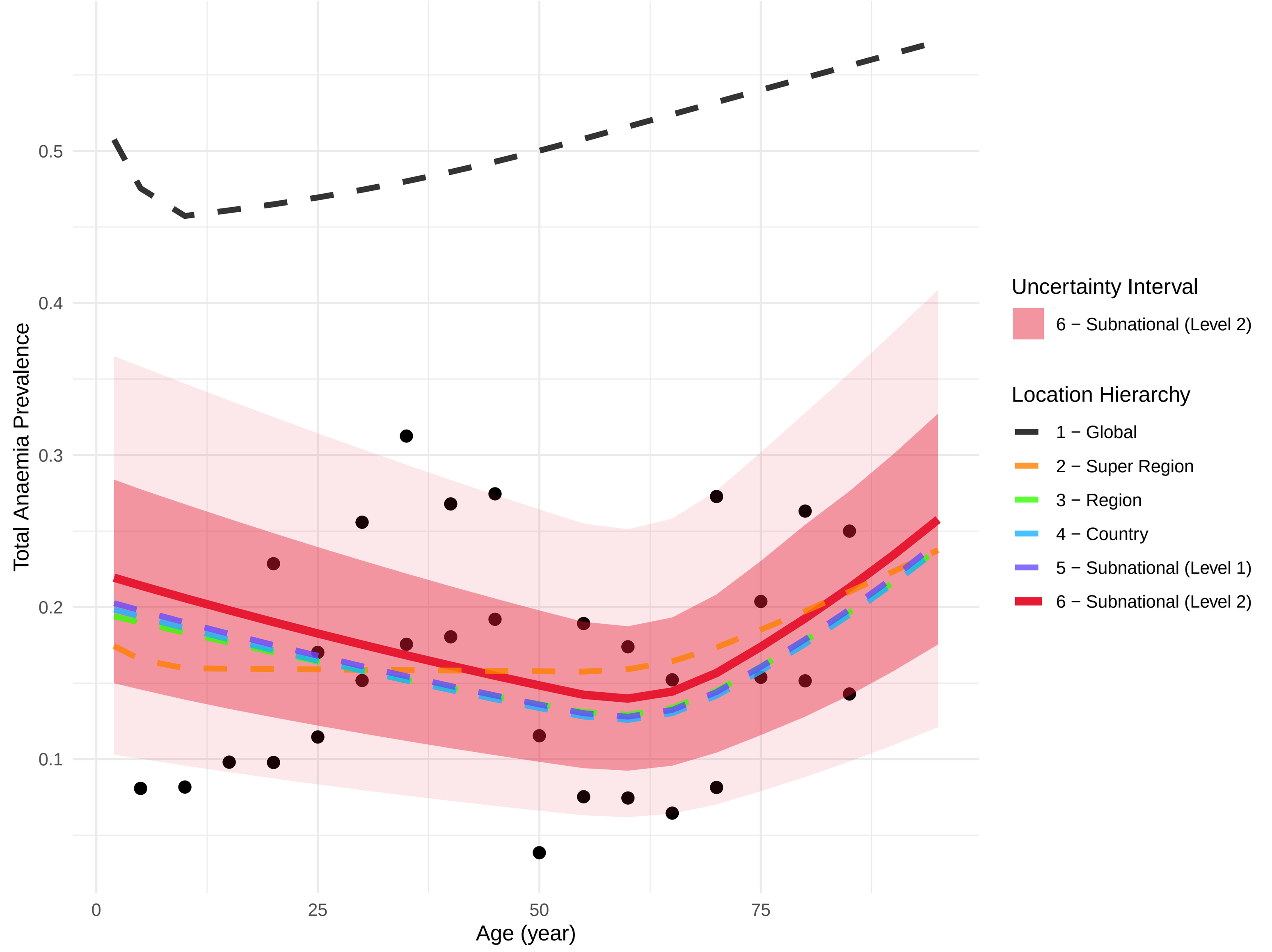

MR−BRT Age/Sex Fits − Non−pregnant Females 2+ Years Old − Total Anaemia Prevalence − West Midlands
WHO−2024 Adjustment Method

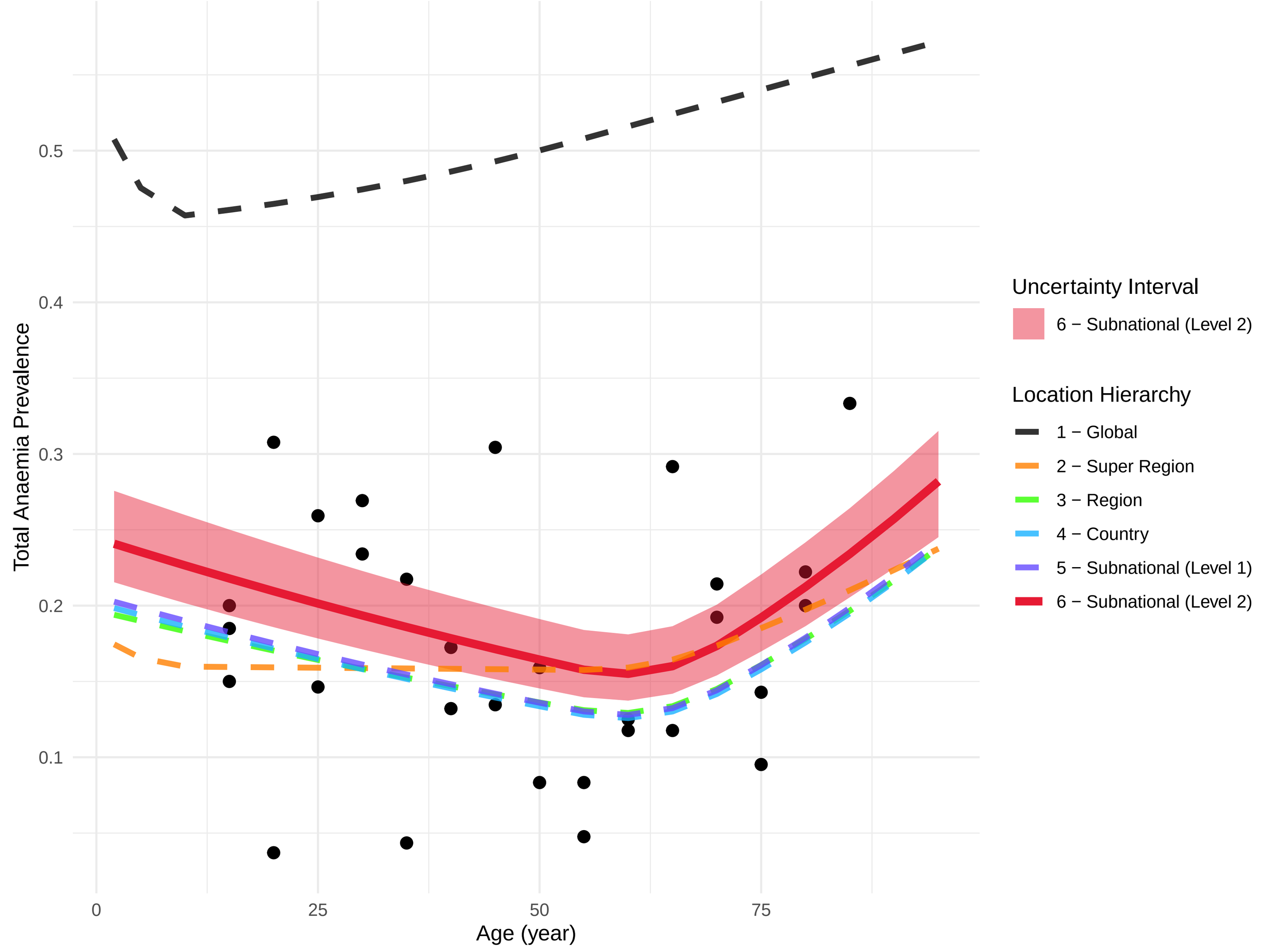

MR−BRT Age/Sex Fits − Non−pregnant Females 2+ Years Old − Total Anaemia Prevalence − Yorkshire and the Humber
WHO−2024 Adjustment Method

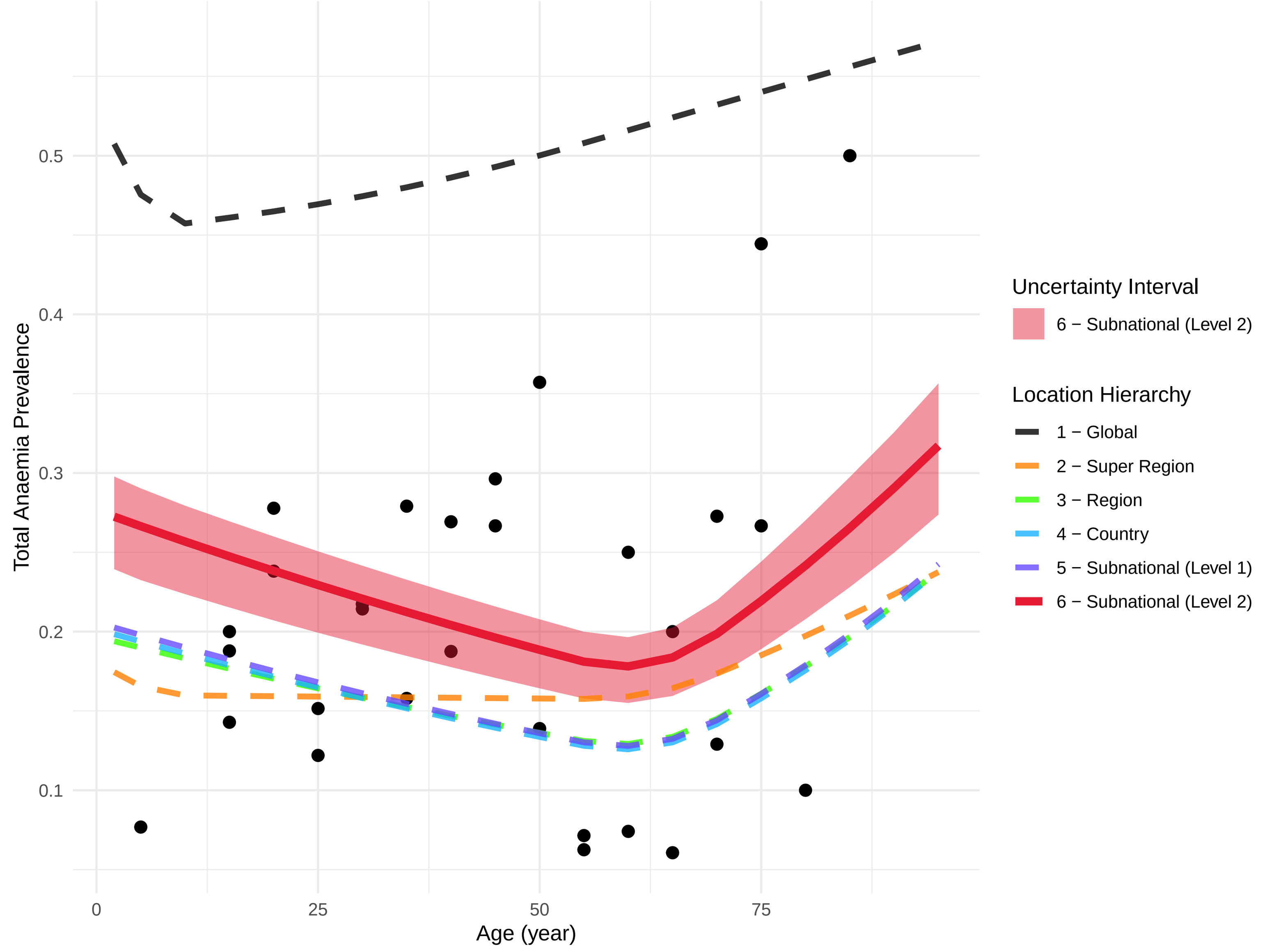

MR−BRT Age/Sex Fits − Non−pregnant Females 2+ Years Old − Total Anaemia Prevalence − Scotland
WHO−2024 Adjustment Method

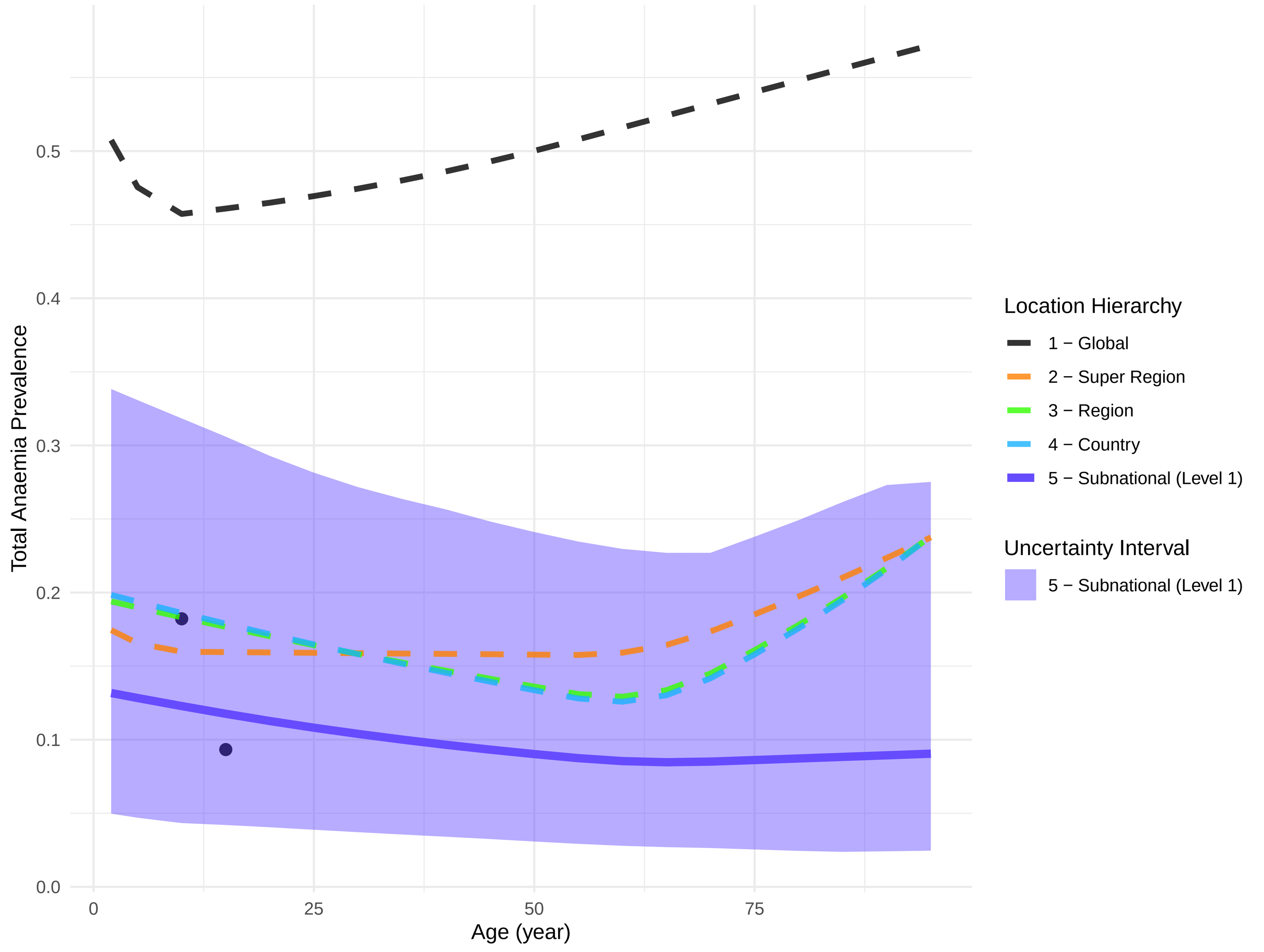

MR−BRT Age/Sex Fits − Non−pregnant Females 2+ Years Old − Total Anaemia Prevalence − Wales
WHO−2024 Adjustment Method

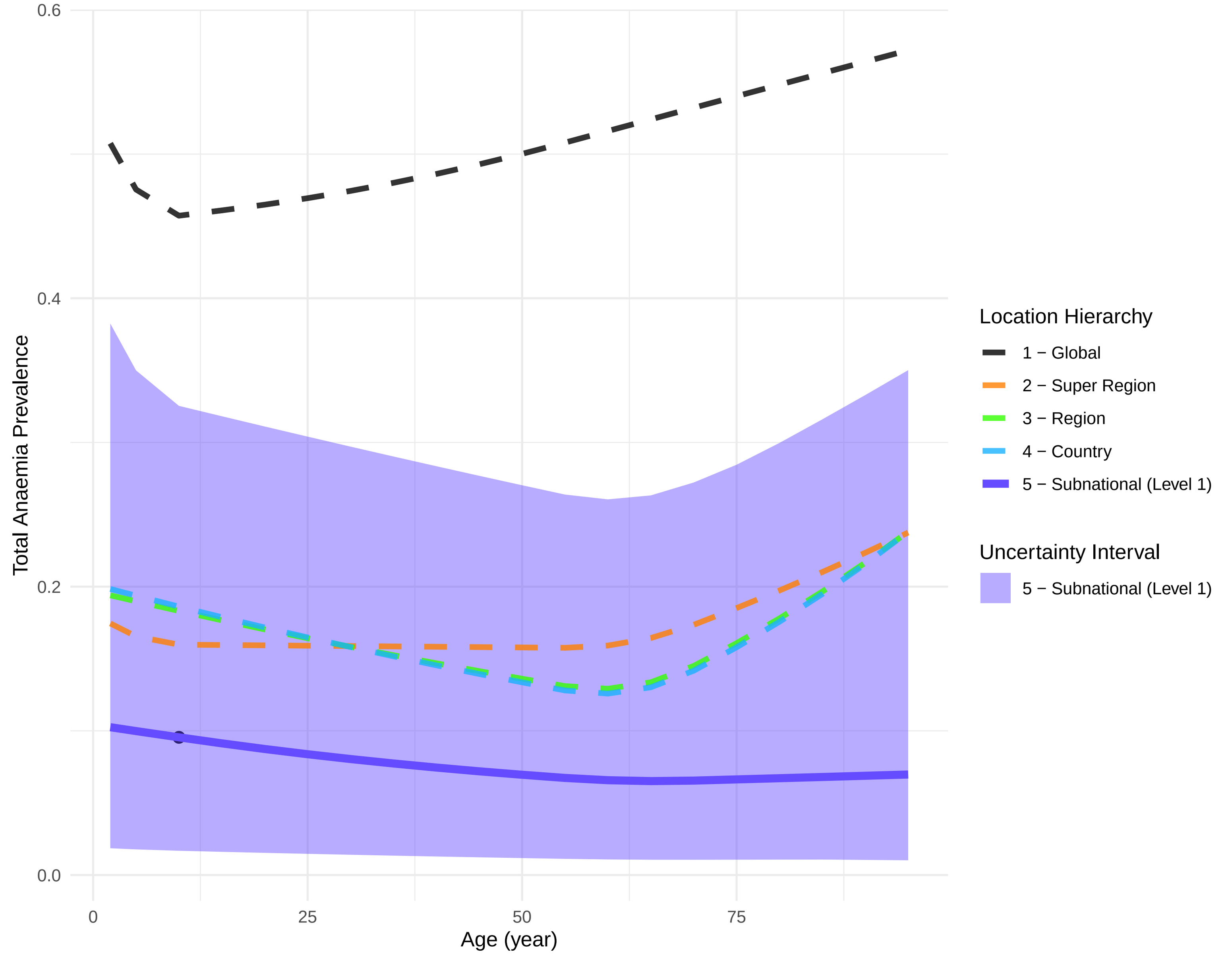

MR−BRT Age/Sex Fits − Non−pregnant Females 2+ Years Old − Total Anaemia Prevalence − Latin America and Caribbean
WHO−2024 Adjustment Method

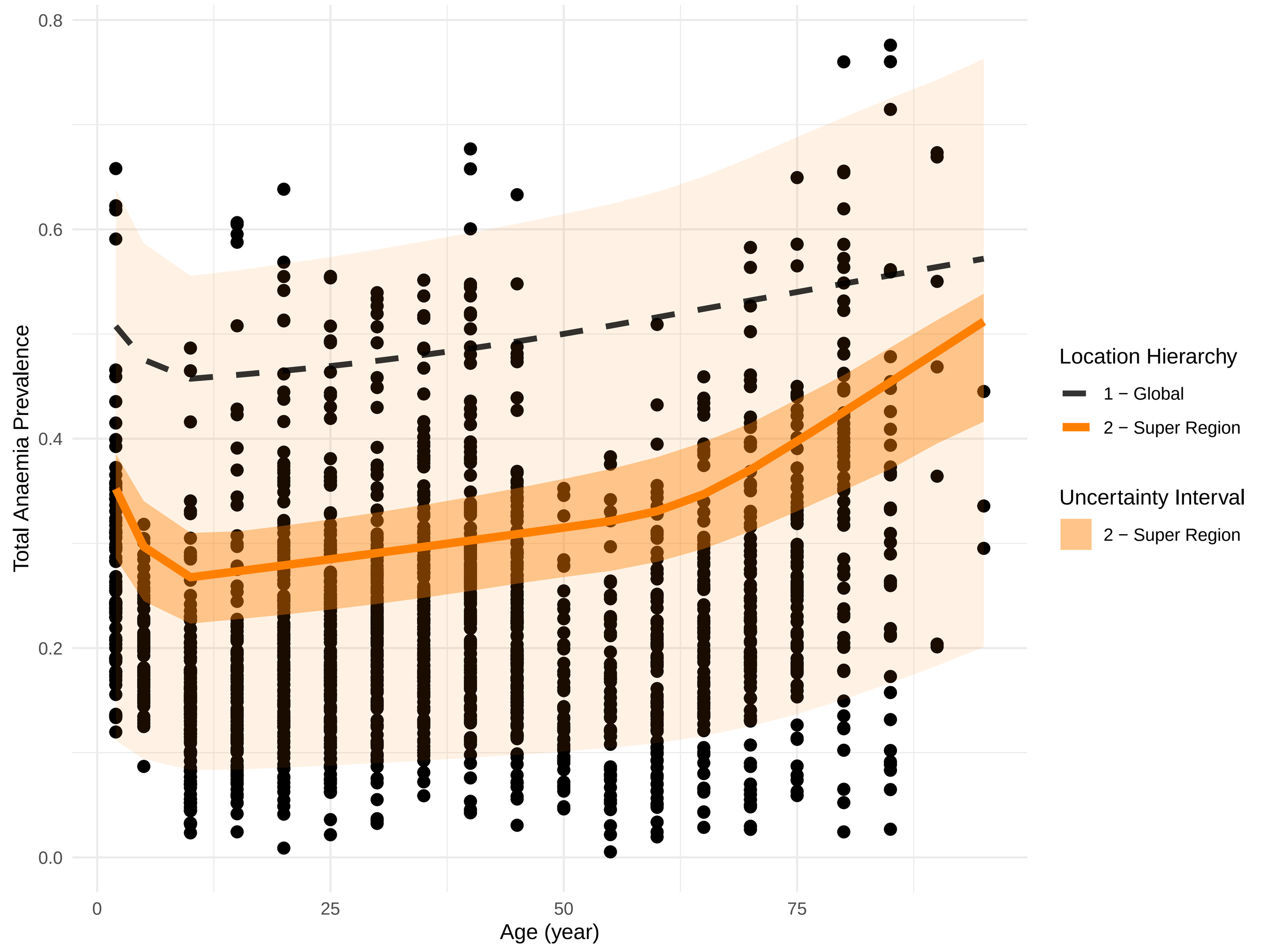

MR−BRT Age/Sex Fits − Non−pregnant Females 2+ Years Old − Total Anaemia Prevalence − Andean Latin America
WHO−2024 Adjustment Method

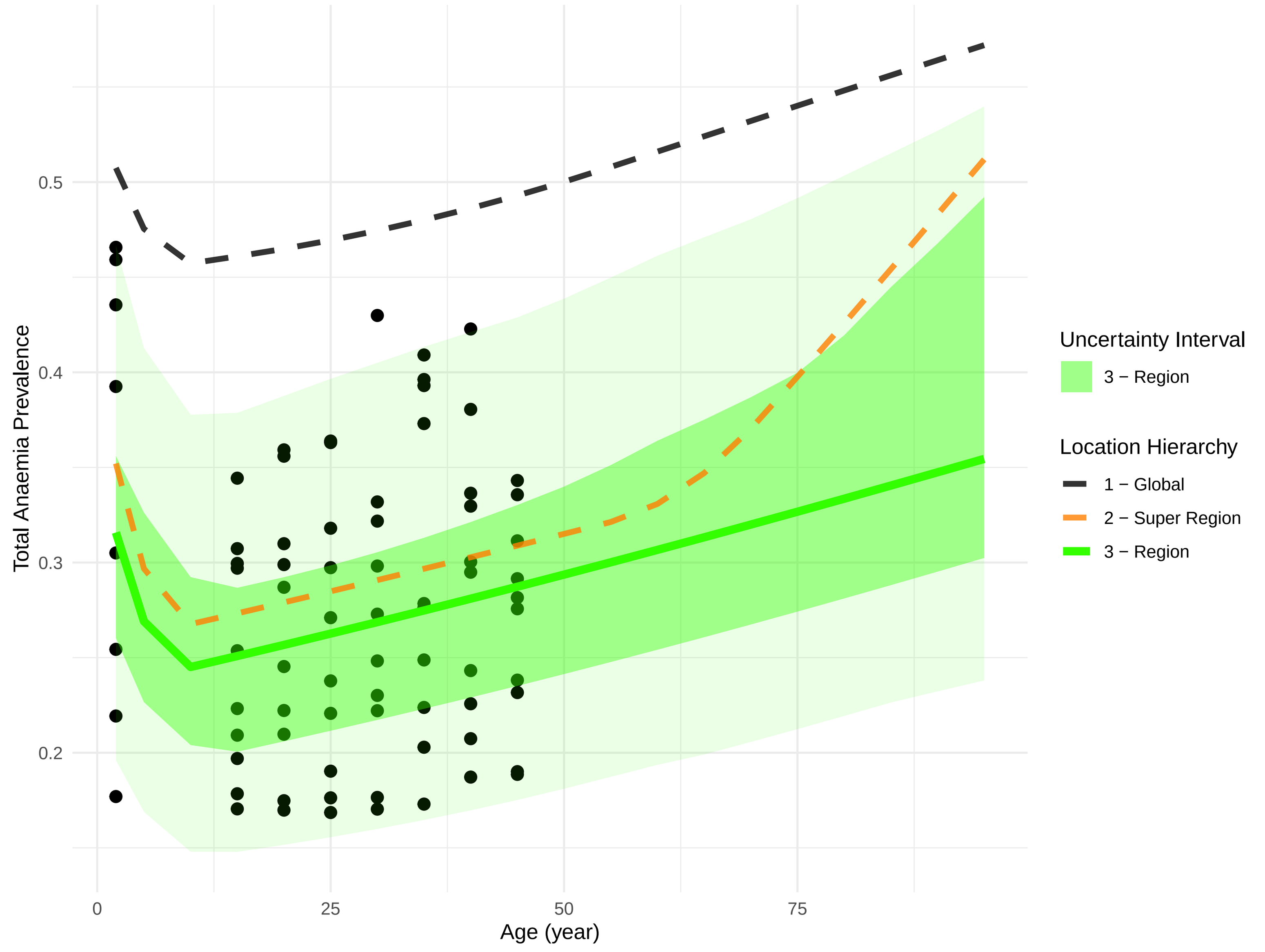

MR−BRT Age/Sex Fits − Non−pregnant Females 2+ Years Old − Total Anaemia Prevalence − Bolivia (Plurinational State of)
WHO−2024 Adjustment Method

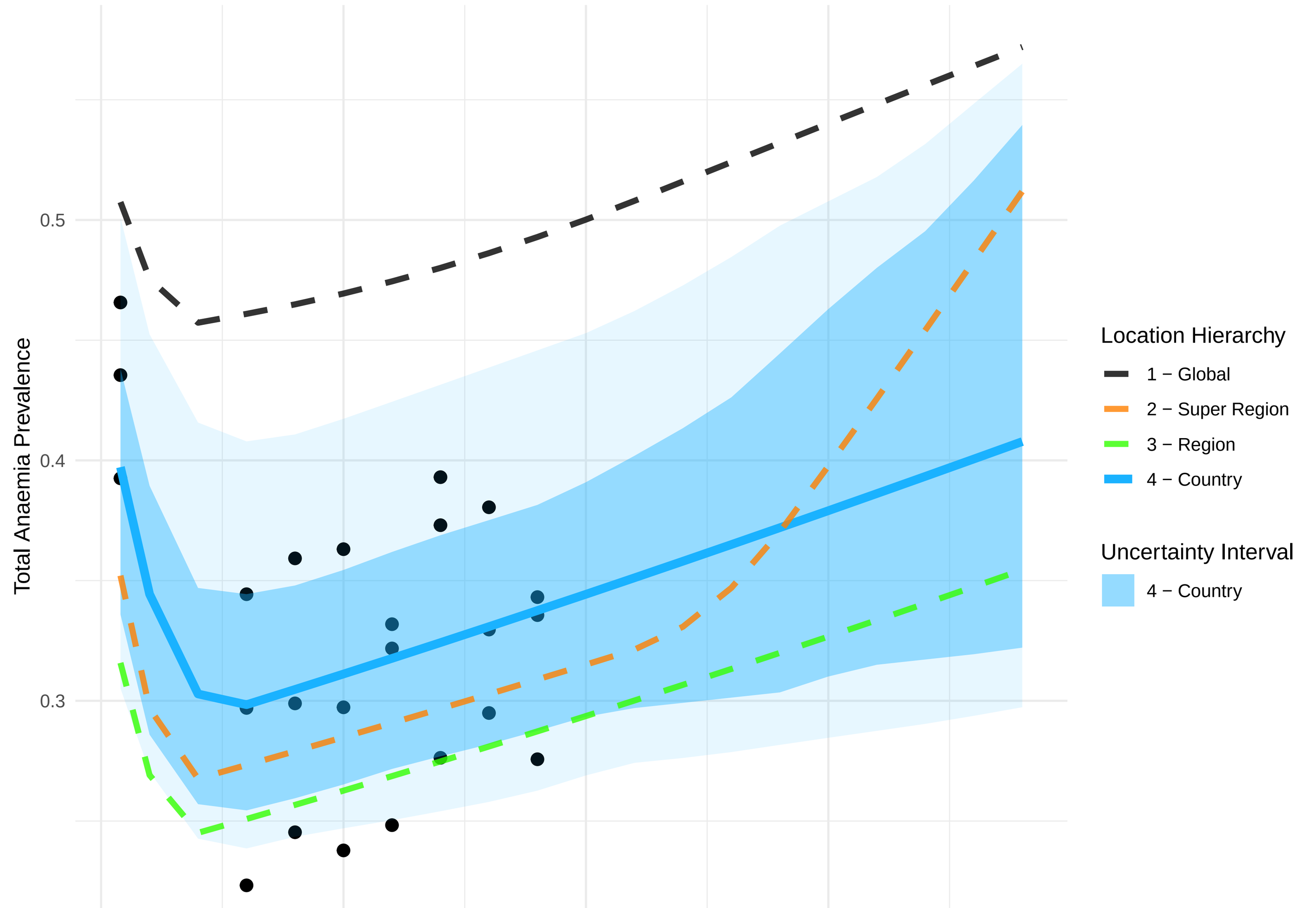

MR−BRT Age/Sex Fits − Non−pregnant Females 2+ Years Old − Total Anaemia Prevalence − Peru
WHO−2024 Adjustment Method

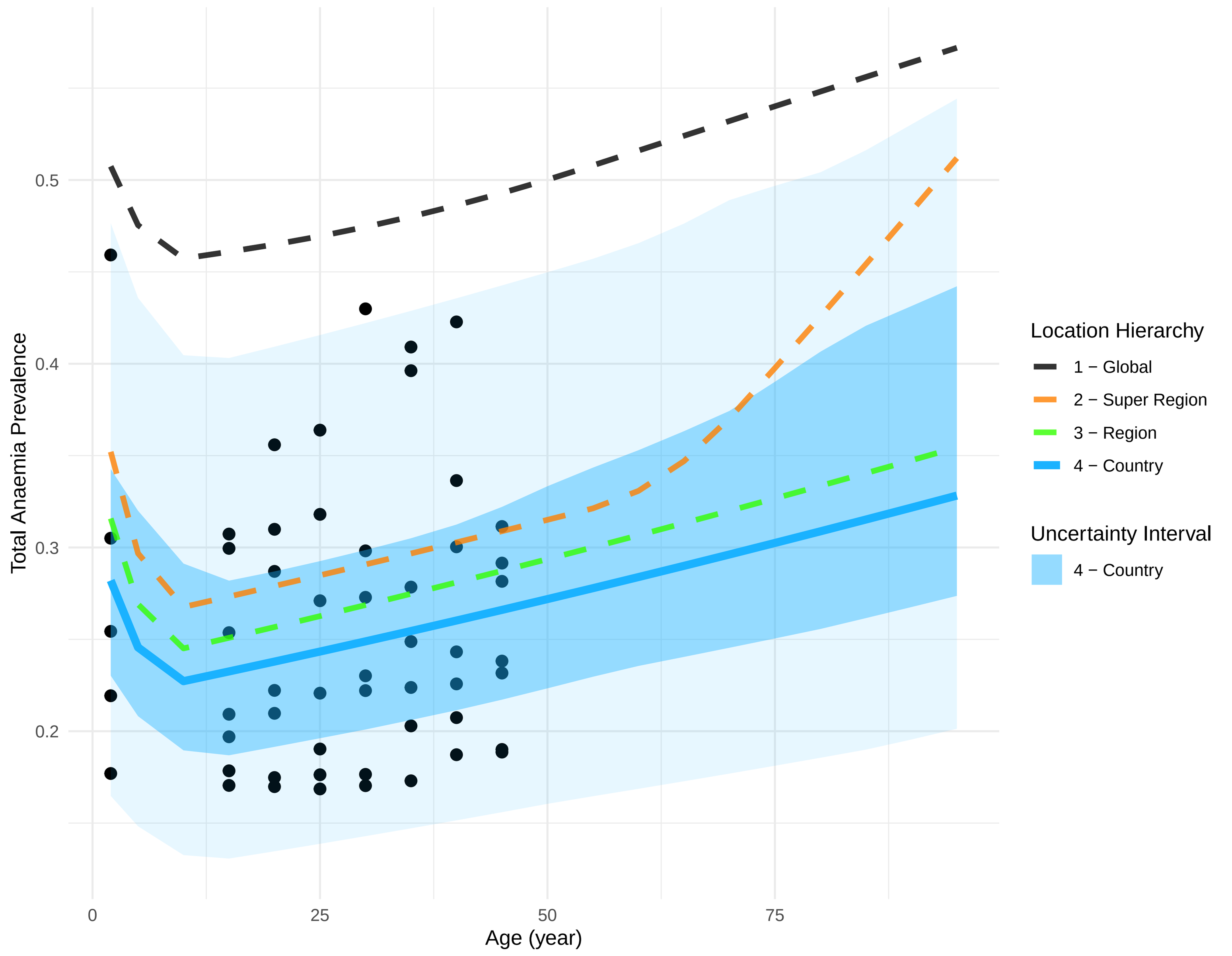

MR−BRT Age/Sex Fits − Non−pregnant Females 2+ Years Old − Total Anaemia Prevalence − Caribbean
WHO−2024 Adjustment Method

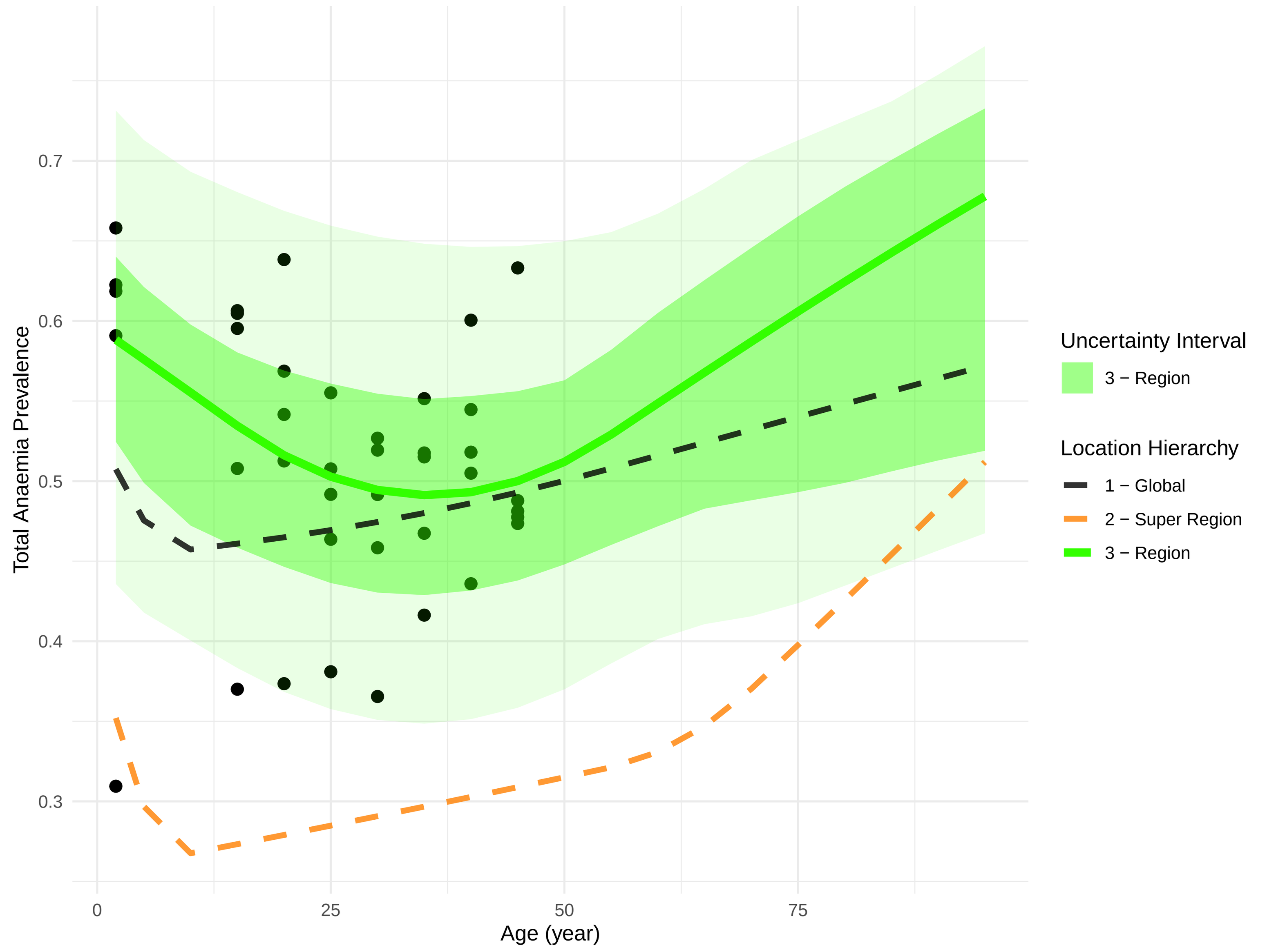

MR−BRT Age/Sex Fits − Non−pregnant Females 2+ Years Old − Total Anaemia Prevalence − Guyana
WHO−2024 Adjustment Method

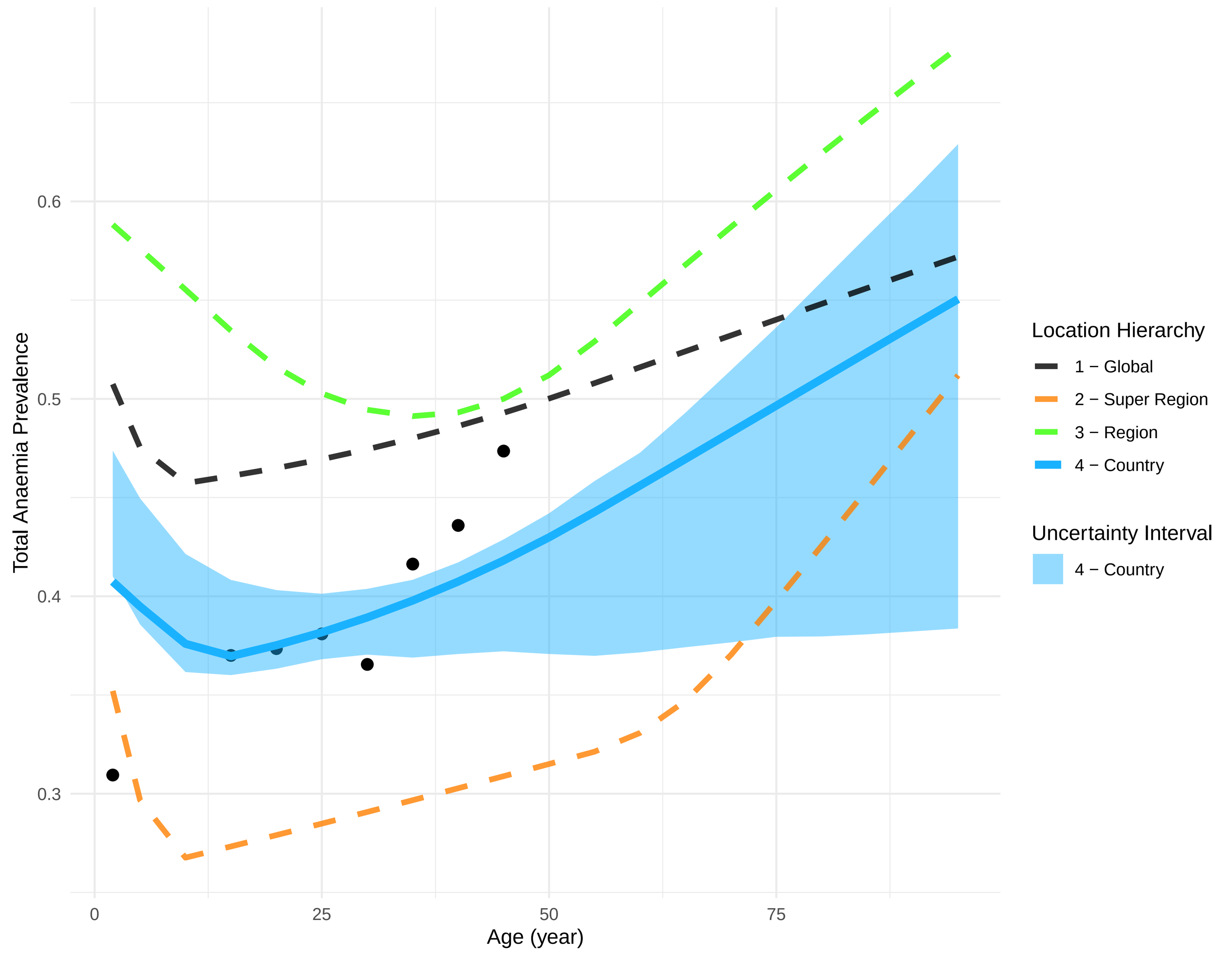

MR−BRT Age/Sex Fits − Non−pregnant Females 2+ Years Old − Total Anaemia Prevalence − Haiti
WHO−2024 Adjustment Method

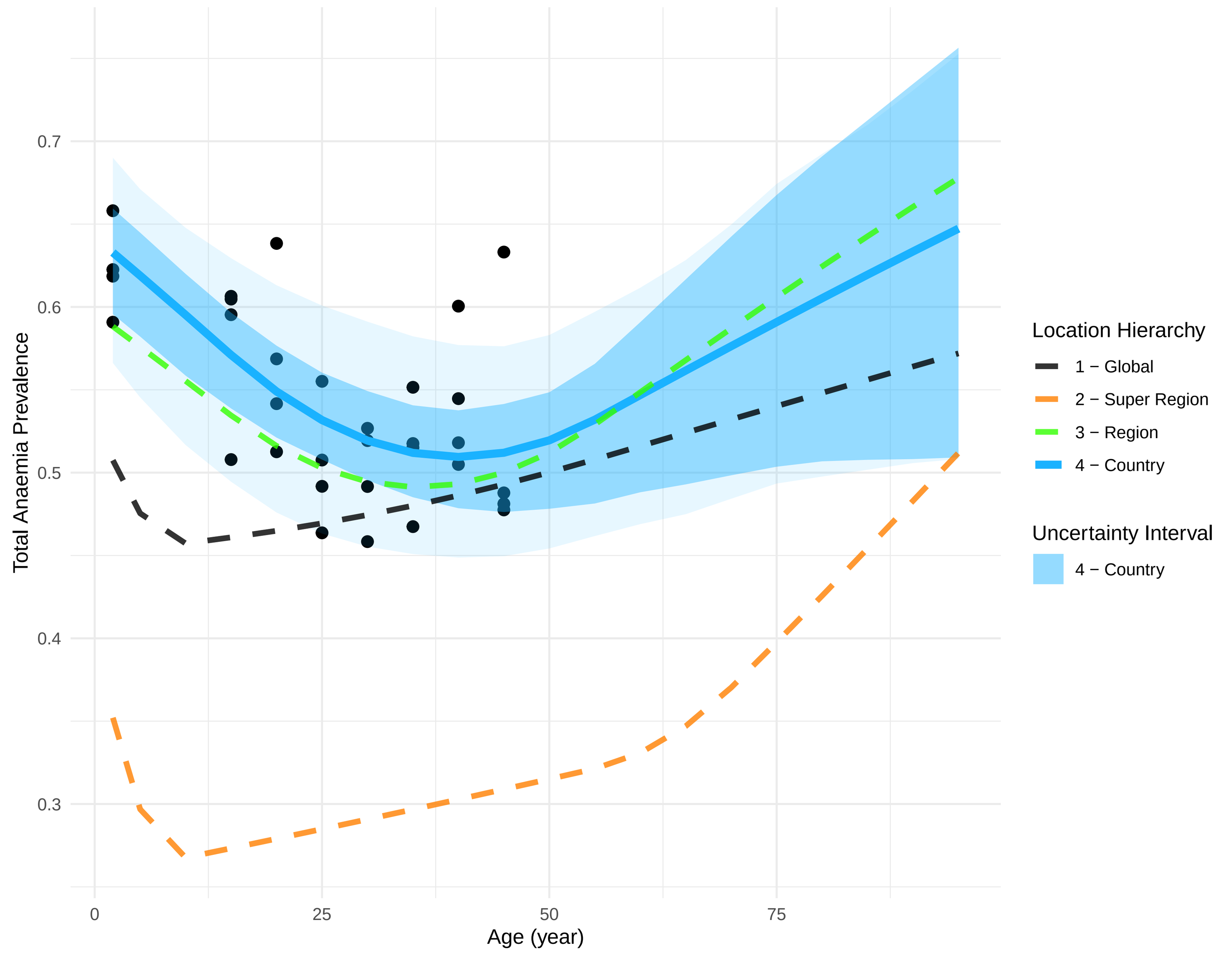

MR−BRT Age/Sex Fits − Non−pregnant Females 2+ Years Old − Total Anaemia Prevalence − Central Latin America
WHO−2024 Adjustment Method

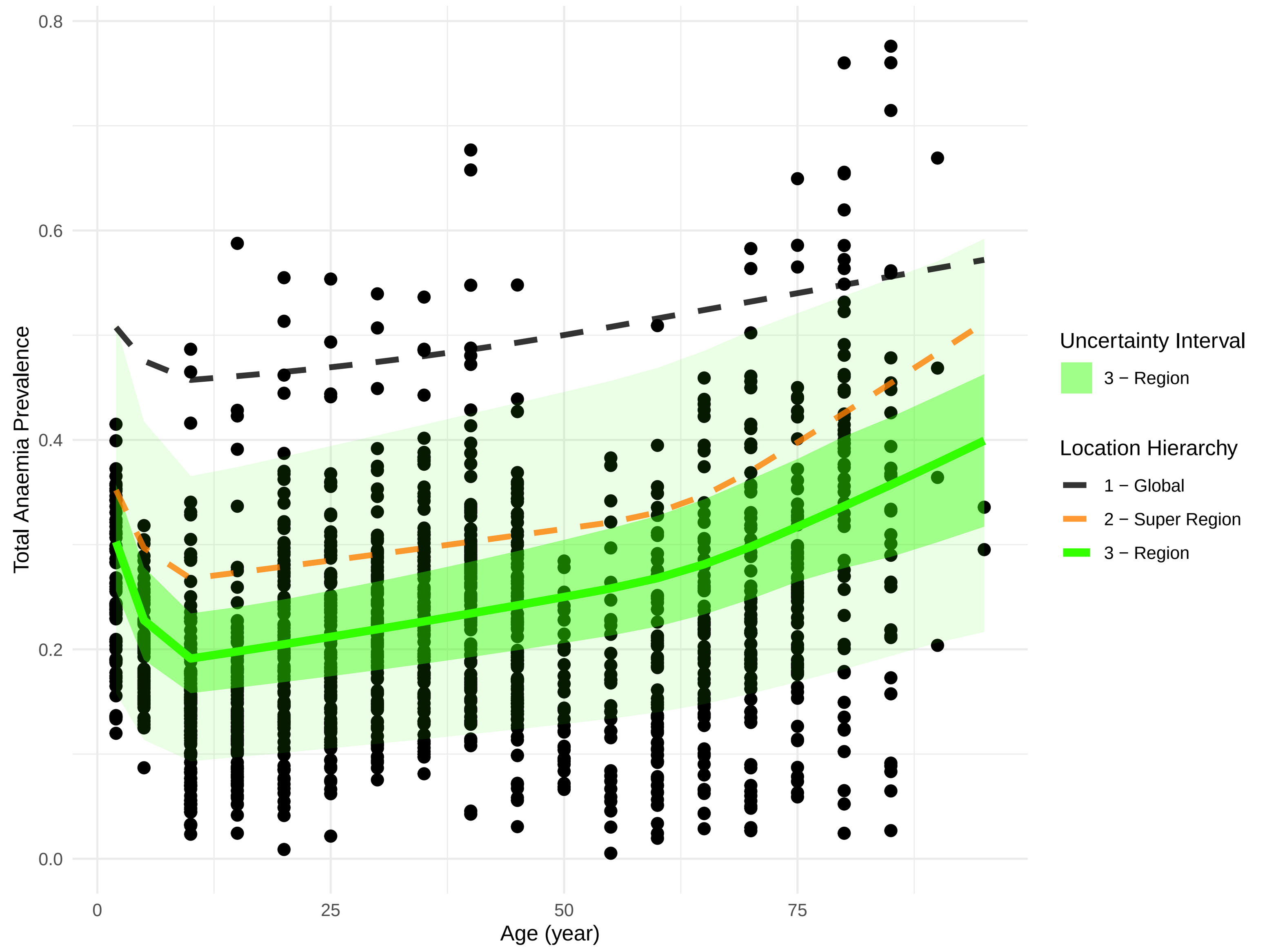

MR−BRT Age/Sex Fits − Non−pregnant Females 2+ Years Old − Total Anaemia Prevalence − El Salvador
WHO−2024 Adjustment Method

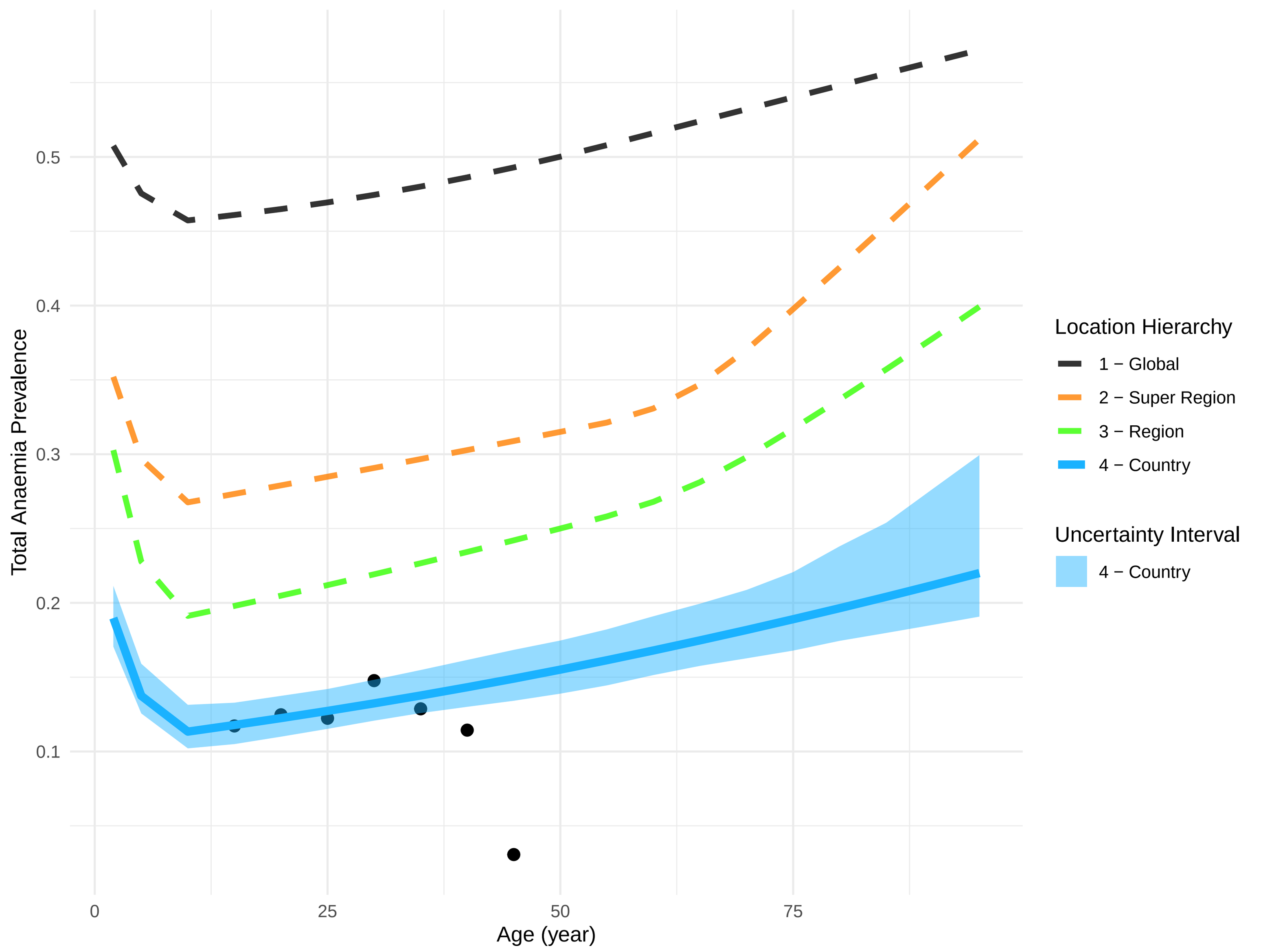

MR−BRT Age/Sex Fits − Non−pregnant Females 2+ Years Old − Total Anaemia Prevalence − Guatemala
WHO−2024 Adjustment Method

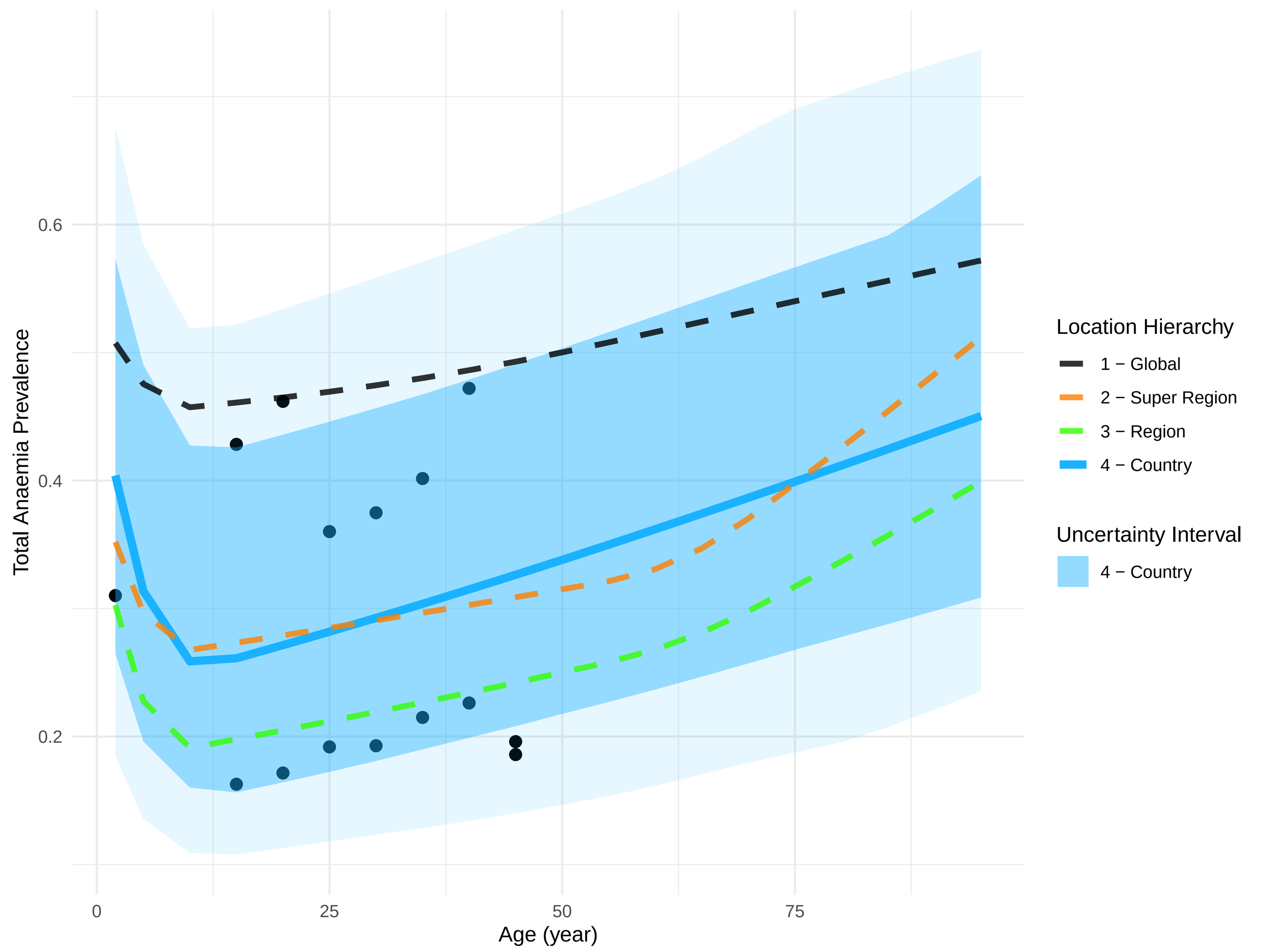

MR−BRT Age/Sex Fits − Non−pregnant Females 2+ Years Old − Total Anaemia Prevalence − Honduras
WHO−2024 Adjustment Method

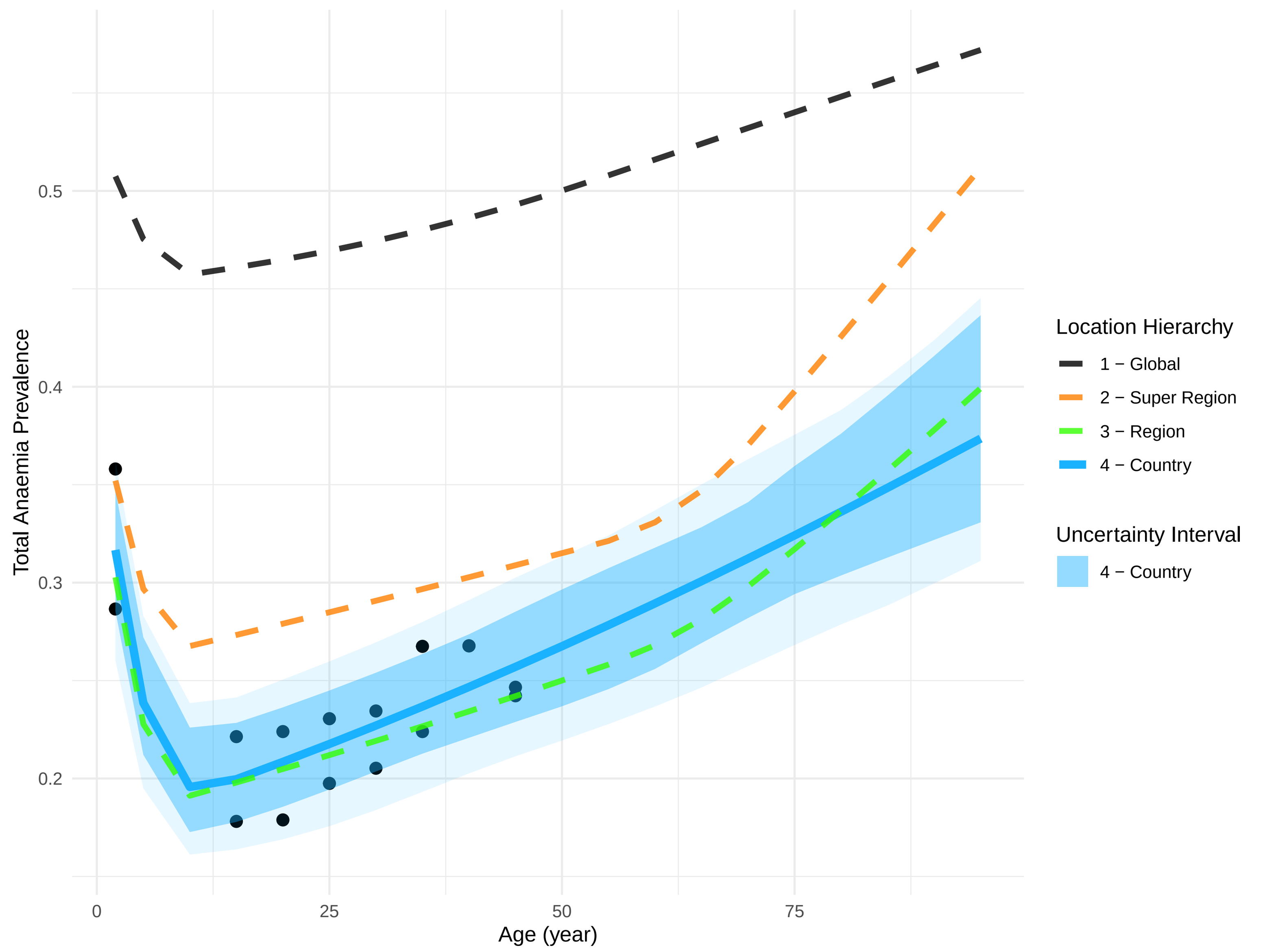

MR−BRT Age/Sex Fits − Non−pregnant Females 2+ Years Old − Total Anaemia Prevalence − Mexico
WHO−2024 Adjustment Method

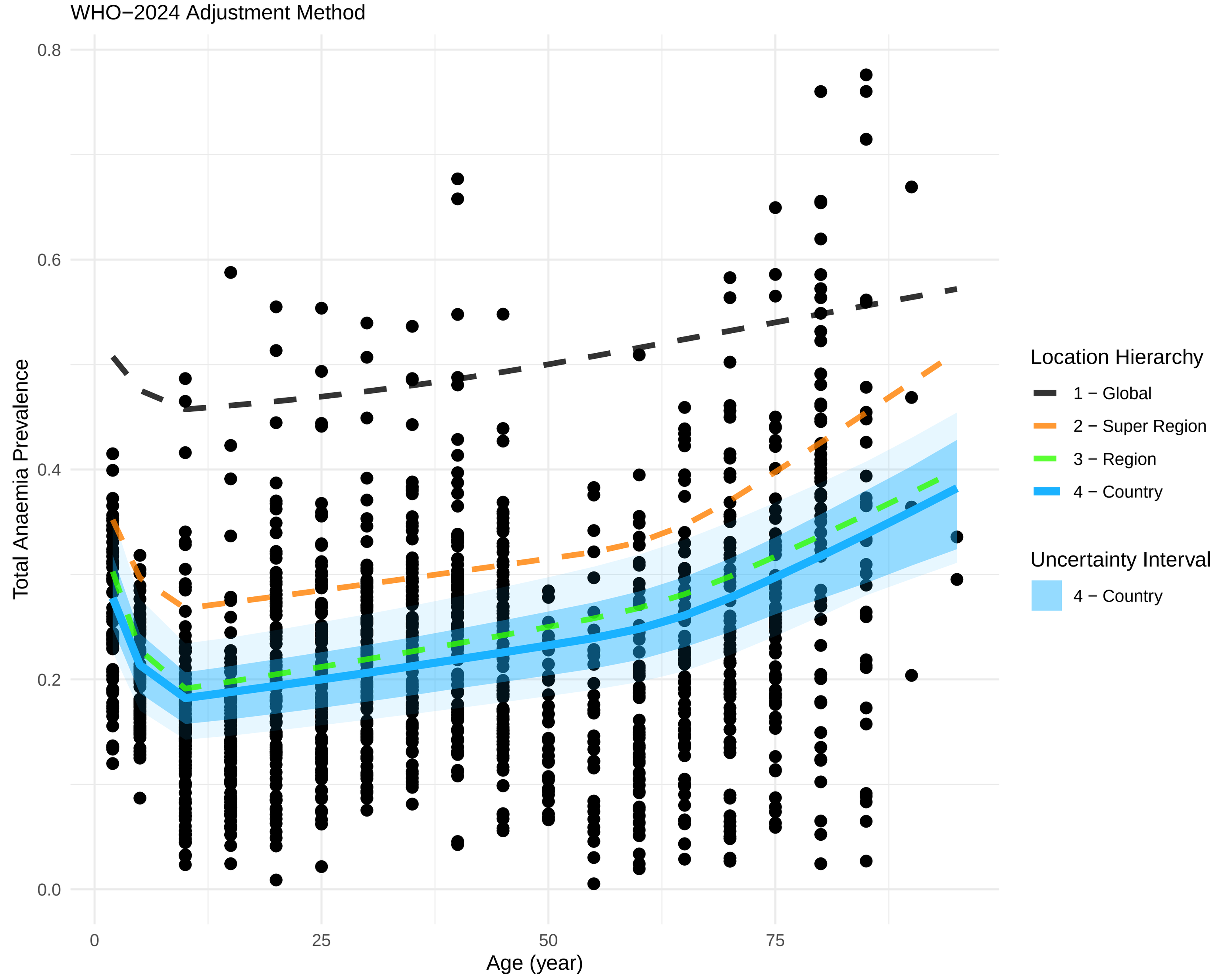

MR−BRT Age/Sex Fits − Non−pregnant Females 2+ Years Old − Total Anaemia Prevalence − Aguascalientes
WHO−2024 Adjustment Method

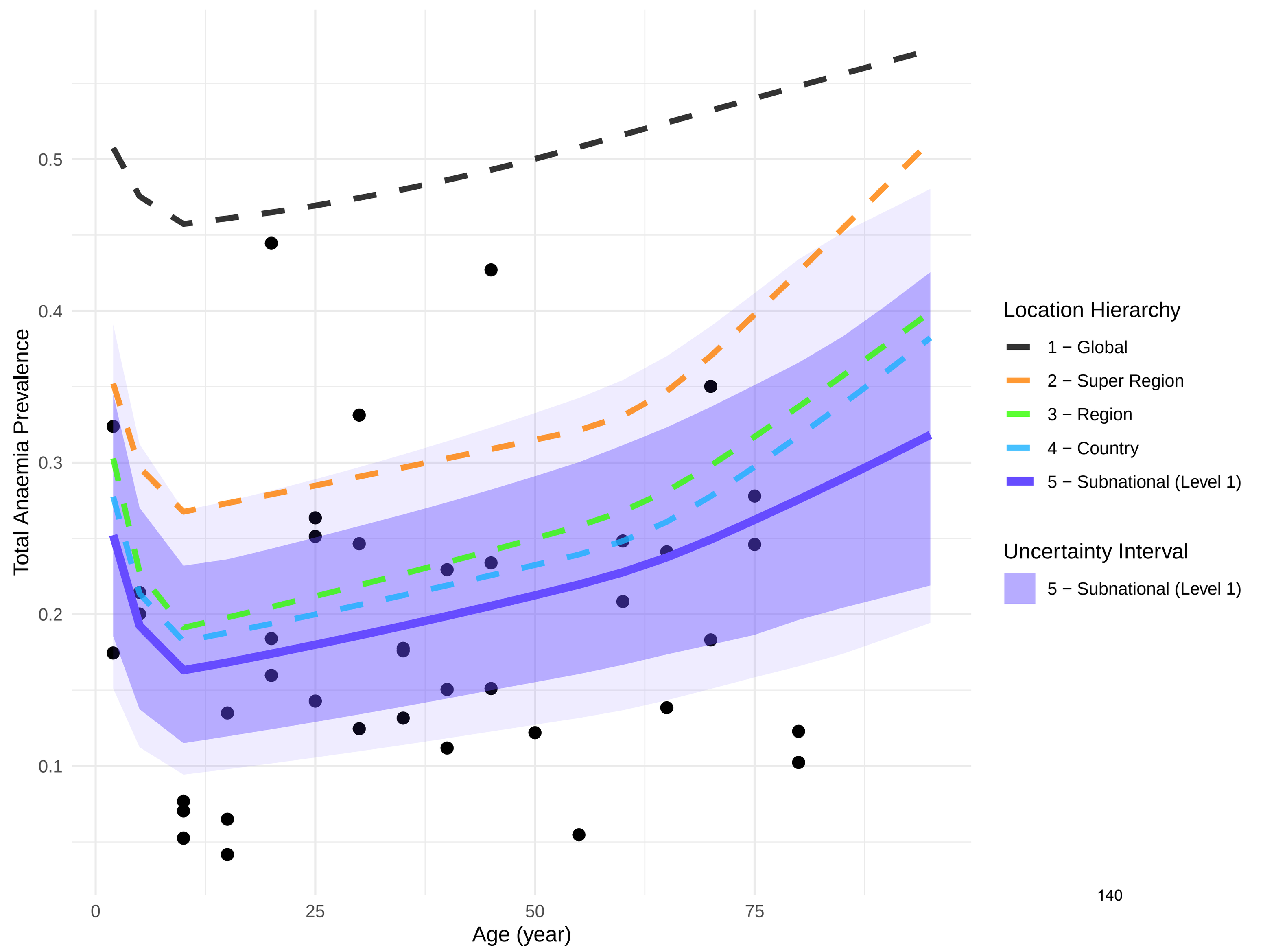

MR−BRT Age/Sex Fits − Non−pregnant Females 2+ Years Old − Total Anaemia Prevalence − Baja California
WHO−2024 Adjustment Method

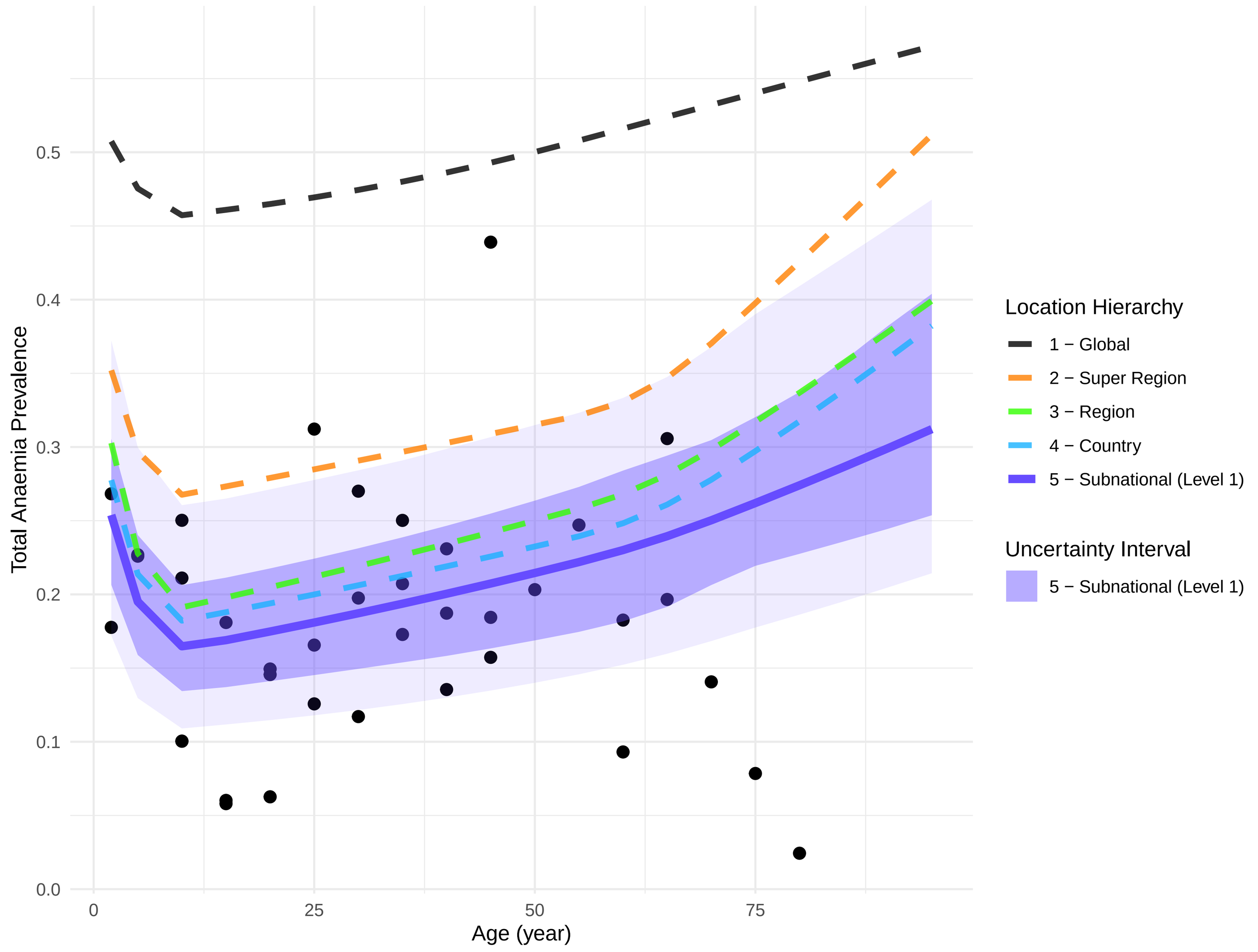

MR−BRT Age/Sex Fits − Non−pregnant Females 2+ Years Old − Total Anaemia Prevalence − Baja California Sur
WHO−2024 Adjustment Method

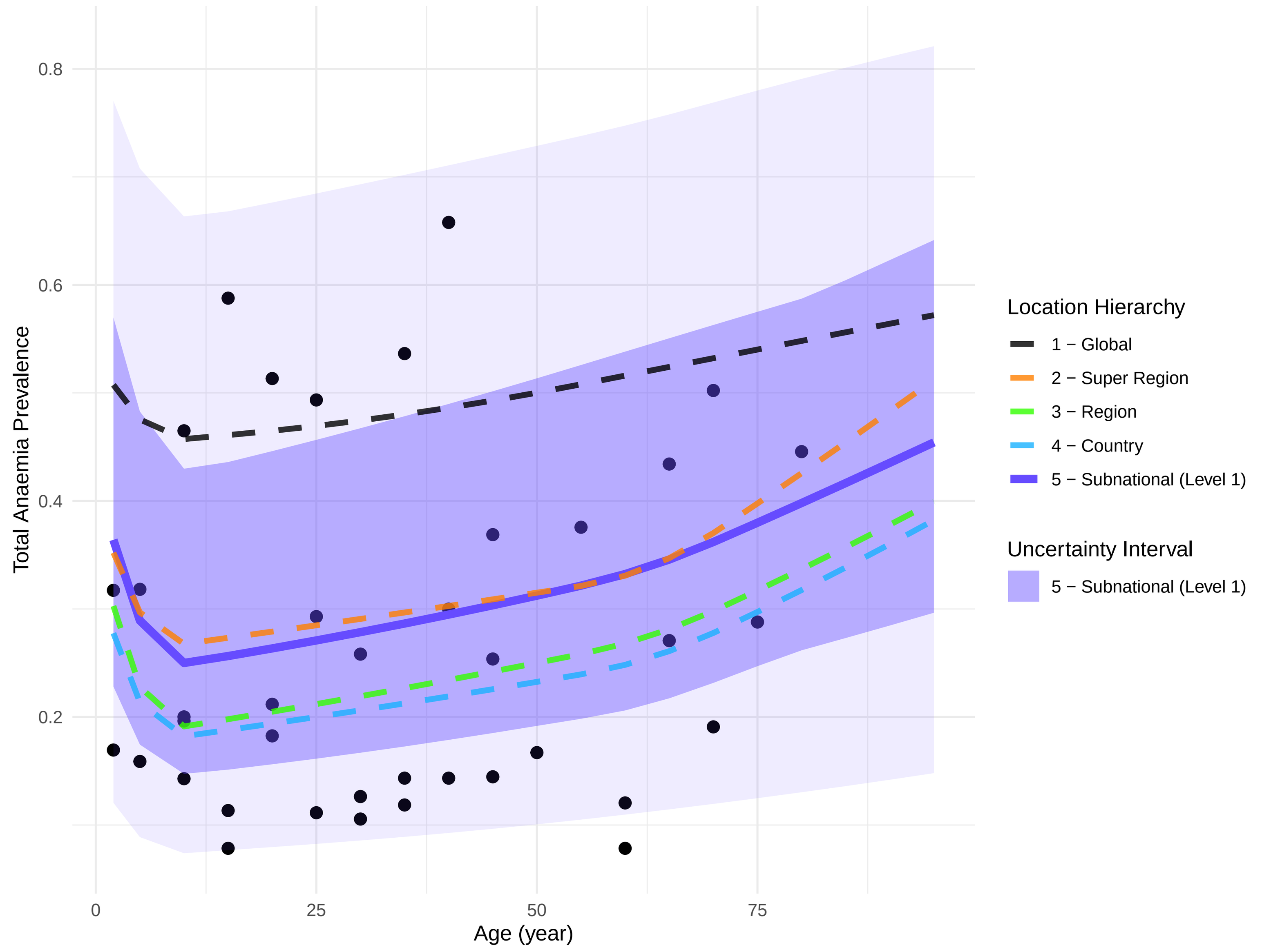

MR−BRT Age/Sex Fits − Non−pregnant Females 2+ Years Old − Total Anaemia Prevalence − Campeche
WHO−2024 Adjustment Method

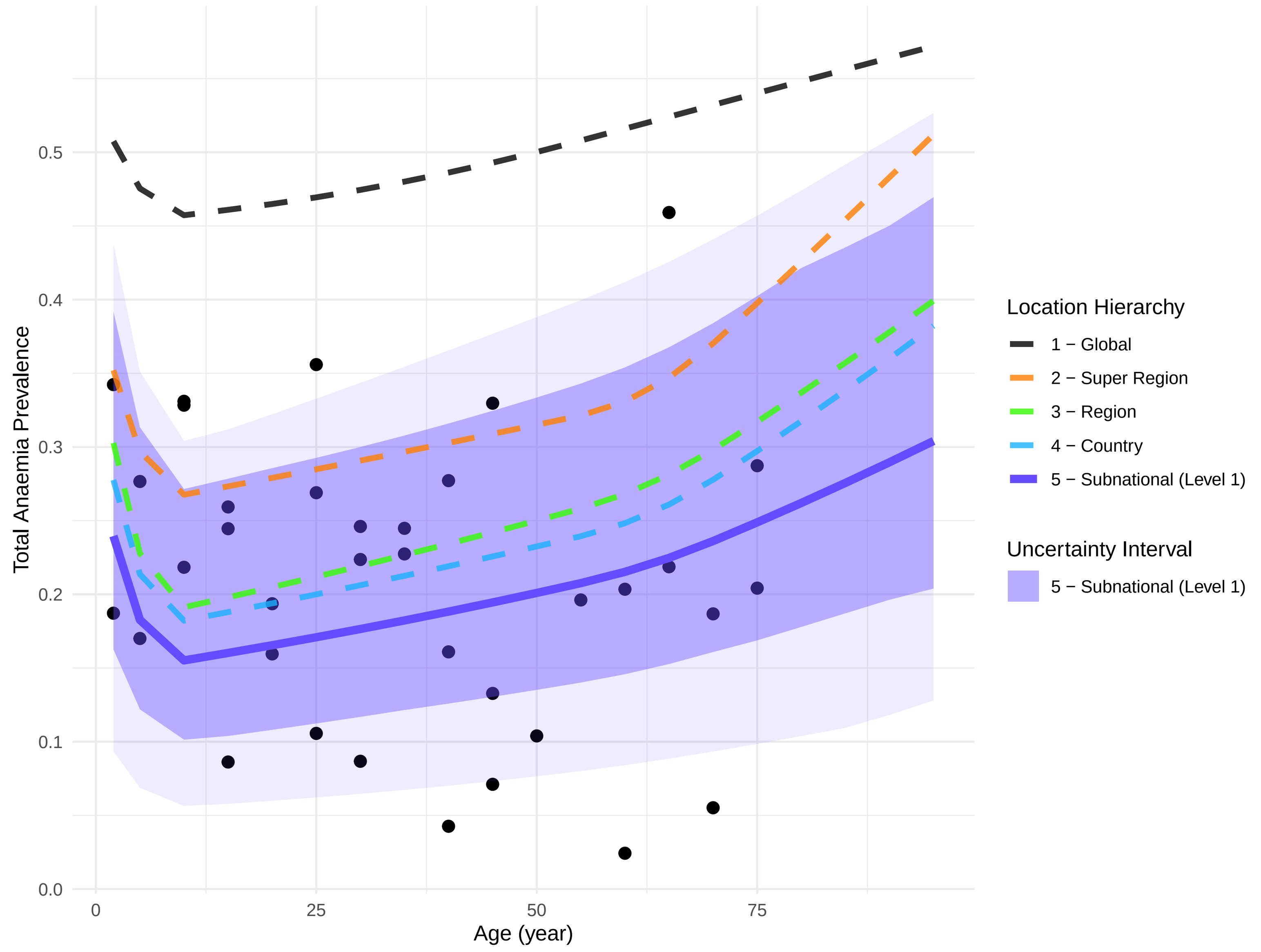

MR−BRT Age/Sex Fits − Non−pregnant Females 2+ Years Old − Total Anaemia Prevalence − Chiapas
WHO−2024 Adjustment Method

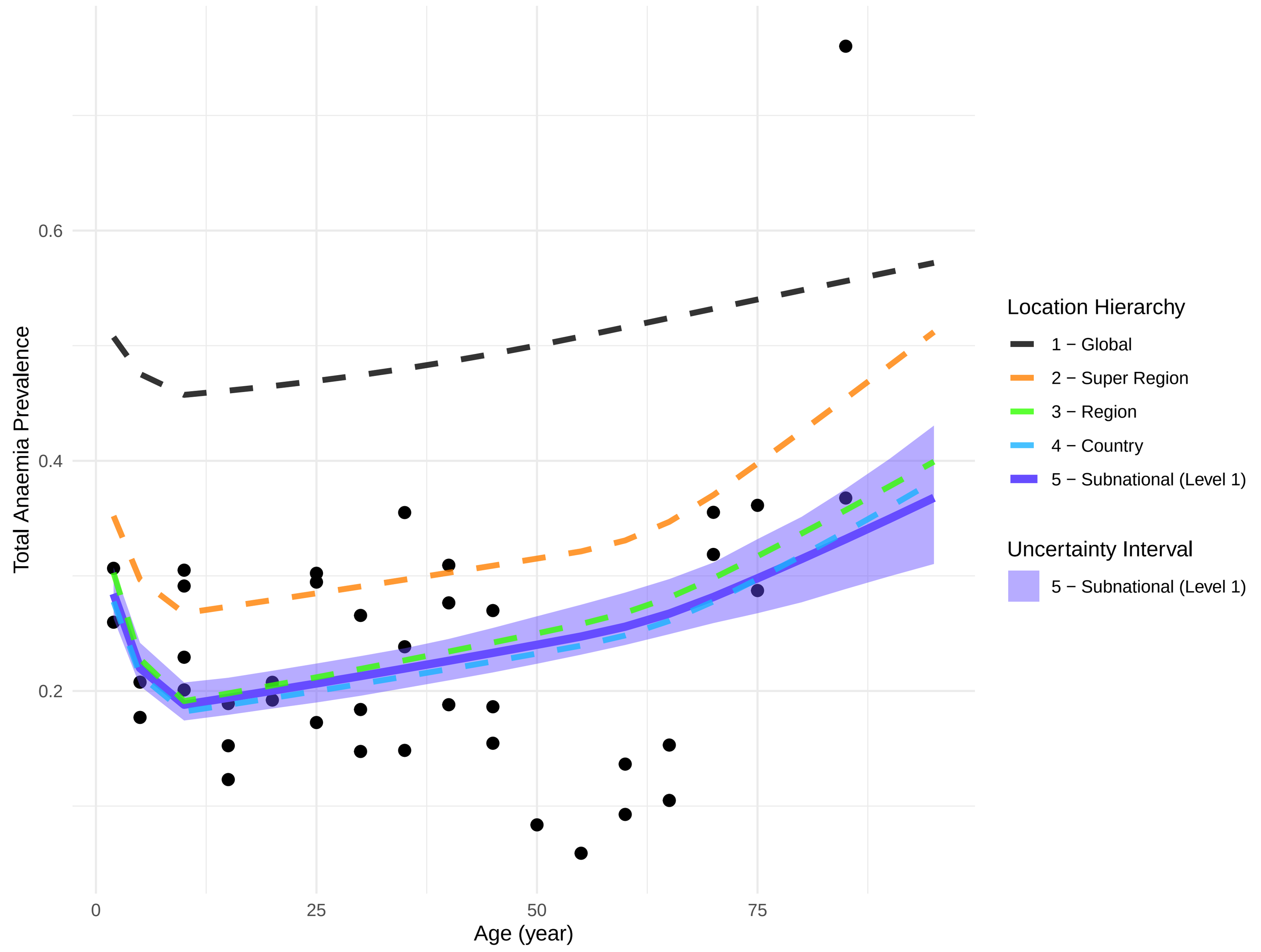

MR−BRT Age/Sex Fits − Non−pregnant Females 2+ Years Old − Total Anaemia Prevalence − Chihuahua
WHO−2024 Adjustment Method

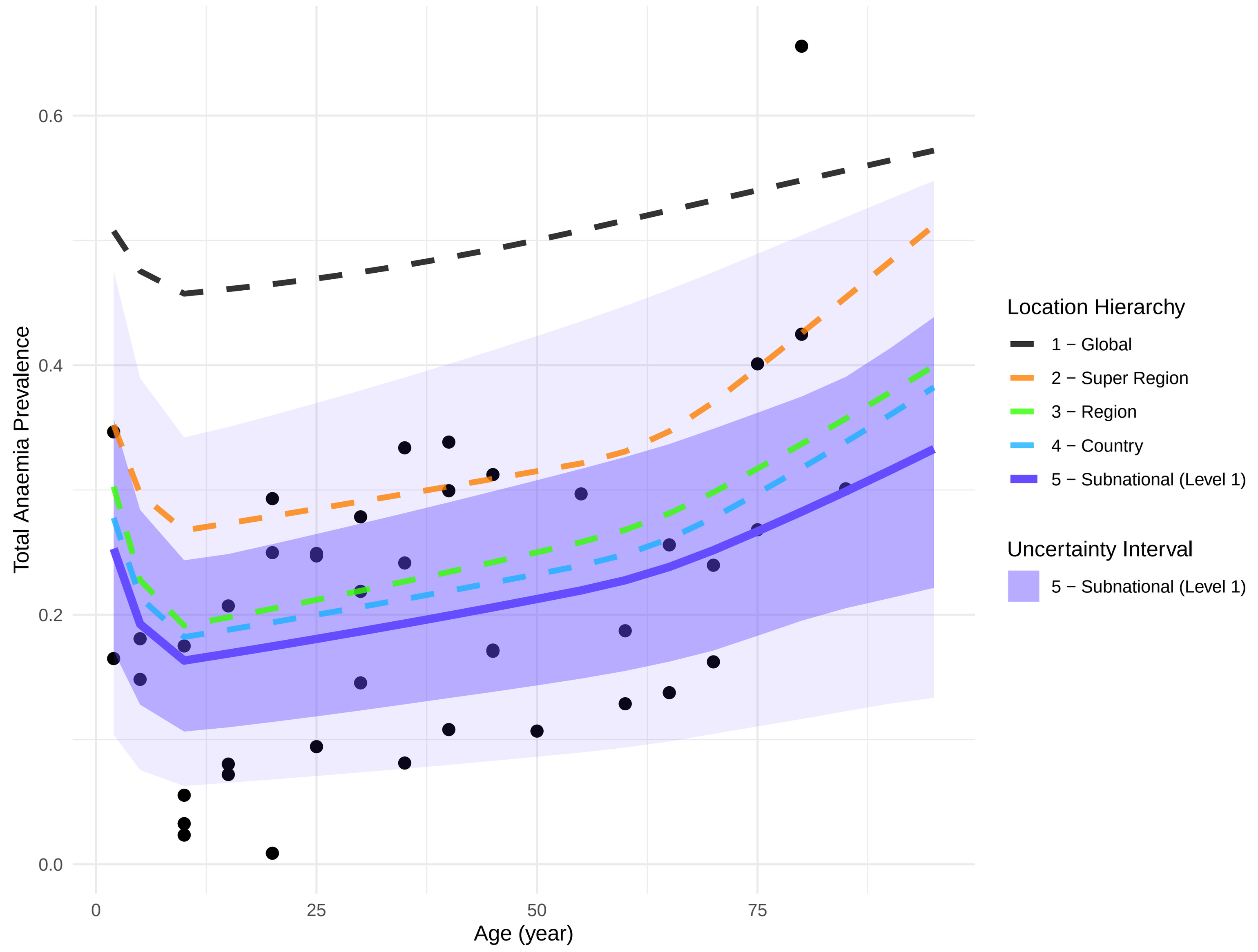

MR−BRT Age/Sex Fits − Non−pregnant Females 2+ Years Old − Total Anaemia Prevalence − Coahuila
WHO−2024 Adjustment Method

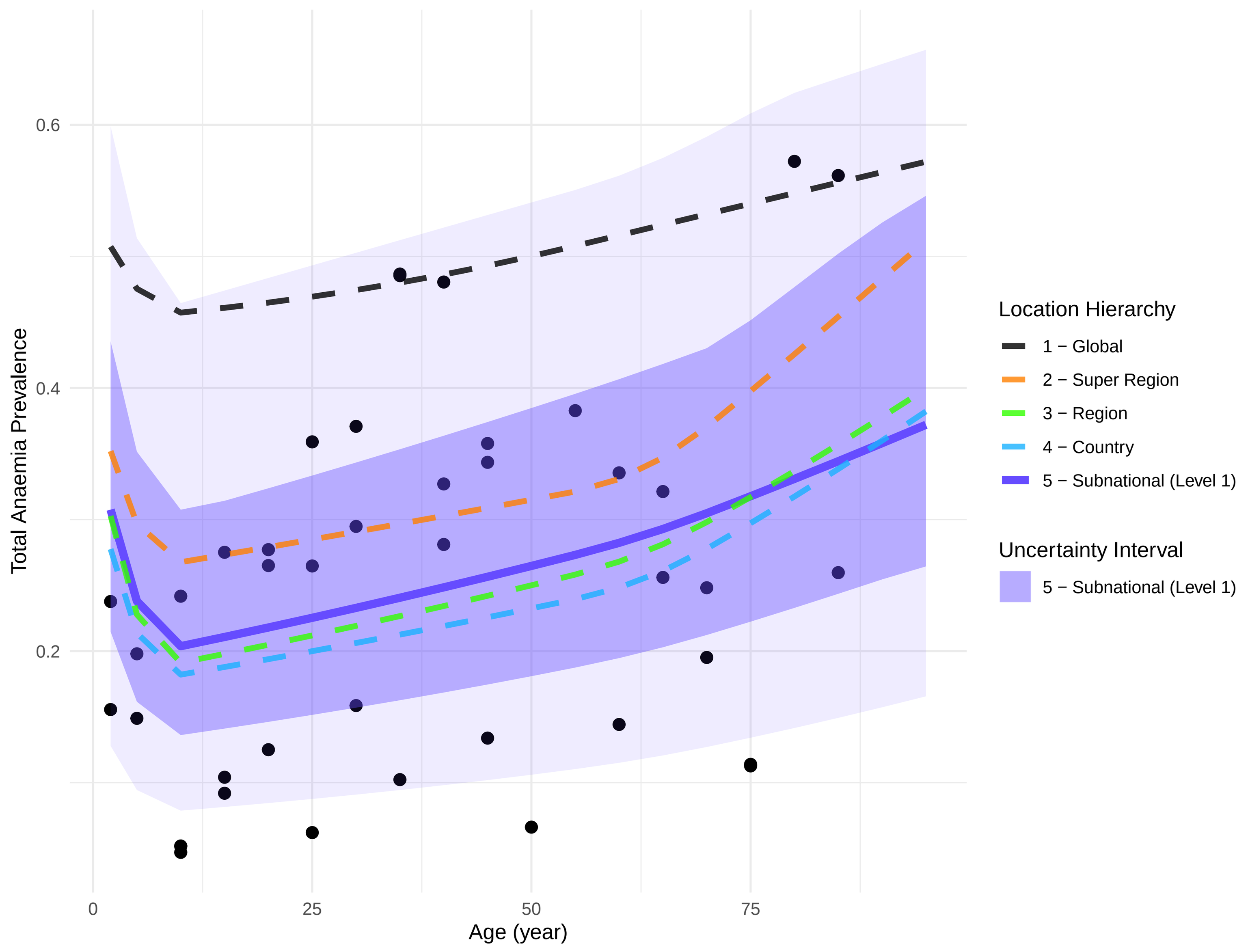

MR−BRT Age/Sex Fits − Non−pregnant Females 2+ Years Old − Total Anaemia Prevalence − Colima
WHO−2024 Adjustment Method

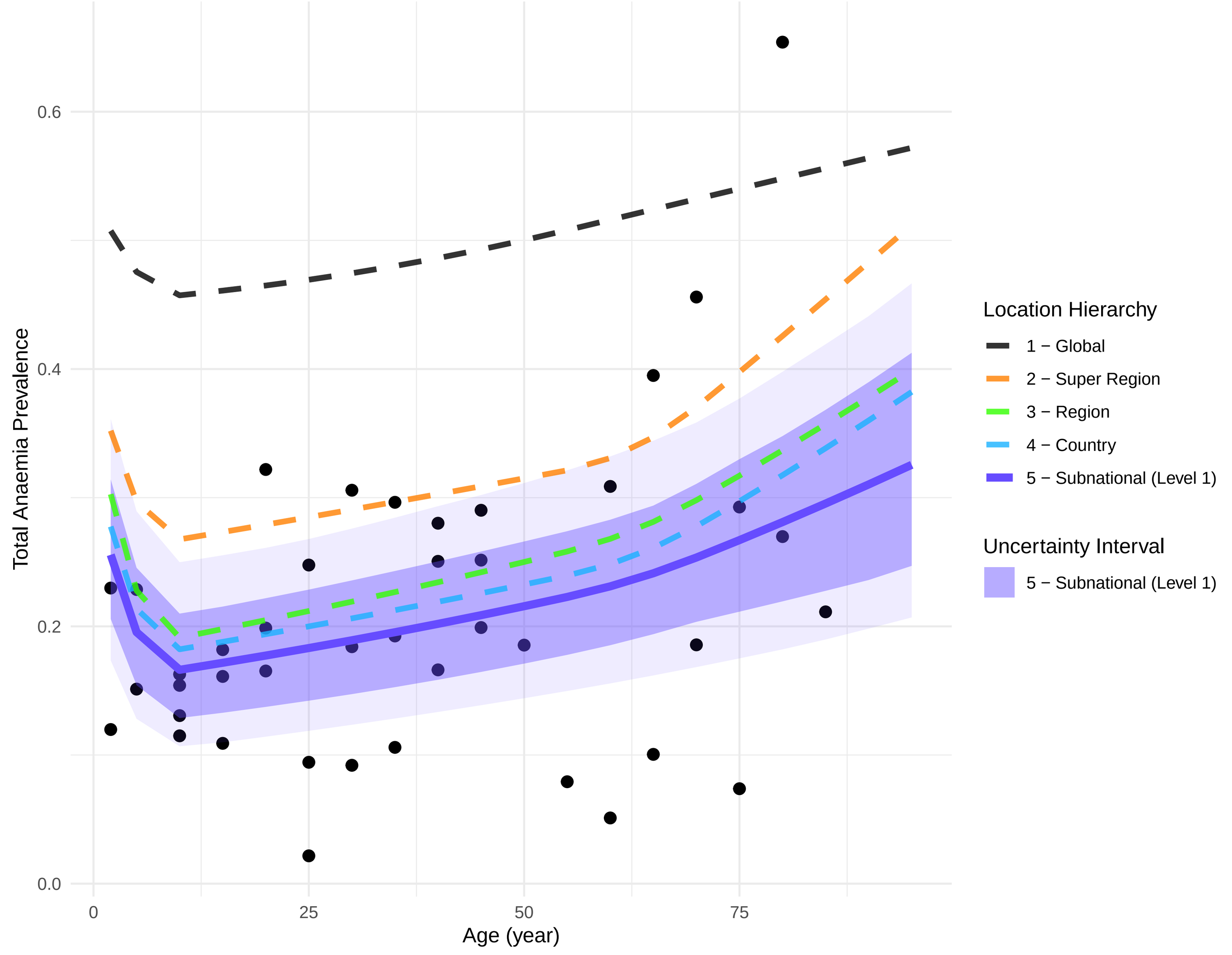

MR−BRT Age/Sex Fits − Non−pregnant Females 2+ Years Old − Total Anaemia Prevalence − Durango
WHO−2024 Adjustment Method

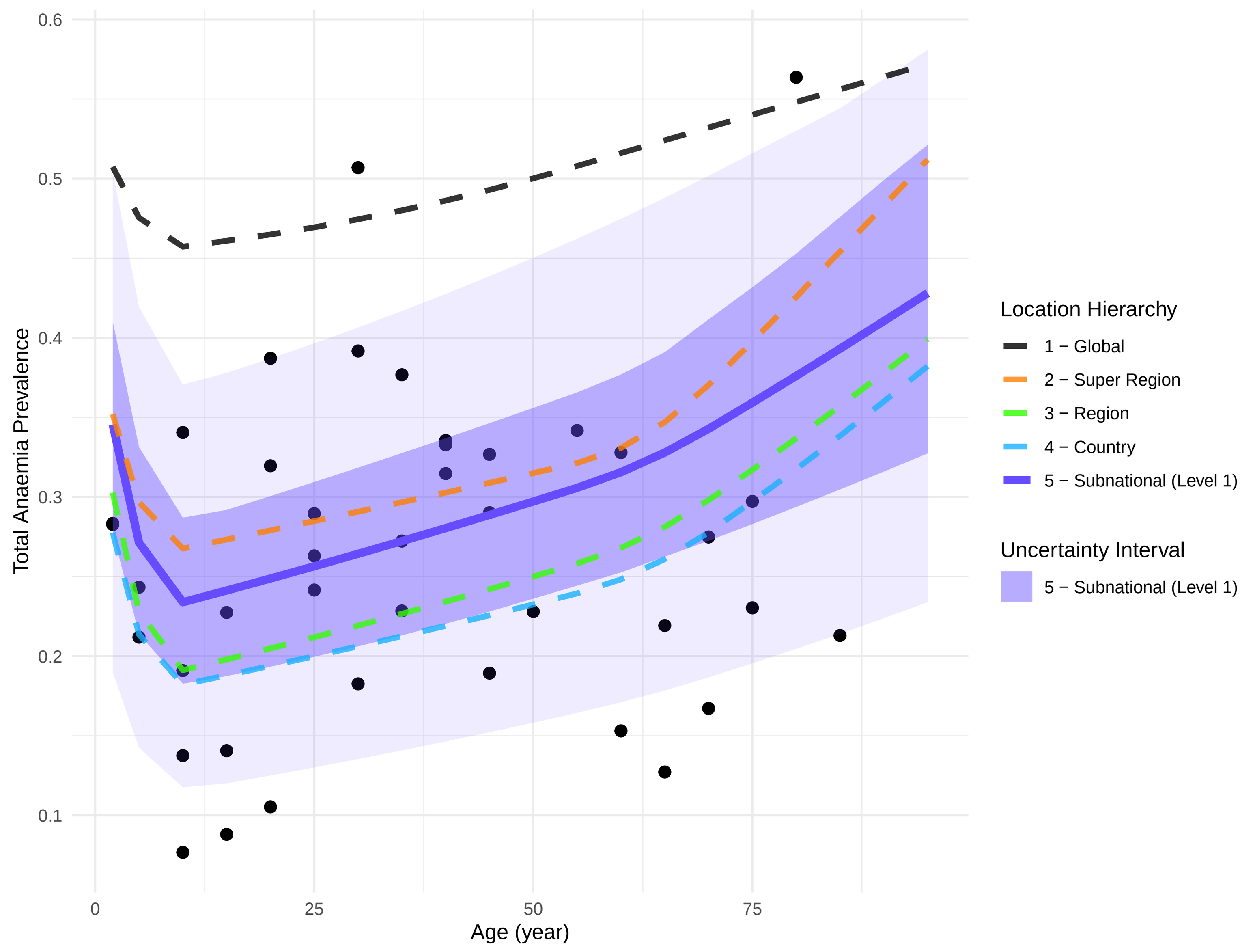

MR−BRT Age/Sex Fits − Non−pregnant Females 2+ Years Old − Total Anaemia Prevalence − Guanajuato
WHO−2024 Adjustment Method

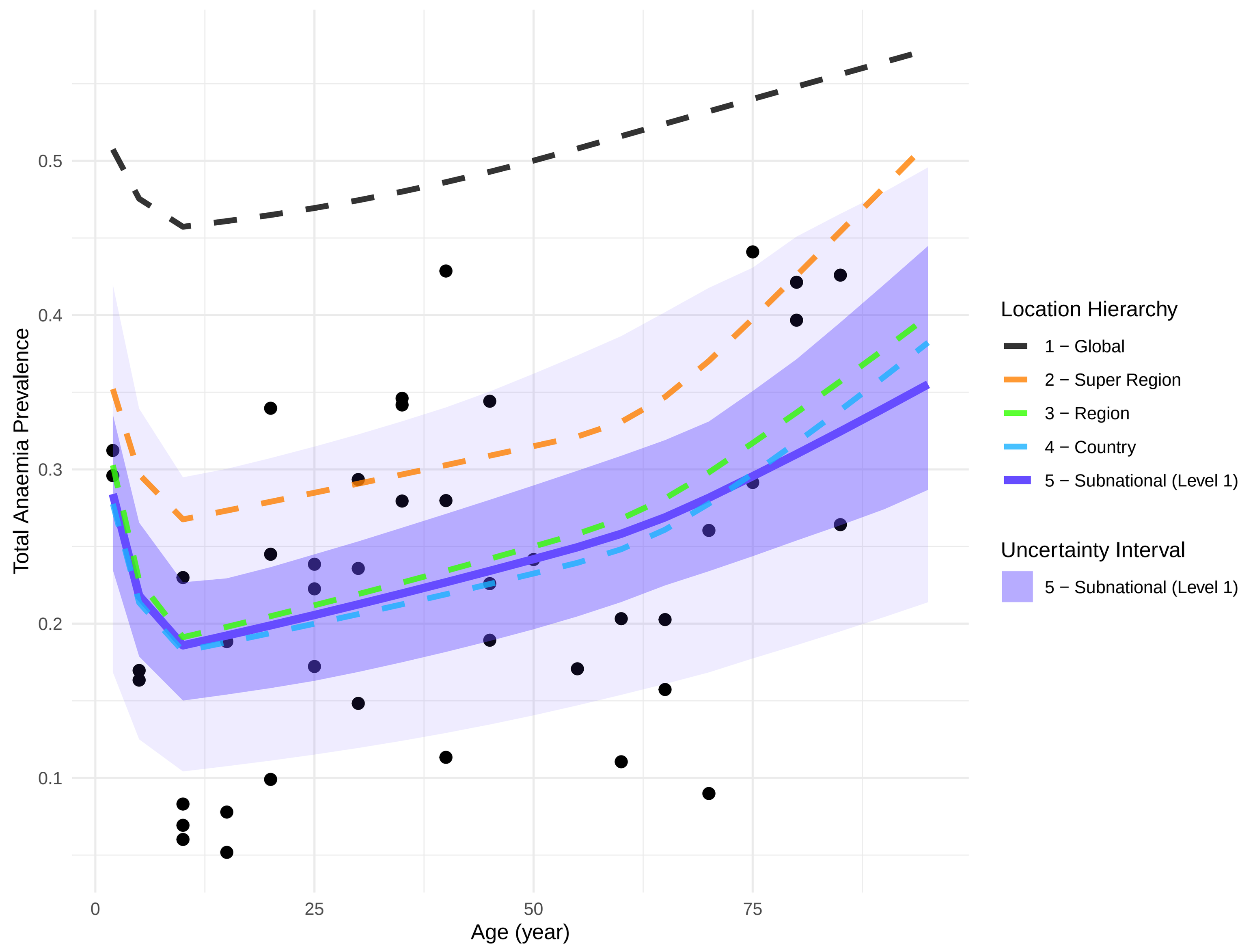

MR−BRT Age/Sex Fits − Non−pregnant Females 2+ Years Old − Total Anaemia Prevalence − Guerrero
WHO−2024 Adjustment Method

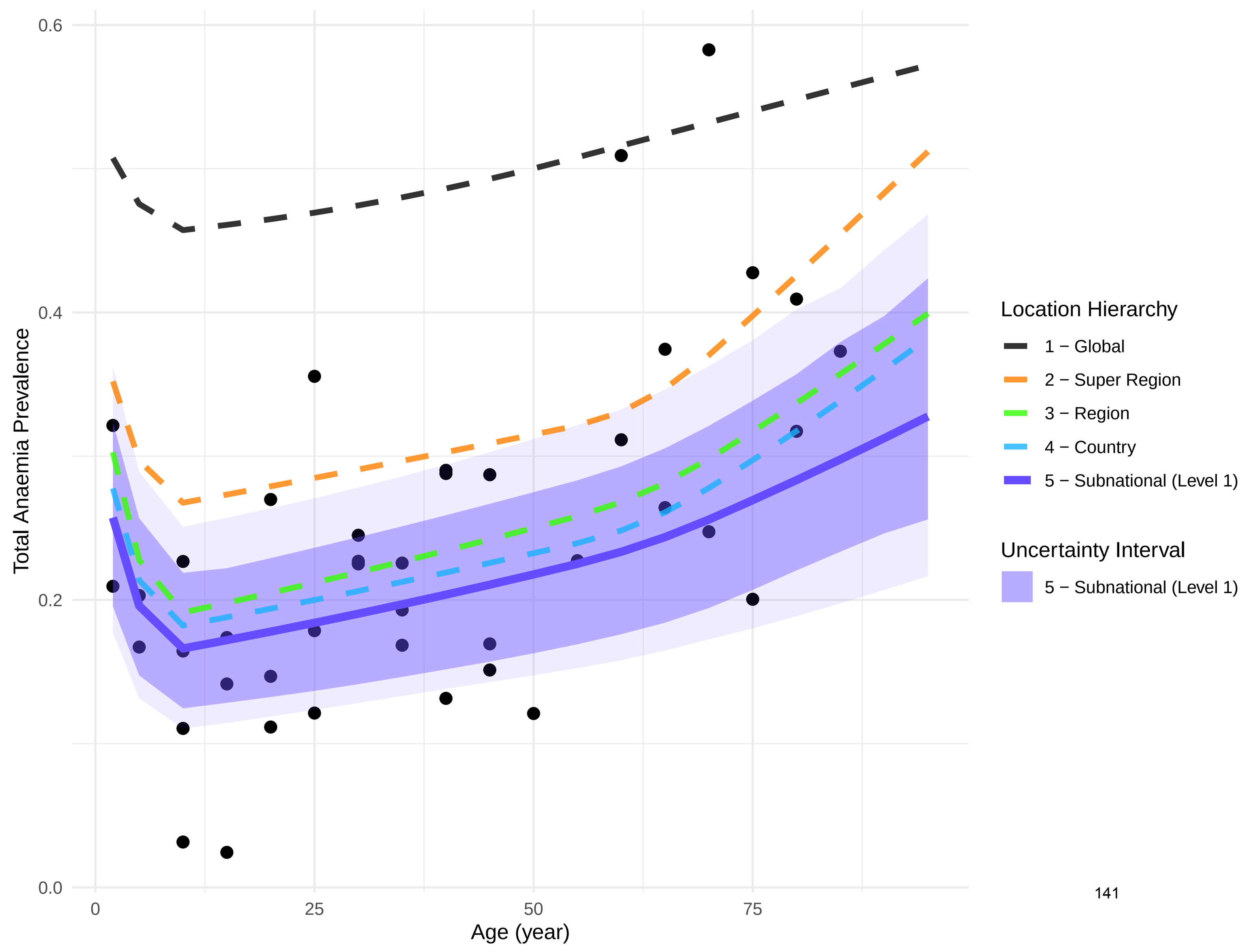

MR−BRT Age/Sex Fits − Non−pregnant Females 2+ Years Old − Total Anaemia Prevalence − Hidalgo
WHO−2024 Adjustment Method

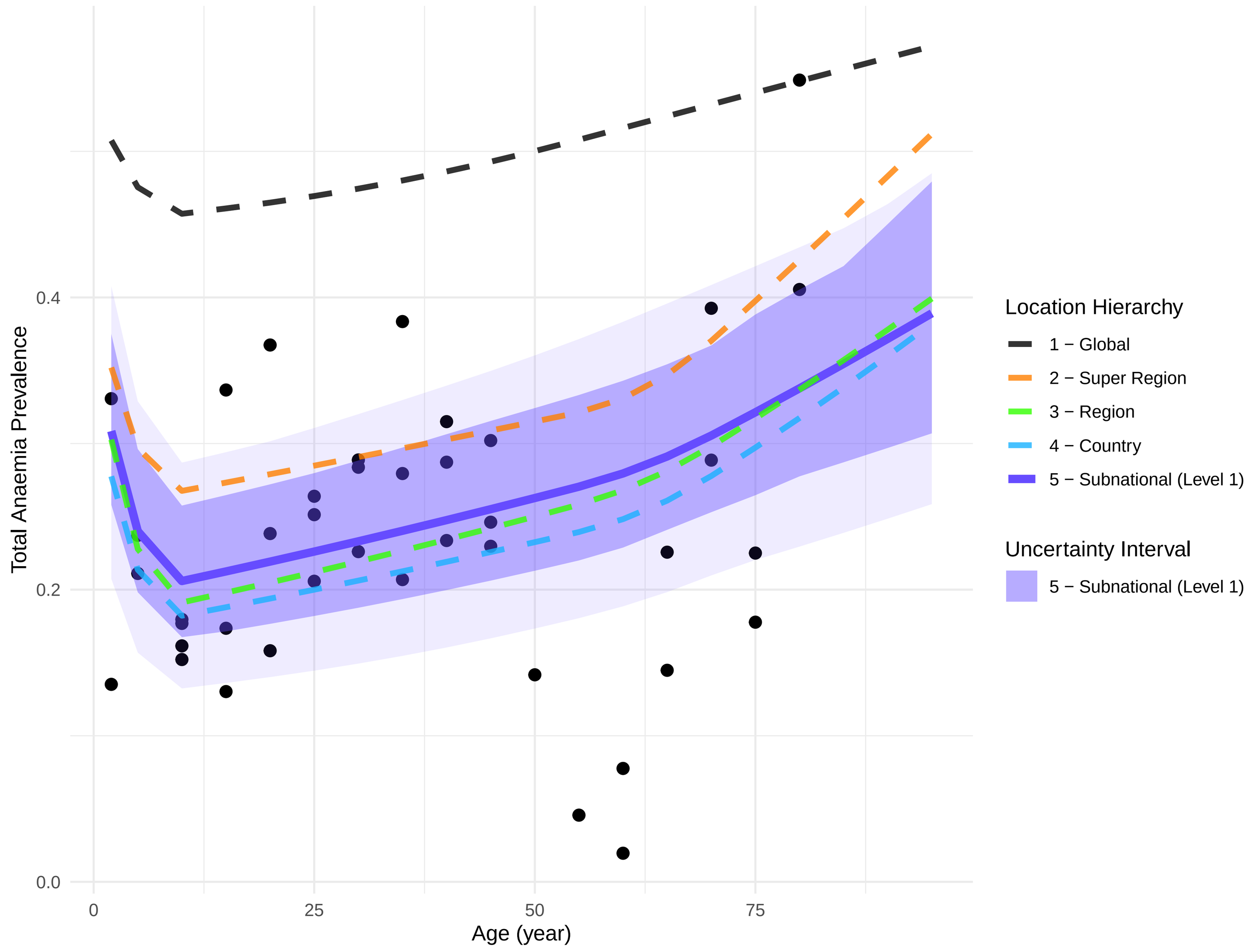

MR−BRT Age/Sex Fits − Non−pregnant Females 2+ Years Old − Total Anaemia Prevalence − Jalisco
WHO−2024 Adjustment Method

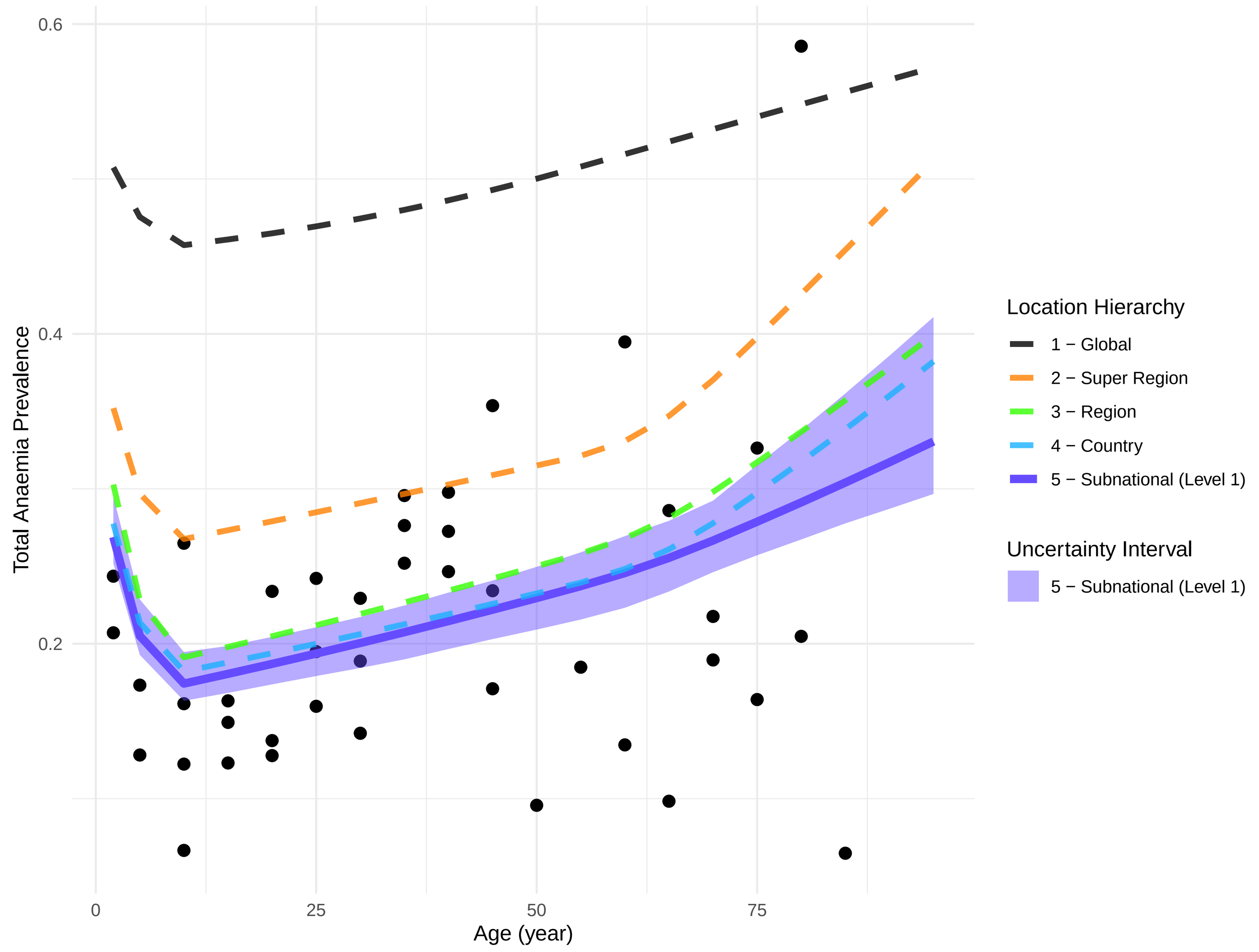

MR−BRT Age/Sex Fits − Non−pregnant Females 2+ Years Old − Total Anaemia Prevalence − México
WHO−2024 Adjustment Method

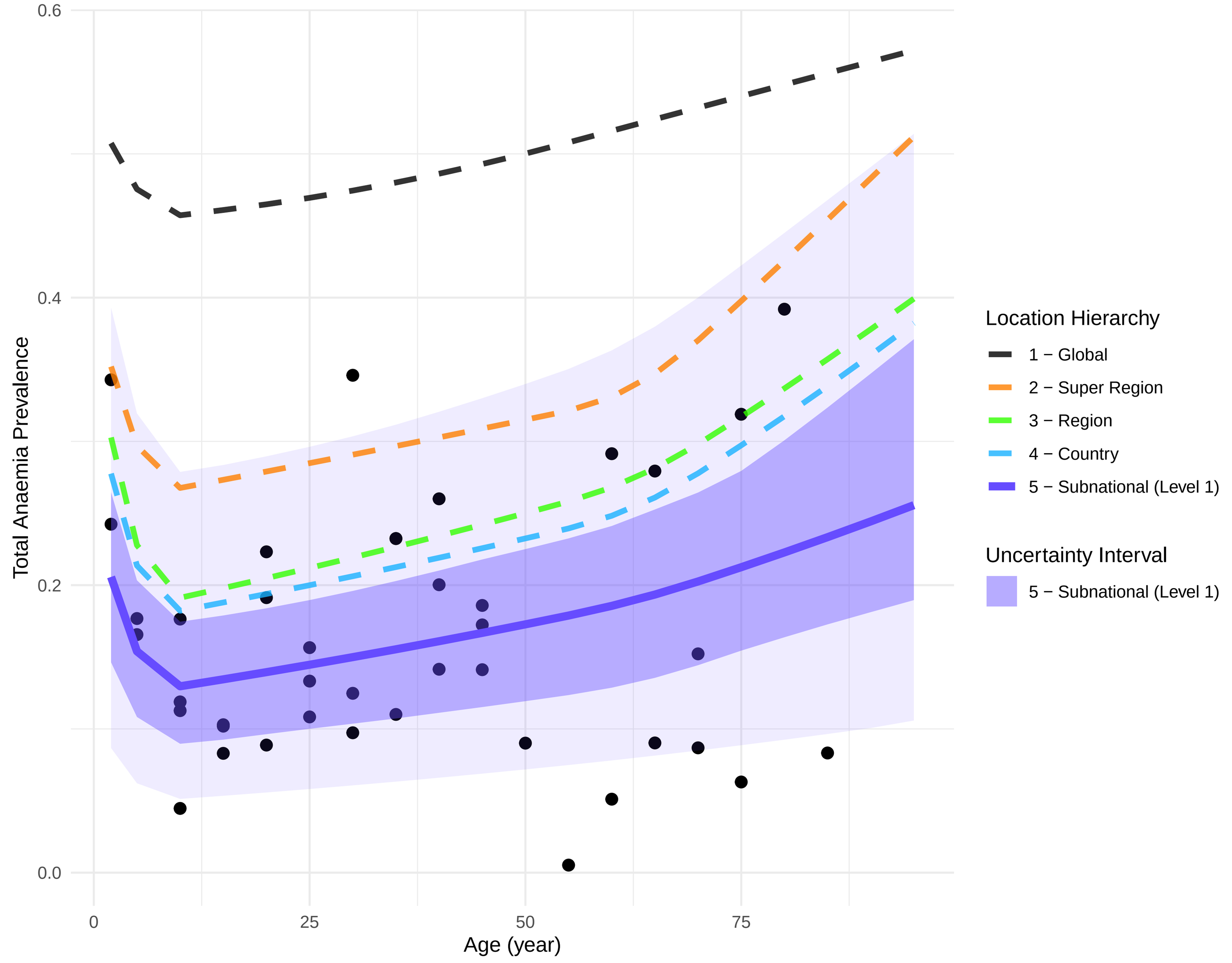

MR−BRT Age/Sex Fits − Non−pregnant Females 2+ Years Old − Total Anaemia Prevalence − Mexico City
WHO−2024 Adjustment Method

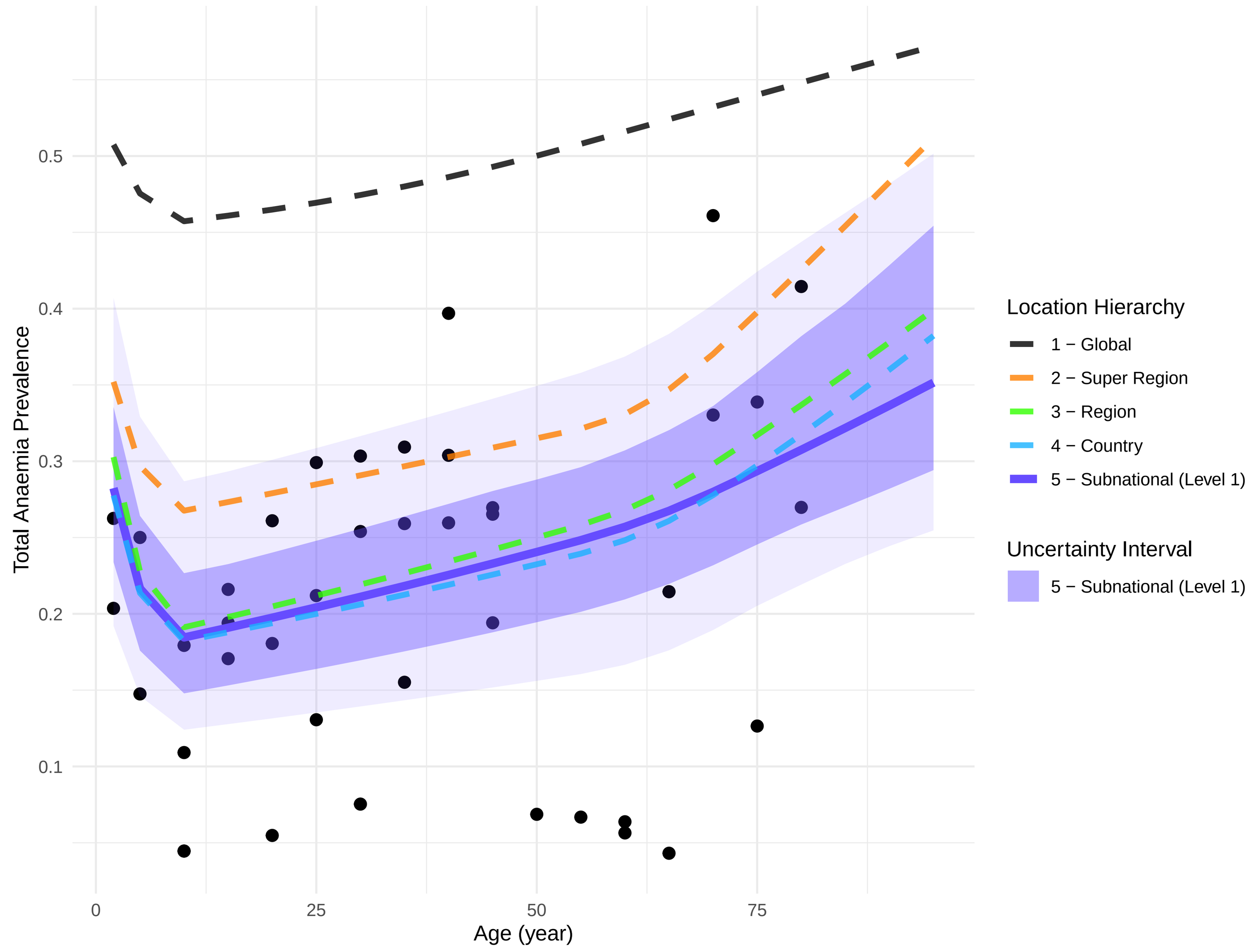

MR−BRT Age/Sex Fits − Non−pregnant Females 2+ Years Old − Total Anaemia Prevalence − Michoacán de Ocampo
WHO−2024 Adjustment Method

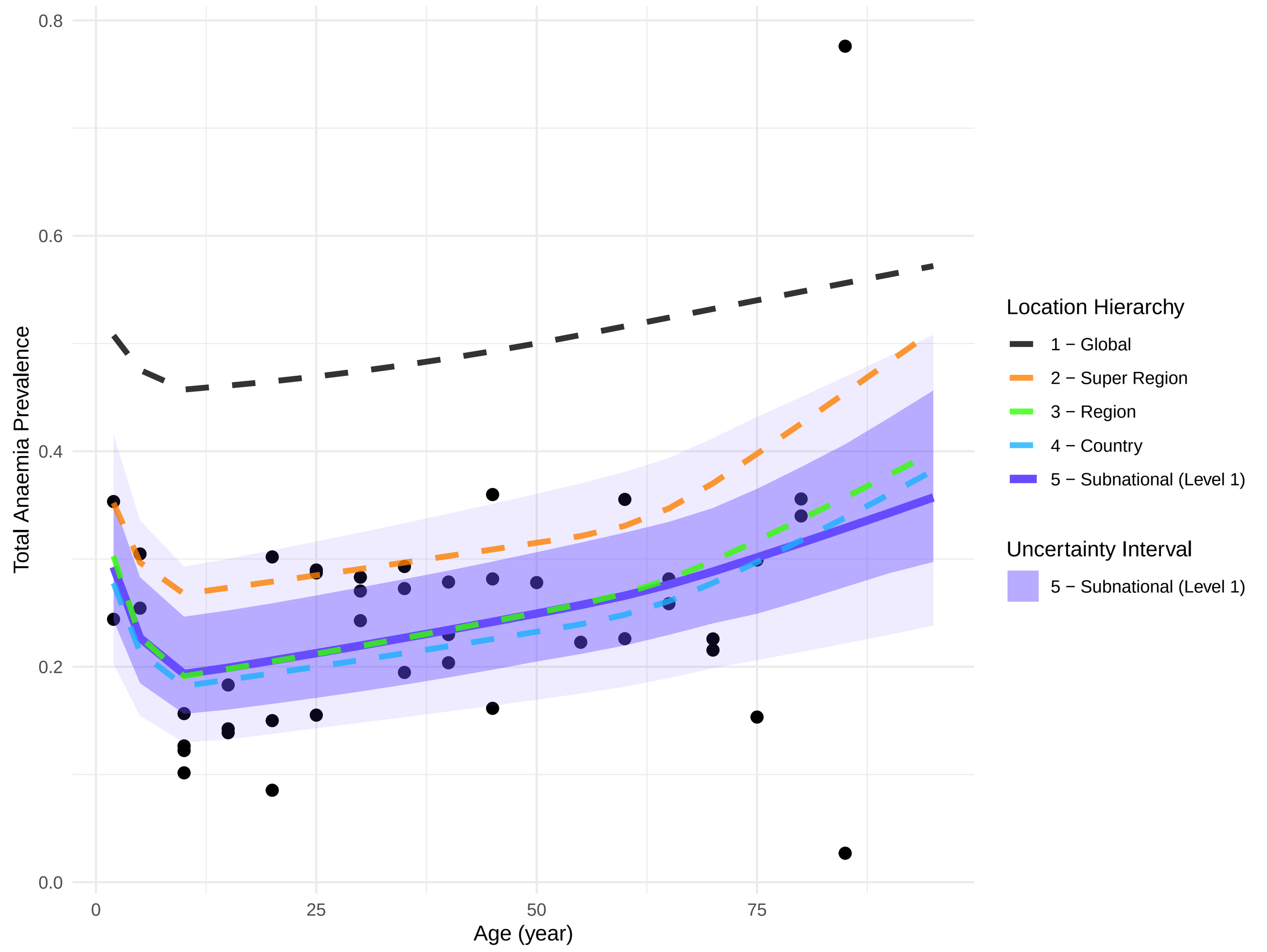

MR−BRT Age/Sex Fits − Non−pregnant Females 2+ Years Old − Total Anaemia Prevalence − Morelos
WHO−2024 Adjustment Method

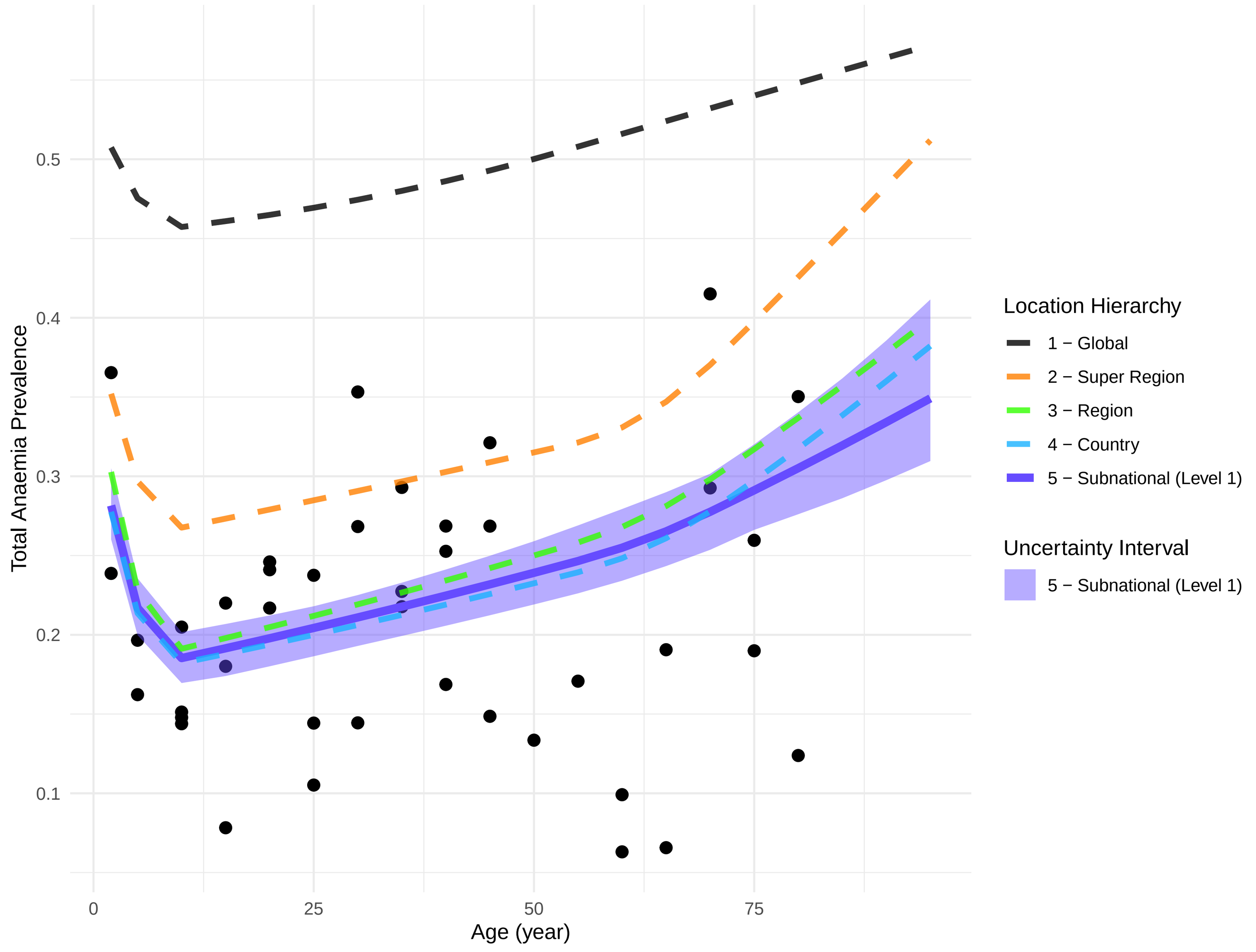

MR−BRT Age/Sex Fits − Non−pregnant Females 2+ Years Old − Total Anaemia Prevalence − Nayarit
WHO−2024 Adjustment Method

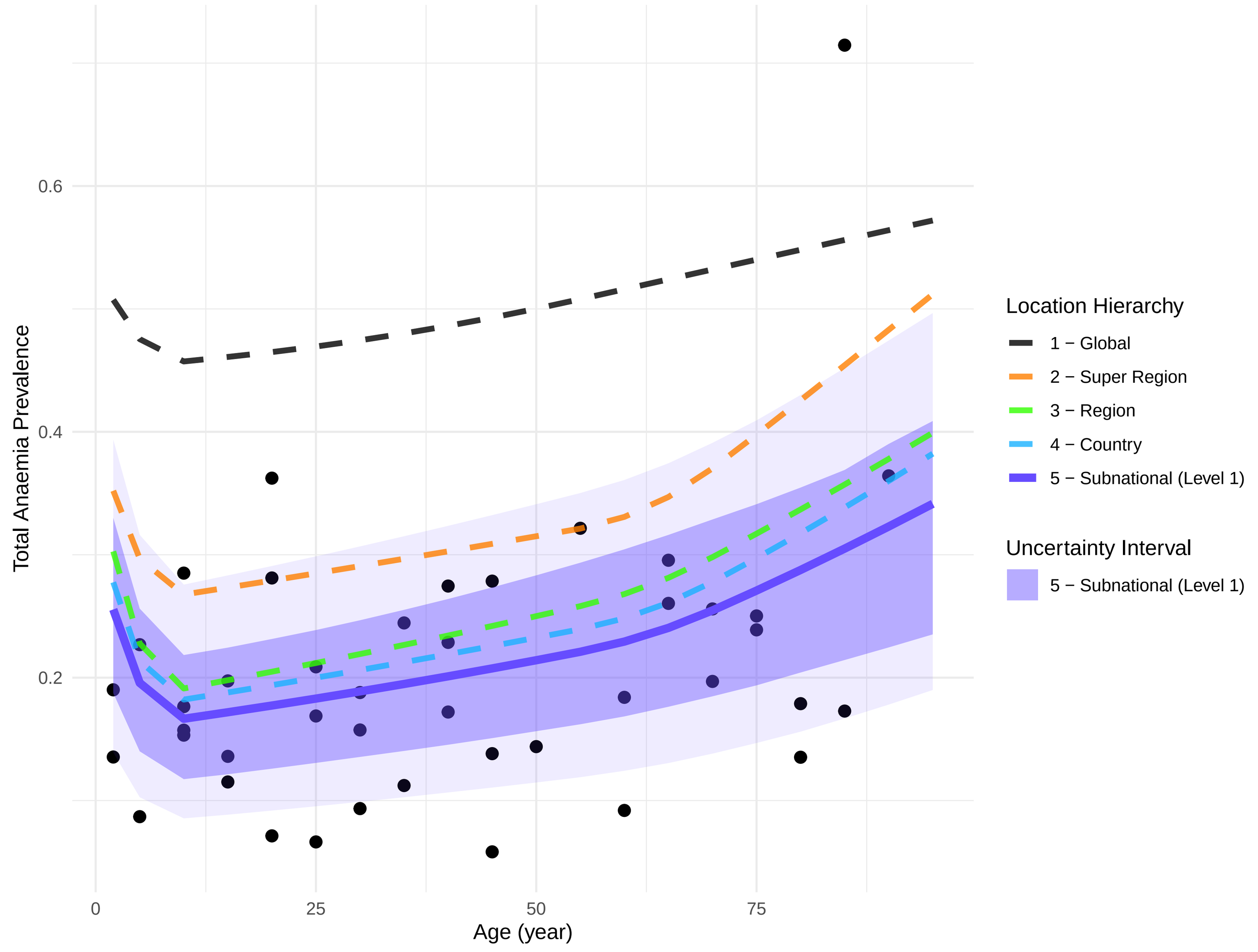

MR−BRT Age/Sex Fits − Non−pregnant Females 2+ Years Old − Total Anaemia Prevalence − Nuevo León
WHO−2024 Adjustment Method

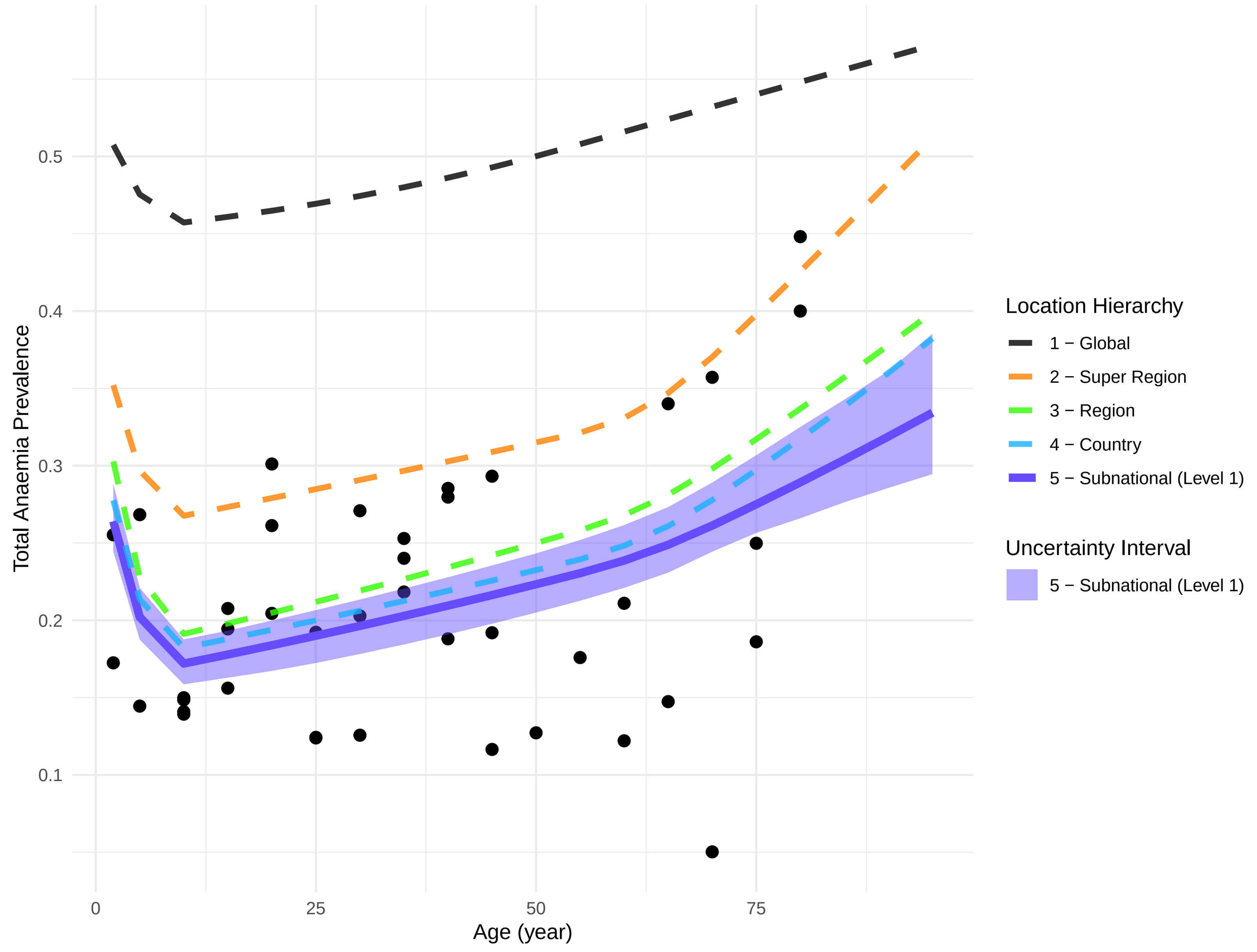

MR−BRT Age/Sex Fits − Non−pregnant Females 2+ Years Old − Total Anaemia Prevalence − Oaxaca
WHO−2024 Adjustment Method

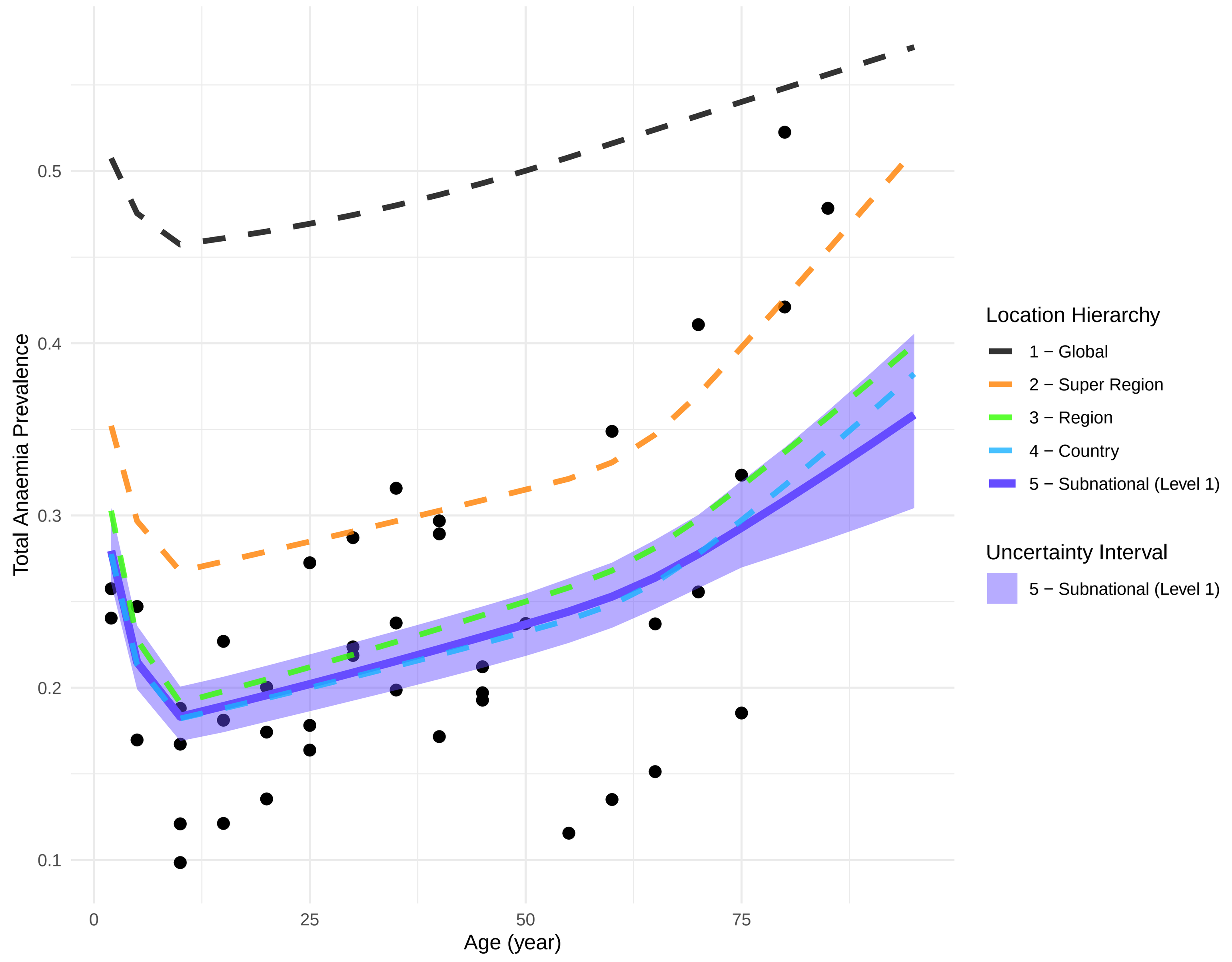

MR−BRT Age/Sex Fits − Non−pregnant Females 2+ Years Old − Total Anaemia Prevalence − Puebla
WHO−2024 Adjustment Method

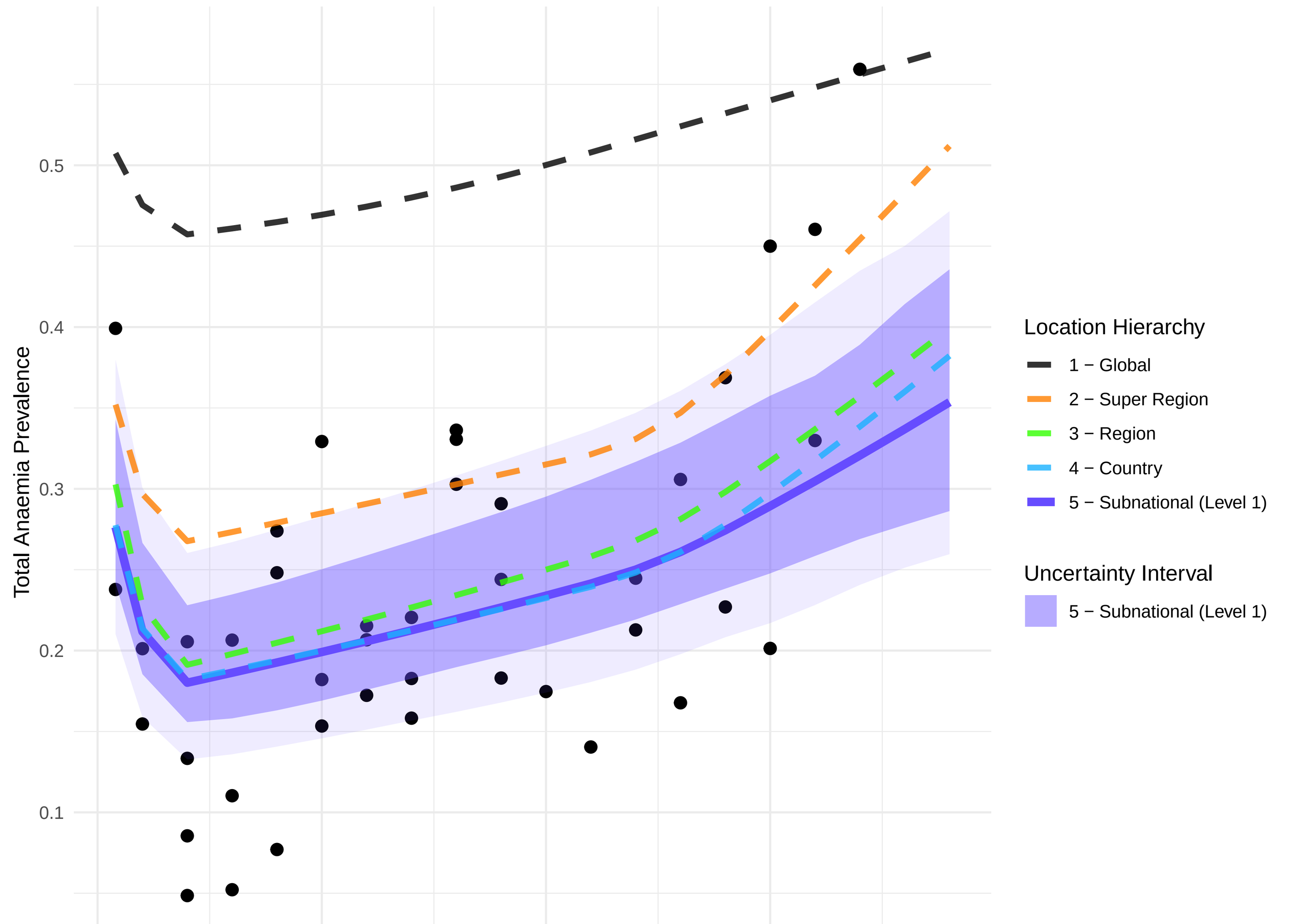

MR−BRT Age/Sex Fits − Non−pregnant Females 2+ Years Old − Total Anaemia Prevalence − Querétaro
WHO−2024 Adjustment Method

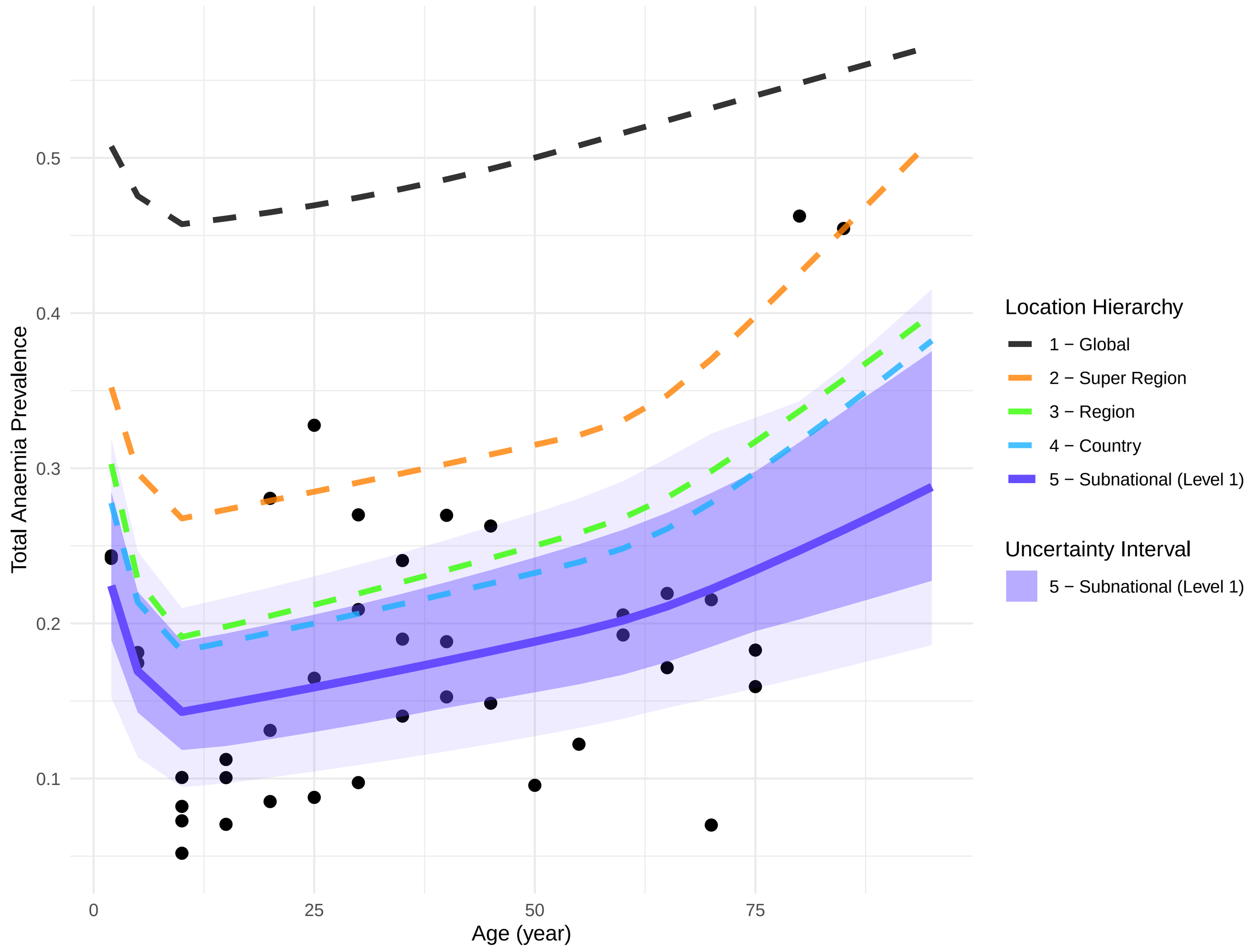

MR−BRT Age/Sex Fits − Non−pregnant Females 2+ Years Old − Total Anaemia Prevalence − Quintana Roo
WHO−2024 Adjustment Method

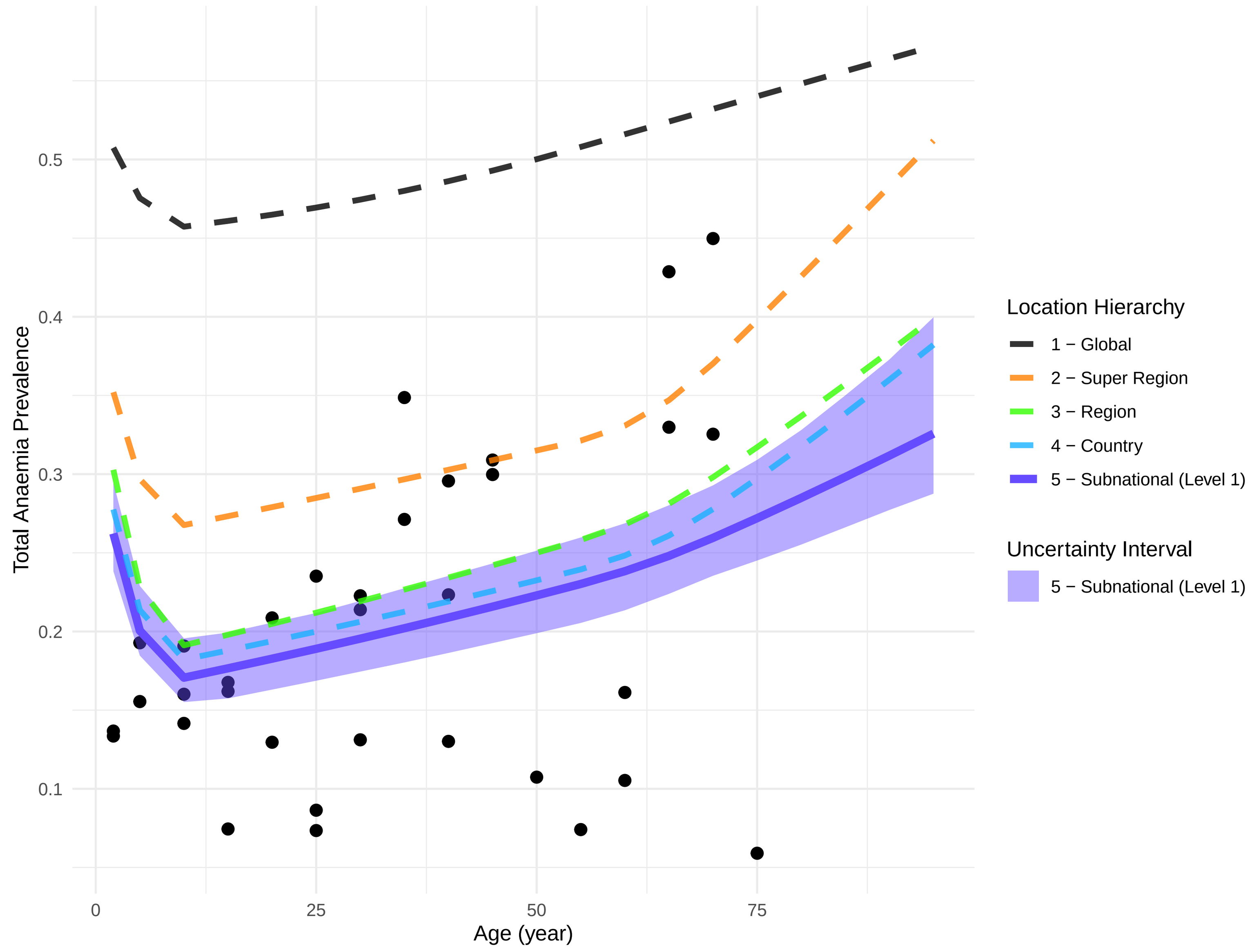

MR−BRT Age/Sex Fits − Non−pregnant Females 2+ Years Old − Total Anaemia Prevalence − San Luis Potosí
WHO−2024 Adjustment Method

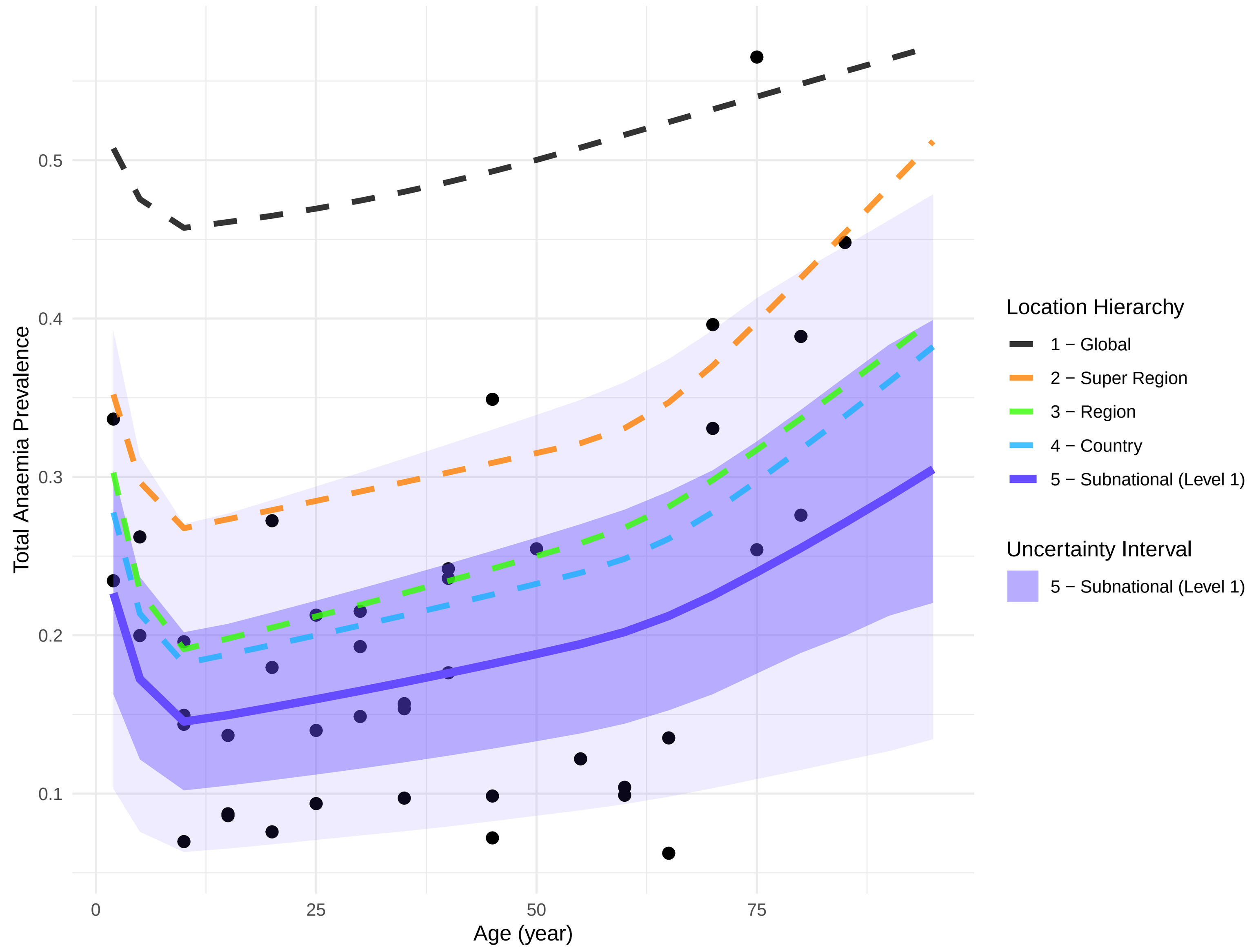

MR−BRT Age/Sex Fits − Non−pregnant Females 2+ Years Old − Total Anaemia Prevalence − Sinaloa
WHO−2024 Adjustment Method

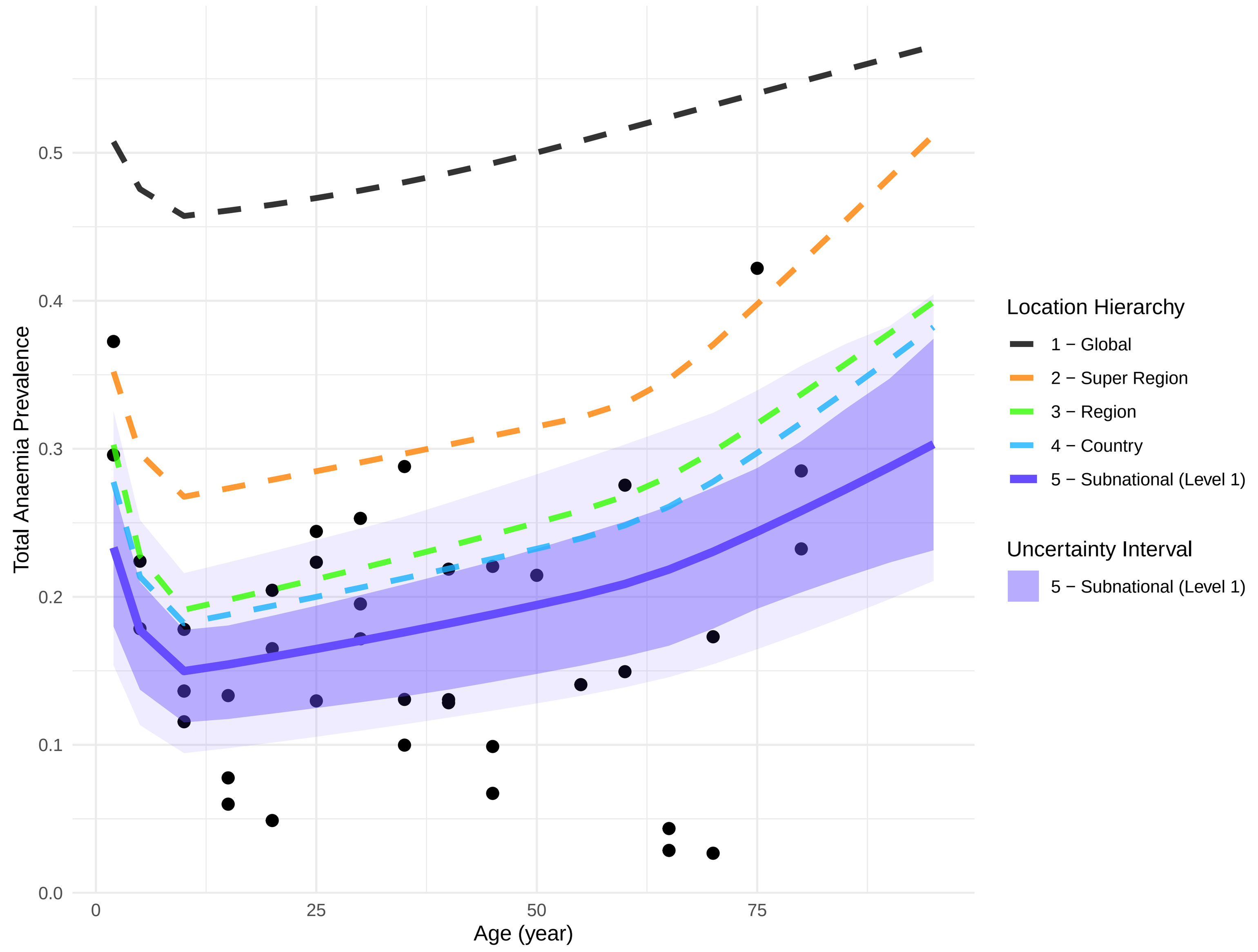

MR−BRT Age/Sex Fits − Non−pregnant Females 2+ Years Old − Total Anaemia Prevalence − Sonora
WHO−2024 Adjustment Method

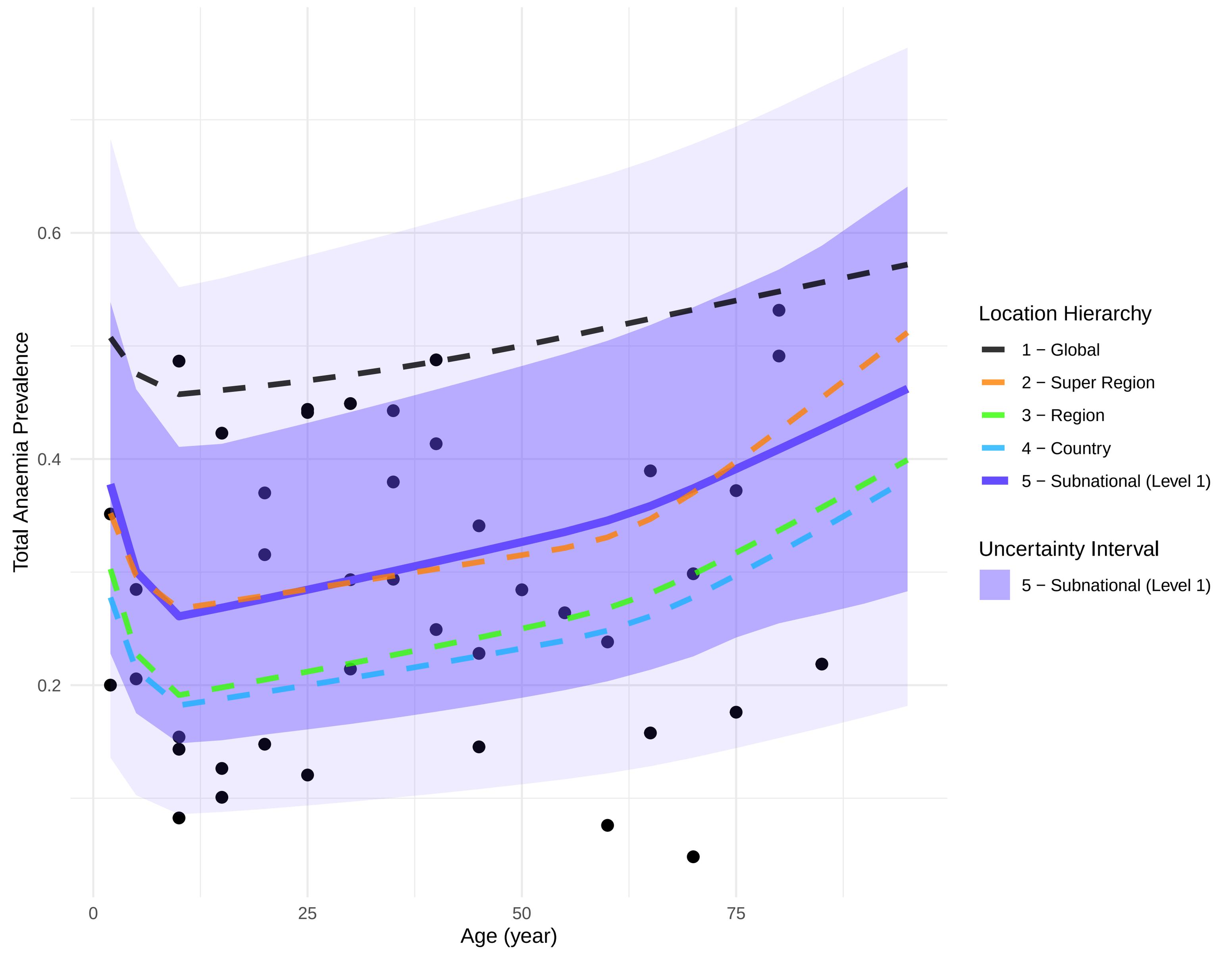

MR−BRT Age/Sex Fits − Non−pregnant Females 2+ Years Old − Total Anaemia Prevalence − Tabasco
WHO−2024 Adjustment Method

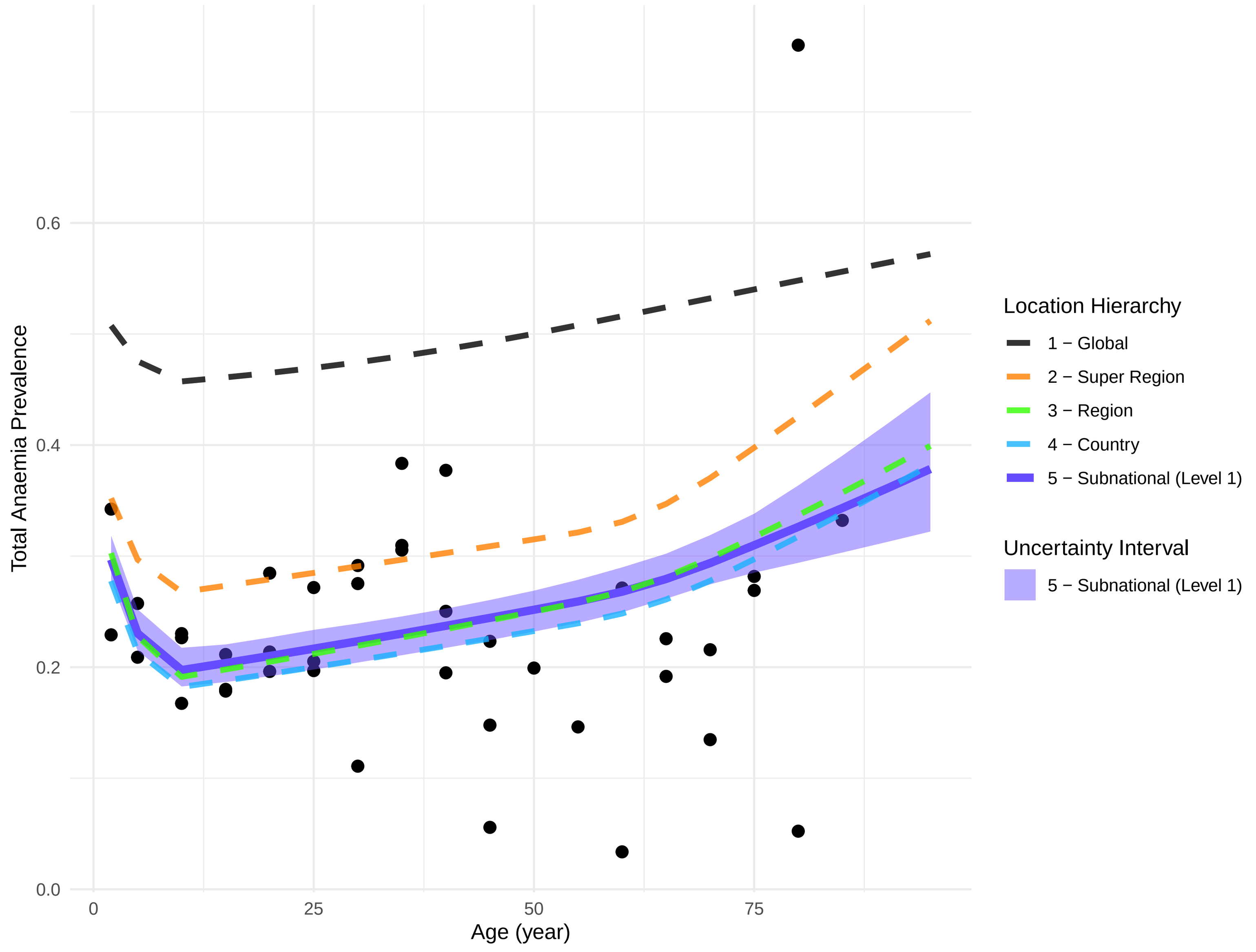

MR−BRT Age/Sex Fits − Non−pregnant Females 2+ Years Old − Total Anaemia Prevalence − Tamaulipas
WHO−2024 Adjustment Method

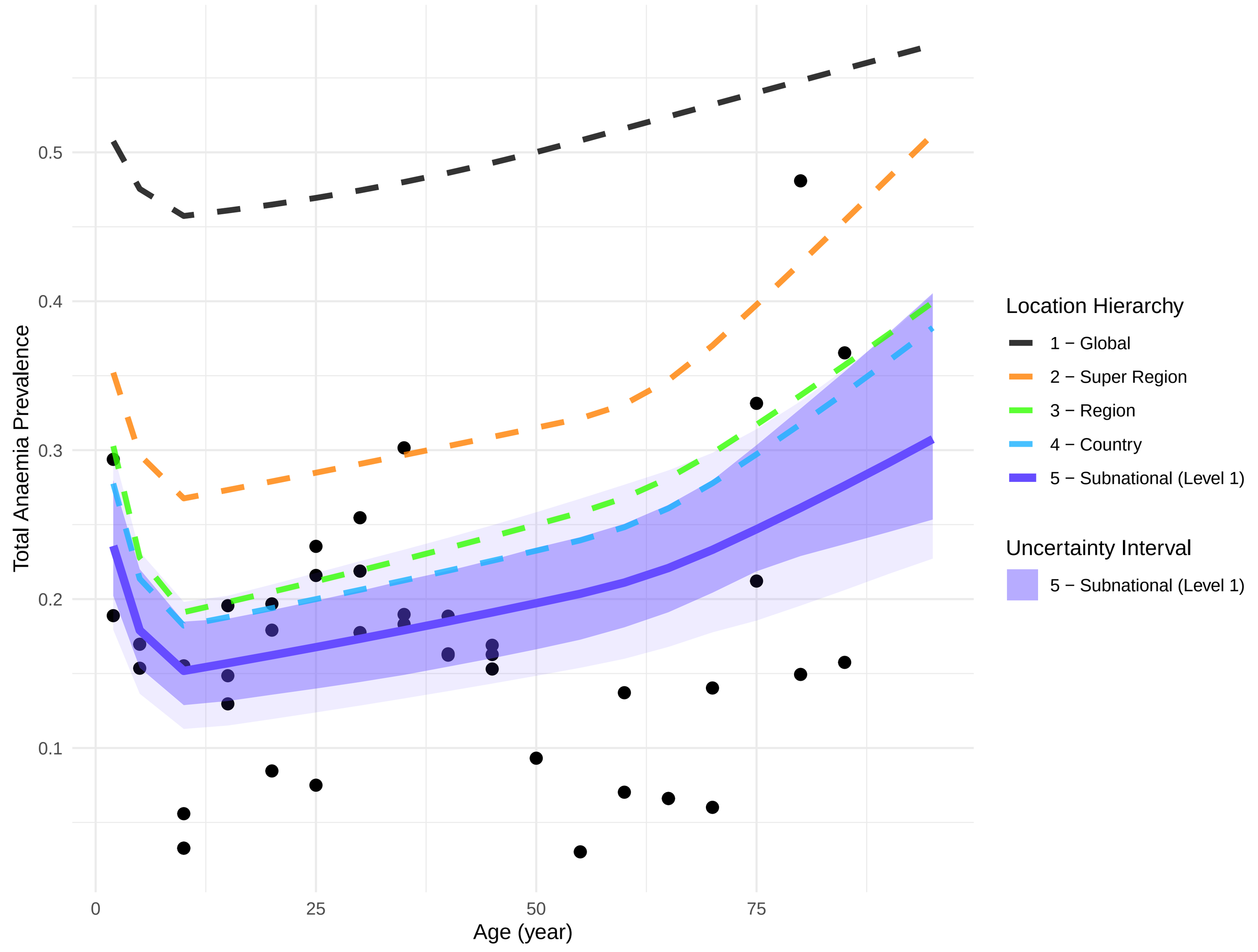

MR−BRT Age/Sex Fits − Non−pregnant Females 2+ Years Old − Total Anaemia Prevalence − Tlaxcala
WHO−2024 Adjustment Method

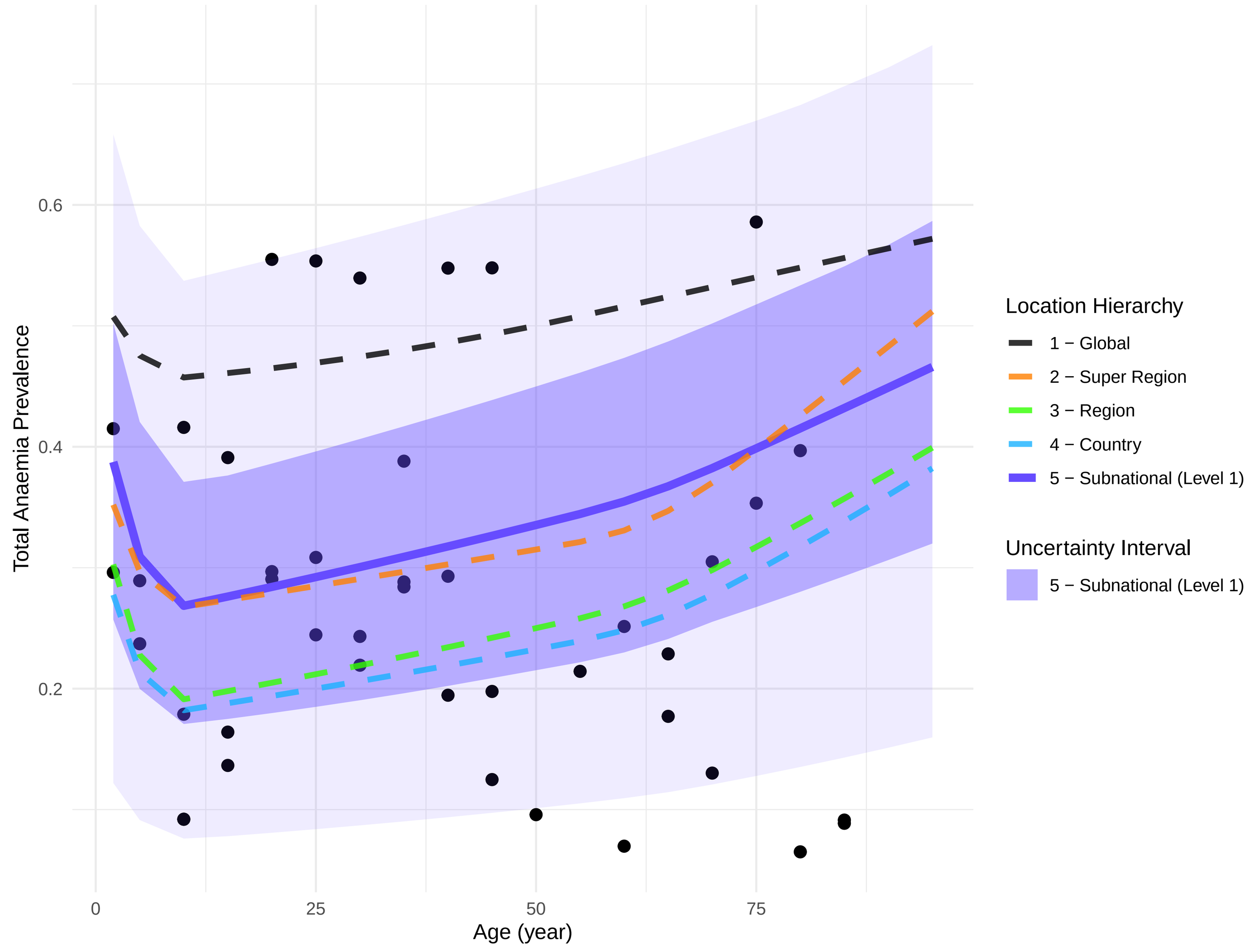

MR−BRT Age/Sex Fits − Non−pregnant Females 2+ Years Old − Total Anaemia Prevalence − Veracruz de Ignacio de la Llave
WHO−2024 Adjustment Method

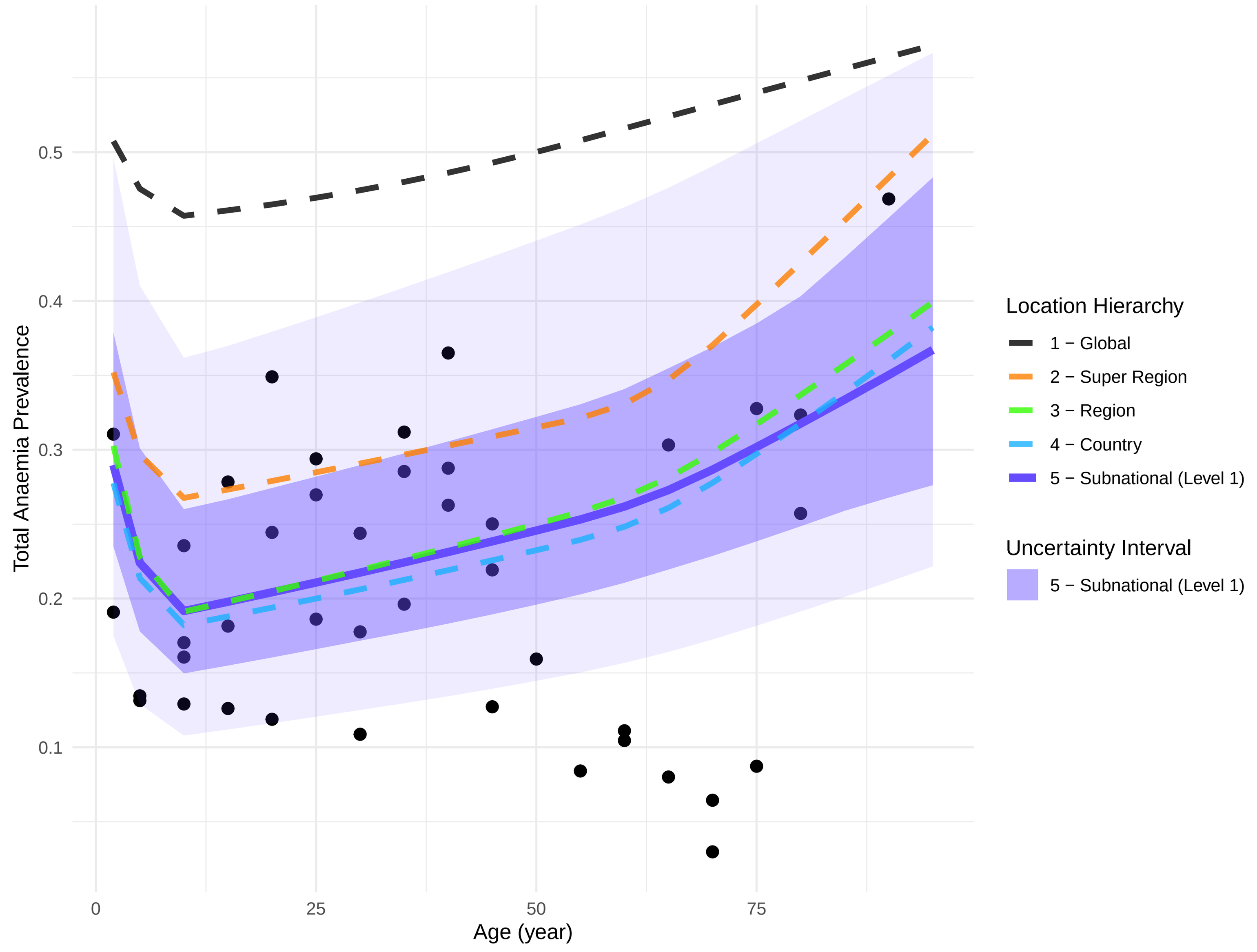

MR−BRT Age/Sex Fits − Non−pregnant Females 2+ Years Old − Total Anaemia Prevalence − Yucatán
WHO−2024 Adjustment Method

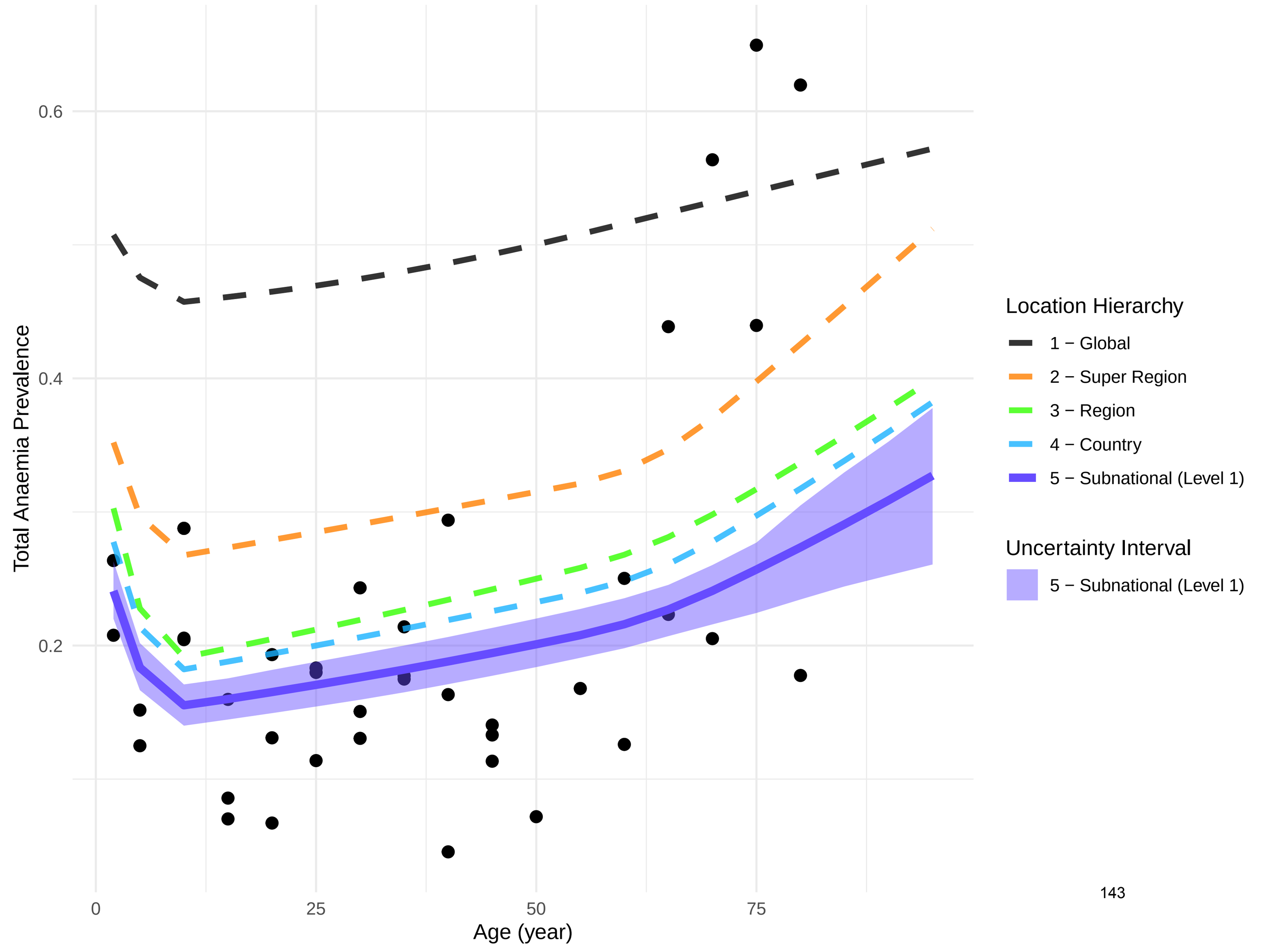

MR−BRT Age/Sex Fits − Non−pregnant Females 2+ Years Old − Total Anaemia Prevalence − Zacatecas
WHO−2024 Adjustment Method

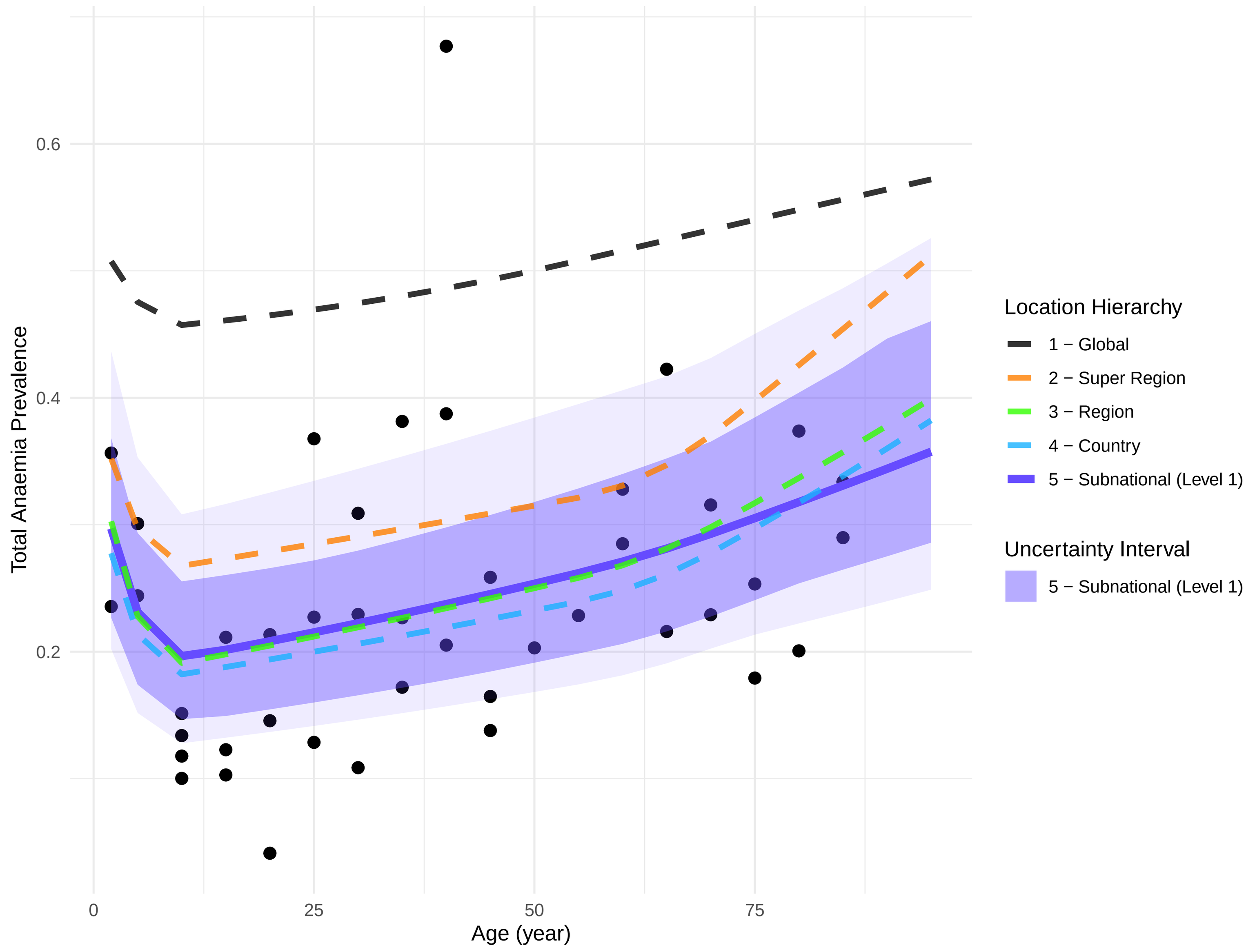

MR−BRT Age/Sex Fits − Non−pregnant Females 2+ Years Old − Total Anaemia Prevalence − Tropical Latin America
WHO−2024 Adjustment Method

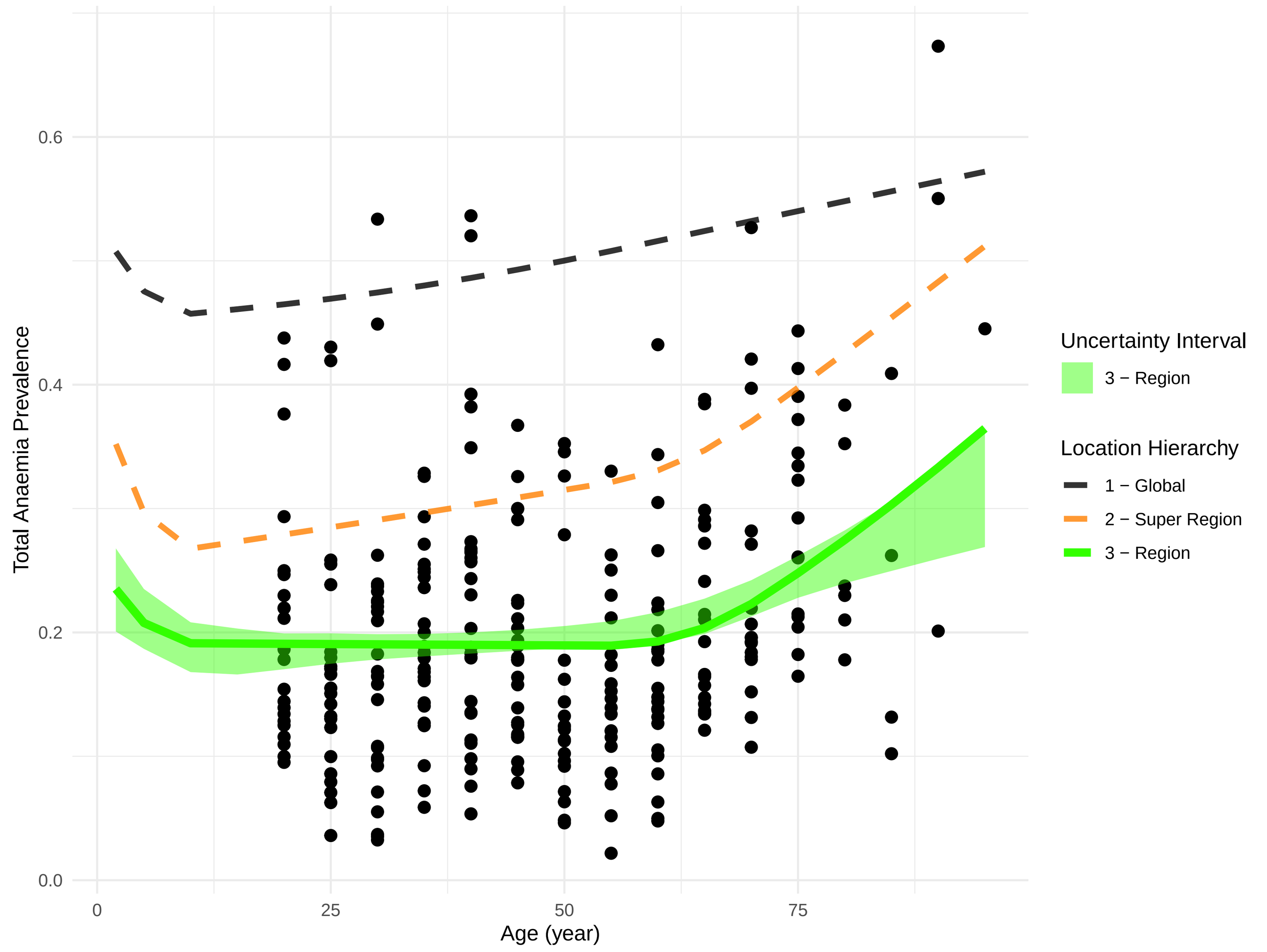

MR−BRT Age/Sex Fits − Non−pregnant Females 2+ Years Old − Total Anaemia Prevalence − Brazil
WHO−2024 Adjustment Method

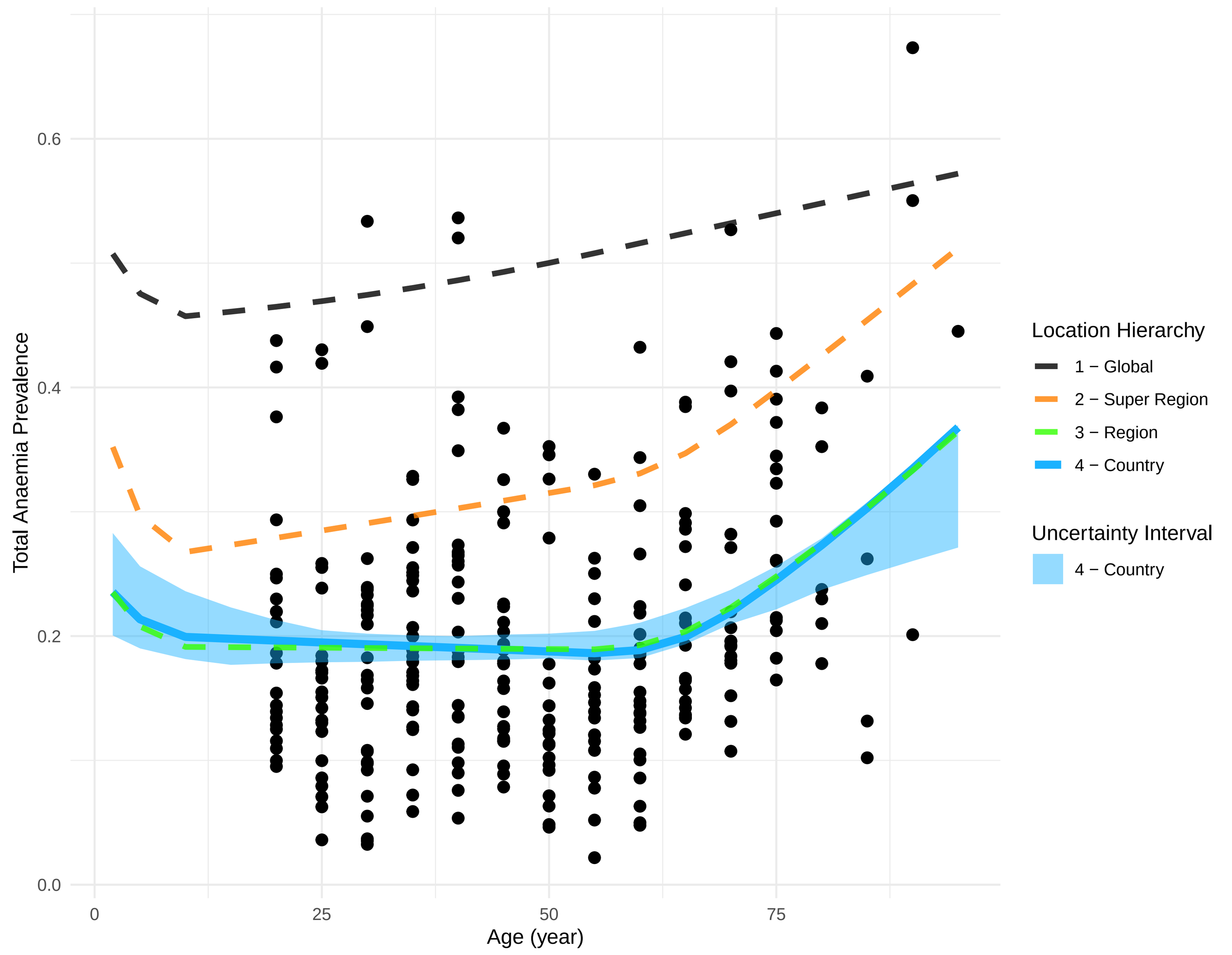

MR−BRT Age/Sex Fits − Non−pregnant Females 2+ Years Old − Total Anaemia Prevalence − Acre
WHO−2024 Adjustment Method

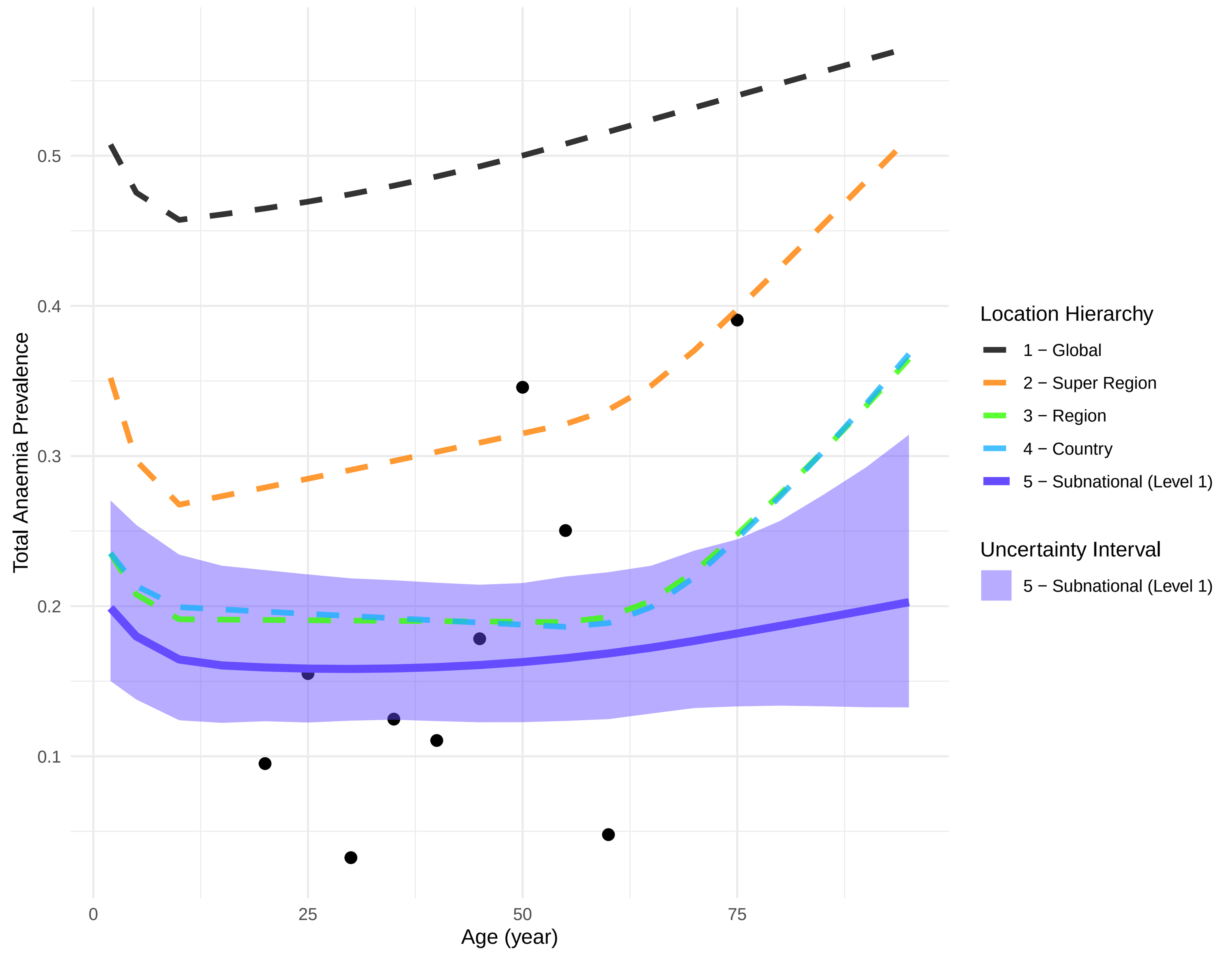

MR−BRT Age/Sex Fits − Non−pregnant Females 2+ Years Old − Total Anaemia Prevalence − Alagoas
WHO−2024 Adjustment Method

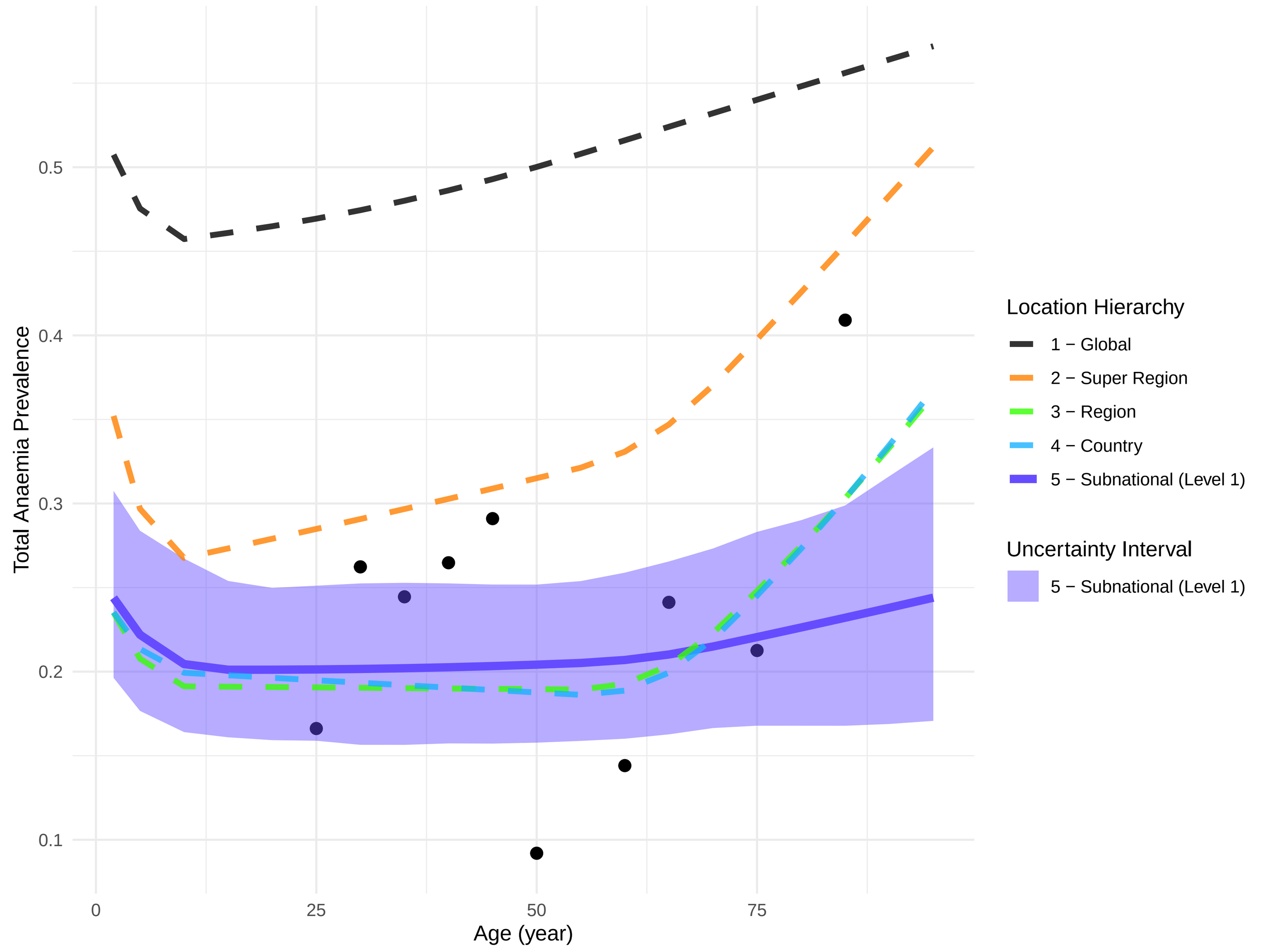

MR−BRT Age/Sex Fits − Non−pregnant Females 2+ Years Old − Total Anaemia Prevalence − Amapá
WHO−2024 Adjustment Method

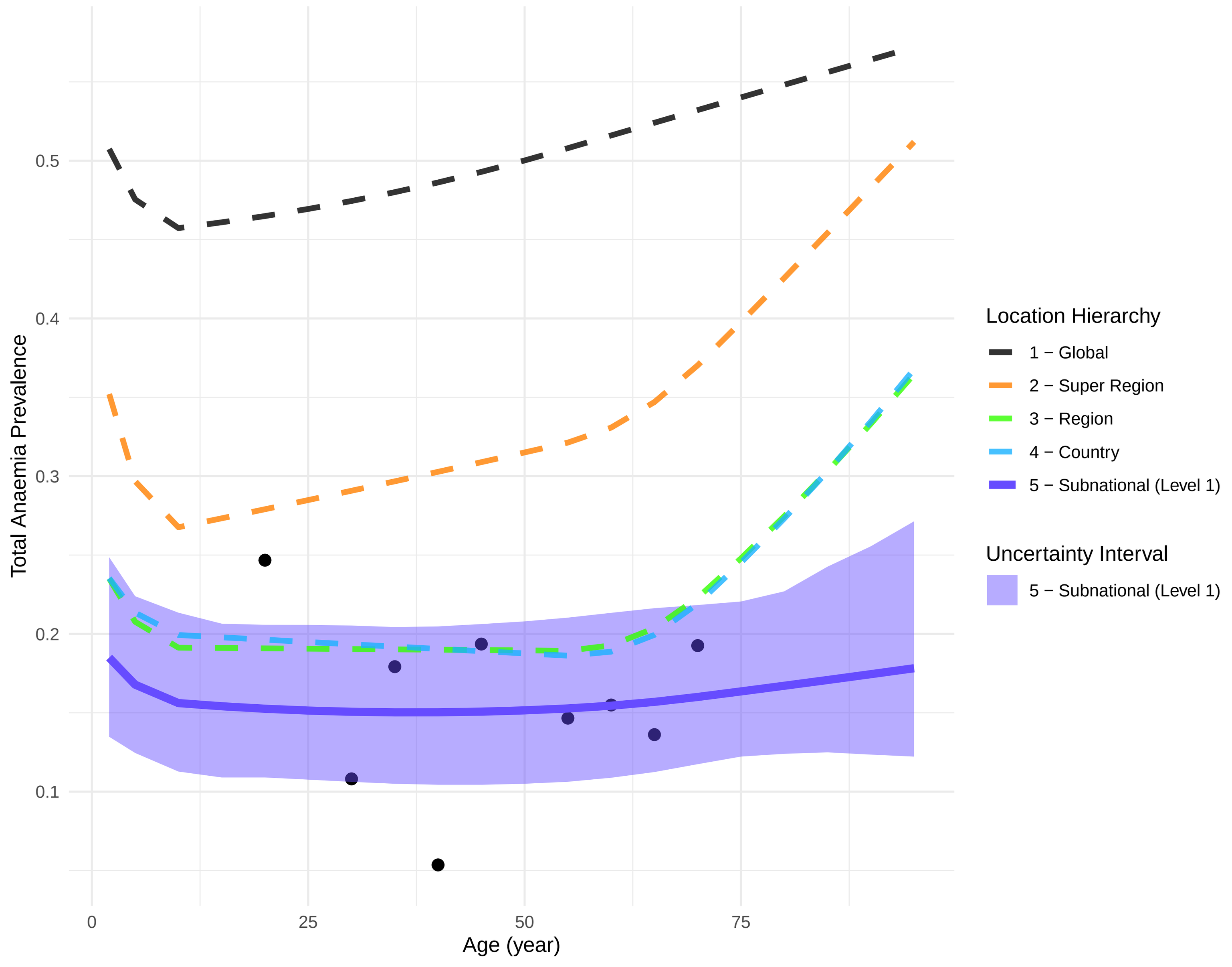

MR−BRT Age/Sex Fits − Non−pregnant Females 2+ Years Old − Total Anaemia Prevalence − Amazonas
WHO−2024 Adjustment Method

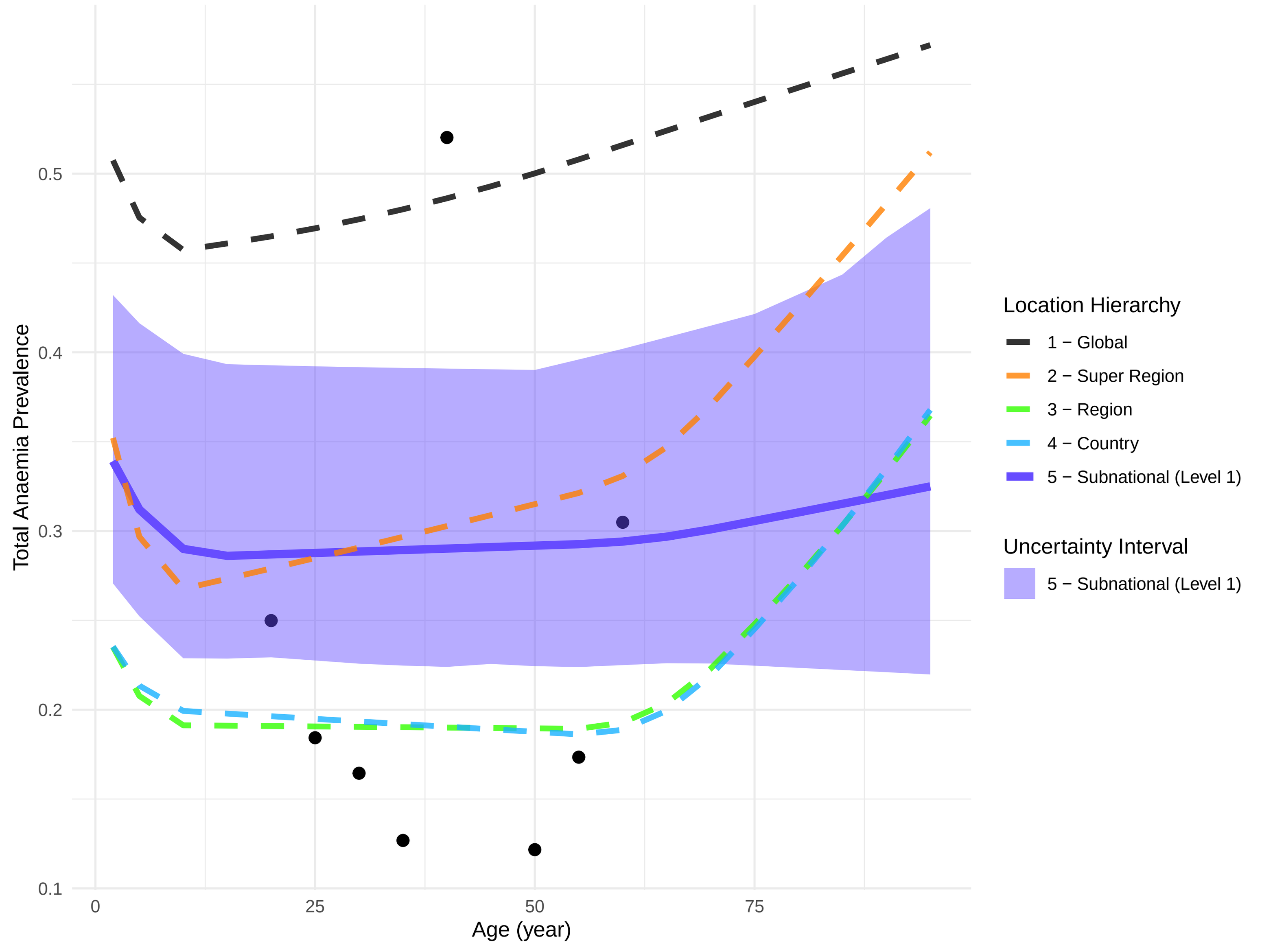

MR−BRT Age/Sex Fits − Non−pregnant Females 2+ Years Old − Total Anaemia Prevalence − Bahia
WHO−2024 Adjustment Method

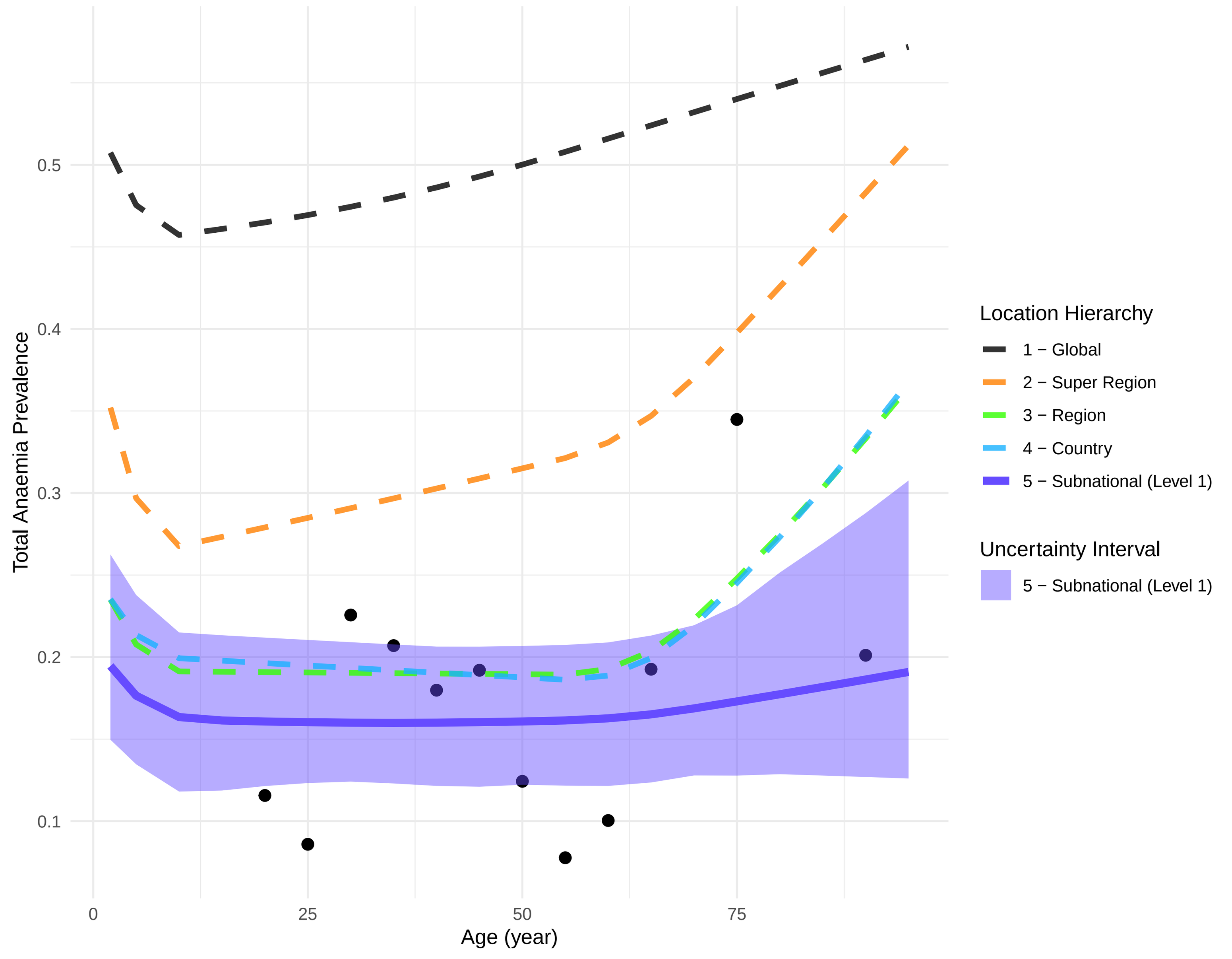

MR−BRT Age/Sex Fits − Non−pregnant Females 2+ Years Old − Total Anaemia Prevalence − Ceará
WHO−2024 Adjustment Method

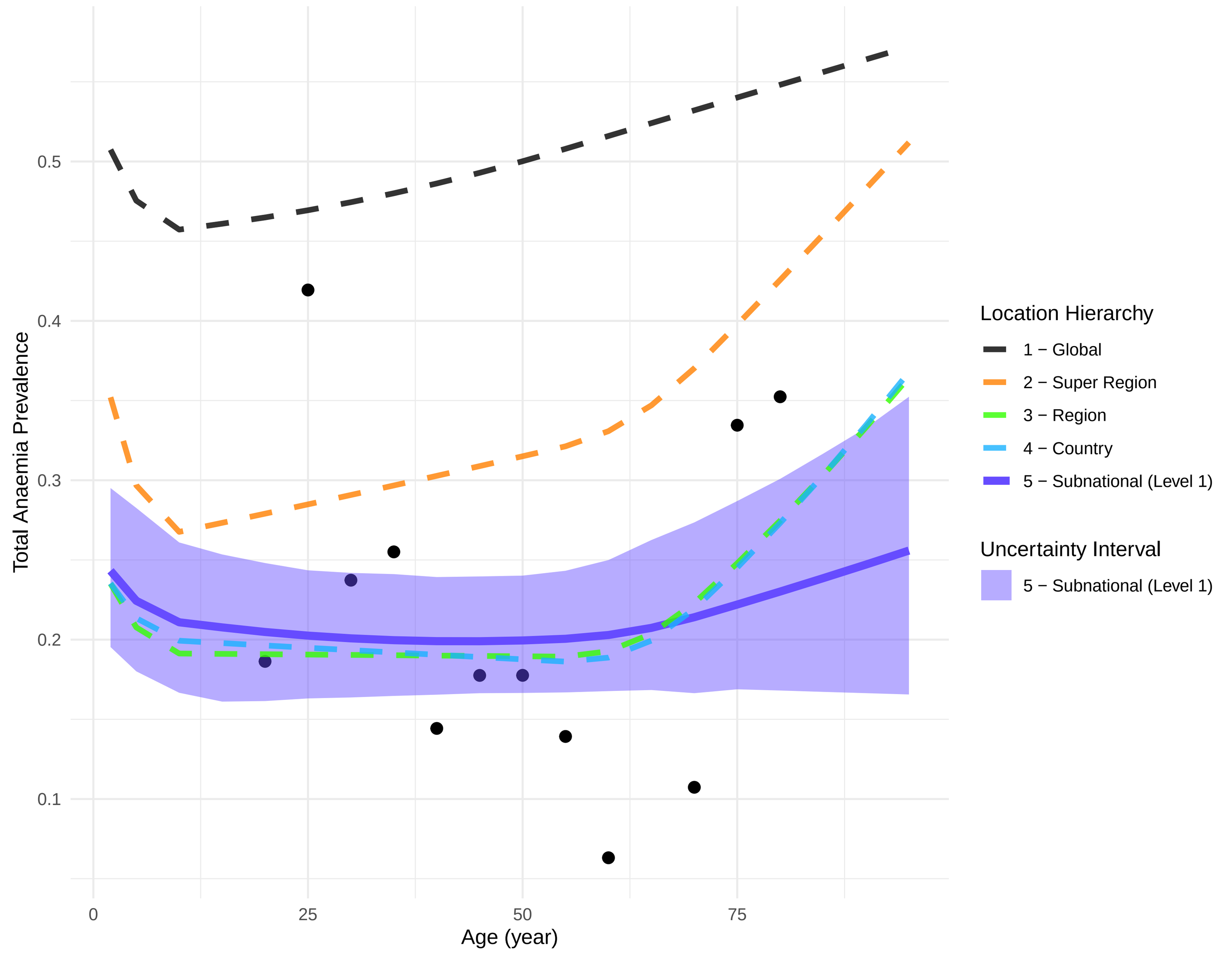

MR−BRT Age/Sex Fits − Non−pregnant Females 2+ Years Old − Total Anaemia Prevalence − Distrito Federal
WHO−2024 Adjustment Method

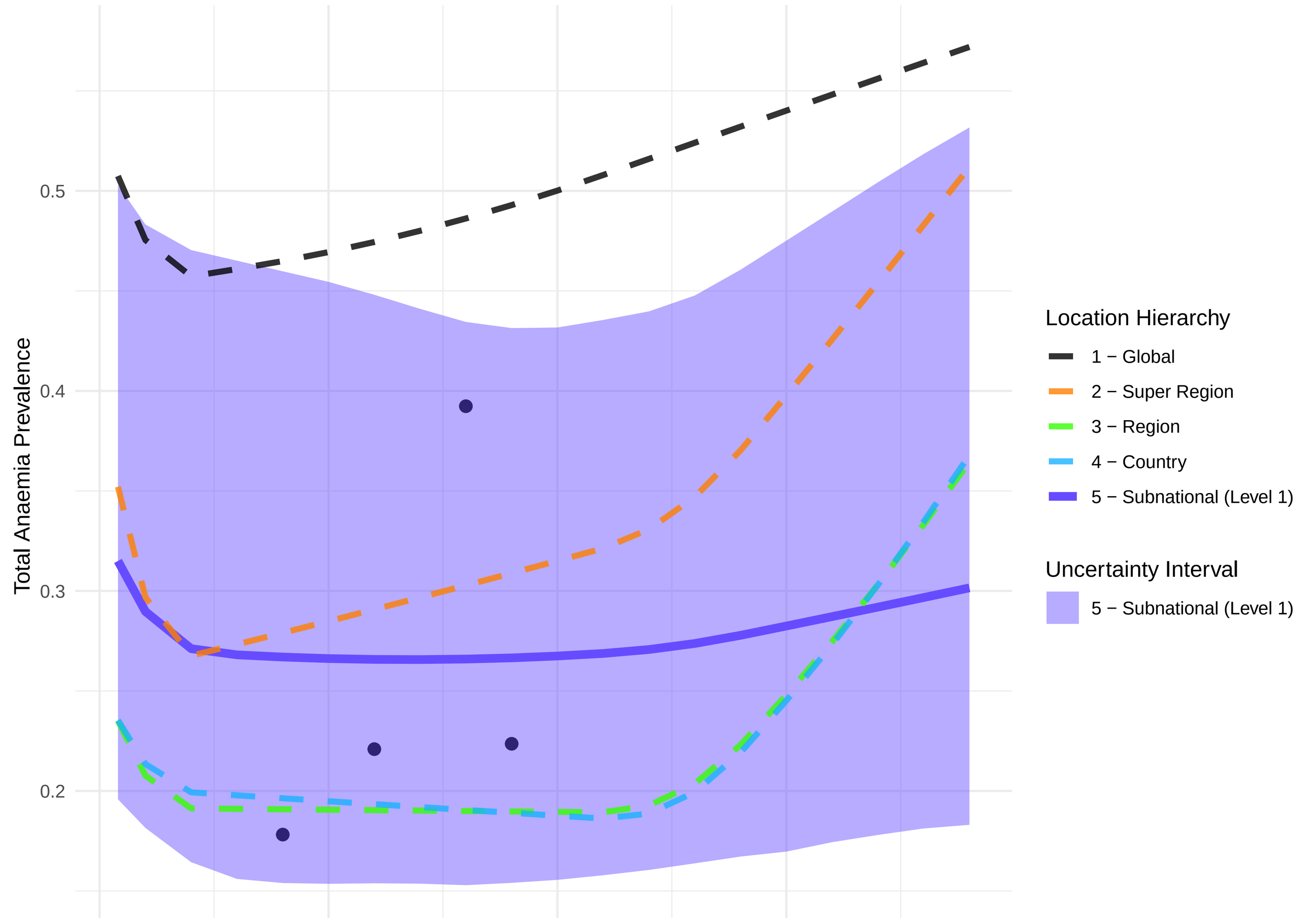

MR−BRT Age/Sex Fits − Non−pregnant Females 2+ Years Old − Total Anaemia Prevalence − Espírito Santo
WHO−2024 Adjustment Method

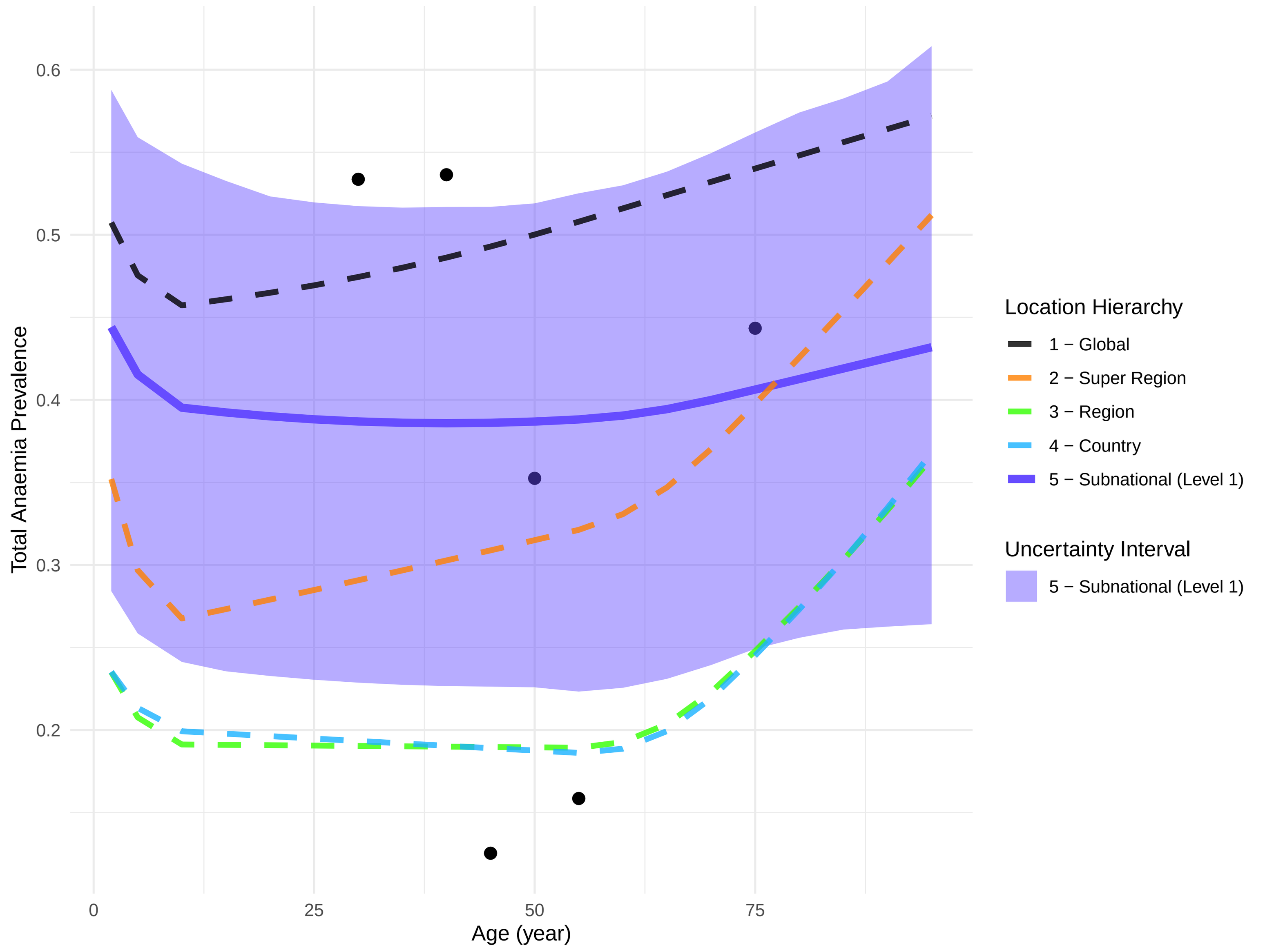

MR−BRT Age/Sex Fits − Non−pregnant Females 2+ Years Old − Total Anaemia Prevalence − Goiás
WHO−2024 Adjustment Method

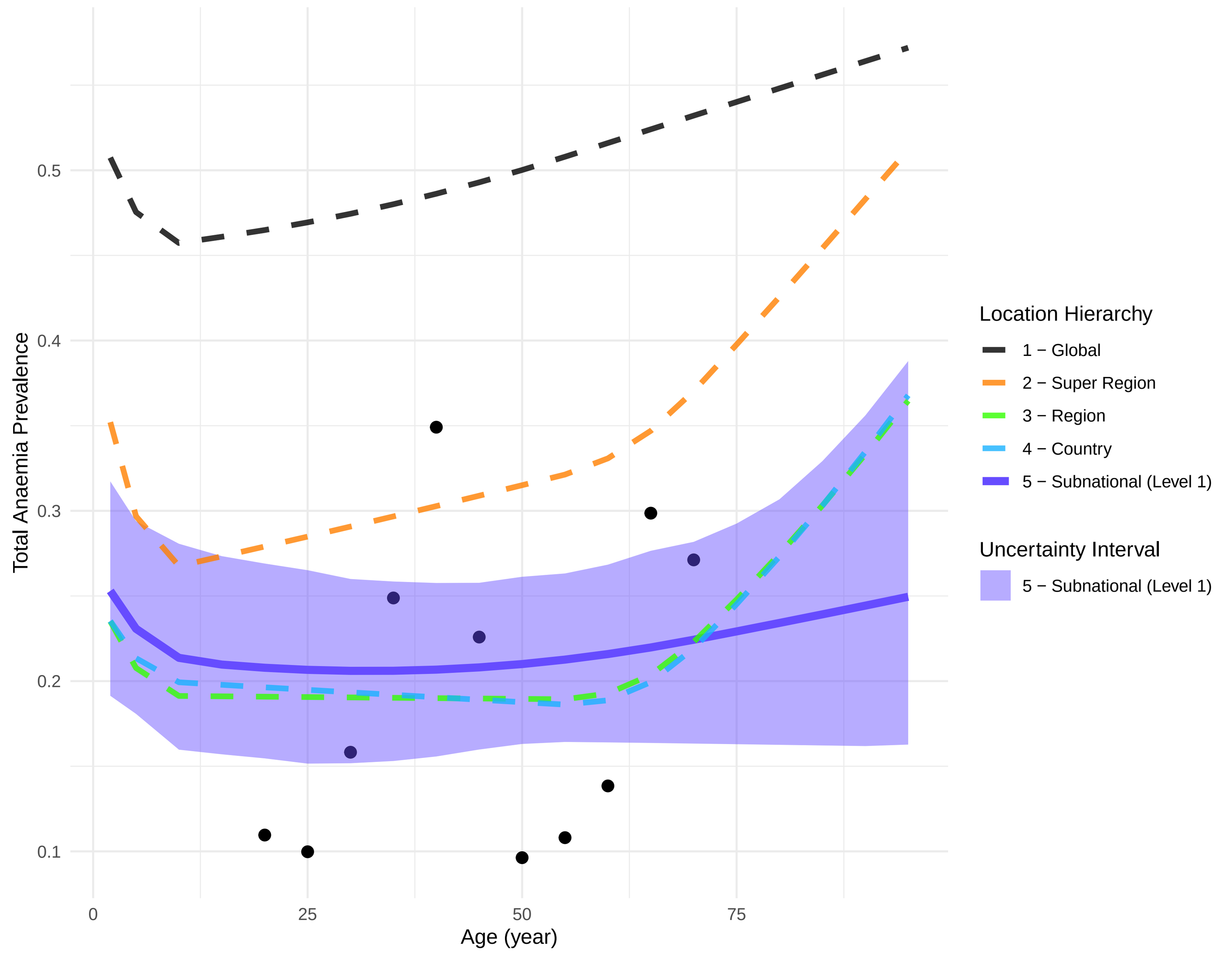

MR−BRT Age/Sex Fits − Non−pregnant Females 2+ Years Old − Total Anaemia Prevalence − Maranhão
WHO−2024 Adjustment Method

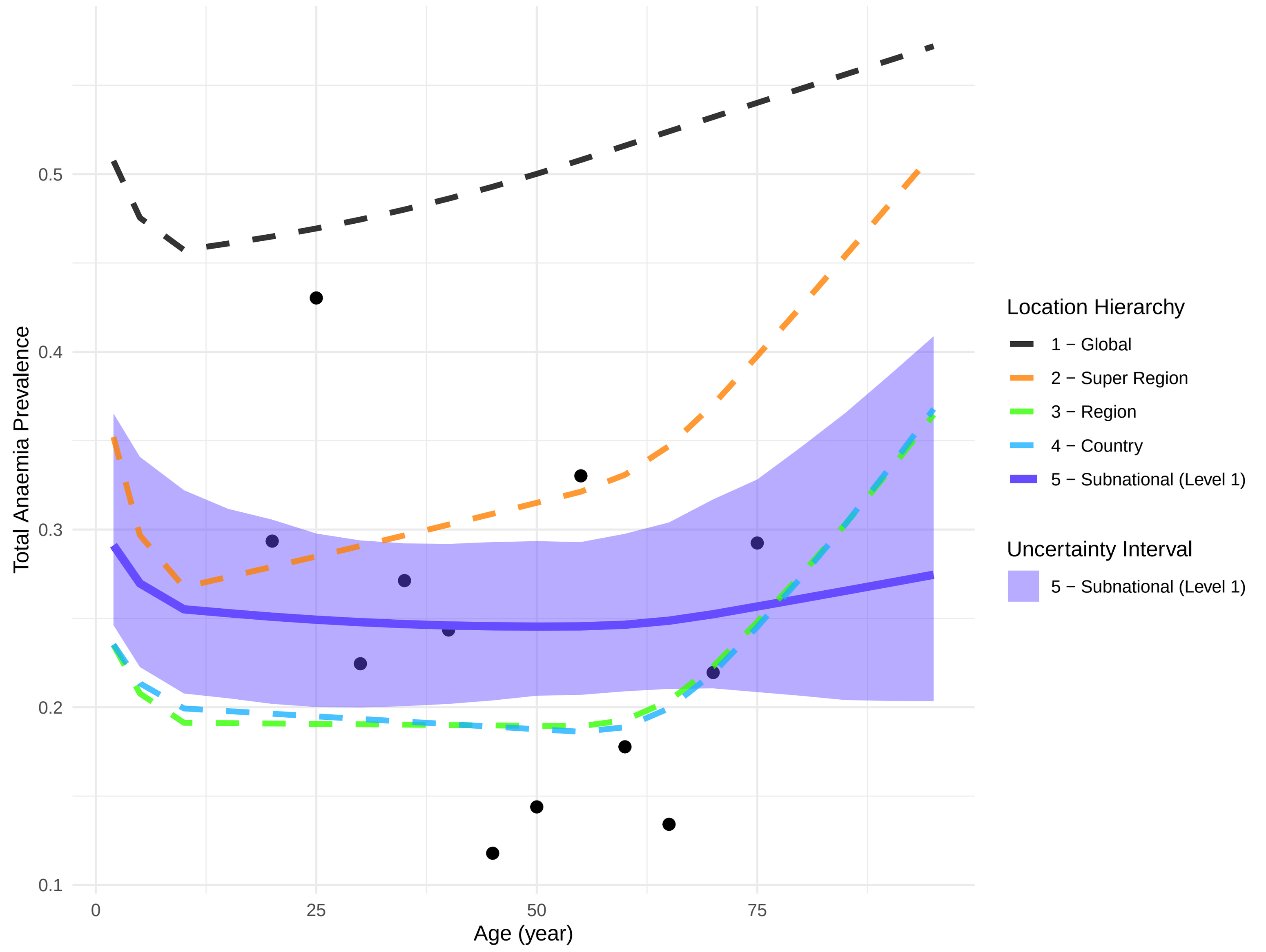

MR−BRT Age/Sex Fits − Non−pregnant Females 2+ Years Old − Total Anaemia Prevalence − Mato Grosso
WHO−2024 Adjustment Method

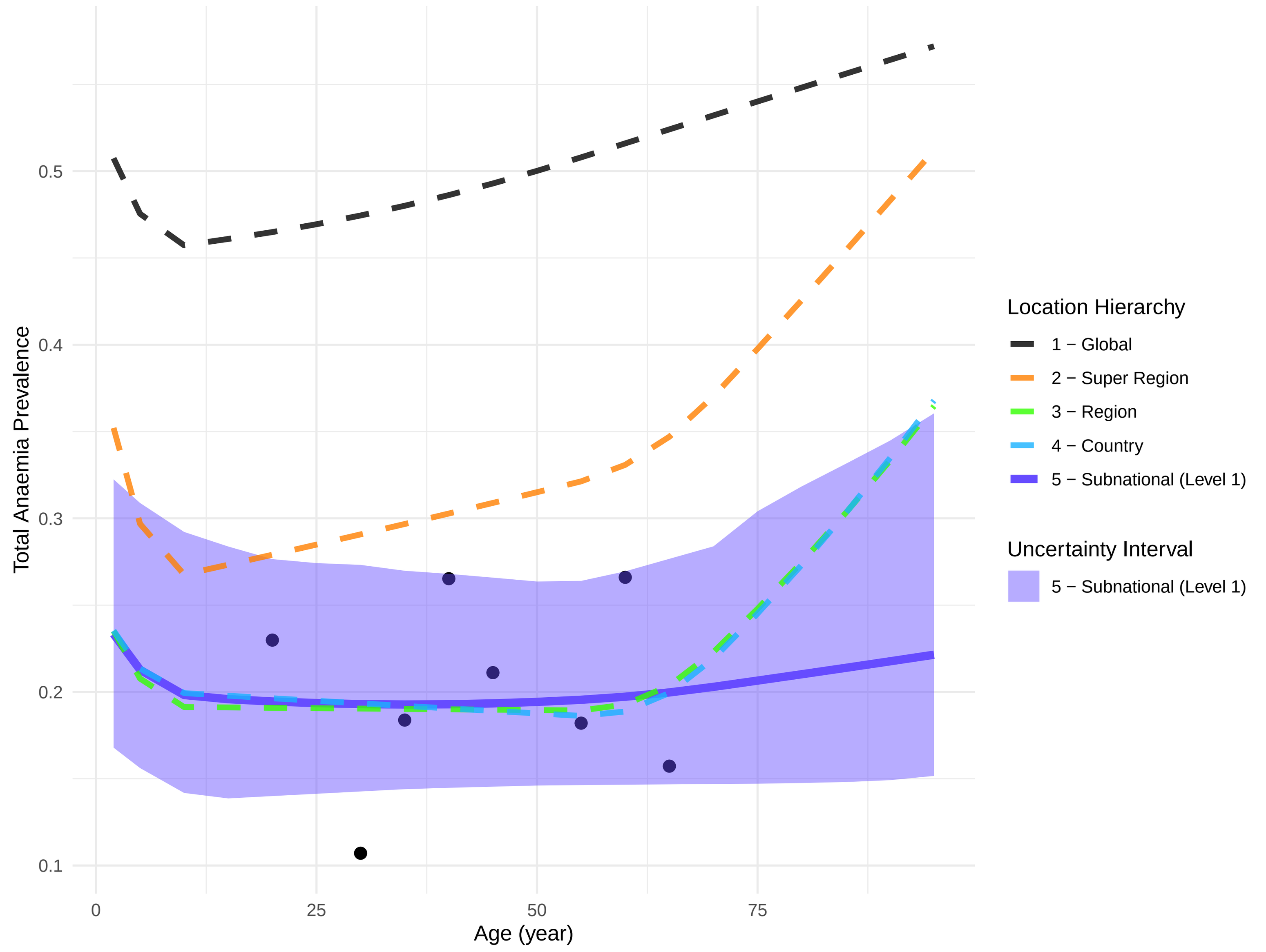

MR−BRT Age/Sex Fits − Non−pregnant Females 2+ Years Old − Total Anaemia Prevalence − Mato Grosso do Sul
WHO−2024 Adjustment Method

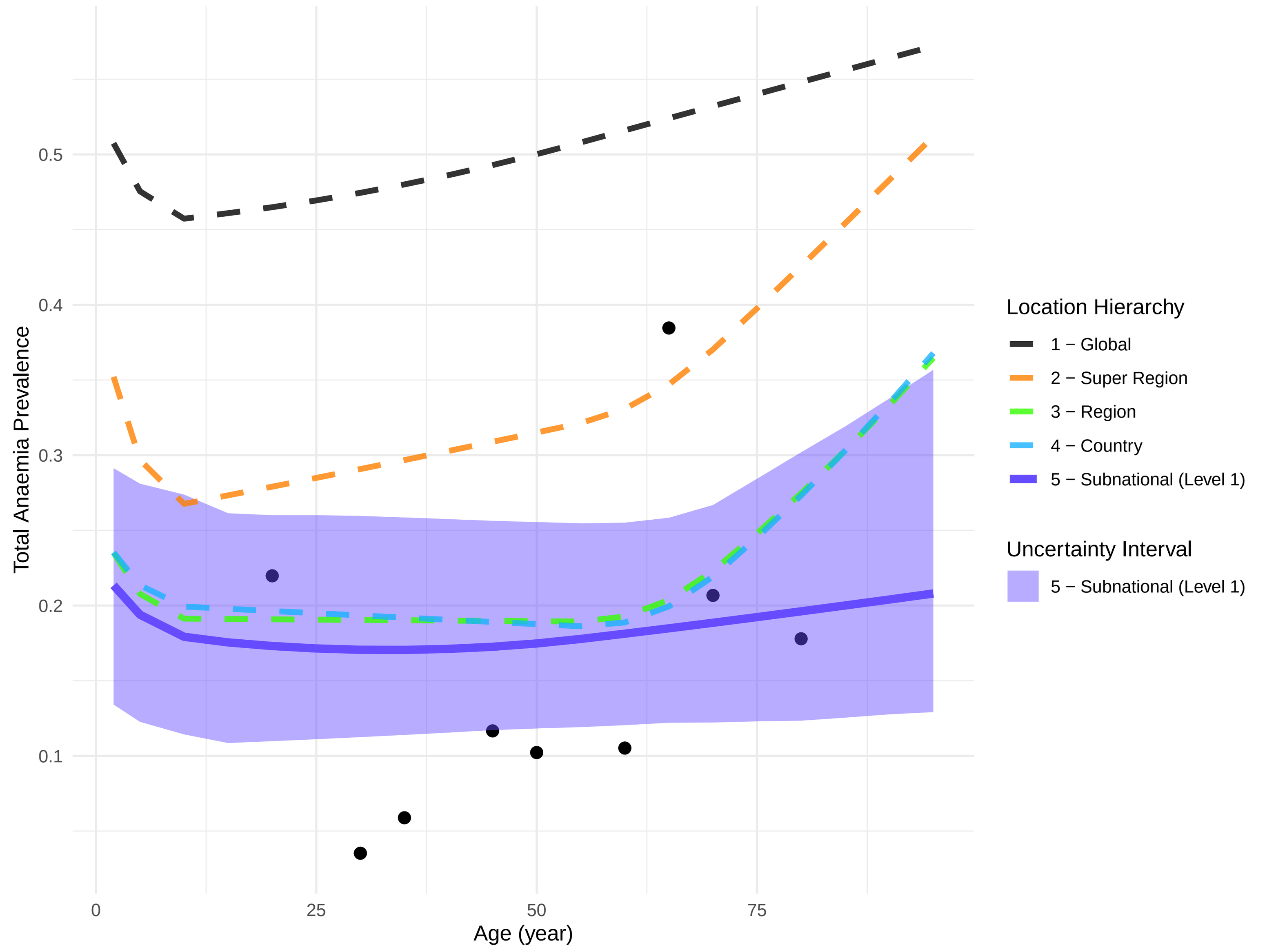

MR−BRT Age/Sex Fits − Non−pregnant Females 2+ Years Old − Total Anaemia Prevalence − Minas Gerais
WHO−2024 Adjustment Method

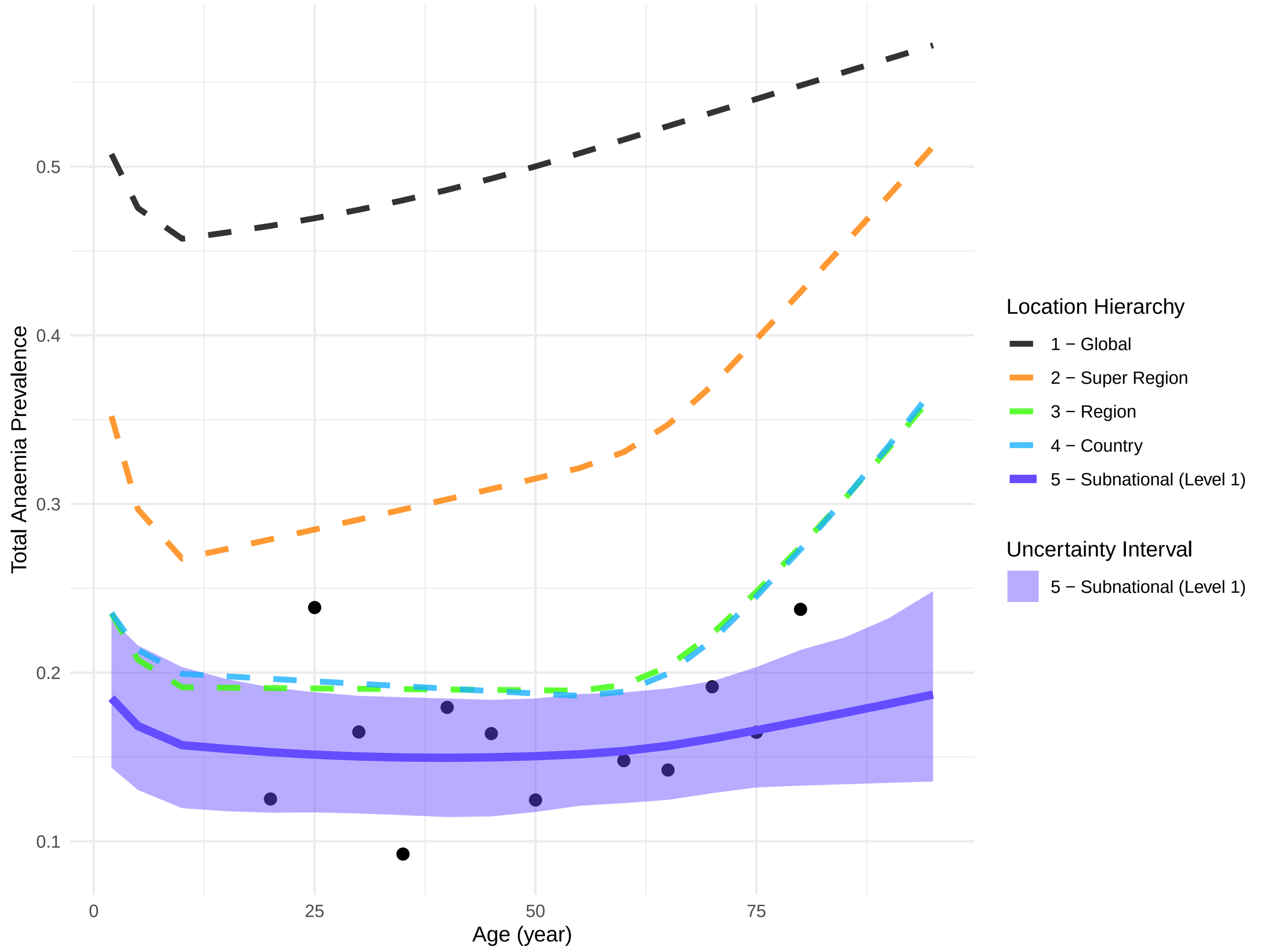

MR−BRT Age/Sex Fits − Non−pregnant Females 2+ Years Old − Total Anaemia Prevalence − Pará
WHO−2024 Adjustment Method

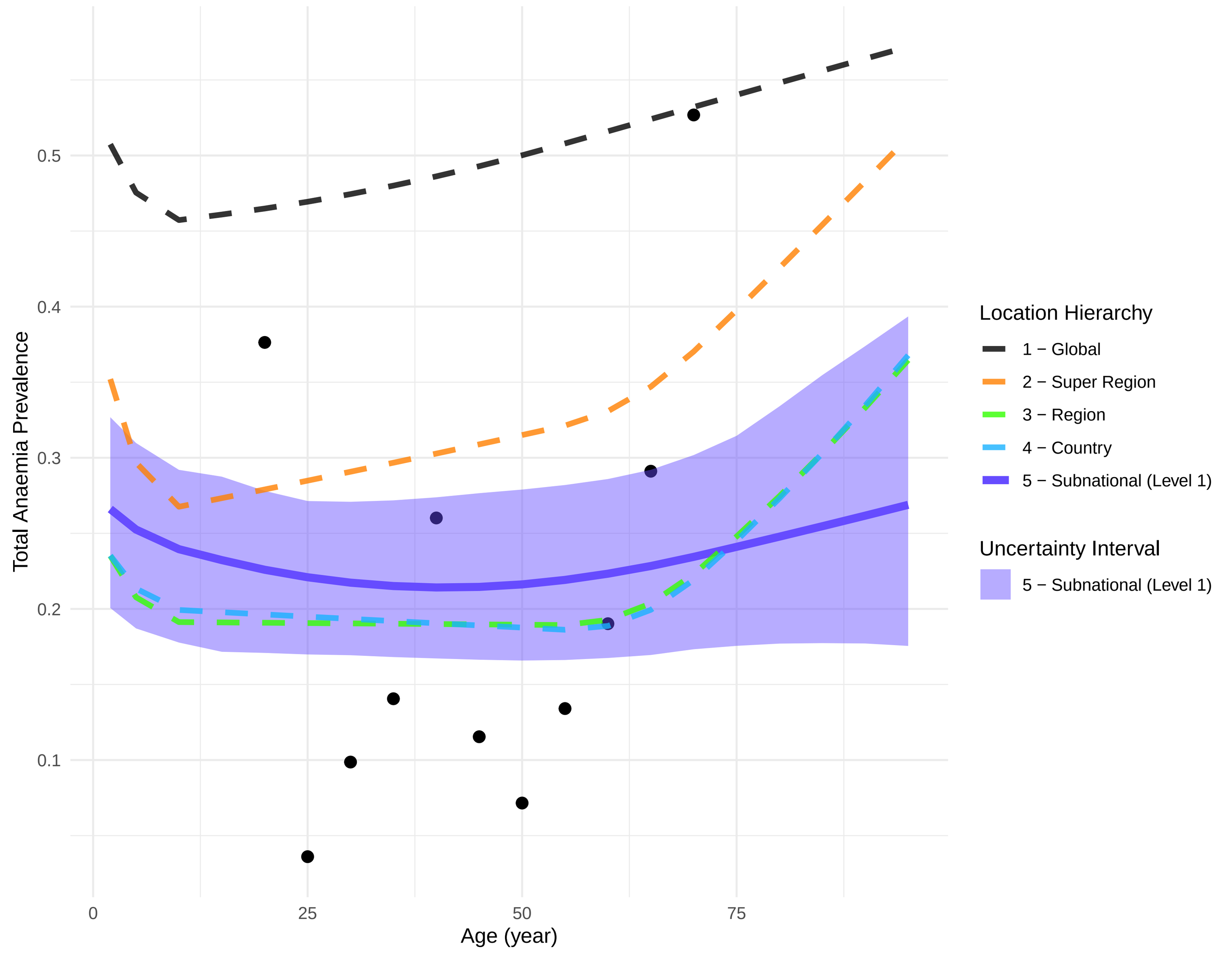

MR−BRT Age/Sex Fits − Non−pregnant Females 2+ Years Old − Total Anaemia Prevalence − Paraíba
WHO−2024 Adjustment Method

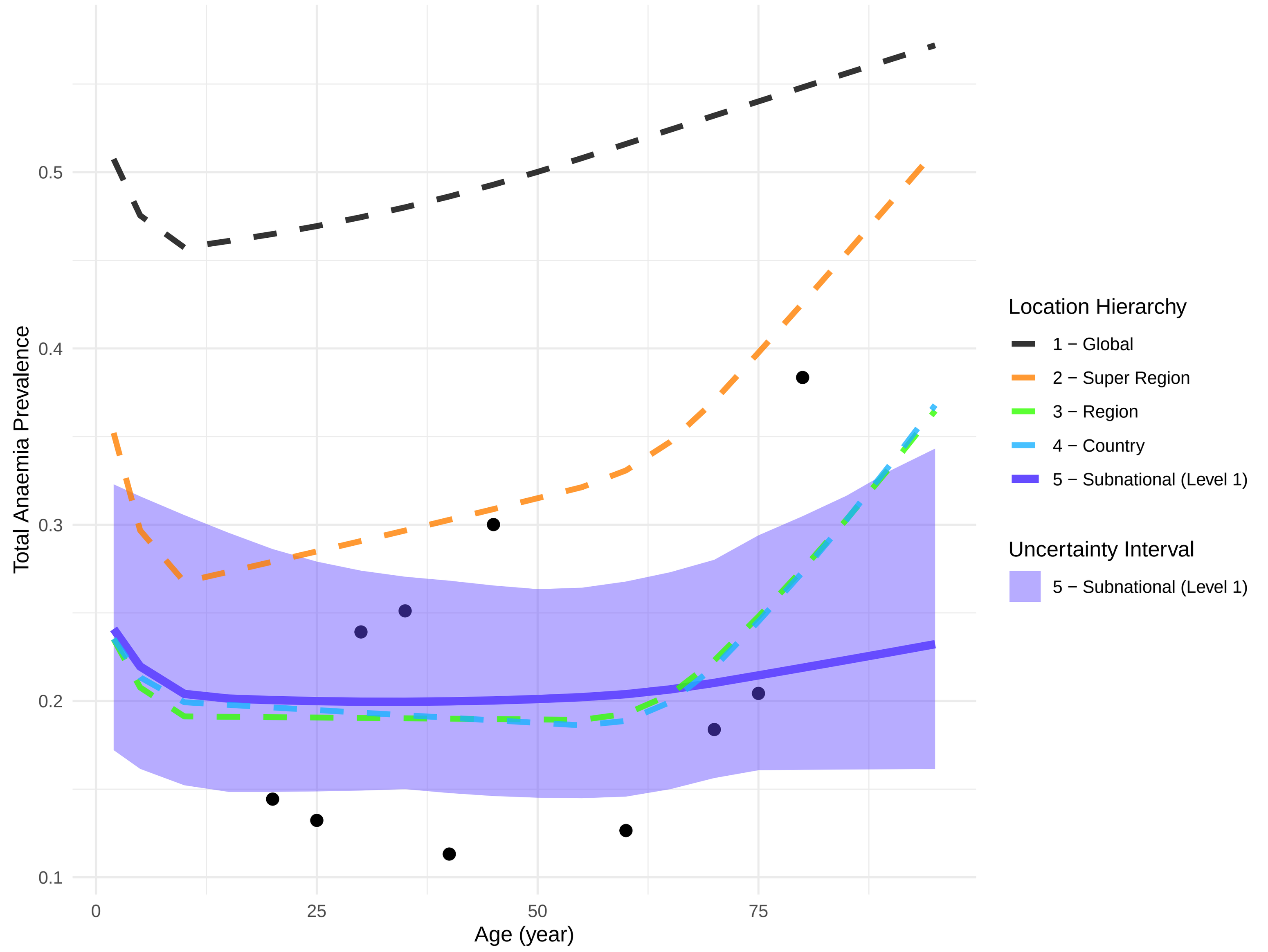

MR−BRT Age/Sex Fits − Non−pregnant Females 2+ Years Old − Total Anaemia Prevalence − Paraná
WHO−2024 Adjustment Method

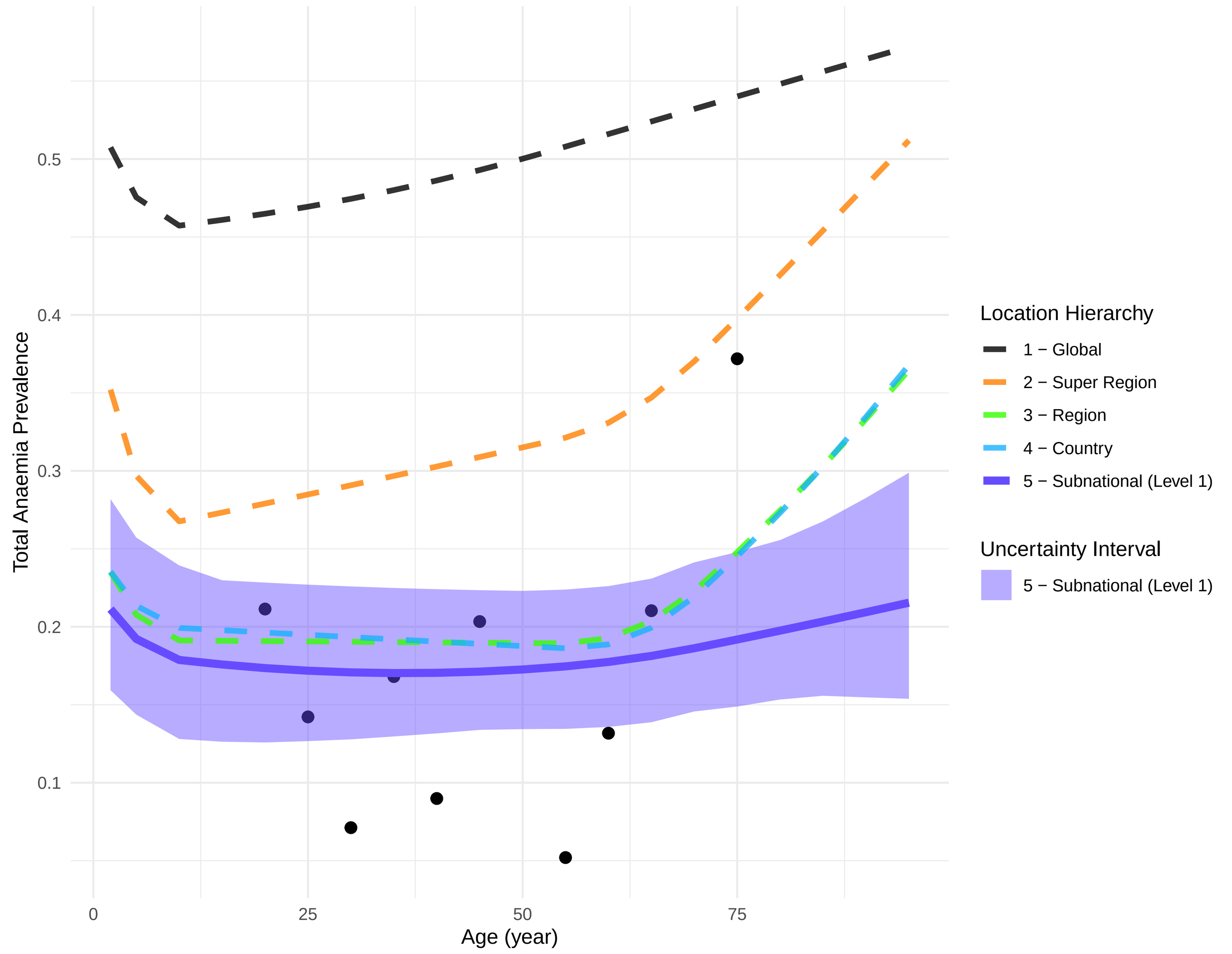

MR−BRT Age/Sex Fits − Non−pregnant Females 2+ Years Old − Total Anaemia Prevalence − Pernambuco
WHO−2024 Adjustment Method

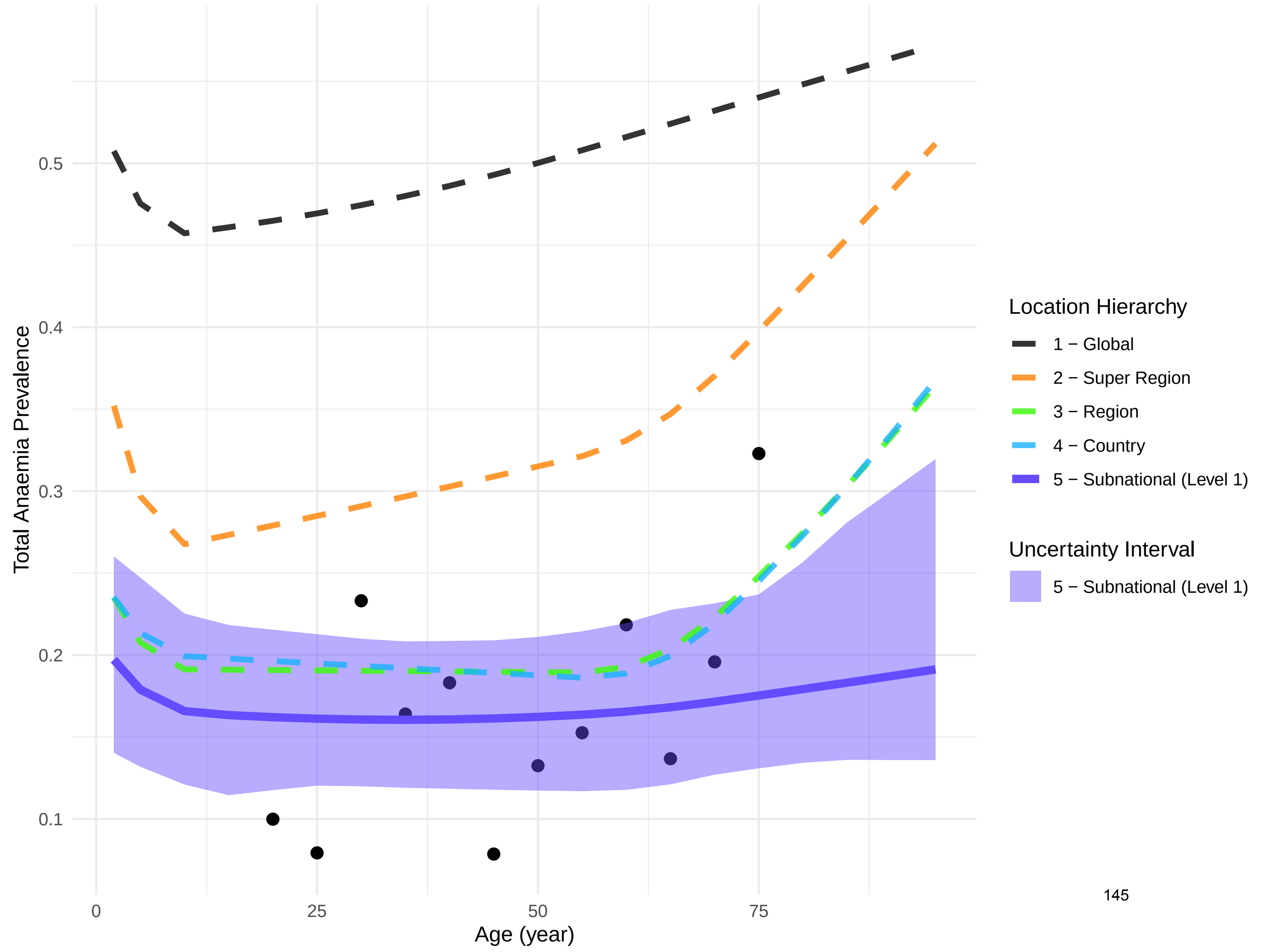

MR−BRT Age/Sex Fits − Non−pregnant Females 2+ Years Old − Total Anaemia Prevalence − Piaui
WHO−2024 Adjustment Method

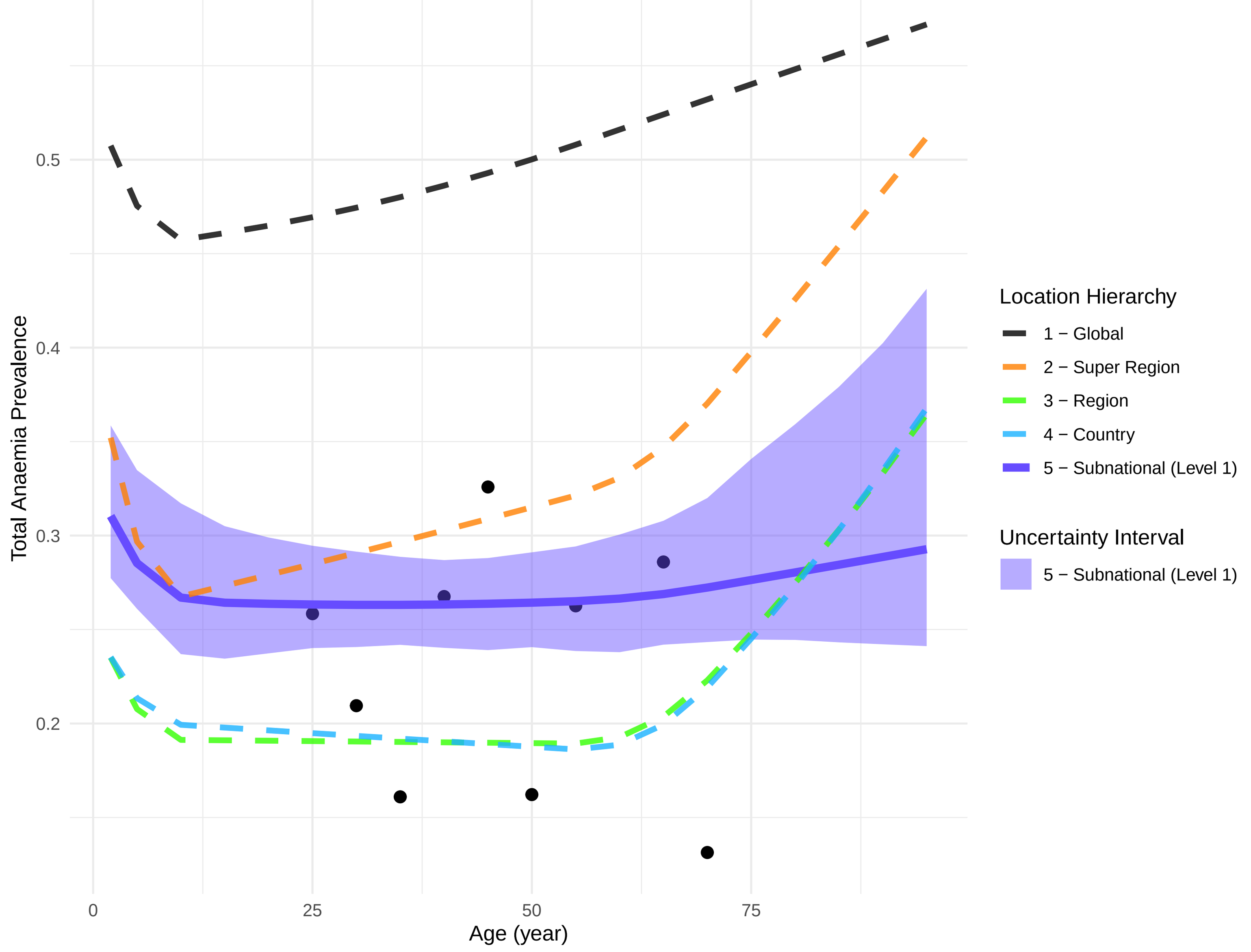

MR−BRT Age/Sex Fits − Non−pregnant Females 2+ Years Old − Total Anaemia Prevalence − Rio de Janeiro
WHO−2024 Adjustment Method

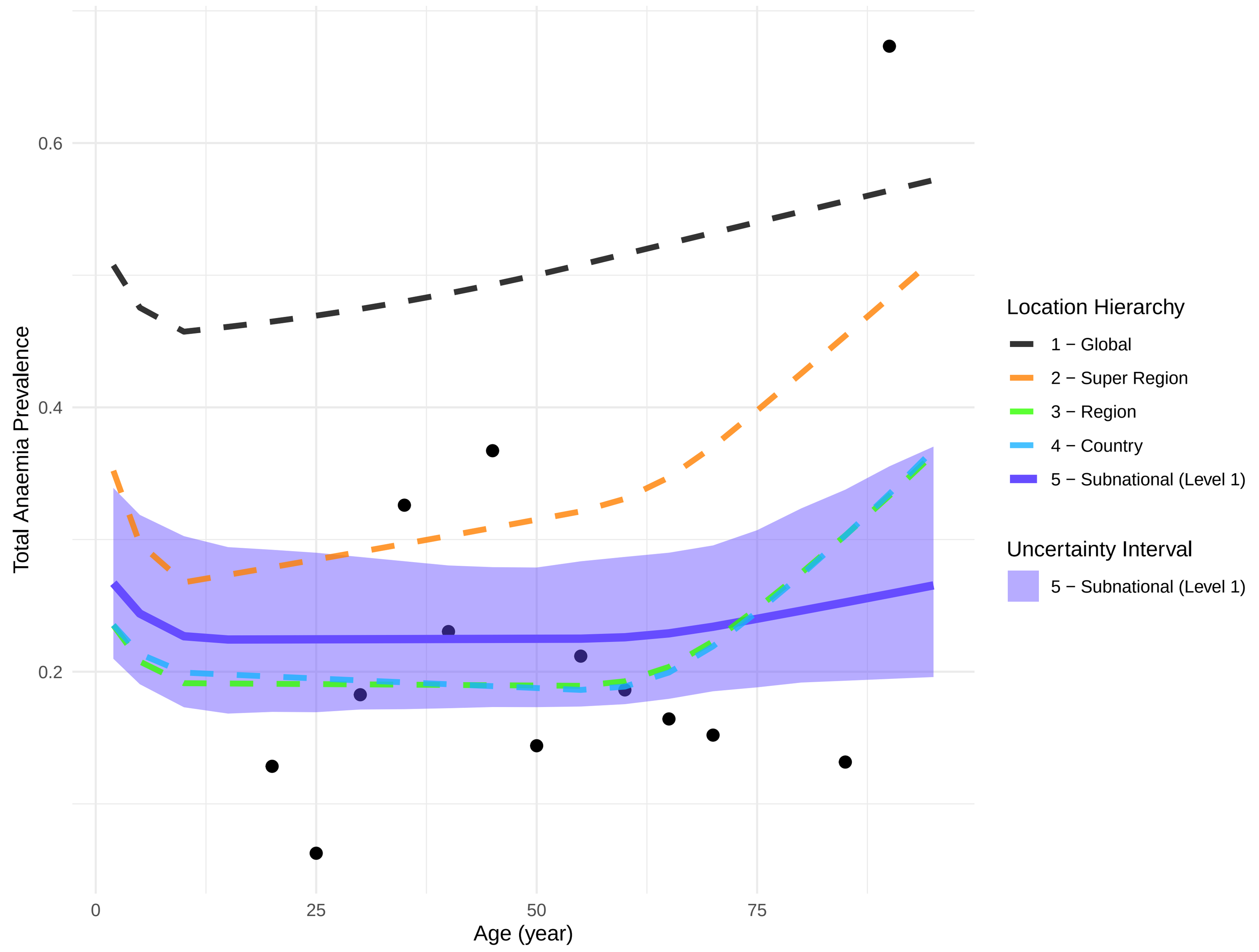

MR−BRT Age/Sex Fits − Non−pregnant Females 2+ Years Old − Total Anaemia Prevalence − Rio Grande do Norte
WHO−2024 Adjustment Method

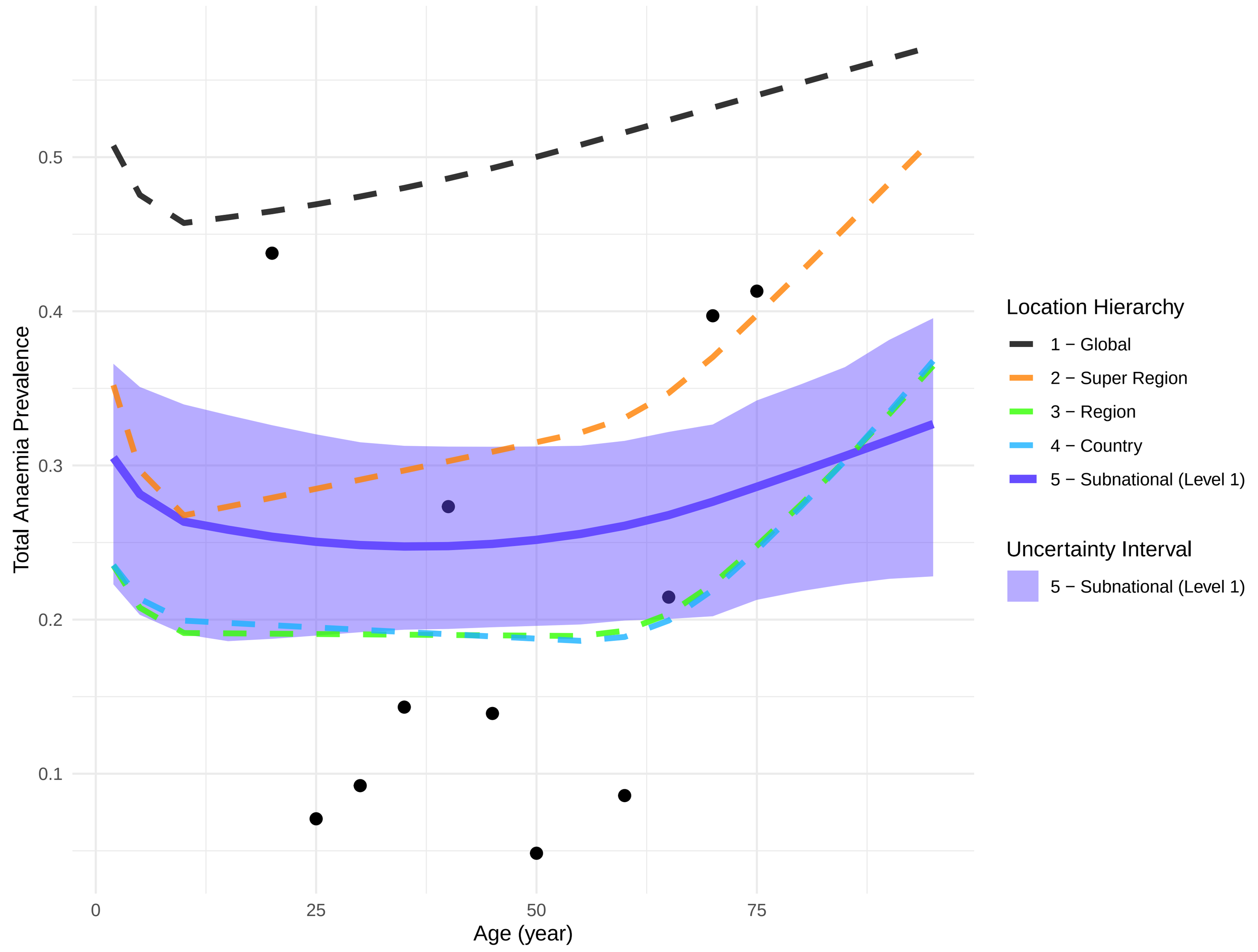

MR−BRT Age/Sex Fits − Non−pregnant Females 2+ Years Old − Total Anaemia Prevalence − Rio Grande do Sul
WHO−2024 Adjustment Method

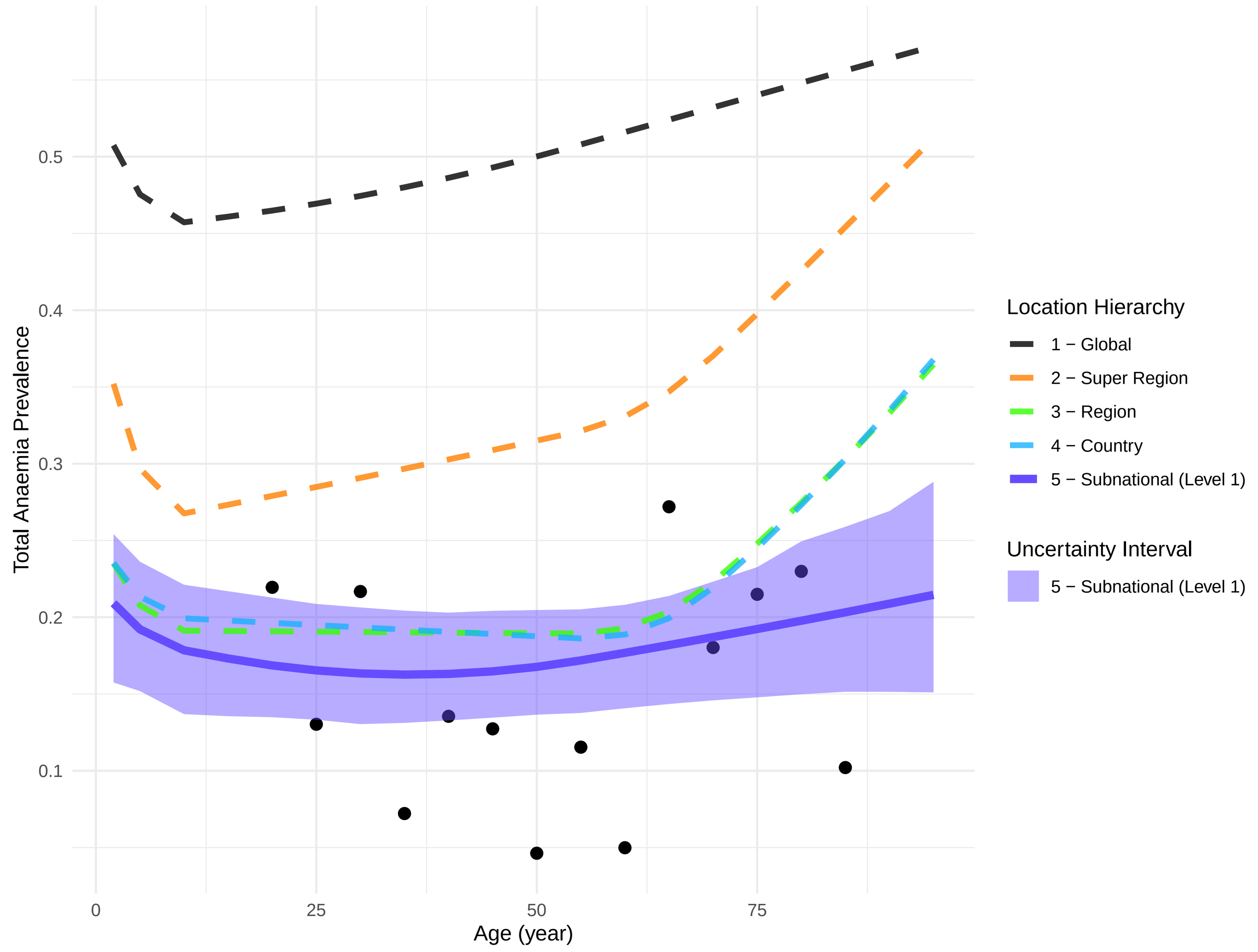

MR−BRT Age/Sex Fits − Non−pregnant Females 2+ Years Old − Total Anaemia Prevalence − Rondônia
WHO−2024 Adjustment Method

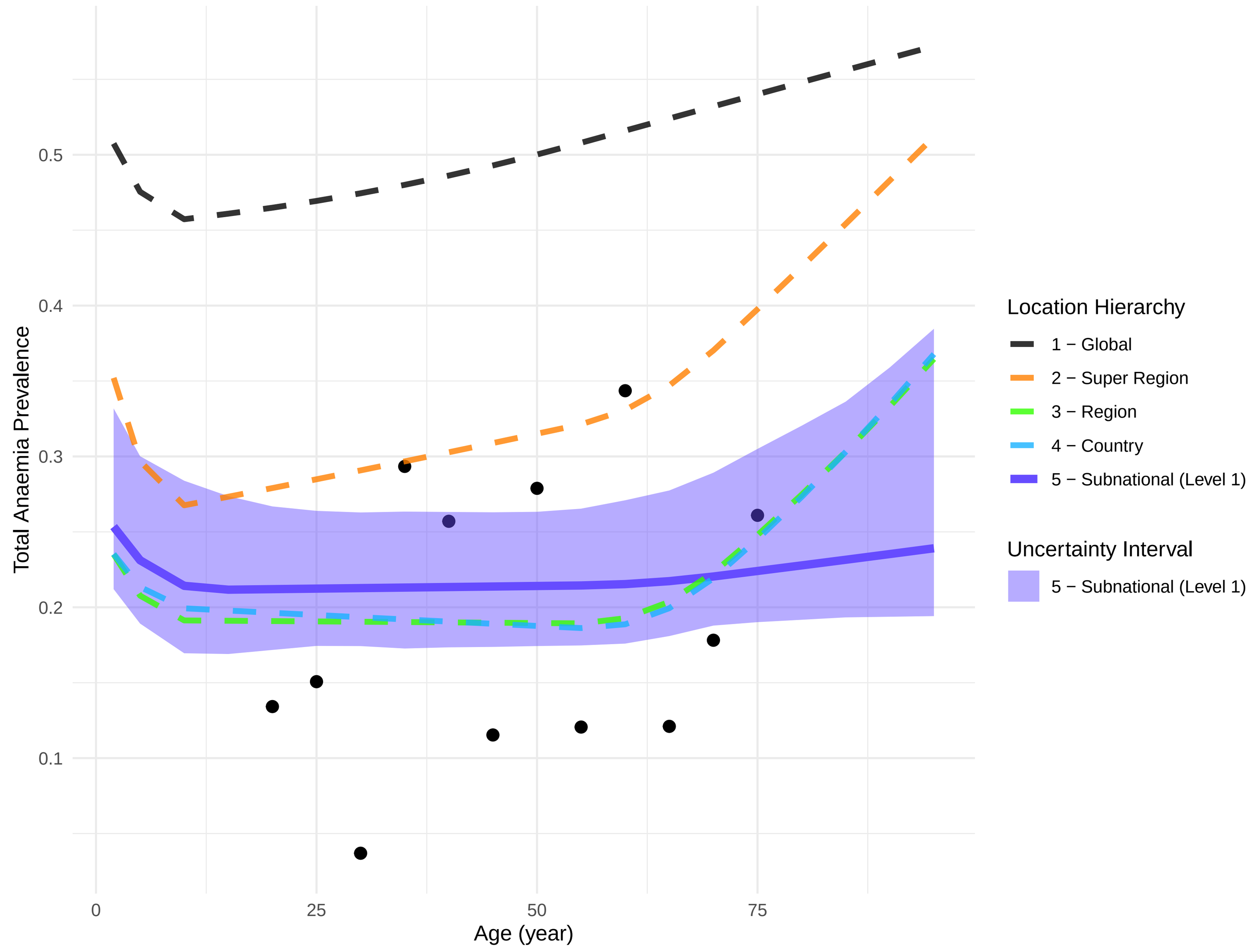

MR−BRT Age/Sex Fits − Non−pregnant Females 2+ Years Old − Total Anaemia Prevalence − Roraima
WHO−2024 Adjustment Method

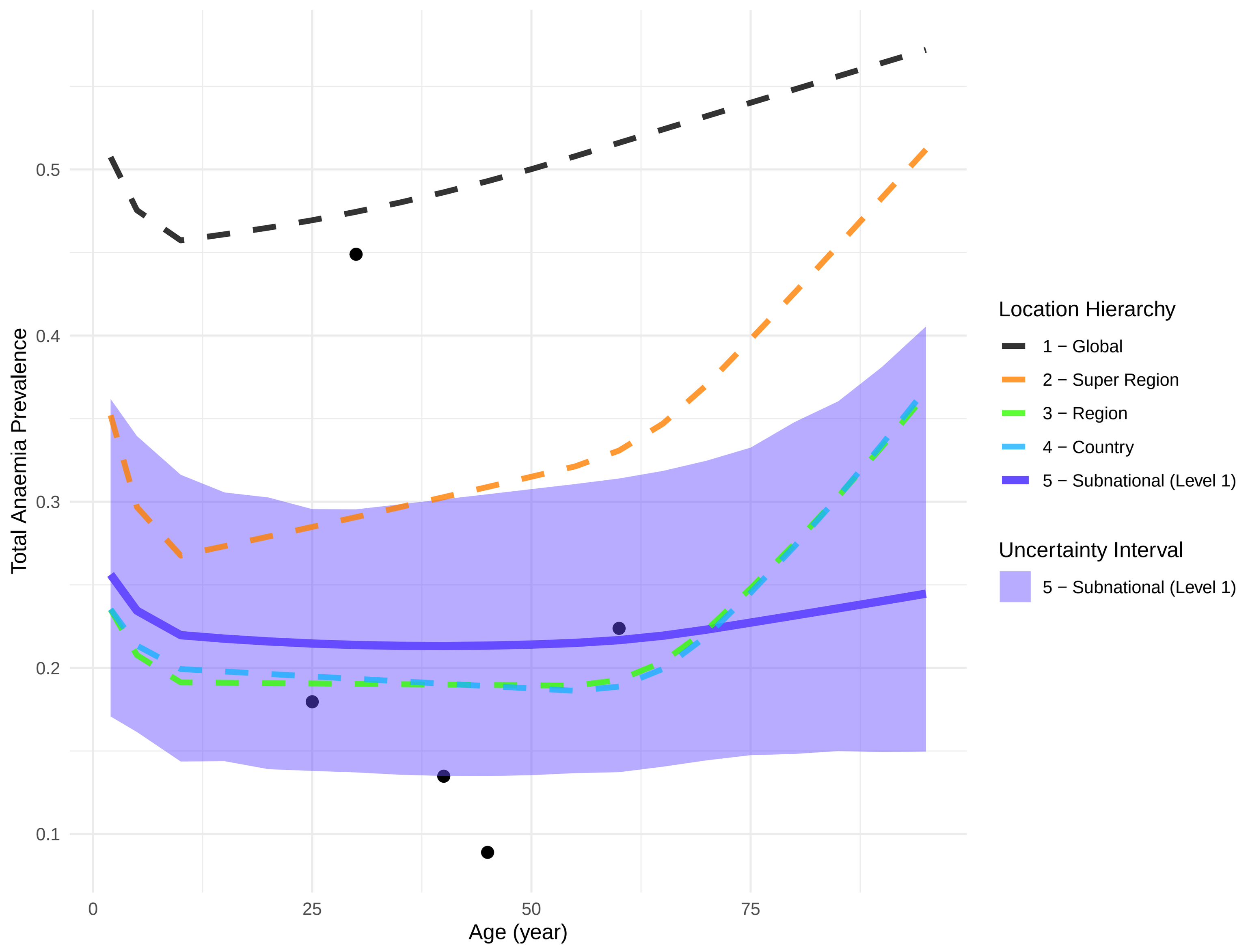

MR−BRT Age/Sex Fits − Non−pregnant Females 2+ Years Old − Total Anaemia Prevalence − Santa Catarina
WHO−2024 Adjustment Method

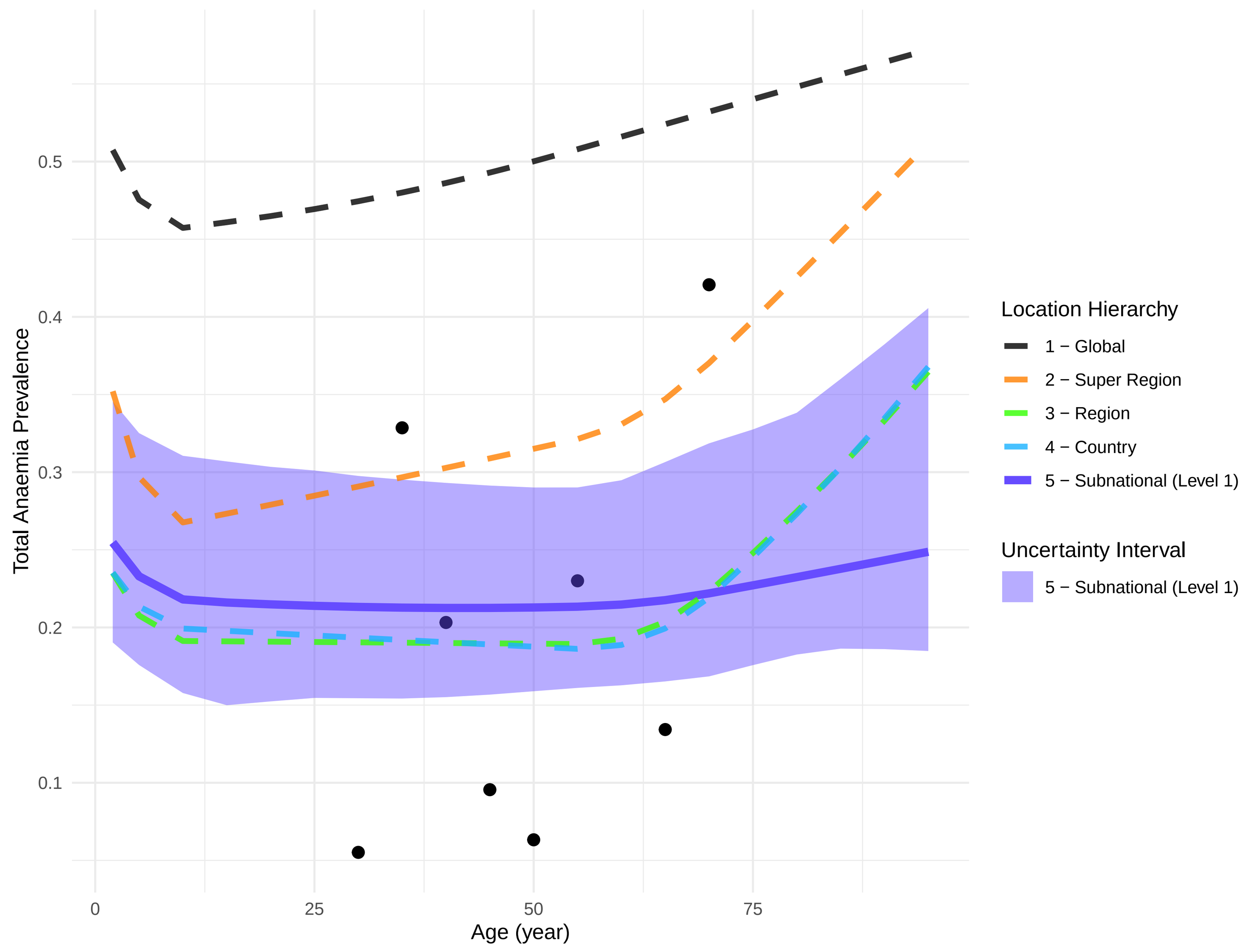

MR−BRT Age/Sex Fits − Non−pregnant Females 2+ Years Old − Total Anaemia Prevalence − São Paulo
WHO−2024 Adjustment Method

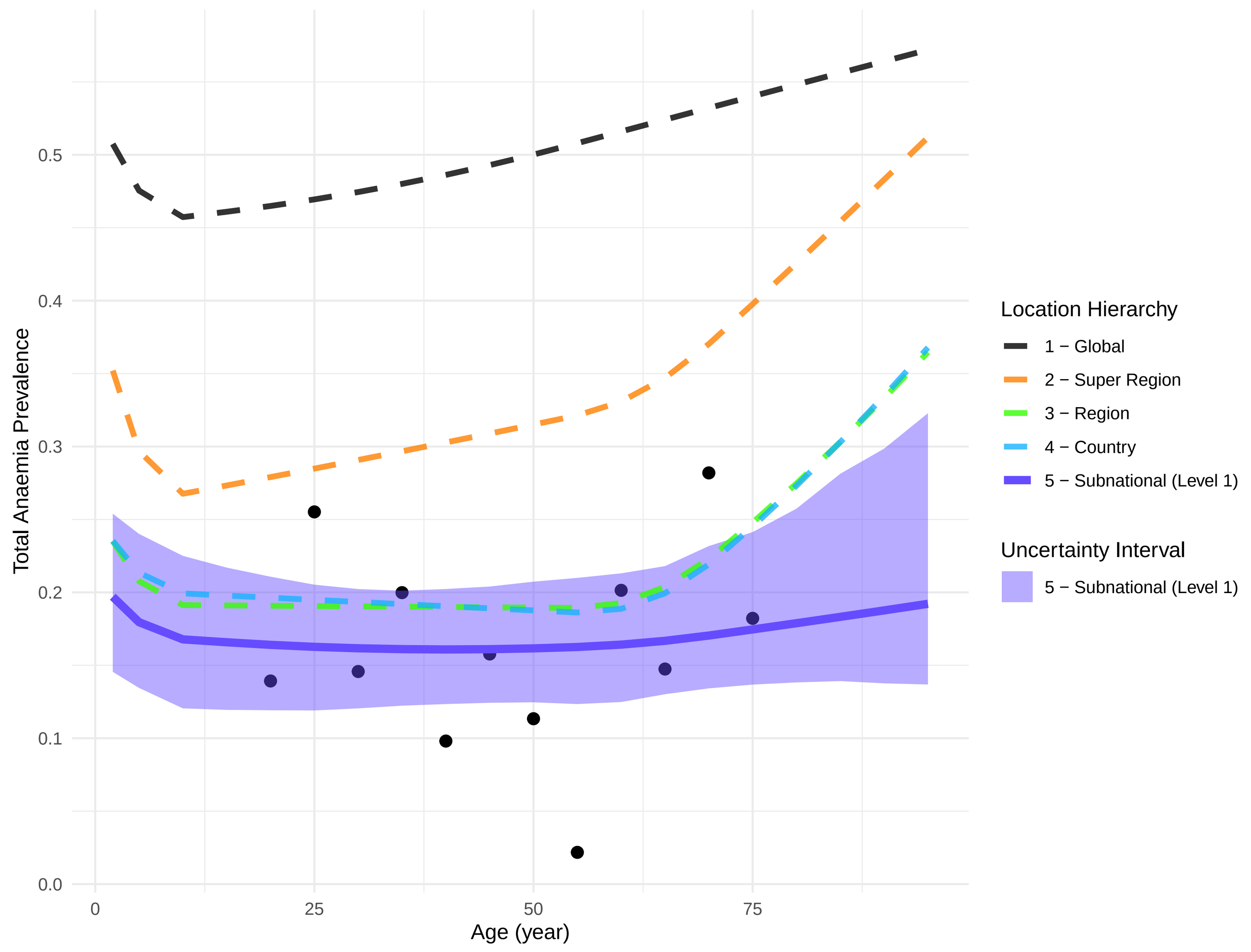

MR−BRT Age/Sex Fits − Non−pregnant Females 2+ Years Old − Total Anaemia Prevalence − Sergipe
WHO−2024 Adjustment Method

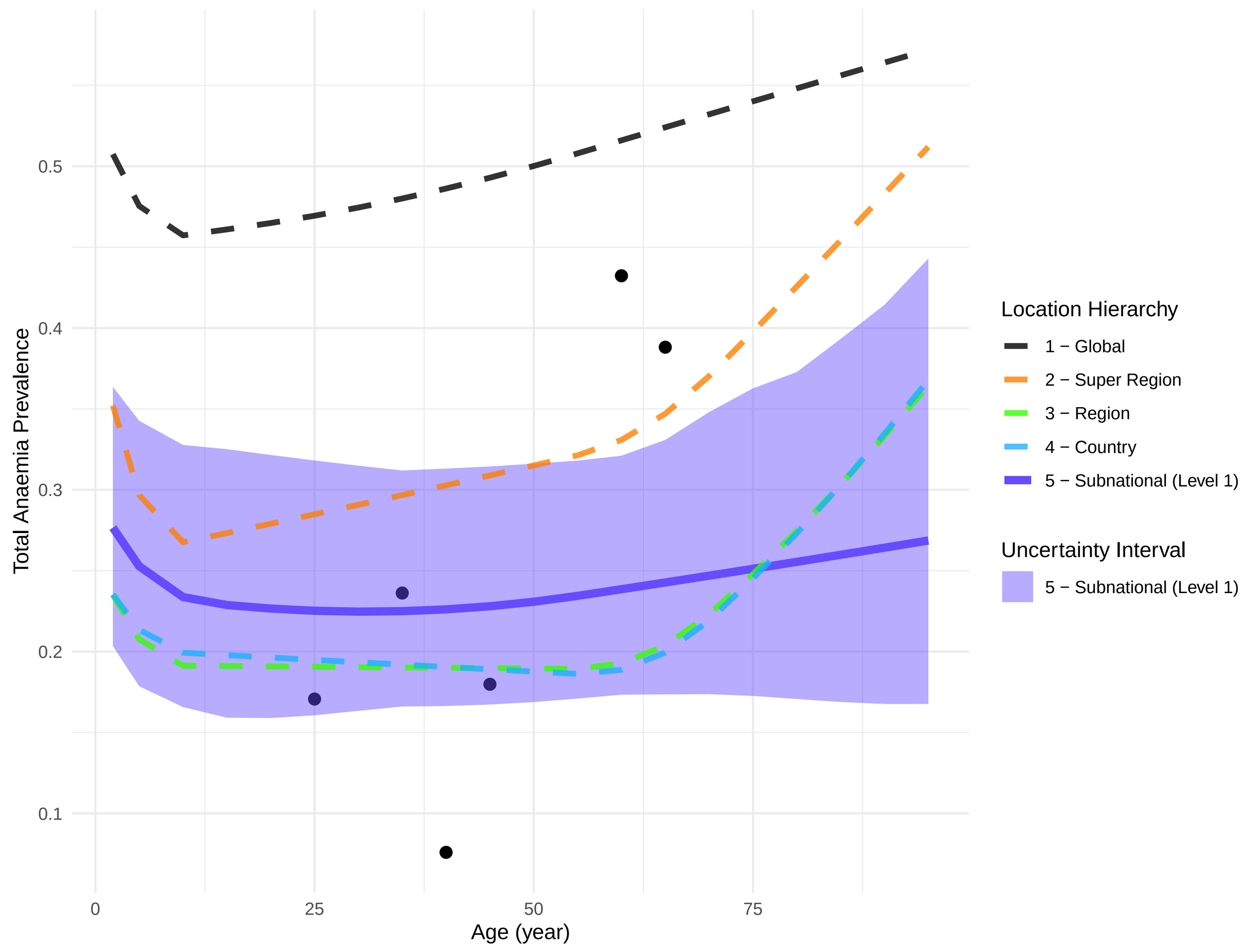

MR−BRT Age/Sex Fits − Non−pregnant Females 2+ Years Old − Total Anaemia Prevalence − Tocantins
WHO−2024 Adjustment Method

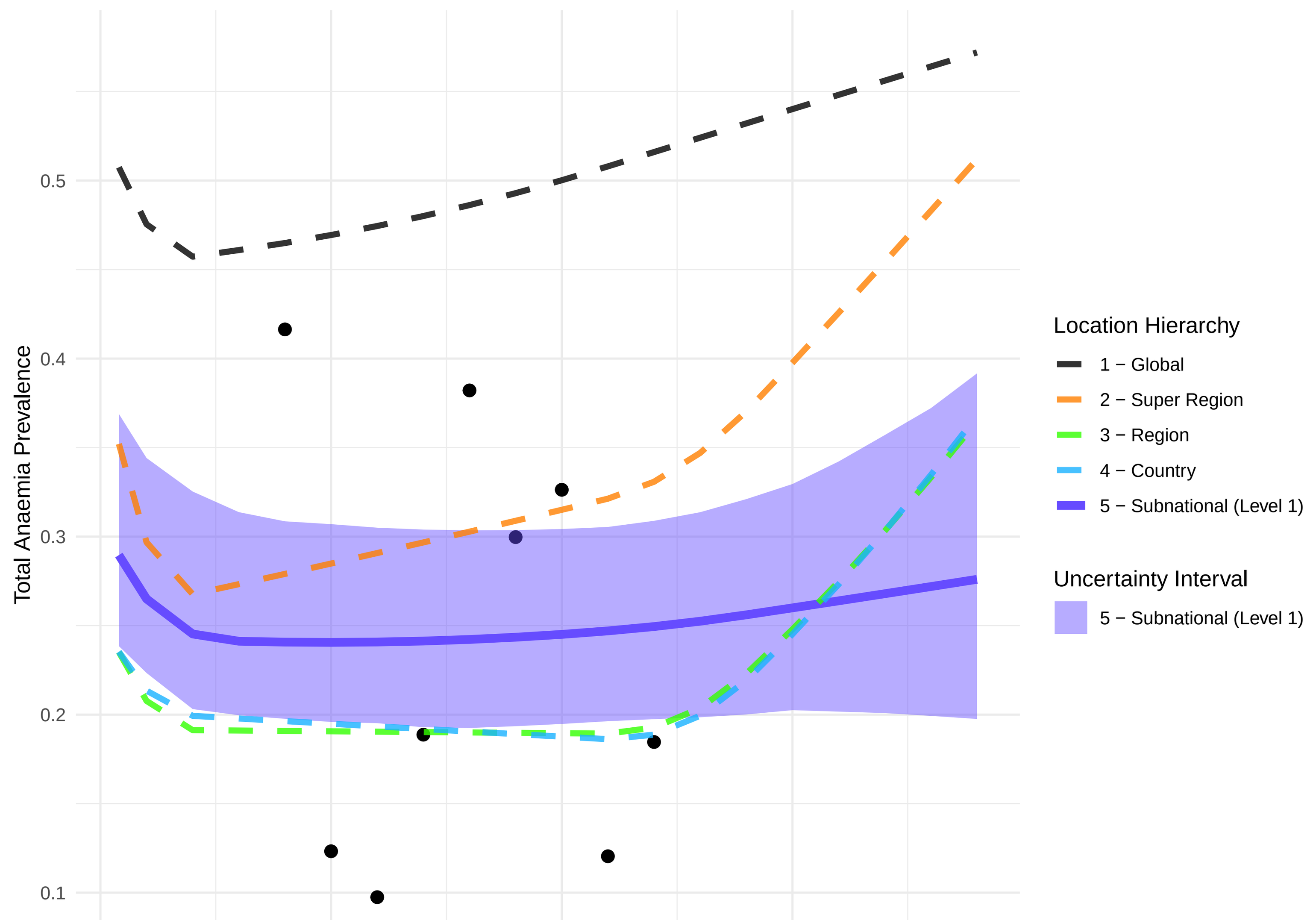

MR−BRT Age/Sex Fits − Non−pregnant Females 2+ Years Old − Total Anaemia Prevalence − North Africa and Middle East
WHO−2024 Adjustment Method

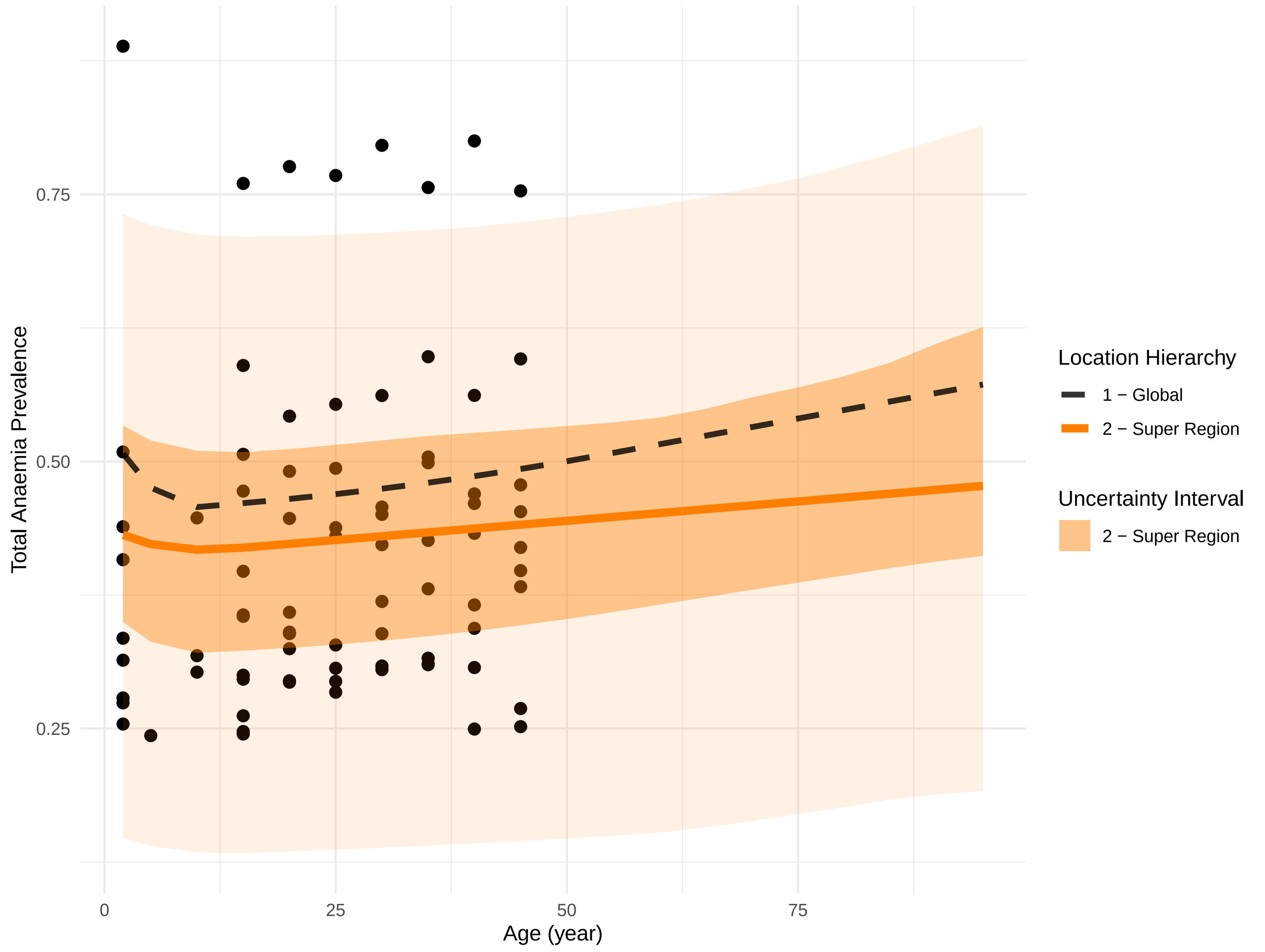

MR−BRT Age/Sex Fits − Non−pregnant Females 2+ Years Old − Total Anaemia Prevalence − North Africa and Middle East
WHO−2024 Adjustment Method

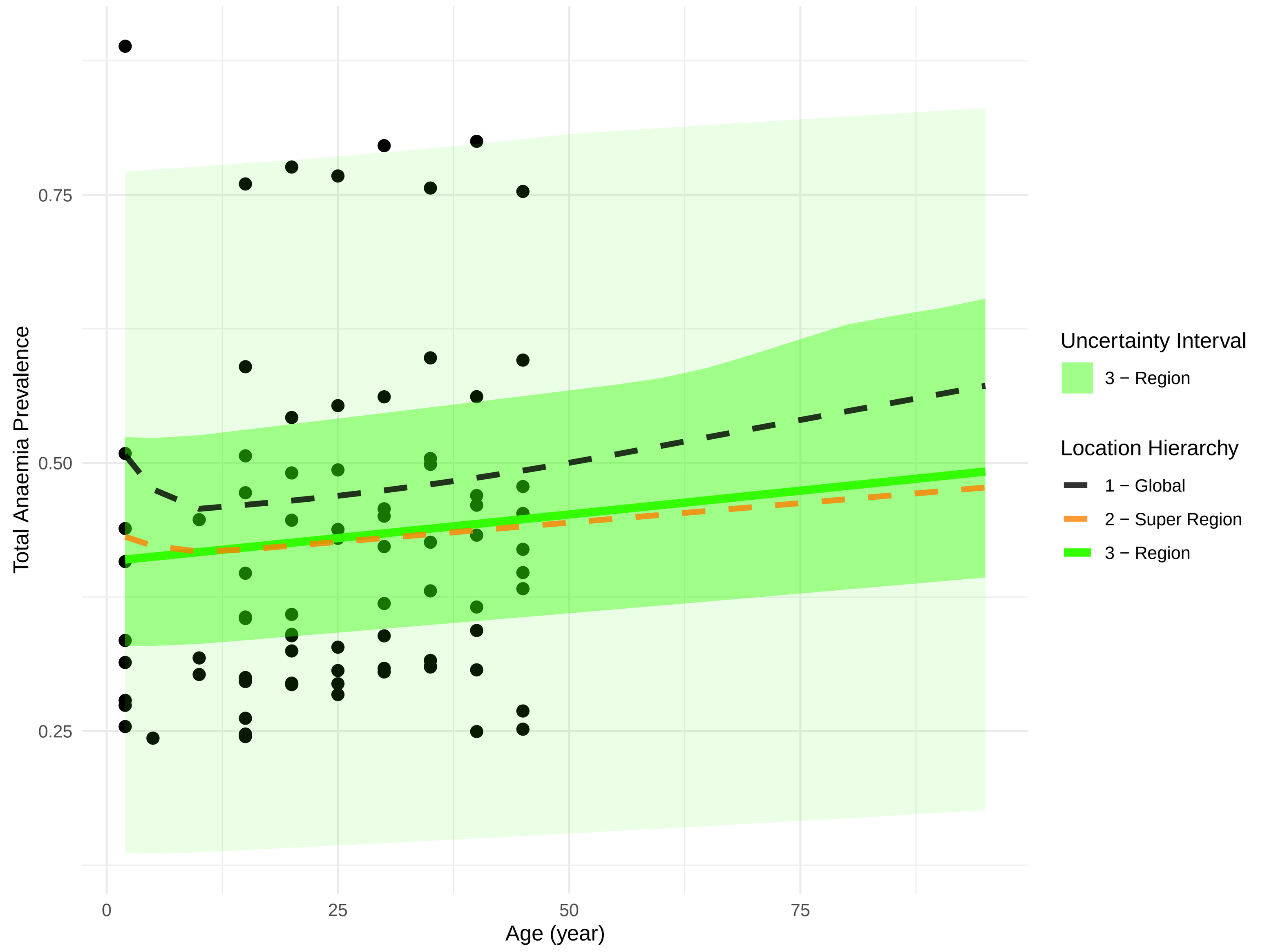

MR−BRT Age/Sex Fits − Non−pregnant Females 2+ Years Old − Total Anaemia Prevalence − Egypt
WHO−2024 Adjustment Method

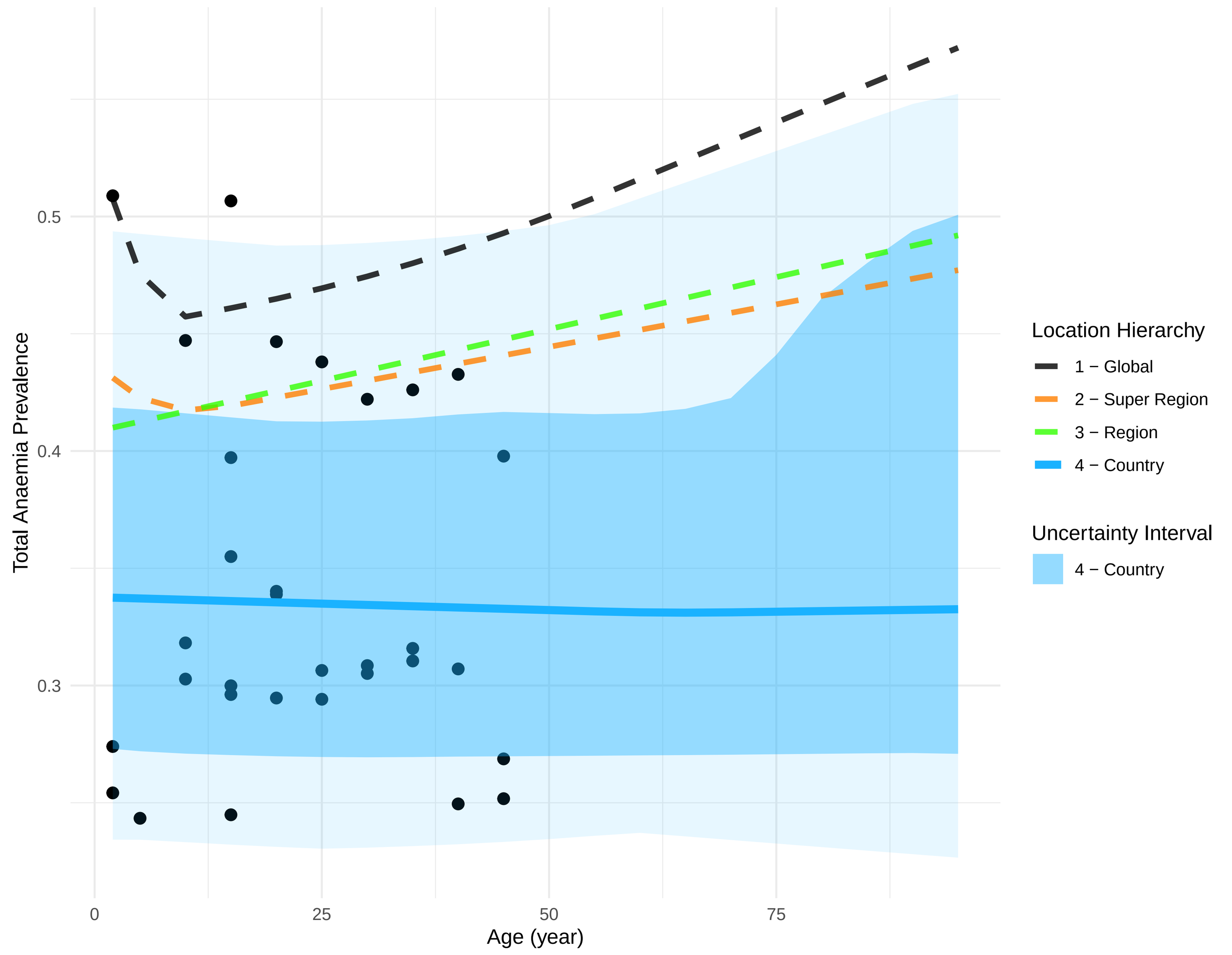

MR−BRT Age/Sex Fits − Non−pregnant Females 2+ Years Old − Total Anaemia Prevalence − Jordan
WHO−2024 Adjustment Method

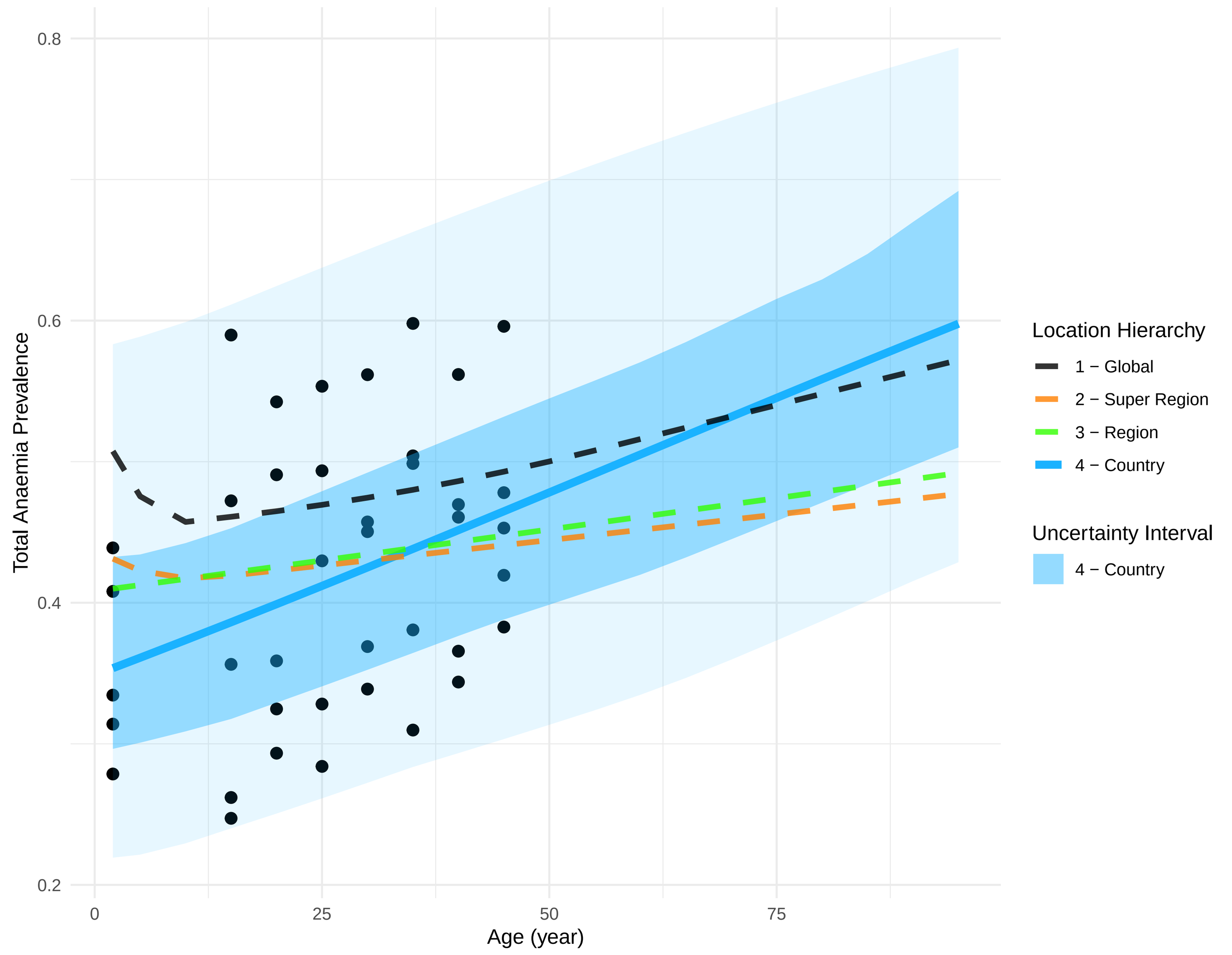

MR−BRT Age/Sex Fits − Non−pregnant Females 2+ Years Old − Total Anaemia Prevalence − Yemen
WHO−2024 Adjustment Method

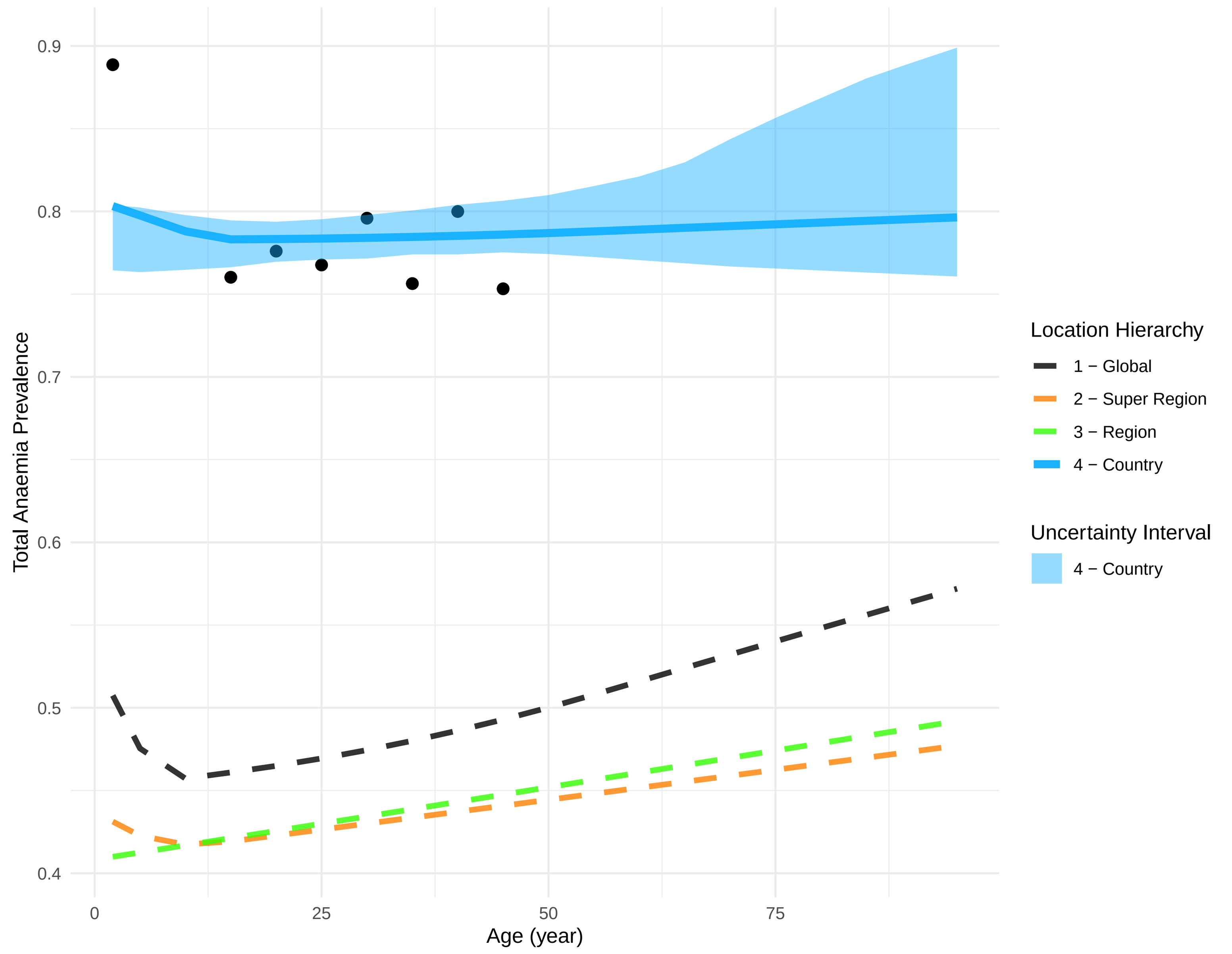

MR−BRT Age/Sex Fits − Non−pregnant Females 2+ Years Old − Total Anaemia Prevalence − South Asia
WHO−2024 Adjustment Method

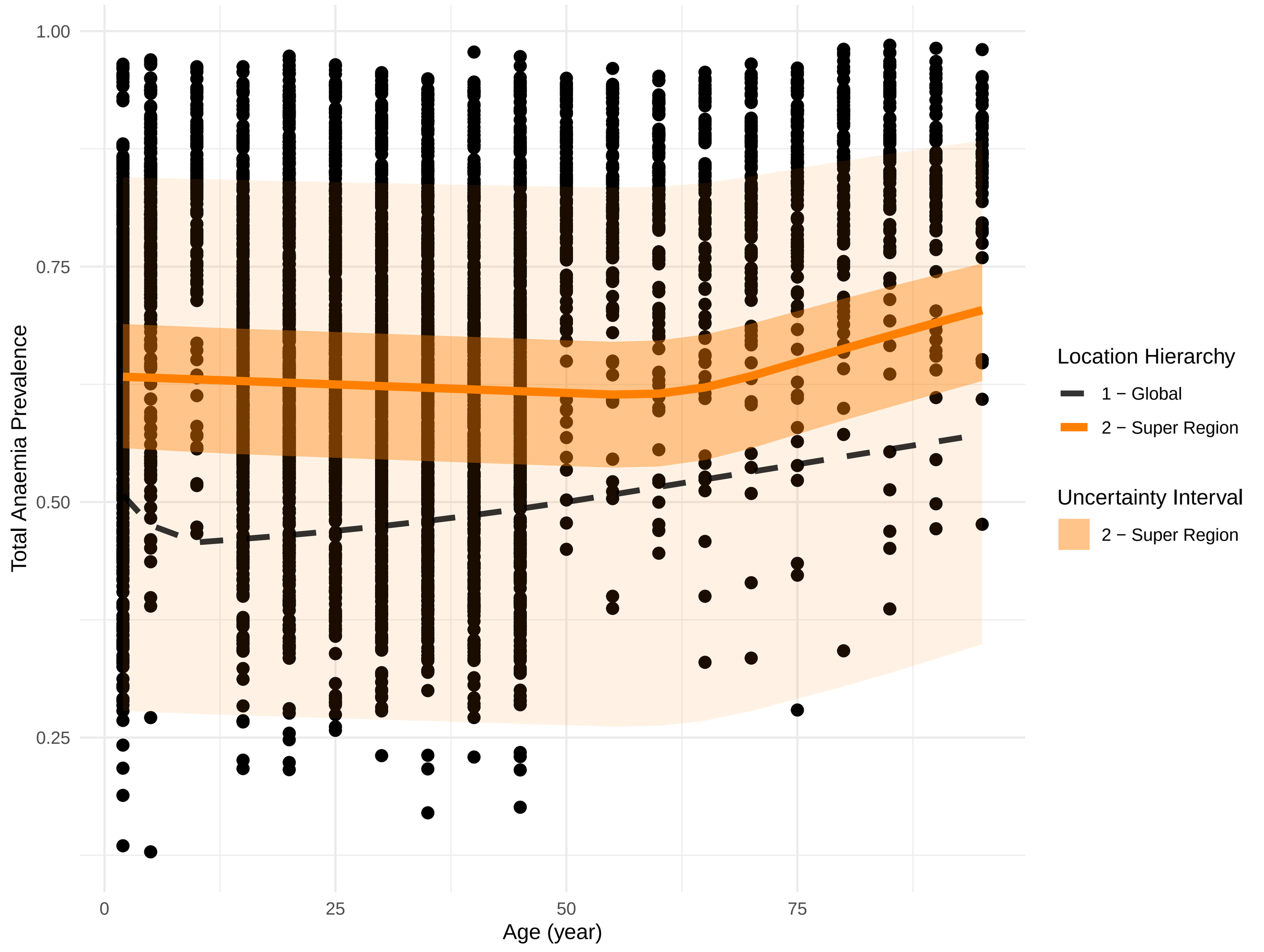

MR−BRT Age/Sex Fits − Non−pregnant Females 2+ Years Old − Total Anaemia Prevalence − South Asia
WHO−2024 Adjustment Method

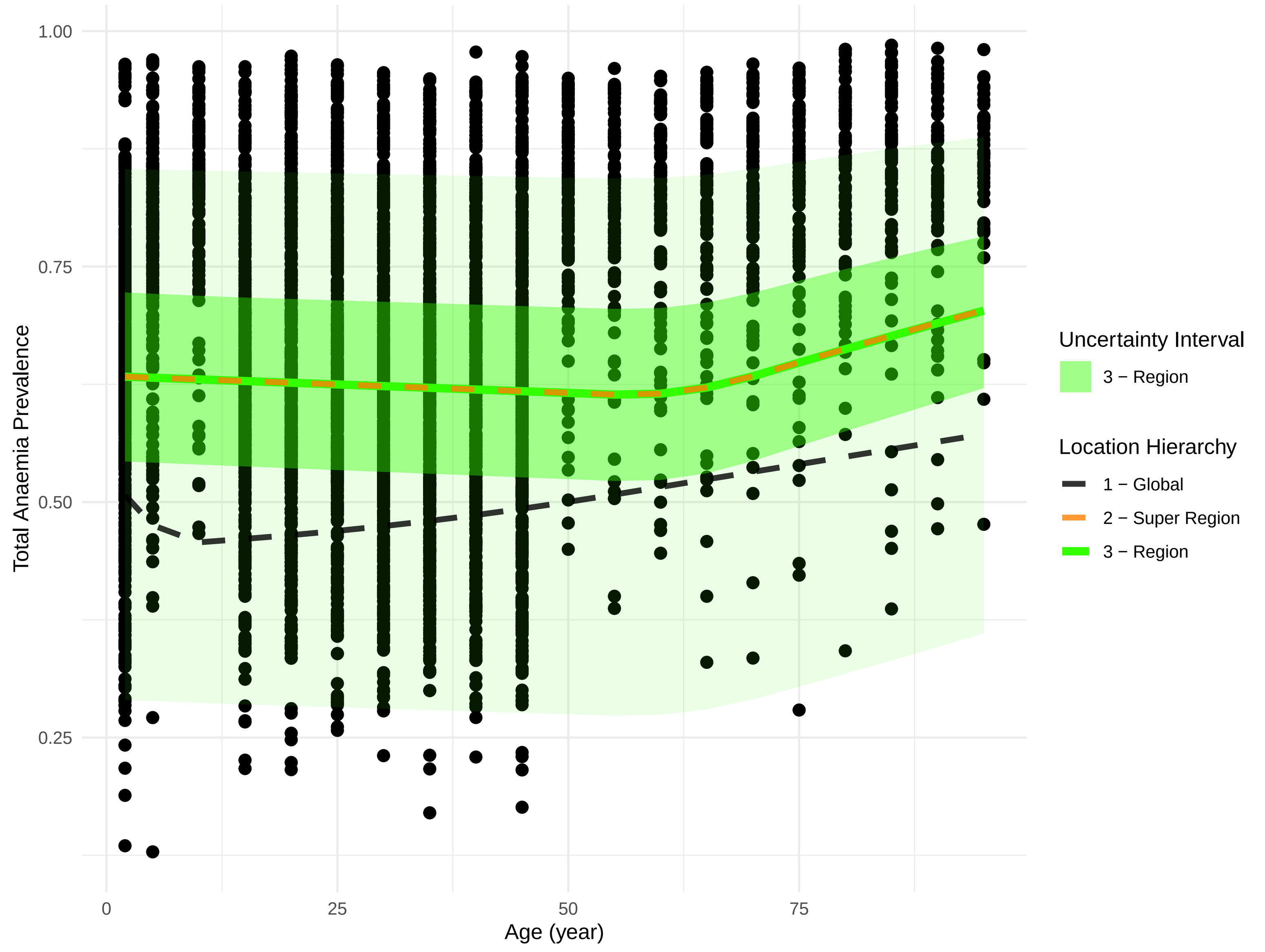

MR−BRT Age/Sex Fits − Non−pregnant Females 2+ Years Old − Total Anaemia Prevalence − Bangladesh
WHO−2024 Adjustment Method

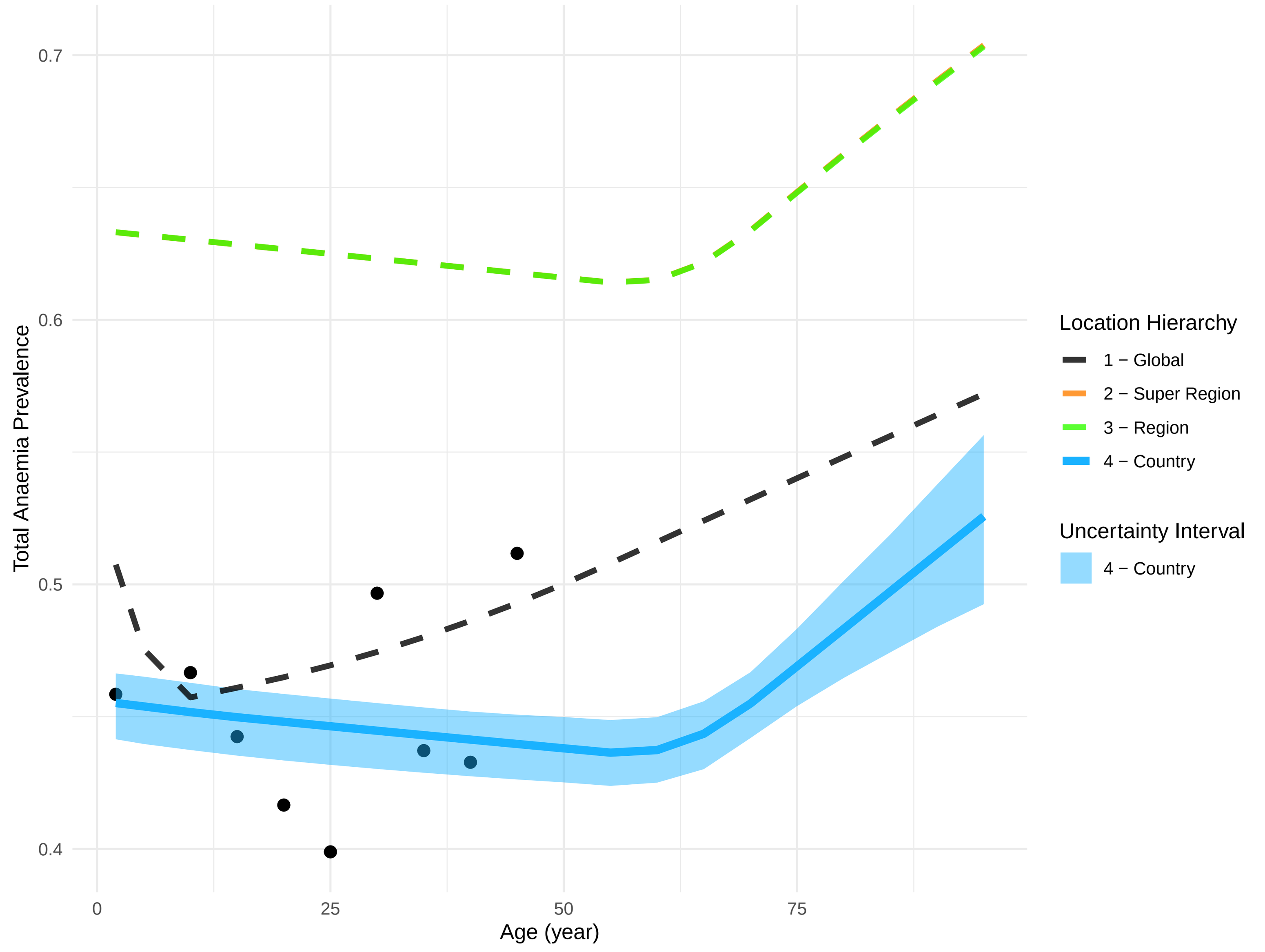

MR−BRT Age/Sex Fits − Non−pregnant Females 2+ Years Old − Total Anaemia Prevalence − India
WHO−2024 Adjustment Method

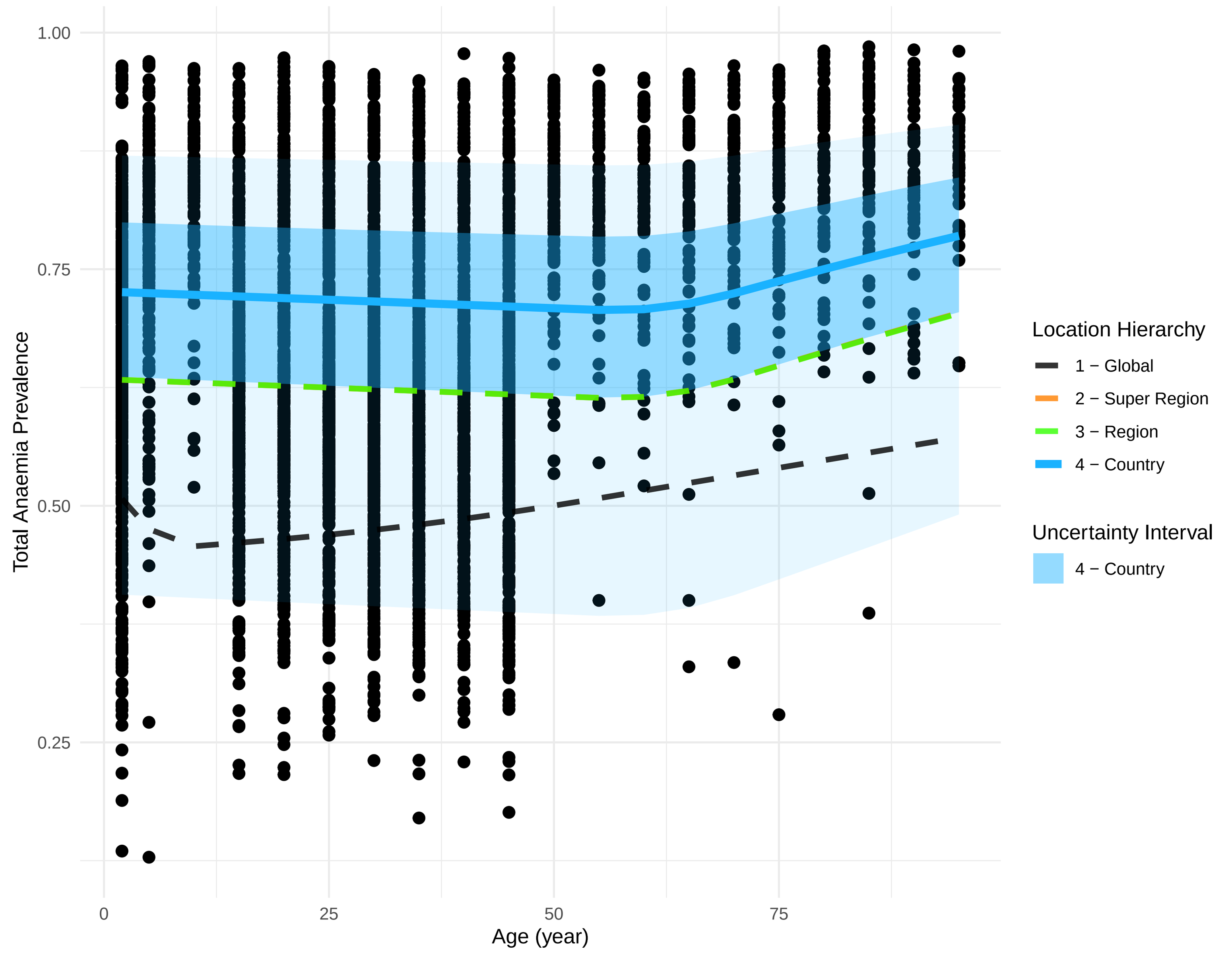

MR−BRT Age/Sex Fits − Non−pregnant Females 2+ Years Old − Total Anaemia Prevalence − Andhra Pradesh
WHO−2024 Adjustment Method

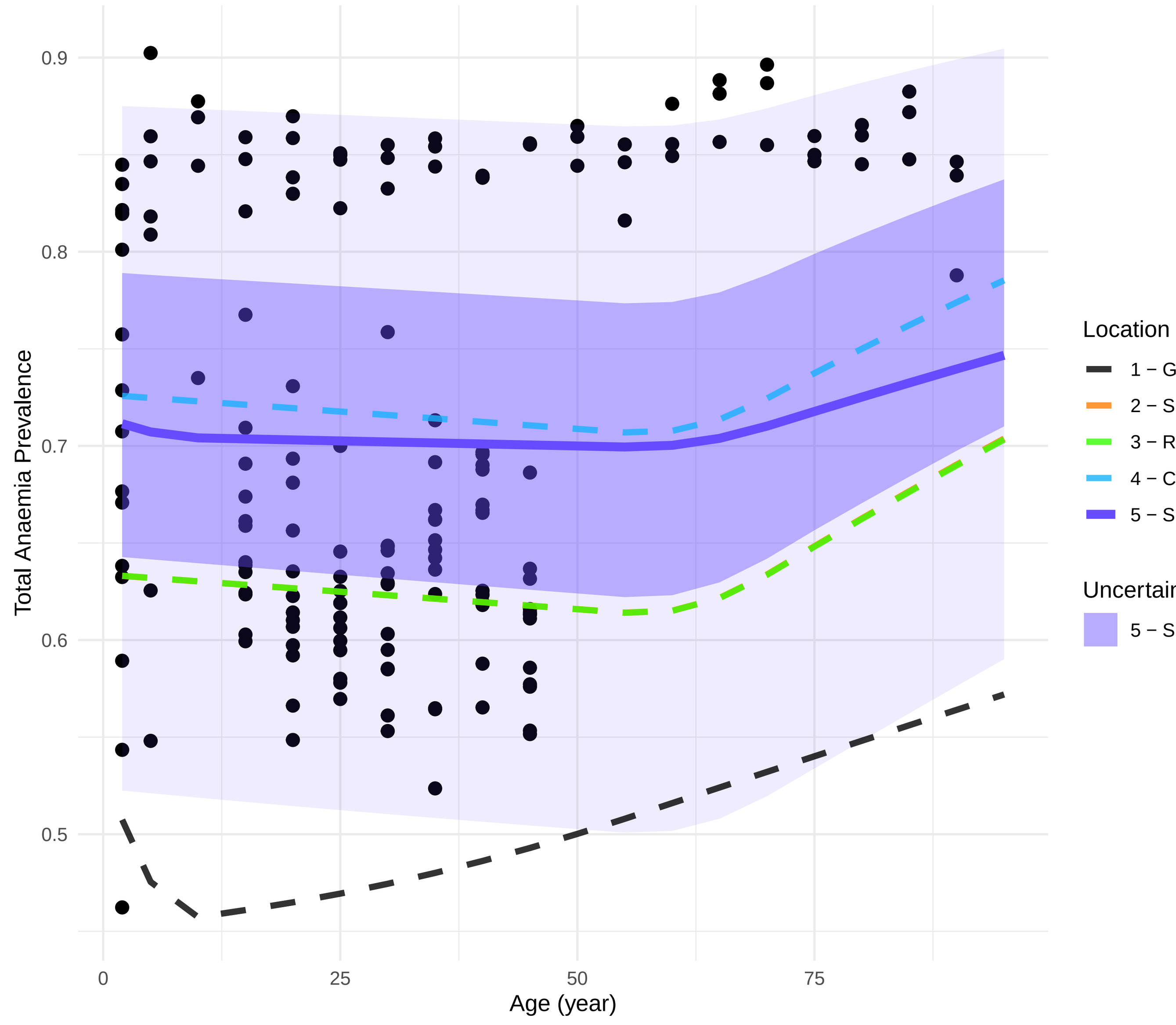

MR−BRT Age/Sex Fits − Non−pregnant Females 2+ Years Old − Total Anaemia Prevalence − Andhra Pradesh, Rural
WHO−2024 Adjustment Method

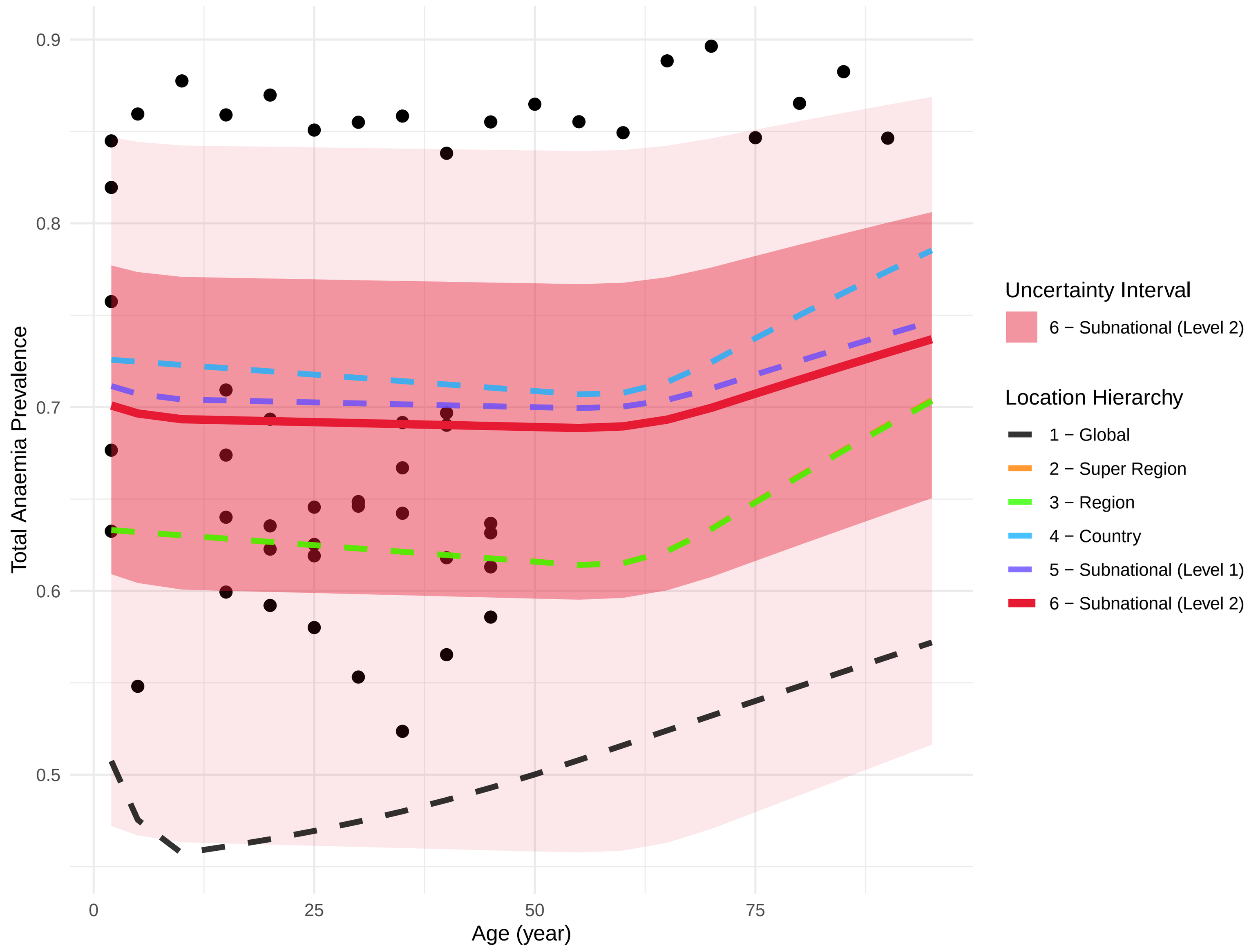

MR−BRT Age/Sex Fits − Non−pregnant Females 2+ Years Old − Total Anaemia Prevalence − Andhra Pradesh, Urban
WHO−2024 Adjustment Method

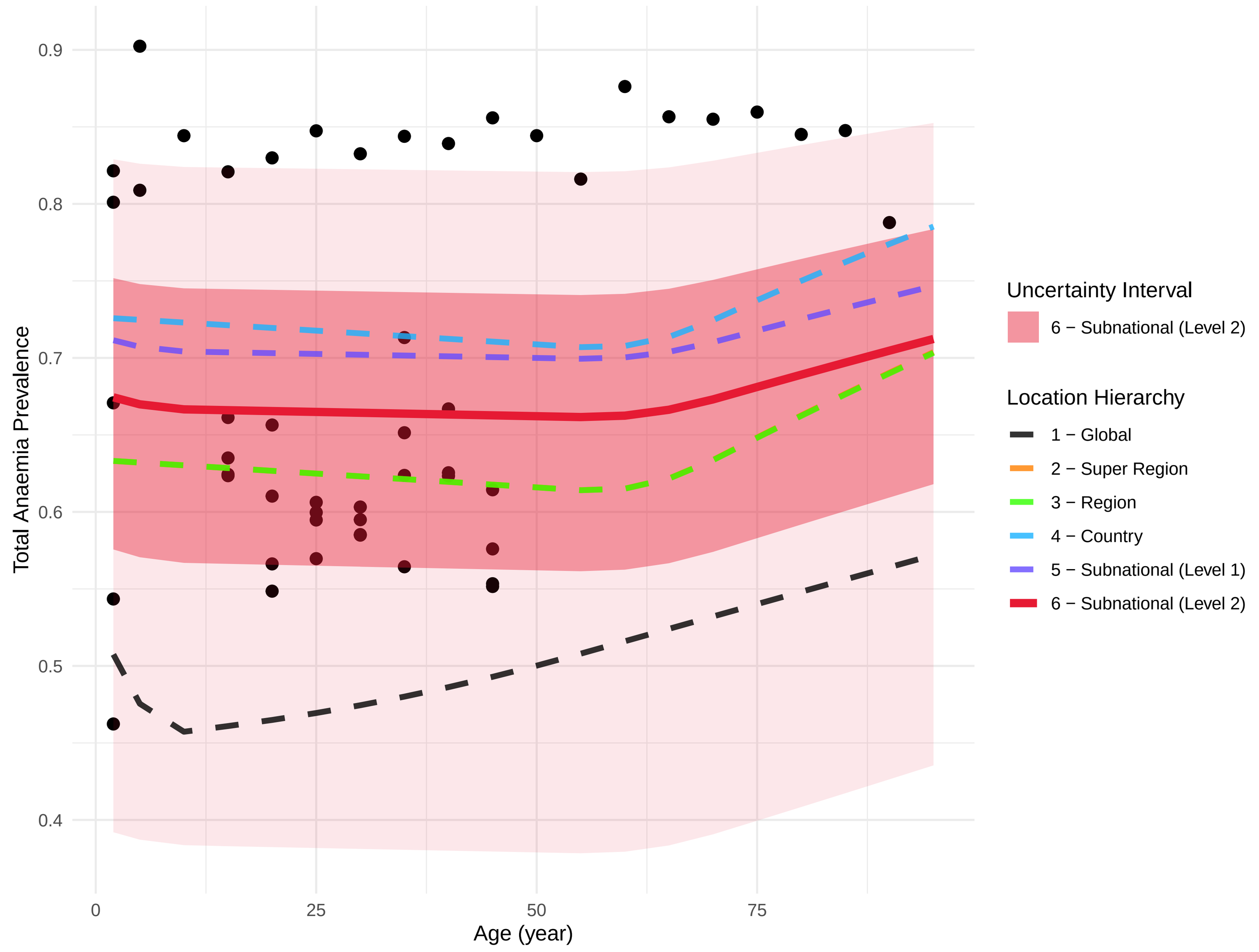

MR−BRT Age/Sex Fits − Non−pregnant Females 2+ Years Old − Total Anaemia Prevalence − Arunachal Pradesh
WHO−2024 Adjustment Method

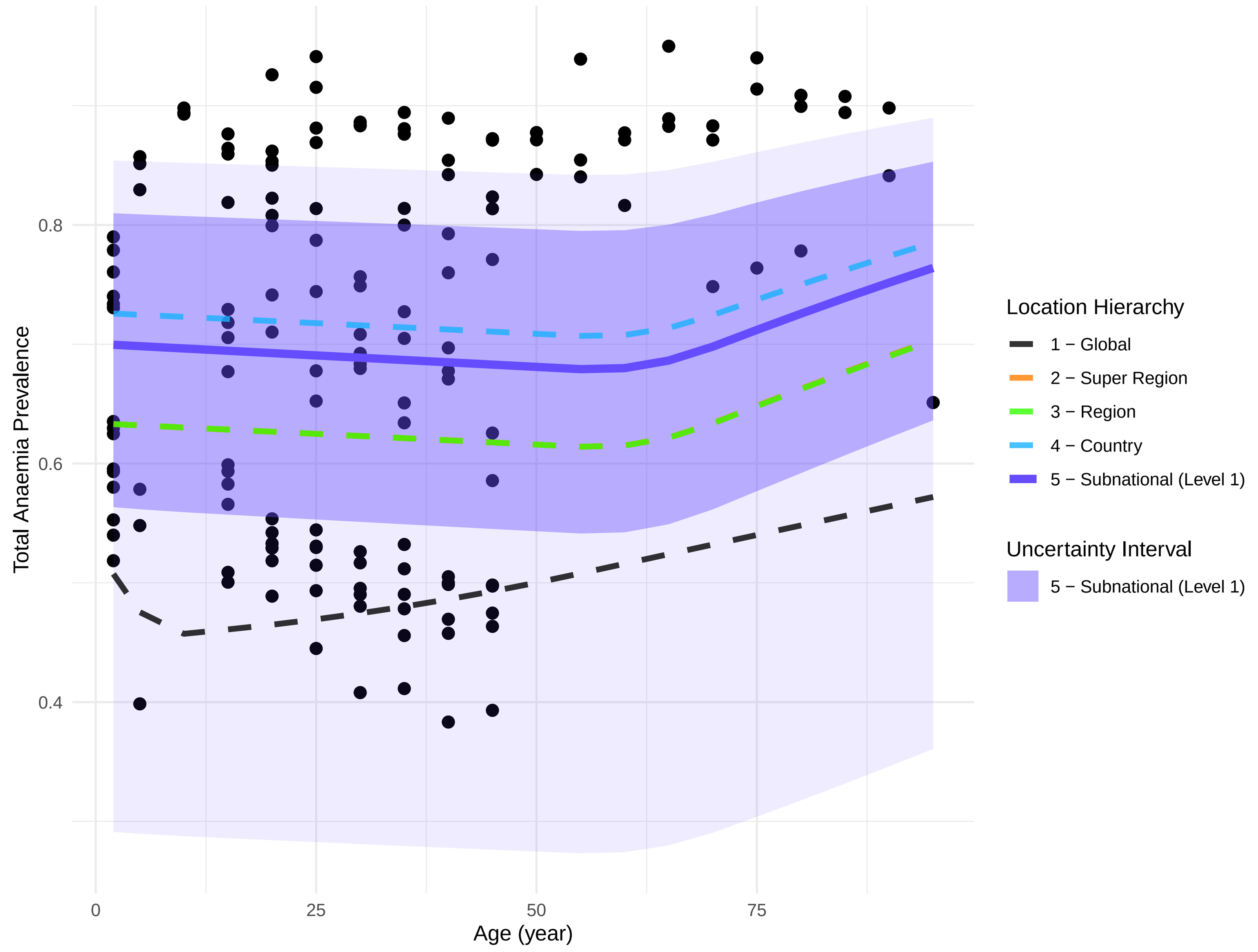

MR−BRT Age/Sex Fits − Non−pregnant Females 2+ Years Old − Total Anaemia Prevalence − Arunachal Pradesh, Rural
WHO−2024 Adjustment Method

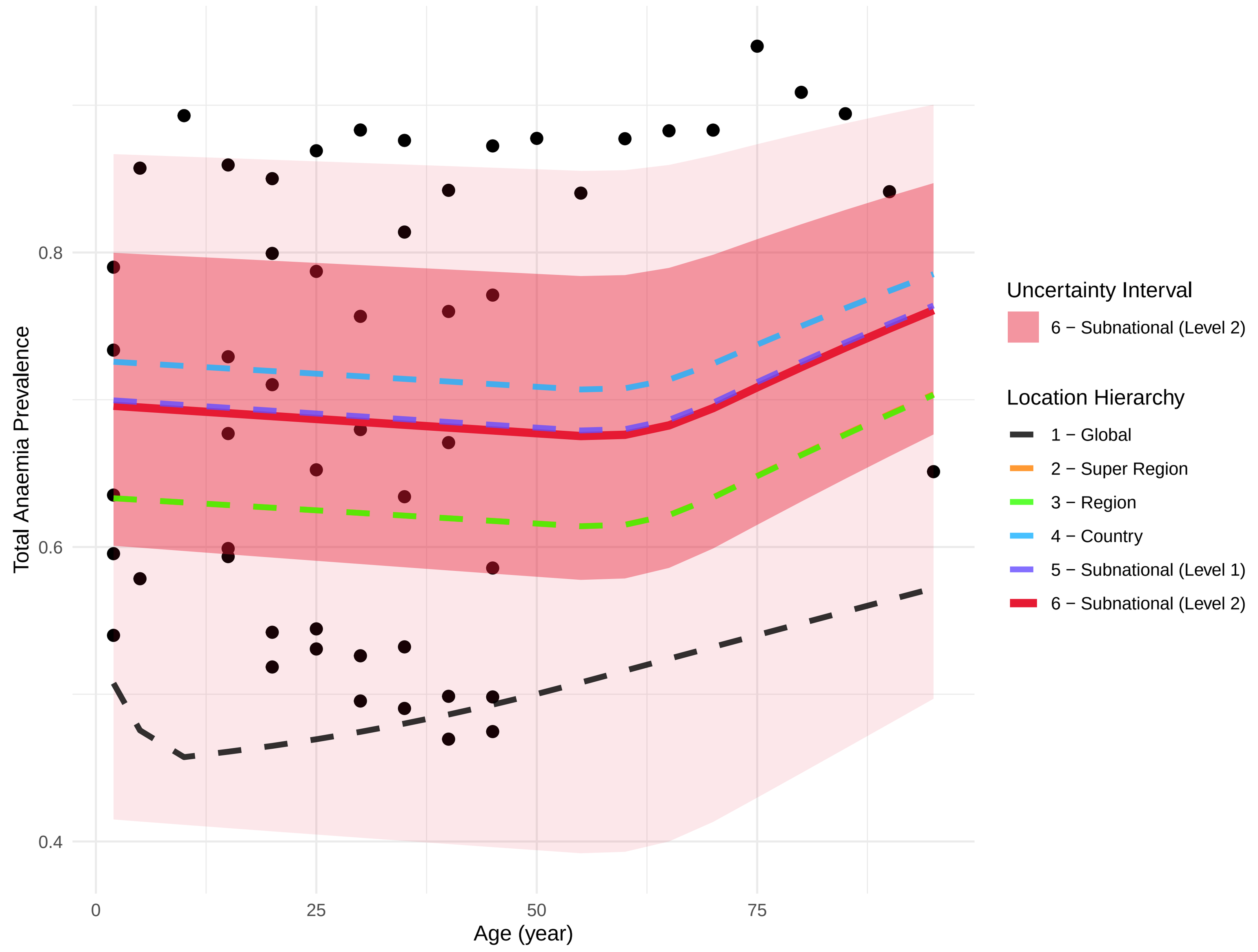

MR−BRT Age/Sex Fits − Non−pregnant Females 2+ Years Old − Total Anaemia Prevalence − Arunachal Pradesh, Urban
WHO−2024 Adjustment Method

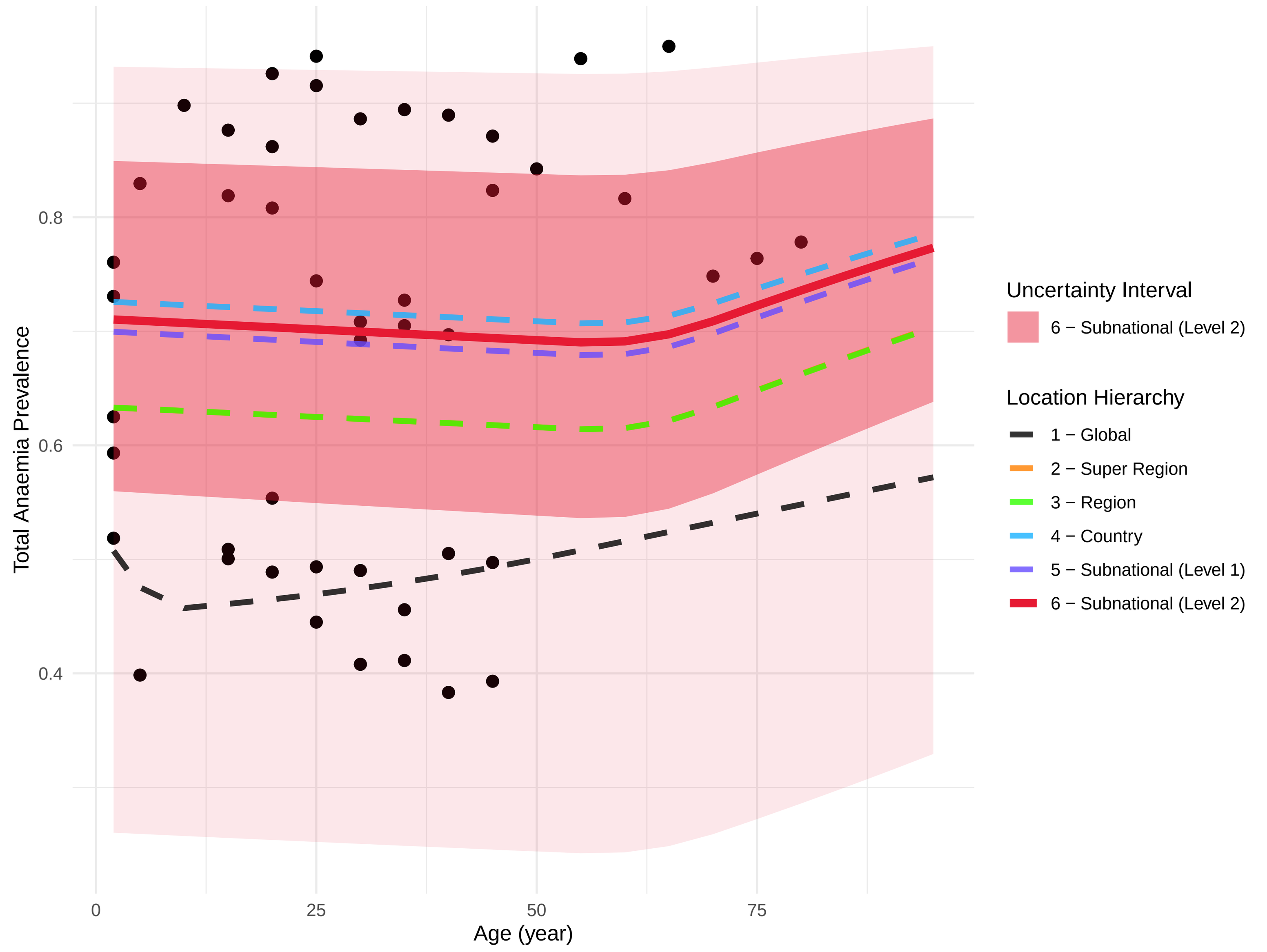

MR−BRT Age/Sex Fits − Non−pregnant Females 2+ Years Old − Total Anaemia Prevalence − Assam
WHO−2024 Adjustment Method

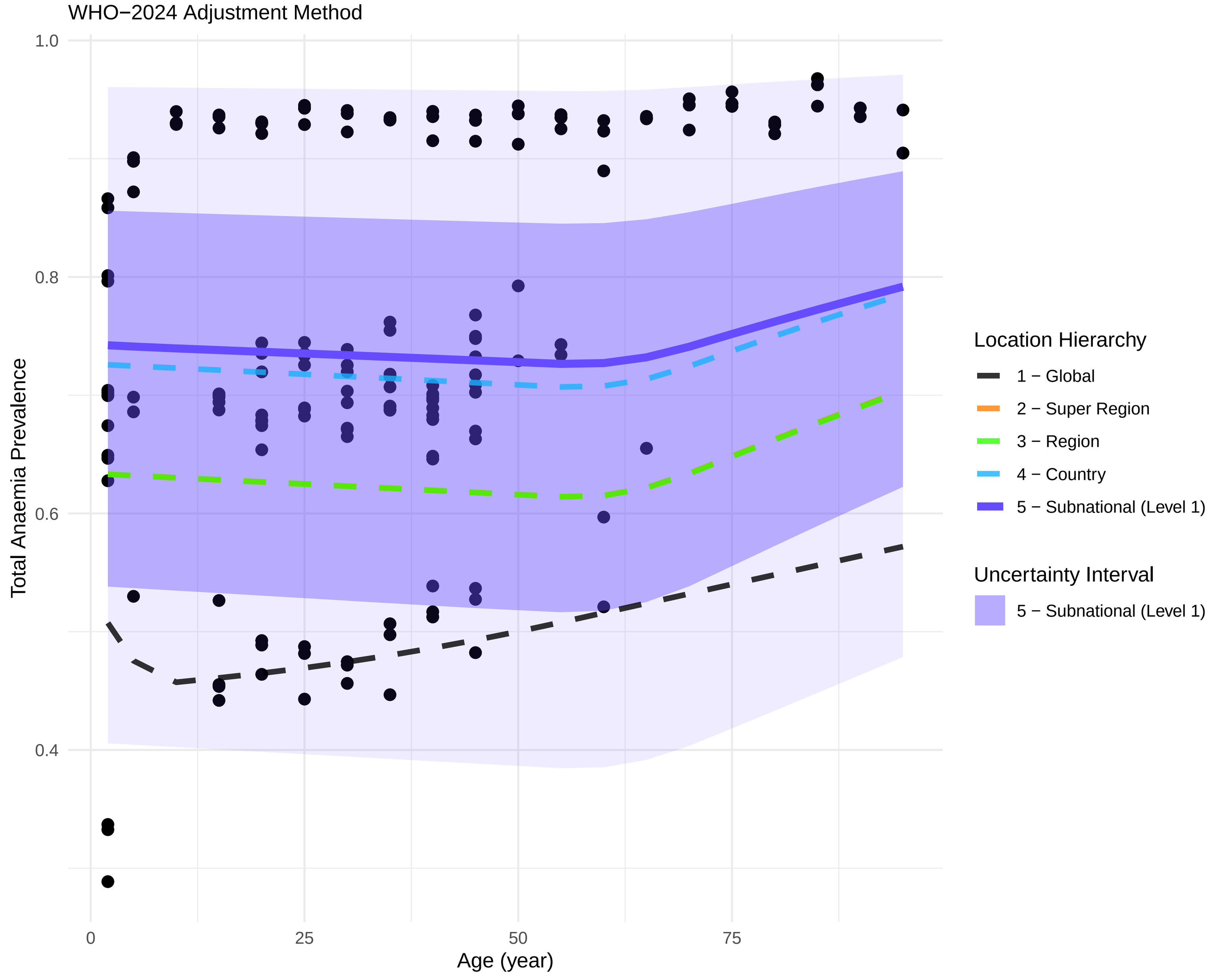

MR−BRT Age/Sex Fits − Non−pregnant Females 2+ Years Old − Total Anaemia Prevalence − Assam, Rural
WHO−2024 Adjustment Method

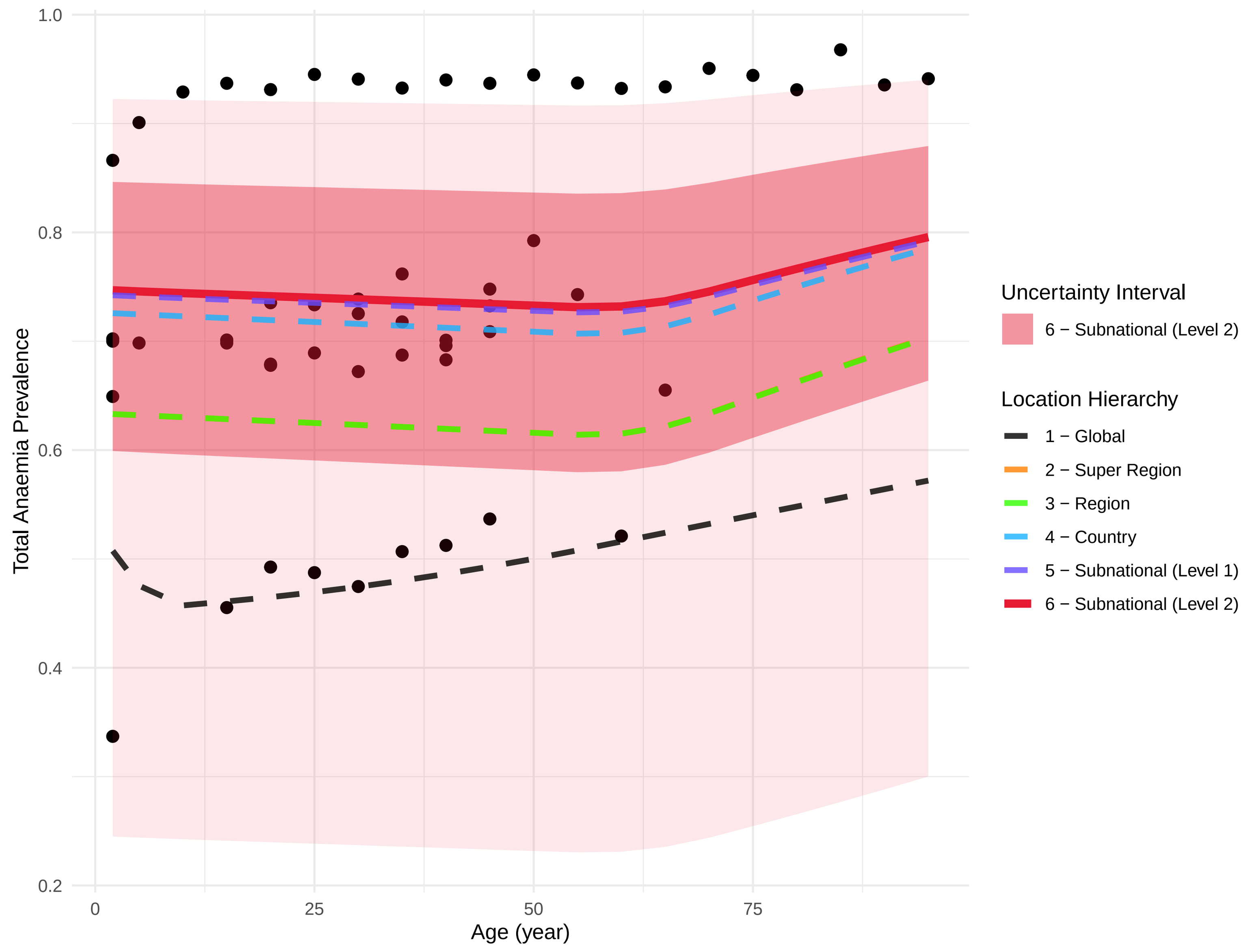

MR−BRT Age/Sex Fits − Non−pregnant Females 2+ Years Old − Total Anaemia Prevalence − Assam, Urban
WHO−2024 Adjustment Method

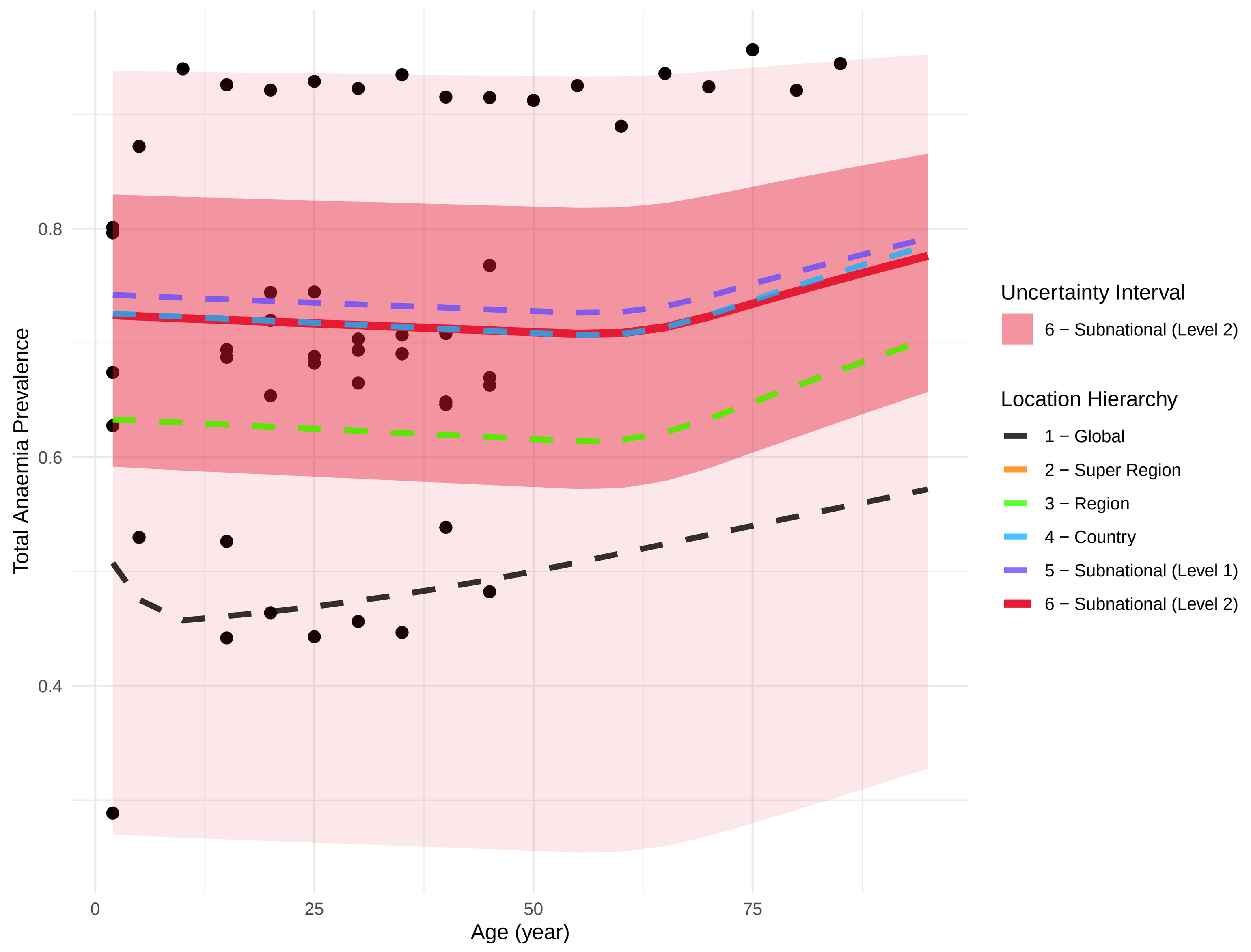

MR−BRT Age/Sex Fits − Non−pregnant Females 2+ Years Old − Total Anaemia Prevalence − Bihar
WHO−2024 Adjustment Method

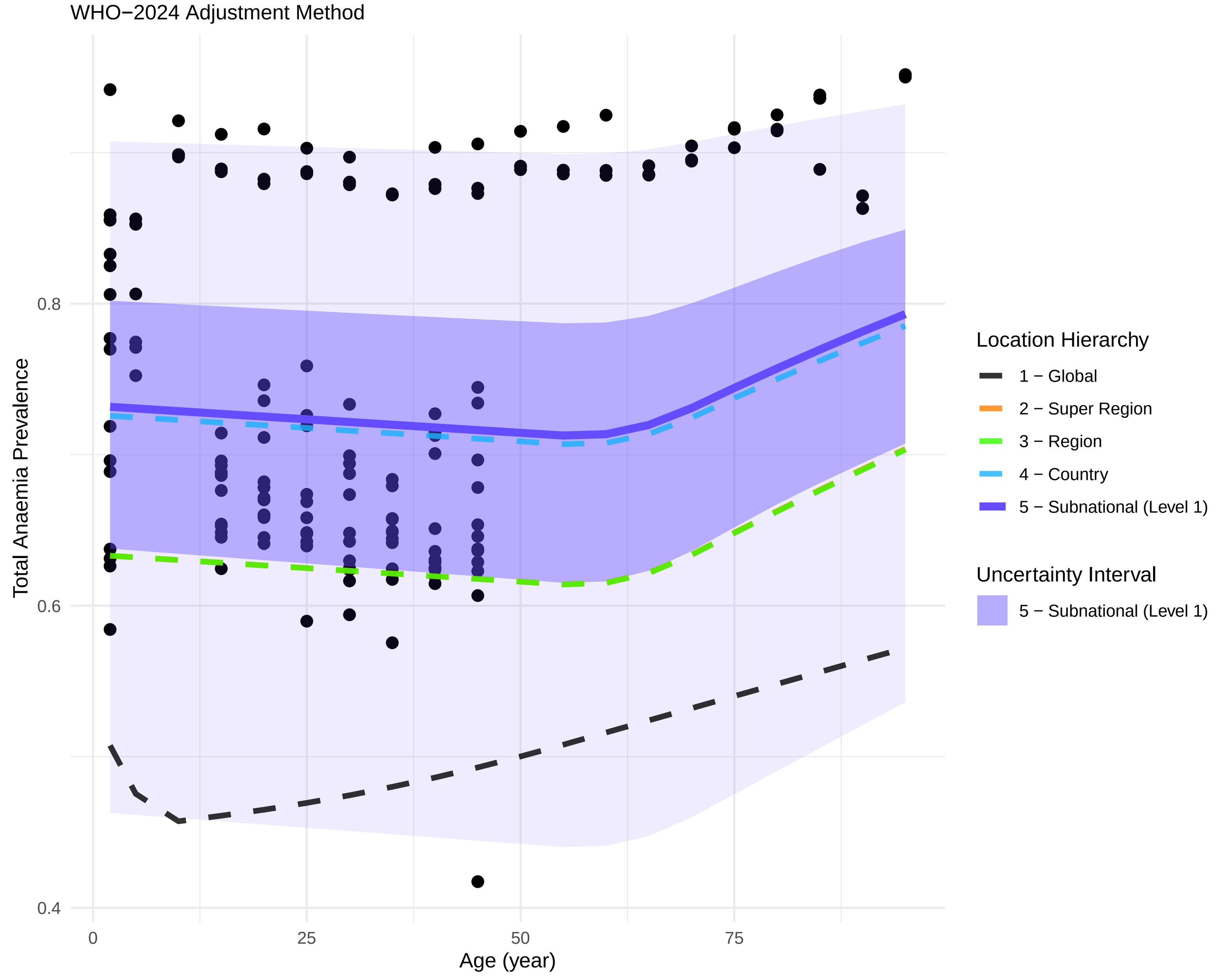

MR−BRT Age/Sex Fits − Non−pregnant Females 2+ Years Old − Total Anaemia Prevalence − Bihar, Rural
WHO−2024 Adjustment Method

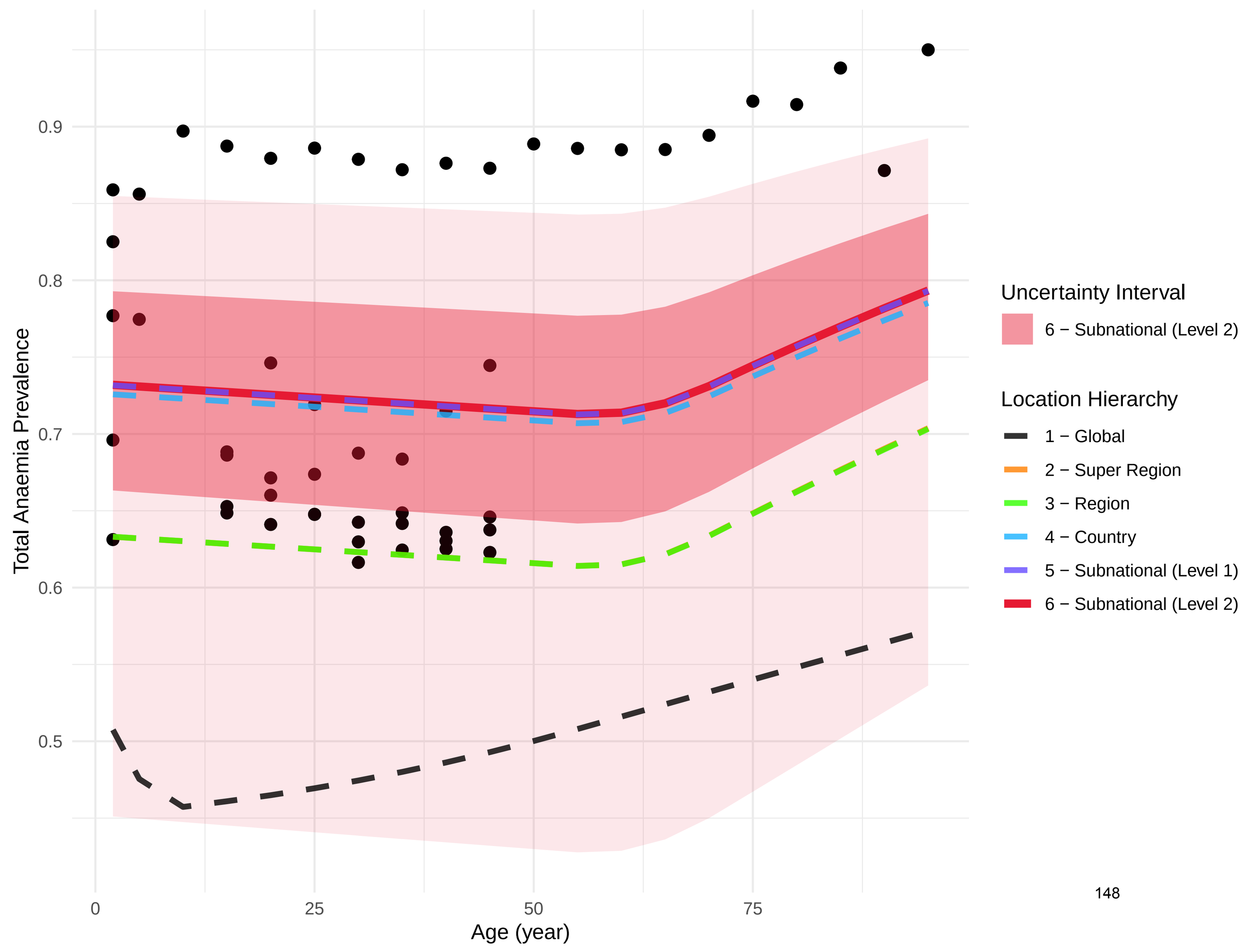

MR−BRT Age/Sex Fits − Non-pregnant Females 2+ Years Old − Total Anaemia Prevalence − Bihar, Urban
WHO−2024 Adjustment Method

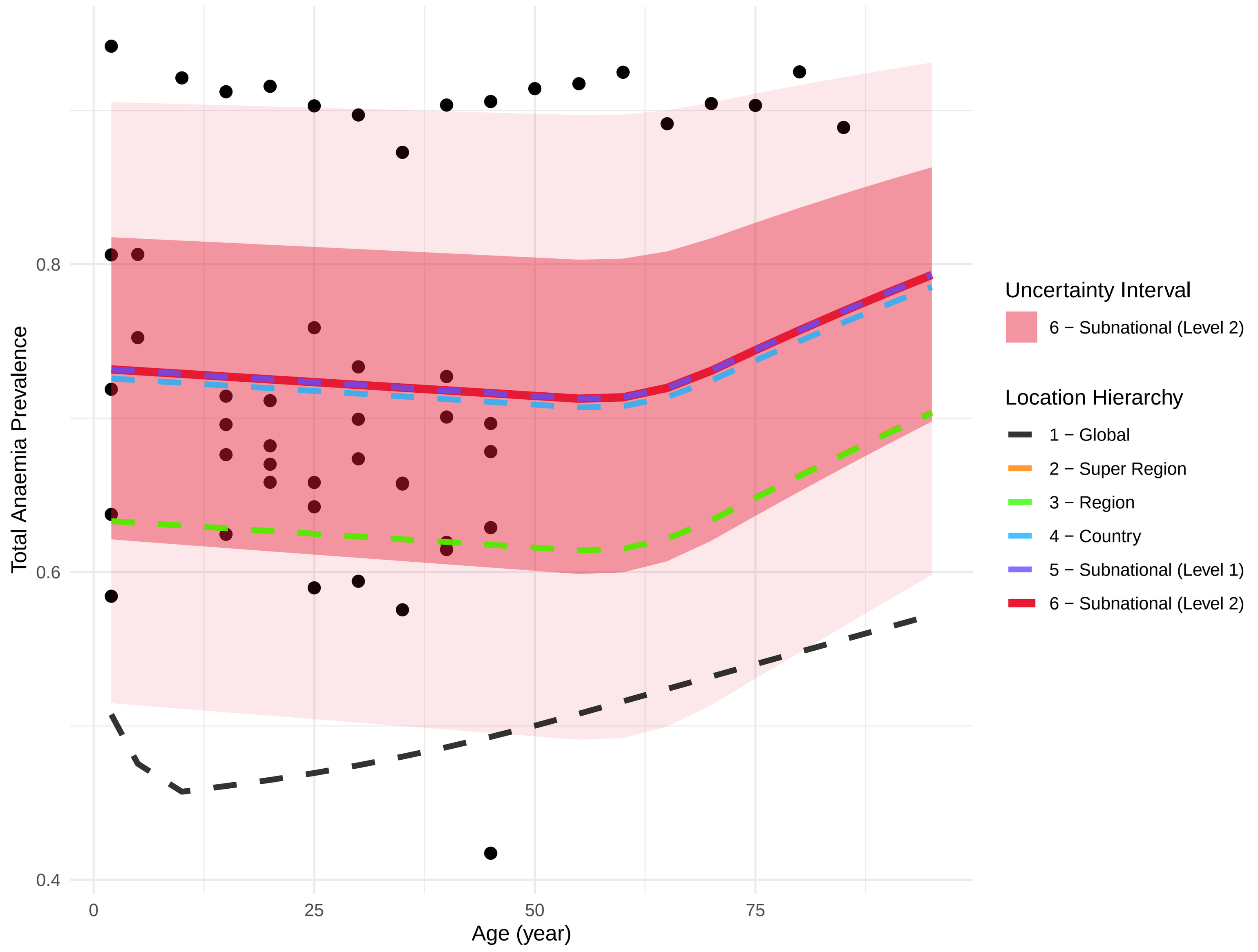

MR−BRT Age/Sex Fits − Non-pregnant Females 2+ Years Old − Total Anaemia Prevalence − Chhattisgarh
WHO−2024 Adjustment Method

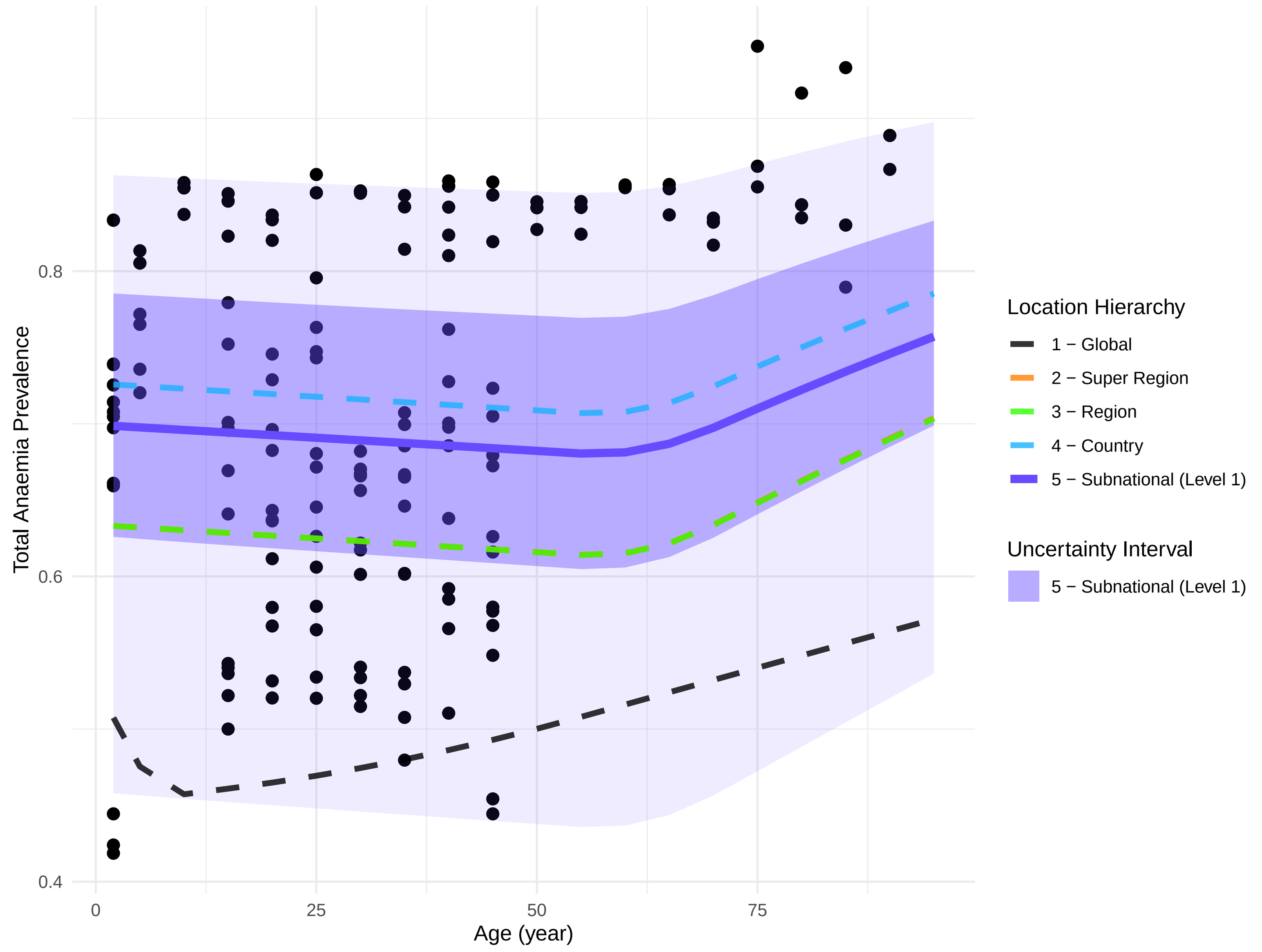

MR−BRT Age/Sex Fits − Non-pregnant Females 2+ Years Old − Total Anaemia Prevalence − Chhattisgarh, Rural
WHO−2024 Adjustment Method

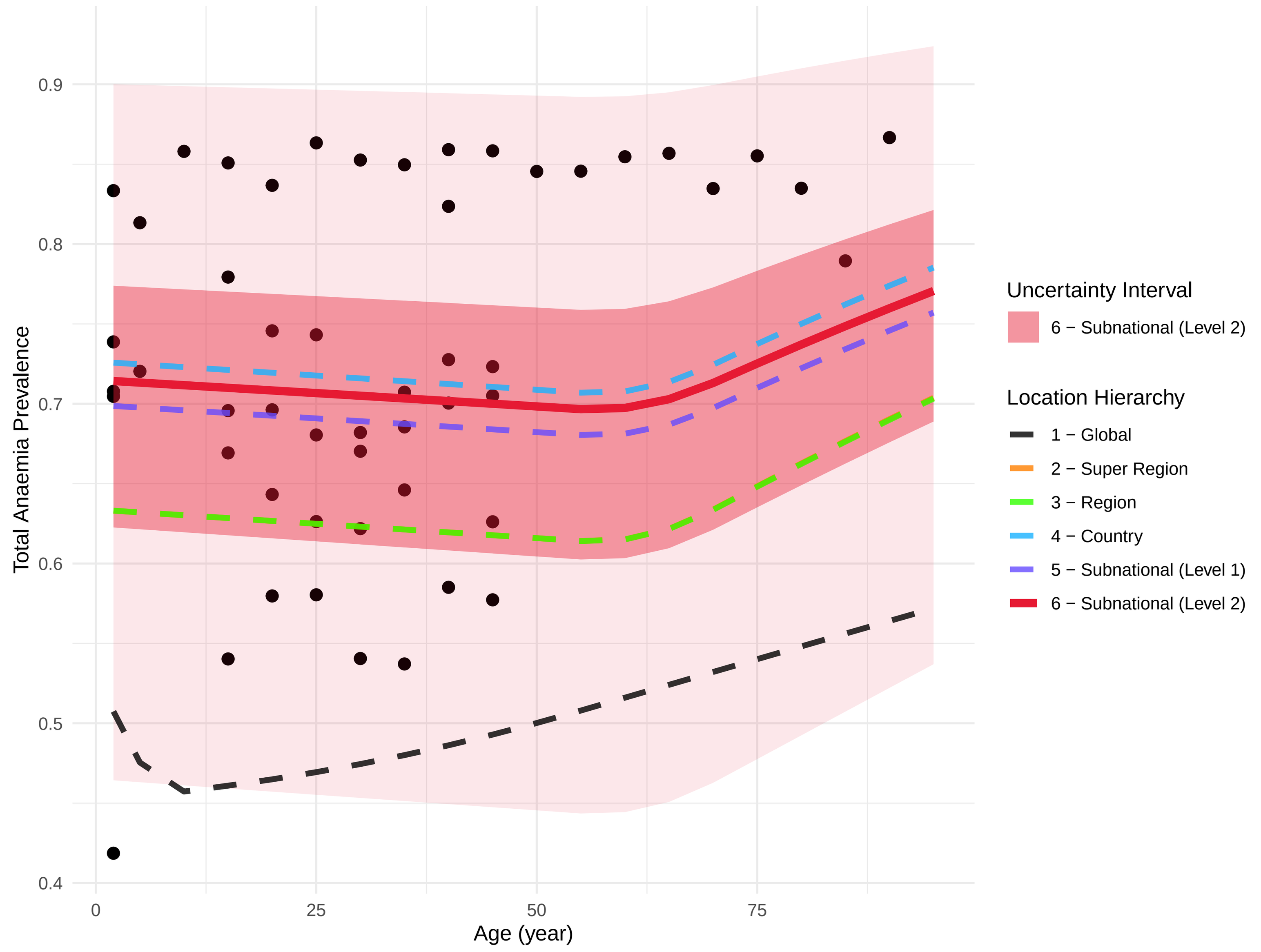

MR−BRT Age/Sex Fits − Non-pregnant Females 2+ Years Old − Total Anaemia Prevalence − Chhattisgarh, Urban
WHO−2024 Adjustment Method

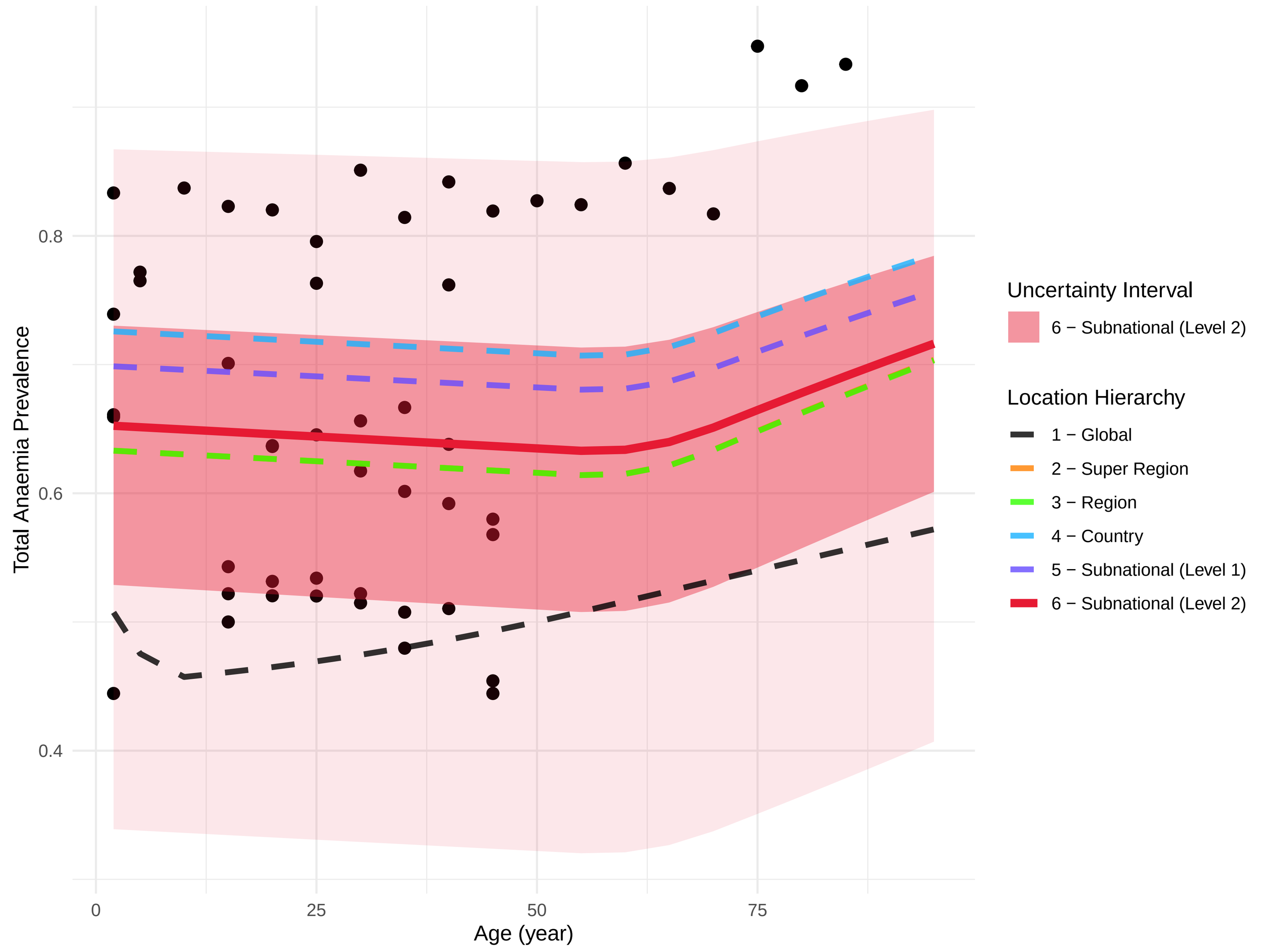

MR−BRT Age/Sex Fits − Non-pregnant Females 2+ Years Old − Total Anaemia Prevalence − Delhi
WHO−2024 Adjustment Method

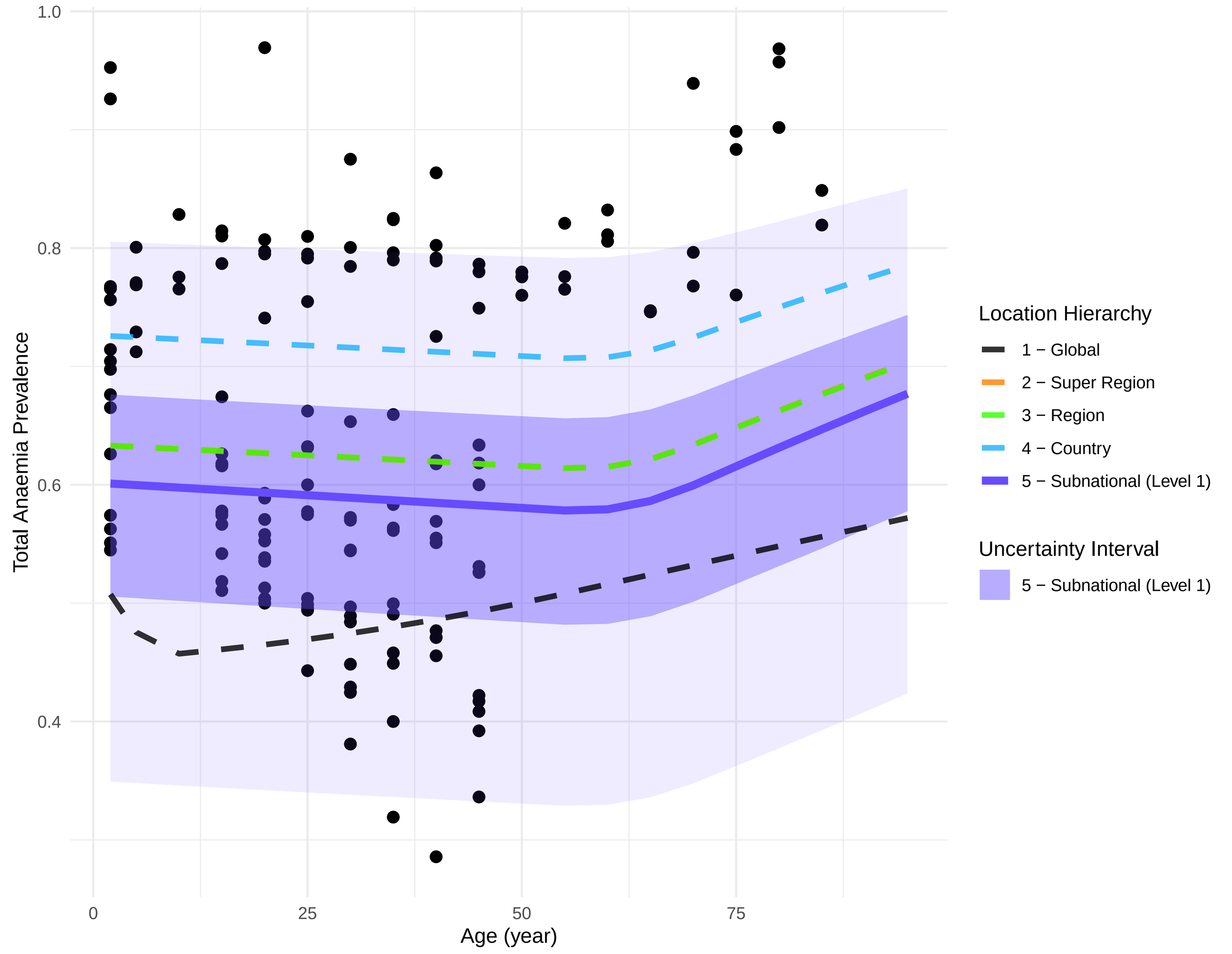

MR−BRT Age/Sex Fits − Non-pregnant Females 2+ Years Old − Total Anaemia Prevalence − Delhi, Rural
WHO−2024 Adjustment Method

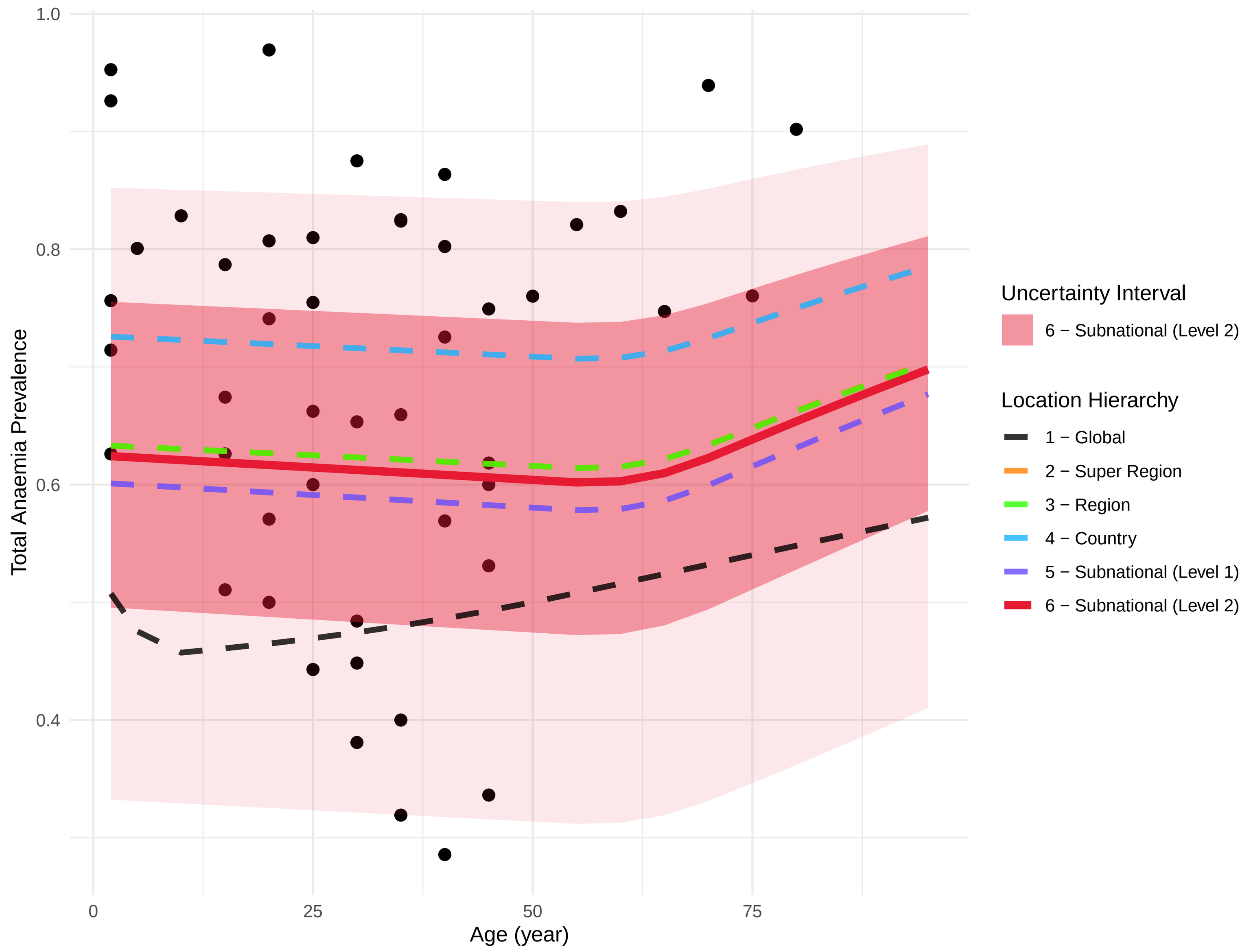

MR−BRT Age/Sex Fits − Non-pregnant Females 2+ Years Old − Total Anaemia Prevalence − Delhi, Urban
WHO−2024 Adjustment Method

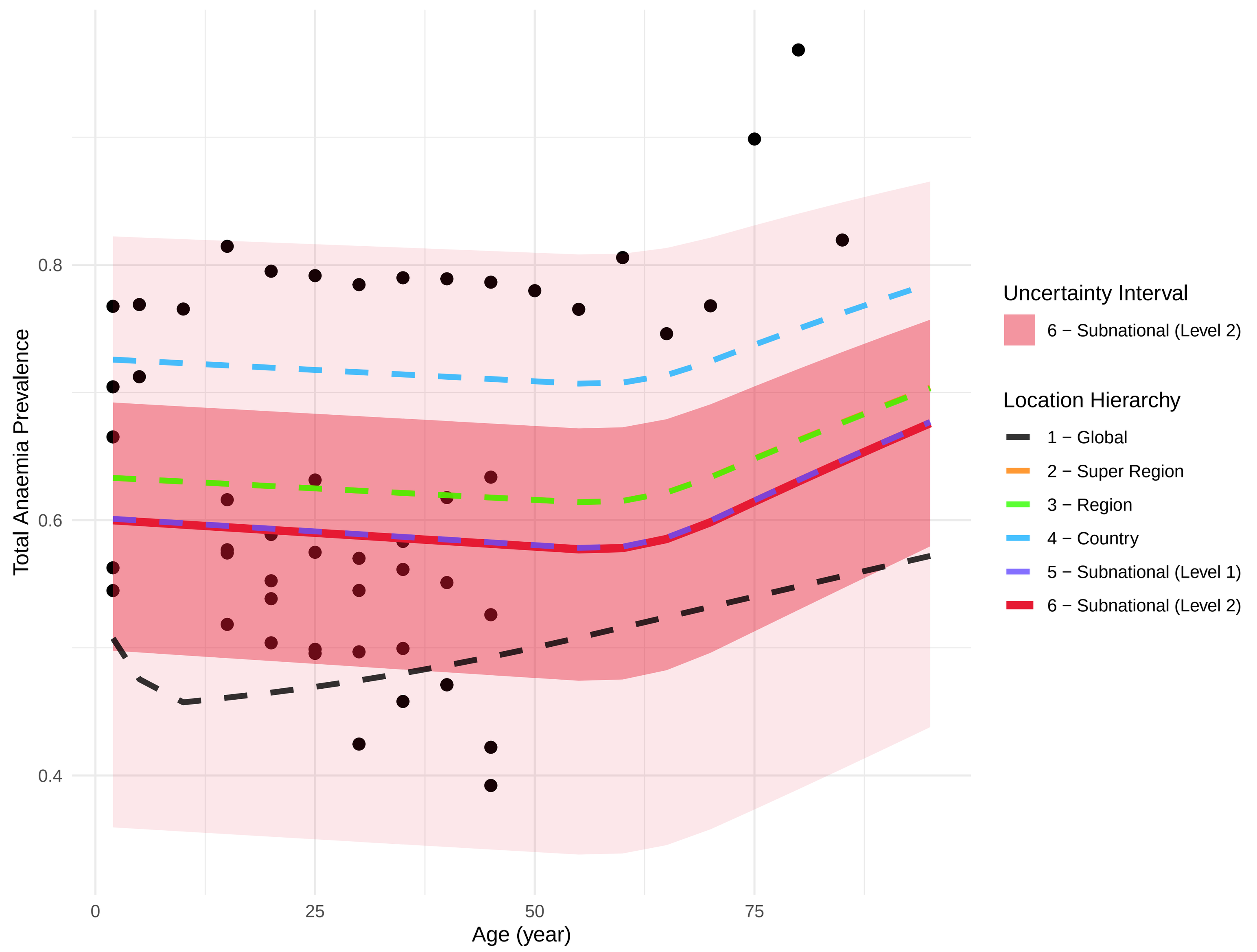

MR−BRT Age/Sex Fits − Non-pregnant Females 2+ Years Old − Total Anaemia Prevalence − Goa
WHO−2024 Adjustment Method

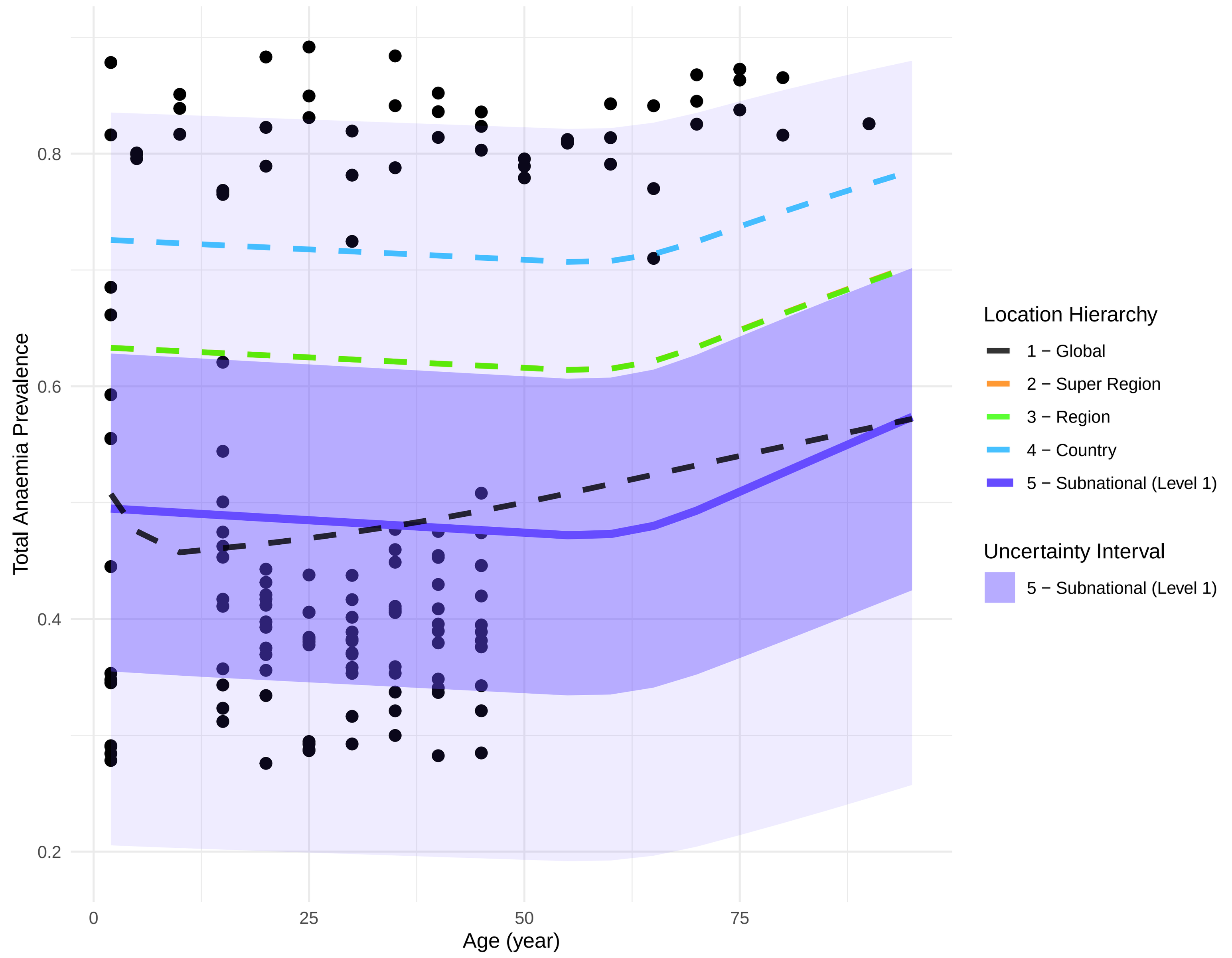

MR−BRT Age/Sex Fits − Non-pregnant Females 2+ Years Old − Total Anaemia Prevalence − Goa, Rural
WHO−2024 Adjustment Method

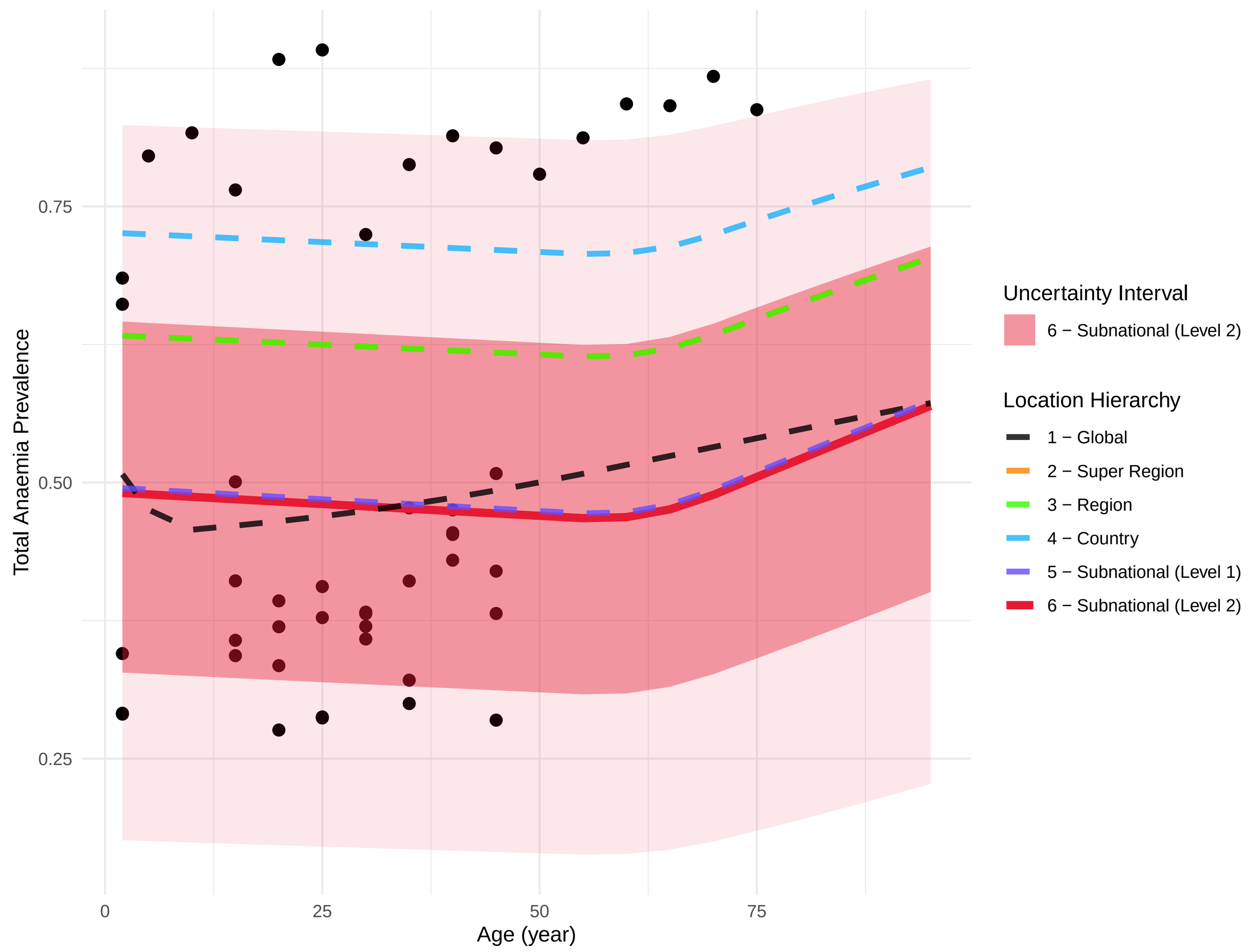

MR−BRT Age/Sex Fits − Non-pregnant Females 2+ Years Old − Total Anaemia Prevalence − Goa, Urban
WHO−2024 Adjustment Method

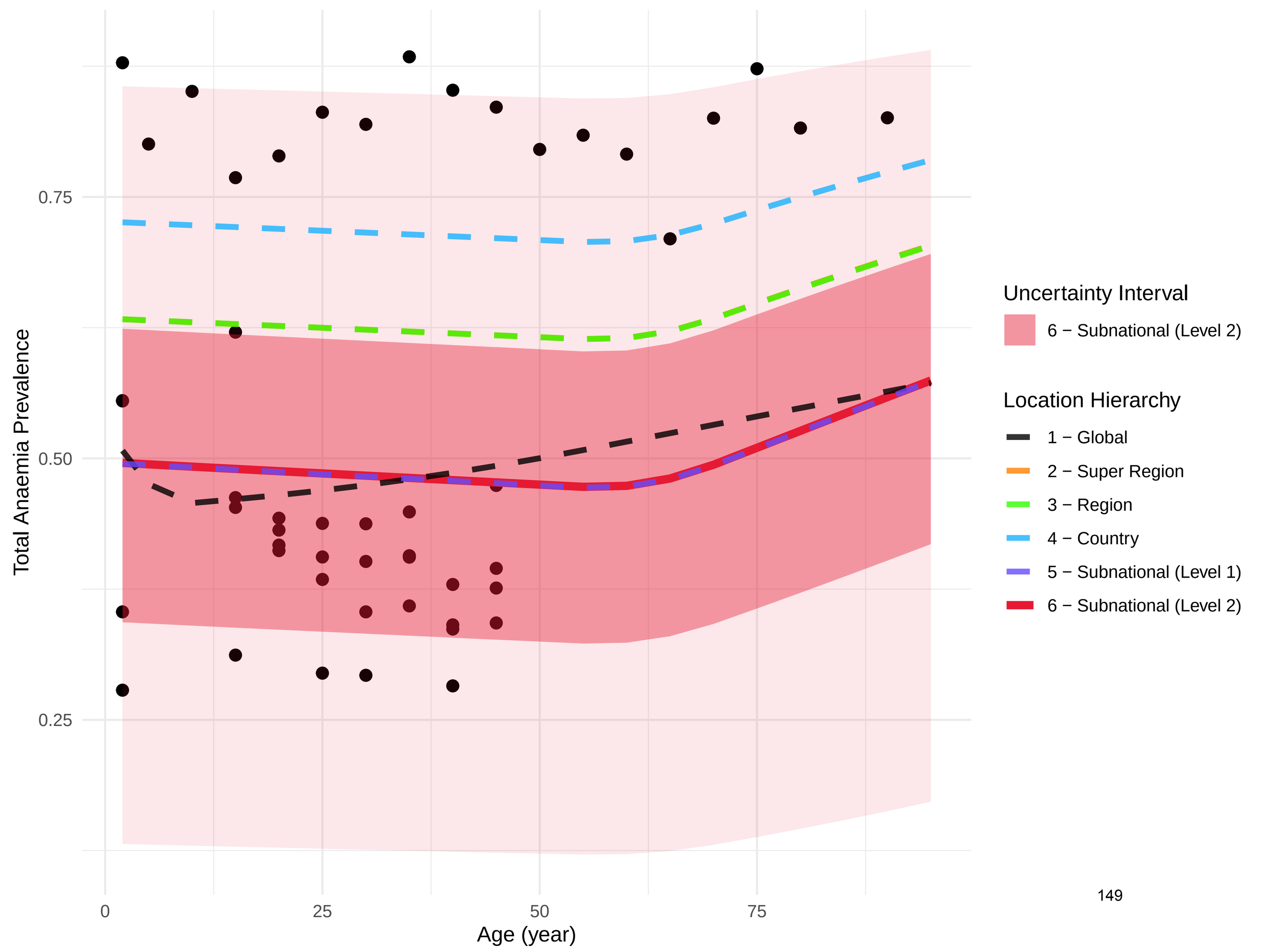

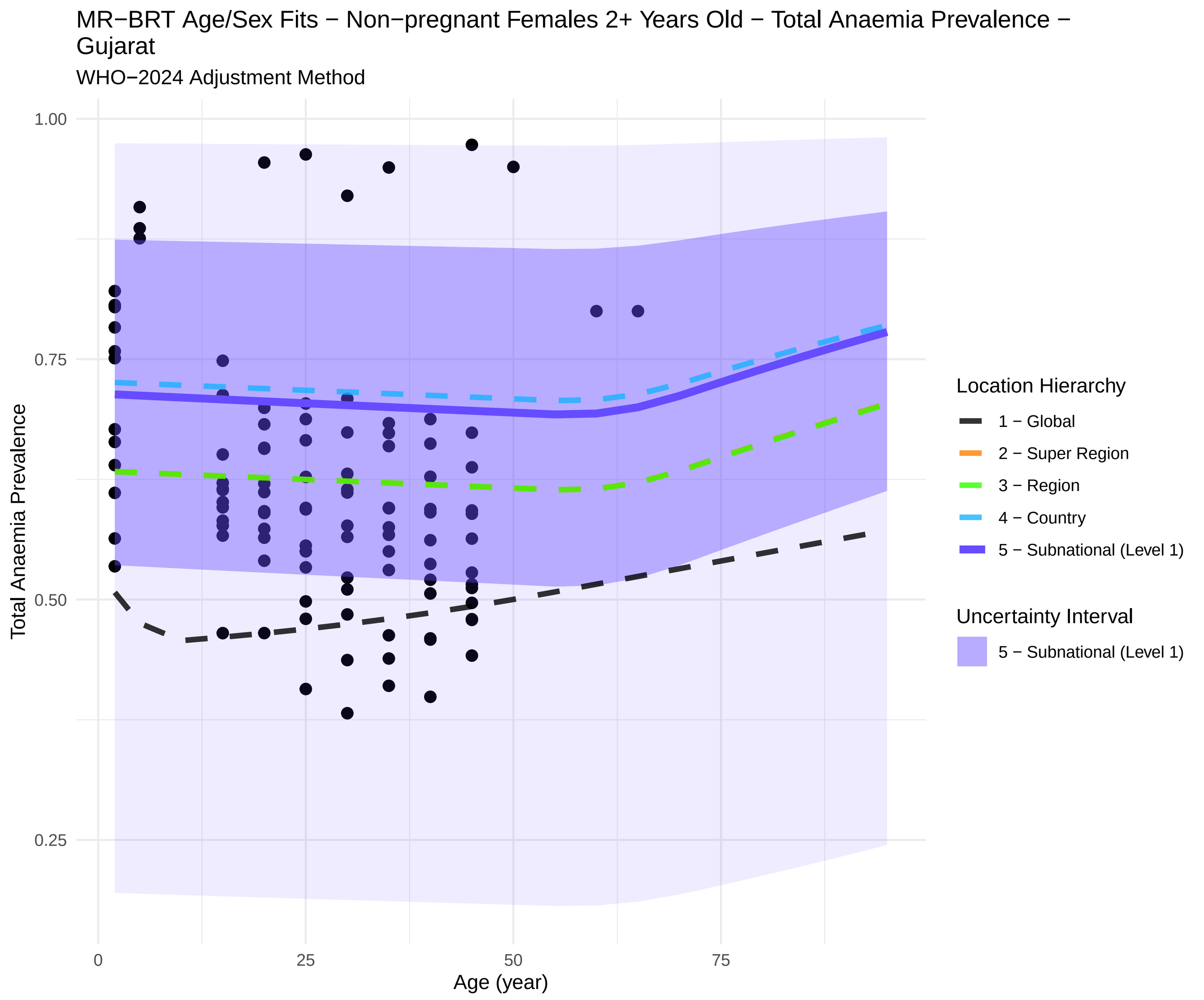
MR−BRT Age/Sex Fits − Non−pregnant Females 2+ Years Old − Total Anaemia Prevalence − Gujarat
WHO−2024 Adjustment Method
Total Anaemia Prevalence
Age (year)
Location Hierarchy
1 − Global
2 − Super Region
3 − Region
4 − Country
5 − Subnational (Level 1)
Uncertainty Interval
5 − Subnational (Level 1)

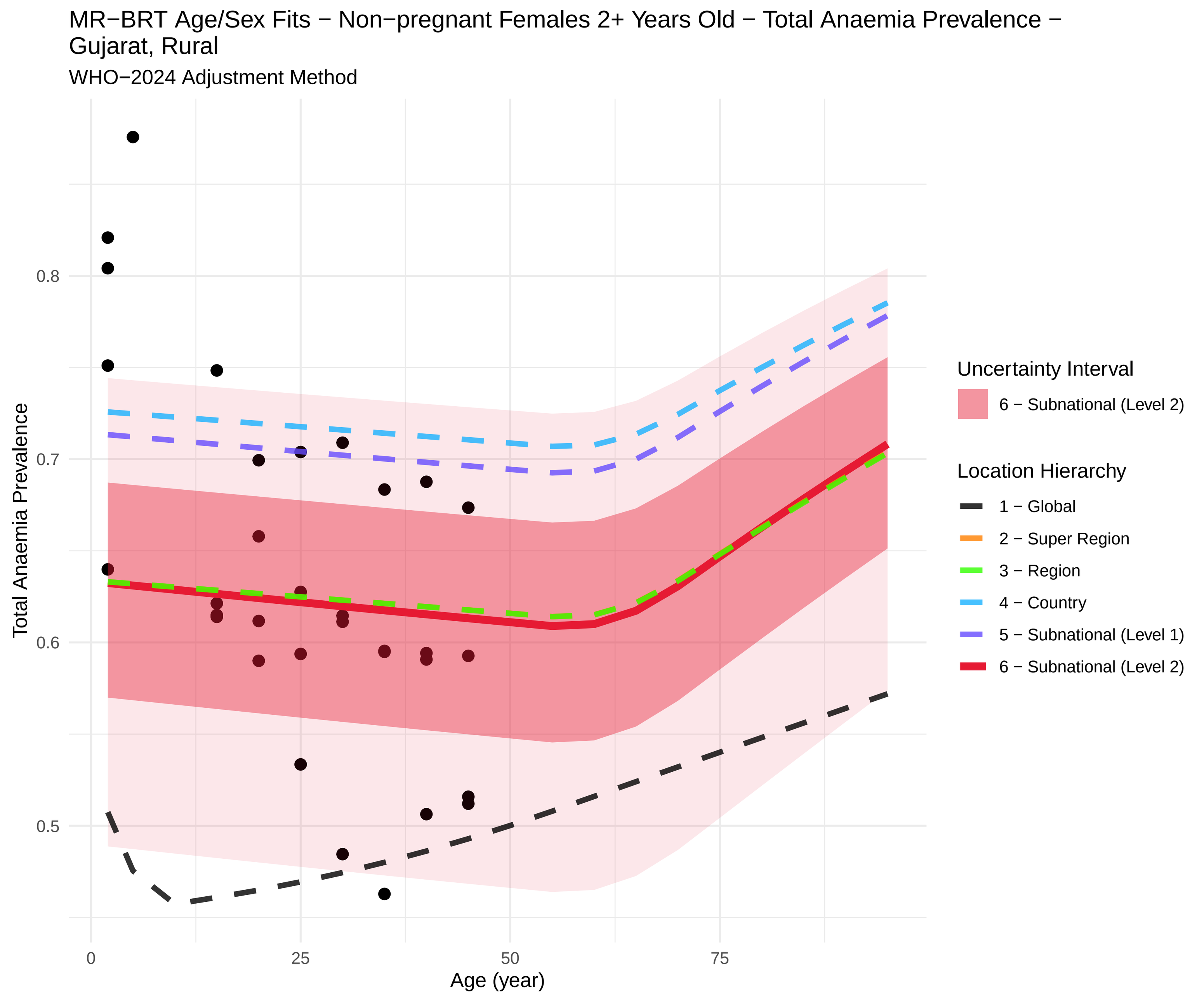
MR−BRT Age/Sex Fits − Non−pregnant Females 2+ Years Old − Total Anaemia Prevalence − Gujarat, Rural
WHO−2024 Adjustment Method
Total Anaemia Prevalence
Age (year)
Uncertainty Interval
6 − Subnational (Level 2)
Location Hierarchy
1 − Global
2 − Super Region
3 − Region
4 − Country
5 − Subnational (Level 1)
6 − Subnational (Level 2)

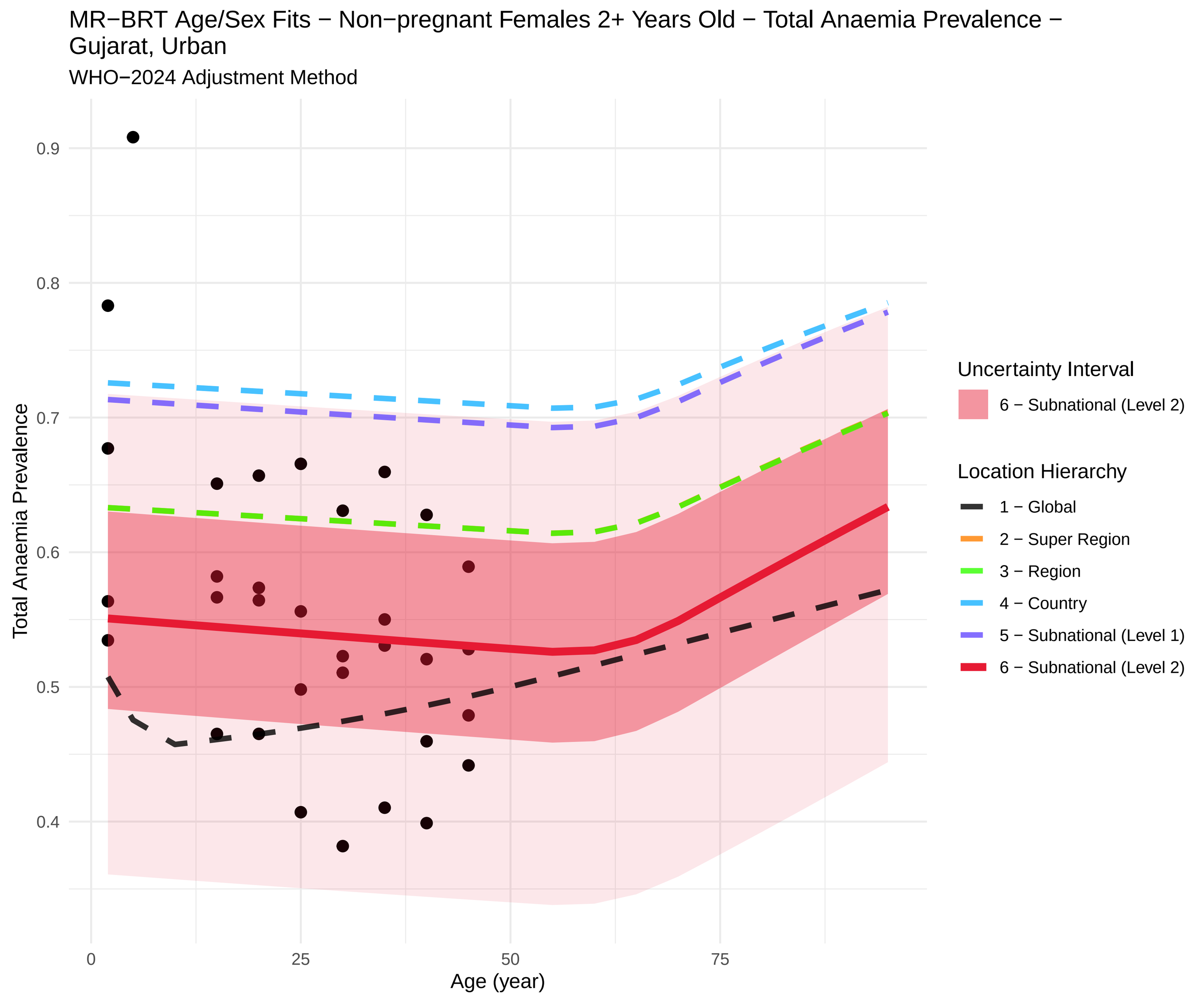
MR−BRT Age/Sex Fits − Non−pregnant Females 2+ Years Old − Total Anaemia Prevalence − Gujarat, Urban
WHO−2024 Adjustment Method
Total Anaemia Prevalence
Age (year)
Uncertainty Interval
6 − Subnational (Level 2)
Location Hierarchy
1 − Global
2 − Super Region
3 − Region
4 − Country
5 − Subnational (Level 1)
6 − Subnational (Level 2)

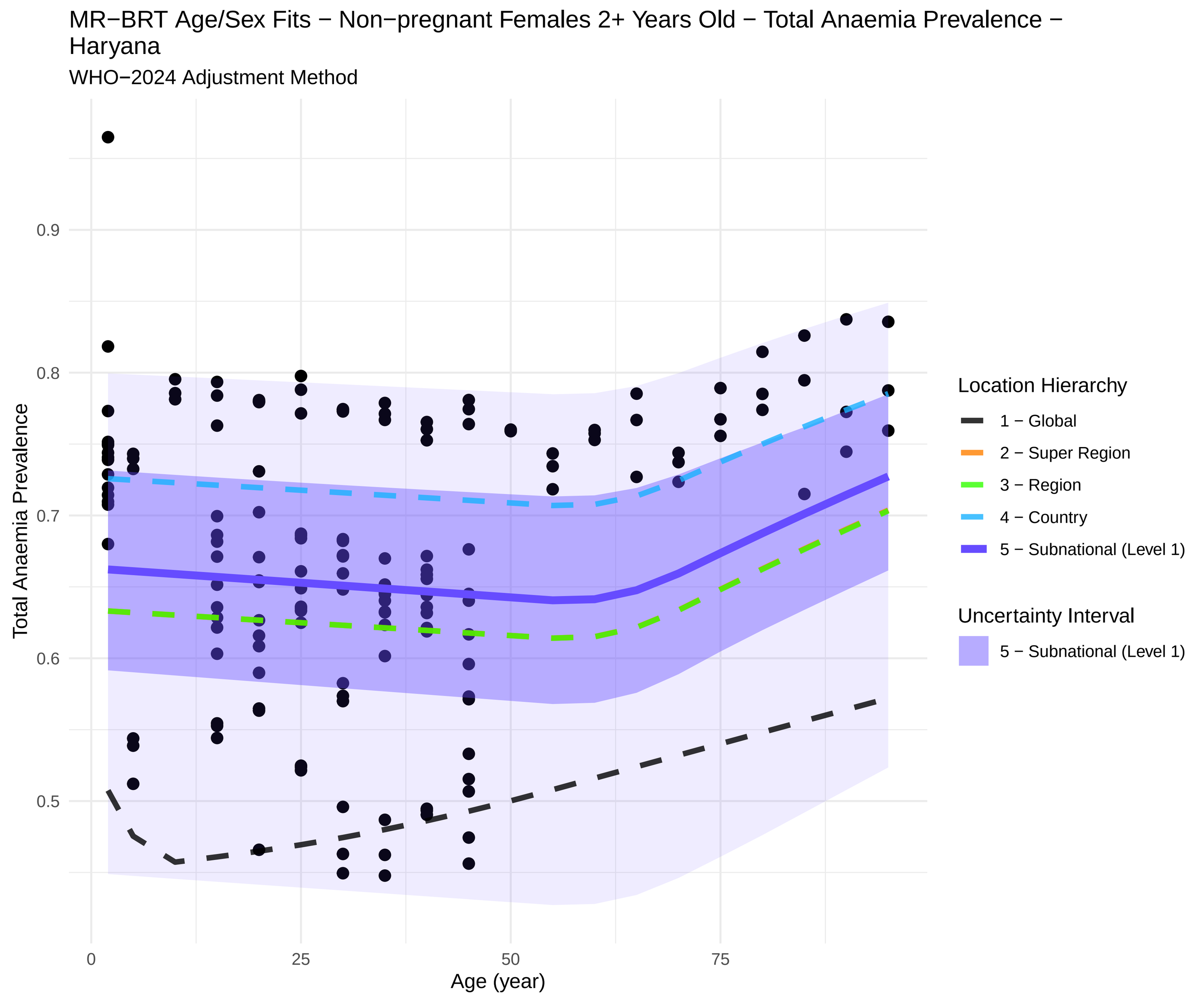
MR−BRT Age/Sex Fits − Non−pregnant Females 2+ Years Old − Total Anaemia Prevalence − Haryana
WHO−2024 Adjustment Method
Total Anaemia Prevalence
Age (year)
Location Hierarchy
1 − Global
2 − Super Region
3 − Region
4 − Country
5 − Subnational (Level 1)
Uncertainty Interval
5 − Subnational (Level 1)

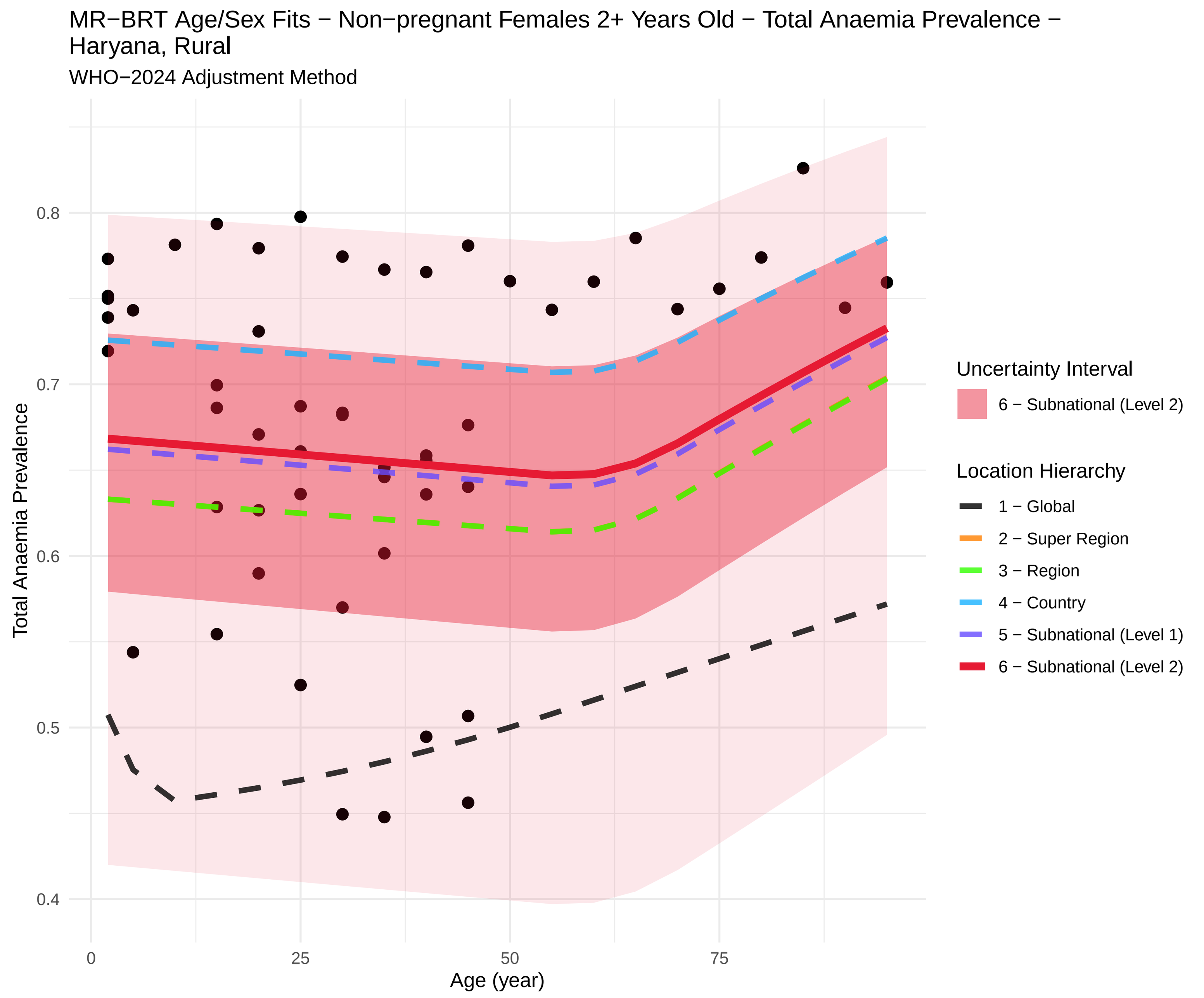
MR−BRT Age/Sex Fits − Non−pregnant Females 2+ Years Old − Total Anaemia Prevalence − Haryana, Rural
WHO−2024 Adjustment Method
Total Anaemia Prevalence
Age (year)
Uncertainty Interval
6 − Subnational (Level 2)
Location Hierarchy
1 − Global
2 − Super Region
3 − Region
4 − Country
5 − Subnational (Level 1)
6 − Subnational (Level 2)

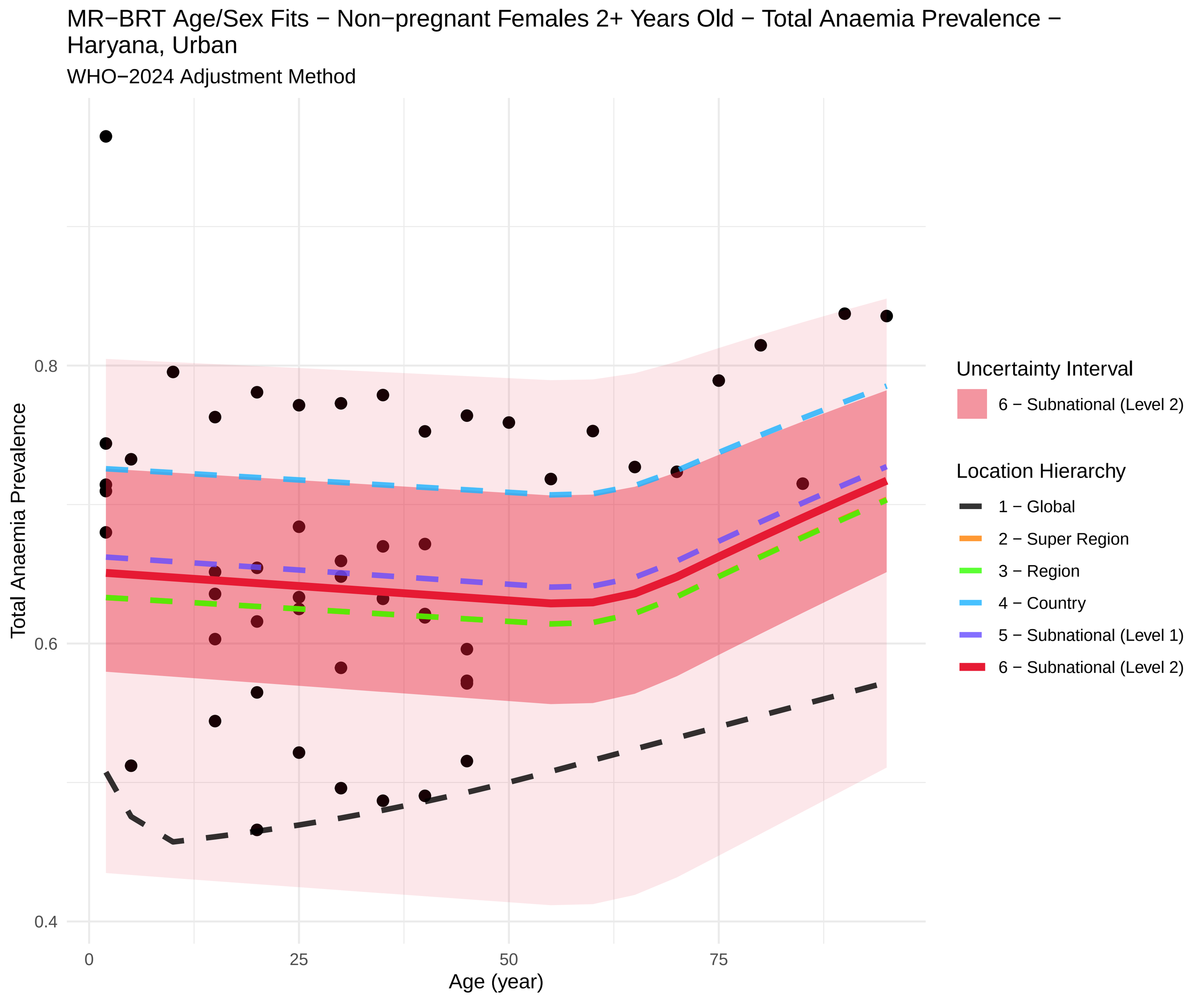
MR−BRT Age/Sex Fits − Non−pregnant Females 2+ Years Old − Total Anaemia Prevalence − Haryana, Urban
WHO−2024 Adjustment Method
Total Anaemia Prevalence
Age (year)
Uncertainty Interval
6 − Subnational (Level 2)
Location Hierarchy
1 − Global
2 − Super Region
3 − Region
4 − Country
5 − Subnational (Level 1)
6 − Subnational (Level 2)

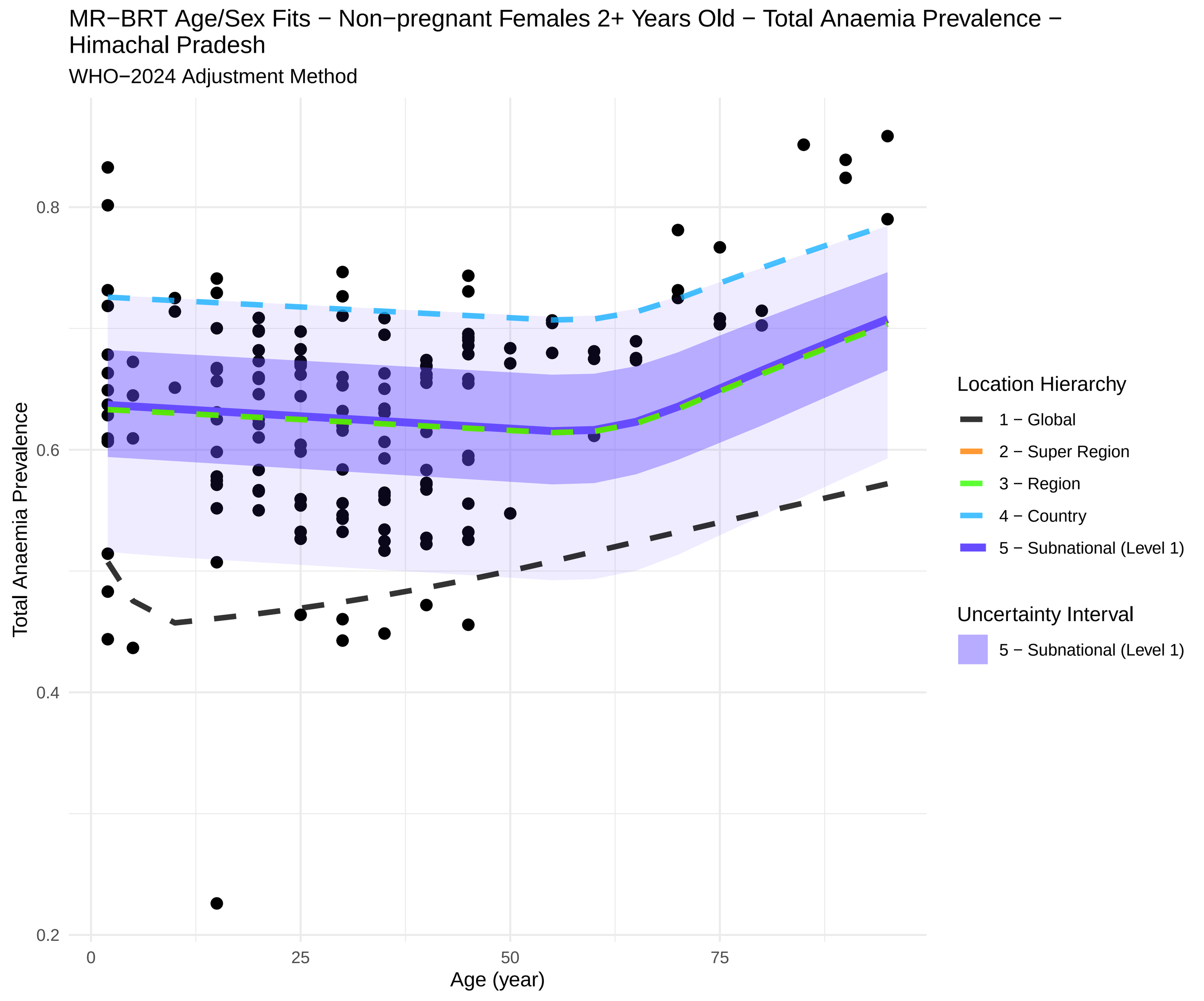
MR−BRT Age/Sex Fits − Non−pregnant Females 2+ Years Old − Total Anaemia Prevalence − Himachal Pradesh
WHO−2024 Adjustment Method
Total Anaemia Prevalence
Age (year)
Location Hierarchy
1 − Global
2 − Super Region
3 − Region
4 − Country
5 − Subnational (Level 1)
Uncertainty Interval
5 − Subnational (Level 1)

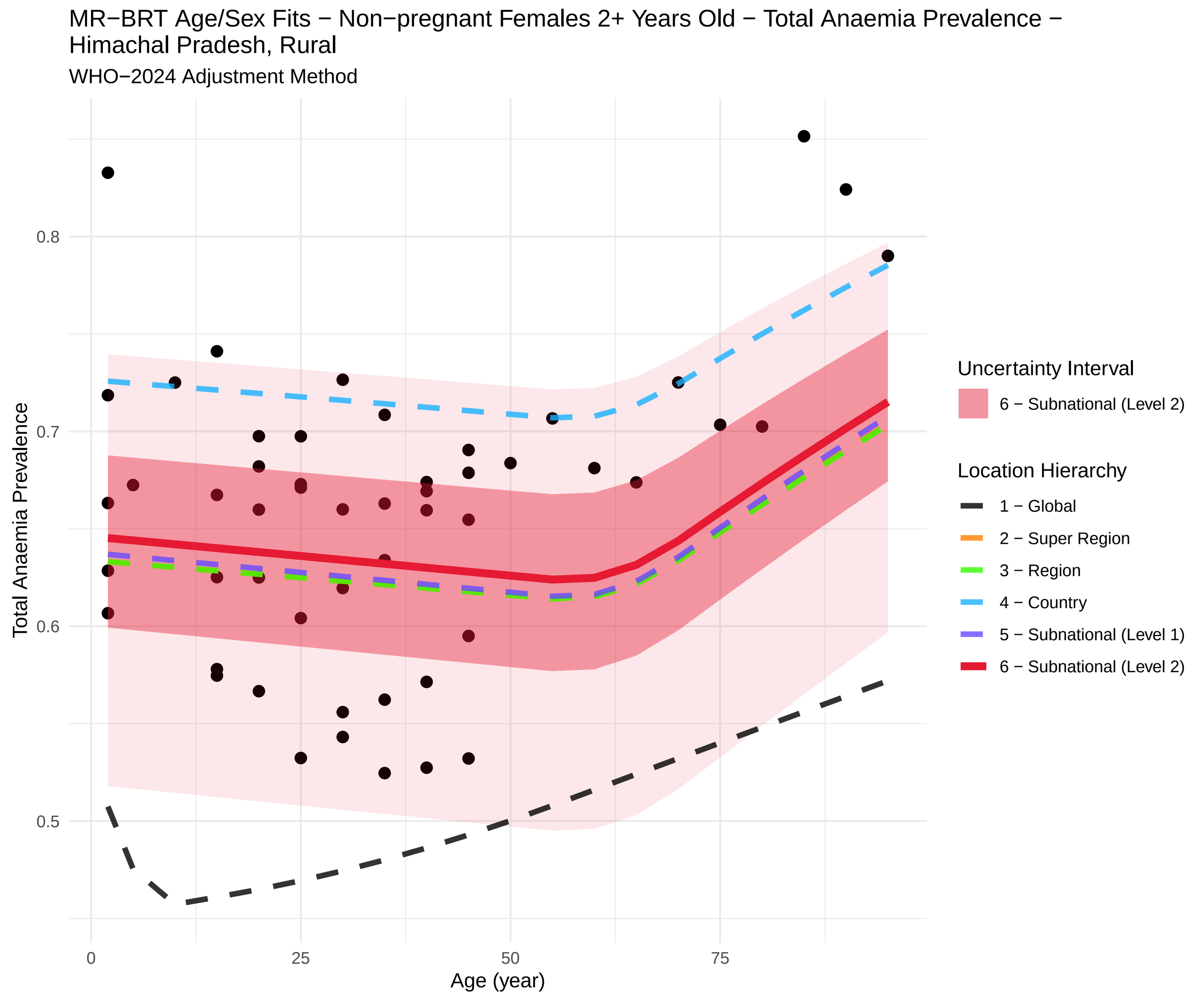
MR−BRT Age/Sex Fits − Non−pregnant Females 2+ Years Old − Total Anaemia Prevalence − Himachal Pradesh, Rural
WHO−2024 Adjustment Method
Total Anaemia Prevalence
Age (year)
Uncertainty Interval
6 − Subnational (Level 2)
Location Hierarchy
1 − Global
2 − Super Region
3 − Region
4 − Country
5 − Subnational (Level 1)
6 − Subnational (Level 2)

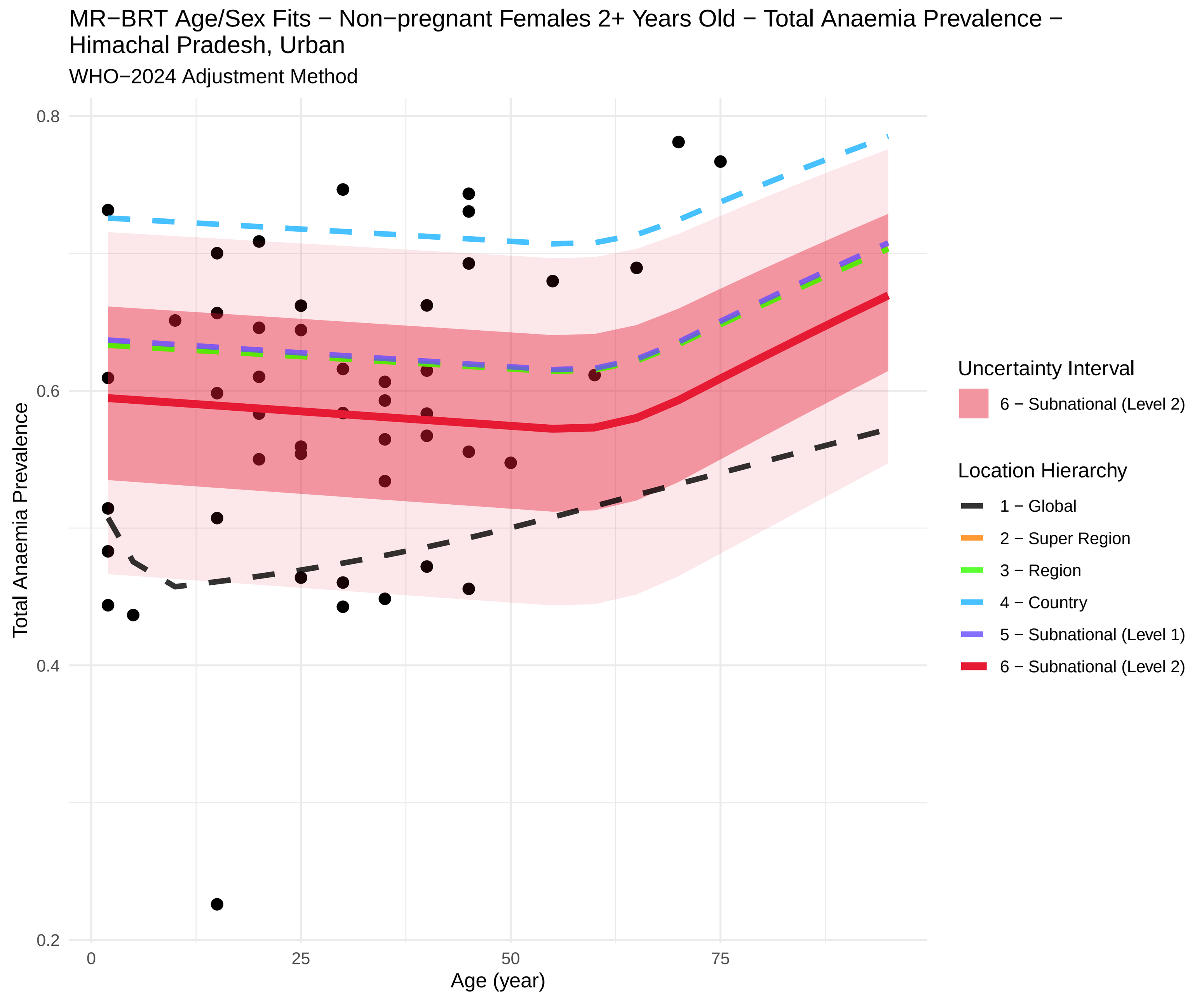
MR−BRT Age/Sex Fits − Non−pregnant Females 2+ Years Old − Total Anaemia Prevalence − Himachal Pradesh, Urban
WHO−2024 Adjustment Method
Total Anaemia Prevalence
Age (year)
Uncertainty Interval
6 − Subnational (Level 2)
Location Hierarchy
1 − Global
2 − Super Region
3 − Region
4 − Country
5 − Subnational (Level 1)
6 − Subnational (Level 2)

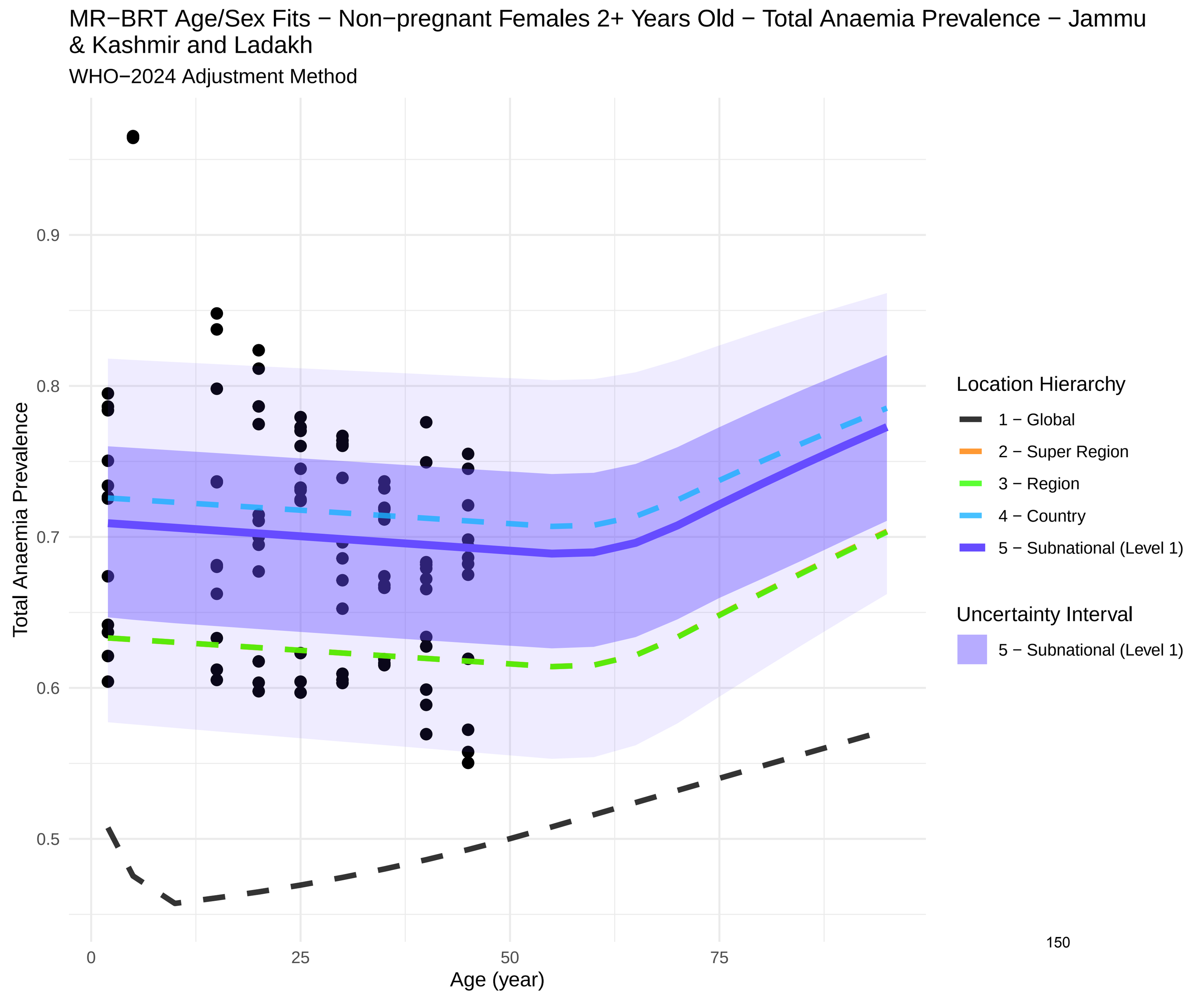
MR−BRT Age/Sex Fits − Non−pregnant Females 2+ Years Old − Total Anaemia Prevalence − Jammu & Kashmir and Ladakh
WHO−2024 Adjustment Method
Total Anaemia Prevalence
Age (year)
Location Hierarchy
1 − Global
2 − Super Region
3 − Region
4 − Country
5 − Subnational (Level 1)
Uncertainty Interval
5 − Subnational (Level 1)

MR−BRT Age/Sex Fits − Non−pregnant Females 2+ Years Old − Total Anaemia Prevalence − Jammu & Kashmir and Ladakh, Rural
WHO−2024 Adjustment Method

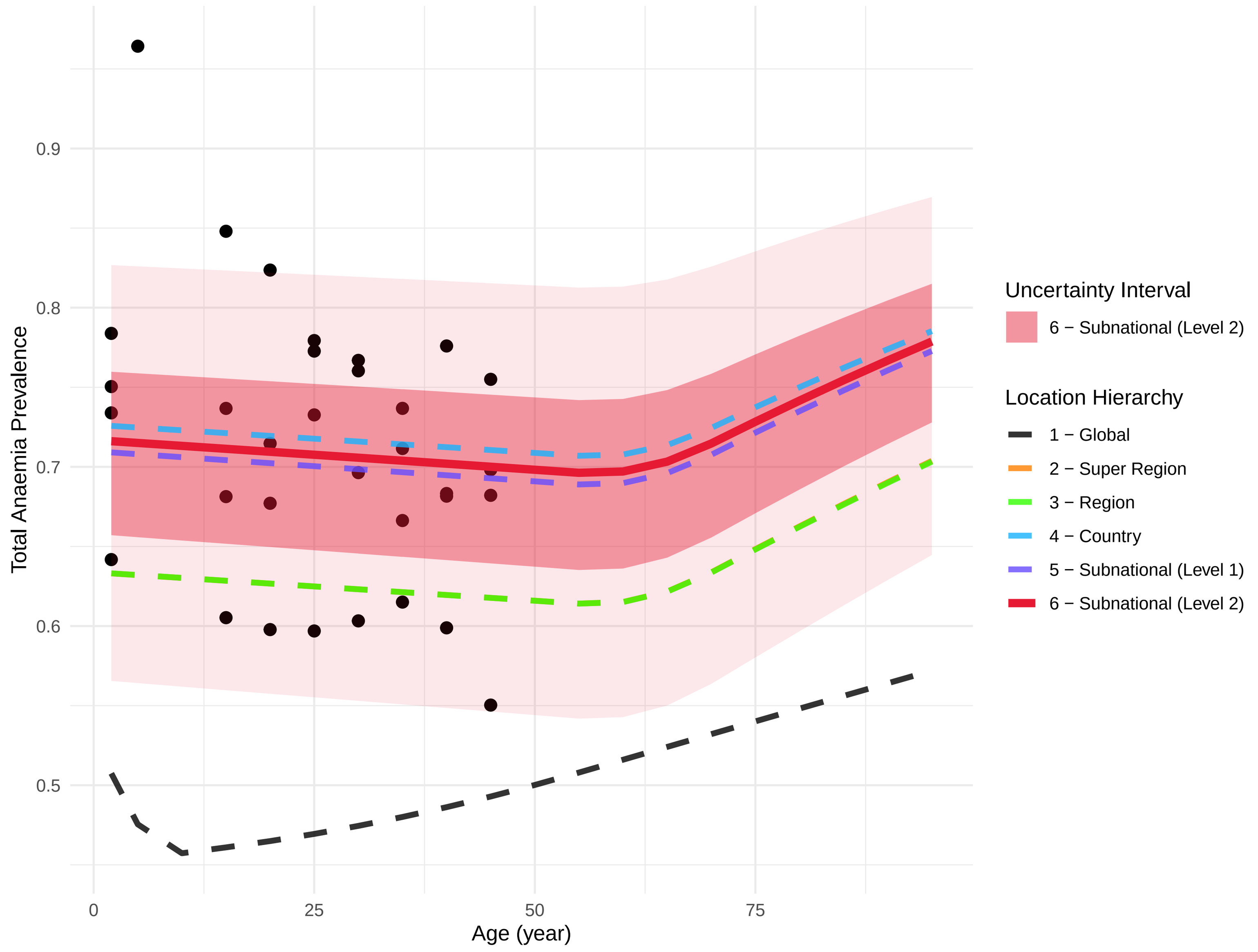

MR−BRT Age/Sex Fits − Non−pregnant Females 2+ Years Old − Total Anaemia Prevalence − Jammu & Kashmir and Ladakh, Urban
WHO−2024 Adjustment Method

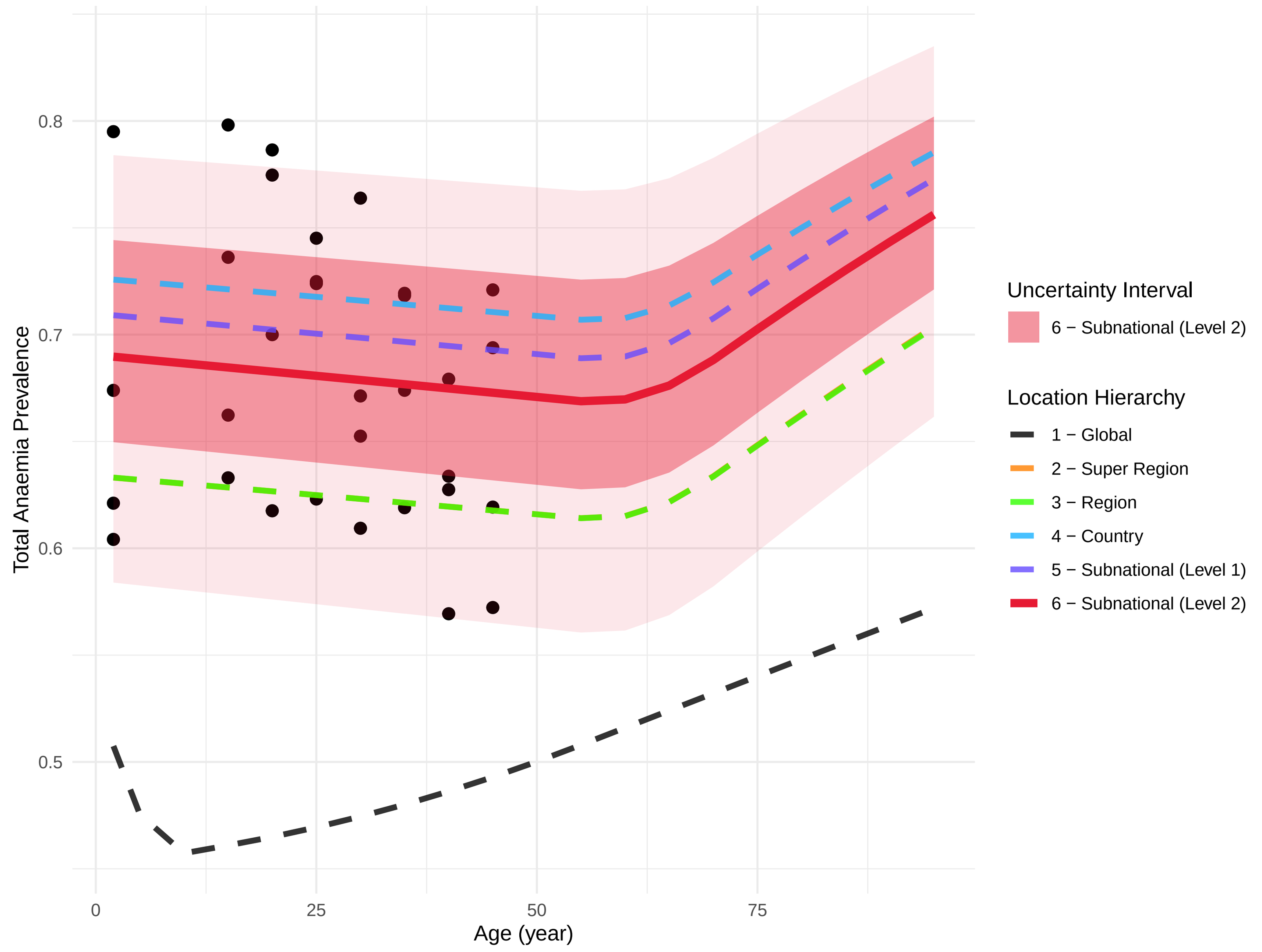

MR−BRT Age/Sex Fits − Non−pregnant Females 2+ Years Old − Total Anaemia Prevalence − Jharkhand
WHO−2024 Adjustment Method

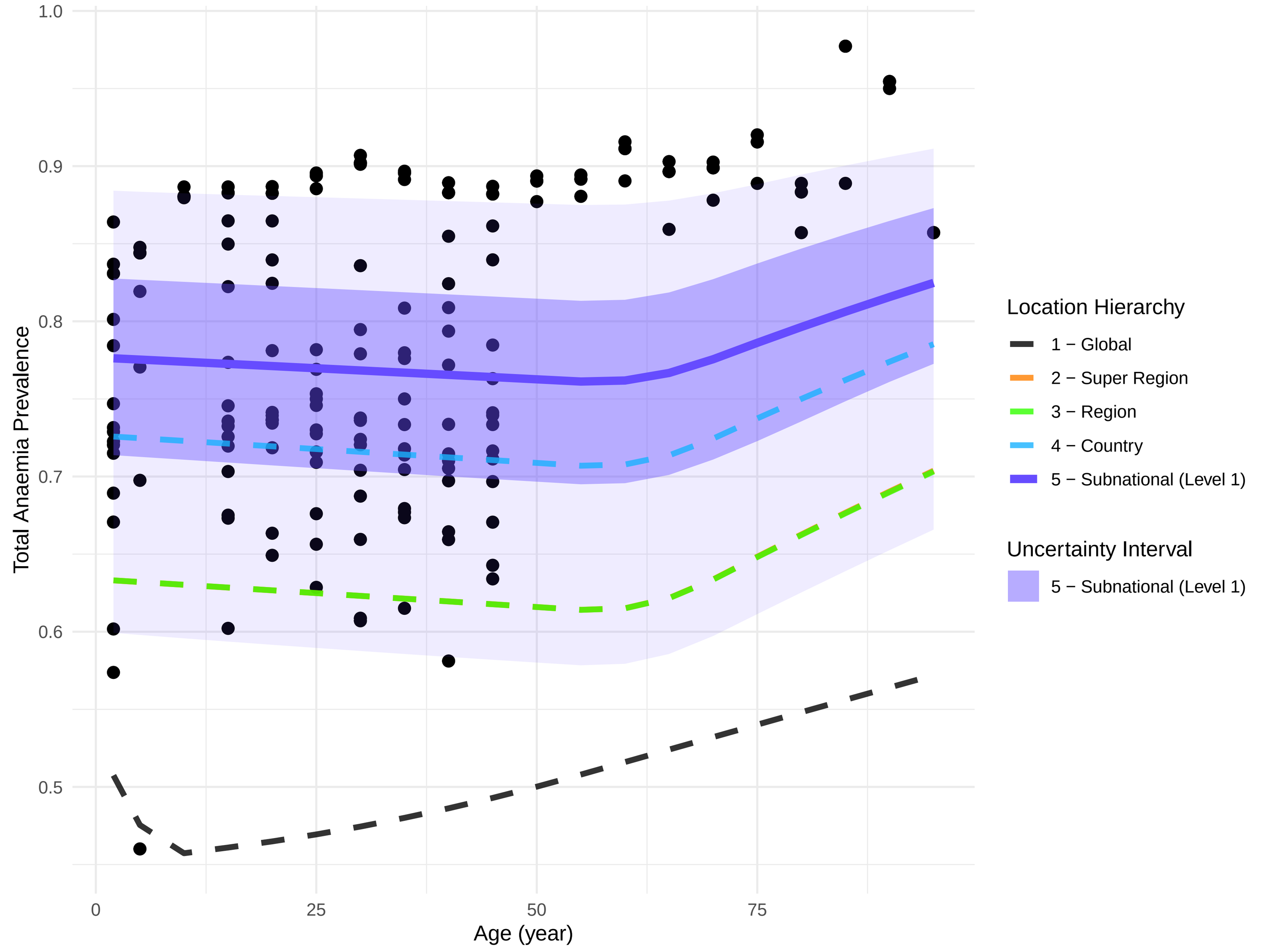

MR−BRT Age/Sex Fits − Non−pregnant Females 2+ Years Old − Total Anaemia Prevalence − Jharkhand, Rural
WHO−2024 Adjustment Method

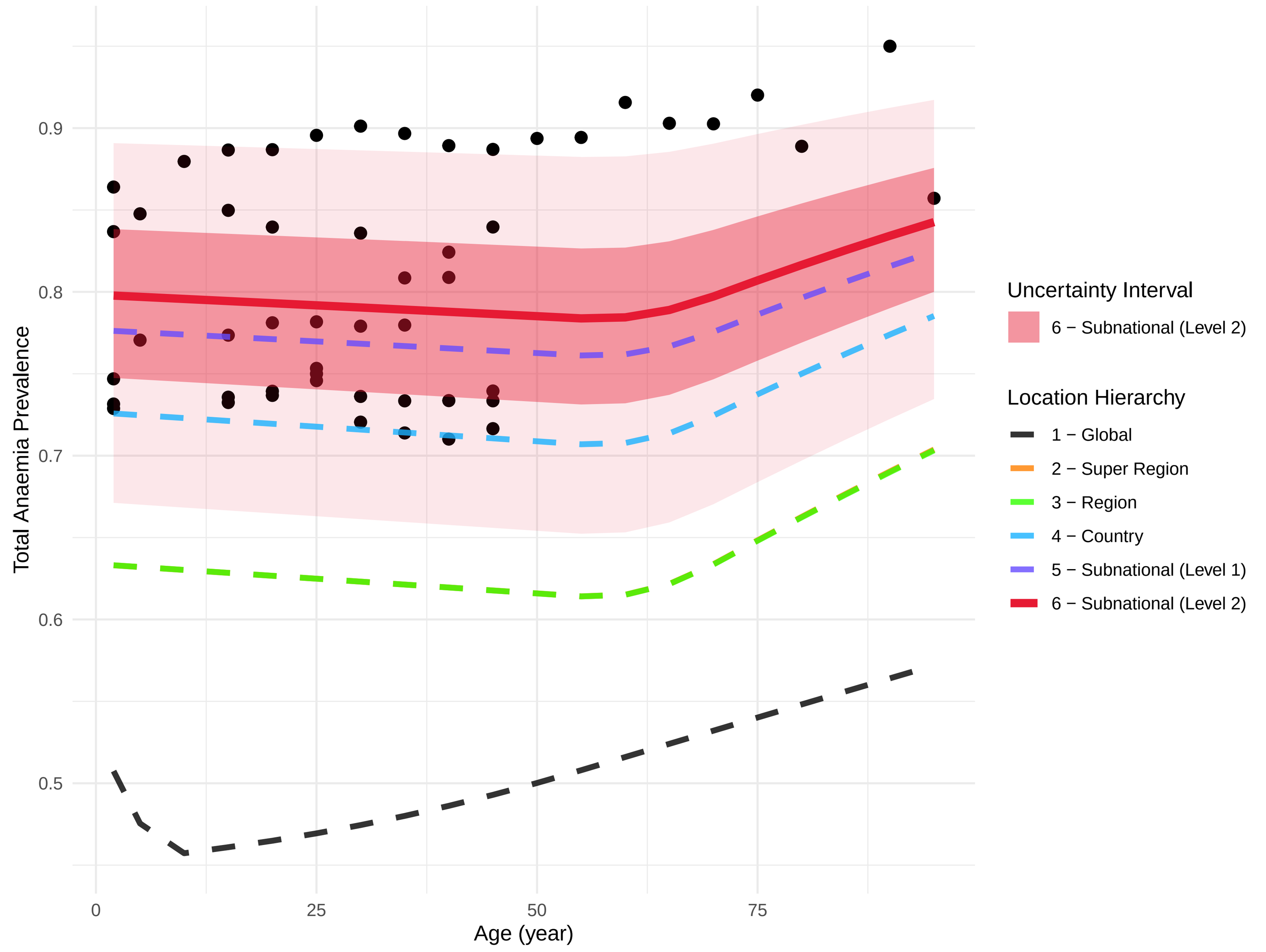

MR−BRT Age/Sex Fits − Non−pregnant Females 2+ Years Old − Total Anaemia Prevalence − Jharkhand, Urban
WHO−2024 Adjustment Method

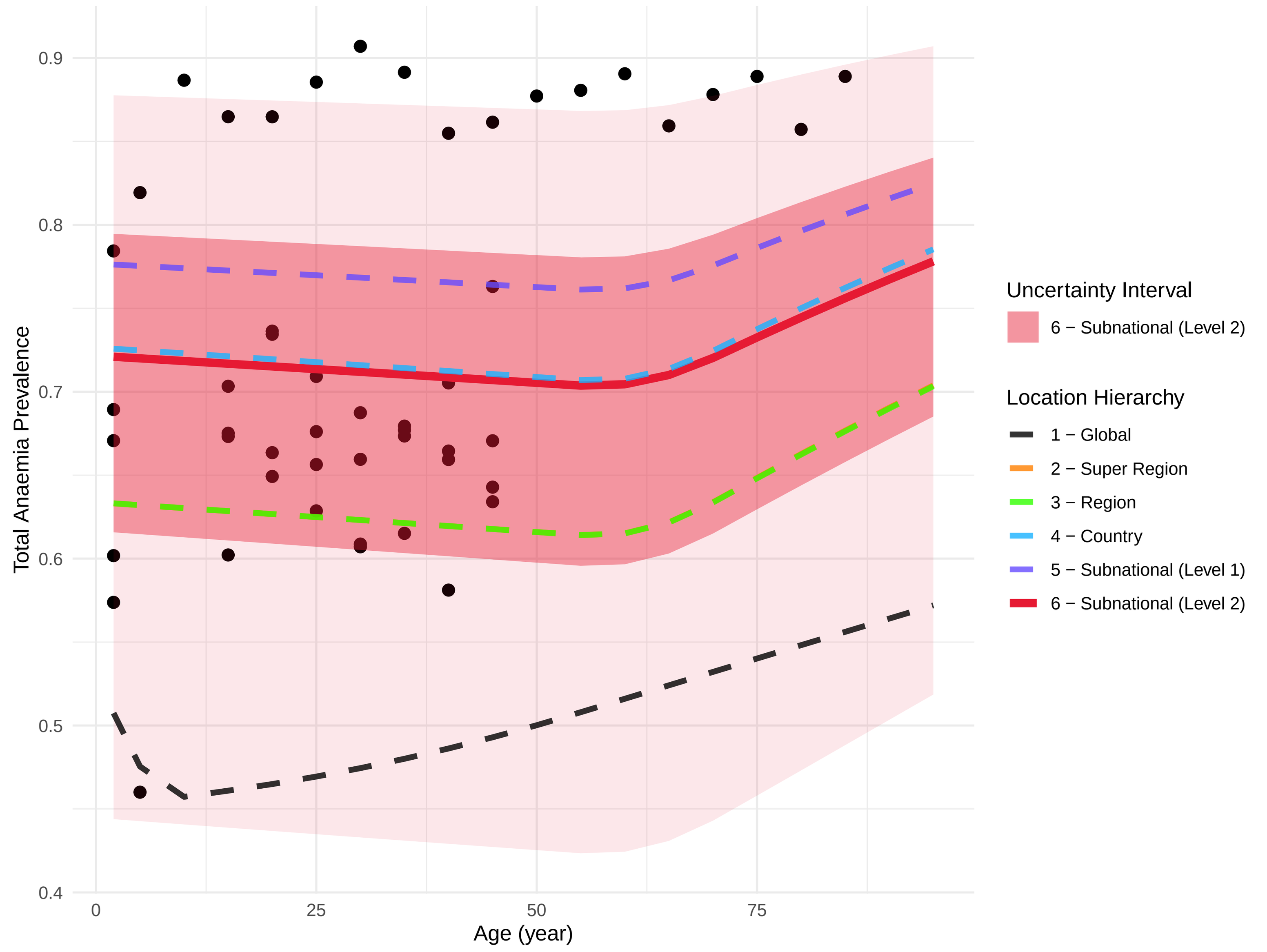

MR−BRT Age/Sex Fits − Non−pregnant Females 2+ Years Old − Total Anaemia Prevalence − Karnataka
WHO−2024 Adjustment Method

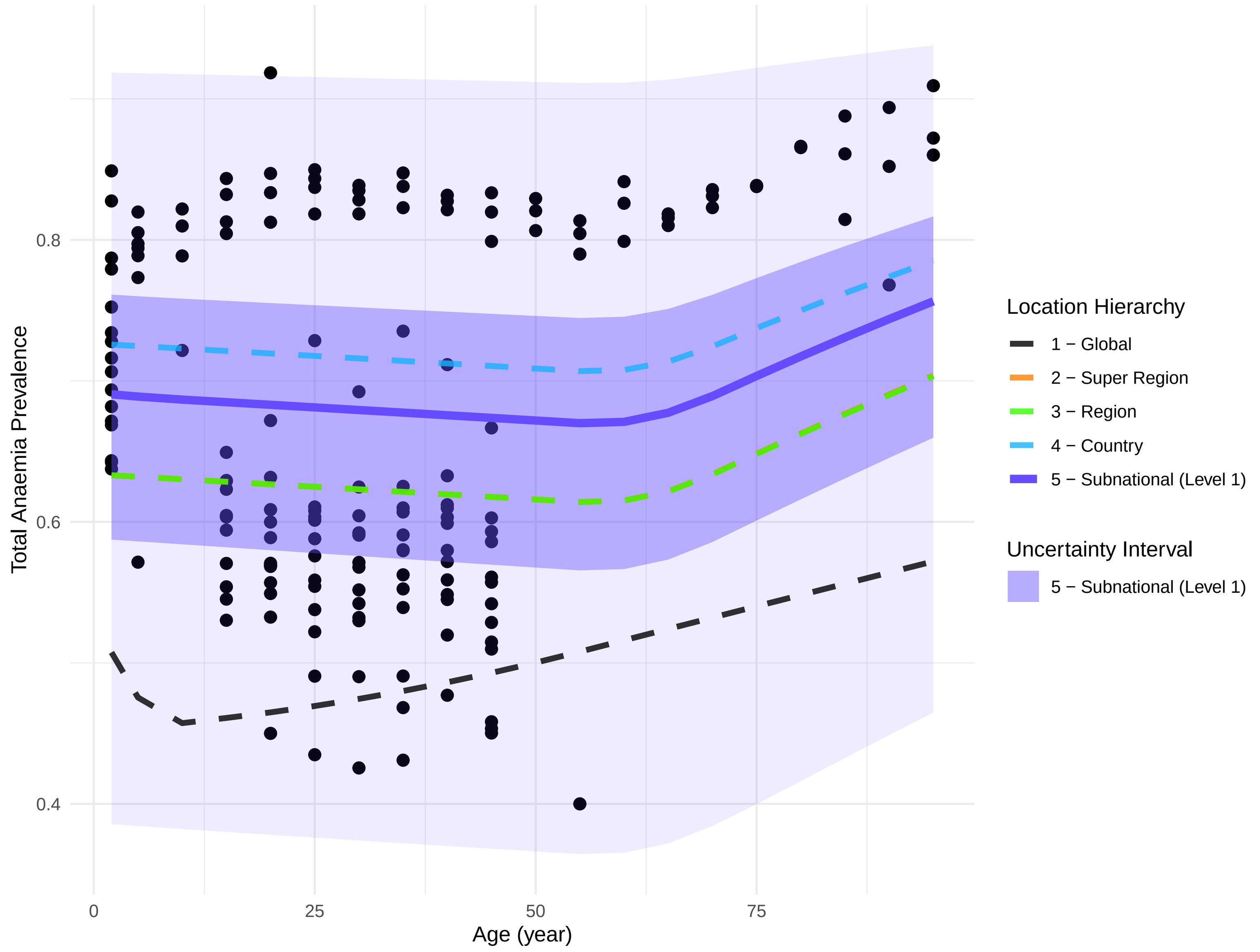

MR−BRT Age/Sex Fits − Non−pregnant Females 2+ Years Old − Total Anaemia Prevalence − Karnataka, Rural
WHO−2024 Adjustment Method

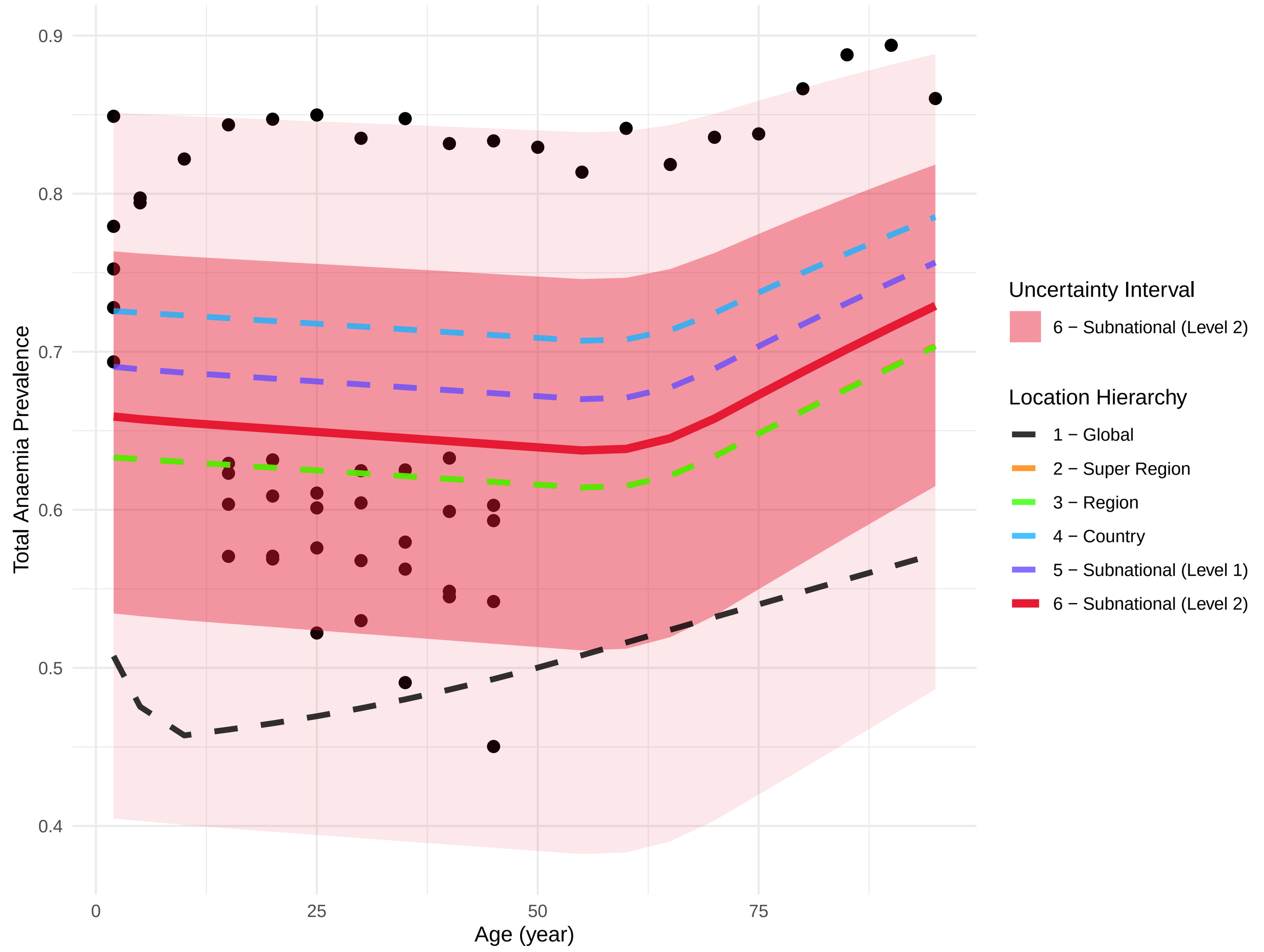

MR−BRT Age/Sex Fits − Non−pregnant Females 2+ Years Old − Total Anaemia Prevalence − Karnataka, Urban
WHO−2024 Adjustment Method

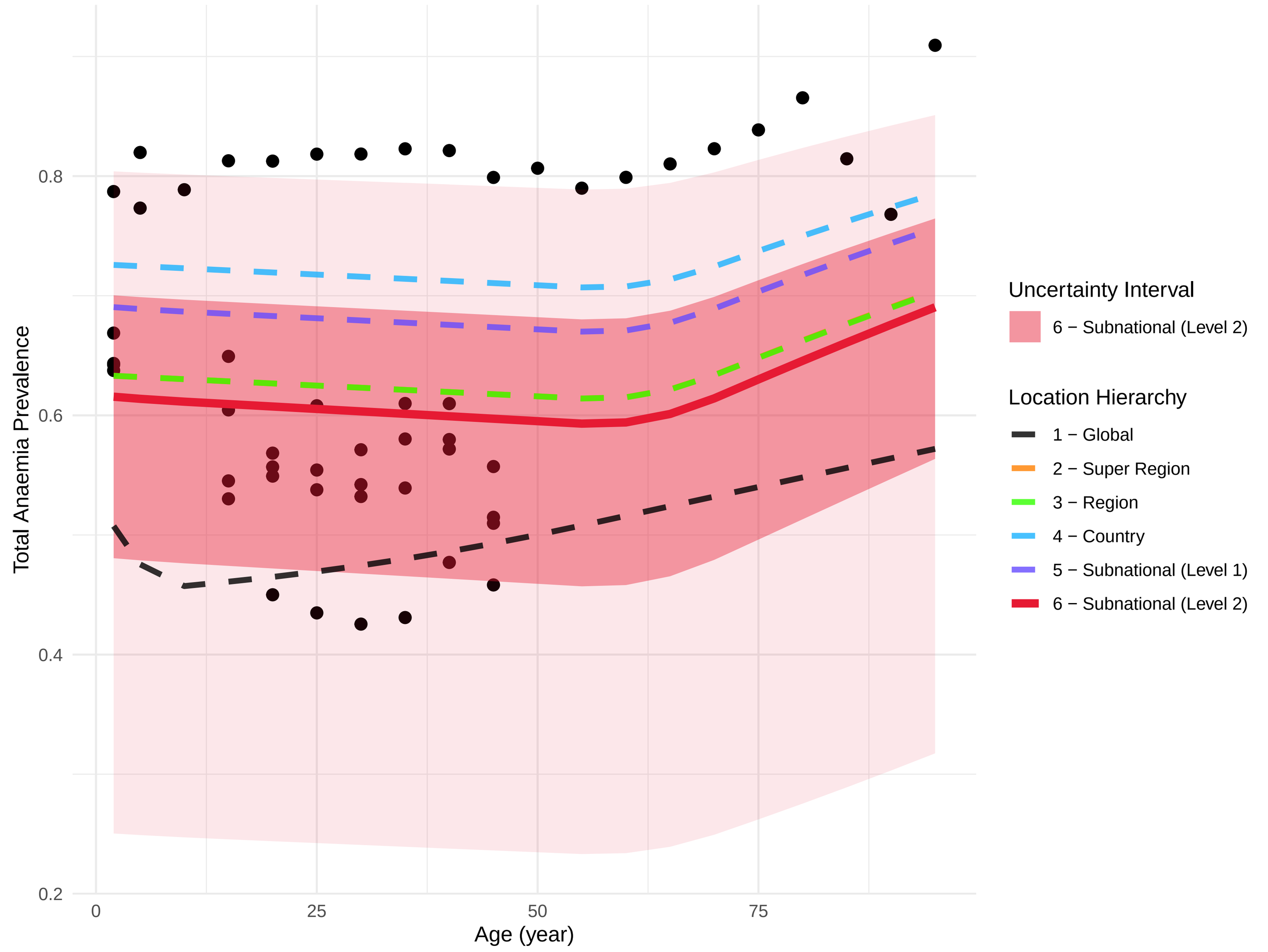

MR−BRT Age/Sex Fits − Non−pregnant Females 2+ Years Old − Total Anaemia Prevalence − Kerala
WHO−2024 Adjustment Method

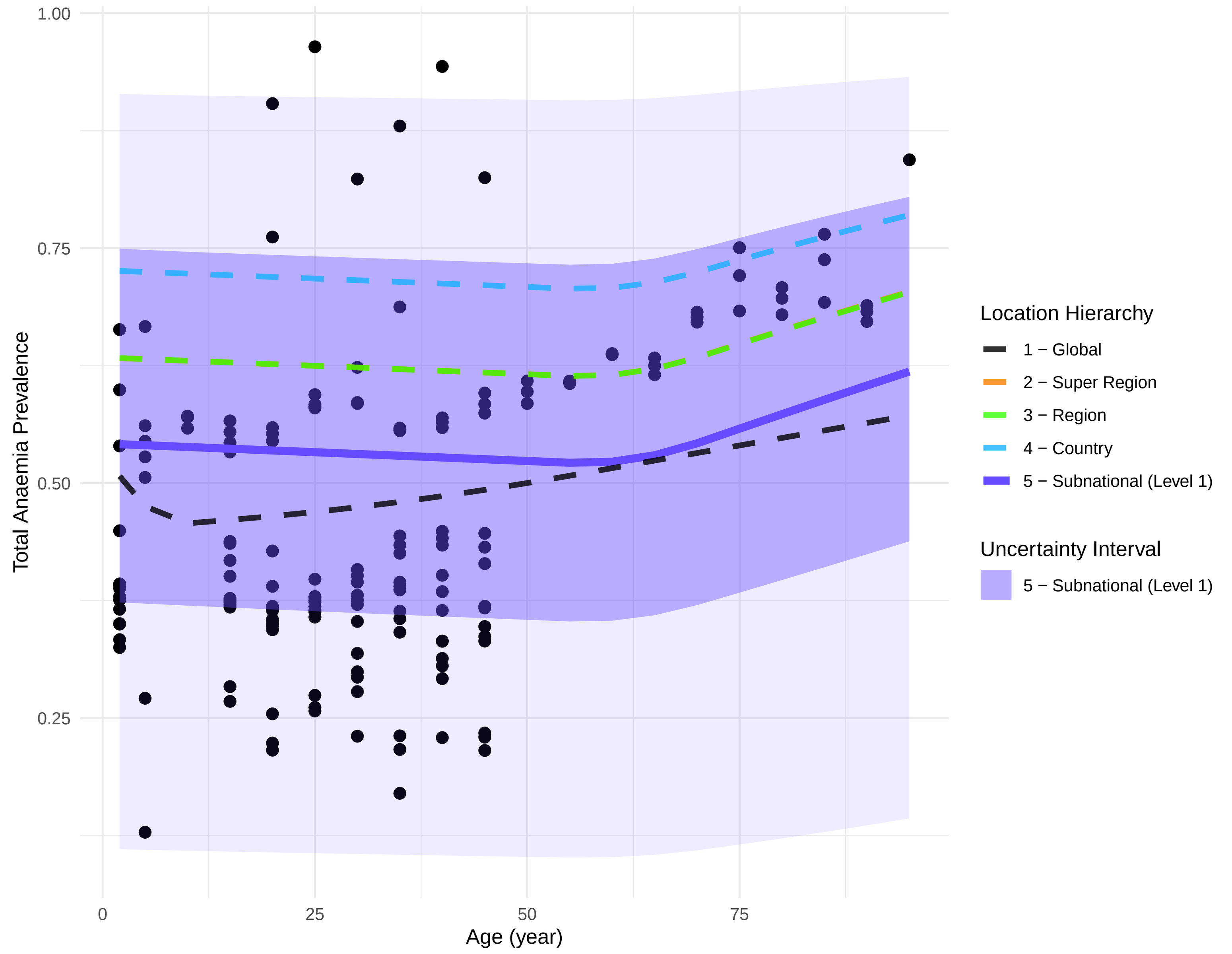

MR−BRT Age/Sex Fits − Non−pregnant Females 2+ Years Old − Total Anaemia Prevalence − Kerala, Rural
WHO−2024 Adjustment Method

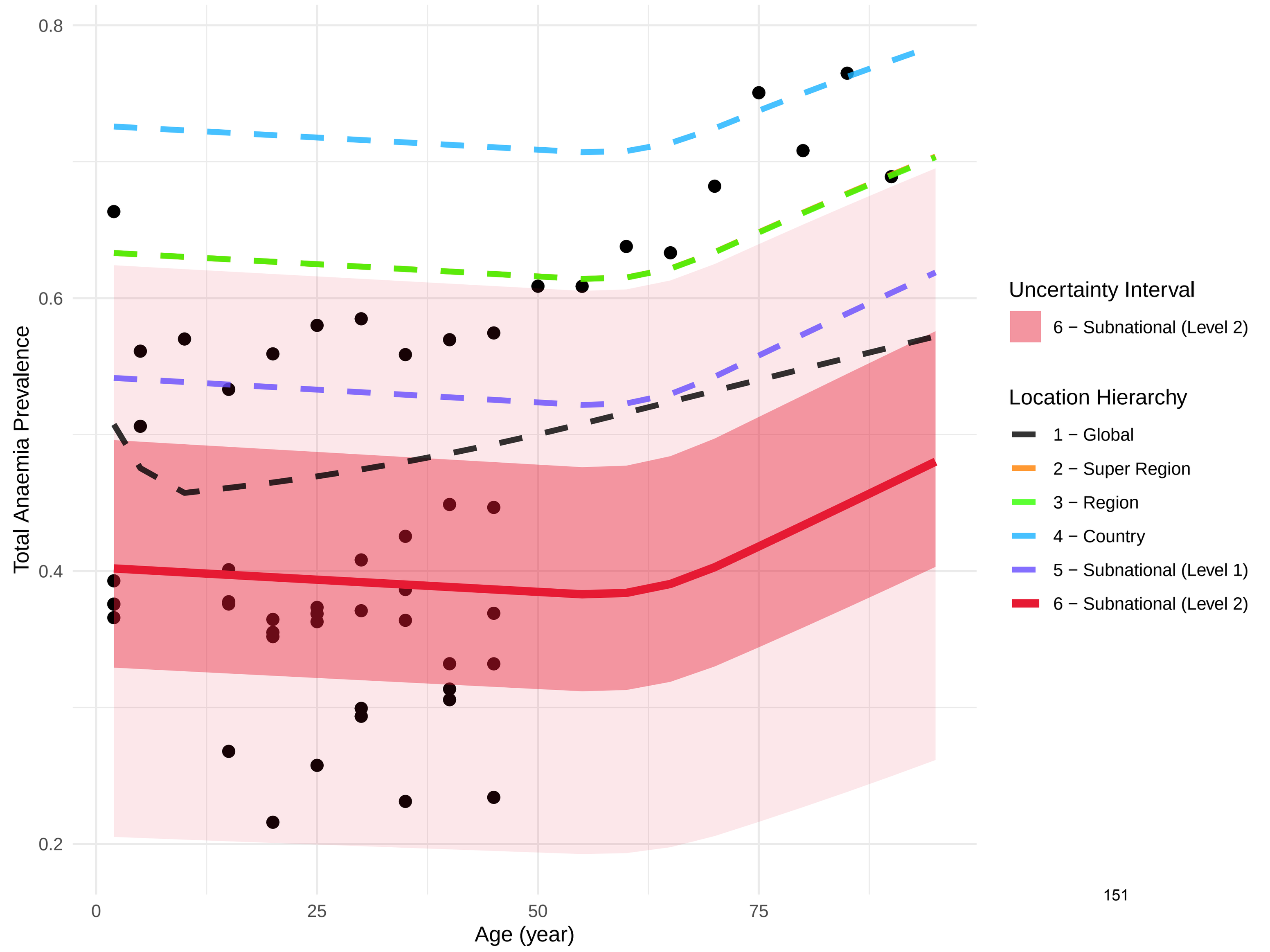

MR−BRT Age/Sex Fits − Non−pregnant Females 2+ Years Old − Total Anaemia Prevalence − Kerala, Urban
WHO−2024 Adjustment Method

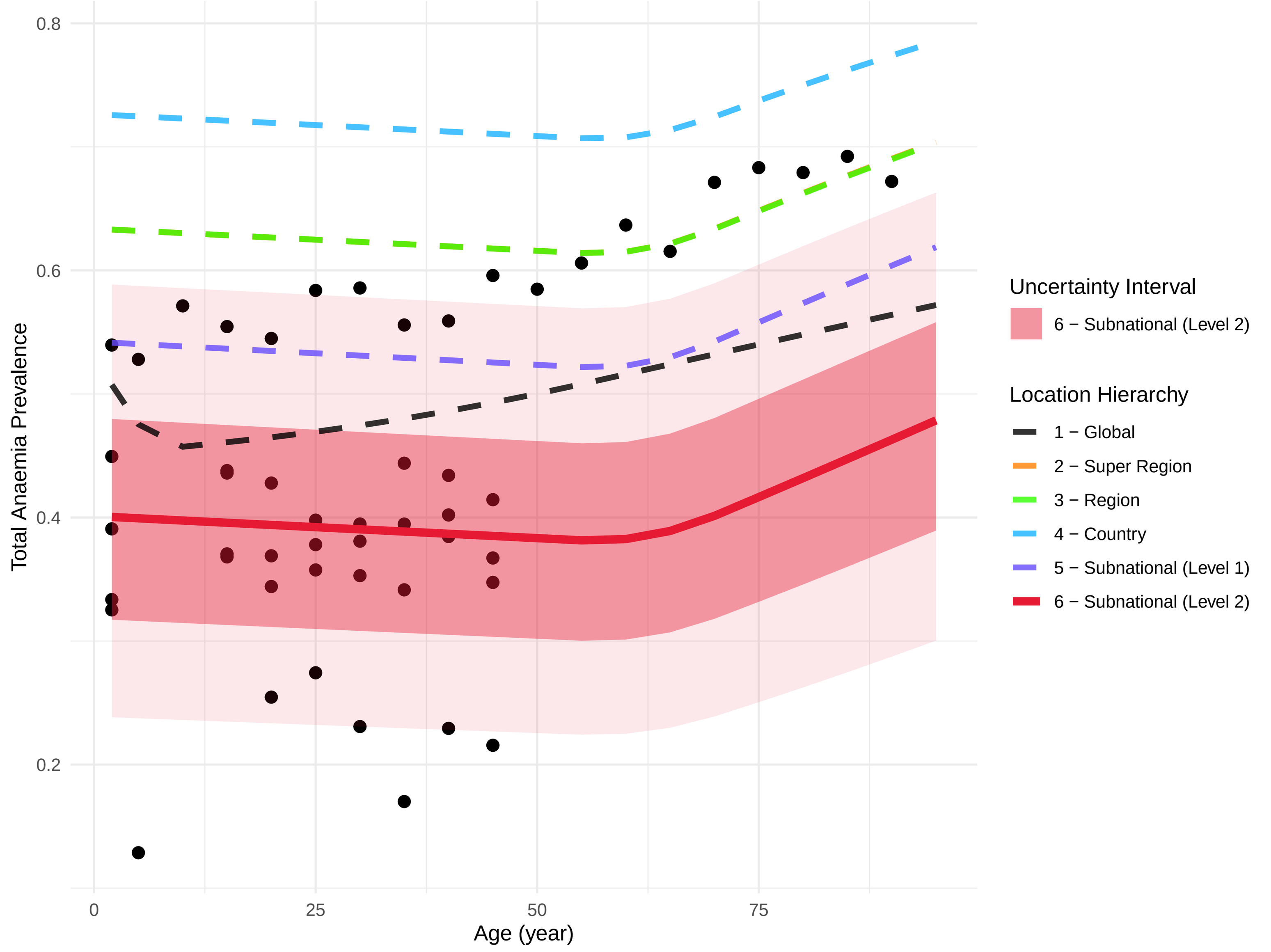

MR−BRT Age/Sex Fits − Non−pregnant Females 2+ Years Old − Total Anaemia Prevalence − Madhya Pradesh
WHO−2024 Adjustment Method

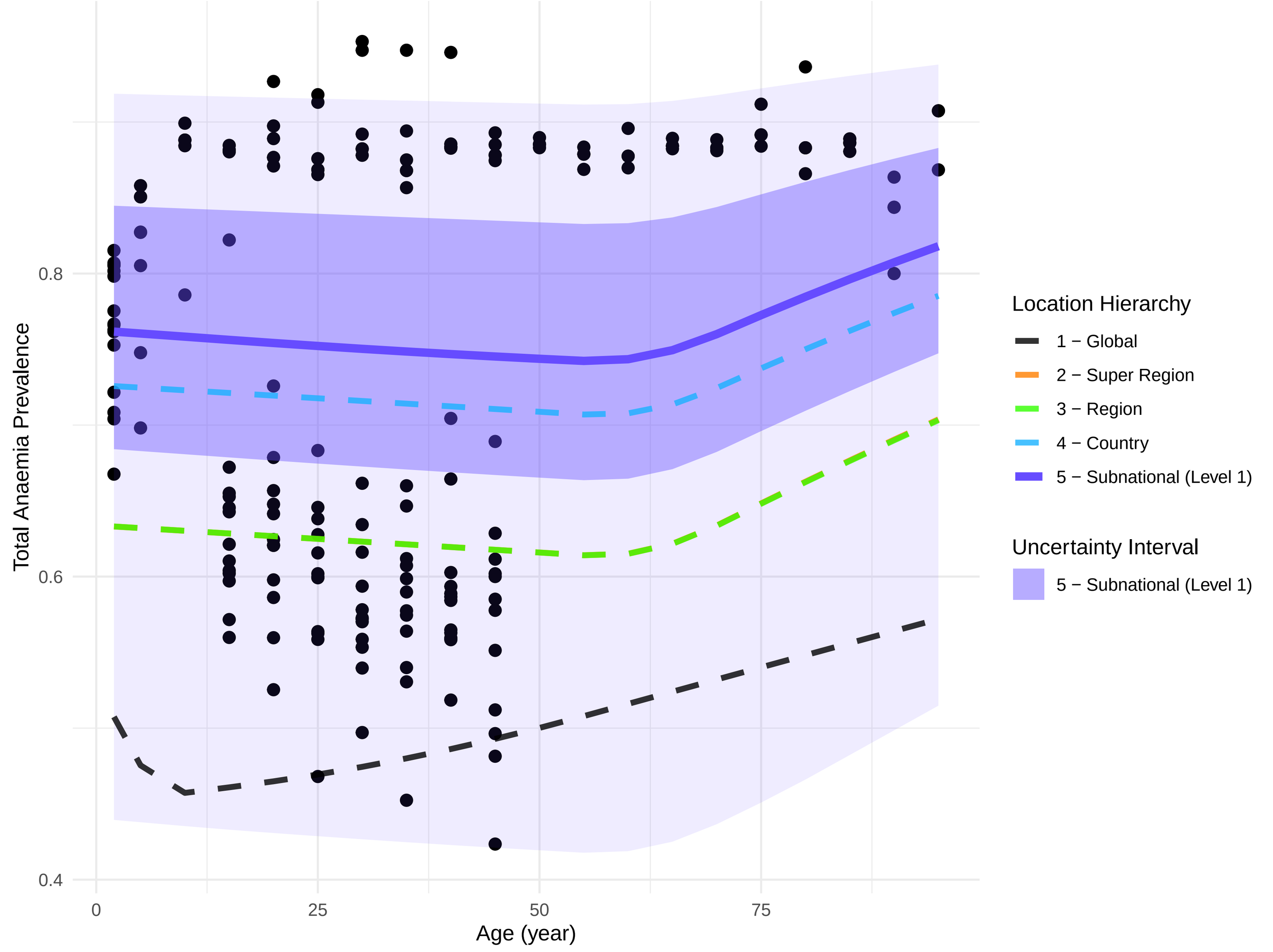

MR−BRT Age/Sex Fits − Non−pregnant Females 2+ Years Old − Total Anaemia Prevalence − Madhya Pradesh, Rural
WHO−2024 Adjustment Method

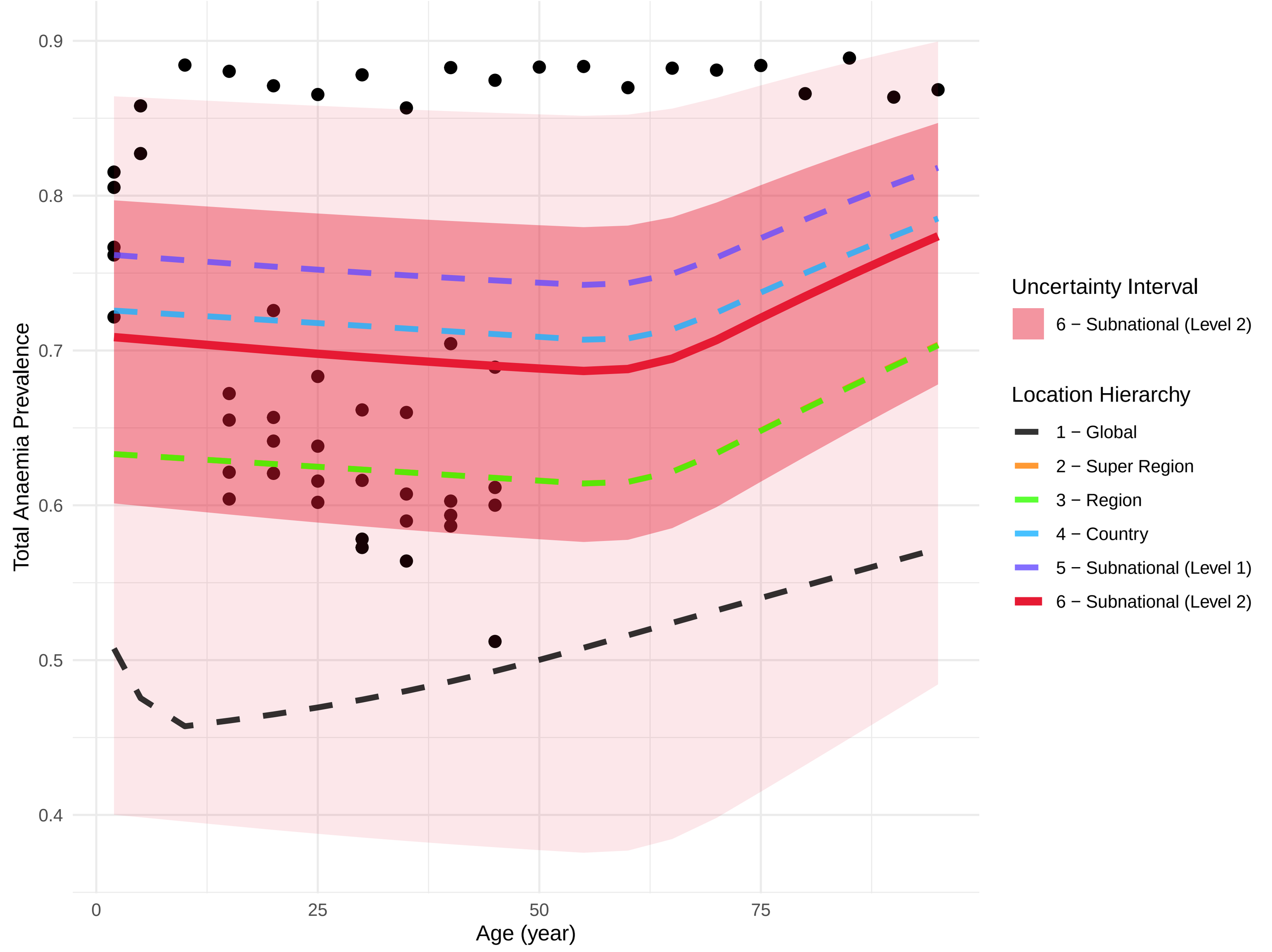

MR−BRT Age/Sex Fits − Non−pregnant Females 2+ Years Old − Total Anaemia Prevalence − Madhya Pradesh, Urban
WHO−2024 Adjustment Method

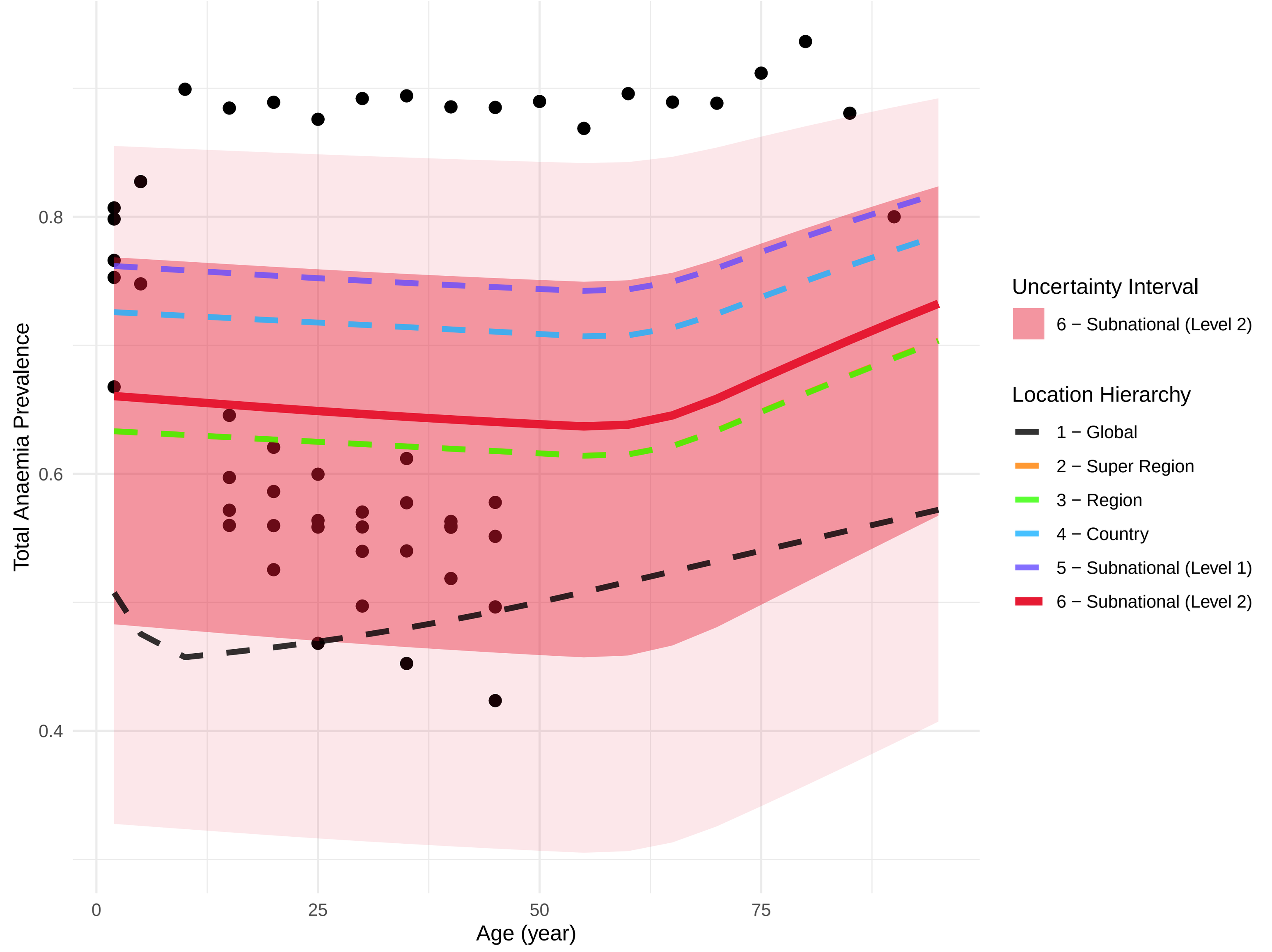

MR−BRT Age/Sex Fits − Non−pregnant Females 2+ Years Old − Total Anaemia Prevalence − Maharashtra
WHO−2024 Adjustment Method

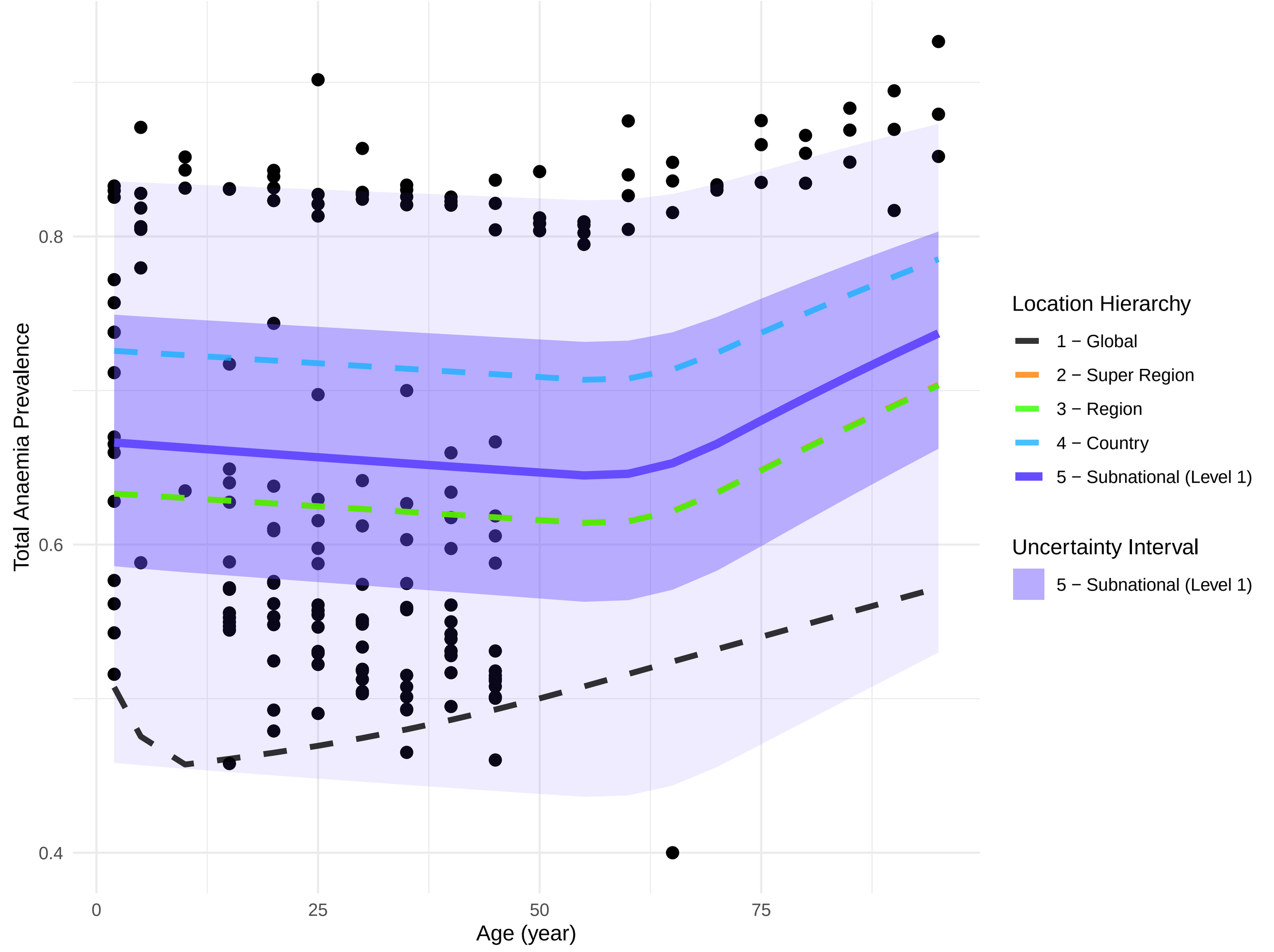

MR−BRT Age/Sex Fits − Non−pregnant Females 2+ Years Old − Total Anaemia Prevalence − Maharashtra, Rural
WHO−2024 Adjustment Method

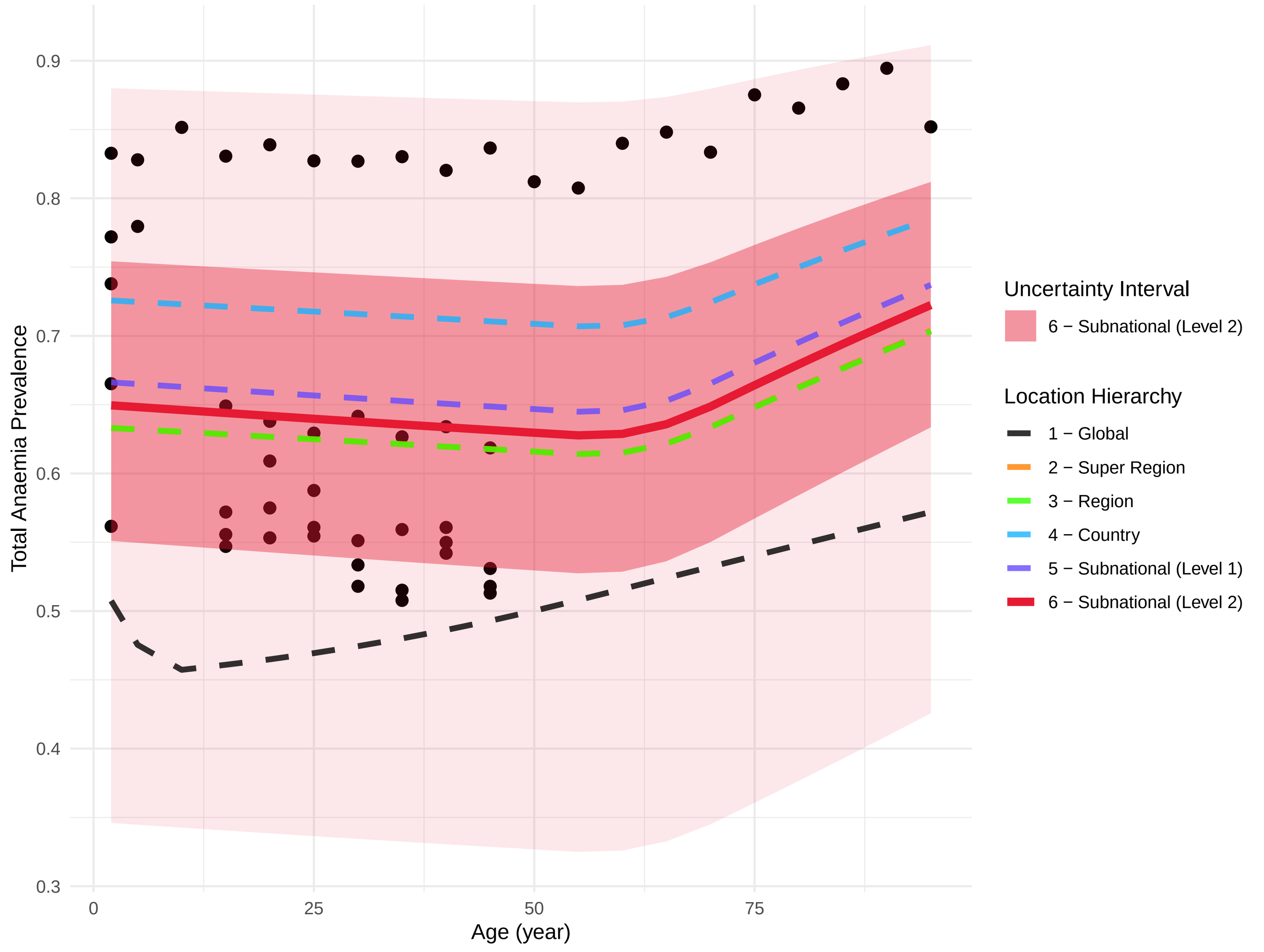

MR−BRT Age/Sex Fits − Non−pregnant Females 2+ Years Old − Total Anaemia Prevalence − Maharashtra, Urban
WHO−2024 Adjustment Method

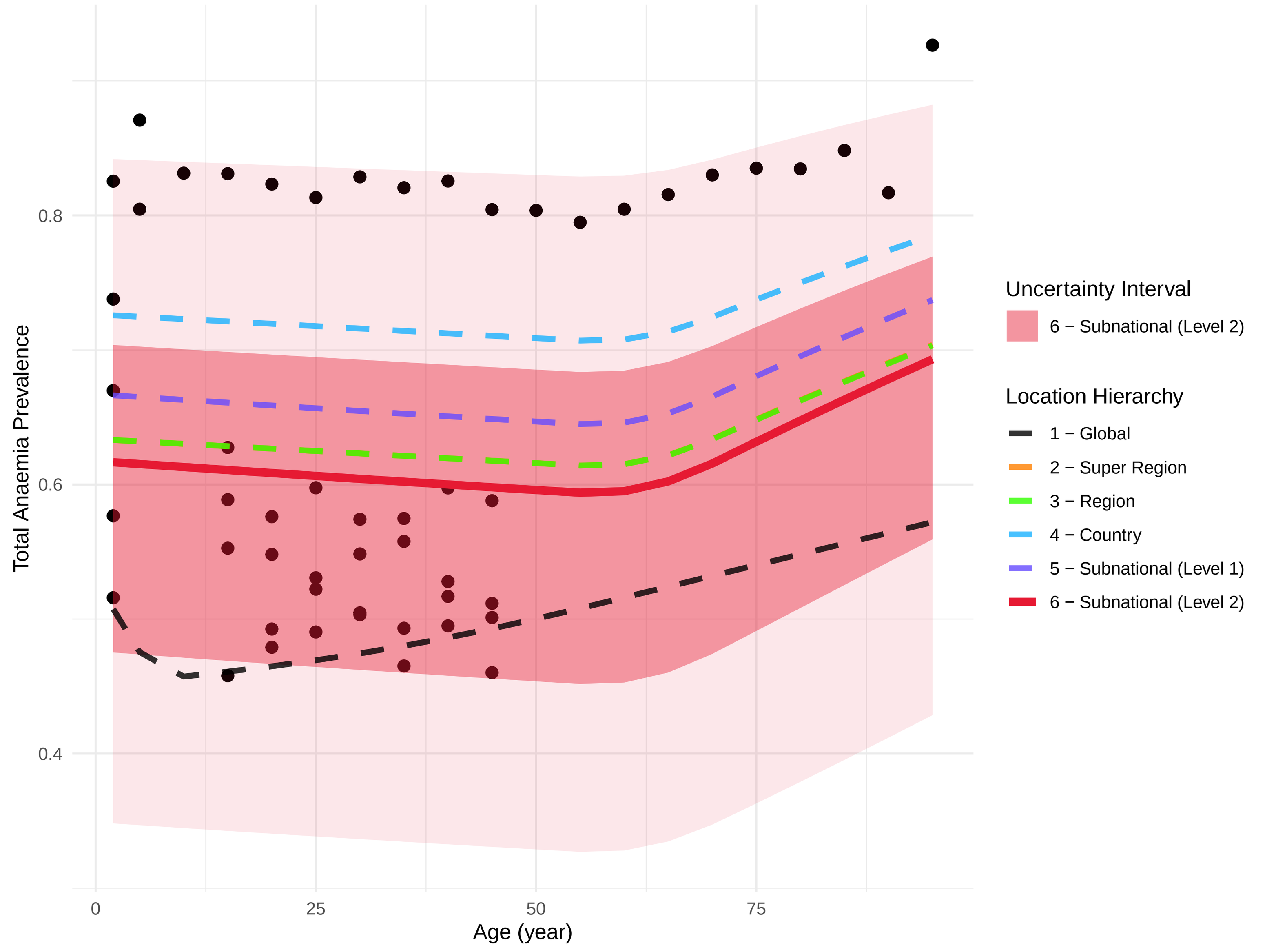

MR−BRT Age/Sex Fits − Non−pregnant Females 2+ Years Old − Total Anaemia Prevalence − Manipur
WHO−2024 Adjustment Method

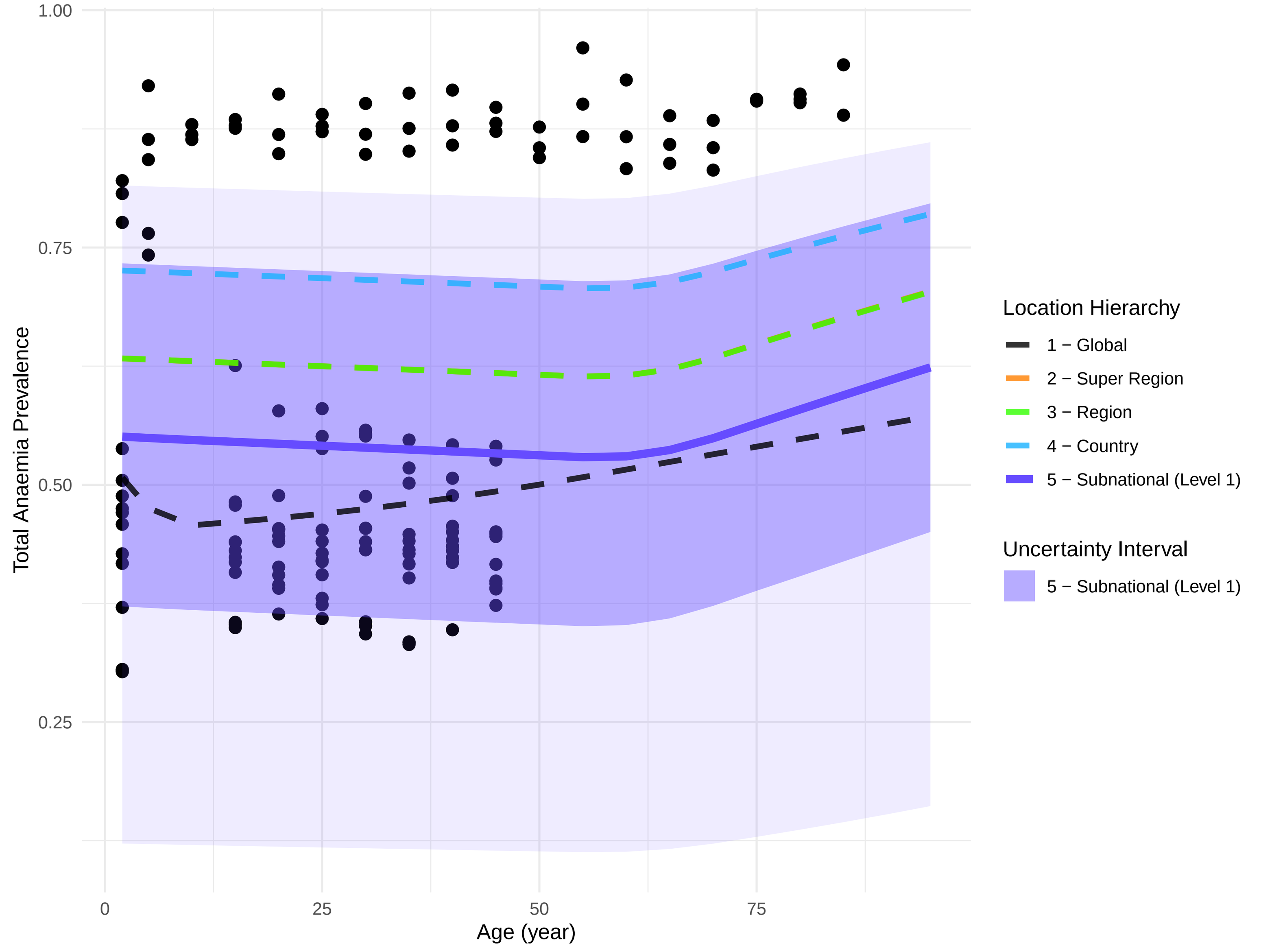

MR−BRT Age/Sex Fits − Non−pregnant Females 2+ Years Old − Total Anaemia Prevalence − Manipur, Rural
WHO−2024 Adjustment Method

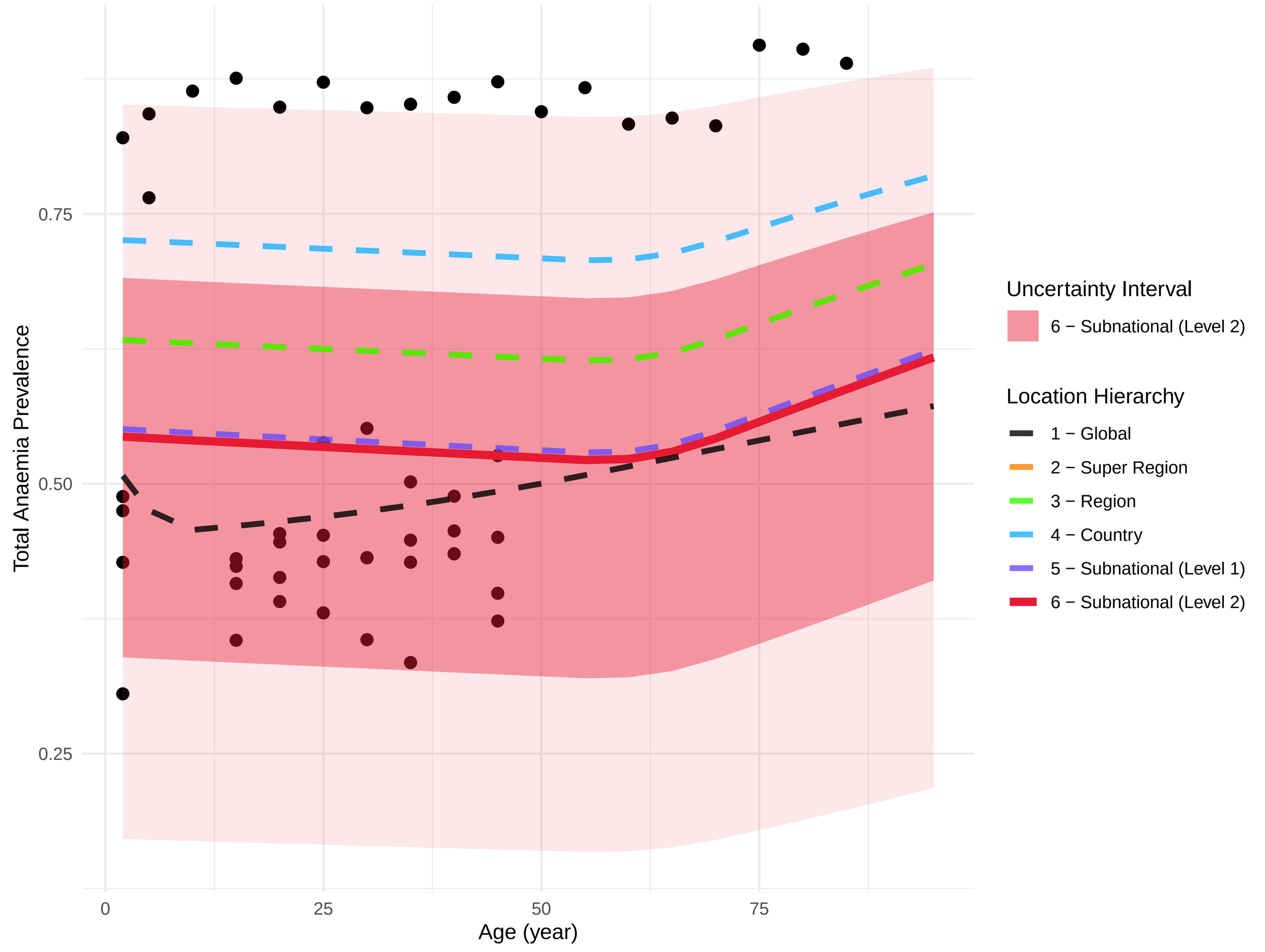

MR−BRT Age/Sex Fits − Non−pregnant Females 2+ Years Old − Total Anaemia Prevalence − Manipur, Urban
WHO−2024 Adjustment Method

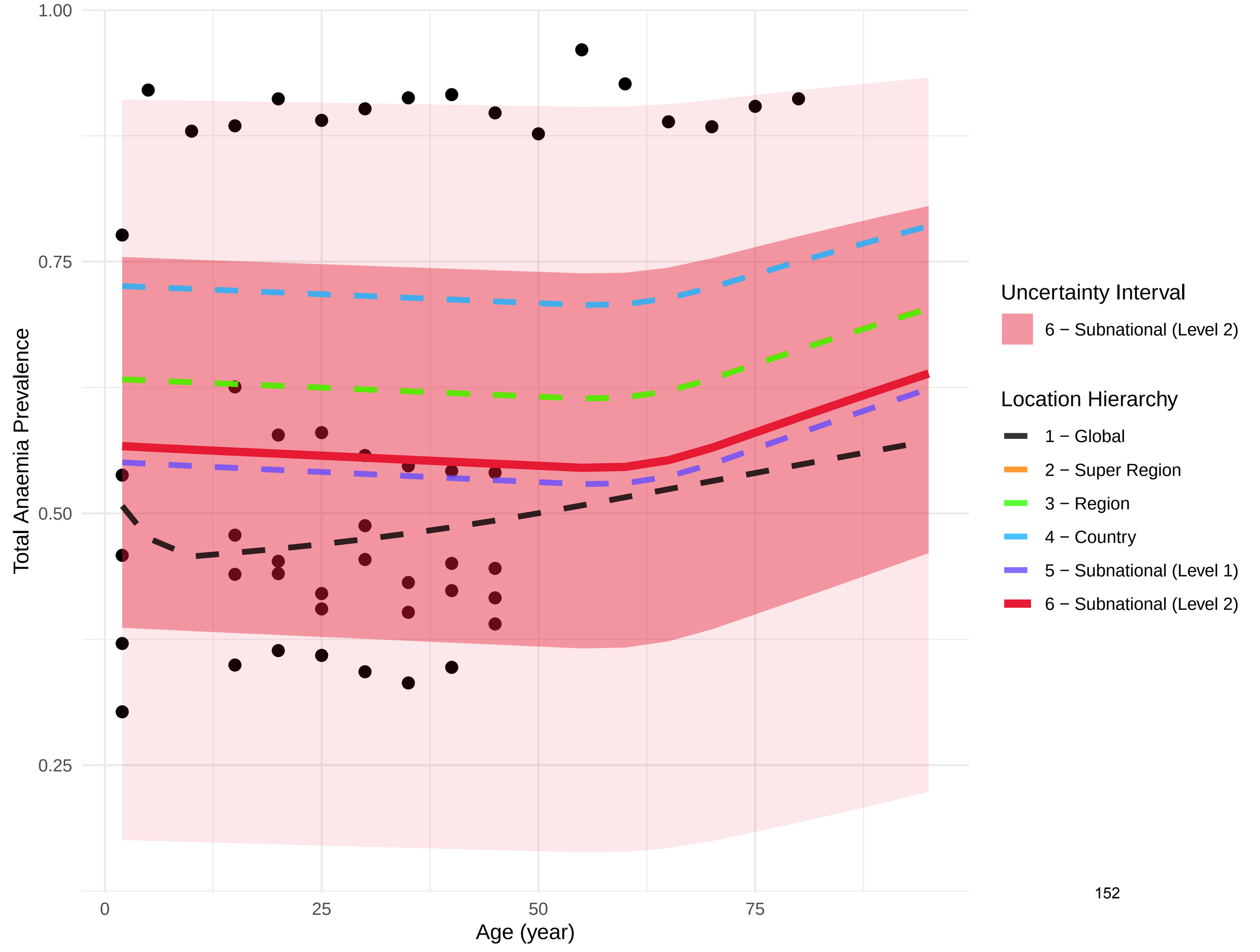

MR−BRT Age/Sex Fits − Non-pregnant Females 2+ Years Old − Total Anaemia Prevalence − Meghalaya

WHO−2024 Adjustment Method

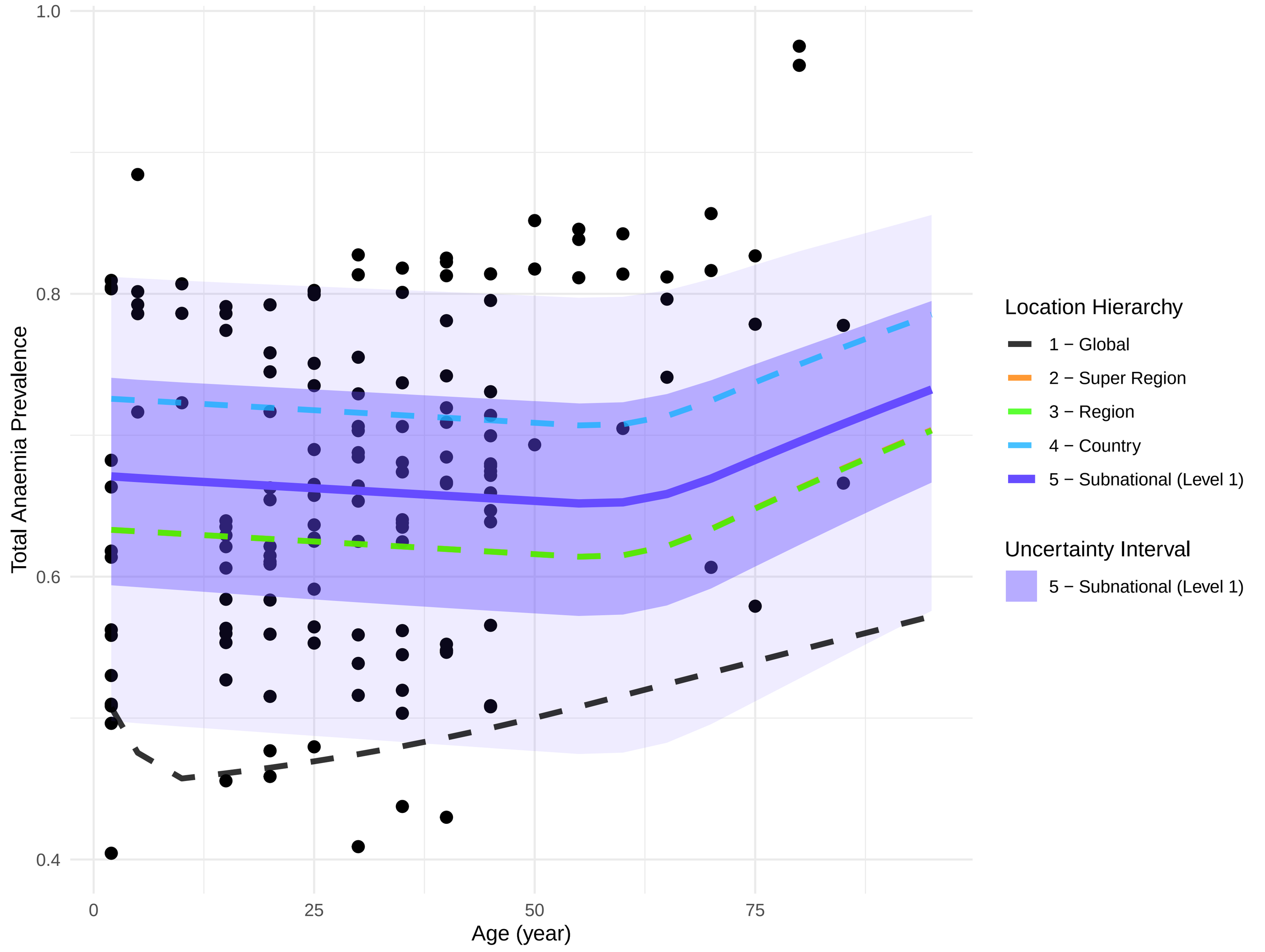

MR−BRT Age/Sex Fits − Non-pregnant Females 2+ Years Old − Total Anaemia Prevalence − Meghalaya, Rural

WHO−2024 Adjustment Method

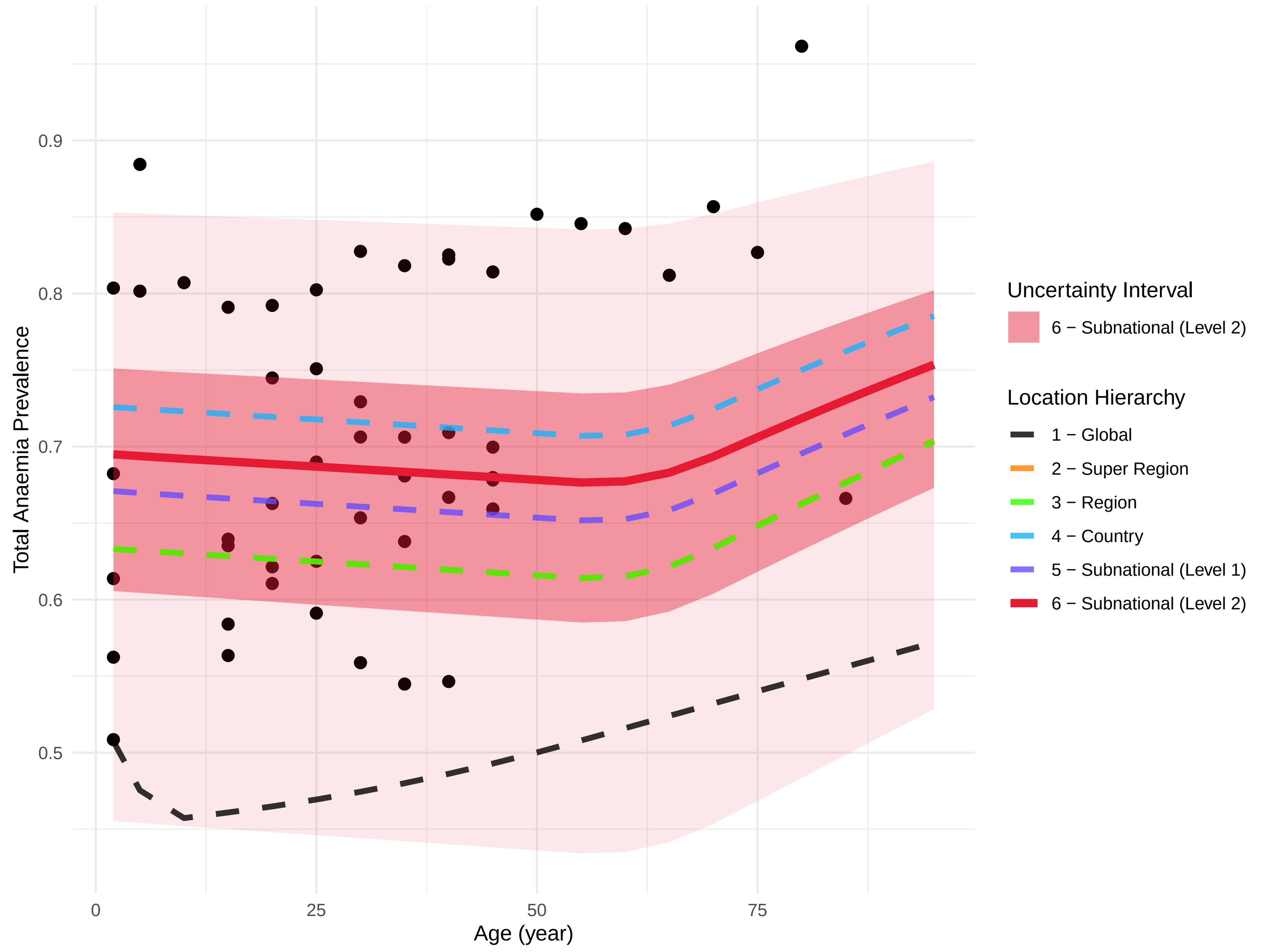

MR−BRT Age/Sex Fits − Non-pregnant Females 2+ Years Old − Total Anaemia Prevalence − Meghalaya, Urban

WHO−2024 Adjustment Method

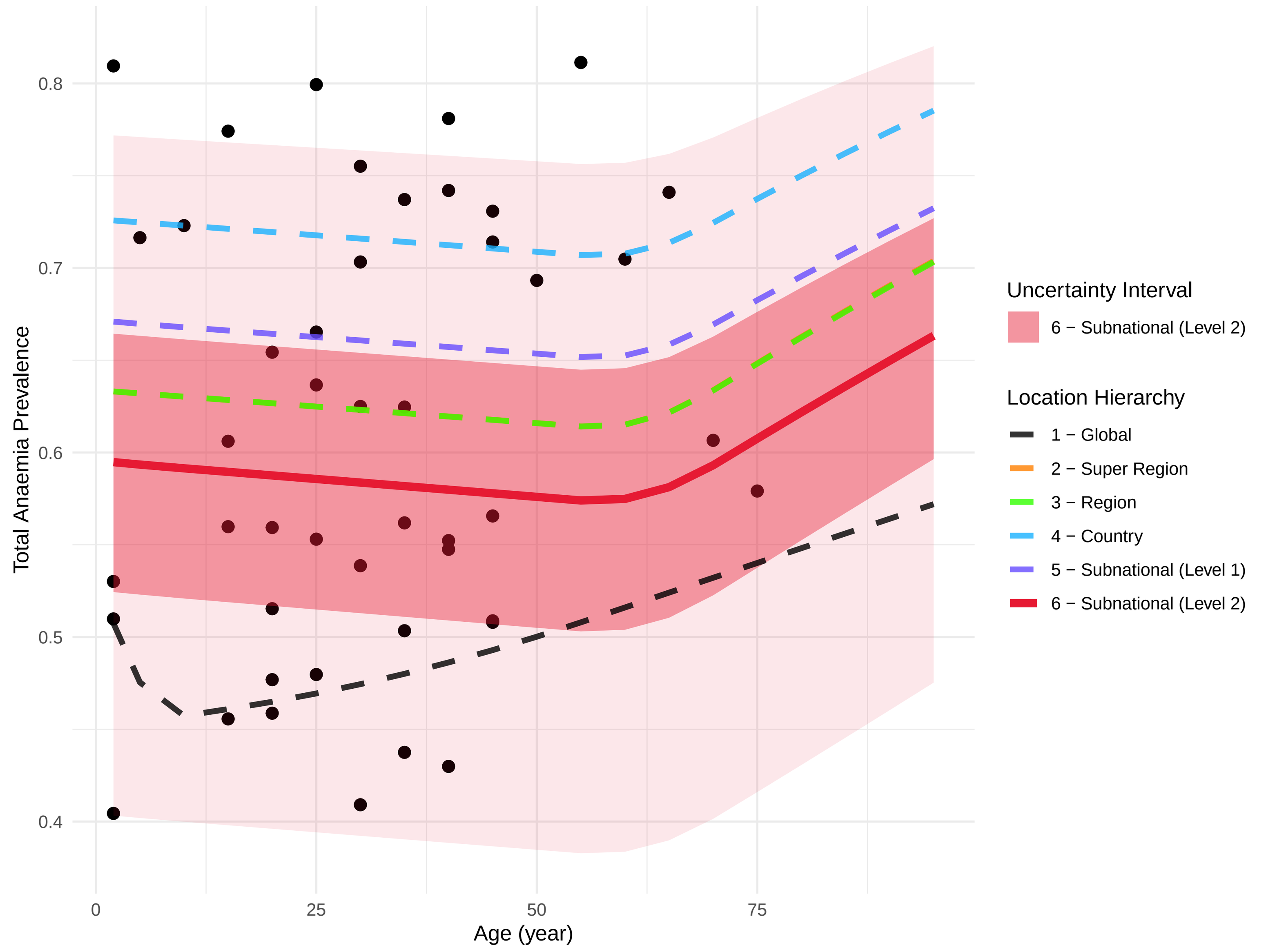

MR−BRT Age/Sex Fits − Non-pregnant Females 2+ Years Old − Total Anaemia Prevalence − Mizoram

WHO−2024 Adjustment Method

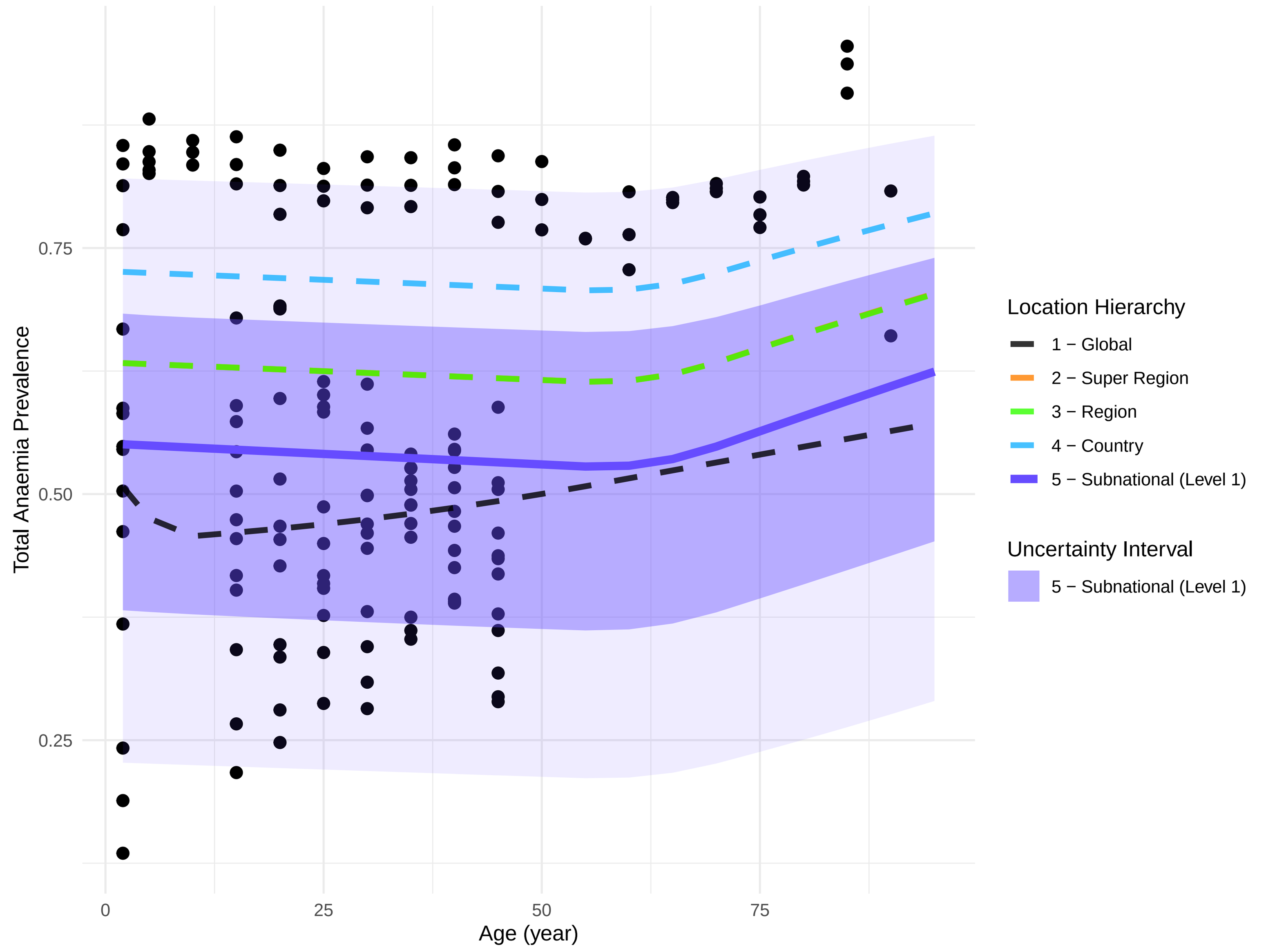

MR−BRT Age/Sex Fits − Non-pregnant Females 2+ Years Old − Total Anaemia Prevalence − Mizoram, Rural

WHO−2024 Adjustment Method

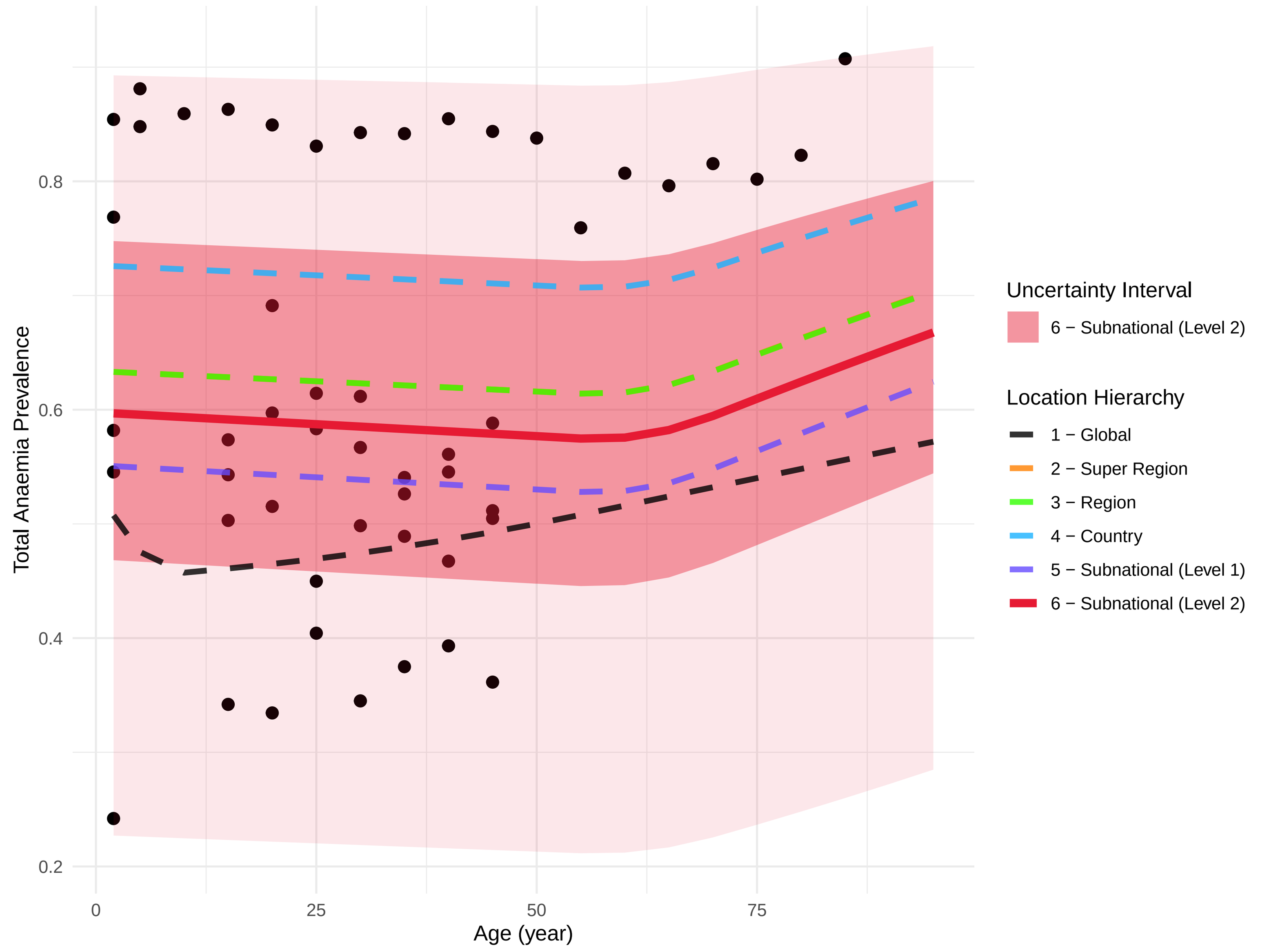

MR−BRT Age/Sex Fits − Non-pregnant Females 2+ Years Old − Total Anaemia Prevalence − Mizoram, Urban

WHO−2024 Adjustment Method

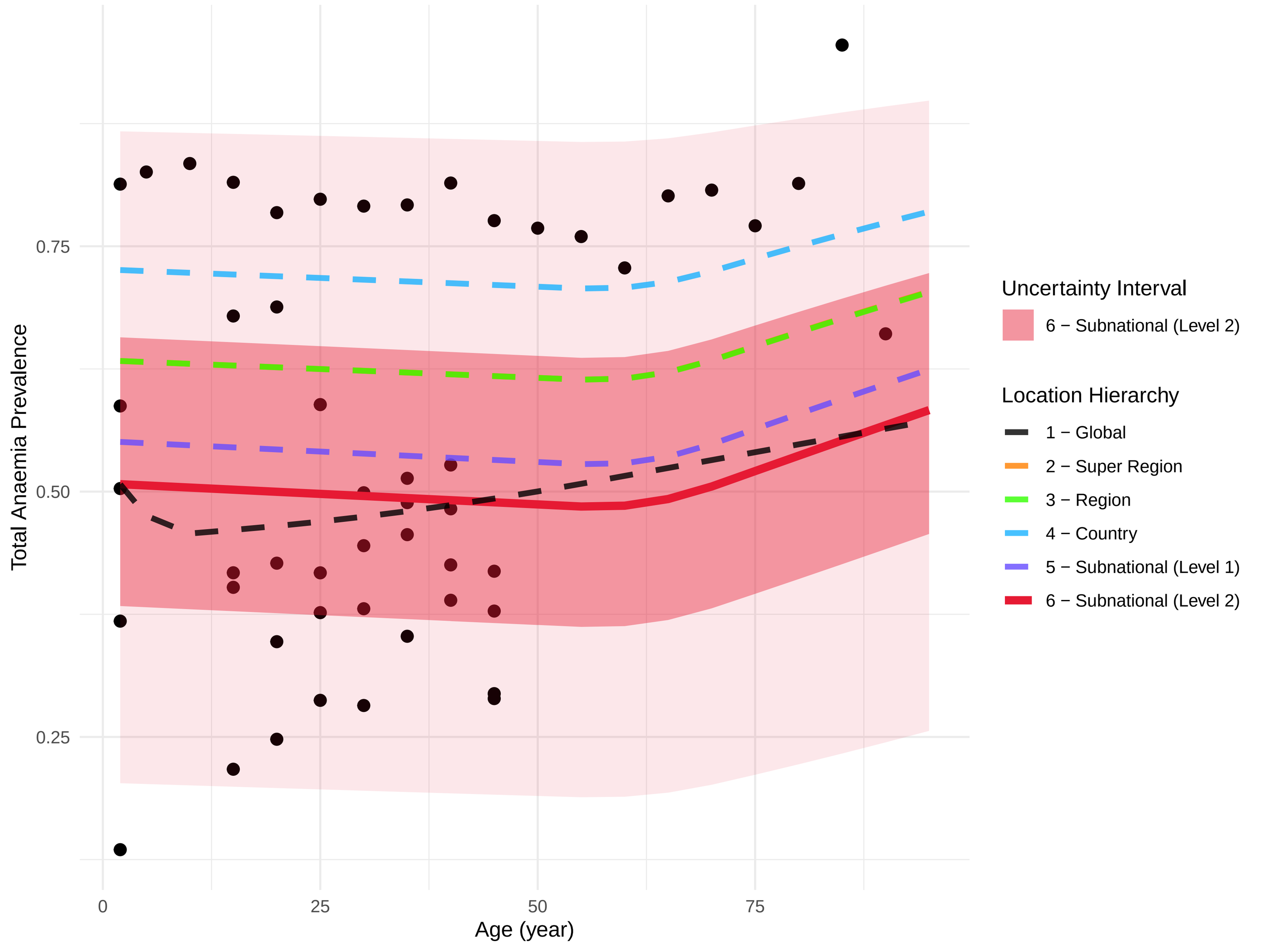

MR−BRT Age/Sex Fits − Non-pregnant Females 2+ Years Old − Total Anaemia Prevalence − Nagaland

WHO−2024 Adjustment Method

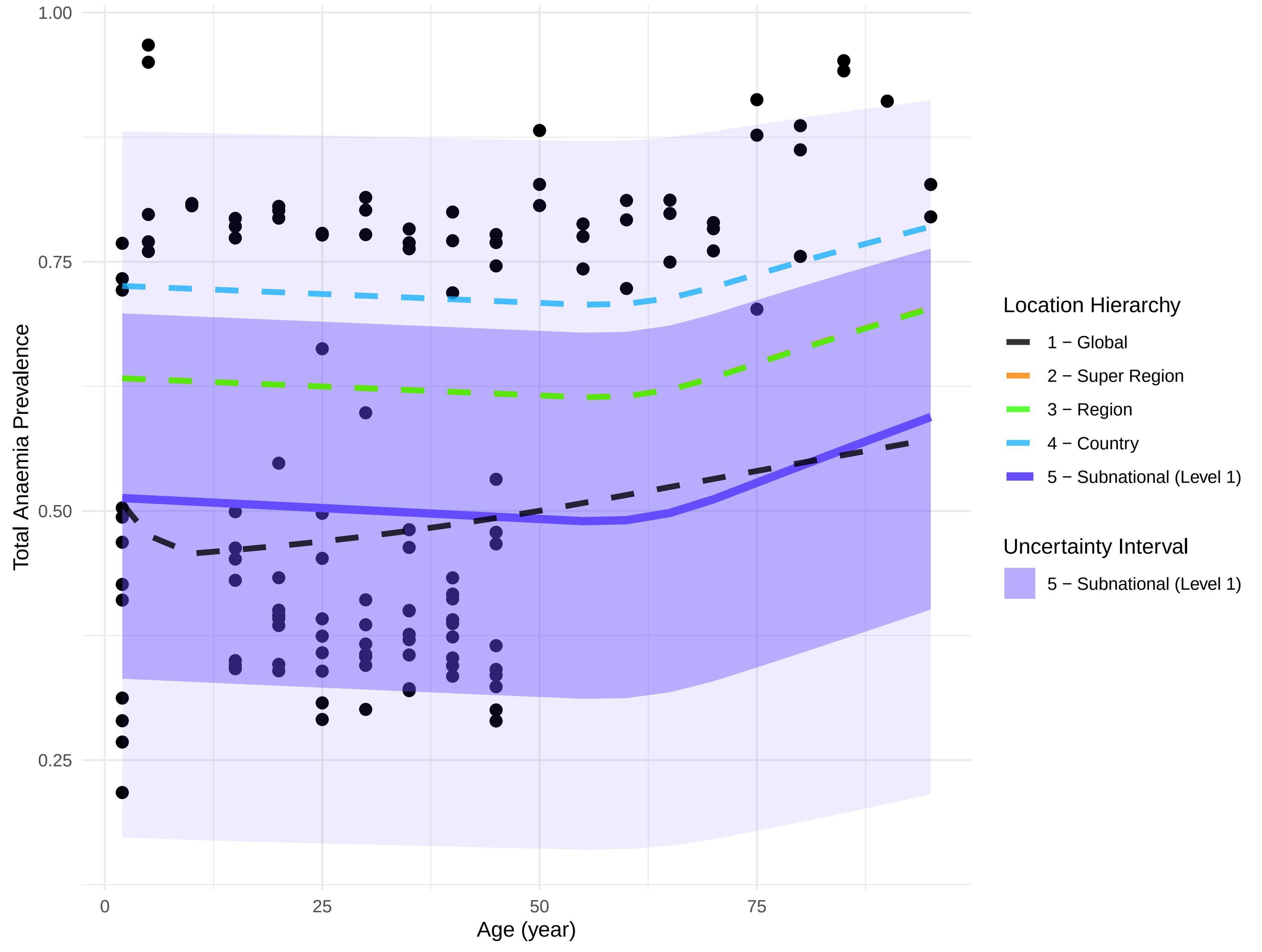

MR−BRT Age/Sex Fits − Non-pregnant Females 2+ Years Old − Total Anaemia Prevalence − Nagaland, Rural

WHO−2024 Adjustment Method

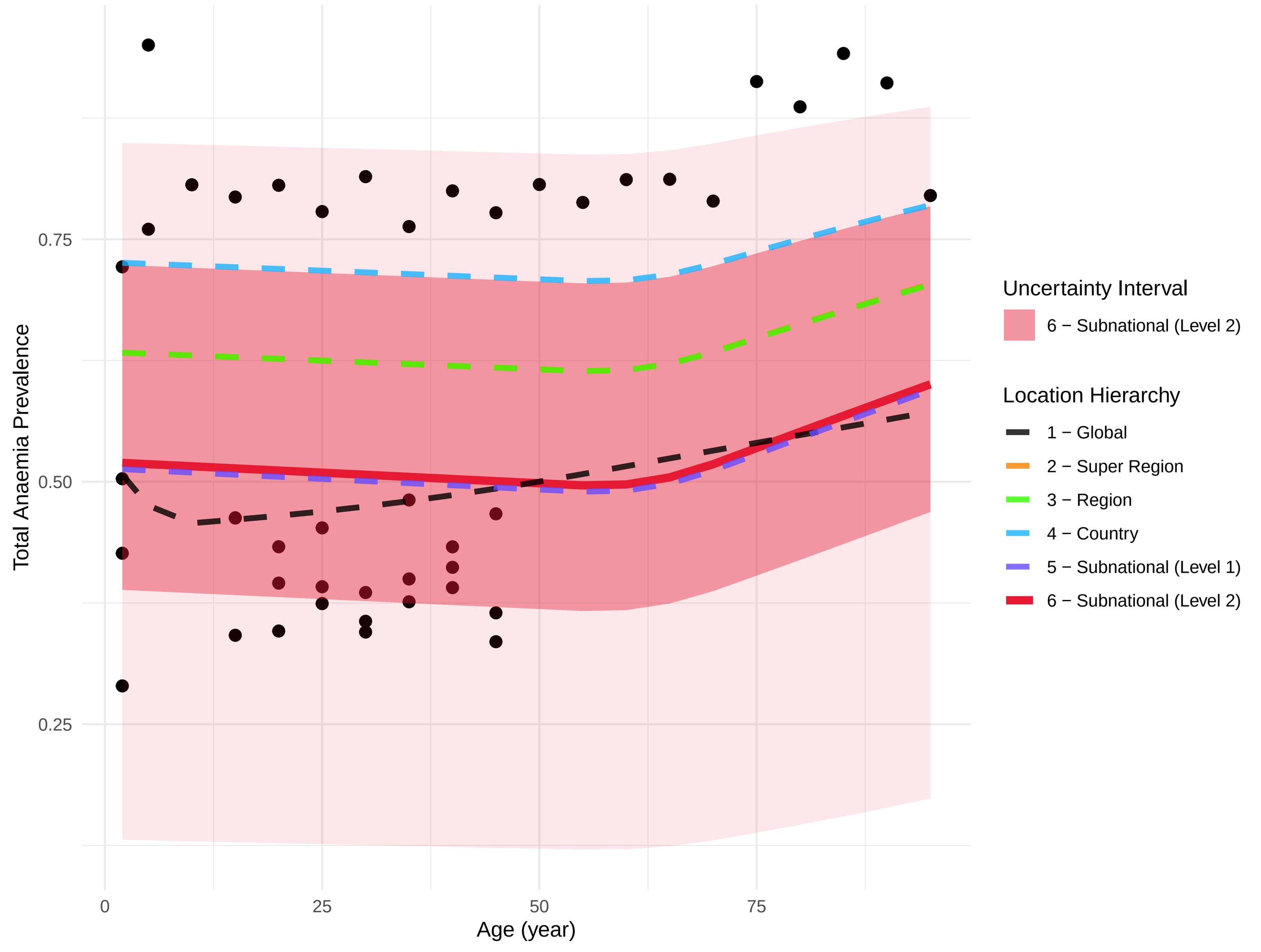

MR−BRT Age/Sex Fits − Non-pregnant Females 2+ Years Old − Total Anaemia Prevalence − Nagaland, Urban

WHO−2024 Adjustment Method

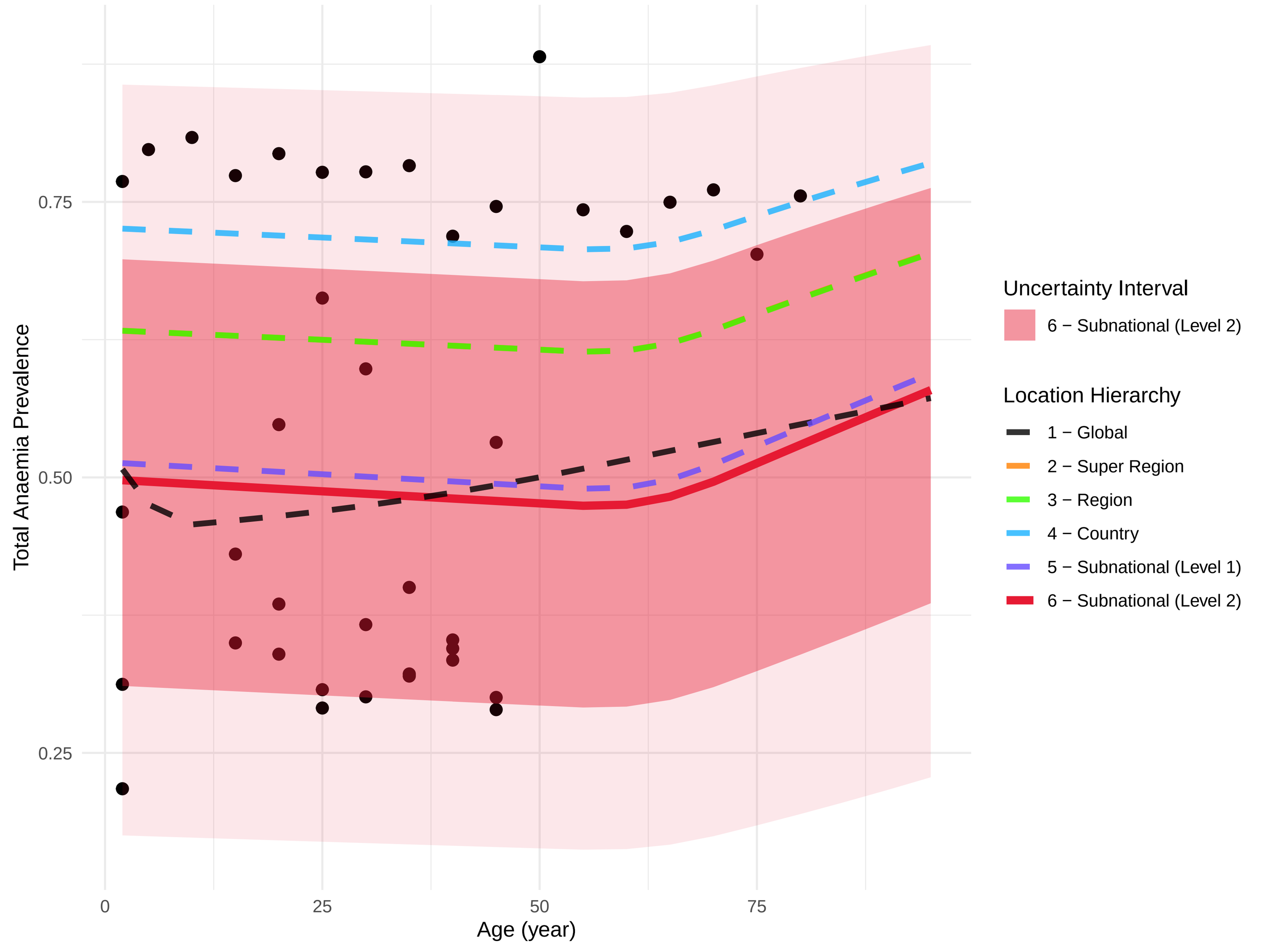

MR−BRT Age/Sex Fits − Non-pregnant Females 2+ Years Old − Total Anaemia Prevalence − Odisha

WHO−2024 Adjustment Method

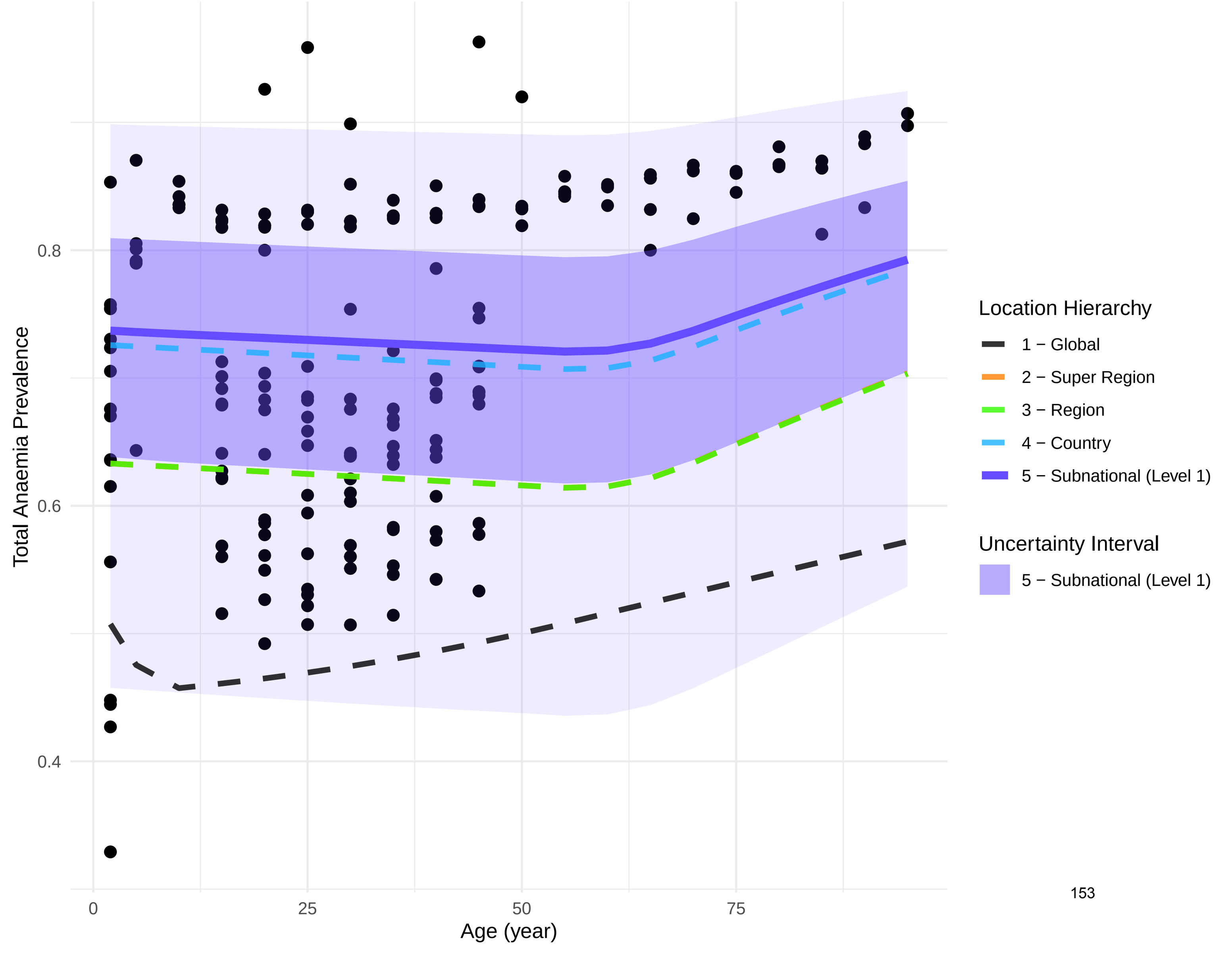

MR−BRT Age/Sex Fits − Non−pregnant Females 2+ Years Old − Total Anaemia Prevalence − Odisha, Rural
WHO−2024 Adjustment Method

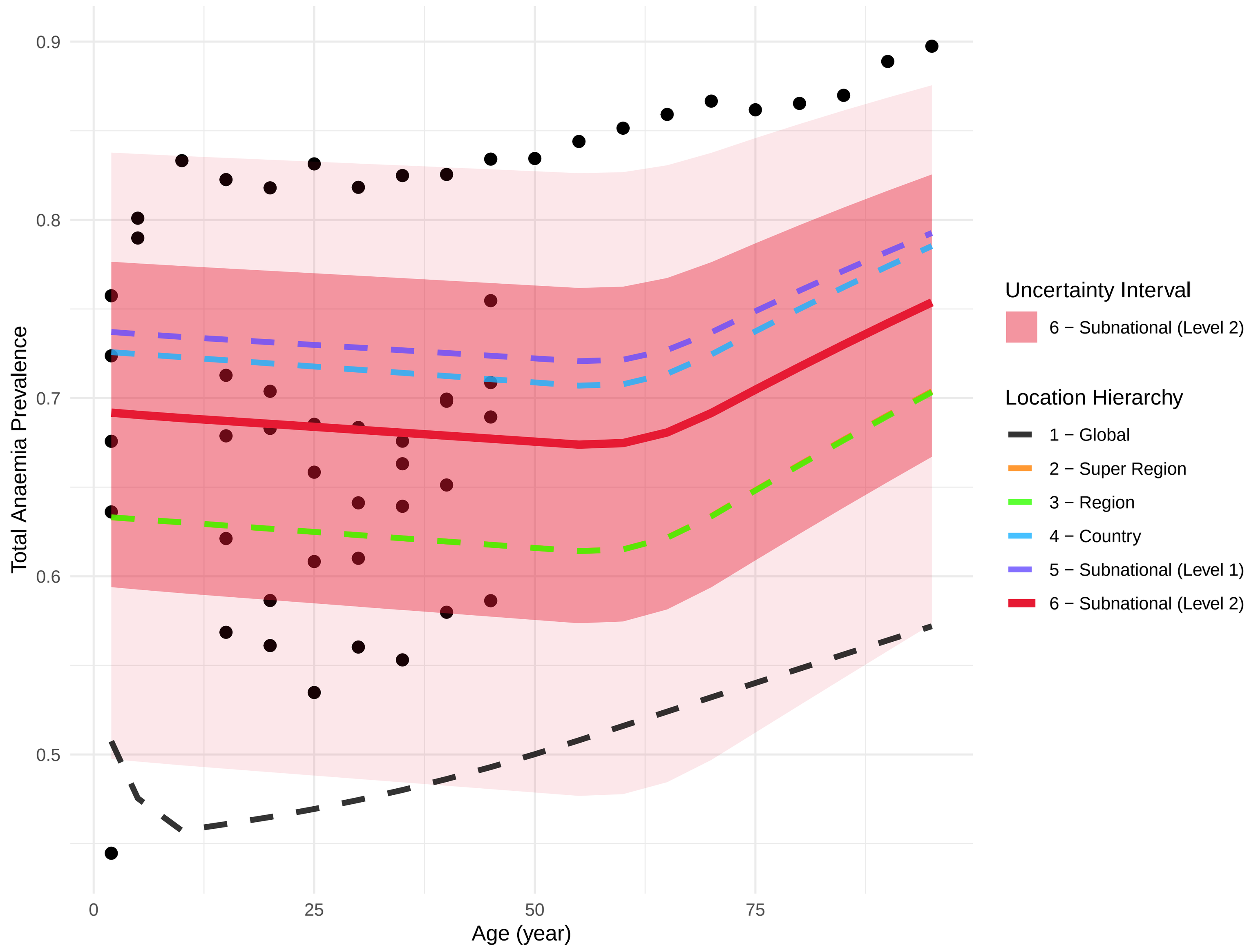

MR−BRT Age/Sex Fits − Non−pregnant Females 2+ Years Old − Total Anaemia Prevalence − Odisha, Urban
WHO−2024 Adjustment Method

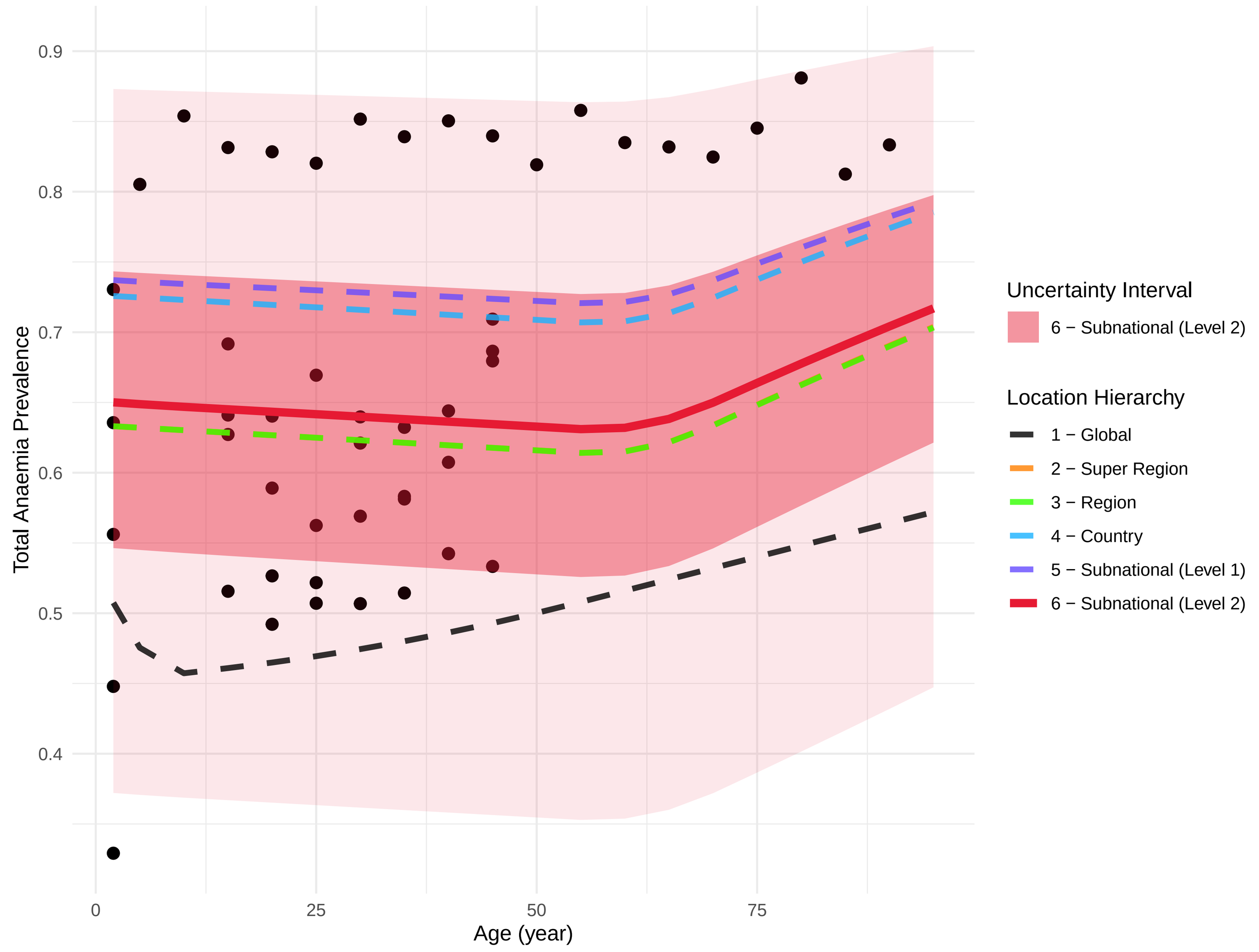

MR−BRT Age/Sex Fits − Non−pregnant Females 2+ Years Old − Total Anaemia Prevalence − Other Union Territories
WHO−2024 Adjustment Method

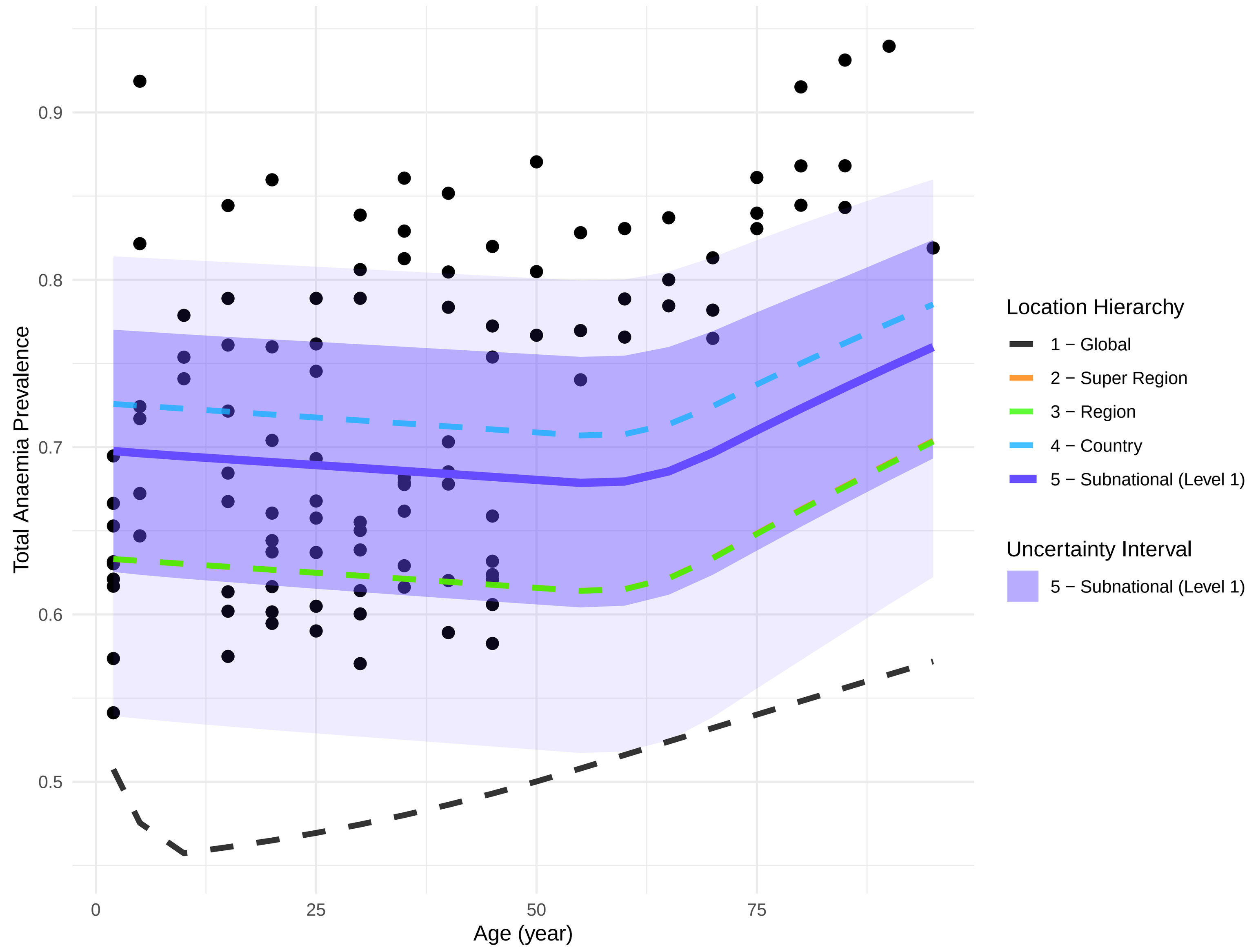

MR−BRT Age/Sex Fits − Non−pregnant Females 2+ Years Old − Total Anaemia Prevalence − Other Union Territories, Rural
WHO−2024 Adjustment Method

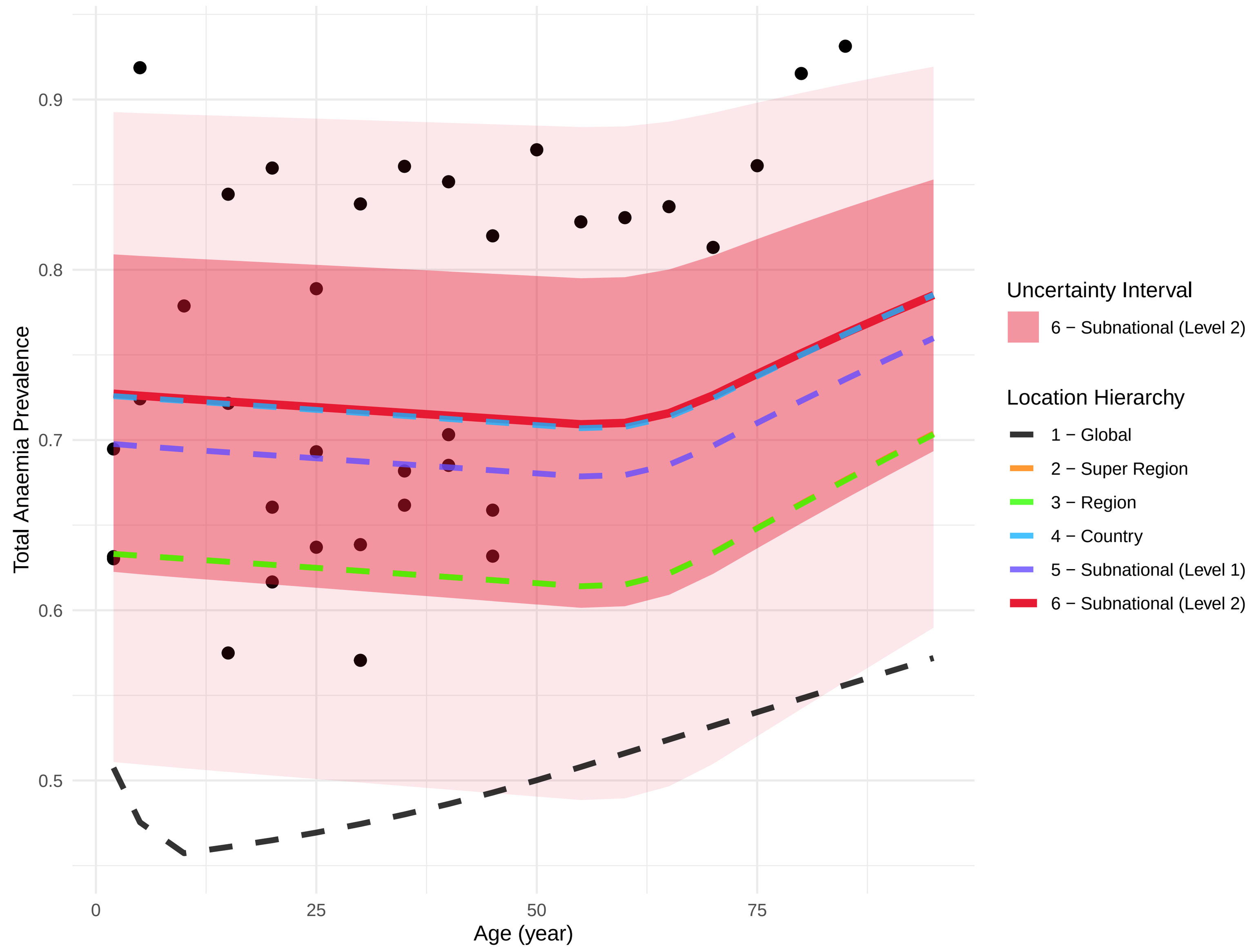

MR−BRT Age/Sex Fits − Non−pregnant Females 2+ Years Old − Total Anaemia Prevalence − Other Union Territories, Urban
WHO−2024 Adjustment Method

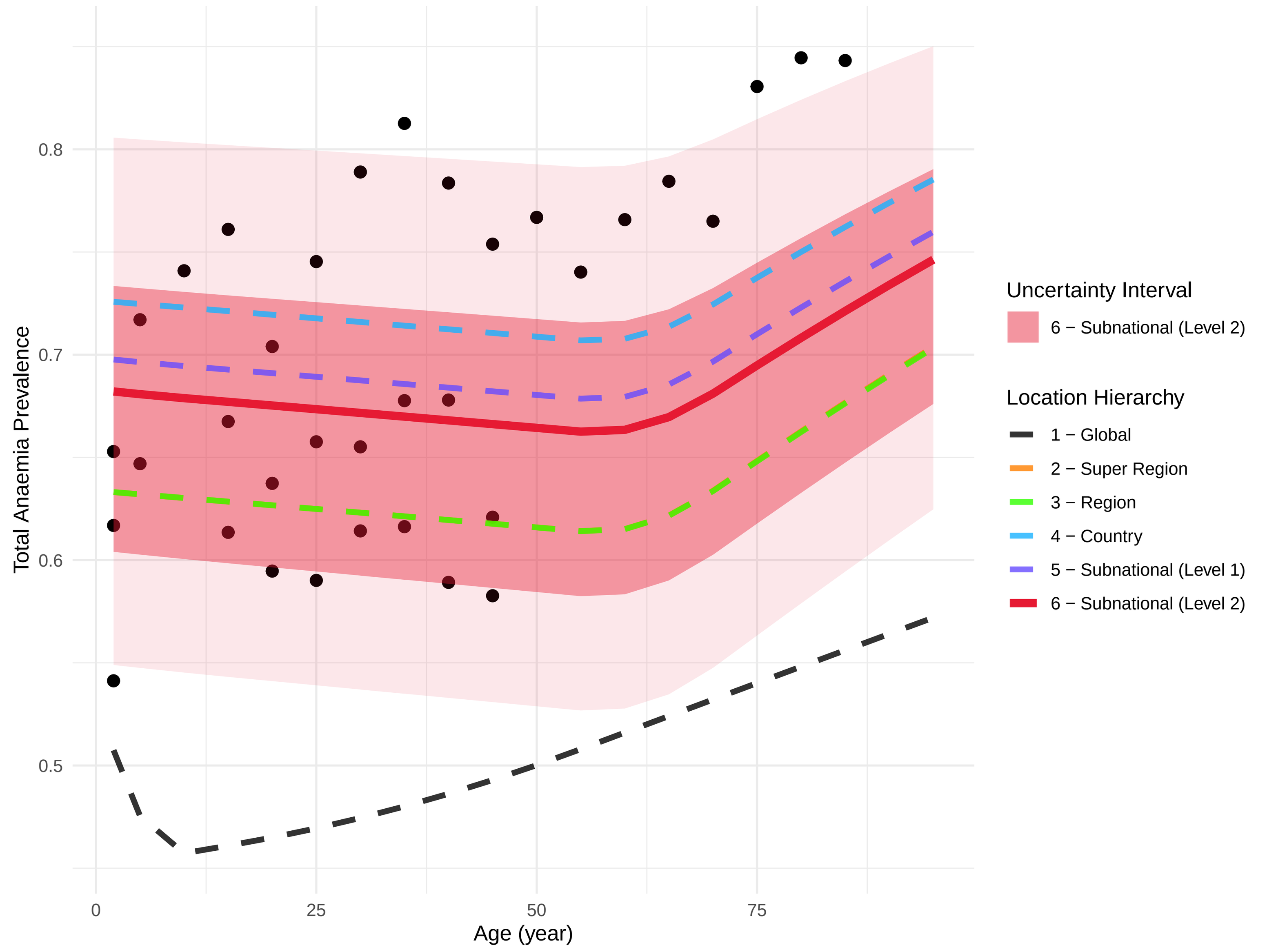

MR−BRT Age/Sex Fits − Non−pregnant Females 2+ Years Old − Total Anaemia Prevalence − Punjab
WHO−2024 Adjustment Method

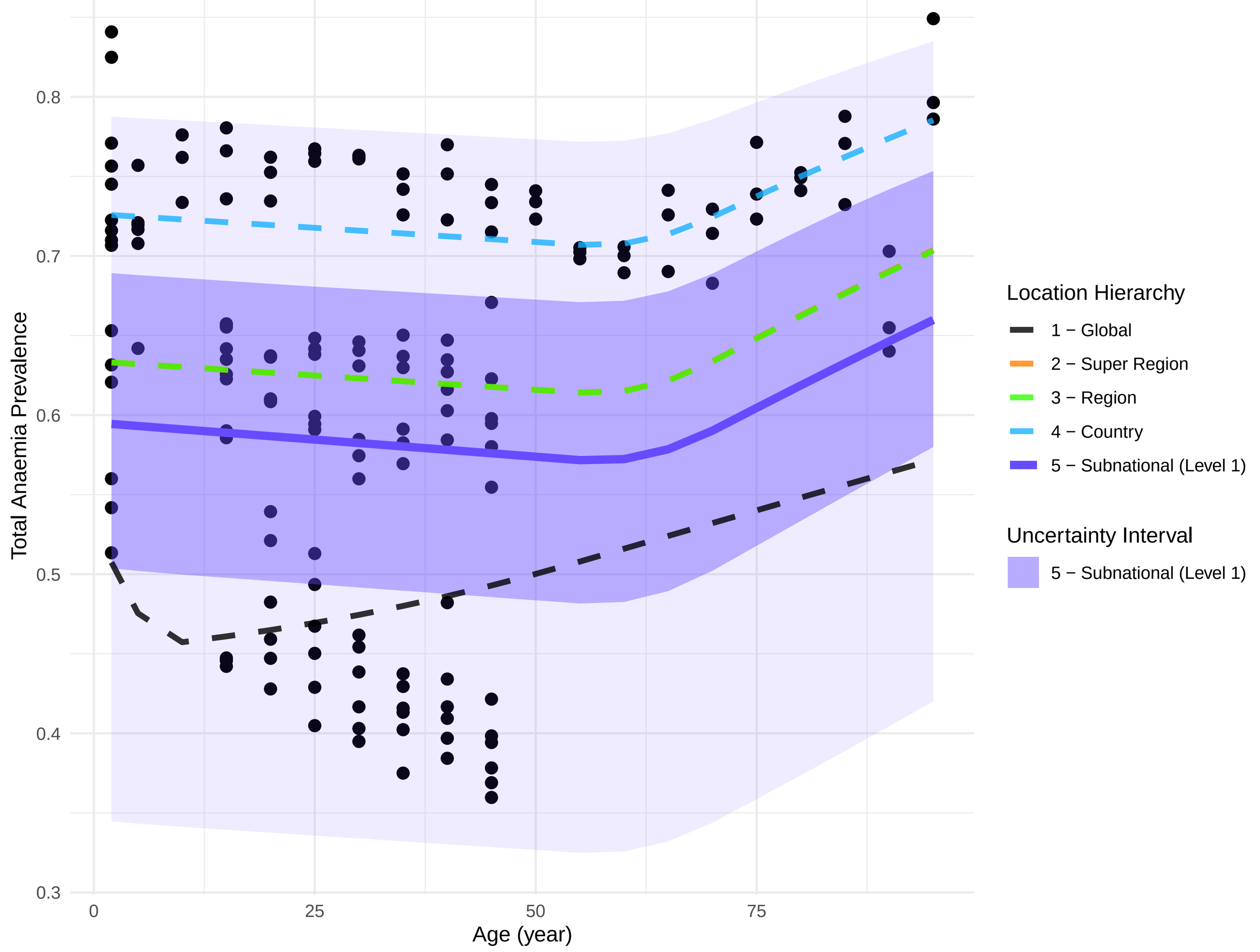

MR−BRT Age/Sex Fits − Non−pregnant Females 2+ Years Old − Total Anaemia Prevalence − Punjab, Rural
WHO−2024 Adjustment Method

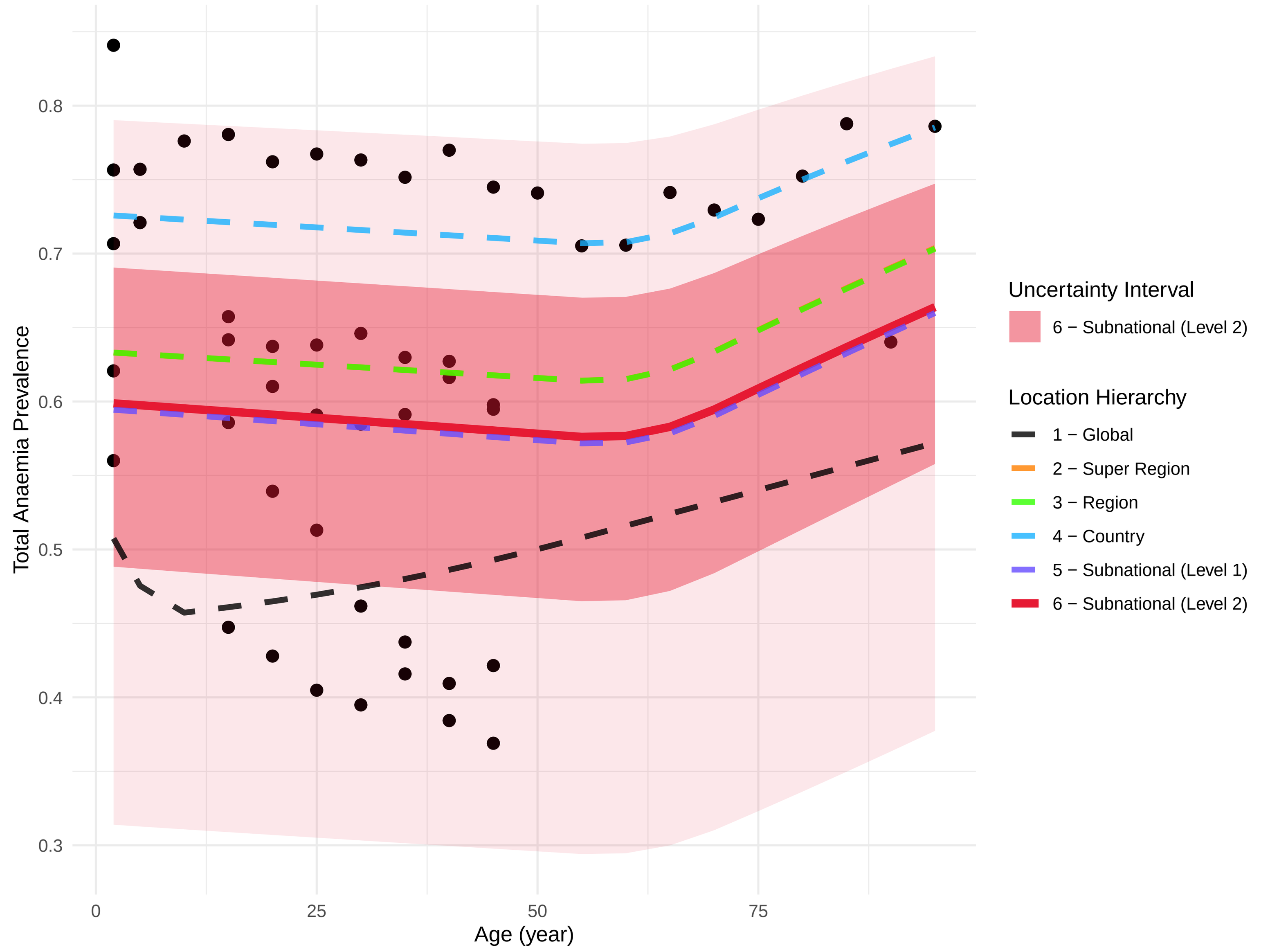

MR−BRT Age/Sex Fits − Non−pregnant Females 2+ Years Old − Total Anaemia Prevalence − Punjab, Urban
WHO−2024 Adjustment Method

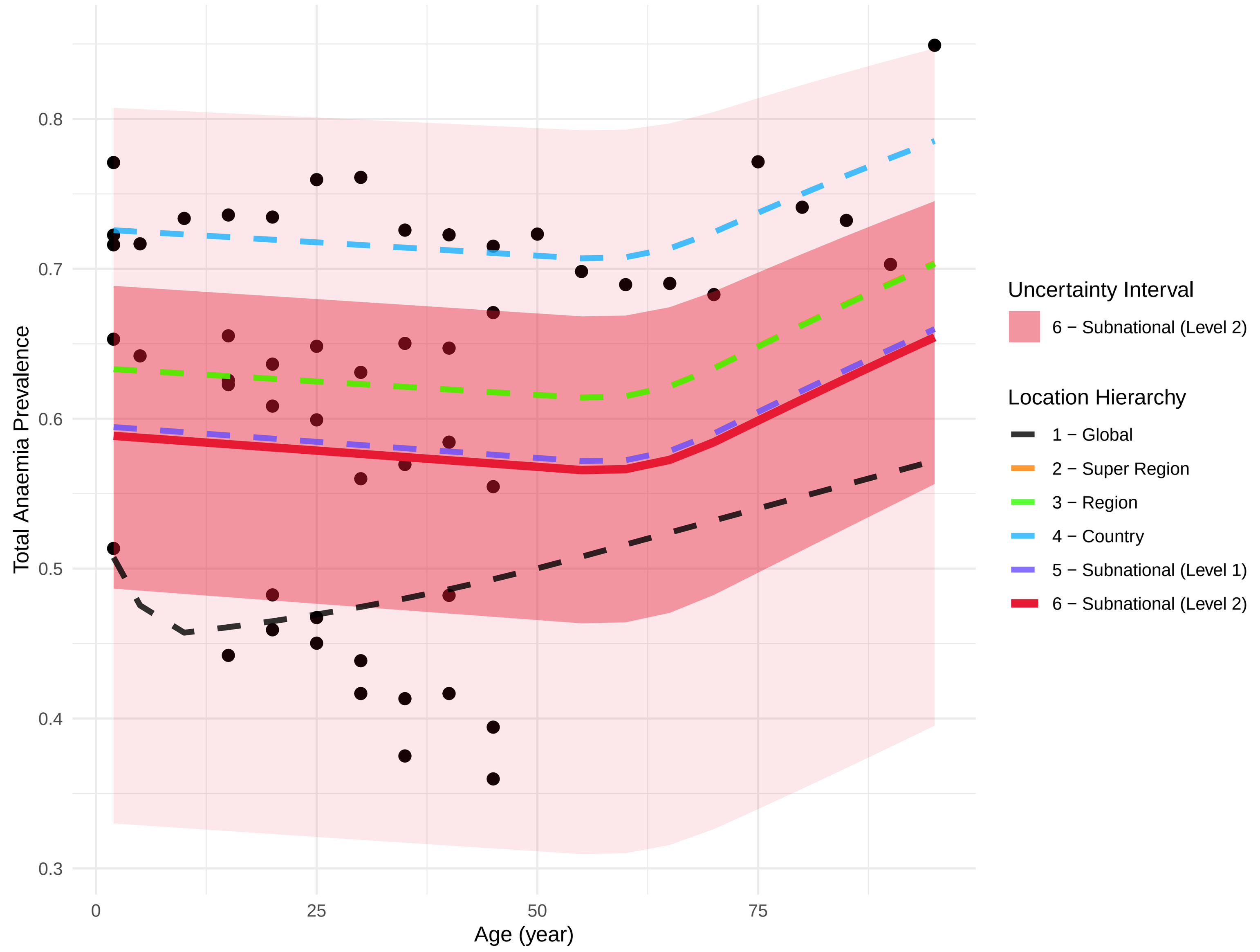

MR−BRT Age/Sex Fits − Non−pregnant Females 2+ Years Old − Total Anaemia Prevalence − Rajasthan
WHO−2024 Adjustment Method

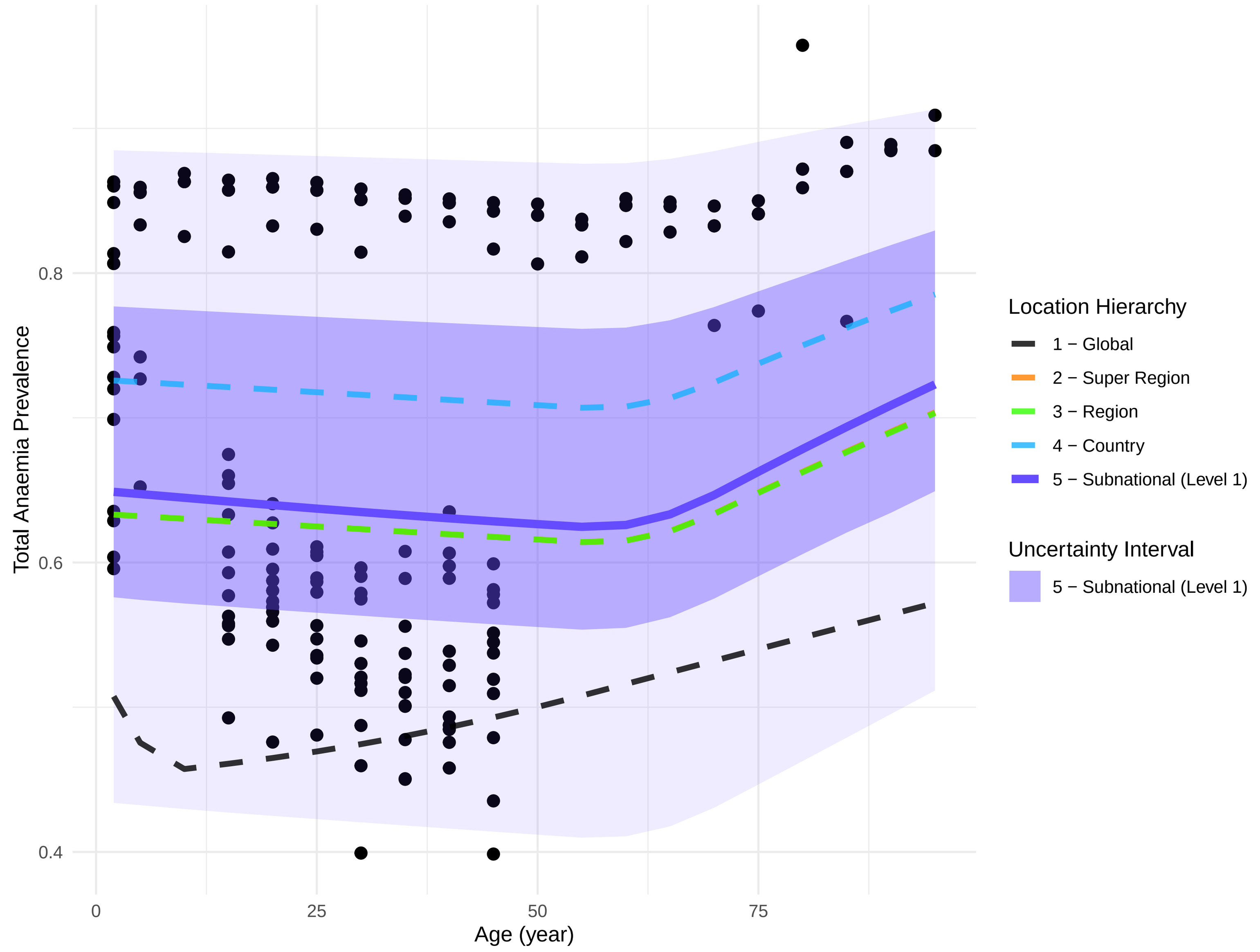

MR−BRT Age/Sex Fits − Non−pregnant Females 2+ Years Old − Total Anaemia Prevalence − Rajasthan, Rural
WHO−2024 Adjustment Method

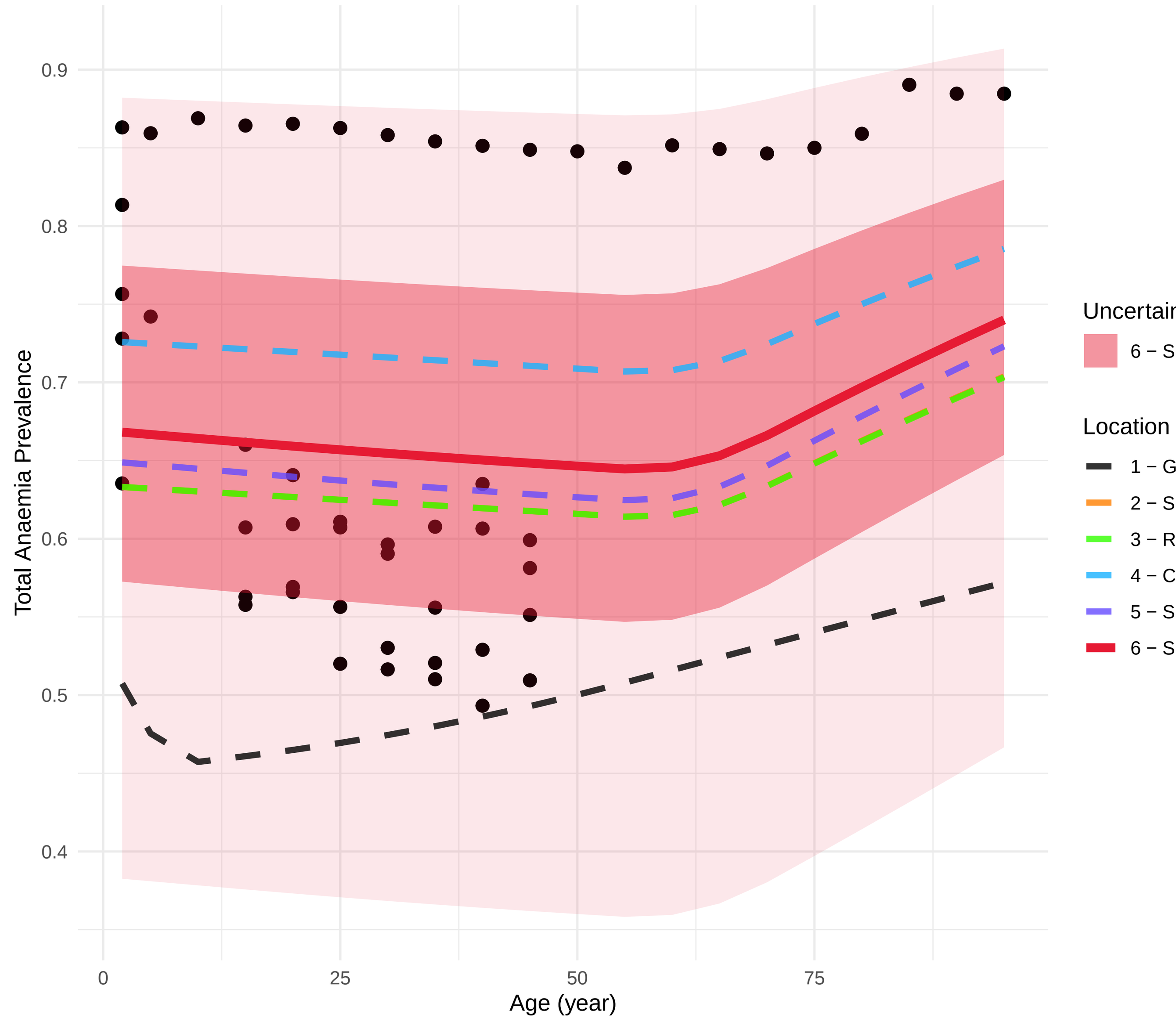

MR−BRT Age/Sex Fits − Non−pregnant Females 2+ Years Old − Total Anaemia Prevalence − Rajasthan, Urban
WHO−2024 Adjustment Method

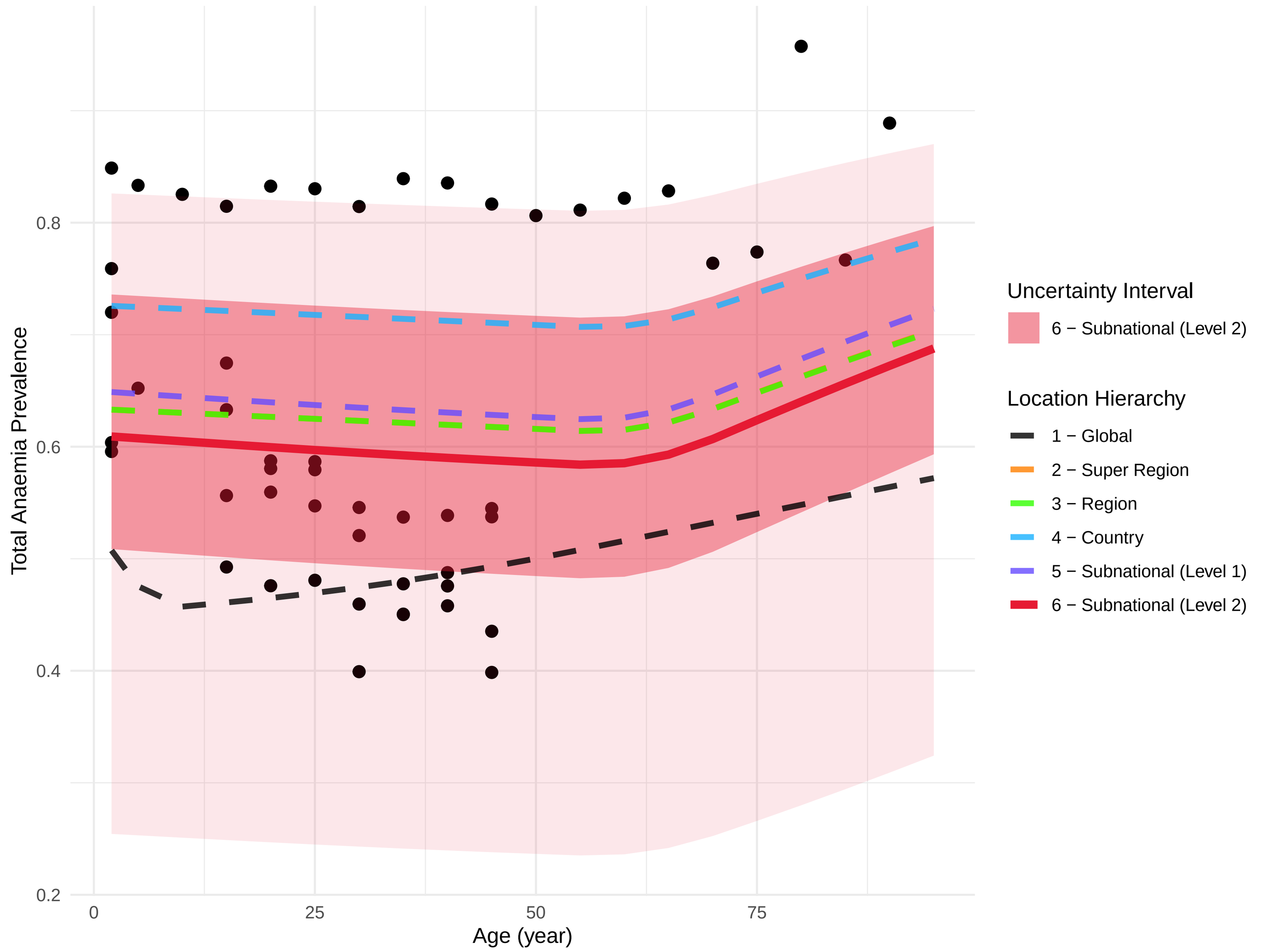

MR−BRT Age/Sex Fits − Non−pregnant Females 2+ Years Old − Total Anaemia Prevalence − Sikkim
WHO−2024 Adjustment Method

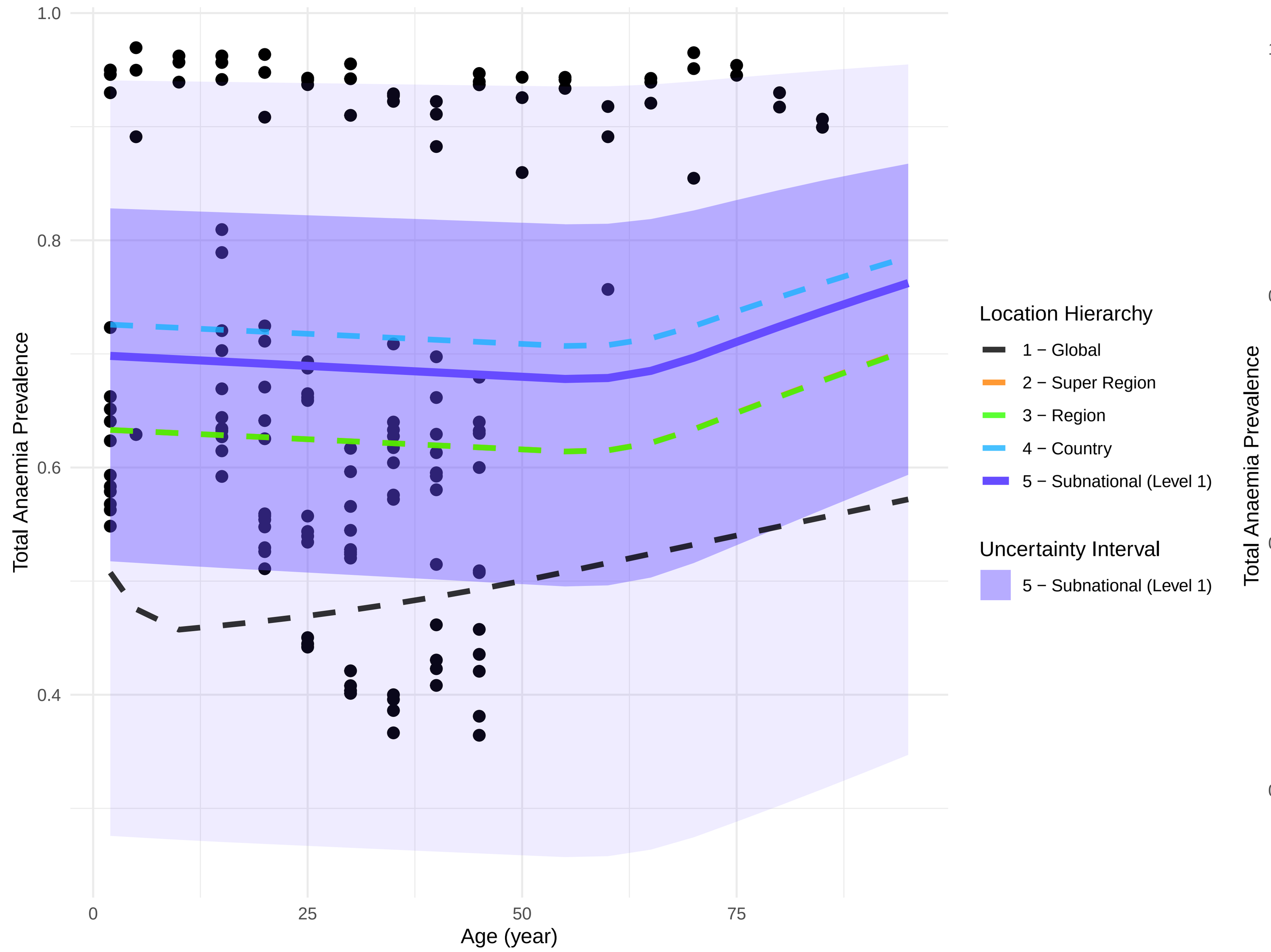

MR−BRT Age/Sex Fits − Non−pregnant Females 2+ Years Old − Total Anaemia Prevalence − Sikkim, Rural
WHO−2024 Adjustment Method

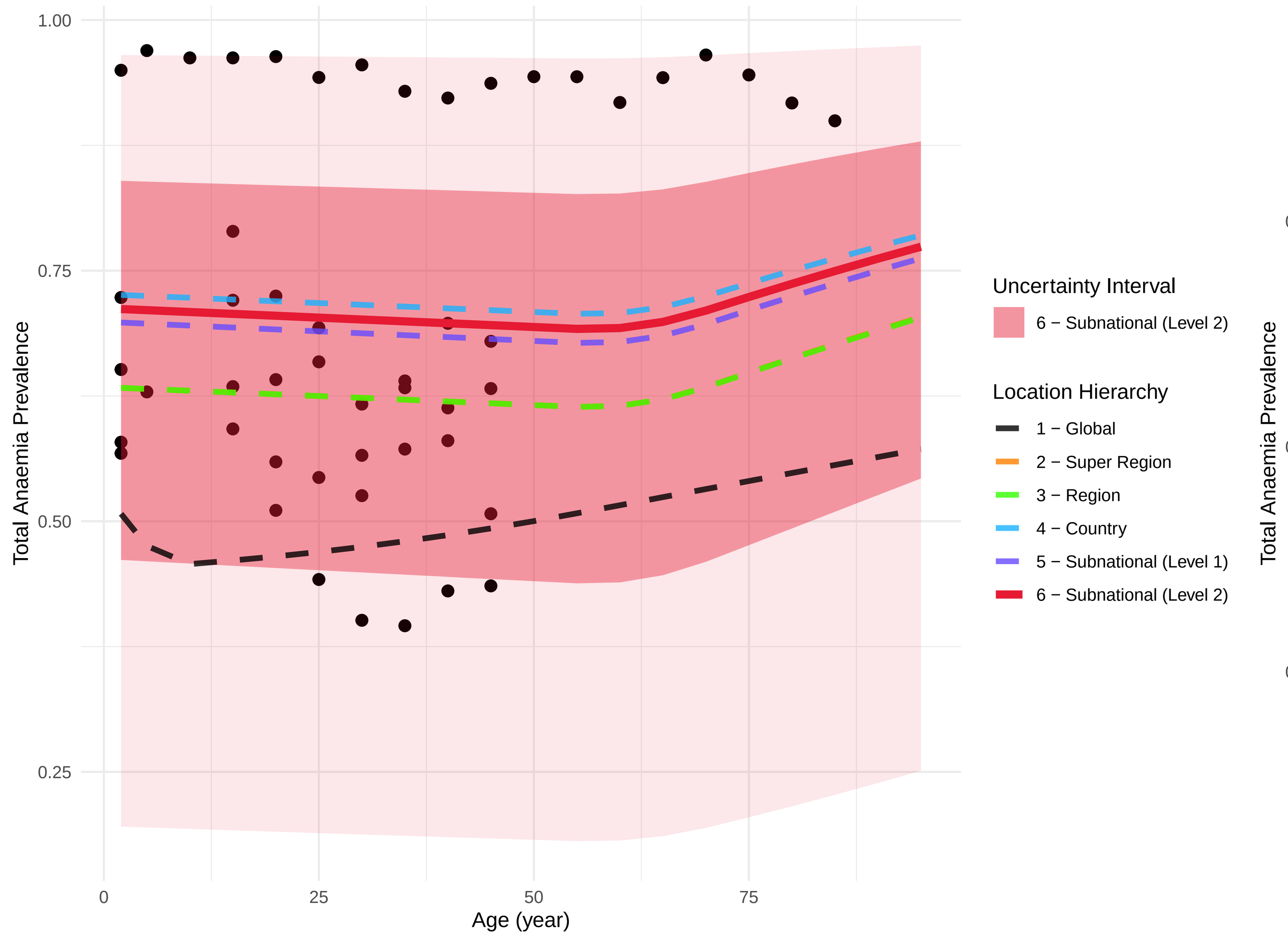

MR−BRT Age/Sex Fits − Non−pregnant Females 2+ Years Old − Total Anaemia Prevalence − Sikkim, Urban
WHO−2024 Adjustment Method

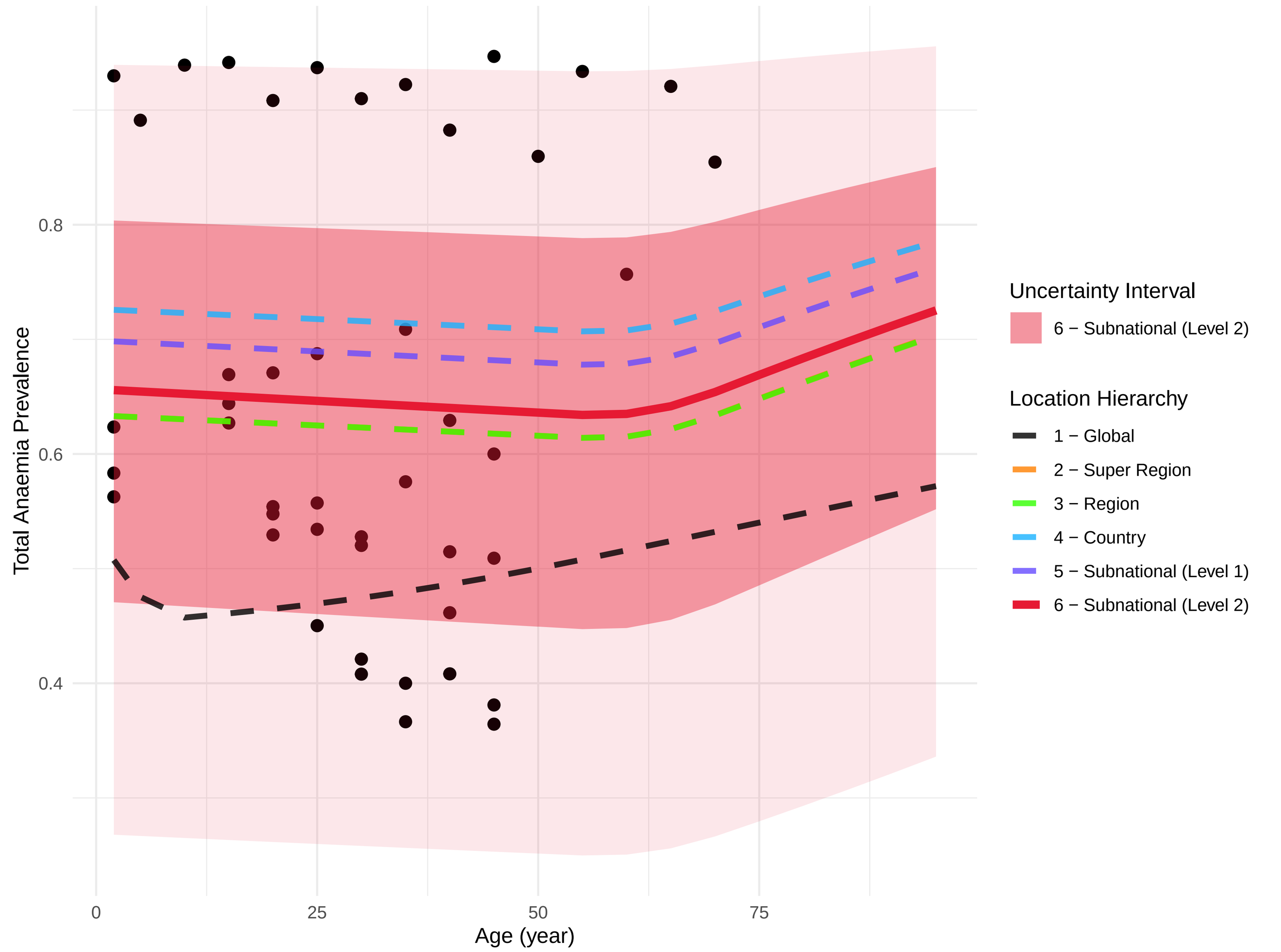

MR−BRT Age/Sex Fits − Non−pregnant Females 2+ Years Old − Total Anaemia Prevalence − Tamil Nadu
WHO−2024 Adjustment Method

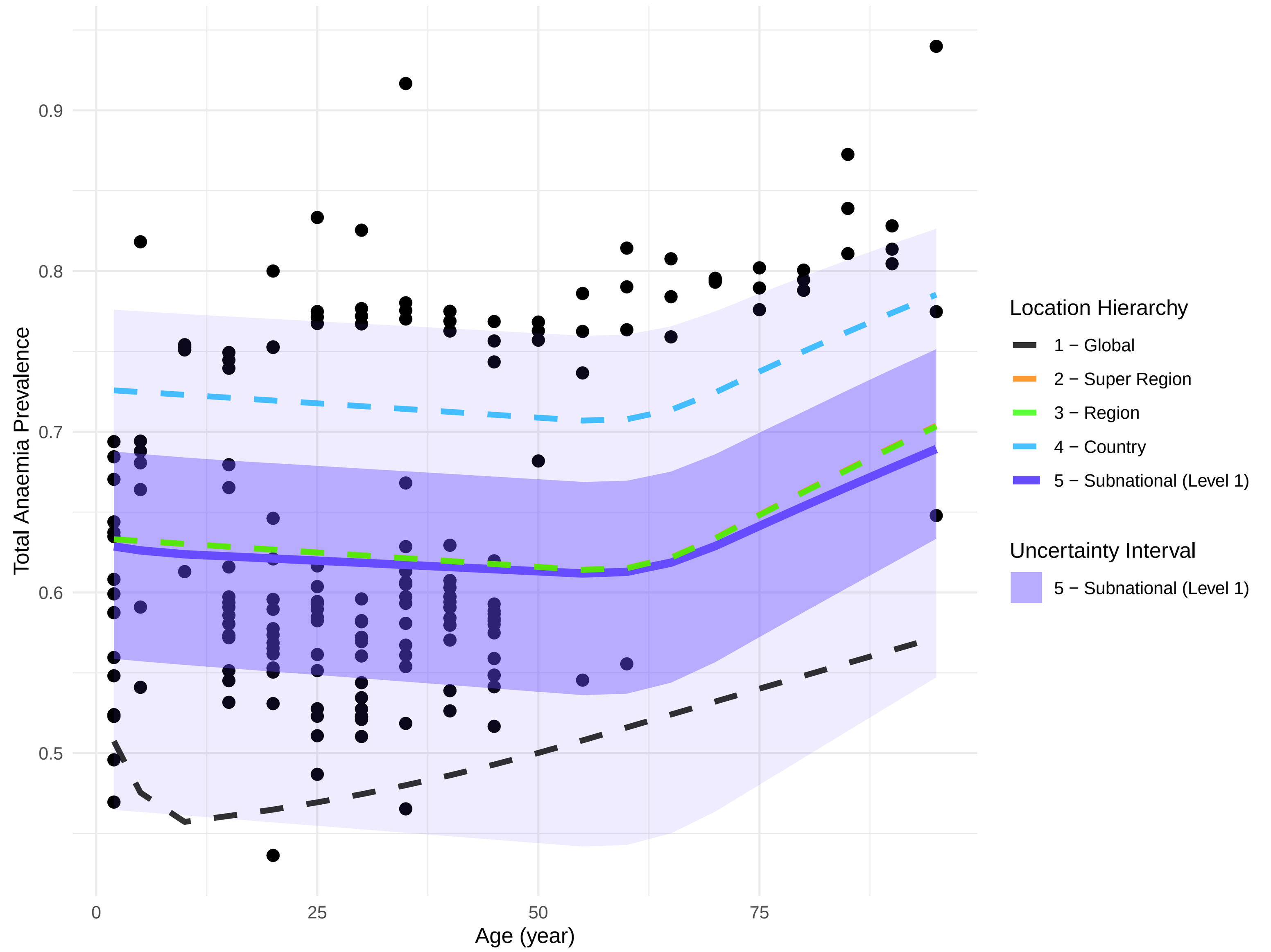

MR−BRT Age/Sex Fits − Non−pregnant Females 2+ Years Old − Total Anaemia Prevalence − Tamil Nadu, Rural
WHO−2024 Adjustment Method

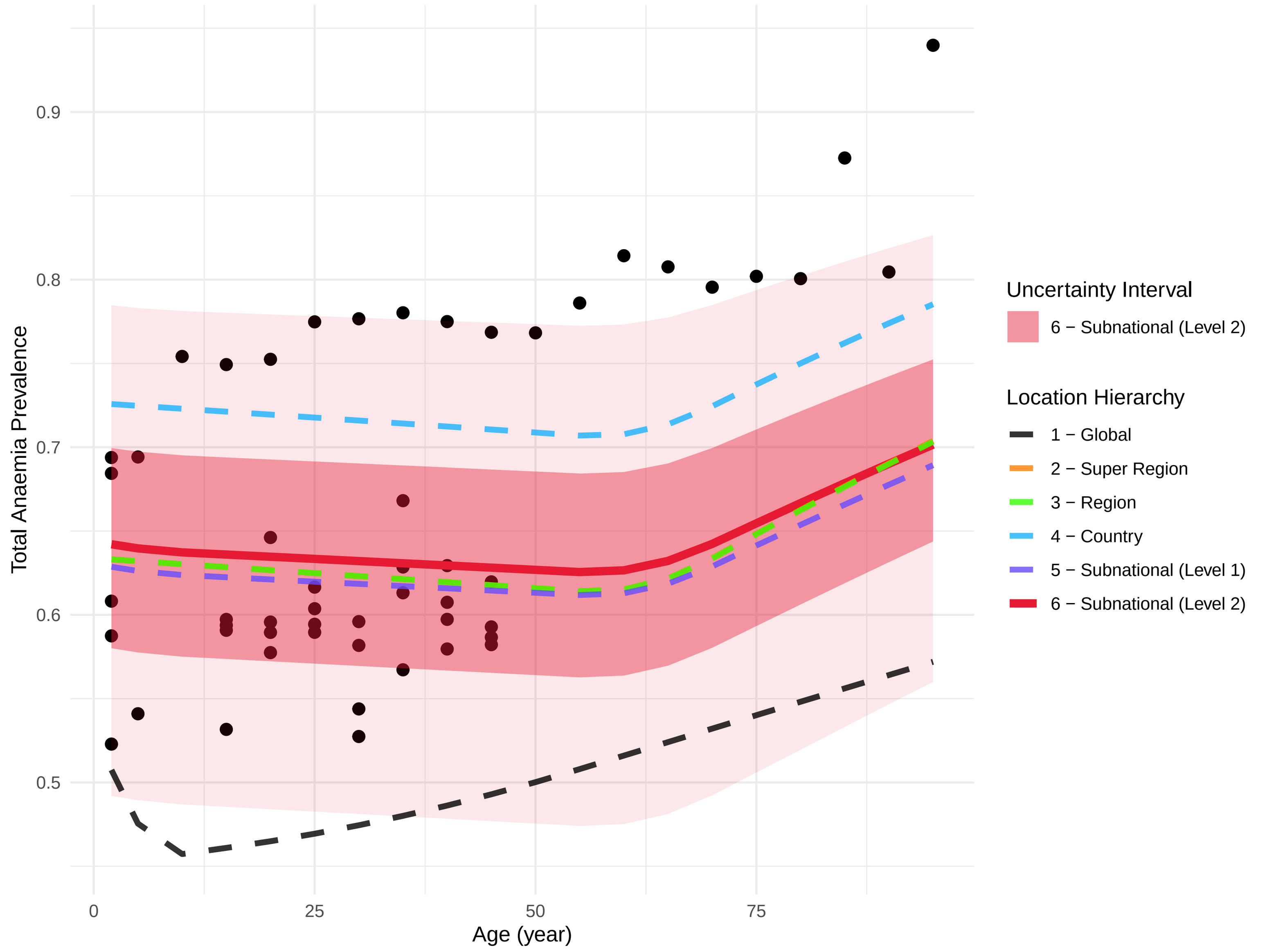

MR−BRT Age/Sex Fits − Non−pregnant Females 2+ Years Old − Total Anaemia Prevalence − Tamil Nadu, Urban
WHO−2024 Adjustment Method

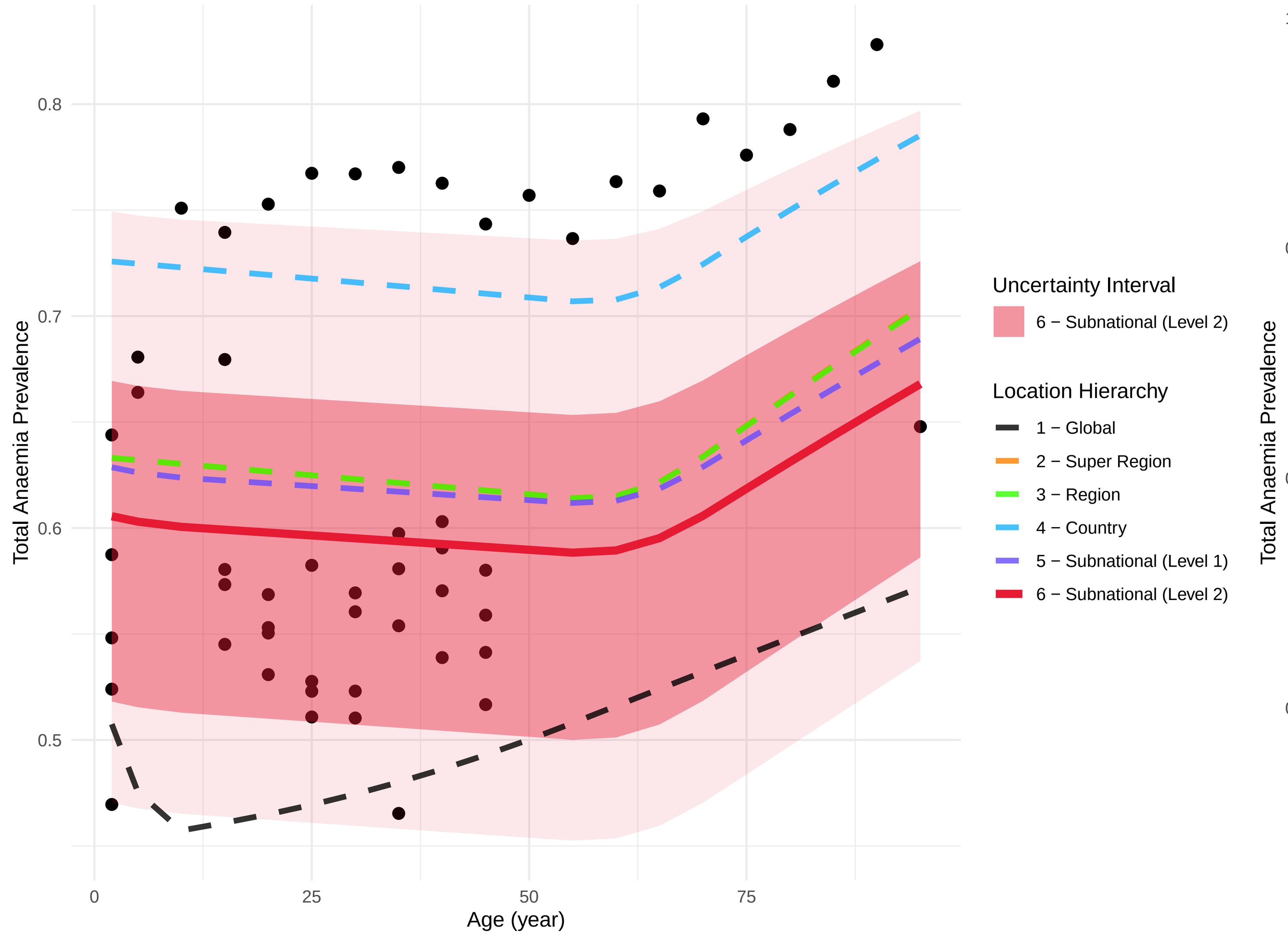

MR−BRT Age/Sex Fits − Non−pregnant Females 2+ Years Old − Total Anaemia Prevalence − Telangana
WHO−2024 Adjustment Method

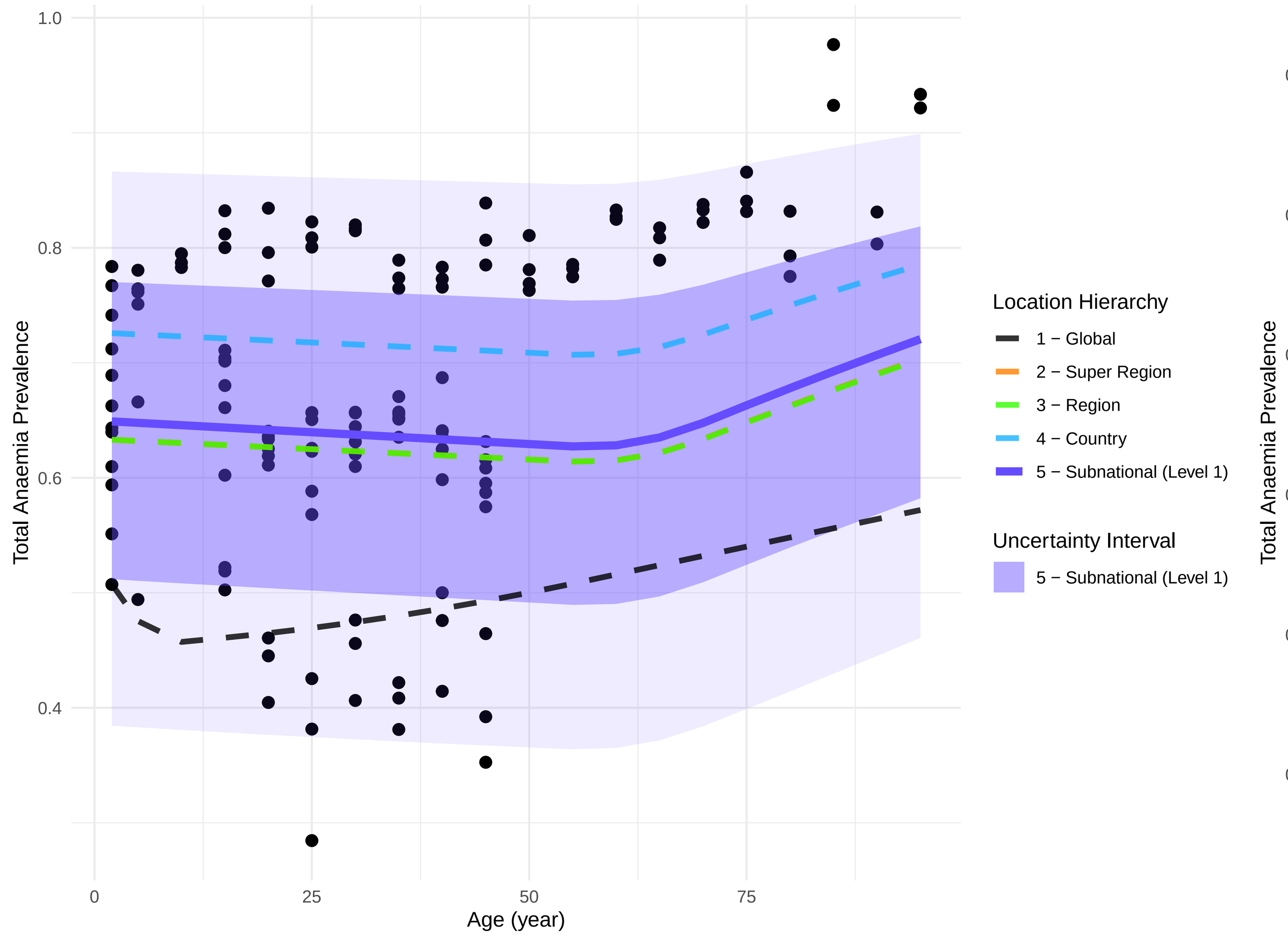

MR−BRT Age/Sex Fits − Non−pregnant Females 2+ Years Old − Total Anaemia Prevalence − Telangana, Rural
WHO−2024 Adjustment Method

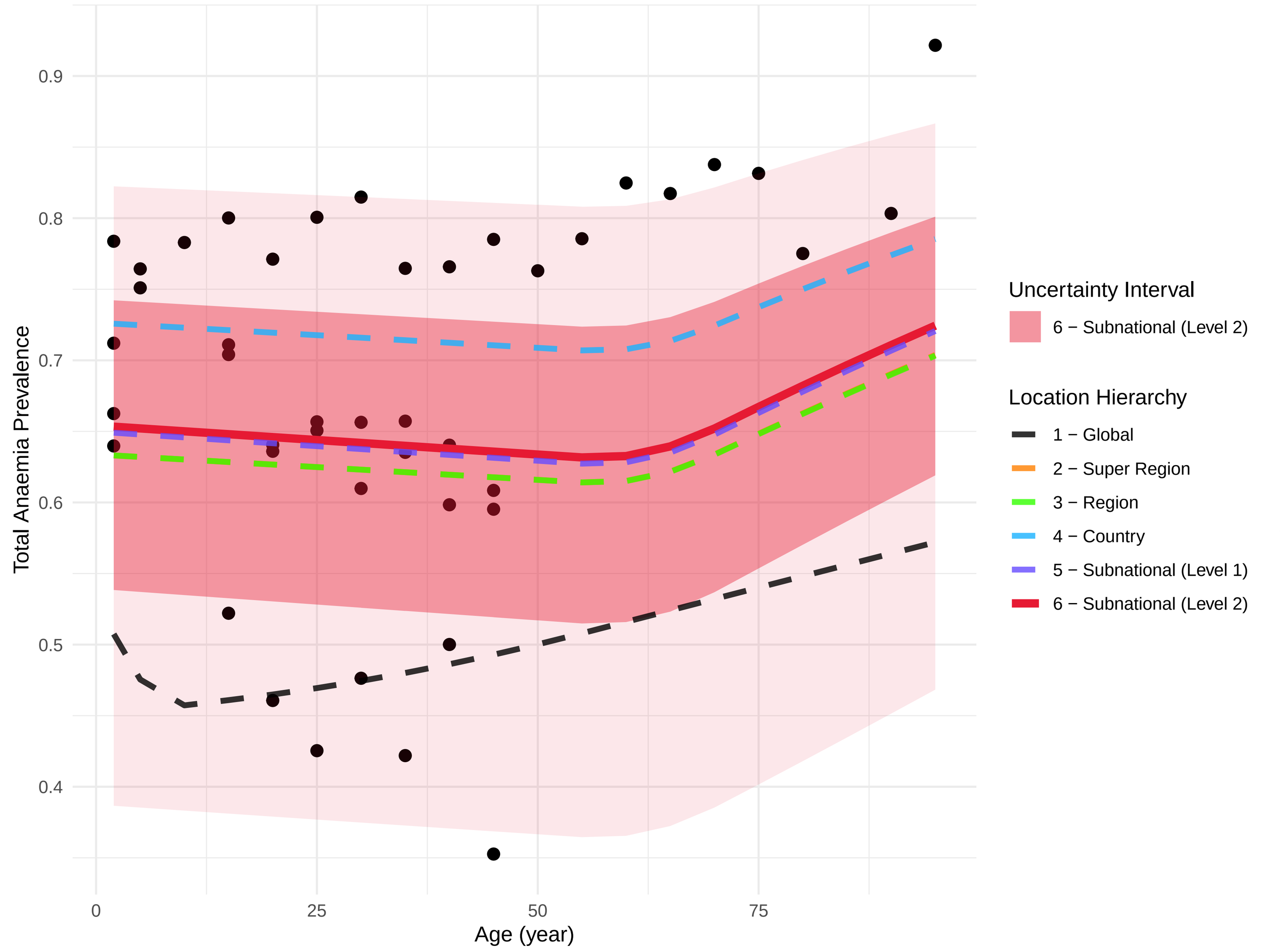

MR−BRT Age/Sex Fits − Non−pregnant Females 2+ Years Old − Total Anaemia Prevalence − Telangana, Urban
WHO−2024 Adjustment Method

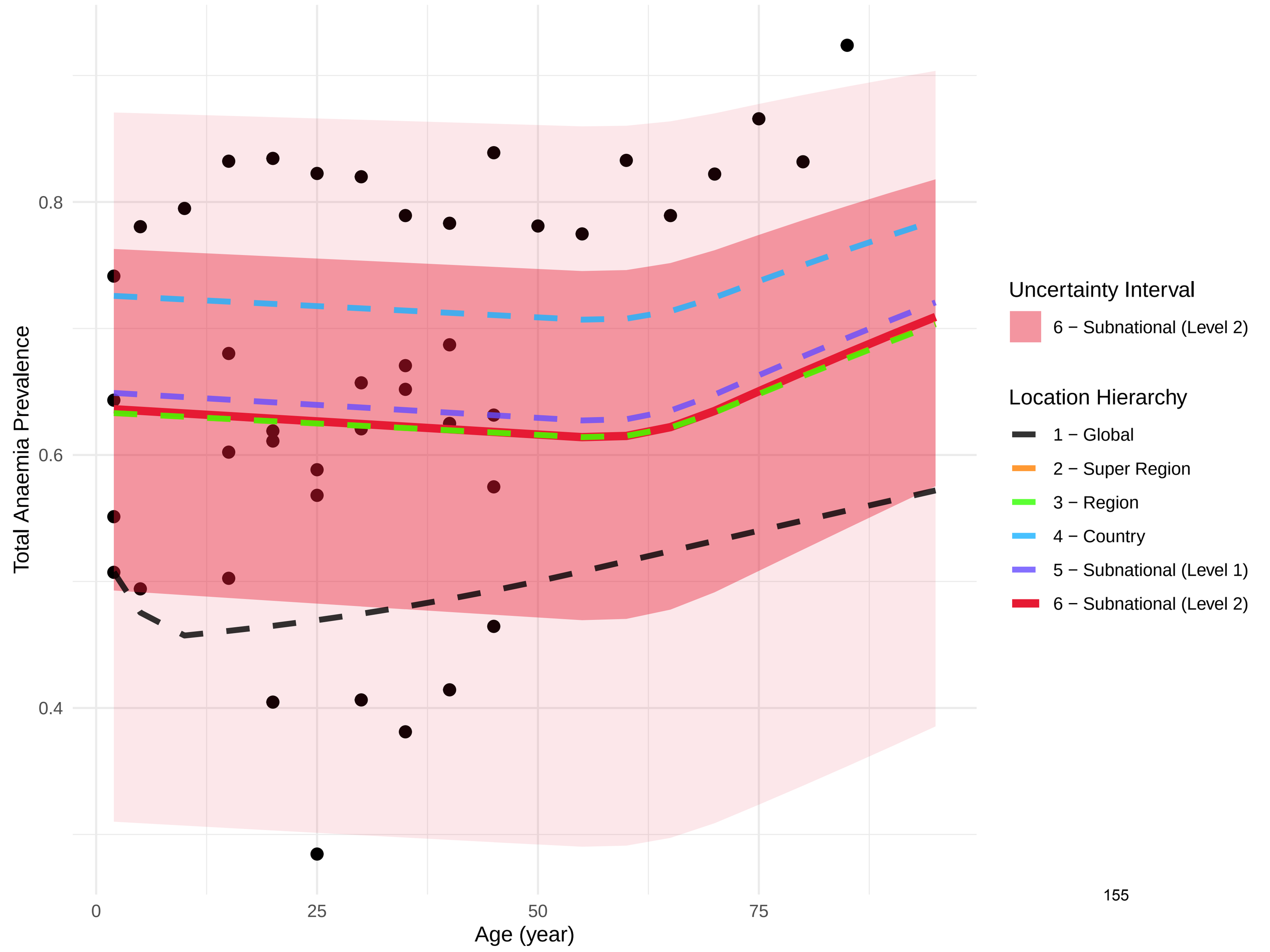

MR−BRT Age/Sex Fits − Non−pregnant Females 2+ Years Old − Total Anaemia Prevalence − Tripura
WHO−2024 Adjustment Method

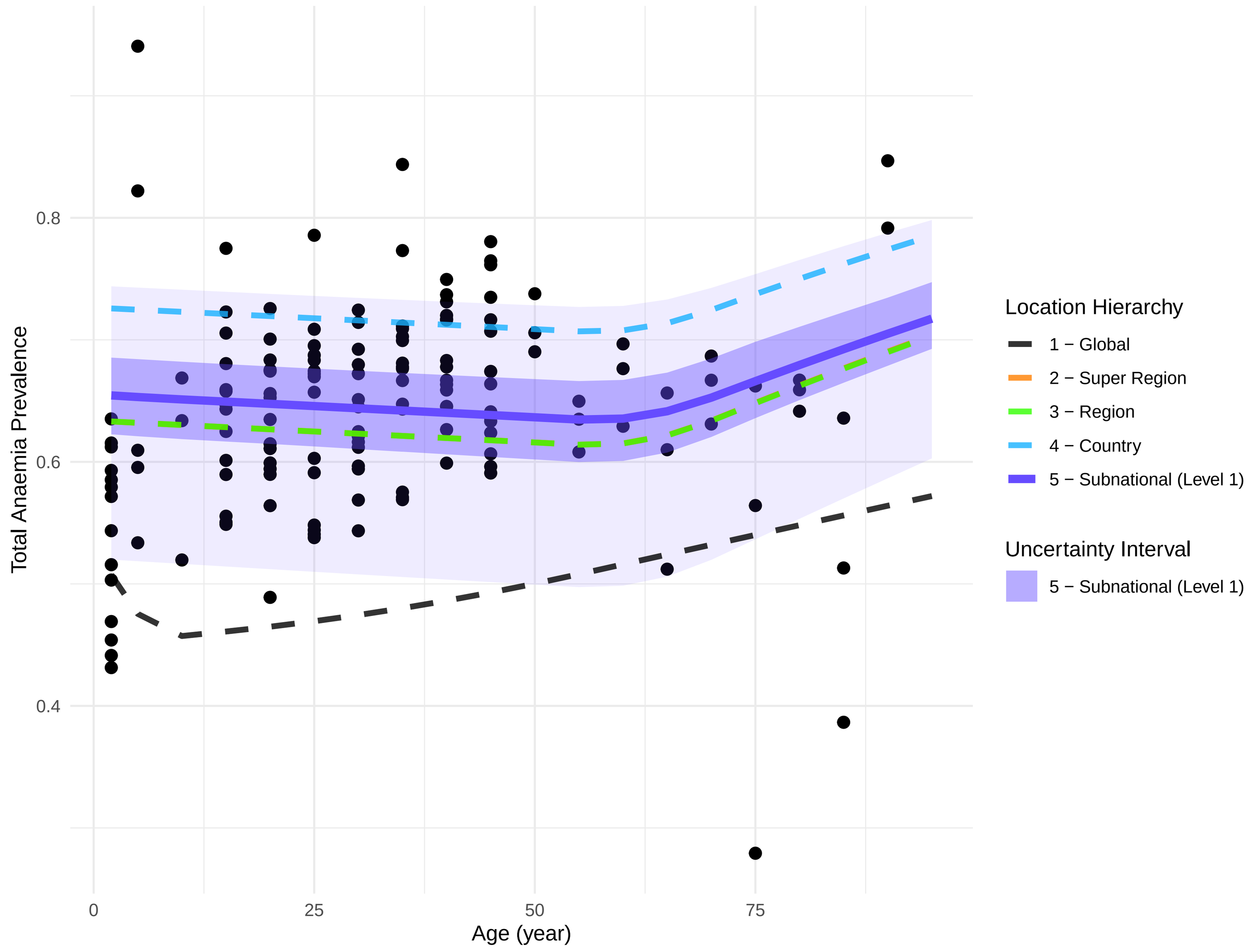

MR−BRT Age/Sex Fits − Non−pregnant Females 2+ Years Old − Total Anaemia Prevalence − Tripura, Rural
WHO−2024 Adjustment Method

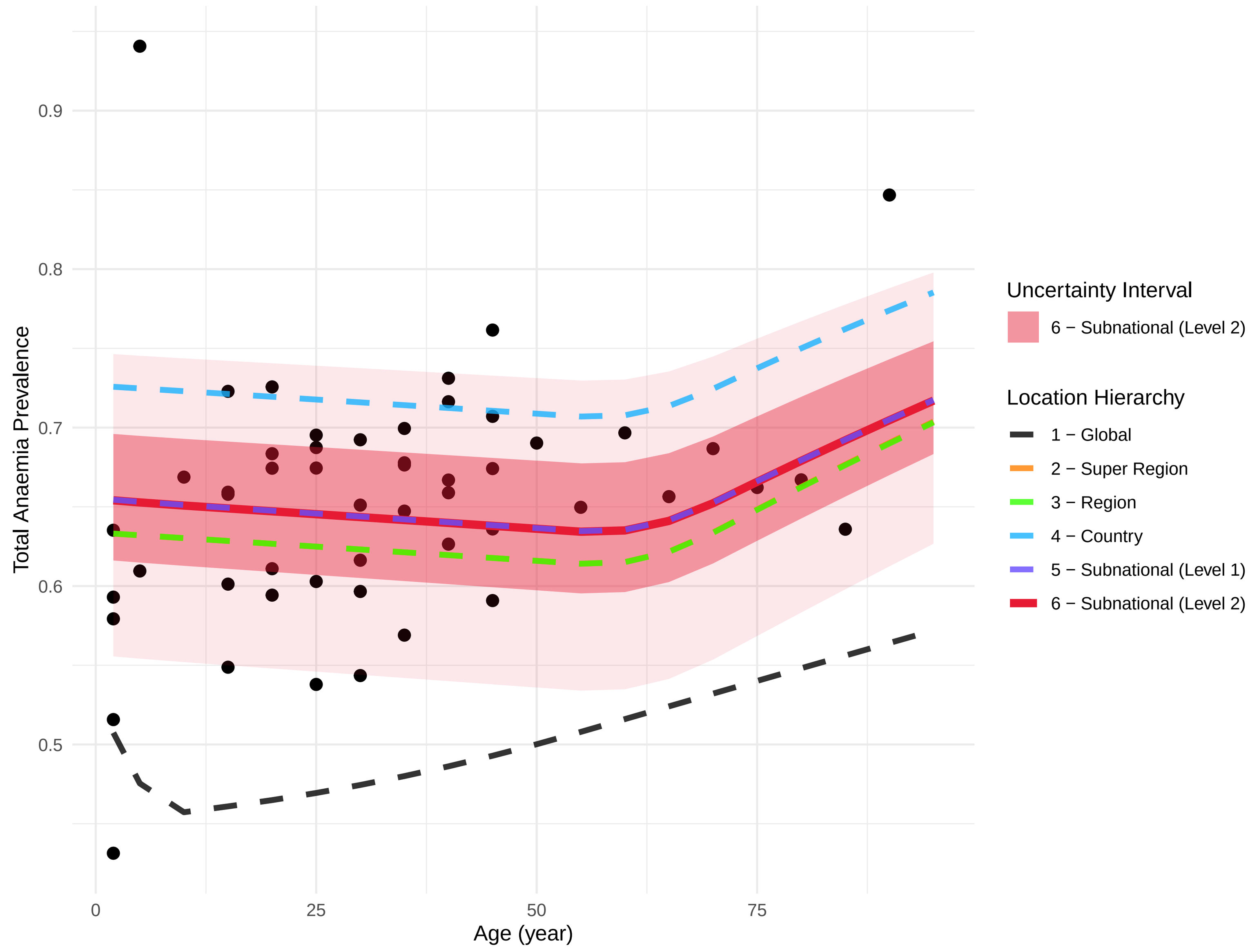

MR−BRT Age/Sex Fits − Non−pregnant Females 2+ Years Old − Total Anaemia Prevalence − Tripura, Urban
WHO−2024 Adjustment Method

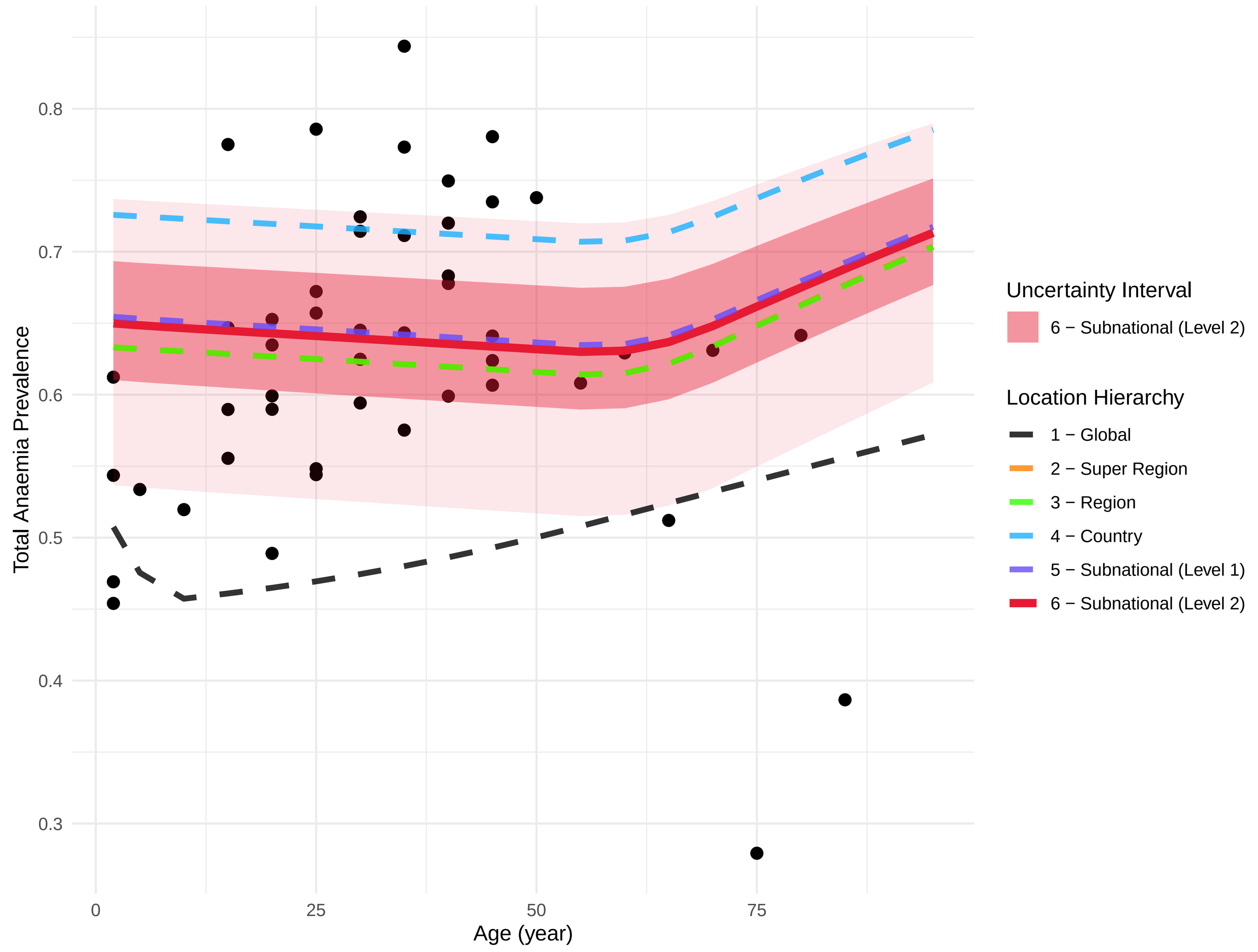

MR−BRT Age/Sex Fits − Non−pregnant Females 2+ Years Old − Total Anaemia Prevalence − Uttar Pradesh
WHO−2024 Adjustment Method

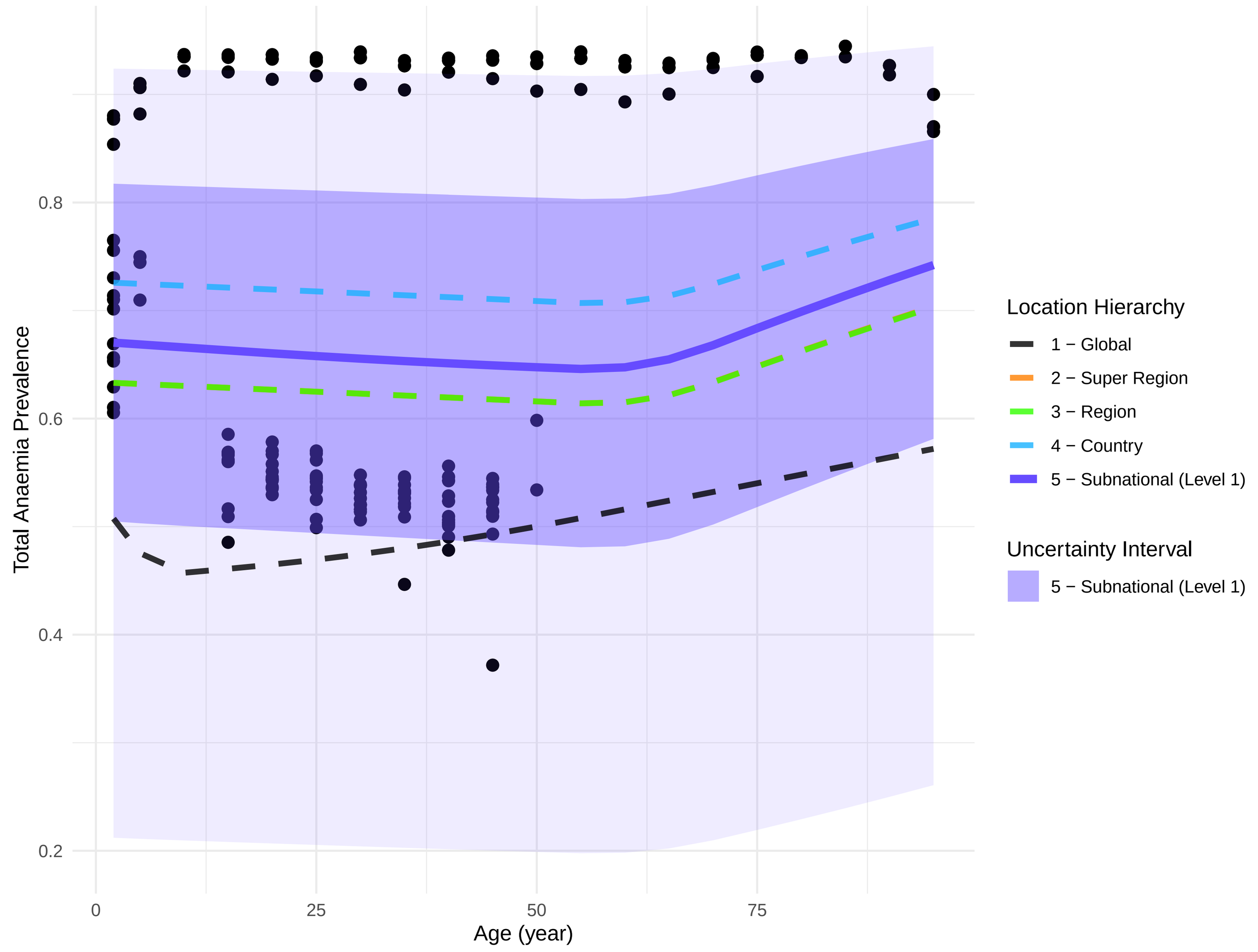

MR−BRT Age/Sex Fits − Non−pregnant Females 2+ Years Old − Total Anaemia Prevalence − Uttar Pradesh, Rural
WHO−2024 Adjustment Method

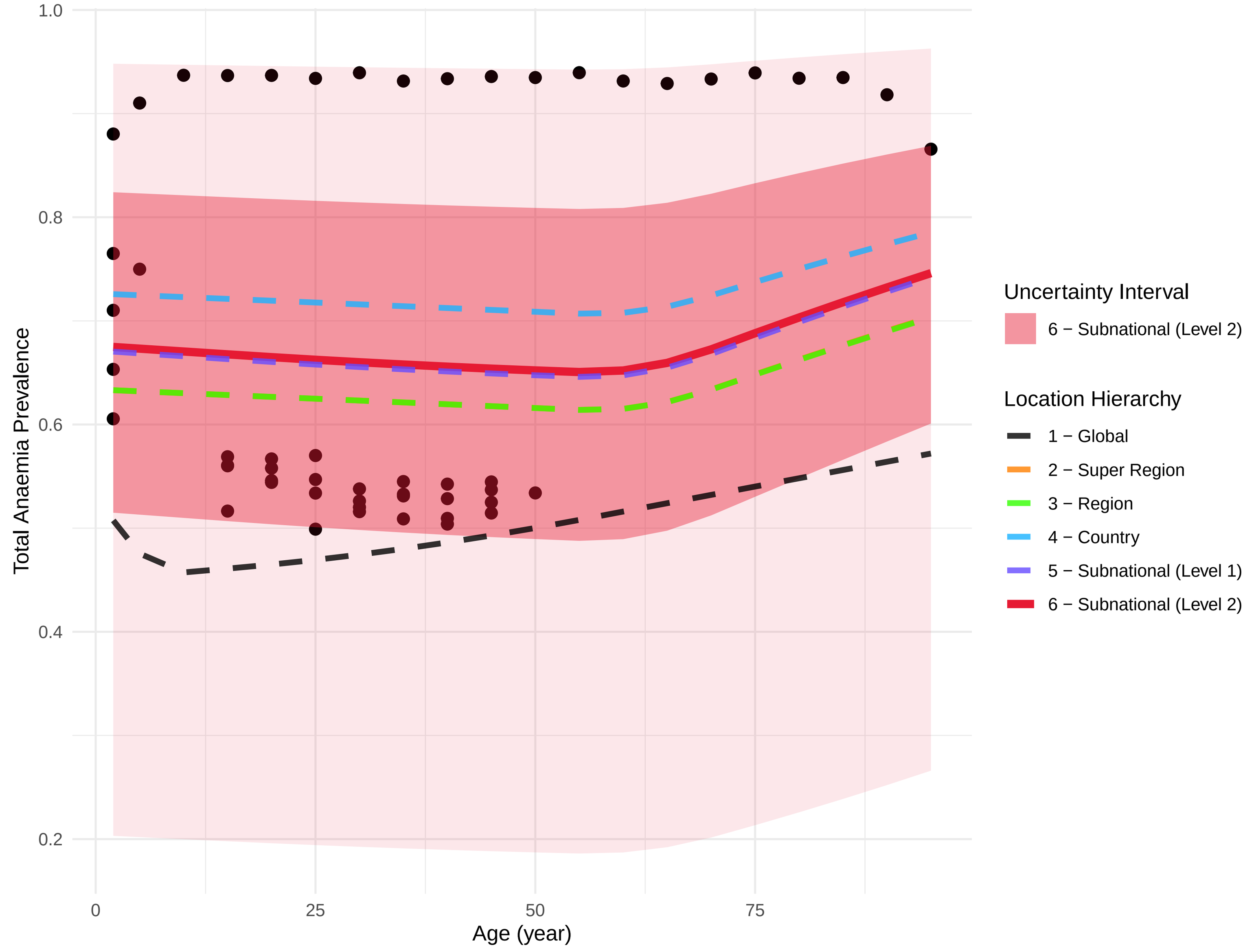

MR−BRT Age/Sex Fits − Non−pregnant Females 2+ Years Old − Total Anaemia Prevalence − Uttar Pradesh, Urban
WHO−2024 Adjustment Method

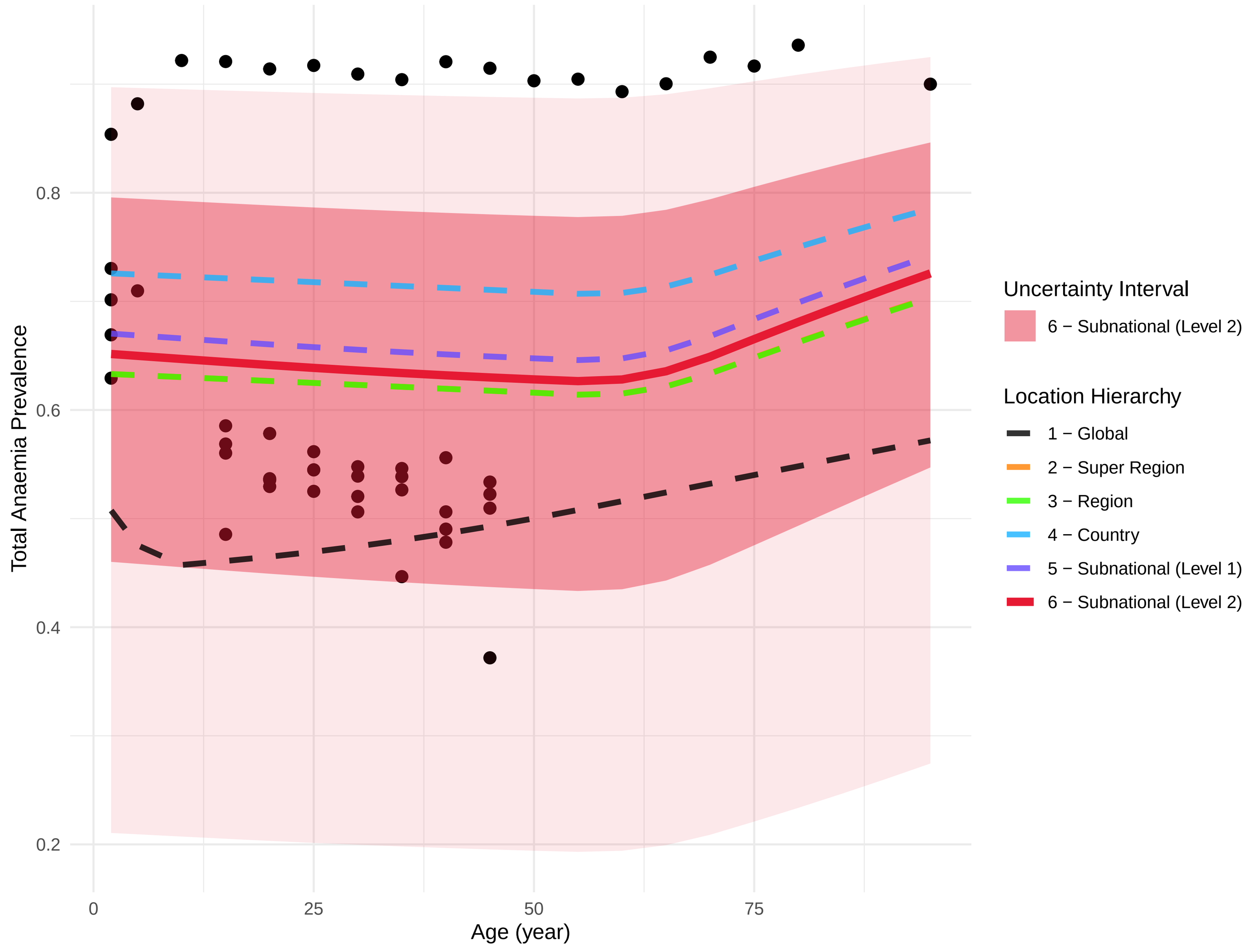

MR−BRT Age/Sex Fits − Non−pregnant Females 2+ Years Old − Total Anaemia Prevalence − Uttarakhand
WHO−2024 Adjustment Method

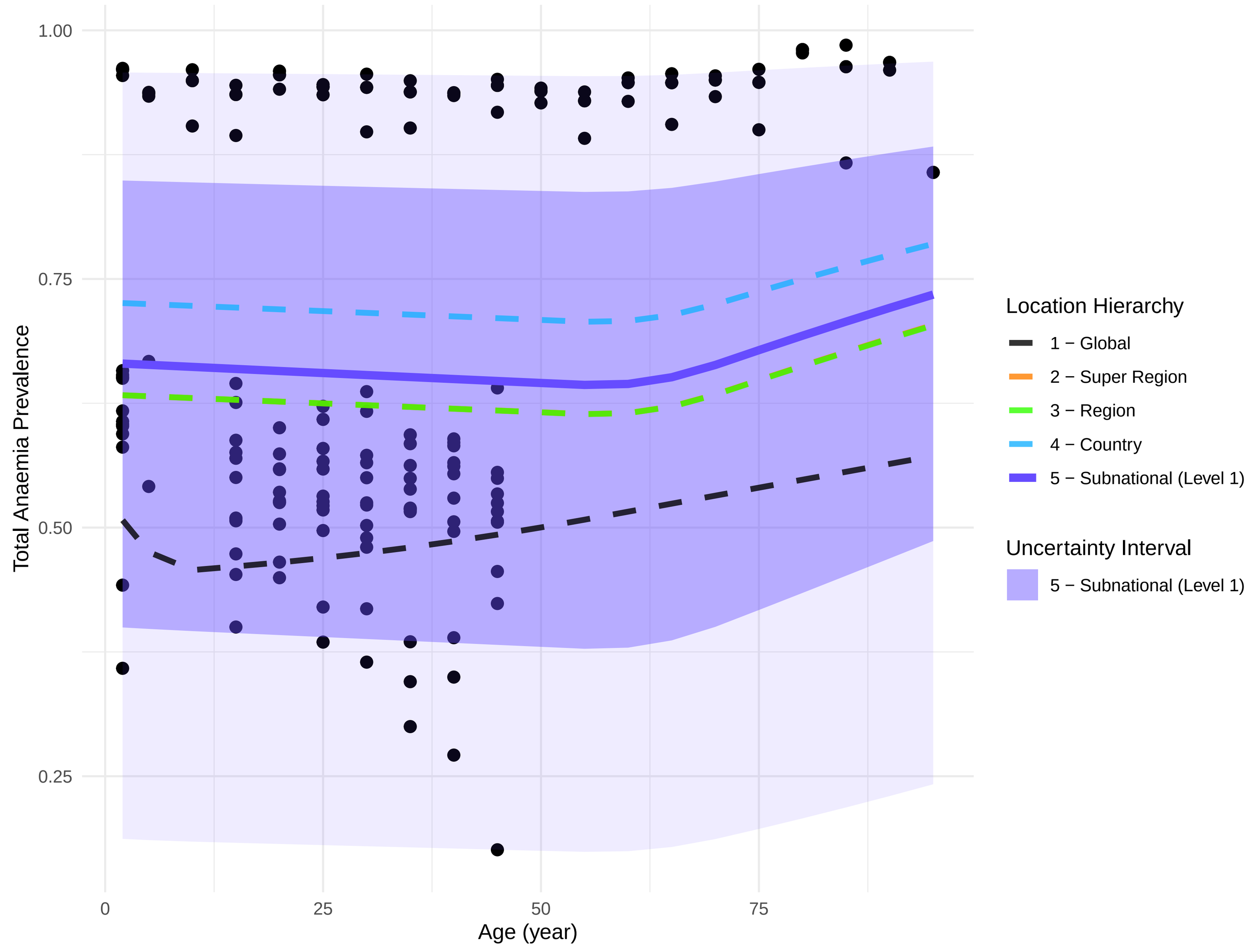

MR−BRT Age/Sex Fits − Non−pregnant Females 2+ Years Old − Total Anaemia Prevalence − Uttarakhand, Rural
WHO−2024 Adjustment Method

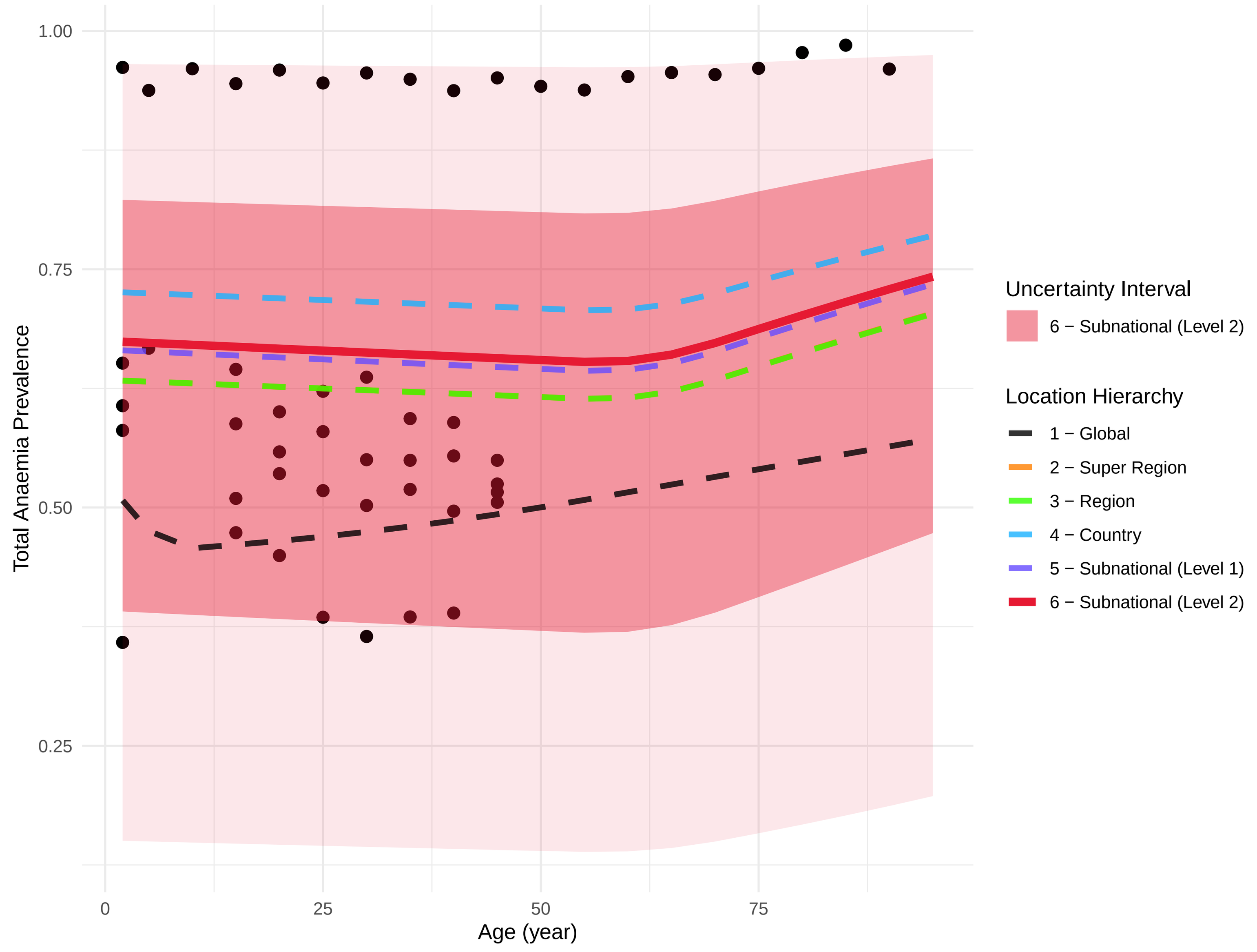

MR−BRT Age/Sex Fits − Non−pregnant Females 2+ Years Old − Total Anaemia Prevalence − Uttarakhand, Urban
WHO−2024 Adjustment Method

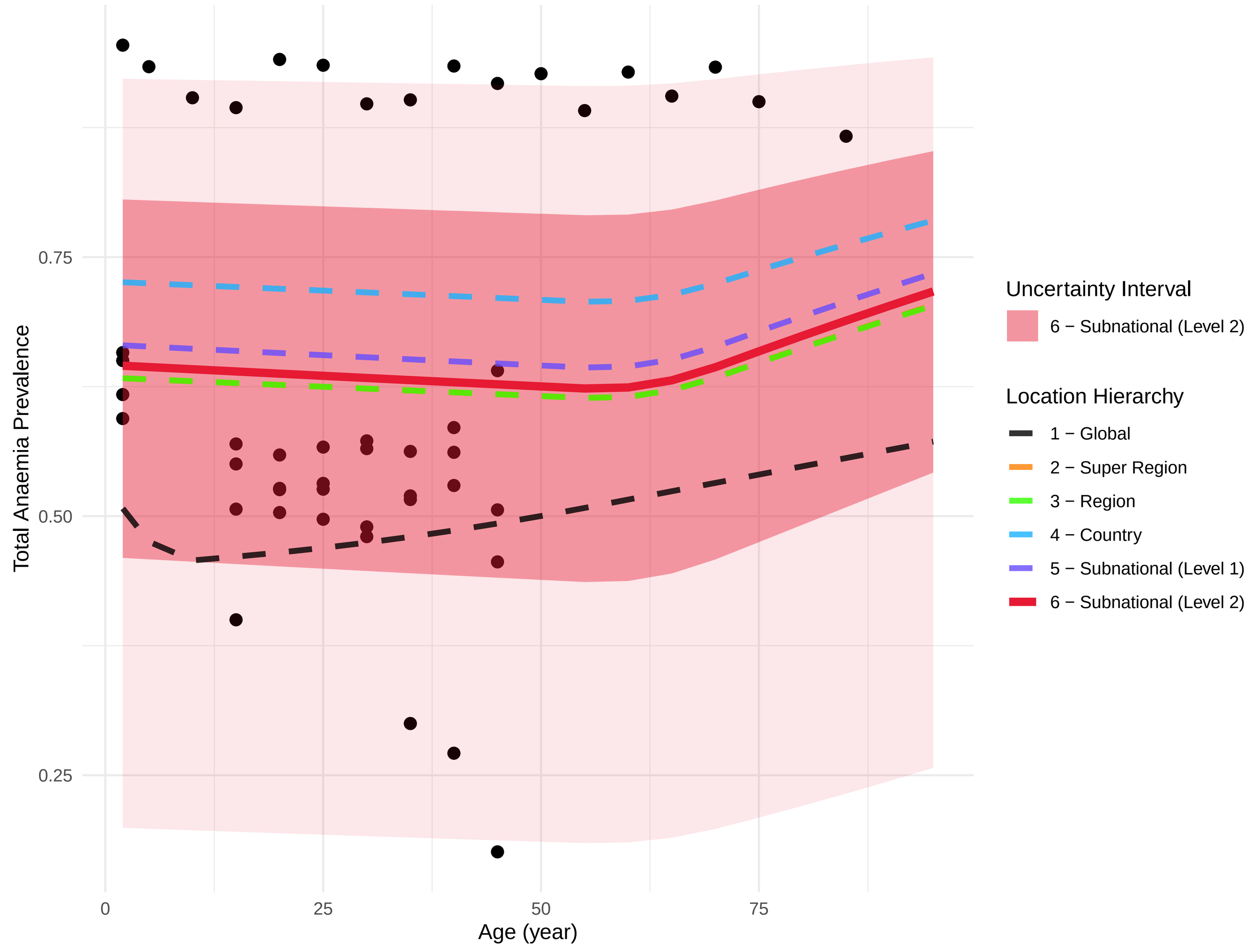

MR−BRT Age/Sex Fits − Non−pregnant Females 2+ Years Old − Total Anaemia Prevalence − West Bengal
WHO−2024 Adjustment Method

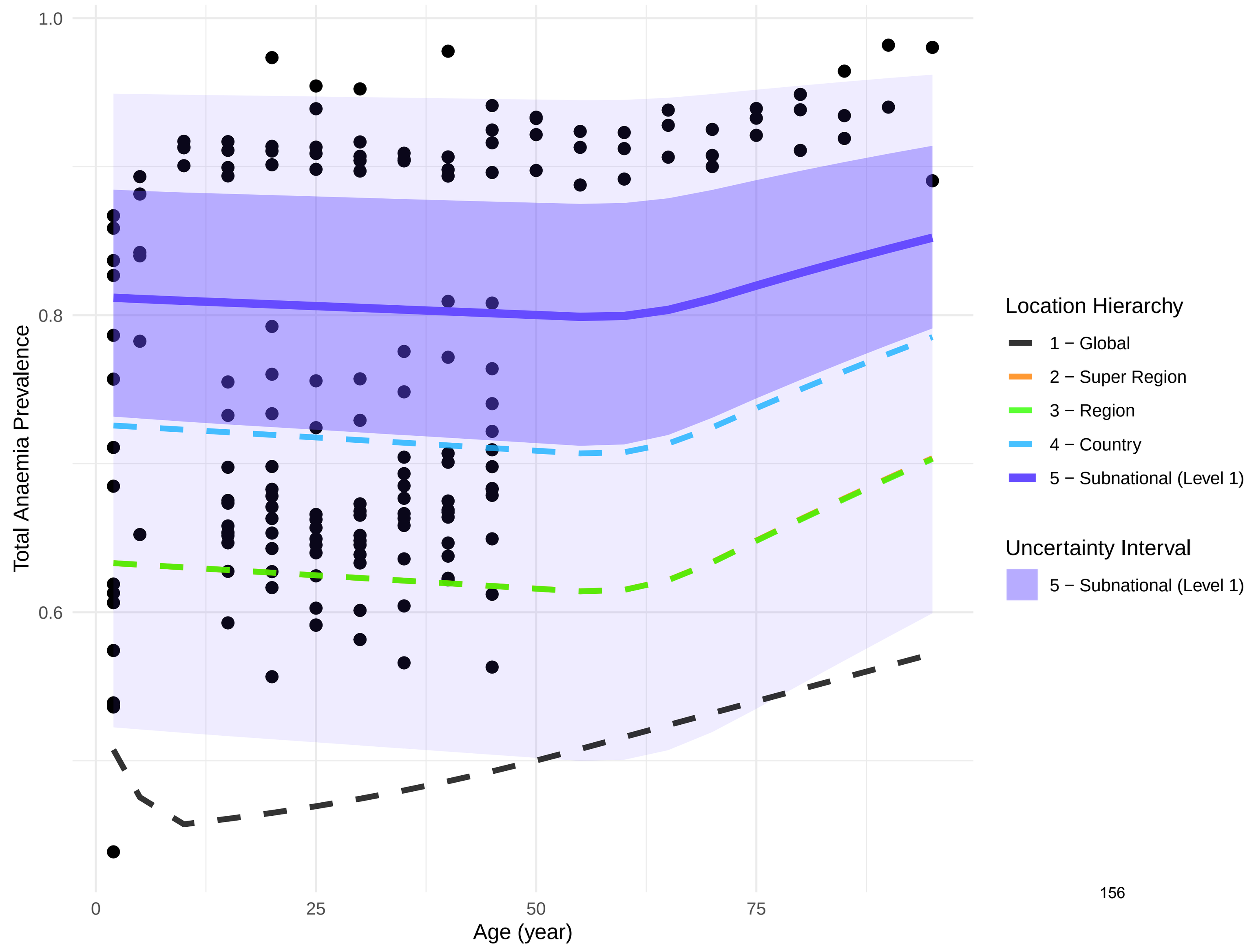

MR−BRT Age/Sex Fits − Non-pregnant Females 2+ Years Old − Total Anaemia Prevalence − West Bengal, Rural
WHO−2024 Adjustment Method

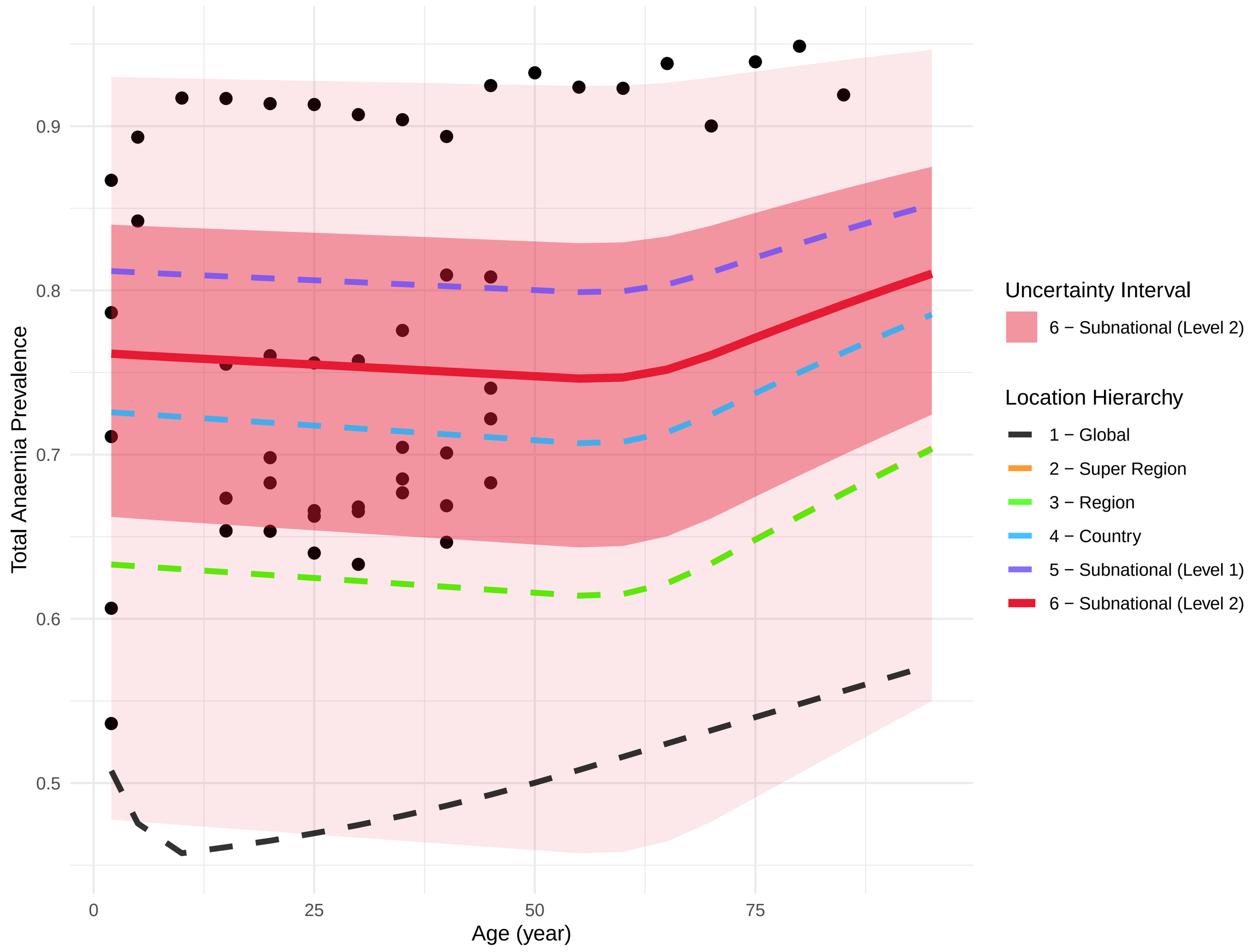

MR−BRT Age/Sex Fits − Non-pregnant Females 2+ Years Old − Total Anaemia Prevalence − West Bengal, Urban
WHO−2024 Adjustment Method

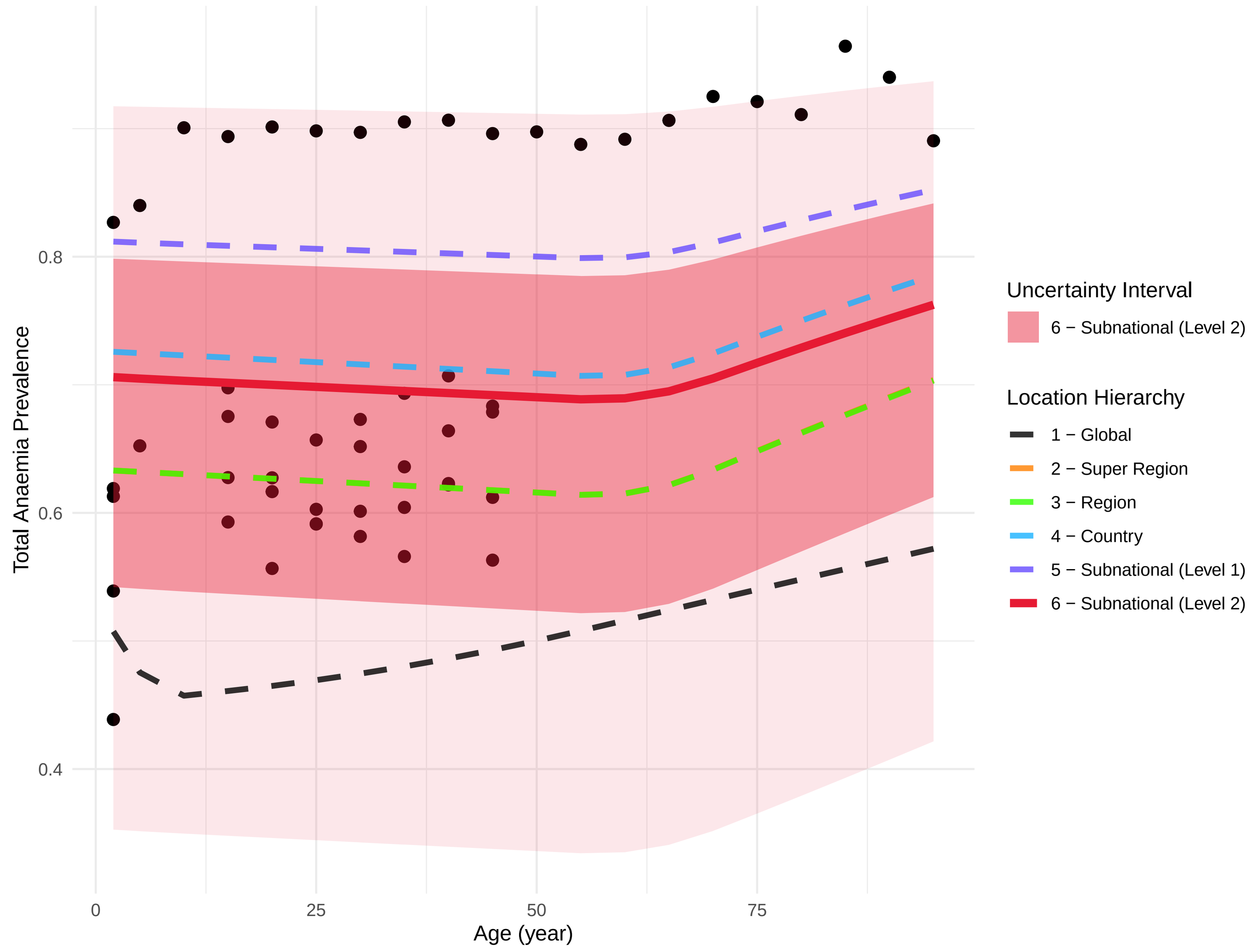

MR−BRT Age/Sex Fits − Non-pregnant Females 2+ Years Old − Total Anaemia Prevalence − Nepal
WHO−2024 Adjustment Method

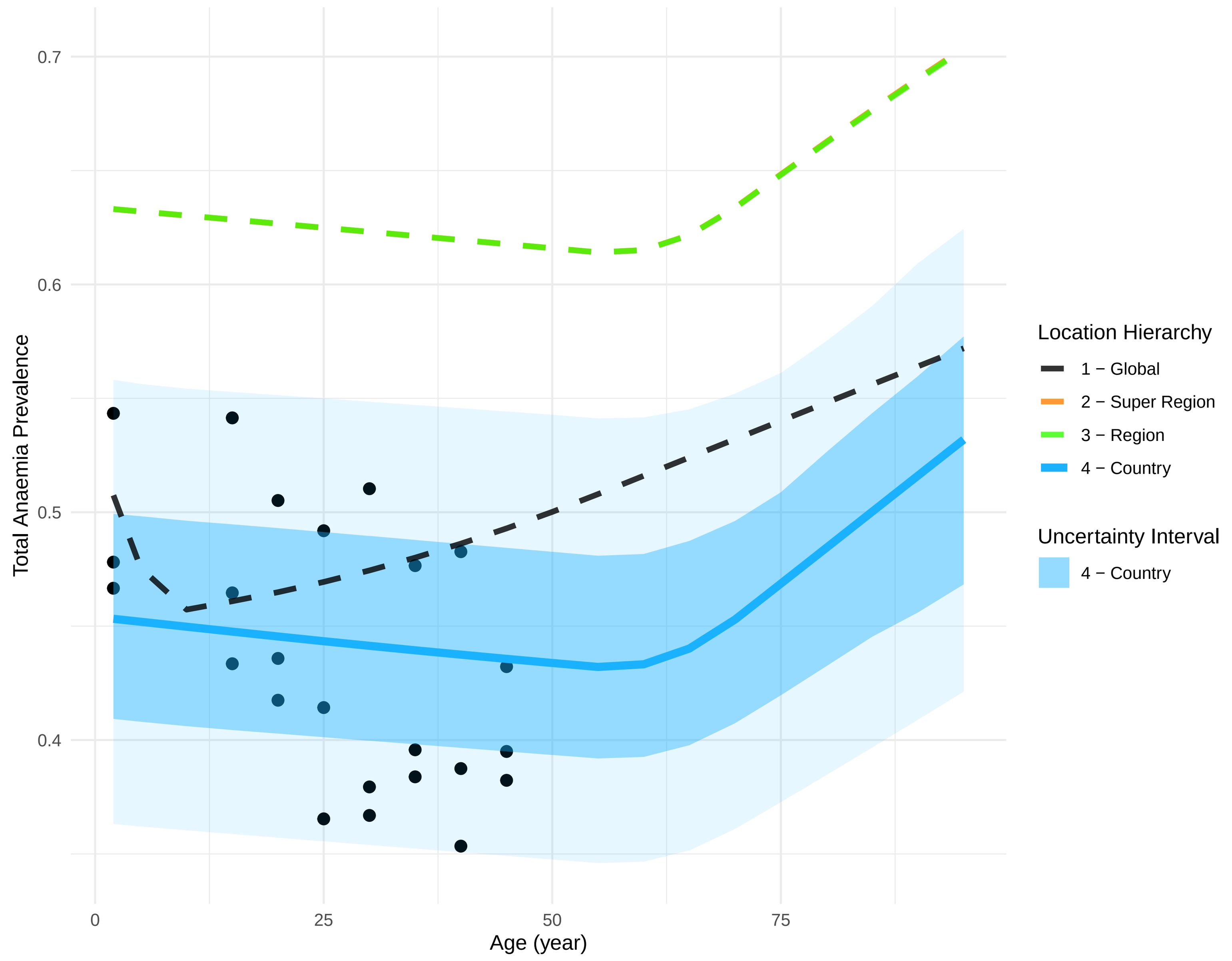

MR−BRT Age/Sex Fits − Non-pregnant Females 2+ Years Old − Total Anaemia Prevalence − Pakistan
WHO−2024 Adjustment Method

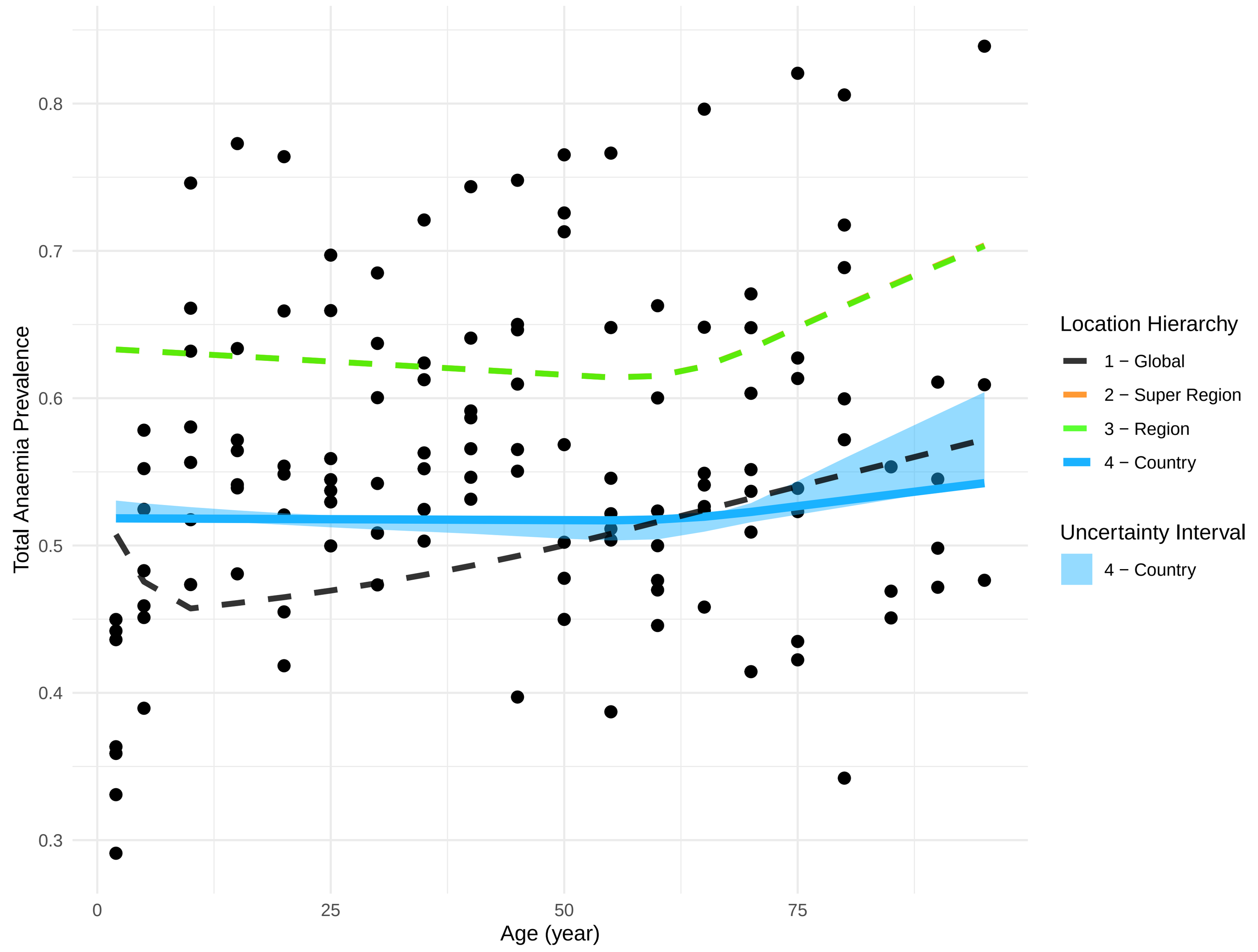

MR−BRT Age/Sex Fits − Non-pregnant Females 2+ Years Old − Total Anaemia Prevalence − Azad Jammu & Kashmir
WHO−2024 Adjustment Method

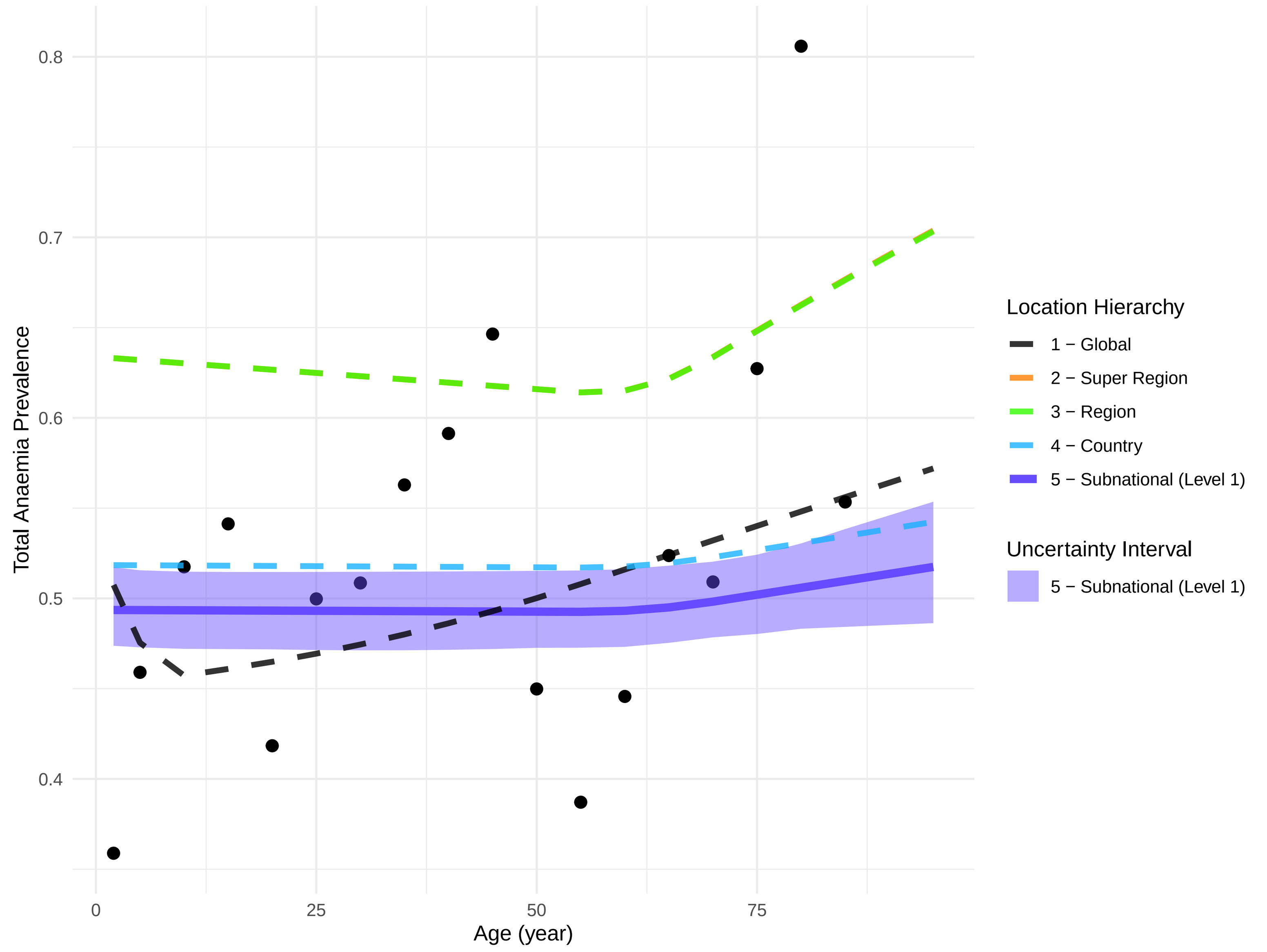

MR−BRT Age/Sex Fits − Non-pregnant Females 2+ Years Old − Total Anaemia Prevalence − Balochistan
WHO−2024 Adjustment Method

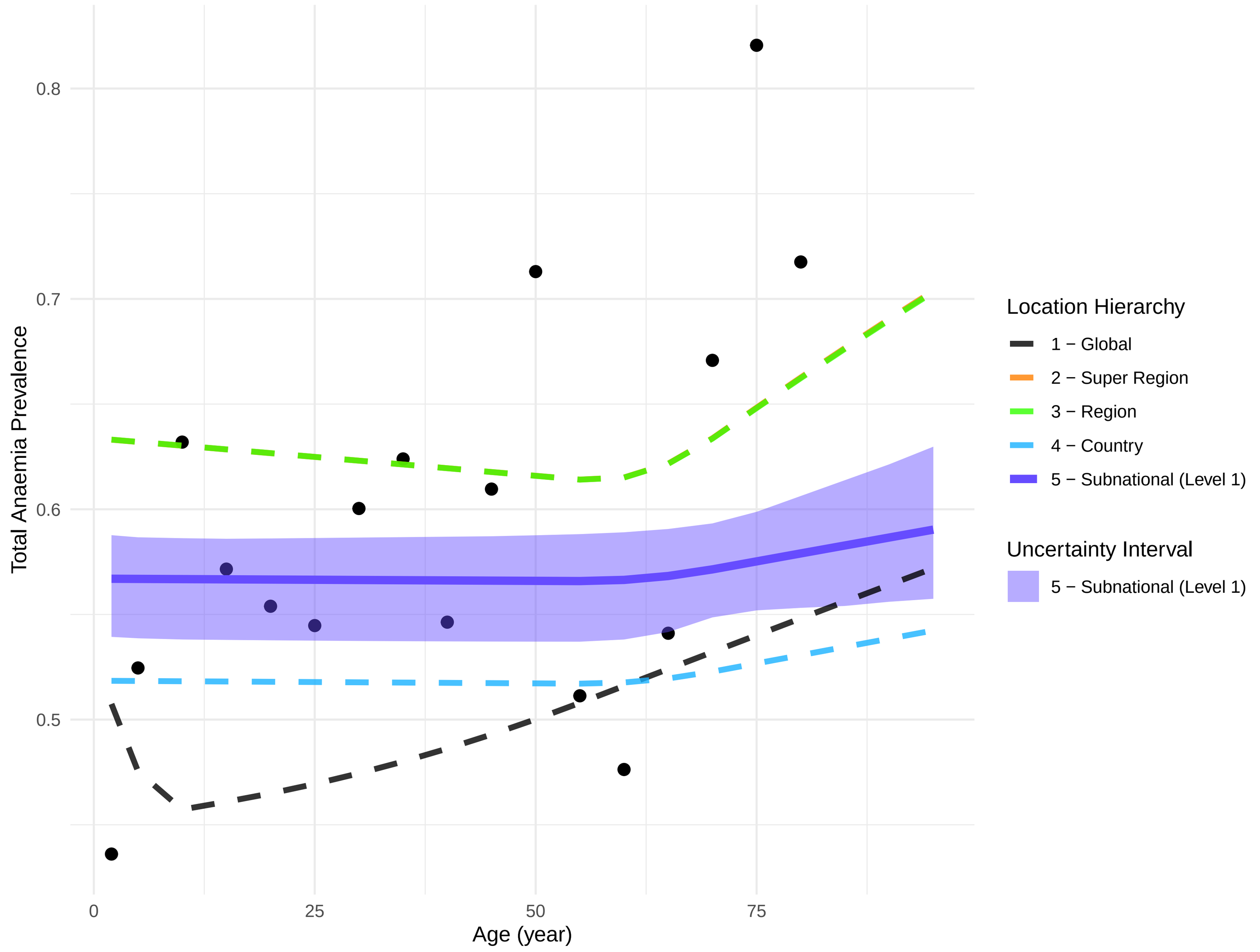

MR−BRT Age/Sex Fits − Non-pregnant Females 2+ Years Old − Total Anaemia Prevalence − Gilgit−Baltistan
WHO−2024 Adjustment Method

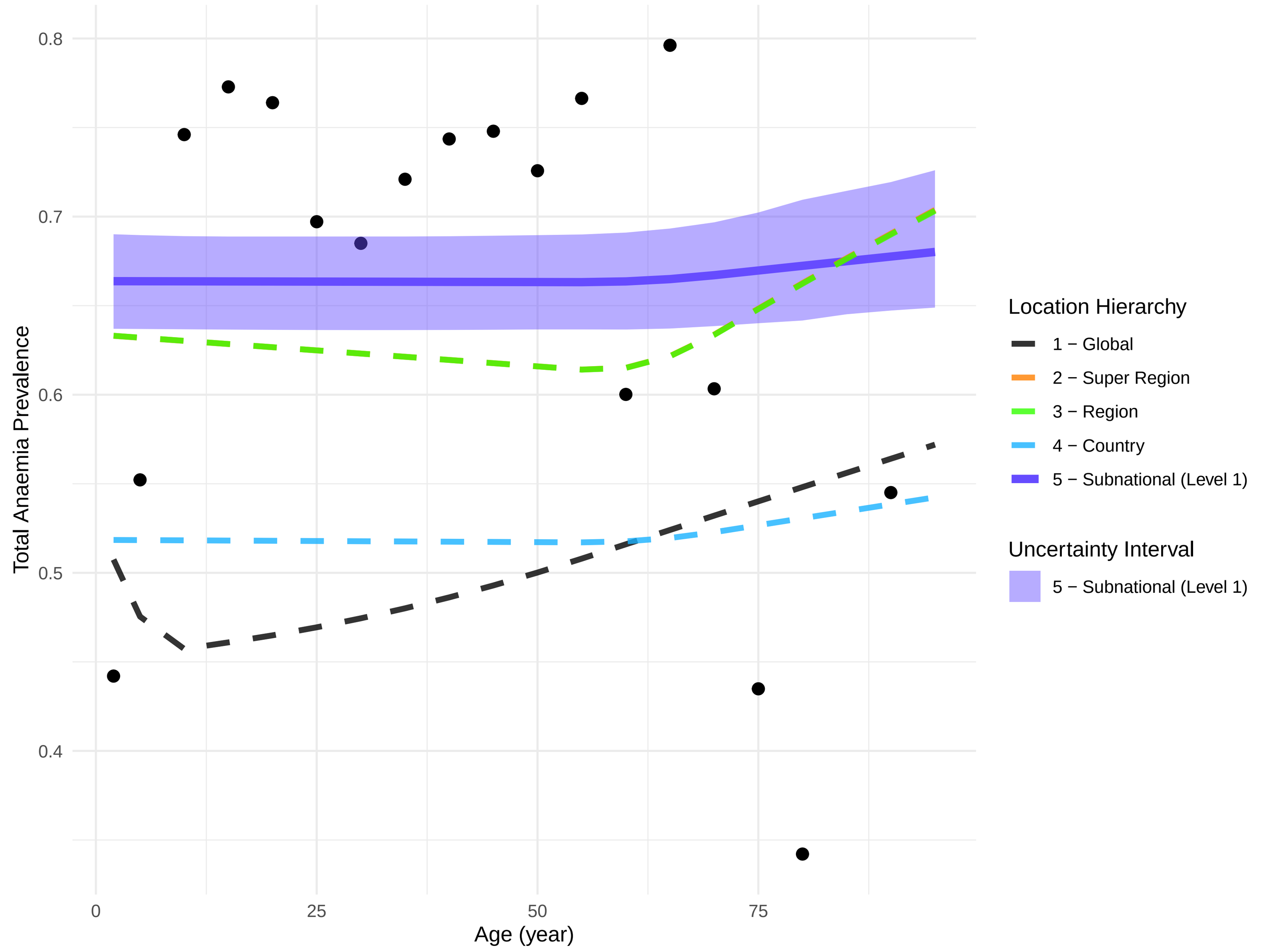

MR−BRT Age/Sex Fits − Non-pregnant Females 2+ Years Old − Total Anaemia Prevalence − Khyber Pakhtunkhwa
WHO−2024 Adjustment Method

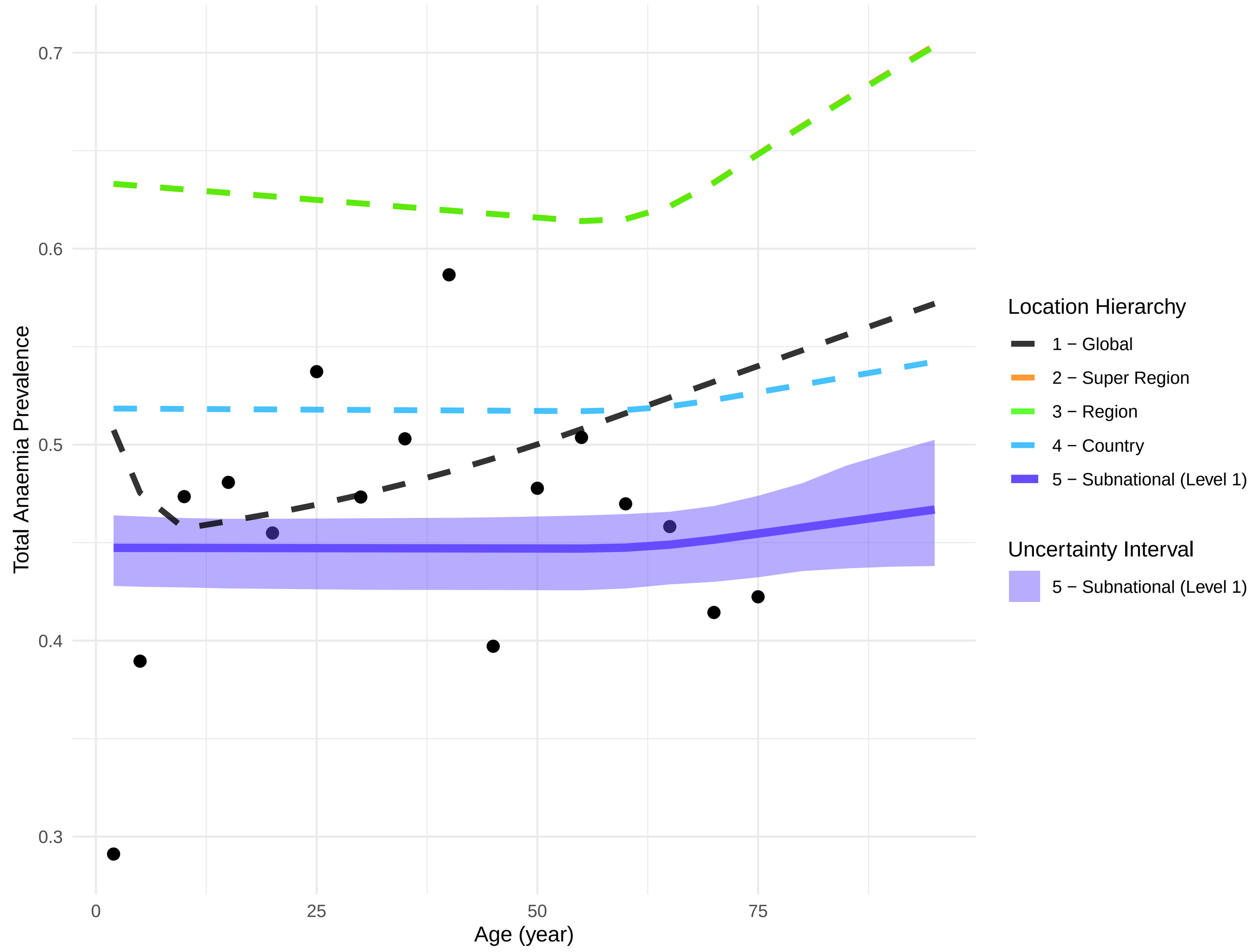

MR−BRT Age/Sex Fits − Non-pregnant Females 2+ Years Old − Total Anaemia Prevalence − Punjab
WHO−2024 Adjustment Method

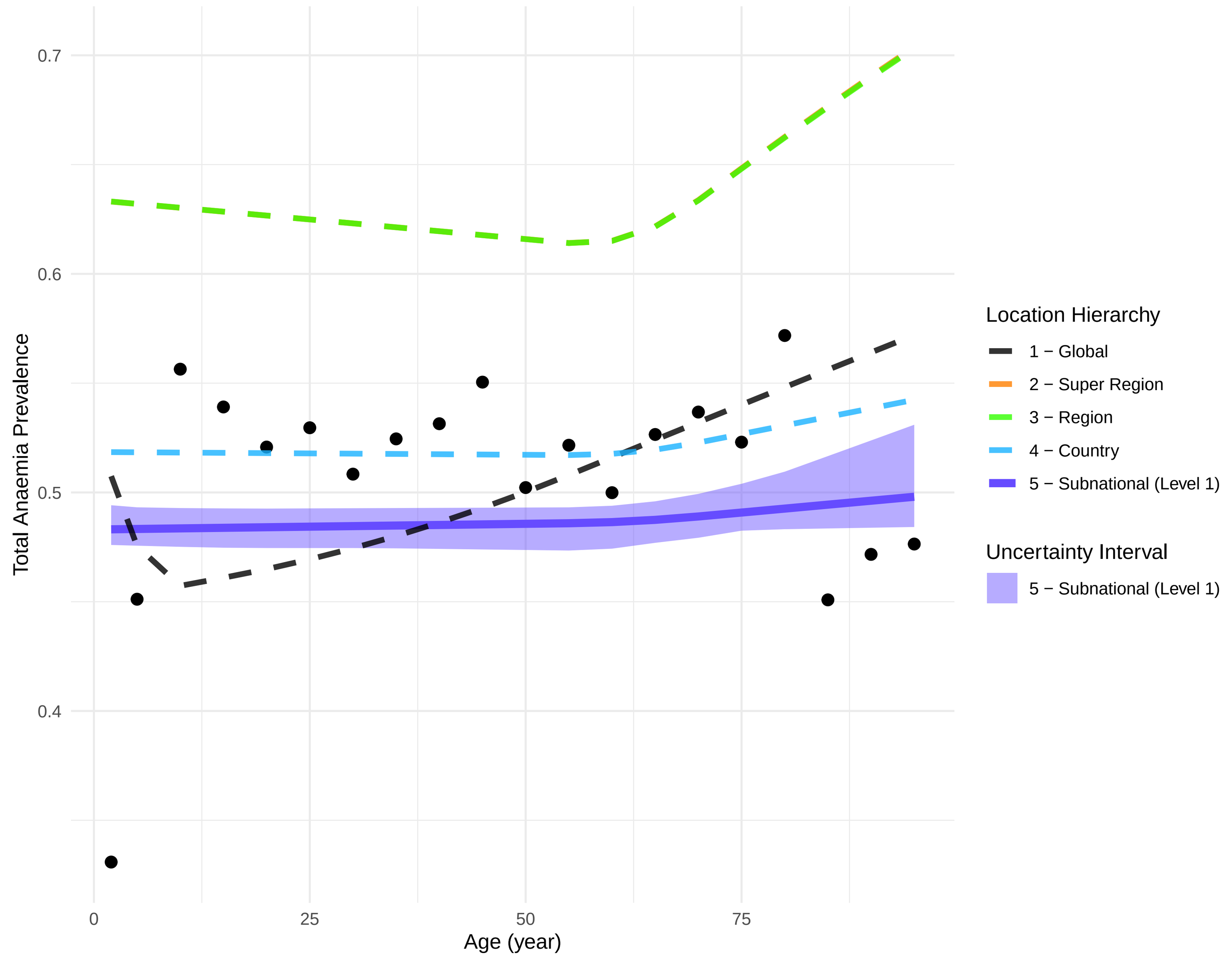

MR−BRT Age/Sex Fits − Non-pregnant Females 2+ Years Old − Total Anaemia Prevalence − Sindh
WHO−2024 Adjustment Method

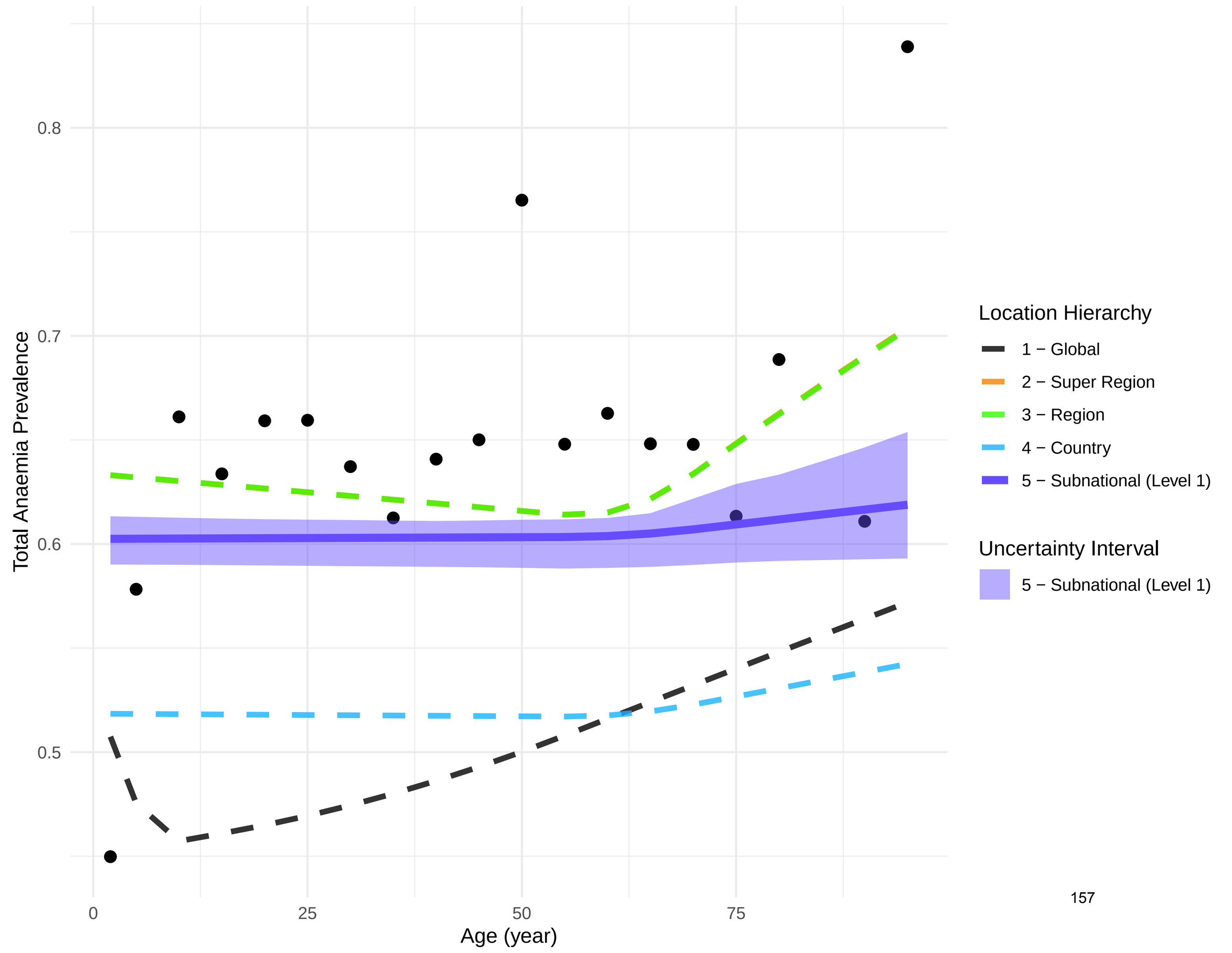

MR−BRT Age/Sex Fits − Non−pregnant Females 2+ Years Old − Total Anaemia Prevalence − Southeast Asia, East Asia, and Oceania
WHO−2024 Adjustment Method

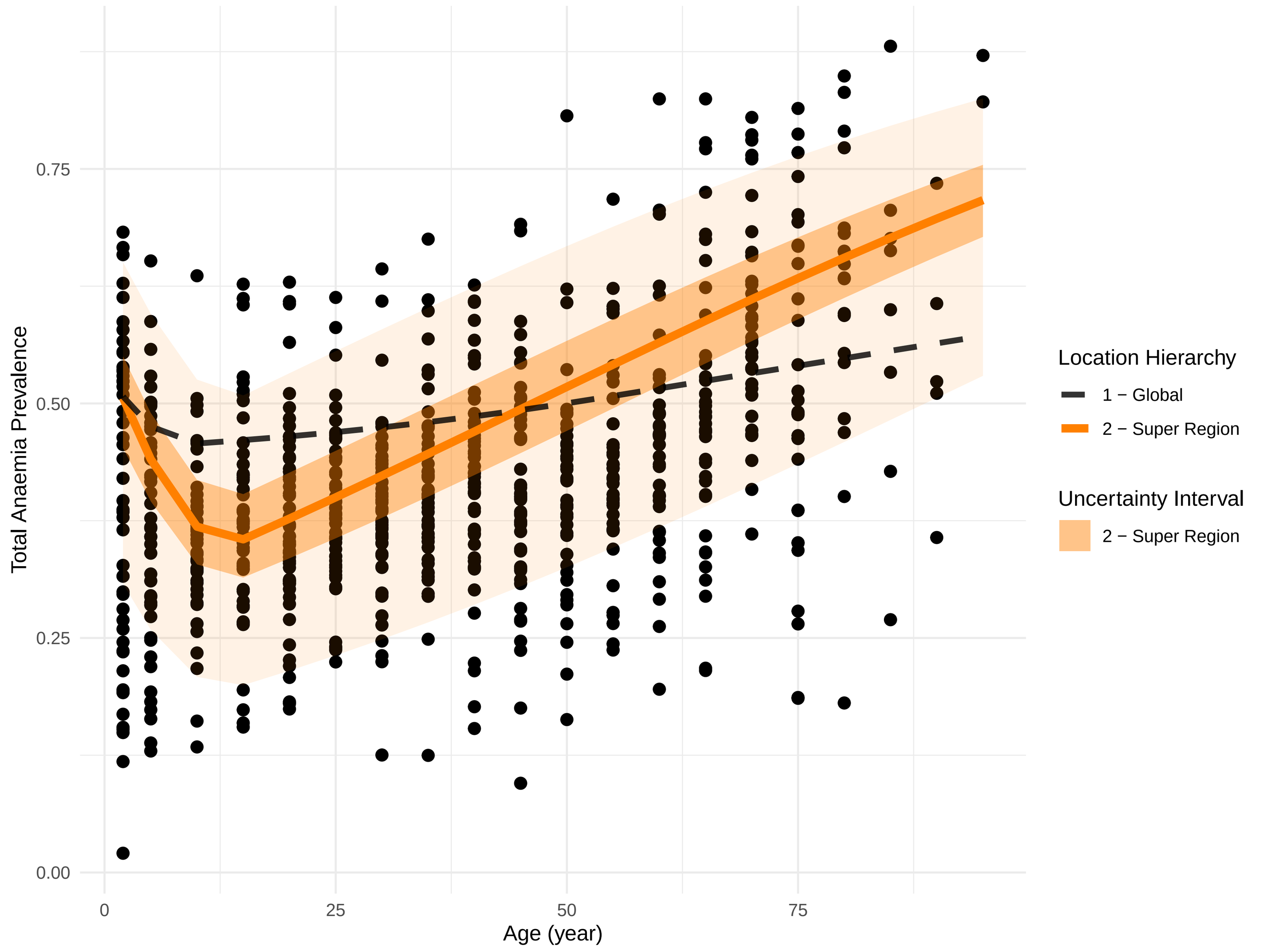

MR−BRT Age/Sex Fits − Non−pregnant Females 2+ Years Old − Total Anaemia Prevalence − Oceania
WHO−2024 Adjustment Method

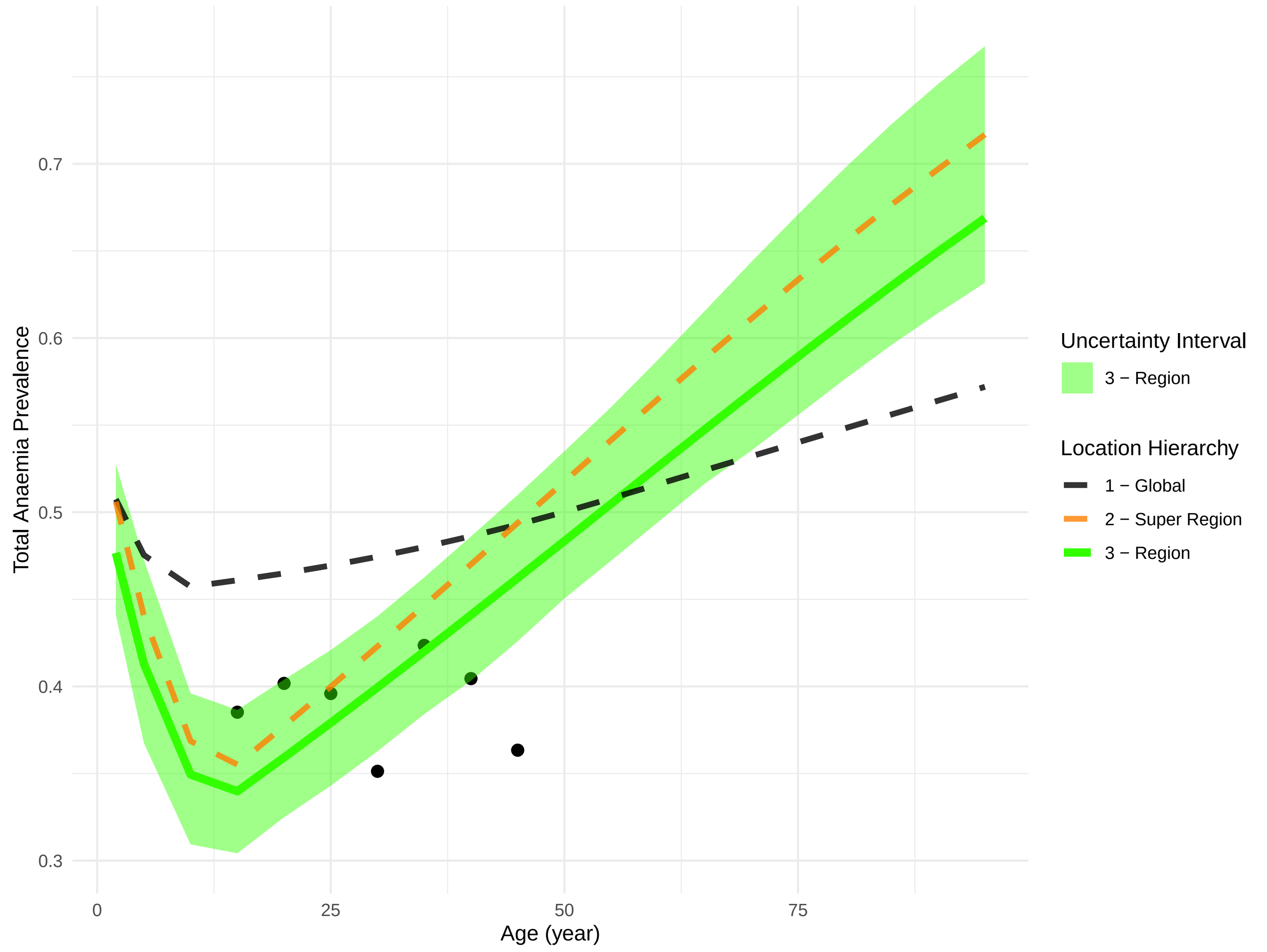

MR−BRT Age/Sex Fits − Non−pregnant Females 2+ Years Old − Total Anaemia Prevalence − Papua New Guinea
WHO−2024 Adjustment Method

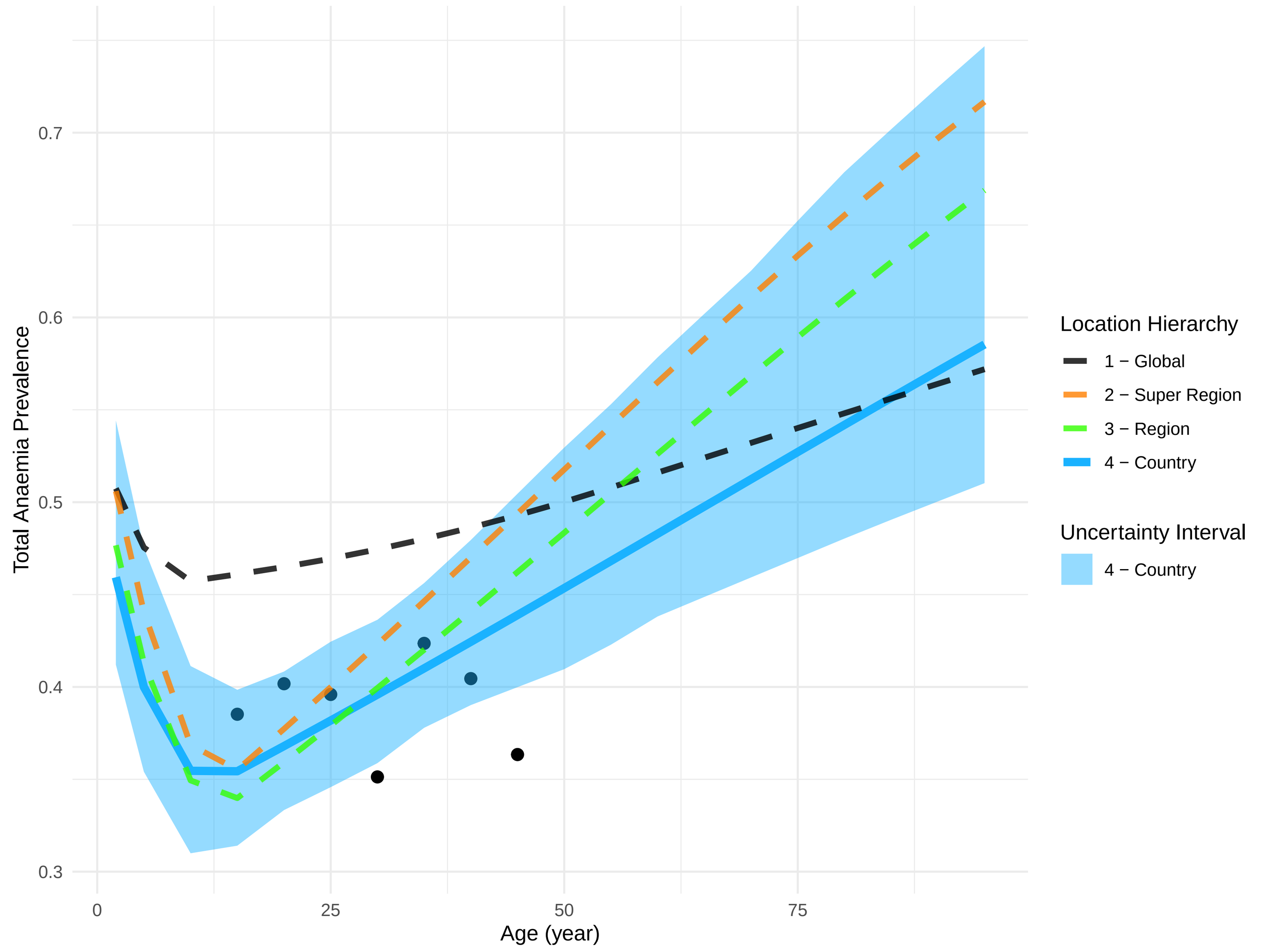

MR−BRT Age/Sex Fits − Non−pregnant Females 2+ Years Old − Total Anaemia Prevalence − Southeast Asia
WHO−2024 Adjustment Method

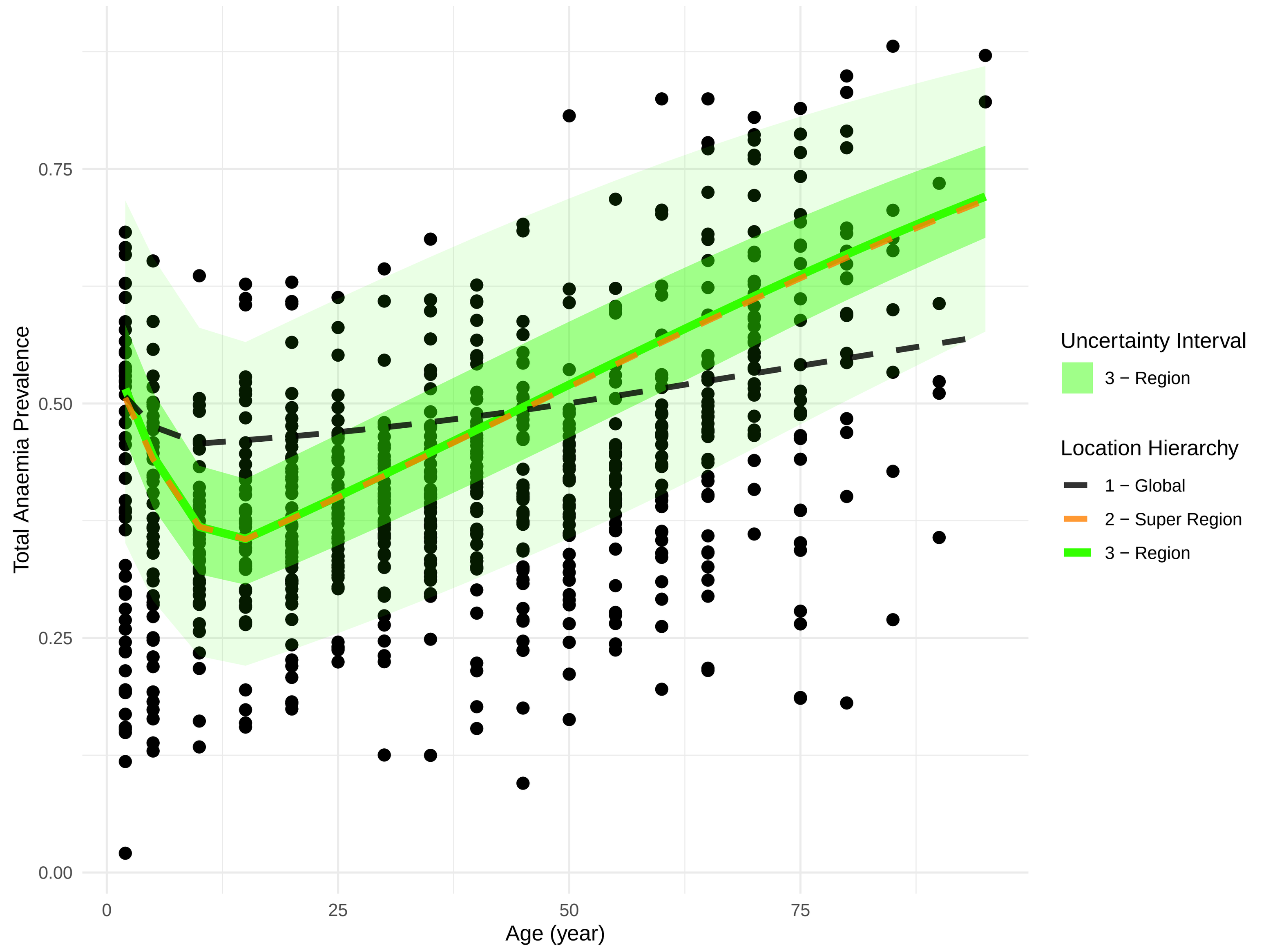

MR−BRT Age/Sex Fits − Non−pregnant Females 2+ Years Old − Total Anaemia Prevalence − Cambodia
WHO−2024 Adjustment Method

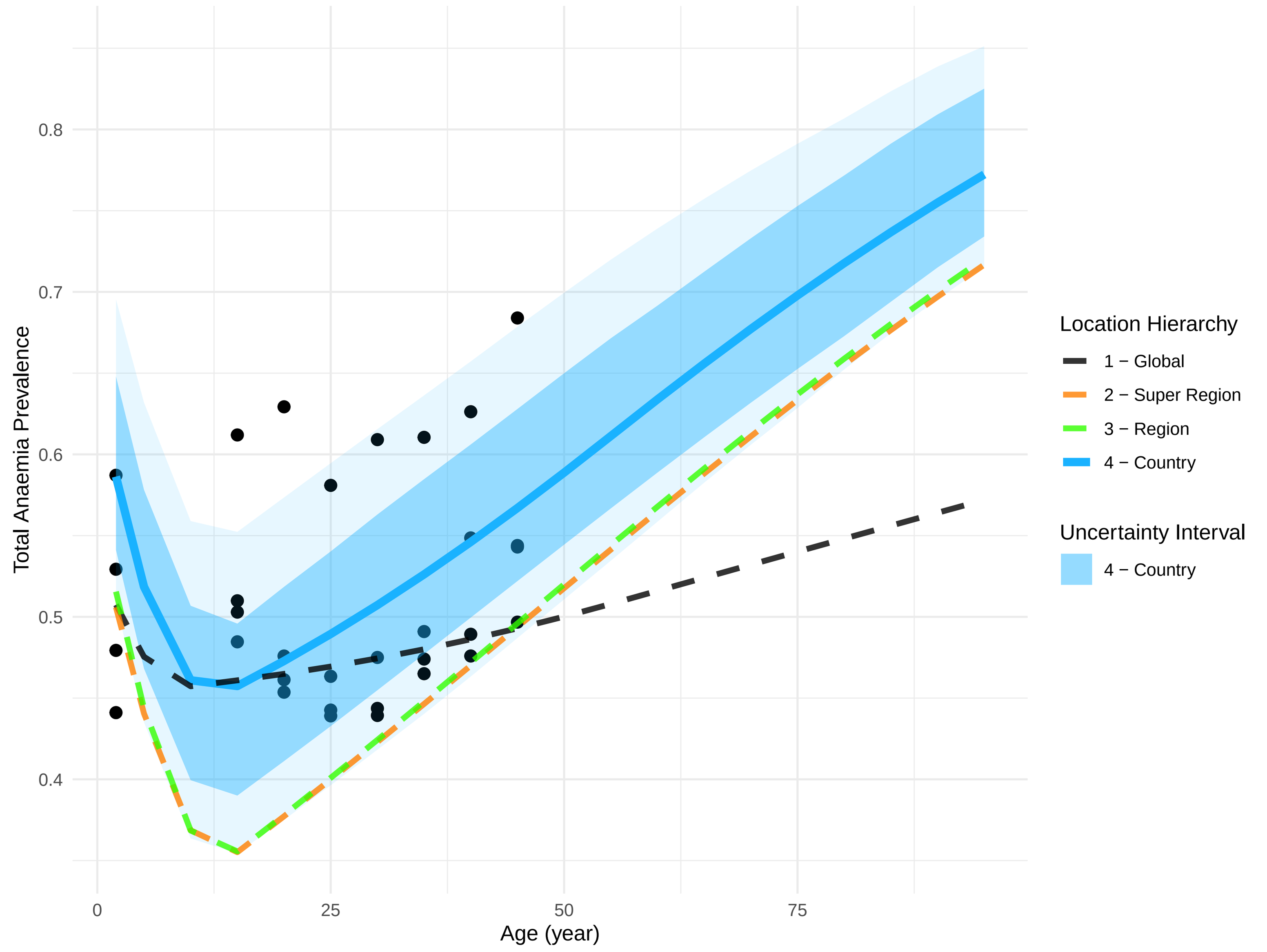

MR−BRT Age/Sex Fits − Non−pregnant Females 2+ Years Old − Total Anaemia Prevalence − Indonesia
WHO−2024 Adjustment Method

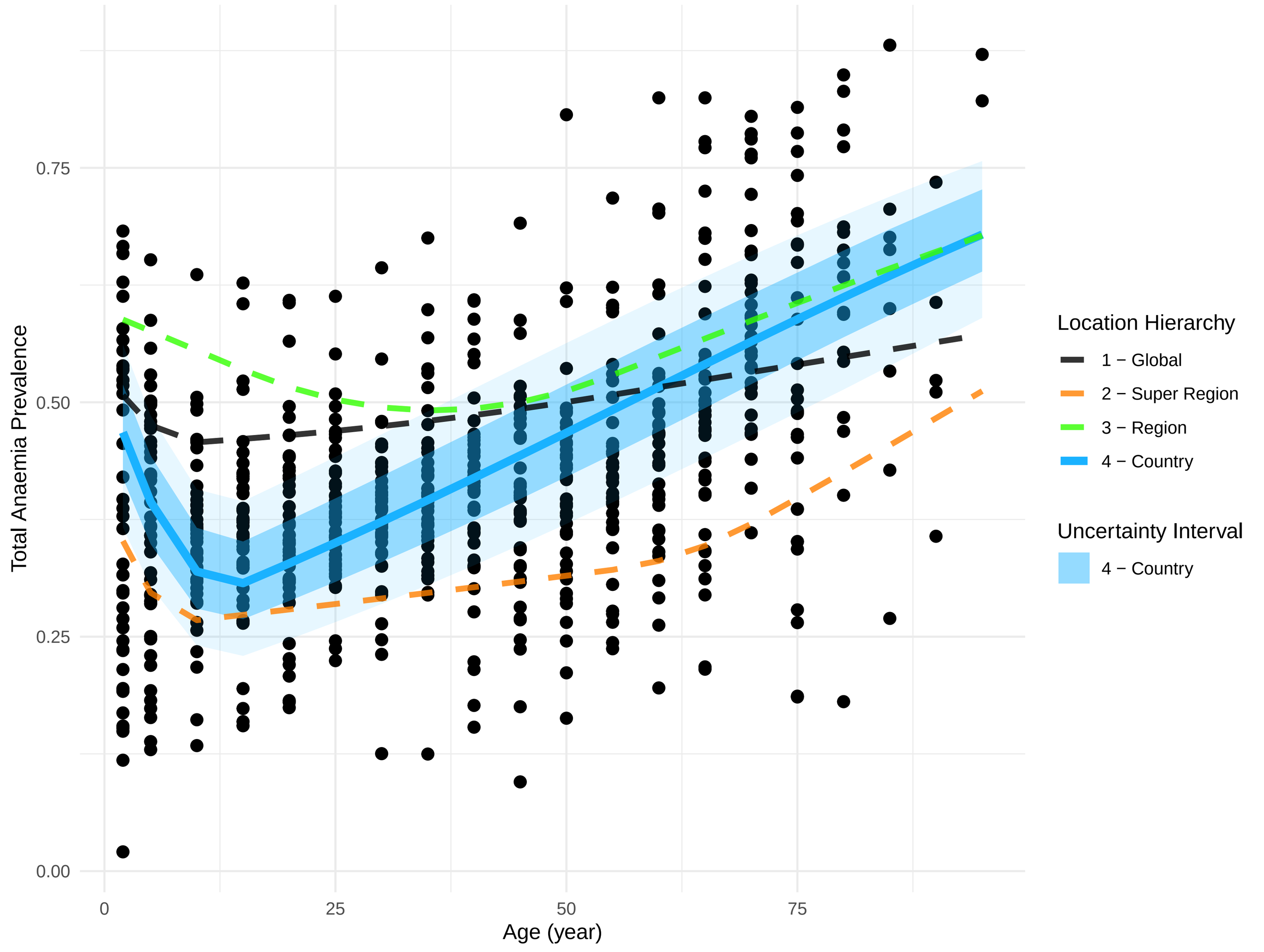

MR−BRT Age/Sex Fits − Non−pregnant Females 2+ Years Old − Total Anaemia Prevalence − Bali
WHO−2024 Adjustment Method

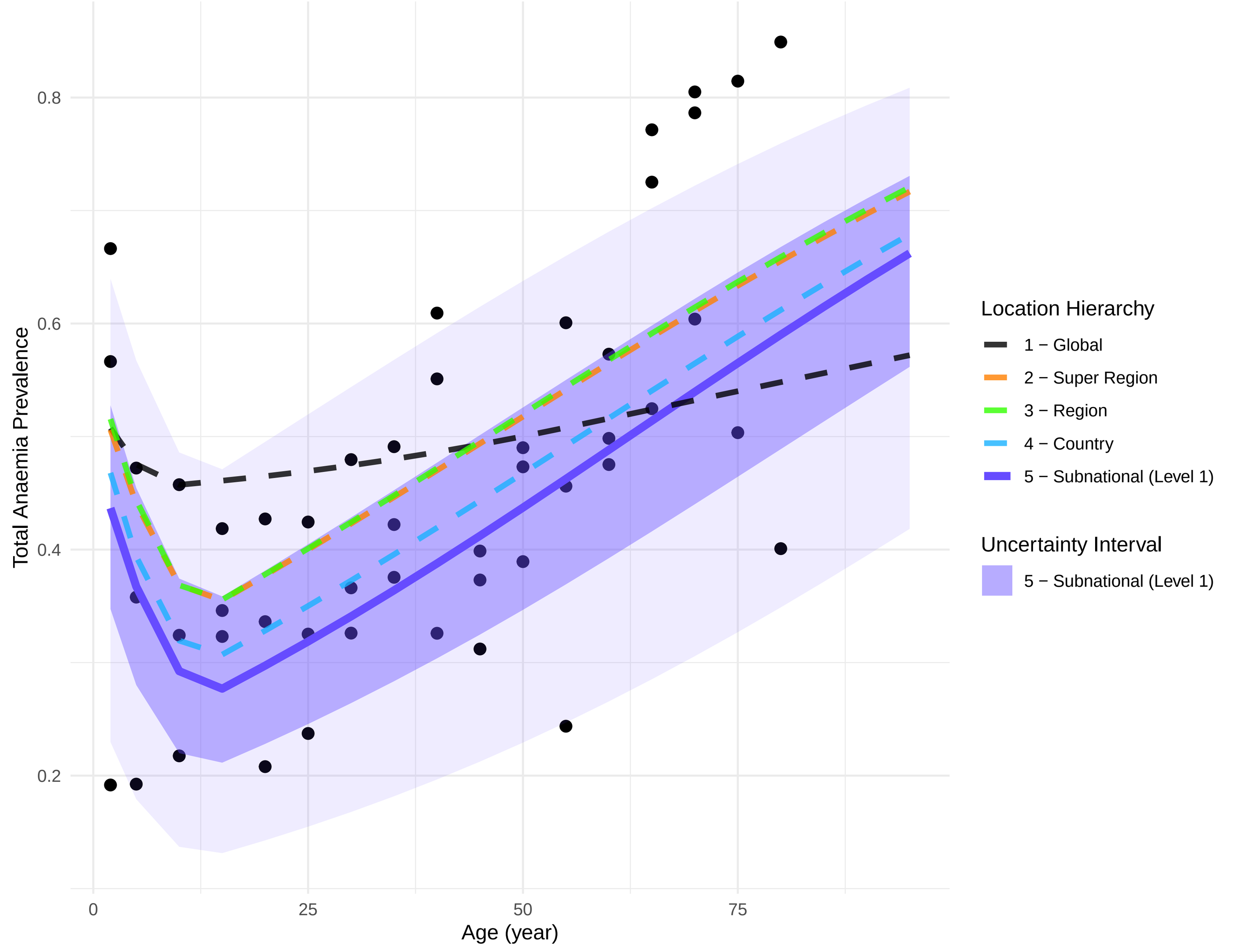

MR−BRT Age/Sex Fits − Non−pregnant Females 2+ Years Old − Total Anaemia Prevalence − Bangka−Belitung Islands
WHO−2024 Adjustment Method

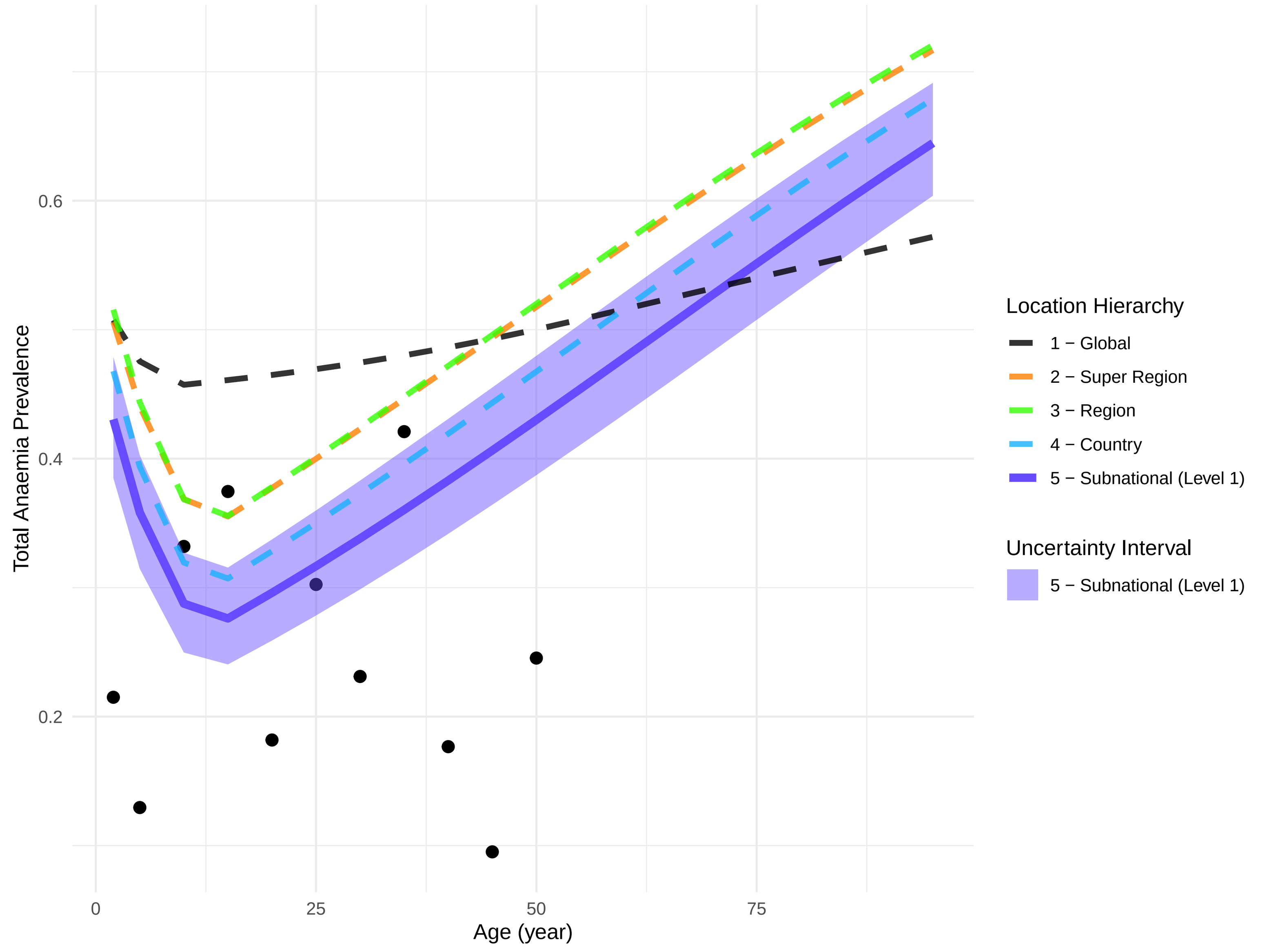

MR−BRT Age/Sex Fits − Non−pregnant Females 2+ Years Old − Total Anaemia Prevalence − Banten
WHO−2024 Adjustment Method

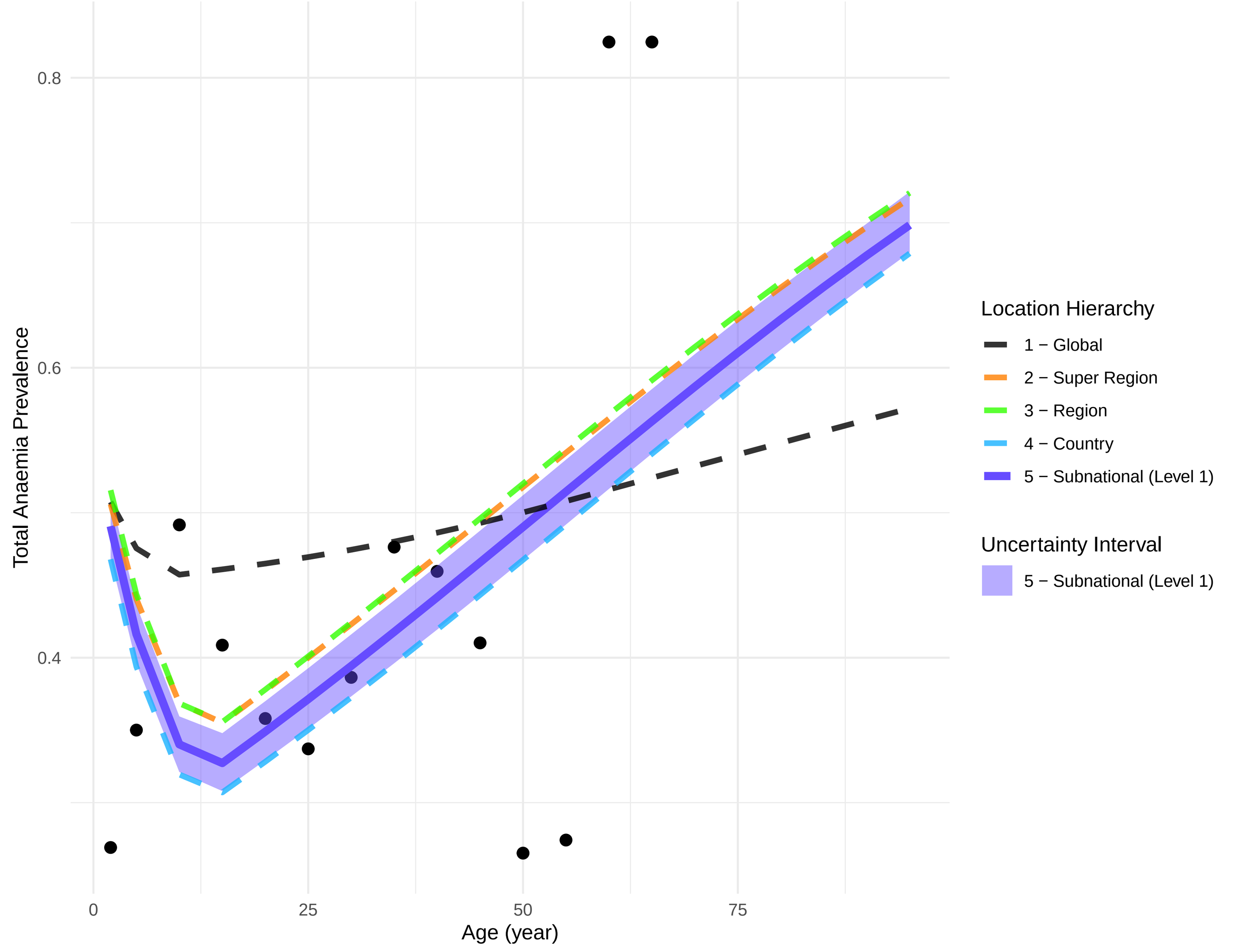

MR−BRT Age/Sex Fits − Non−pregnant Females 2+ Years Old − Total Anaemia Prevalence − Jakarta
WHO−2024 Adjustment Method

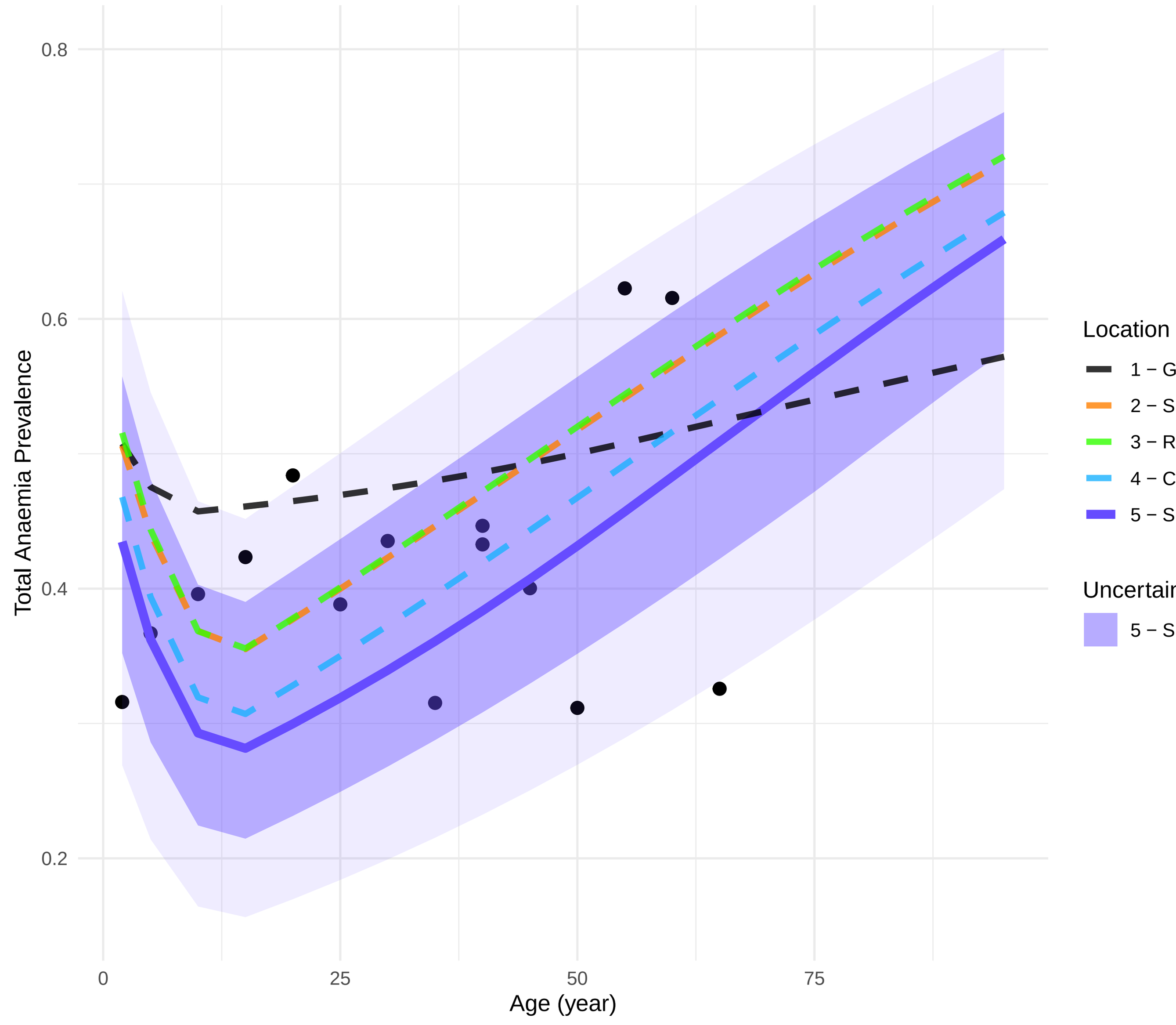

MR−BRT Age/Sex Fits − Non−pregnant Females 2+ Years Old − Total Anaemia Prevalence − West Java

WHO−2024 Adjustment Method

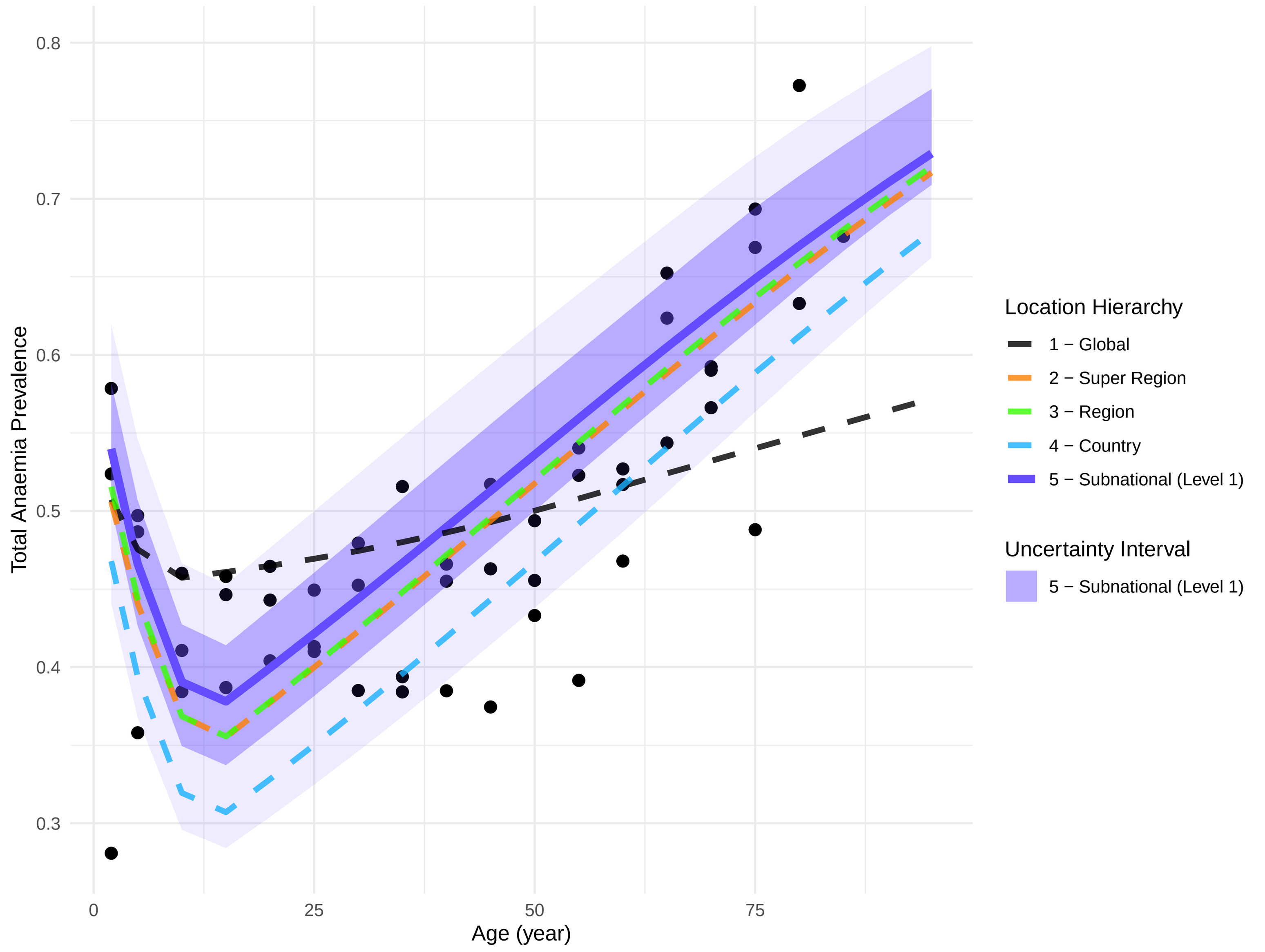

MR−BRT Age/Sex Fits − Non−pregnant Females 2+ Years Old − Total Anaemia Prevalence − Central Java

WHO−2024 Adjustment Method

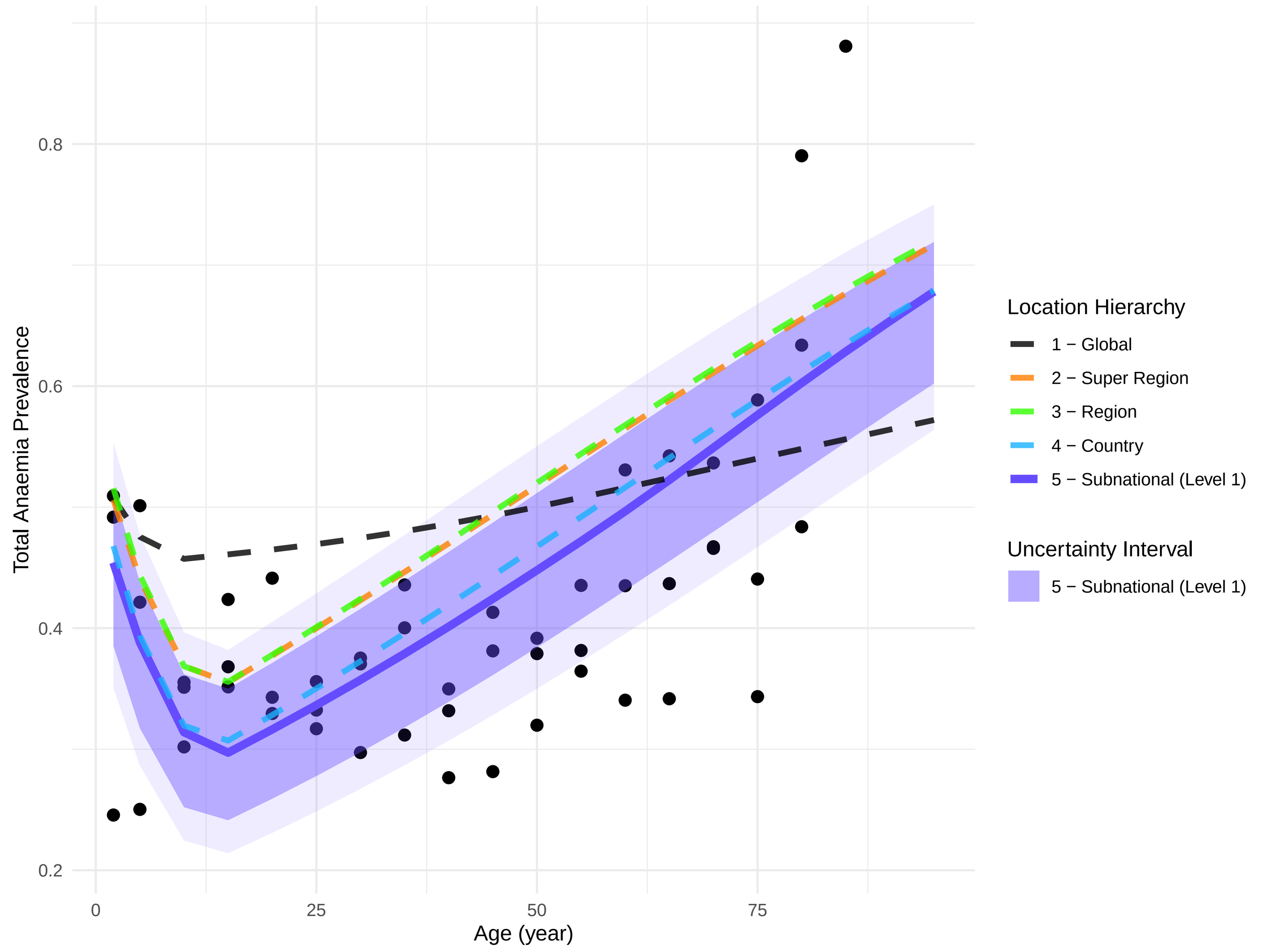

MR−BRT Age/Sex Fits − Non−pregnant Females 2+ Years Old − Total Anaemia Prevalence − East Java

WHO−2024 Adjustment Method

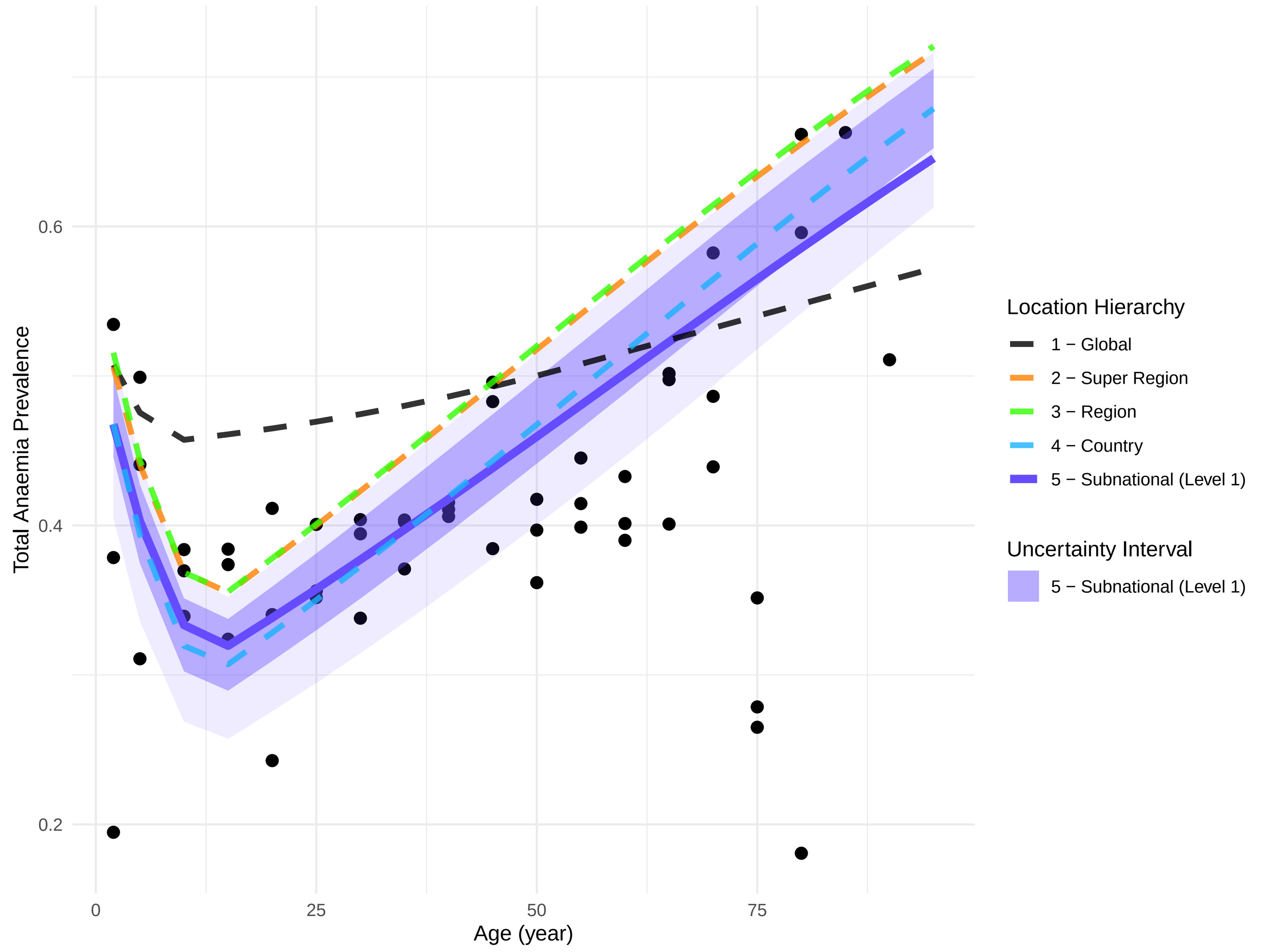

MR−BRT Age/Sex Fits − Non−pregnant Females 2+ Years Old − Total Anaemia Prevalence − South Kalimantan

WHO−2024 Adjustment Method

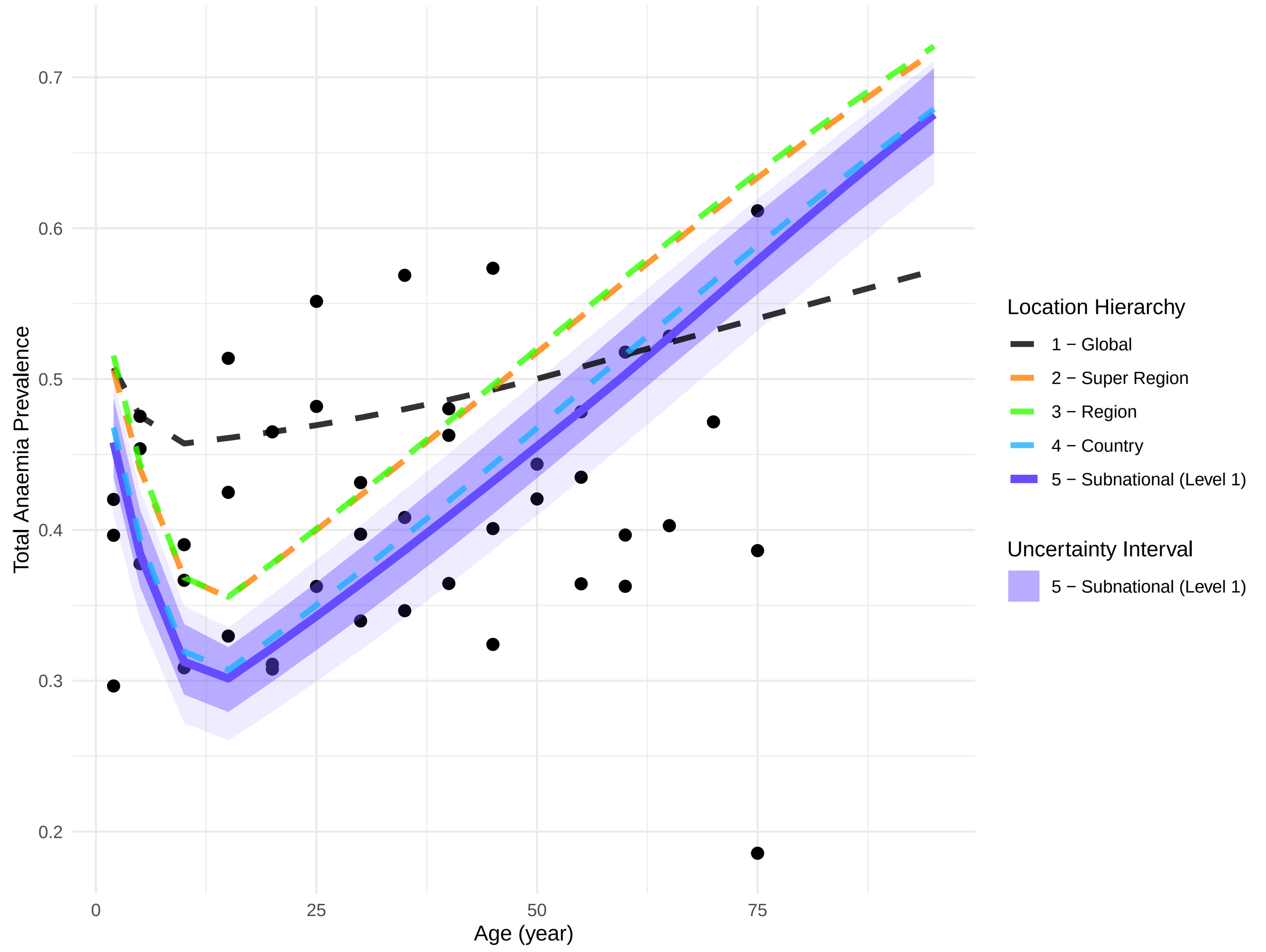

MR−BRT Age/Sex Fits − Non−pregnant Females 2+ Years Old − Total Anaemia Prevalence − Central Kalimantan

WHO−2024 Adjustment Method

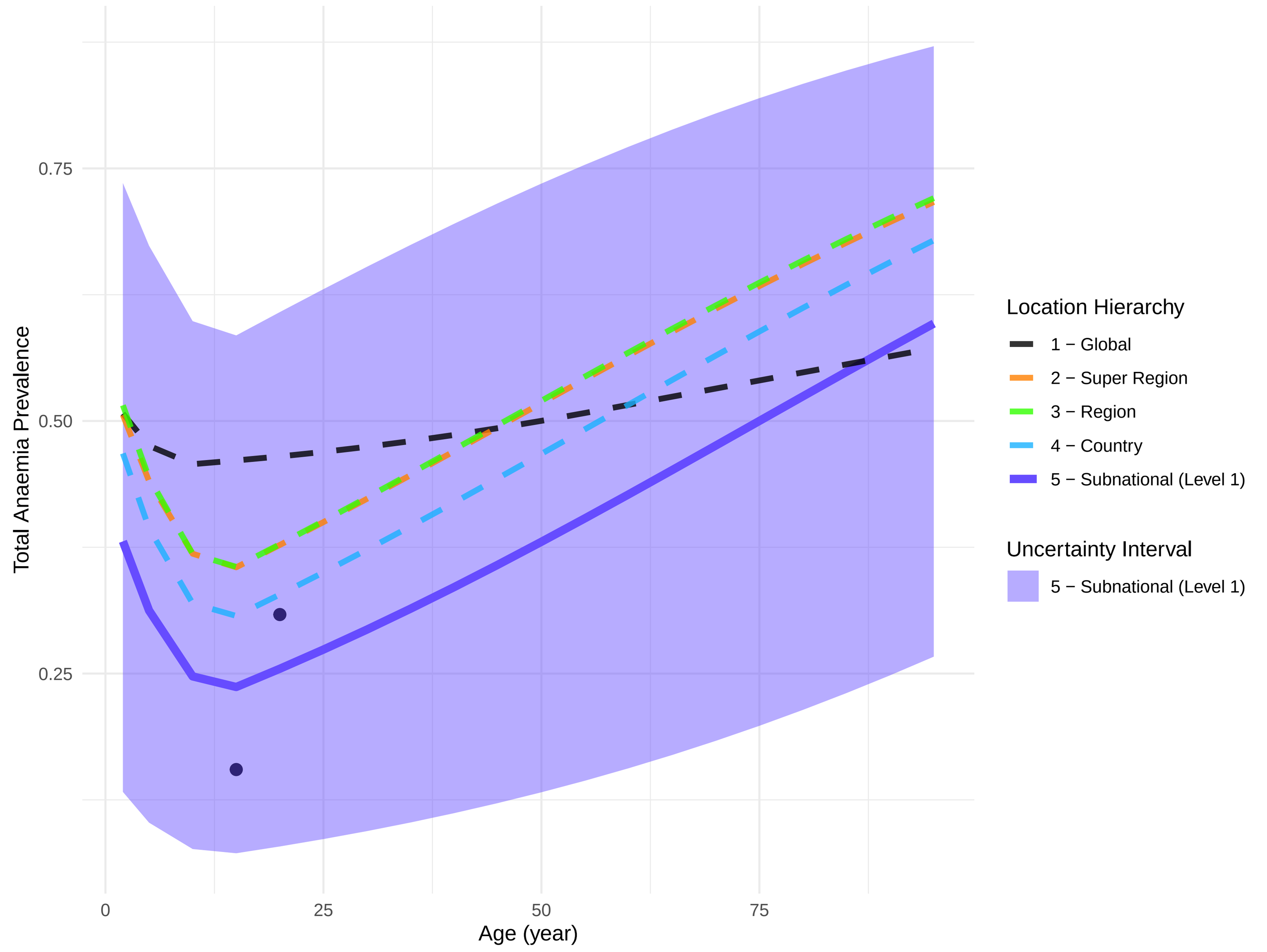

MR−BRT Age/Sex Fits − Non−pregnant Females 2+ Years Old − Total Anaemia Prevalence − East Kalimantan

WHO−2024 Adjustment Method

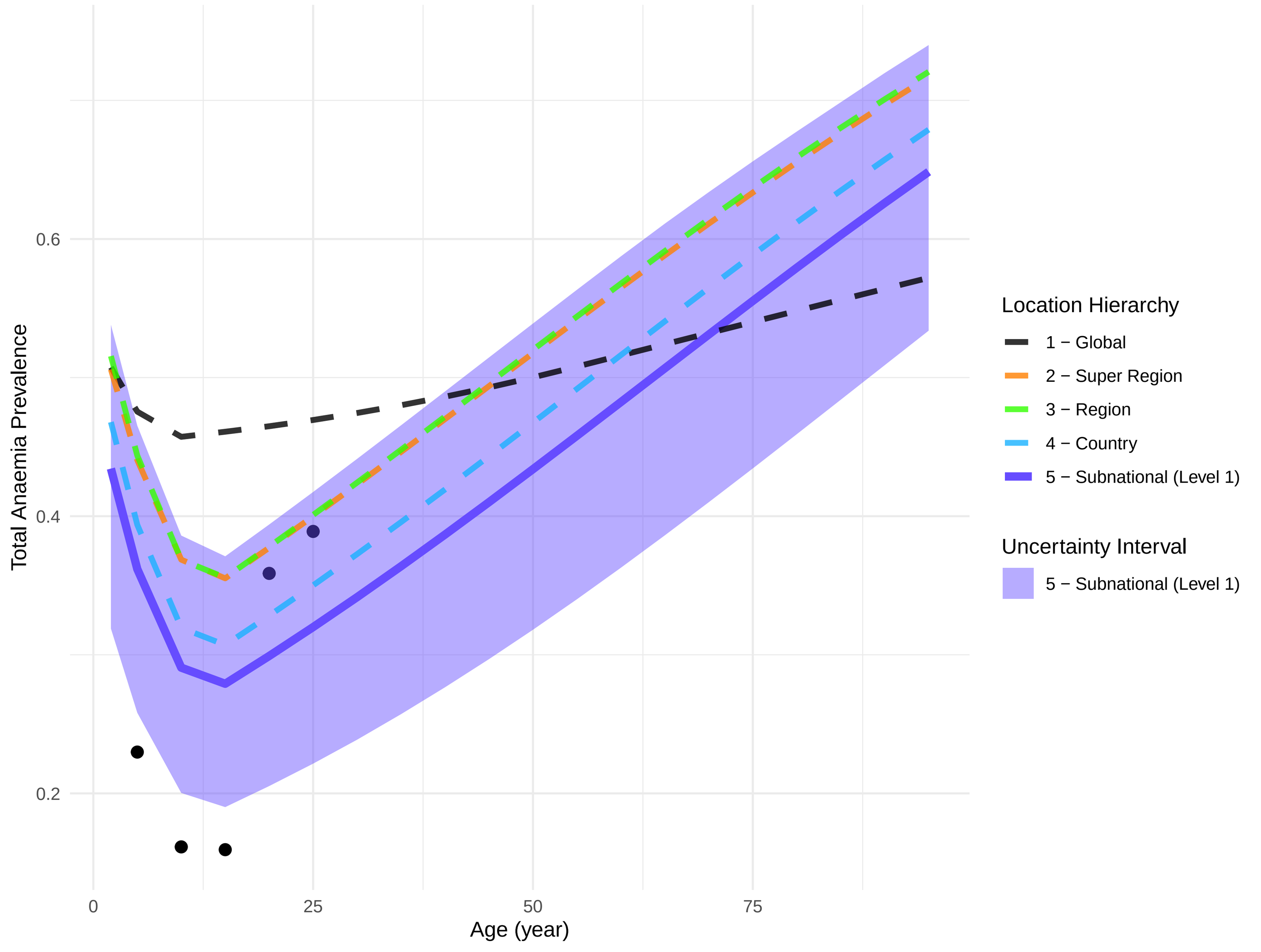

MR−BRT Age/Sex Fits − Non−pregnant Females 2+ Years Old − Total Anaemia Prevalence − Riau Islands

WHO−2024 Adjustment Method

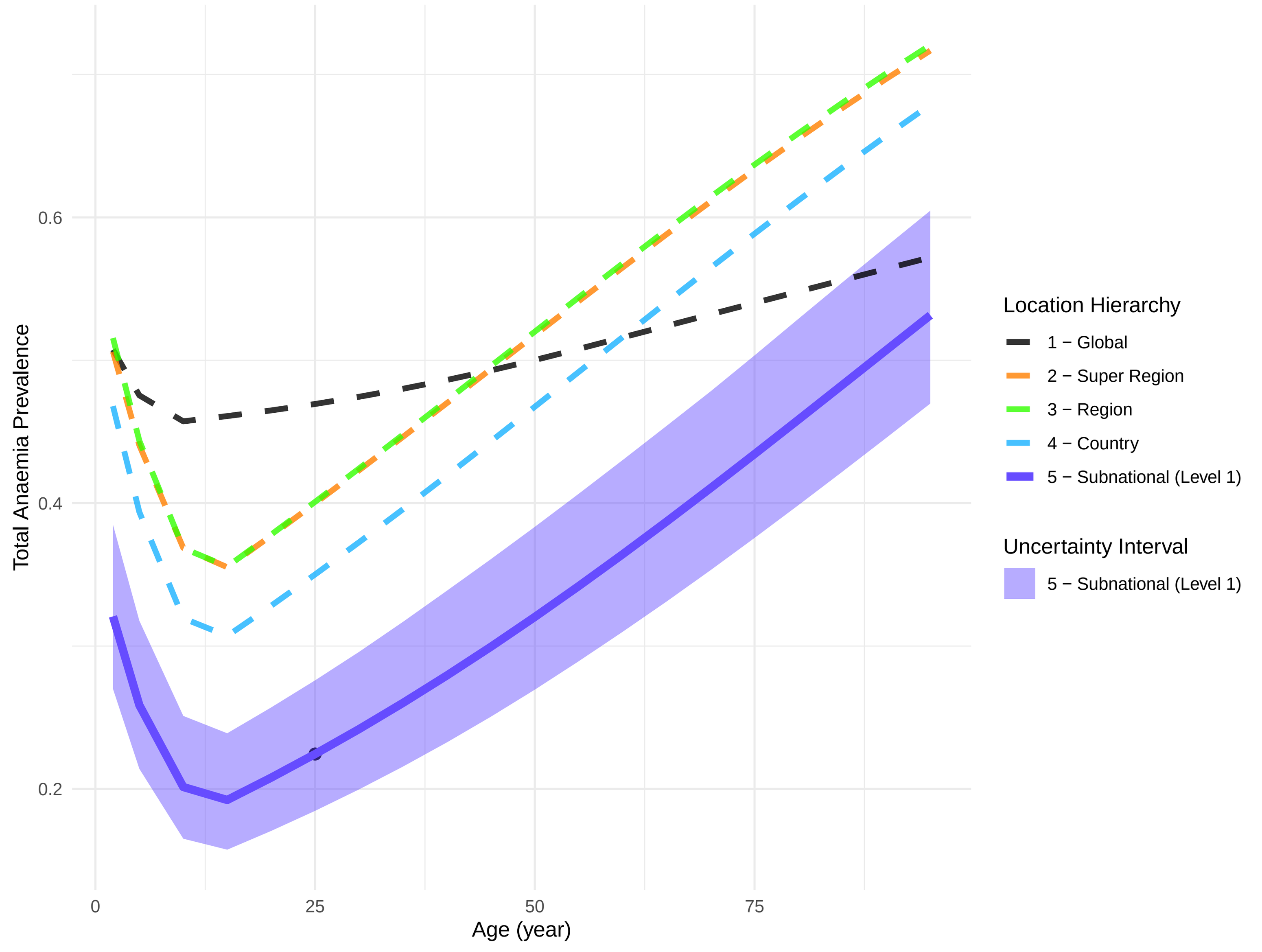

MR−BRT Age/Sex Fits − Non−pregnant Females 2+ Years Old − Total Anaemia Prevalence − Lampung

WHO−2024 Adjustment Method

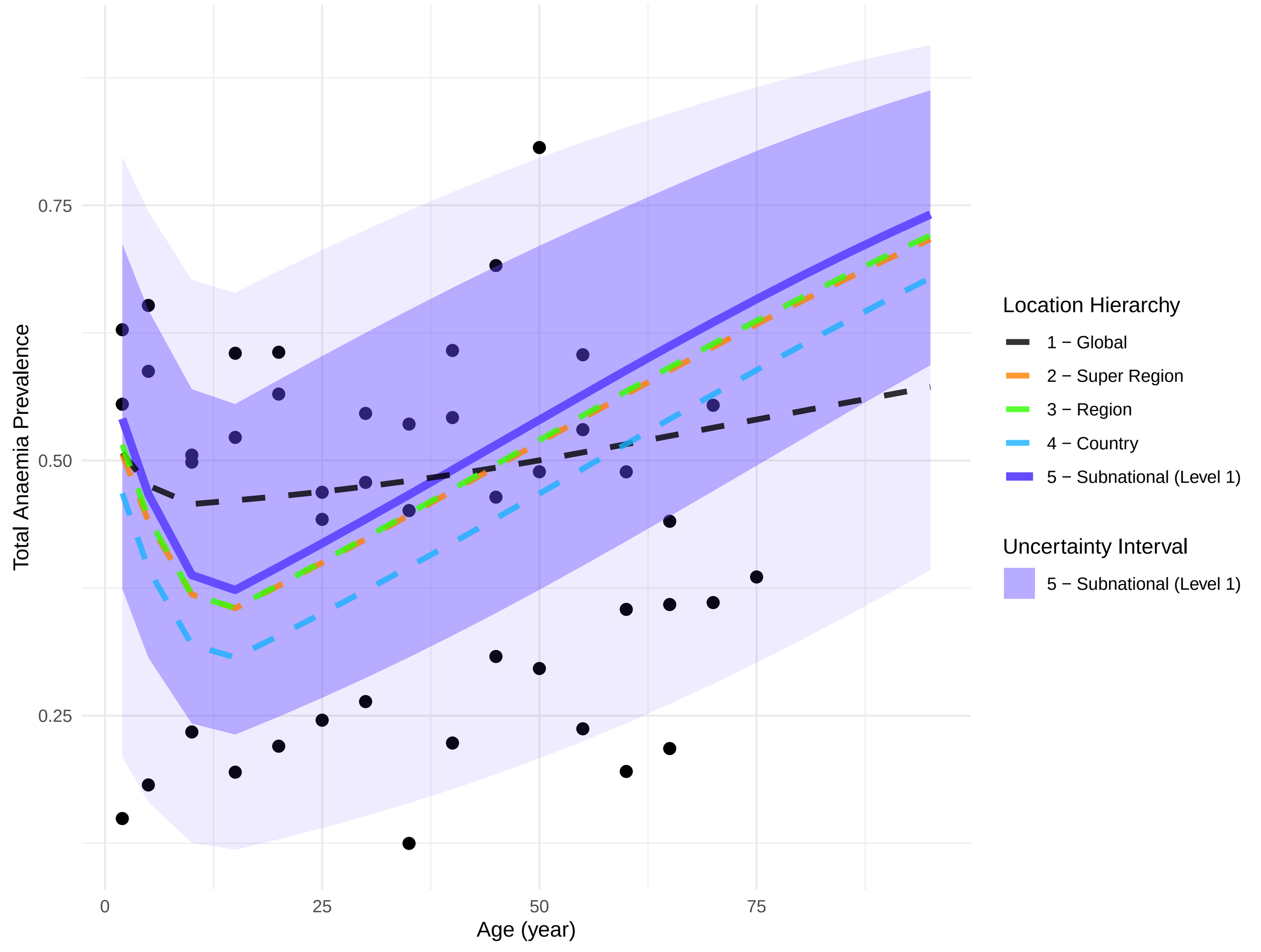

MR−BRT Age/Sex Fits − Non−pregnant Females 2+ Years Old − Total Anaemia Prevalence − West Nusa Tenggara

WHO−2024 Adjustment Method

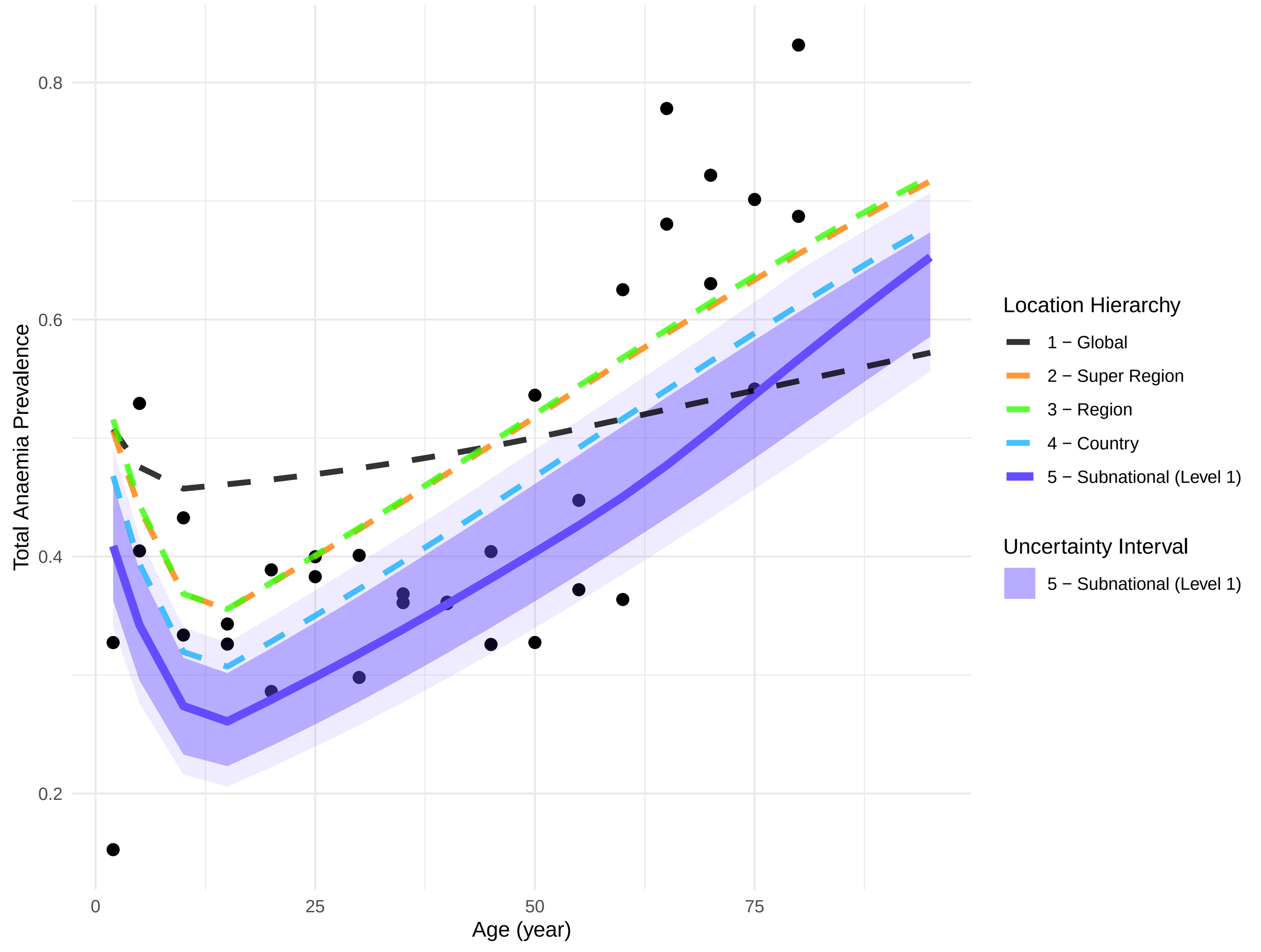

MR−BRT Age/Sex Fits − Non−pregnant Females 2+ Years Old − Total Anaemia Prevalence − Riau

WHO−2024 Adjustment Method

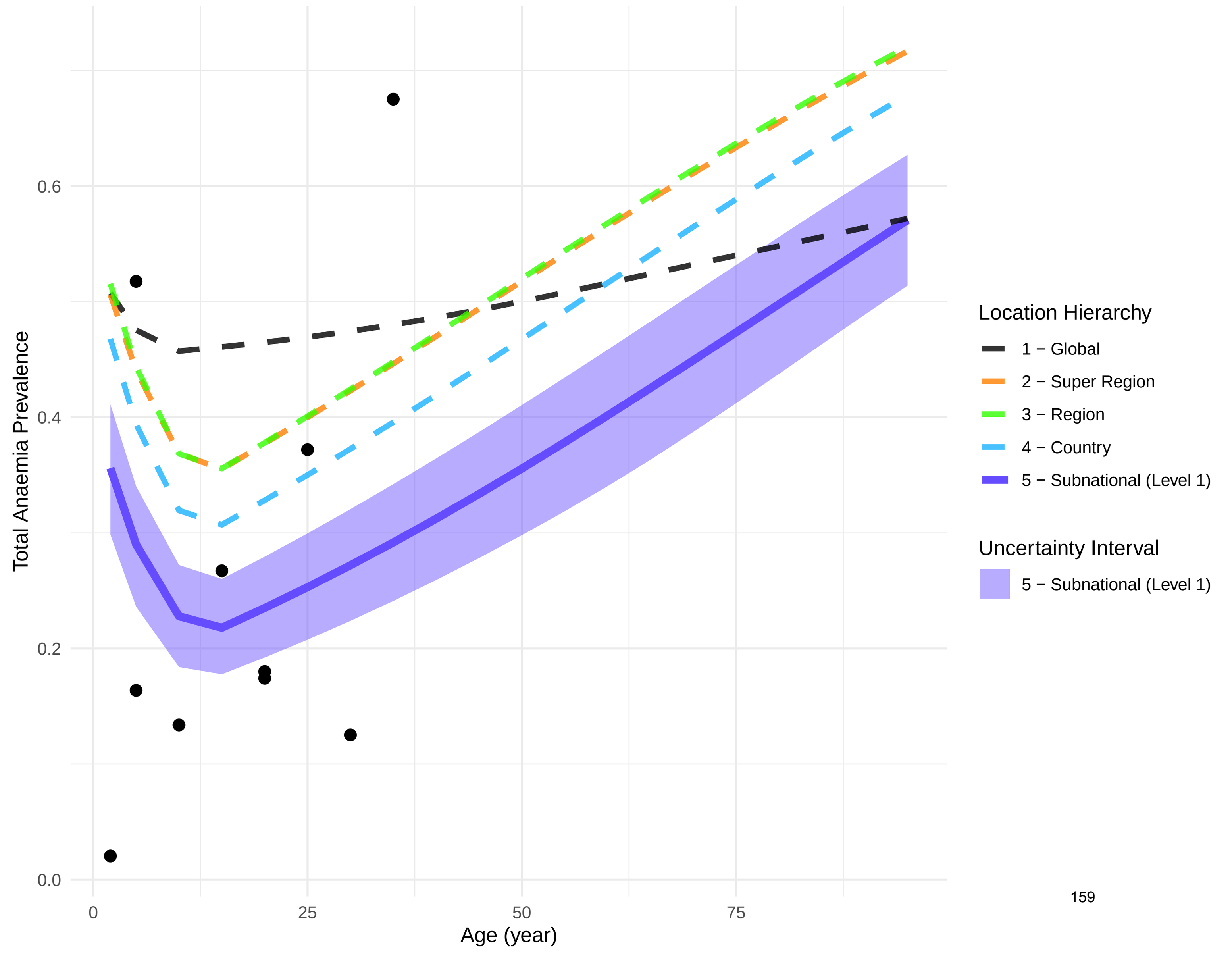

MR−BRT Age/Sex Fits − Non-pregnant Females 2+ Years Old − Total Anaemia Prevalence − West Sulawesi
WHO−2024 Adjustment Method

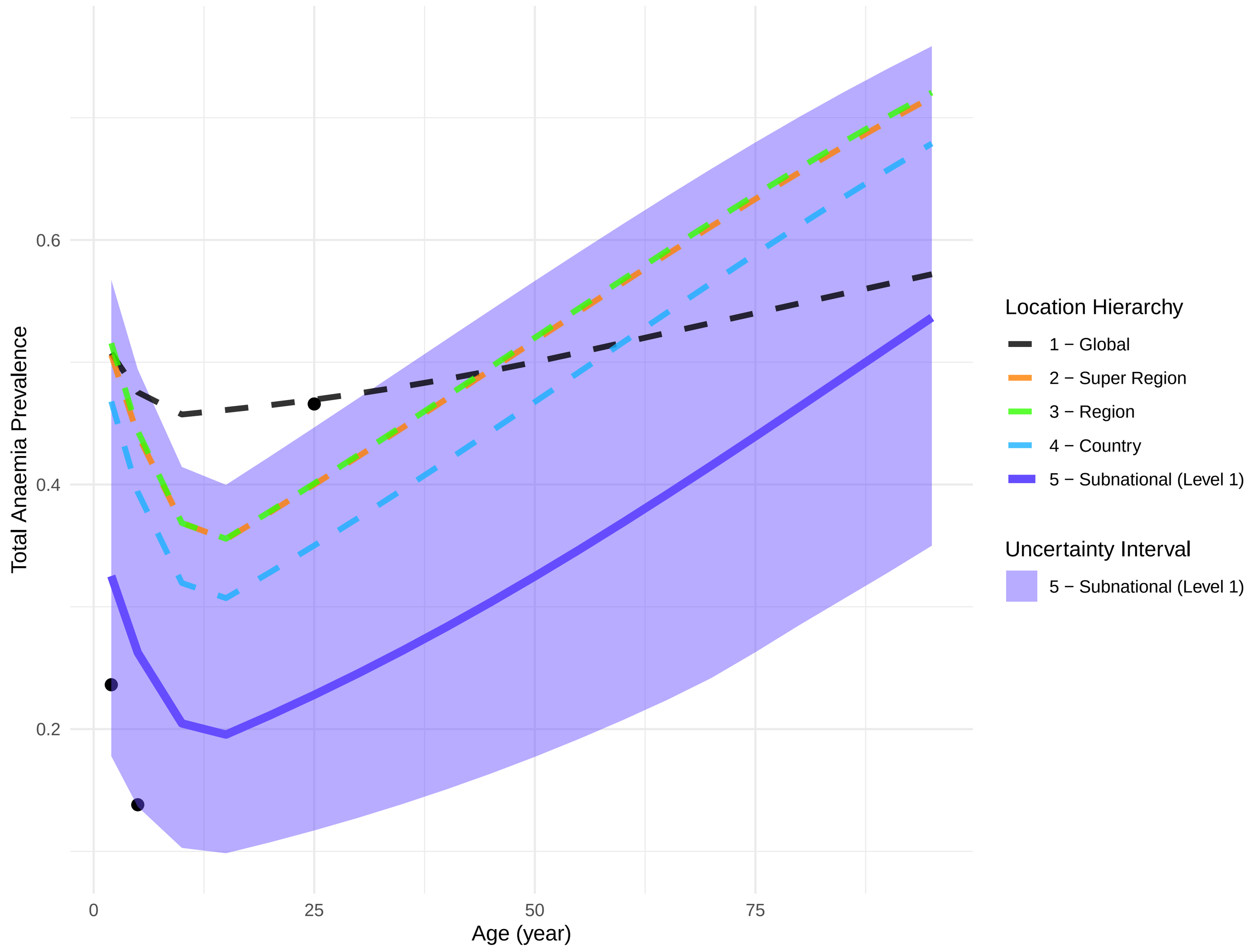

MR−BRT Age/Sex Fits − Non-pregnant Females 2+ Years Old − Total Anaemia Prevalence − South Sulawesi
WHO−2024 Adjustment Method

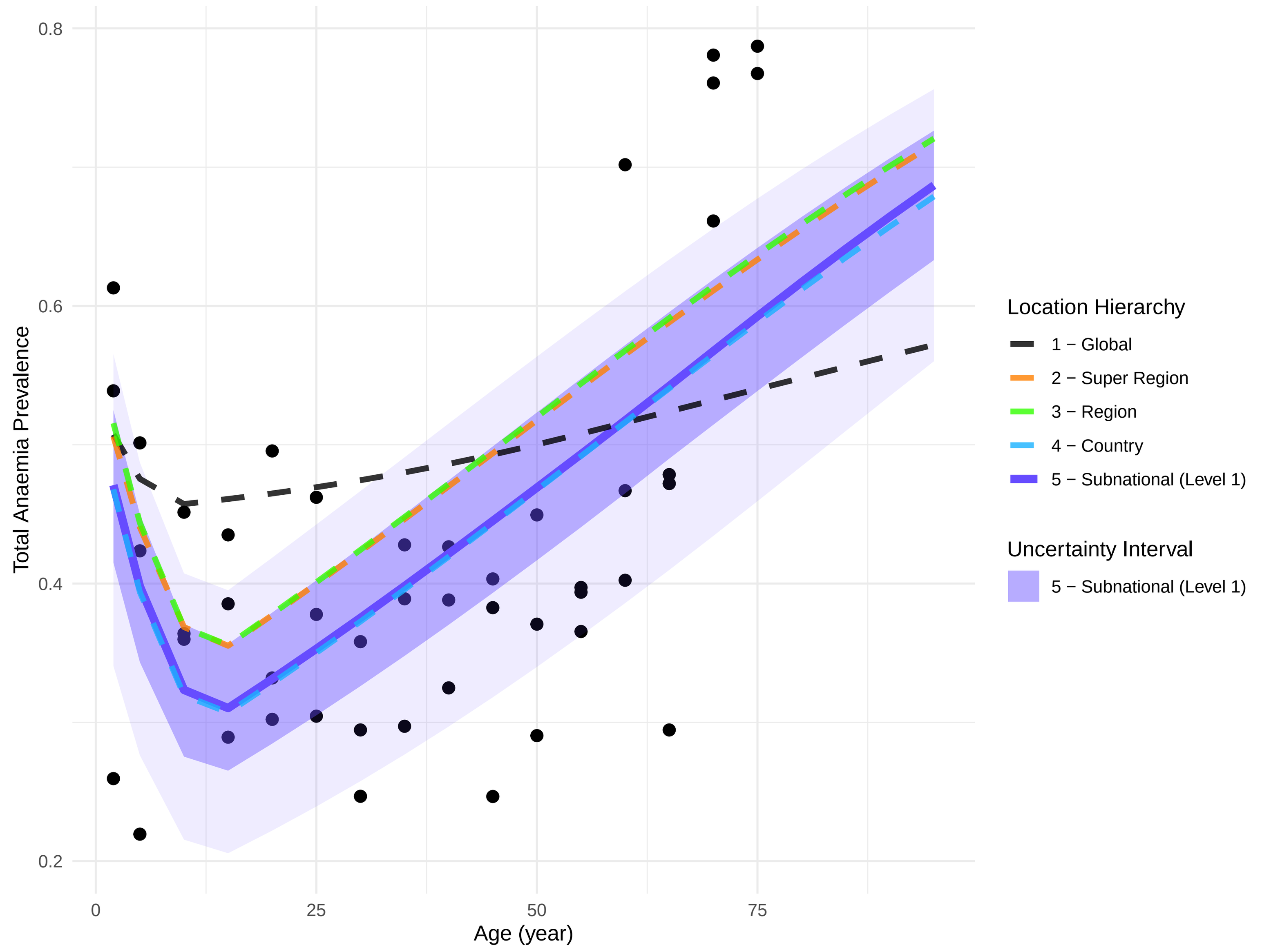

MR−BRT Age/Sex Fits − Non-pregnant Females 2+ Years Old − Total Anaemia Prevalence − West Sumatra
WHO−2024 Adjustment Method

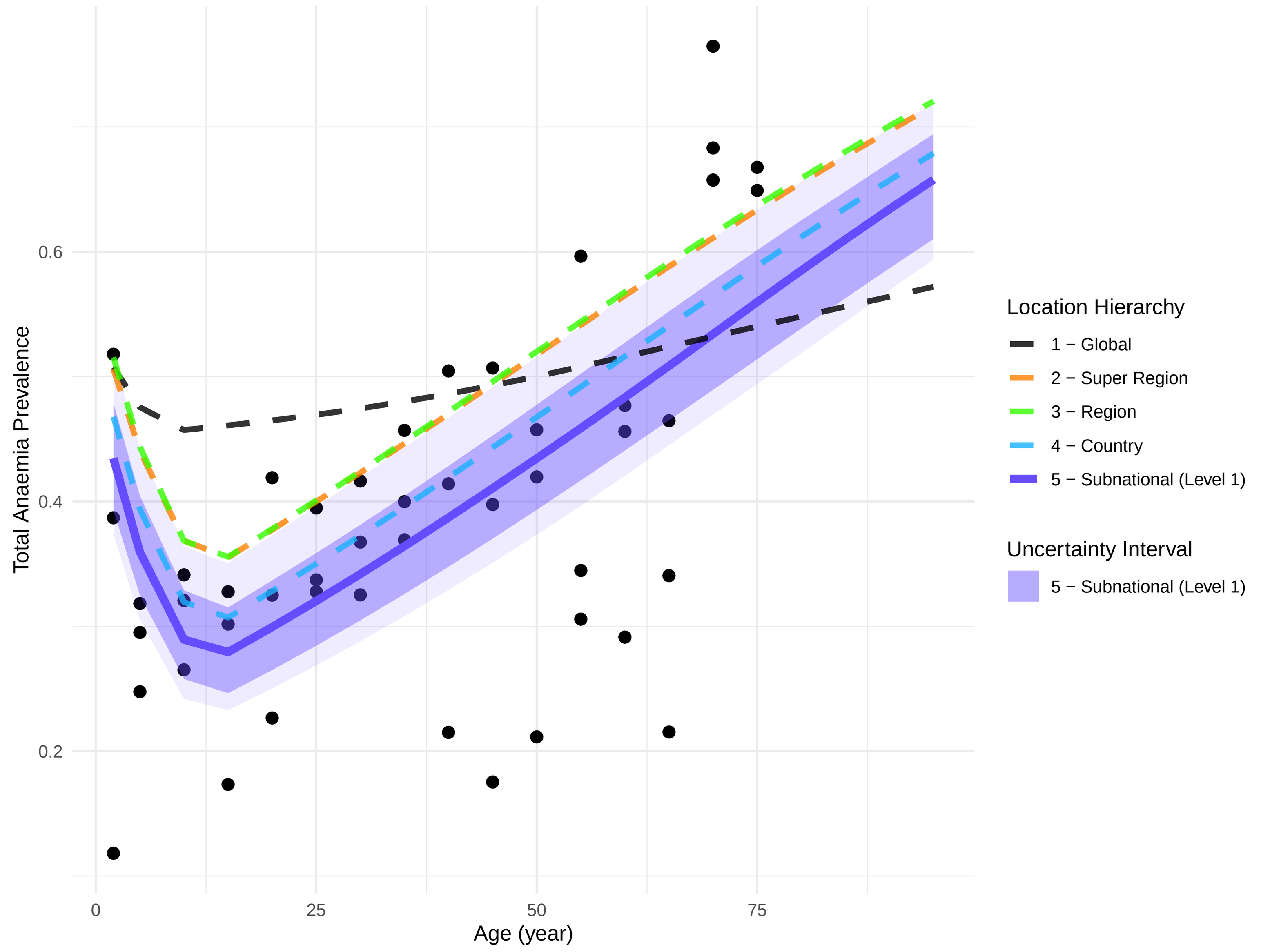

MR−BRT Age/Sex Fits − Non-pregnant Females 2+ Years Old − Total Anaemia Prevalence − South Sumatra
WHO−2024 Adjustment Method

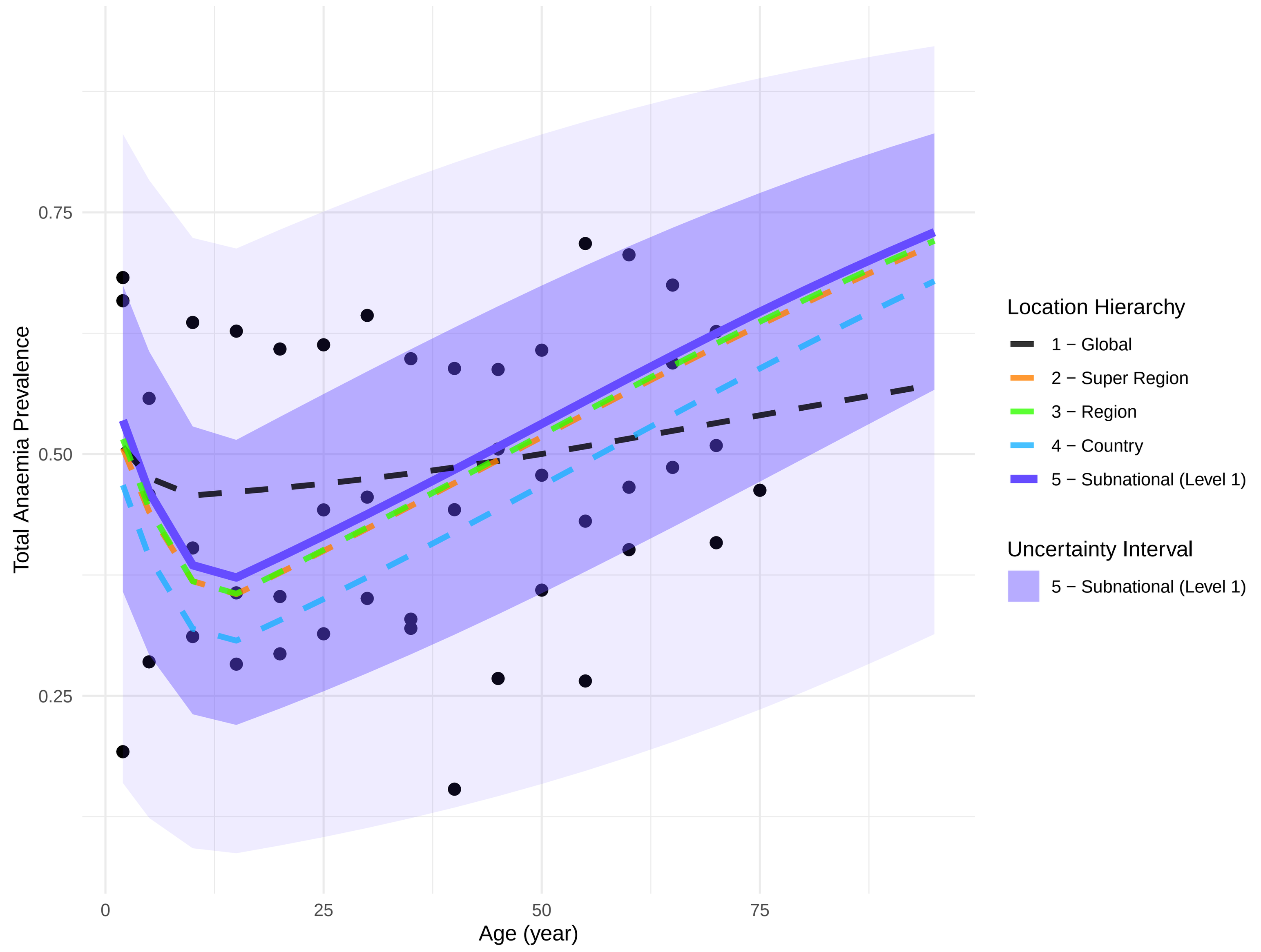

MR−BRT Age/Sex Fits − Non-pregnant Females 2+ Years Old − Total Anaemia Prevalence − North Sumatra
WHO−2024 Adjustment Method

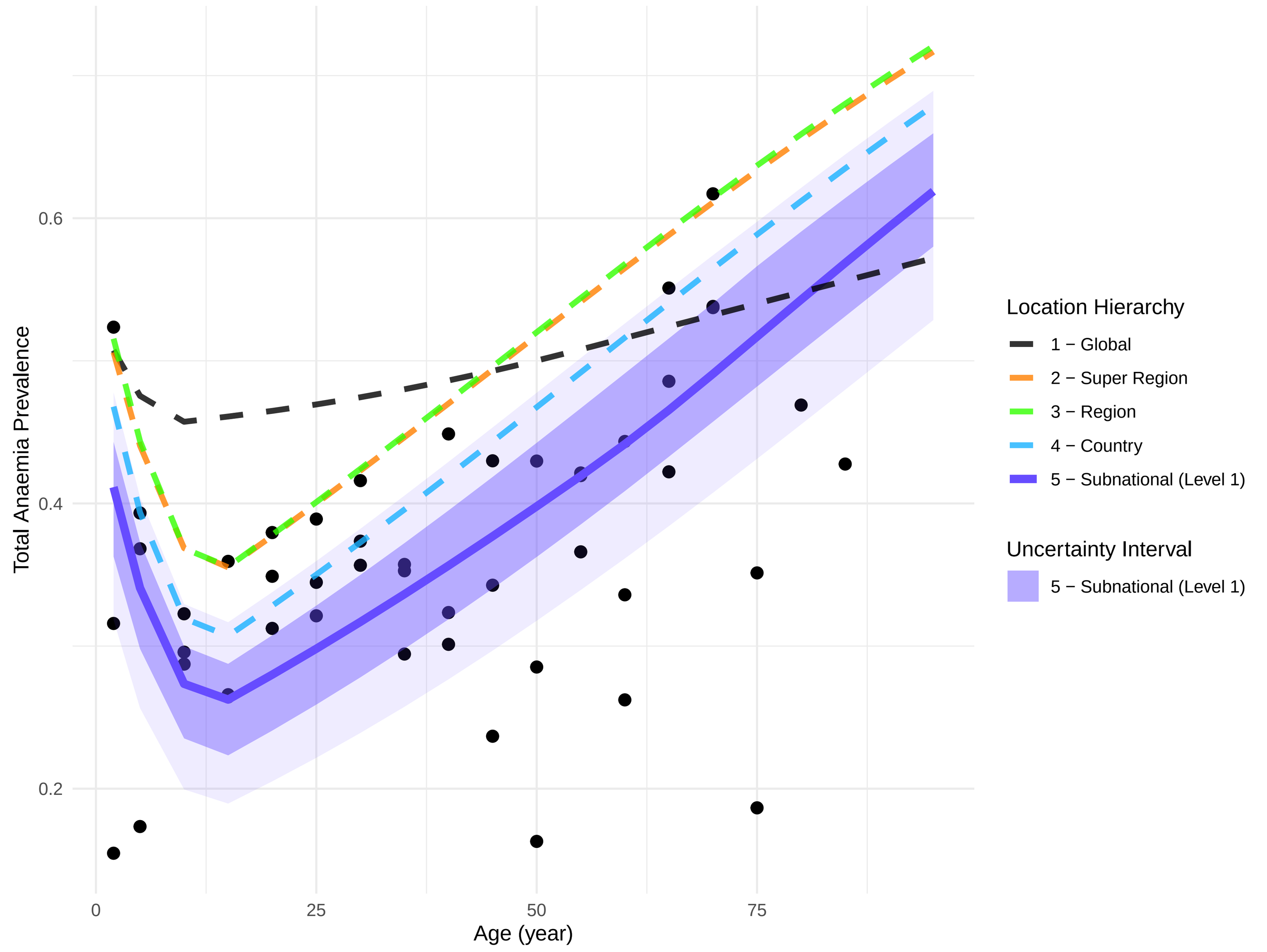

MR−BRT Age/Sex Fits − Non-pregnant Females 2+ Years Old − Total Anaemia Prevalence − Yogyakarta
WHO−2024 Adjustment Method

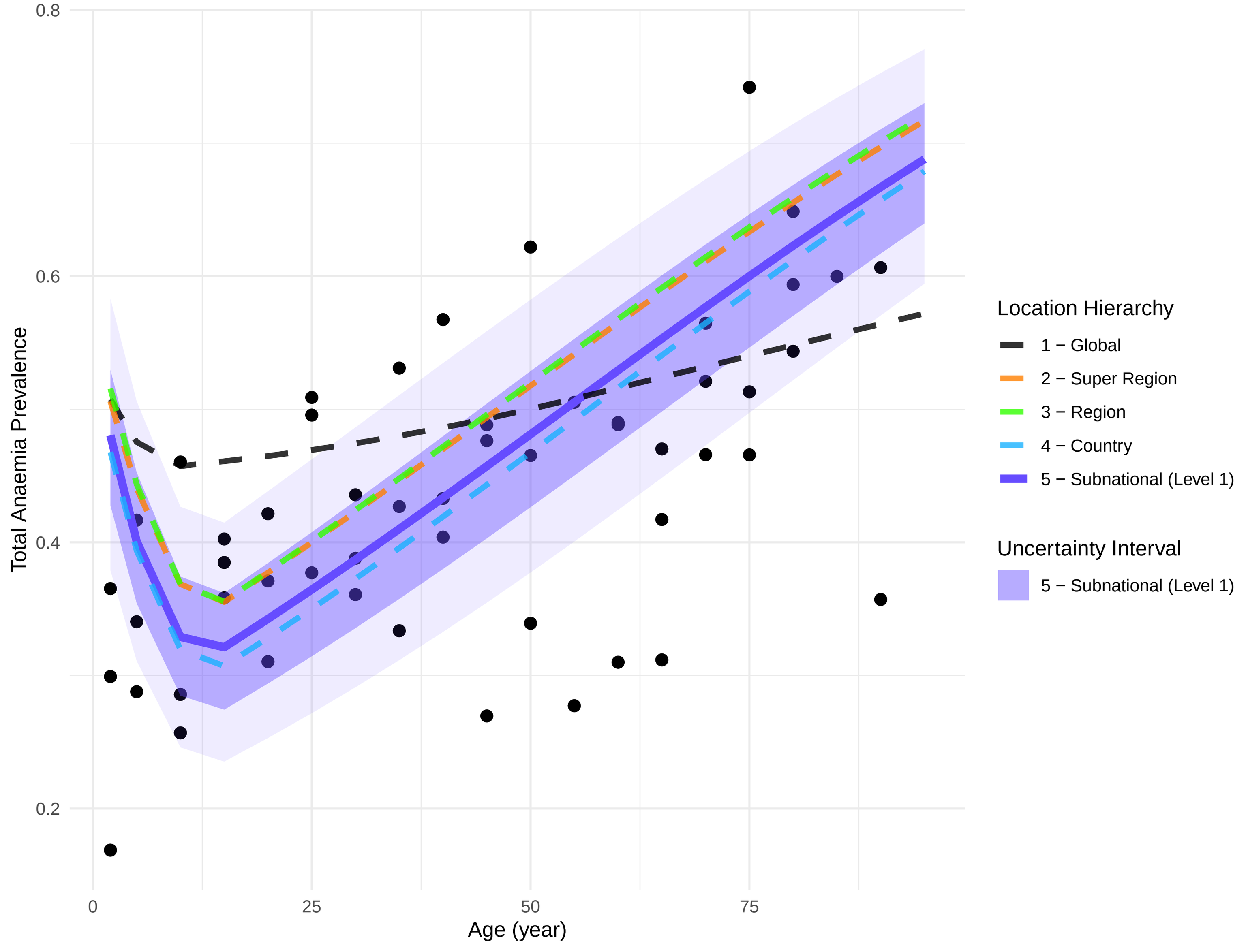

MR−BRT Age/Sex Fits − Non-pregnant Females 2+ Years Old − Total Anaemia Prevalence − Myanmar
WHO−2024 Adjustment Method

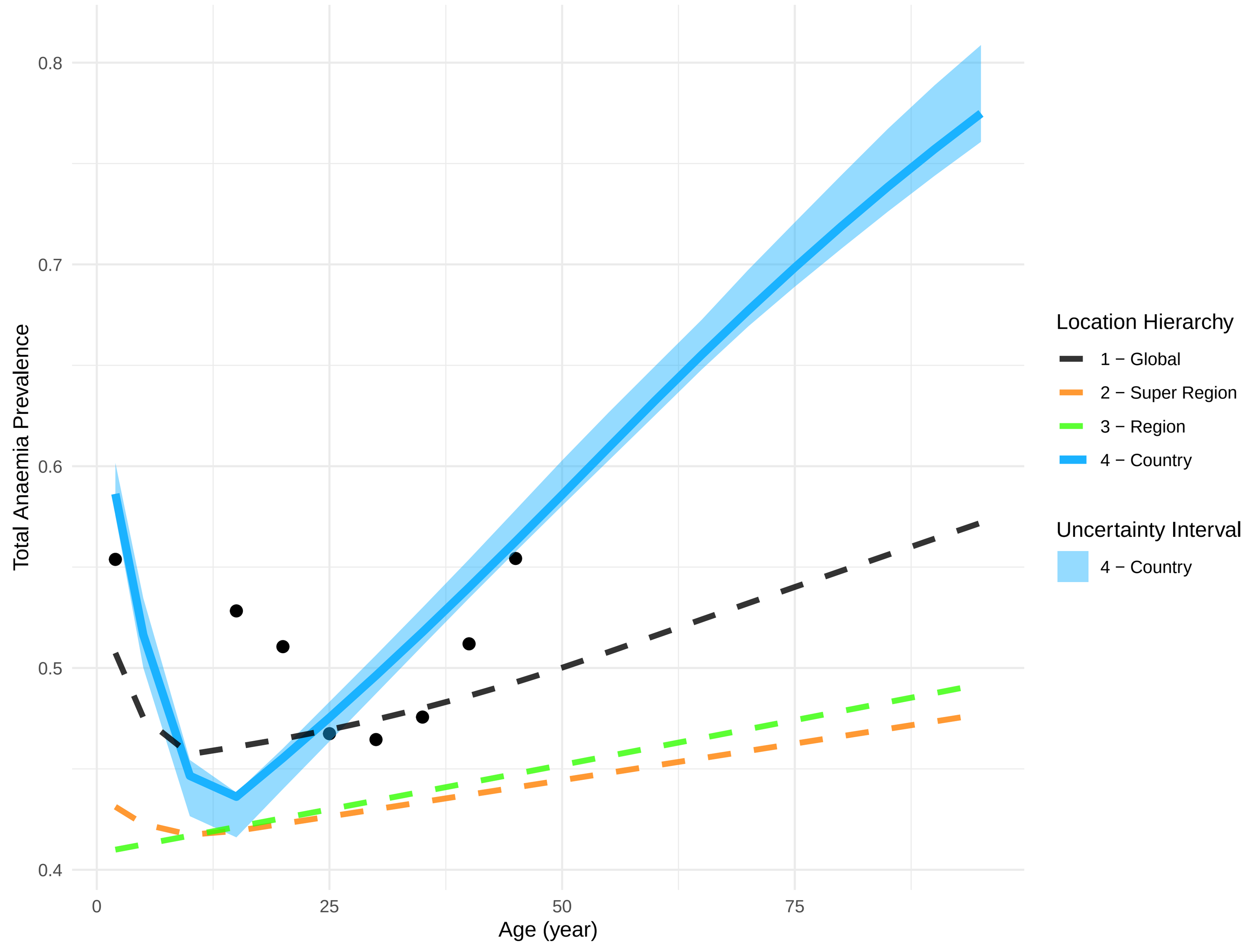

MR−BRT Age/Sex Fits − Non-pregnant Females 2+ Years Old − Total Anaemia Prevalence − Timor-Leste
WHO−2024 Adjustment Method

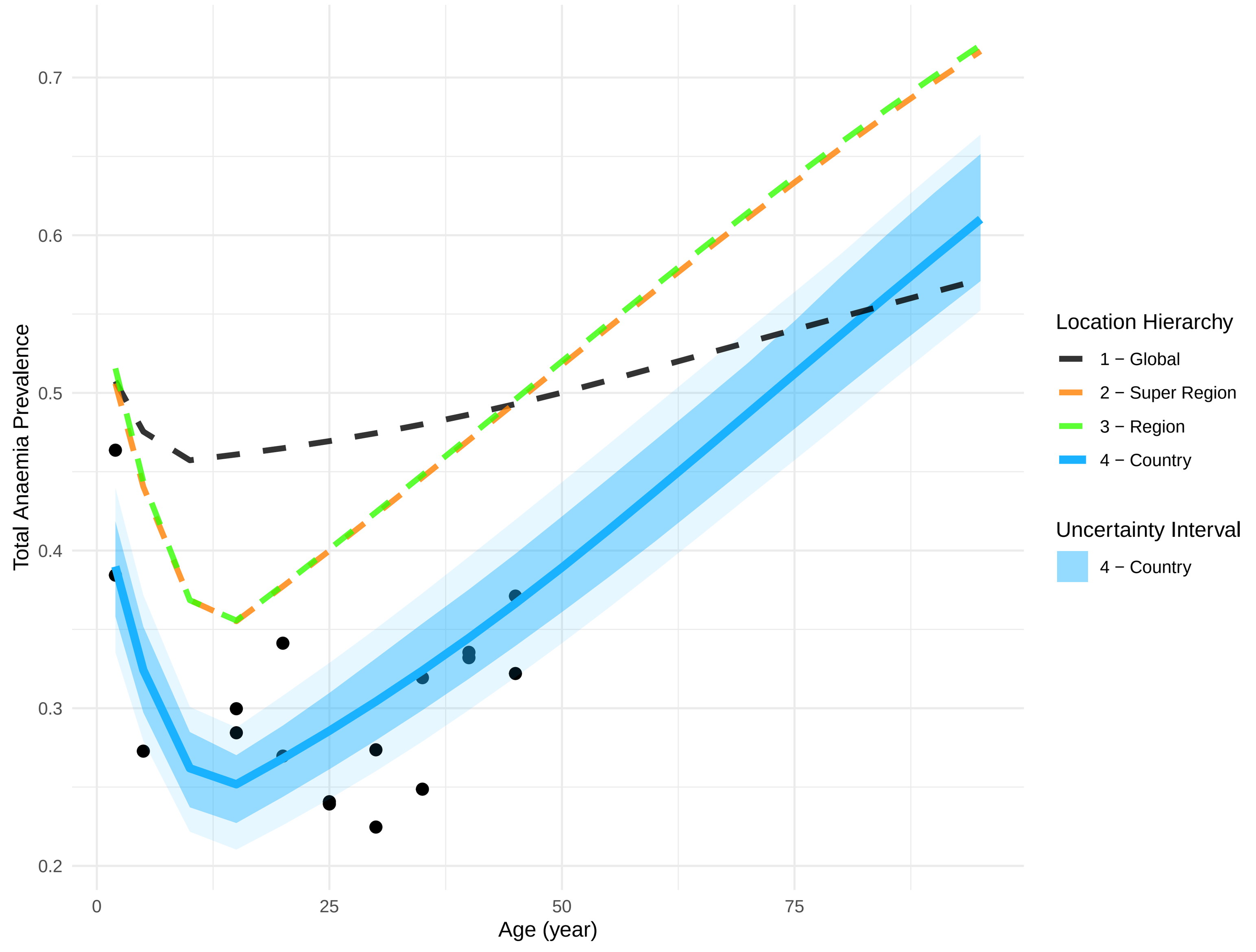

MR−BRT Age/Sex Fits − Non-pregnant Females 2+ Years Old − Total Anaemia Prevalence − Sub-Saharan Africa
WHO−2024 Adjustment Method

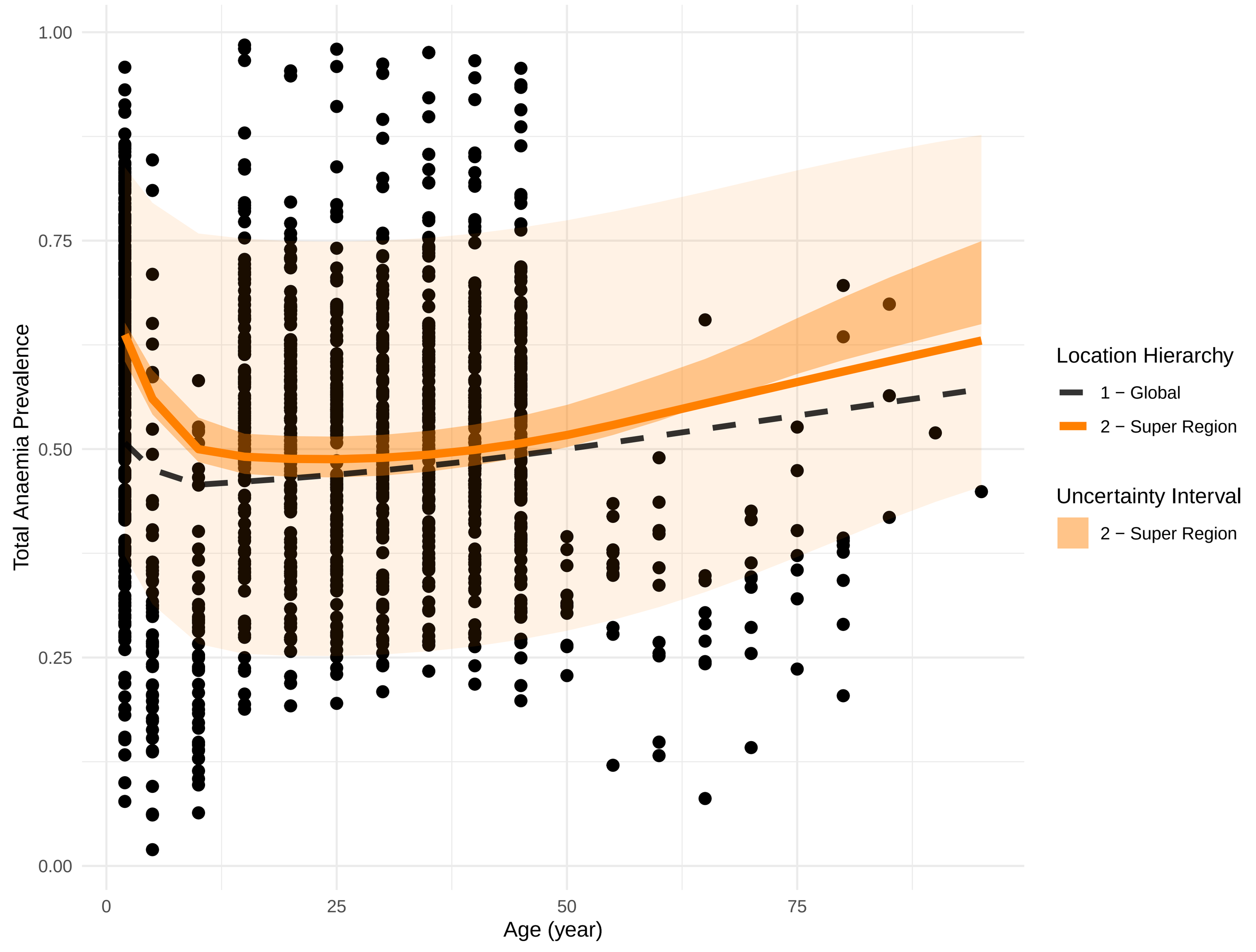

MR−BRT Age/Sex Fits − Non-pregnant Females 2+ Years Old − Total Anaemia Prevalence − Central Sub-Saharan Africa
WHO−2024 Adjustment Method

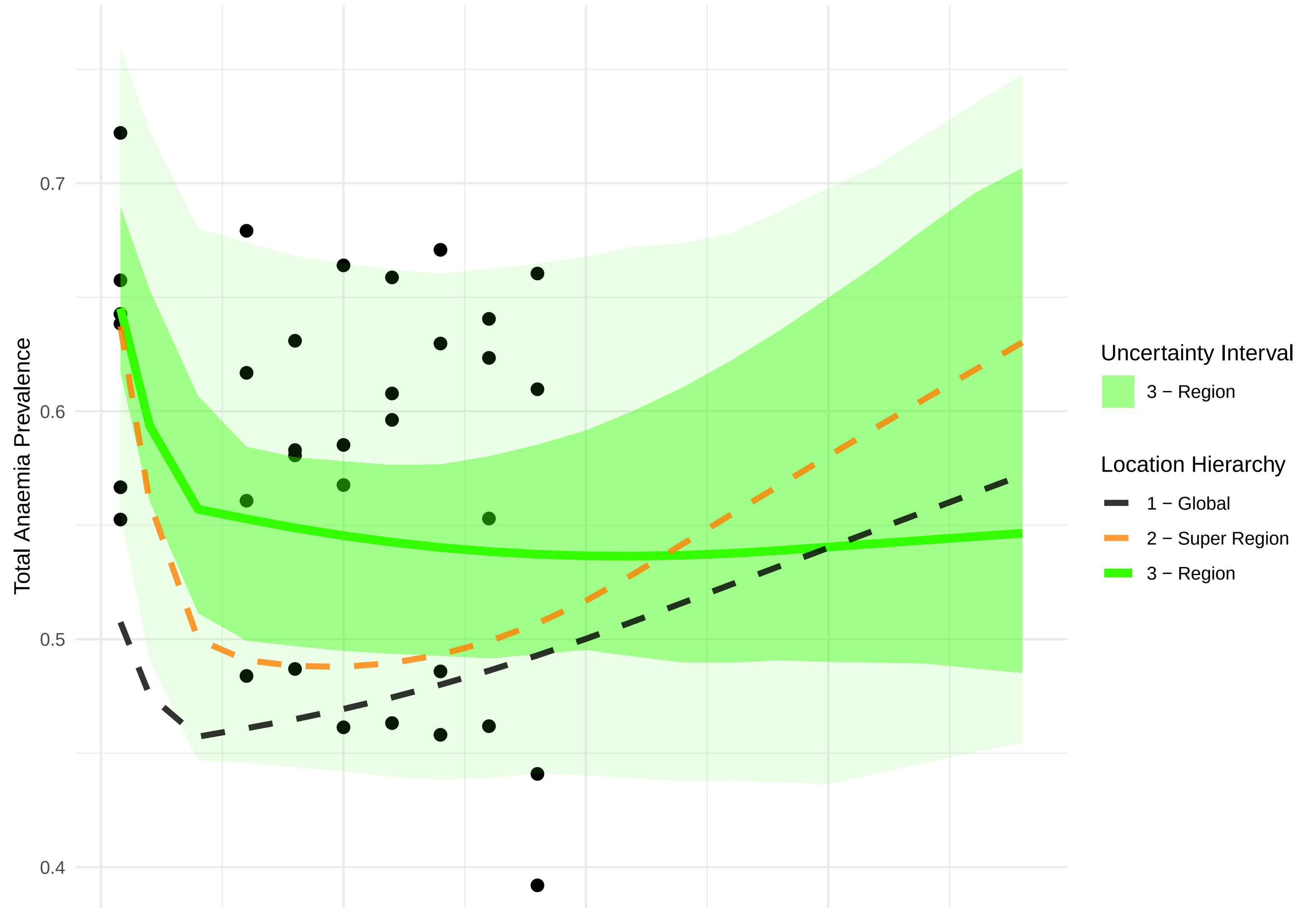

MR−BRT Age/Sex Fits − Non−pregnant Females 2+ Years Old − Total Anaemia Prevalence − Angola
WHO−2024 Adjustment Method

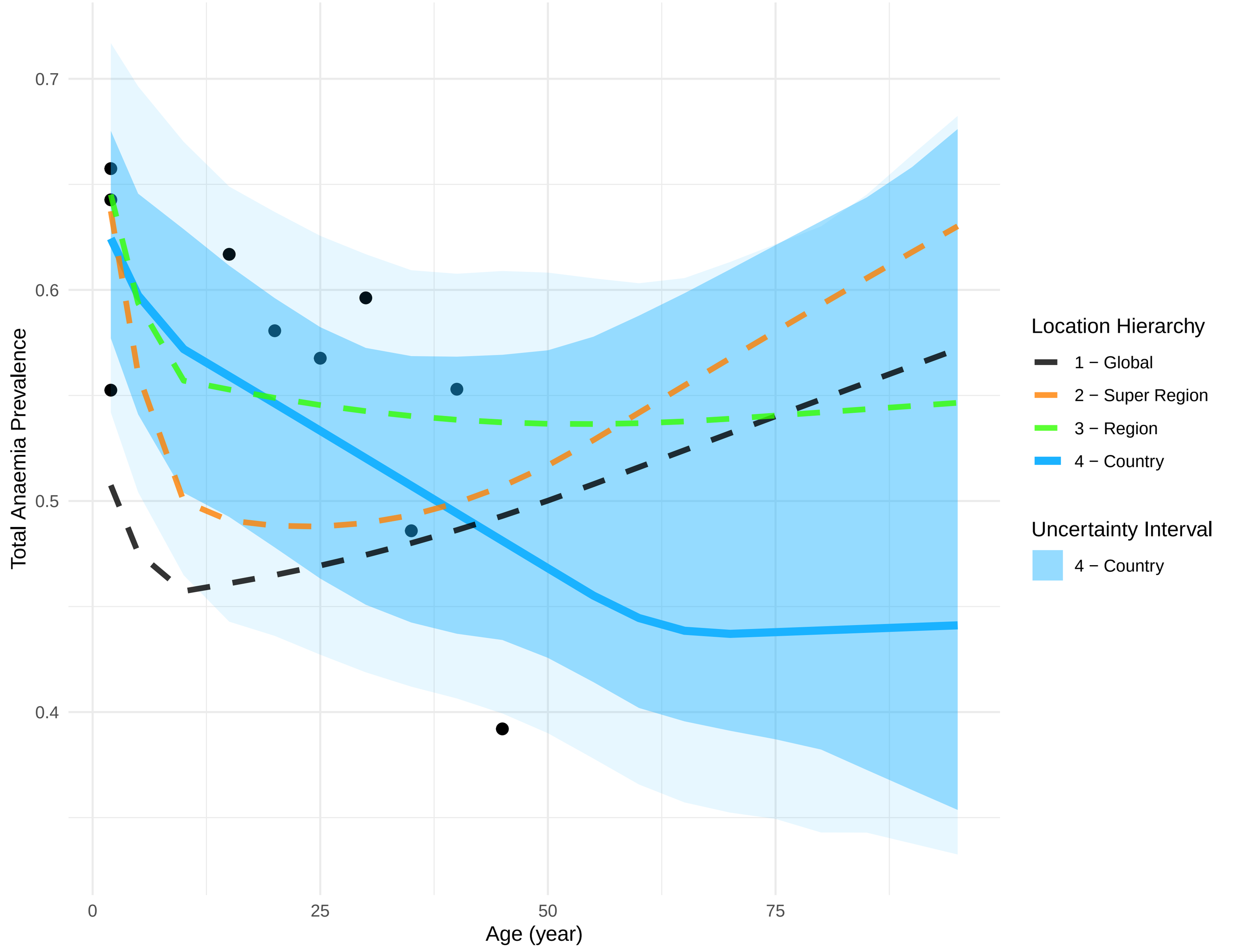

MR−BRT Age/Sex Fits − Non−pregnant Females 2+ Years Old − Total Anaemia Prevalence − Democratic Republic of the Congo
WHO−2024 Adjustment Method

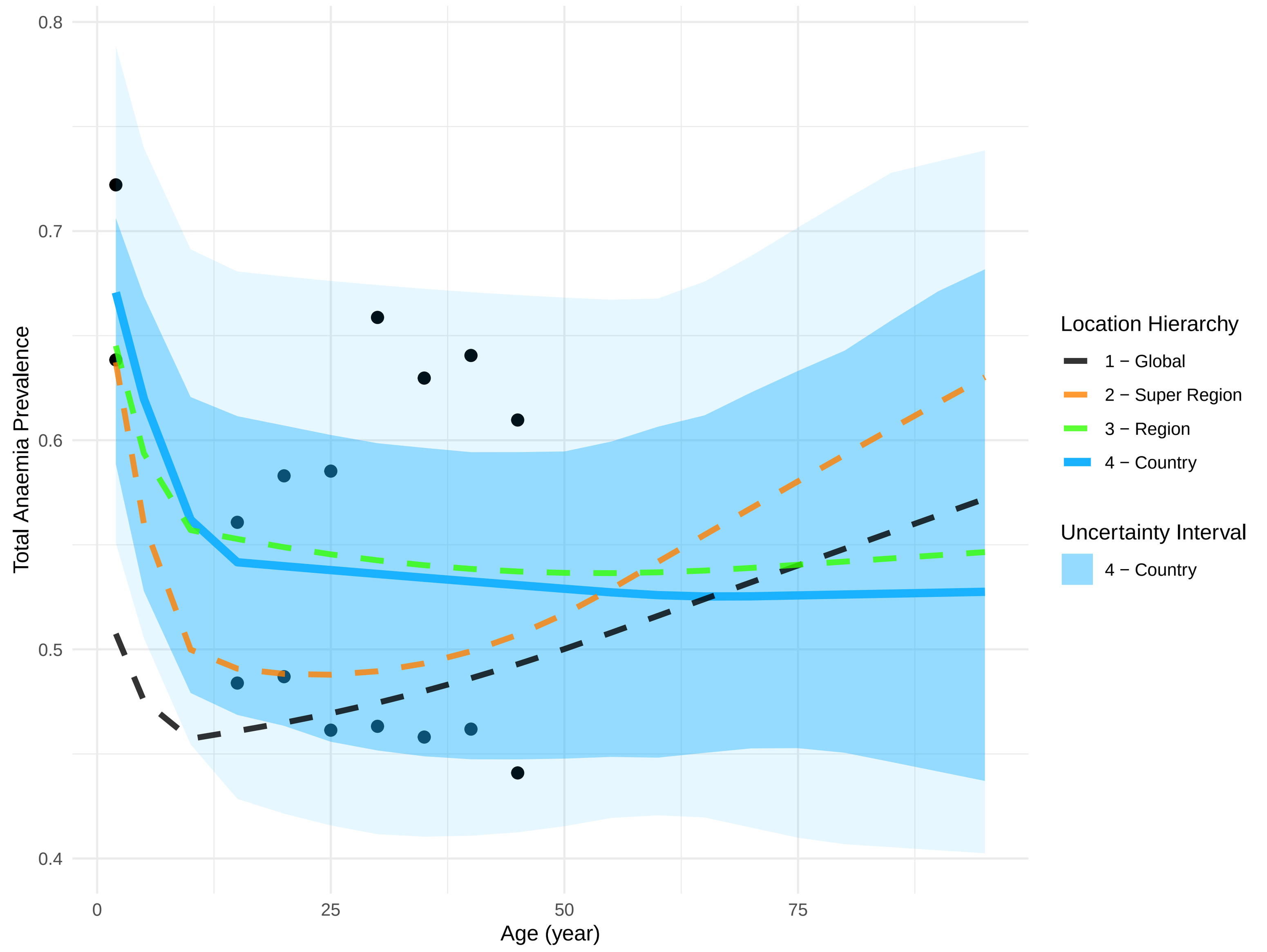

MR−BRT Age/Sex Fits − Non−pregnant Females 2+ Years Old − Total Anaemia Prevalence − Gabon
WHO−2024 Adjustment Method

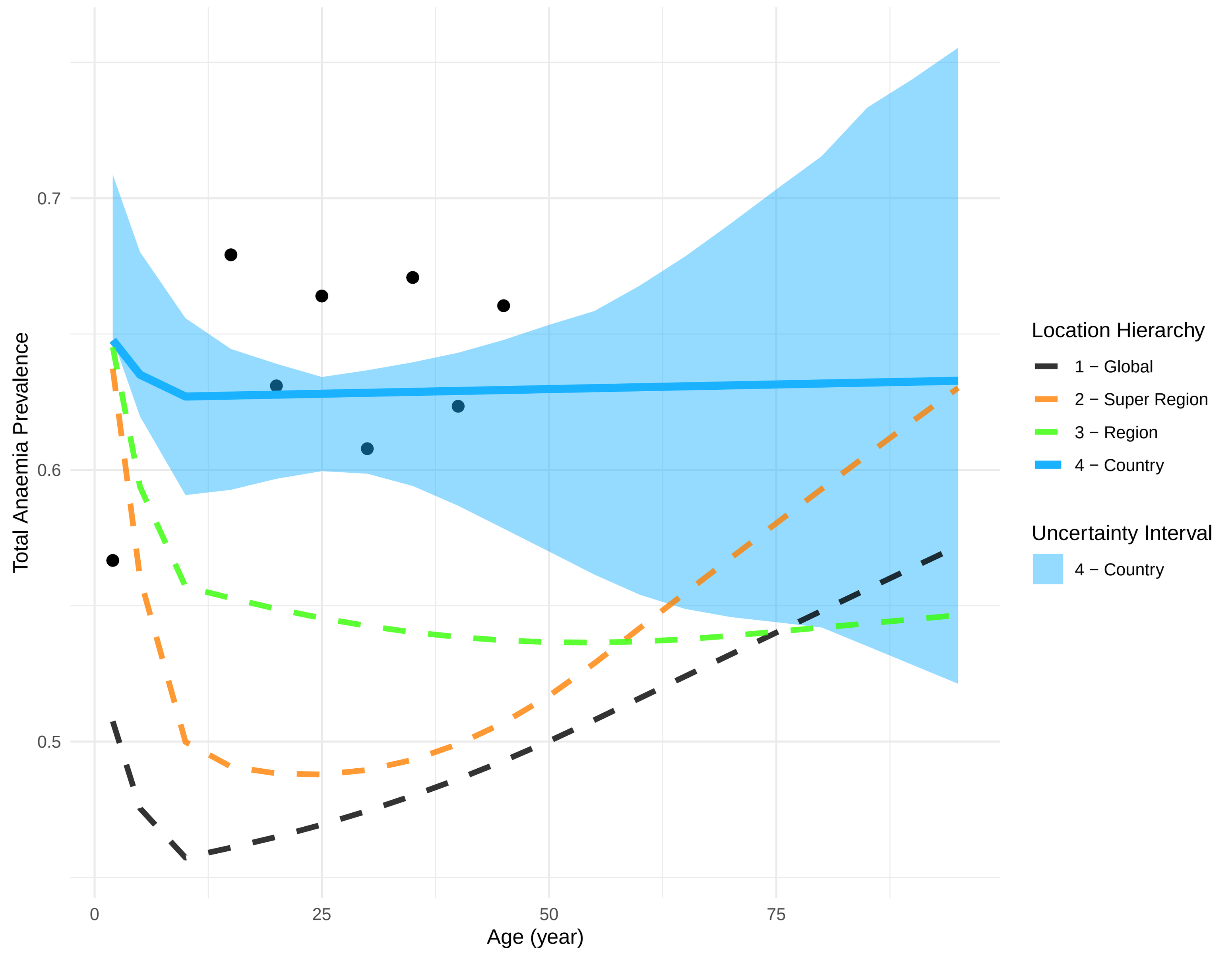

MR−BRT Age/Sex Fits − Non−pregnant Females 2+ Years Old − Total Anaemia Prevalence − Eastern Sub−Saharan Africa
WHO−2024 Adjustment Method

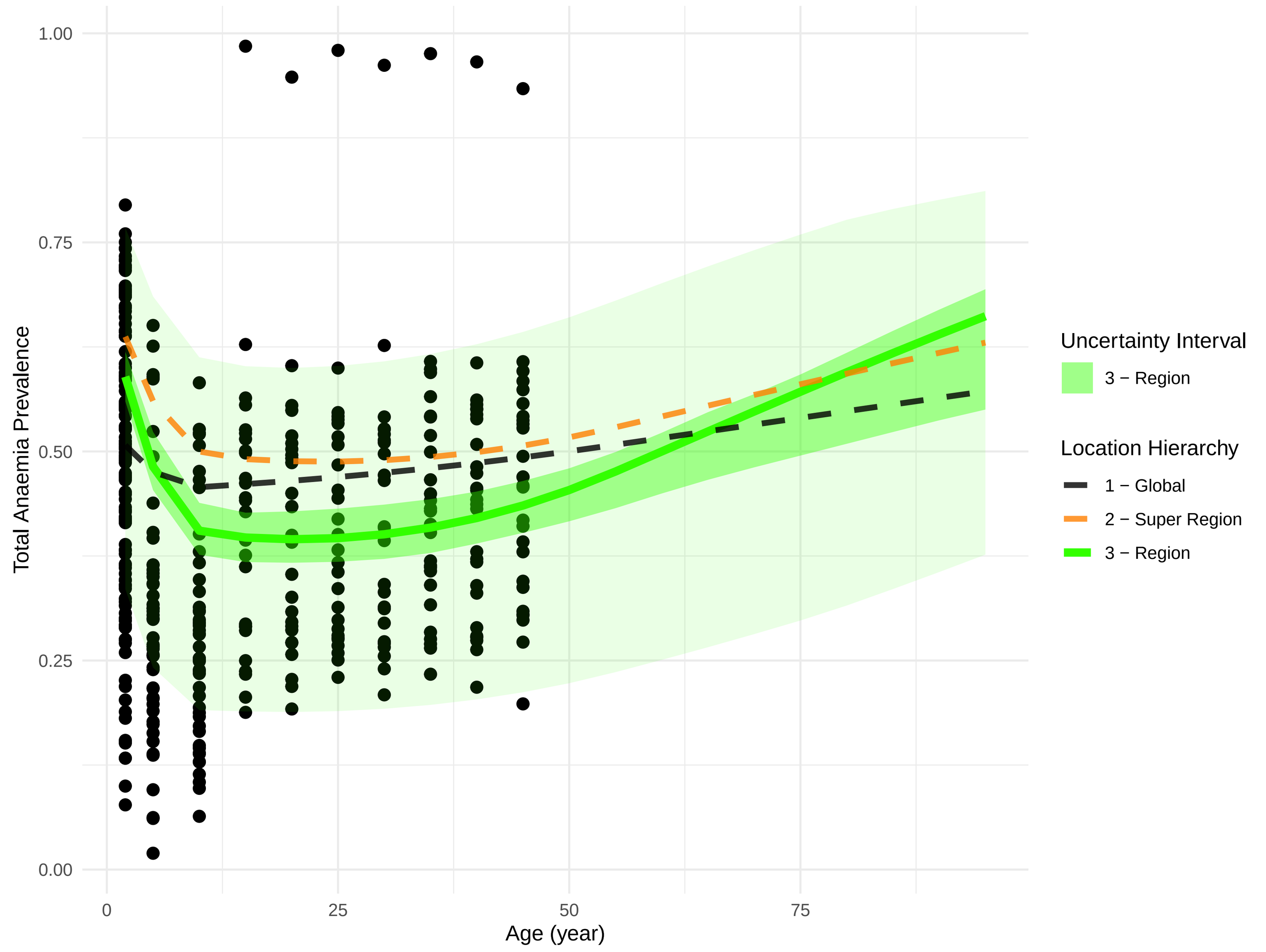

MR−BRT Age/Sex Fits − Non−pregnant Females 2+ Years Old − Total Anaemia Prevalence − Burundi
WHO−2024 Adjustment Method

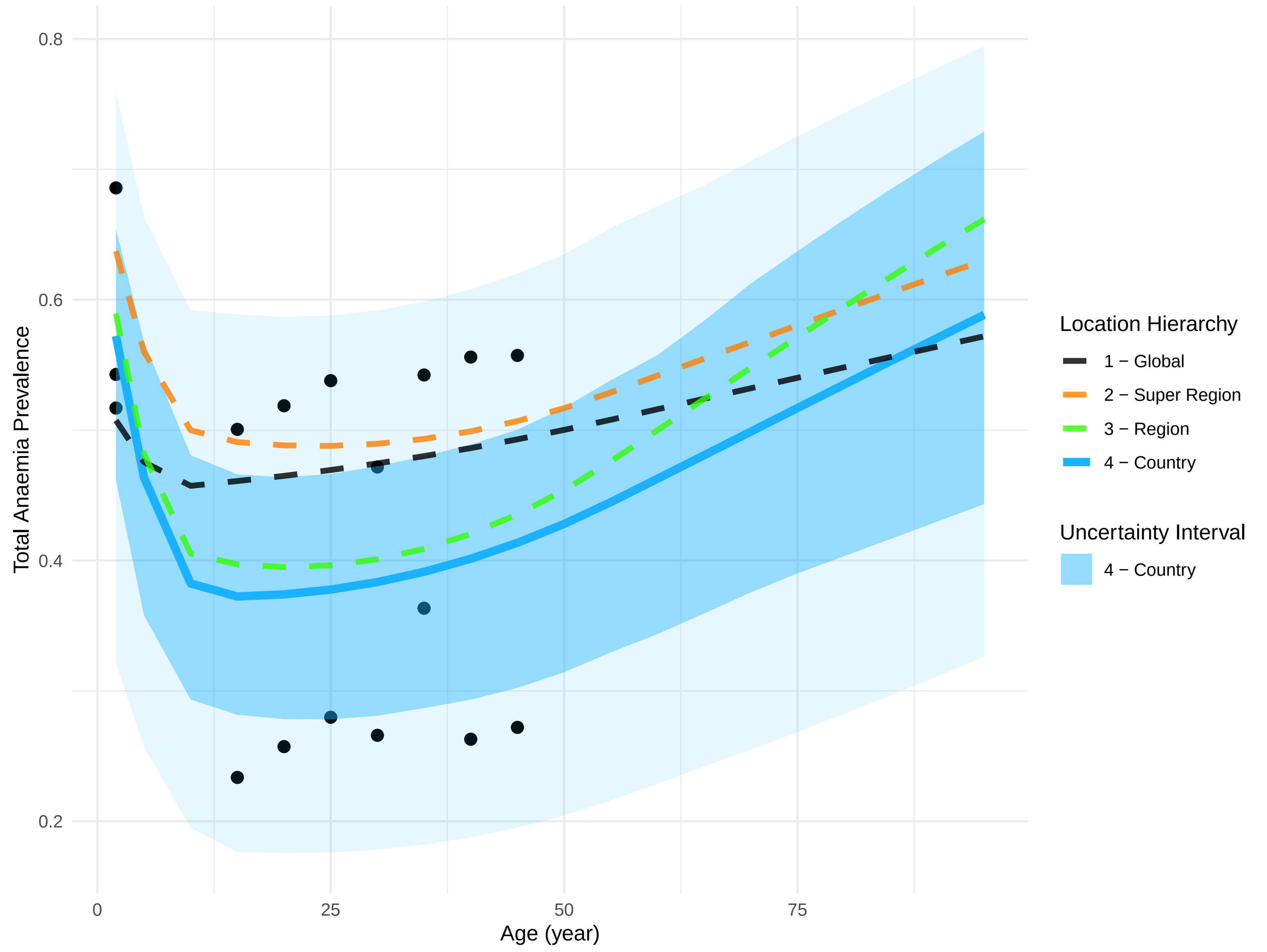

MR−BRT Age/Sex Fits − Non−pregnant Females 2+ Years Old − Total Anaemia Prevalence − Ethiopia
WHO−2024 Adjustment Method

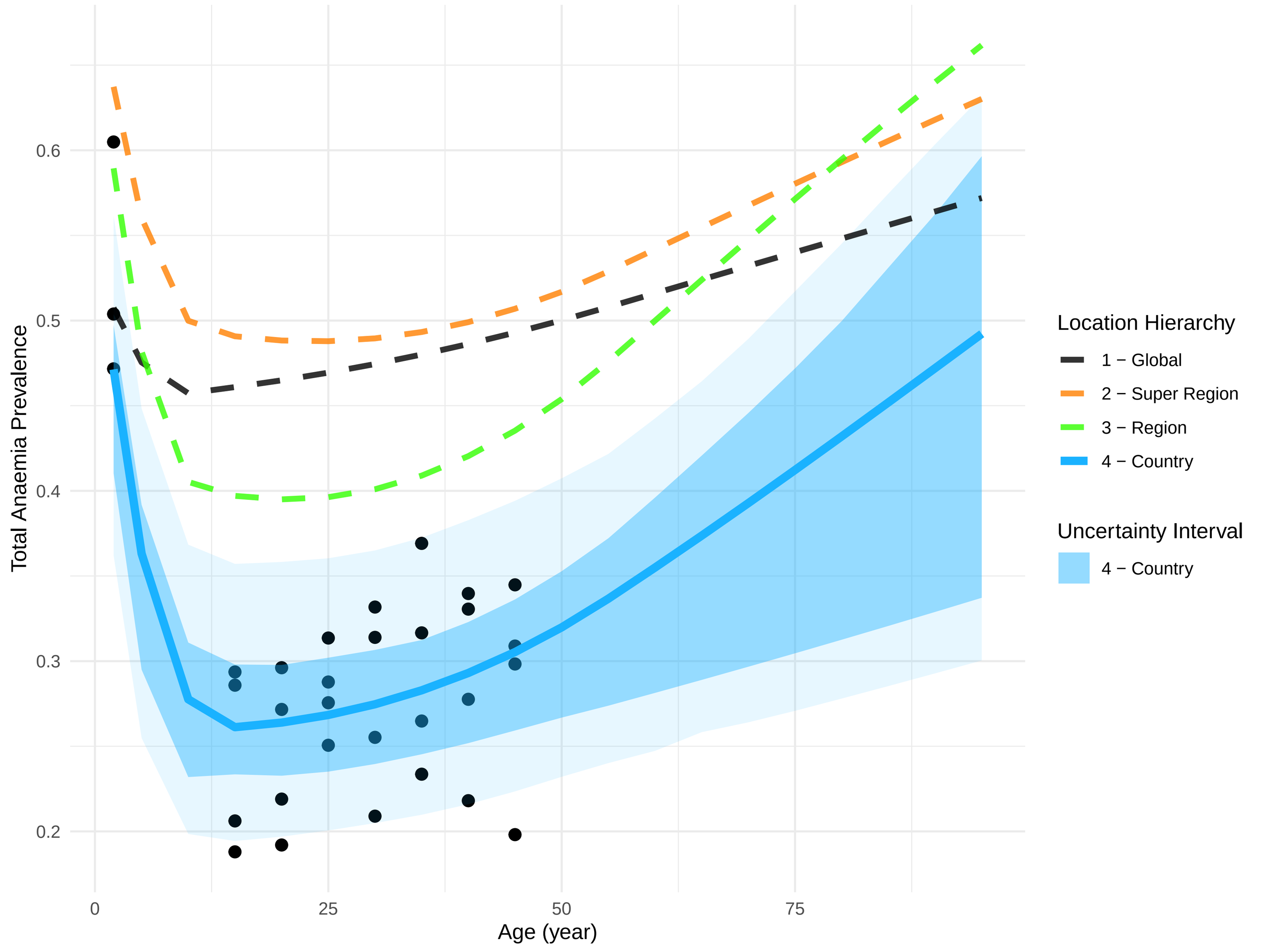

MR−BRT Age/Sex Fits − Non−pregnant Females 2+ Years Old − Total Anaemia Prevalence − Kenya
WHO−2024 Adjustment Method

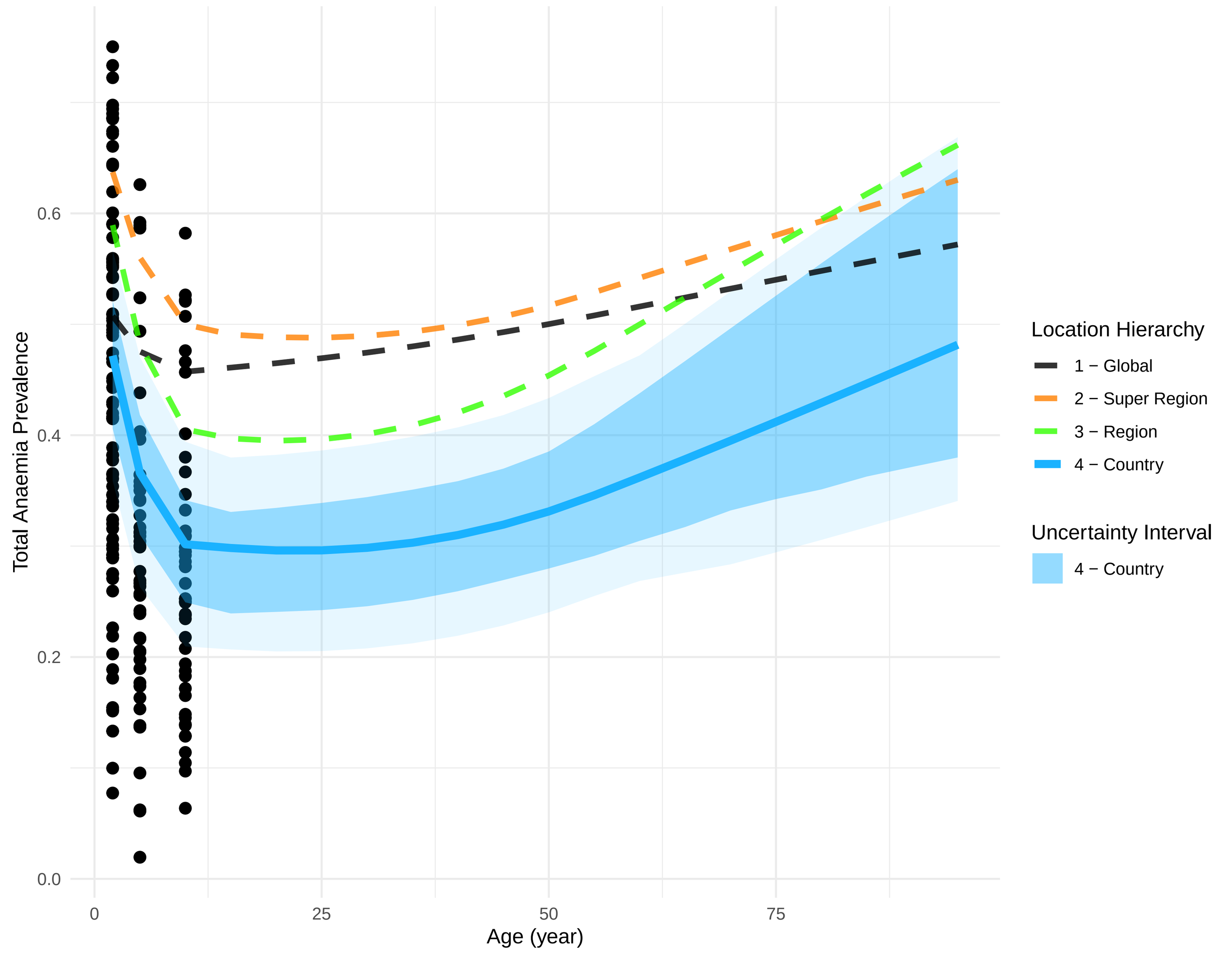

MR−BRT Age/Sex Fits − Non−pregnant Females 2+ Years Old − Total Anaemia Prevalence − Baringo
WHO−2024 Adjustment Method

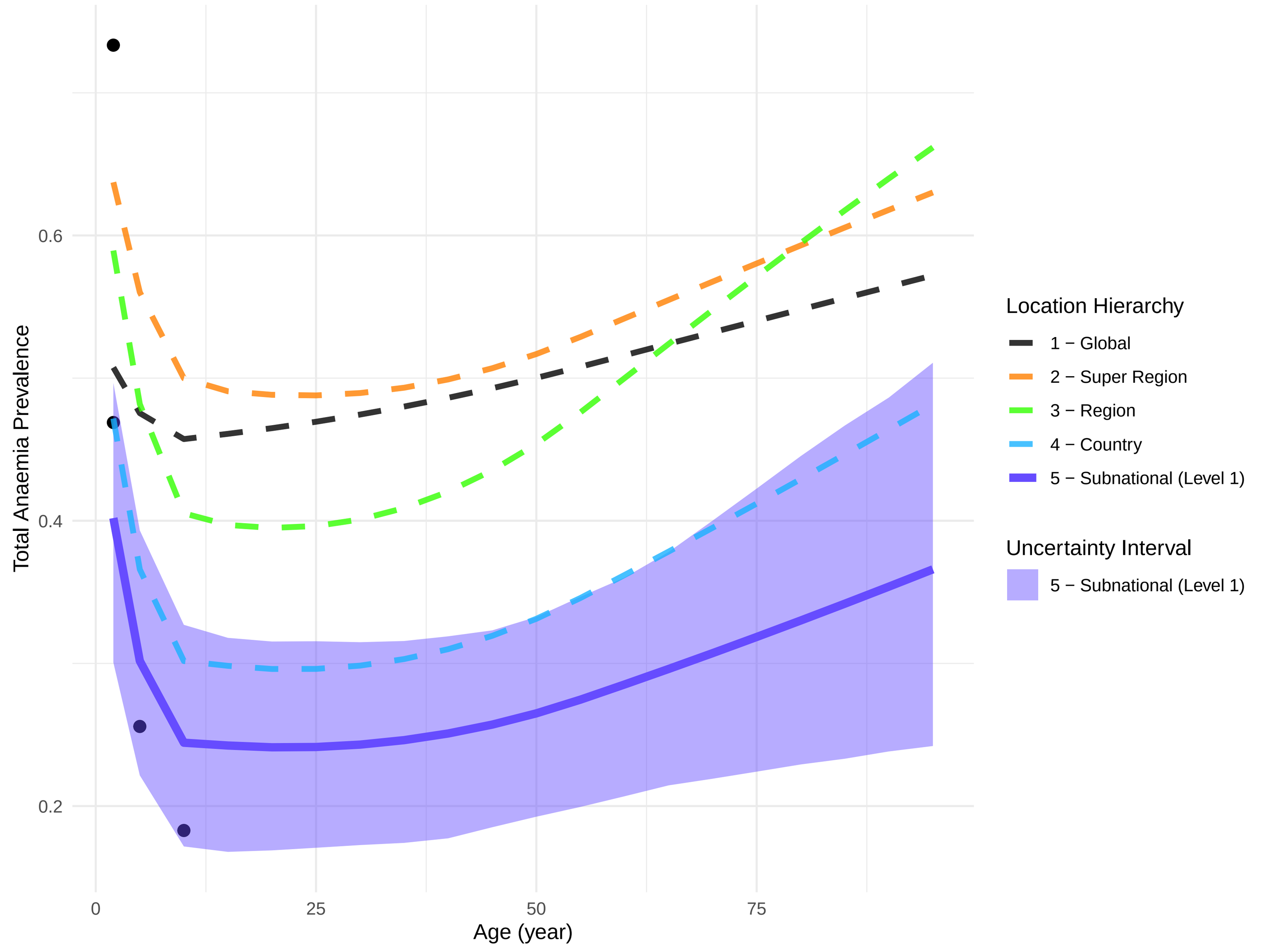

MR−BRT Age/Sex Fits − Non−pregnant Females 2+ Years Old − Total Anaemia Prevalence − Bomet
WHO−2024 Adjustment Method

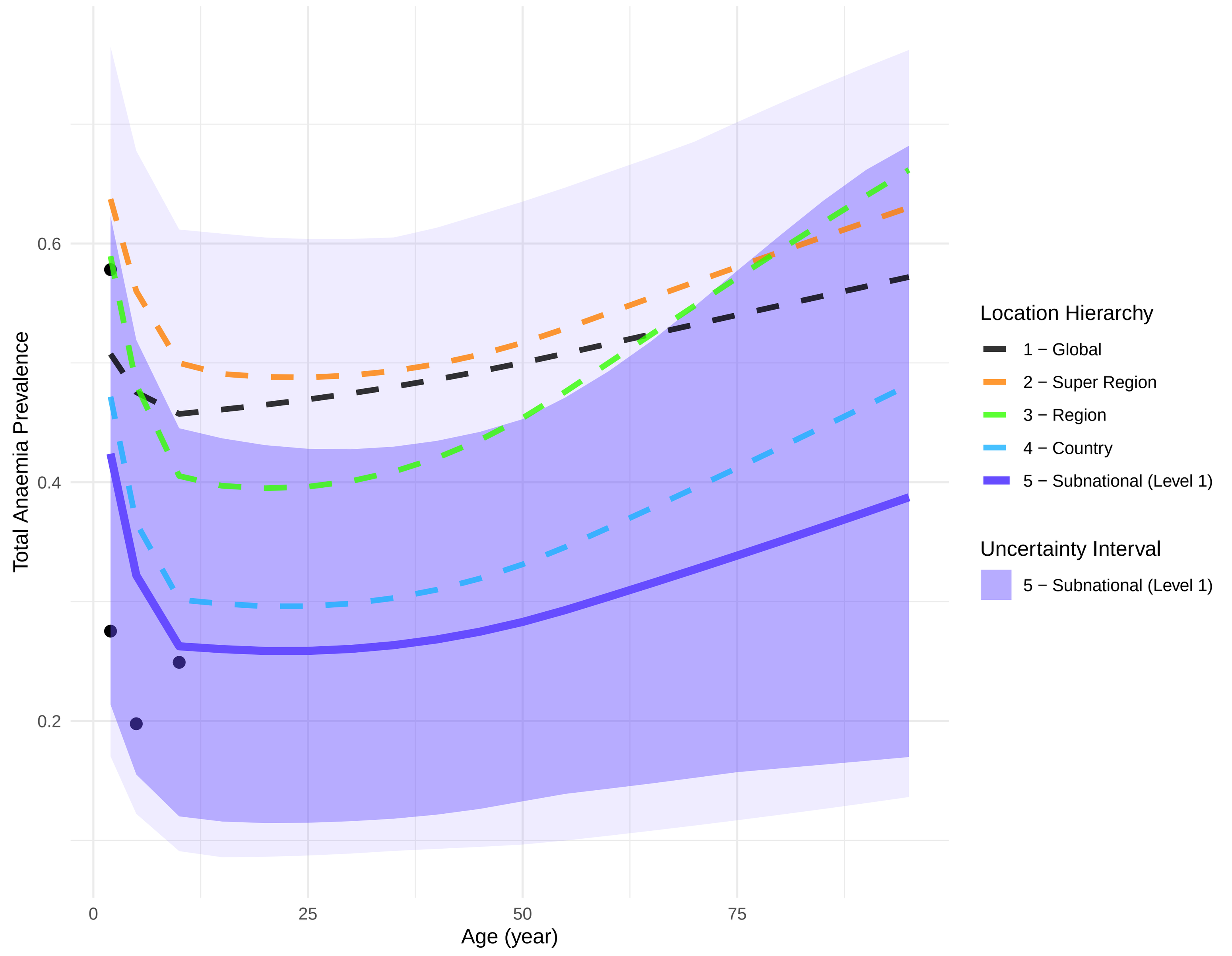

MR−BRT Age/Sex Fits − Non−pregnant Females 2+ Years Old − Total Anaemia Prevalence − Bungoma
WHO−2024 Adjustment Method

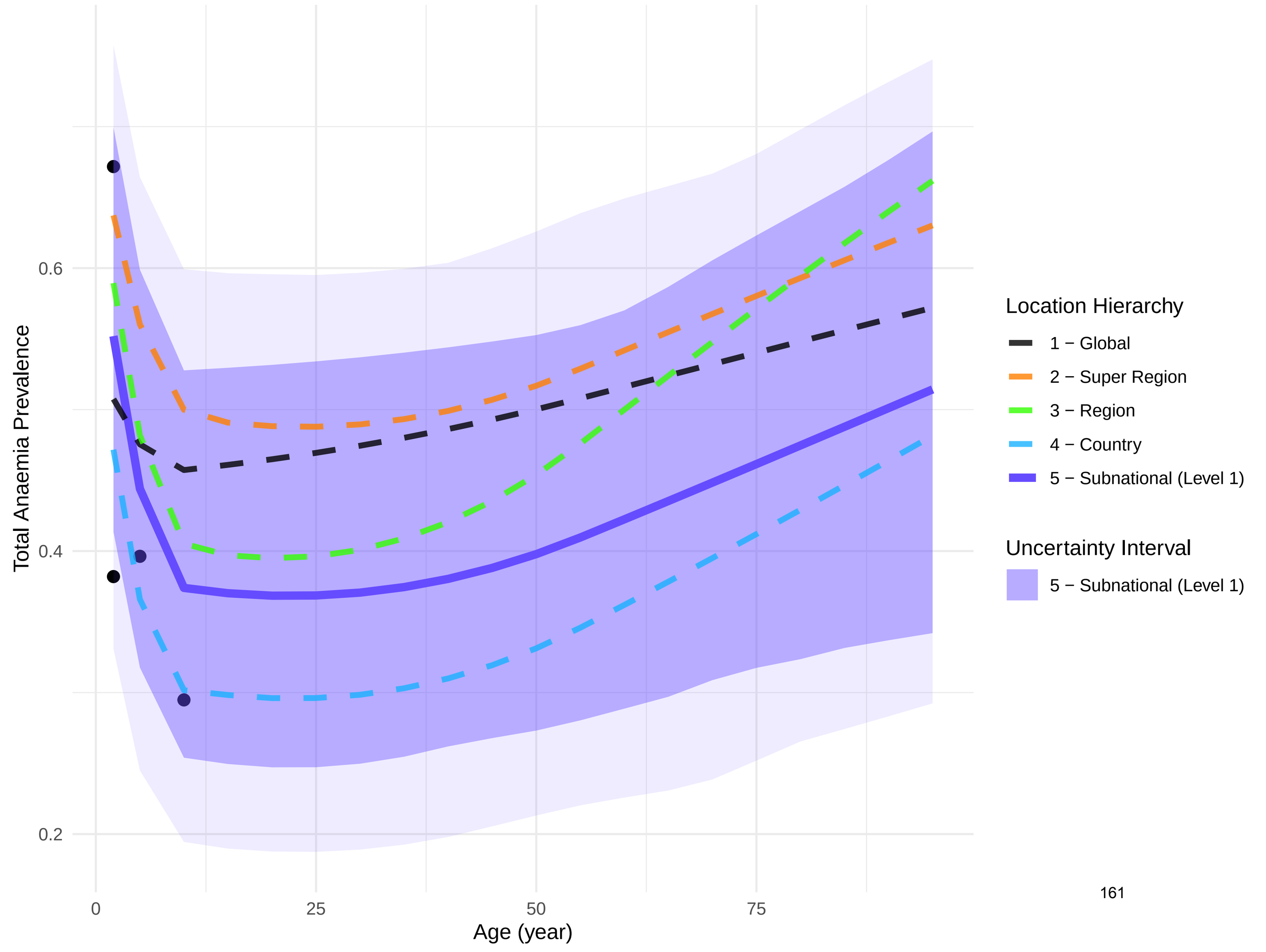

MR−BRT Age/Sex Fits − Non−pregnant Females 2+ Years Old − Total Anaemia Prevalence − Busia
WHO−2024 Adjustment Method

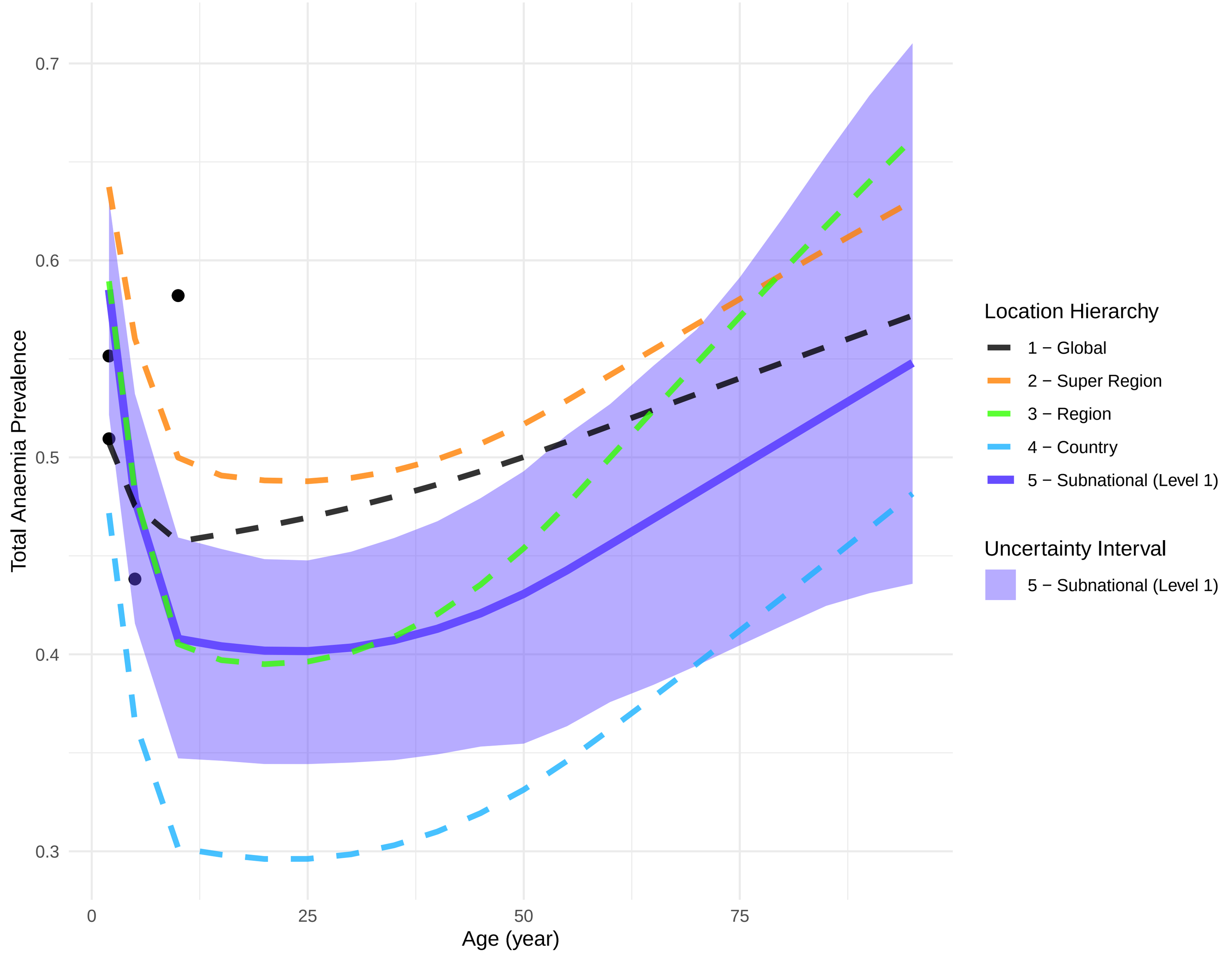

MR−BRT Age/Sex Fits − Non−pregnant Females 2+ Years Old − Total Anaemia Prevalence − Elgeyo−Marakwet
WHO−2024 Adjustment Method

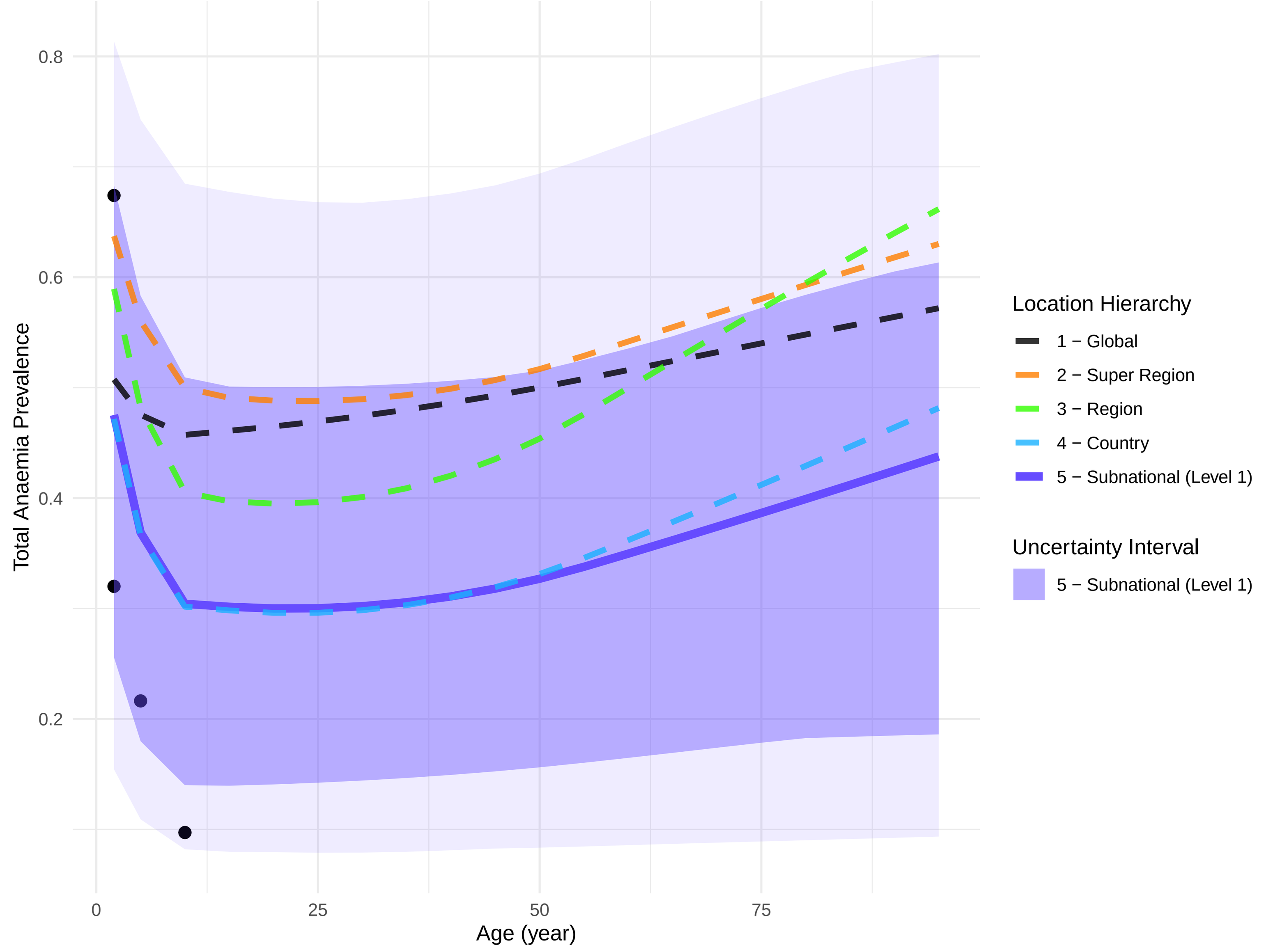

MR−BRT Age/Sex Fits − Non−pregnant Females 2+ Years Old − Total Anaemia Prevalence − Embu
WHO−2024 Adjustment Method

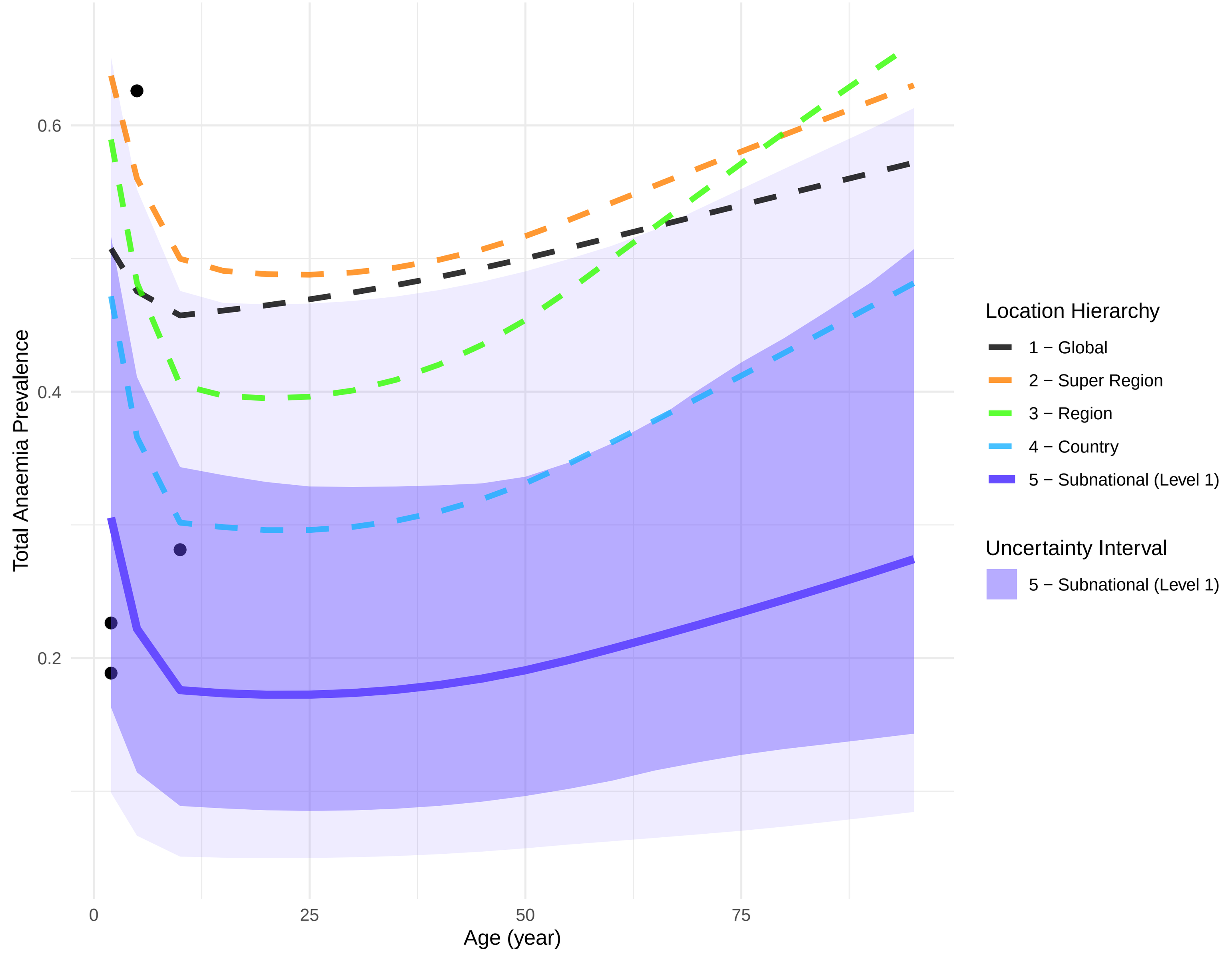

MR−BRT Age/Sex Fits − Non−pregnant Females 2+ Years Old − Total Anaemia Prevalence − Garissa
WHO−2024 Adjustment Method

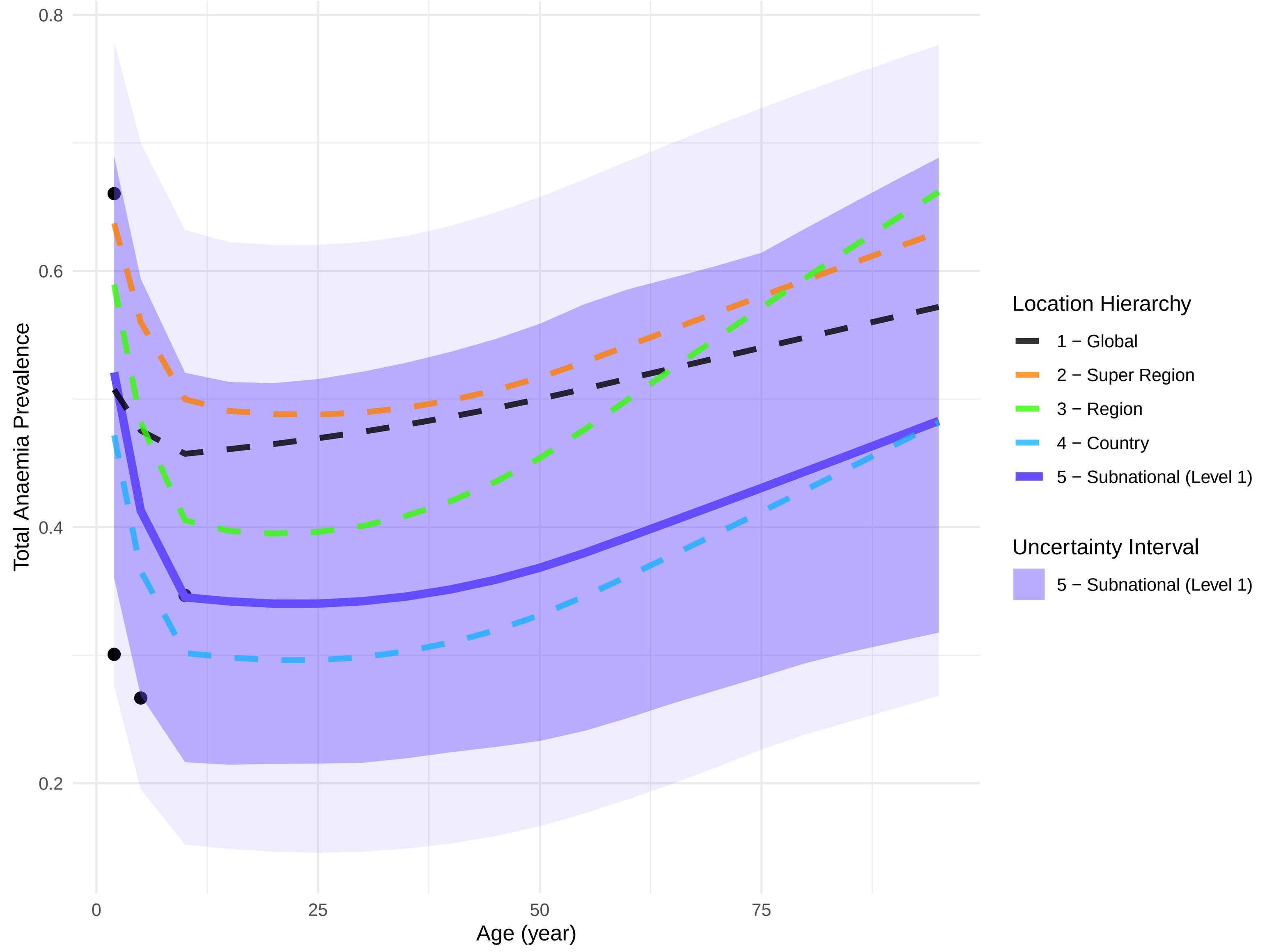

MR−BRT Age/Sex Fits − Non−pregnant Females 2+ Years Old − Total Anaemia Prevalence − Homa Bay
WHO−2024 Adjustment Method

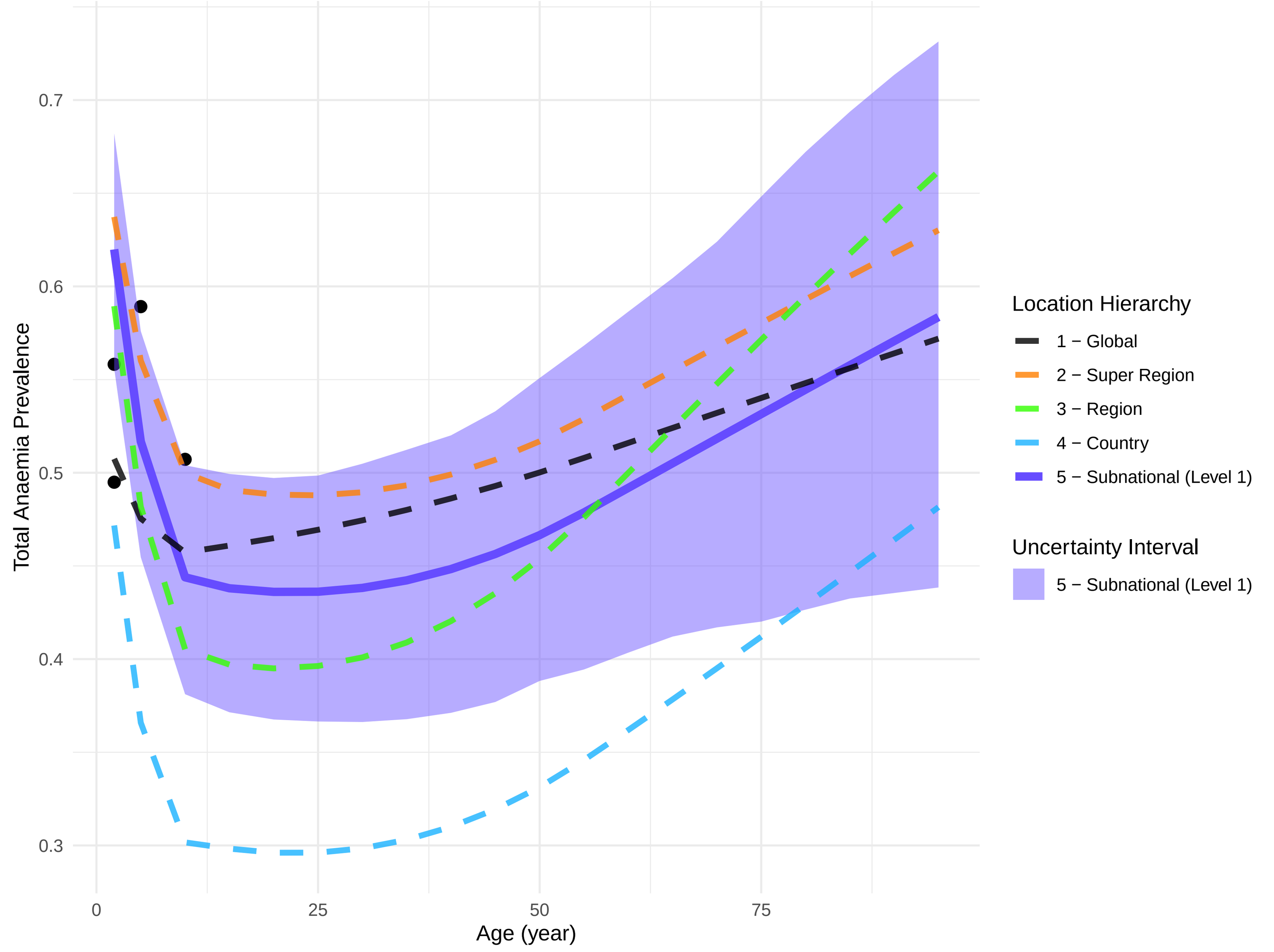

MR−BRT Age/Sex Fits − Non−pregnant Females 2+ Years Old − Total Anaemia Prevalence − Isiolo
WHO−2024 Adjustment Method

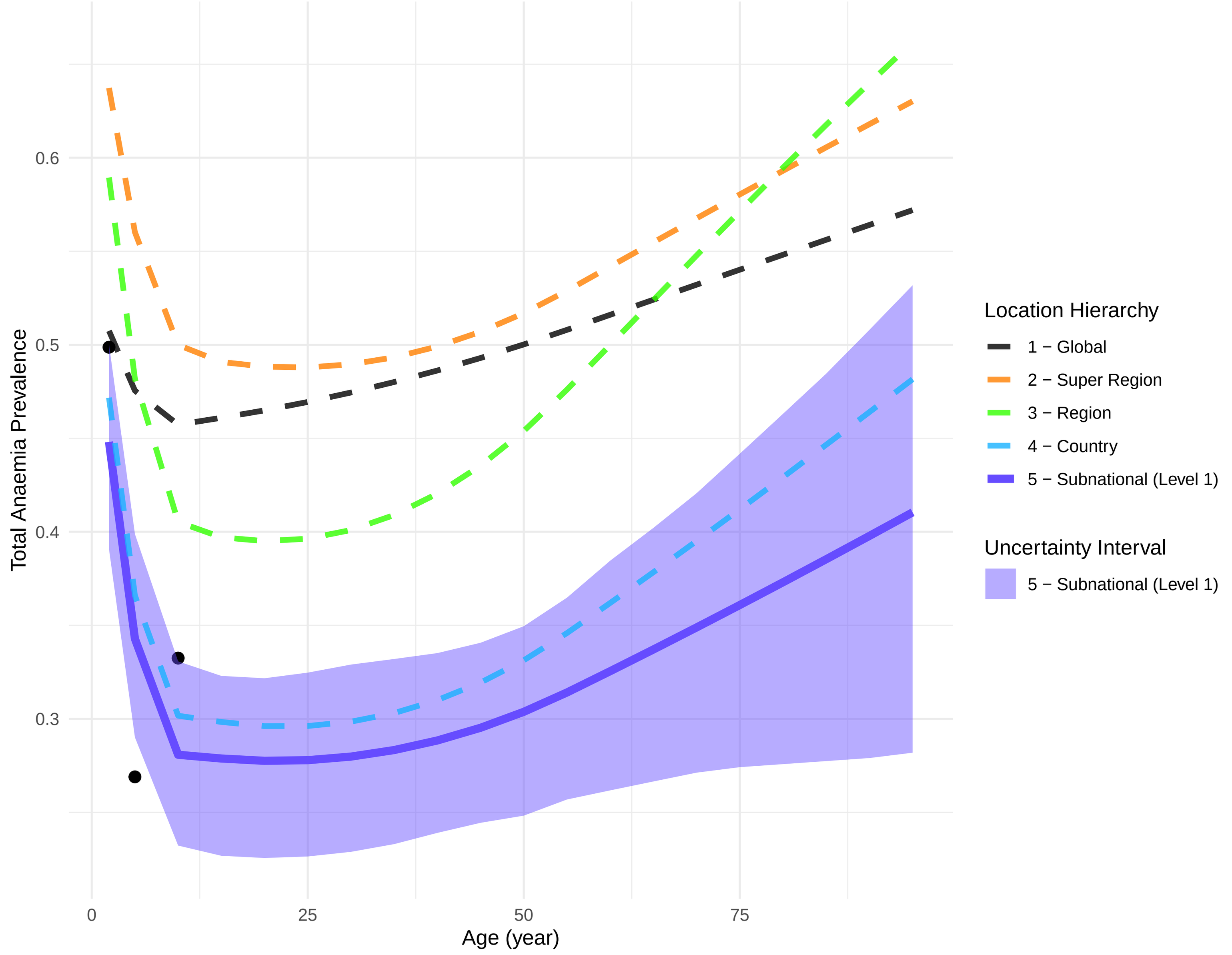

MR−BRT Age/Sex Fits − Non−pregnant Females 2+ Years Old − Total Anaemia Prevalence − Kajiado
WHO−2024 Adjustment Method

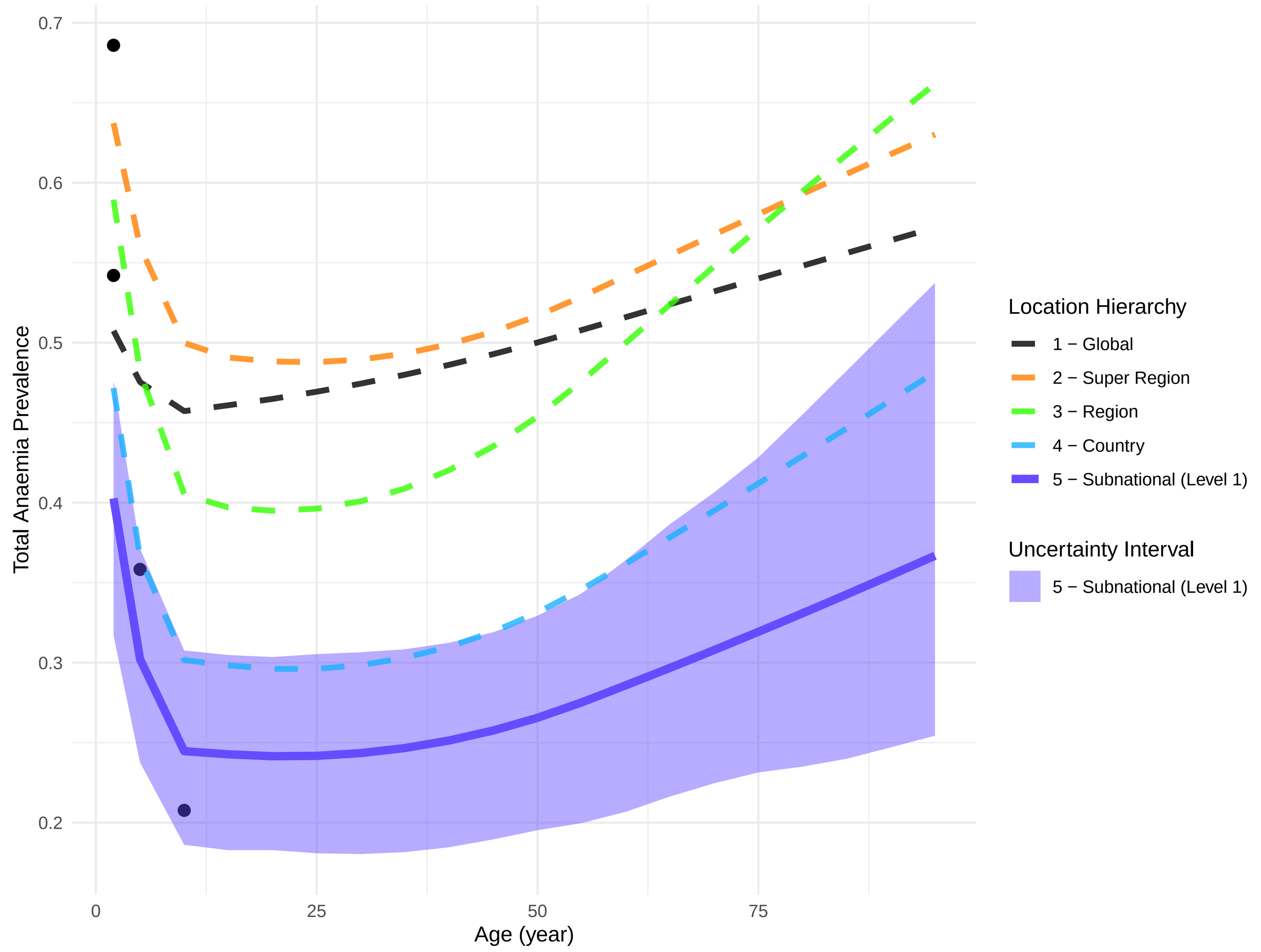

MR−BRT Age/Sex Fits − Non−pregnant Females 2+ Years Old − Total Anaemia Prevalence − Kakamega
WHO−2024 Adjustment Method

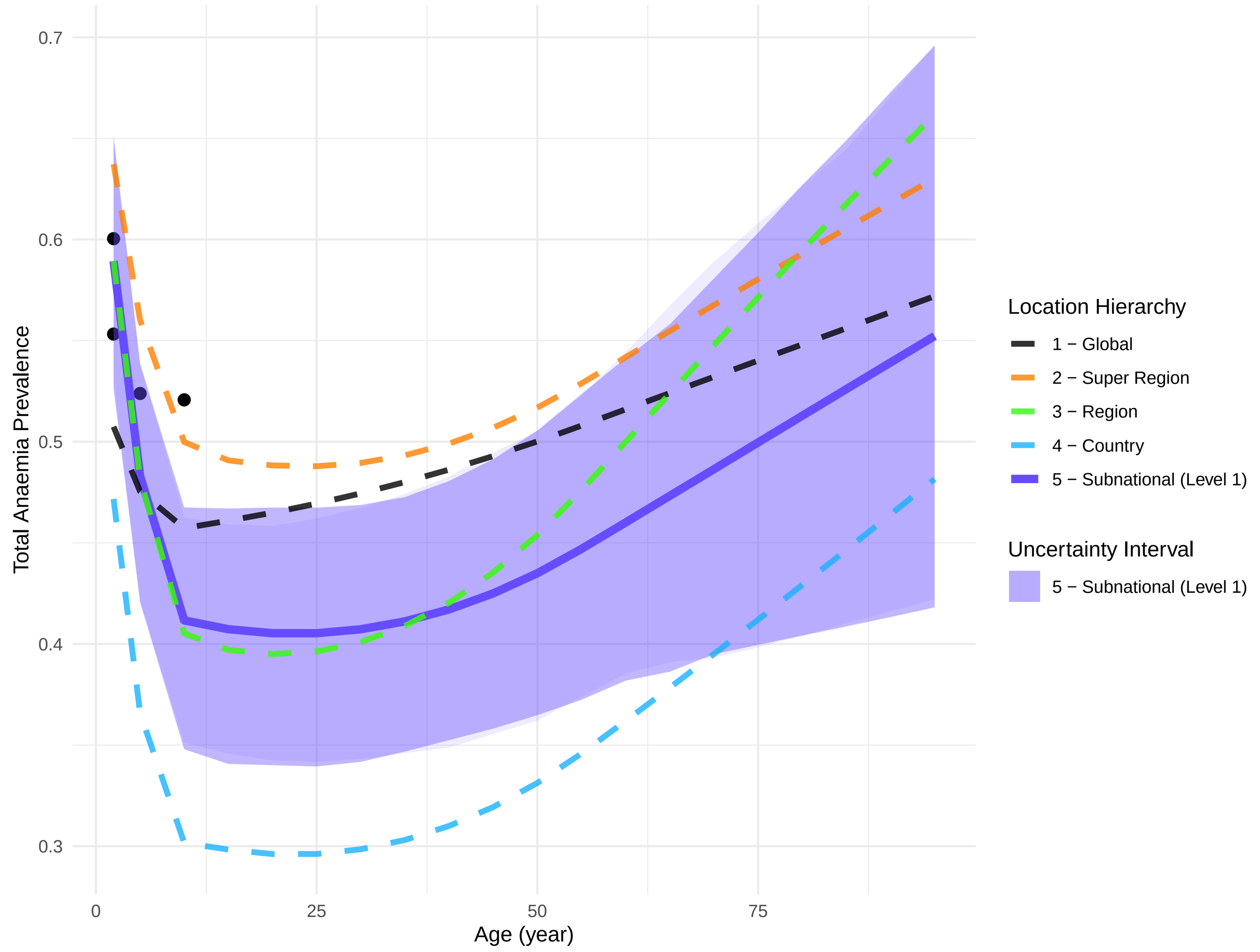

MR−BRT Age/Sex Fits − Non−pregnant Females 2+ Years Old − Total Anaemia Prevalence − Kericho
WHO−2024 Adjustment Method

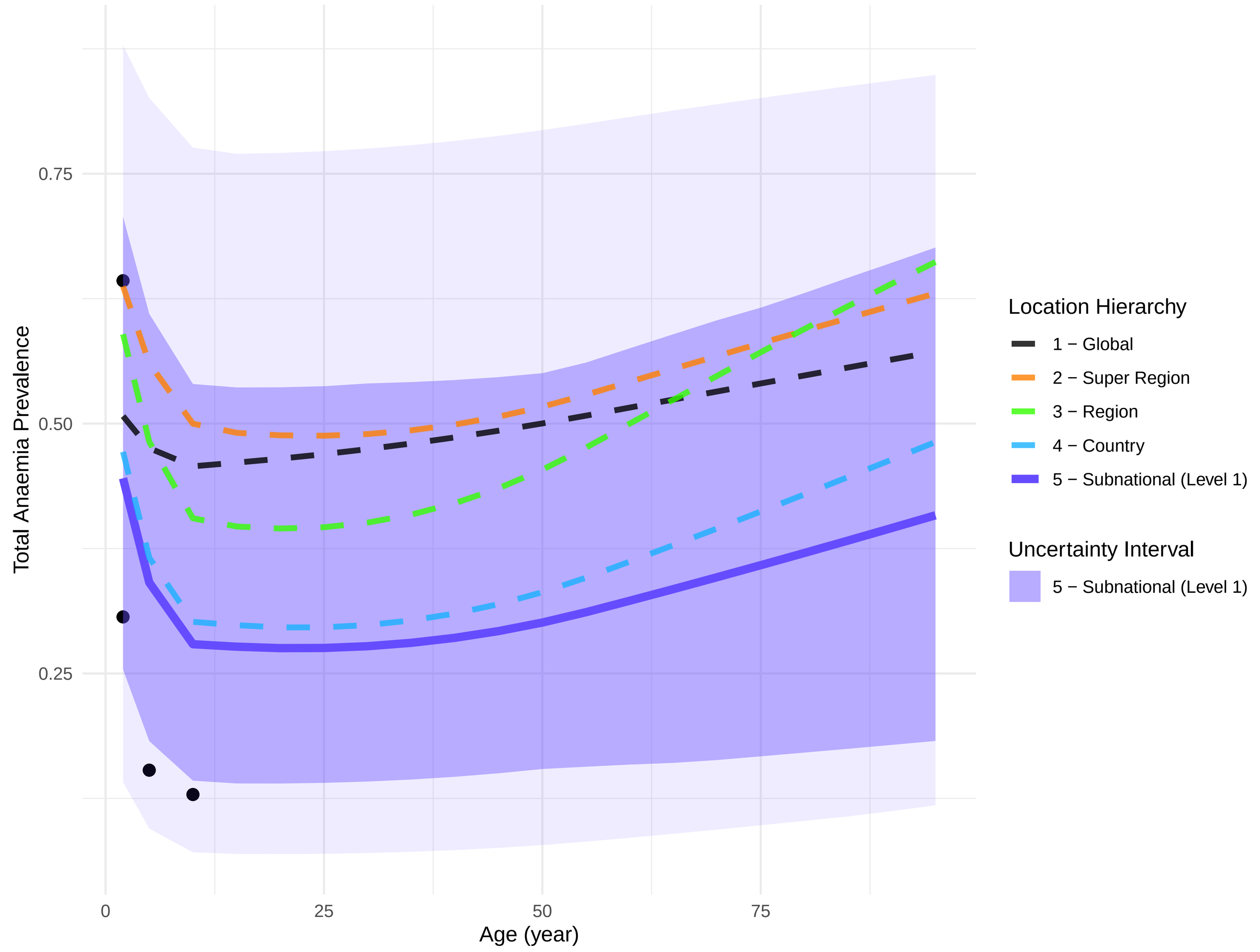

MR−BRT Age/Sex Fits − Non−pregnant Females 2+ Years Old − Total Anaemia Prevalence − Kiambu
WHO−2024 Adjustment Method

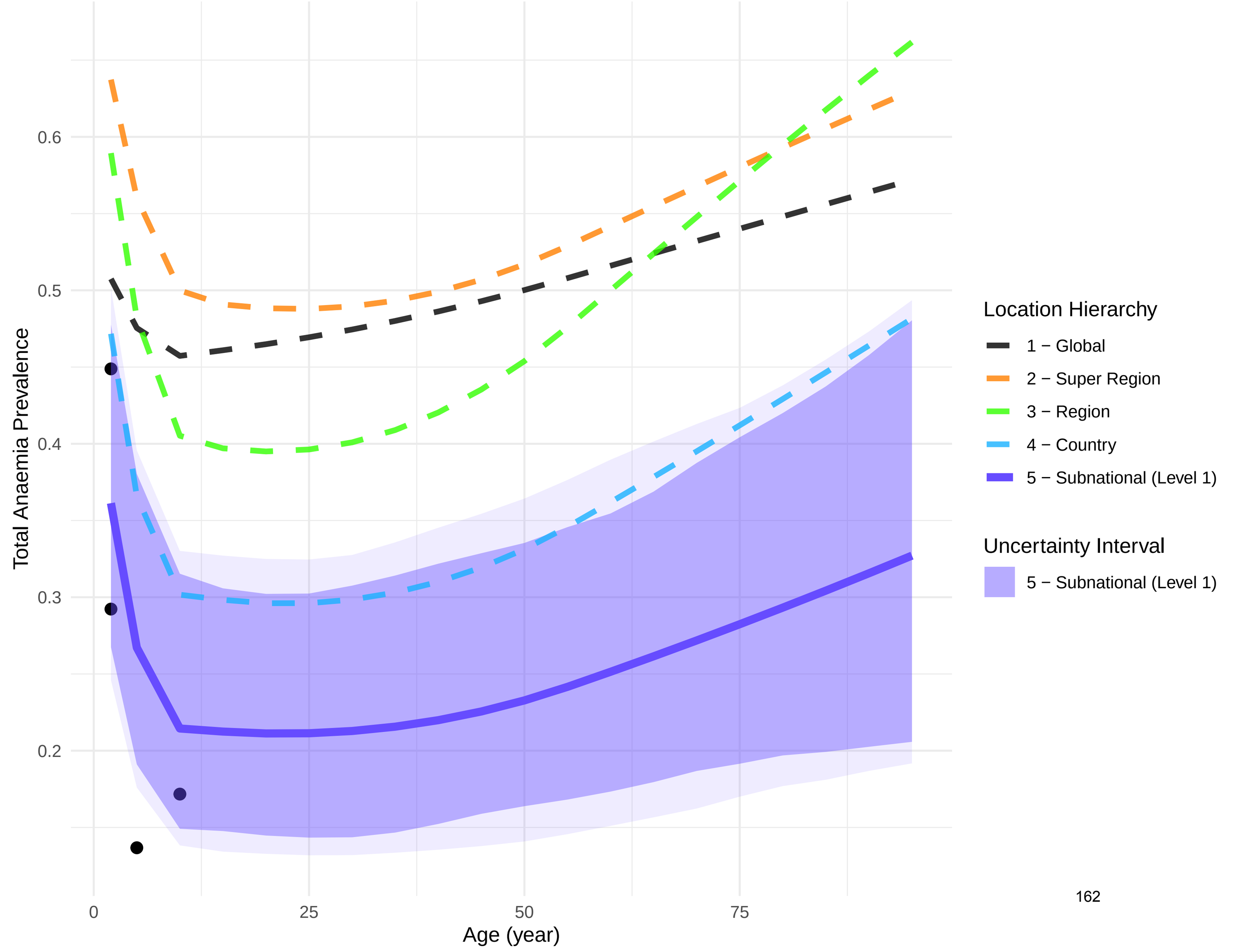

MR−BRT Age/Sex Fits − Non−pregnant Females 2+ Years Old − Total Anaemia Prevalence − Kilifi
WHO−2024 Adjustment Method

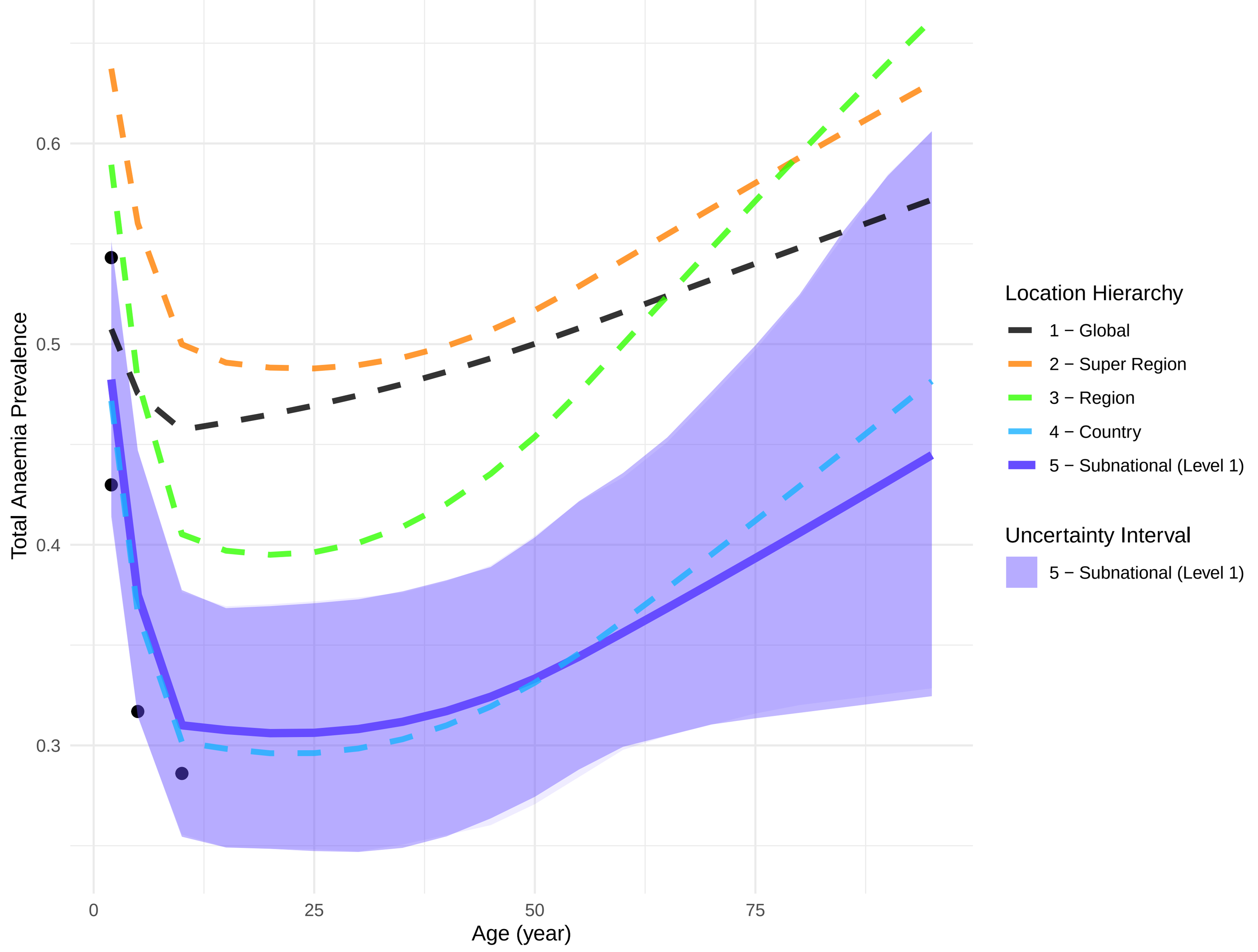

MR−BRT Age/Sex Fits − Non−pregnant Females 2+ Years Old − Total Anaemia Prevalence − Kirinyaga
WHO−2024 Adjustment Method

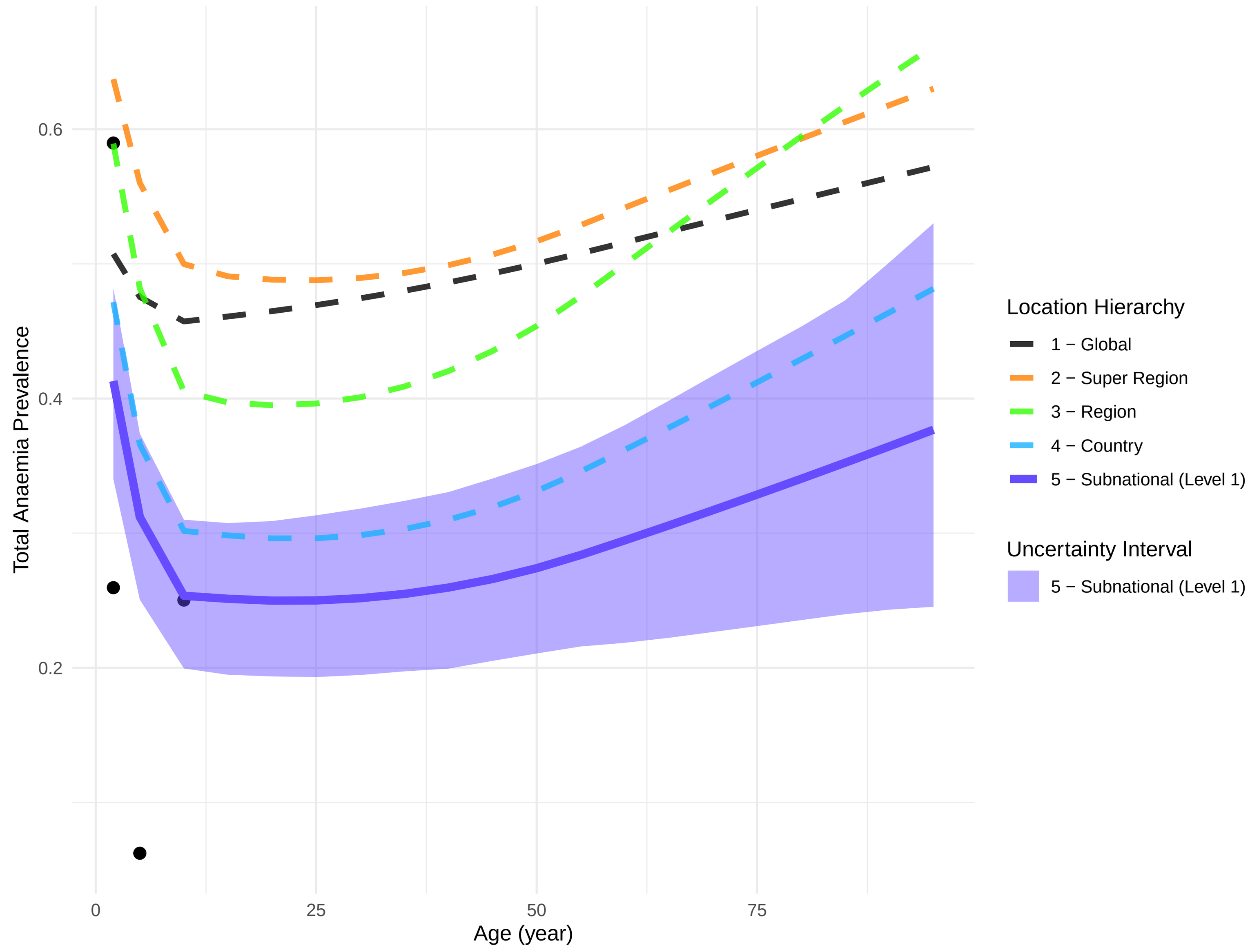

MR−BRT Age/Sex Fits − Non−pregnant Females 2+ Years Old − Total Anaemia Prevalence − Kisii
WHO−2024 Adjustment Method

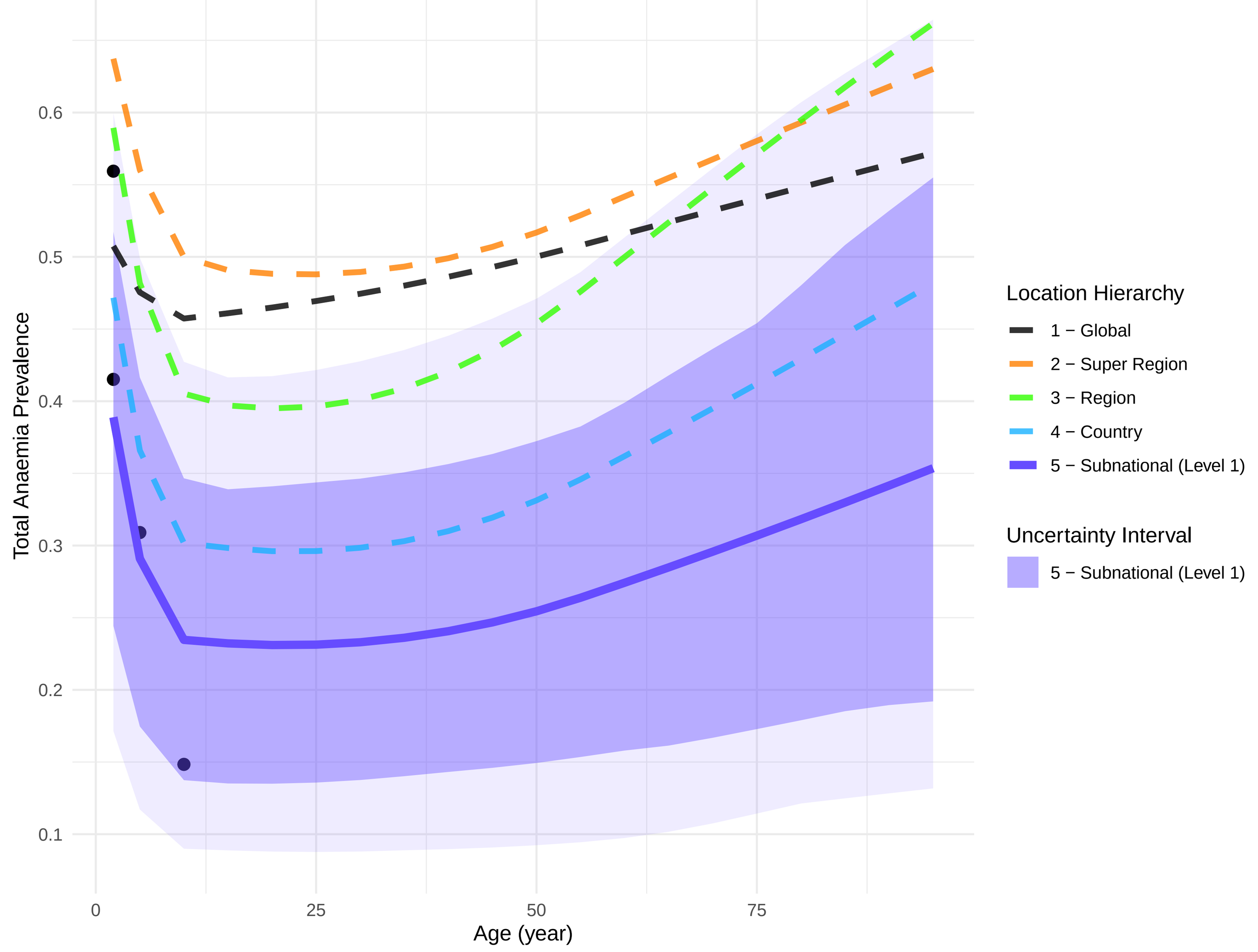

MR−BRT Age/Sex Fits − Non−pregnant Females 2+ Years Old − Total Anaemia Prevalence − Kisumu
WHO−2024 Adjustment Method

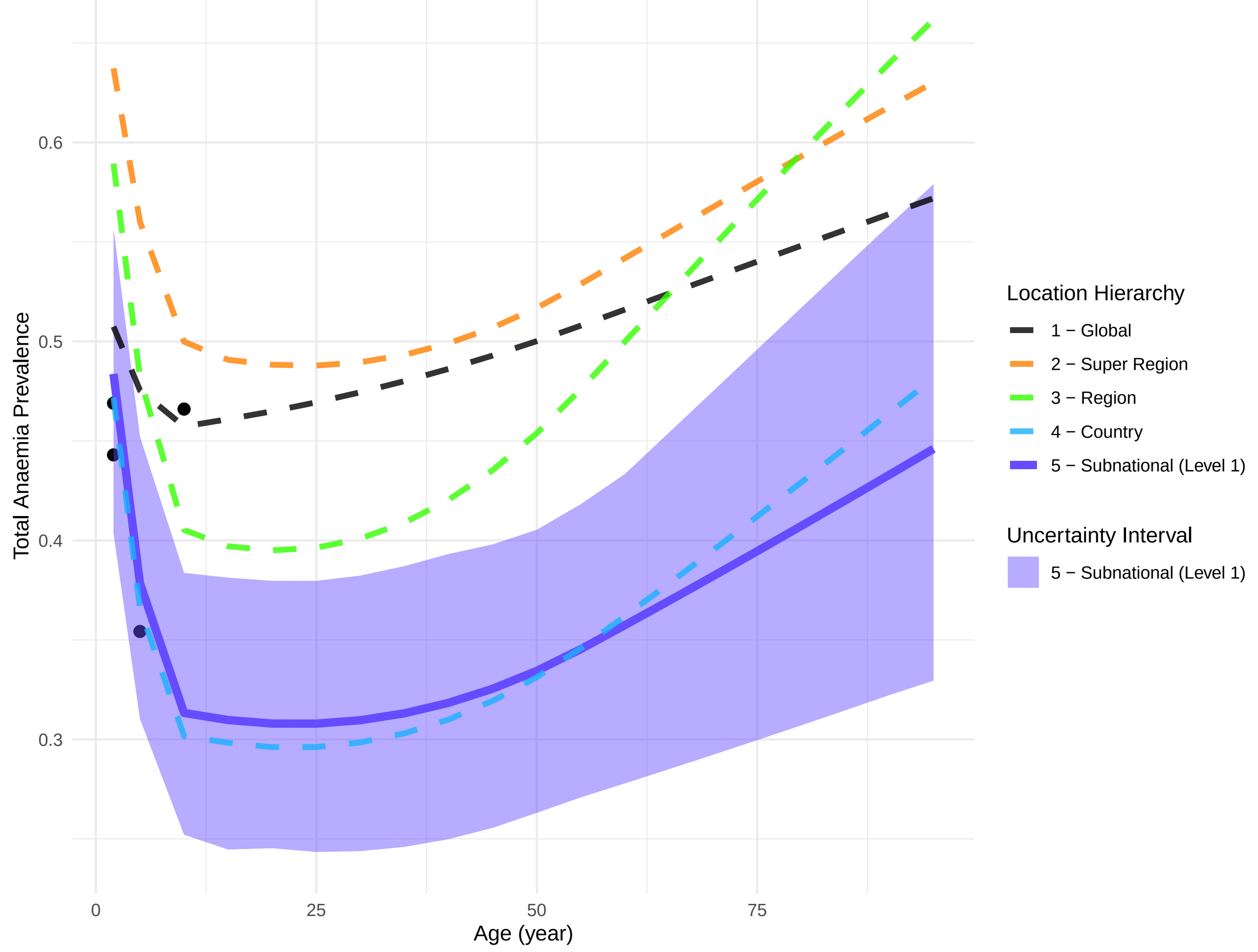

MR−BRT Age/Sex Fits − Non−pregnant Females 2+ Years Old − Total Anaemia Prevalence − Kitui
WHO−2024 Adjustment Method

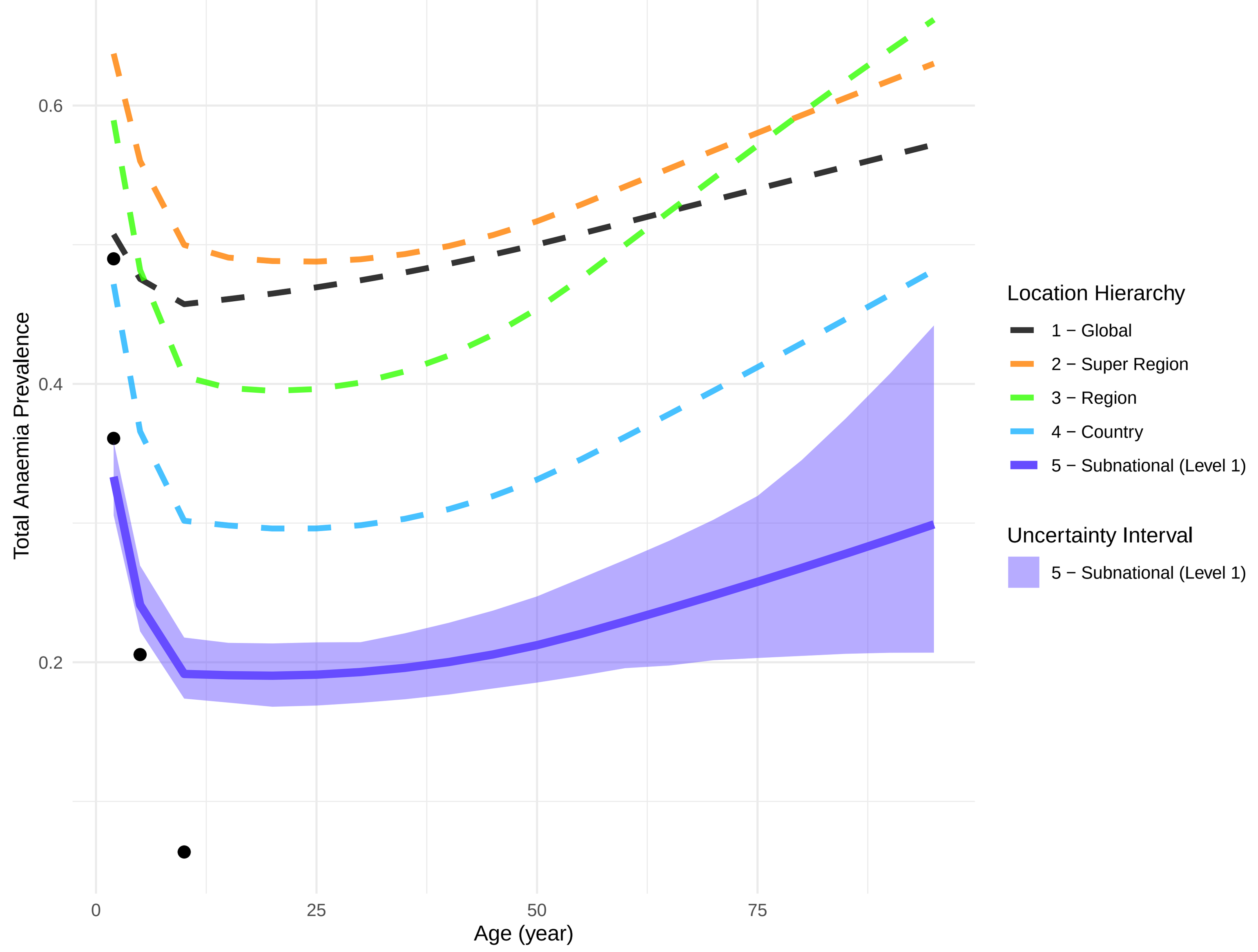

MR−BRT Age/Sex Fits − Non−pregnant Females 2+ Years Old − Total Anaemia Prevalence − Kwale
WHO−2024 Adjustment Method

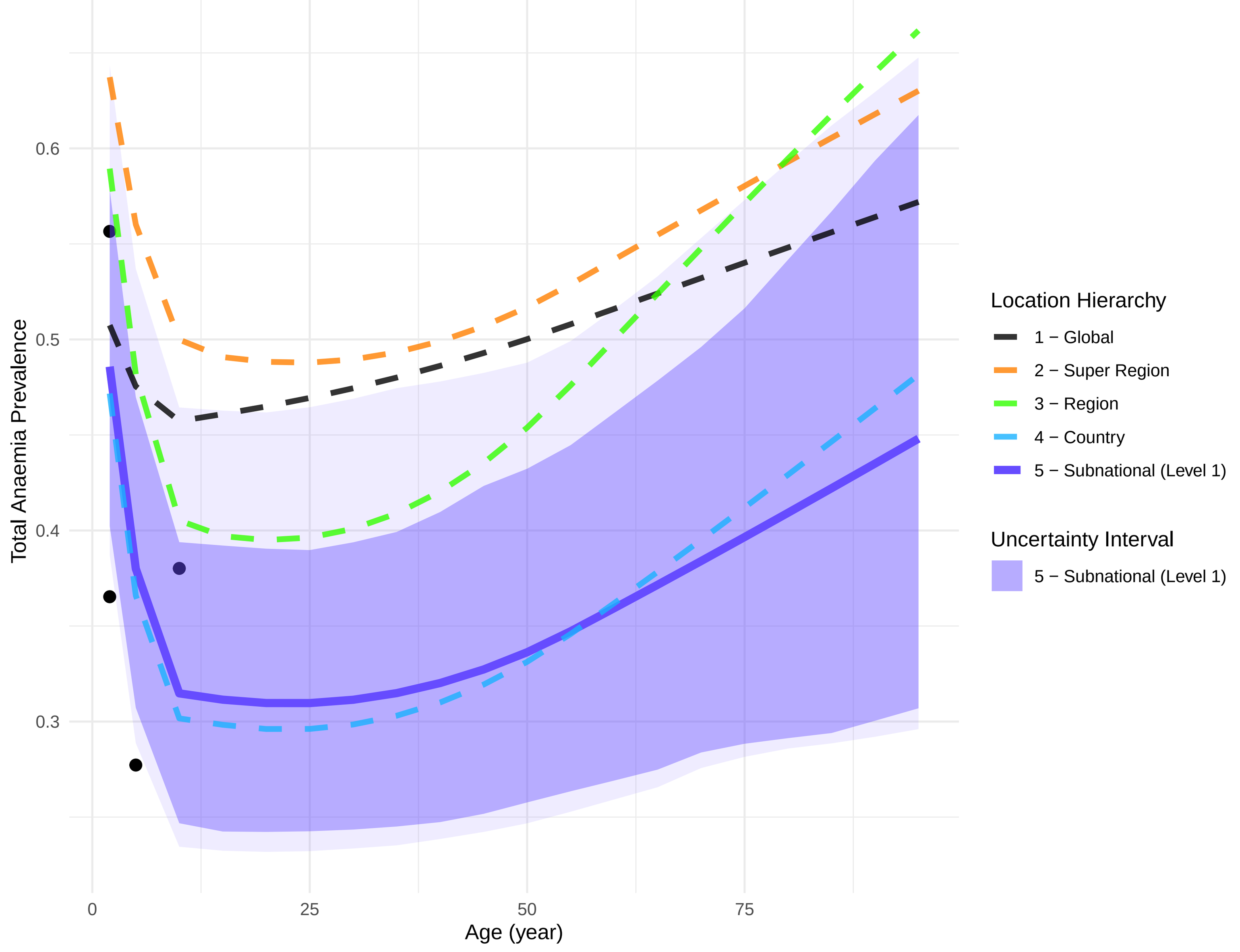

MR−BRT Age/Sex Fits − Non−pregnant Females 2+ Years Old − Total Anaemia Prevalence − Laikipia
WHO−2024 Adjustment Method

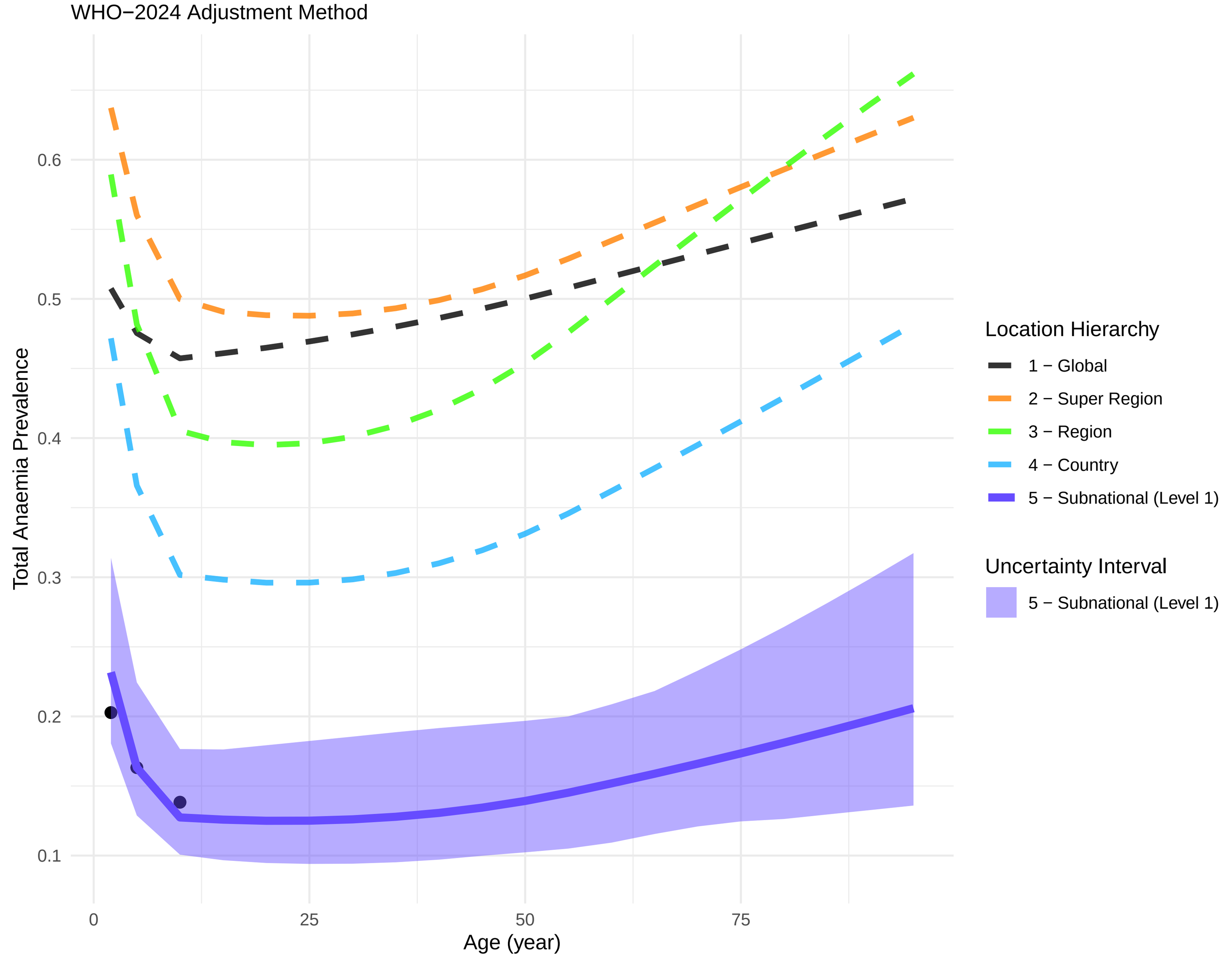

MR−BRT Age/Sex Fits − Non−pregnant Females 2+ Years Old − Total Anaemia Prevalence − Lamu
WHO−2024 Adjustment Method

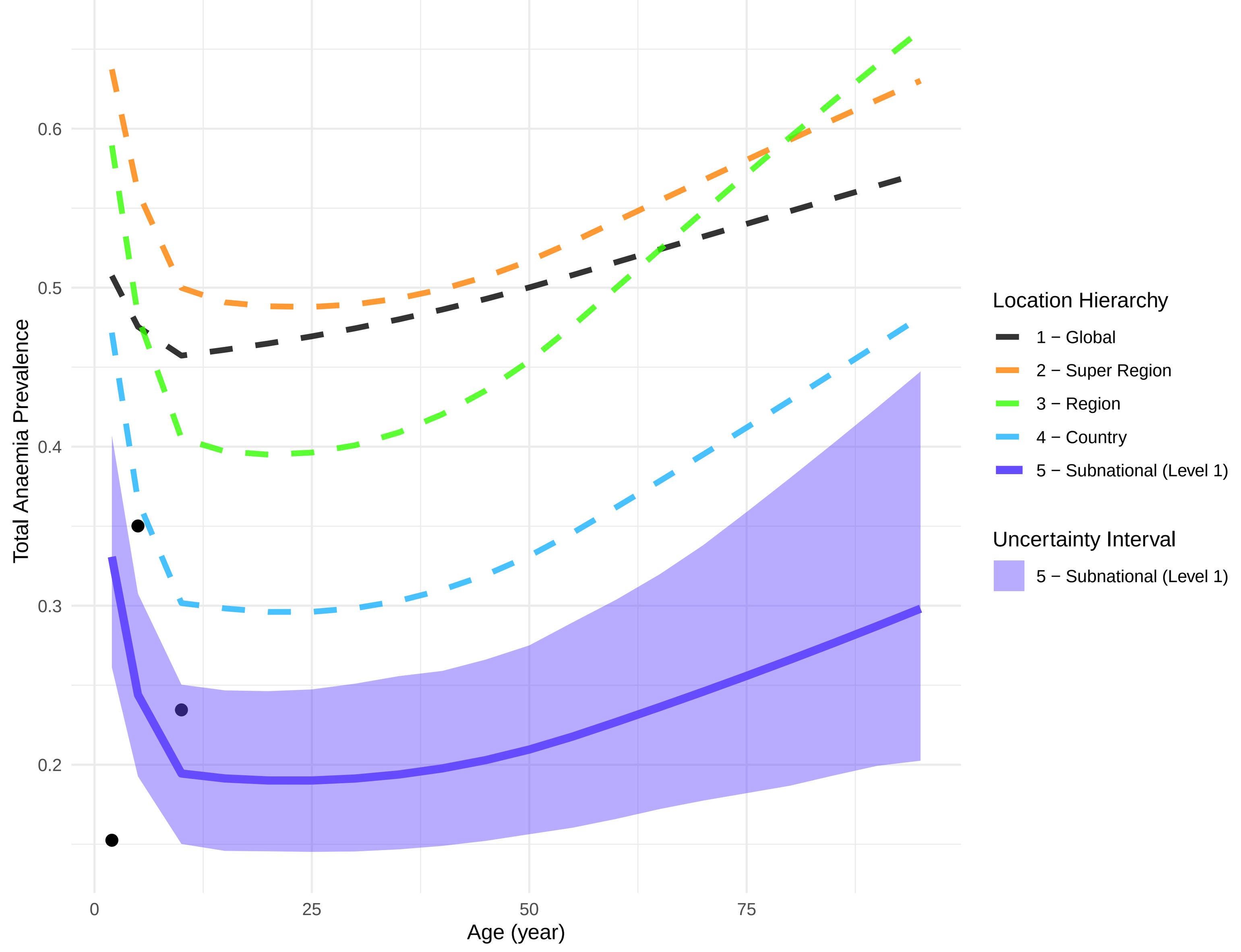

MR−BRT Age/Sex Fits − Non−pregnant Females 2+ Years Old − Total Anaemia Prevalence − Machakos
WHO−2024 Adjustment Method

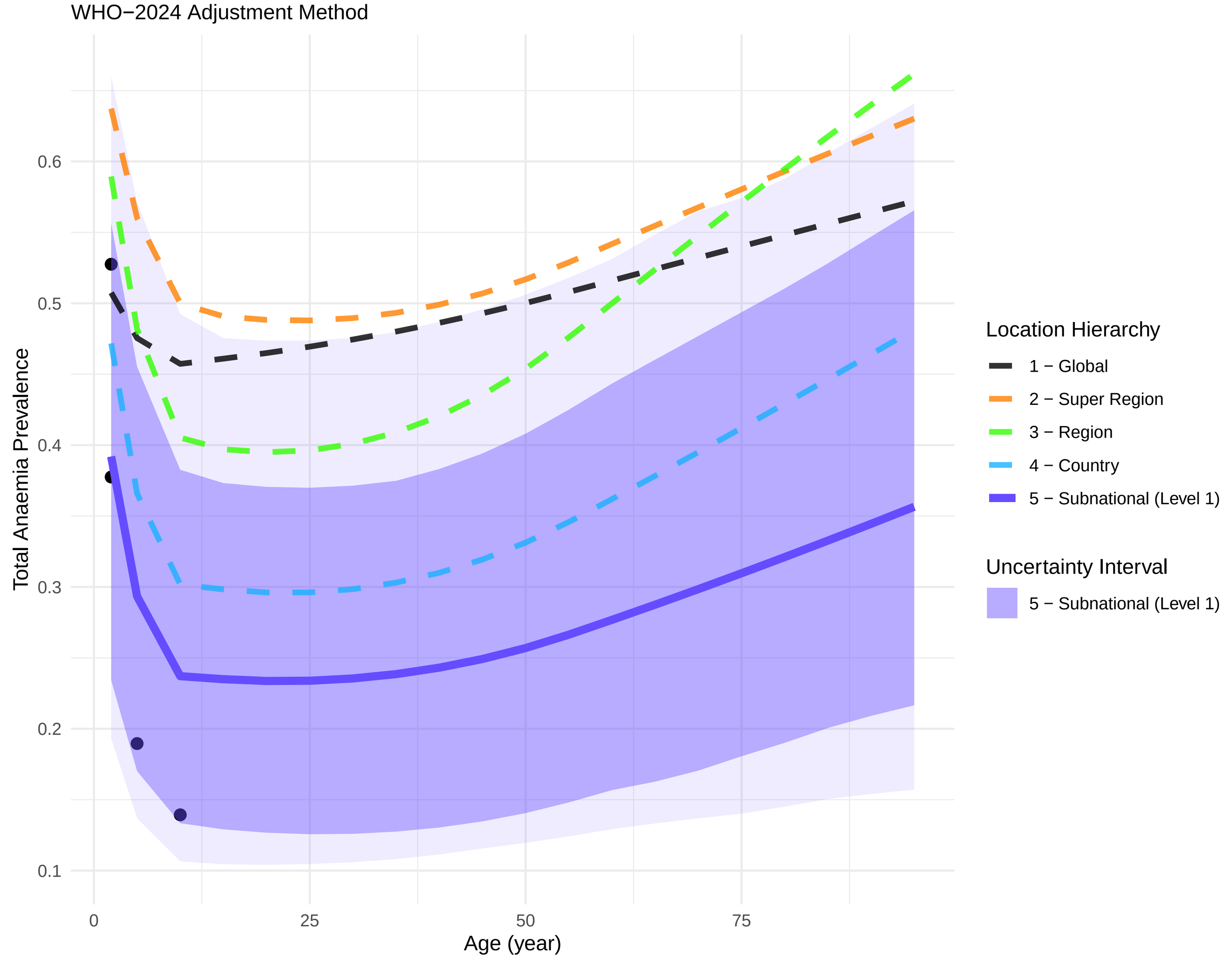

MR−BRT Age/Sex Fits − Non−pregnant Females 2+ Years Old − Total Anaemia Prevalence − Makueni
WHO−2024 Adjustment Method

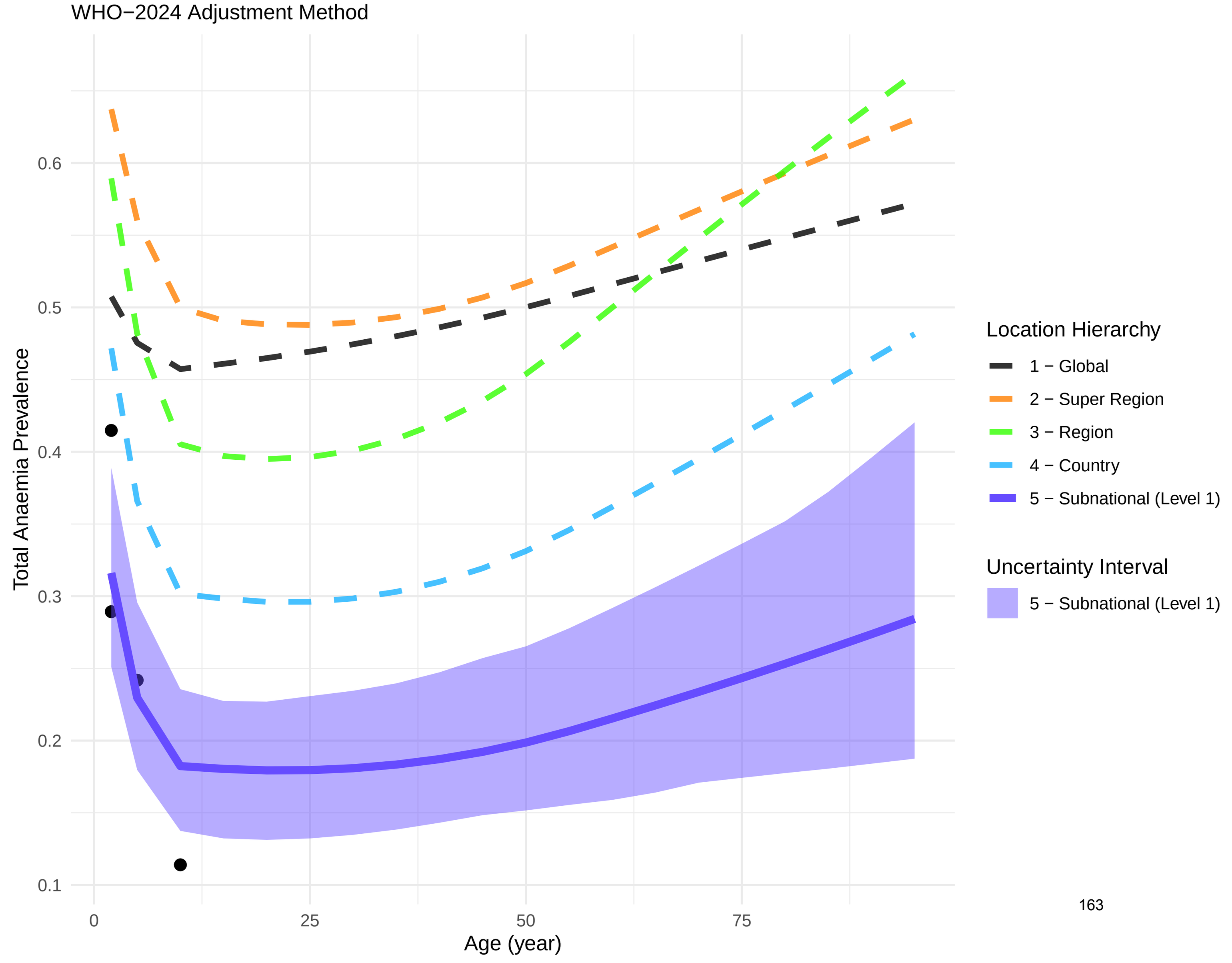

MR−BRT Age/Sex Fits − Non−pregnant Females 2+ Years Old − Total Anaemia Prevalence − Mandera
WHO−2024 Adjustment Method

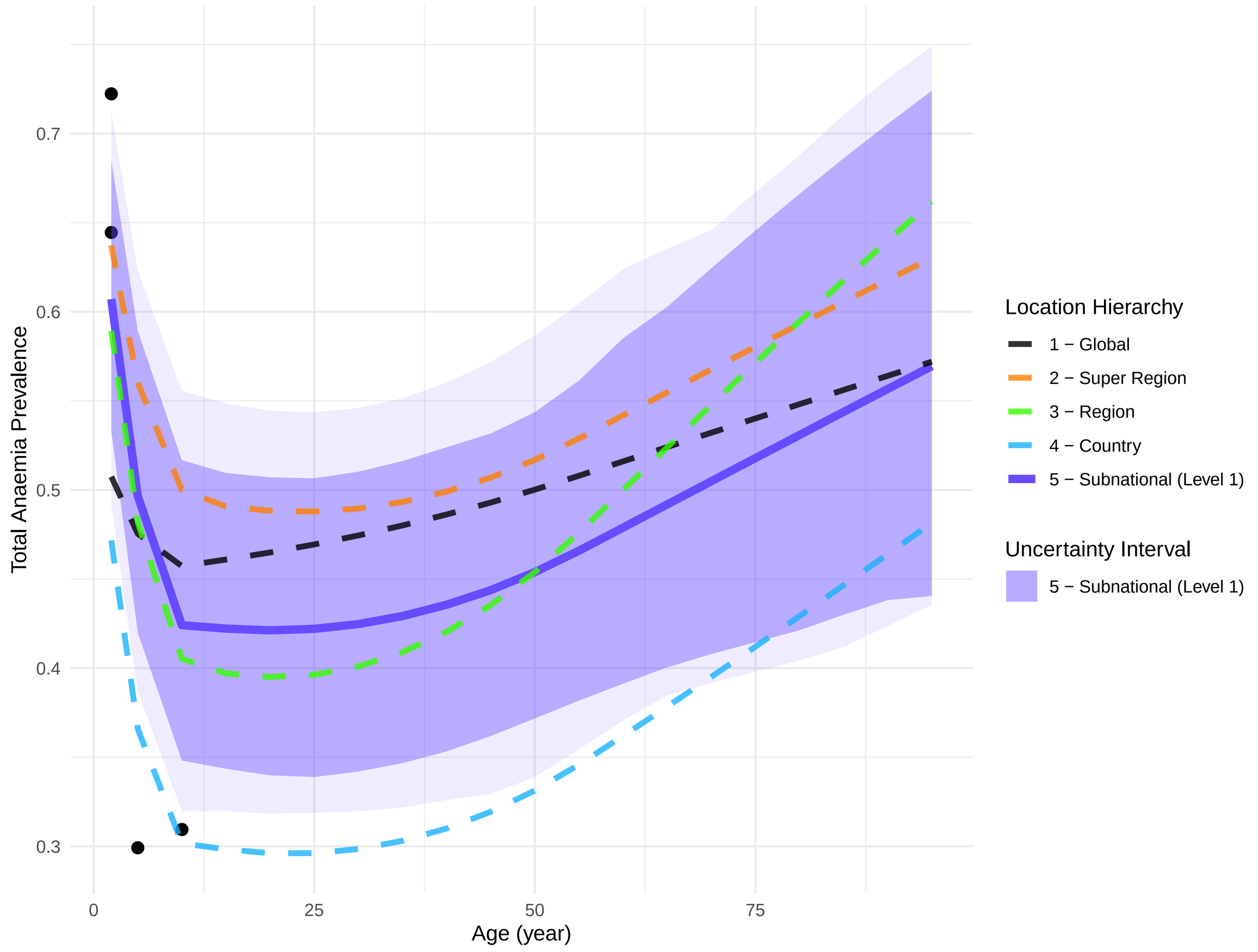

MR−BRT Age/Sex Fits − Non−pregnant Females 2+ Years Old − Total Anaemia Prevalence − Marsabit
WHO−2024 Adjustment Method

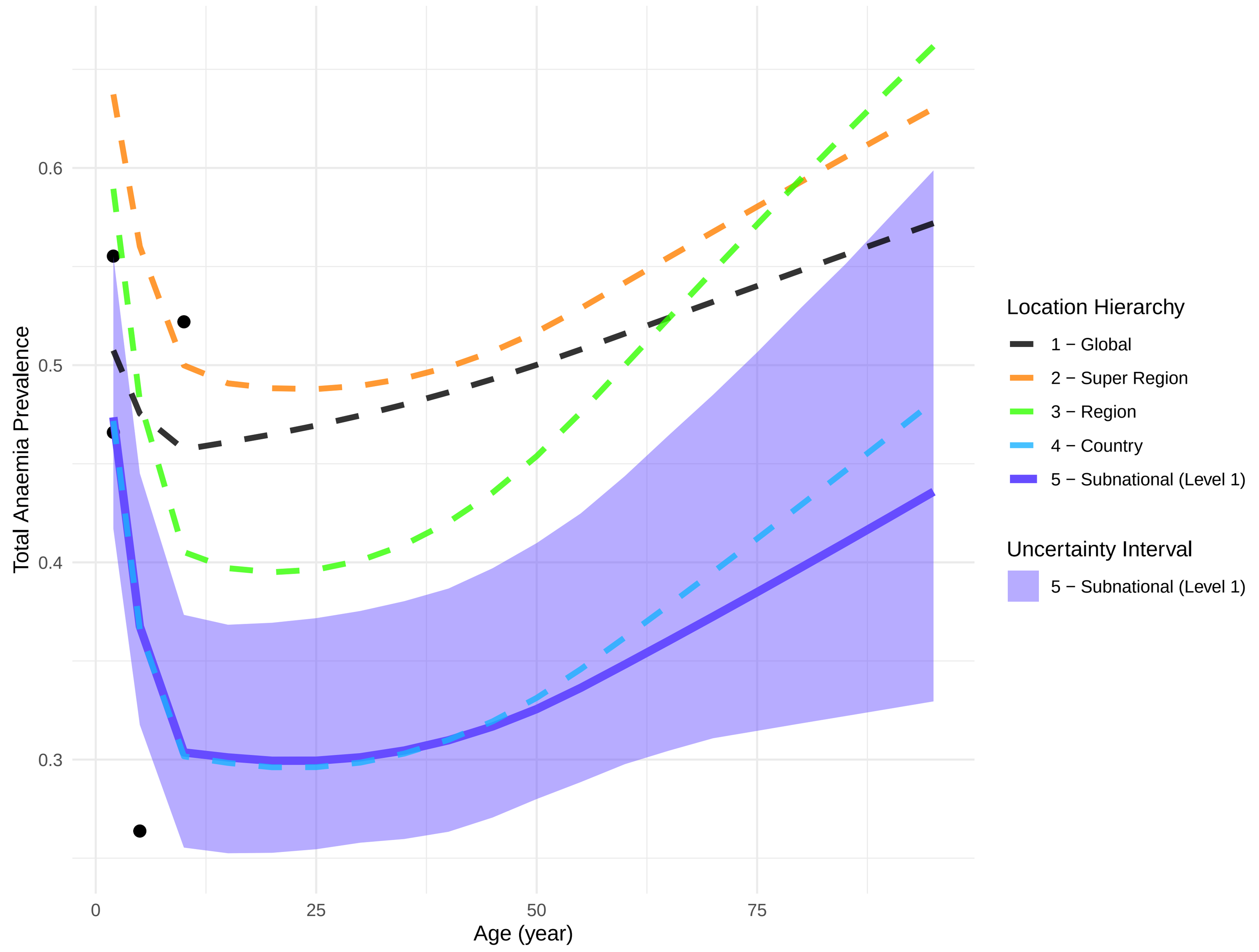

MR−BRT Age/Sex Fits − Non−pregnant Females 2+ Years Old − Total Anaemia Prevalence − Meru
WHO−2024 Adjustment Method

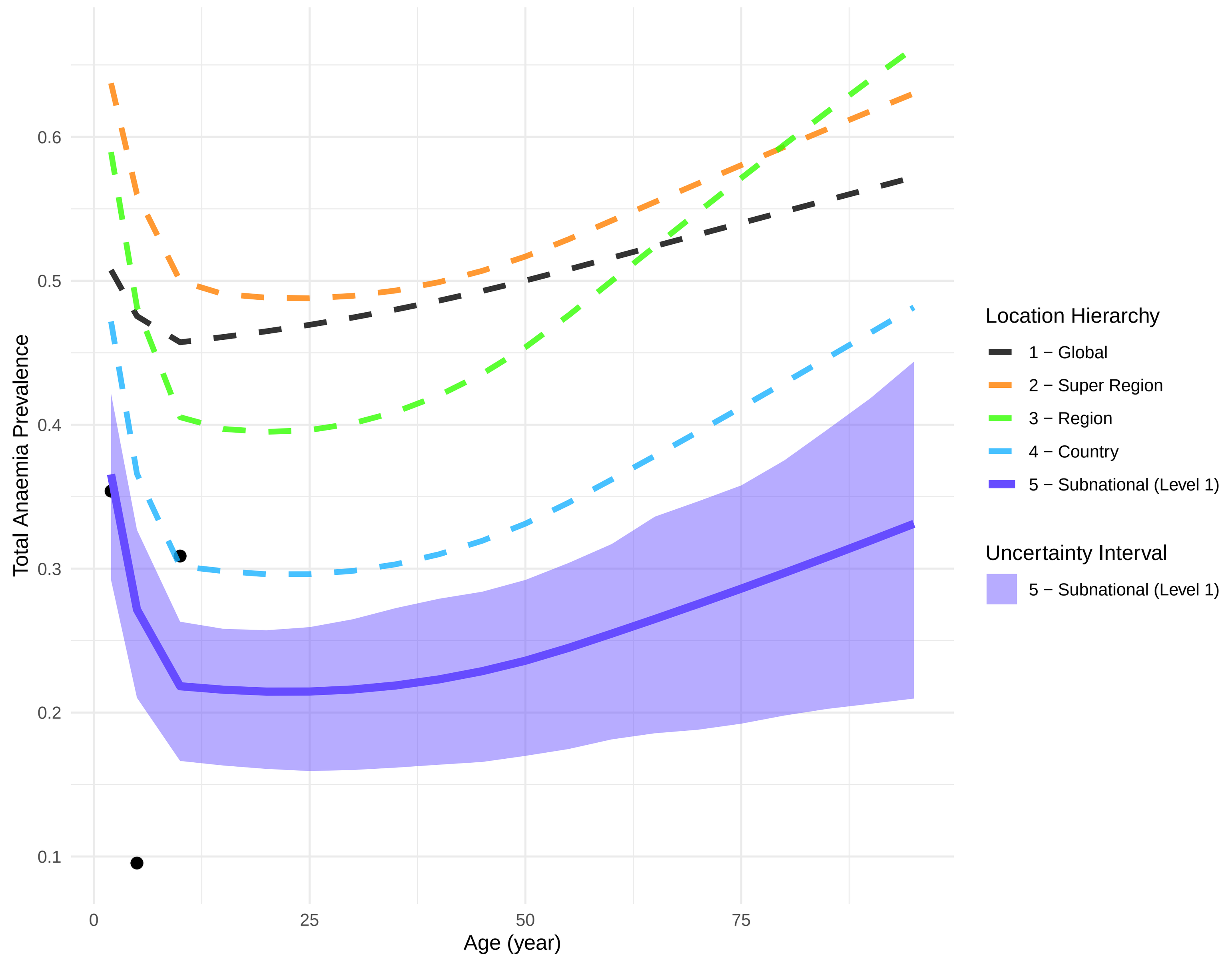

MR−BRT Age/Sex Fits − Non−pregnant Females 2+ Years Old − Total Anaemia Prevalence − Migori
WHO−2024 Adjustment Method

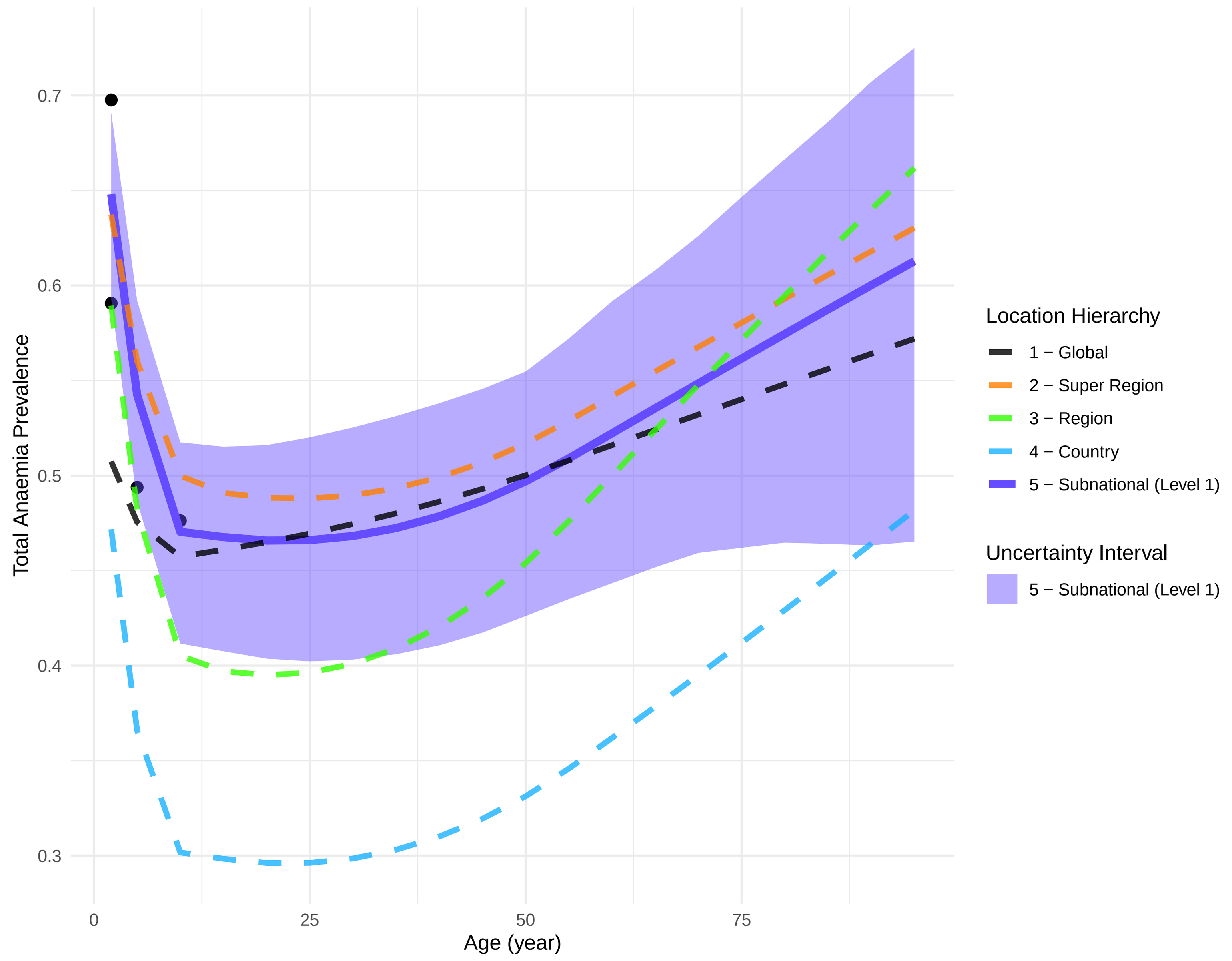

MR−BRT Age/Sex Fits − Non−pregnant Females 2+ Years Old − Total Anaemia Prevalence − Mombasa
WHO−2024 Adjustment Method

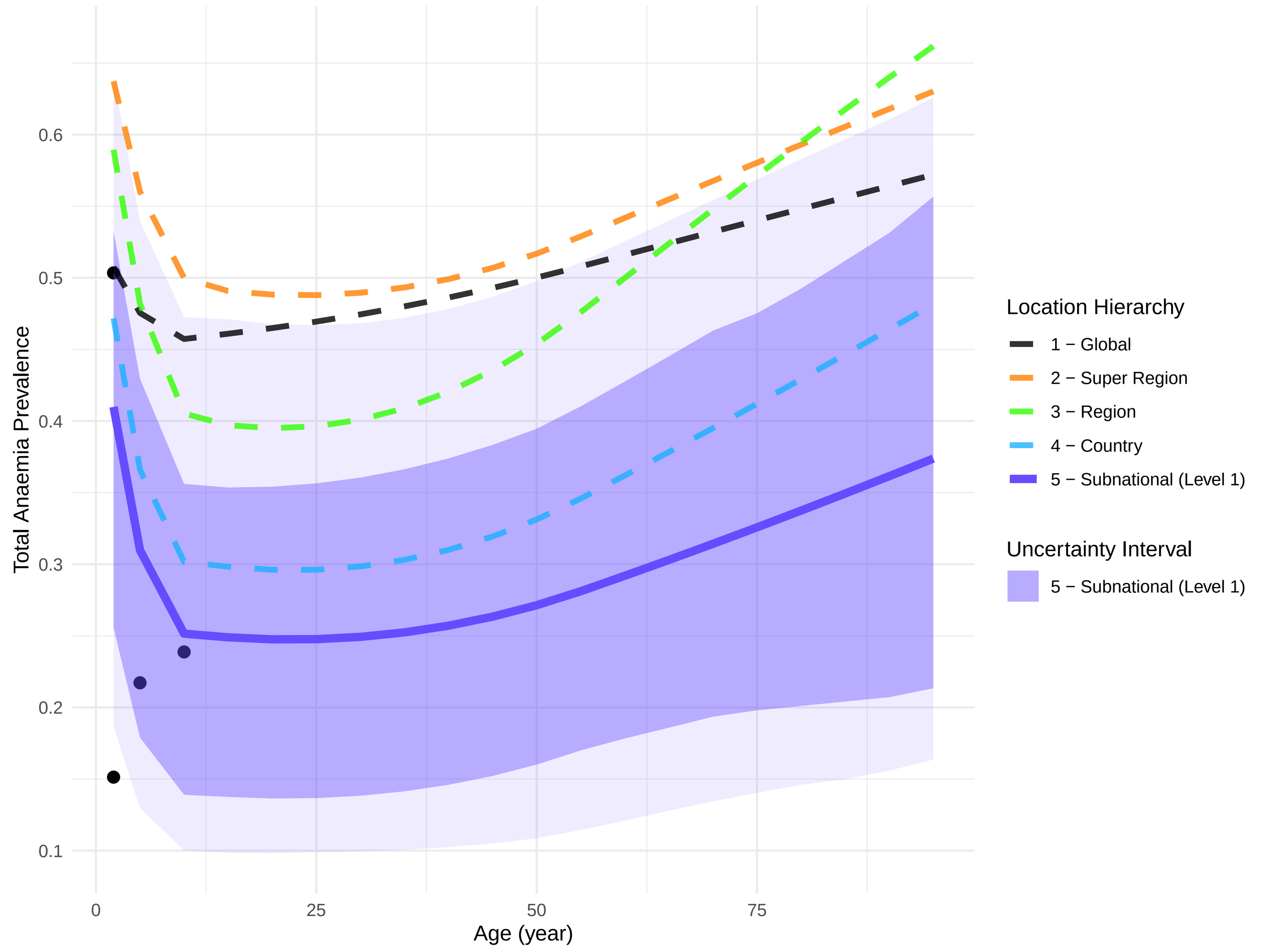

MR−BRT Age/Sex Fits − Non−pregnant Females 2+ Years Old − Total Anaemia Prevalence − Murang'a
WHO−2024 Adjustment Method

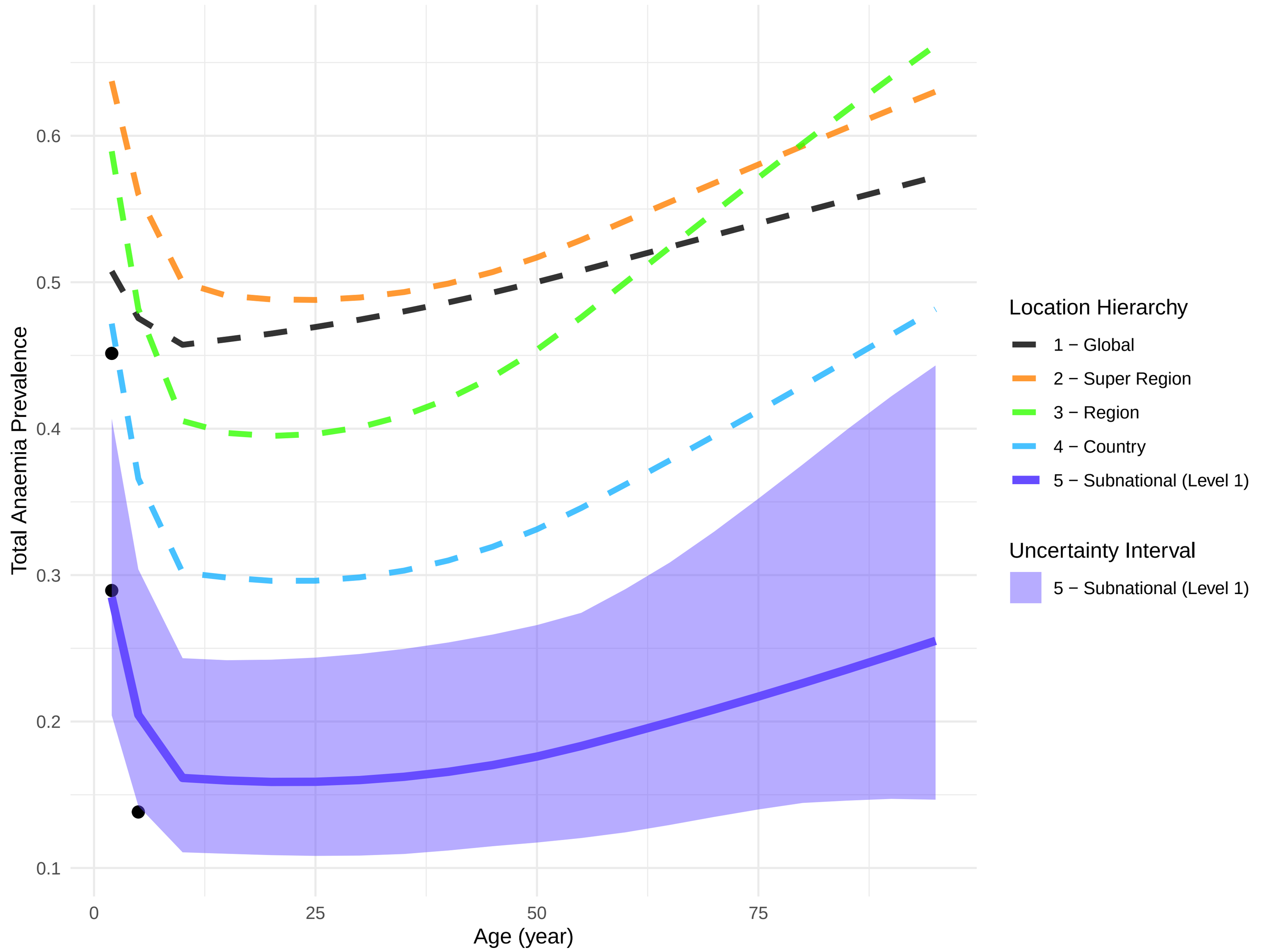

MR−BRT Age/Sex Fits − Non−pregnant Females 2+ Years Old − Total Anaemia Prevalence − Nairobi
WHO−2024 Adjustment Method

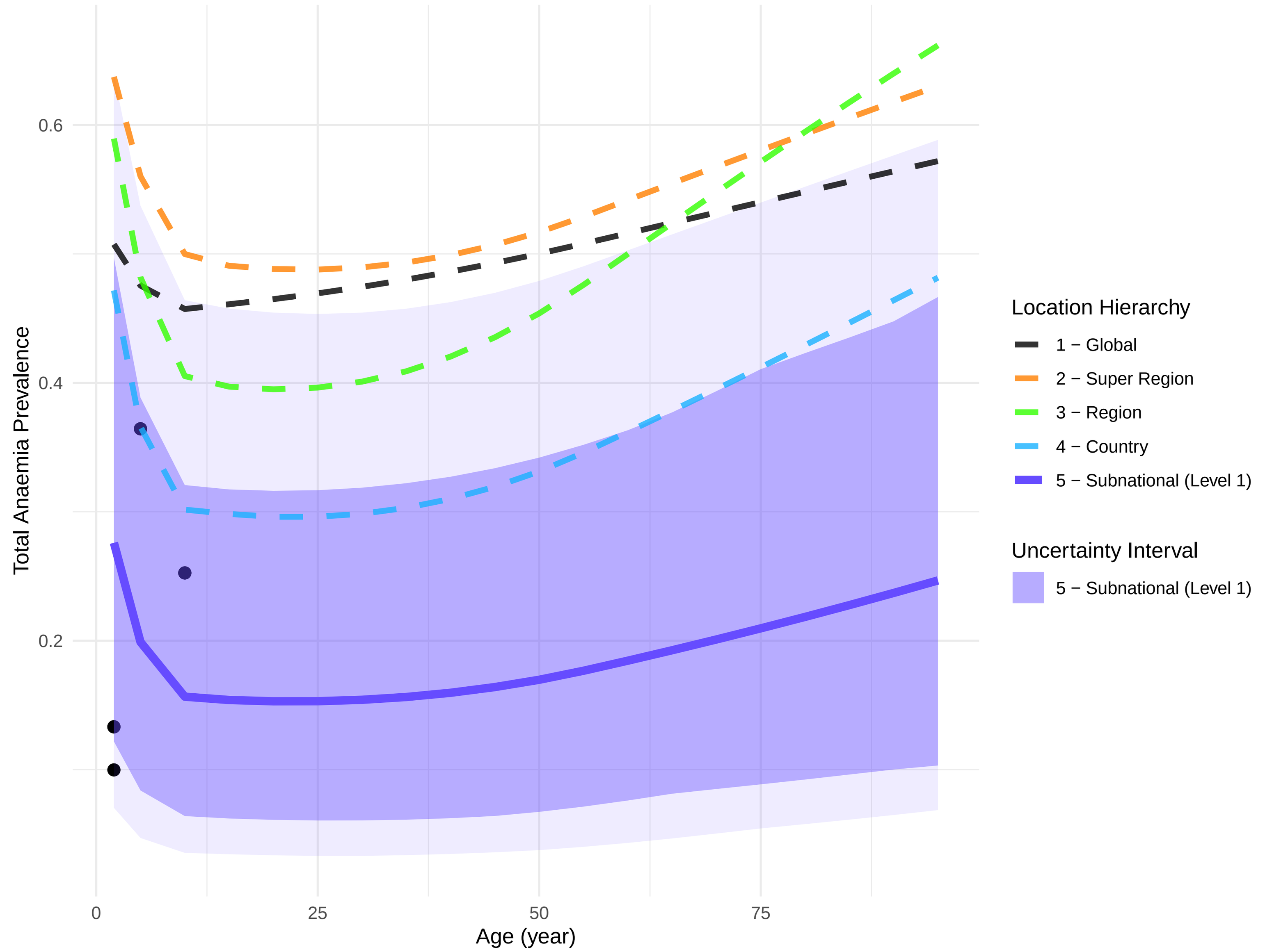

MR−BRT Age/Sex Fits − Non−pregnant Females 2+ Years Old − Total Anaemia Prevalence − Nakuru
WHO−2024 Adjustment Method

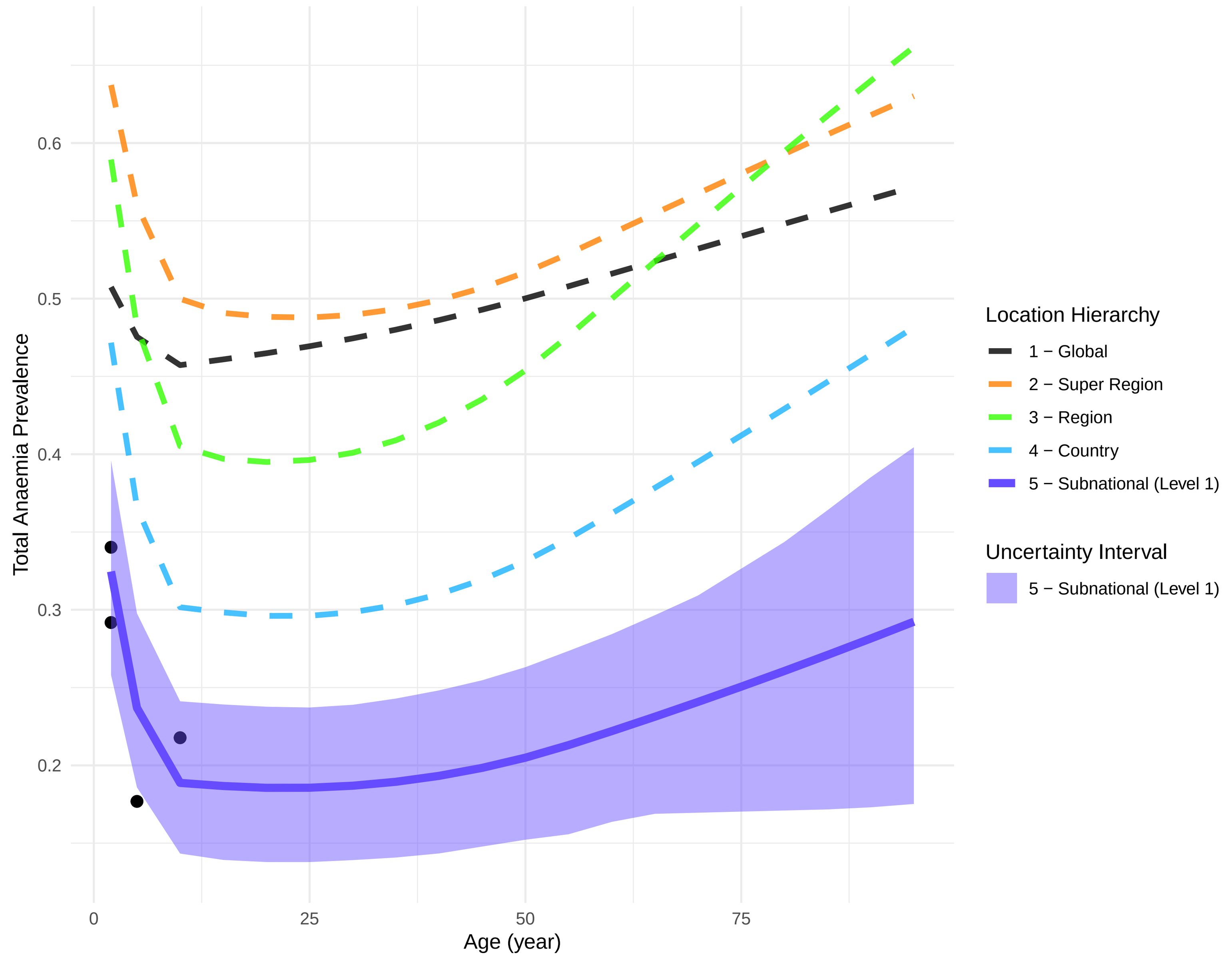

MR−BRT Age/Sex Fits − Non−pregnant Females 2+ Years Old − Total Anaemia Prevalence − Nandi
WHO−2024 Adjustment Method

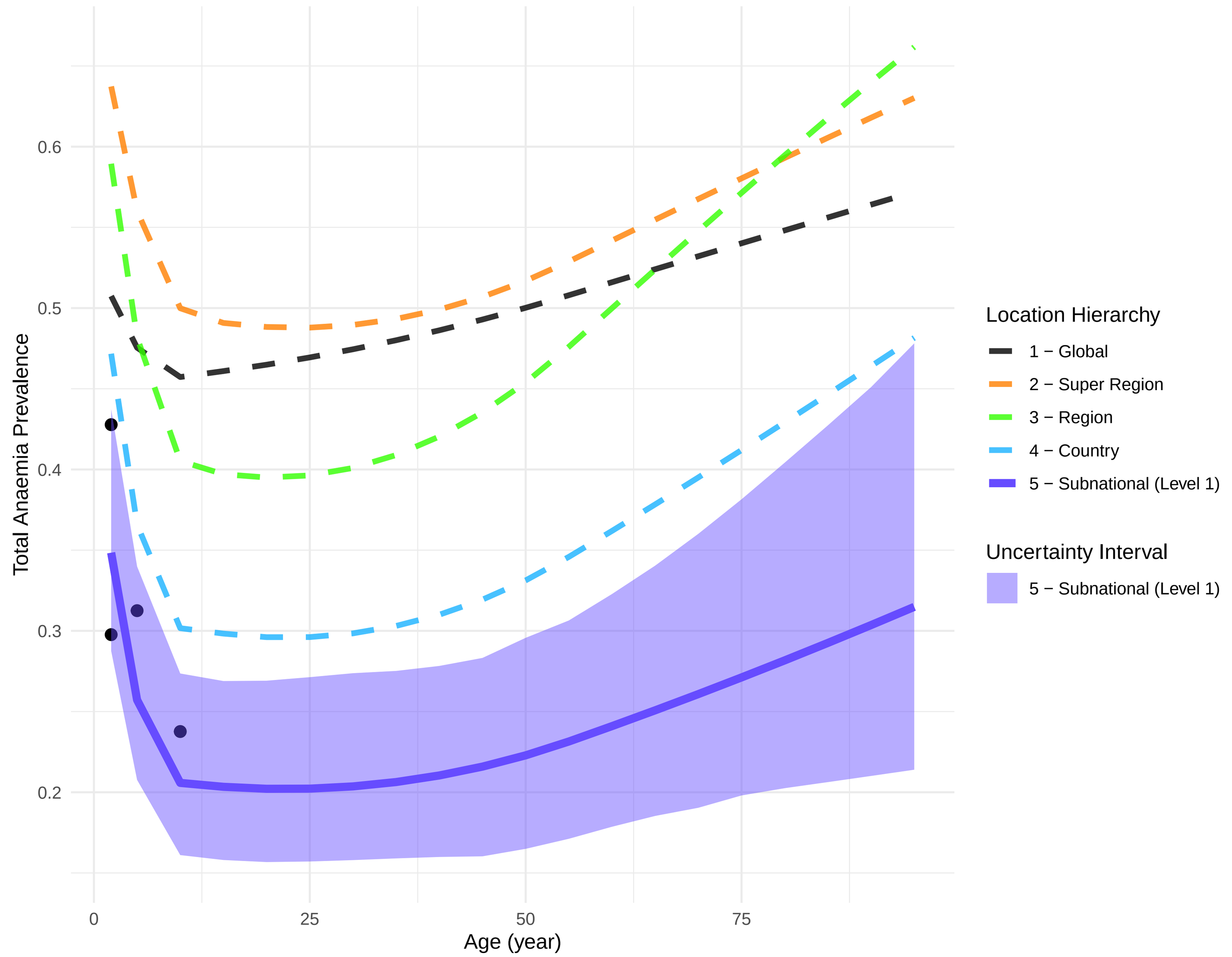

MR−BRT Age/Sex Fits − Non−pregnant Females 2+ Years Old − Total Anaemia Prevalence − Narok
WHO−2024 Adjustment Method

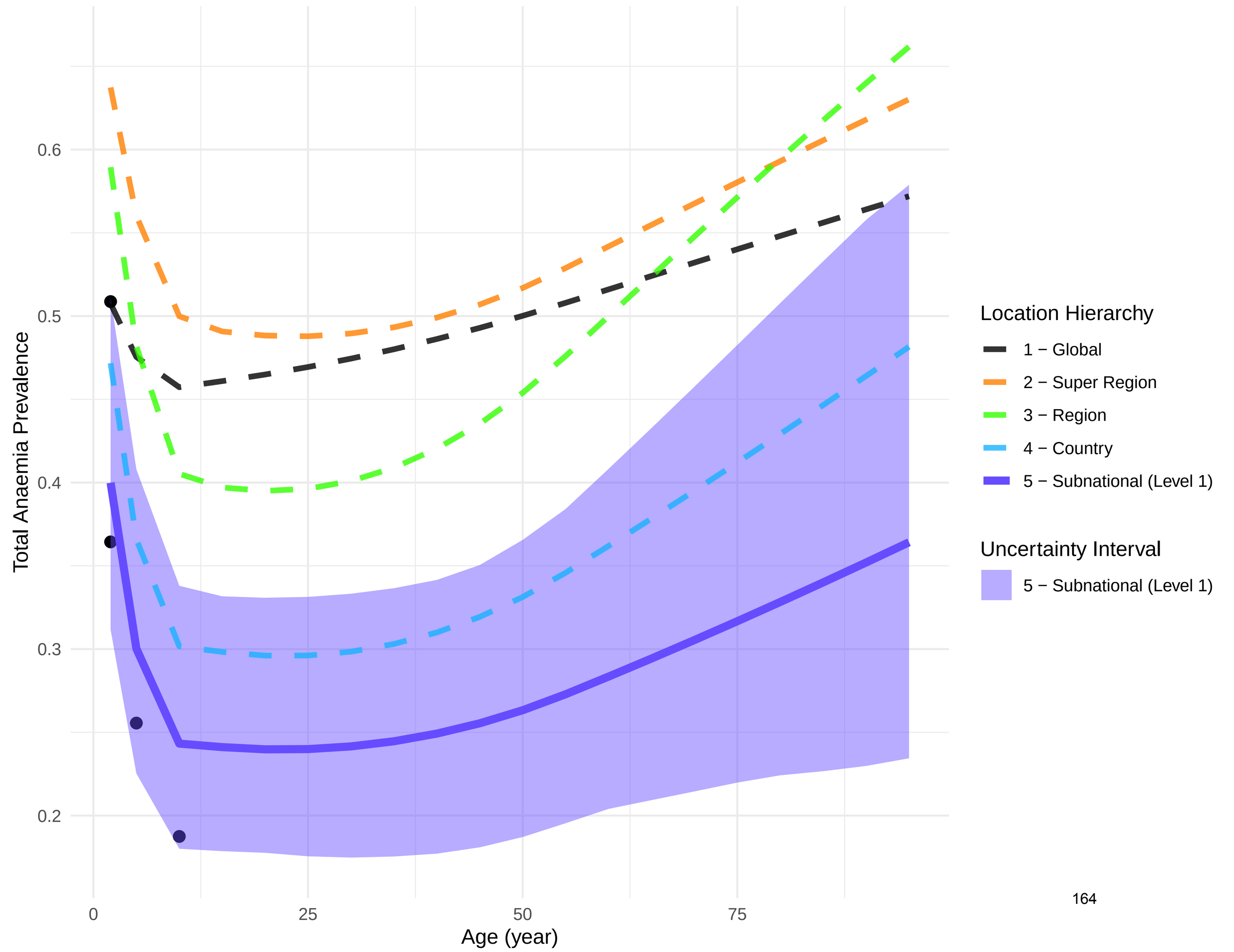

MR−BRT Age/Sex Fits − Non−pregnant Females 2+ Years Old − Total Anaemia Prevalence − Nyamira
WHO−2024 Adjustment Method

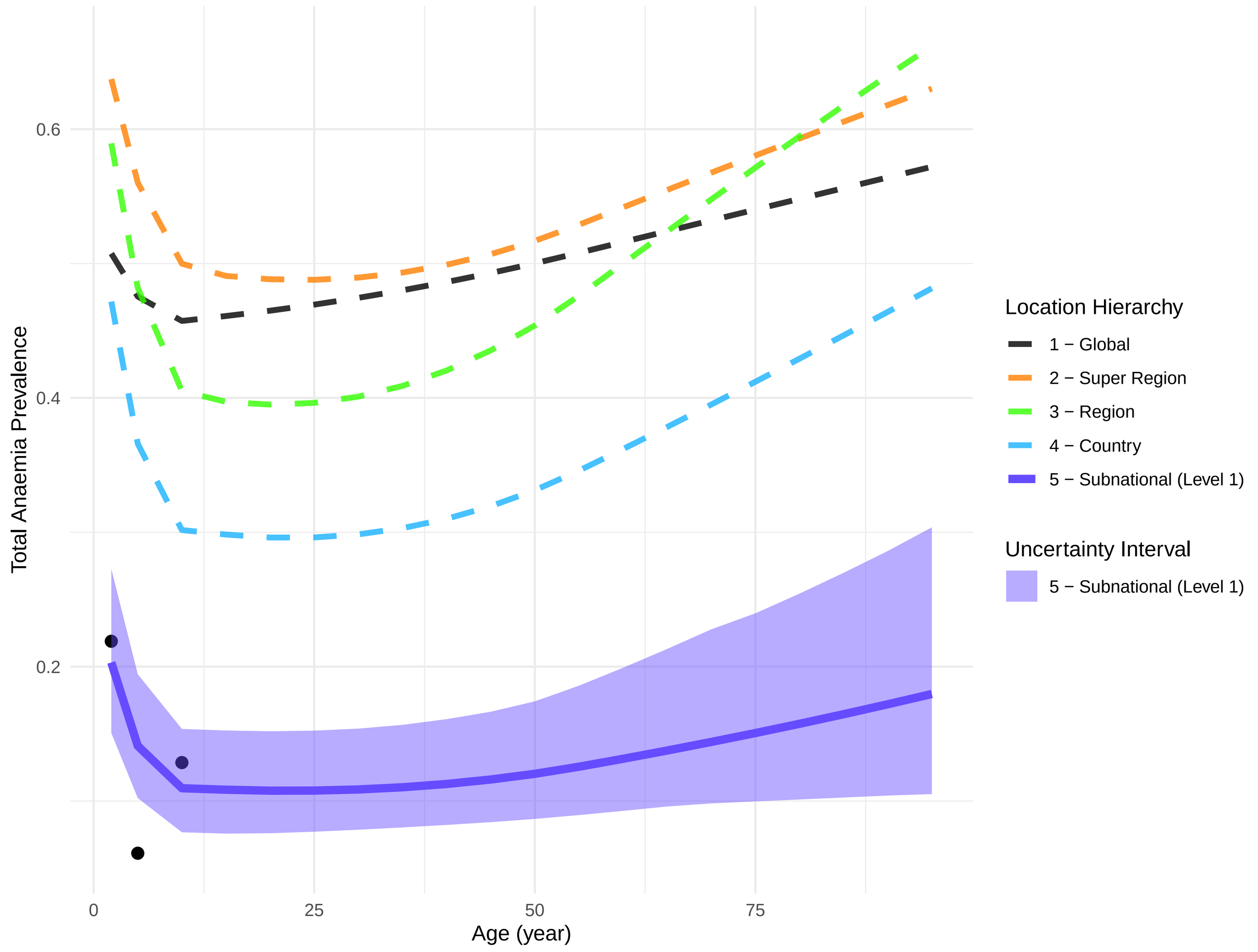

MR−BRT Age/Sex Fits − Non−pregnant Females 2+ Years Old − Total Anaemia Prevalence − Nyandarua
WHO−2024 Adjustment Method

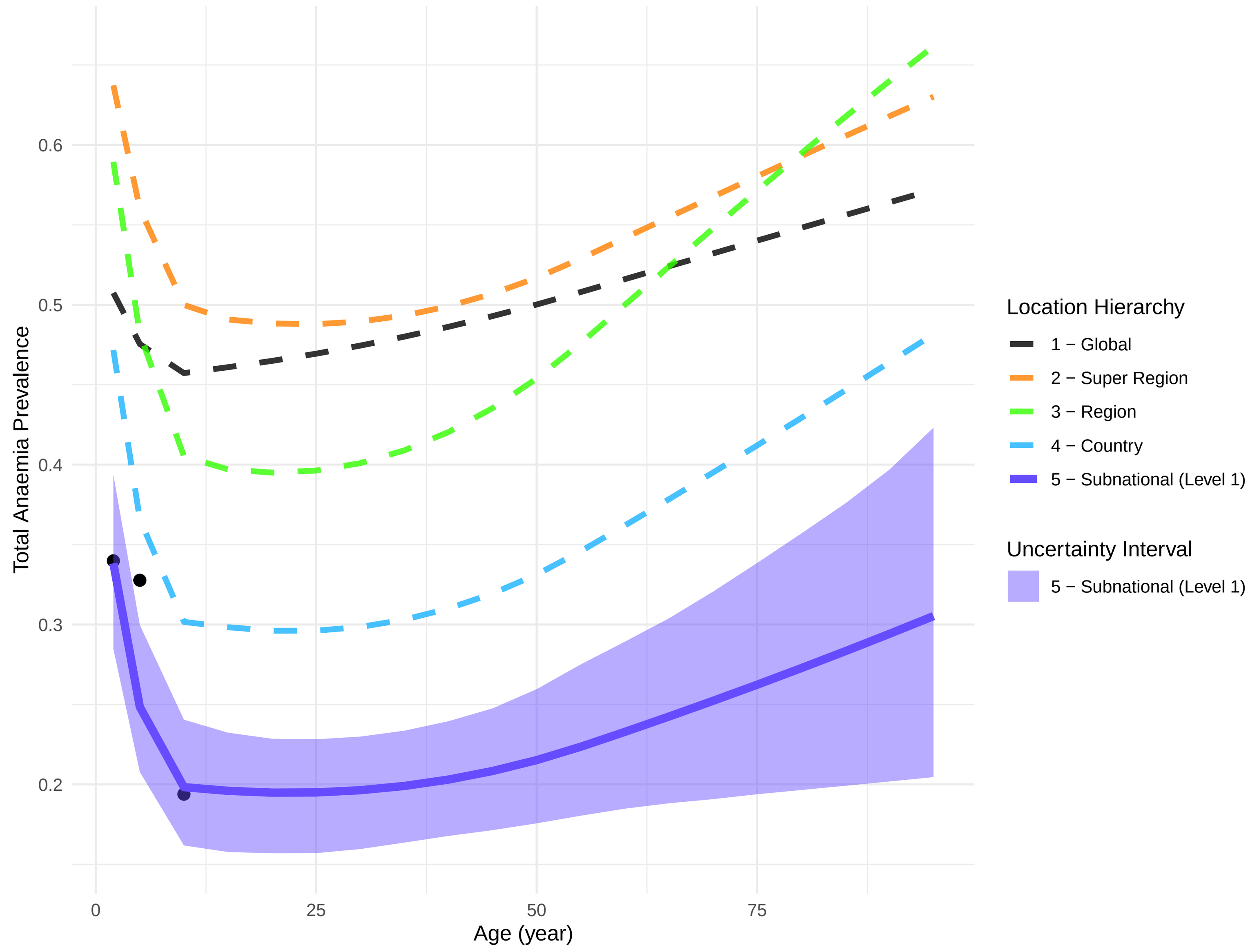

MR−BRT Age/Sex Fits − Non−pregnant Females 2+ Years Old − Total Anaemia Prevalence − Nyeri
WHO−2024 Adjustment Method

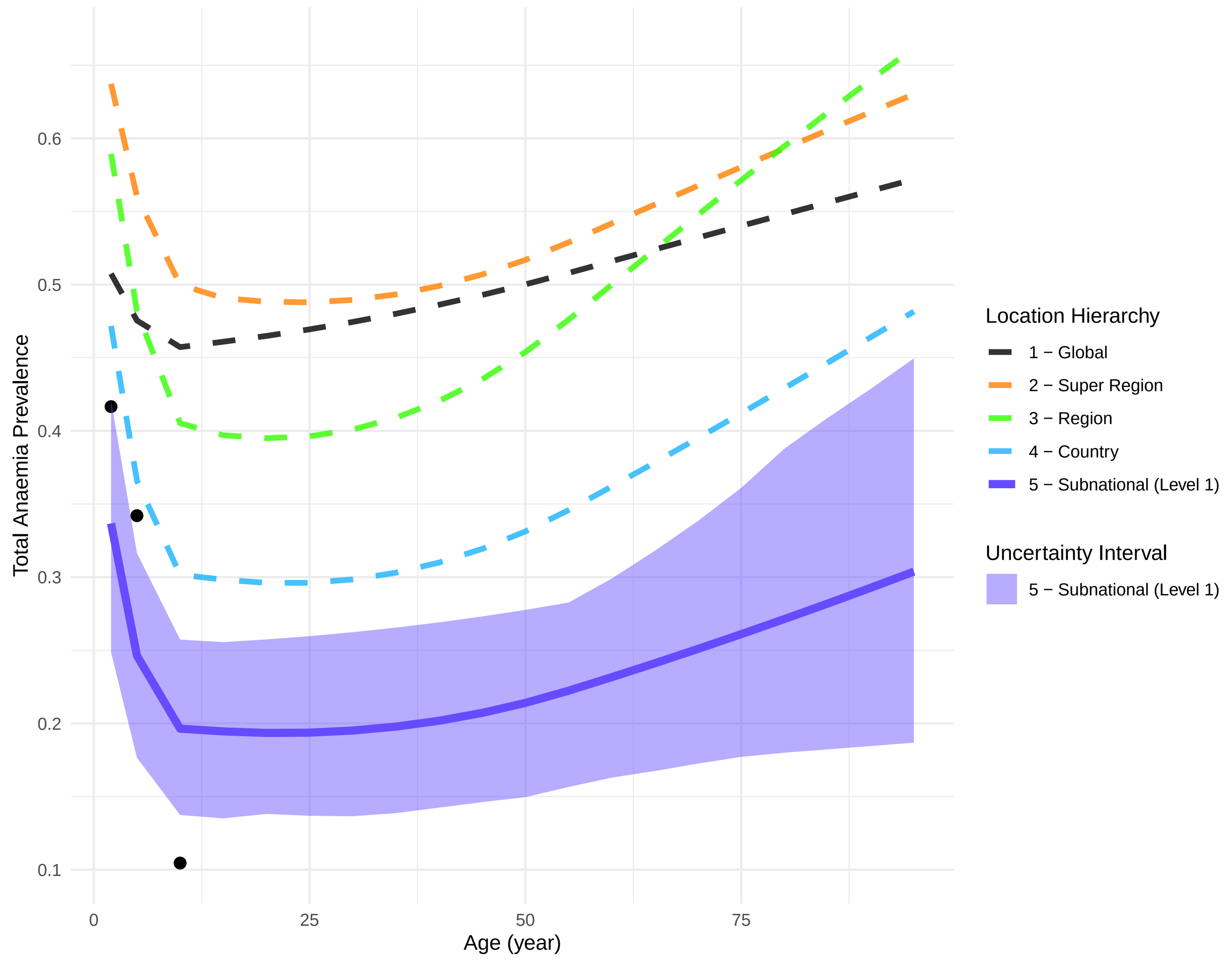

MR−BRT Age/Sex Fits − Non−pregnant Females 2+ Years Old − Total Anaemia Prevalence − Samburu
WHO−2024 Adjustment Method

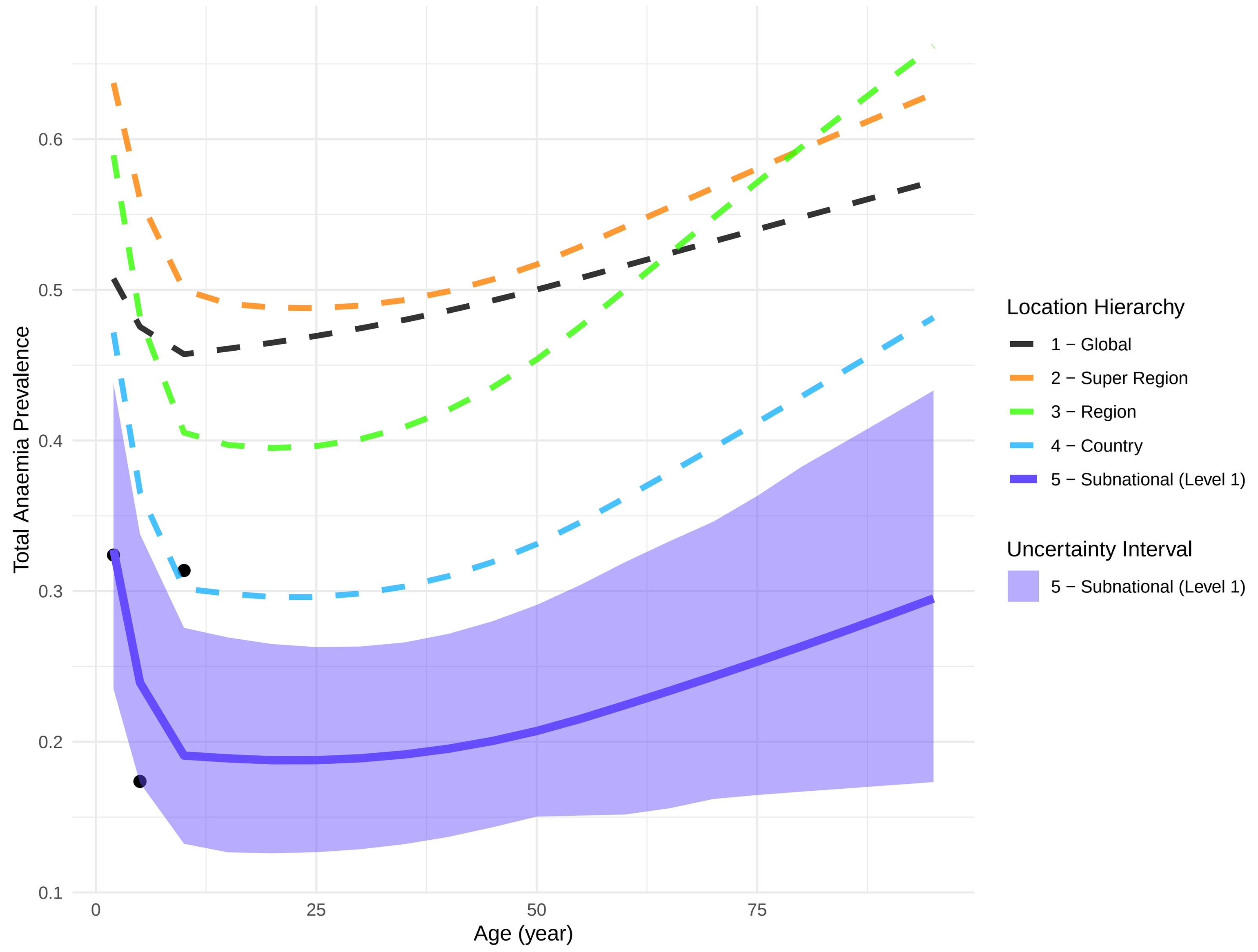

MR−BRT Age/Sex Fits − Non−pregnant Females 2+ Years Old − Total Anaemia Prevalence − Siaya
WHO−2024 Adjustment Method

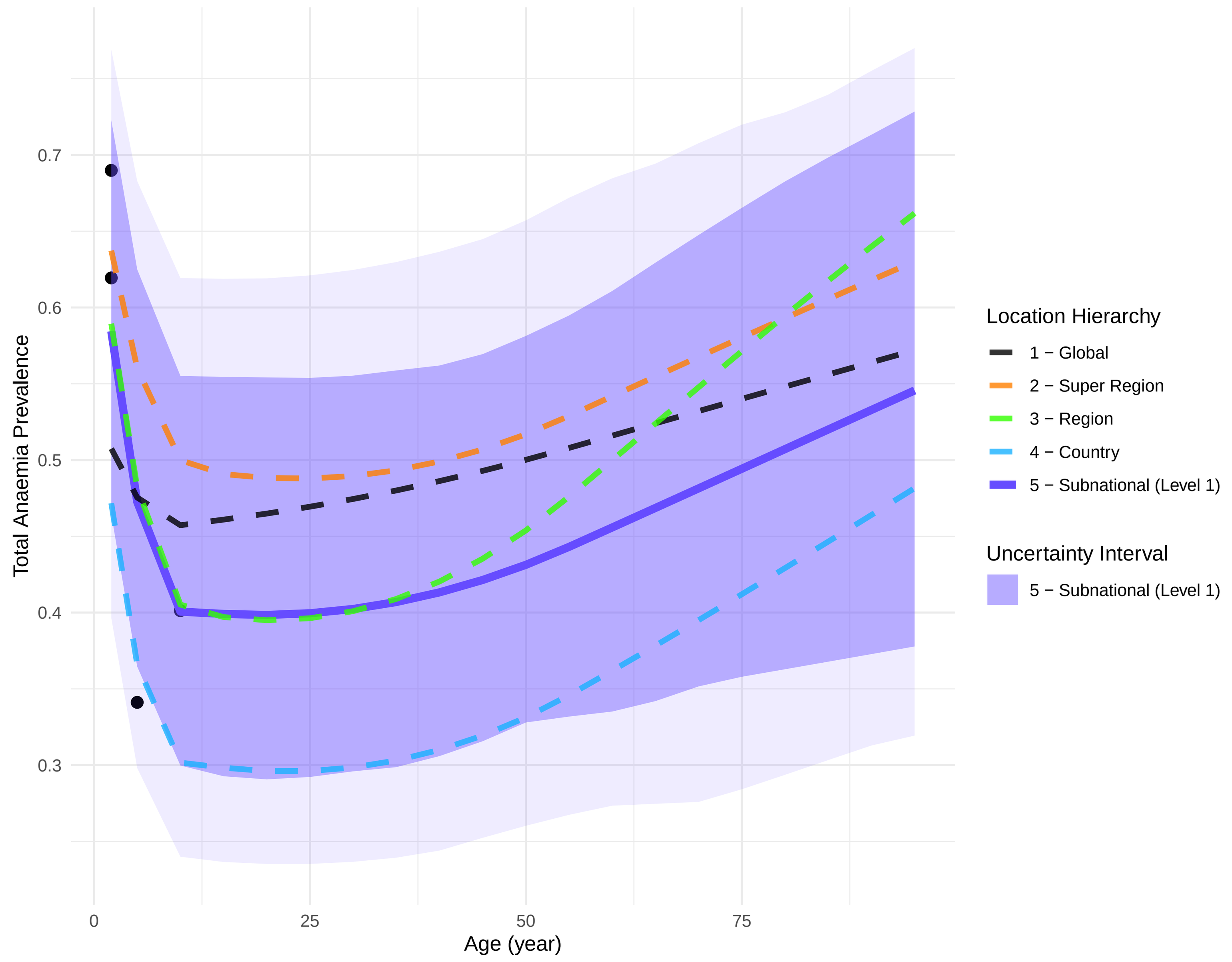

MR−BRT Age/Sex Fits − Non−pregnant Females 2+ Years Old − Total Anaemia Prevalence − Taita Taveta
WHO−2024 Adjustment Method

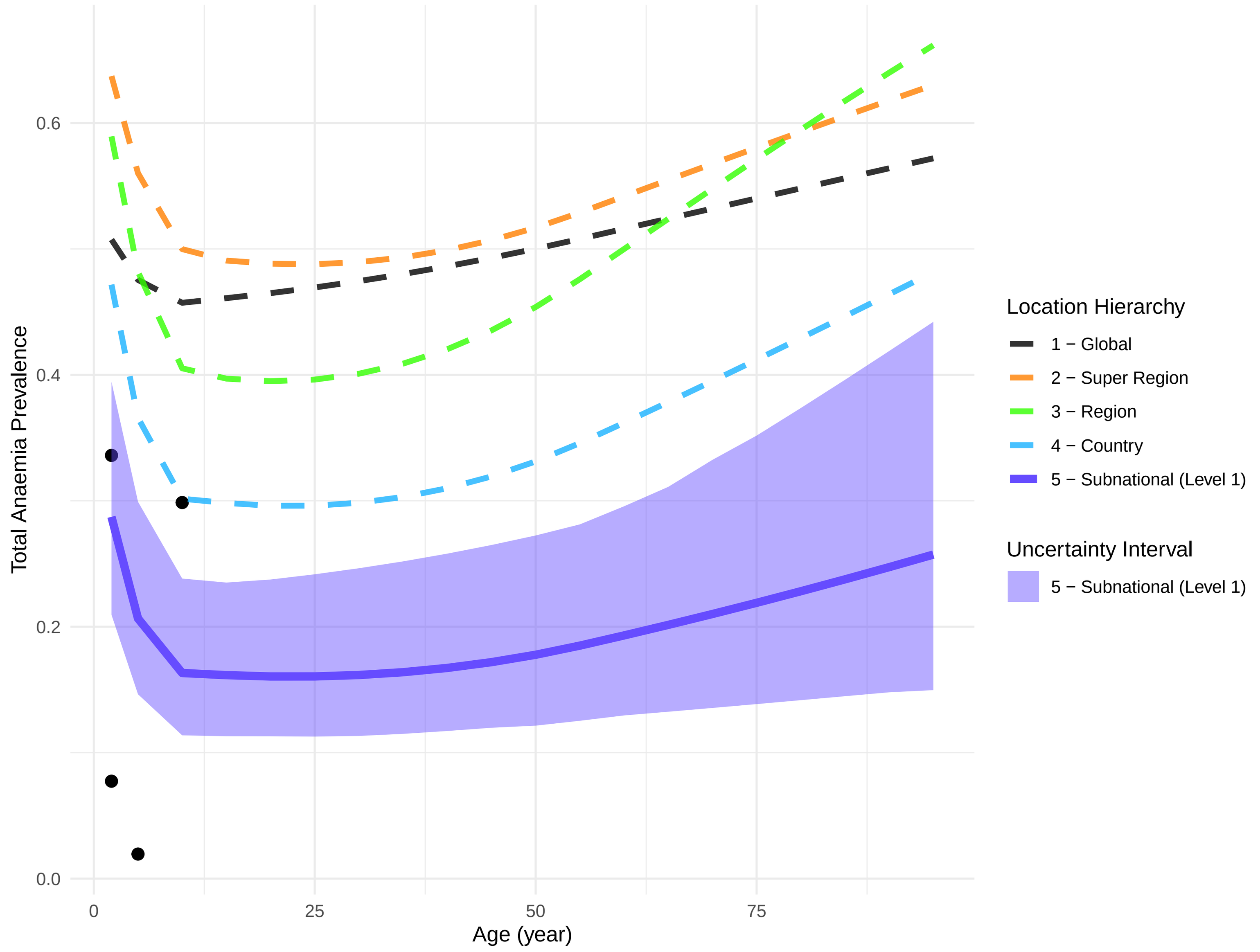

MR−BRT Age/Sex Fits − Non−pregnant Females 2+ Years Old − Total Anaemia Prevalence − Tana River
WHO−2024 Adjustment Method

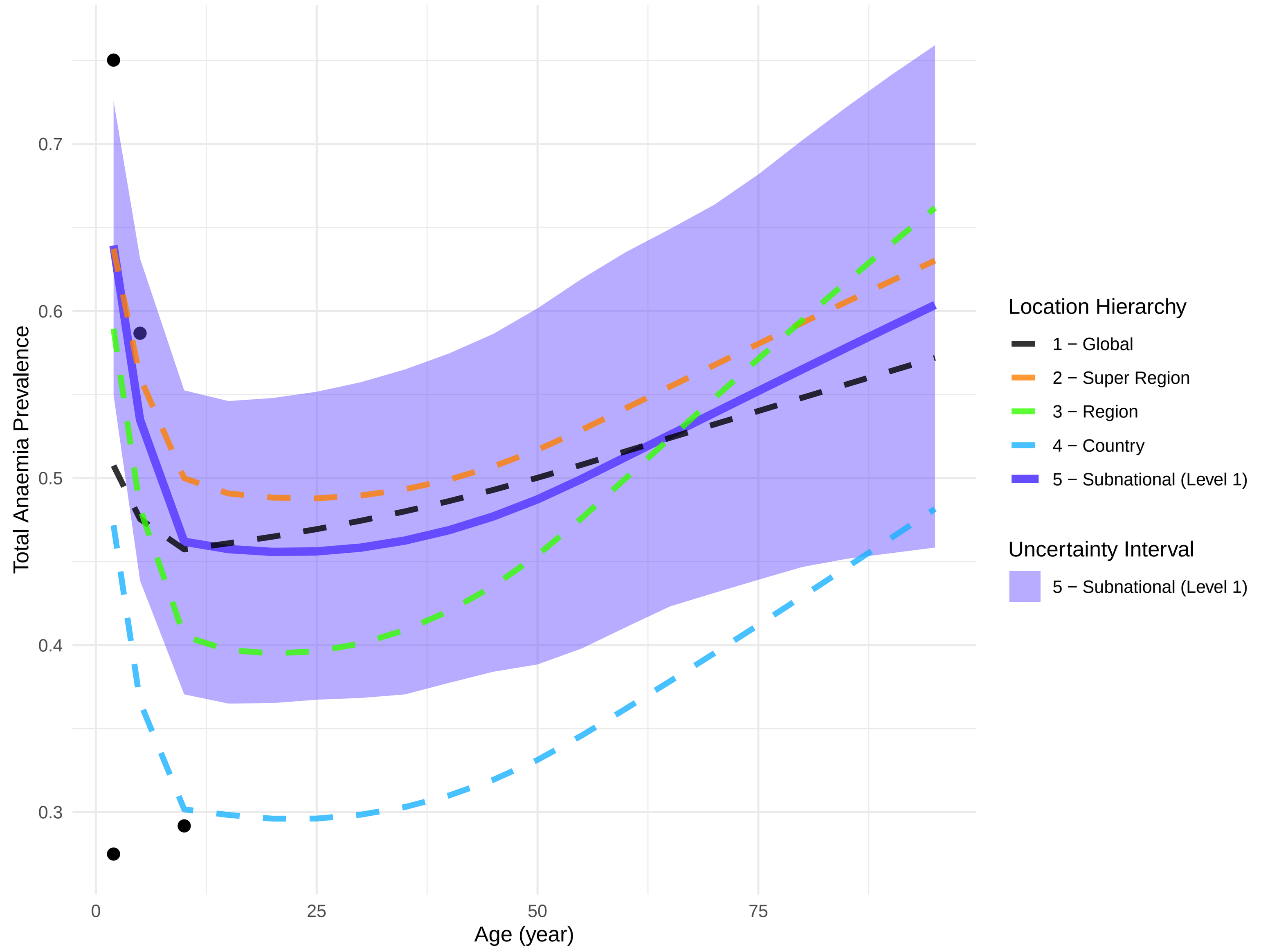

MR−BRT Age/Sex Fits − Non−pregnant Females 2+ Years Old − Total Anaemia Prevalence − Tharaka Nithi
WHO−2024 Adjustment Method

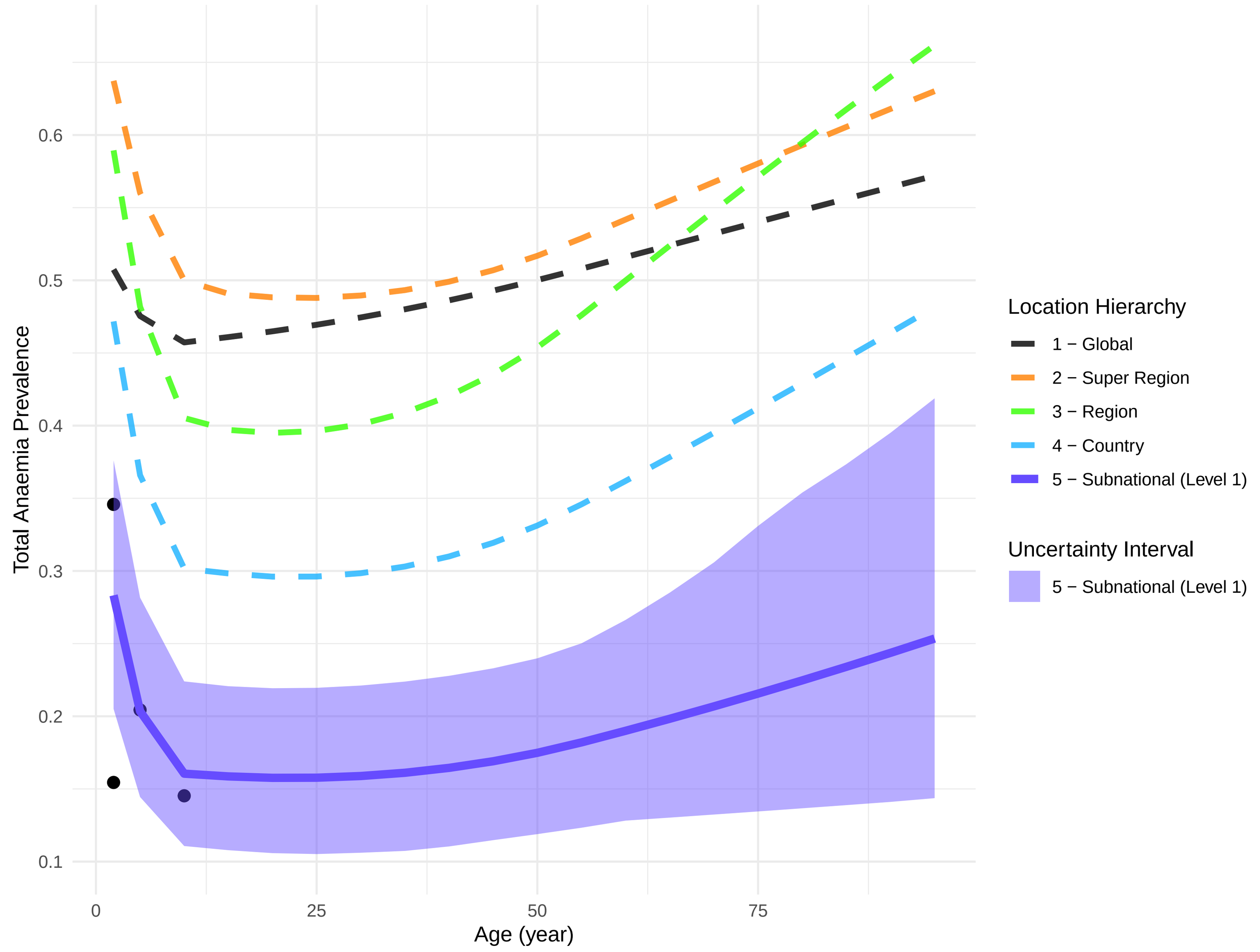

MR−BRT Age/Sex Fits − Non−pregnant Females 2+ Years Old − Total Anaemia Prevalence − Trans Nzoia
WHO−2024 Adjustment Method

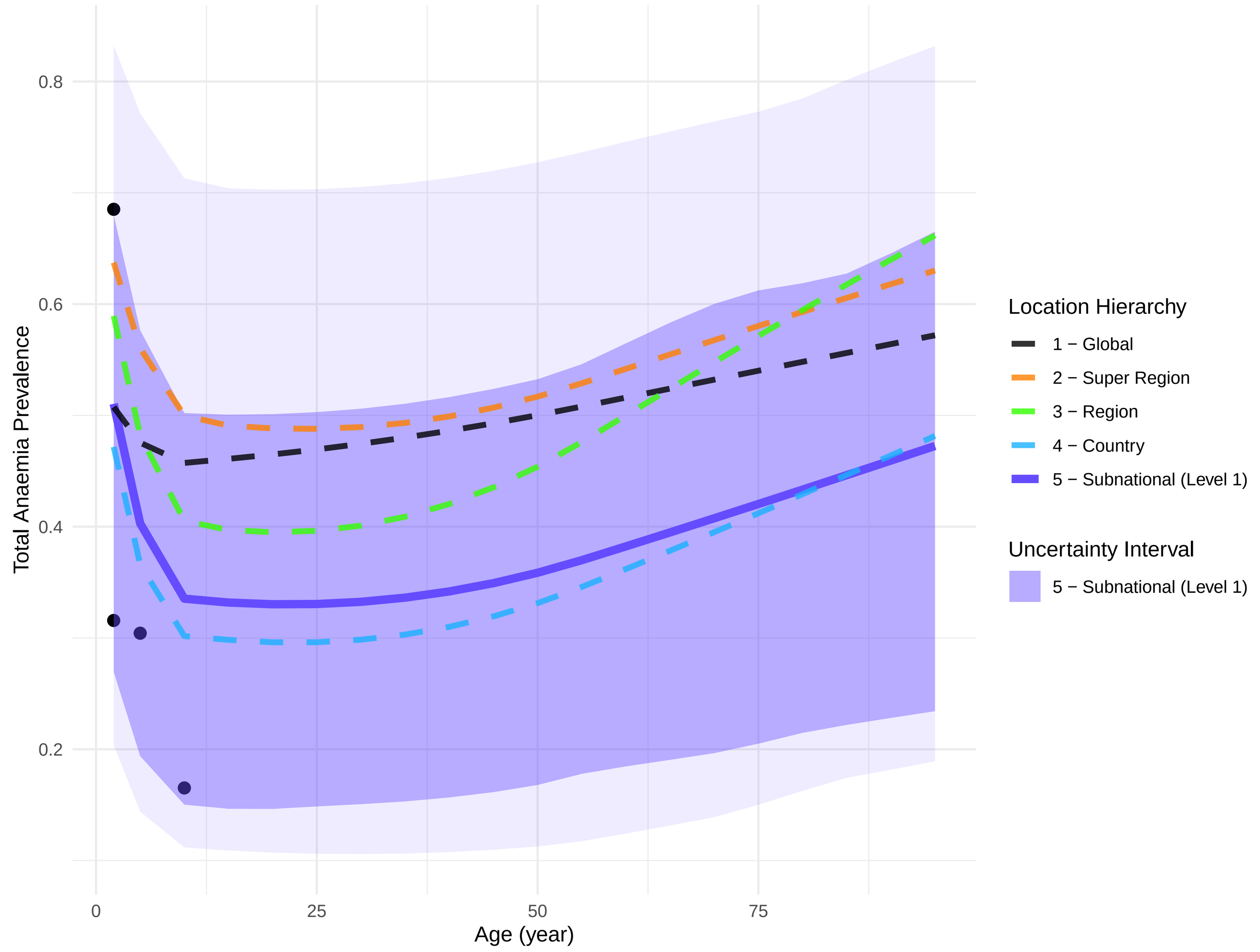

MR−BRT Age/Sex Fits − Non−pregnant Females 2+ Years Old − Total Anaemia Prevalence − Turkana
WHO−2024 Adjustment Method

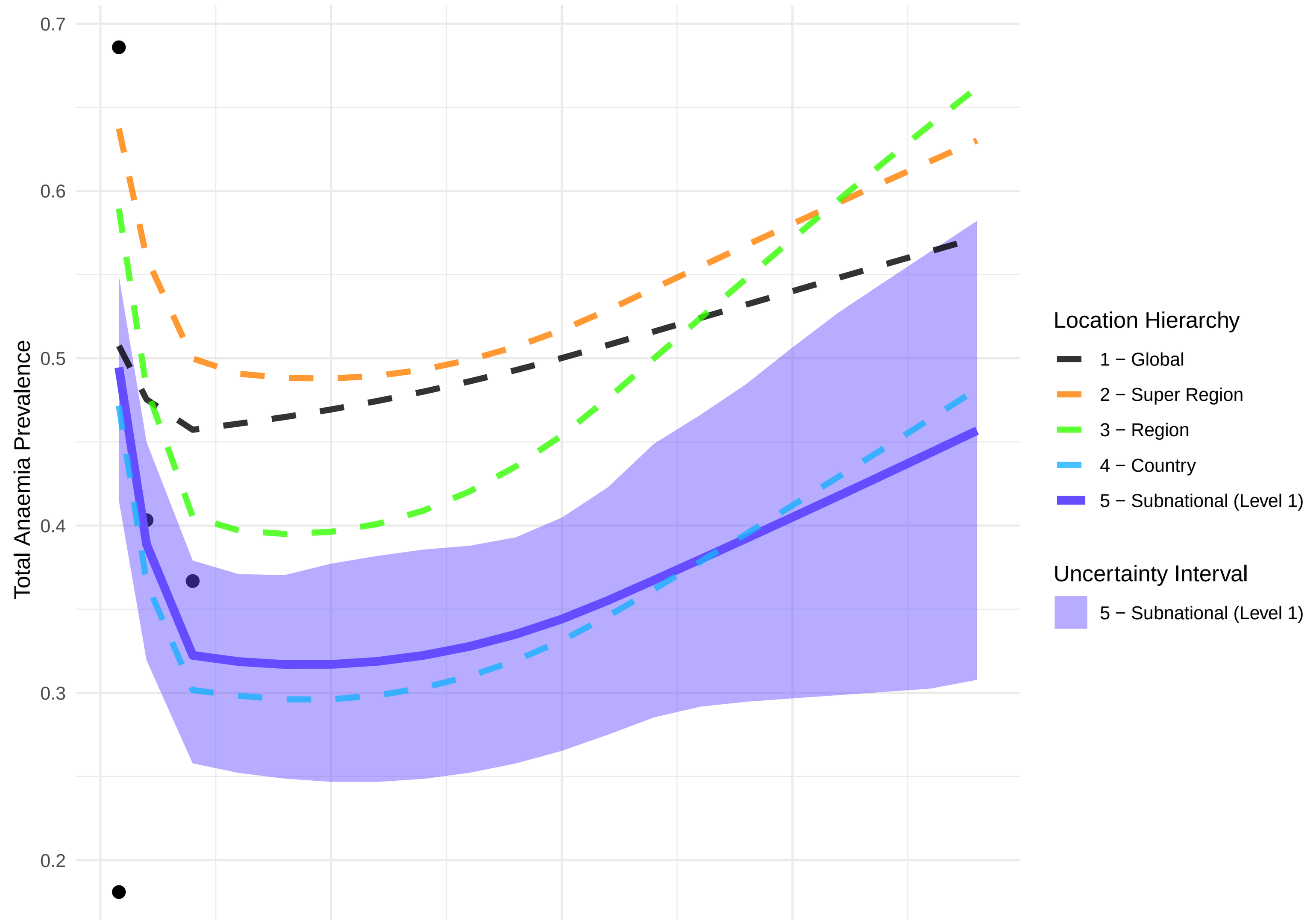

MR−BRT Age/Sex Fits − Non−pregnant Females 2+ Years Old − Total Anaemia Prevalence − Uasin Gishu
WHO−2024 Adjustment Method

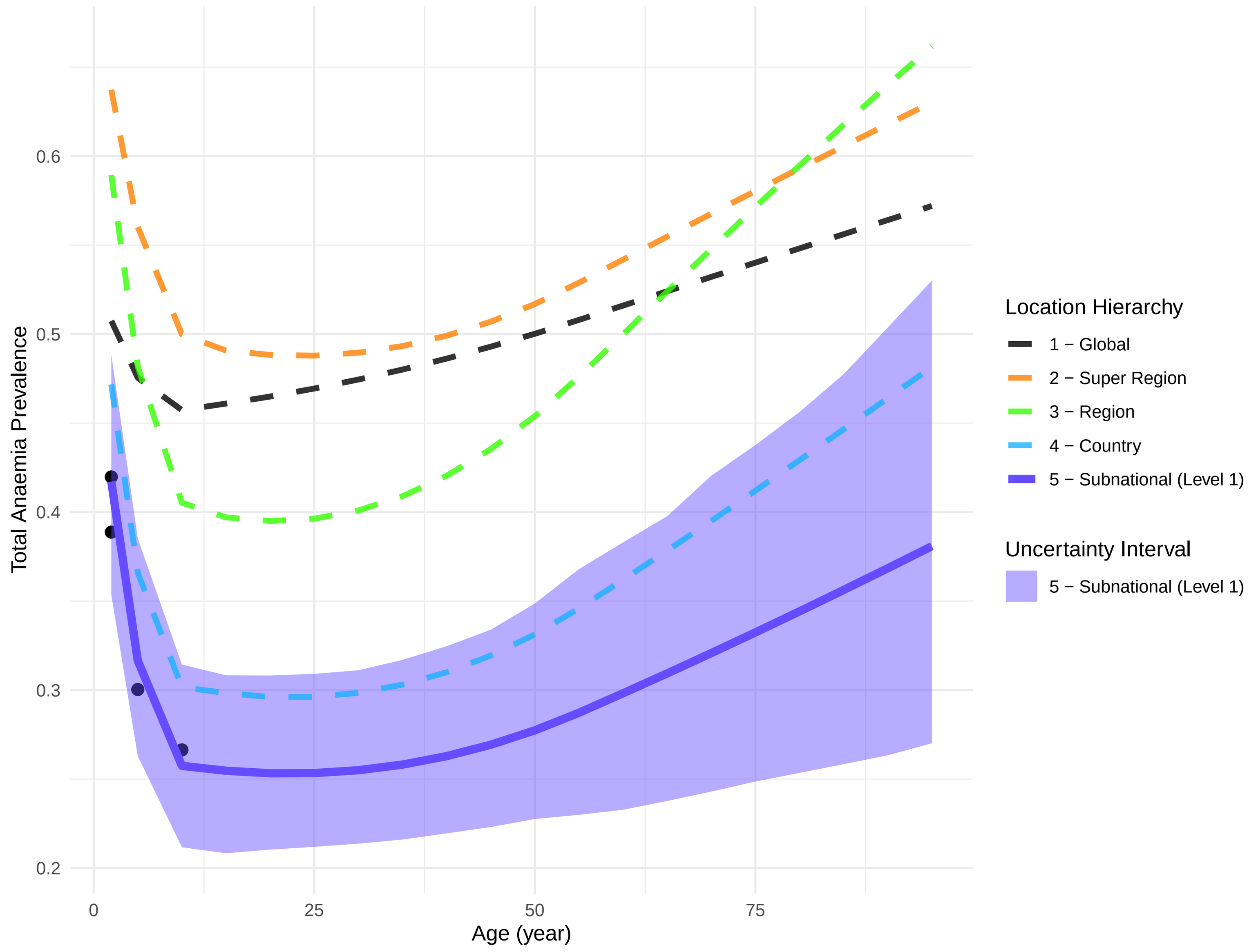

MR−BRT Age/Sex Fits − Non−pregnant Females 2+ Years Old − Total Anaemia Prevalence − Vihiga
WHO−2024 Adjustment Method

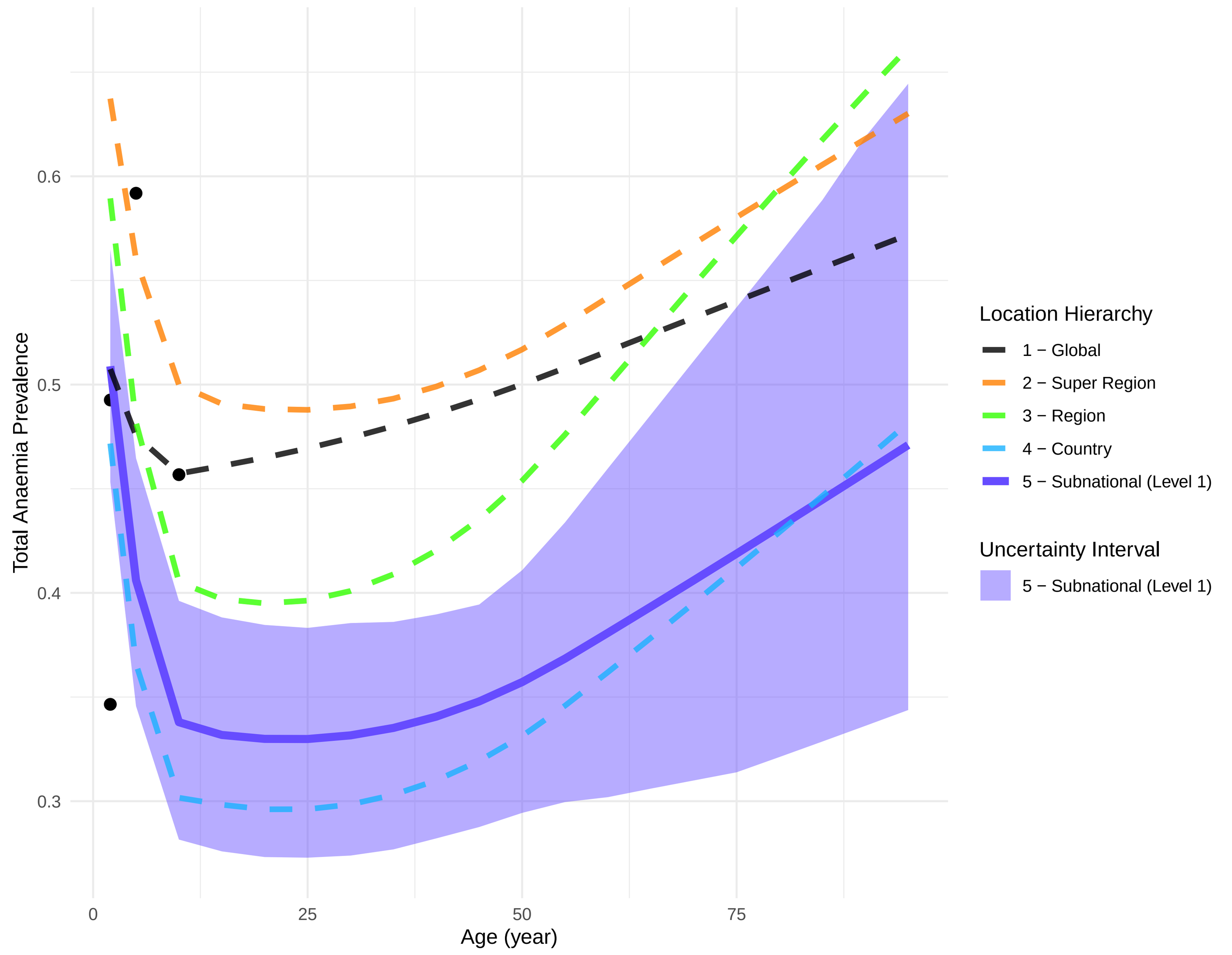

MR−BRT Age/Sex Fits − Non−pregnant Females 2+ Years Old − Total Anaemia Prevalence − Wajir
WHO−2024 Adjustment Method

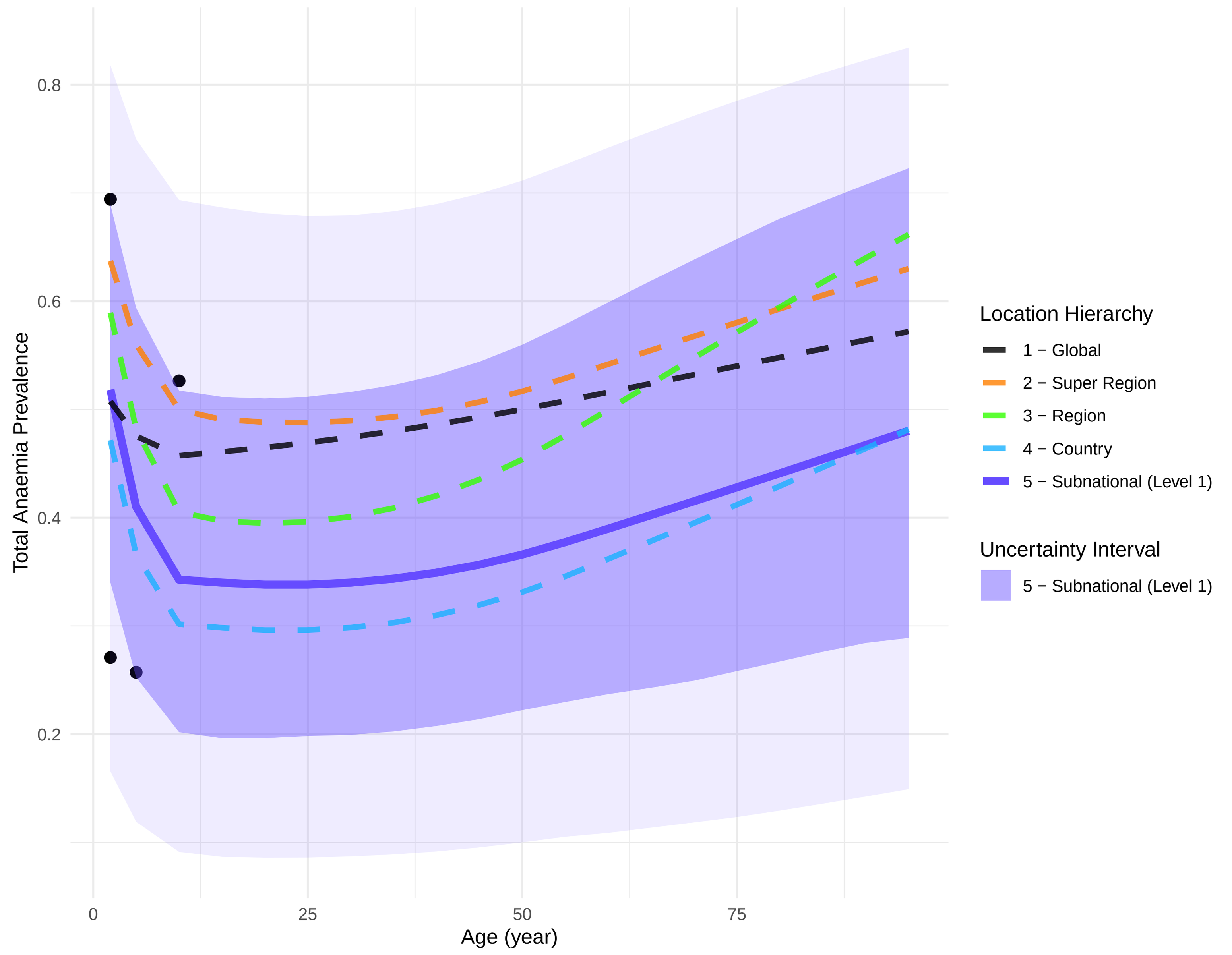

MR−BRT Age/Sex Fits − Non−pregnant Females 2+ Years Old − Total Anaemia Prevalence − West Pokot
WHO−2024 Adjustment Method

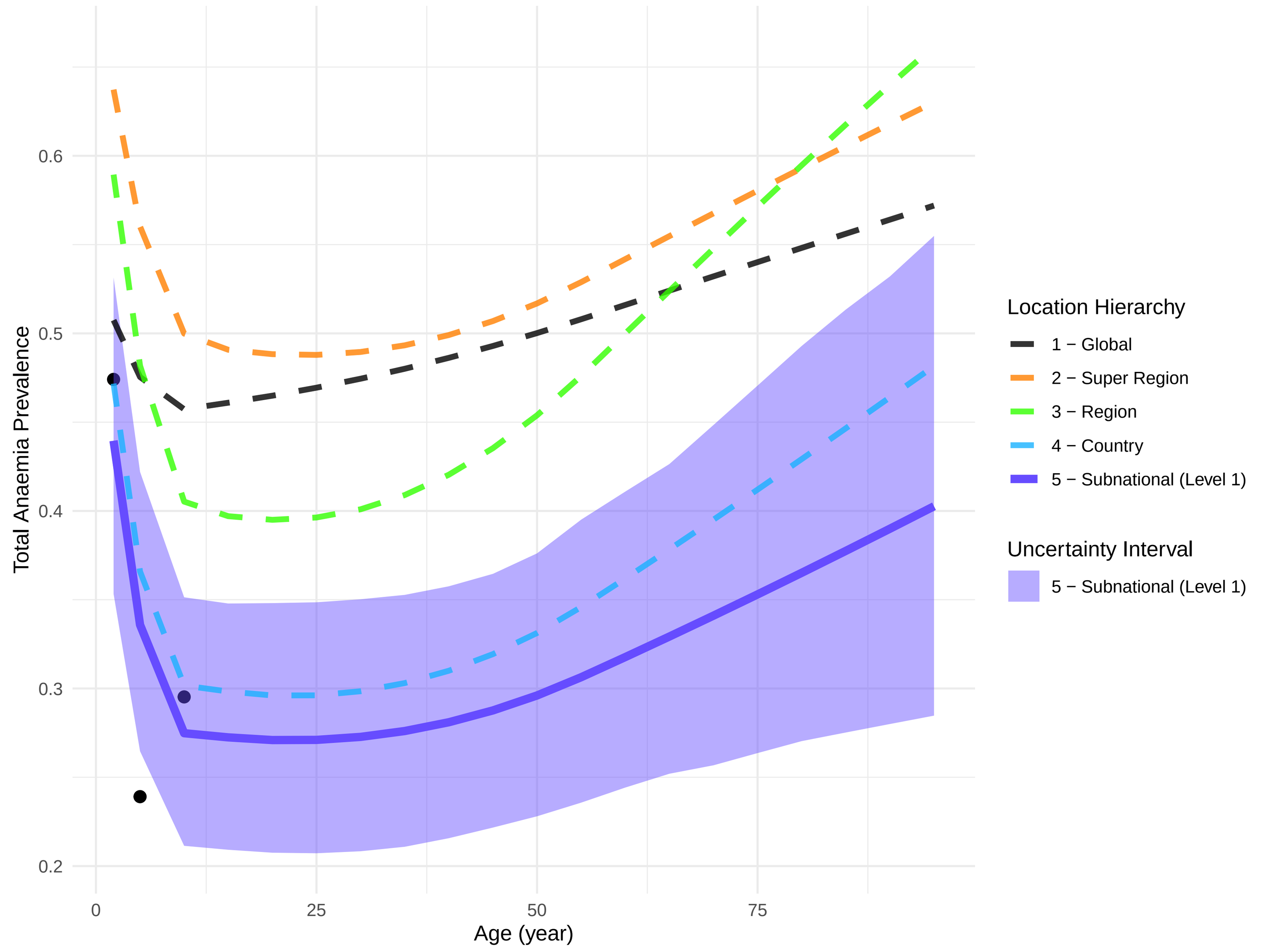

MR−BRT Age/Sex Fits − Non−pregnant Females 2+ Years Old − Total Anaemia Prevalence − Madagascar
WHO−2024 Adjustment Method

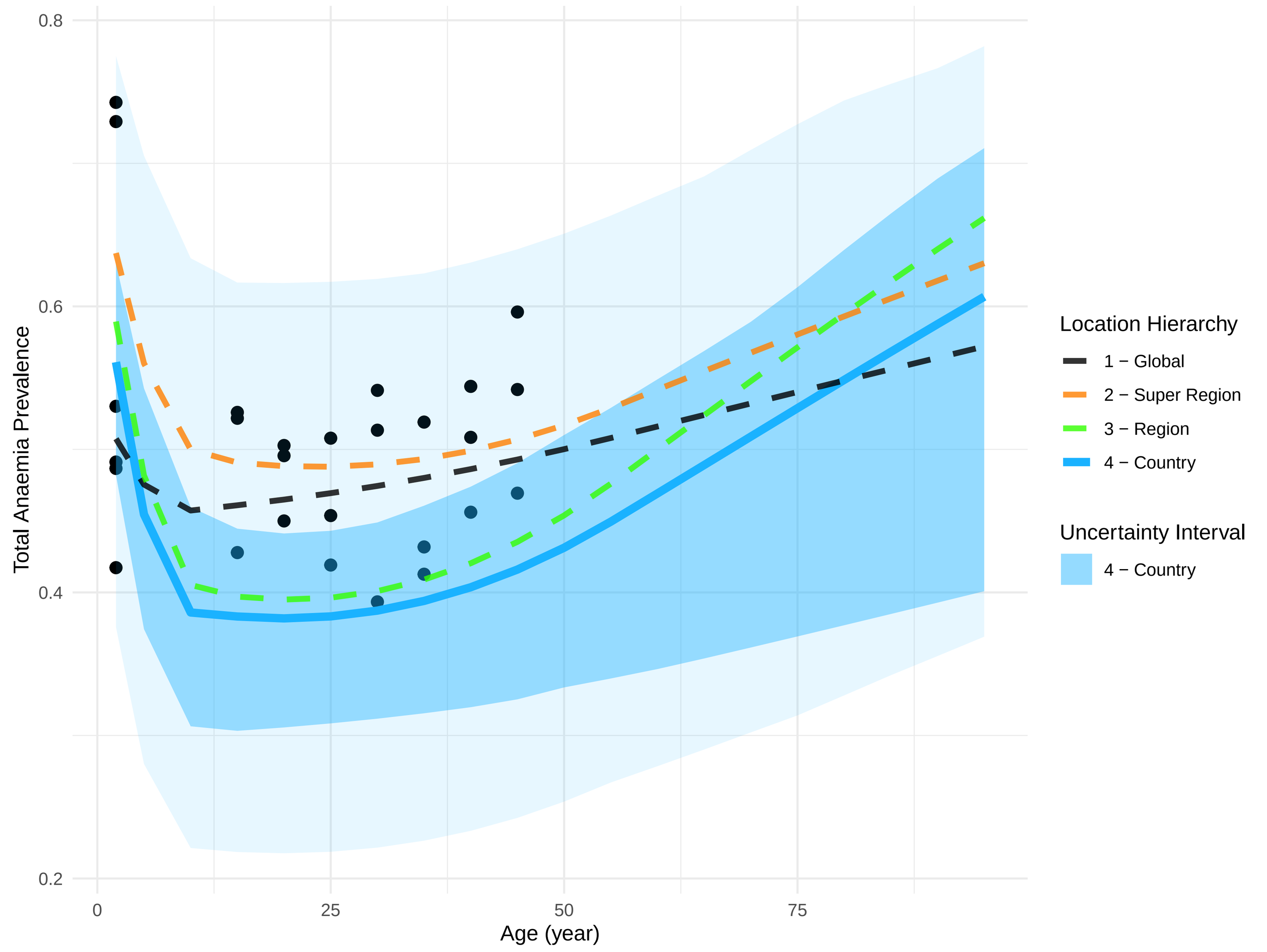

MR−BRT Age/Sex Fits − Non−pregnant Females 2+ Years Old − Total Anaemia Prevalence − Malawi
WHO−2024 Adjustment Method

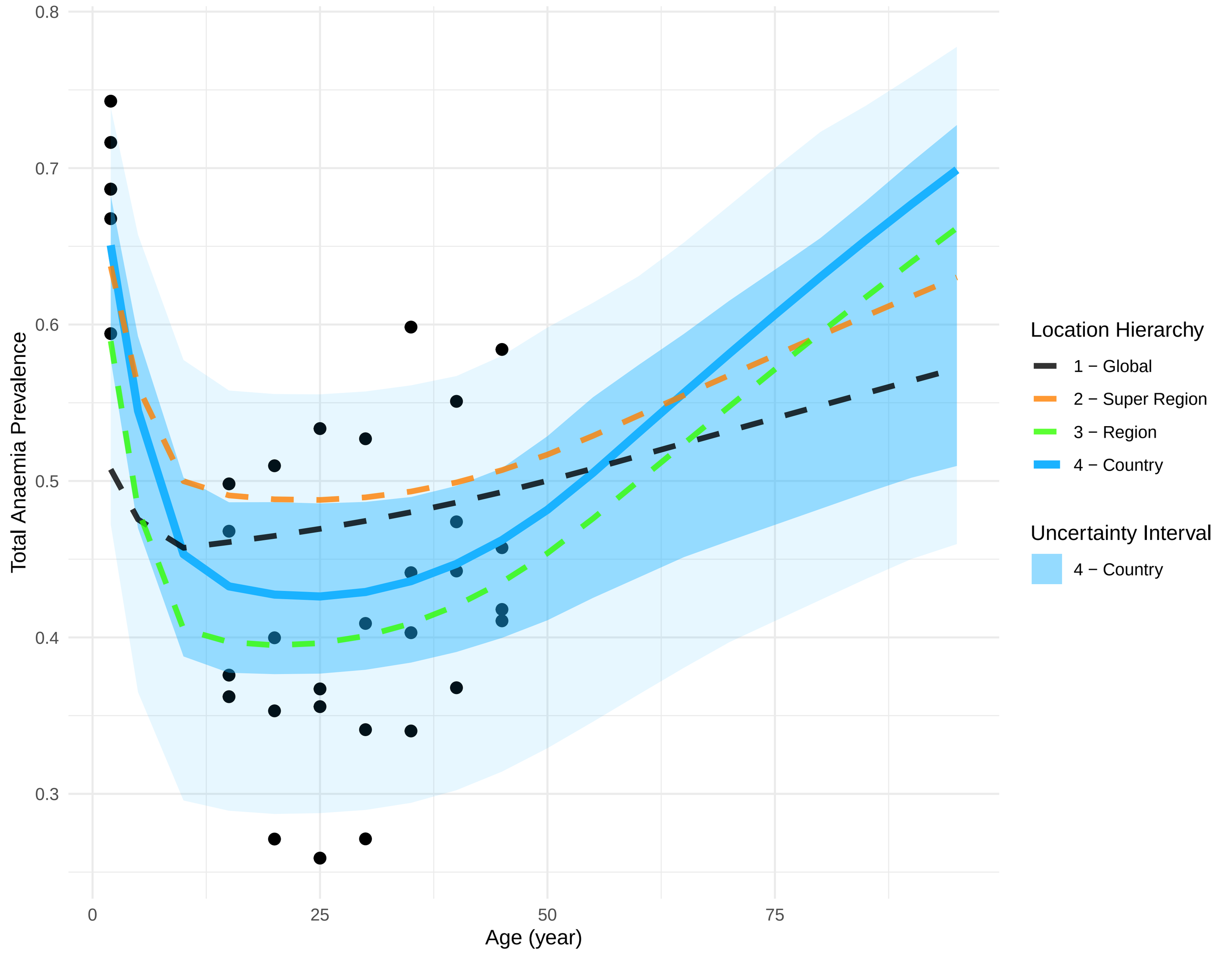

MR−BRT Age/Sex Fits − Non−pregnant Females 2+ Years Old − Total Anaemia Prevalence − Mozambique
WHO−2024 Adjustment Method

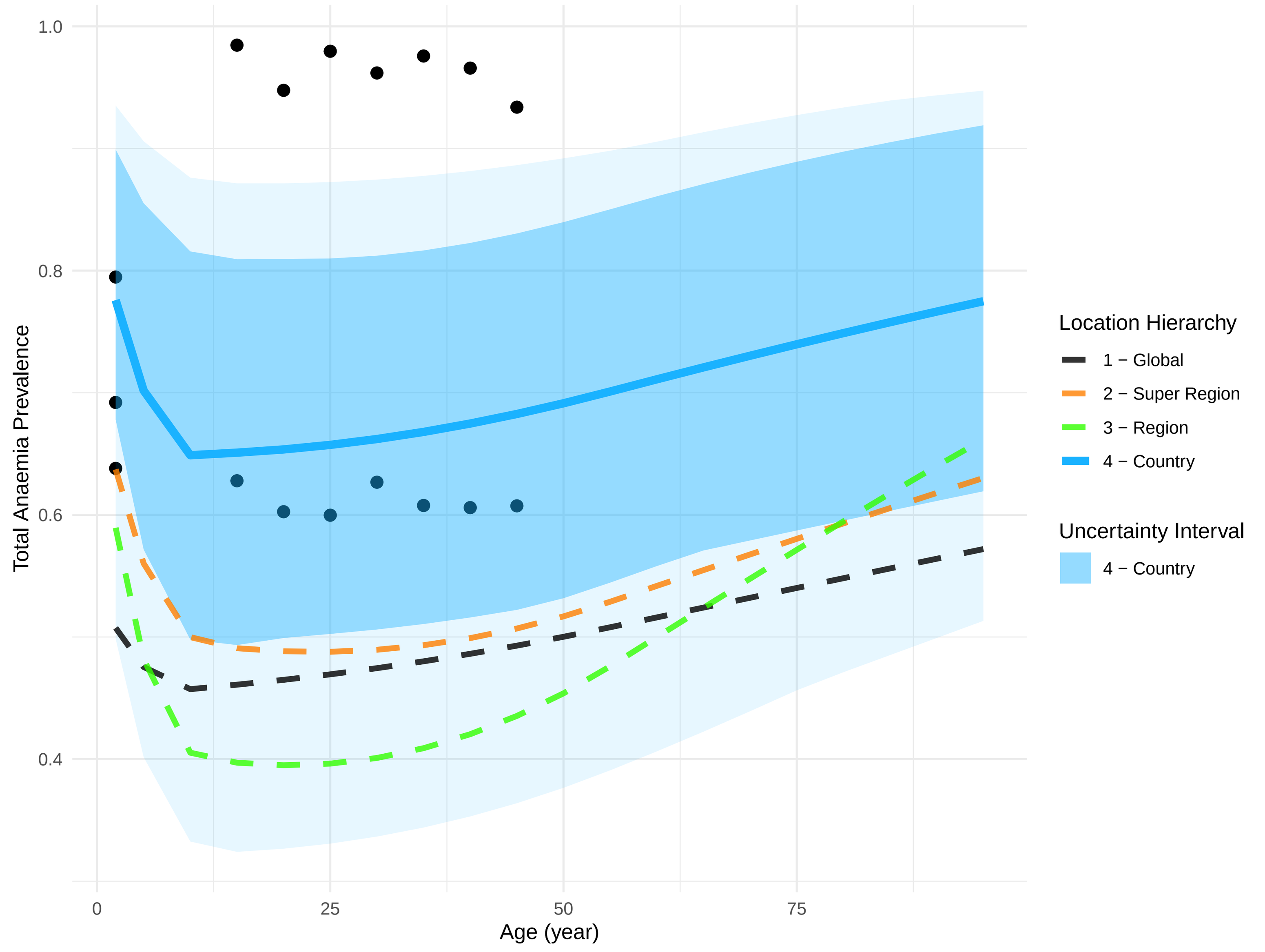

MR−BRT Age/Sex Fits − Non−pregnant Females 2+ Years Old − Total Anaemia Prevalence − Rwanda
WHO−2024 Adjustment Method

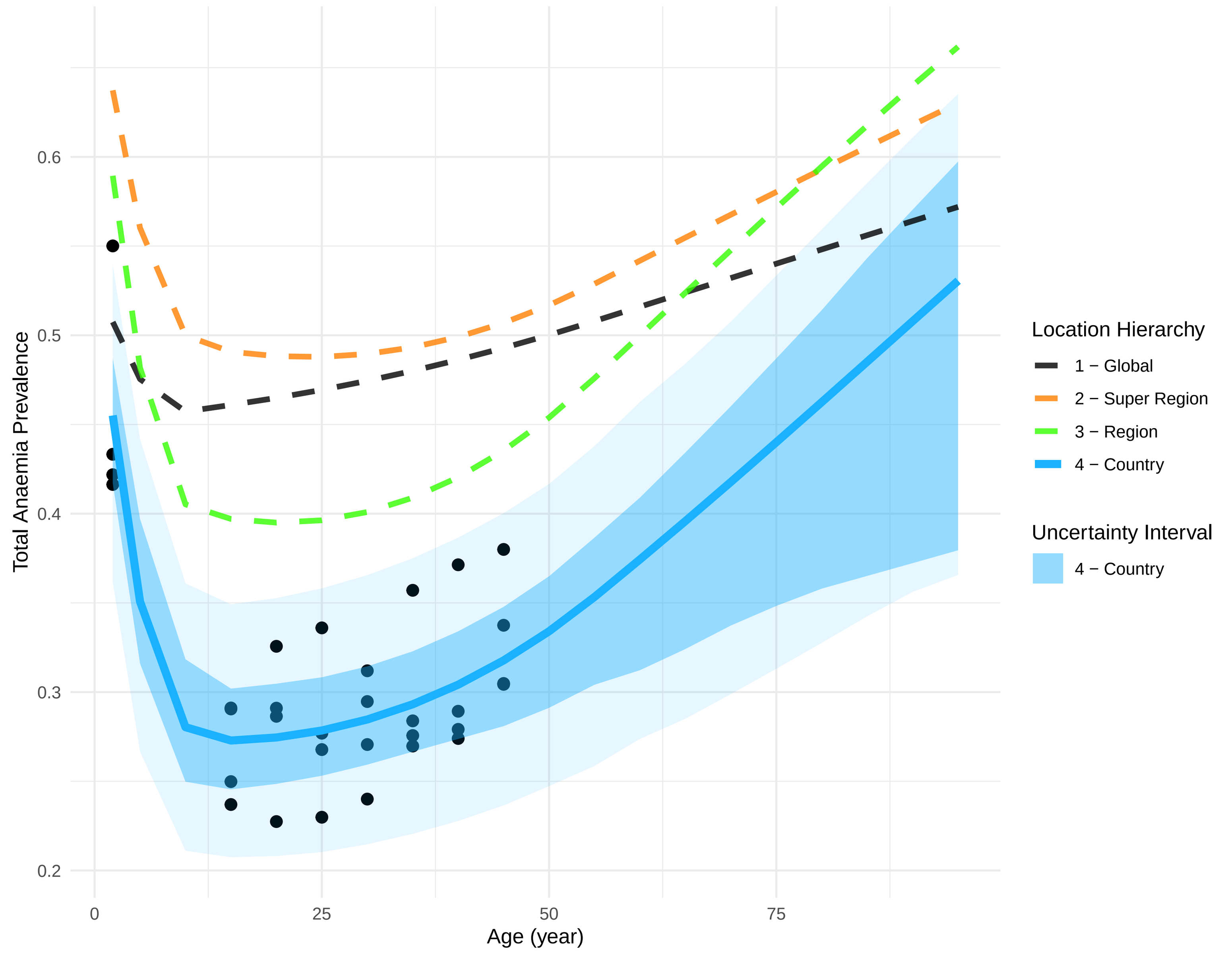

MR−BRT Age/Sex Fits − Non−pregnant Females 2+ Years Old − Total Anaemia Prevalence − Uganda
WHO−2024 Adjustment Method

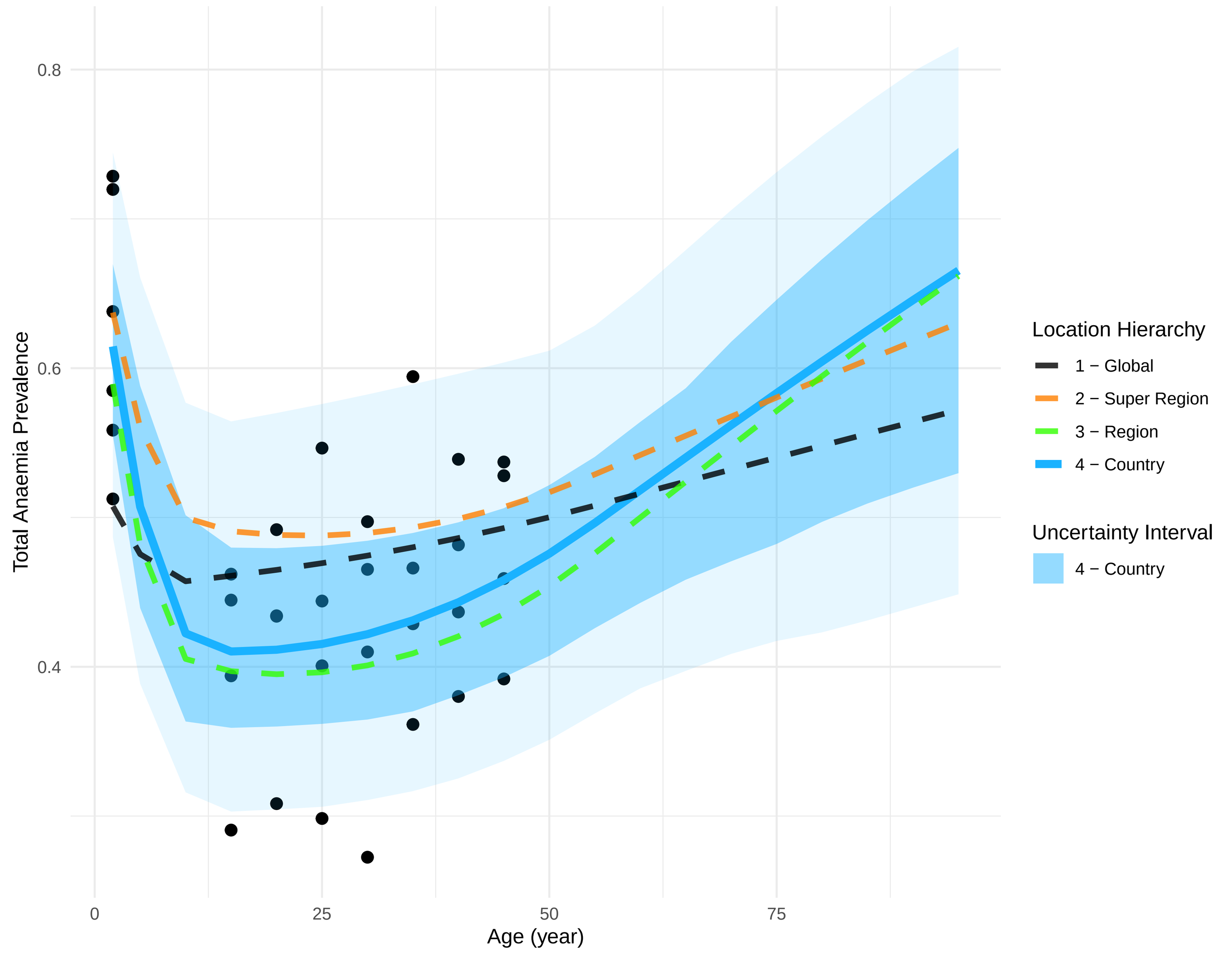

MR−BRT Age/Sex Fits − Non−pregnant Females 2+ Years Old − Total Anaemia Prevalence − United Republic of Tanzania
WHO−2024 Adjustment Method

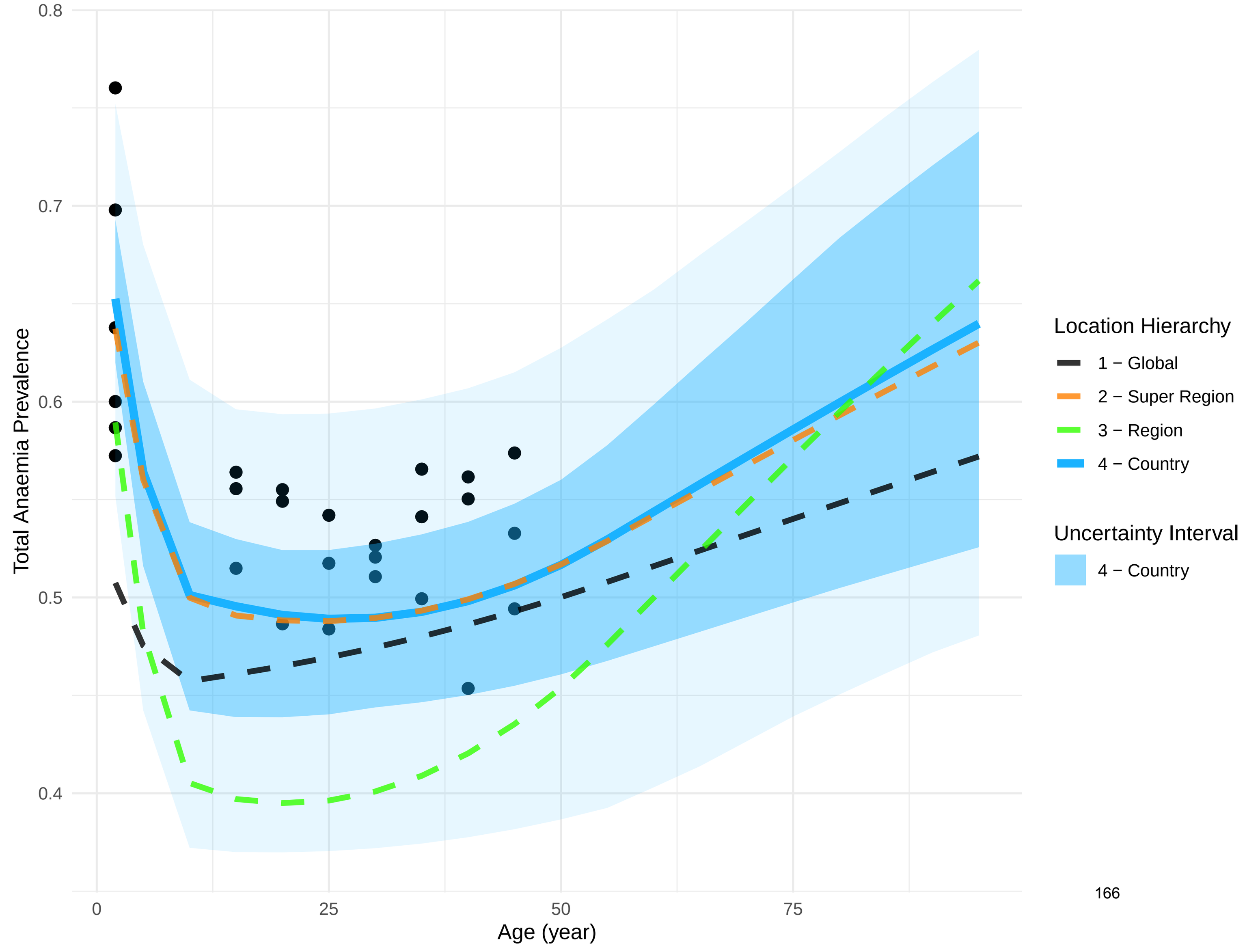

MR−BRT Age/Sex Fits − Non−pregnant Females 2+ Years Old − Total Anaemia Prevalence − Zambia
WHO−2024 Adjustment Method

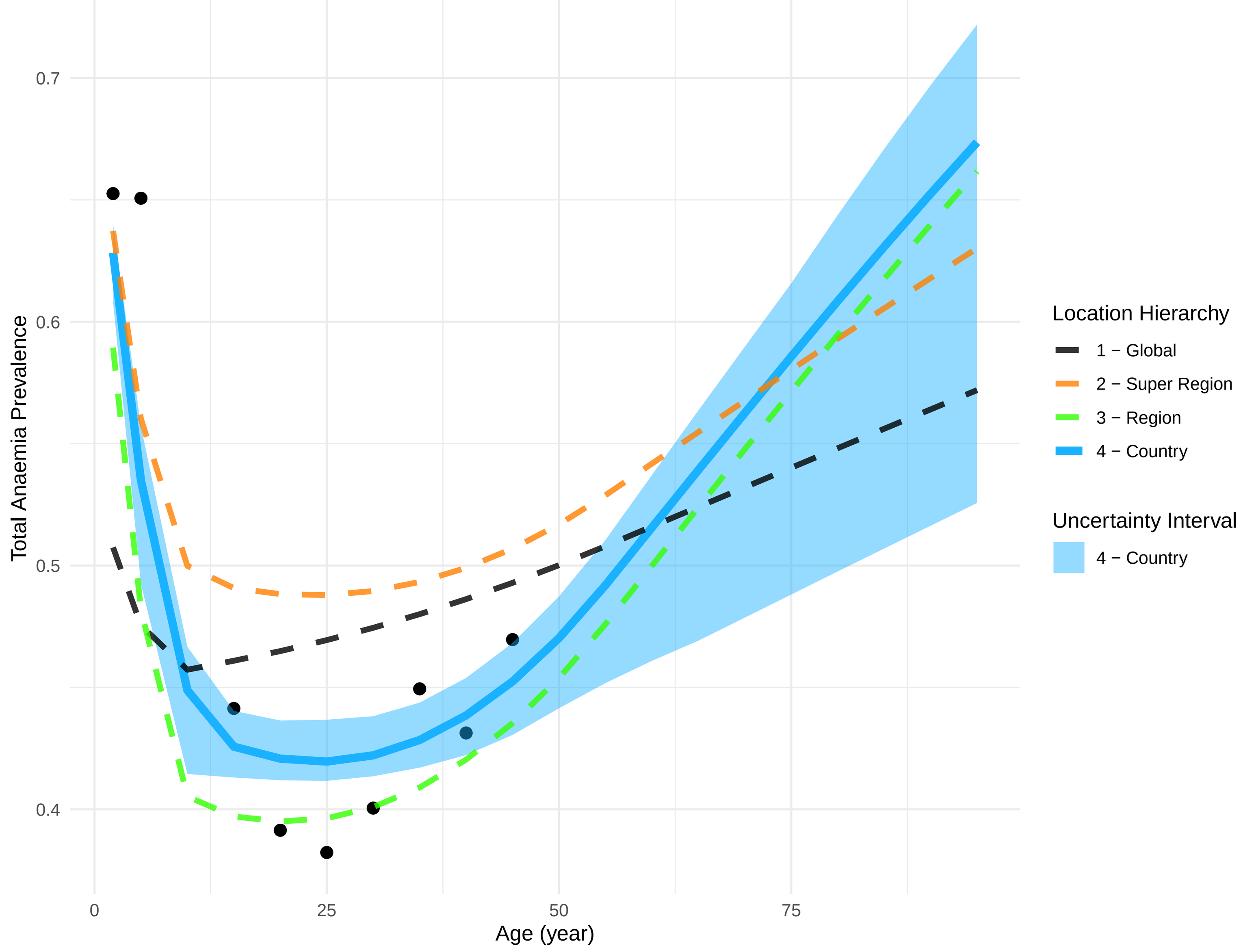

MR−BRT Age/Sex Fits − Non−pregnant Females 2+ Years Old − Total Anaemia Prevalence − Southern Sub−Saharan Africa
WHO−2024 Adjustment Method

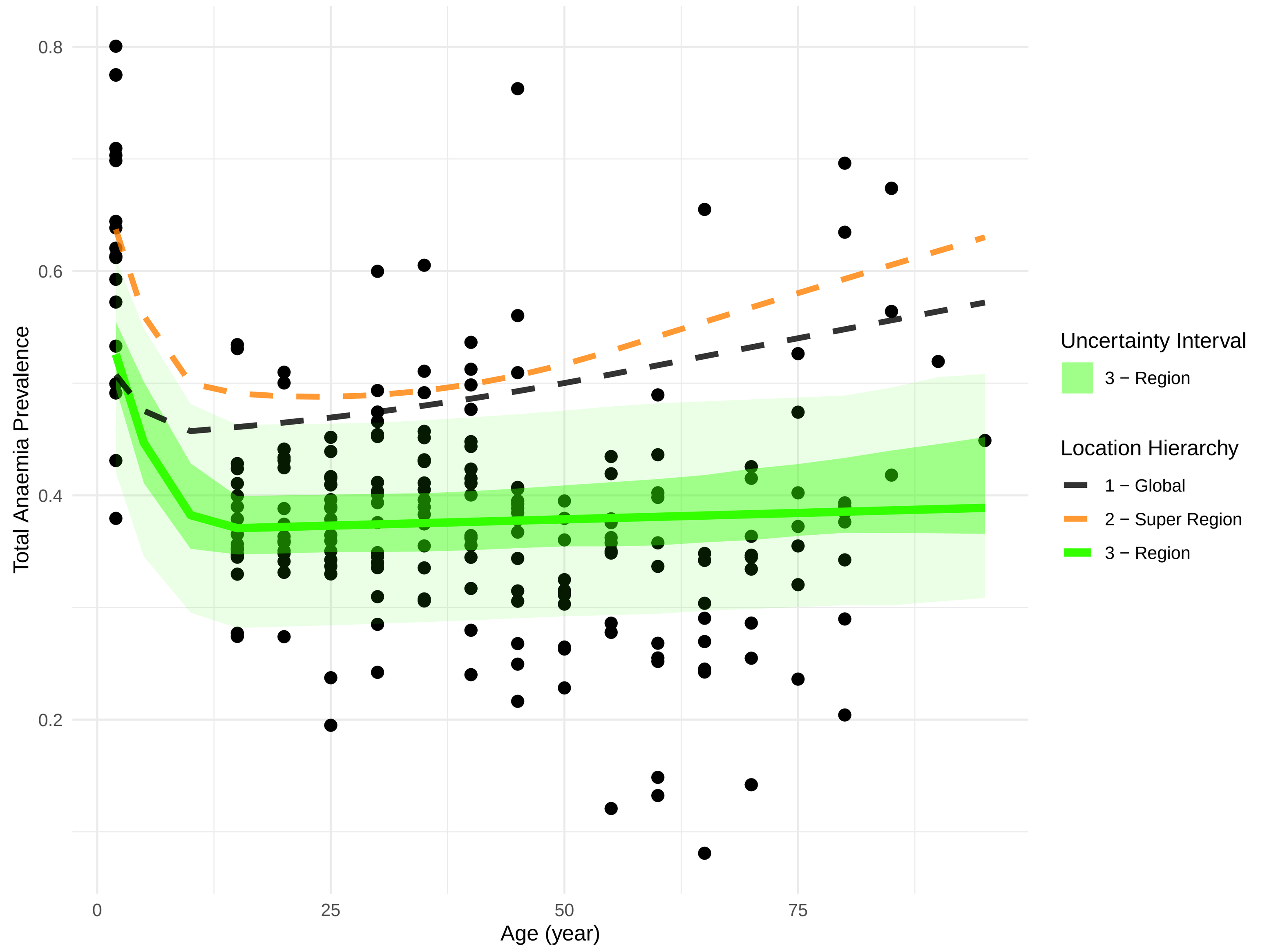

MR−BRT Age/Sex Fits − Non−pregnant Females 2+ Years Old − Total Anaemia Prevalence − Eswatini
WHO−2024 Adjustment Method

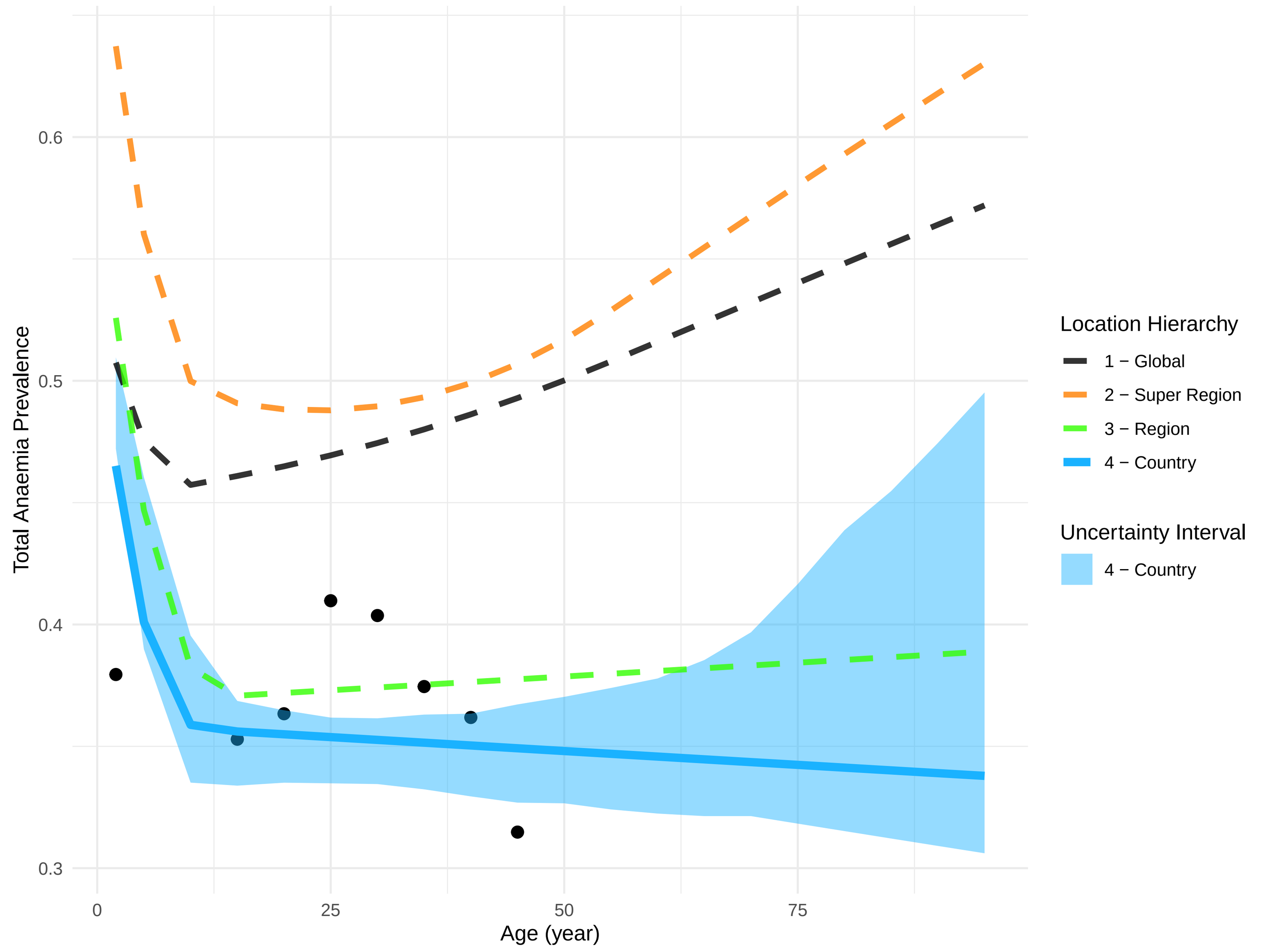

MR−BRT Age/Sex Fits − Non−pregnant Females 2+ Years Old − Total Anaemia Prevalence − Lesotho
WHO−2024 Adjustment Method

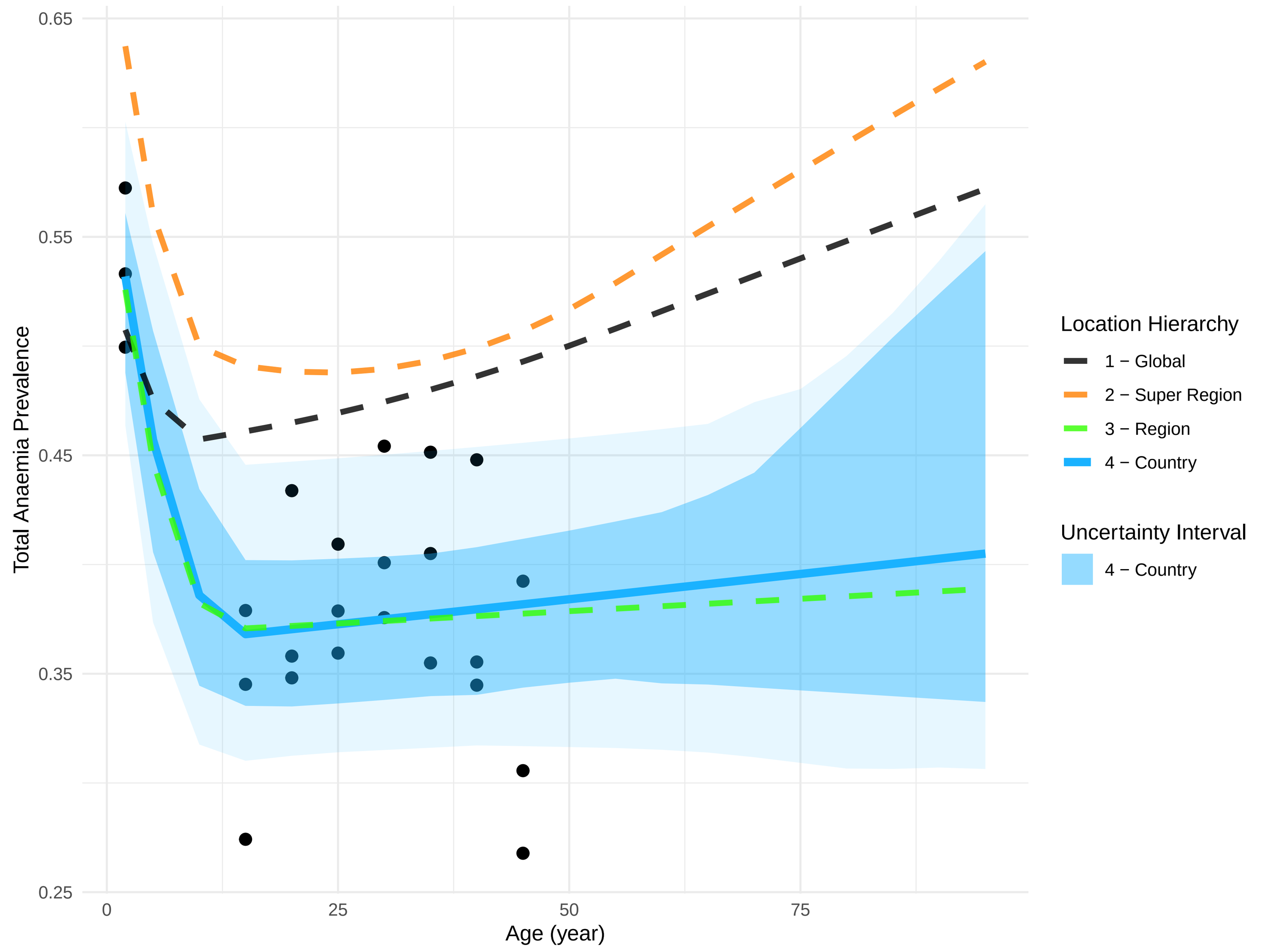

MR−BRT Age/Sex Fits − Non−pregnant Females 2+ Years Old − Total Anaemia Prevalence − Namibia
WHO−2024 Adjustment Method

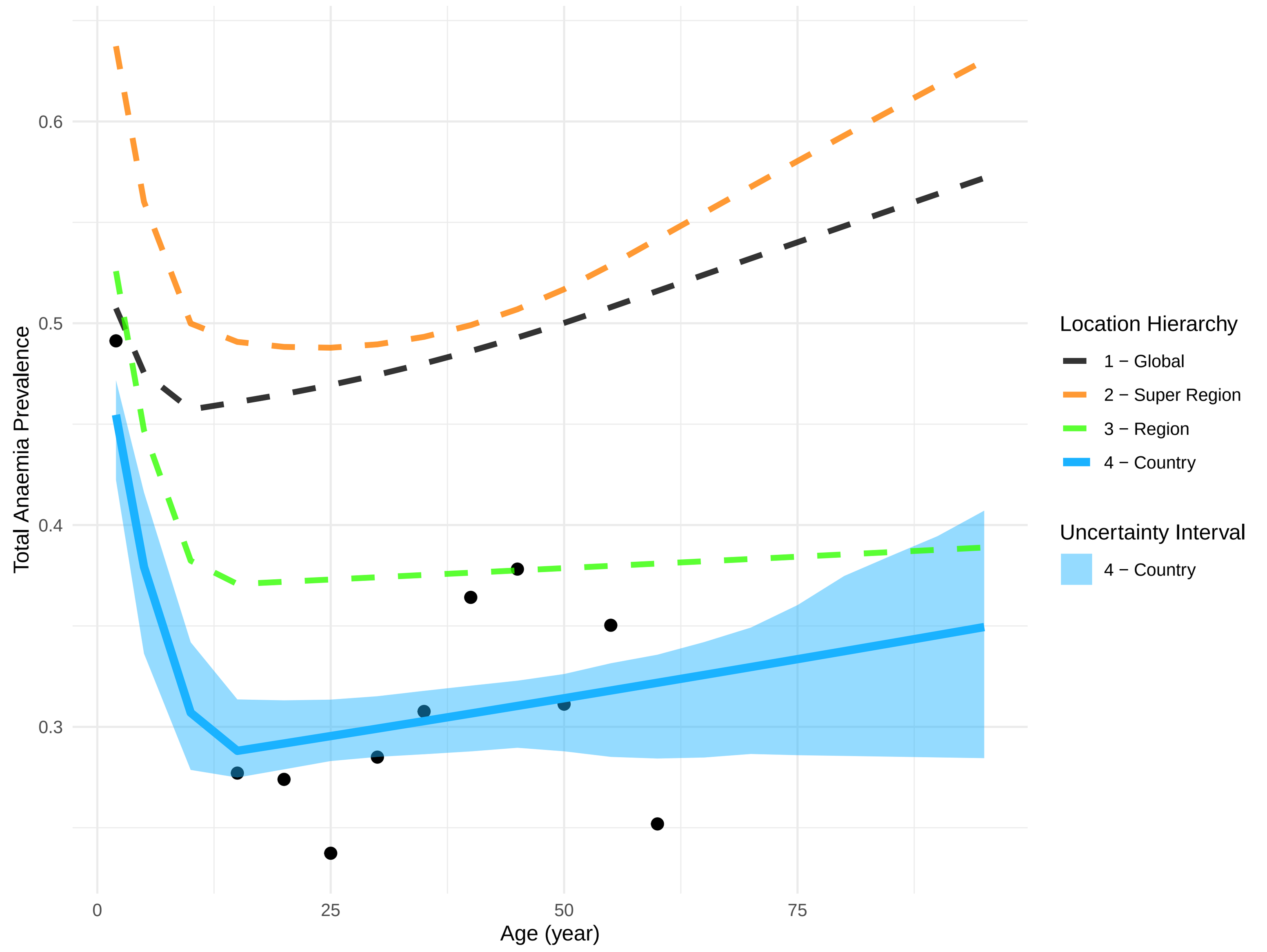

MR−BRT Age/Sex Fits − Non−pregnant Females 2+ Years Old − Total Anaemia Prevalence − South Africa
WHO−2024 Adjustment Method

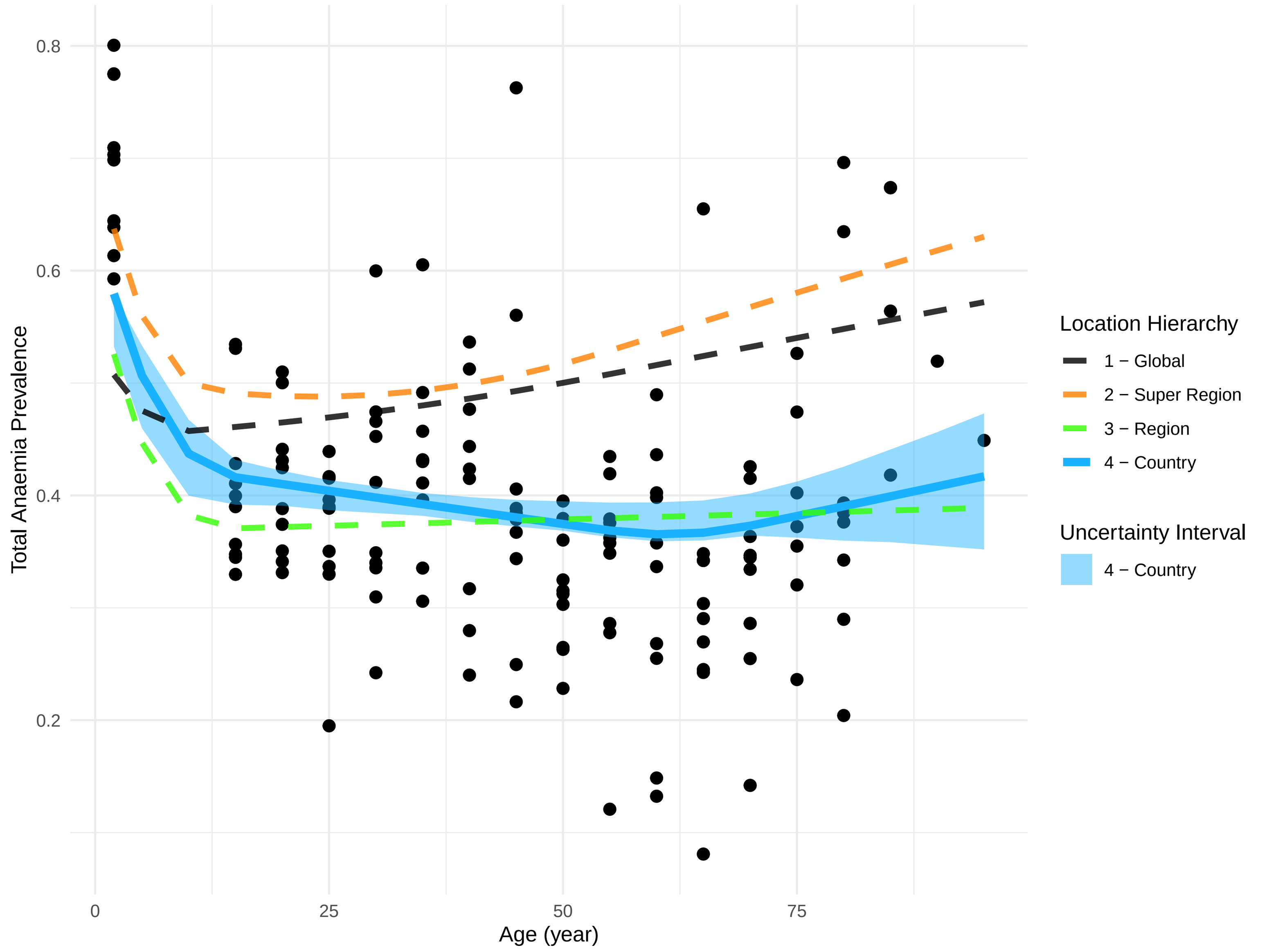

MR−BRT Age/Sex Fits − Non−pregnant Females 2+ Years Old − Total Anaemia Prevalence − Eastern Cape
WHO−2024 Adjustment Method

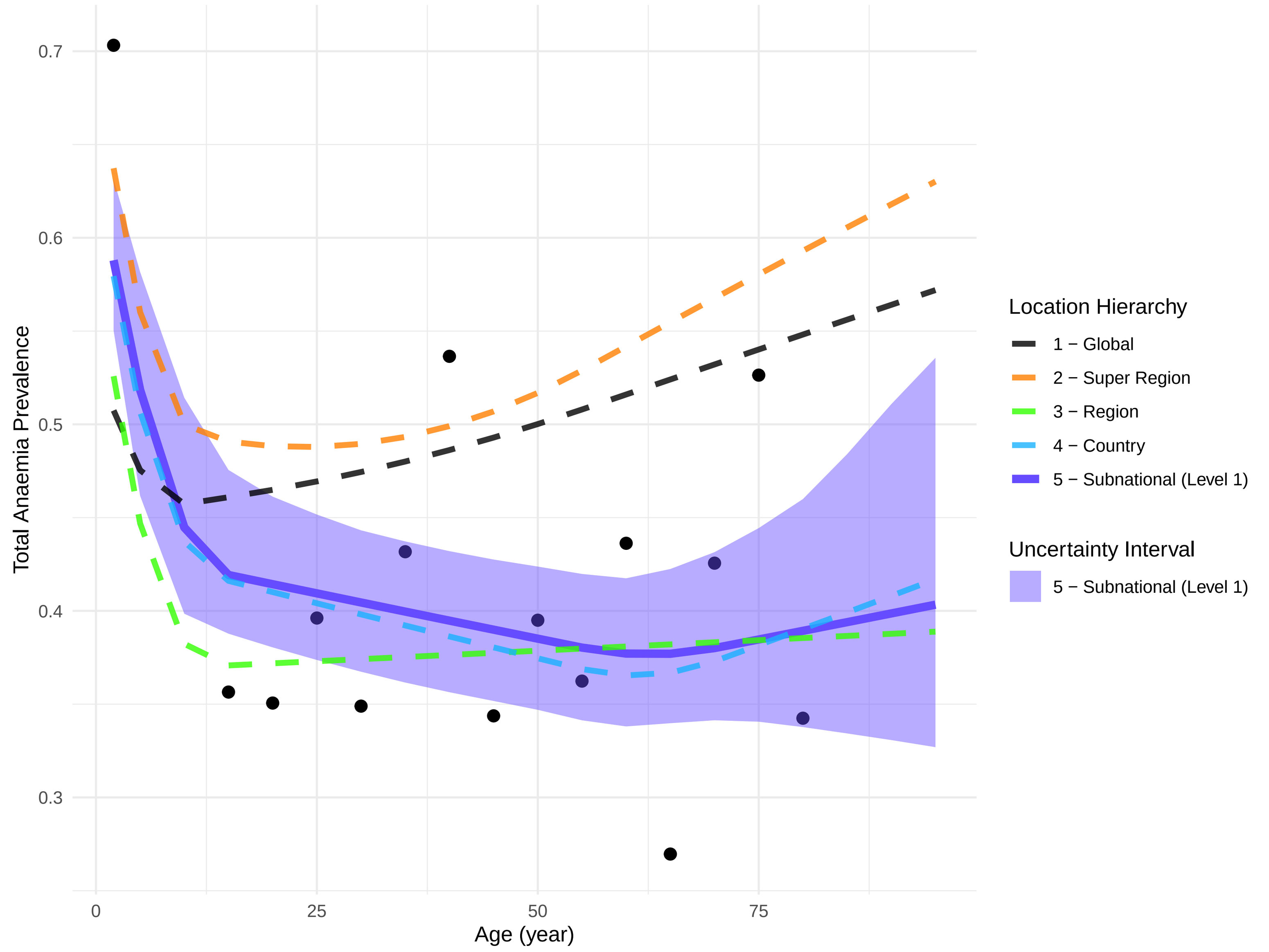

MR−BRT Age/Sex Fits − Non−pregnant Females 2+ Years Old − Total Anaemia Prevalence − Free State
WHO−2024 Adjustment Method

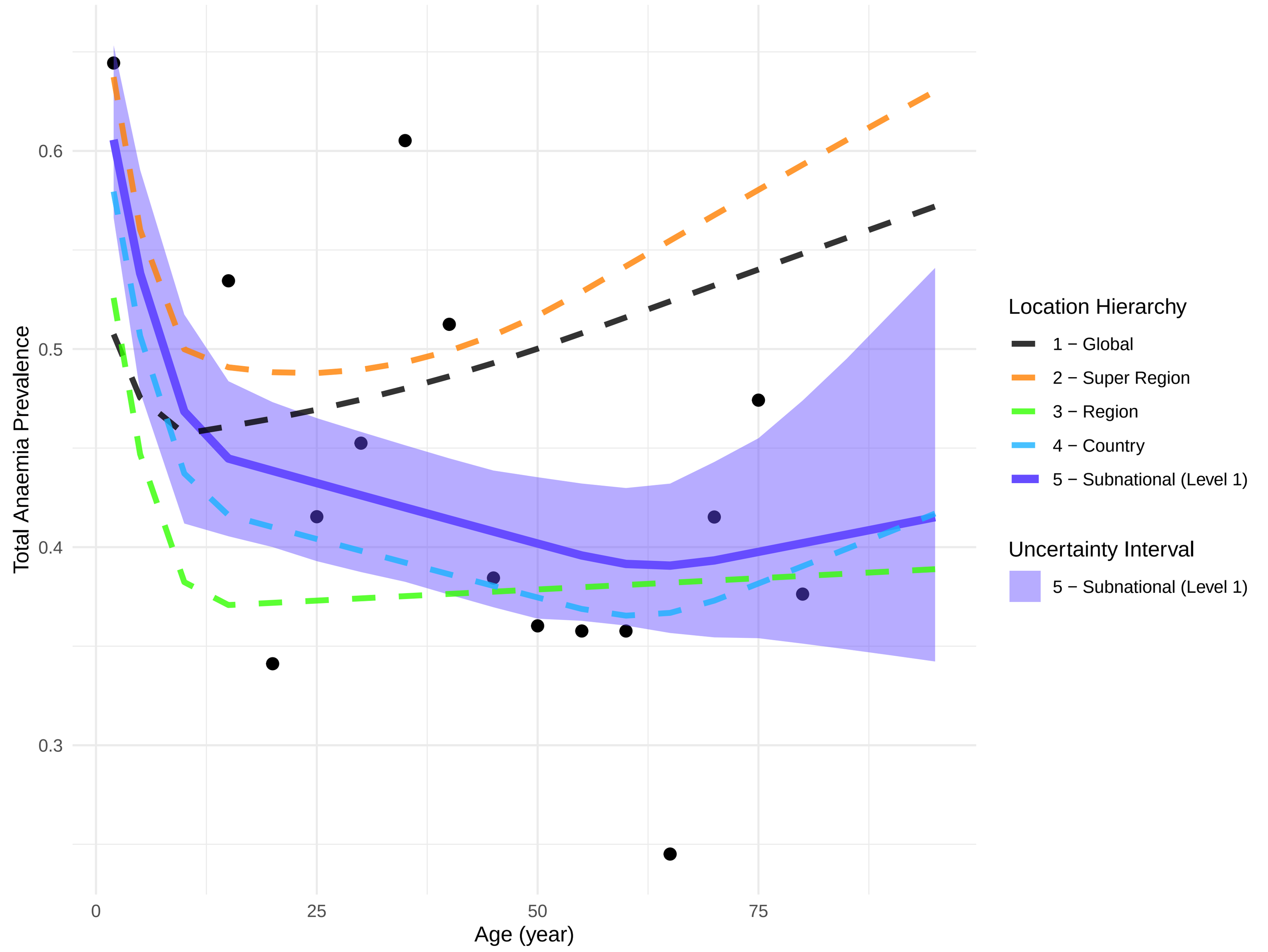

MR−BRT Age/Sex Fits − Non−pregnant Females 2+ Years Old − Total Anaemia Prevalence − Gauteng
WHO−2024 Adjustment Method

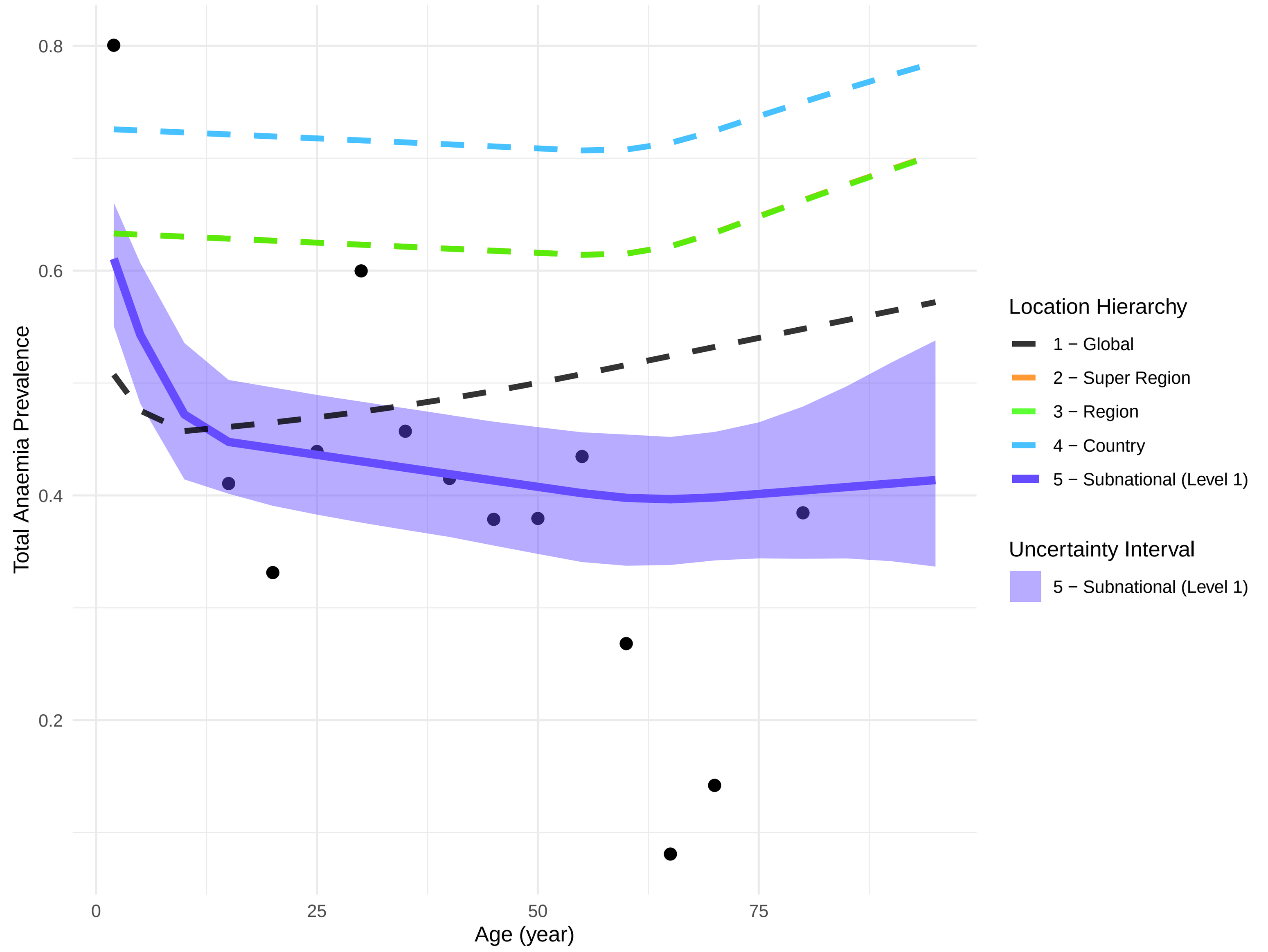

MR−BRT Age/Sex Fits − Non−pregnant Females 2+ Years Old − Total Anaemia Prevalence − KwaZulu−Natal
WHO−2024 Adjustment Method

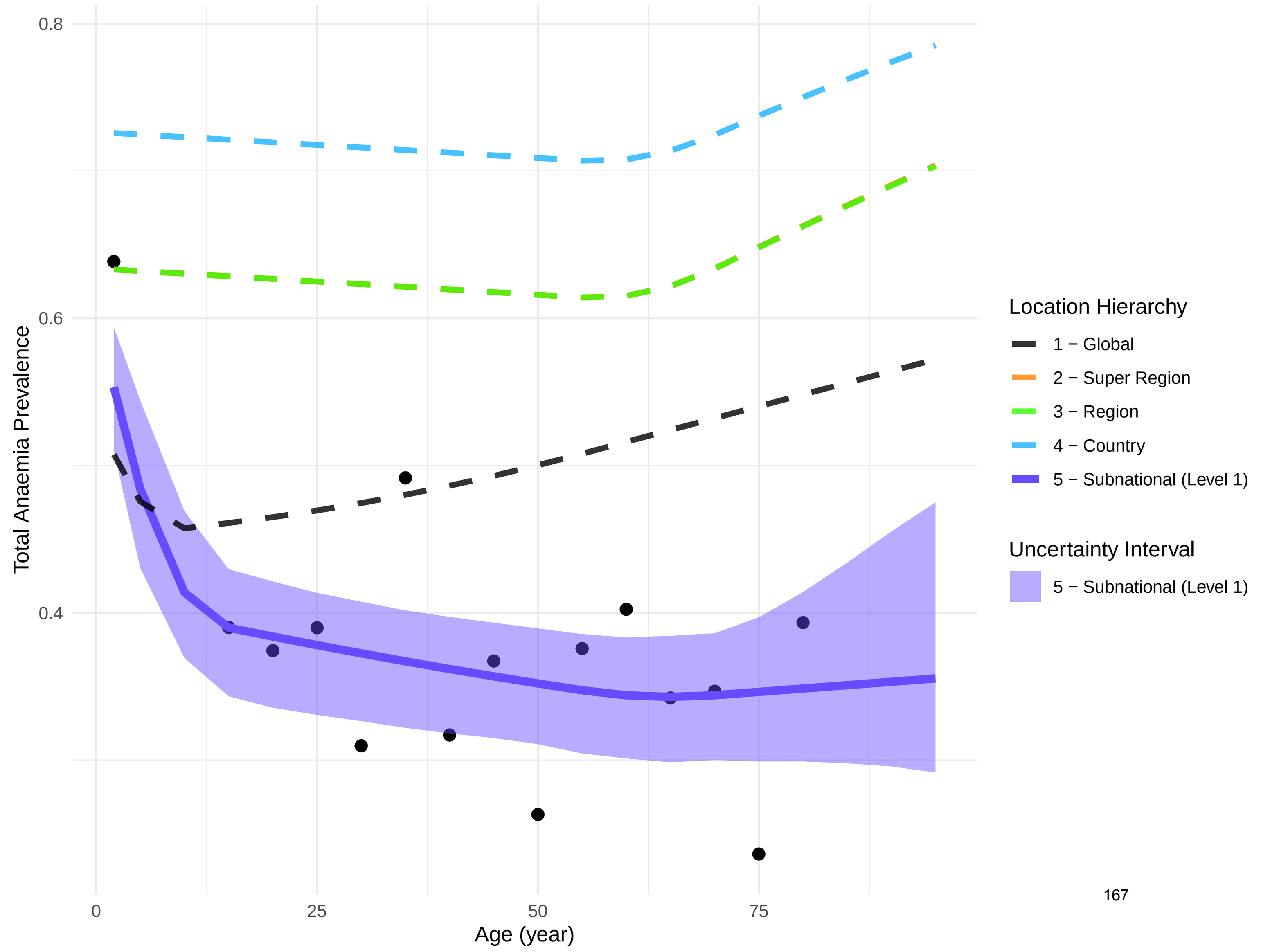

MR−BRT Age/Sex Fits − Non−pregnant Females 2+ Years Old − Total Anaemia Prevalence − Limpopo
WHO−2024 Adjustment Method

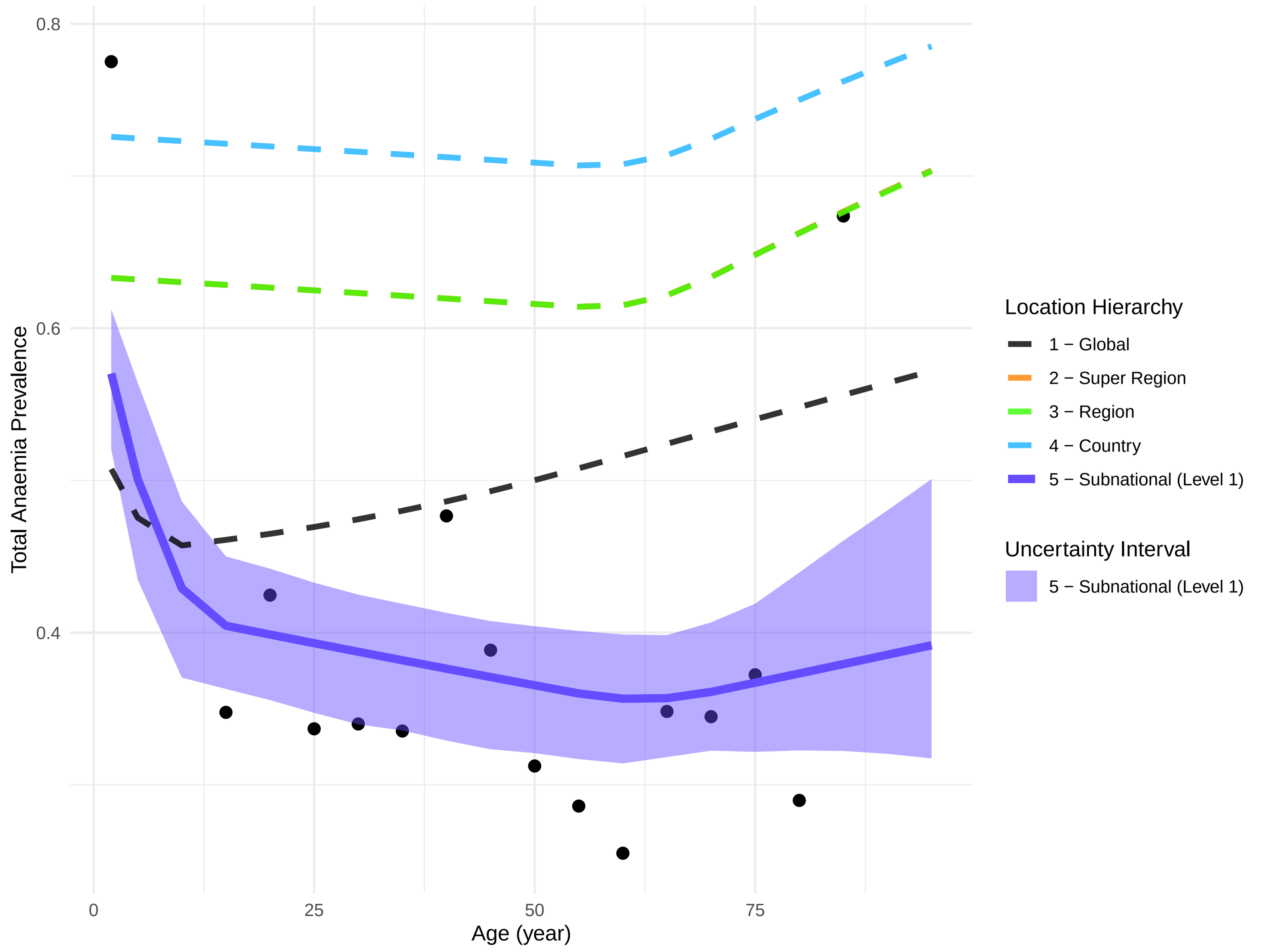

MR−BRT Age/Sex Fits − Non−pregnant Females 2+ Years Old − Total Anaemia Prevalence − Mpumalanga
WHO−2024 Adjustment Method

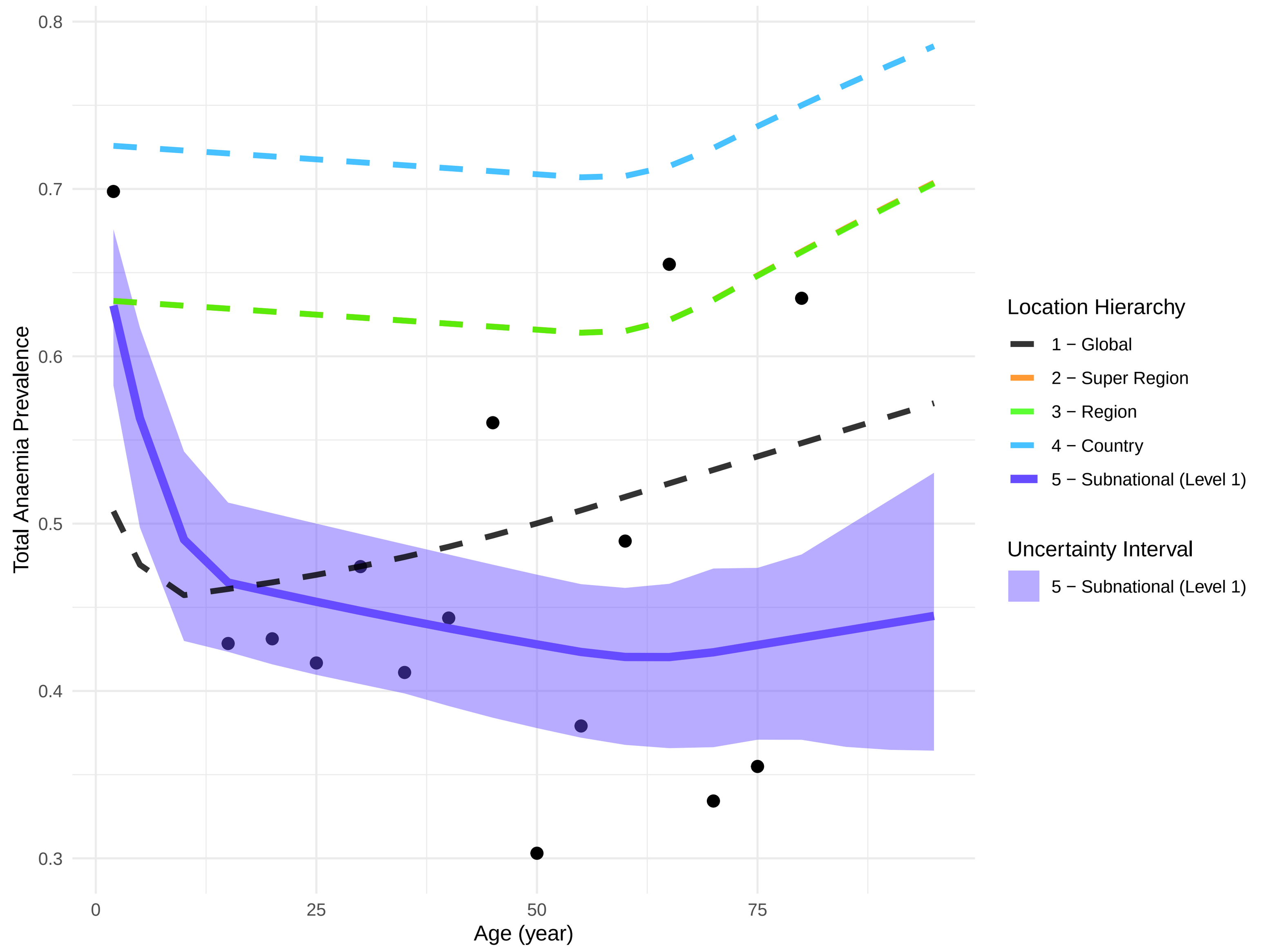

MR−BRT Age/Sex Fits − Non−pregnant Females 2+ Years Old − Total Anaemia Prevalence − North−West
WHO−2024 Adjustment Method

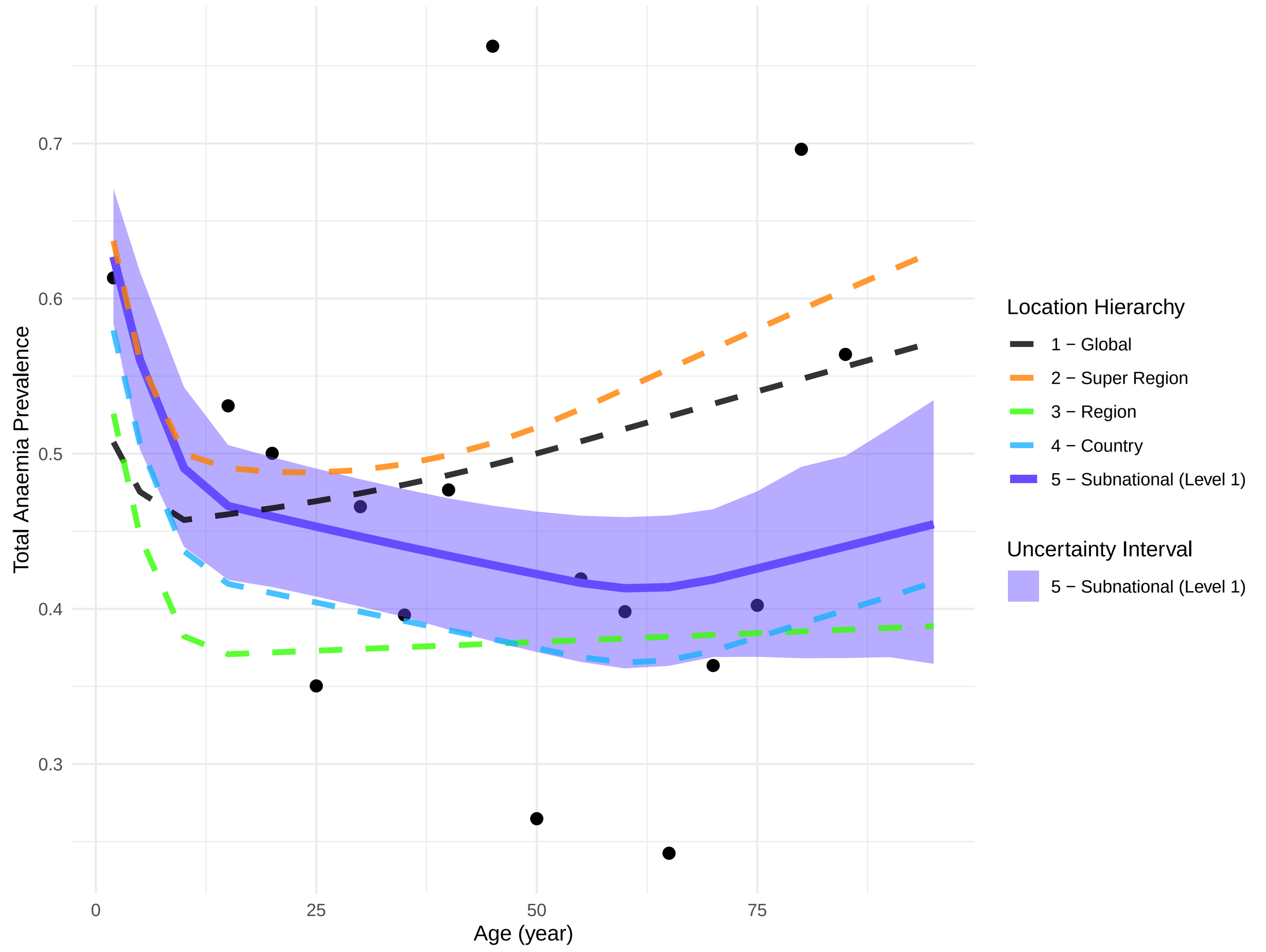

MR−BRT Age/Sex Fits − Non−pregnant Females 2+ Years Old − Total Anaemia Prevalence − Northern Cape
WHO−2024 Adjustment Method

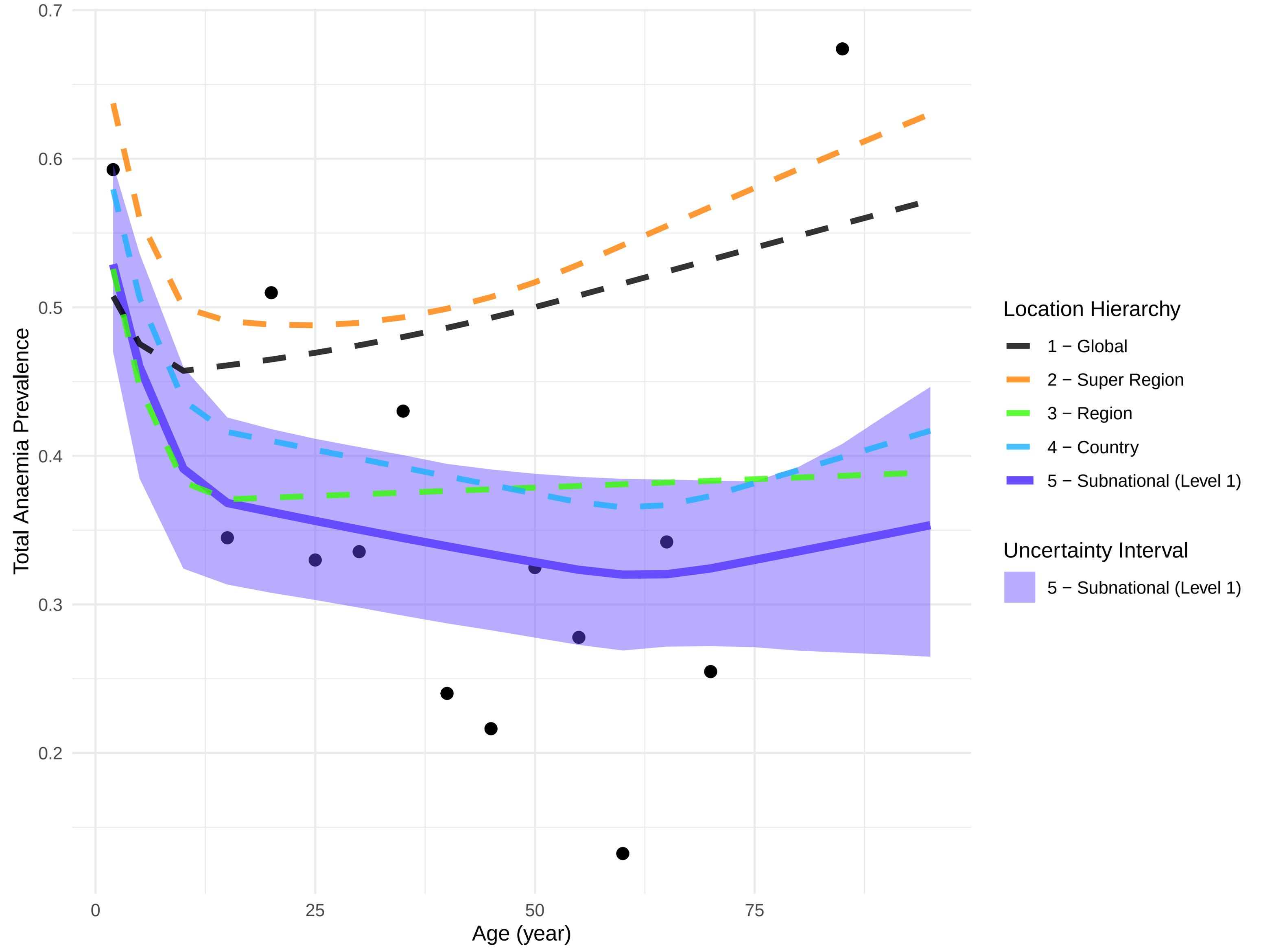

MR−BRT Age/Sex Fits − Non−pregnant Females 2+ Years Old − Total Anaemia Prevalence − Western Cape
WHO−2024 Adjustment Method

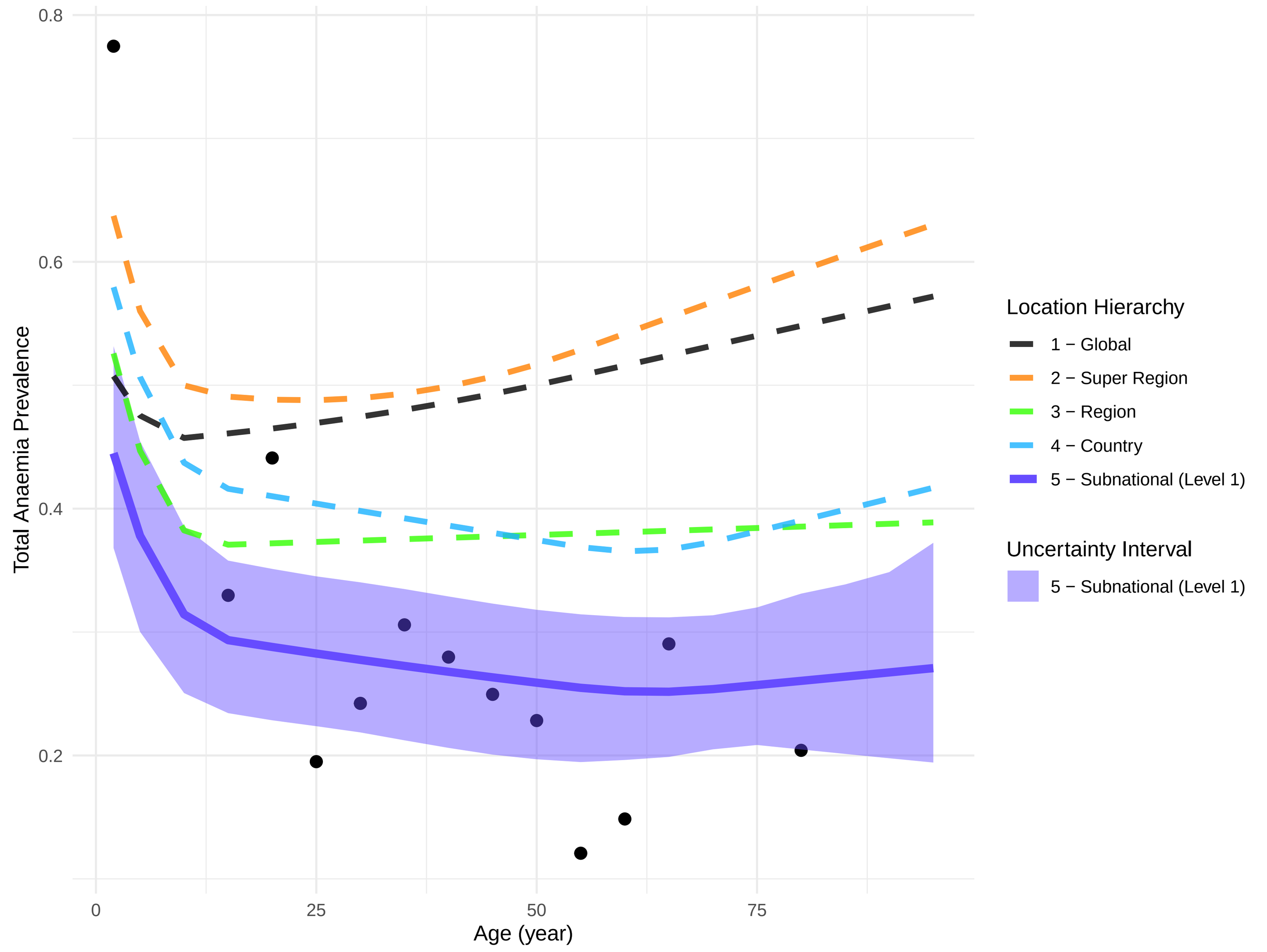

MR−BRT Age/Sex Fits − Non−pregnant Females 2+ Years Old − Total Anaemia Prevalence − Zimbabwe
WHO−2024 Adjustment Method

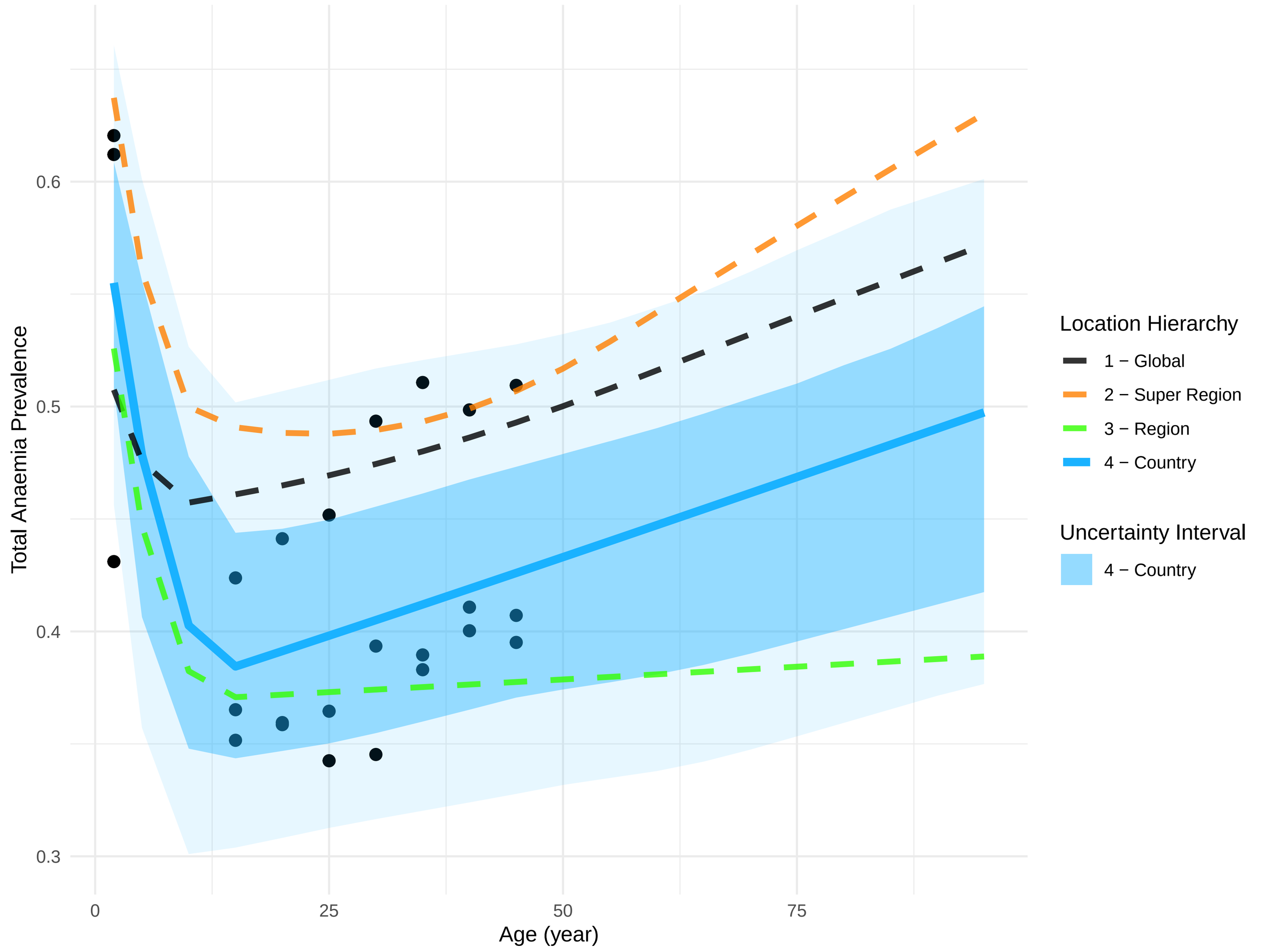

MR−BRT Age/Sex Fits − Non−pregnant Females 2+ Years Old − Total Anaemia Prevalence − Western Sub−Saharan Africa
WHO−2024 Adjustment Method

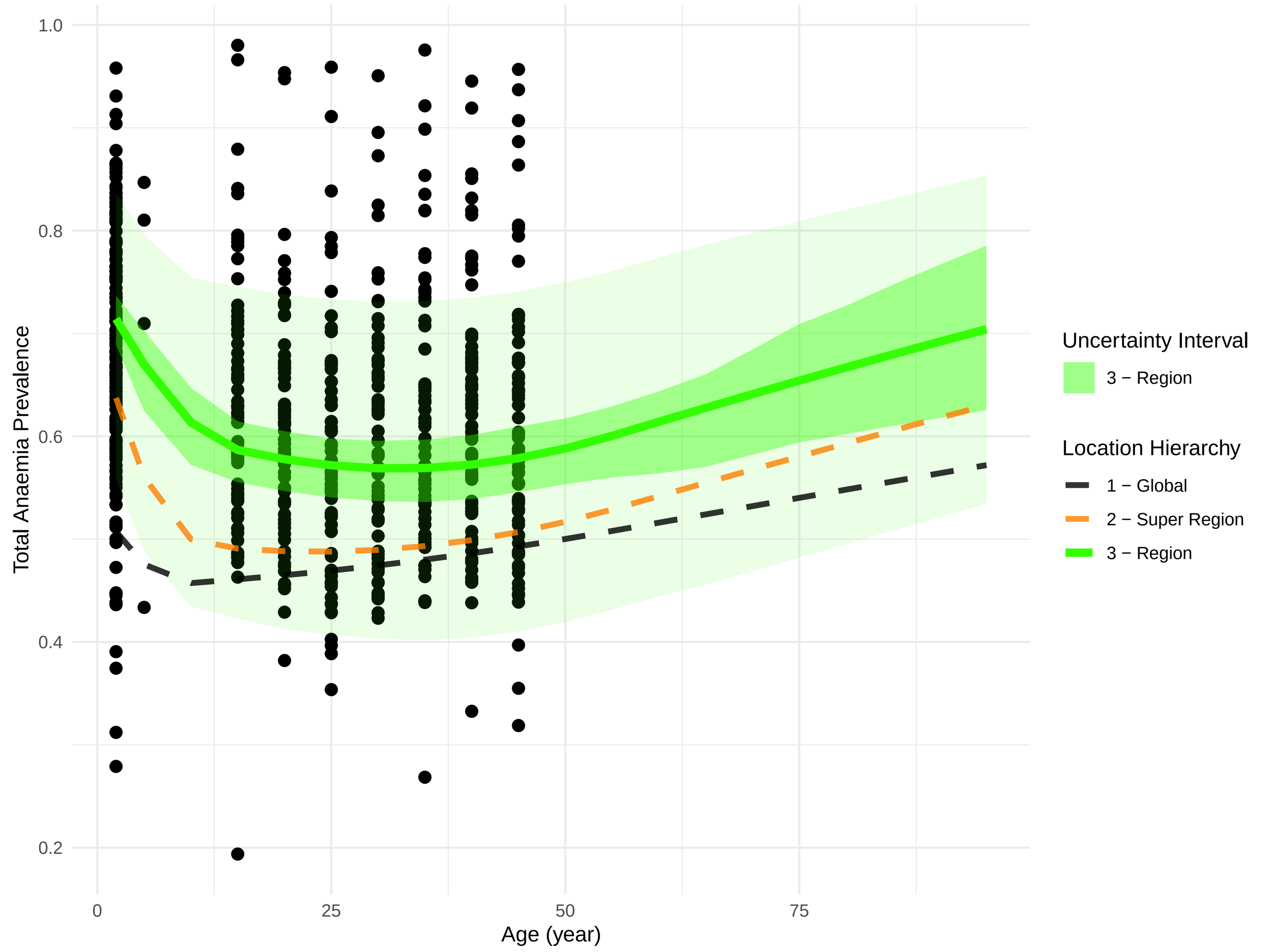

MR−BRT Age/Sex Fits − Non−pregnant Females 2+ Years Old − Total Anaemia Prevalence − Benin
WHO−2024 Adjustment Method

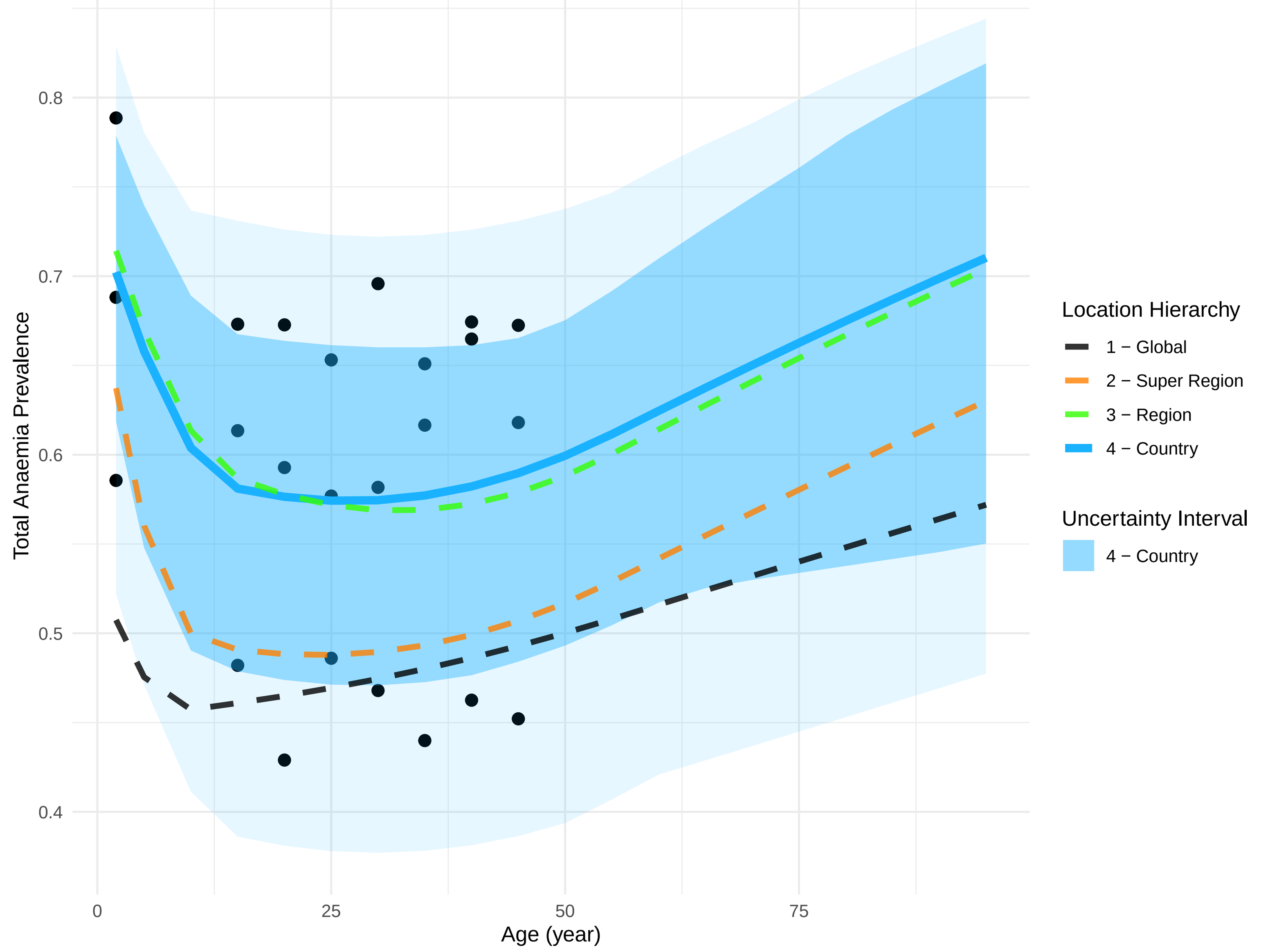

MR−BRT Age/Sex Fits − Non−pregnant Females 2+ Years Old − Total Anaemia Prevalence − Burkina Faso
WHO−2024 Adjustment Method

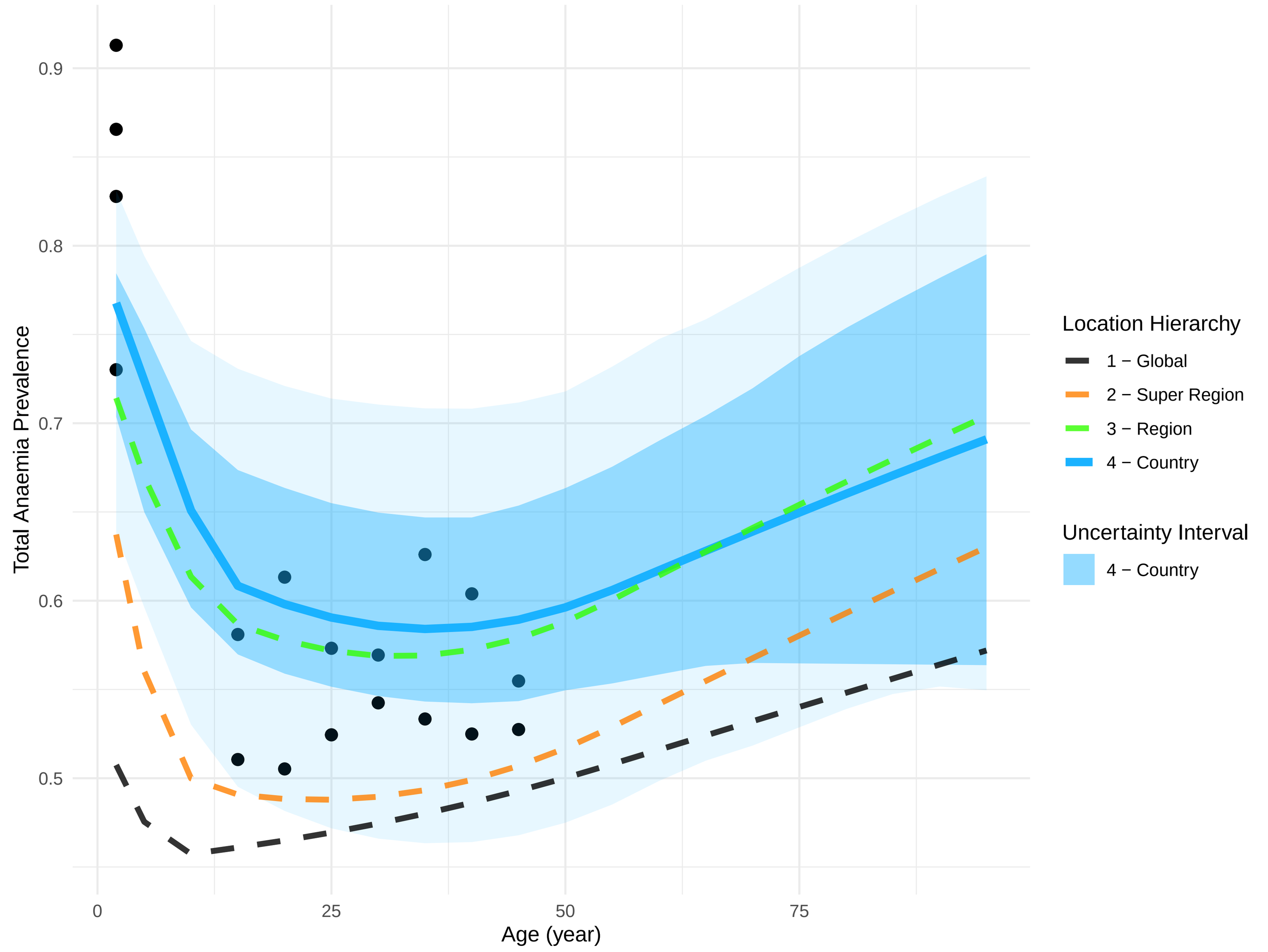

MR−BRT Age/Sex Fits − Non−pregnant Females 2+ Years Old − Total Anaemia Prevalence − Cameroon
WHO−2024 Adjustment Method

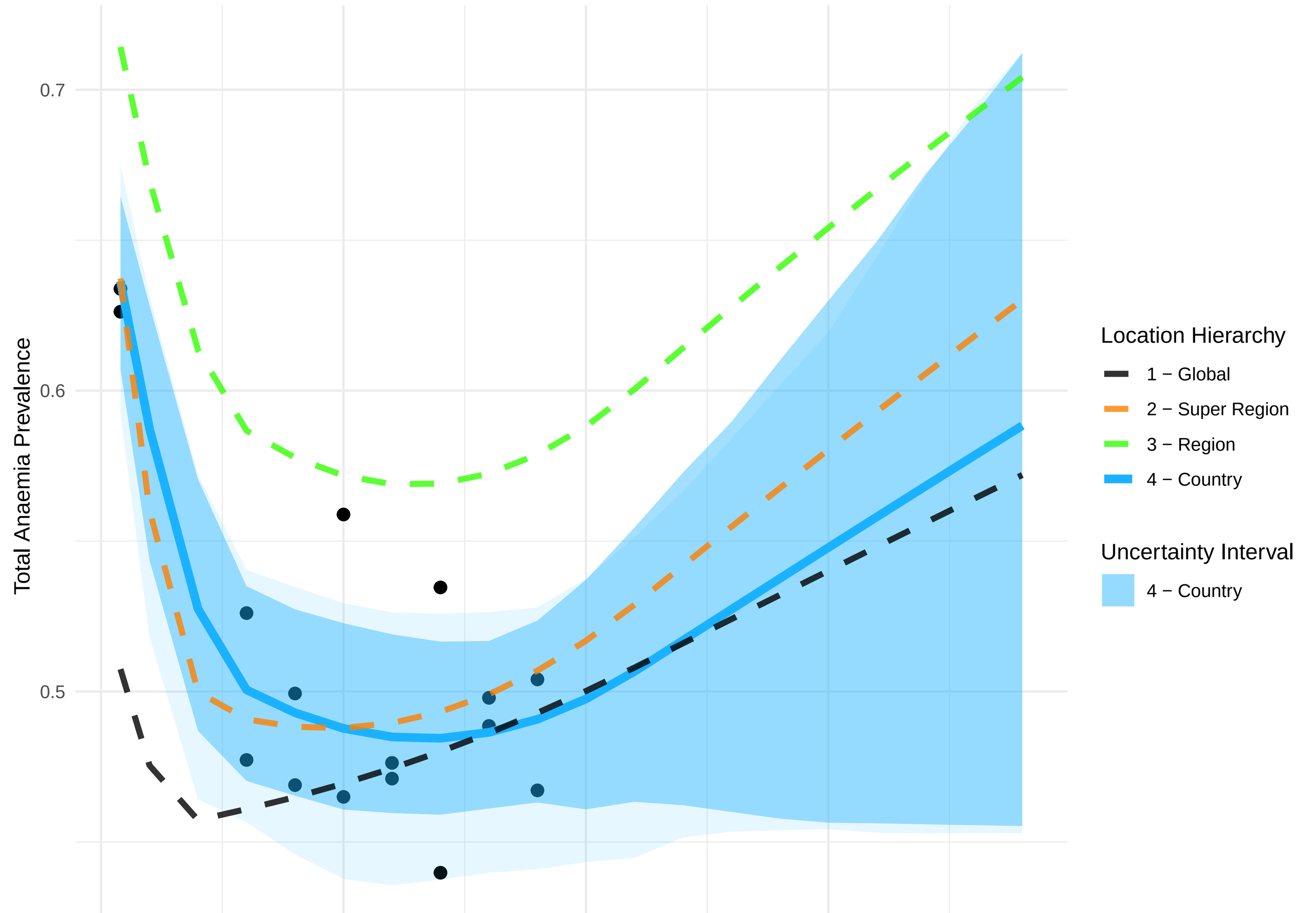

MR−BRT Age/Sex Fits − Non−pregnant Females 2+ Years Old − Total Anaemia Prevalence − Côte d'Ivoire
WHO−2024 Adjustment Method

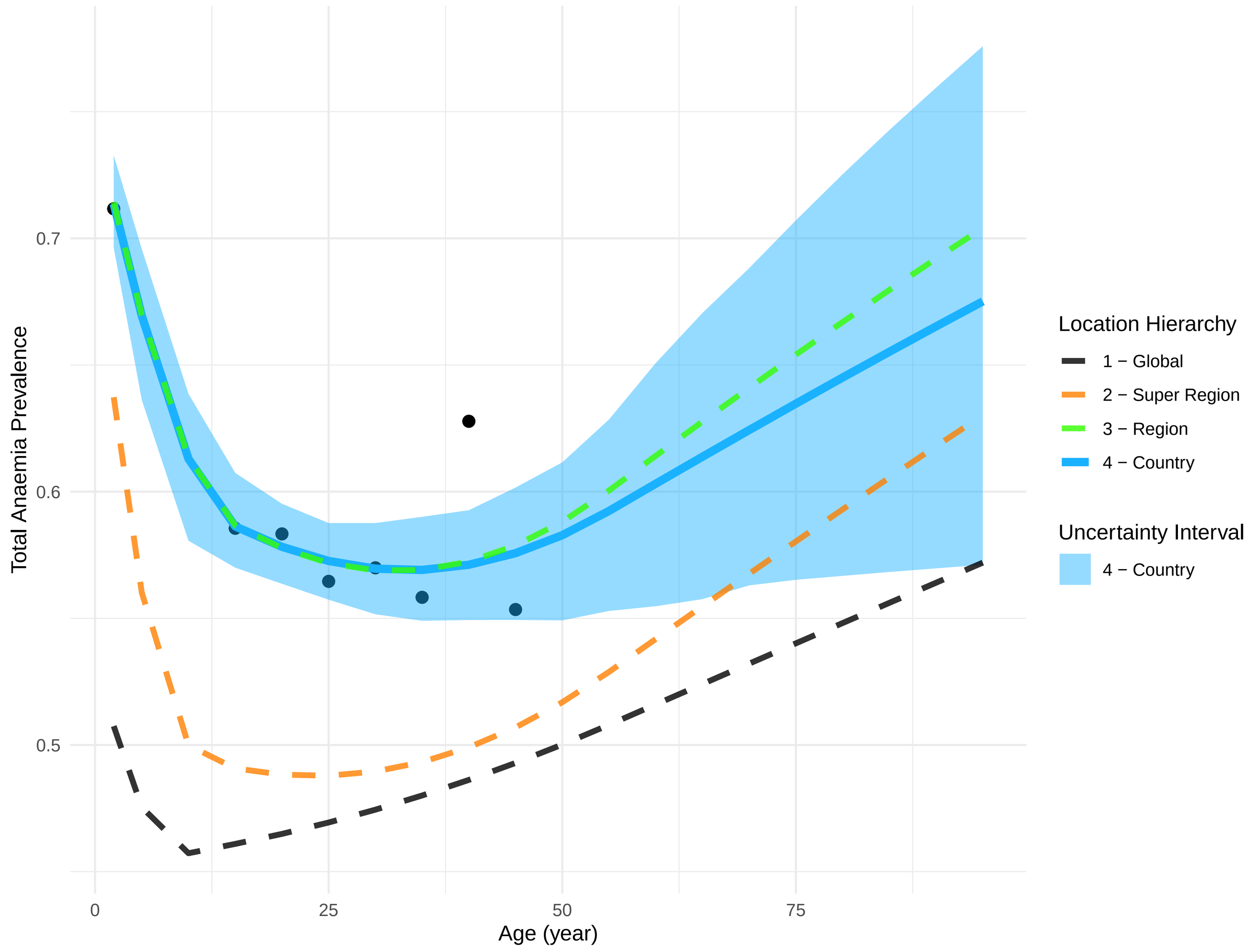

MR−BRT Age/Sex Fits − Non−pregnant Females 2+ Years Old − Total Anaemia Prevalence − Gambia
WHO−2024 Adjustment Method

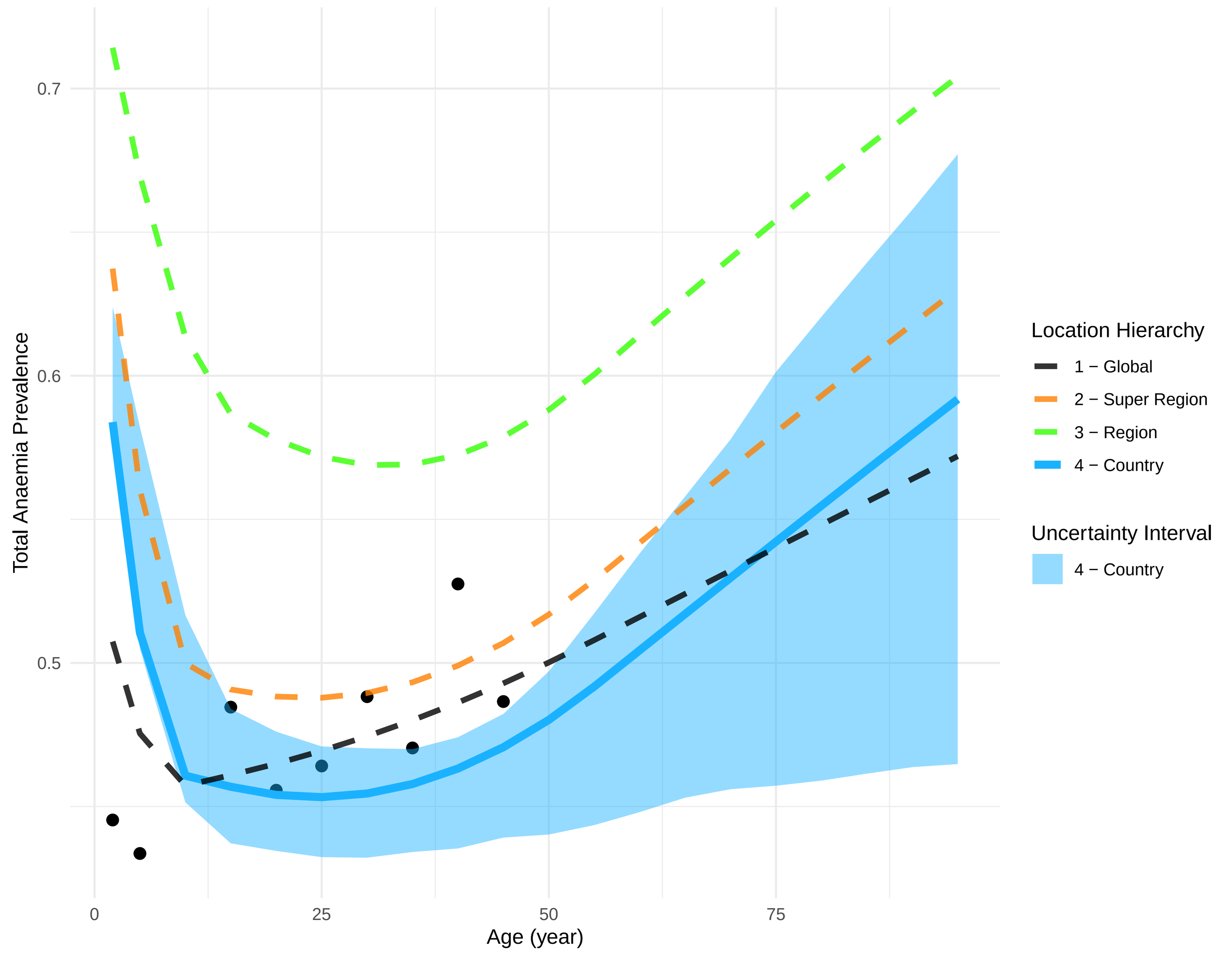

MR−BRT Age/Sex Fits − Non−pregnant Females 2+ Years Old − Total Anaemia Prevalence − Ghana
WHO−2024 Adjustment Method

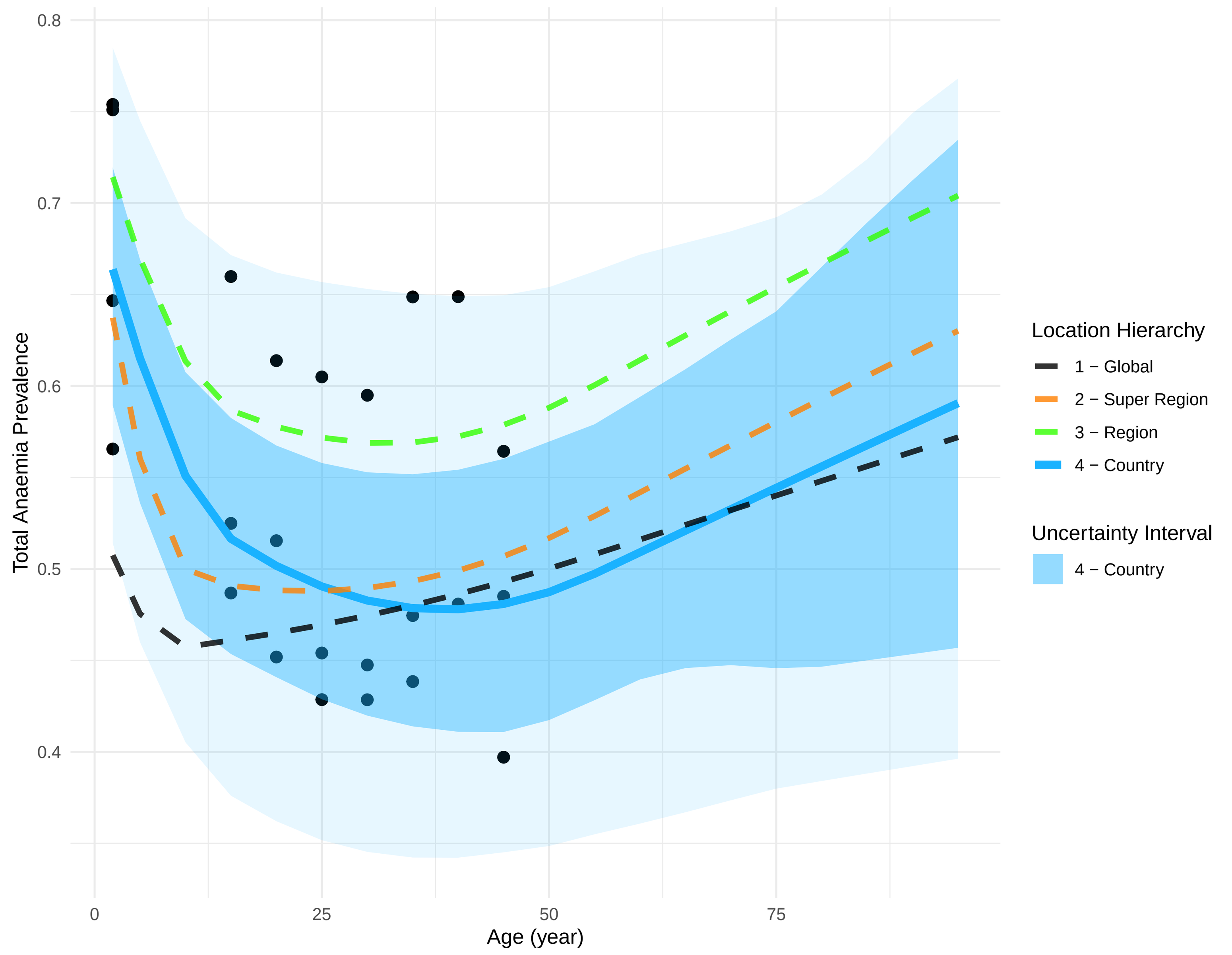

MR−BRT Age/Sex Fits − Non−pregnant Females 2+ Years Old − Total Anaemia Prevalence − Guinea
WHO−2024 Adjustment Method

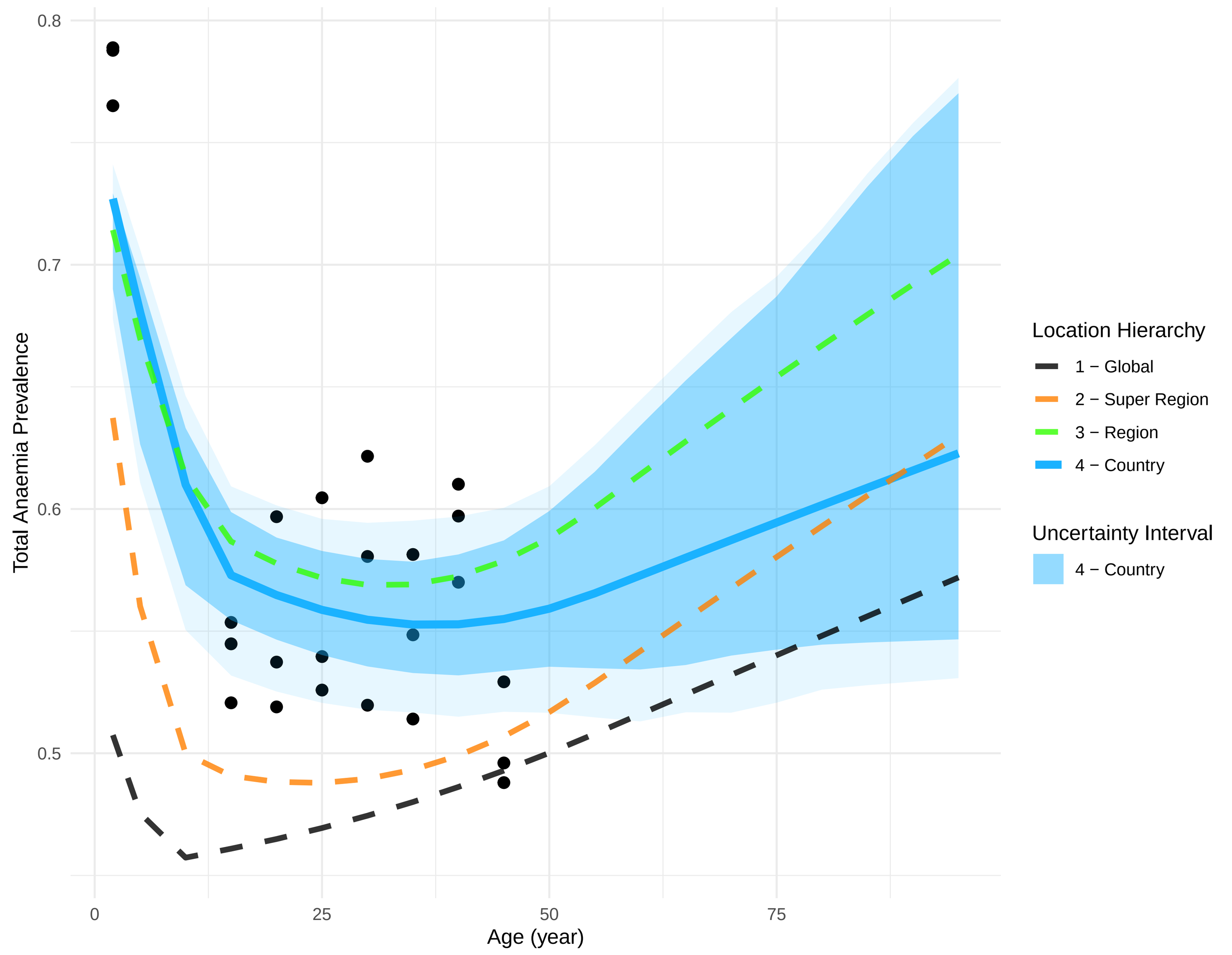

MR−BRT Age/Sex Fits − Non−pregnant Females 2+ Years Old − Total Anaemia Prevalence − Liberia
WHO−2024 Adjustment Method

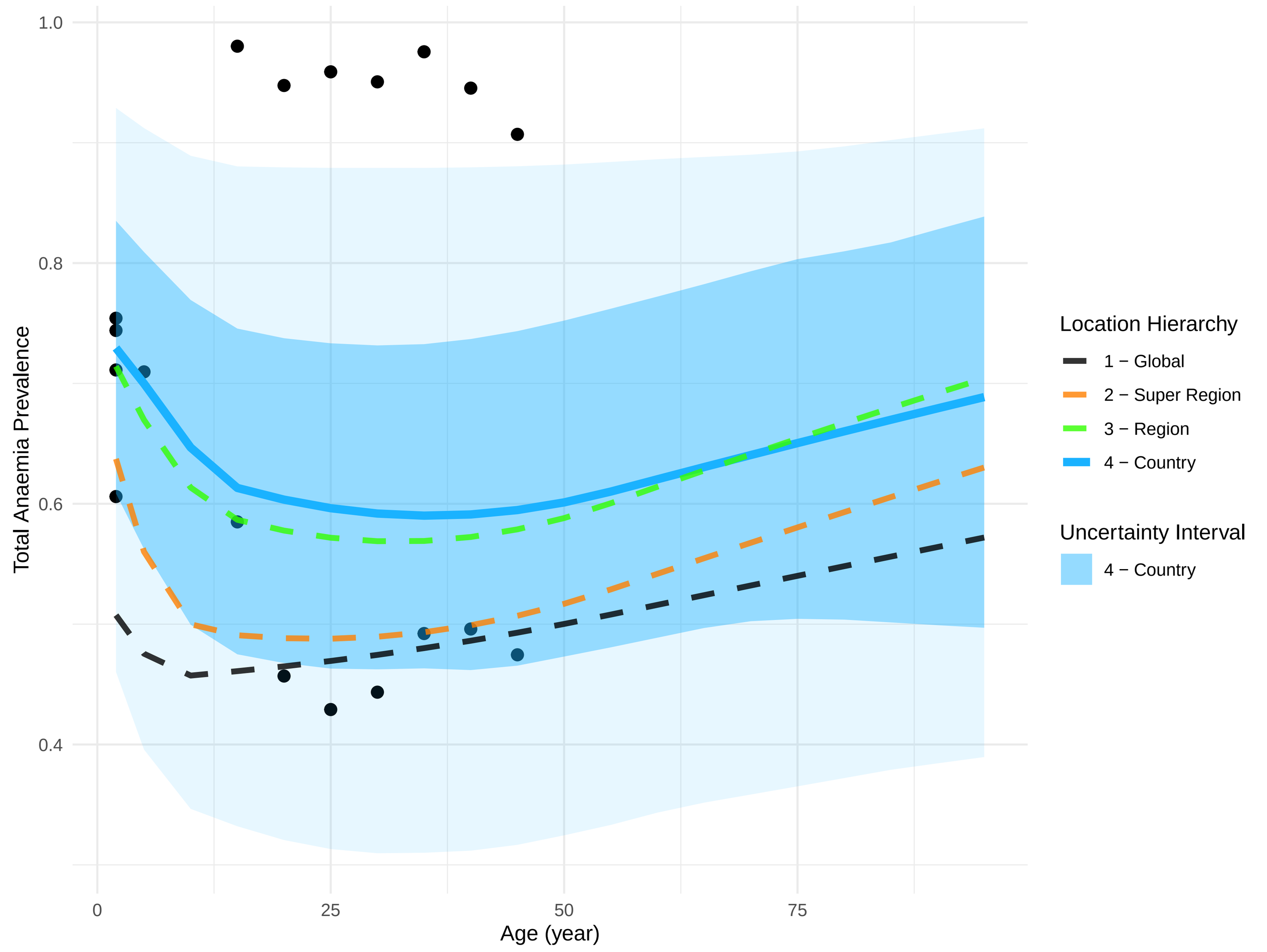

MR−BRT Age/Sex Fits − Non−pregnant Females 2+ Years Old − Total Anaemia Prevalence − Mali
WHO−2024 Adjustment Method

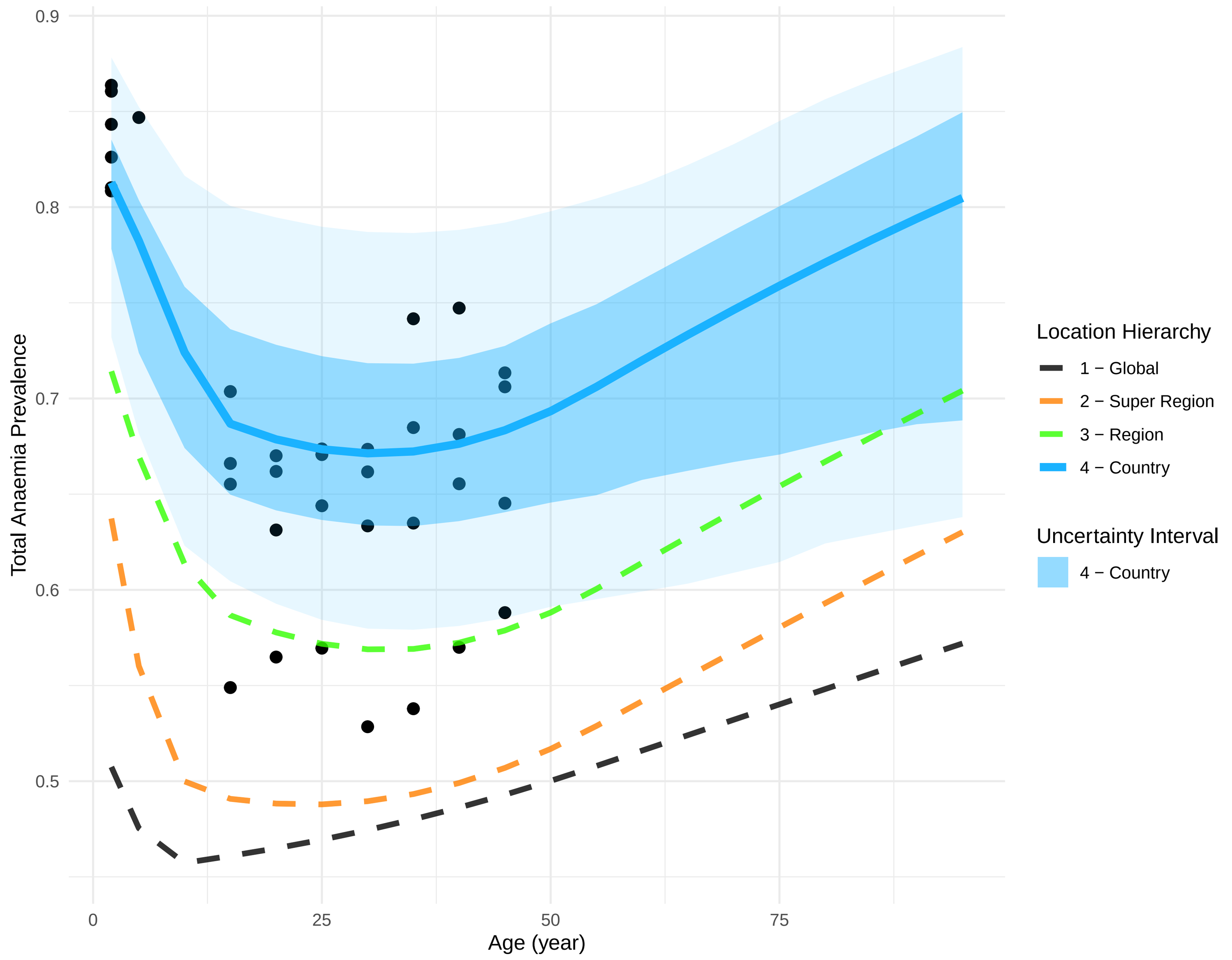

MR−BRT Age/Sex Fits − Non−pregnant Females 2+ Years Old − Total Anaemia Prevalence − Niger
WHO−2024 Adjustment Method

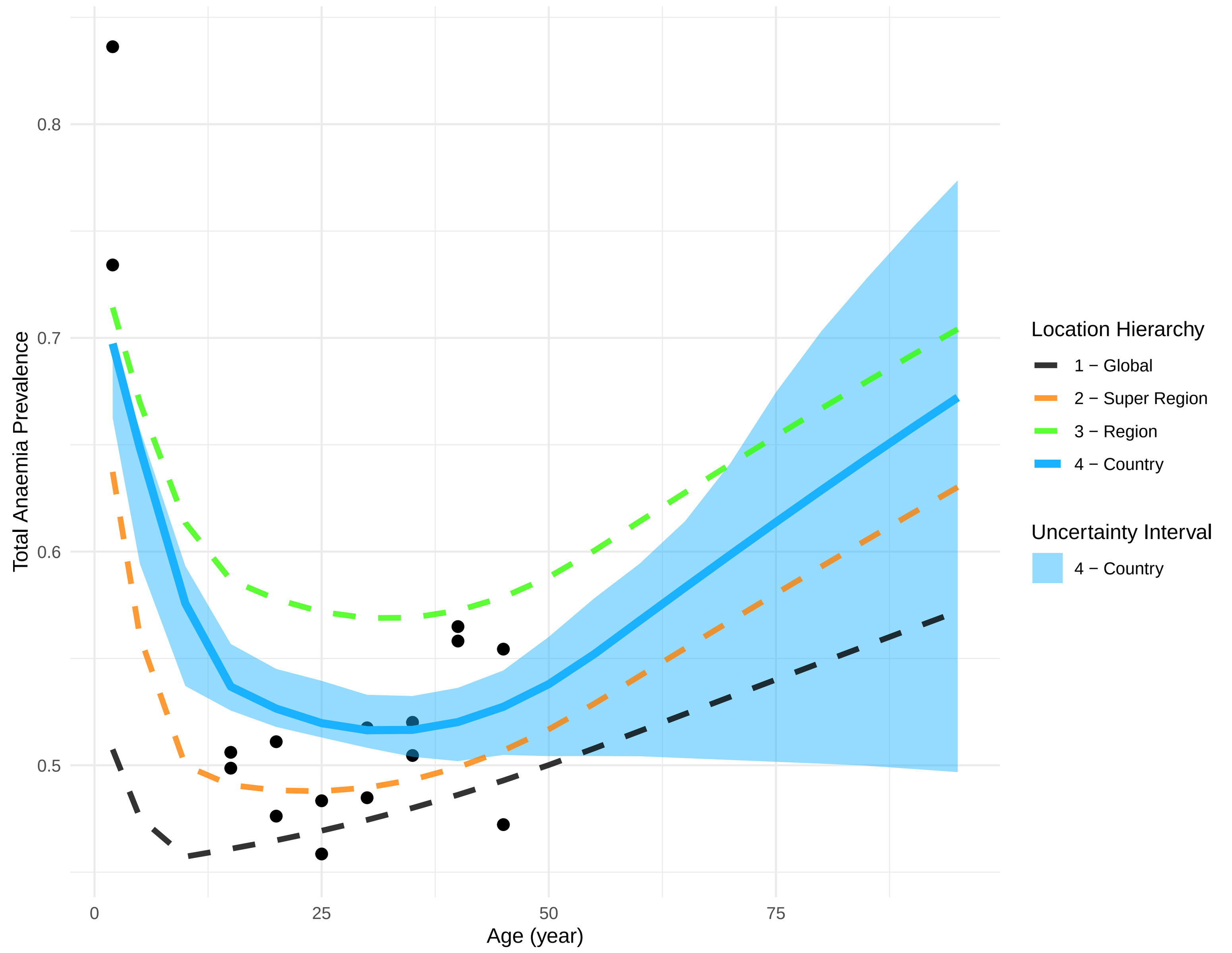

MR−BRT Age/Sex Fits − Non−pregnant Females 2+ Years Old − Total Anaemia Prevalence − Nigeria
WHO−2024 Adjustment Method

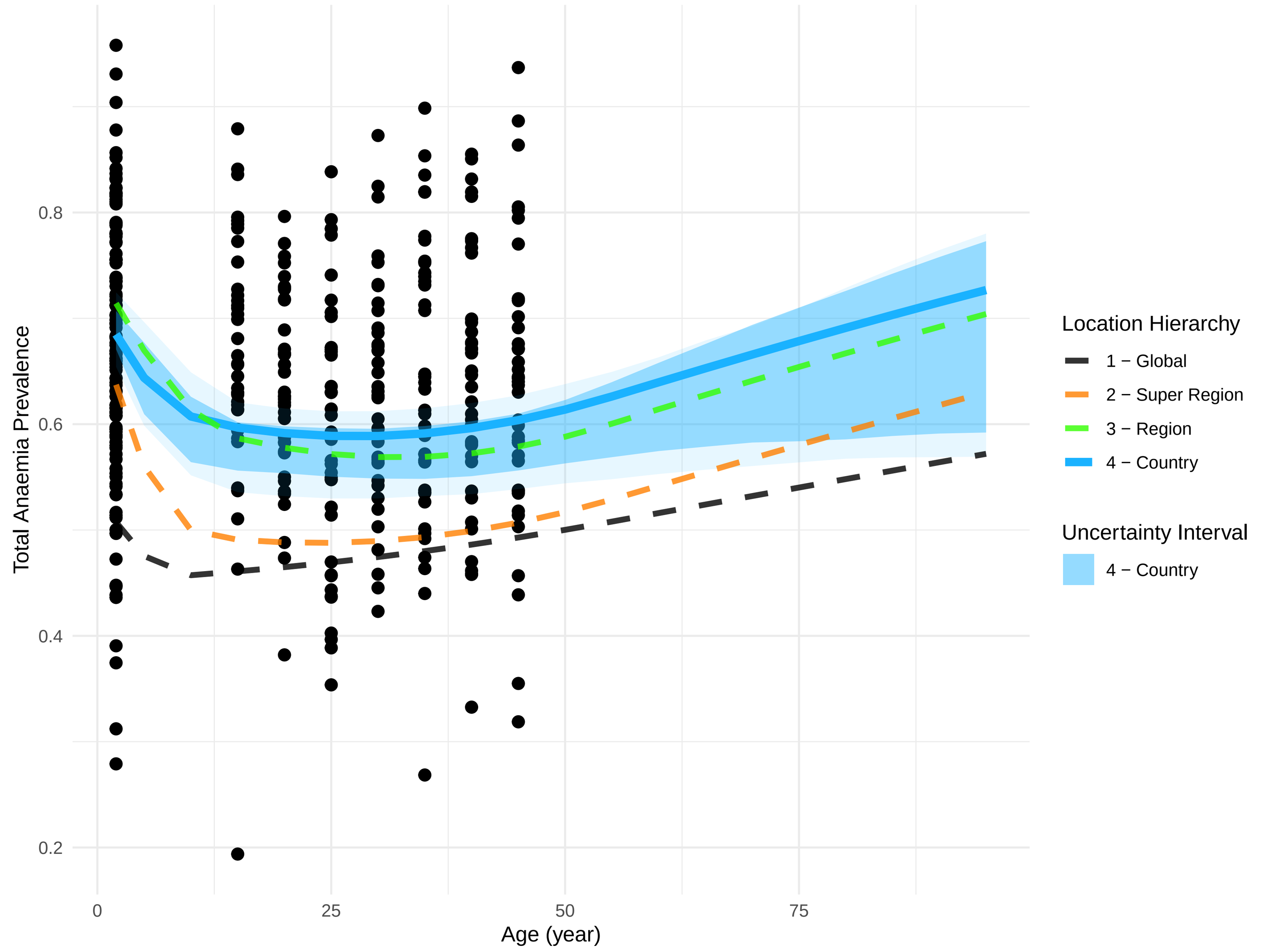

MR−BRT Age/Sex Fits − Non−pregnant Females 2+ Years Old − Total Anaemia Prevalence − Abia
WHO−2024 Adjustment Method

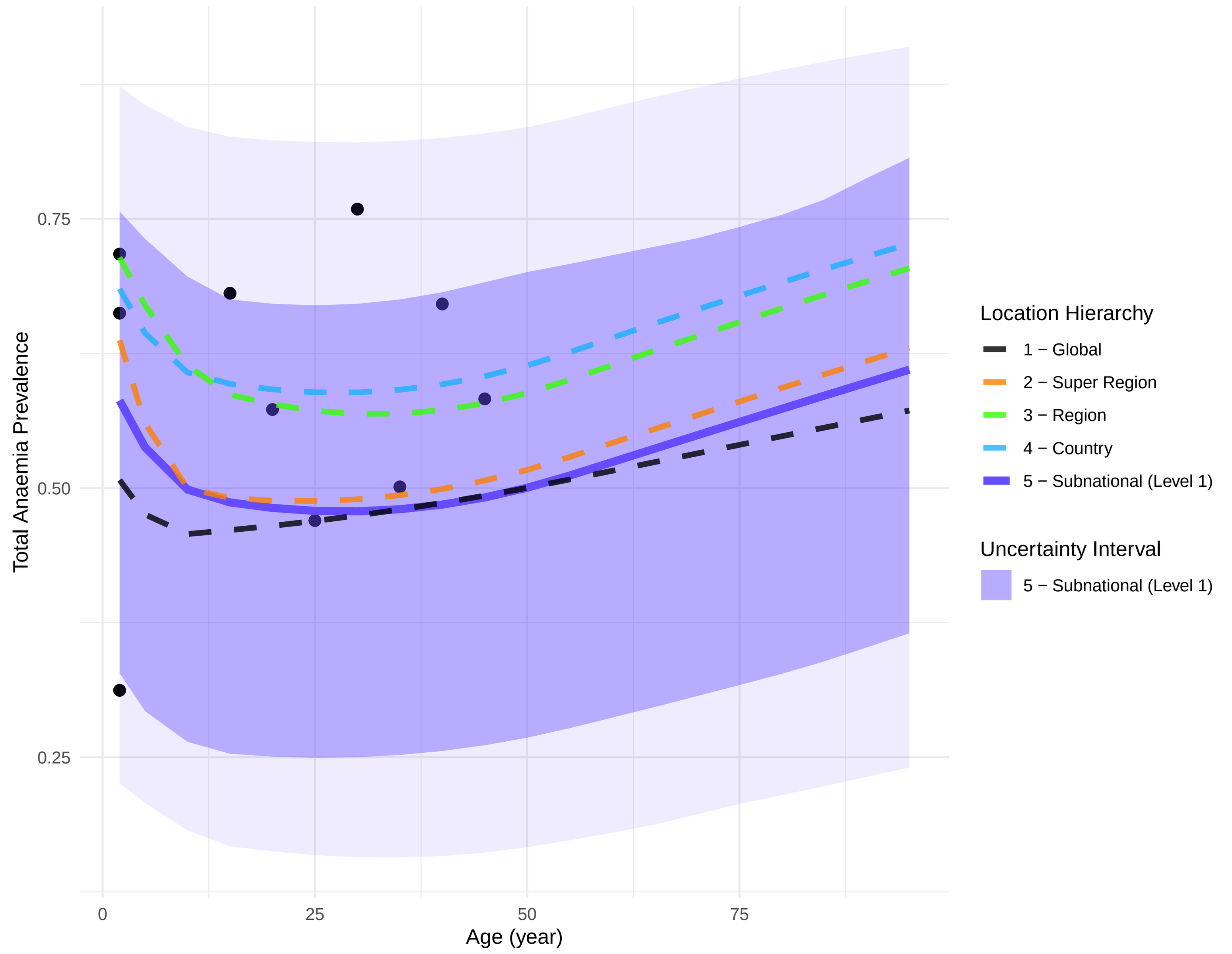

MR−BRT Age/Sex Fits − Non−pregnant Females 2+ Years Old − Total Anaemia Prevalence − Adamawa
WHO−2024 Adjustment Method

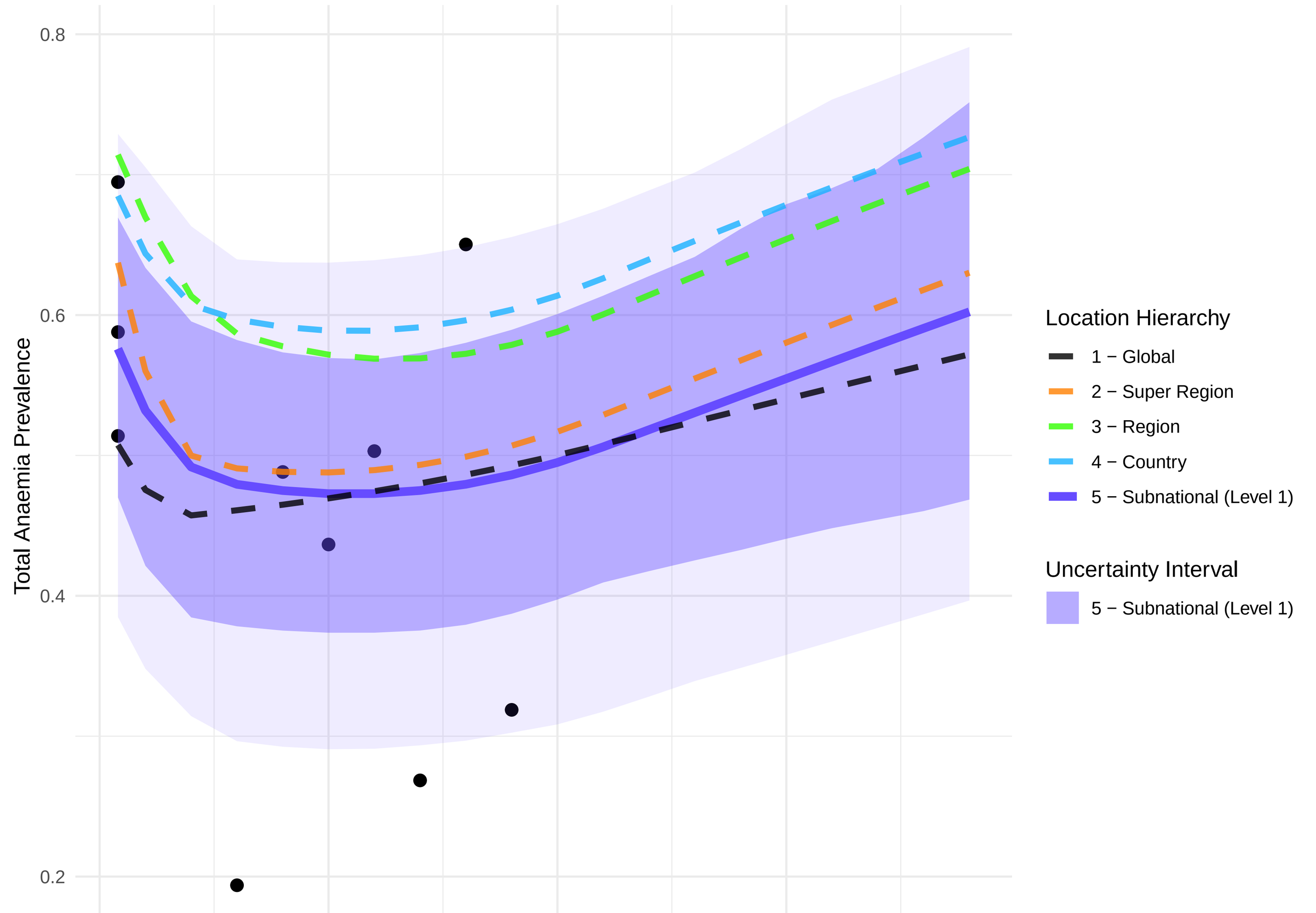

MR−BRT Age/Sex Fits − Non−pregnant Females 2+ Years Old − Total Anaemia Prevalence − Akwa Ibom
WHO−2024 Adjustment Method

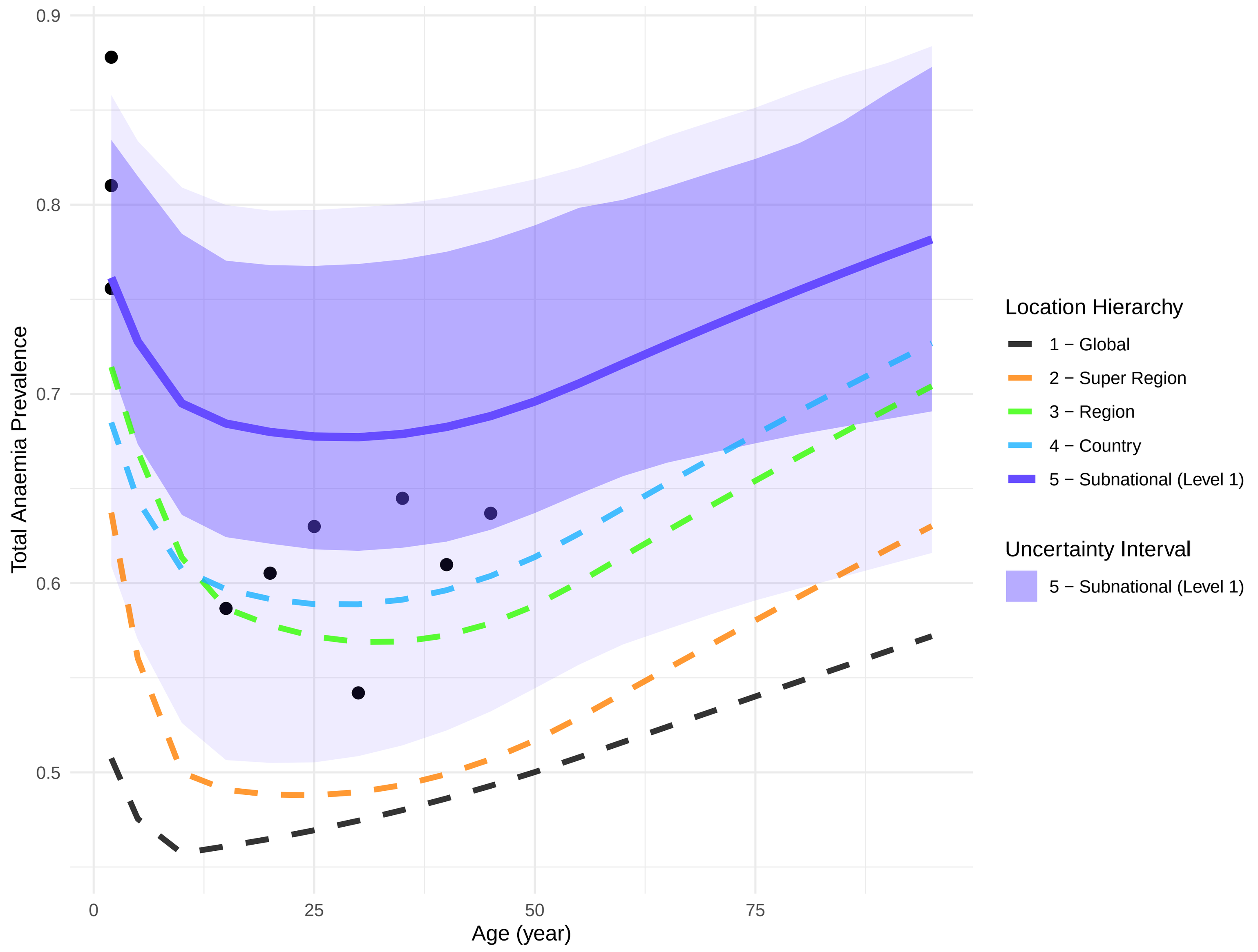

MR−BRT Age/Sex Fits − Non−pregnant Females 2+ Years Old − Total Anaemia Prevalence − Anambra
WHO−2024 Adjustment Method

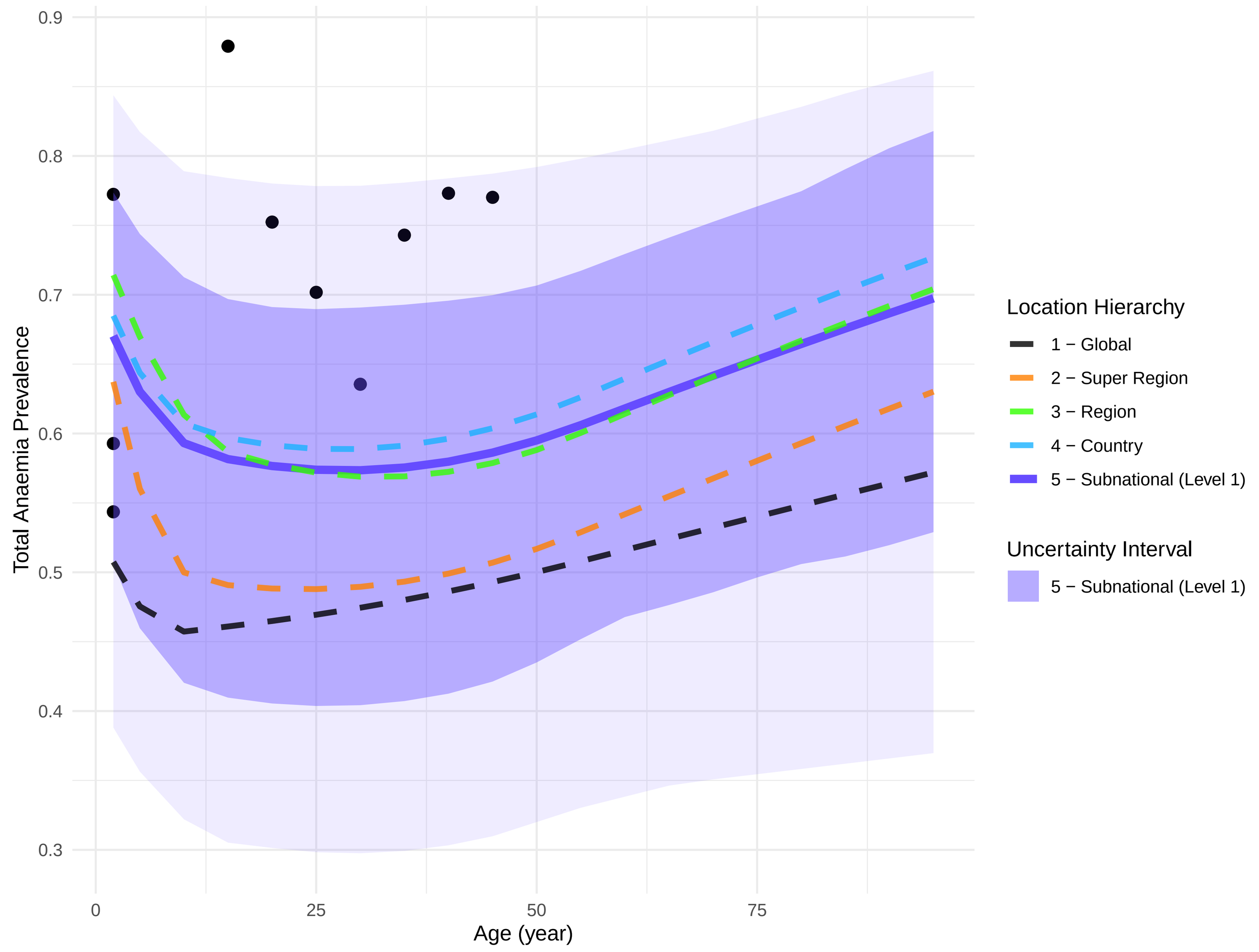

MR−BRT Age/Sex Fits − Non−pregnant Females 2+ Years Old − Total Anaemia Prevalence − Bauchi
WHO−2024 Adjustment Method

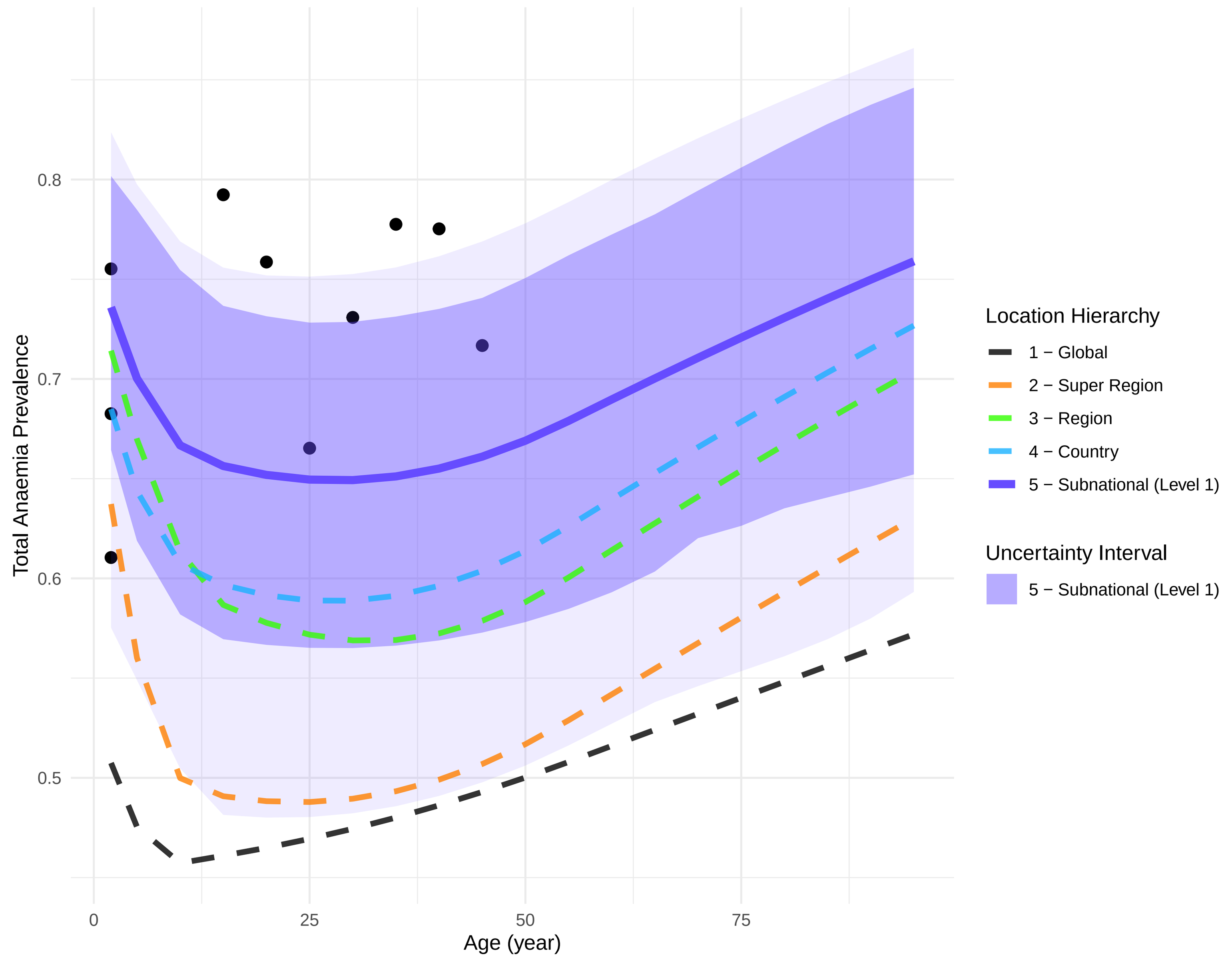

MR−BRT Age/Sex Fits − Non−pregnant Females 2+ Years Old − Total Anaemia Prevalence − Bayelsa
WHO−2024 Adjustment Method

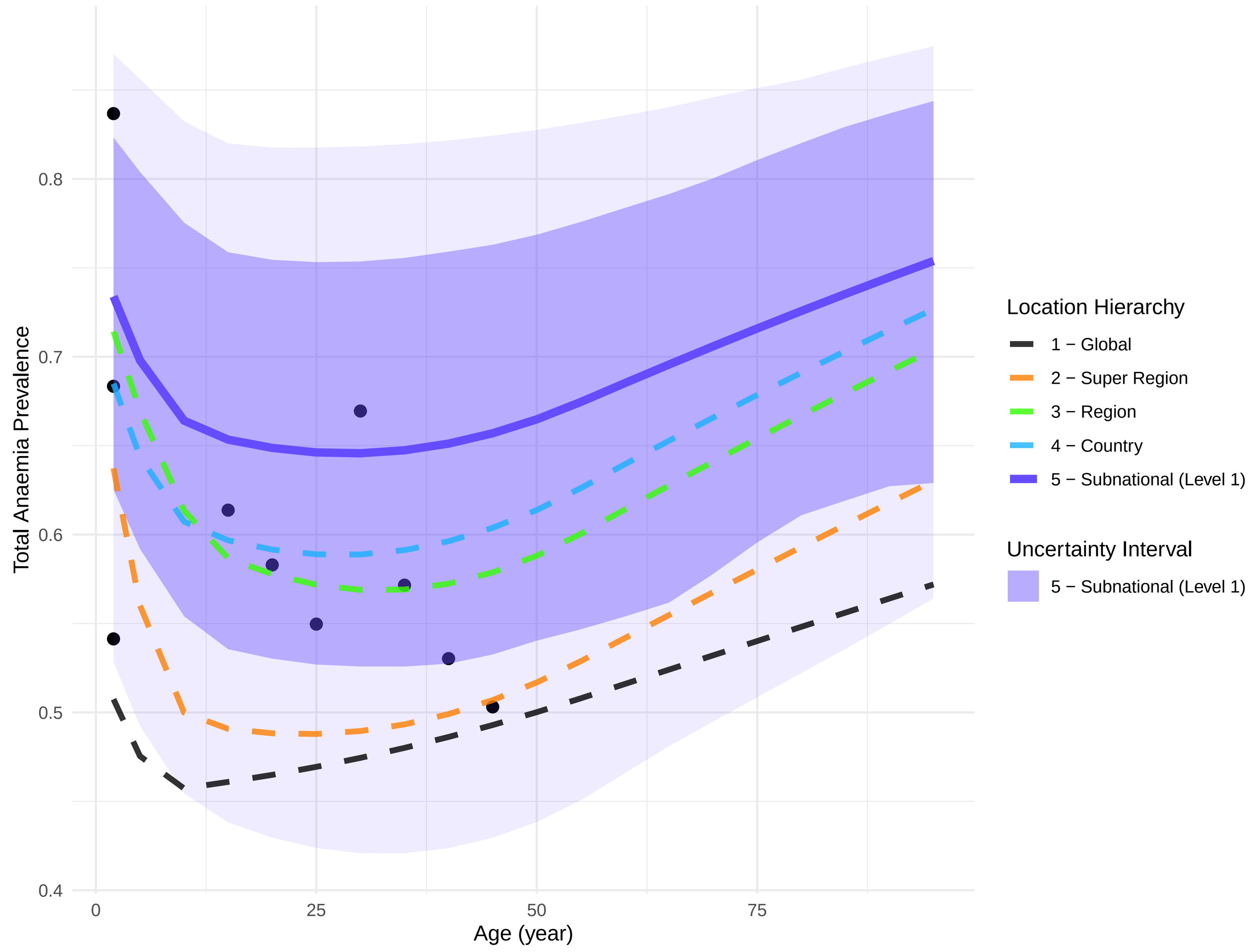

MR−BRT Age/Sex Fits − Non−pregnant Females 2+ Years Old − Total Anaemia Prevalence − Benue
WHO−2024 Adjustment Method

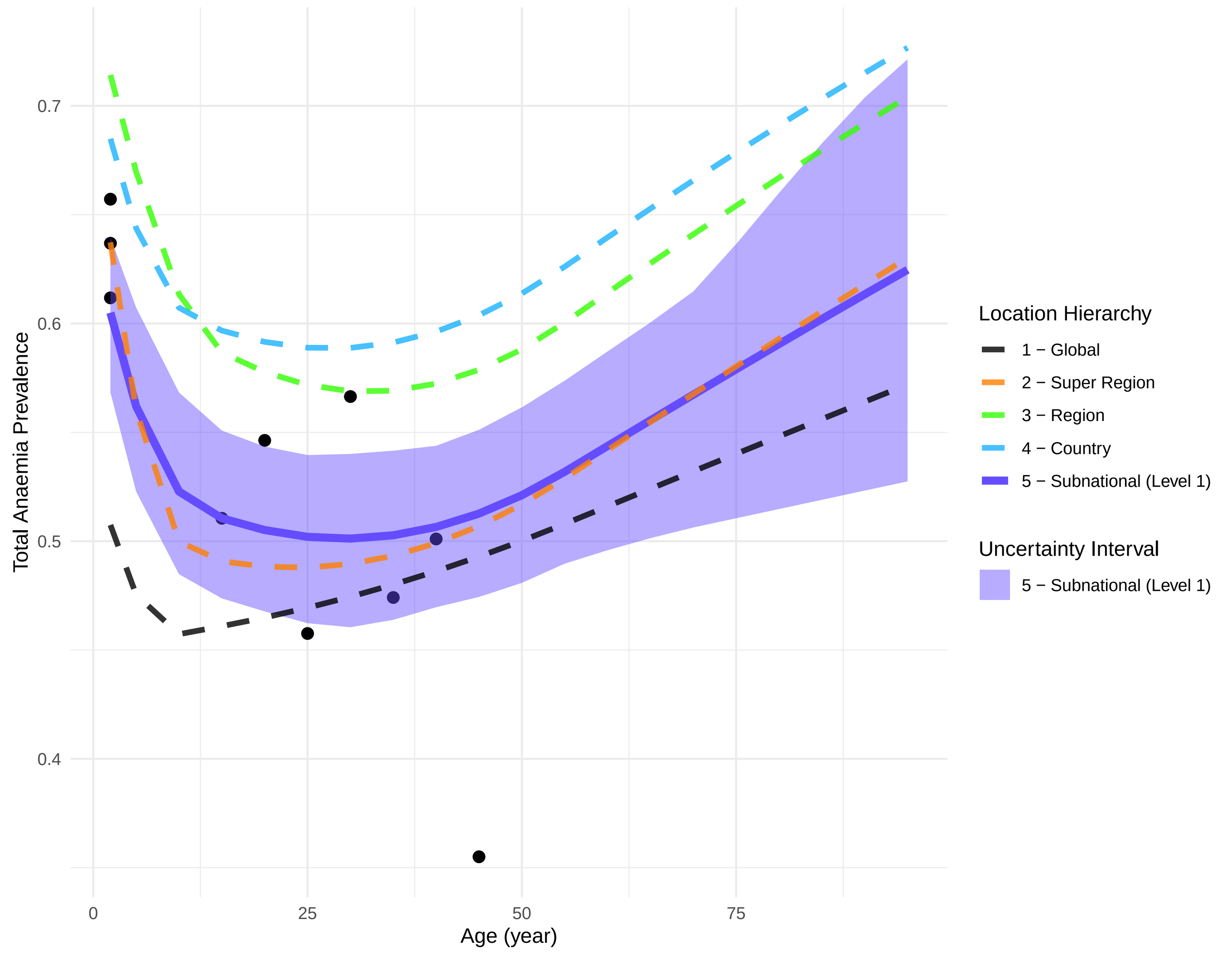

MR−BRT Age/Sex Fits − Non−pregnant Females 2+ Years Old − Total Anaemia Prevalence − Borno
WHO−2024 Adjustment Method

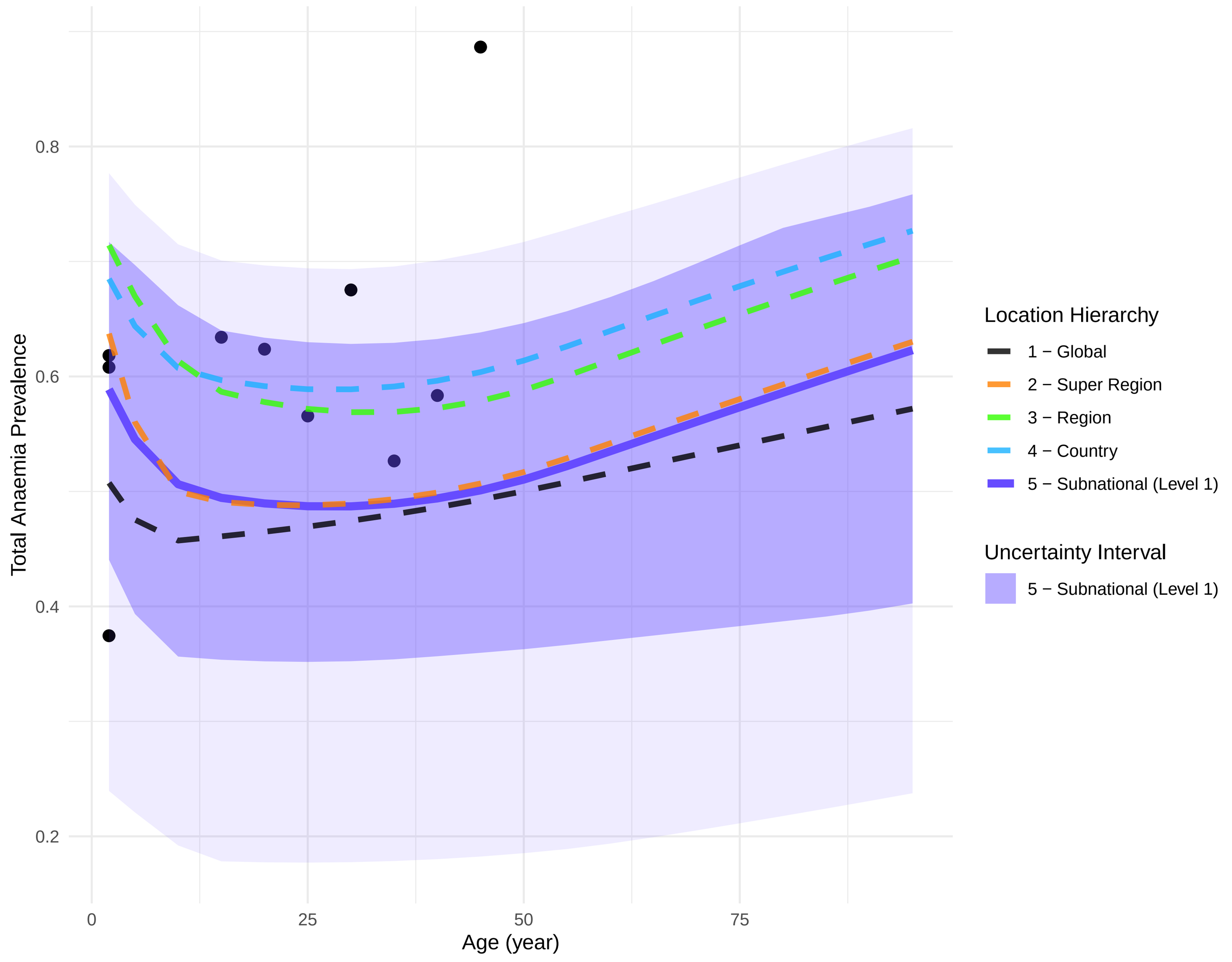

MR−BRT Age/Sex Fits − Non−pregnant Females 2+ Years Old − Total Anaemia Prevalence − Cross River
WHO−2024 Adjustment Method

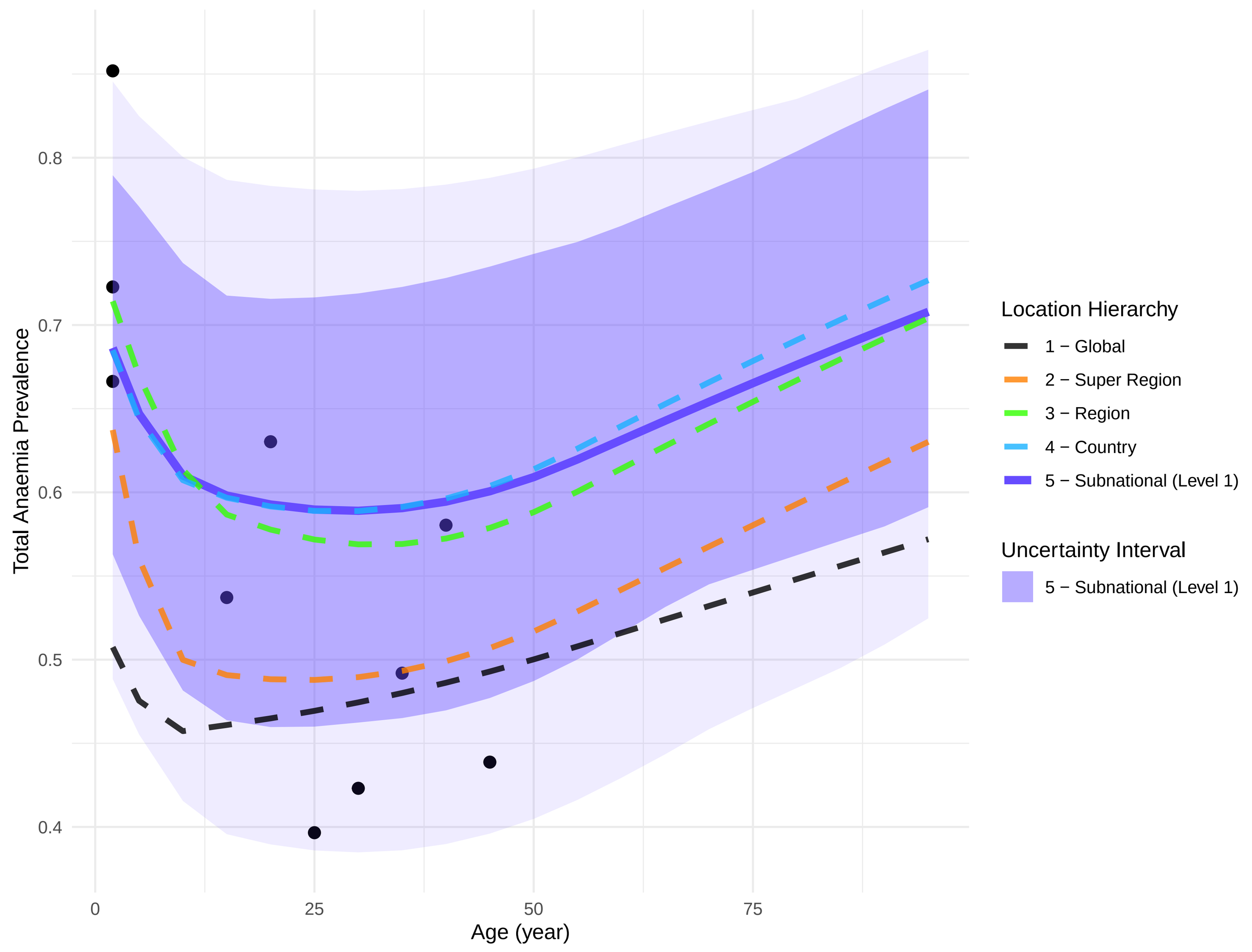

MR−BRT Age/Sex Fits − Non−pregnant Females 2+ Years Old − Total Anaemia Prevalence − Delta
WHO−2024 Adjustment Method

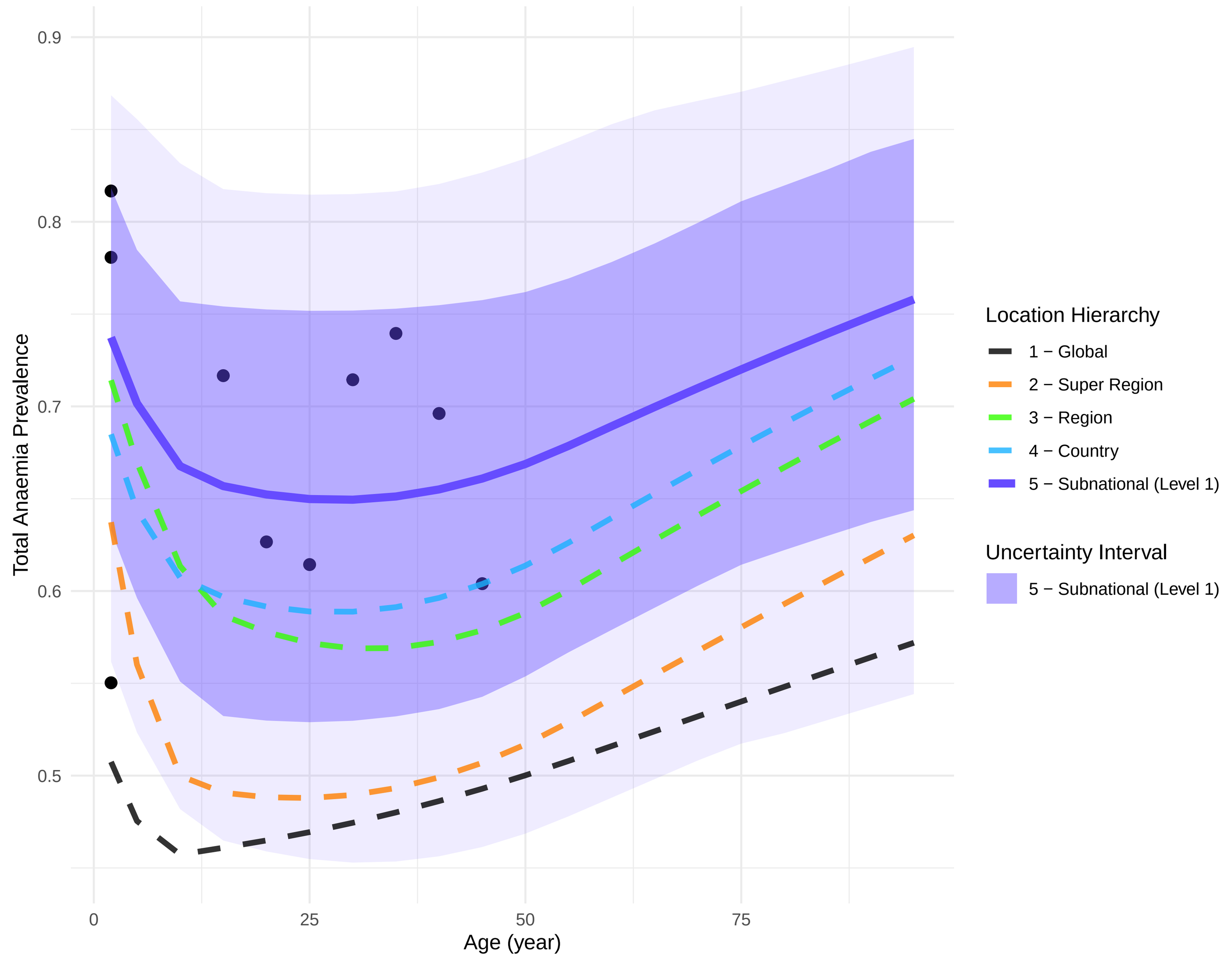

MR−BRT Age/Sex Fits − Non−pregnant Females 2+ Years Old − Total Anaemia Prevalence − Ebonyi
WHO−2024 Adjustment Method

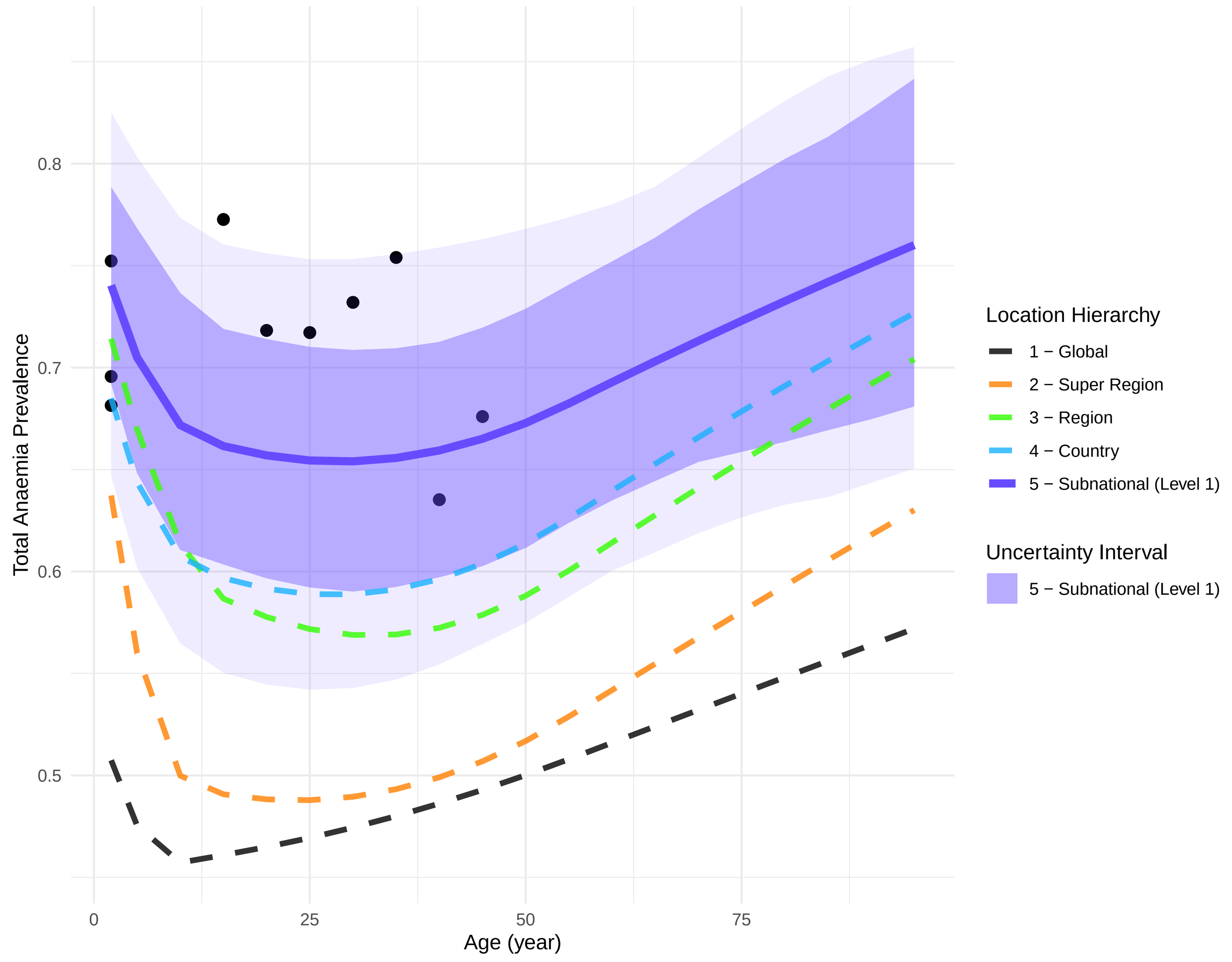

MR−BRT Age/Sex Fits − Non−pregnant Females 2+ Years Old − Total Anaemia Prevalence − Edo
WHO−2024 Adjustment Method

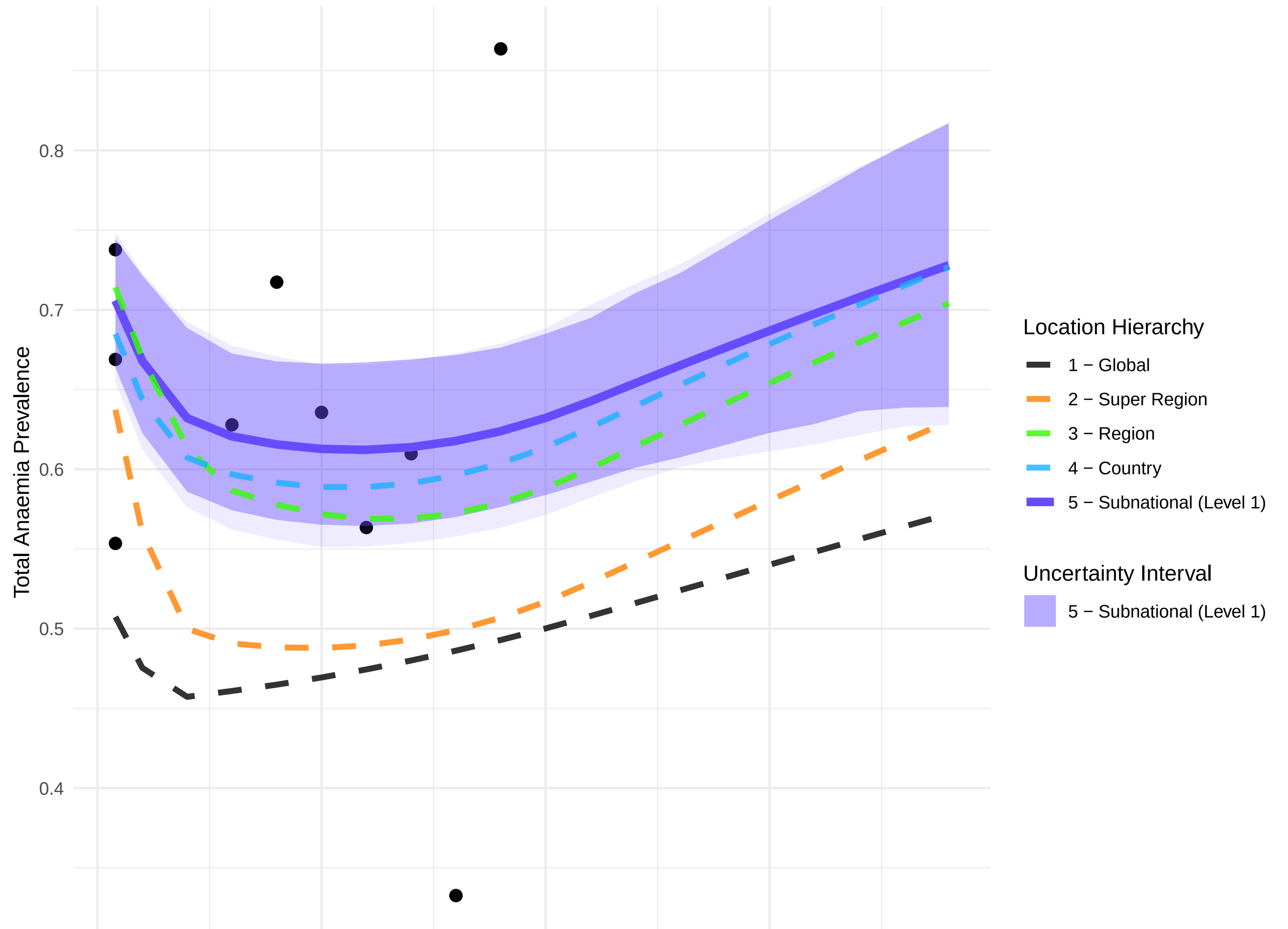

MR−BRT Age/Sex Fits − Non−pregnant Females 2+ Years Old − Total Anaemia Prevalence − Ekiti
WHO−2024 Adjustment Method

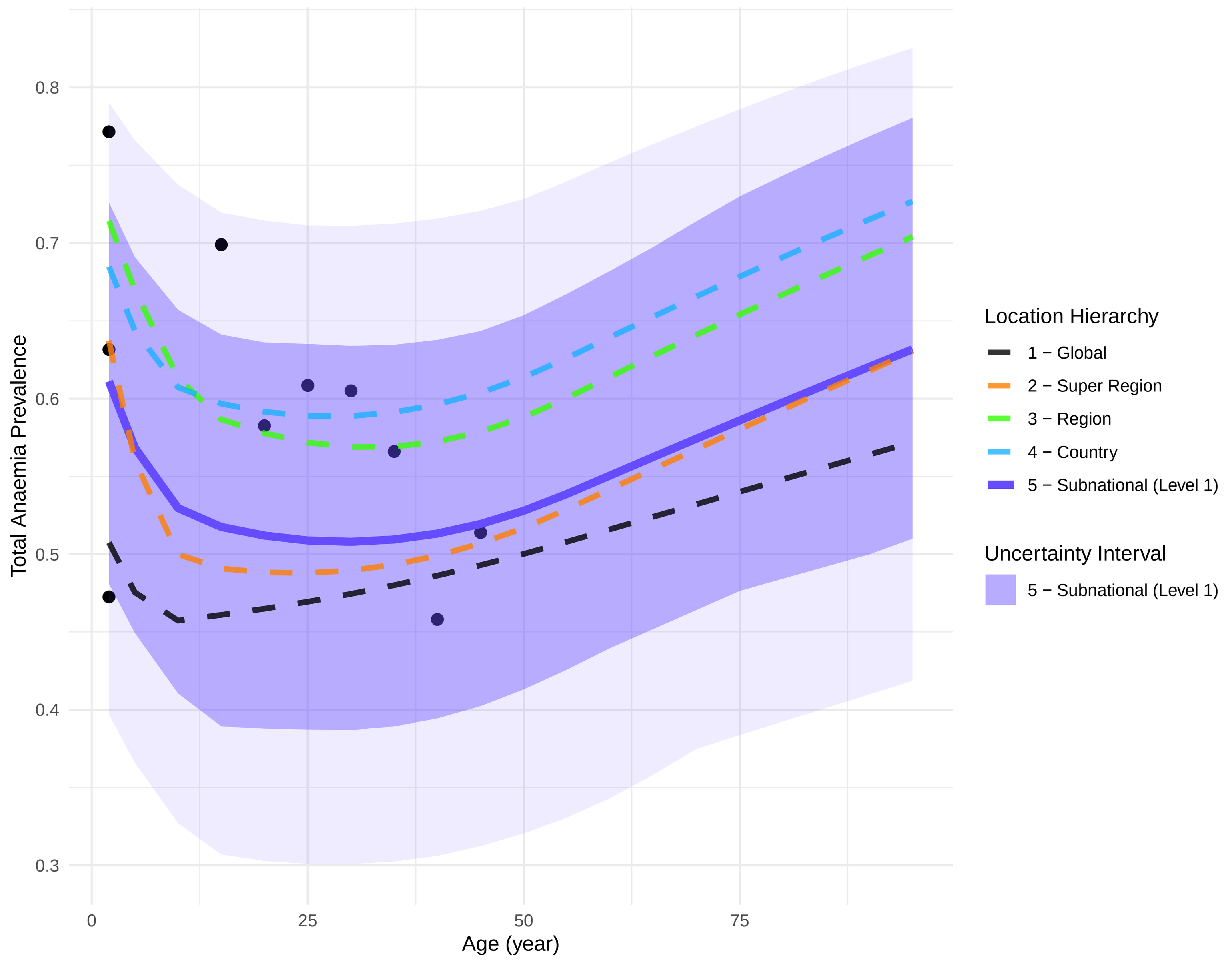

MR−BRT Age/Sex Fits − Non−pregnant Females 2+ Years Old − Total Anaemia Prevalence − Enugu
WHO−2024 Adjustment Method

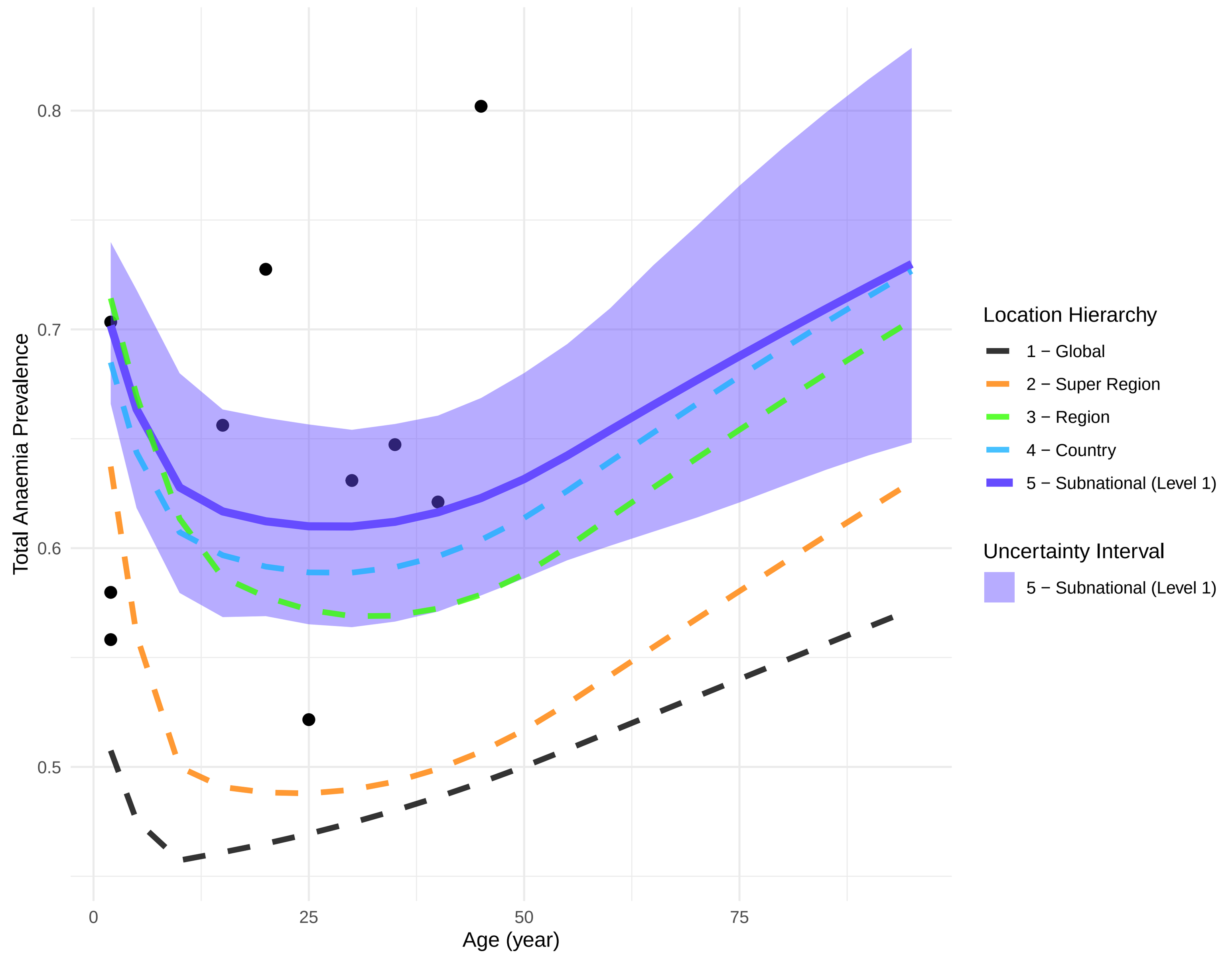

MR−BRT Age/Sex Fits − Non−pregnant Females 2+ Years Old − Total Anaemia Prevalence − FCT (Abuja)
WHO−2024 Adjustment Method

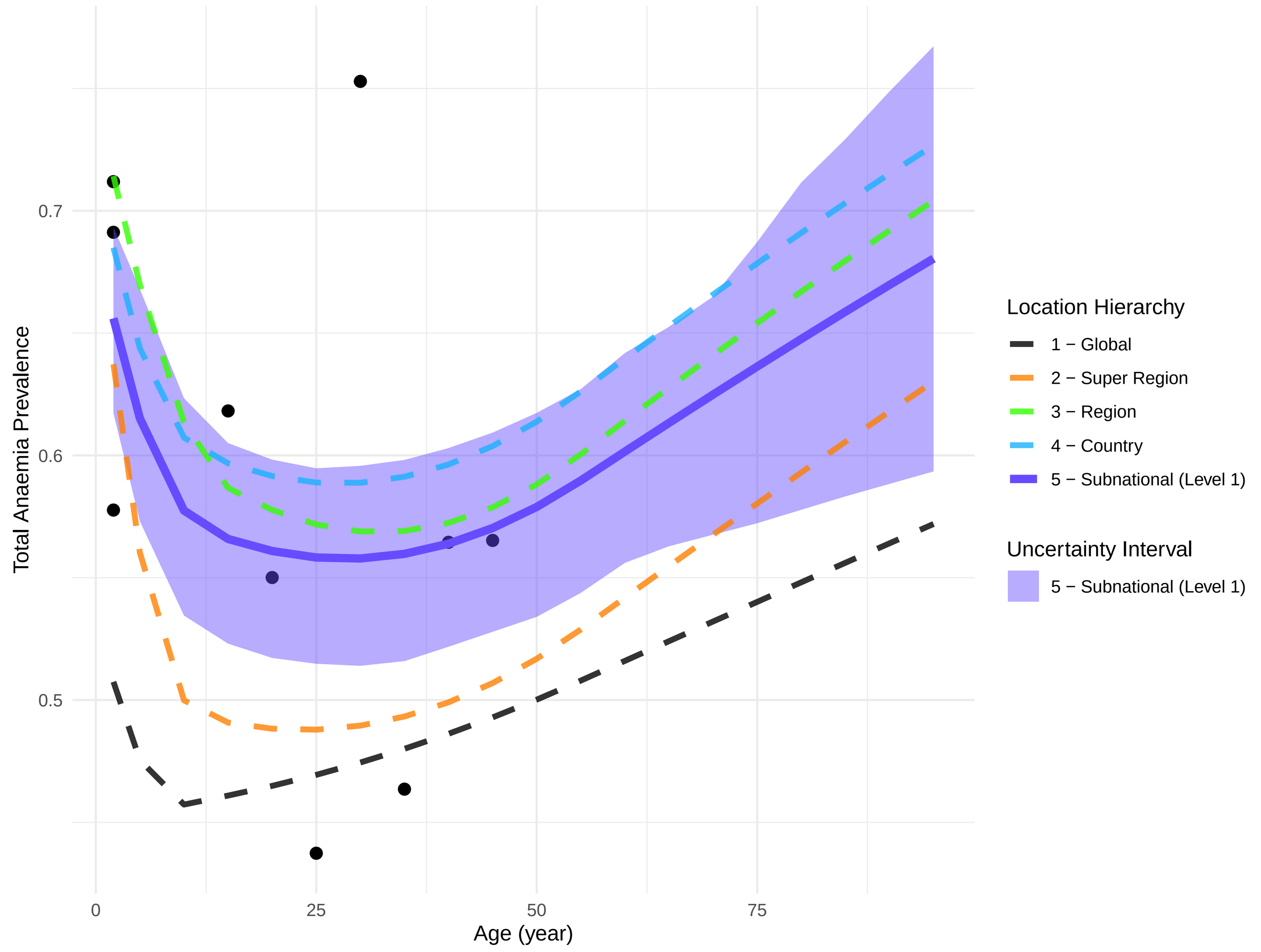

MR−BRT Age/Sex Fits − Non−pregnant Females 2+ Years Old − Total Anaemia Prevalence − Gombe
WHO−2024 Adjustment Method

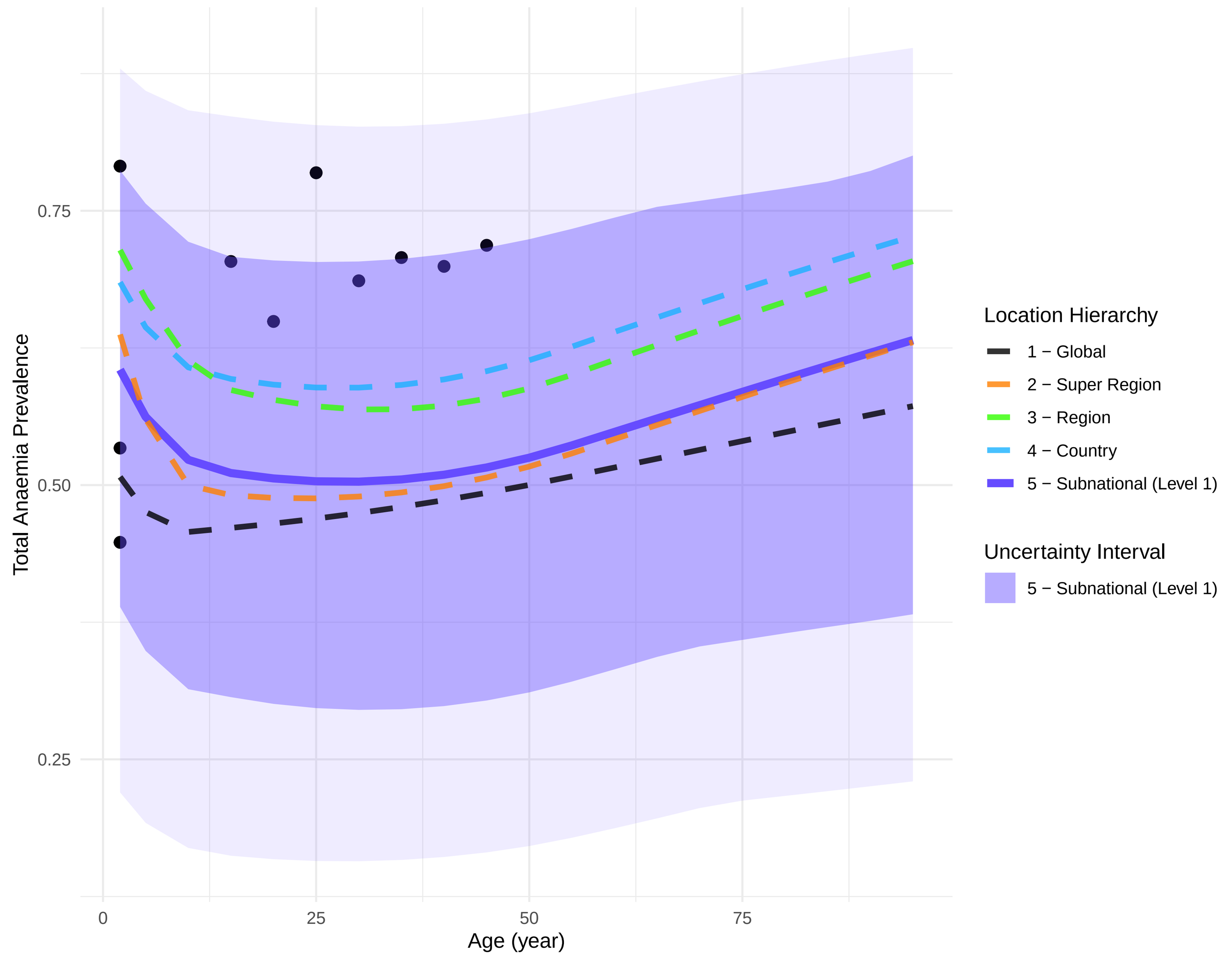

MR−BRT Age/Sex Fits − Non−pregnant Females 2+ Years Old − Total Anaemia Prevalence − Imo
WHO−2024 Adjustment Method

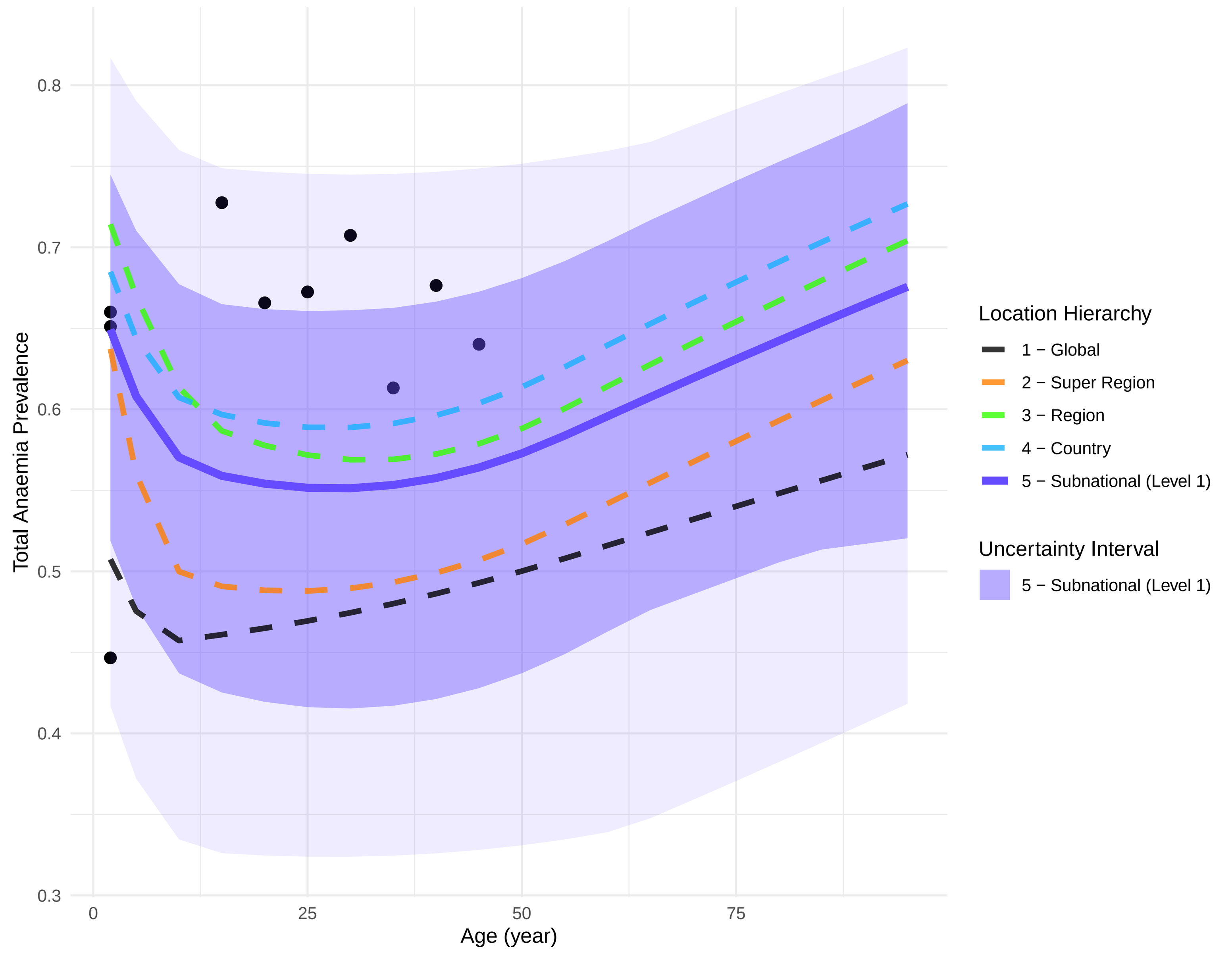

MR−BRT Age/Sex Fits − Non−pregnant Females 2+ Years Old − Total Anaemia Prevalence − Jigawa
WHO−2024 Adjustment Method

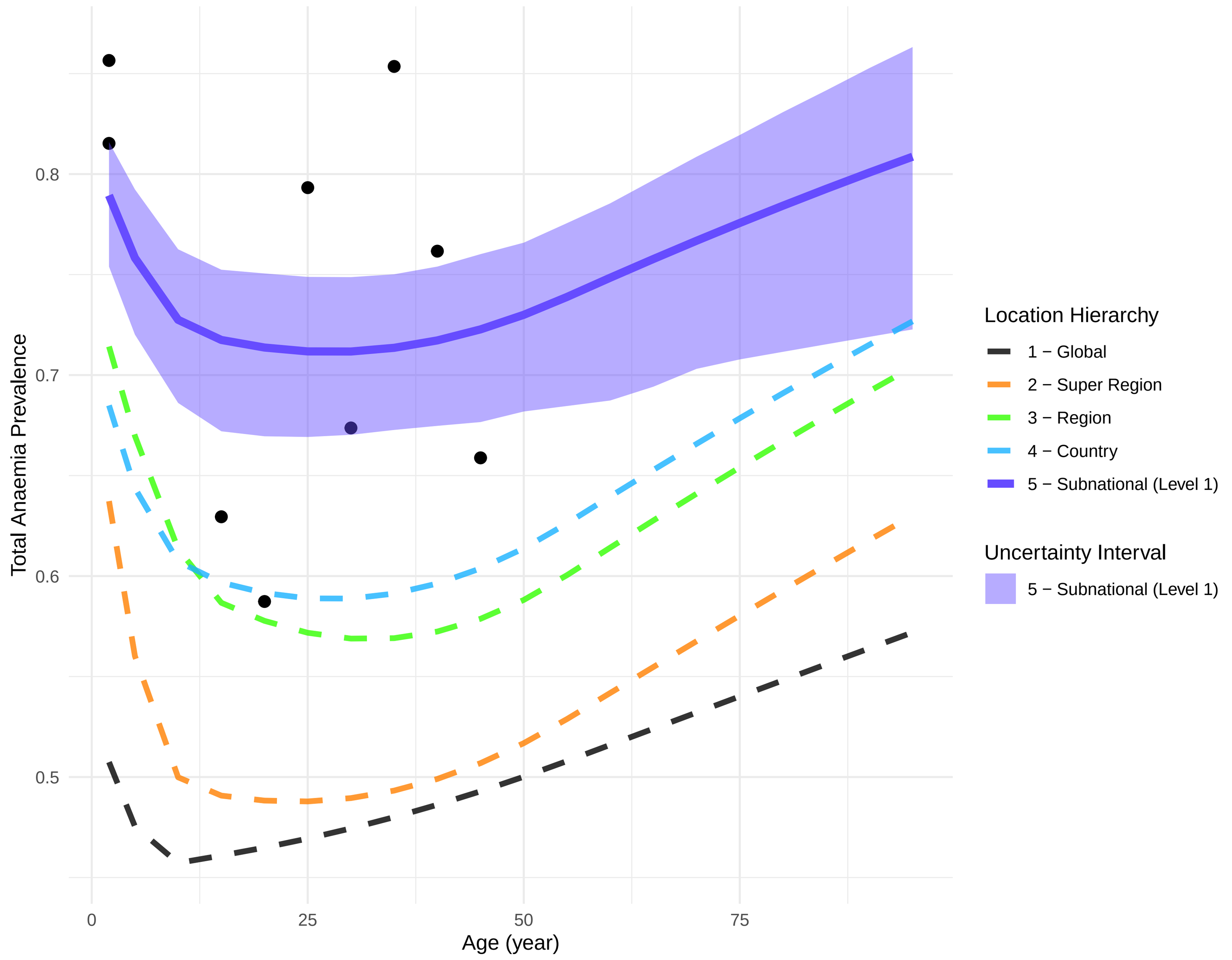

MR−BRT Age/Sex Fits − Non−pregnant Females 2+ Years Old − Total Anaemia Prevalence − Kaduna
WHO−2024 Adjustment Method

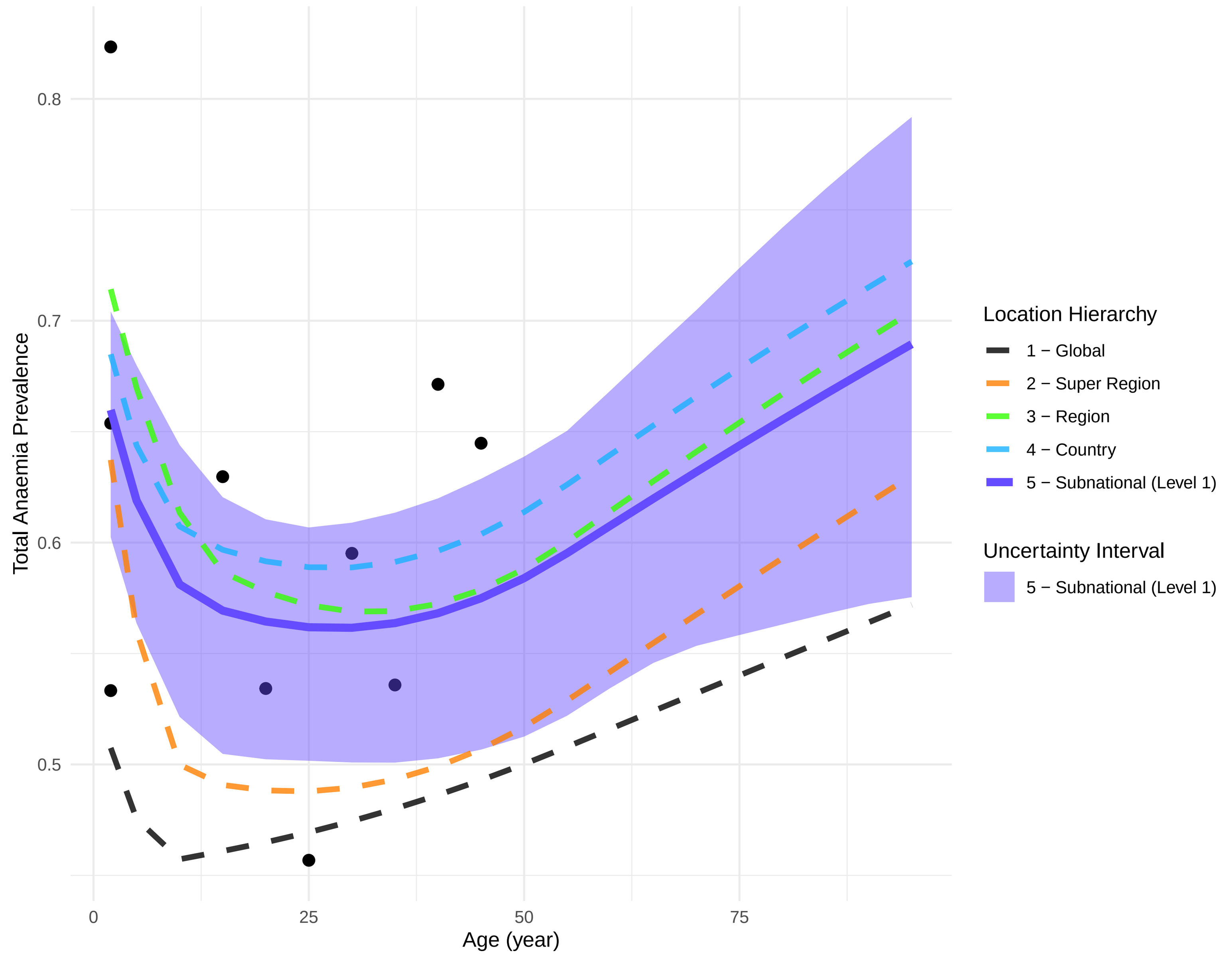

MR−BRT Age/Sex Fits − Non−pregnant Females 2+ Years Old − Total Anaemia Prevalence − Kano
WHO−2024 Adjustment Method

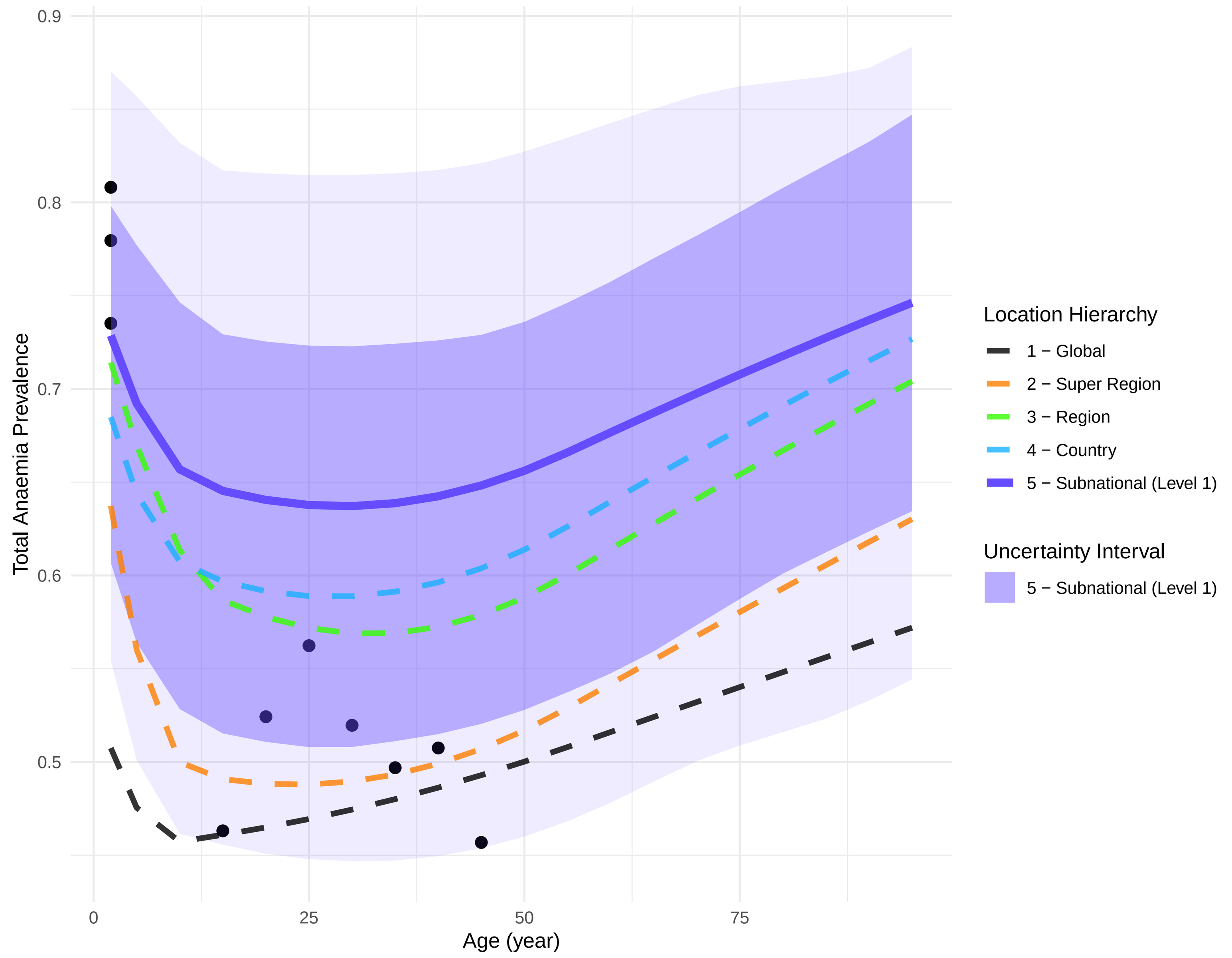

MR−BRT Age/Sex Fits − Non−pregnant Females 2+ Years Old − Total Anaemia Prevalence − Katsina
WHO−2024 Adjustment Method

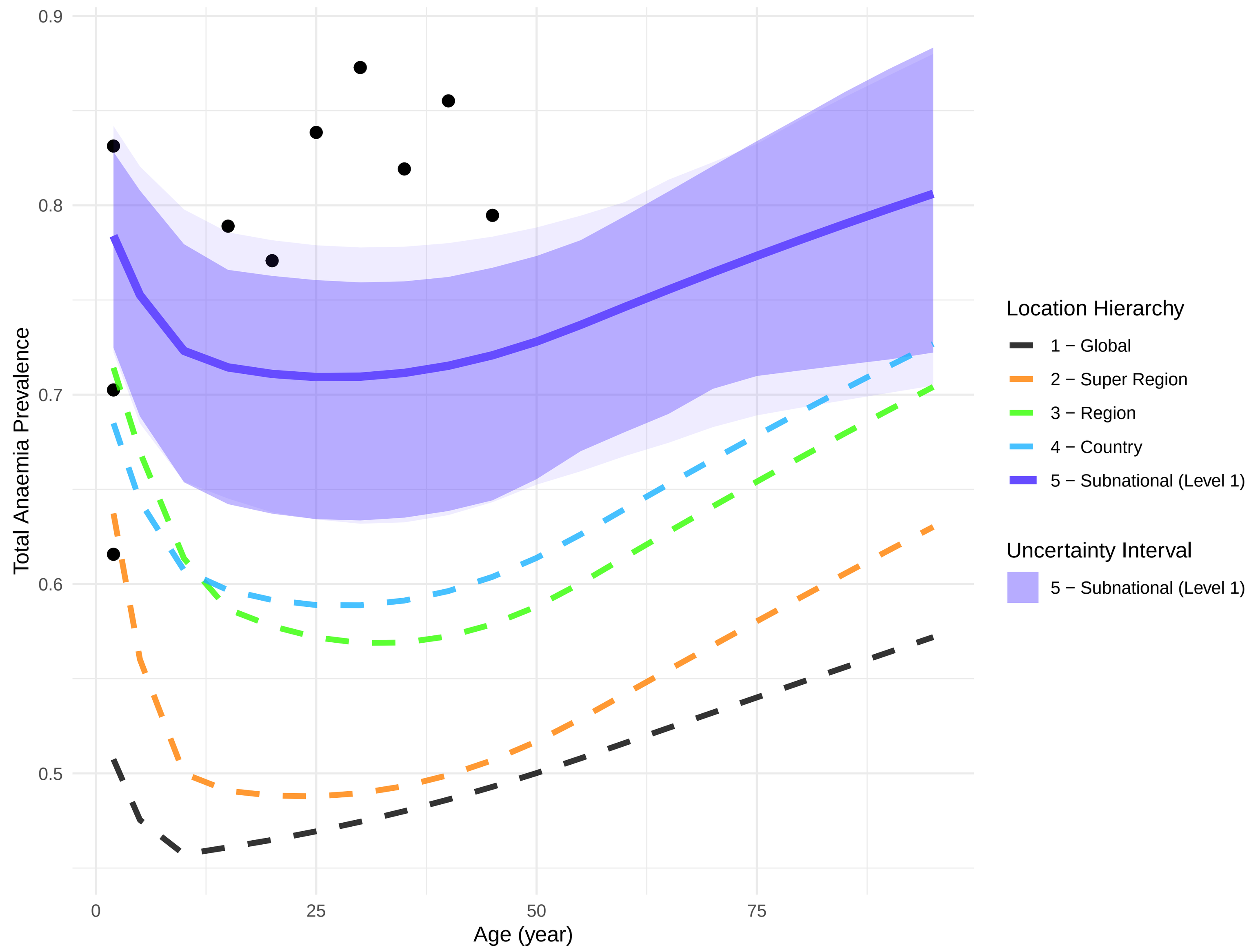

MR−BRT Age/Sex Fits − Non−pregnant Females 2+ Years Old − Total Anaemia Prevalence − Kebbi
WHO−2024 Adjustment Method

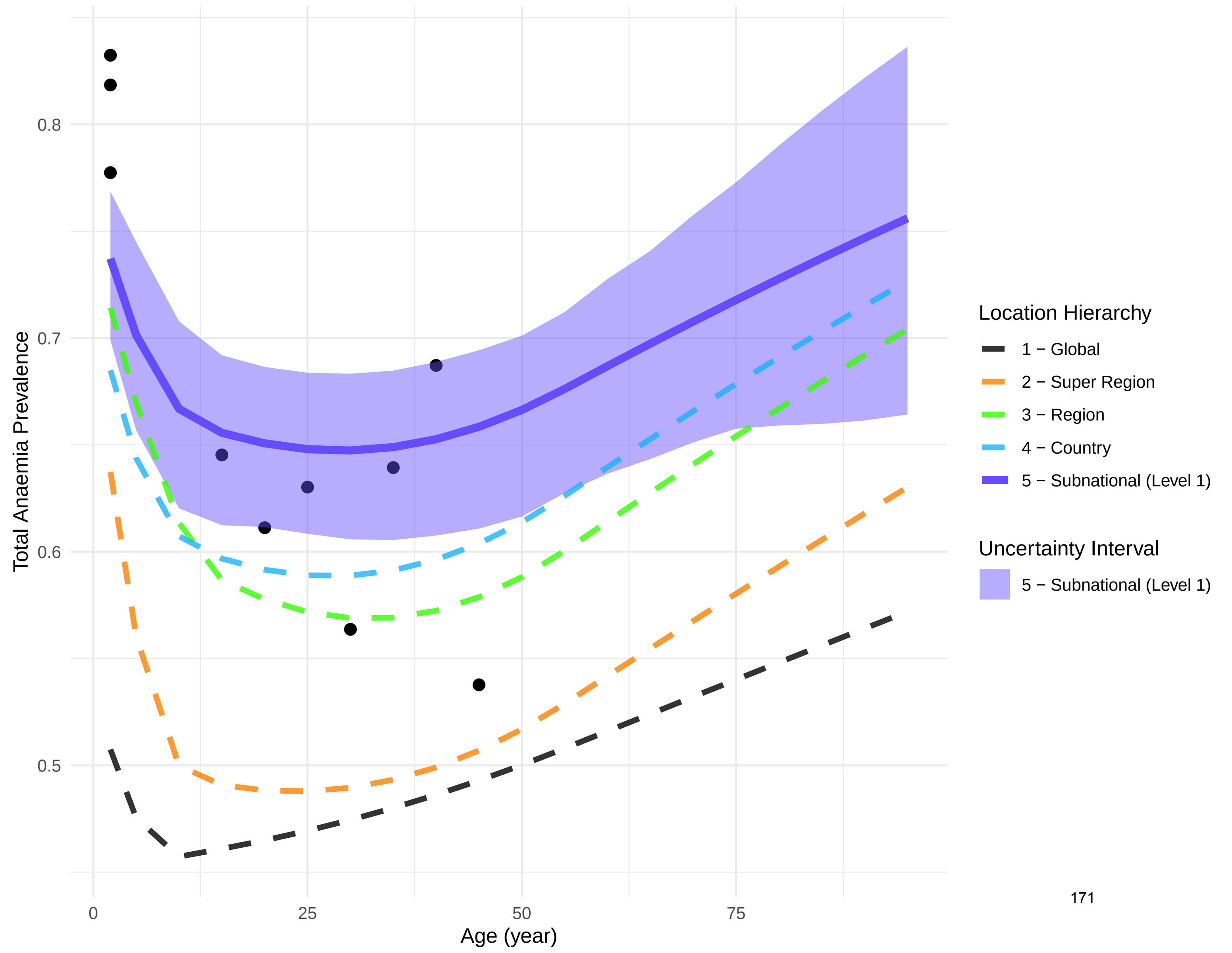

MR−BRT Age/Sex Fits − Non−pregnant Females 2+ Years Old − Total Anaemia Prevalence − Kogi
WHO−2024 Adjustment Method

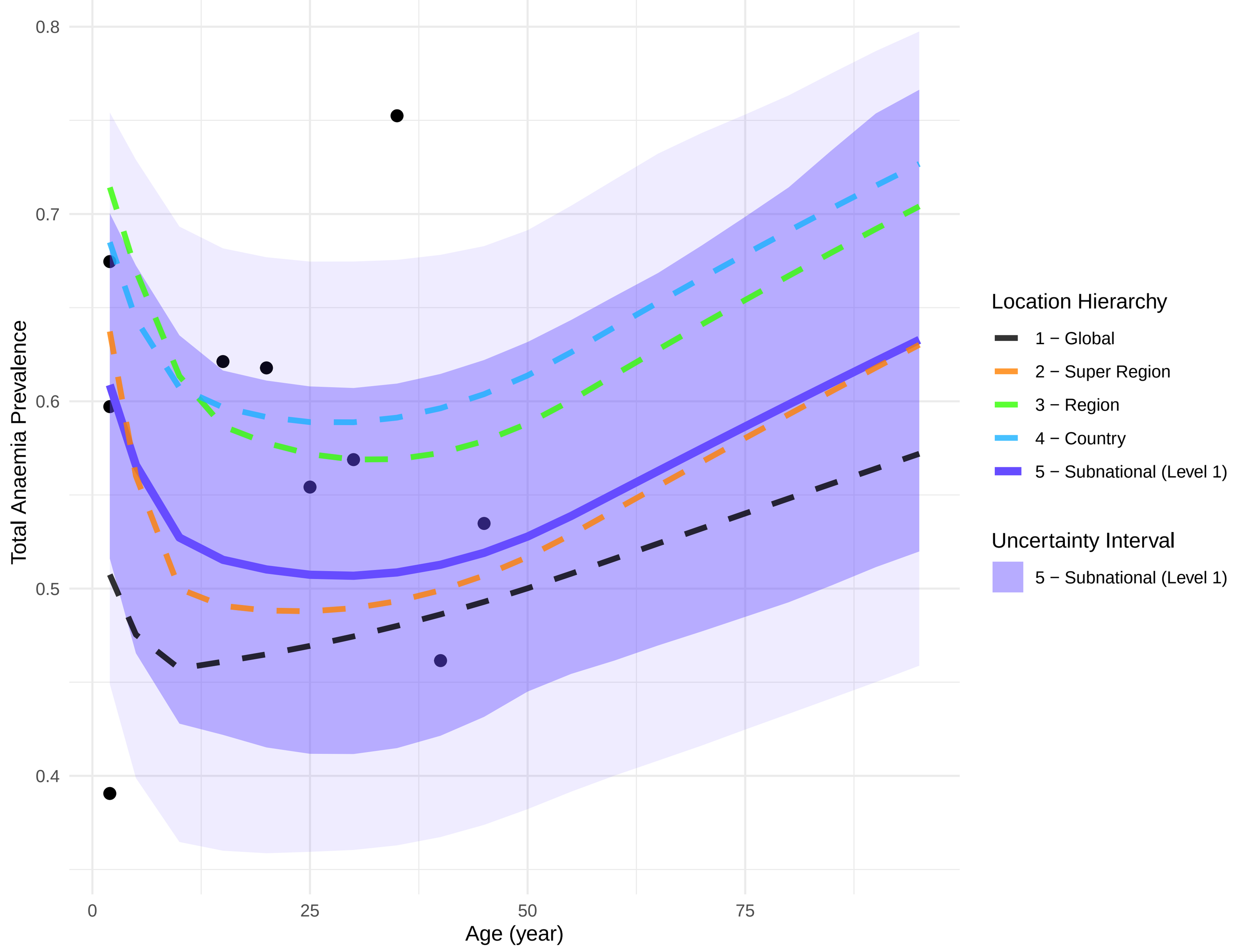

MR−BRT Age/Sex Fits − Non−pregnant Females 2+ Years Old − Total Anaemia Prevalence − Kwara
WHO−2024 Adjustment Method

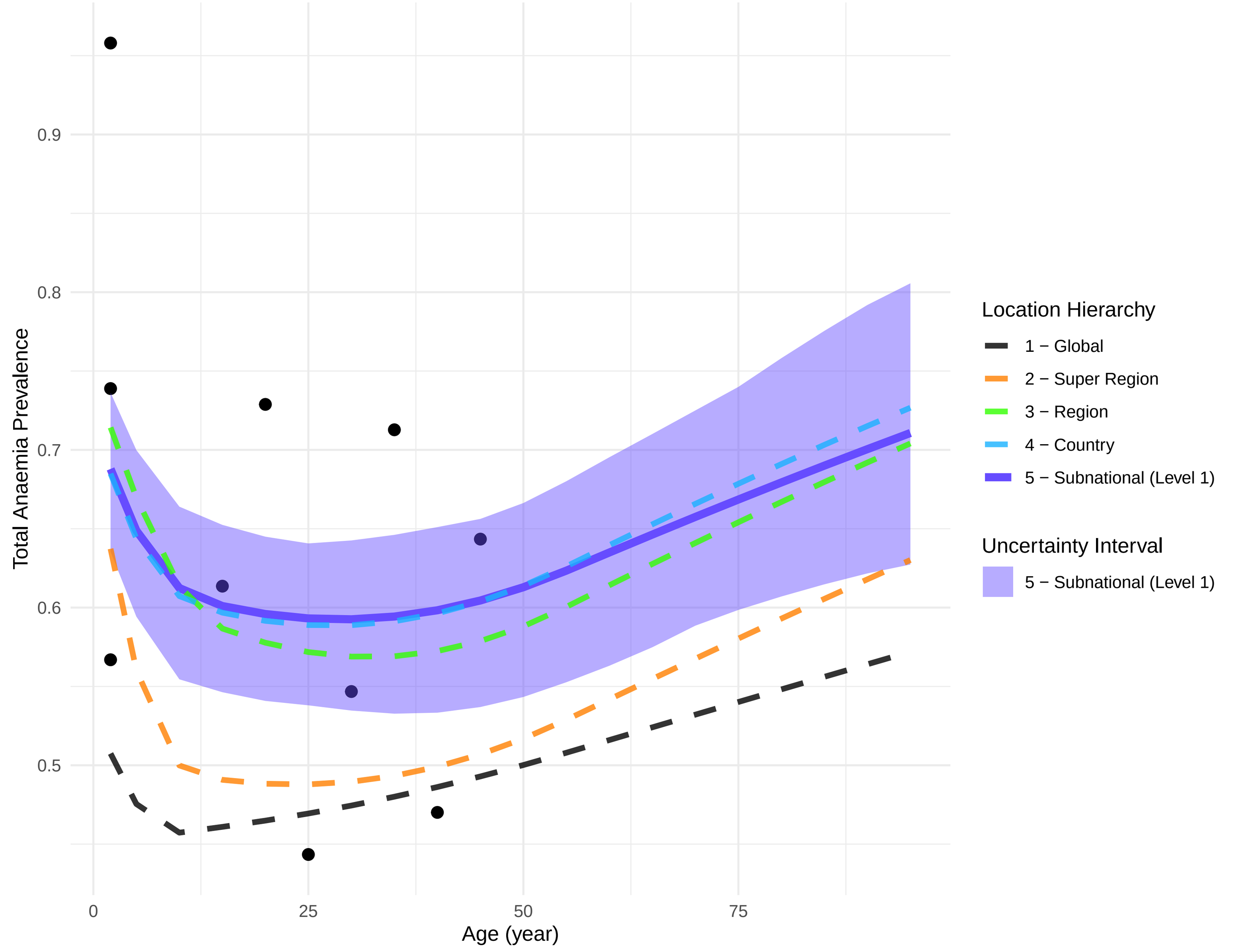

MR−BRT Age/Sex Fits − Non−pregnant Females 2+ Years Old − Total Anaemia Prevalence − Lagos
WHO−2024 Adjustment Method

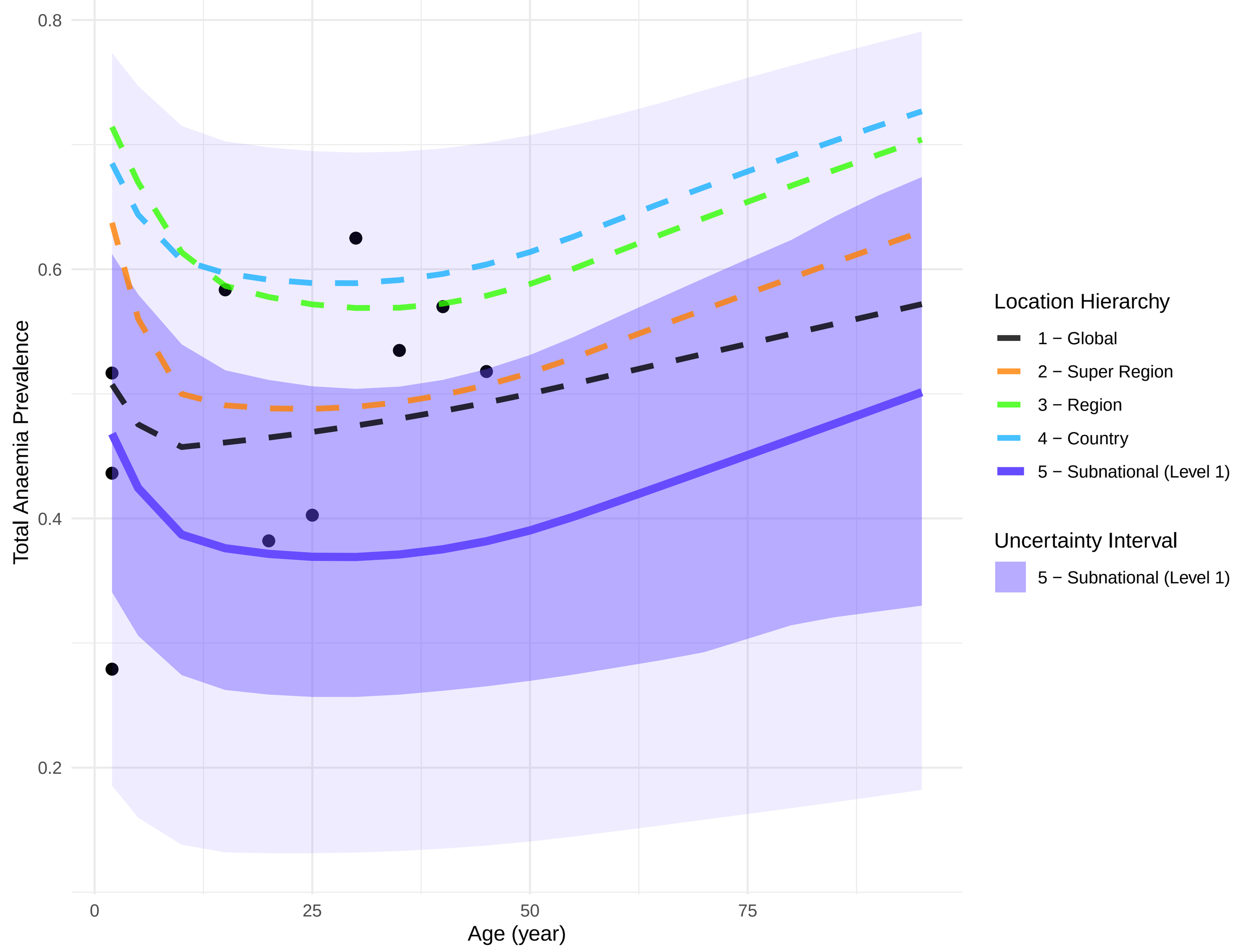

MR−BRT Age/Sex Fits − Non−pregnant Females 2+ Years Old − Total Anaemia Prevalence − Nasarawa
WHO−2024 Adjustment Method

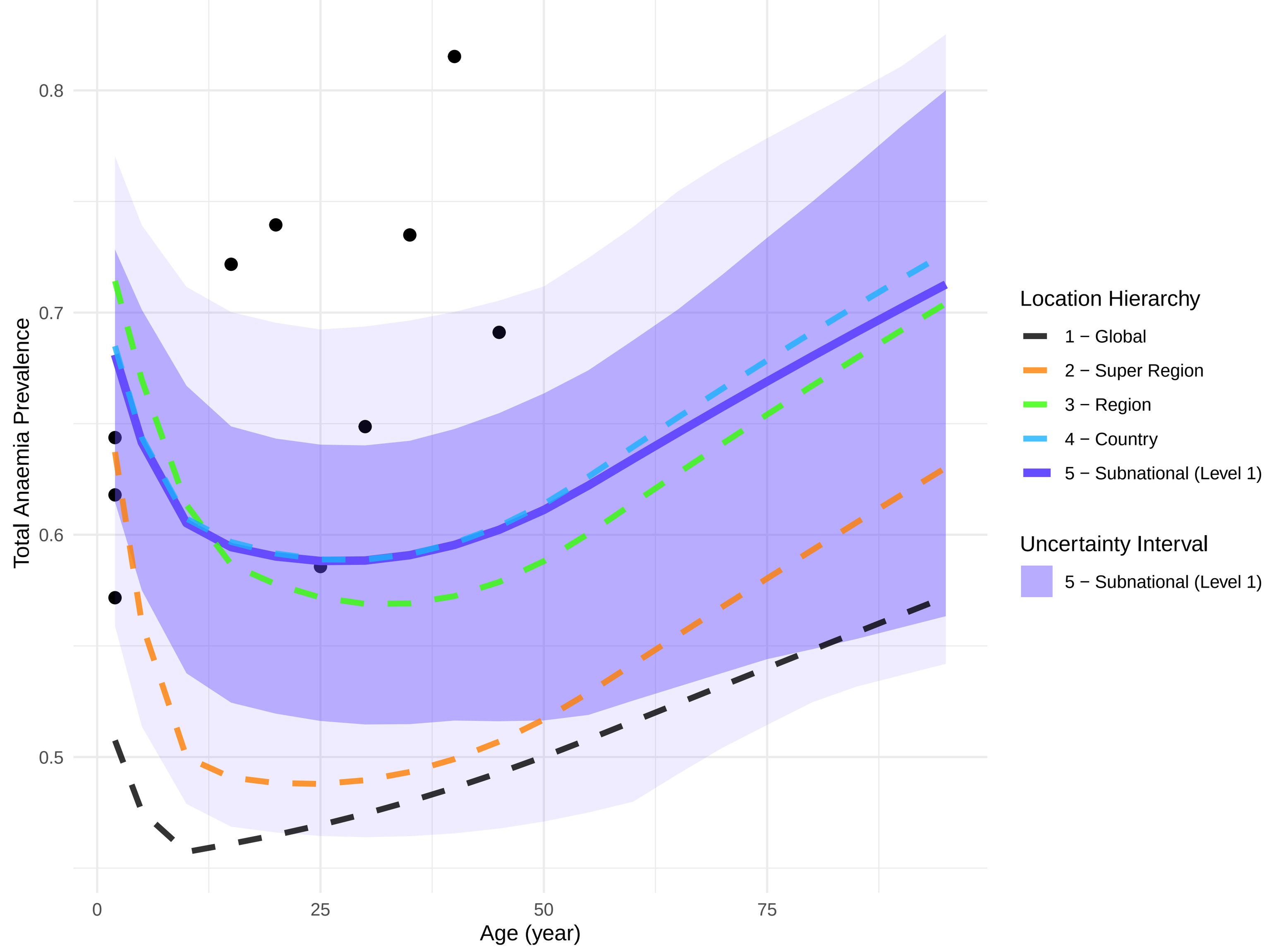

MR−BRT Age/Sex Fits − Non−pregnant Females 2+ Years Old − Total Anaemia Prevalence − Niger
WHO−2024 Adjustment Method

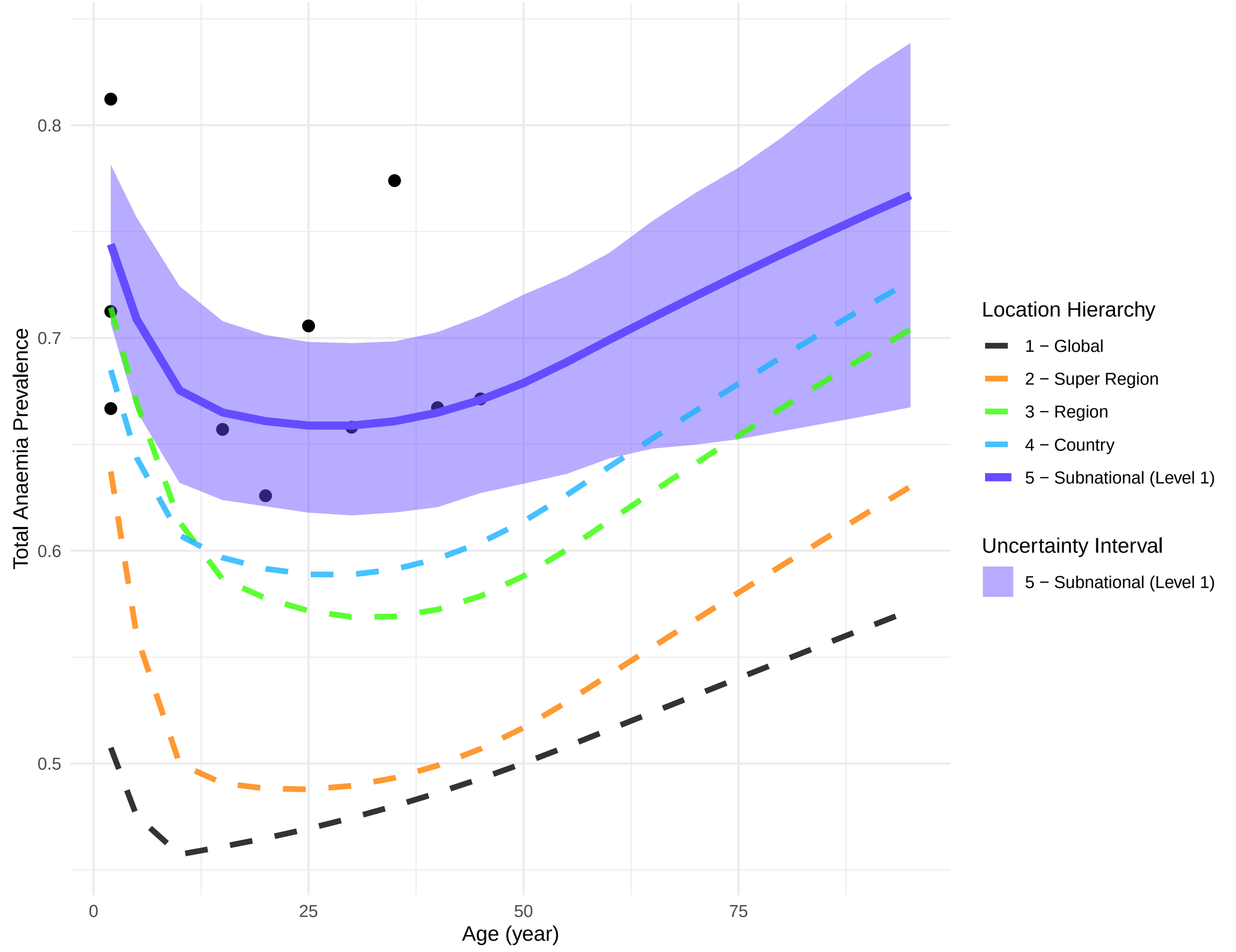

MR−BRT Age/Sex Fits − Non−pregnant Females 2+ Years Old − Total Anaemia Prevalence − Ogun
WHO−2024 Adjustment Method

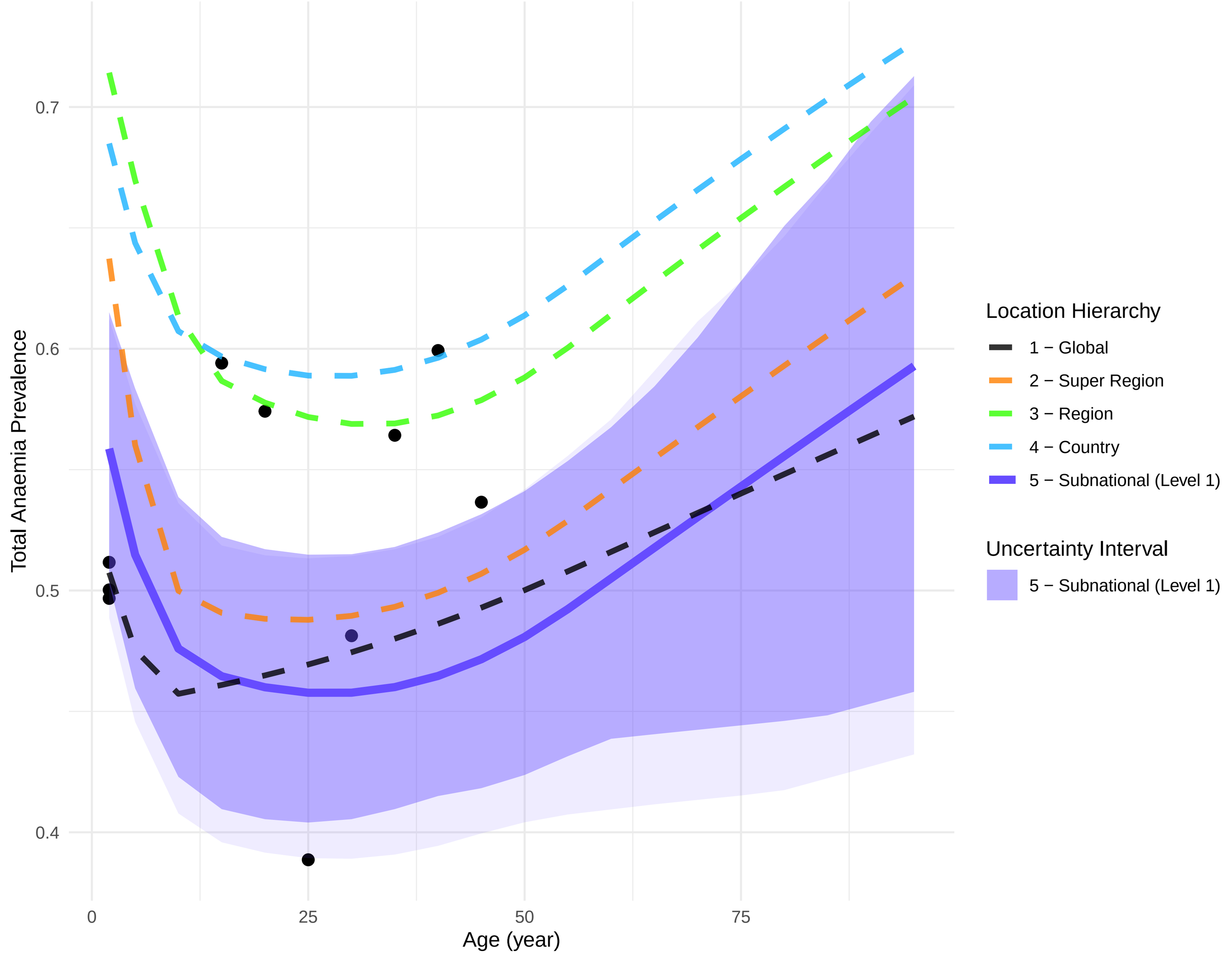

MR−BRT Age/Sex Fits − Non−pregnant Females 2+ Years Old − Total Anaemia Prevalence − Ondo
WHO−2024 Adjustment Method

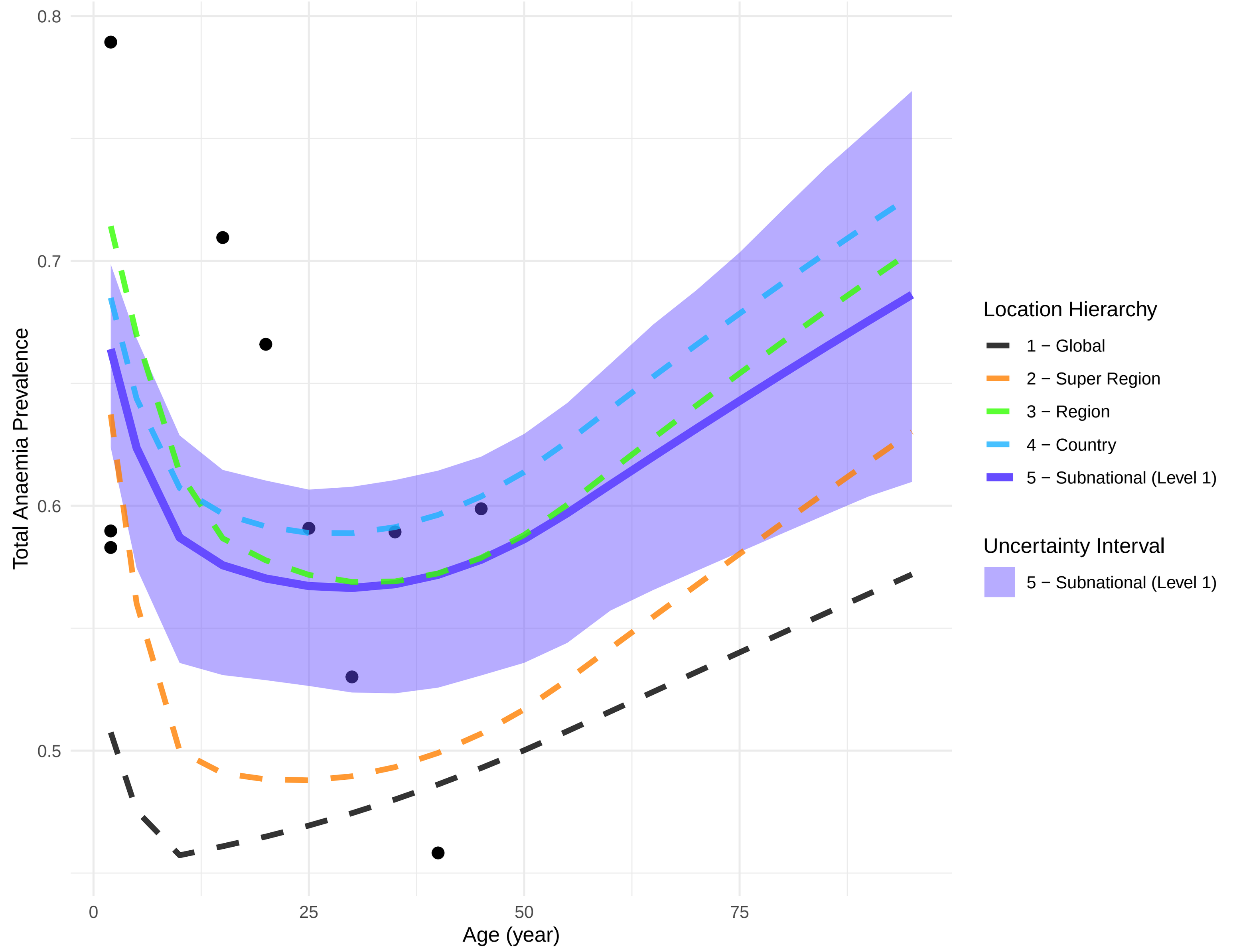

MR−BRT Age/Sex Fits − Non−pregnant Females 2+ Years Old − Total Anaemia Prevalence − Osun
WHO−2024 Adjustment Method

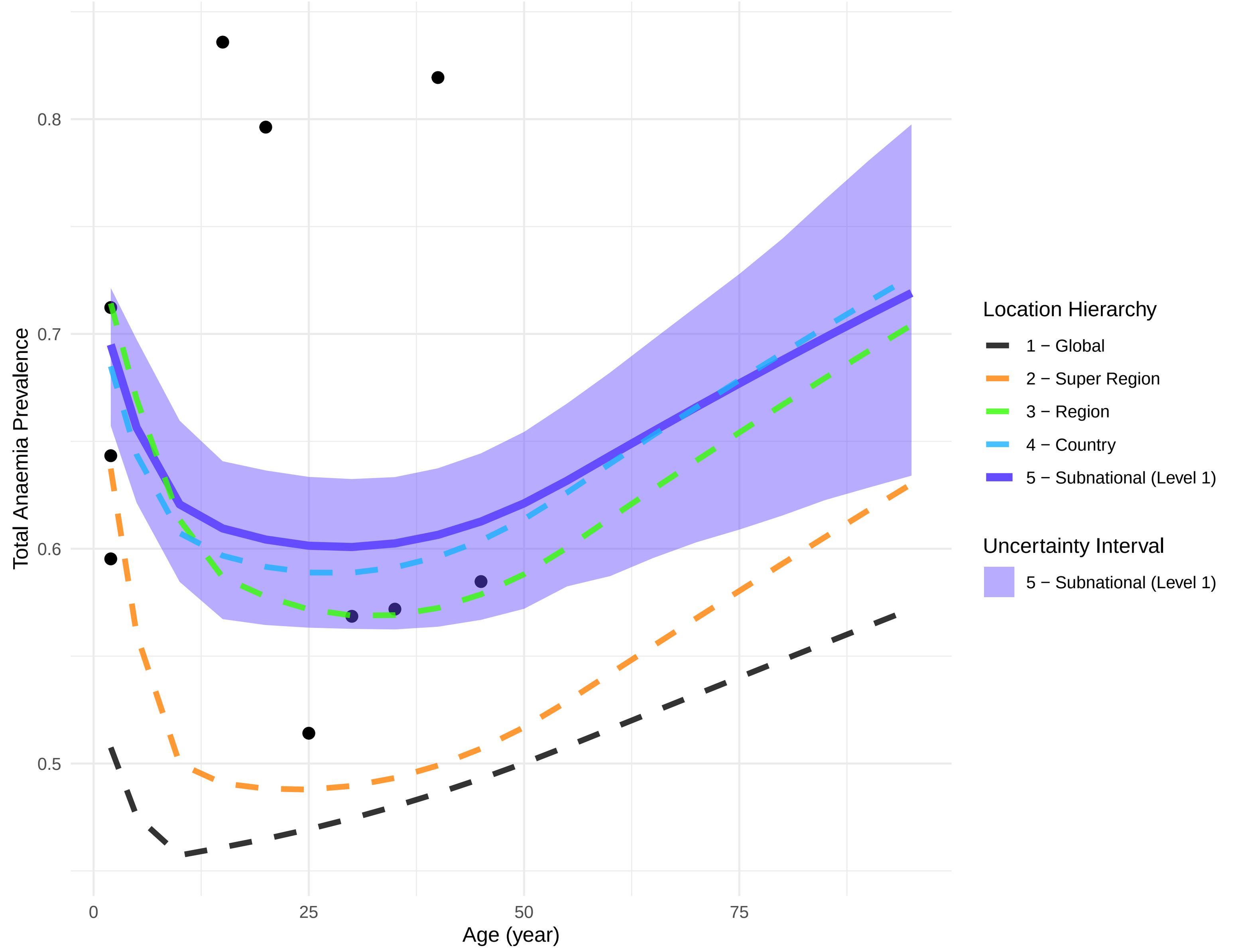

MR−BRT Age/Sex Fits − Non−pregnant Females 2+ Years Old − Total Anaemia Prevalence − Oyo
WHO−2024 Adjustment Method

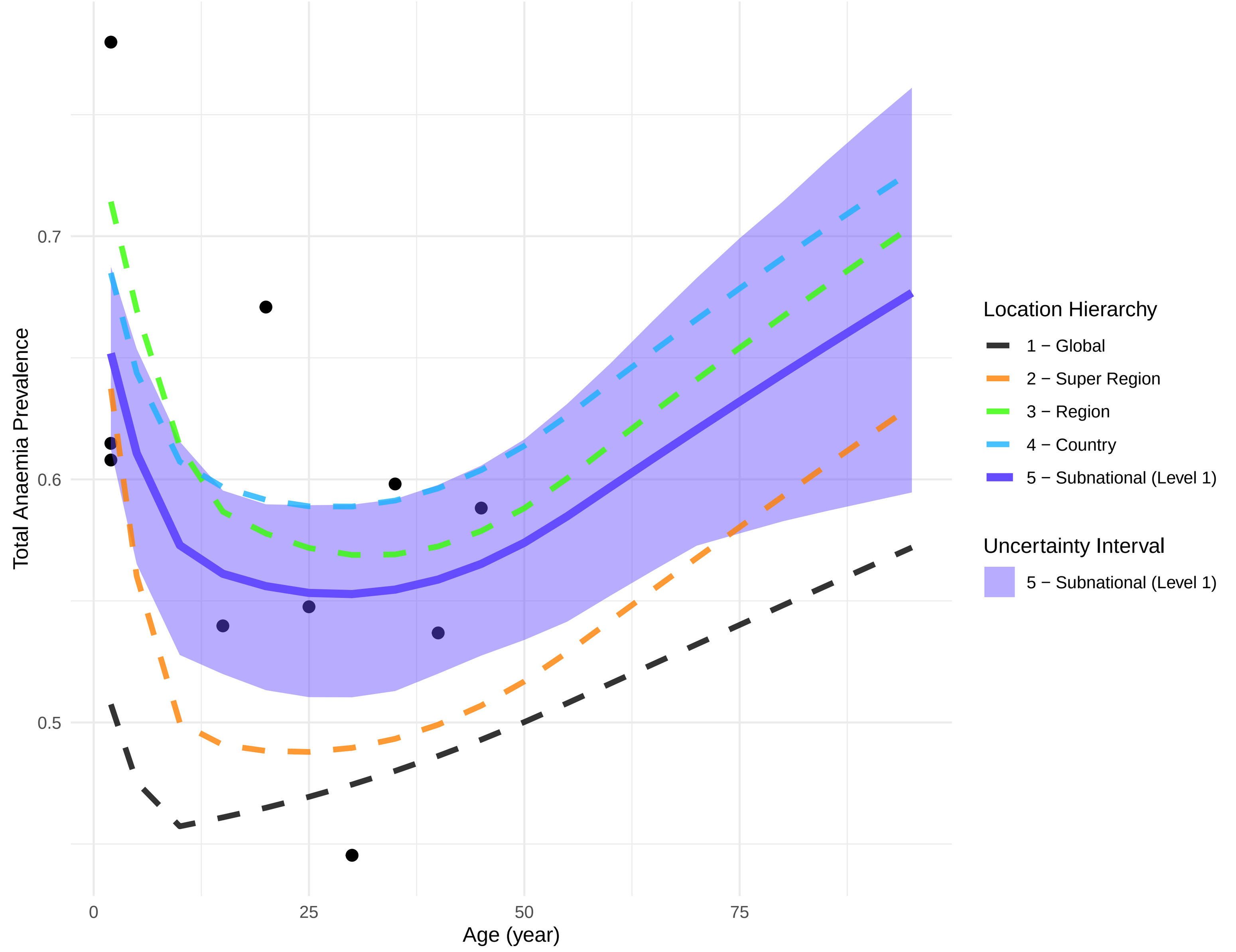

MR−BRT Age/Sex Fits − Non−pregnant Females 2+ Years Old − Total Anaemia Prevalence − Plateau
WHO−2024 Adjustment Method

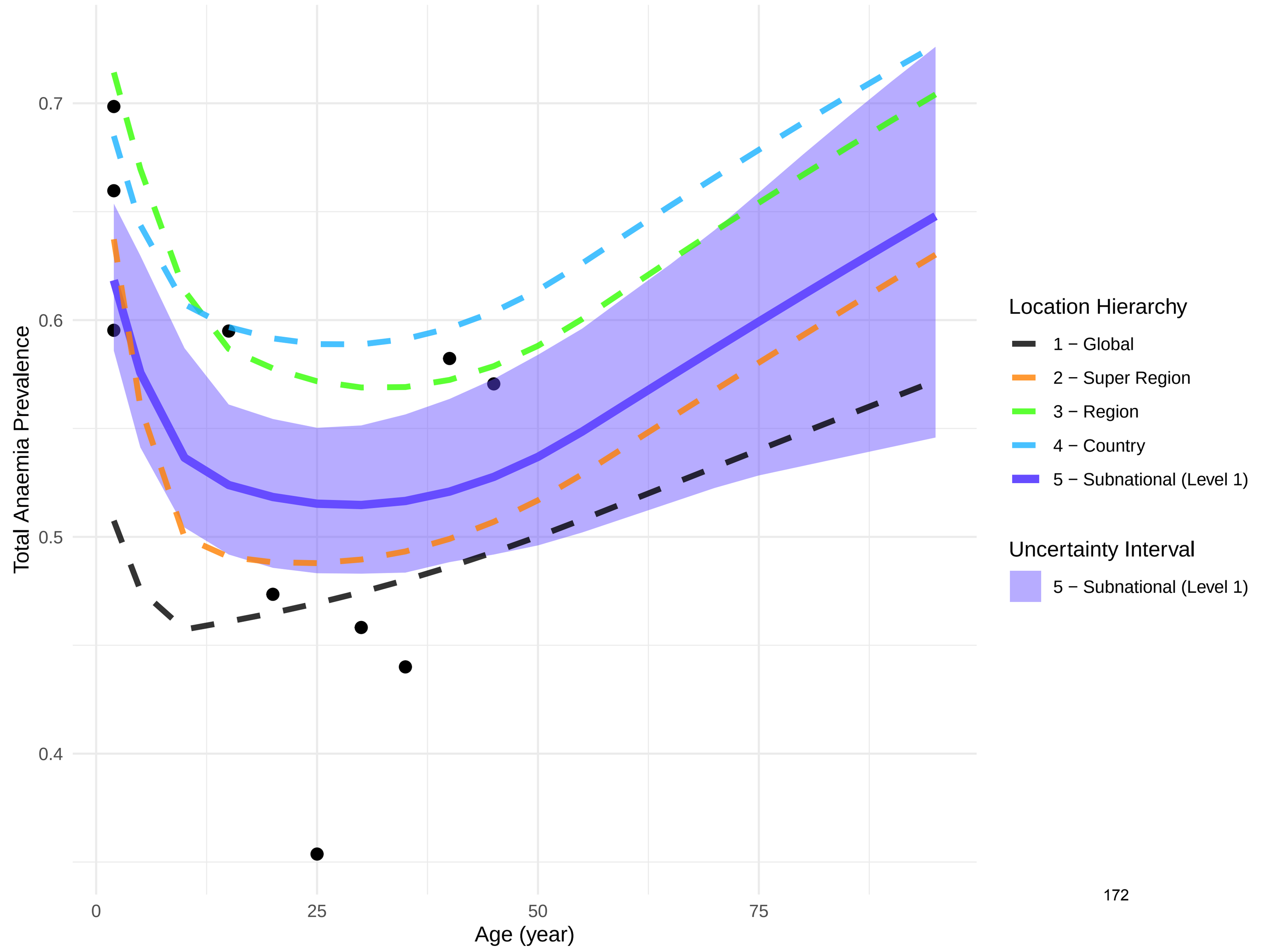

MR−BRT Age/Sex Fits − Non−pregnant Females 2+ Years Old − Total Anaemia Prevalence − Rivers
WHO−2024 Adjustment Method

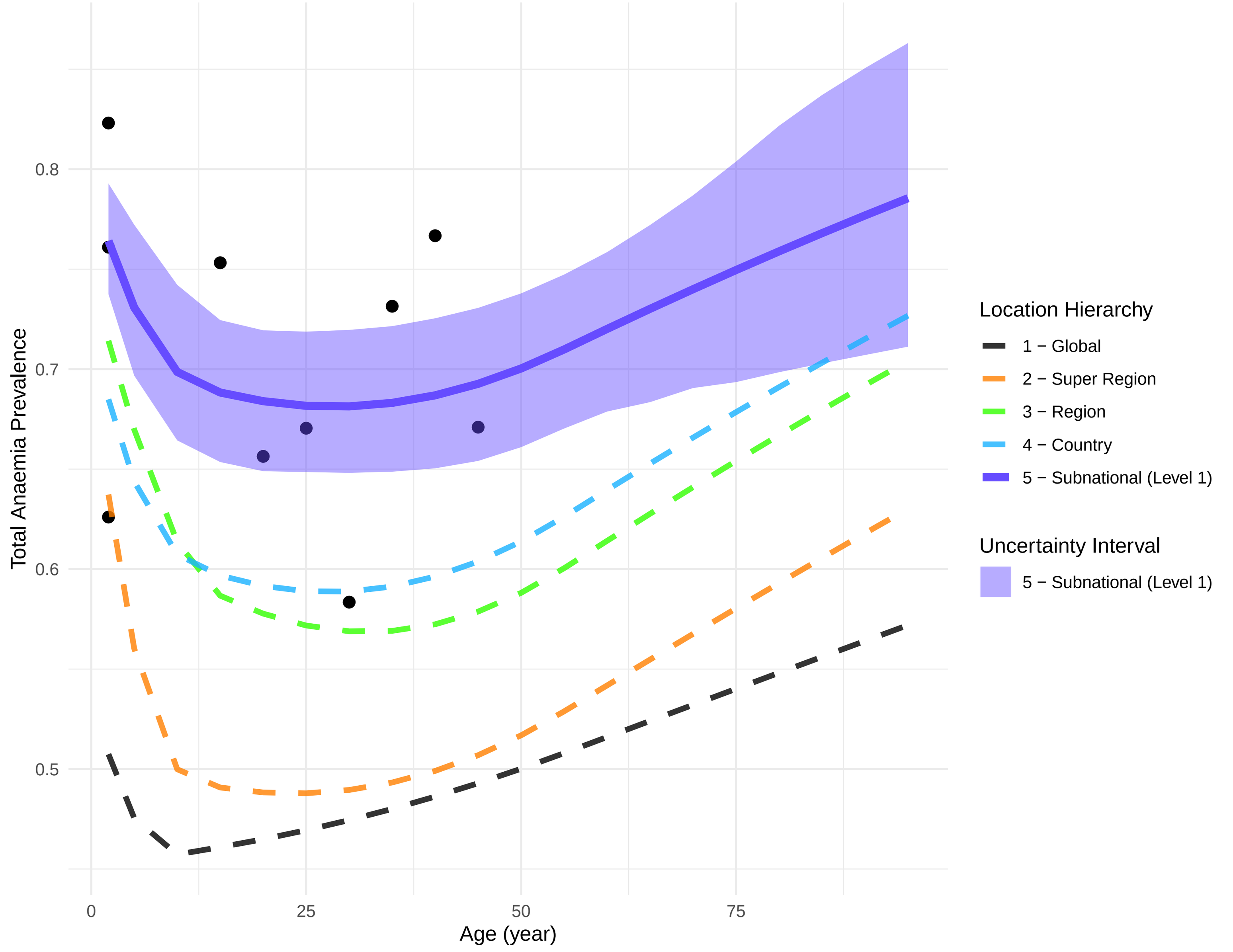

MR−BRT Age/Sex Fits − Non−pregnant Females 2+ Years Old − Total Anaemia Prevalence − Sokoto
WHO−2024 Adjustment Method

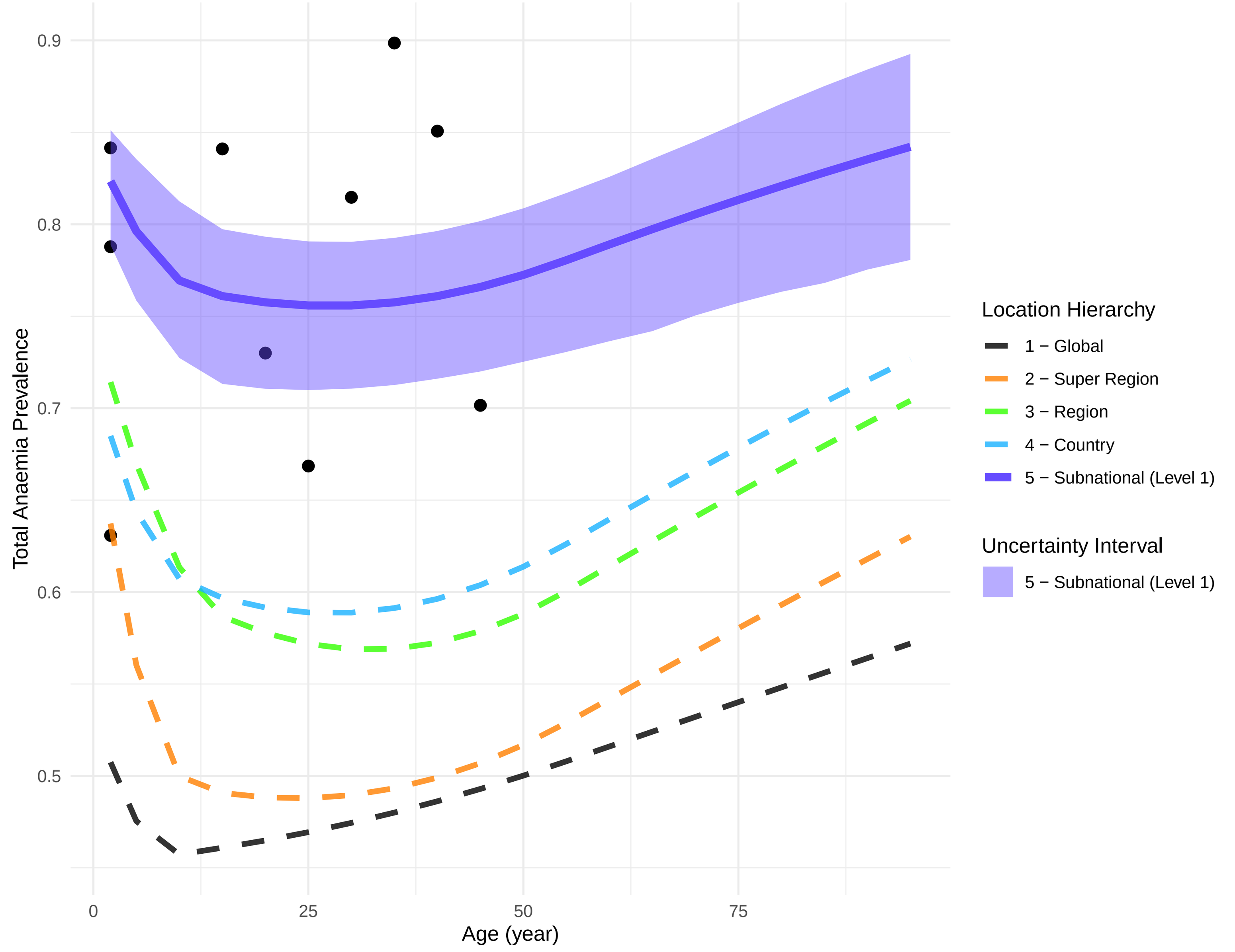

MR−BRT Age/Sex Fits − Non−pregnant Females 2+ Years Old − Total Anaemia Prevalence − Taraba
WHO−2024 Adjustment Method

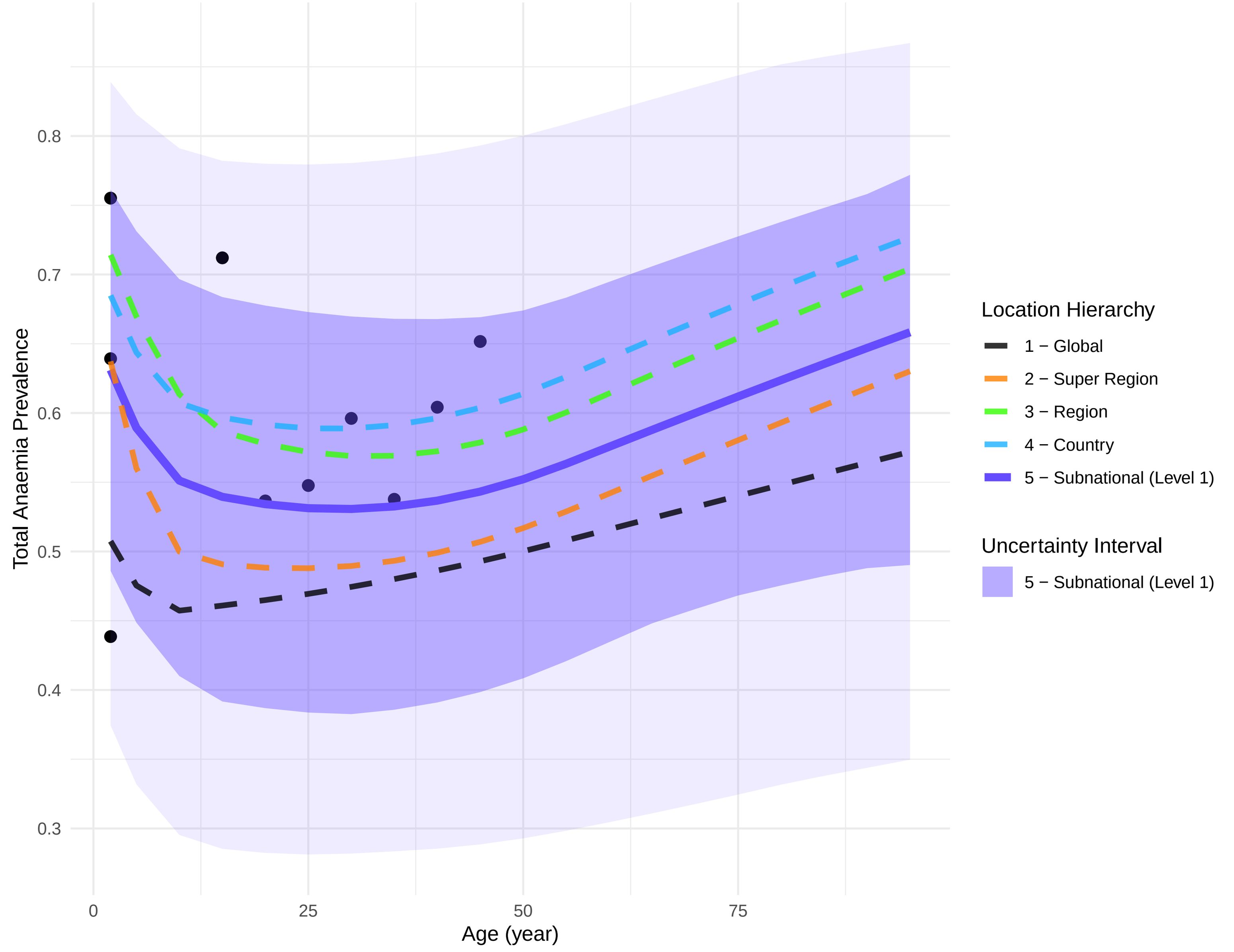

MR−BRT Age/Sex Fits − Non−pregnant Females 2+ Years Old − Total Anaemia Prevalence − Yobe
WHO−2024 Adjustment Method

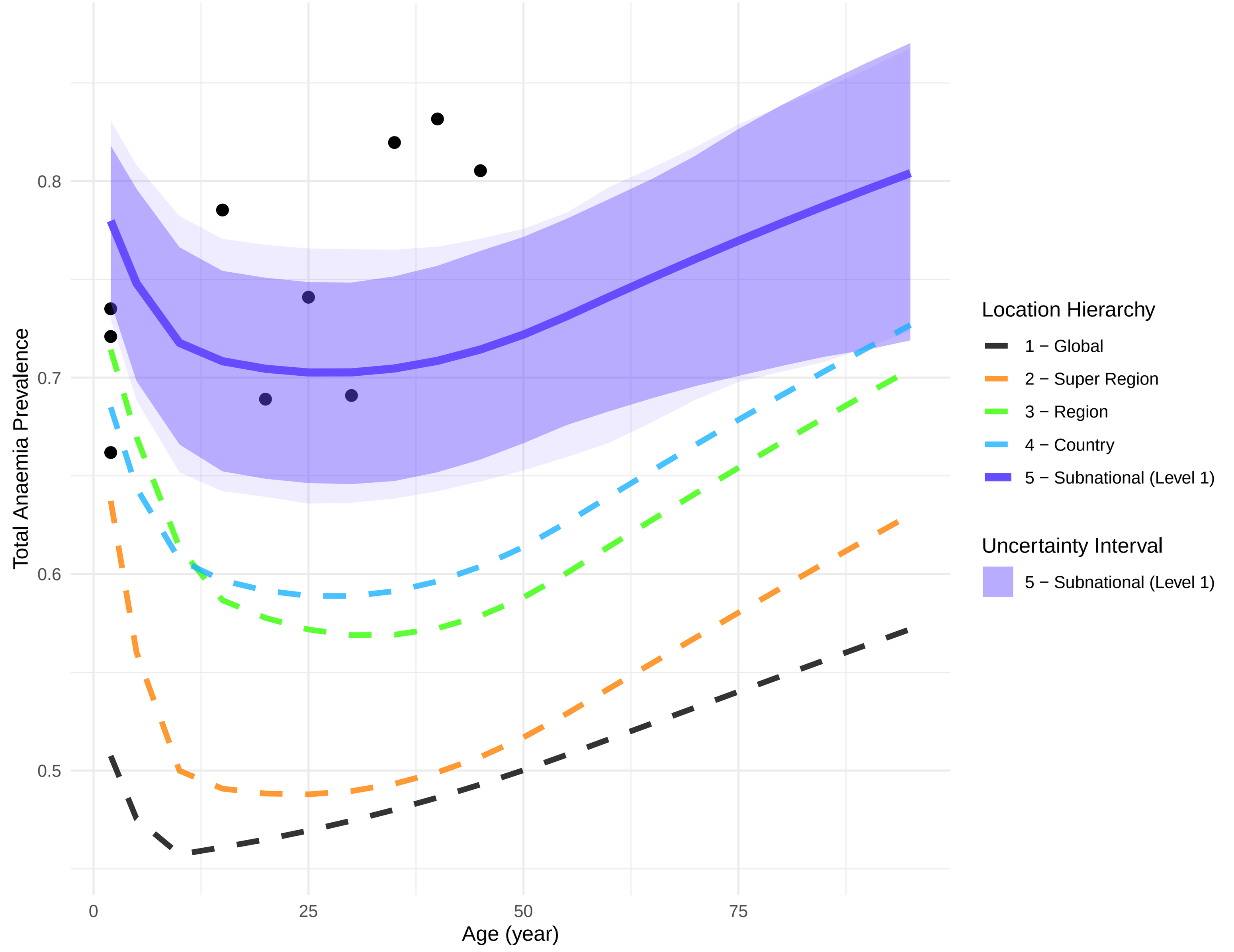

MR−BRT Age/Sex Fits − Non−pregnant Females 2+ Years Old − Total Anaemia Prevalence − Zamfara
WHO−2024 Adjustment Method

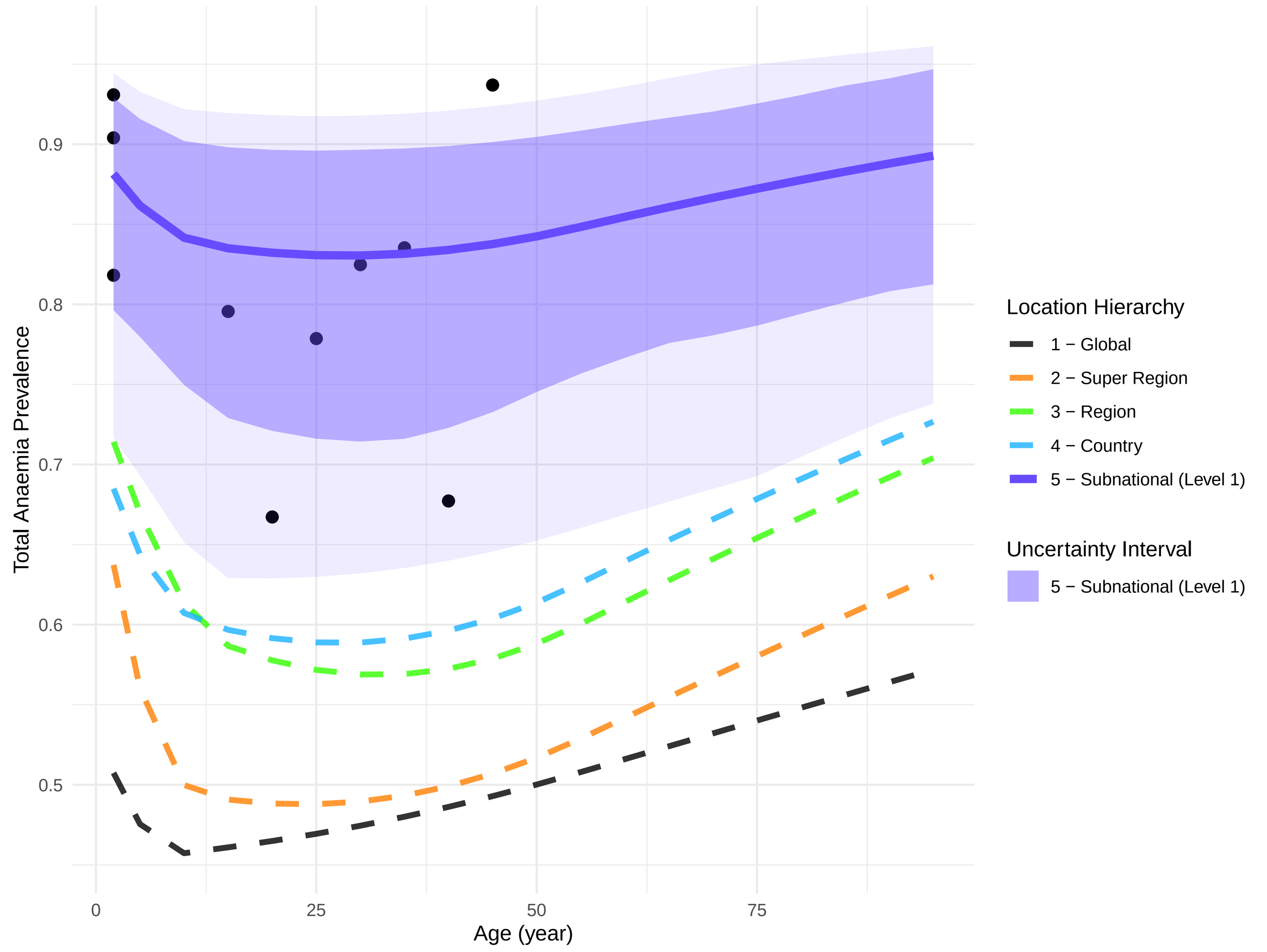

MR−BRT Age/Sex Fits − Non−pregnant Females 2+ Years Old − Total Anaemia Prevalence − Senegal
WHO−2024 Adjustment Method

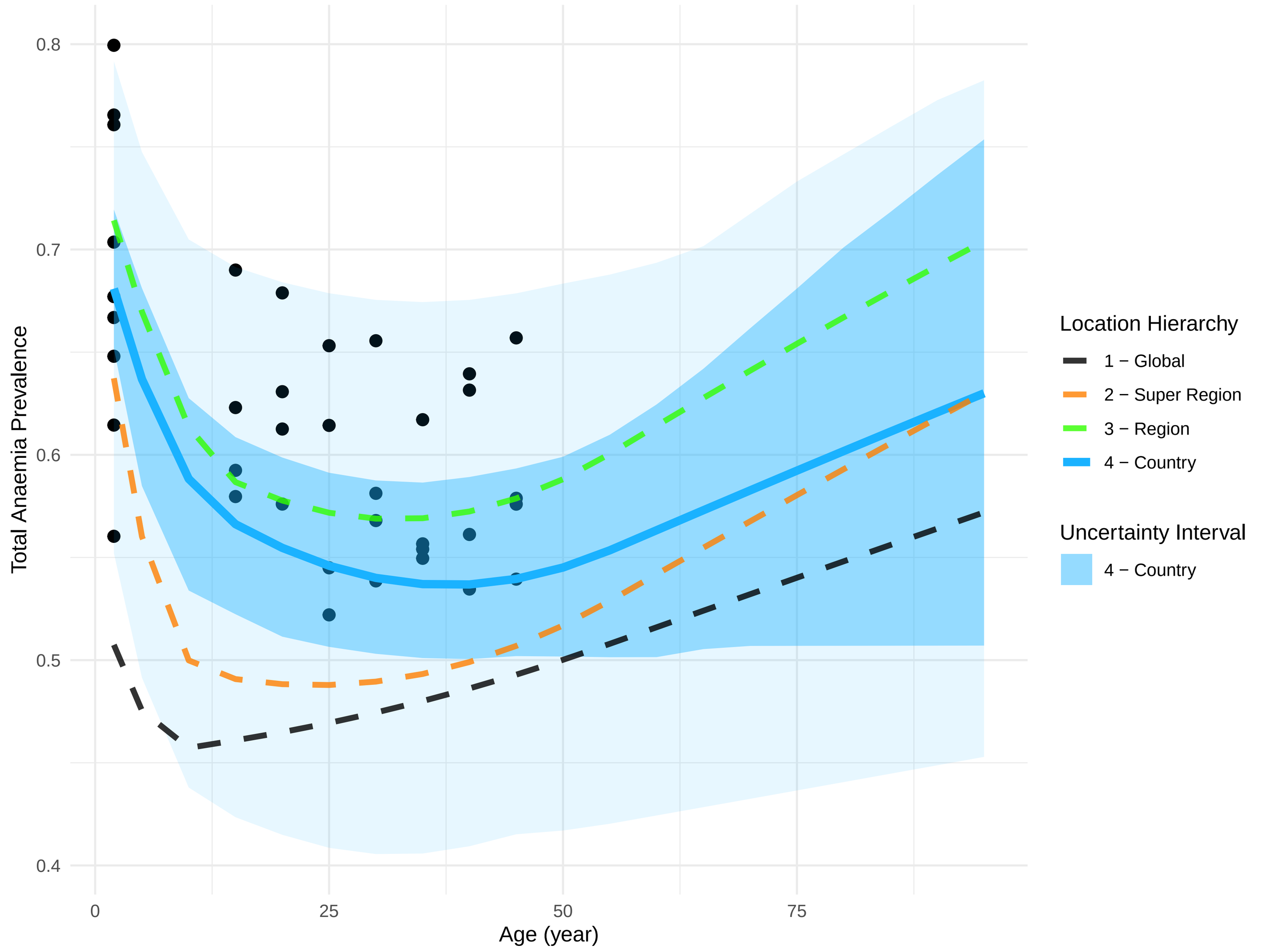

MR−BRT Age/Sex Fits − Non−pregnant Females 2+ Years Old − Total Anaemia Prevalence − Sierra Leone
WHO−2024 Adjustment Method

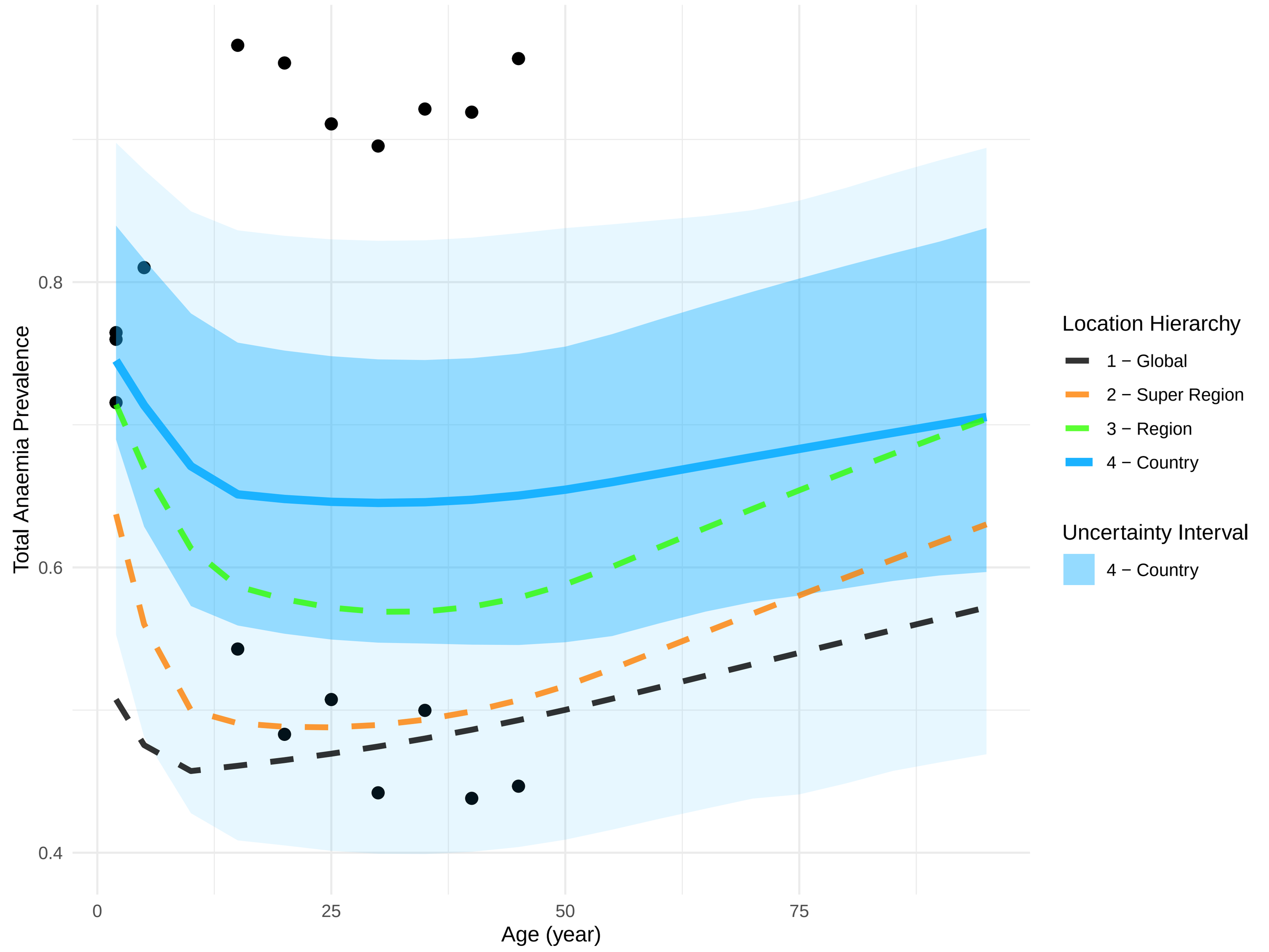

MR−BRT Age/Sex Fits − Non−pregnant Females 2+ Years Old − Total Anaemia Prevalence − Togo
WHO−2024 Adjustment Method

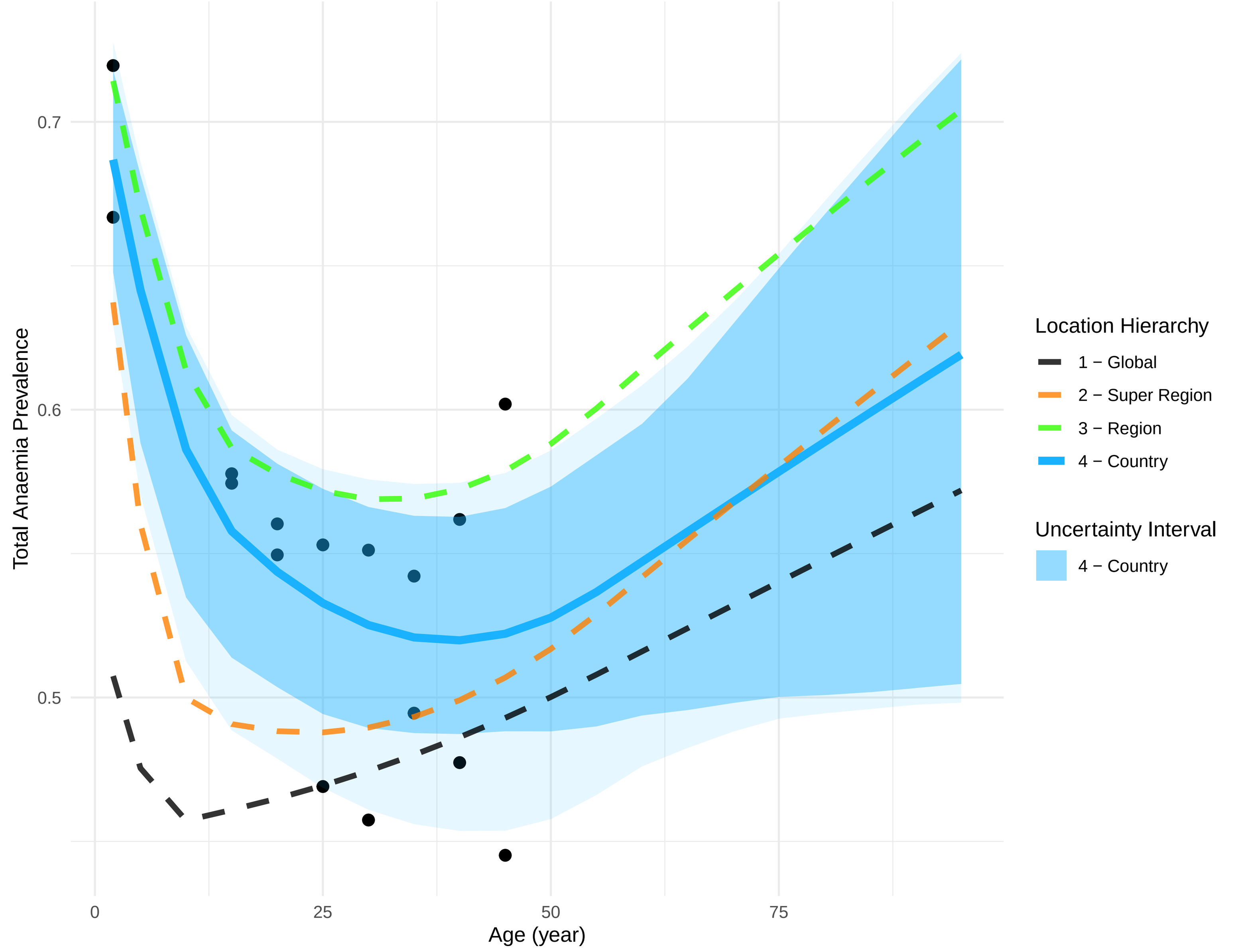

MR−BRT Age/Sex Fits − Males 2+ Years Old − Total Anaemia Prevalence − Global
WHO−2001 Adjustment Method

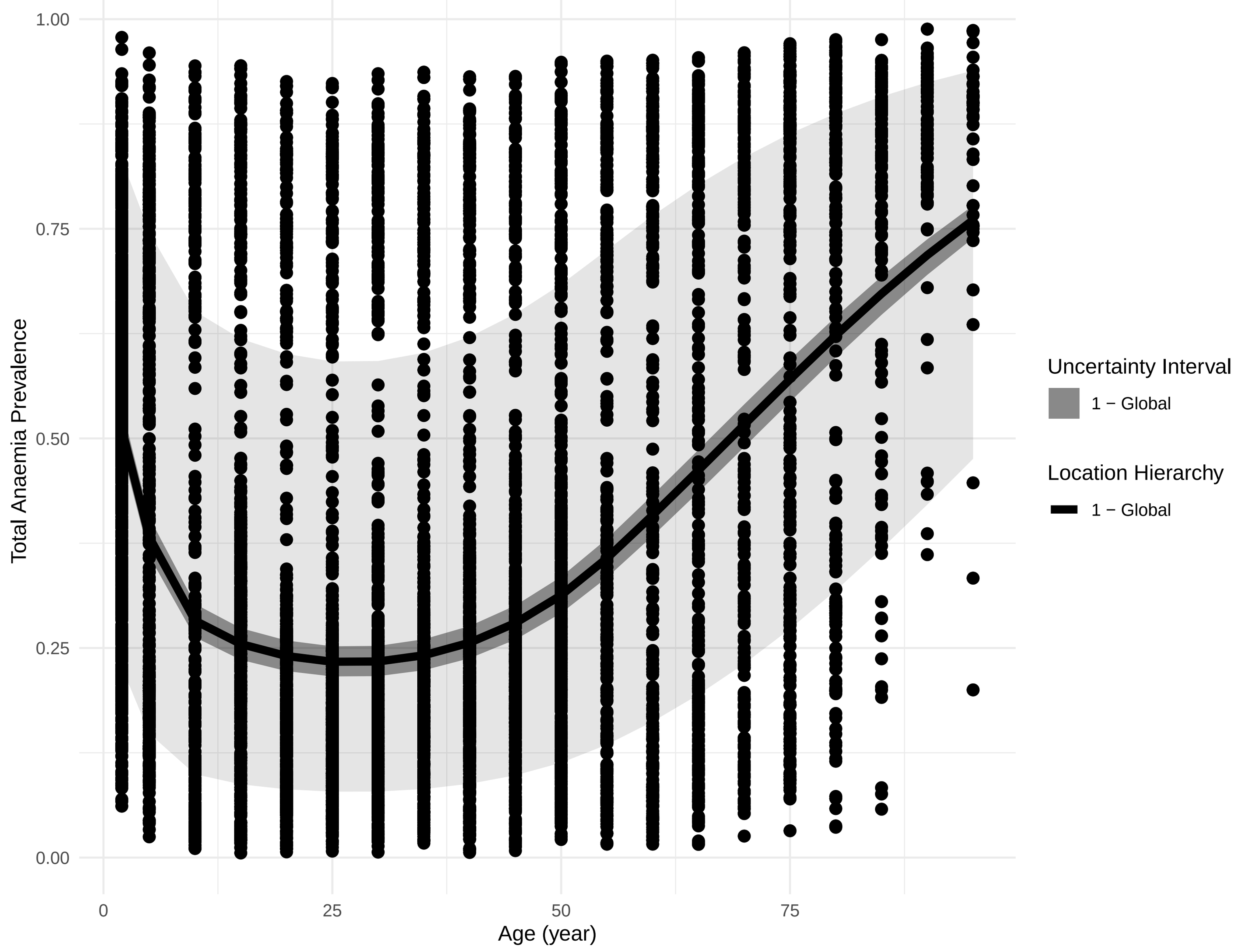

MR−BRT Age/Sex Fits − Males 2+ Years Old − Total Anaemia Prevalence − Central Europe, Eastern Europe, and Central Asia
WHO−2001 Adjustment Method

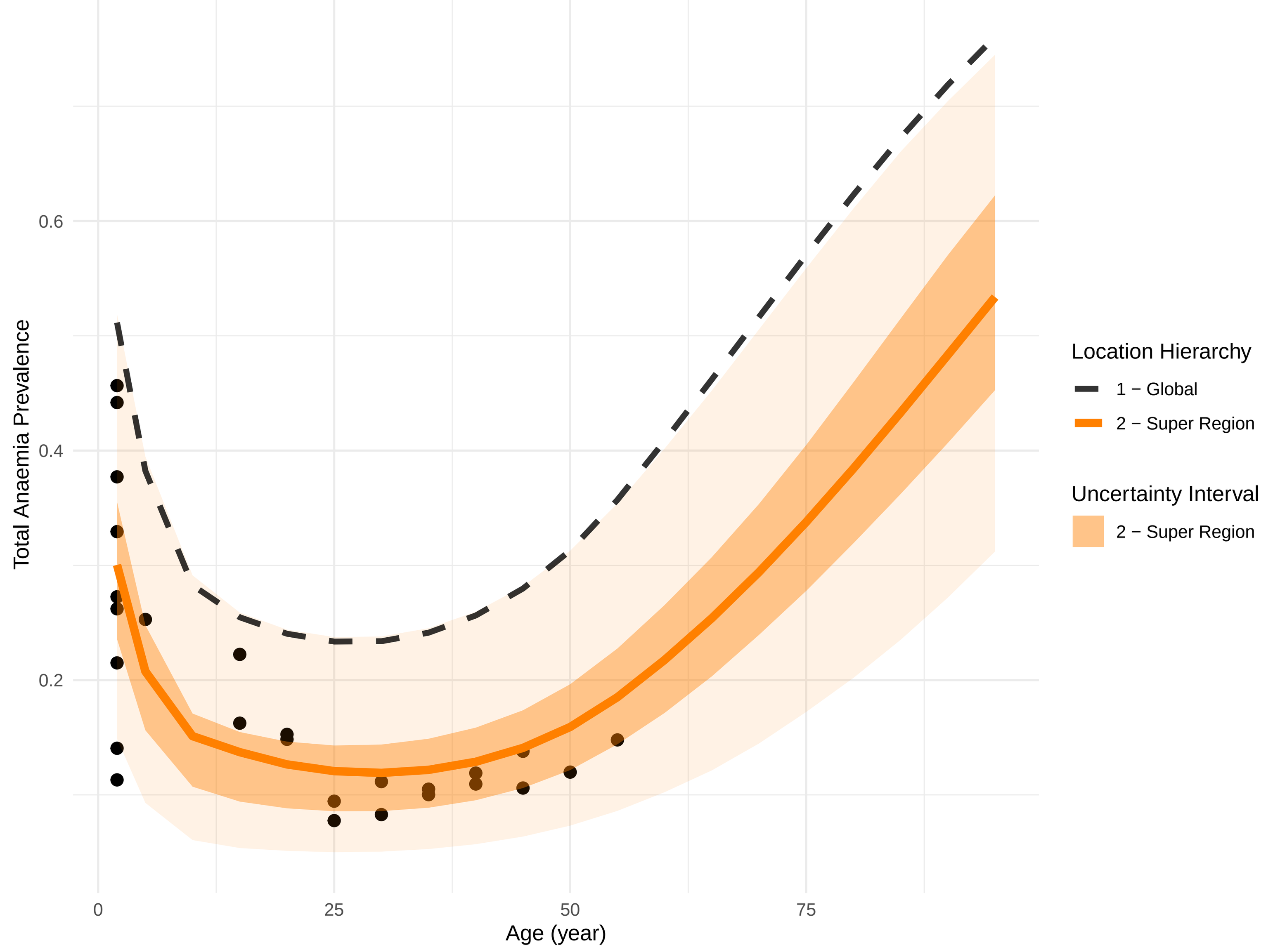

MR−BRT Age/Sex Fits − Males 2+ Years Old − Total Anaemia Prevalence − Central Asia
WHO−2001 Adjustment Method

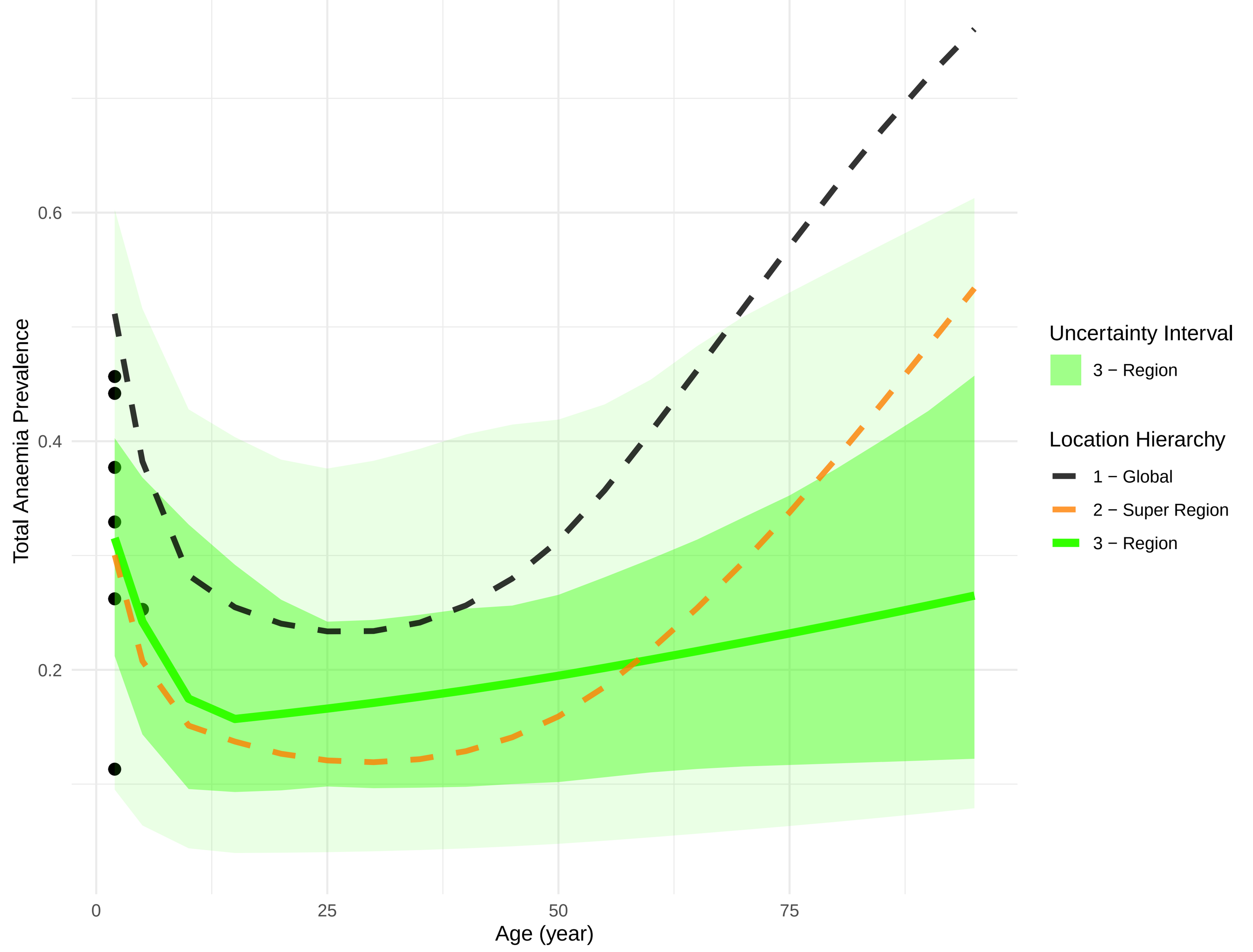

MR−BRT Age/Sex Fits − Males 2+ Years Old − Total Anaemia Prevalence − Armenia
WHO−2001 Adjustment Method

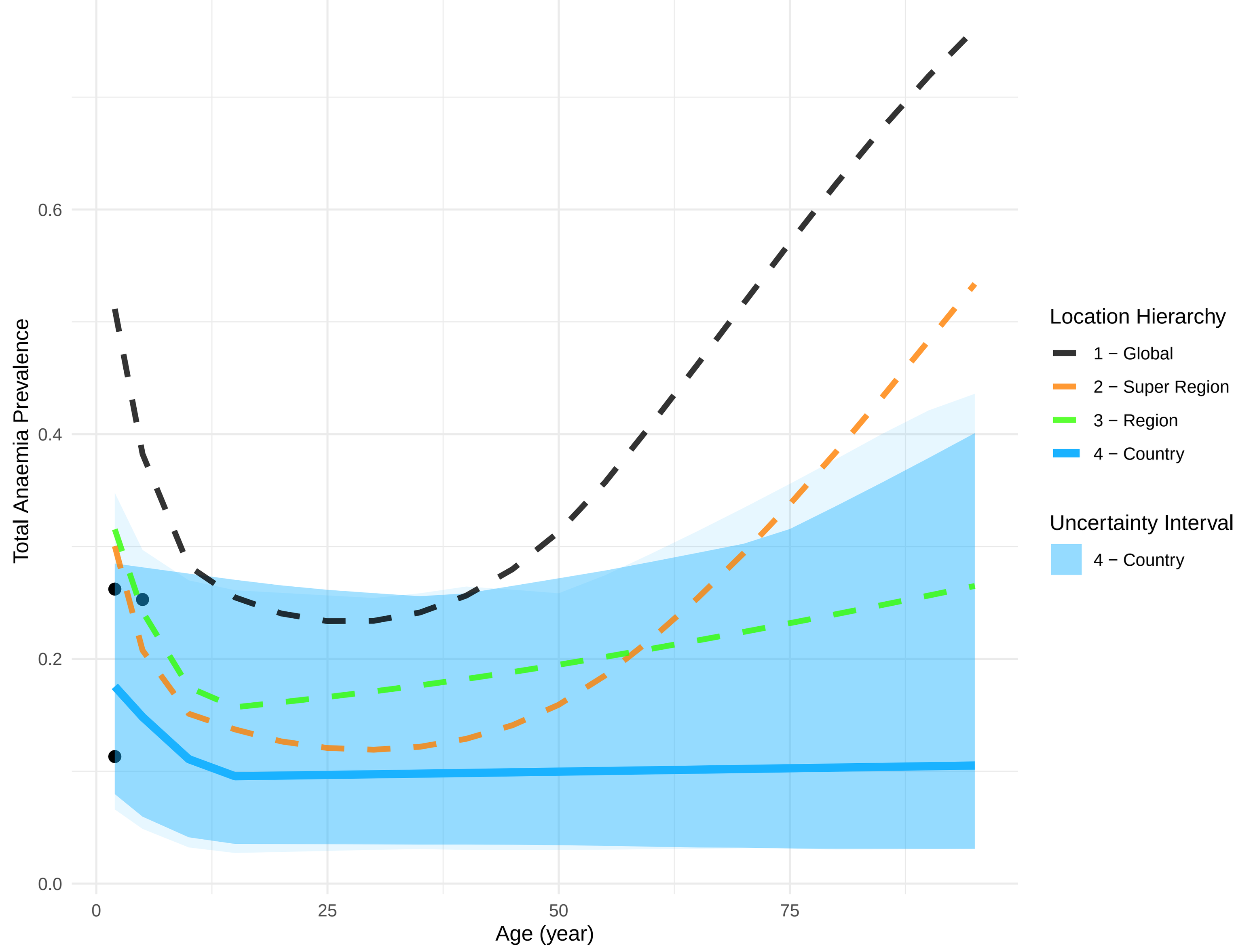

MR−BRT Age/Sex Fits − Males 2+ Years Old − Total Anaemia Prevalence − Azerbaijan
WHO−2001 Adjustment Method

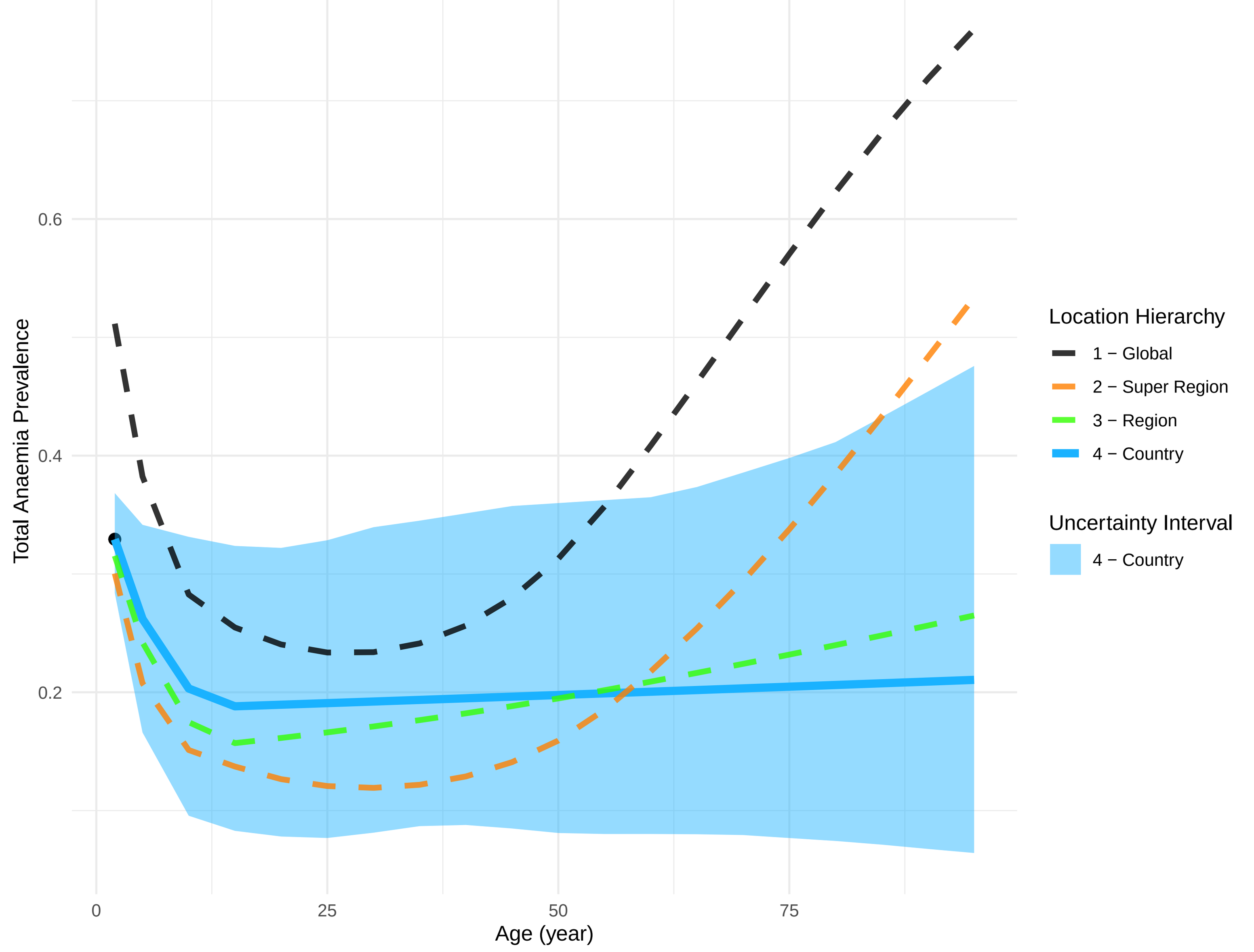

MR−BRT Age/Sex Fits − Males 2+ Years Old − Total Anaemia Prevalence − Kyrgyzstan
WHO−2001 Adjustment Method

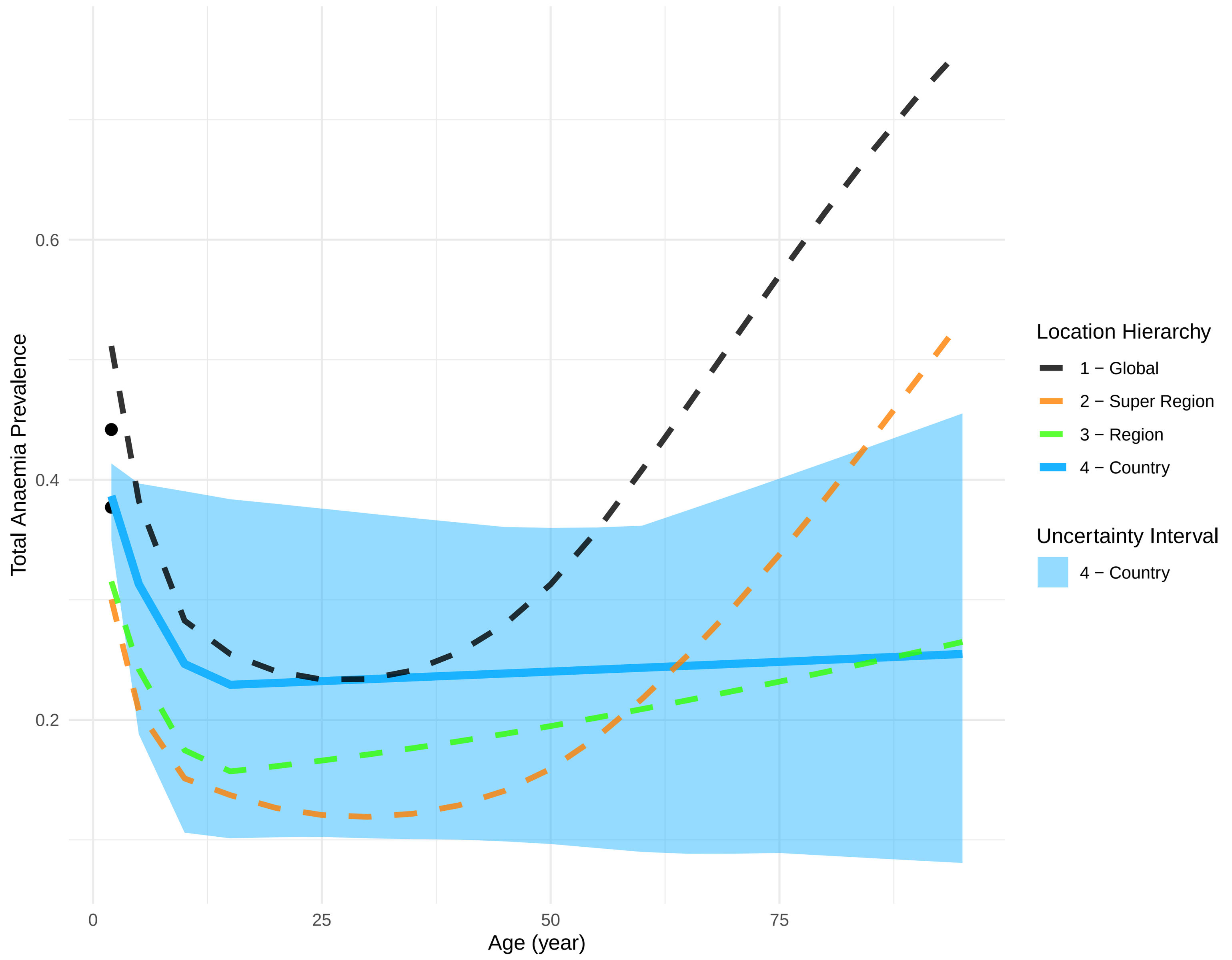

MR−BRT Age/Sex Fits − Males 2+ Years Old − Total Anaemia Prevalence − Uzbekistan
WHO−2001 Adjustment Method

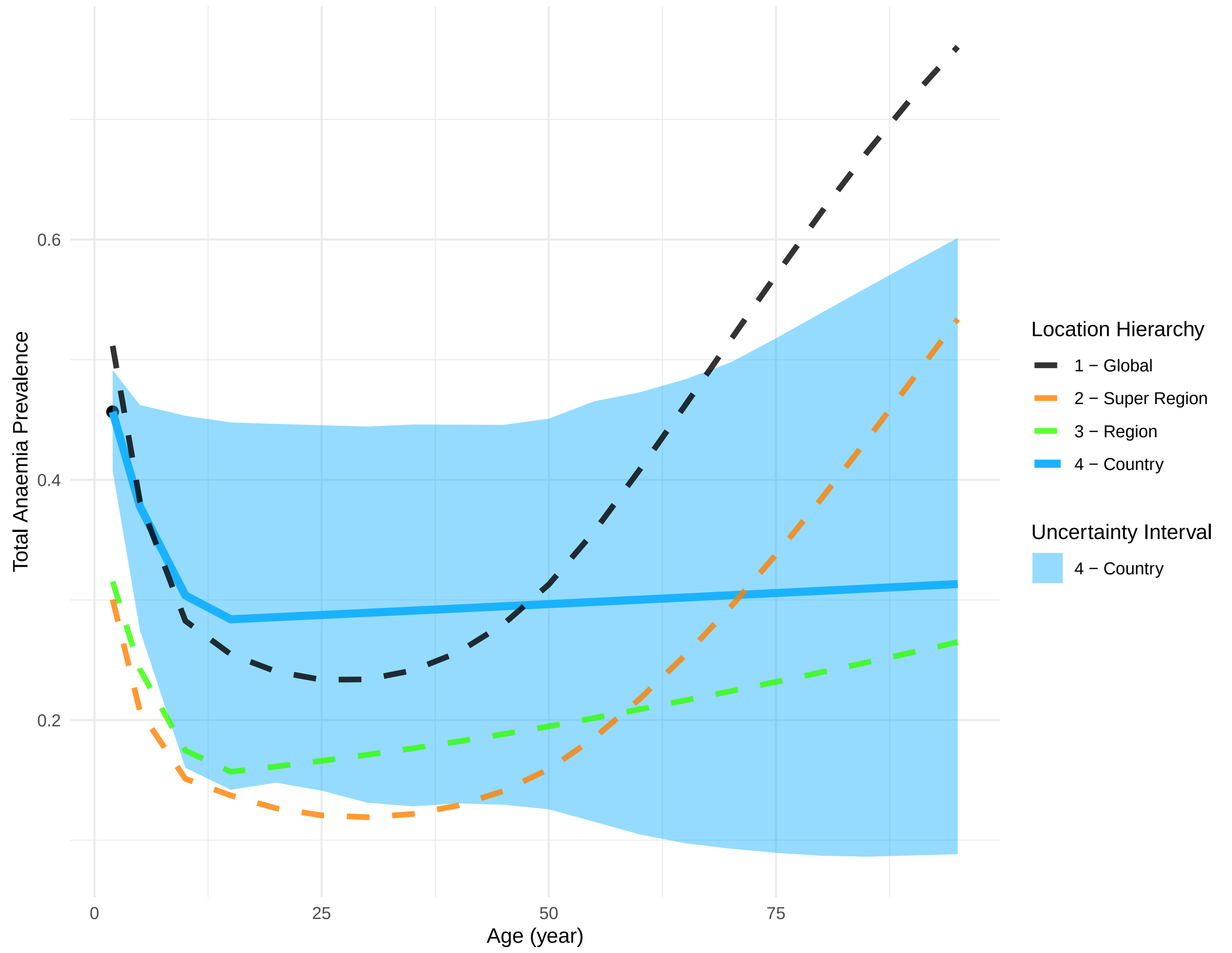

MR−BRT Age/Sex Fits − Males 2+ Years Old − Total Anaemia Prevalence − Central Europe
WHO−2001 Adjustment Method

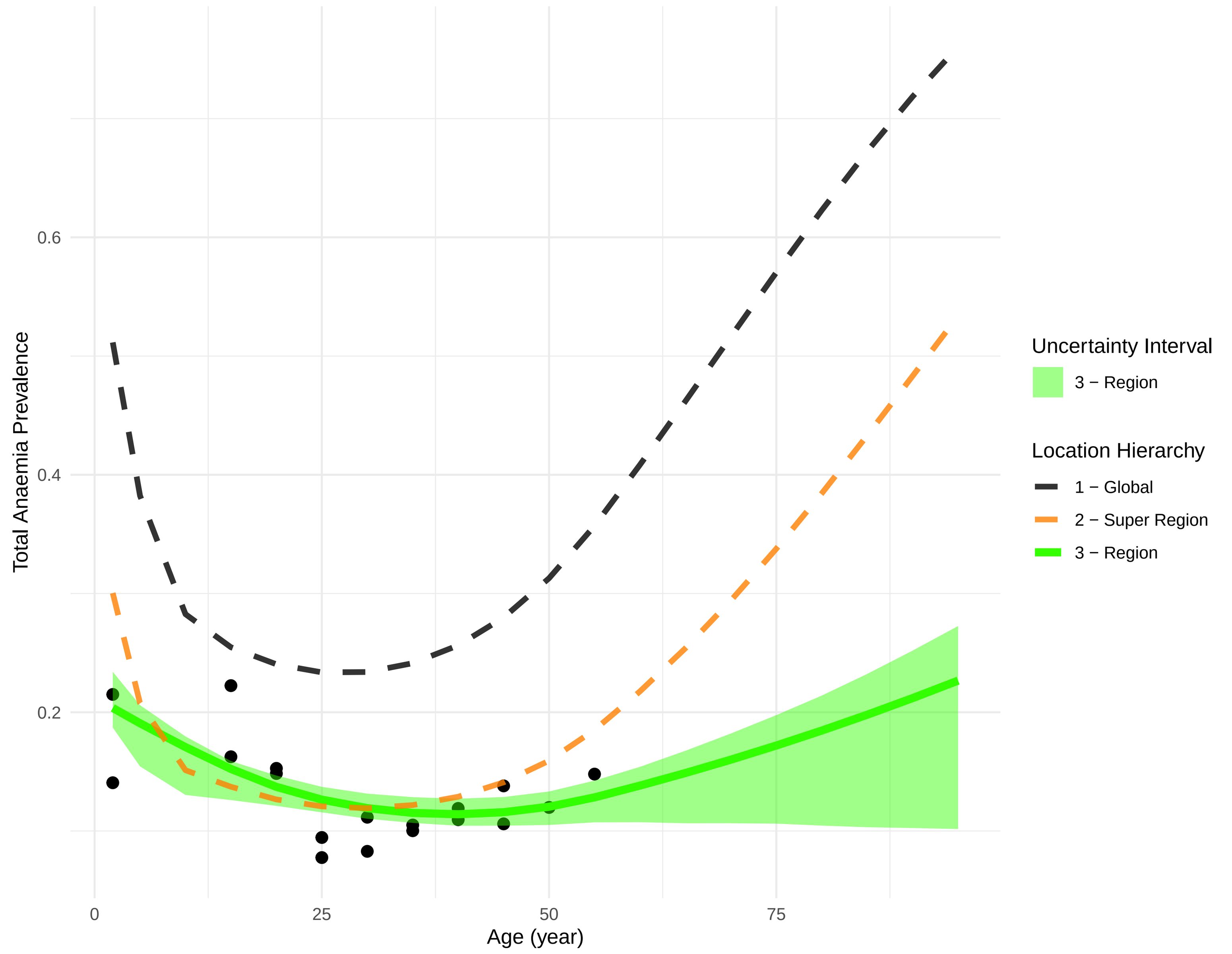

MR−BRT Age/Sex Fits − Males 2+ Years Old − Total Anaemia Prevalence − Albania
WHO−2001 Adjustment Method

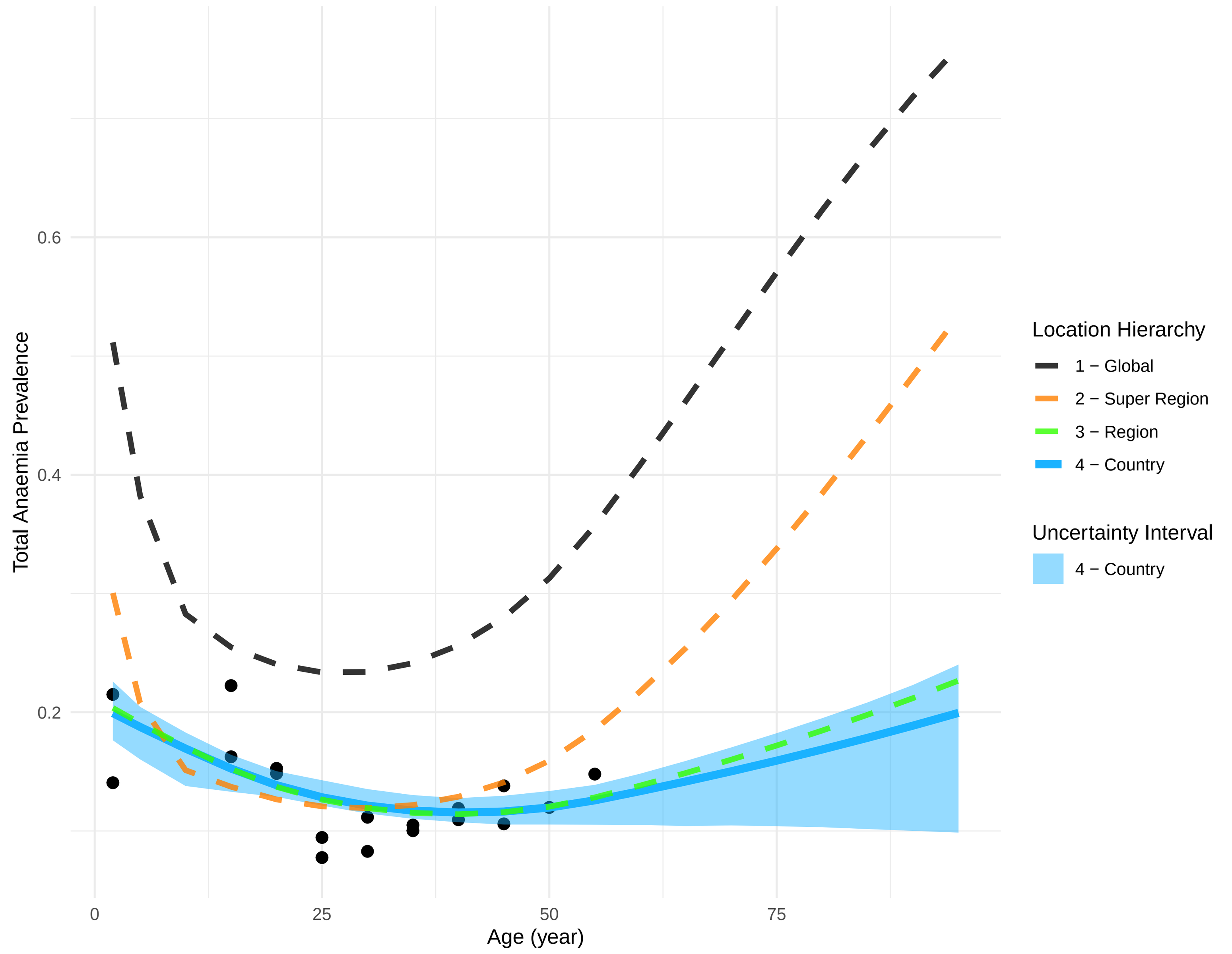

MR−BRT Age/Sex Fits − Males 2+ Years Old − Total Anaemia Prevalence − Eastern Europe
WHO−2001 Adjustment Method

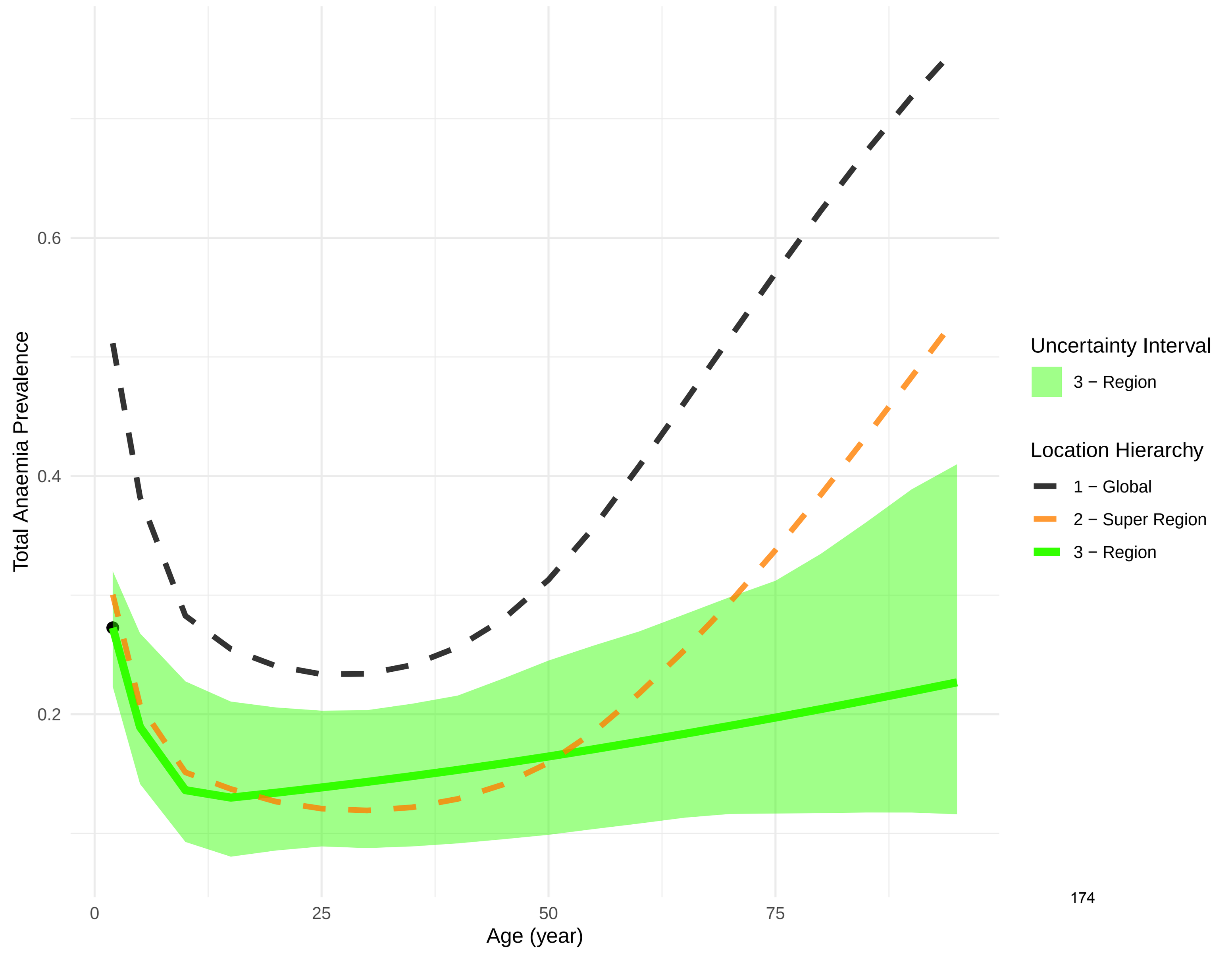

MR−BRT Age/Sex Fits − Males 2+ Years Old − Total Anaemia Prevalence − Republic of Moldova
WHO−2001 Adjustment Method

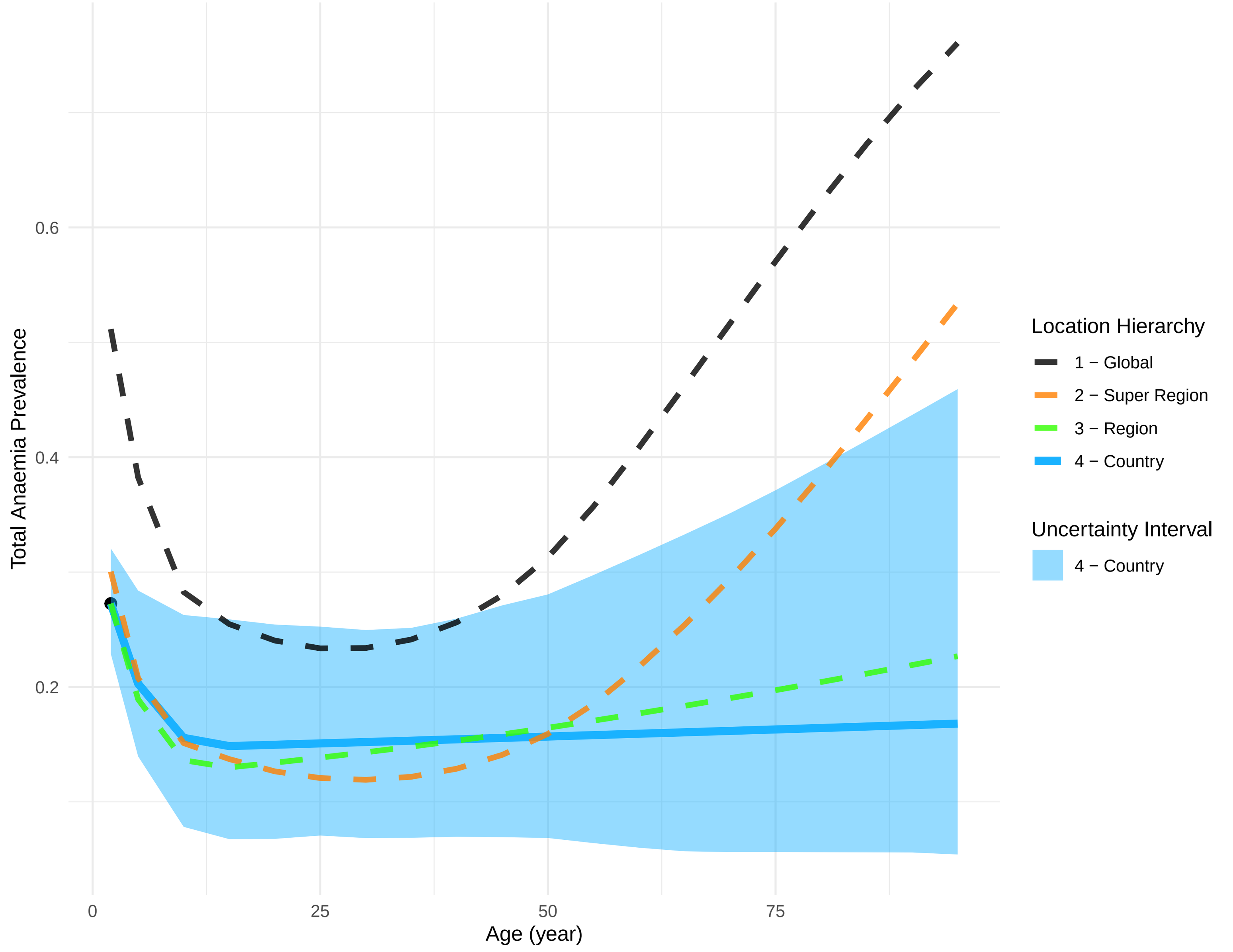

MR−BRT Age/Sex Fits − Males 2+ Years Old − Total Anaemia Prevalence − High−income
WHO−2001 Adjustment Method

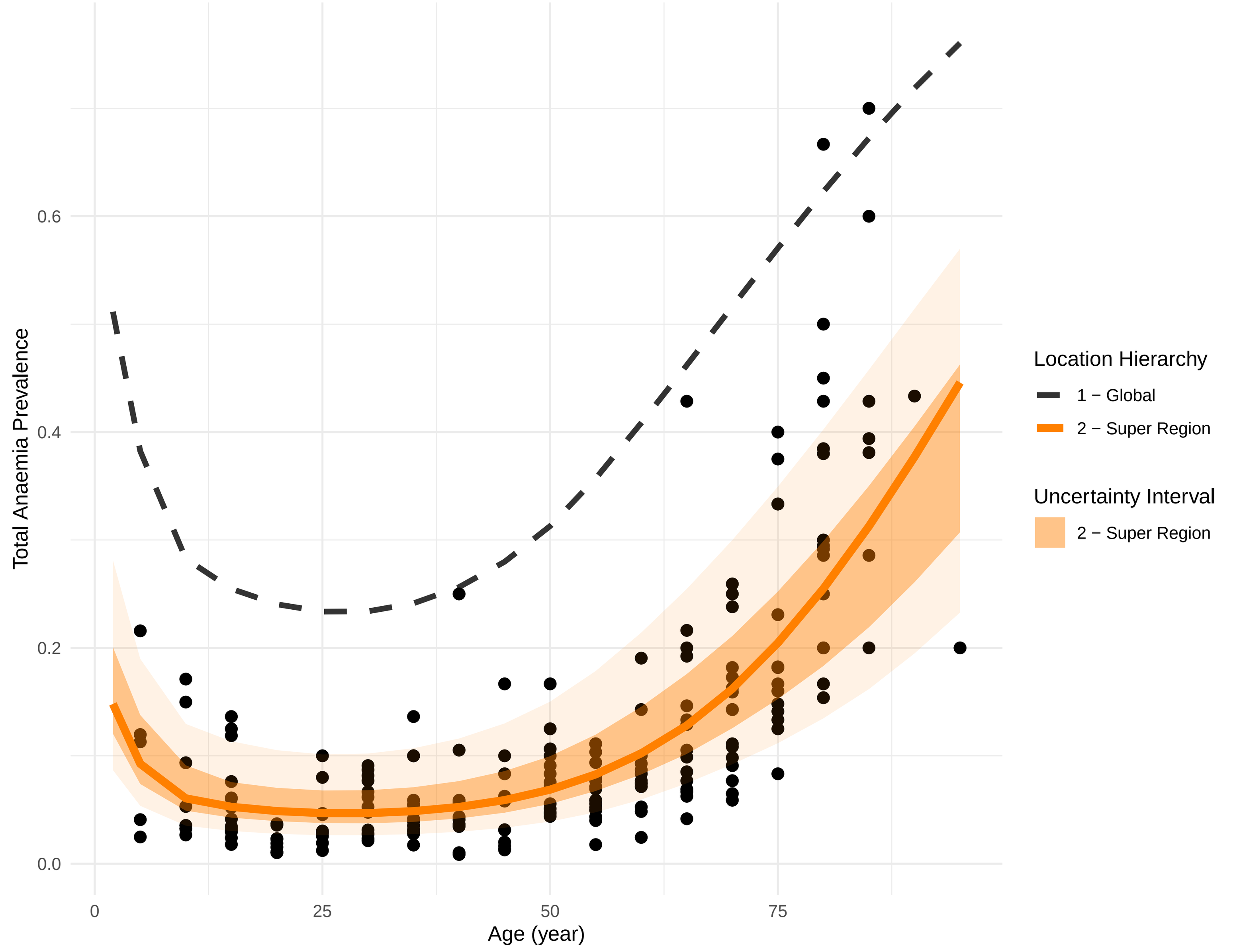

MR−BRT Age/Sex Fits − Males 2+ Years Old − Total Anaemia Prevalence − Western Europe
WHO−2001 Adjustment Method

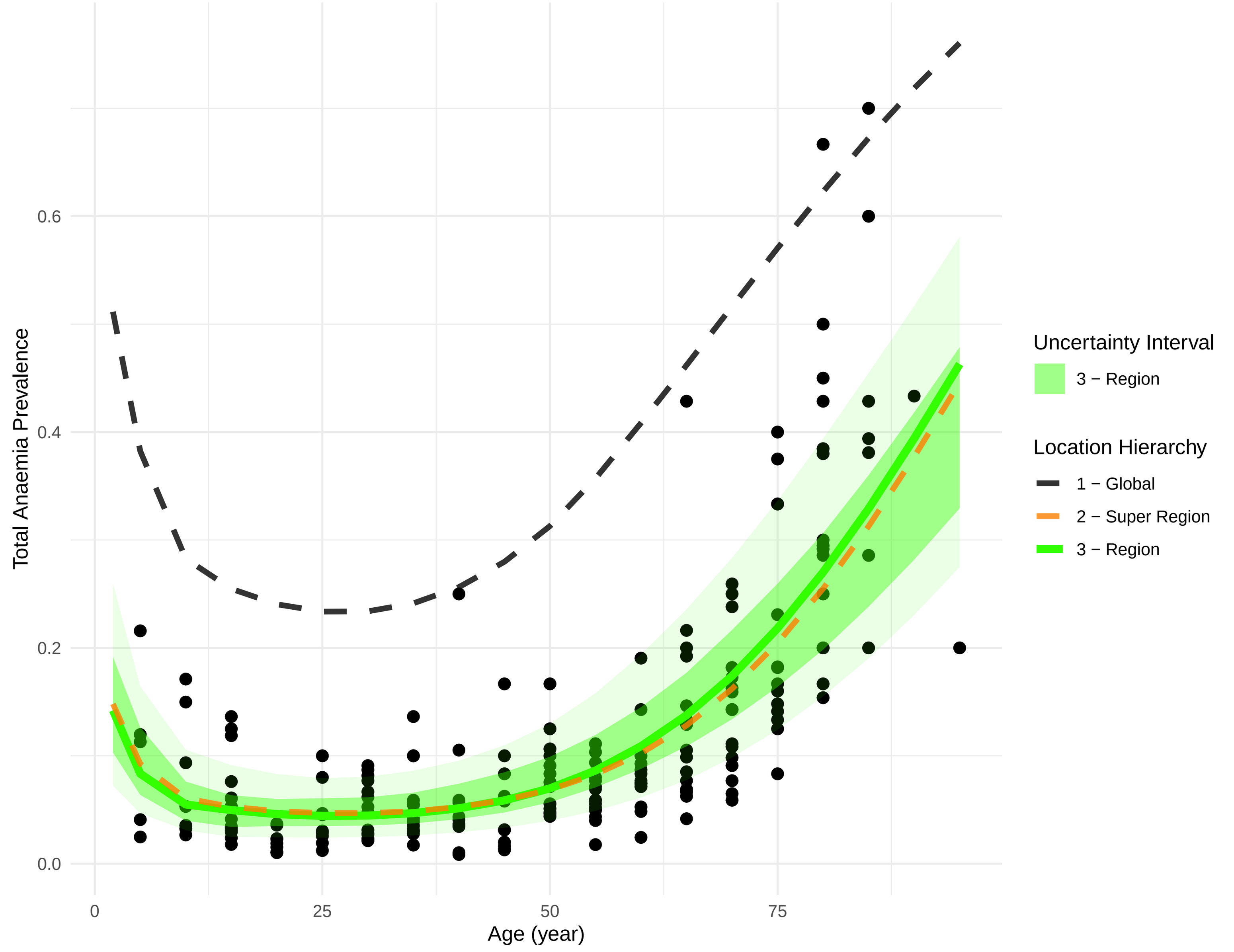

MR−BRT Age/Sex Fits − Males 2+ Years Old − Total Anaemia Prevalence − United Kingdom
WHO−2001 Adjustment Method

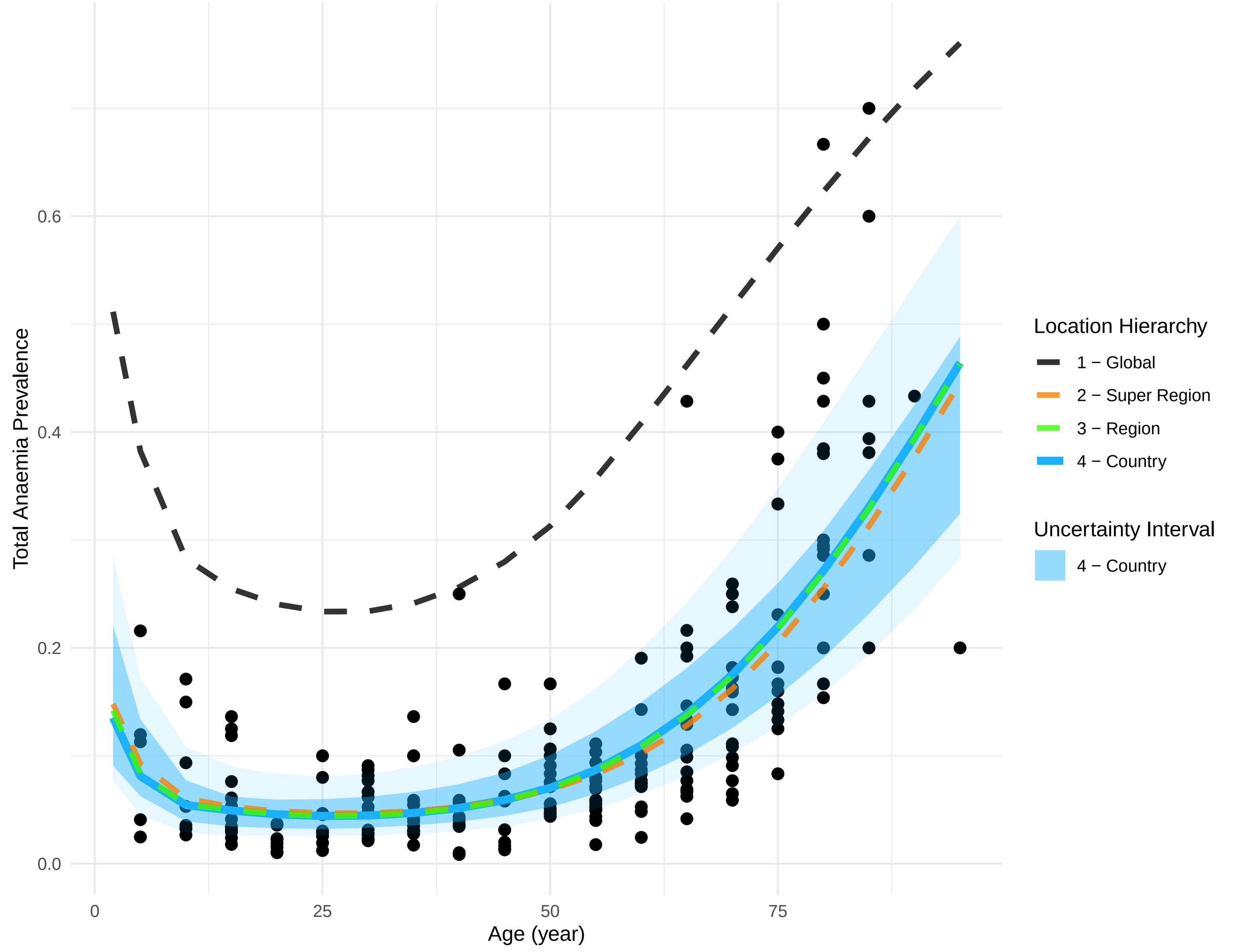

MR−BRT Age/Sex Fits − Males 2+ Years Old − Total Anaemia Prevalence − England
WHO−2001 Adjustment Method

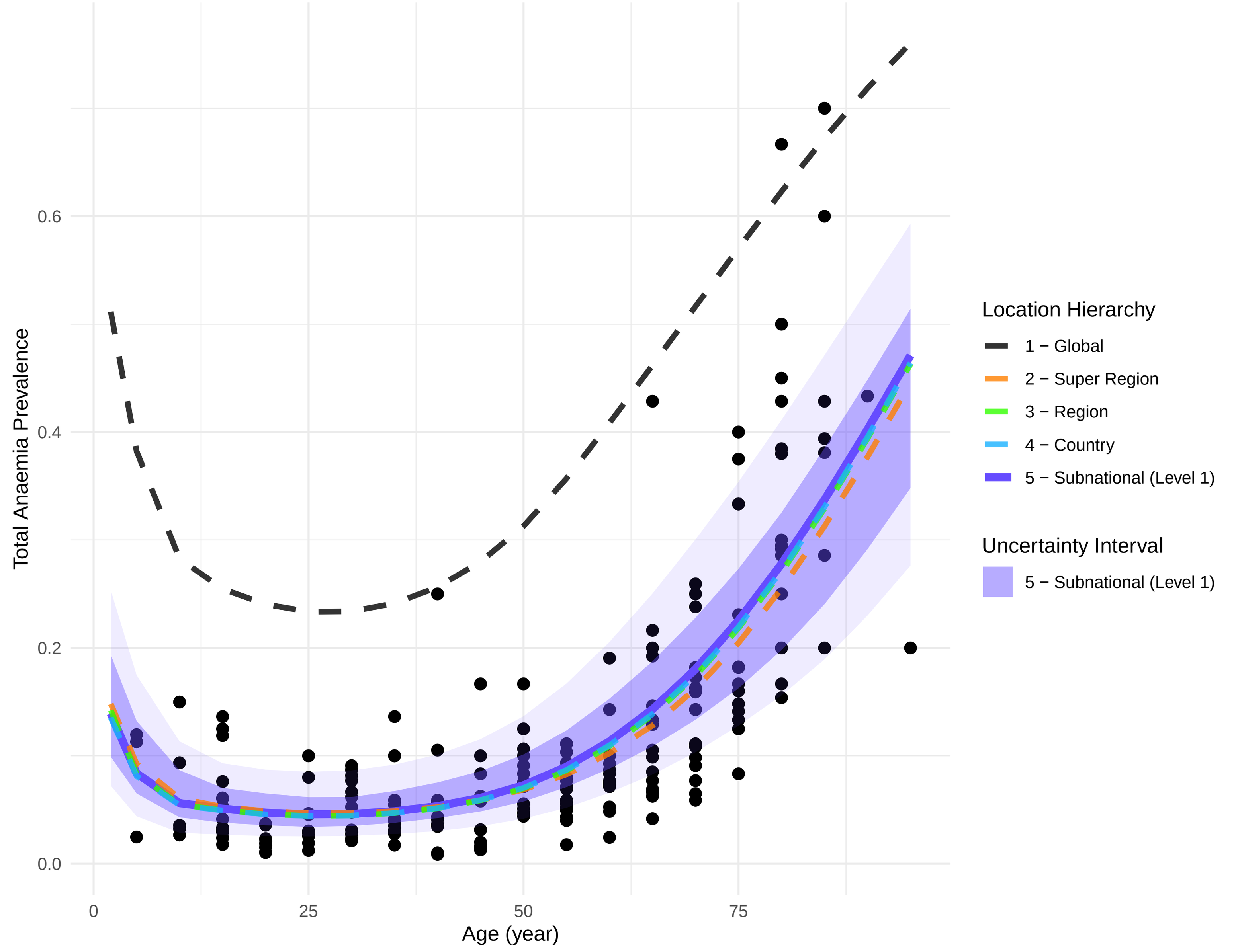

MR−BRT Age/Sex Fits − Males 2+ Years Old − Total Anaemia Prevalence − East Midlands
WHO−2001 Adjustment Method

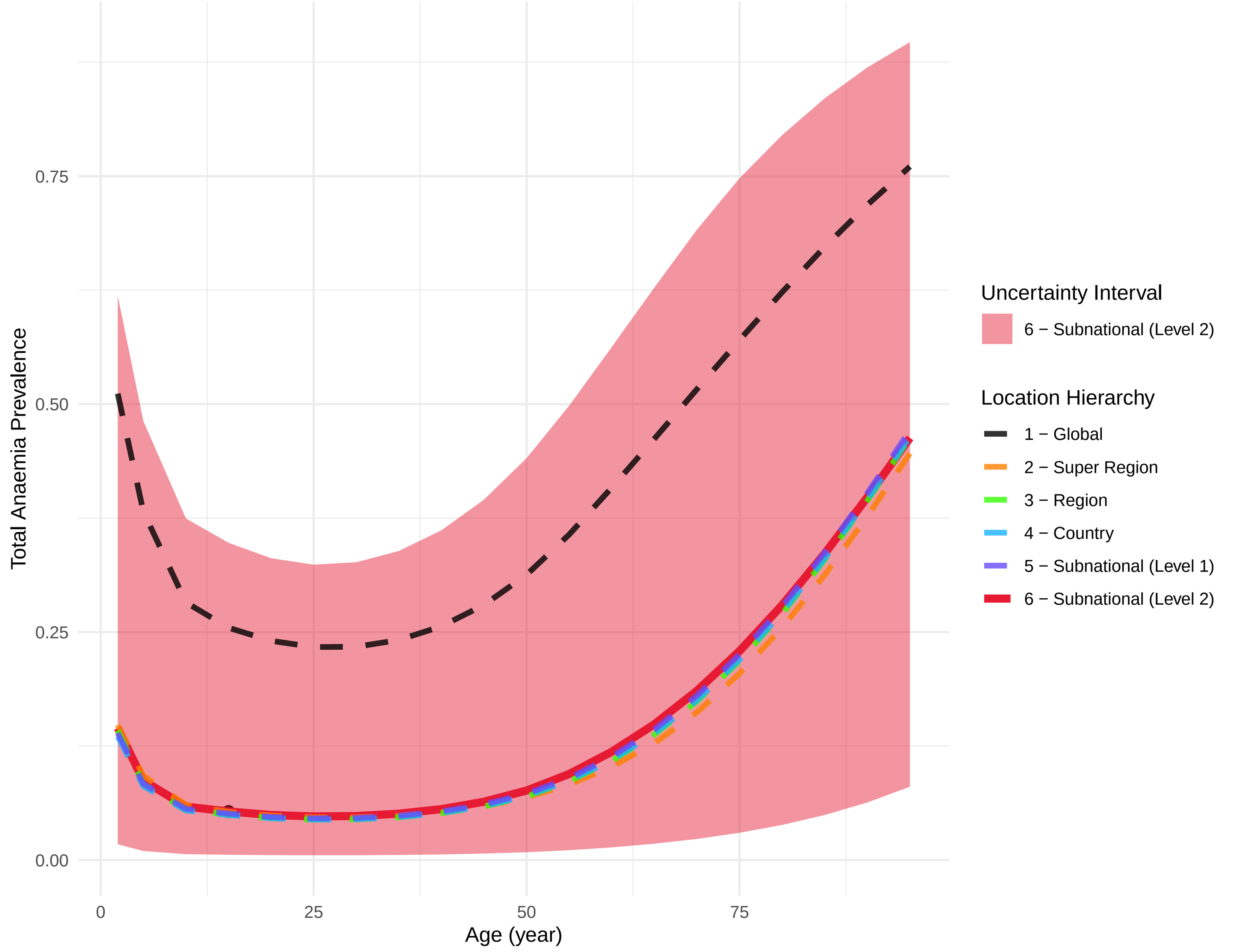

MR−BRT Age/Sex Fits − Males 2+ Years Old − Total Anaemia Prevalence − East of England
WHO−2001 Adjustment Method

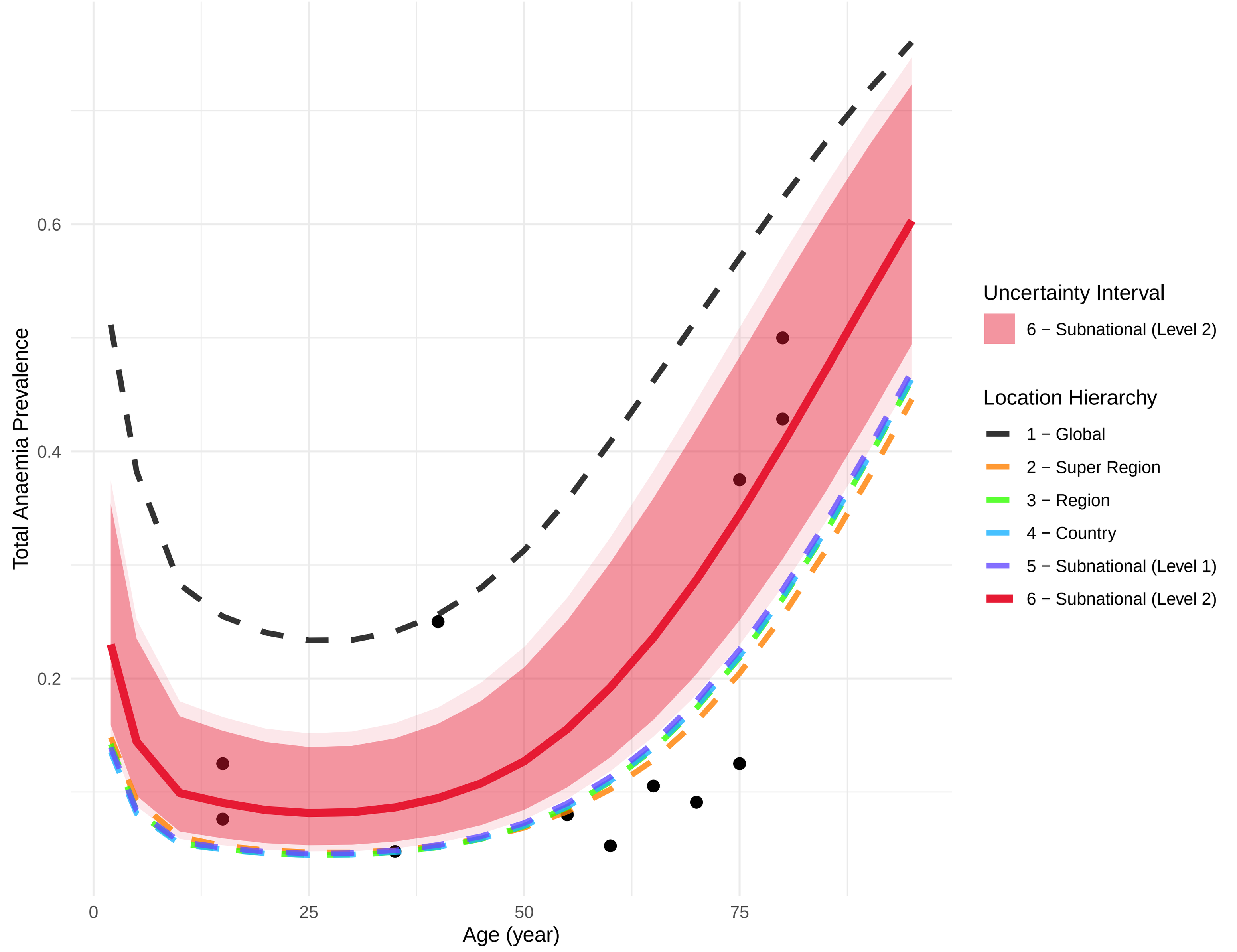

MR−BRT Age/Sex Fits − Males 2+ Years Old − Total Anaemia Prevalence − Greater London
WHO−2001 Adjustment Method

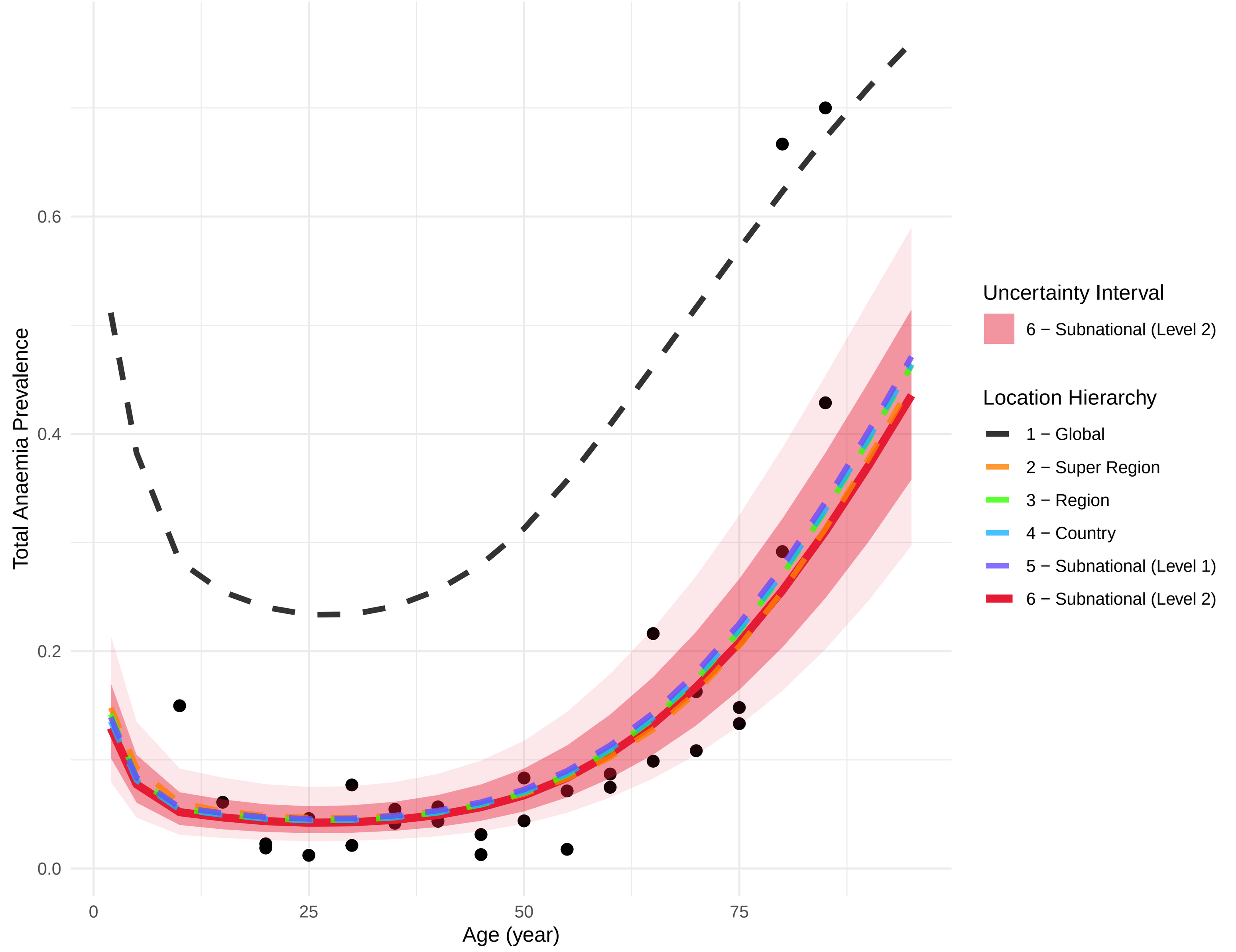

MR−BRT Age/Sex Fits − Males 2+ Years Old − Total Anaemia Prevalence − North West England
WHO−2001 Adjustment Method

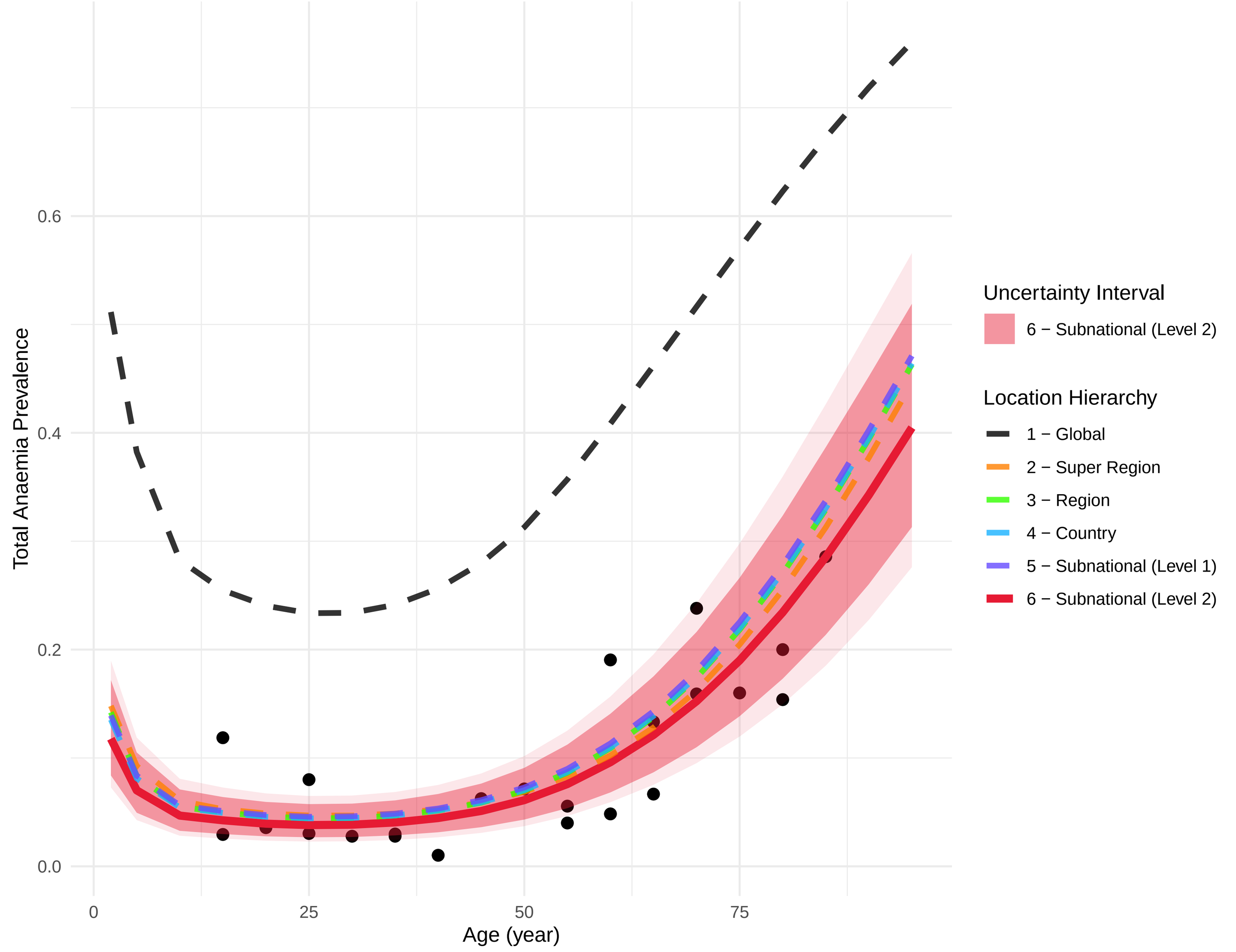

MR−BRT Age/Sex Fits − Males 2+ Years Old − Total Anaemia Prevalence − South East England
WHO−2001 Adjustment Method

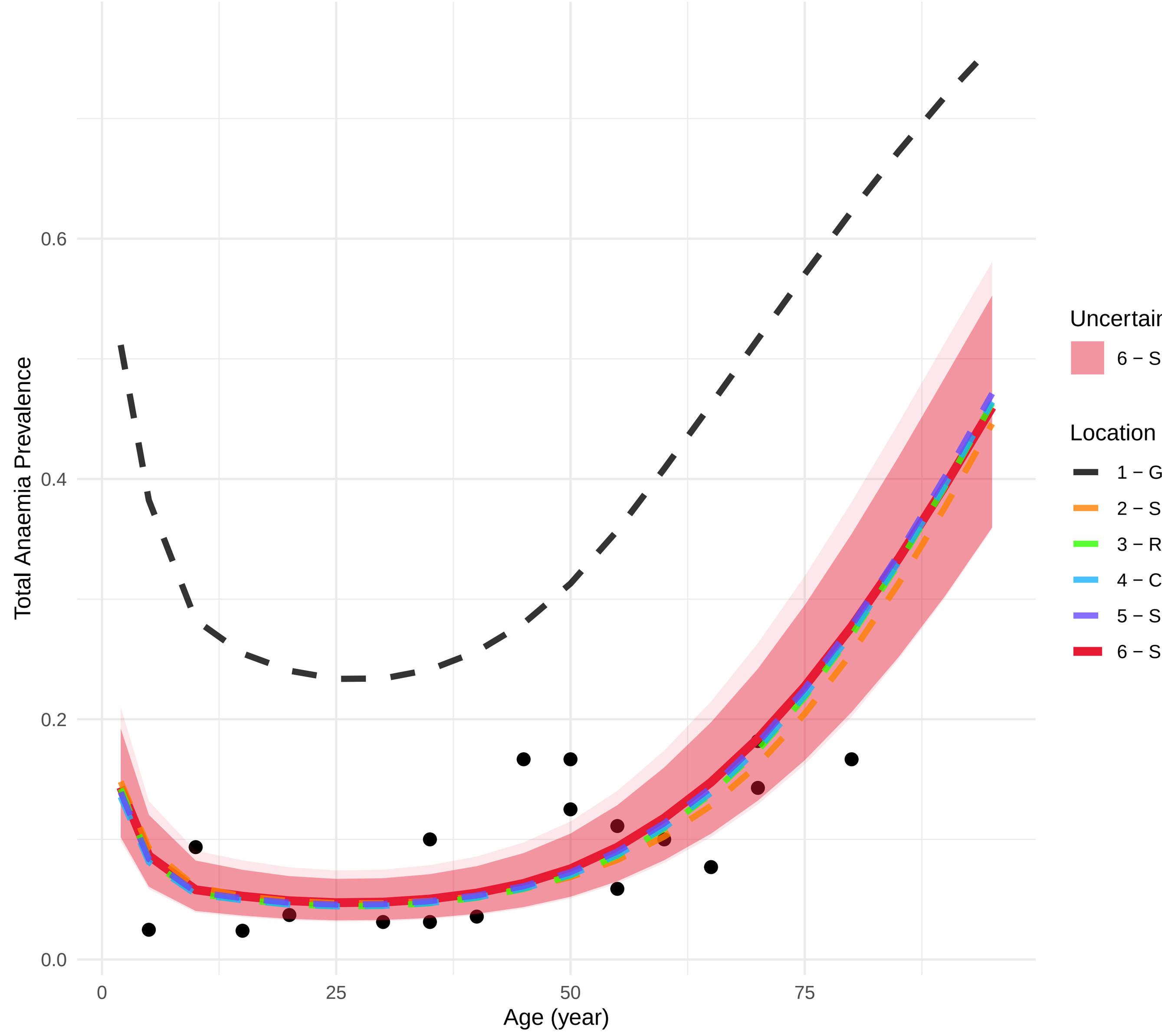

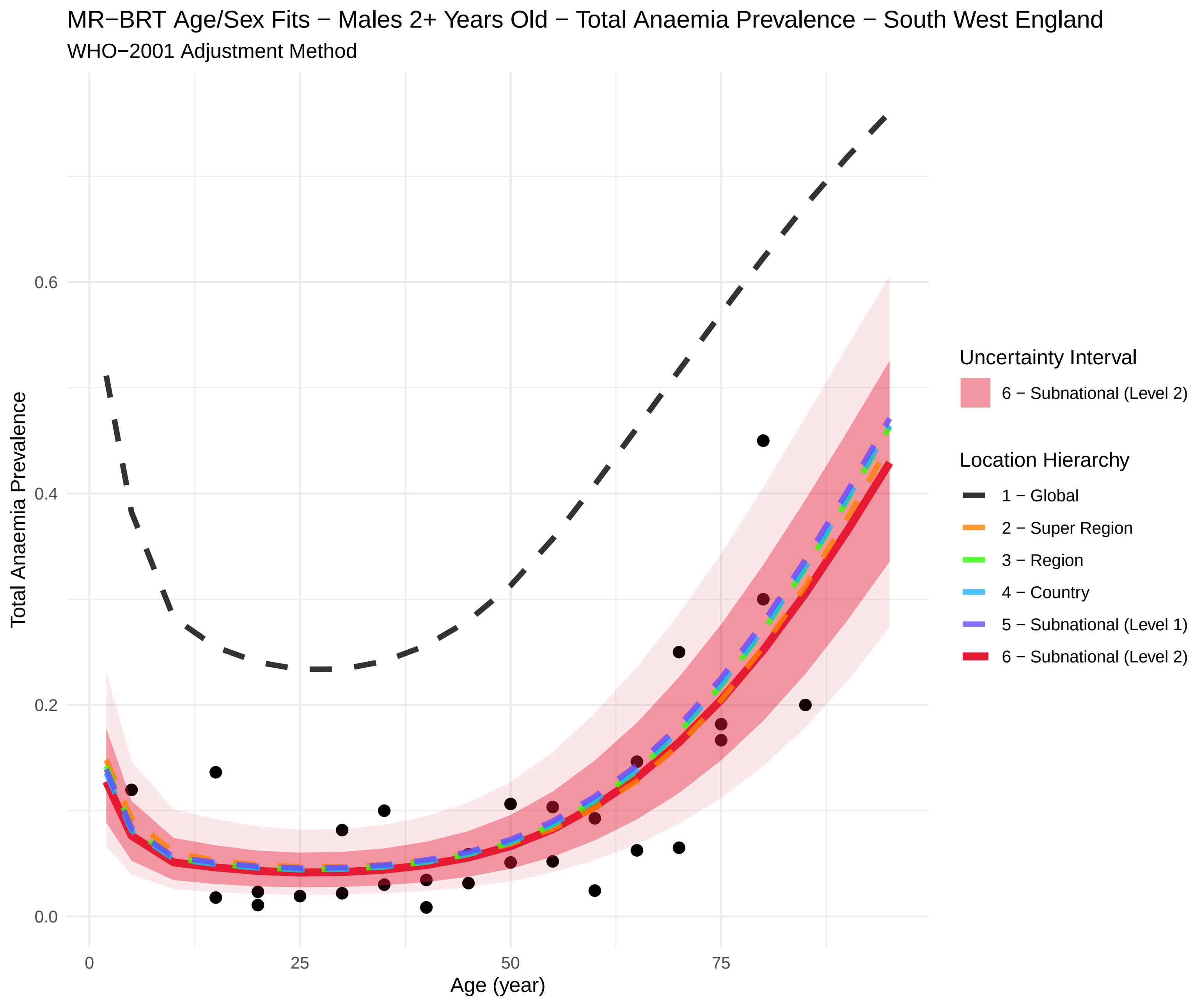
MR−BRT Age/Sex Fits − Males 2+ Years Old − Total Anaemia Prevalence − South West England
WHO−2001 Adjustment Method
Total Anaemia Prevalence
Age (year)
Uncertainty Interval
6 − Subnational (Level 2)
Location Hierarchy
1 − Global
2 − Super Region
3 − Region
4 − Country
5 − Subnational (Level 1)
6 − Subnational (Level 2)

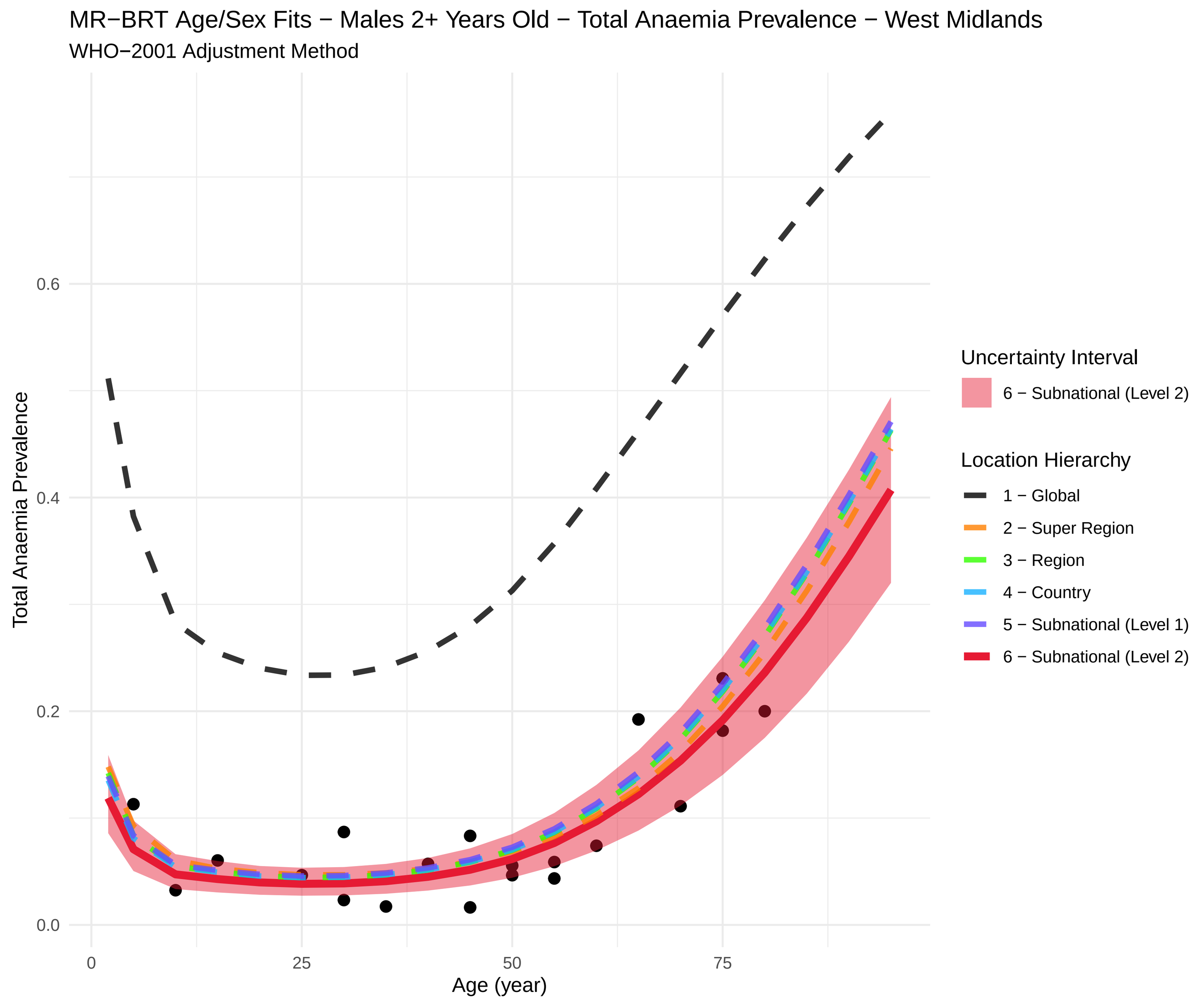
MR−BRT Age/Sex Fits − Males 2+ Years Old − Total Anaemia Prevalence − West Midlands
WHO−2001 Adjustment Method
Total Anaemia Prevalence
Age (year)
Uncertainty Interval
6 − Subnational (Level 2)
Location Hierarchy
1 − Global
2 − Super Region
3 − Region
4 − Country
5 − Subnational (Level 1)
6 − Subnational (Level 2)

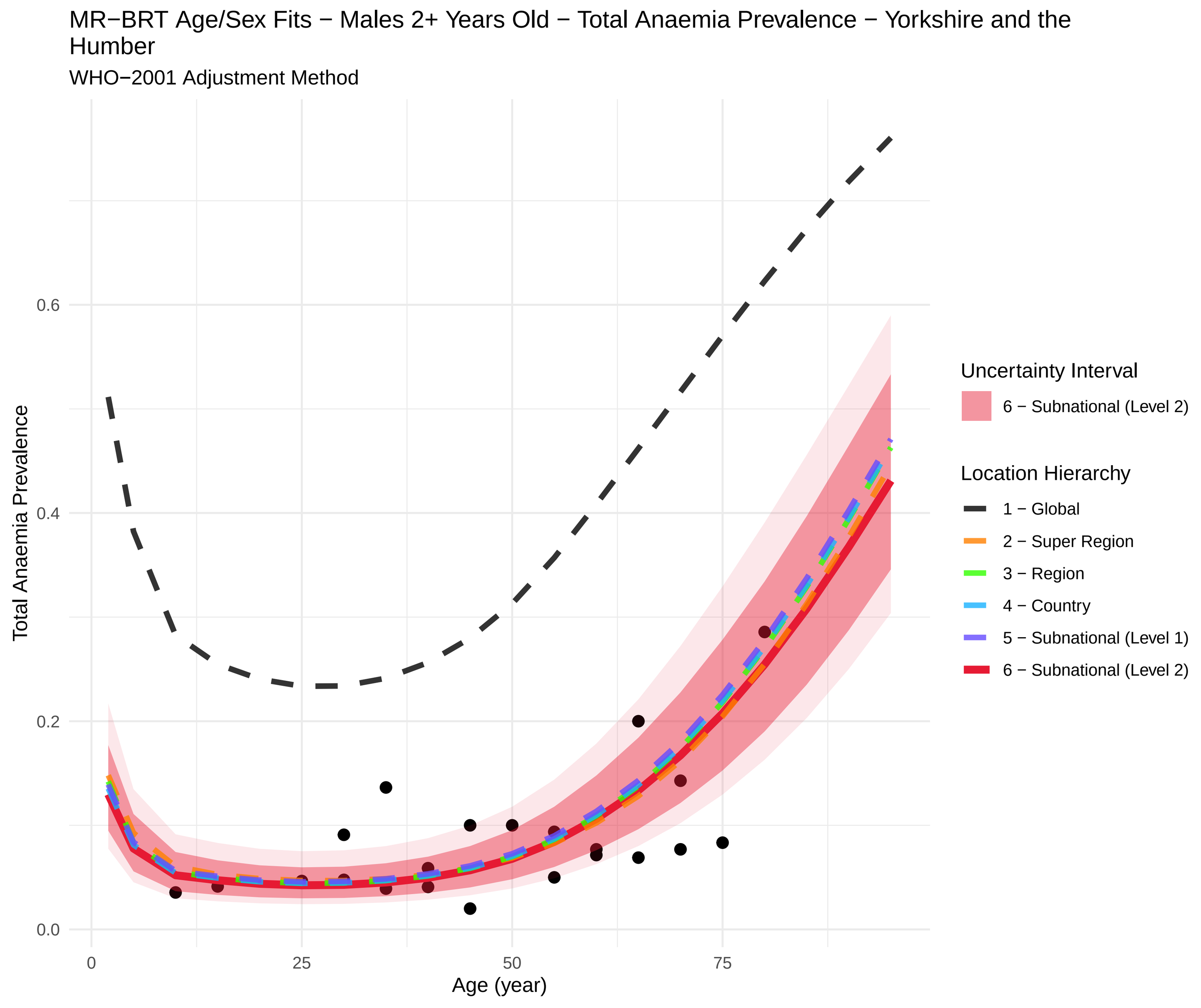
MR−BRT Age/Sex Fits − Males 2+ Years Old − Total Anaemia Prevalence − Yorkshire and the Humber
WHO−2001 Adjustment Method
Total Anaemia Prevalence
Age (year)
Uncertainty Interval
6 − Subnational (Level 2)
Location Hierarchy
1 − Global
2 − Super Region
3 − Region
4 − Country
5 − Subnational (Level 1)
6 − Subnational (Level 2)

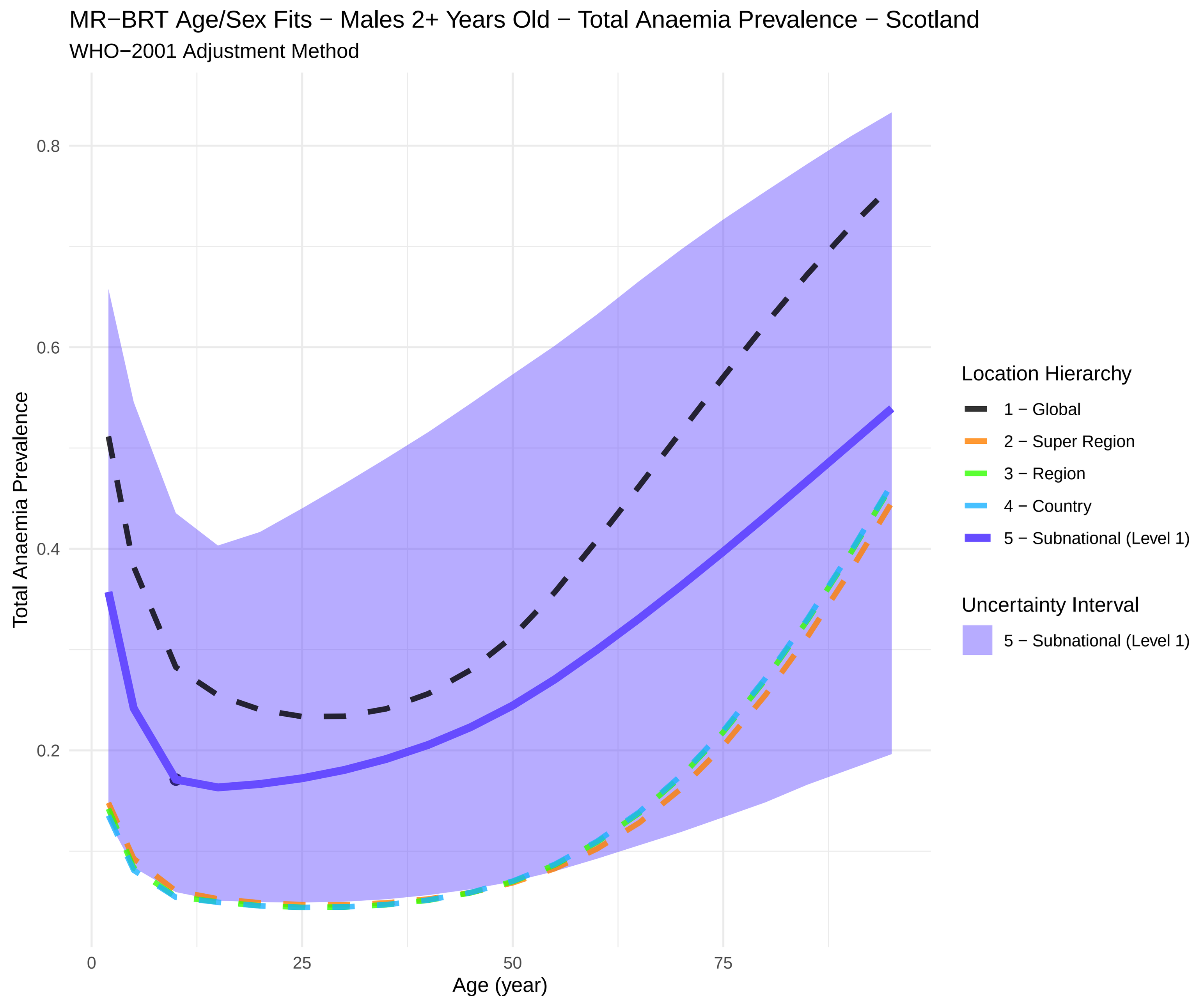
MR−BRT Age/Sex Fits − Males 2+ Years Old − Total Anaemia Prevalence − Scotland
WHO−2001 Adjustment Method
Total Anaemia Prevalence
Age (year)
Location Hierarchy
1 − Global
2 − Super Region
3 − Region
4 − Country
5 − Subnational (Level 1)
Uncertainty Interval
5 − Subnational (Level 1)

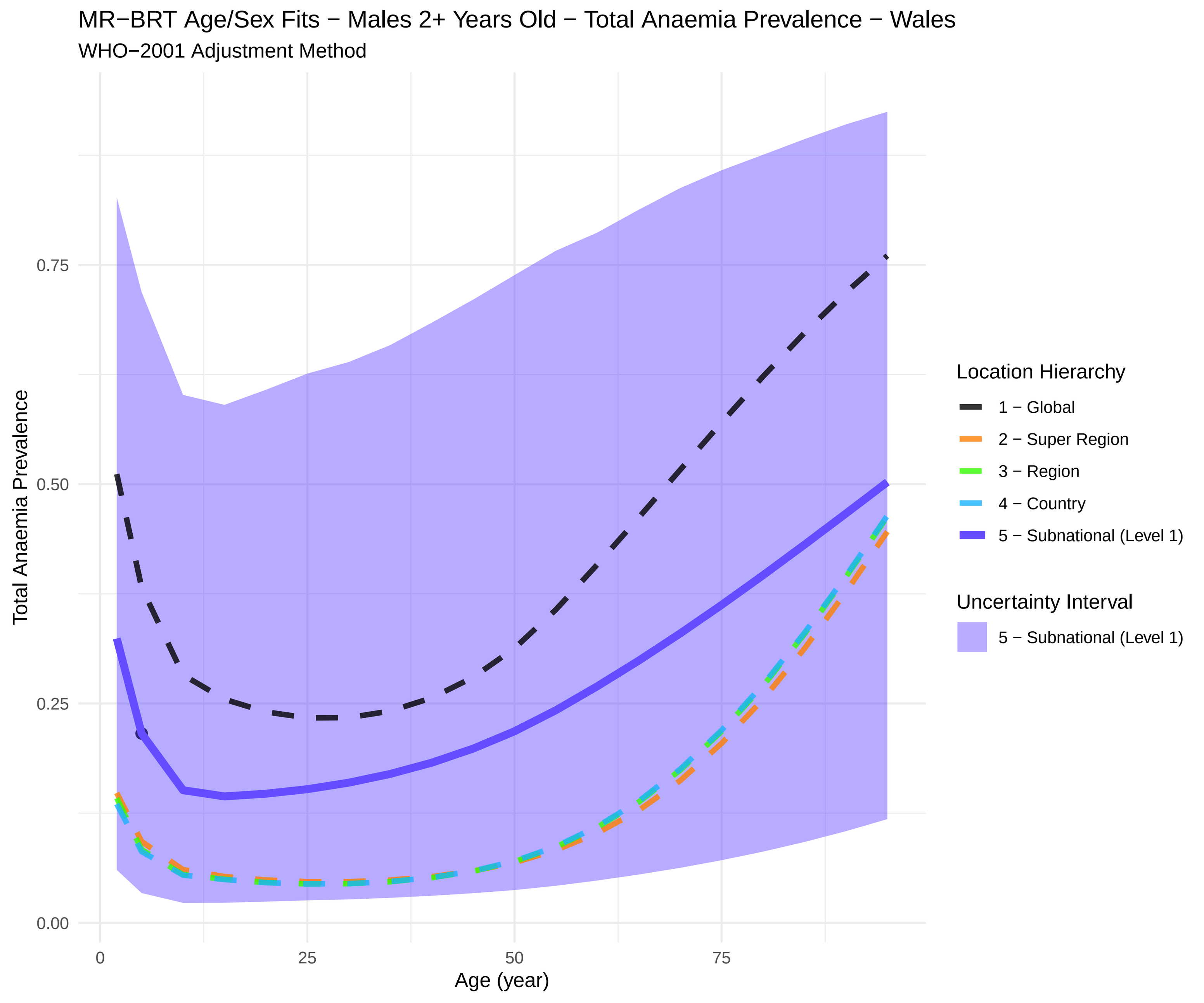
MR−BRT Age/Sex Fits − Males 2+ Years Old − Total Anaemia Prevalence − Wales
WHO−2001 Adjustment Method
Total Anaemia Prevalence
Age (year)
Location Hierarchy
1 − Global
2 − Super Region
3 − Region
4 − Country
5 − Subnational (Level 1)
Uncertainty Interval
5 − Subnational (Level 1)

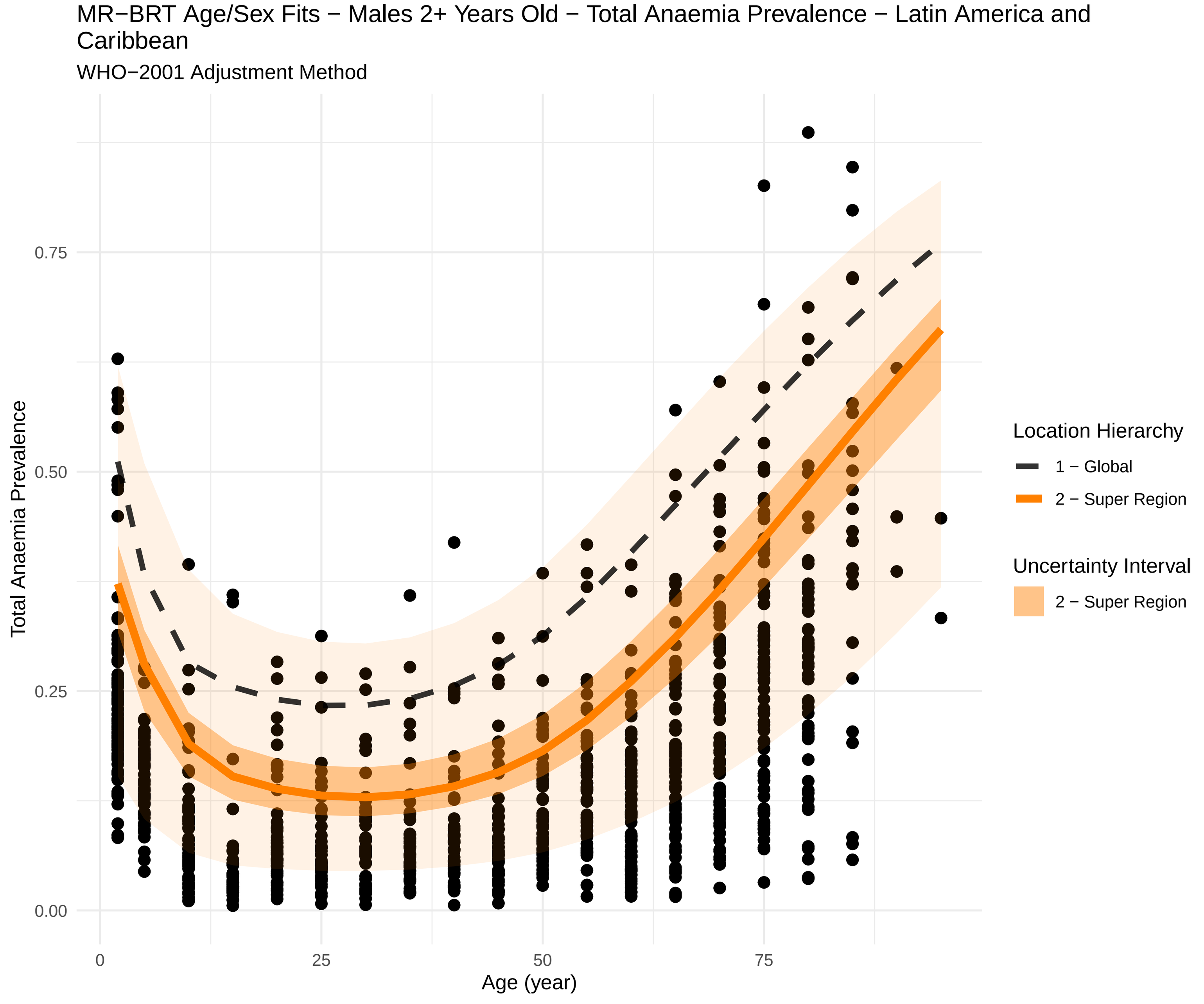
MR−BRT Age/Sex Fits − Males 2+ Years Old − Total Anaemia Prevalence − Latin America and Caribbean
WHO−2001 Adjustment Method
Total Anaemia Prevalence
Age (year)
Location Hierarchy
1 − Global
2 − Super Region
Uncertainty Interval
2 − Super Region

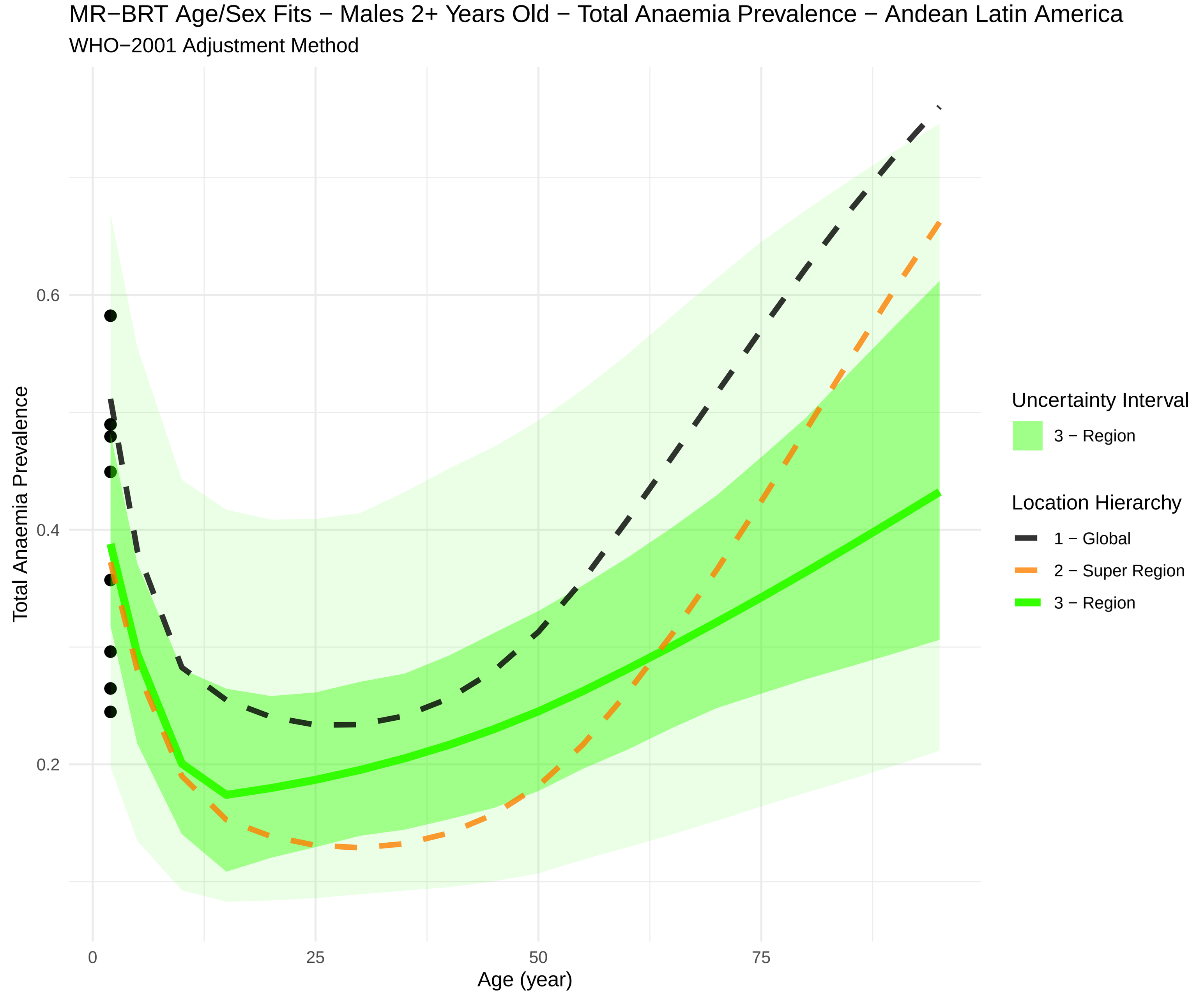
MR−BRT Age/Sex Fits − Males 2+ Years Old − Total Anaemia Prevalence − Andean Latin America
WHO−2001 Adjustment Method
Total Anaemia Prevalence
Age (year)
Uncertainty Interval
3 − Region
Location Hierarchy
1 − Global
2 − Super Region
3 − Region

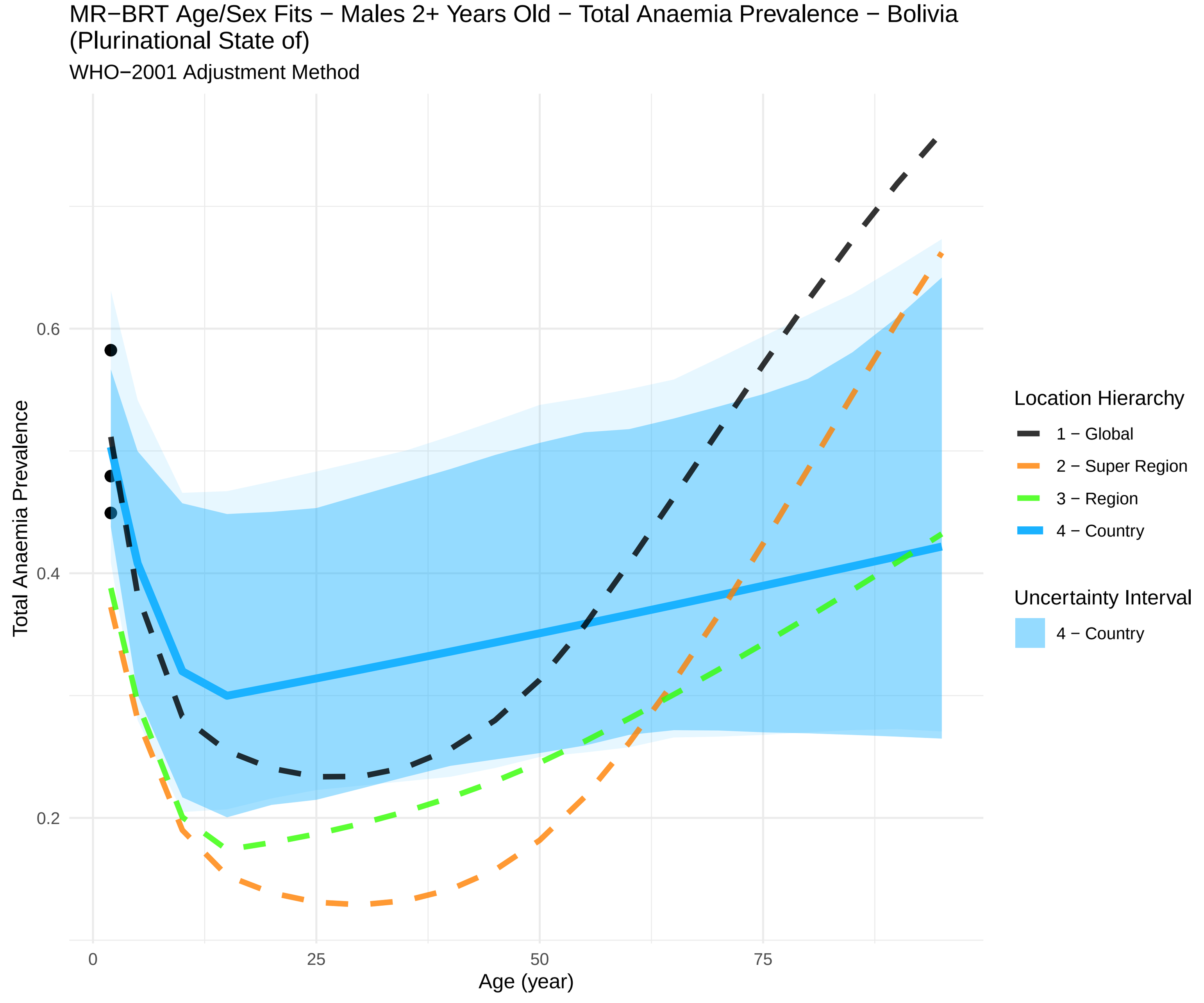
MR−BRT Age/Sex Fits − Males 2+ Years Old − Total Anaemia Prevalence − Bolivia (Plurinational State of)
WHO−2001 Adjustment Method
Total Anaemia Prevalence
Age (year)
Location Hierarchy
1 − Global
2 − Super Region
3 − Region
4 − Country
Uncertainty Interval
4 − Country

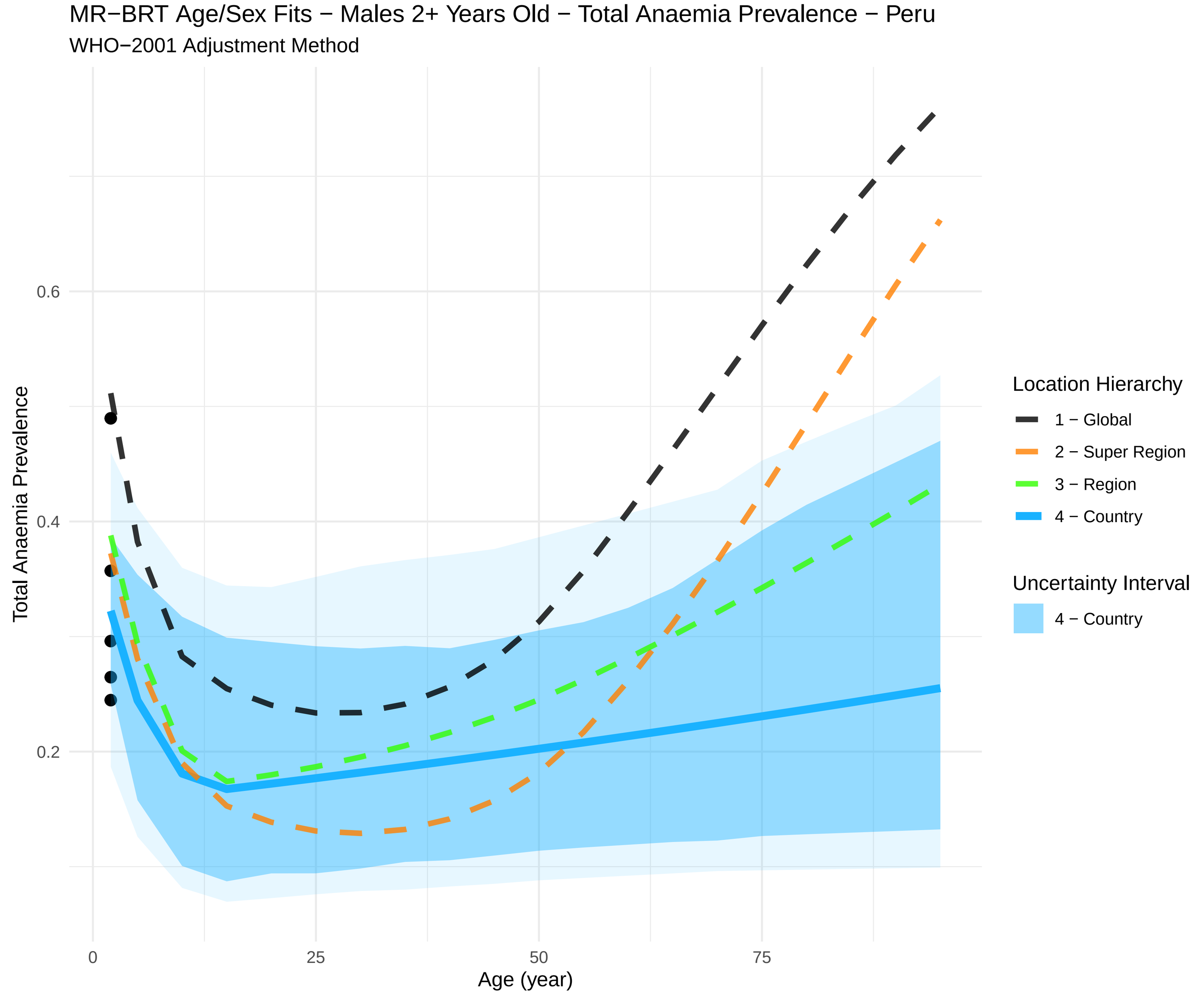
MR−BRT Age/Sex Fits − Males 2+ Years Old − Total Anaemia Prevalence − Peru
WHO−2001 Adjustment Method
Total Anaemia Prevalence
Age (year)
Location Hierarchy
1 − Global
2 − Super Region
3 − Region
4 − Country
Uncertainty Interval
4 − Country

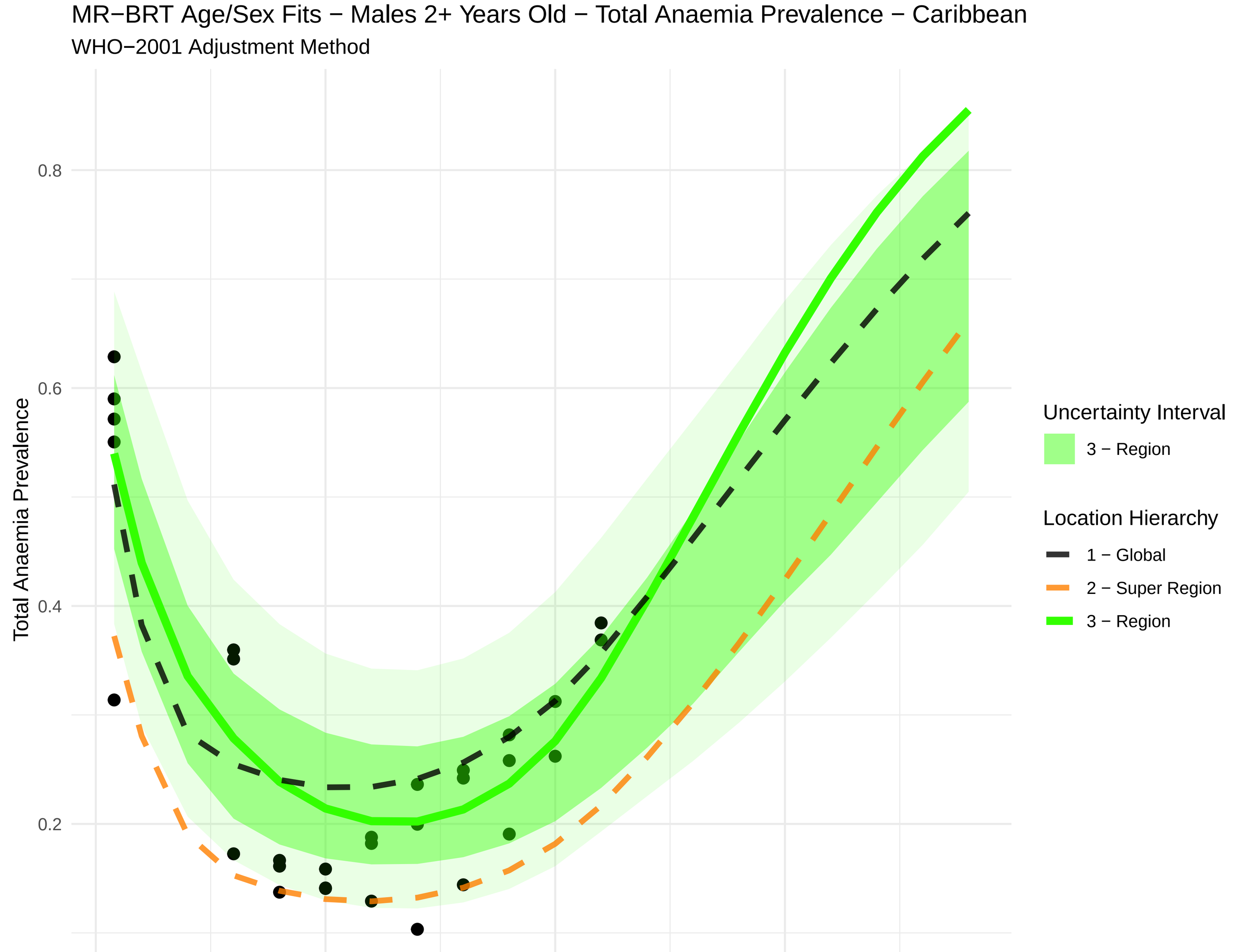
MR−BRT Age/Sex Fits − Males 2+ Years Old − Total Anaemia Prevalence − Caribbean
WHO−2001 Adjustment Method
Total Anaemia Prevalence
Age (year)
Uncertainty Interval
3 − Region
Location Hierarchy
1 − Global
2 − Super Region
3 − Region

MR−BRT Age/Sex Fits − Males 2+ Years Old − Total Anaemia Prevalence − Guyana
WHO−2001 Adjustment Method

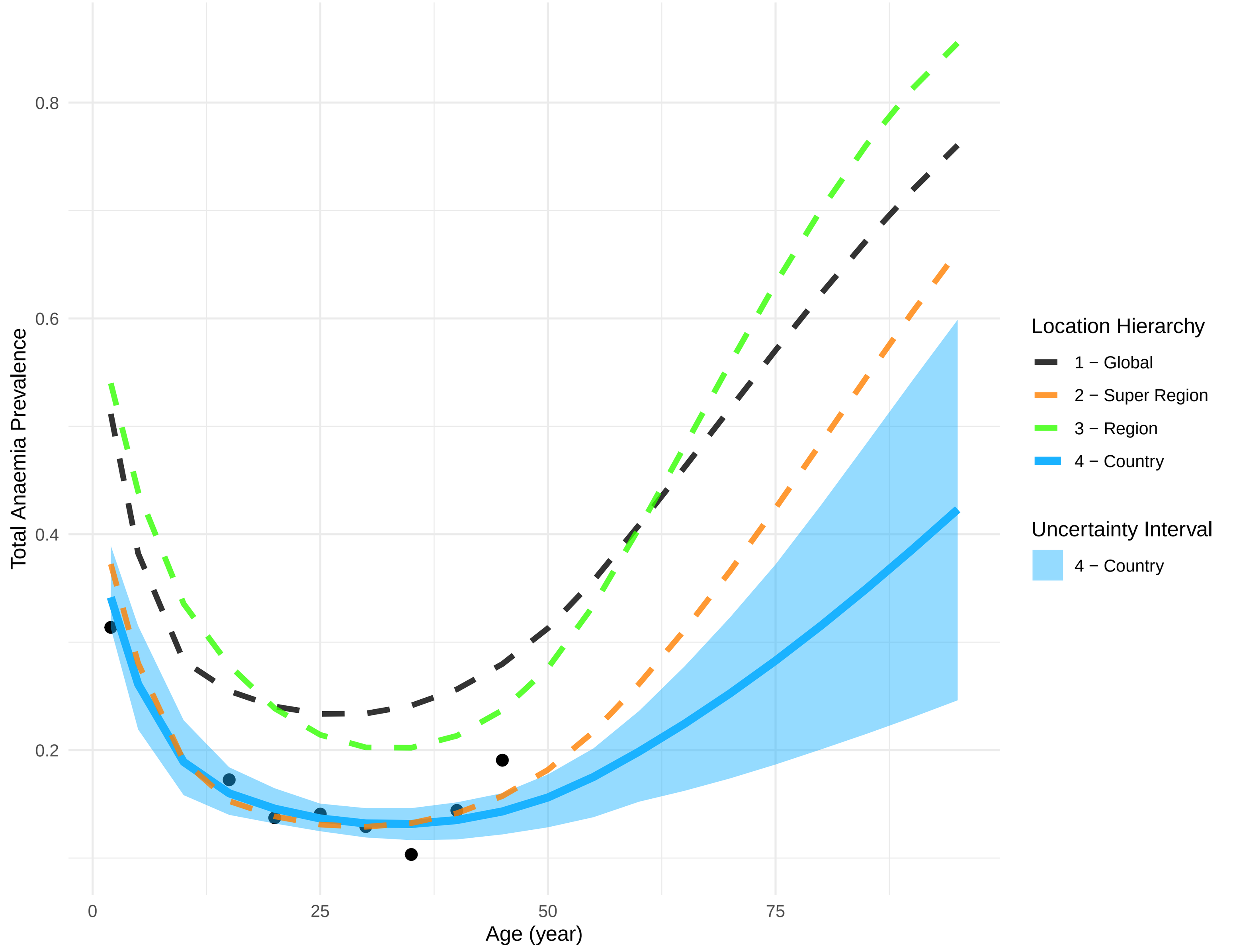

MR−BRT Age/Sex Fits − Males 2+ Years Old − Total Anaemia Prevalence − Haiti
WHO−2001 Adjustment Method

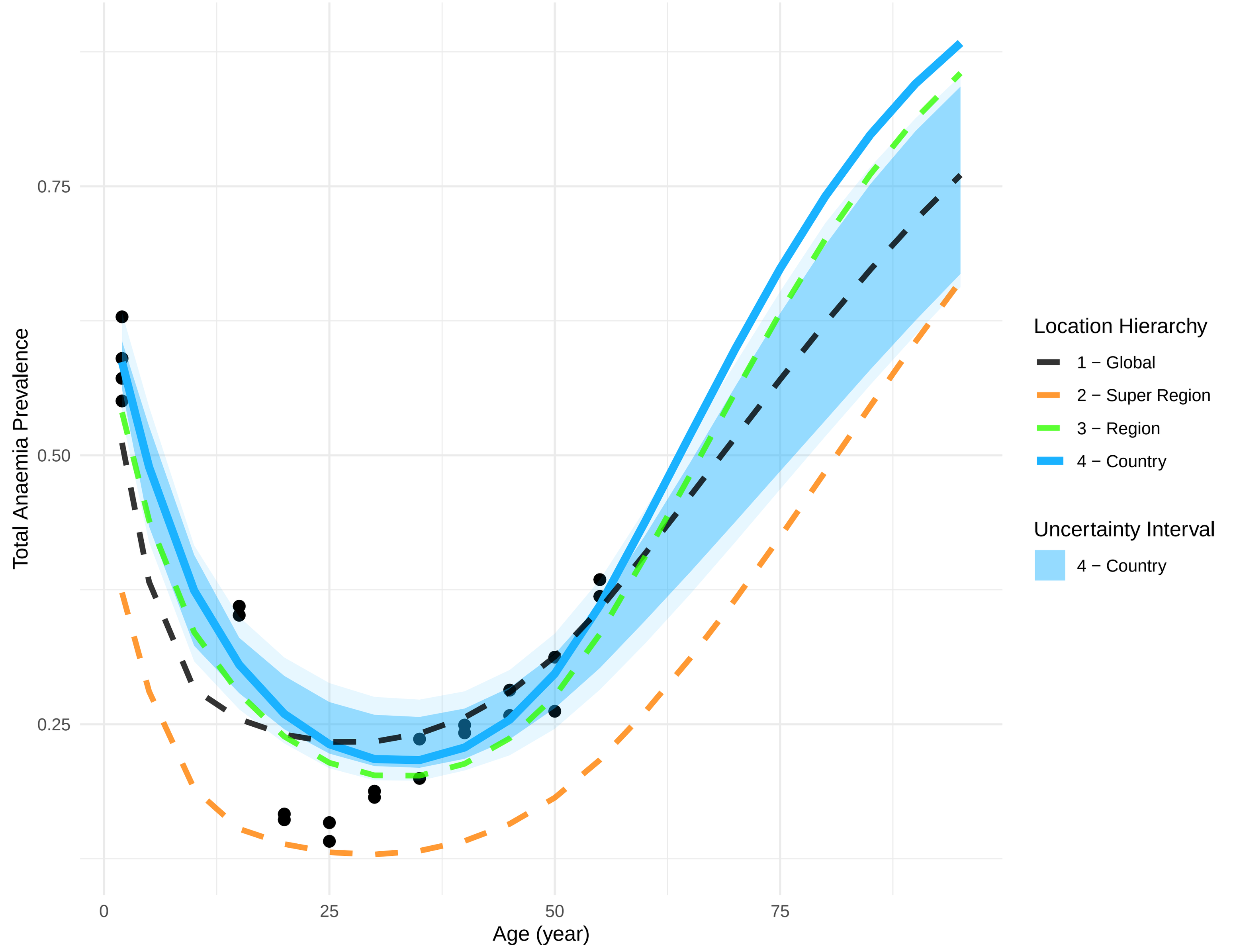

MR−BRT Age/Sex Fits − Males 2+ Years Old − Total Anaemia Prevalence − Central Latin America
WHO−2001 Adjustment Method

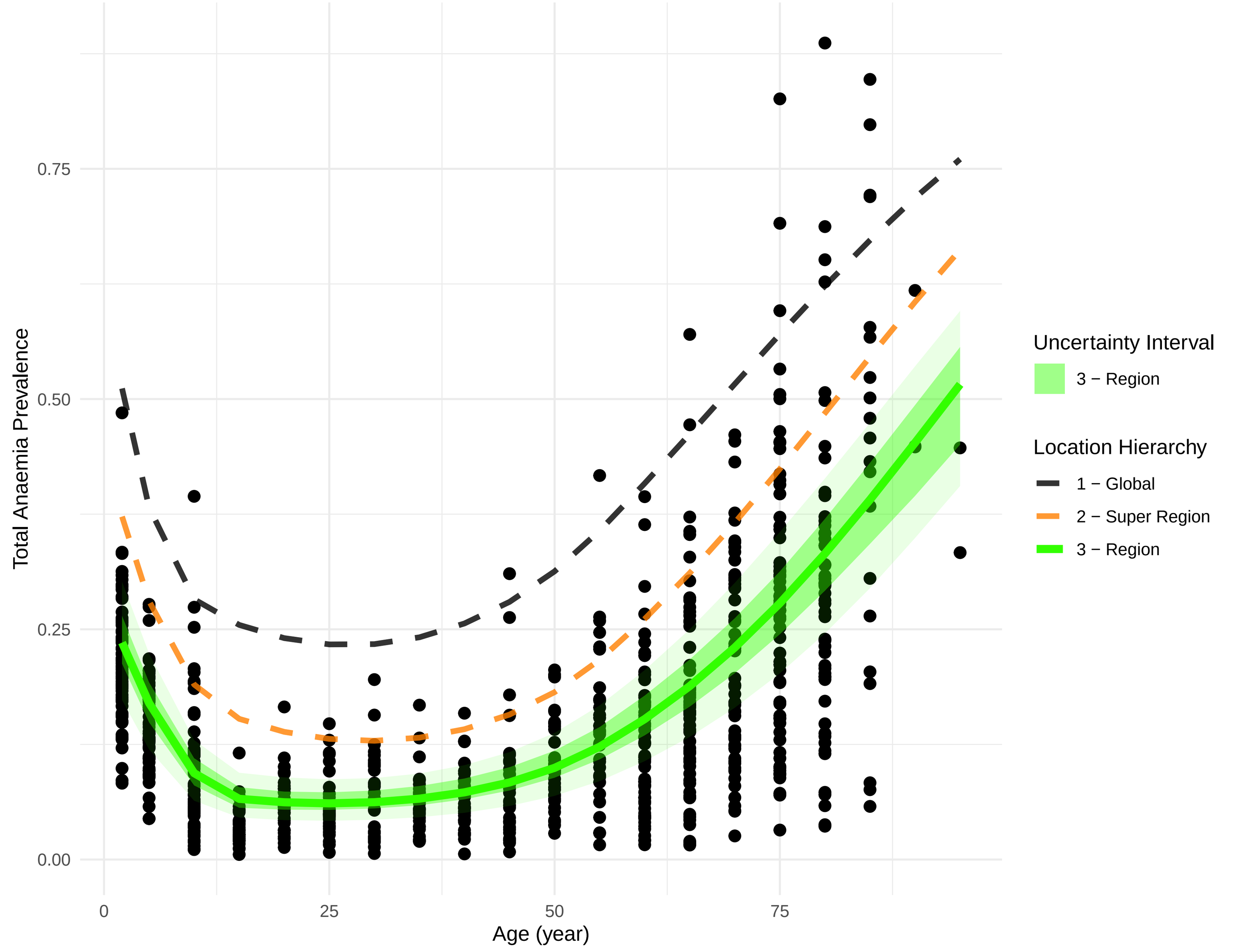

MR−BRT Age/Sex Fits − Males 2+ Years Old − Total Anaemia Prevalence − Guatemala
WHO−2001 Adjustment Method

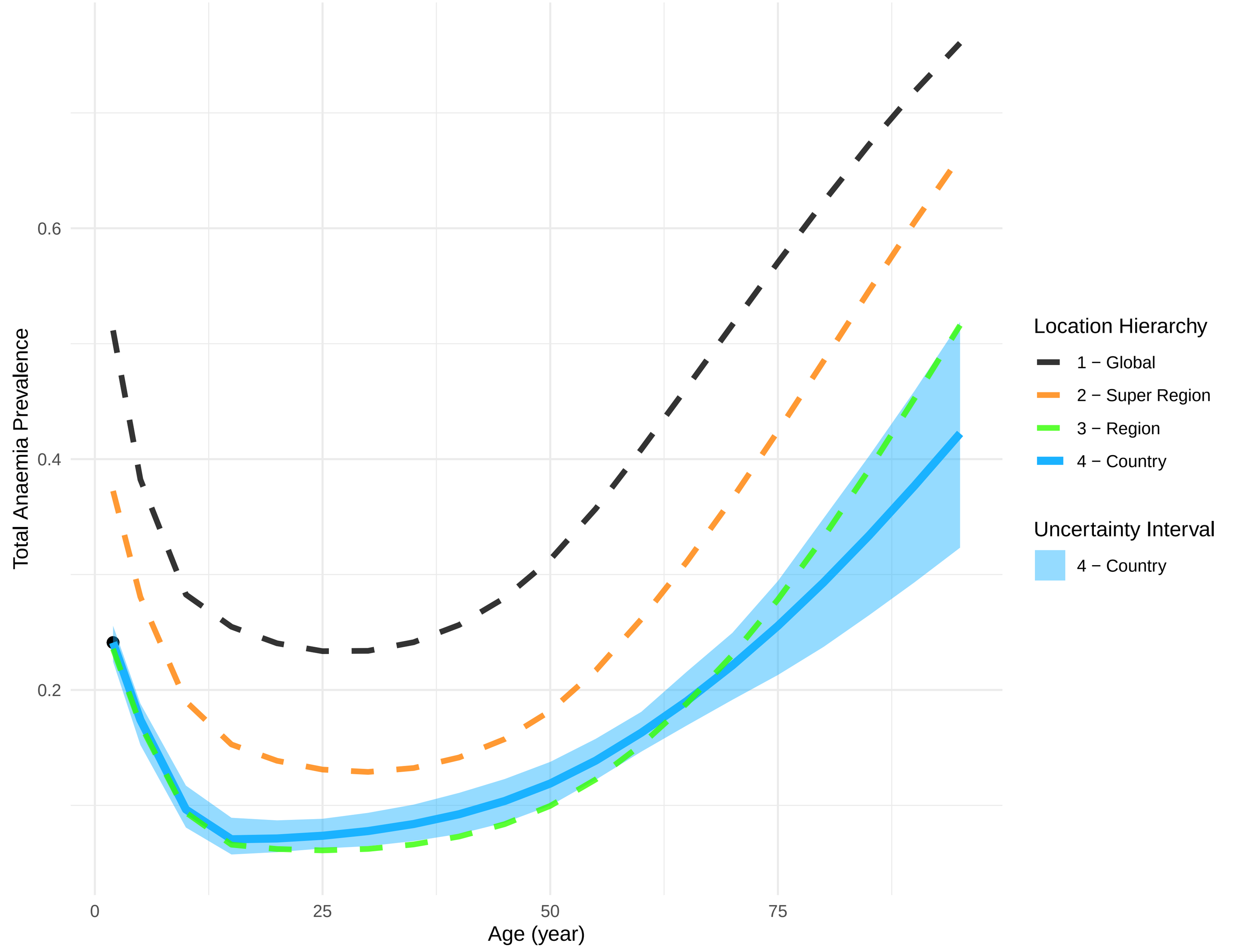

MR−BRT Age/Sex Fits − Males 2+ Years Old − Total Anaemia Prevalence − Honduras
WHO−2001 Adjustment Method

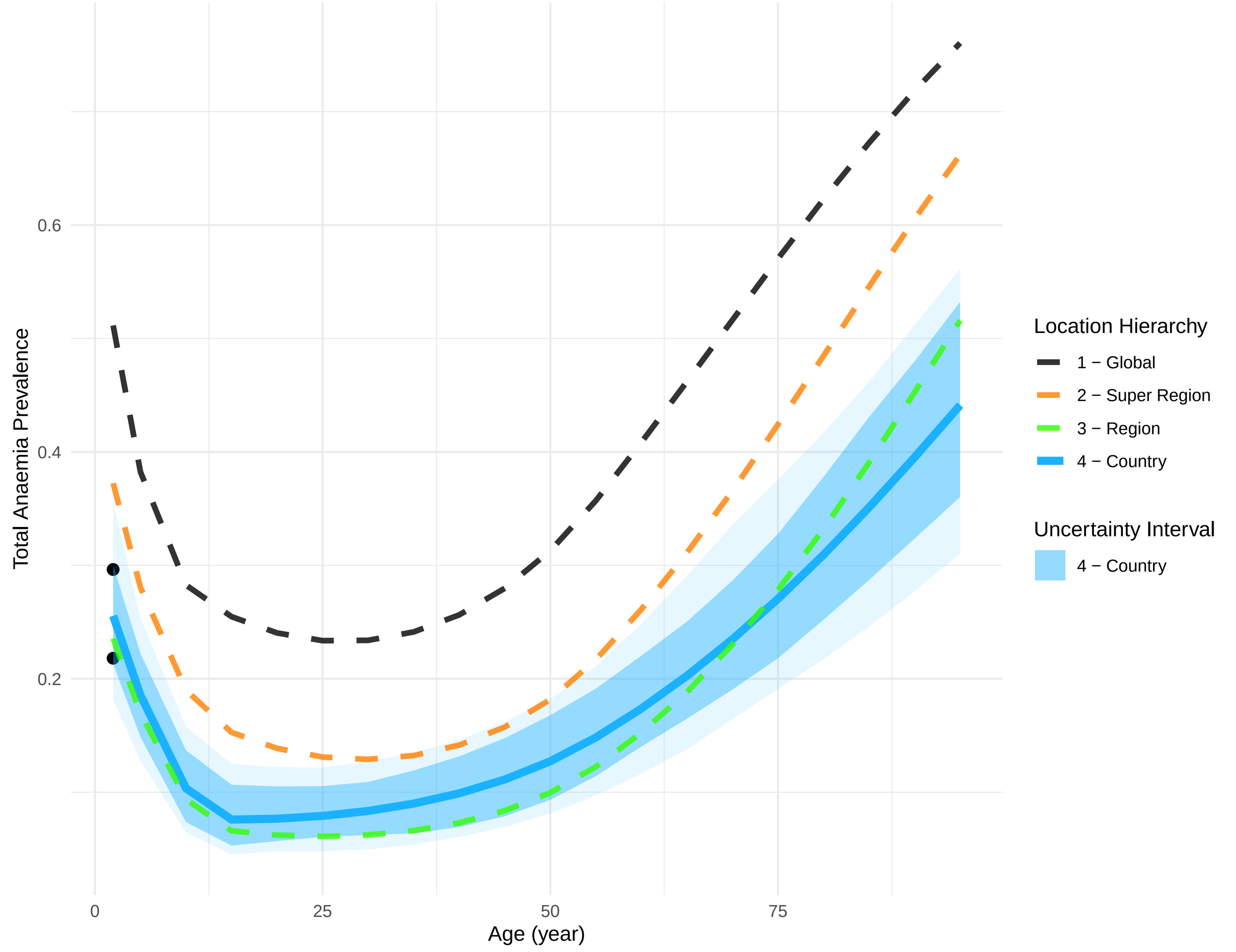

MR−BRT Age/Sex Fits − Males 2+ Years Old − Total Anaemia Prevalence − Mexico
WHO−2001 Adjustment Method

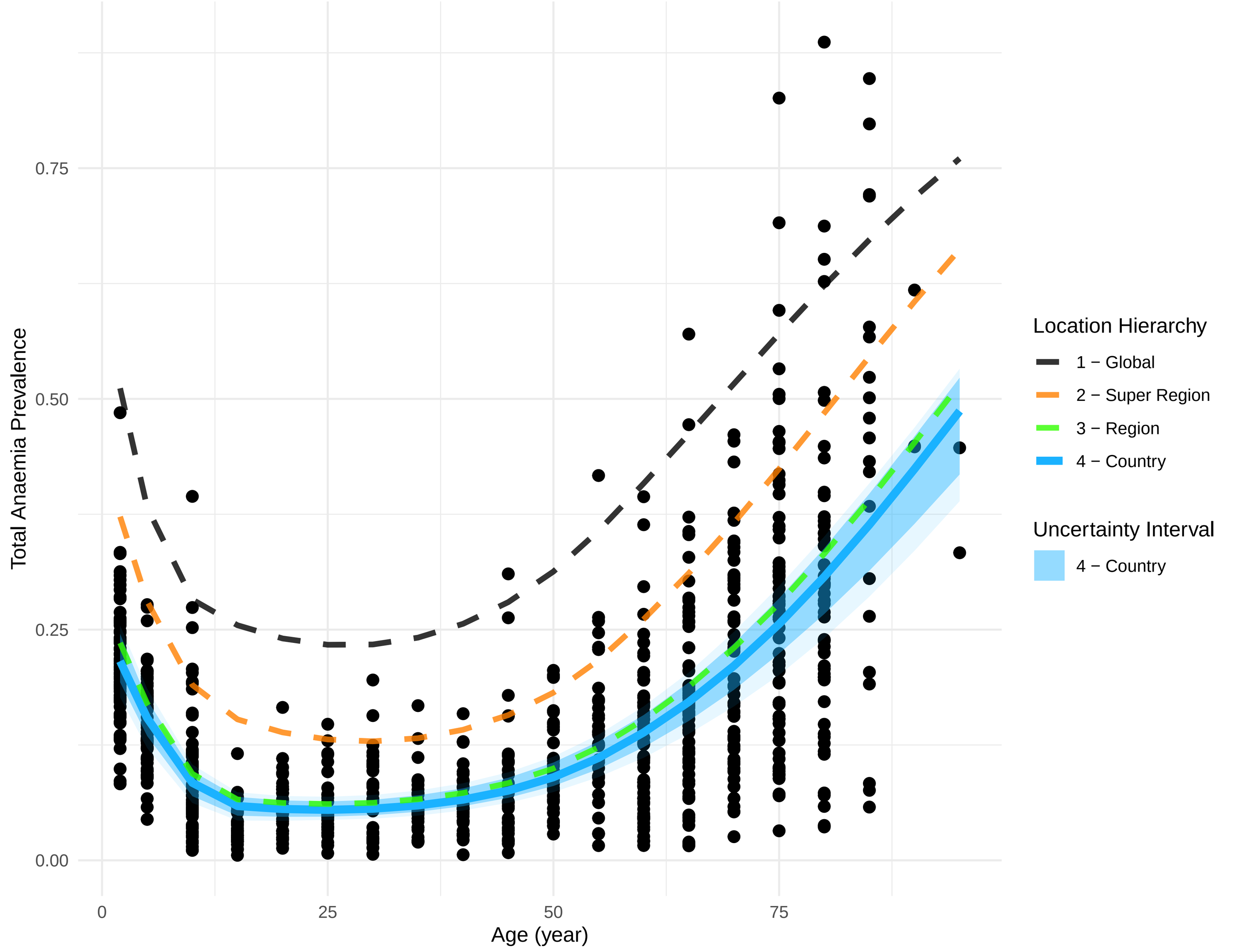

MR−BRT Age/Sex Fits − Males 2+ Years Old − Total Anaemia Prevalence − Aguascalientes
WHO−2001 Adjustment Method

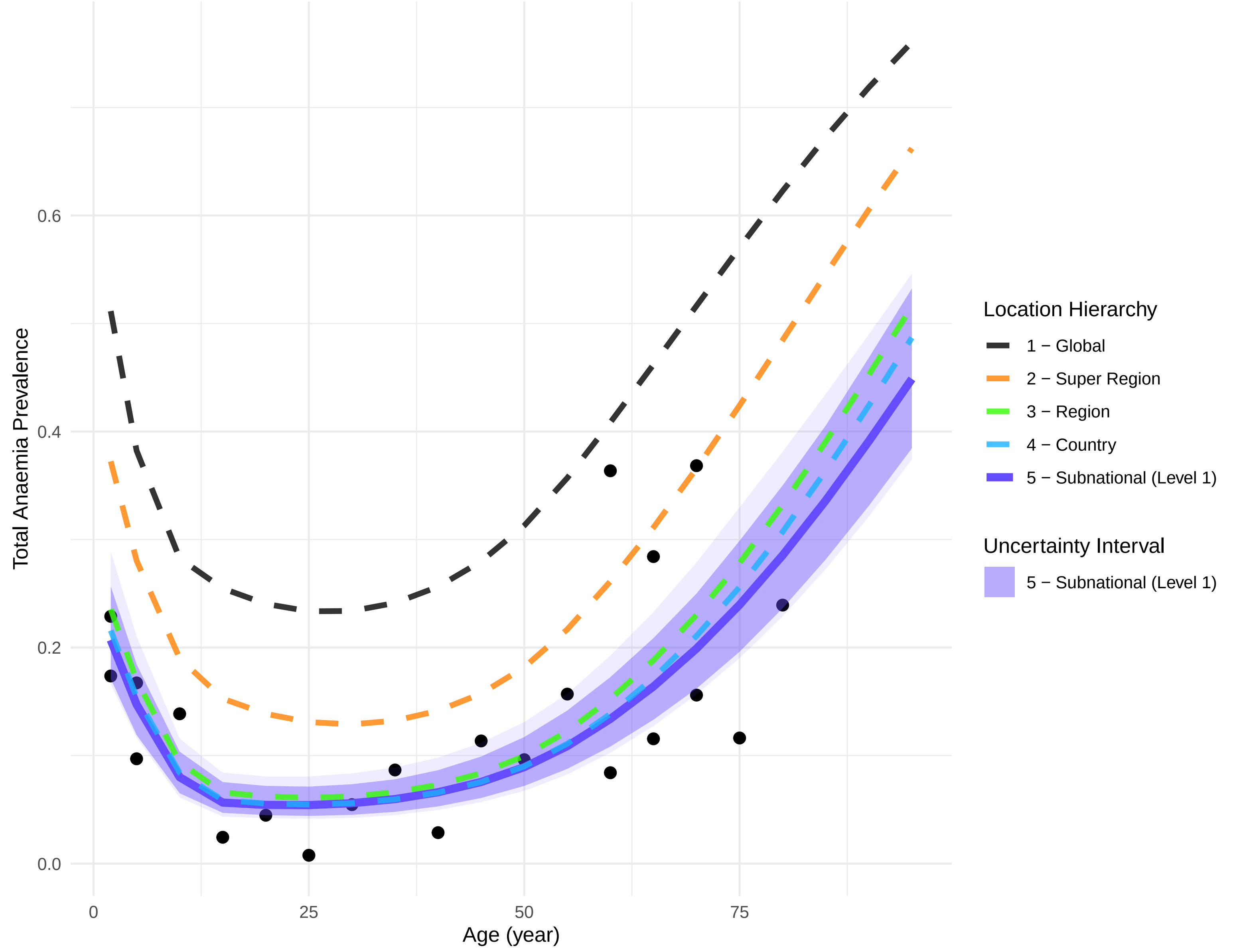

MR−BRT Age/Sex Fits − Males 2+ Years Old − Total Anaemia Prevalence − Baja California
WHO−2001 Adjustment Method

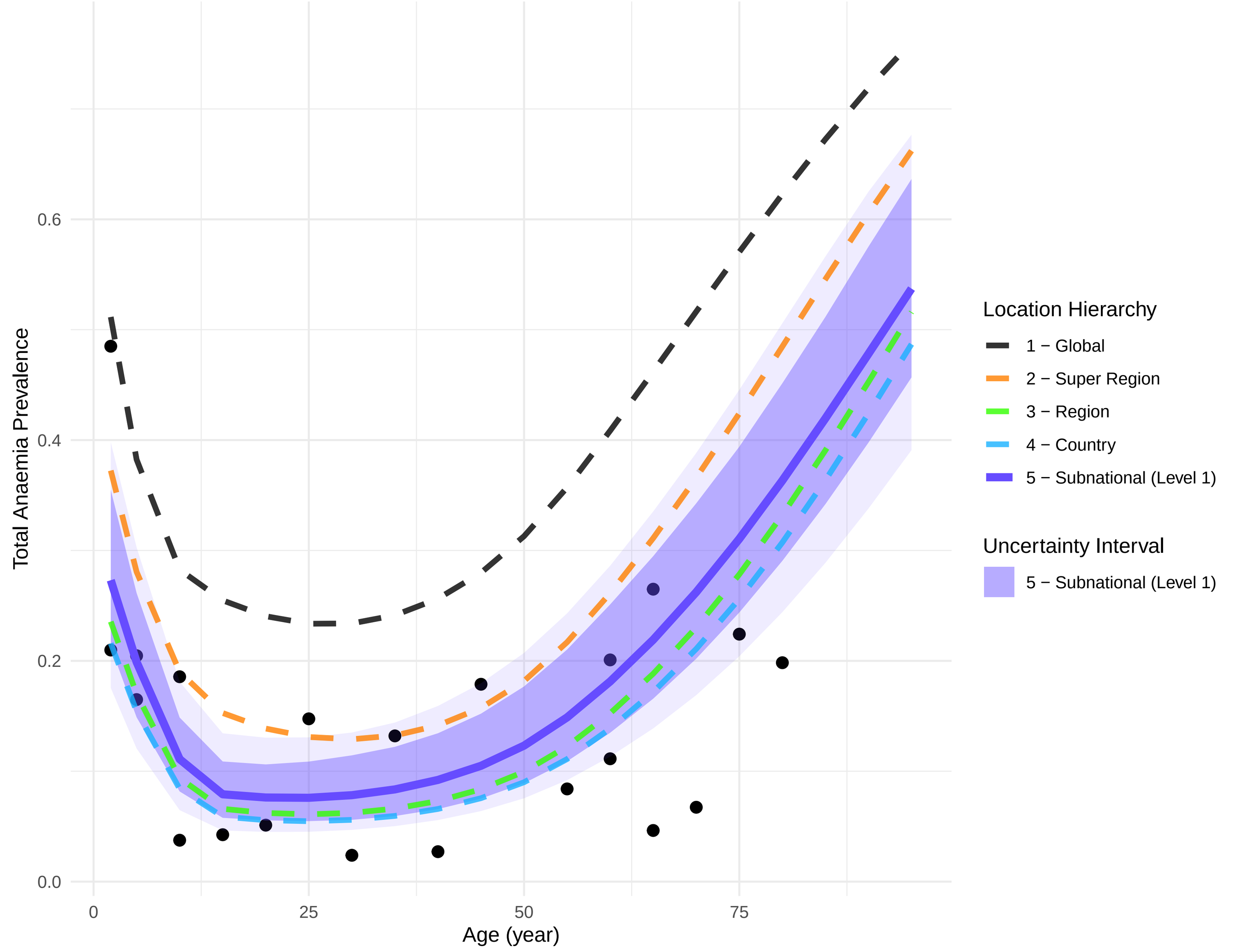

MR−BRT Age/Sex Fits − Males 2+ Years Old − Total Anaemia Prevalence − Baja California Sur
WHO−2001 Adjustment Method

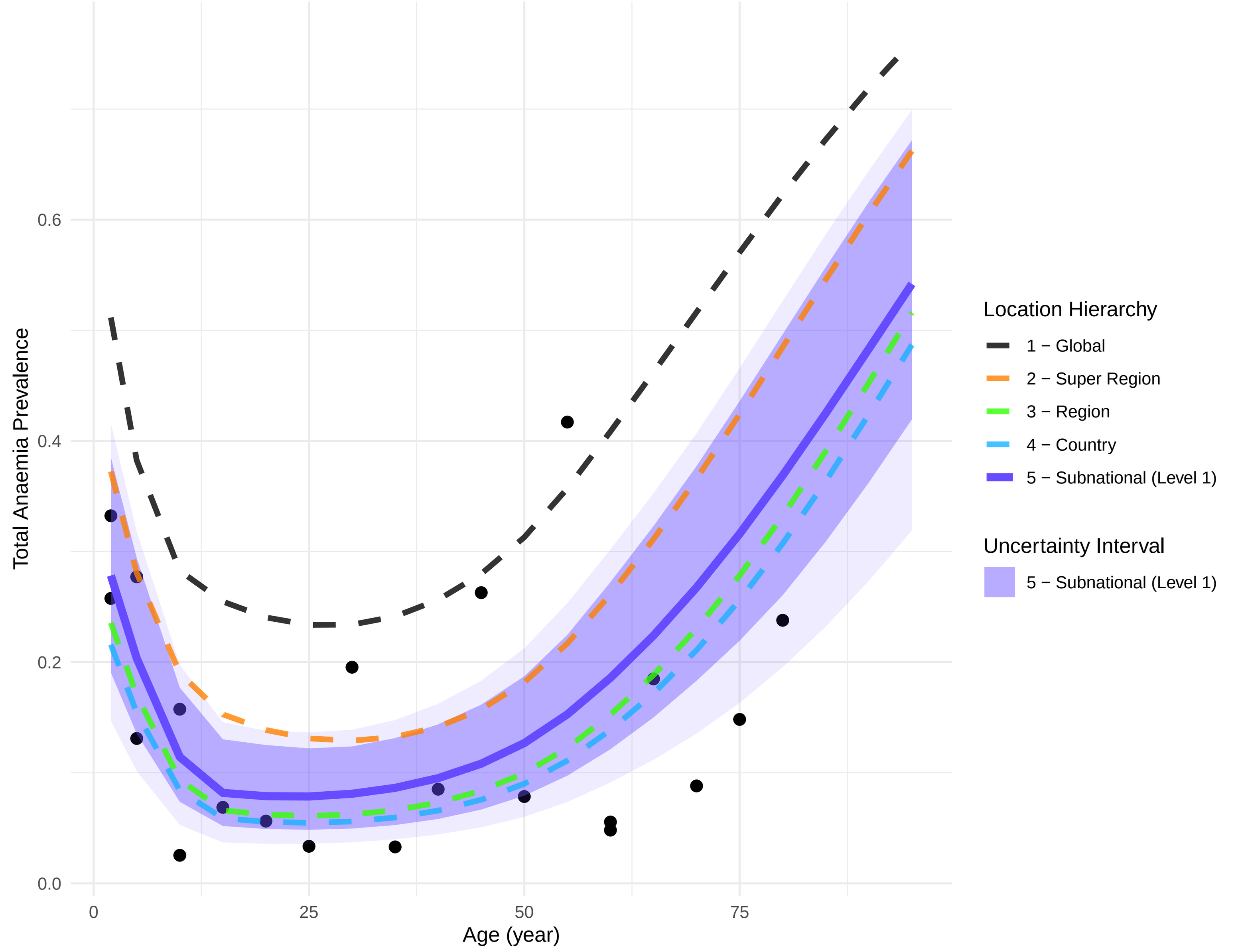

MR−BRT Age/Sex Fits − Males 2+ Years Old − Total Anaemia Prevalence − Campeche
WHO−2001 Adjustment Method

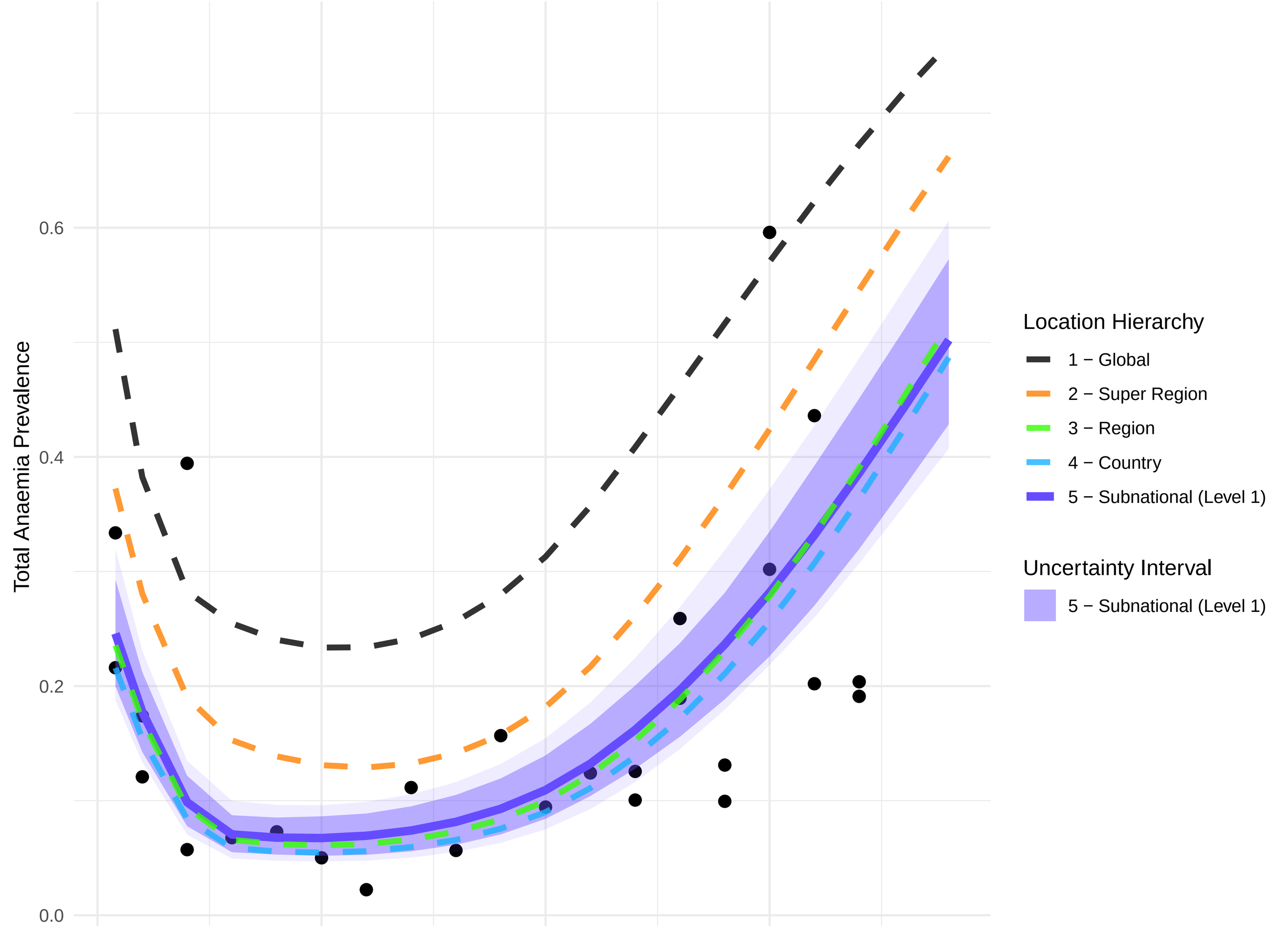

MR−BRT Age/Sex Fits − Males 2+ Years Old − Total Anaemia Prevalence − Chiapas
WHO−2001 Adjustment Method

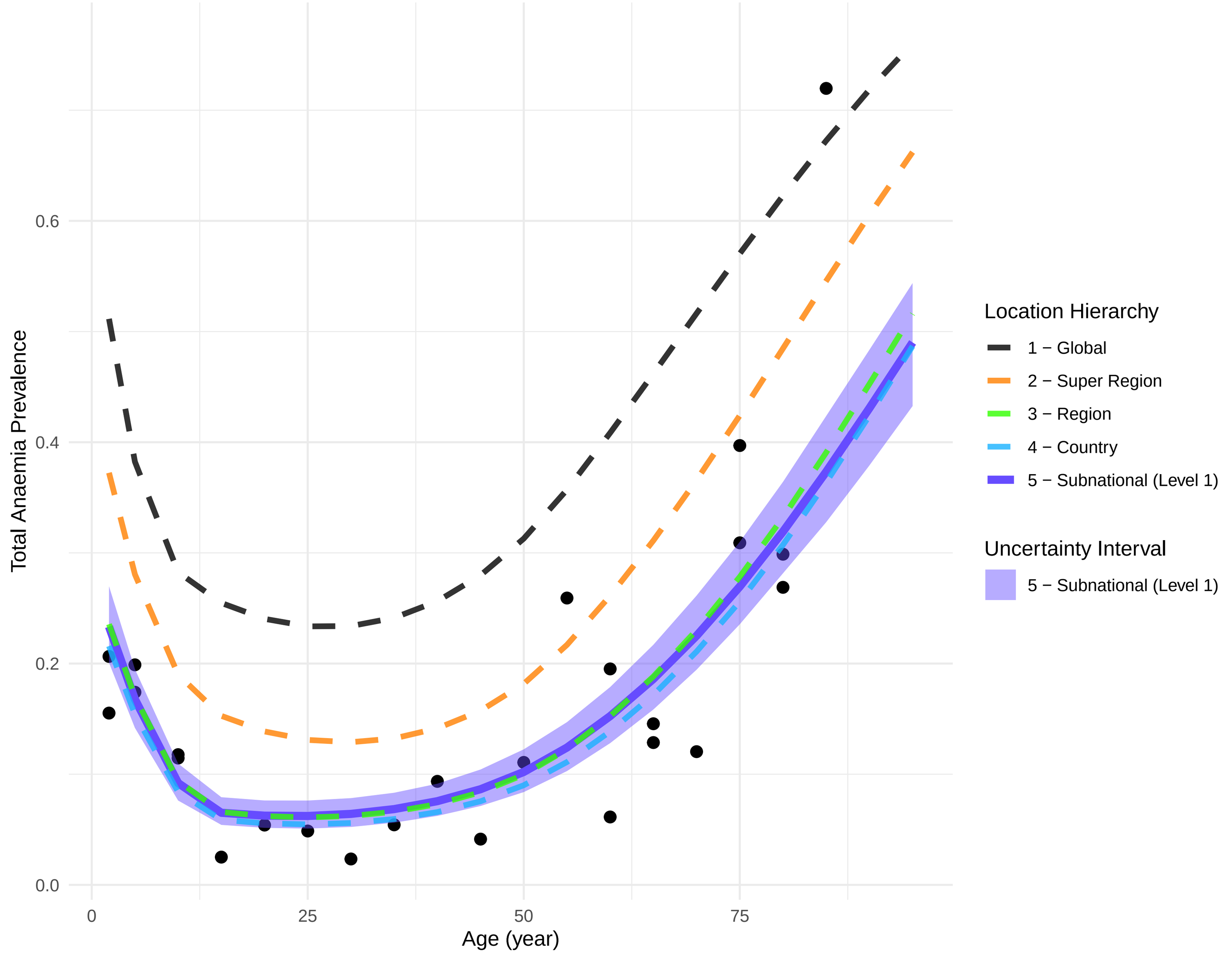

MR−BRT Age/Sex Fits − Males 2+ Years Old − Total Anaemia Prevalence − Chihuahua
WHO−2001 Adjustment Method

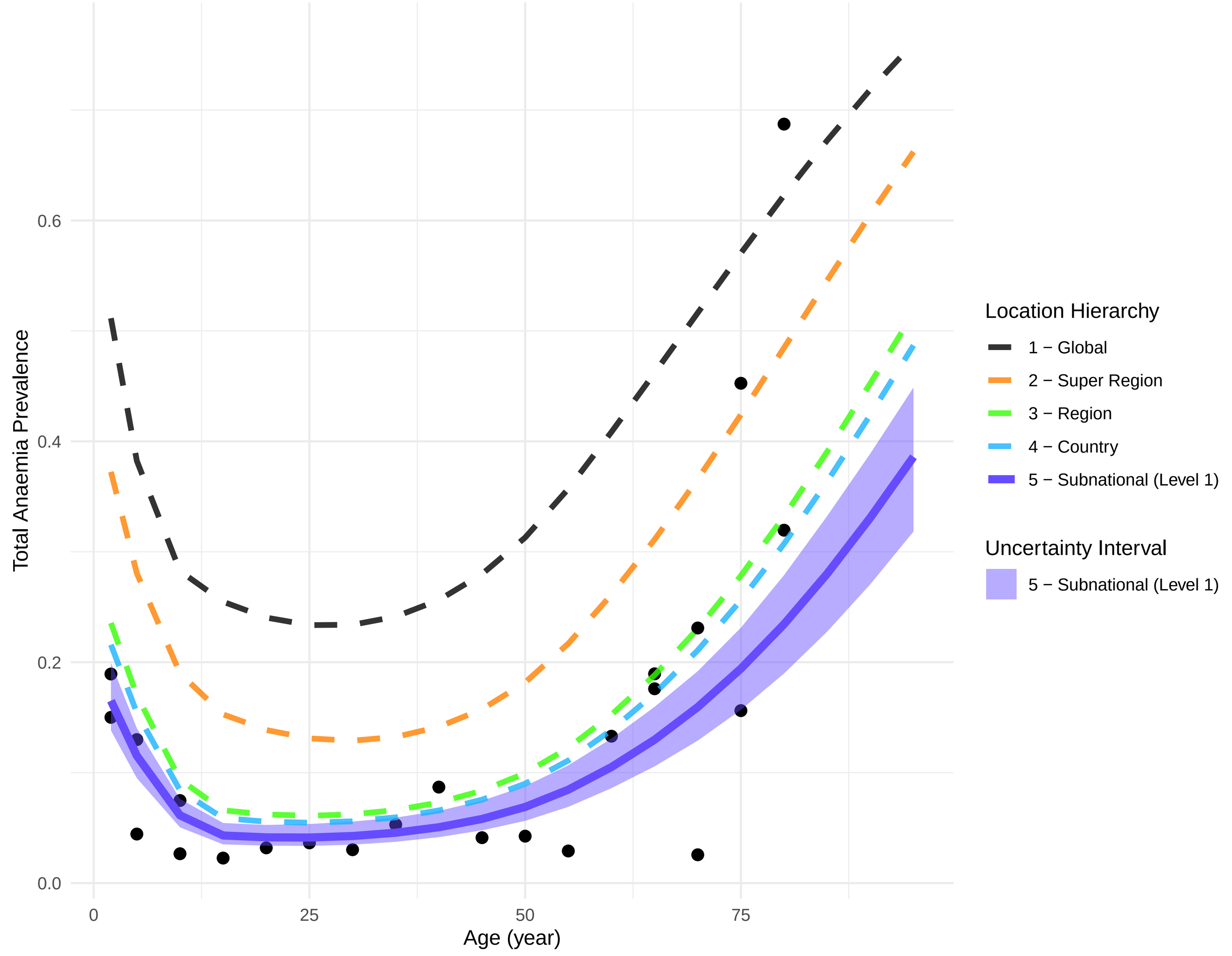

MR−BRT Age/Sex Fits − Males 2+ Years Old − Total Anaemia Prevalence − Coahuila
WHO−2001 Adjustment Method

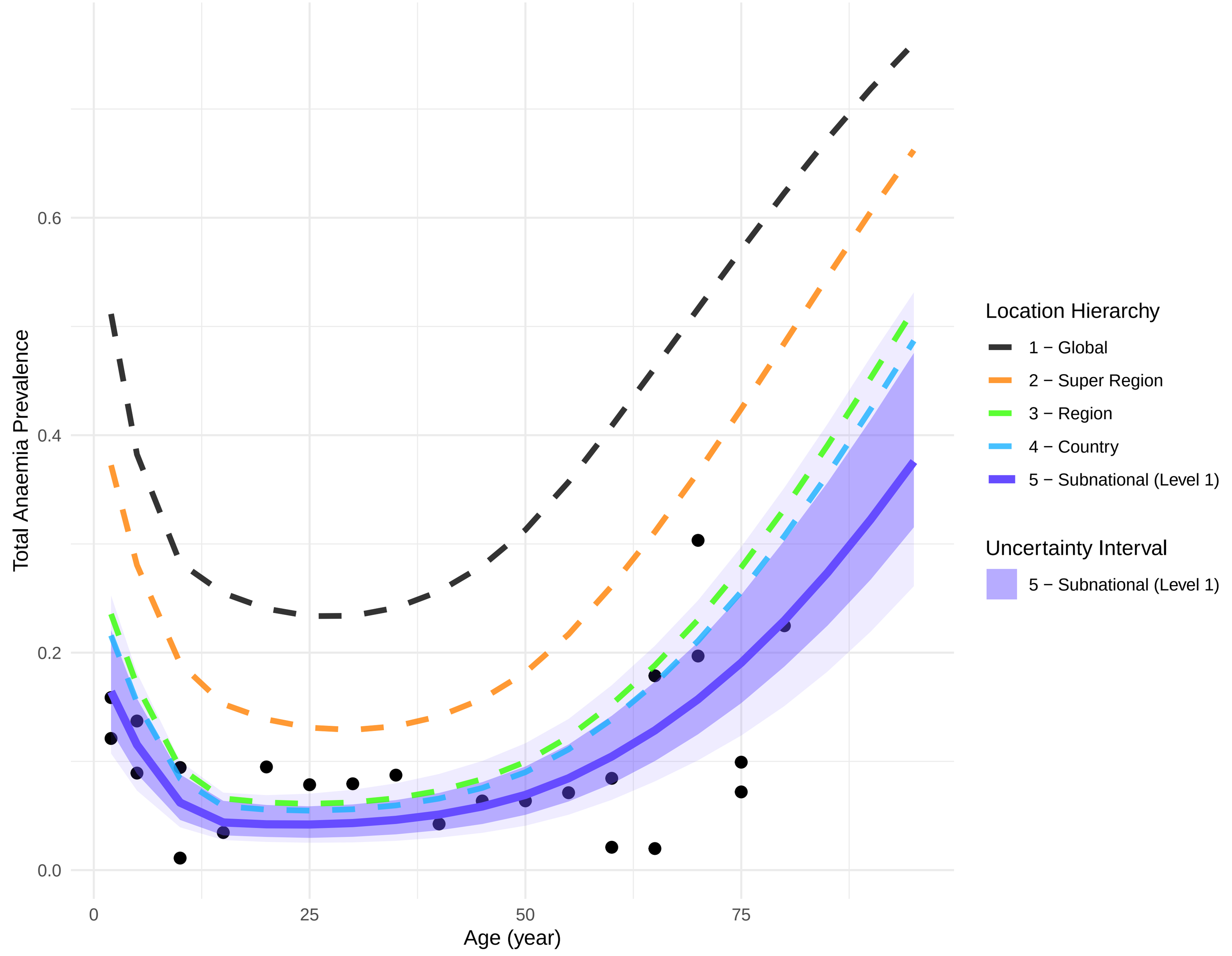

MR−BRT Age/Sex Fits − Males 2+ Years Old − Total Anaemia Prevalence − Colima
WHO−2001 Adjustment Method

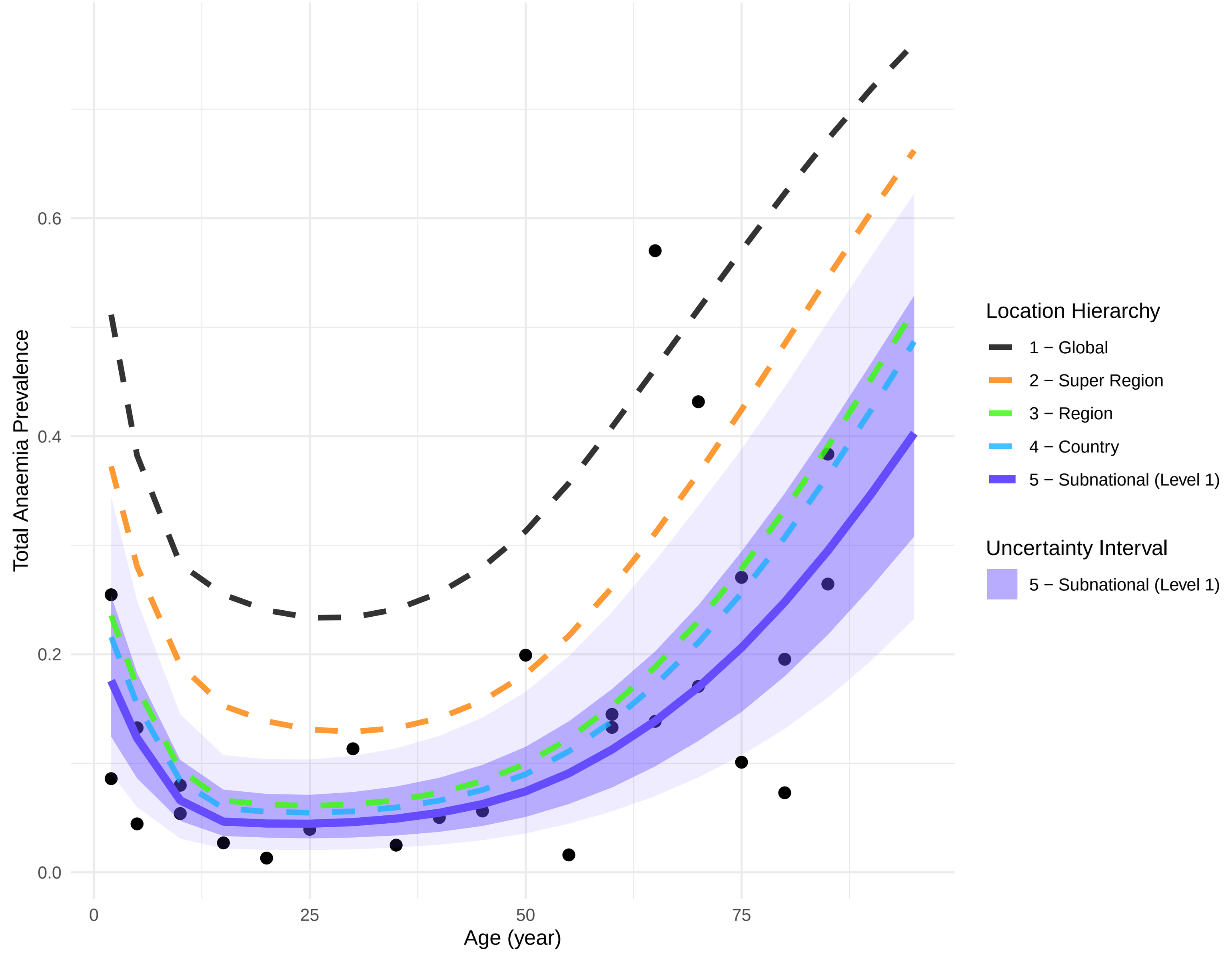

MR−BRT Age/Sex Fits − Males 2+ Years Old − Total Anaemia Prevalence − Durango
WHO−2001 Adjustment Method

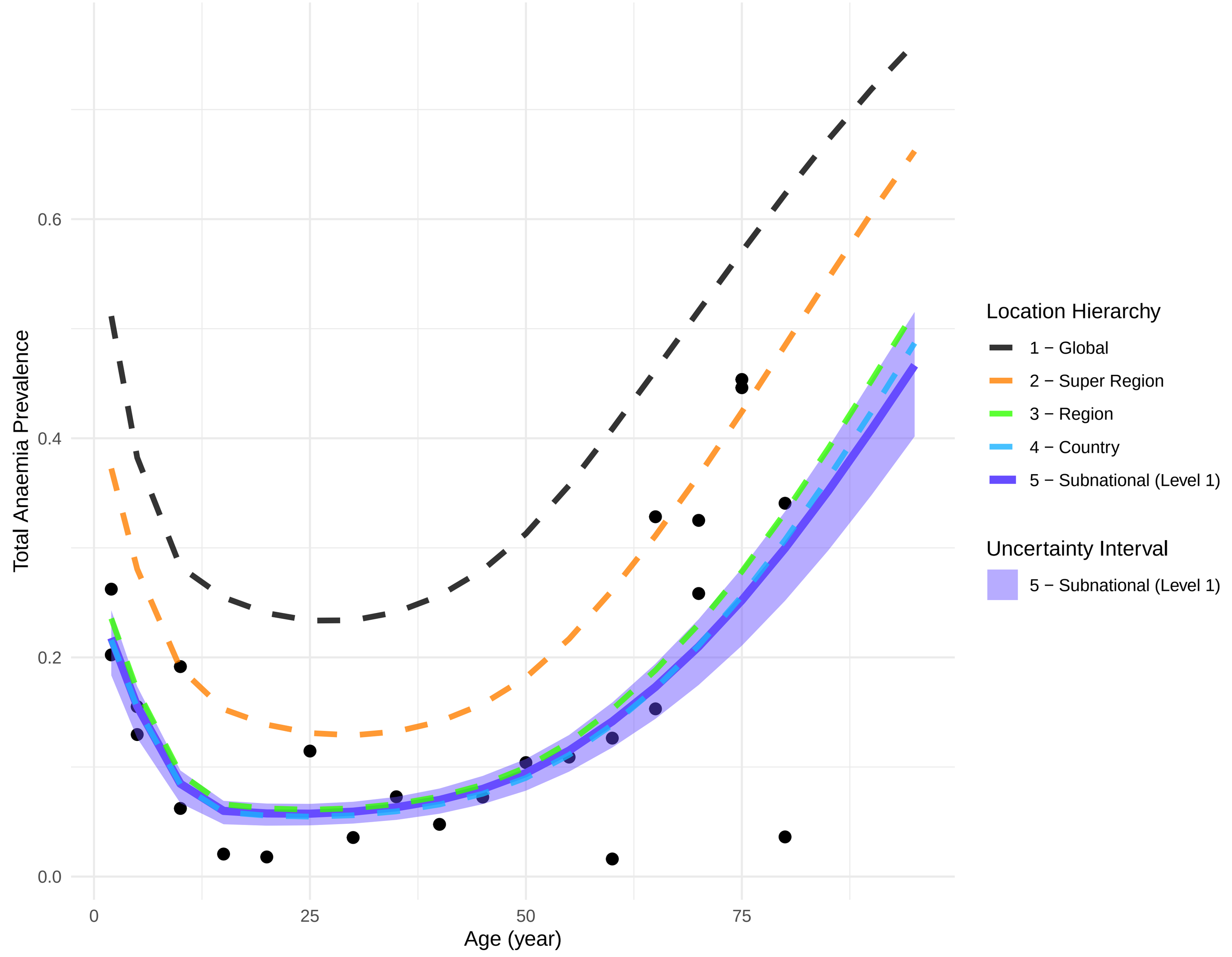

MR−BRT Age/Sex Fits − Males 2+ Years Old − Total Anaemia Prevalence − Guanajuato
WHO−2001 Adjustment Method

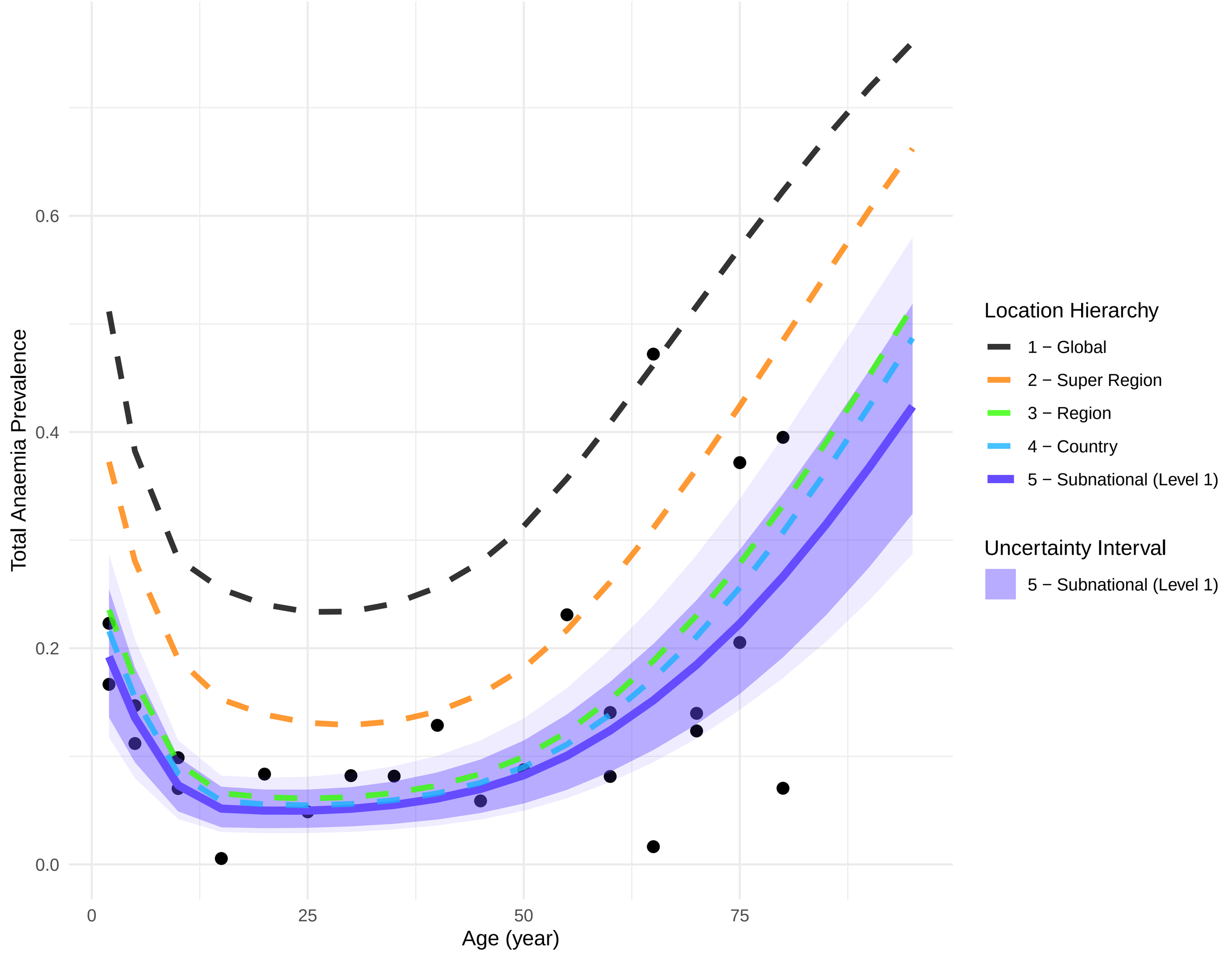

MR−BRT Age/Sex Fits − Males 2+ Years Old − Total Anaemia Prevalence − Guerrero
WHO−2001 Adjustment Method

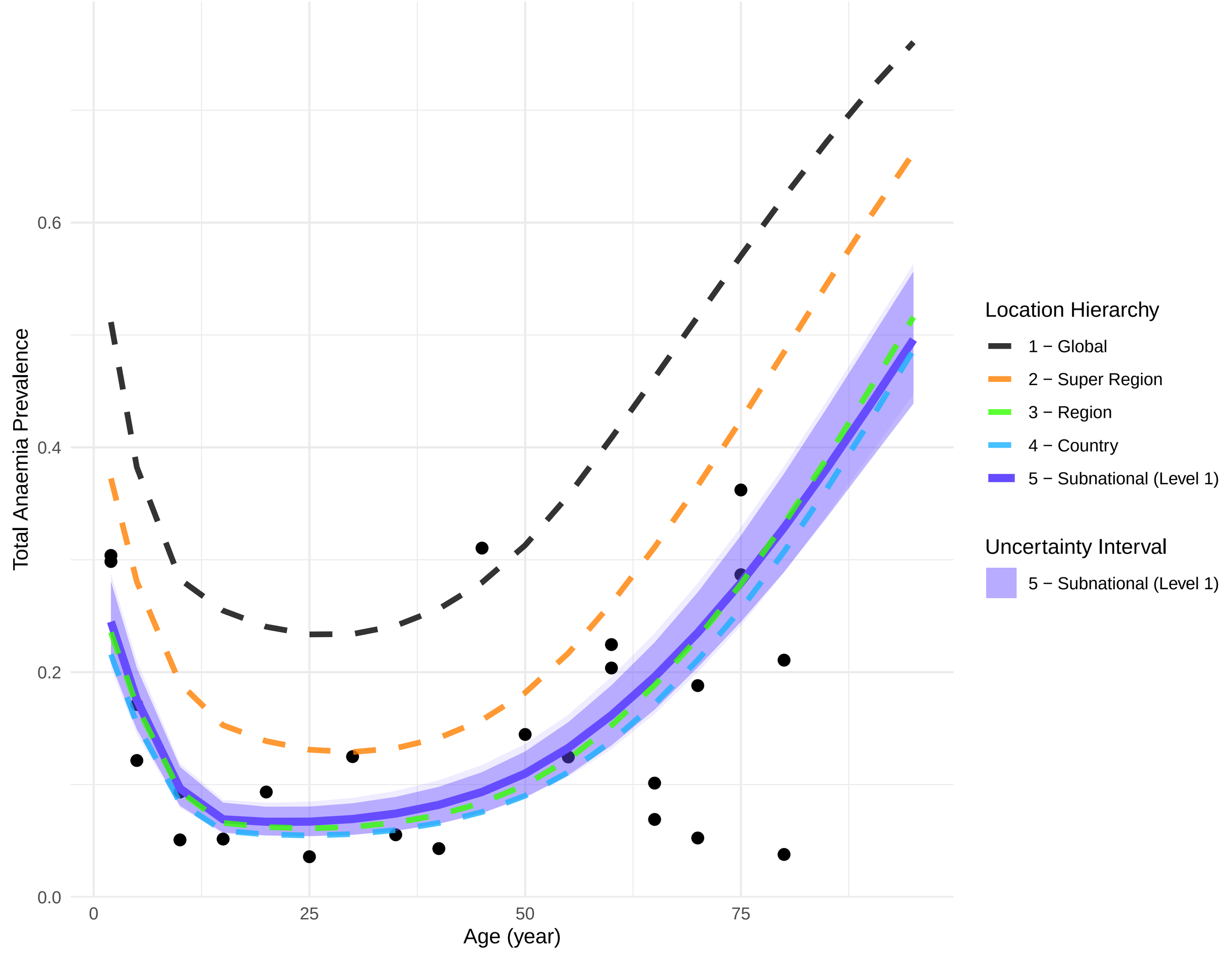

MR−BRT Age/Sex Fits − Males 2+ Years Old − Total Anaemia Prevalence − Hidalgo
WHO−2001 Adjustment Method

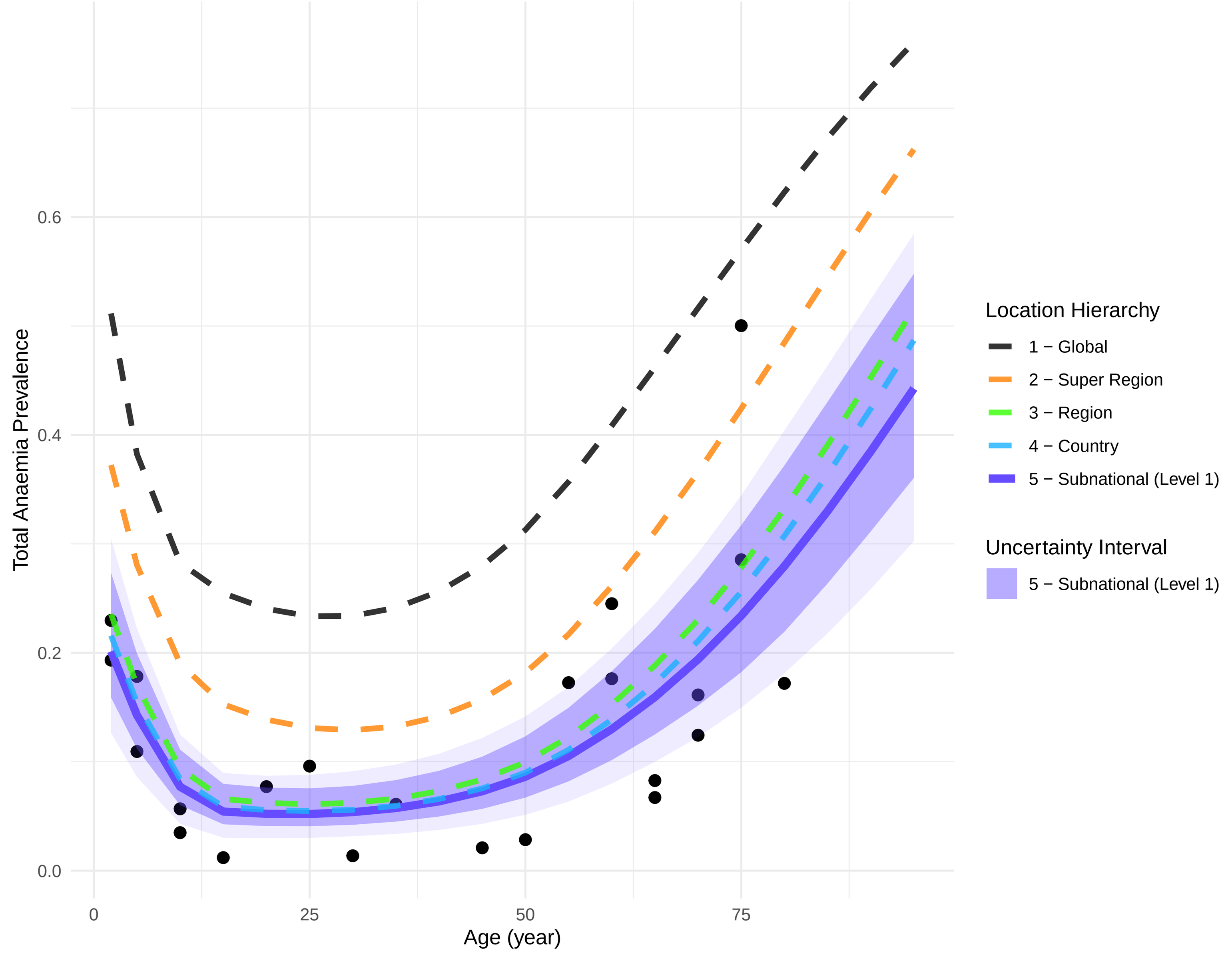

MR−BRT Age/Sex Fits − Males 2+ Years Old − Total Anaemia Prevalence − Jalisco
WHO−2001 Adjustment Method

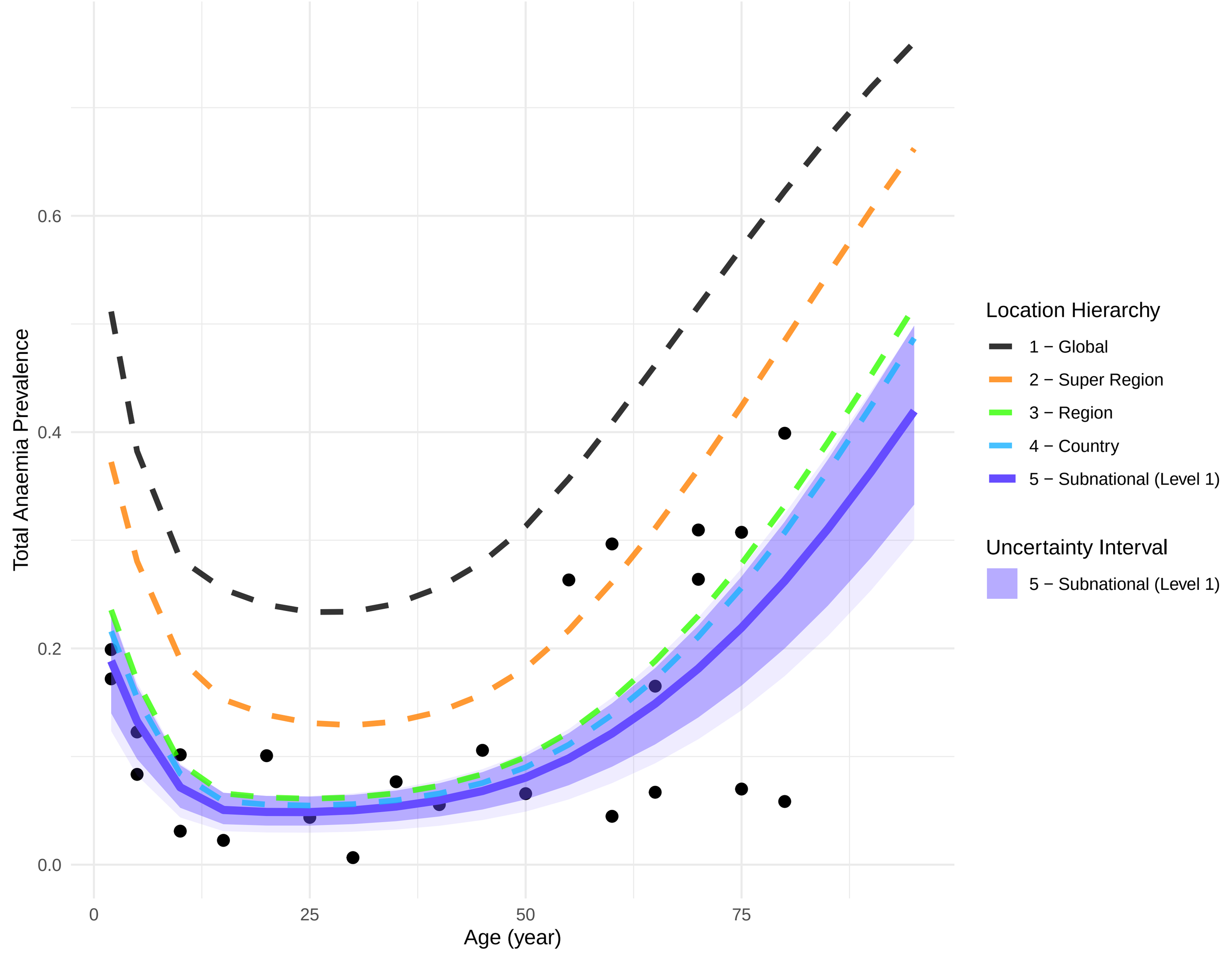

MR−BRT Age/Sex Fits − Males 2+ Years Old − Total Anaemia Prevalence − México
WHO−2001 Adjustment Method

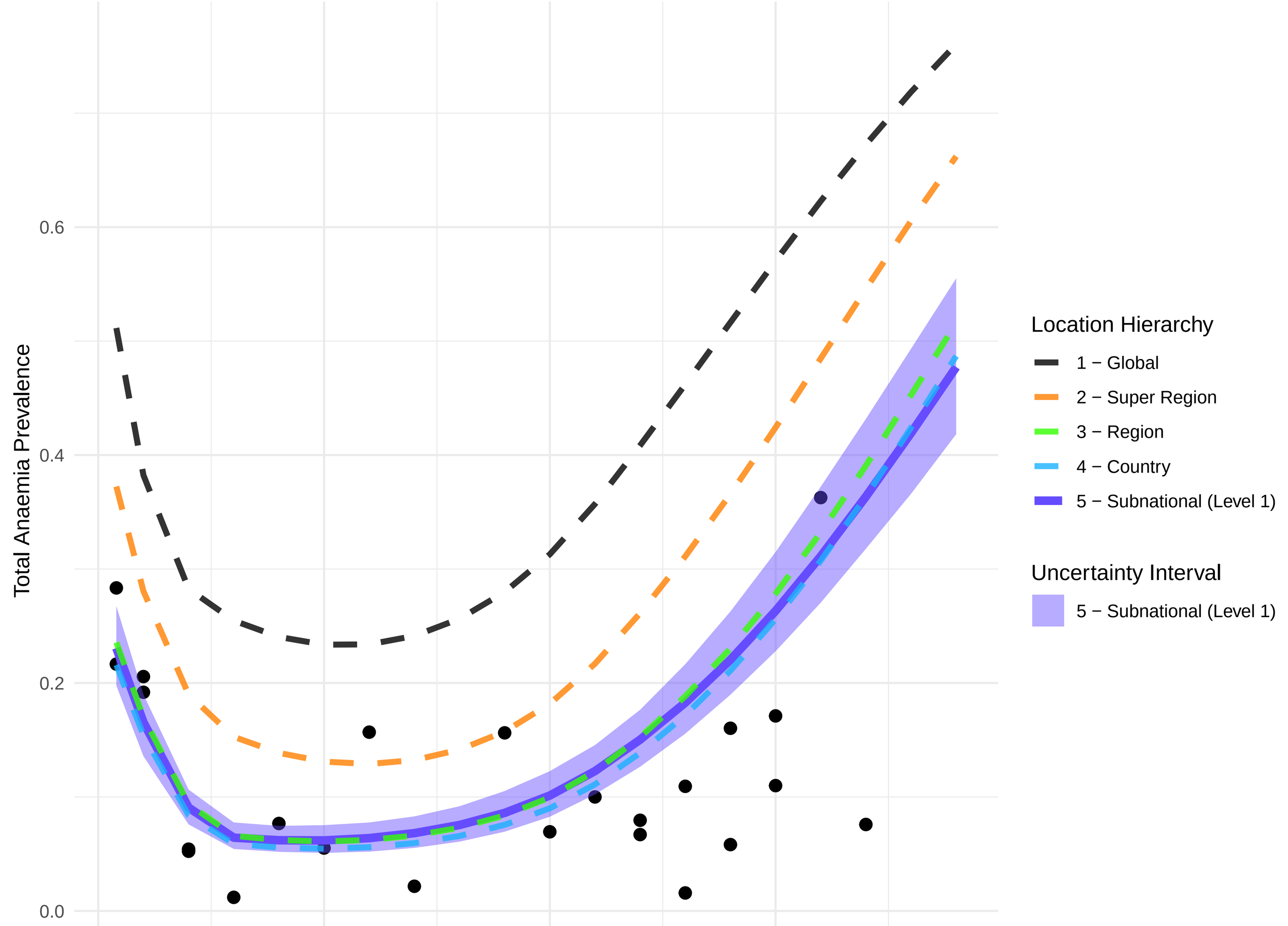

MR−BRT Age/Sex Fits − Males 2+ Years Old − Total Anaemia Prevalence − Mexico City
WHO−2001 Adjustment Method

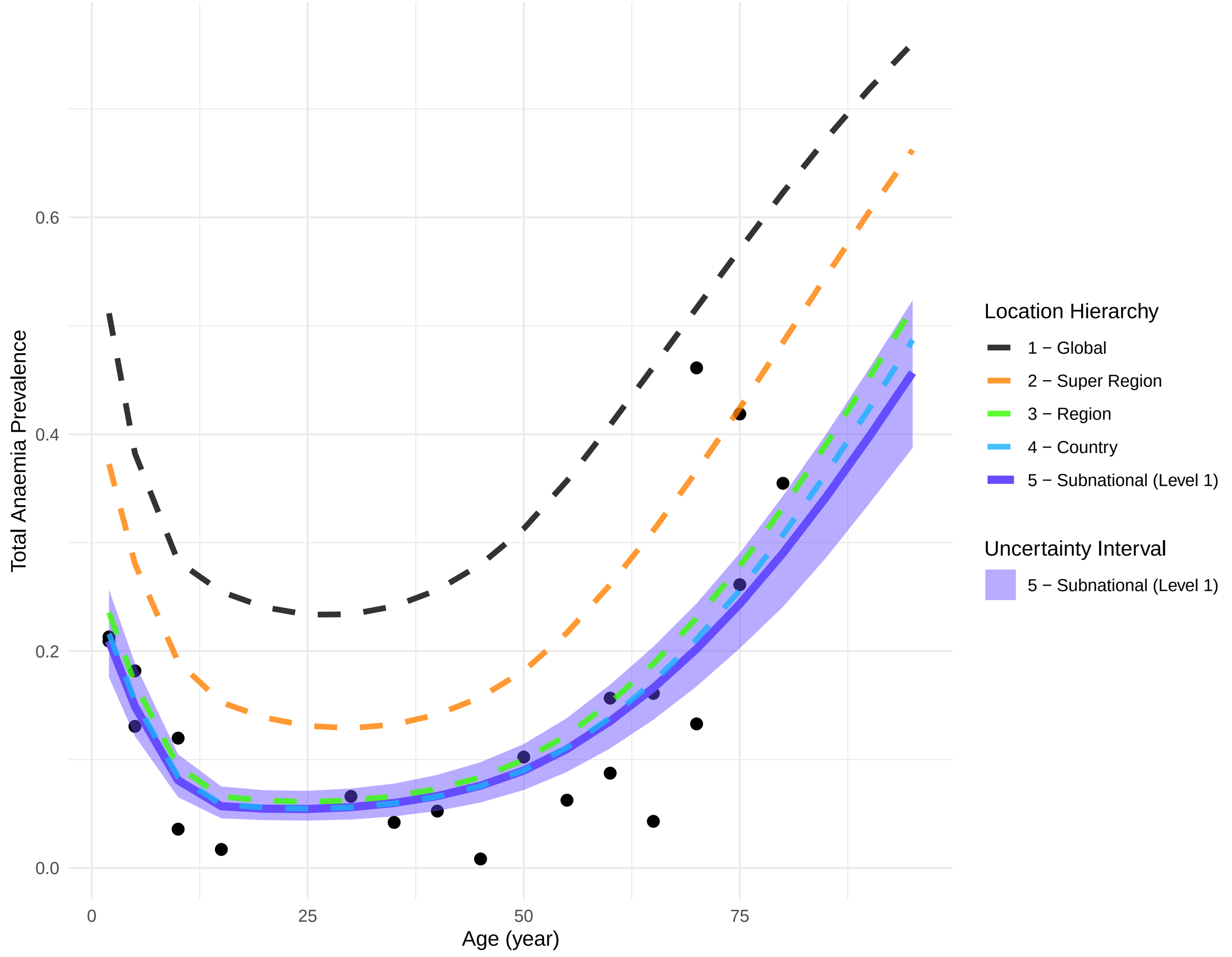

MR−BRT Age/Sex Fits − Males 2+ Years Old − Total Anaemia Prevalence − Michoacán de Ocampo
WHO−2001 Adjustment Method

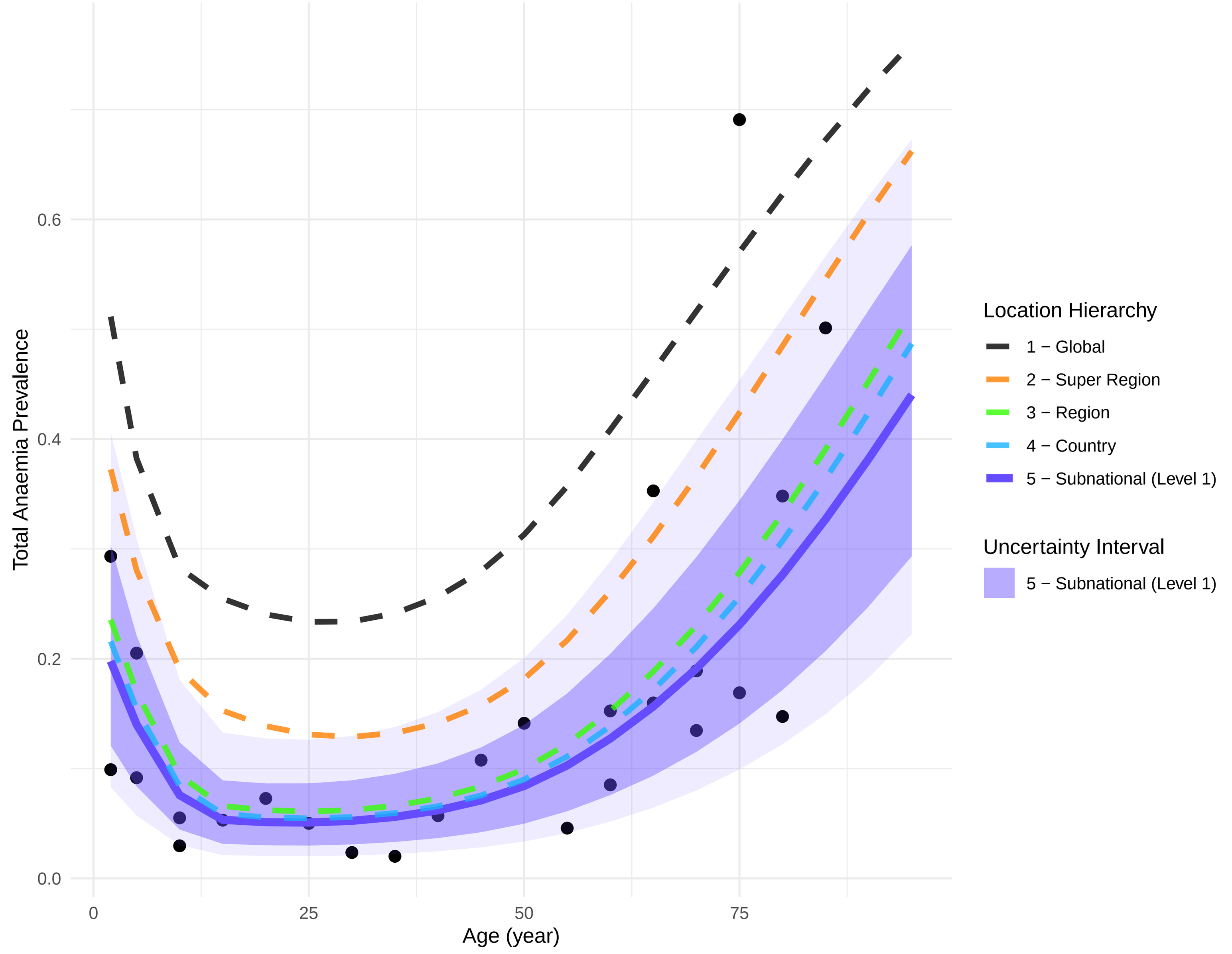

MR−BRT Age/Sex Fits − Males 2+ Years Old − Total Anaemia Prevalence − Morelos
WHO−2001 Adjustment Method

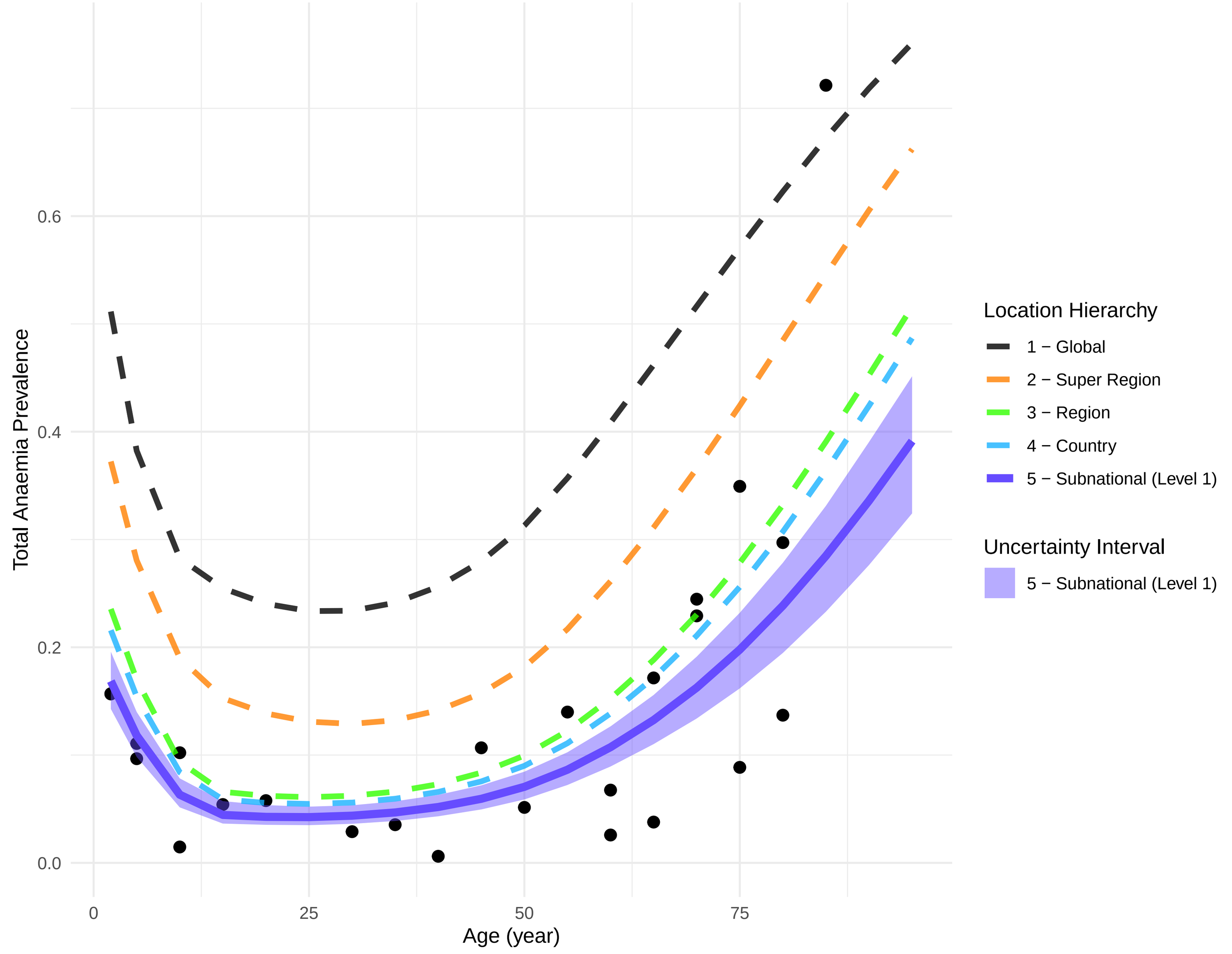

MR−BRT Age/Sex Fits − Males 2+ Years Old − Total Anaemia Prevalence − Nayarit
WHO−2001 Adjustment Method

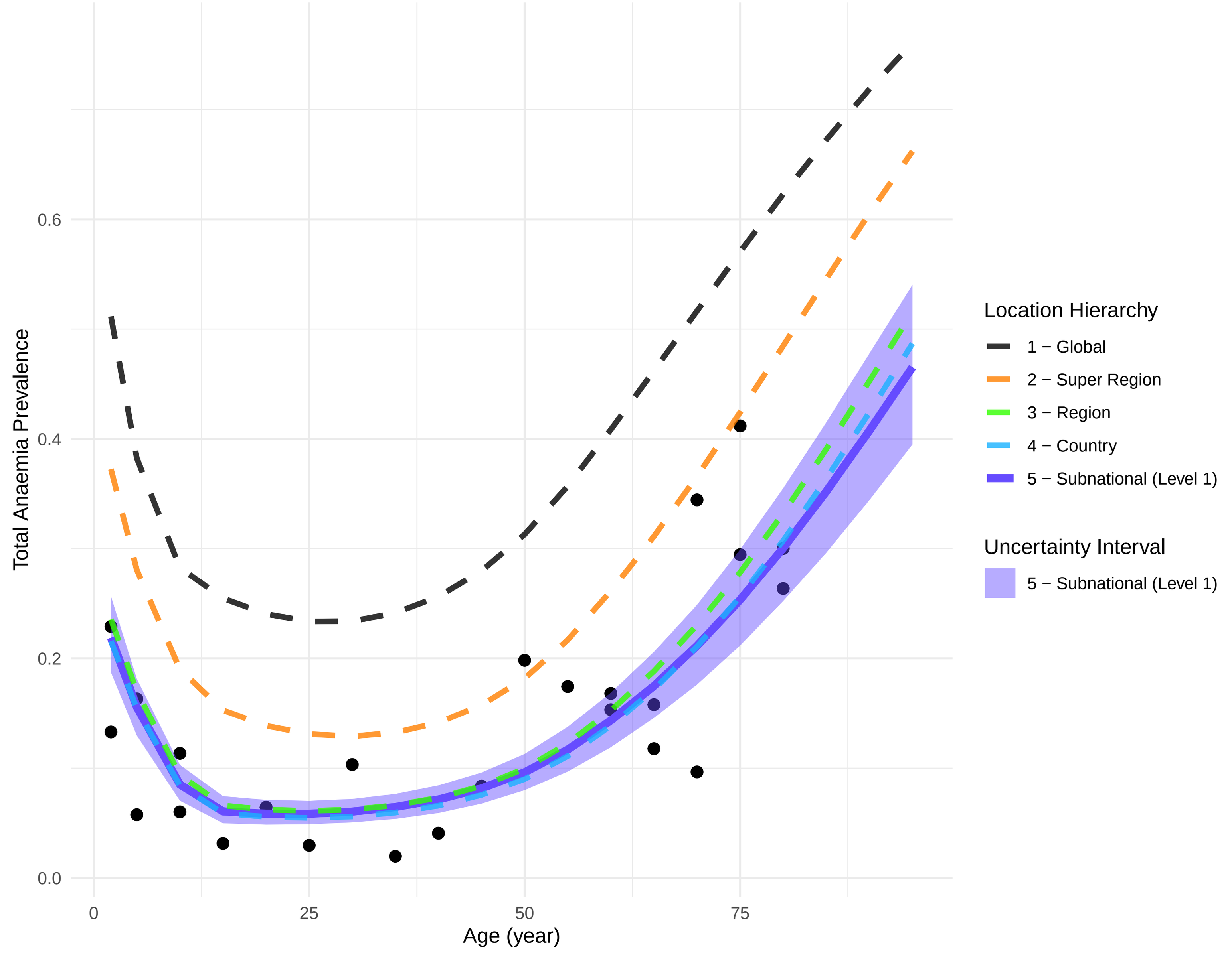

MR−BRT Age/Sex Fits − Males 2+ Years Old − Total Anaemia Prevalence − Nuevo León
WHO−2001 Adjustment Method

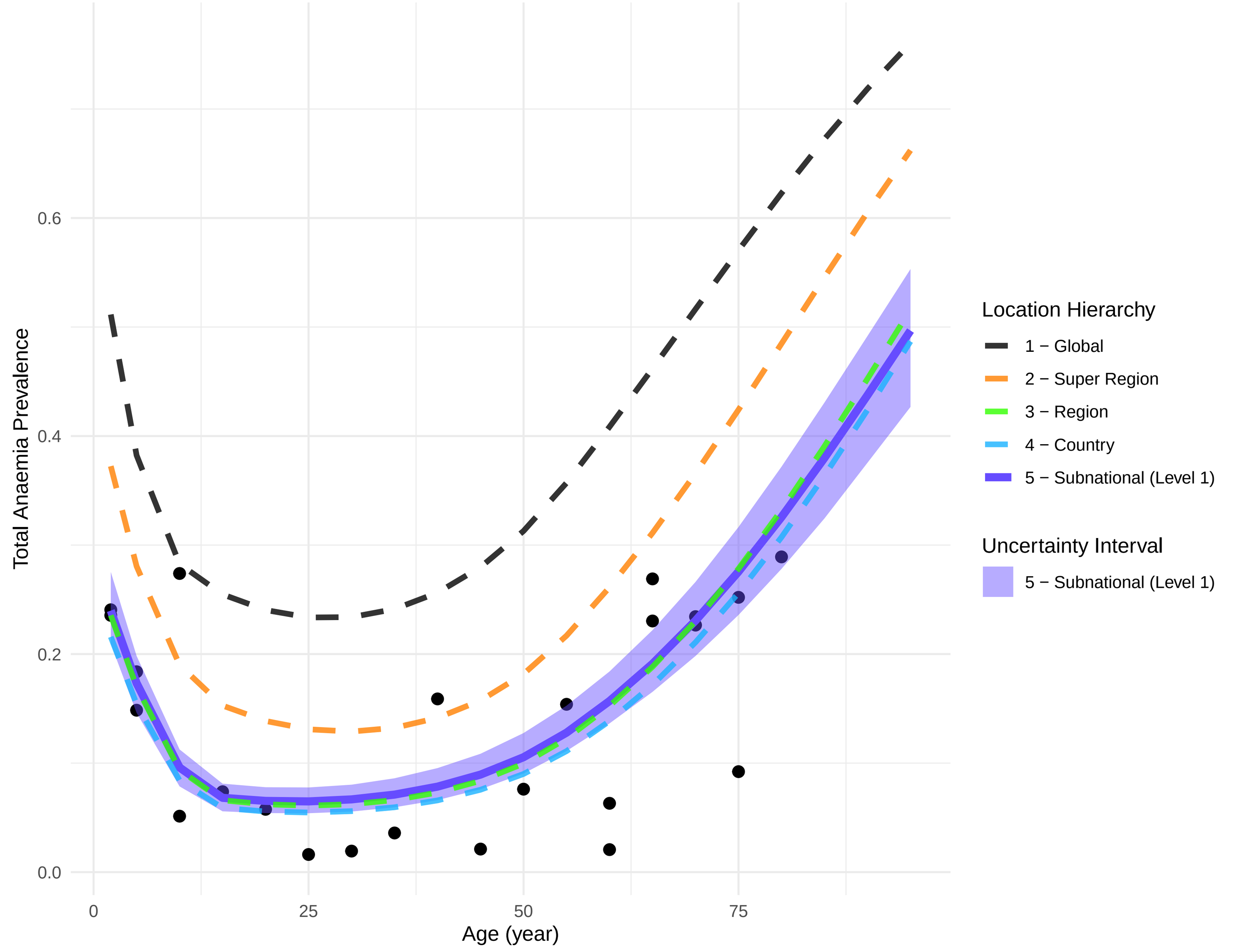

MR−BRT Age/Sex Fits − Males 2+ Years Old − Total Anaemia Prevalence − Oaxaca
WHO−2001 Adjustment Method

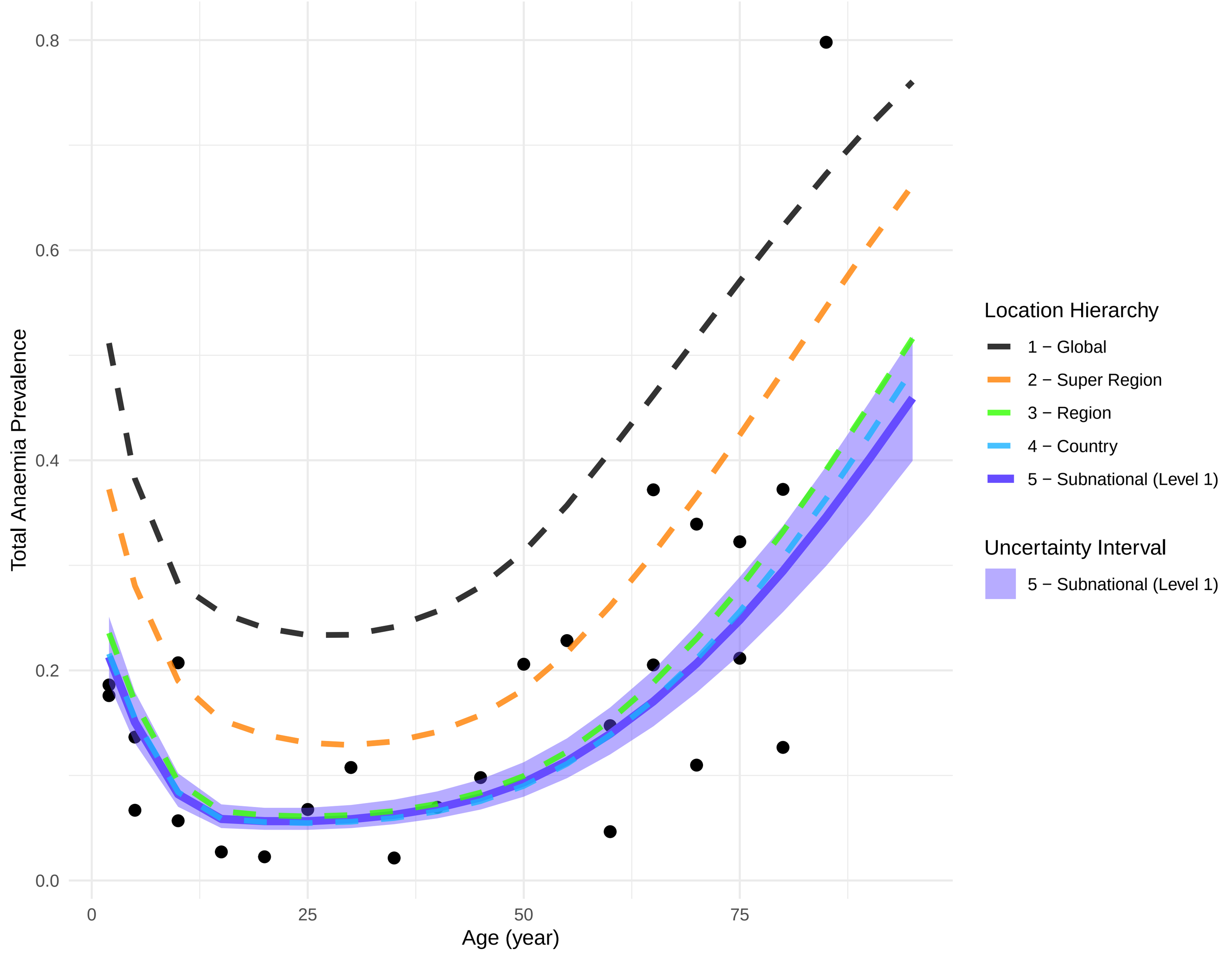

MR−BRT Age/Sex Fits − Males 2+ Years Old − Total Anaemia Prevalence − Puebla
WHO−2001 Adjustment Method

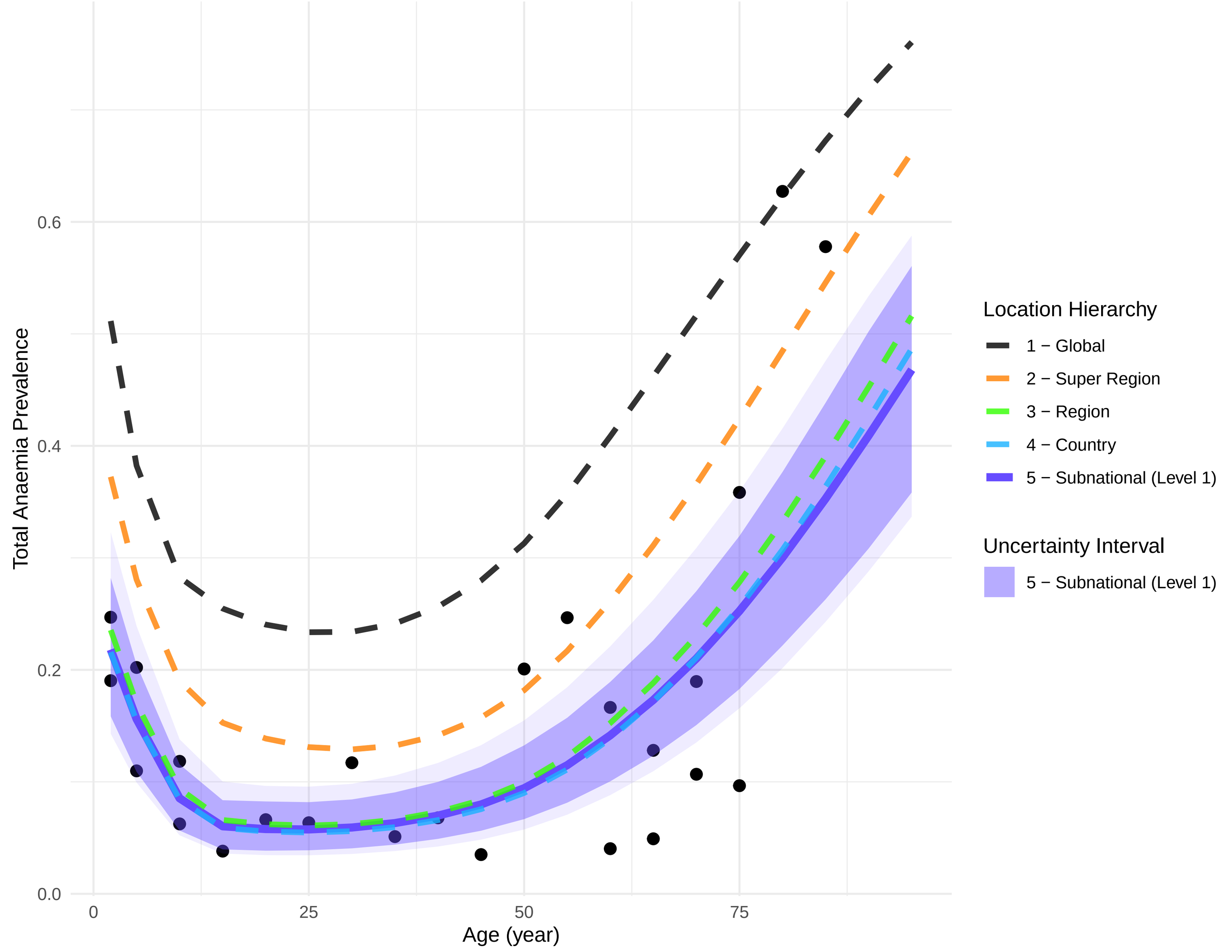

MR−BRT Age/Sex Fits − Males 2+ Years Old − Total Anaemia Prevalence − Querétaro
WHO−2001 Adjustment Method

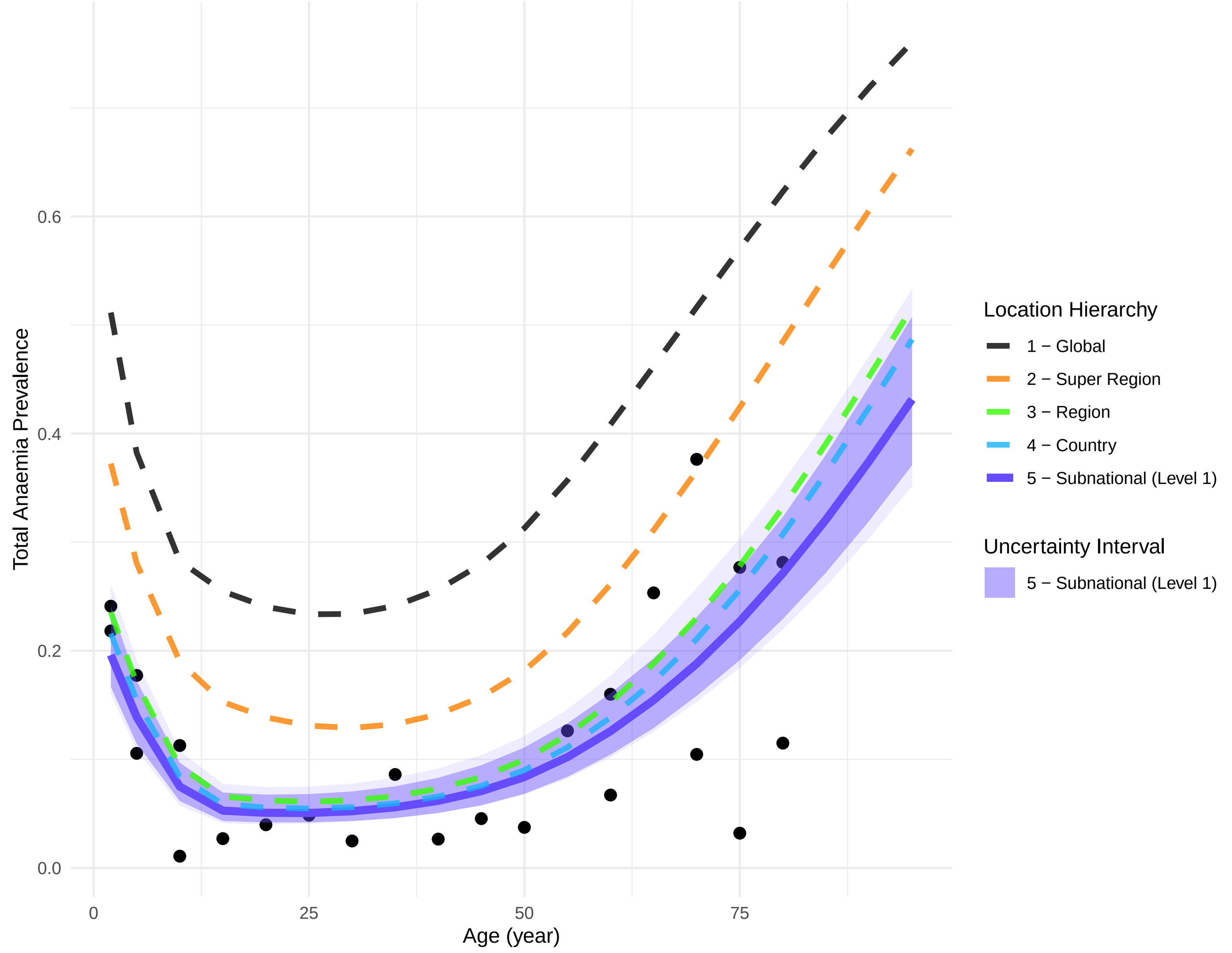

MR−BRT Age/Sex Fits − Males 2+ Years Old − Total Anaemia Prevalence − Quintana Roo
WHO−2001 Adjustment Method

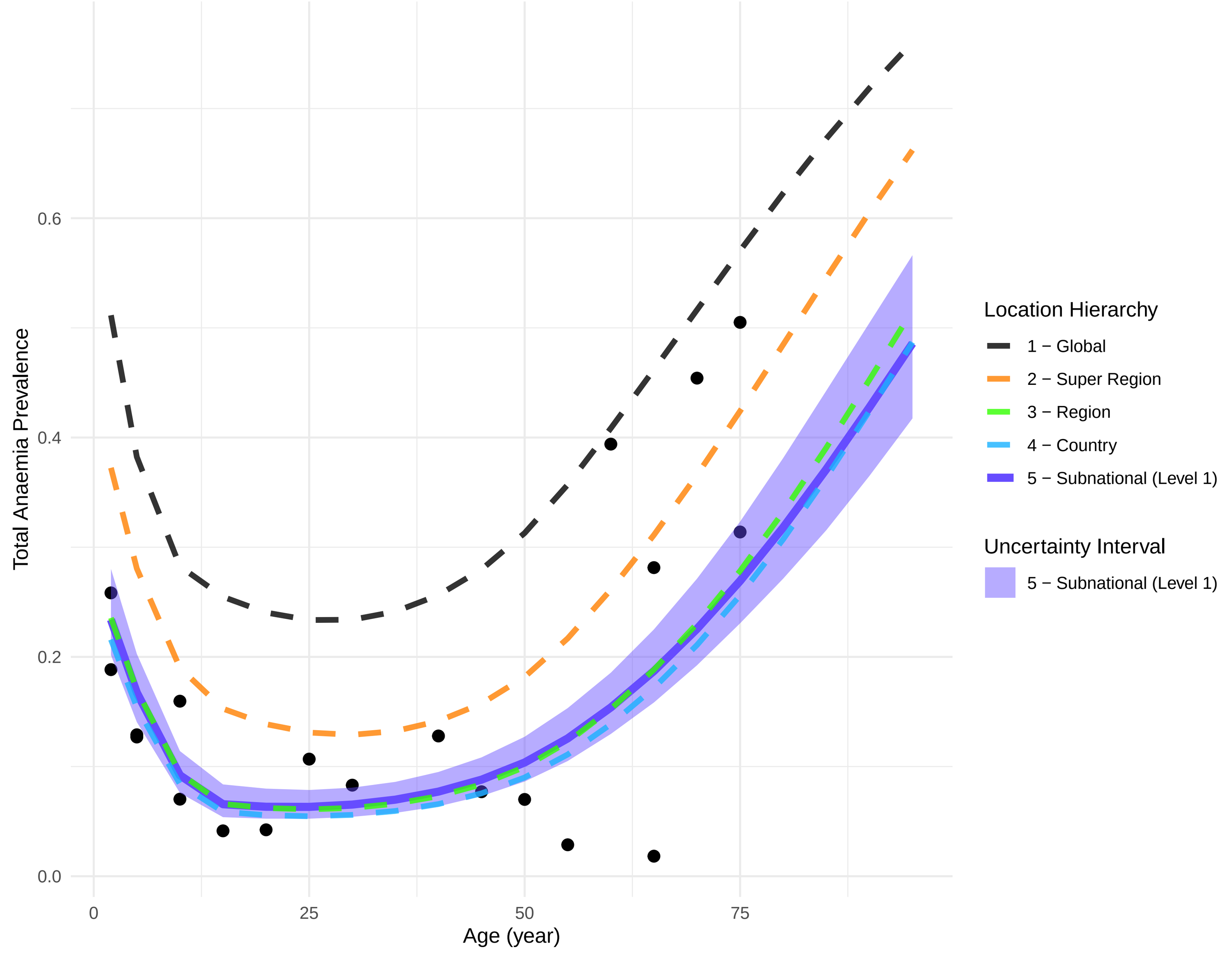

MR−BRT Age/Sex Fits − Males 2+ Years Old − Total Anaemia Prevalence − San Luis Potosí
WHO−2001 Adjustment Method

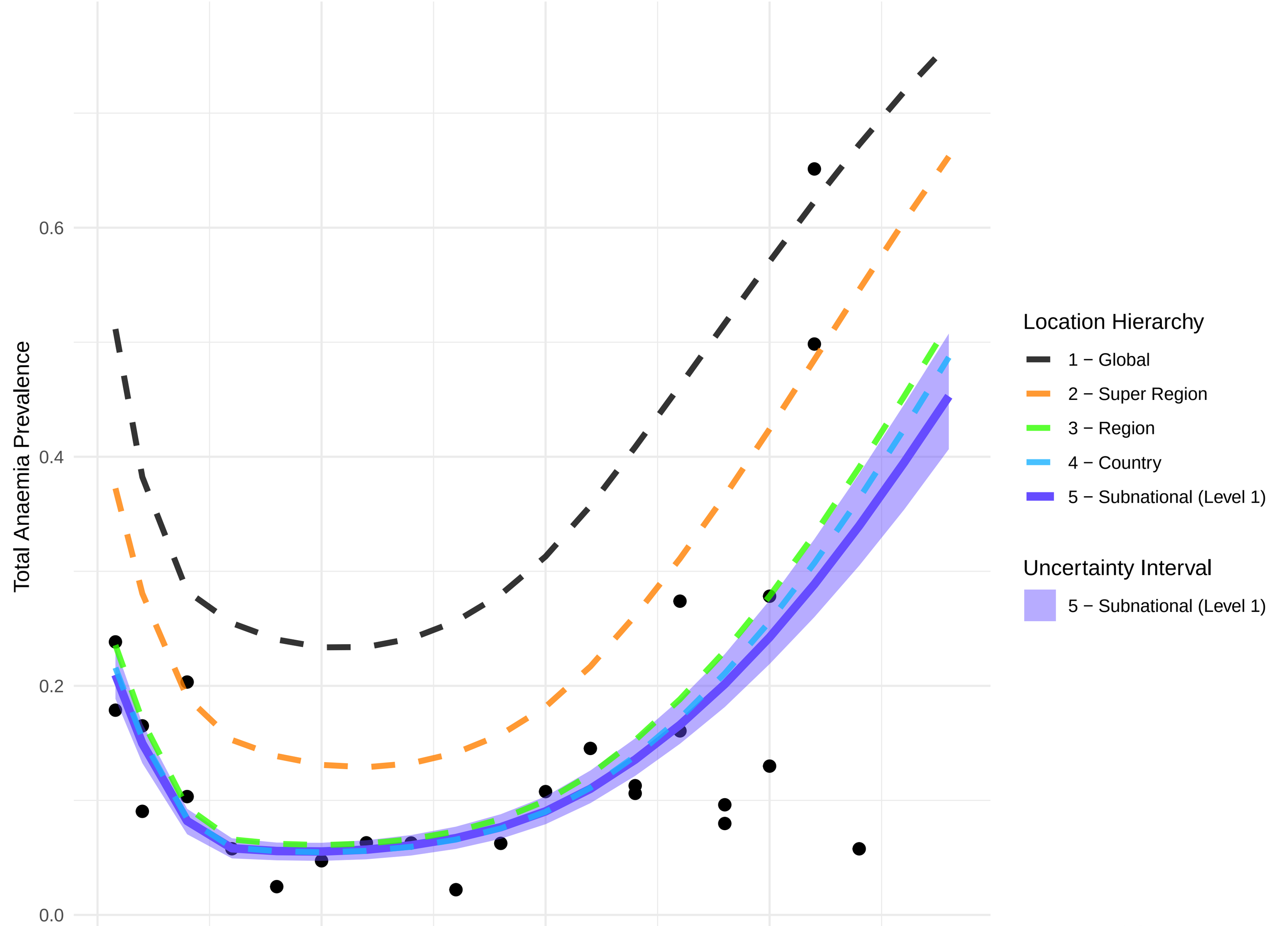

MR−BRT Age/Sex Fits − Males 2+ Years Old − Total Anaemia Prevalence − Sinaloa
WHO−2001 Adjustment Method

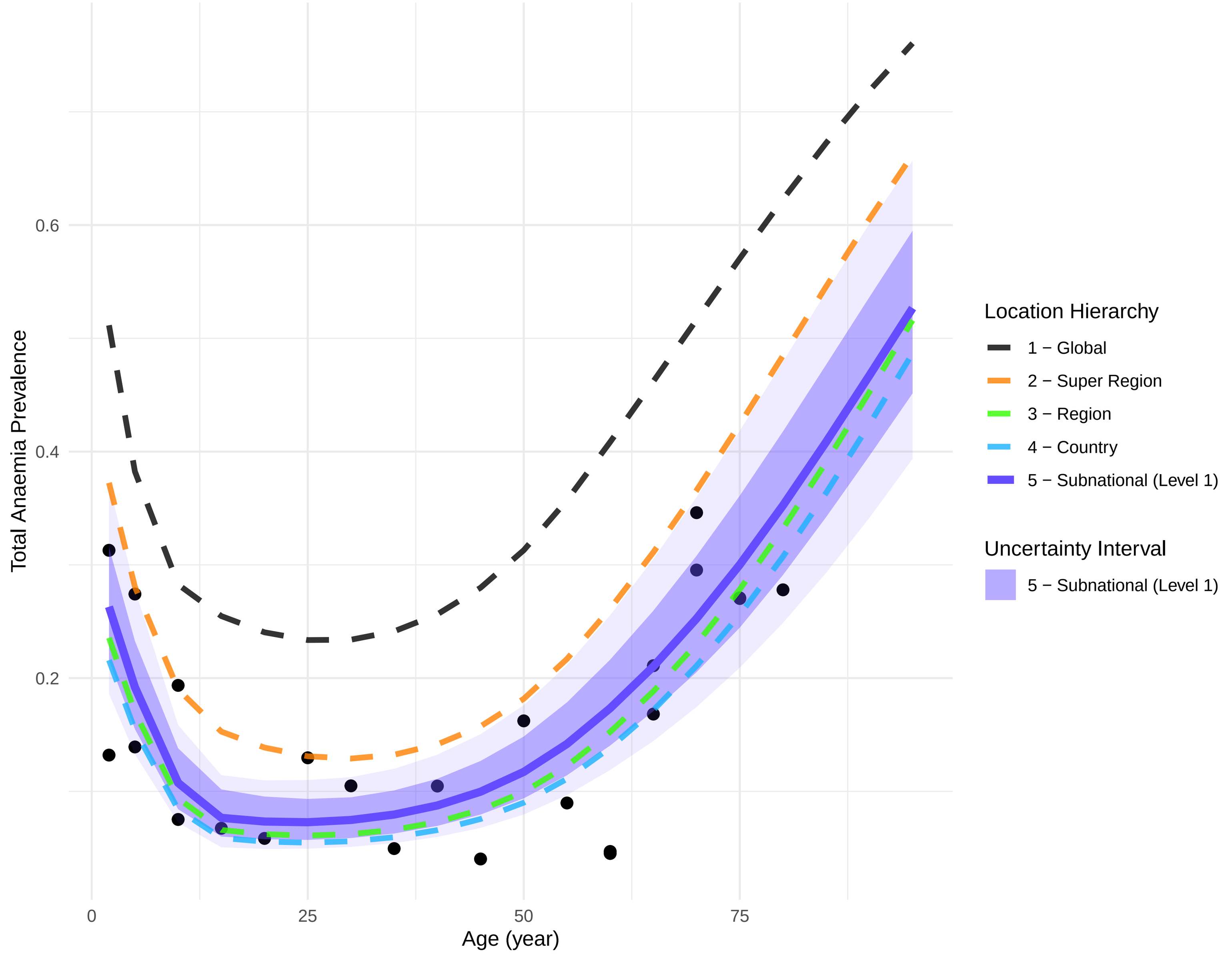

MR−BRT Age/Sex Fits − Males 2+ Years Old − Total Anaemia Prevalence − Sonora
WHO−2001 Adjustment Method

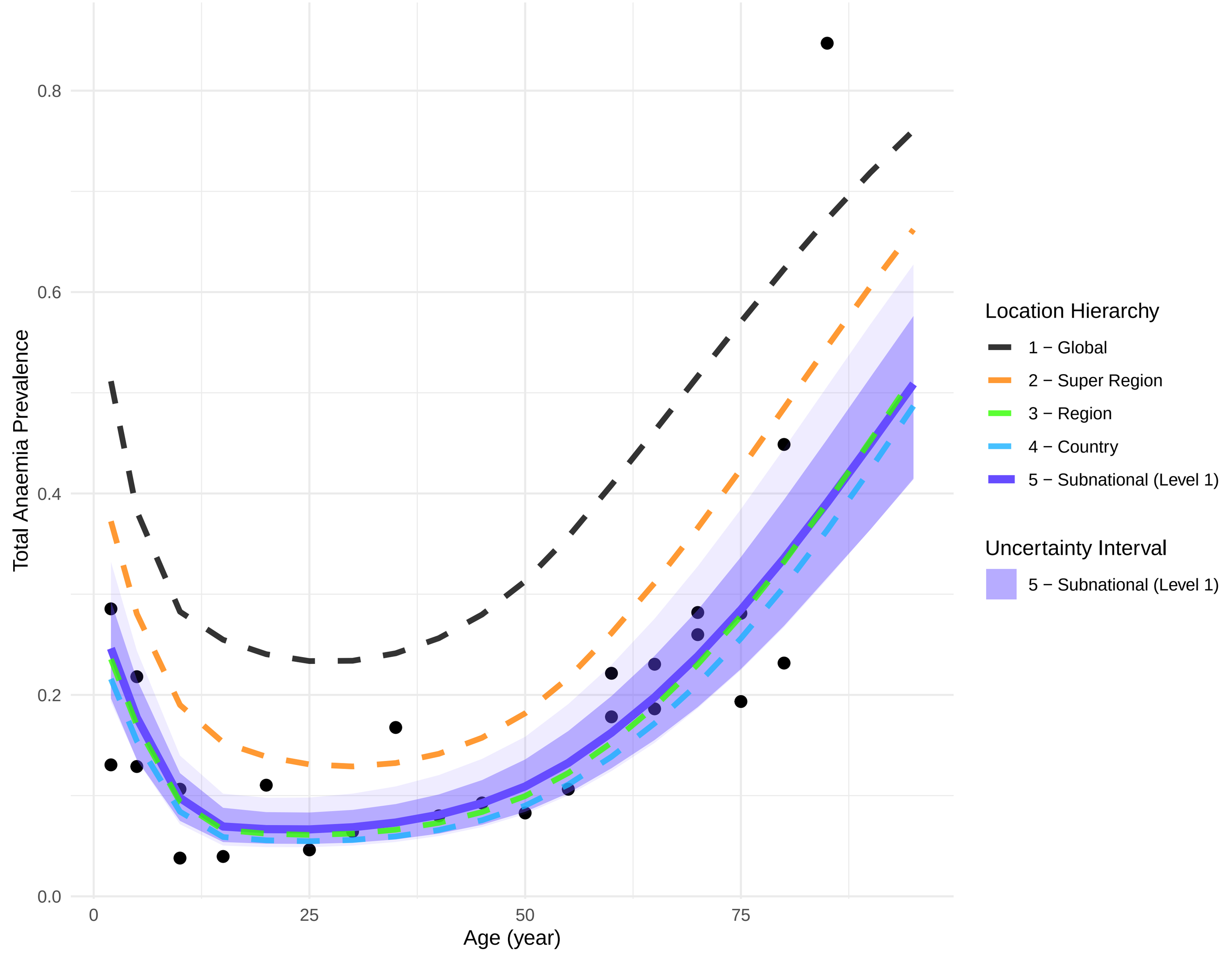

MR−BRT Age/Sex Fits − Males 2+ Years Old − Total Anaemia Prevalence − Tabasco
WHO−2001 Adjustment Method

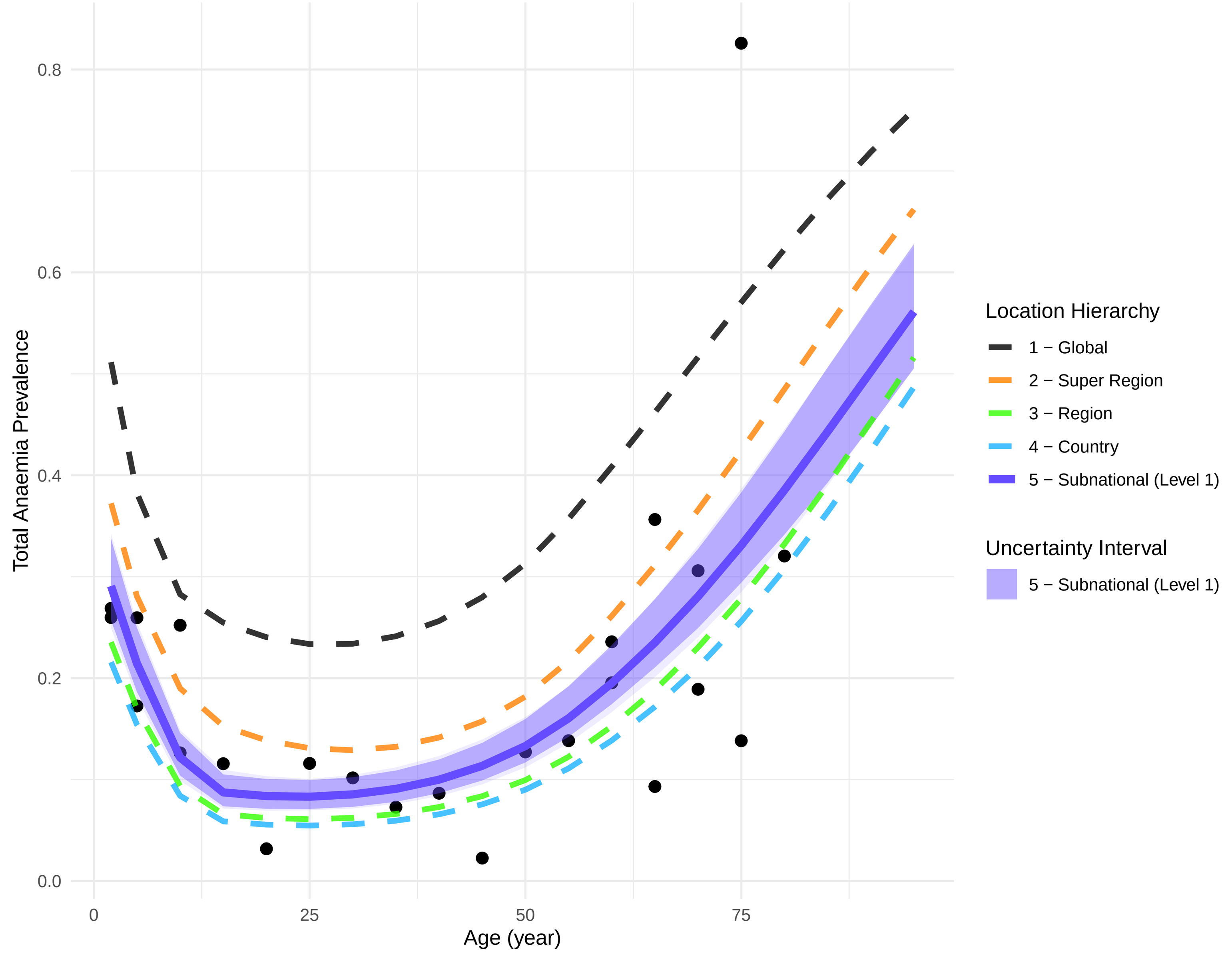

MR−BRT Age/Sex Fits − Males 2+ Years Old − Total Anaemia Prevalence − Tamaulipas
WHO−2001 Adjustment Method

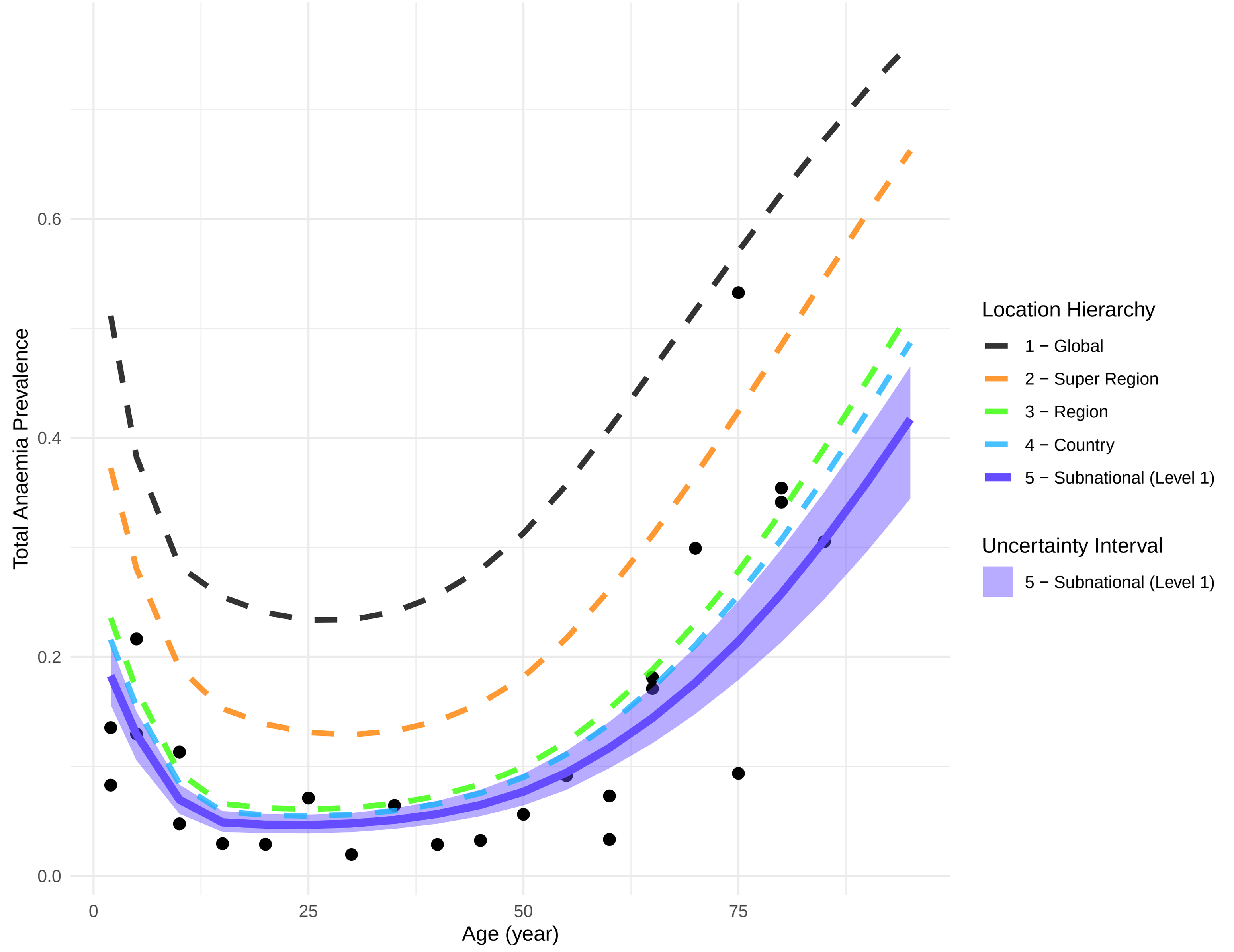

MR−BRT Age/Sex Fits − Males 2+ Years Old − Total Anaemia Prevalence − Tlaxcala
WHO−2001 Adjustment Method

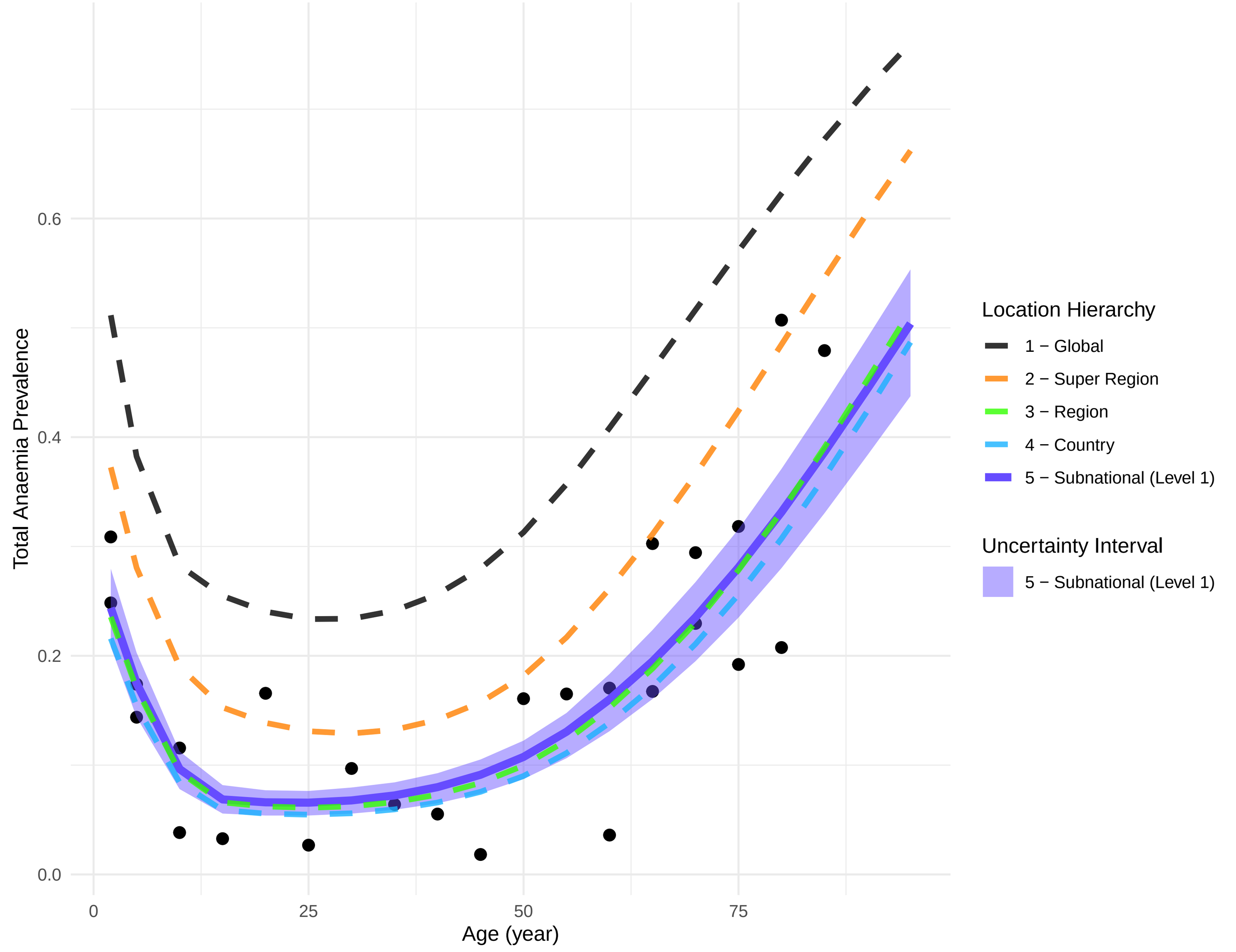

MR−BRT Age/Sex Fits − Males 2+ Years Old − Total Anaemia Prevalence − Veracruz de Ignacio de la Llave
WHO−2001 Adjustment Method

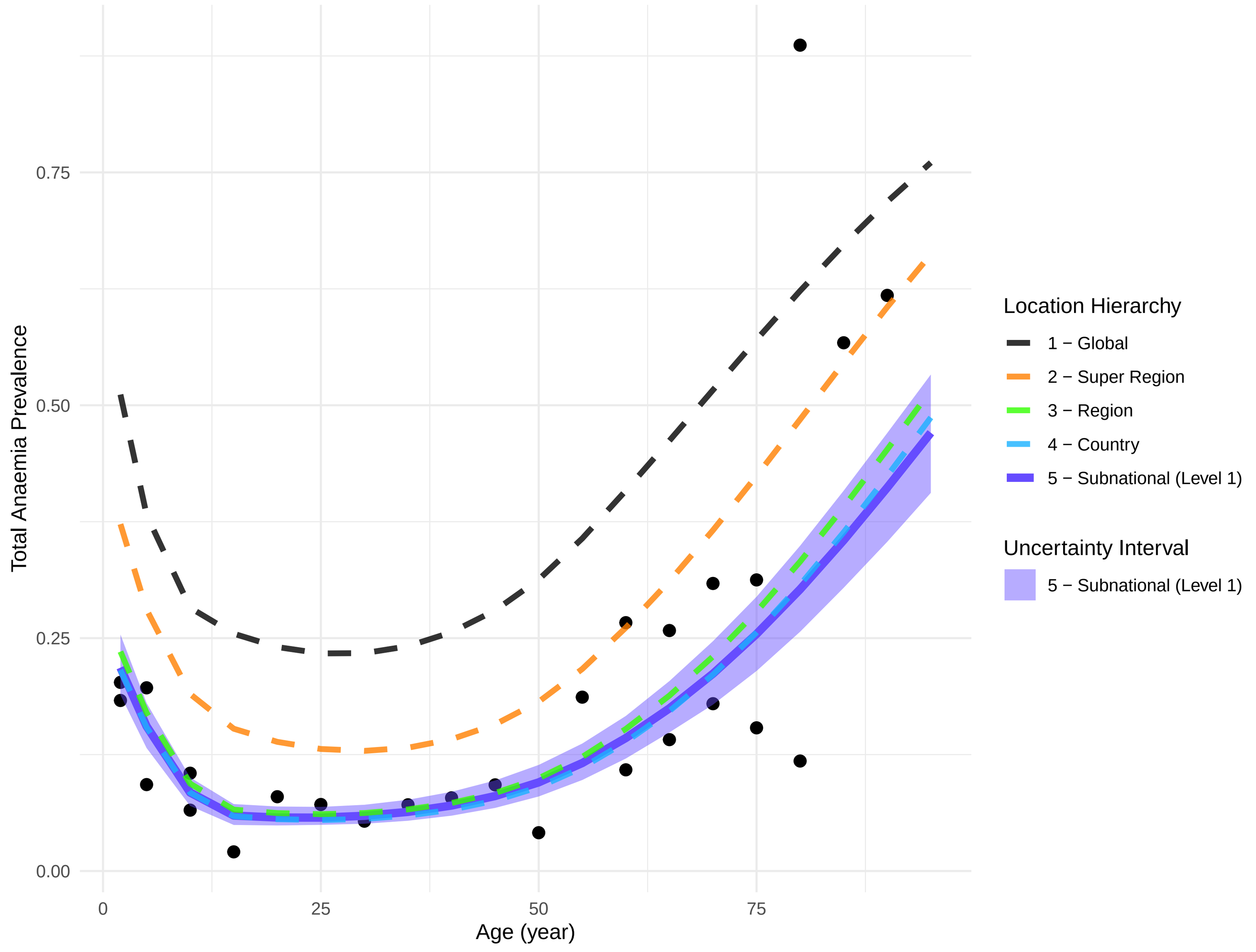

MR−BRT Age/Sex Fits − Males 2+ Years Old − Total Anaemia Prevalence − Yucatán
WHO−2001 Adjustment Method

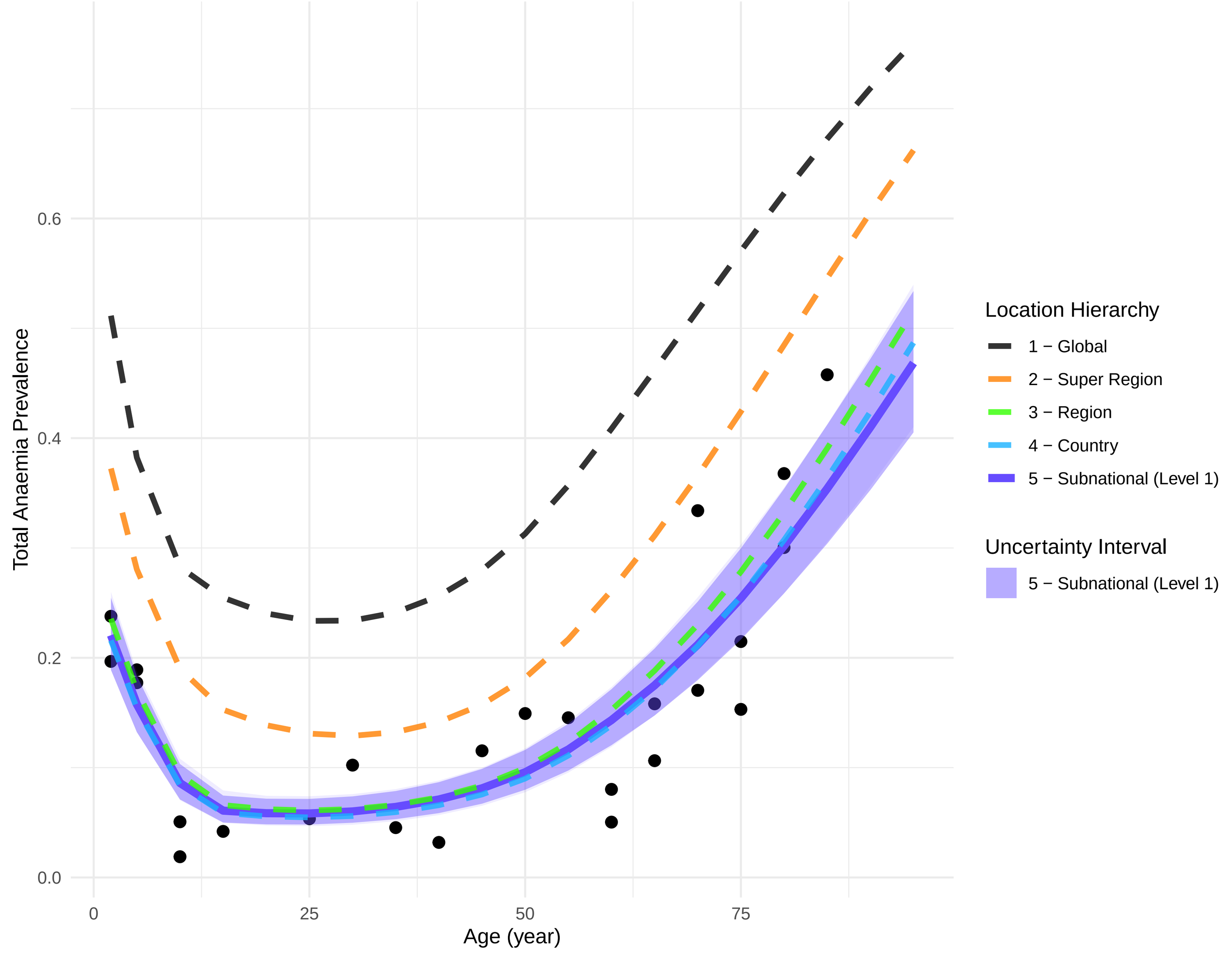

MR−BRT Age/Sex Fits − Males 2+ Years Old − Total Anaemia Prevalence − Zacatecas
WHO−2001 Adjustment Method

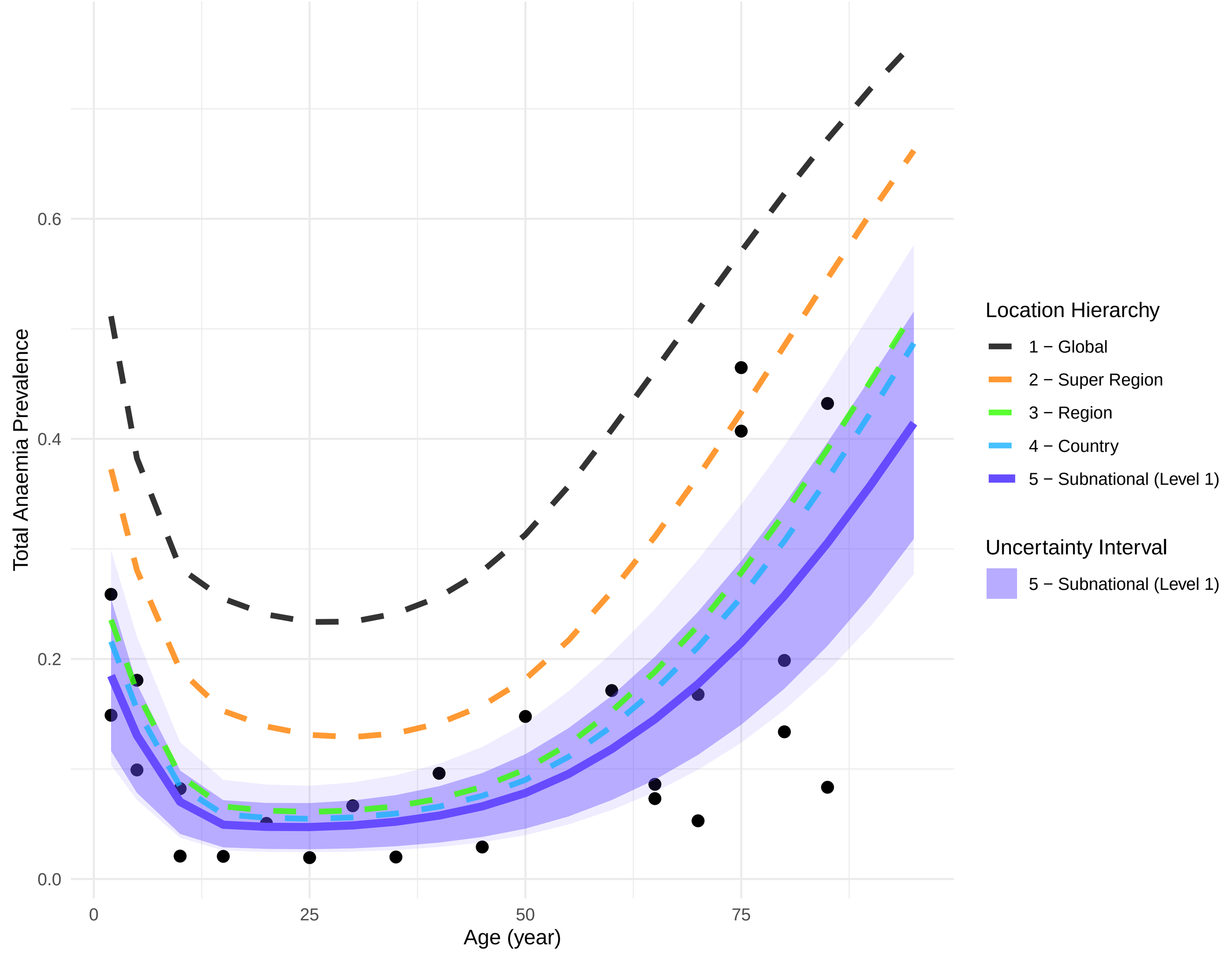

MR−BRT Age/Sex Fits − Males 2+ Years Old − Total Anaemia Prevalence − Tropical Latin America
WHO−2001 Adjustment Method

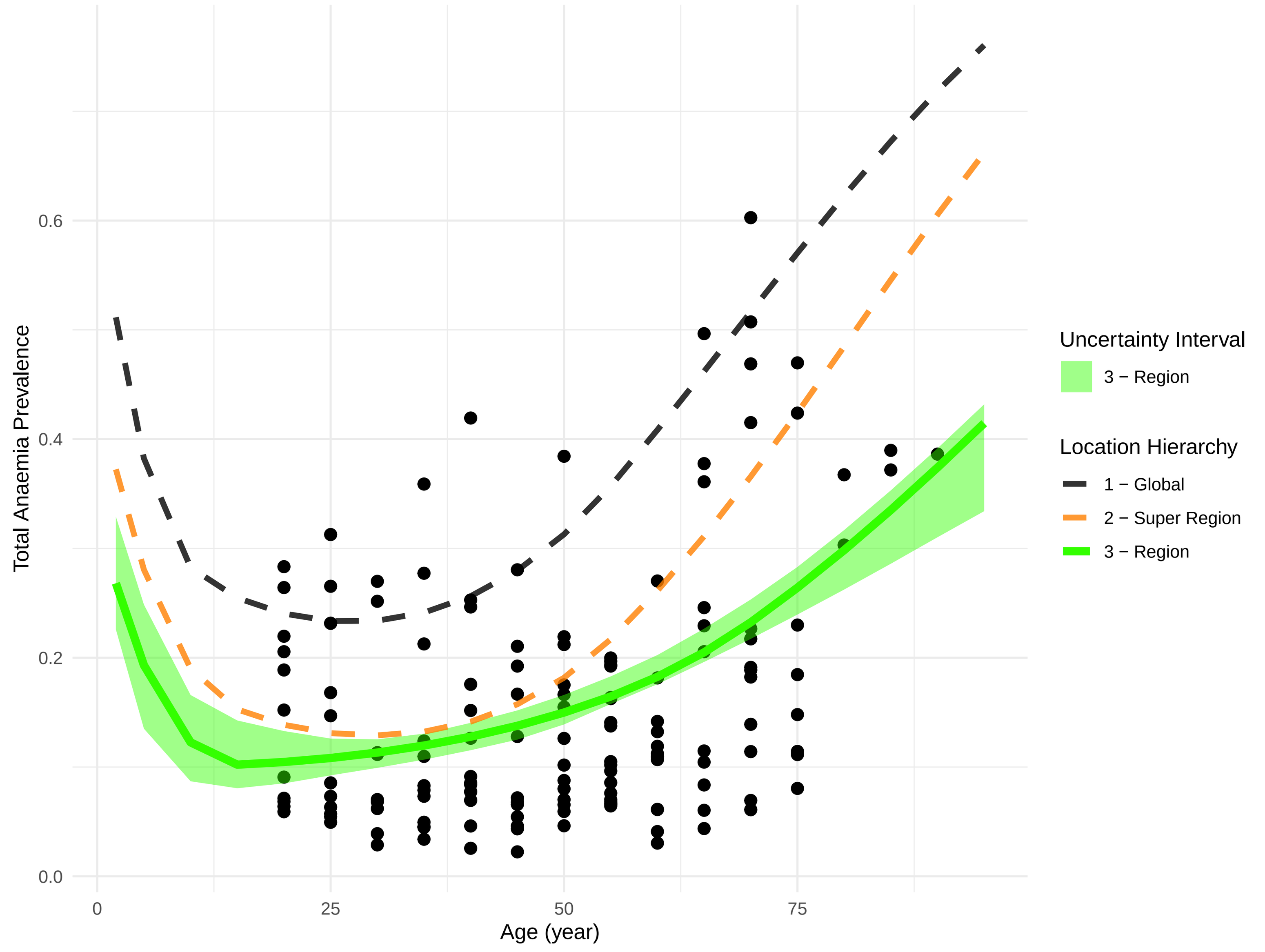

MR−BRT Age/Sex Fits − Males 2+ Years Old − Total Anaemia Prevalence − Brazil
WHO−2001 Adjustment Method

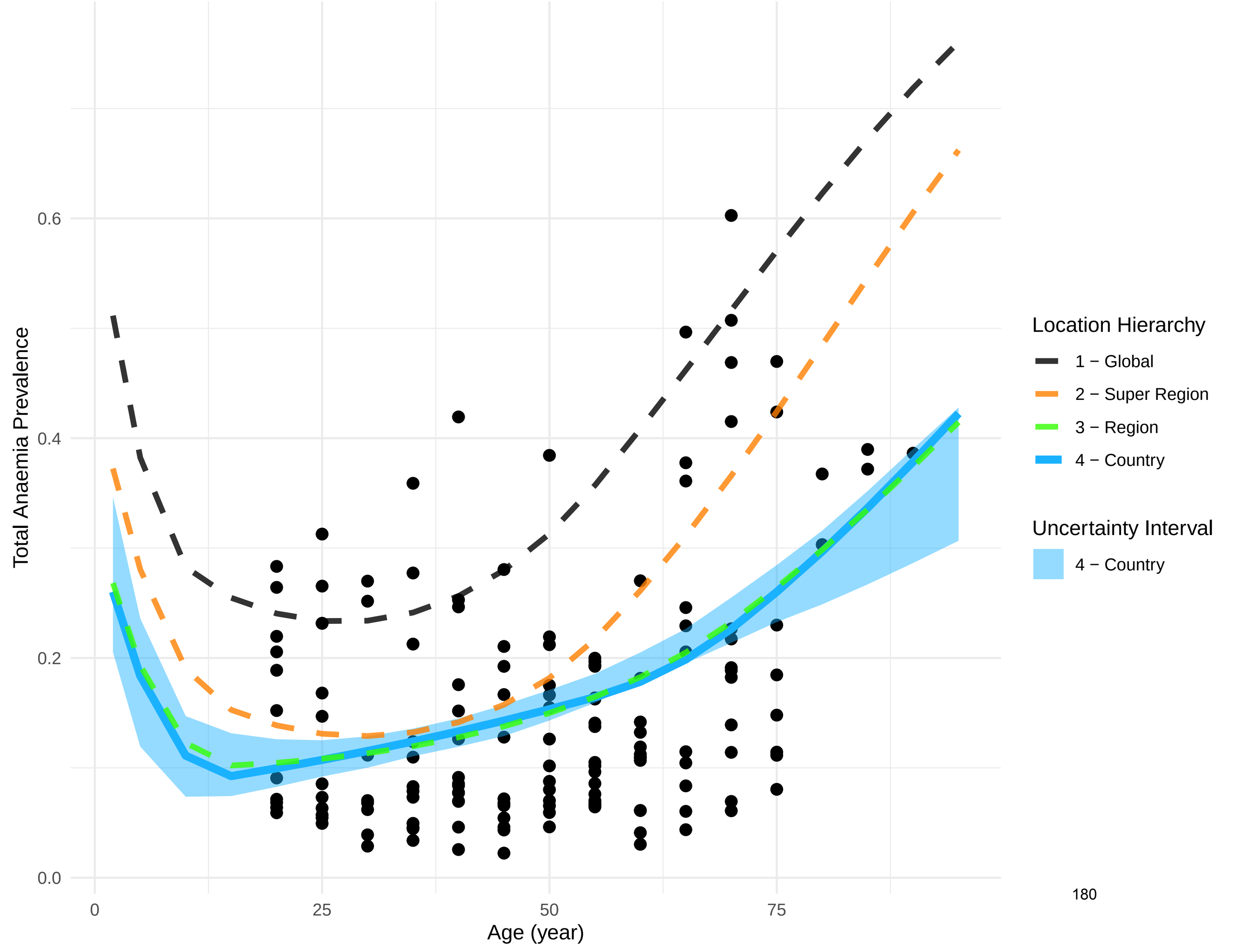

MR−BRT Age/Sex Fits − Males 2+ Years Old − Total Anaemia Prevalence − Acre
WHO−2001 Adjustment Method

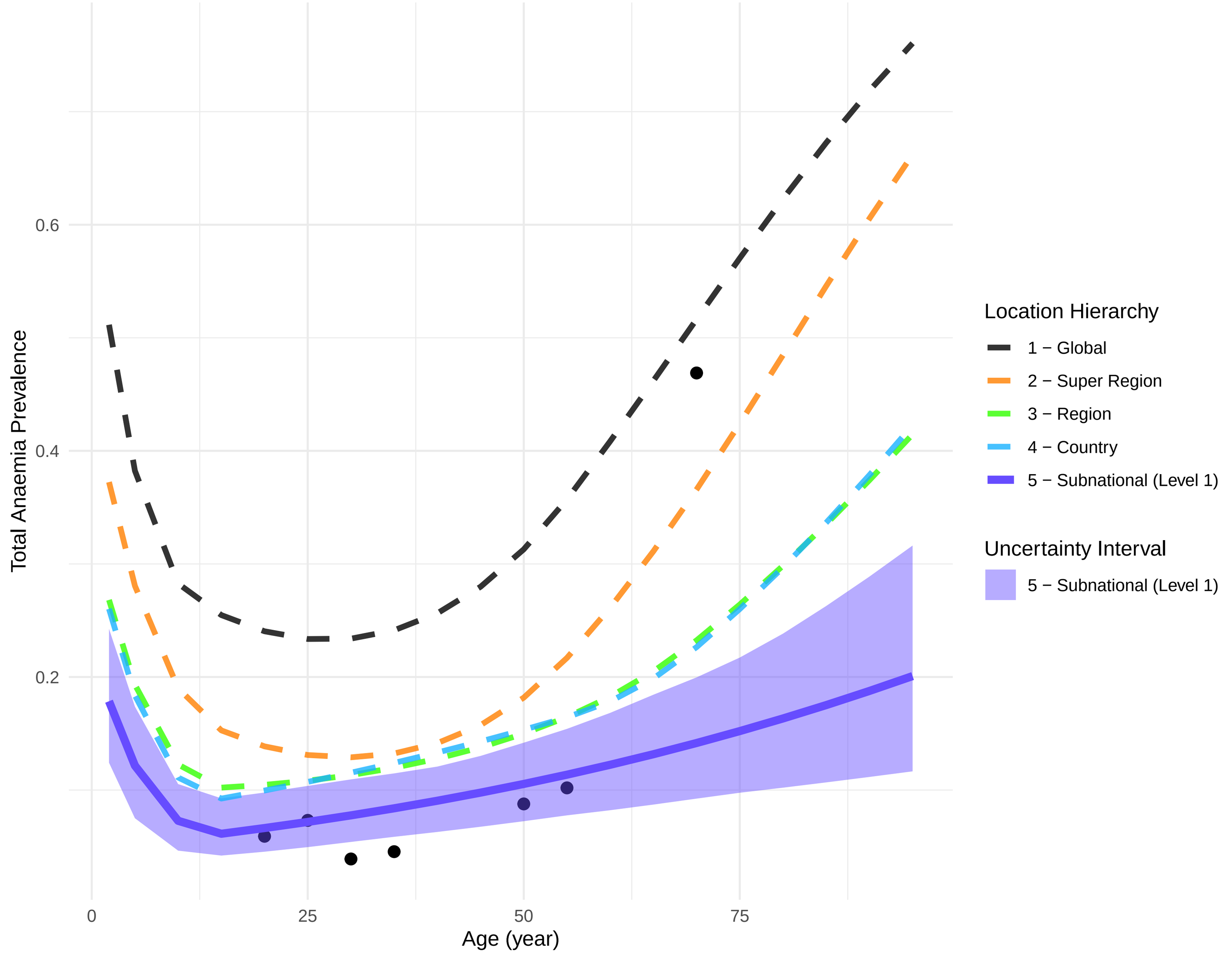

MR−BRT Age/Sex Fits − Males 2+ Years Old − Total Anaemia Prevalence − Alagoas
WHO−2001 Adjustment Method

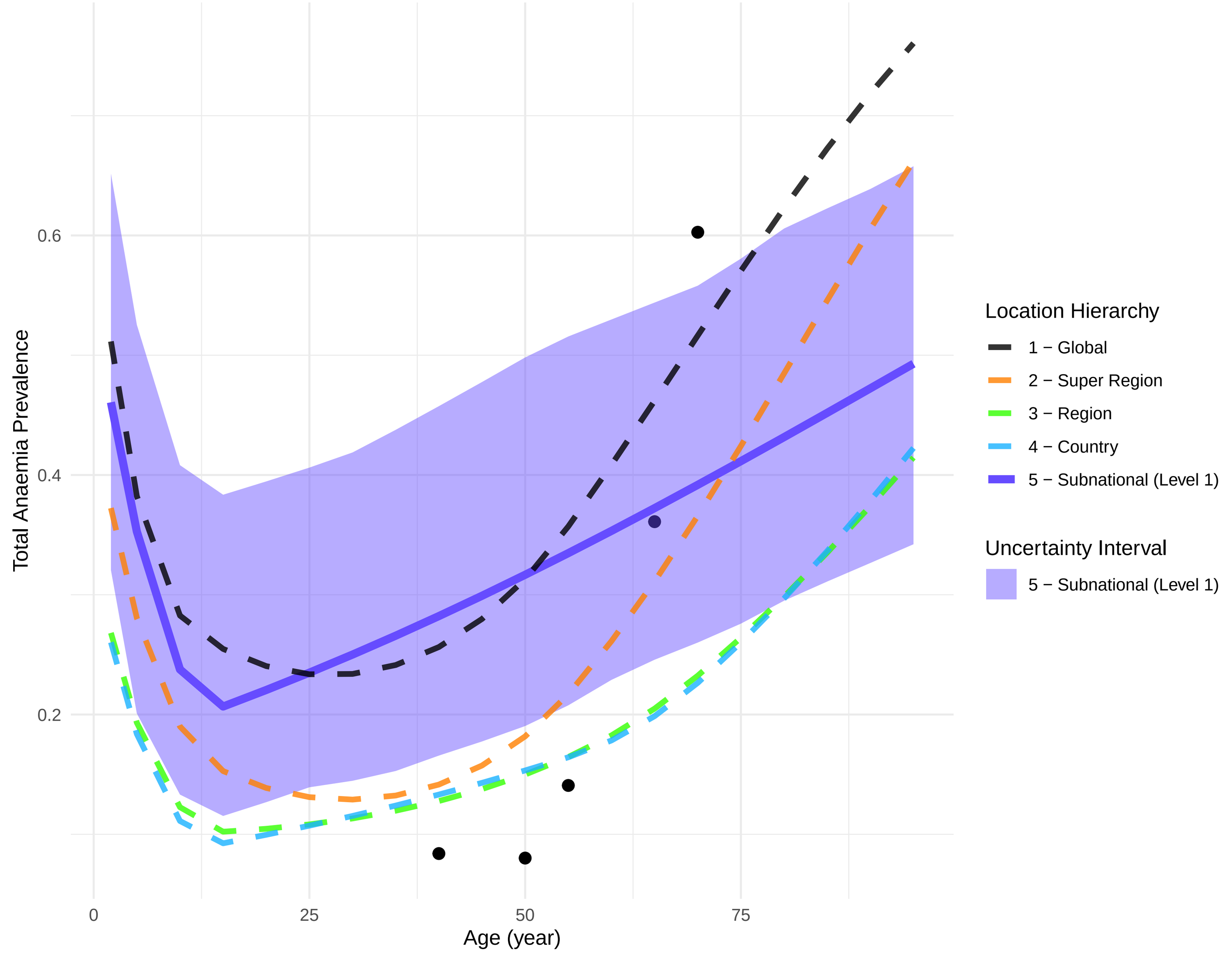

MR−BRT Age/Sex Fits − Males 2+ Years Old − Total Anaemia Prevalence − Amapá
WHO−2001 Adjustment Method

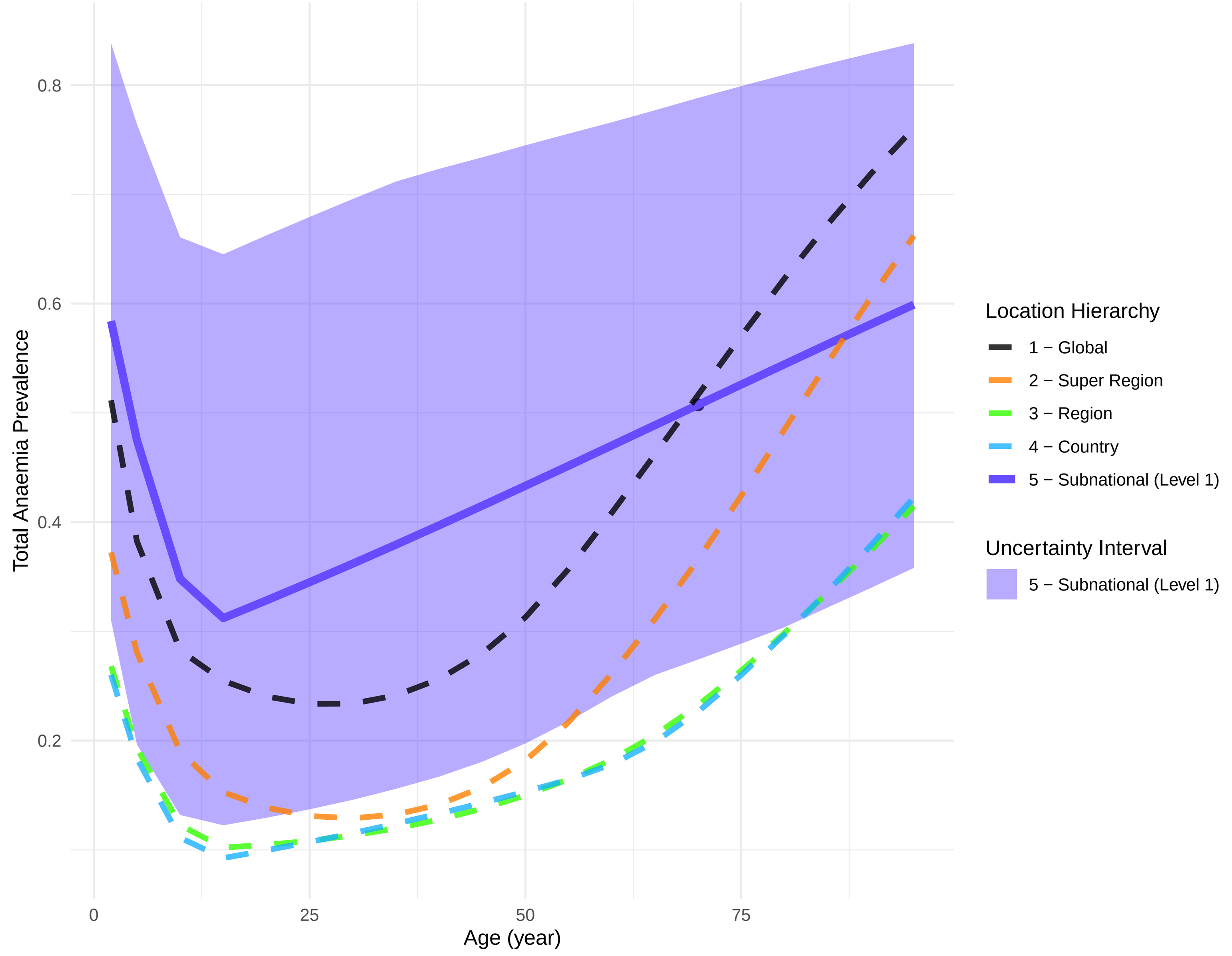

MR−BRT Age/Sex Fits − Males 2+ Years Old − Total Anaemia Prevalence − Amazonas
WHO−2001 Adjustment Method

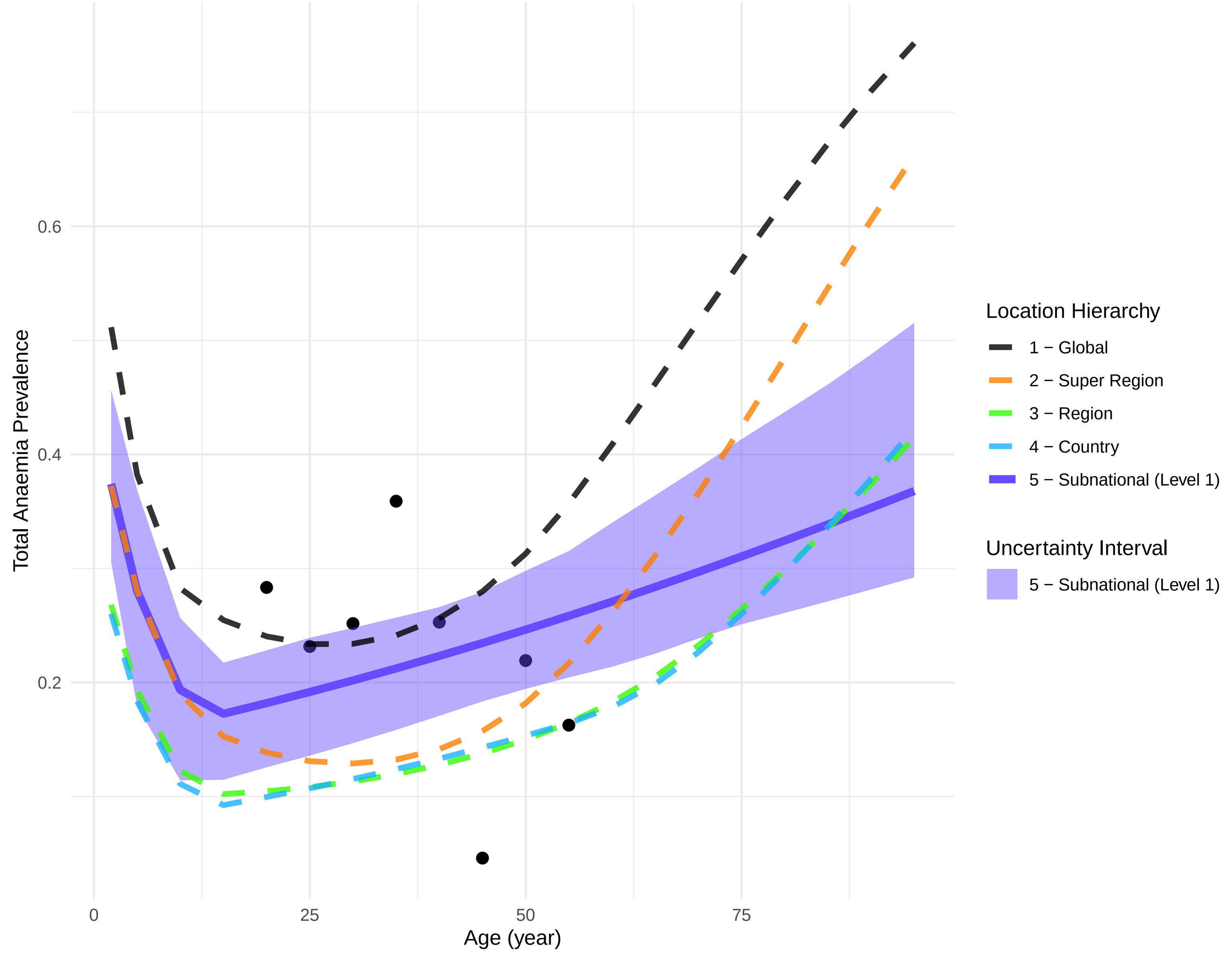

MR−BRT Age/Sex Fits − Males 2+ Years Old − Total Anaemia Prevalence − Bahia
WHO−2001 Adjustment Method

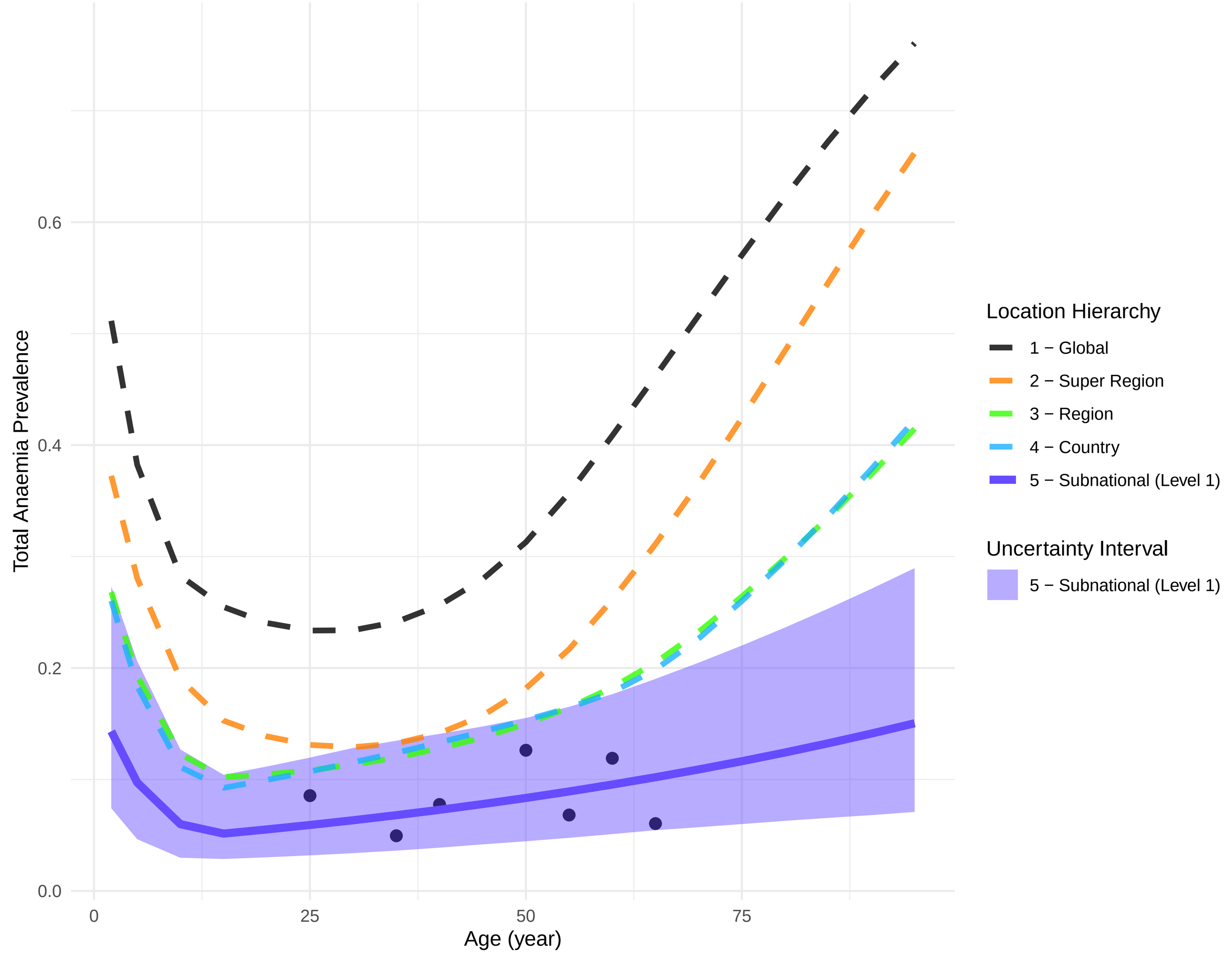

MR−BRT Age/Sex Fits − Males 2+ Years Old − Total Anaemia Prevalence − Ceará
WHO−2001 Adjustment Method

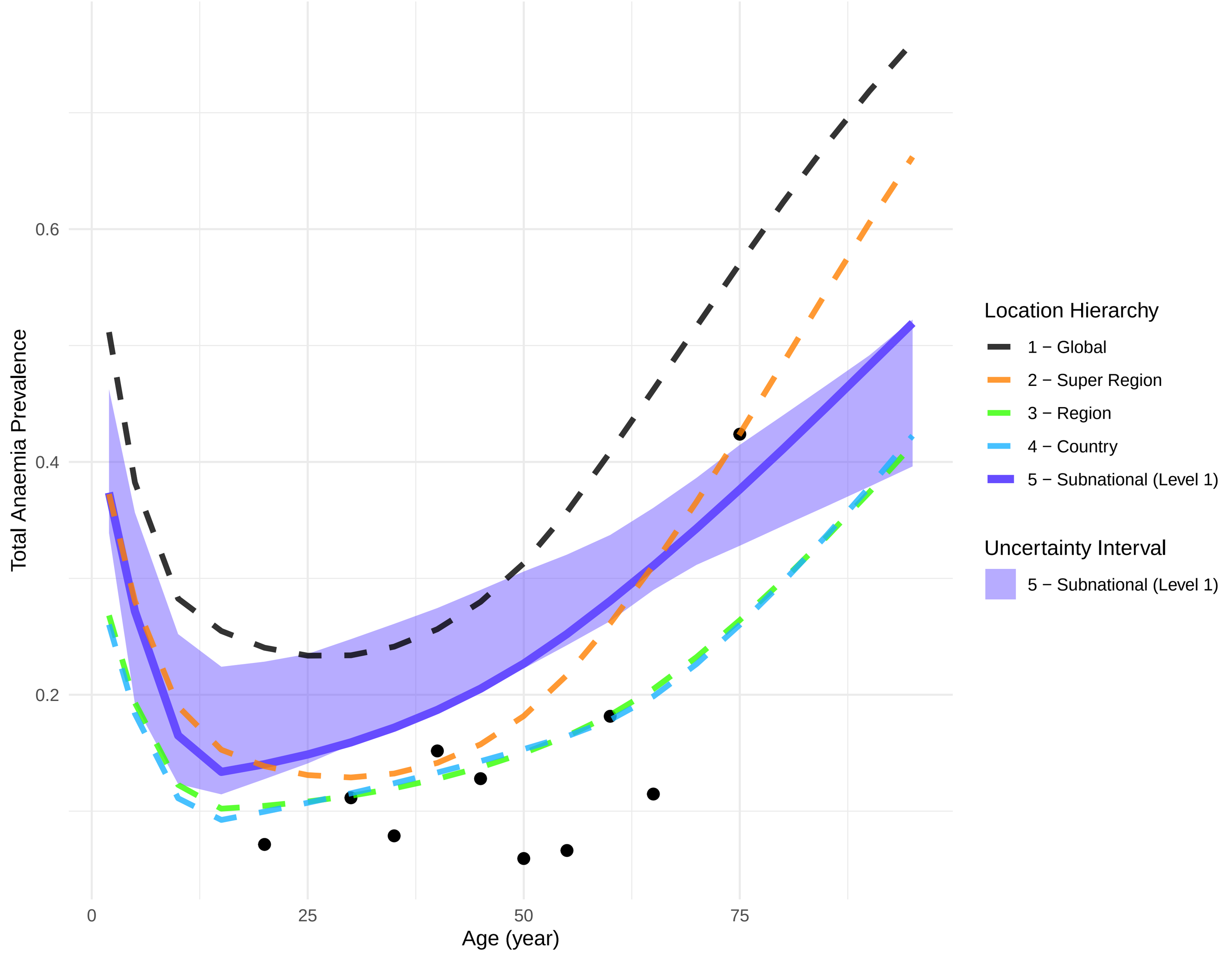

MR−BRT Age/Sex Fits − Males 2+ Years Old − Total Anaemia Prevalence − Distrito Federal
WHO−2001 Adjustment Method

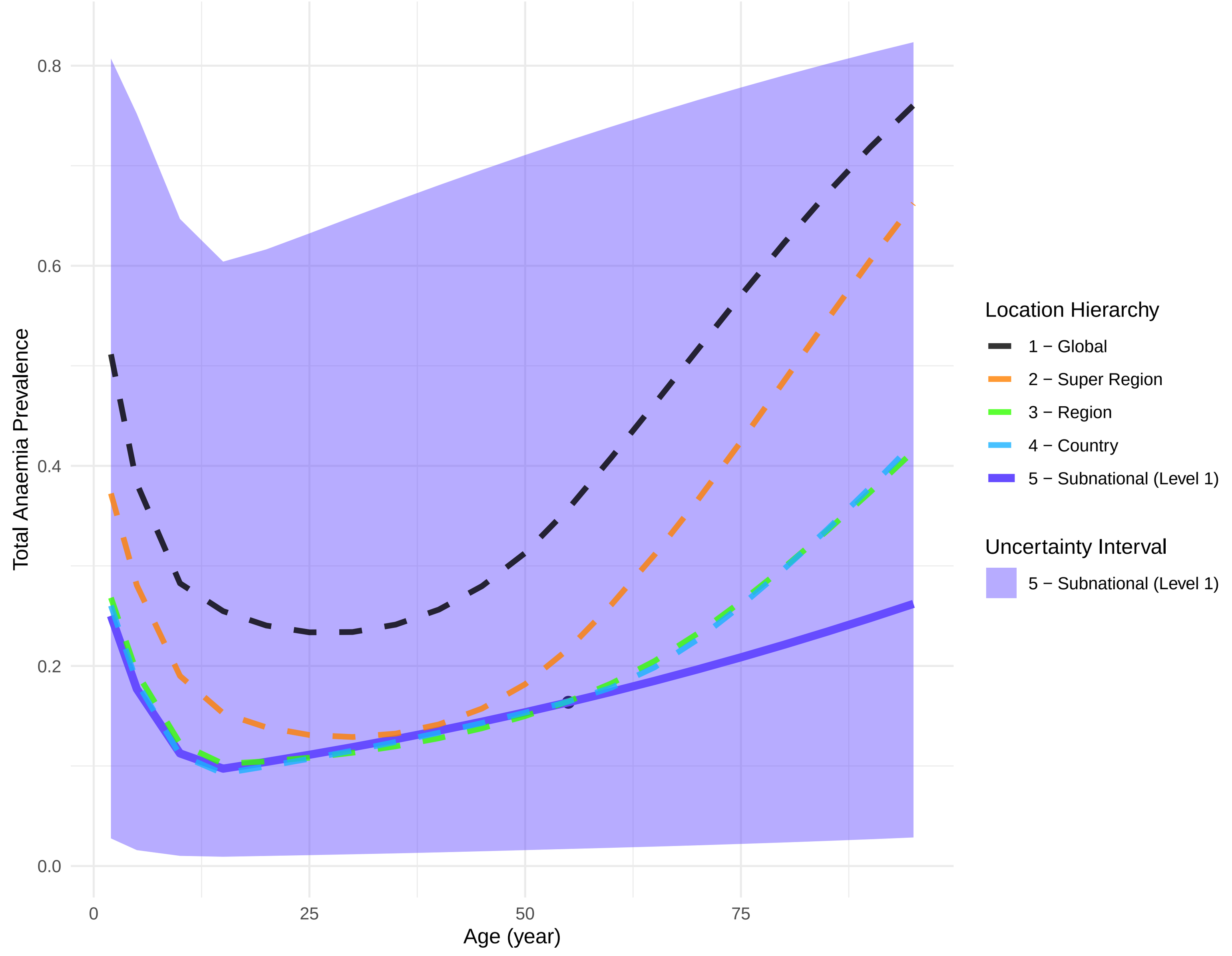

MR−BRT Age/Sex Fits − Males 2+ Years Old − Total Anaemia Prevalence − Espírito Santo
WHO−2001 Adjustment Method

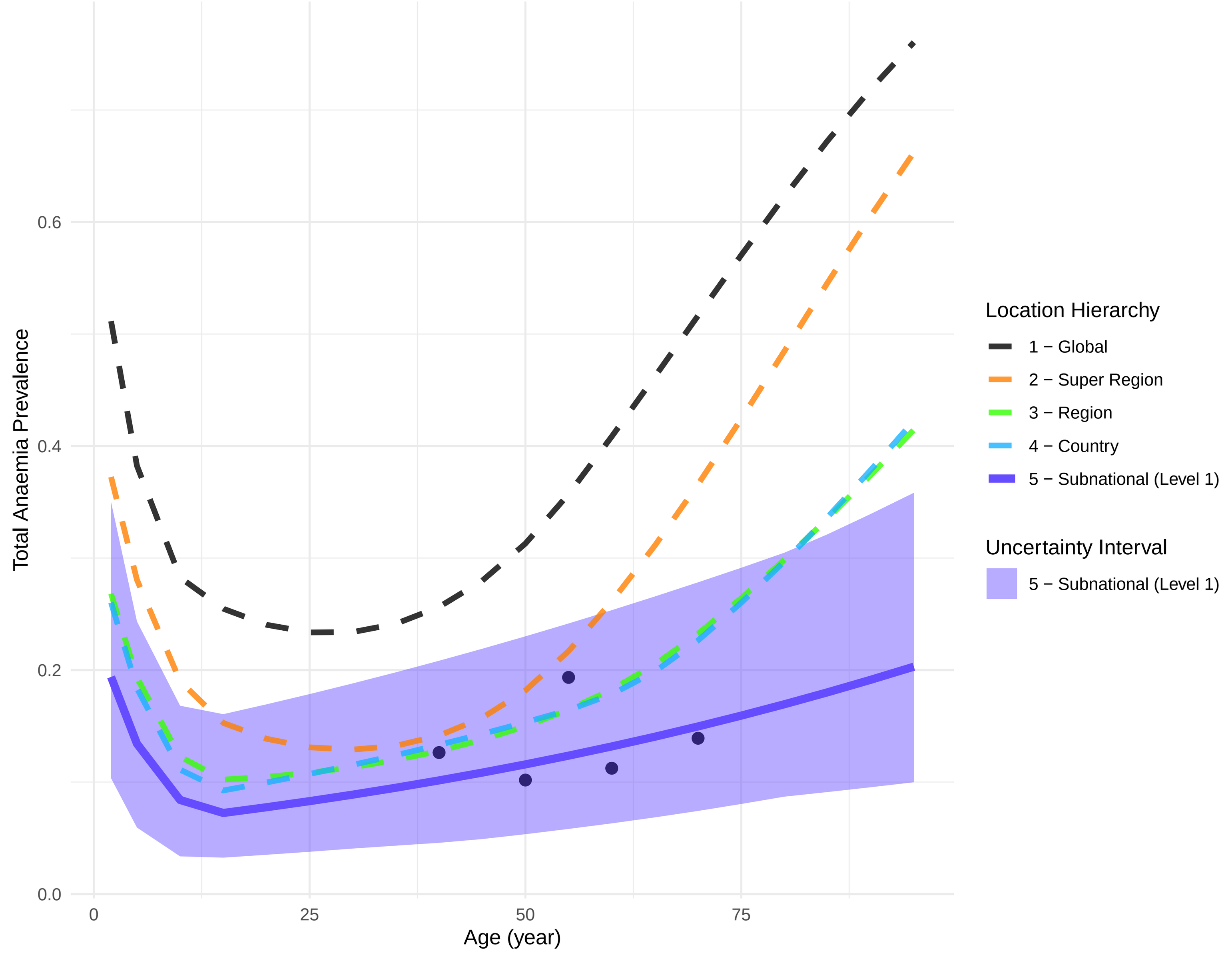

MR−BRT Age/Sex Fits − Males 2+ Years Old − Total Anaemia Prevalence − Goiás
WHO−2001 Adjustment Method

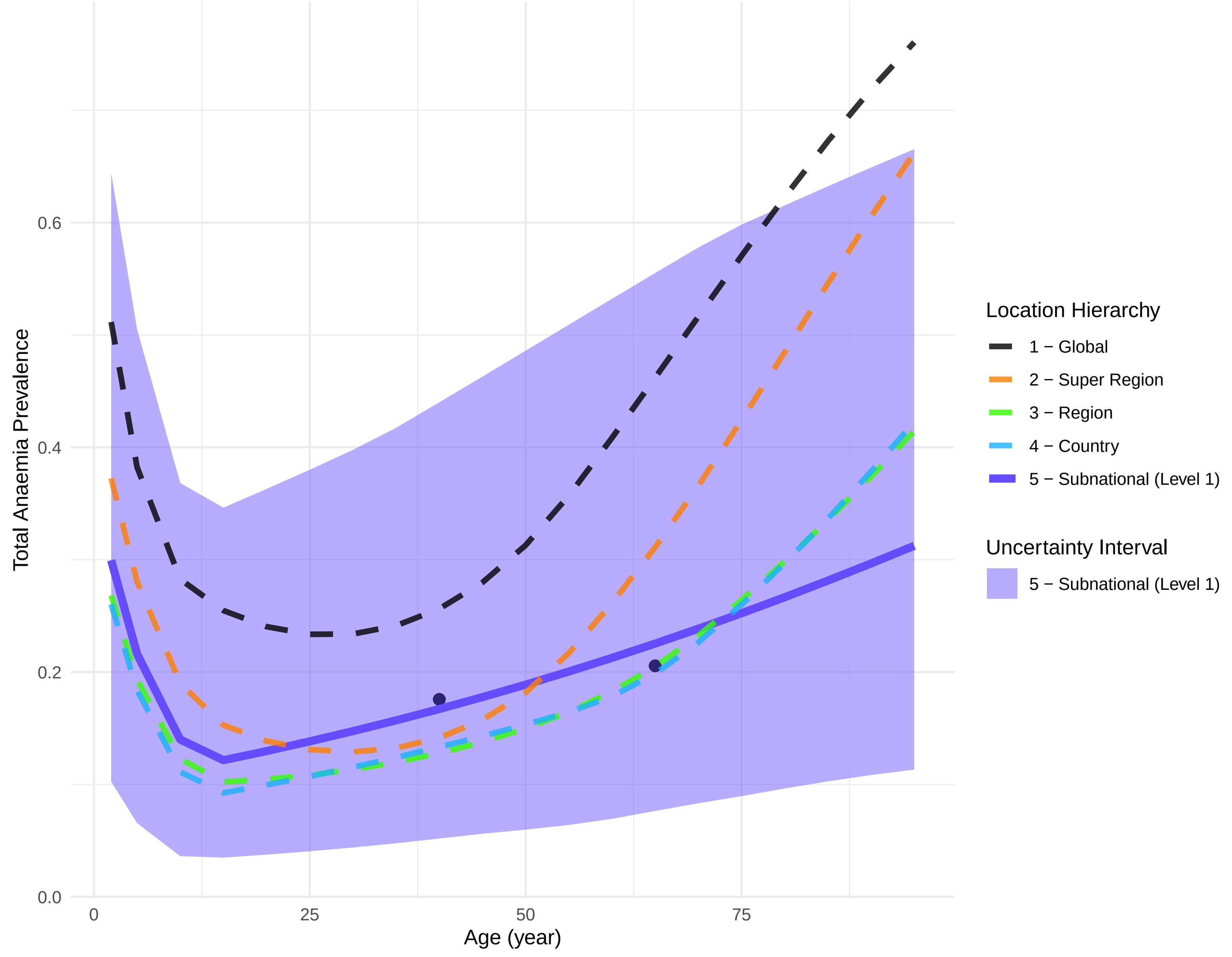

MR−BRT Age/Sex Fits − Males 2+ Years Old − Total Anaemia Prevalence − Maranhão
WHO−2001 Adjustment Method

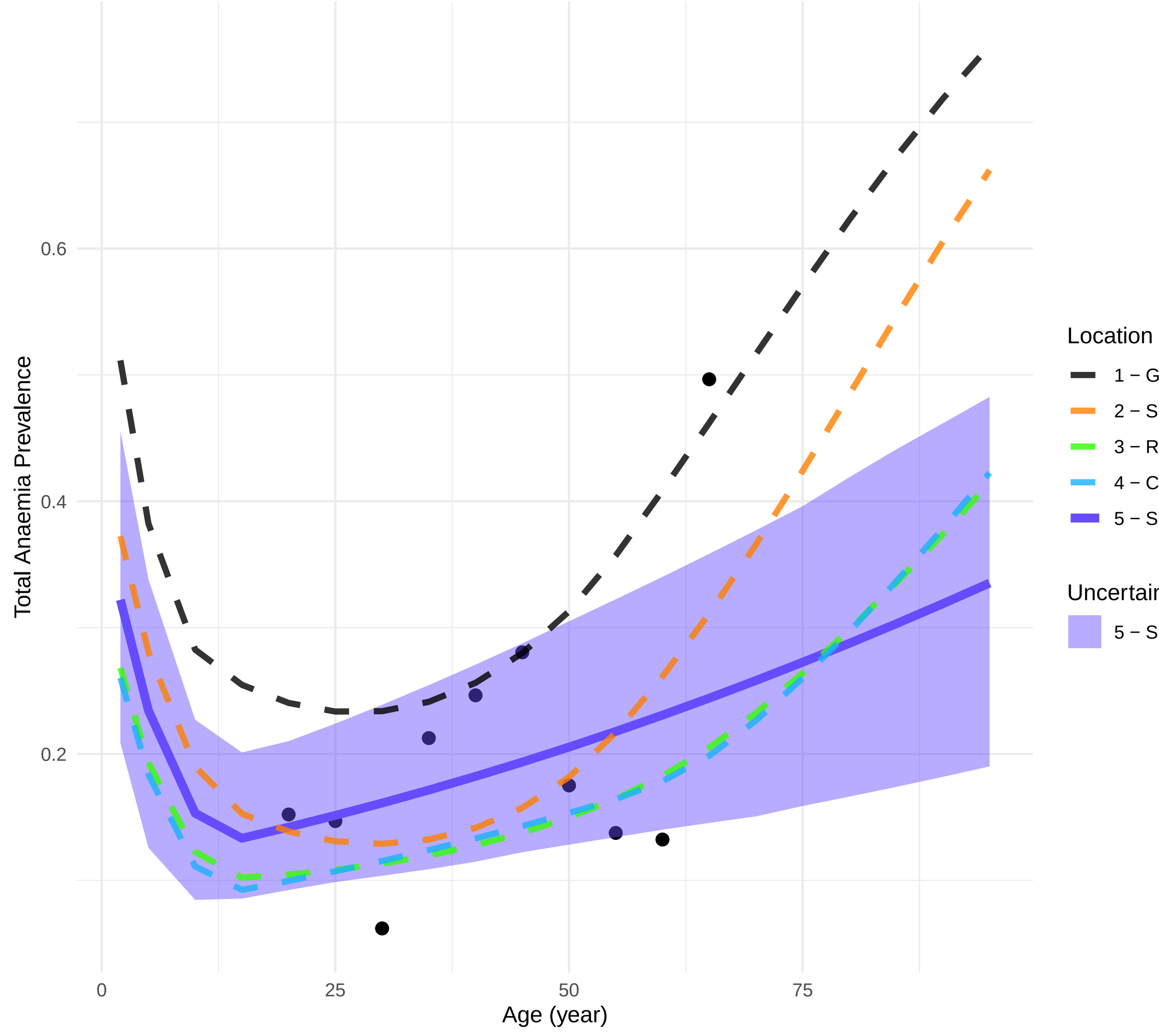

MR−BRT Age/Sex Fits − Males 2+ Years Old − Total Anaemia Prevalence − Mato Grosso
WHO−2001 Adjustment Method

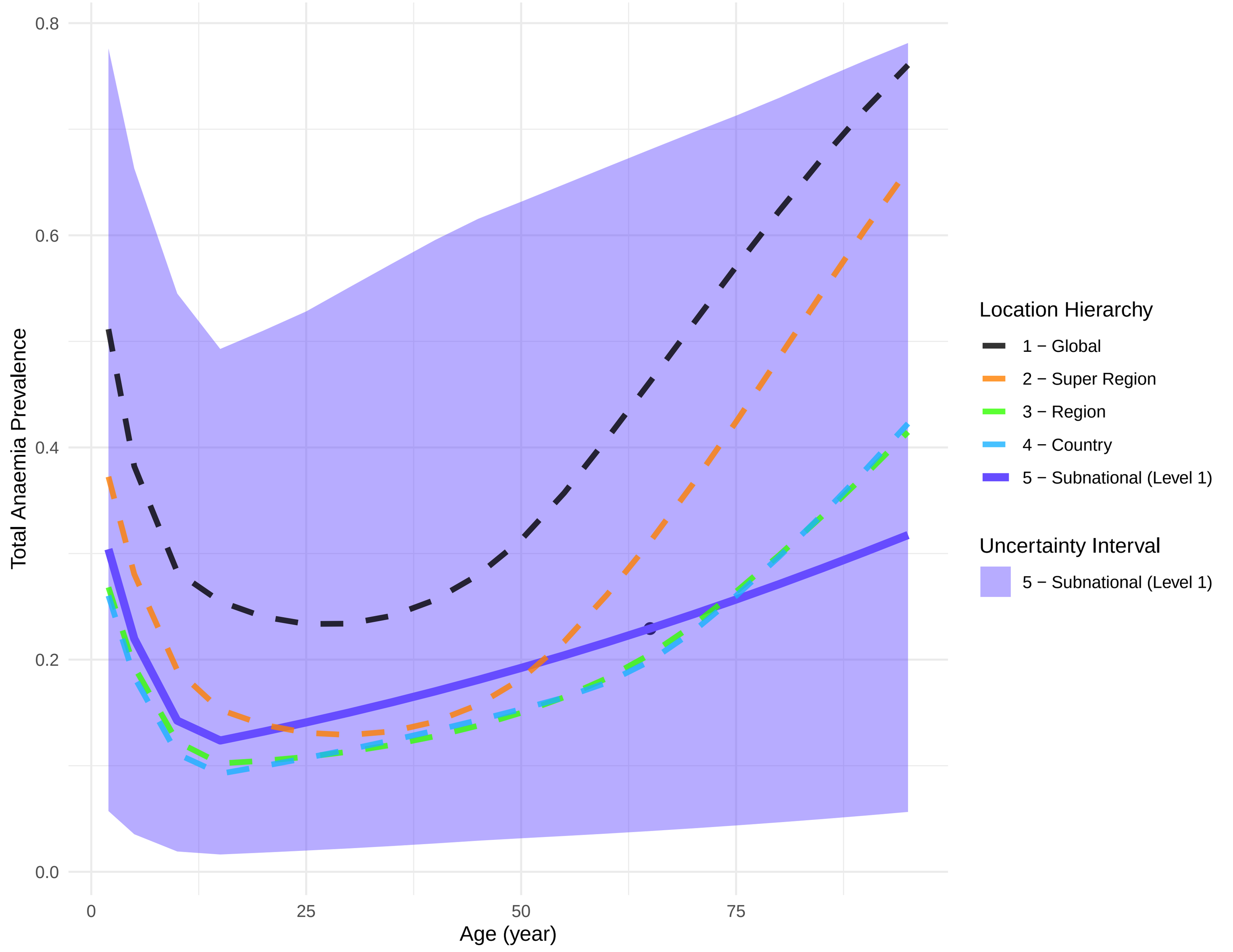

MR−BRT Age/Sex Fits − Males 2+ Years Old − Total Anaemia Prevalence − Mato Grosso do Sul
WHO−2001 Adjustment Method

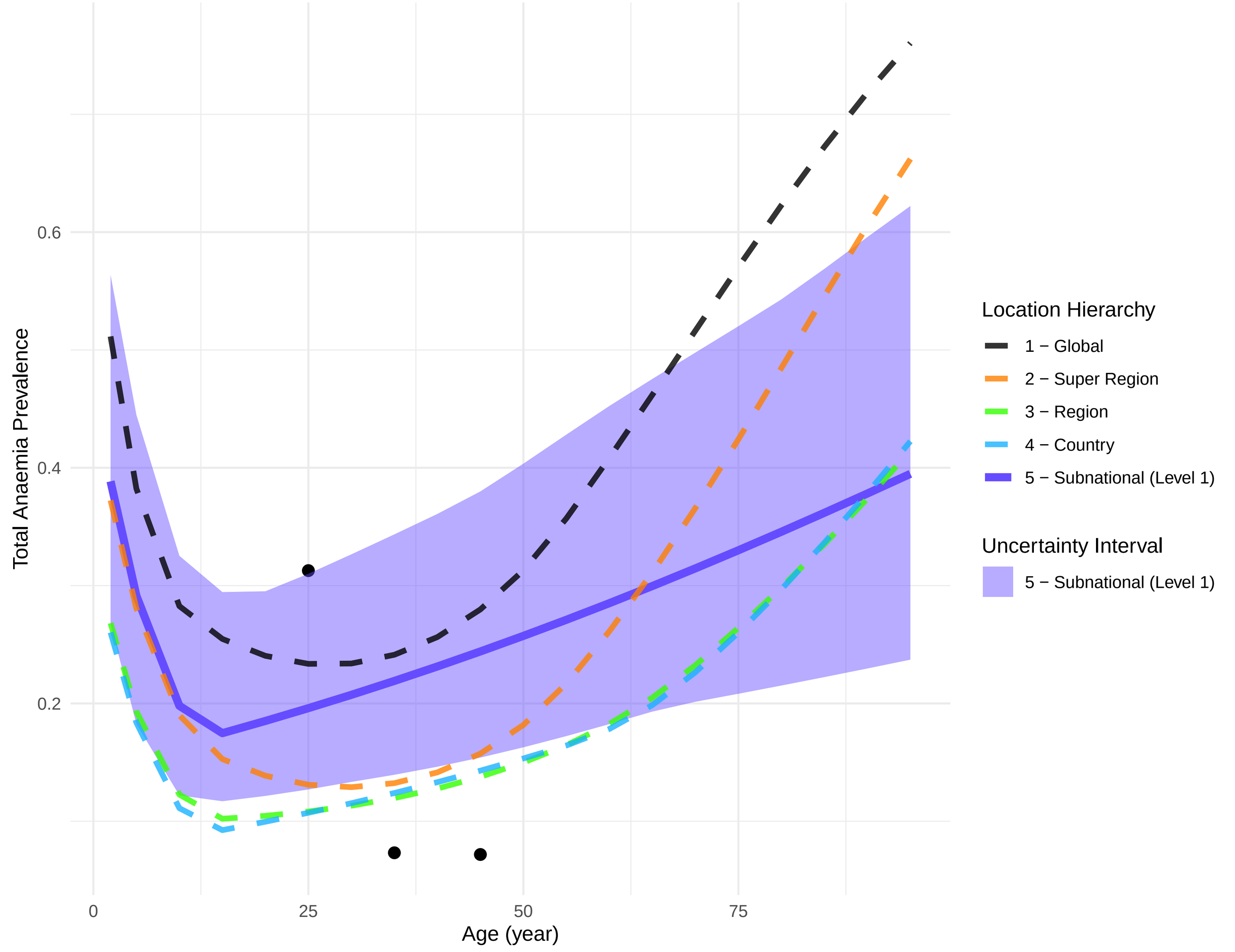

MR−BRT Age/Sex Fits − Males 2+ Years Old − Total Anaemia Prevalence − Minas Gerais
WHO−2001 Adjustment Method

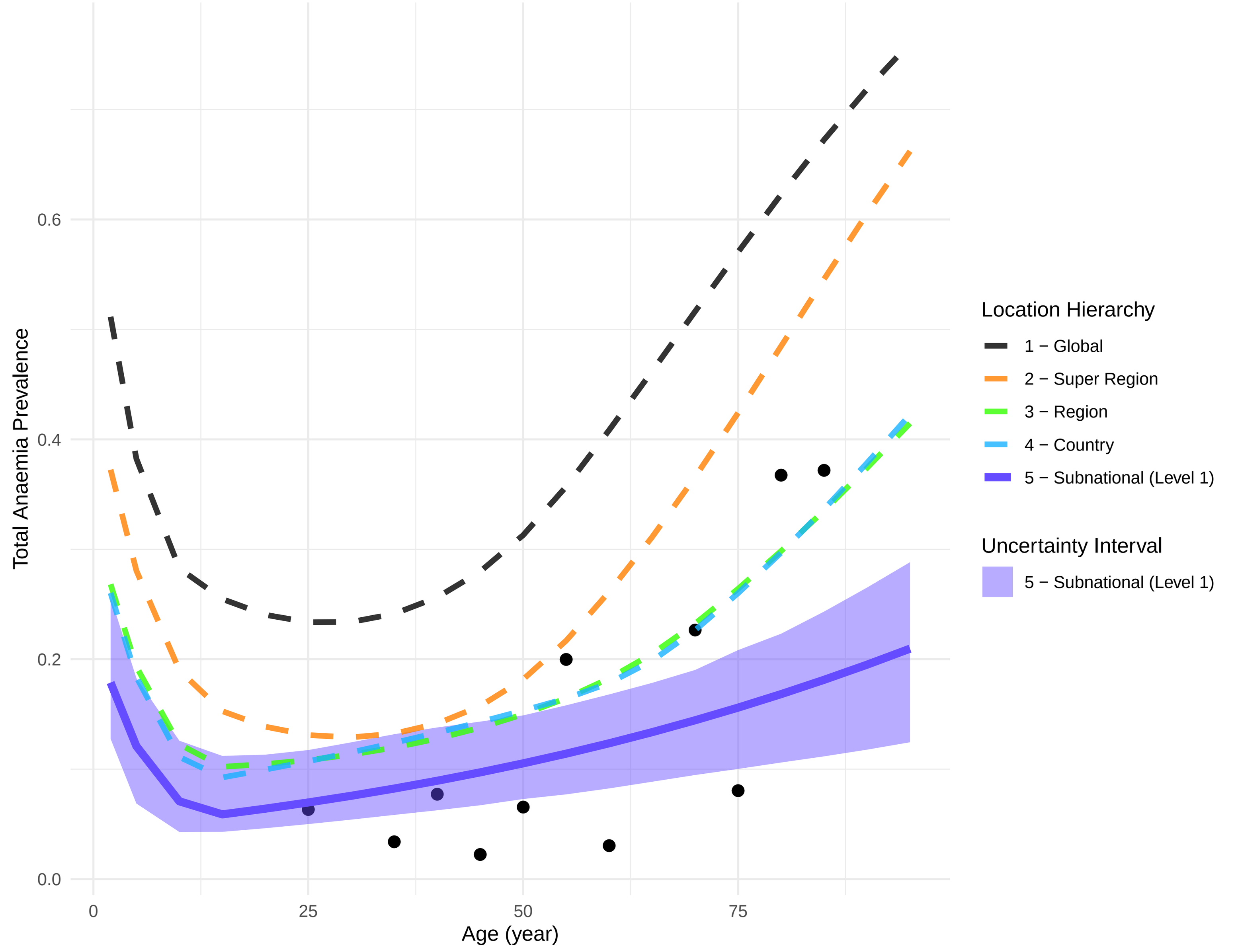

MR−BRT Age/Sex Fits − Males 2+ Years Old − Total Anaemia Prevalence − Pará
WHO−2001 Adjustment Method

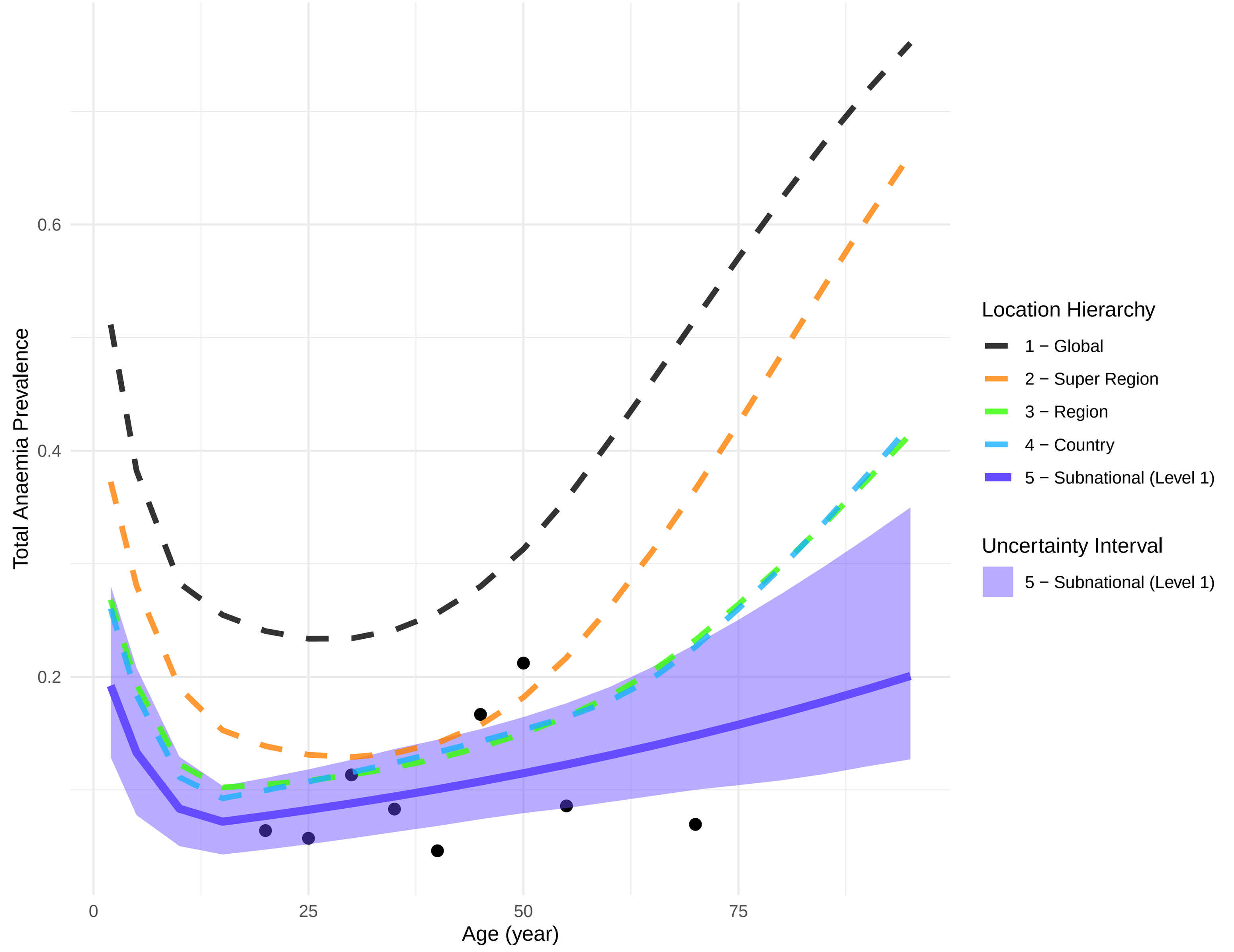

MR−BRT Age/Sex Fits − Males 2+ Years Old − Total Anaemia Prevalence − Paraíba
WHO−2001 Adjustment Method

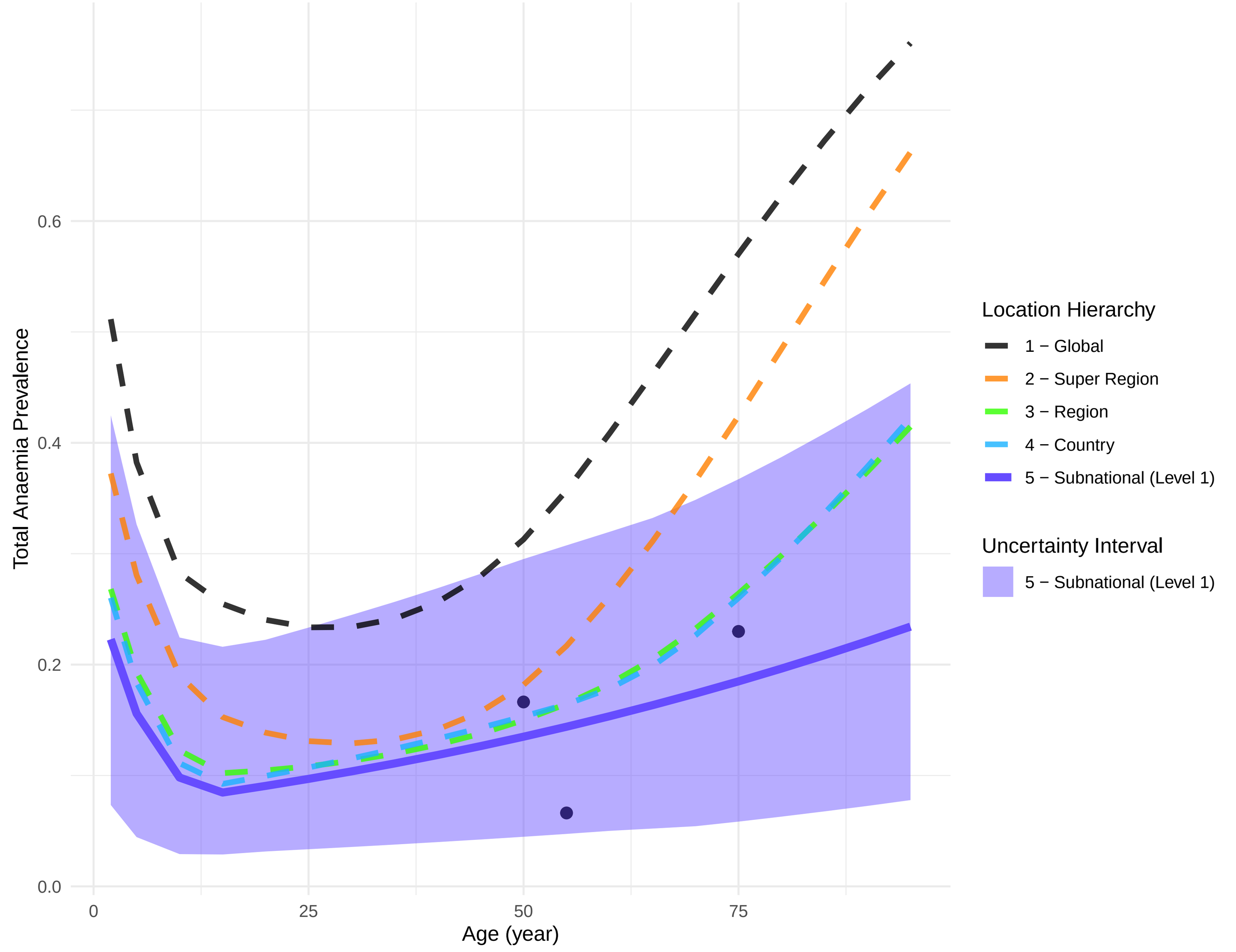

MR−BRT Age/Sex Fits − Males 2+ Years Old − Total Anaemia Prevalence − Paraná
WHO−2001 Adjustment Method

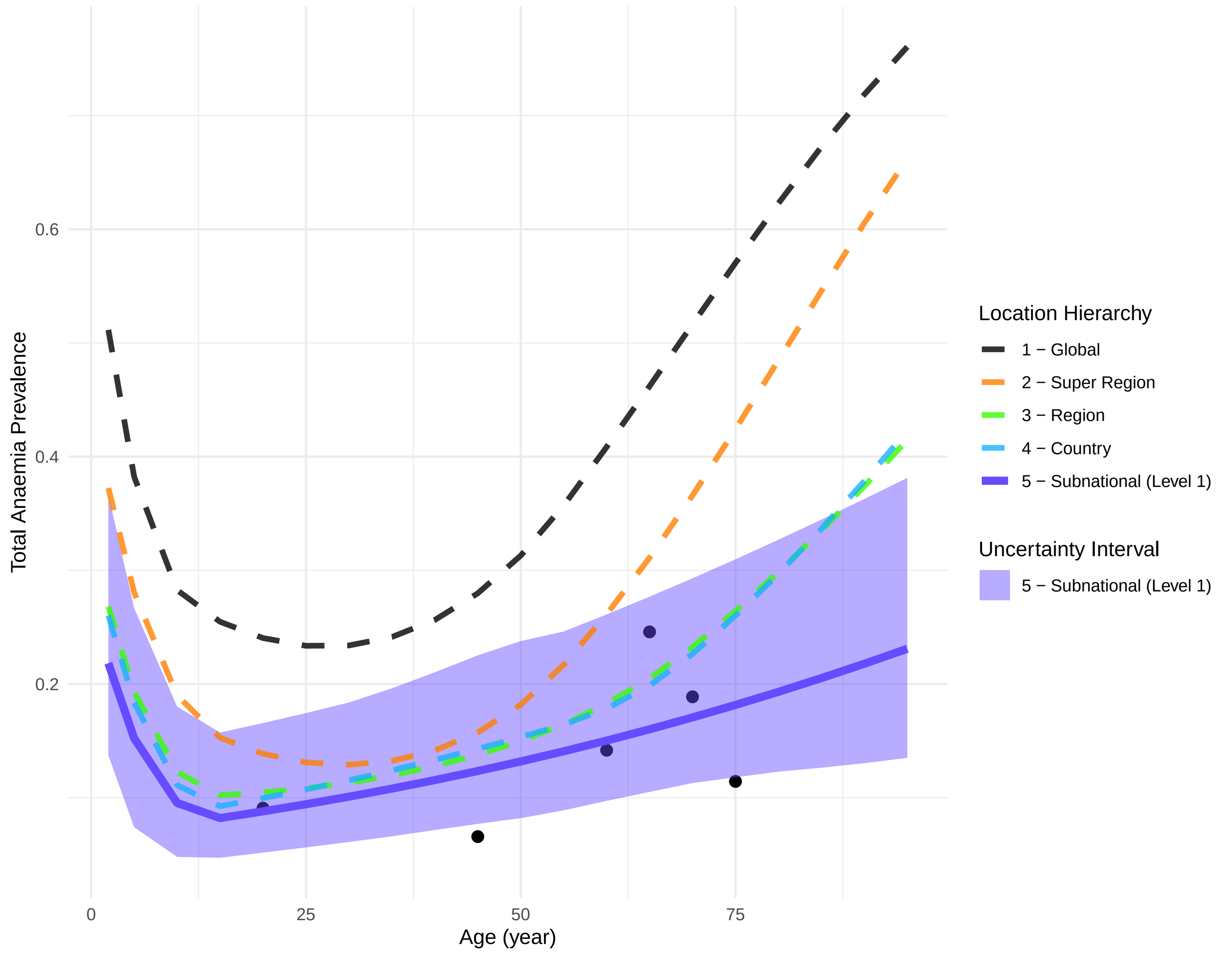

MR−BRT Age/Sex Fits − Males 2+ Years Old − Total Anaemia Prevalence − Pernambuco
WHO−2001 Adjustment Method

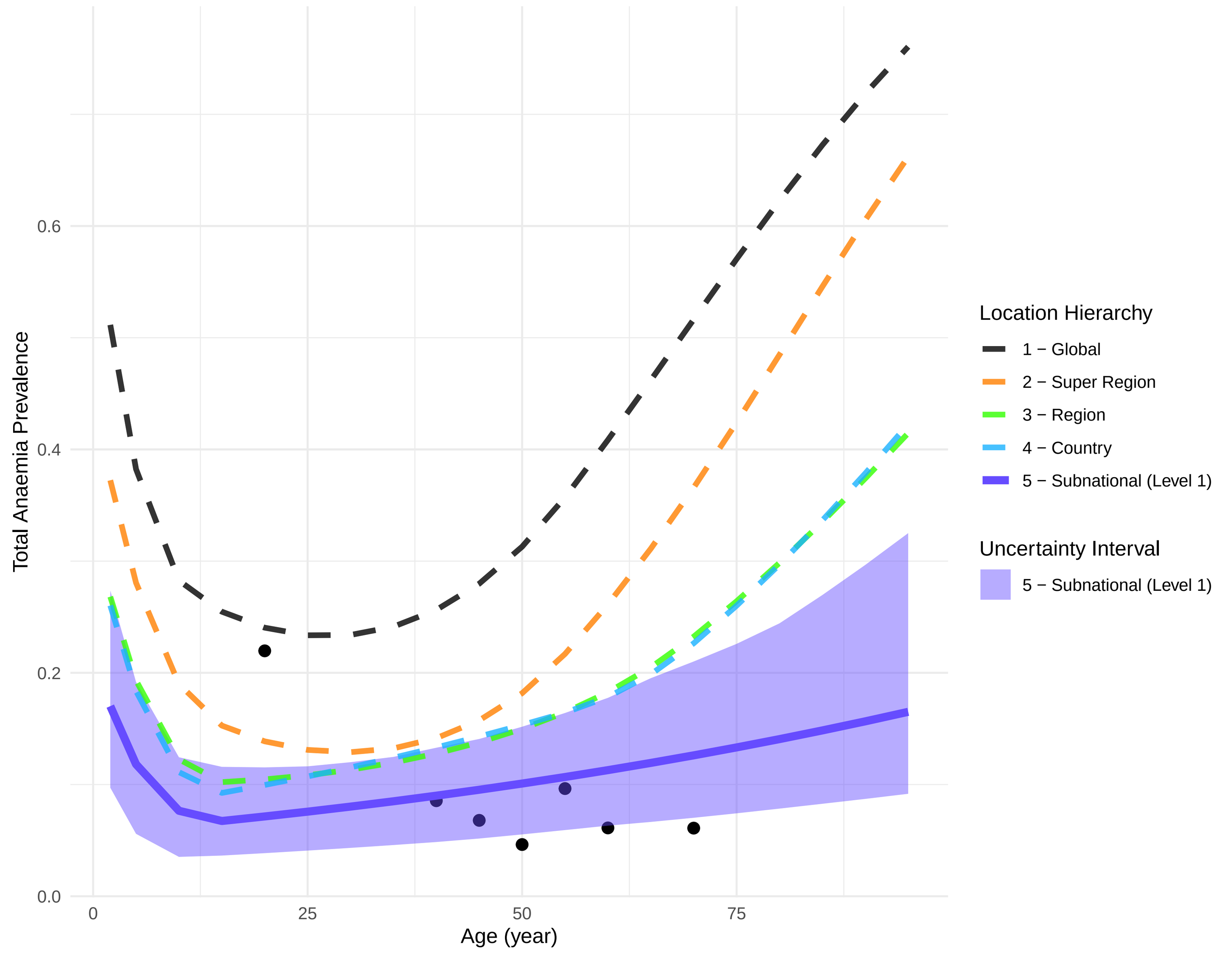

MR−BRT Age/Sex Fits − Males 2+ Years Old − Total Anaemia Prevalence − Piaui
WHO−2001 Adjustment Method

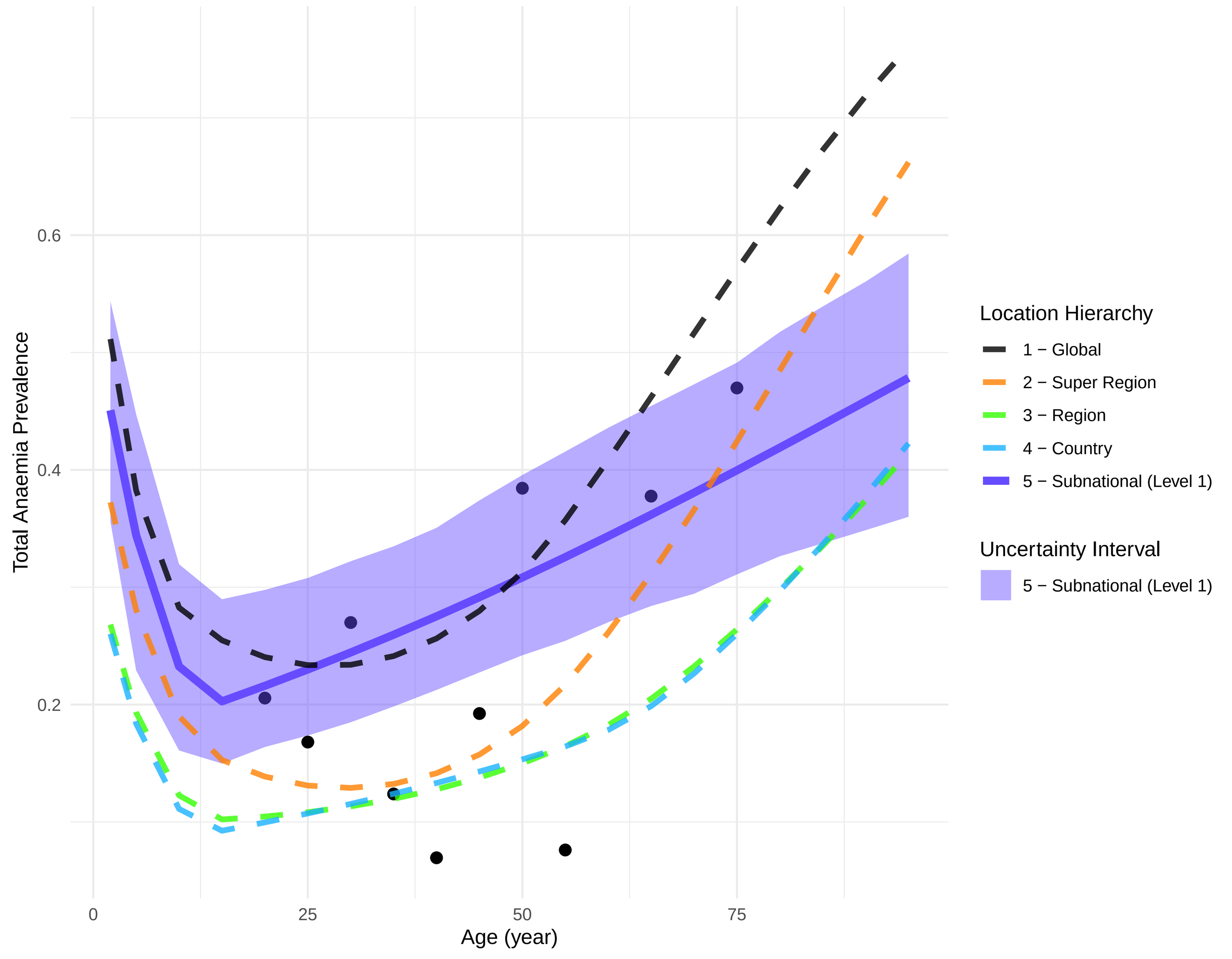

MR−BRT Age/Sex Fits − Males 2+ Years Old − Total Anaemia Prevalence − Rio de Janeiro
WHO−2001 Adjustment Method

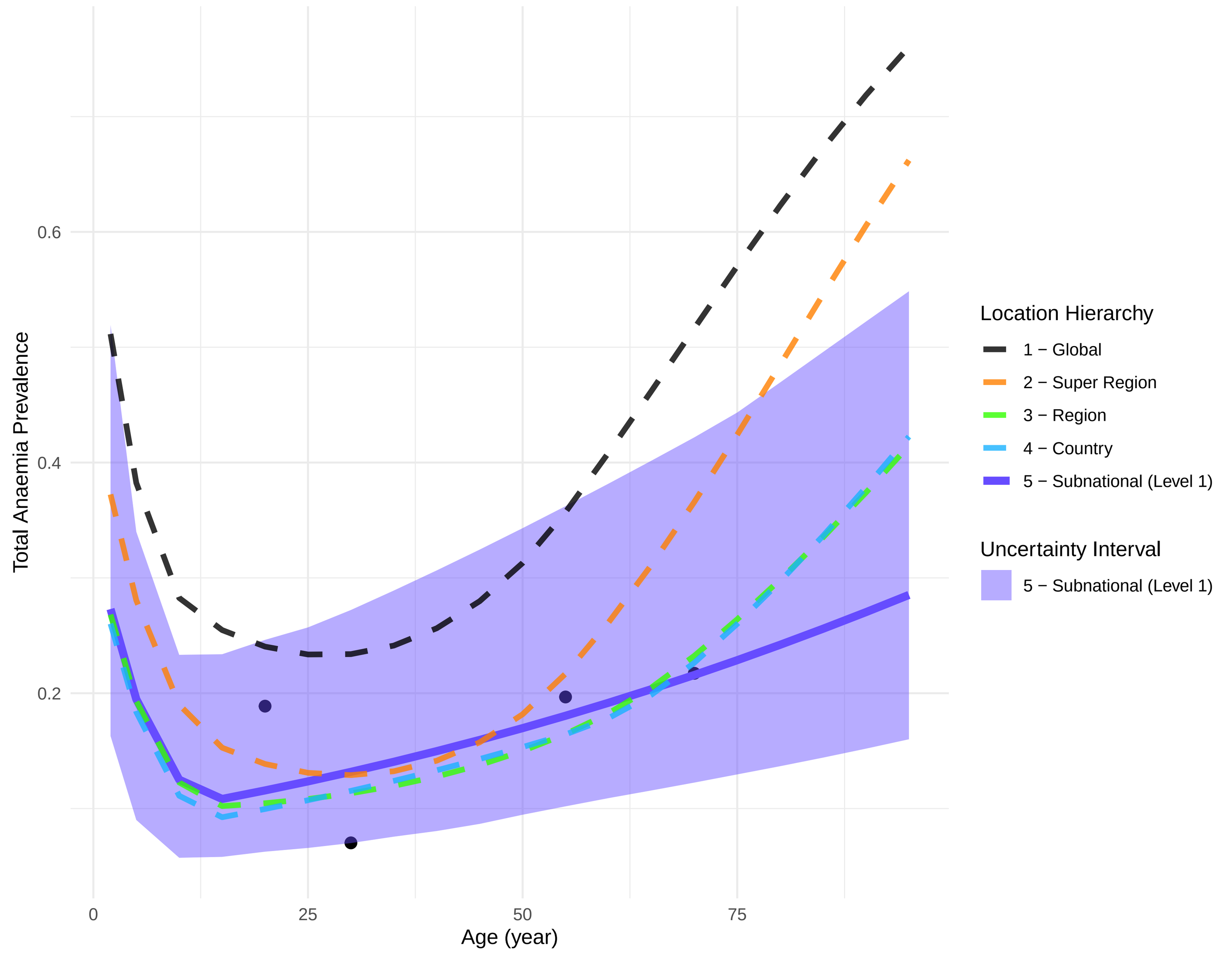

MR−BRT Age/Sex Fits − Males 2+ Years Old − Total Anaemia Prevalence − Rio Grande do Norte
WHO−2001 Adjustment Method

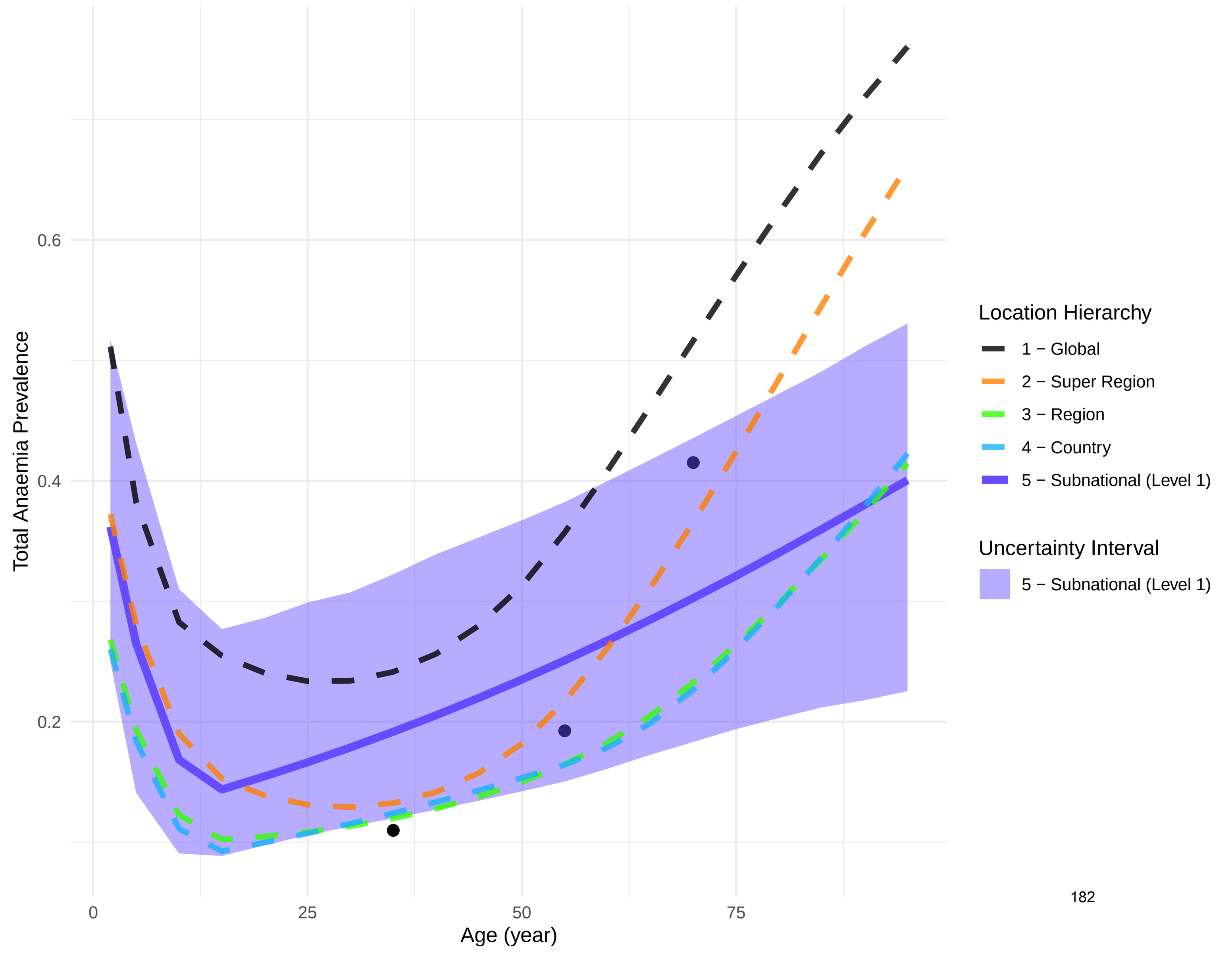

MR−BRT Age/Sex Fits − Males 2+ Years Old − Total Anaemia Prevalence − Rio Grande do Sul
WHO−2001 Adjustment Method

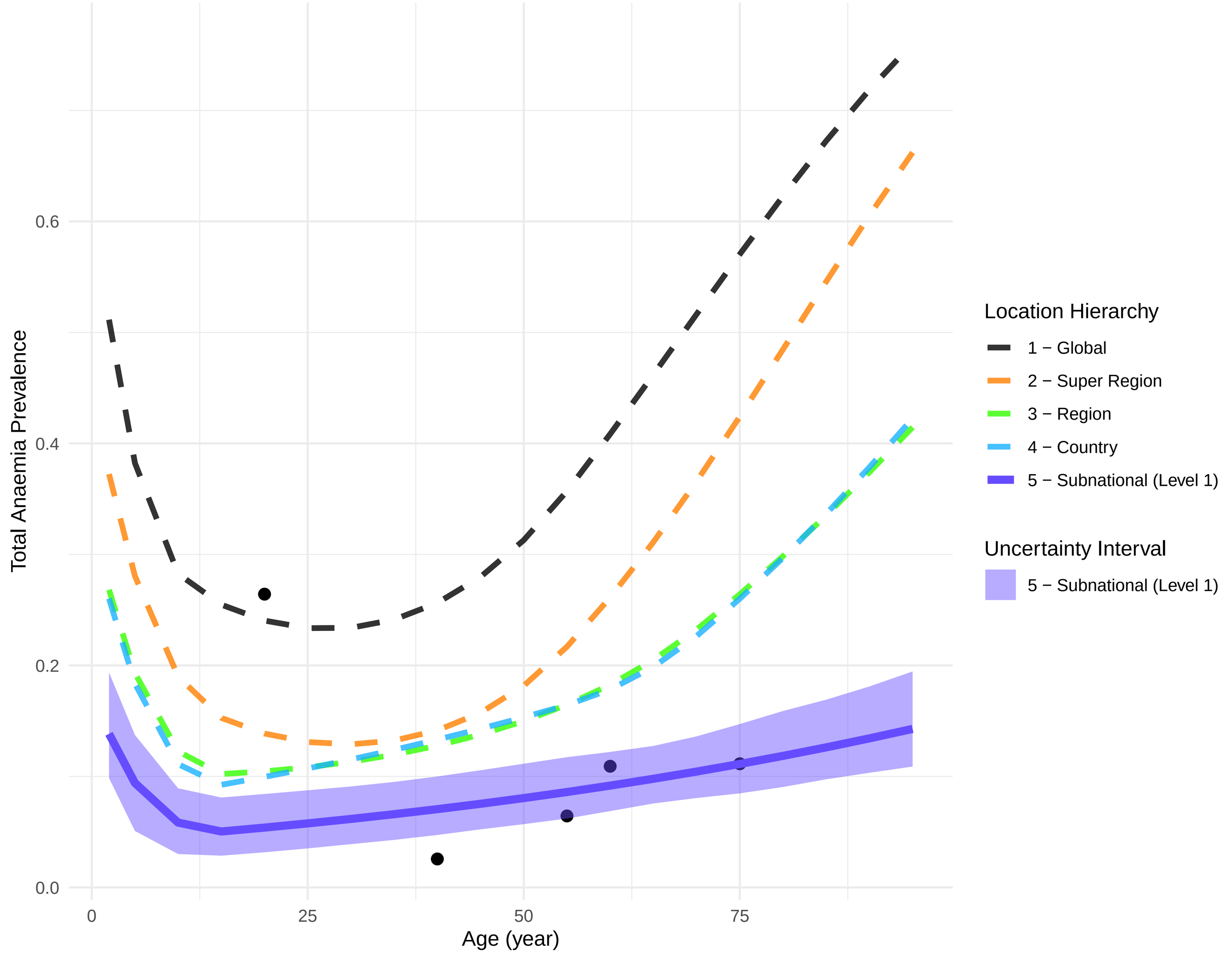

MR−BRT Age/Sex Fits − Males 2+ Years Old − Total Anaemia Prevalence − Rondônia
WHO−2001 Adjustment Method

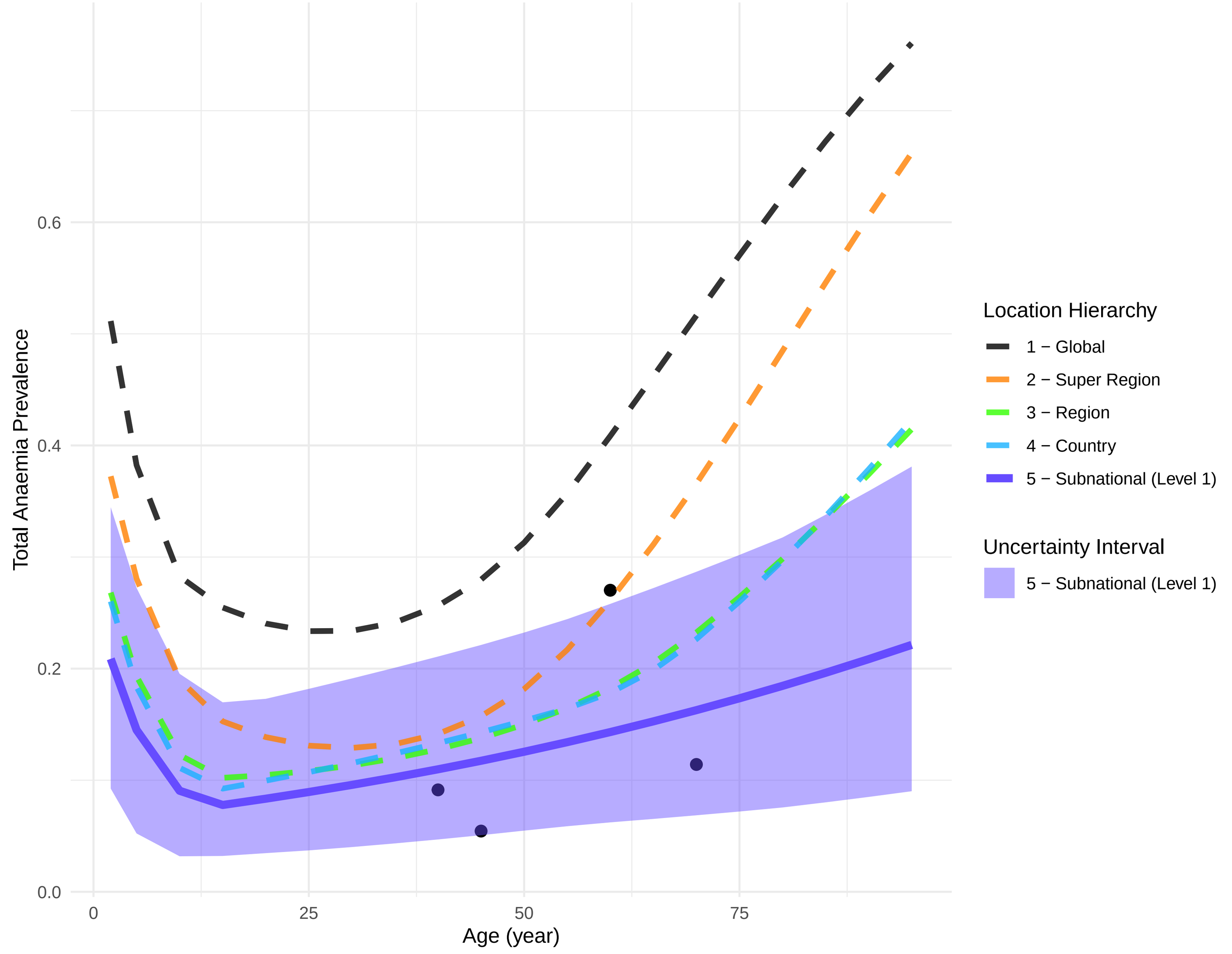

MR−BRT Age/Sex Fits − Males 2+ Years Old − Total Anaemia Prevalence − Roraima
WHO−2001 Adjustment Method

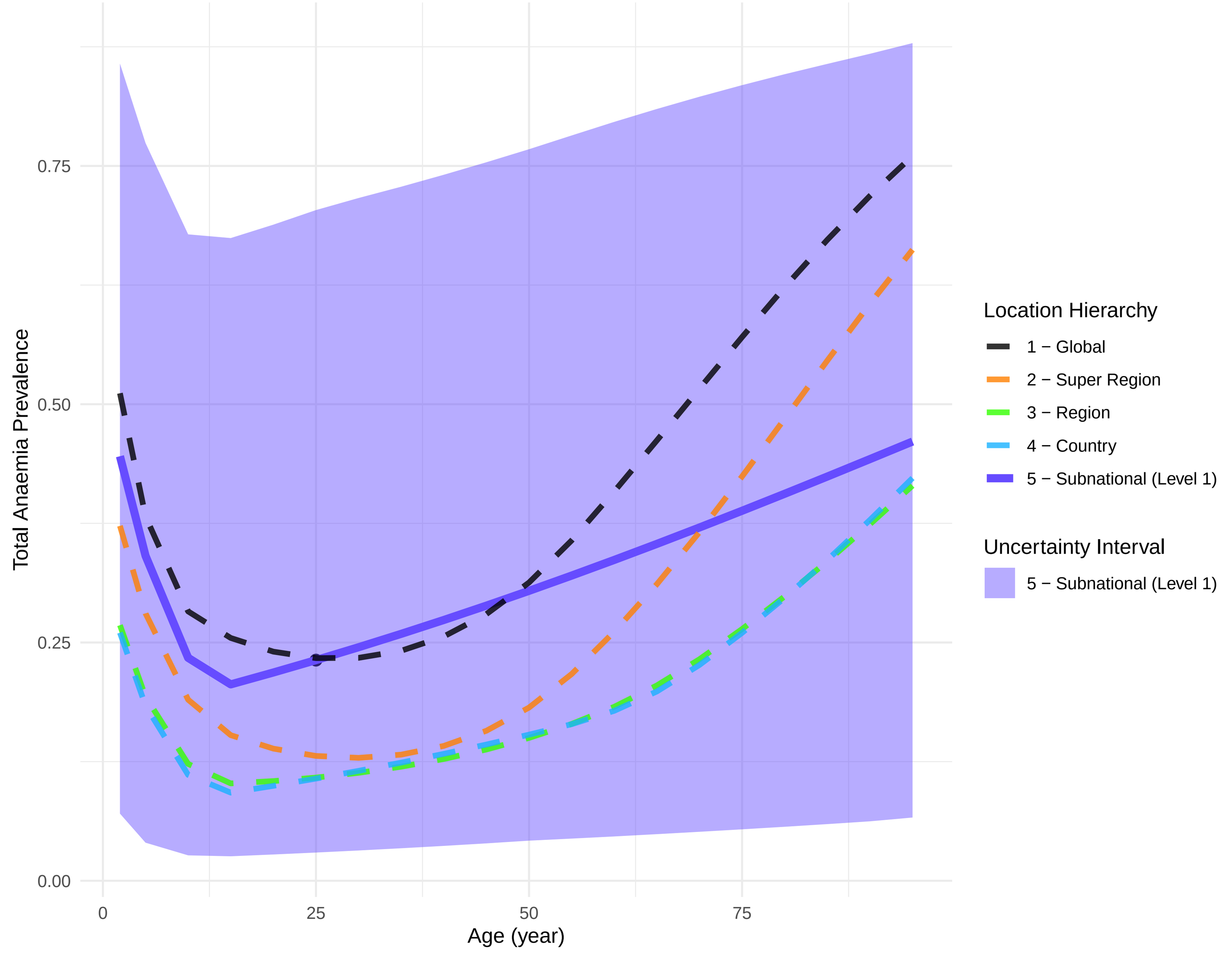

MR−BRT Age/Sex Fits − Males 2+ Years Old − Total Anaemia Prevalence − Santa Catarina
WHO−2001 Adjustment Method

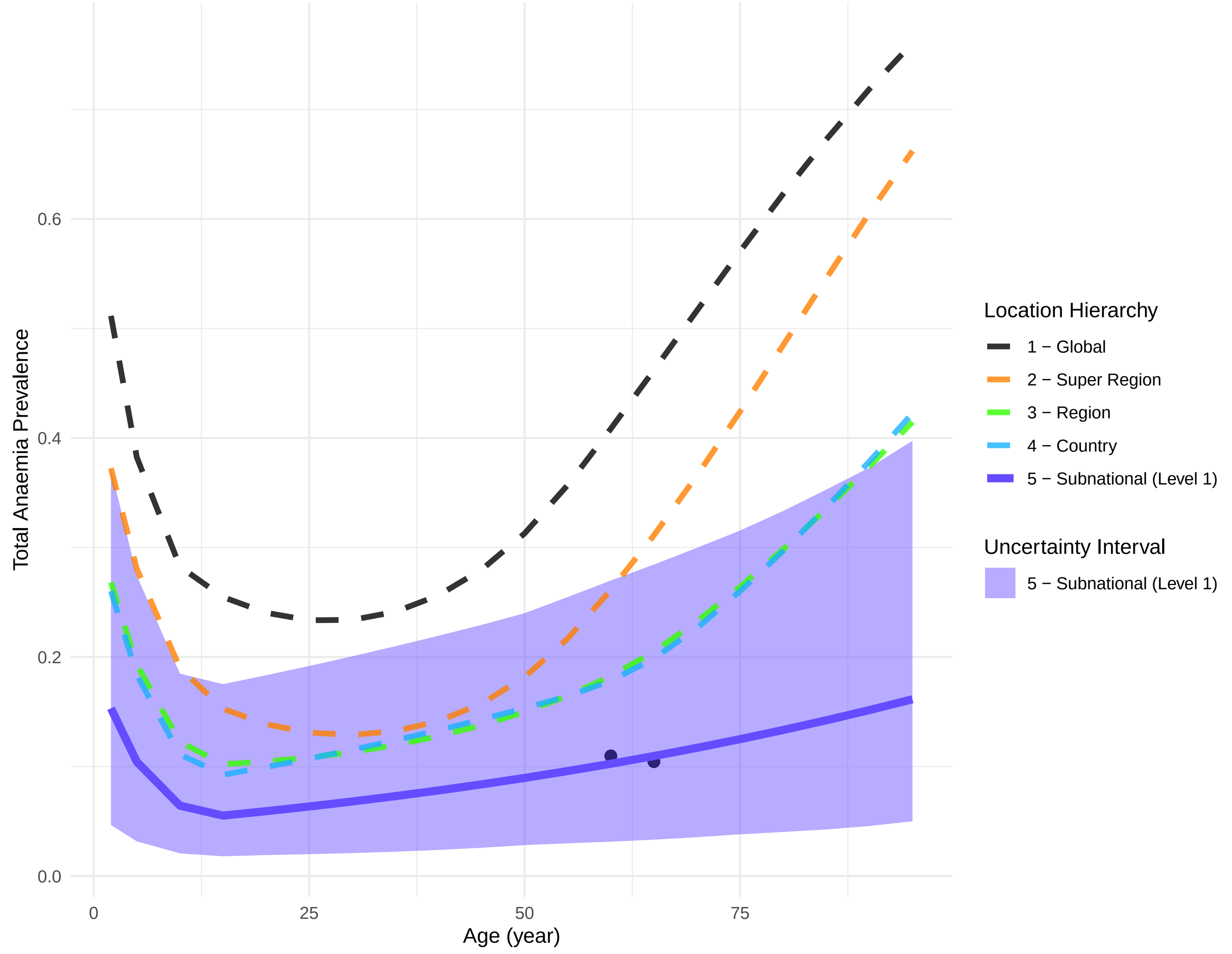

MR−BRT Age/Sex Fits − Males 2+ Years Old − Total Anaemia Prevalence − São Paulo
WHO−2001 Adjustment Method

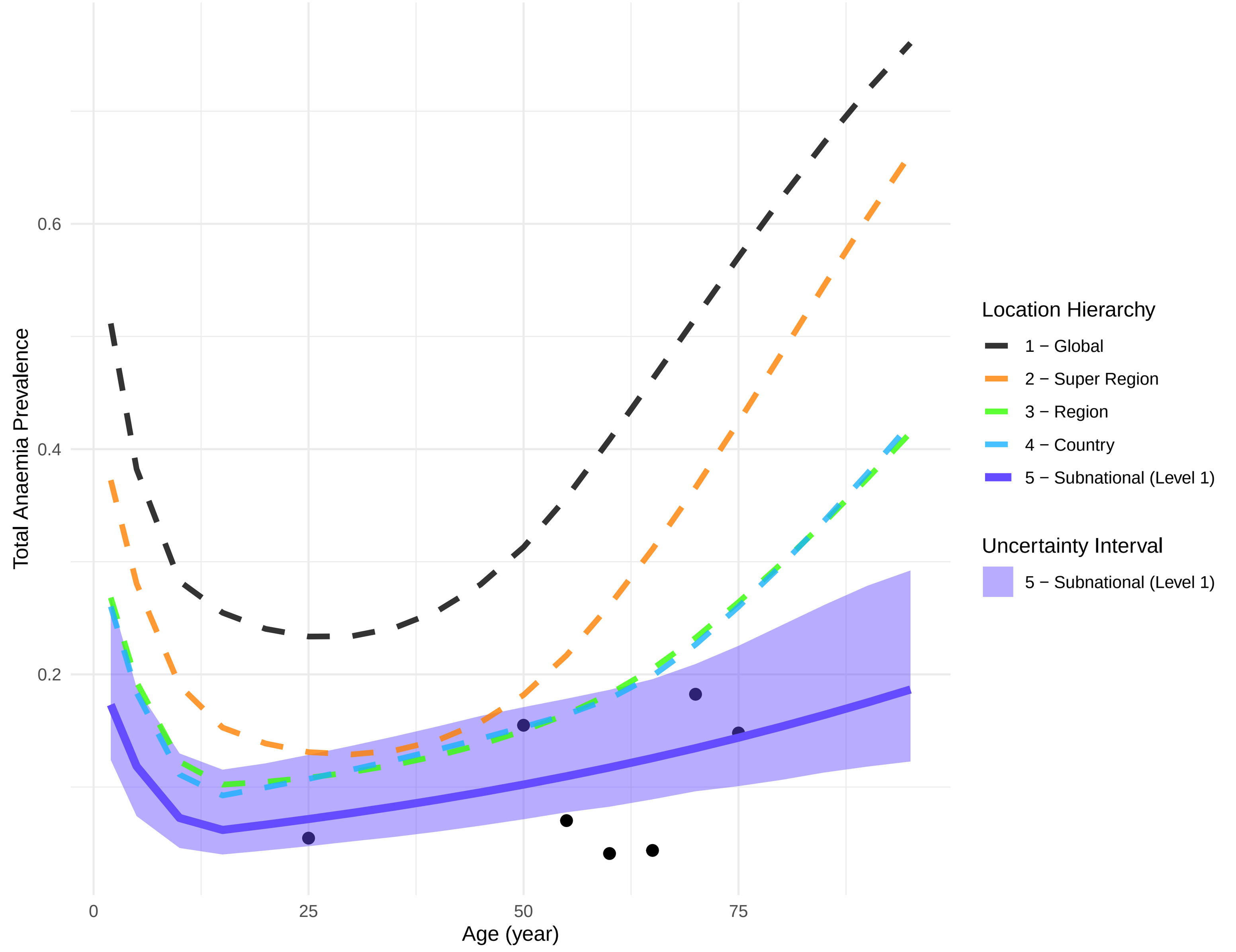

MR−BRT Age/Sex Fits − Males 2+ Years Old − Total Anaemia Prevalence − Sergipe
WHO−2001 Adjustment Method

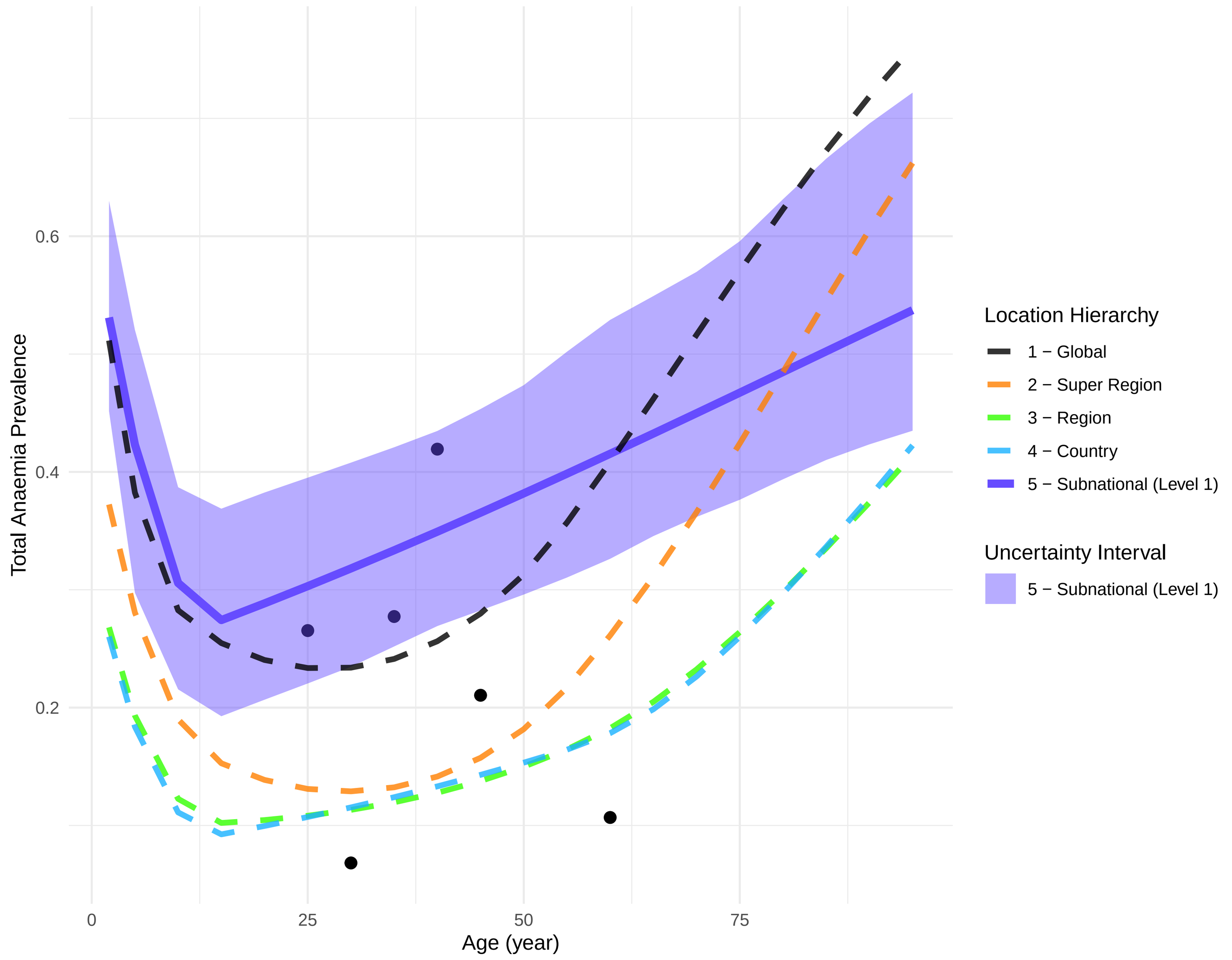

MR−BRT Age/Sex Fits − Males 2+ Years Old − Total Anaemia Prevalence − North Africa and Middle East
WHO−2001 Adjustment Method

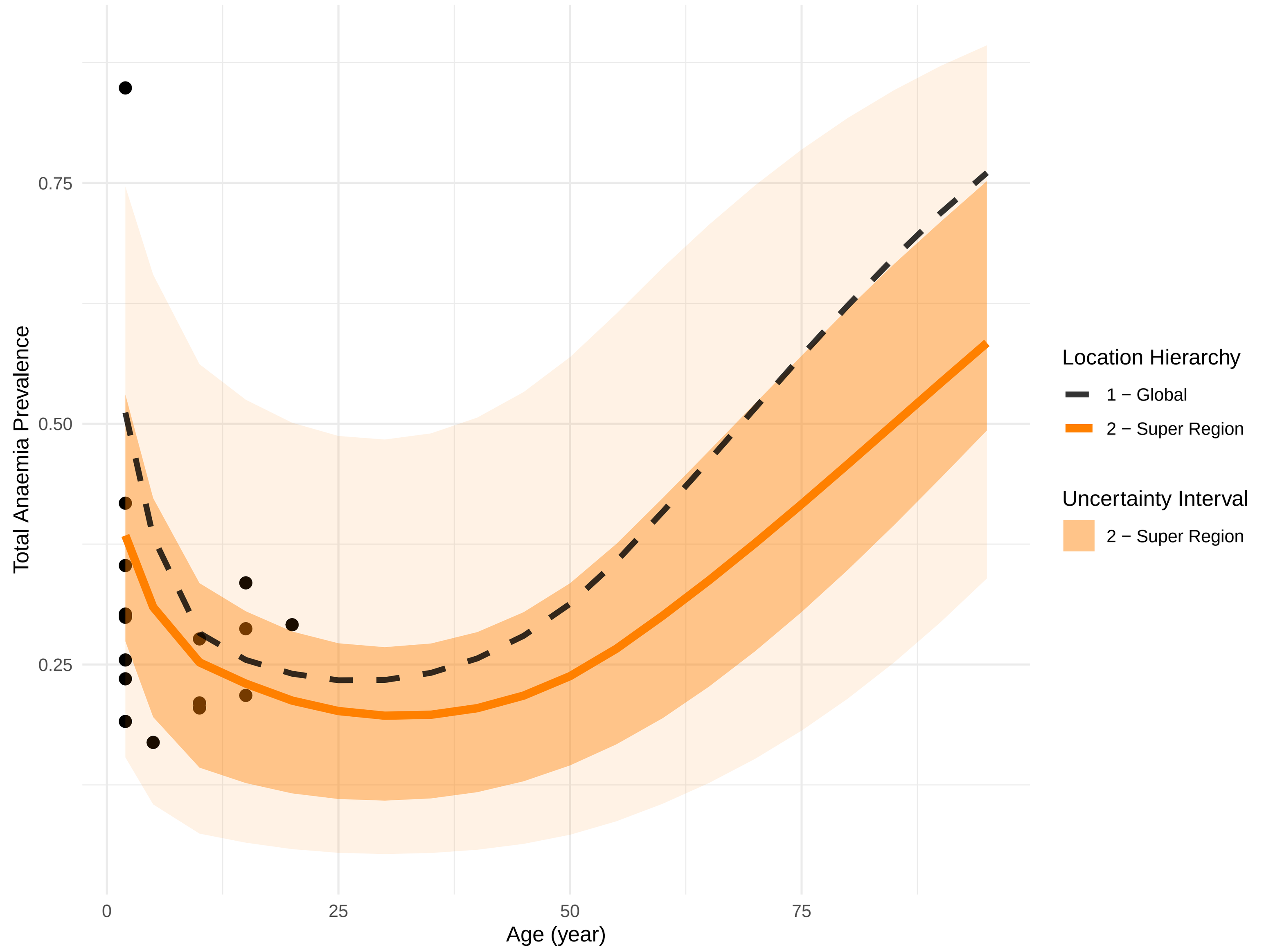

MR−BRT Age/Sex Fits − Males 2+ Years Old − Total Anaemia Prevalence − North Africa and Middle East
WHO−2001 Adjustment Method

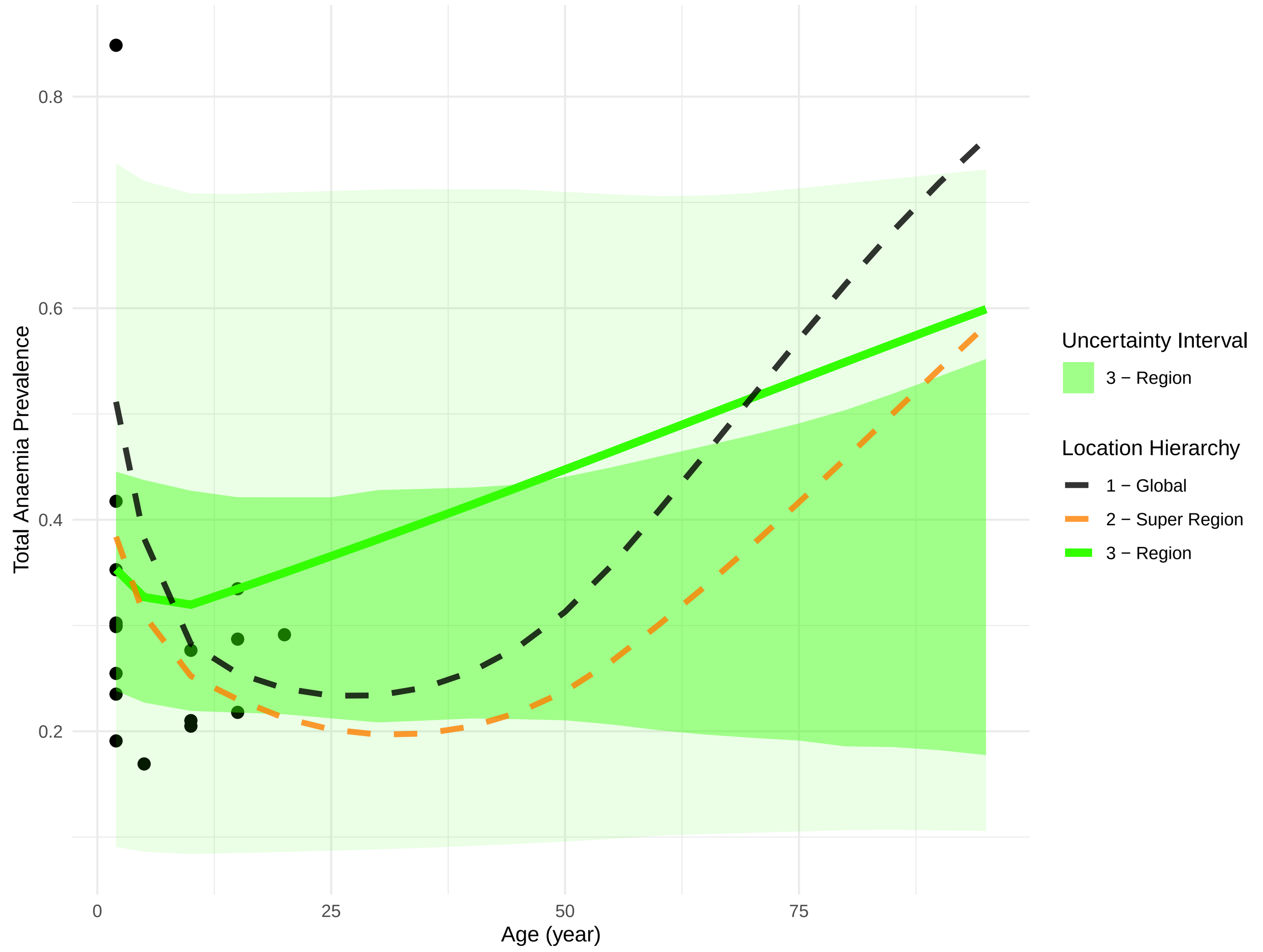

MR−BRT Age/Sex Fits − Males 2+ Years Old − Total Anaemia Prevalence − Egypt
WHO−2001 Adjustment Method

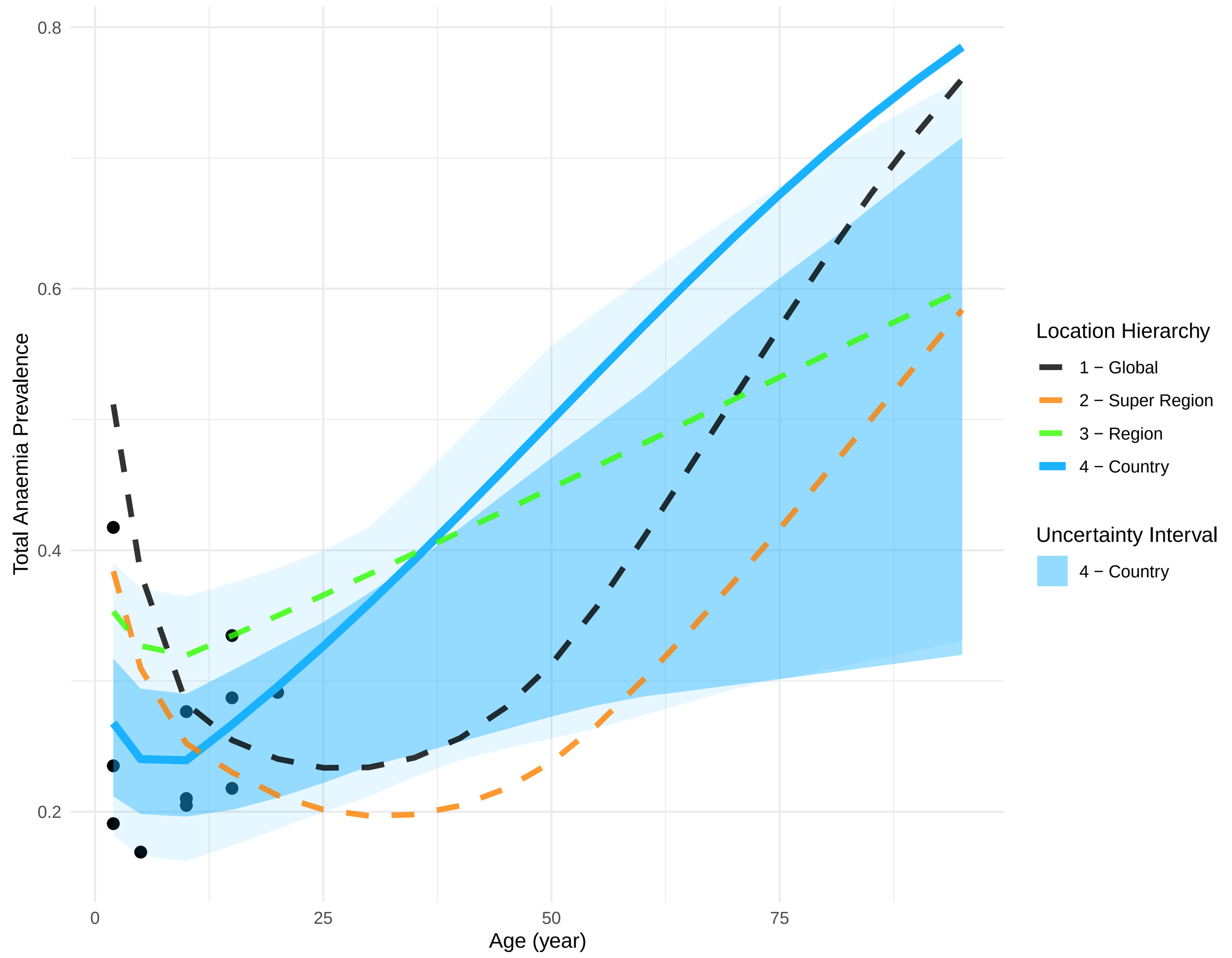

MR−BRT Age/Sex Fits − Males 2+ Years Old − Total Anaemia Prevalence − Jordan
WHO−2001 Adjustment Method

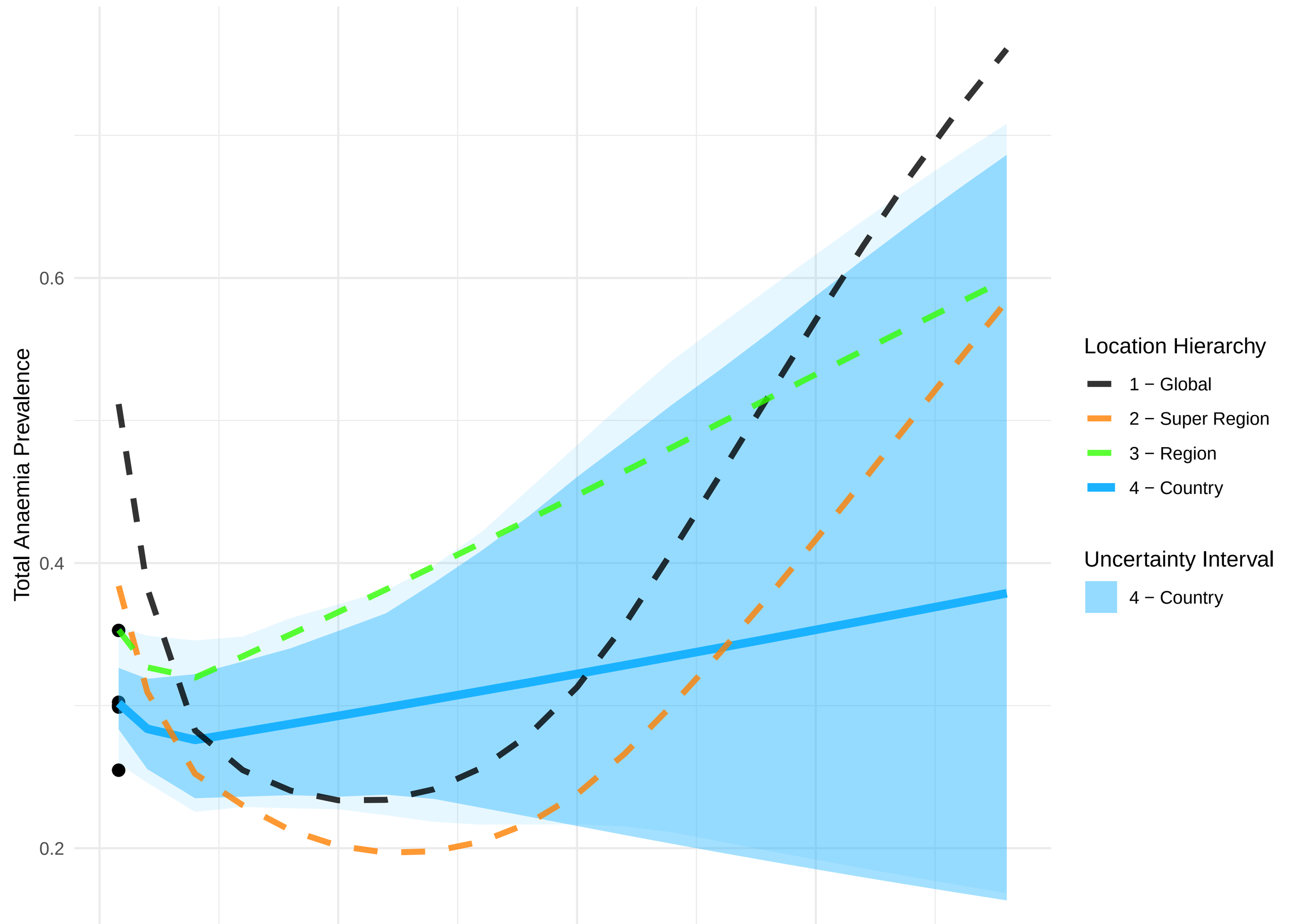

MR−BRT Age/Sex Fits − Males 2+ Years Old − Total Anaemia Prevalence − Yemen
WHO−2001 Adjustment Method

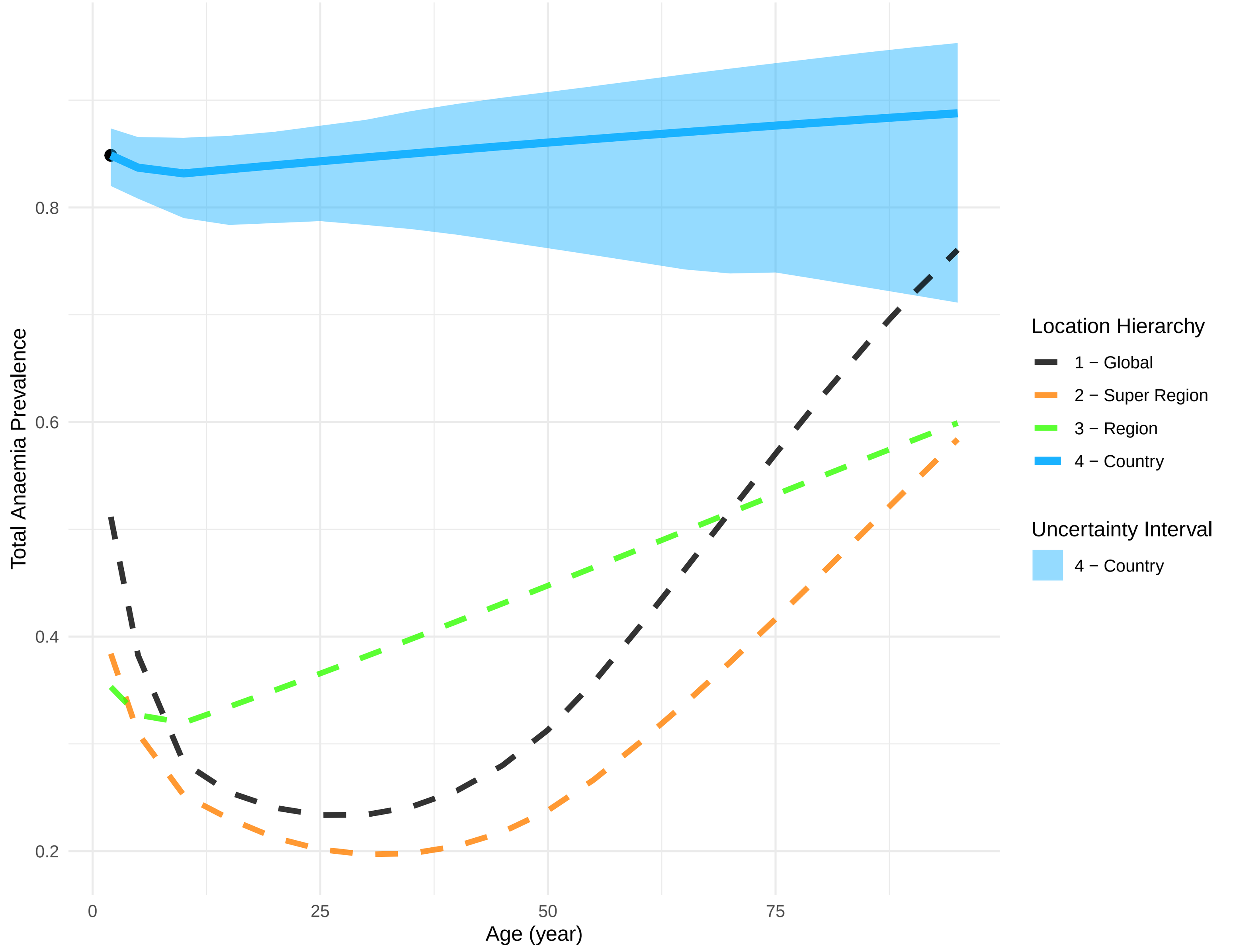

MR−BRT Age/Sex Fits − Males 2+ Years Old − Total Anaemia Prevalence − South Asia
WHO−2001 Adjustment Method

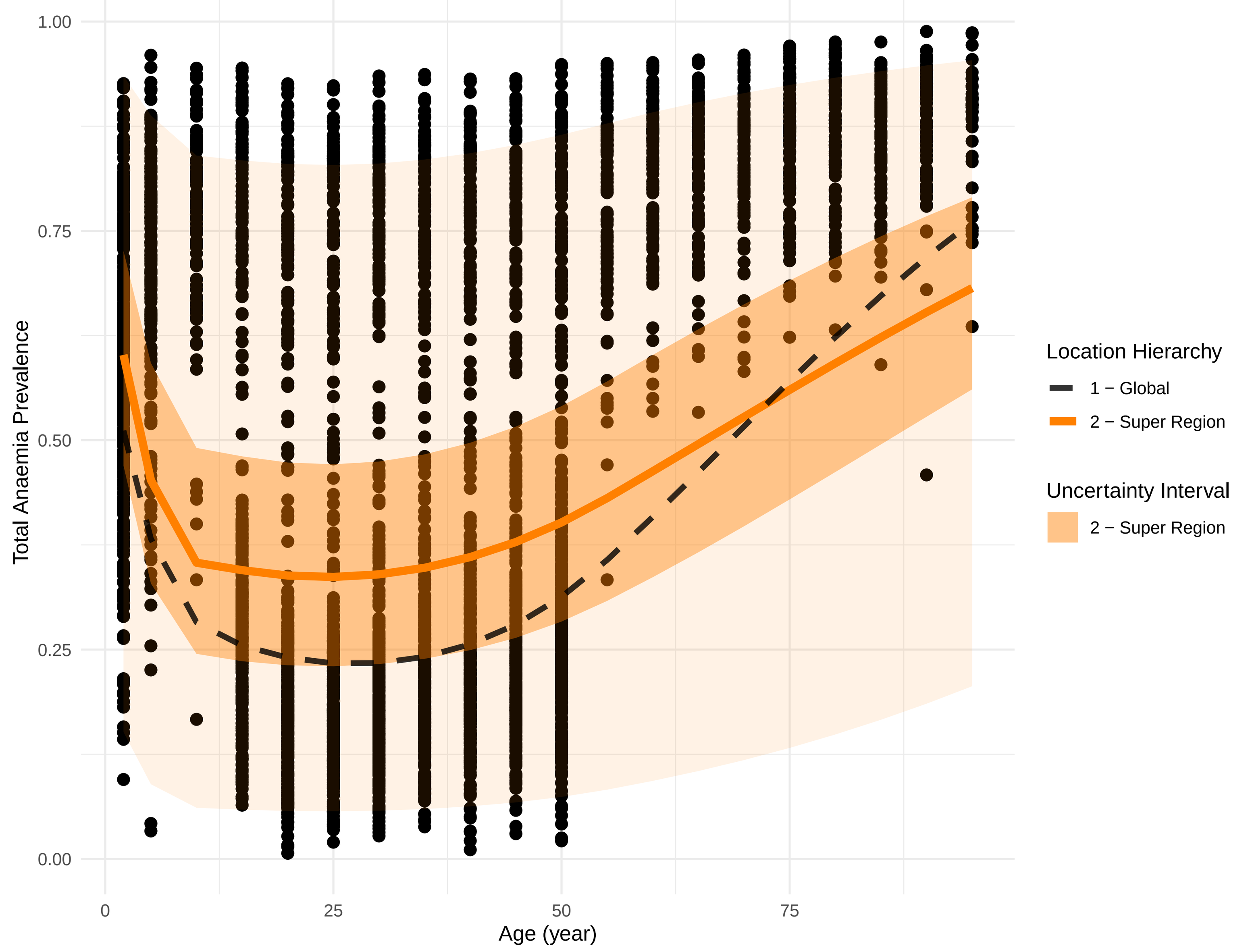

MR−BRT Age/Sex Fits − Males 2+ Years Old − Total Anaemia Prevalence − South Asia
WHO−2001 Adjustment Method

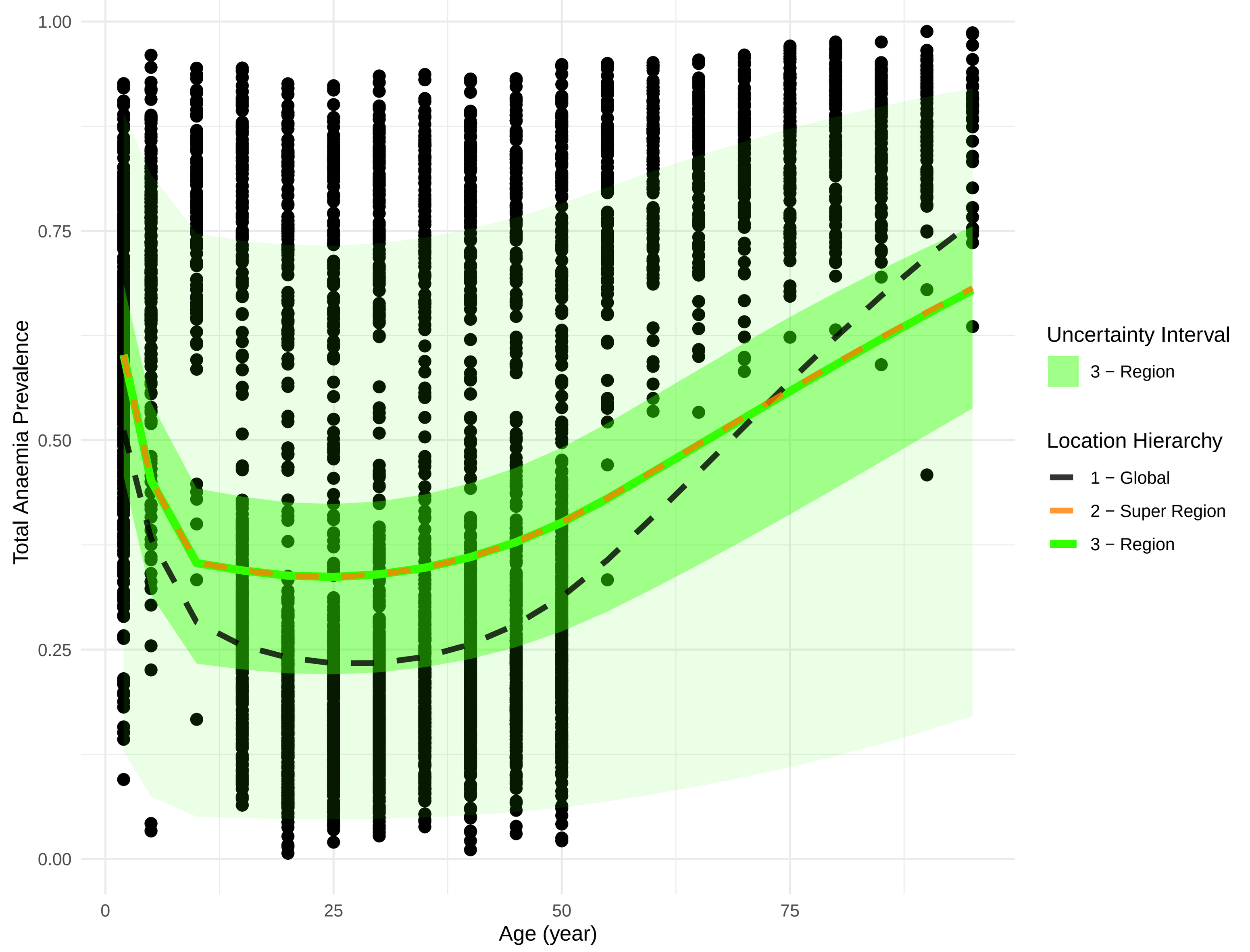

MR−BRT Age/Sex Fits − Males 2+ Years Old − Total Anaemia Prevalence − Bangladesh
WHO−2001 Adjustment Method

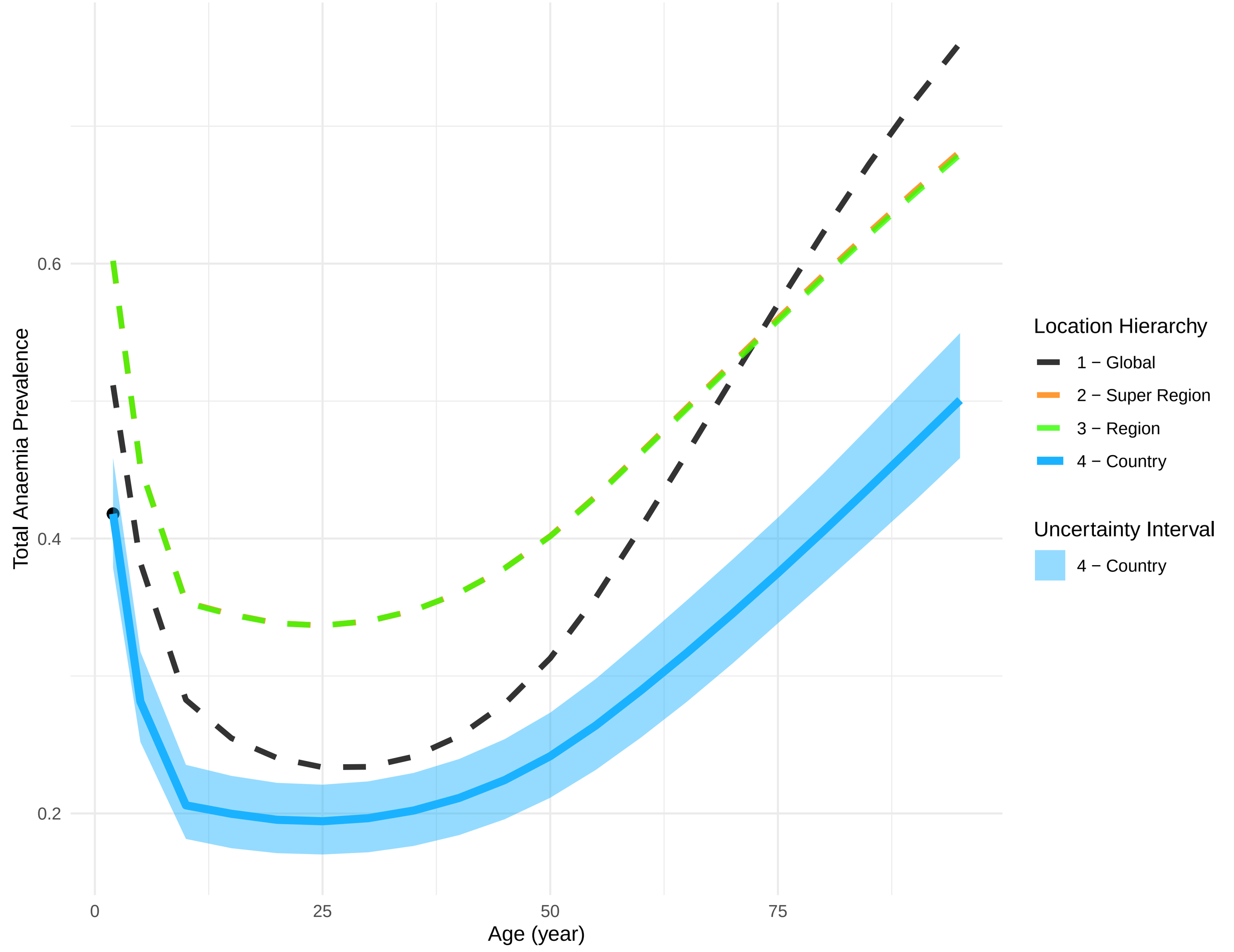

MR−BRT Age/Sex Fits − Males 2+ Years Old − Total Anaemia Prevalence − India
WHO−2001 Adjustment Method

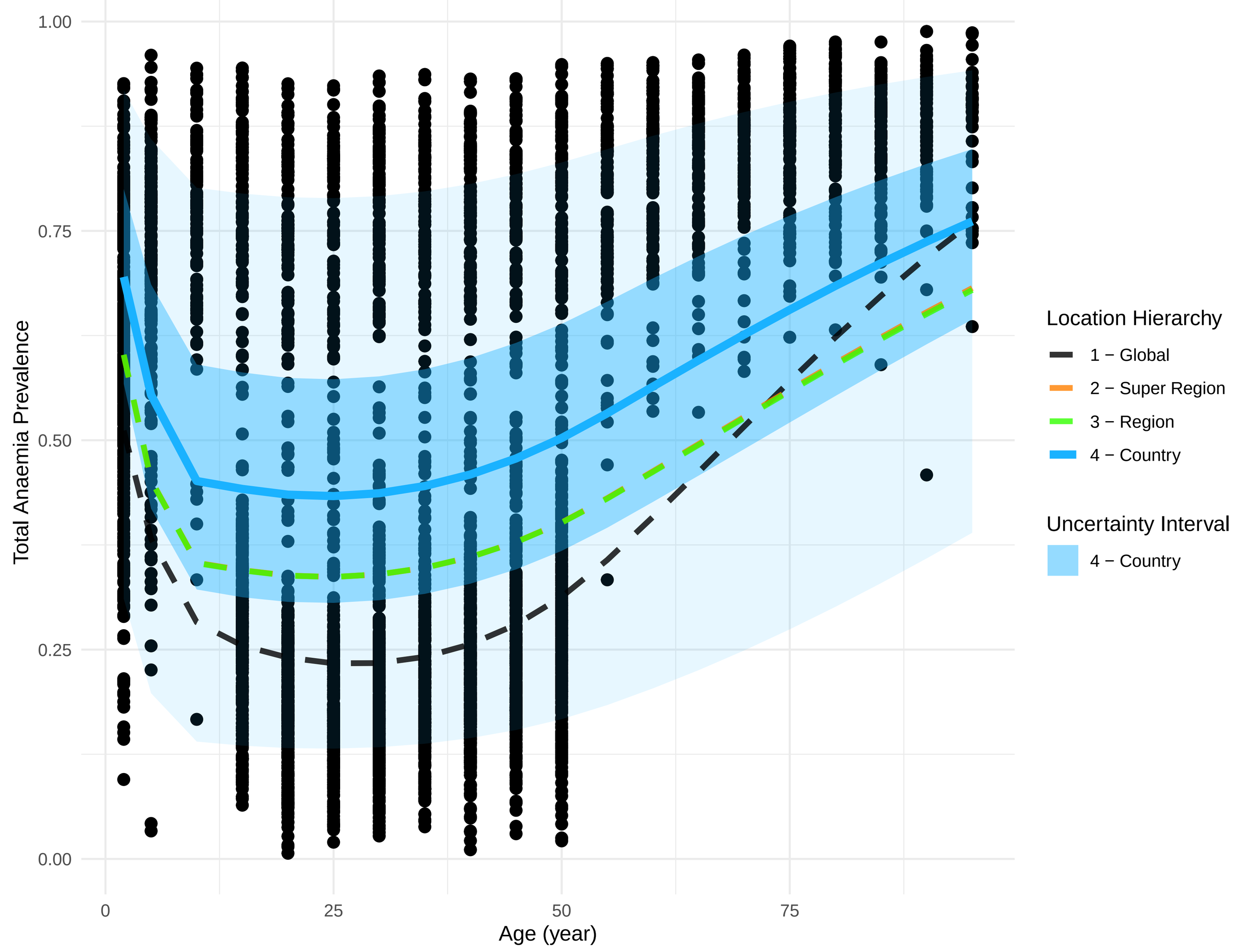

MR−BRT Age/Sex Fits − Males 2+ Years Old − Total Anaemia Prevalence − Andhra Pradesh
WHO−2001 Adjustment Method

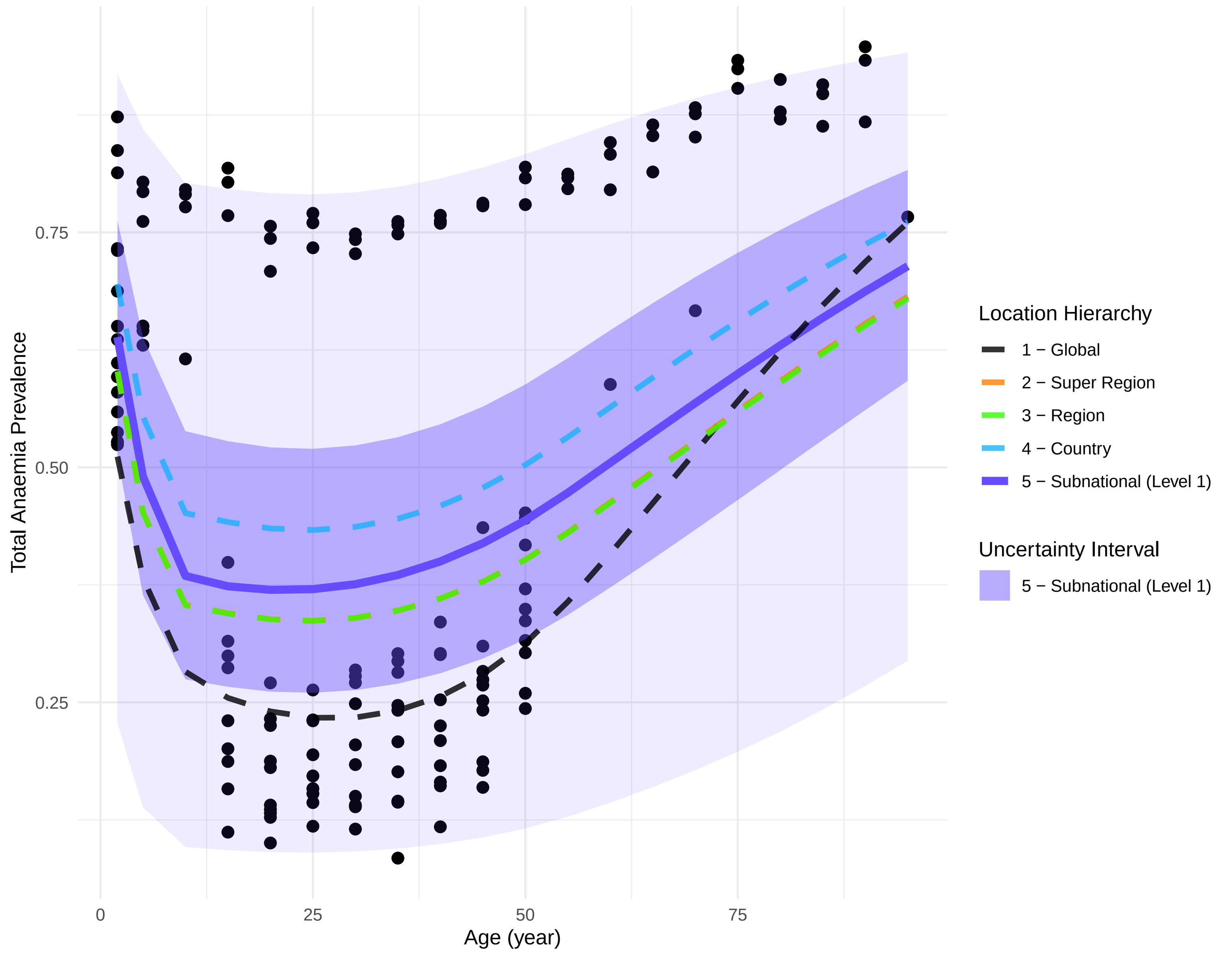

MR−BRT Age/Sex Fits − Males 2+ Years Old − Total Anaemia Prevalence − Andhra Pradesh, Rural
WHO−2001 Adjustment Method

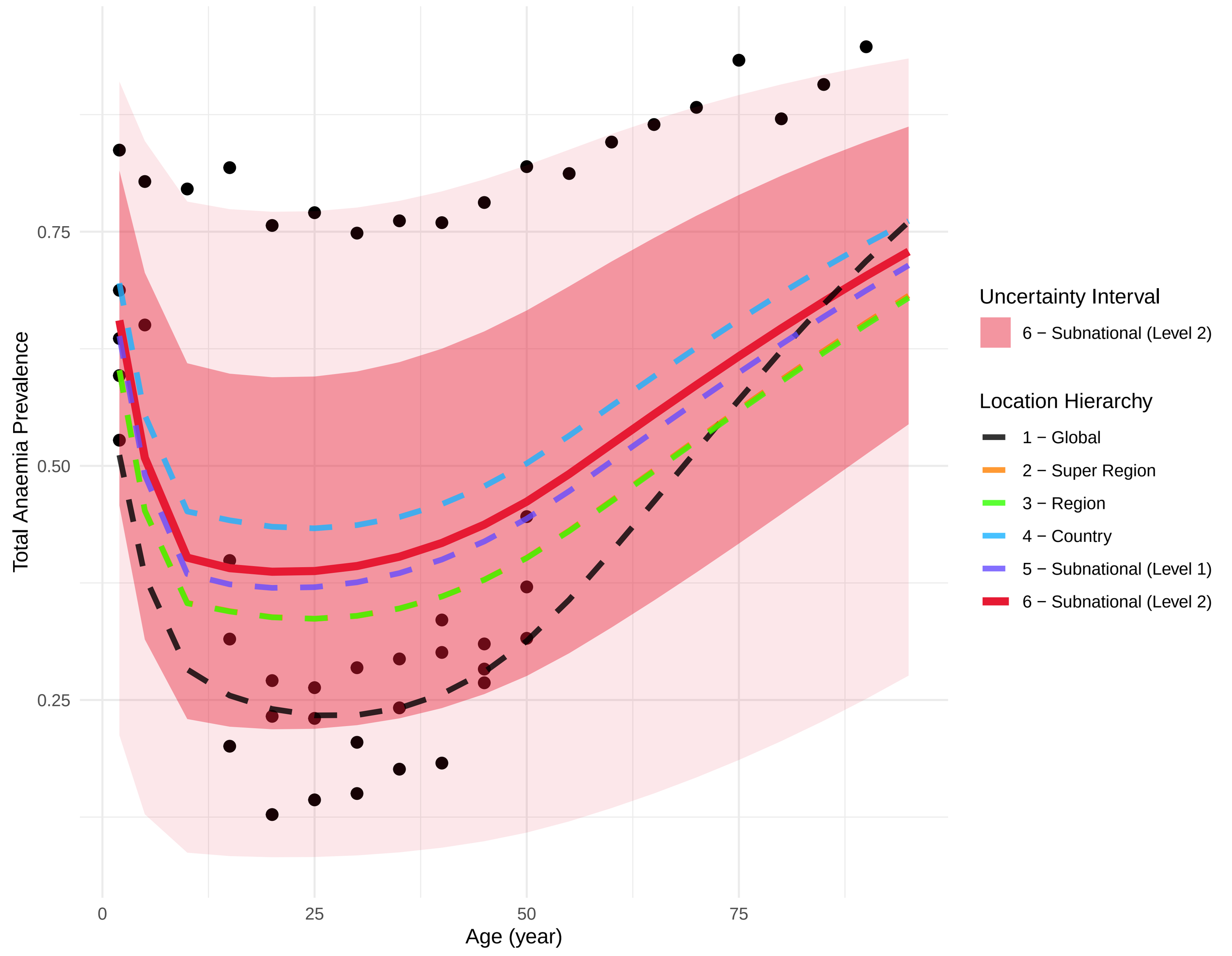

MR−BRT Age/Sex Fits − Males 2+ Years Old − Total Anaemia Prevalence − Andhra Pradesh, Urban
WHO−2001 Adjustment Method

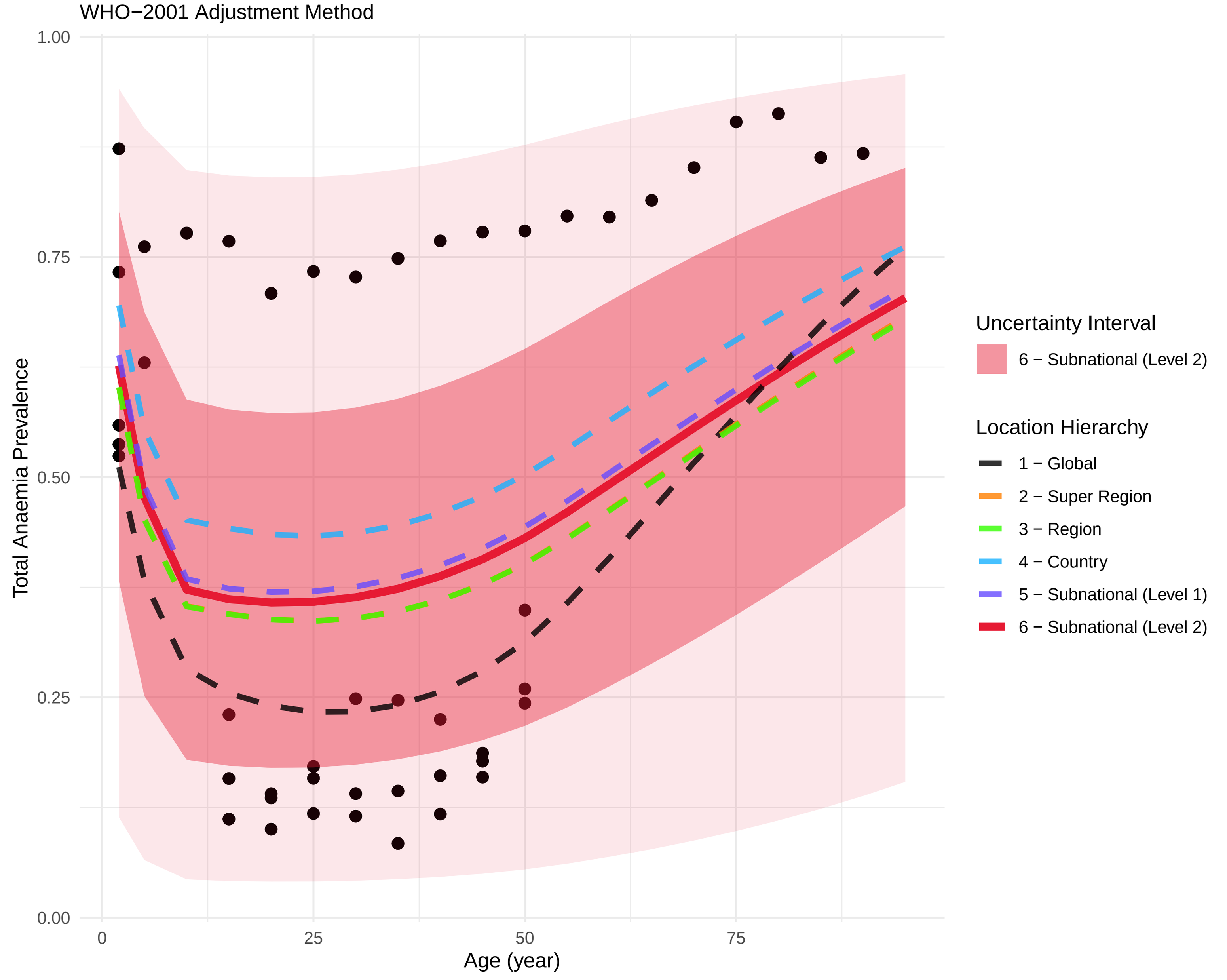

MR−BRT Age/Sex Fits − Males 2+ Years Old − Total Anaemia Prevalence − Arunachal Pradesh
WHO−2001 Adjustment Method

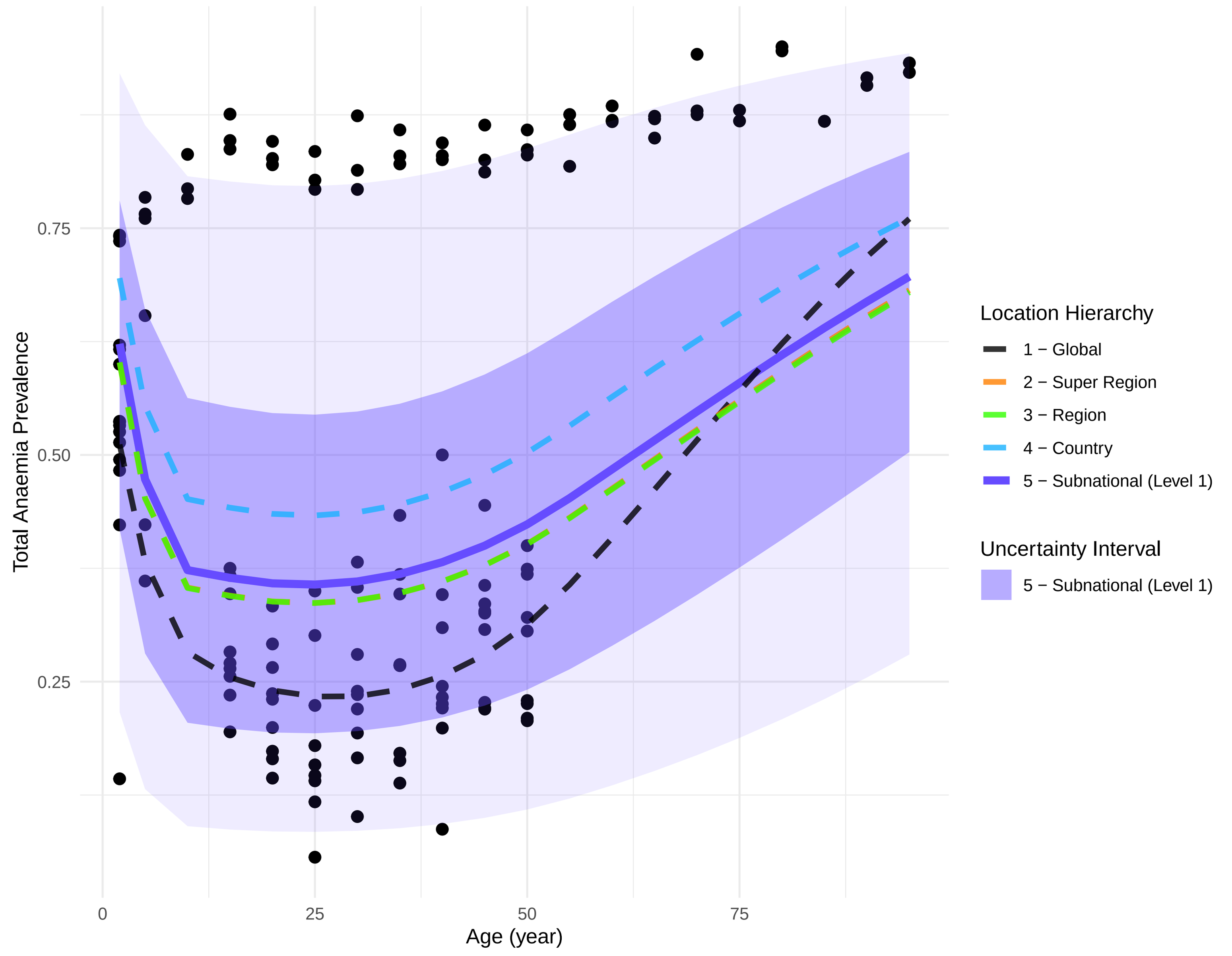

MR−BRT Age/Sex Fits − Males 2+ Years Old − Total Anaemia Prevalence − Arunachal Pradesh, Rural
WHO−2001 Adjustment Method

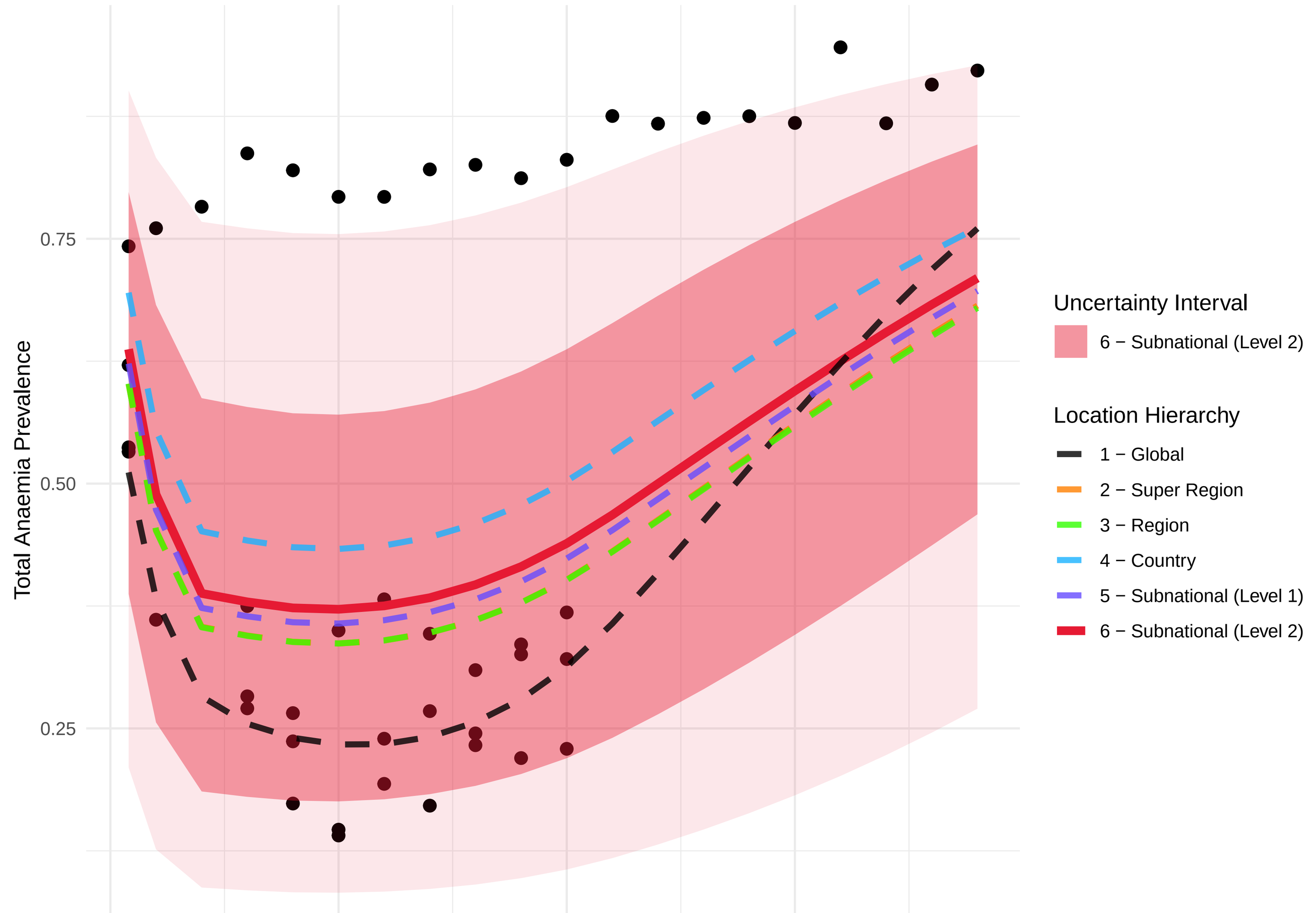

MR−BRT Age/Sex Fits − Males 2+ Years Old − Total Anaemia Prevalence − Arunachal Pradesh, Urban
WHO−2001 Adjustment Method

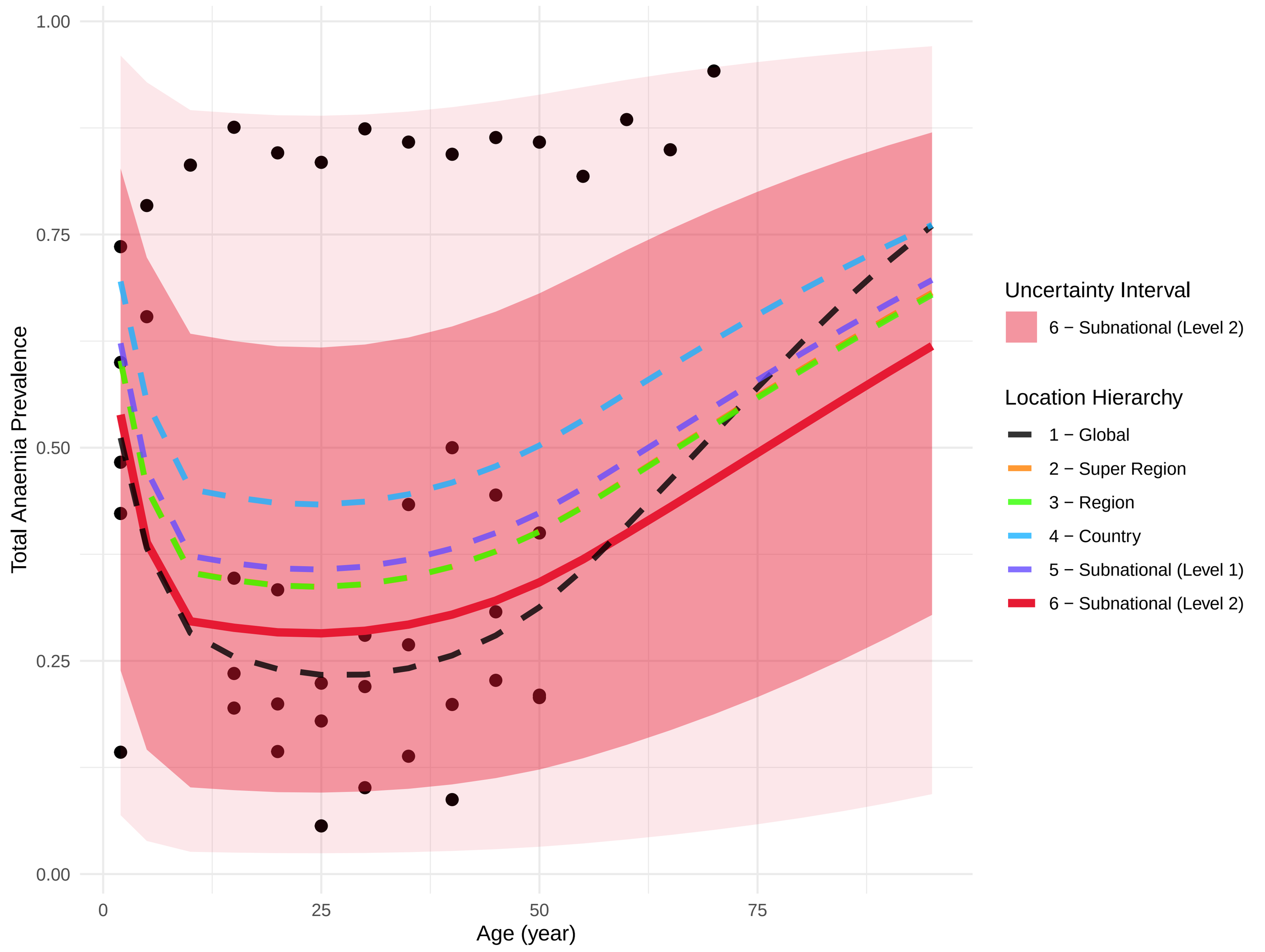

MR−BRT Age/Sex Fits − Males 2+ Years Old − Total Anaemia Prevalence − Assam
WHO−2001 Adjustment Method

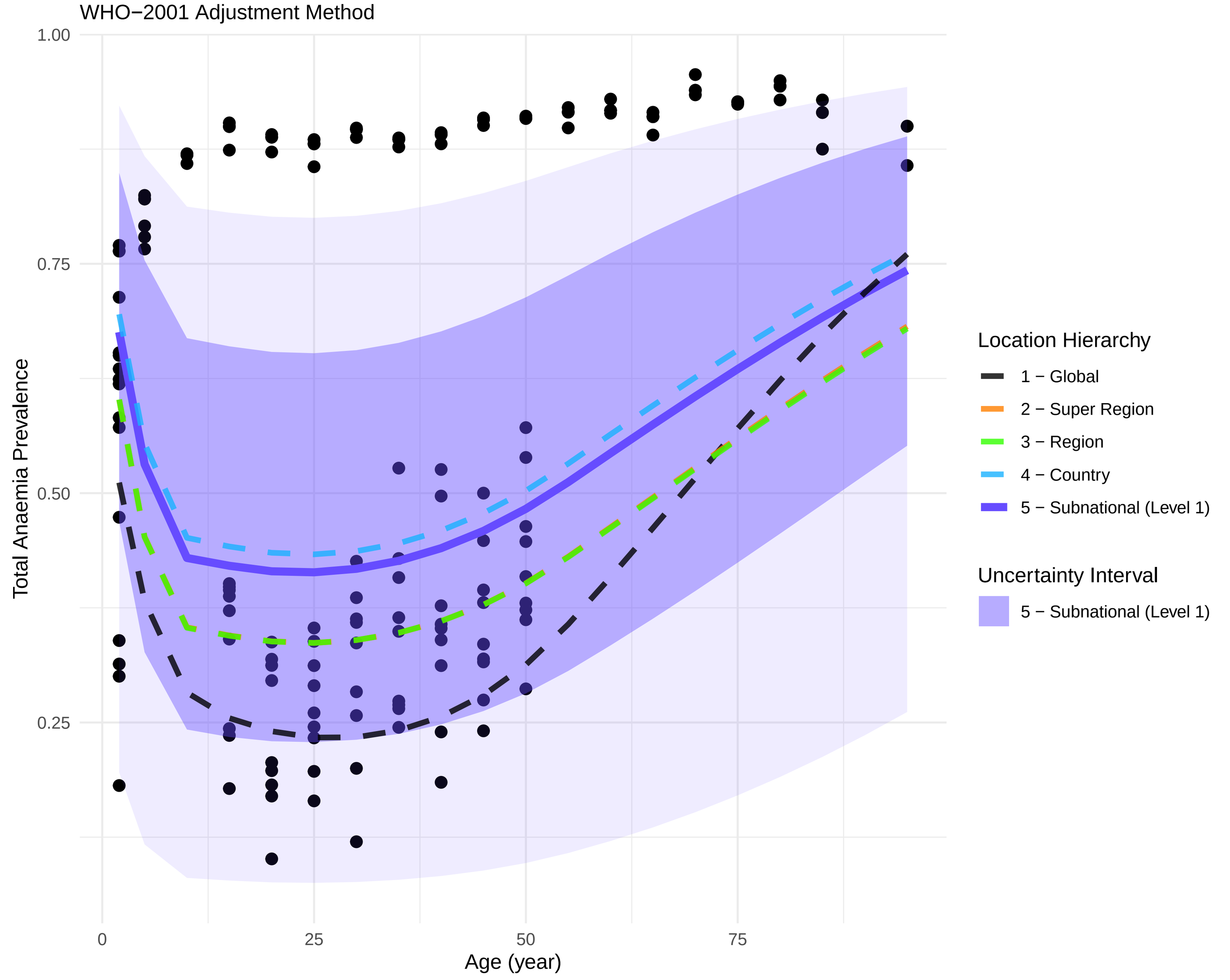

MR−BRT Age/Sex Fits − Males 2+ Years Old − Total Anaemia Prevalence − Assam, Rural
WHO−2001 Adjustment Method

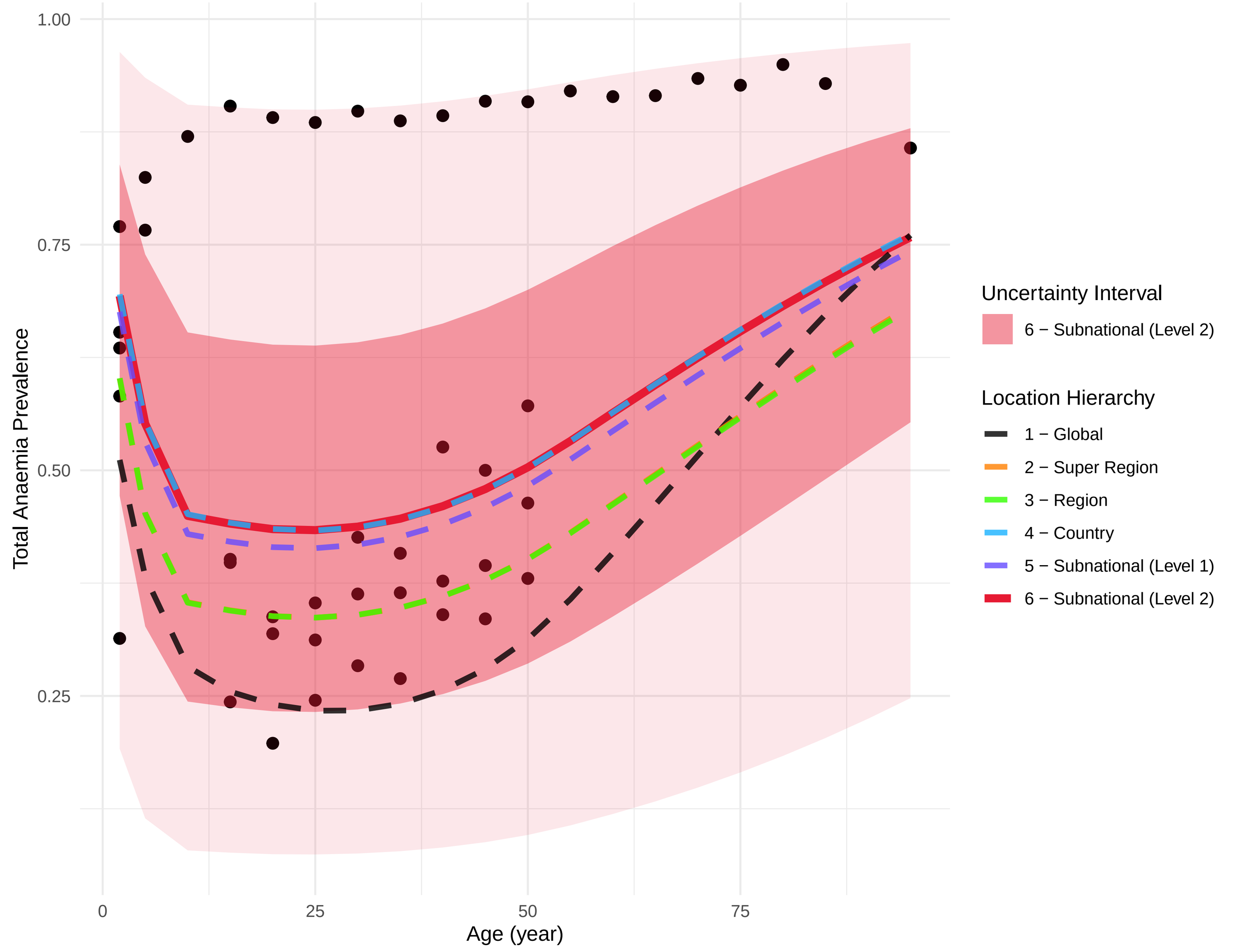

MR−BRT Age/Sex Fits − Males 2+ Years Old − Total Anaemia Prevalence − Assam, Urban
WHO−2001 Adjustment Method

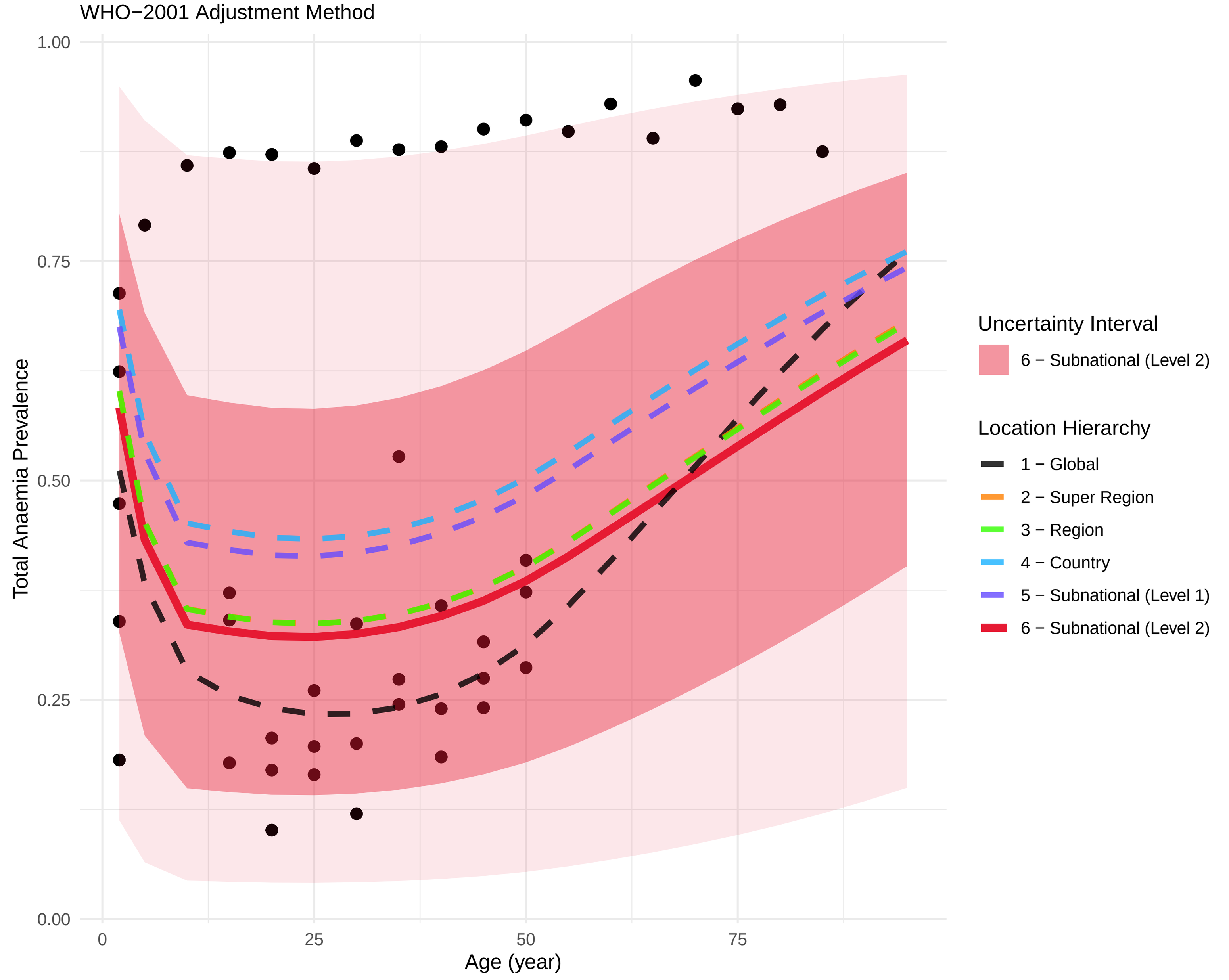

MR−BRT Age/Sex Fits − Males 2+ Years Old − Total Anaemia Prevalence − Bihar
WHO−2001 Adjustment Method

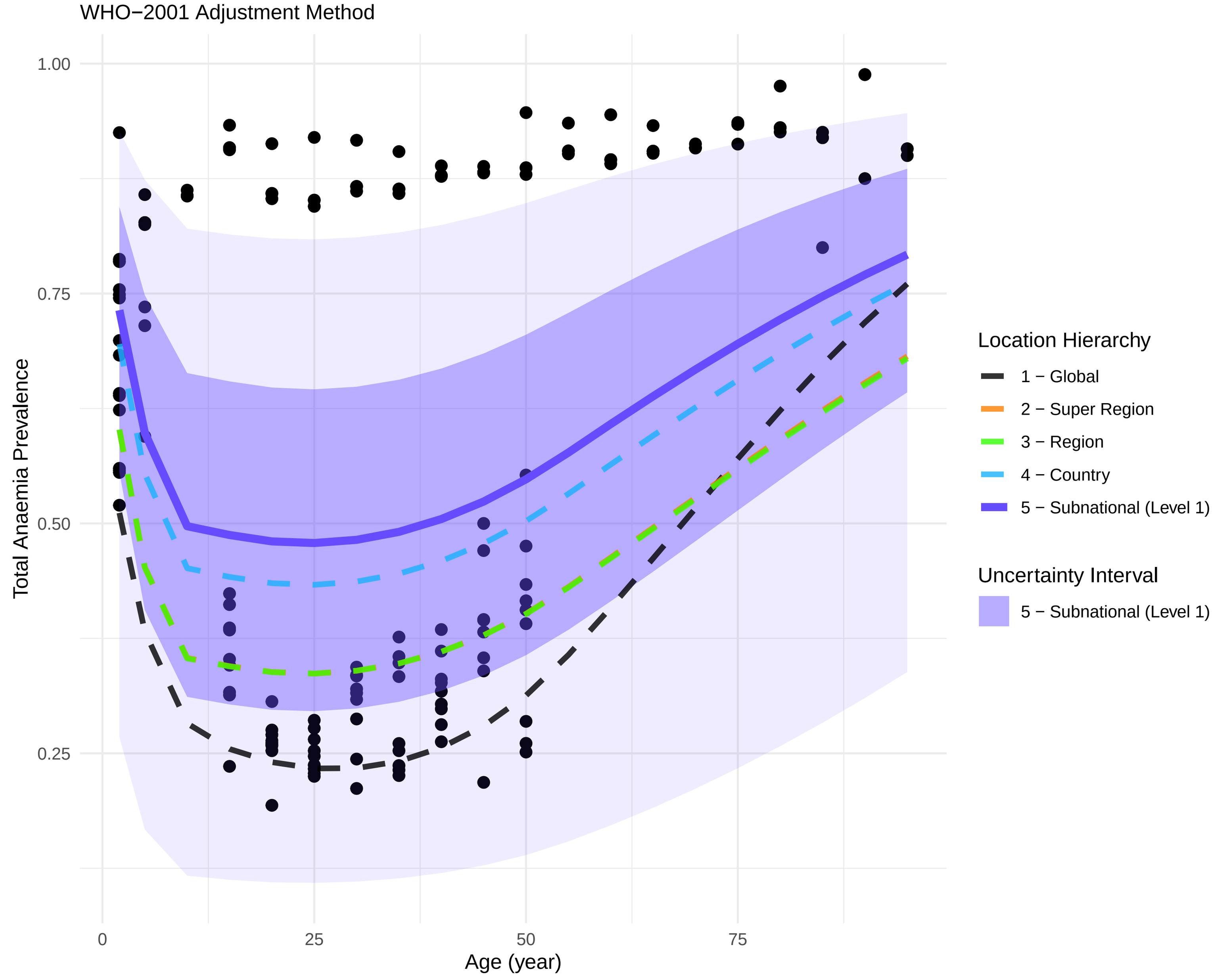

MR−BRT Age/Sex Fits − Males 2+ Years Old − Total Anaemia Prevalence − Bihar, Rural
WHO−2001 Adjustment Method

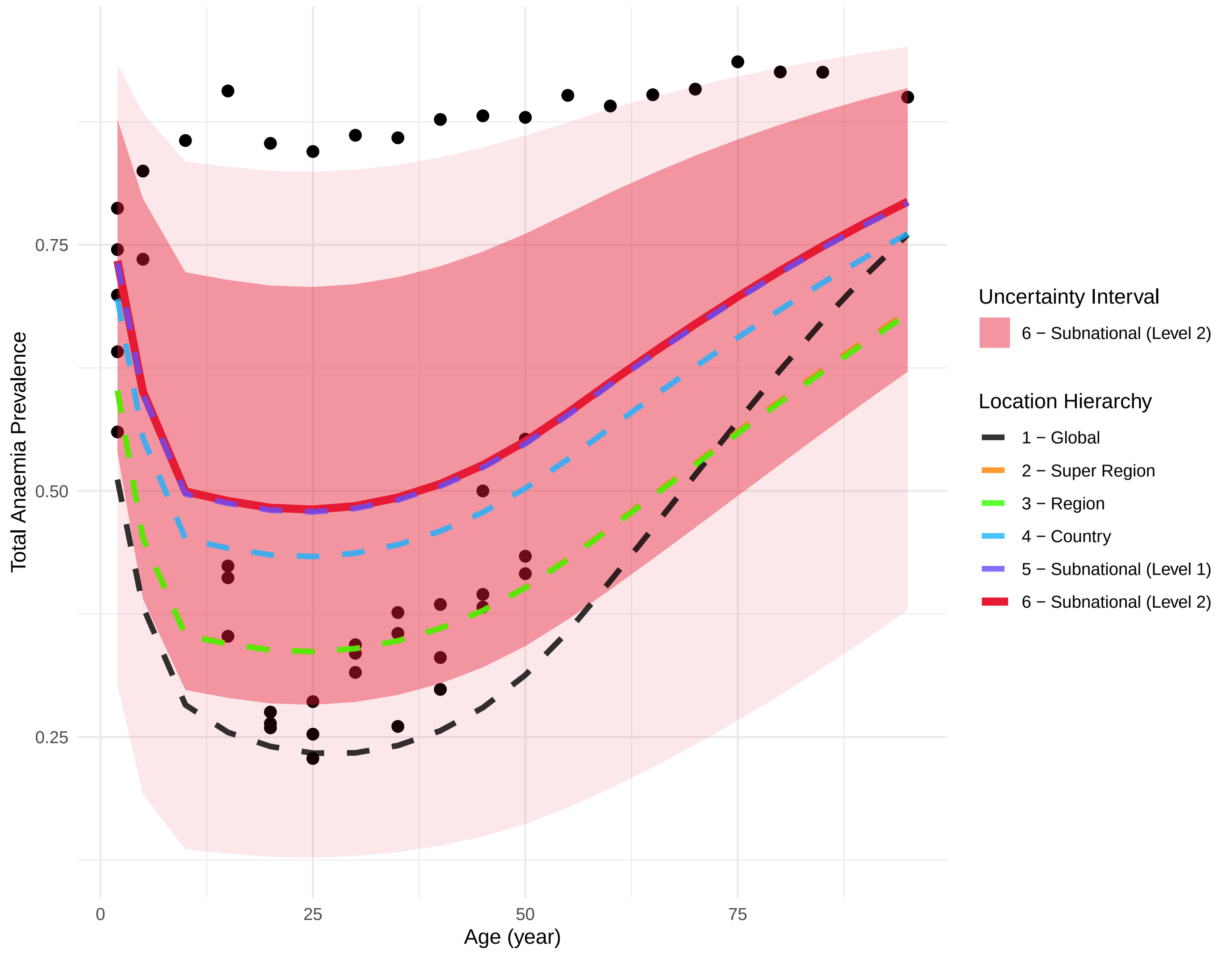

MR−BRT Age/Sex Fits − Males 2+ Years Old − Total Anaemia Prevalence − Bihar, Urban
WHO−2001 Adjustment Method

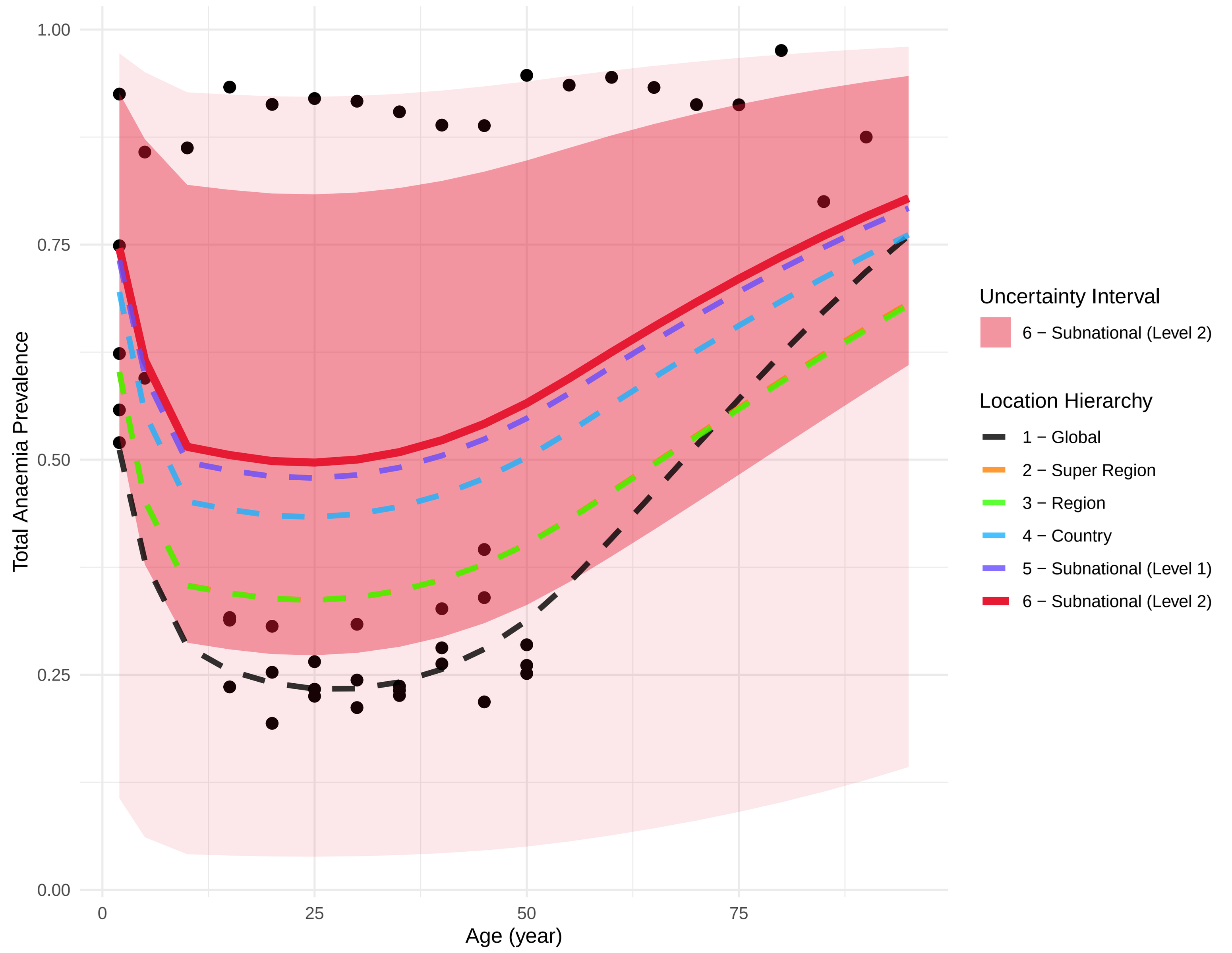

MR−BRT Age/Sex Fits − Males 2+ Years Old − Total Anaemia Prevalence − Chhattisgarh
WHO−2001 Adjustment Method

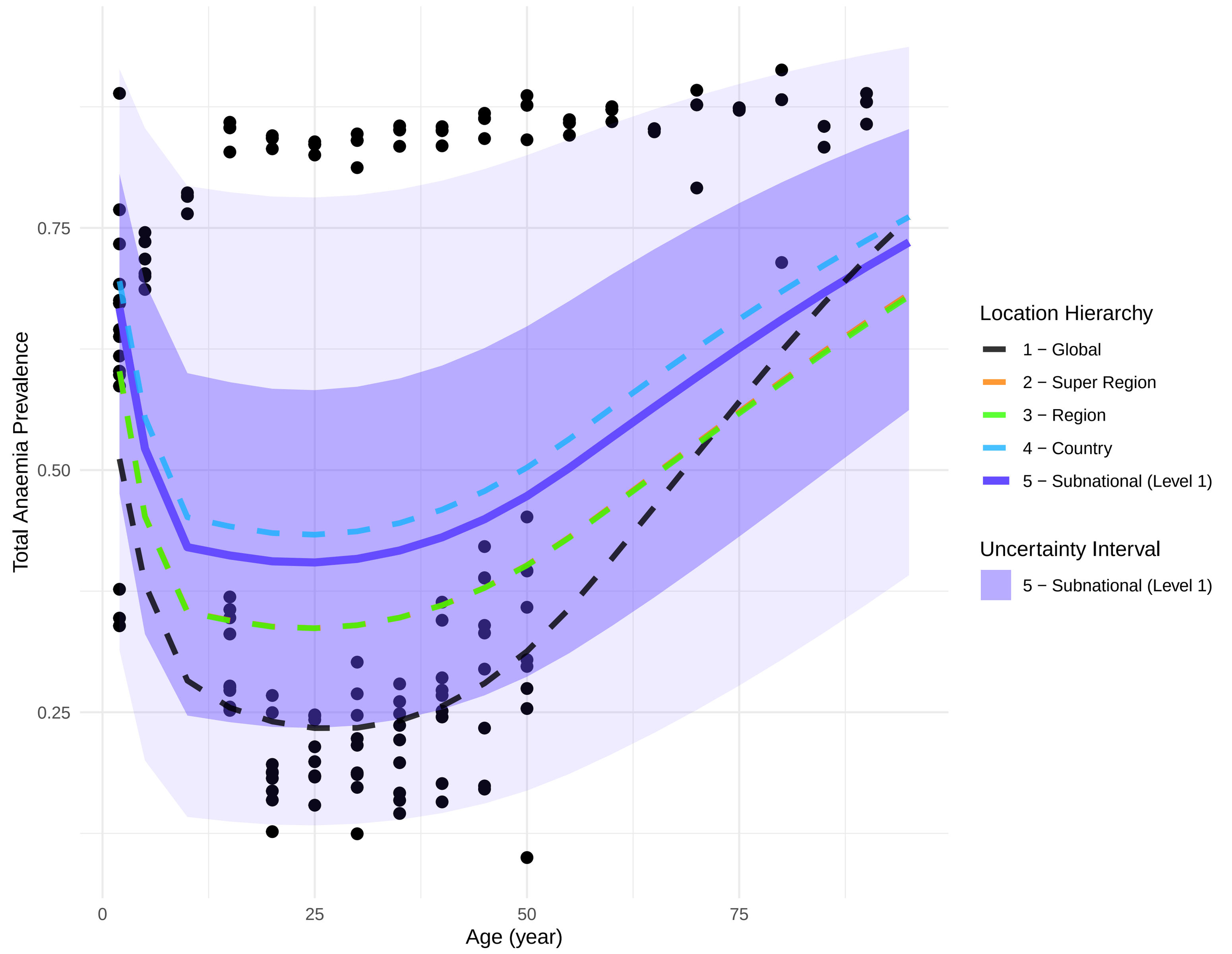

MR−BRT Age/Sex Fits − Males 2+ Years Old − Total Anaemia Prevalence − Chhattisgarh, Rural
WHO−2001 Adjustment Method

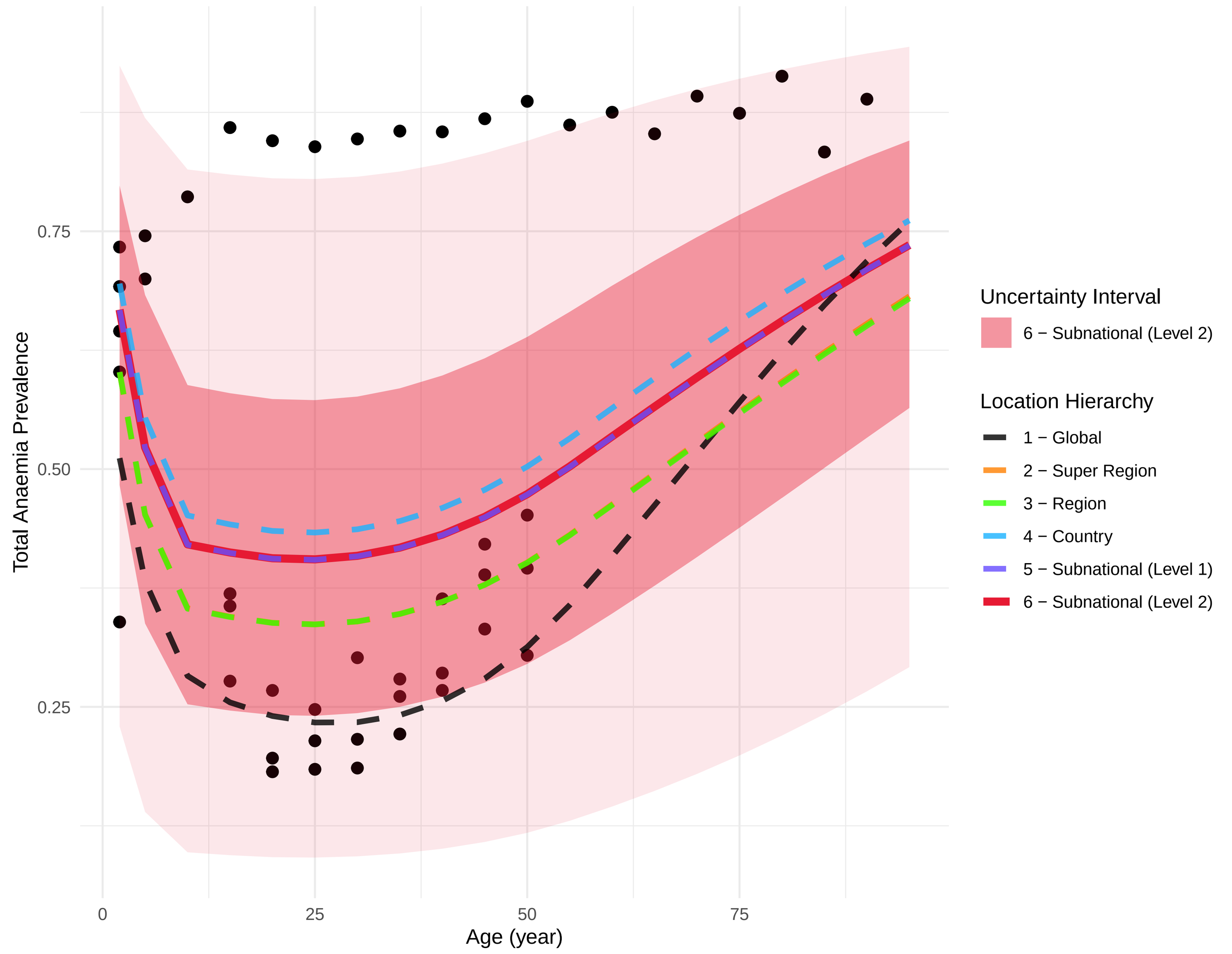

MR−BRT Age/Sex Fits − Males 2+ Years Old − Total Anaemia Prevalence − Chhattisgarh, Urban
WHO−2001 Adjustment Method

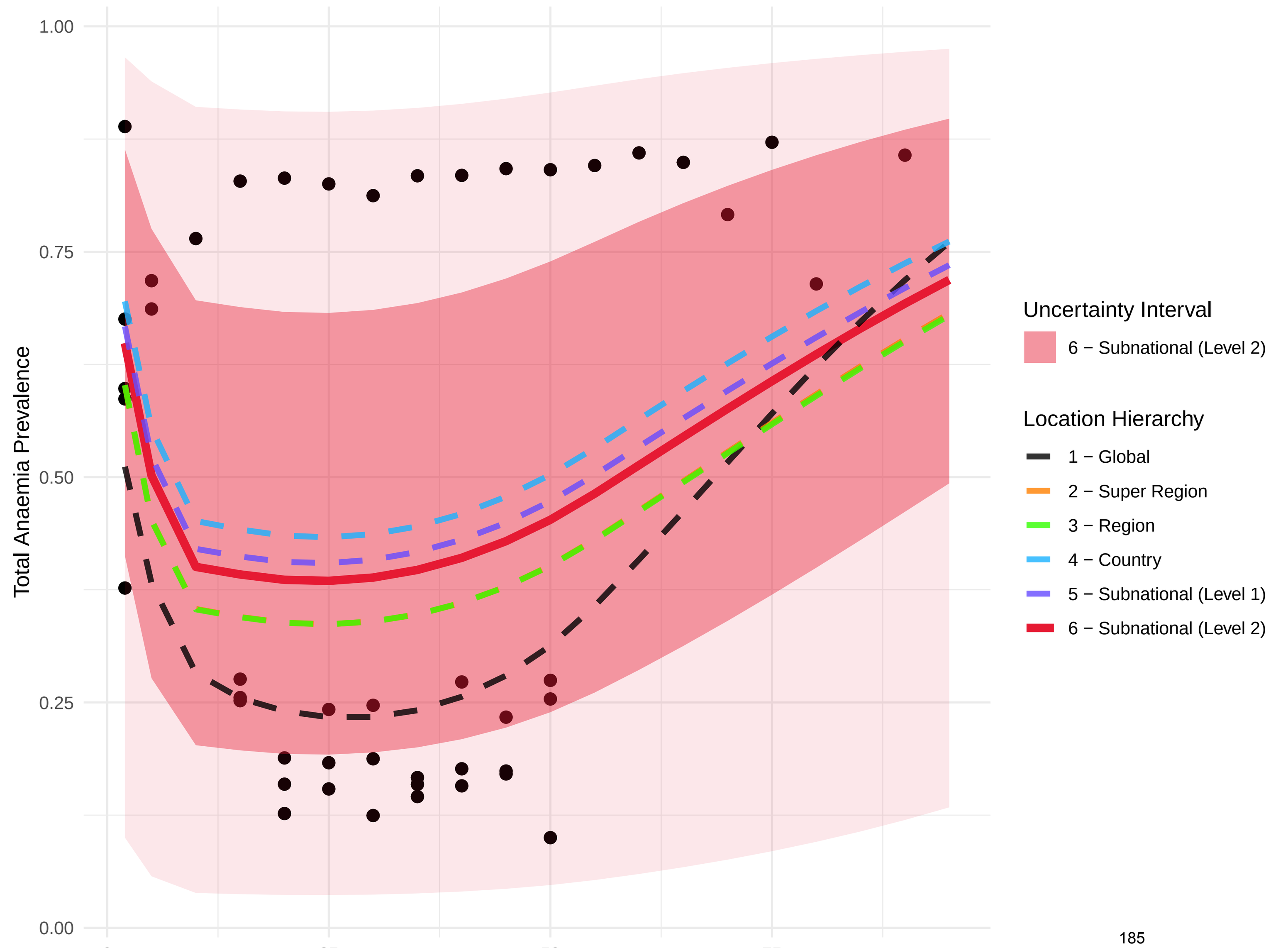

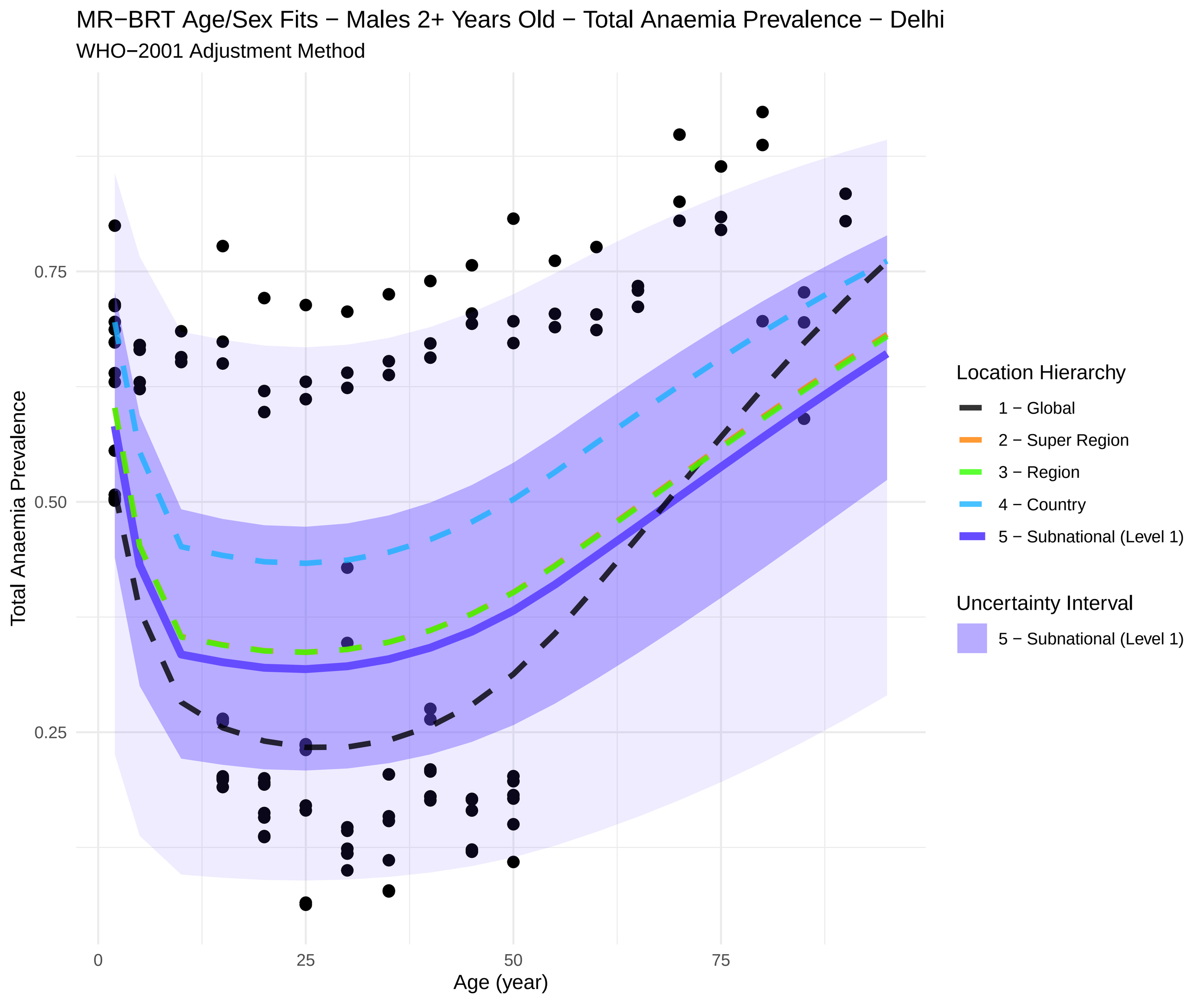
MR−BRT Age/Sex Fits − Males 2+ Years Old − Total Anaemia Prevalence − Delhi
WHO−2001 Adjustment Method
Total Anaemia Prevalence
Age (year)
Location Hierarchy
1 − Global
2 − Super Region
3 − Region
4 − Country
5 − Subnational (Level 1)
Uncertainty Interval
5 − Subnational (Level 1)

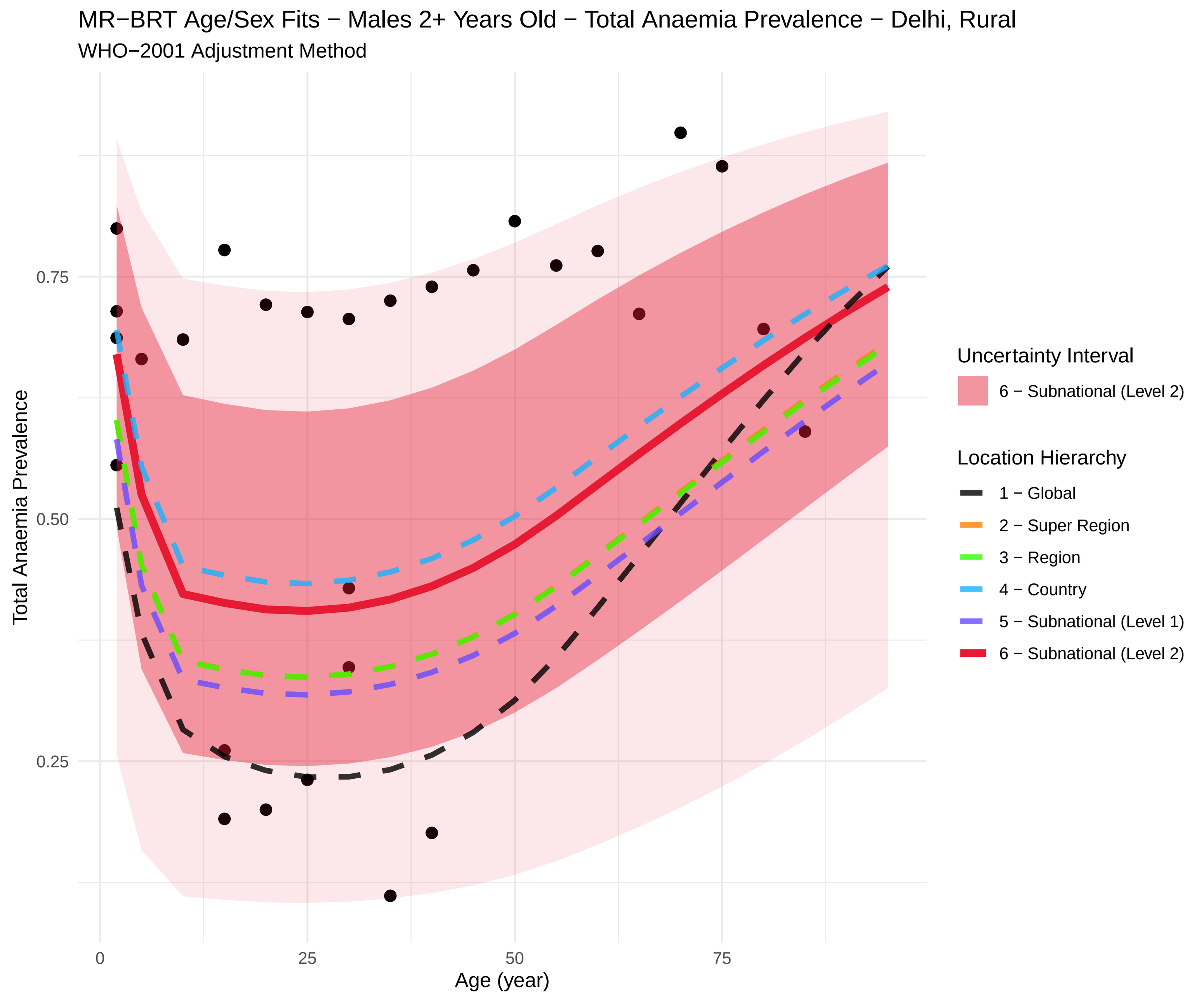
MR−BRT Age/Sex Fits − Males 2+ Years Old − Total Anaemia Prevalence − Delhi, Rural
WHO−2001 Adjustment Method
Total Anaemia Prevalence
Age (year)
Uncertainty Interval
6 − Subnational (Level 2)
Location Hierarchy
1 − Global
2 − Super Region
3 − Region
4 − Country
5 − Subnational (Level 1)
6 − Subnational (Level 2)

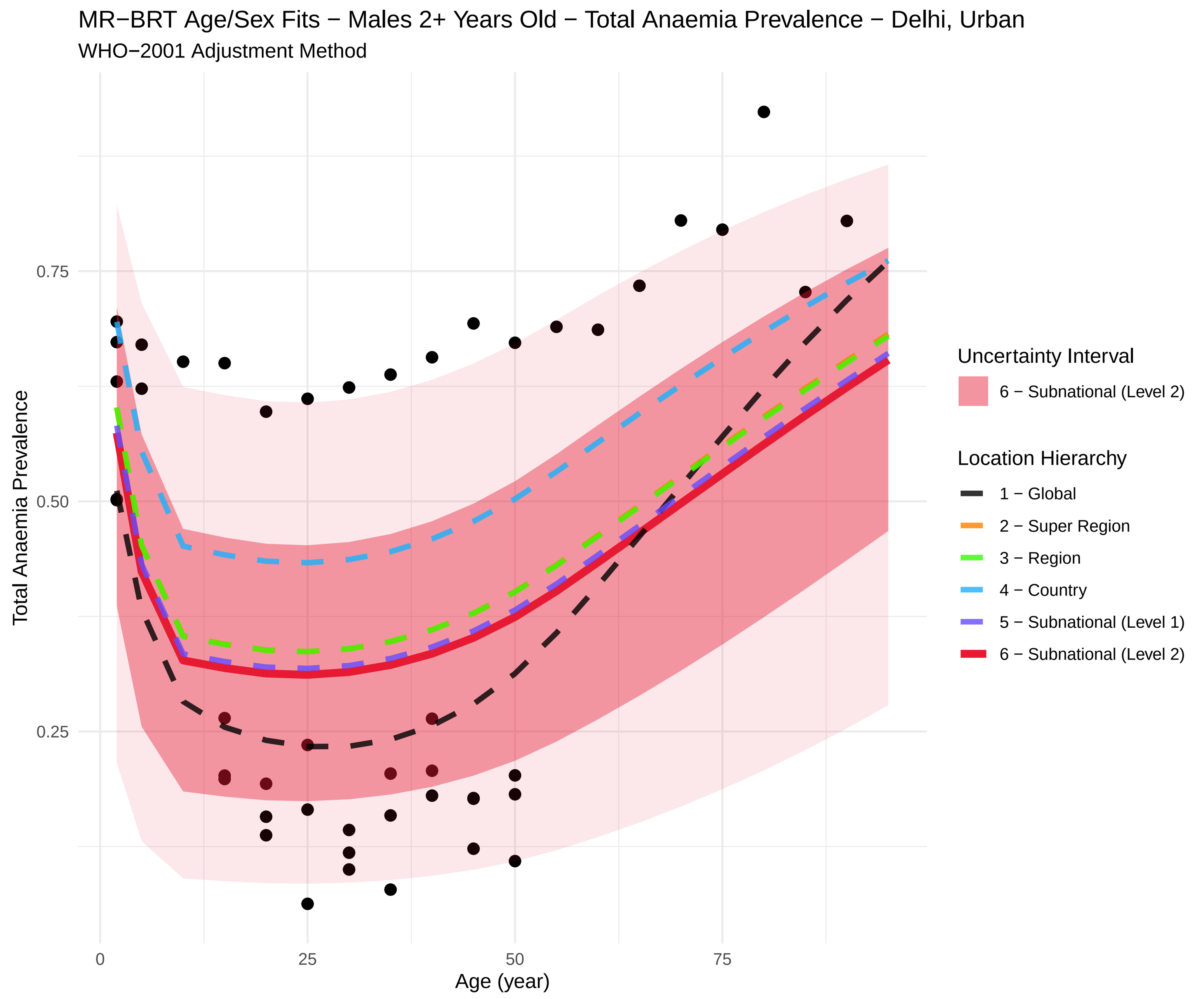
MR−BRT Age/Sex Fits − Males 2+ Years Old − Total Anaemia Prevalence − Delhi, Urban
WHO−2001 Adjustment Method
Total Anaemia Prevalence
Age (year)
Uncertainty Interval
6 − Subnational (Level 2)
Location Hierarchy
1 − Global
2 − Super Region
3 − Region
4 − Country
5 − Subnational (Level 1)
6 − Subnational (Level 2)

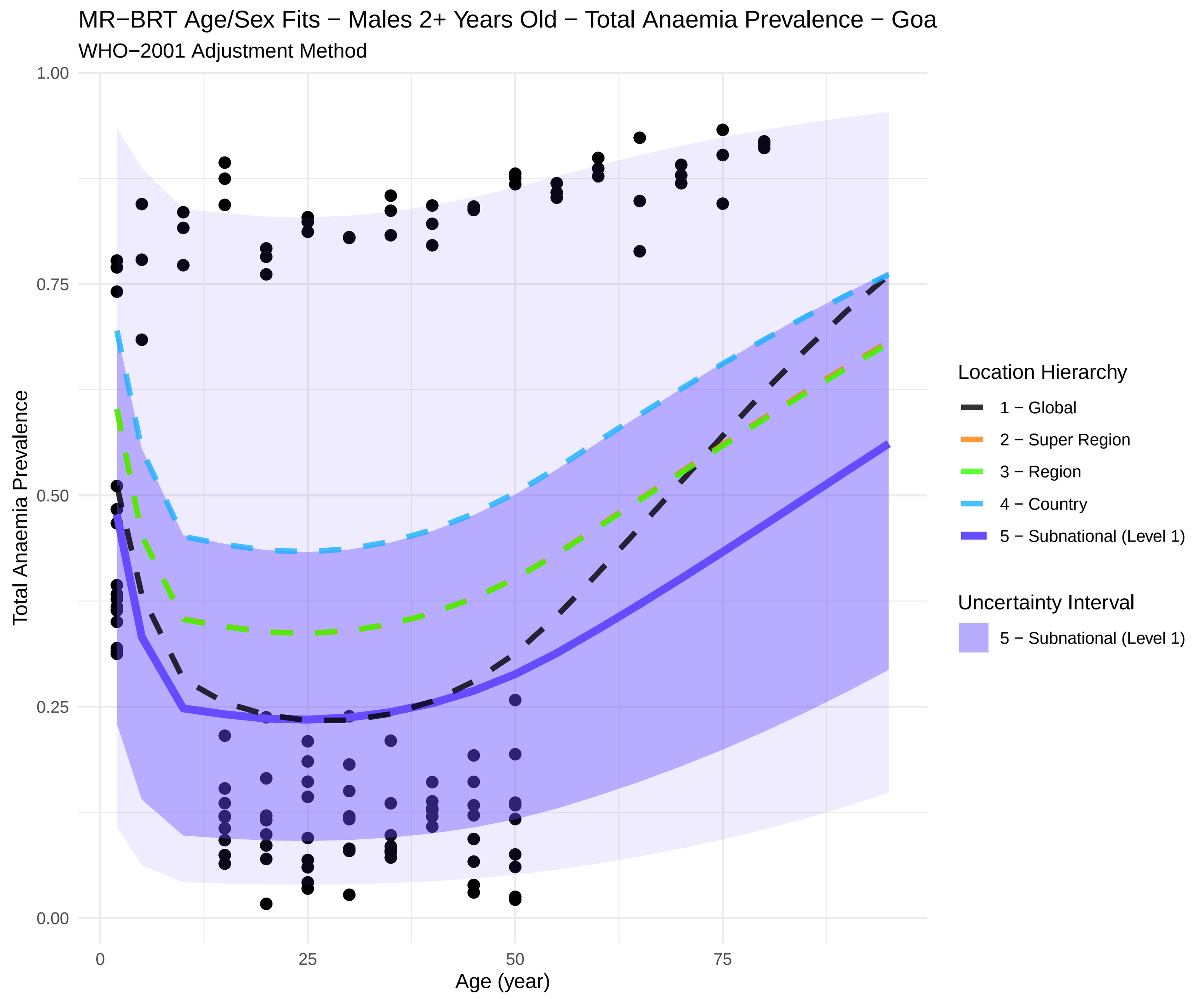
MR−BRT Age/Sex Fits − Males 2+ Years Old − Total Anaemia Prevalence − Goa
WHO−2001 Adjustment Method
Total Anaemia Prevalence
Age (year)
Location Hierarchy
1 − Global
2 − Super Region
3 − Region
4 − Country
5 − Subnational (Level 1)
Uncertainty Interval
5 − Subnational (Level 1)

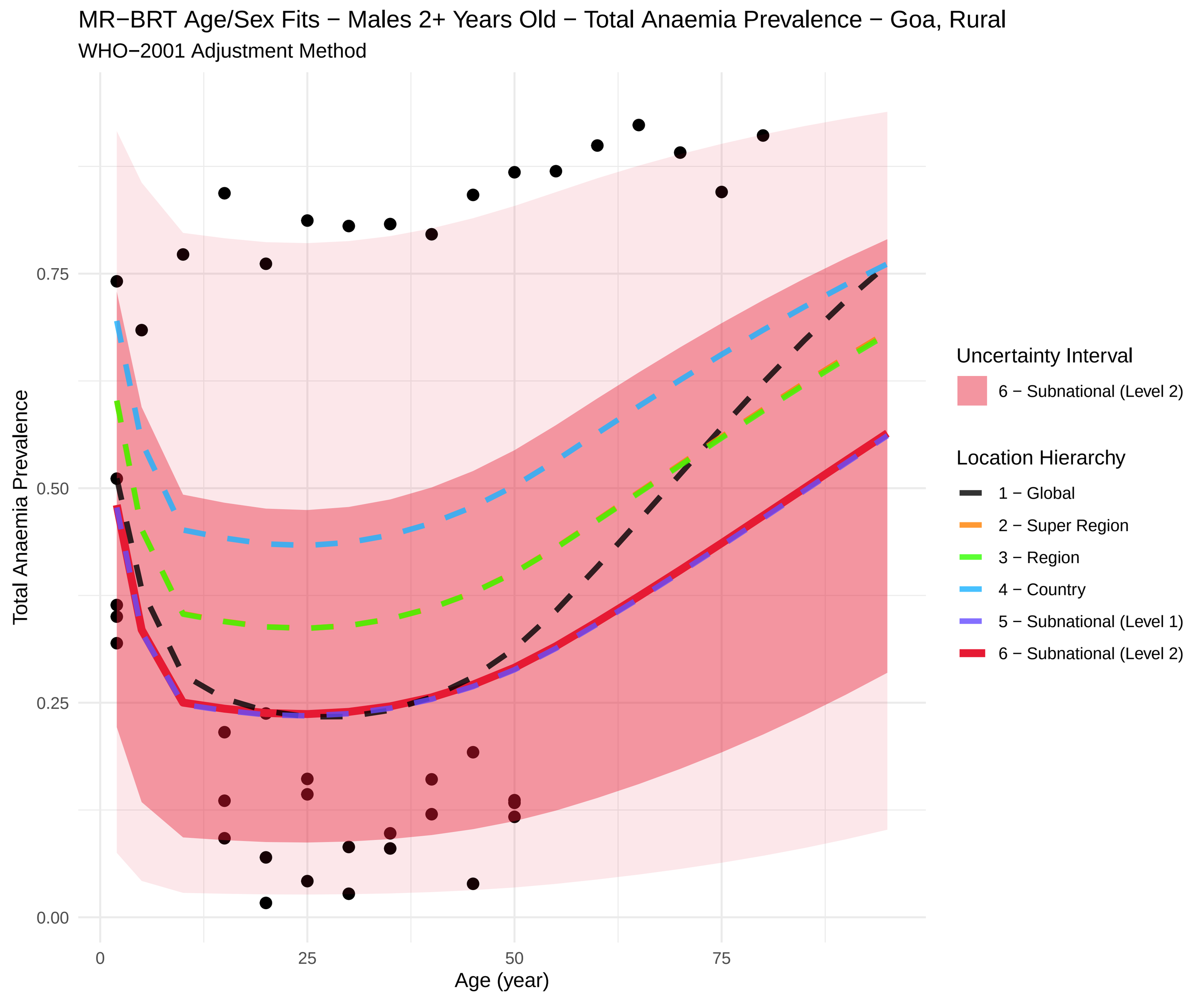
MR−BRT Age/Sex Fits − Males 2+ Years Old − Total Anaemia Prevalence − Goa, Rural
WHO−2001 Adjustment Method
Total Anaemia Prevalence
Age (year)
Uncertainty Interval
6 − Subnational (Level 2)
Location Hierarchy
1 − Global
2 − Super Region
3 − Region
4 − Country
5 − Subnational (Level 1)
6 − Subnational (Level 2)

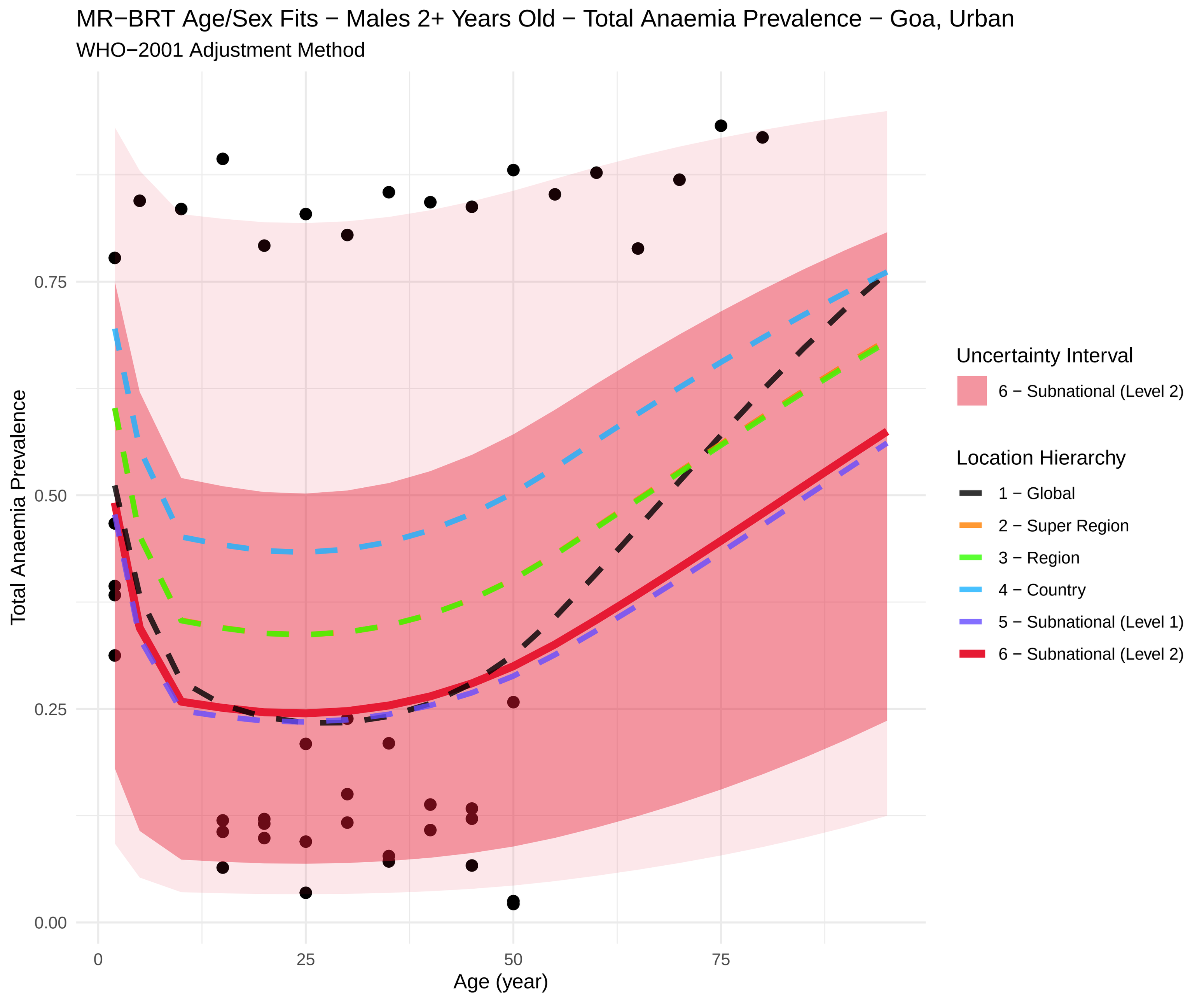
MR−BRT Age/Sex Fits − Males 2+ Years Old − Total Anaemia Prevalence − Goa, Urban
WHO−2001 Adjustment Method
Total Anaemia Prevalence
Age (year)
Uncertainty Interval
6 − Subnational (Level 2)
Location Hierarchy
1 − Global
2 − Super Region
3 − Region
4 − Country
5 − Subnational (Level 1)
6 − Subnational (Level 2)

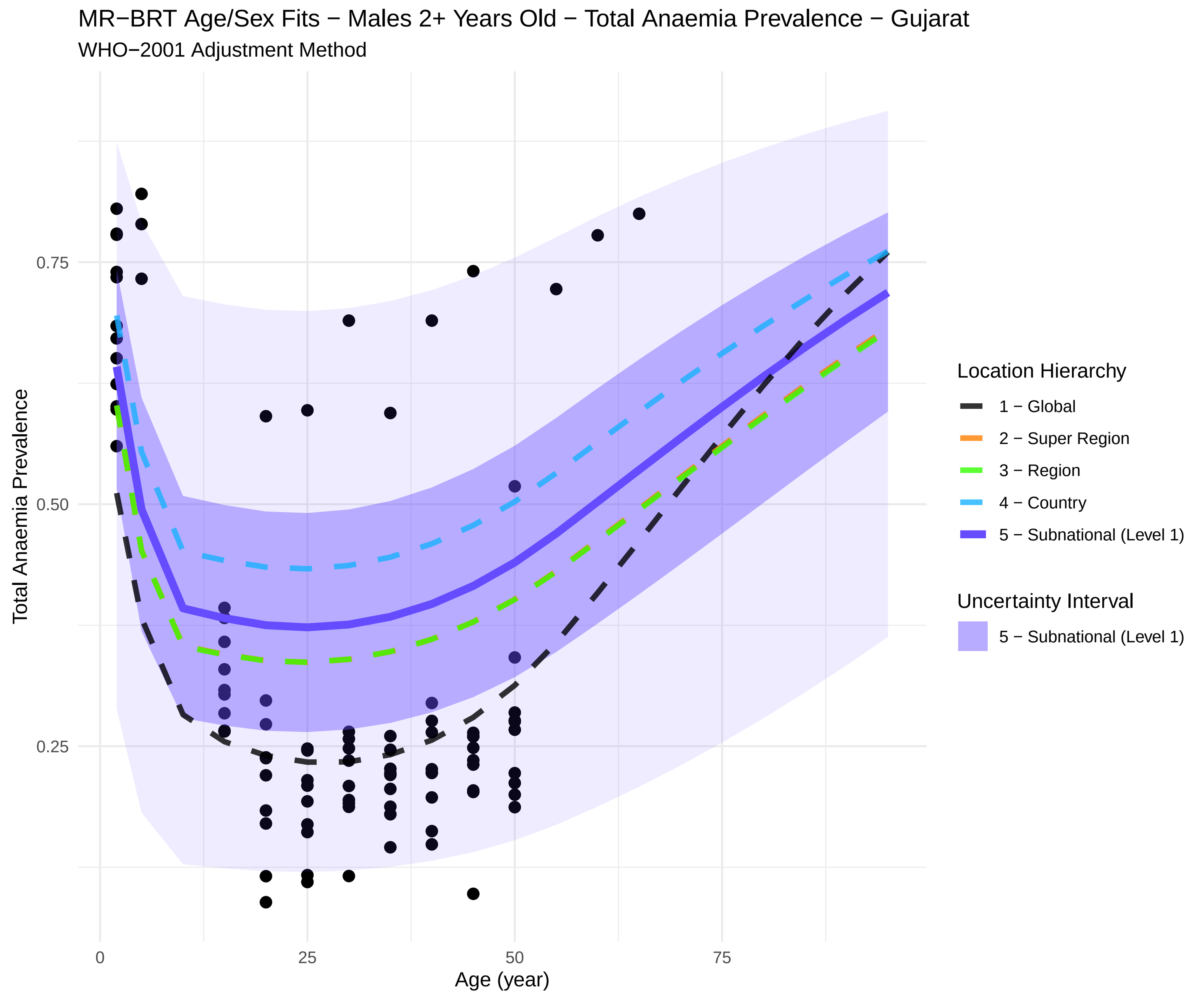
MR−BRT Age/Sex Fits − Males 2+ Years Old − Total Anaemia Prevalence − Gujarat
WHO−2001 Adjustment Method
Total Anaemia Prevalence
Age (year)
Location Hierarchy
1 − Global
2 − Super Region
3 − Region
4 − Country
5 − Subnational (Level 1)
Uncertainty Interval
5 − Subnational (Level 1)

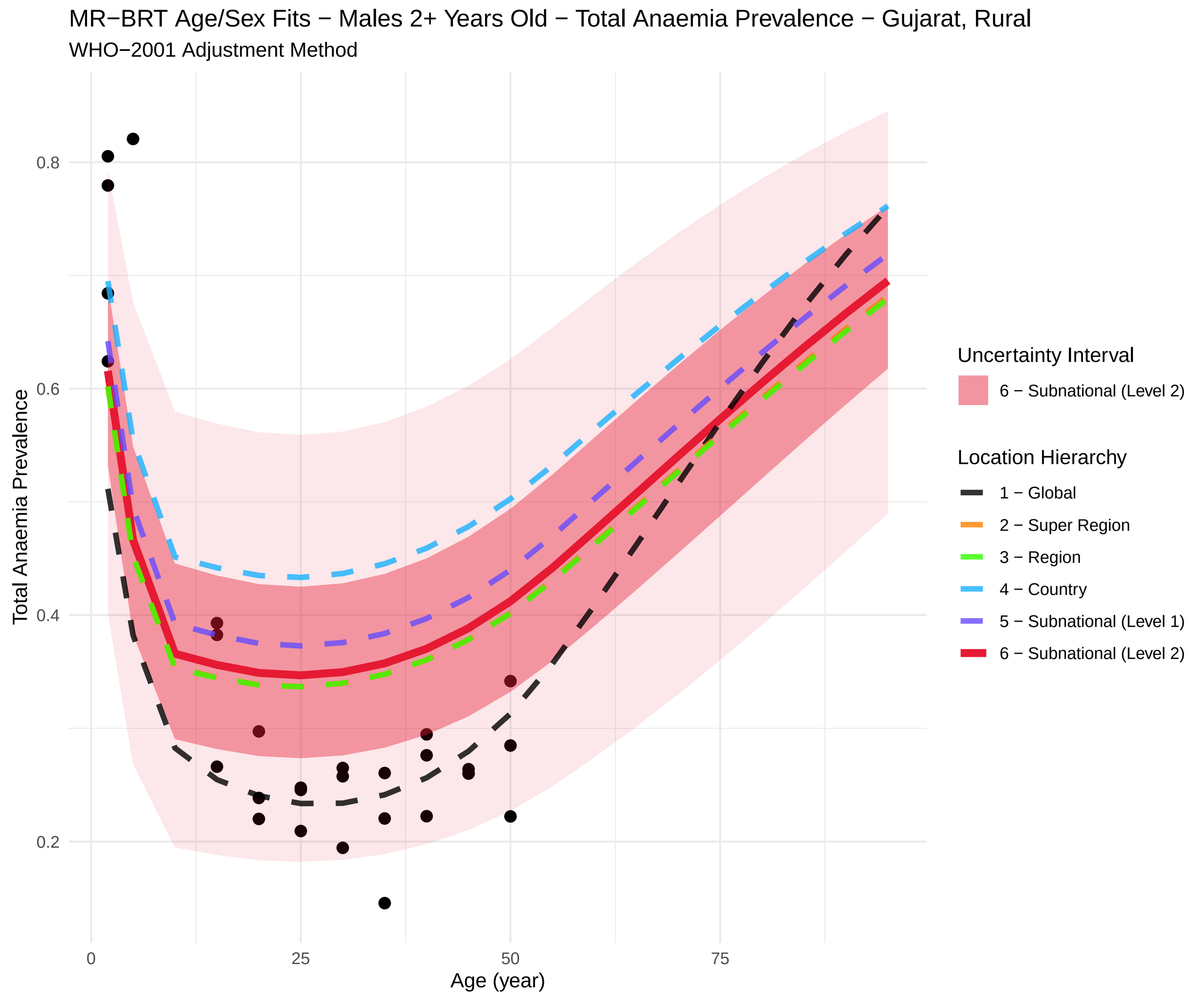
MR−BRT Age/Sex Fits − Males 2+ Years Old − Total Anaemia Prevalence − Gujarat, Rural
WHO−2001 Adjustment Method
Total Anaemia Prevalence
Age (year)
Uncertainty Interval
6 − Subnational (Level 2)
Location Hierarchy
1 − Global
2 − Super Region
3 − Region
4 − Country
5 − Subnational (Level 1)
6 − Subnational (Level 2)

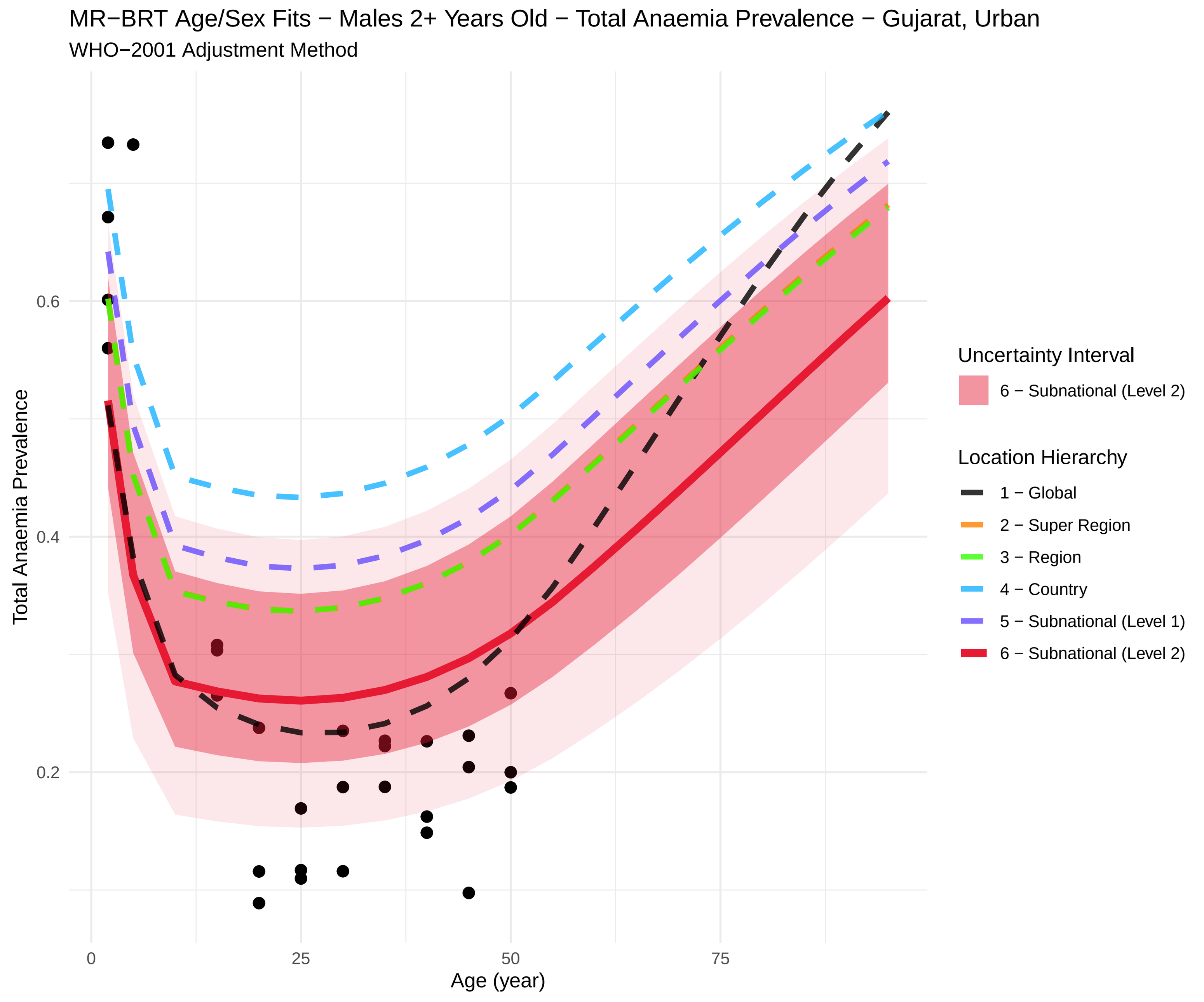
MR−BRT Age/Sex Fits − Males 2+ Years Old − Total Anaemia Prevalence − Gujarat, Urban
WHO−2001 Adjustment Method
Total Anaemia Prevalence
Age (year)
Uncertainty Interval
6 − Subnational (Level 2)
Location Hierarchy
1 − Global
2 − Super Region
3 − Region
4 − Country
5 − Subnational (Level 1)
6 − Subnational (Level 2)

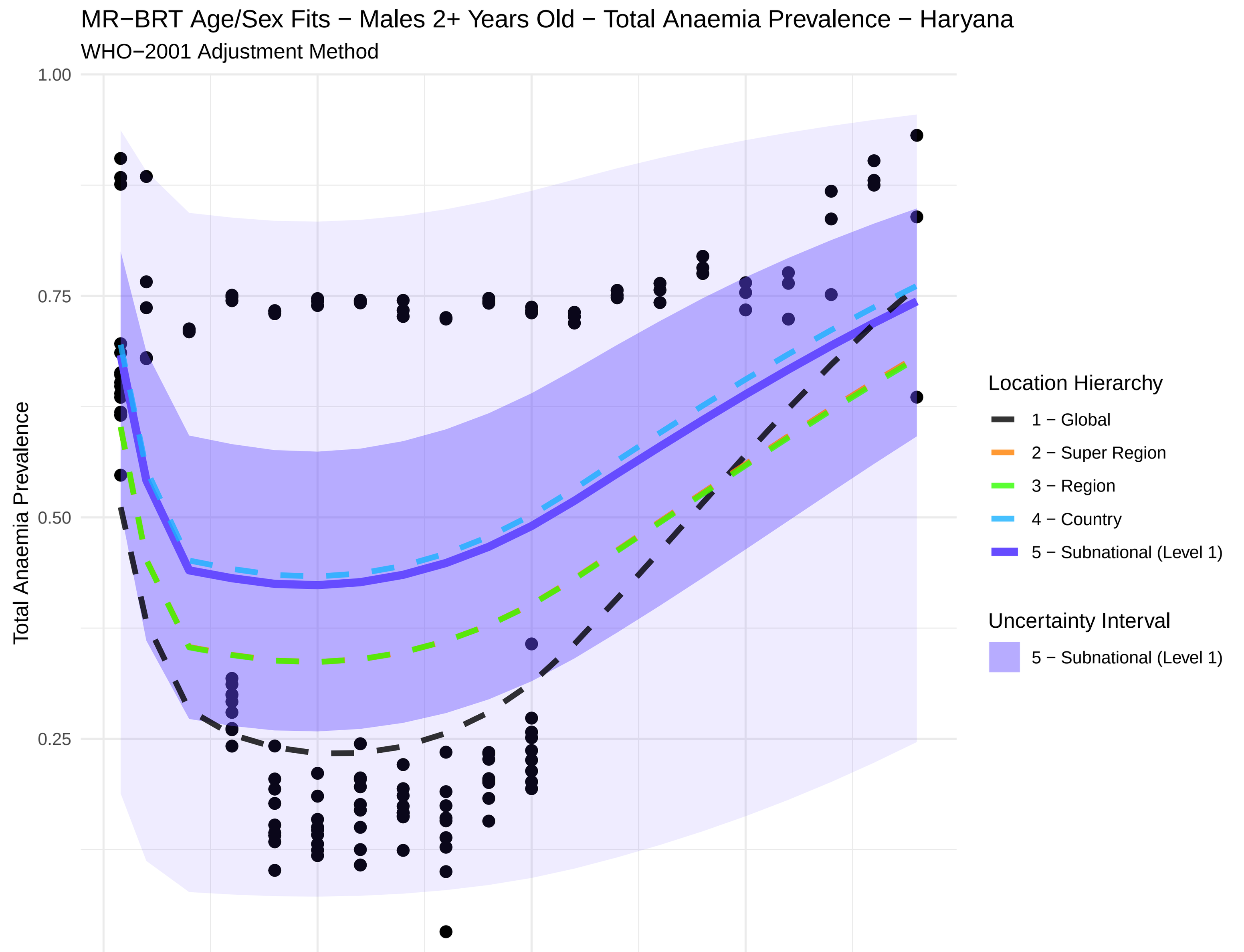
MR−BRT Age/Sex Fits − Males 2+ Years Old − Total Anaemia Prevalence − Haryana
WHO−2001 Adjustment Method
Total Anaemia Prevalence
Age (year)
Location Hierarchy
1 − Global
2 − Super Region
3 − Region
4 − Country
5 − Subnational (Level 1)
Uncertainty Interval
5 − Subnational (Level 1)

MR−BRT Age/Sex Fits − Males 2+ Years Old − Total Anaemia Prevalence − Haryana, Rural
WHO−2001 Adjustment Method

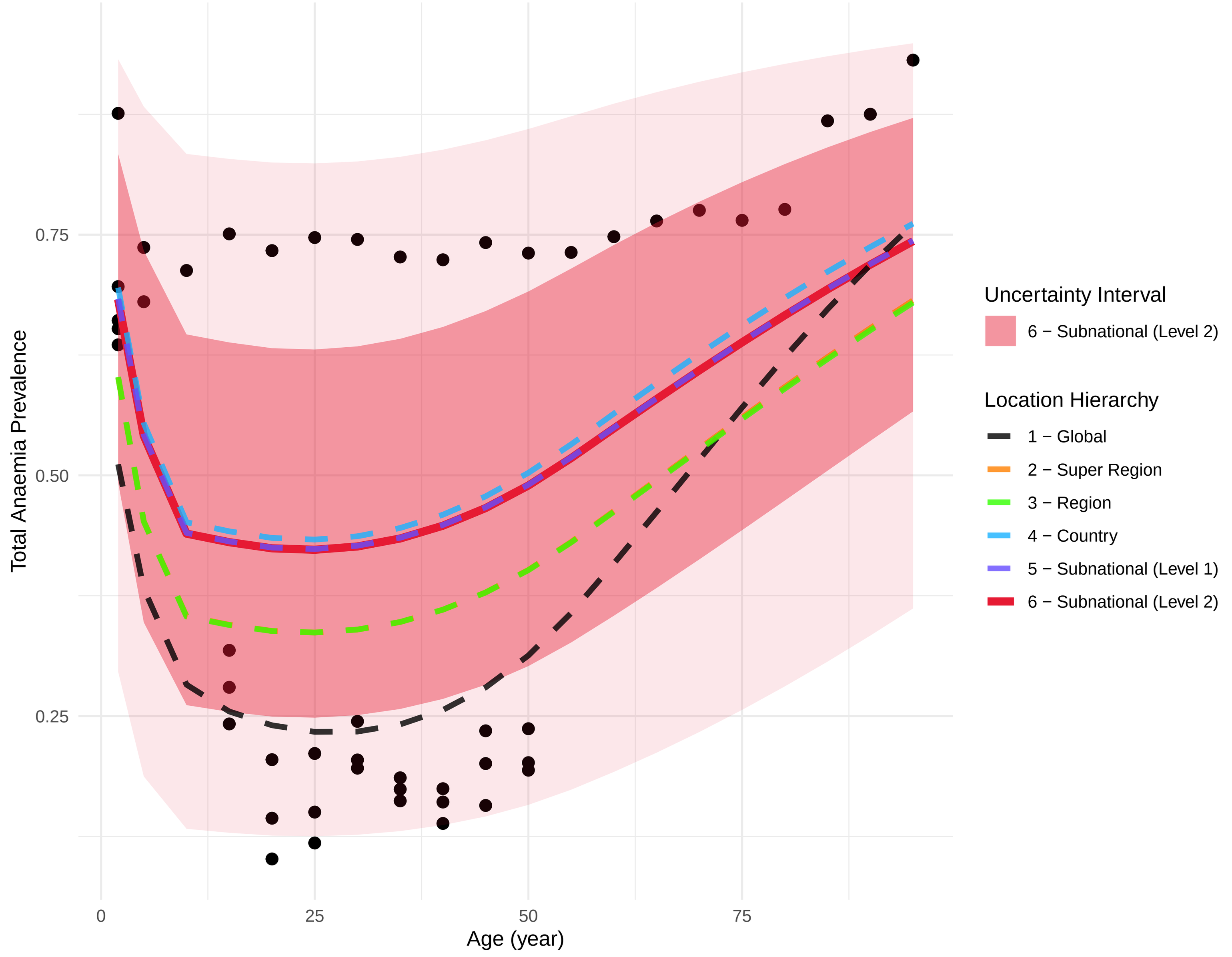

MR−BRT Age/Sex Fits − Males 2+ Years Old − Total Anaemia Prevalence − Haryana, Urban
WHO−2001 Adjustment Method

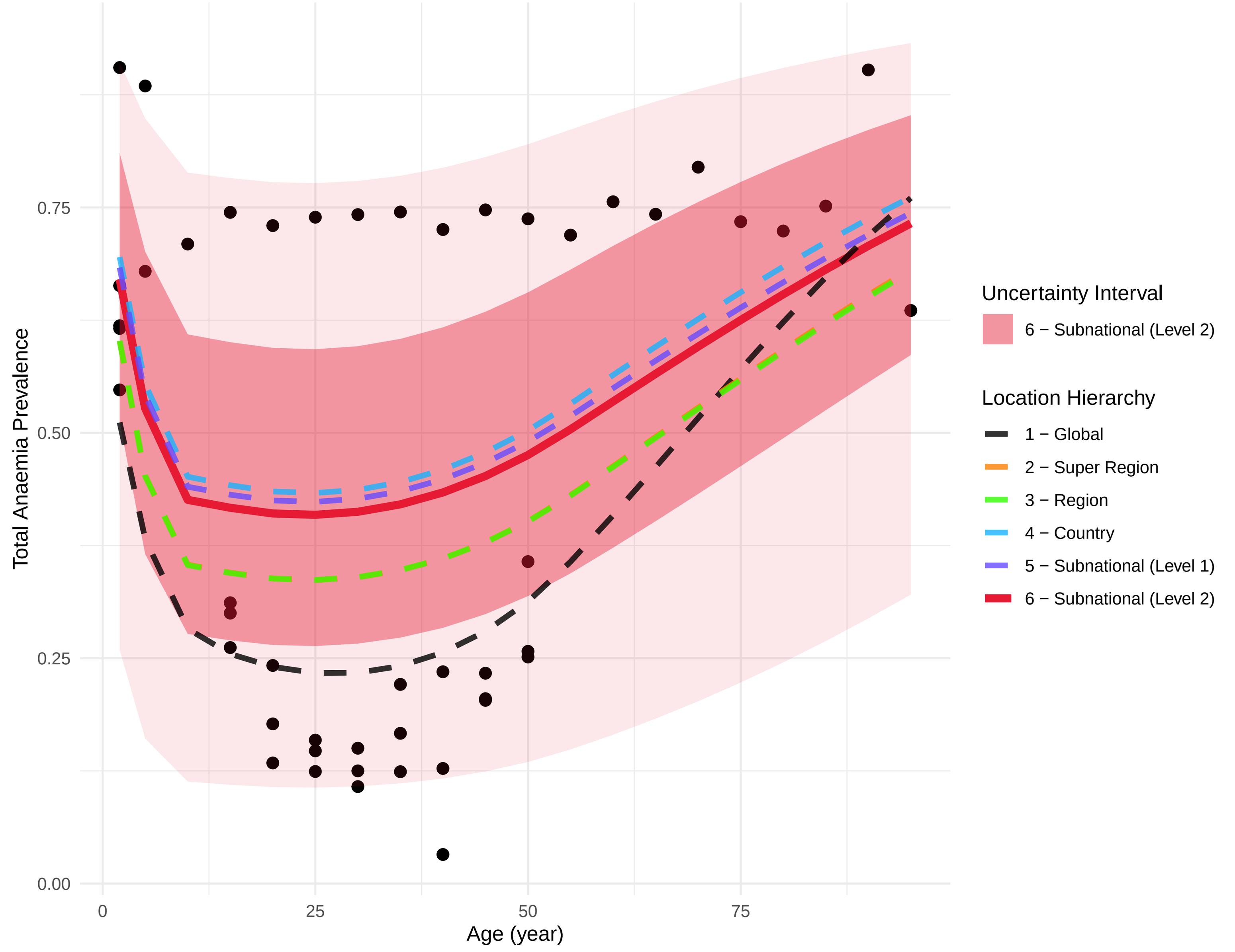

MR−BRT Age/Sex Fits − Males 2+ Years Old − Total Anaemia Prevalence − Himachal Pradesh
WHO−2001 Adjustment Method

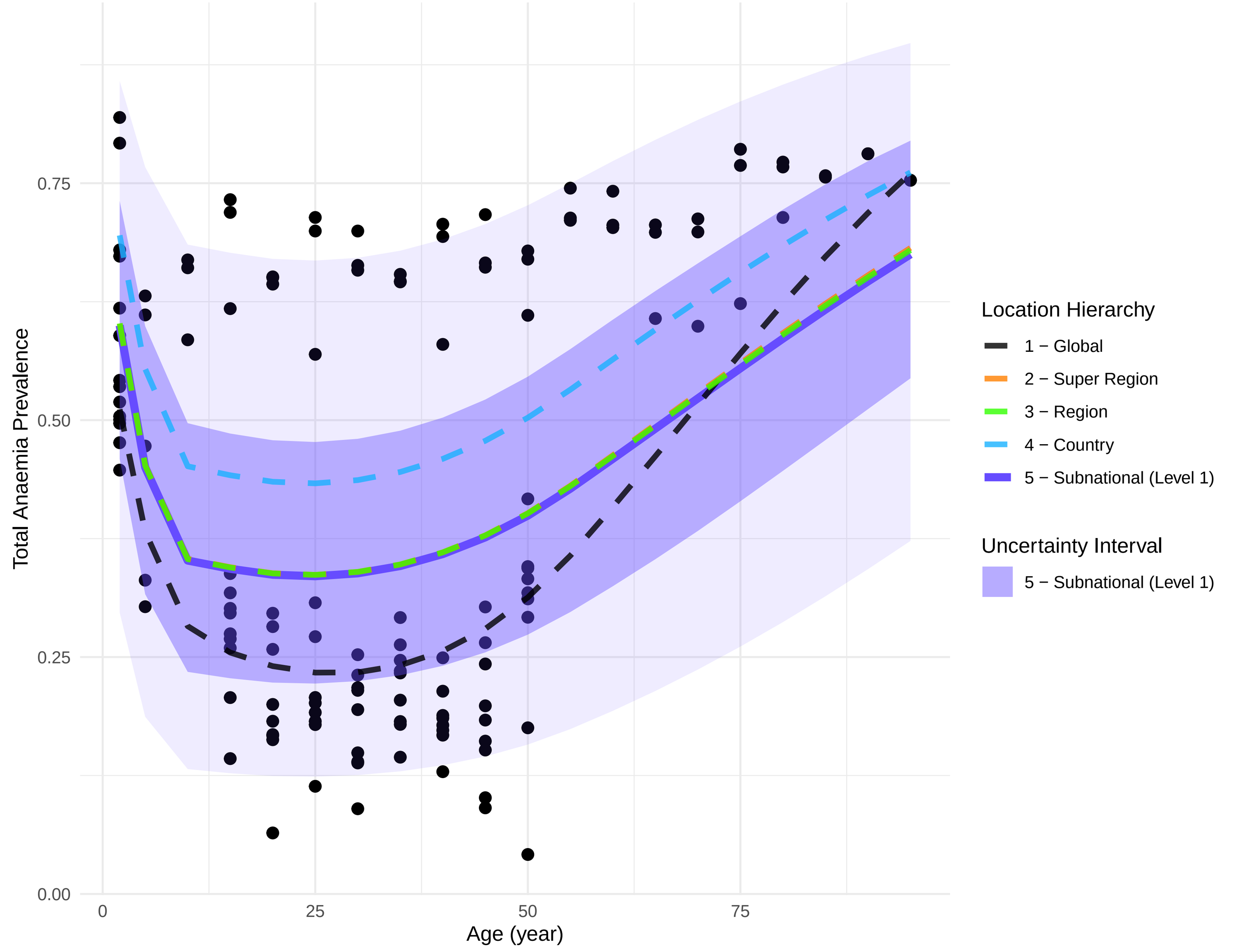

MR−BRT Age/Sex Fits − Males 2+ Years Old − Total Anaemia Prevalence − Himachal Pradesh, Rural
WHO−2001 Adjustment Method

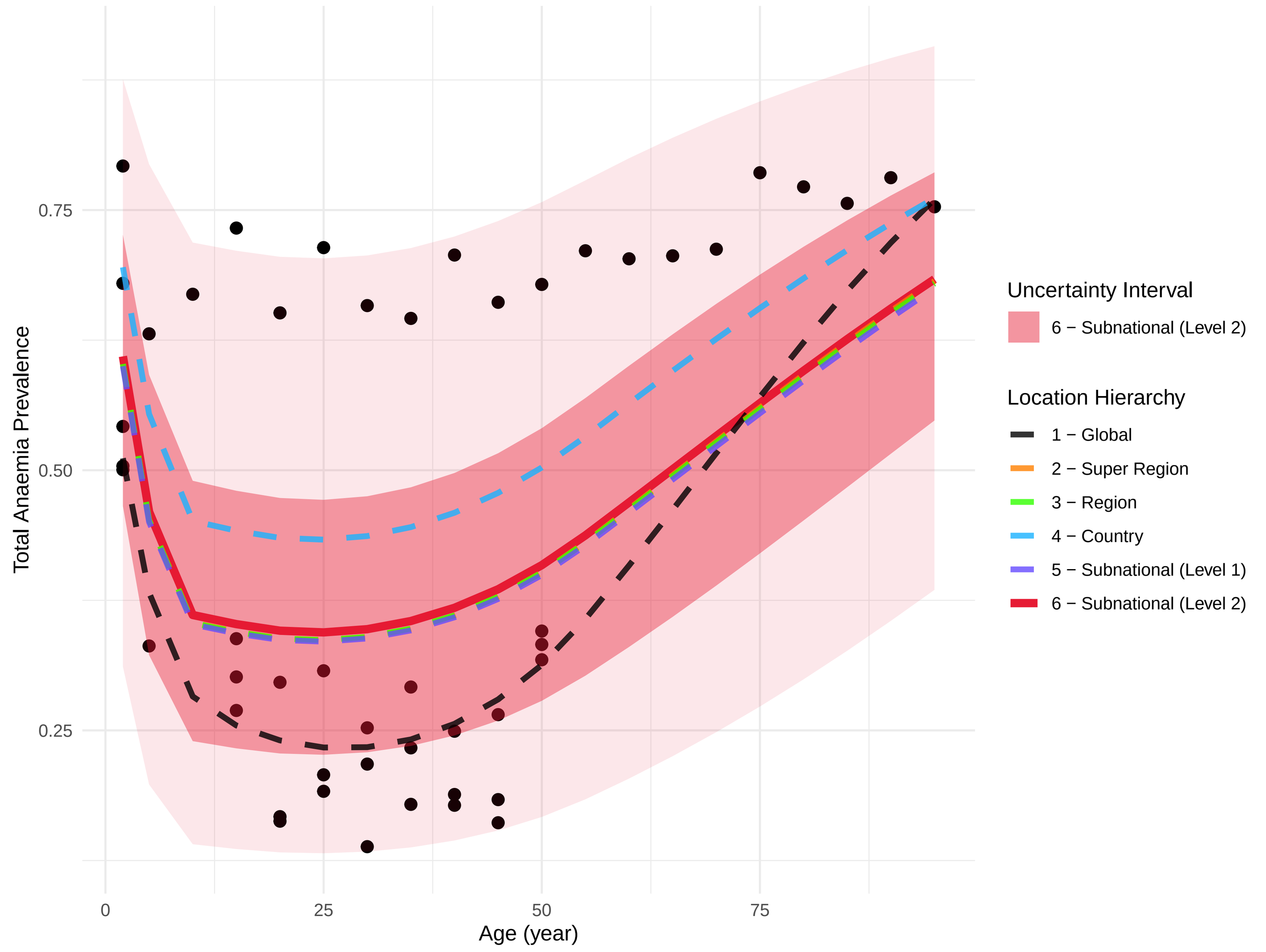

MR−BRT Age/Sex Fits − Males 2+ Years Old − Total Anaemia Prevalence − Himachal Pradesh, Urban
WHO−2001 Adjustment Method

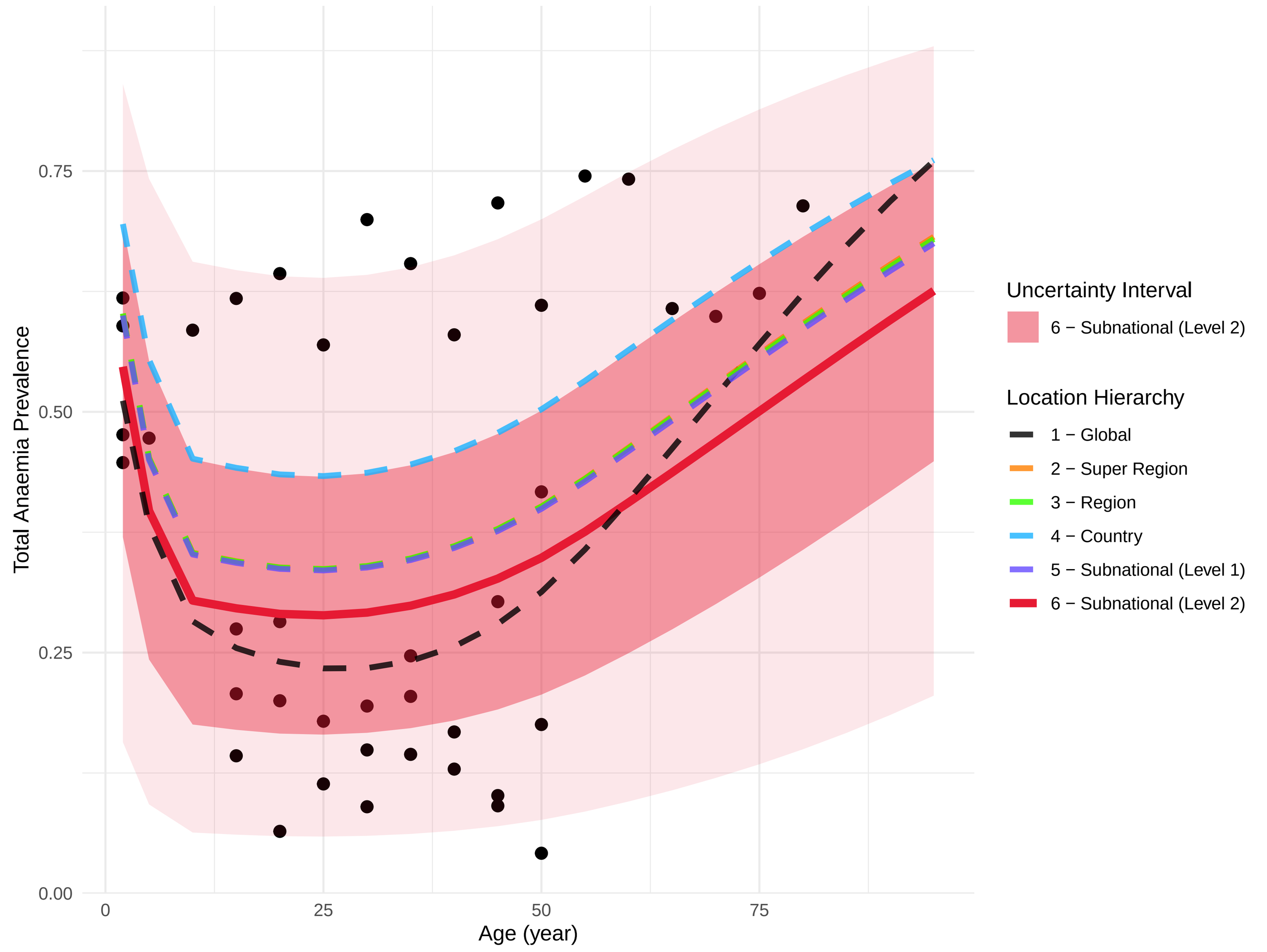

MR−BRT Age/Sex Fits − Males 2+ Years Old − Total Anaemia Prevalence − Jammu & Kashmir and Ladakh
WHO−2001 Adjustment Method

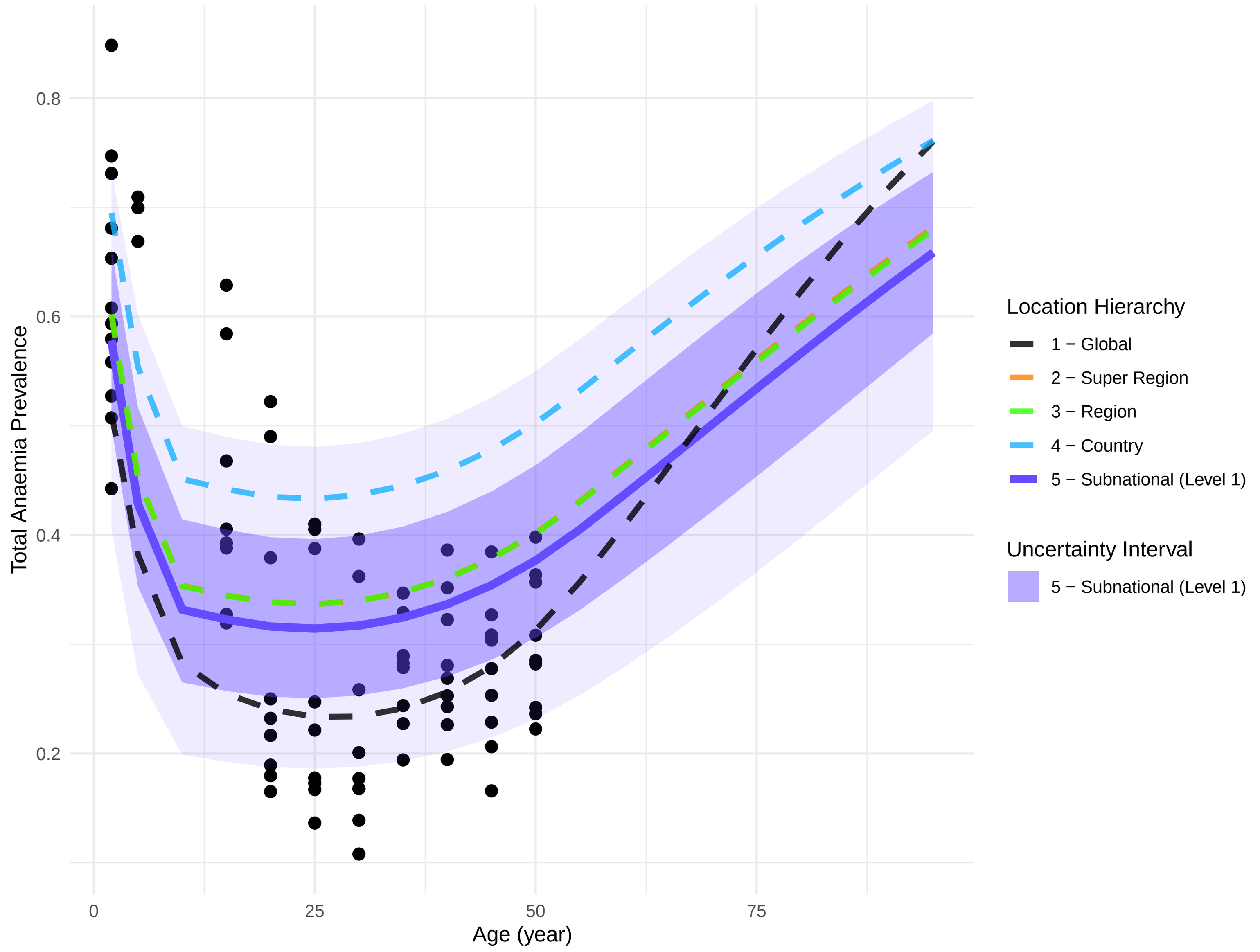

MR−BRT Age/Sex Fits − Males 2+ Years Old − Total Anaemia Prevalence − Jammu & Kashmir and Ladakh, Rural
WHO−2001 Adjustment Method

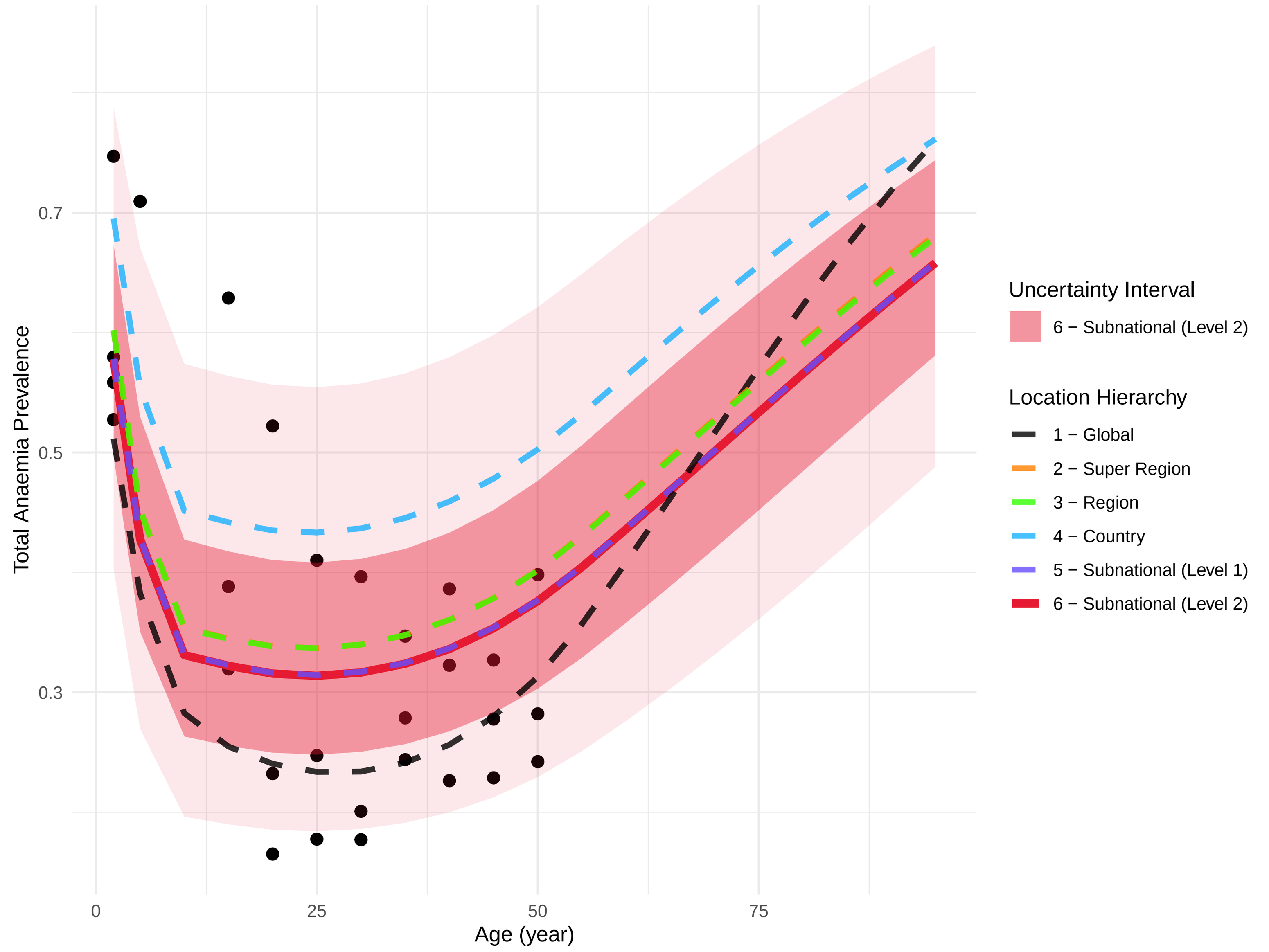

MR−BRT Age/Sex Fits − Males 2+ Years Old − Total Anaemia Prevalence − Jammu & Kashmir and Ladakh, Urban
WHO−2001 Adjustment Method

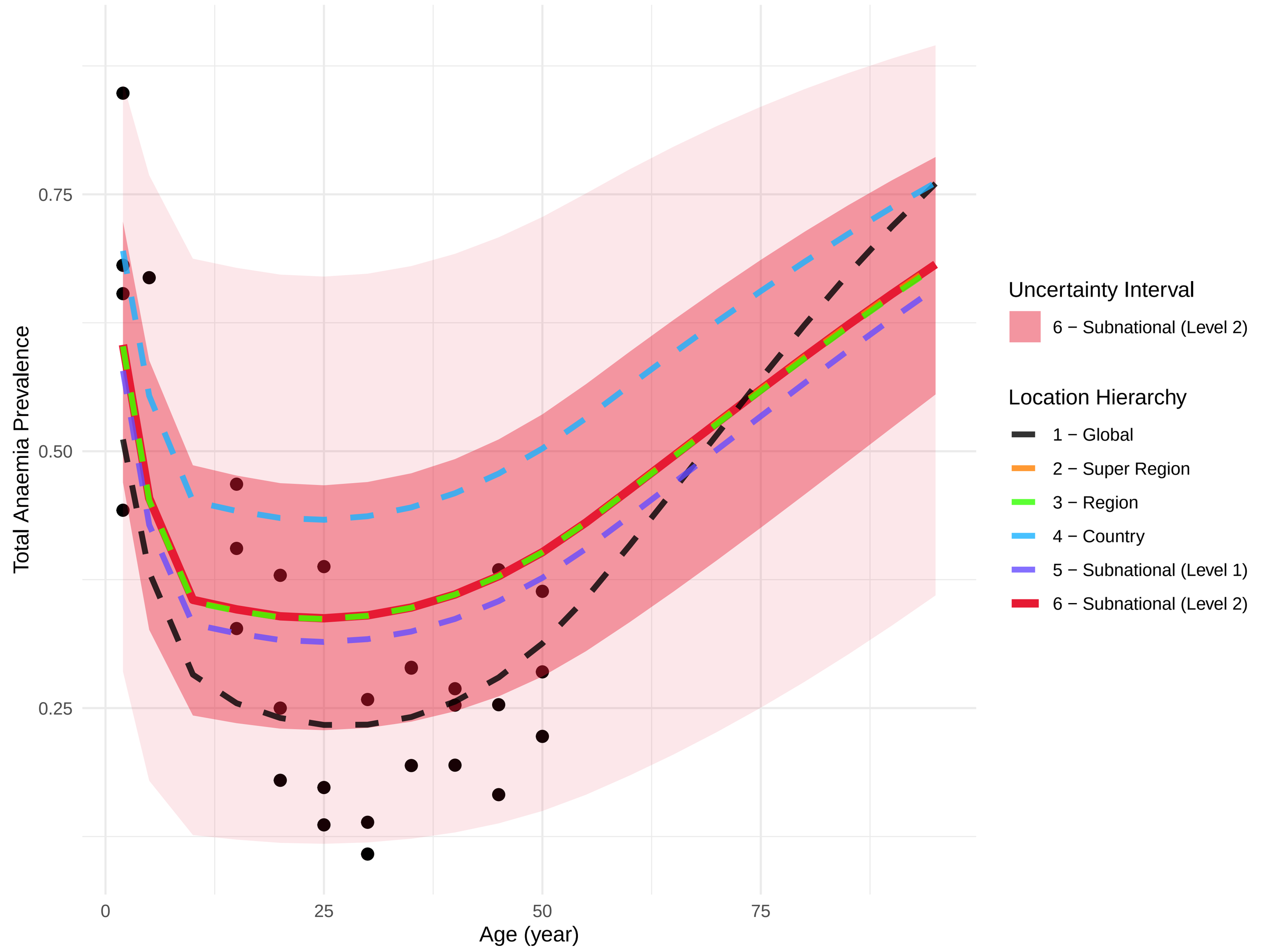

MR−BRT Age/Sex Fits − Males 2+ Years Old − Total Anaemia Prevalence − Jharkhand
WHO−2001 Adjustment Method

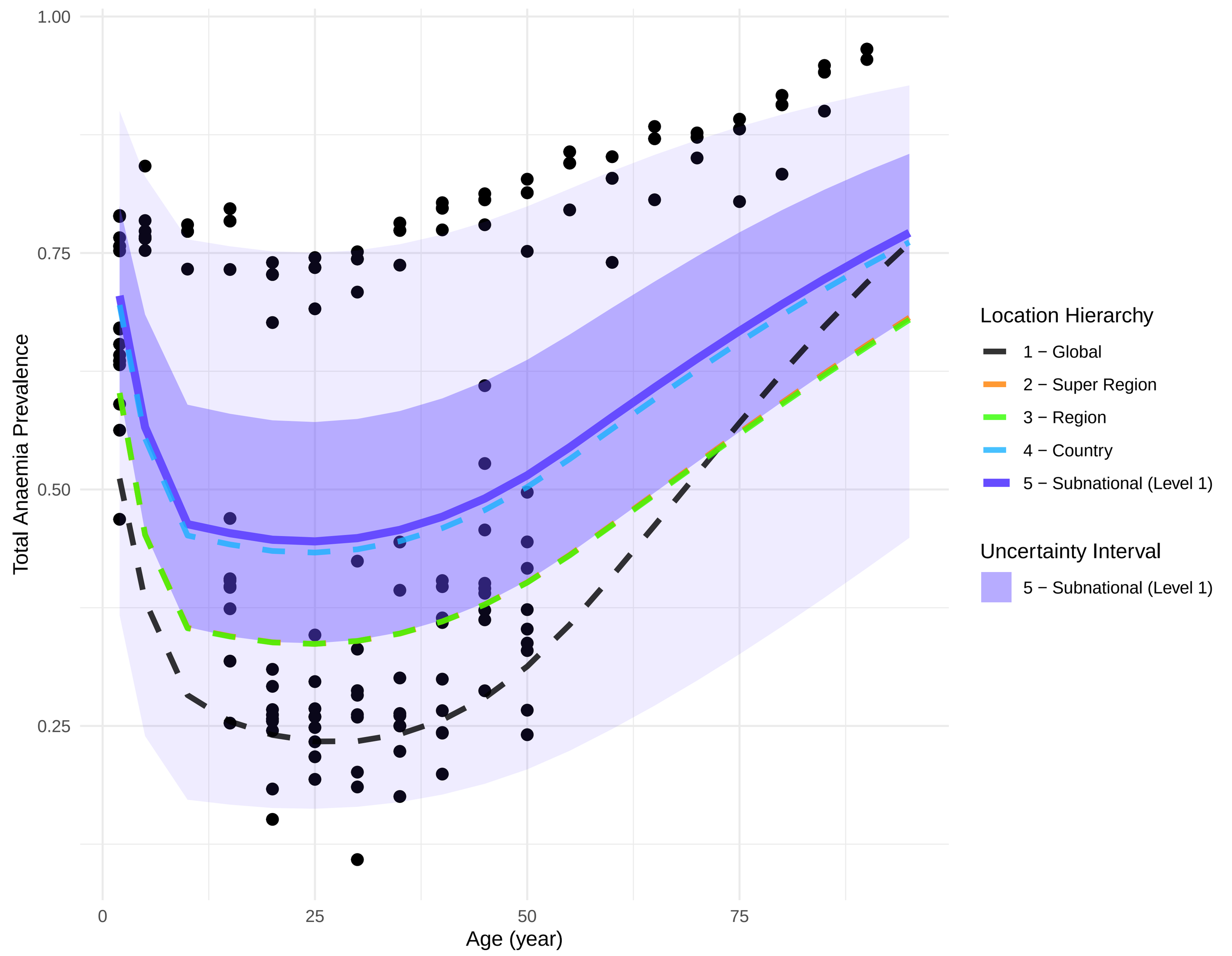

MR−BRT Age/Sex Fits − Males 2+ Years Old − Total Anaemia Prevalence − Jharkhand, Rural
WHO−2001 Adjustment Method

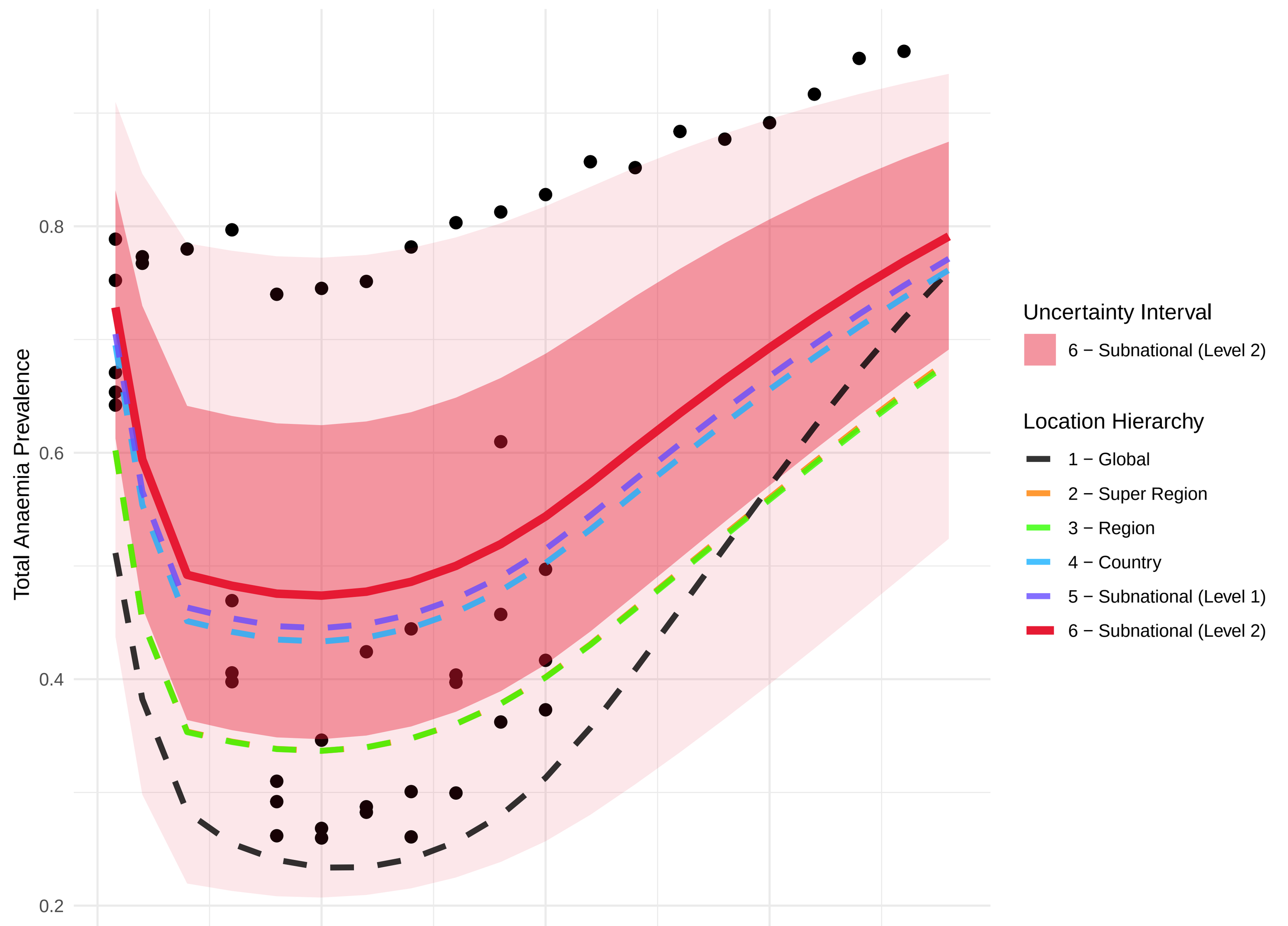

MR−BRT Age/Sex Fits − Males 2+ Years Old − Total Anaemia Prevalence − Jharkhand, Urban
WHO−2001 Adjustment Method

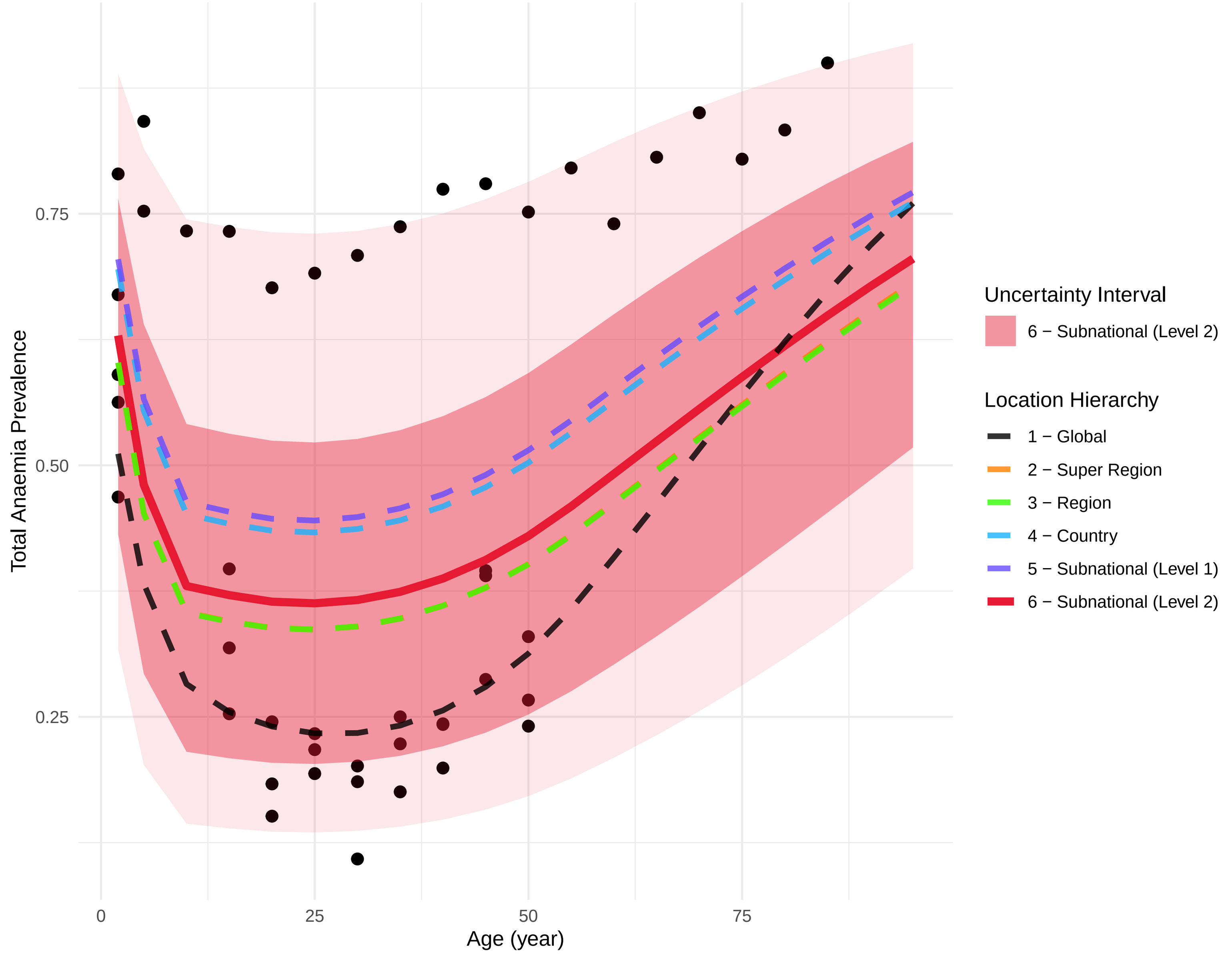

MR−BRT Age/Sex Fits − Males 2+ Years Old − Total Anaemia Prevalence − Karnataka
WHO−2001 Adjustment Method

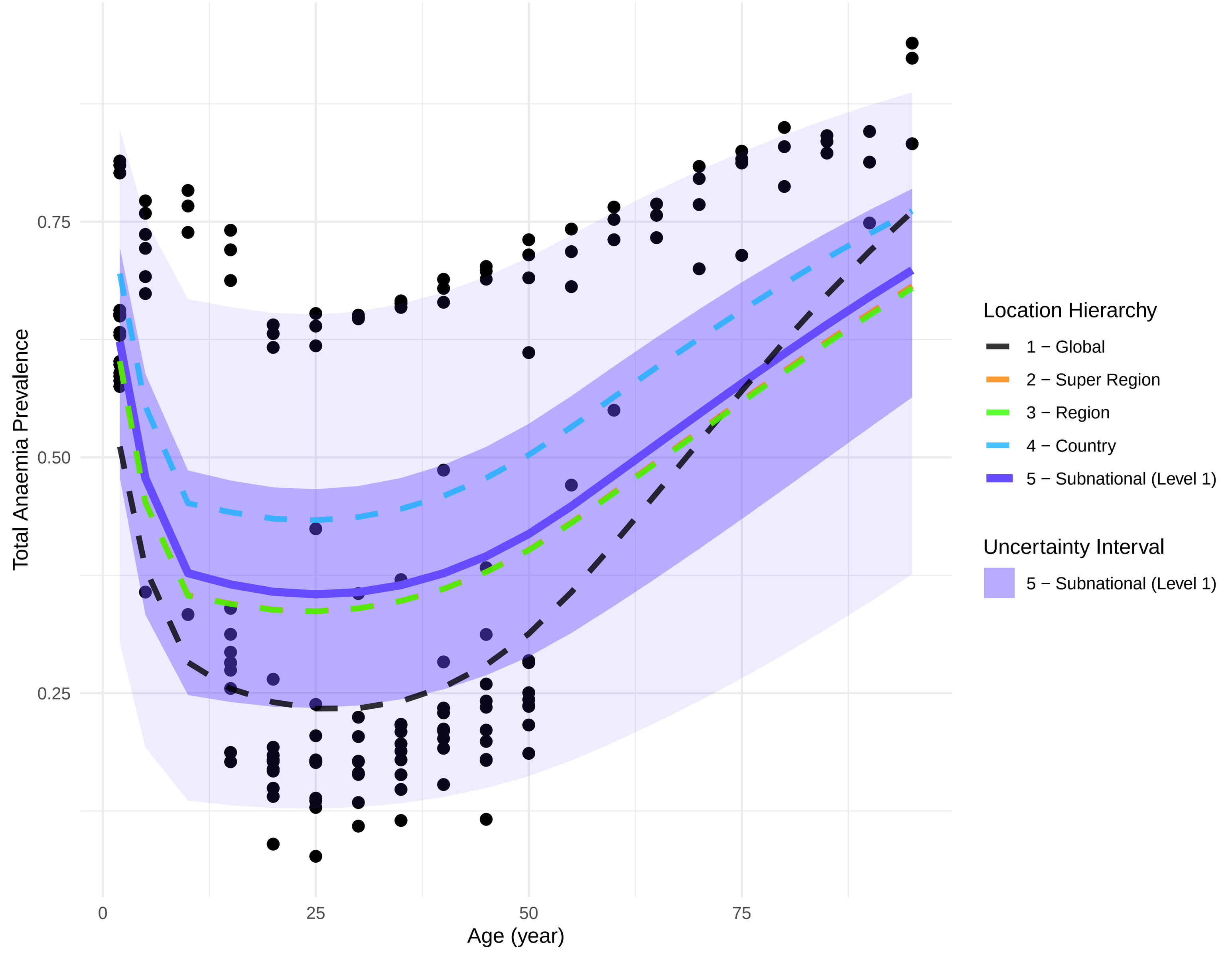

MR−BRT Age/Sex Fits − Males 2+ Years Old − Total Anaemia Prevalence − Karnataka, Rural
WHO−2001 Adjustment Method

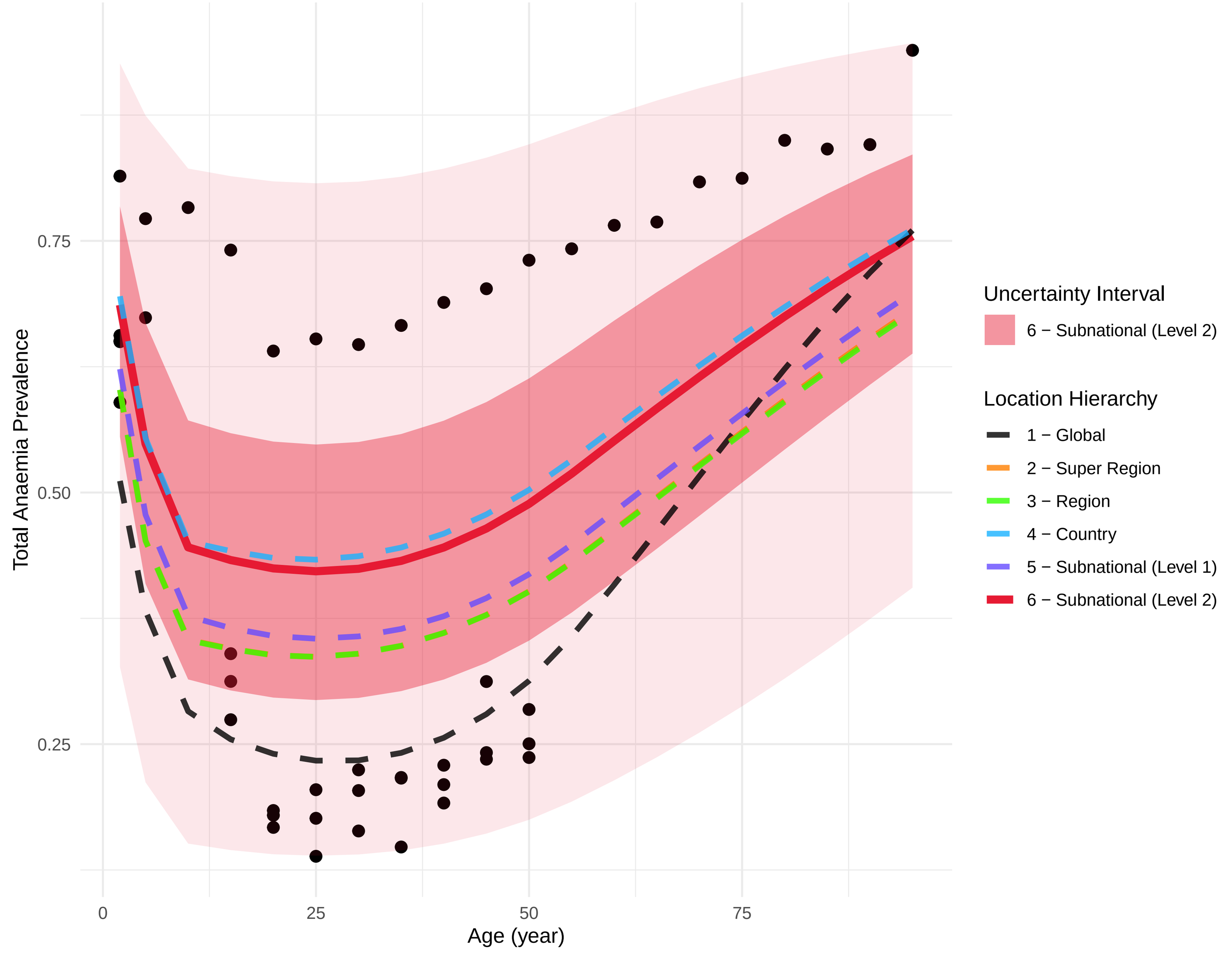

MR−BRT Age/Sex Fits − Males 2+ Years Old − Total Anaemia Prevalence − Karnataka, Urban
WHO−2001 Adjustment Method

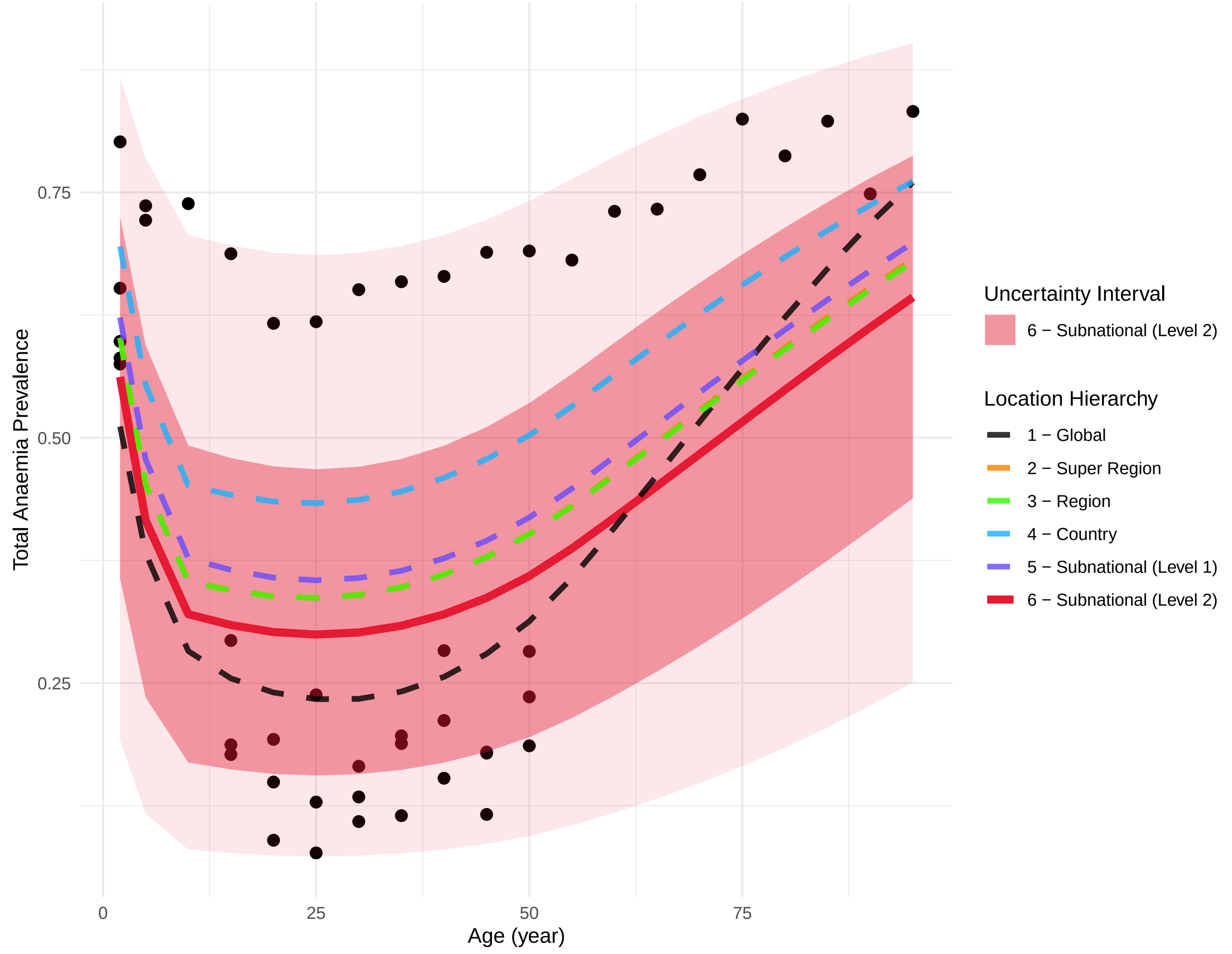

MR−BRT Age/Sex Fits − Males 2+ Years Old − Total Anaemia Prevalence − Kerala
WHO−2001 Adjustment Method

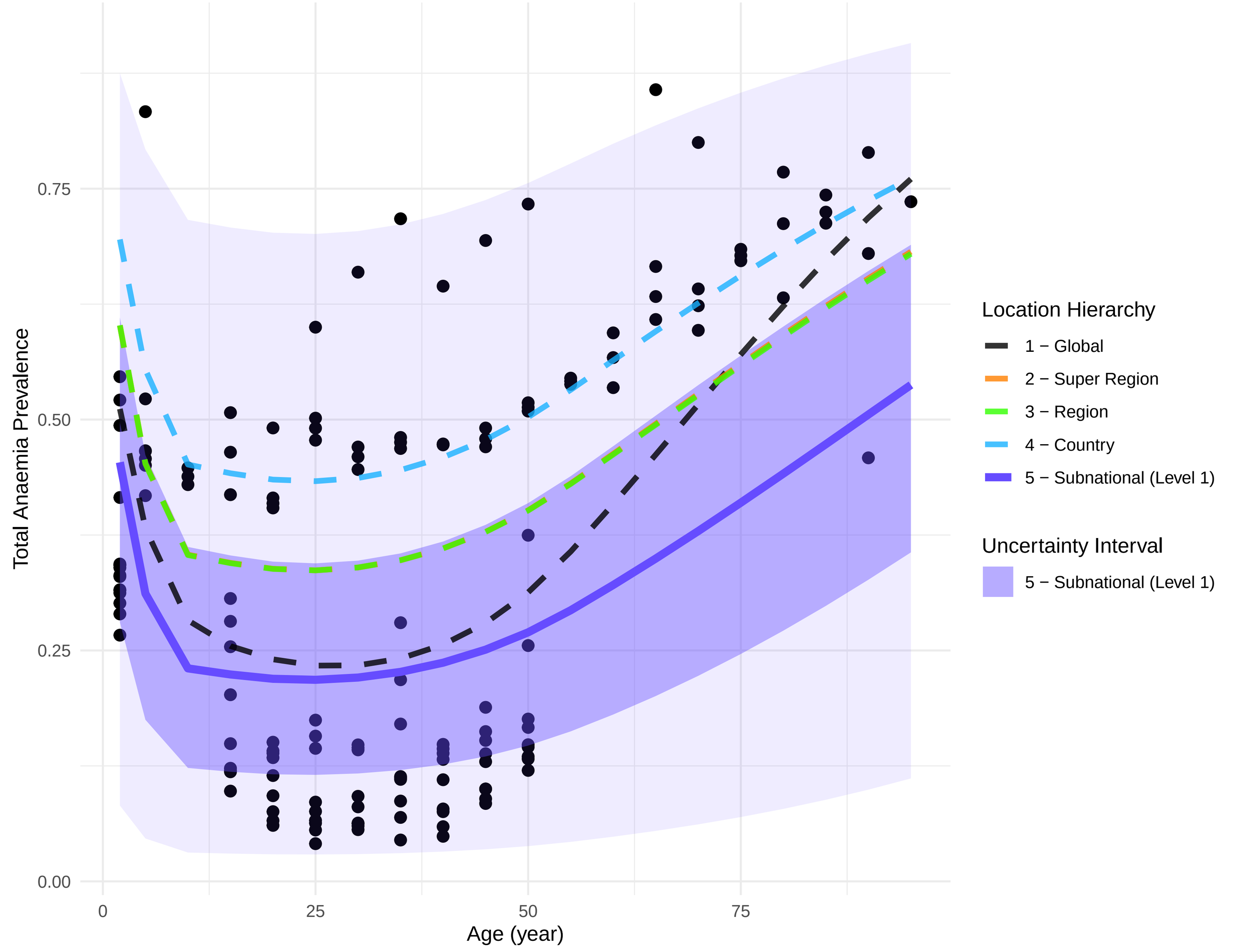

MR−BRT Age/Sex Fits − Males 2+ Years Old − Total Anaemia Prevalence − Kerala, Rural
WHO−2001 Adjustment Method

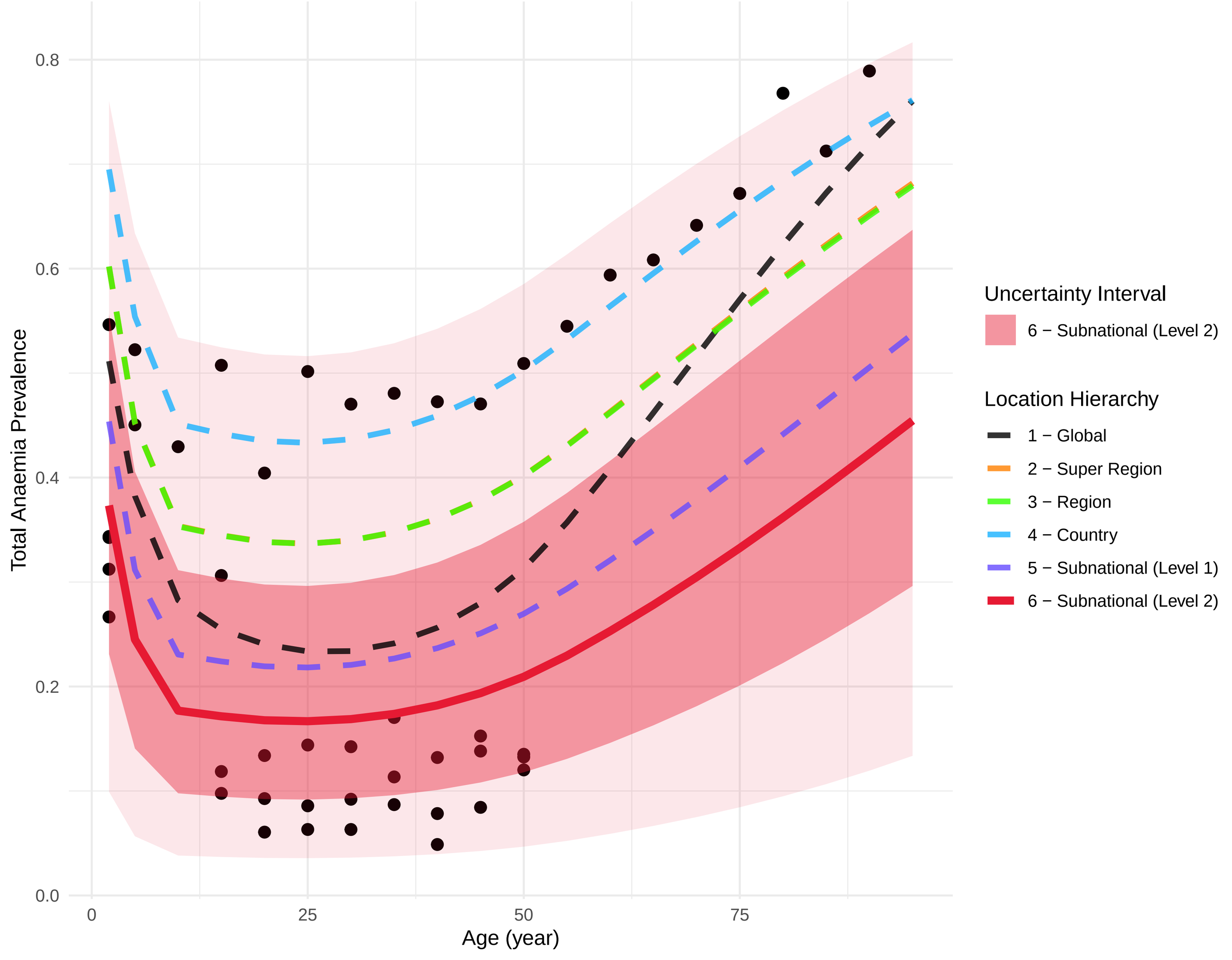

MR−BRT Age/Sex Fits − Males 2+ Years Old − Total Anaemia Prevalence − Kerala, Urban
WHO−2001 Adjustment Method

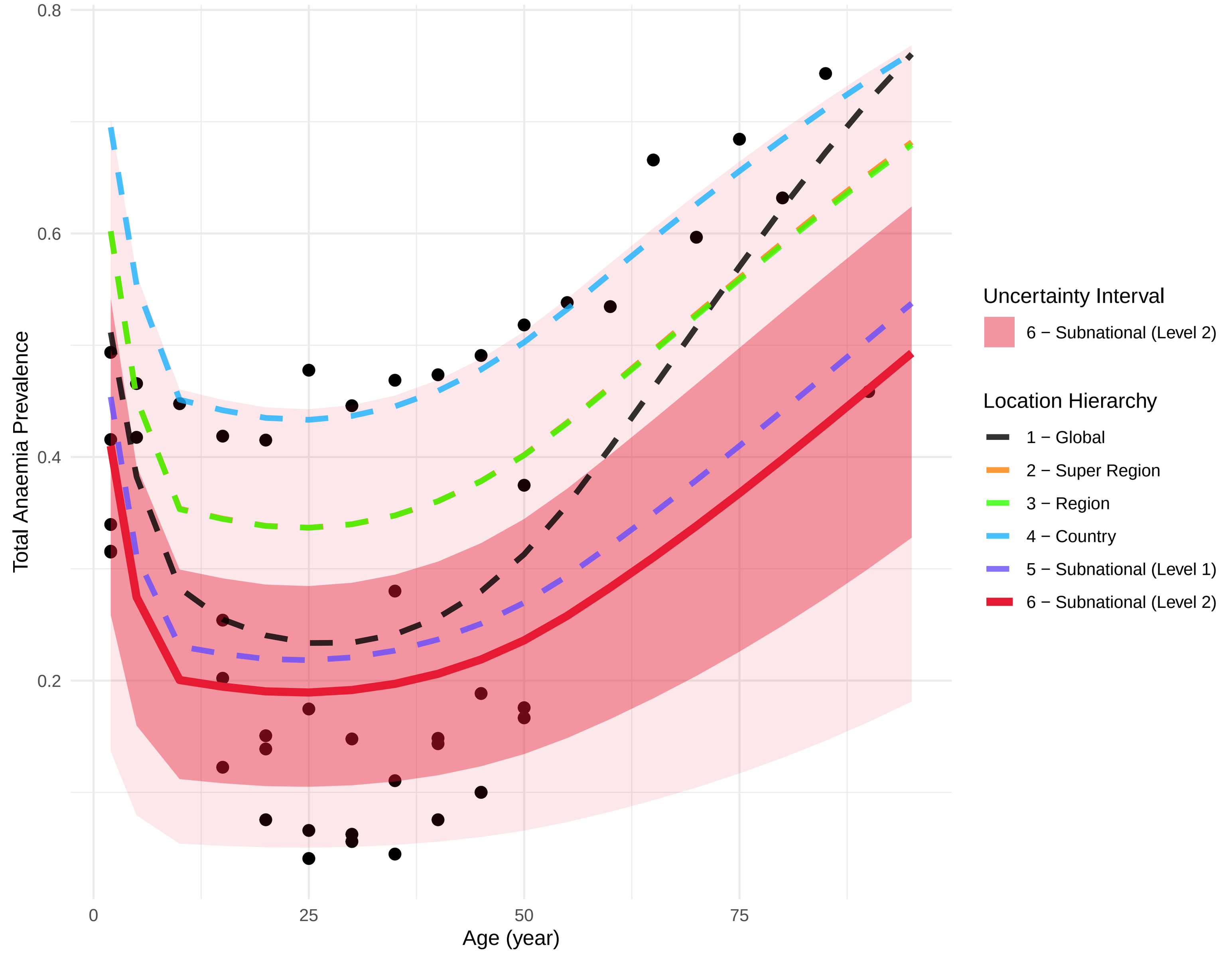

MR−BRT Age/Sex Fits − Males 2+ Years Old − Total Anaemia Prevalence − Madhya Pradesh
WHO−2001 Adjustment Method

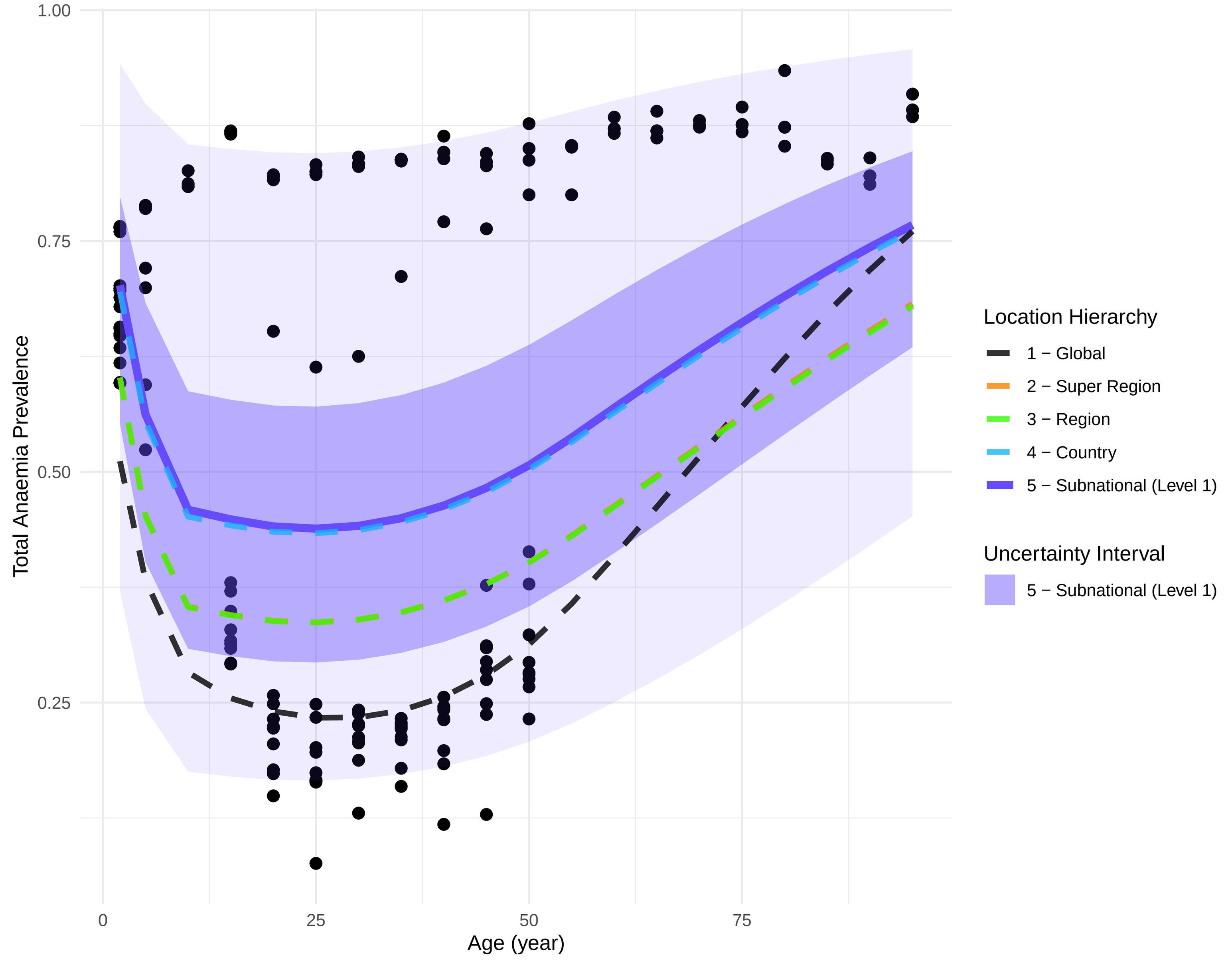

MR−BRT Age/Sex Fits − Males 2+ Years Old − Total Anaemia Prevalence − Madhya Pradesh, Rural
WHO−2001 Adjustment Method

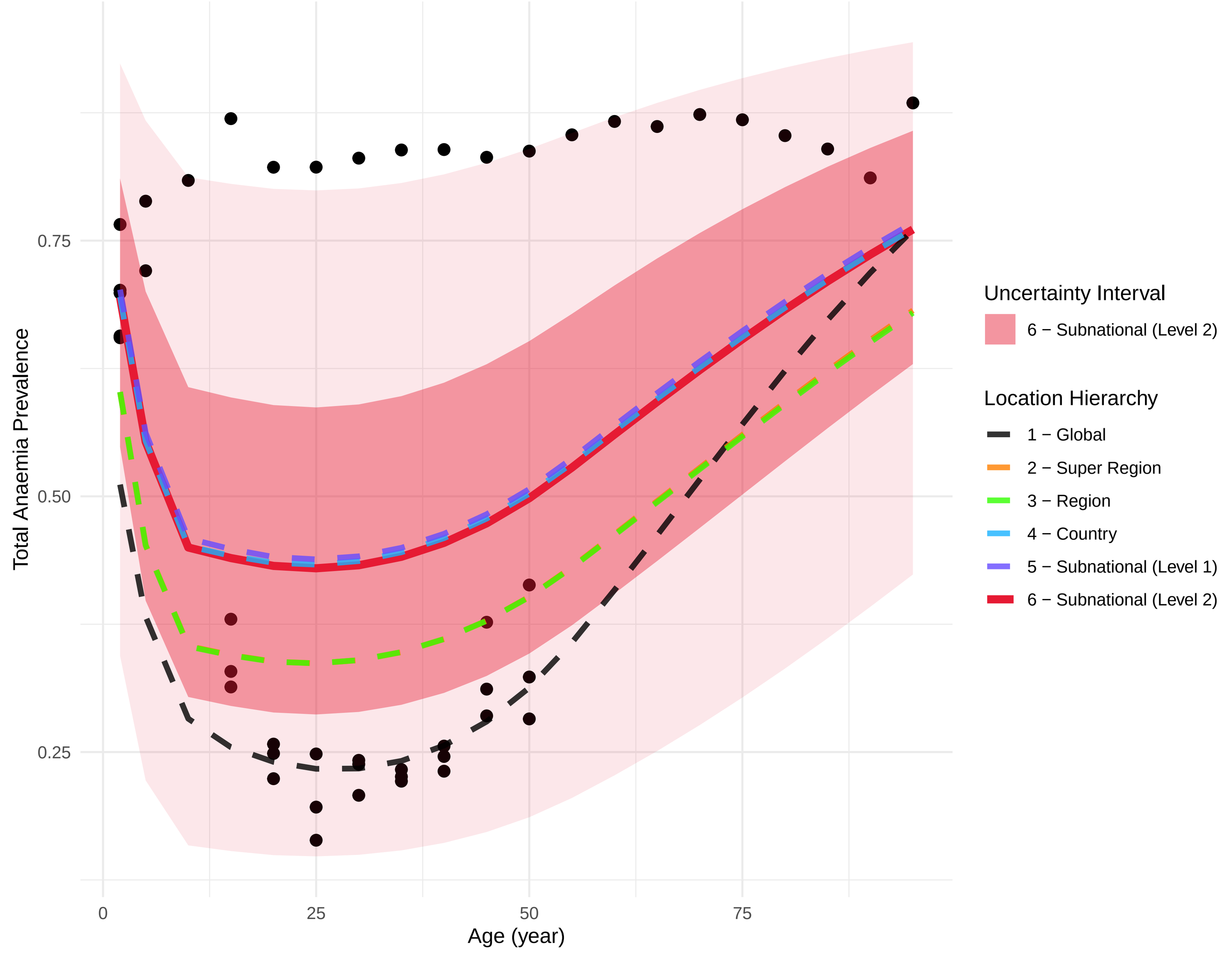

MR−BRT Age/Sex Fits − Males 2+ Years Old − Total Anaemia Prevalence − Madhya Pradesh, Urban
WHO−2001 Adjustment Method

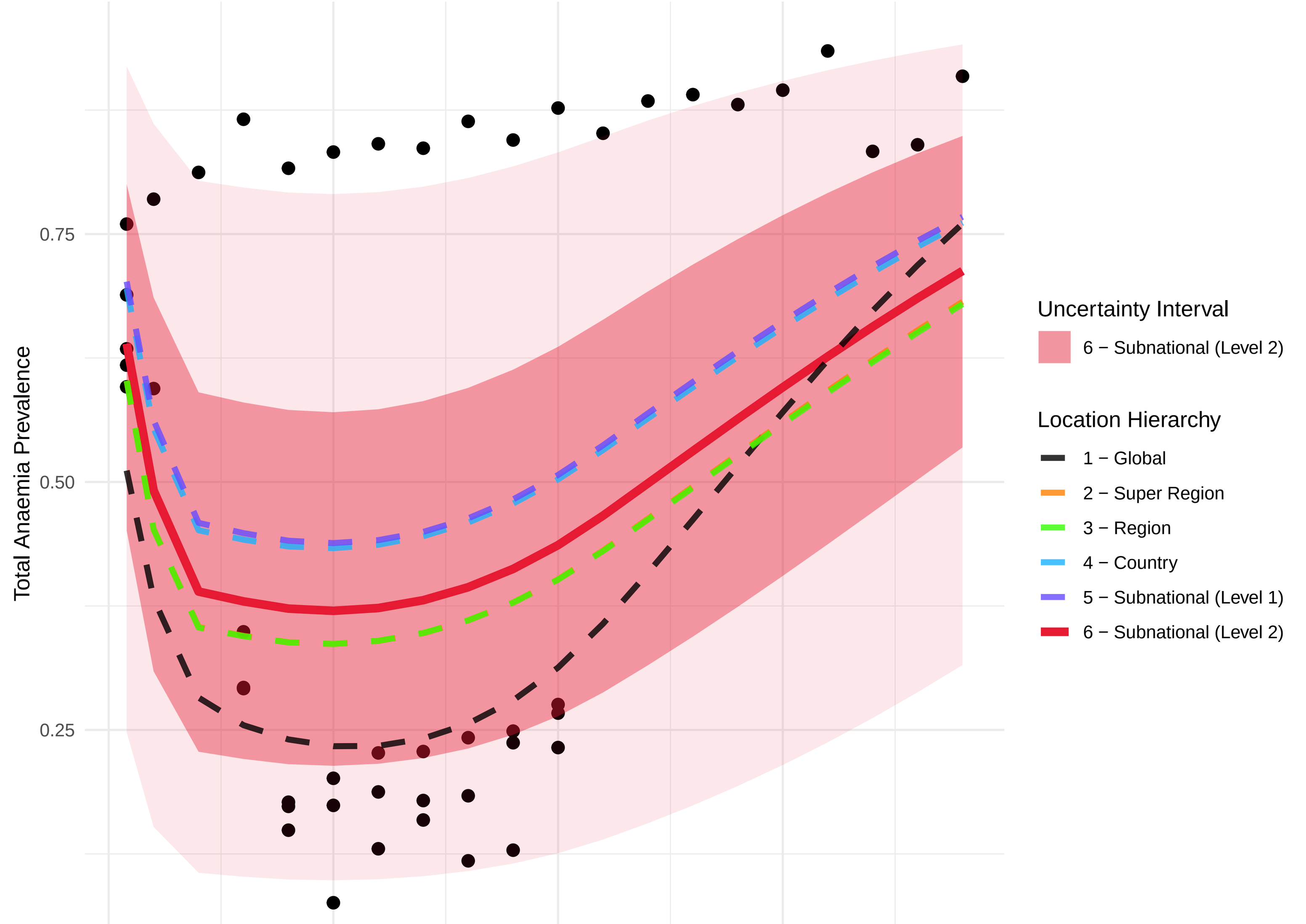

MR−BRT Age/Sex Fits − Males 2+ Years Old − Total Anaemia Prevalence − Maharashtra
WHO−2001 Adjustment Method

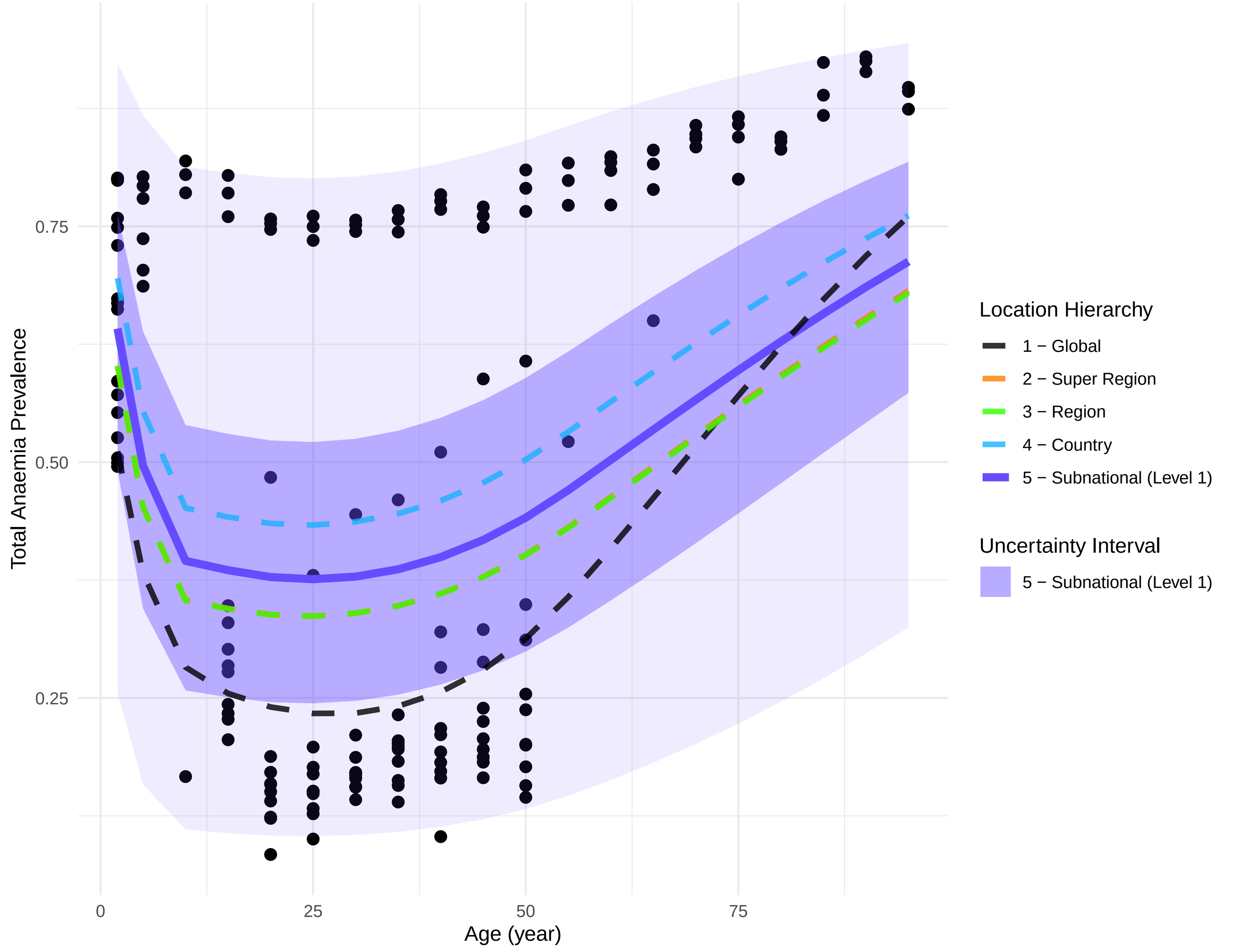

MR−BRT Age/Sex Fits − Males 2+ Years Old − Total Anaemia Prevalence − Maharashtra, Rural
WHO−2001 Adjustment Method

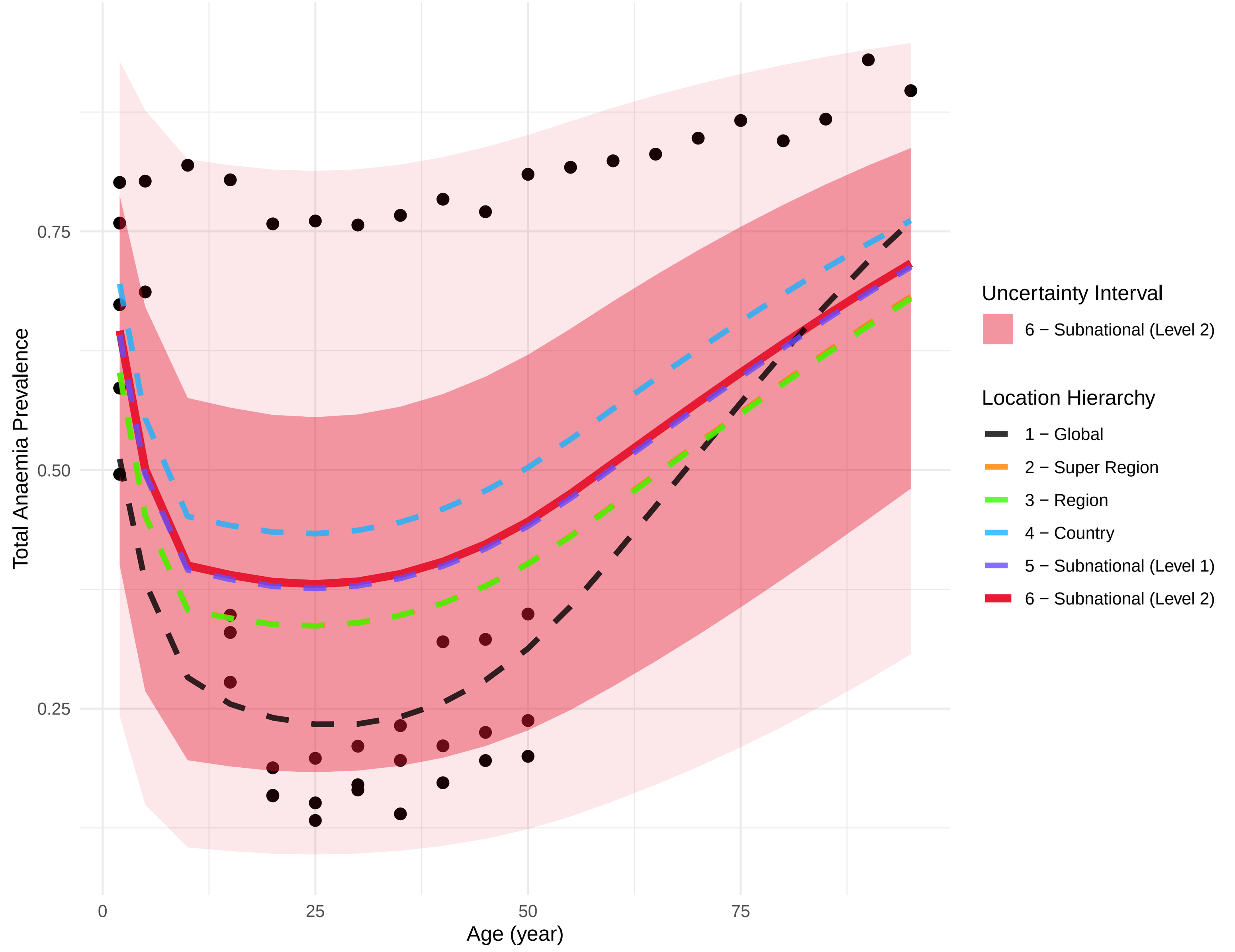

MR−BRT Age/Sex Fits − Males 2+ Years Old − Total Anaemia Prevalence − Maharashtra, Urban
WHO−2001 Adjustment Method

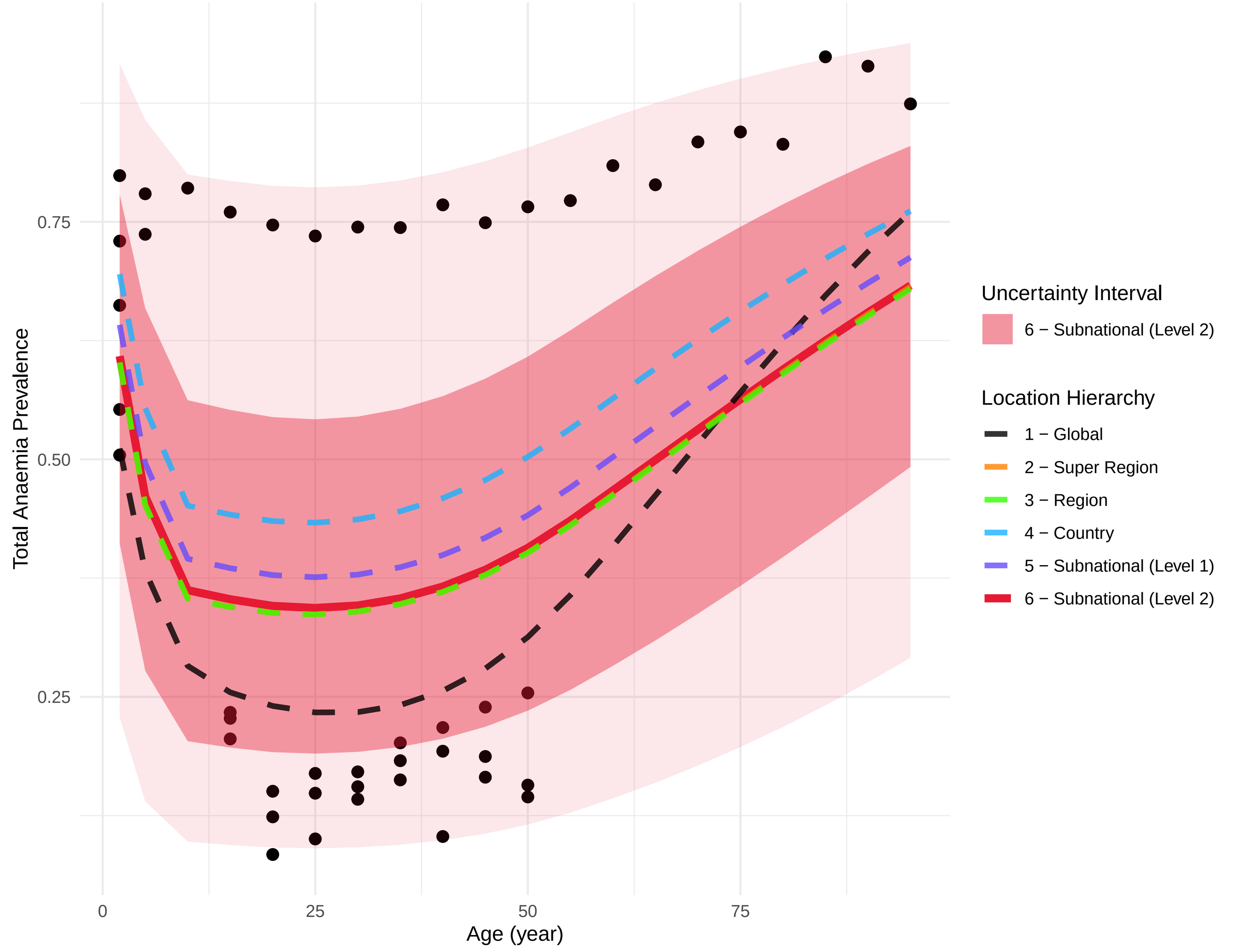

MR−BRT Age/Sex Fits − Males 2+ Years Old − Total Anaemia Prevalence − Manipur
WHO−2001 Adjustment Method

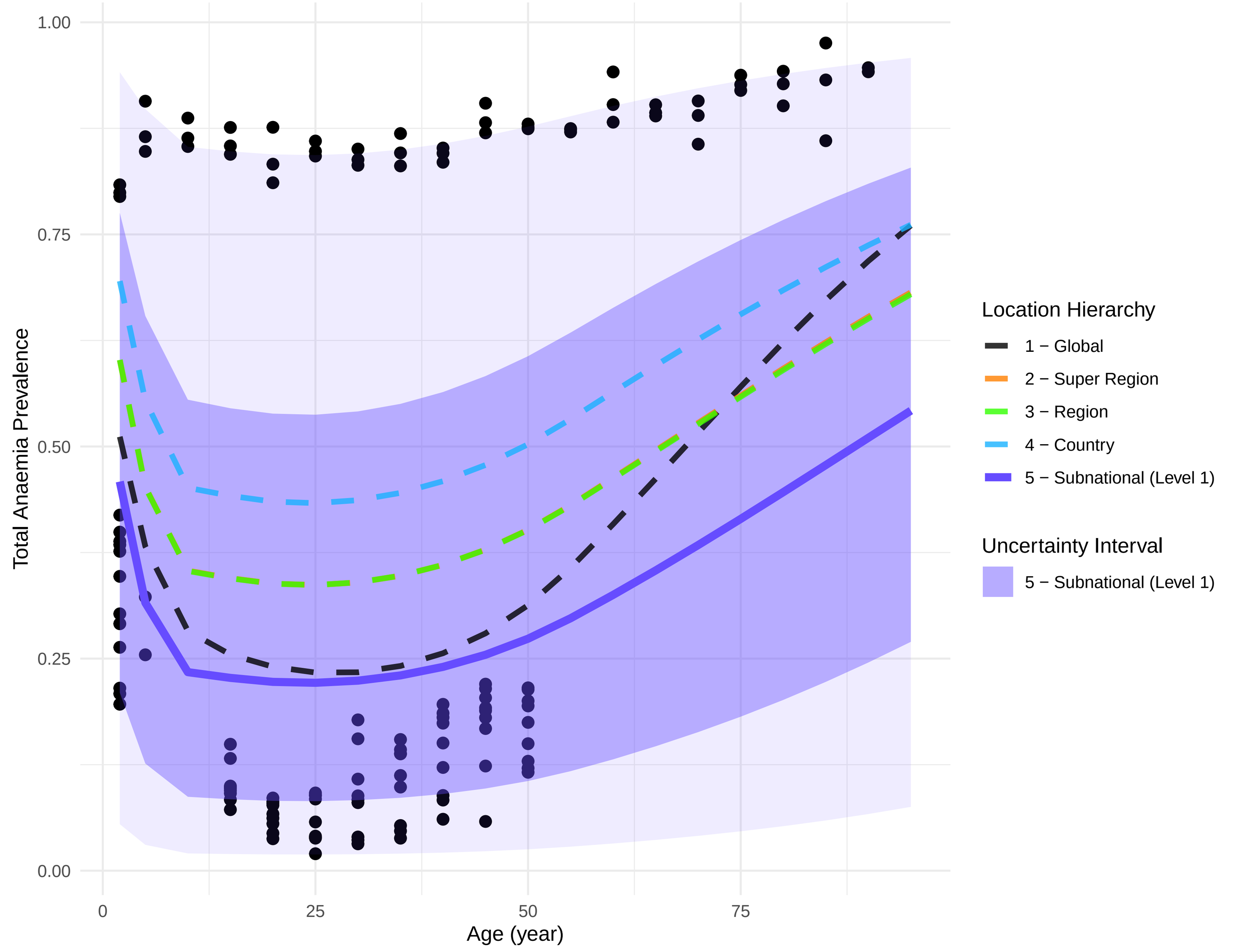

MR−BRT Age/Sex Fits − Males 2+ Years Old − Total Anaemia Prevalence − Manipur, Rural
WHO−2001 Adjustment Method

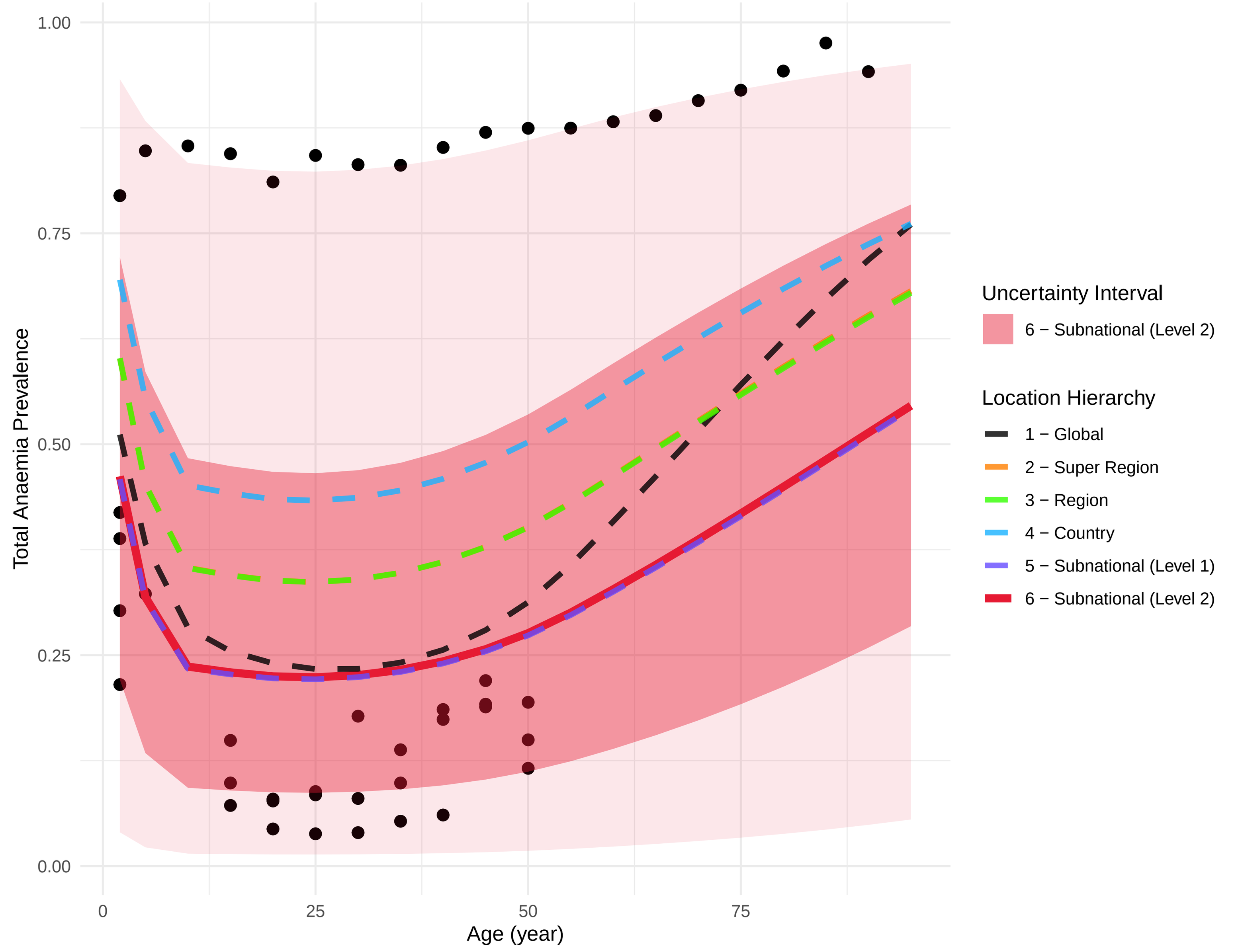

MR−BRT Age/Sex Fits − Males 2+ Years Old − Total Anaemia Prevalence − Manipur, Urban
WHO−2001 Adjustment Method

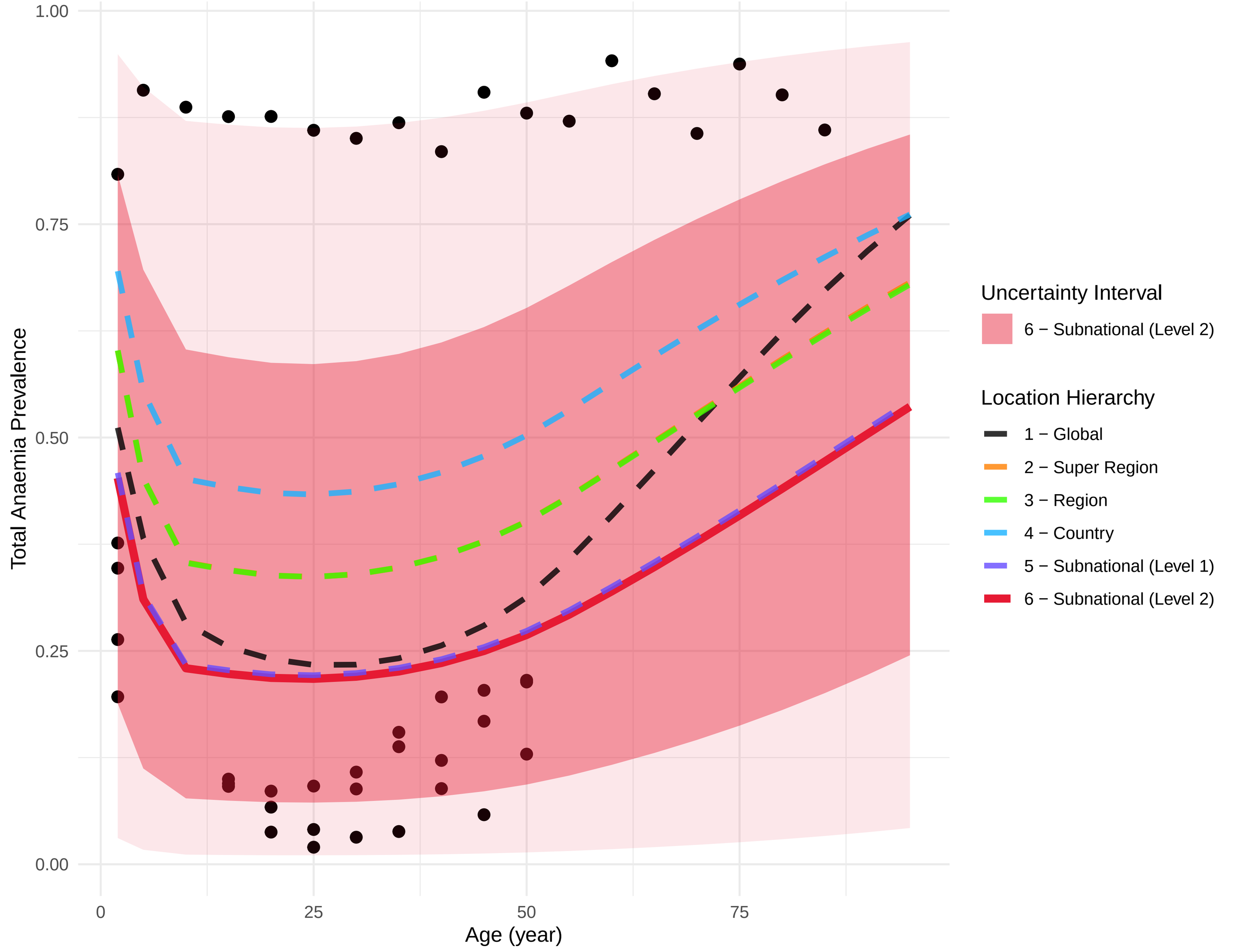

MR−BRT Age/Sex Fits − Males 2+ Years Old − Total Anaemia Prevalence − Meghalaya
WHO−2001 Adjustment Method

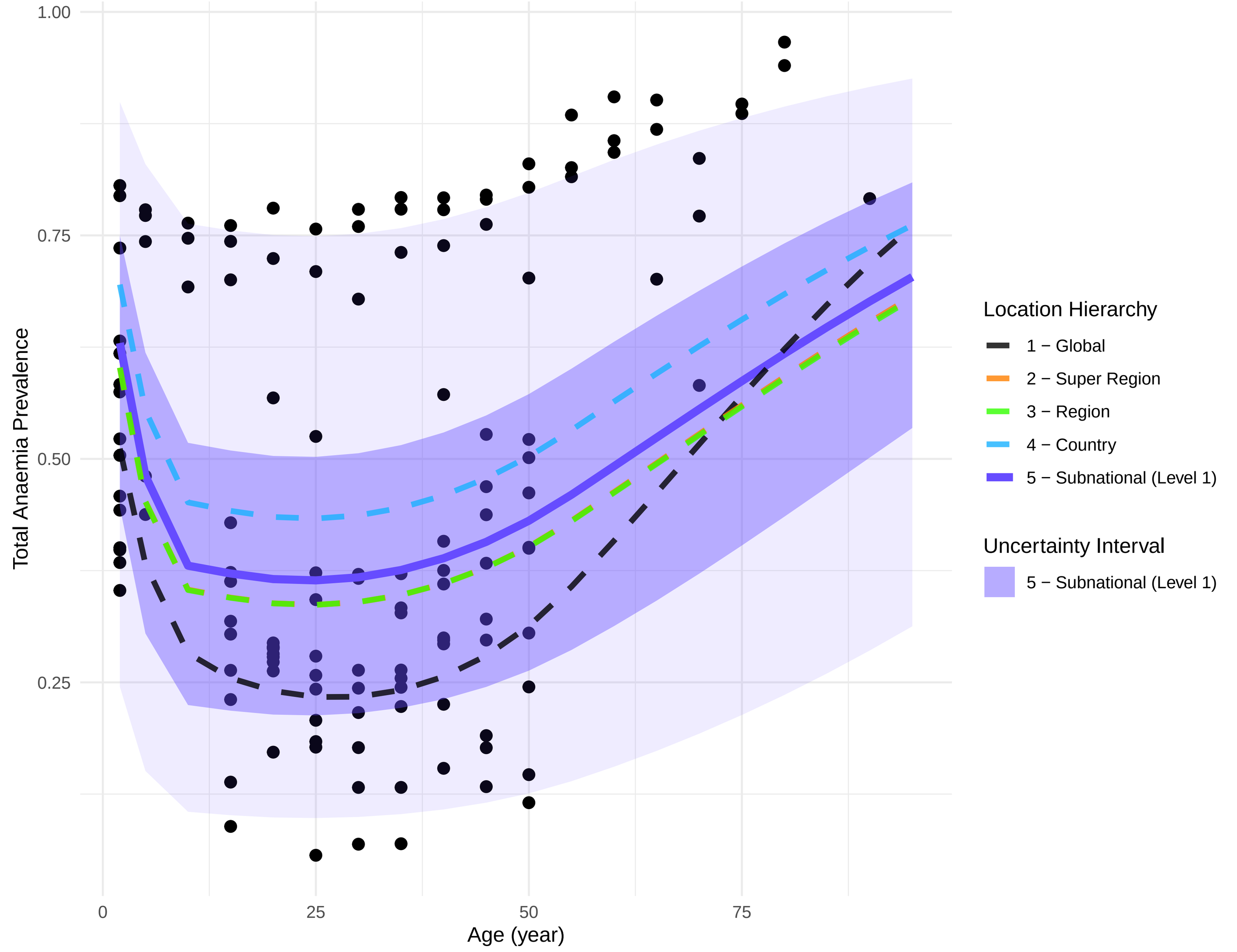

MR−BRT Age/Sex Fits − Males 2+ Years Old − Total Anaemia Prevalence − Meghalaya, Rural
WHO−2001 Adjustment Method

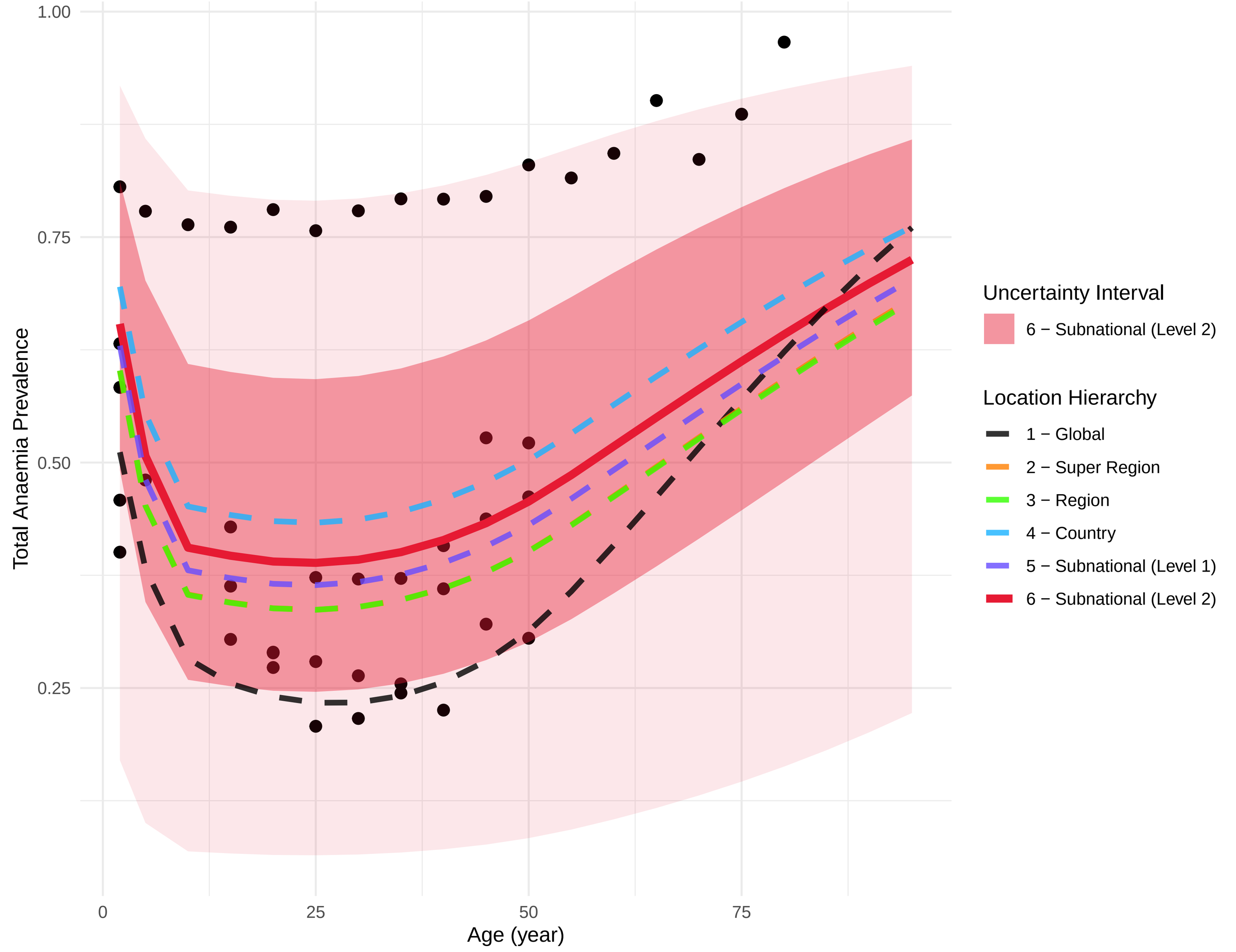

MR−BRT Age/Sex Fits − Males 2+ Years Old − Total Anaemia Prevalence − Meghalaya, Urban
WHO−2001 Adjustment Method

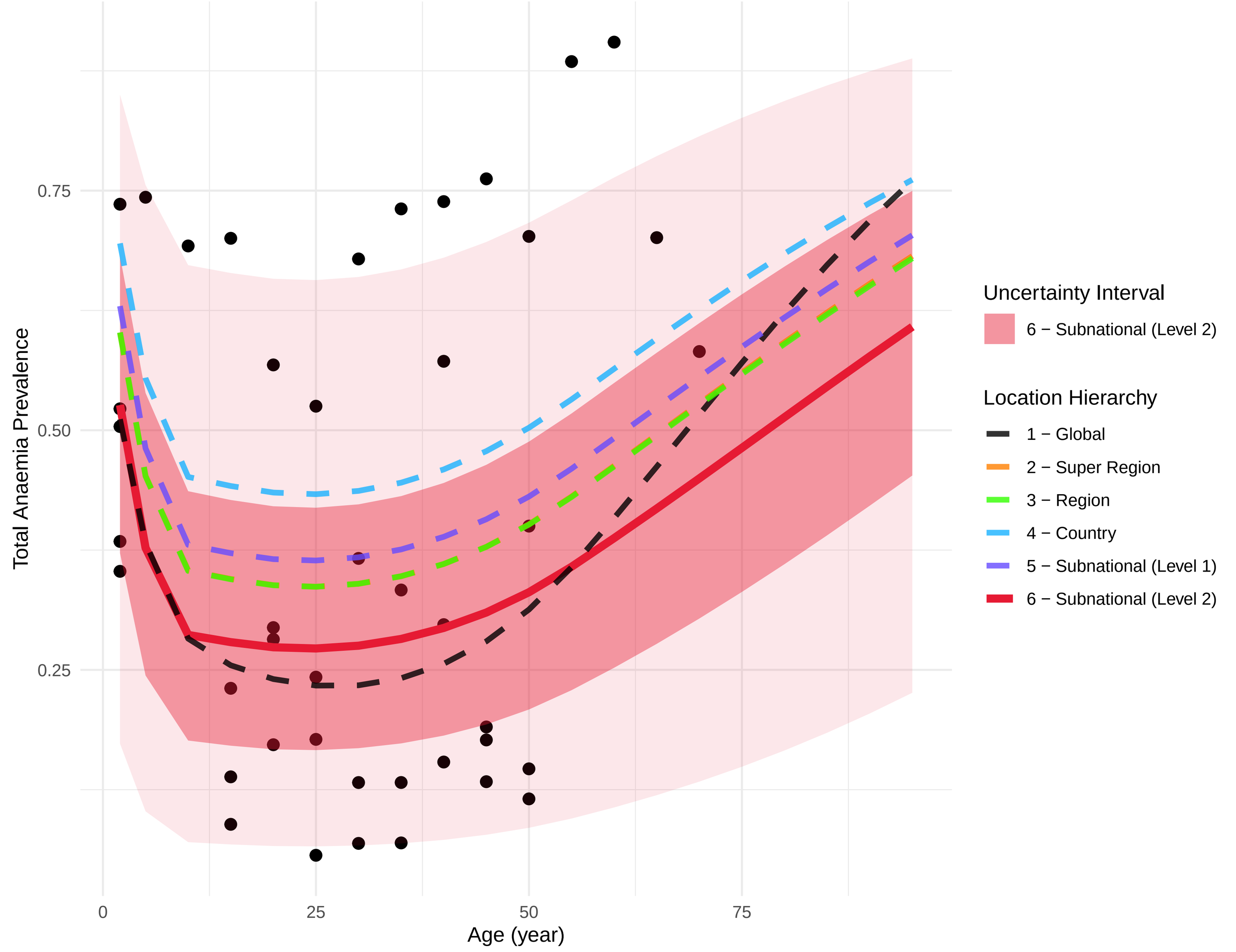

MR−BRT Age/Sex Fits − Males 2+ Years Old − Total Anaemia Prevalence − Mizoram
WHO−2001 Adjustment Method

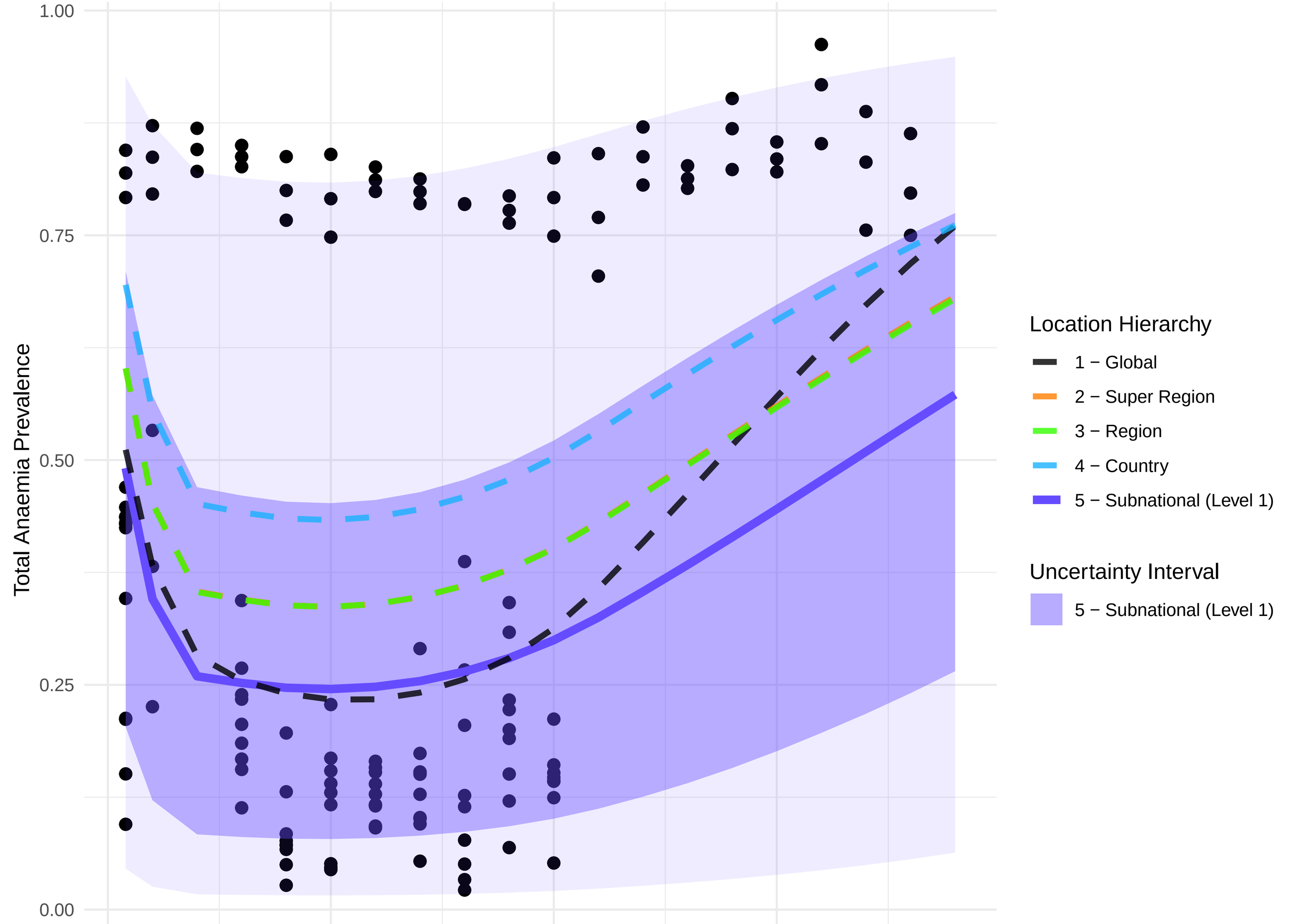

MR−BRT Age/Sex Fits − Males 2+ Years Old − Total Anaemia Prevalence − Mizoram, Rural
WHO−2001 Adjustment Method

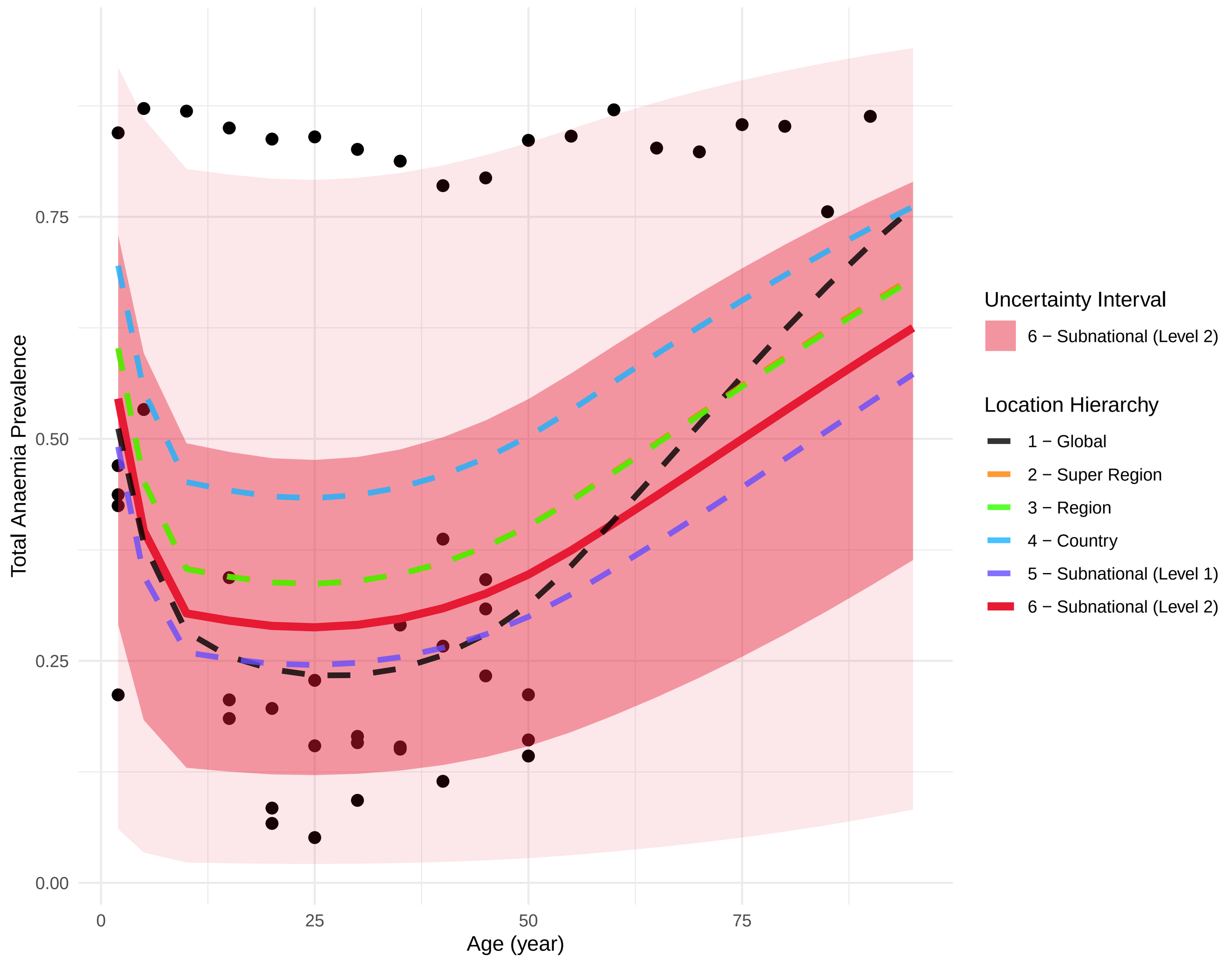

MR−BRT Age/Sex Fits − Males 2+ Years Old − Total Anaemia Prevalence − Mizoram, Urban
WHO−2001 Adjustment Method

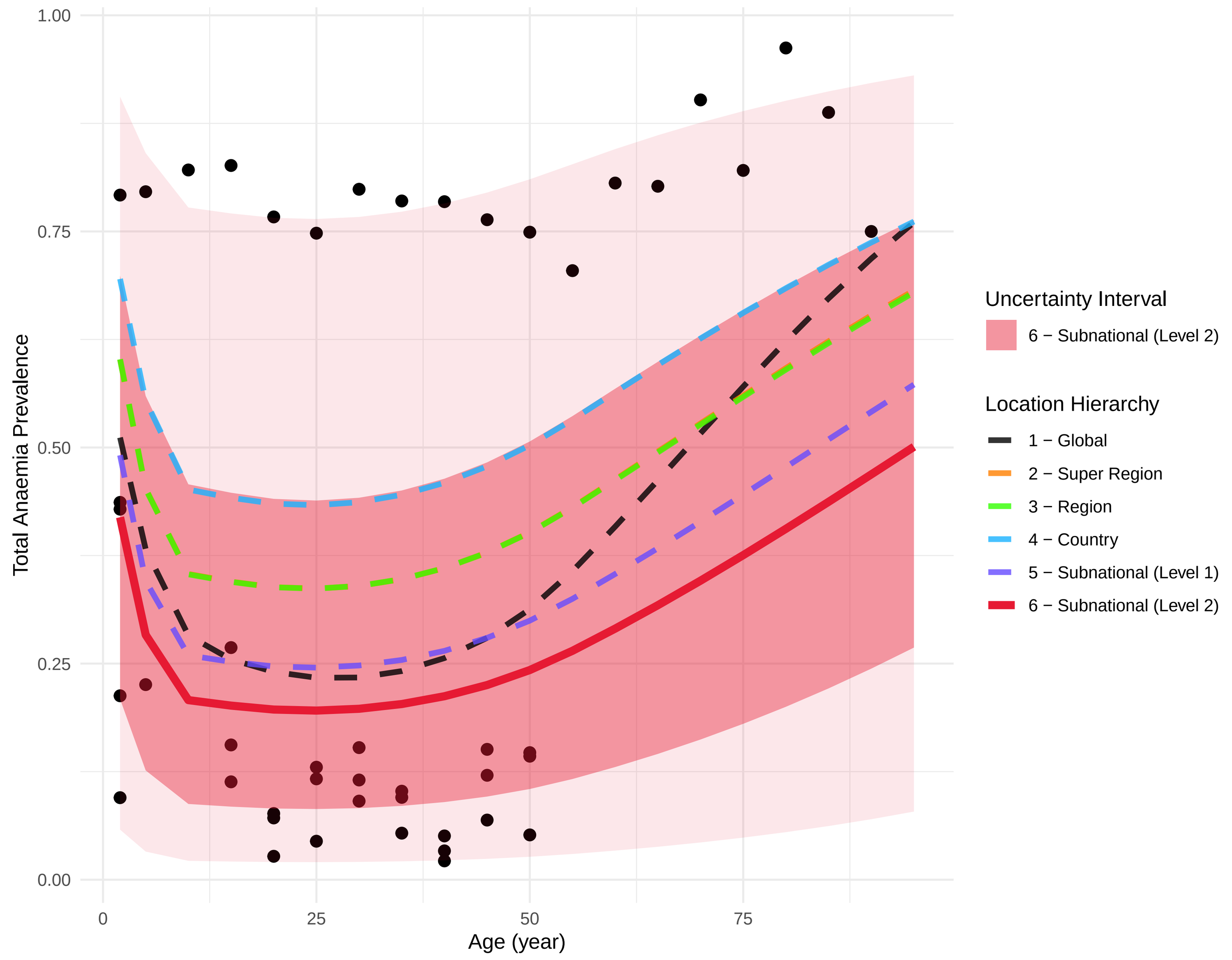

MR−BRT Age/Sex Fits − Males 2+ Years Old − Total Anaemia Prevalence − Nagaland
WHO−2001 Adjustment Method

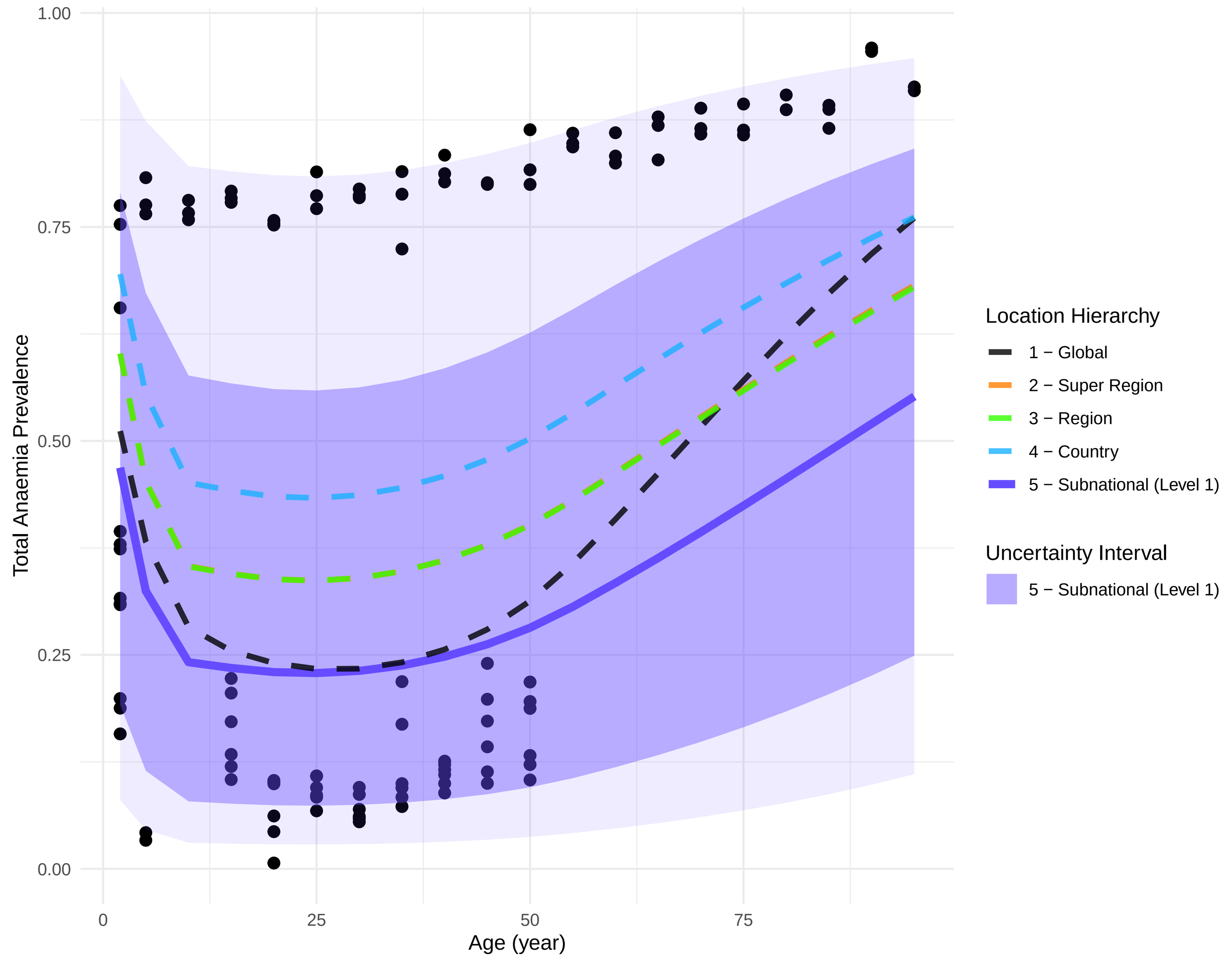

MR−BRT Age/Sex Fits − Males 2+ Years Old − Total Anaemia Prevalence − Nagaland, Rural
WHO−2001 Adjustment Method

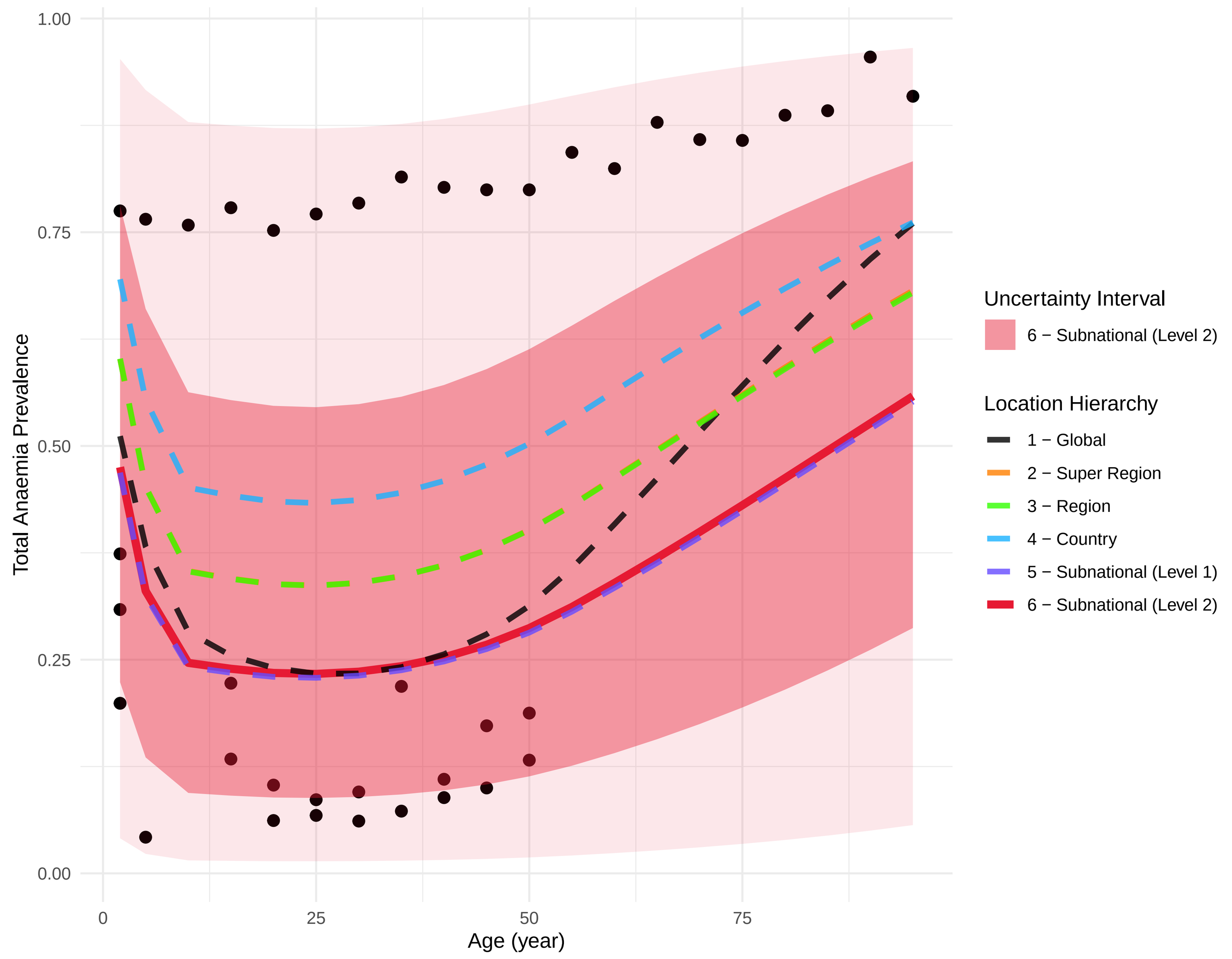

MR−BRT Age/Sex Fits − Males 2+ Years Old − Total Anaemia Prevalence − Nagaland, Urban
WHO−2001 Adjustment Method

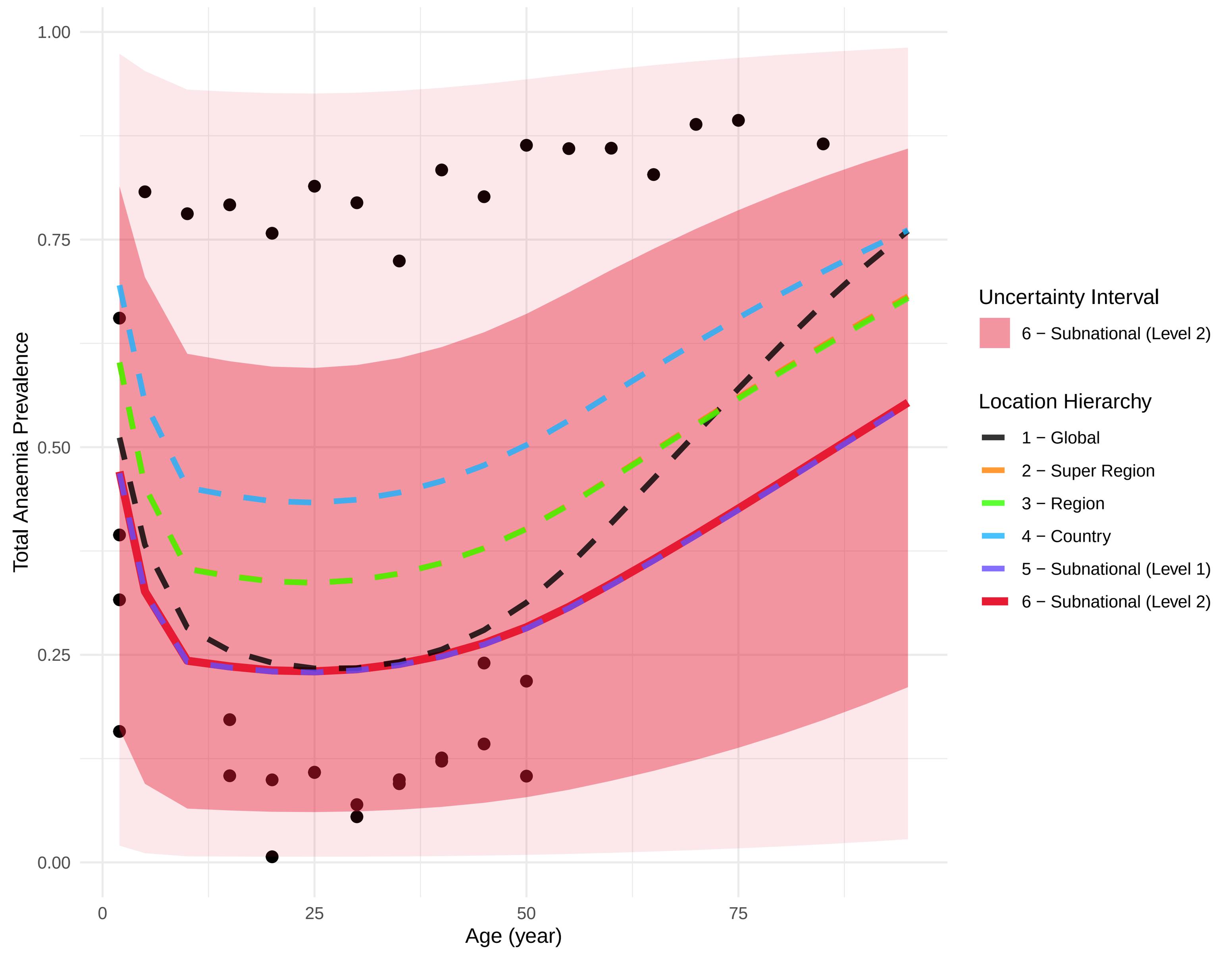

MR−BRT Age/Sex Fits − Males 2+ Years Old − Total Anaemia Prevalence − Odisha
WHO−2001 Adjustment Method

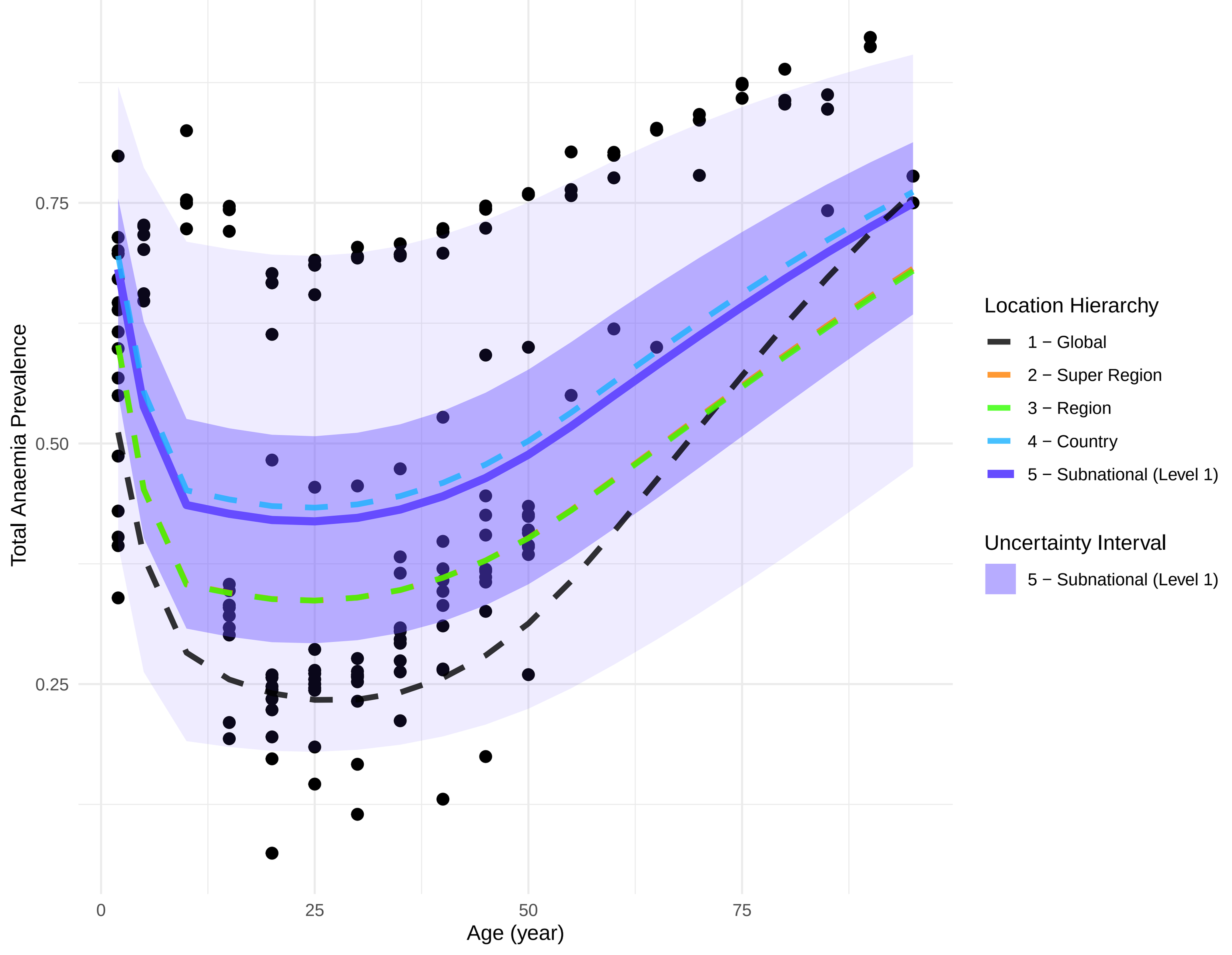

MR−BRT Age/Sex Fits − Males 2+ Years Old − Total Anaemia Prevalence − Odisha, Rural
WHO−2001 Adjustment Method

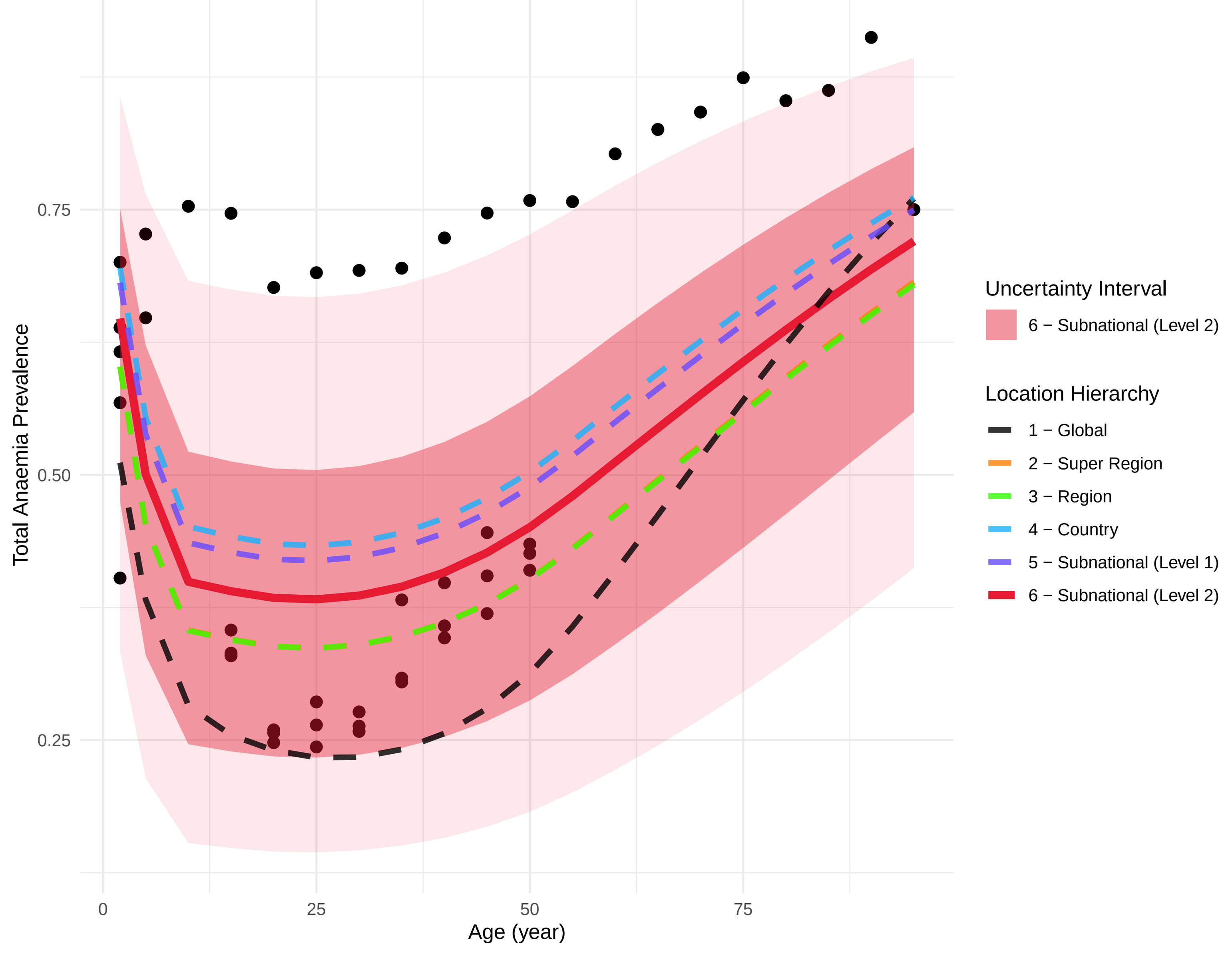

MR−BRT Age/Sex Fits − Males 2+ Years Old − Total Anaemia Prevalence − Odisha, Urban
WHO−2001 Adjustment Method

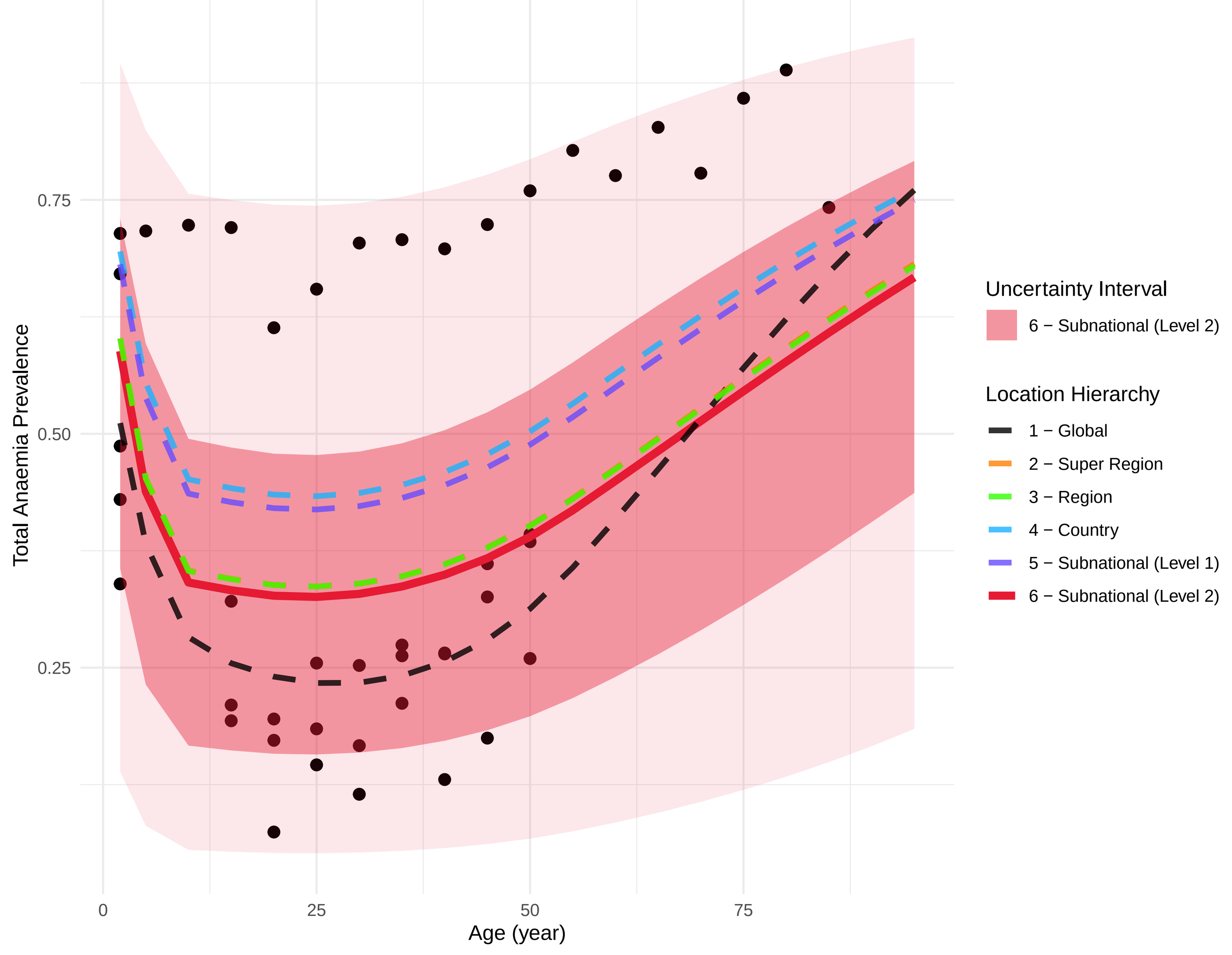

MR−BRT Age/Sex Fits − Males 2+ Years Old − Total Anaemia Prevalence − Other Union Territories
WHO−2001 Adjustment Method

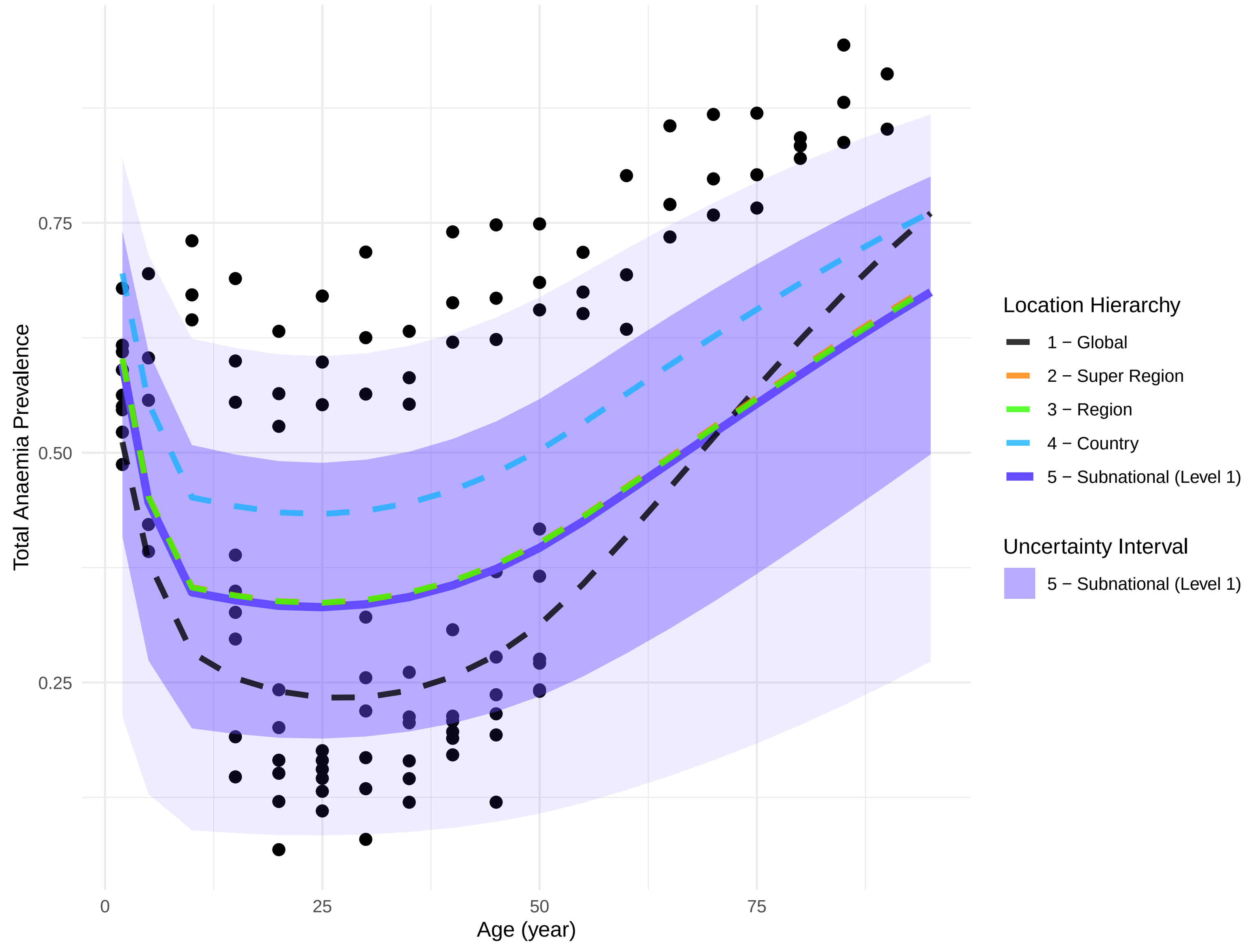

MR−BRT Age/Sex Fits − Males 2+ Years Old − Total Anaemia Prevalence − Other Union Territories, Rural
WHO−2001 Adjustment Method

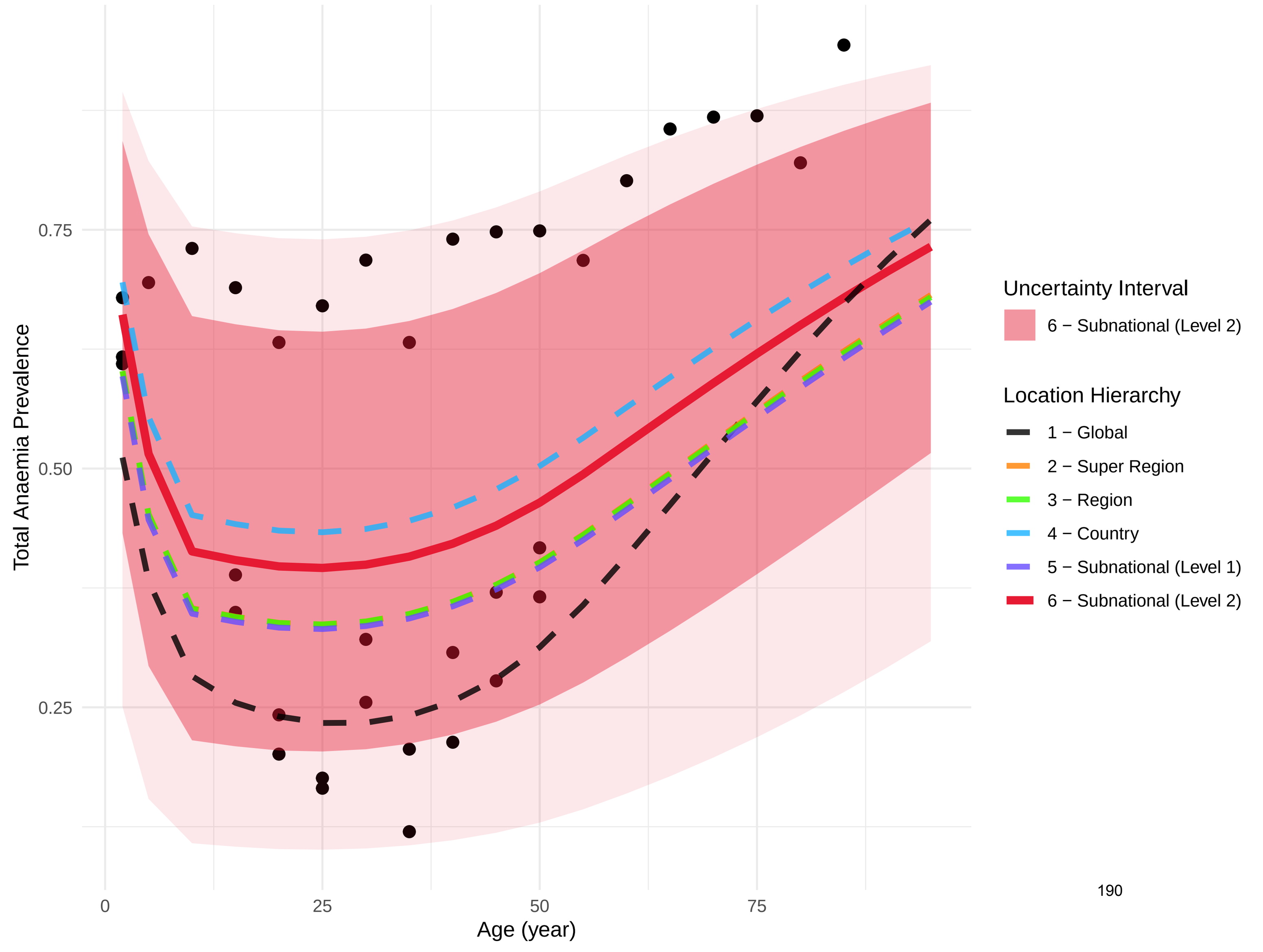

MR−BRT Age/Sex Fits − Males 2+ Years Old − Total Anaemia Prevalence − Other Union Territories, Urban
WHO−2001 Adjustment Method

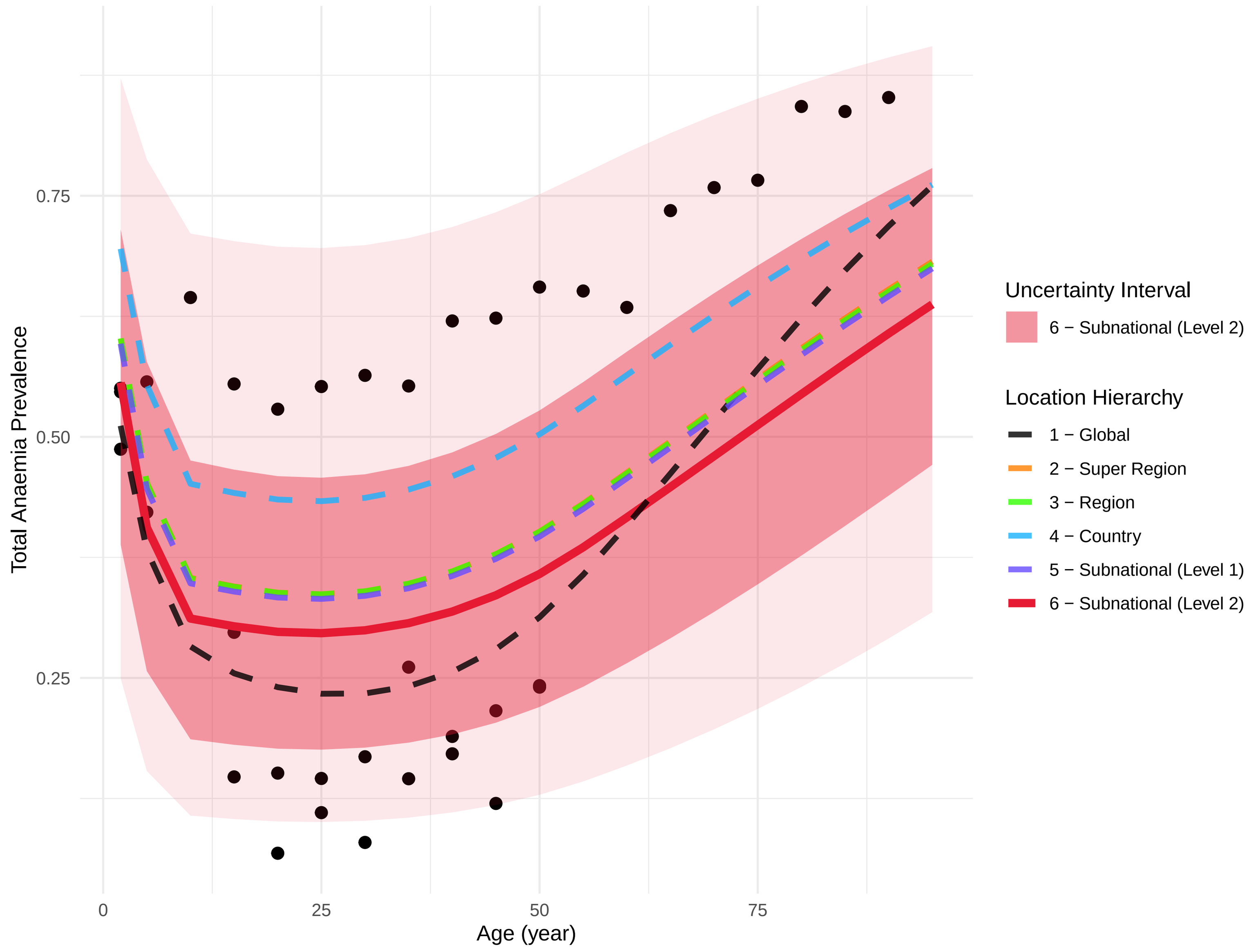

MR−BRT Age/Sex Fits − Males 2+ Years Old − Total Anaemia Prevalence − Punjab
WHO−2001 Adjustment Method

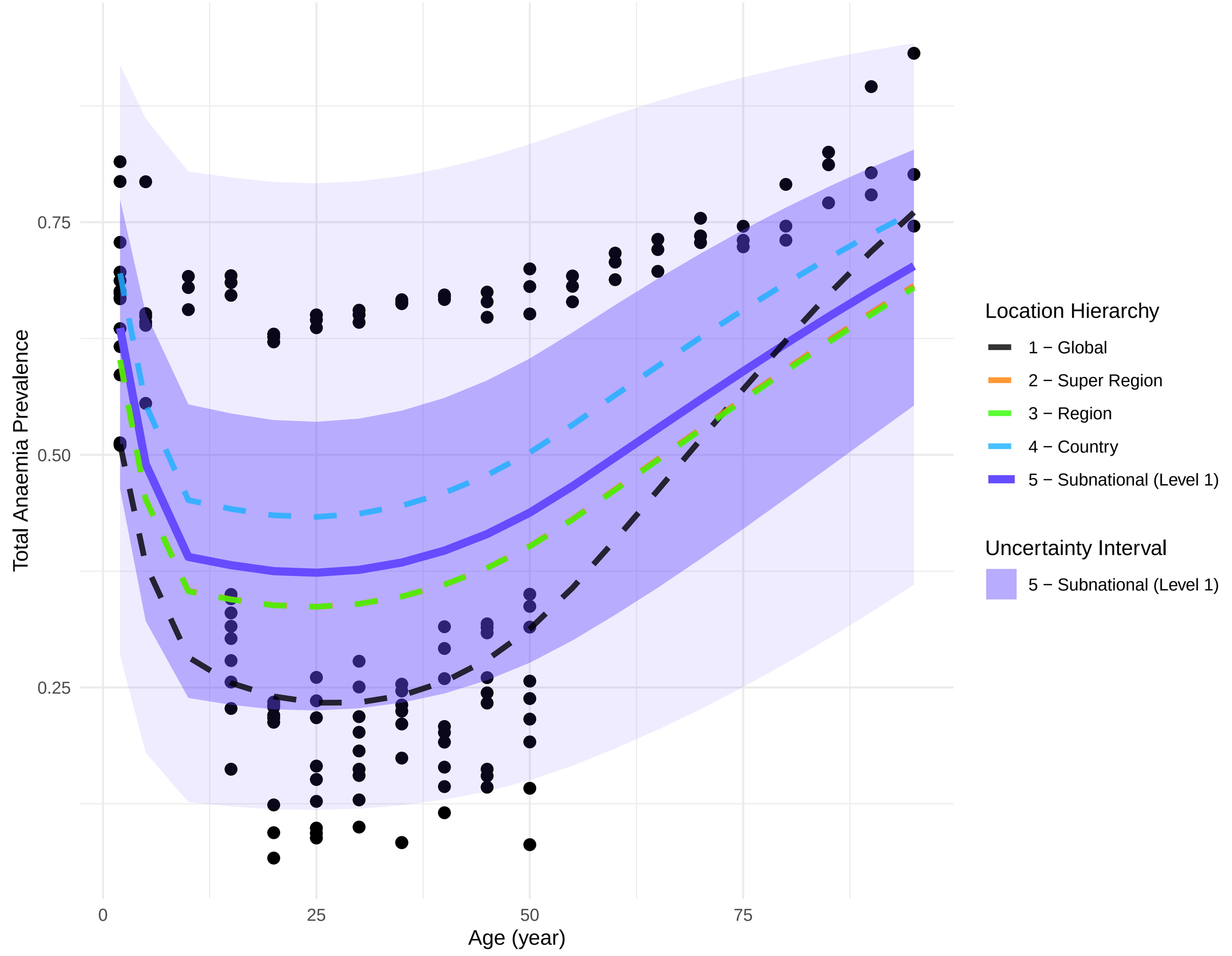

MR−BRT Age/Sex Fits − Males 2+ Years Old − Total Anaemia Prevalence − Punjab, Rural
WHO−2001 Adjustment Method

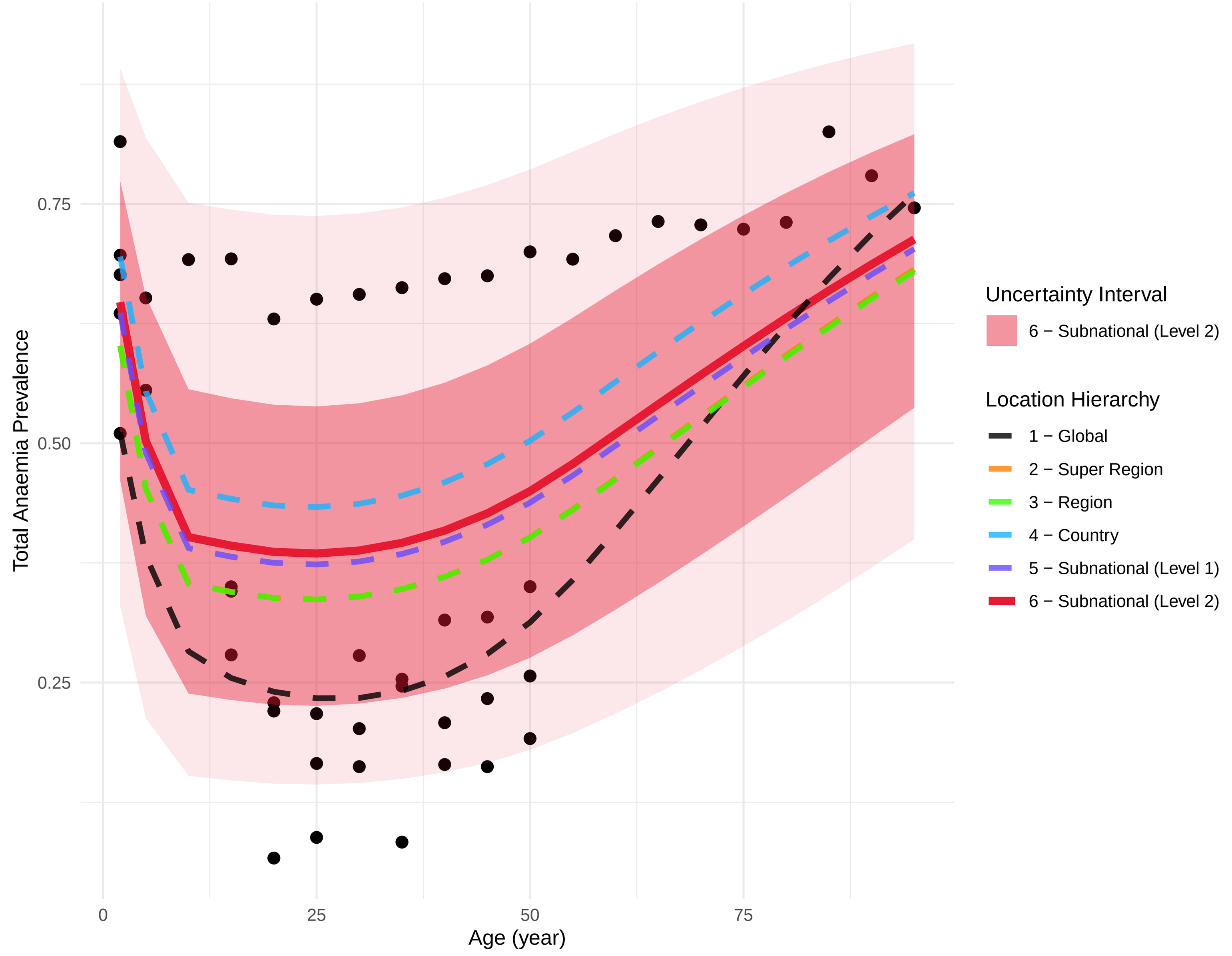

MR−BRT Age/Sex Fits − Males 2+ Years Old − Total Anaemia Prevalence − Punjab, Urban
WHO−2001 Adjustment Method

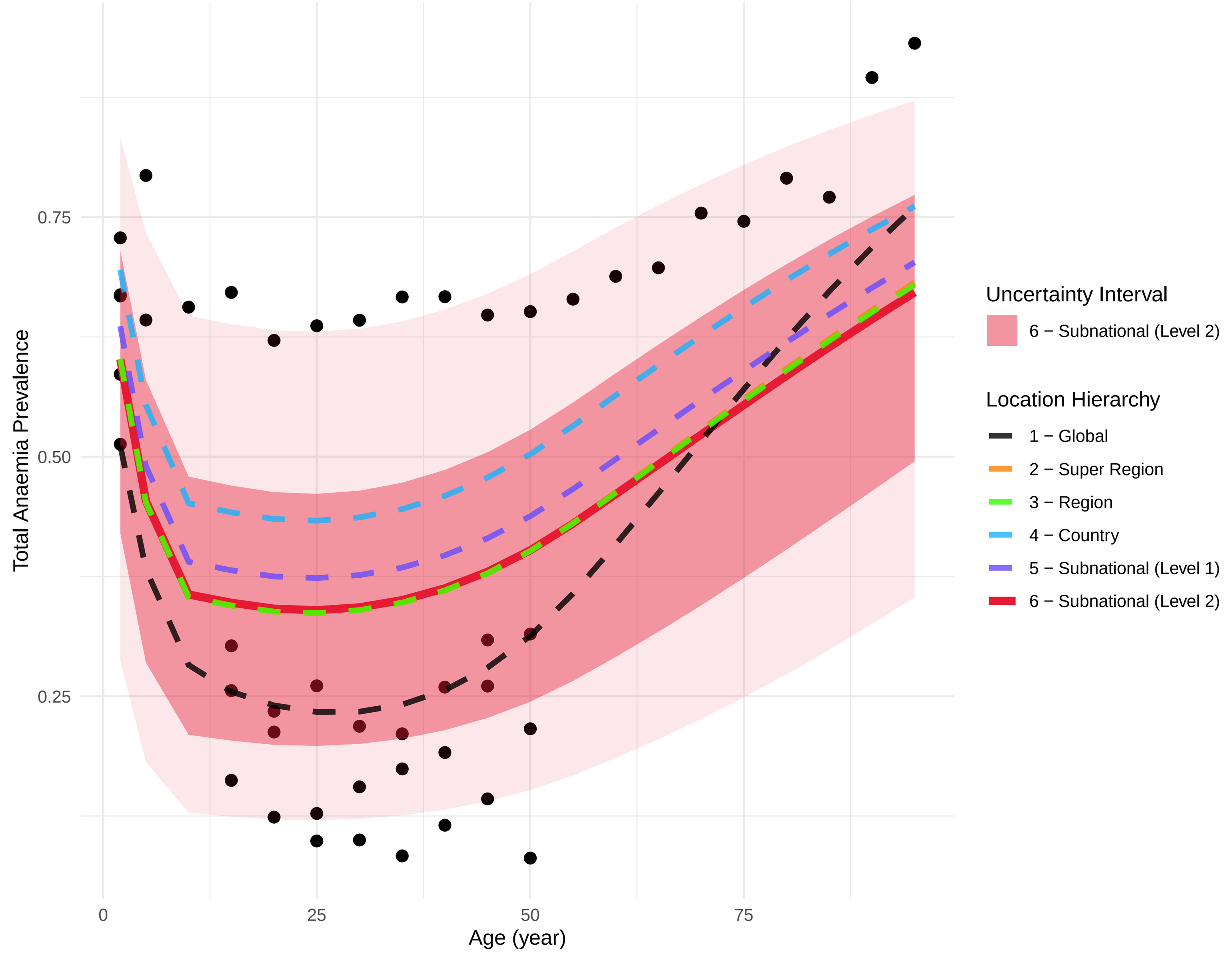

MR−BRT Age/Sex Fits − Males 2+ Years Old − Total Anaemia Prevalence − Rajasthan
WHO−2001 Adjustment Method

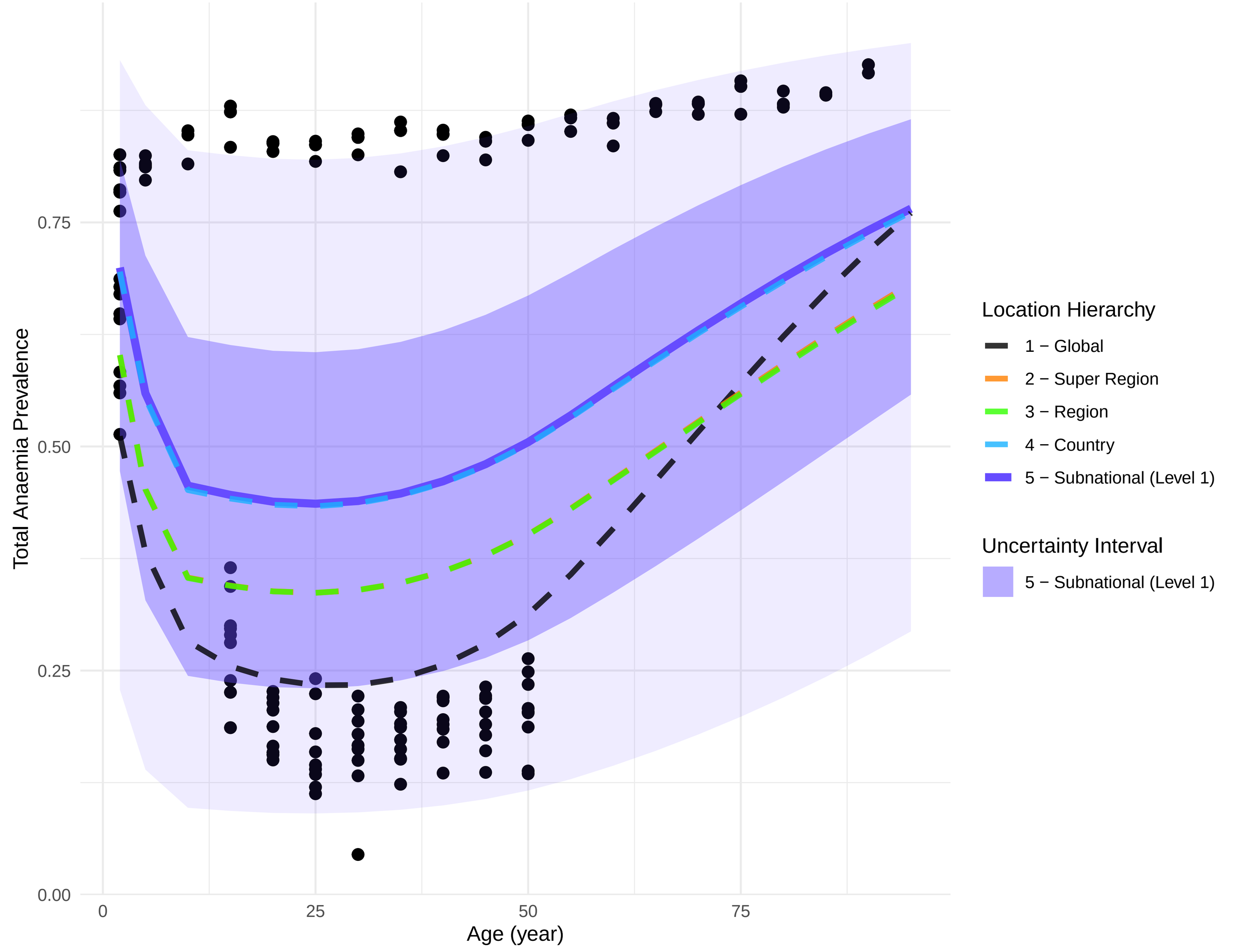

MR−BRT Age/Sex Fits − Males 2+ Years Old − Total Anaemia Prevalence − Rajasthan, Rural
WHO−2001 Adjustment Method

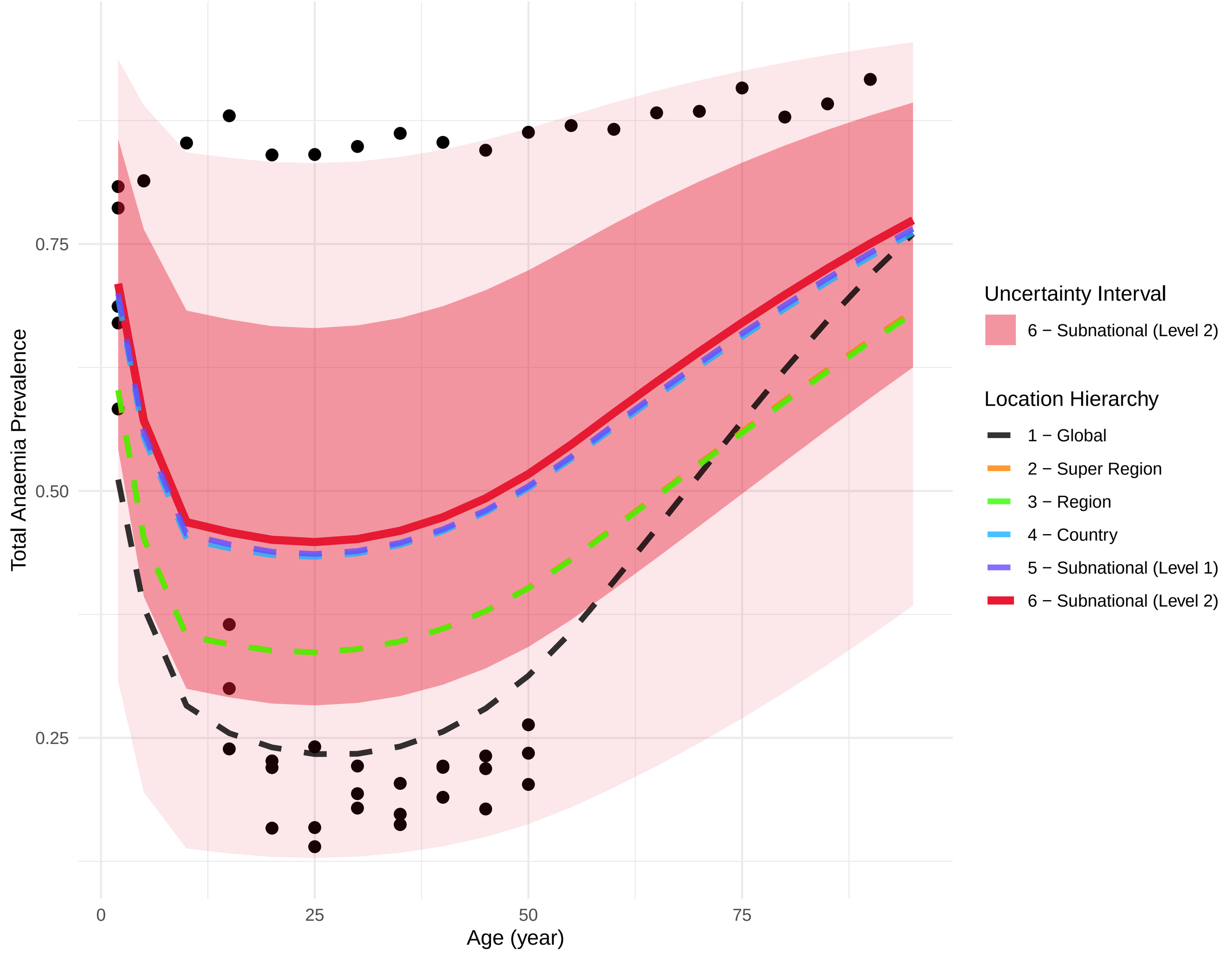

MR−BRT Age/Sex Fits − Males 2+ Years Old − Total Anaemia Prevalence − Rajasthan, Urban
WHO−2001 Adjustment Method

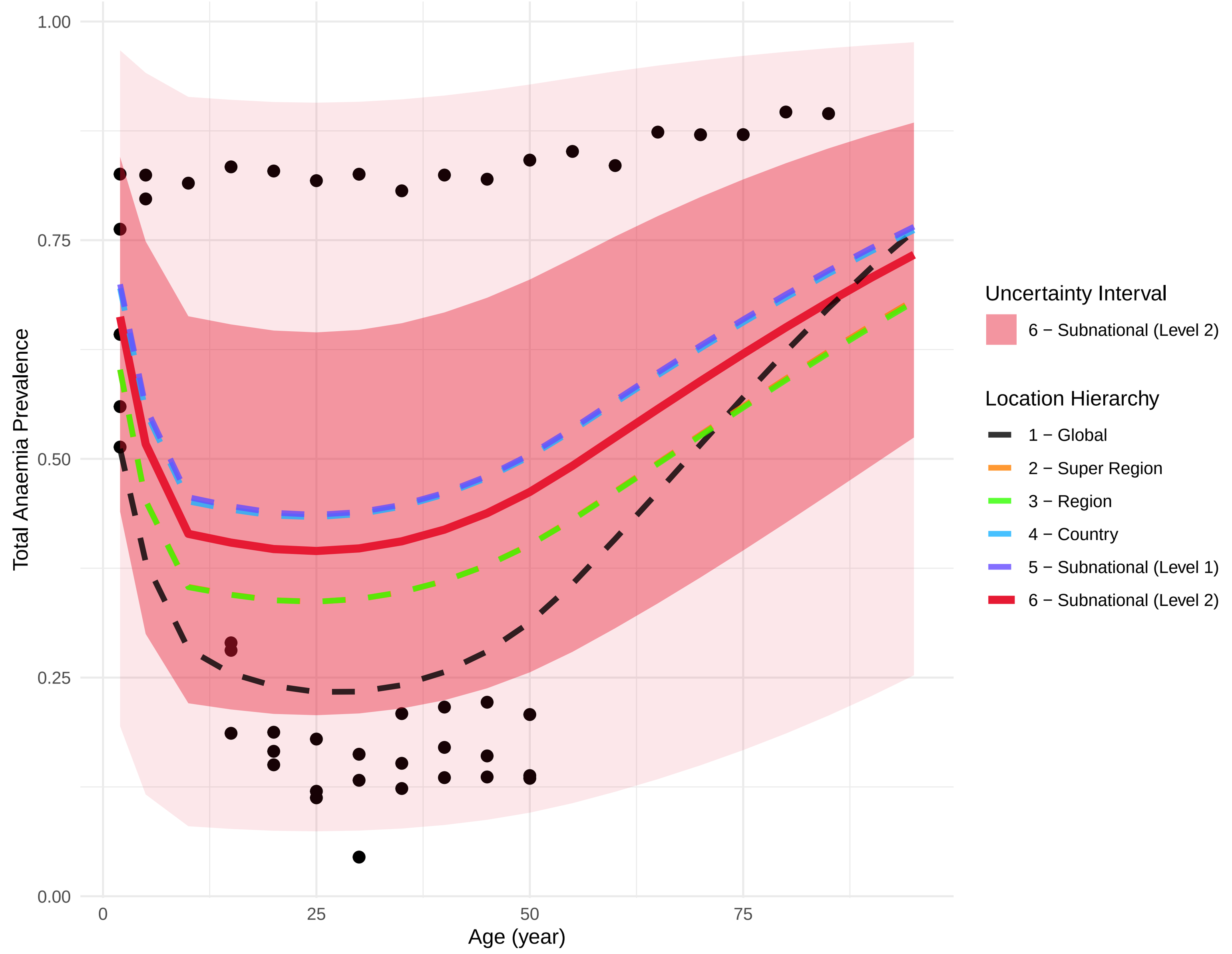

MR−BRT Age/Sex Fits − Males 2+ Years Old − Total Anaemia Prevalence − Sikkim
WHO−2001 Adjustment Method

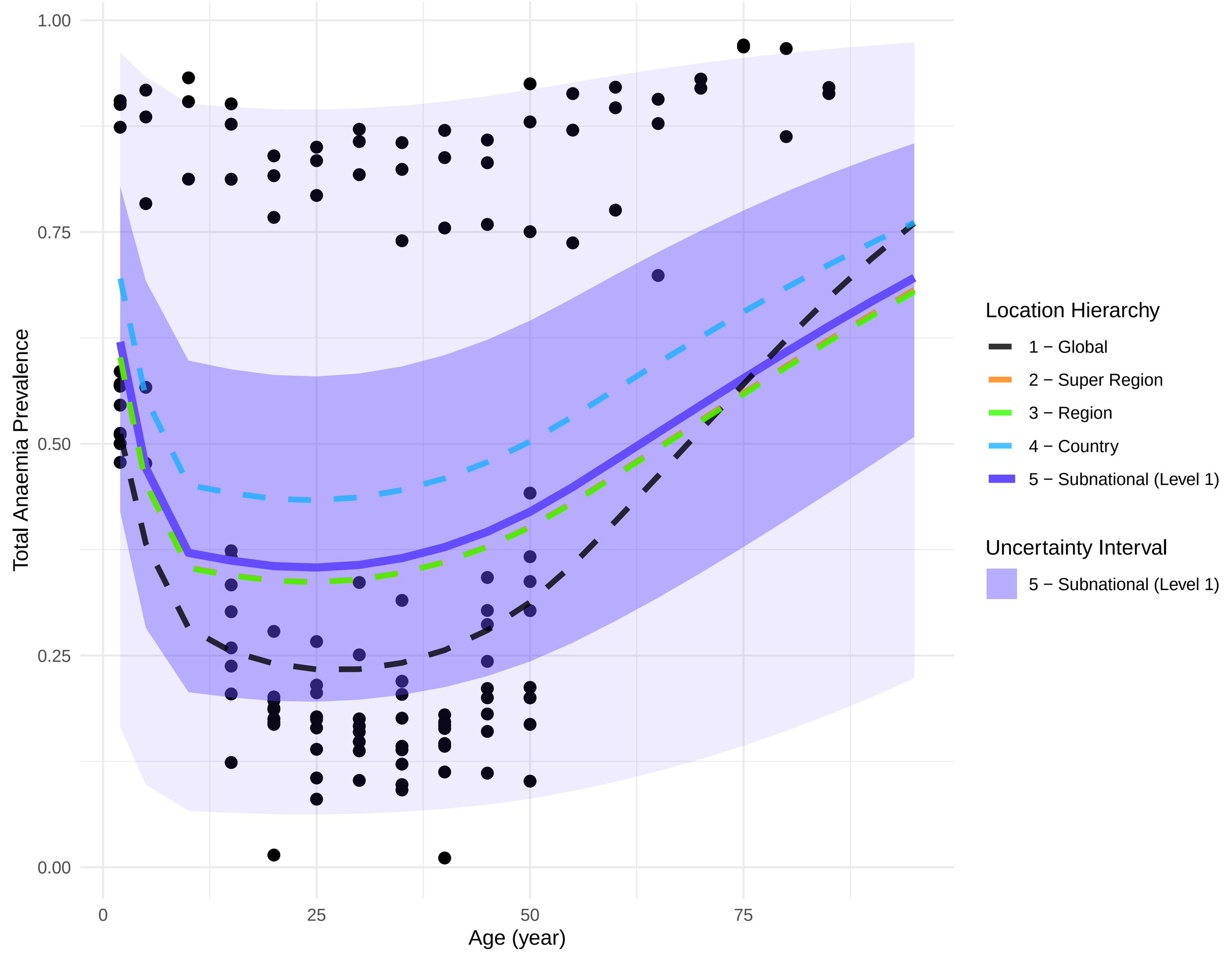

MR−BRT Age/Sex Fits − Males 2+ Years Old − Total Anaemia Prevalence − Sikkim, Rural
WHO−2001 Adjustment Method

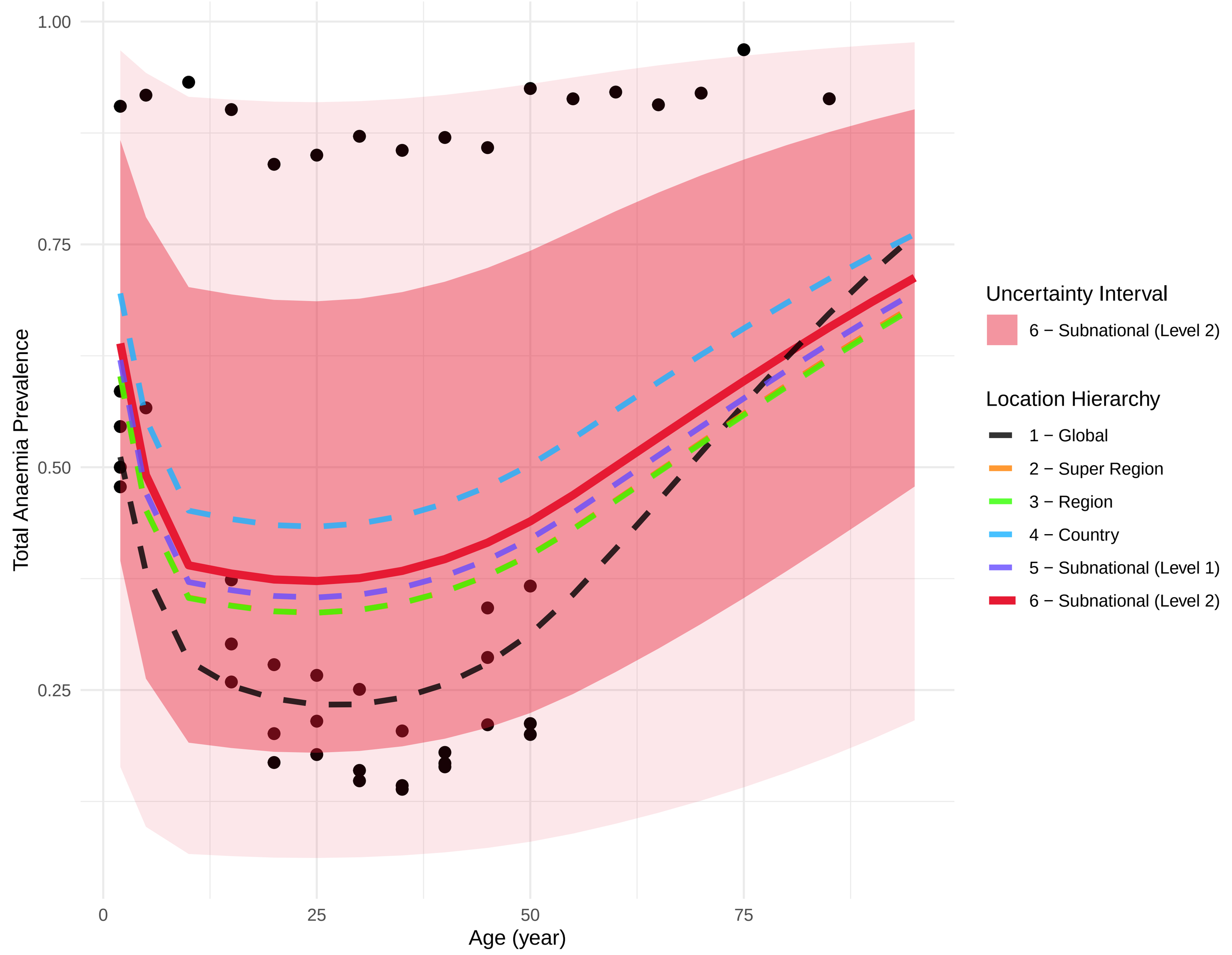

MR−BRT Age/Sex Fits − Males 2+ Years Old − Total Anaemia Prevalence − Sikkim, Urban
WHO−2001 Adjustment Method

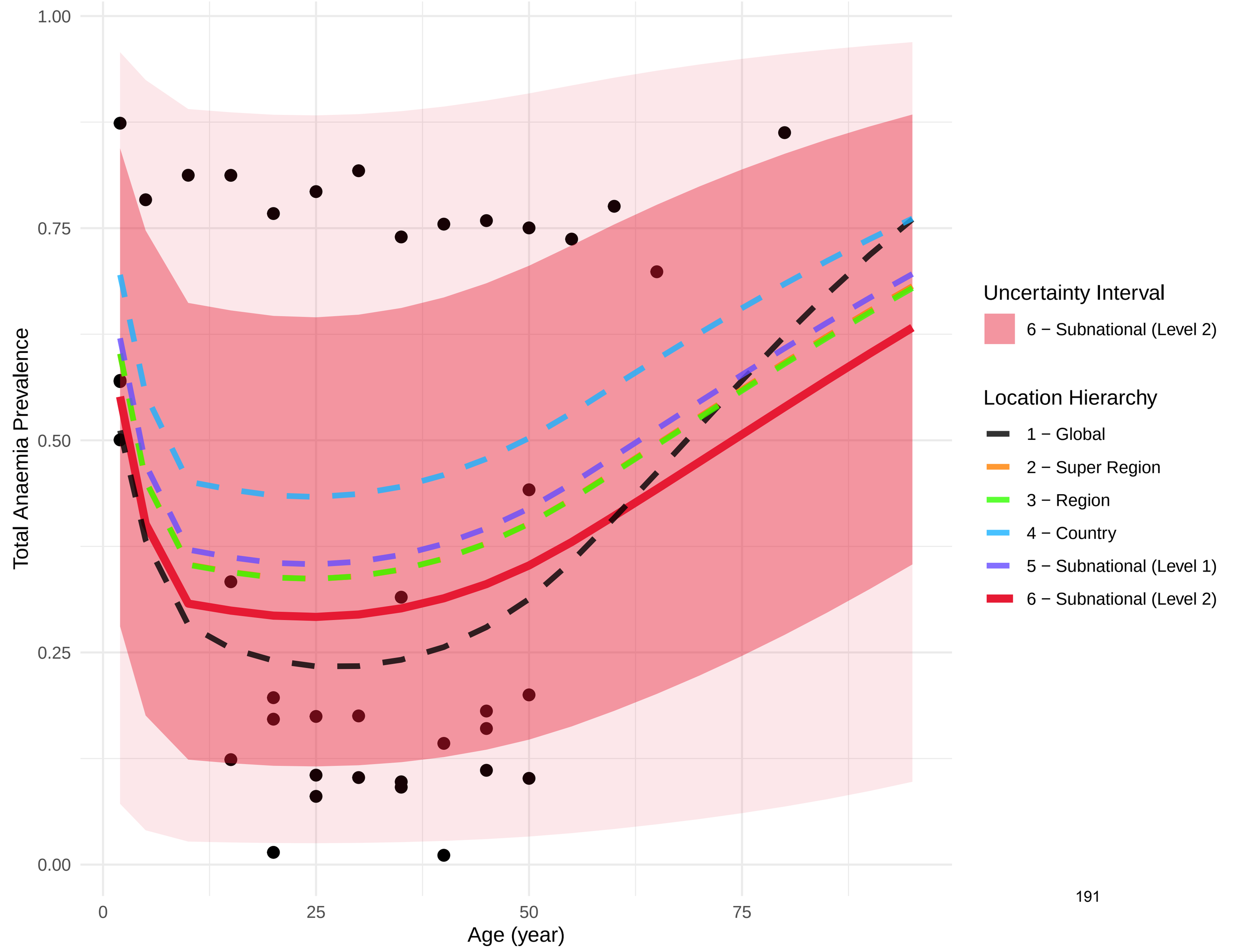

MR−BRT Age/Sex Fits − Males 2+ Years Old − Total Anaemia Prevalence − Tamil Nadu
WHO−2001 Adjustment Method

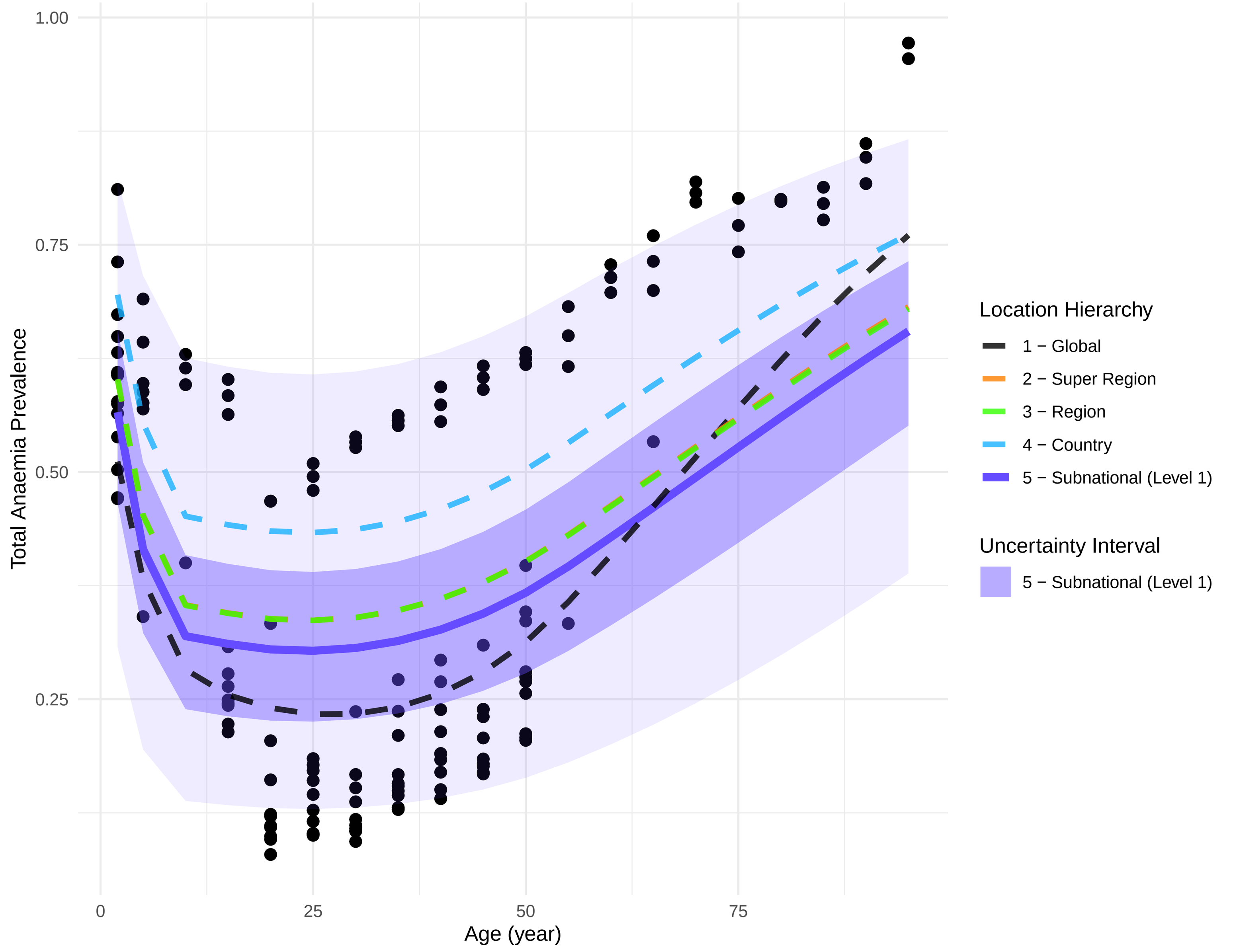

MR−BRT Age/Sex Fits − Males 2+ Years Old − Total Anaemia Prevalence − Tamil Nadu, Rural
WHO−2001 Adjustment Method

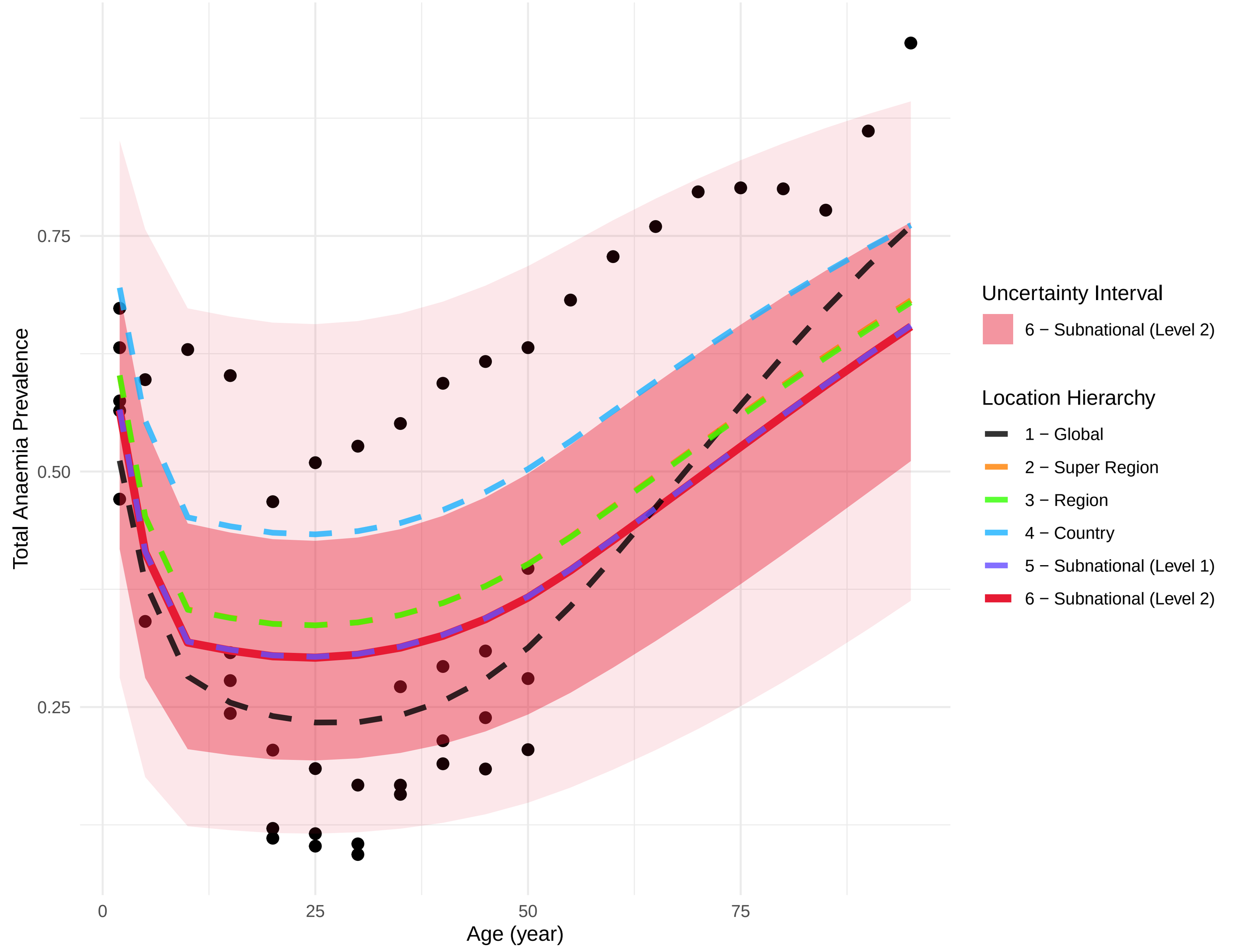

MR−BRT Age/Sex Fits − Males 2+ Years Old − Total Anaemia Prevalence − Tamil Nadu, Urban
WHO−2001 Adjustment Method

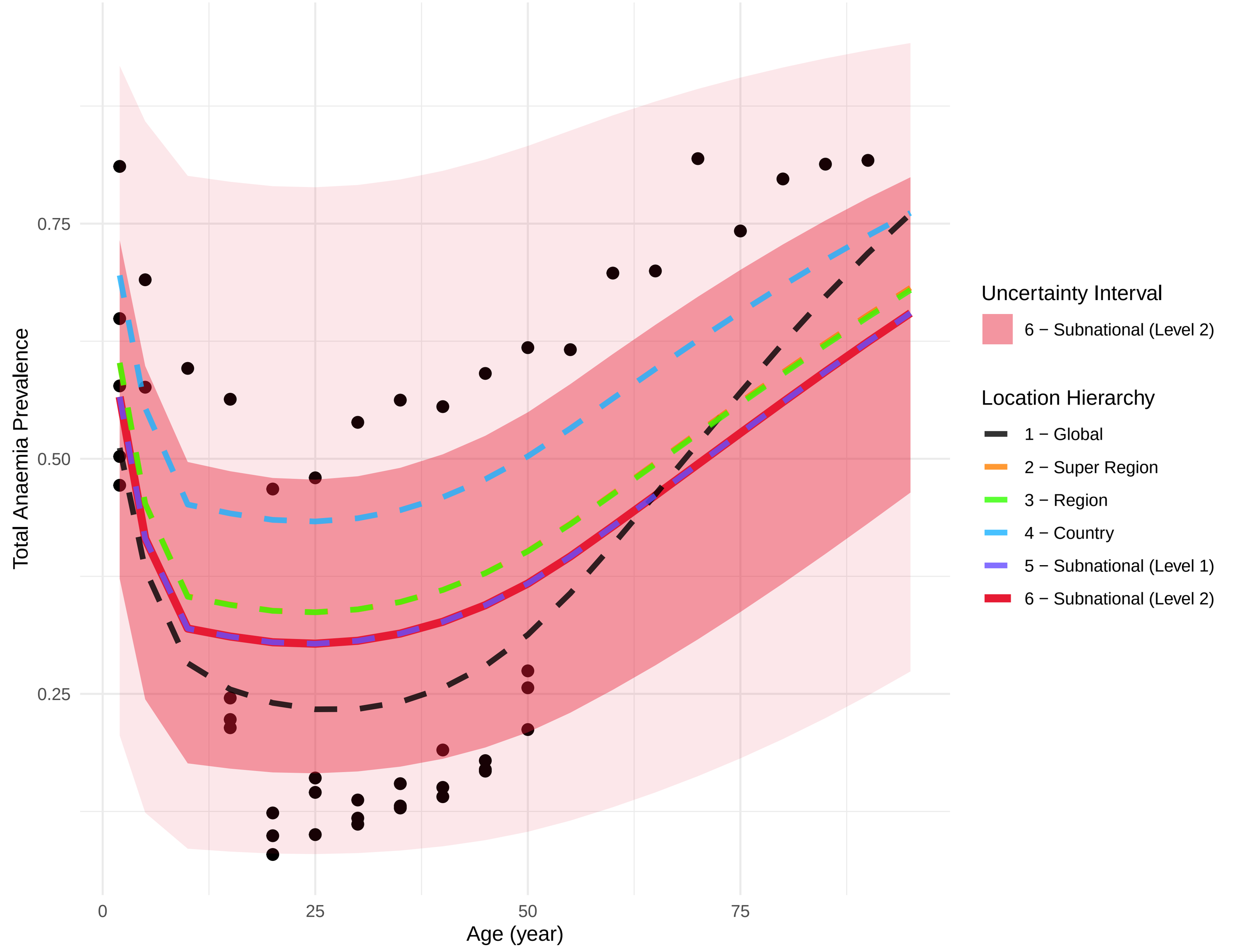

MR−BRT Age/Sex Fits − Males 2+ Years Old − Total Anaemia Prevalence − Telangana
WHO−2001 Adjustment Method

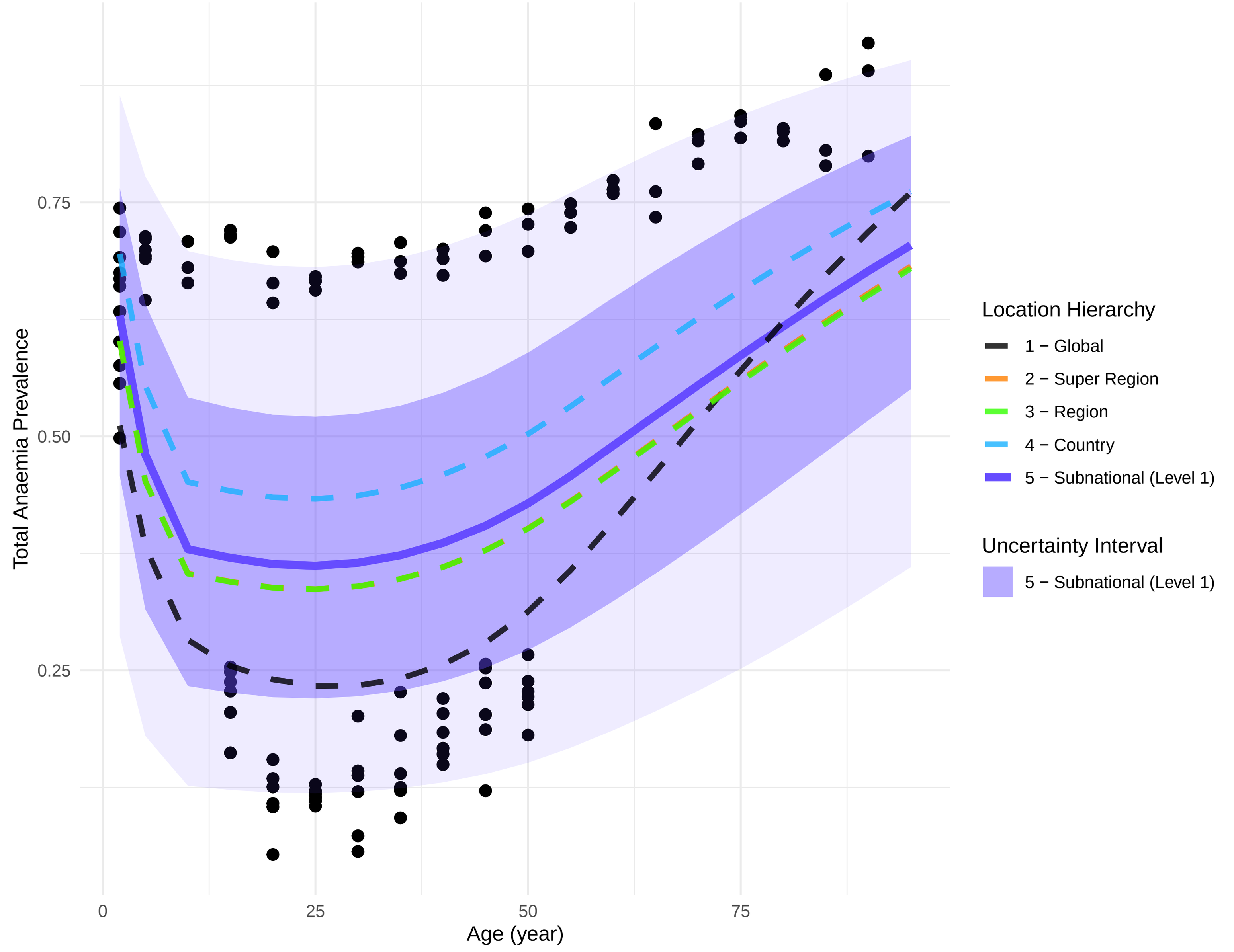

MR−BRT Age/Sex Fits − Males 2+ Years Old − Total Anaemia Prevalence − Telangana, Rural
WHO−2001 Adjustment Method

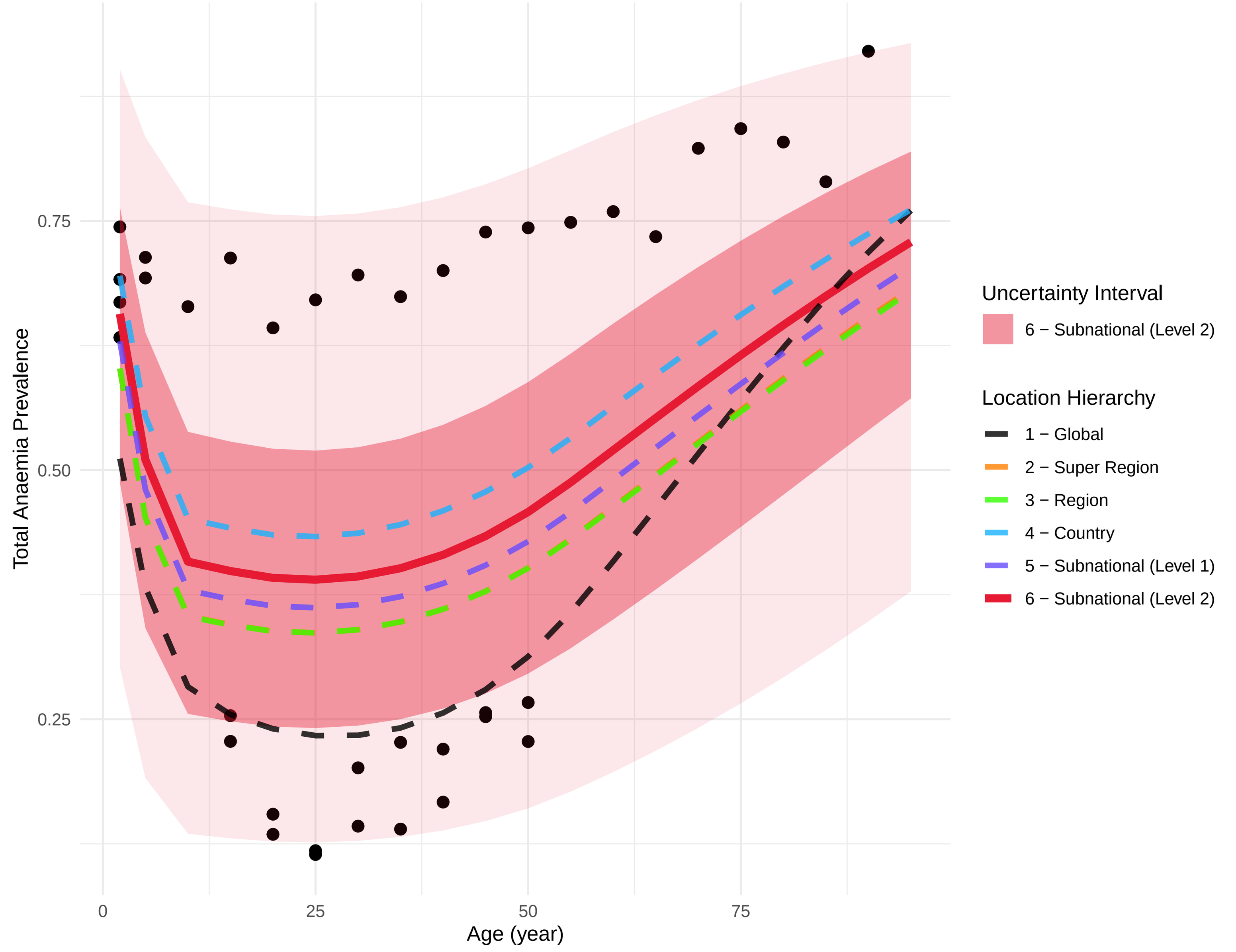

MR−BRT Age/Sex Fits − Males 2+ Years Old − Total Anaemia Prevalence − Telangana, Urban
WHO−2001 Adjustment Method

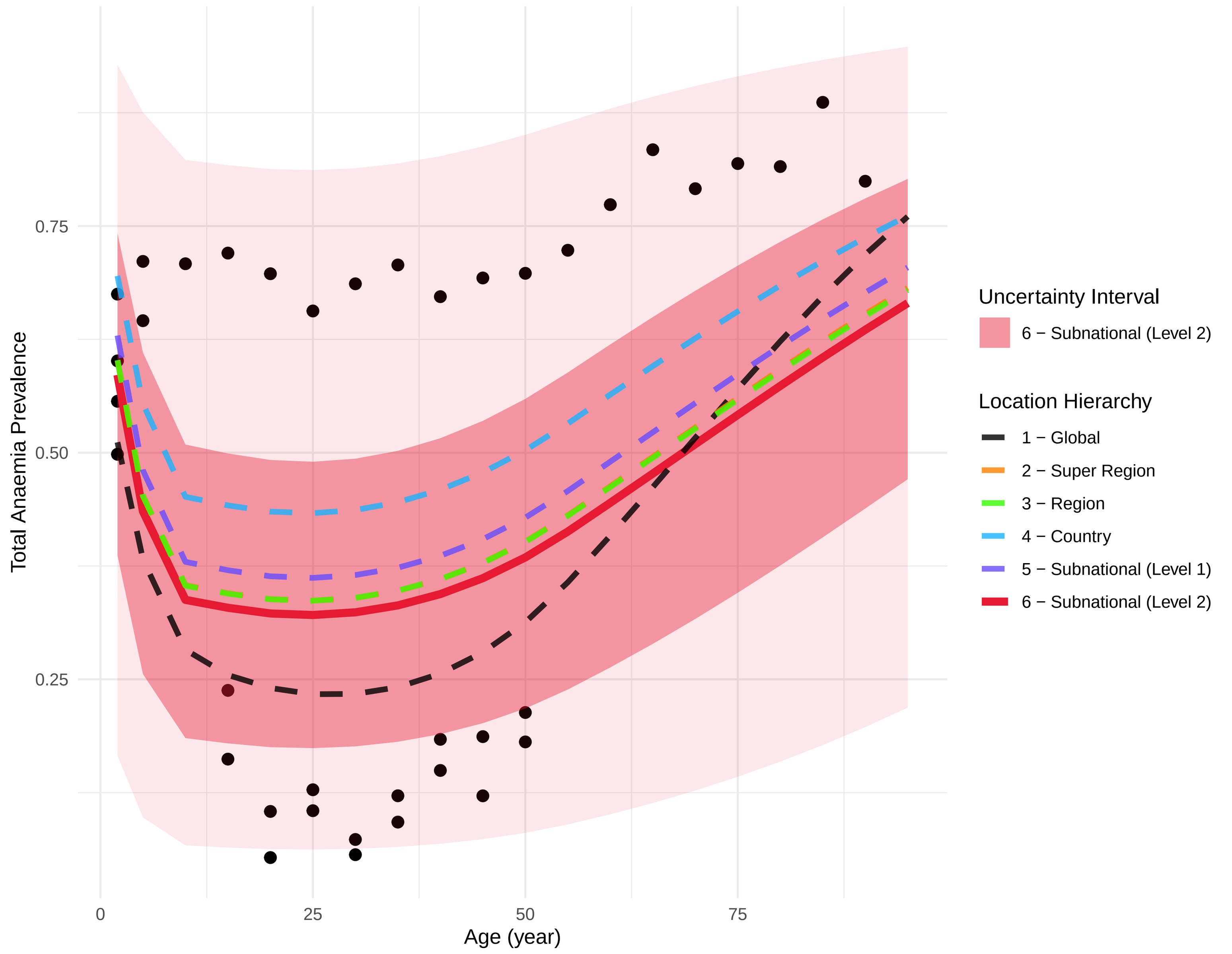

MR−BRT Age/Sex Fits − Males 2+ Years Old − Total Anaemia Prevalence − Tripura
WHO−2001 Adjustment Method

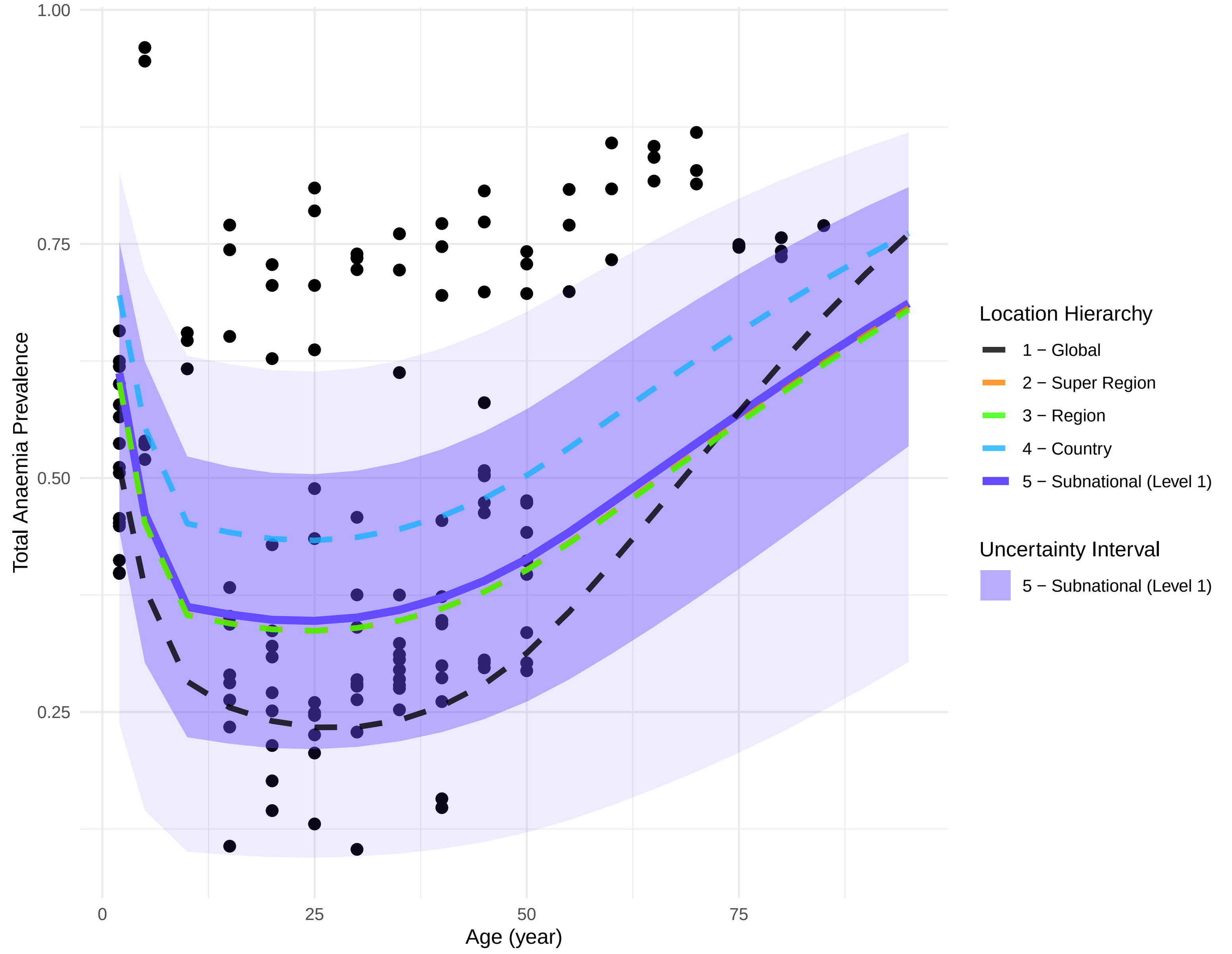

MR−BRT Age/Sex Fits − Males 2+ Years Old − Total Anaemia Prevalence − Tripura, Rural
WHO−2001 Adjustment Method

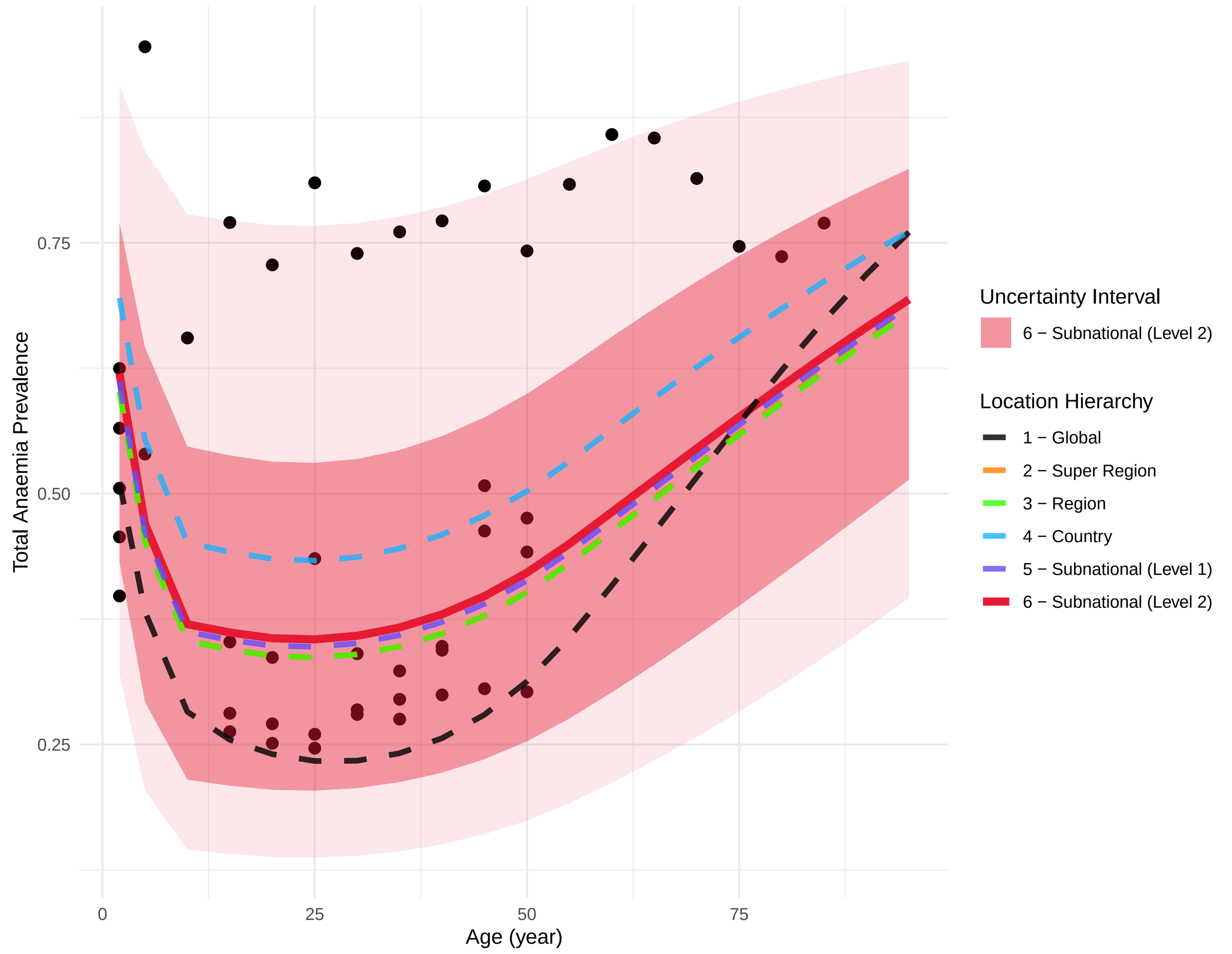

MR−BRT Age/Sex Fits − Males 2+ Years Old − Total Anaemia Prevalence − Tripura, Urban
WHO−2001 Adjustment Method

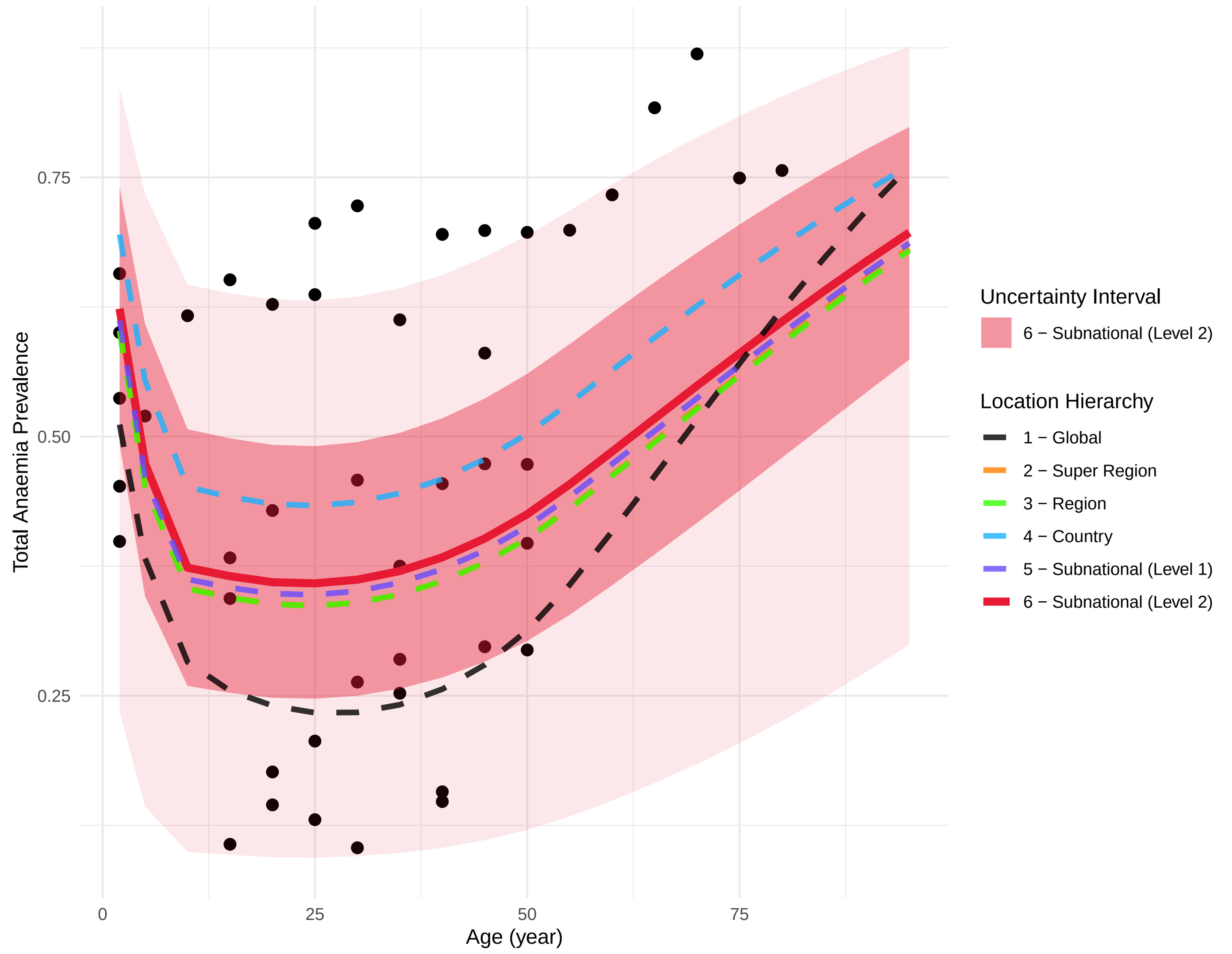

MR−BRT Age/Sex Fits − Males 2+ Years Old − Total Anaemia Prevalence − Uttar Pradesh
WHO−2001 Adjustment Method

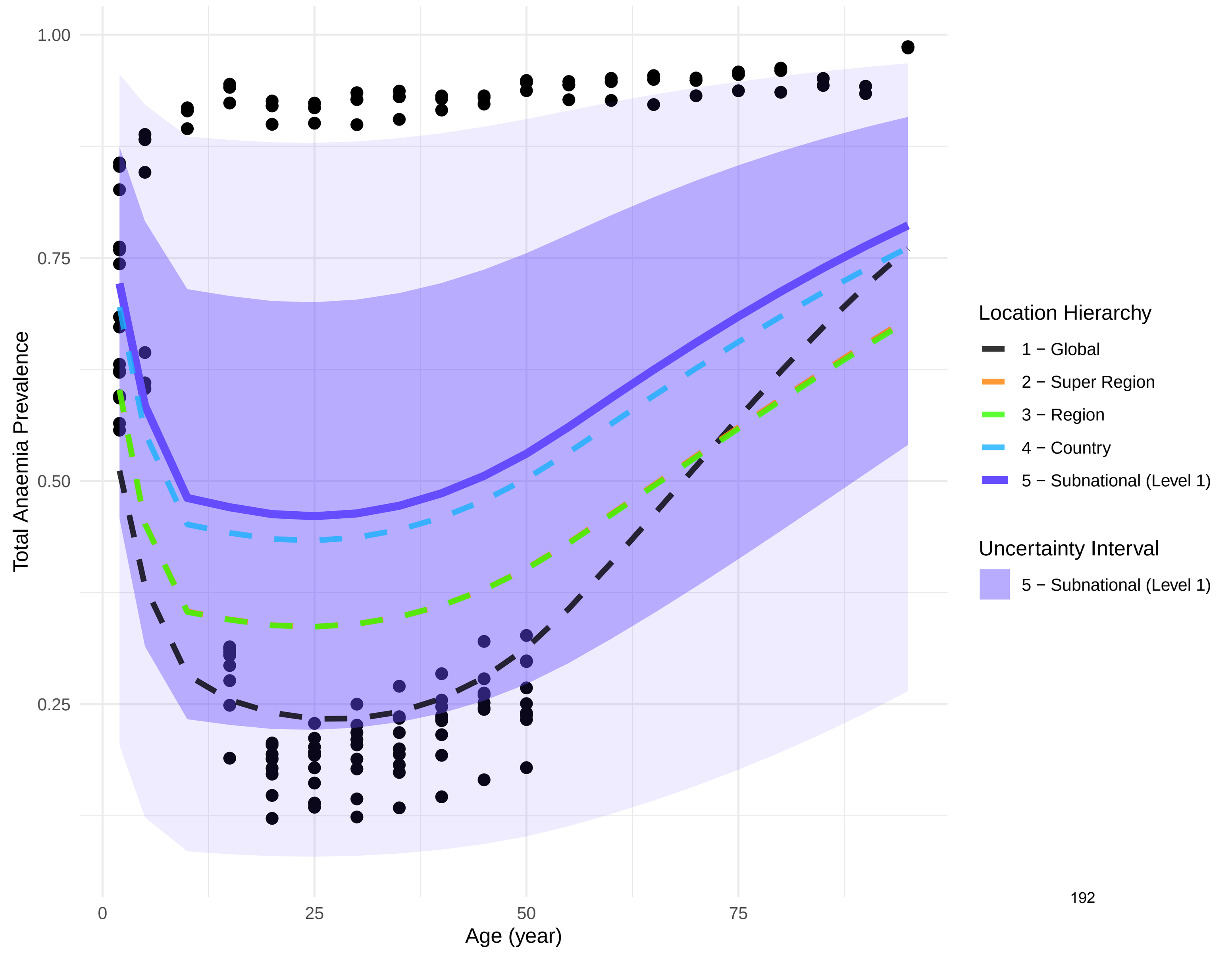

MR−BRT Age/Sex Fits − Males 2+ Years Old − Total Anaemia Prevalence − Uttar Pradesh, Rural

WHO−2001 Adjustment Method

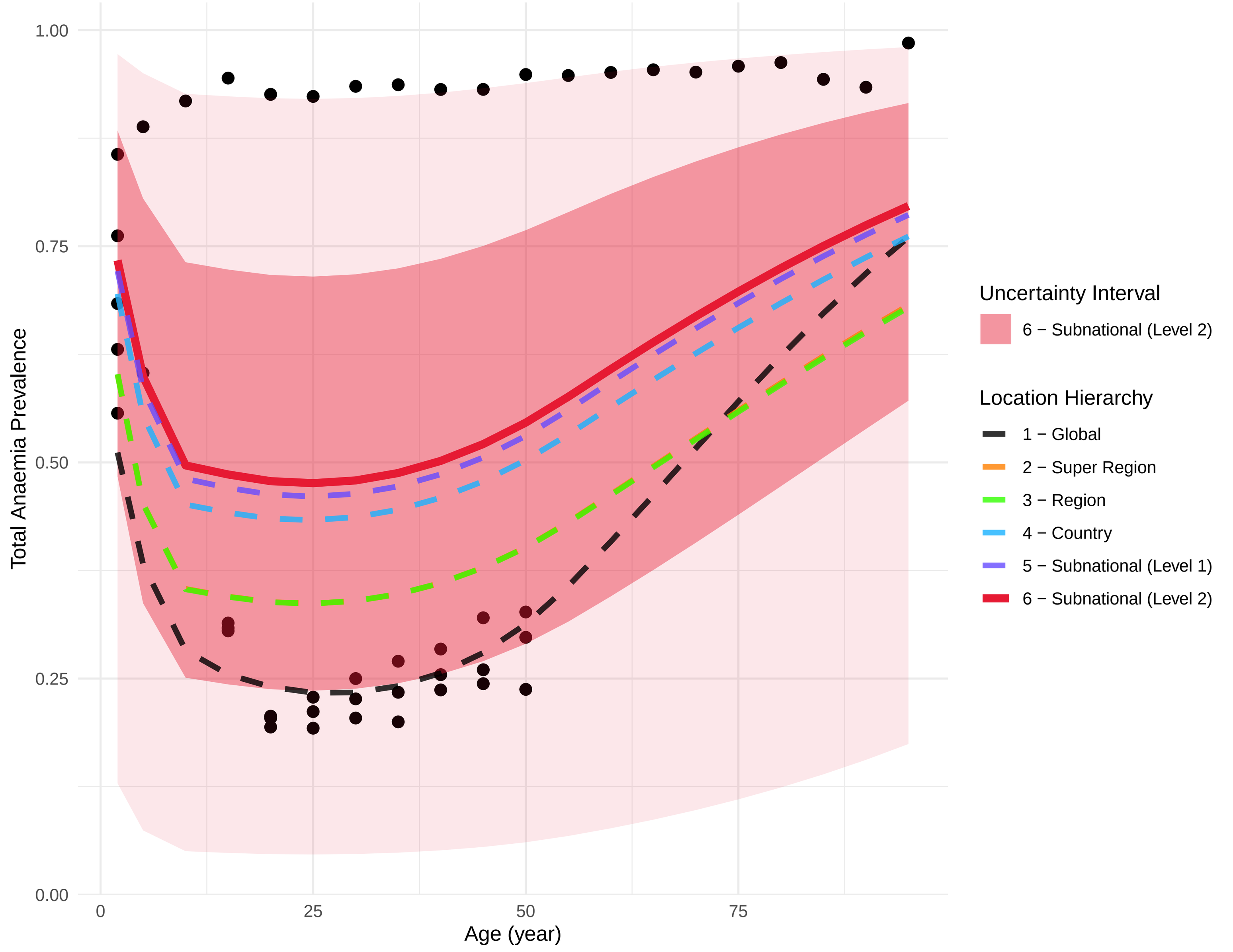

MR−BRT Age/Sex Fits − Males 2+ Years Old − Total Anaemia Prevalence − Uttar Pradesh, Urban

WHO−2001 Adjustment Method

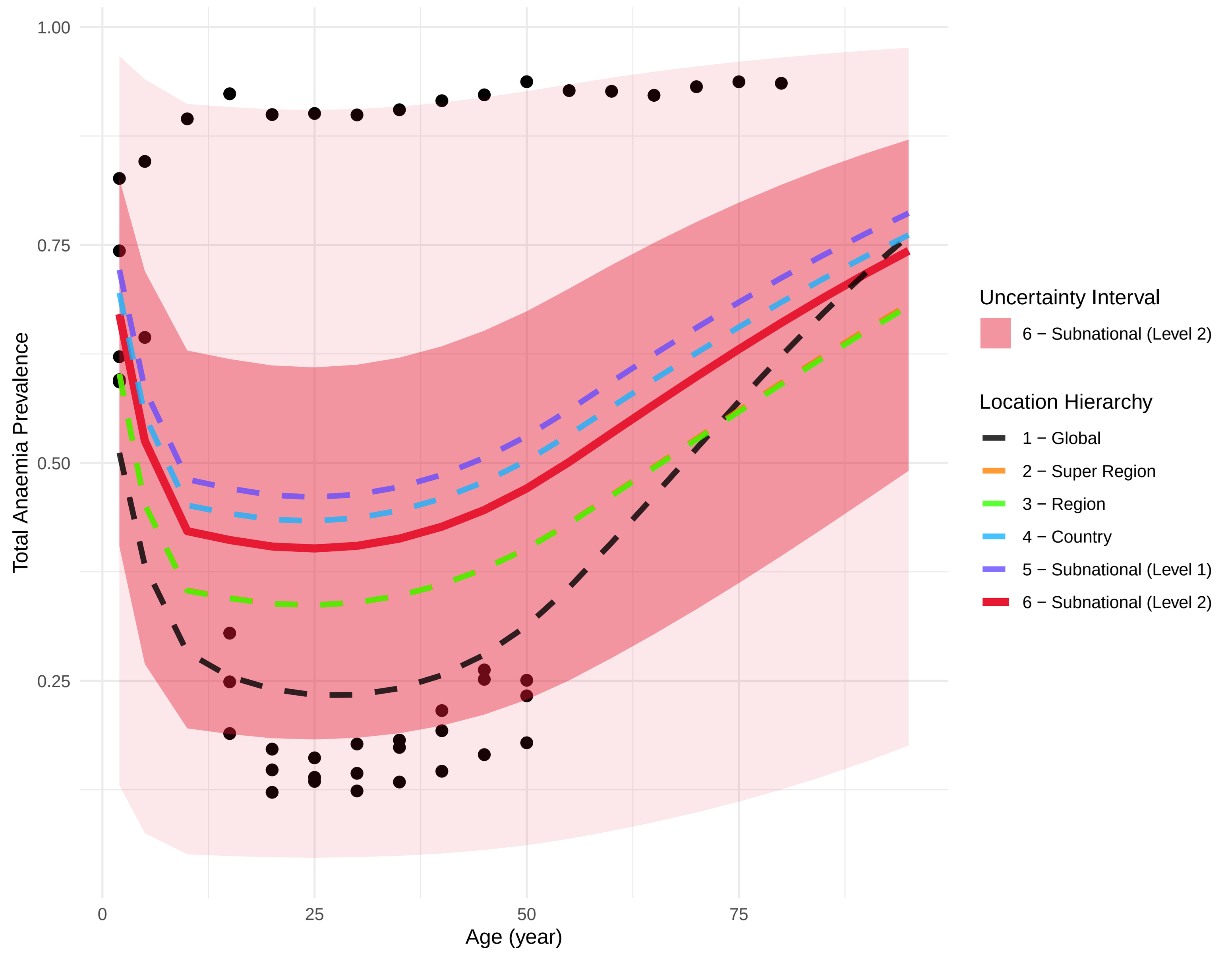

MR−BRT Age/Sex Fits − Males 2+ Years Old − Total Anaemia Prevalence − Uttarakhand

WHO−2001 Adjustment Method

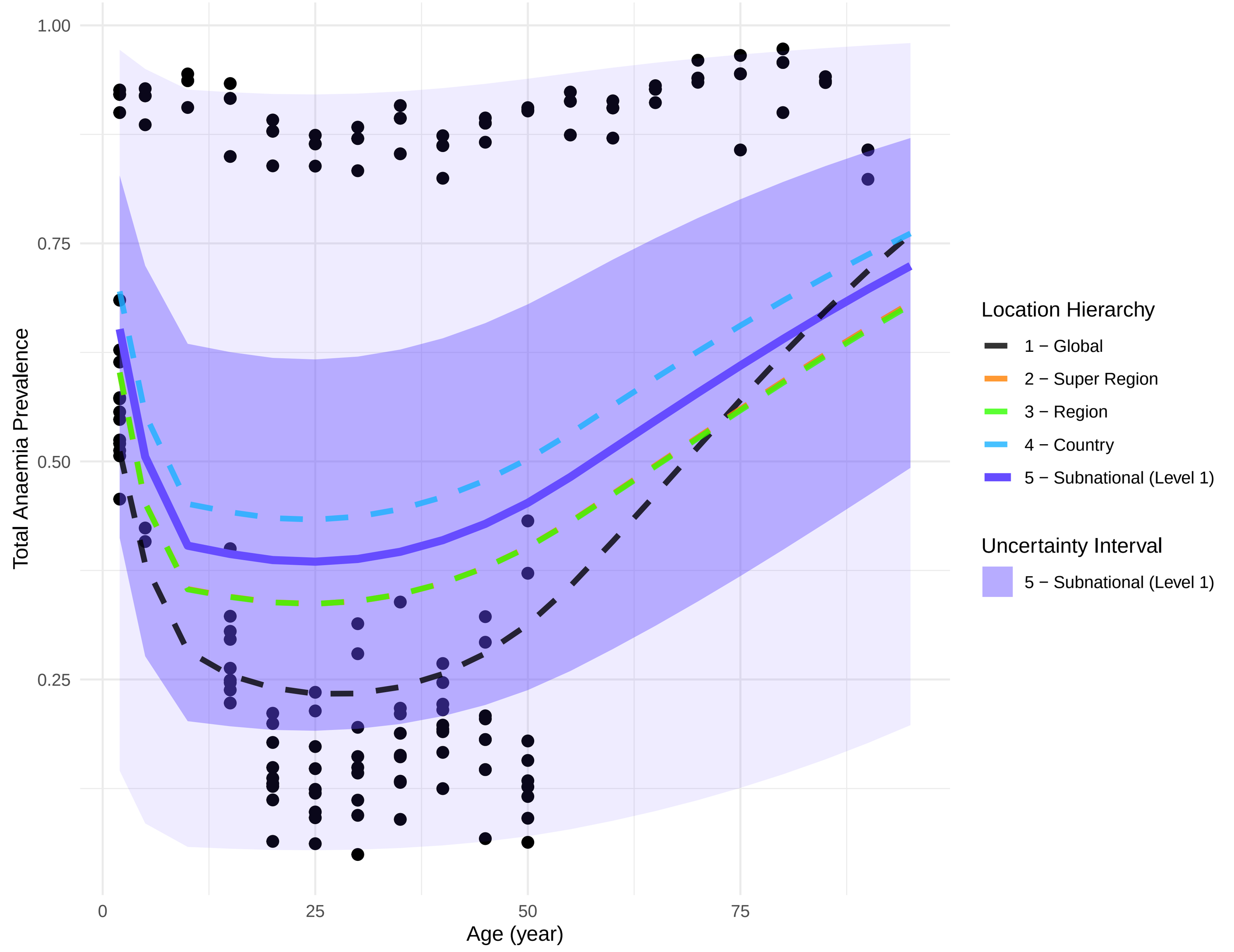

MR−BRT Age/Sex Fits − Males 2+ Years Old − Total Anaemia Prevalence − Uttarakhand, Rural

WHO−2001 Adjustment Method

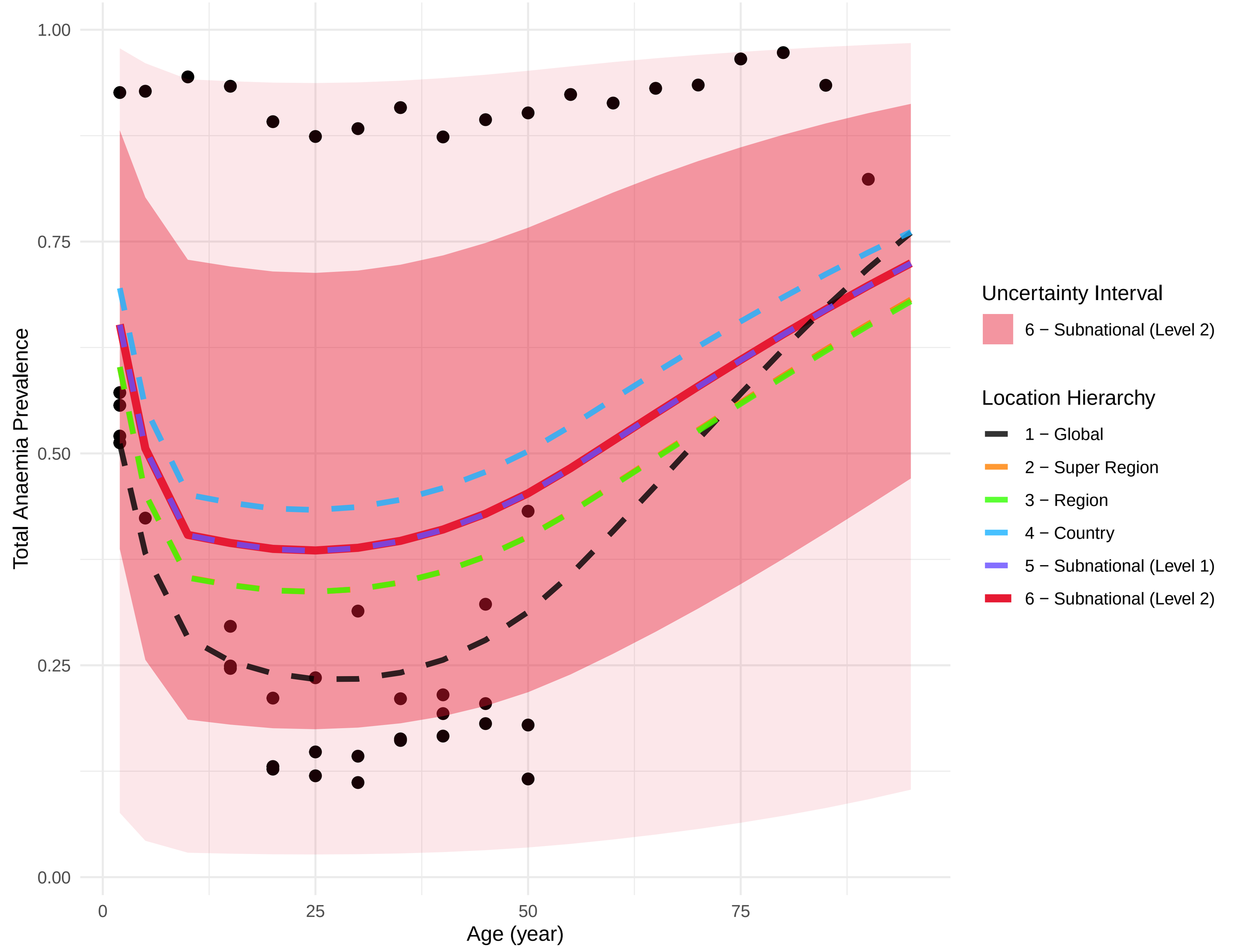

MR−BRT Age/Sex Fits − Males 2+ Years Old − Total Anaemia Prevalence − Uttarakhand, Urban

WHO−2001 Adjustment Method

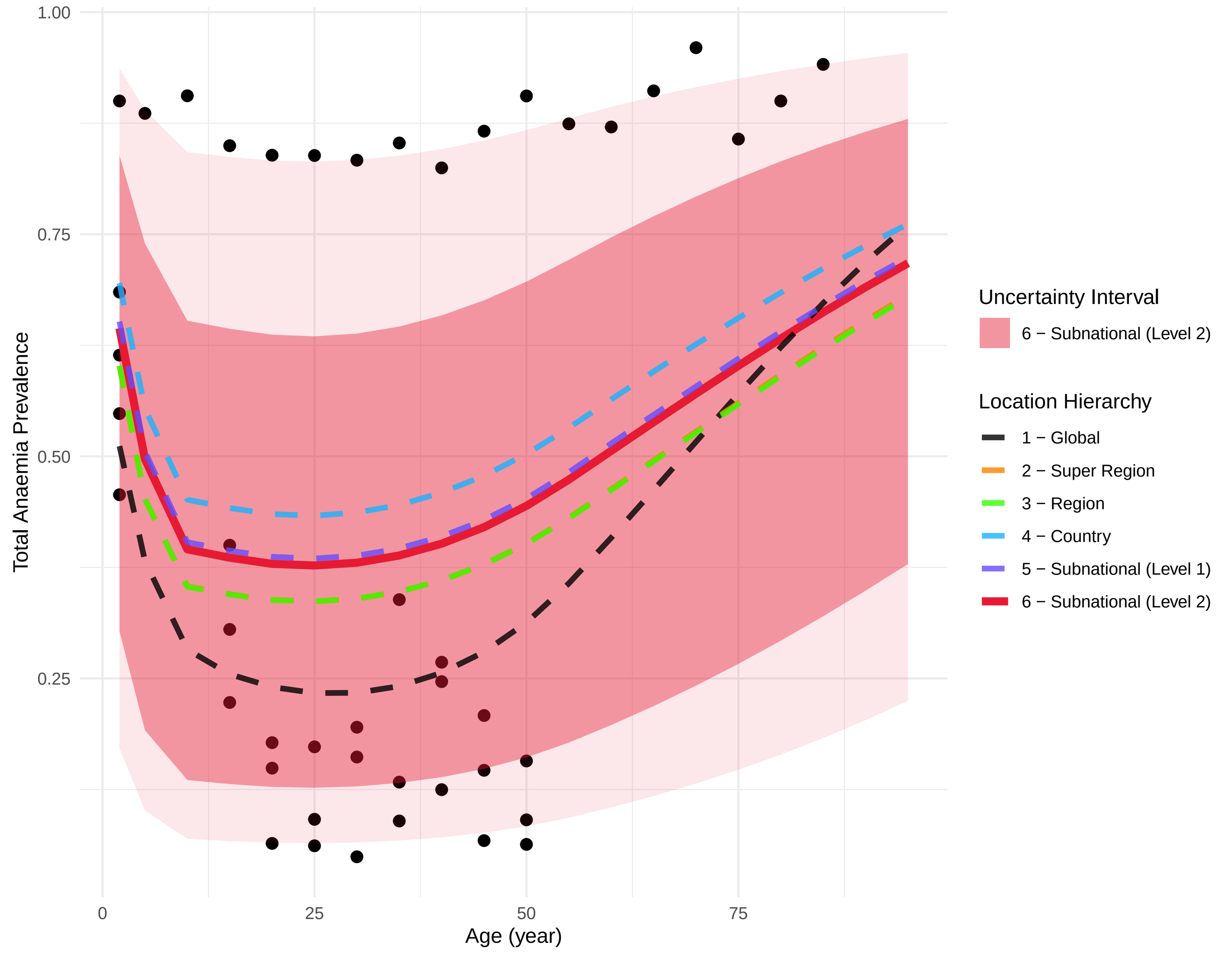

MR−BRT Age/Sex Fits − Males 2+ Years Old − Total Anaemia Prevalence − West Bengal

WHO−2001 Adjustment Method

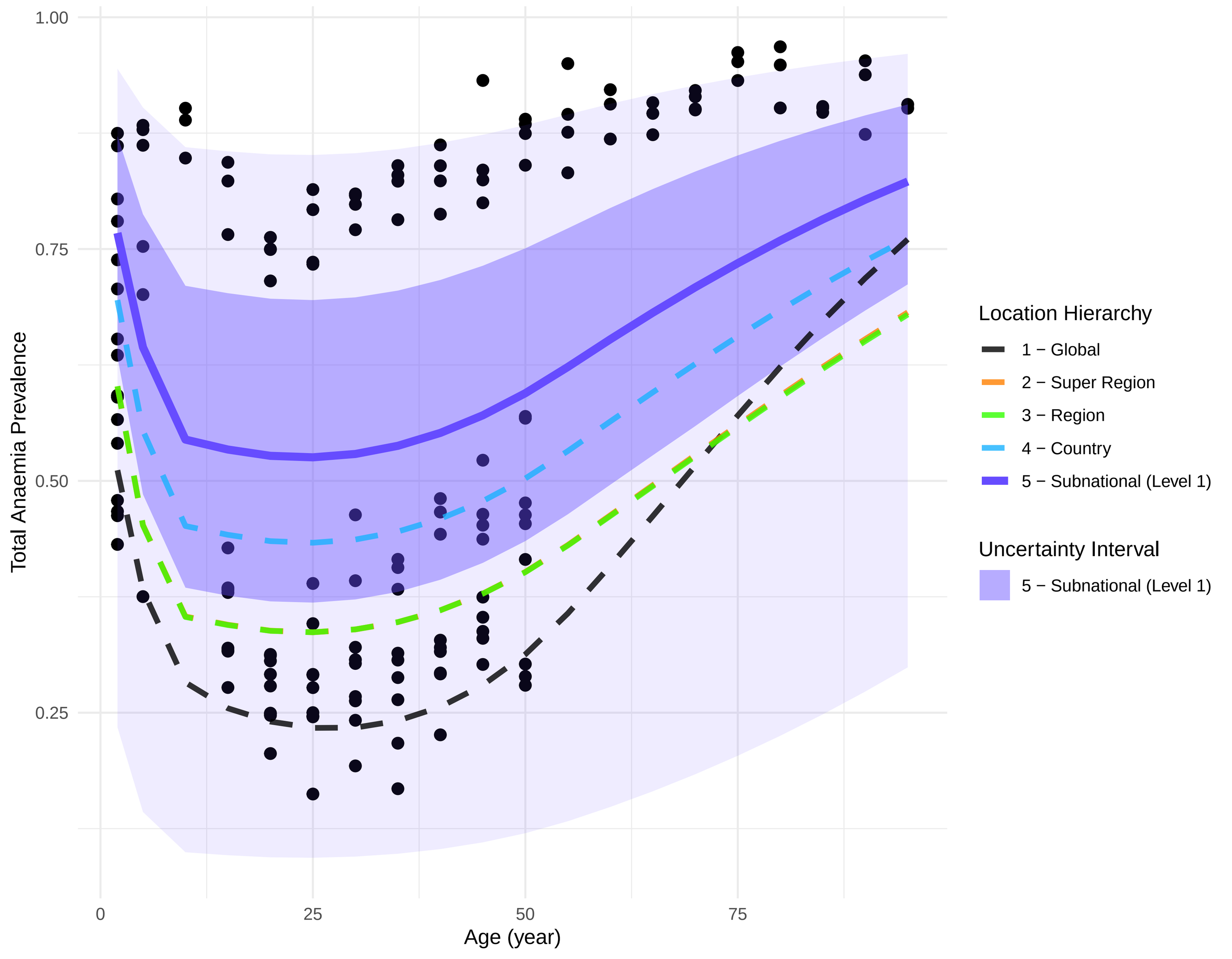

MR−BRT Age/Sex Fits − Males 2+ Years Old − Total Anaemia Prevalence − West Bengal, Rural

WHO−2001 Adjustment Method

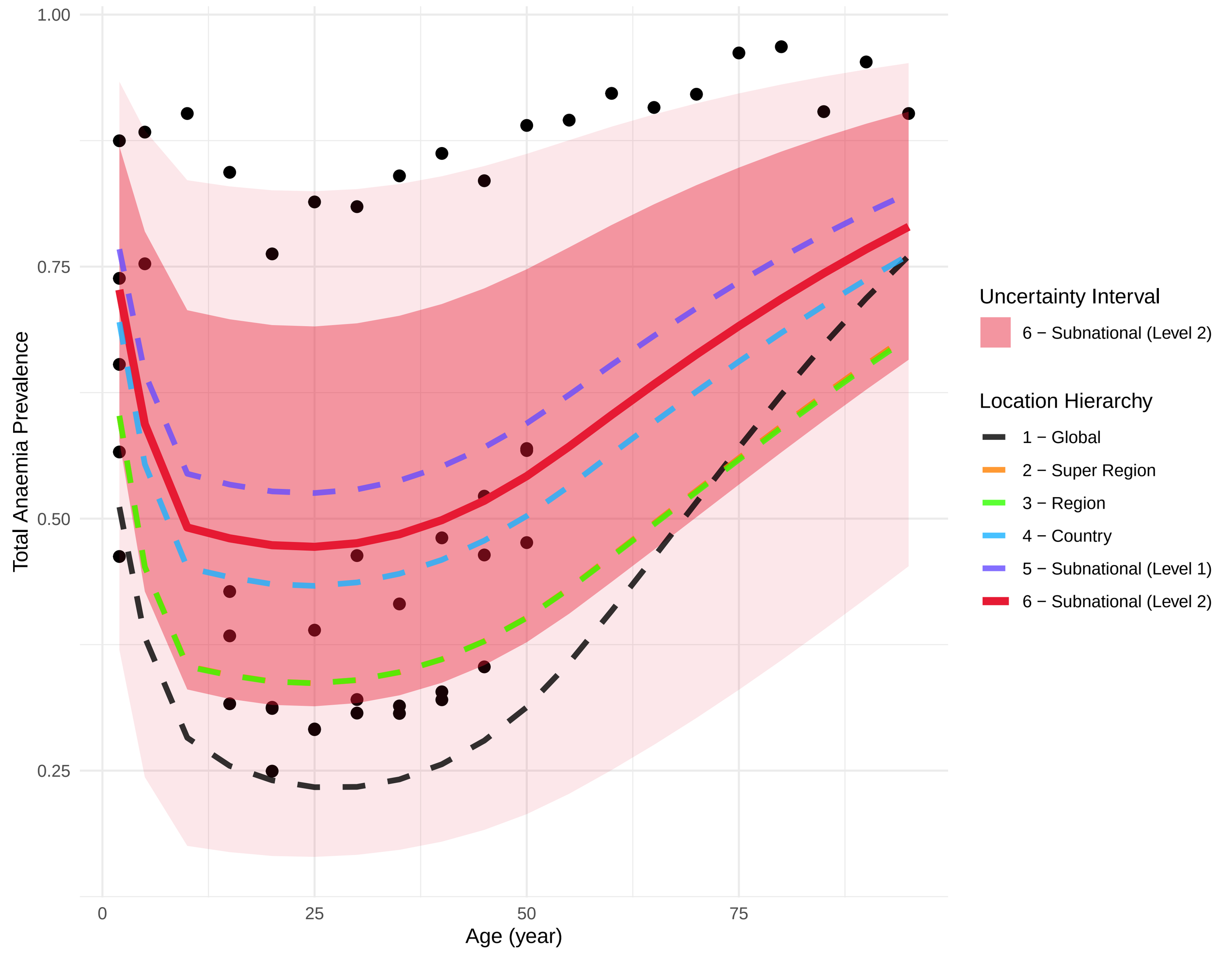

MR−BRT Age/Sex Fits − Males 2+ Years Old − Total Anaemia Prevalence − West Bengal, Urban

WHO−2001 Adjustment Method

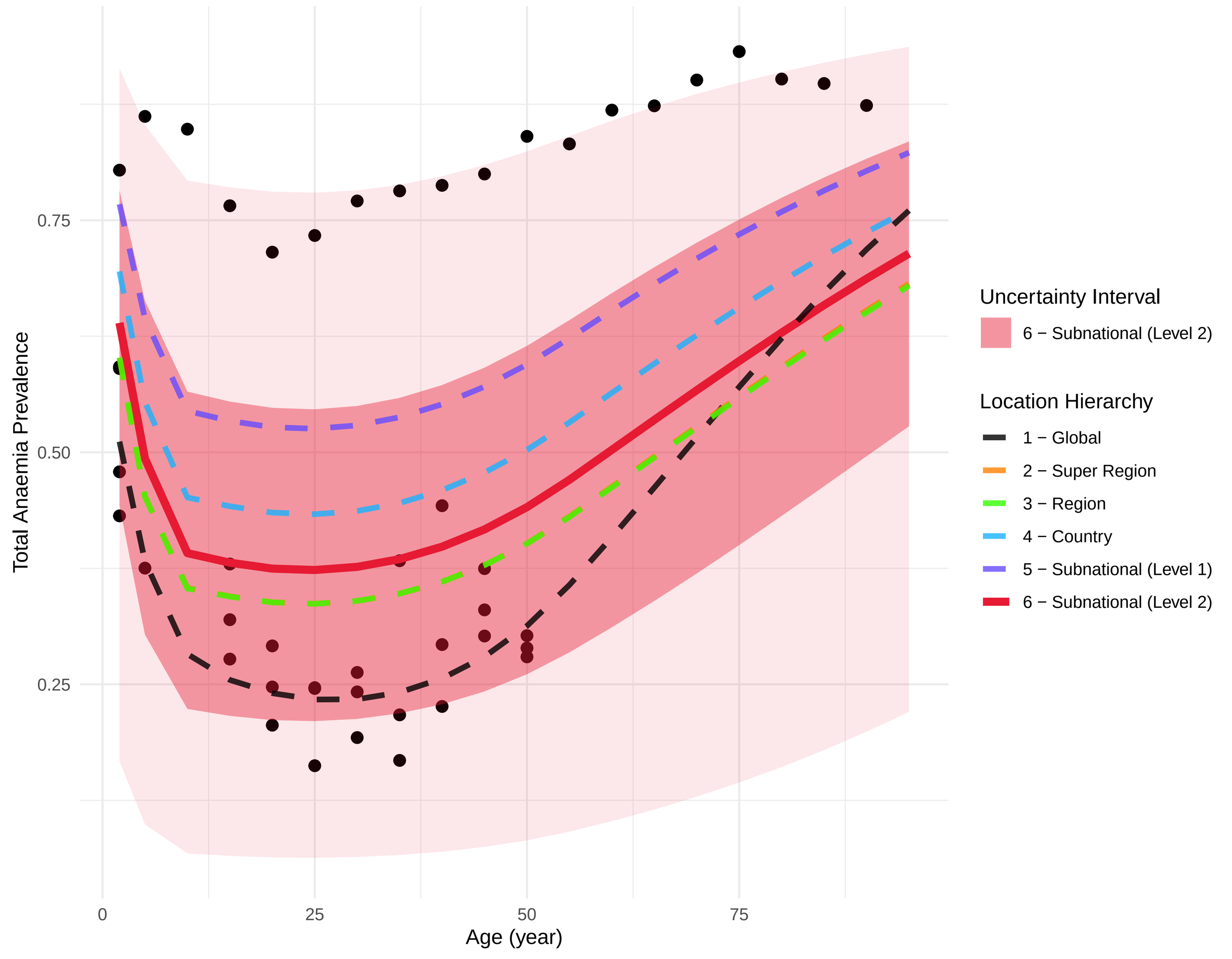

MR−BRT Age/Sex Fits − Males 2+ Years Old − Total Anaemia Prevalence − Nepal

WHO−2001 Adjustment Method

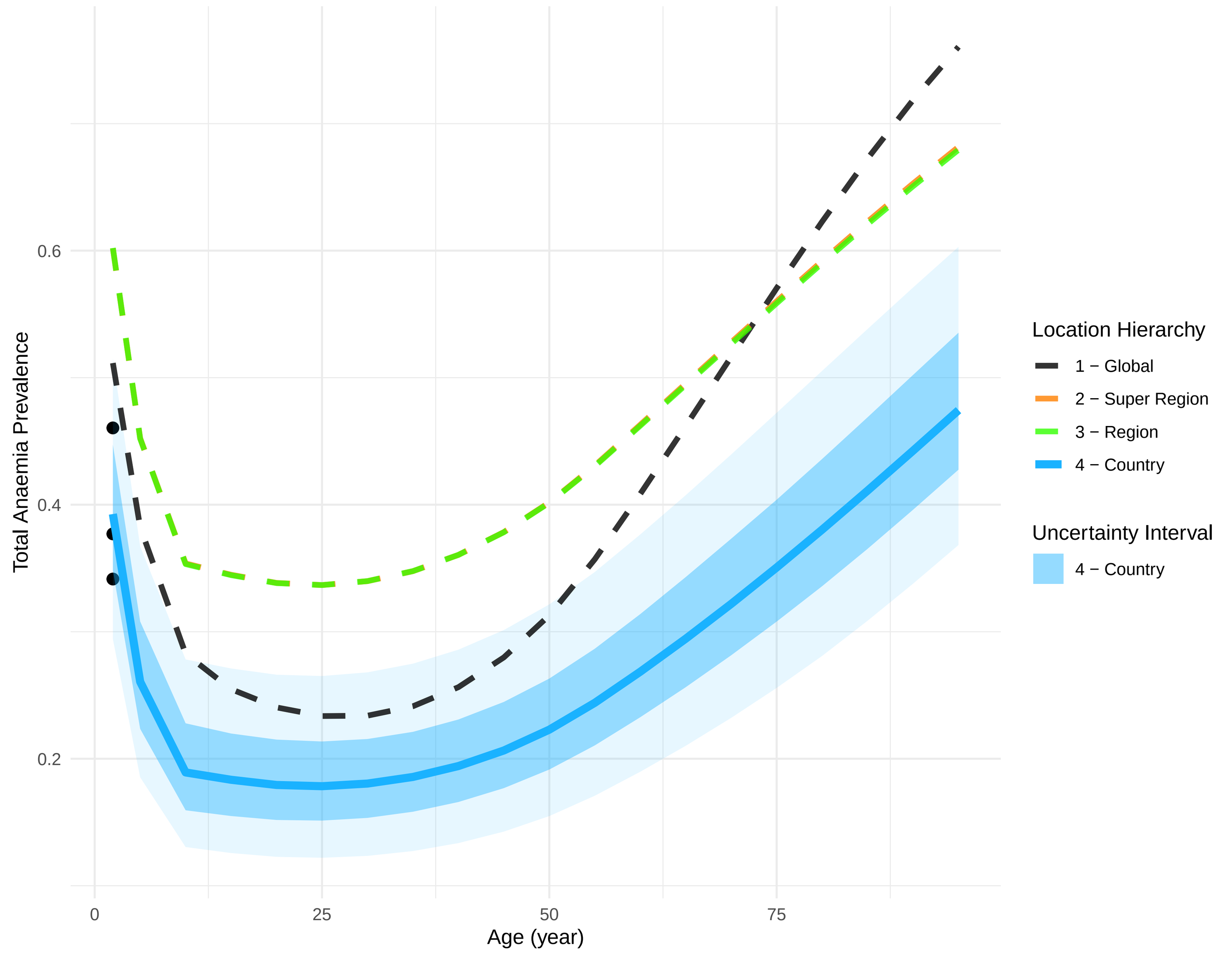

MR−BRT Age/Sex Fits − Males 2+ Years Old − Total Anaemia Prevalence − Southeast Asia, East Asia, and Oceania

WHO−2001 Adjustment Method

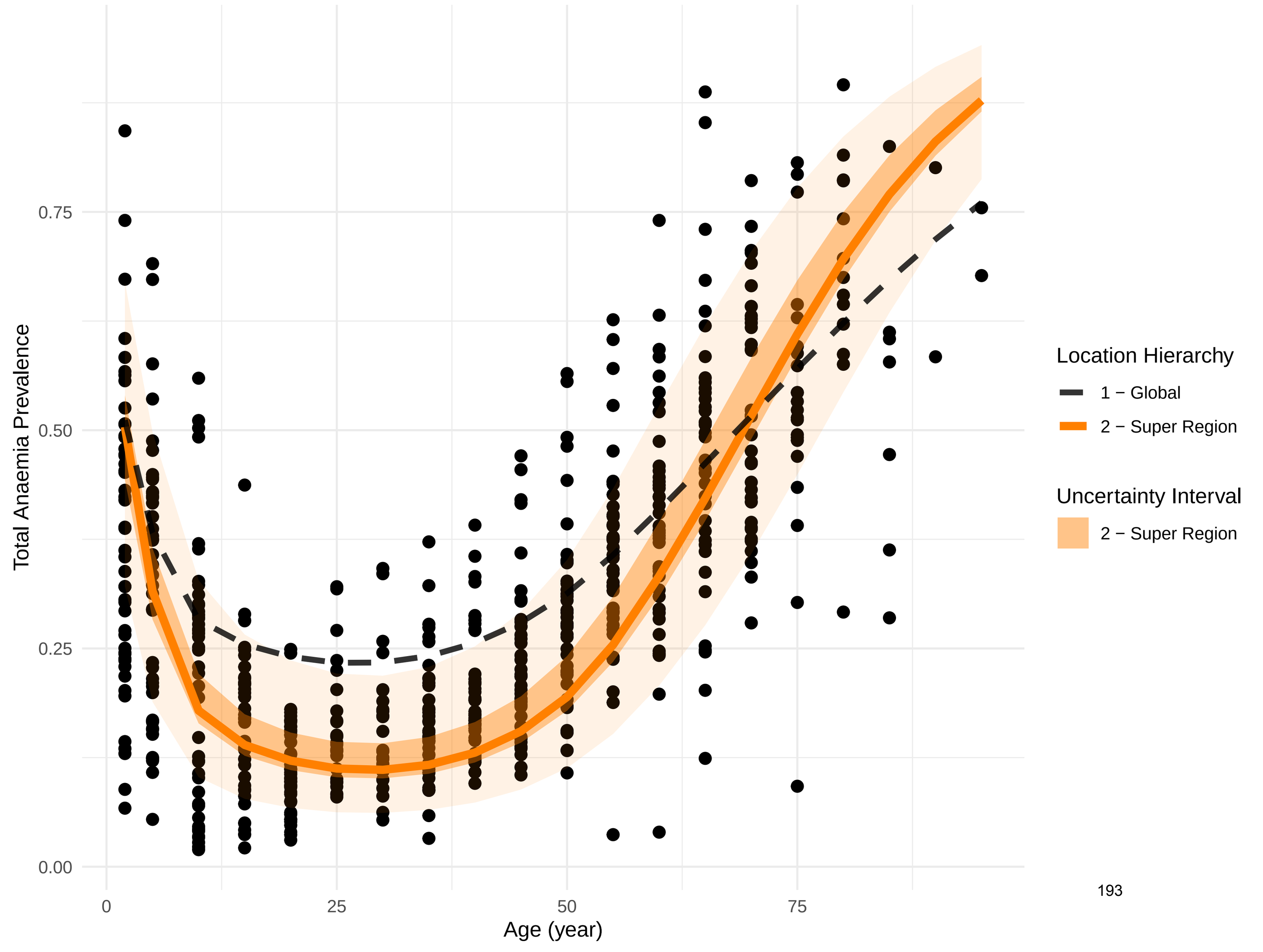

MR−BRT Age/Sex Fits − Males 2+ Years Old − Total Anaemia Prevalence − Southeast Asia
WHO−2001 Adjustment Method

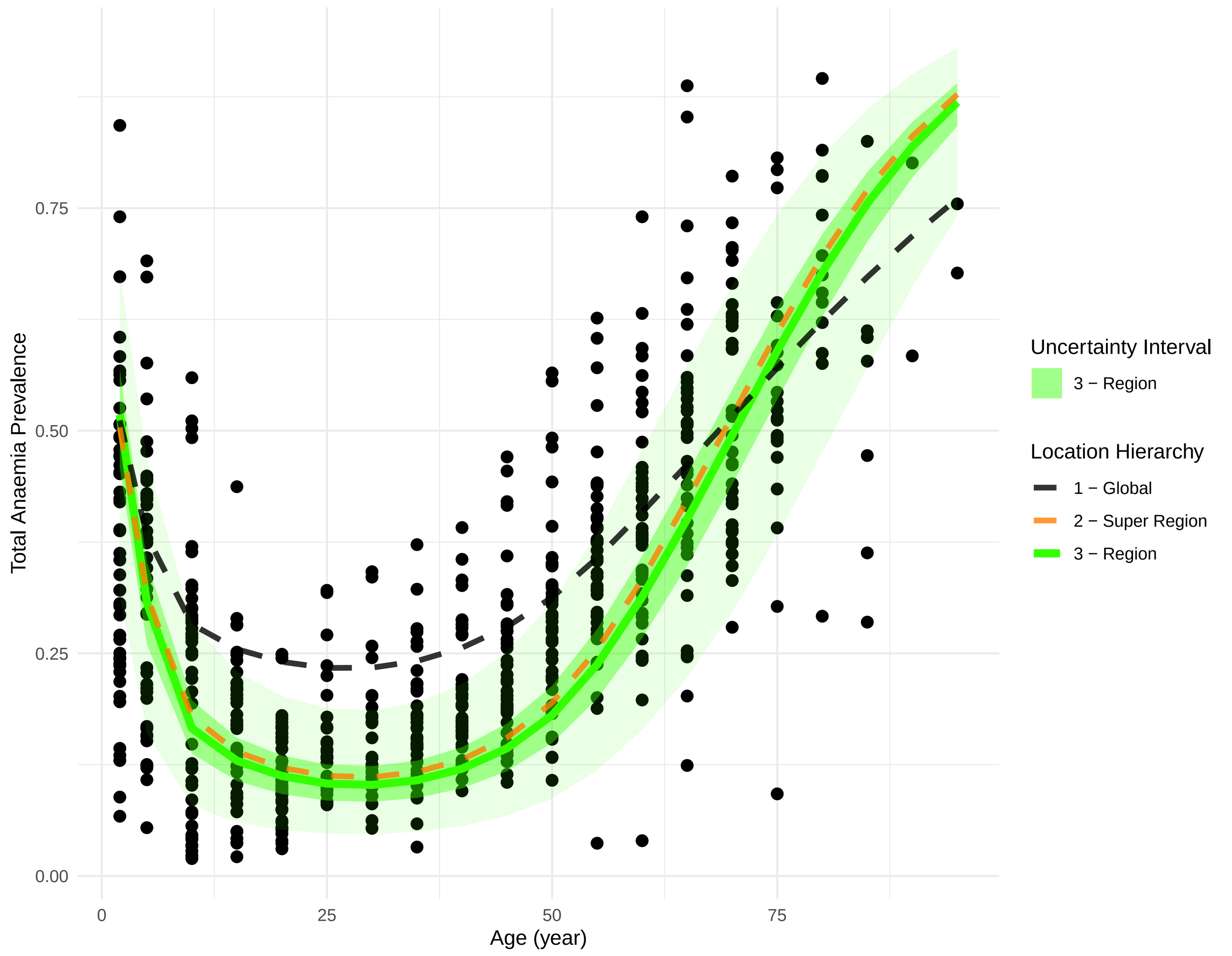

MR−BRT Age/Sex Fits − Males 2+ Years Old − Total Anaemia Prevalence − Cambodia
WHO−2001 Adjustment Method

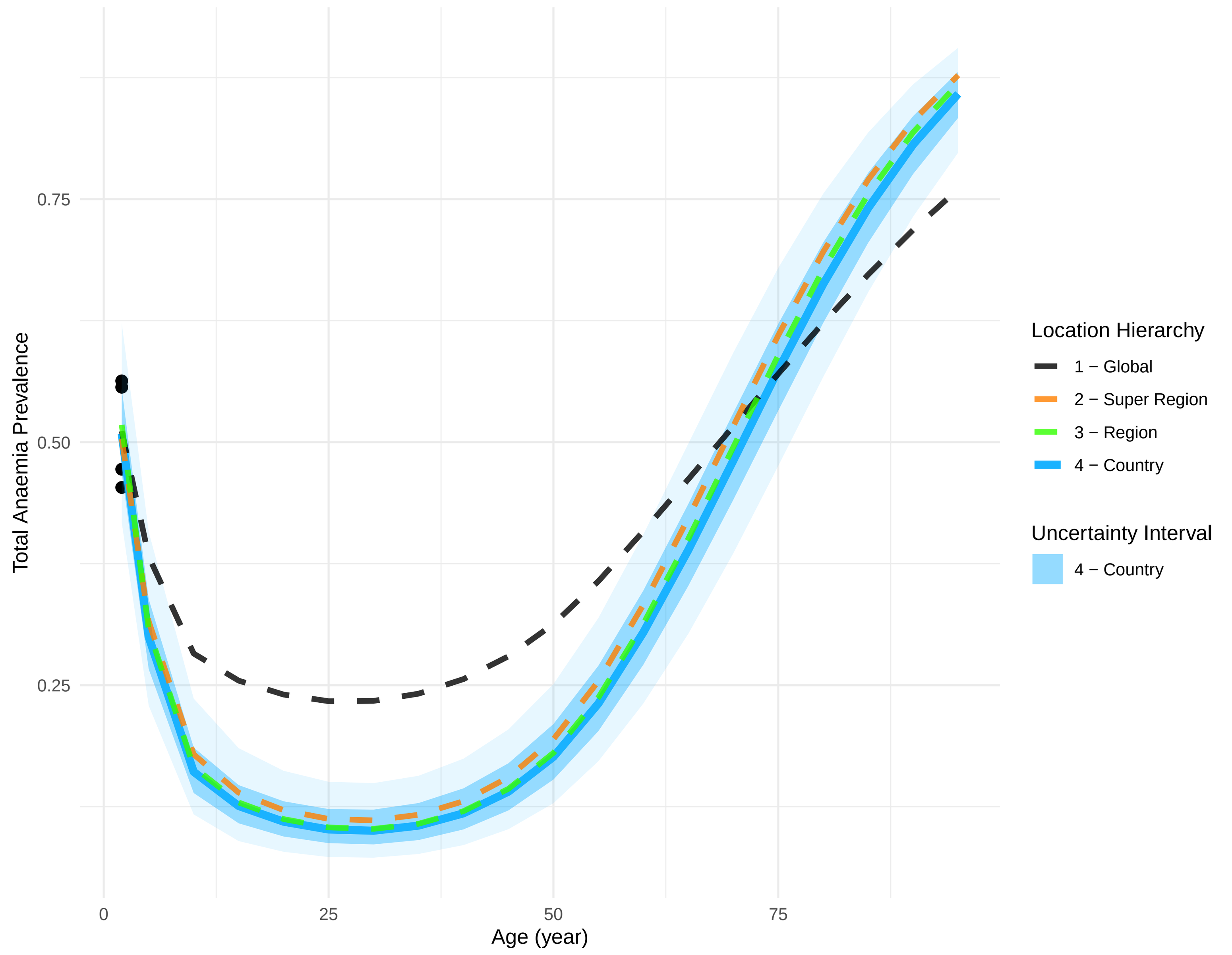

MR−BRT Age/Sex Fits − Males 2+ Years Old − Total Anaemia Prevalence − Indonesia
WHO−2001 Adjustment Method

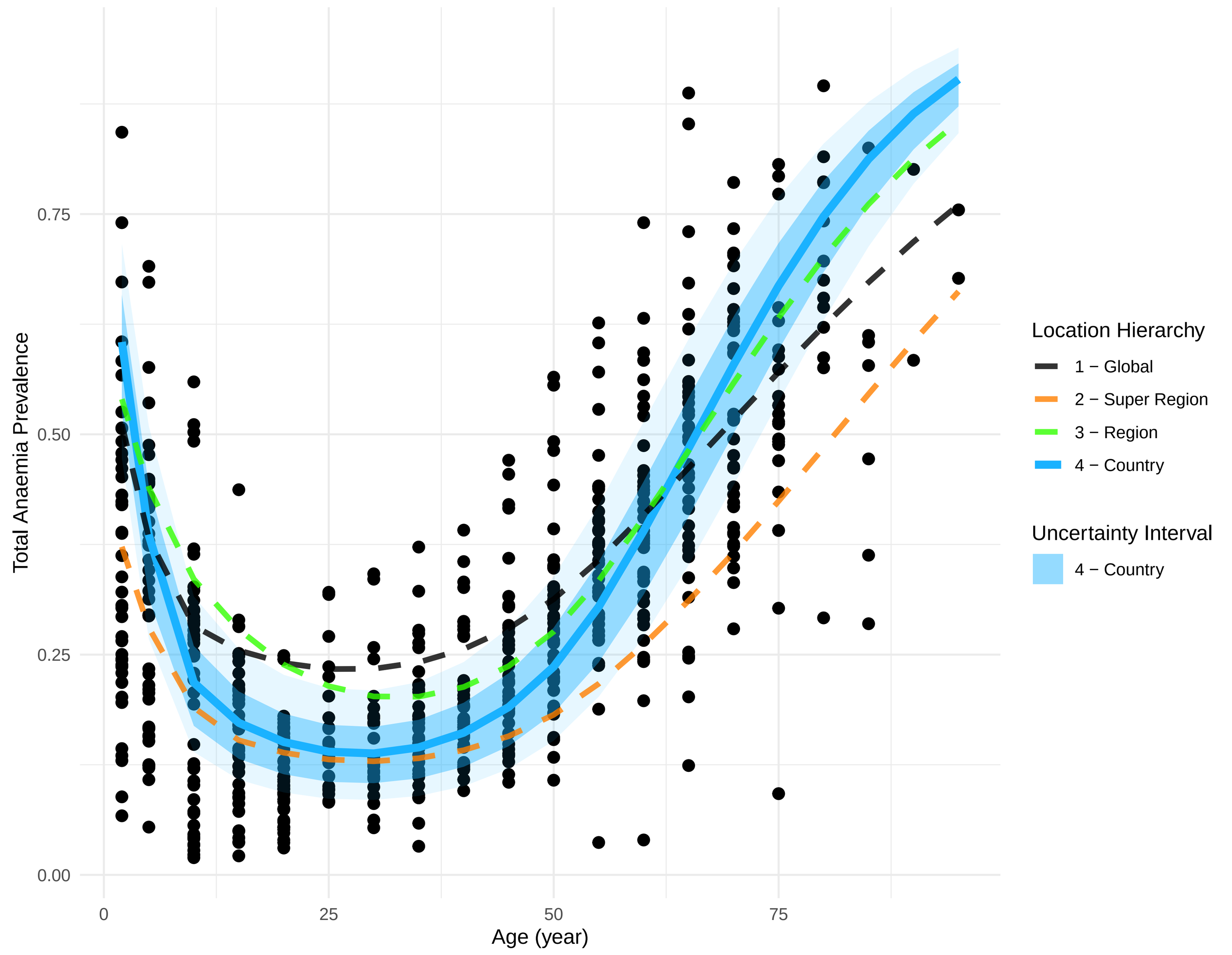

MR−BRT Age/Sex Fits − Males 2+ Years Old − Total Anaemia Prevalence − Bali
WHO−2001 Adjustment Method

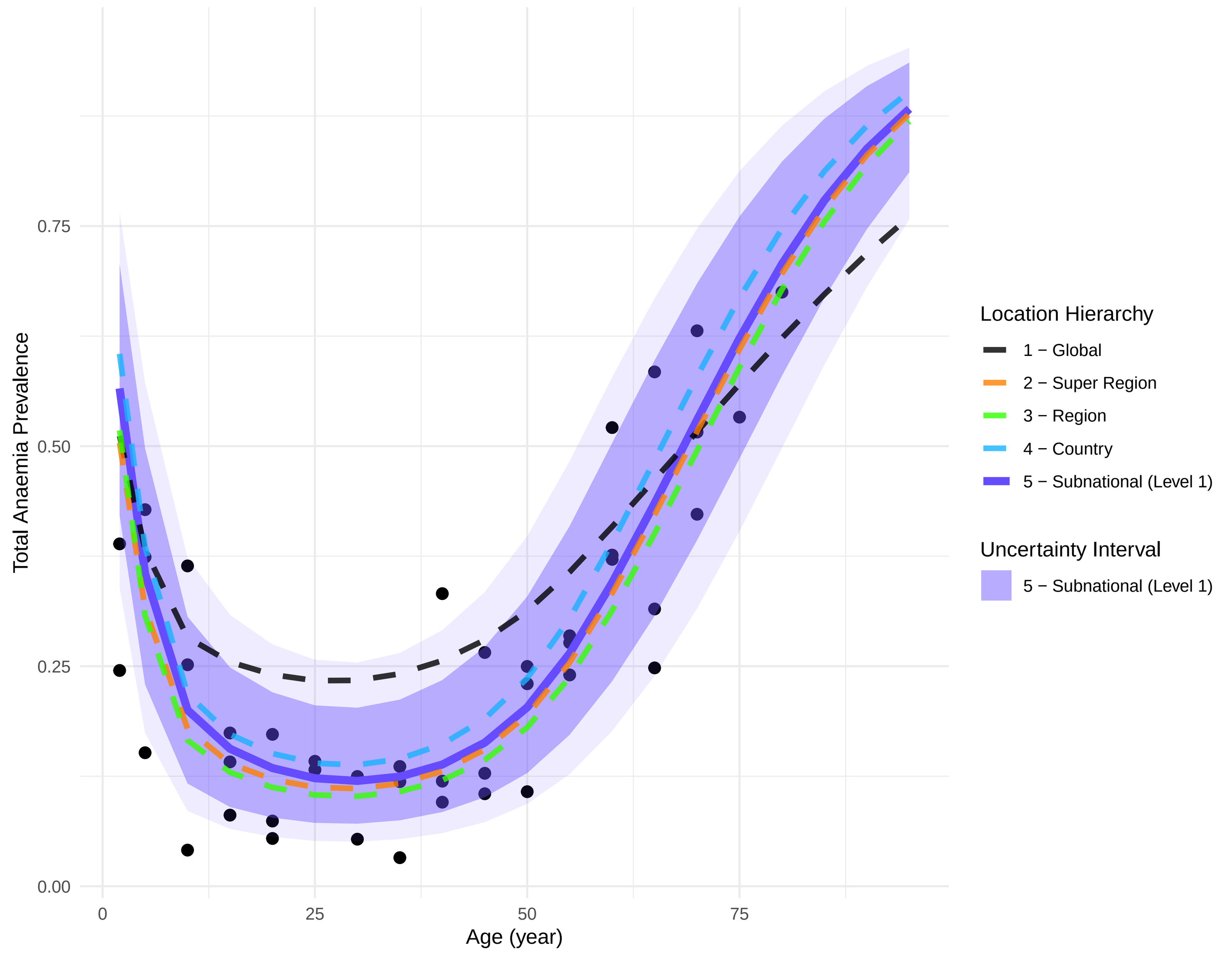

MR−BRT Age/Sex Fits − Males 2+ Years Old − Total Anaemia Prevalence − Bangka−Belitung Islands
WHO−2001 Adjustment Method

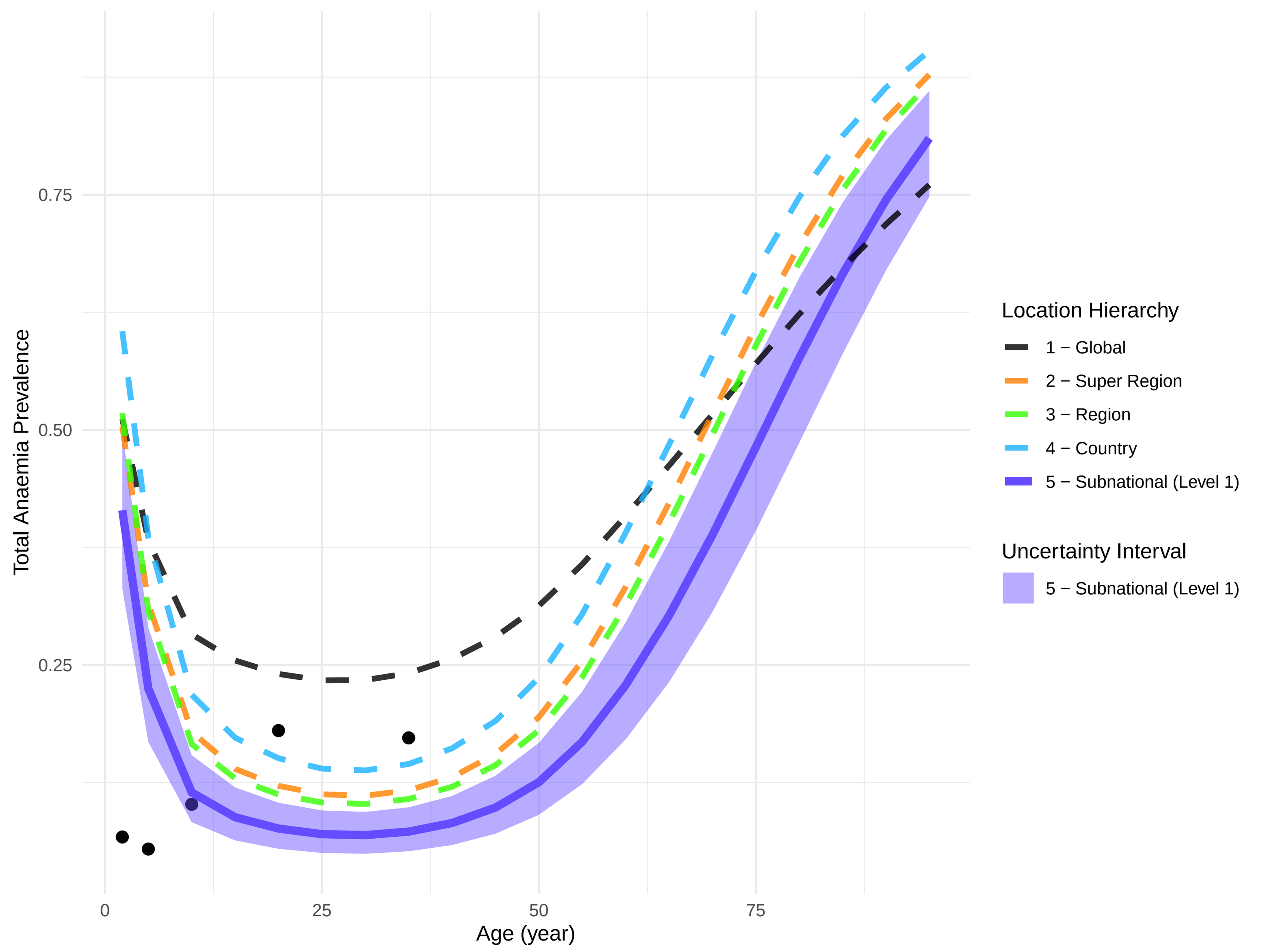

MR−BRT Age/Sex Fits − Males 2+ Years Old − Total Anaemia Prevalence − Banten
WHO−2001 Adjustment Method

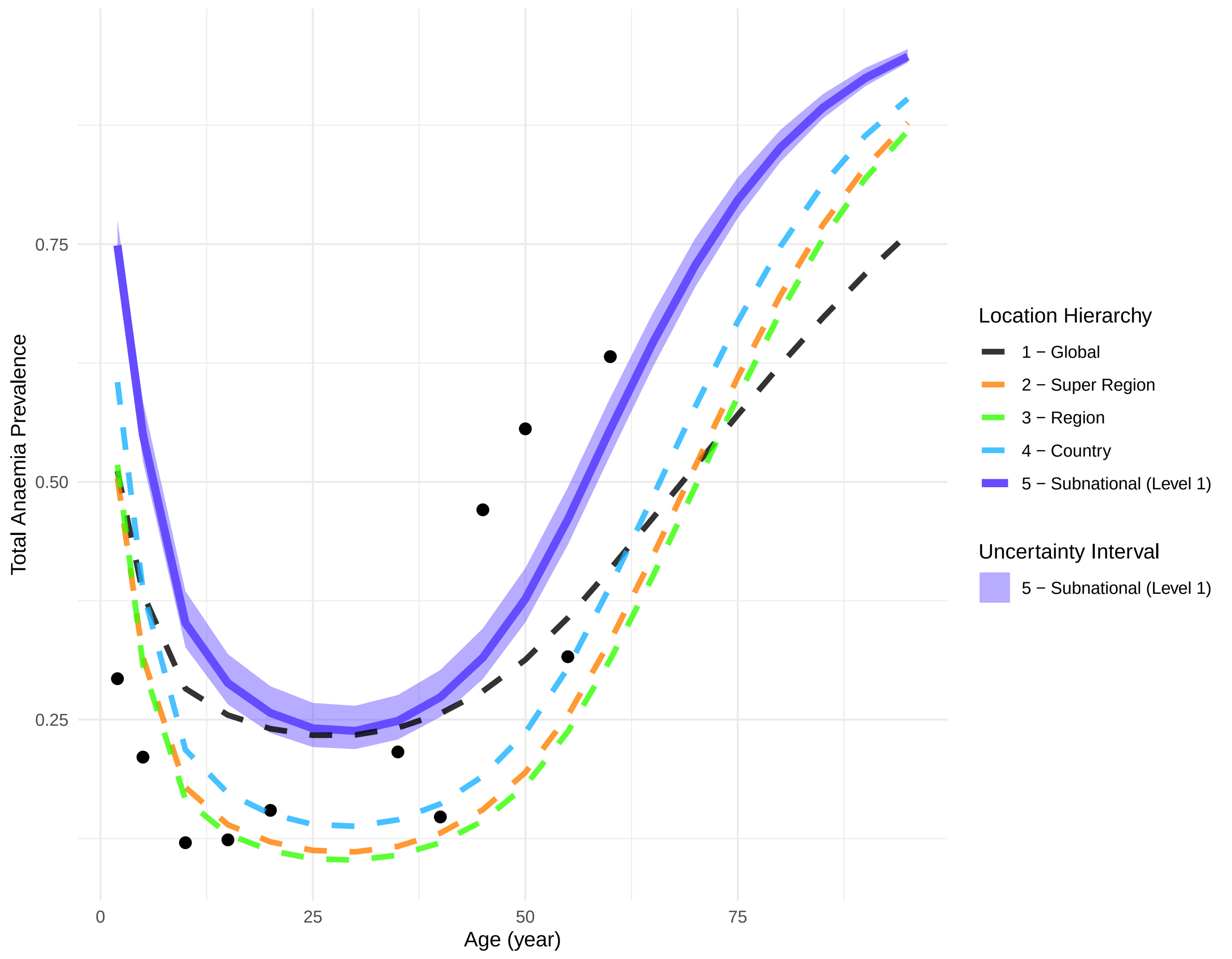

MR−BRT Age/Sex Fits − Males 2+ Years Old − Total Anaemia Prevalence − Jakarta
WHO−2001 Adjustment Method

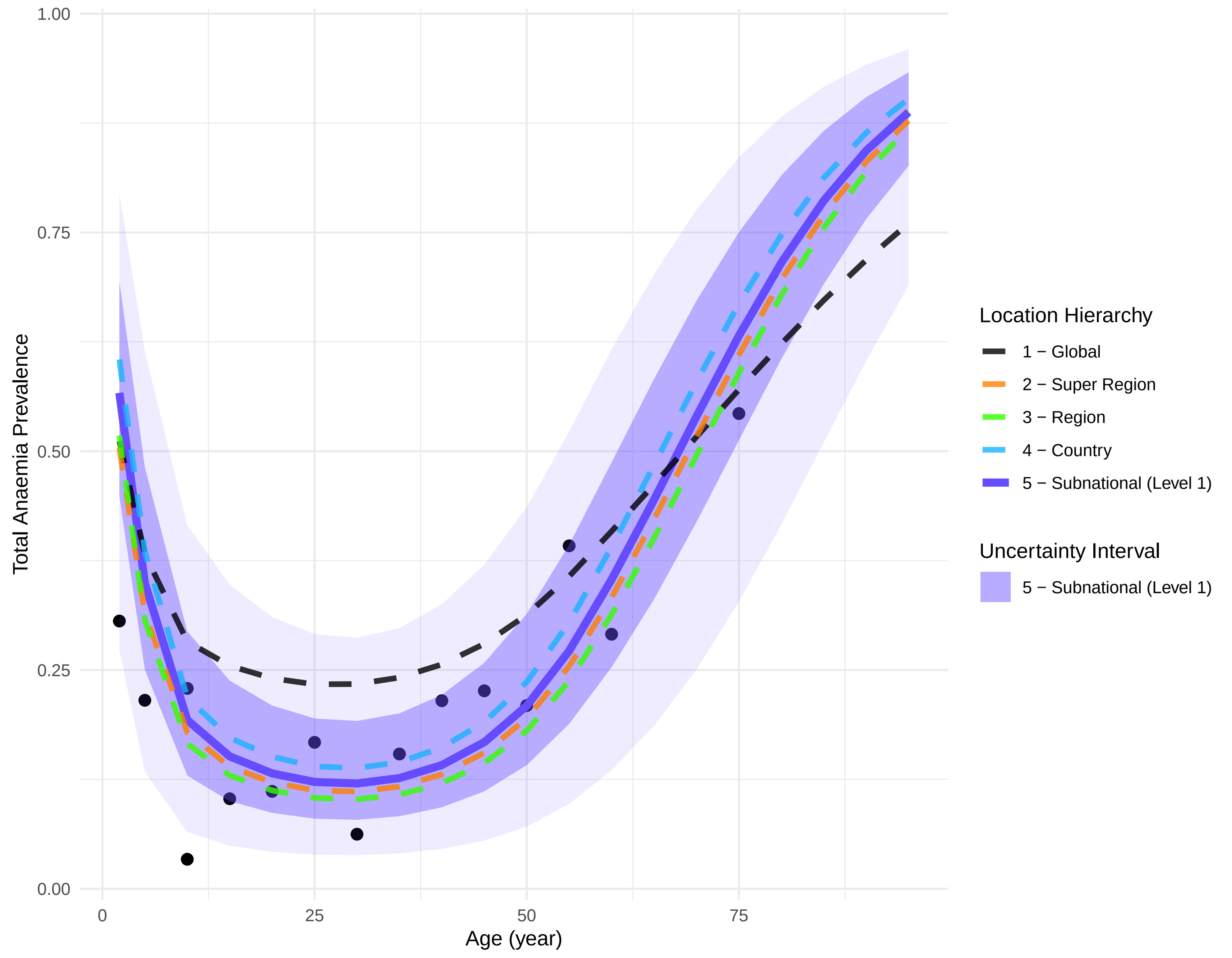

MR−BRT Age/Sex Fits − Males 2+ Years Old − Total Anaemia Prevalence − West Java
WHO−2001 Adjustment Method

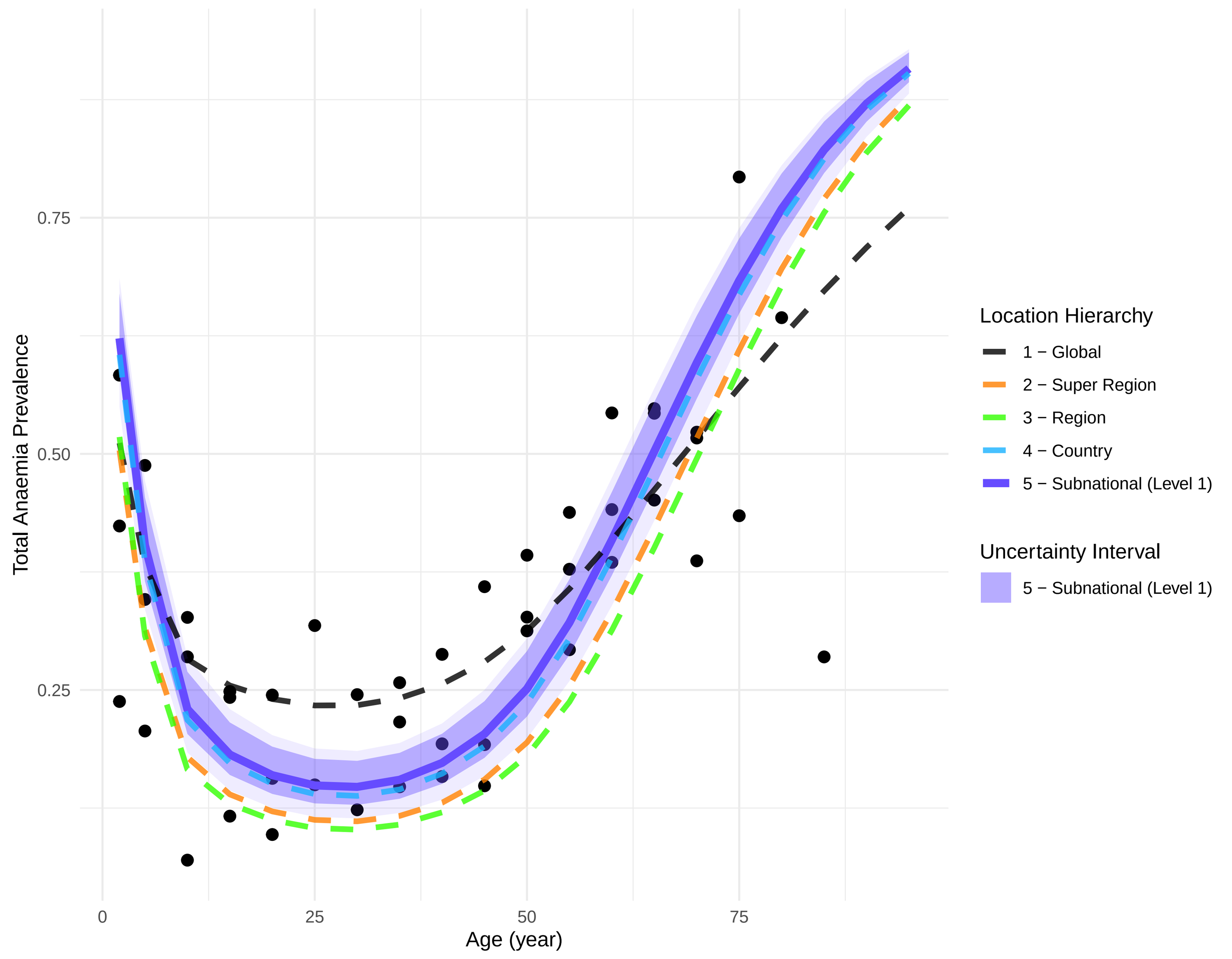

MR−BRT Age/Sex Fits − Males 2+ Years Old − Total Anaemia Prevalence − Central Java
WHO−2001 Adjustment Method

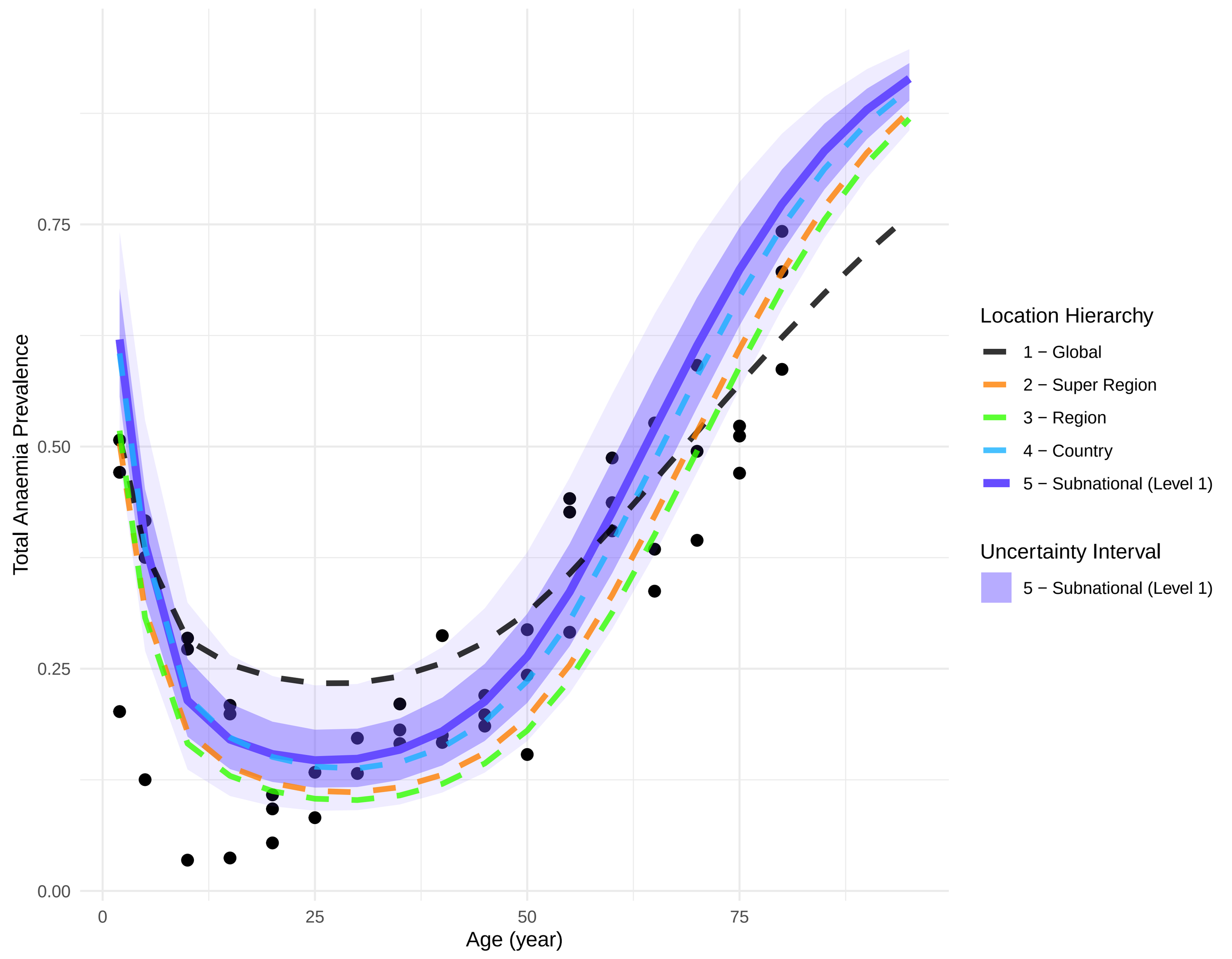

MR−BRT Age/Sex Fits − Males 2+ Years Old − Total Anaemia Prevalence − East Java
WHO−2001 Adjustment Method

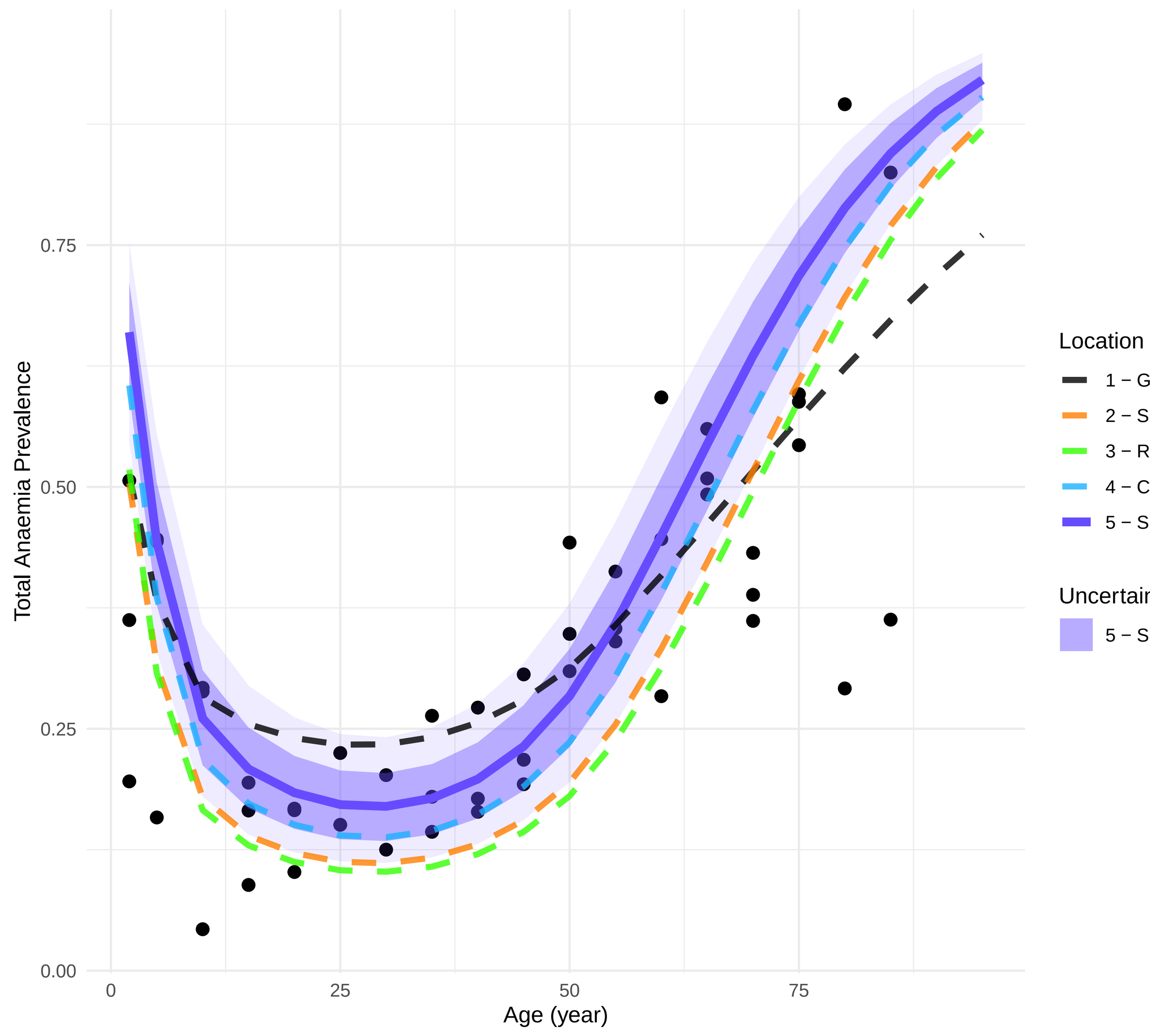

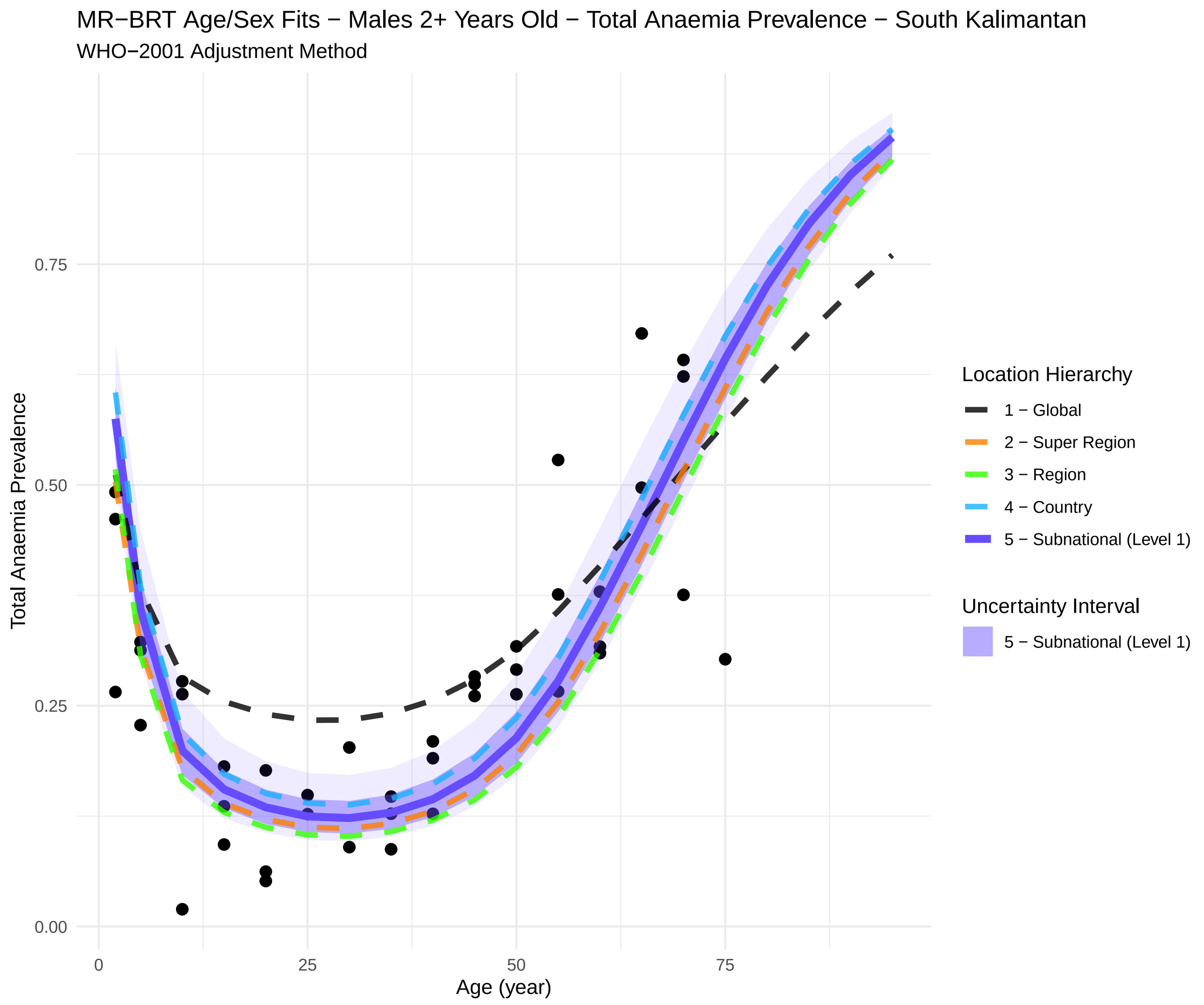
MR−BRT Age/Sex Fits − Males 2+ Years Old − Total Anaemia Prevalence − South Kalimantan
WHO−2001 Adjustment Method
Total Anaemia Prevalence
Age (year)
Location Hierarchy
1 − Global
2 − Super Region
3 − Region
4 − Country
5 − Subnational (Level 1)
Uncertainty Interval
5 − Subnational (Level 1)

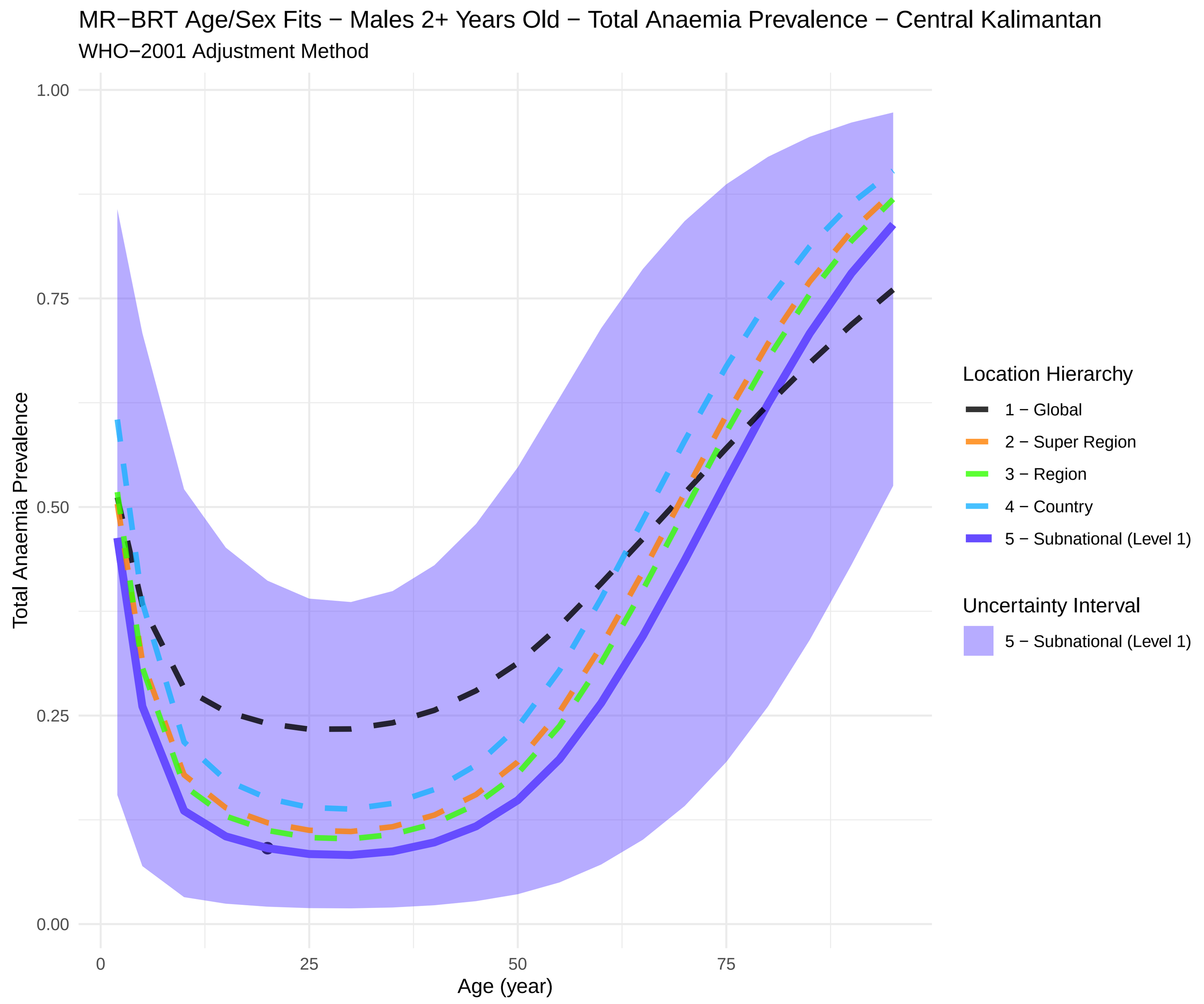
MR−BRT Age/Sex Fits − Males 2+ Years Old − Total Anaemia Prevalence − Central Kalimantan
WHO−2001 Adjustment Method
Total Anaemia Prevalence
Age (year)
Location Hierarchy
1 − Global
2 − Super Region
3 − Region
4 − Country
5 − Subnational (Level 1)
Uncertainty Interval
5 − Subnational (Level 1)

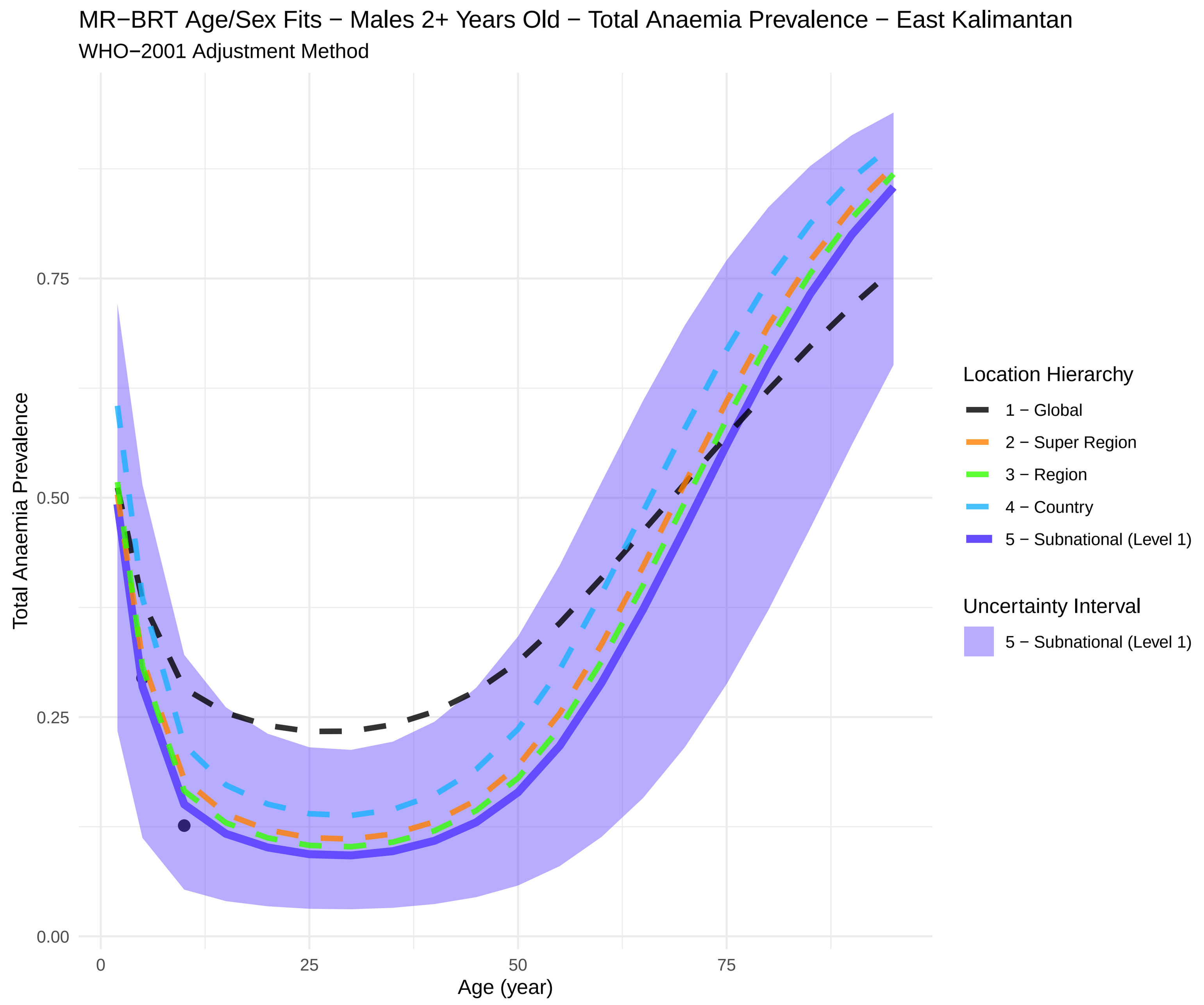
MR−BRT Age/Sex Fits − Males 2+ Years Old − Total Anaemia Prevalence − East Kalimantan
WHO−2001 Adjustment Method
Total Anaemia Prevalence
Age (year)
Location Hierarchy
1 − Global
2 − Super Region
3 − Region
4 − Country
5 − Subnational (Level 1)
Uncertainty Interval
5 − Subnational (Level 1)

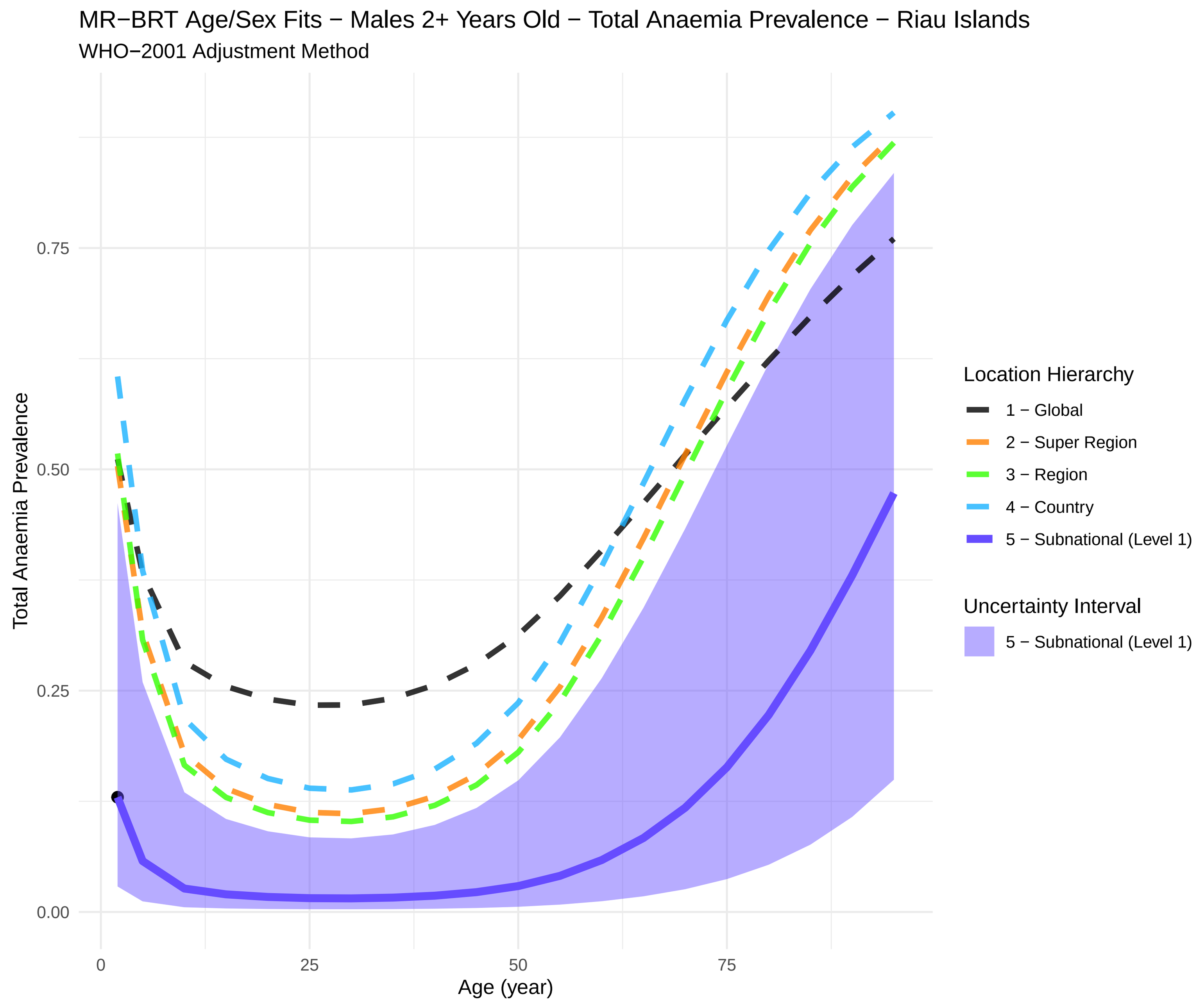
MR−BRT Age/Sex Fits − Males 2+ Years Old − Total Anaemia Prevalence − Riau Islands
WHO−2001 Adjustment Method
Total Anaemia Prevalence
Age (year)
Location Hierarchy
1 − Global
2 − Super Region
3 − Region
4 − Country
5 − Subnational (Level 1)
Uncertainty Interval
5 − Subnational (Level 1)

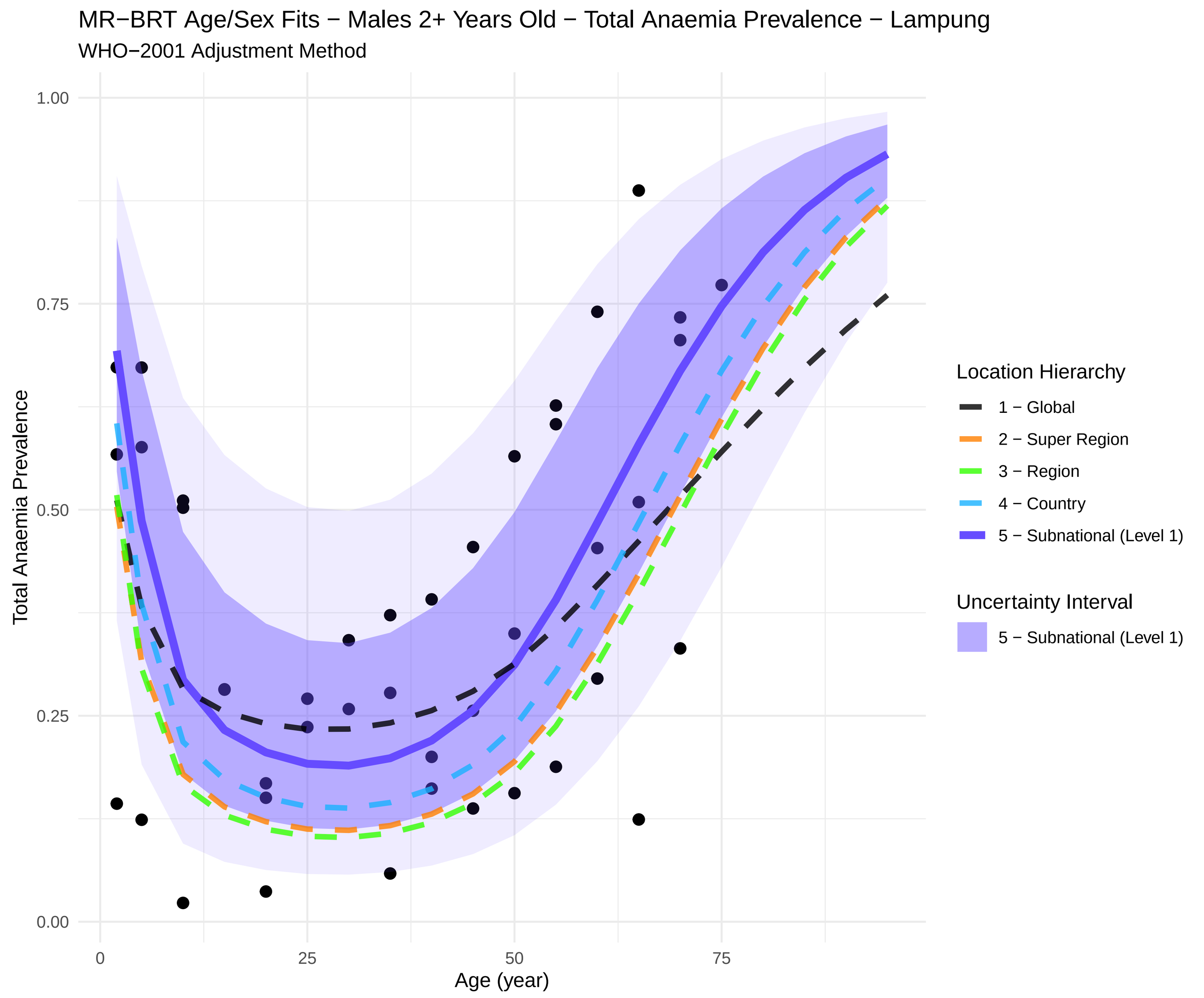
MR−BRT Age/Sex Fits − Males 2+ Years Old − Total Anaemia Prevalence − Lampung
WHO−2001 Adjustment Method
Total Anaemia Prevalence
Age (year)
Location Hierarchy
1 − Global
2 − Super Region
3 − Region
4 − Country
5 − Subnational (Level 1)
Uncertainty Interval
5 − Subnational (Level 1)

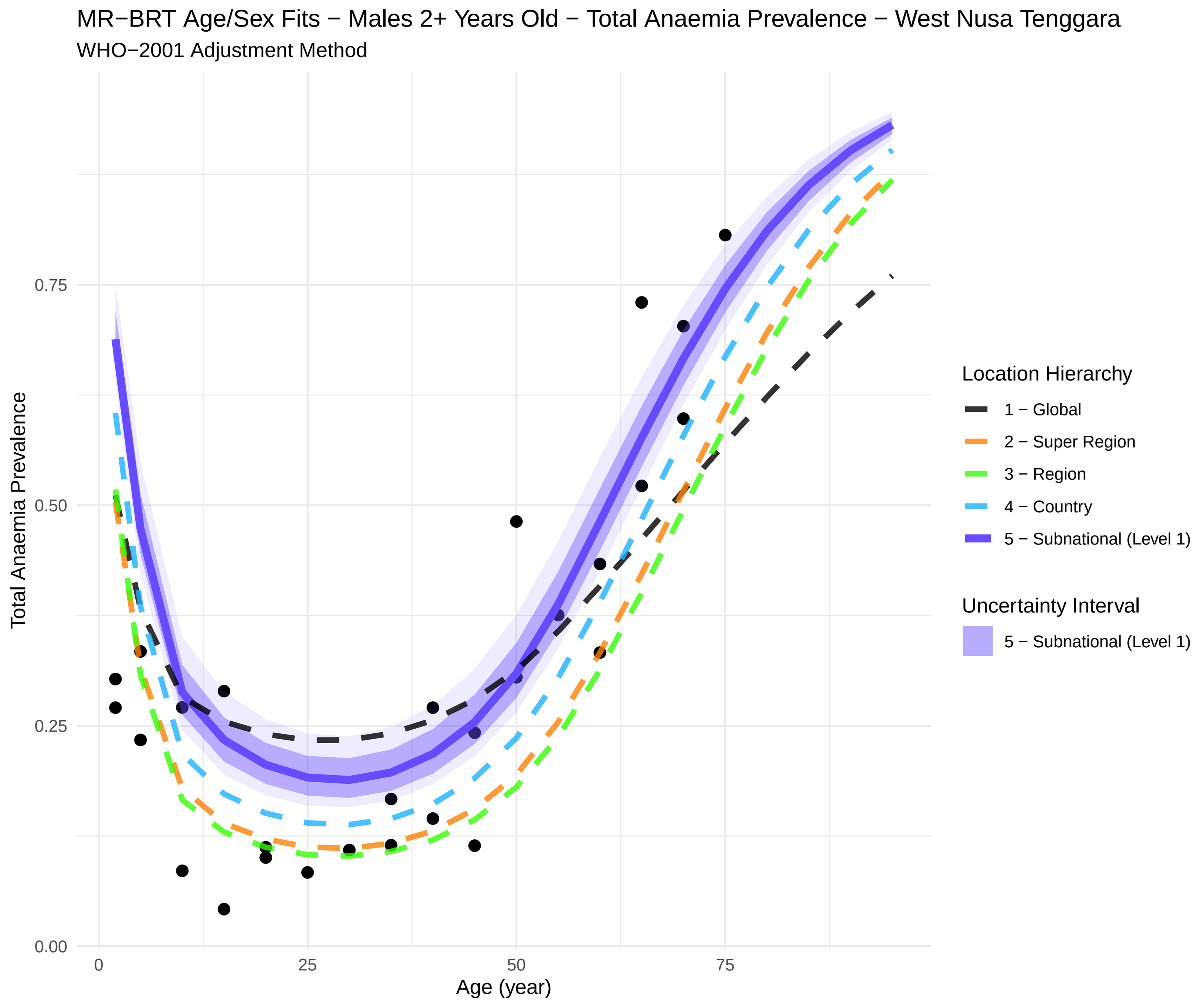
MR−BRT Age/Sex Fits − Males 2+ Years Old − Total Anaemia Prevalence − West Nusa Tenggara
WHO−2001 Adjustment Method
Total Anaemia Prevalence
Age (year)
Location Hierarchy
1 − Global
2 − Super Region
3 − Region
4 − Country
5 − Subnational (Level 1)
Uncertainty Interval
5 − Subnational (Level 1)

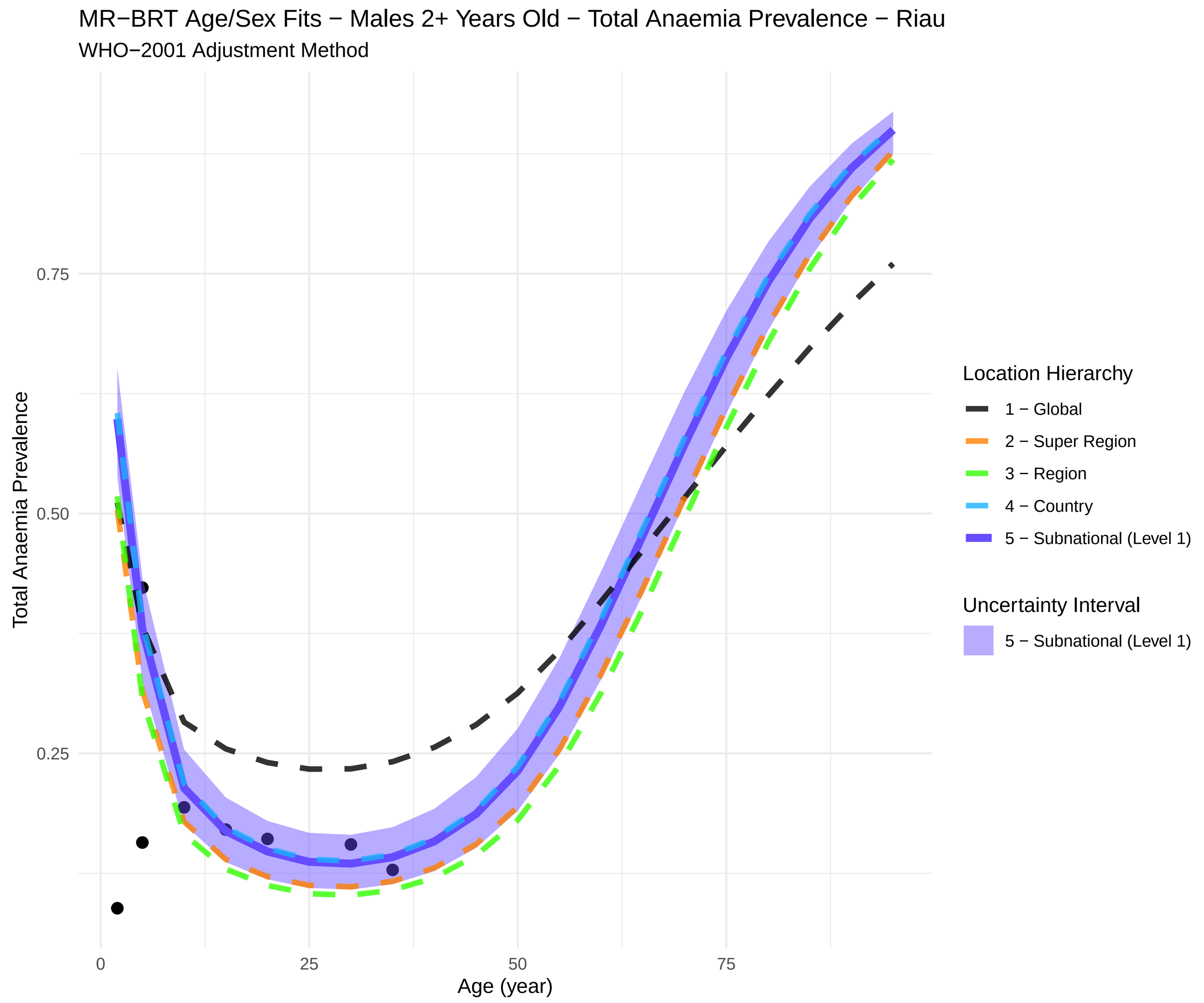
MR−BRT Age/Sex Fits − Males 2+ Years Old − Total Anaemia Prevalence − Riau
WHO−2001 Adjustment Method
Total Anaemia Prevalence
Age (year)
Location Hierarchy
1 − Global
2 − Super Region
3 − Region
4 − Country
5 − Subnational (Level 1)
Uncertainty Interval
5 − Subnational (Level 1)

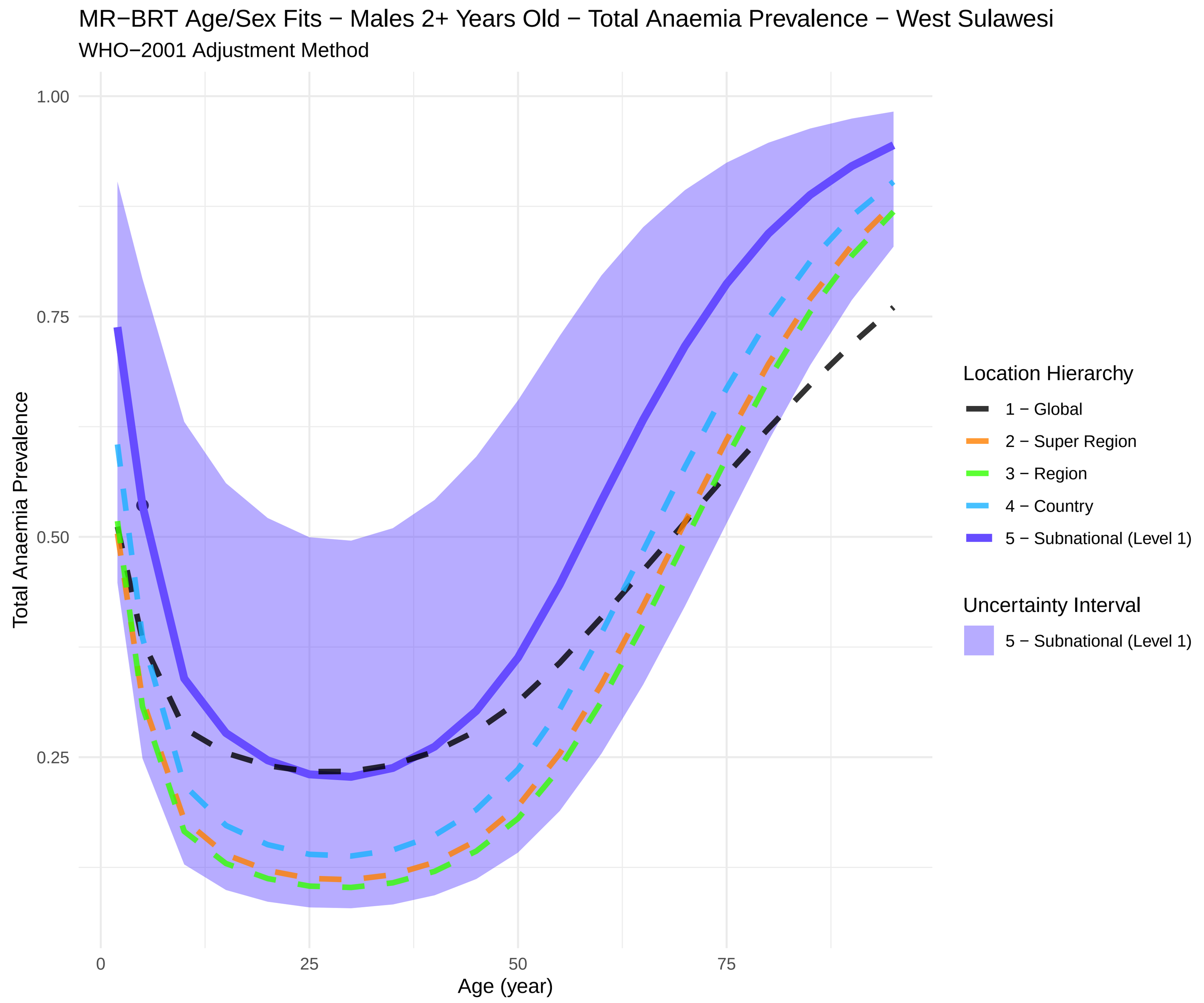
MR−BRT Age/Sex Fits − Males 2+ Years Old − Total Anaemia Prevalence − West Sulawesi
WHO−2001 Adjustment Method
Total Anaemia Prevalence
Age (year)
Location Hierarchy
1 − Global
2 − Super Region
3 − Region
4 − Country
5 − Subnational (Level 1)
Uncertainty Interval
5 − Subnational (Level 1)

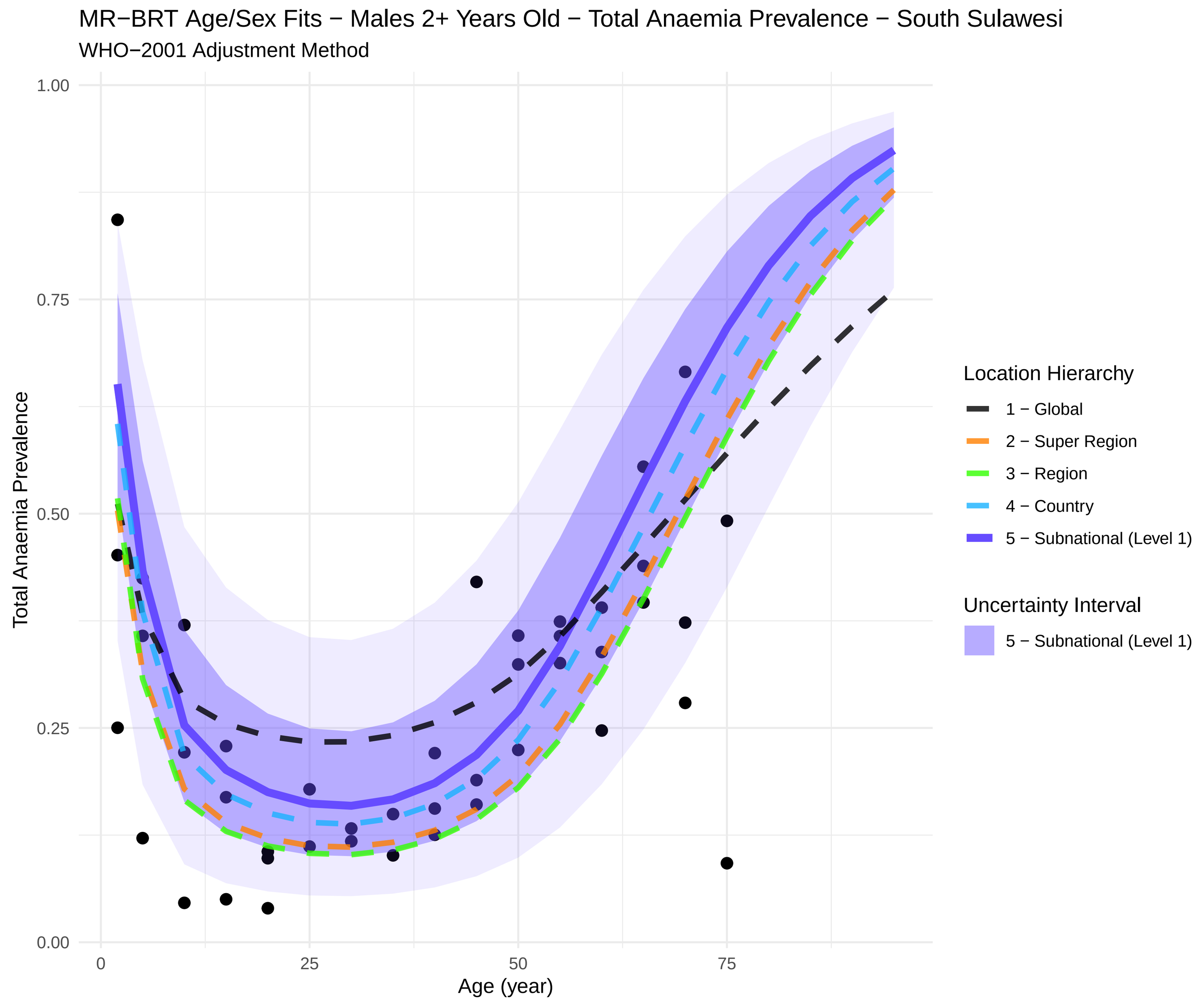
MR−BRT Age/Sex Fits − Males 2+ Years Old − Total Anaemia Prevalence − South Sulawesi
WHO−2001 Adjustment Method
Total Anaemia Prevalence
Age (year)
Location Hierarchy
1 − Global
2 − Super Region
3 − Region
4 − Country
5 − Subnational (Level 1)
Uncertainty Interval
5 − Subnational (Level 1)

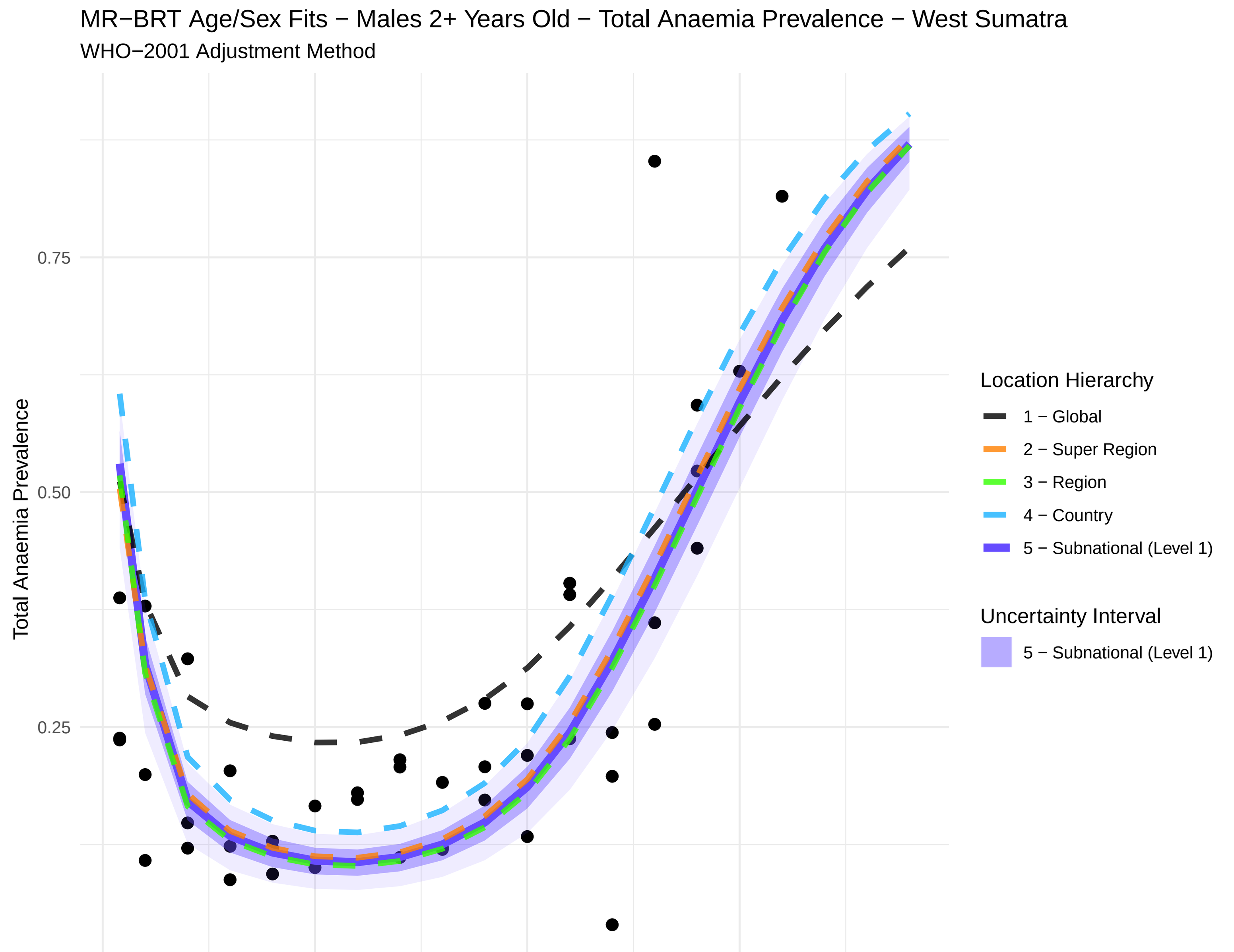
MR−BRT Age/Sex Fits − Males 2+ Years Old − Total Anaemia Prevalence − West Sumatra
WHO−2001 Adjustment Method
Total Anaemia Prevalence
Age (year)
Location Hierarchy
1 − Global
2 − Super Region
3 − Region
4 − Country
5 − Subnational (Level 1)
Uncertainty Interval
5 − Subnational (Level 1)

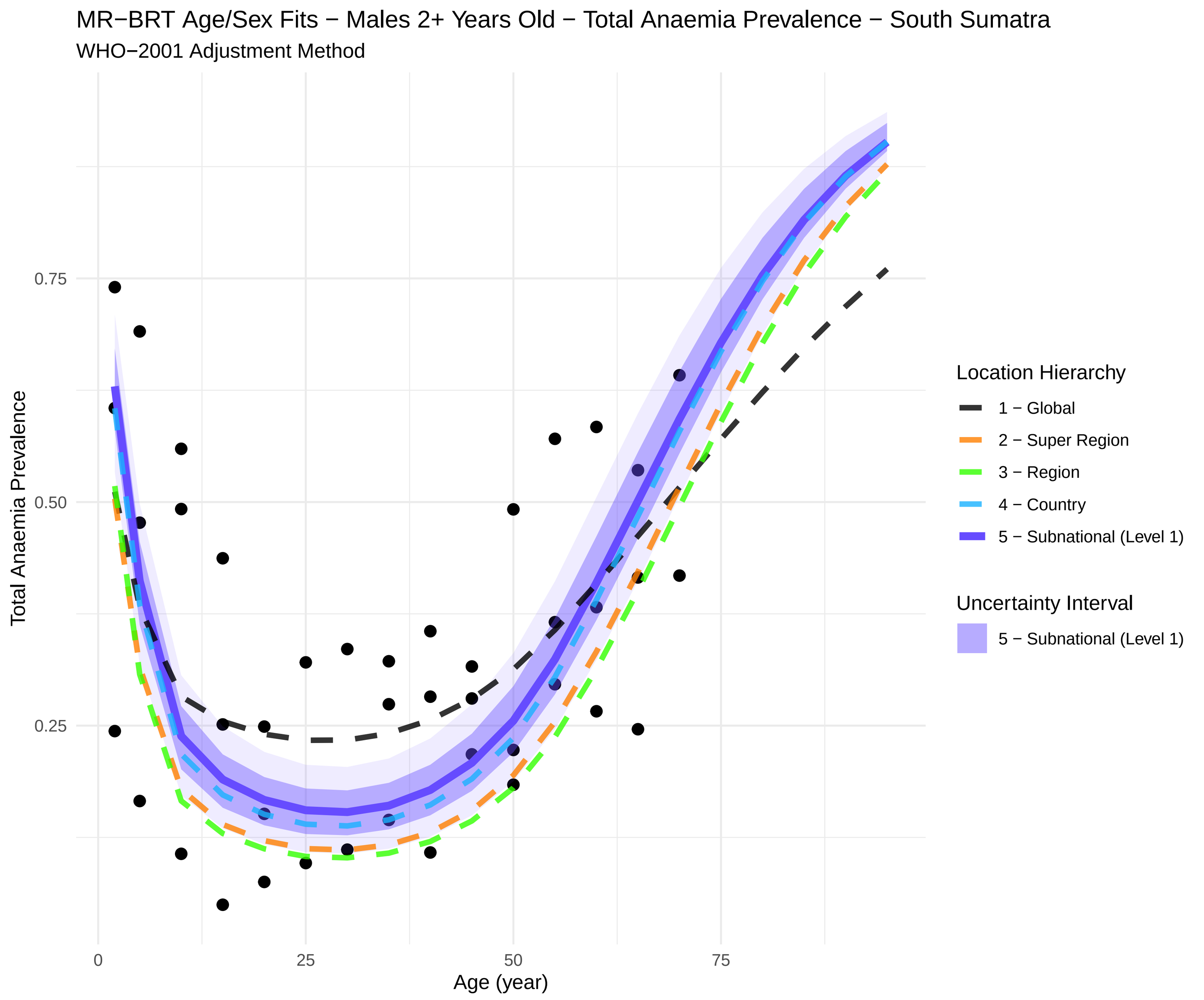
MR−BRT Age/Sex Fits − Males 2+ Years Old − Total Anaemia Prevalence − South Sumatra
WHO−2001 Adjustment Method
Total Anaemia Prevalence
Age (year)
Location Hierarchy
1 − Global
2 − Super Region
3 − Region
4 − Country
5 − Subnational (Level 1)
Uncertainty Interval
5 − Subnational (Level 1)

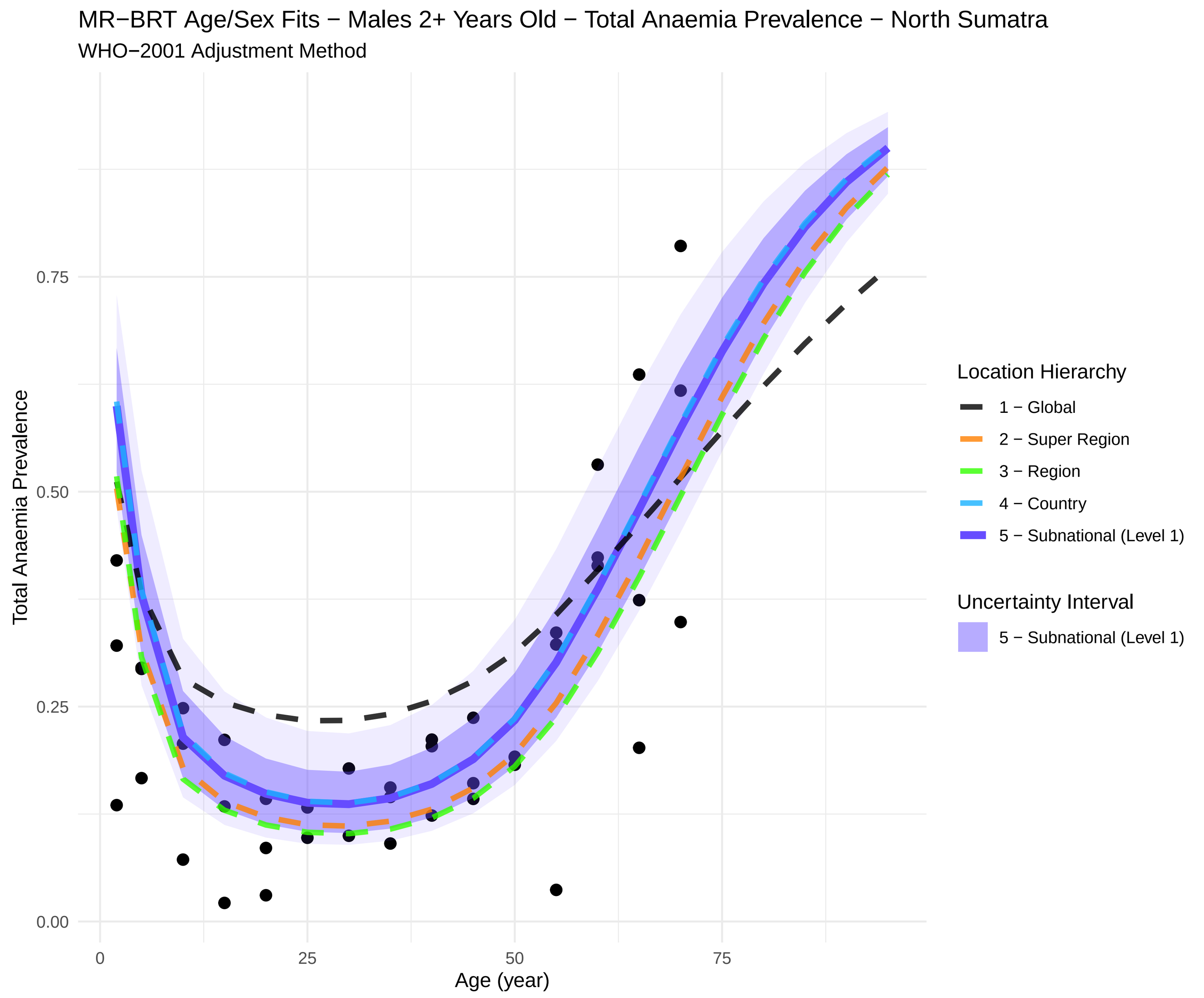
MR−BRT Age/Sex Fits − Males 2+ Years Old − Total Anaemia Prevalence − North Sumatra
WHO−2001 Adjustment Method
Total Anaemia Prevalence
Age (year)
Location Hierarchy
1 − Global
2 − Super Region
3 − Region
4 − Country
5 − Subnational (Level 1)
Uncertainty Interval
5 − Subnational (Level 1)

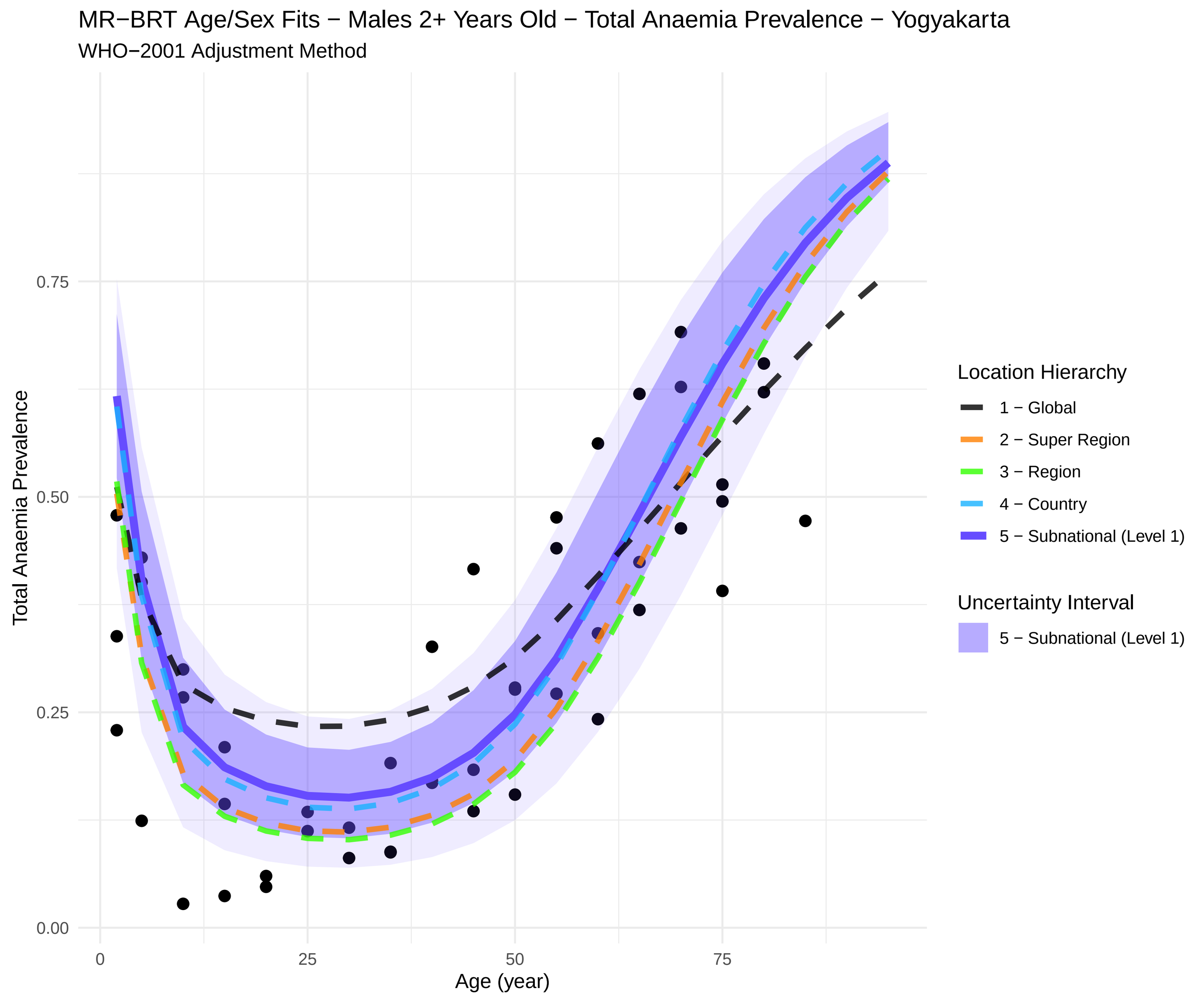
MR−BRT Age/Sex Fits − Males 2+ Years Old − Total Anaemia Prevalence − Yogyakarta
WHO−2001 Adjustment Method
Total Anaemia Prevalence
Age (year)
Location Hierarchy
1 − Global
2 − Super Region
3 − Region
4 − Country
5 − Subnational (Level 1)
Uncertainty Interval
5 − Subnational (Level 1)

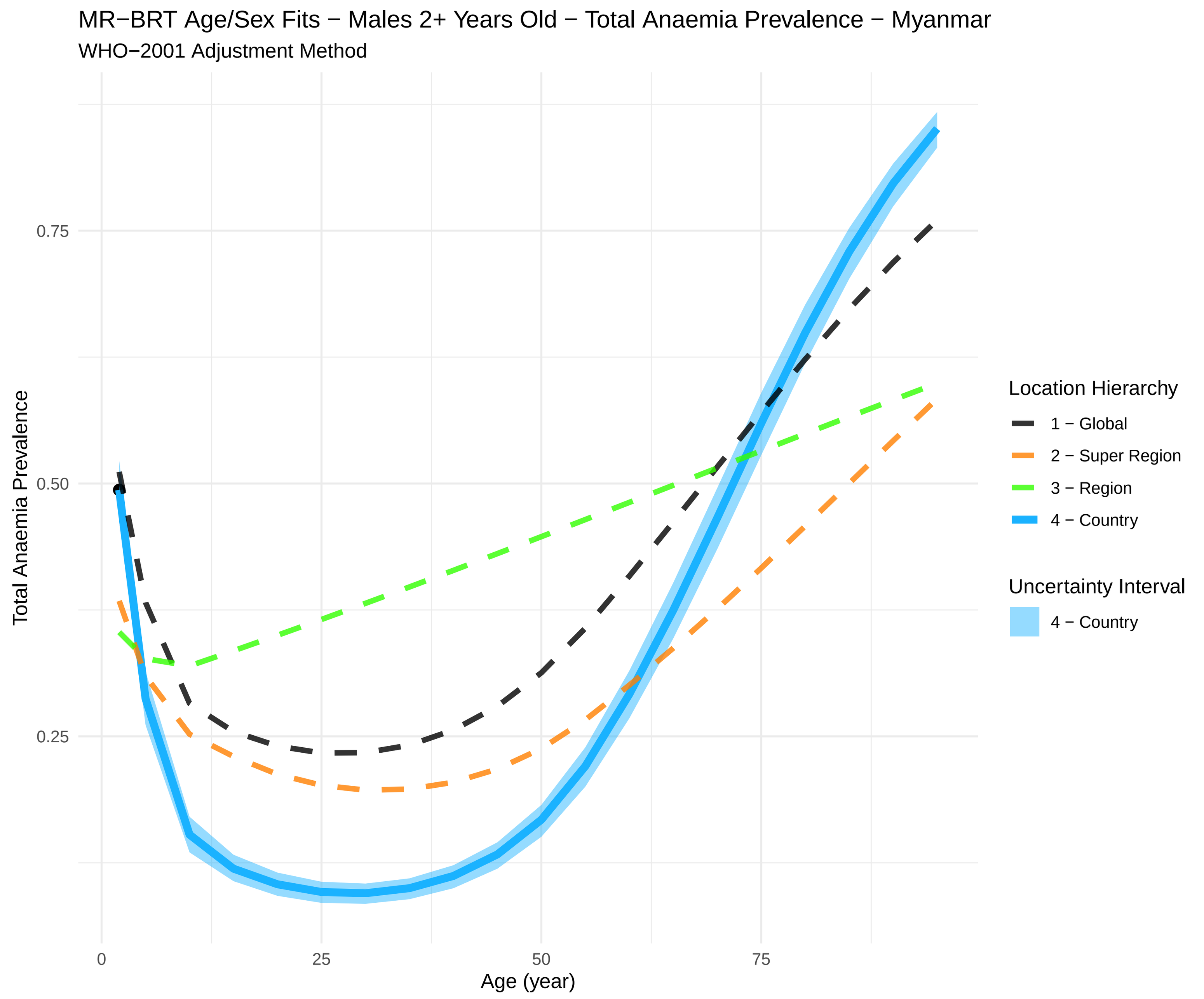
MR−BRT Age/Sex Fits − Males 2+ Years Old − Total Anaemia Prevalence − Myanmar
WHO−2001 Adjustment Method
Total Anaemia Prevalence
Age (year)
Location Hierarchy
1 − Global
2 − Super Region
3 − Region
4 − Country
Uncertainty Interval
4 − Country

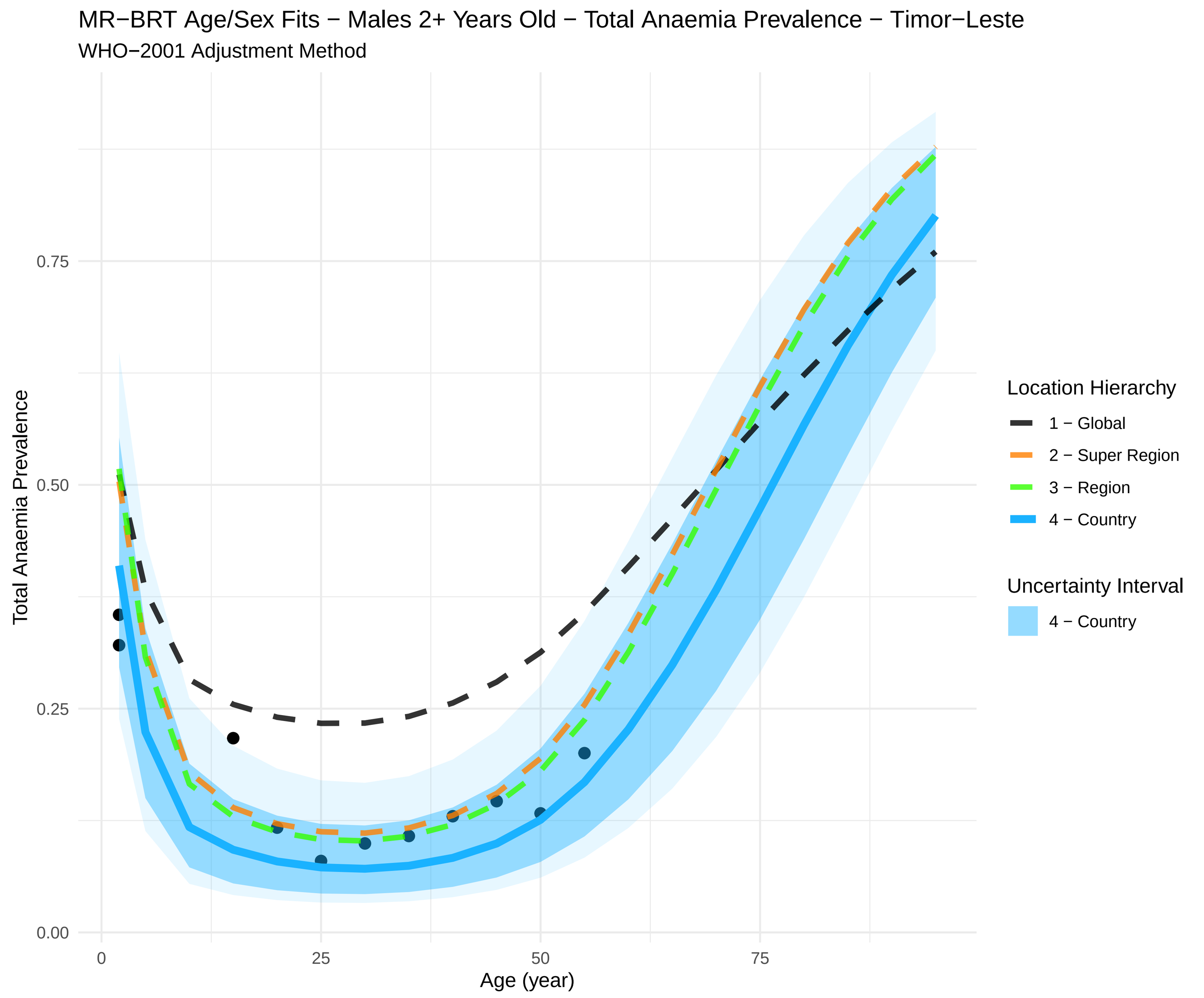
MR−BRT Age/Sex Fits − Males 2+ Years Old − Total Anaemia Prevalence − Timor−Leste
WHO−2001 Adjustment Method
Total Anaemia Prevalence
Age (year)
Location Hierarchy
1 − Global
2 − Super Region
3 − Region
4 − Country
Uncertainty Interval
4 − Country

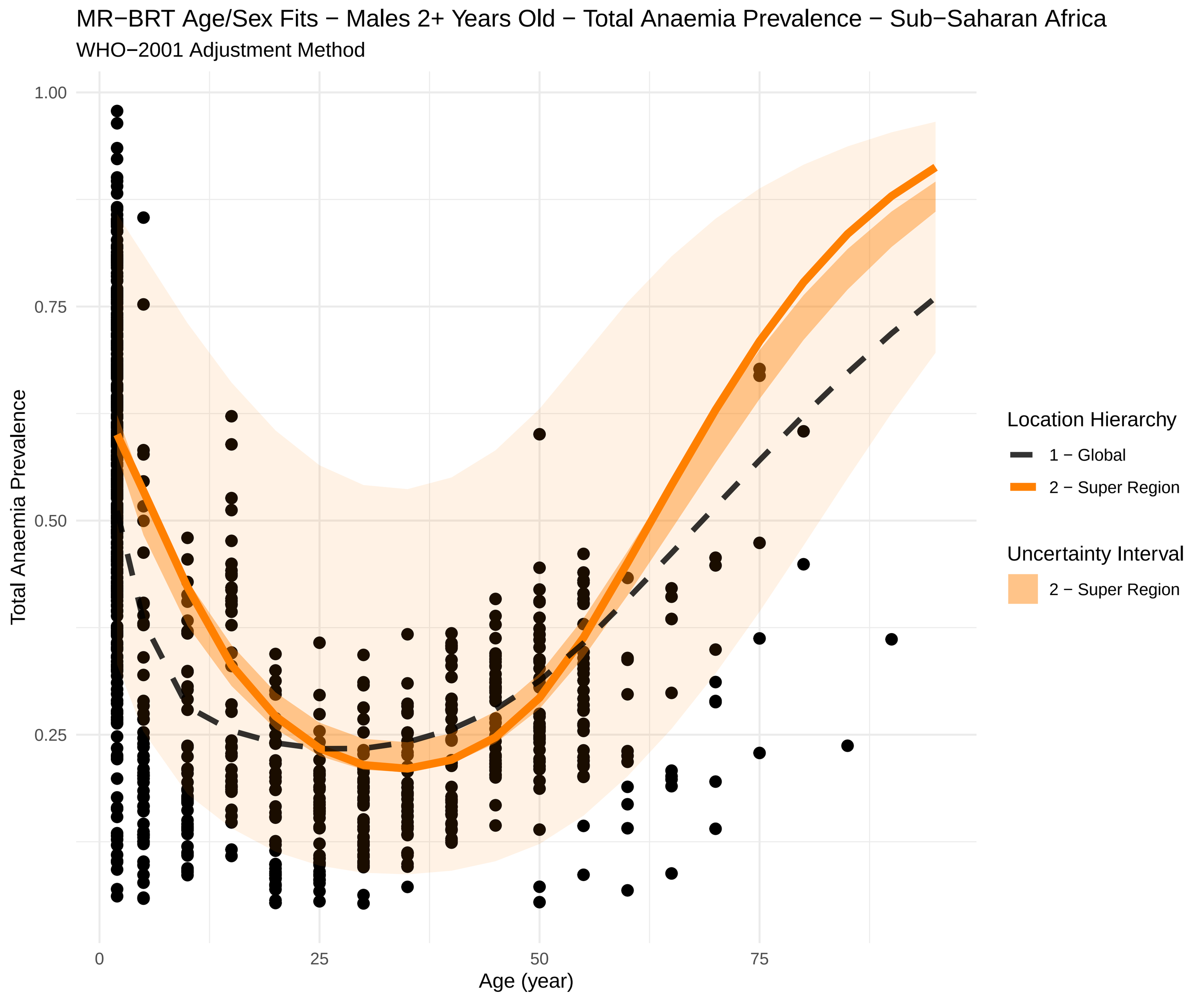
MR−BRT Age/Sex Fits − Males 2+ Years Old − Total Anaemia Prevalence − Sub−Saharan Africa
WHO−2001 Adjustment Method
Total Anaemia Prevalence
Age (year)
Location Hierarchy
1 − Global
2 − Super Region
Uncertainty Interval
2 − Super Region

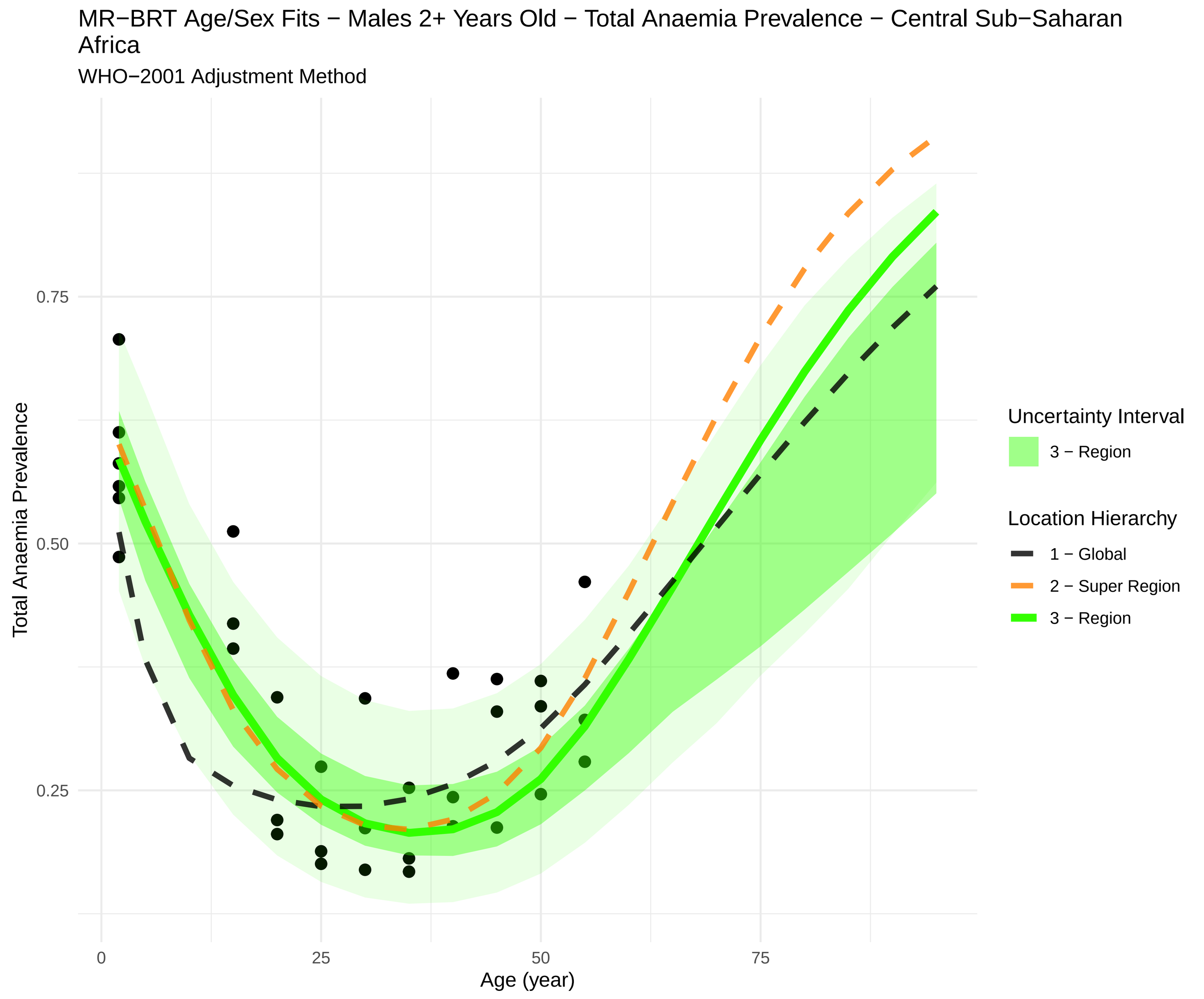
MR−BRT Age/Sex Fits − Males 2+ Years Old − Total Anaemia Prevalence − Central Sub−Saharan Africa
WHO−2001 Adjustment Method
Total Anaemia Prevalence
Age (year)
Uncertainty Interval
3 − Region
Location Hierarchy
1 − Global
2 − Super Region
3 − Region

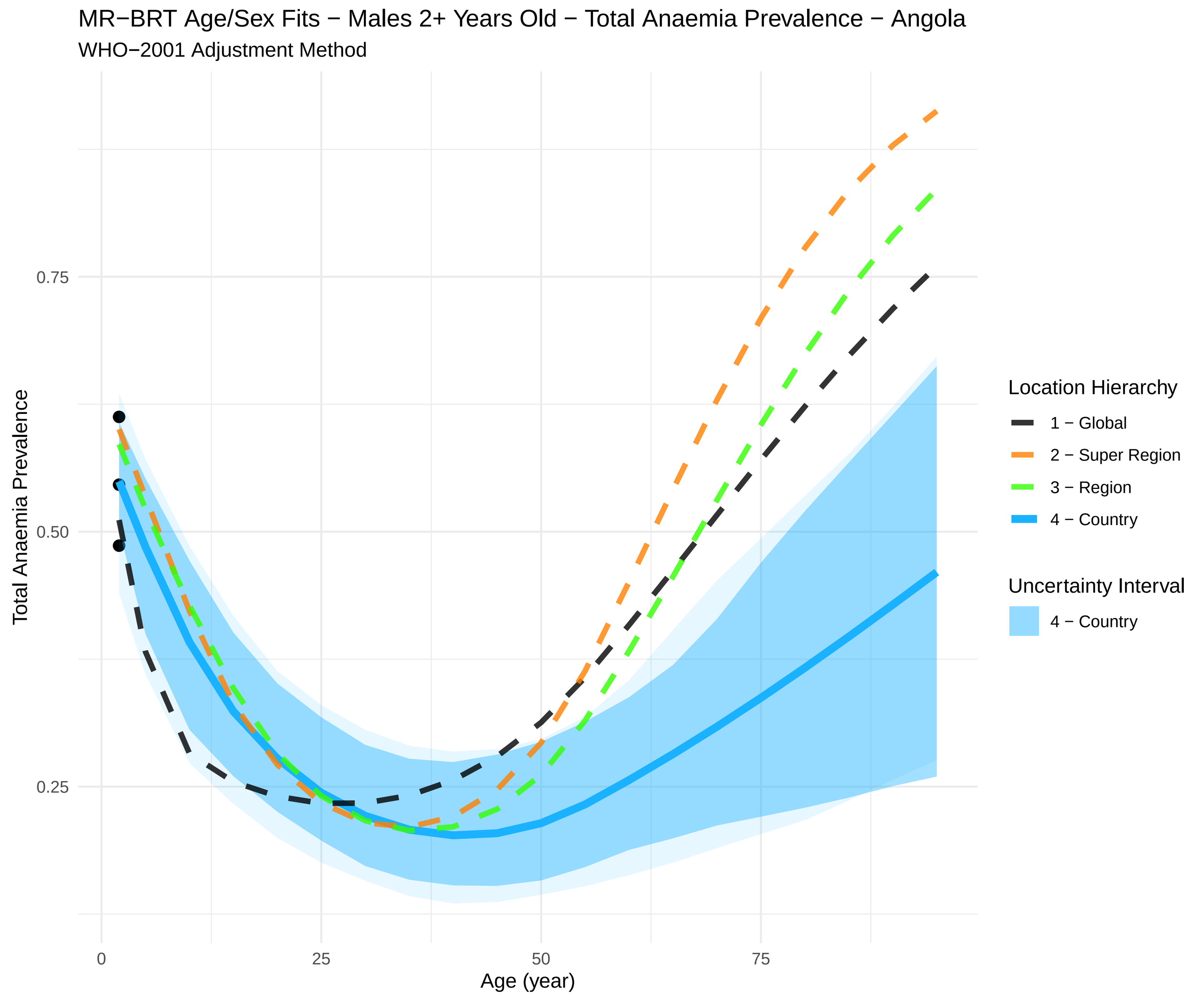
MR−BRT Age/Sex Fits − Males 2+ Years Old − Total Anaemia Prevalence − Angola
WHO−2001 Adjustment Method
Total Anaemia Prevalence
Age (year)
Location Hierarchy
1 − Global
2 − Super Region
3 − Region
4 − Country
Uncertainty Interval
4 − Country

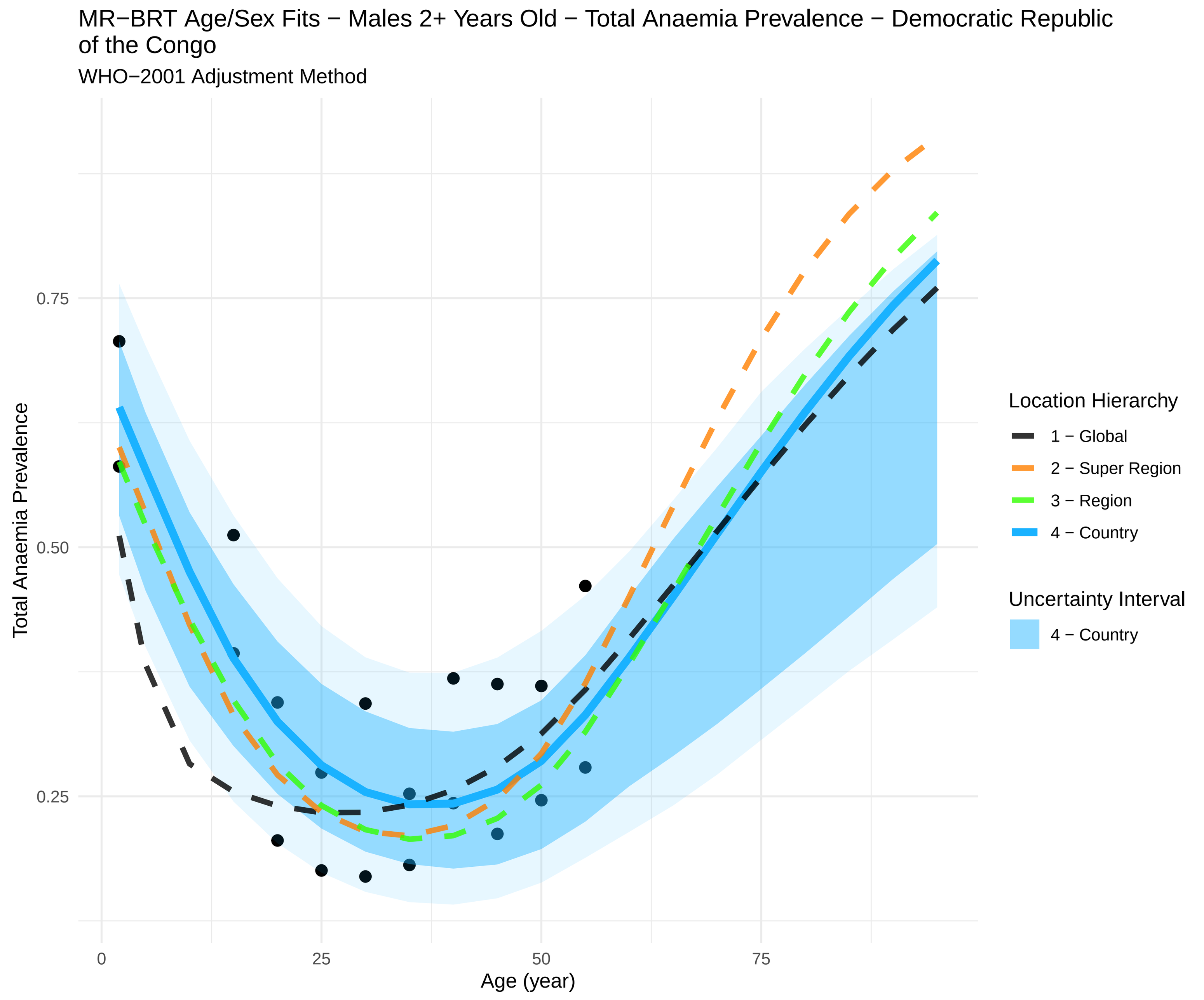
MR−BRT Age/Sex Fits − Males 2+ Years Old − Total Anaemia Prevalence − Democratic Republic of the Congo
WHO−2001 Adjustment Method
Total Anaemia Prevalence
Age (year)
Location Hierarchy
1 − Global
2 − Super Region
3 − Region
4 − Country
Uncertainty Interval
4 − Country

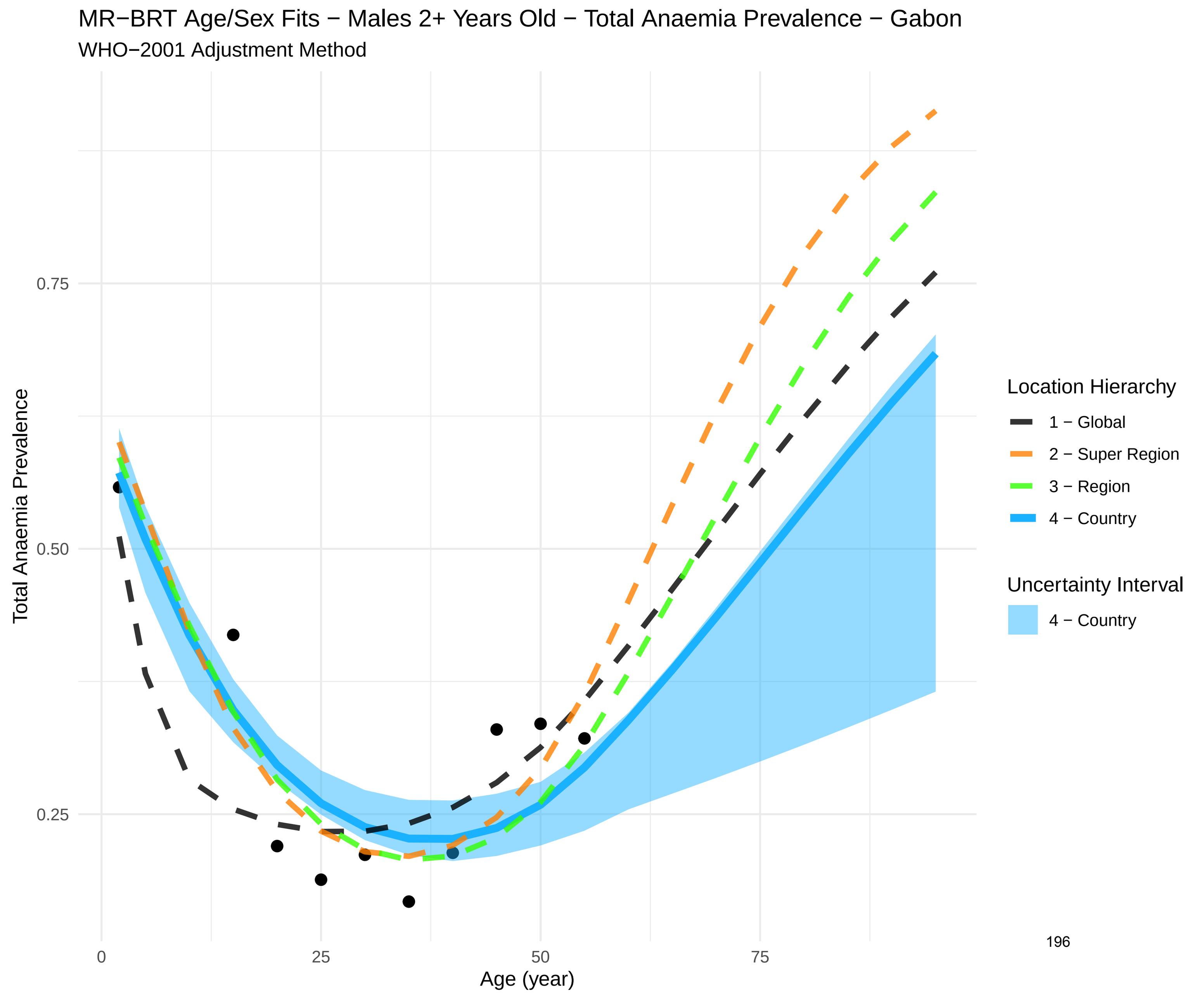
MR−BRT Age/Sex Fits − Males 2+ Years Old − Total Anaemia Prevalence − Gabon
WHO−2001 Adjustment Method
Total Anaemia Prevalence
Age (year)
Location Hierarchy
1 − Global
2 − Super Region
3 − Region
4 − Country
Uncertainty Interval
4 − Country

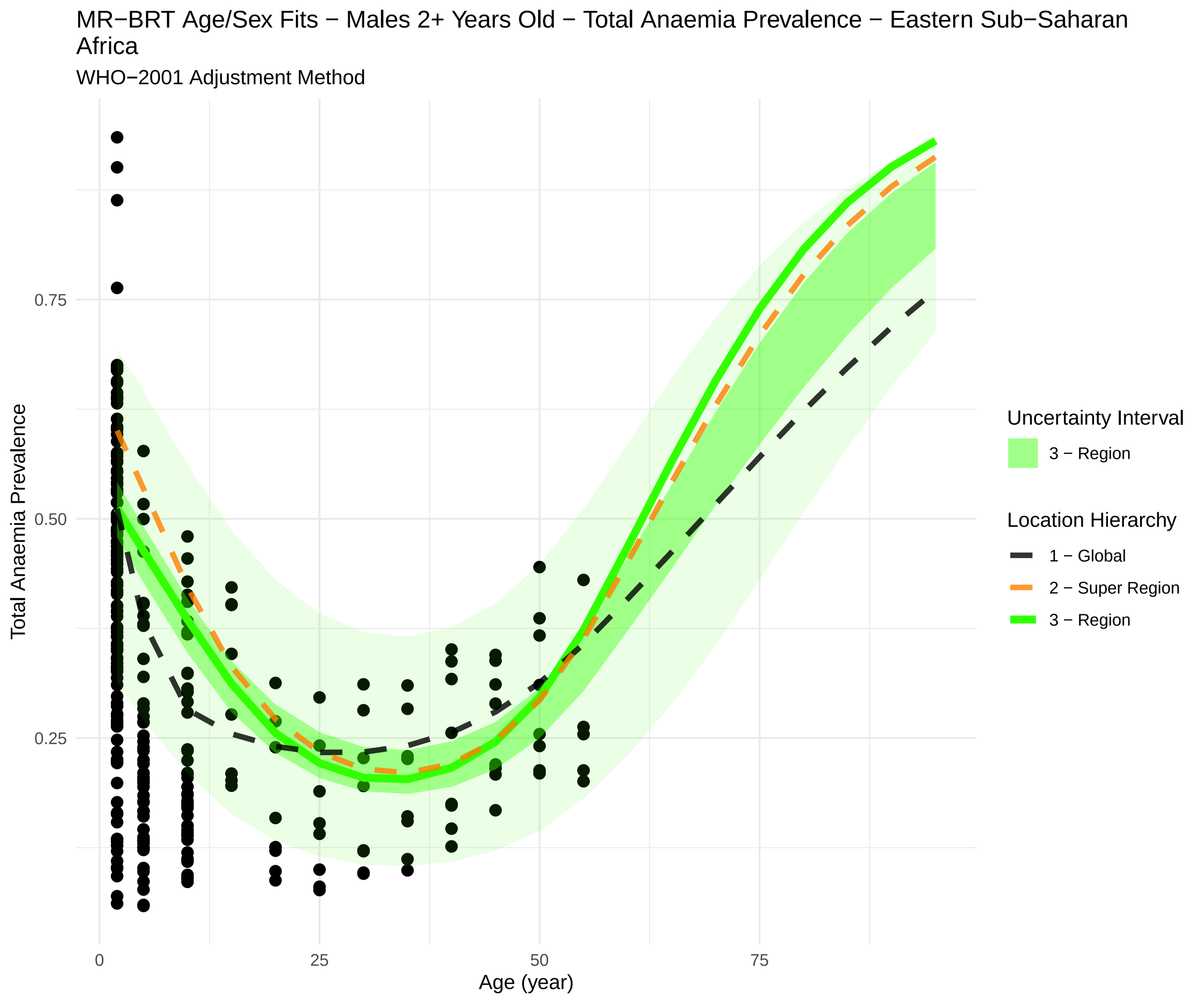
MR−BRT Age/Sex Fits − Males 2+ Years Old − Total Anaemia Prevalence − Eastern Sub−Saharan Africa
WHO−2001 Adjustment Method
Total Anaemia Prevalence
Age (year)
Uncertainty Interval
3 − Region
Location Hierarchy
1 − Global
2 − Super Region
3 − Region

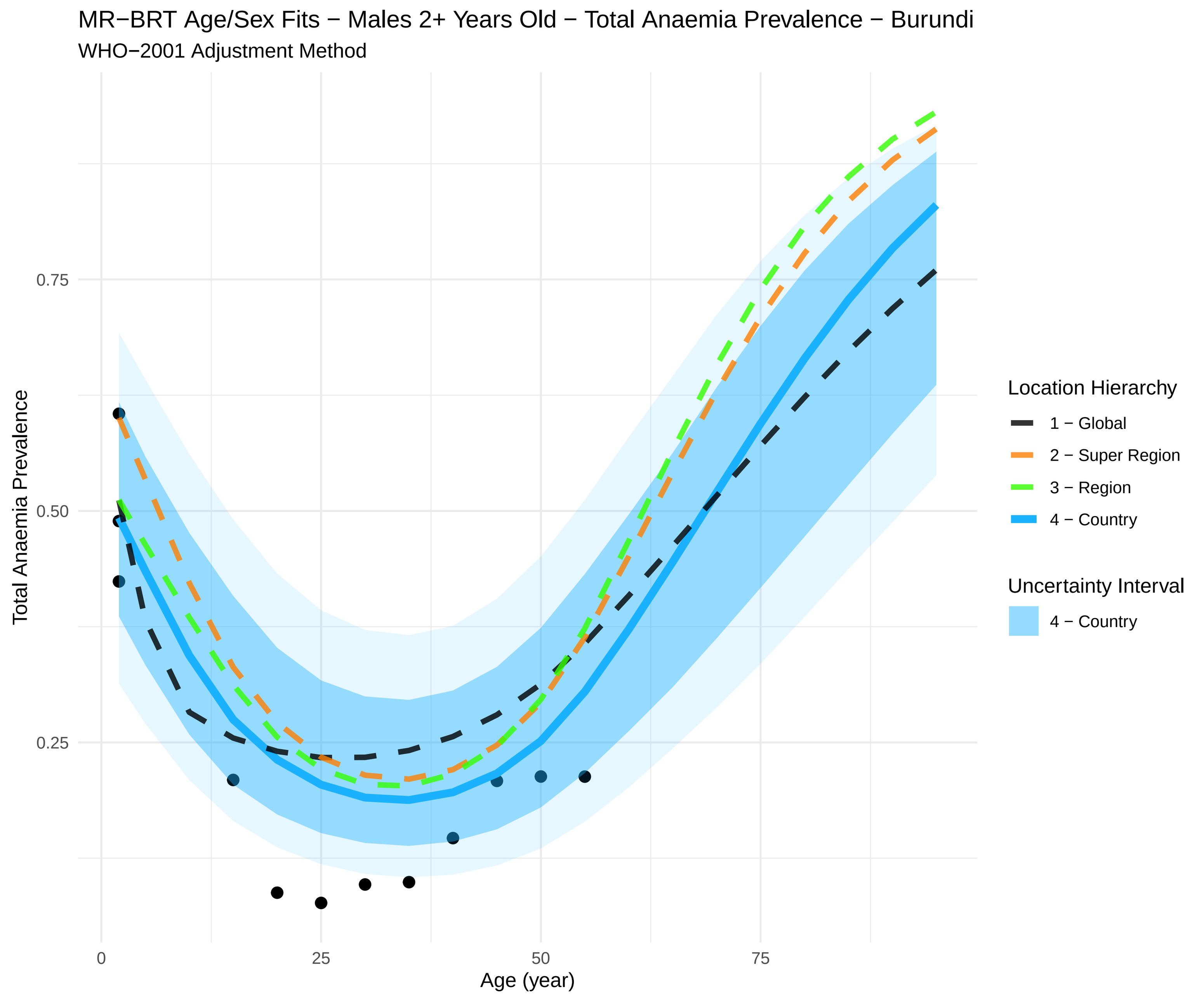
MR−BRT Age/Sex Fits − Males 2+ Years Old − Total Anaemia Prevalence − Burundi
WHO−2001 Adjustment Method
Total Anaemia Prevalence
Age (year)
Location Hierarchy
1 − Global
2 − Super Region
3 − Region
4 − Country
Uncertainty Interval
4 − Country

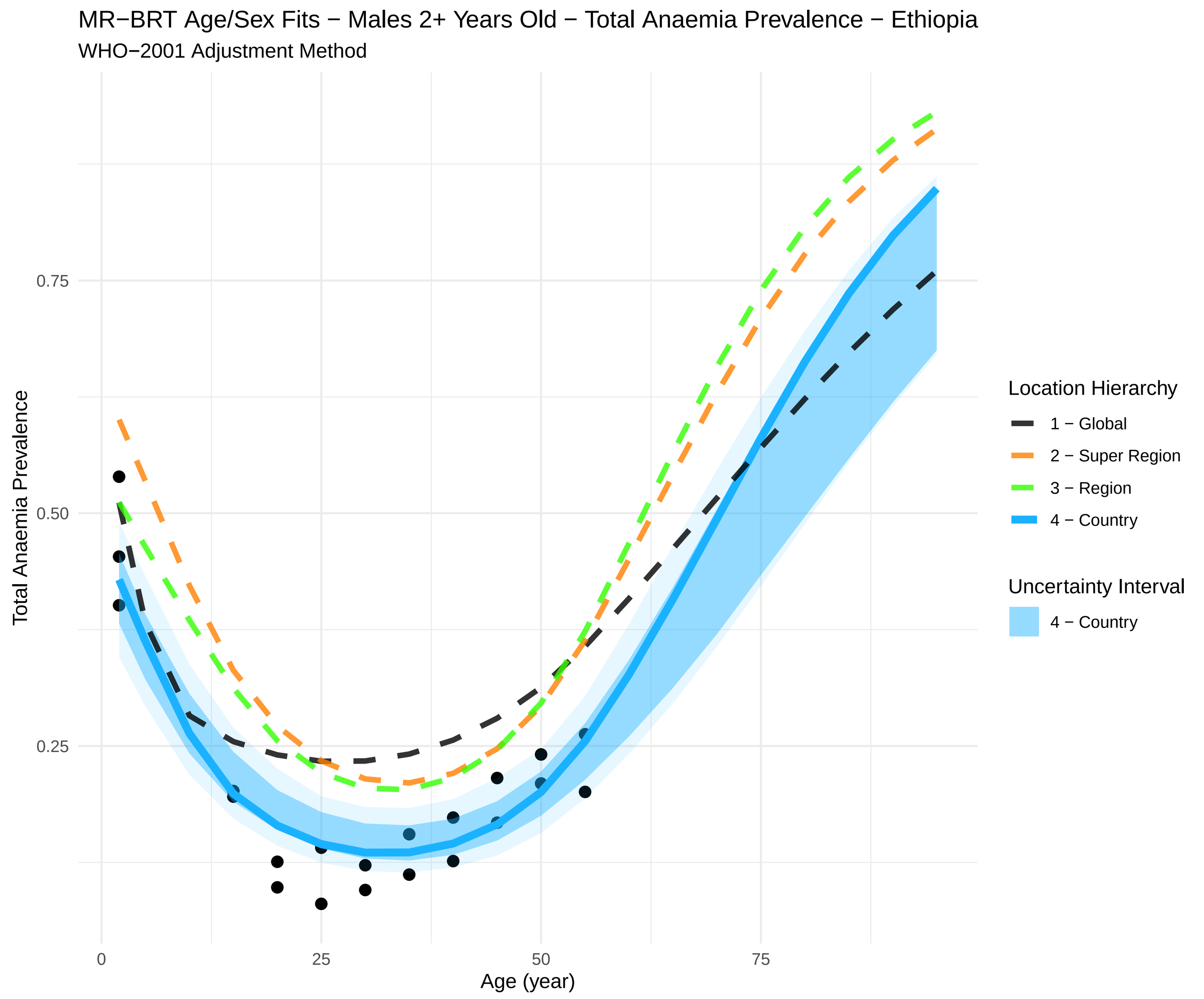
MR−BRT Age/Sex Fits − Males 2+ Years Old − Total Anaemia Prevalence − Ethiopia
WHO−2001 Adjustment Method
Total Anaemia Prevalence
Age (year)
Location Hierarchy
1 − Global
2 − Super Region
3 − Region
4 − Country
Uncertainty Interval
4 − Country

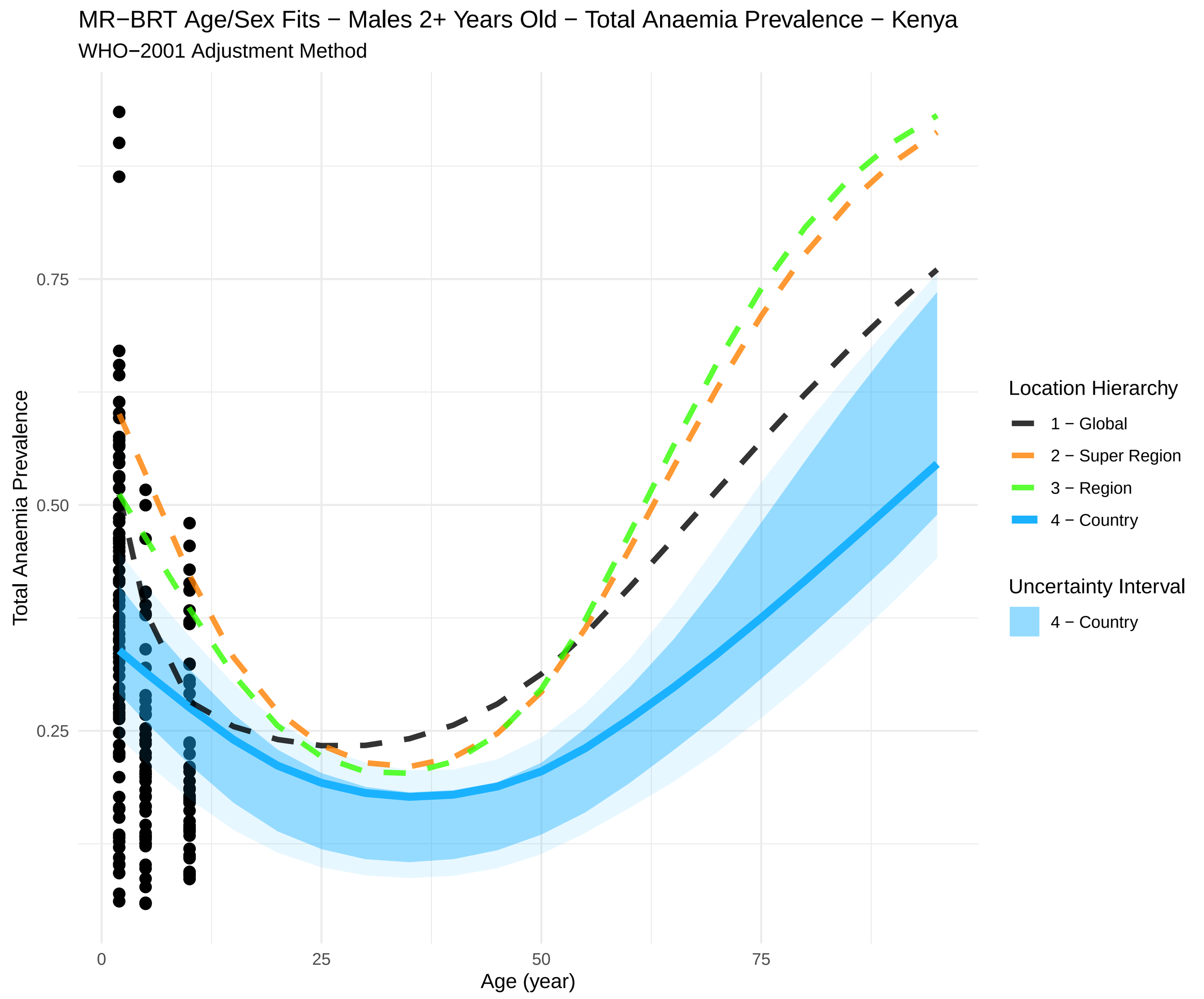
MR−BRT Age/Sex Fits − Males 2+ Years Old − Total Anaemia Prevalence − Kenya
WHO−2001 Adjustment Method
Total Anaemia Prevalence
Age (year)
Location Hierarchy
1 − Global
2 − Super Region
3 − Region
4 − Country
Uncertainty Interval
4 − Country

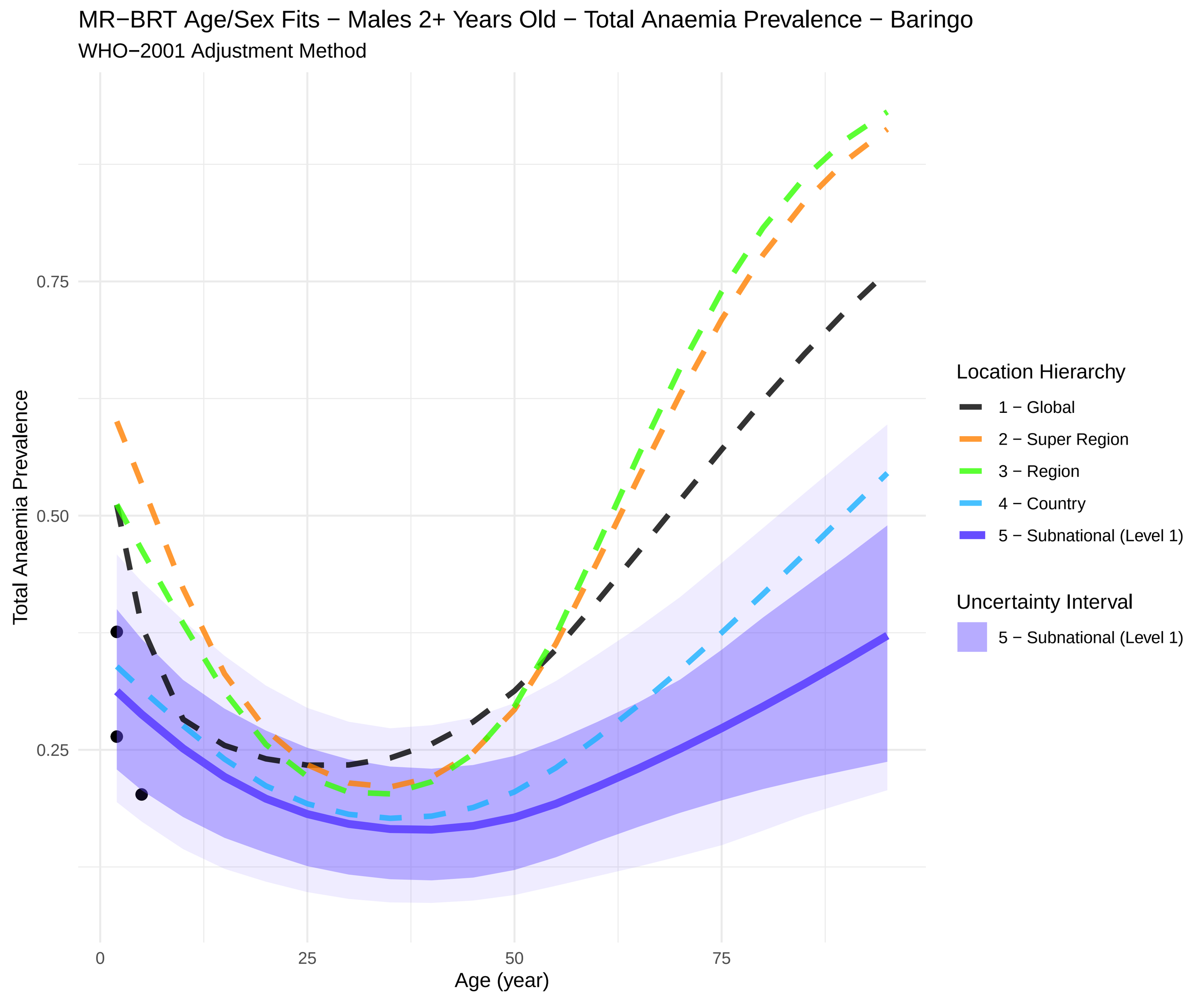
MR−BRT Age/Sex Fits − Males 2+ Years Old − Total Anaemia Prevalence − Baringo
WHO−2001 Adjustment Method
Total Anaemia Prevalence
Age (year)
Location Hierarchy
1 − Global
2 − Super Region
3 − Region
4 − Country
5 − Subnational (Level 1)
Uncertainty Interval
5 − Subnational (Level 1)

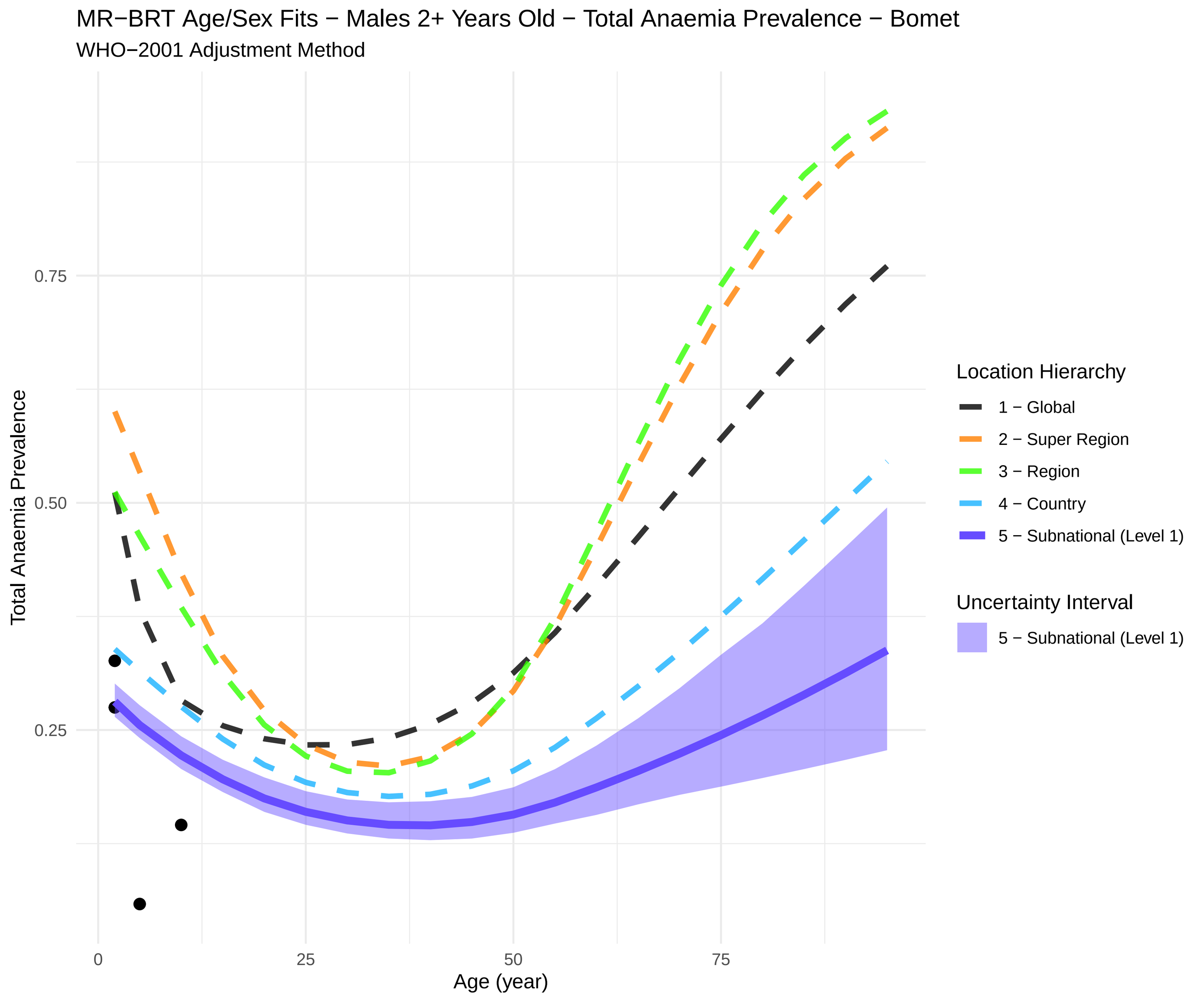
MR−BRT Age/Sex Fits − Males 2+ Years Old − Total Anaemia Prevalence − Bomet
WHO−2001 Adjustment Method
Total Anaemia Prevalence
Age (year)
Location Hierarchy
1 − Global
2 − Super Region
3 − Region
4 − Country
5 − Subnational (Level 1)
Uncertainty Interval
5 − Subnational (Level 1)

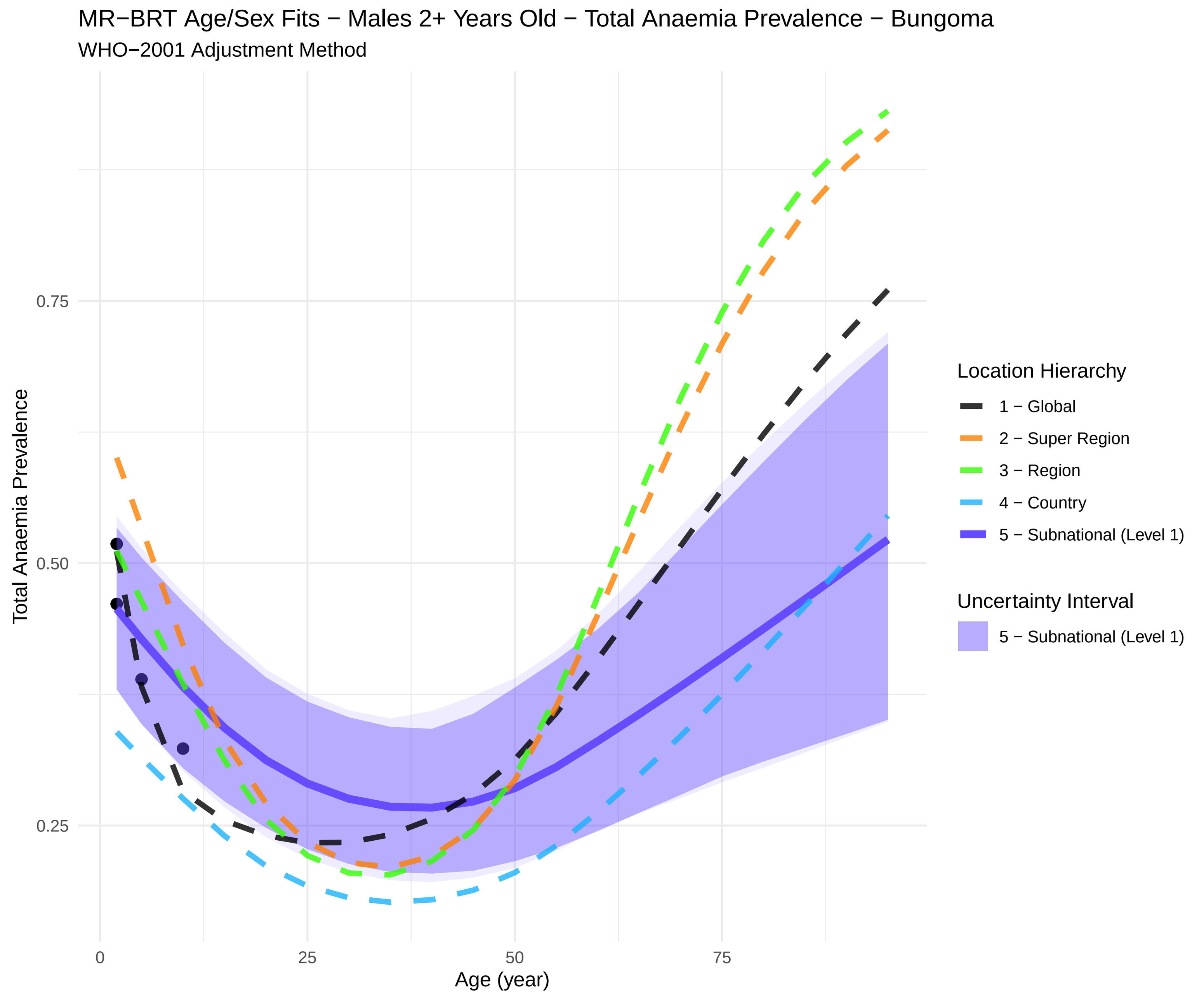
MR−BRT Age/Sex Fits − Males 2+ Years Old − Total Anaemia Prevalence − Bungoma
WHO−2001 Adjustment Method
Total Anaemia Prevalence
Age (year)
Location Hierarchy
1 − Global
2 − Super Region
3 − Region
4 − Country
5 − Subnational (Level 1)
Uncertainty Interval
5 − Subnational (Level 1)

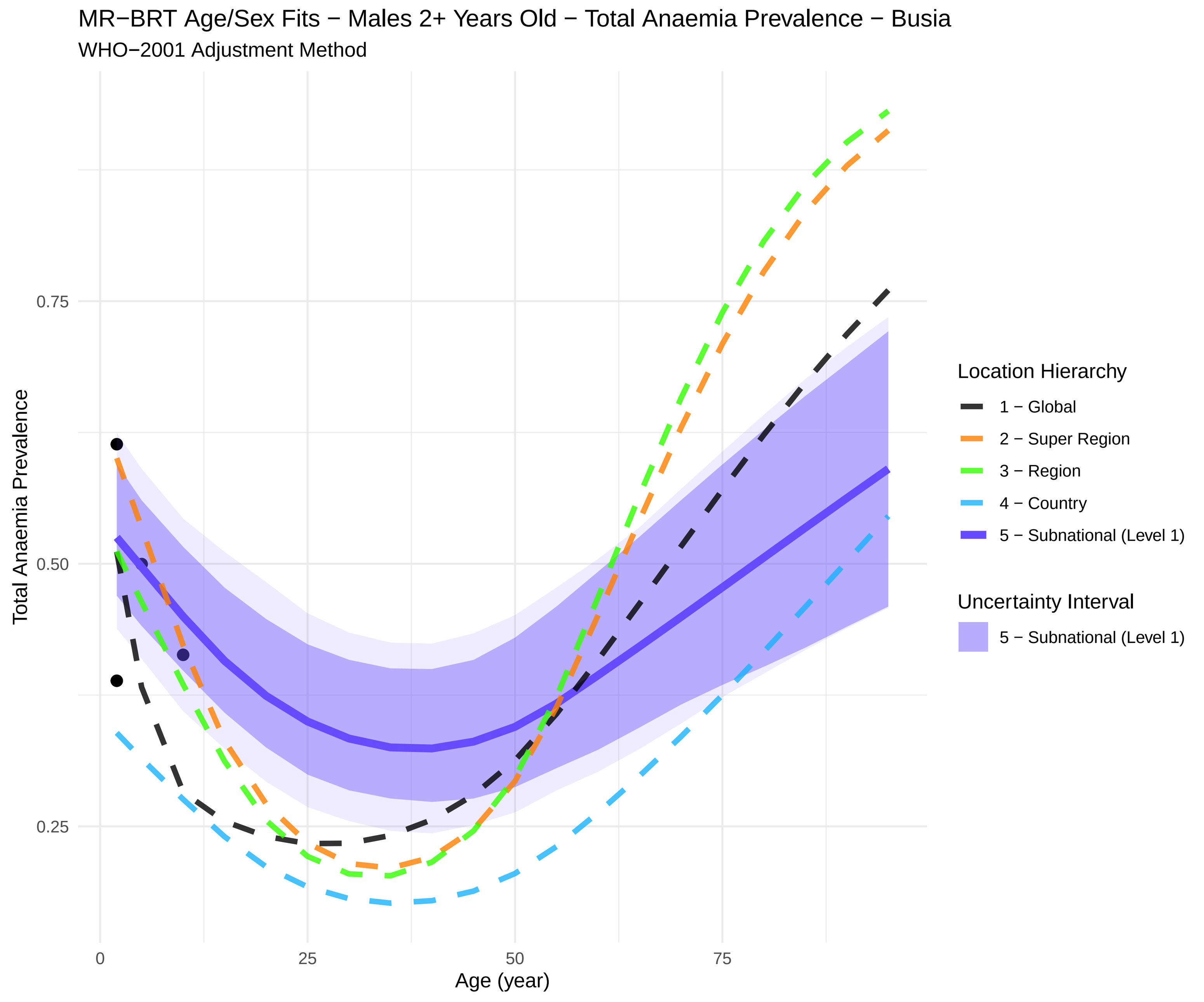
MR−BRT Age/Sex Fits − Males 2+ Years Old − Total Anaemia Prevalence − Busia
WHO−2001 Adjustment Method
Total Anaemia Prevalence
Age (year)
Location Hierarchy
1 − Global
2 − Super Region
3 − Region
4 − Country
5 − Subnational (Level 1)
Uncertainty Interval
5 − Subnational (Level 1)

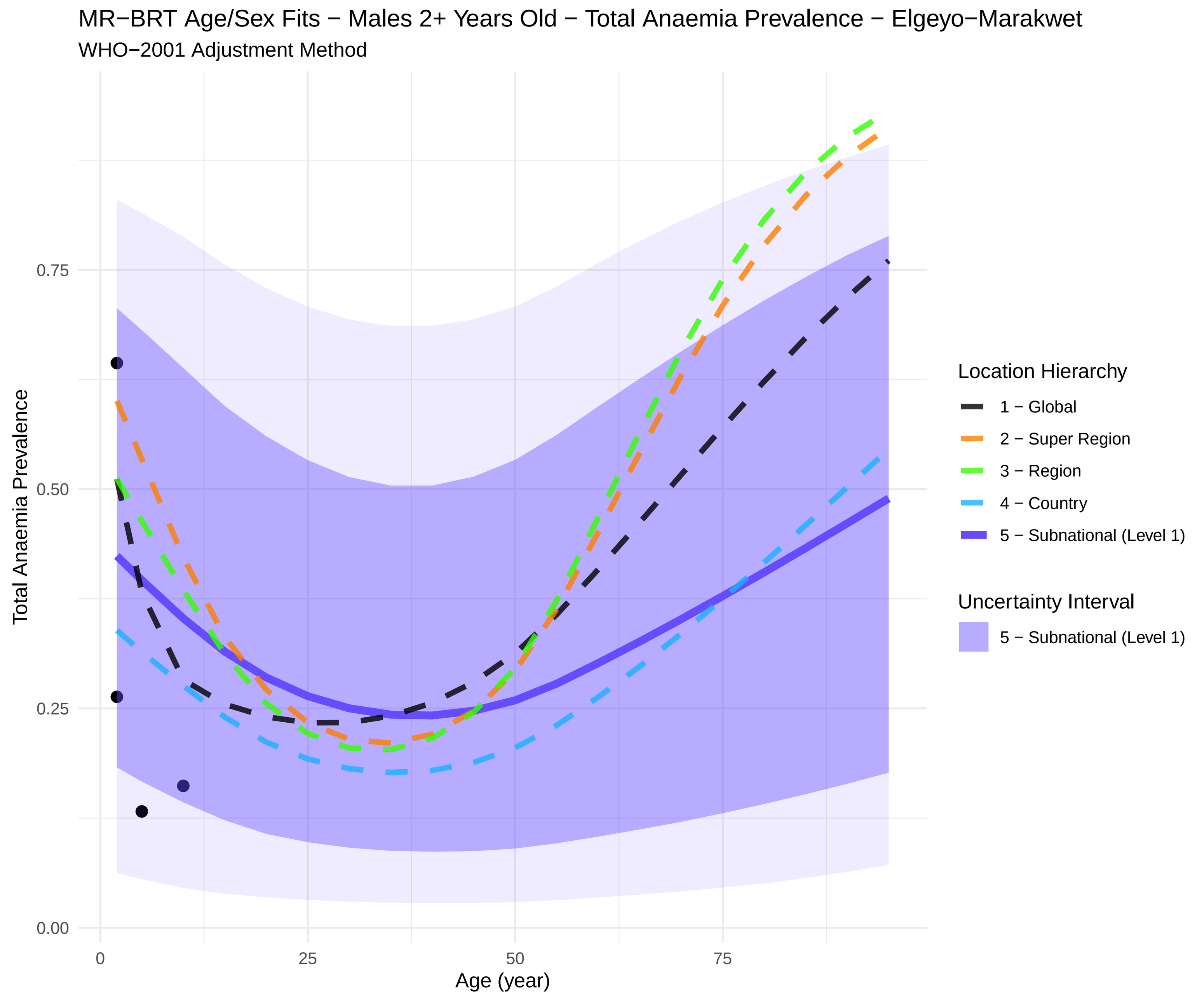
MR−BRT Age/Sex Fits − Males 2+ Years Old − Total Anaemia Prevalence − Elgeyo−Marakwet
WHO−2001 Adjustment Method
Total Anaemia Prevalence
Age (year)
Location Hierarchy
1 − Global
2 − Super Region
3 − Region
4 − Country
5 − Subnational (Level 1)
Uncertainty Interval
5 − Subnational (Level 1)

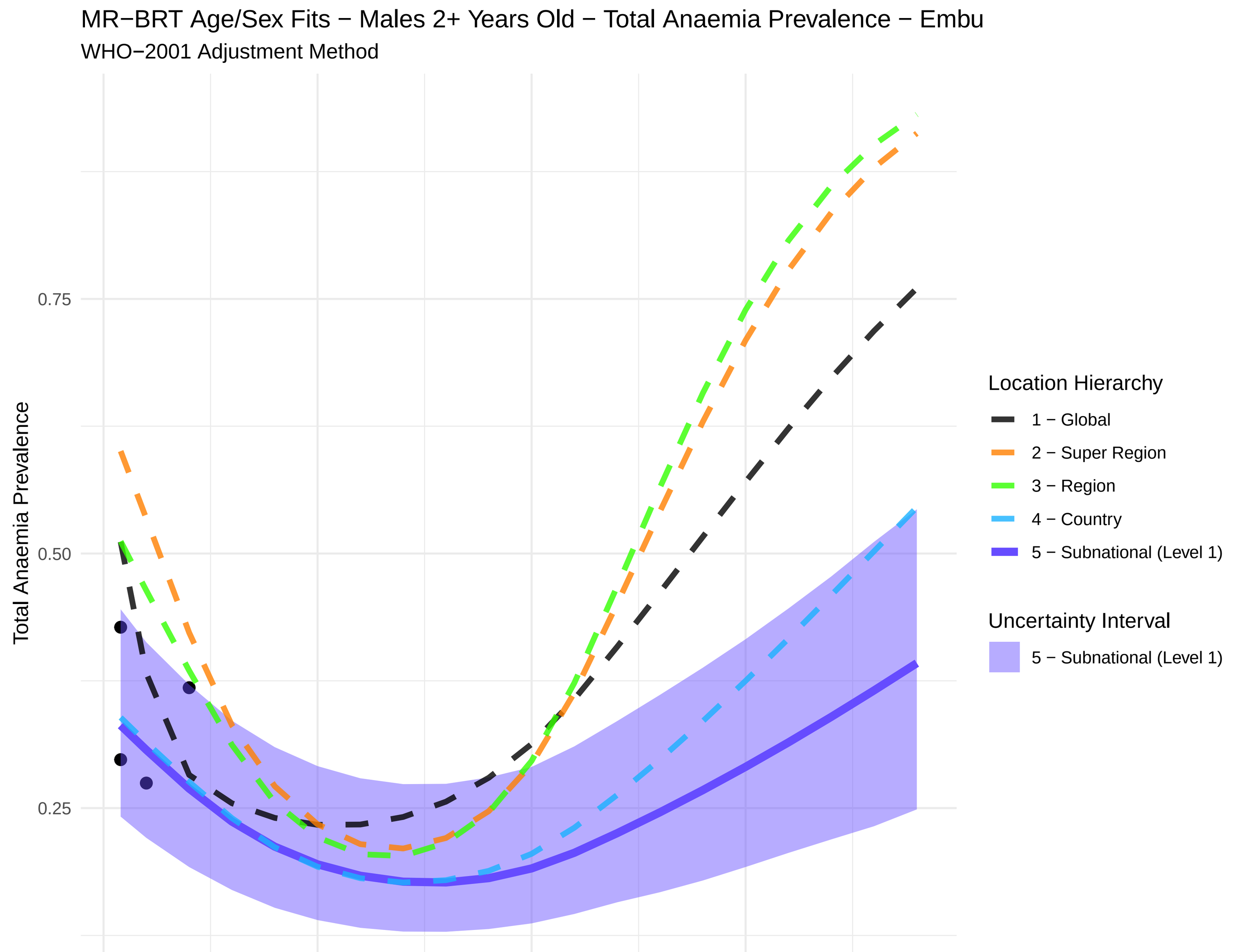
MR−BRT Age/Sex Fits − Males 2+ Years Old − Total Anaemia Prevalence − Embu
WHO−2001 Adjustment Method
Total Anaemia Prevalence
Age (year)
Location Hierarchy
1 − Global
2 − Super Region
3 − Region
4 − Country
5 − Subnational (Level 1)
Uncertainty Interval
5 − Subnational (Level 1)

MR−BRT Age/Sex Fits − Males 2+ Years Old − Total Anaemia Prevalence − Garissa
WHO−2001 Adjustment Method

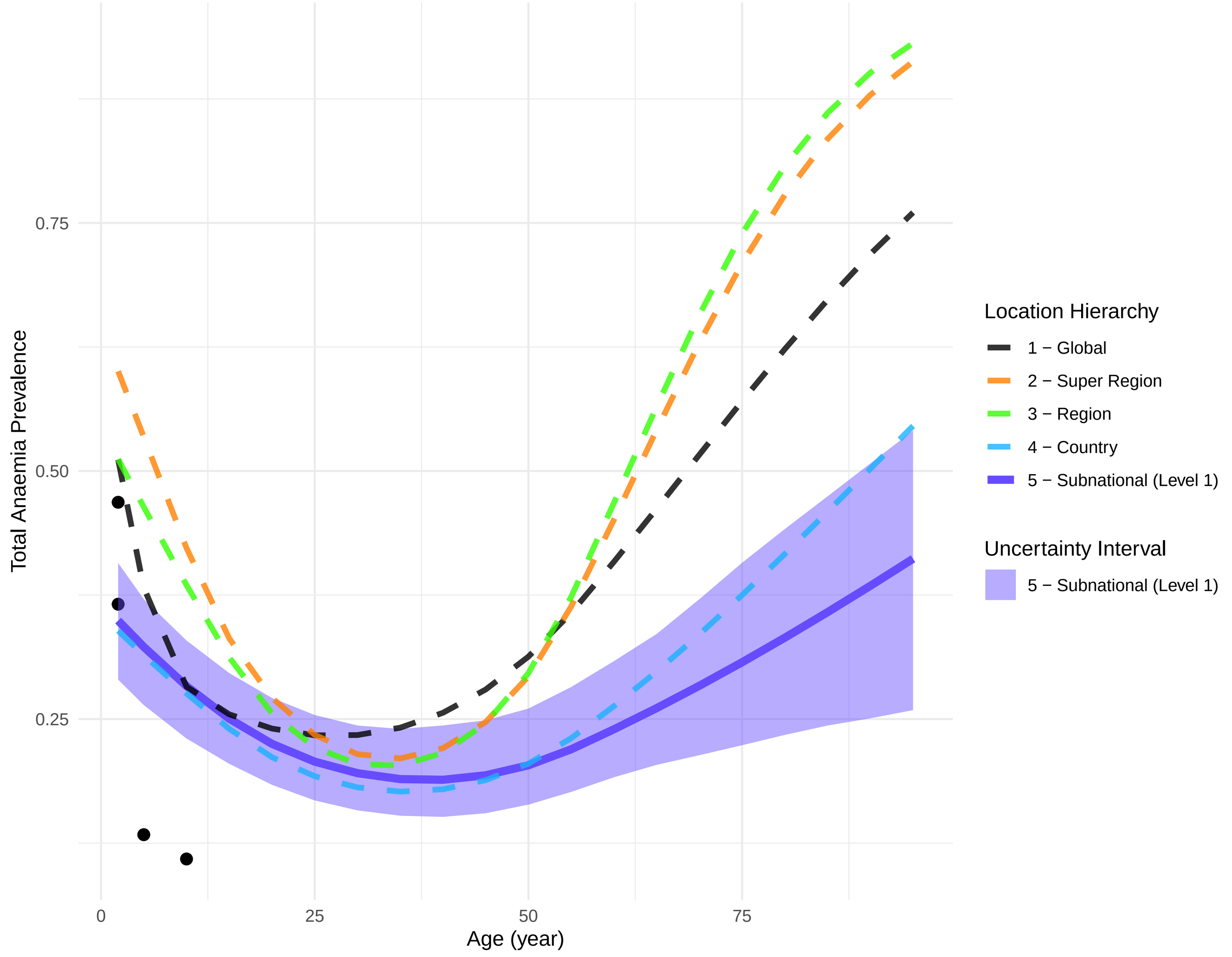

MR−BRT Age/Sex Fits − Males 2+ Years Old − Total Anaemia Prevalence − Homa Bay
WHO−2001 Adjustment Method

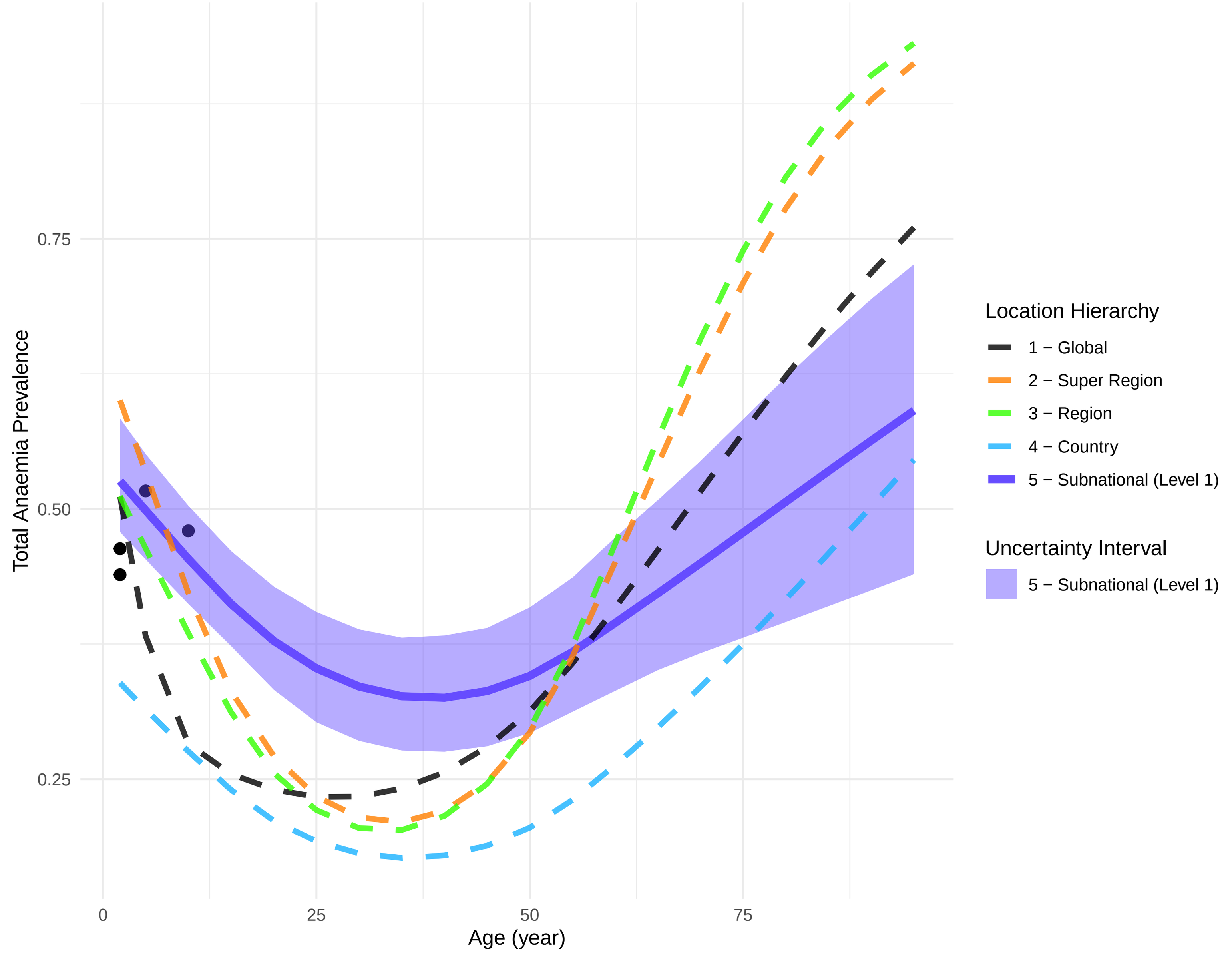

MR−BRT Age/Sex Fits − Males 2+ Years Old − Total Anaemia Prevalence − Isiolo
WHO−2001 Adjustment Method

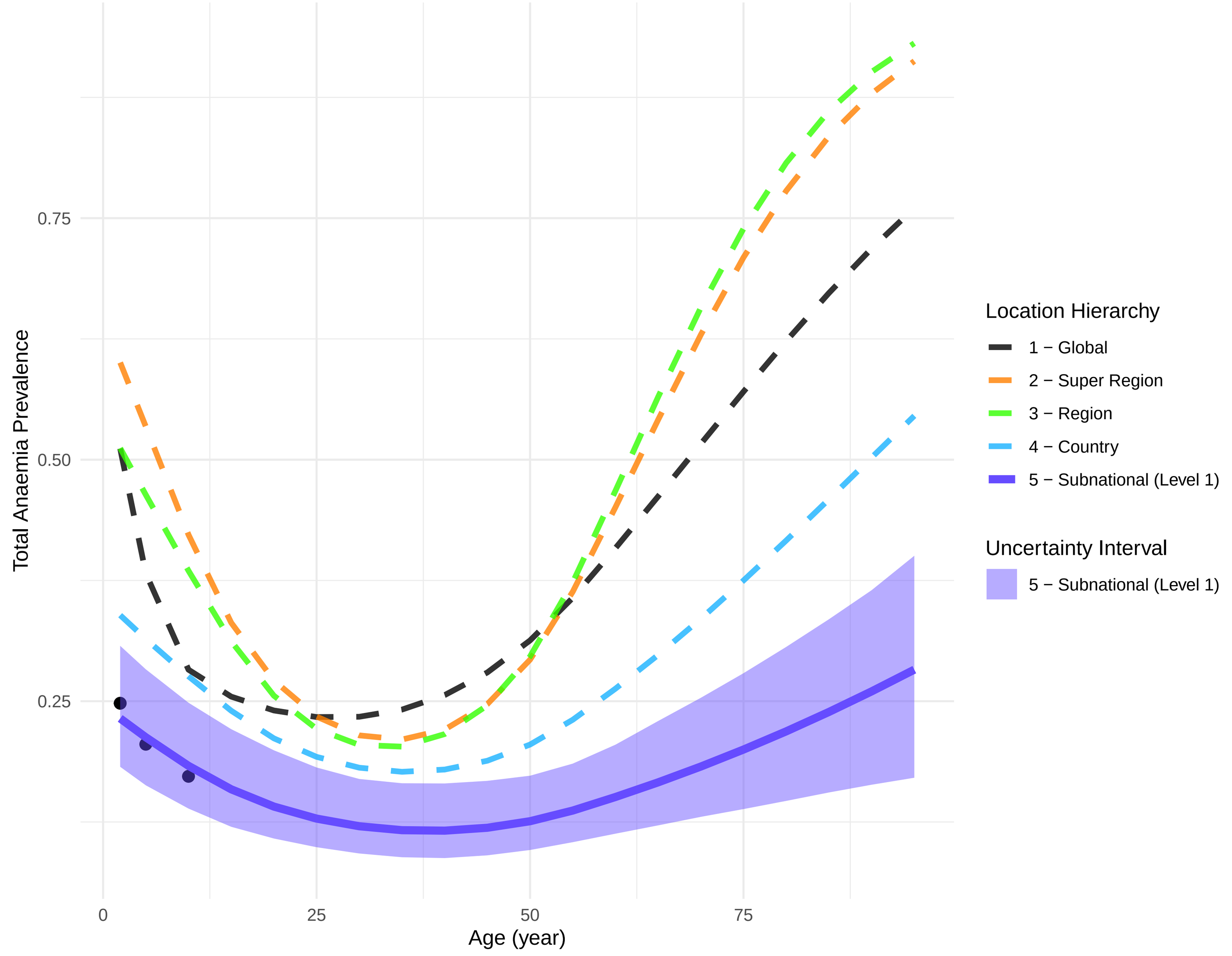

MR−BRT Age/Sex Fits − Males 2+ Years Old − Total Anaemia Prevalence − Kajiado
WHO−2001 Adjustment Method

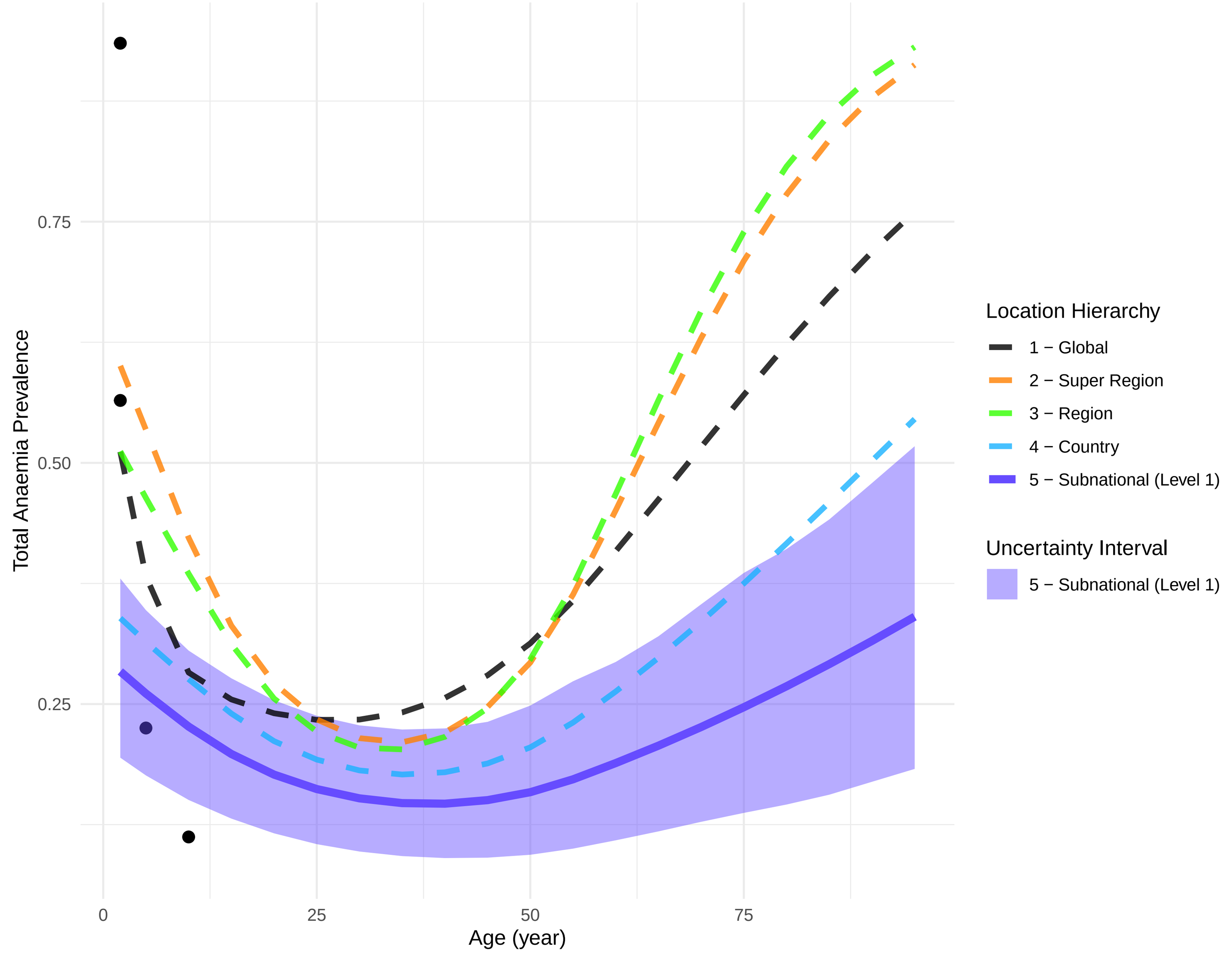

MR−BRT Age/Sex Fits − Males 2+ Years Old − Total Anaemia Prevalence − Kakamega
WHO−2001 Adjustment Method

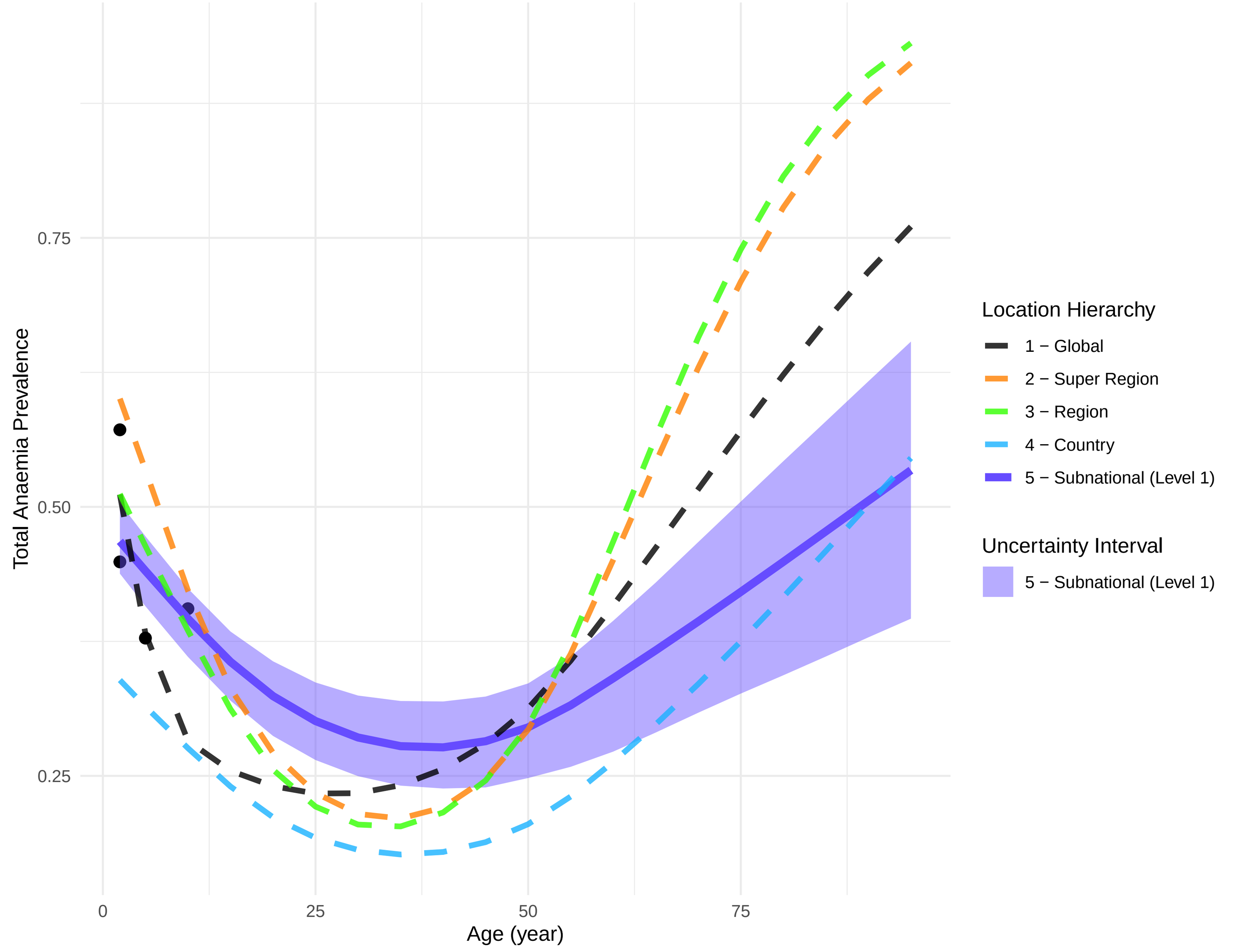

MR−BRT Age/Sex Fits − Males 2+ Years Old − Total Anaemia Prevalence − Kericho
WHO−2001 Adjustment Method

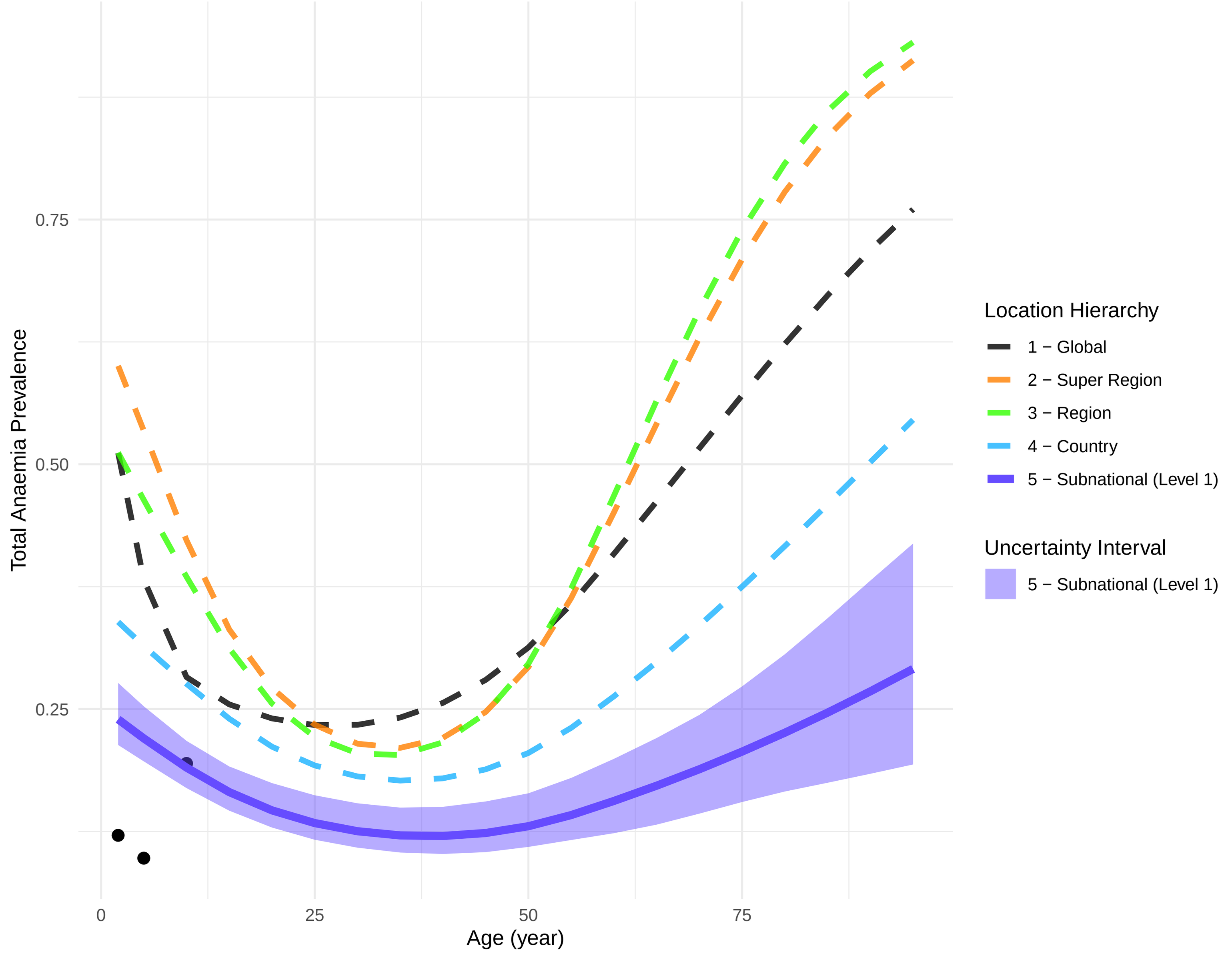

MR−BRT Age/Sex Fits − Males 2+ Years Old − Total Anaemia Prevalence − Kiambu
WHO−2001 Adjustment Method

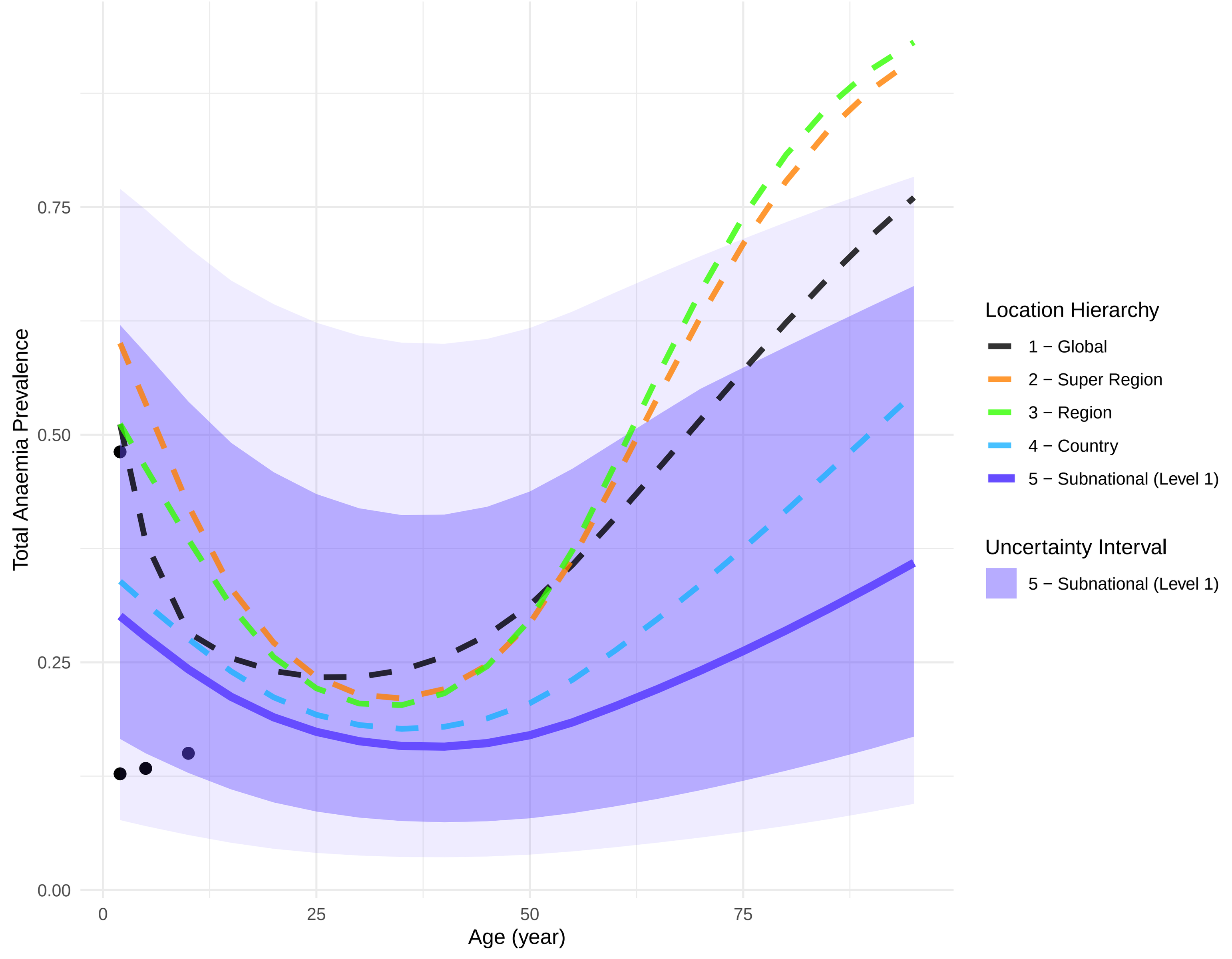

MR−BRT Age/Sex Fits − Males 2+ Years Old − Total Anaemia Prevalence − Kilifi
WHO−2001 Adjustment Method

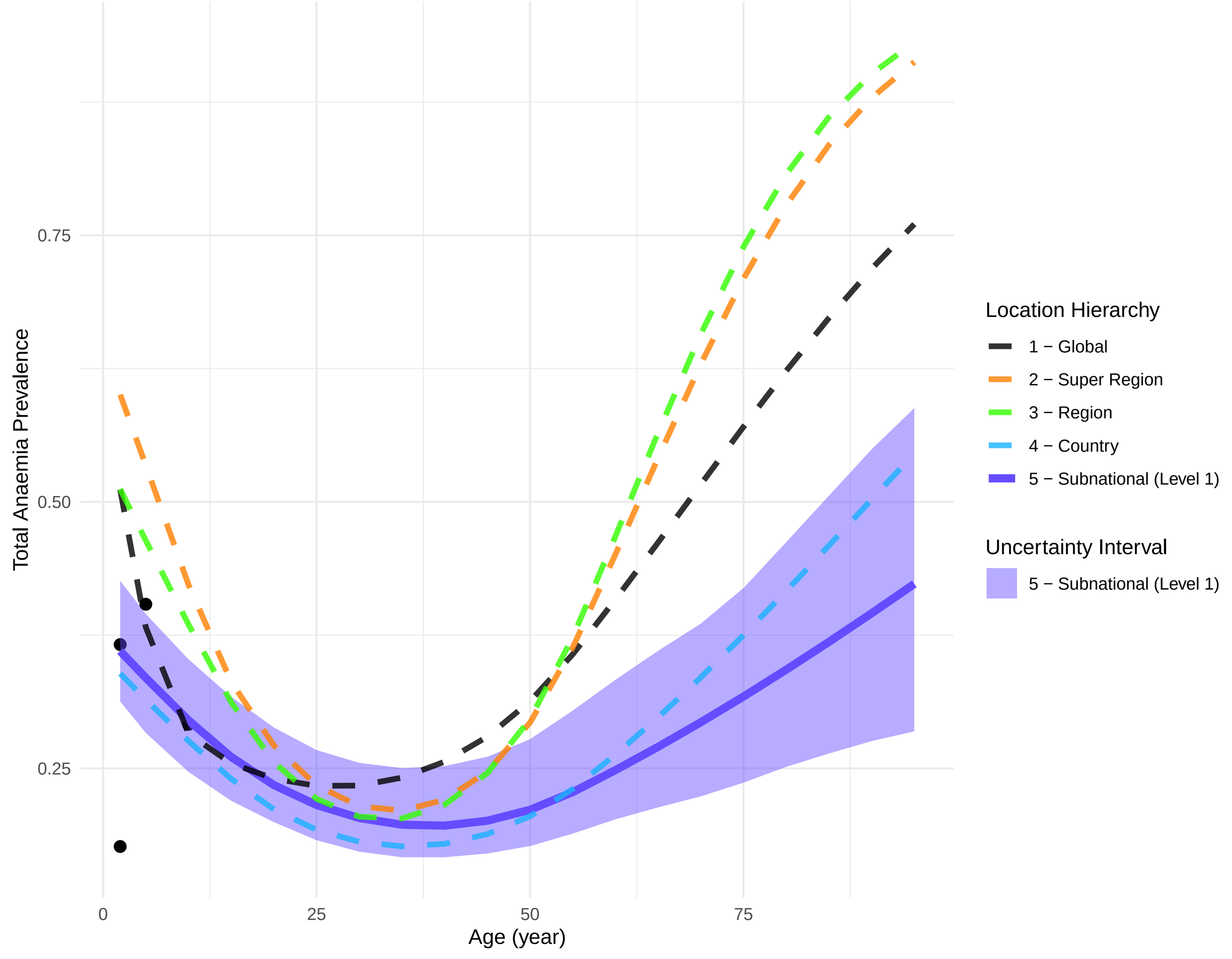

MR−BRT Age/Sex Fits − Males 2+ Years Old − Total Anaemia Prevalence − Kirinyaga
WHO−2001 Adjustment Method

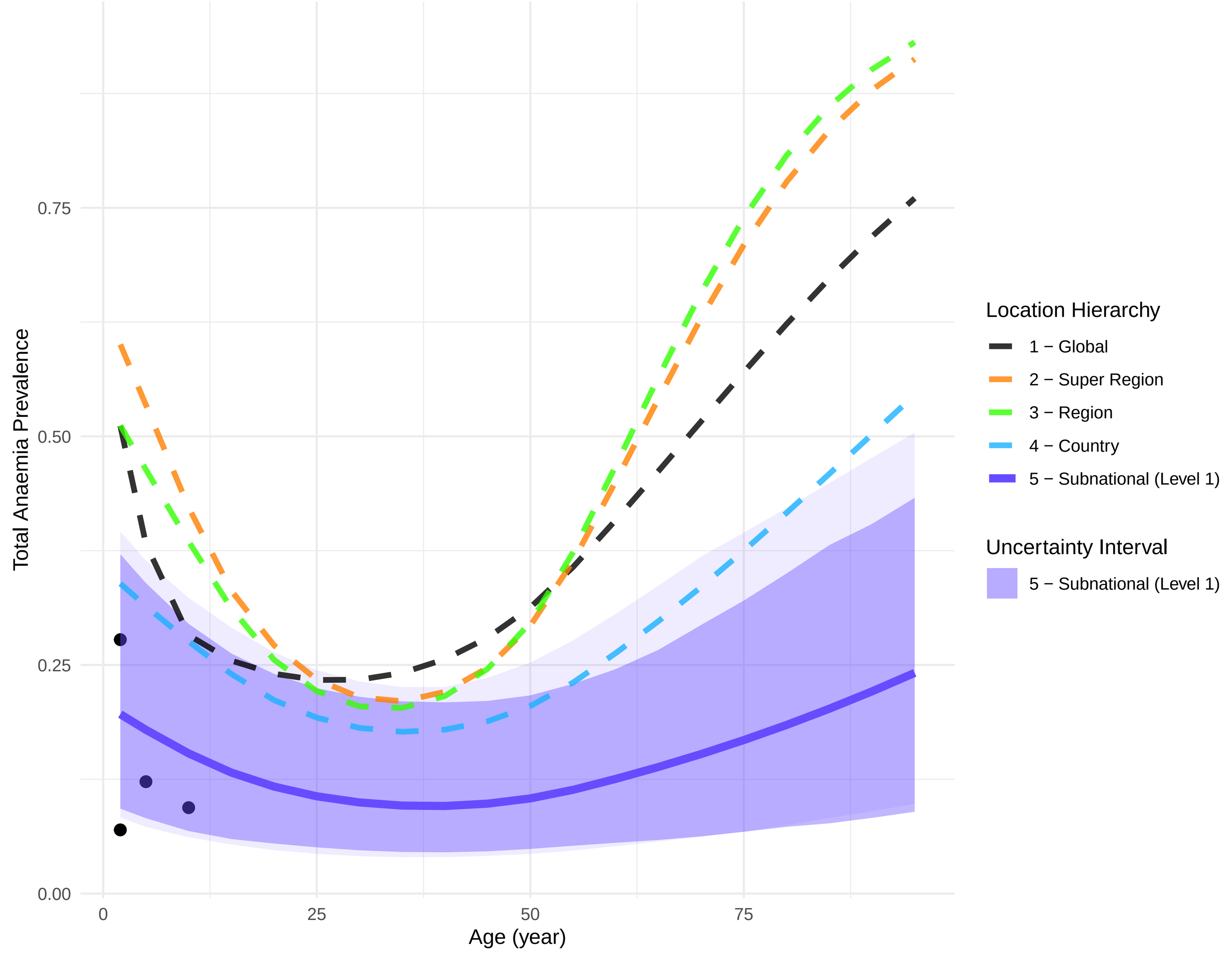

MR−BRT Age/Sex Fits − Males 2+ Years Old − Total Anaemia Prevalence − Kisii
WHO−2001 Adjustment Method

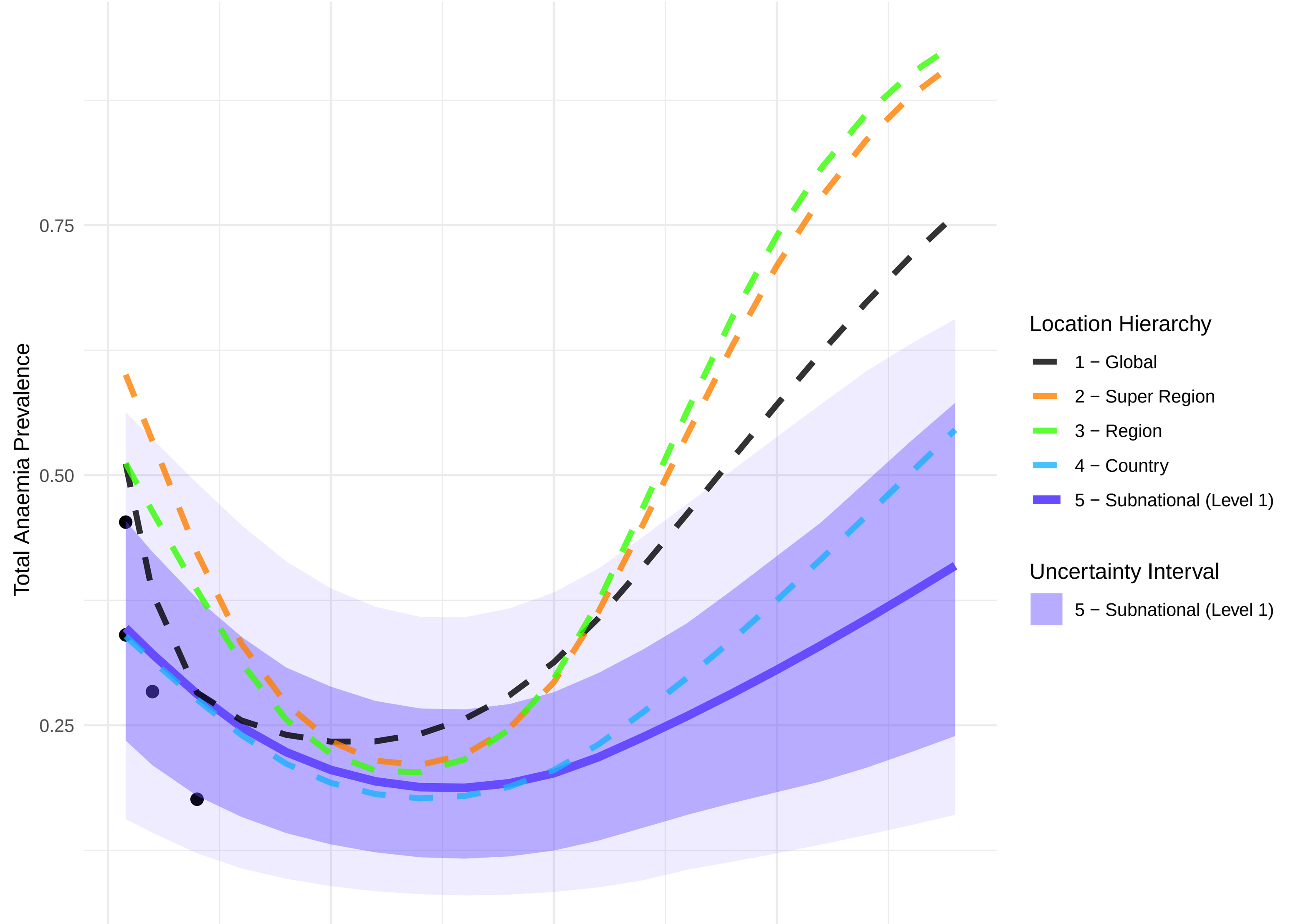

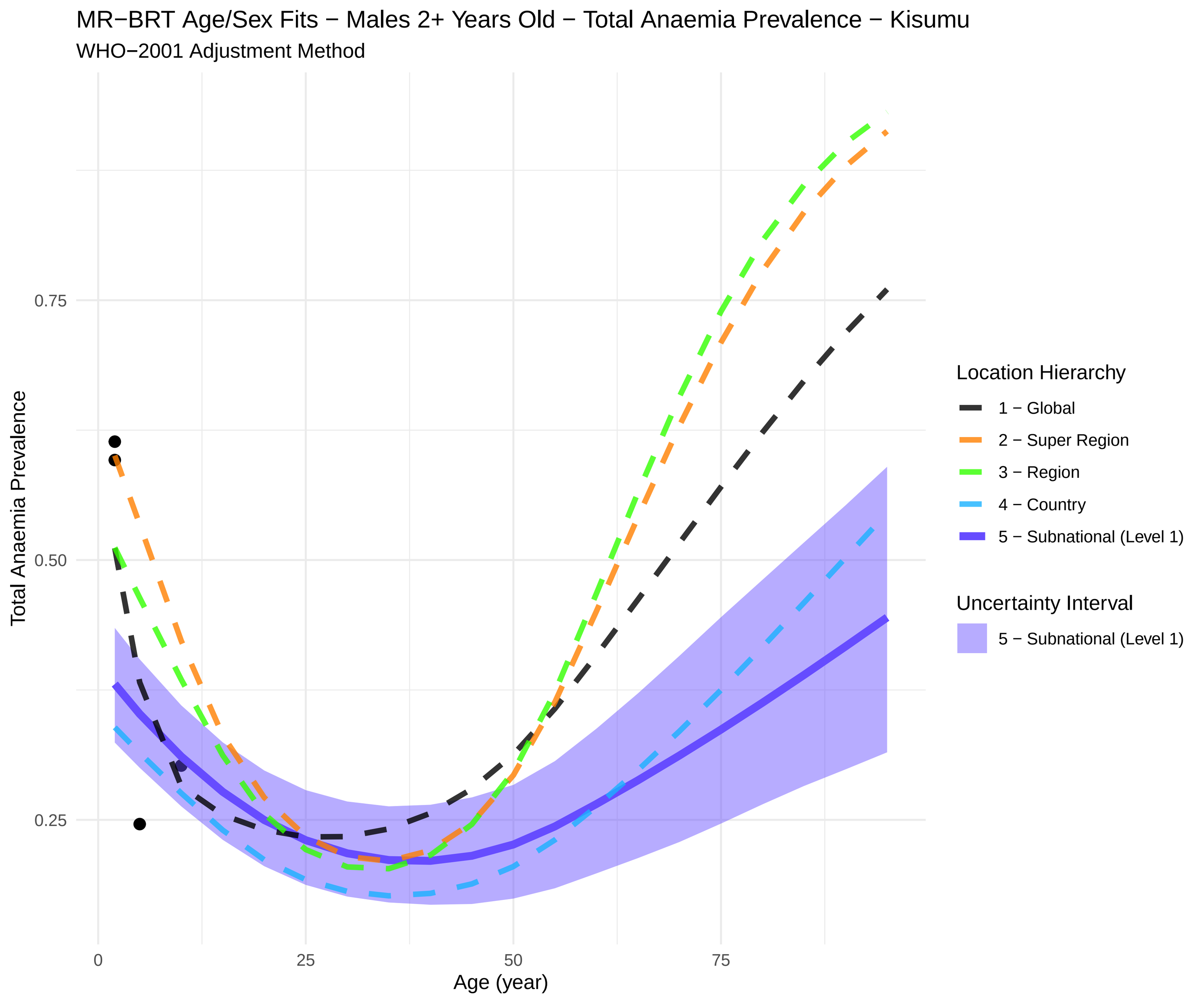
MR−BRT Age/Sex Fits − Males 2+ Years Old − Total Anaemia Prevalence − Kisumu
WHO−2001 Adjustment Method
Total Anaemia Prevalence
0.25
0.50
0.75
0
25
50
75
Age (year)
Location Hierarchy
1 − Global
2 − Super Region
3 − Region
4 − Country
5 − Subnational (Level 1)
Uncertainty Interval
5 − Subnational (Level 1)

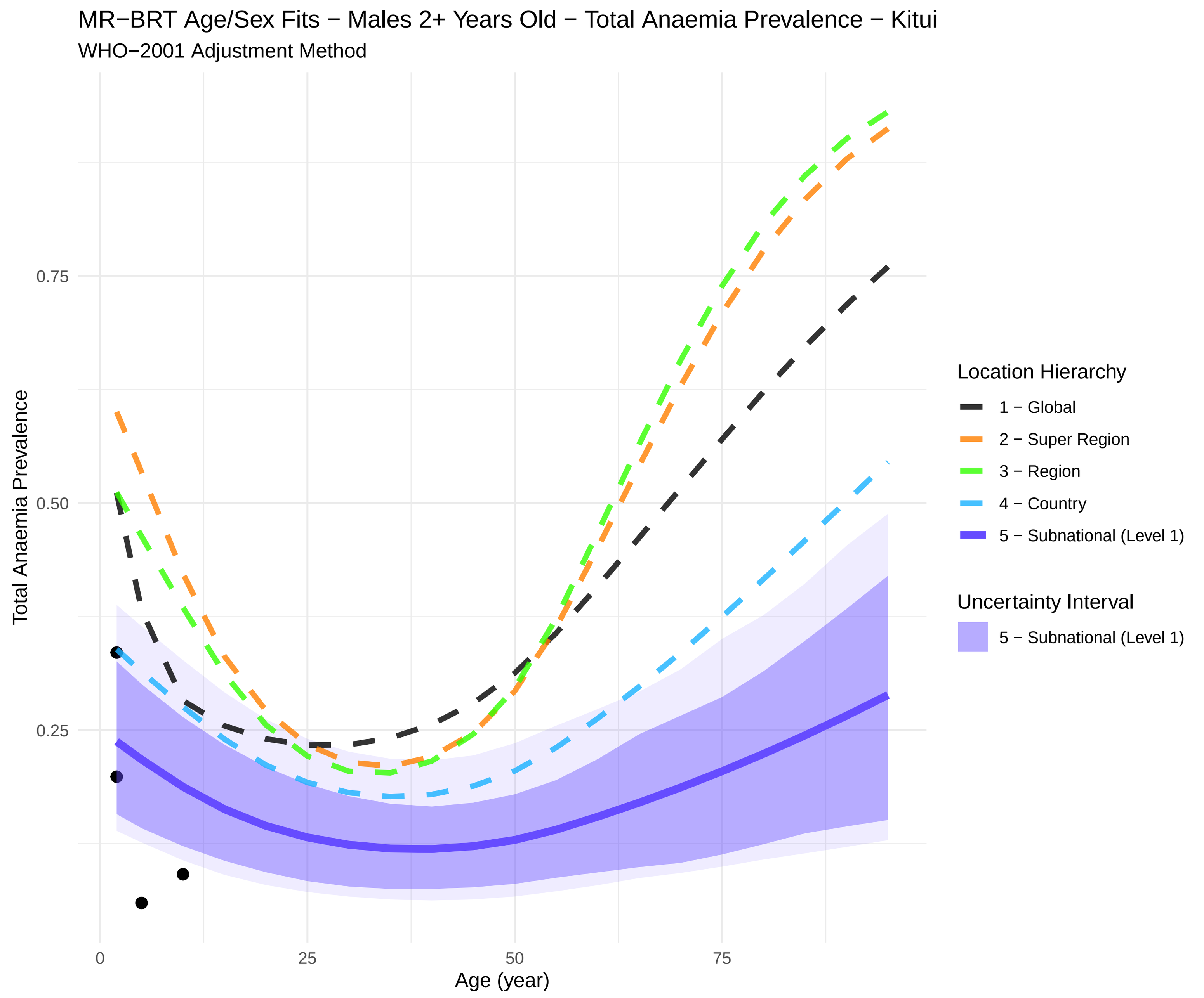
MR−BRT Age/Sex Fits − Males 2+ Years Old − Total Anaemia Prevalence − Kitui
WHO−2001 Adjustment Method
Total Anaemia Prevalence
0.25
0.50
0.75
0
25
50
75
Age (year)
Location Hierarchy
1 − Global
2 − Super Region
3 − Region
4 − Country
5 − Subnational (Level 1)
Uncertainty Interval
5 − Subnational (Level 1)

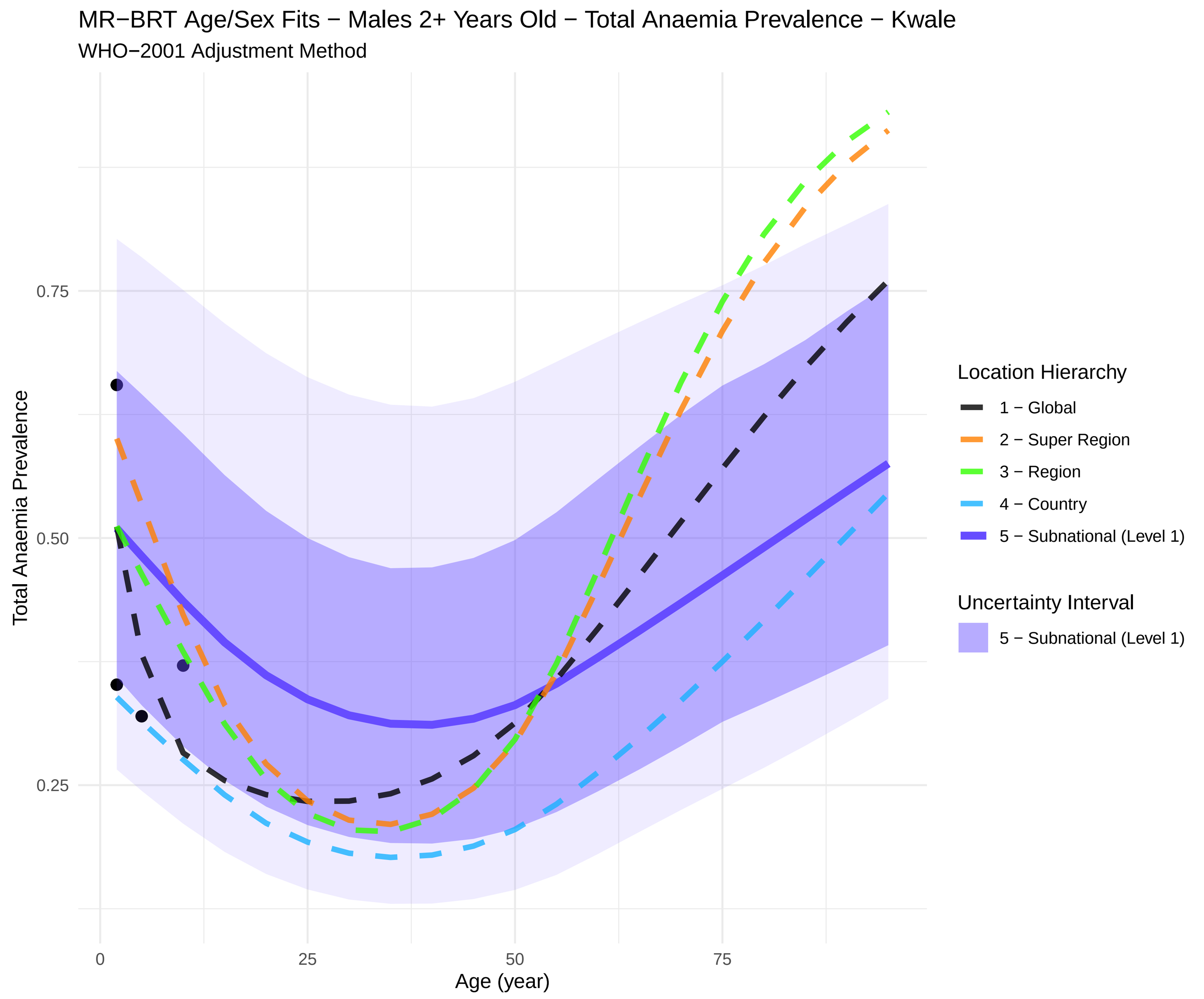
MR−BRT Age/Sex Fits − Males 2+ Years Old − Total Anaemia Prevalence − Kwale
WHO−2001 Adjustment Method
Total Anaemia Prevalence
0.25
0.50
0.75
0
25
50
75
Age (year)
Location Hierarchy
1 − Global
2 − Super Region
3 − Region
4 − Country
5 − Subnational (Level 1)
Uncertainty Interval
5 − Subnational (Level 1)

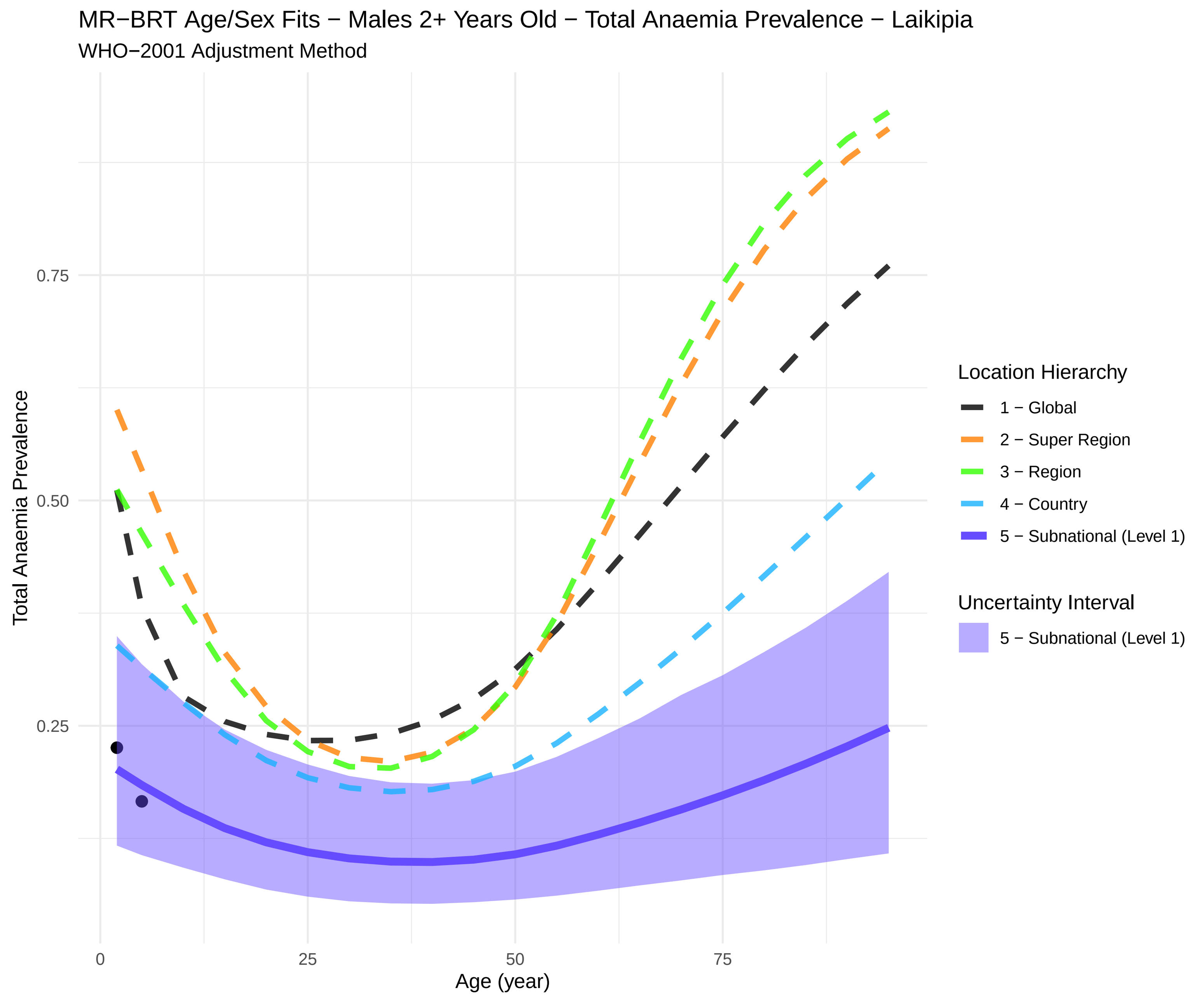
MR−BRT Age/Sex Fits − Males 2+ Years Old − Total Anaemia Prevalence − Laikipia
WHO−2001 Adjustment Method
Total Anaemia Prevalence
0.25
0.50
0.75
0
25
50
75
Age (year)
Location Hierarchy
1 − Global
2 − Super Region
3 − Region
4 − Country
5 − Subnational (Level 1)
Uncertainty Interval
5 − Subnational (Level 1)

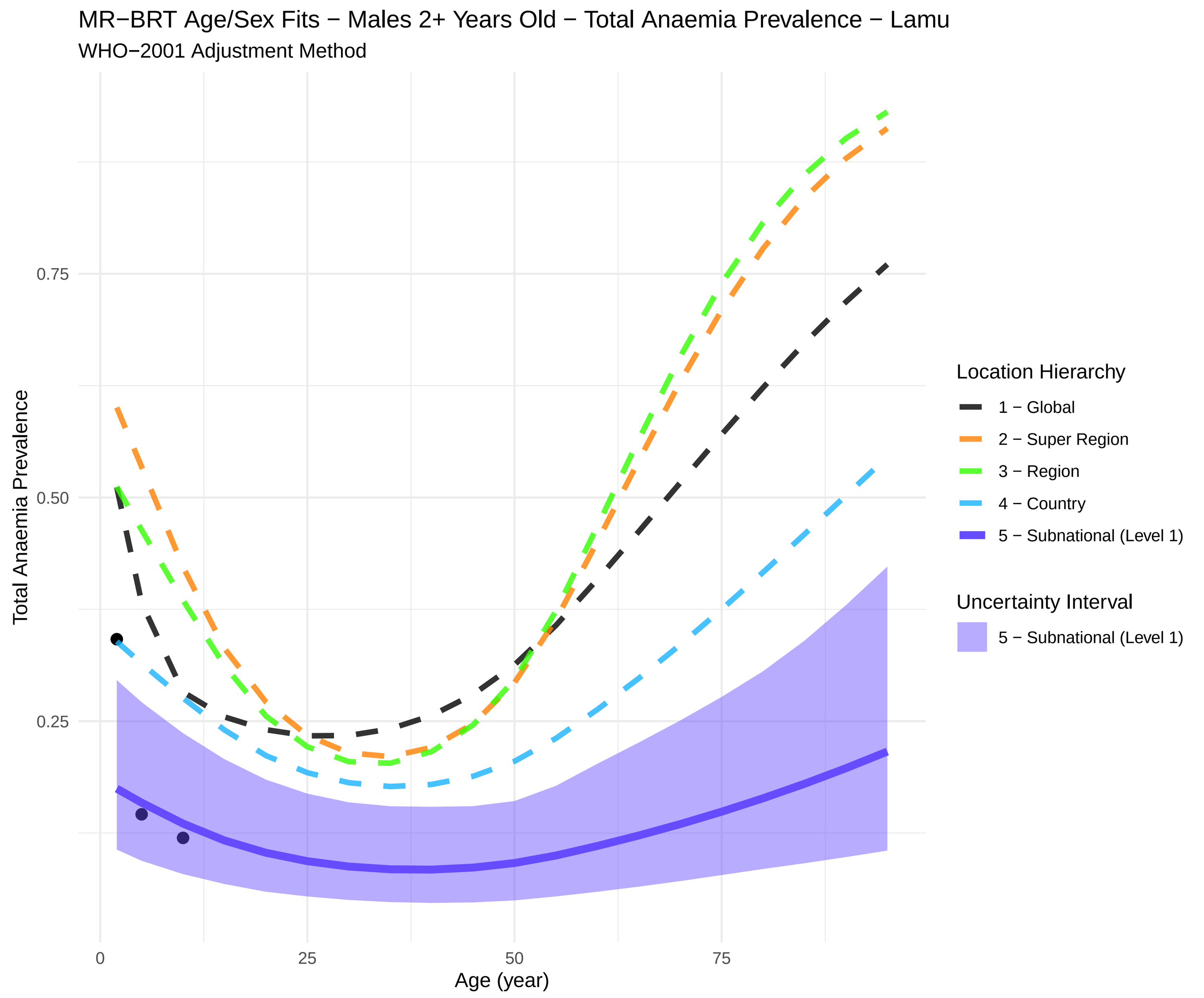
MR−BRT Age/Sex Fits − Males 2+ Years Old − Total Anaemia Prevalence − Lamu
WHO−2001 Adjustment Method
Total Anaemia Prevalence
0.25
0.50
0.75
0
25
50
75
Age (year)
Location Hierarchy
1 − Global
2 − Super Region
3 − Region
4 − Country
5 − Subnational (Level 1)
Uncertainty Interval
5 − Subnational (Level 1)

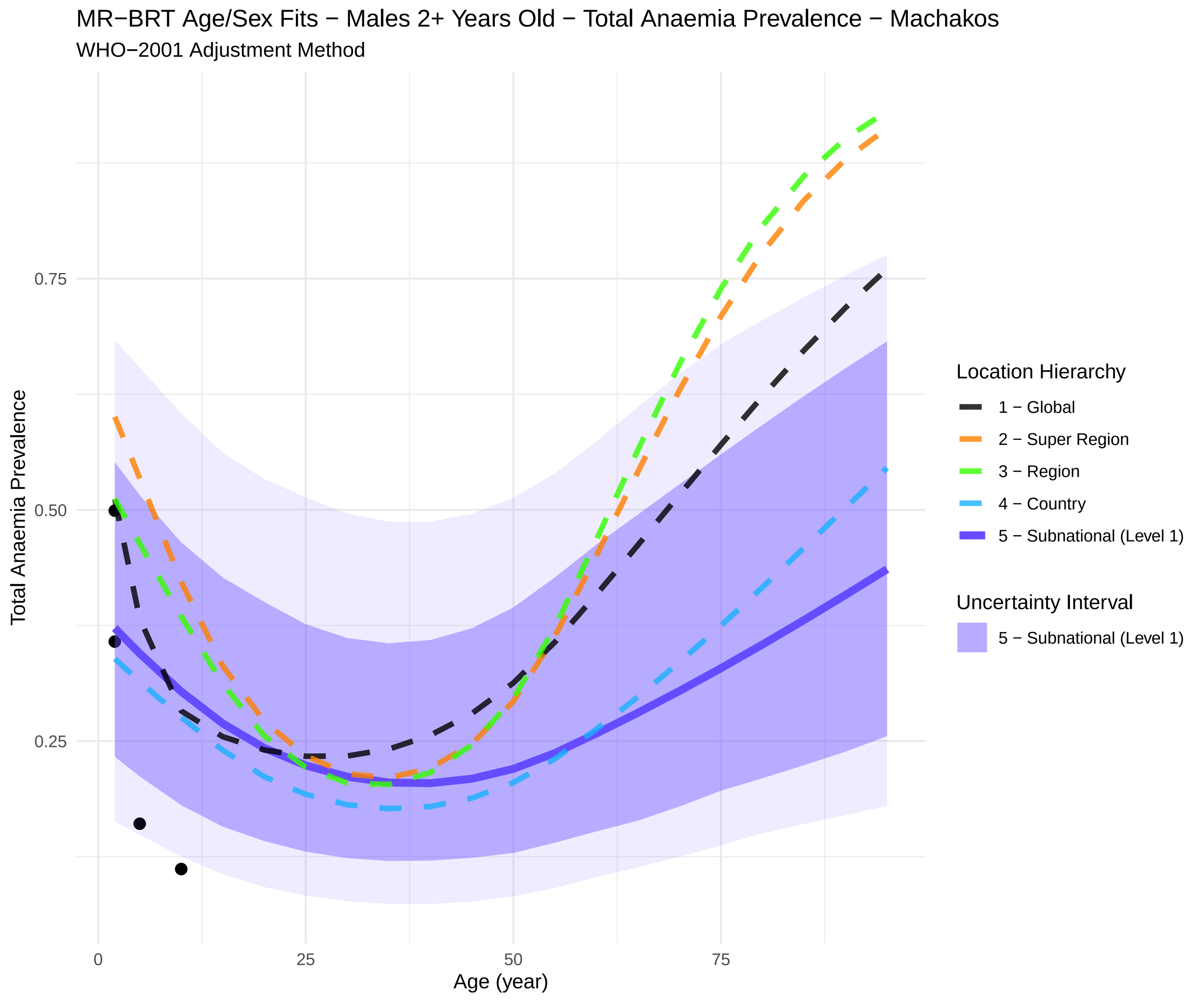
MR−BRT Age/Sex Fits − Males 2+ Years Old − Total Anaemia Prevalence − Machakos
WHO−2001 Adjustment Method
Total Anaemia Prevalence
0.25
0.50
0.75
0
25
50
75
Age (year)
Location Hierarchy
1 − Global
2 − Super Region
3 − Region
4 − Country
5 − Subnational (Level 1)
Uncertainty Interval
5 − Subnational (Level 1)

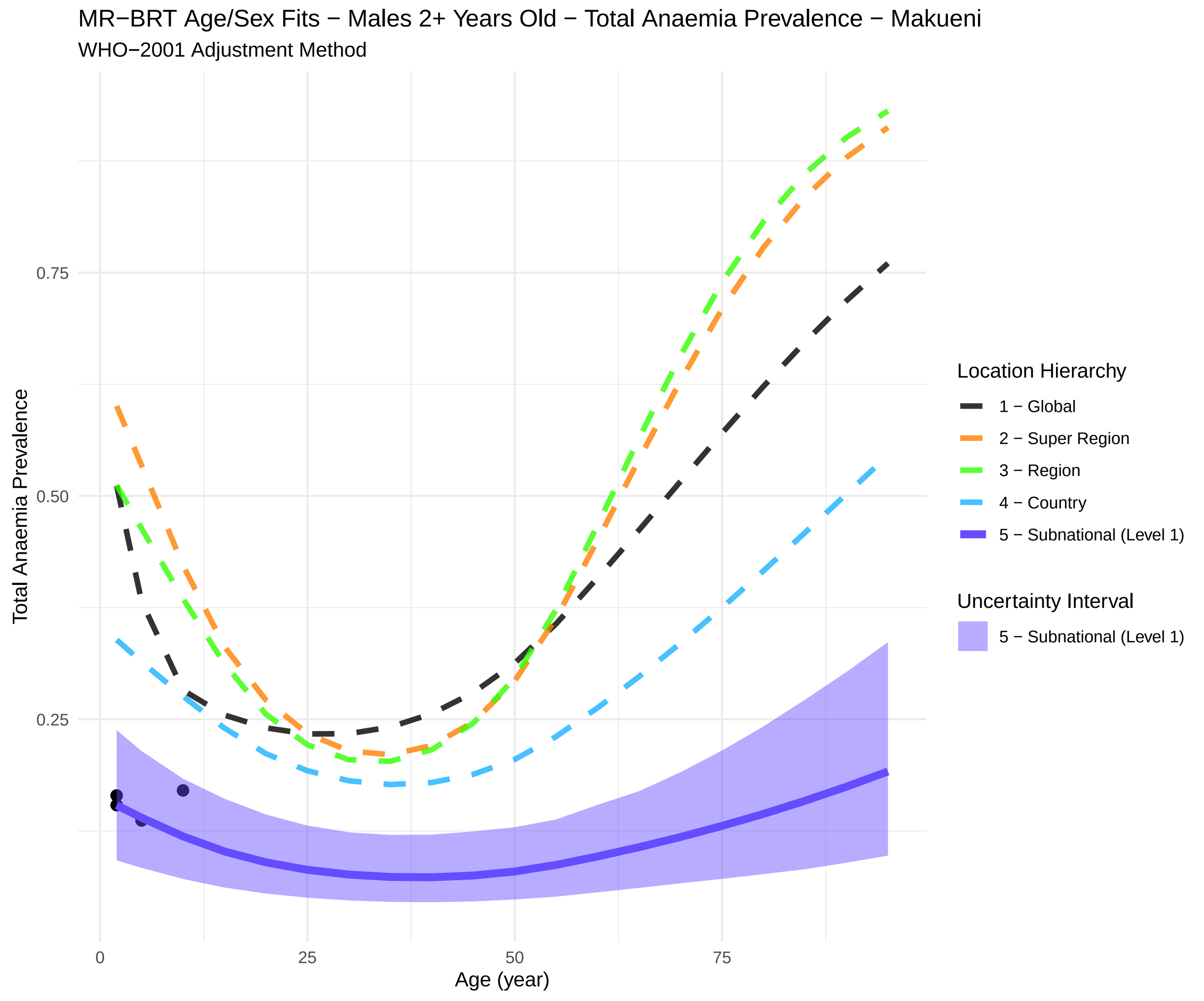
MR−BRT Age/Sex Fits − Males 2+ Years Old − Total Anaemia Prevalence − Makueni
WHO−2001 Adjustment Method
Total Anaemia Prevalence
0.25
0.50
0.75
0
25
50
75
Age (year)
Location Hierarchy
1 − Global
2 − Super Region
3 − Region
4 − Country
5 − Subnational (Level 1)
Uncertainty Interval
5 − Subnational (Level 1)

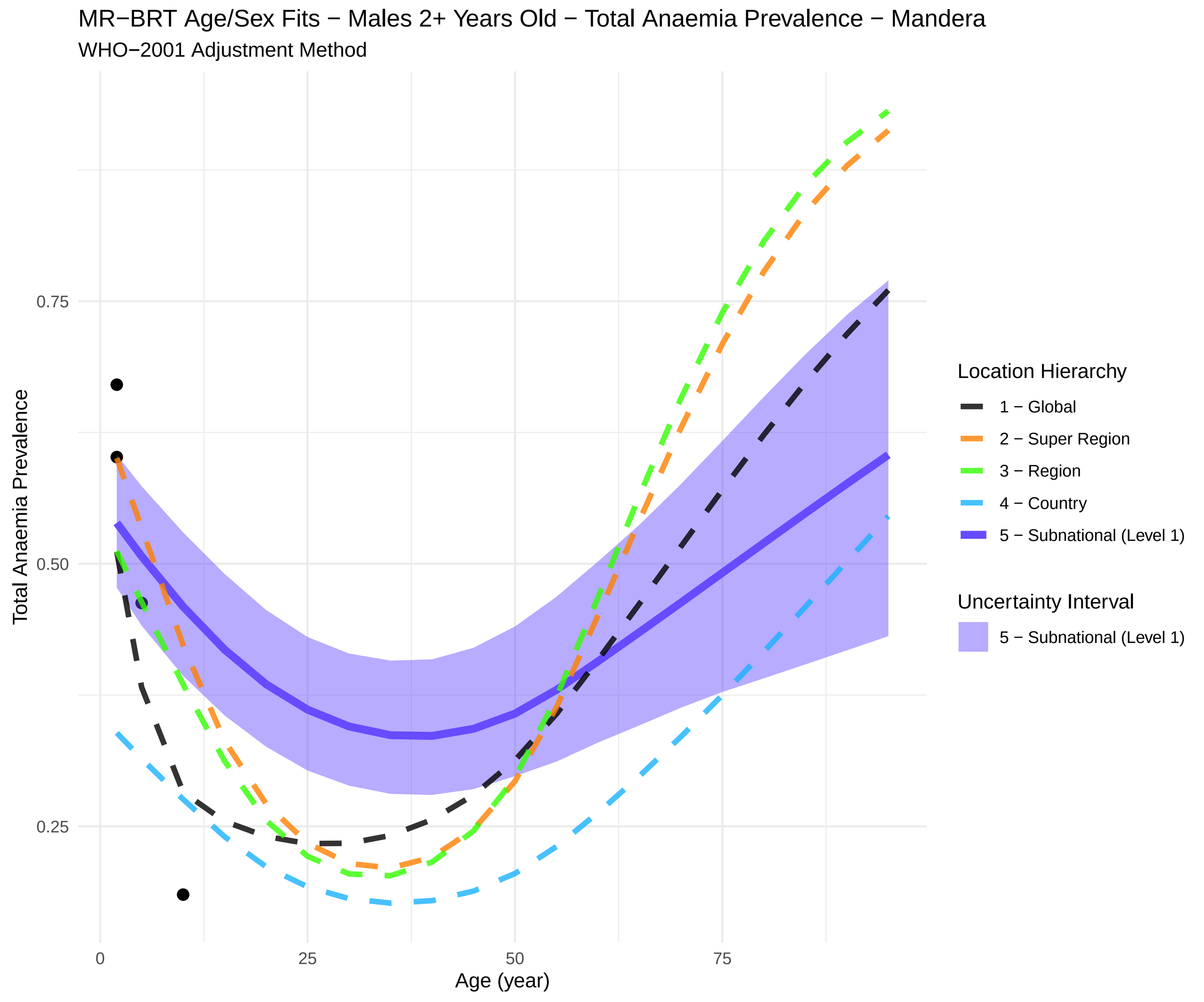
MR−BRT Age/Sex Fits − Males 2+ Years Old − Total Anaemia Prevalence − Mandera
WHO−2001 Adjustment Method
Total Anaemia Prevalence
0.25
0.50
0.75
0
25
50
75
Age (year)
Location Hierarchy
1 − Global
2 − Super Region
3 − Region
4 − Country
5 − Subnational (Level 1)
Uncertainty Interval
5 − Subnational (Level 1)

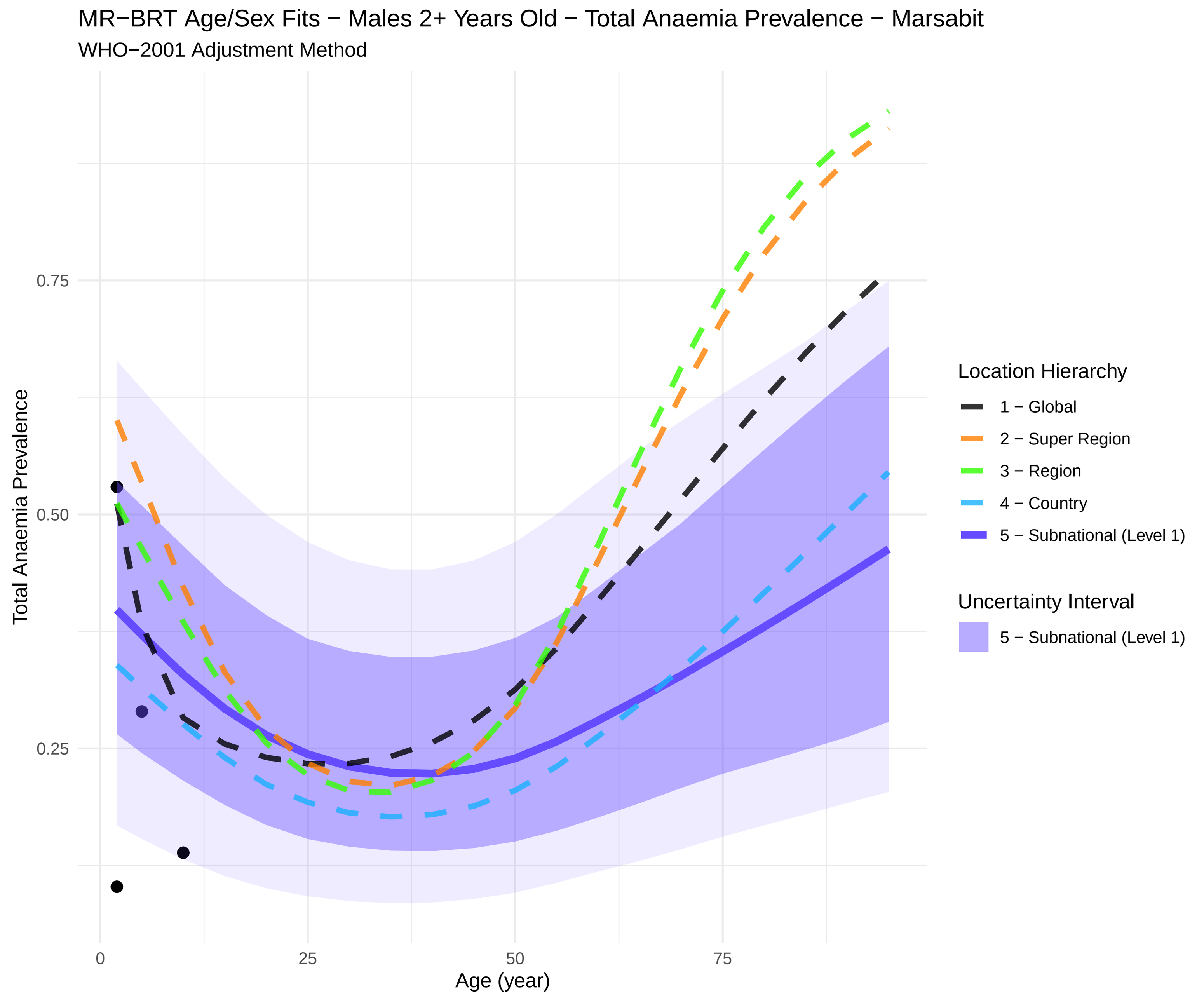
MR−BRT Age/Sex Fits − Males 2+ Years Old − Total Anaemia Prevalence − Marsabit
WHO−2001 Adjustment Method
Total Anaemia Prevalence
0.25
0.50
0.75
0
25
50
75
Age (year)
Location Hierarchy
1 − Global
2 − Super Region
3 − Region
4 − Country
5 − Subnational (Level 1)
Uncertainty Interval
5 − Subnational (Level 1)

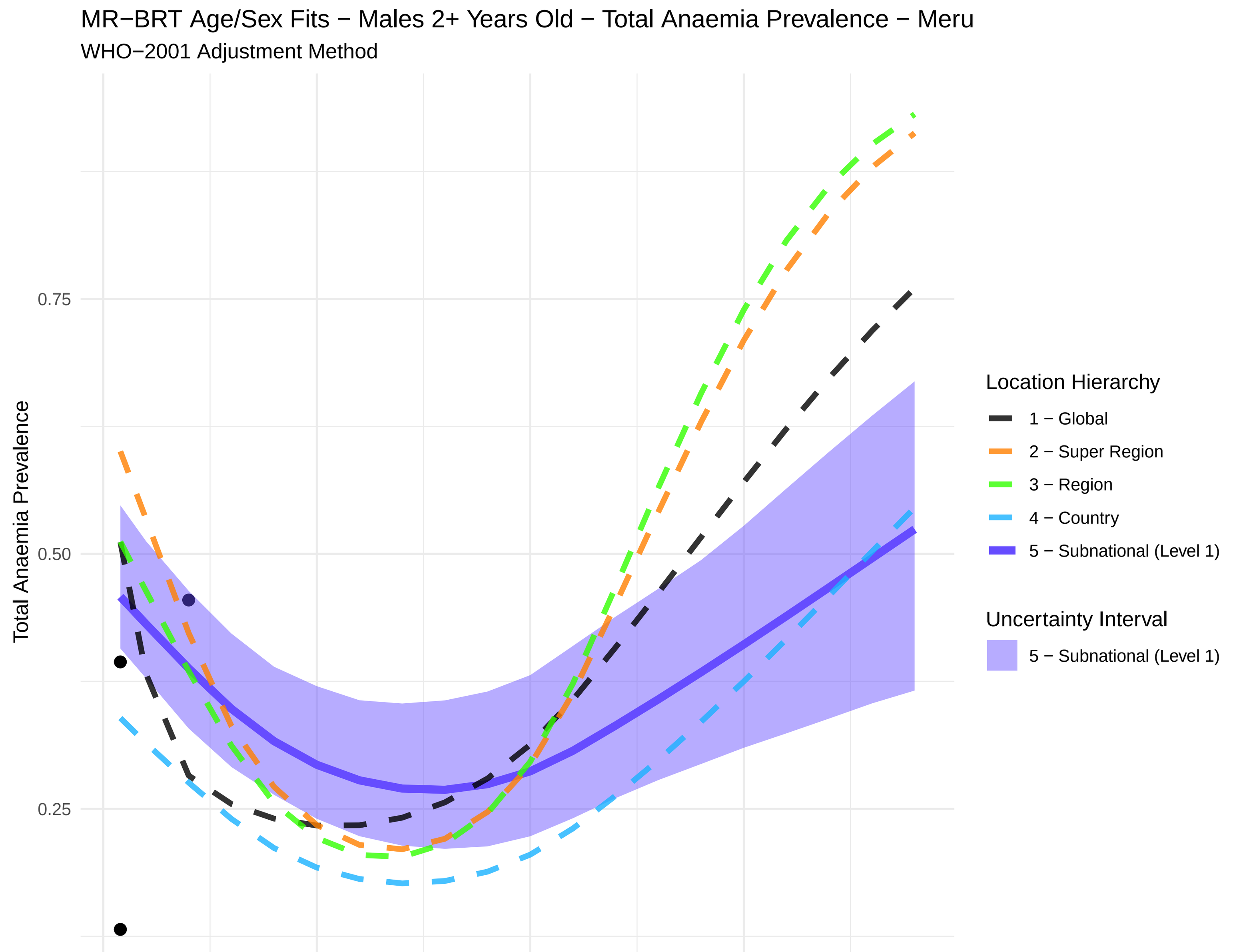
MR−BRT Age/Sex Fits − Males 2+ Years Old − Total Anaemia Prevalence − Meru
WHO−2001 Adjustment Method
Total Anaemia Prevalence
0.25
0.50
0.75
0
25
50
75
Age (year)
Location Hierarchy
1 − Global
2 − Super Region
3 − Region
4 − Country
5 − Subnational (Level 1)
Uncertainty Interval
5 − Subnational (Level 1)

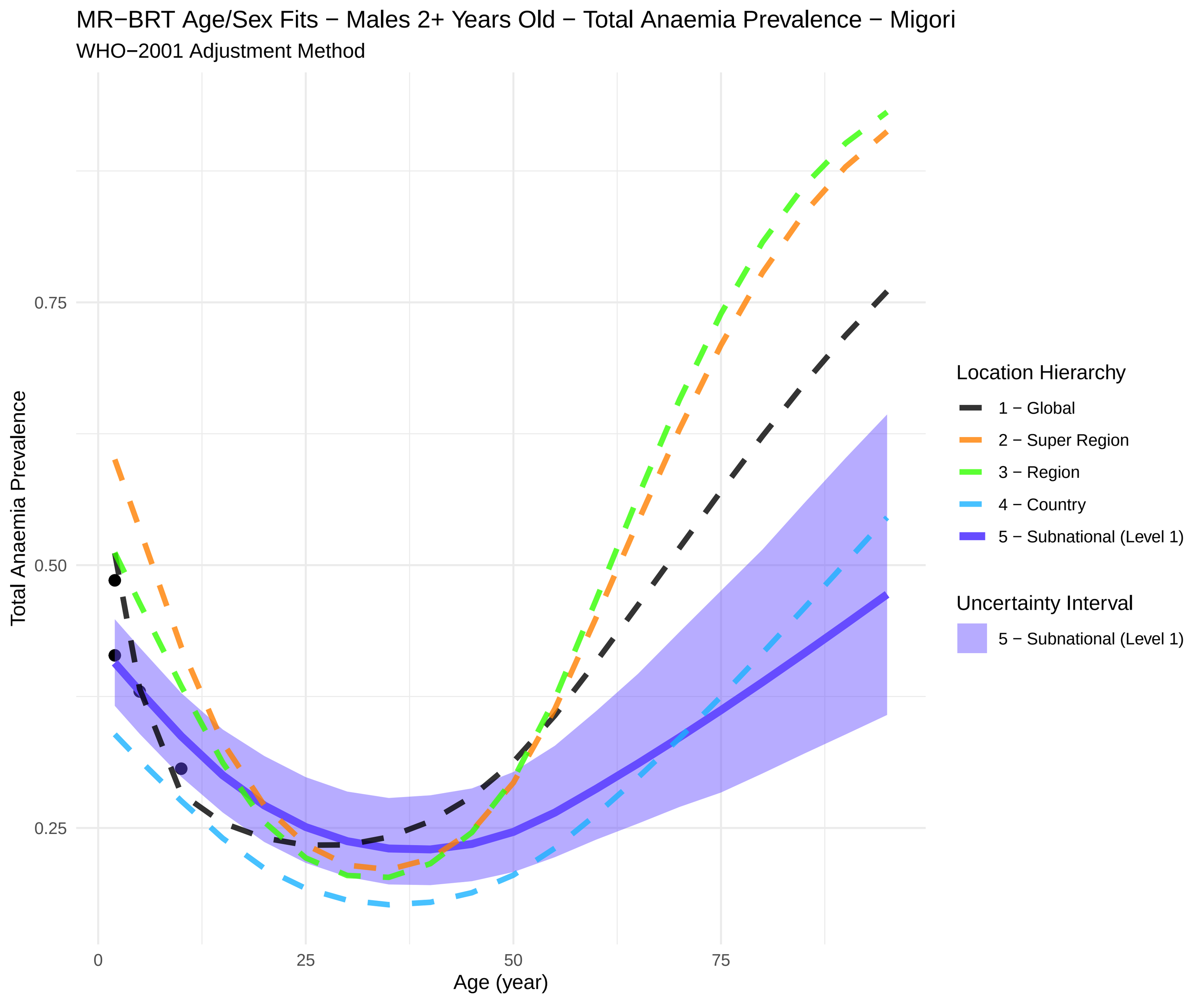
MR−BRT Age/Sex Fits − Males 2+ Years Old − Total Anaemia Prevalence − Migori
WHO−2001 Adjustment Method
Total Anaemia Prevalence
Age (year)
Location Hierarchy
1 − Global
2 − Super Region
3 − Region
4 − Country
5 − Subnational (Level 1)
Uncertainty Interval
5 − Subnational (Level 1)

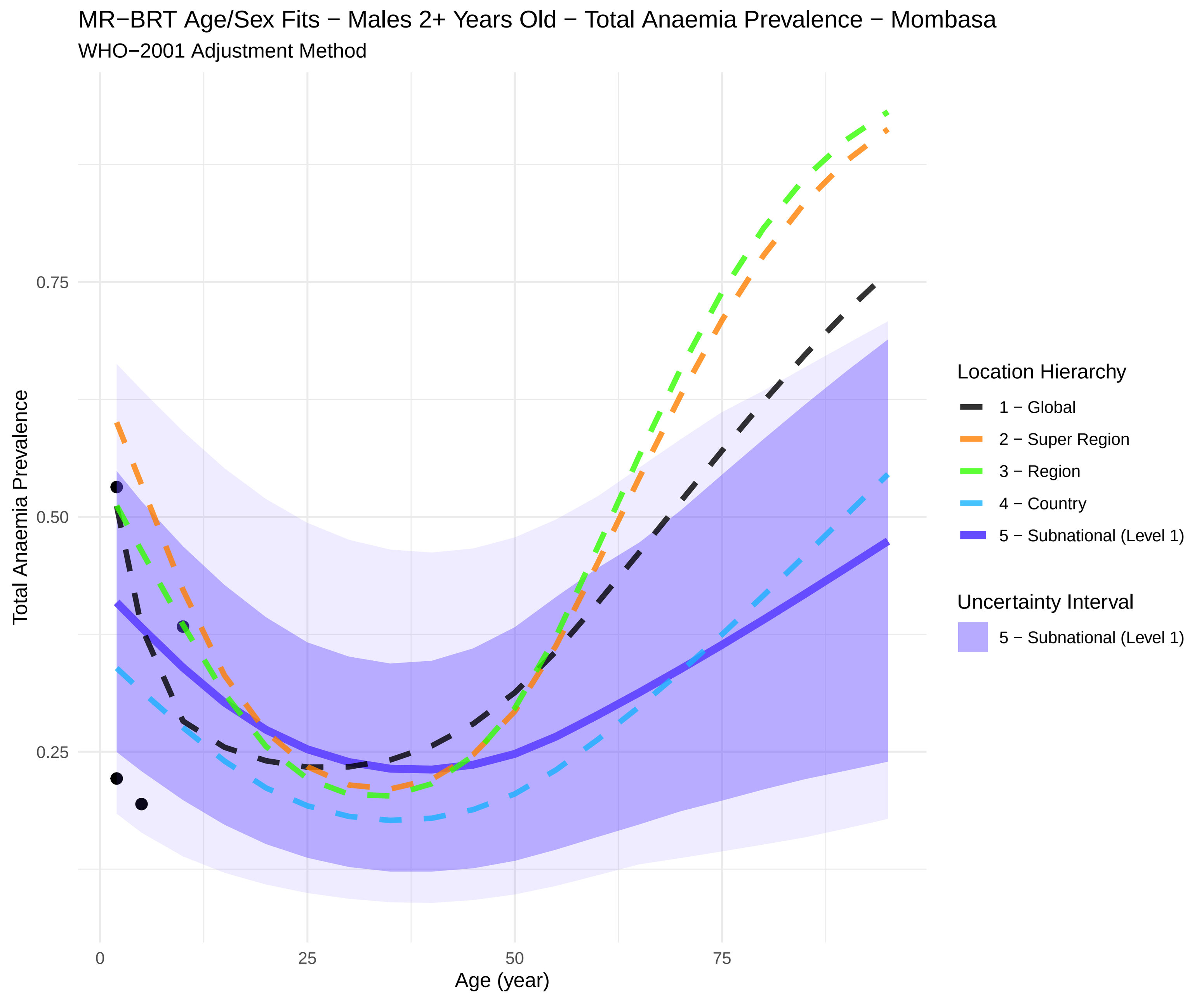
MR−BRT Age/Sex Fits − Males 2+ Years Old − Total Anaemia Prevalence − Mombasa
WHO−2001 Adjustment Method
Total Anaemia Prevalence
Age (year)
Location Hierarchy
1 − Global
2 − Super Region
3 − Region
4 − Country
5 − Subnational (Level 1)
Uncertainty Interval
5 − Subnational (Level 1)

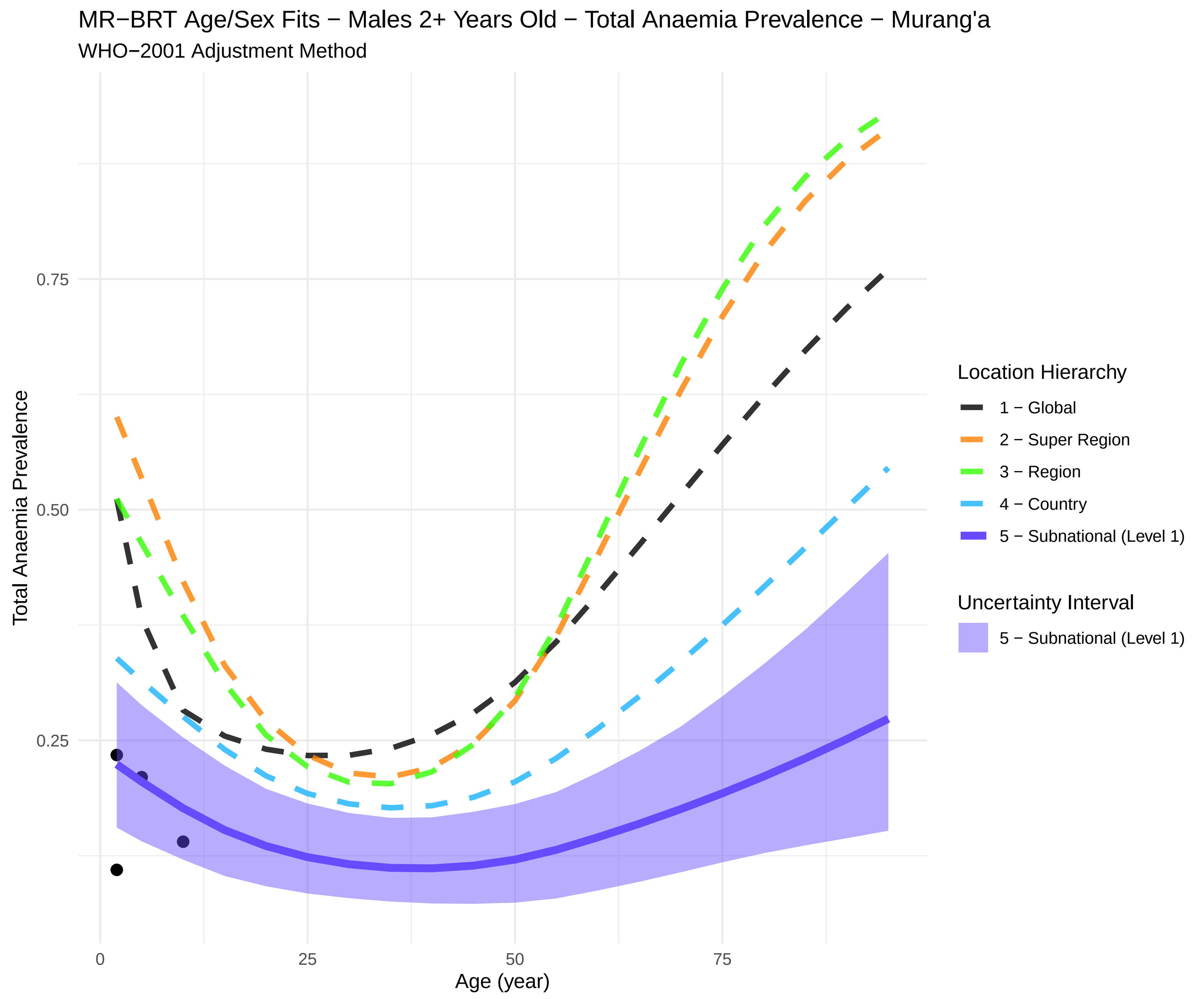
MR−BRT Age/Sex Fits − Males 2+ Years Old − Total Anaemia Prevalence − Murang'a
WHO−2001 Adjustment Method
Total Anaemia Prevalence
Age (year)
Location Hierarchy
1 − Global
2 − Super Region
3 − Region
4 − Country
5 − Subnational (Level 1)
Uncertainty Interval
5 − Subnational (Level 1)

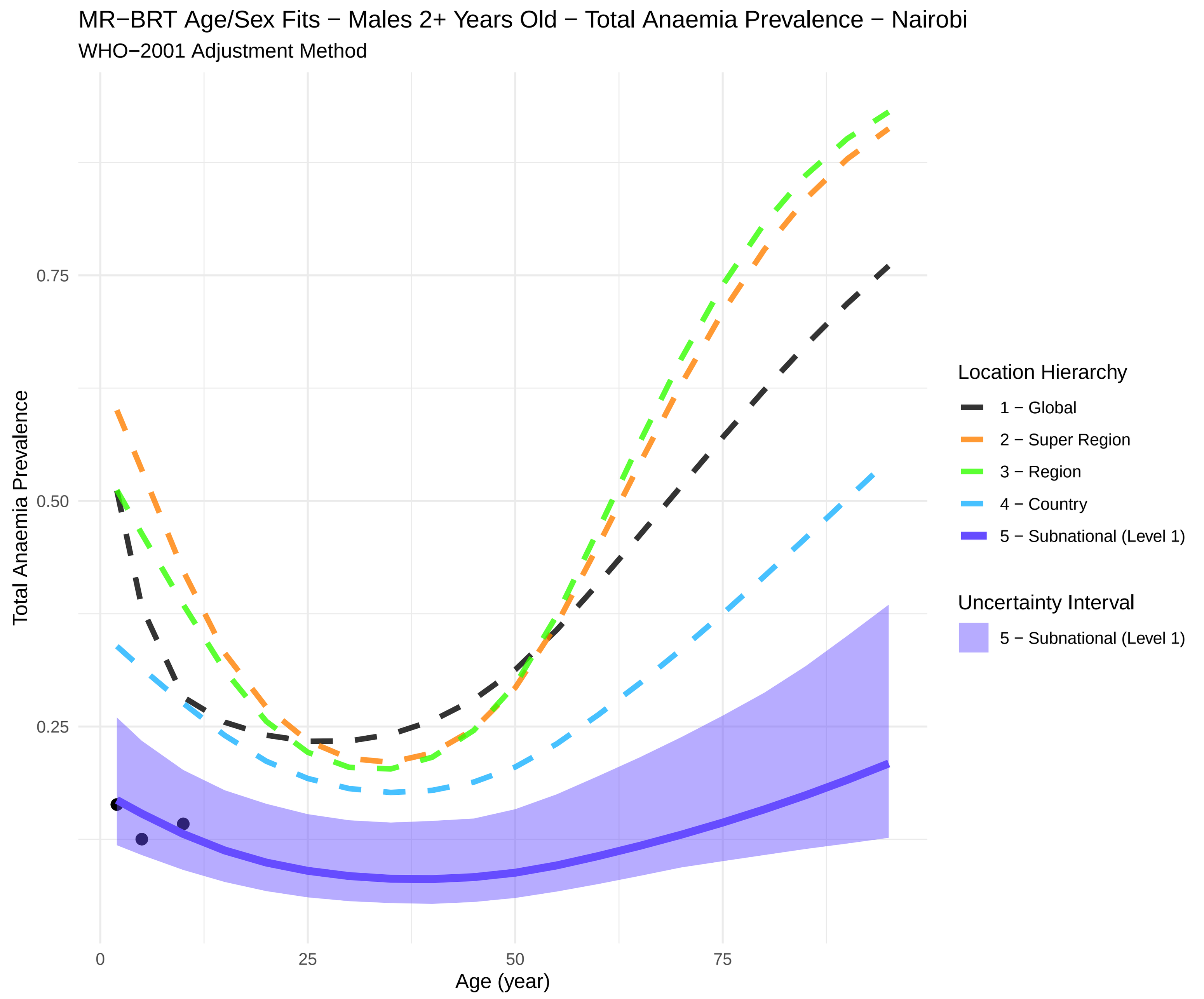
MR−BRT Age/Sex Fits − Males 2+ Years Old − Total Anaemia Prevalence − Nairobi
WHO−2001 Adjustment Method
Total Anaemia Prevalence
Age (year)
Location Hierarchy
1 − Global
2 − Super Region
3 − Region
4 − Country
5 − Subnational (Level 1)
Uncertainty Interval
5 − Subnational (Level 1)

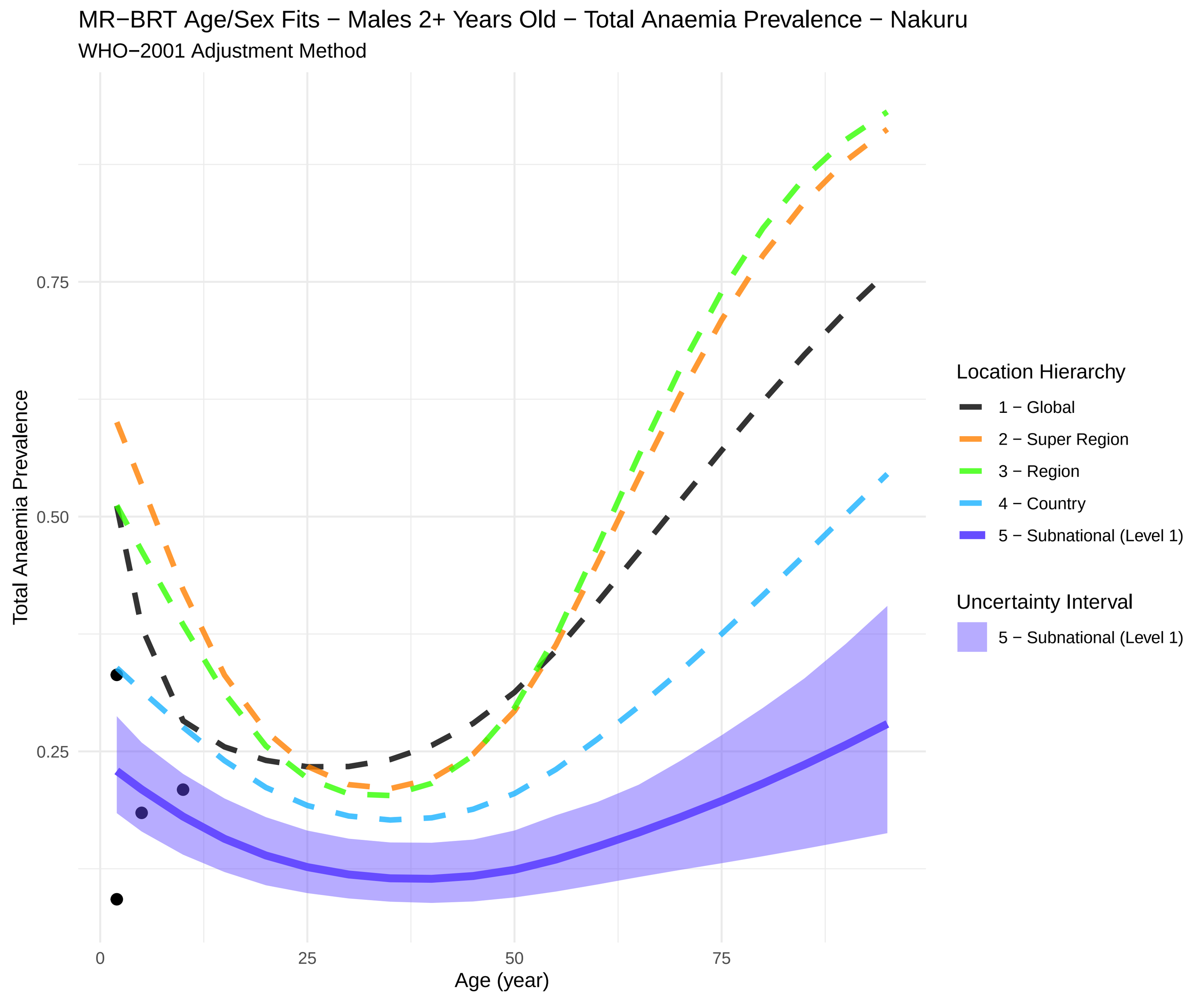
MR−BRT Age/Sex Fits − Males 2+ Years Old − Total Anaemia Prevalence − Nakuru
WHO−2001 Adjustment Method
Total Anaemia Prevalence
Age (year)
Location Hierarchy
1 − Global
2 − Super Region
3 − Region
4 − Country
5 − Subnational (Level 1)
Uncertainty Interval
5 − Subnational (Level 1)

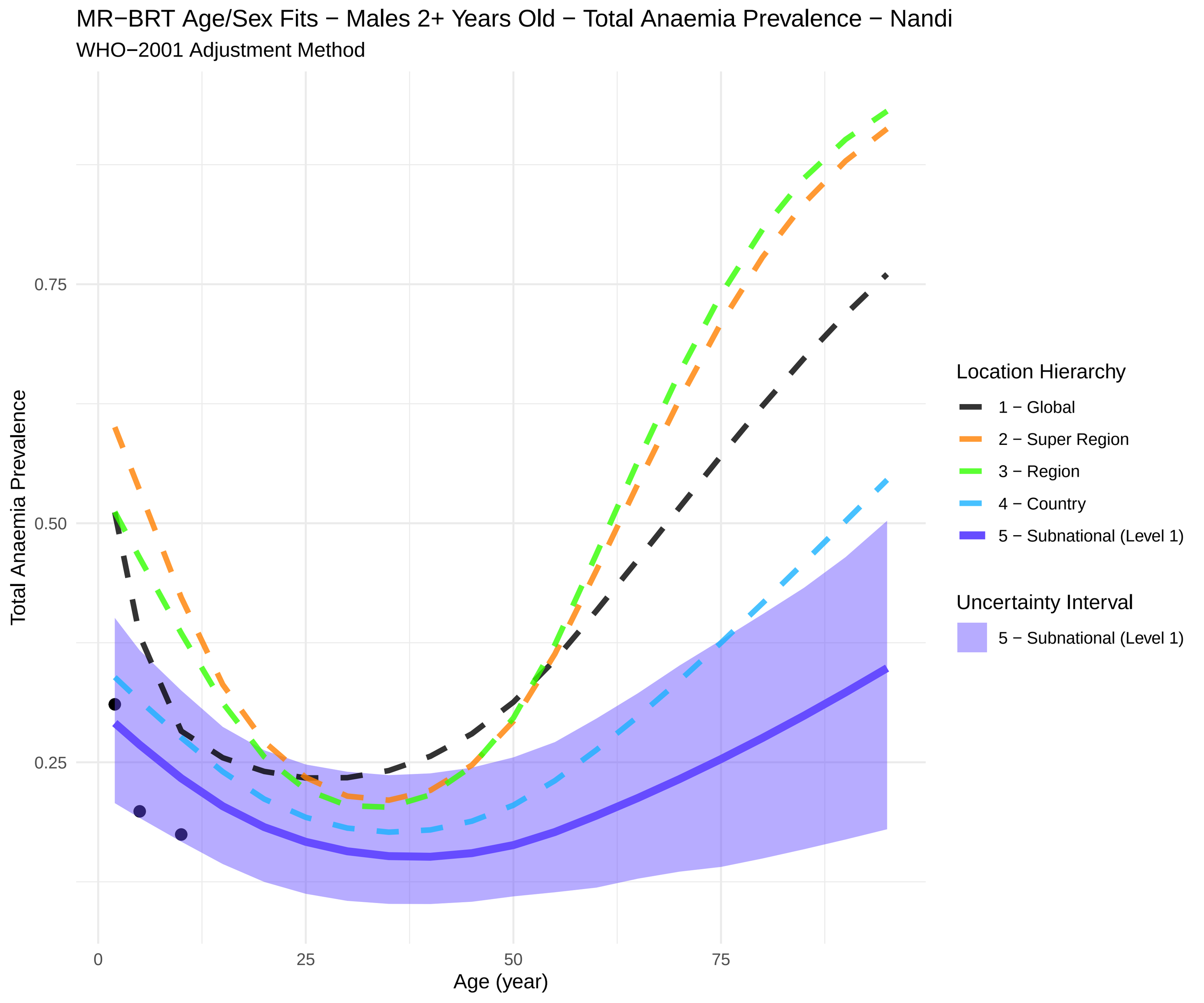
MR−BRT Age/Sex Fits − Males 2+ Years Old − Total Anaemia Prevalence − Nandi
WHO−2001 Adjustment Method
Total Anaemia Prevalence
Age (year)
Location Hierarchy
1 − Global
2 − Super Region
3 − Region
4 − Country
5 − Subnational (Level 1)
Uncertainty Interval
5 − Subnational (Level 1)

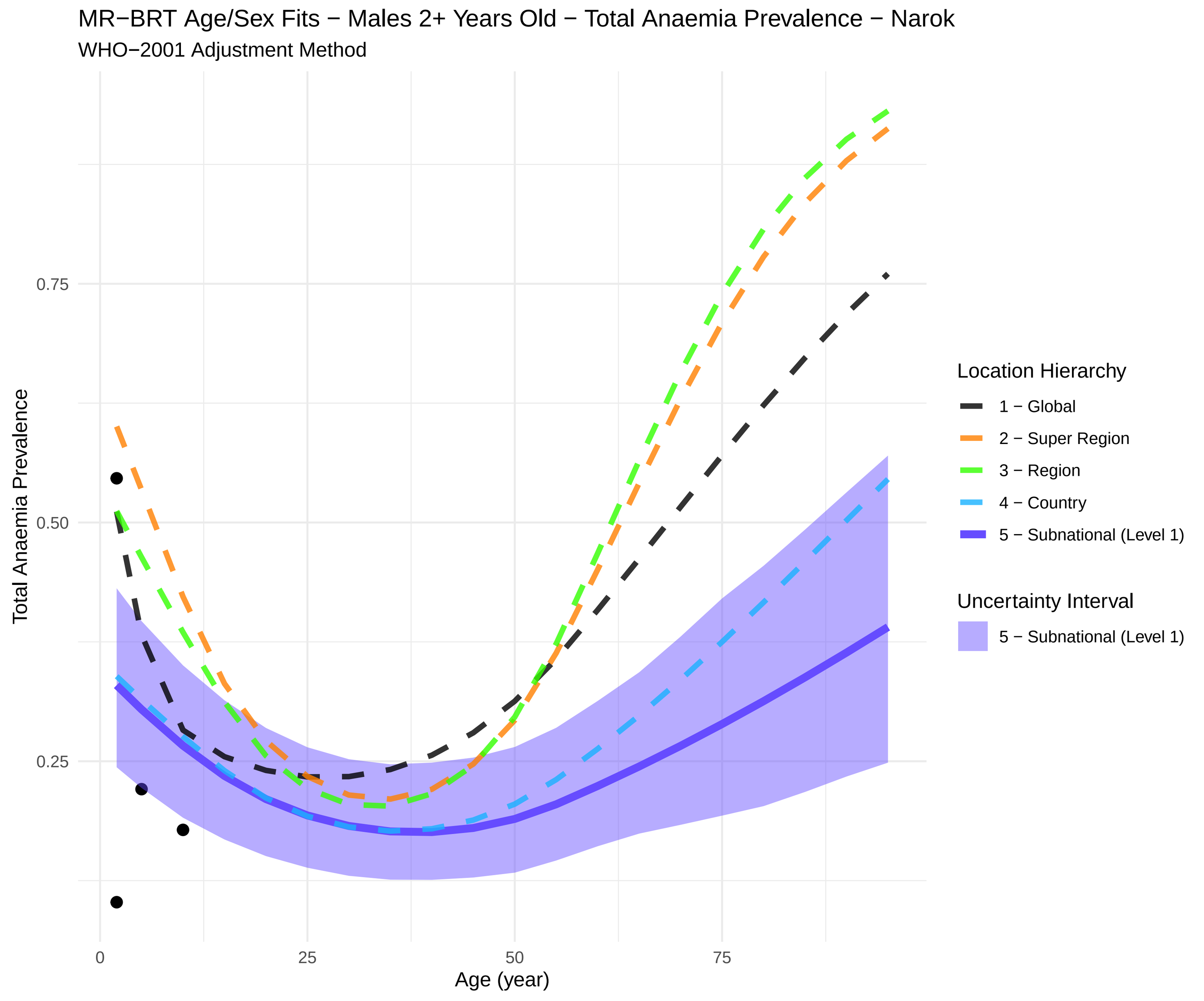
MR−BRT Age/Sex Fits − Males 2+ Years Old − Total Anaemia Prevalence − Narok
WHO−2001 Adjustment Method
Total Anaemia Prevalence
Age (year)
Location Hierarchy
1 − Global
2 − Super Region
3 − Region
4 − Country
5 − Subnational (Level 1)
Uncertainty Interval
5 − Subnational (Level 1)

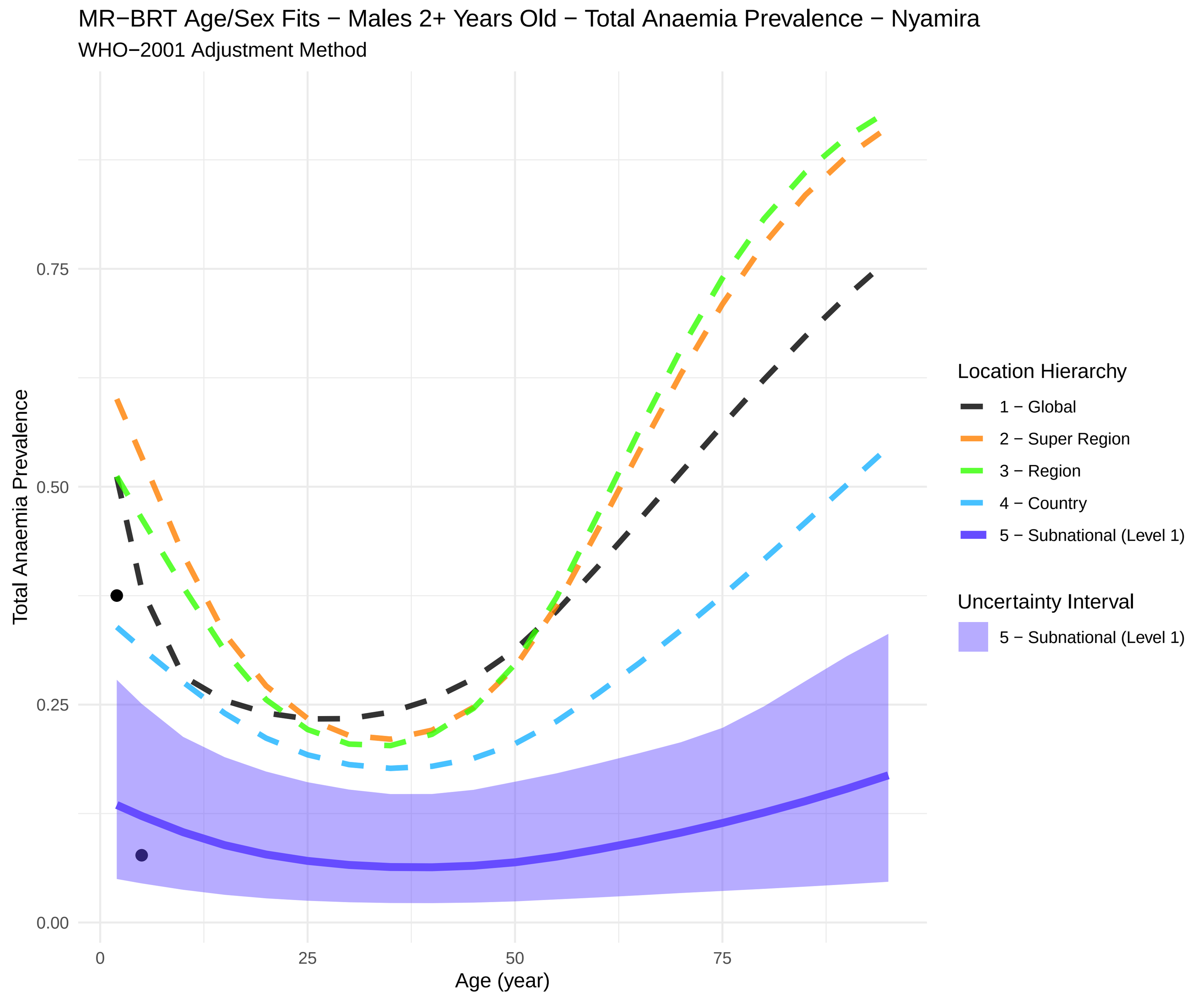
MR−BRT Age/Sex Fits − Males 2+ Years Old − Total Anaemia Prevalence − Nyamira
WHO−2001 Adjustment Method
Total Anaemia Prevalence
Age (year)
Location Hierarchy
1 − Global
2 − Super Region
3 − Region
4 − Country
5 − Subnational (Level 1)
Uncertainty Interval
5 − Subnational (Level 1)

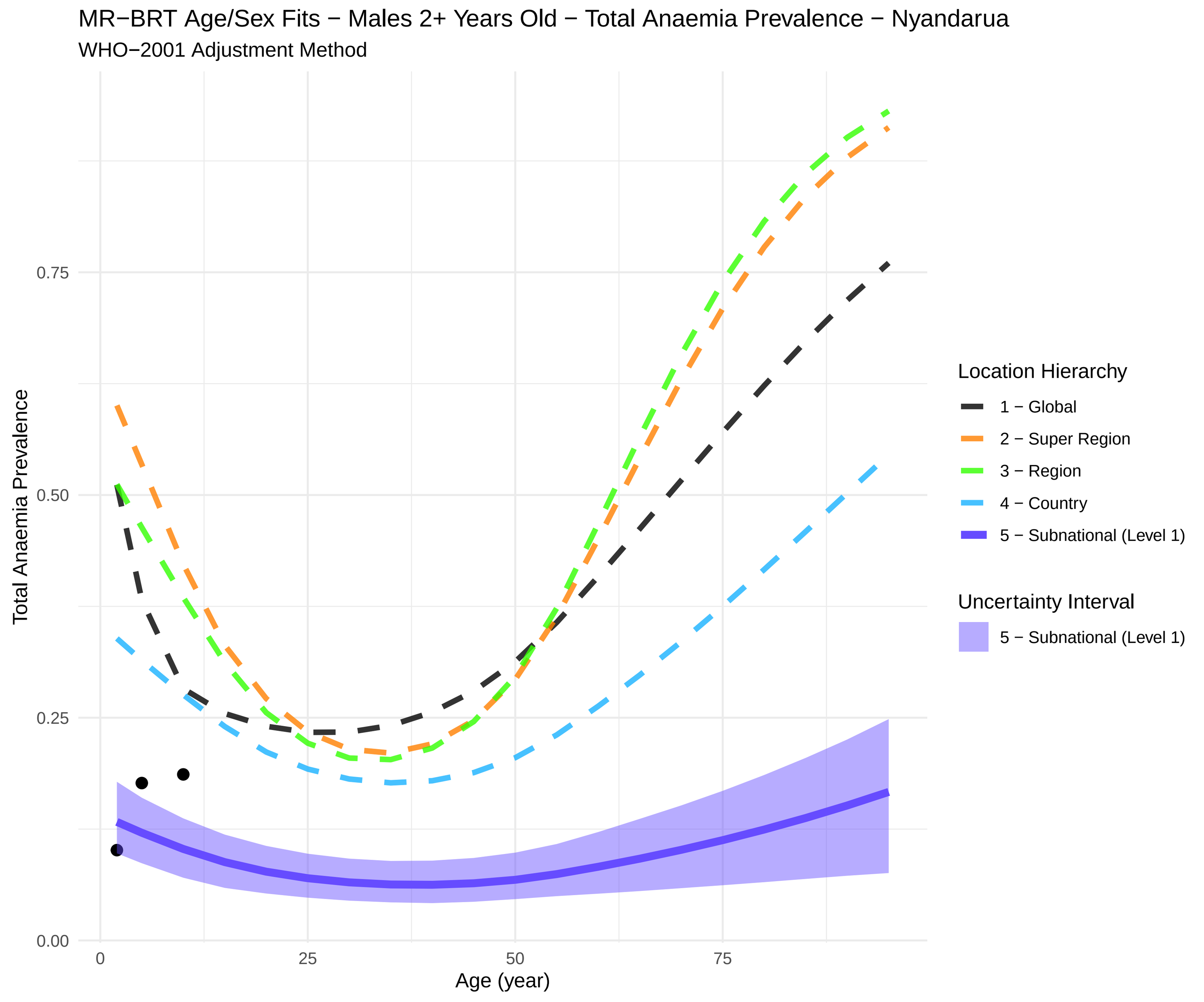
MR−BRT Age/Sex Fits − Males 2+ Years Old − Total Anaemia Prevalence − Nyandarua
WHO−2001 Adjustment Method
Total Anaemia Prevalence
Age (year)
Location Hierarchy
1 − Global
2 − Super Region
3 − Region
4 − Country
5 − Subnational (Level 1)
Uncertainty Interval
5 − Subnational (Level 1)

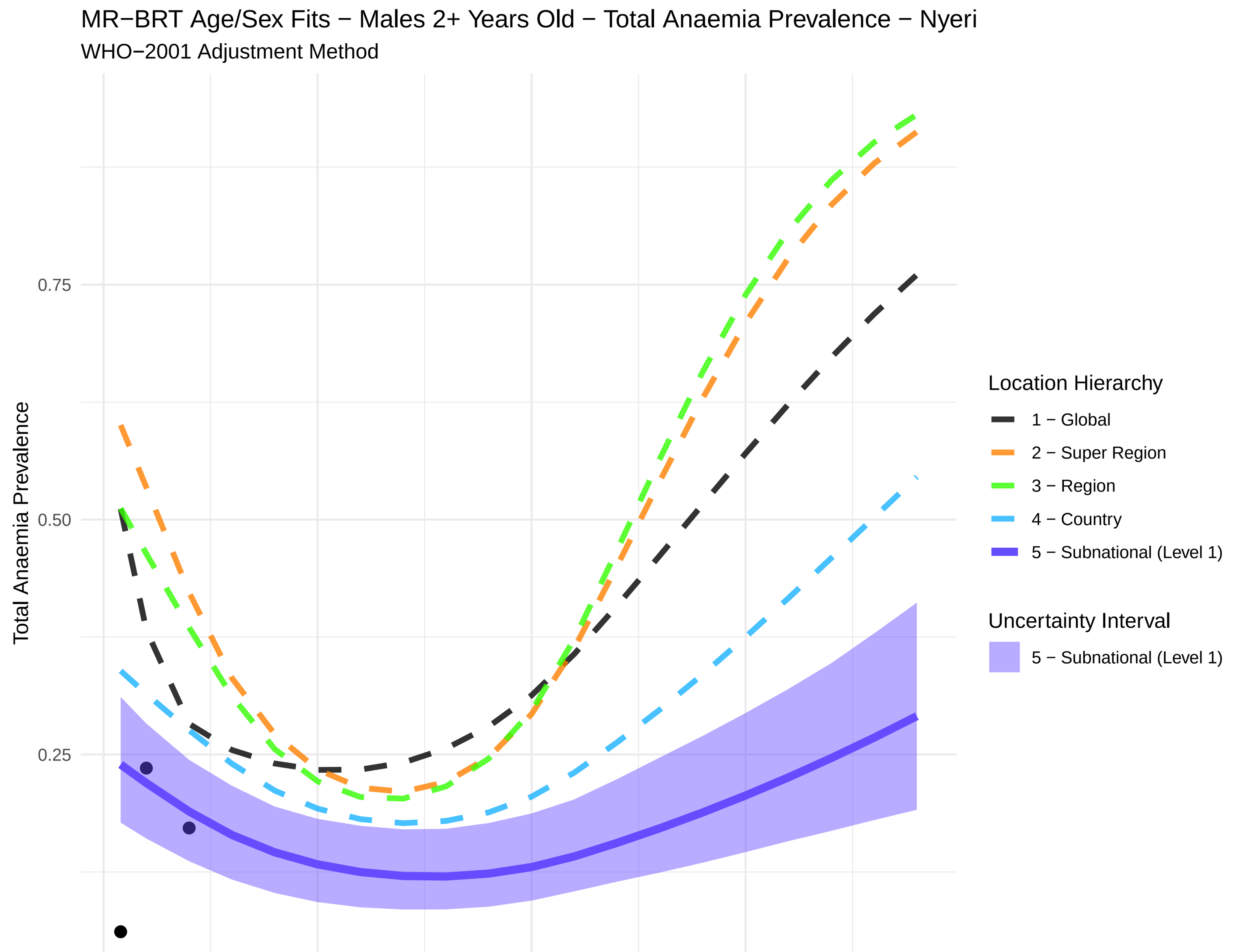
MR−BRT Age/Sex Fits − Males 2+ Years Old − Total Anaemia Prevalence − Nyeri
WHO−2001 Adjustment Method
Total Anaemia Prevalence
Age (year)
Location Hierarchy
1 − Global
2 − Super Region
3 − Region
4 − Country
5 − Subnational (Level 1)
Uncertainty Interval
5 − Subnational (Level 1)

MR−BRT Age/Sex Fits − Males 2+ Years Old − Total Anaemia Prevalence − Samburu
WHO−2001 Adjustment Method

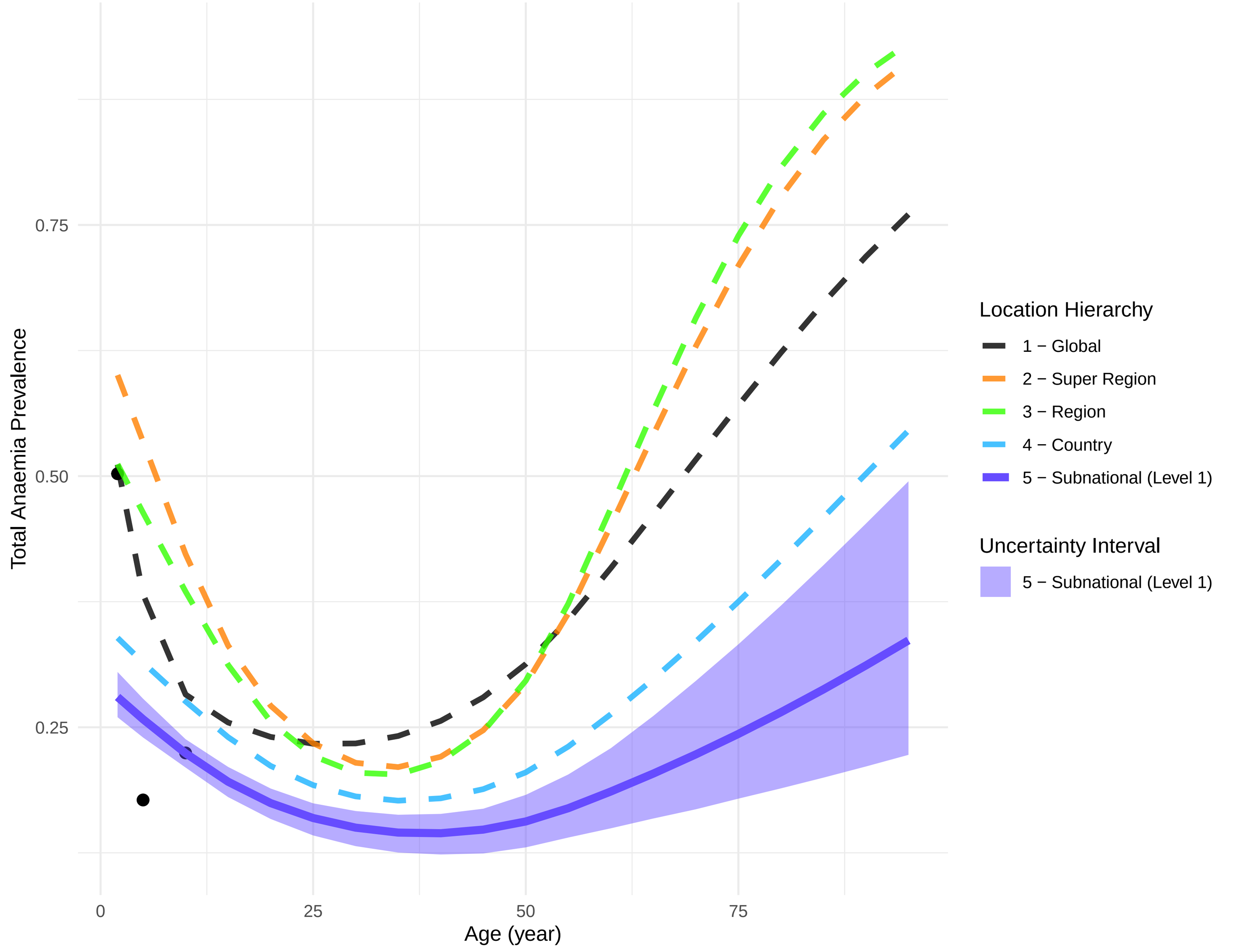

MR−BRT Age/Sex Fits − Males 2+ Years Old − Total Anaemia Prevalence − Siaya
WHO−2001 Adjustment Method

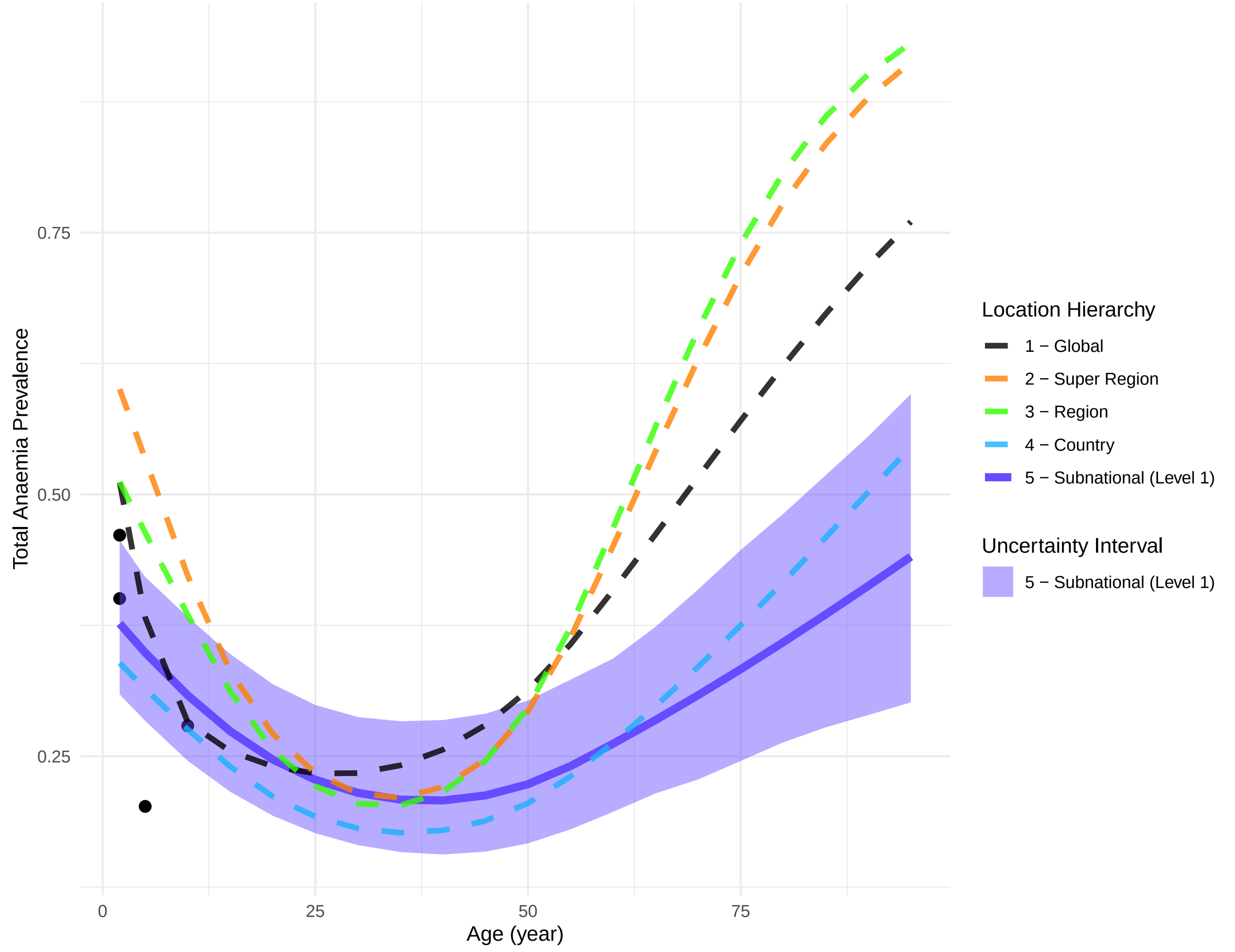

MR−BRT Age/Sex Fits − Males 2+ Years Old − Total Anaemia Prevalence − Taita Taveta
WHO−2001 Adjustment Method

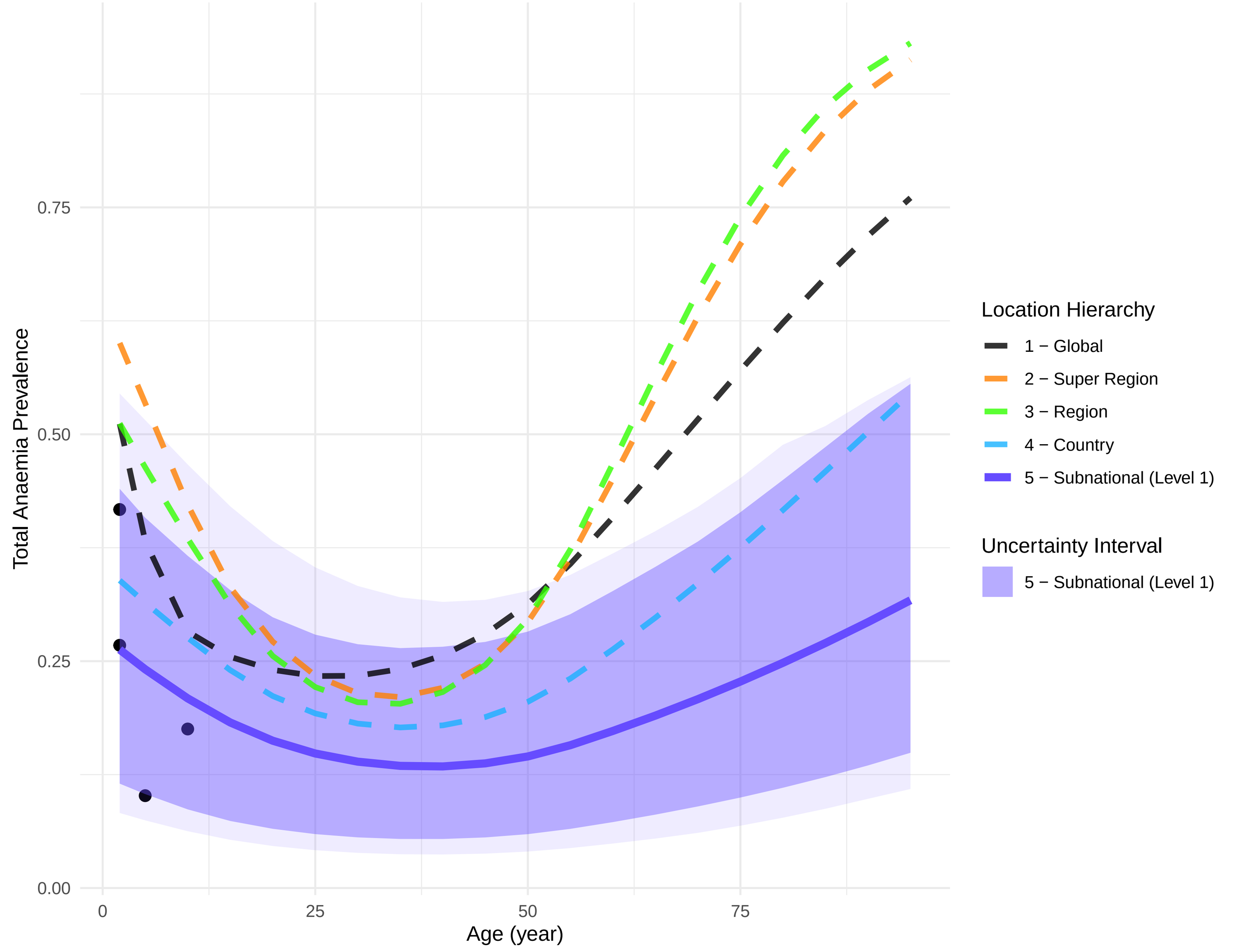

MR−BRT Age/Sex Fits − Males 2+ Years Old − Total Anaemia Prevalence − Tana River
WHO−2001 Adjustment Method

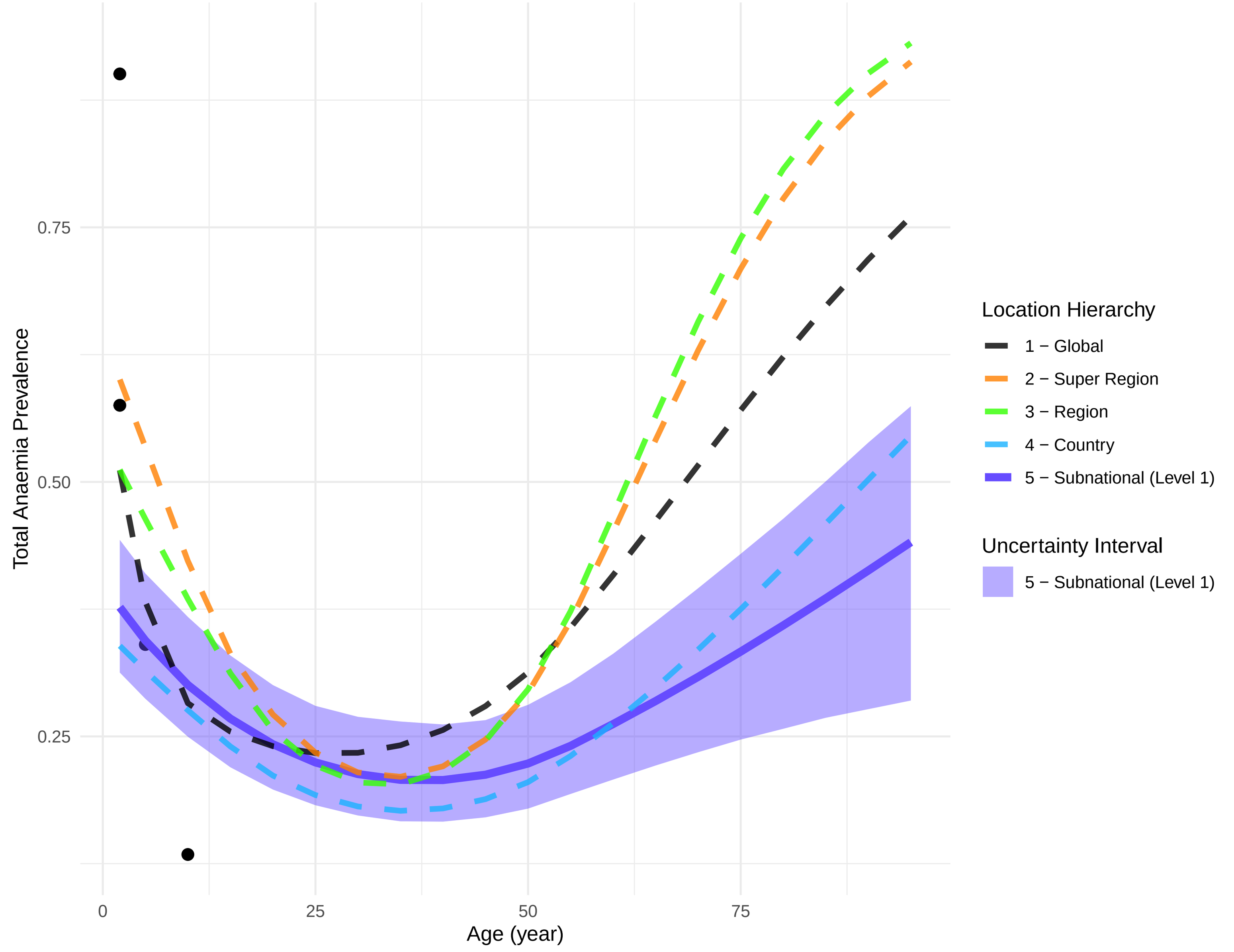

MR−BRT Age/Sex Fits − Males 2+ Years Old − Total Anaemia Prevalence − Tharaka Nithi
WHO−2001 Adjustment Method

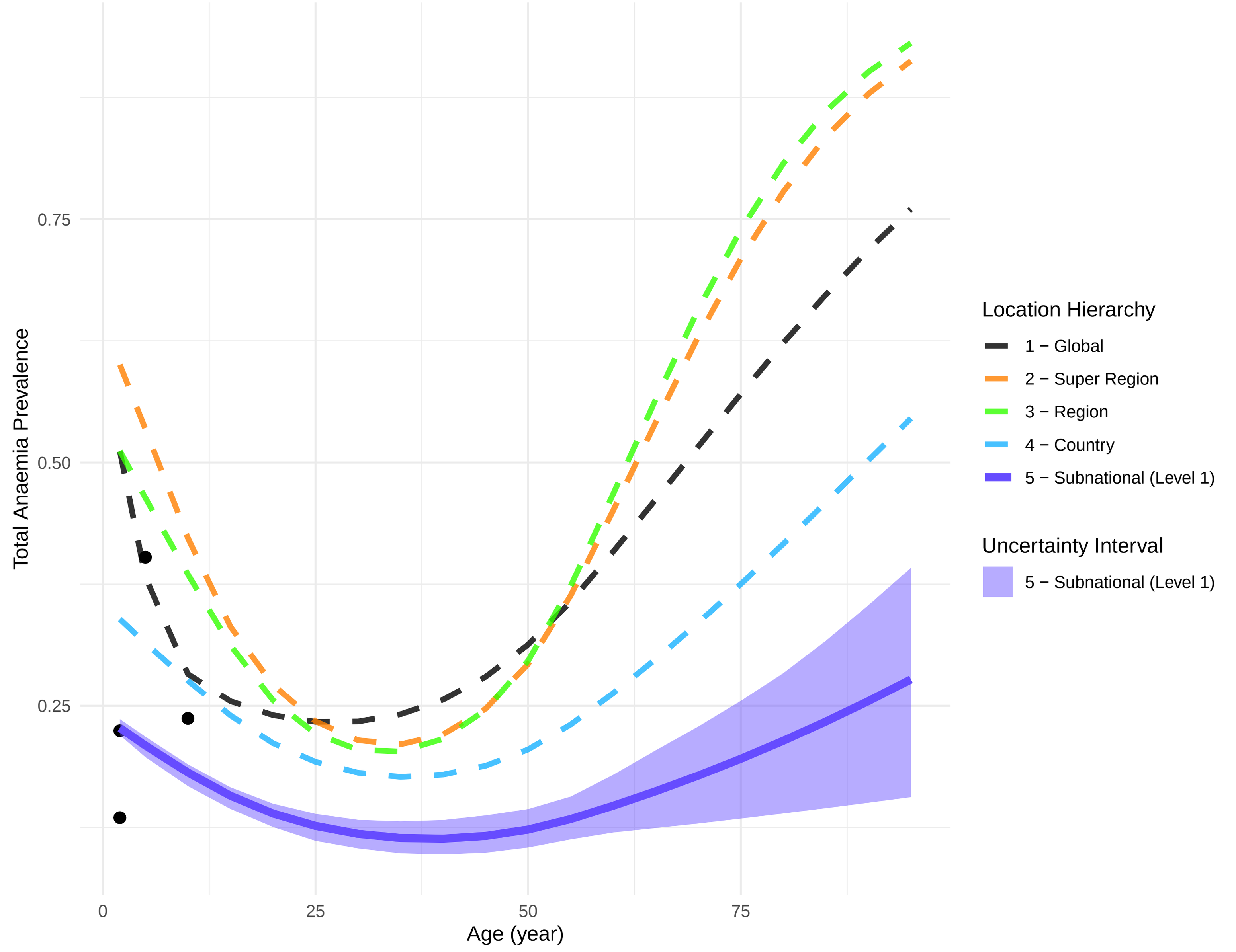

MR−BRT Age/Sex Fits − Males 2+ Years Old − Total Anaemia Prevalence − Trans Nzoia
WHO−2001 Adjustment Method

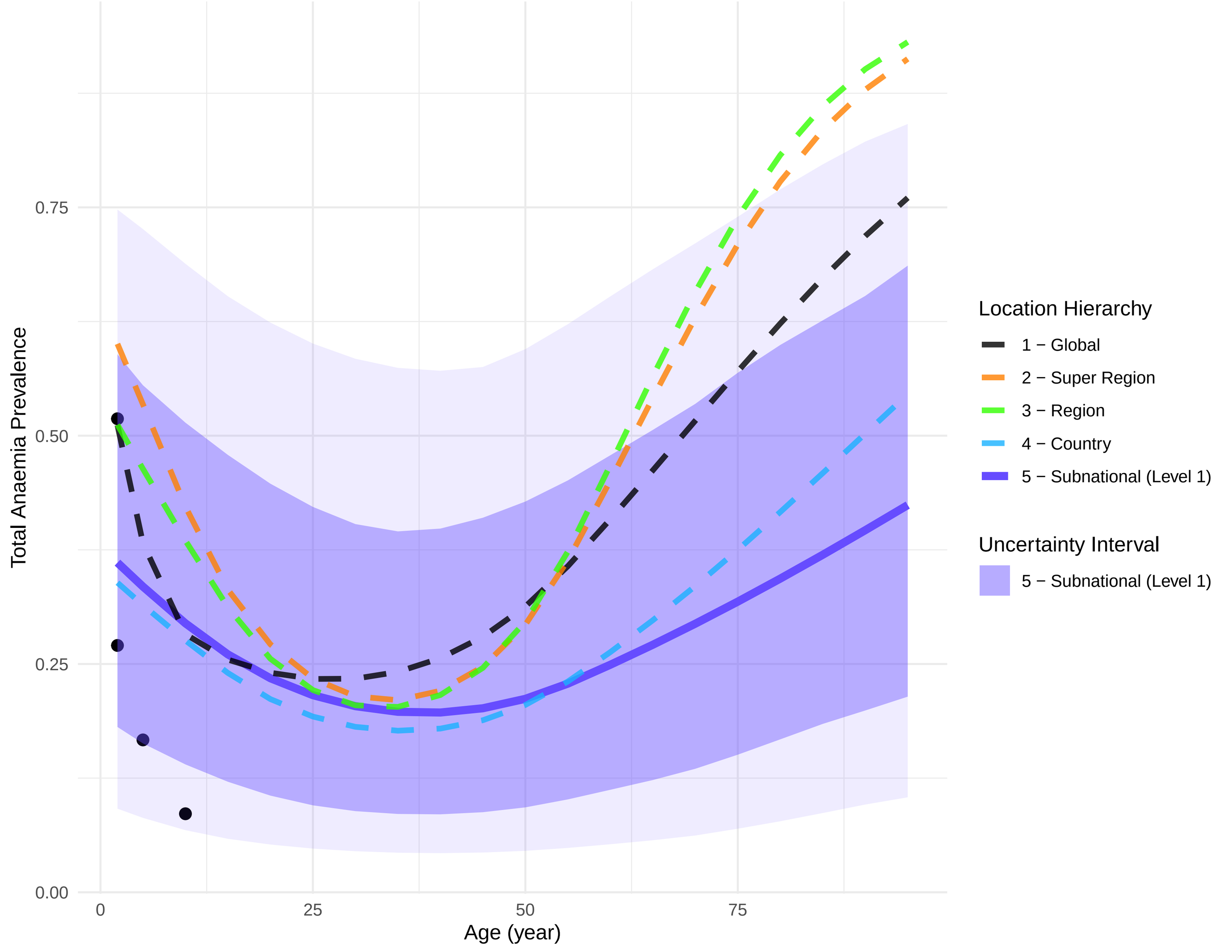

MR−BRT Age/Sex Fits − Males 2+ Years Old − Total Anaemia Prevalence − Turkana
WHO−2001 Adjustment Method

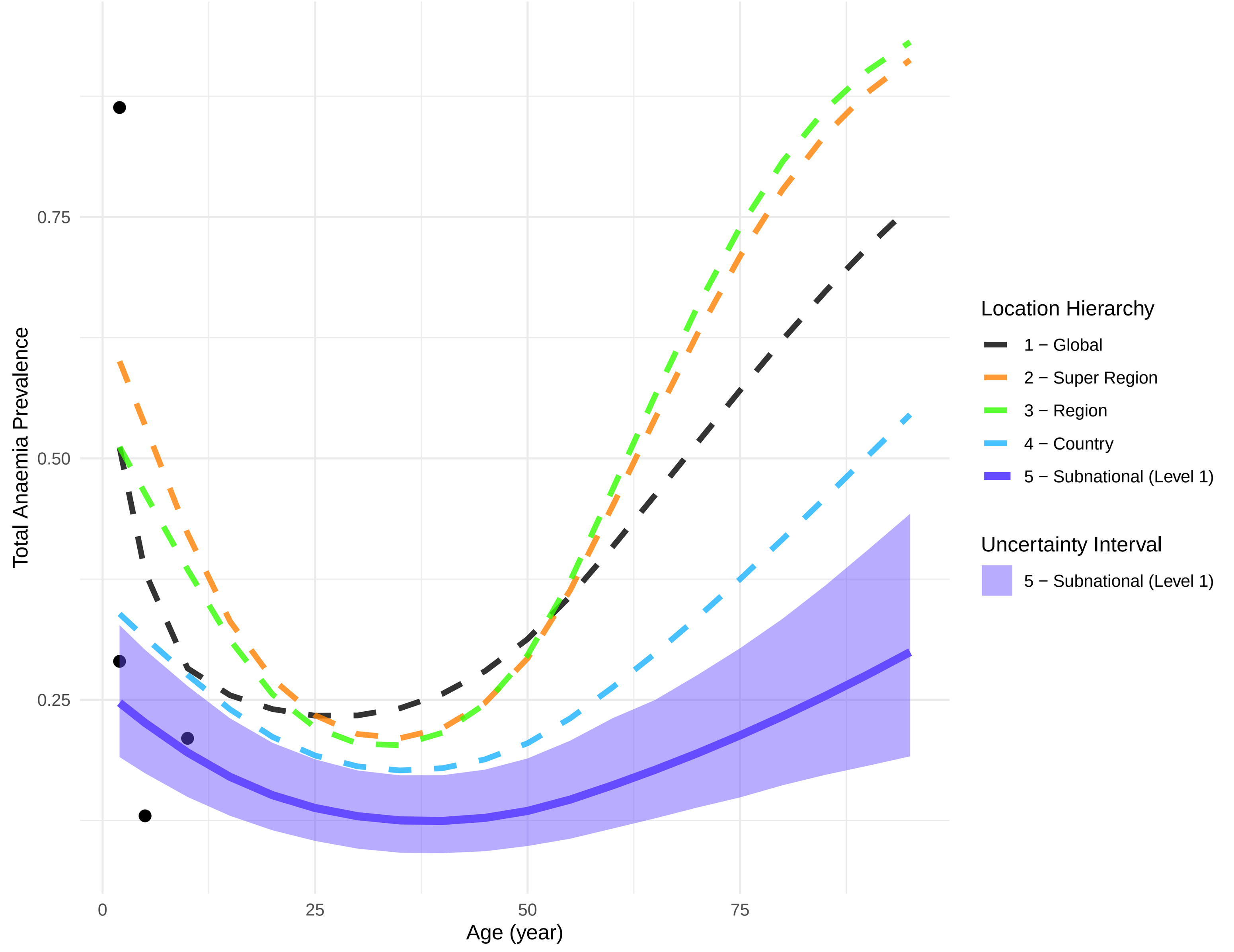

MR−BRT Age/Sex Fits − Males 2+ Years Old − Total Anaemia Prevalence − Uasin Gishu
WHO−2001 Adjustment Method

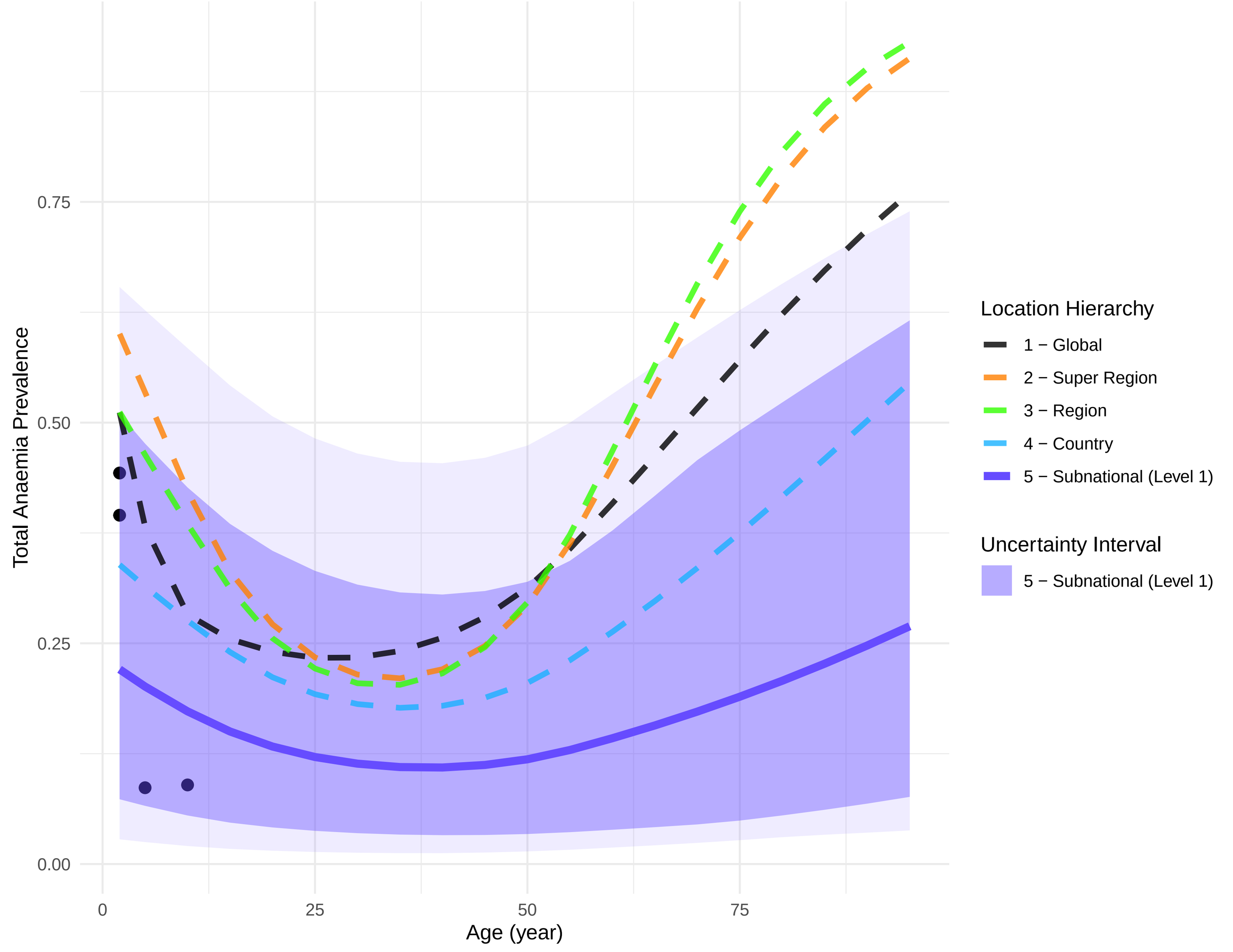

MR−BRT Age/Sex Fits − Males 2+ Years Old − Total Anaemia Prevalence − Vihiga
WHO−2001 Adjustment Method

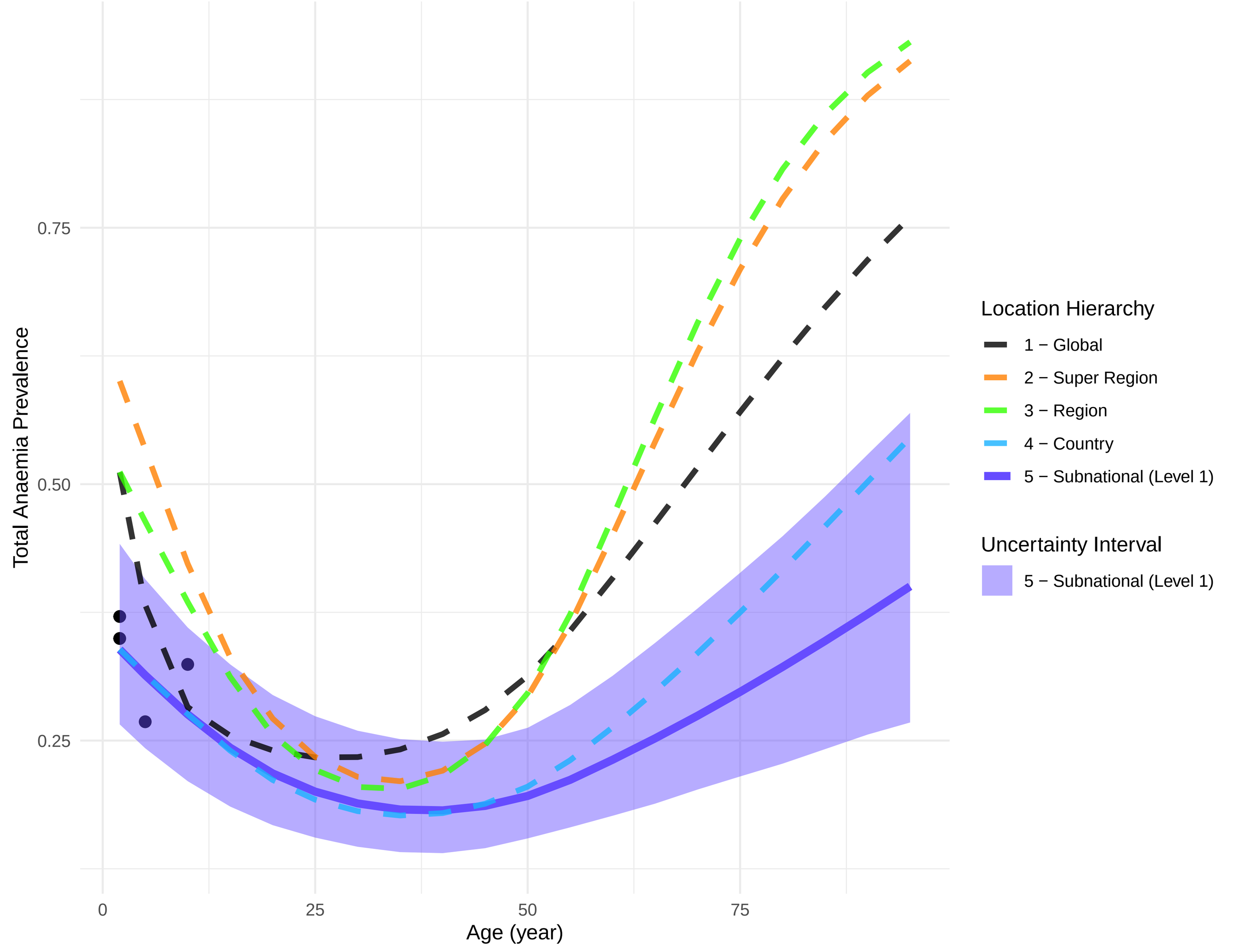

MR−BRT Age/Sex Fits − Males 2+ Years Old − Total Anaemia Prevalence − Wajir
WHO−2001 Adjustment Method

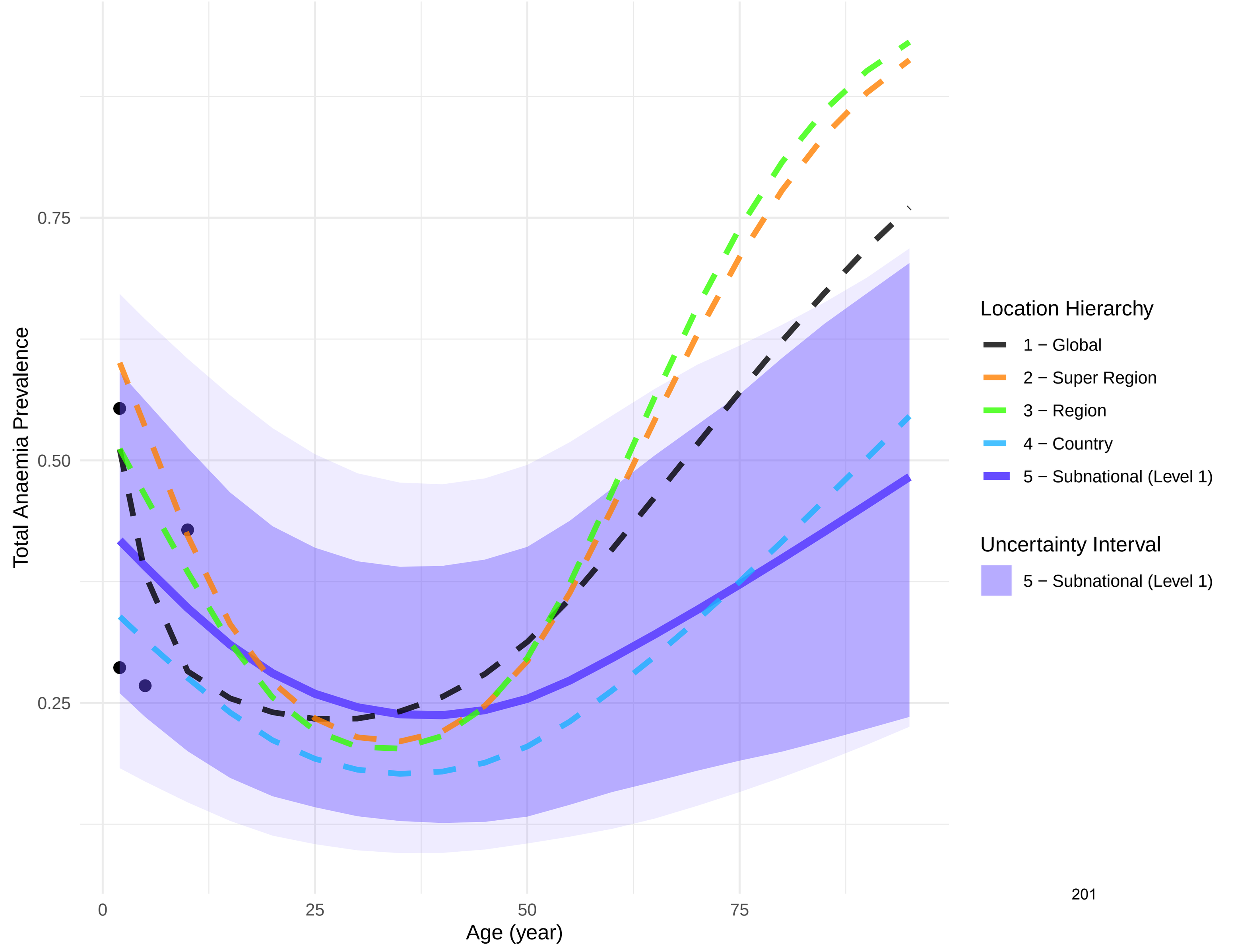

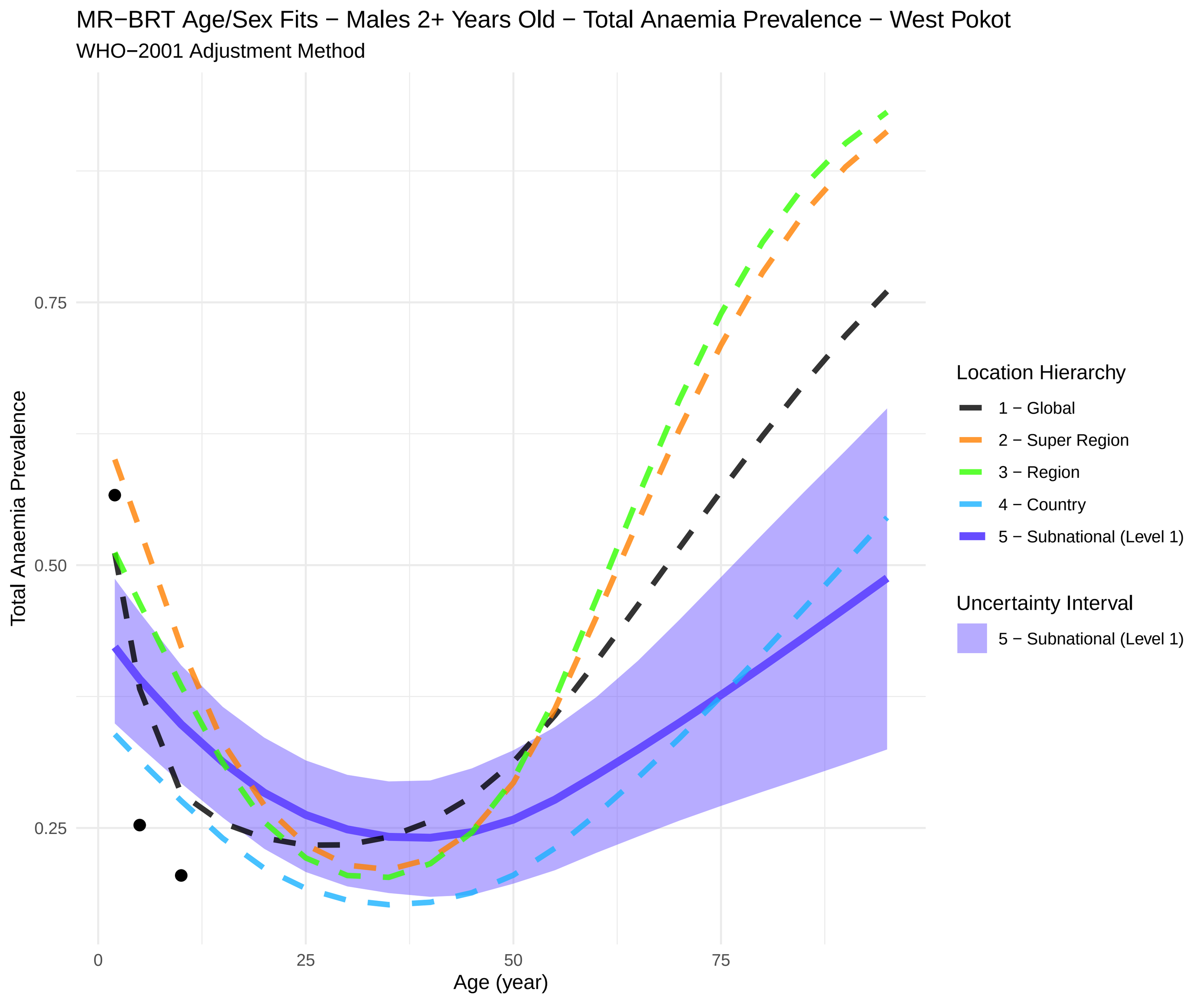
MR−BRT Age/Sex Fits − Males 2+ Years Old − Total Anaemia Prevalence − West Pokot
WHO−2001 Adjustment Method
Total Anaemia Prevalence
Age (year)
Location Hierarchy
1 − Global
2 − Super Region
3 − Region
4 − Country
5 − Subnational (Level 1)
Uncertainty Interval
5 − Subnational (Level 1)

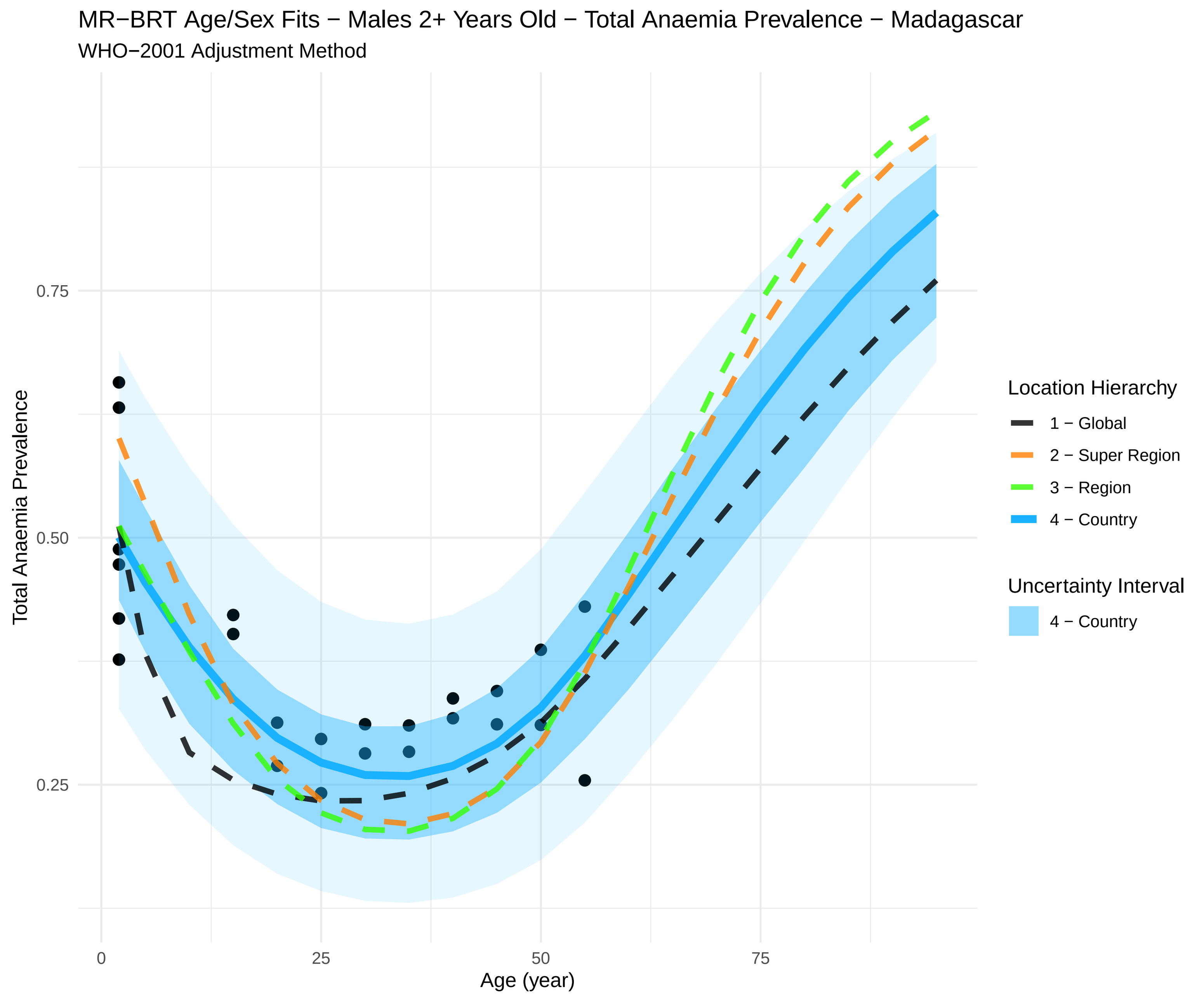
MR−BRT Age/Sex Fits − Males 2+ Years Old − Total Anaemia Prevalence − Madagascar
WHO−2001 Adjustment Method
Total Anaemia Prevalence
Age (year)
Location Hierarchy
1 − Global
2 − Super Region
3 − Region
4 − Country
Uncertainty Interval
4 − Country

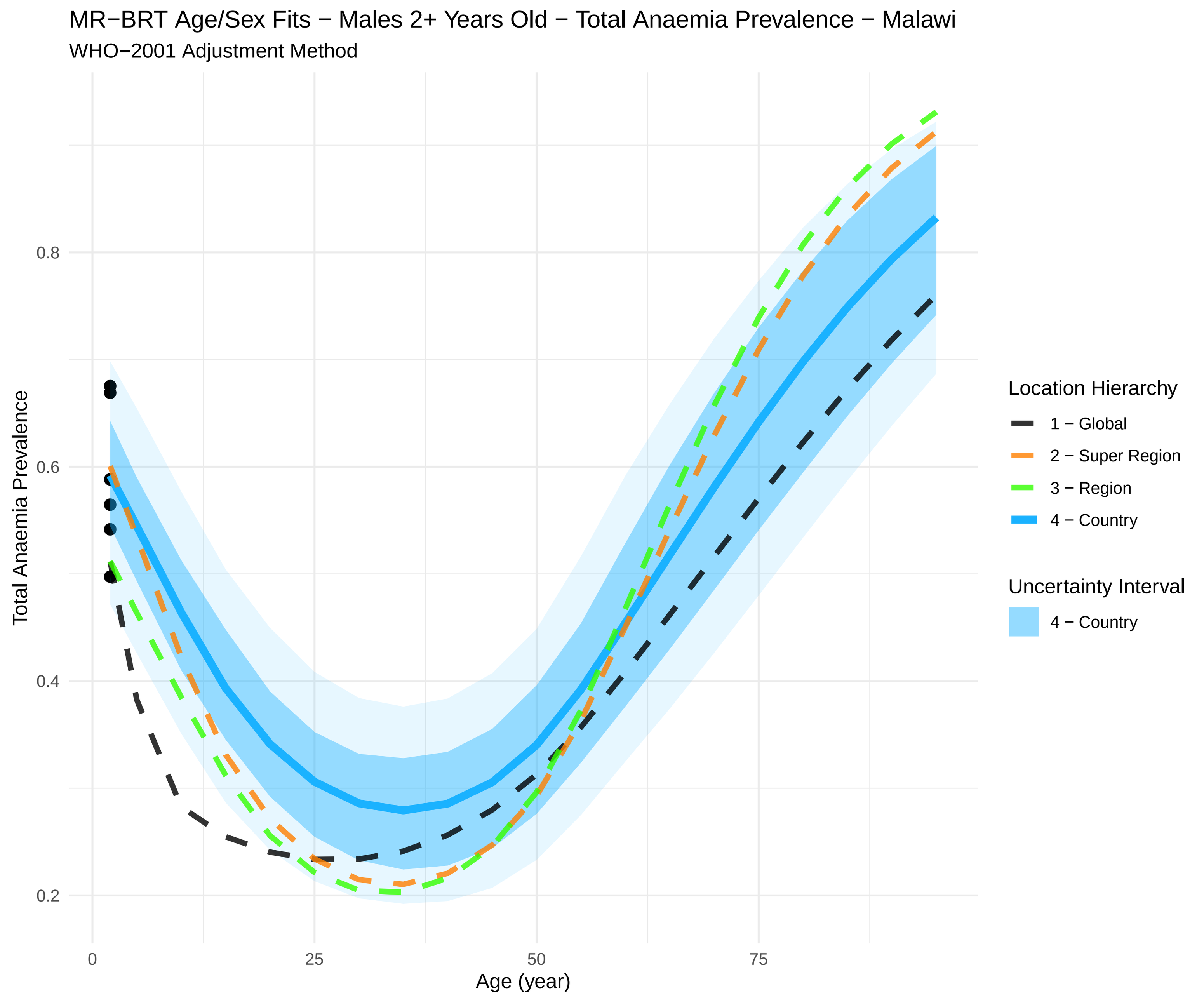
MR−BRT Age/Sex Fits − Males 2+ Years Old − Total Anaemia Prevalence − Malawi
WHO−2001 Adjustment Method
Total Anaemia Prevalence
Age (year)
Location Hierarchy
1 − Global
2 − Super Region
3 − Region
4 − Country
Uncertainty Interval
4 − Country

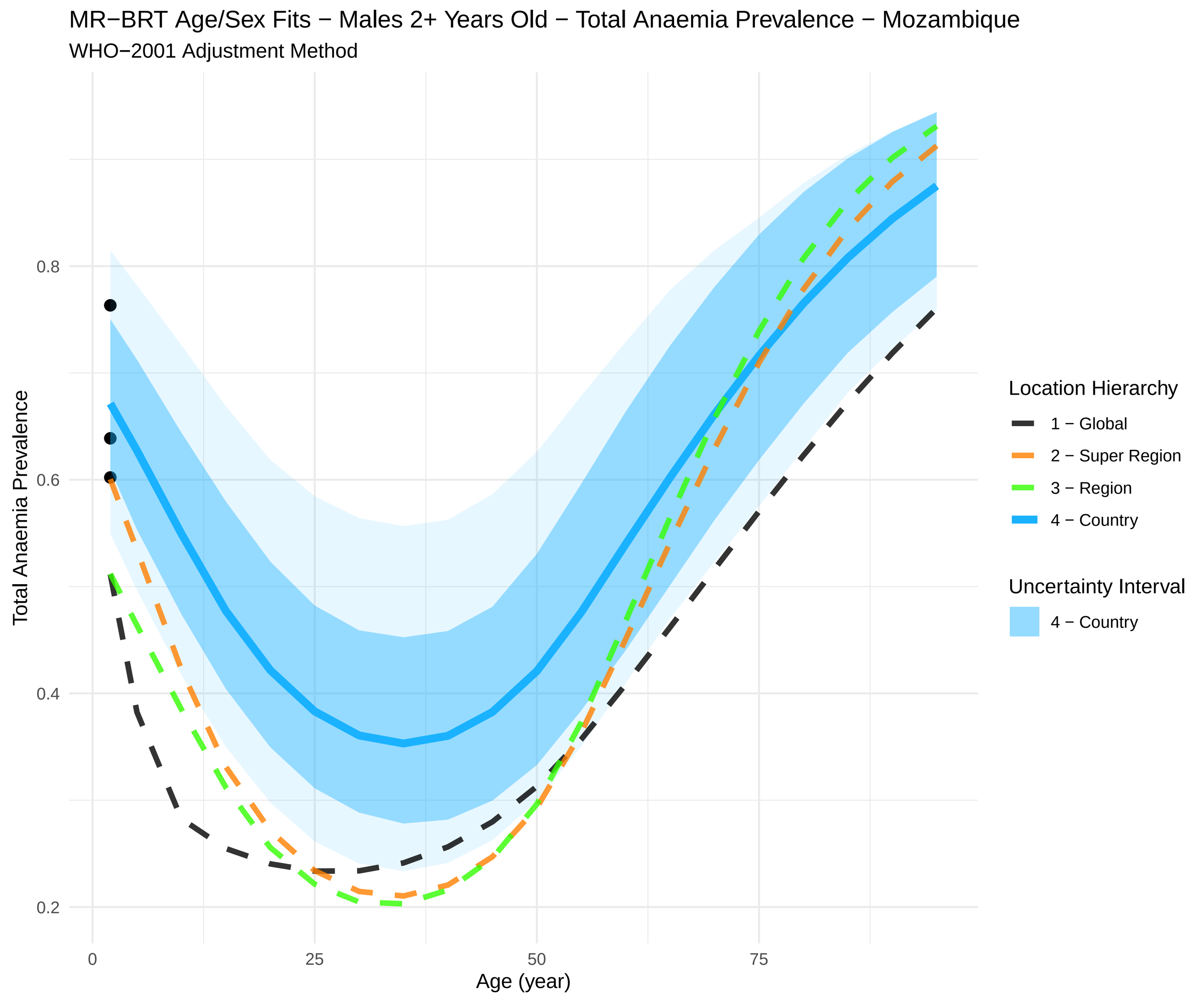
MR−BRT Age/Sex Fits − Males 2+ Years Old − Total Anaemia Prevalence − Mozambique
WHO−2001 Adjustment Method
Total Anaemia Prevalence
Age (year)
Location Hierarchy
1 − Global
2 − Super Region
3 − Region
4 − Country
Uncertainty Interval
4 − Country

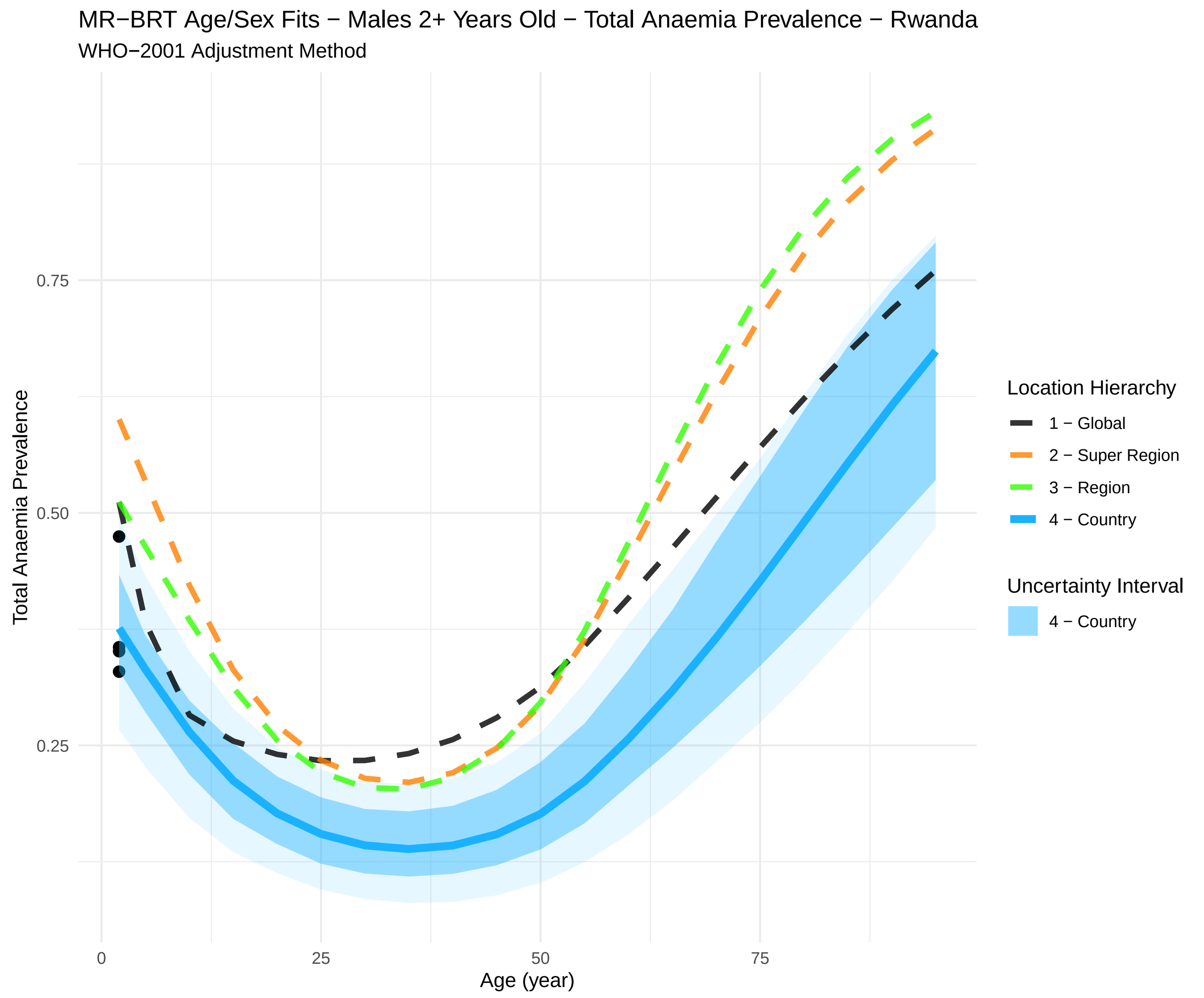
MR−BRT Age/Sex Fits − Males 2+ Years Old − Total Anaemia Prevalence − Rwanda
WHO−2001 Adjustment Method
Total Anaemia Prevalence
Age (year)
Location Hierarchy
1 − Global
2 − Super Region
3 − Region
4 − Country
Uncertainty Interval
4 − Country

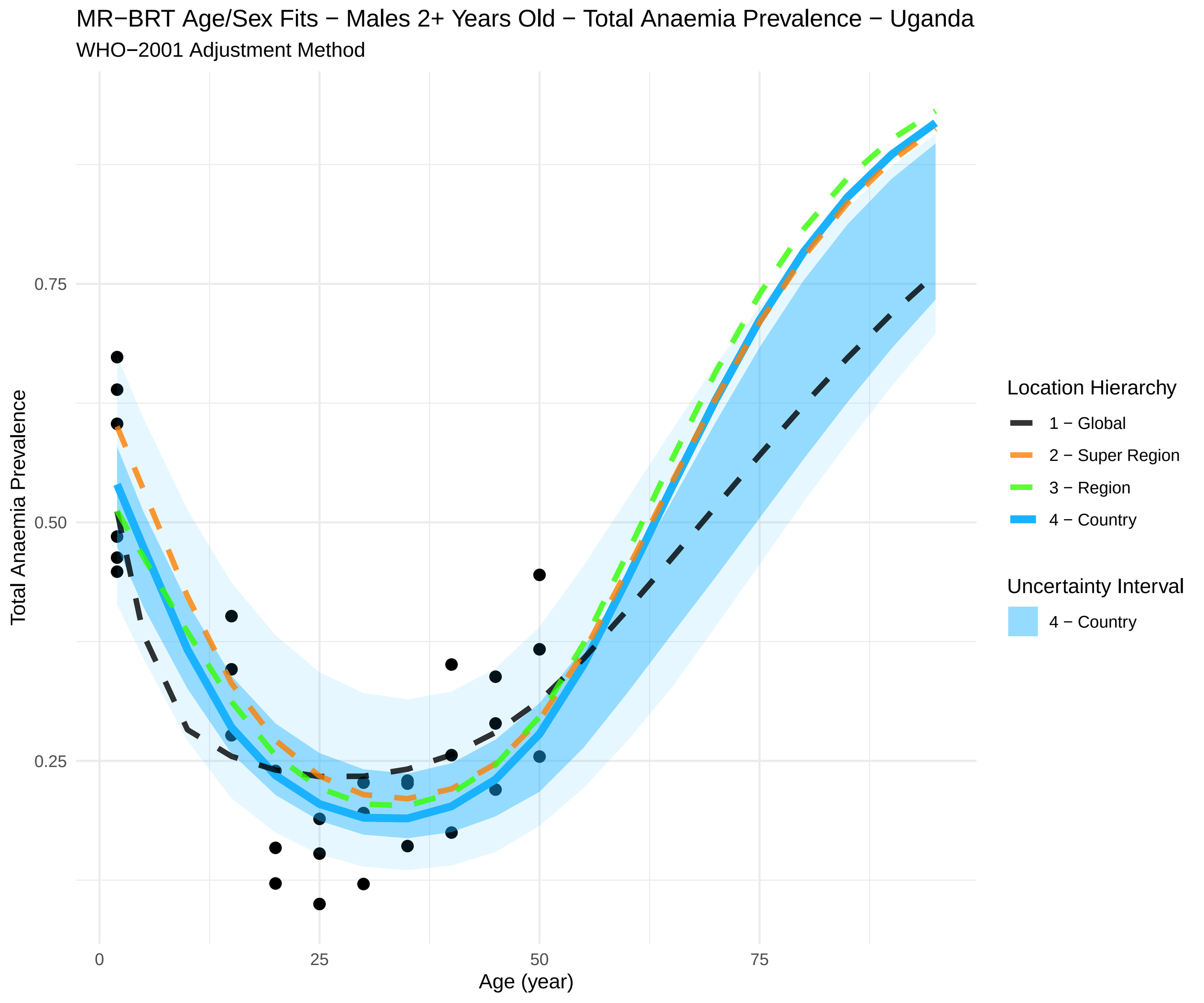
MR−BRT Age/Sex Fits − Males 2+ Years Old − Total Anaemia Prevalence − Uganda
WHO−2001 Adjustment Method
Total Anaemia Prevalence
Age (year)
Location Hierarchy
1 − Global
2 − Super Region
3 − Region
4 − Country
Uncertainty Interval
4 − Country

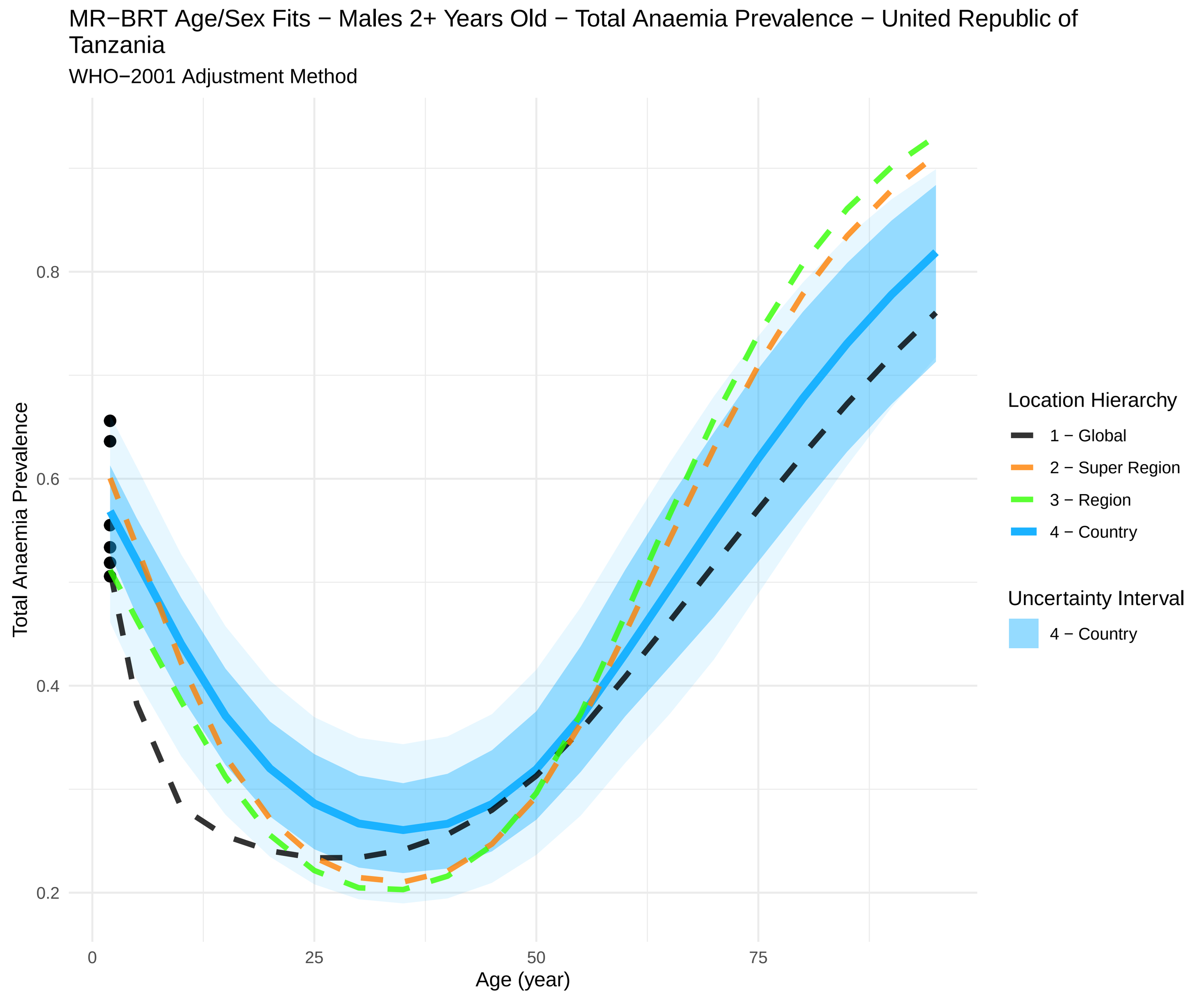
MR−BRT Age/Sex Fits − Males 2+ Years Old − Total Anaemia Prevalence − United Republic of Tanzania
WHO−2001 Adjustment Method
Total Anaemia Prevalence
Age (year)
Location Hierarchy
1 − Global
2 − Super Region
3 − Region
4 − Country
Uncertainty Interval
4 − Country

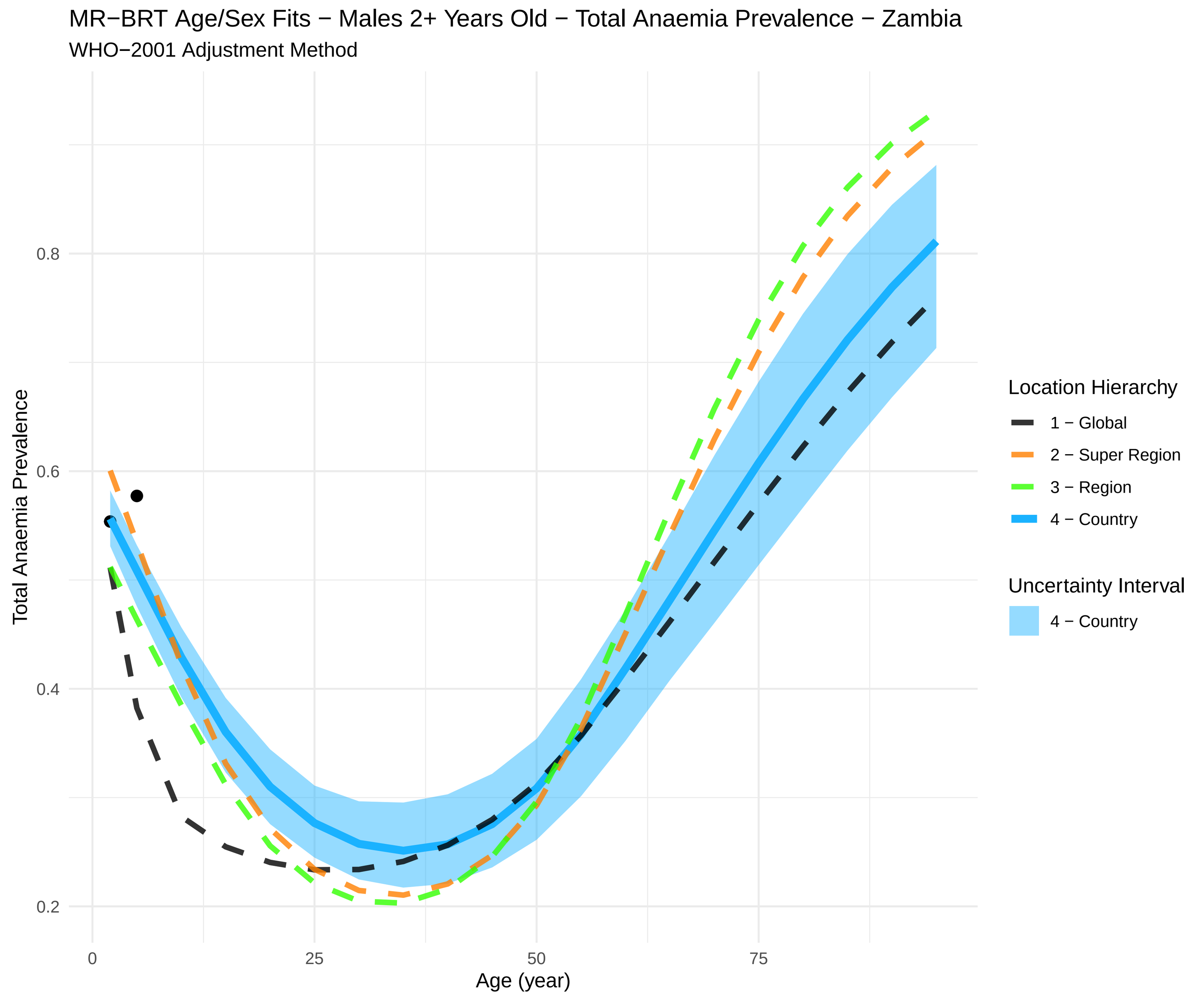
MR−BRT Age/Sex Fits − Males 2+ Years Old − Total Anaemia Prevalence − Zambia
WHO−2001 Adjustment Method
Total Anaemia Prevalence
Age (year)
Location Hierarchy
1 − Global
2 − Super Region
3 − Region
4 − Country
Uncertainty Interval
4 − Country

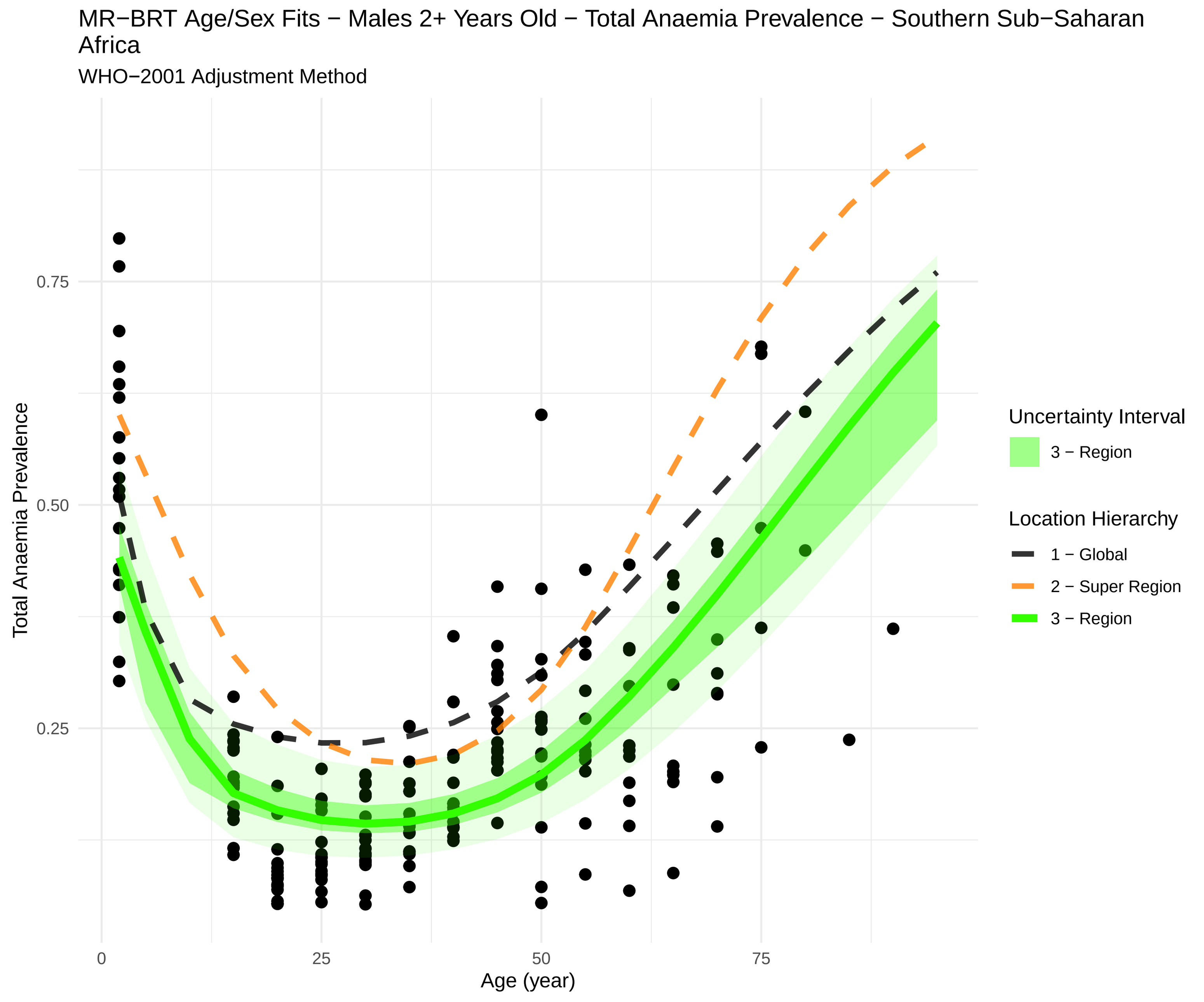
MR−BRT Age/Sex Fits − Males 2+ Years Old − Total Anaemia Prevalence − Southern Sub−Saharan Africa
WHO−2001 Adjustment Method
Total Anaemia Prevalence
Age (year)
Uncertainty Interval
3 − Region
Location Hierarchy
1 − Global
2 − Super Region
3 − Region

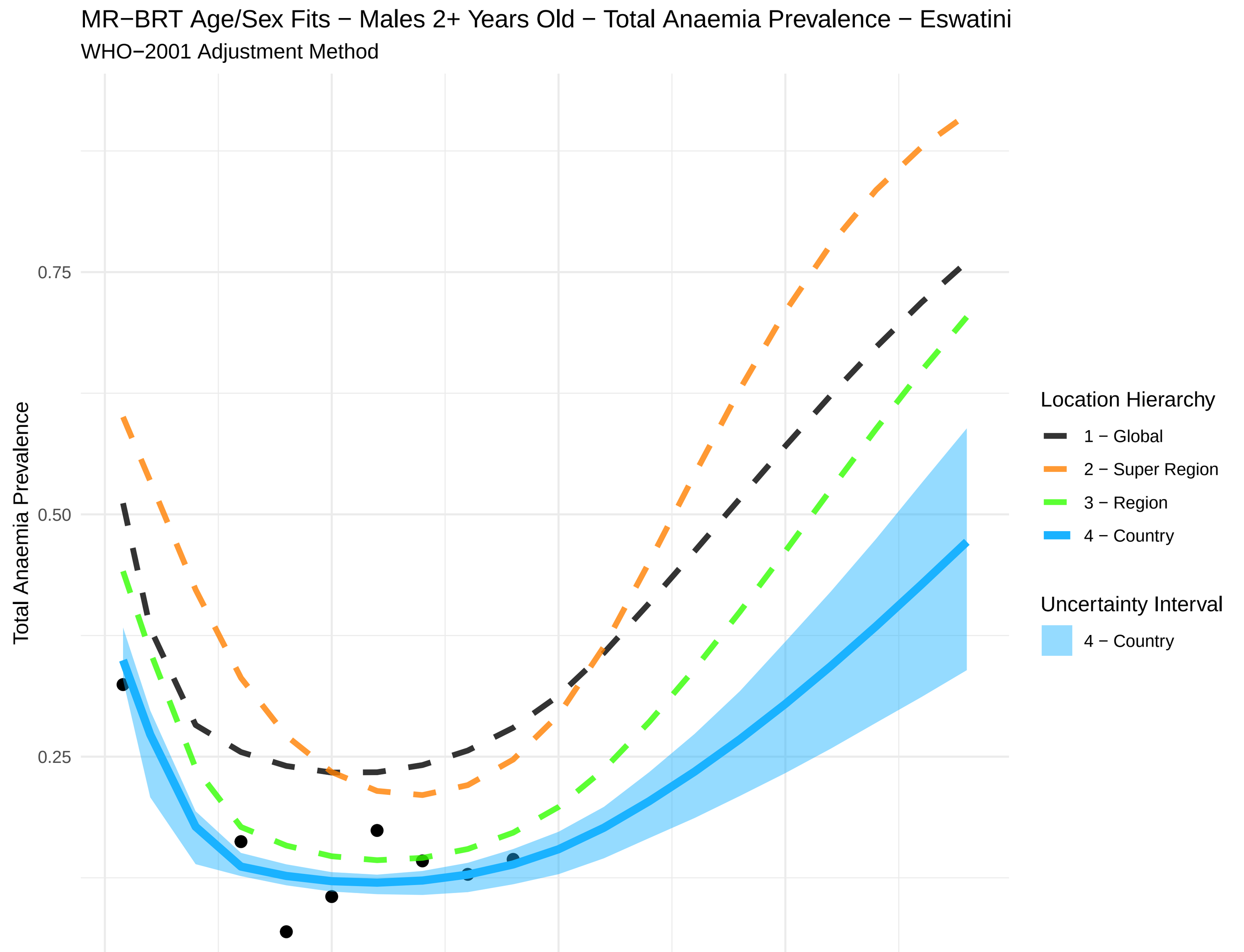
MR−BRT Age/Sex Fits − Males 2+ Years Old − Total Anaemia Prevalence − Eswatini
WHO−2001 Adjustment Method
Total Anaemia Prevalence
Age (year)
Location Hierarchy
1 − Global
2 − Super Region
3 − Region
4 − Country
Uncertainty Interval
4 − Country

MR−BRT Age/Sex Fits − Males 2+ Years Old − Total Anaemia Prevalence − Lesotho
WHO−2001 Adjustment Method

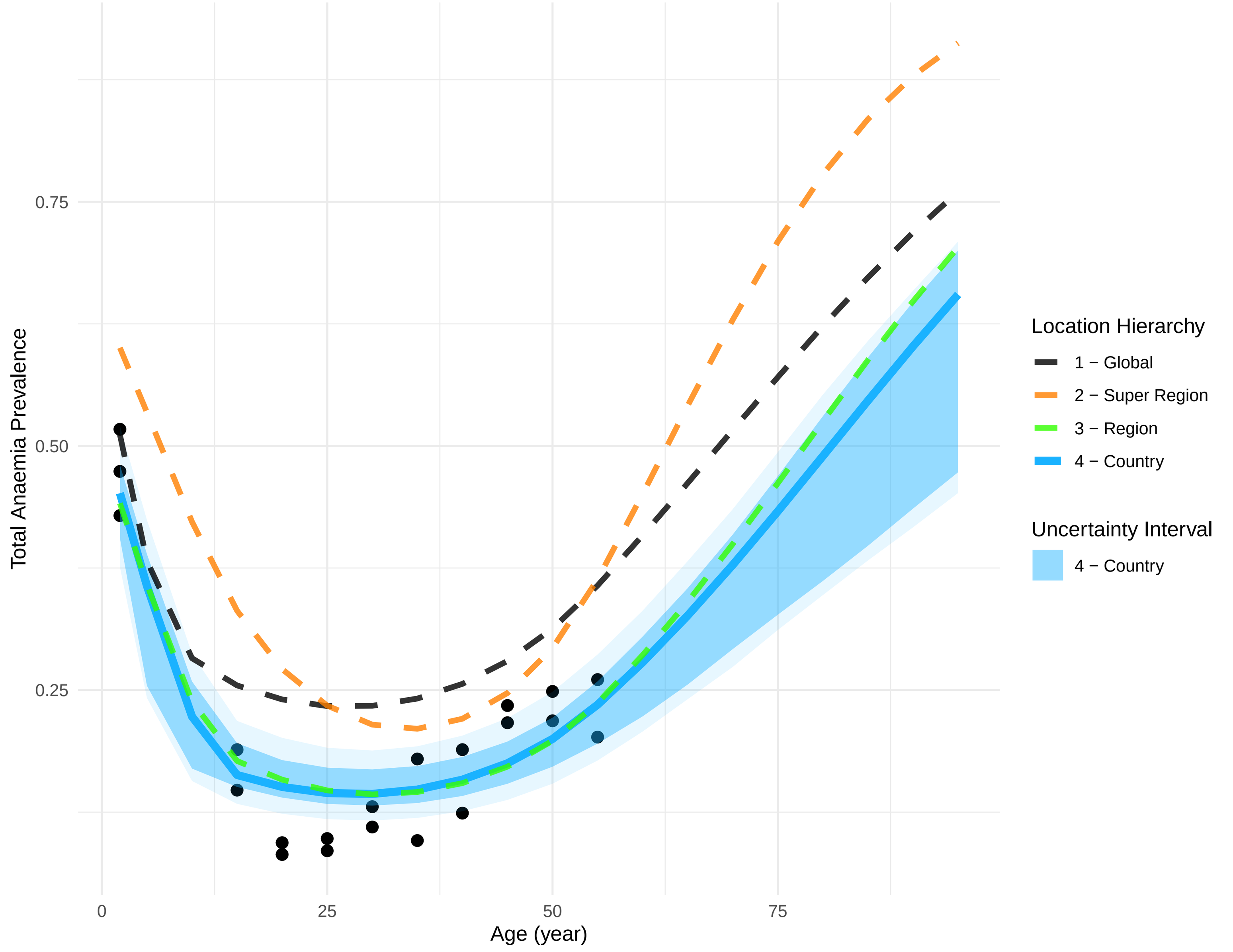

MR−BRT Age/Sex Fits − Males 2+ Years Old − Total Anaemia Prevalence − Namibia
WHO−2001 Adjustment Method

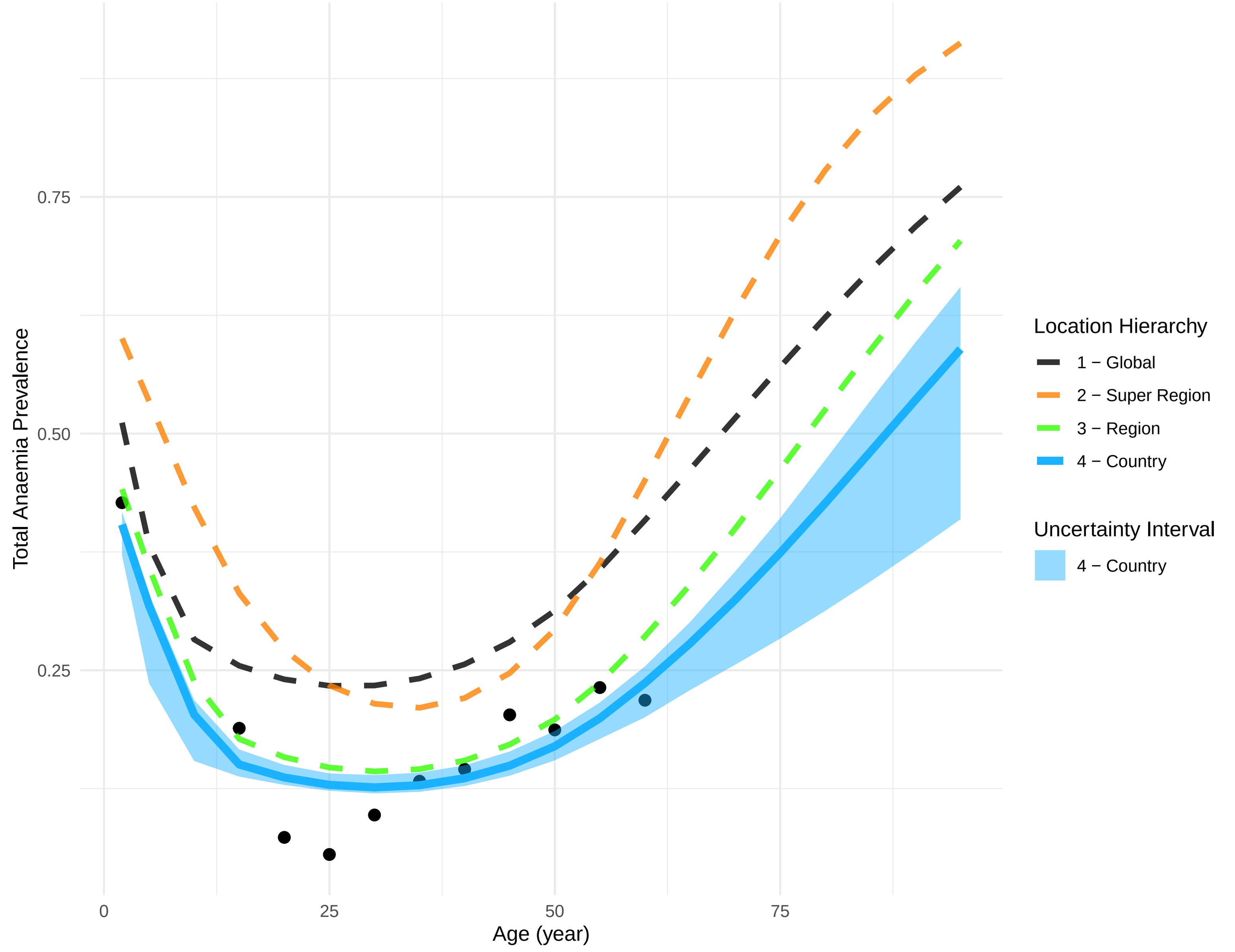

MR−BRT Age/Sex Fits − Males 2+ Years Old − Total Anaemia Prevalence − South Africa
WHO−2001 Adjustment Method

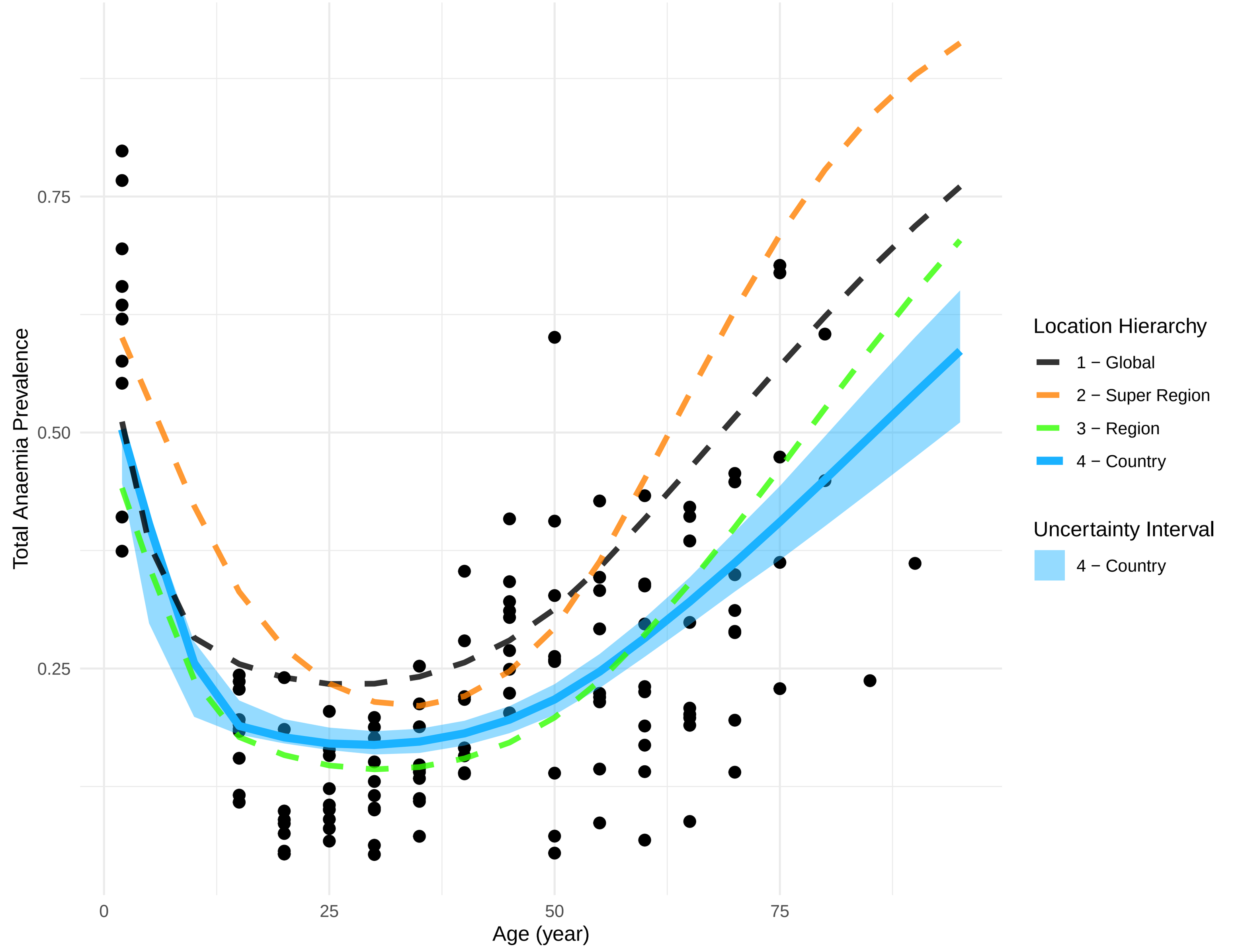

MR−BRT Age/Sex Fits − Males 2+ Years Old − Total Anaemia Prevalence − Eastern Cape
WHO−2001 Adjustment Method

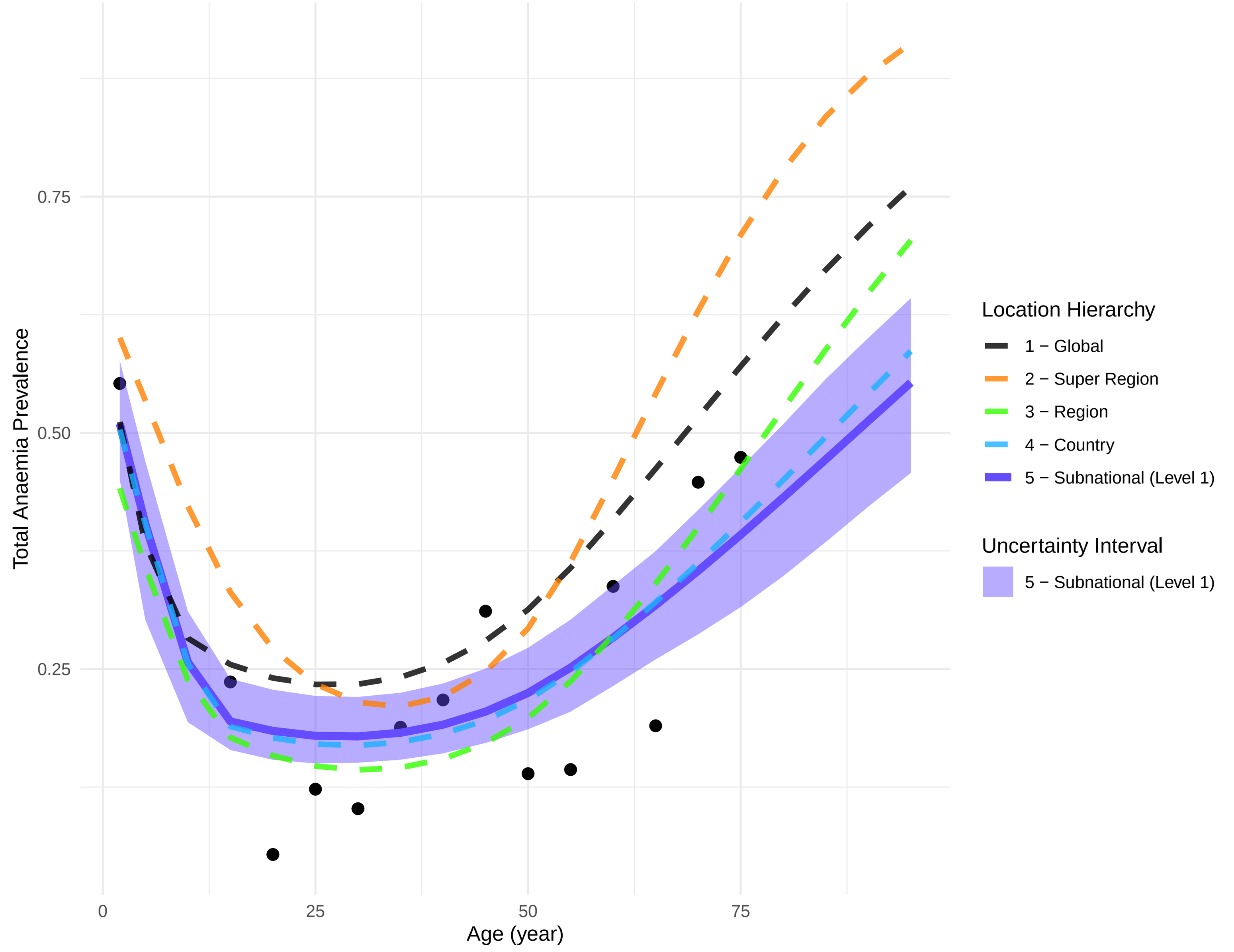

MR−BRT Age/Sex Fits − Males 2+ Years Old − Total Anaemia Prevalence − Free State
WHO−2001 Adjustment Method

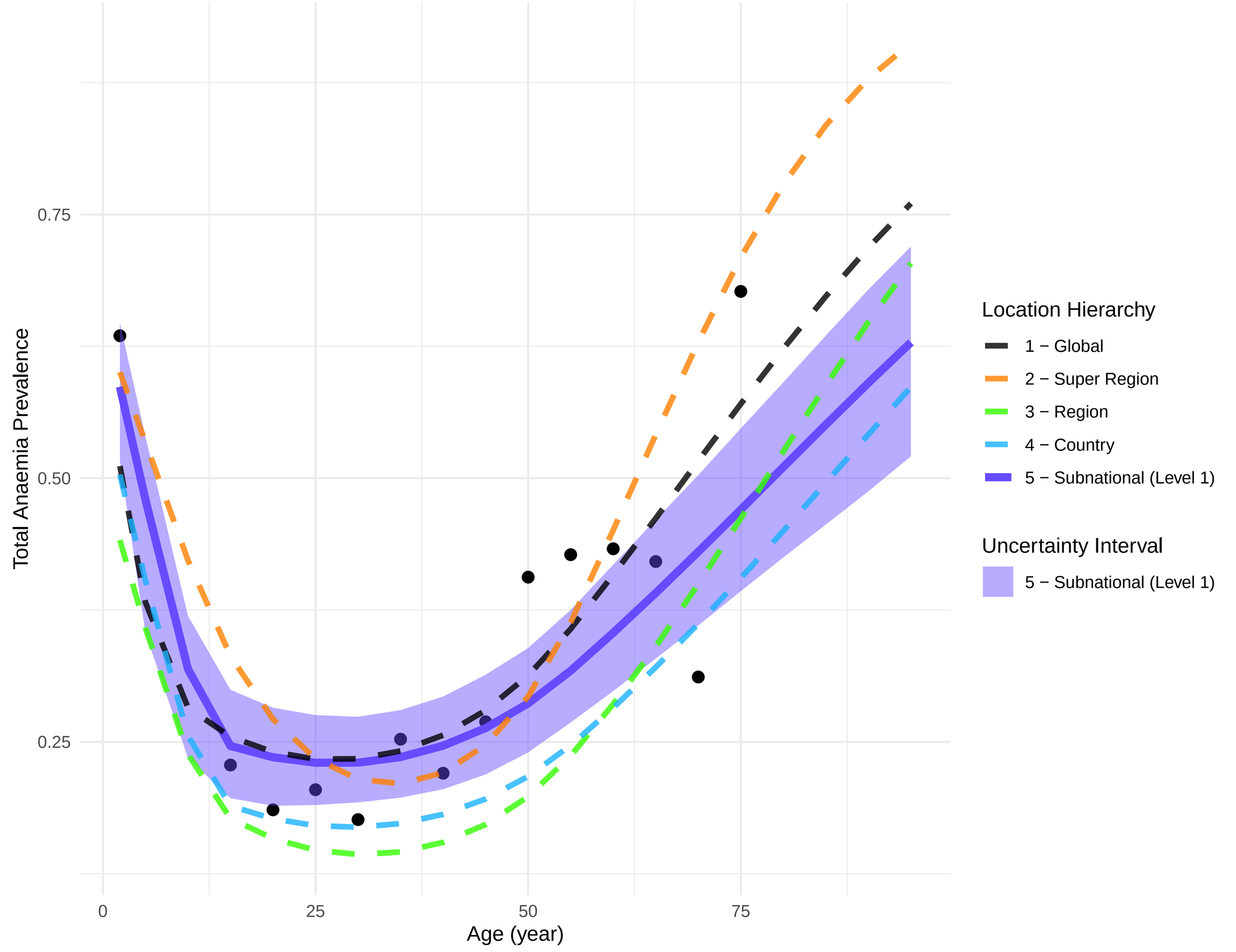

MR−BRT Age/Sex Fits − Males 2+ Years Old − Total Anaemia Prevalence − Gauteng
WHO−2001 Adjustment Method

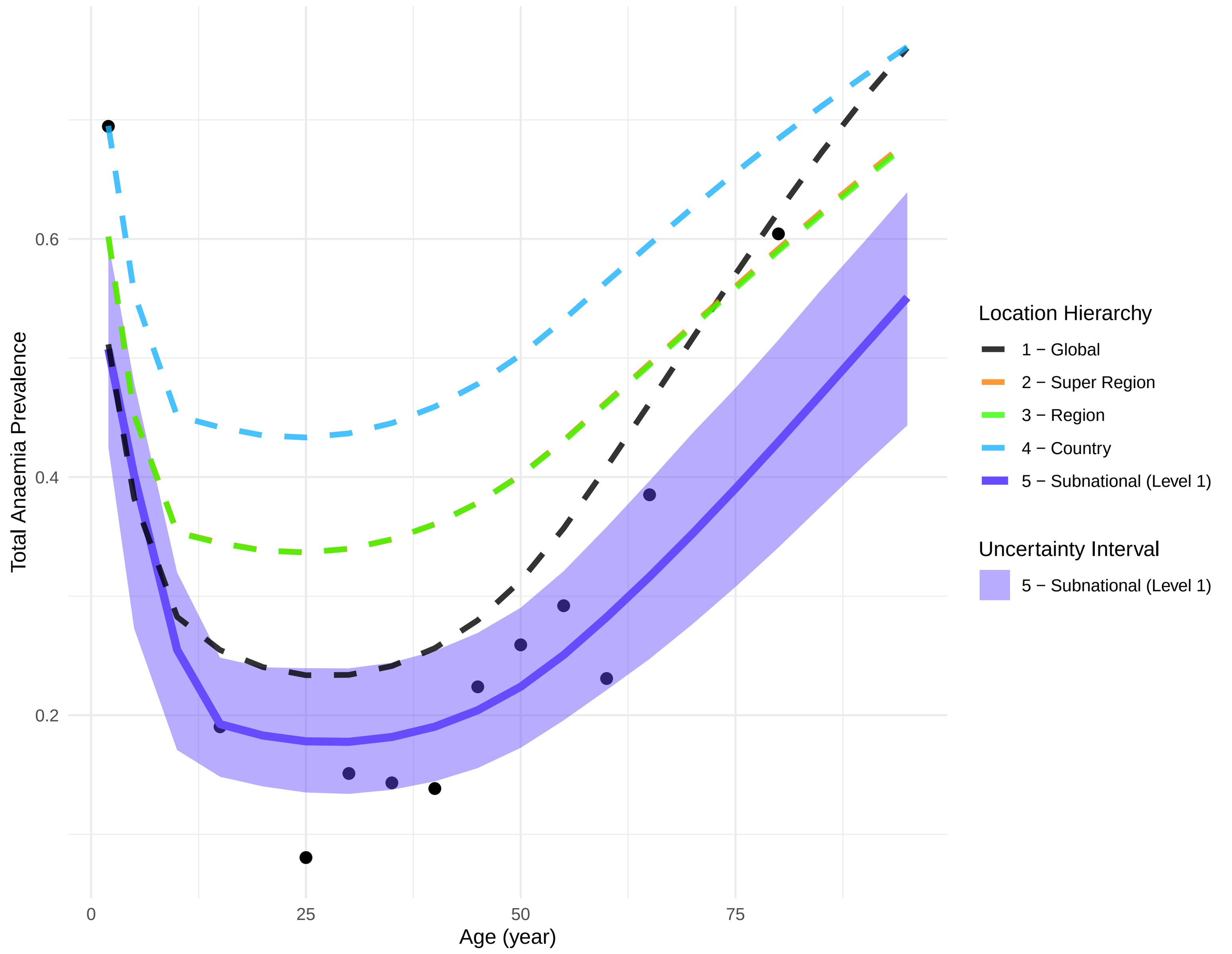

MR−BRT Age/Sex Fits − Males 2+ Years Old − Total Anaemia Prevalence − KwaZulu−Natal
WHO−2001 Adjustment Method

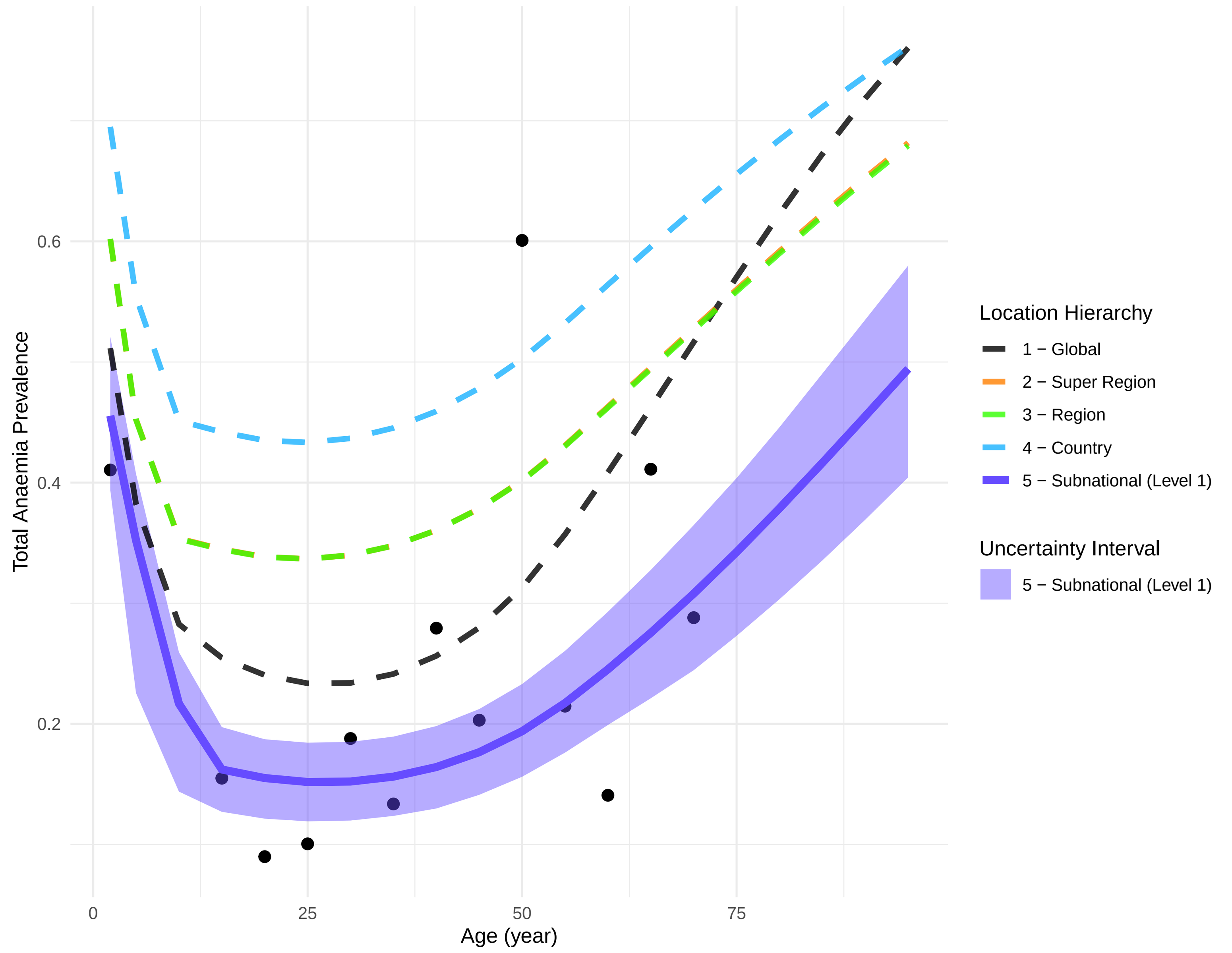

MR−BRT Age/Sex Fits − Males 2+ Years Old − Total Anaemia Prevalence − Limpopo
WHO−2001 Adjustment Method

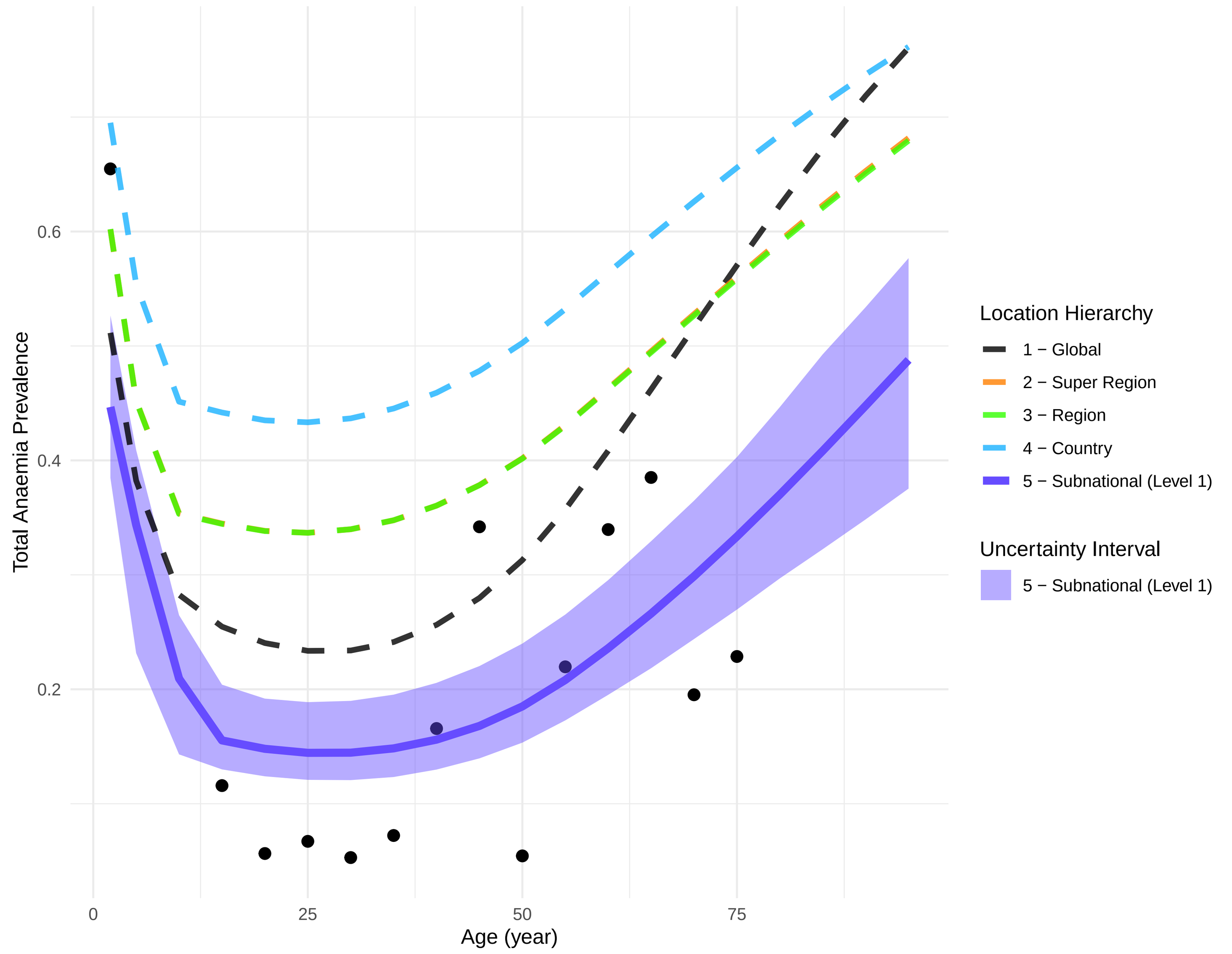

MR−BRT Age/Sex Fits − Males 2+ Years Old − Total Anaemia Prevalence − Mpumalanga
WHO−2001 Adjustment Method

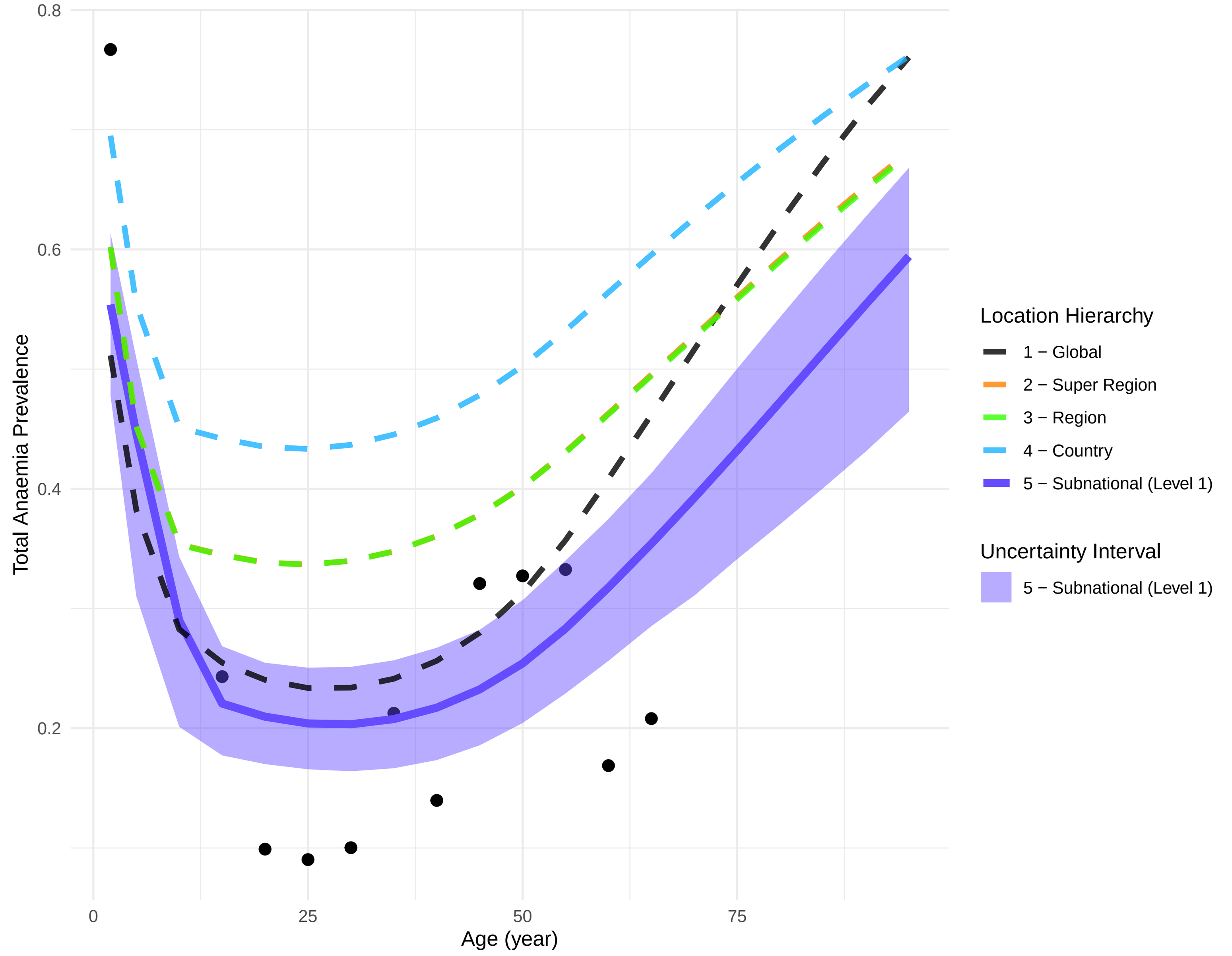

MR−BRT Age/Sex Fits − Males 2+ Years Old − Total Anaemia Prevalence − North−West
WHO−2001 Adjustment Method

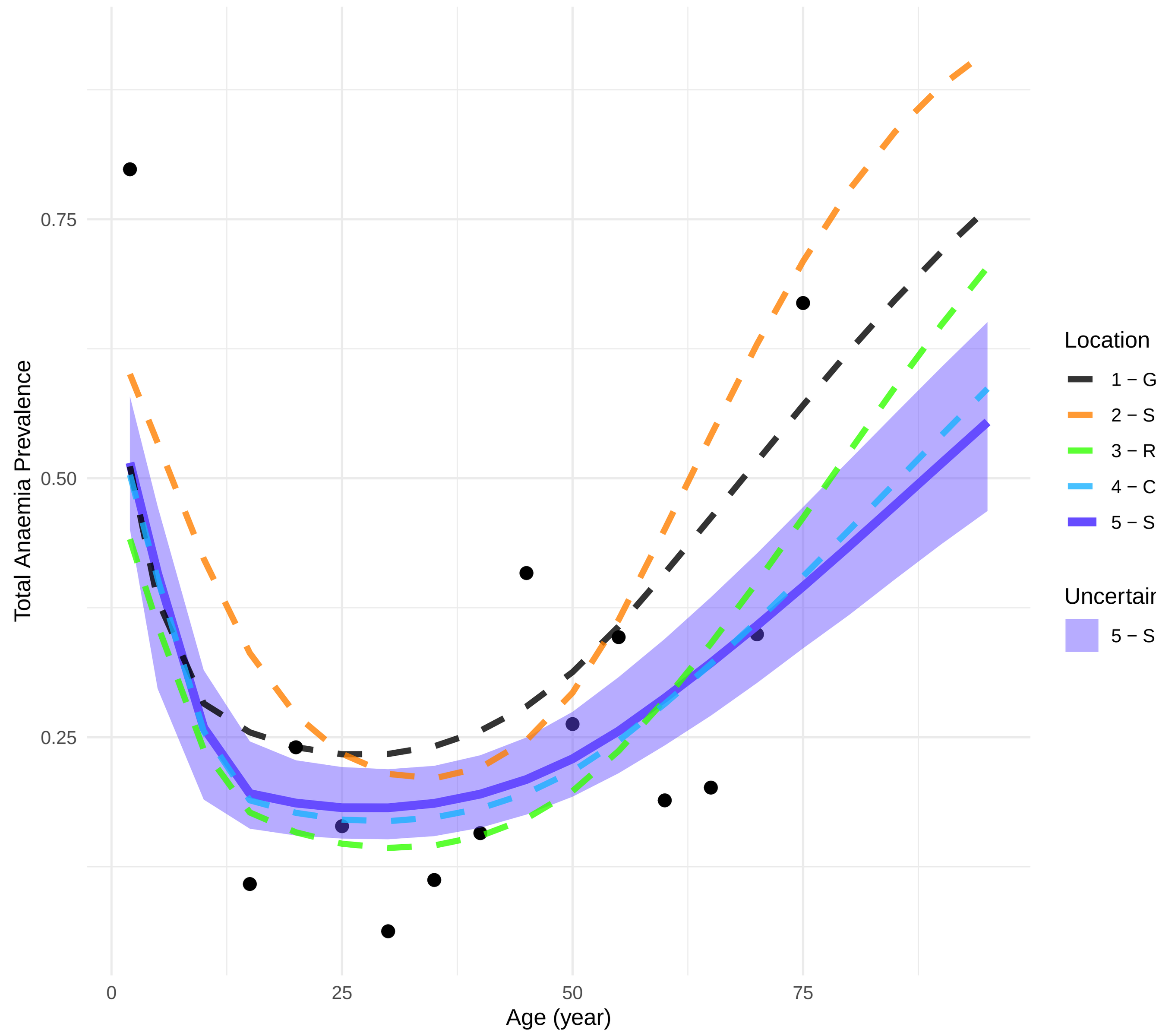

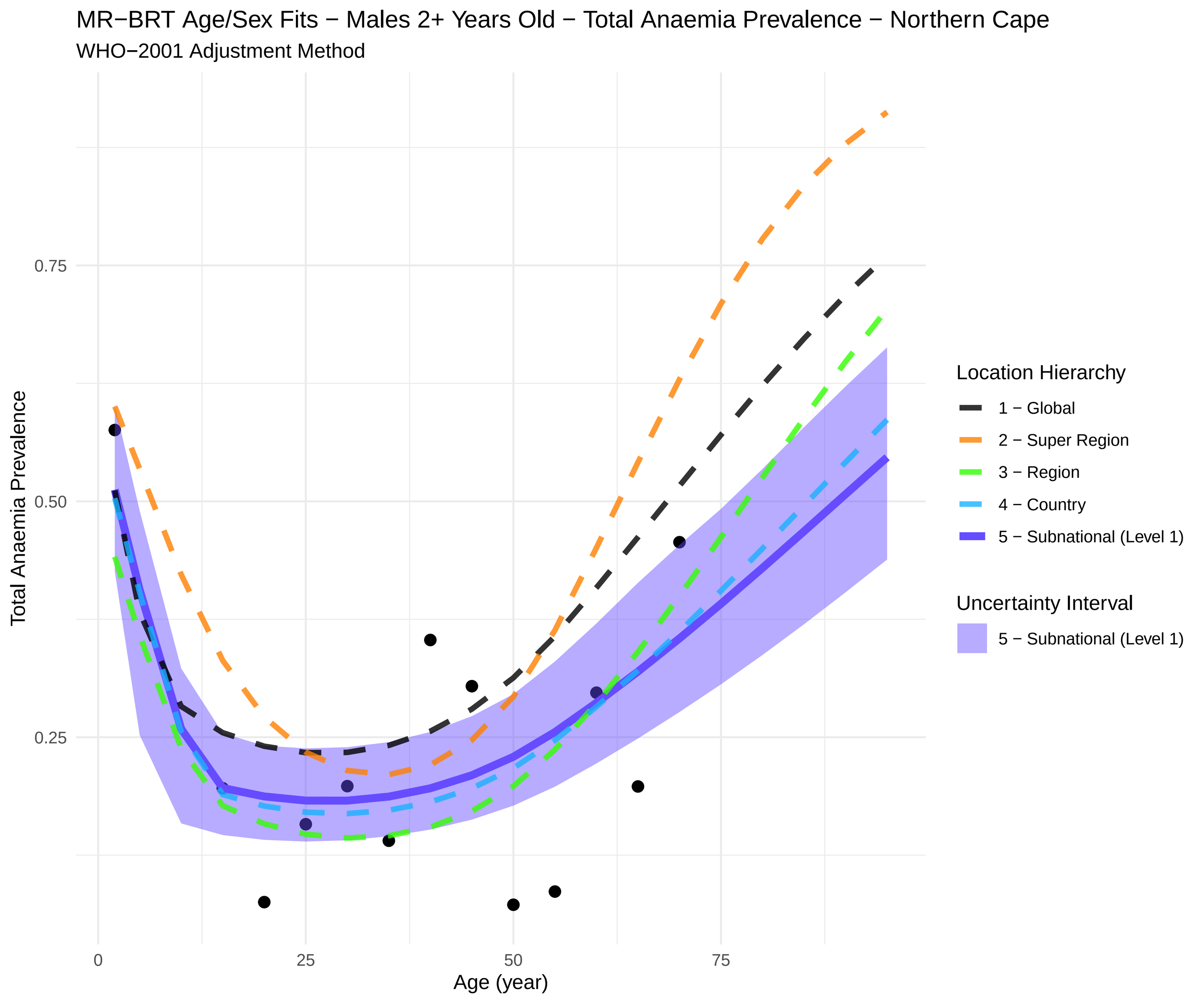
MR−BRT Age/Sex Fits − Males 2+ Years Old − Total Anaemia Prevalence − Northern Cape
WHO−2001 Adjustment Method
Total Anaemia Prevalence
Age (year)
Location Hierarchy
1 − Global
2 − Super Region
3 − Region
4 − Country
5 − Subnational (Level 1)
Uncertainty Interval
5 − Subnational (Level 1)

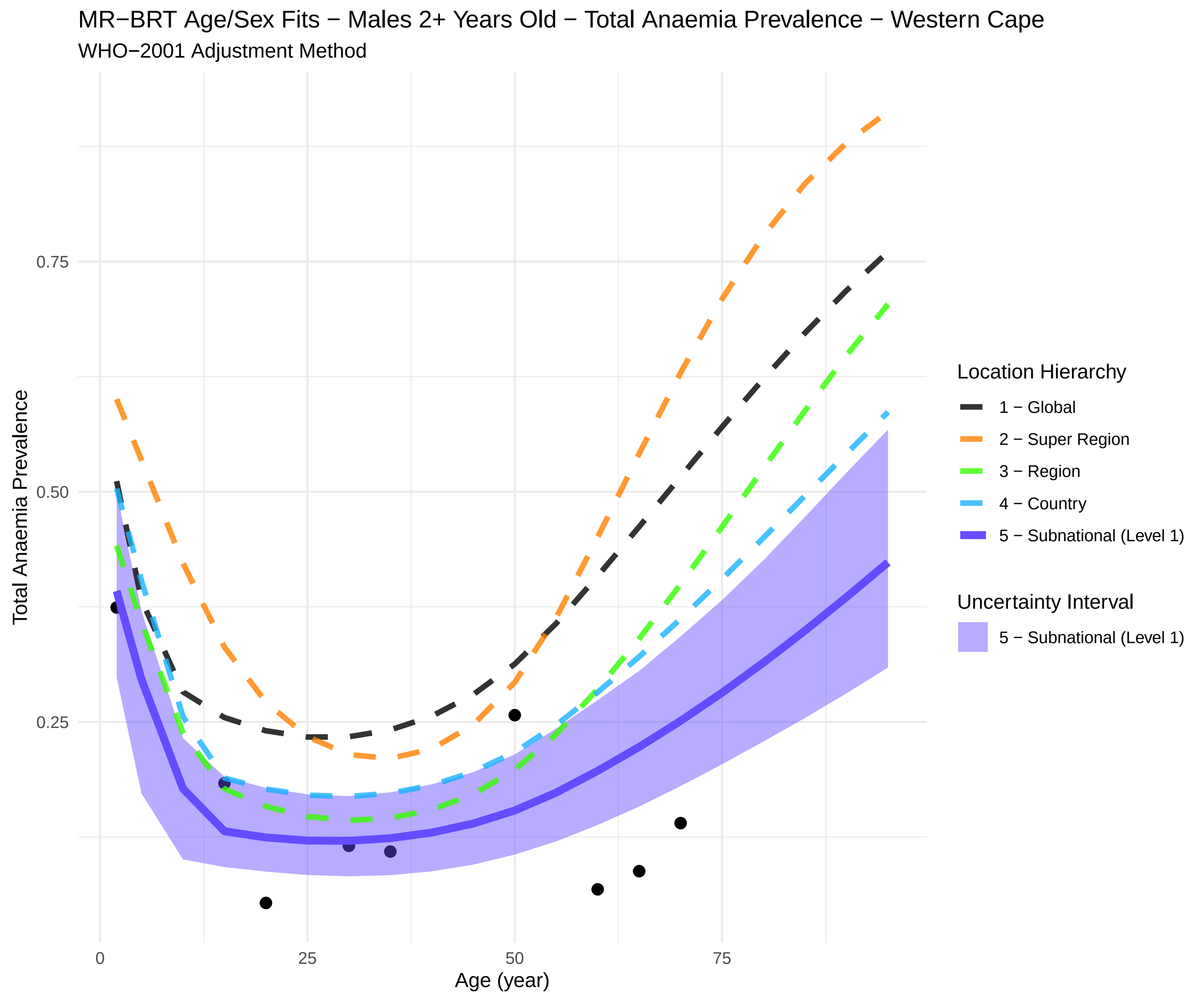
MR−BRT Age/Sex Fits − Males 2+ Years Old − Total Anaemia Prevalence − Western Cape
WHO−2001 Adjustment Method
Total Anaemia Prevalence
Age (year)
Location Hierarchy
1 − Global
2 − Super Region
3 − Region
4 − Country
5 − Subnational (Level 1)
Uncertainty Interval
5 − Subnational (Level 1)

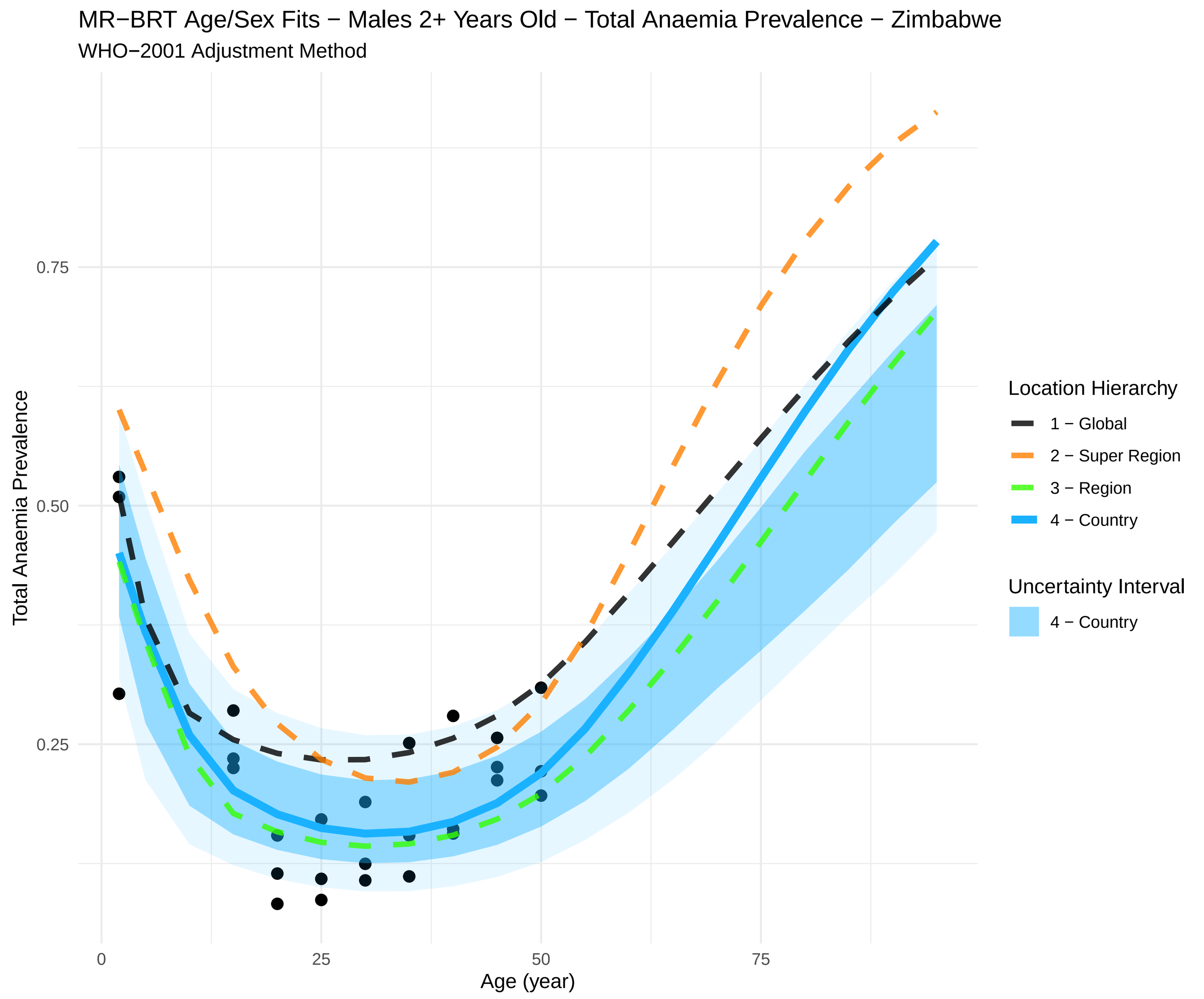
MR−BRT Age/Sex Fits − Males 2+ Years Old − Total Anaemia Prevalence − Zimbabwe
WHO−2001 Adjustment Method
Total Anaemia Prevalence
Age (year)
Location Hierarchy
1 − Global
2 − Super Region
3 − Region
4 − Country
Uncertainty Interval
4 − Country

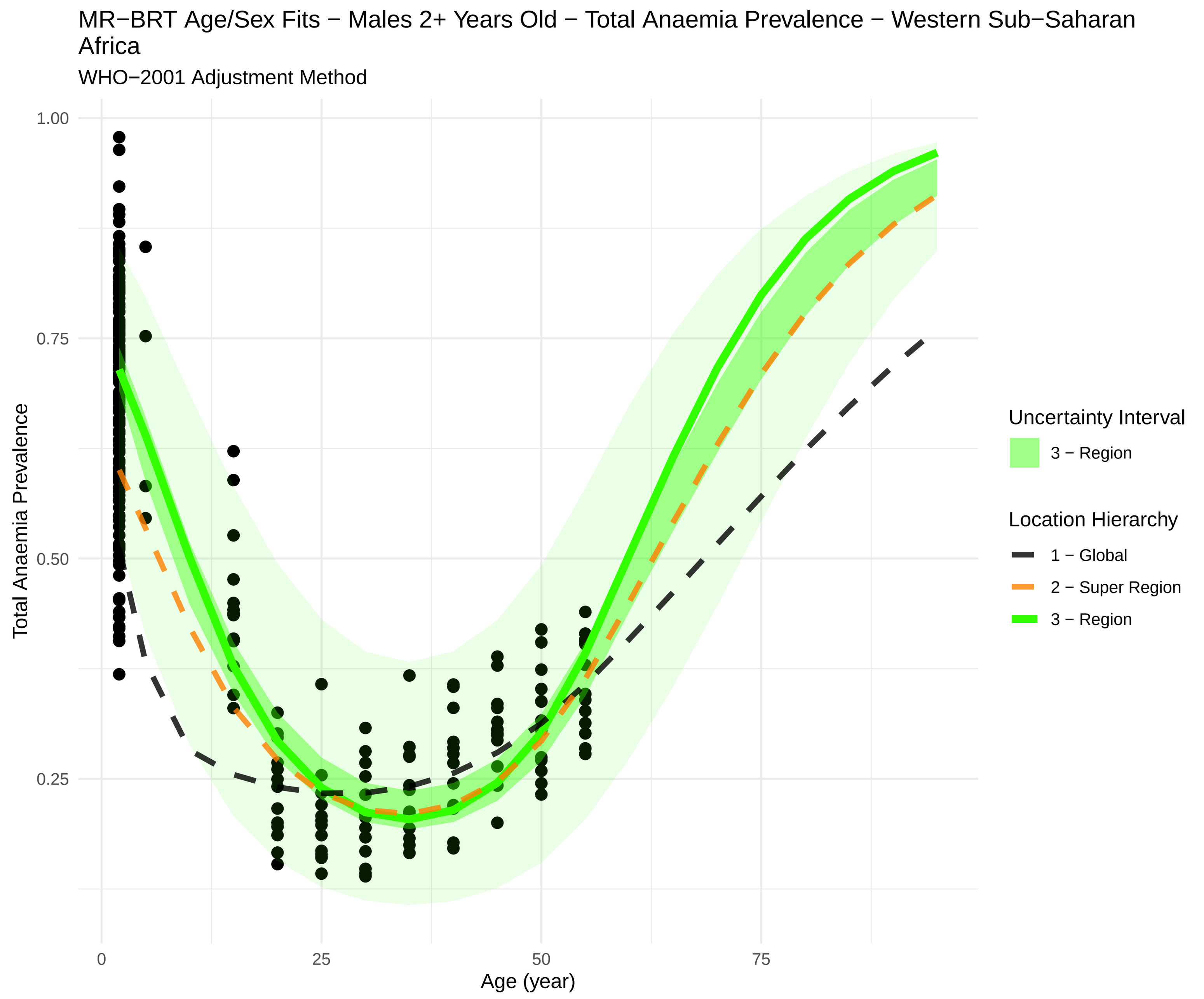
MR−BRT Age/Sex Fits − Males 2+ Years Old − Total Anaemia Prevalence − Western Sub−Saharan Africa
WHO−2001 Adjustment Method
Total Anaemia Prevalence
Age (year)
Uncertainty Interval
3 − Region
Location Hierarchy
1 − Global
2 − Super Region
3 − Region

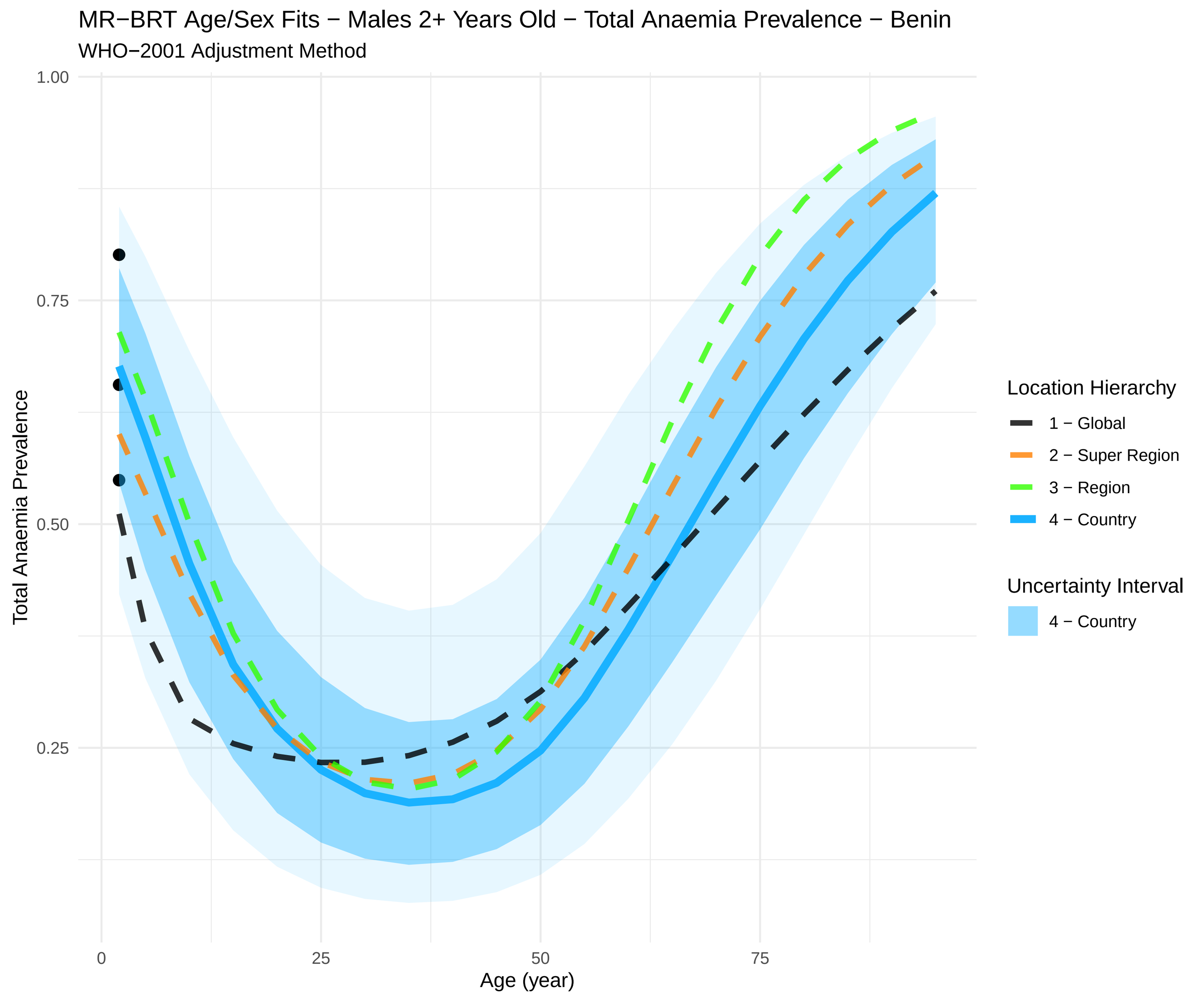
MR−BRT Age/Sex Fits − Males 2+ Years Old − Total Anaemia Prevalence − Benin
WHO−2001 Adjustment Method
Total Anaemia Prevalence
Age (year)
Location Hierarchy
1 − Global
2 − Super Region
3 − Region
4 − Country
Uncertainty Interval
4 − Country

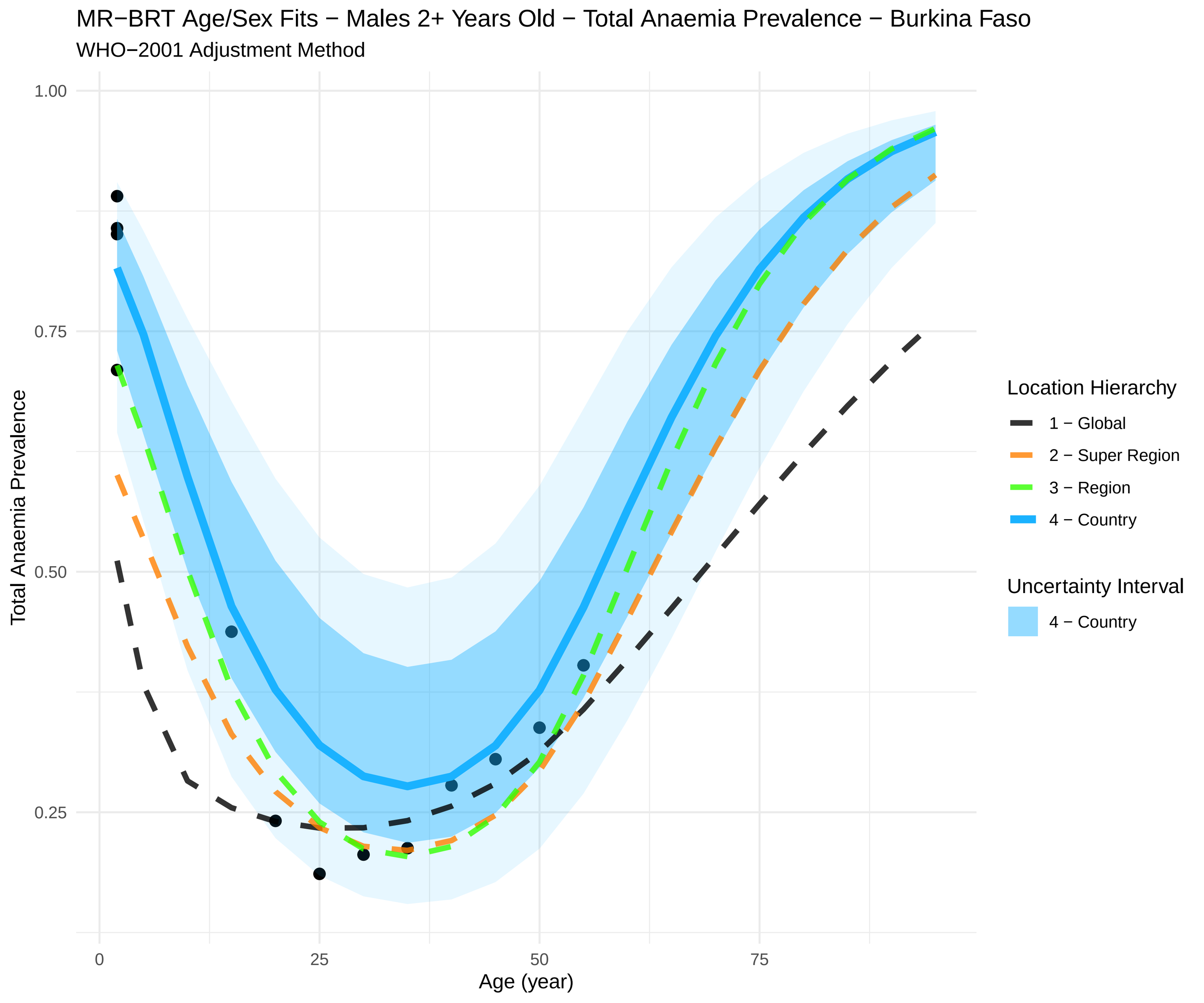
MR−BRT Age/Sex Fits − Males 2+ Years Old − Total Anaemia Prevalence − Burkina Faso
WHO−2001 Adjustment Method
Total Anaemia Prevalence
Age (year)
Location Hierarchy
1 − Global
2 − Super Region
3 − Region
4 − Country
Uncertainty Interval
4 − Country

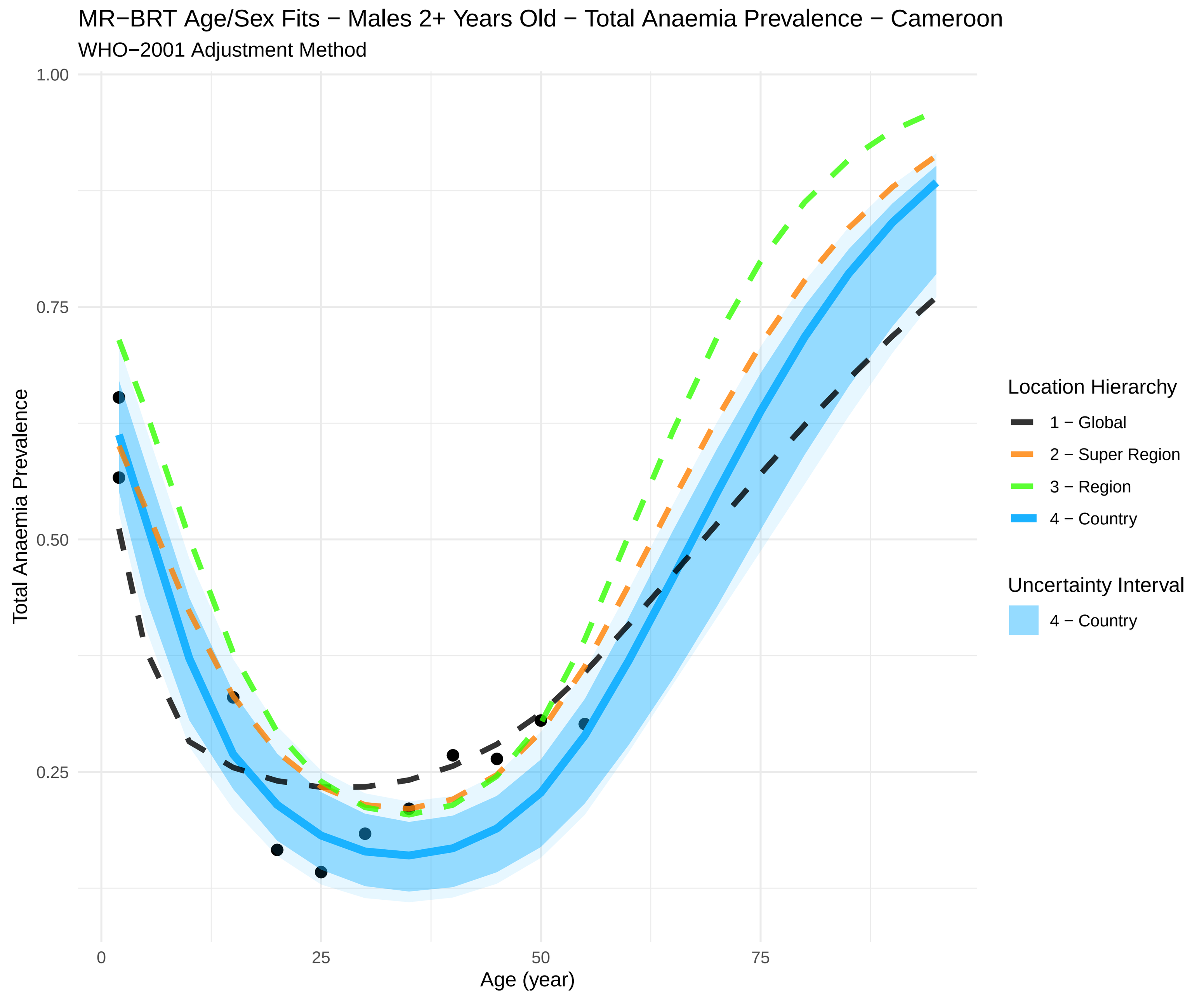
MR−BRT Age/Sex Fits − Males 2+ Years Old − Total Anaemia Prevalence − Cameroon
WHO−2001 Adjustment Method
Total Anaemia Prevalence
Age (year)
Location Hierarchy
1 − Global
2 − Super Region
3 − Region
4 − Country
Uncertainty Interval
4 − Country

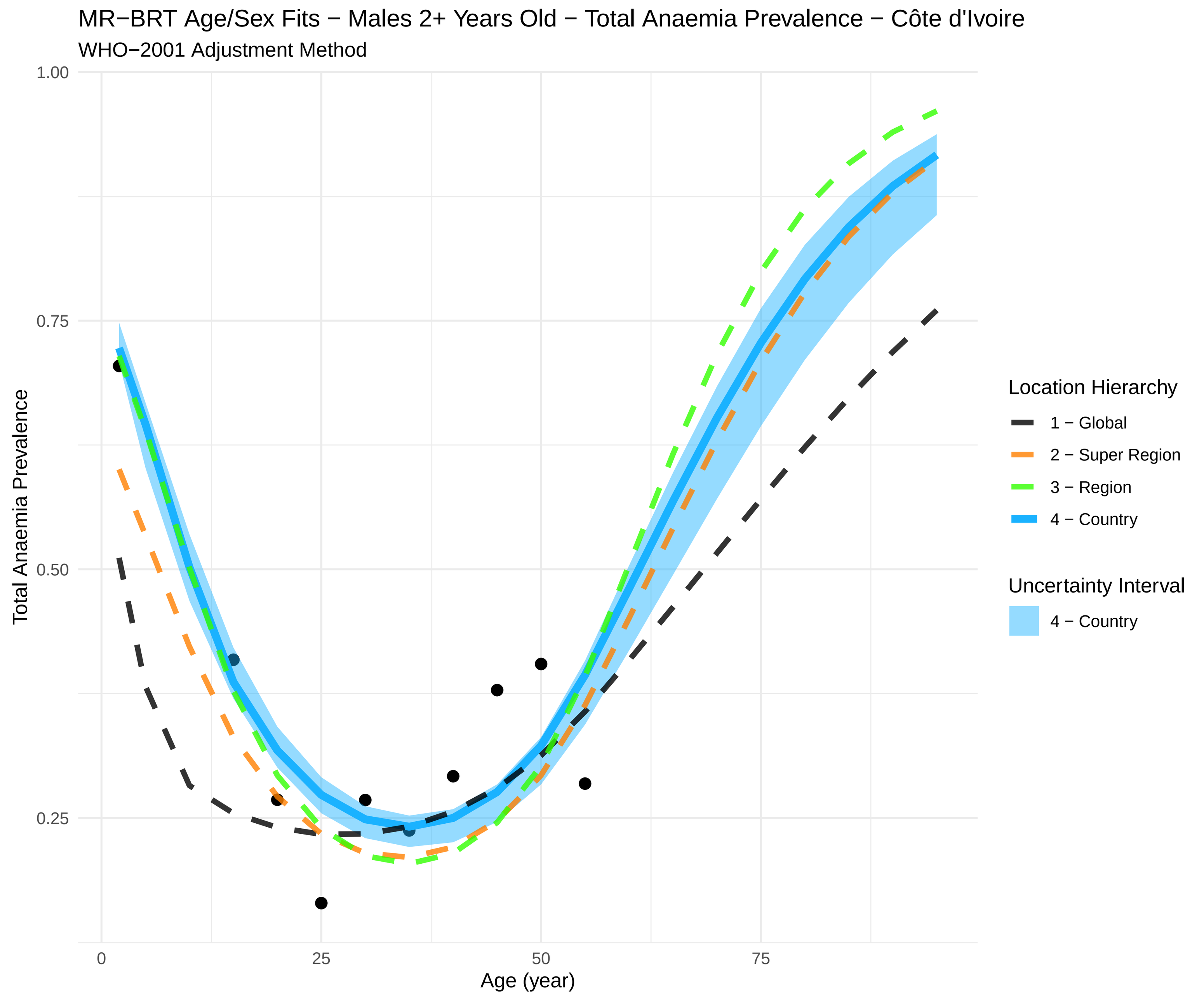
MR−BRT Age/Sex Fits − Males 2+ Years Old − Total Anaemia Prevalence − Côte d'Ivoire
WHO−2001 Adjustment Method
Total Anaemia Prevalence
Age (year)
Location Hierarchy
1 − Global
2 − Super Region
3 − Region
4 − Country
Uncertainty Interval
4 − Country

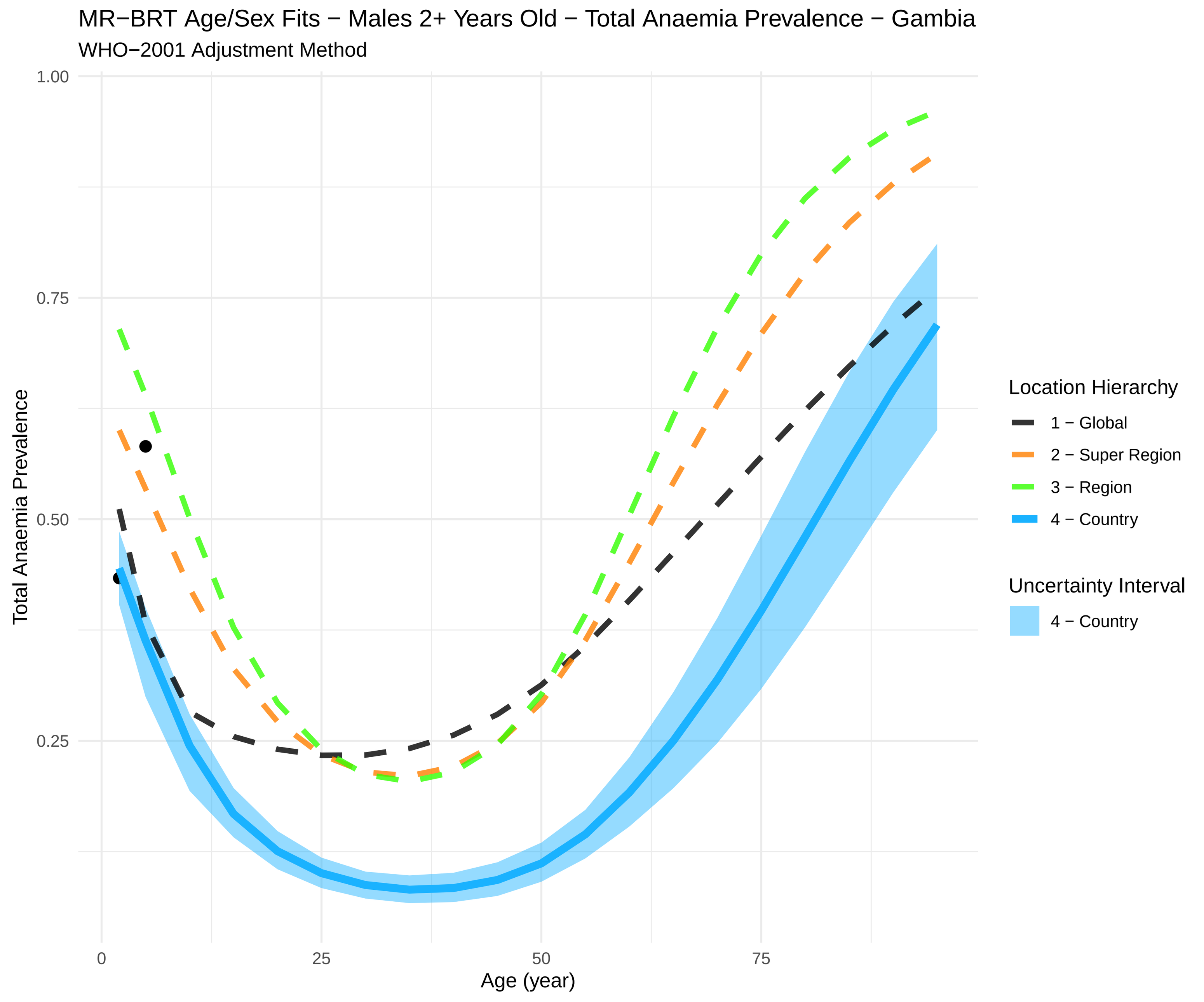
MR−BRT Age/Sex Fits − Males 2+ Years Old − Total Anaemia Prevalence − Gambia
WHO−2001 Adjustment Method
Total Anaemia Prevalence
Age (year)
Location Hierarchy
1 − Global
2 − Super Region
3 − Region
4 − Country
Uncertainty Interval
4 − Country

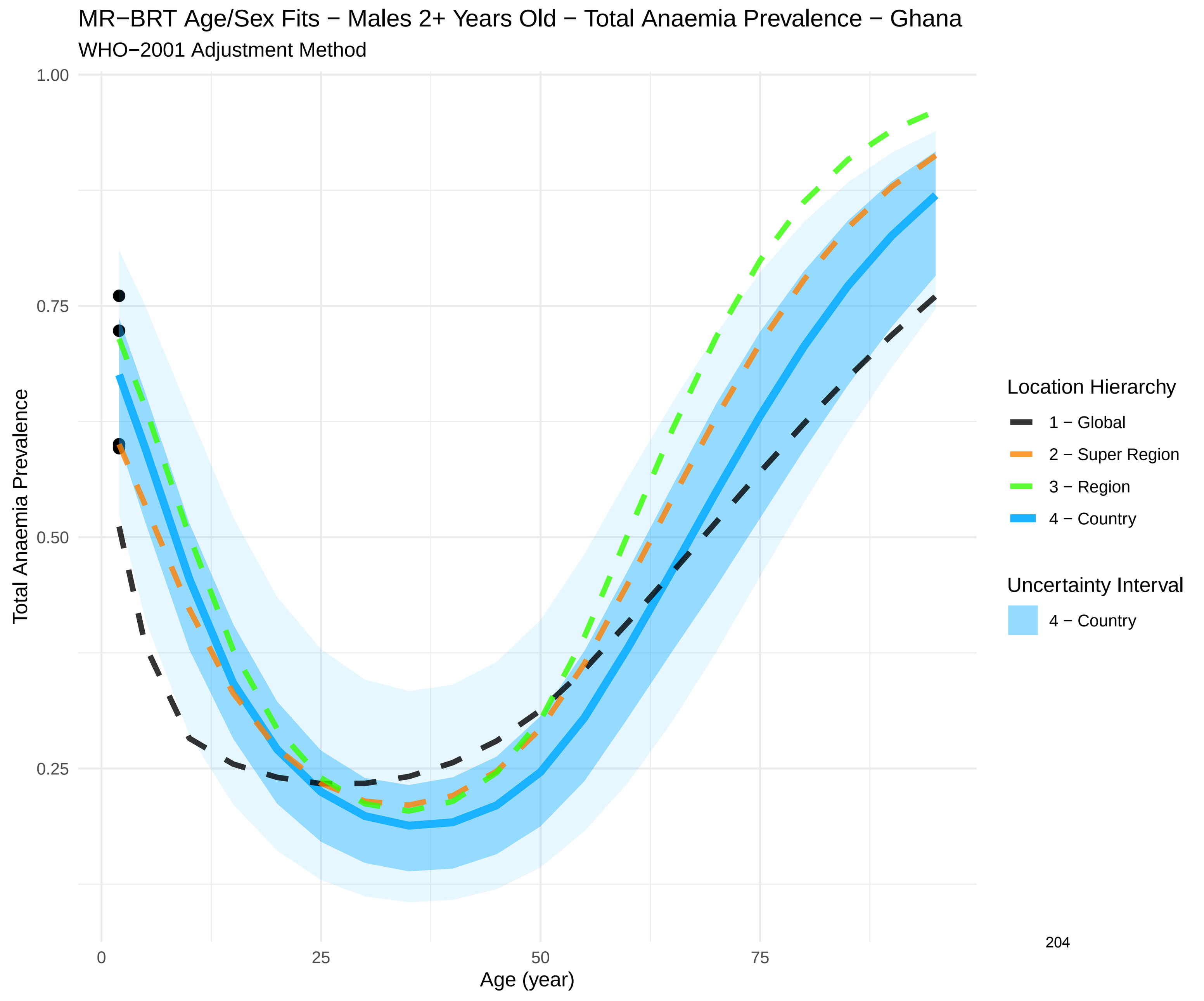
MR−BRT Age/Sex Fits − Males 2+ Years Old − Total Anaemia Prevalence − Ghana
WHO−2001 Adjustment Method
Total Anaemia Prevalence
Age (year)
Location Hierarchy
1 − Global
2 − Super Region
3 − Region
4 − Country
Uncertainty Interval
4 − Country

MR−BRT Age/Sex Fits − Males 2+ Years Old − Total Anaemia Prevalence − Guinea
WHO−2001 Adjustment Method

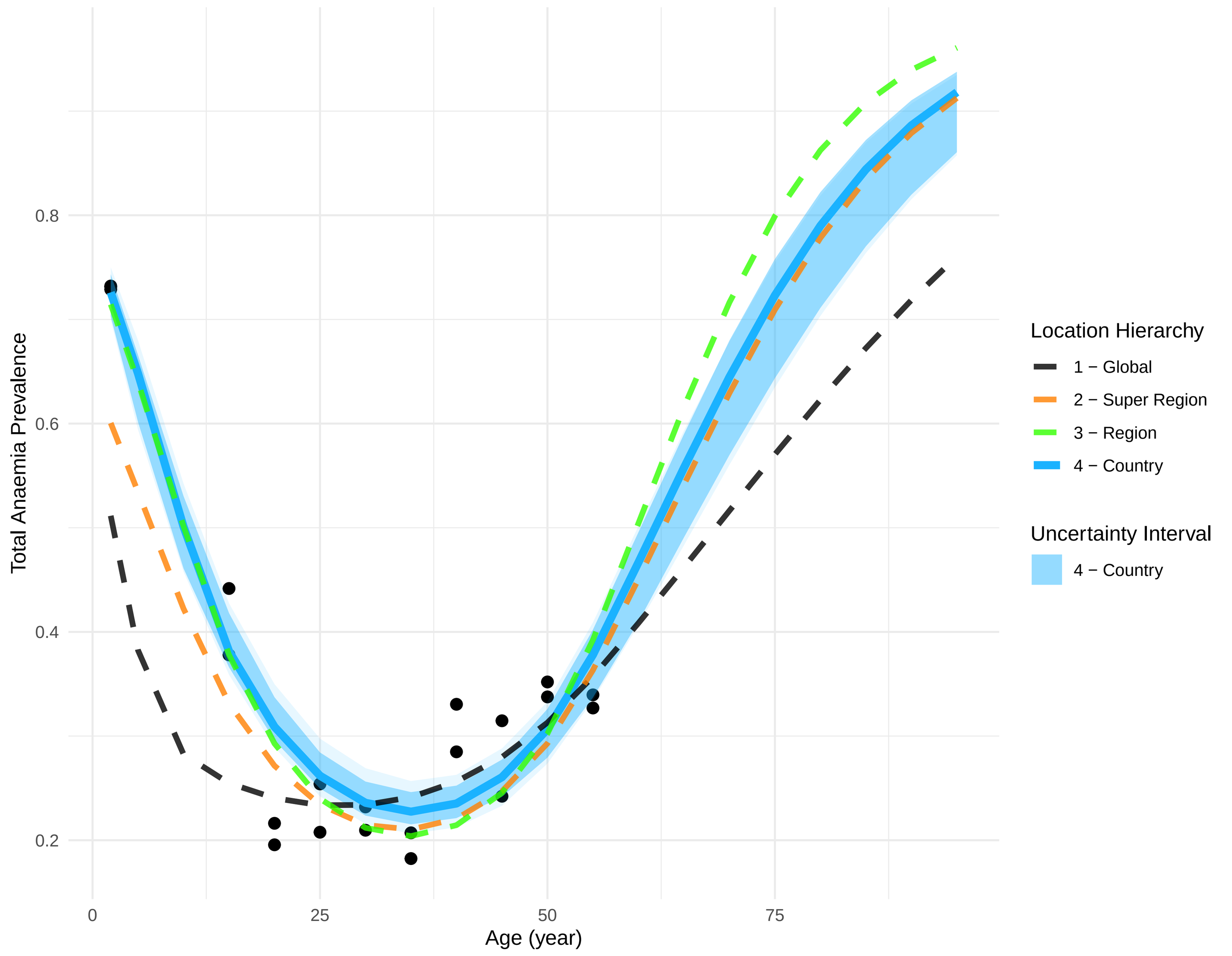

MR−BRT Age/Sex Fits − Males 2+ Years Old − Total Anaemia Prevalence − Liberia
WHO−2001 Adjustment Method

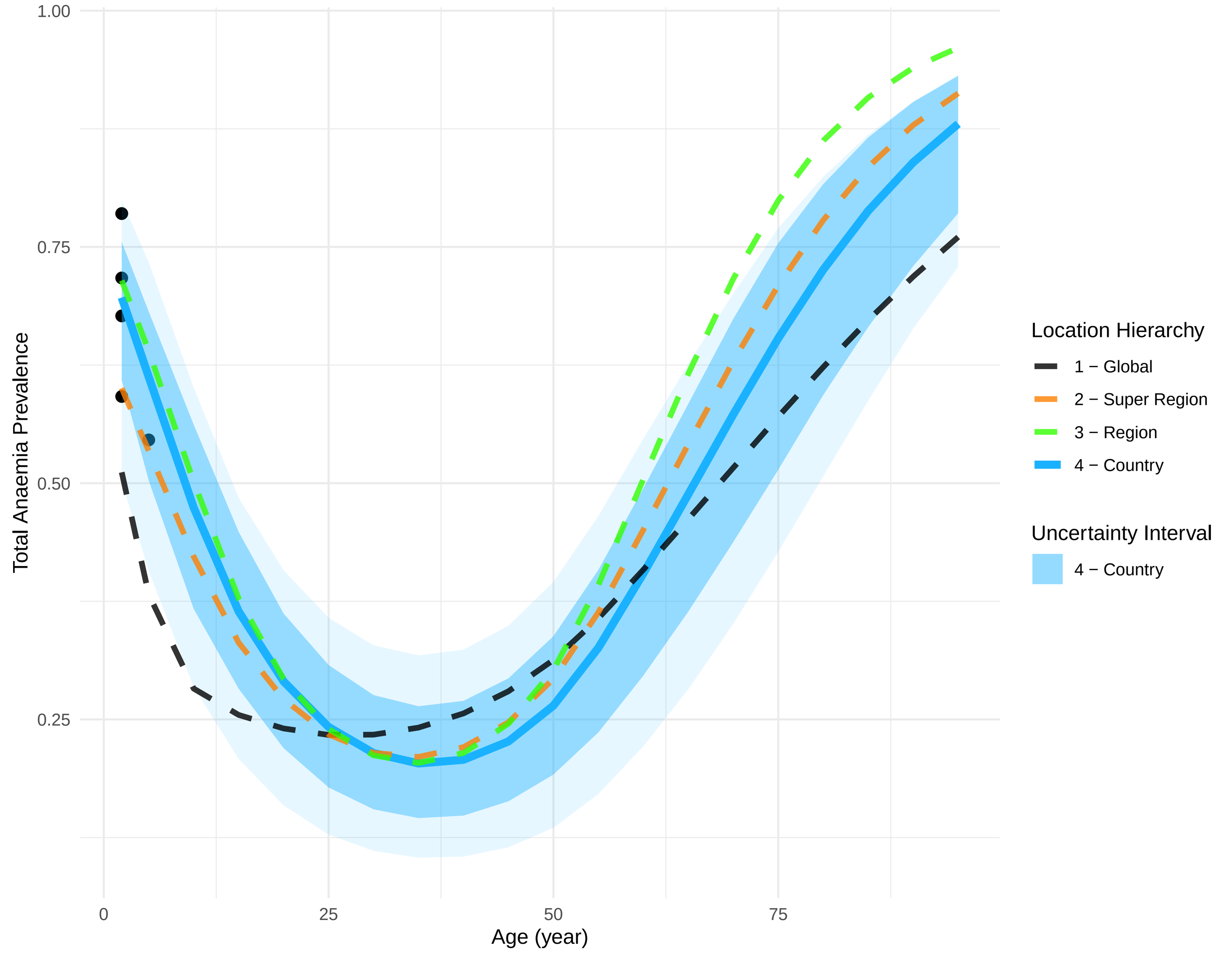

MR−BRT Age/Sex Fits − Males 2+ Years Old − Total Anaemia Prevalence − Mali
WHO−2001 Adjustment Method

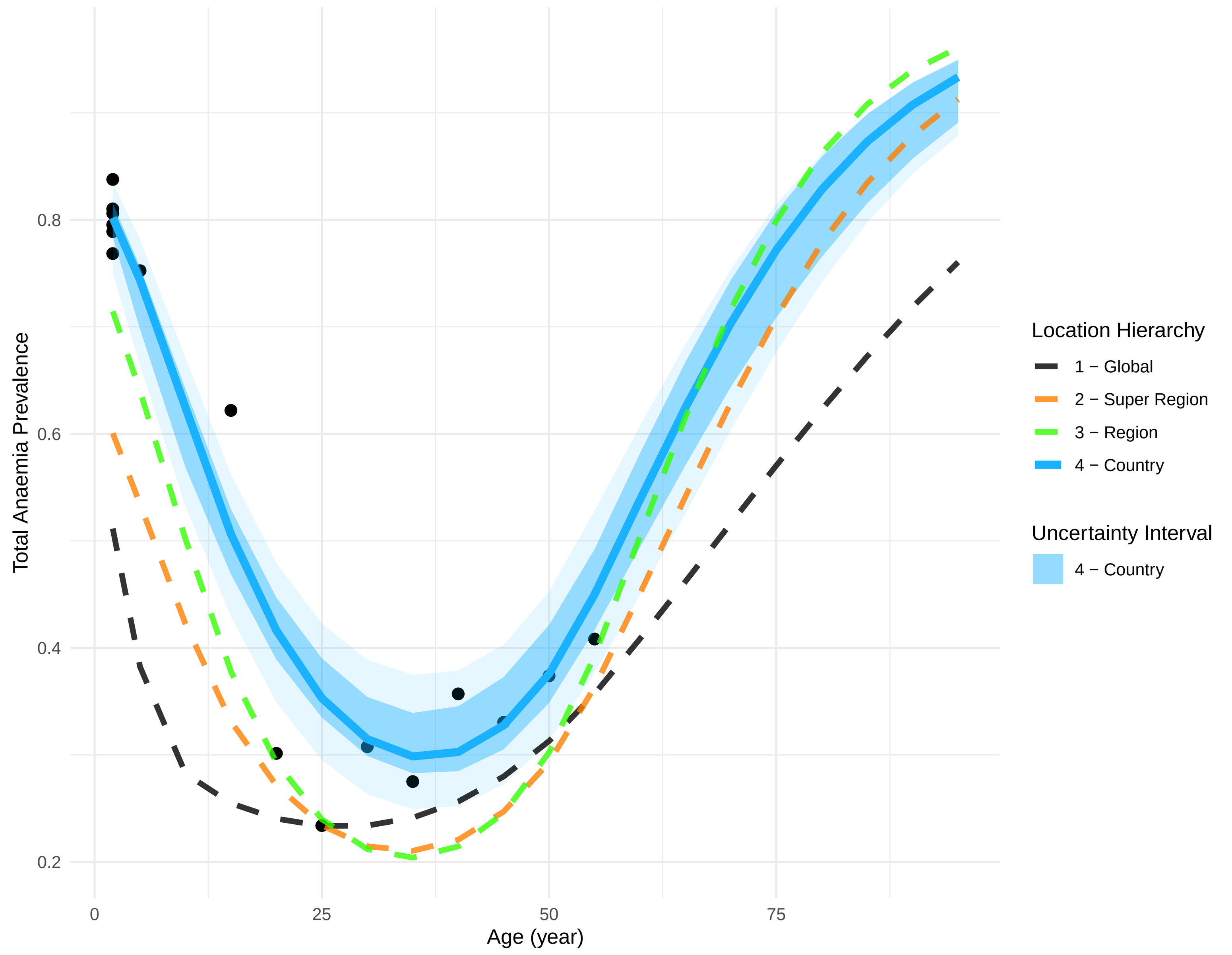

MR−BRT Age/Sex Fits − Males 2+ Years Old − Total Anaemia Prevalence − Niger
WHO−2001 Adjustment Method

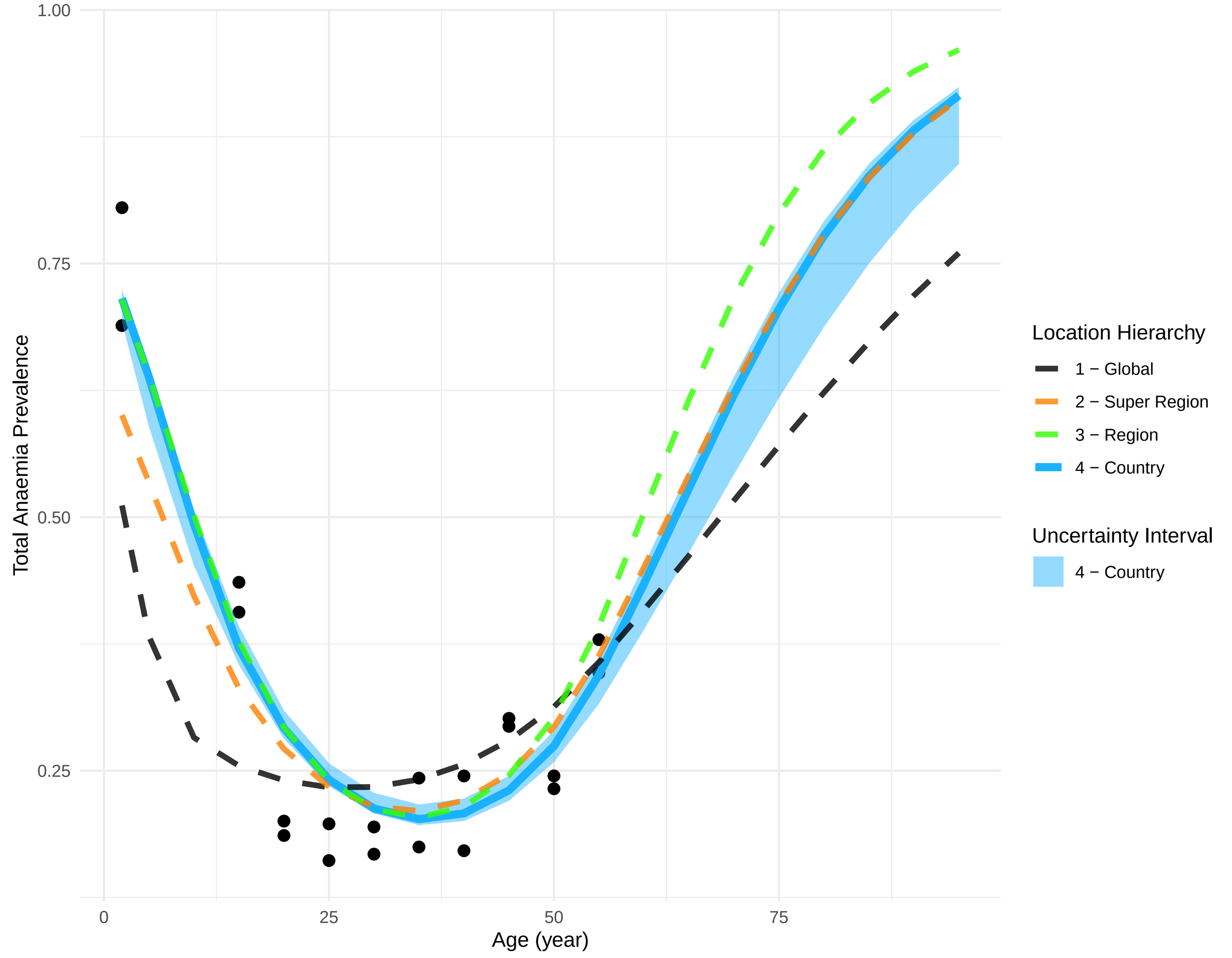

MR−BRT Age/Sex Fits − Males 2+ Years Old − Total Anaemia Prevalence − Nigeria
WHO−2001 Adjustment Method

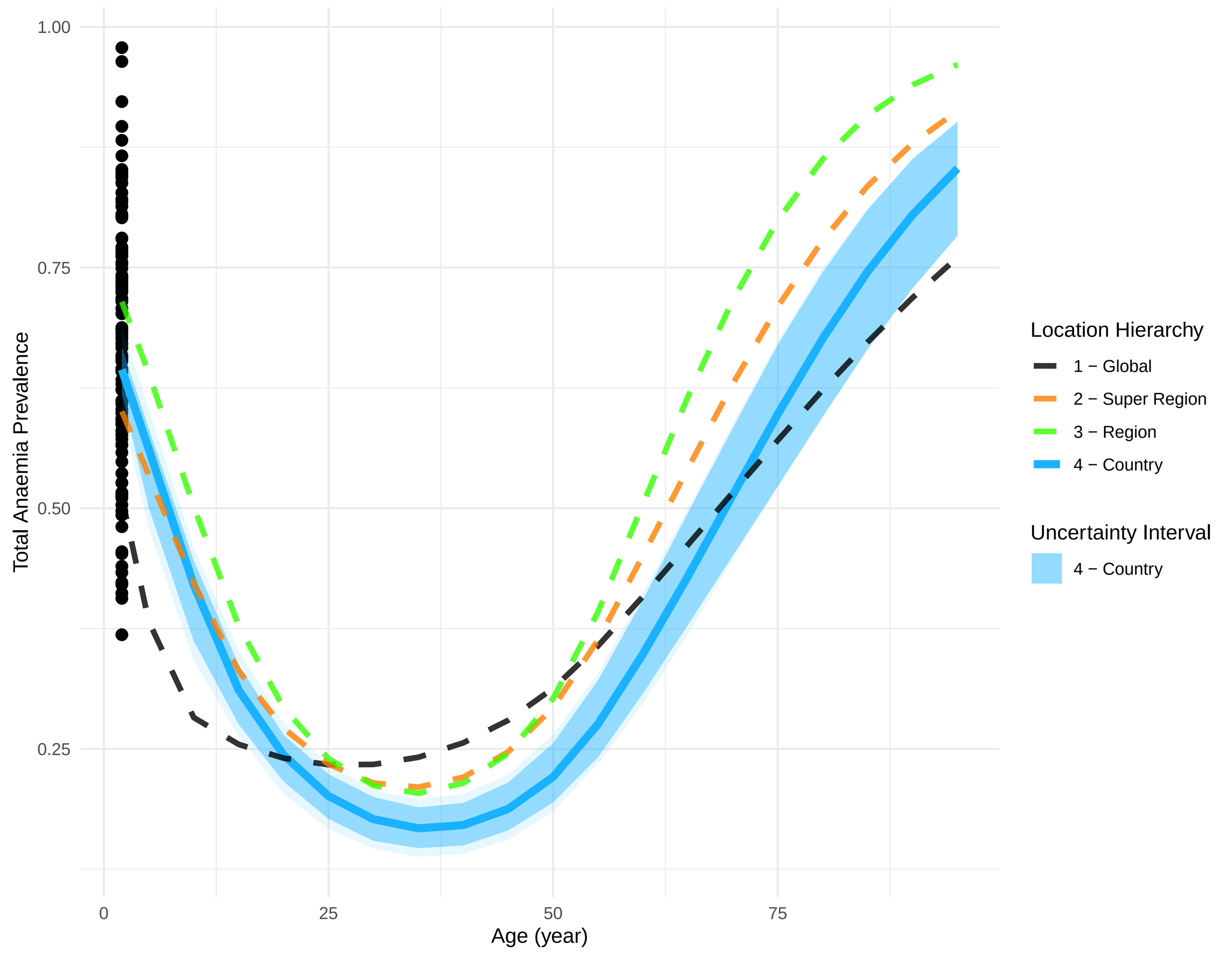

MR−BRT Age/Sex Fits − Males 2+ Years Old − Total Anaemia Prevalence − Abia
WHO−2001 Adjustment Method

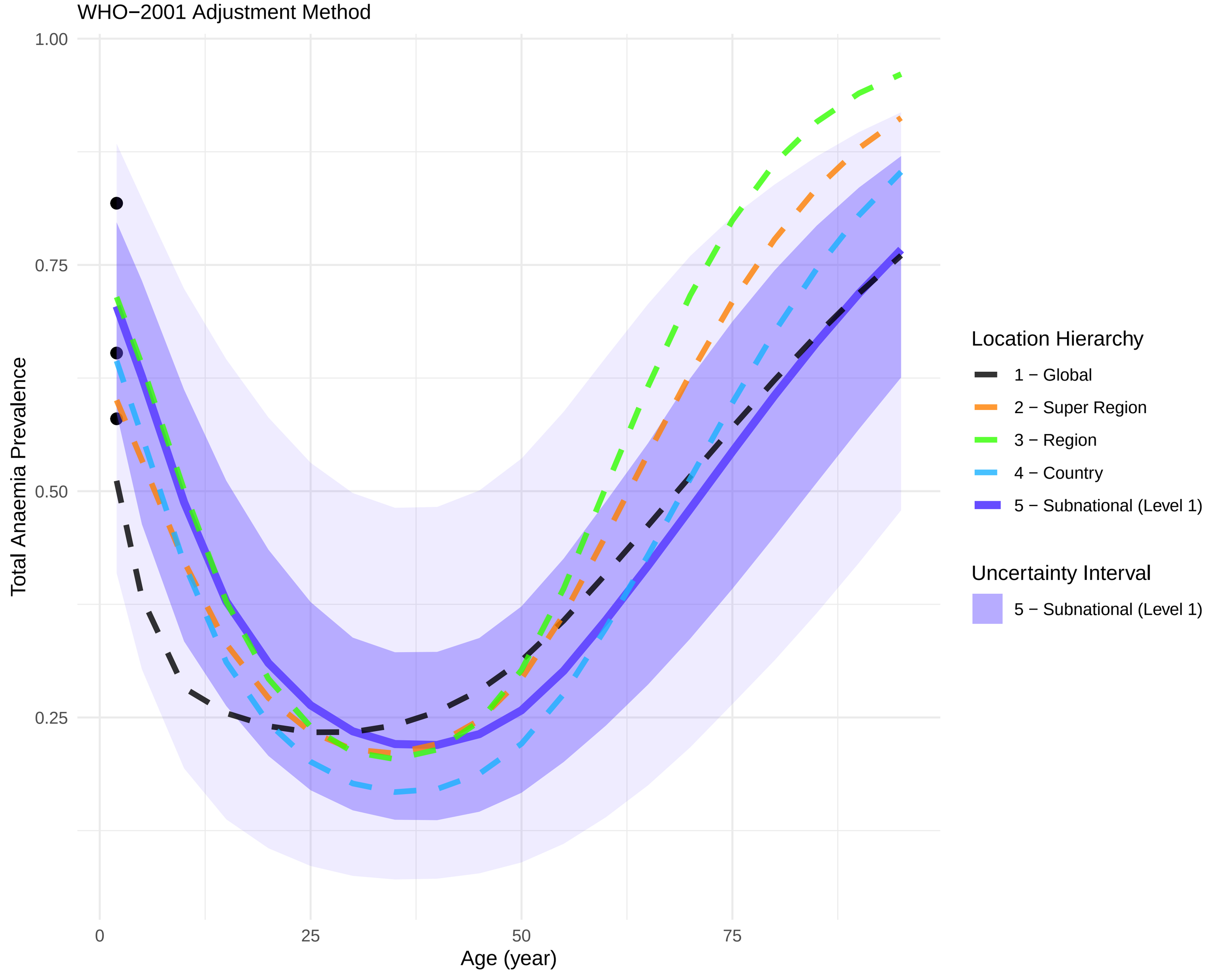

MR−BRT Age/Sex Fits − Males 2+ Years Old − Total Anaemia Prevalence − Adamawa
WHO−2001 Adjustment Method

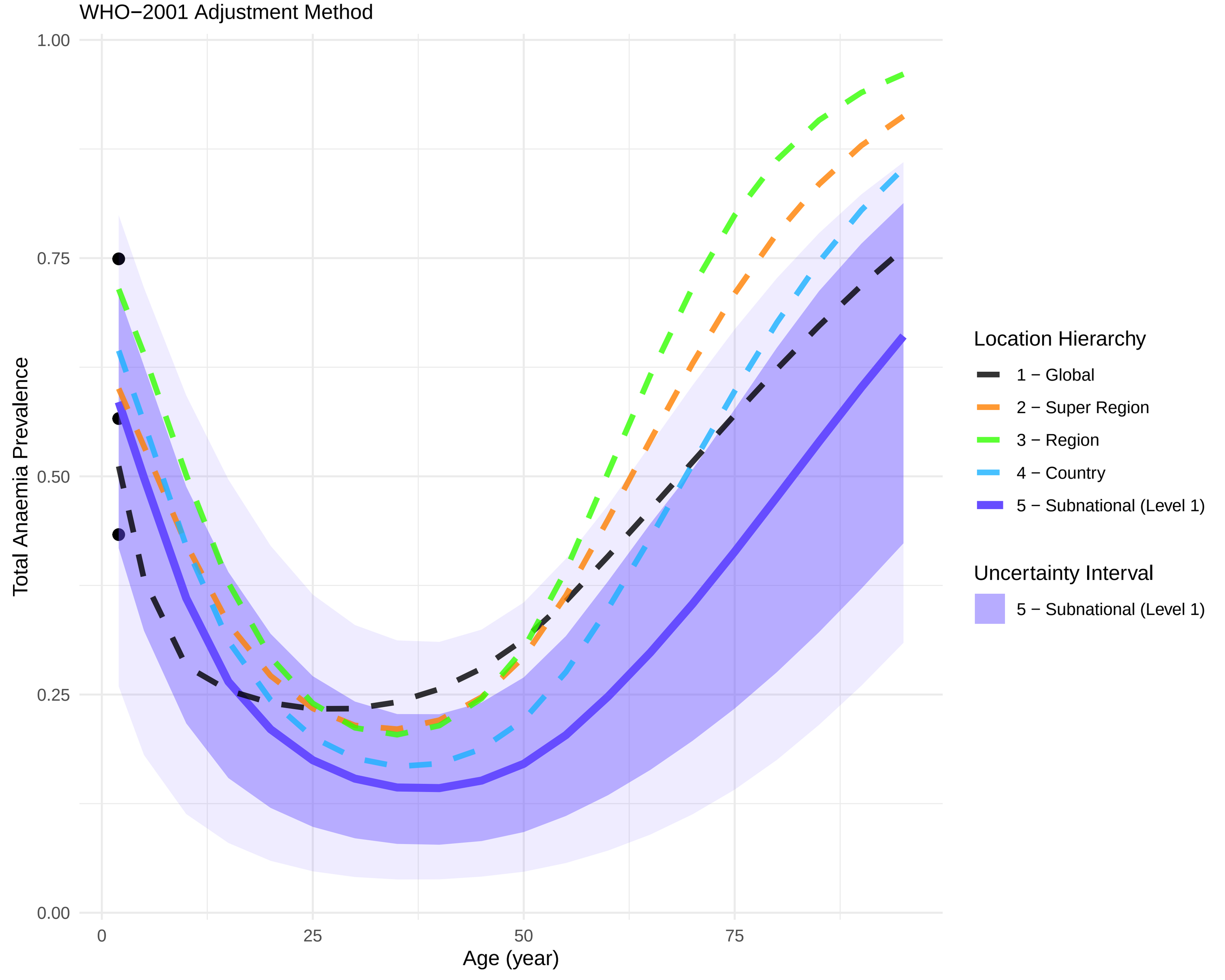

MR−BRT Age/Sex Fits − Males 2+ Years Old − Total Anaemia Prevalence − Akwa Ibom
WHO−2001 Adjustment Method

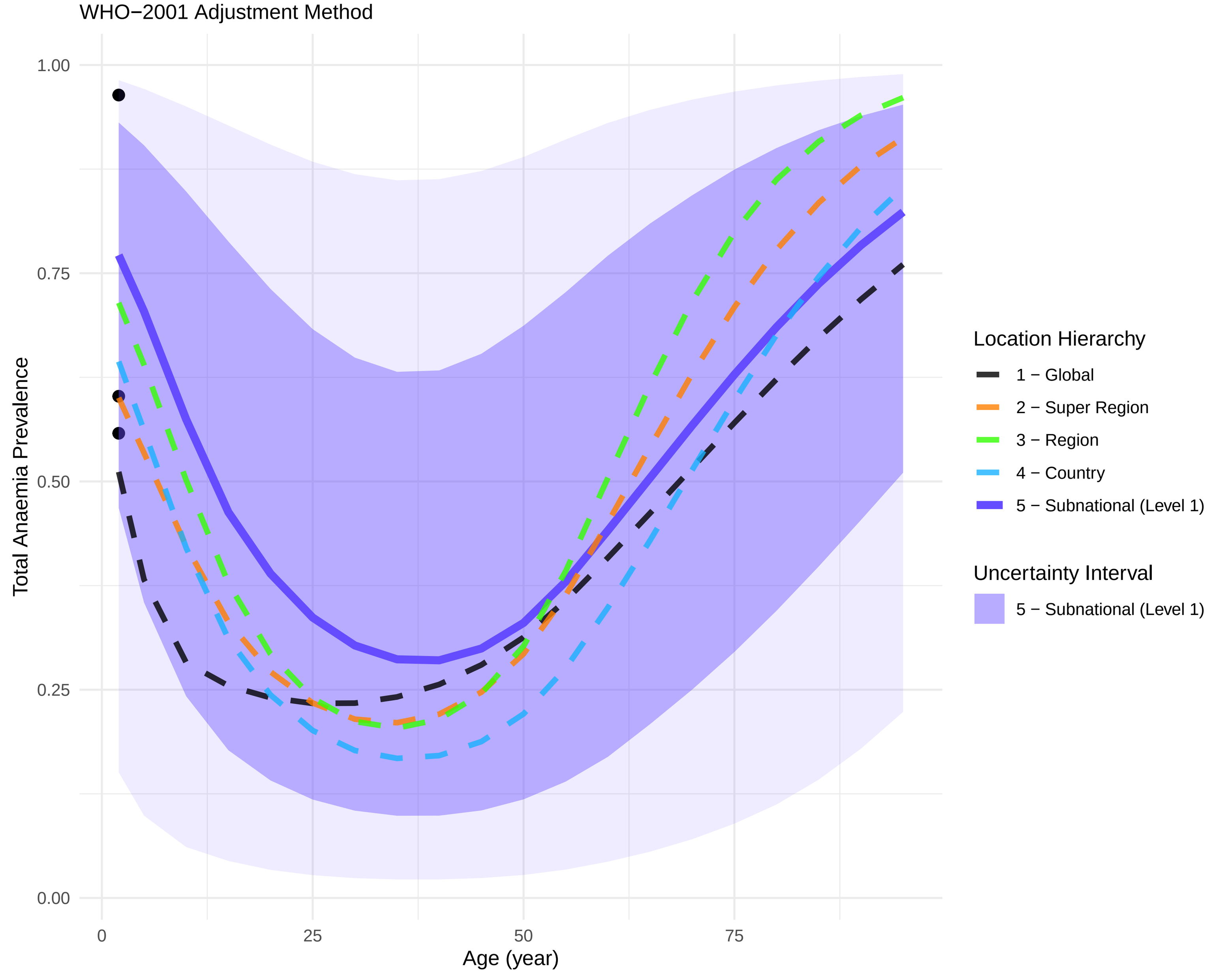

MR−BRT Age/Sex Fits − Males 2+ Years Old − Total Anaemia Prevalence − Anambra
WHO−2001 Adjustment Method

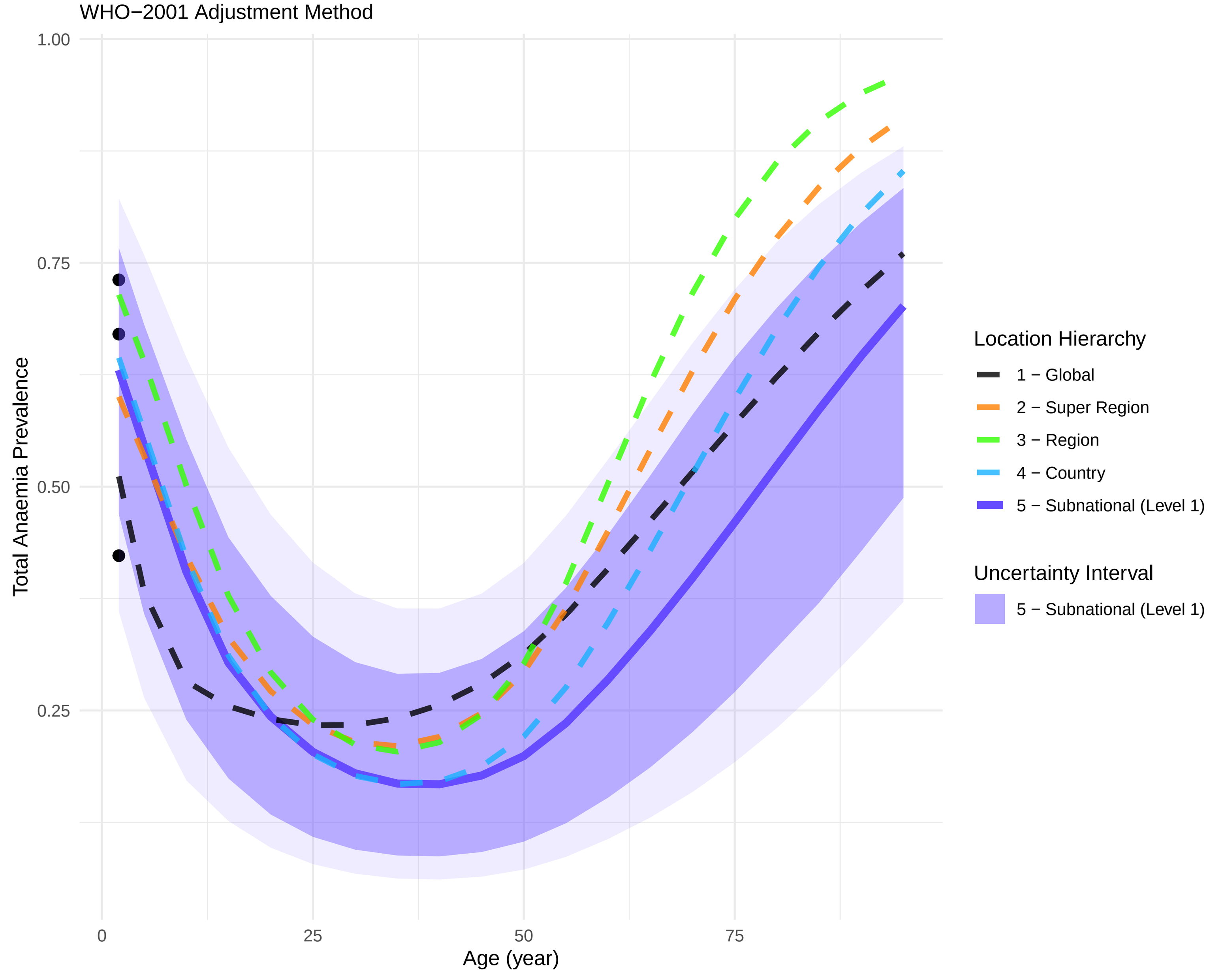

MR−BRT Age/Sex Fits − Males 2+ Years Old − Total Anaemia Prevalence − Bauchi
WHO−2001 Adjustment Method

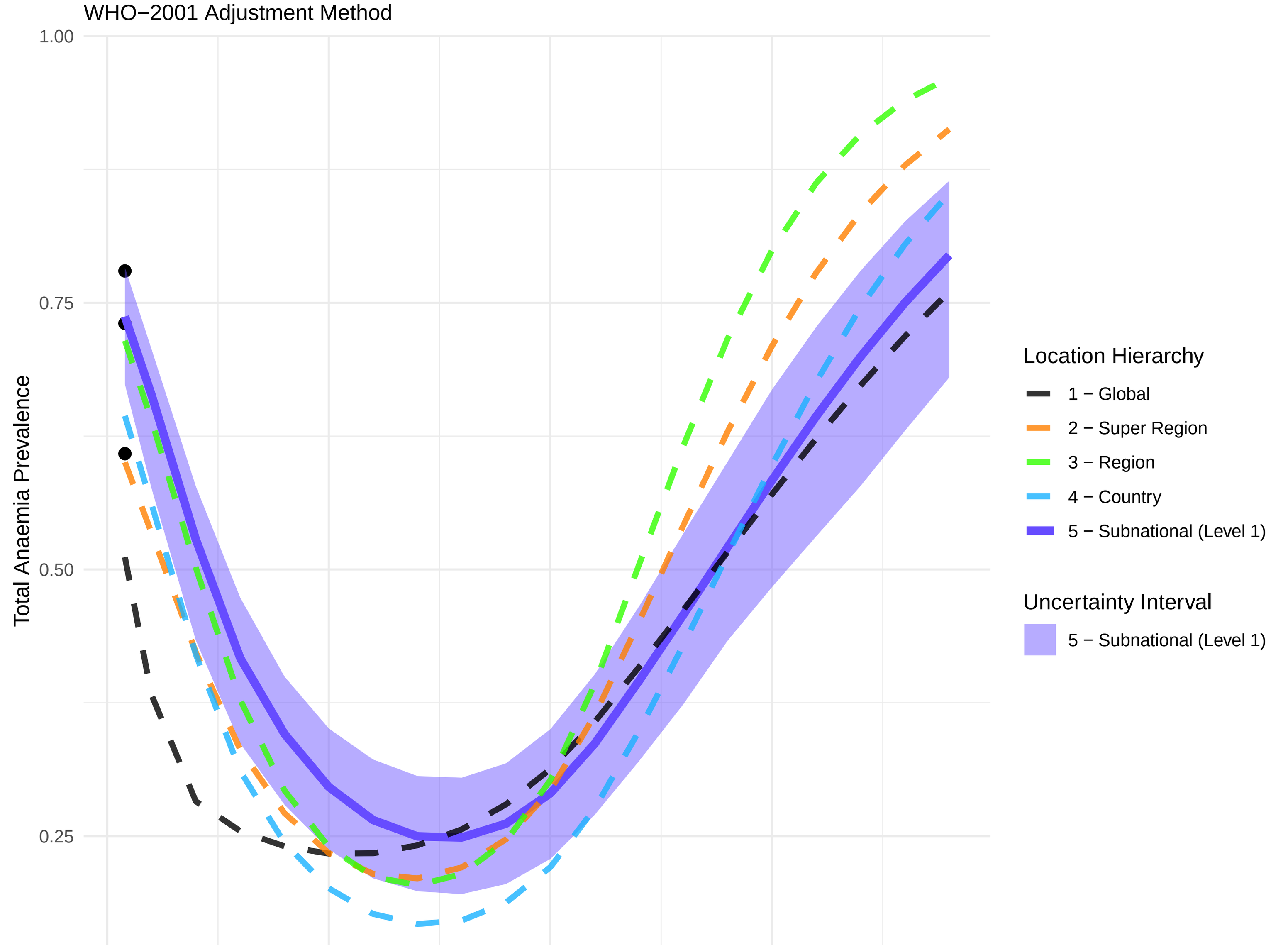

MR−BRT Age/Sex Fits − Males 2+ Years Old − Total Anaemia Prevalence − Bayelsa
WHO−2001 Adjustment Method

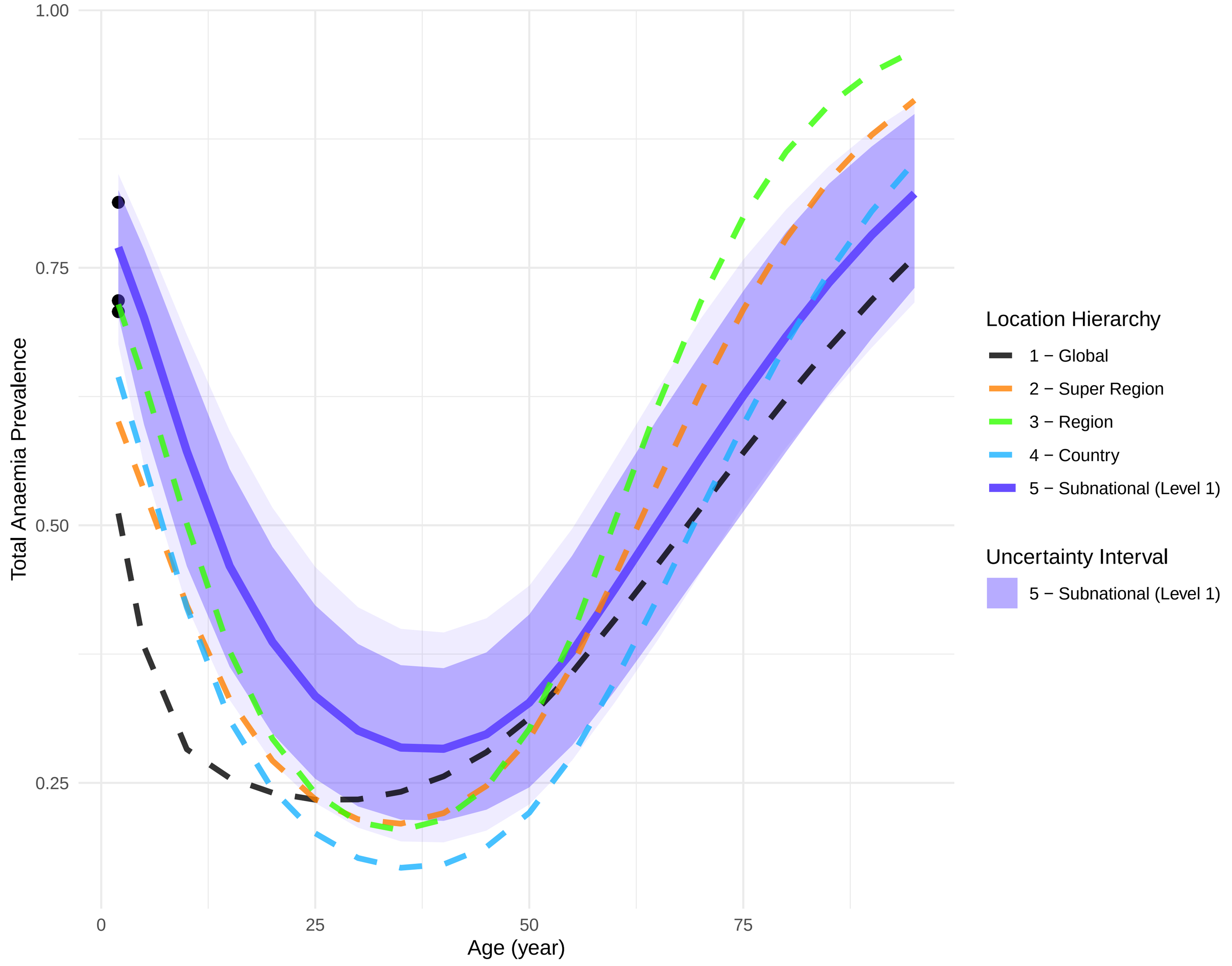

MR−BRT Age/Sex Fits − Males 2+ Years Old − Total Anaemia Prevalence − Benue
WHO−2001 Adjustment Method

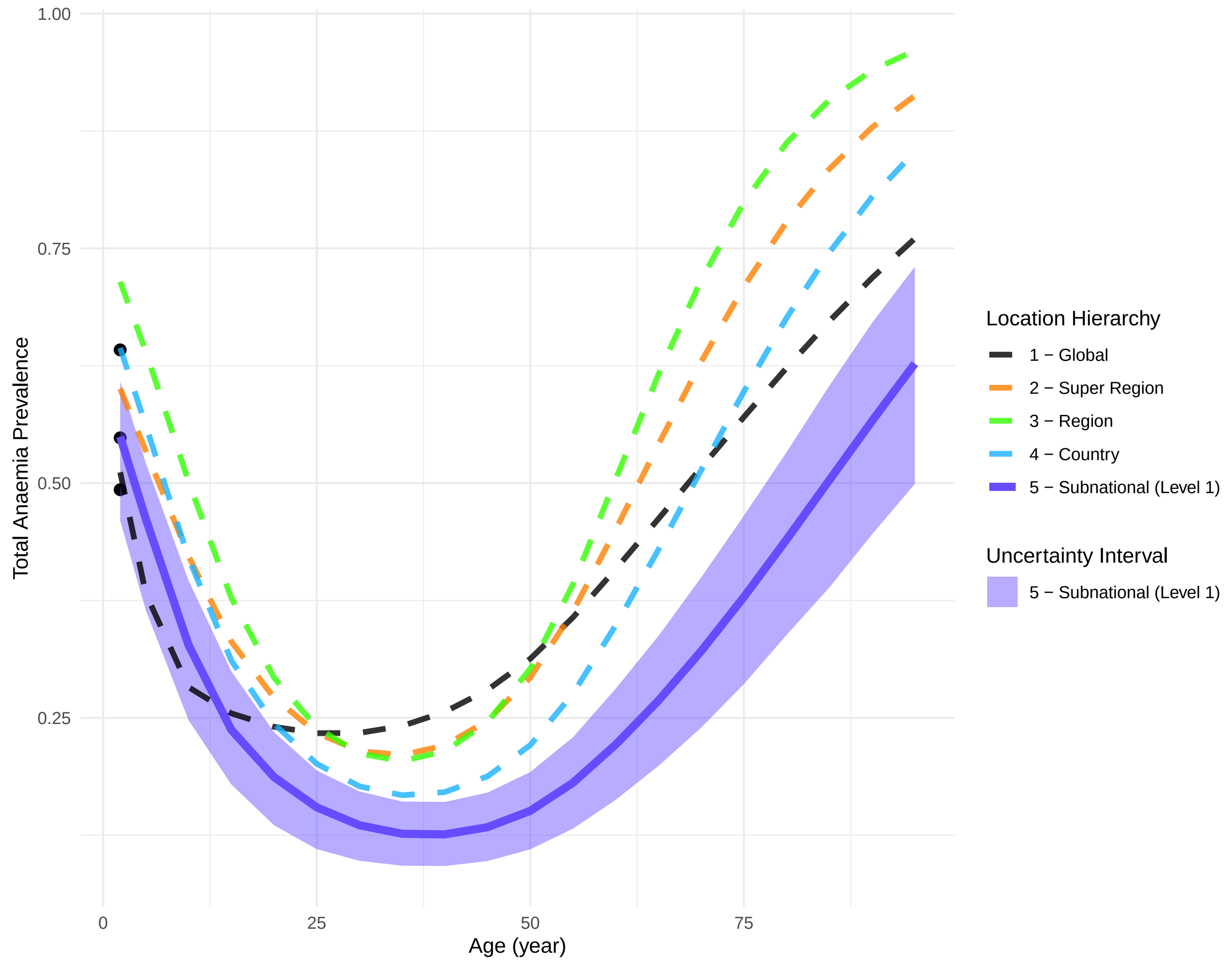

MR−BRT Age/Sex Fits − Males 2+ Years Old − Total Anaemia Prevalence − Borno
WHO−2001 Adjustment Method

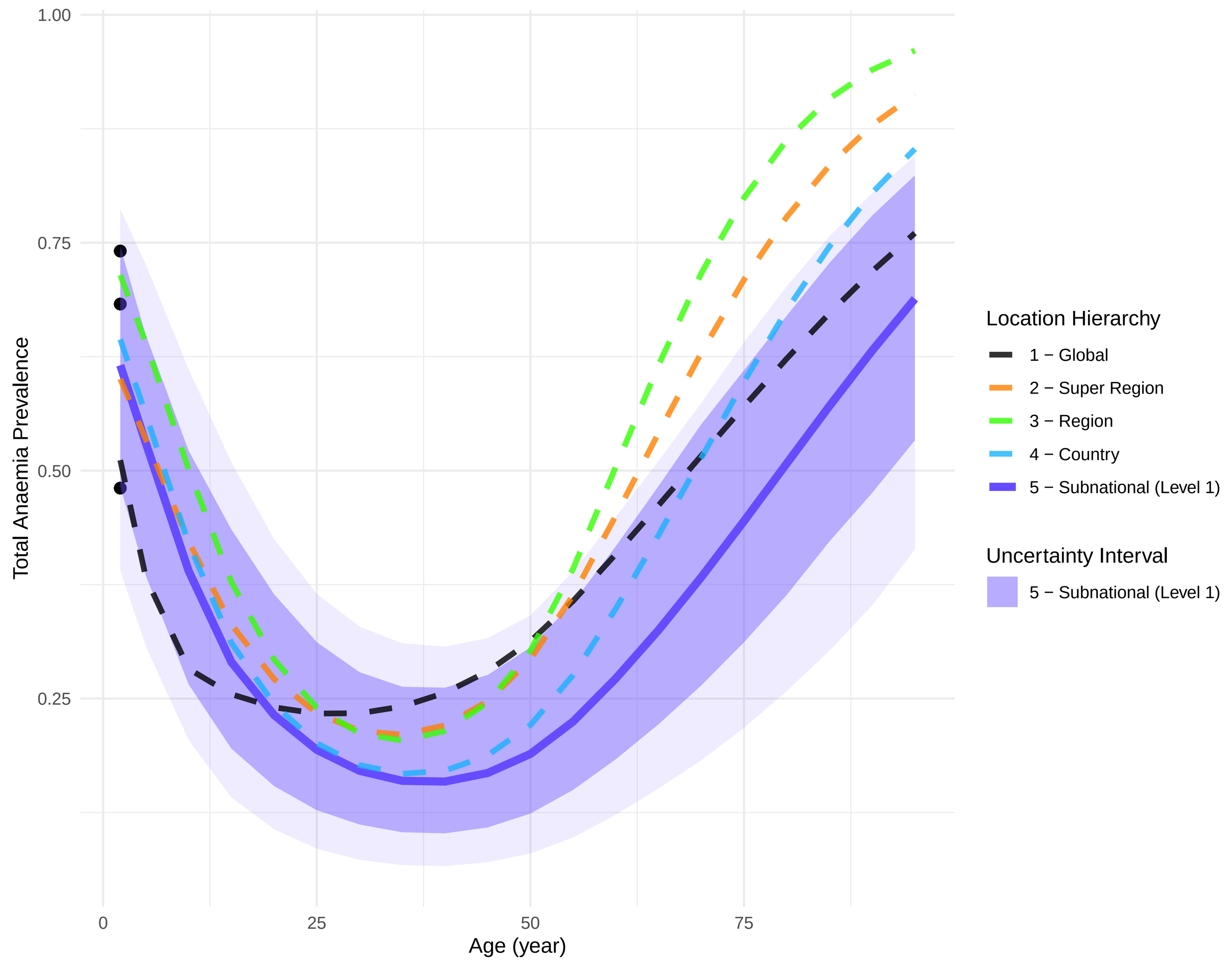

MR−BRT Age/Sex Fits − Males 2+ Years Old − Total Anaemia Prevalence − Cross River
WHO−2001 Adjustment Method

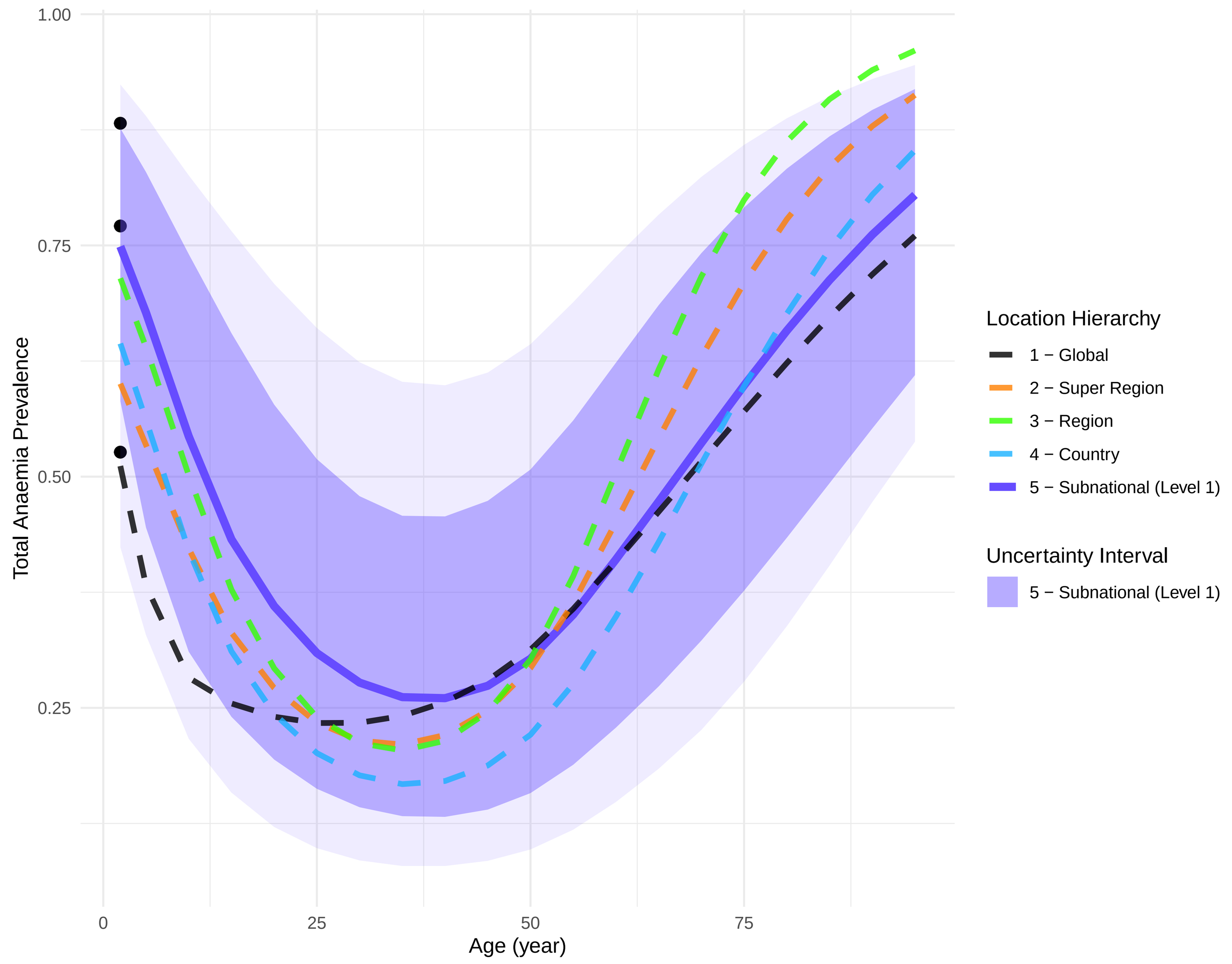

MR−BRT Age/Sex Fits − Males 2+ Years Old − Total Anaemia Prevalence − Delta
WHO−2001 Adjustment Method

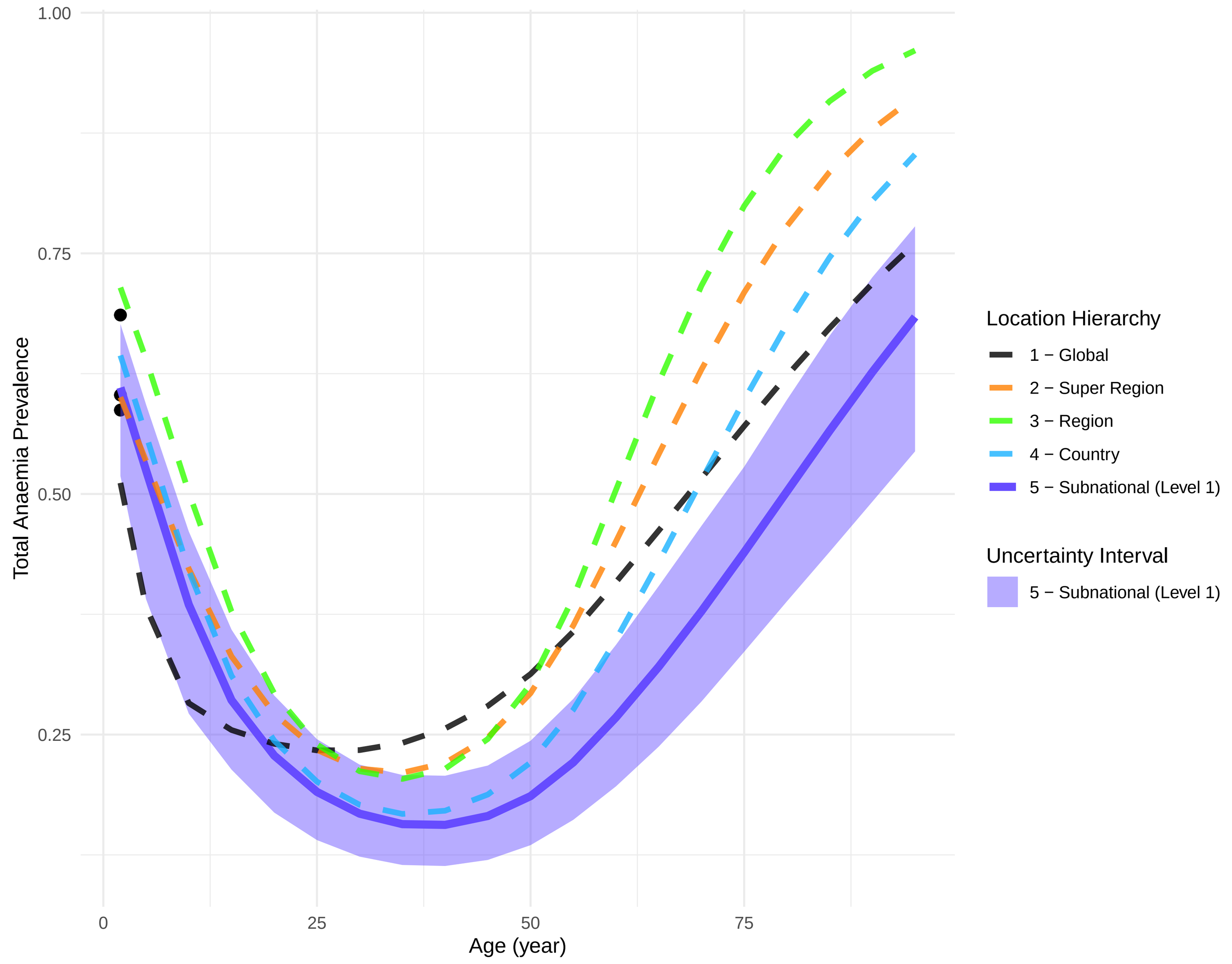

MR−BRT Age/Sex Fits − Males 2+ Years Old − Total Anaemia Prevalence − Ebonyi
WHO−2001 Adjustment Method

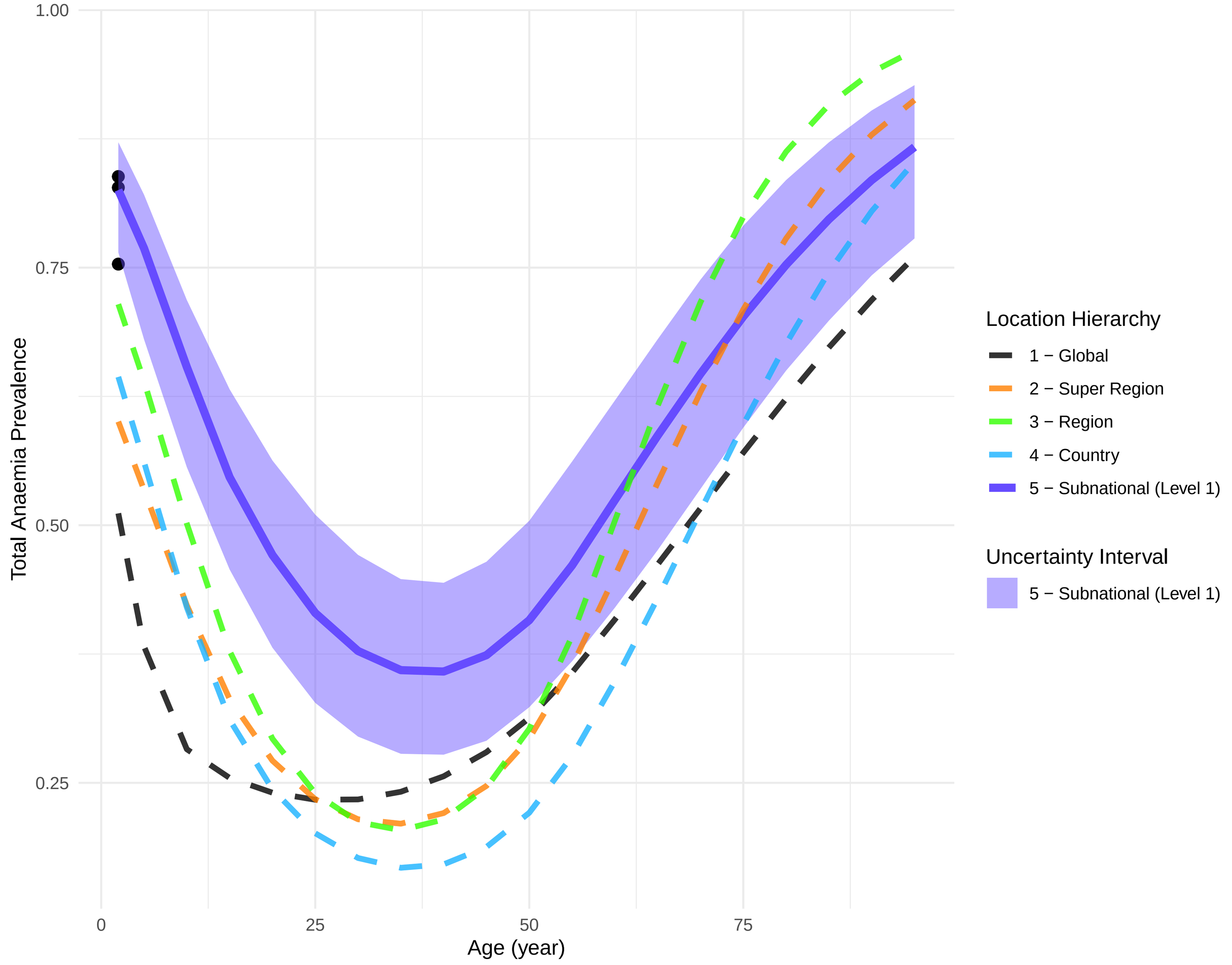

MR−BRT Age/Sex Fits − Males 2+ Years Old − Total Anaemia Prevalence − Edo
WHO−2001 Adjustment Method

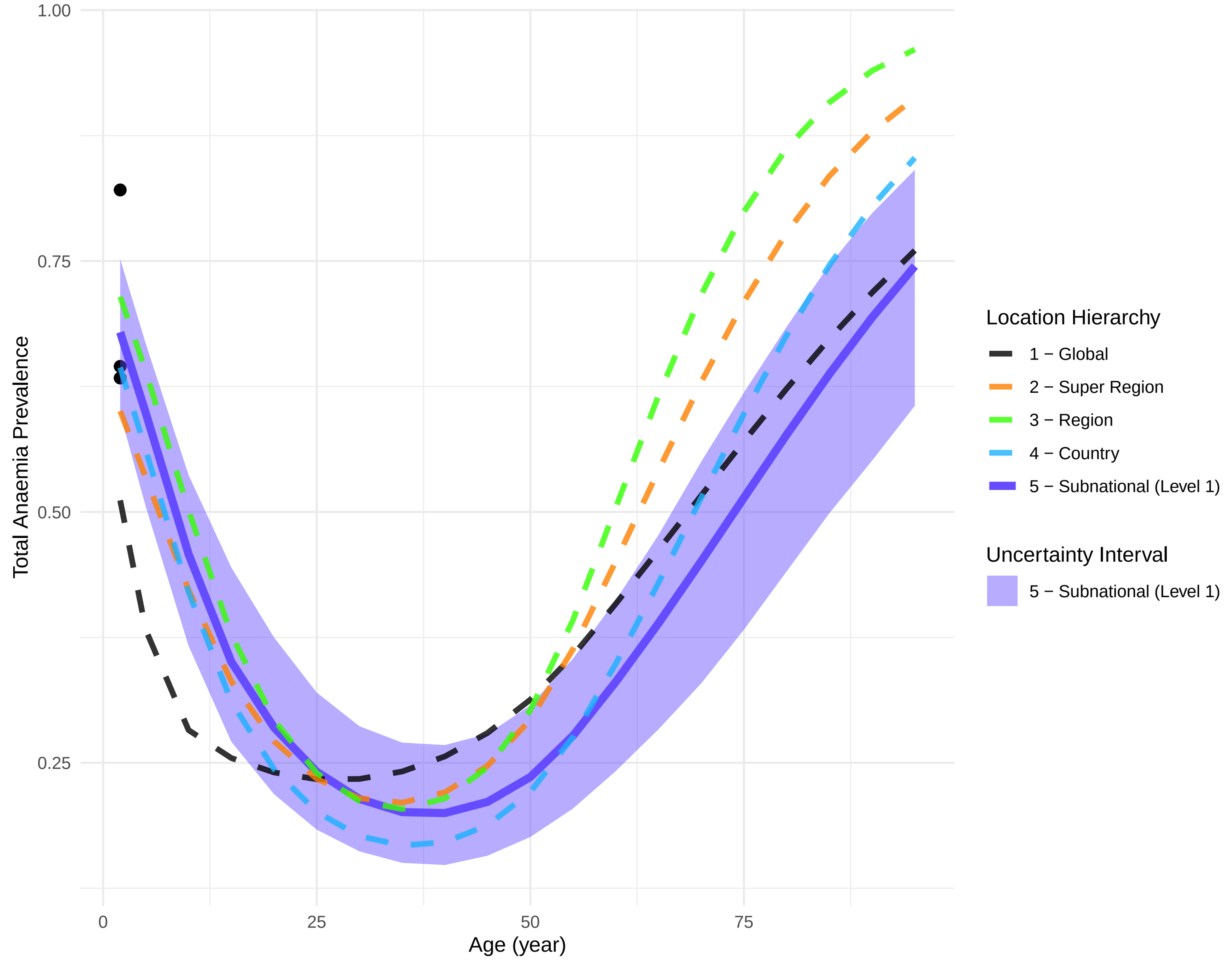

MR−BRT Age/Sex Fits − Males 2+ Years Old − Total Anaemia Prevalence − Ekiti
WHO−2001 Adjustment Method

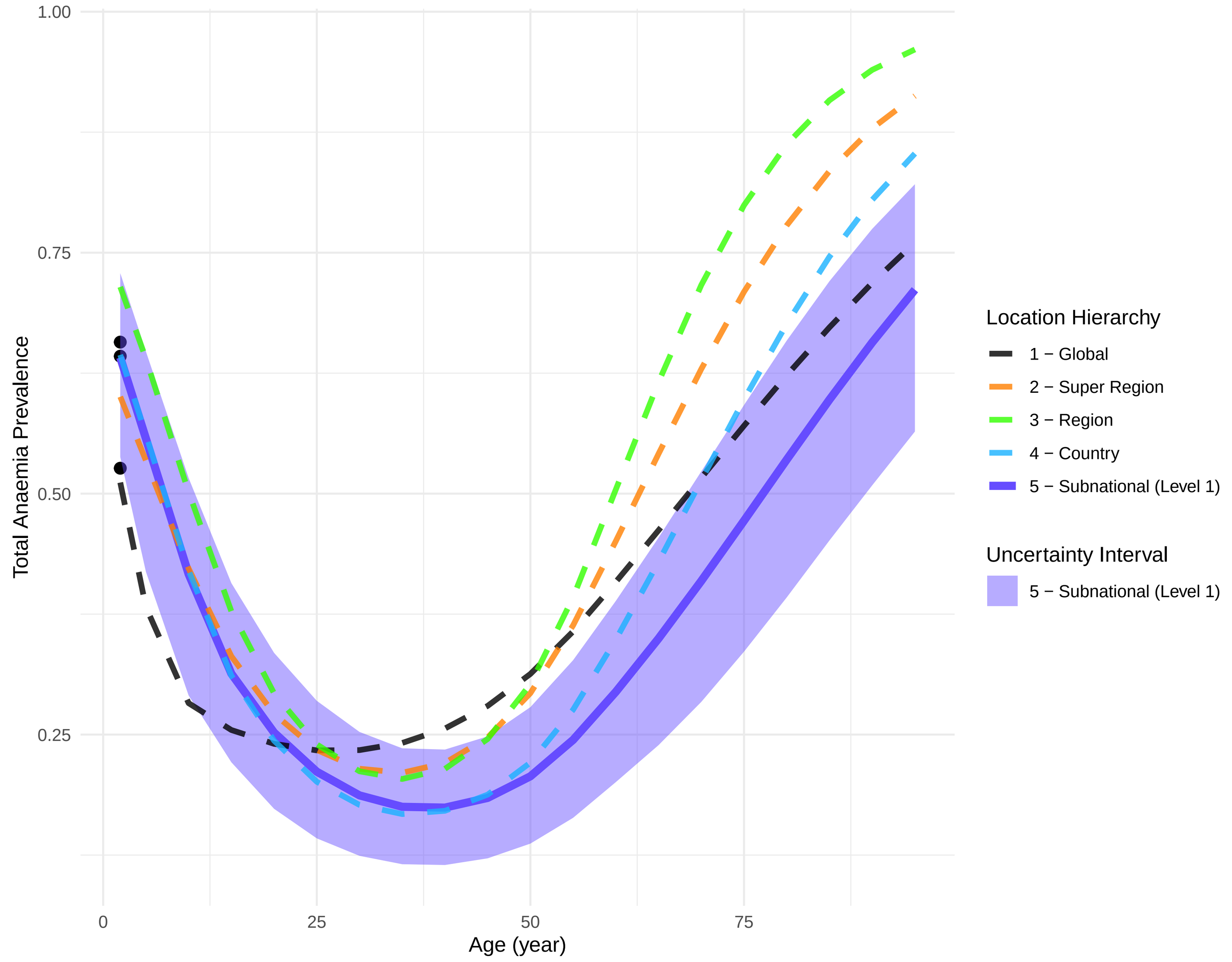

MR−BRT Age/Sex Fits − Males 2+ Years Old − Total Anaemia Prevalence − Enugu
WHO−2001 Adjustment Method

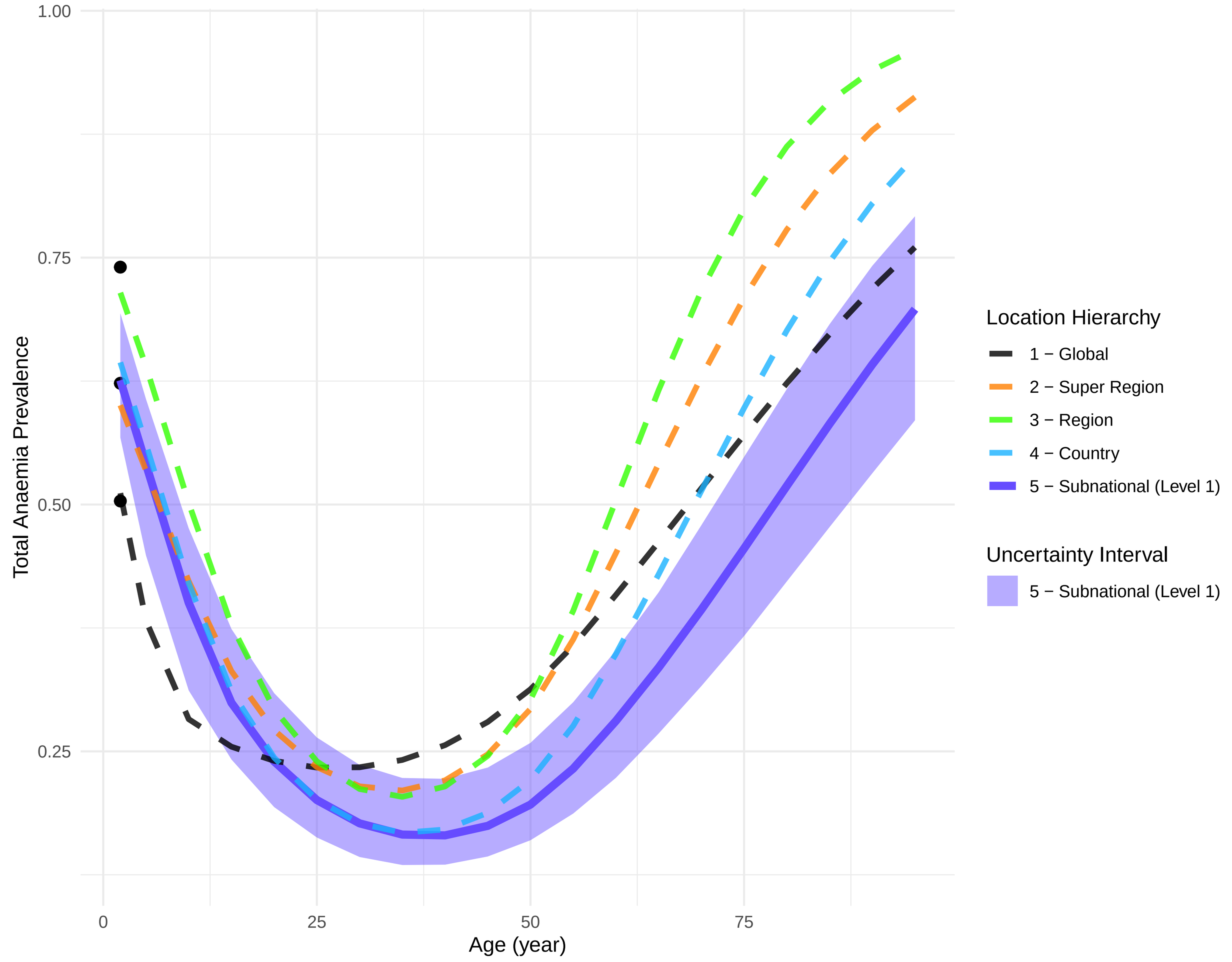

MR−BRT Age/Sex Fits − Males 2+ Years Old − Total Anaemia Prevalence − FCT (Abuja)
WHO−2001 Adjustment Method

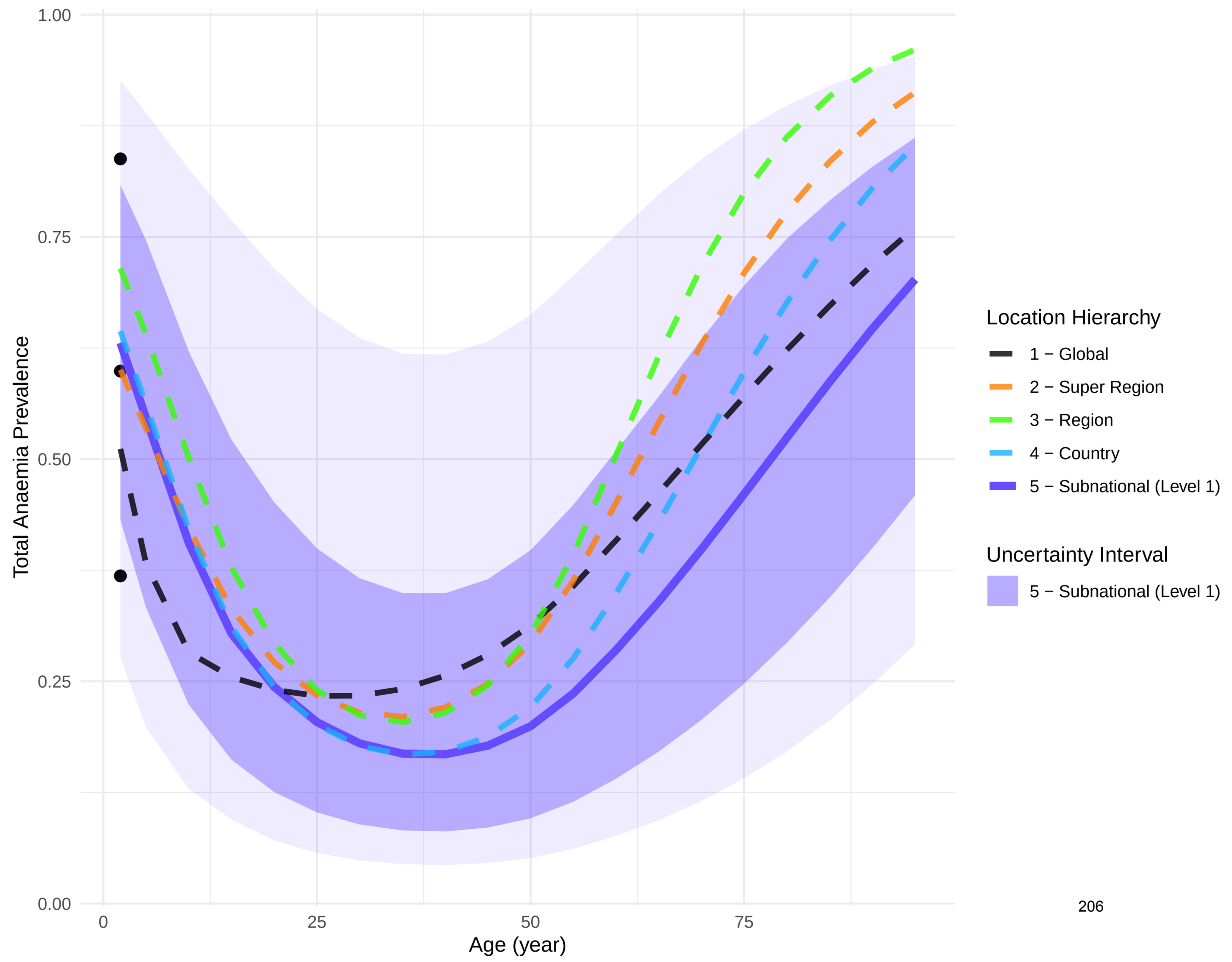

MR−BRT Age/Sex Fits − Males 2+ Years Old − Total Anaemia Prevalence − Gombe
WHO−2001 Adjustment Method

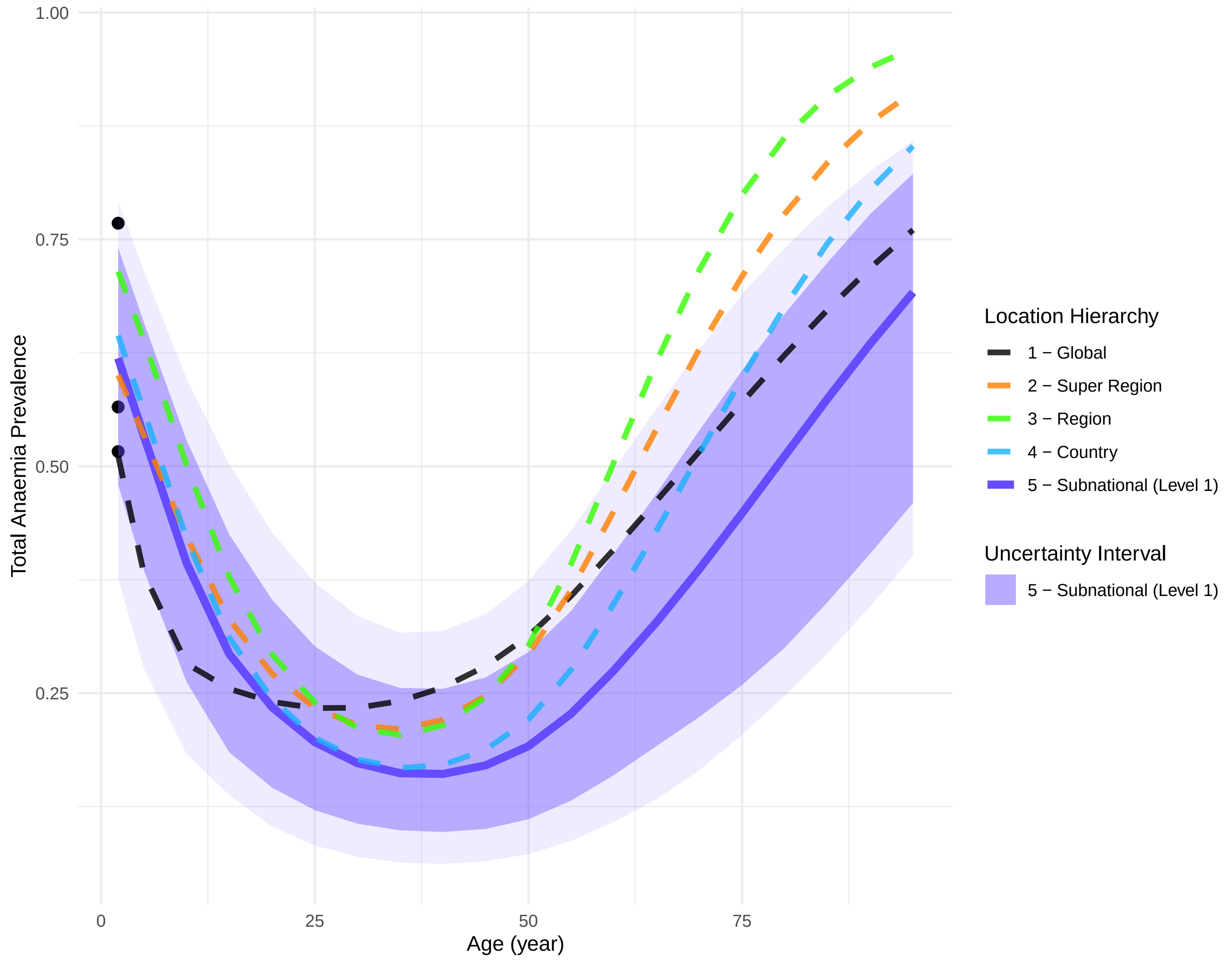

MR−BRT Age/Sex Fits − Males 2+ Years Old − Total Anaemia Prevalence − Imo
WHO−2001 Adjustment Method

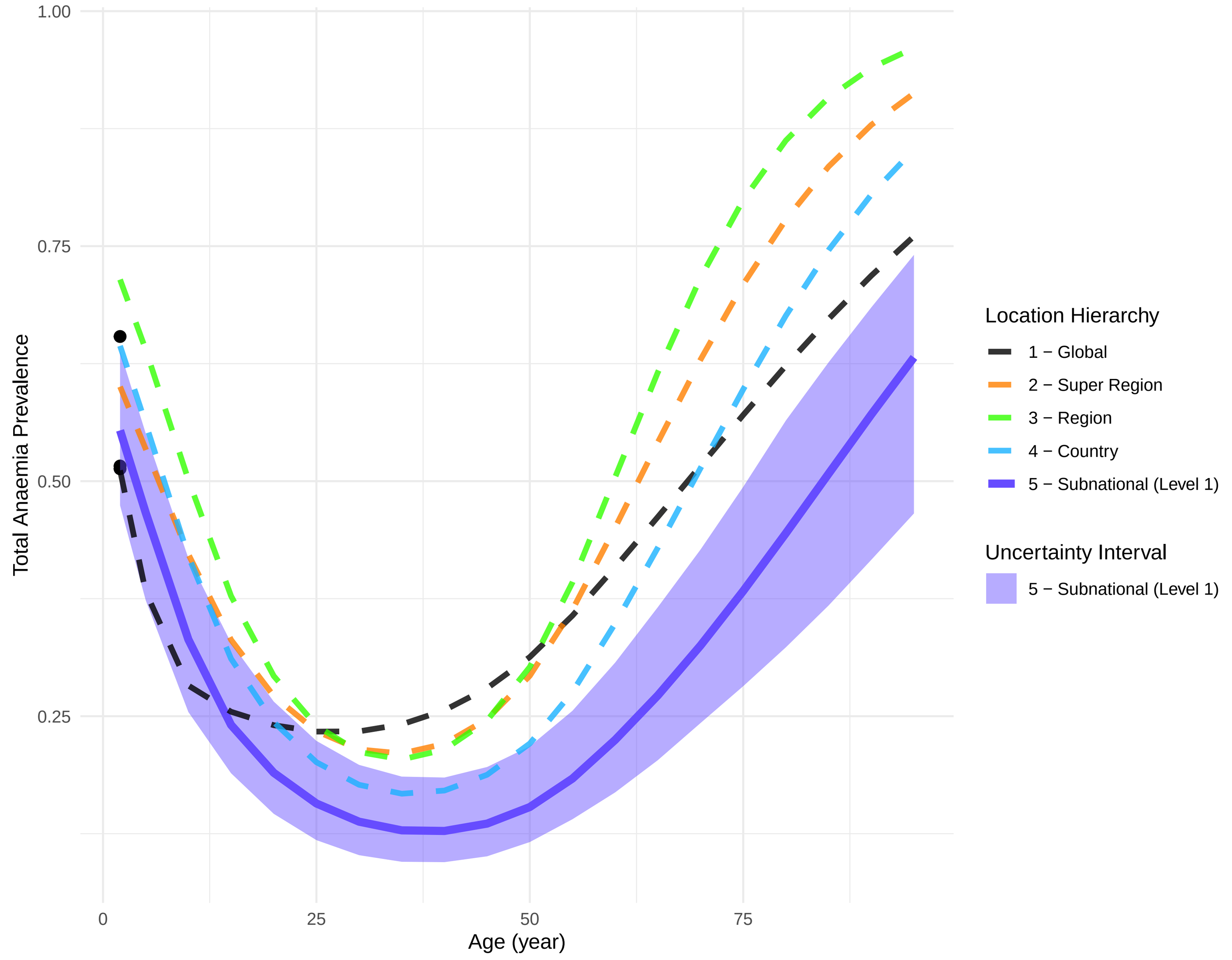

MR−BRT Age/Sex Fits − Males 2+ Years Old − Total Anaemia Prevalence − Jigawa
WHO−2001 Adjustment Method

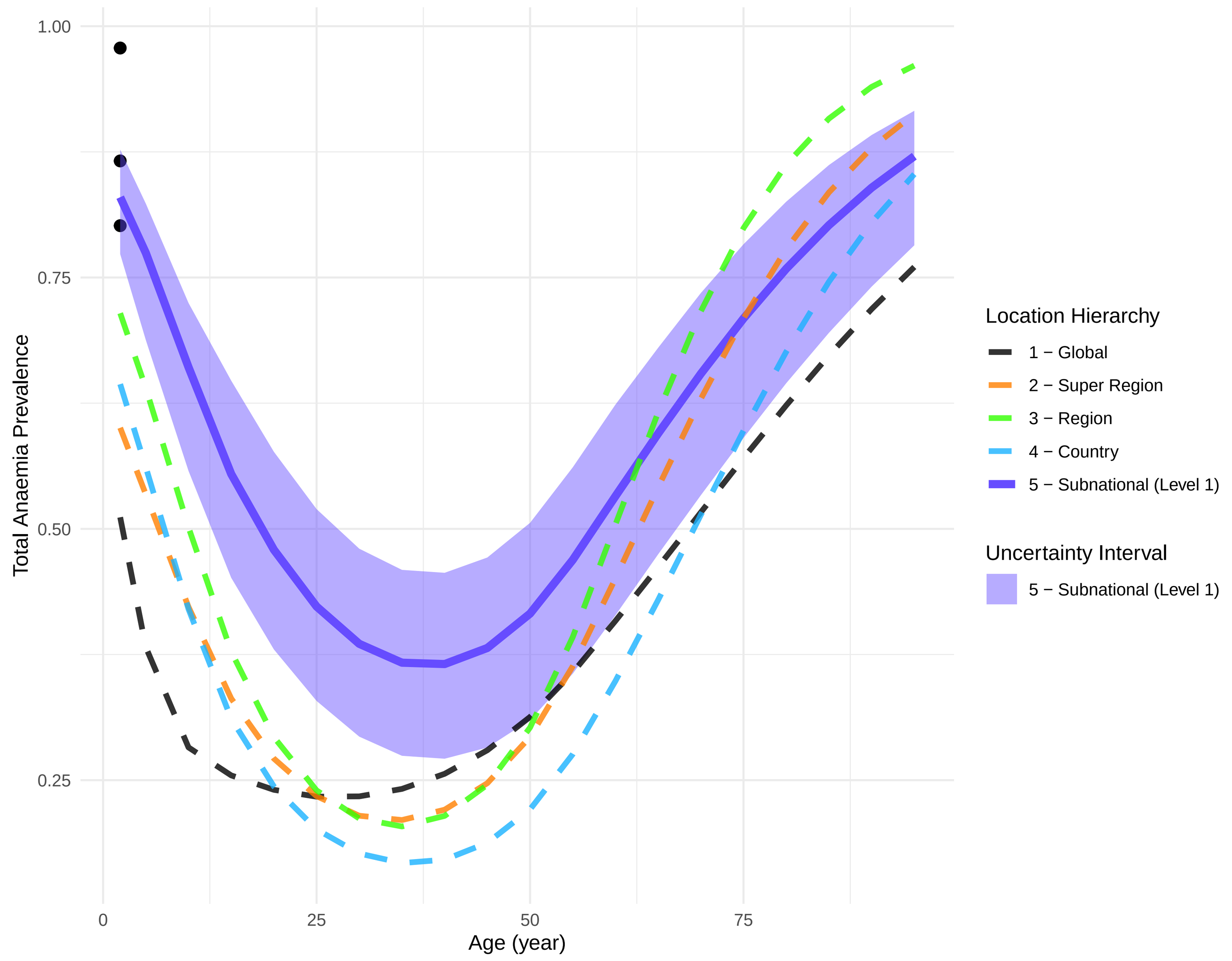

MR−BRT Age/Sex Fits − Males 2+ Years Old − Total Anaemia Prevalence − Kaduna
WHO−2001 Adjustment Method

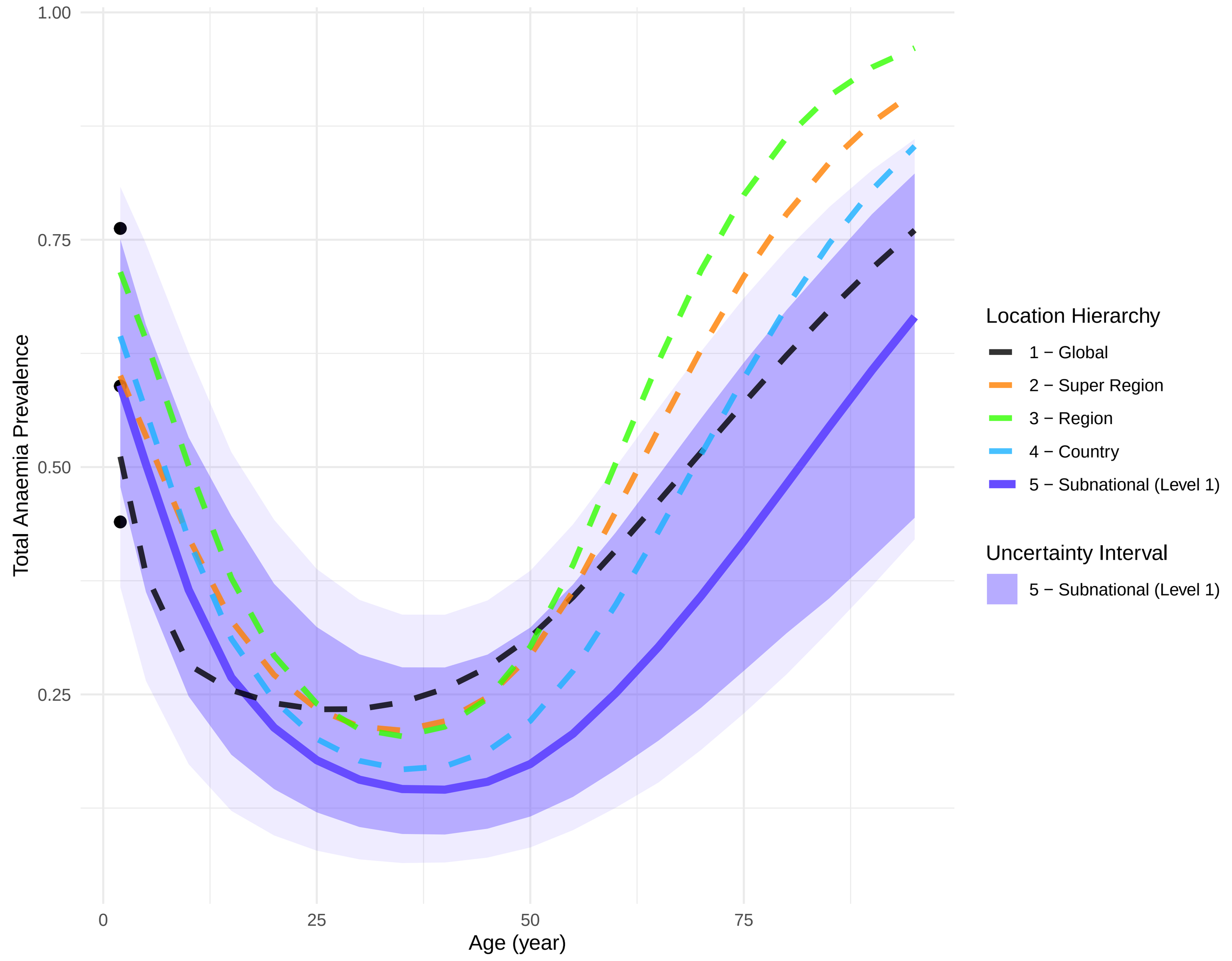

MR−BRT Age/Sex Fits − Males 2+ Years Old − Total Anaemia Prevalence − Kano
WHO−2001 Adjustment Method

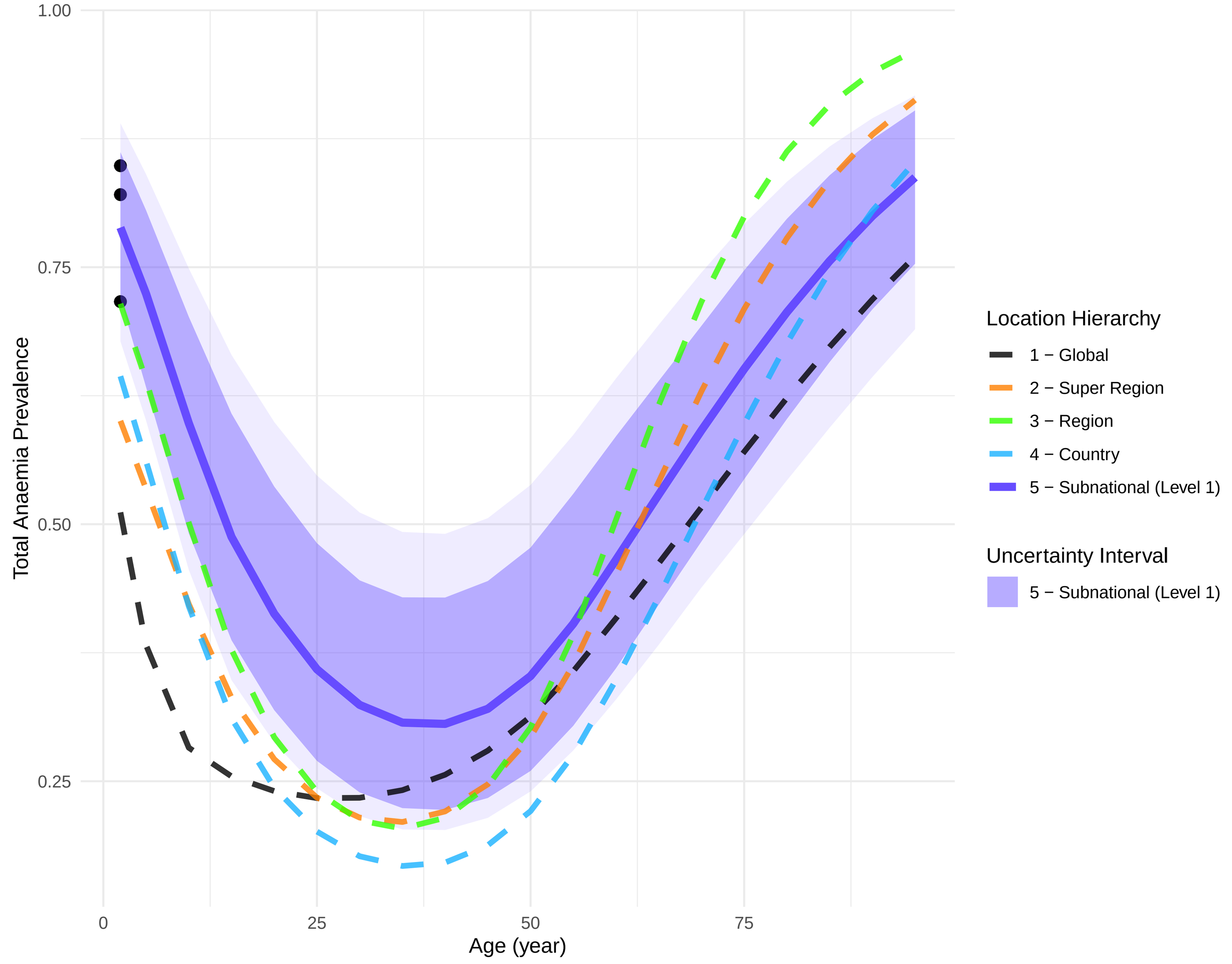

MR−BRT Age/Sex Fits − Males 2+ Years Old − Total Anaemia Prevalence − Katsina
WHO−2001 Adjustment Method

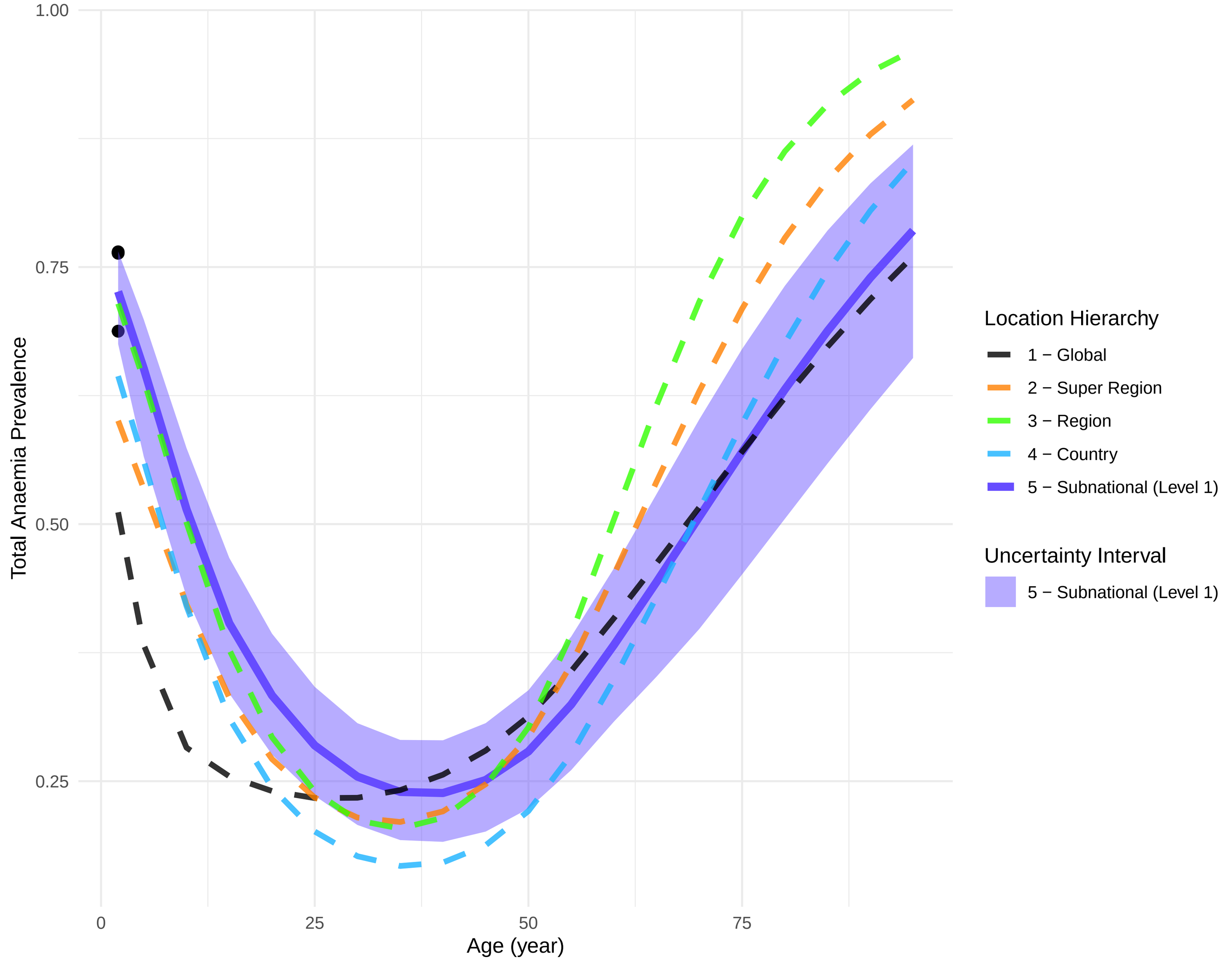

MR−BRT Age/Sex Fits − Males 2+ Years Old − Total Anaemia Prevalence − Kebbi
WHO−2001 Adjustment Method

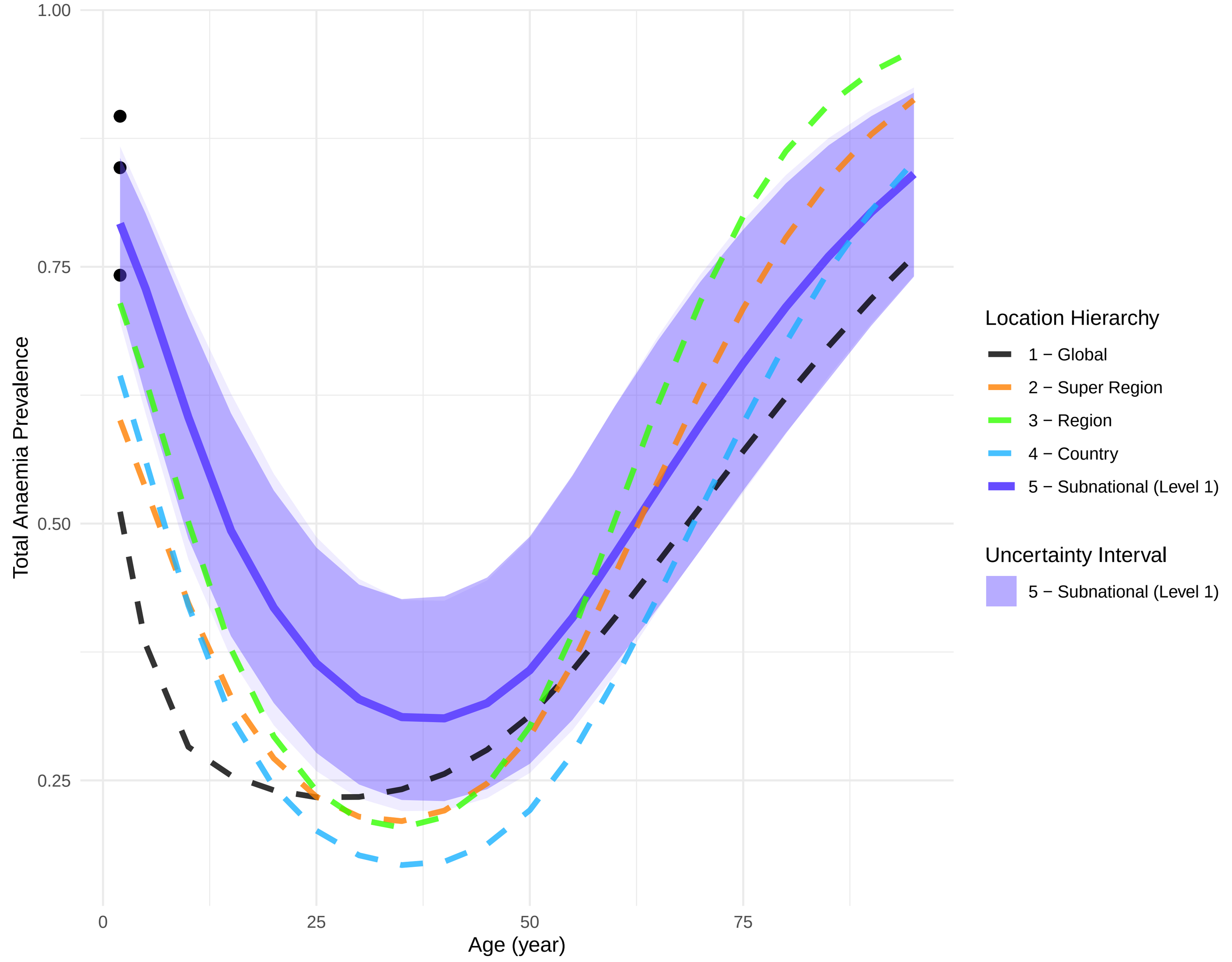

MR−BRT Age/Sex Fits − Males 2+ Years Old − Total Anaemia Prevalence − Kogi
WHO−2001 Adjustment Method

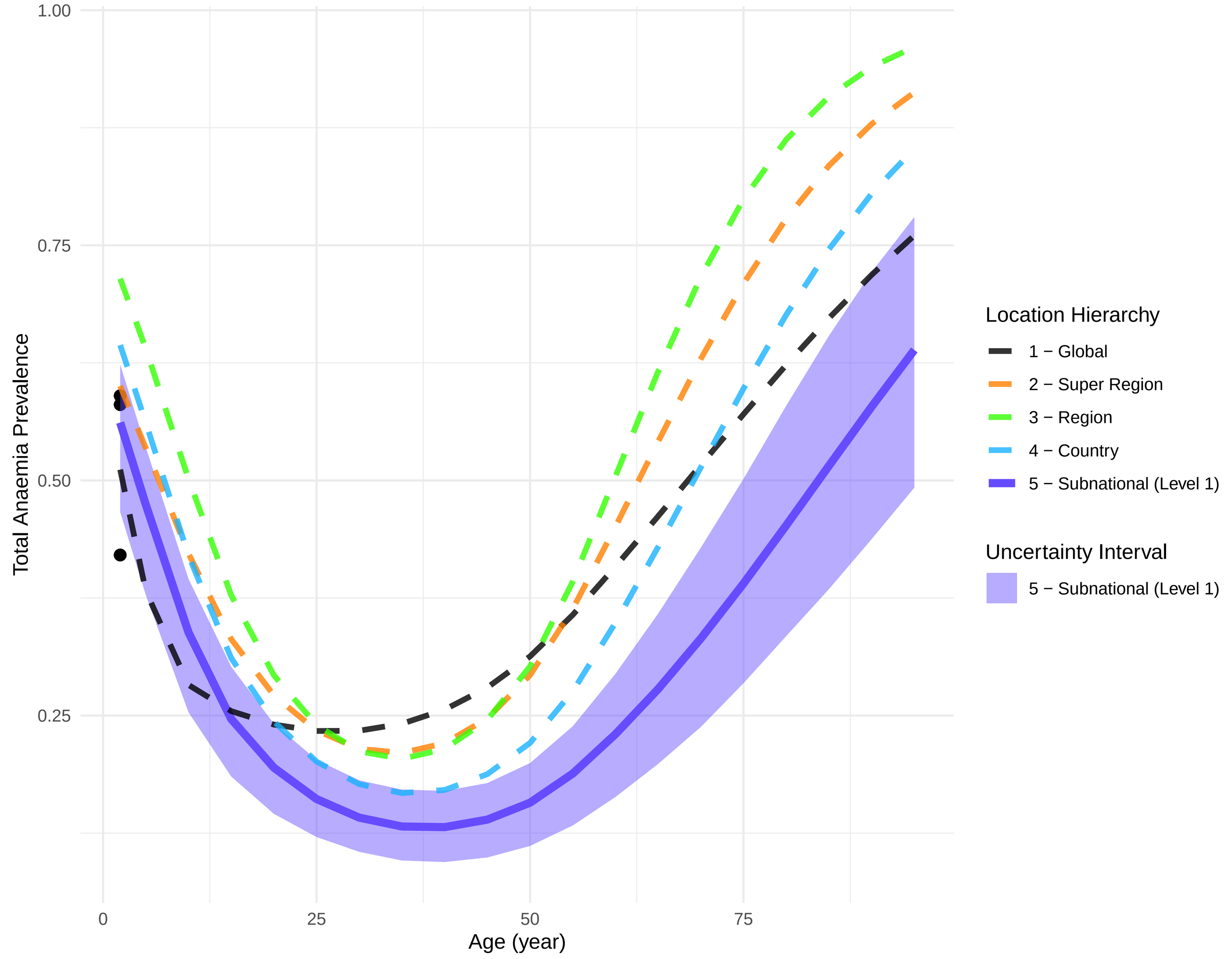

MR−BRT Age/Sex Fits − Males 2+ Years Old − Total Anaemia Prevalence − Kwara
WHO−2001 Adjustment Method

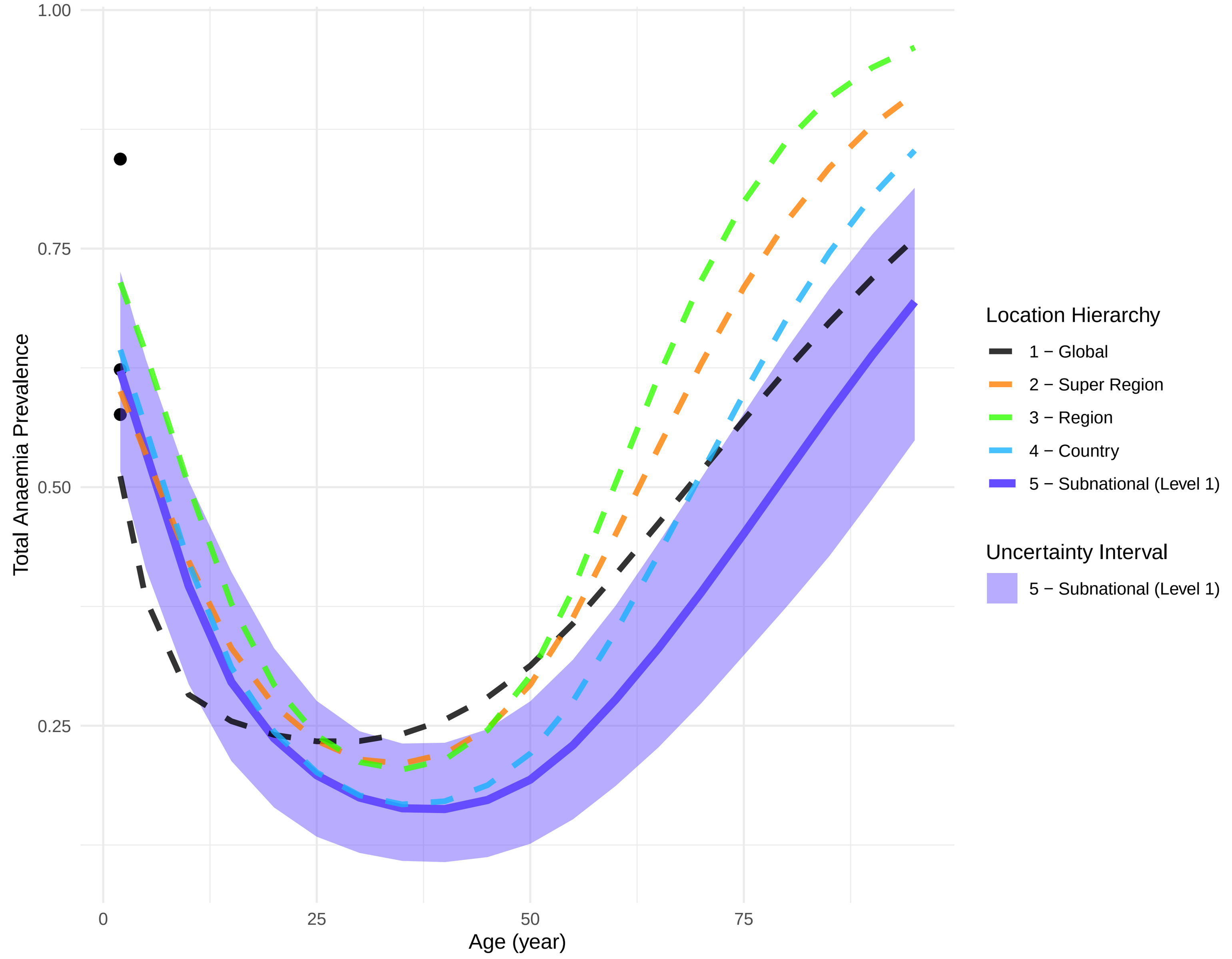

MR−BRT Age/Sex Fits − Males 2+ Years Old − Total Anaemia Prevalence − Lagos
WHO−2001 Adjustment Method

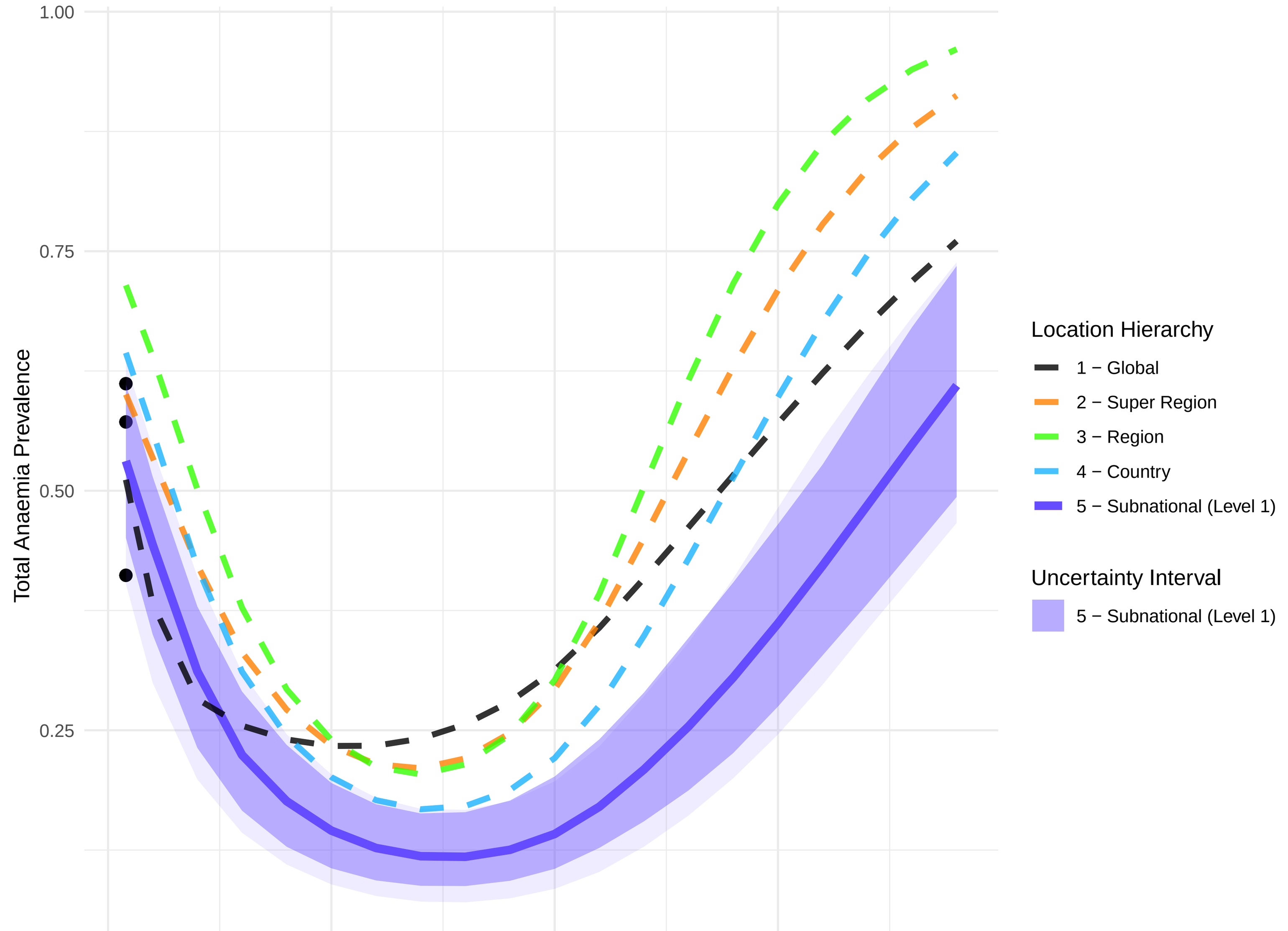

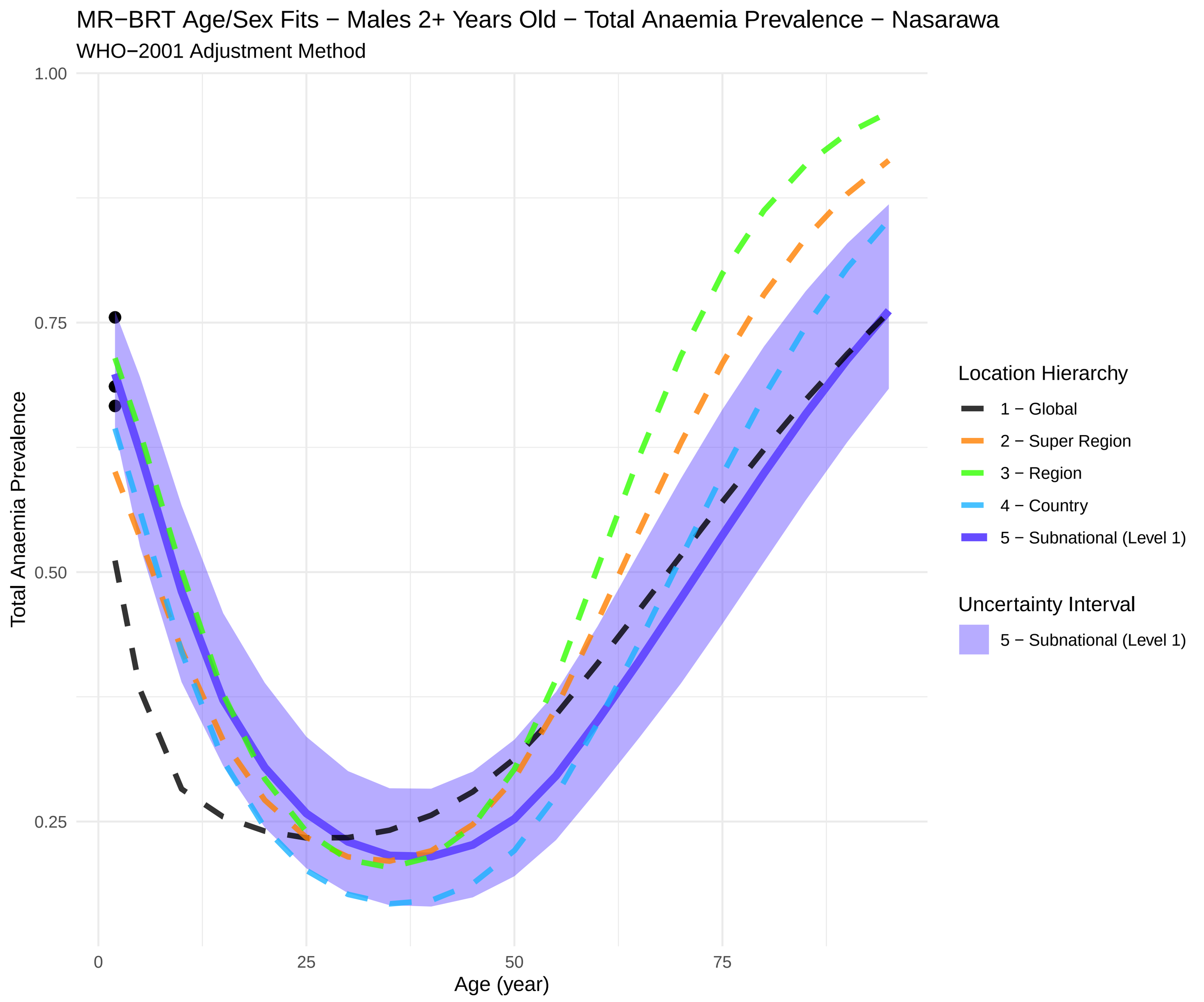
MR−BRT Age/Sex Fits − Males 2+ Years Old − Total Anaemia Prevalence − Nasarawa
WHO−2001 Adjustment Method
Total Anaemia Prevalence
Age (year)
Location Hierarchy
1 − Global
2 − Super Region
3 − Region
4 − Country
5 − Subnational (Level 1)
Uncertainty Interval
5 − Subnational (Level 1)

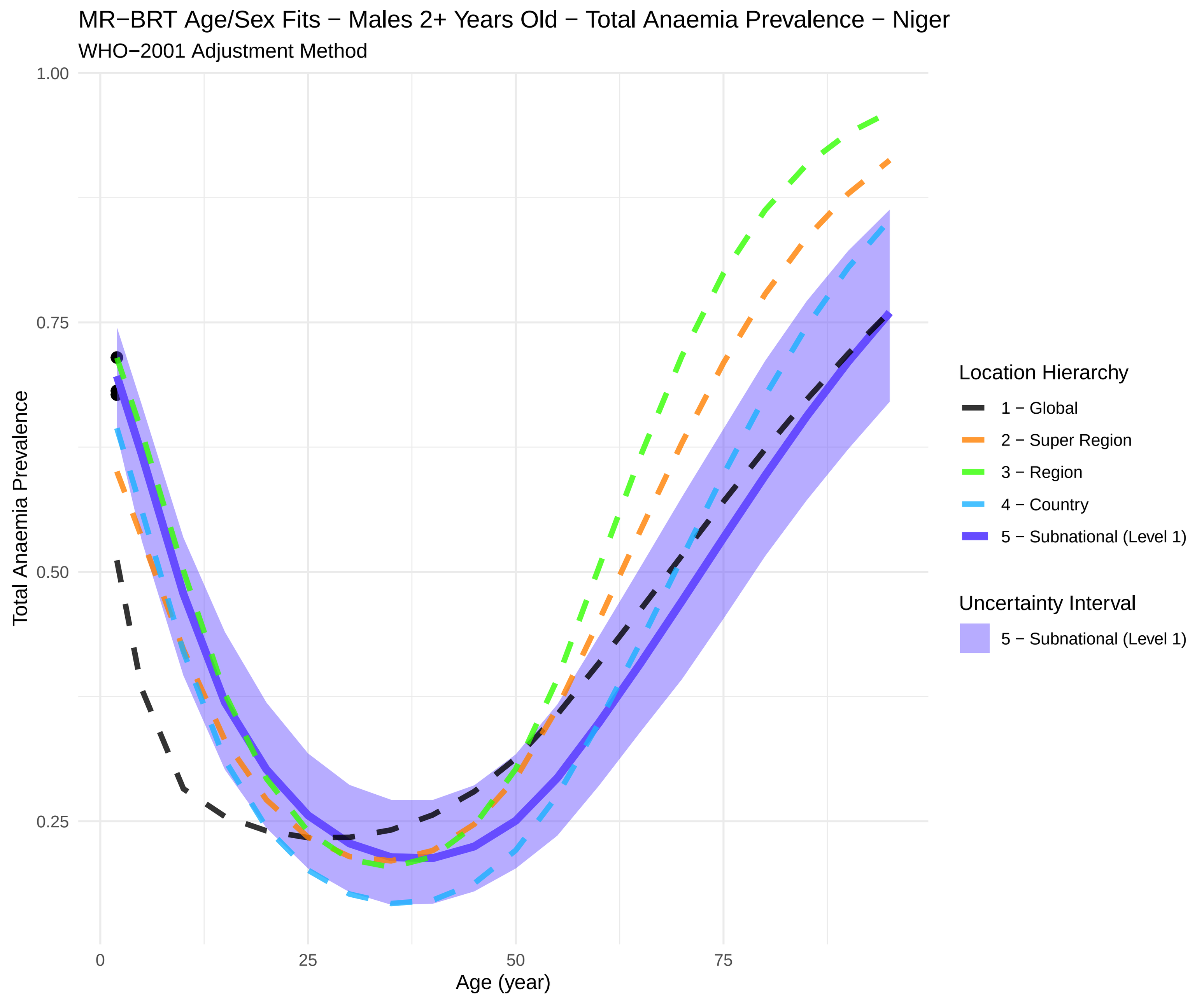
MR−BRT Age/Sex Fits − Males 2+ Years Old − Total Anaemia Prevalence − Niger
WHO−2001 Adjustment Method
Total Anaemia Prevalence
Age (year)
Location Hierarchy
1 − Global
2 − Super Region
3 − Region
4 − Country
5 − Subnational (Level 1)
Uncertainty Interval
5 − Subnational (Level 1)

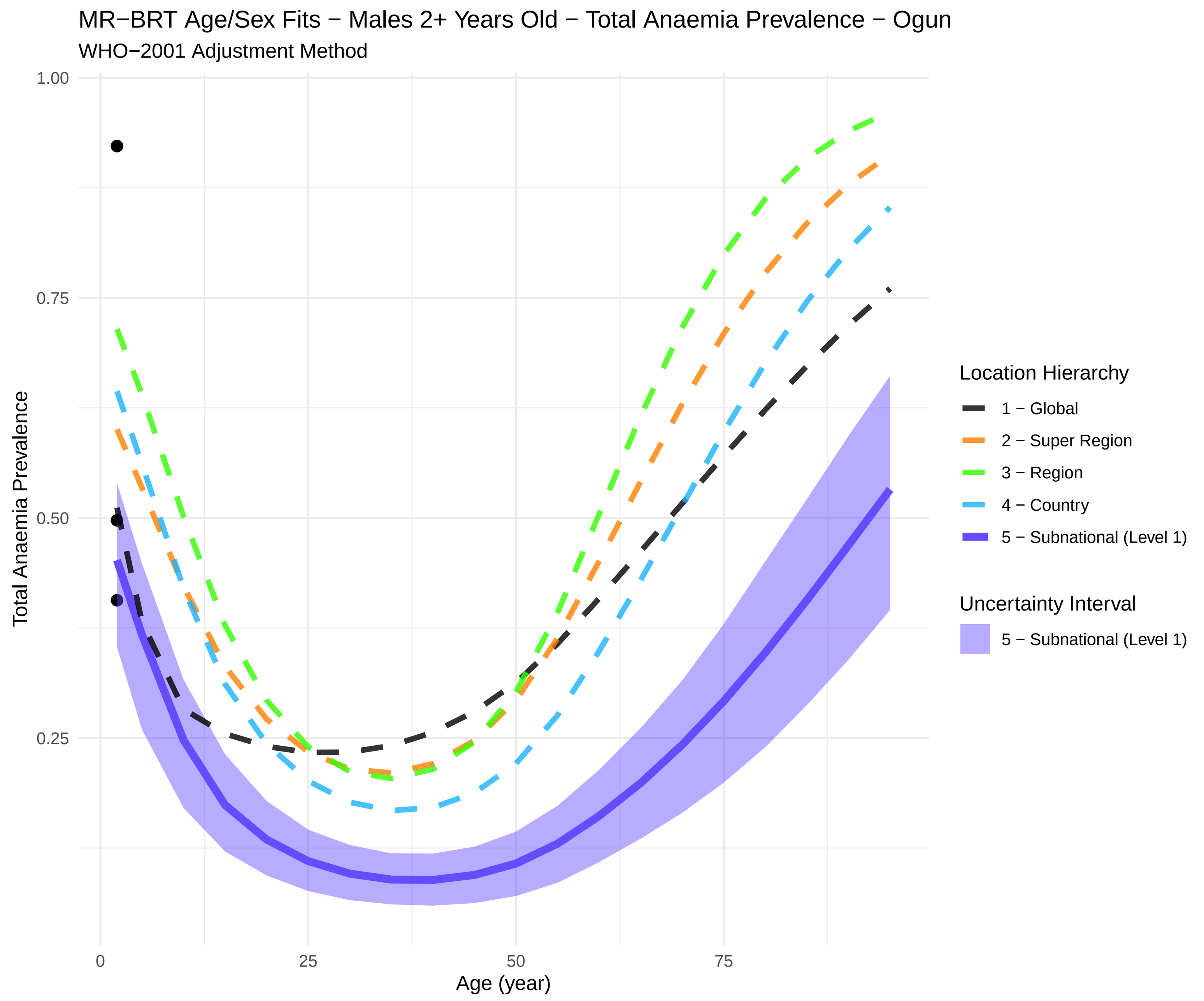
MR−BRT Age/Sex Fits − Males 2+ Years Old − Total Anaemia Prevalence − Ogun
WHO−2001 Adjustment Method
Total Anaemia Prevalence
Age (year)
Location Hierarchy
1 − Global
2 − Super Region
3 − Region
4 − Country
5 − Subnational (Level 1)
Uncertainty Interval
5 − Subnational (Level 1)

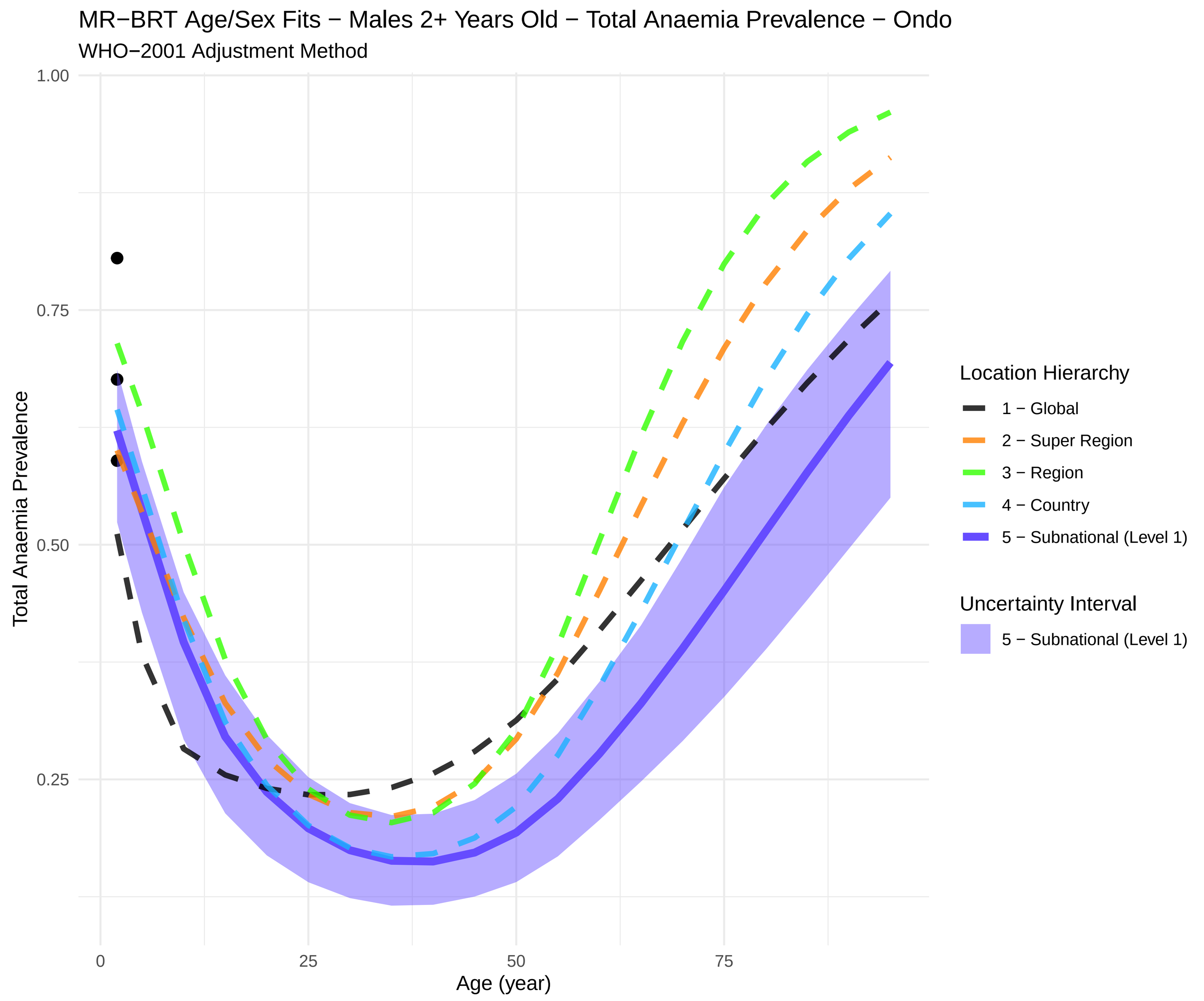
MR−BRT Age/Sex Fits − Males 2+ Years Old − Total Anaemia Prevalence − Ondo
WHO−2001 Adjustment Method
Total Anaemia Prevalence
Age (year)
Location Hierarchy
1 − Global
2 − Super Region
3 − Region
4 − Country
5 − Subnational (Level 1)
Uncertainty Interval
5 − Subnational (Level 1)

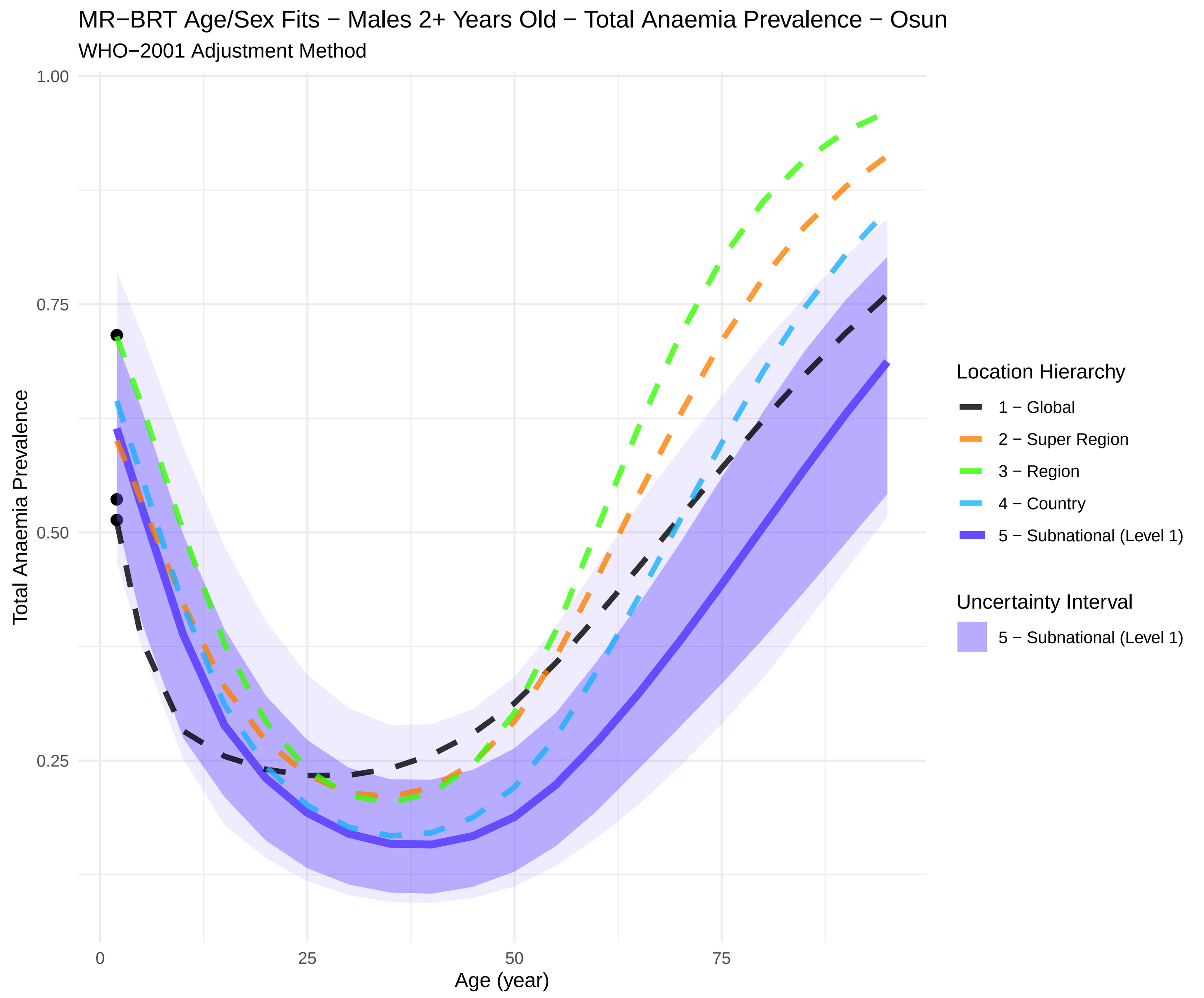
MR−BRT Age/Sex Fits − Males 2+ Years Old − Total Anaemia Prevalence − Osun
WHO−2001 Adjustment Method
Total Anaemia Prevalence
Age (year)
Location Hierarchy
1 − Global
2 − Super Region
3 − Region
4 − Country
5 − Subnational (Level 1)
Uncertainty Interval
5 − Subnational (Level 1)

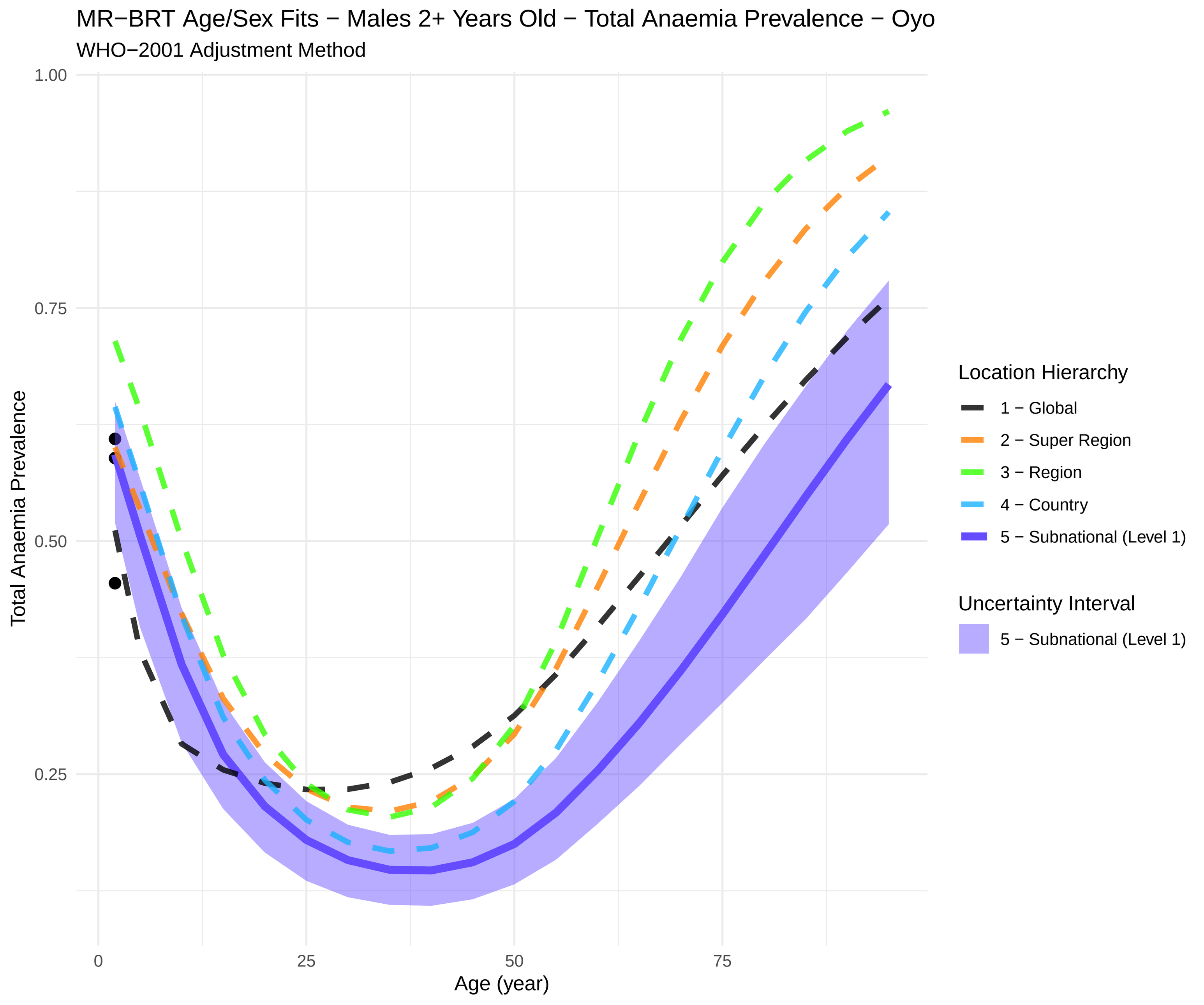
MR−BRT Age/Sex Fits − Males 2+ Years Old − Total Anaemia Prevalence − Oyo
WHO−2001 Adjustment Method
Total Anaemia Prevalence
Age (year)
Location Hierarchy
1 − Global
2 − Super Region
3 − Region
4 − Country
5 − Subnational (Level 1)
Uncertainty Interval
5 − Subnational (Level 1)

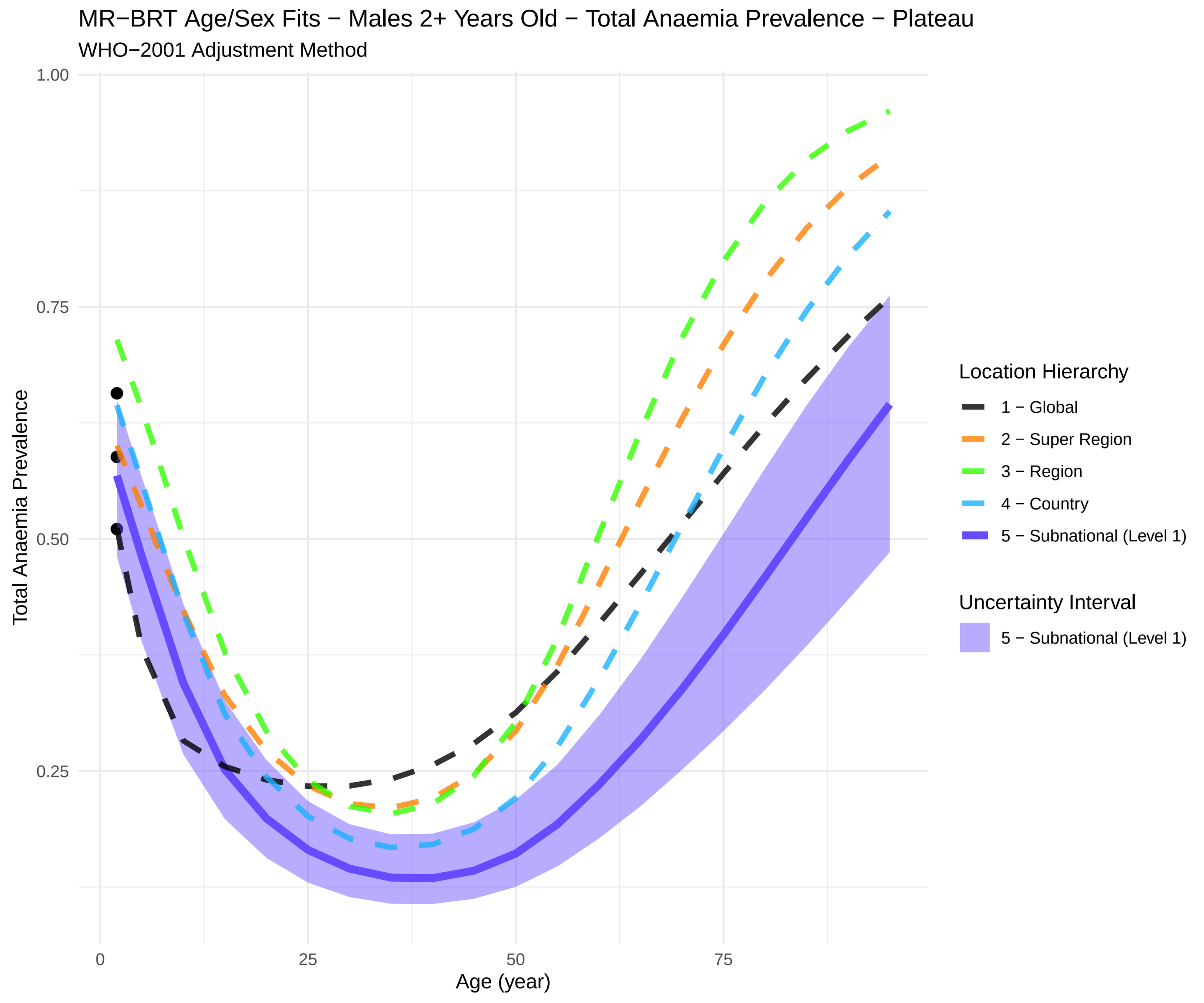
MR−BRT Age/Sex Fits − Males 2+ Years Old − Total Anaemia Prevalence − Plateau
WHO−2001 Adjustment Method
Total Anaemia Prevalence
Age (year)
Location Hierarchy
1 − Global
2 − Super Region
3 − Region
4 − Country
5 − Subnational (Level 1)
Uncertainty Interval
5 − Subnational (Level 1)

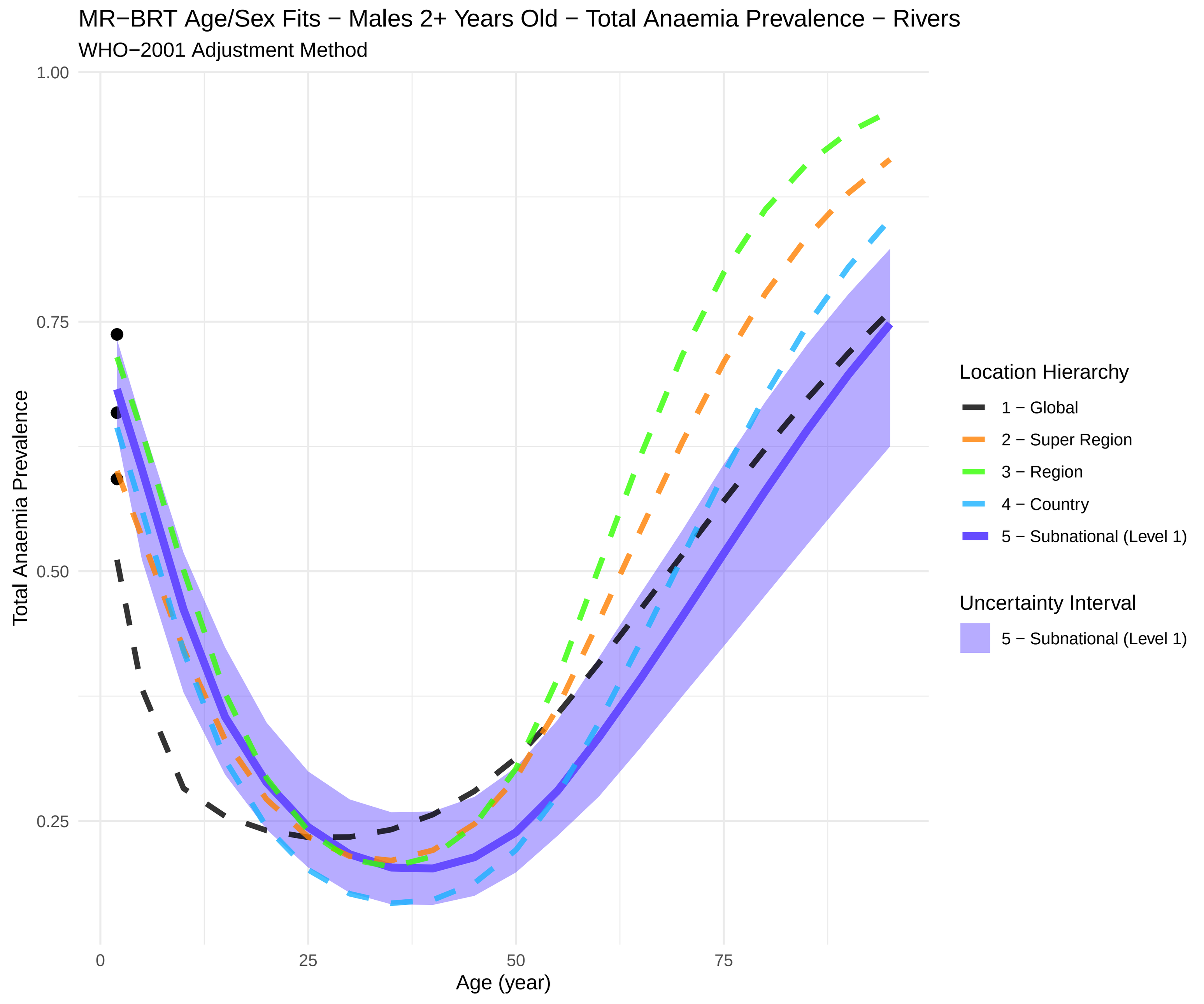
MR−BRT Age/Sex Fits − Males 2+ Years Old − Total Anaemia Prevalence − Rivers
WHO−2001 Adjustment Method
Total Anaemia Prevalence
Age (year)
Location Hierarchy
1 − Global
2 − Super Region
3 − Region
4 − Country
5 − Subnational (Level 1)
Uncertainty Interval
5 − Subnational (Level 1)

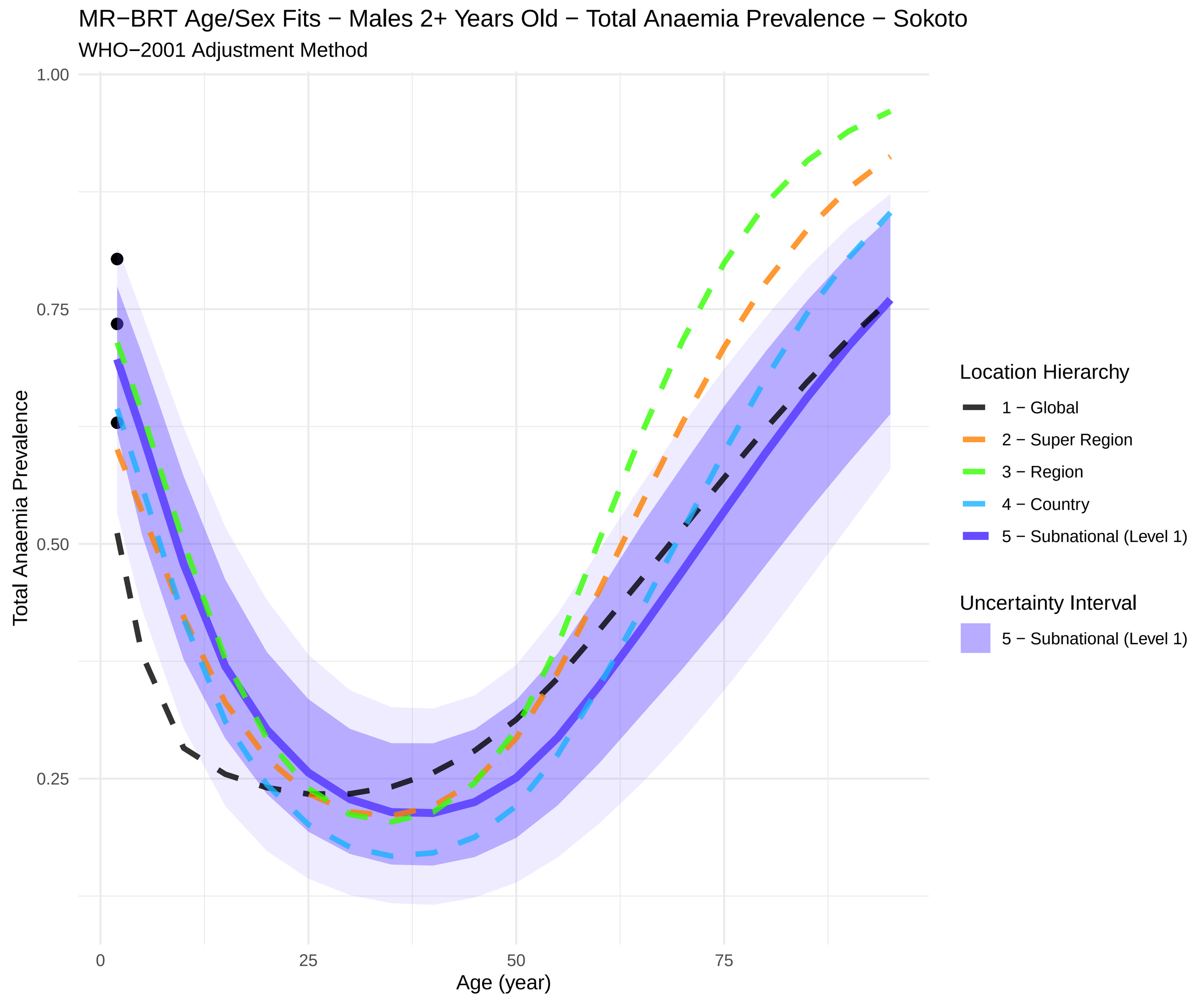
MR−BRT Age/Sex Fits − Males 2+ Years Old − Total Anaemia Prevalence − Sokoto
WHO−2001 Adjustment Method
Total Anaemia Prevalence
Age (year)
Location Hierarchy
1 − Global
2 − Super Region
3 − Region
4 − Country
5 − Subnational (Level 1)
Uncertainty Interval
5 − Subnational (Level 1)

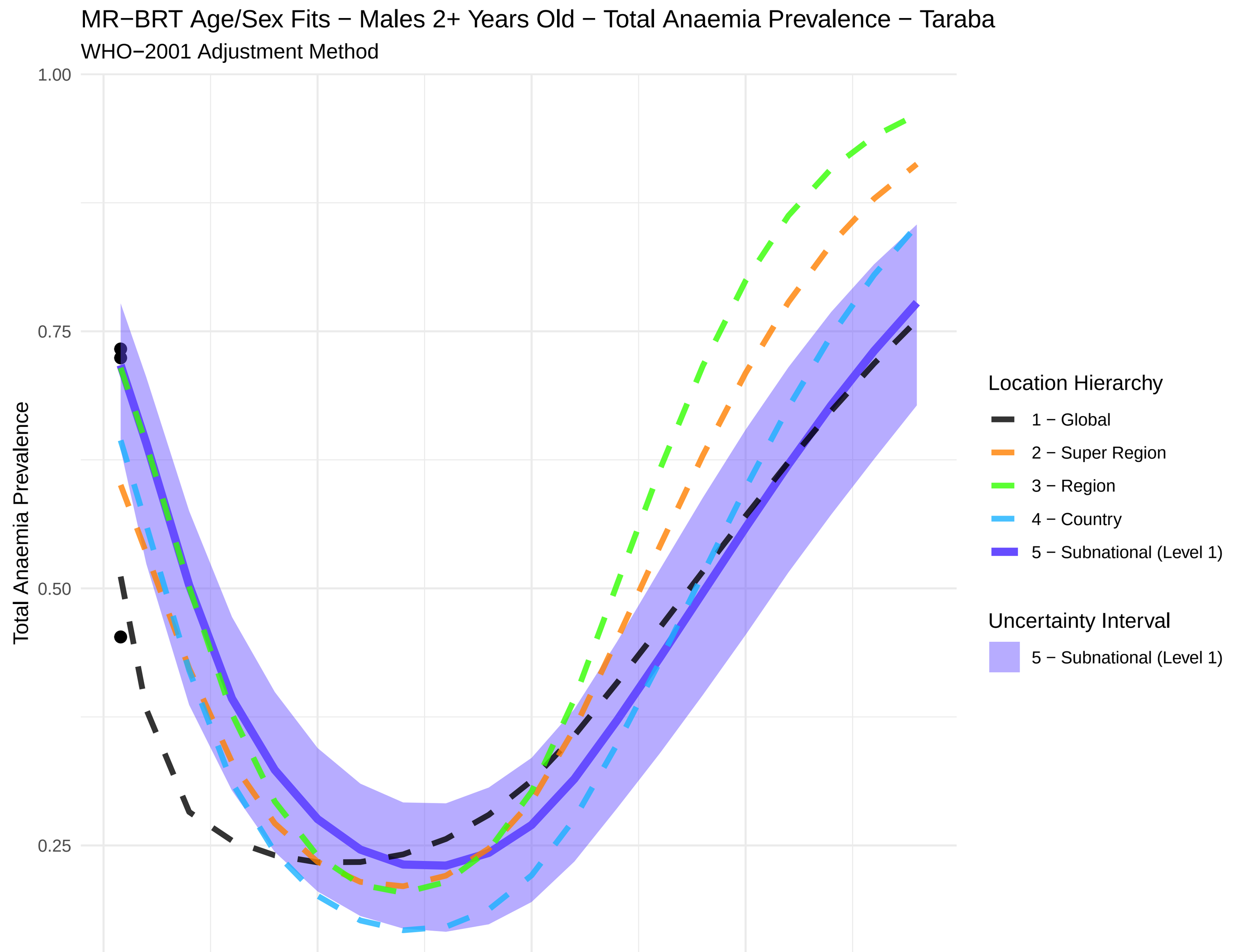
MR−BRT Age/Sex Fits − Males 2+ Years Old − Total Anaemia Prevalence − Taraba
WHO−2001 Adjustment Method
Total Anaemia Prevalence
Age (year)
Location Hierarchy
1 − Global
2 − Super Region
3 − Region
4 − Country
5 − Subnational (Level 1)
Uncertainty Interval
5 − Subnational (Level 1)

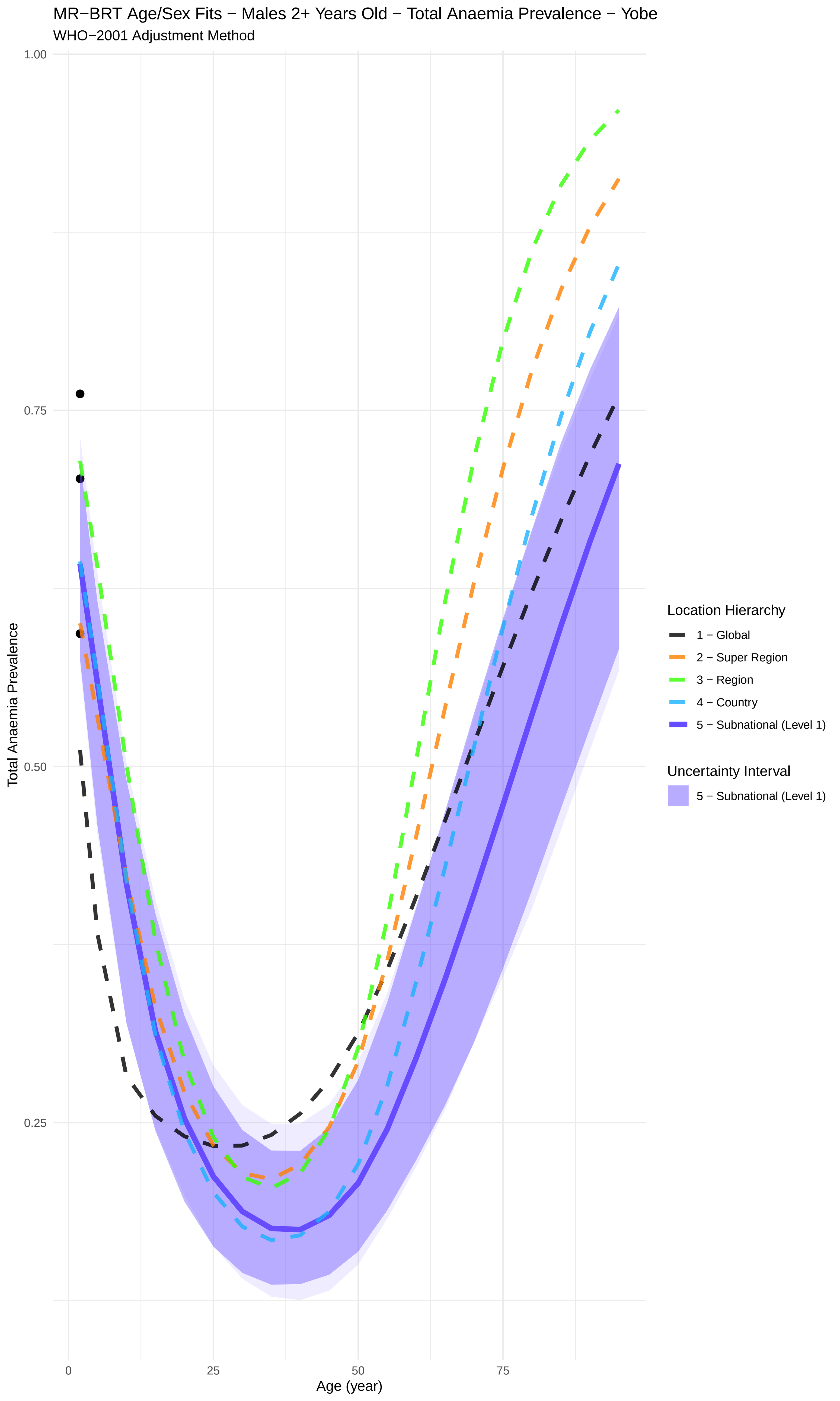
MR−BRT Age/Sex Fits − Males 2+ Years Old − Total Anaemia Prevalence − Yobe
WHO−2001 Adjustment Method
Total Anaemia Prevalence
Age (year)
Location Hierarchy
1 − Global
2 − Super Region
3 − Region
4 − Country
5 − Subnational (Level 1)
Uncertainty Interval
5 − Subnational (Level 1)

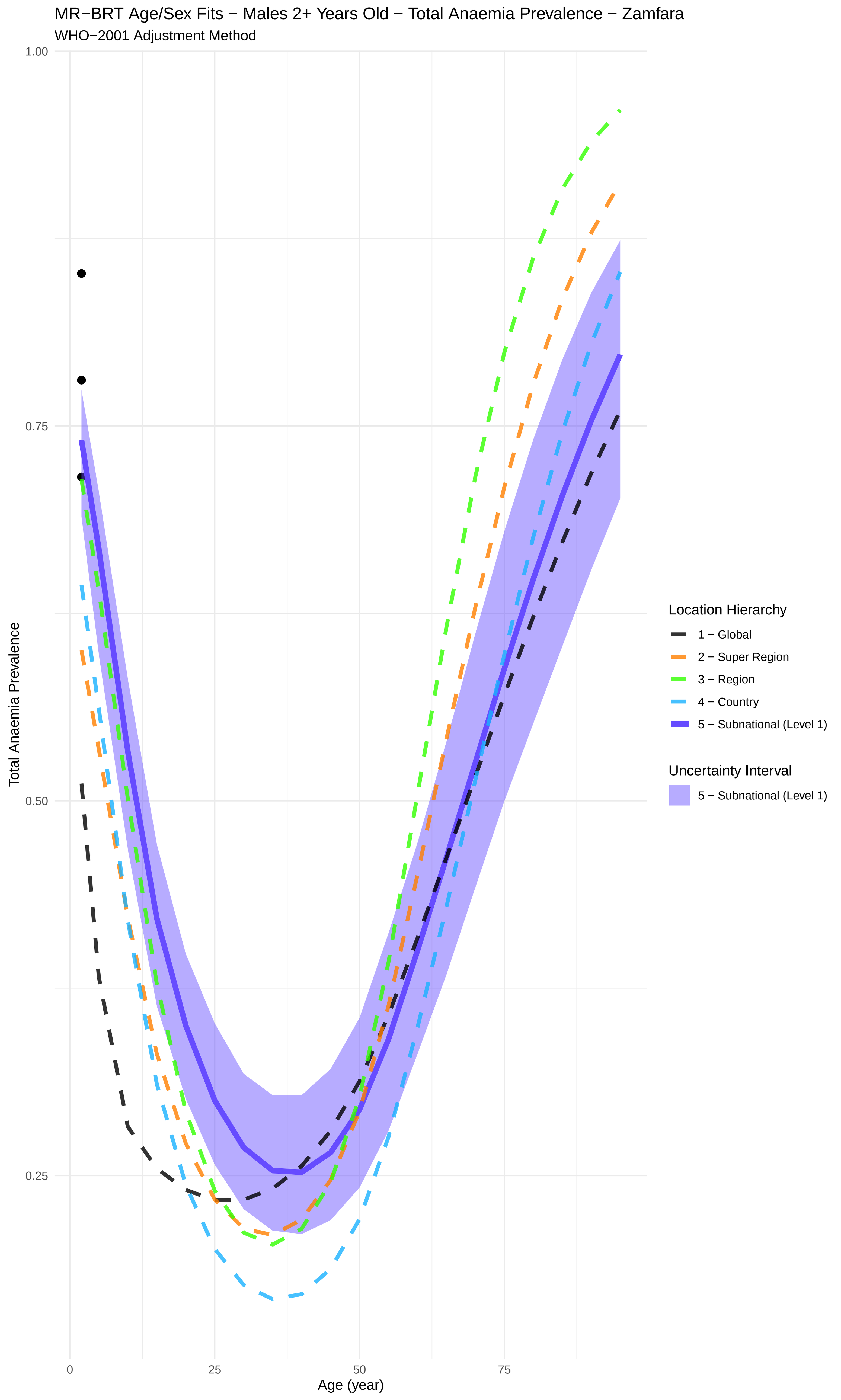
MR−BRT Age/Sex Fits − Males 2+ Years Old − Total Anaemia Prevalence − Zamfara
WHO−2001 Adjustment Method
Total Anaemia Prevalence
Age (year)
Location Hierarchy
1 − Global
2 − Super Region
3 − Region
4 − Country
5 − Subnational (Level 1)
Uncertainty Interval
5 − Subnational (Level 1)

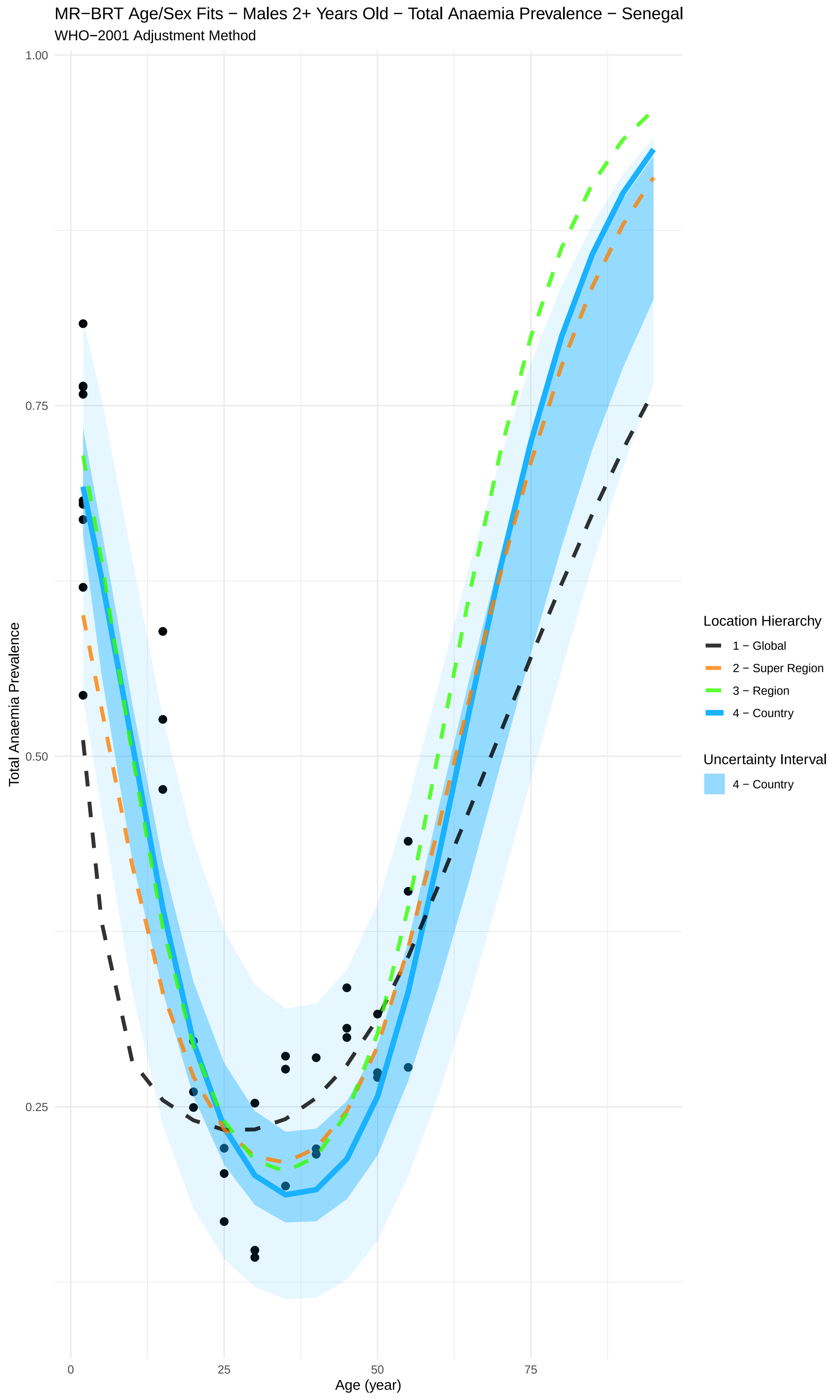
MR−BRT Age/Sex Fits − Males 2+ Years Old − Total Anaemia Prevalence − Senegal
WHO−2001 Adjustment Method
Total Anaemia Prevalence
Age (year)
Location Hierarchy
1 − Global
2 − Super Region
3 − Region
4 − Country
Uncertainty Interval
4 − Country

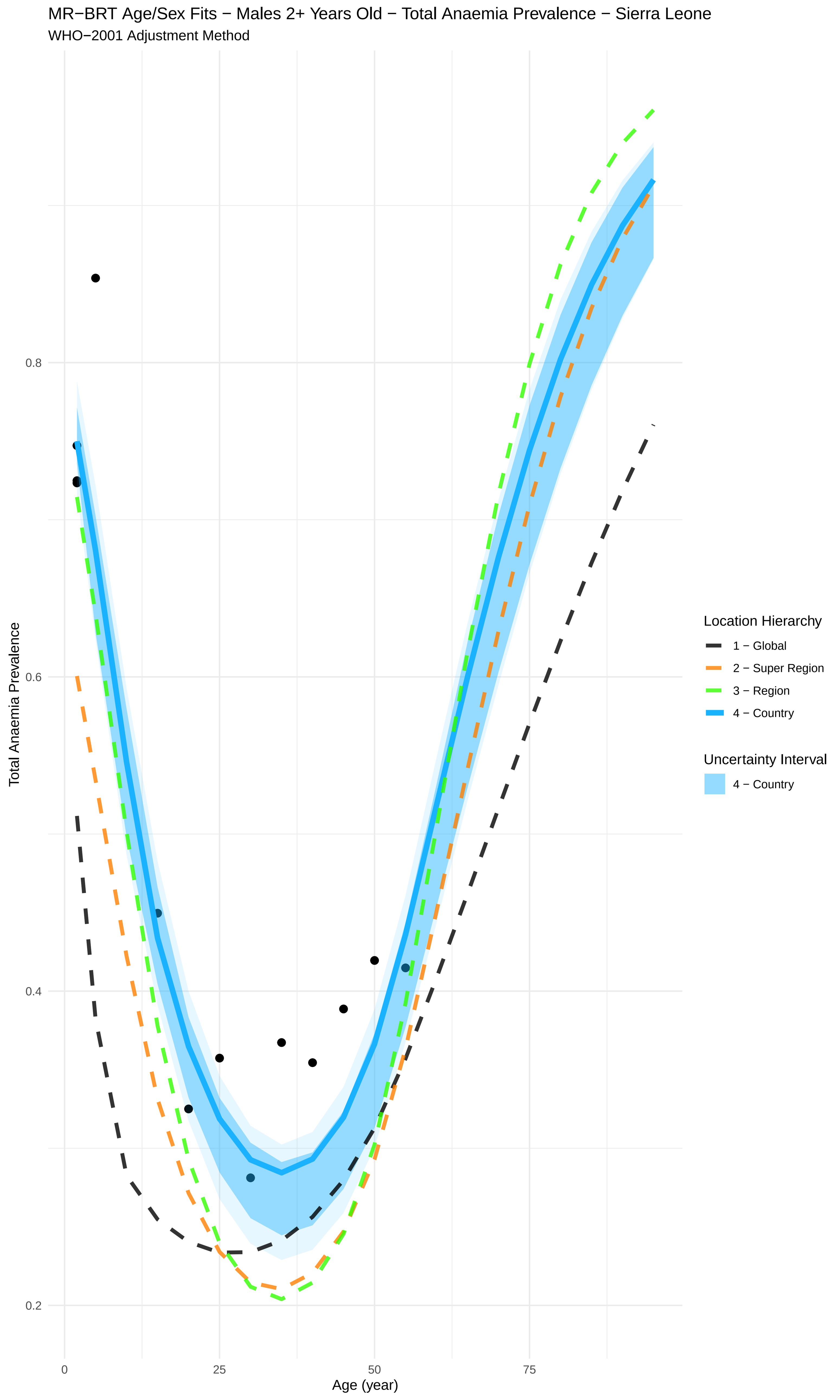
MR−BRT Age/Sex Fits − Males 2+ Years Old − Total Anaemia Prevalence − Sierra Leone
WHO−2001 Adjustment Method
Total Anaemia Prevalence
Age (year)
Location Hierarchy
1 − Global
2 − Super Region
3 − Region
4 − Country
Uncertainty Interval
4 − Country

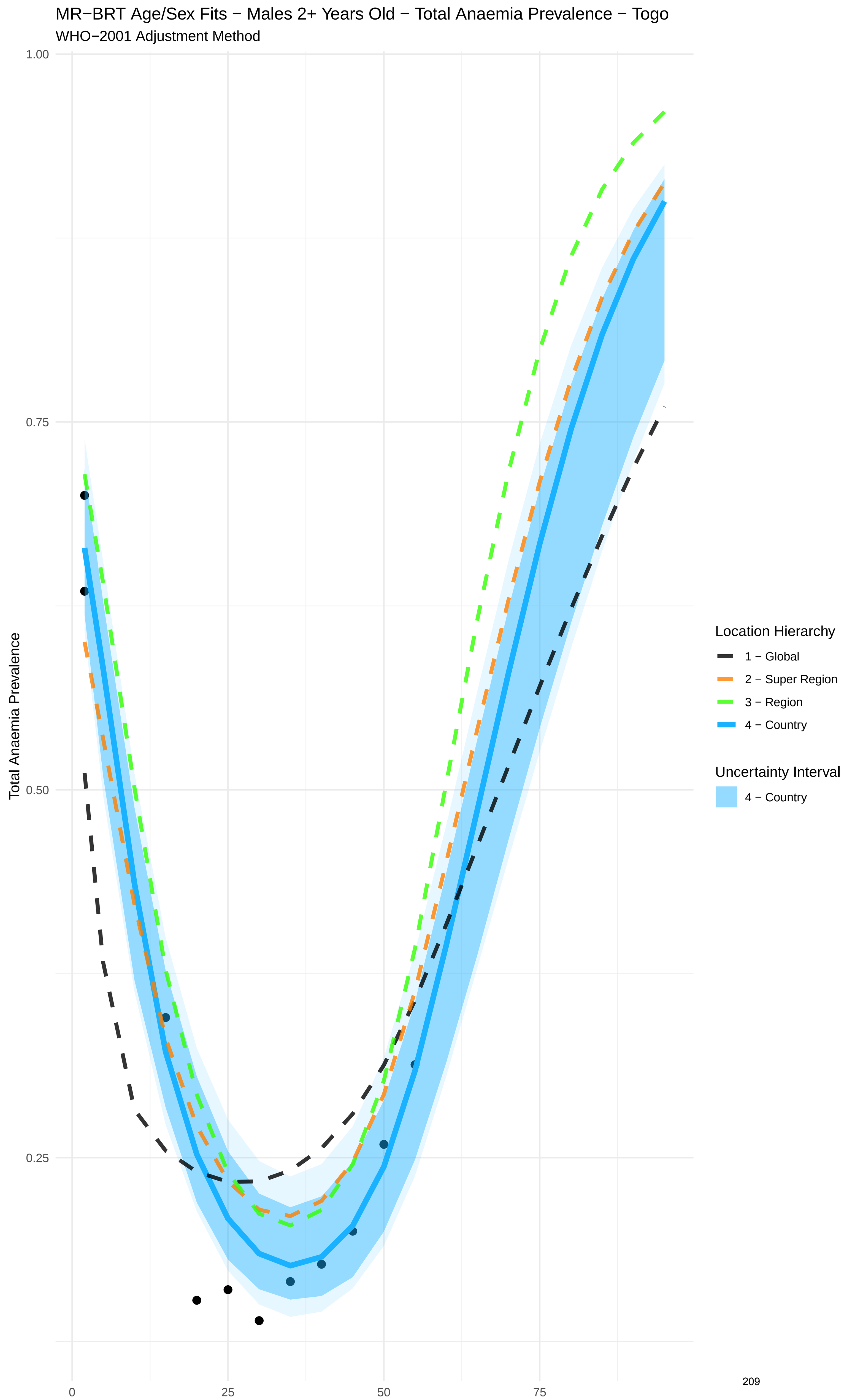
MR−BRT Age/Sex Fits − Males 2+ Years Old − Total Anaemia Prevalence − Togo
WHO−2001 Adjustment Method
Total Anaemia Prevalence
Age (year)
Location Hierarchy
1 − Global
2 − Super Region
3 − Region
4 − Country
Uncertainty Interval
4 − Country

MR−BRT Age/Sex Fits − Non−pregnant Females 2+ Years Old − Total Anaemia Prevalence − Global
WHO−2001 Adjustment Method

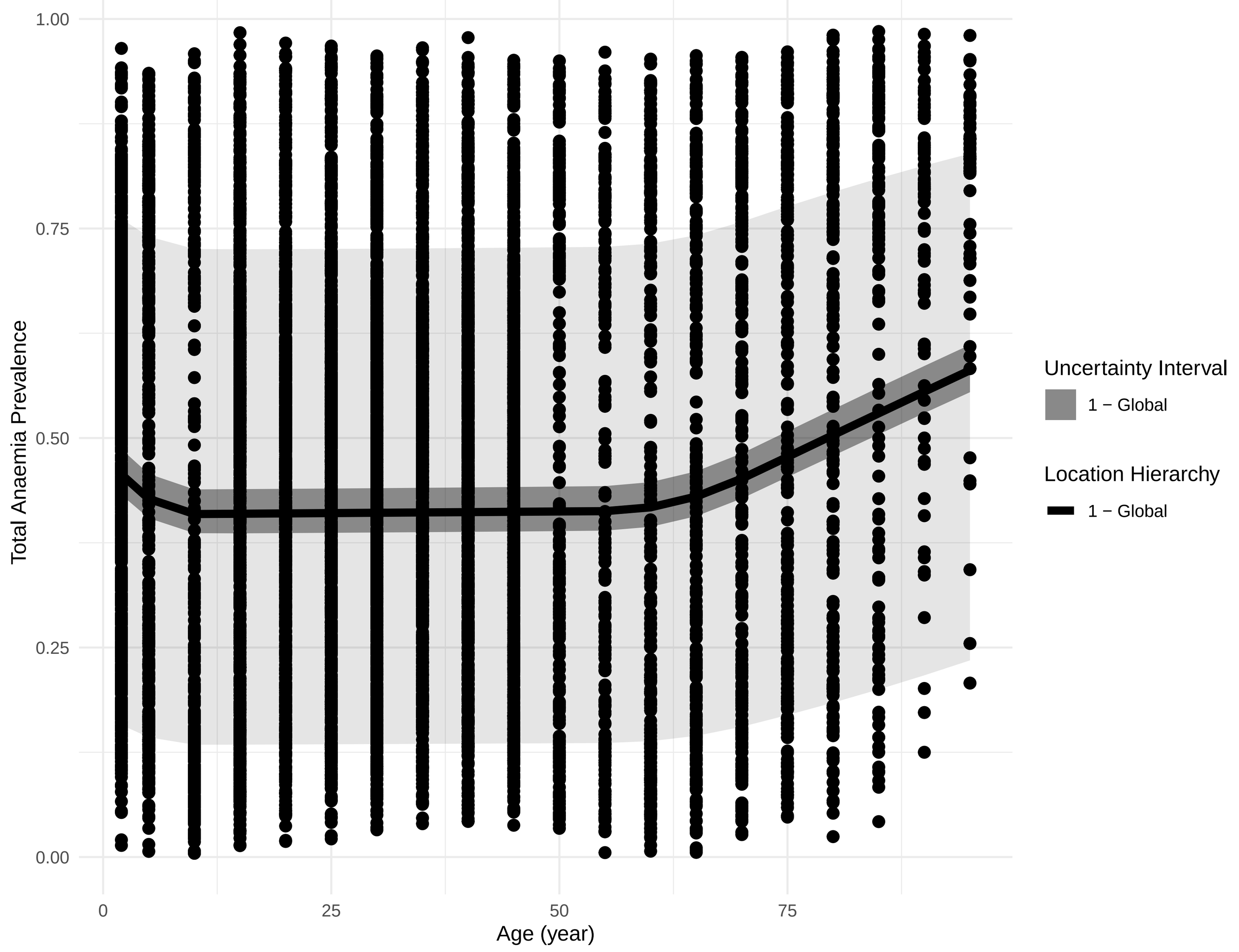

MR−BRT Age/Sex Fits − Non−pregnant Females 2+ Years Old − Total Anaemia Prevalence − Central Europe, Eastern Europe, and Central Asia
WHO−2001 Adjustment Method

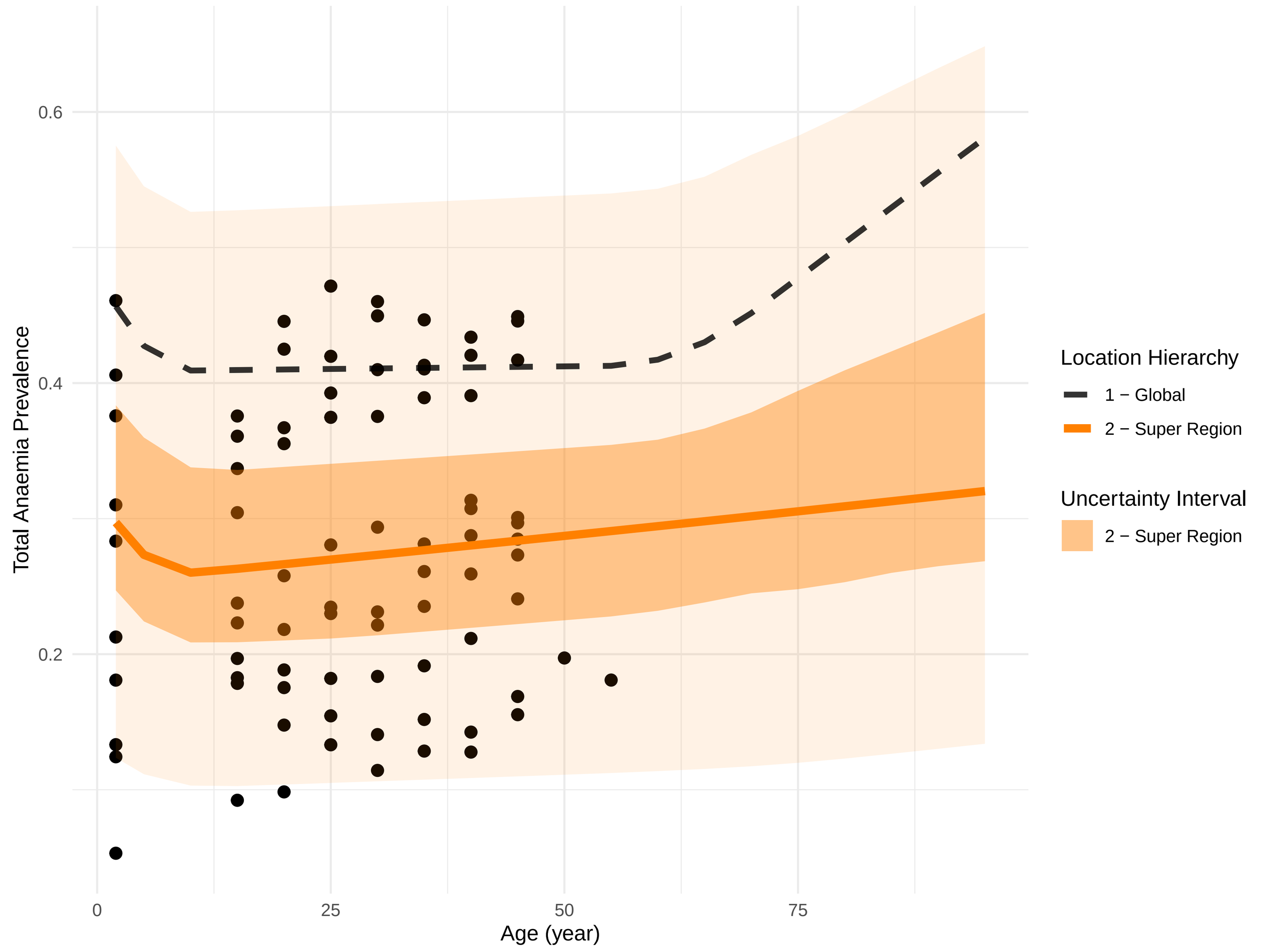

MR−BRT Age/Sex Fits − Non−pregnant Females 2+ Years Old − Total Anaemia Prevalence − Central Asia
WHO−2001 Adjustment Method

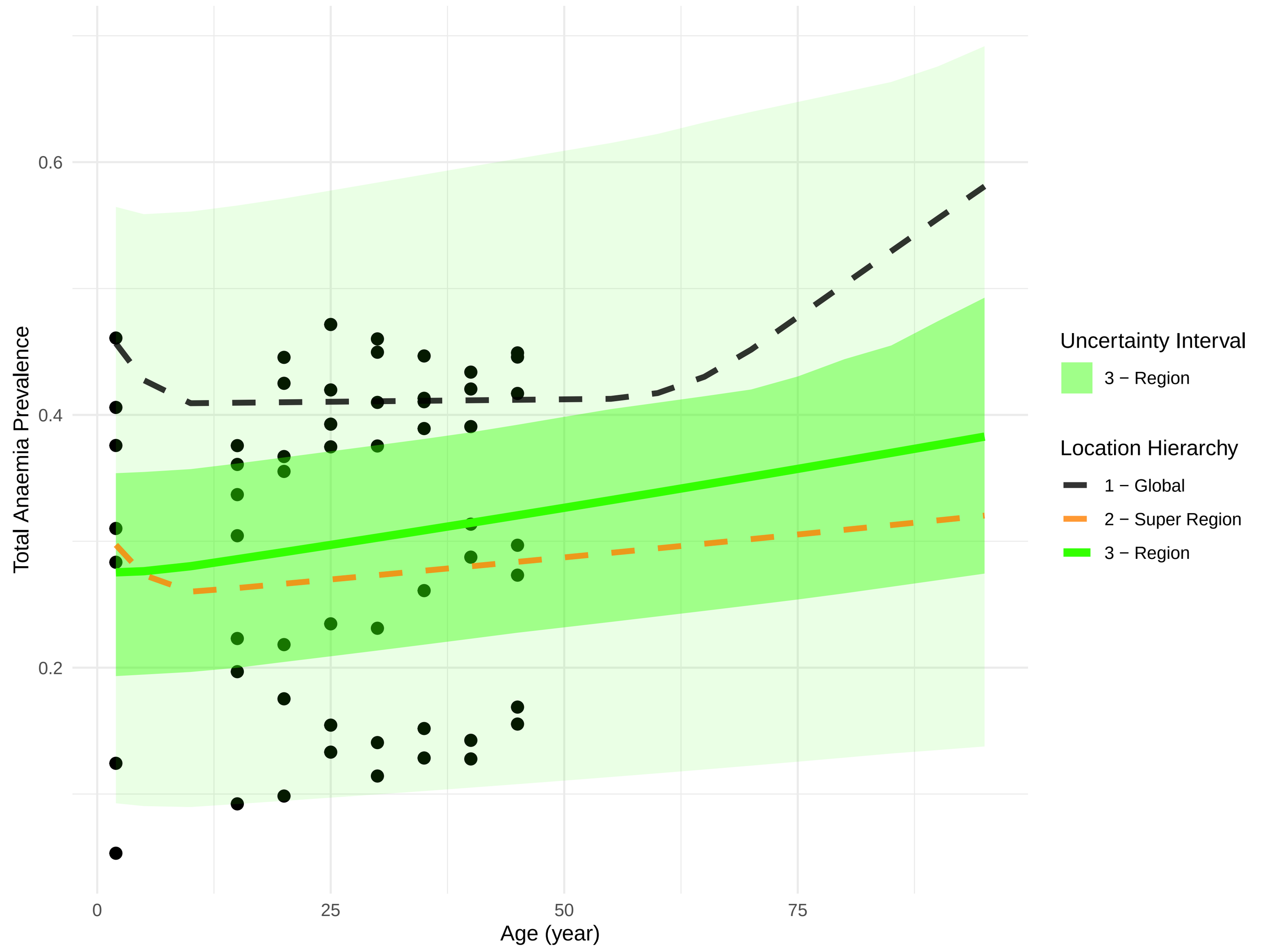

MR−BRT Age/Sex Fits − Non−pregnant Females 2+ Years Old − Total Anaemia Prevalence − Armenia
WHO−2001 Adjustment Method

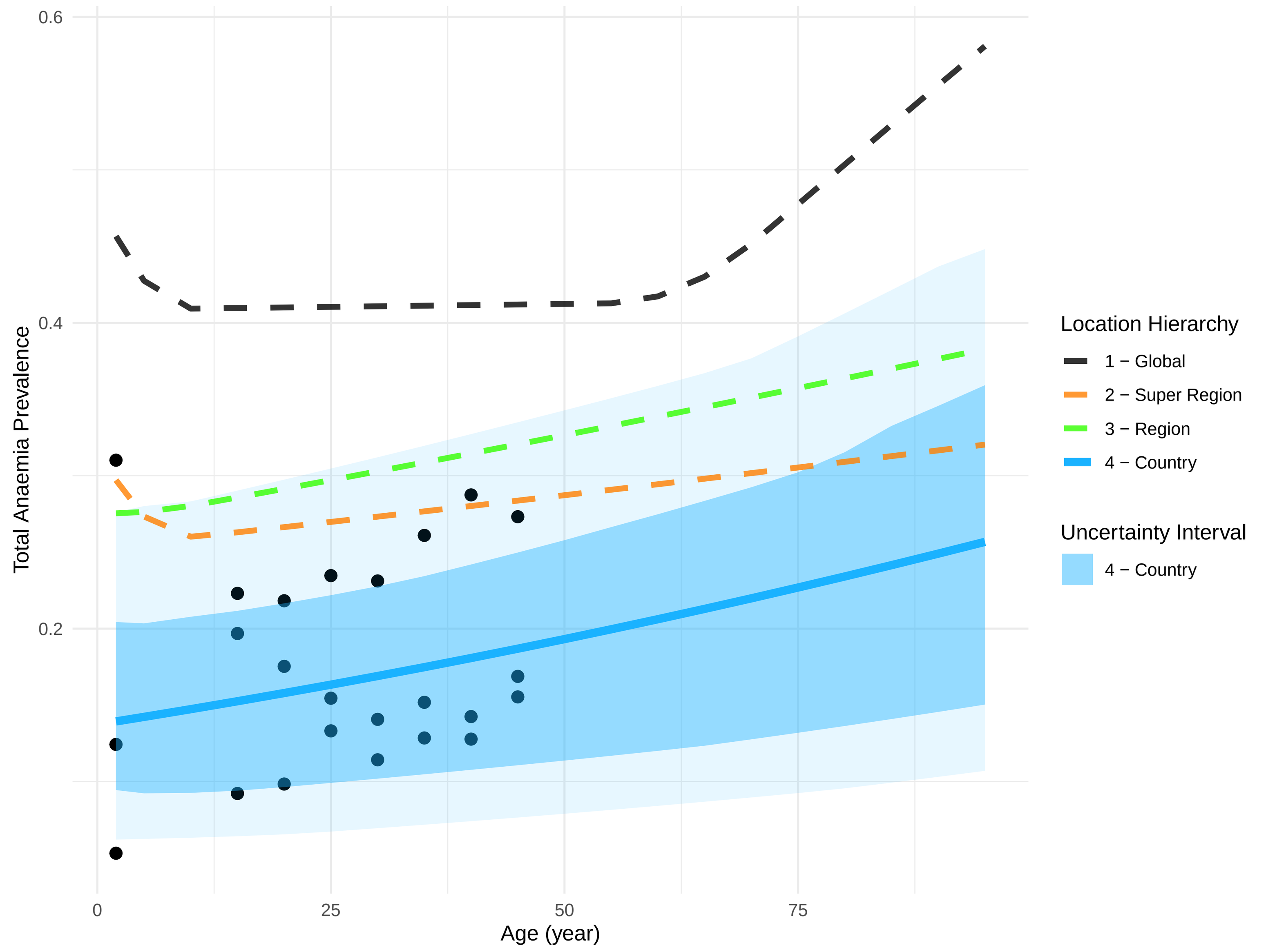

MR−BRT Age/Sex Fits − Non−pregnant Females 2+ Years Old − Total Anaemia Prevalence − Azerbaijan
WHO−2001 Adjustment Method

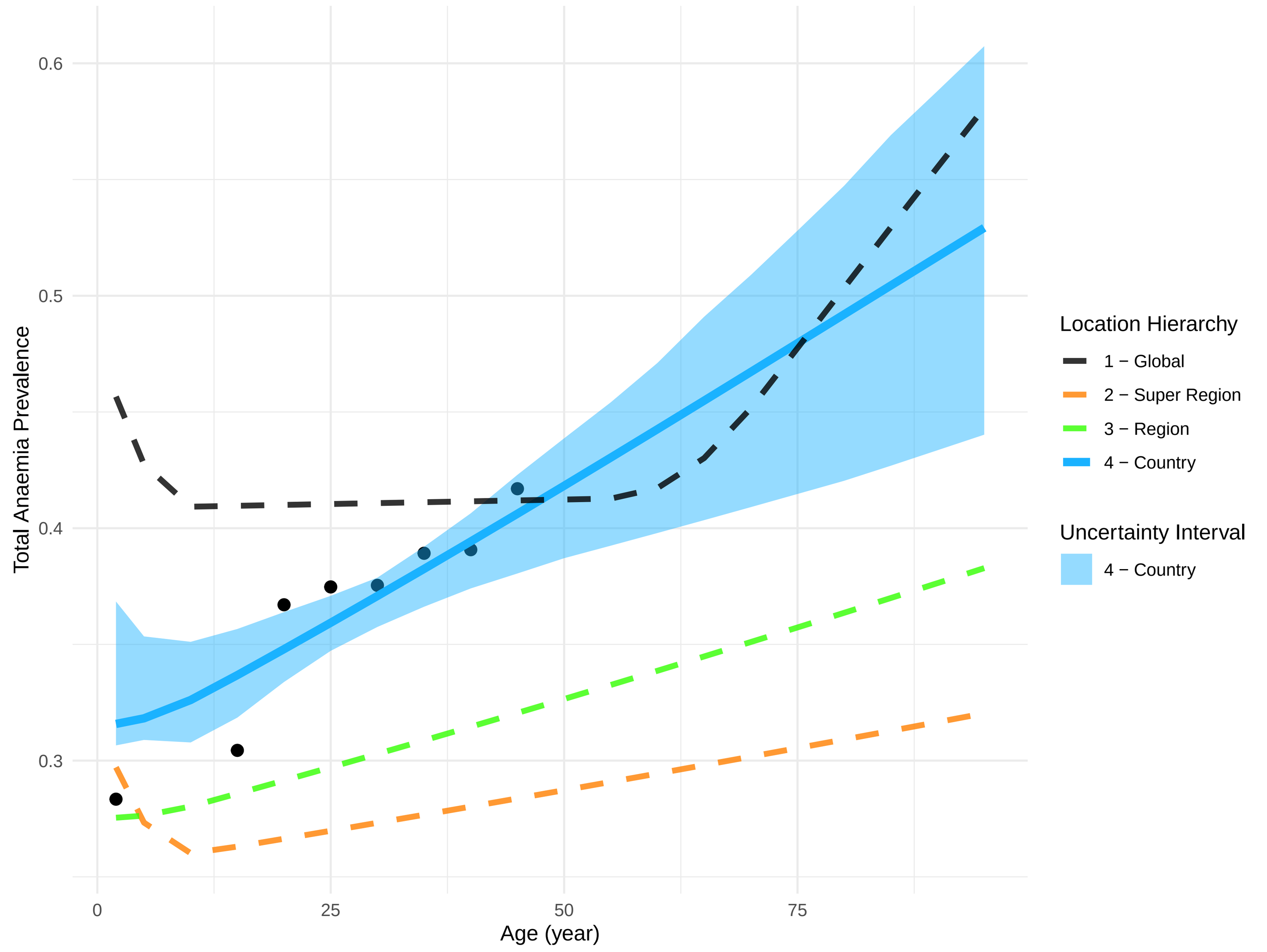

MR−BRT Age/Sex Fits − Non−pregnant Females 2+ Years Old − Total Anaemia Prevalence − Kyrgyzstan
WHO−2001 Adjustment Method

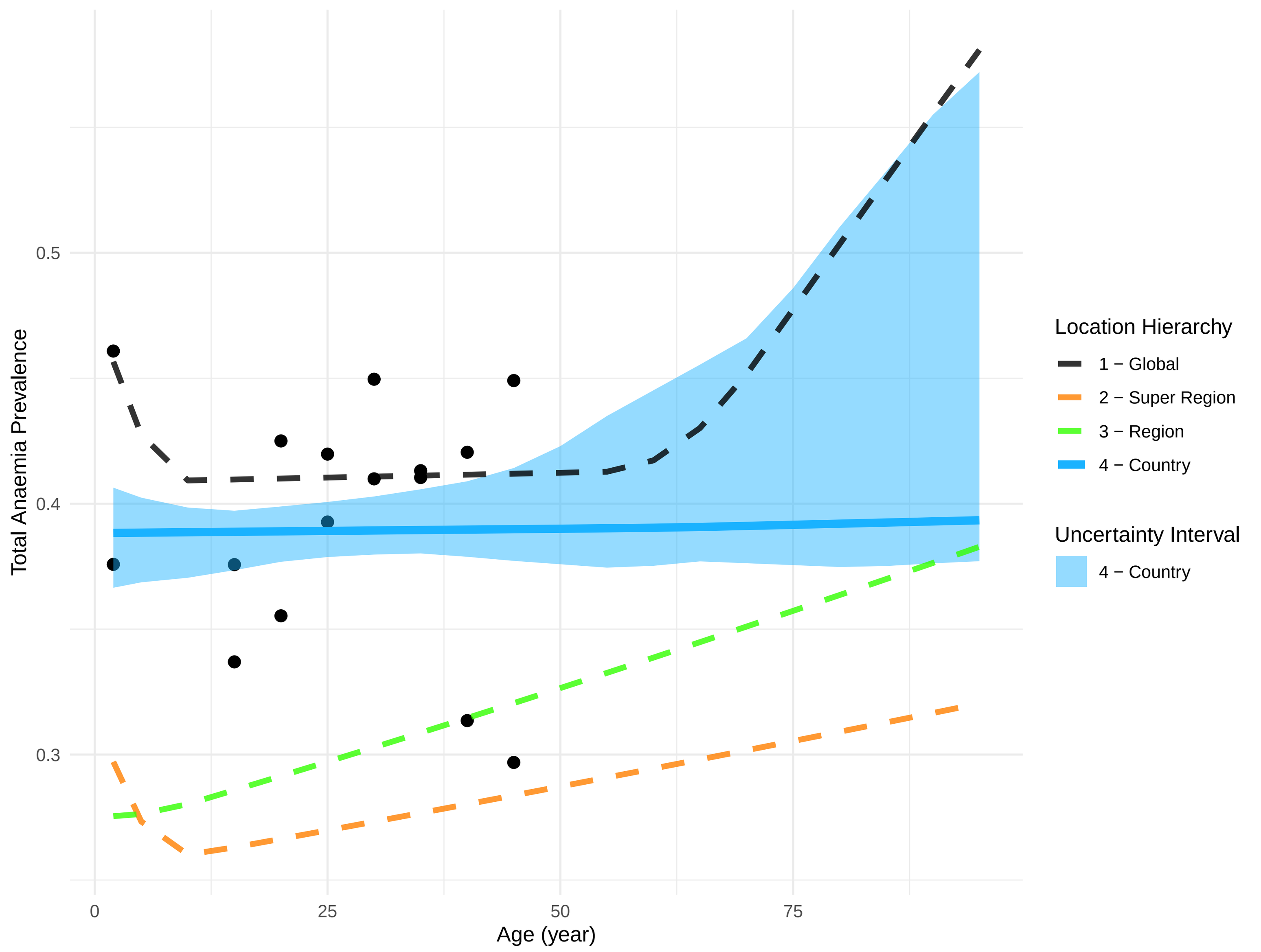

MR−BRT Age/Sex Fits − Non−pregnant Females 2+ Years Old − Total Anaemia Prevalence − Tajikistan
WHO−2001 Adjustment Method

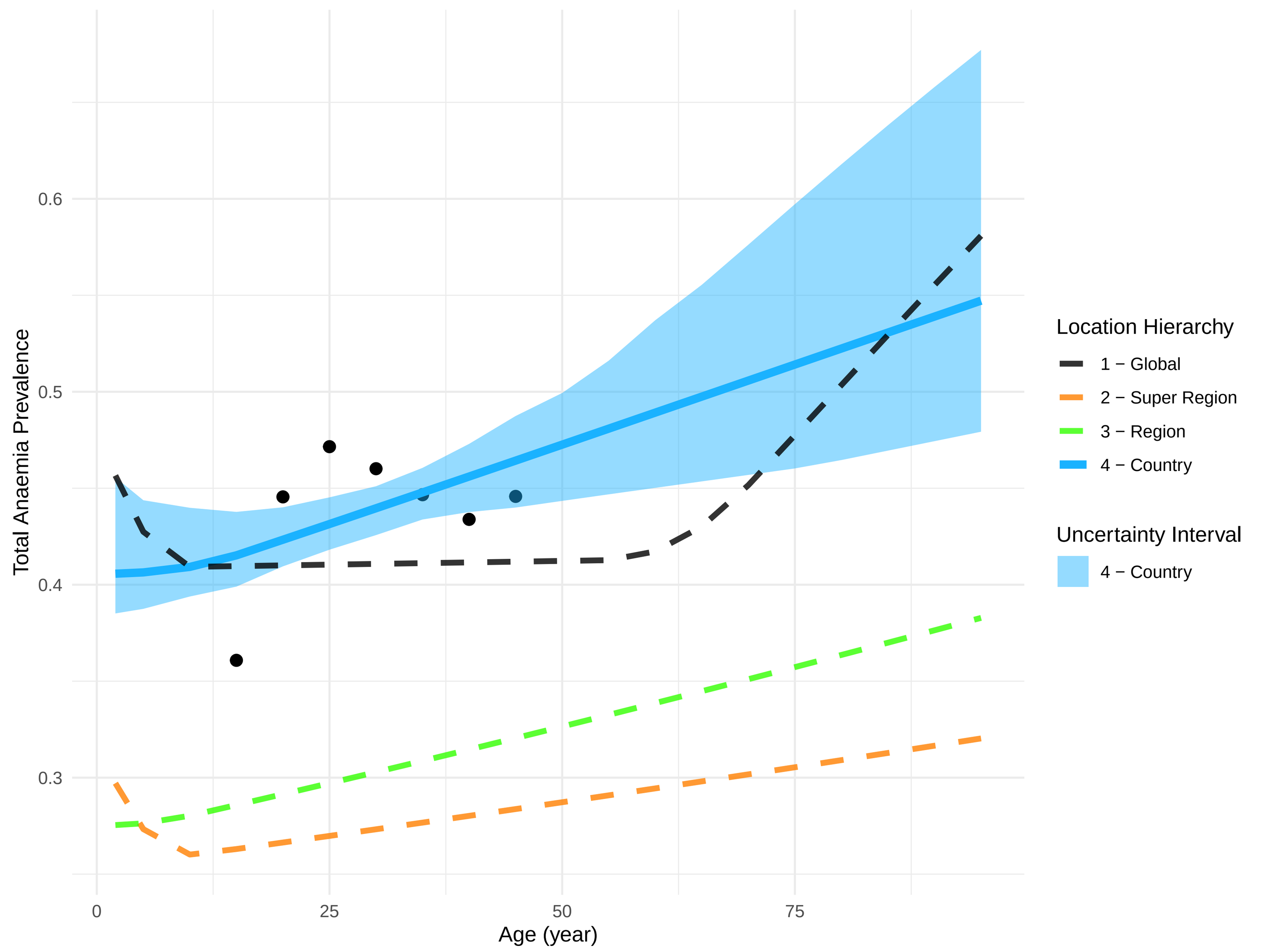

MR−BRT Age/Sex Fits − Non−pregnant Females 2+ Years Old − Total Anaemia Prevalence − Uzbekistan
WHO−2001 Adjustment Method

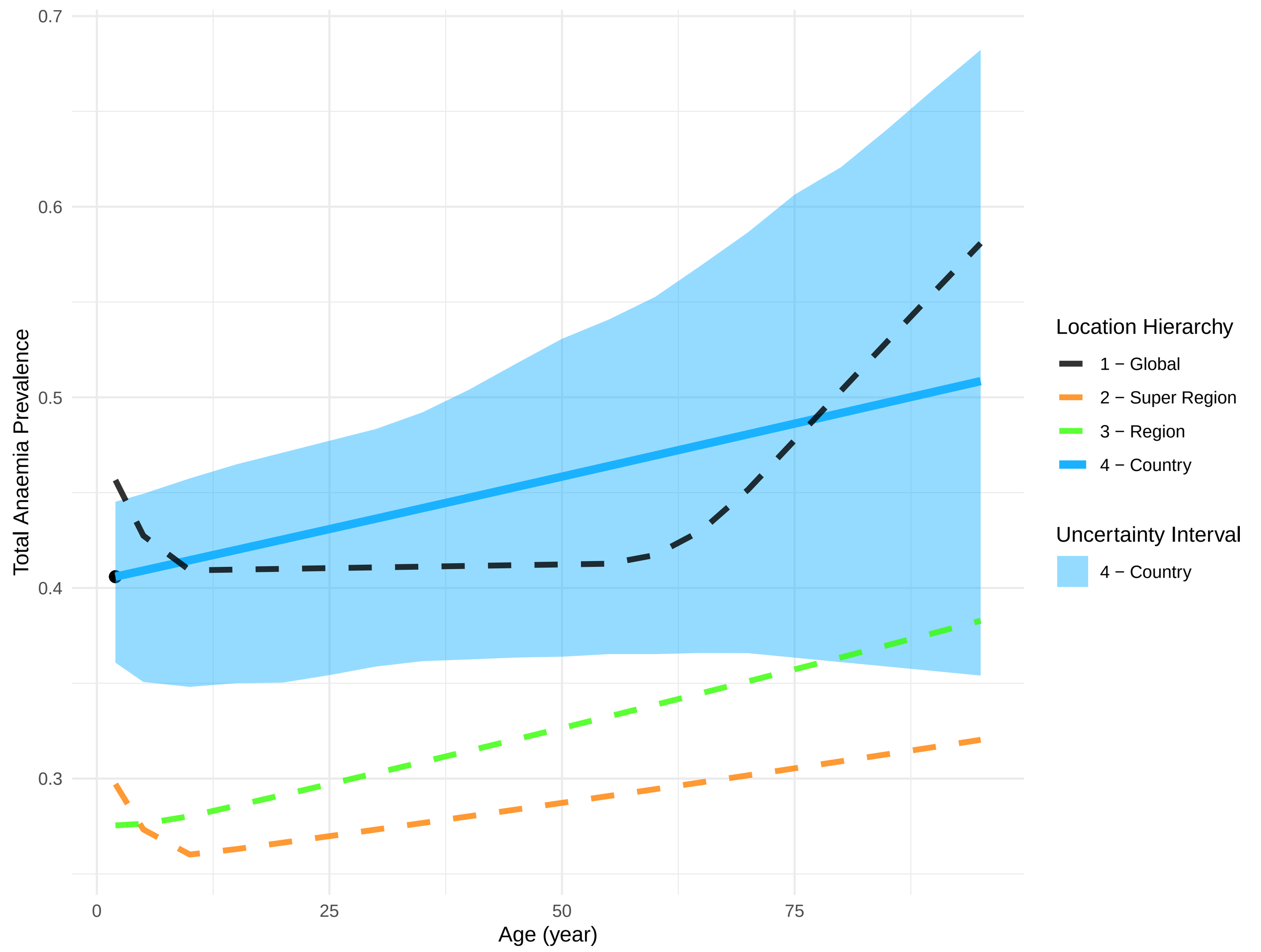

MR−BRT Age/Sex Fits − Non−pregnant Females 2+ Years Old − Total Anaemia Prevalence − Central Europe
WHO−2001 Adjustment Method

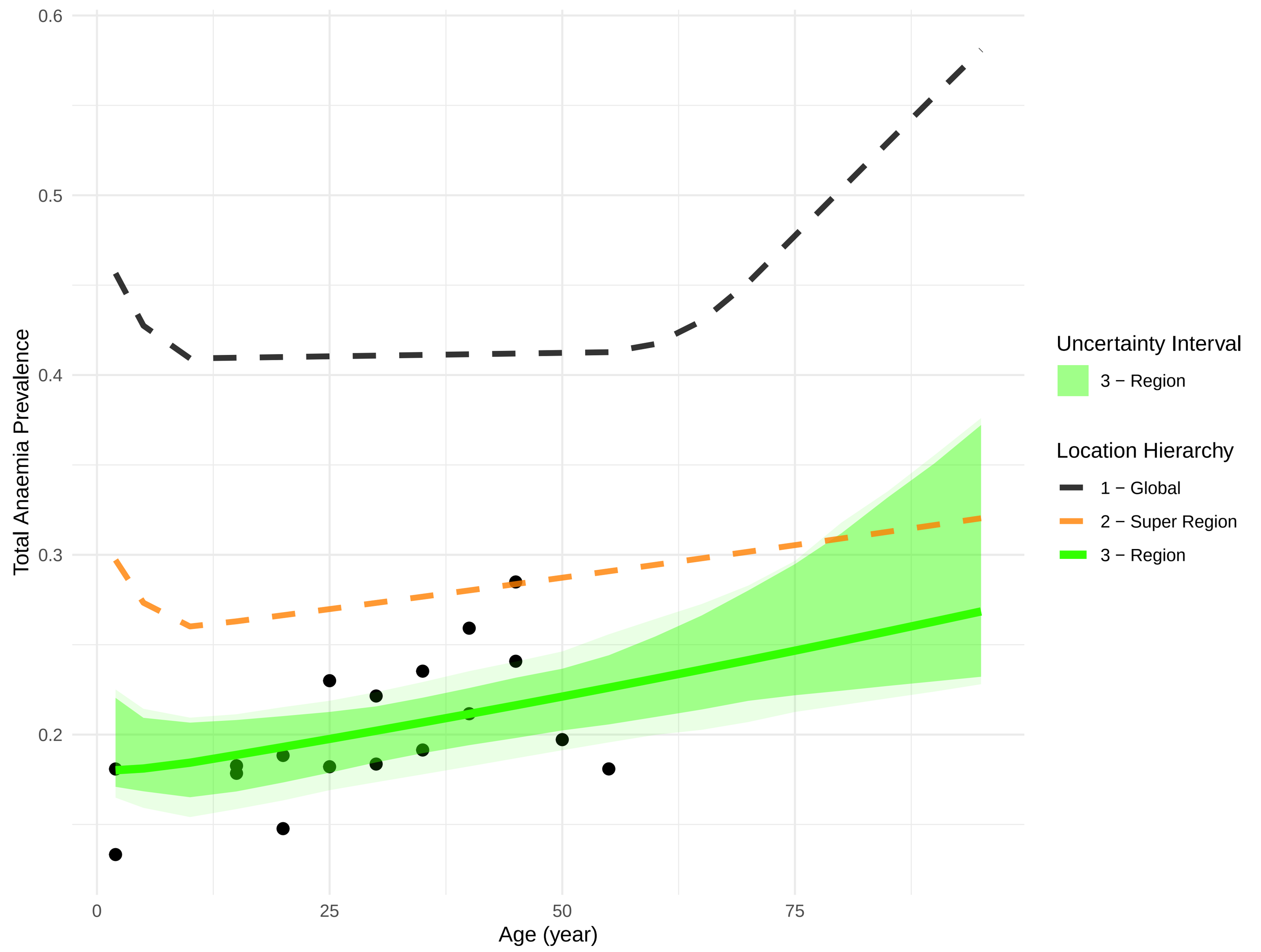

MR−BRT Age/Sex Fits − Non−pregnant Females 2+ Years Old − Total Anaemia Prevalence − Albania
WHO−2001 Adjustment Method

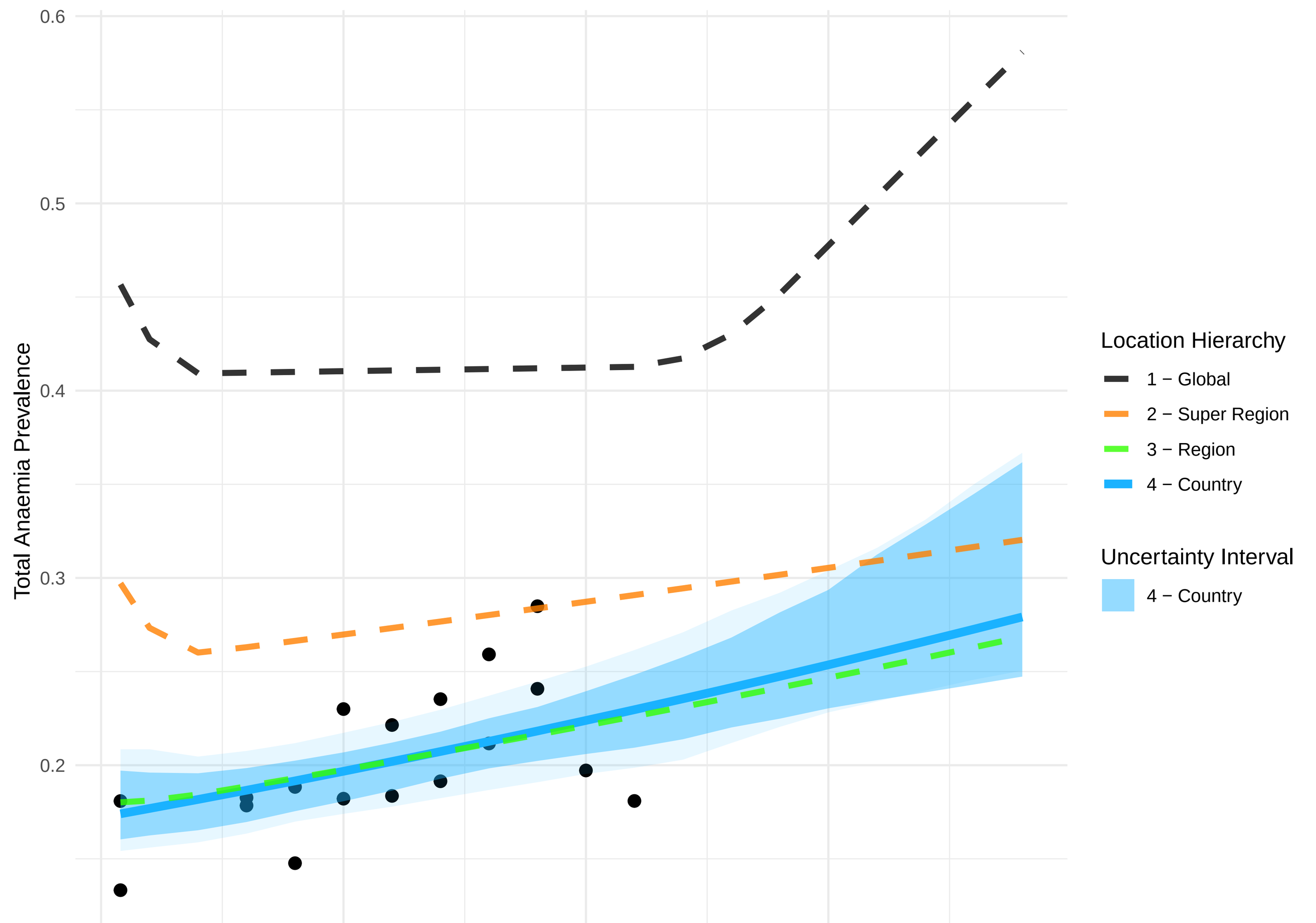

MR−BRT Age/Sex Fits − Non−pregnant Females 2+ Years Old − Total Anaemia Prevalence − Eastern Europe
WHO−2001 Adjustment Method

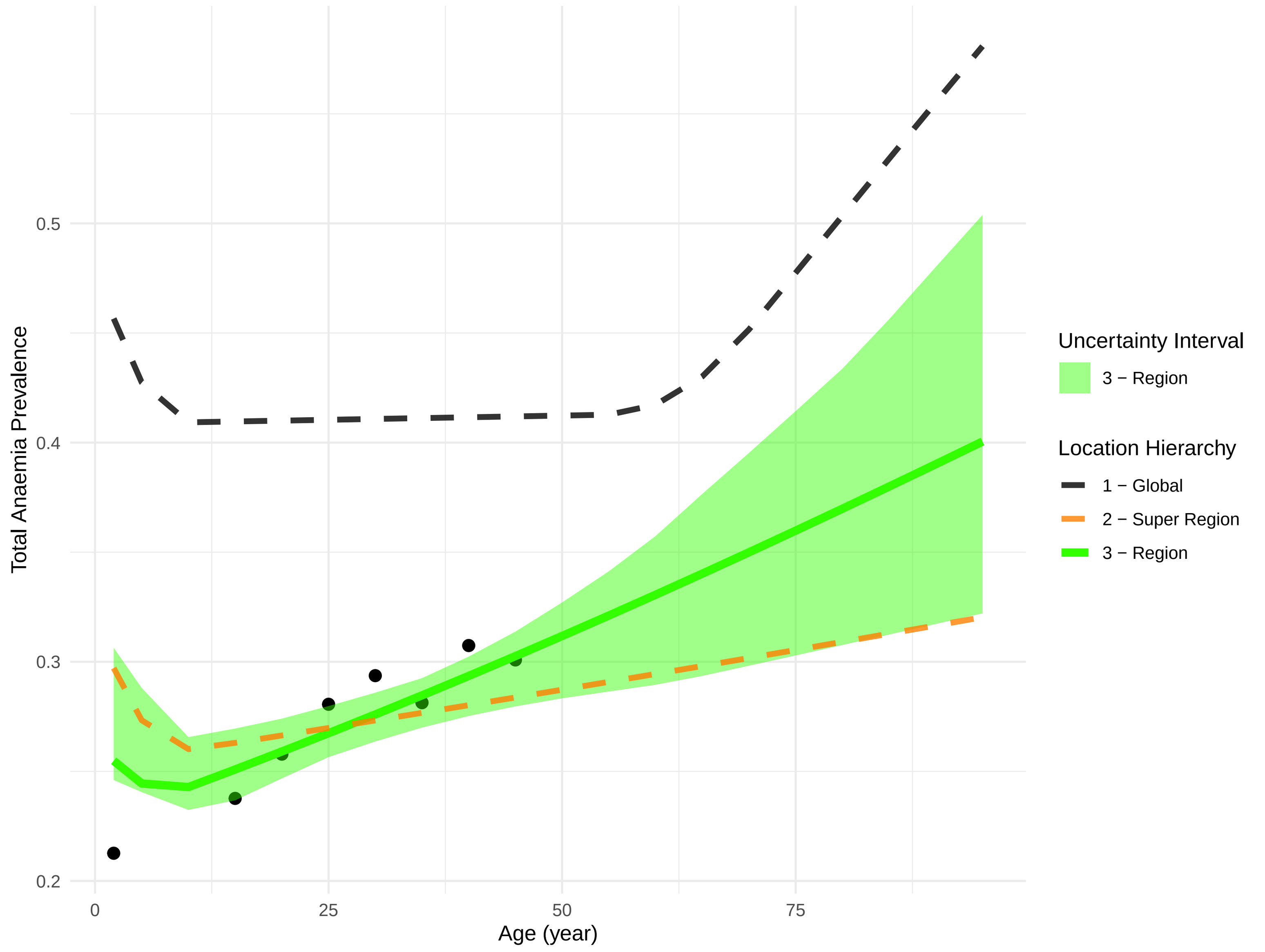

MR−BRT Age/Sex Fits − Non−pregnant Females 2+ Years Old − Total Anaemia Prevalence − Republic of Moldova
WHO−2001 Adjustment Method

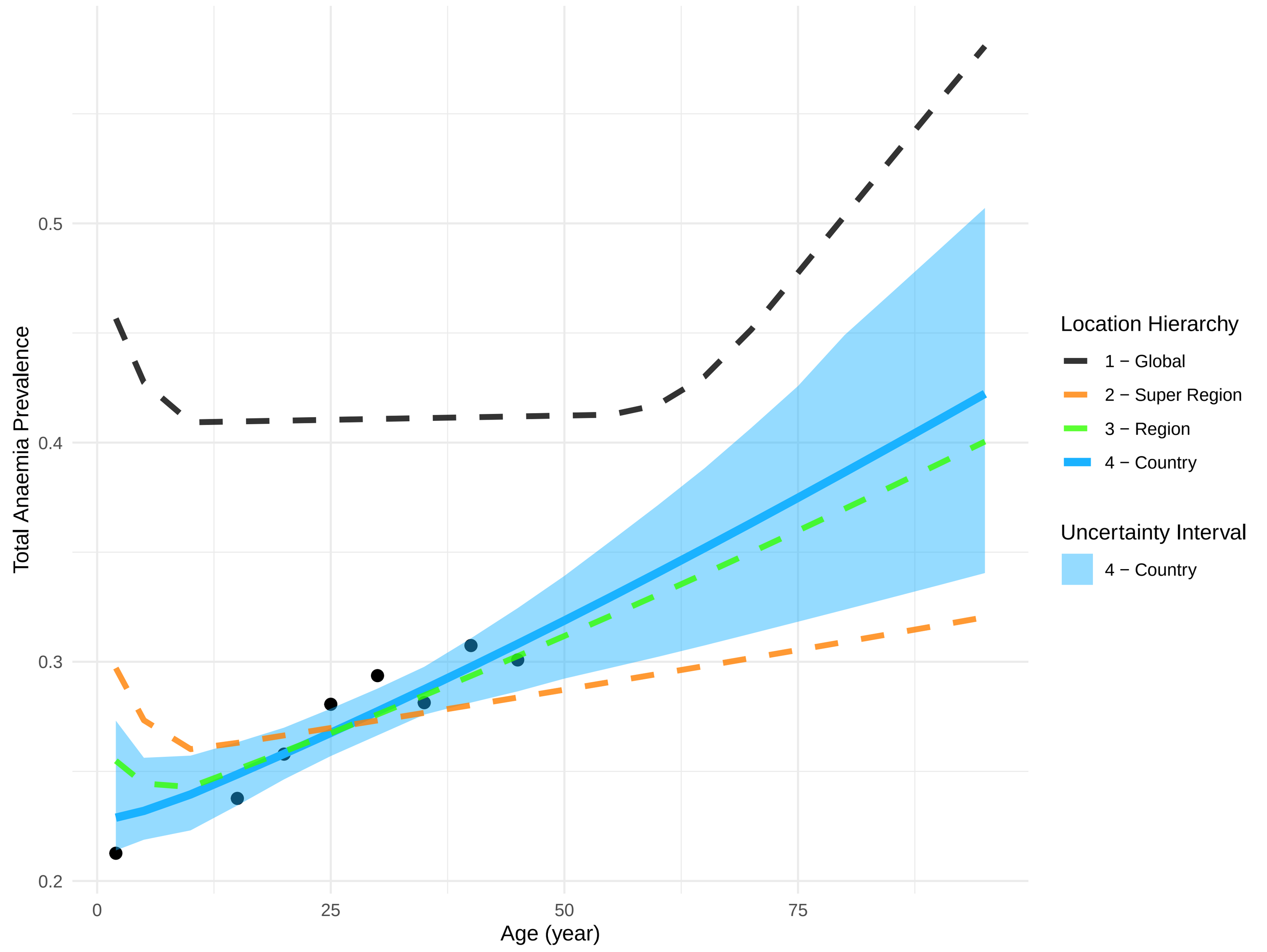

MR−BRT Age/Sex Fits − Non−pregnant Females 2+ Years Old − Total Anaemia Prevalence − High−income
WHO−2001 Adjustment Method

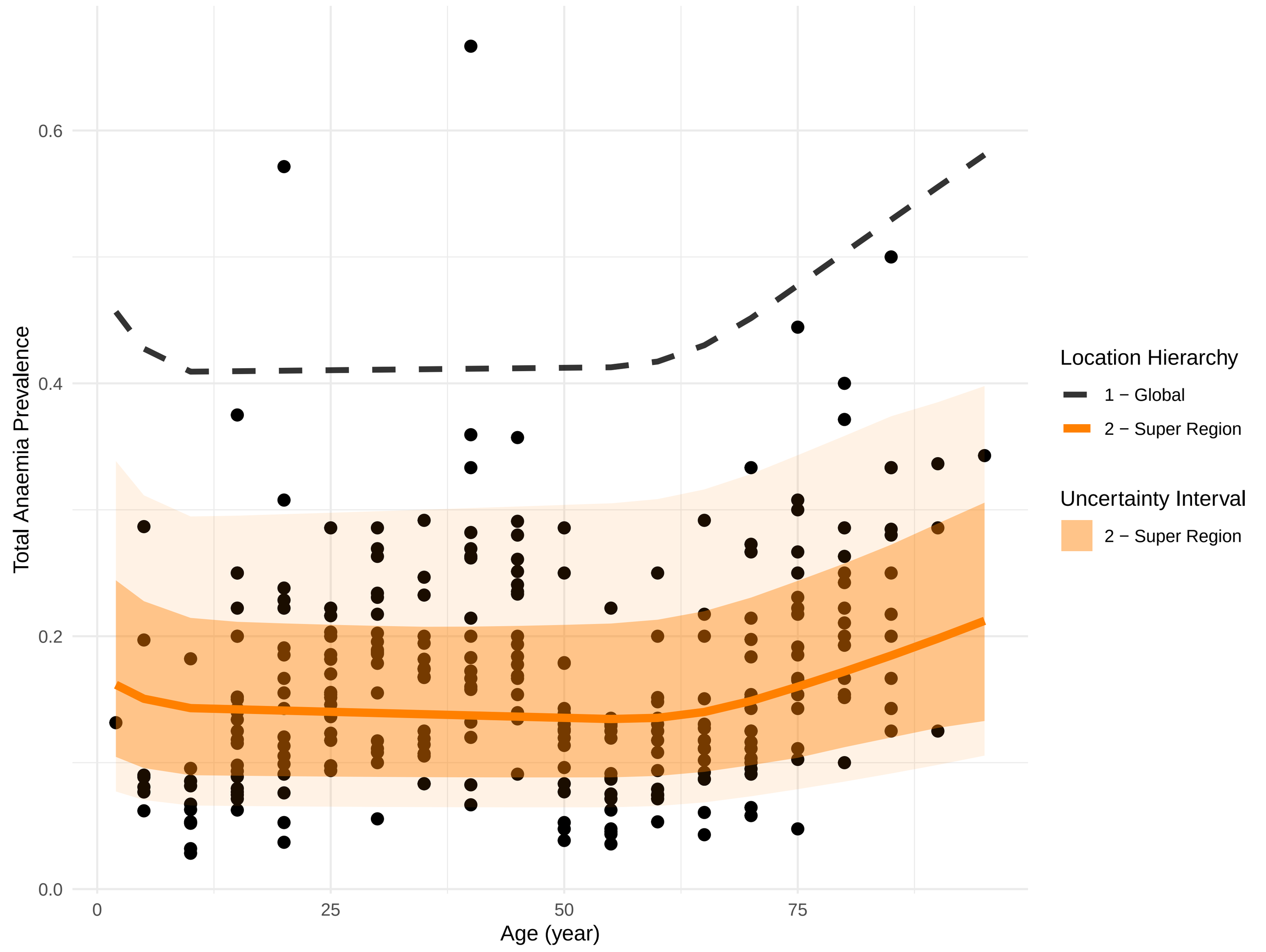

MR−BRT Age/Sex Fits − Non−pregnant Females 2+ Years Old − Total Anaemia Prevalence − Western Europe
WHO−2001 Adjustment Method

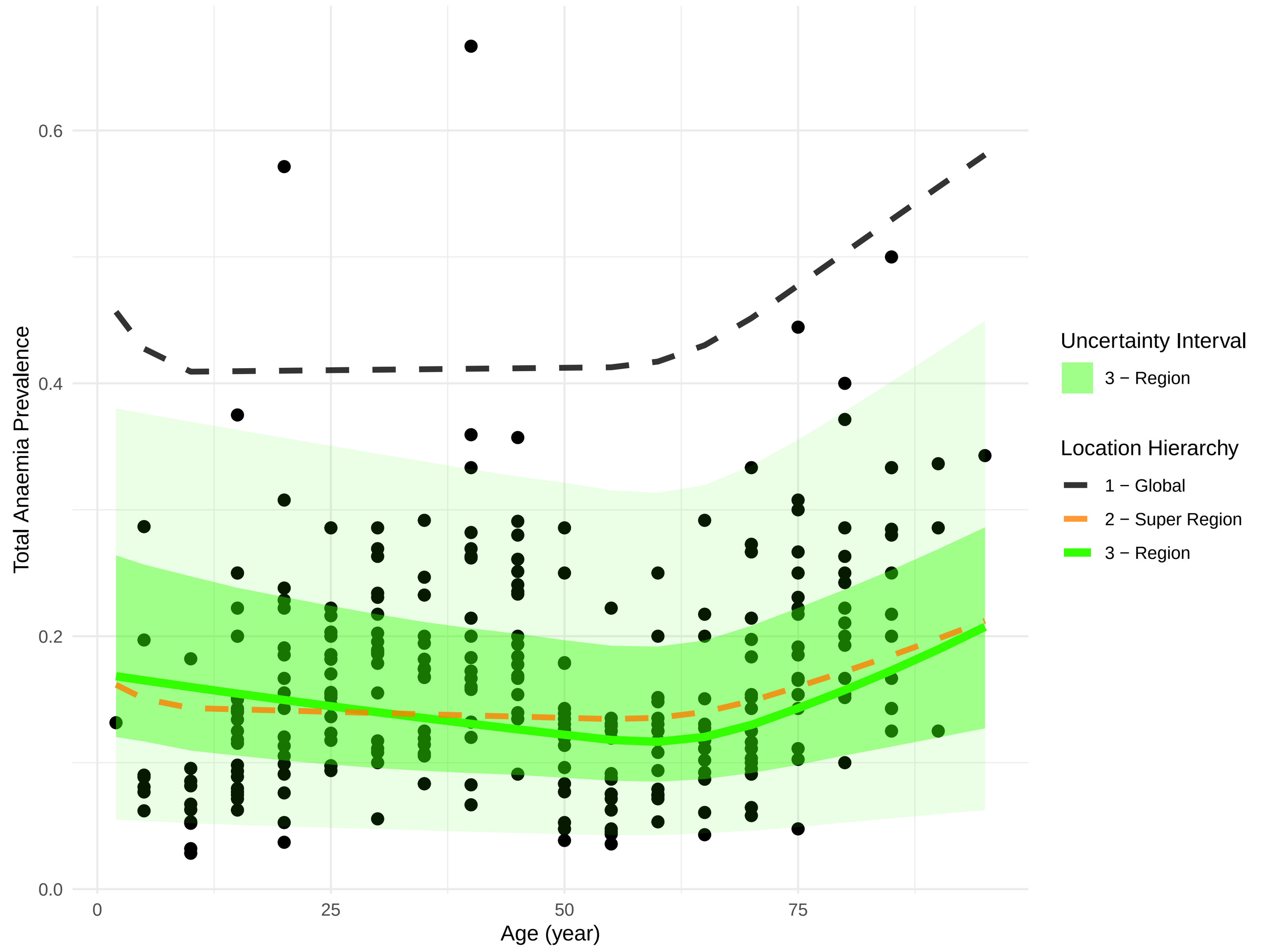

MR−BRT Age/Sex Fits − Non−pregnant Females 2+ Years Old − Total Anaemia Prevalence − United Kingdom
WHO−2001 Adjustment Method

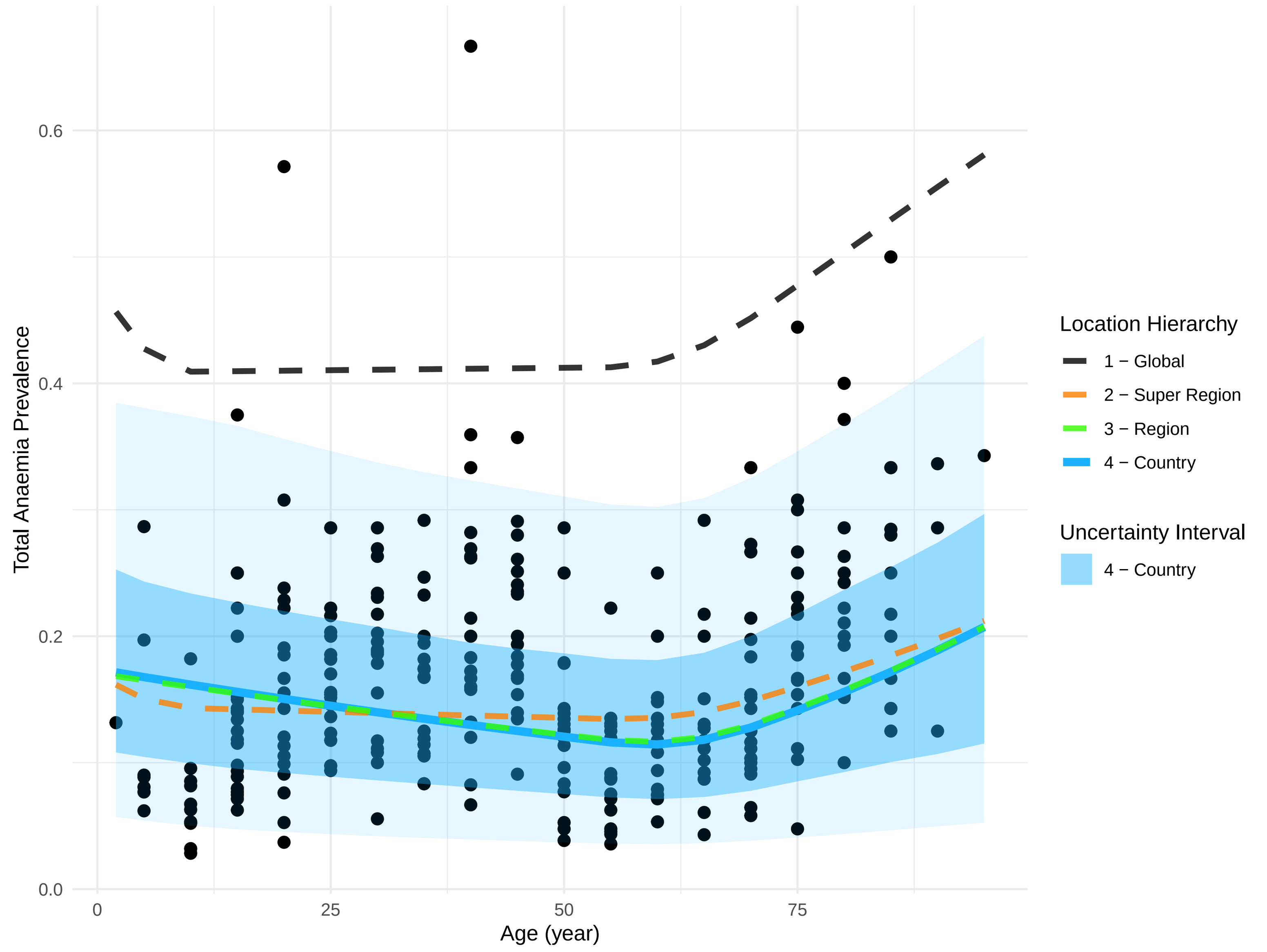

MR−BRT Age/Sex Fits − Non−pregnant Females 2+ Years Old − Total Anaemia Prevalence − England
WHO−2001 Adjustment Method

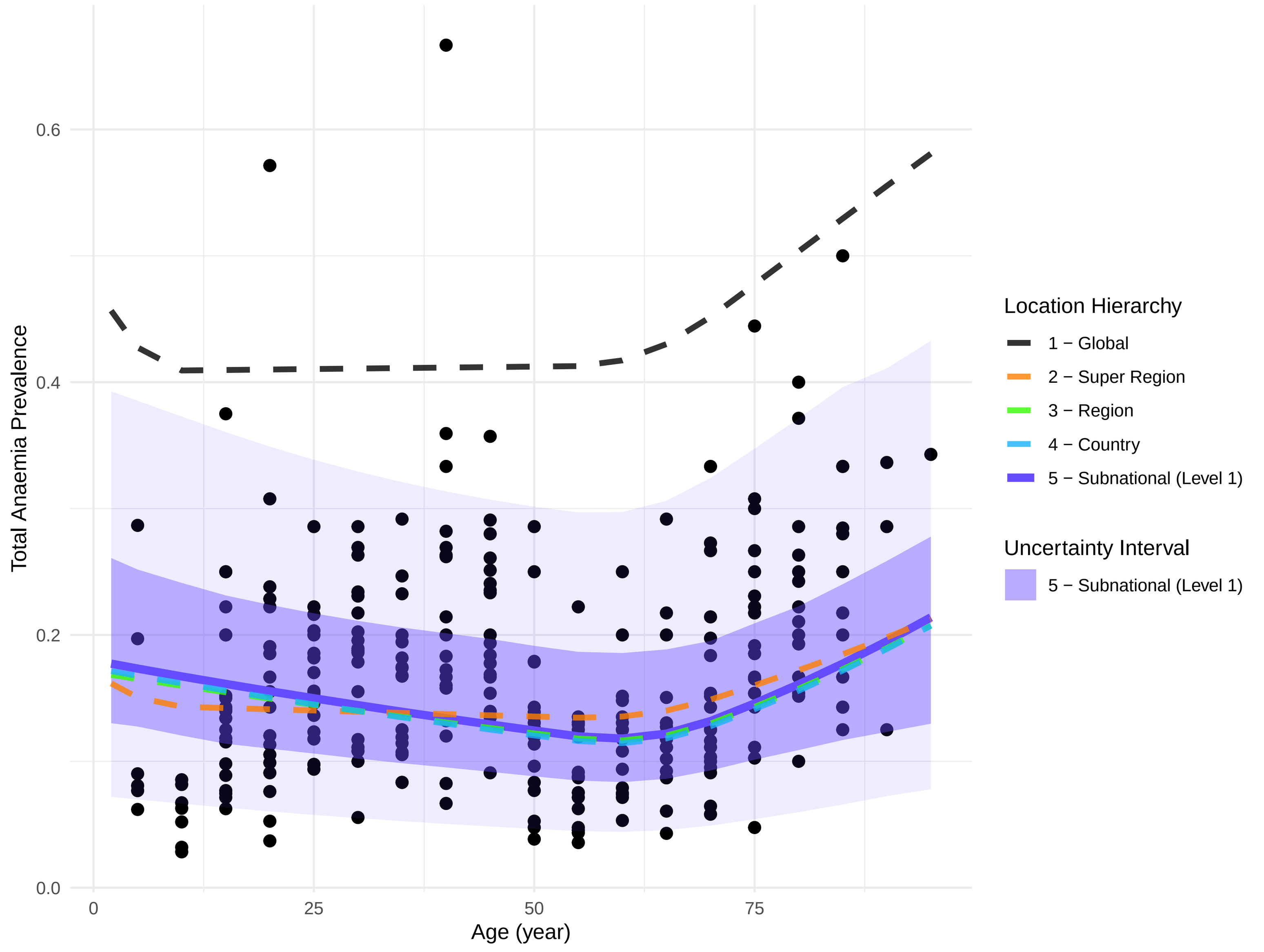

MR−BRT Age/Sex Fits − Non−pregnant Females 2+ Years Old − Total Anaemia Prevalence − East Midlands
WHO−2001 Adjustment Method

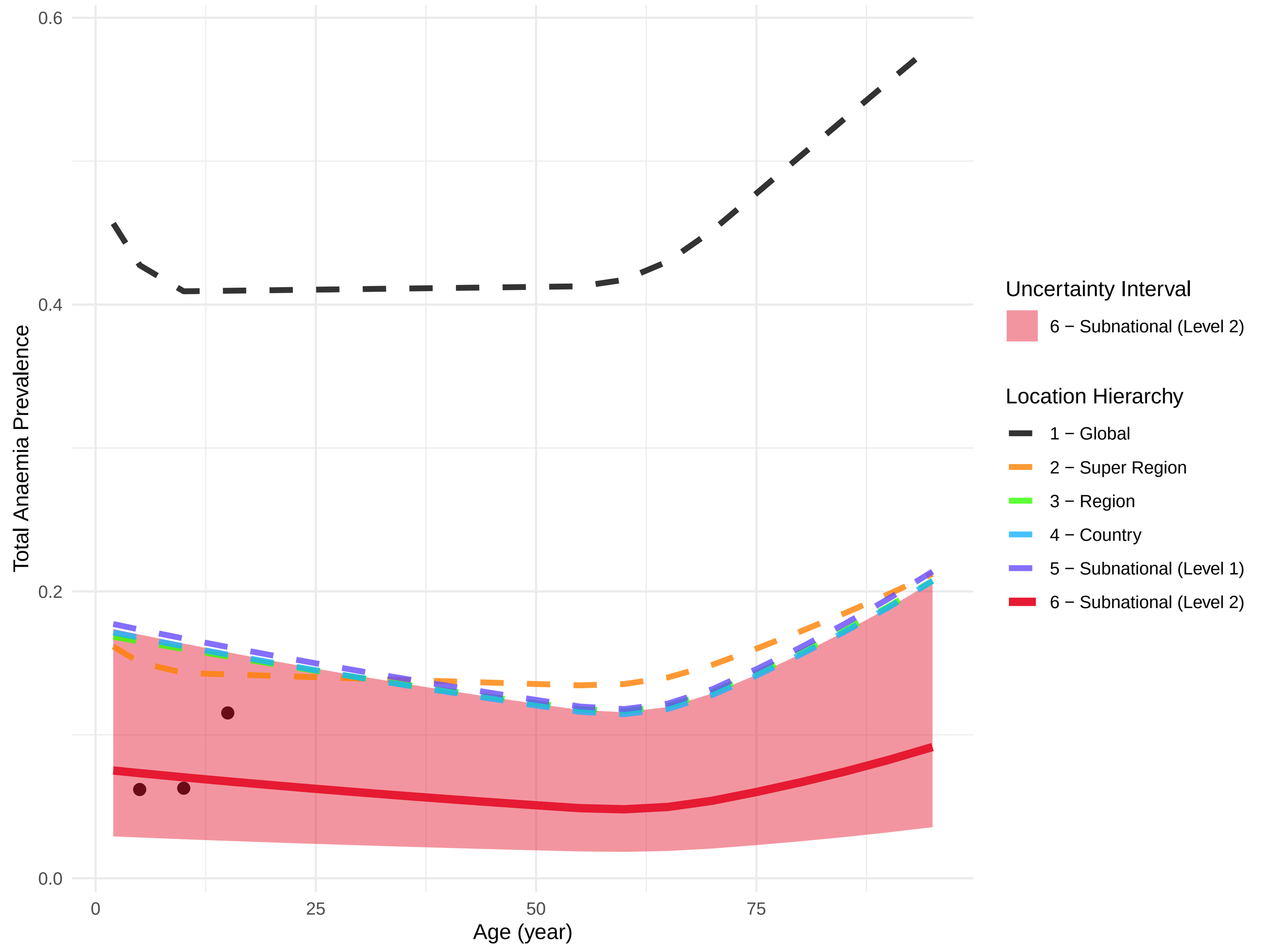

MR−BRT Age/Sex Fits − Non−pregnant Females 2+ Years Old − Total Anaemia Prevalence − East of England
WHO−2001 Adjustment Method

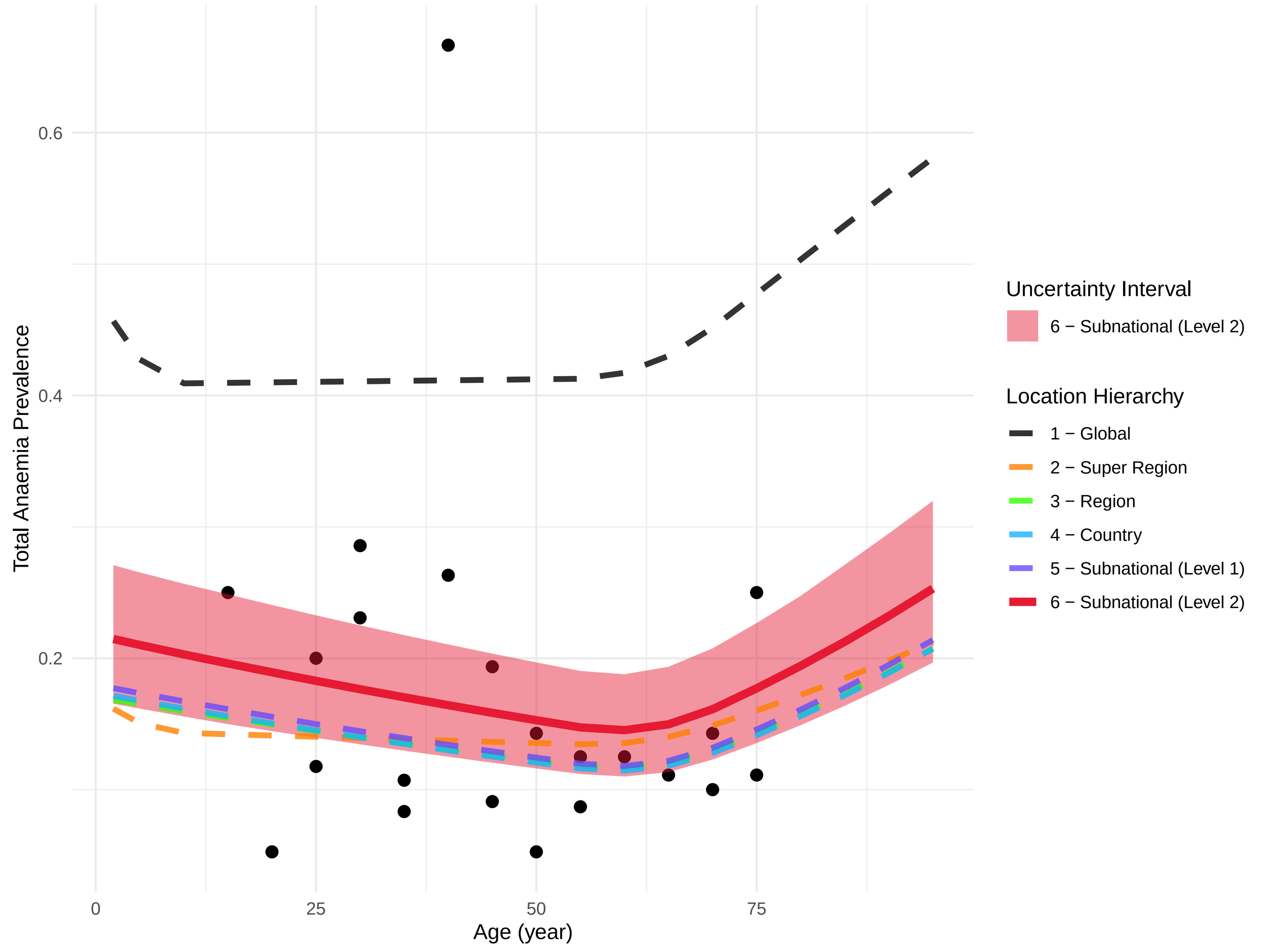

MR−BRT Age/Sex Fits − Non−pregnant Females 2+ Years Old − Total Anaemia Prevalence − Greater London
WHO−2001 Adjustment Method

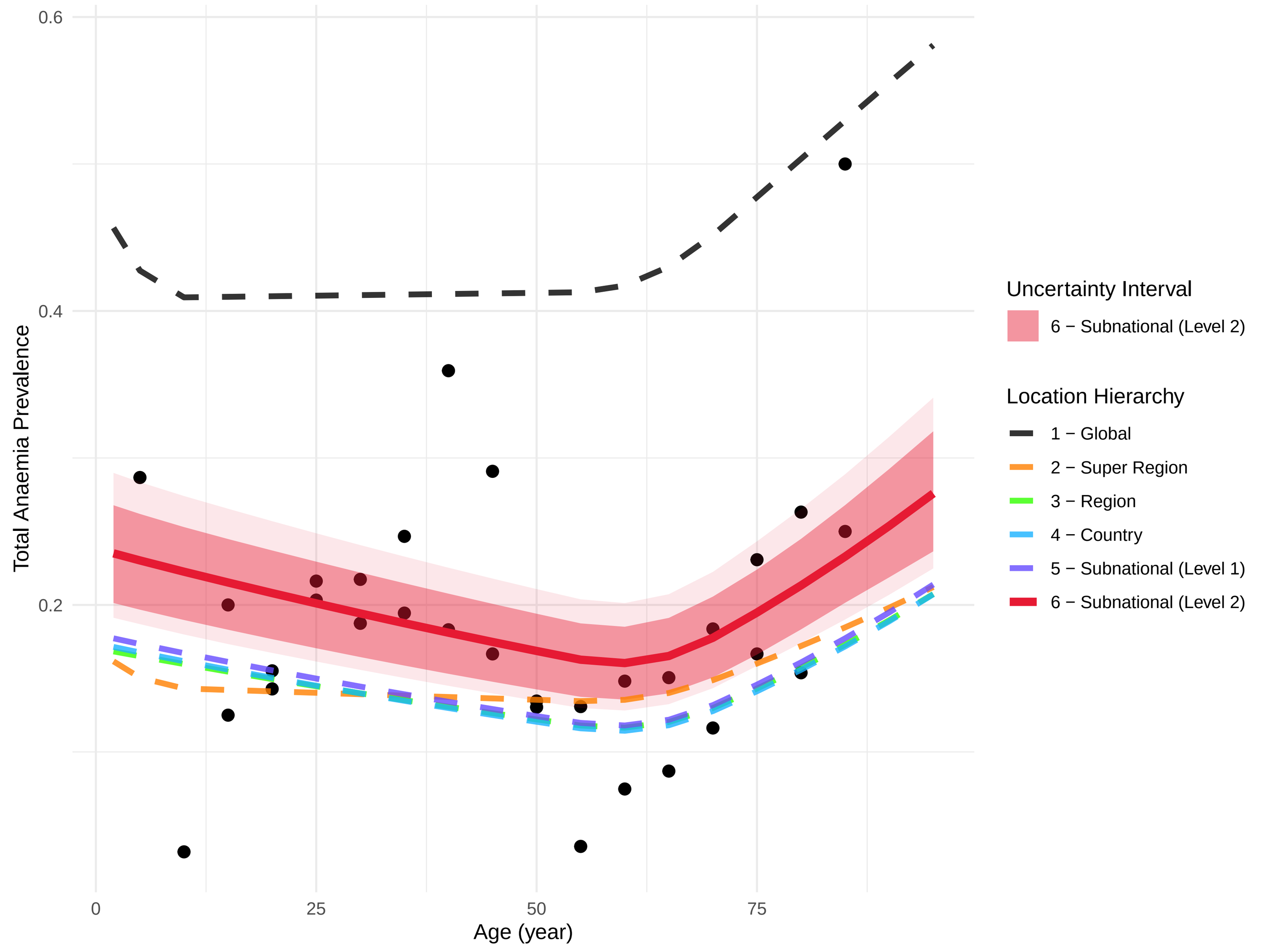

MR−BRT Age/Sex Fits − Non−pregnant Females 2+ Years Old − Total Anaemia Prevalence − North East England
WHO−2001 Adjustment Method

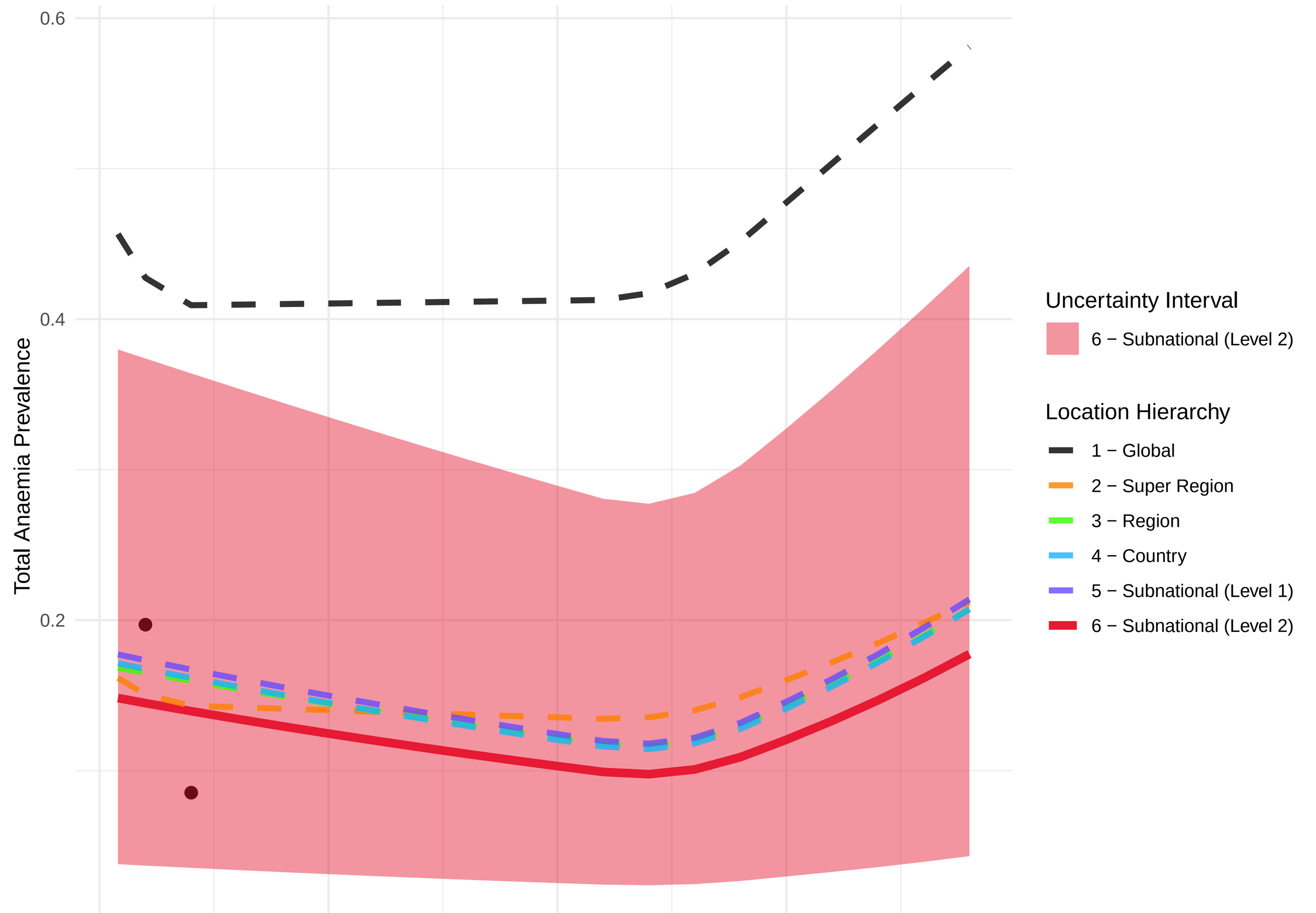

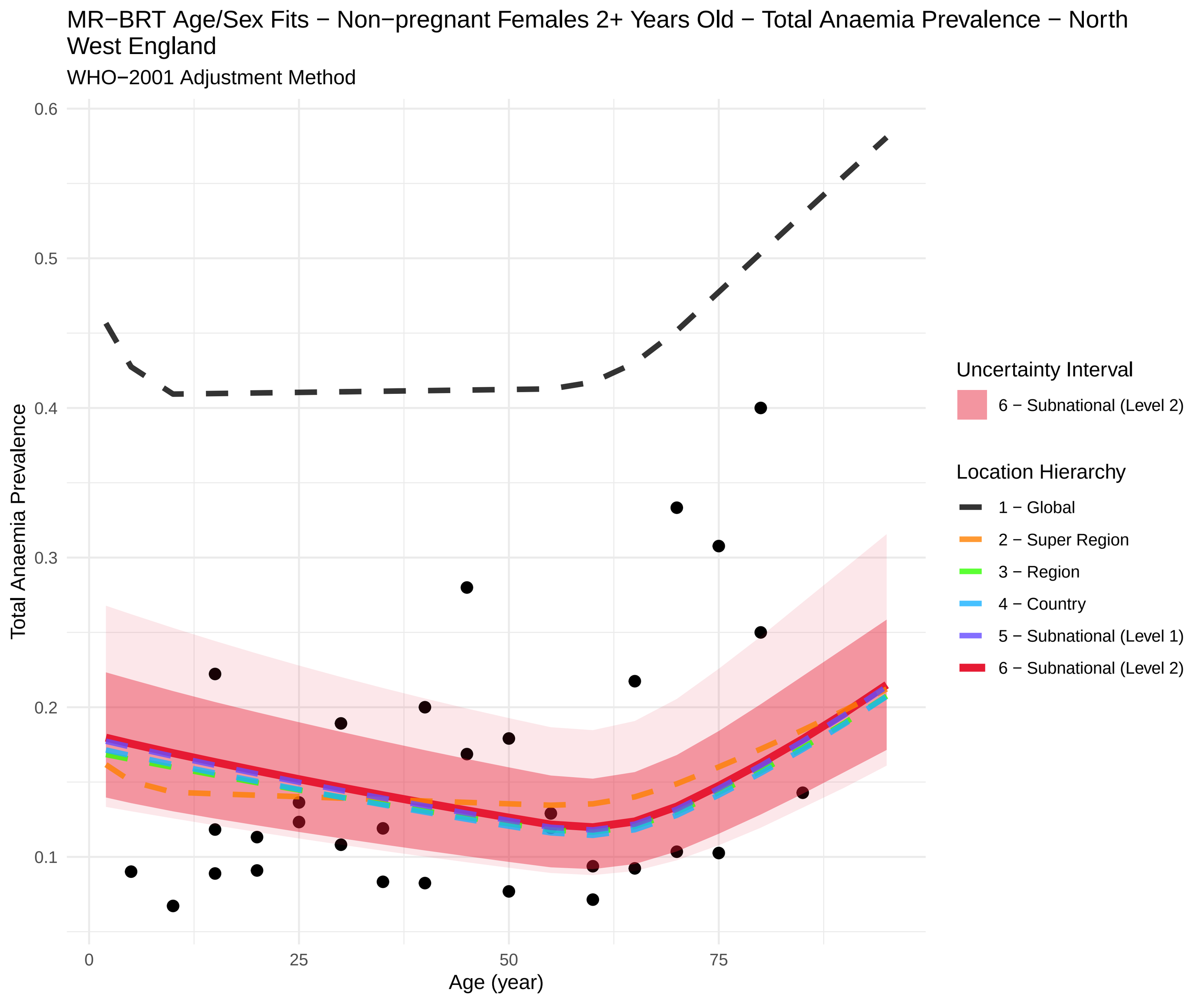
MR−BRT Age/Sex Fits − Non−pregnant Females 2+ Years Old − Total Anaemia Prevalence − North West England
WHO−2001 Adjustment Method
Total Anaemia Prevalence
Age (year)
Uncertainty Interval
6 − Subnational (Level 2)
Location Hierarchy
1 − Global
2 − Super Region
3 − Region
4 − Country
5 − Subnational (Level 1)
6 − Subnational (Level 2)

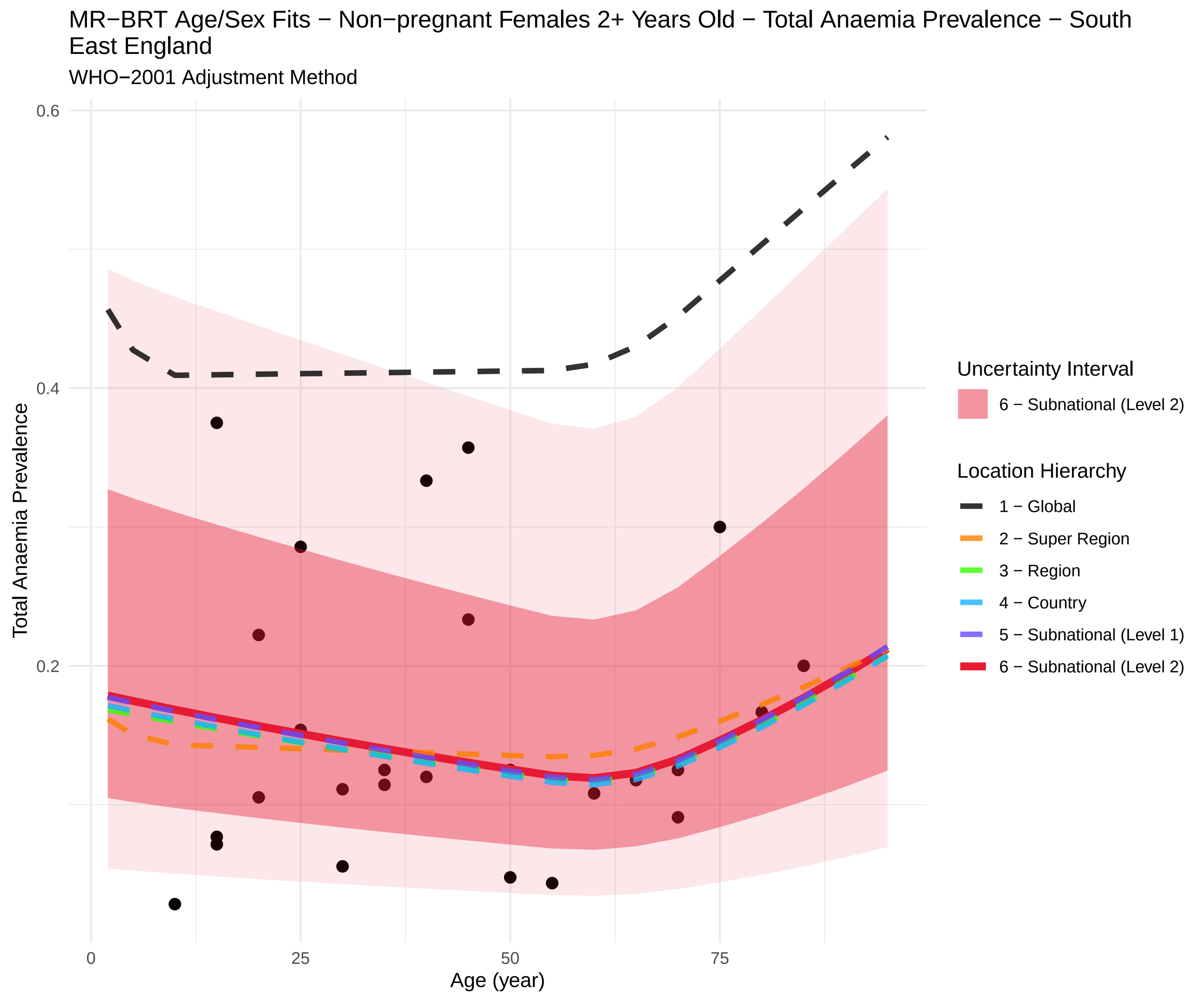
MR−BRT Age/Sex Fits − Non−pregnant Females 2+ Years Old − Total Anaemia Prevalence − South East England
WHO−2001 Adjustment Method
Total Anaemia Prevalence
Age (year)
Uncertainty Interval
6 − Subnational (Level 2)
Location Hierarchy
1 − Global
2 − Super Region
3 − Region
4 − Country
5 − Subnational (Level 1)
6 − Subnational (Level 2)

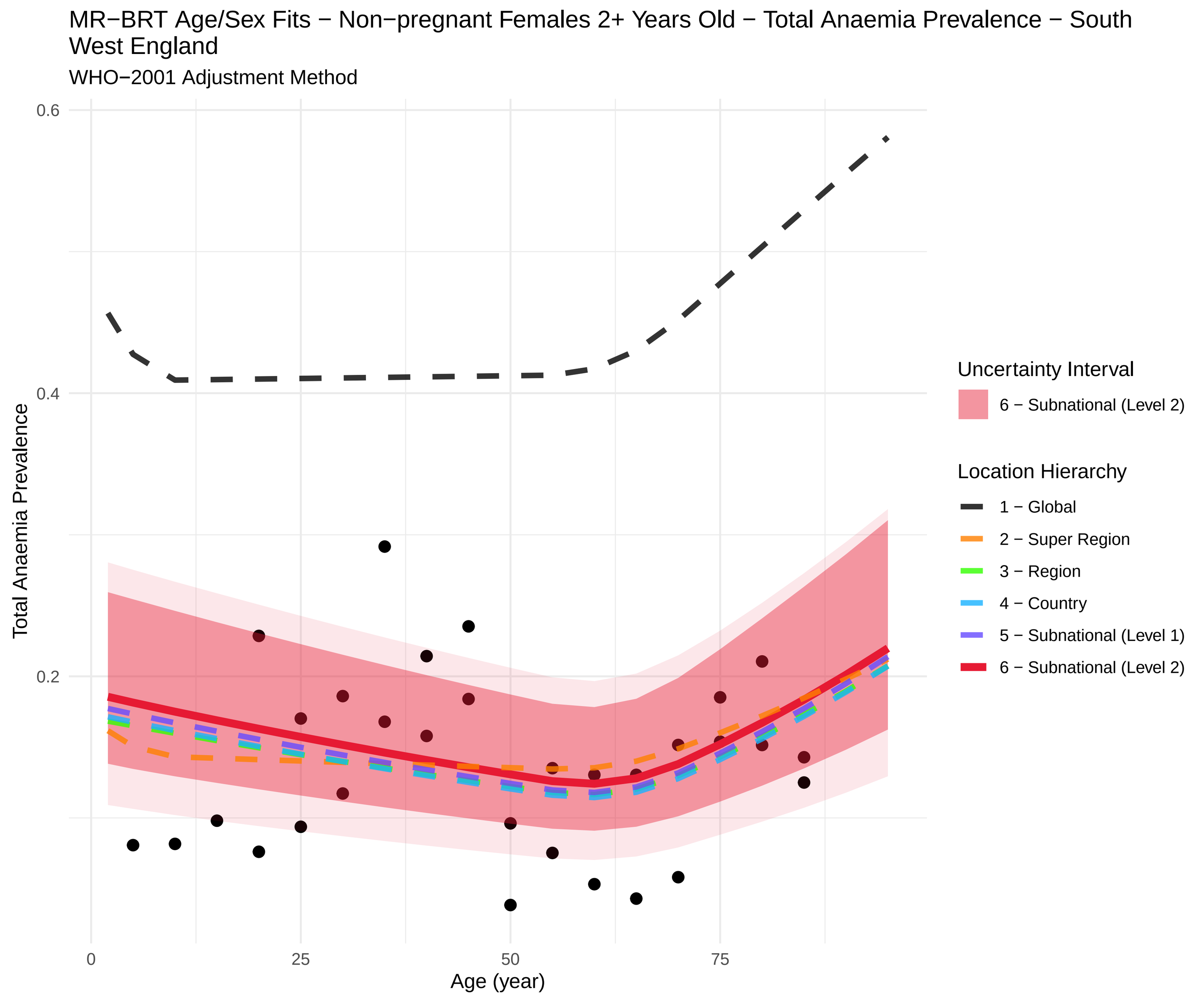
MR−BRT Age/Sex Fits − Non−pregnant Females 2+ Years Old − Total Anaemia Prevalence − South West England
WHO−2001 Adjustment Method
Total Anaemia Prevalence
Age (year)
Uncertainty Interval
6 − Subnational (Level 2)
Location Hierarchy
1 − Global
2 − Super Region
3 − Region
4 − Country
5 − Subnational (Level 1)
6 − Subnational (Level 2)

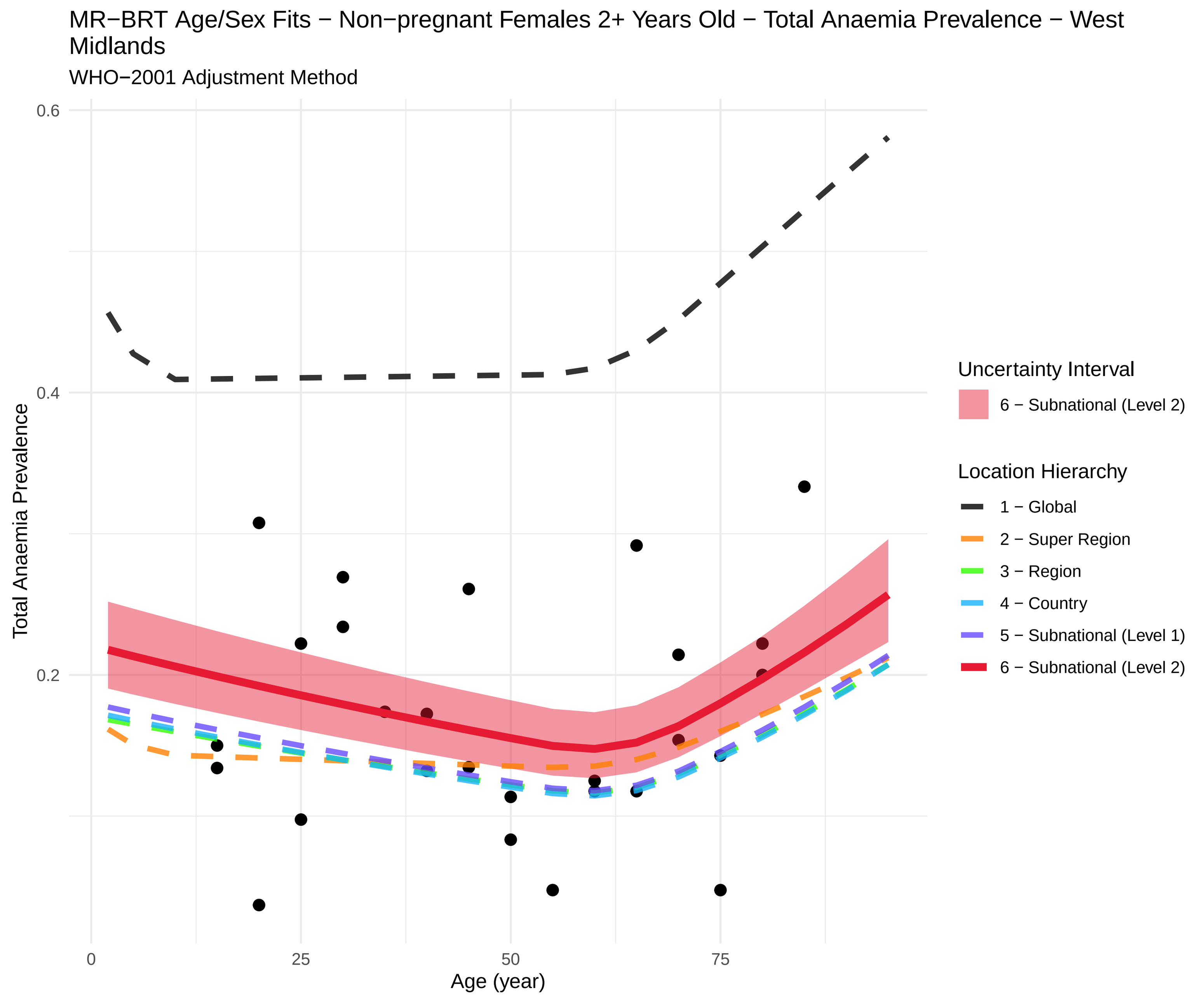
MR−BRT Age/Sex Fits − Non−pregnant Females 2+ Years Old − Total Anaemia Prevalence − West Midlands
WHO−2001 Adjustment Method
Total Anaemia Prevalence
Age (year)
Uncertainty Interval
6 − Subnational (Level 2)
Location Hierarchy
1 − Global
2 − Super Region
3 − Region
4 − Country
5 − Subnational (Level 1)
6 − Subnational (Level 2)

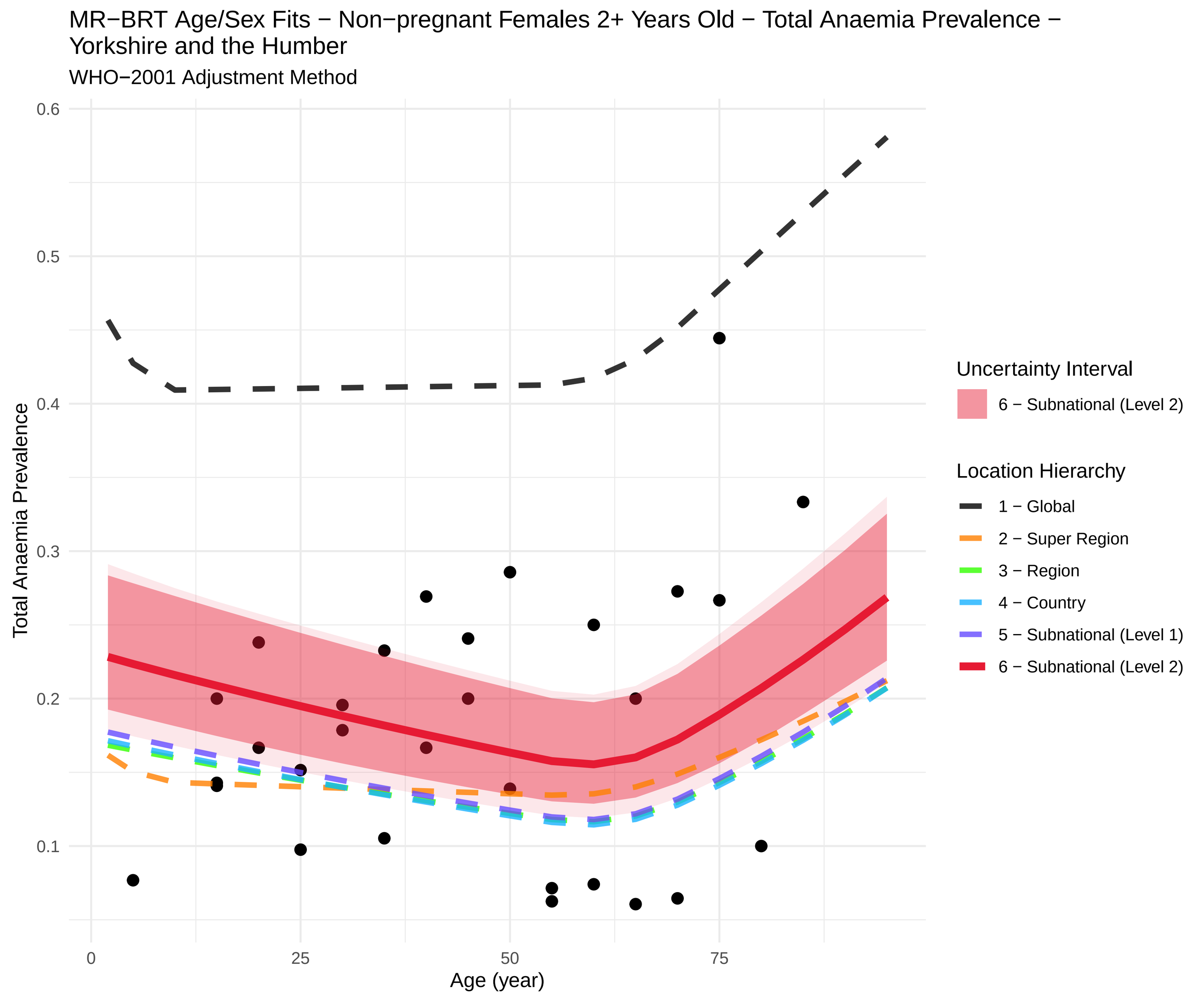
MR−BRT Age/Sex Fits − Non−pregnant Females 2+ Years Old − Total Anaemia Prevalence − Yorkshire and the Humber
WHO−2001 Adjustment Method
Total Anaemia Prevalence
Age (year)
Uncertainty Interval
6 − Subnational (Level 2)
Location Hierarchy
1 − Global
2 − Super Region
3 − Region
4 − Country
5 − Subnational (Level 1)
6 − Subnational (Level 2)

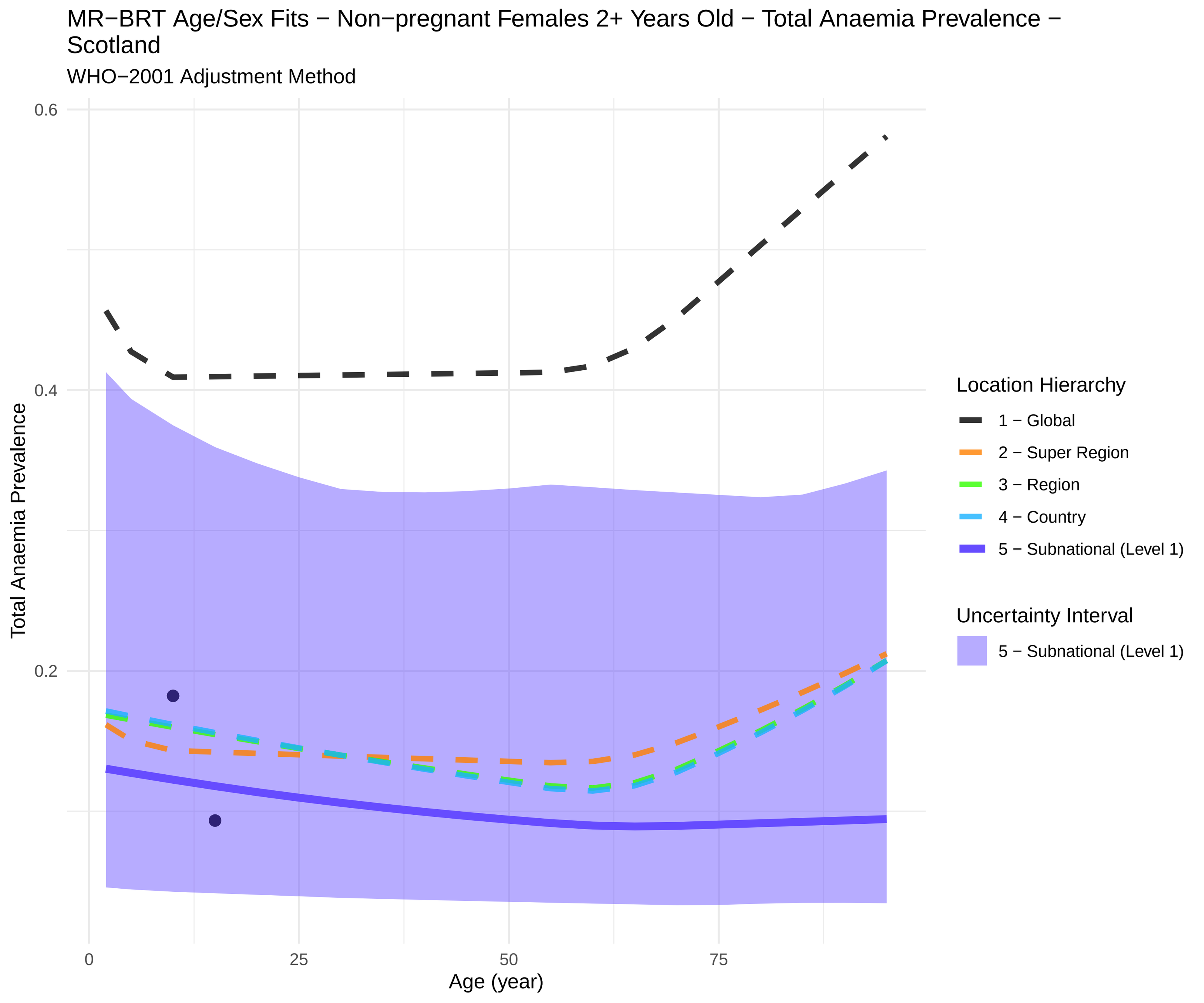
MR−BRT Age/Sex Fits − Non−pregnant Females 2+ Years Old − Total Anaemia Prevalence − Scotland
WHO−2001 Adjustment Method
Total Anaemia Prevalence
Age (year)
Location Hierarchy
1 − Global
2 − Super Region
3 − Region
4 − Country
5 − Subnational (Level 1)
Uncertainty Interval
5 − Subnational (Level 1)

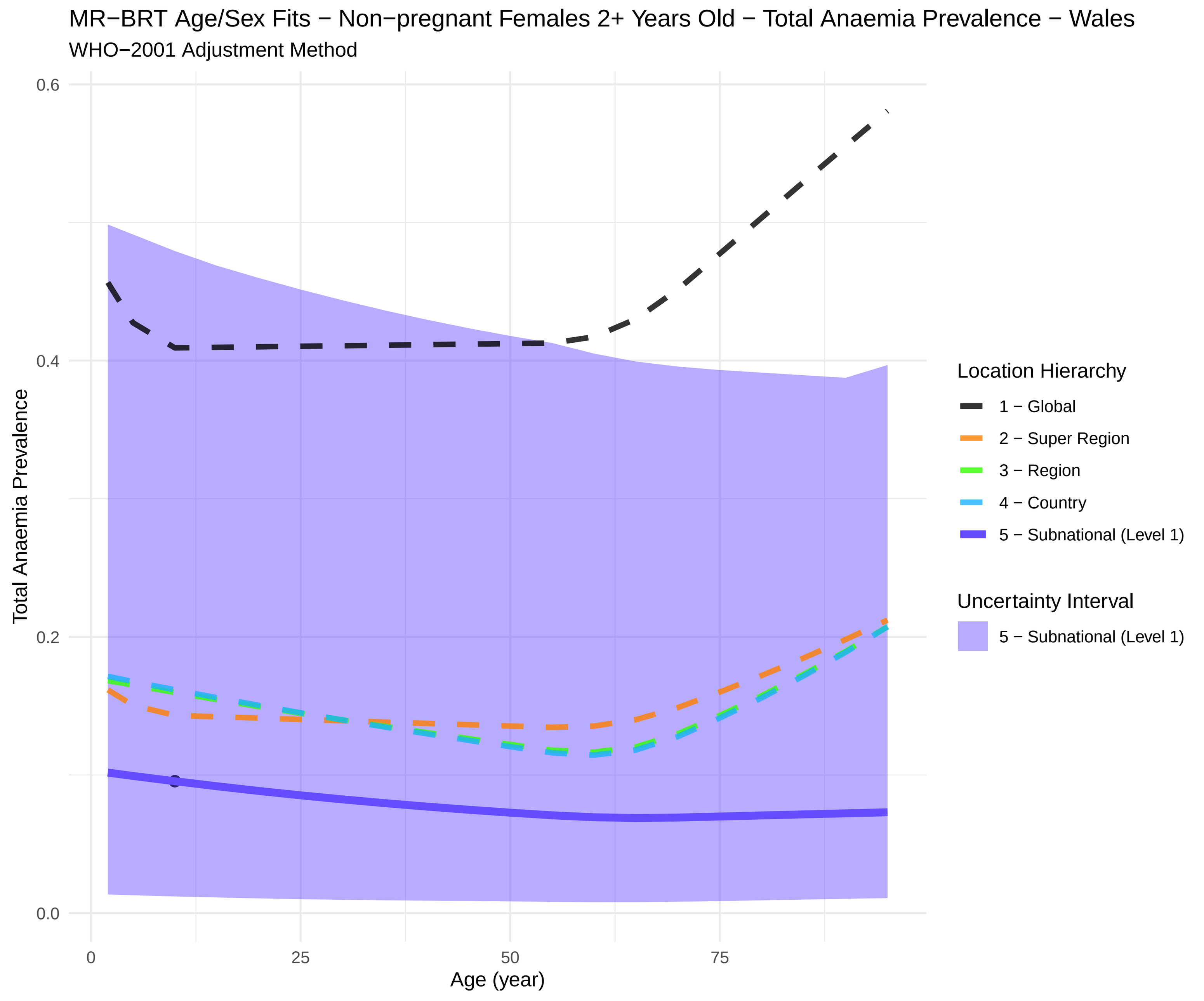
MR−BRT Age/Sex Fits − Non−pregnant Females 2+ Years Old − Total Anaemia Prevalence − Wales
WHO−2001 Adjustment Method
Total Anaemia Prevalence
Age (year)
Location Hierarchy
1 − Global
2 − Super Region
3 − Region
4 − Country
5 − Subnational (Level 1)
Uncertainty Interval
5 − Subnational (Level 1)

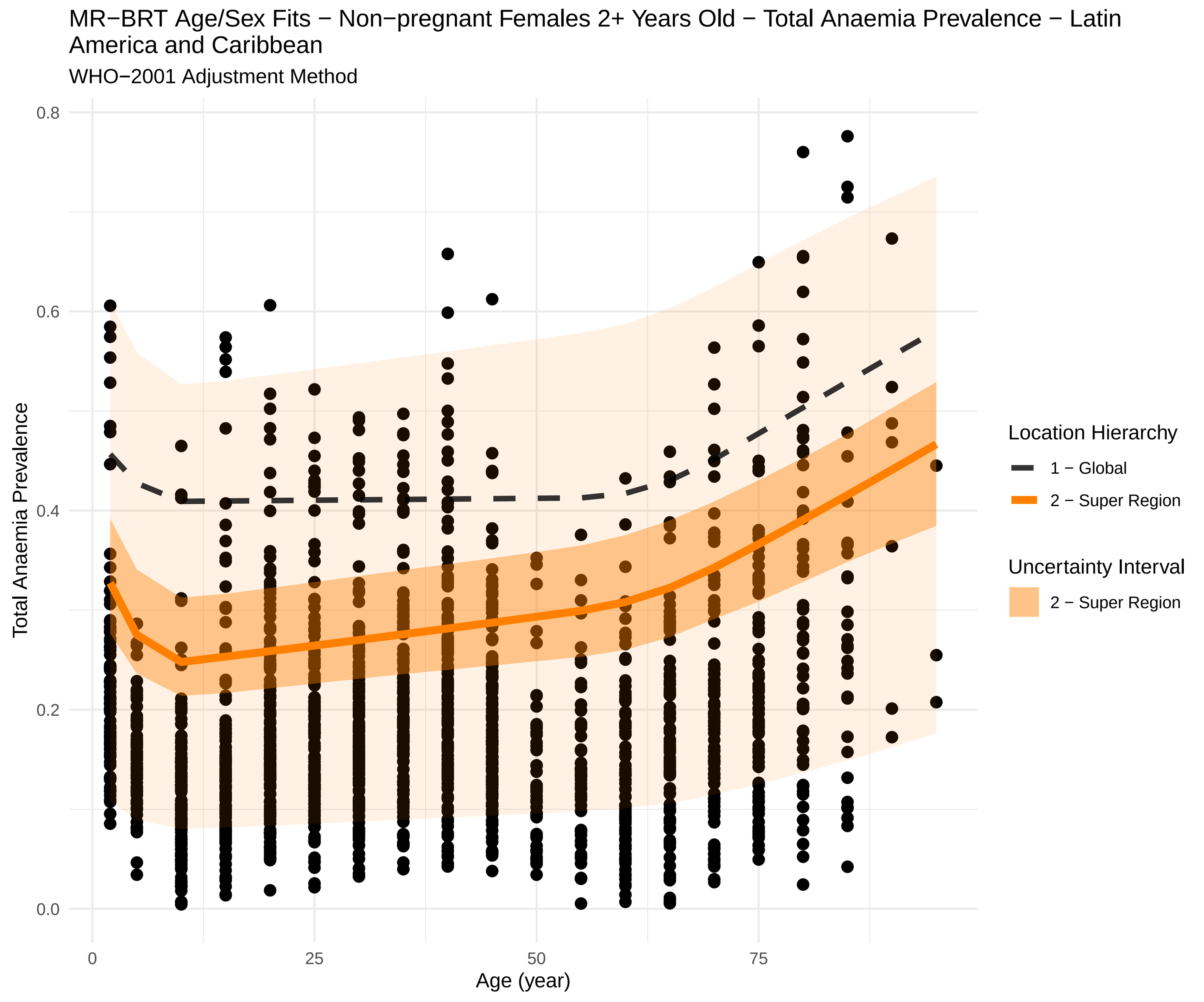
MR−BRT Age/Sex Fits − Non−pregnant Females 2+ Years Old − Total Anaemia Prevalence − Latin America and Caribbean
WHO−2001 Adjustment Method
Total Anaemia Prevalence
Age (year)
Location Hierarchy
1 − Global
2 − Super Region
Uncertainty Interval
2 − Super Region

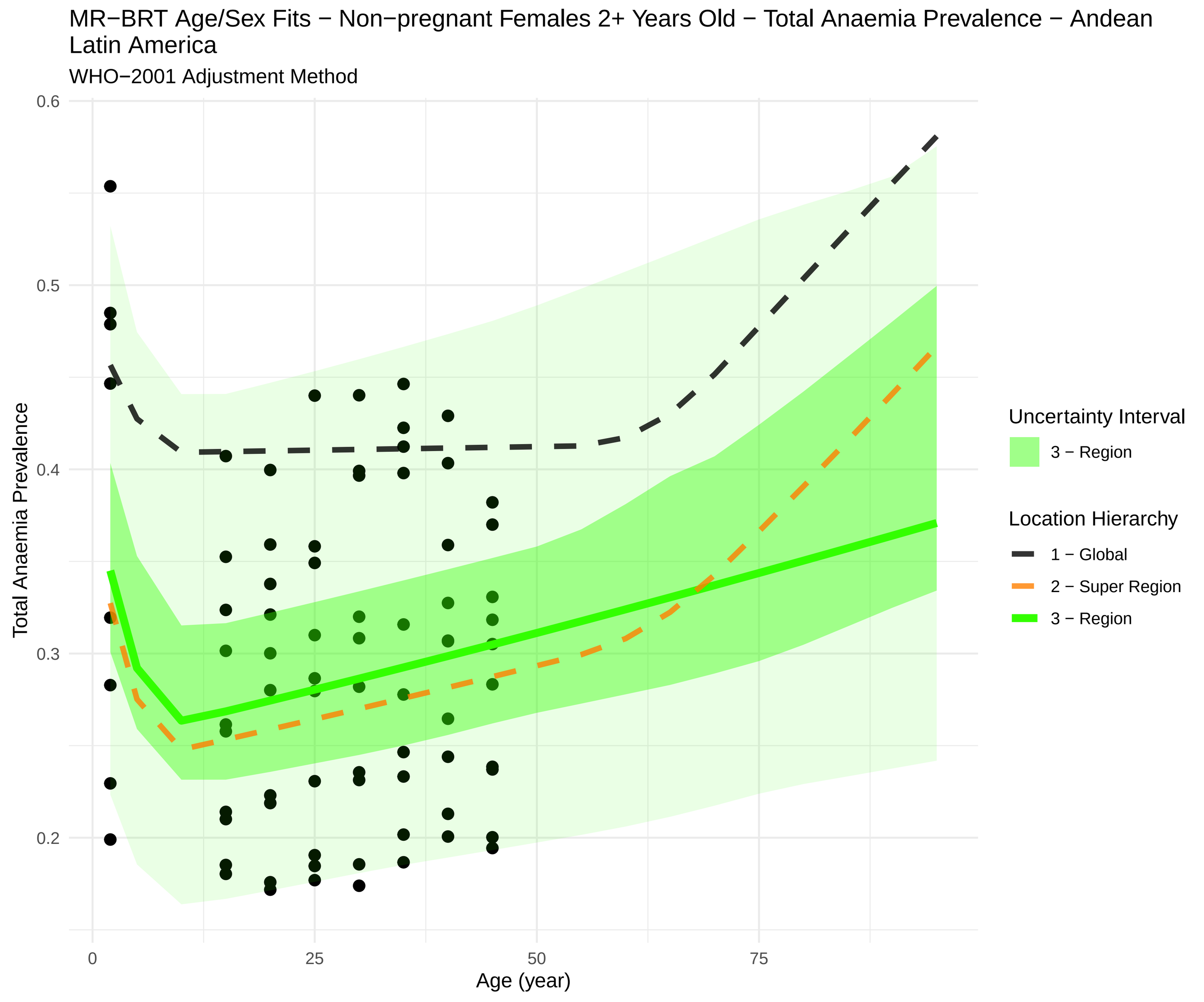
MR−BRT Age/Sex Fits − Non−pregnant Females 2+ Years Old − Total Anaemia Prevalence − Andean Latin America
WHO−2001 Adjustment Method
Total Anaemia Prevalence
Age (year)
Uncertainty Interval
3 − Region
Location Hierarchy
1 − Global
2 − Super Region
3 − Region

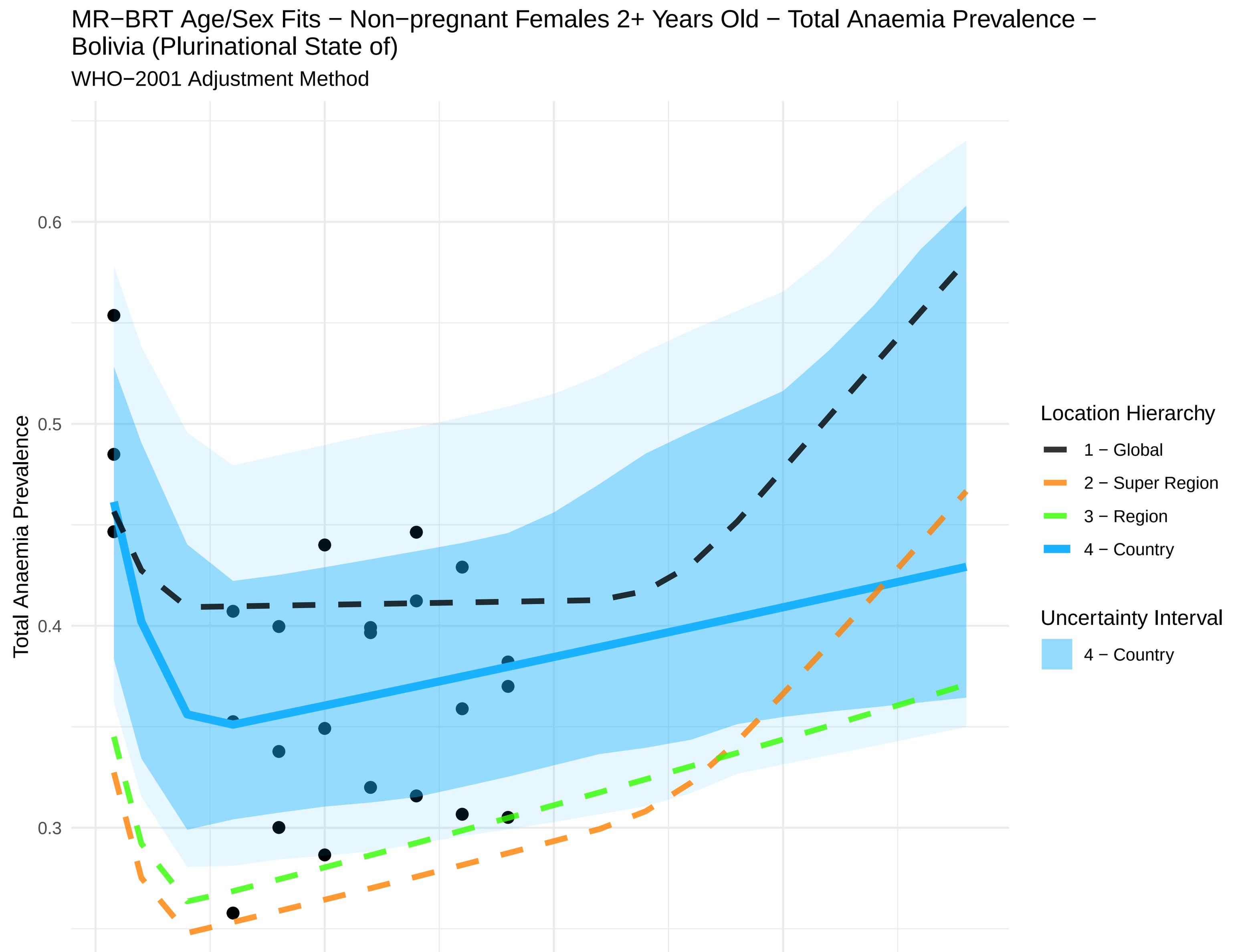
MR−BRT Age/Sex Fits − Non−pregnant Females 2+ Years Old − Total Anaemia Prevalence − Bolivia (Plurinational State of)
WHO−2001 Adjustment Method
Total Anaemia Prevalence
Age (year)
Location Hierarchy
1 − Global
2 − Super Region
3 − Region
4 − Country
Uncertainty Interval
4 − Country

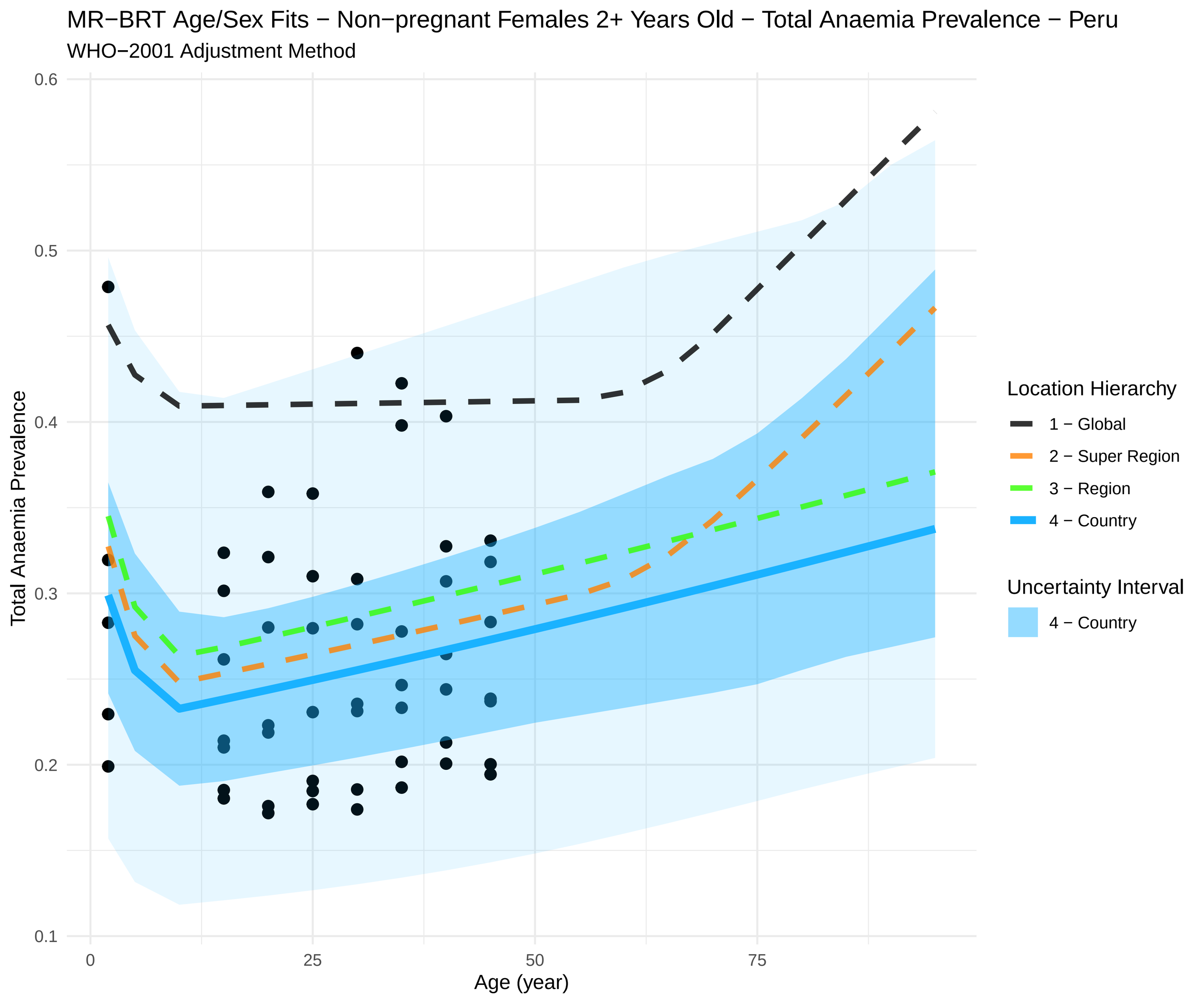
MR−BRT Age/Sex Fits − Non−pregnant Females 2+ Years Old − Total Anaemia Prevalence − Peru
WHO−2001 Adjustment Method
Total Anaemia Prevalence
Age (year)
Location Hierarchy
1 − Global
2 − Super Region
3 − Region
4 − Country
Uncertainty Interval
4 − Country

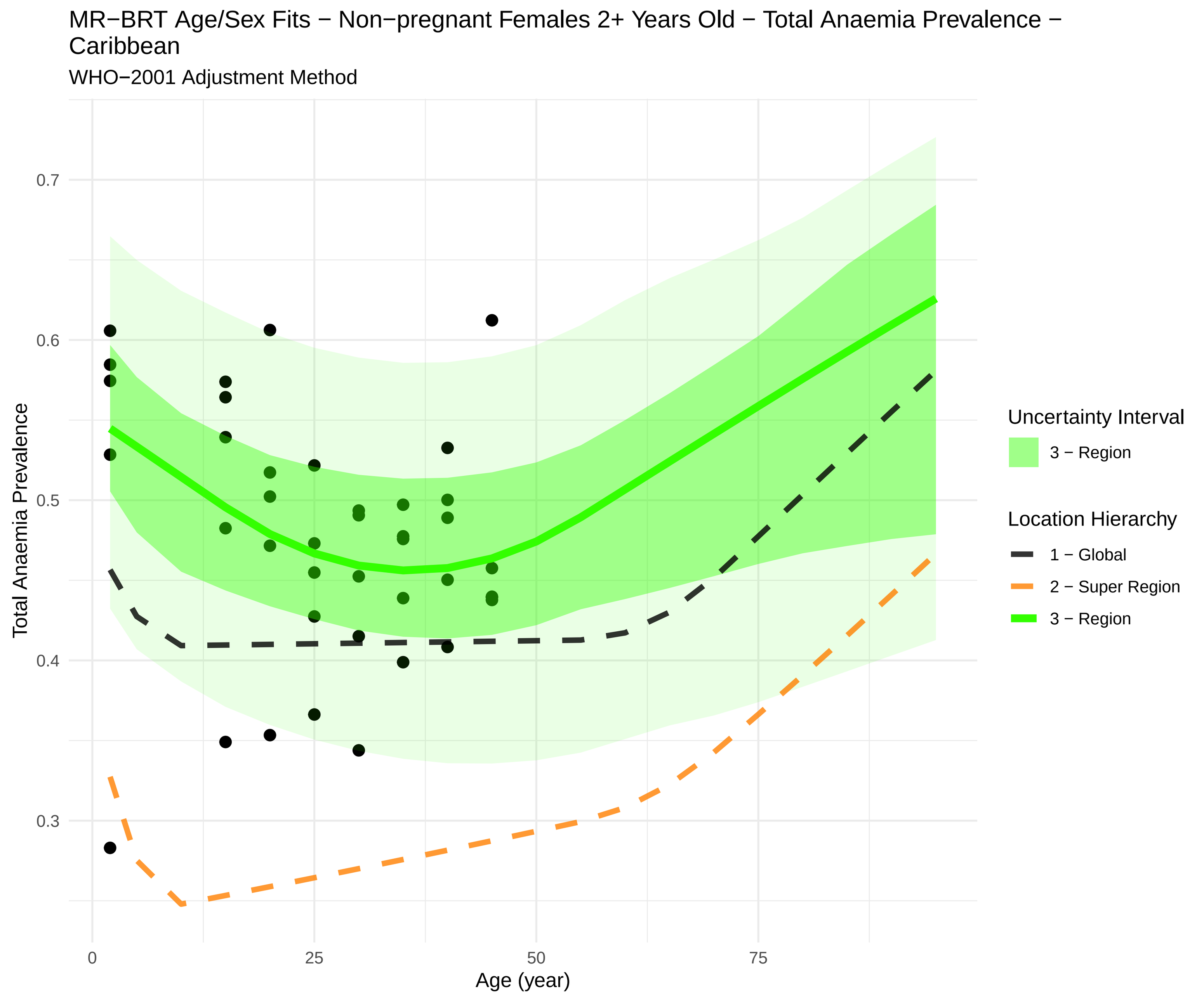
MR−BRT Age/Sex Fits − Non−pregnant Females 2+ Years Old − Total Anaemia Prevalence − Caribbean
WHO−2001 Adjustment Method
Total Anaemia Prevalence
Age (year)
Uncertainty Interval
3 − Region
Location Hierarchy
1 − Global
2 − Super Region
3 − Region

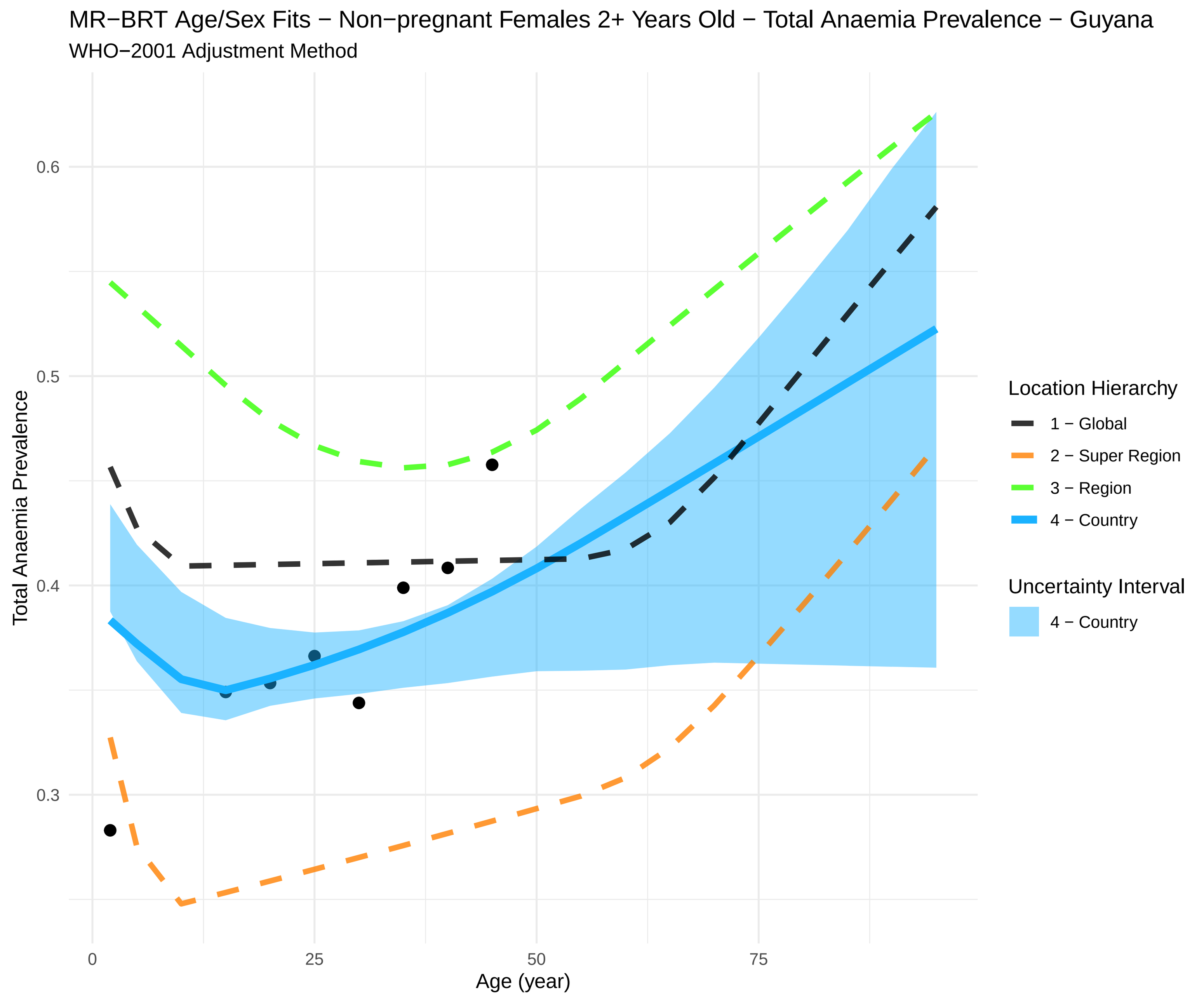
MR−BRT Age/Sex Fits − Non−pregnant Females 2+ Years Old − Total Anaemia Prevalence − Guyana
WHO−2001 Adjustment Method
Total Anaemia Prevalence
Age (year)
Location Hierarchy
1 − Global
2 − Super Region
3 − Region
4 − Country
Uncertainty Interval
4 − Country

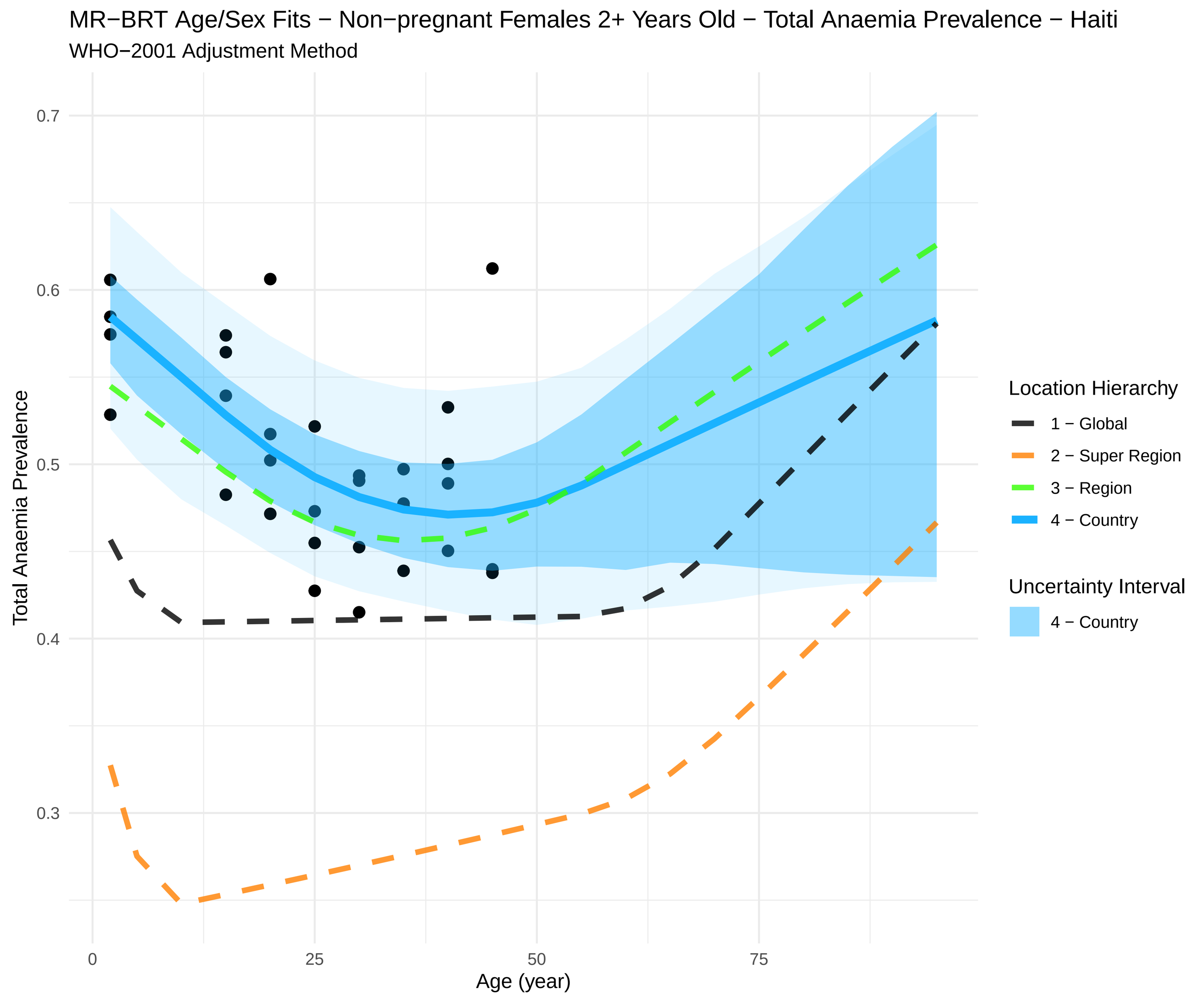
MR−BRT Age/Sex Fits − Non−pregnant Females 2+ Years Old − Total Anaemia Prevalence − Haiti
WHO−2001 Adjustment Method
Total Anaemia Prevalence
Age (year)
Location Hierarchy
1 − Global
2 − Super Region
3 − Region
4 − Country
Uncertainty Interval
4 − Country

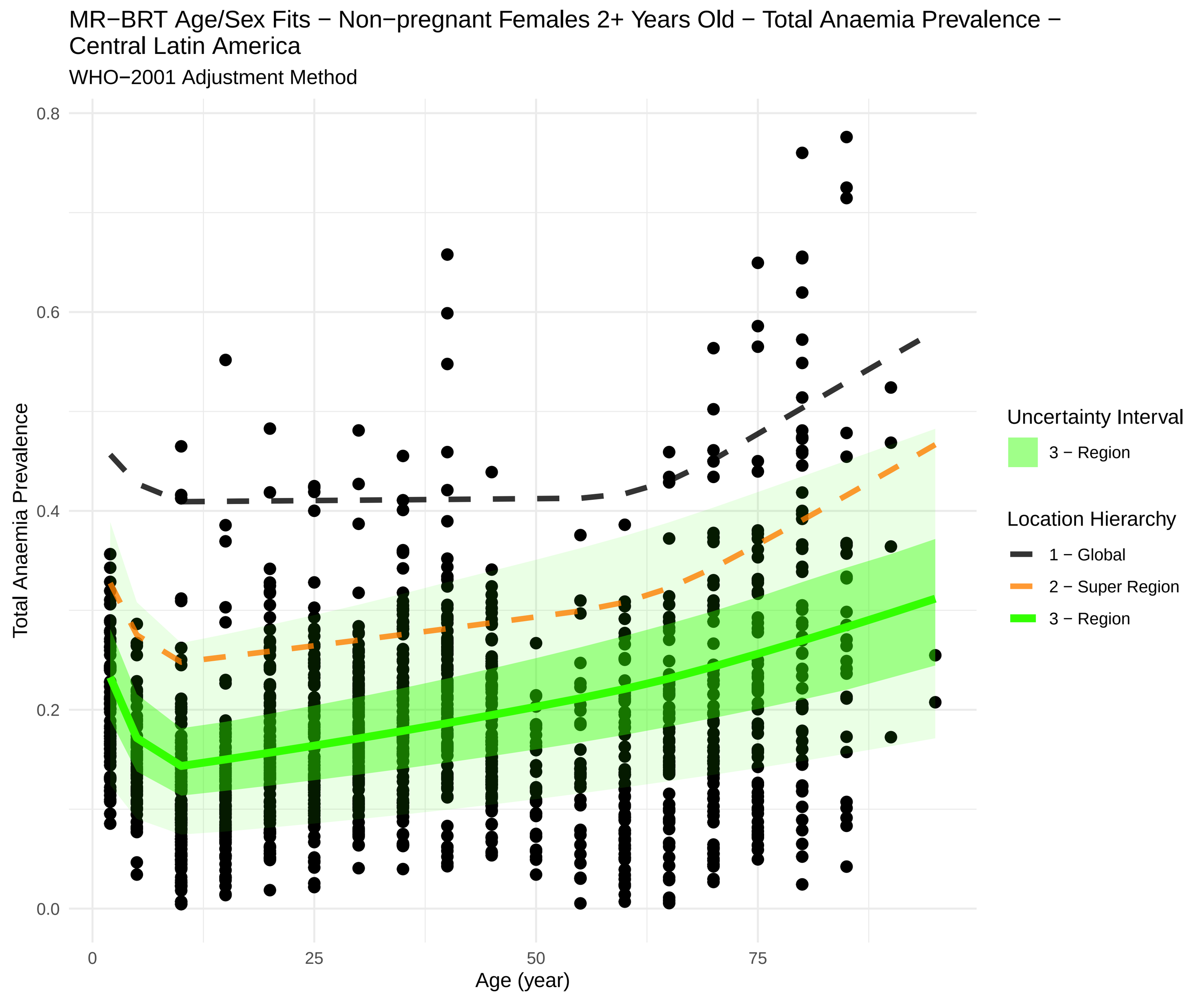
MR−BRT Age/Sex Fits − Non−pregnant Females 2+ Years Old − Total Anaemia Prevalence − Central Latin America
WHO−2001 Adjustment Method
Total Anaemia Prevalence
Age (year)
Uncertainty Interval
3 − Region
Location Hierarchy
1 − Global
2 − Super Region
3 − Region

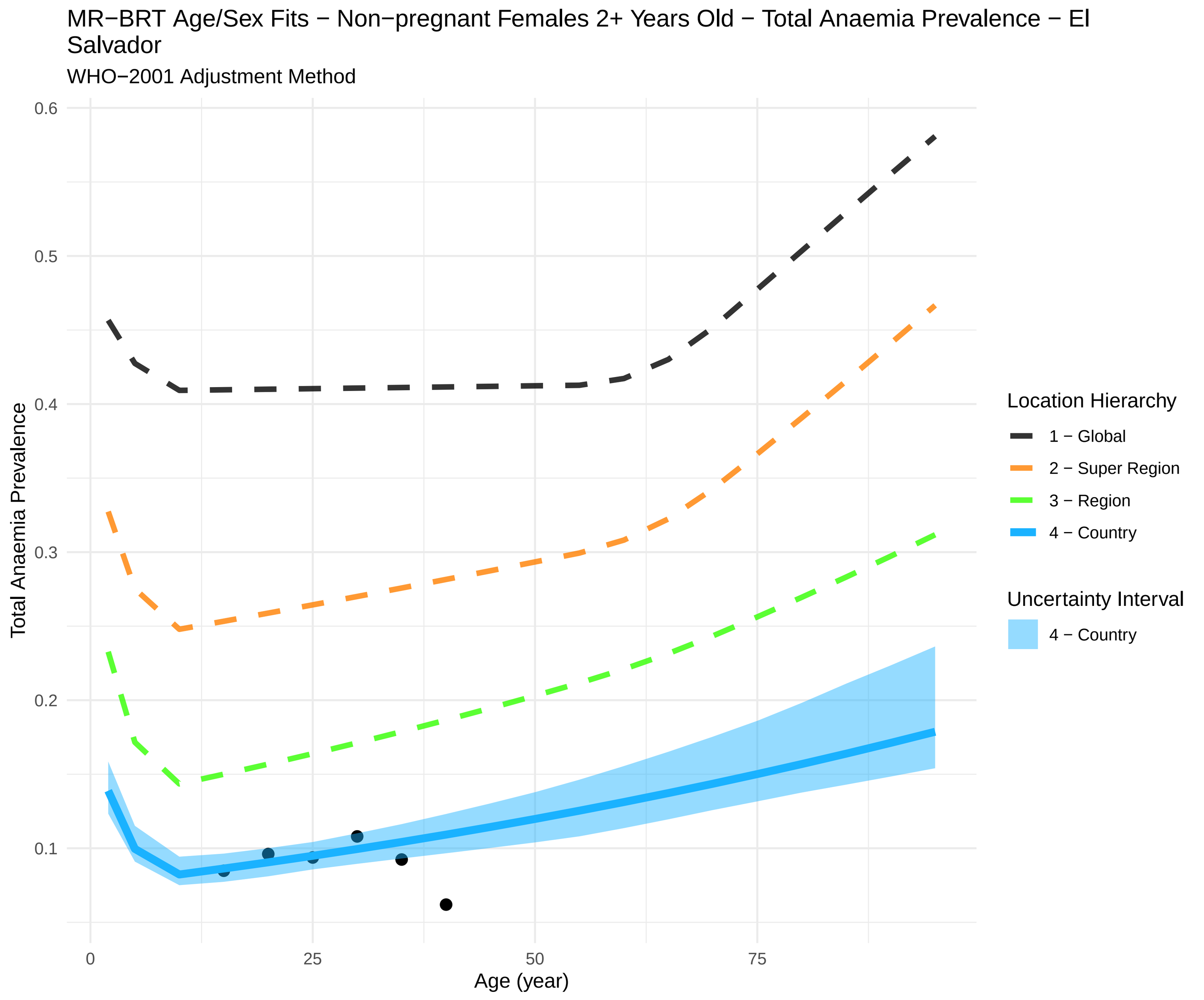
MR−BRT Age/Sex Fits − Non−pregnant Females 2+ Years Old − Total Anaemia Prevalence − El Salvador
WHO−2001 Adjustment Method
Total Anaemia Prevalence
Age (year)
Location Hierarchy
1 − Global
2 − Super Region
3 − Region
4 − Country
Uncertainty Interval
4 − Country

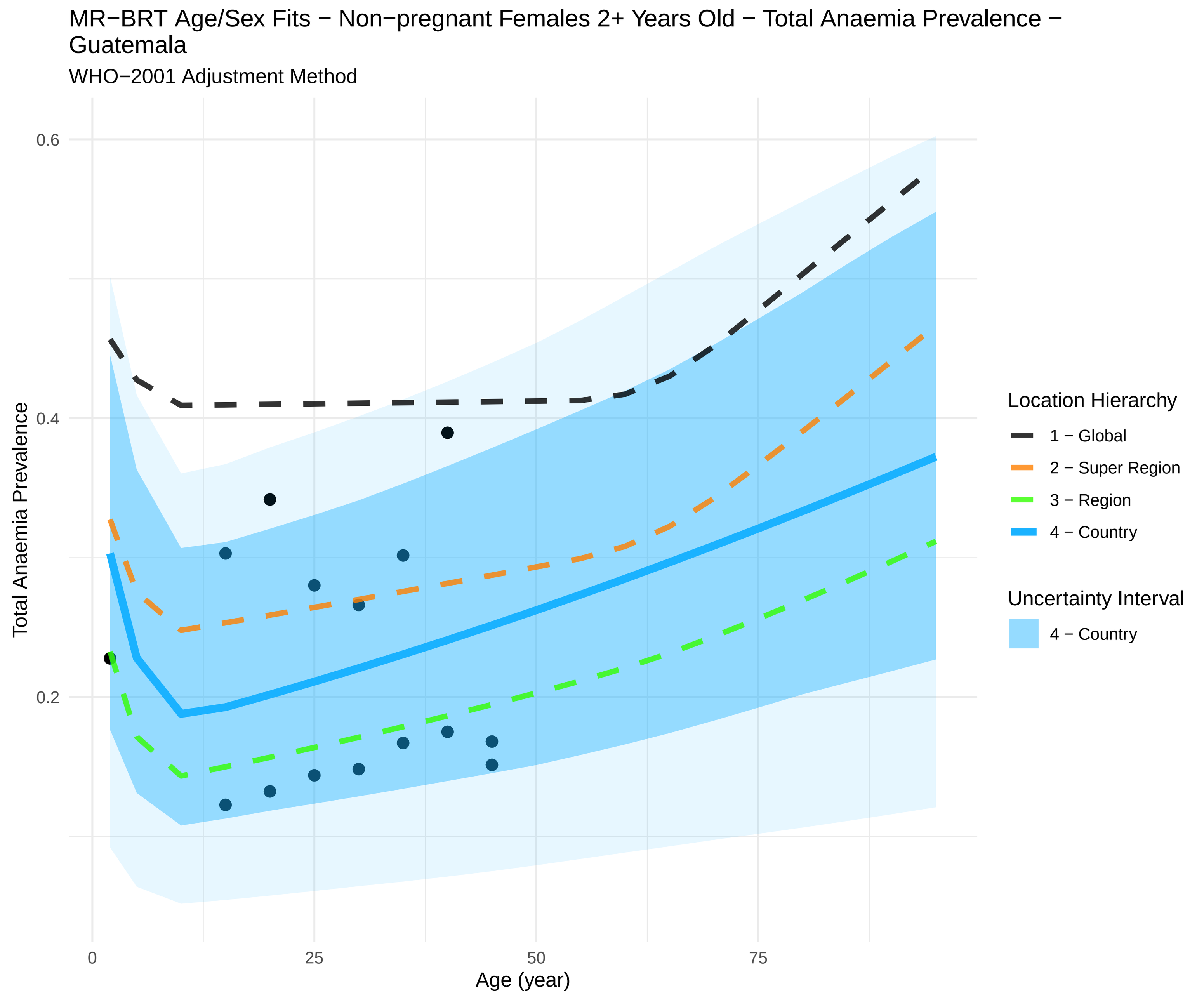
MR−BRT Age/Sex Fits − Non−pregnant Females 2+ Years Old − Total Anaemia Prevalence − Guatemala
WHO−2001 Adjustment Method
Total Anaemia Prevalence
Age (year)
Location Hierarchy
1 − Global
2 − Super Region
3 − Region
4 − Country
Uncertainty Interval
4 − Country

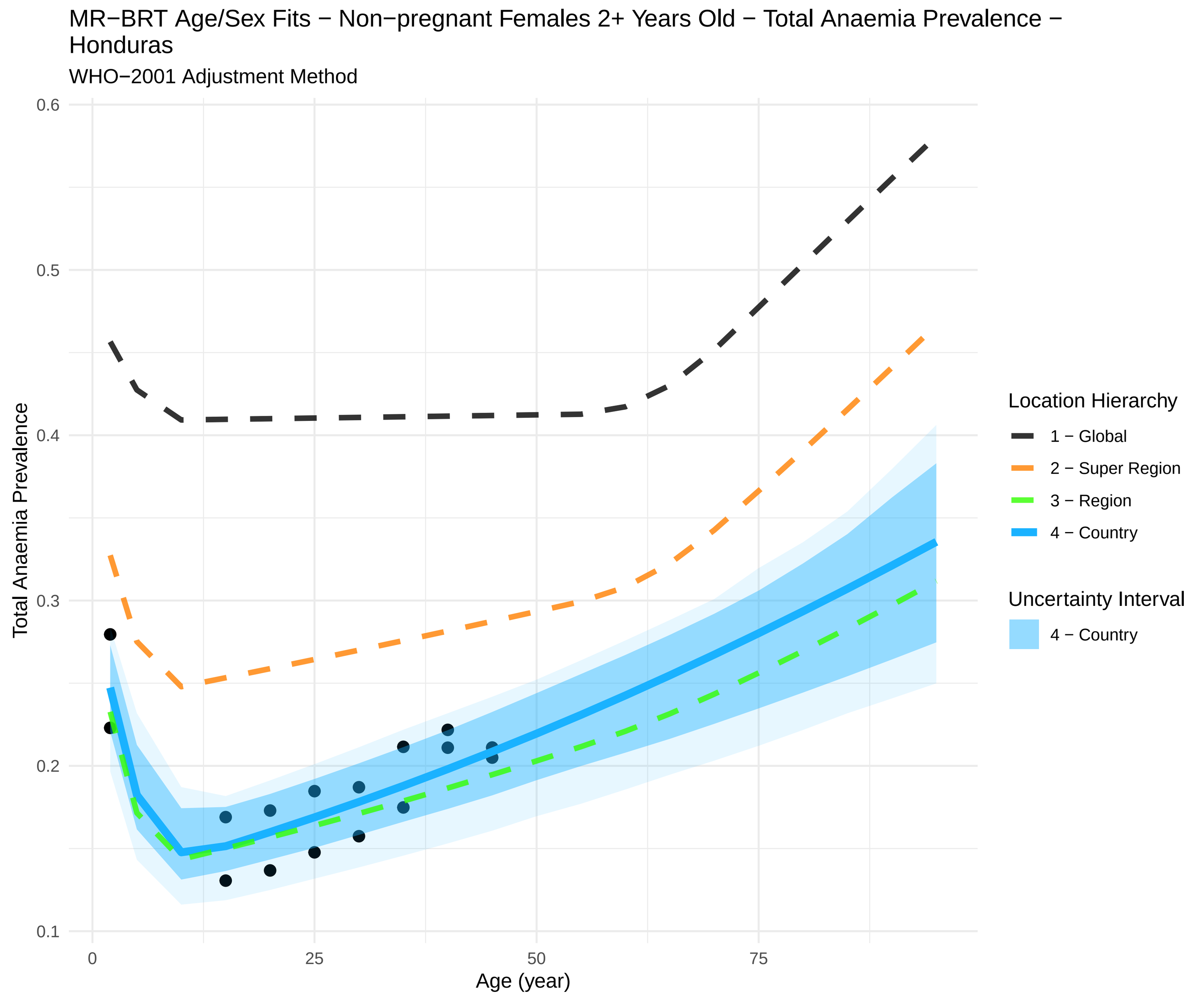
MR−BRT Age/Sex Fits − Non−pregnant Females 2+ Years Old − Total Anaemia Prevalence − Honduras
WHO−2001 Adjustment Method
Total Anaemia Prevalence
Age (year)
Location Hierarchy
1 − Global
2 − Super Region
3 − Region
4 − Country
Uncertainty Interval
4 − Country

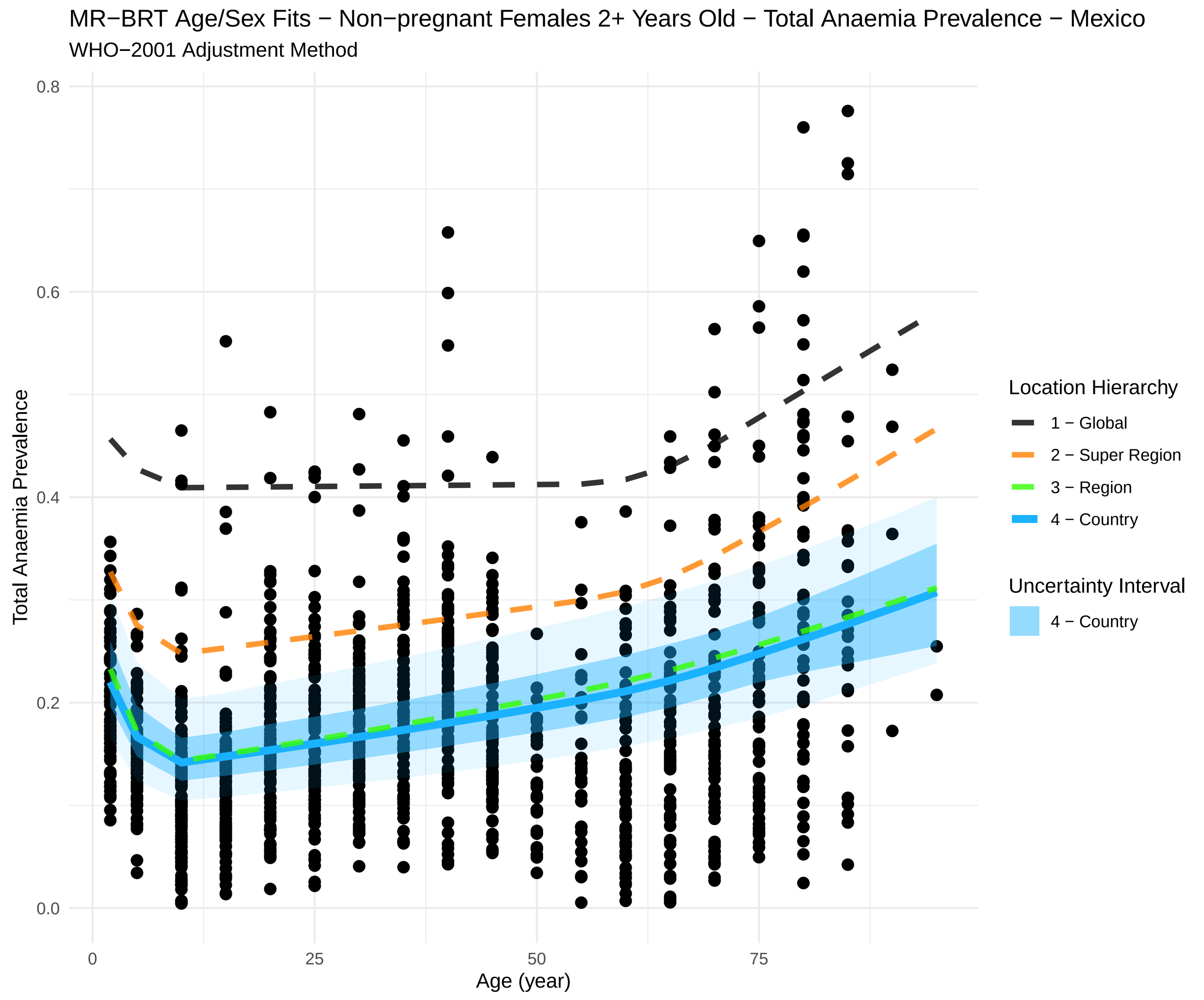
MR−BRT Age/Sex Fits − Non−pregnant Females 2+ Years Old − Total Anaemia Prevalence − Mexico
WHO−2001 Adjustment Method
Total Anaemia Prevalence
Age (year)
Location Hierarchy
1 − Global
2 − Super Region
3 − Region
4 − Country
Uncertainty Interval
4 − Country

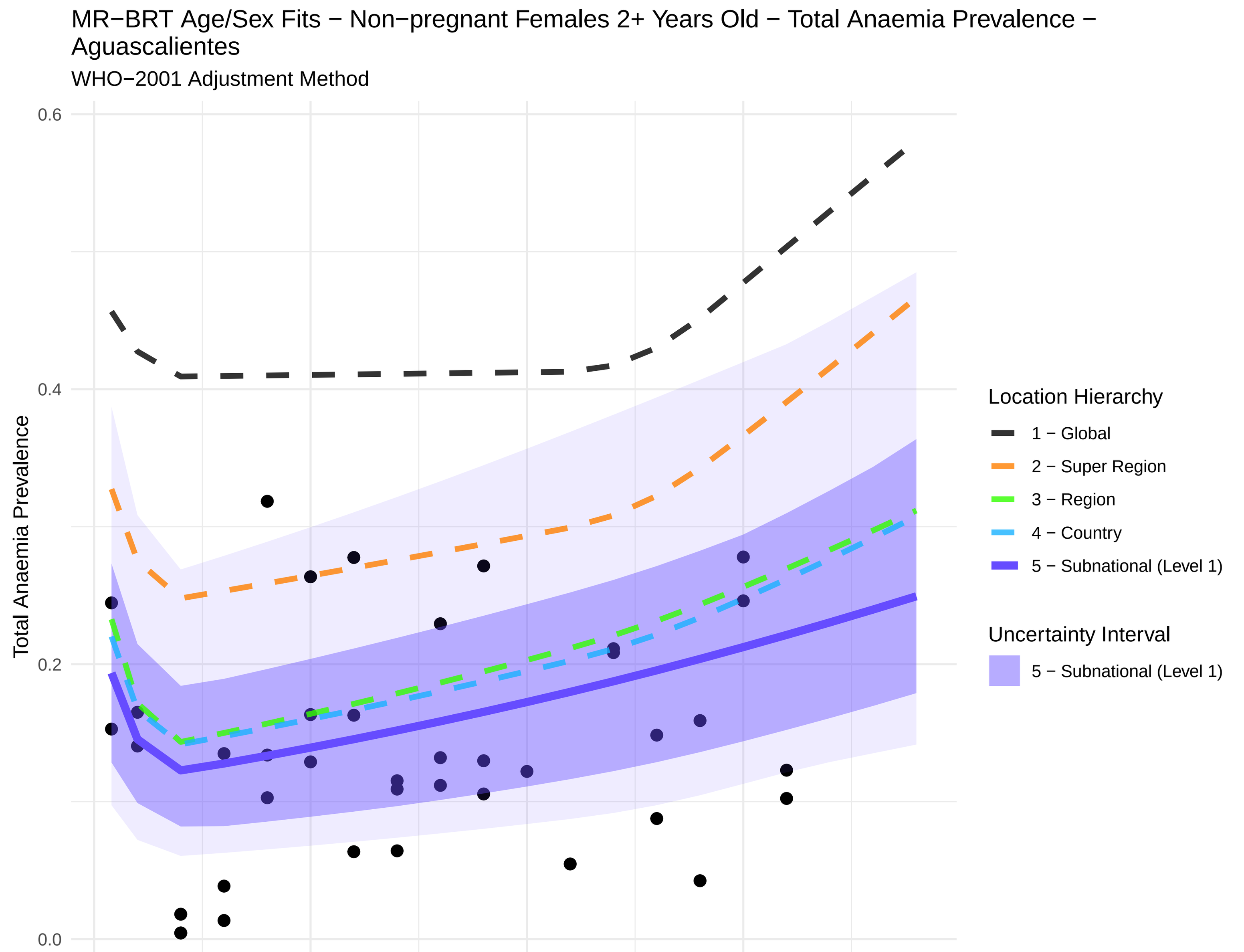
MR−BRT Age/Sex Fits − Non−pregnant Females 2+ Years Old − Total Anaemia Prevalence − Aguascalientes
WHO−2001 Adjustment Method
Total Anaemia Prevalence
Age (year)
Location Hierarchy
1 − Global
2 − Super Region
3 − Region
4 − Country
5 − Subnational (Level 1)
Uncertainty Interval
5 − Subnational (Level 1)

MR−BRT Age/Sex Fits − Non−pregnant Females 2+ Years Old − Total Anaemia Prevalence − Baja California
WHO−2001 Adjustment Method

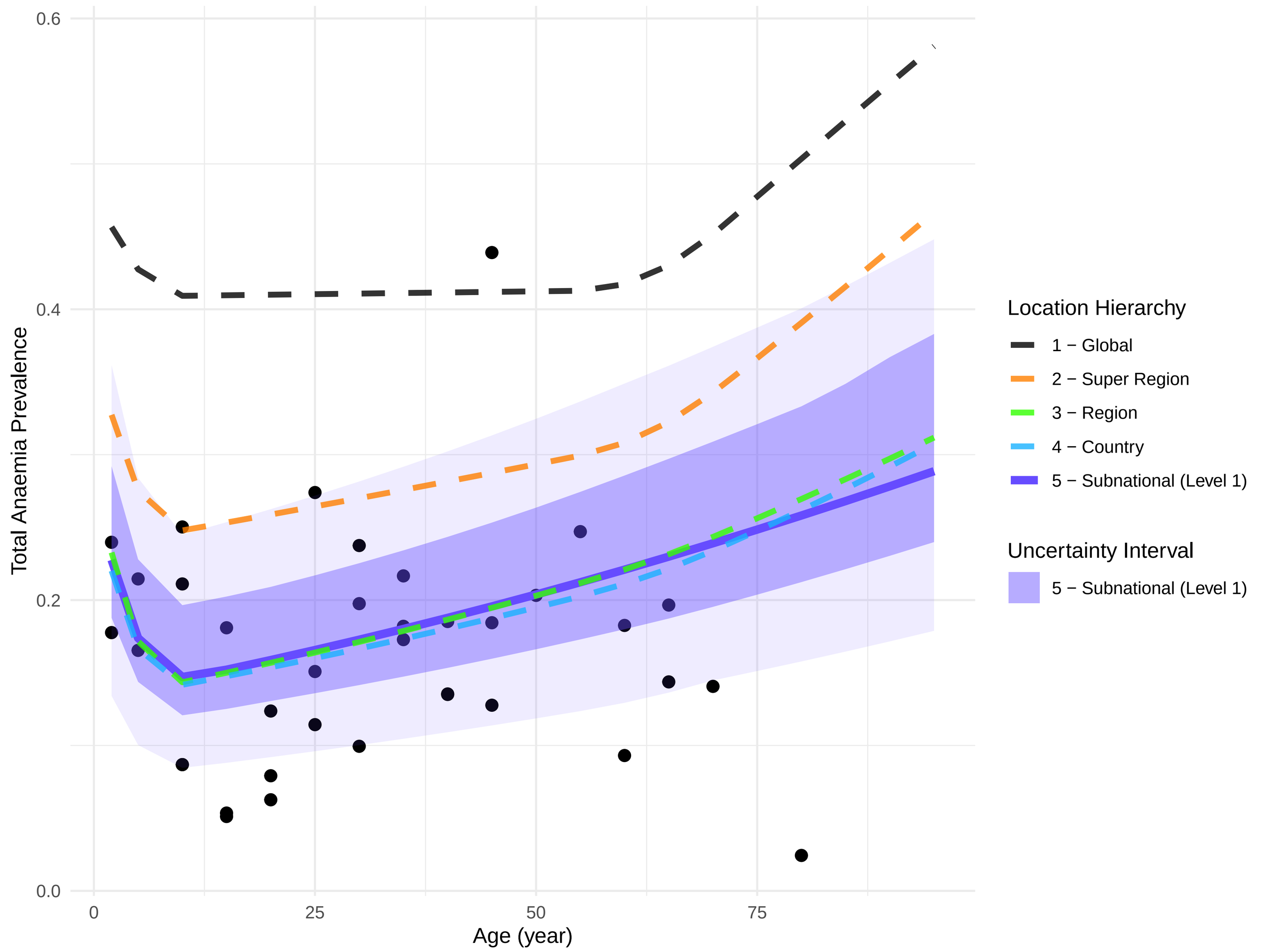

MR−BRT Age/Sex Fits − Non−pregnant Females 2+ Years Old − Total Anaemia Prevalence − Baja California Sur
WHO−2001 Adjustment Method

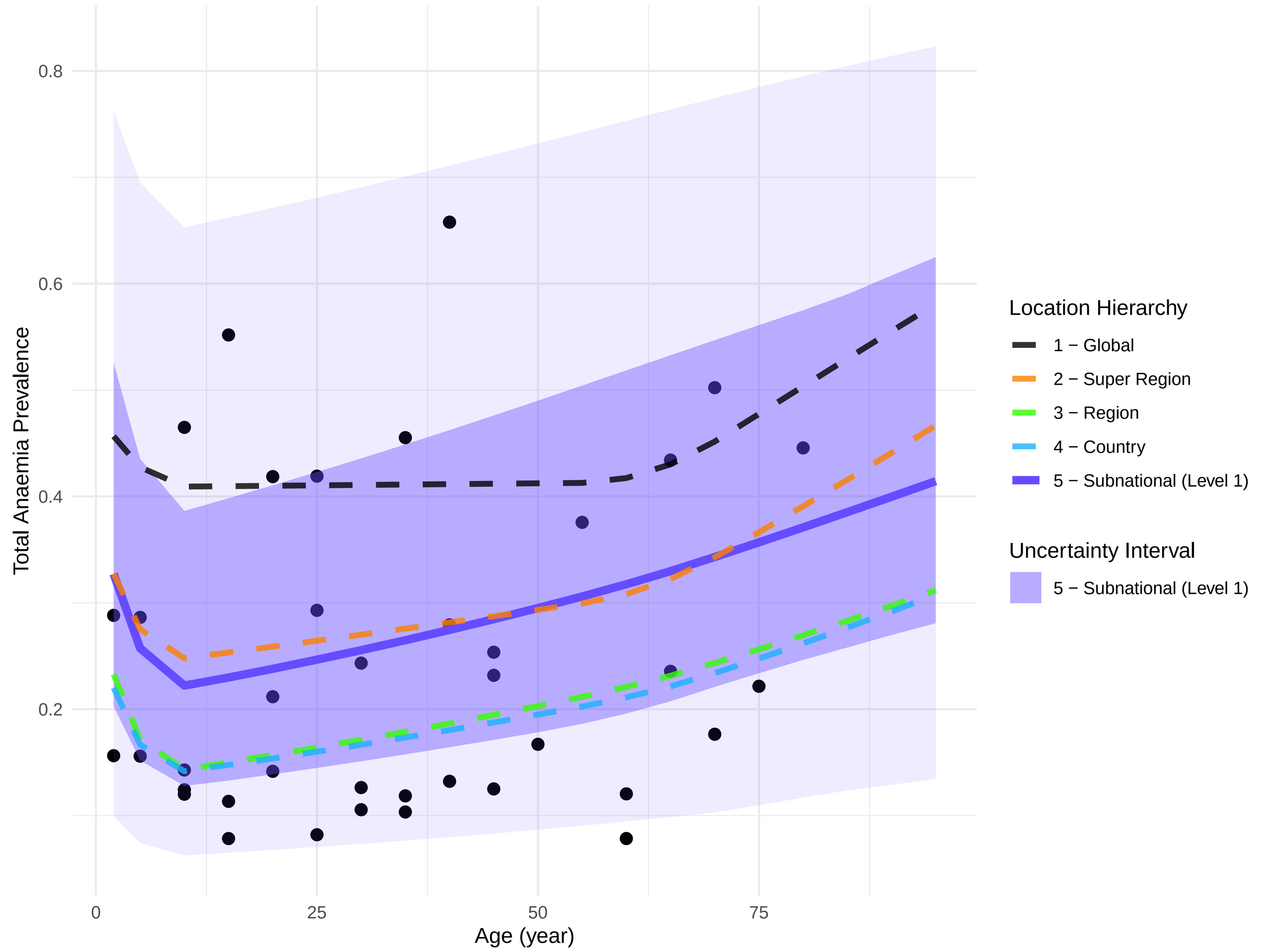

MR−BRT Age/Sex Fits − Non−pregnant Females 2+ Years Old − Total Anaemia Prevalence − Campeche
WHO−2001 Adjustment Method

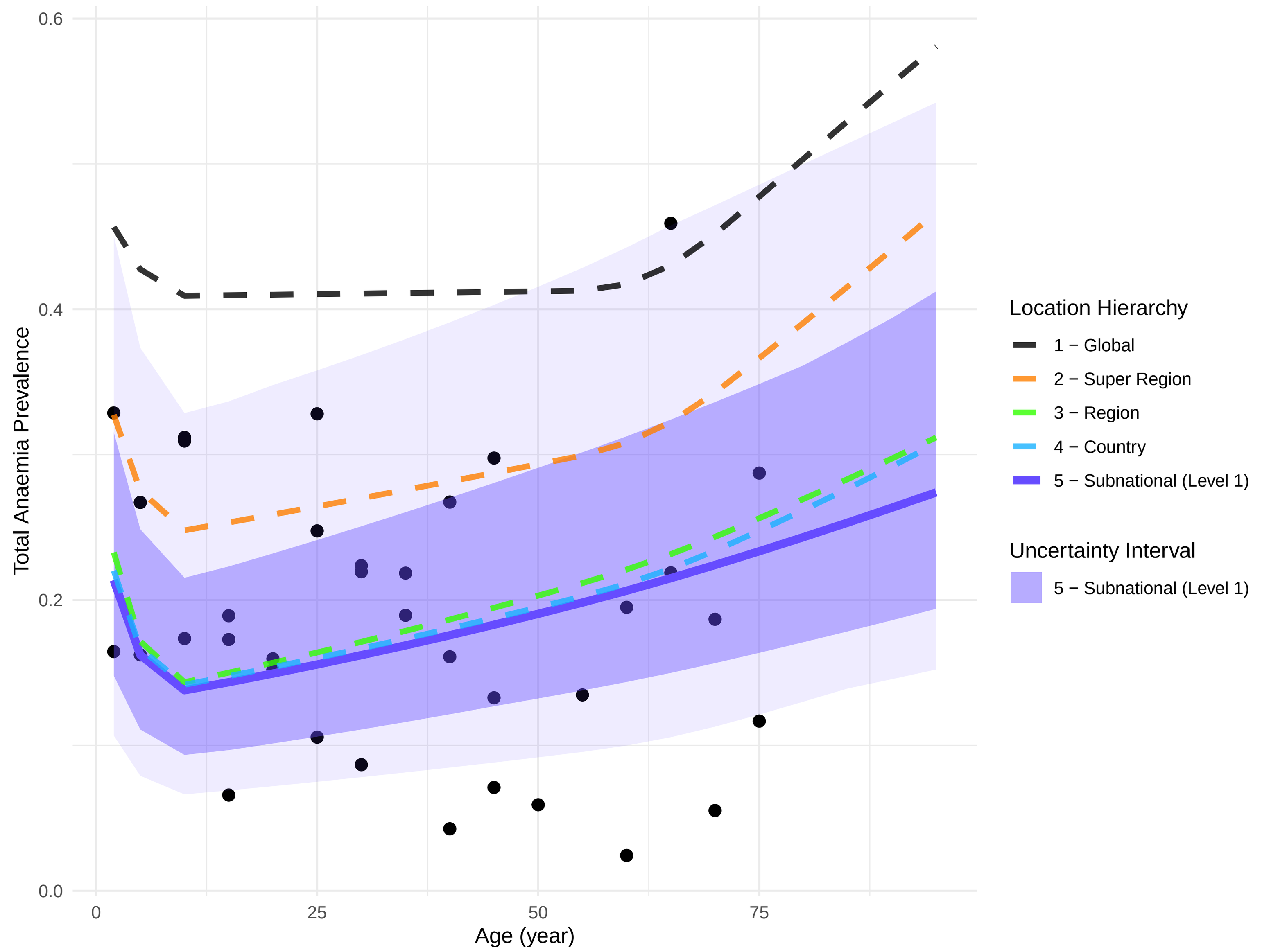

MR−BRT Age/Sex Fits − Non−pregnant Females 2+ Years Old − Total Anaemia Prevalence − Chiapas
WHO−2001 Adjustment Method

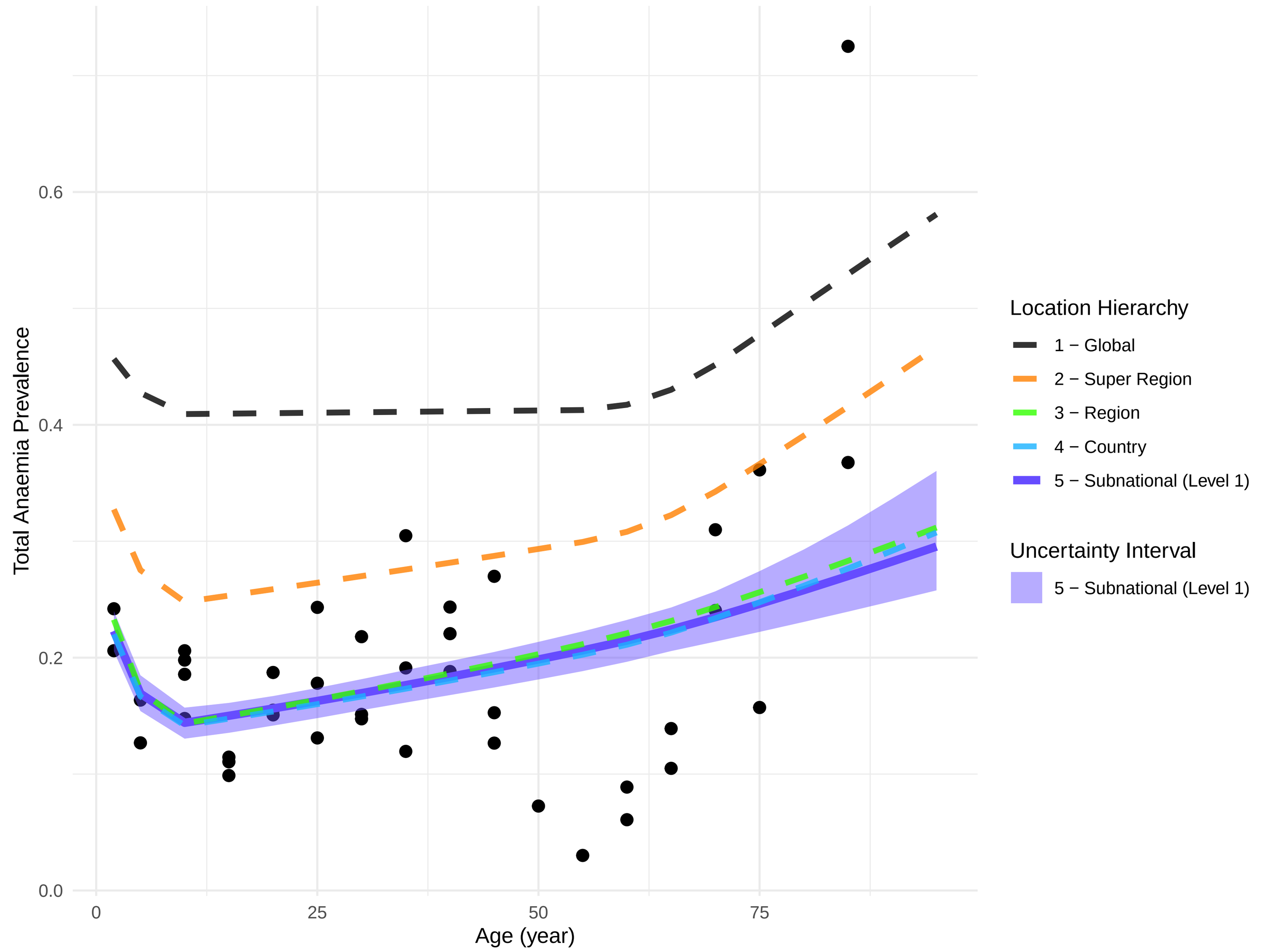

MR−BRT Age/Sex Fits − Non−pregnant Females 2+ Years Old − Total Anaemia Prevalence − Chihuahua
WHO−2001 Adjustment Method

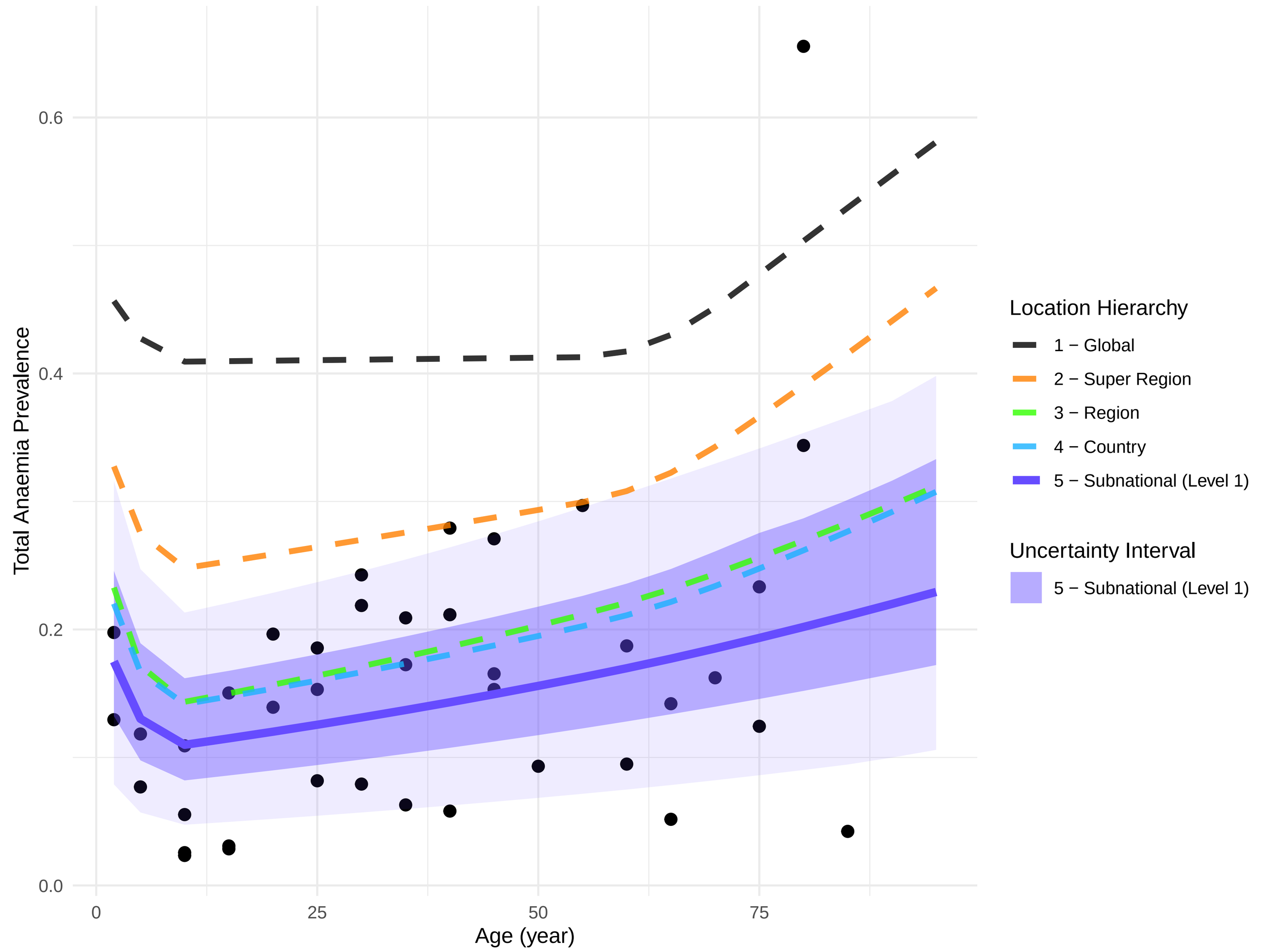

MR−BRT Age/Sex Fits − Non−pregnant Females 2+ Years Old − Total Anaemia Prevalence − Coahuila
WHO−2001 Adjustment Method

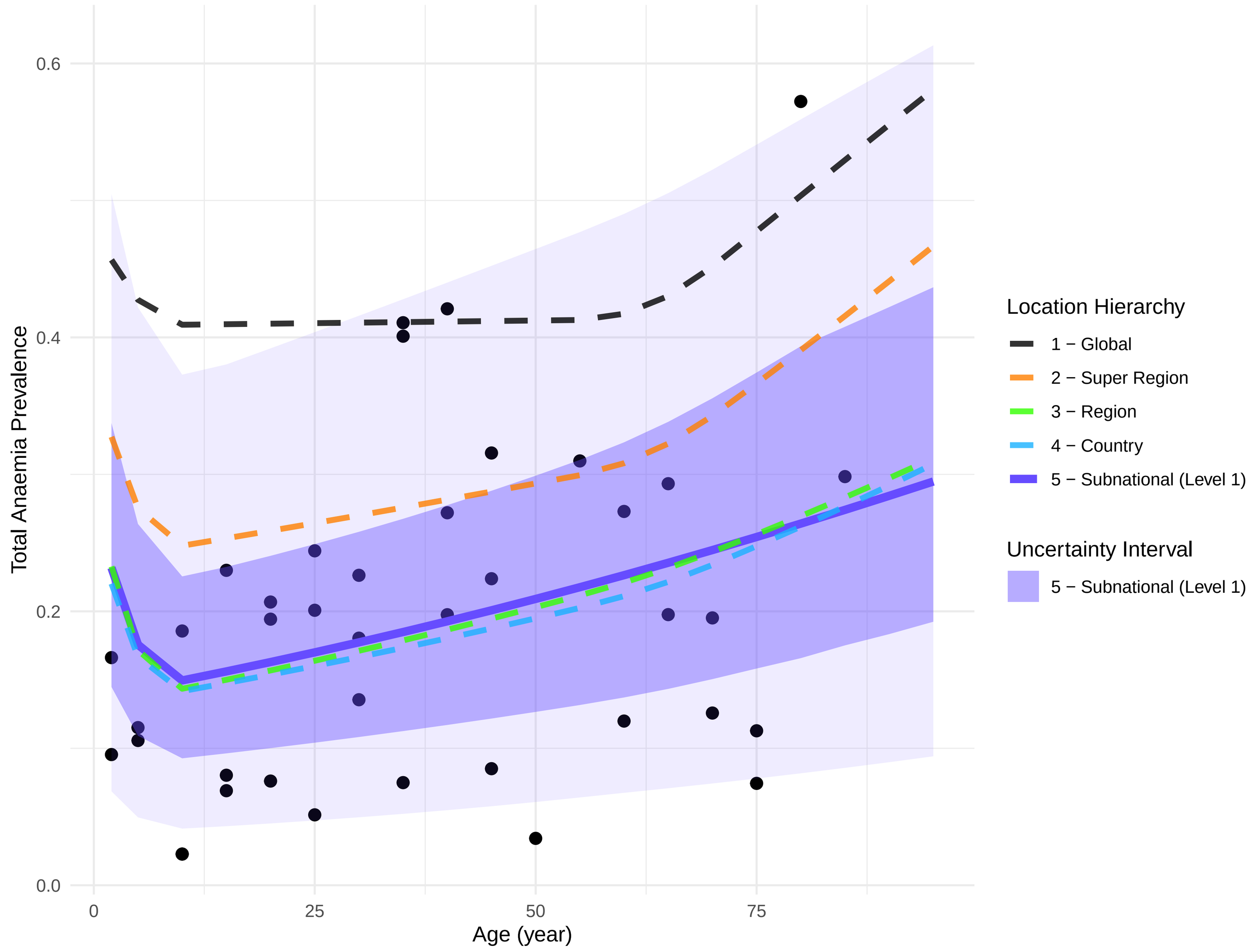

MR−BRT Age/Sex Fits − Non−pregnant Females 2+ Years Old − Total Anaemia Prevalence − Colima
WHO−2001 Adjustment Method

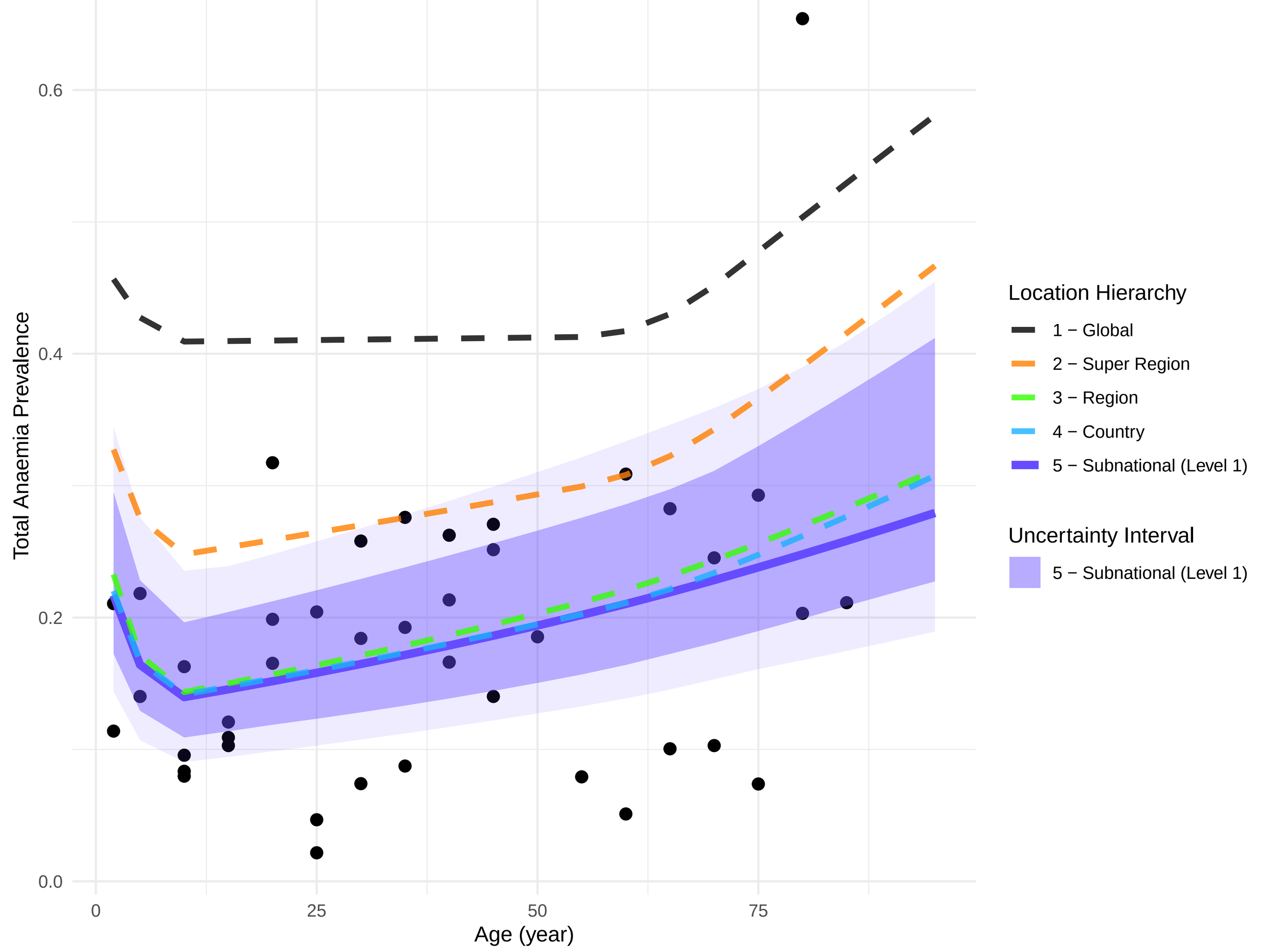

MR−BRT Age/Sex Fits − Non−pregnant Females 2+ Years Old − Total Anaemia Prevalence − Durango
WHO−2001 Adjustment Method

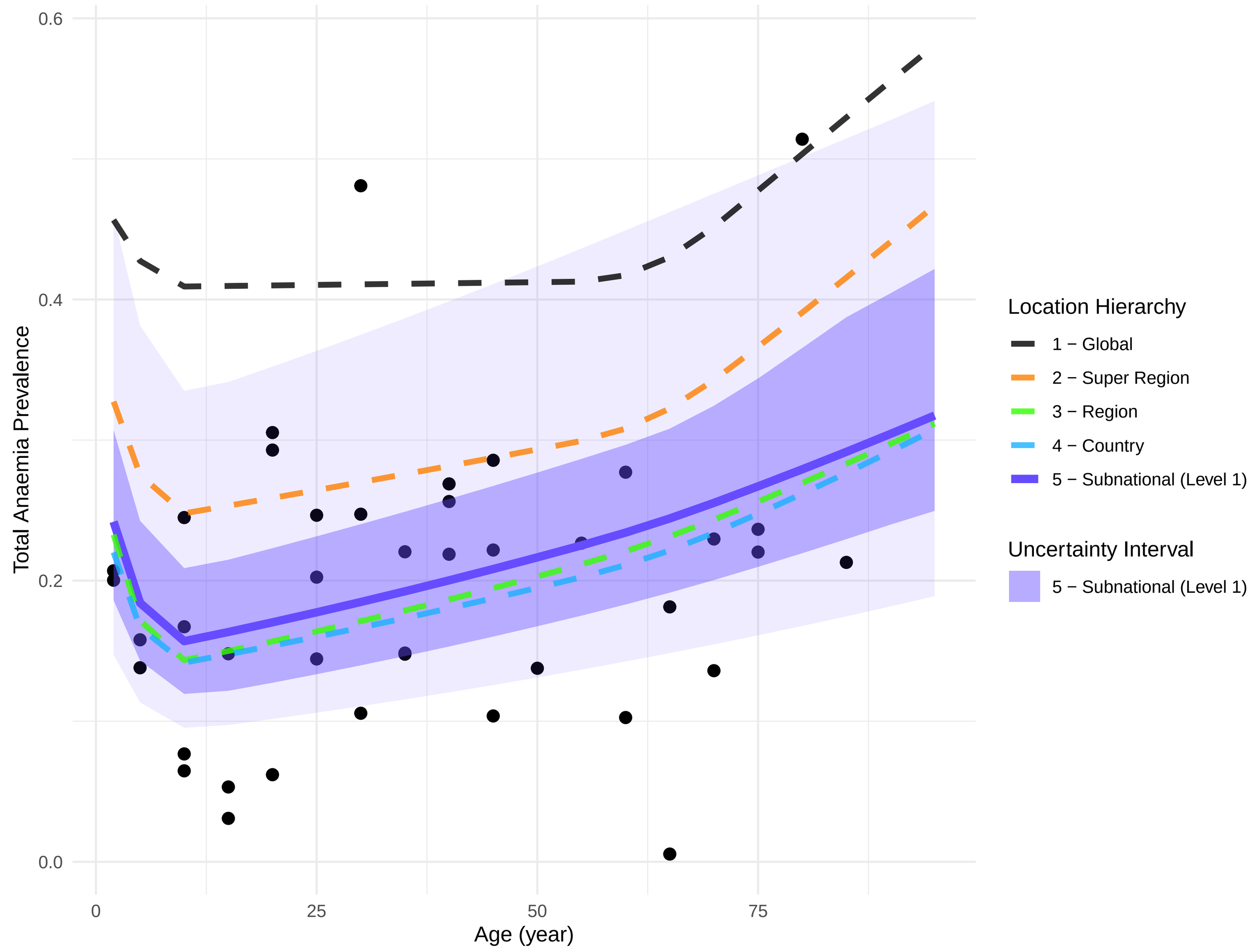

MR−BRT Age/Sex Fits − Non−pregnant Females 2+ Years Old − Total Anaemia Prevalence − Guanajuato
WHO−2001 Adjustment Method

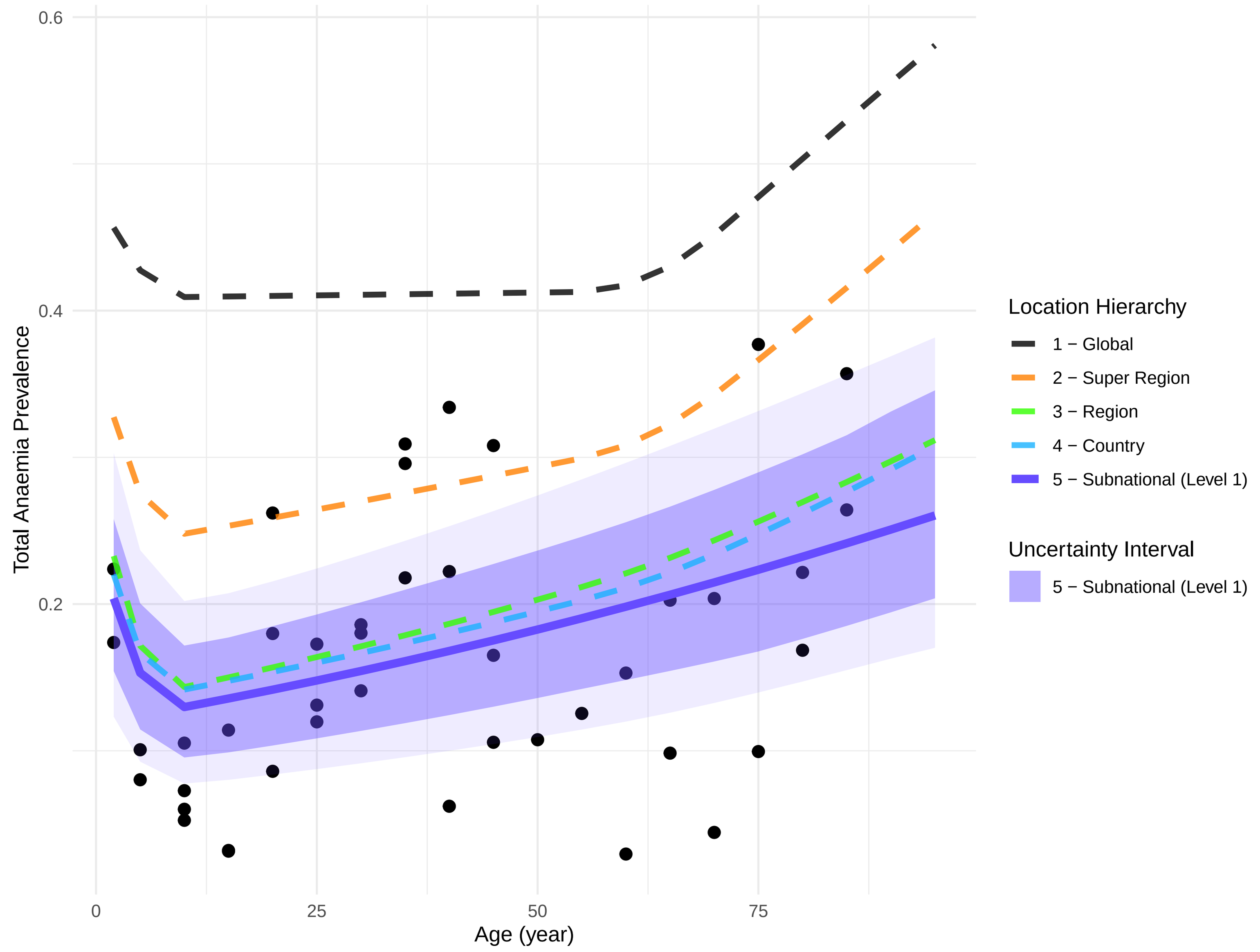

MR−BRT Age/Sex Fits − Non−pregnant Females 2+ Years Old − Total Anaemia Prevalence − Guerrero
WHO−2001 Adjustment Method

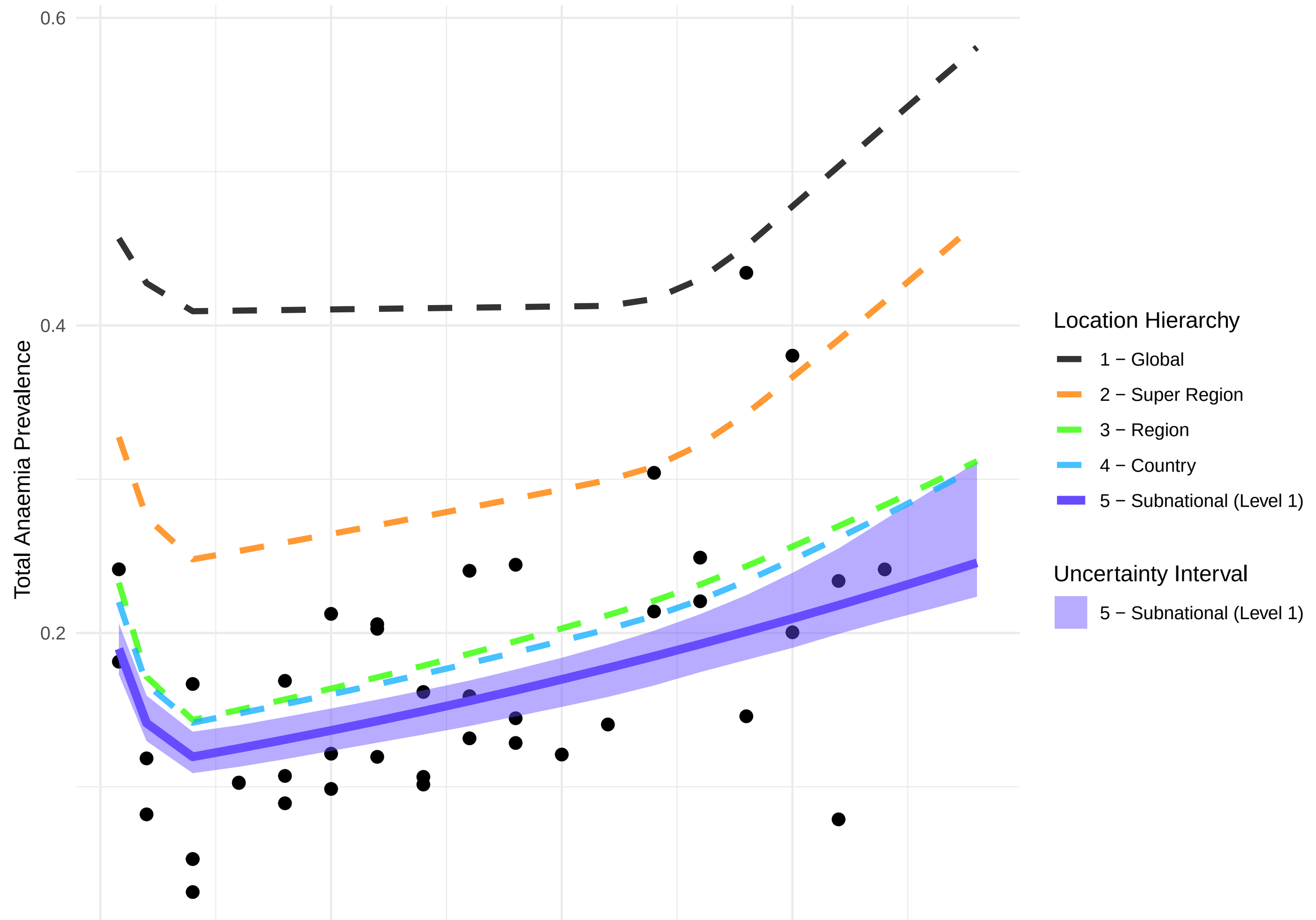

MR−BRT Age/Sex Fits − Non−pregnant Females 2+ Years Old − Total Anaemia Prevalence − Hidalgo
WHO−2001 Adjustment Method

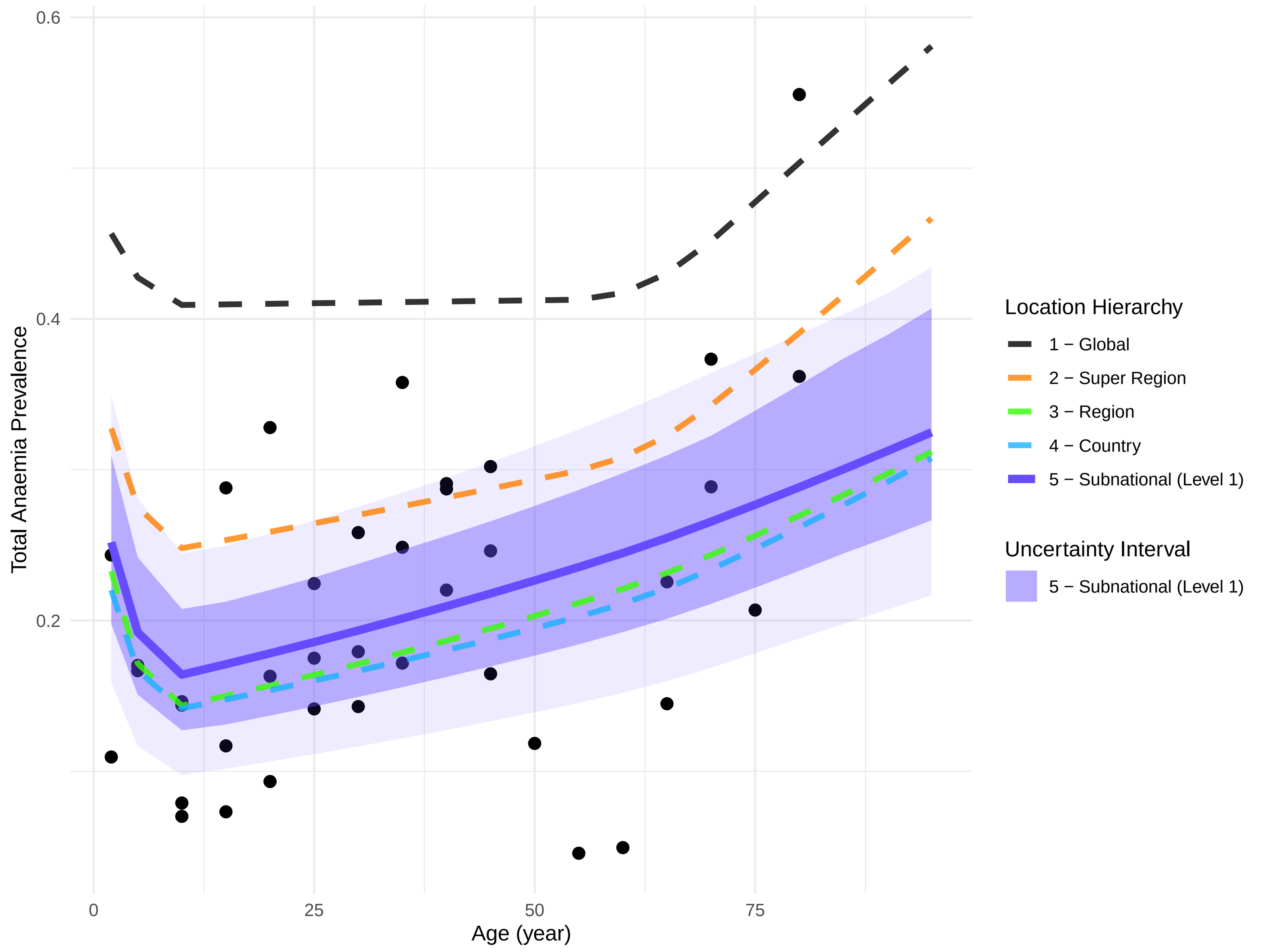

MR−BRT Age/Sex Fits − Non−pregnant Females 2+ Years Old − Total Anaemia Prevalence − Jalisco
WHO−2001 Adjustment Method

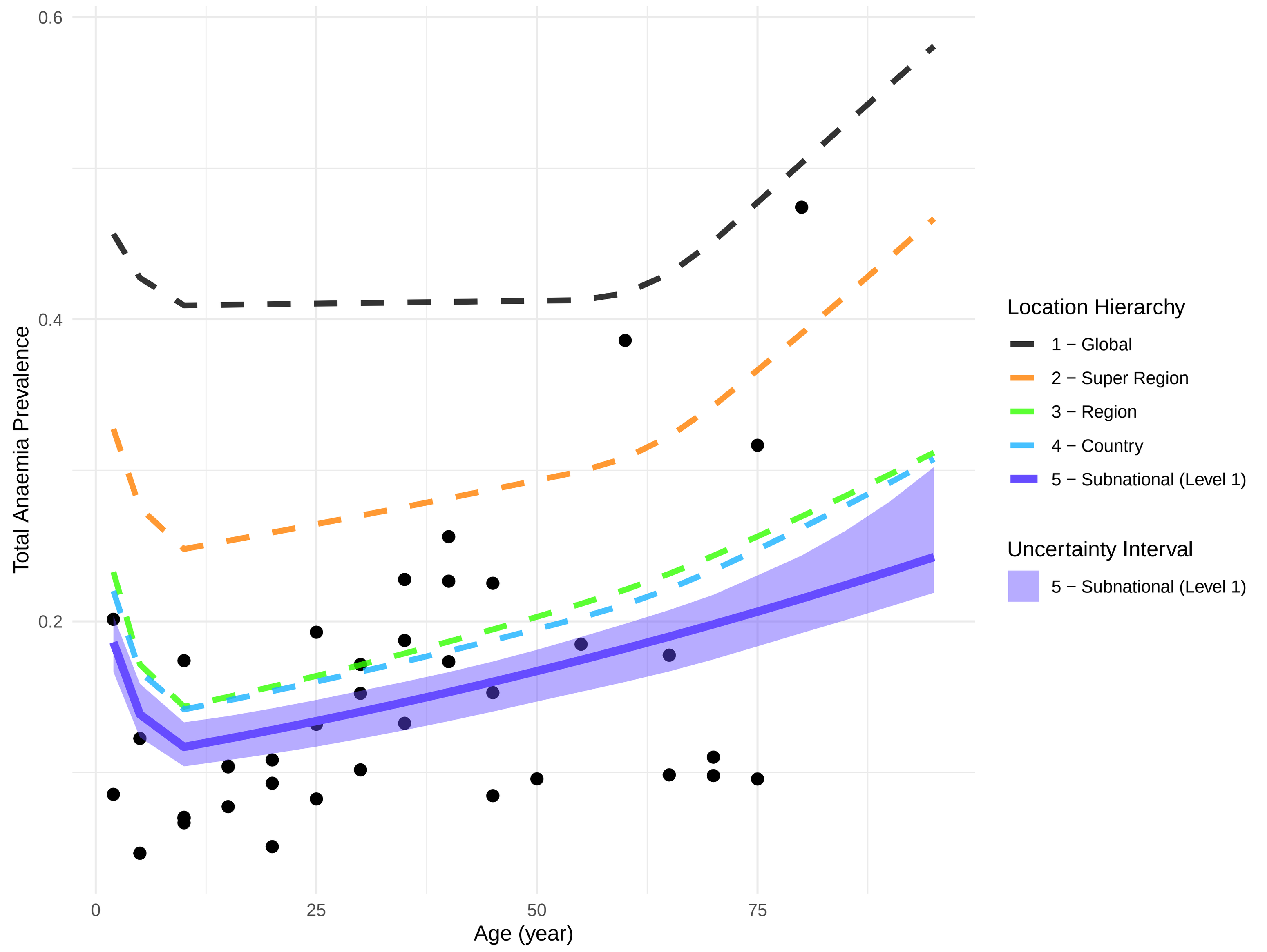

MR−BRT Age/Sex Fits − Non−pregnant Females 2+ Years Old − Total Anaemia Prevalence − México
WHO−2001 Adjustment Method

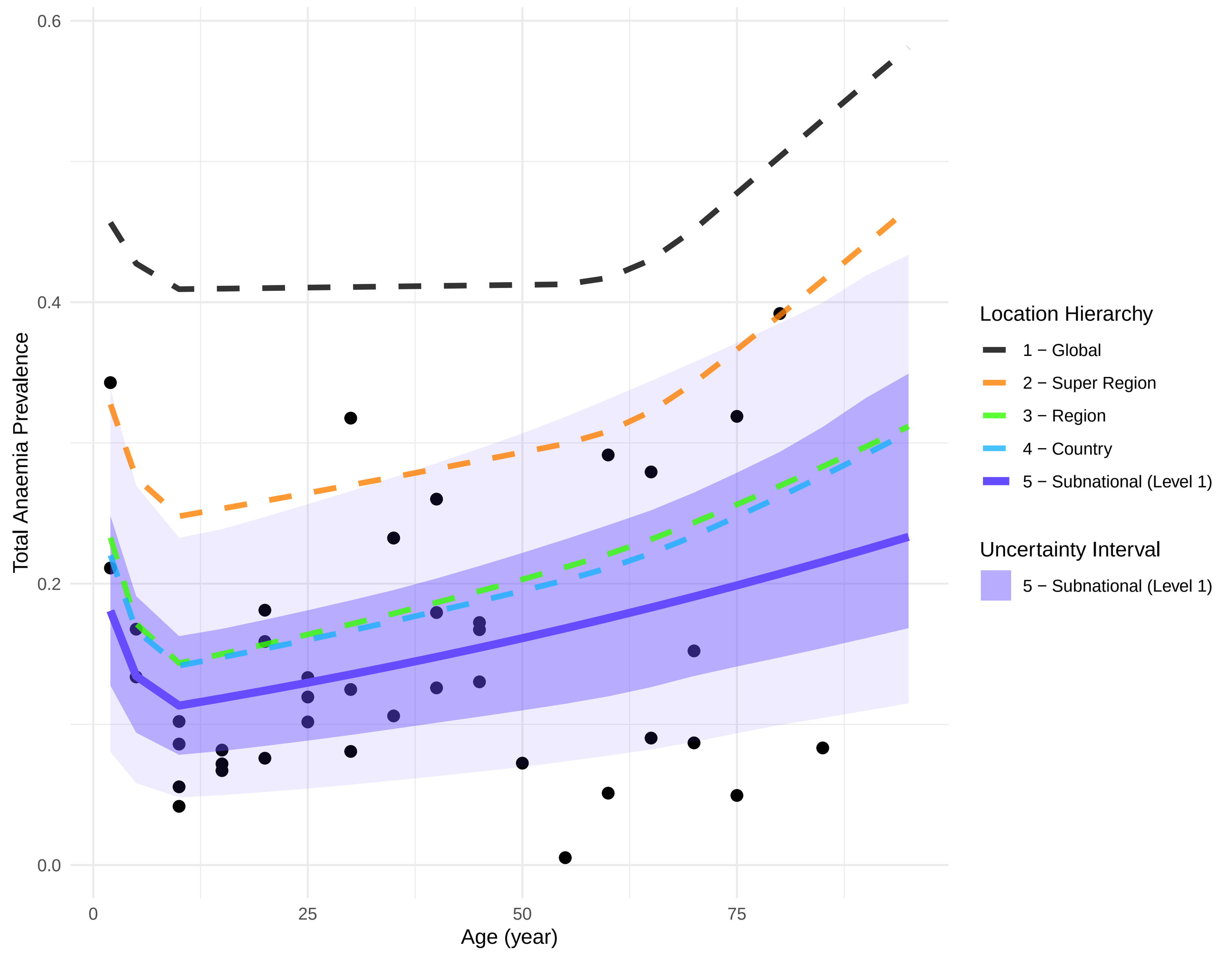

MR−BRT Age/Sex Fits − Non−pregnant Females 2+ Years Old − Total Anaemia Prevalence − Mexico City
WHO−2001 Adjustment Method

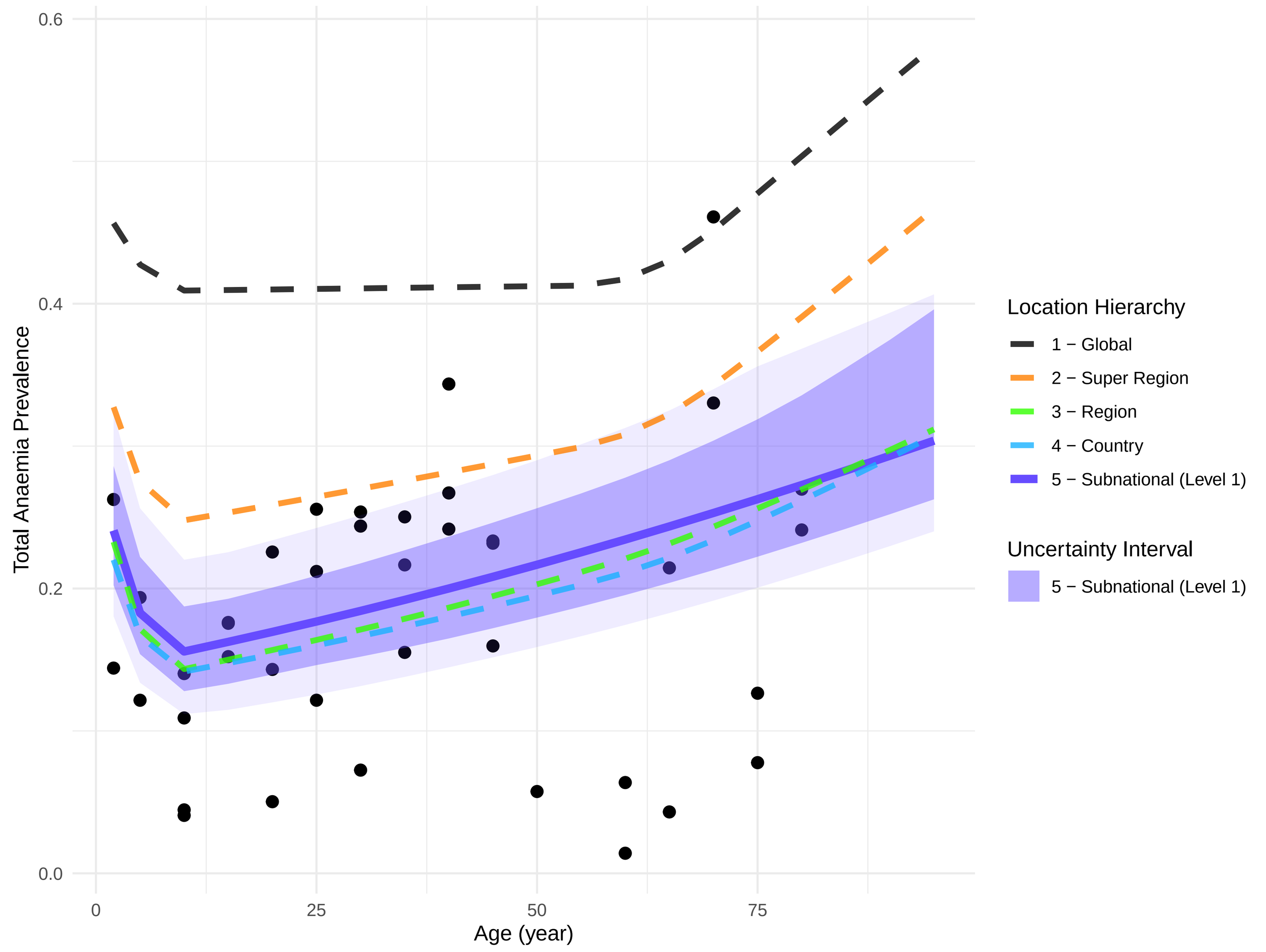

MR−BRT Age/Sex Fits − Non−pregnant Females 2+ Years Old − Total Anaemia Prevalence − Michoacán de Ocampo
WHO−2001 Adjustment Method

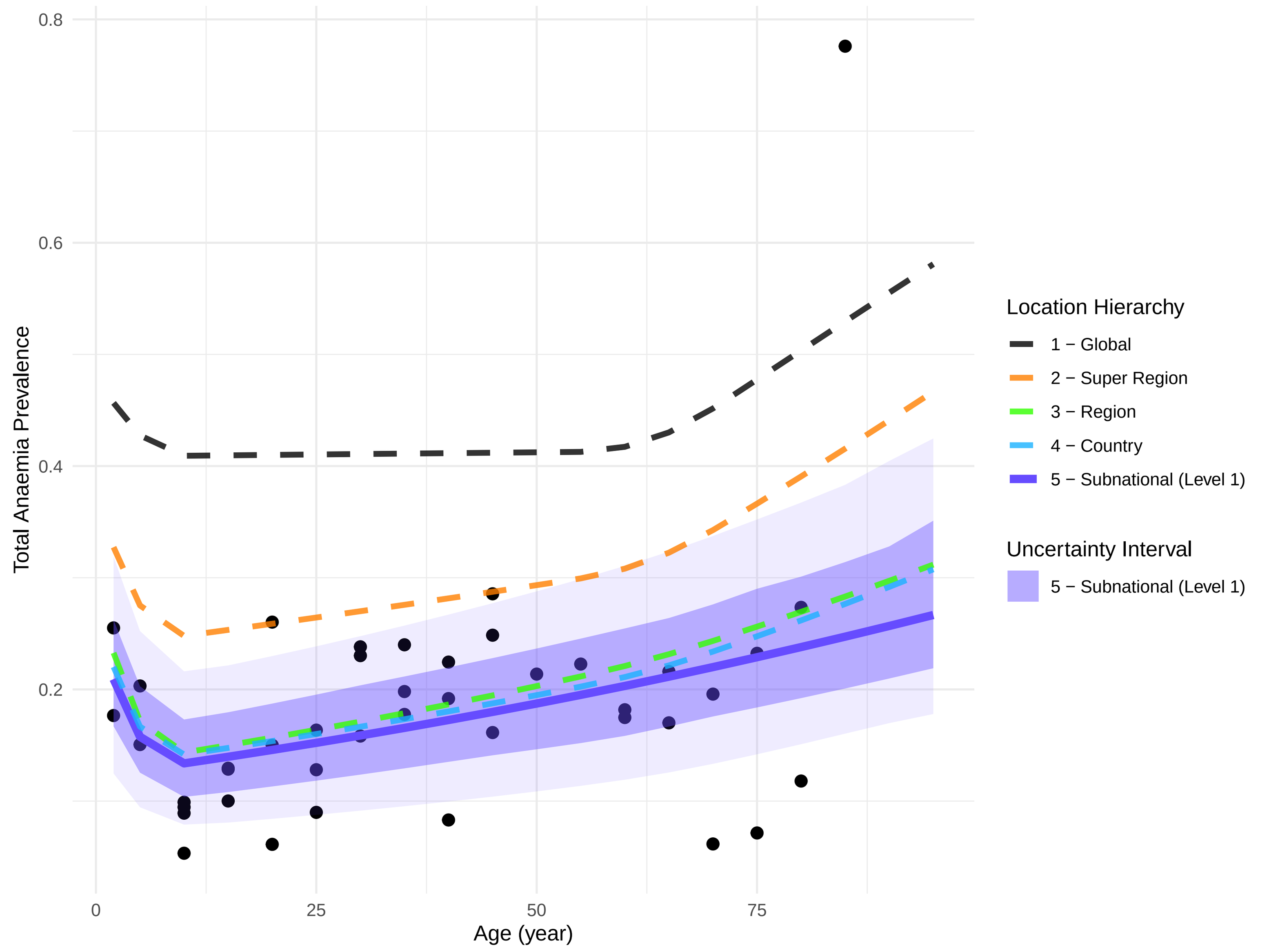

MR−BRT Age/Sex Fits − Non−pregnant Females 2+ Years Old − Total Anaemia Prevalence − Morelos
WHO−2001 Adjustment Method

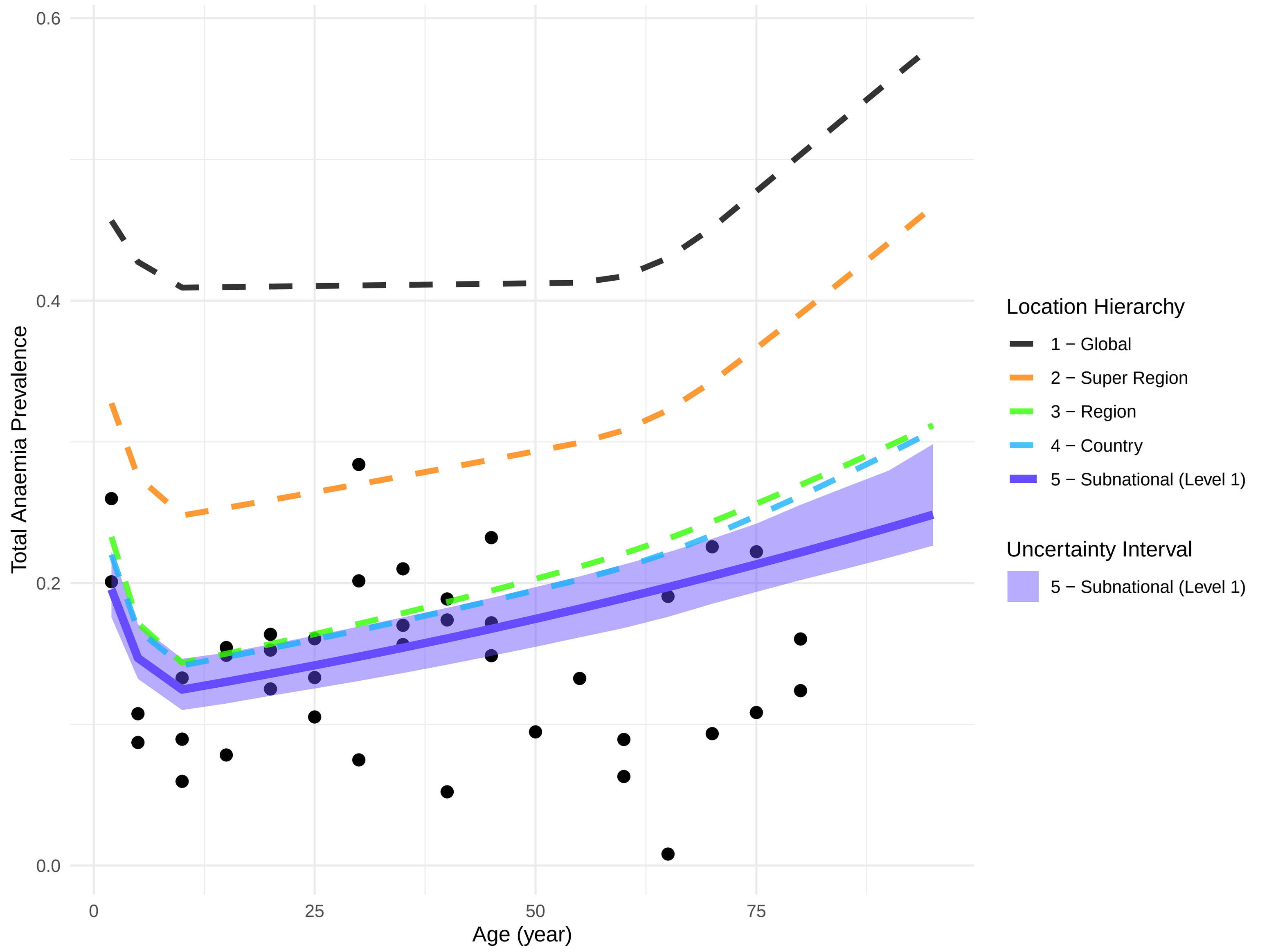

MR−BRT Age/Sex Fits − Non−pregnant Females 2+ Years Old − Total Anaemia Prevalence − Nayarit
WHO−2001 Adjustment Method

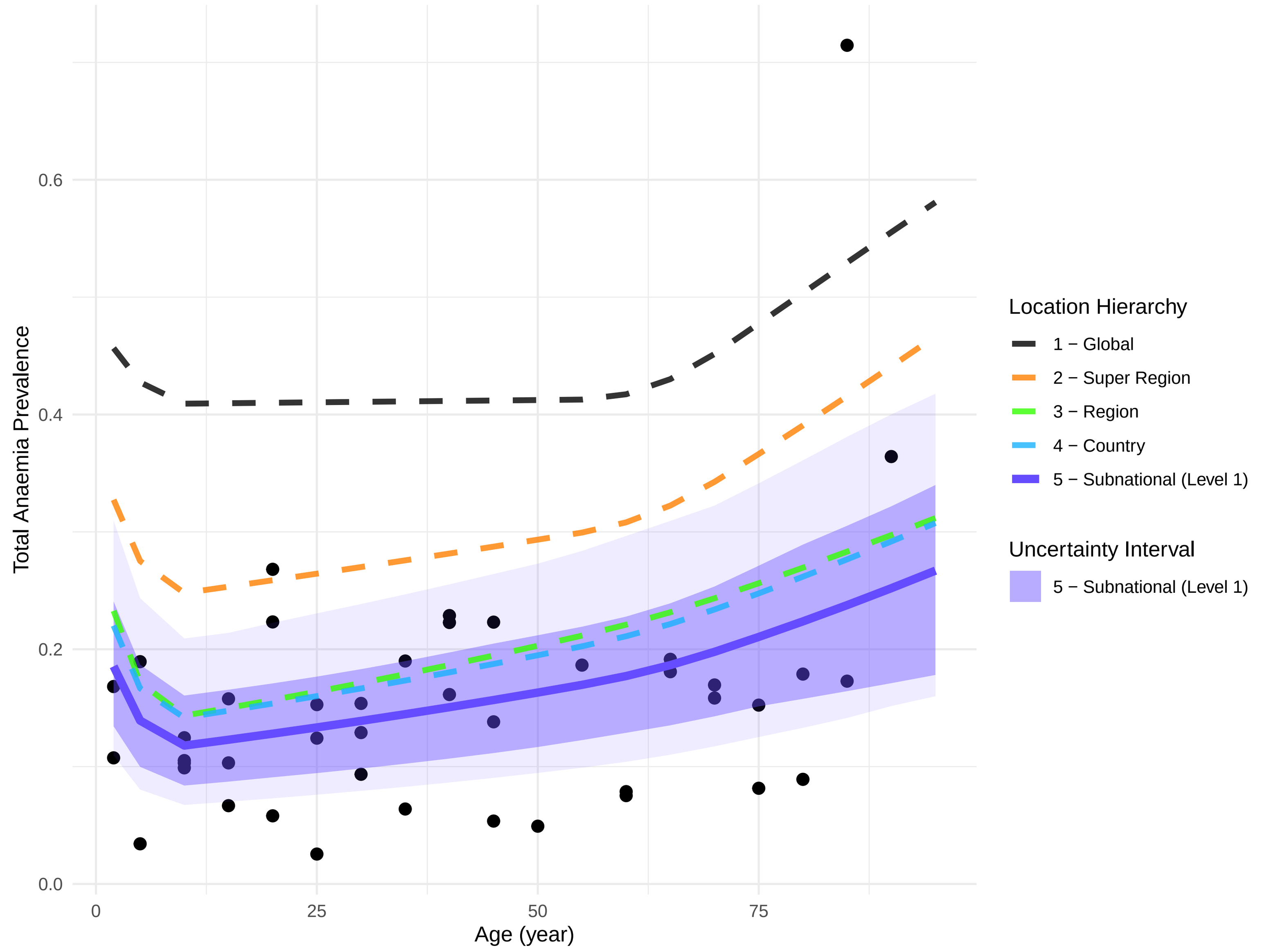

MR−BRT Age/Sex Fits − Non−pregnant Females 2+ Years Old − Total Anaemia Prevalence − Nuevo León
WHO−2001 Adjustment Method

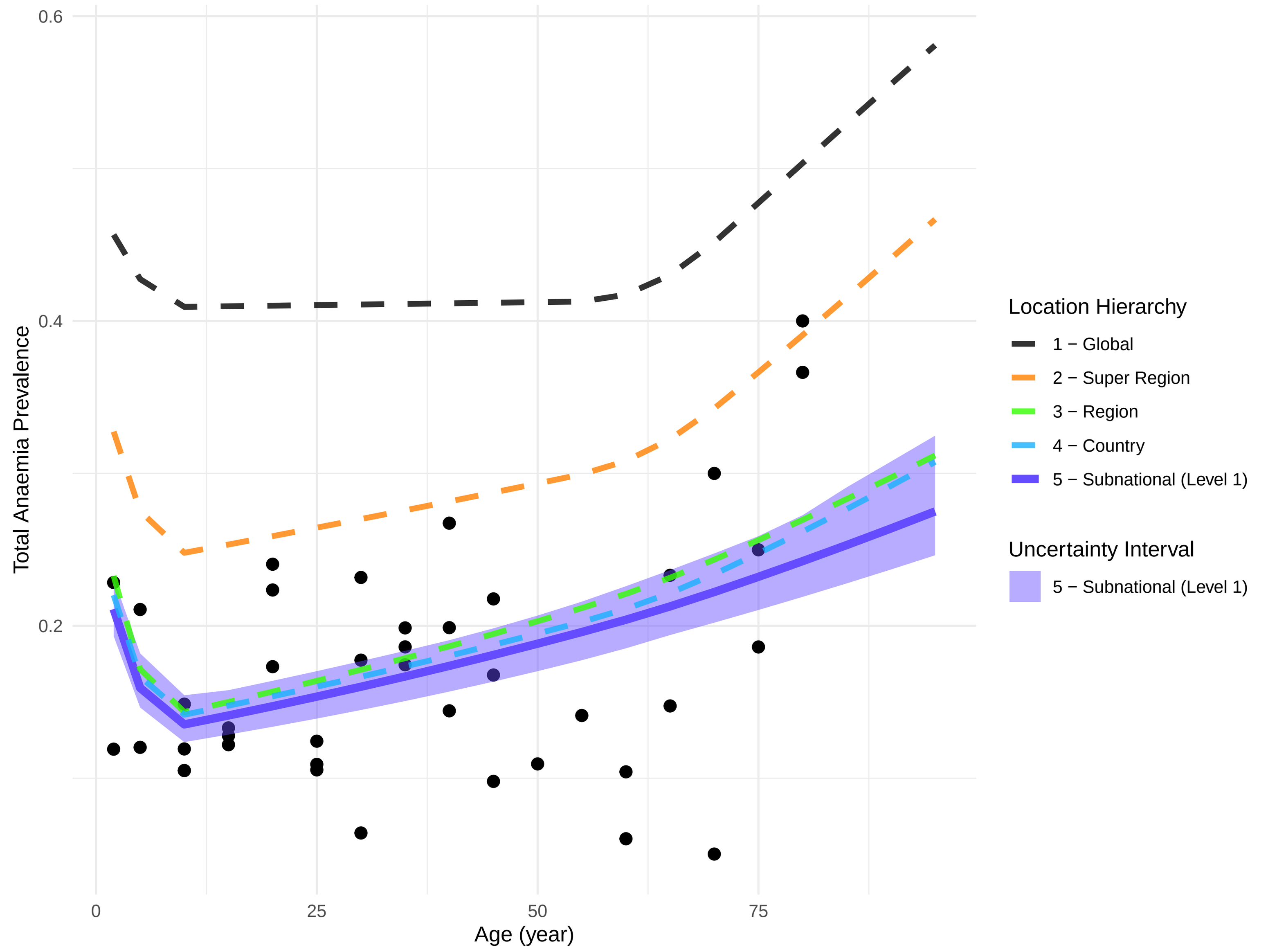

MR−BRT Age/Sex Fits − Non−pregnant Females 2+ Years Old − Total Anaemia Prevalence − Oaxaca
WHO−2001 Adjustment Method

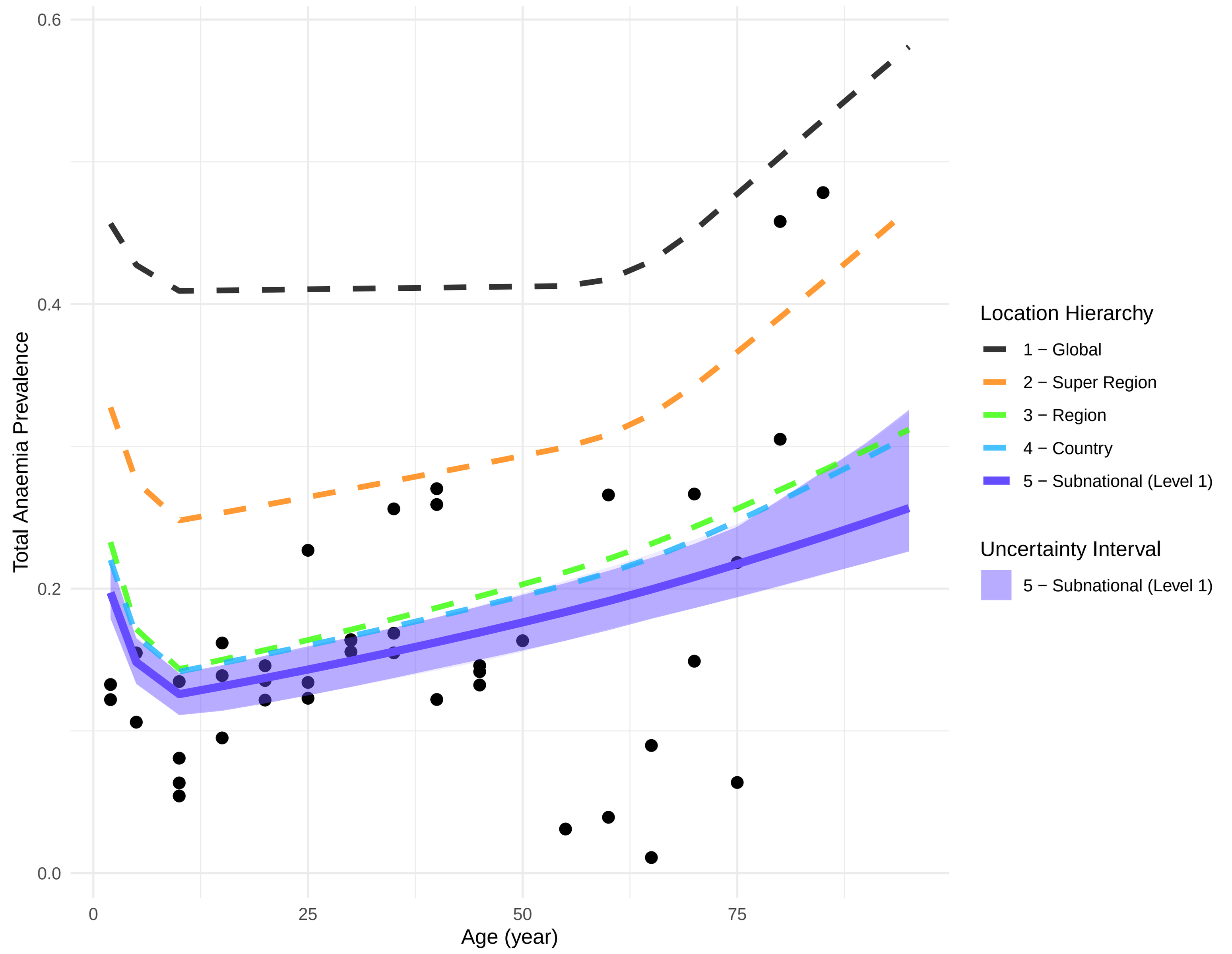

MR−BRT Age/Sex Fits − Non−pregnant Females 2+ Years Old − Total Anaemia Prevalence − Puebla
WHO−2001 Adjustment Method

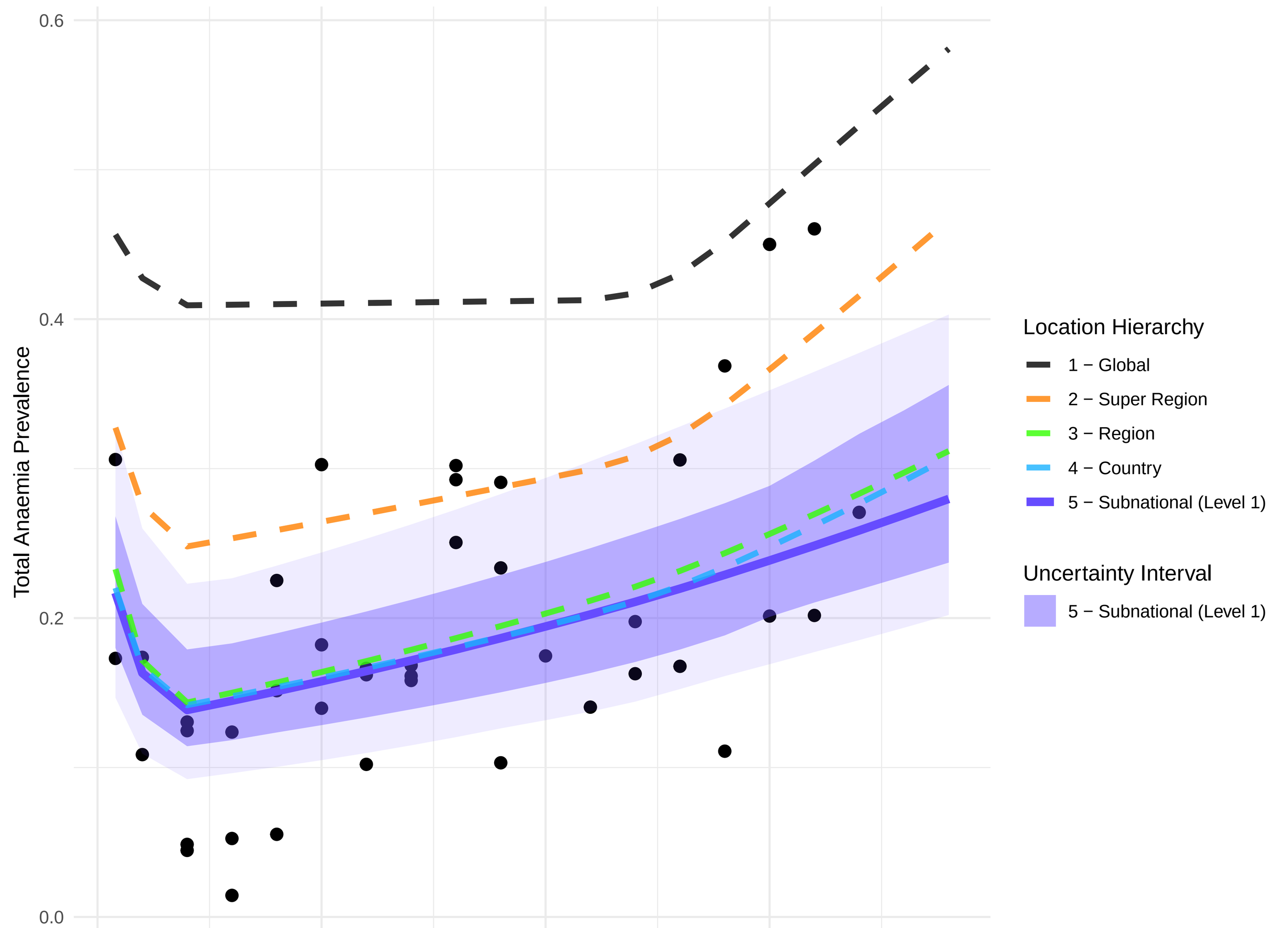

MR−BRT Age/Sex Fits − Non−pregnant Females 2+ Years Old − Total Anaemia Prevalence − Querétaro
WHO−2001 Adjustment Method

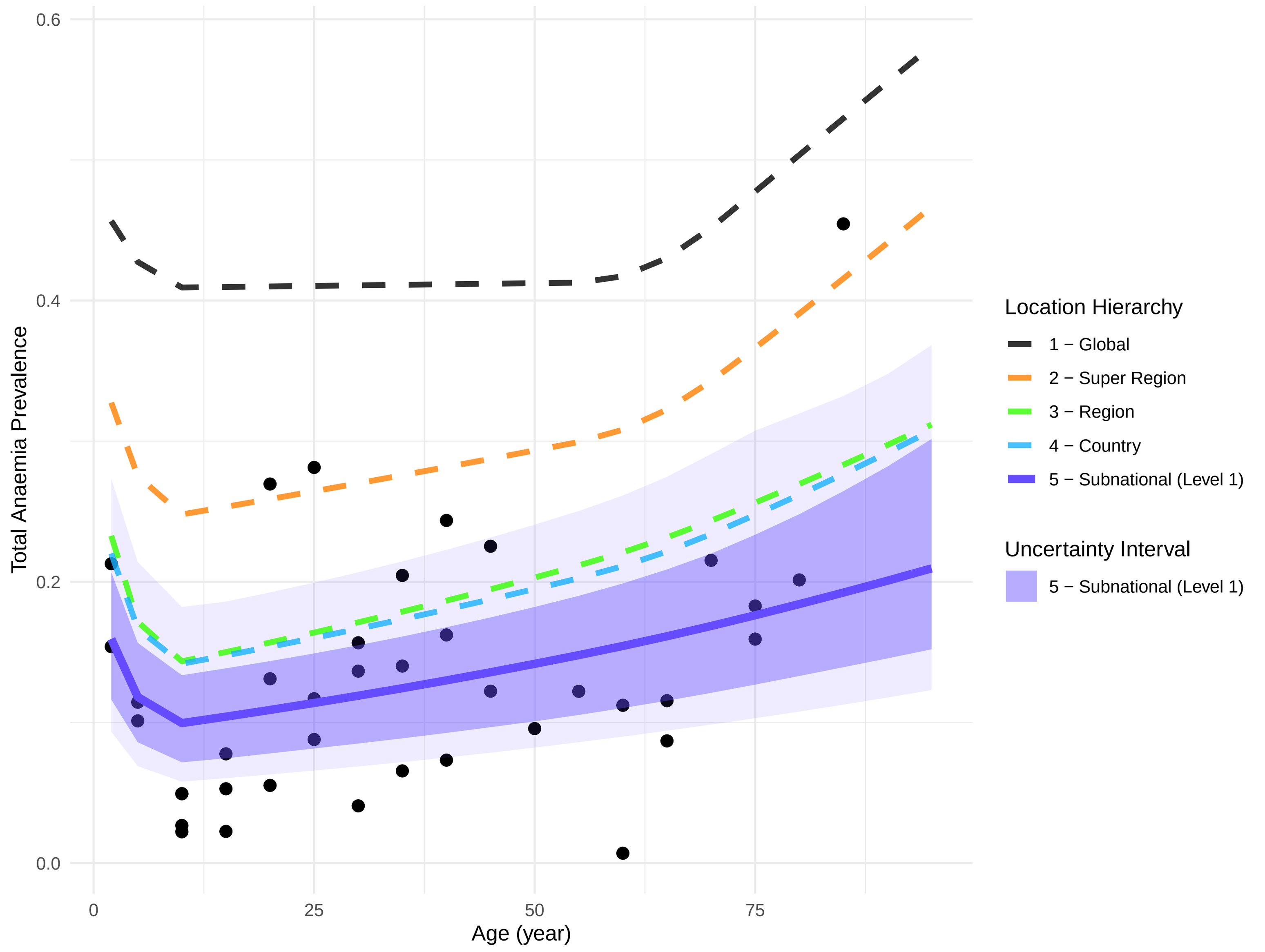

MR−BRT Age/Sex Fits − Non−pregnant Females 2+ Years Old − Total Anaemia Prevalence − Quintana Roo
WHO−2001 Adjustment Method

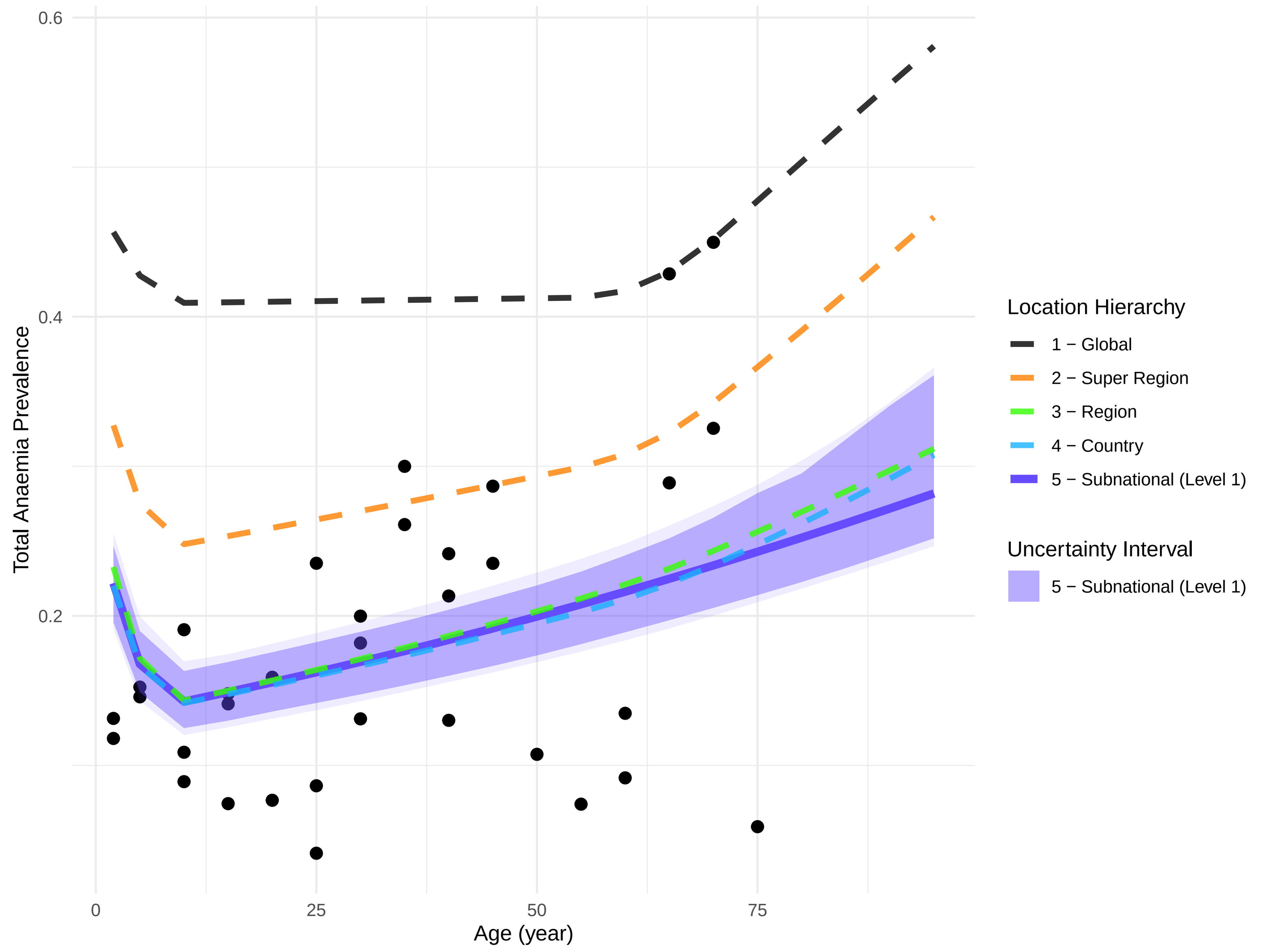

MR−BRT Age/Sex Fits − Non−pregnant Females 2+ Years Old − Total Anaemia Prevalence − San Luis Potosí
WHO−2001 Adjustment Method

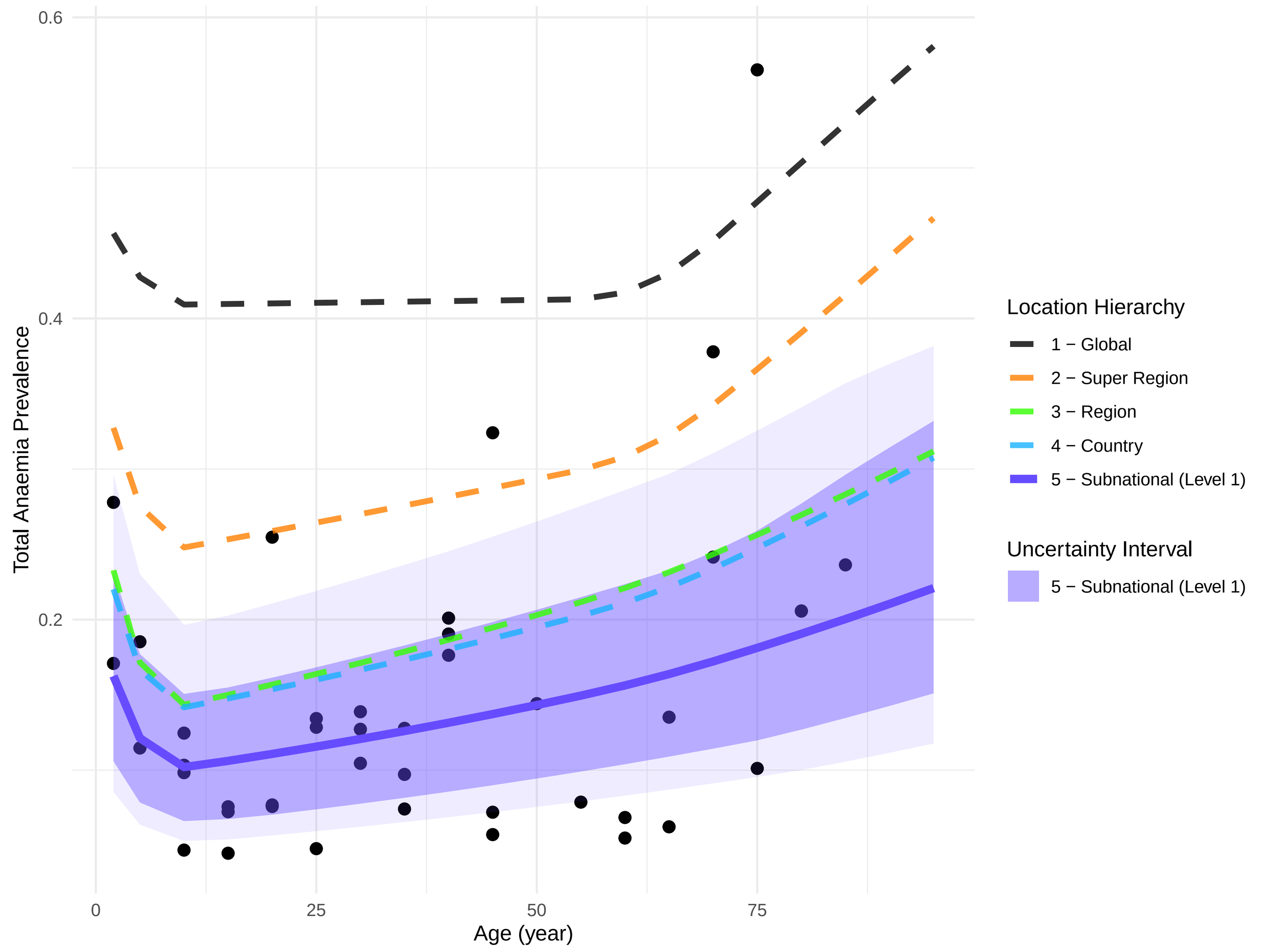

MR−BRT Age/Sex Fits − Non−pregnant Females 2+ Years Old − Total Anaemia Prevalence − Sinaloa
WHO−2001 Adjustment Method

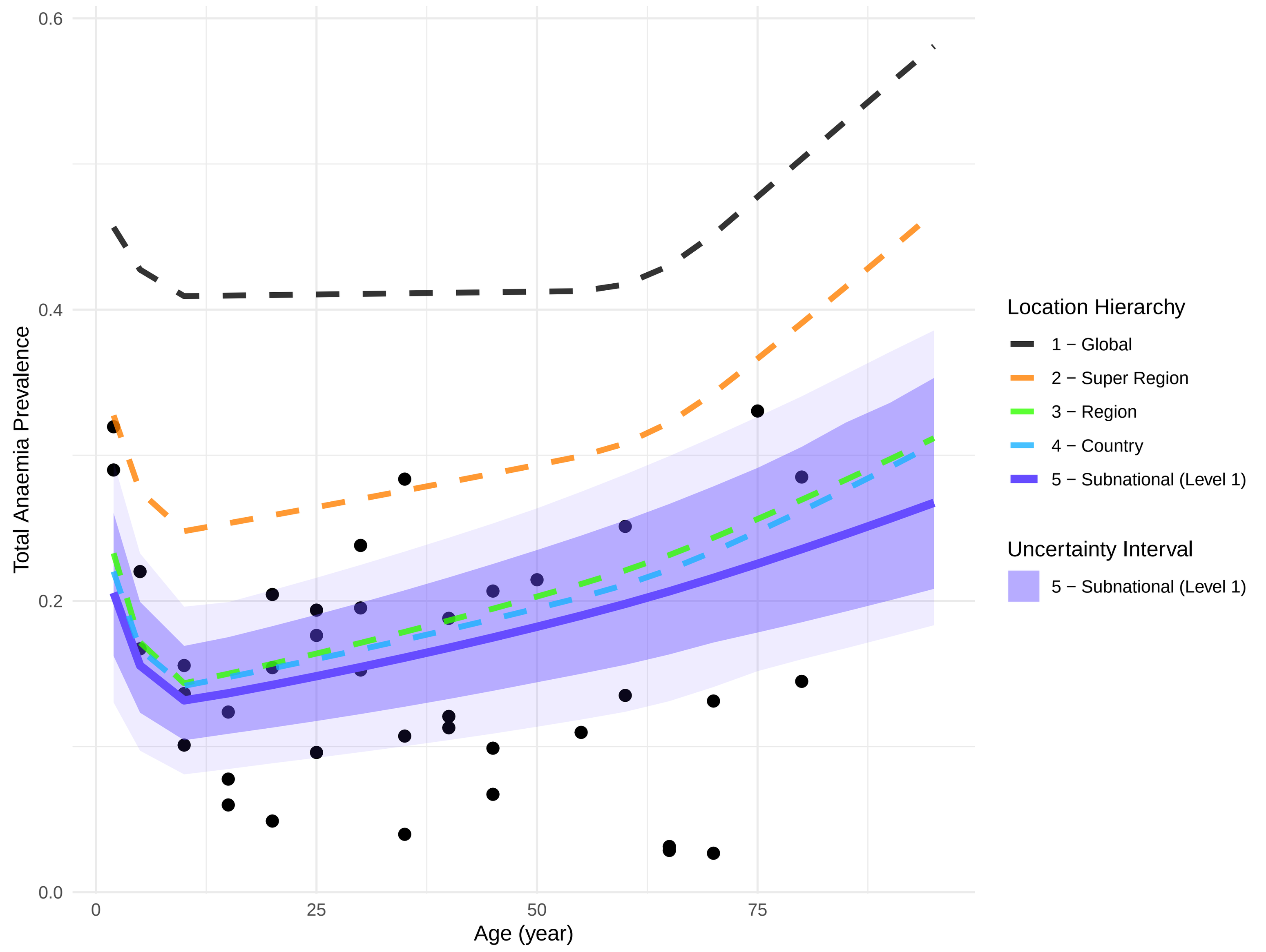

MR−BRT Age/Sex Fits − Non−pregnant Females 2+ Years Old − Total Anaemia Prevalence − Sonora
WHO−2001 Adjustment Method

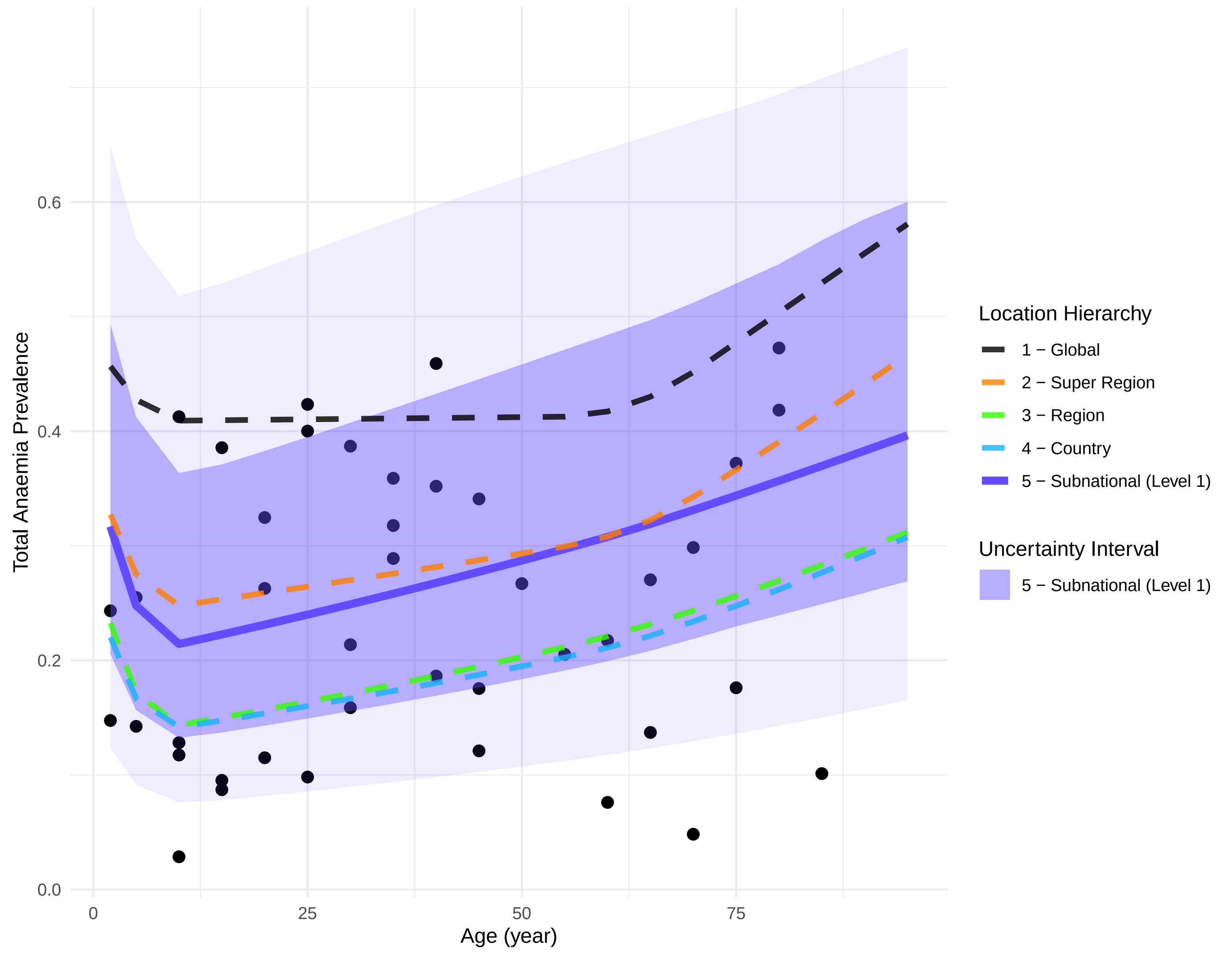

MR−BRT Age/Sex Fits − Non−pregnant Females 2+ Years Old − Total Anaemia Prevalence − Tabasco
WHO−2001 Adjustment Method

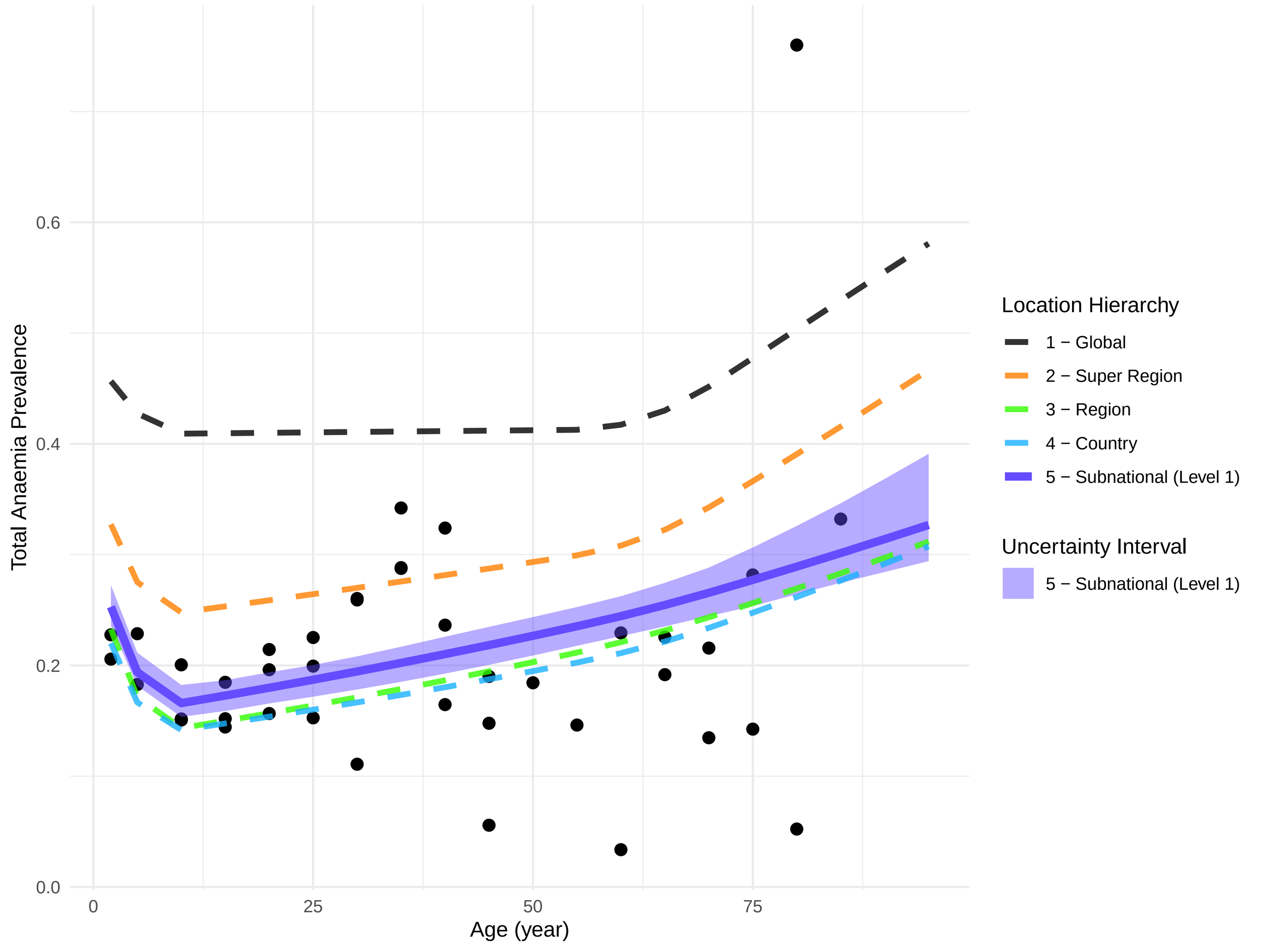

MR−BRT Age/Sex Fits − Non−pregnant Females 2+ Years Old − Total Anaemia Prevalence − Tamaulipas
WHO−2001 Adjustment Method

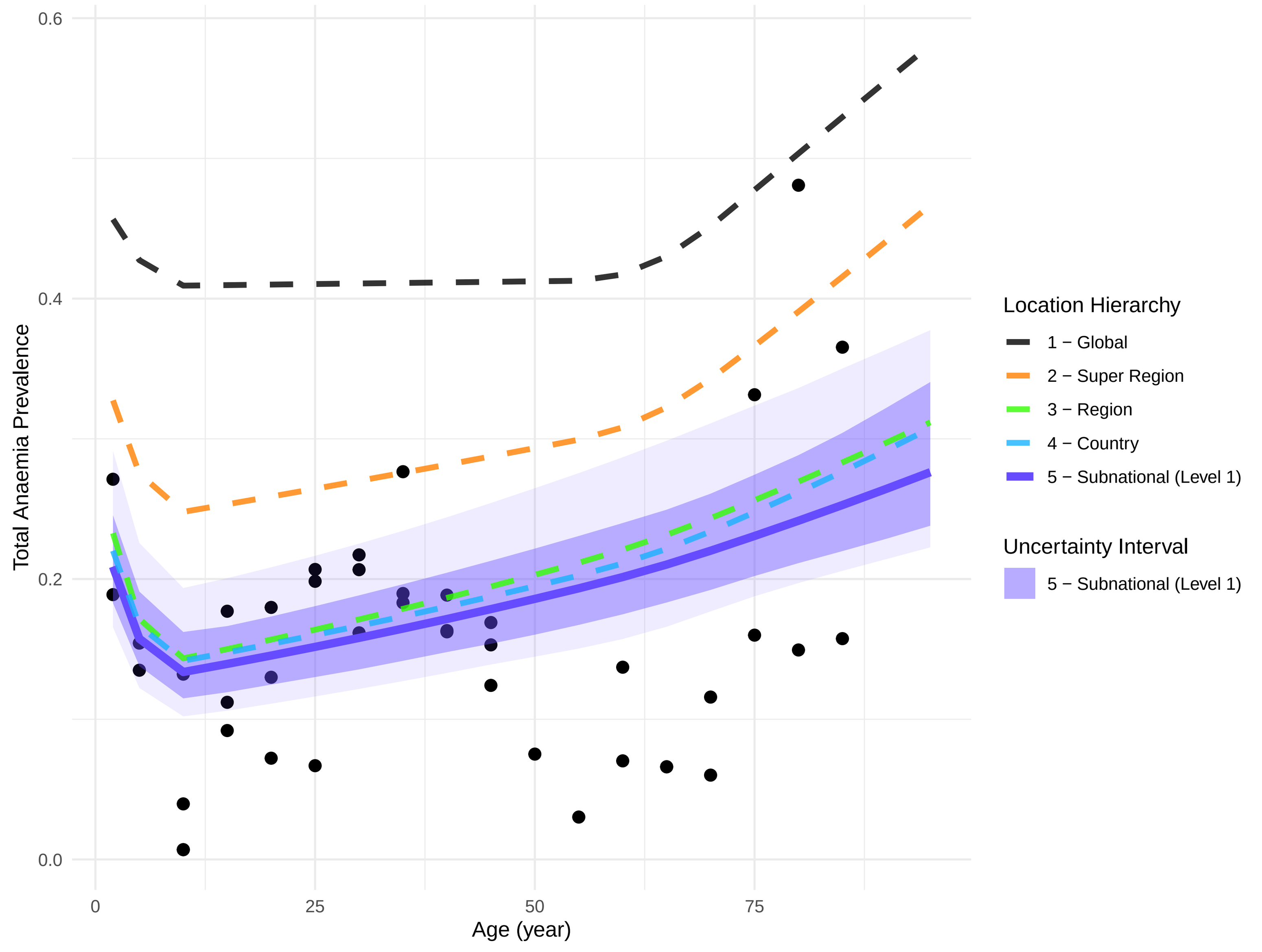

MR−BRT Age/Sex Fits − Non−pregnant Females 2+ Years Old − Total Anaemia Prevalence − Tlaxcala
WHO−2001 Adjustment Method

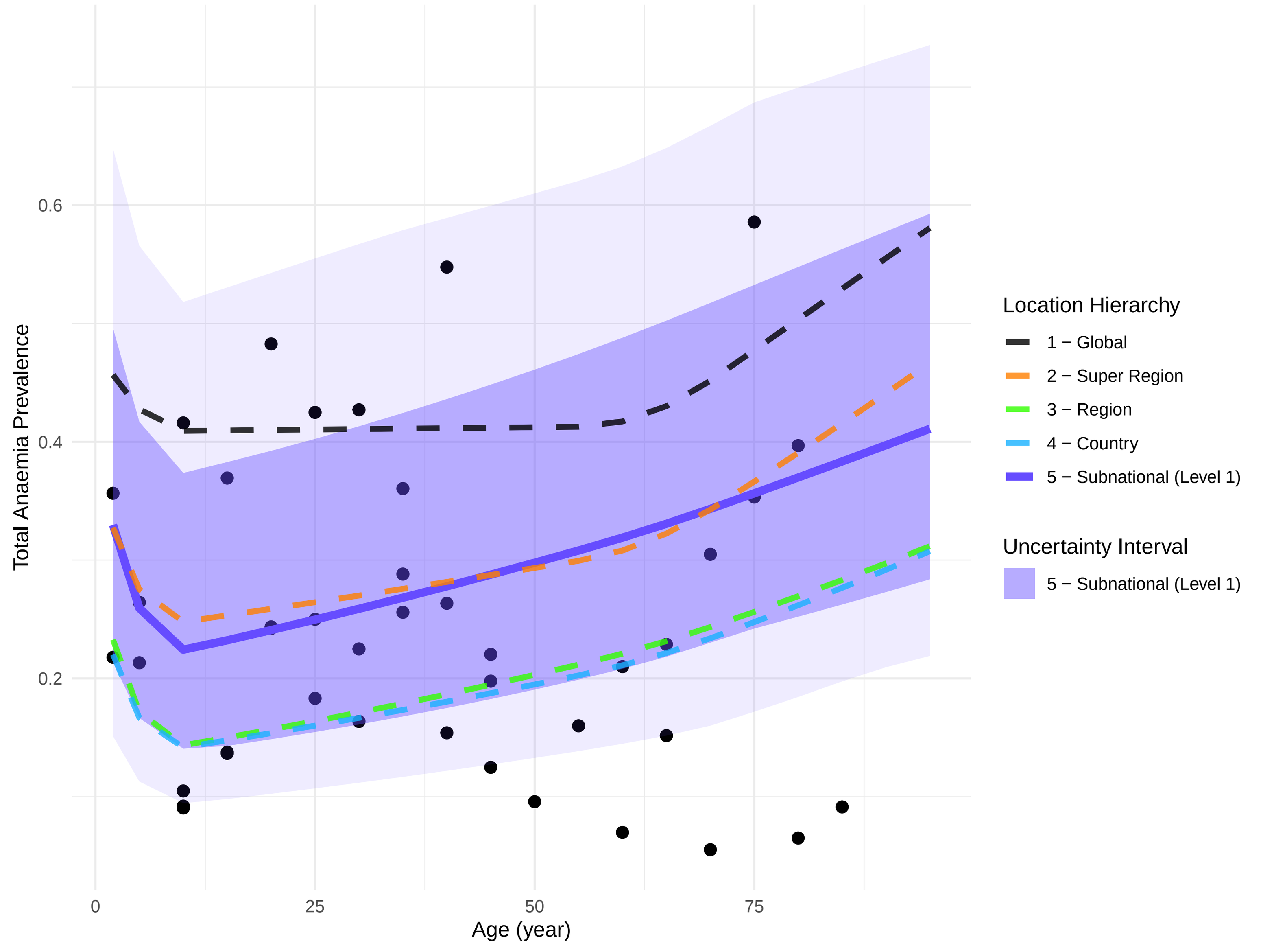

MR−BRT Age/Sex Fits − Non−pregnant Females 2+ Years Old − Total Anaemia Prevalence − Veracruz de Ignacio de la Llave
WHO−2001 Adjustment Method

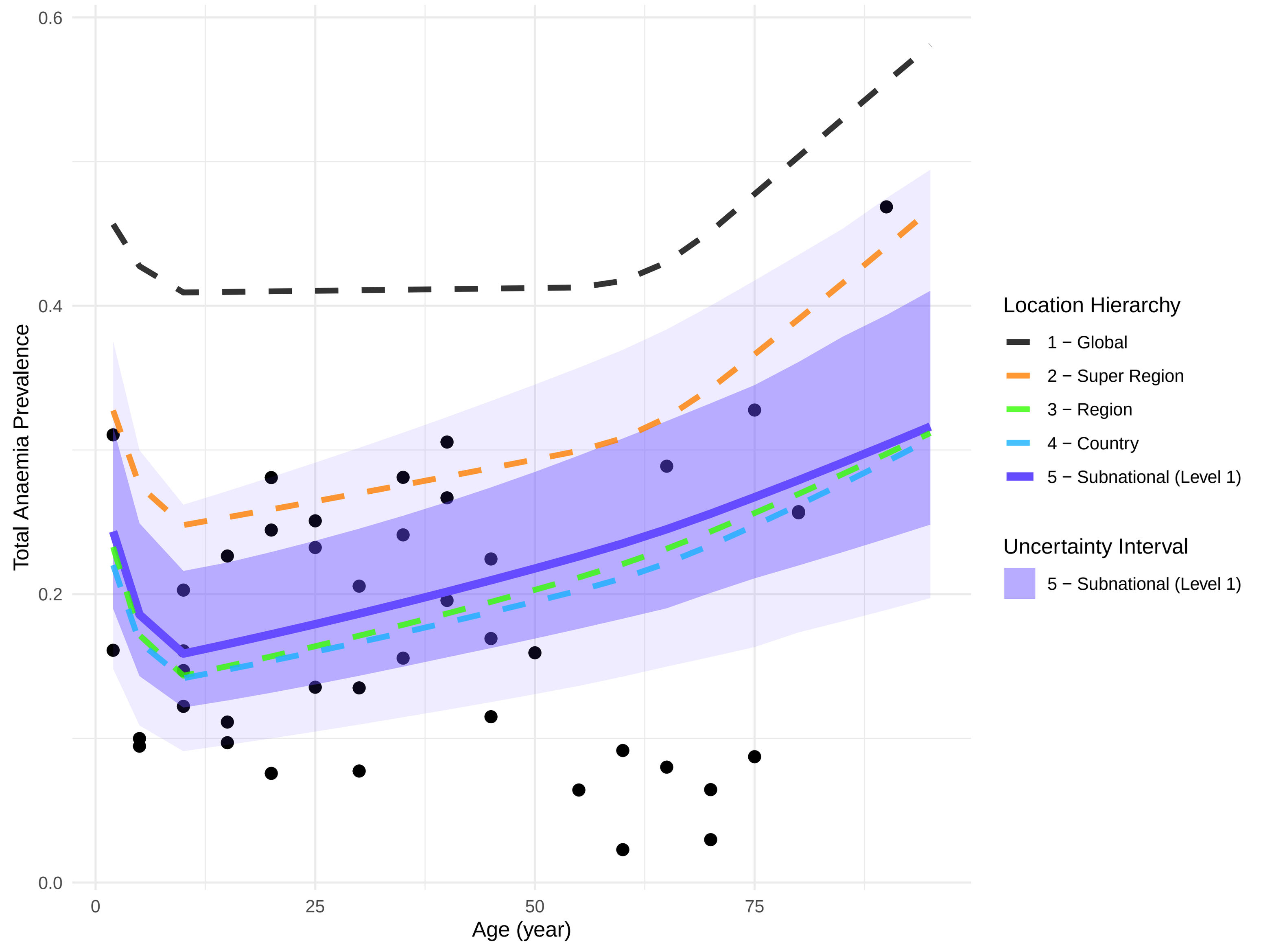

MR−BRT Age/Sex Fits − Non−pregnant Females 2+ Years Old − Total Anaemia Prevalence − Yucatán
WHO−2001 Adjustment Method

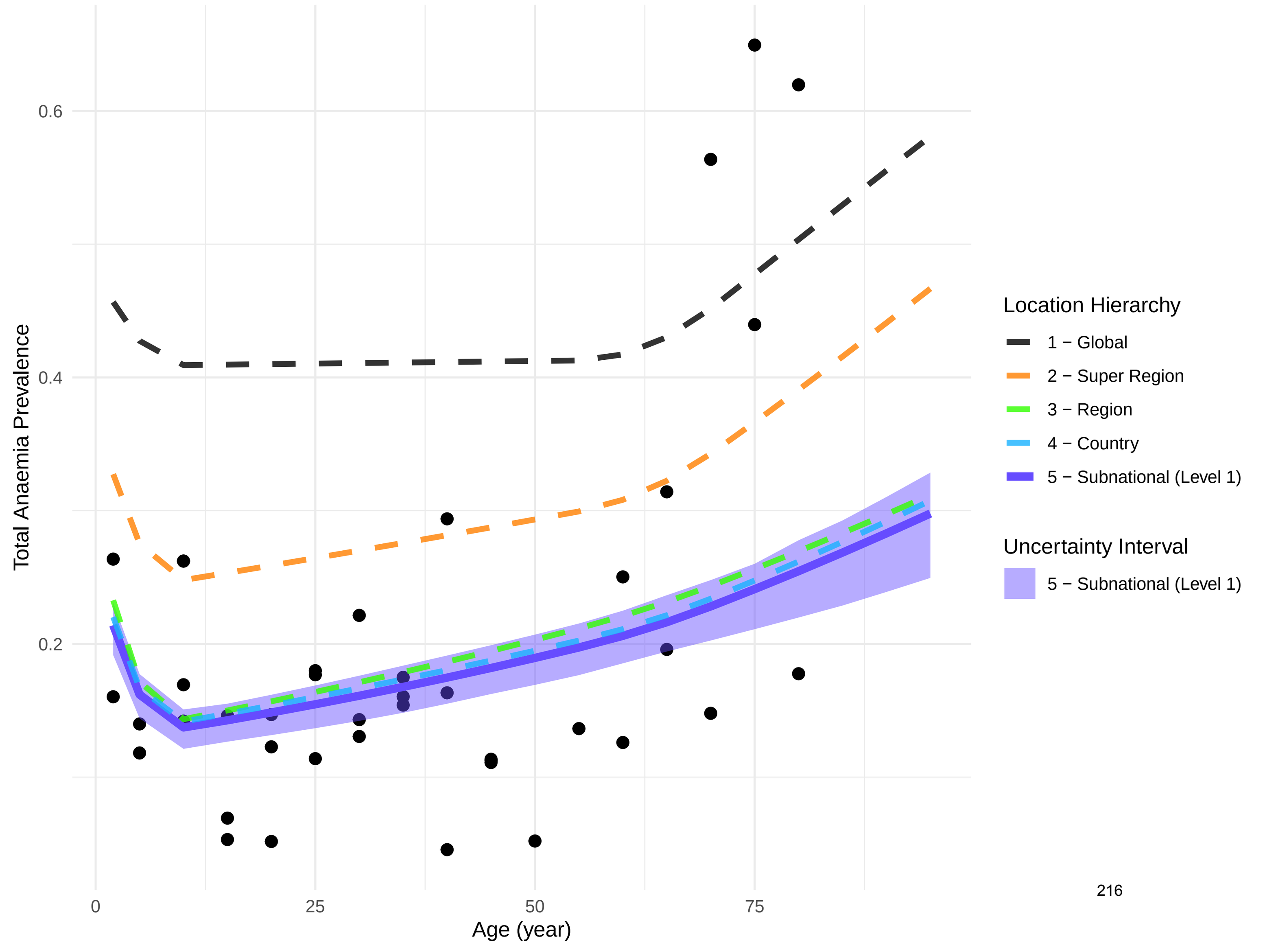

MR−BRT Age/Sex Fits − Non−pregnant Females 2+ Years Old − Total Anaemia Prevalence − Zacatecas
WHO−2001 Adjustment Method

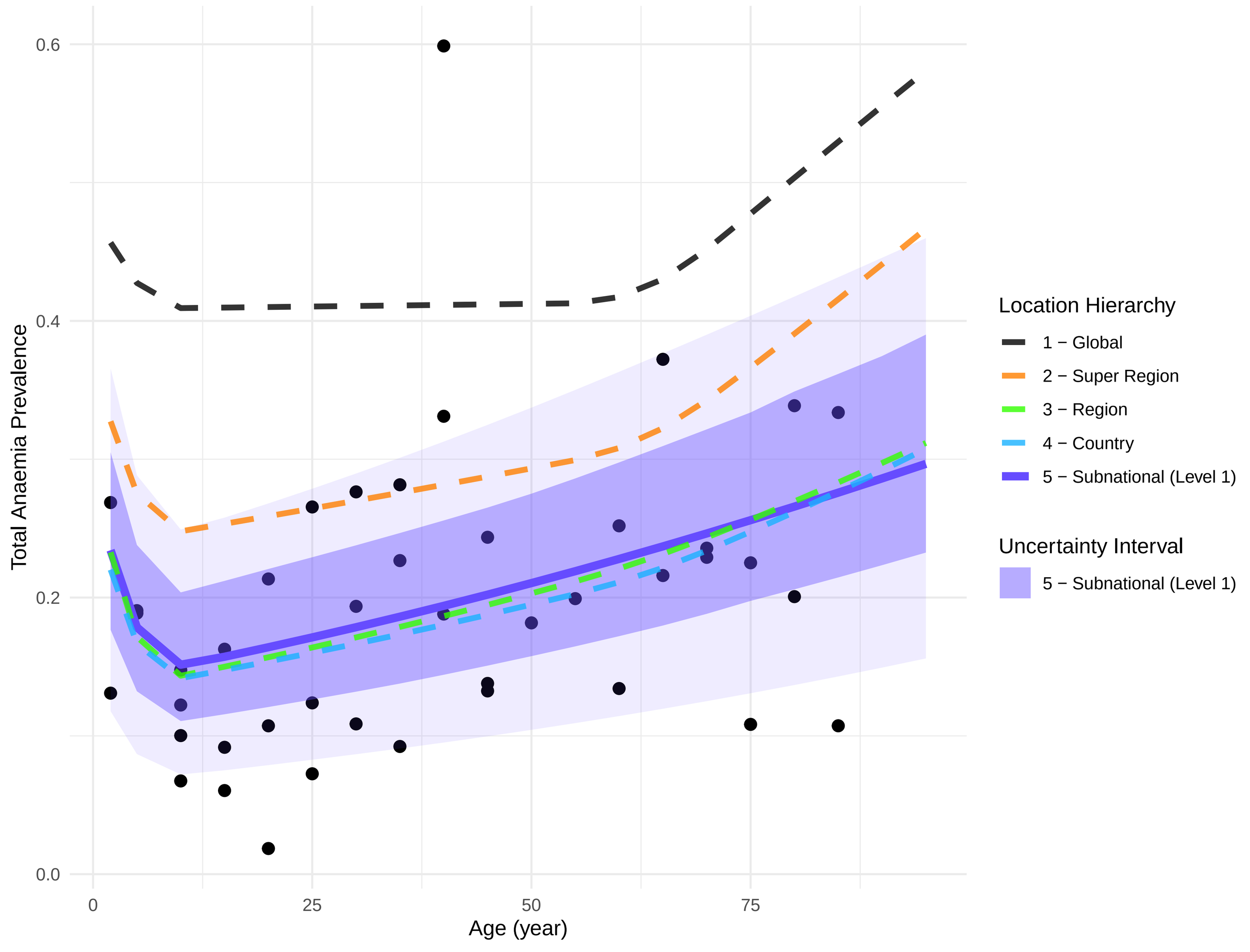

MR−BRT Age/Sex Fits − Non−pregnant Females 2+ Years Old − Total Anaemia Prevalence − Tropical Latin America
WHO−2001 Adjustment Method

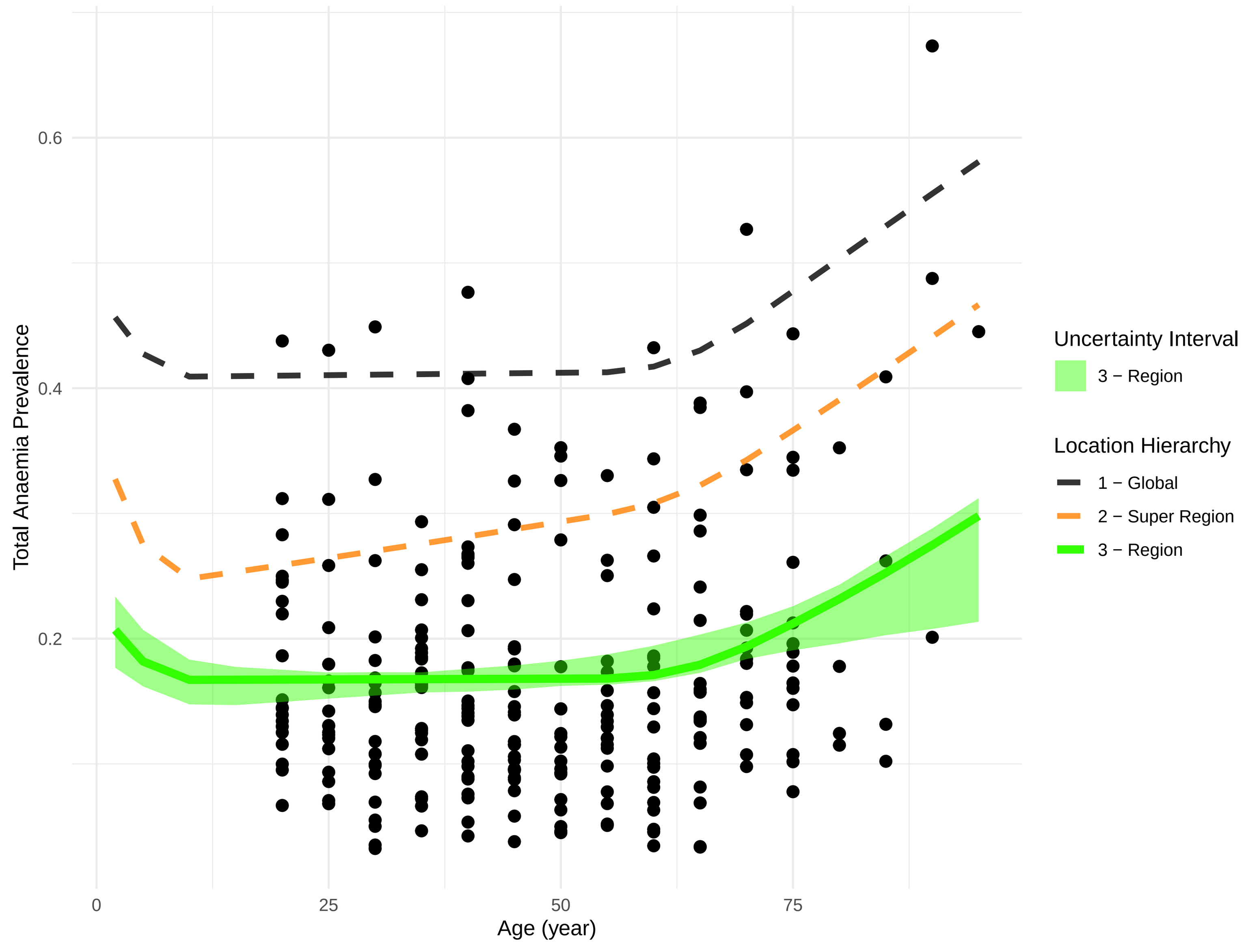

MR−BRT Age/Sex Fits − Non−pregnant Females 2+ Years Old − Total Anaemia Prevalence − Brazil
WHO−2001 Adjustment Method

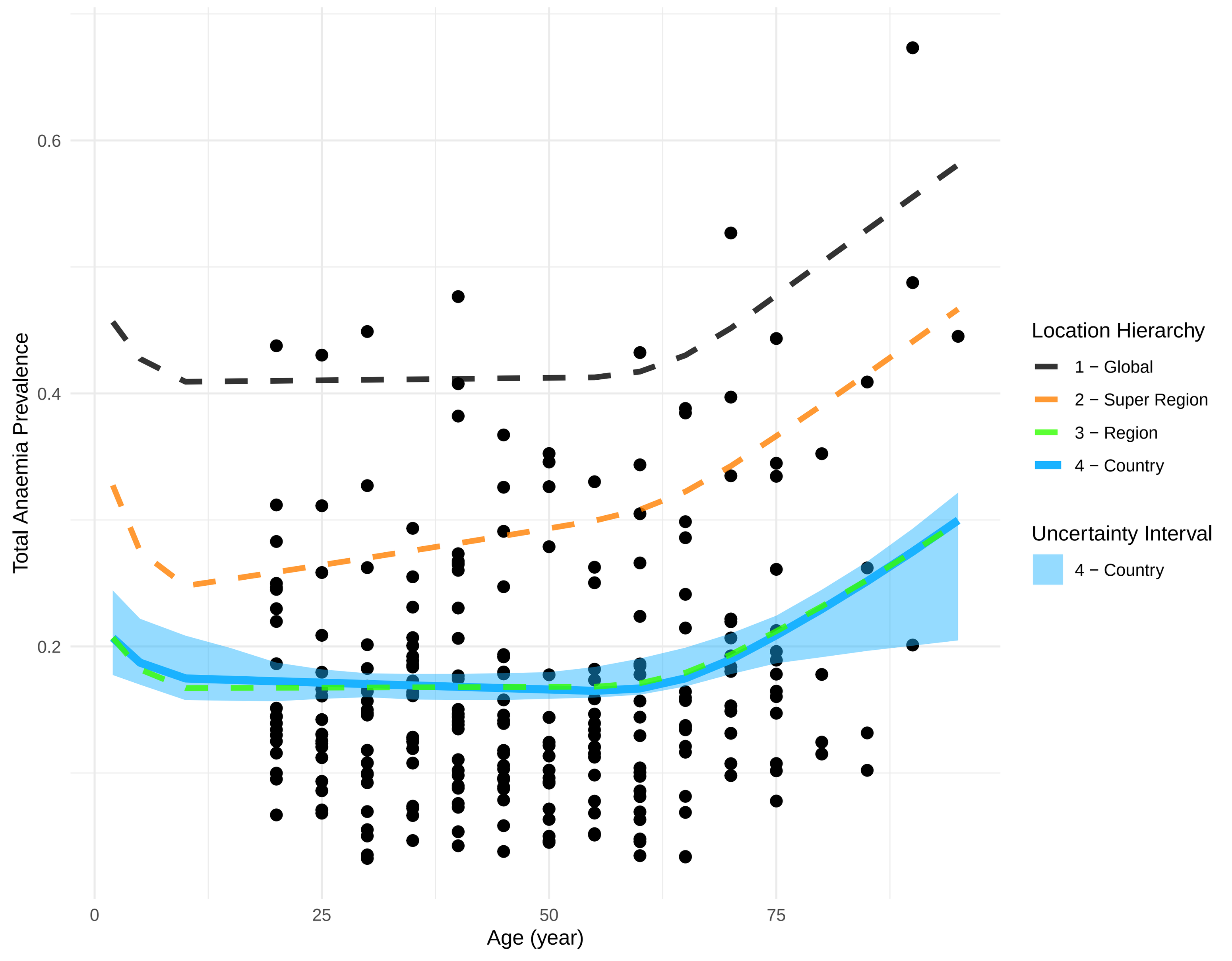

MR−BRT Age/Sex Fits − Non−pregnant Females 2+ Years Old − Total Anaemia Prevalence − Acre
WHO−2001 Adjustment Method

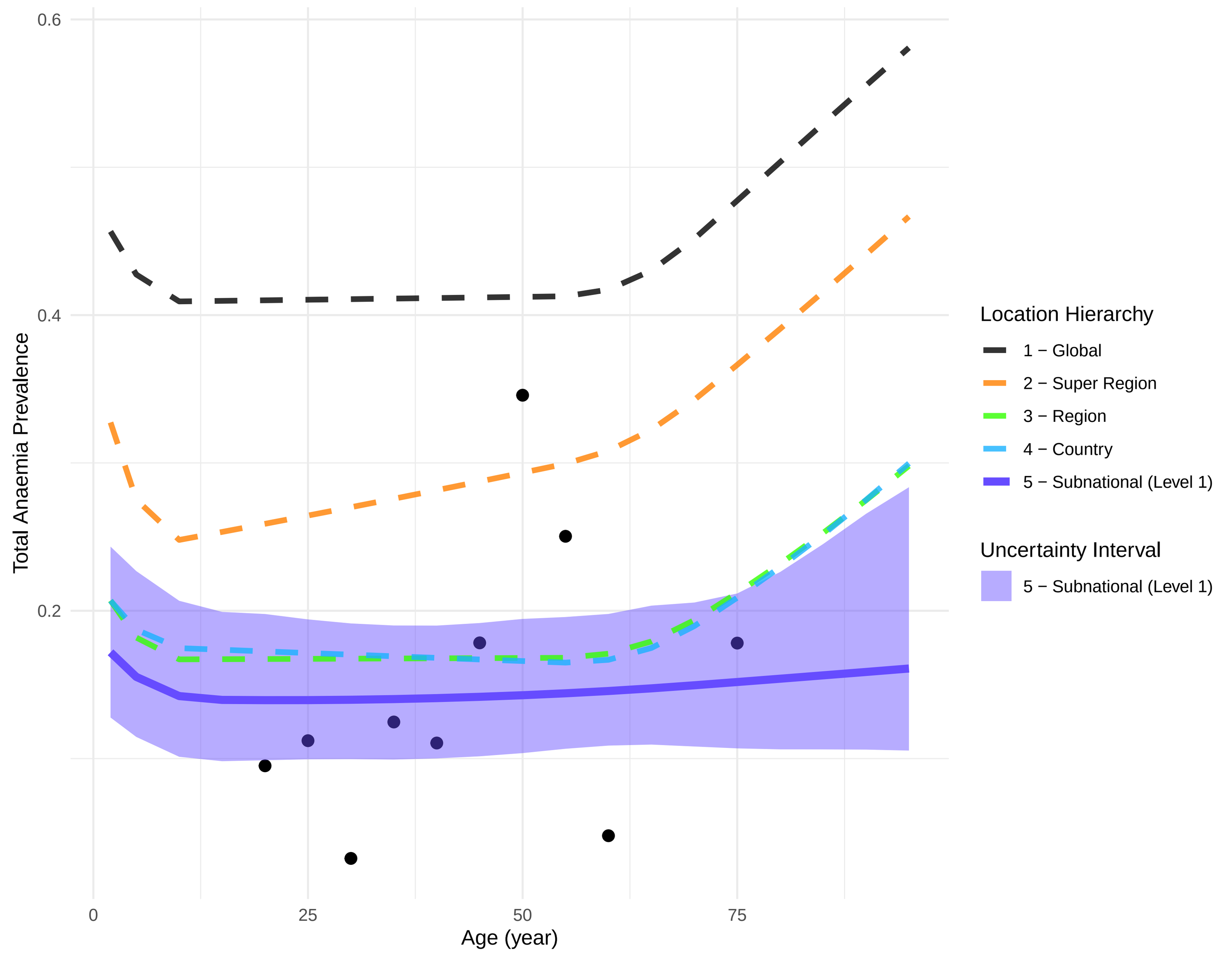

MR−BRT Age/Sex Fits − Non−pregnant Females 2+ Years Old − Total Anaemia Prevalence − Alagoas
WHO−2001 Adjustment Method

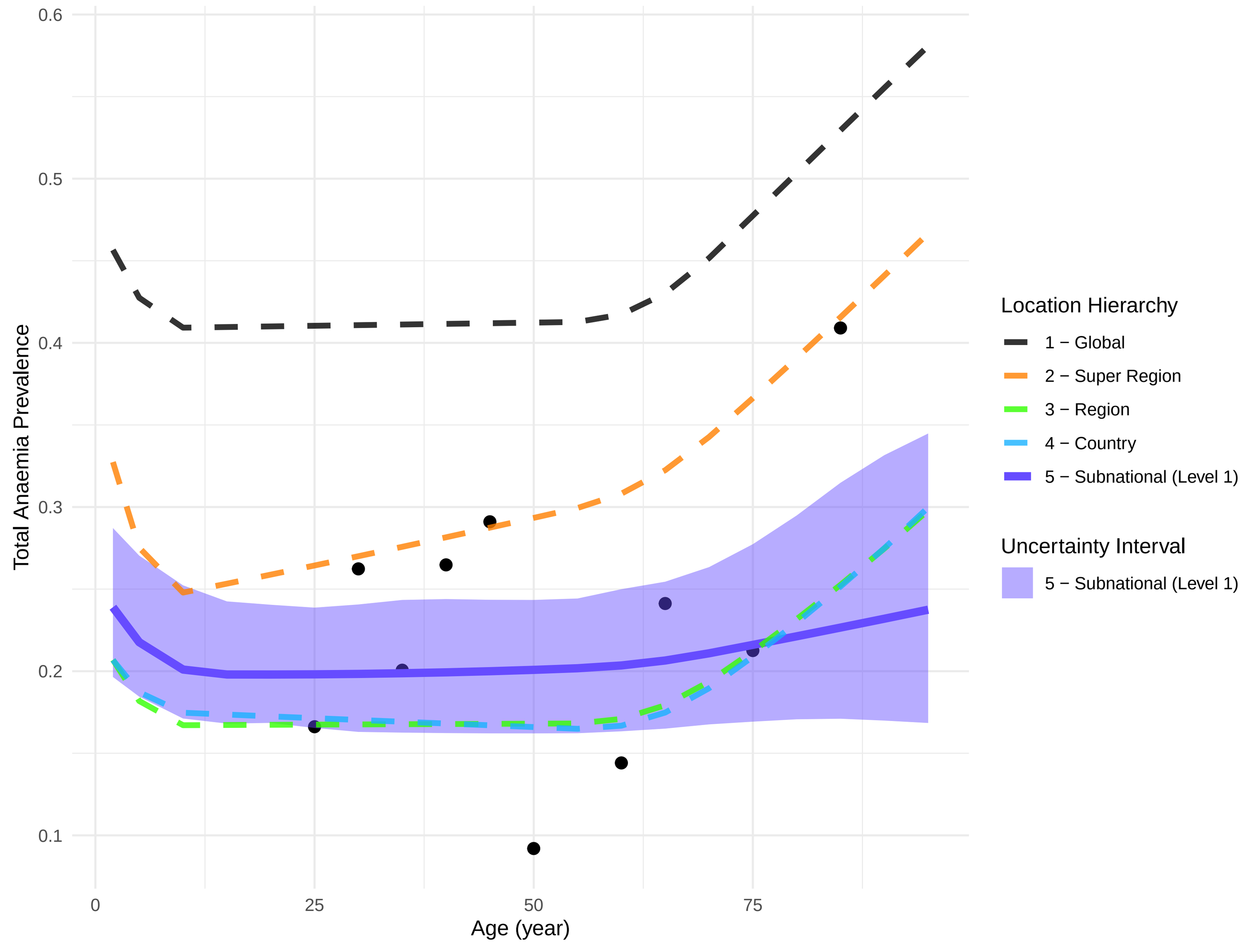

MR−BRT Age/Sex Fits − Non−pregnant Females 2+ Years Old − Total Anaemia Prevalence − Amapá
WHO−2001 Adjustment Method

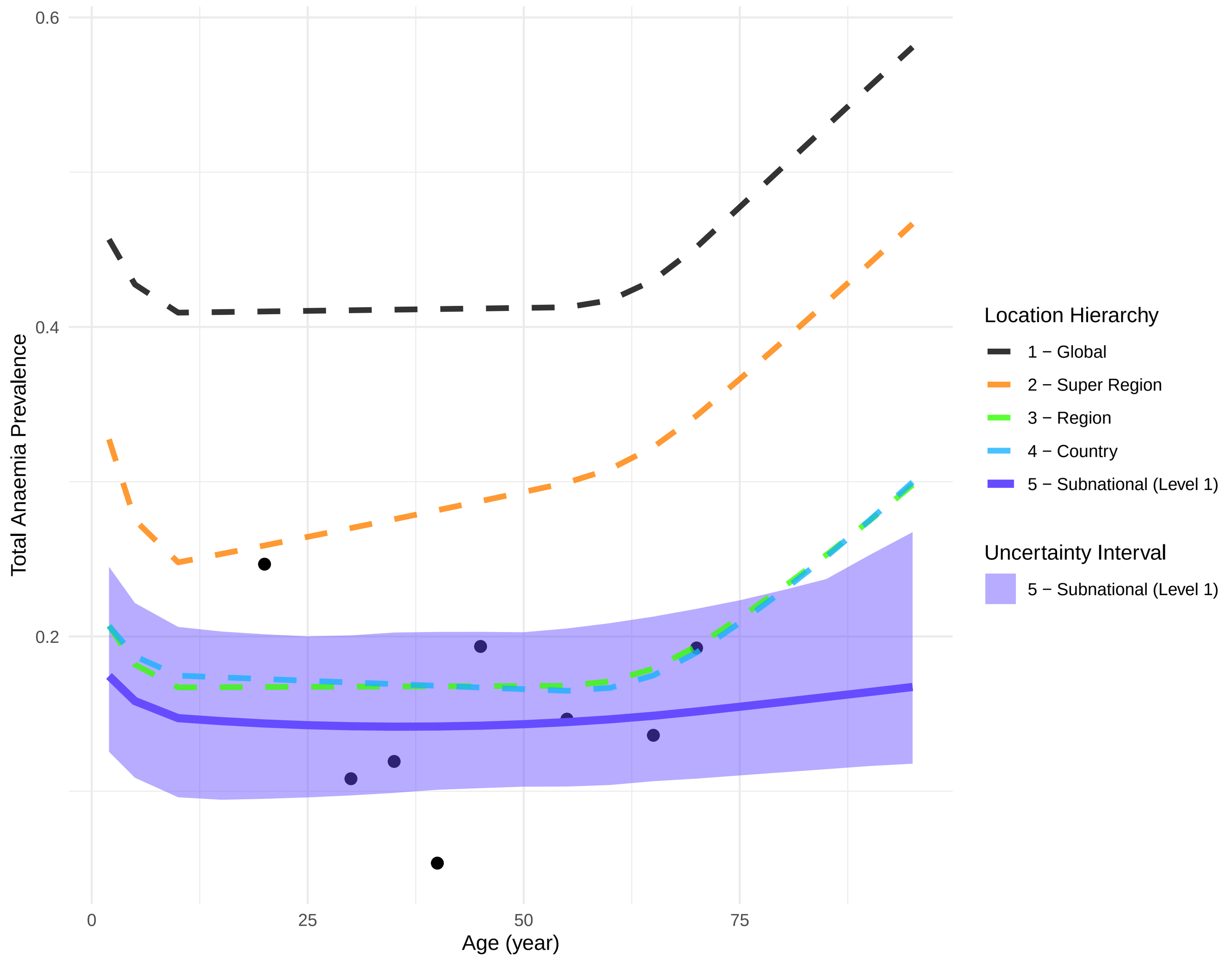

MR−BRT Age/Sex Fits − Non−pregnant Females 2+ Years Old − Total Anaemia Prevalence − Amazonas
WHO−2001 Adjustment Method

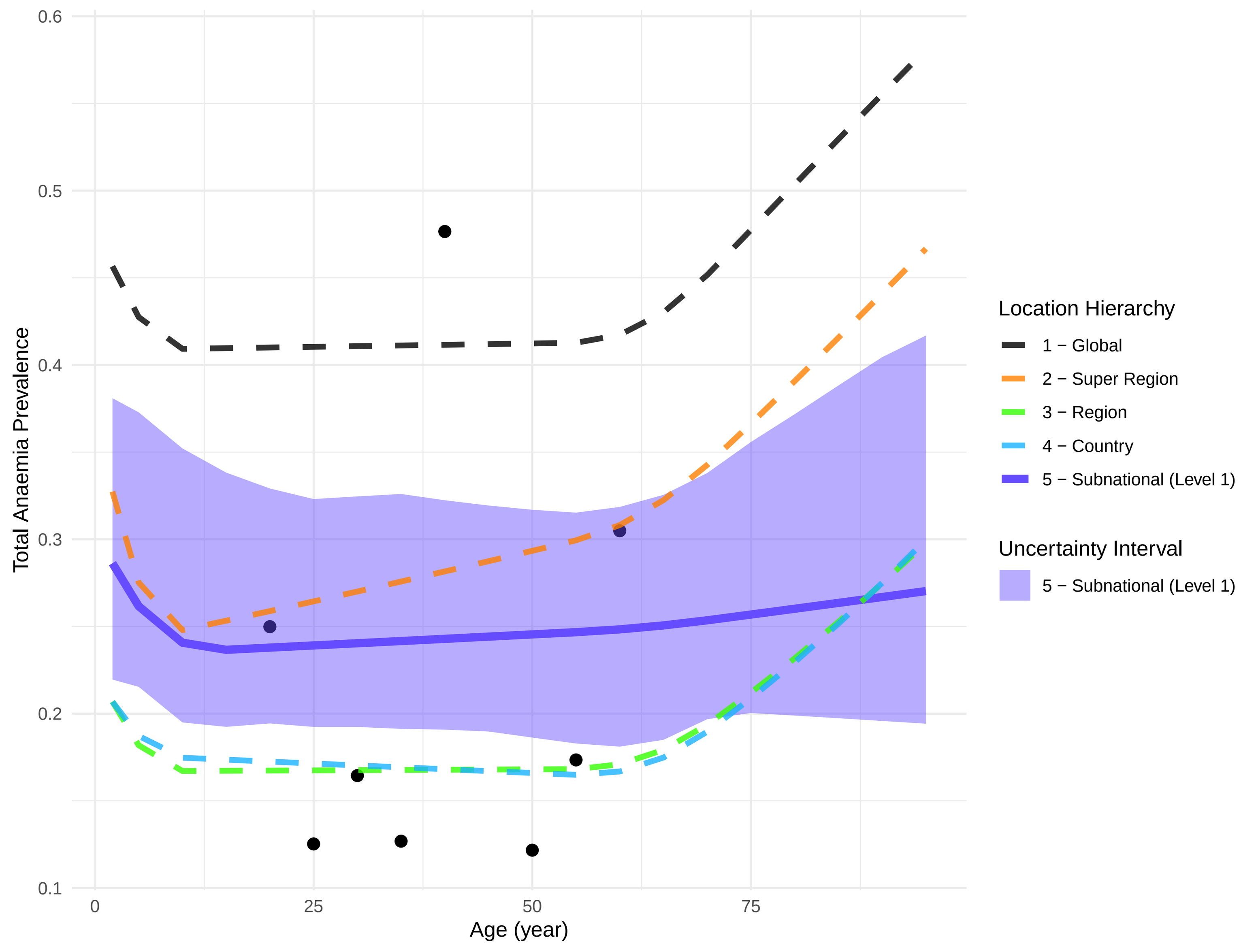

MR−BRT Age/Sex Fits − Non−pregnant Females 2+ Years Old − Total Anaemia Prevalence − Bahia
WHO−2001 Adjustment Method

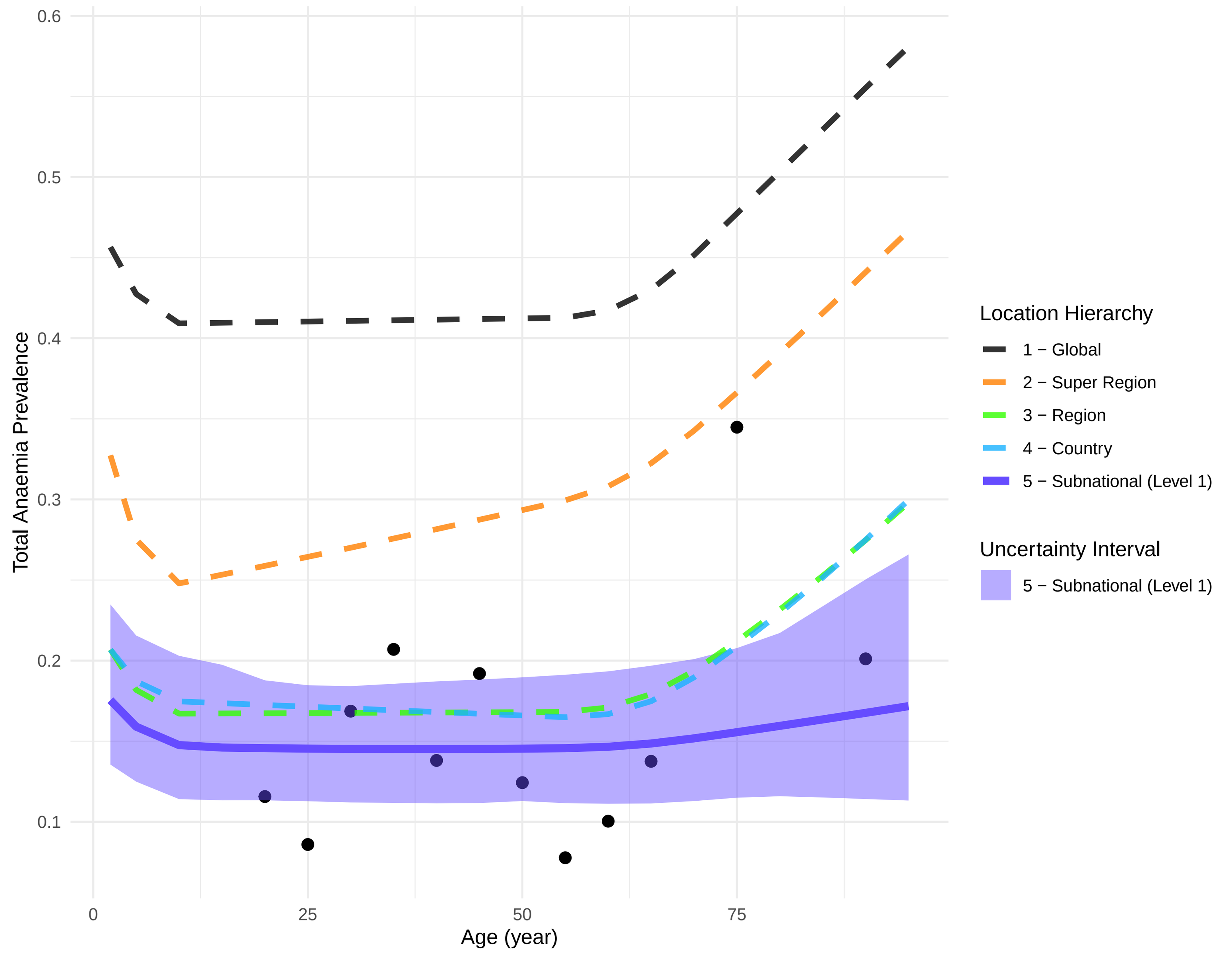

MR−BRT Age/Sex Fits − Non−pregnant Females 2+ Years Old − Total Anaemia Prevalence − Ceará
WHO−2001 Adjustment Method

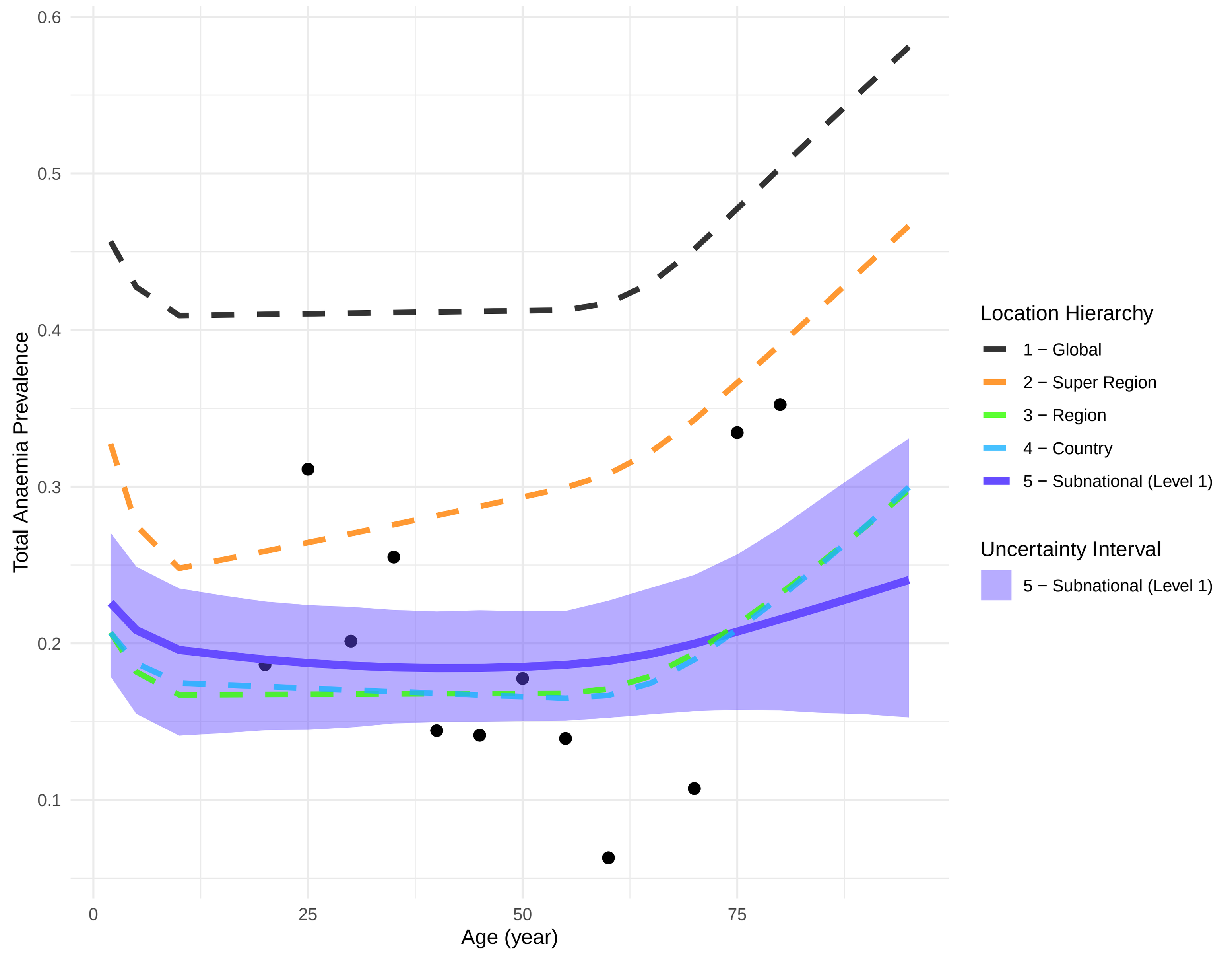

MR−BRT Age/Sex Fits − Non−pregnant Females 2+ Years Old − Total Anaemia Prevalence − Distrito Federal
WHO−2001 Adjustment Method

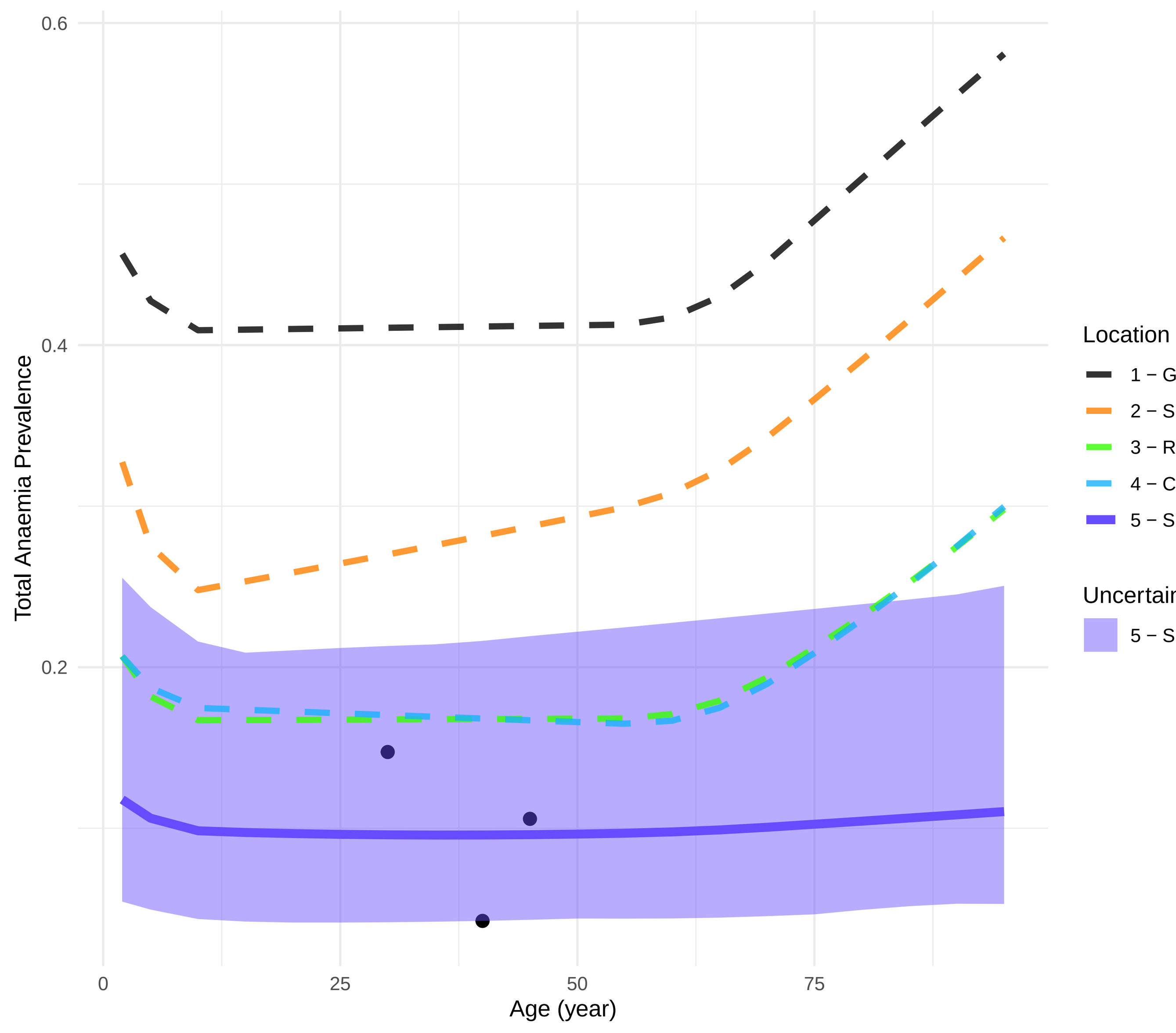

MR−BRT Age/Sex Fits − Non−pregnant Females 2+ Years Old − Total Anaemia Prevalence − Espírito Santo
WHO−2001 Adjustment Method

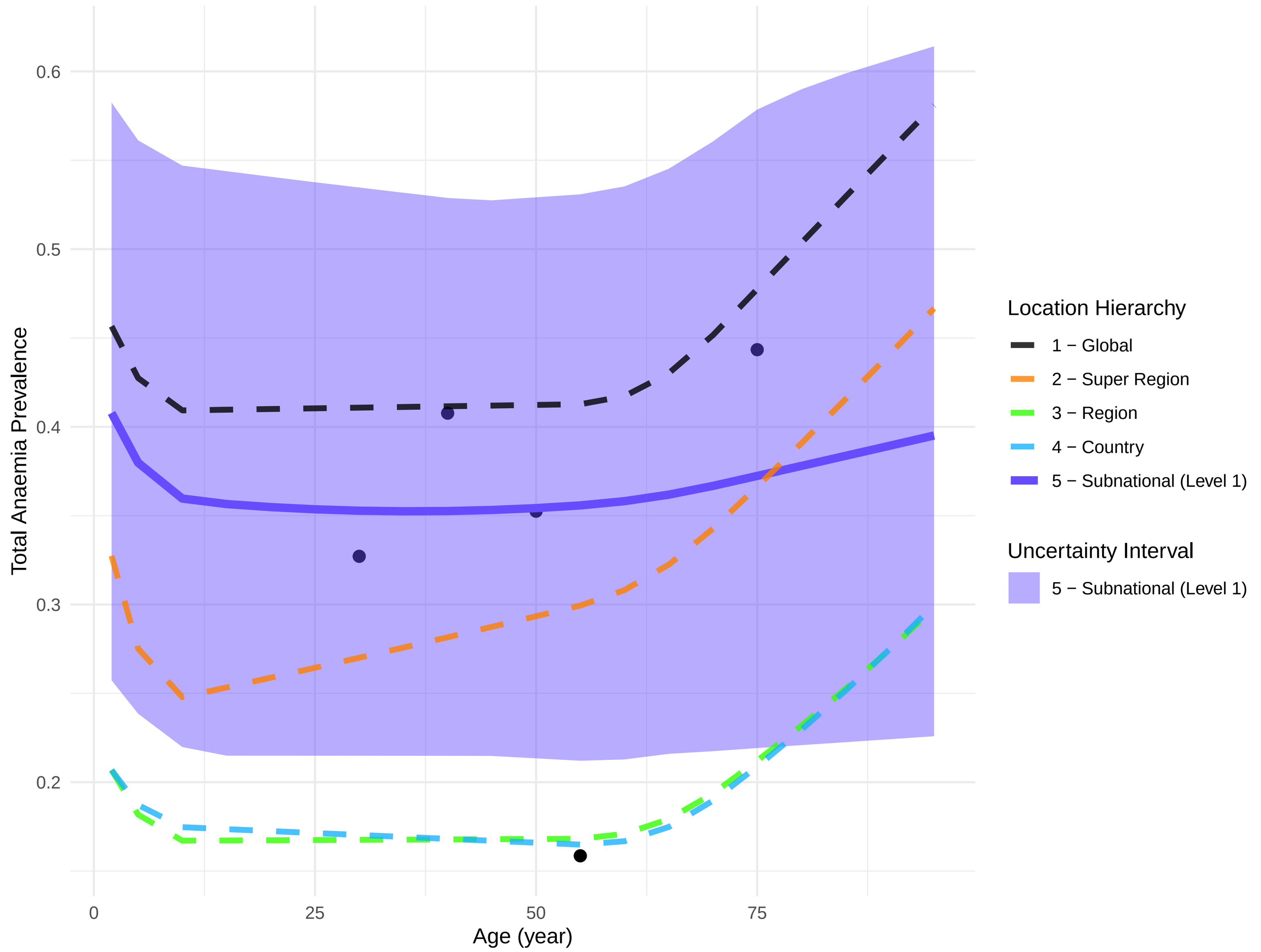

MR−BRT Age/Sex Fits − Non−pregnant Females 2+ Years Old − Total Anaemia Prevalence − Goiás
WHO−2001 Adjustment Method

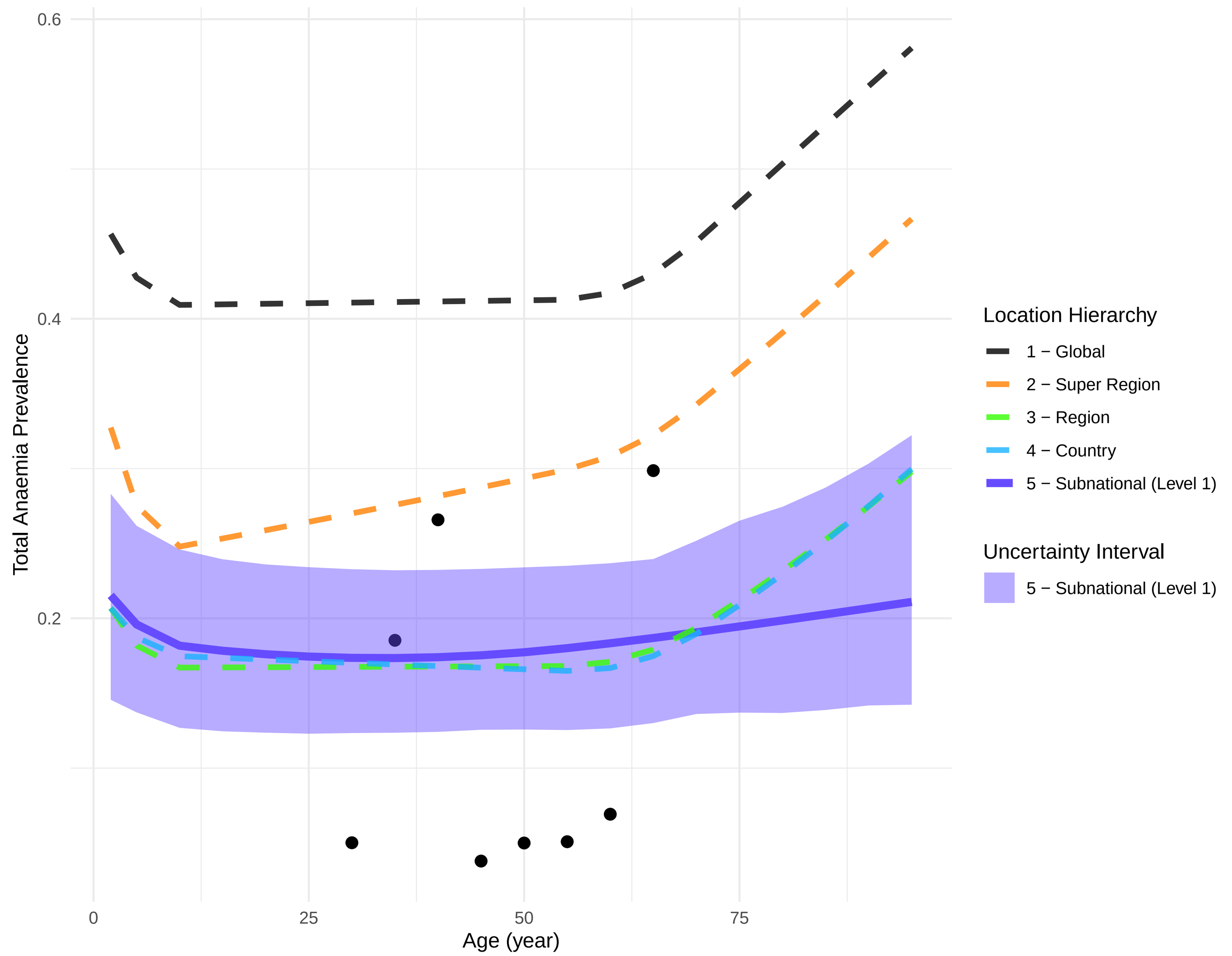

MR−BRT Age/Sex Fits − Non−pregnant Females 2+ Years Old − Total Anaemia Prevalence − Maranhão
WHO−2001 Adjustment Method

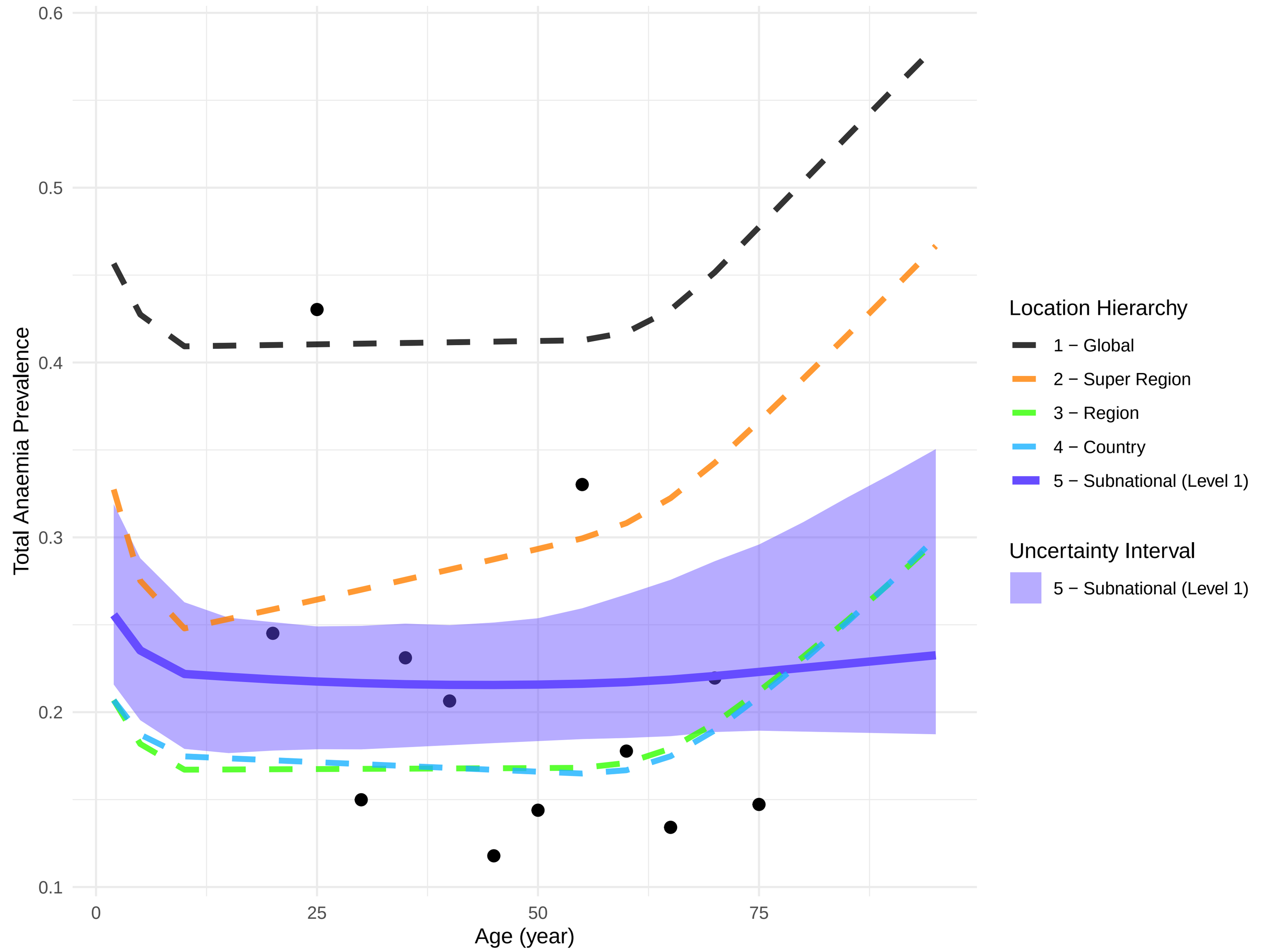

MR−BRT Age/Sex Fits − Non−pregnant Females 2+ Years Old − Total Anaemia Prevalence − Mato Grosso
WHO−2001 Adjustment Method

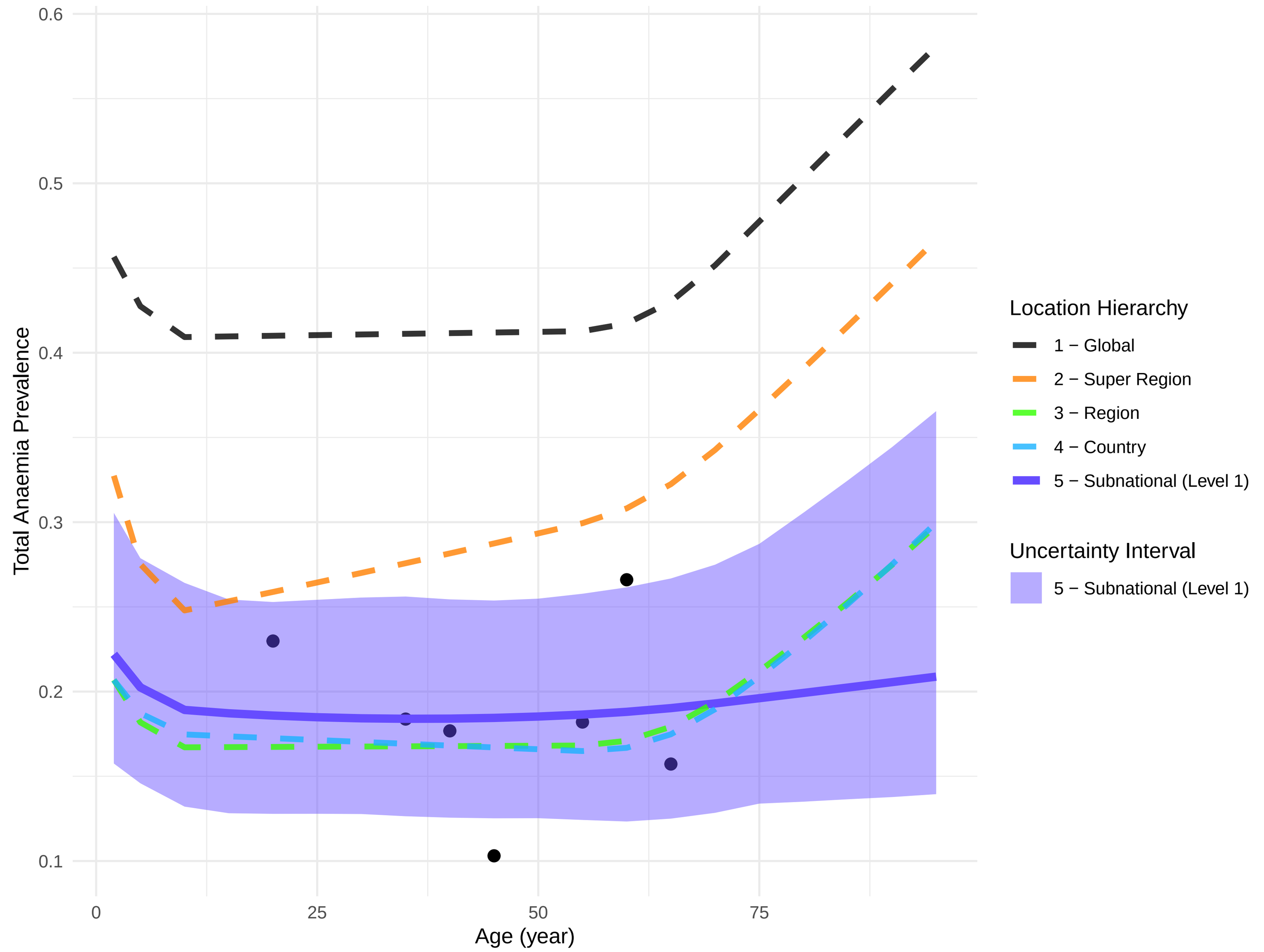

MR−BRT Age/Sex Fits − Non−pregnant Females 2+ Years Old − Total Anaemia Prevalence − Mato Grosso do Sul
WHO−2001 Adjustment Method

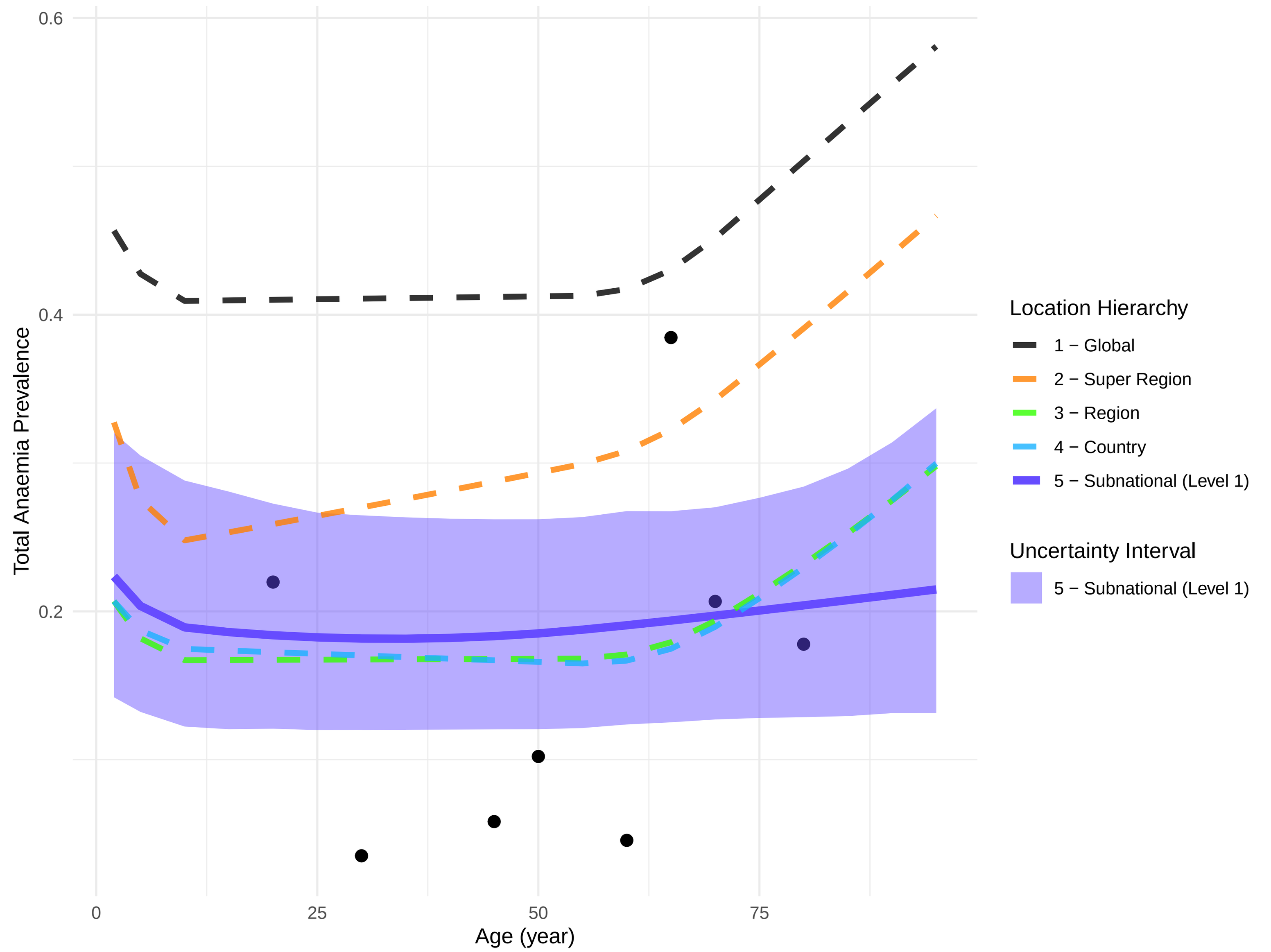

MR−BRT Age/Sex Fits − Non−pregnant Females 2+ Years Old − Total Anaemia Prevalence − Minas Gerais
WHO−2001 Adjustment Method

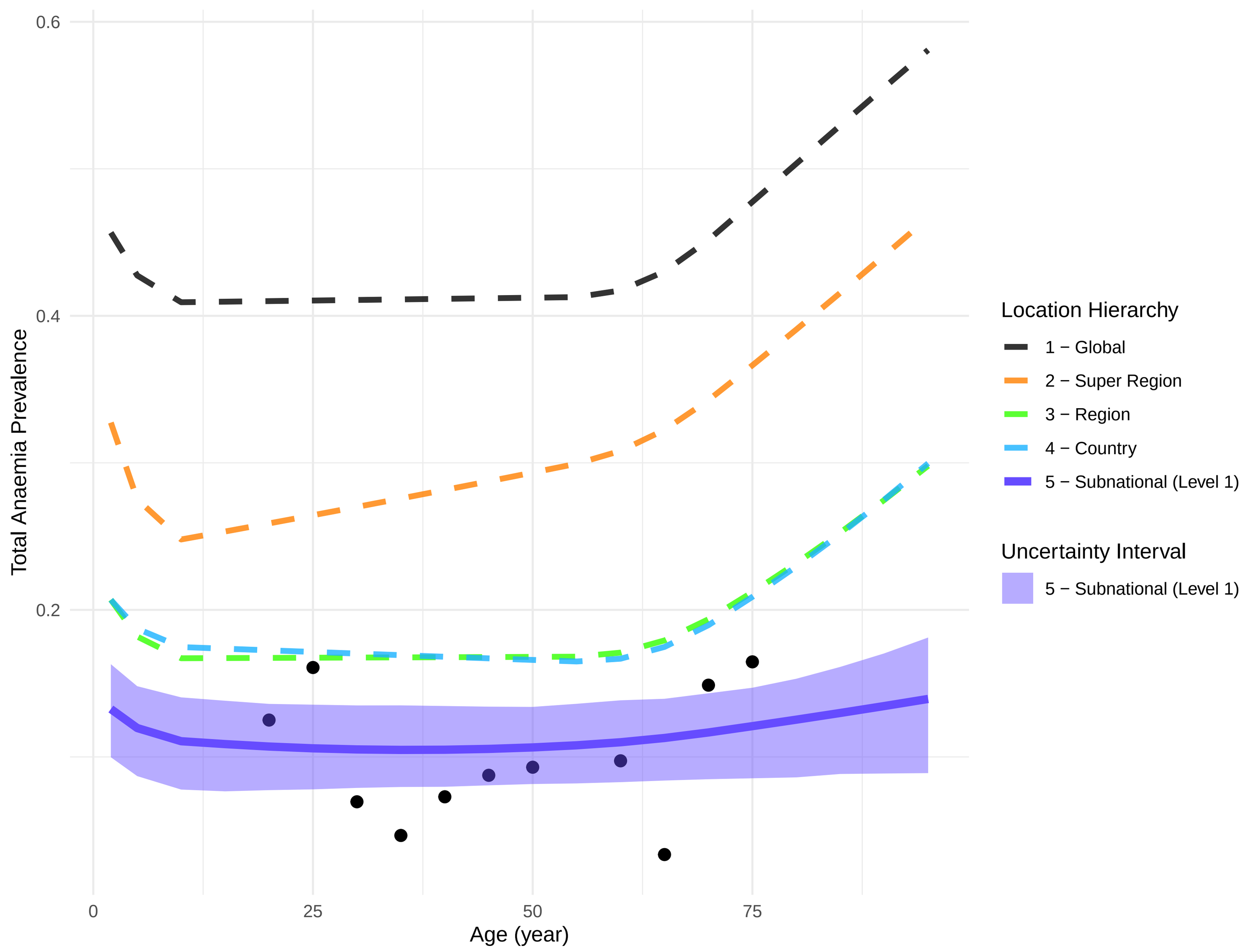

MR−BRT Age/Sex Fits − Non−pregnant Females 2+ Years Old − Total Anaemia Prevalence − Pará
WHO−2001 Adjustment Method

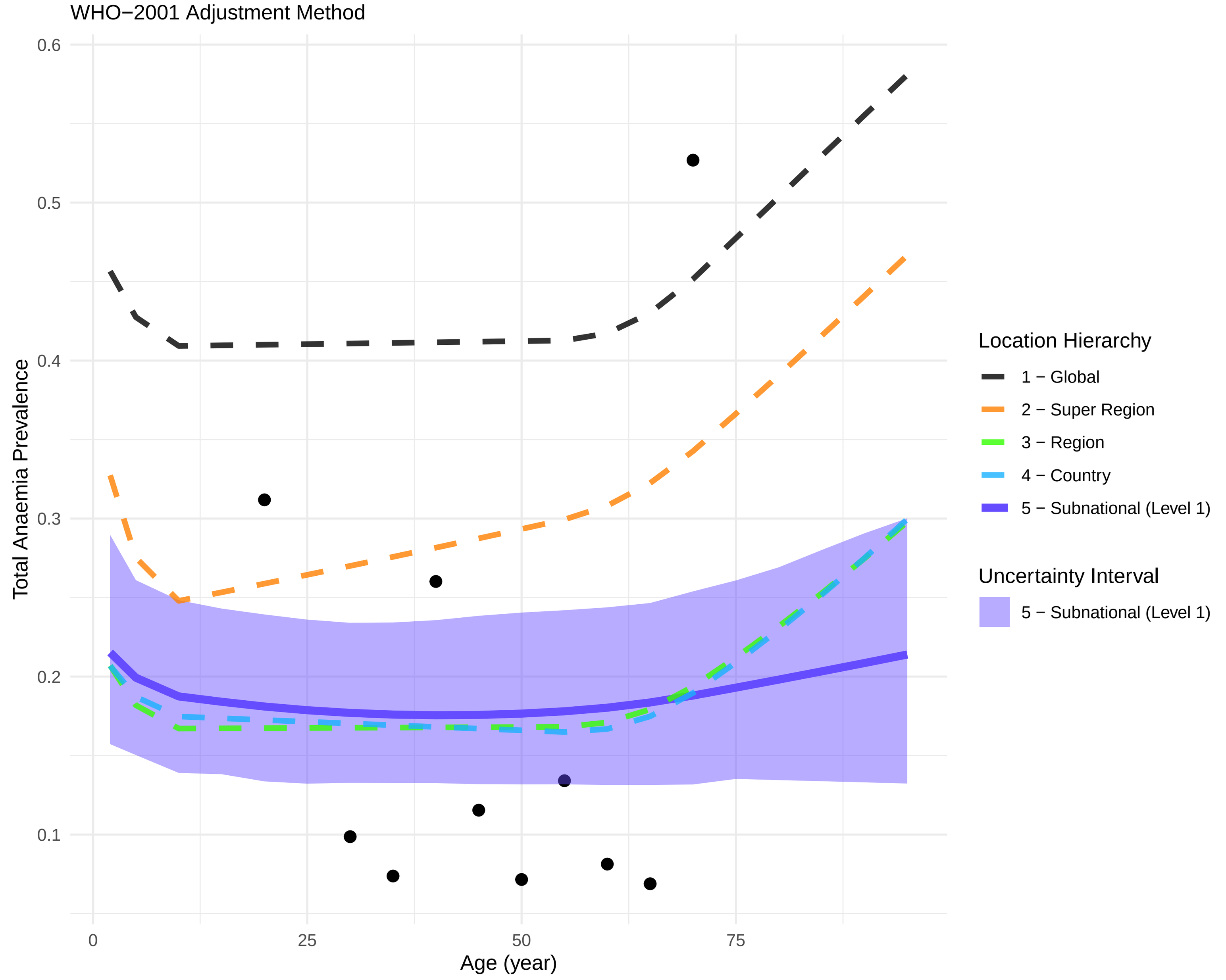

MR−BRT Age/Sex Fits − Non−pregnant Females 2+ Years Old − Total Anaemia Prevalence − Paraíba
WHO−2001 Adjustment Method

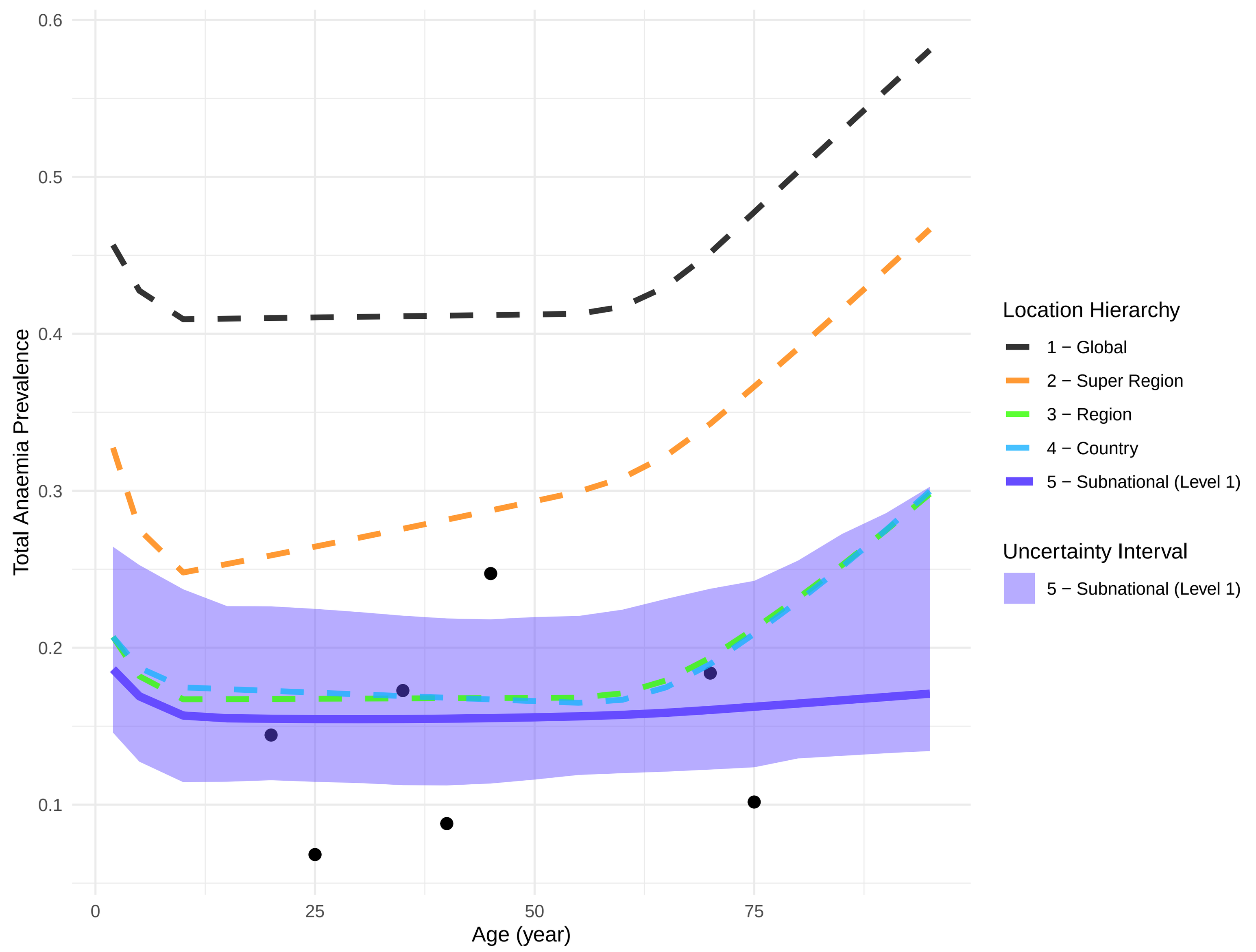

MR−BRT Age/Sex Fits − Non−pregnant Females 2+ Years Old − Total Anaemia Prevalence − Paraná
WHO−2001 Adjustment Method

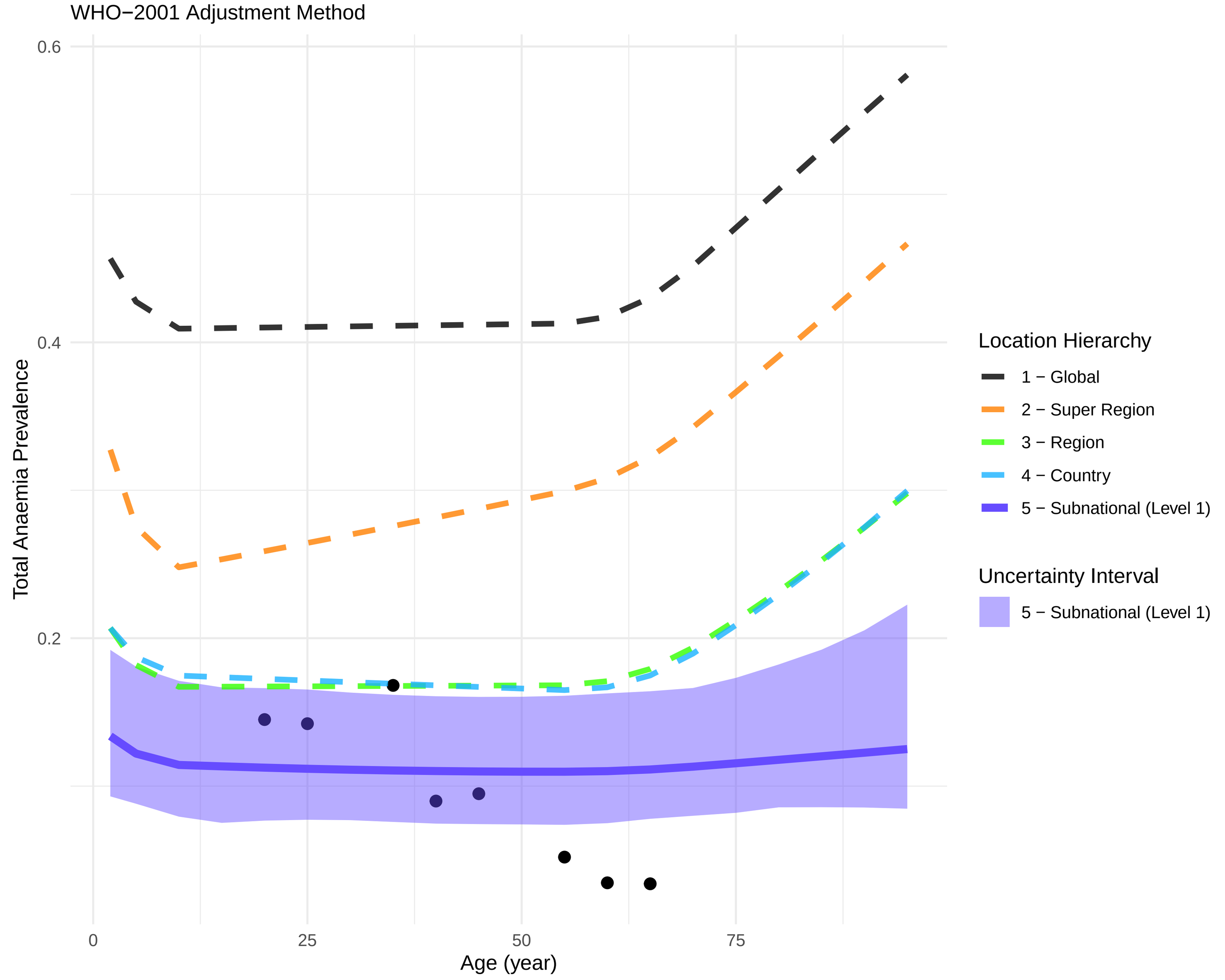

MR−BRT Age/Sex Fits − Non−pregnant Females 2+ Years Old − Total Anaemia Prevalence − Pernambuco
WHO−2001 Adjustment Method

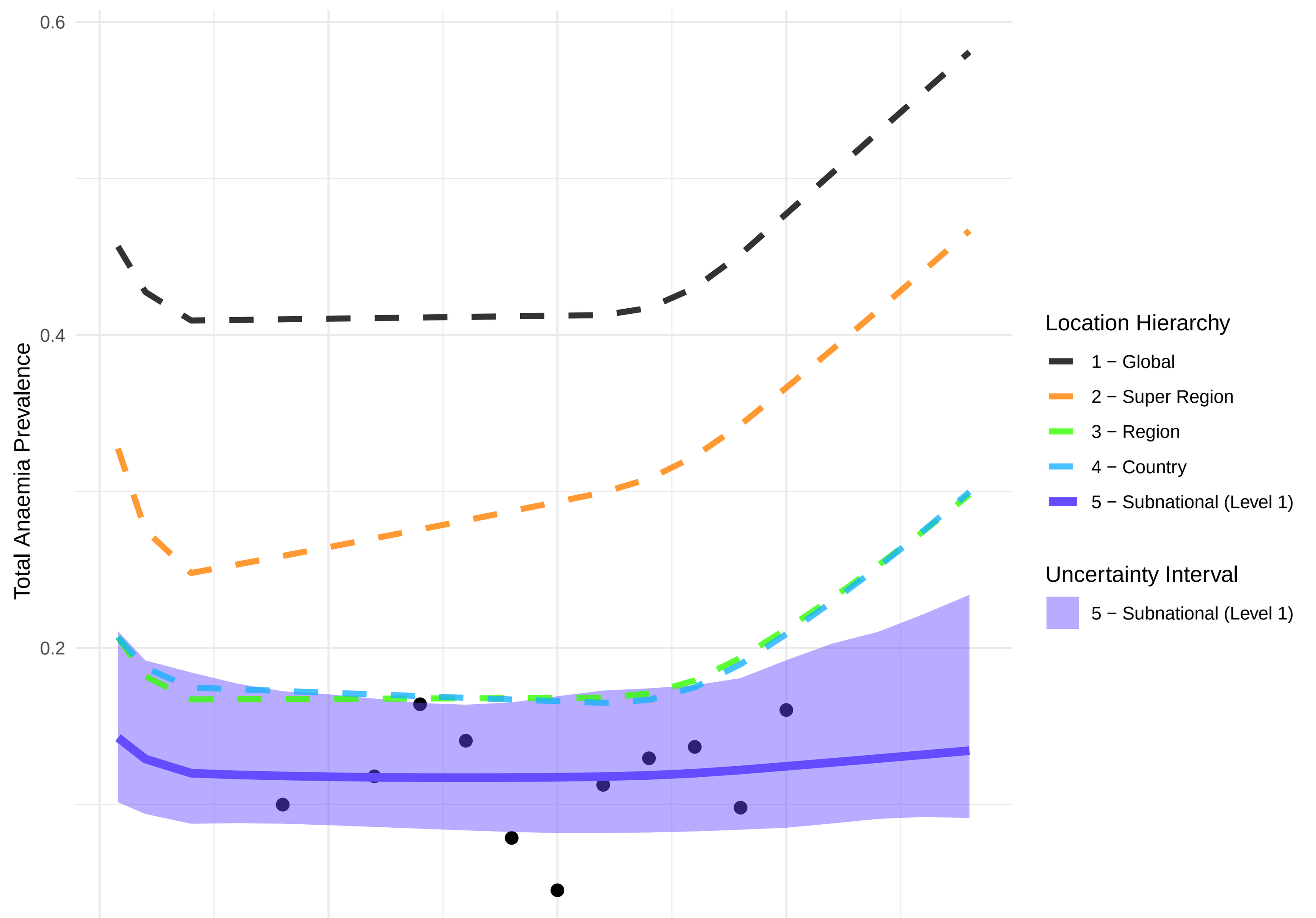

MR−BRT Age/Sex Fits − Non−pregnant Females 2+ Years Old − Total Anaemia Prevalence − Piaui
WHO−2001 Adjustment Method

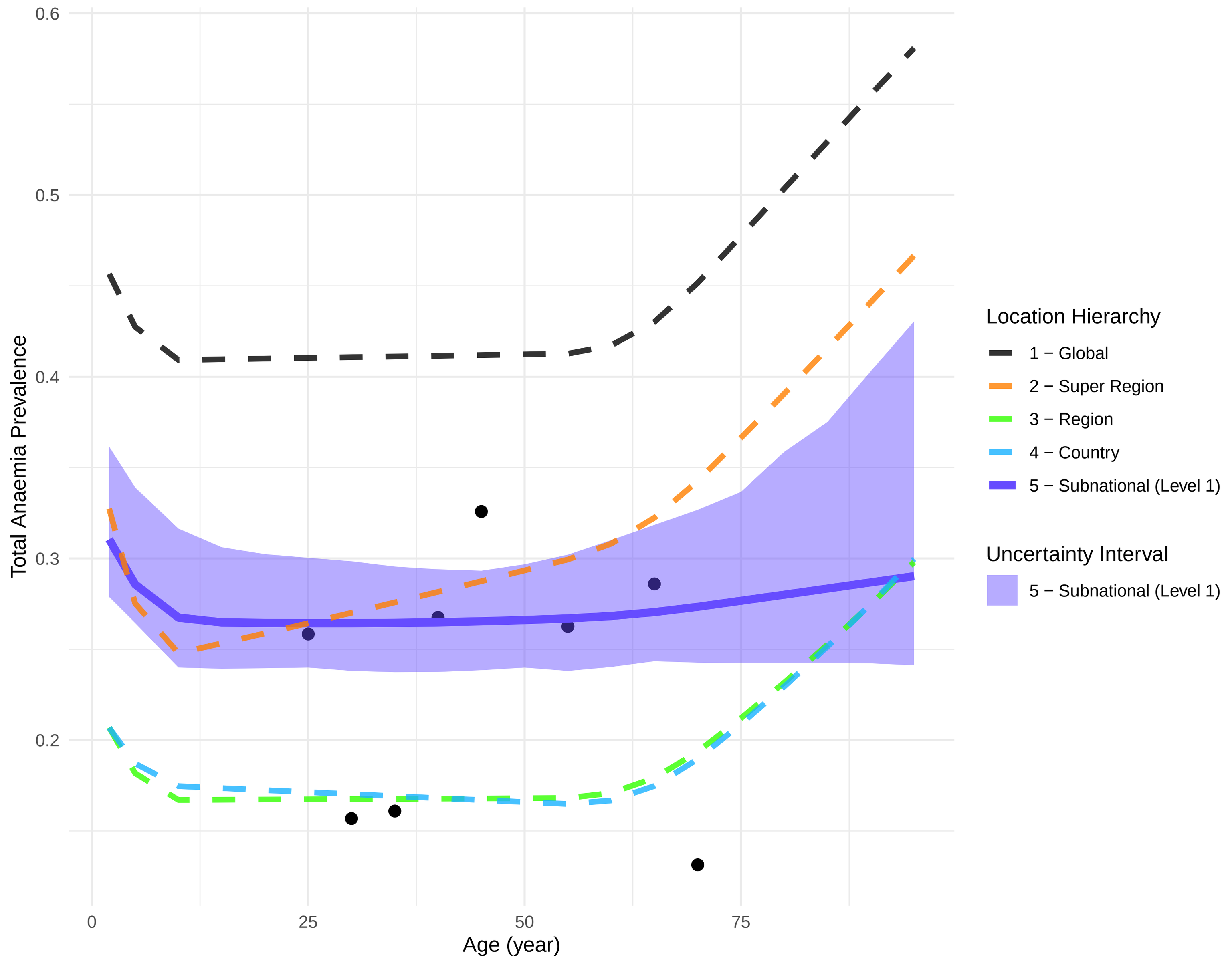

MR−BRT Age/Sex Fits − Non−pregnant Females 2+ Years Old − Total Anaemia Prevalence − Rio de Janeiro
WHO−2001 Adjustment Method

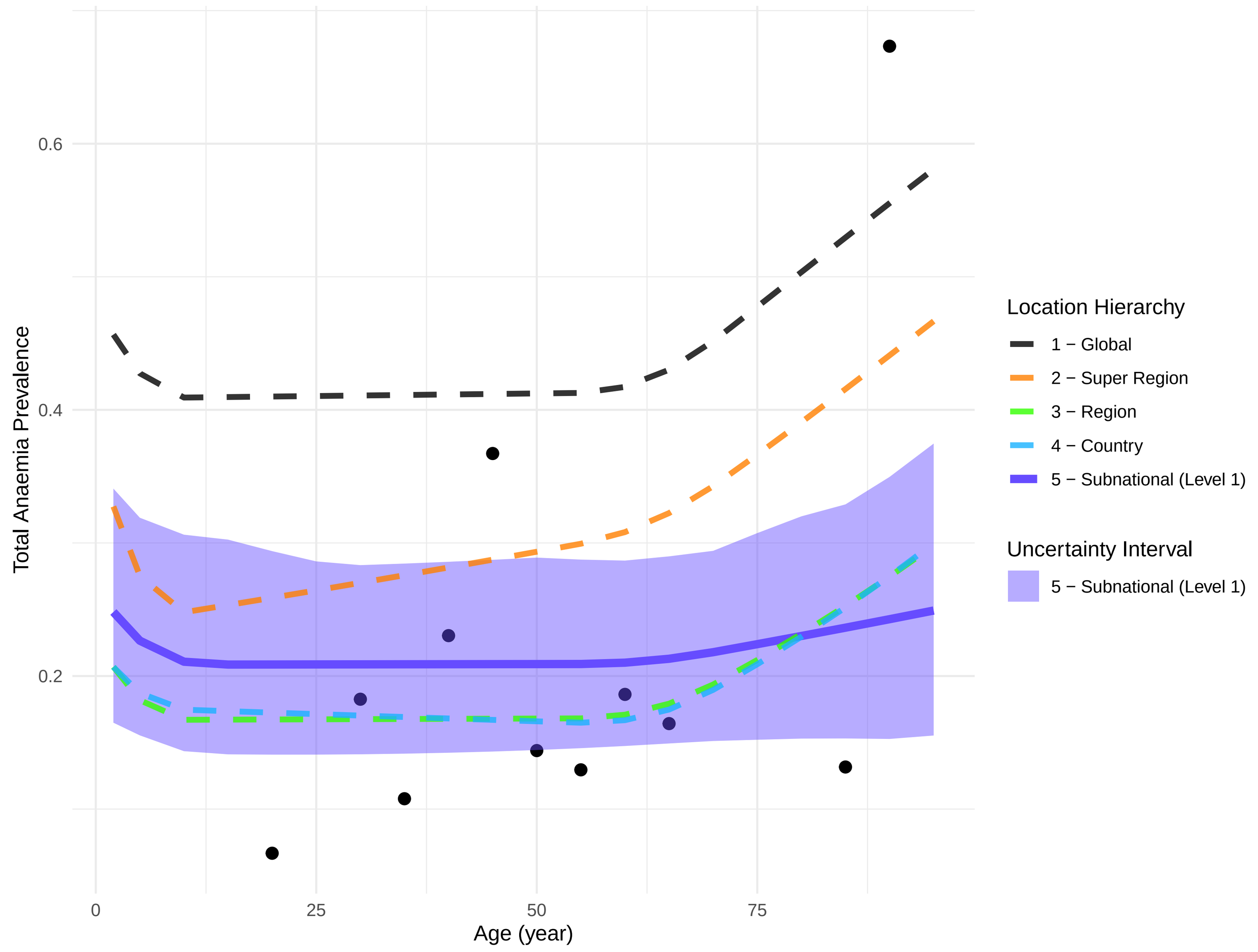

MR−BRT Age/Sex Fits − Non−pregnant Females 2+ Years Old − Total Anaemia Prevalence − Rio Grande do Norte
WHO−2001 Adjustment Method

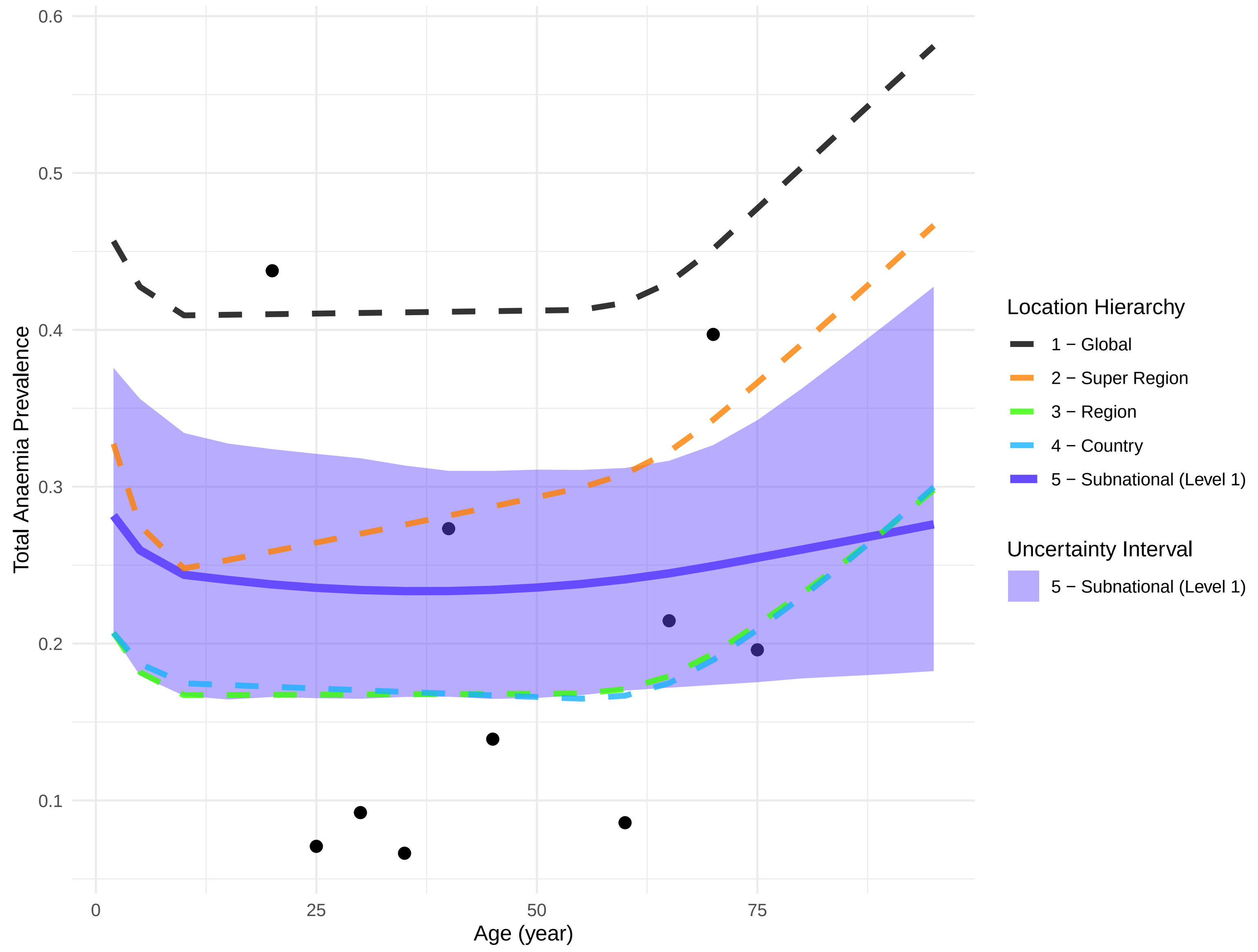

MR−BRT Age/Sex Fits − Non−pregnant Females 2+ Years Old − Total Anaemia Prevalence − Rio Grande do Sul
WHO−2001 Adjustment Method

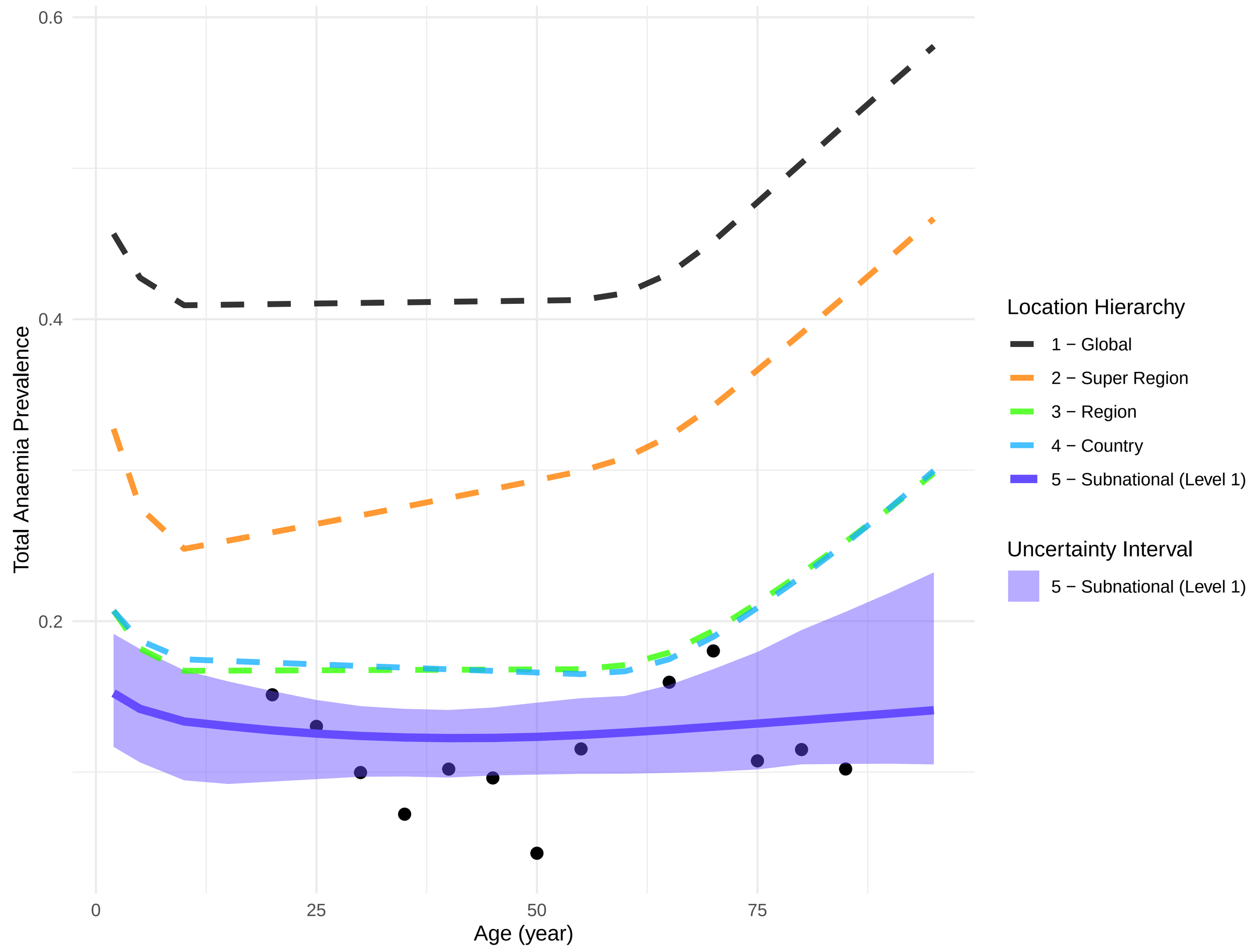

MR−BRT Age/Sex Fits − Non−pregnant Females 2+ Years Old − Total Anaemia Prevalence − Rondônia
WHO−2001 Adjustment Method

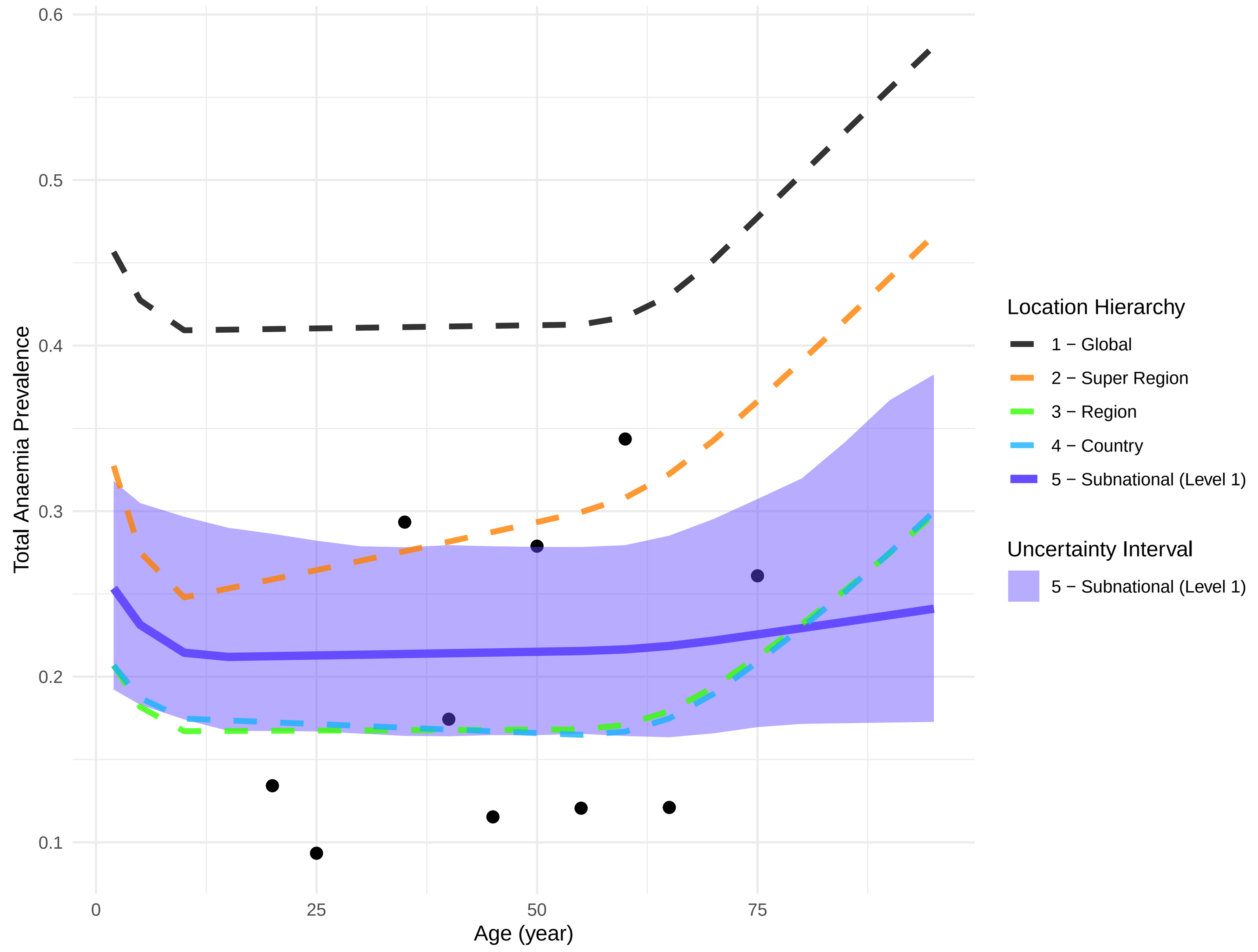

MR−BRT Age/Sex Fits − Non−pregnant Females 2+ Years Old − Total Anaemia Prevalence − Roraima
WHO−2001 Adjustment Method

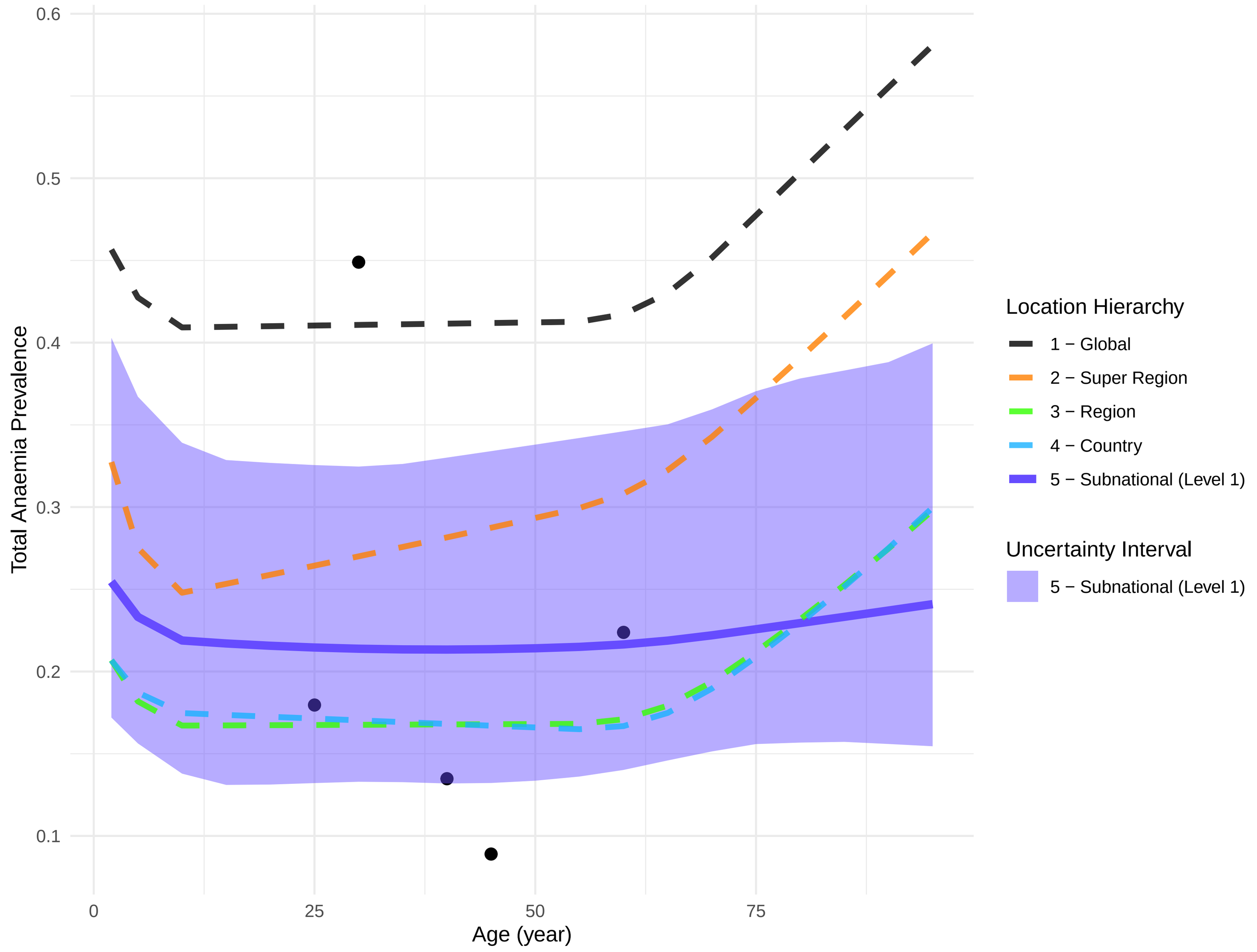

MR−BRT Age/Sex Fits − Non−pregnant Females 2+ Years Old − Total Anaemia Prevalence − Santa Catarina
WHO−2001 Adjustment Method

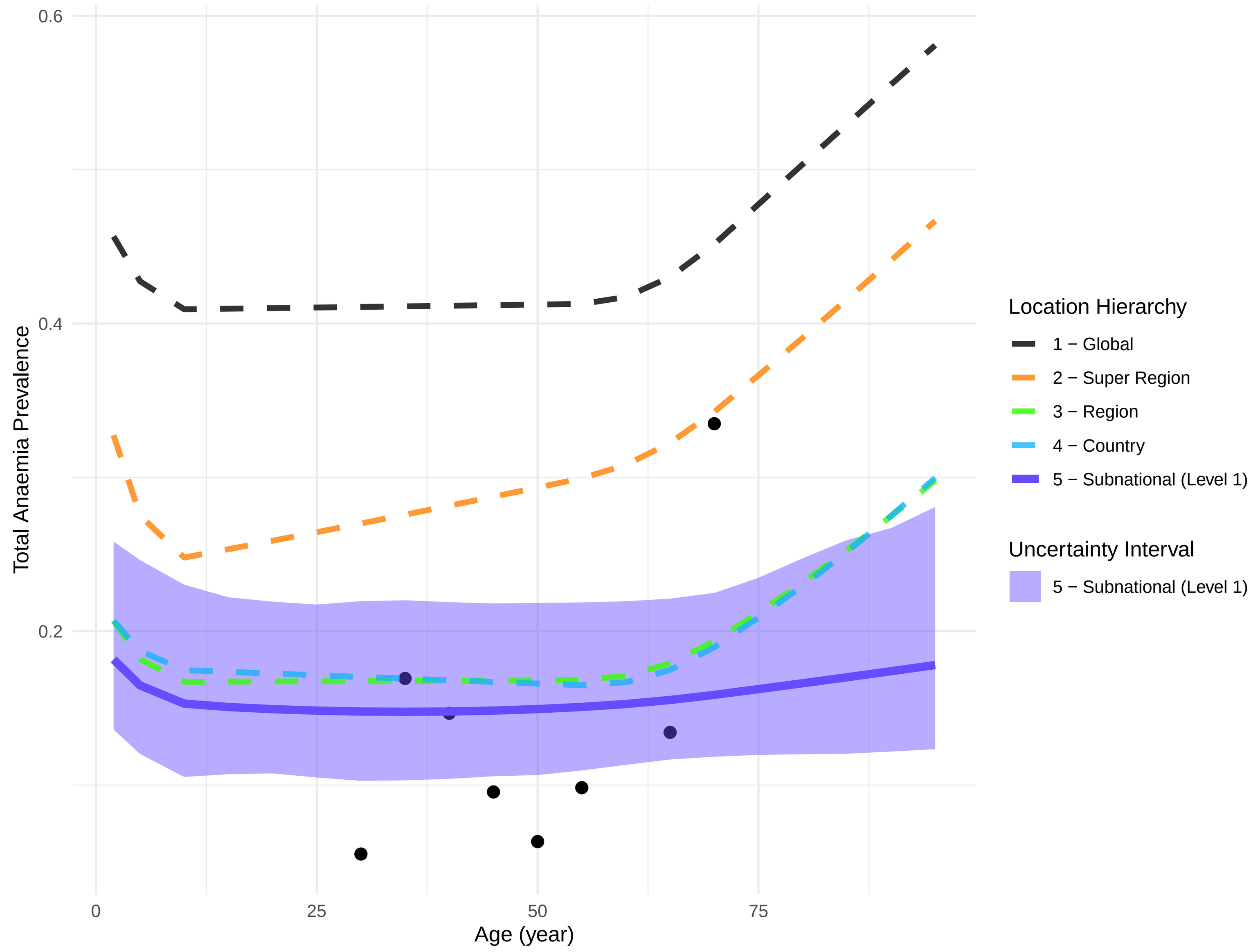

MR−BRT Age/Sex Fits − Non−pregnant Females 2+ Years Old − Total Anaemia Prevalence − São Paulo
WHO−2001 Adjustment Method

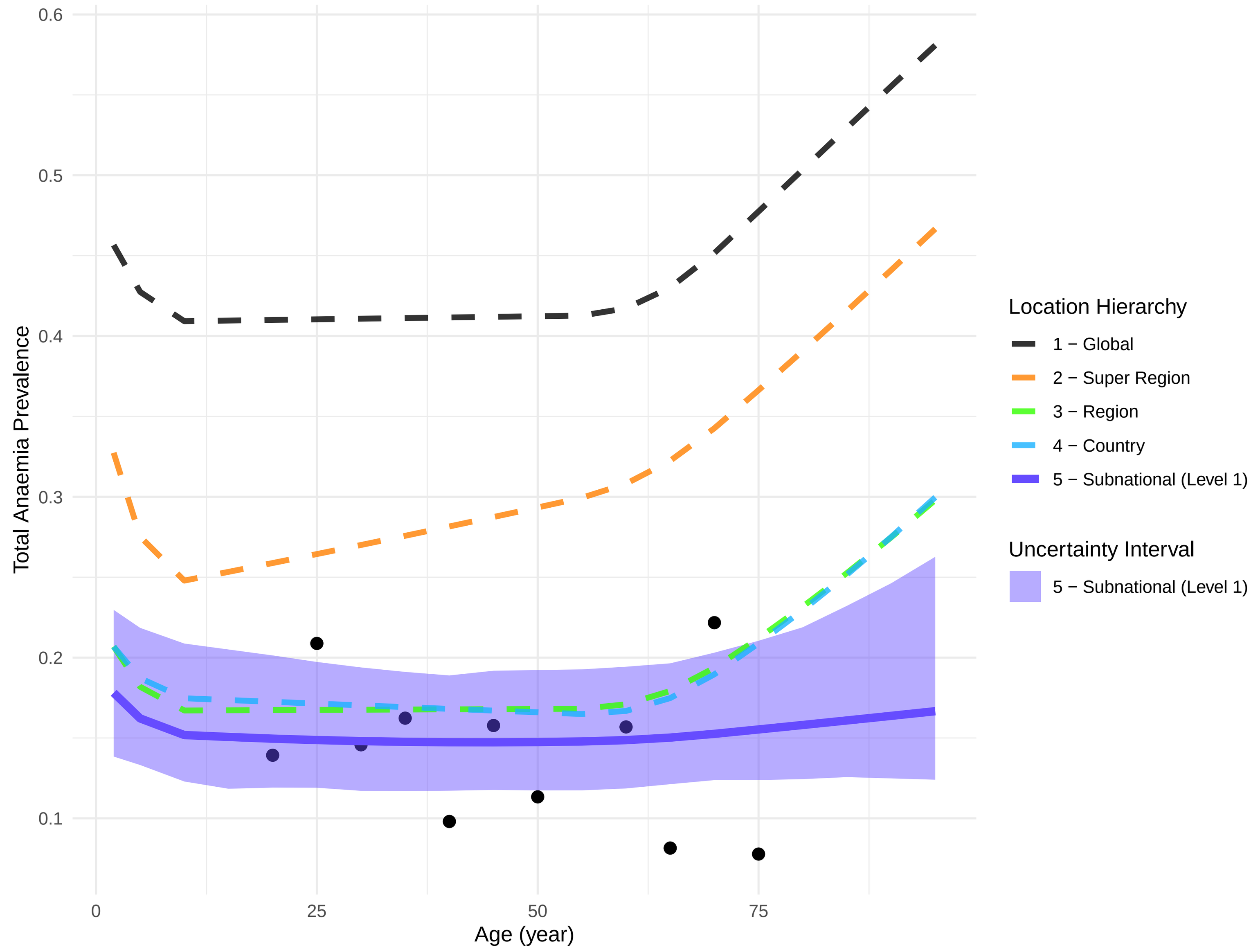

MR−BRT Age/Sex Fits − Non−pregnant Females 2+ Years Old − Total Anaemia Prevalence − Sergipe
WHO−2001 Adjustment Method

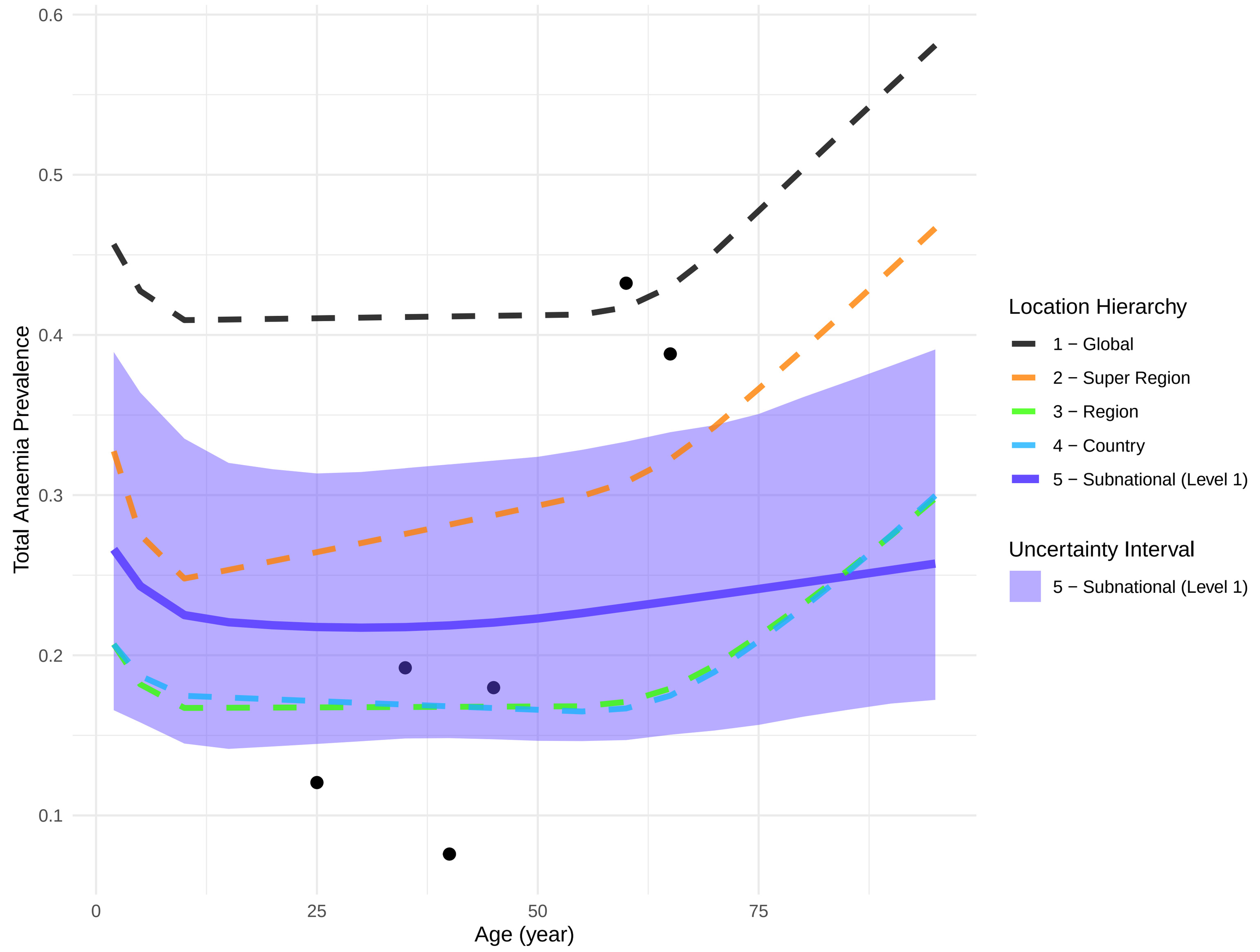

MR−BRT Age/Sex Fits − Non−pregnant Females 2+ Years Old − Total Anaemia Prevalence − Tocantins
WHO−2001 Adjustment Method

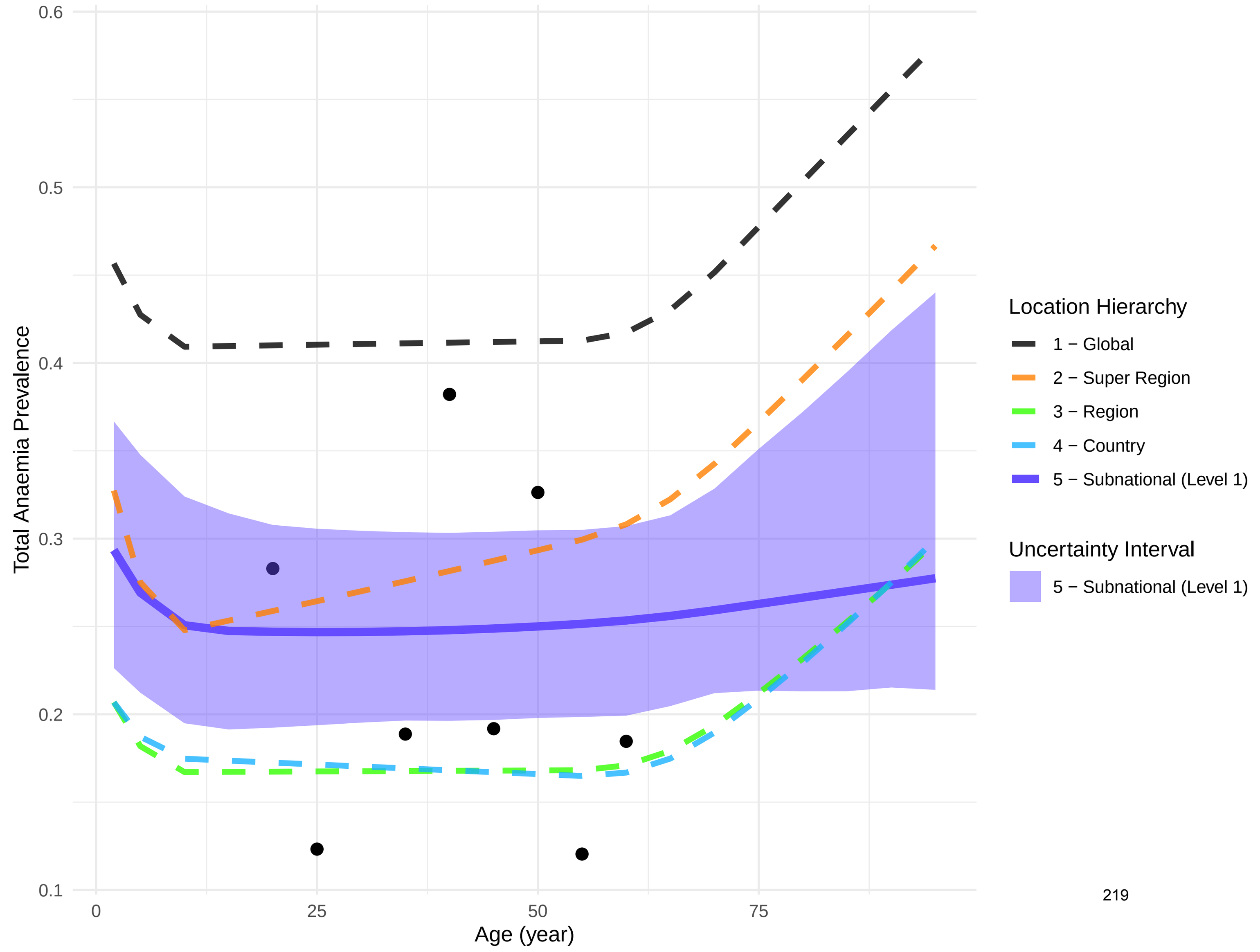

MR−BRT Age/Sex Fits − Non−pregnant Females 2+ Years Old − Total Anaemia Prevalence − North Africa and Middle East
WHO−2001 Adjustment Method

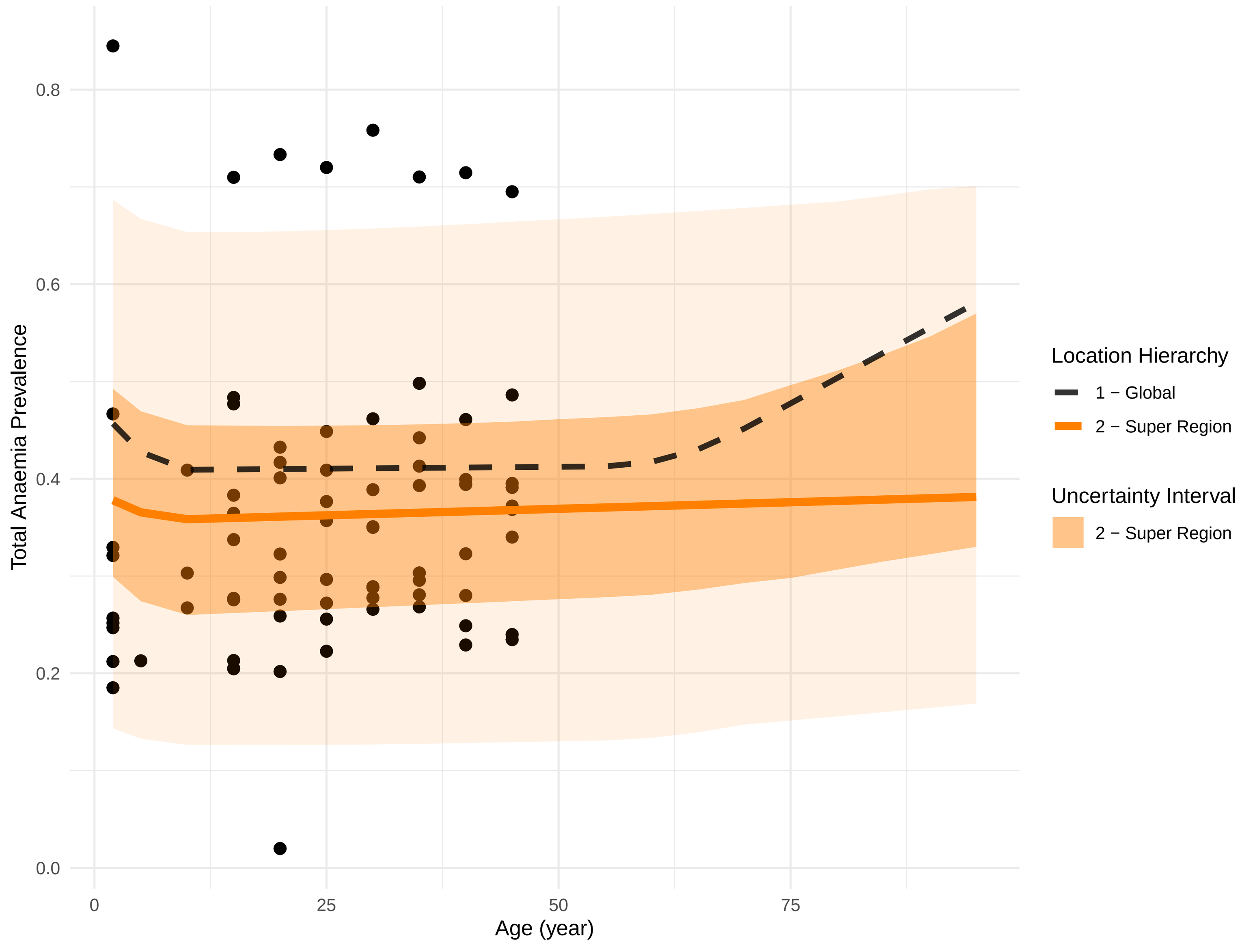

MR−BRT Age/Sex Fits − Non−pregnant Females 2+ Years Old − Total Anaemia Prevalence − North Africa and Middle East
WHO−2001 Adjustment Method

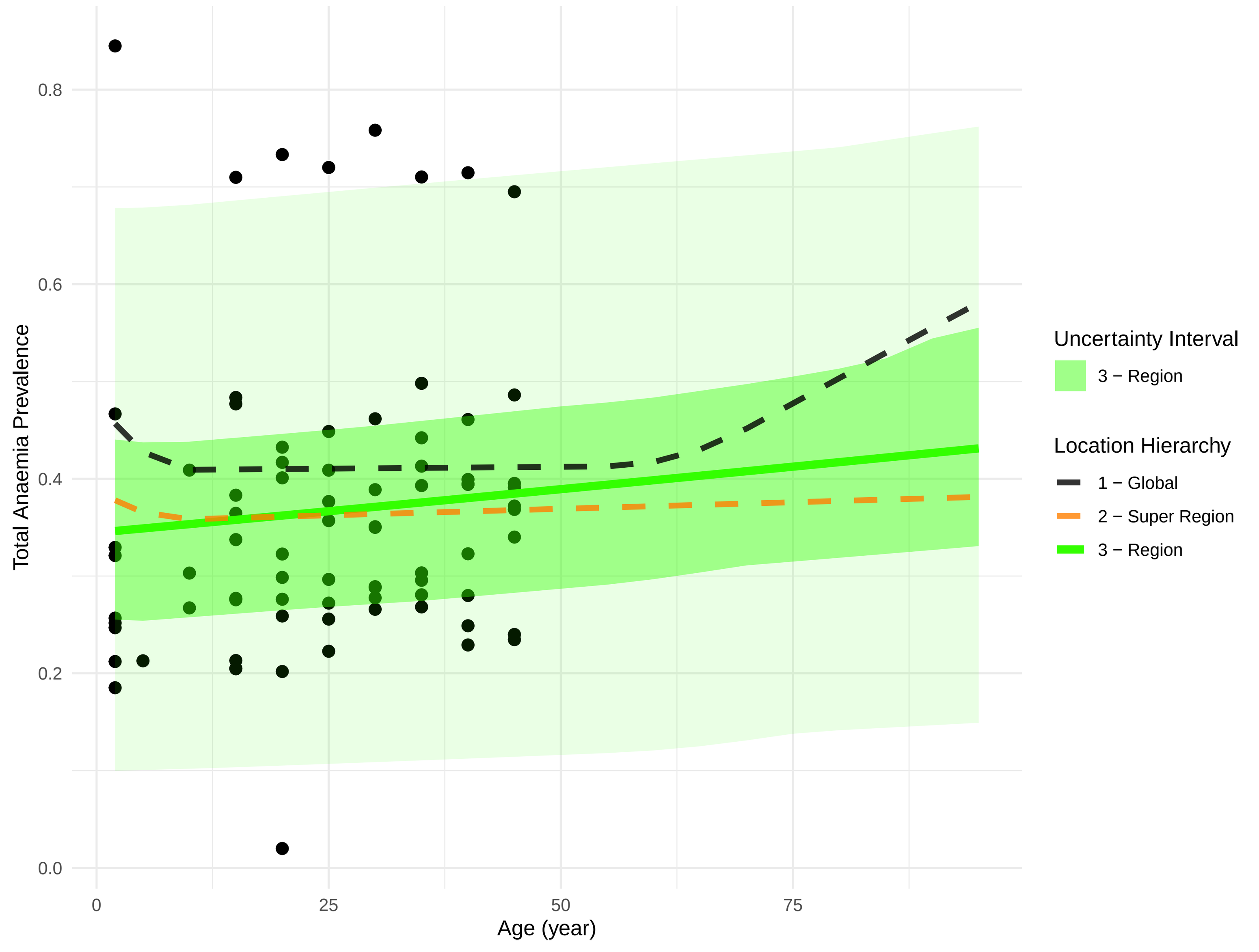

MR−BRT Age/Sex Fits − Non−pregnant Females 2+ Years Old − Total Anaemia Prevalence − Egypt
WHO−2001 Adjustment Method

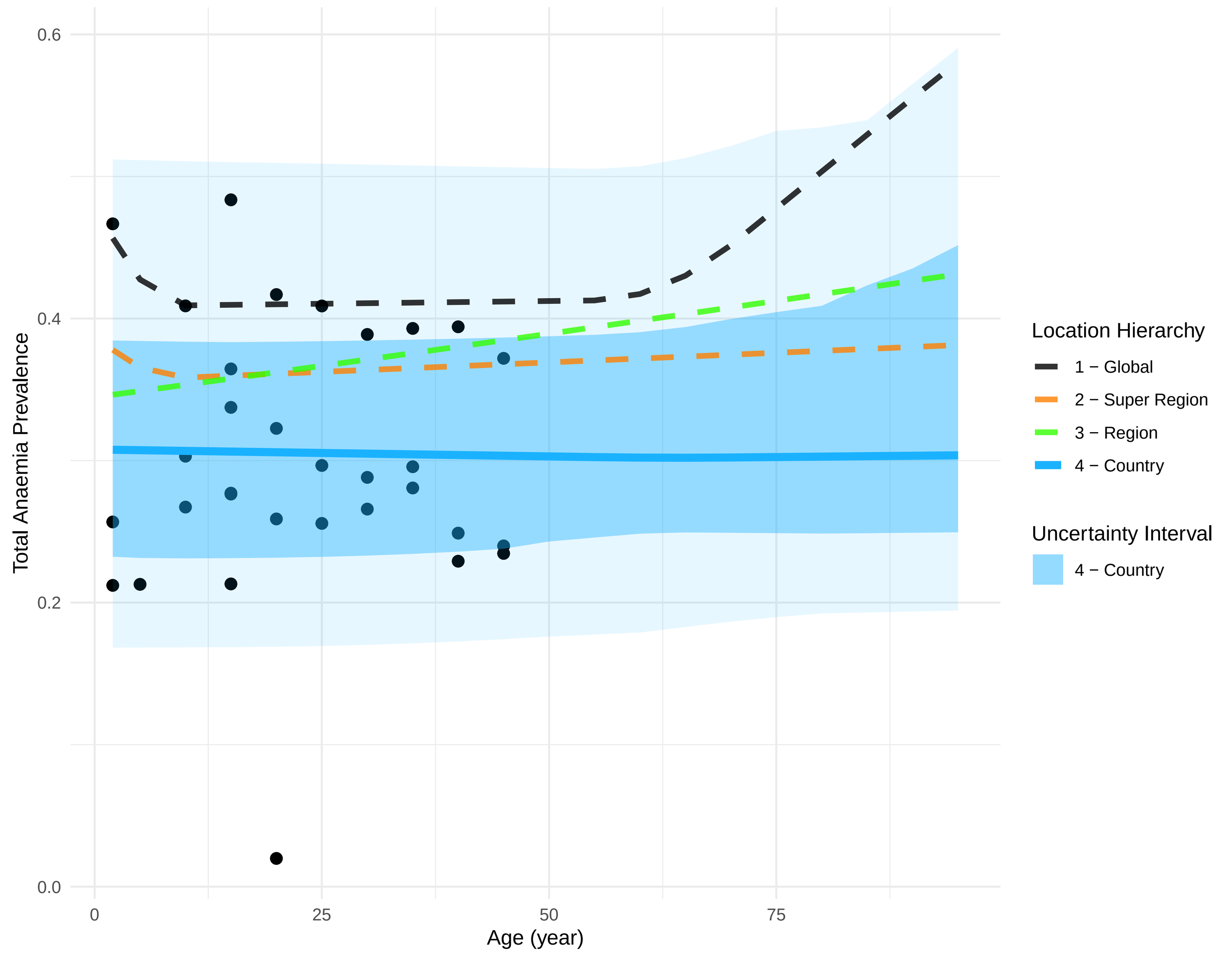

MR−BRT Age/Sex Fits − Non−pregnant Females 2+ Years Old − Total Anaemia Prevalence − Jordan
WHO−2001 Adjustment Method

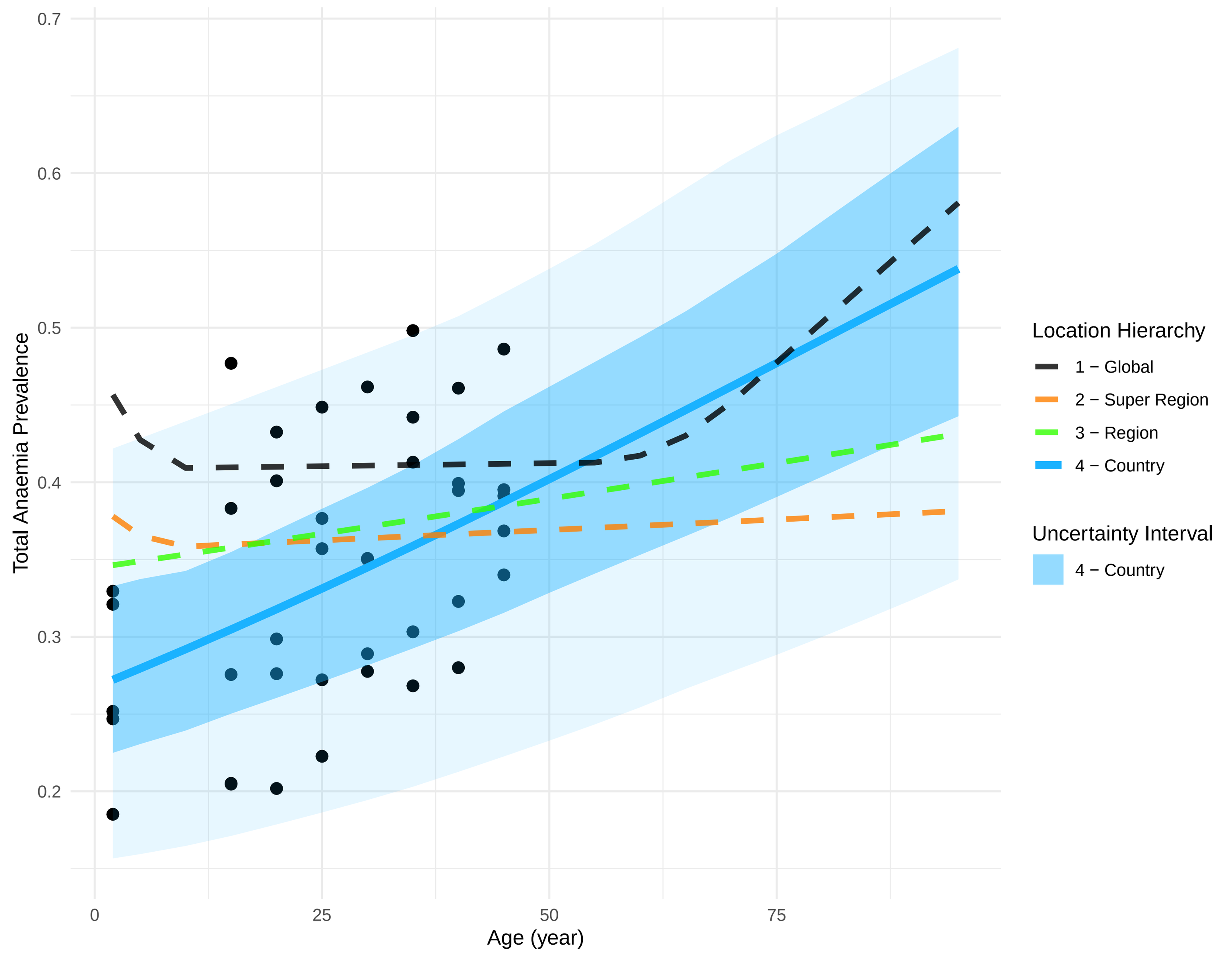

MR−BRT Age/Sex Fits − Non−pregnant Females 2+ Years Old − Total Anaemia Prevalence − Yemen
WHO−2001 Adjustment Method

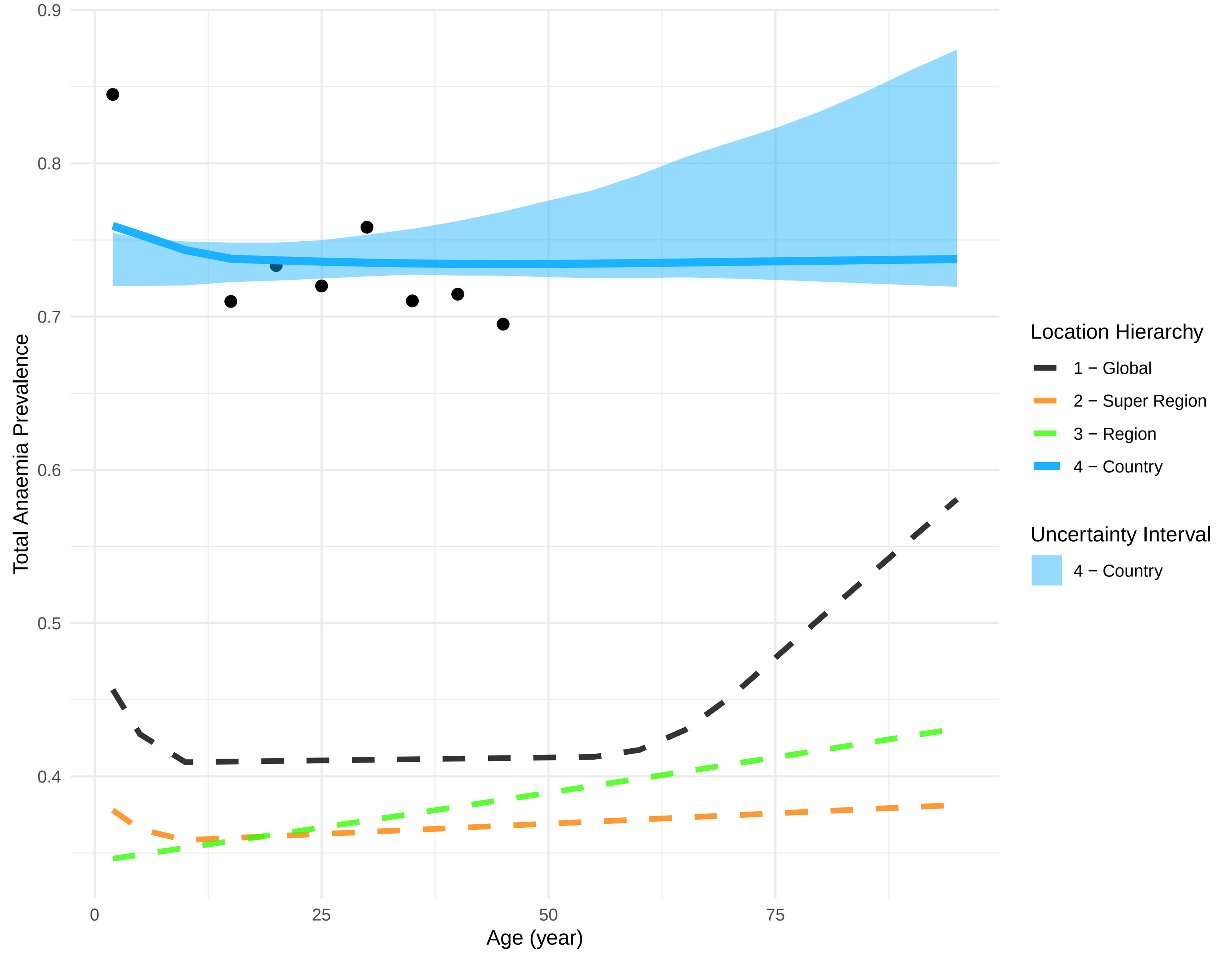

MR−BRT Age/Sex Fits − Non−pregnant Females 2+ Years Old − Total Anaemia Prevalence − South Asia
WHO−2001 Adjustment Method

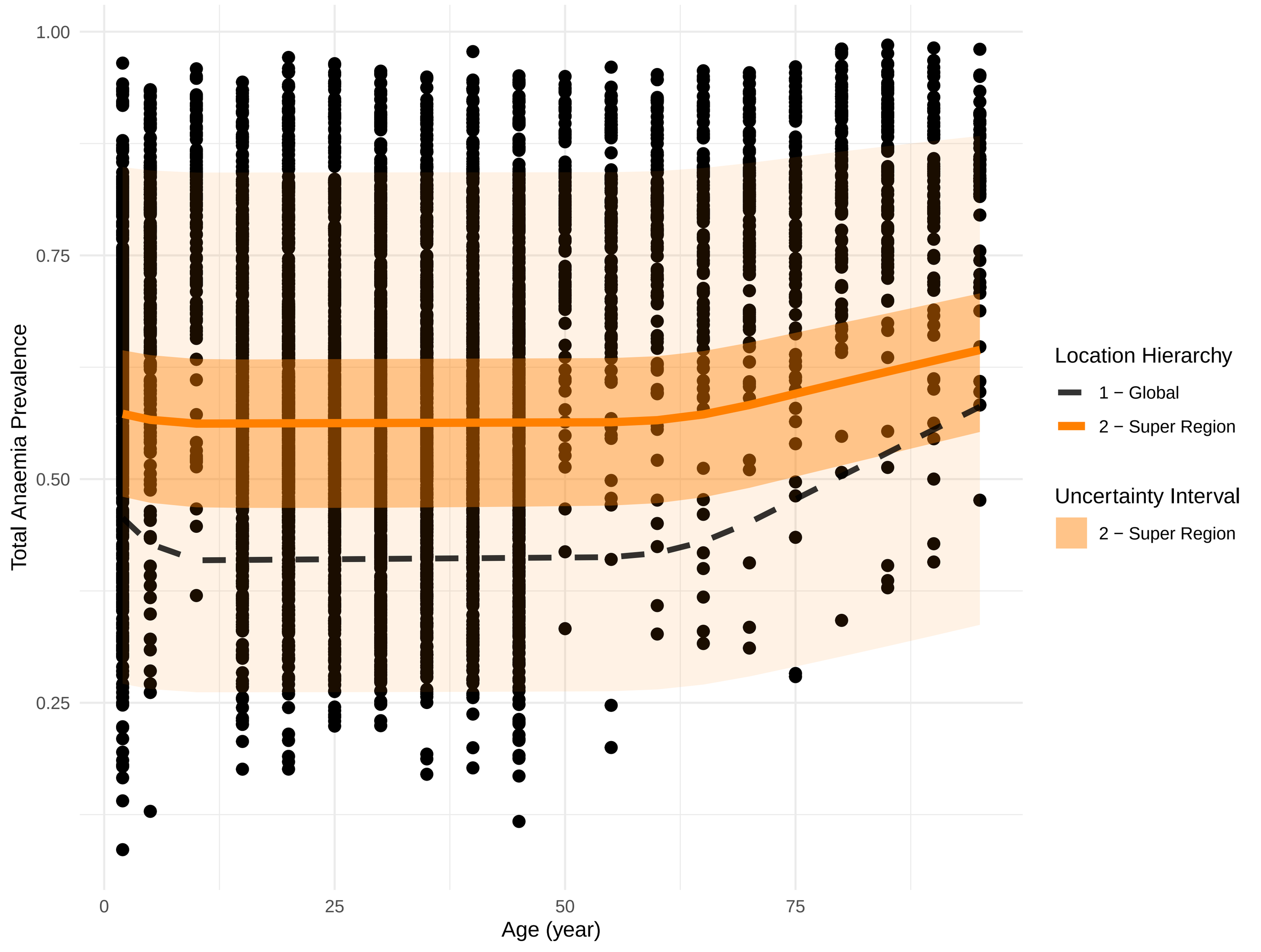

MR−BRT Age/Sex Fits − Non−pregnant Females 2+ Years Old − Total Anaemia Prevalence − South Asia
WHO−2001 Adjustment Method

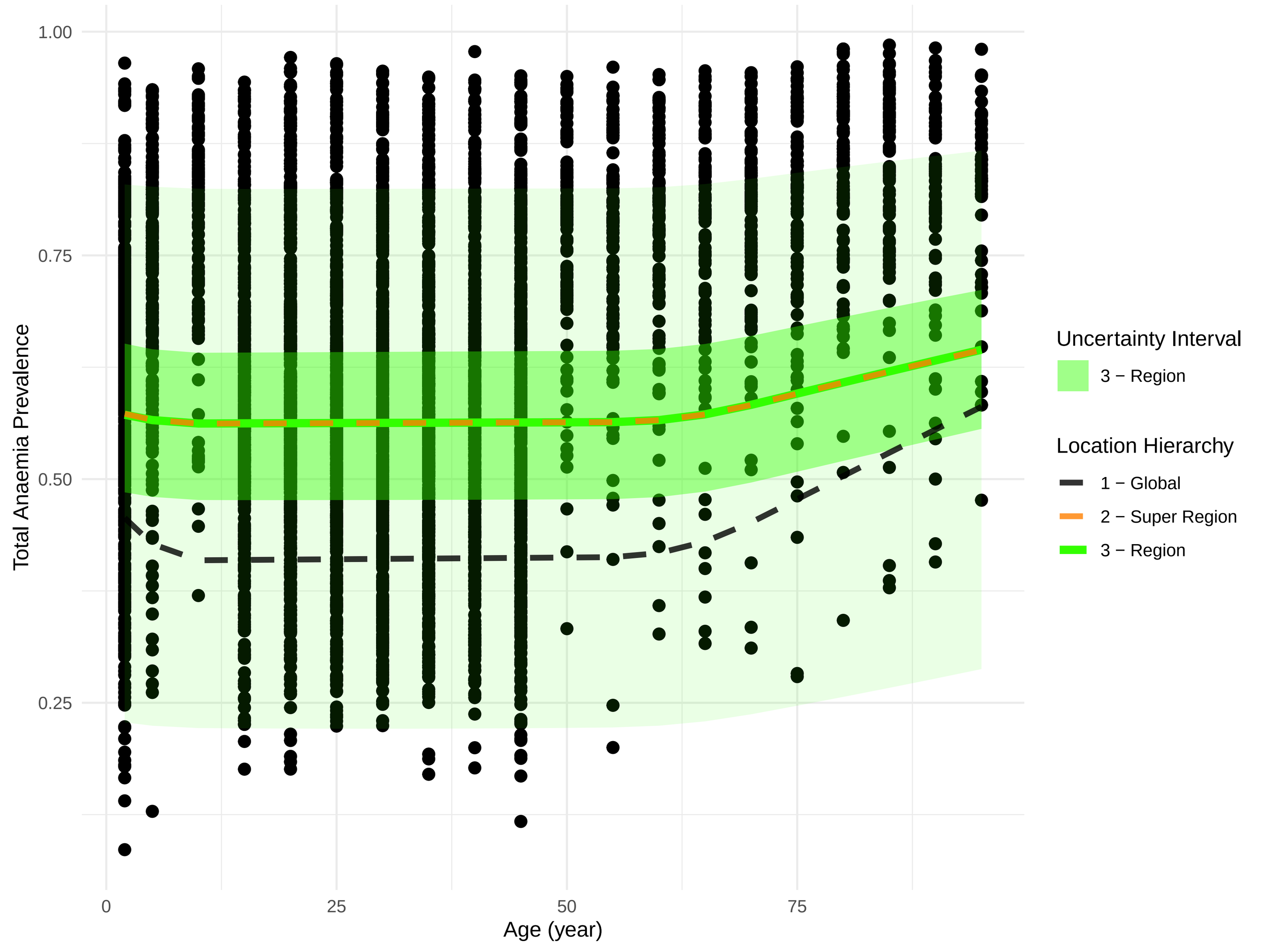

MR−BRT Age/Sex Fits − Non−pregnant Females 2+ Years Old − Total Anaemia Prevalence − Bangladesh
WHO−2001 Adjustment Method

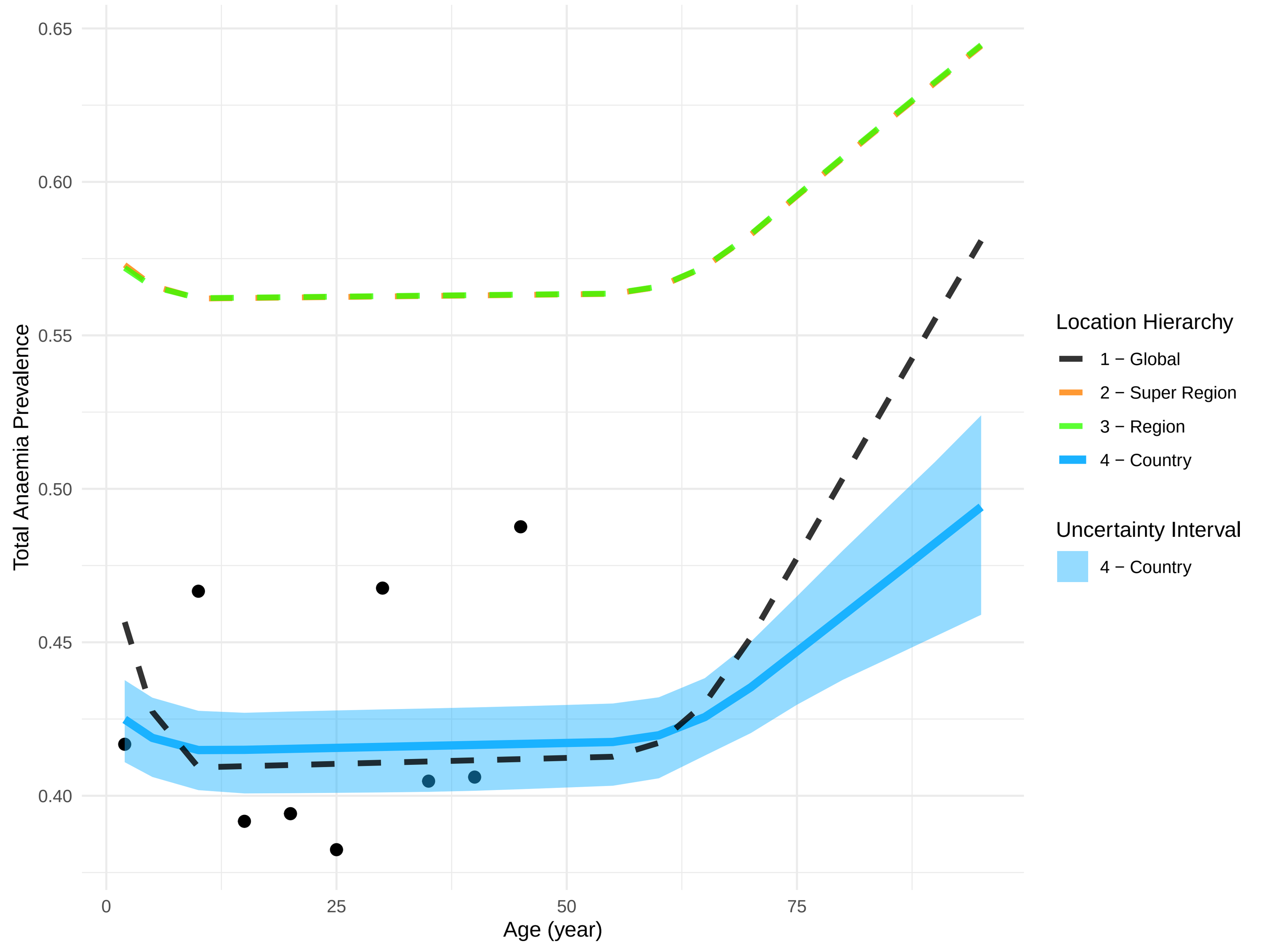

MR−BRT Age/Sex Fits − Non−pregnant Females 2+ Years Old − Total Anaemia Prevalence − India
WHO−2001 Adjustment Method

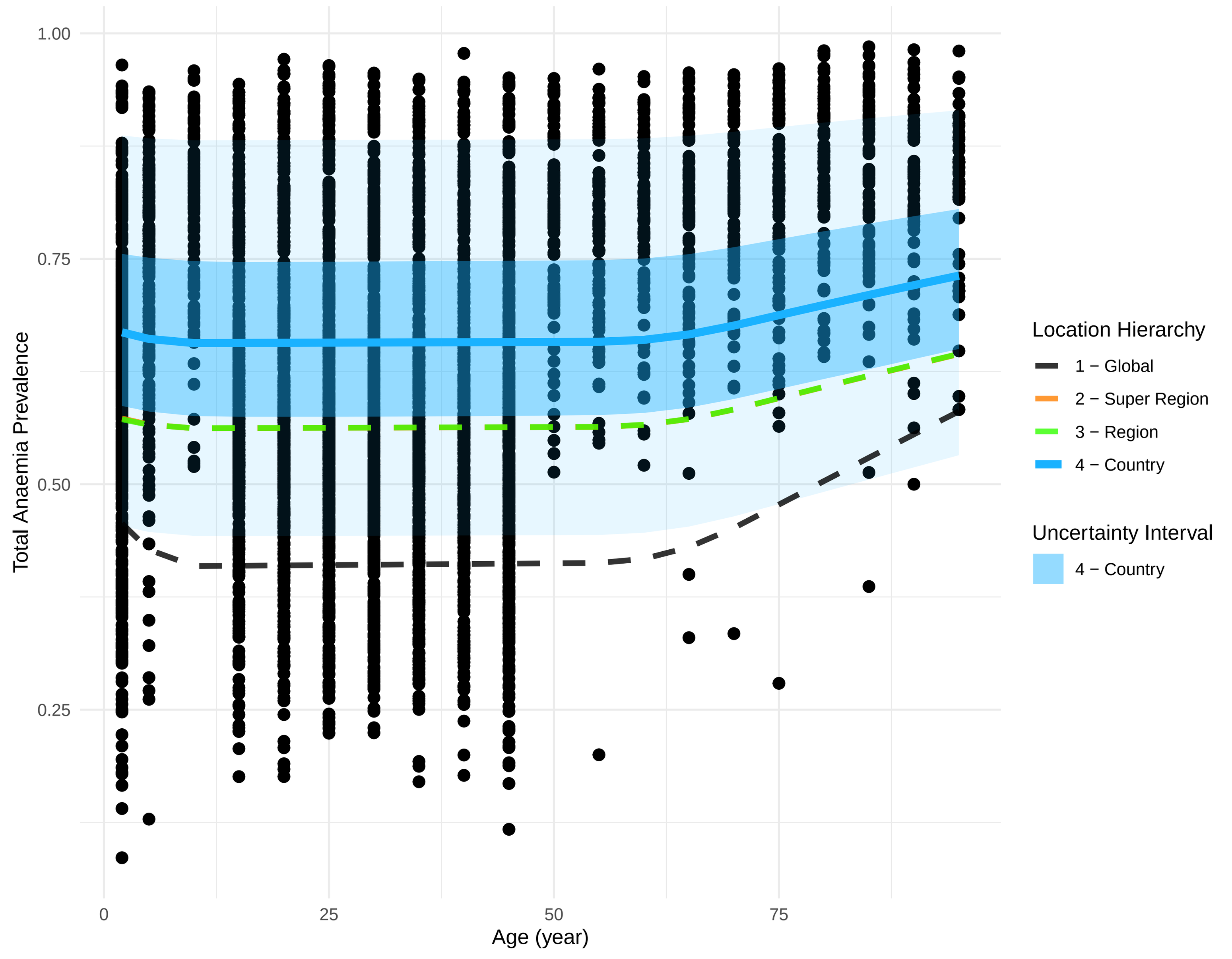

MR−BRT Age/Sex Fits − Non−pregnant Females 2+ Years Old − Total Anaemia Prevalence − Andhra Pradesh
WHO−2001 Adjustment Method

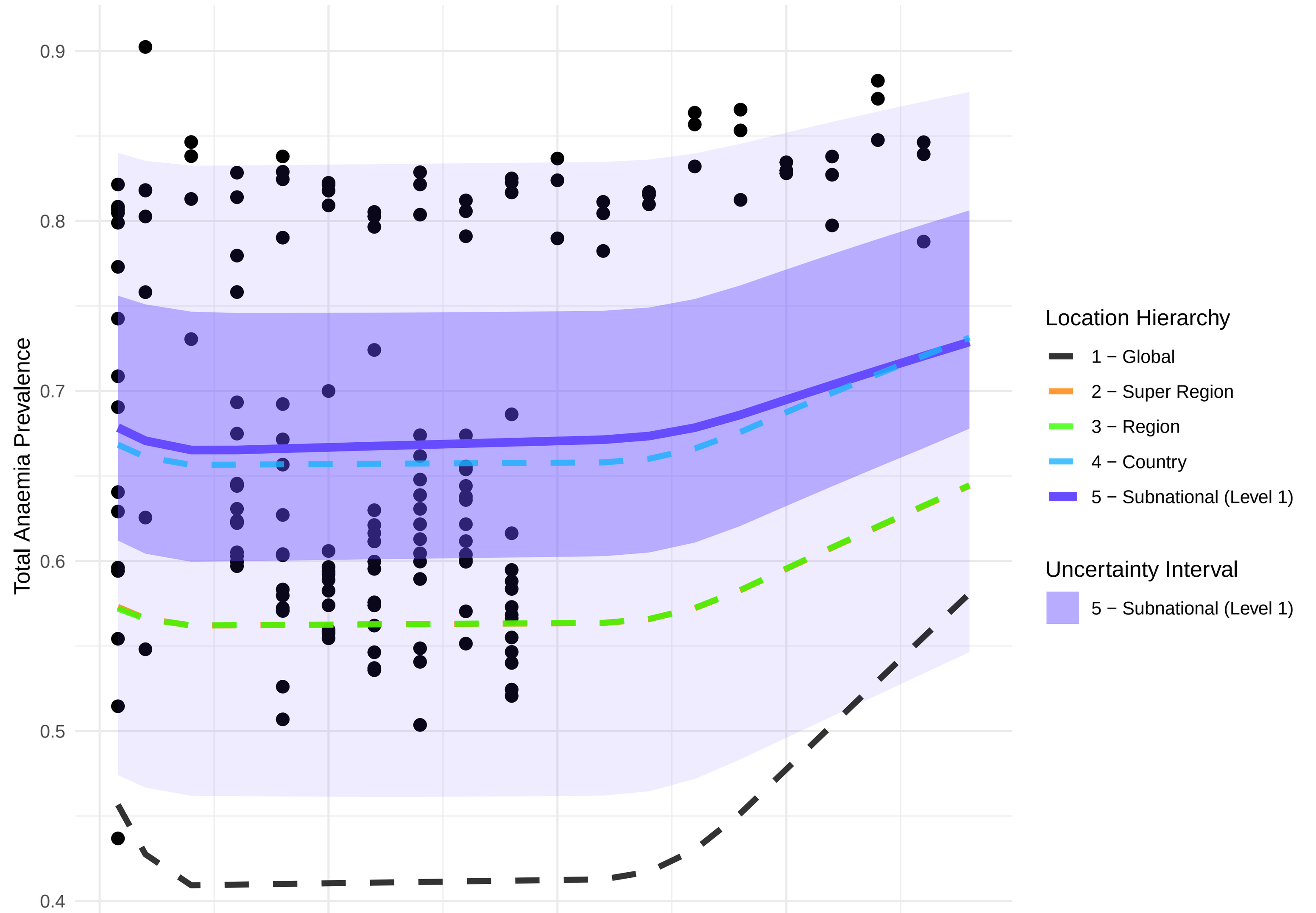

MR−BRT Age/Sex Fits − Non−pregnant Females 2+ Years Old − Total Anaemia Prevalence − Andhra Pradesh, Rural
WHO−2001 Adjustment Method

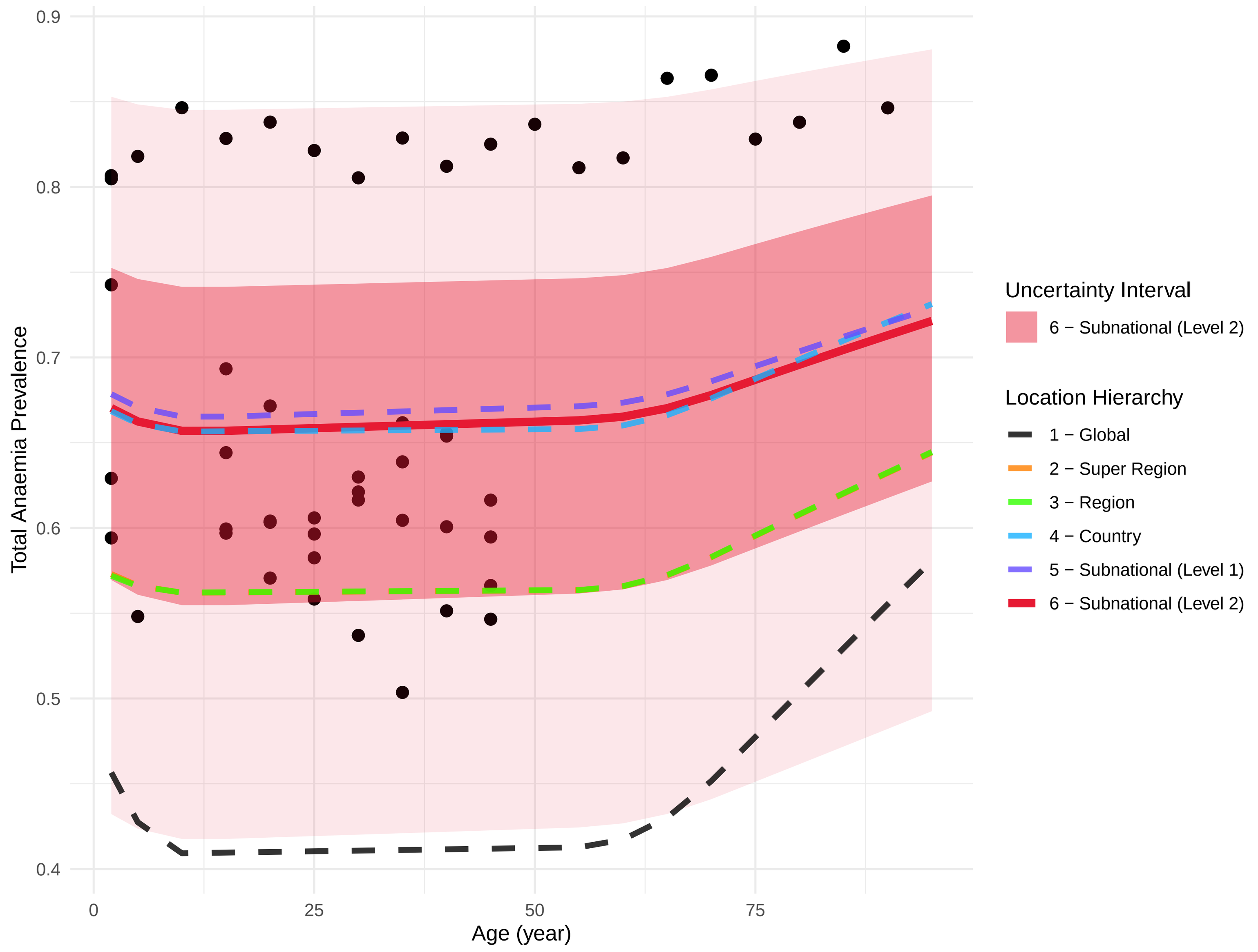

MR−BRT Age/Sex Fits − Non−pregnant Females 2+ Years Old − Total Anaemia Prevalence − Andhra Pradesh, Urban
WHO−2001 Adjustment Method

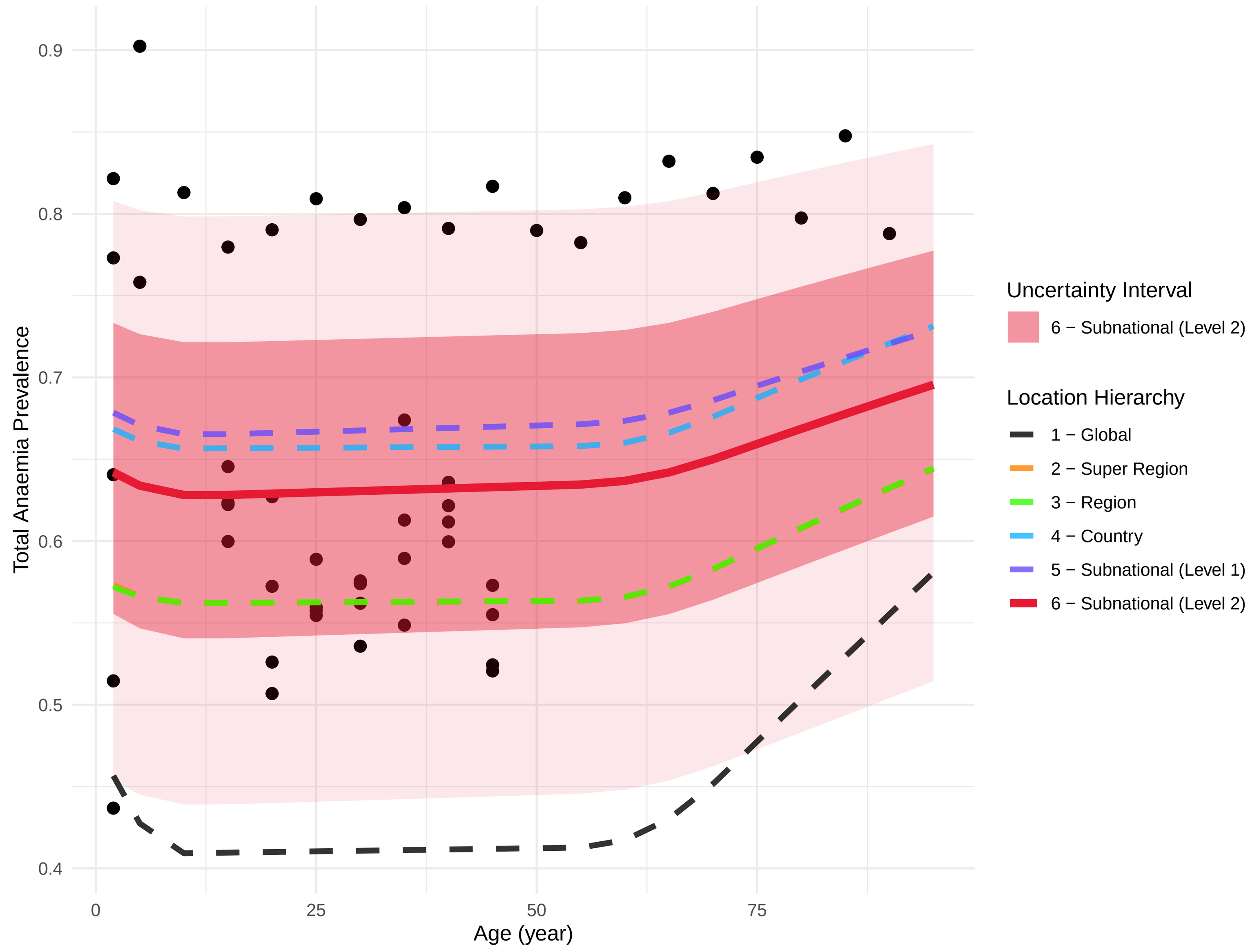

MR−BRT Age/Sex Fits − Non−pregnant Females 2+ Years Old − Total Anaemia Prevalence − Arunachal Pradesh
WHO−2001 Adjustment Method

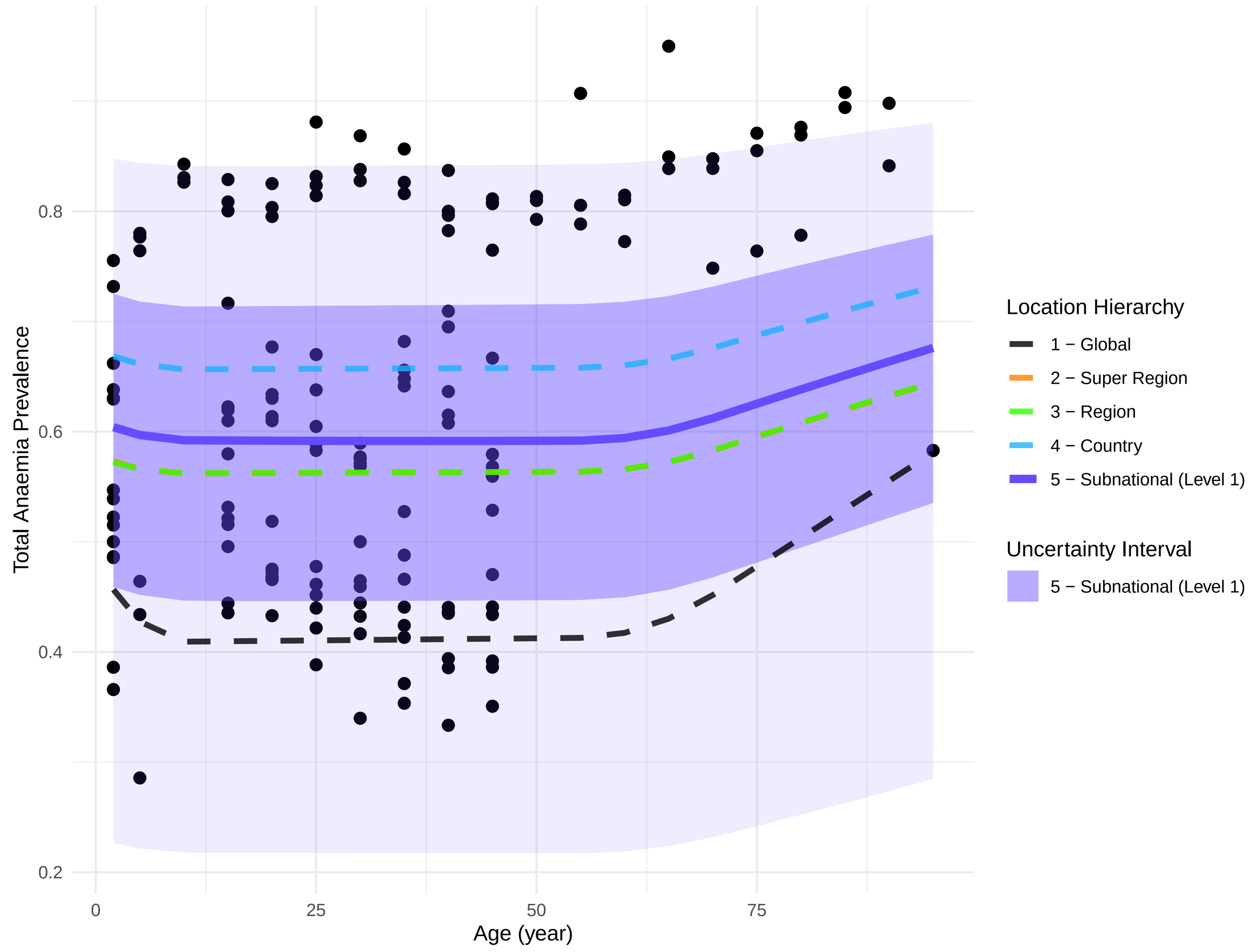

MR−BRT Age/Sex Fits − Non−pregnant Females 2+ Years Old − Total Anaemia Prevalence − Arunachal Pradesh, Rural
WHO−2001 Adjustment Method

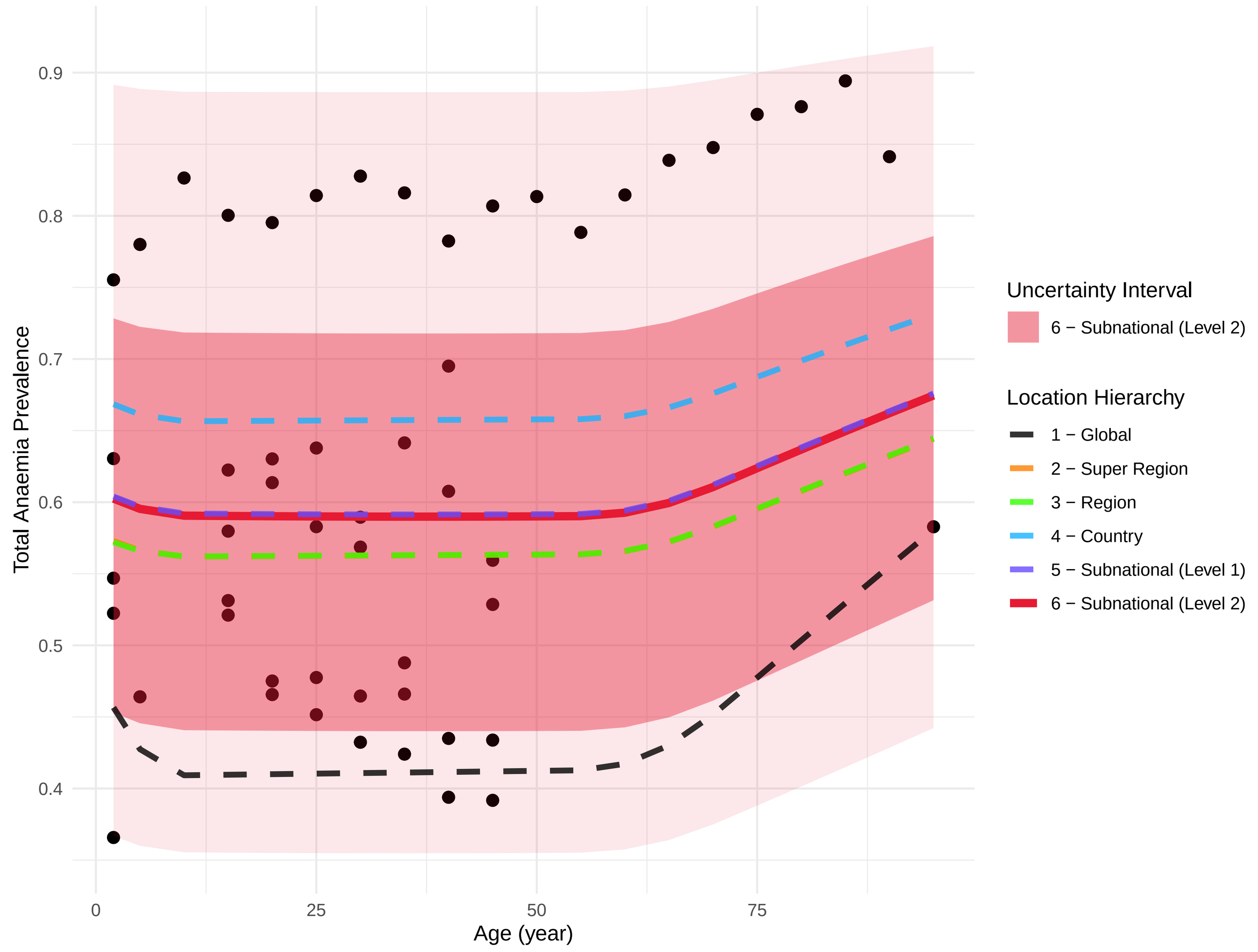

MR−BRT Age/Sex Fits − Non−pregnant Females 2+ Years Old − Total Anaemia Prevalence − Arunachal Pradesh, Urban
WHO−2001 Adjustment Method

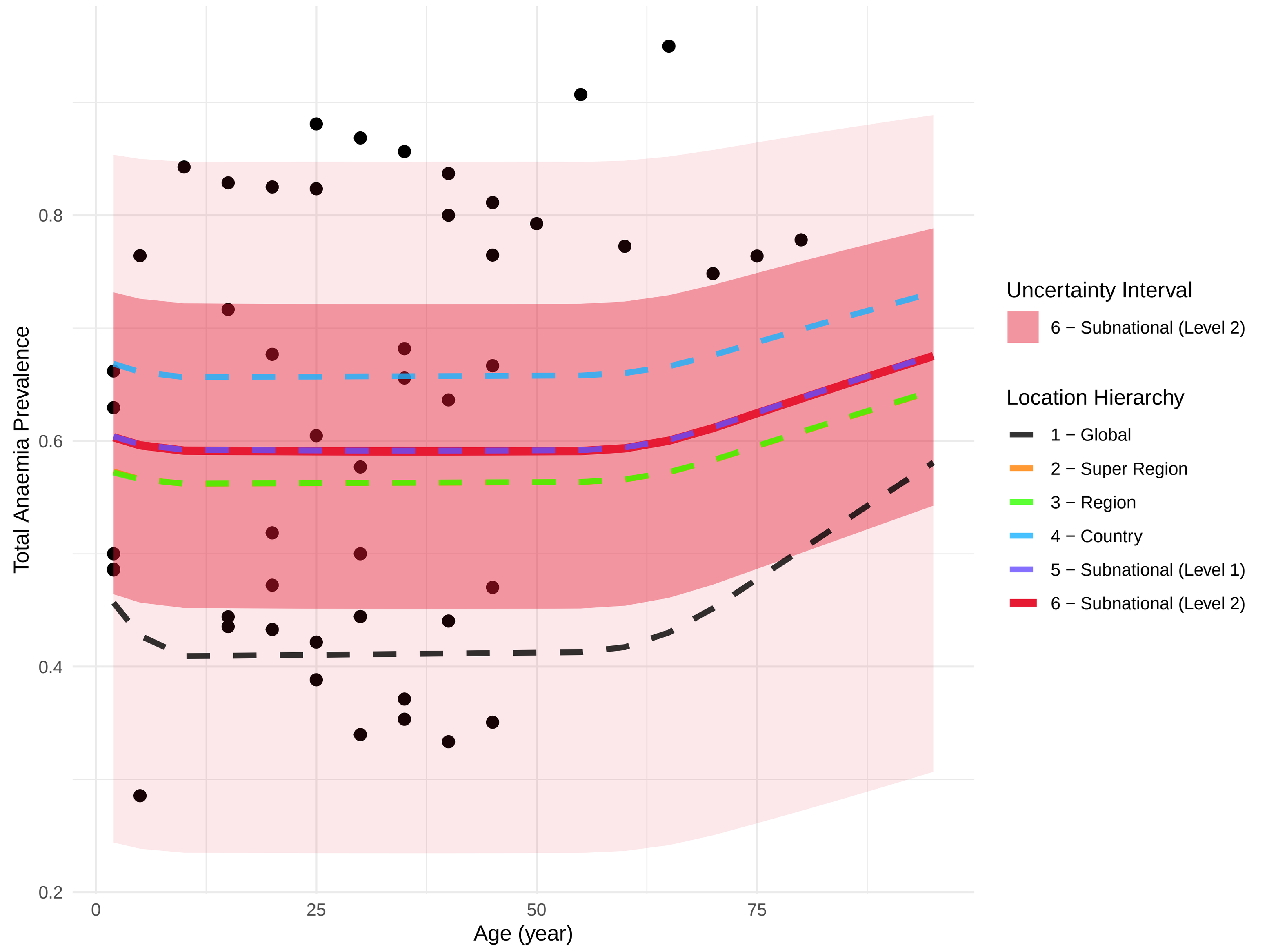

MR−BRT Age/Sex Fits − Non−pregnant Females 2+ Years Old − Total Anaemia Prevalence − Assam
WHO−2001 Adjustment Method

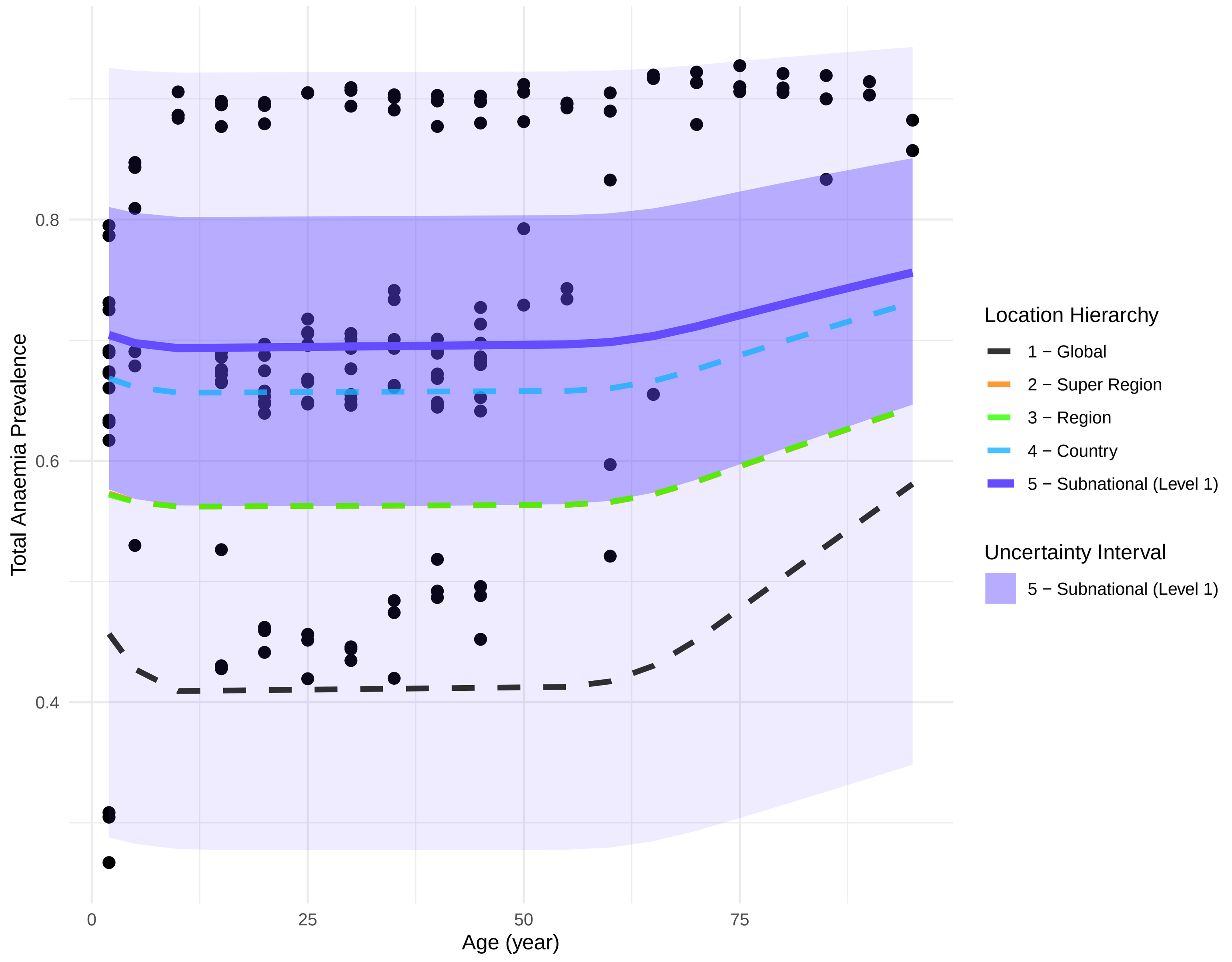

MR−BRT Age/Sex Fits − Non−pregnant Females 2+ Years Old − Total Anaemia Prevalence − Assam, Rural
WHO−2001 Adjustment Method

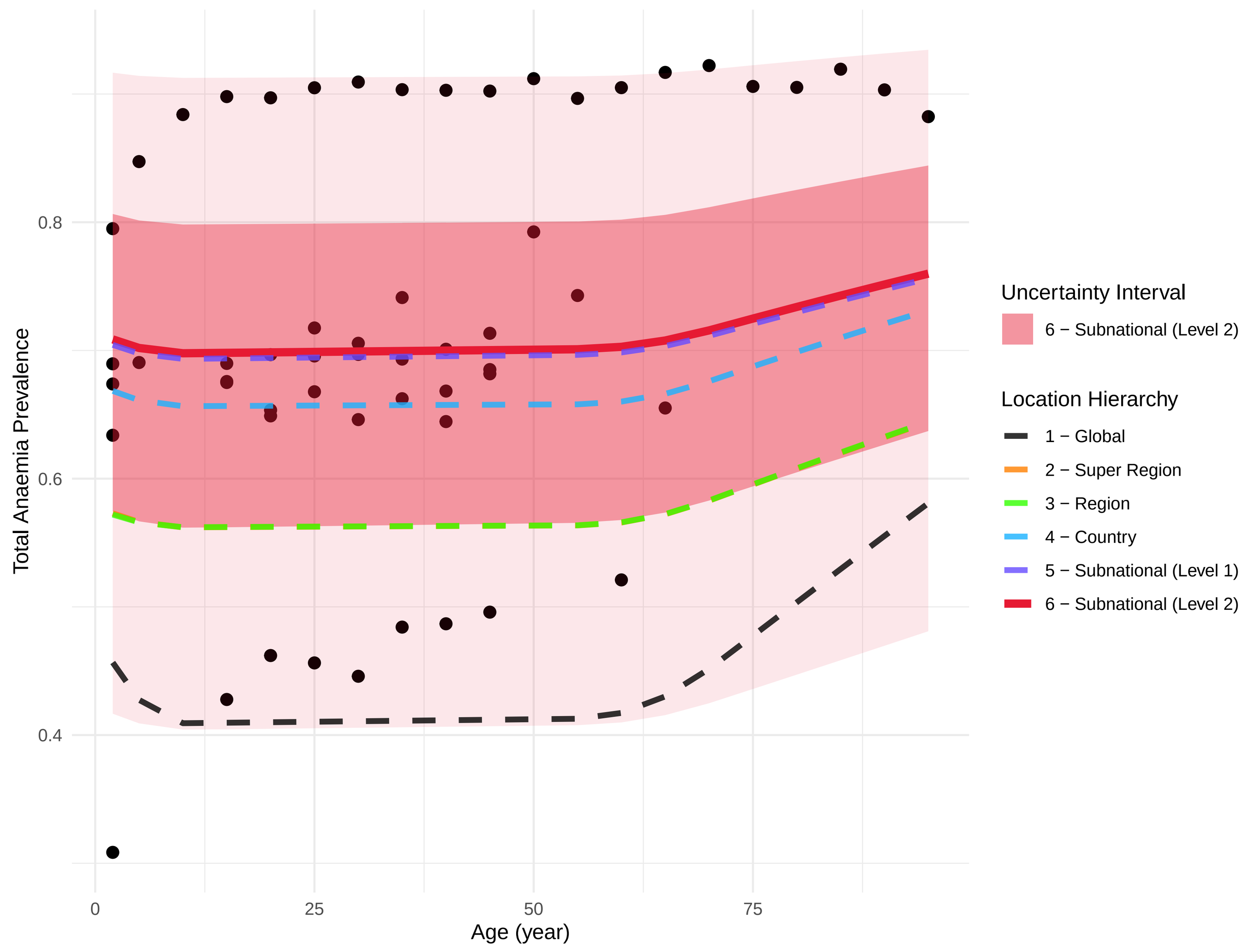

MR−BRT Age/Sex Fits − Non−pregnant Females 2+ Years Old − Total Anaemia Prevalence − Assam, Urban
WHO−2001 Adjustment Method

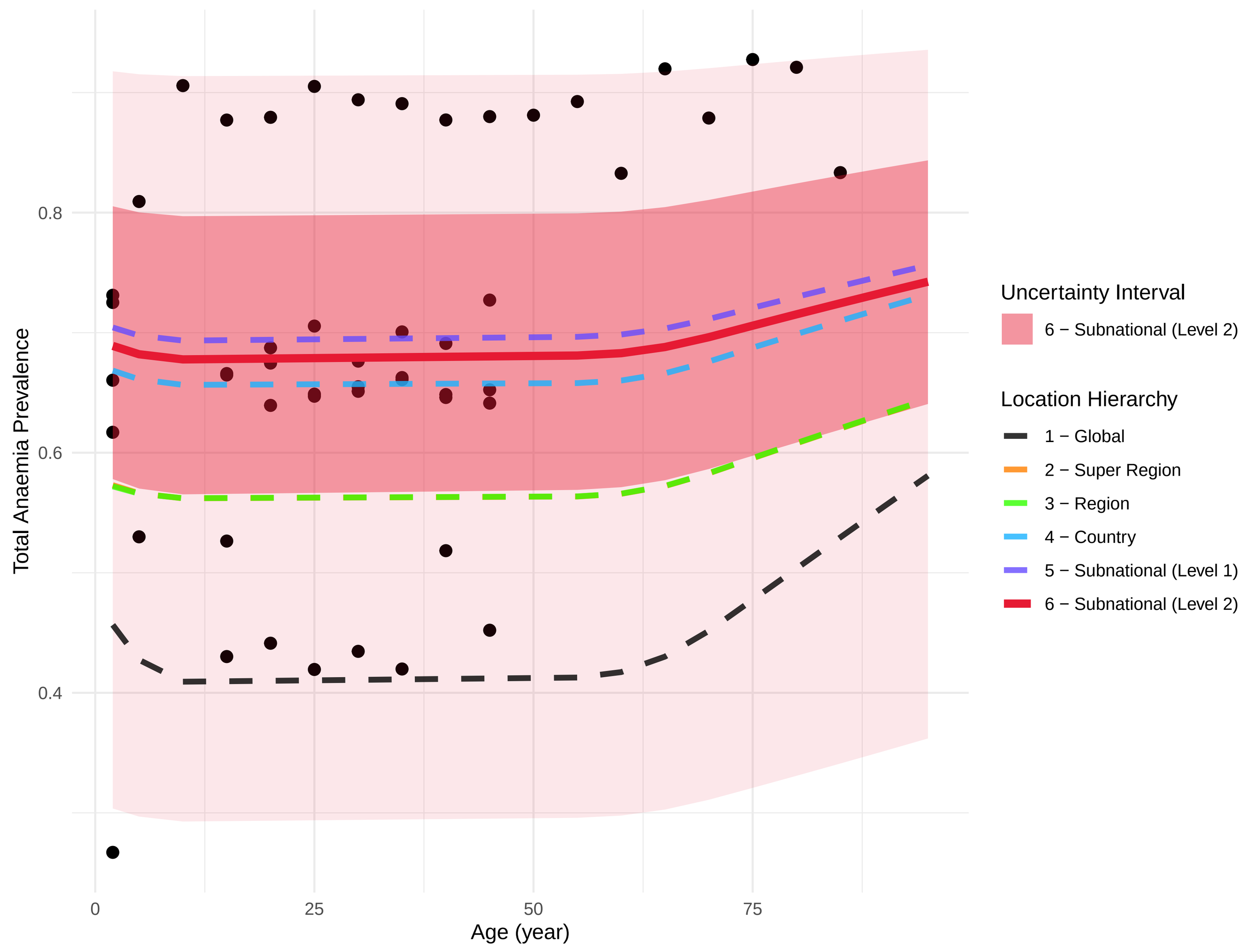

MR−BRT Age/Sex Fits − Non−pregnant Females 2+ Years Old − Total Anaemia Prevalence − Bihar
WHO−2001 Adjustment Method

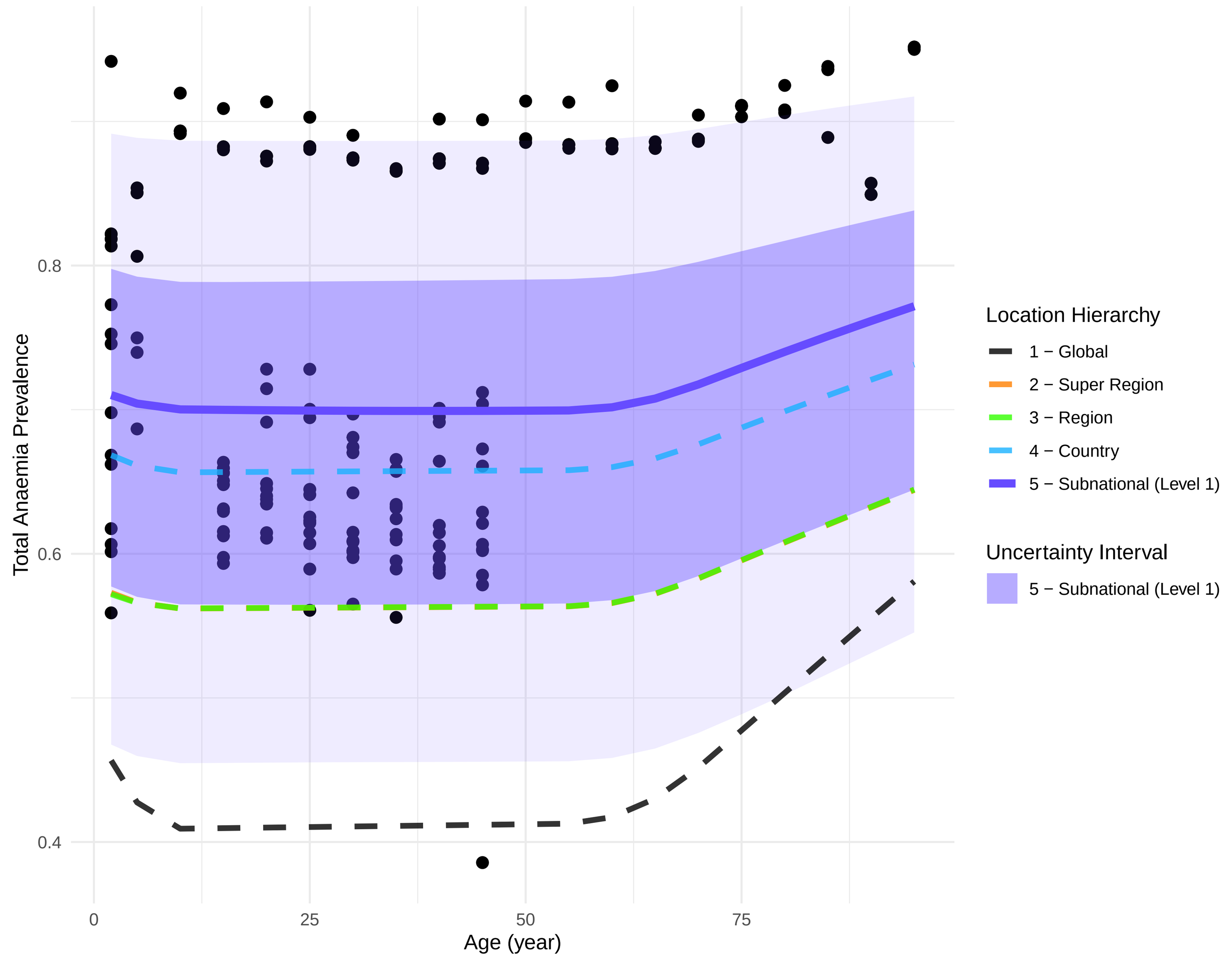

MR−BRT Age/Sex Fits − Non−pregnant Females 2+ Years Old − Total Anaemia Prevalence − Bihar, Rural
WHO−2001 Adjustment Method

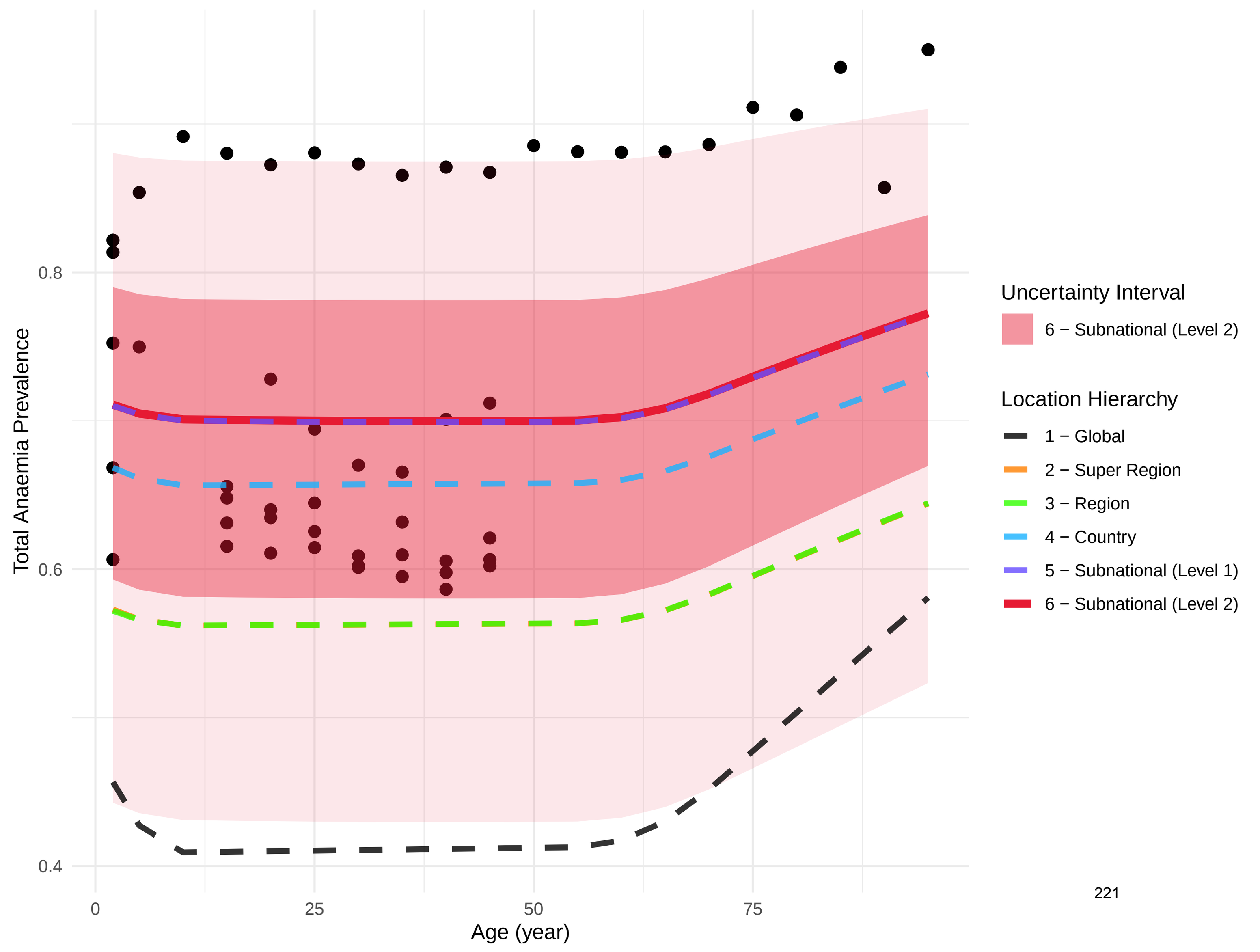

MR−BRT Age/Sex Fits − Non−pregnant Females 2+ Years Old − Total Anaemia Prevalence − Bihar, Urban

WHO−2001 Adjustment Method

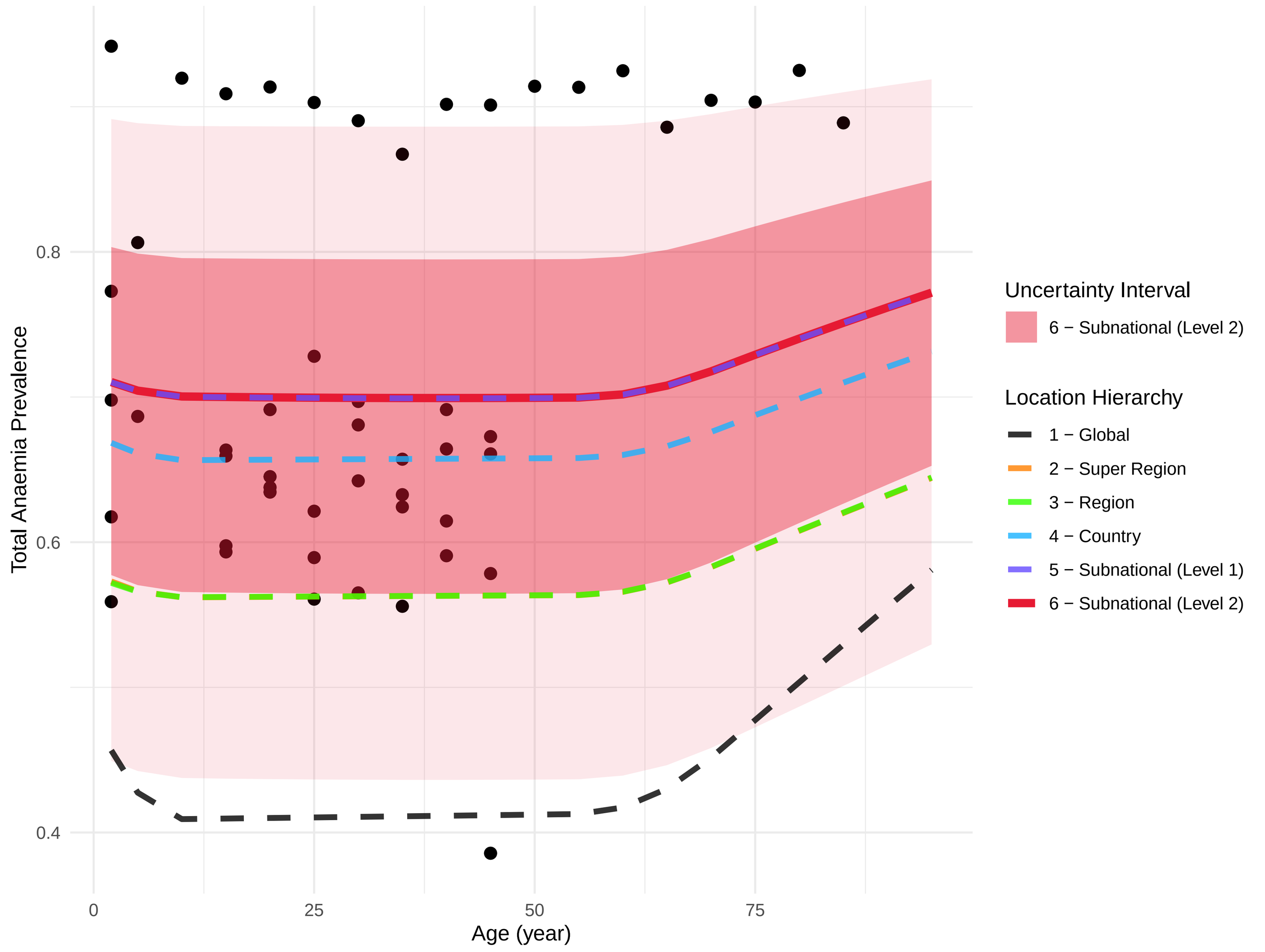

MR−BRT Age/Sex Fits − Non−pregnant Females 2+ Years Old − Total Anaemia Prevalence − Chhattisgarh

WHO−2001 Adjustment Method

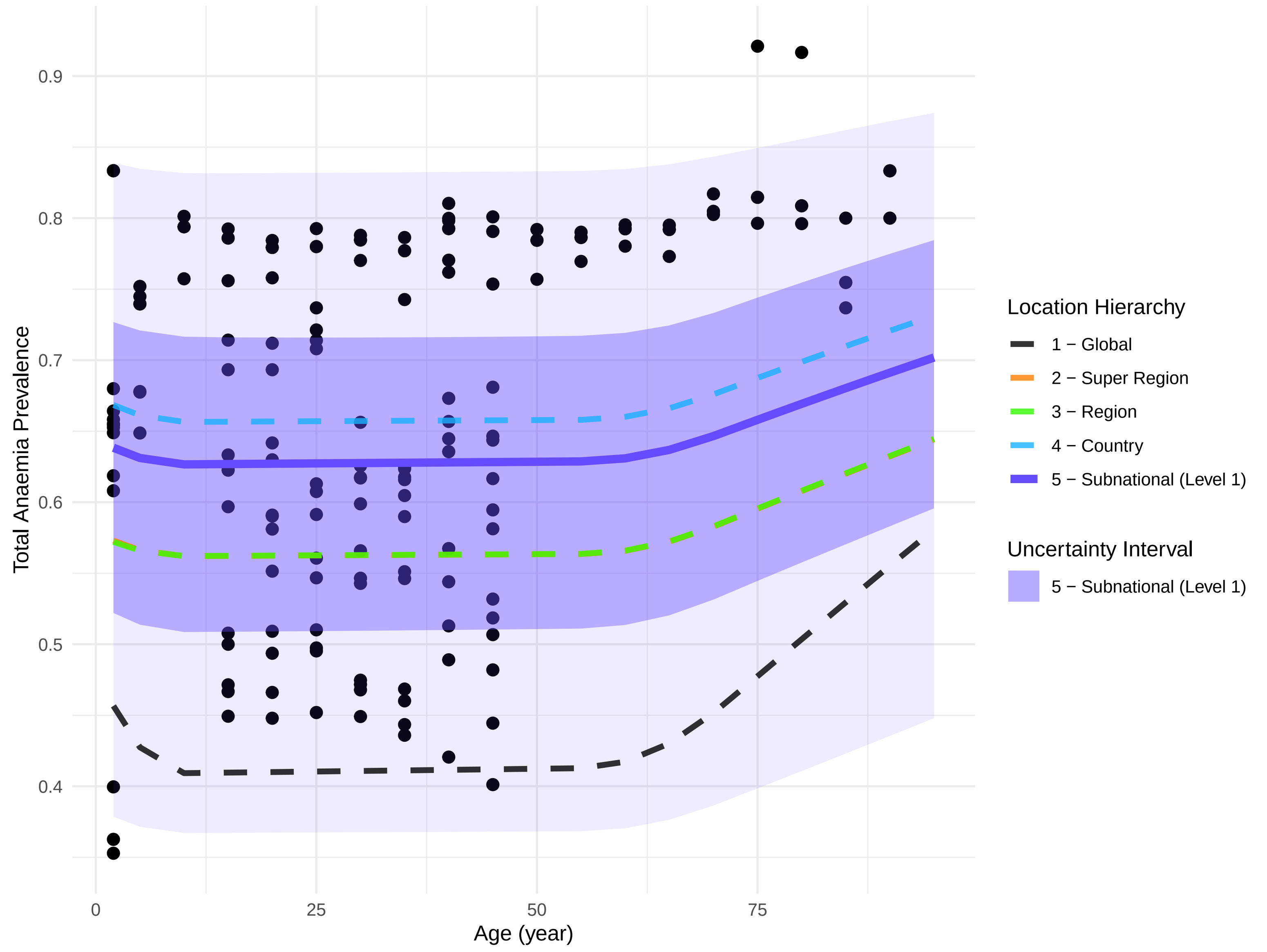

MR−BRT Age/Sex Fits − Non−pregnant Females 2+ Years Old − Total Anaemia Prevalence − Chhattisgarh, Rural

WHO−2001 Adjustment Method

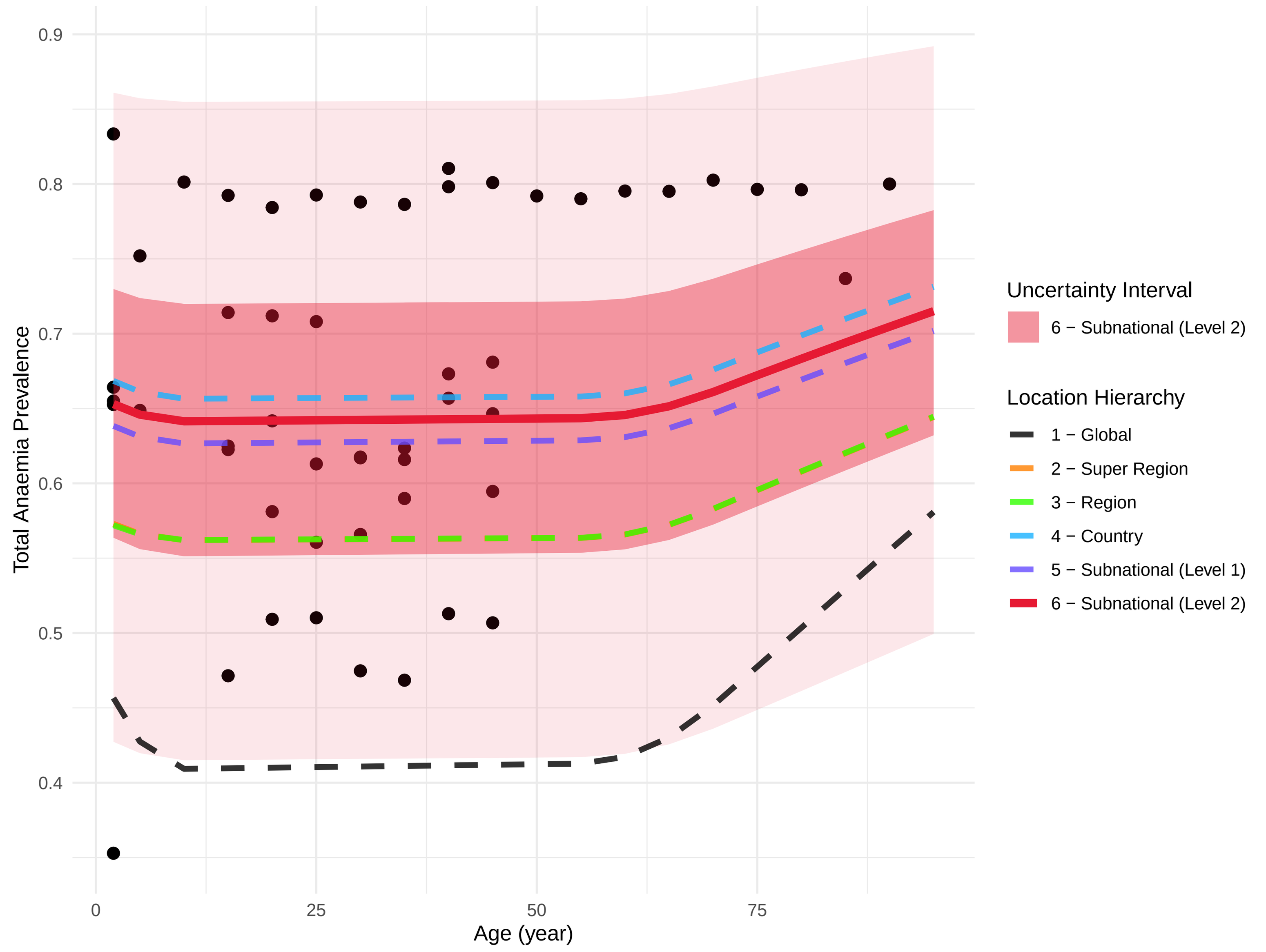

MR−BRT Age/Sex Fits − Non−pregnant Females 2+ Years Old − Total Anaemia Prevalence − Chhattisgarh, Urban

WHO−2001 Adjustment Method

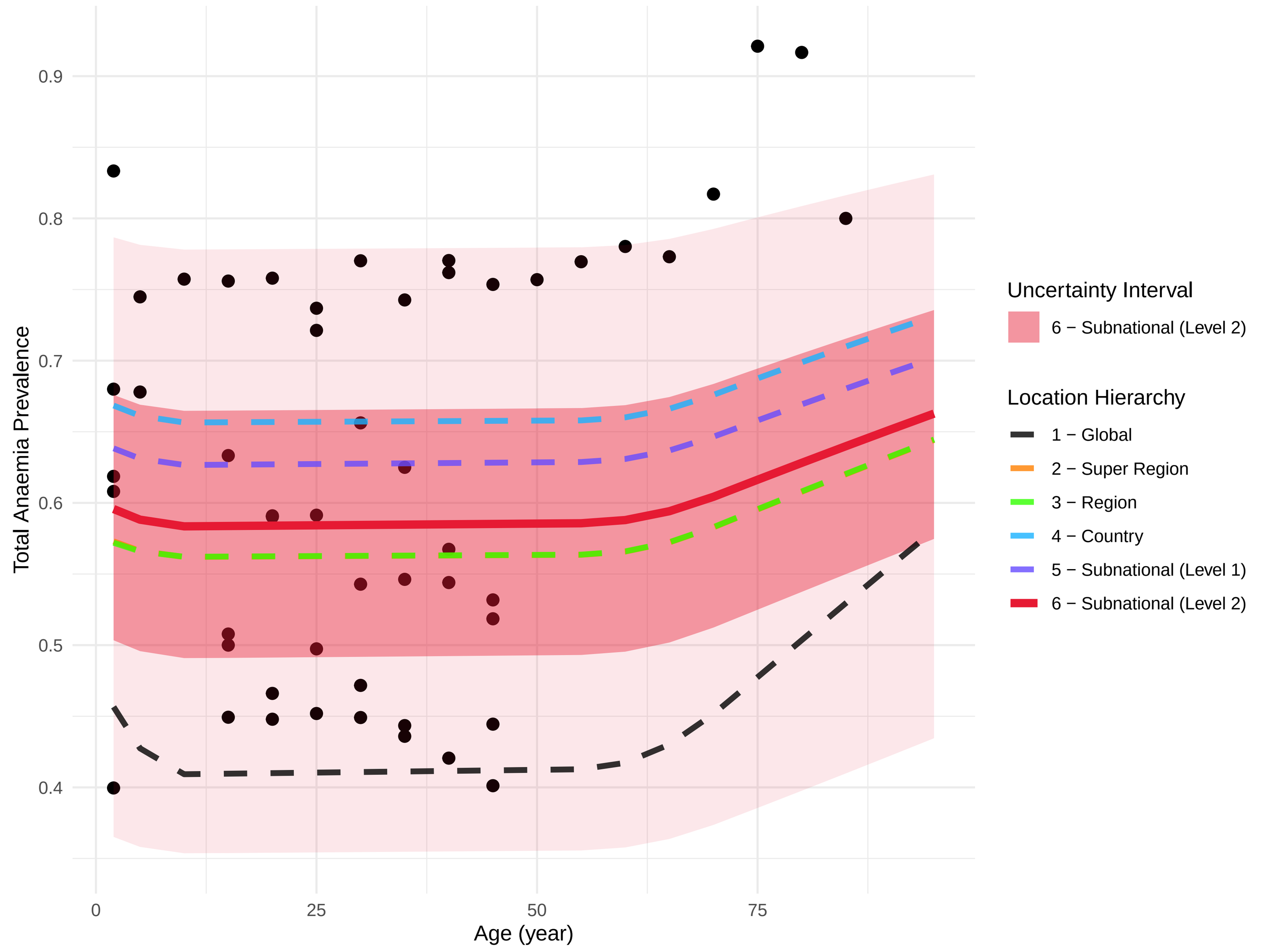

MR−BRT Age/Sex Fits − Non−pregnant Females 2+ Years Old − Total Anaemia Prevalence − Delhi

WHO−2001 Adjustment Method

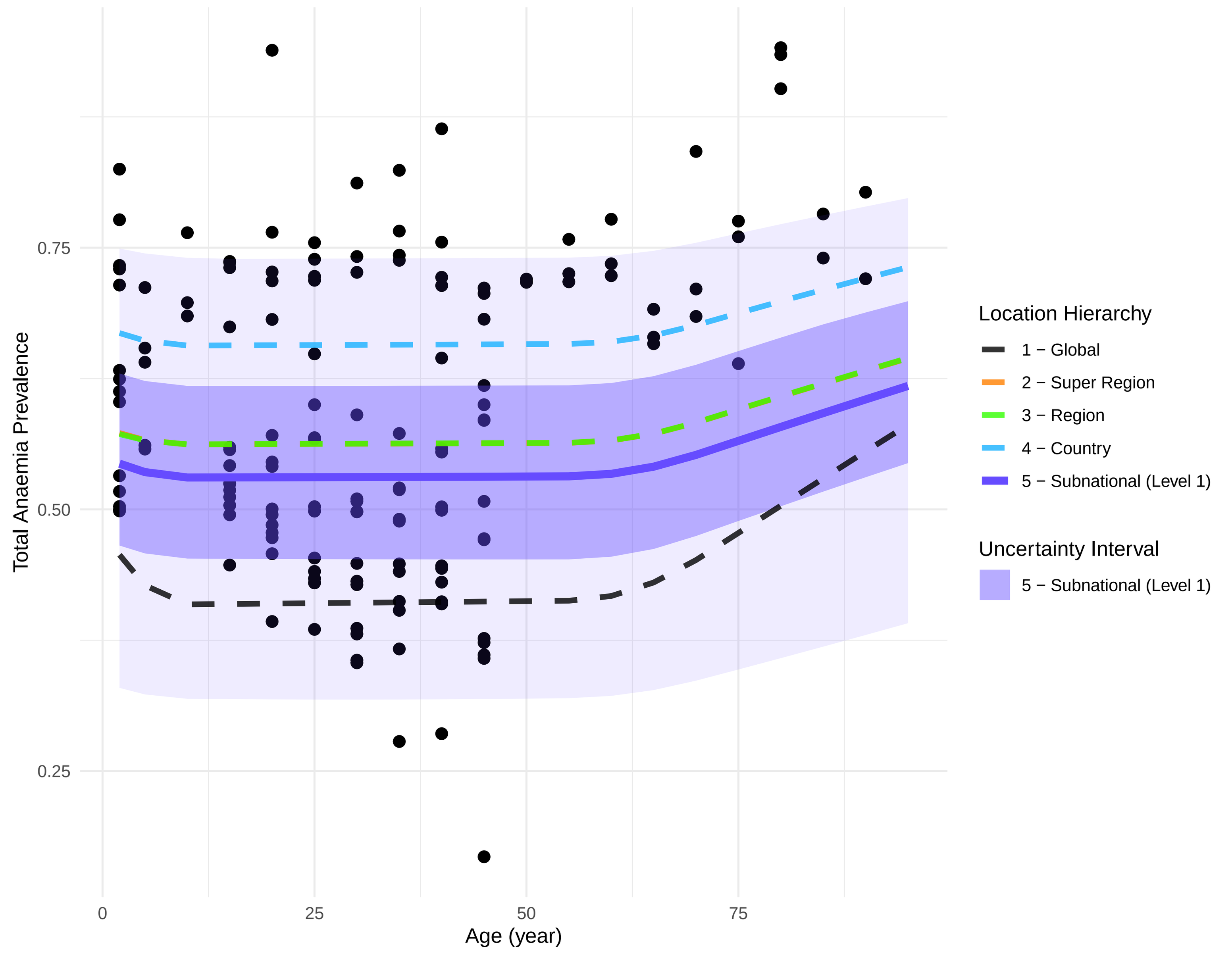

MR−BRT Age/Sex Fits − Non−pregnant Females 2+ Years Old − Total Anaemia Prevalence − Delhi, Rural

WHO−2001 Adjustment Method

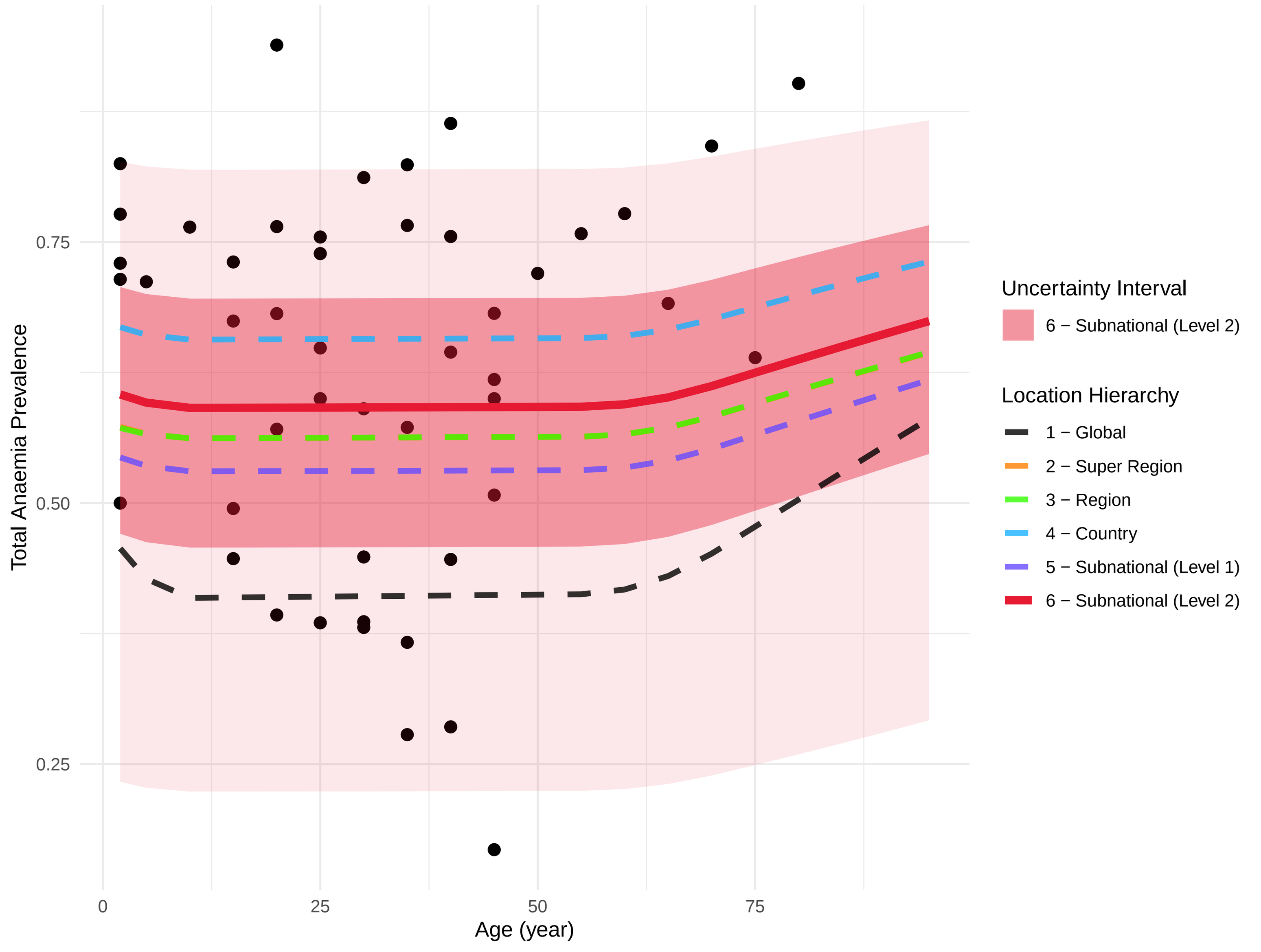

MR−BRT Age/Sex Fits − Non−pregnant Females 2+ Years Old − Total Anaemia Prevalence − Delhi, Urban

WHO−2001 Adjustment Method

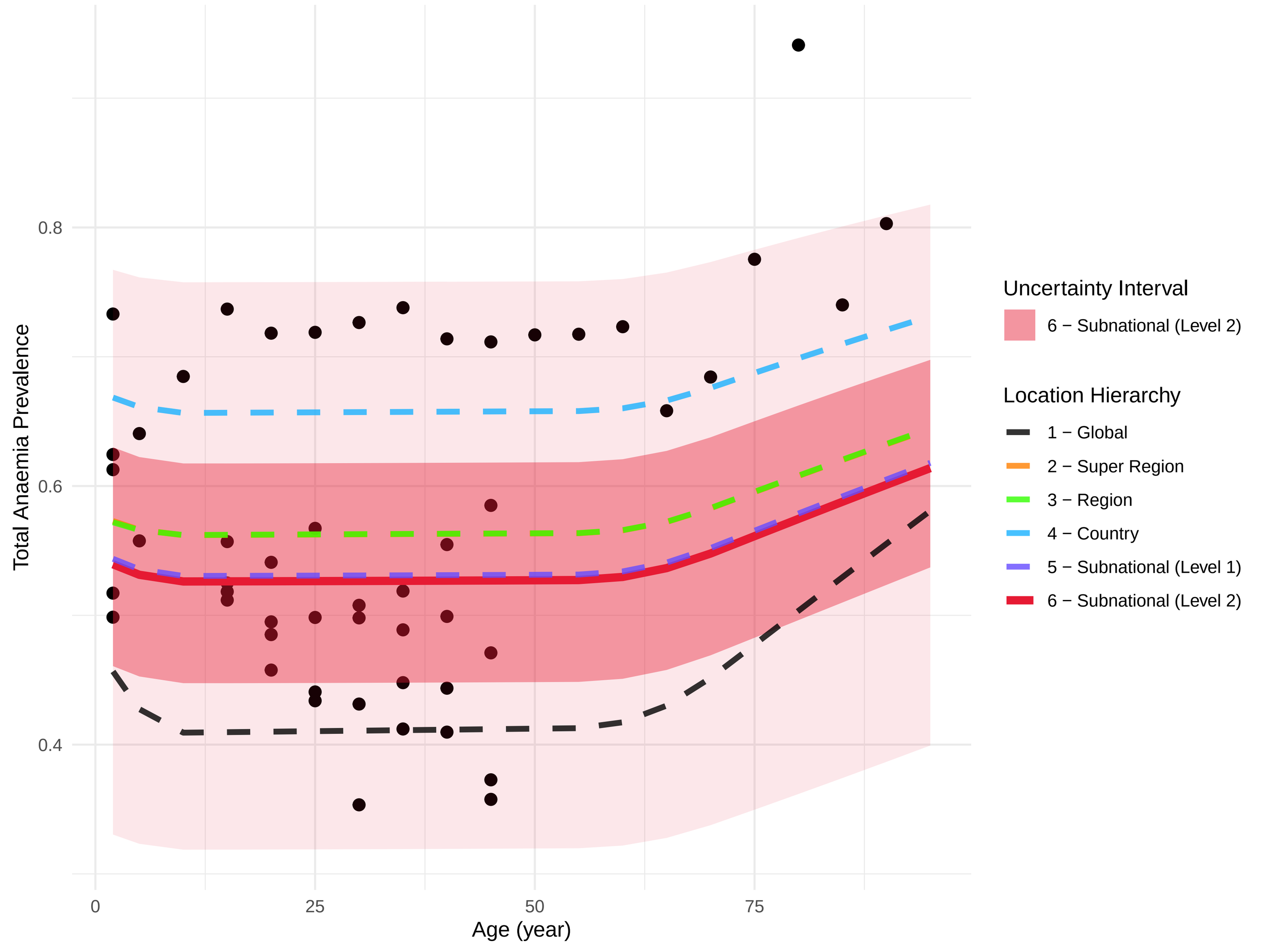

MR−BRT Age/Sex Fits − Non−pregnant Females 2+ Years Old − Total Anaemia Prevalence − Goa

WHO−2001 Adjustment Method

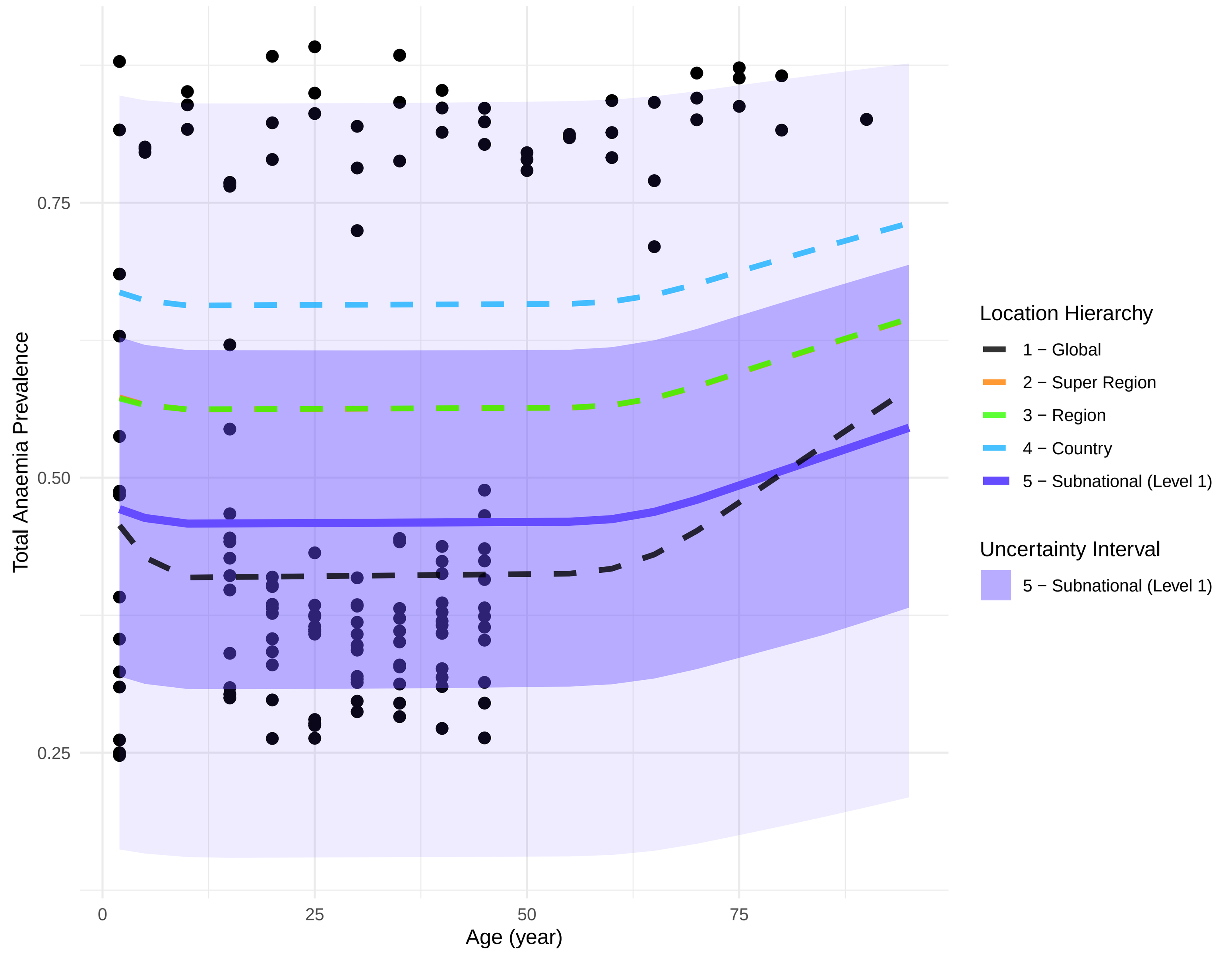

MR−BRT Age/Sex Fits − Non−pregnant Females 2+ Years Old − Total Anaemia Prevalence − Goa, Rural

WHO−2001 Adjustment Method

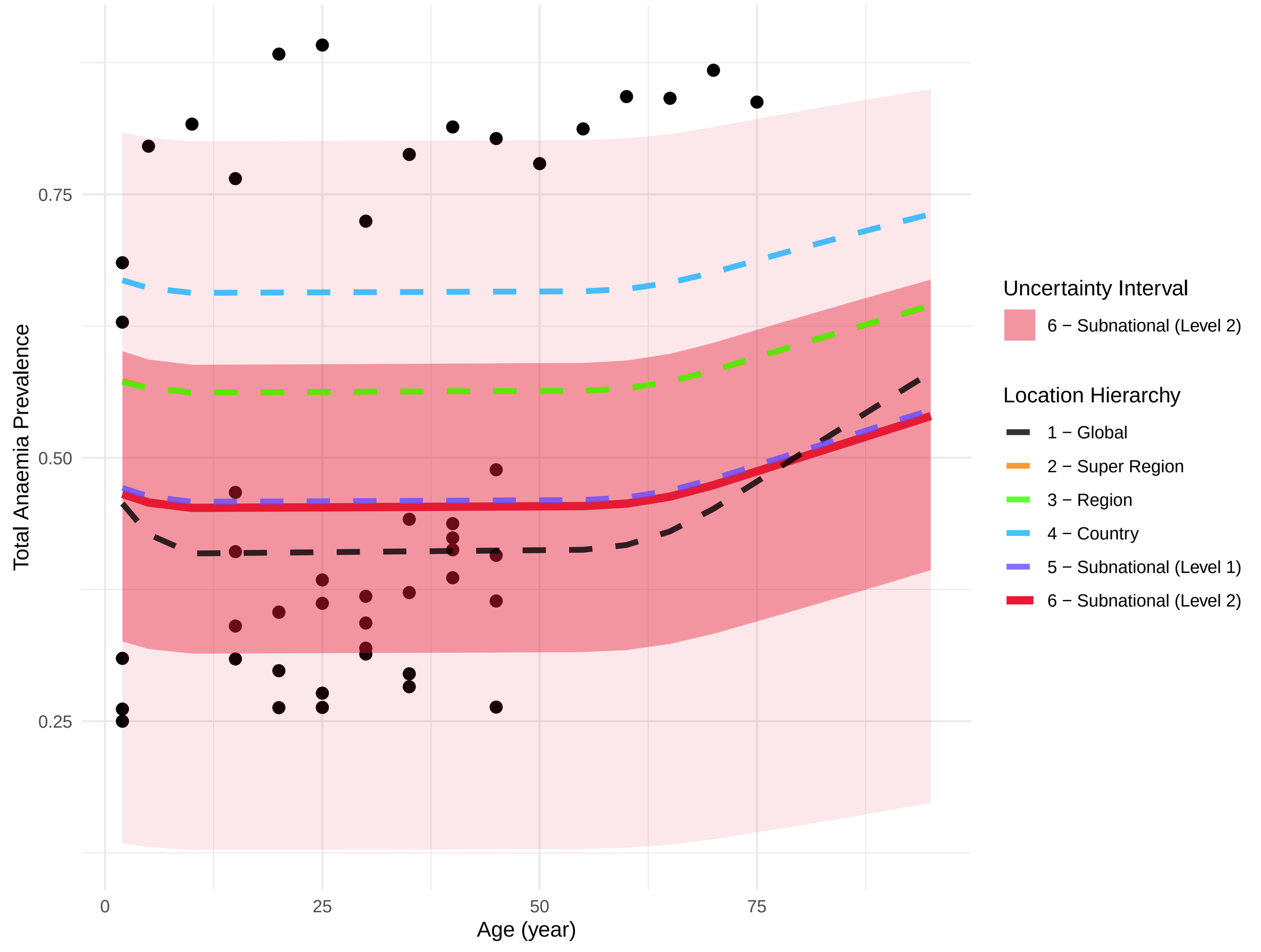

MR−BRT Age/Sex Fits − Non−pregnant Females 2+ Years Old − Total Anaemia Prevalence − Goa, Urban

WHO−2001 Adjustment Method

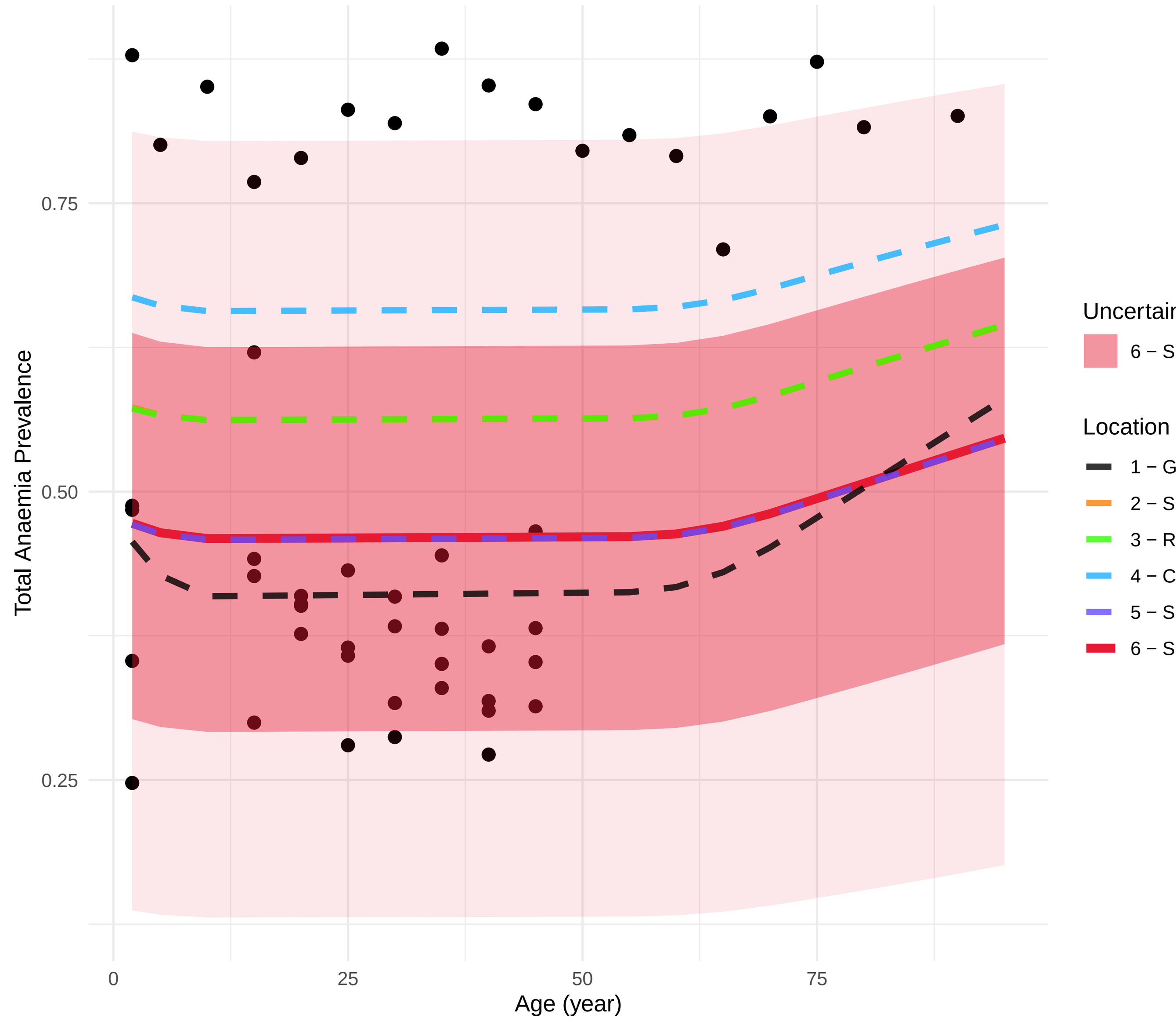

MR−BRT Age/Sex Fits − Non−pregnant Females 2+ Years Old − Total Anaemia Prevalence − Gujarat
WHO−2001 Adjustment Method

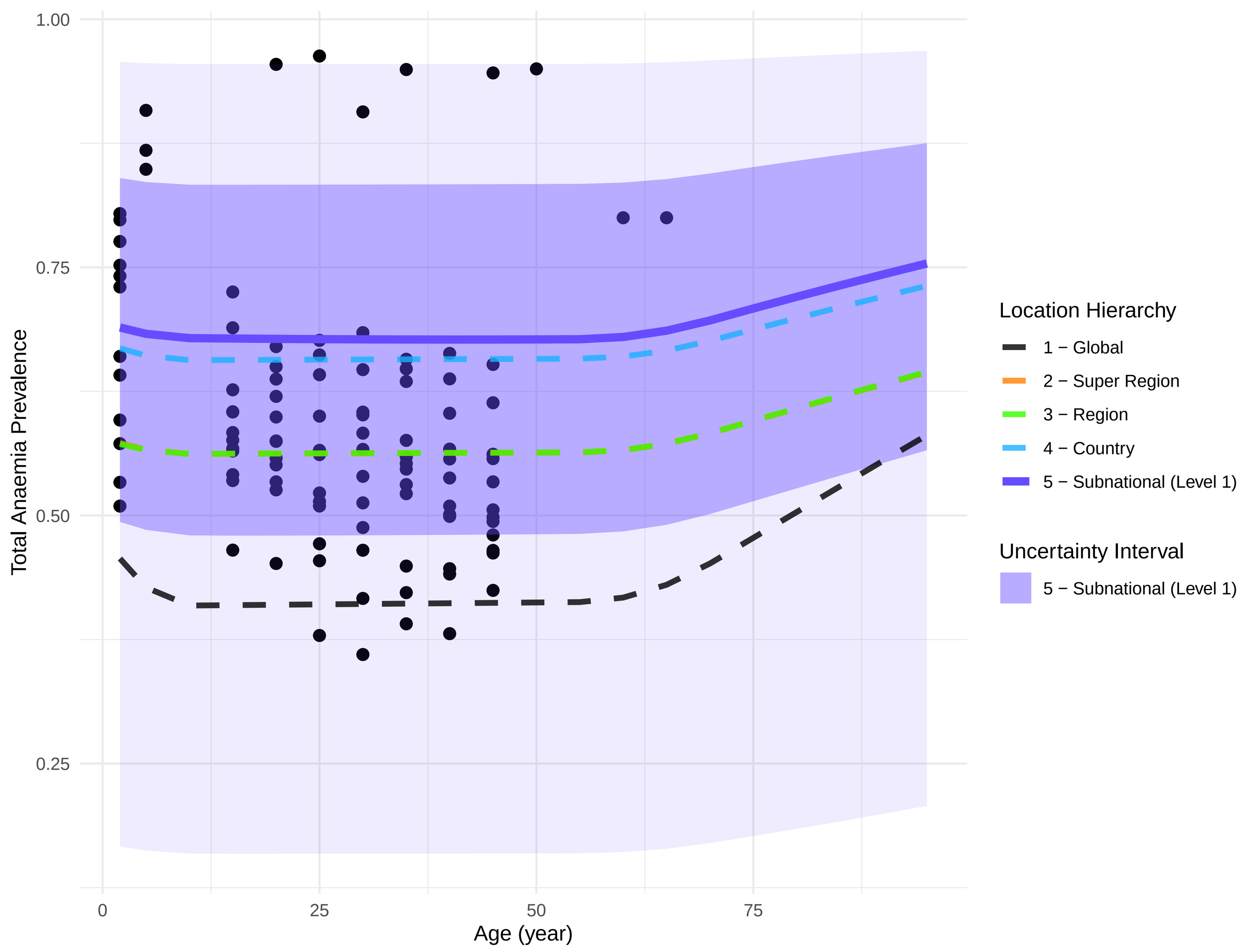

MR−BRT Age/Sex Fits − Non−pregnant Females 2+ Years Old − Total Anaemia Prevalence − Gujarat, Rural
WHO−2001 Adjustment Method

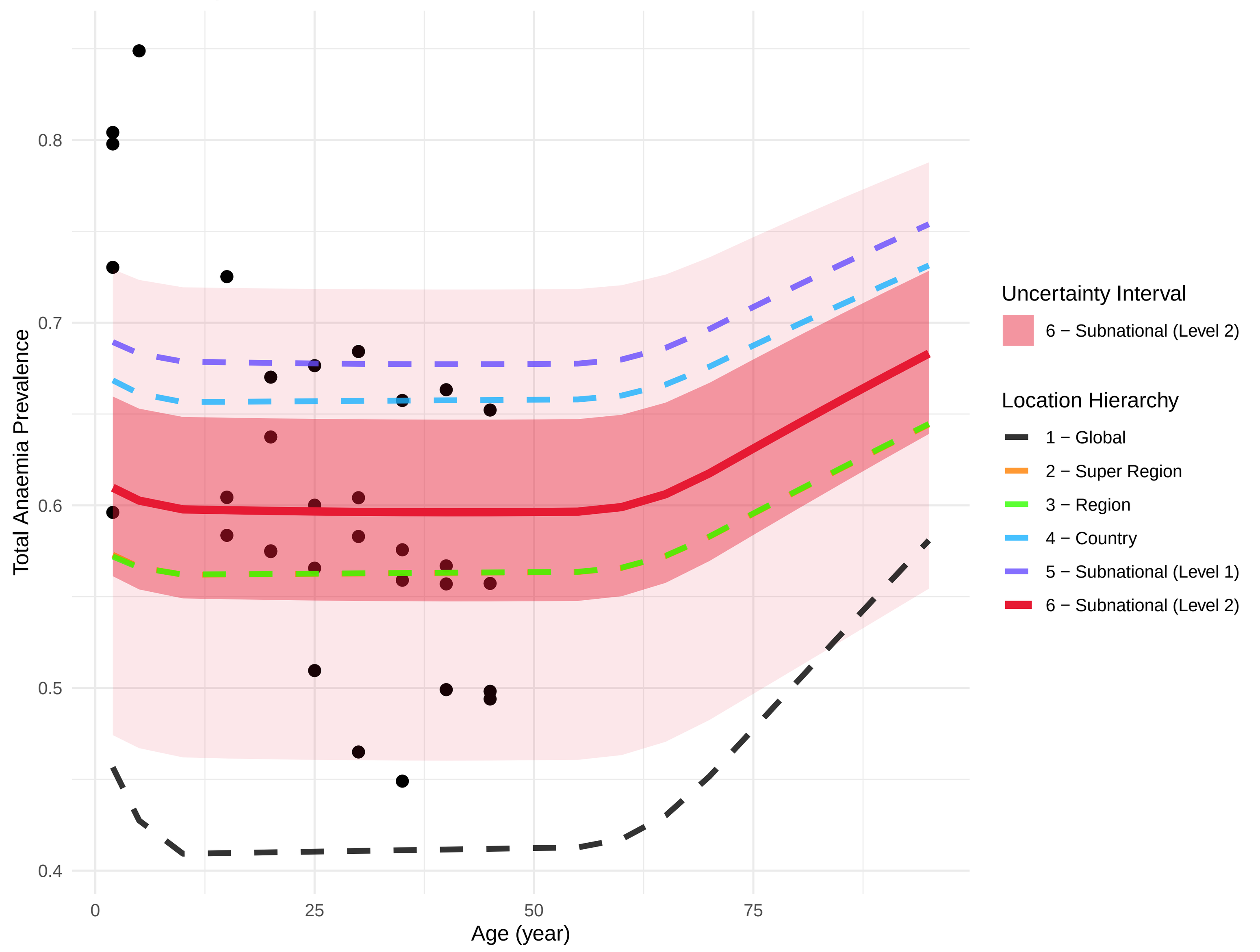

MR−BRT Age/Sex Fits − Non−pregnant Females 2+ Years Old − Total Anaemia Prevalence − Gujarat, Urban
WHO−2001 Adjustment Method

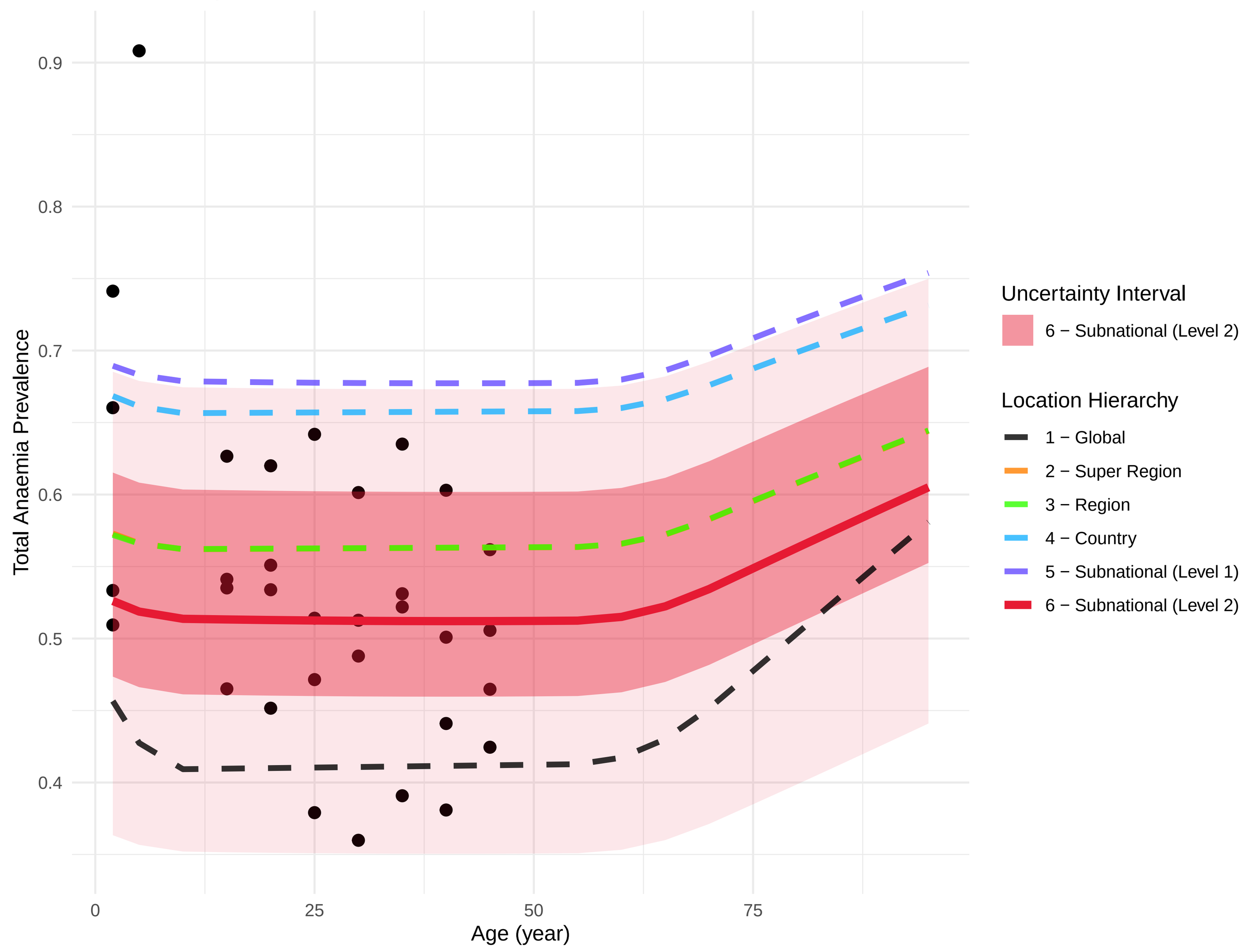

MR−BRT Age/Sex Fits − Non−pregnant Females 2+ Years Old − Total Anaemia Prevalence − Haryana
WHO−2001 Adjustment Method

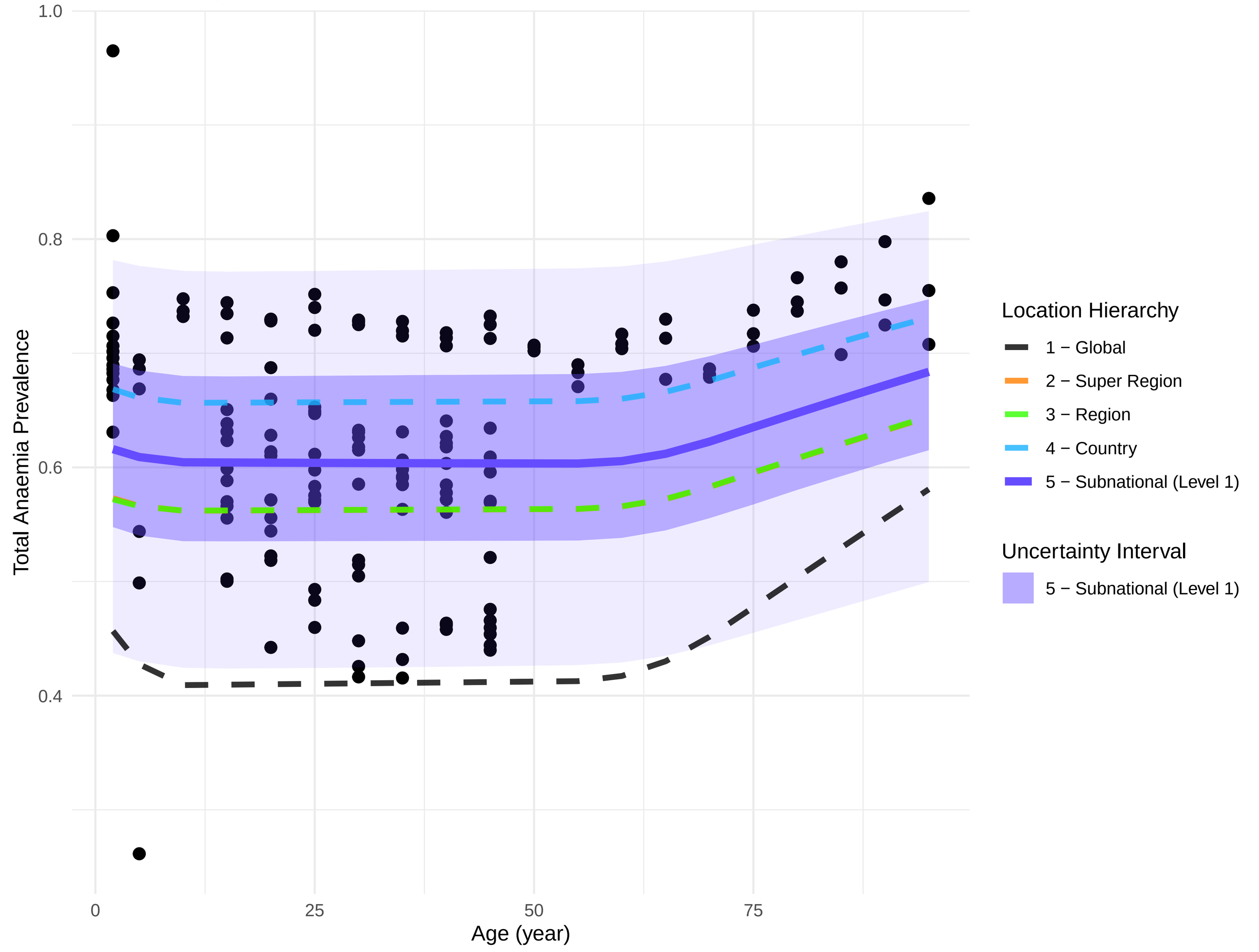

MR−BRT Age/Sex Fits − Non−pregnant Females 2+ Years Old − Total Anaemia Prevalence − Haryana, Rural
WHO−2001 Adjustment Method

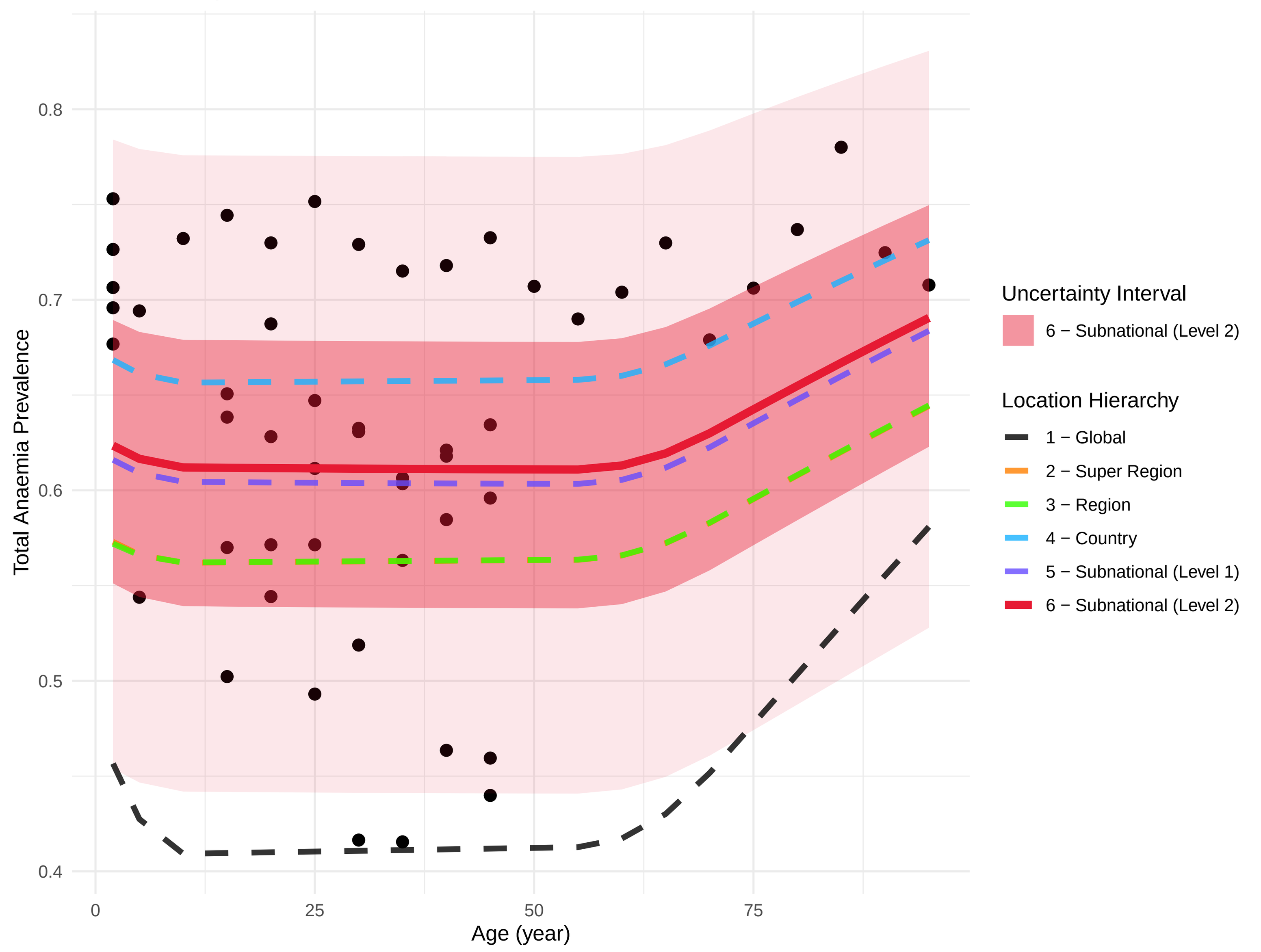

MR−BRT Age/Sex Fits − Non−pregnant Females 2+ Years Old − Total Anaemia Prevalence − Haryana, Urban
WHO−2001 Adjustment Method

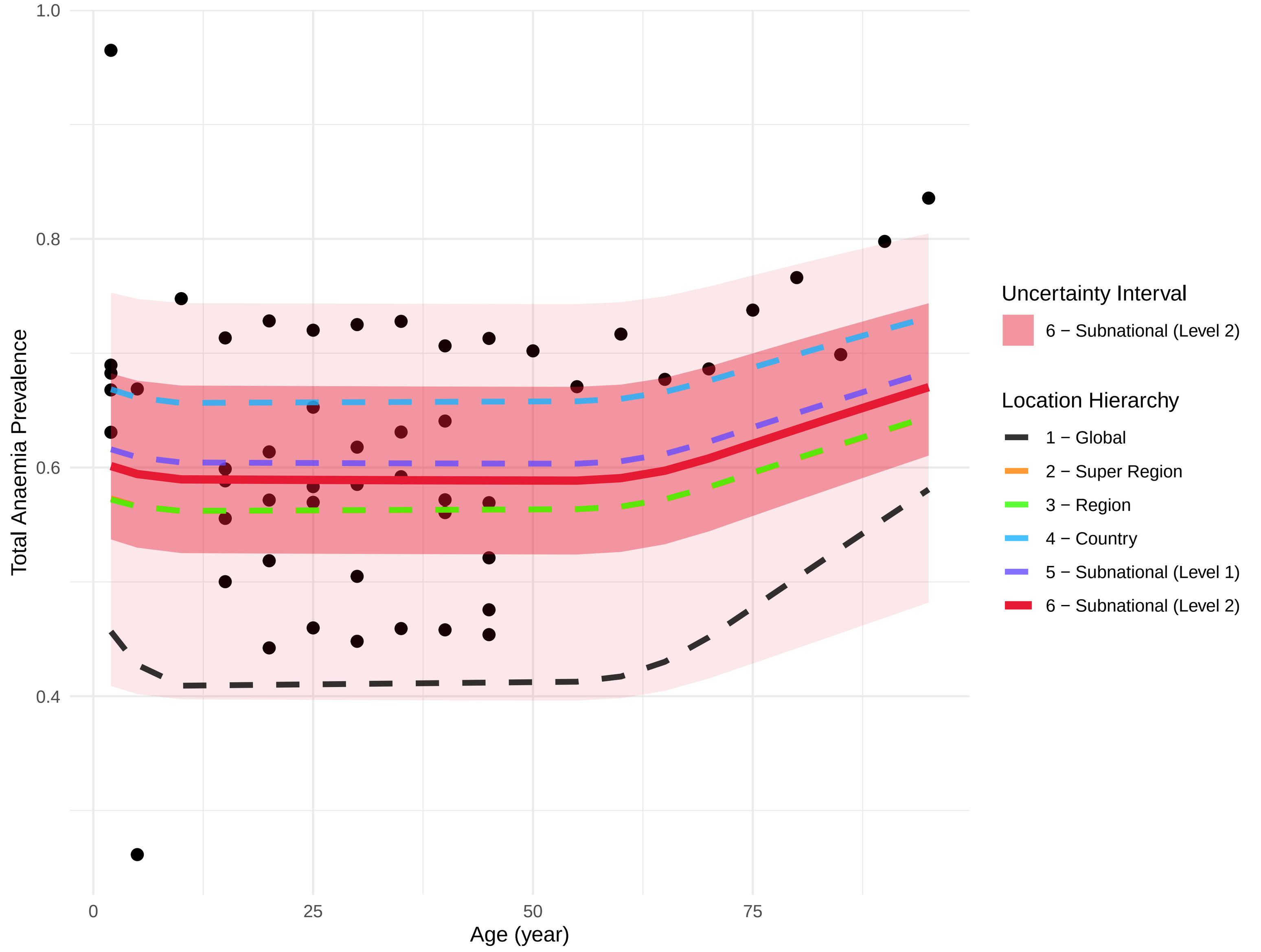

MR−BRT Age/Sex Fits − Non−pregnant Females 2+ Years Old − Total Anaemia Prevalence − Himachal Pradesh
WHO−2001 Adjustment Method

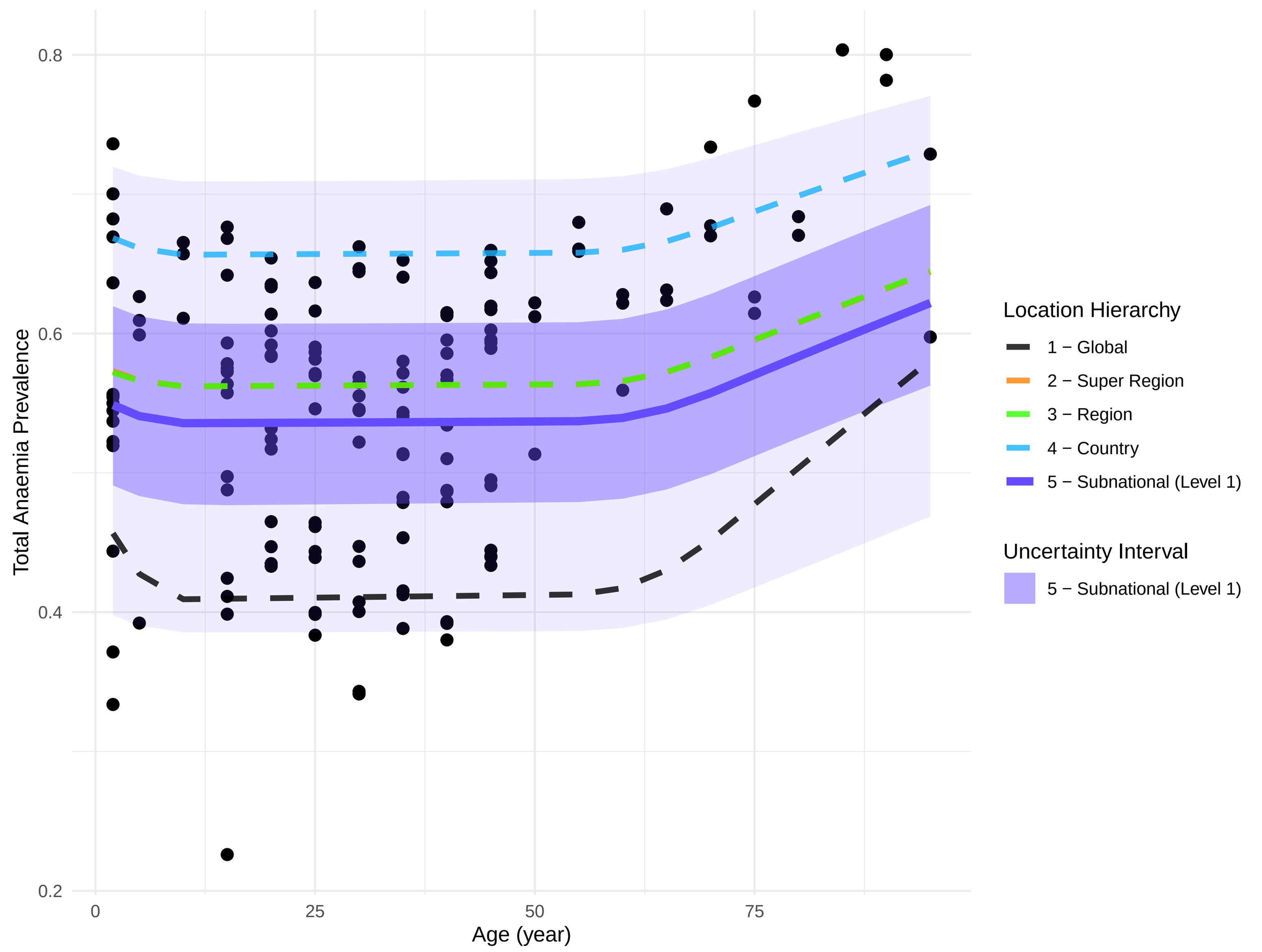

MR−BRT Age/Sex Fits − Non−pregnant Females 2+ Years Old − Total Anaemia Prevalence − Himachal Pradesh, Rural
WHO−2001 Adjustment Method

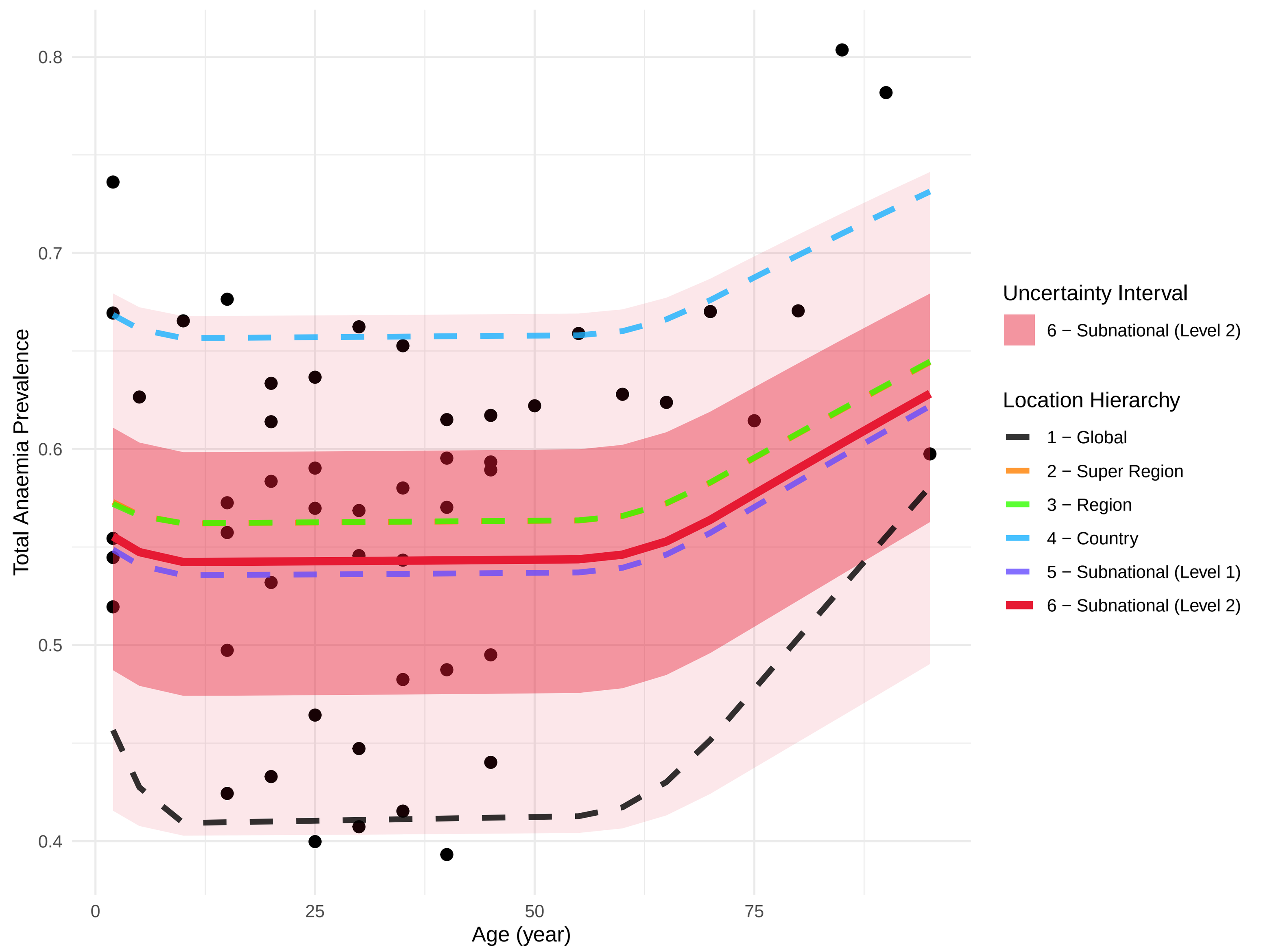

MR−BRT Age/Sex Fits − Non−pregnant Females 2+ Years Old − Total Anaemia Prevalence − Himachal Pradesh, Urban
WHO−2001 Adjustment Method

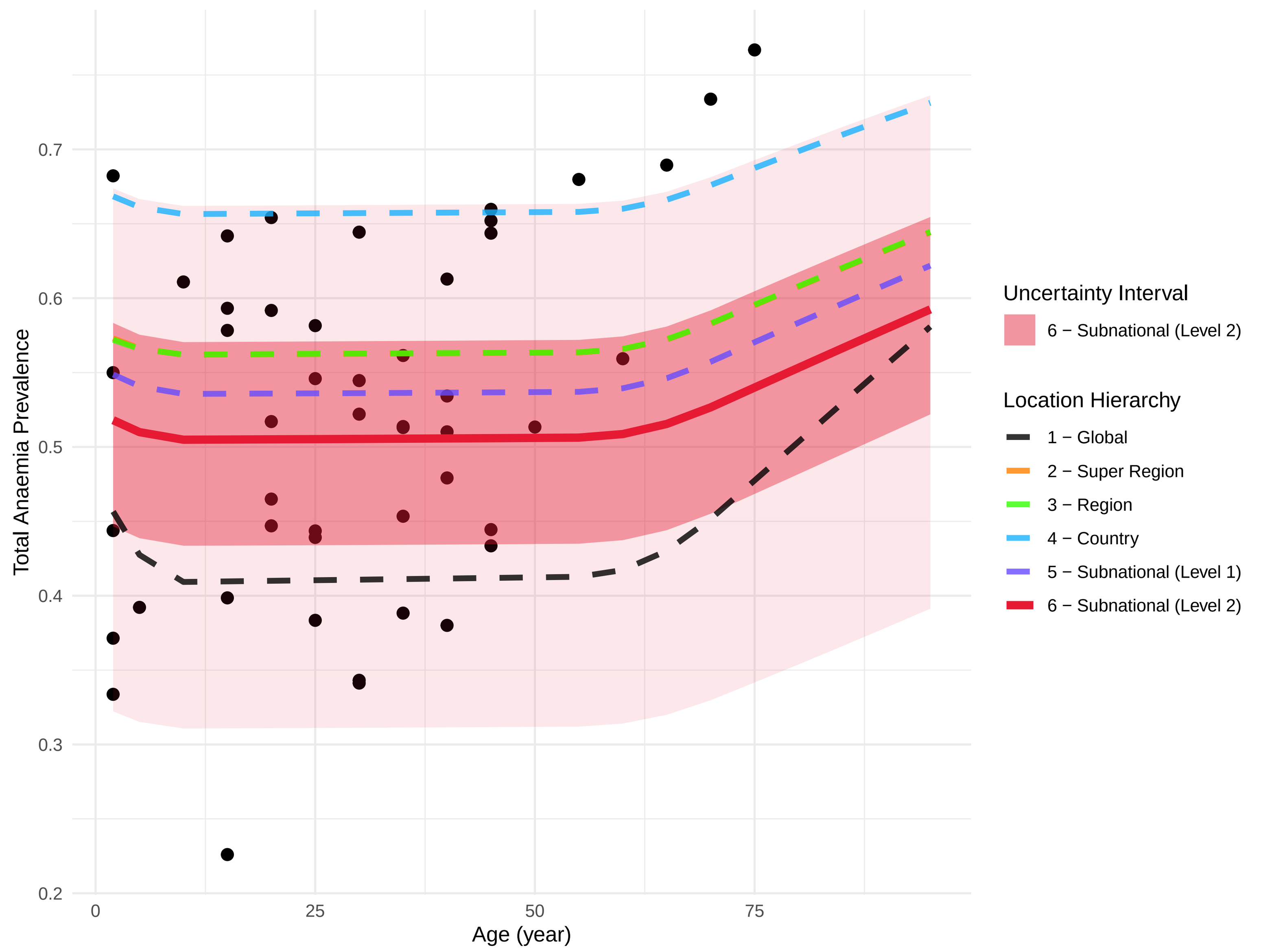

MR−BRT Age/Sex Fits − Non−pregnant Females 2+ Years Old − Total Anaemia Prevalence − Jammu & Kashmir and Ladakh
WHO−2001 Adjustment Method

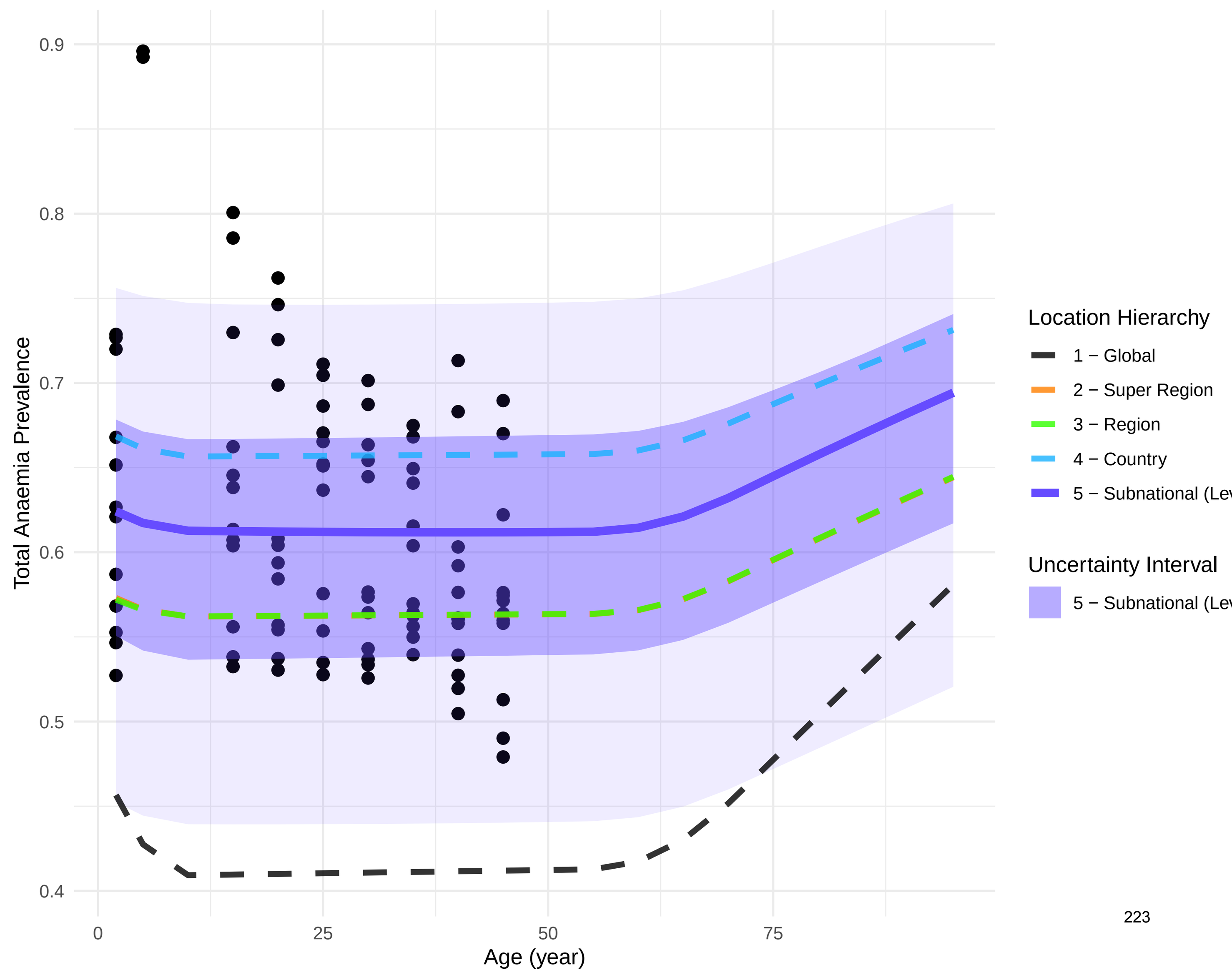

MR−BRT Age/Sex Fits − Non−pregnant Females 2+ Years Old − Total Anaemia Prevalence − Jammu & Kashmir and Ladakh, Rural
WHO−2001 Adjustment Method

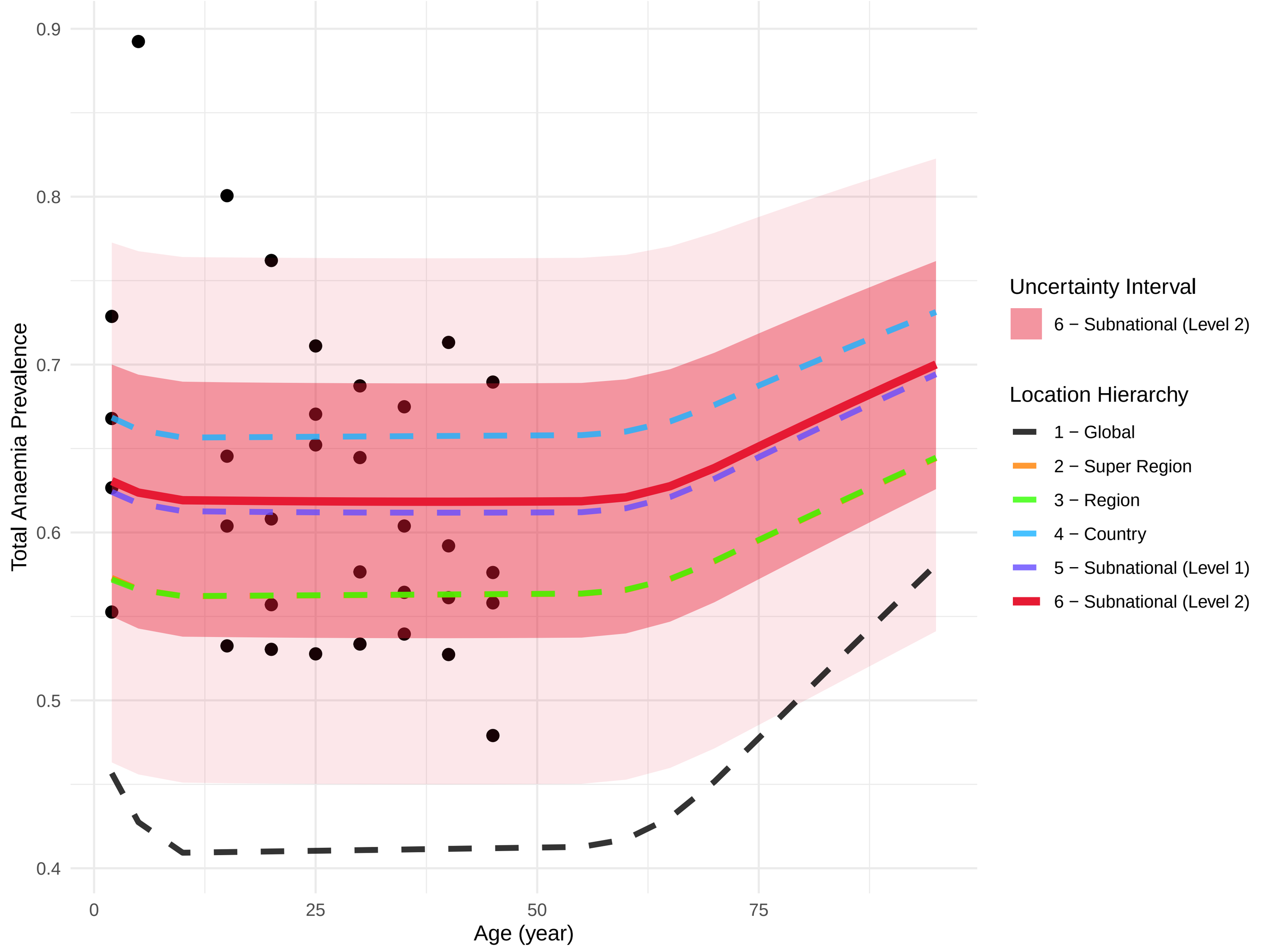

MR−BRT Age/Sex Fits − Non−pregnant Females 2+ Years Old − Total Anaemia Prevalence − Jammu & Kashmir and Ladakh, Urban
WHO−2001 Adjustment Method

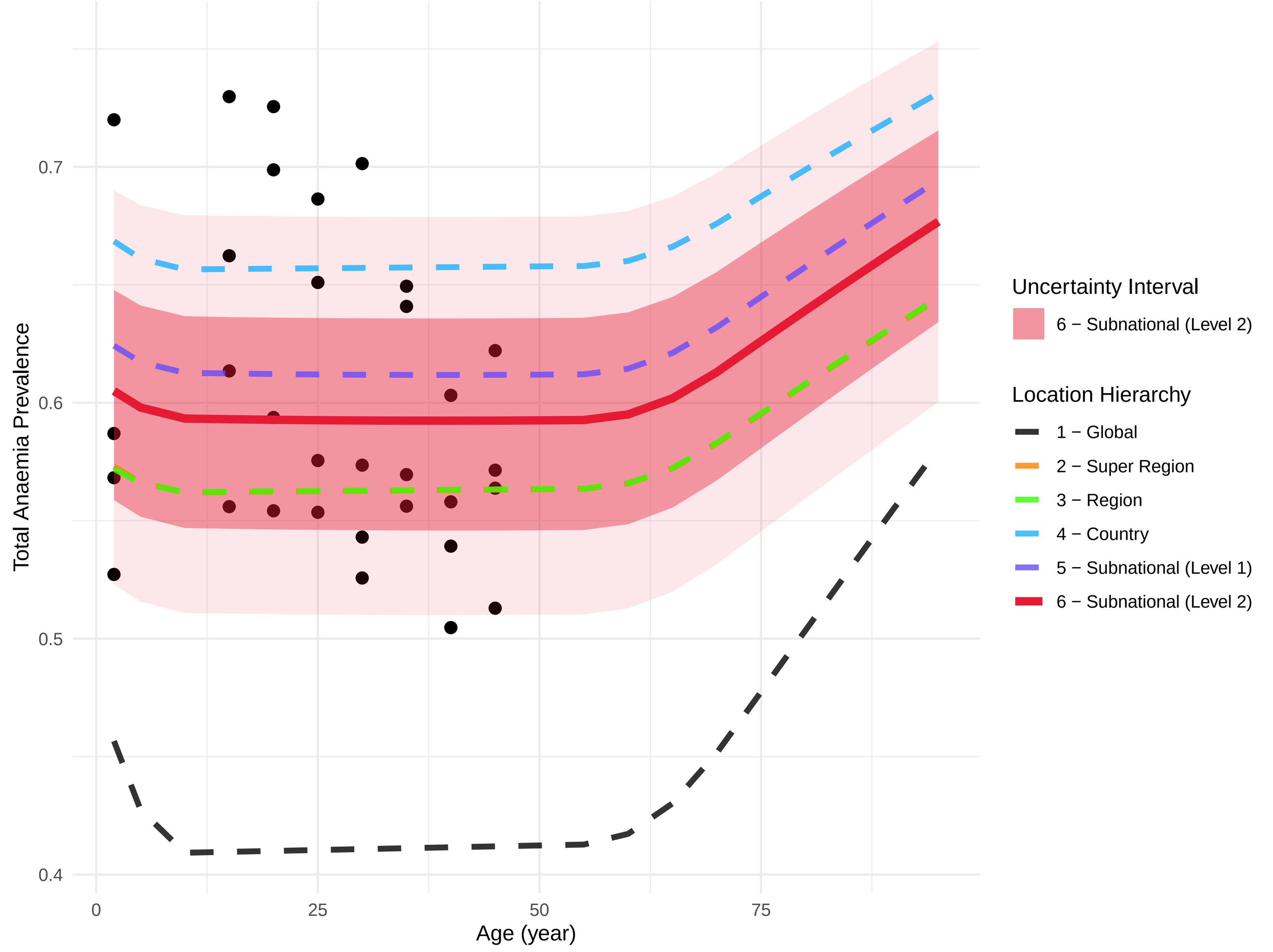

MR−BRT Age/Sex Fits − Non−pregnant Females 2+ Years Old − Total Anaemia Prevalence − Jharkhand
WHO−2001 Adjustment Method

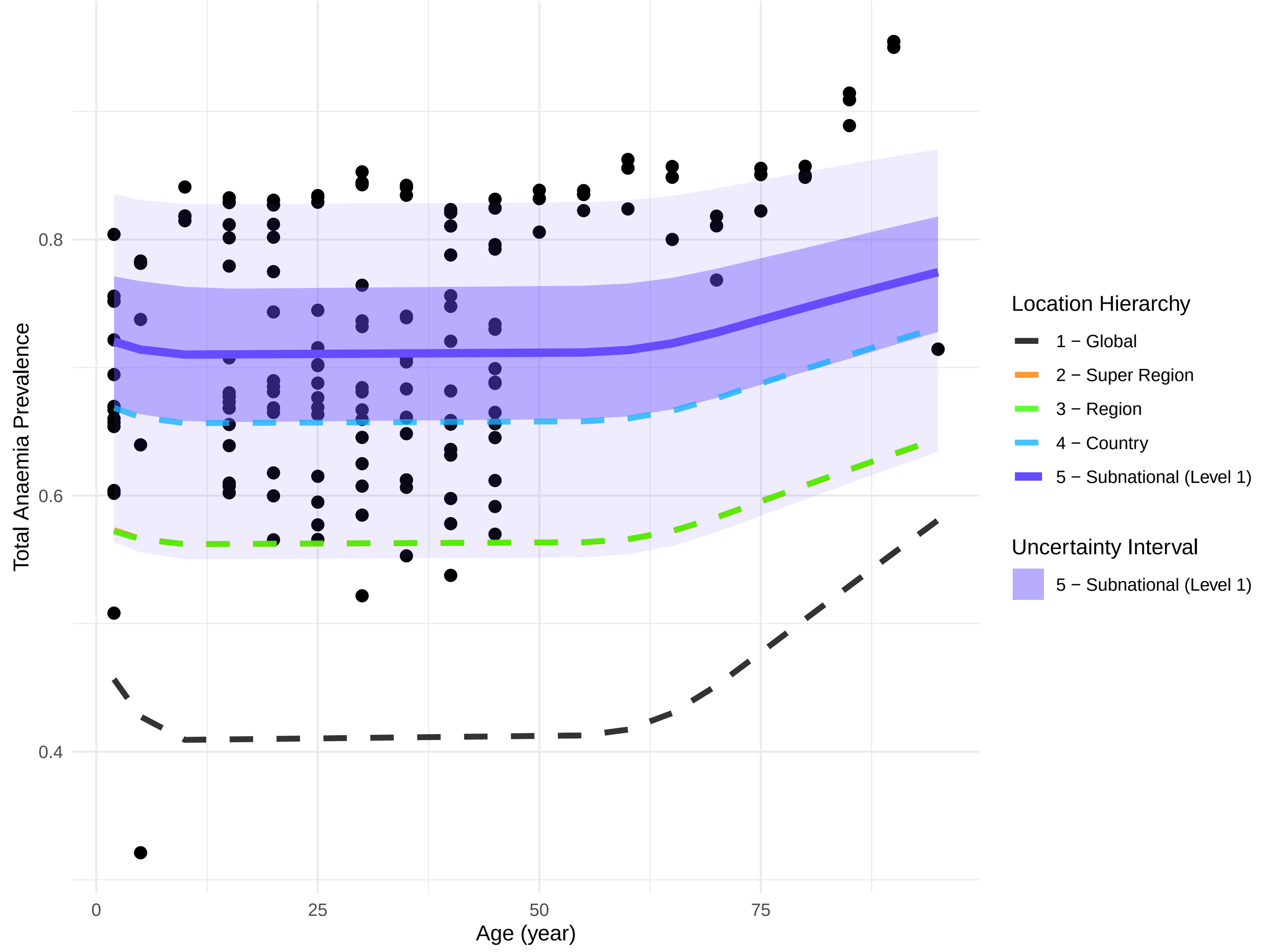

MR−BRT Age/Sex Fits − Non−pregnant Females 2+ Years Old − Total Anaemia Prevalence − Jharkhand, Rural
WHO−2001 Adjustment Method

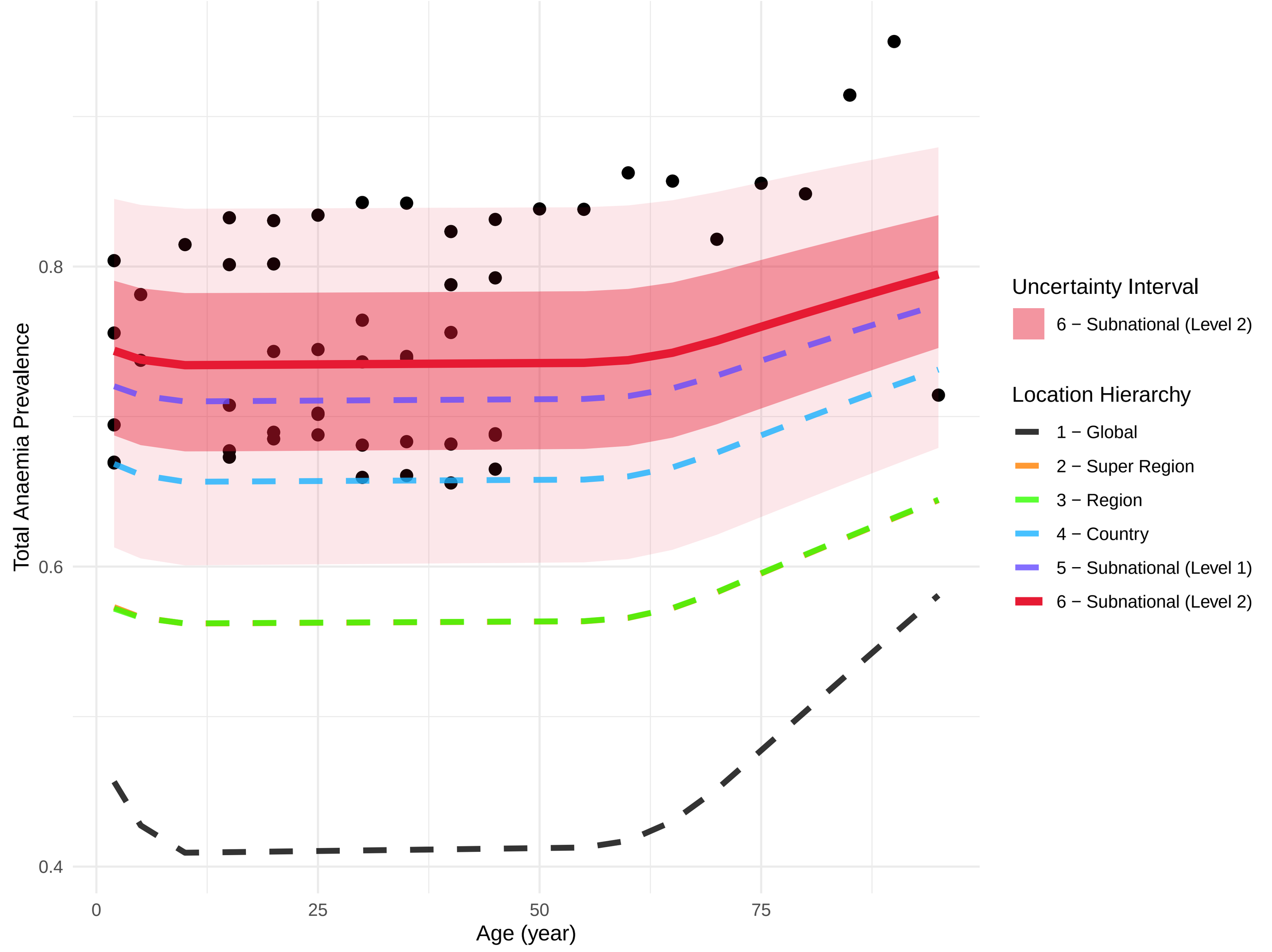

MR−BRT Age/Sex Fits − Non−pregnant Females 2+ Years Old − Total Anaemia Prevalence − Jharkhand, Urban
WHO−2001 Adjustment Method

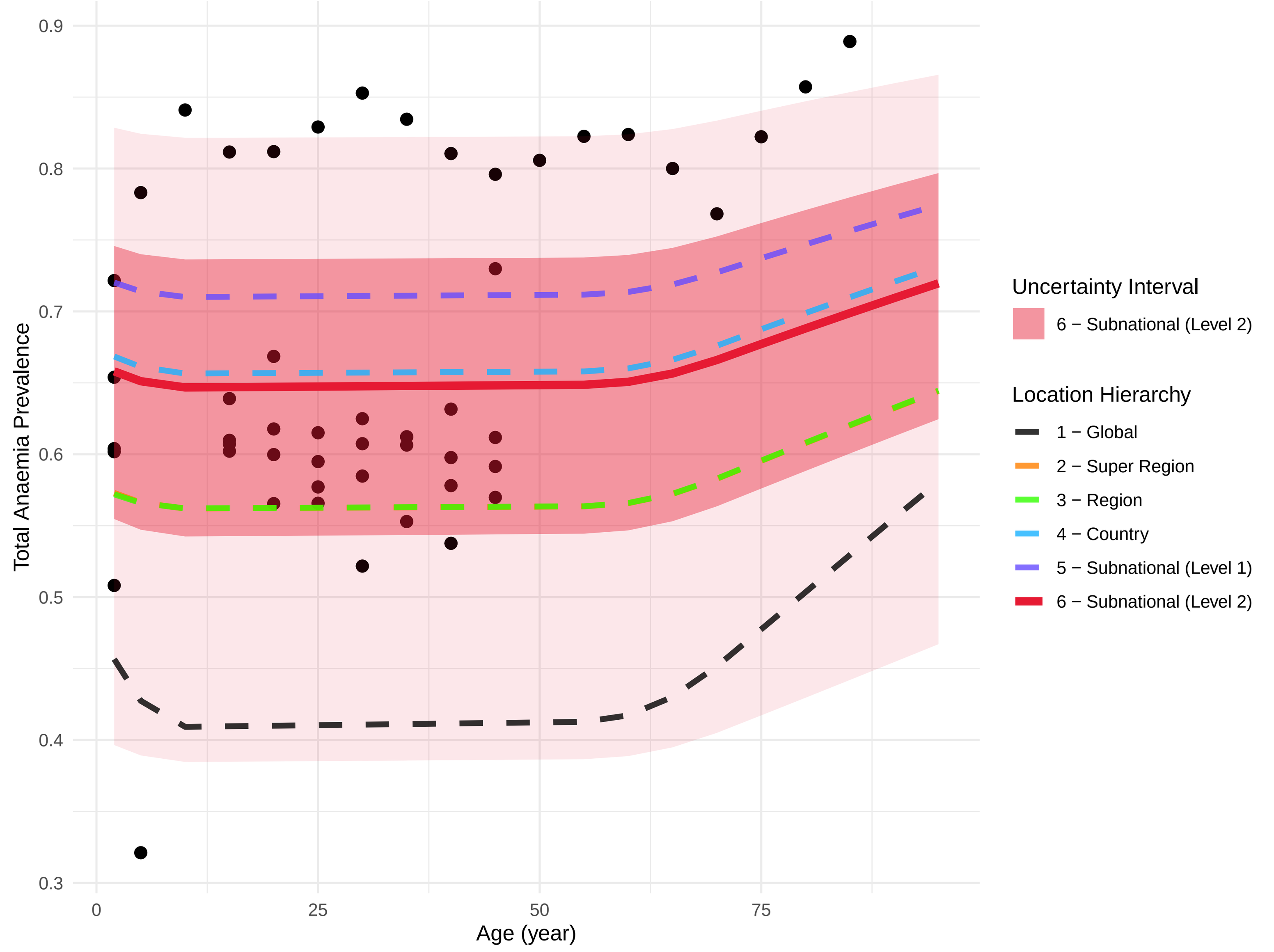

MR−BRT Age/Sex Fits − Non−pregnant Females 2+ Years Old − Total Anaemia Prevalence − Karnataka
WHO−2001 Adjustment Method

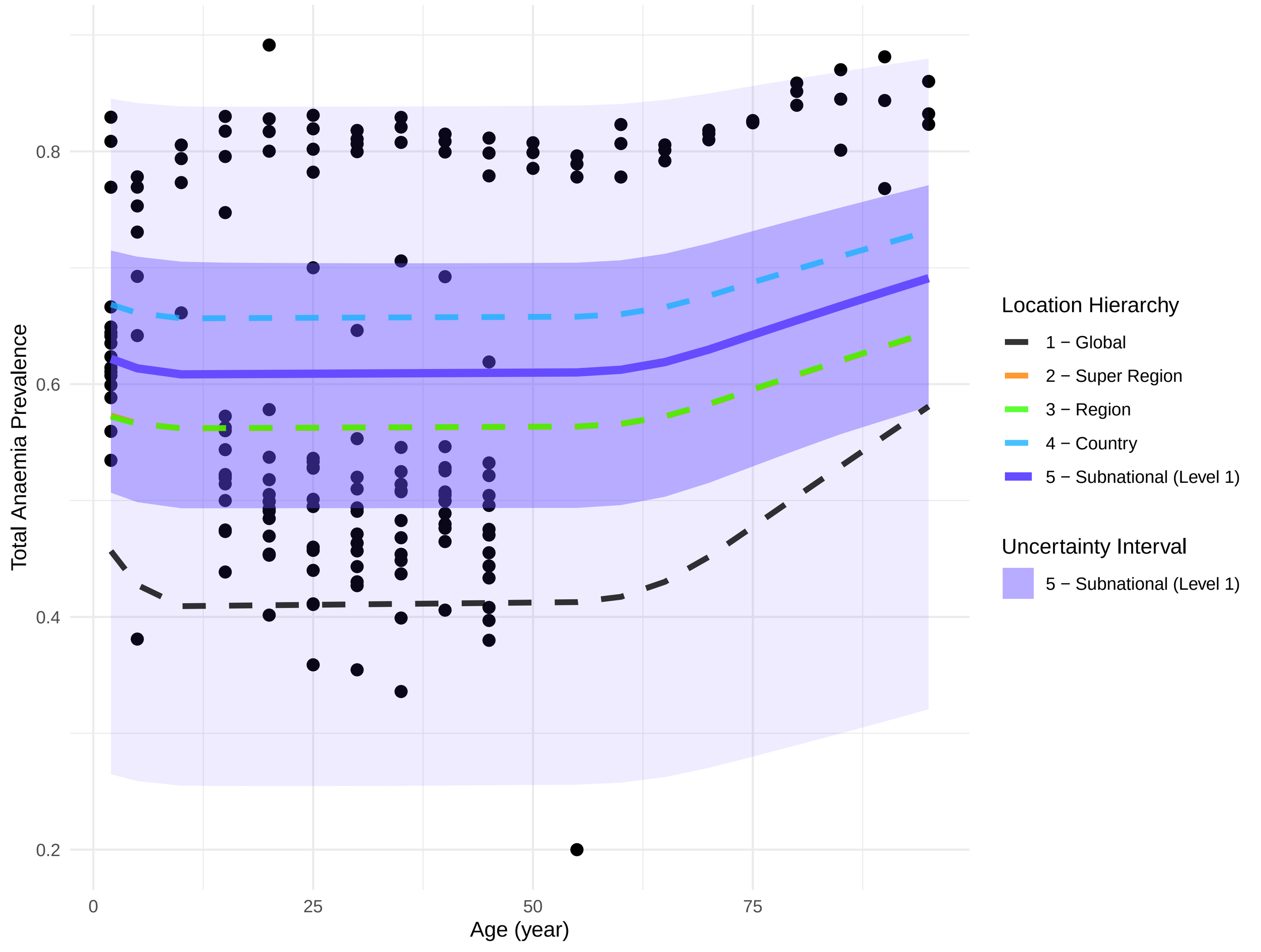

MR−BRT Age/Sex Fits − Non−pregnant Females 2+ Years Old − Total Anaemia Prevalence − Karnataka, Rural
WHO−2001 Adjustment Method

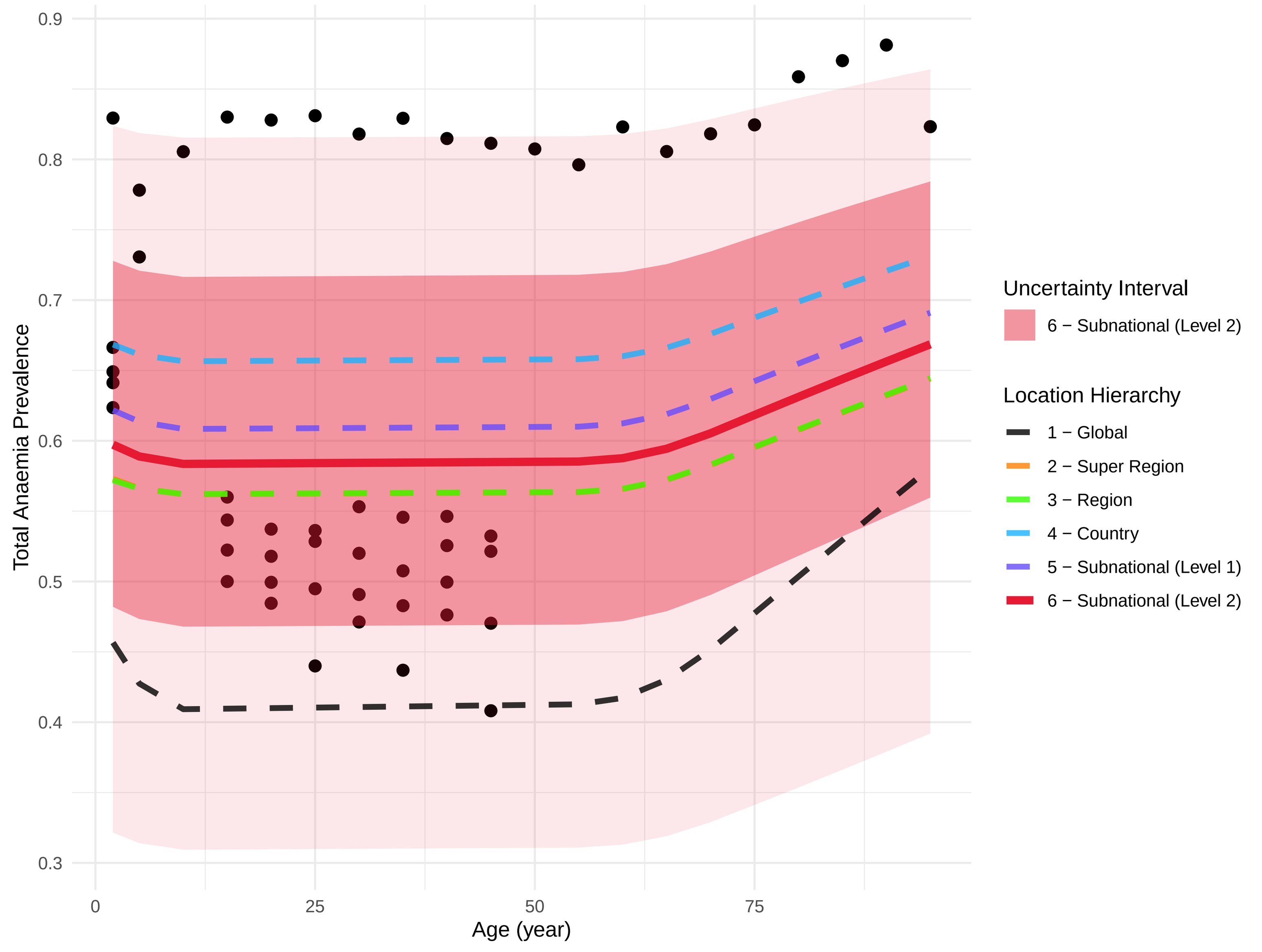

MR−BRT Age/Sex Fits − Non−pregnant Females 2+ Years Old − Total Anaemia Prevalence − Karnataka, Urban
WHO−2001 Adjustment Method

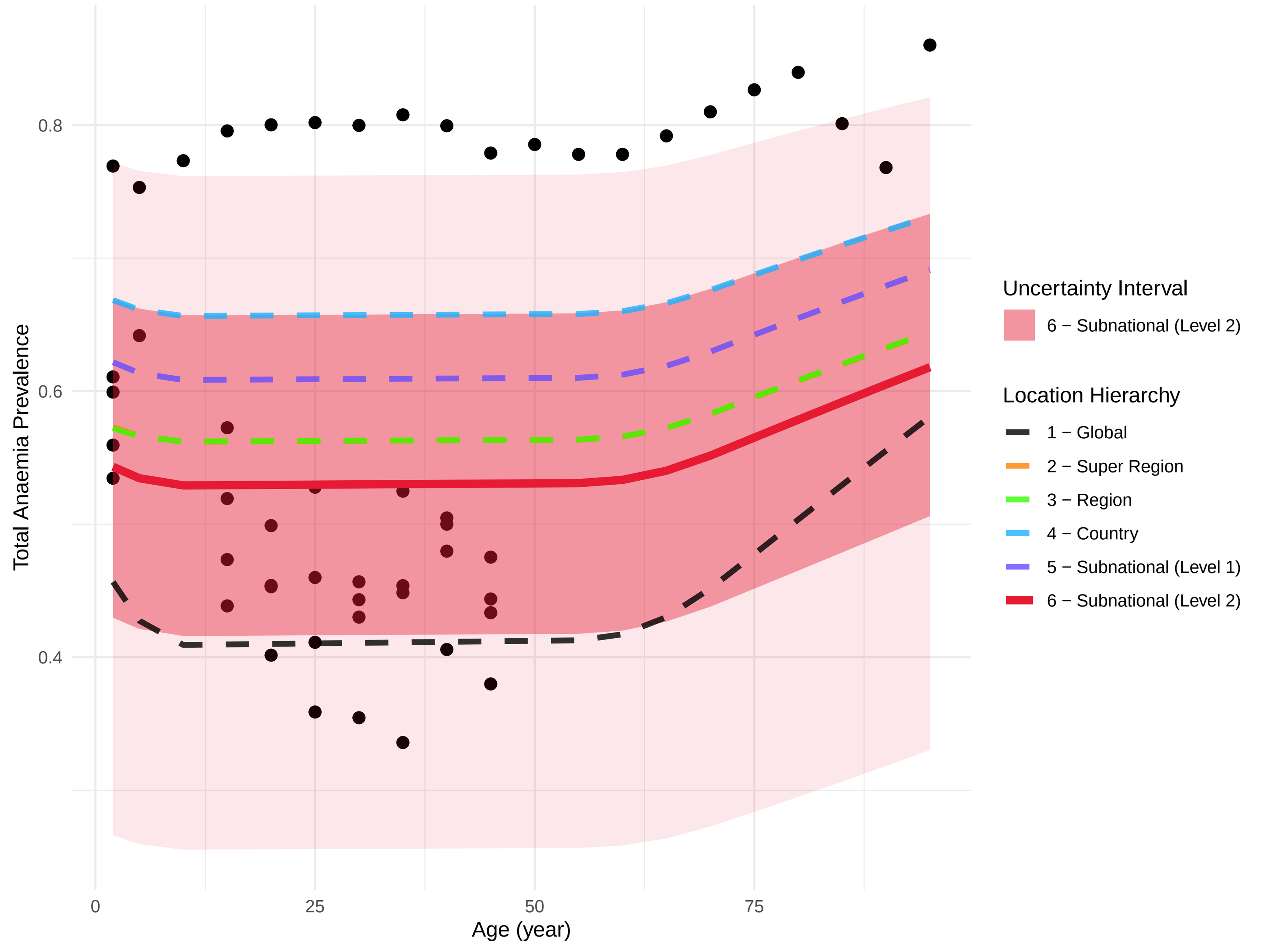

MR−BRT Age/Sex Fits − Non−pregnant Females 2+ Years Old − Total Anaemia Prevalence − Kerala
WHO−2001 Adjustment Method

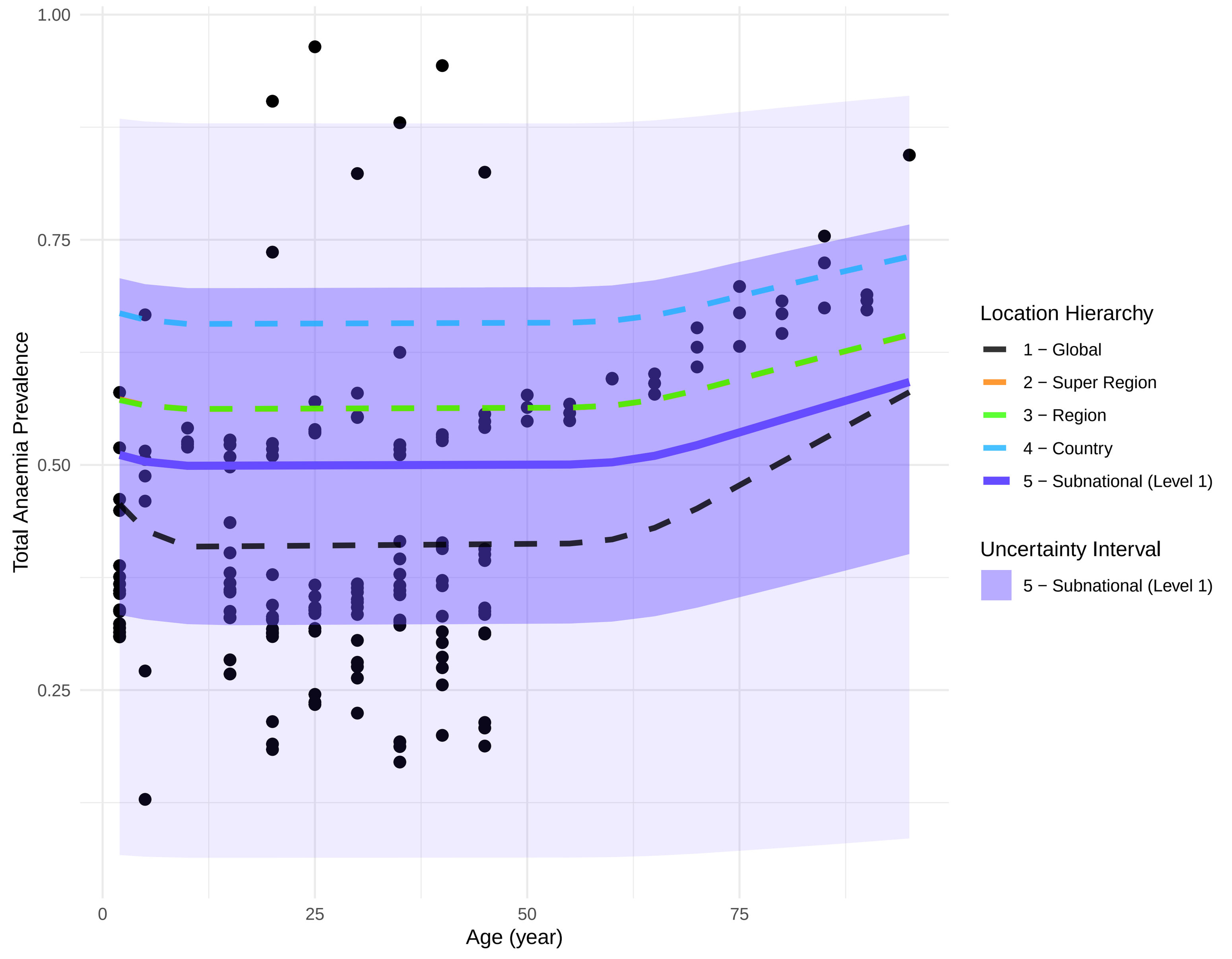

MR−BRT Age/Sex Fits − Non−pregnant Females 2+ Years Old − Total Anaemia Prevalence − Kerala, Rural
WHO−2001 Adjustment Method

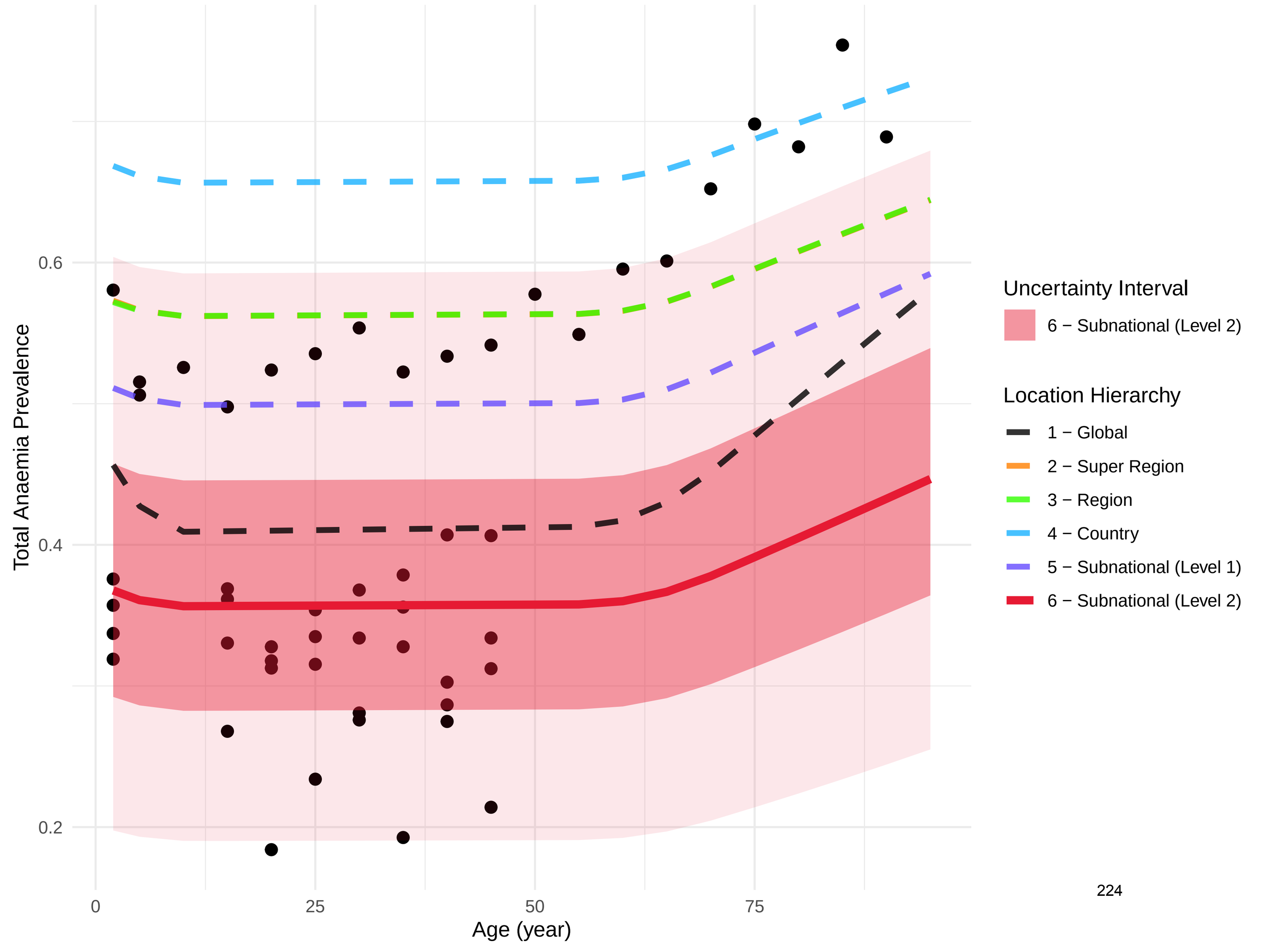

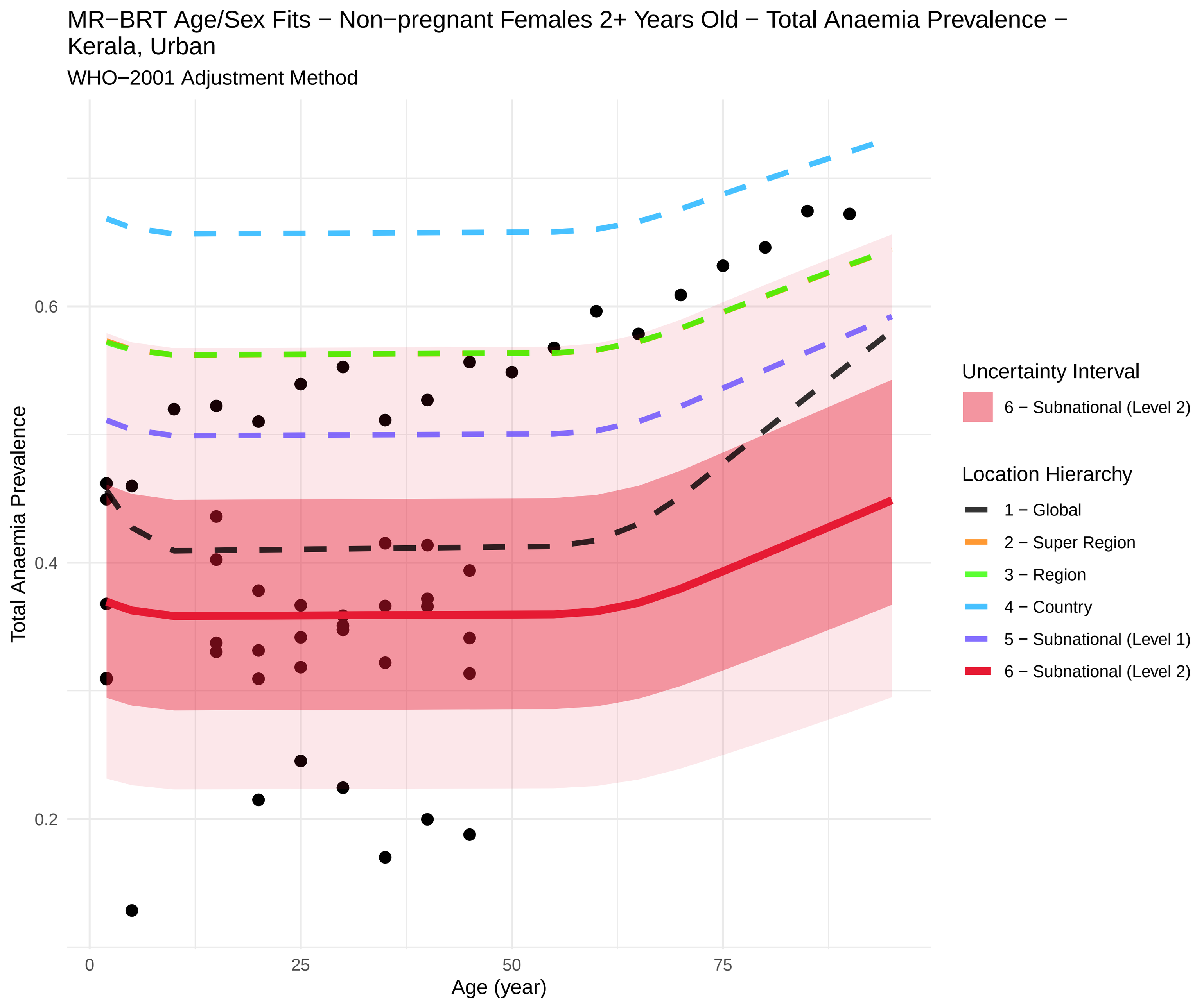
MR−BRT Age/Sex Fits − Non-pregnant Females 2+ Years Old − Total Anaemia Prevalence − Kerala, Urban
WHO−2001 Adjustment Method
Total Anaemia Prevalence
Age (year)
Uncertainty Interval
6 − Subnational (Level 2)
Location Hierarchy
1 − Global
2 − Super Region
3 − Region
4 − Country
5 − Subnational (Level 1)
6 − Subnational (Level 2)

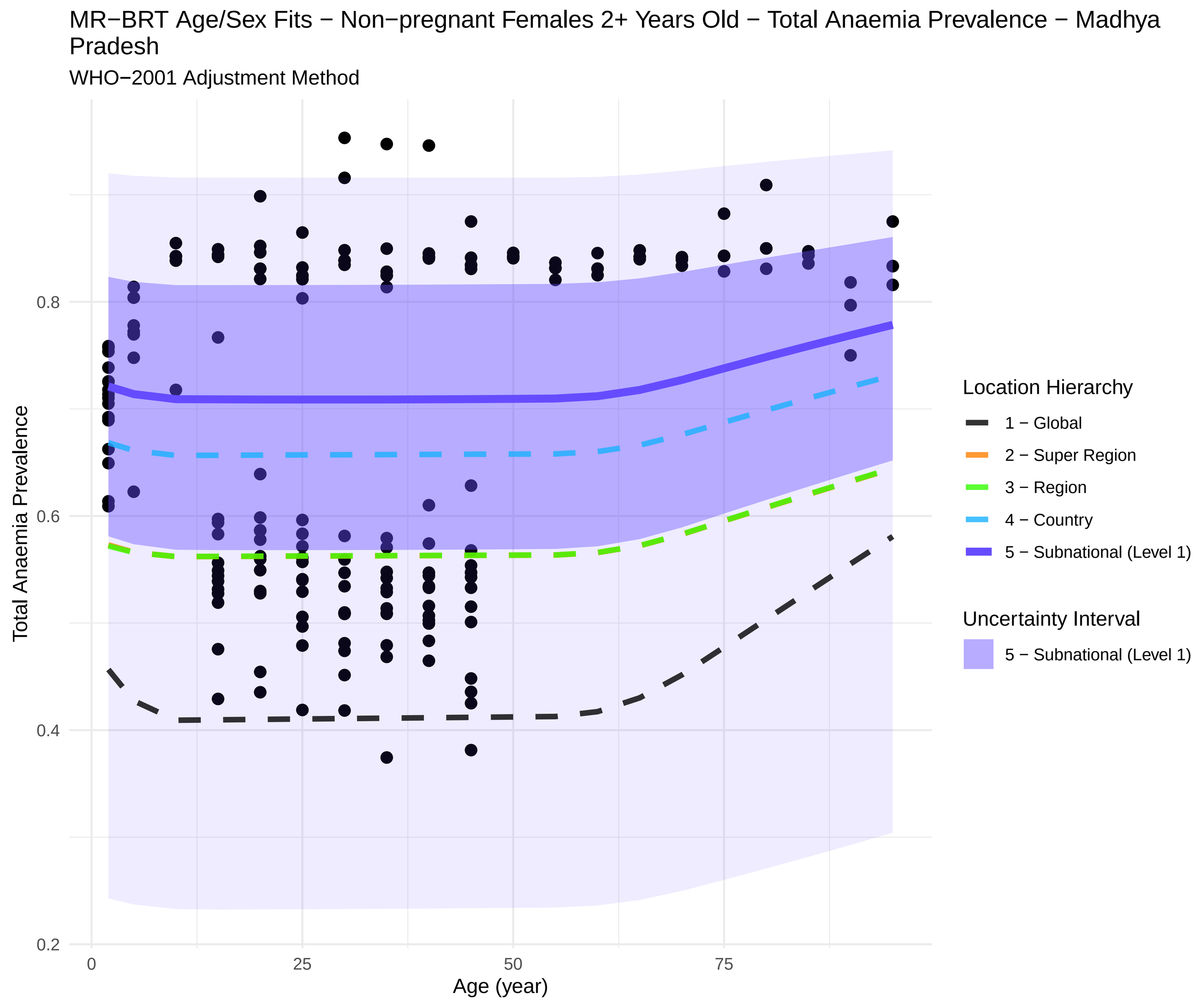
MR−BRT Age/Sex Fits − Non-pregnant Females 2+ Years Old − Total Anaemia Prevalence − Madhya Pradesh
WHO−2001 Adjustment Method
Total Anaemia Prevalence
Age (year)
Location Hierarchy
1 − Global
2 − Super Region
3 − Region
4 − Country
5 − Subnational (Level 1)
Uncertainty Interval
5 − Subnational (Level 1)

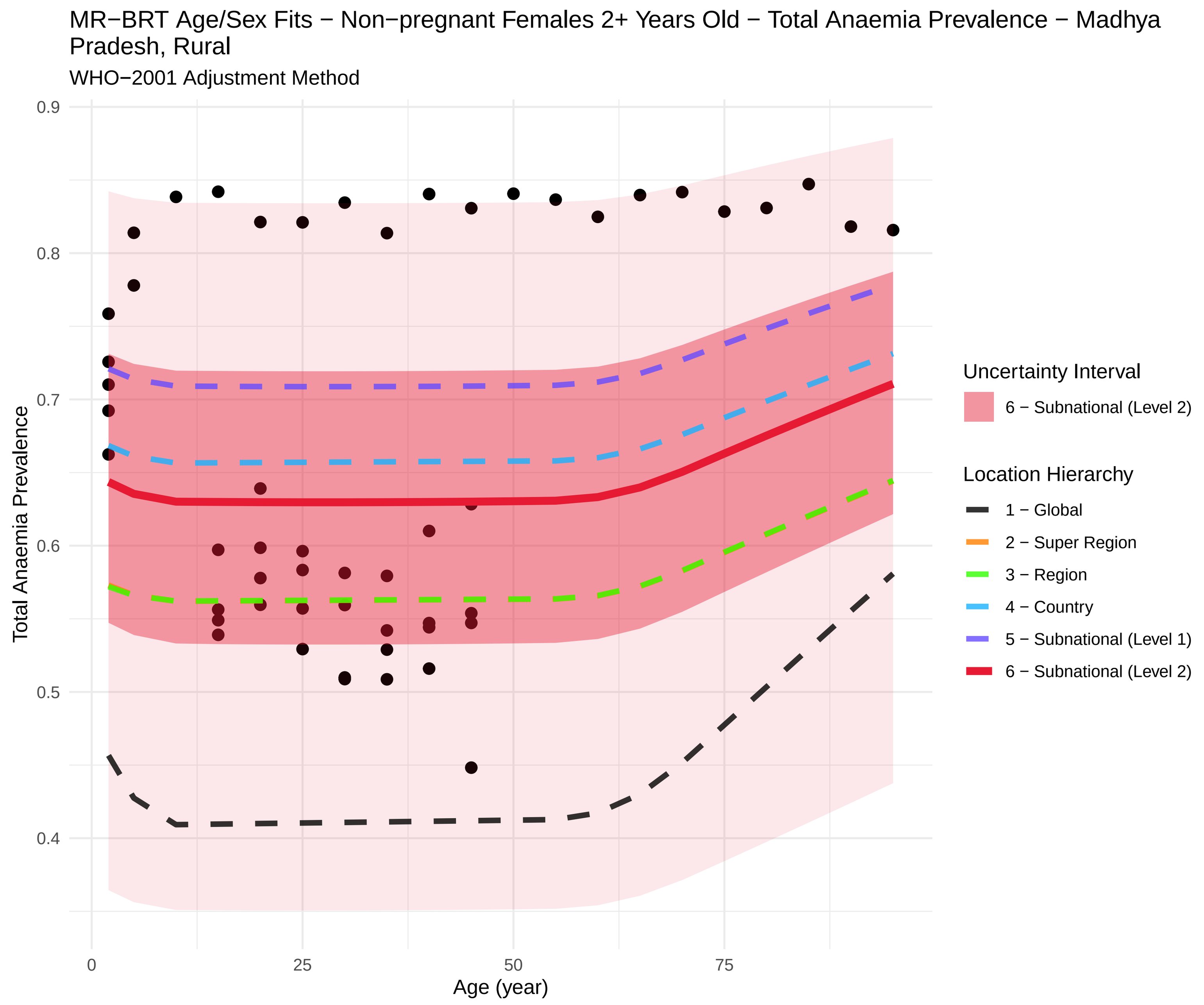
MR−BRT Age/Sex Fits − Non-pregnant Females 2+ Years Old − Total Anaemia Prevalence − Madhya Pradesh, Rural
WHO−2001 Adjustment Method
Total Anaemia Prevalence
Age (year)
Uncertainty Interval
6 − Subnational (Level 2)
Location Hierarchy
1 − Global
2 − Super Region
3 − Region
4 − Country
5 − Subnational (Level 1)
6 − Subnational (Level 2)

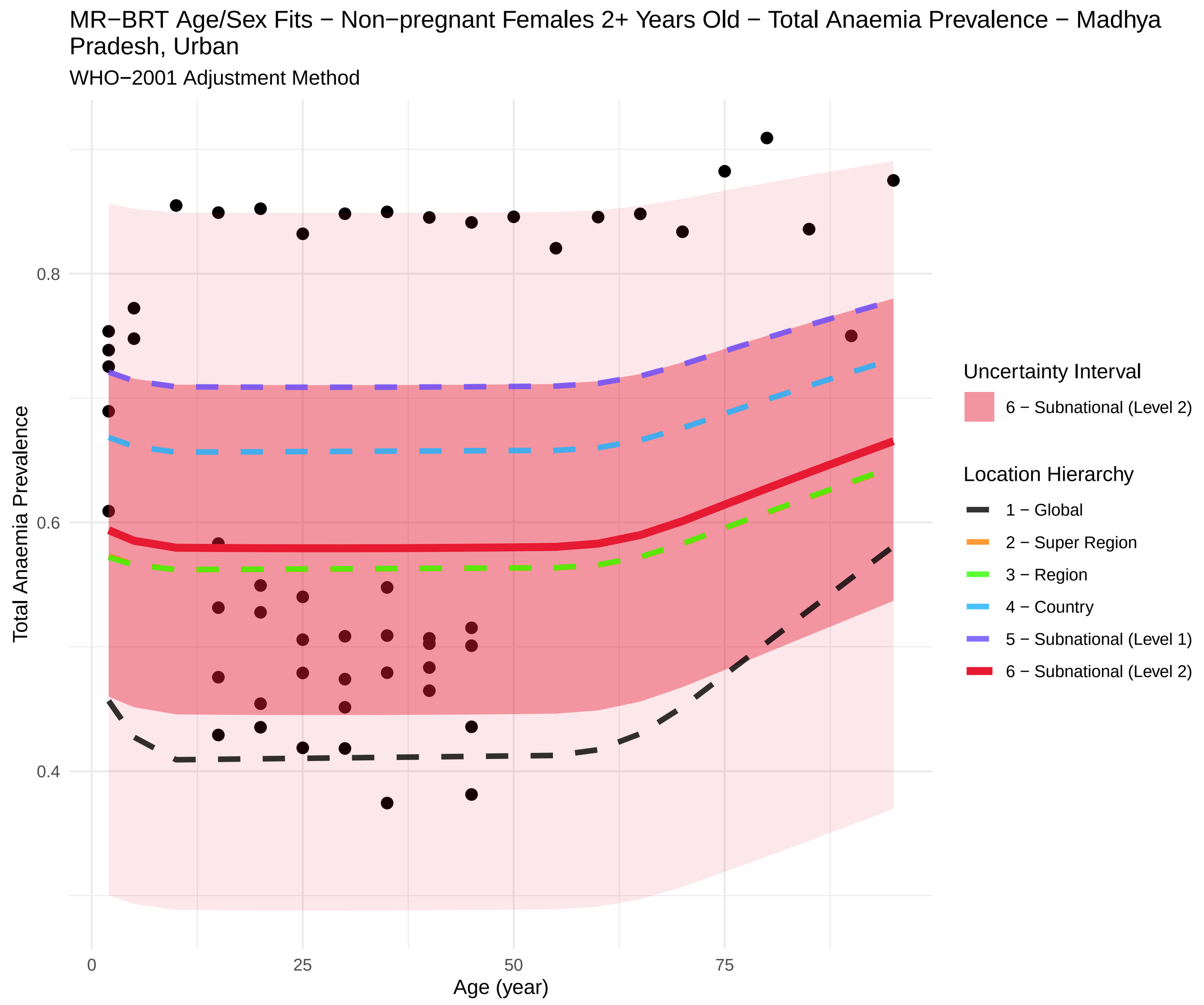
MR−BRT Age/Sex Fits − Non-pregnant Females 2+ Years Old − Total Anaemia Prevalence − Madhya Pradesh, Urban
WHO−2001 Adjustment Method
Total Anaemia Prevalence
Age (year)
Uncertainty Interval
6 − Subnational (Level 2)
Location Hierarchy
1 − Global
2 − Super Region
3 − Region
4 − Country
5 − Subnational (Level 1)
6 − Subnational (Level 2)

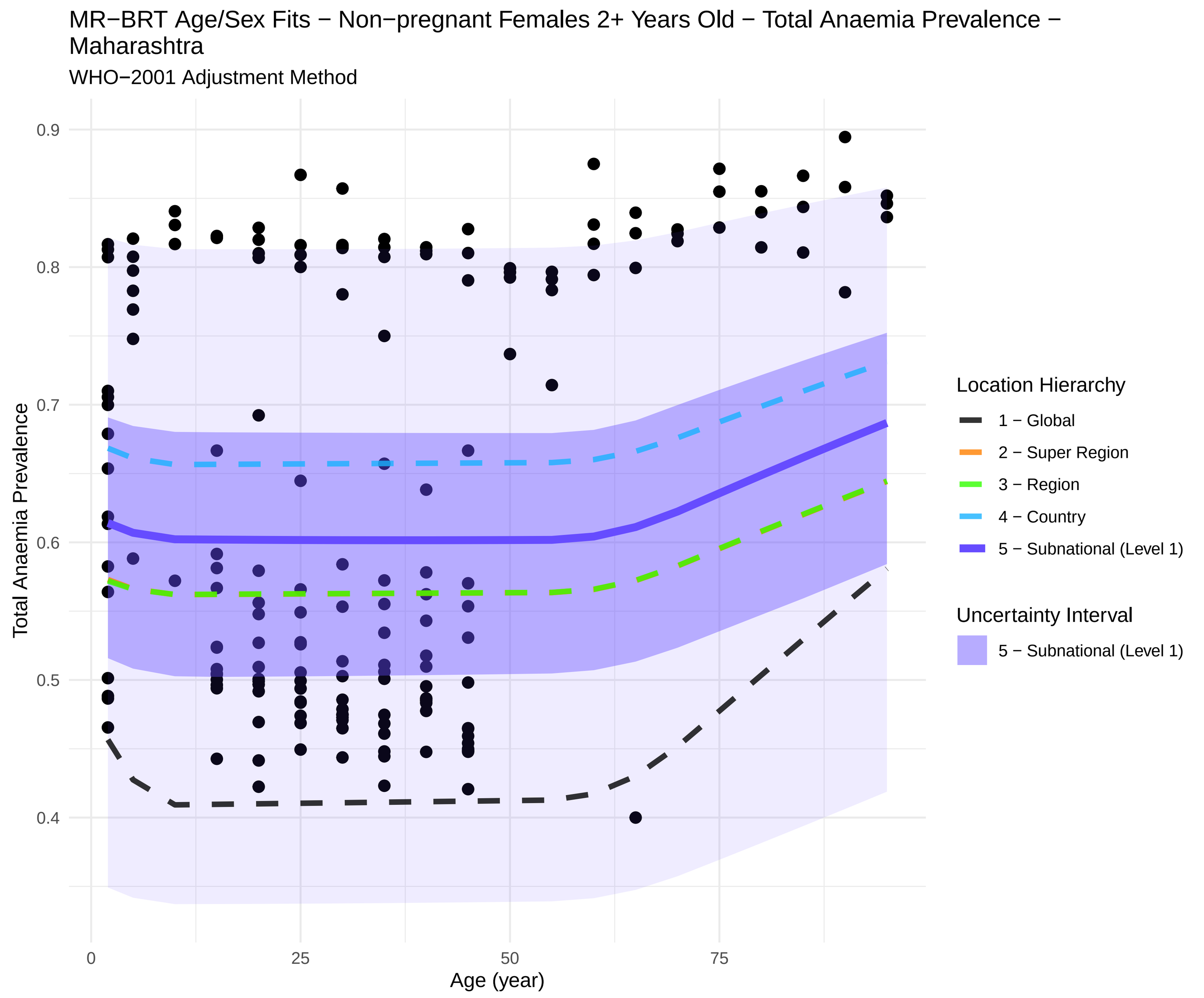
MR−BRT Age/Sex Fits − Non-pregnant Females 2+ Years Old − Total Anaemia Prevalence − Maharashtra
WHO−2001 Adjustment Method
Total Anaemia Prevalence
Age (year)
Location Hierarchy
1 − Global
2 − Super Region
3 − Region
4 − Country
5 − Subnational (Level 1)
Uncertainty Interval
5 − Subnational (Level 1)

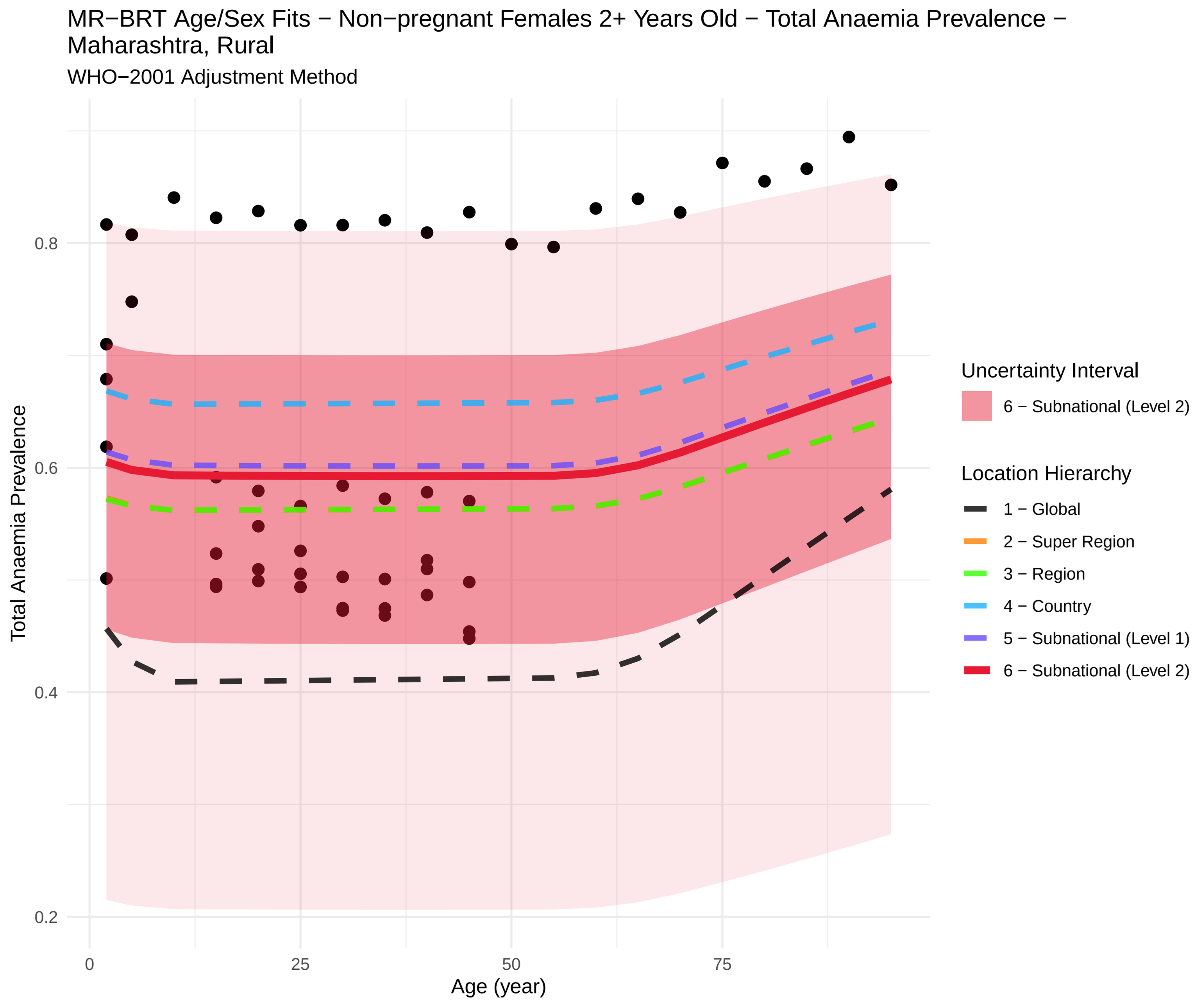
MR−BRT Age/Sex Fits − Non-pregnant Females 2+ Years Old − Total Anaemia Prevalence − Maharashtra, Rural
WHO−2001 Adjustment Method
Total Anaemia Prevalence
Age (year)
Uncertainty Interval
6 − Subnational (Level 2)
Location Hierarchy
1 − Global
2 − Super Region
3 − Region
4 − Country
5 − Subnational (Level 1)
6 − Subnational (Level 2)

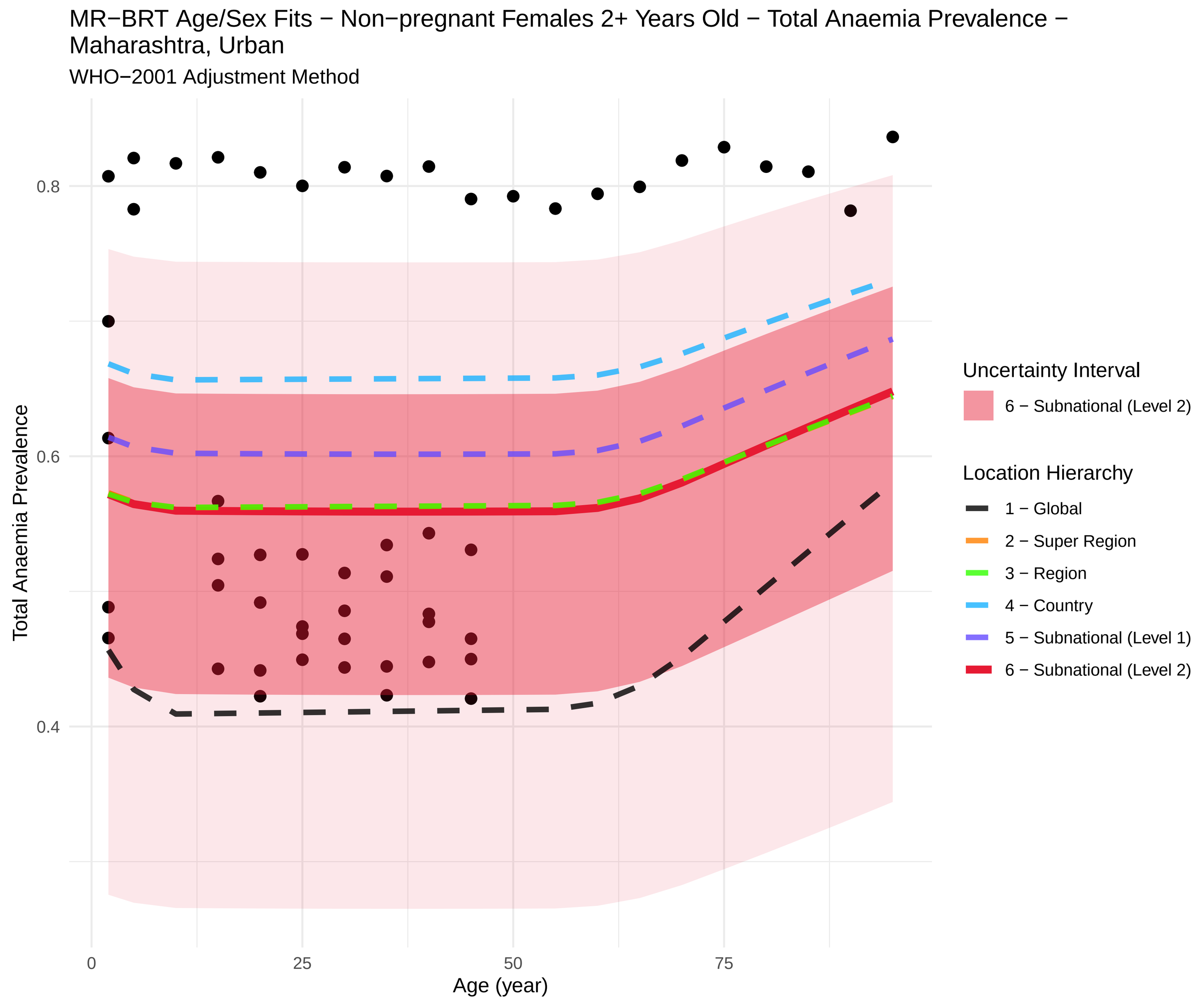
MR−BRT Age/Sex Fits − Non-pregnant Females 2+ Years Old − Total Anaemia Prevalence − Maharashtra, Urban
WHO−2001 Adjustment Method
Total Anaemia Prevalence
Age (year)
Uncertainty Interval
6 − Subnational (Level 2)
Location Hierarchy
1 − Global
2 − Super Region
3 − Region
4 − Country
5 − Subnational (Level 1)
6 − Subnational (Level 2)

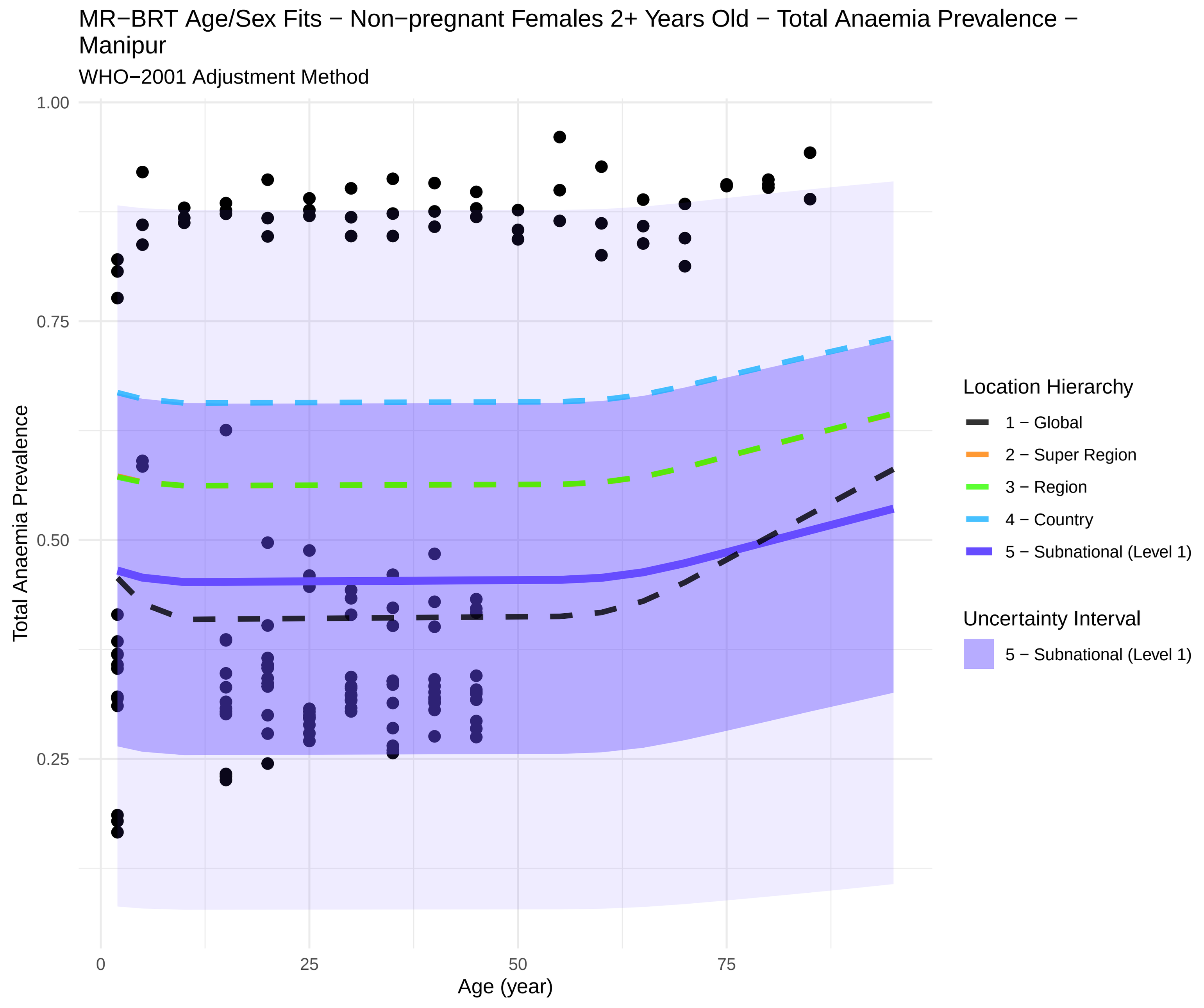
MR−BRT Age/Sex Fits − Non-pregnant Females 2+ Years Old − Total Anaemia Prevalence − Manipur
WHO−2001 Adjustment Method
Total Anaemia Prevalence
Age (year)
Location Hierarchy
1 − Global
2 − Super Region
3 − Region
4 − Country
5 − Subnational (Level 1)
Uncertainty Interval
5 − Subnational (Level 1)

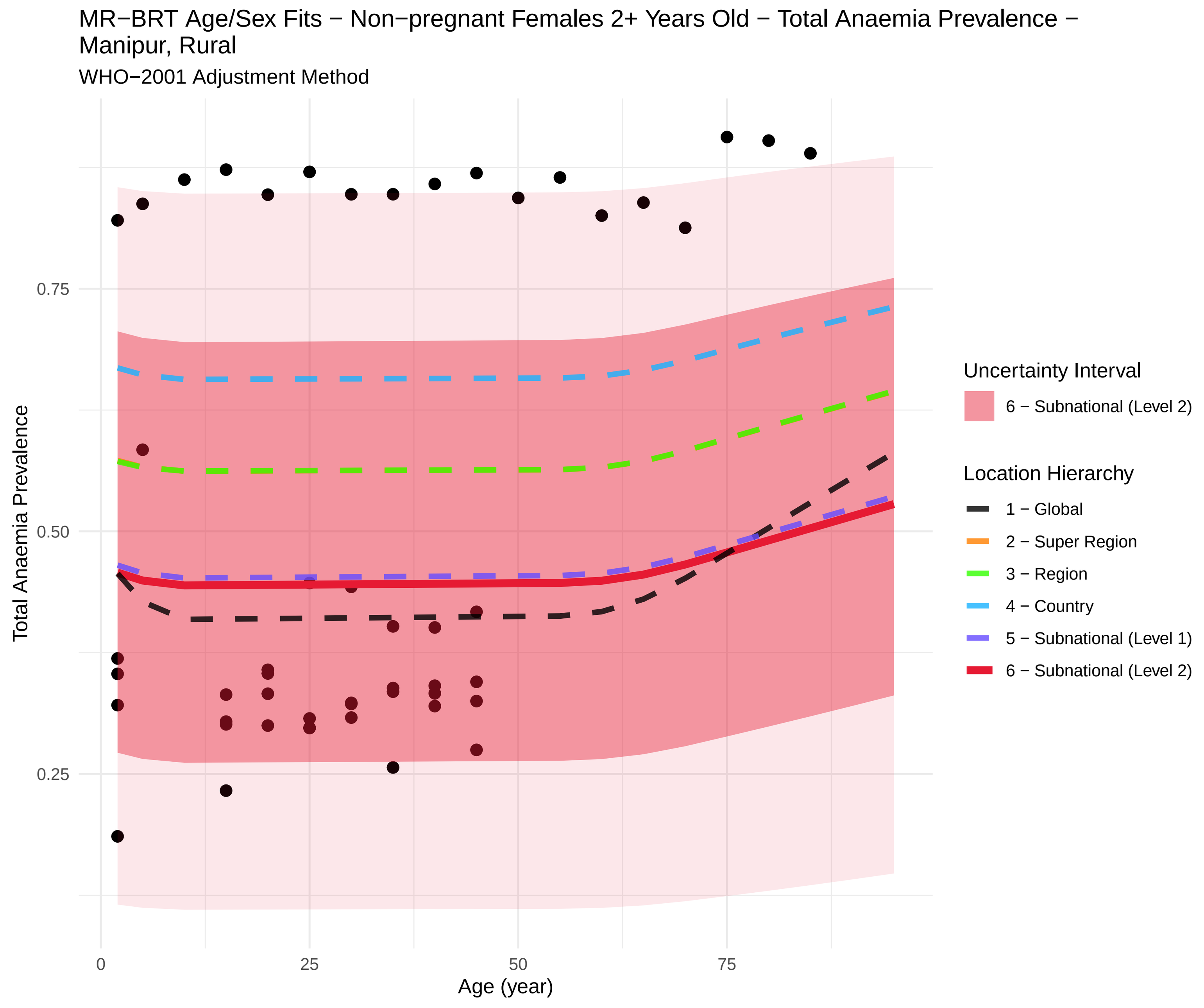
MR−BRT Age/Sex Fits − Non-pregnant Females 2+ Years Old − Total Anaemia Prevalence − Manipur, Rural
WHO−2001 Adjustment Method
Total Anaemia Prevalence
Age (year)
Uncertainty Interval
6 − Subnational (Level 2)
Location Hierarchy
1 − Global
2 − Super Region
3 − Region
4 − Country
5 − Subnational (Level 1)
6 − Subnational (Level 2)

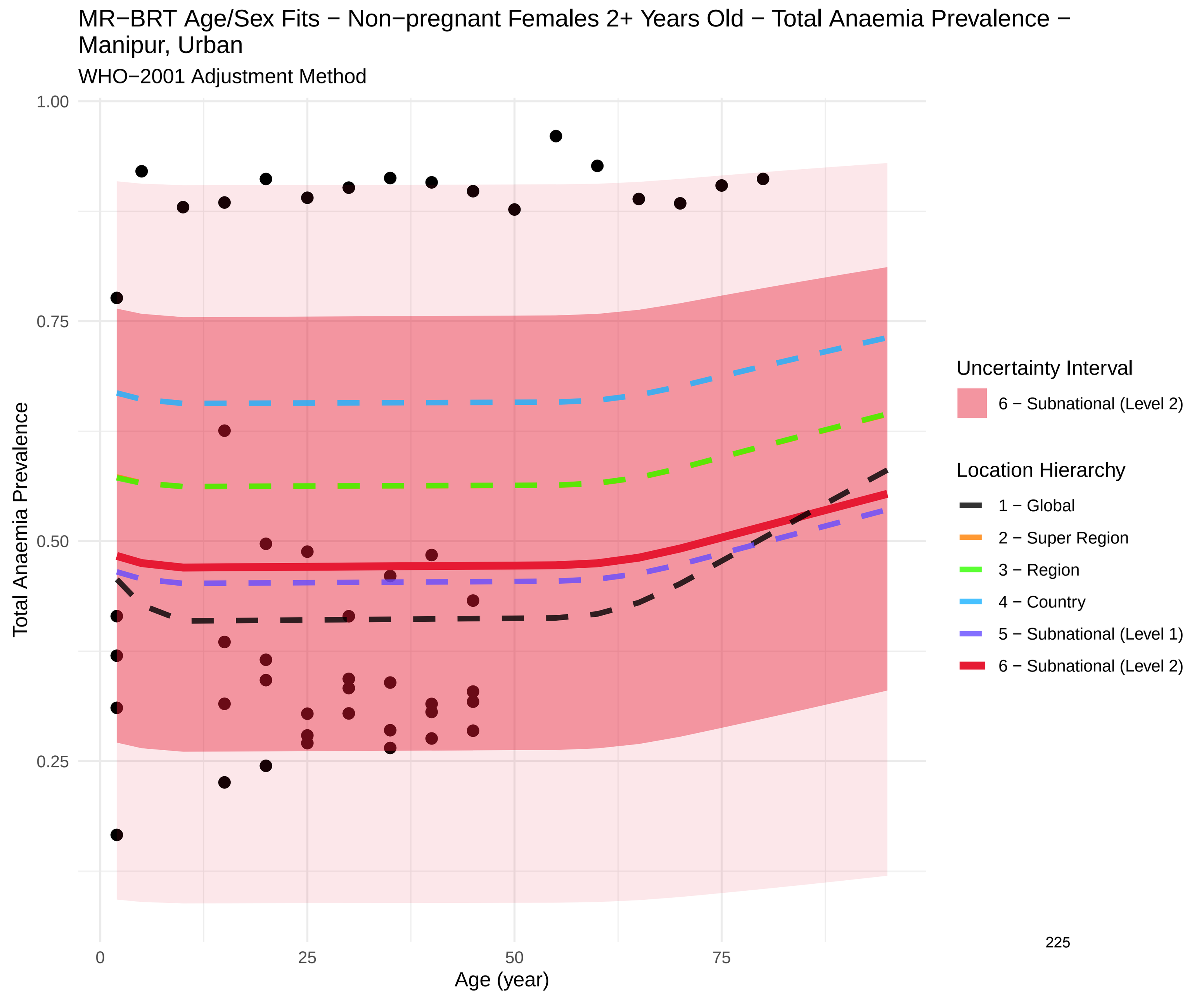
MR−BRT Age/Sex Fits − Non-pregnant Females 2+ Years Old − Total Anaemia Prevalence − Manipur, Urban
WHO−2001 Adjustment Method
Total Anaemia Prevalence
Age (year)
Uncertainty Interval
6 − Subnational (Level 2)
Location Hierarchy
1 − Global
2 − Super Region
3 − Region
4 − Country
5 − Subnational (Level 1)
6 − Subnational (Level 2)

MR−BRT Age/Sex Fits − Non−pregnant Females 2+ Years Old − Total Anaemia Prevalence − Meghalaya
WHO−2001 Adjustment Method

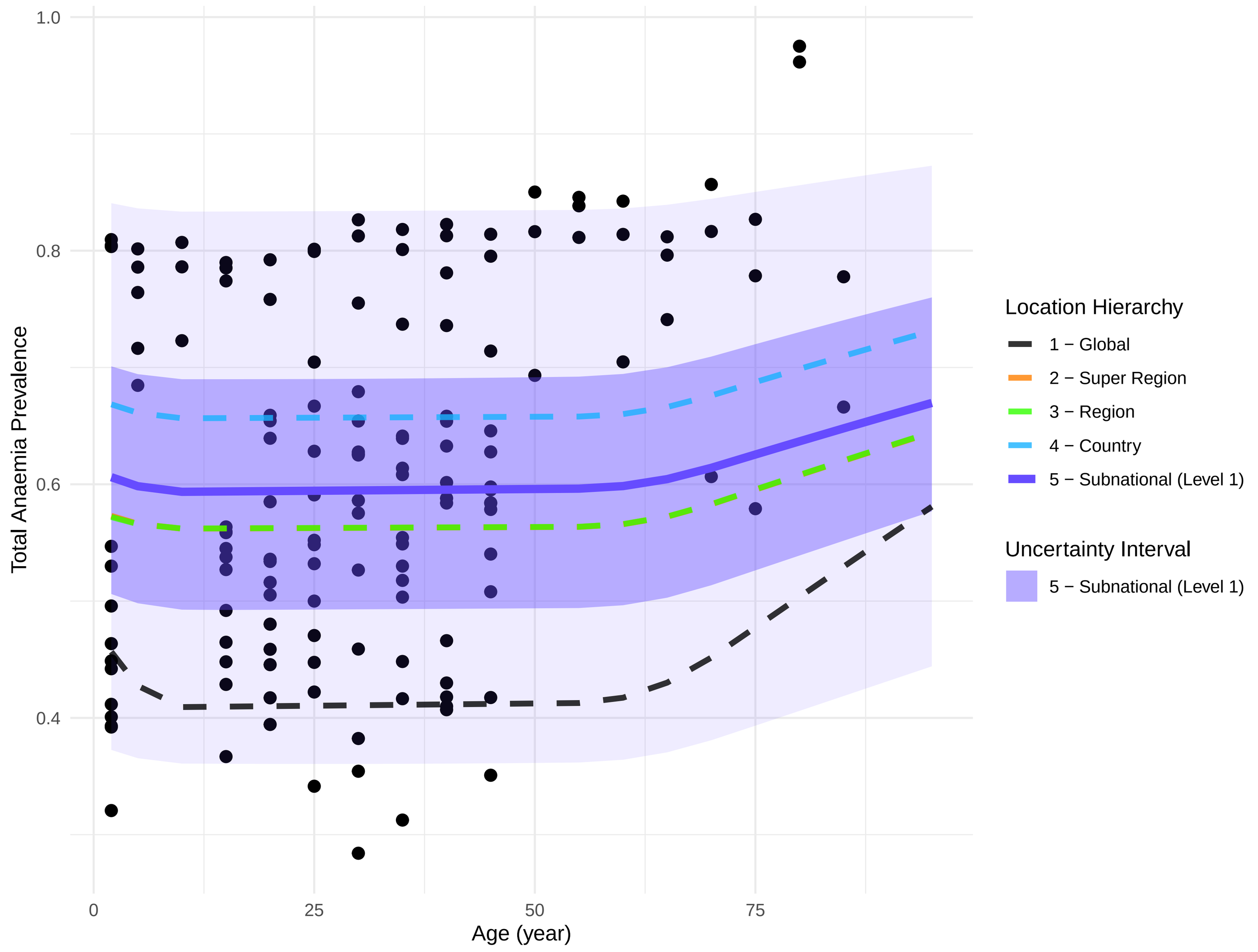

MR−BRT Age/Sex Fits − Non−pregnant Females 2+ Years Old − Total Anaemia Prevalence − Meghalaya, Rural
WHO−2001 Adjustment Method

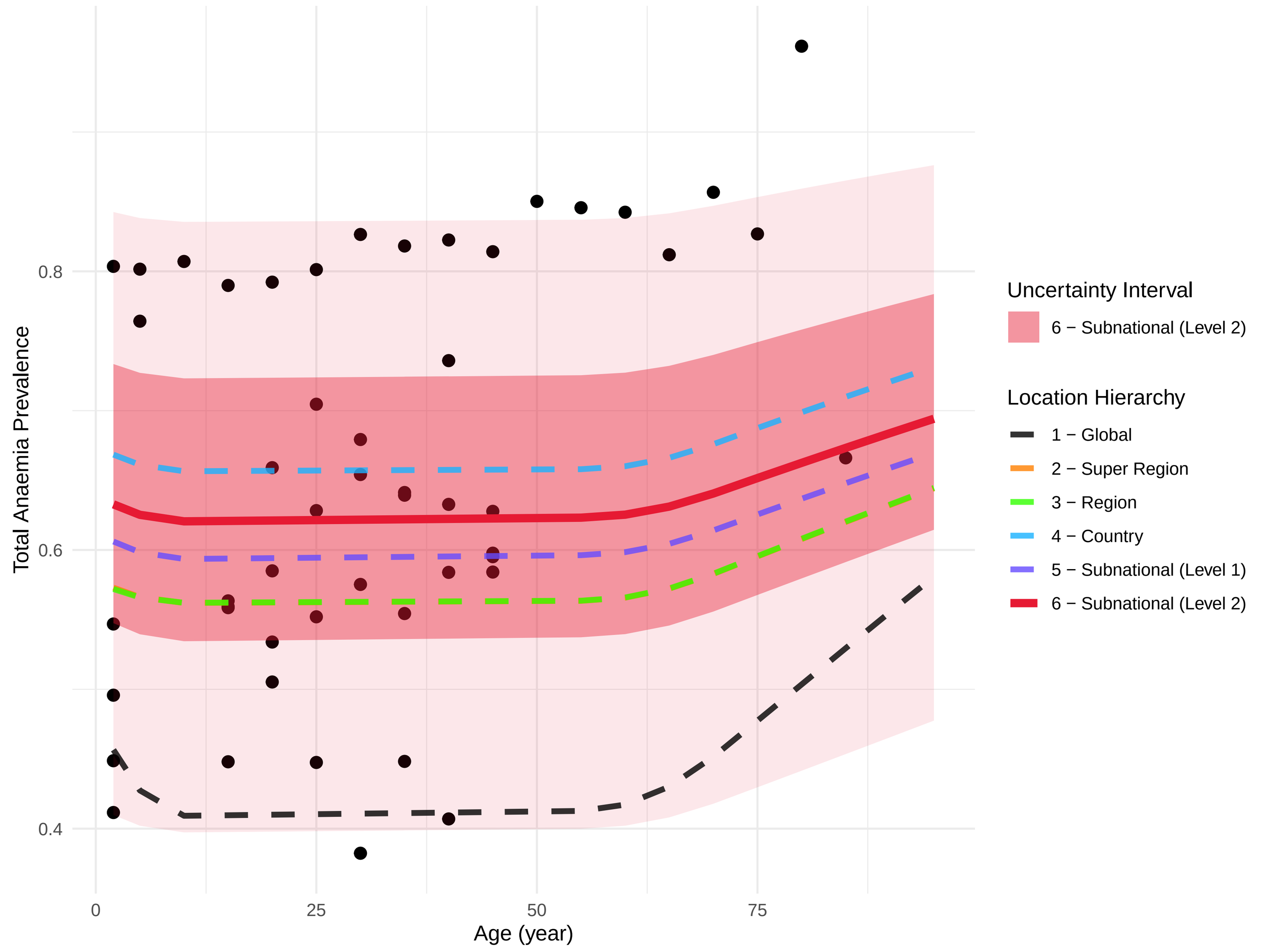

MR−BRT Age/Sex Fits − Non−pregnant Females 2+ Years Old − Total Anaemia Prevalence − Meghalaya, Urban
WHO−2001 Adjustment Method

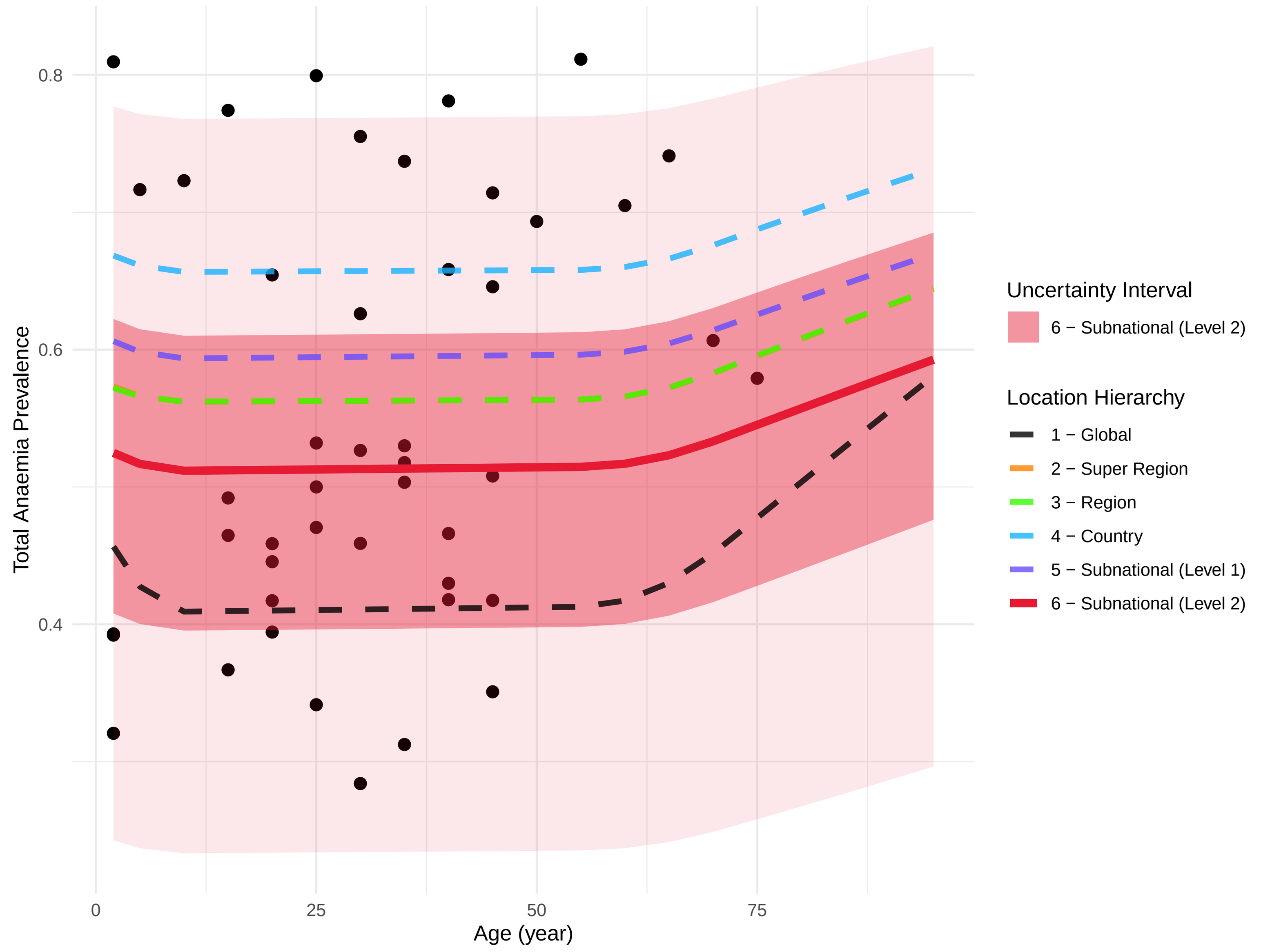

MR−BRT Age/Sex Fits − Non−pregnant Females 2+ Years Old − Total Anaemia Prevalence − Mizoram
WHO−2001 Adjustment Method

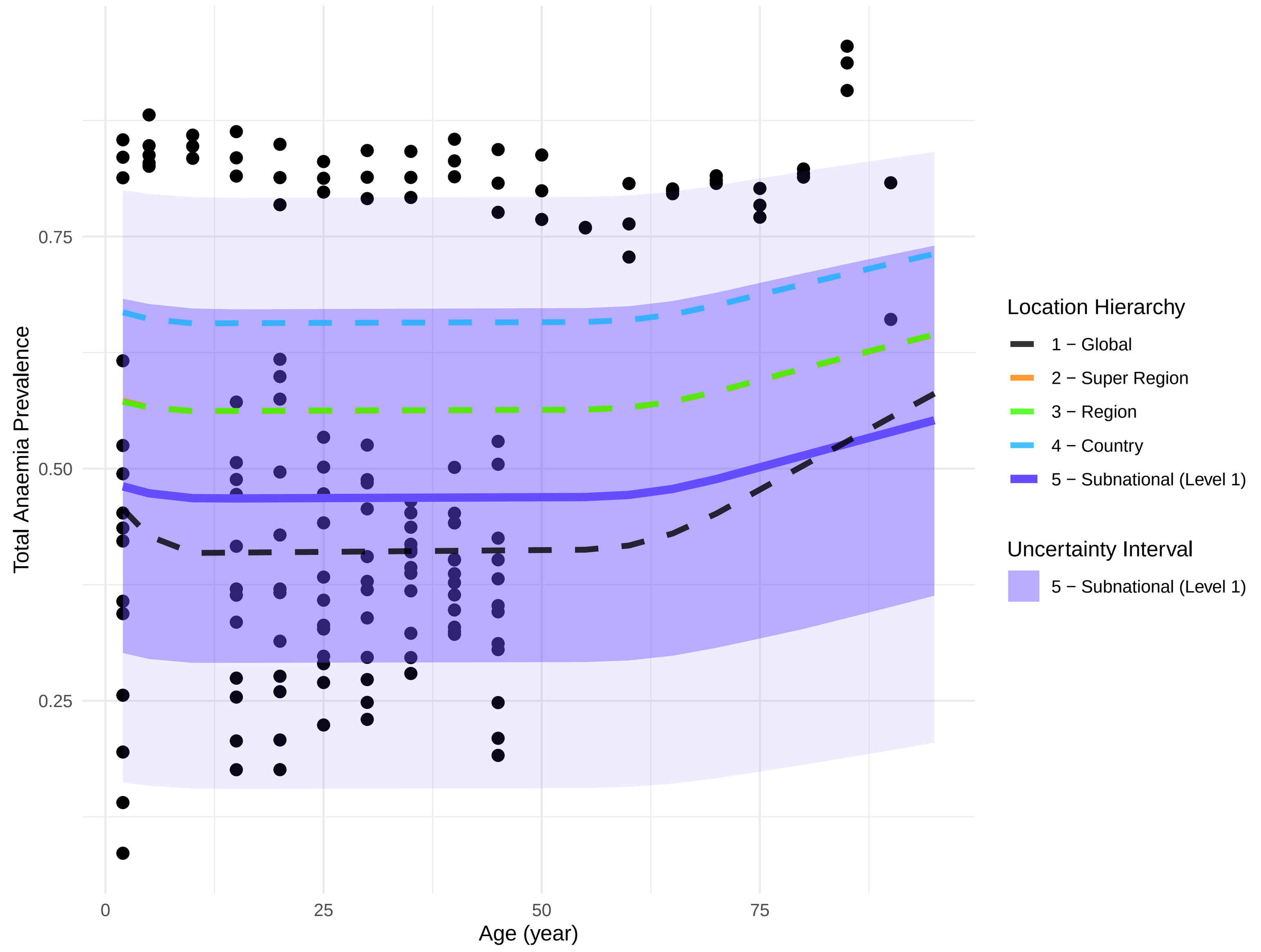

MR−BRT Age/Sex Fits − Non−pregnant Females 2+ Years Old − Total Anaemia Prevalence − Mizoram, Rural
WHO−2001 Adjustment Method

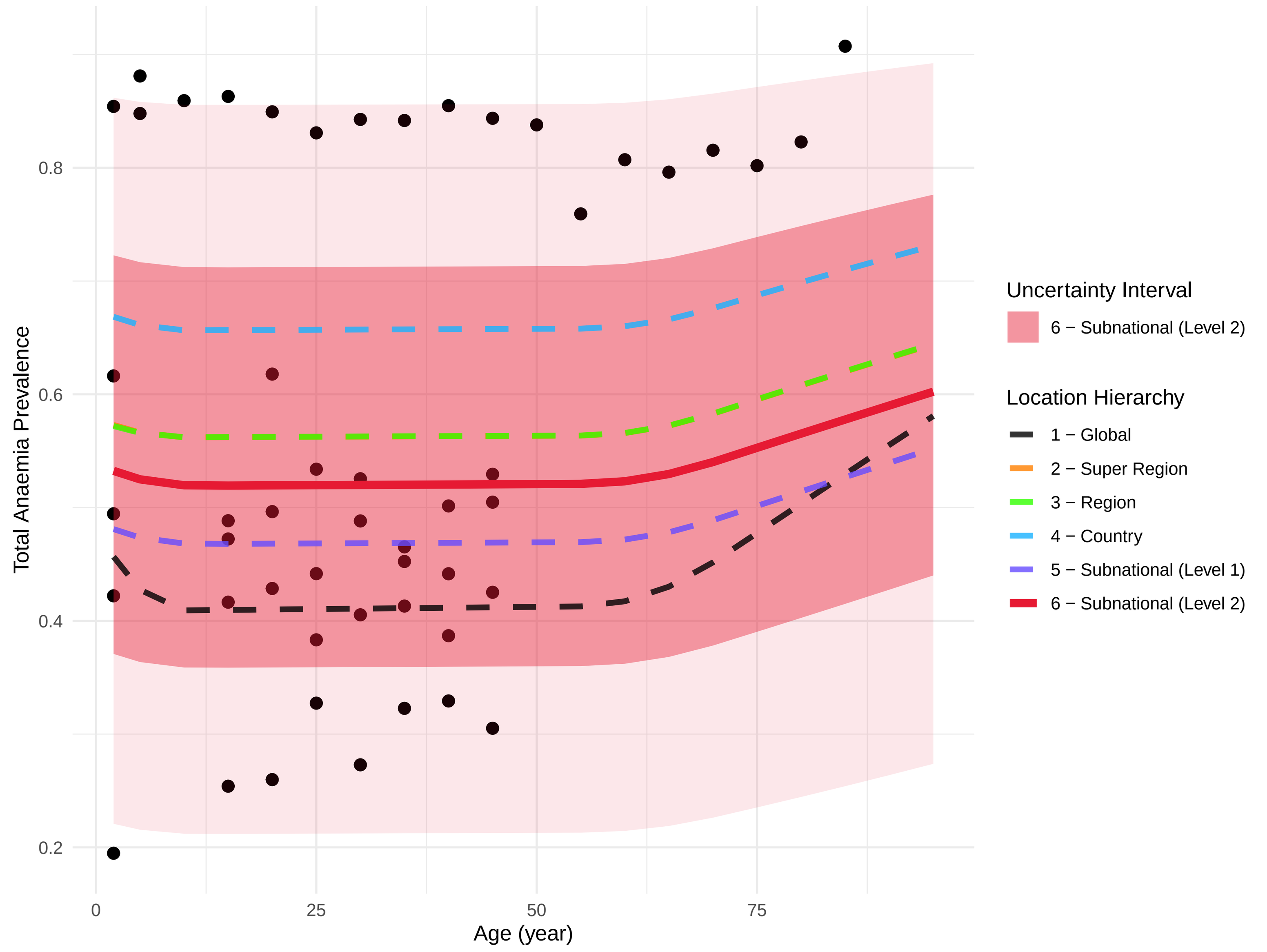

MR−BRT Age/Sex Fits − Non−pregnant Females 2+ Years Old − Total Anaemia Prevalence − Mizoram, Urban
WHO−2001 Adjustment Method

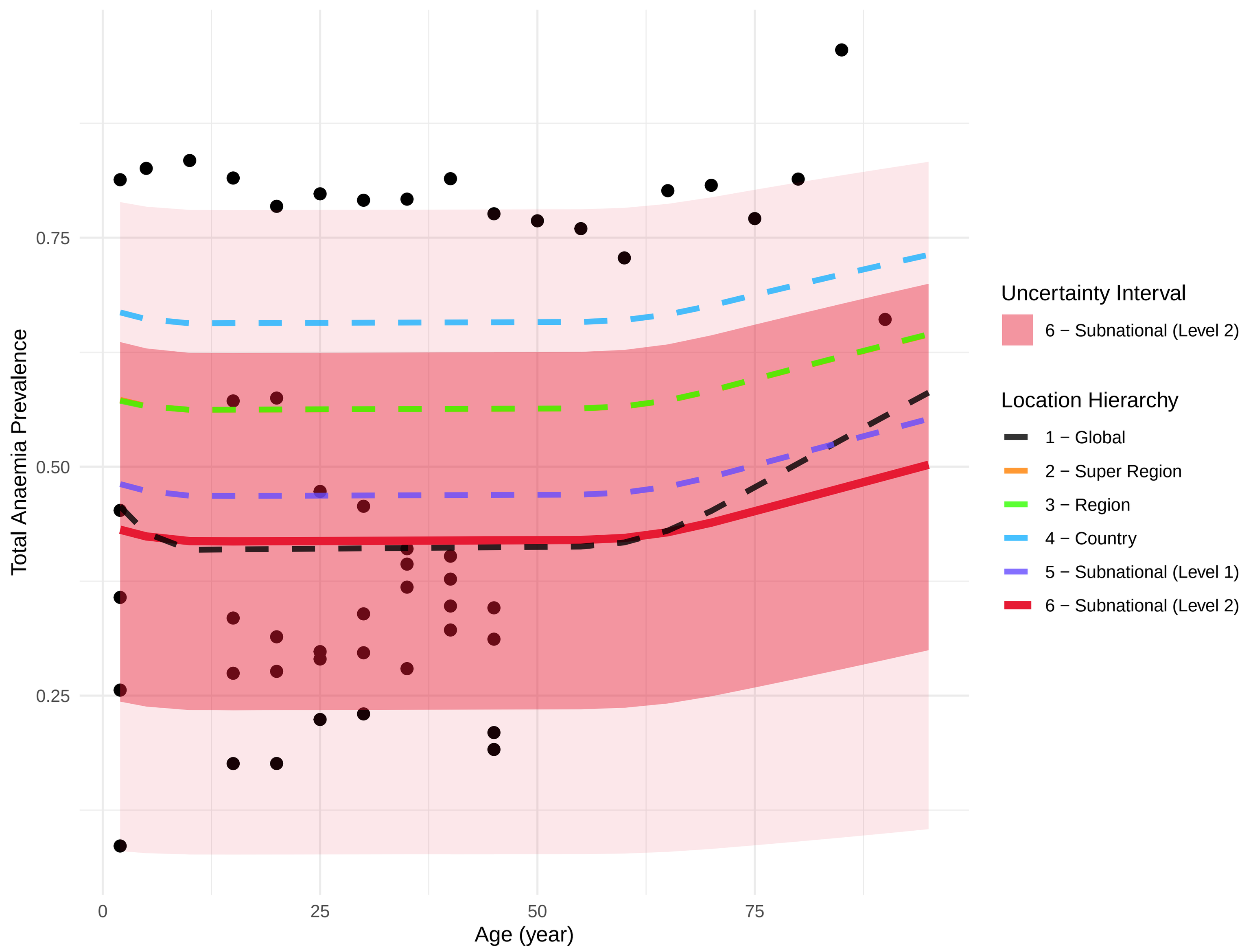

MR−BRT Age/Sex Fits − Non−pregnant Females 2+ Years Old − Total Anaemia Prevalence − Nagaland
WHO−2001 Adjustment Method

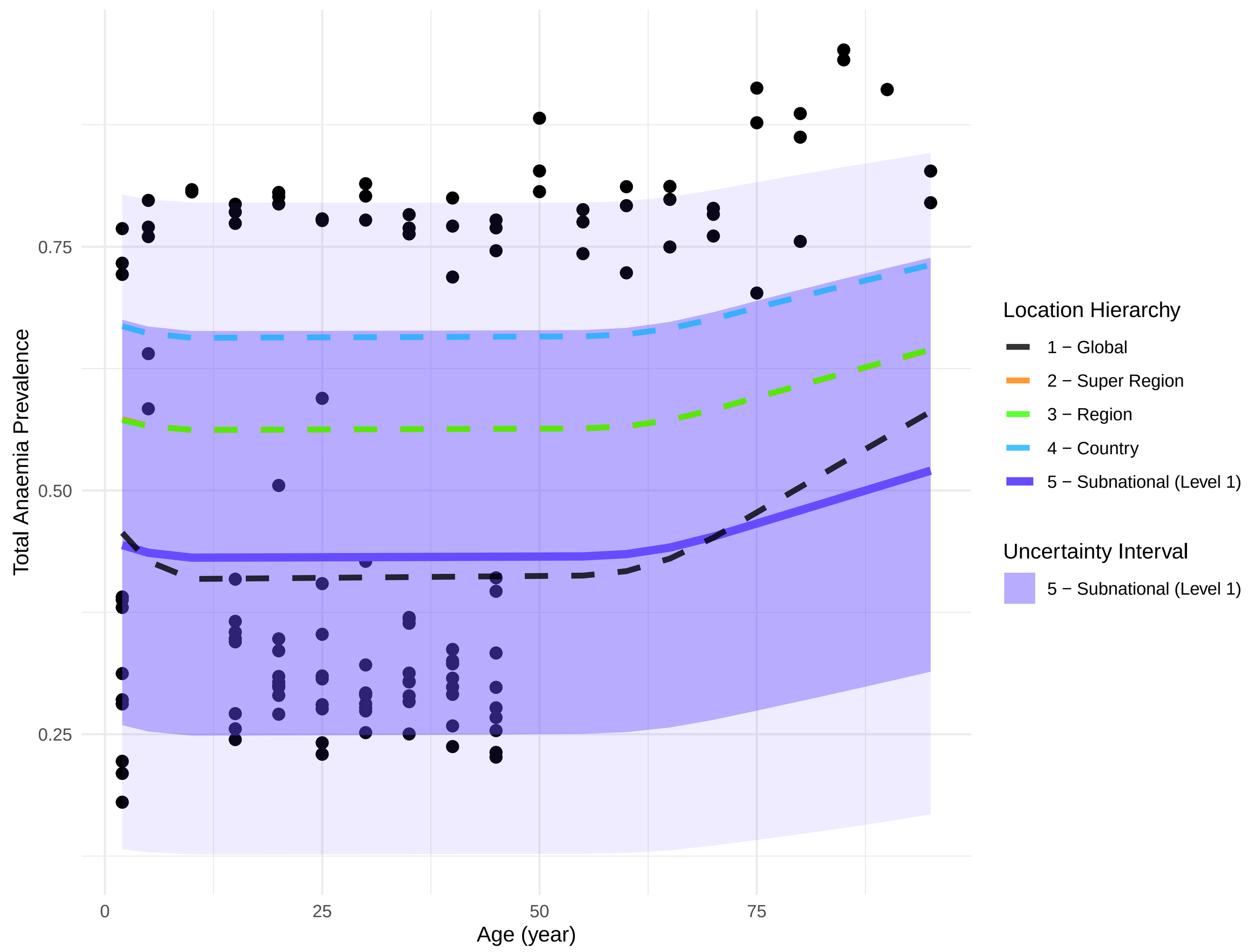

MR−BRT Age/Sex Fits − Non−pregnant Females 2+ Years Old − Total Anaemia Prevalence − Nagaland, Rural
WHO−2001 Adjustment Method

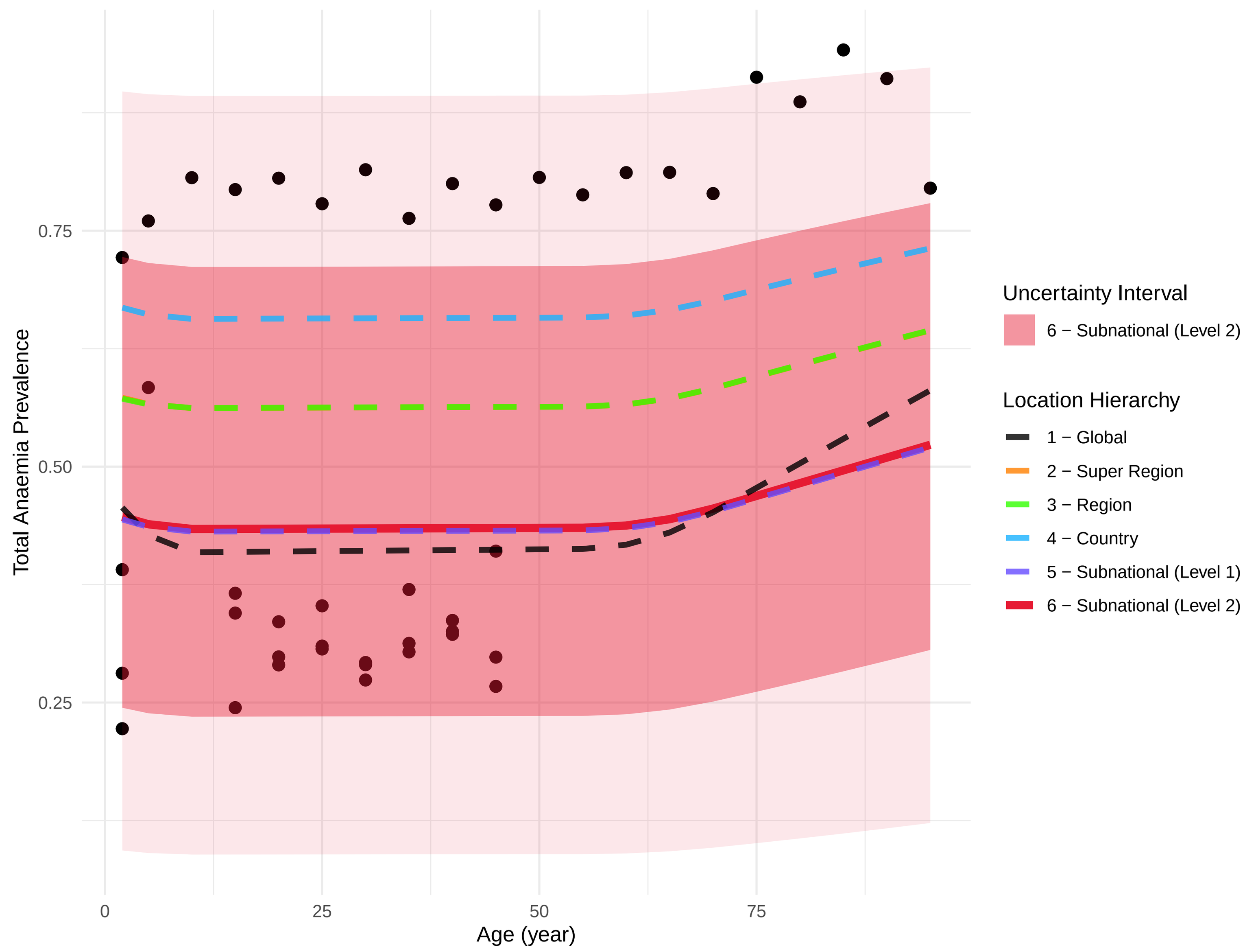

MR−BRT Age/Sex Fits − Non−pregnant Females 2+ Years Old − Total Anaemia Prevalence − Nagaland, Urban
WHO−2001 Adjustment Method

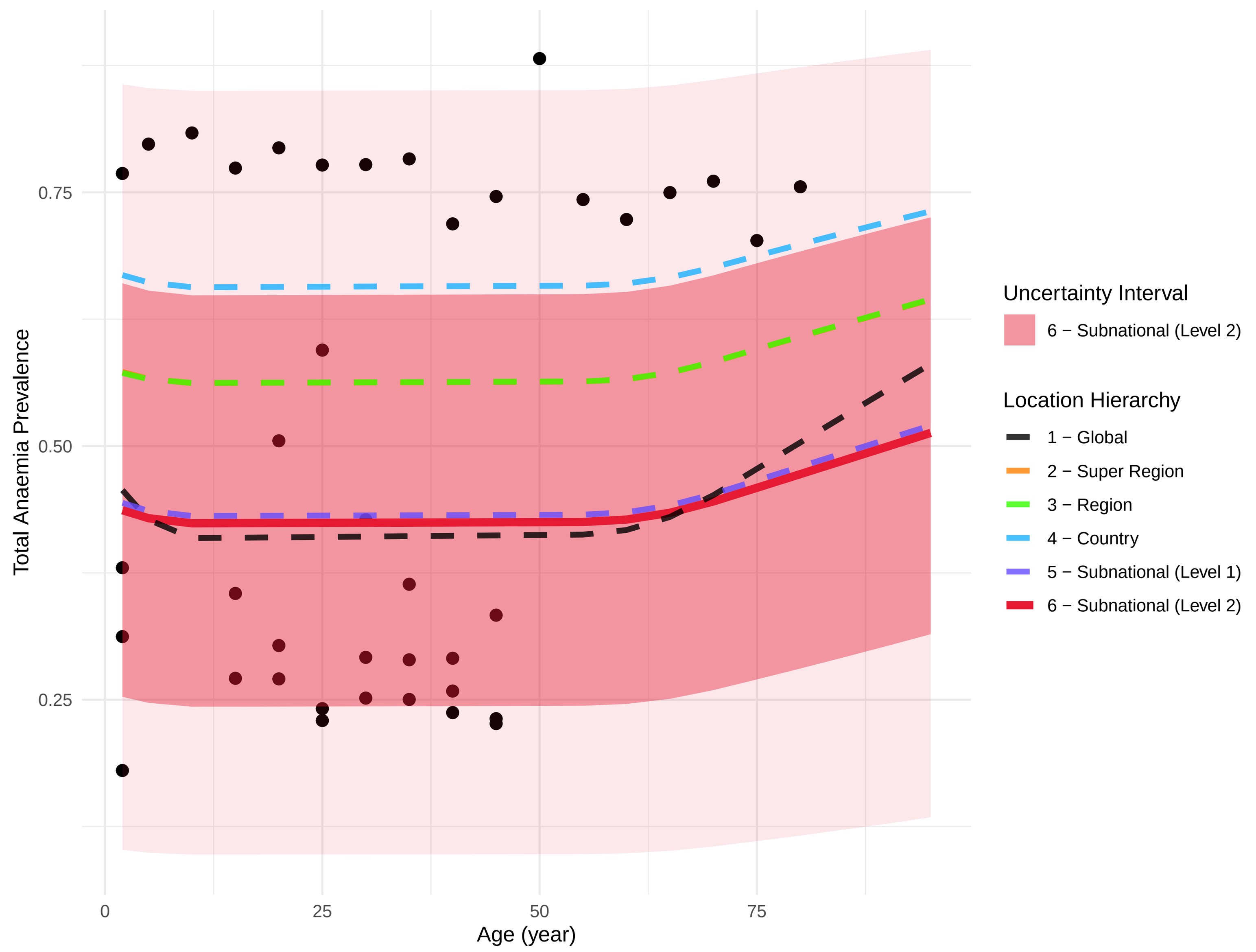

MR−BRT Age/Sex Fits − Non−pregnant Females 2+ Years Old − Total Anaemia Prevalence − Odisha
WHO−2001 Adjustment Method

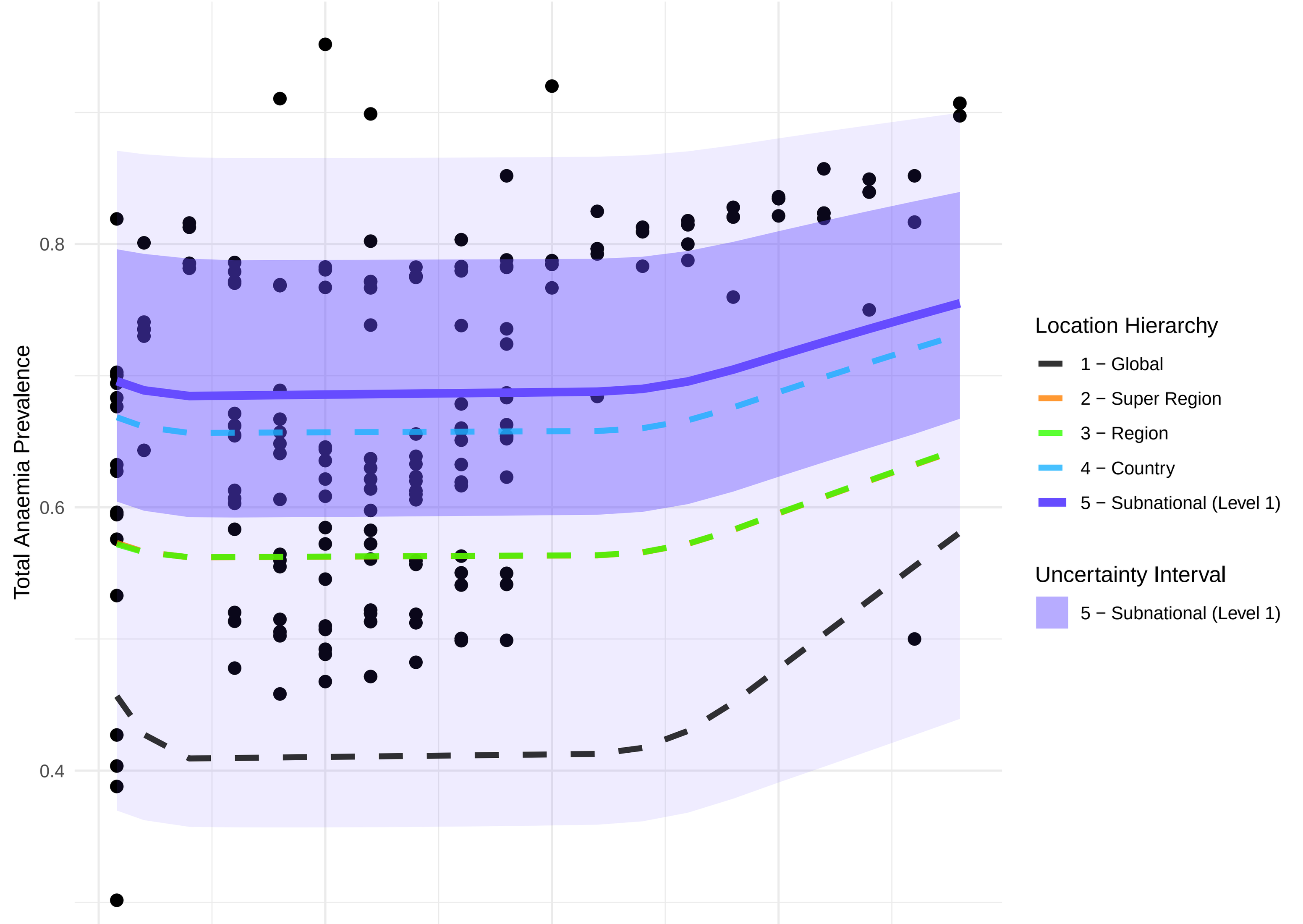

MR−BRT Age/Sex Fits − Non−pregnant Females 2+ Years Old − Total Anaemia Prevalence − Odisha, Rural
WHO−2001 Adjustment Method

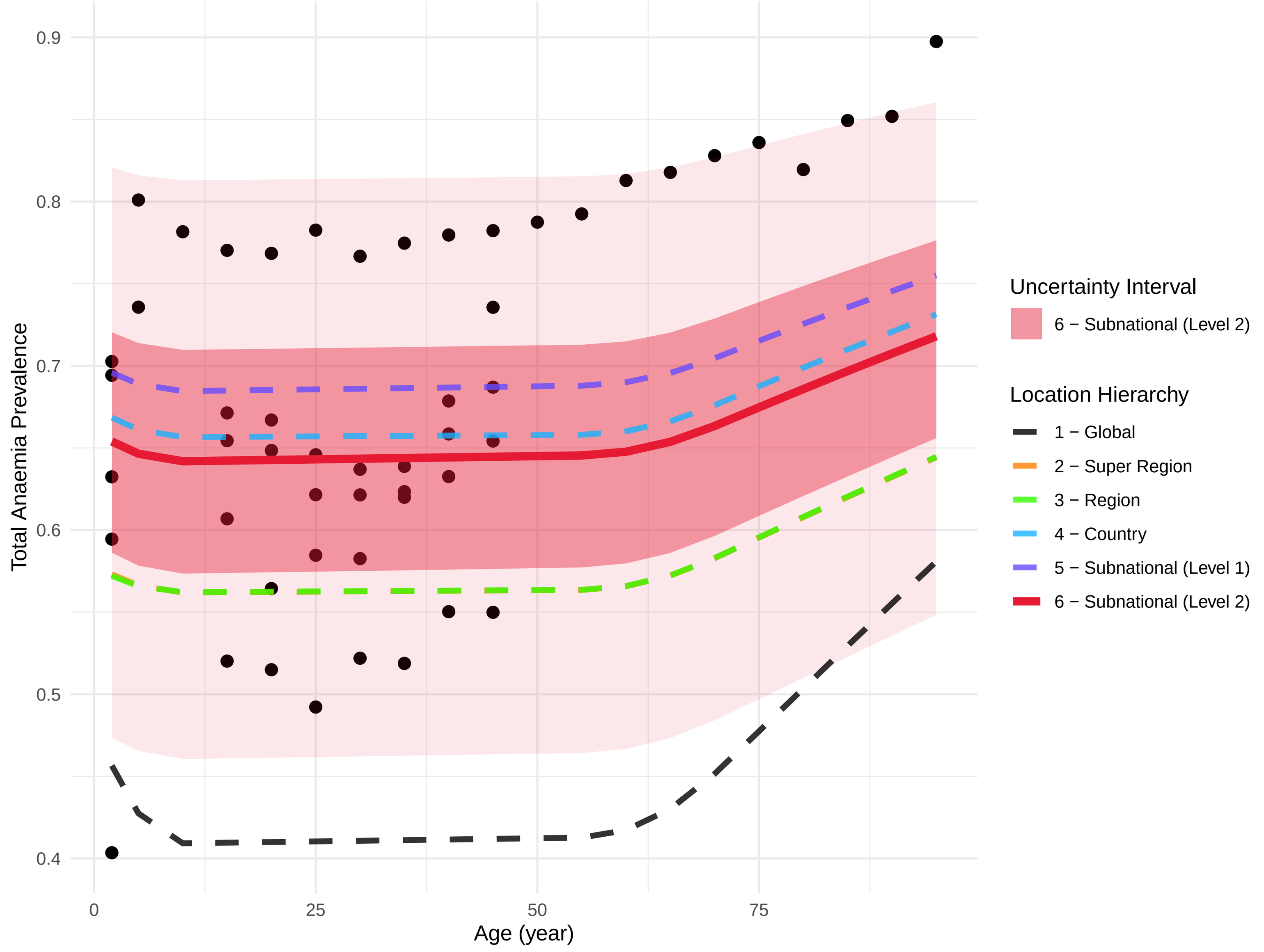

MR−BRT Age/Sex Fits − Non−pregnant Females 2+ Years Old − Total Anaemia Prevalence − Odisha, Urban
WHO−2001 Adjustment Method

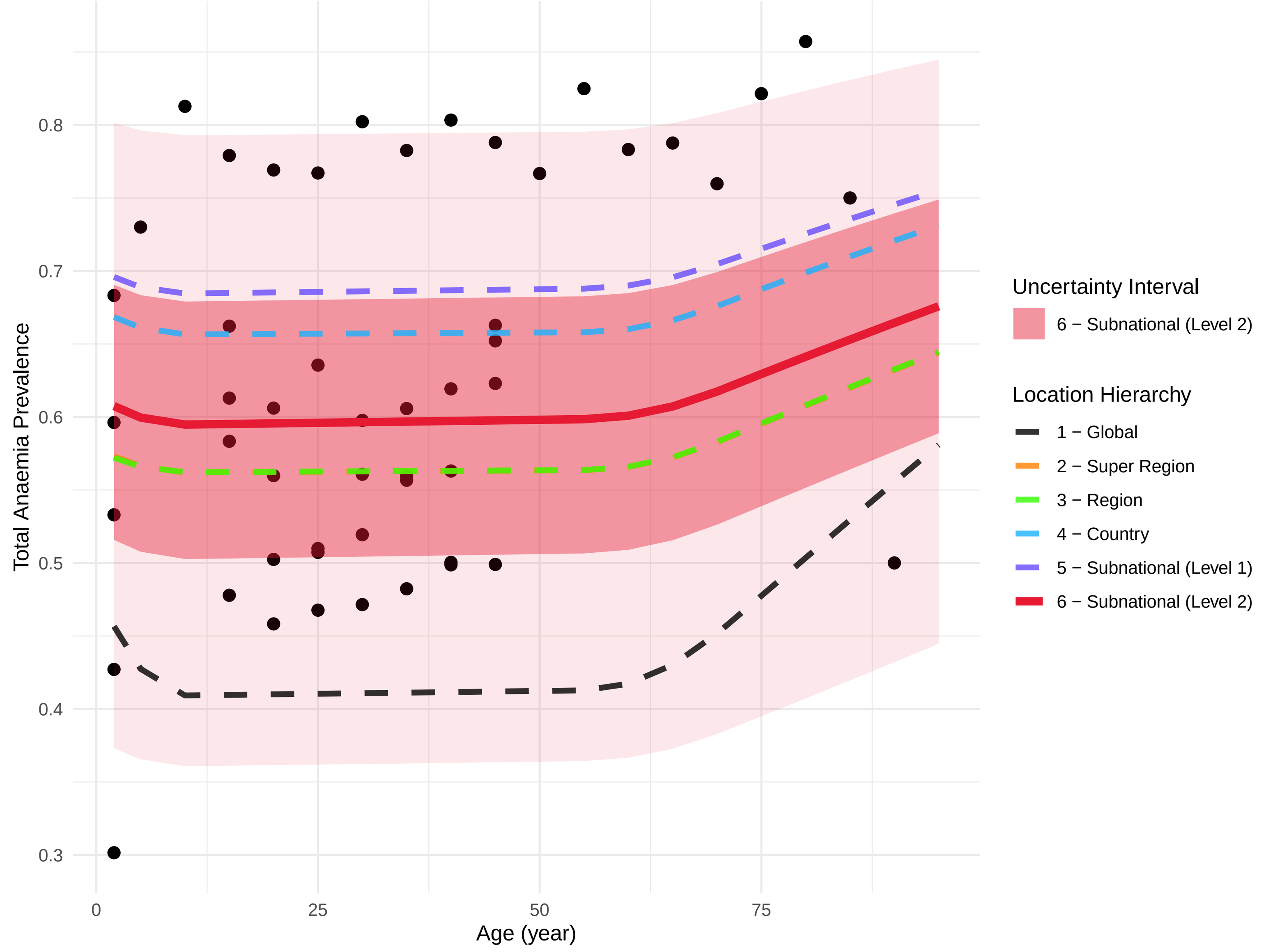

MR−BRT Age/Sex Fits − Non−pregnant Females 2+ Years Old − Total Anaemia Prevalence − Other Union Territories
WHO−2001 Adjustment Method

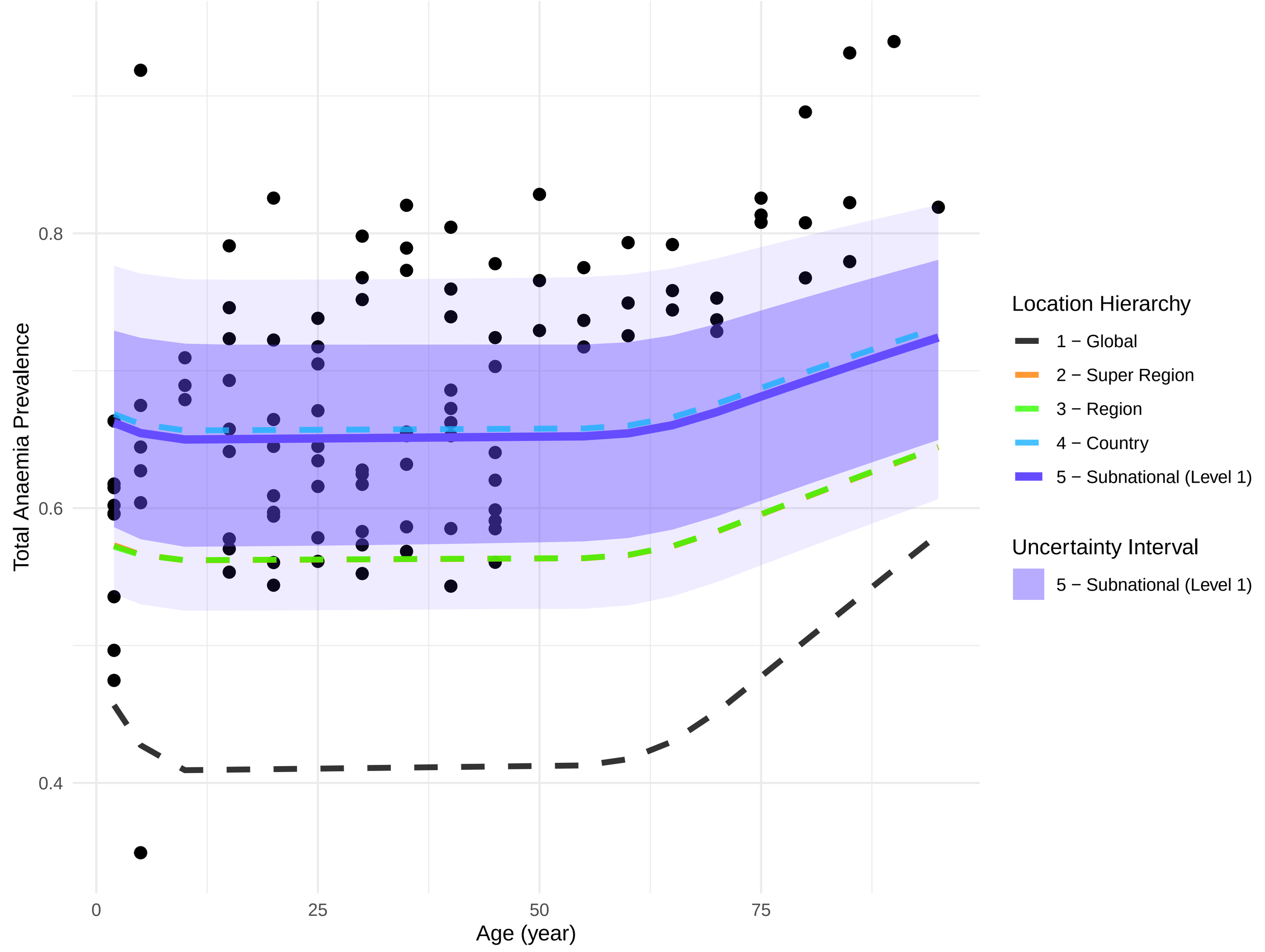

MR−BRT Age/Sex Fits − Non−pregnant Females 2+ Years Old − Total Anaemia Prevalence − Other Union Territories, Rural
WHO−2001 Adjustment Method

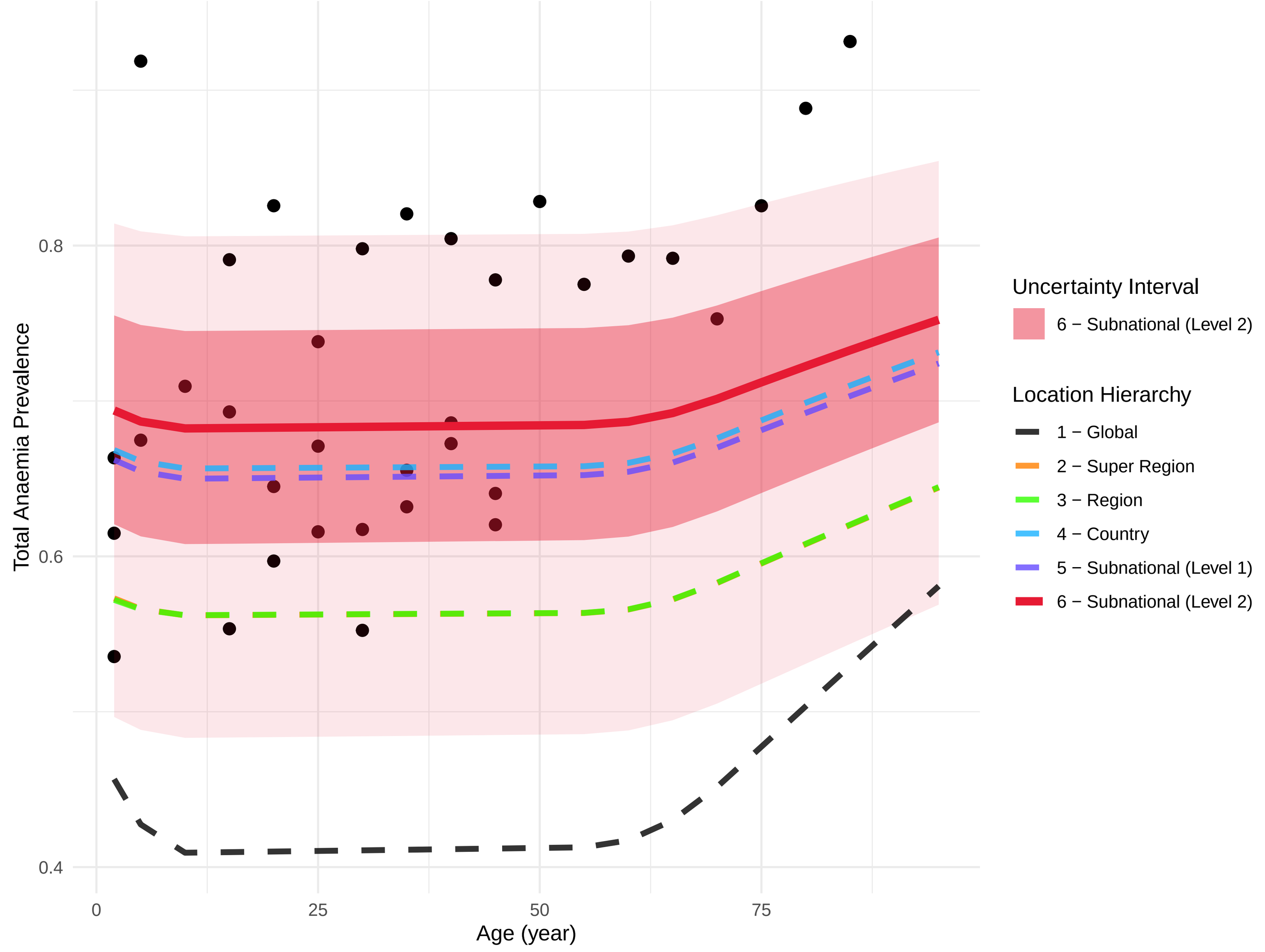

MR−BRT Age/Sex Fits − Non−pregnant Females 2+ Years Old − Total Anaemia Prevalence − Other Union Territories, Urban
WHO−2001 Adjustment Method

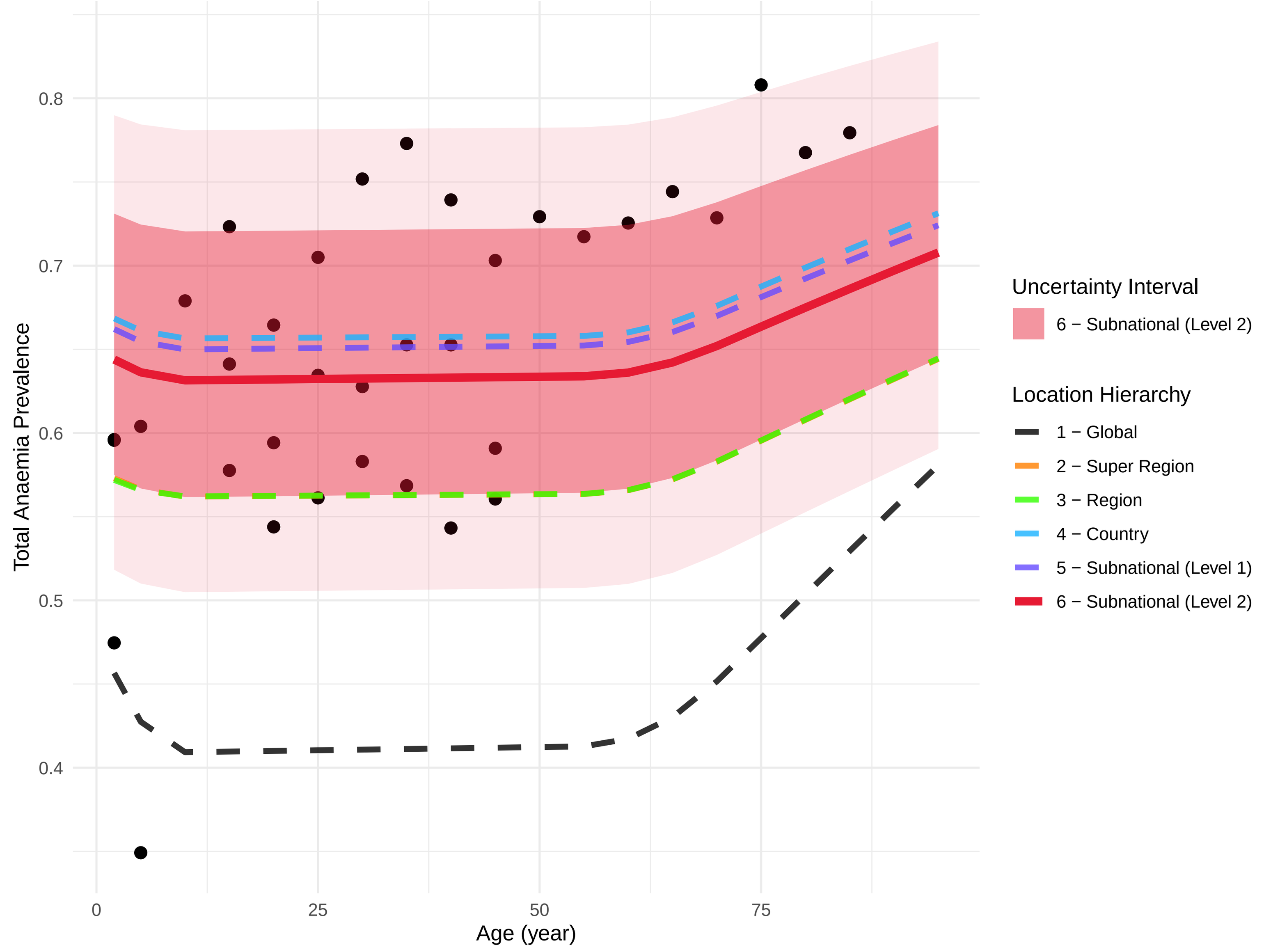

MR−BRT Age/Sex Fits − Non−pregnant Females 2+ Years Old − Total Anaemia Prevalence − Punjab
WHO−2001 Adjustment Method

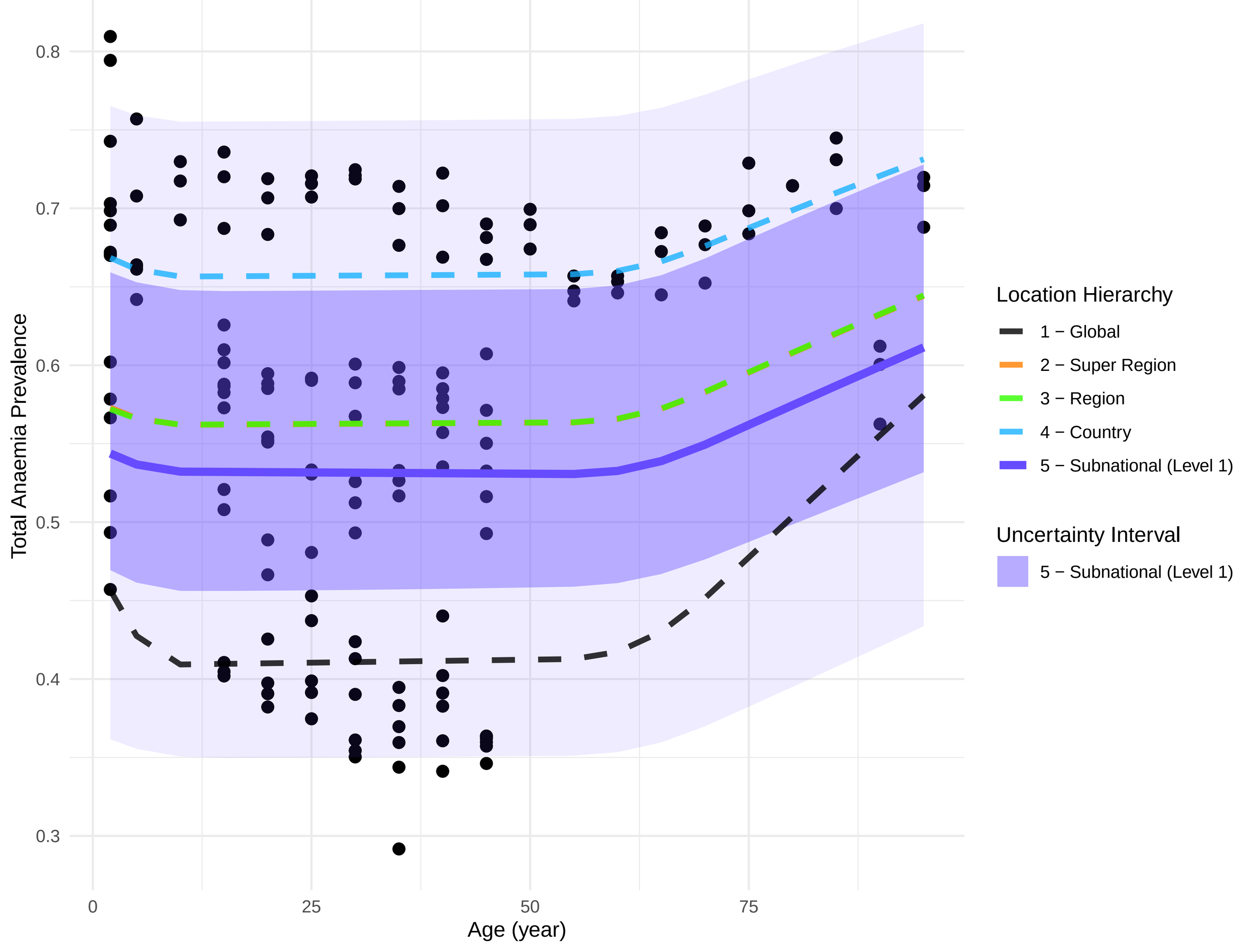

MR−BRT Age/Sex Fits − Non−pregnant Females 2+ Years Old − Total Anaemia Prevalence − Punjab, Rural
WHO−2001 Adjustment Method

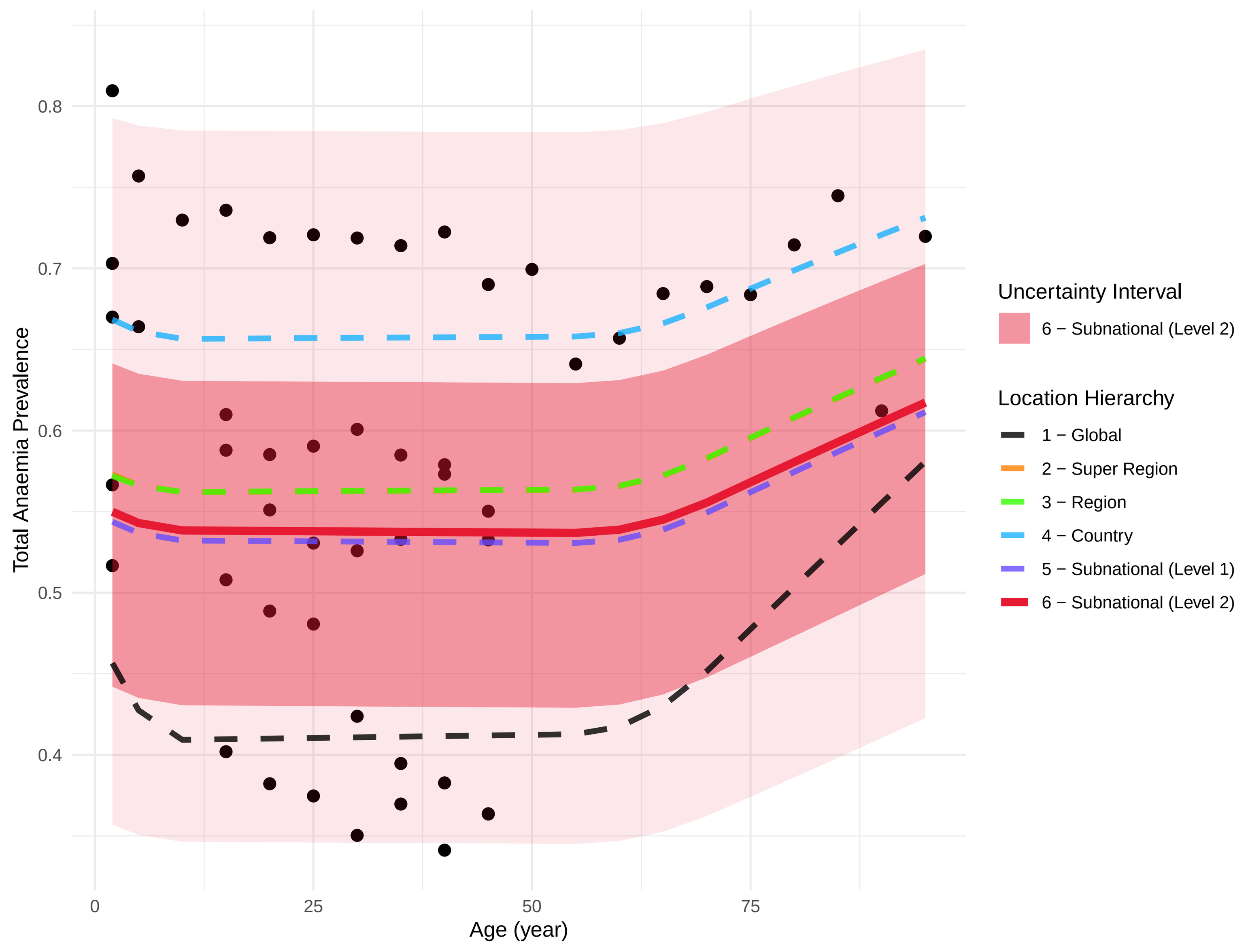

MR−BRT Age/Sex Fits − Non−pregnant Females 2+ Years Old − Total Anaemia Prevalence − Punjab, Urban
WHO−2001 Adjustment Method

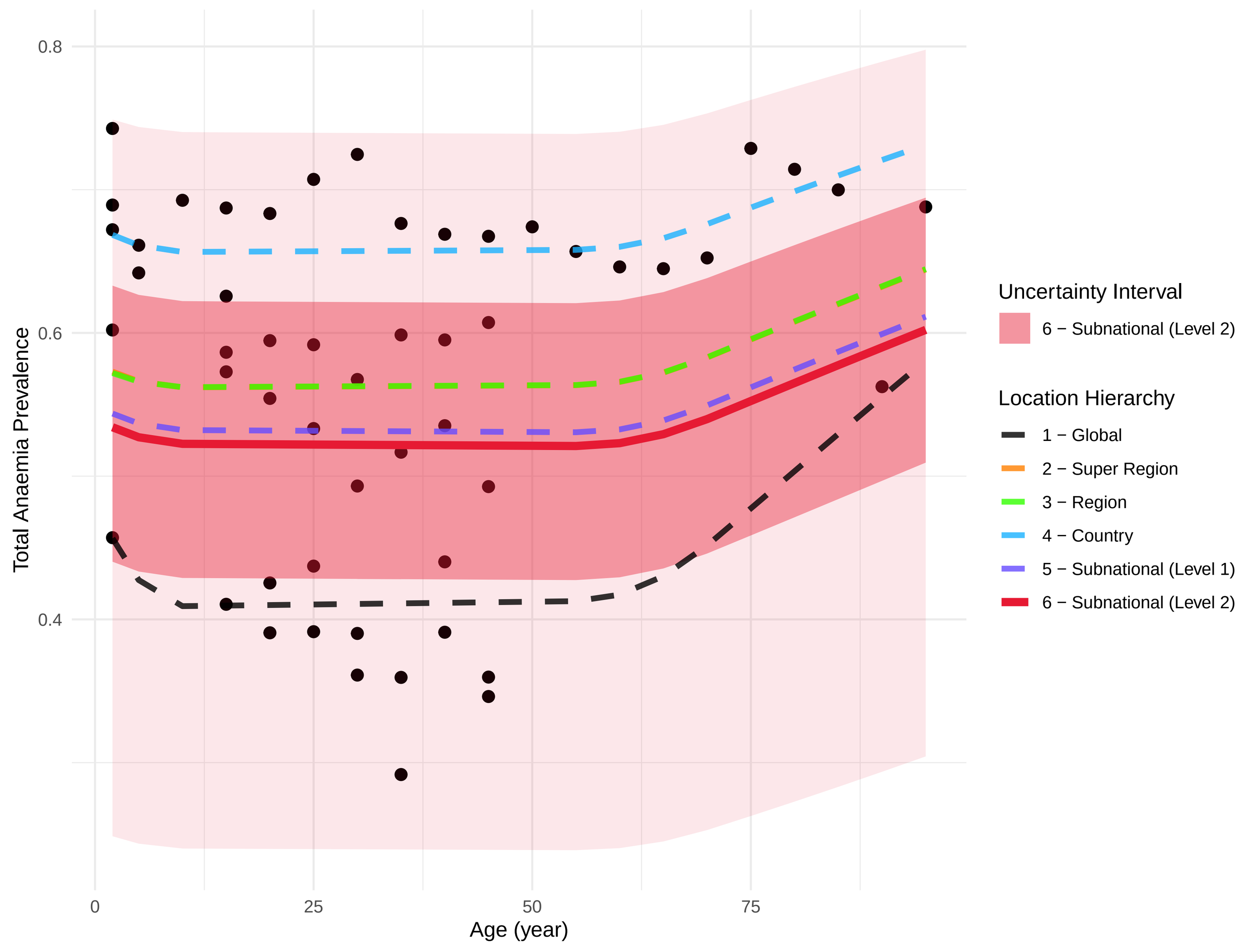

MR−BRT Age/Sex Fits − Non−pregnant Females 2+ Years Old − Total Anaemia Prevalence − Rajasthan
WHO−2001 Adjustment Method

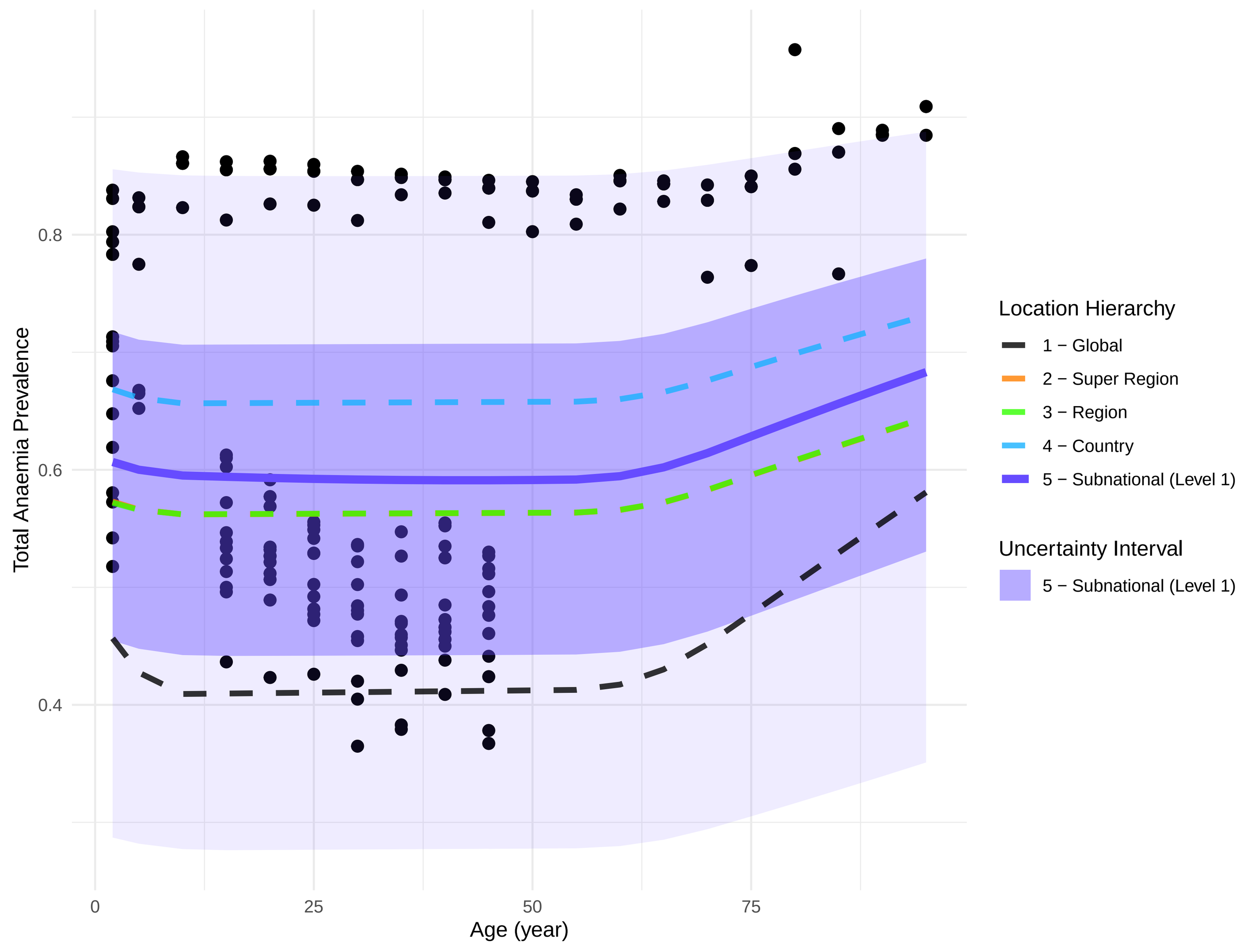

MR−BRT Age/Sex Fits − Non−pregnant Females 2+ Years Old − Total Anaemia Prevalence − Rajasthan, Rural
WHO−2001 Adjustment Method

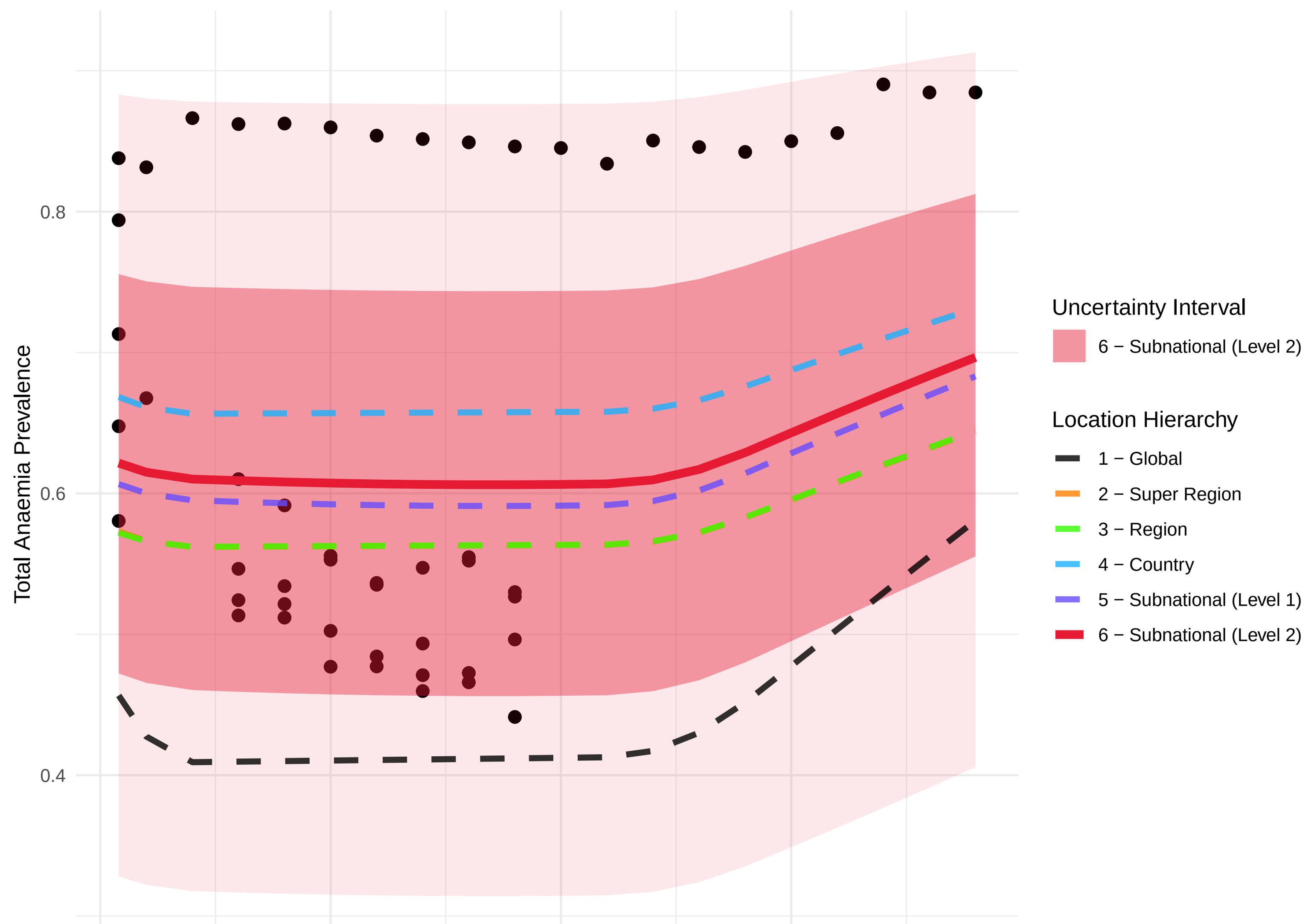

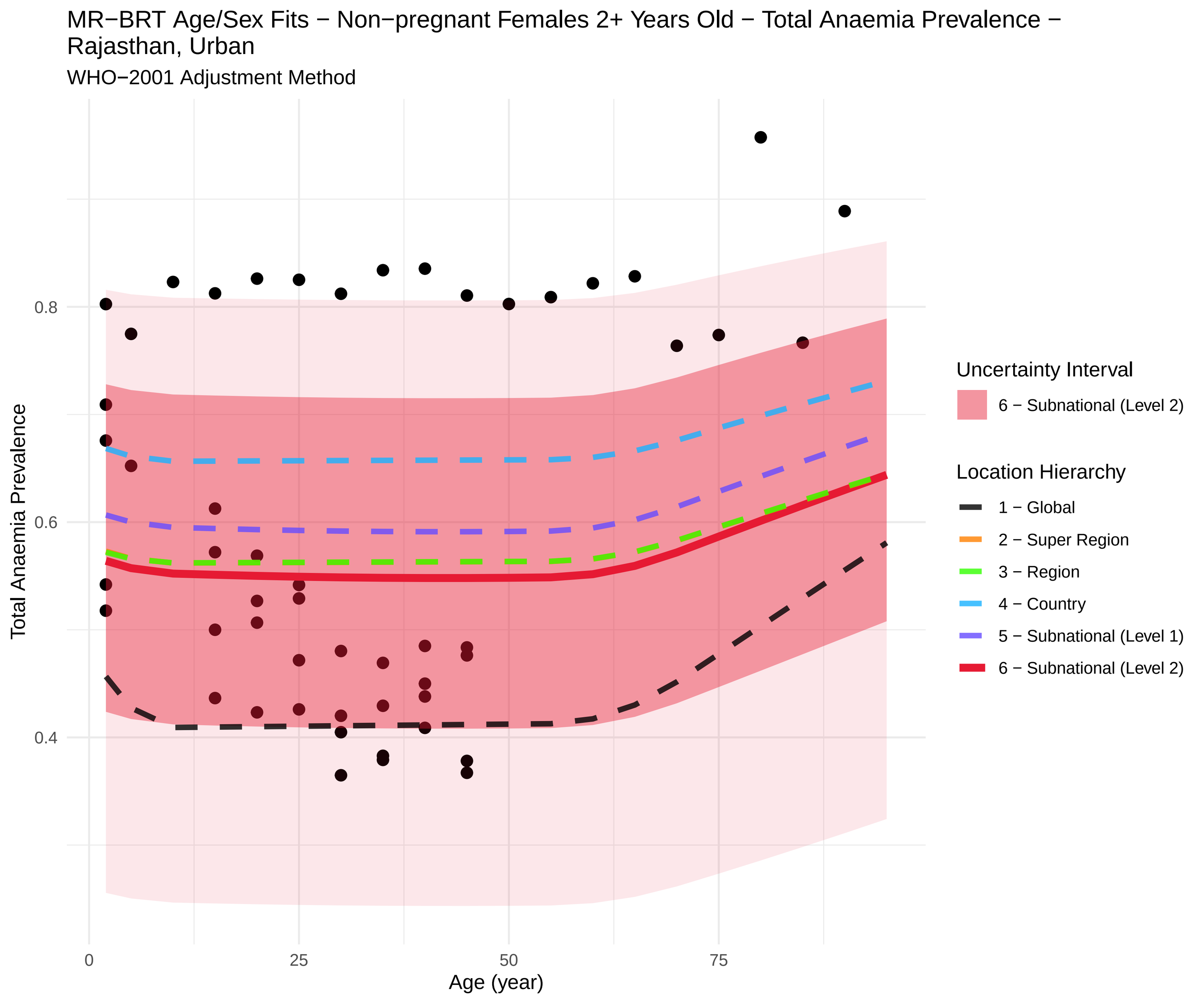
MR−BRT Age/Sex Fits − Non-pregnant Females 2+ Years Old − Total Anaemia Prevalence − Rajasthan, Urban
WHO−2001 Adjustment Method
Total Anaemia Prevalence
Age (year)
Uncertainty Interval
6 − Subnational (Level 2)
Location Hierarchy
1 − Global
2 − Super Region
3 − Region
4 − Country
5 − Subnational (Level 1)
6 − Subnational (Level 2)

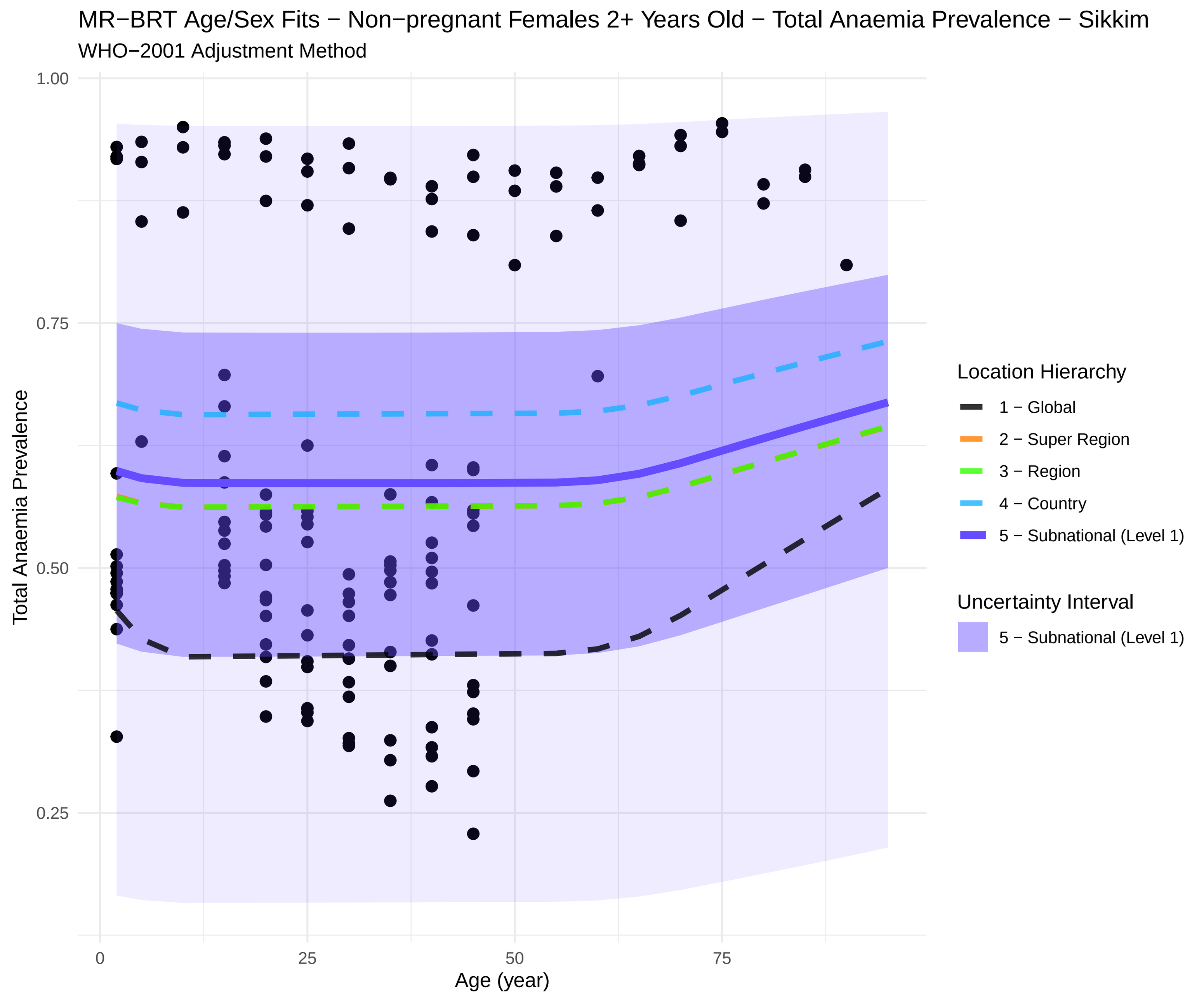
MR−BRT Age/Sex Fits − Non-pregnant Females 2+ Years Old − Total Anaemia Prevalence − Sikkim
WHO−2001 Adjustment Method
Total Anaemia Prevalence
Age (year)
Location Hierarchy
1 − Global
2 − Super Region
3 − Region
4 − Country
5 − Subnational (Level 1)
Uncertainty Interval
5 − Subnational (Level 1)

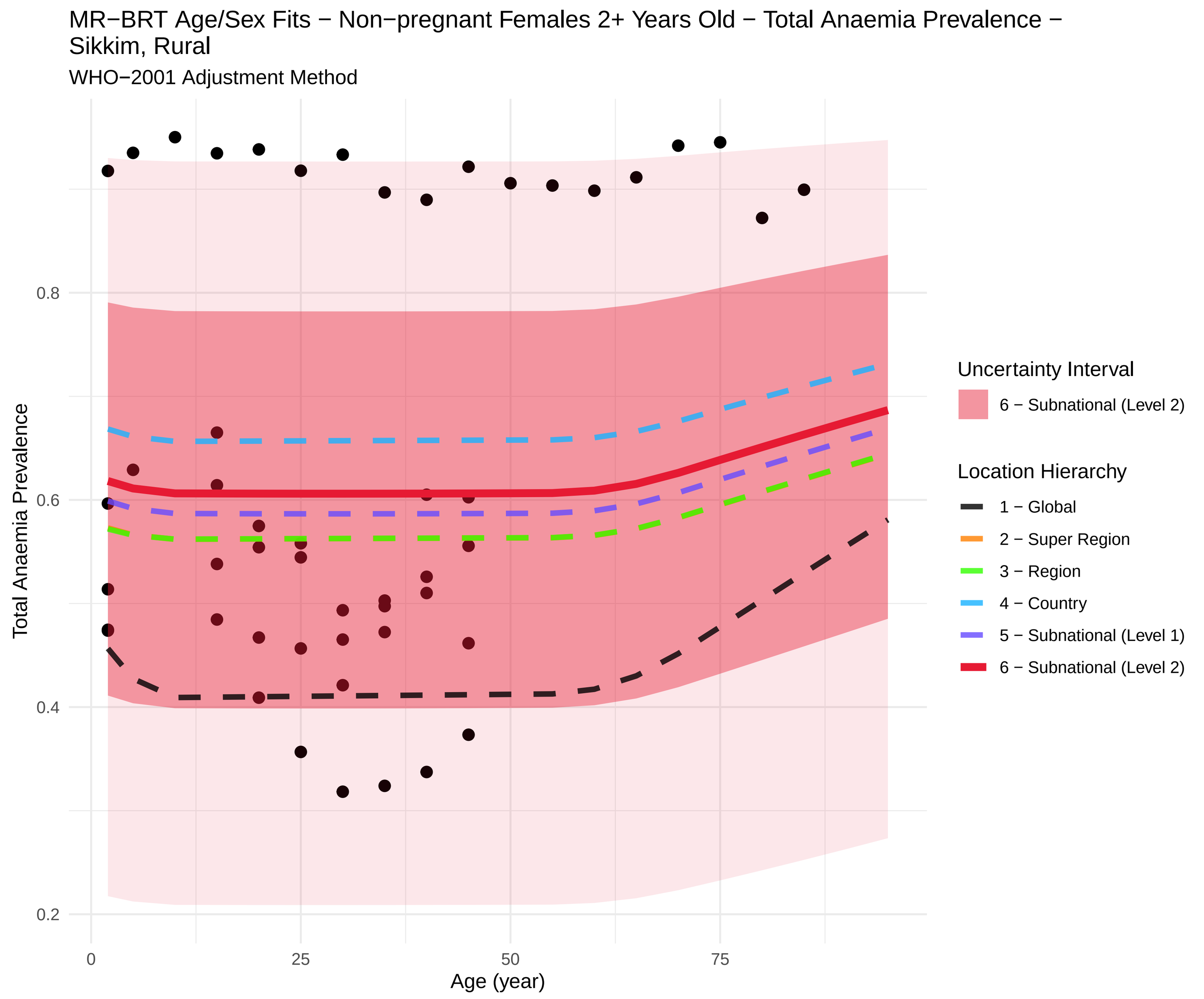
MR−BRT Age/Sex Fits − Non-pregnant Females 2+ Years Old − Total Anaemia Prevalence − Sikkim, Rural
WHO−2001 Adjustment Method
Total Anaemia Prevalence
Age (year)
Uncertainty Interval
6 − Subnational (Level 2)
Location Hierarchy
1 − Global
2 − Super Region
3 − Region
4 − Country
5 − Subnational (Level 1)
6 − Subnational (Level 2)

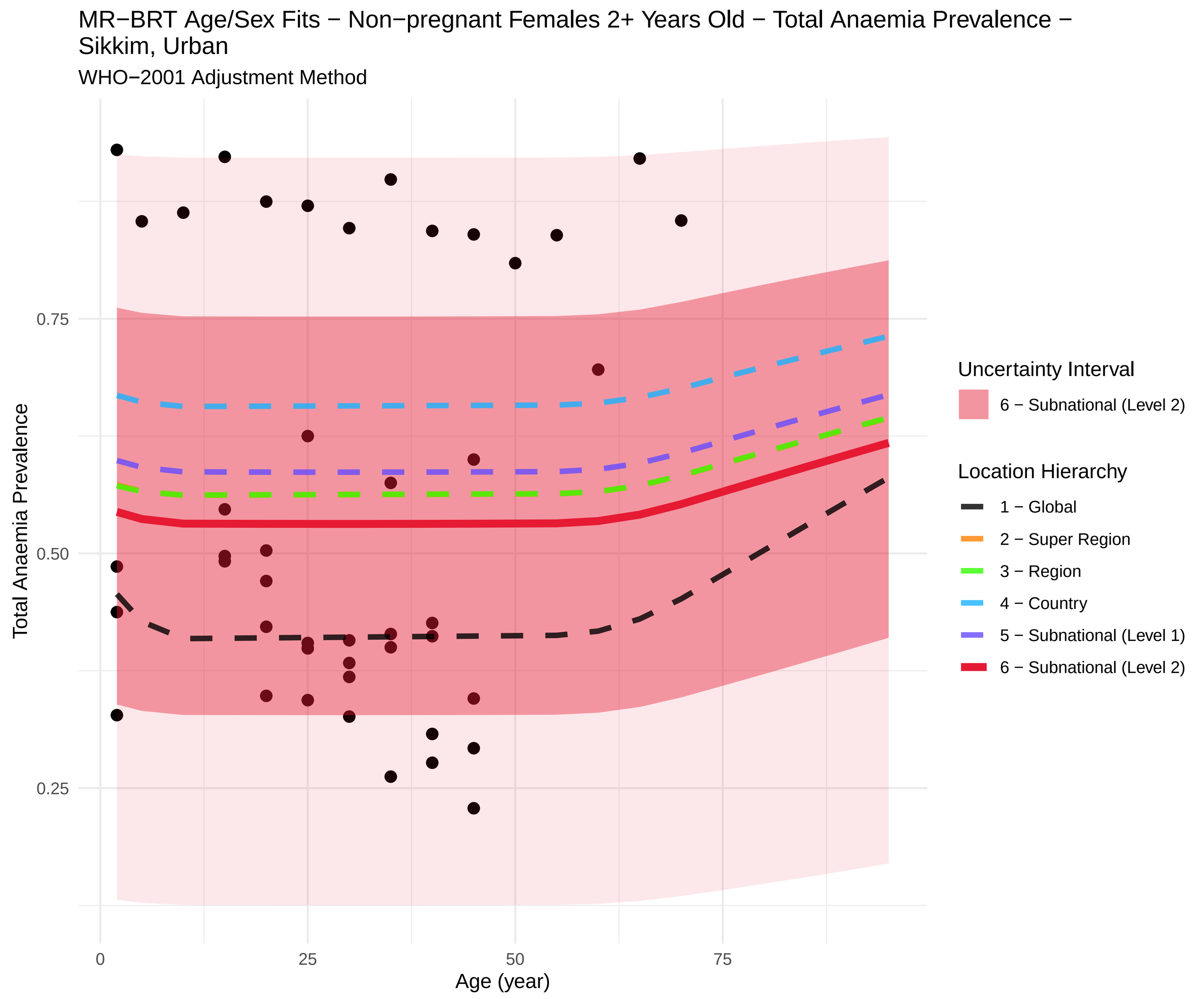
MR−BRT Age/Sex Fits − Non-pregnant Females 2+ Years Old − Total Anaemia Prevalence − Sikkim, Urban
WHO−2001 Adjustment Method
Total Anaemia Prevalence
Age (year)
Uncertainty Interval
6 − Subnational (Level 2)
Location Hierarchy
1 − Global
2 − Super Region
3 − Region
4 − Country
5 − Subnational (Level 1)
6 − Subnational (Level 2)

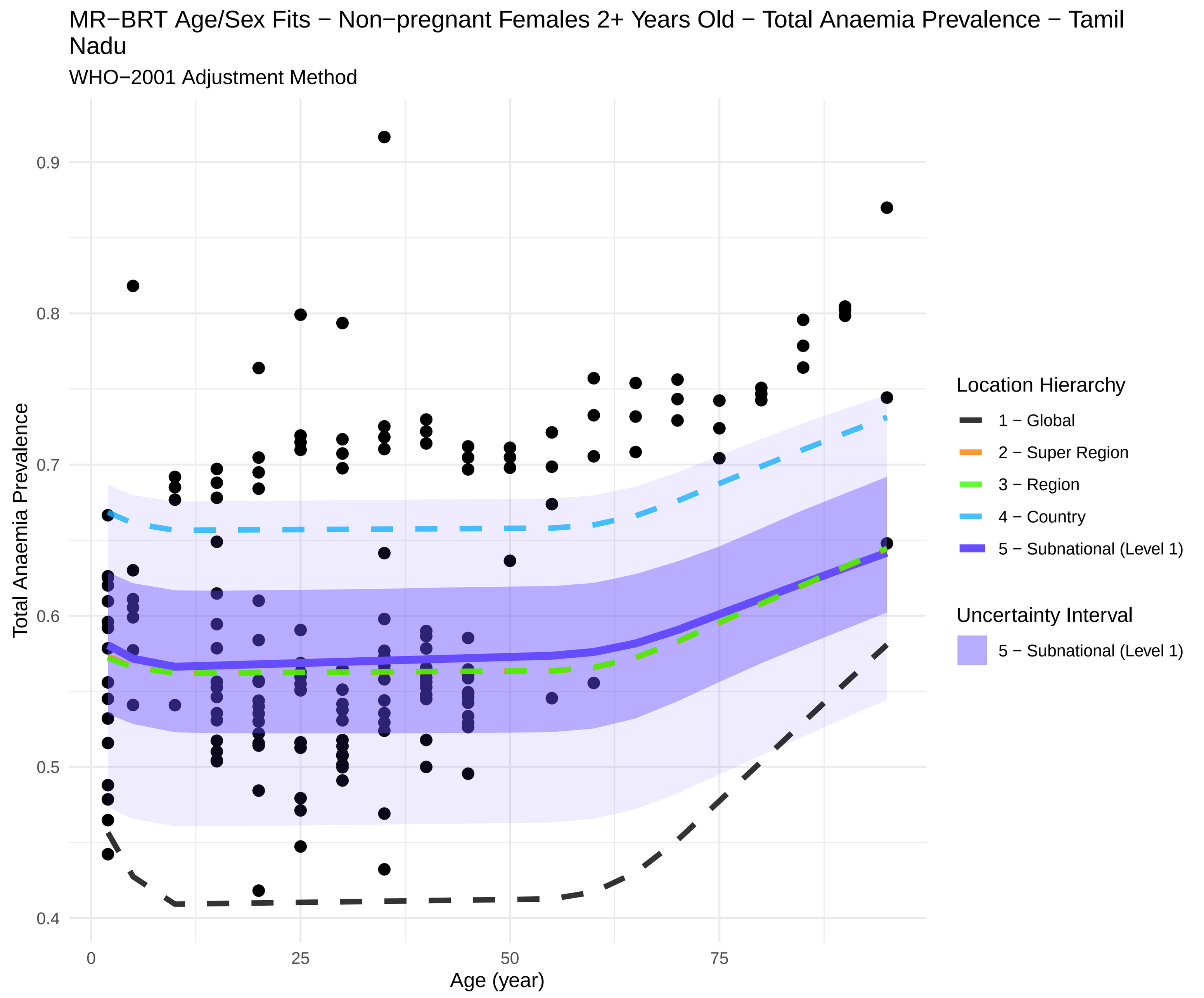
MR−BRT Age/Sex Fits − Non-pregnant Females 2+ Years Old − Total Anaemia Prevalence − Tamil Nadu
WHO−2001 Adjustment Method
Total Anaemia Prevalence
Age (year)
Location Hierarchy
1 − Global
2 − Super Region
3 − Region
4 − Country
5 − Subnational (Level 1)
Uncertainty Interval
5 − Subnational (Level 1)

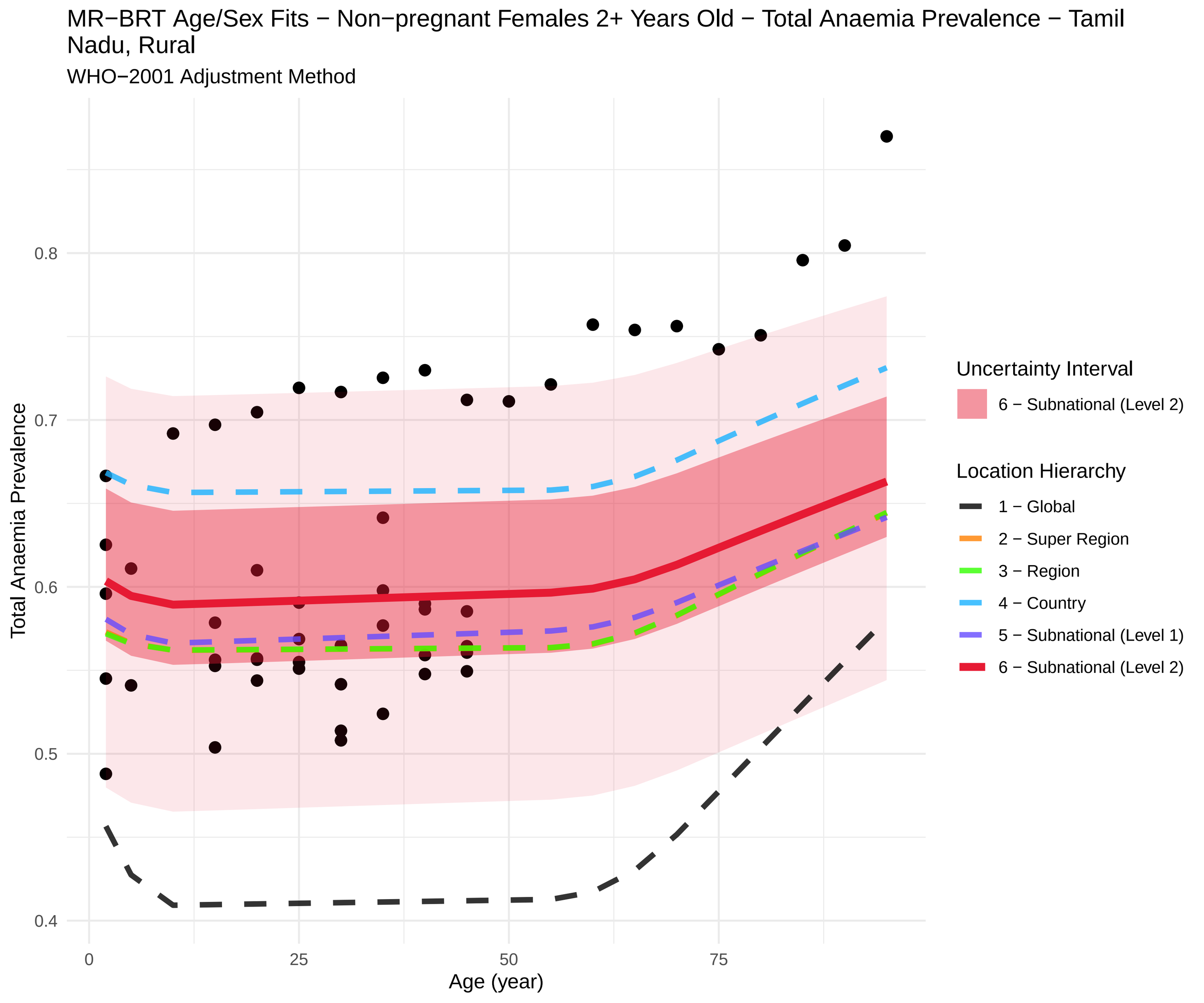
MR−BRT Age/Sex Fits − Non-pregnant Females 2+ Years Old − Total Anaemia Prevalence − Tamil Nadu, Rural
WHO−2001 Adjustment Method
Total Anaemia Prevalence
Age (year)
Uncertainty Interval
6 − Subnational (Level 2)
Location Hierarchy
1 − Global
2 − Super Region
3 − Region
4 − Country
5 − Subnational (Level 1)
6 − Subnational (Level 2)

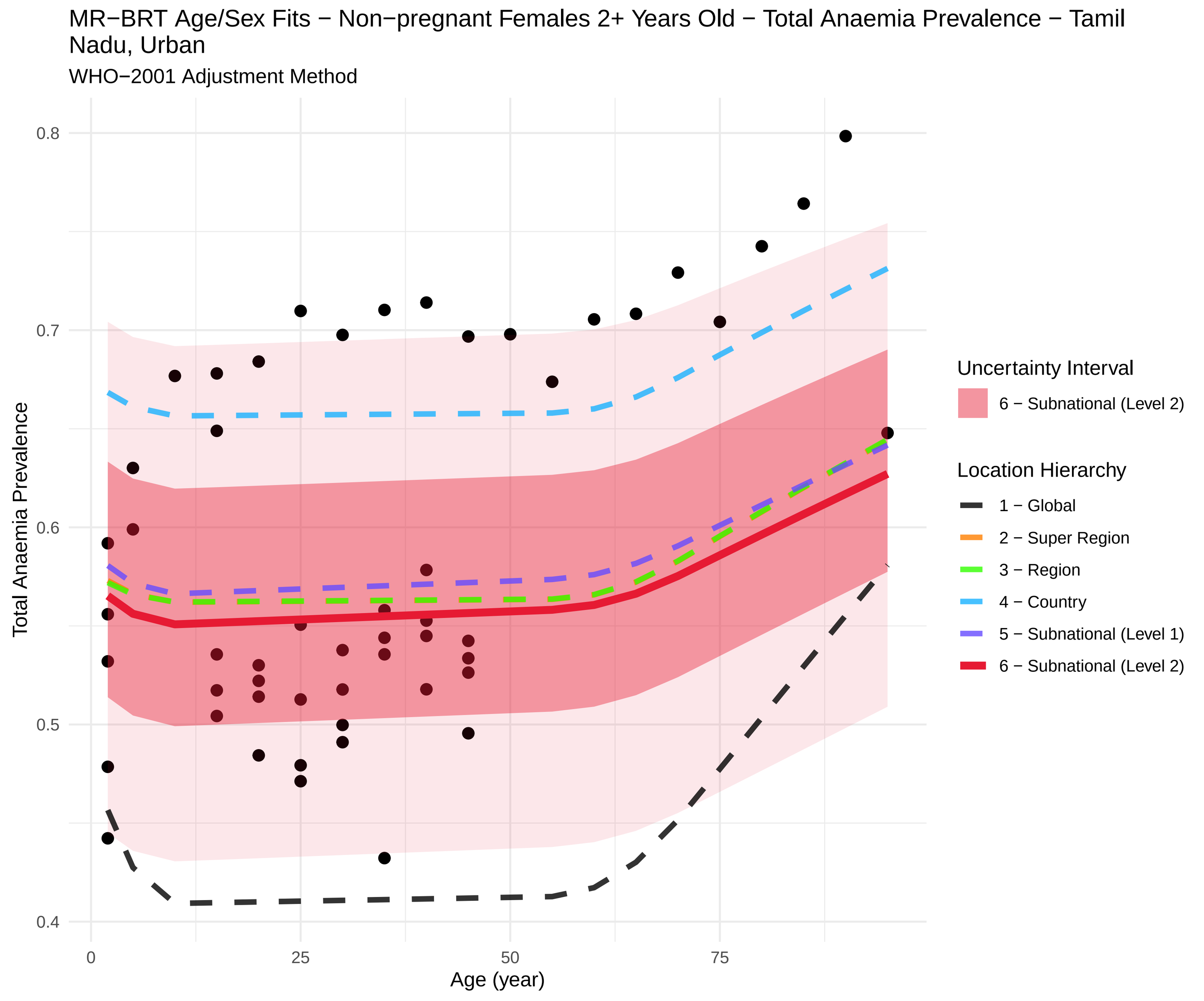
MR−BRT Age/Sex Fits − Non-pregnant Females 2+ Years Old − Total Anaemia Prevalence − Tamil Nadu, Urban
WHO−2001 Adjustment Method
Total Anaemia Prevalence
Age (year)
Uncertainty Interval
6 − Subnational (Level 2)
Location Hierarchy
1 − Global
2 − Super Region
3 − Region
4 − Country
5 − Subnational (Level 1)
6 − Subnational (Level 2)

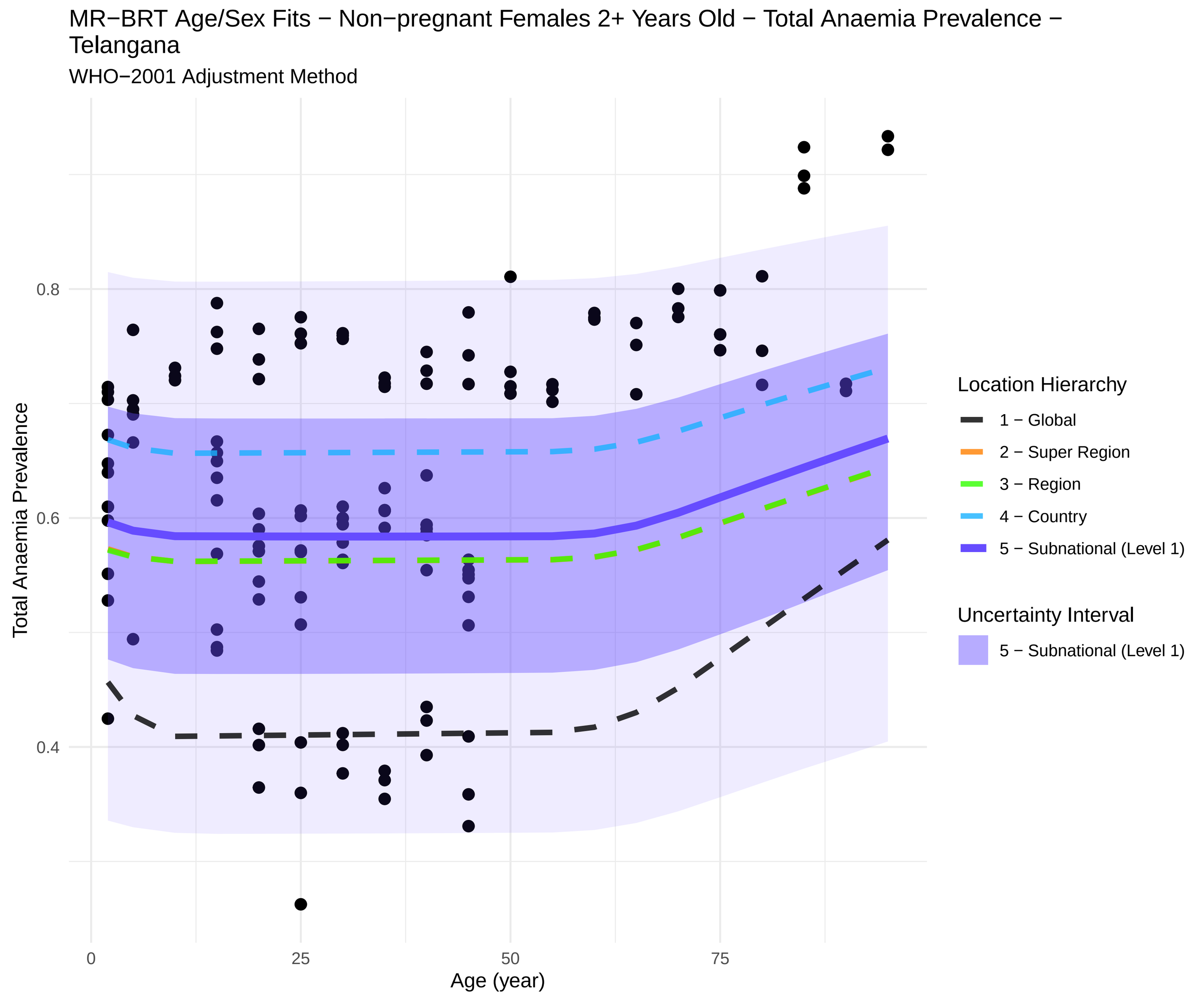
MR−BRT Age/Sex Fits − Non-pregnant Females 2+ Years Old − Total Anaemia Prevalence − Telangana
WHO−2001 Adjustment Method
Total Anaemia Prevalence
Age (year)
Location Hierarchy
1 − Global
2 − Super Region
3 − Region
4 − Country
5 − Subnational (Level 1)
Uncertainty Interval
5 − Subnational (Level 1)

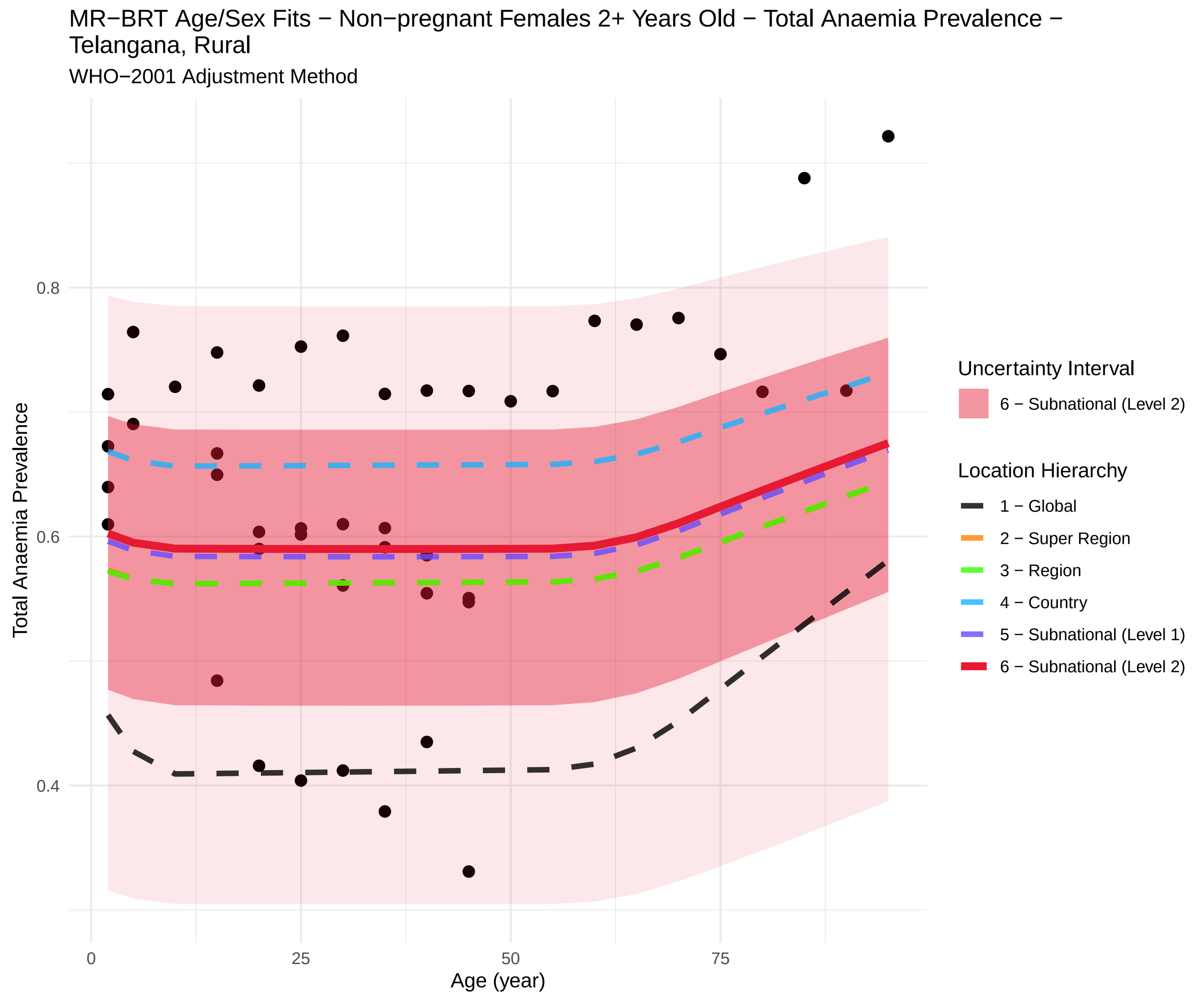
MR−BRT Age/Sex Fits − Non-pregnant Females 2+ Years Old − Total Anaemia Prevalence − Telangana, Rural
WHO−2001 Adjustment Method
Total Anaemia Prevalence
Age (year)
Uncertainty Interval
6 − Subnational (Level 2)
Location Hierarchy
1 − Global
2 − Super Region
3 − Region
4 − Country
5 − Subnational (Level 1)
6 − Subnational (Level 2)

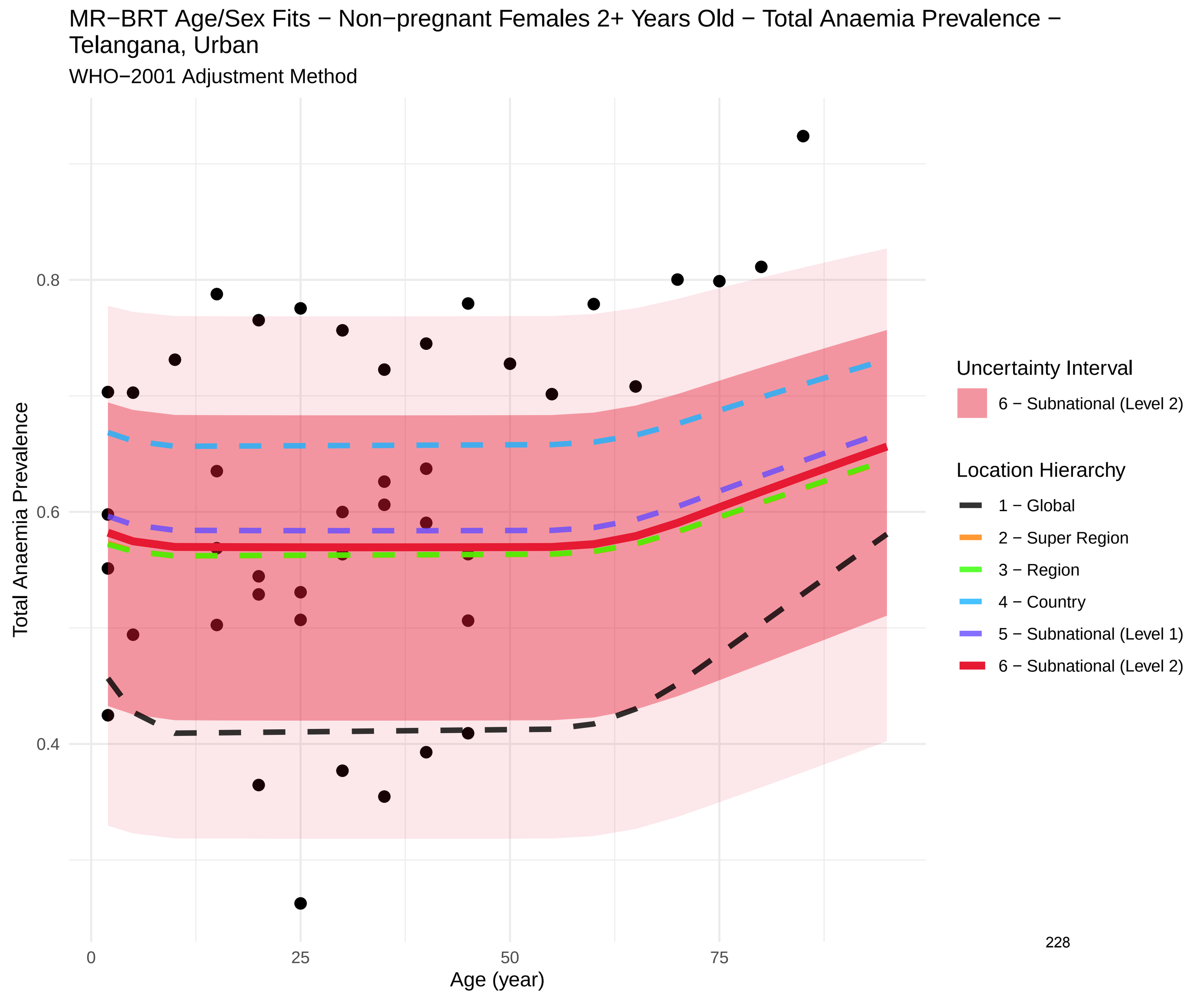
MR−BRT Age/Sex Fits − Non-pregnant Females 2+ Years Old − Total Anaemia Prevalence − Telangana, Urban
WHO−2001 Adjustment Method
Total Anaemia Prevalence
Age (year)
Uncertainty Interval
6 − Subnational (Level 2)
Location Hierarchy
1 − Global
2 − Super Region
3 − Region
4 − Country
5 − Subnational (Level 1)
6 − Subnational (Level 2)

MR−BRT Age/Sex Fits − Non−pregnant Females 2+ Years Old − Total Anaemia Prevalence − Tripura
WHO−2001 Adjustment Method

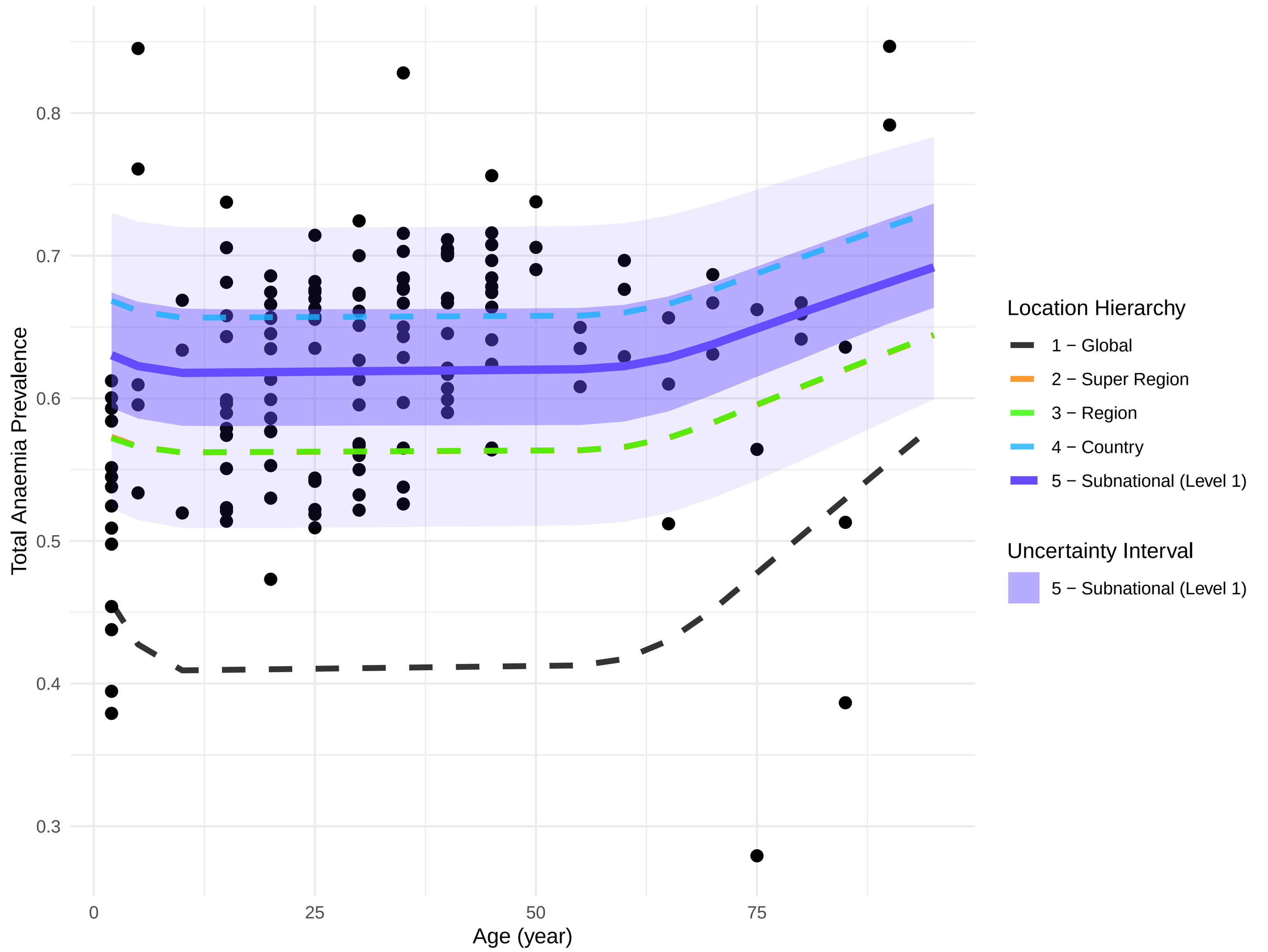

MR−BRT Age/Sex Fits − Non−pregnant Females 2+ Years Old − Total Anaemia Prevalence − Tripura, Rural
WHO−2001 Adjustment Method

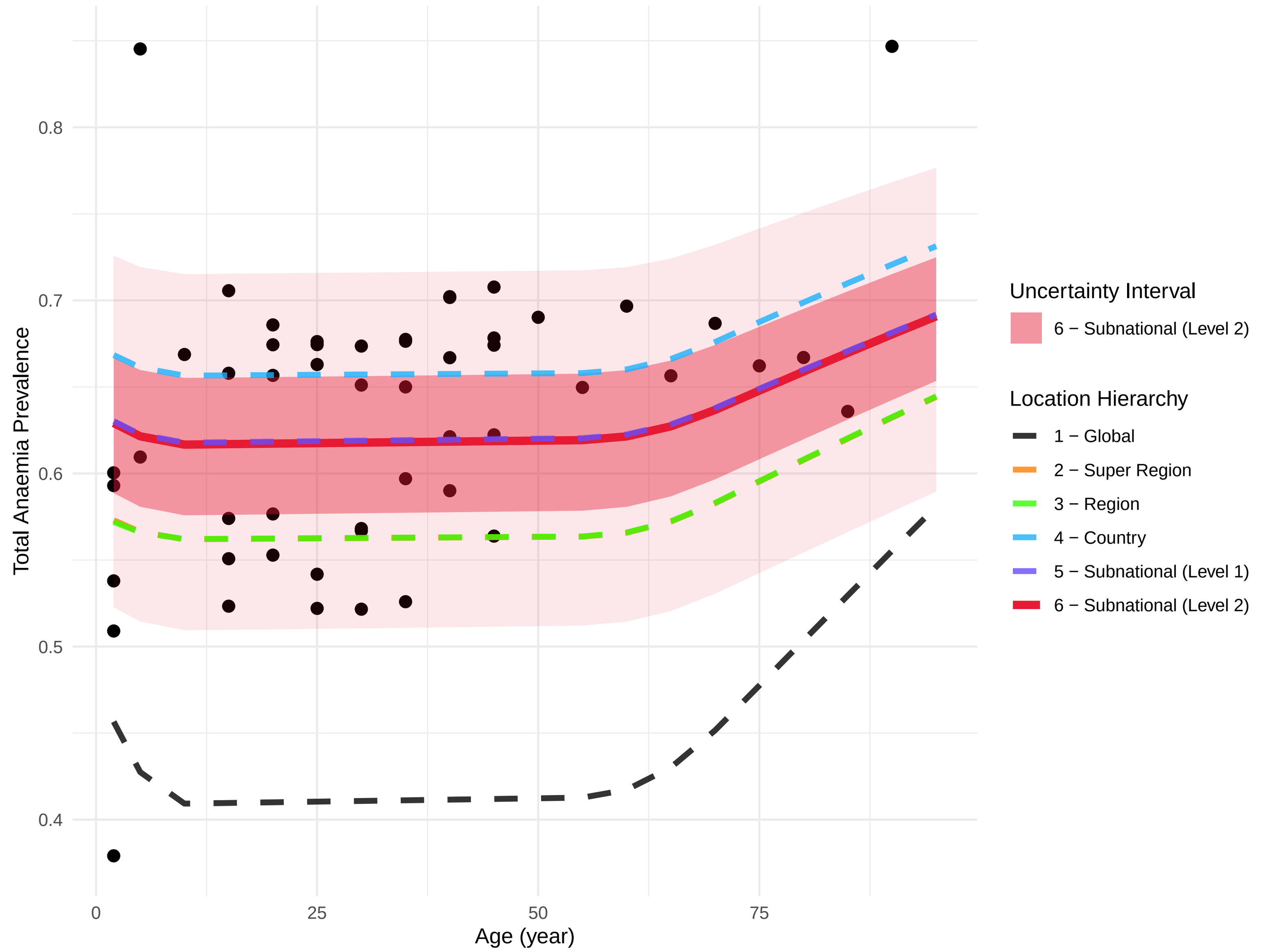

MR−BRT Age/Sex Fits − Non−pregnant Females 2+ Years Old − Total Anaemia Prevalence − Tripura, Urban
WHO−2001 Adjustment Method

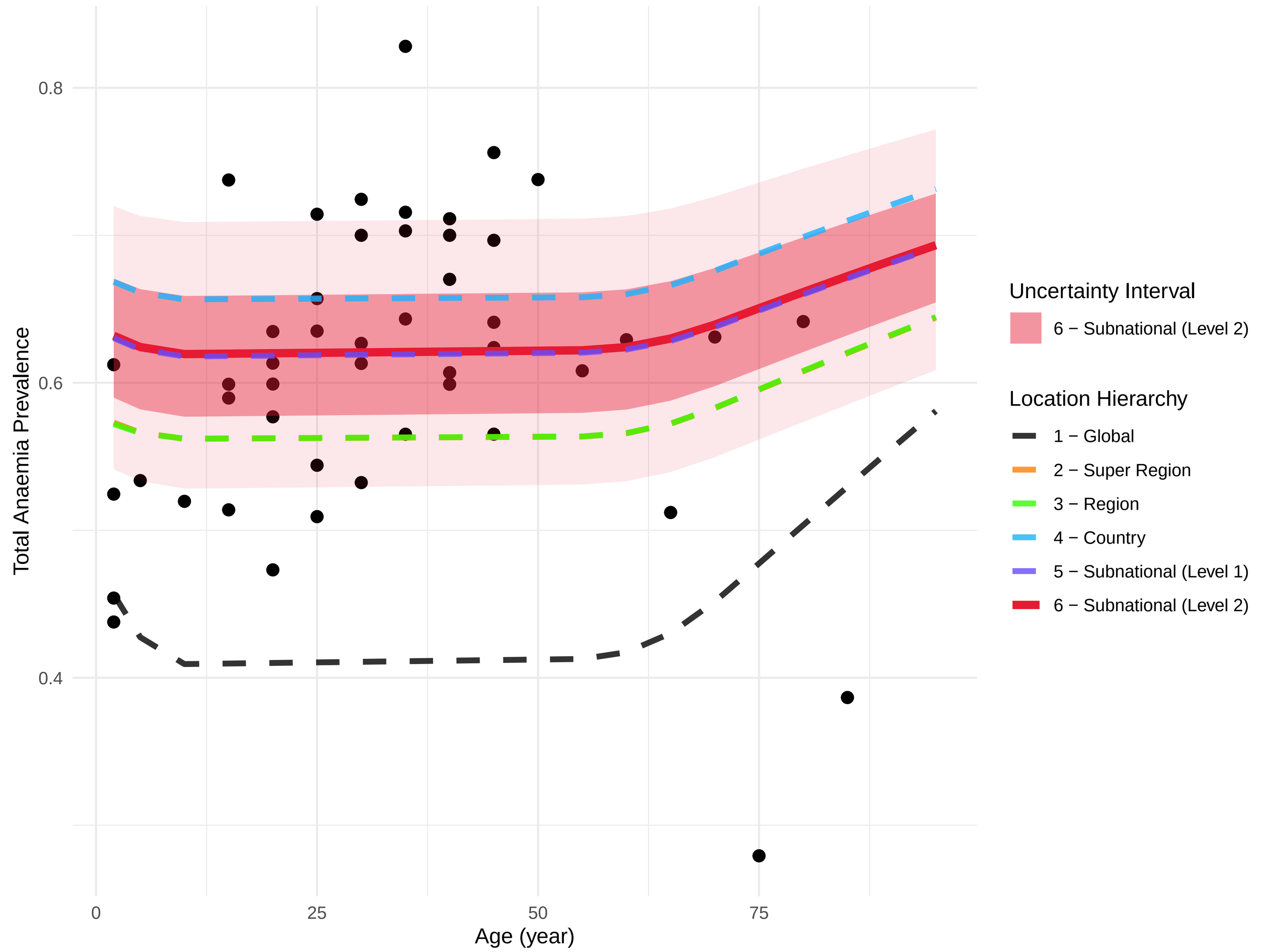

MR−BRT Age/Sex Fits − Non−pregnant Females 2+ Years Old − Total Anaemia Prevalence − Uttar Pradesh
WHO−2001 Adjustment Method

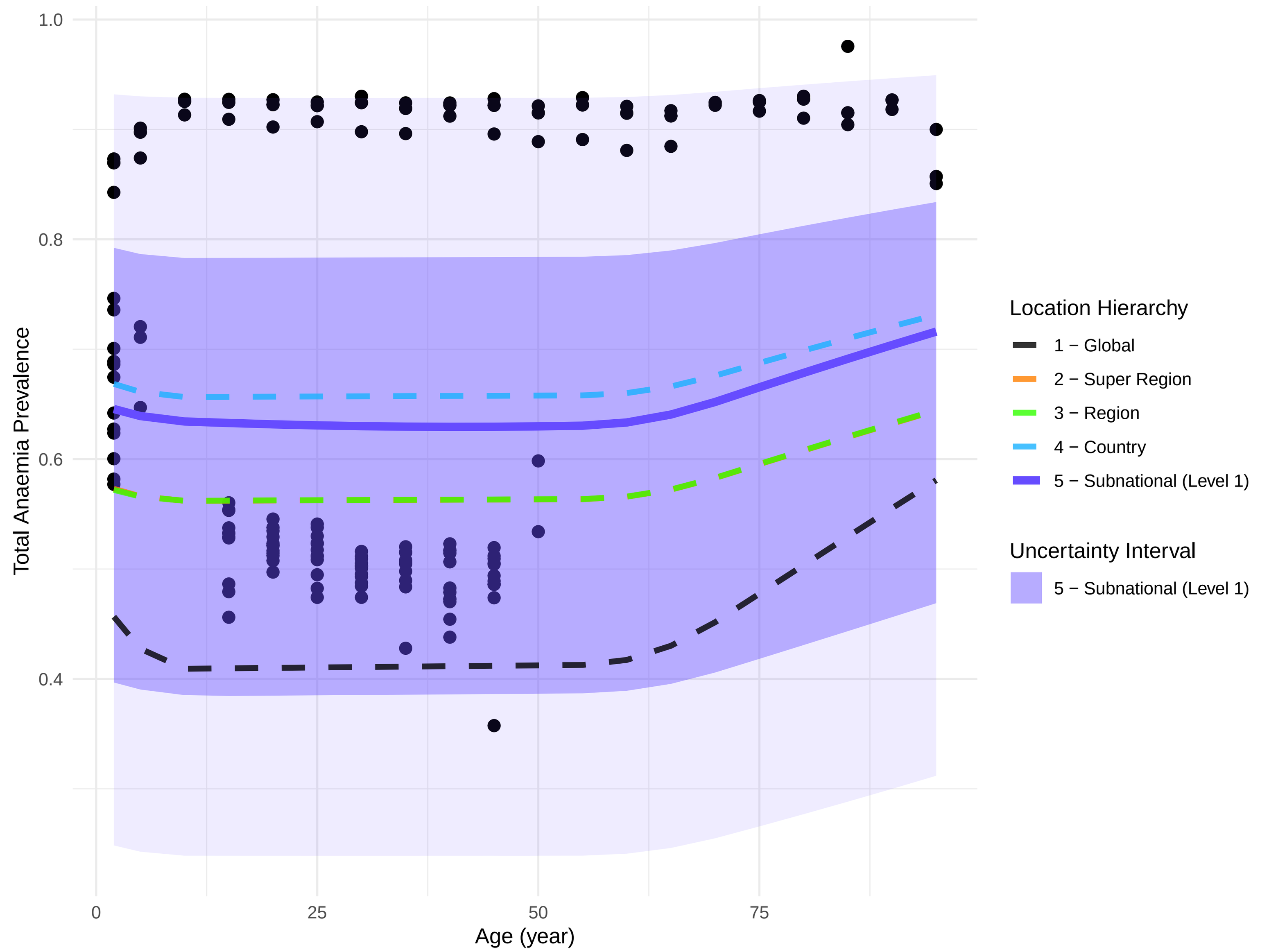

MR−BRT Age/Sex Fits − Non−pregnant Females 2+ Years Old − Total Anaemia Prevalence − Uttar Pradesh, Rural
WHO−2001 Adjustment Method

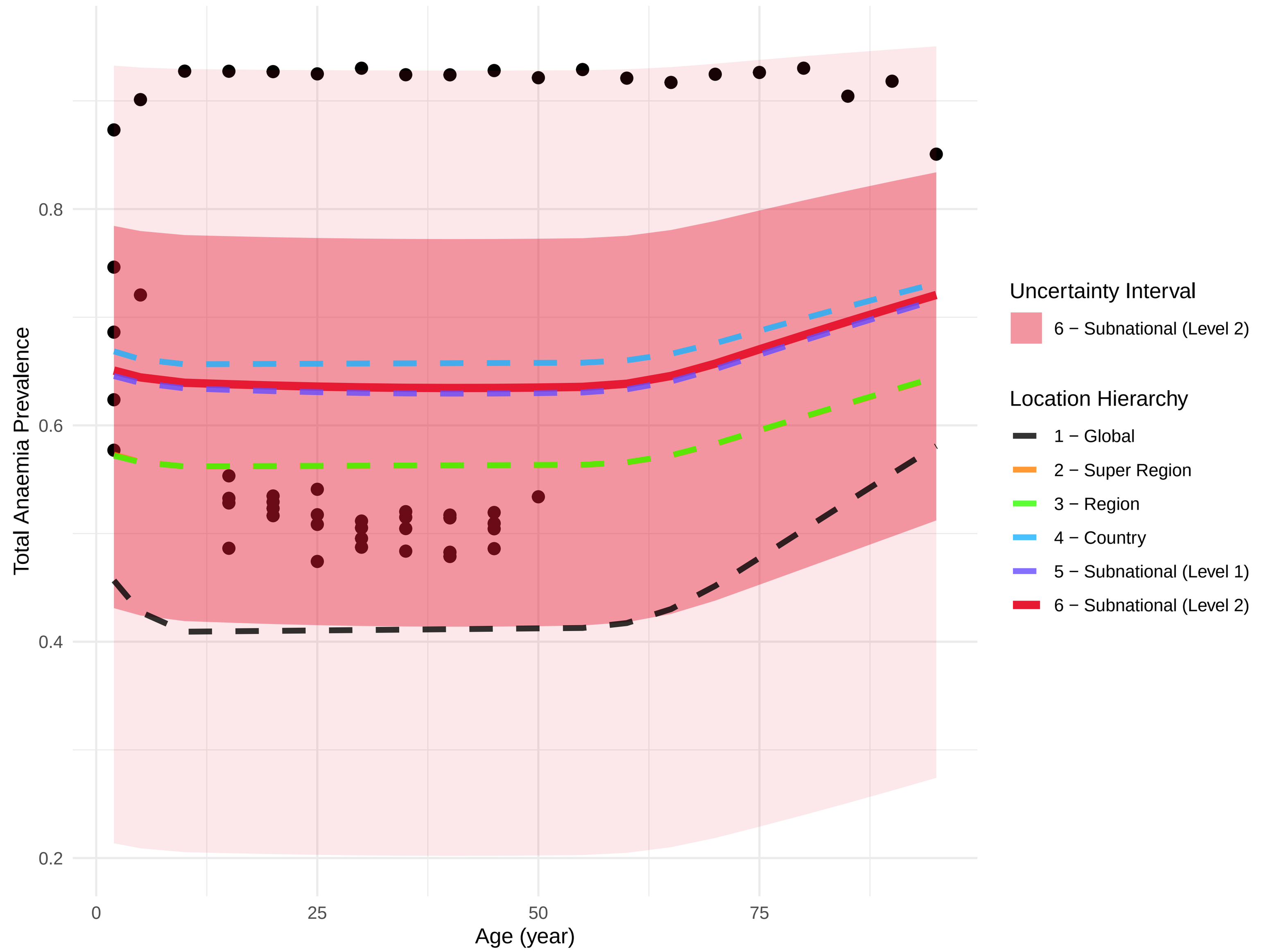

MR−BRT Age/Sex Fits − Non−pregnant Females 2+ Years Old − Total Anaemia Prevalence − Uttar Pradesh, Urban
WHO−2001 Adjustment Method

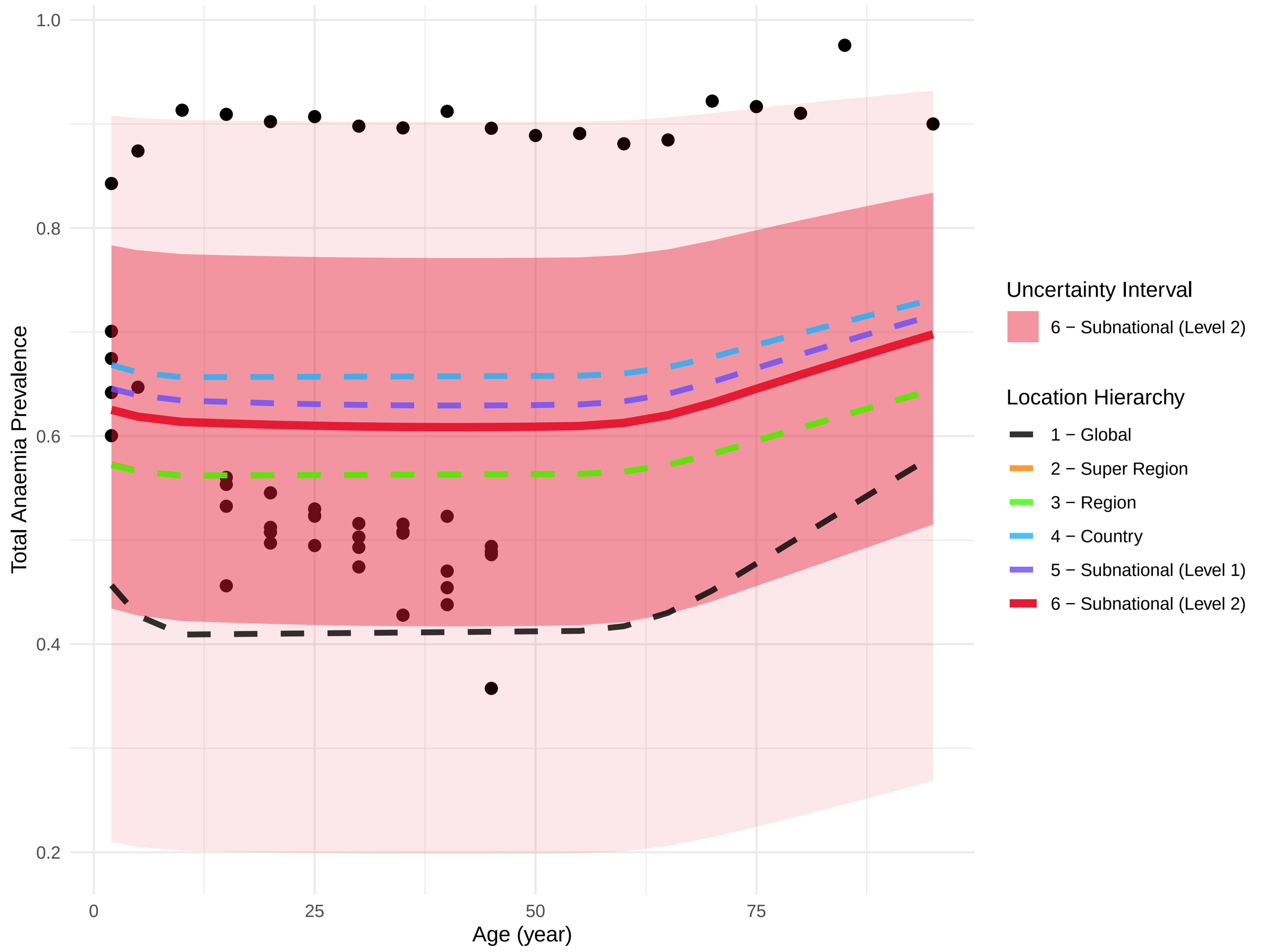

MR−BRT Age/Sex Fits − Non−pregnant Females 2+ Years Old − Total Anaemia Prevalence − Uttarakhand
WHO−2001 Adjustment Method

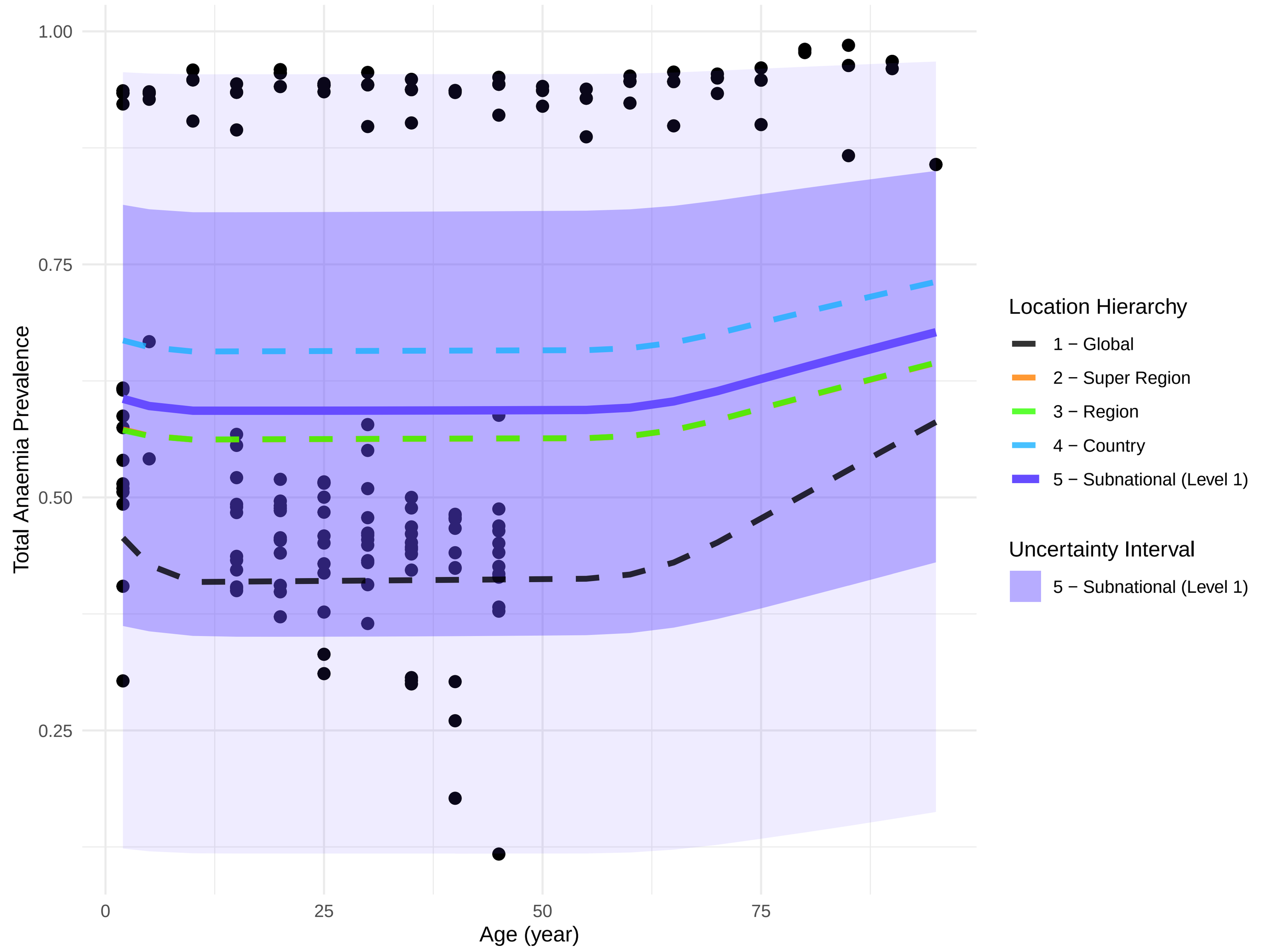

MR−BRT Age/Sex Fits − Non−pregnant Females 2+ Years Old − Total Anaemia Prevalence − Uttarakhand, Rural
WHO−2001 Adjustment Method

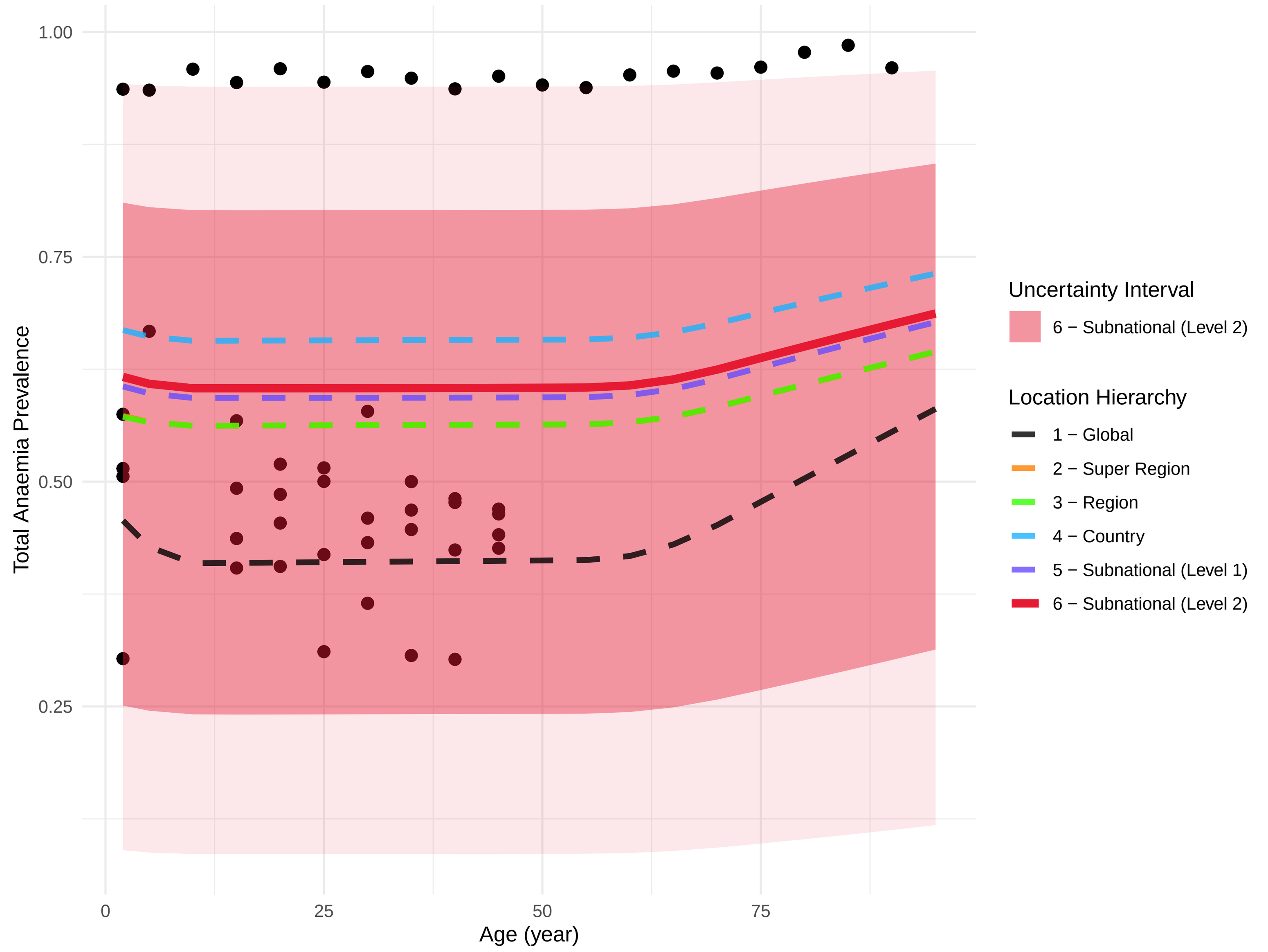

MR−BRT Age/Sex Fits − Non−pregnant Females 2+ Years Old − Total Anaemia Prevalence − Uttarakhand, Urban
WHO−2001 Adjustment Method

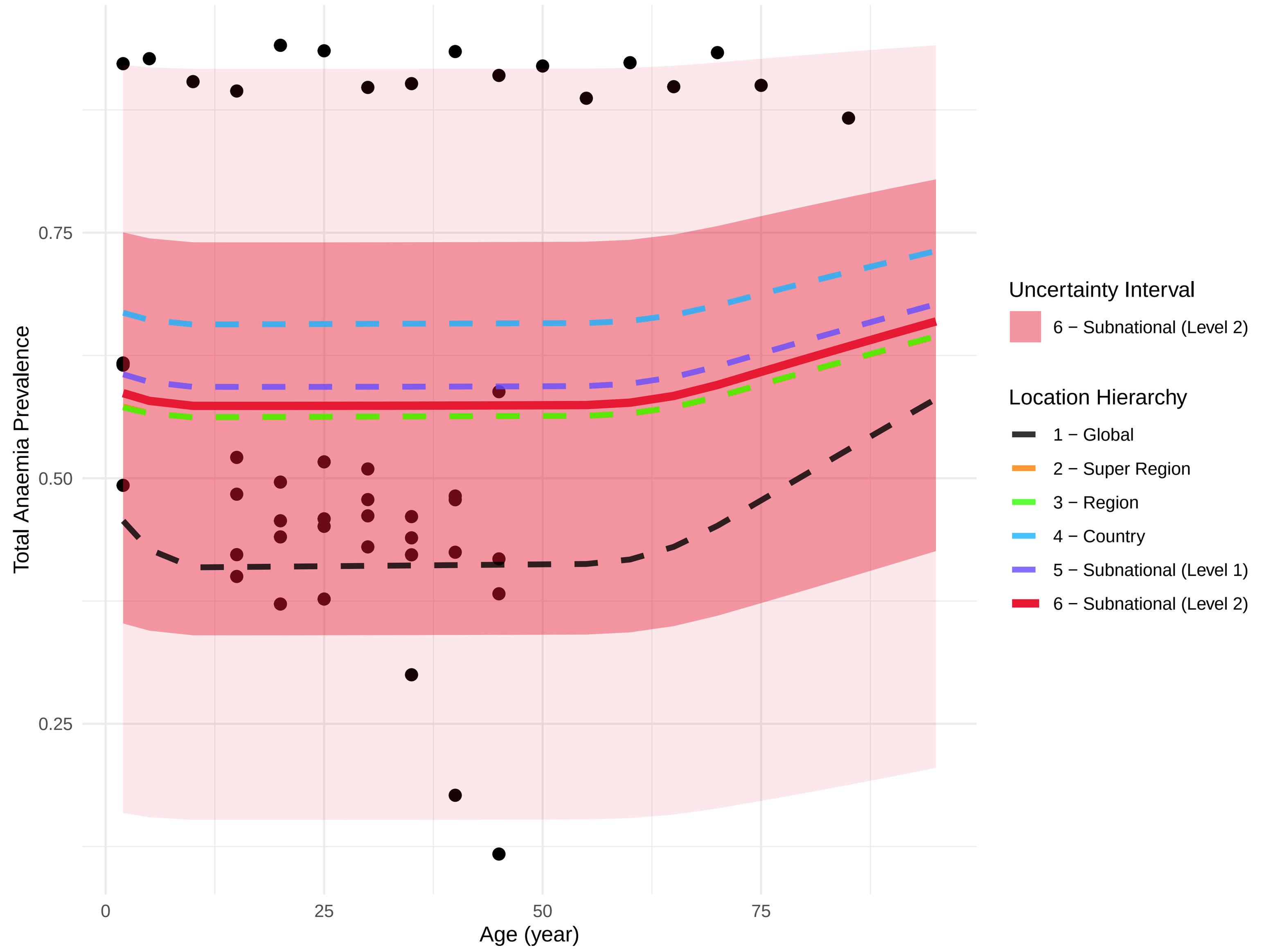

MR−BRT Age/Sex Fits − Non−pregnant Females 2+ Years Old − Total Anaemia Prevalence − West Bengal
WHO−2001 Adjustment Method

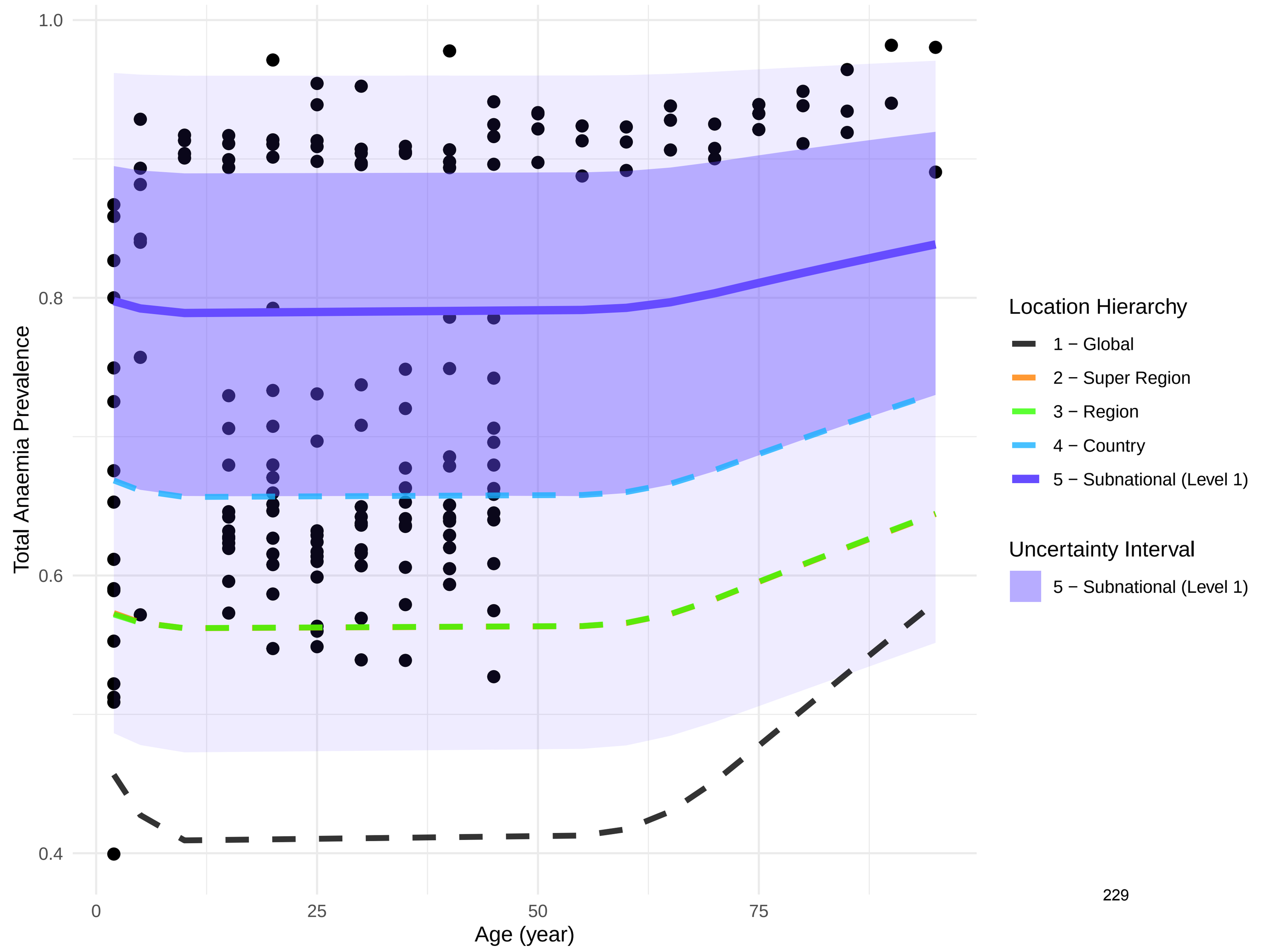

MR−BRT Age/Sex Fits − Non−pregnant Females 2+ Years Old − Total Anaemia Prevalence − West Bengal, Rural
WHO−2001 Adjustment Method

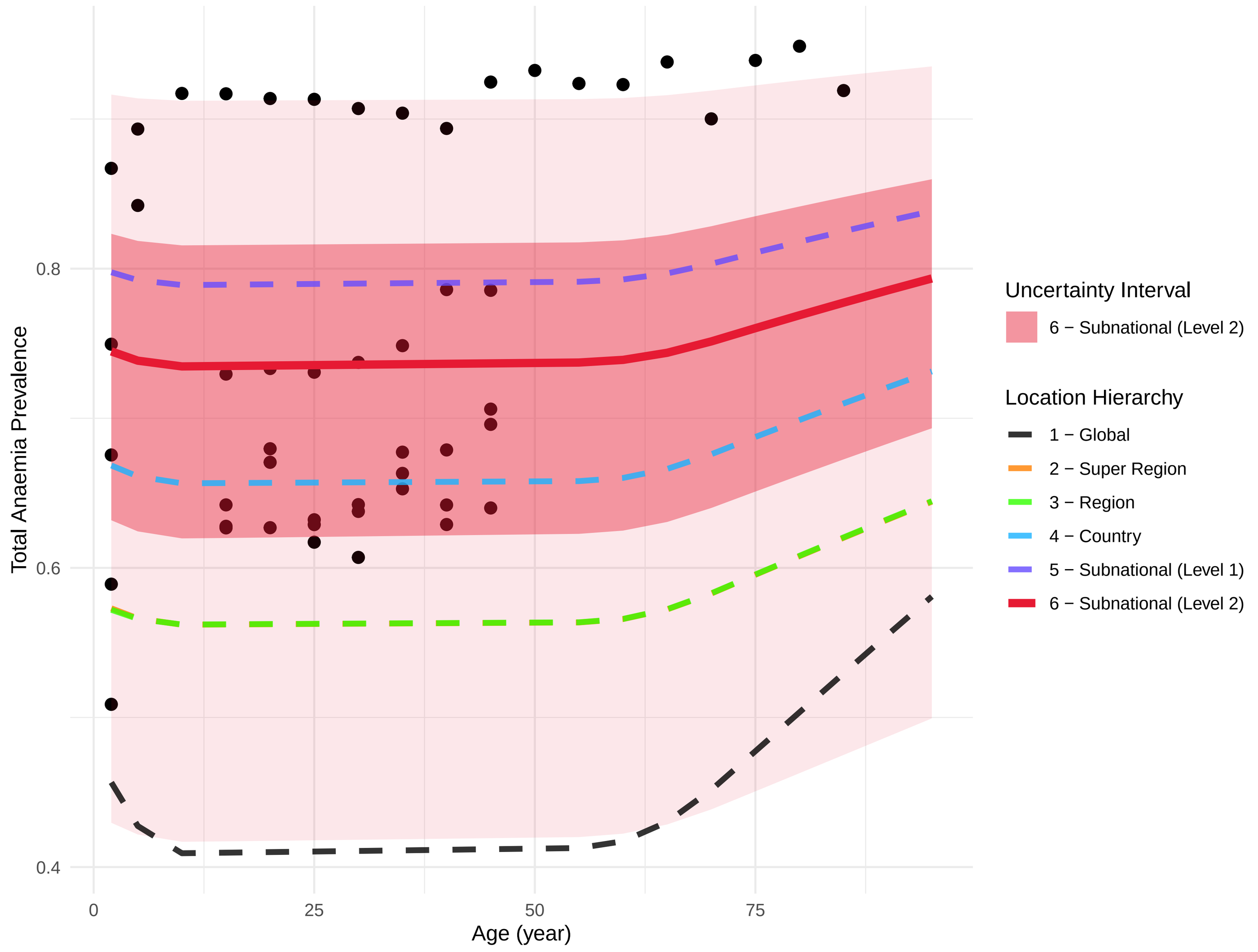

MR−BRT Age/Sex Fits − Non−pregnant Females 2+ Years Old − Total Anaemia Prevalence − West Bengal, Urban
WHO−2001 Adjustment Method

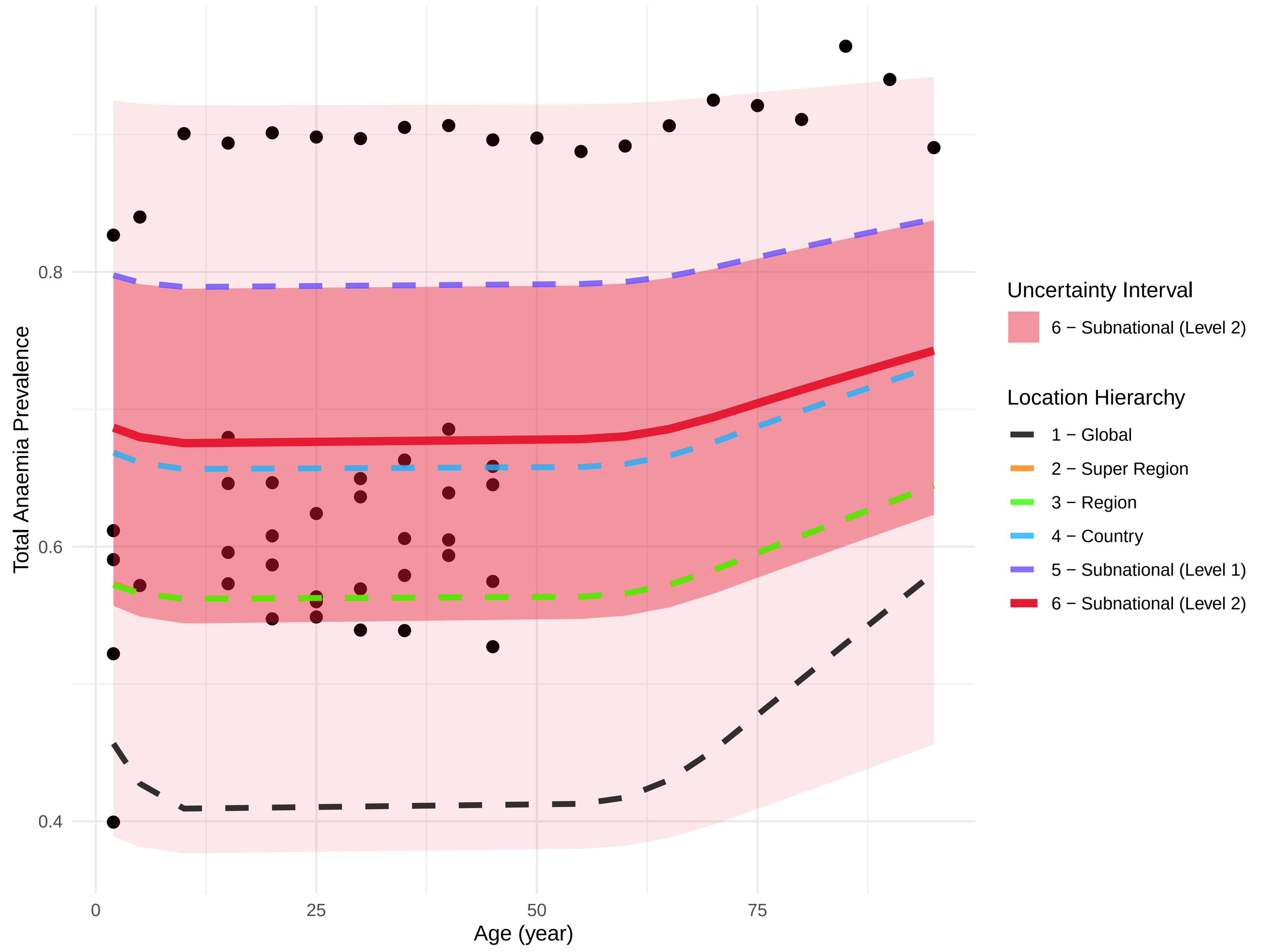

MR−BRT Age/Sex Fits − Non−pregnant Females 2+ Years Old − Total Anaemia Prevalence − Nepal
WHO−2001 Adjustment Method

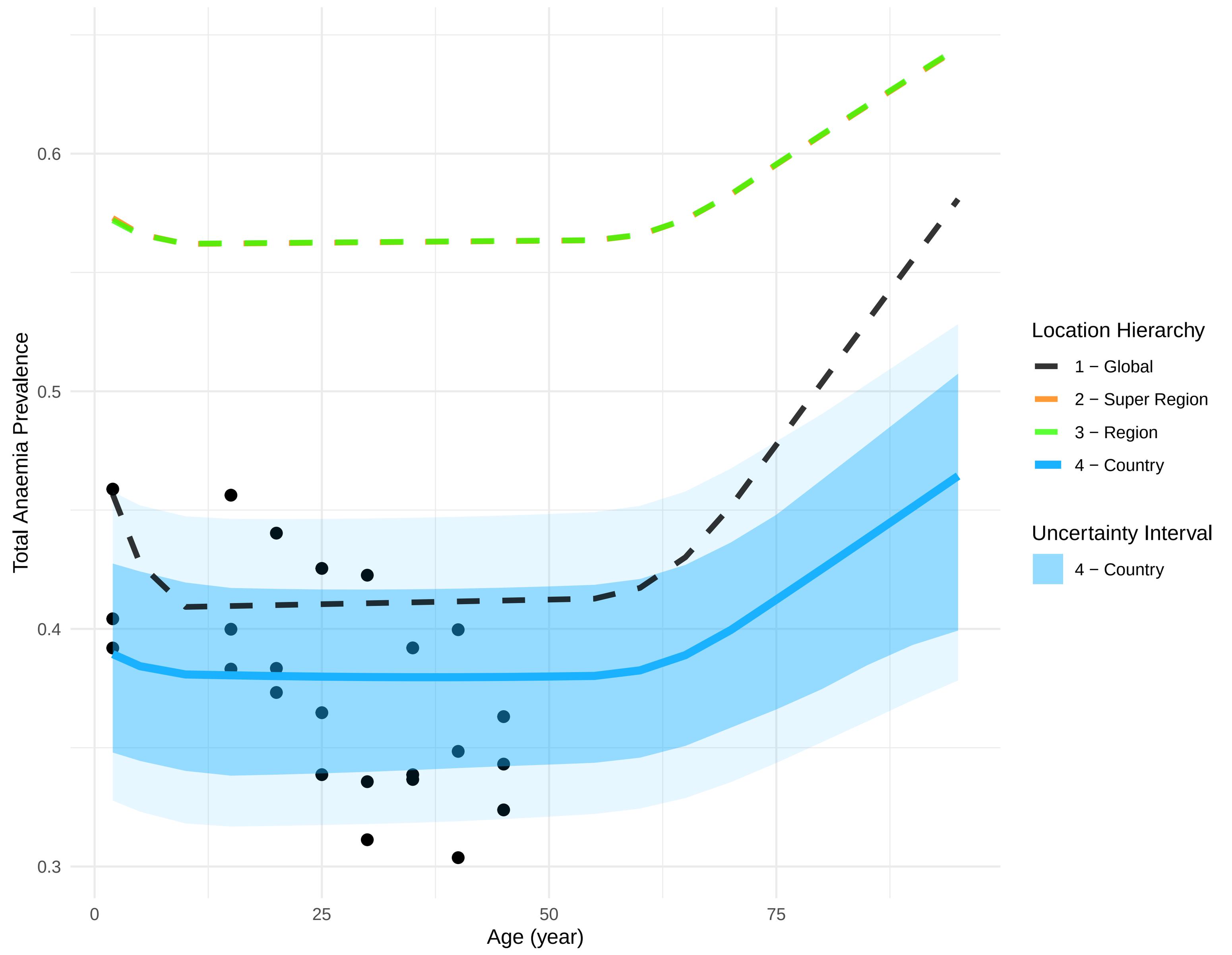

MR−BRT Age/Sex Fits − Non−pregnant Females 2+ Years Old − Total Anaemia Prevalence − Pakistan
WHO−2001 Adjustment Method

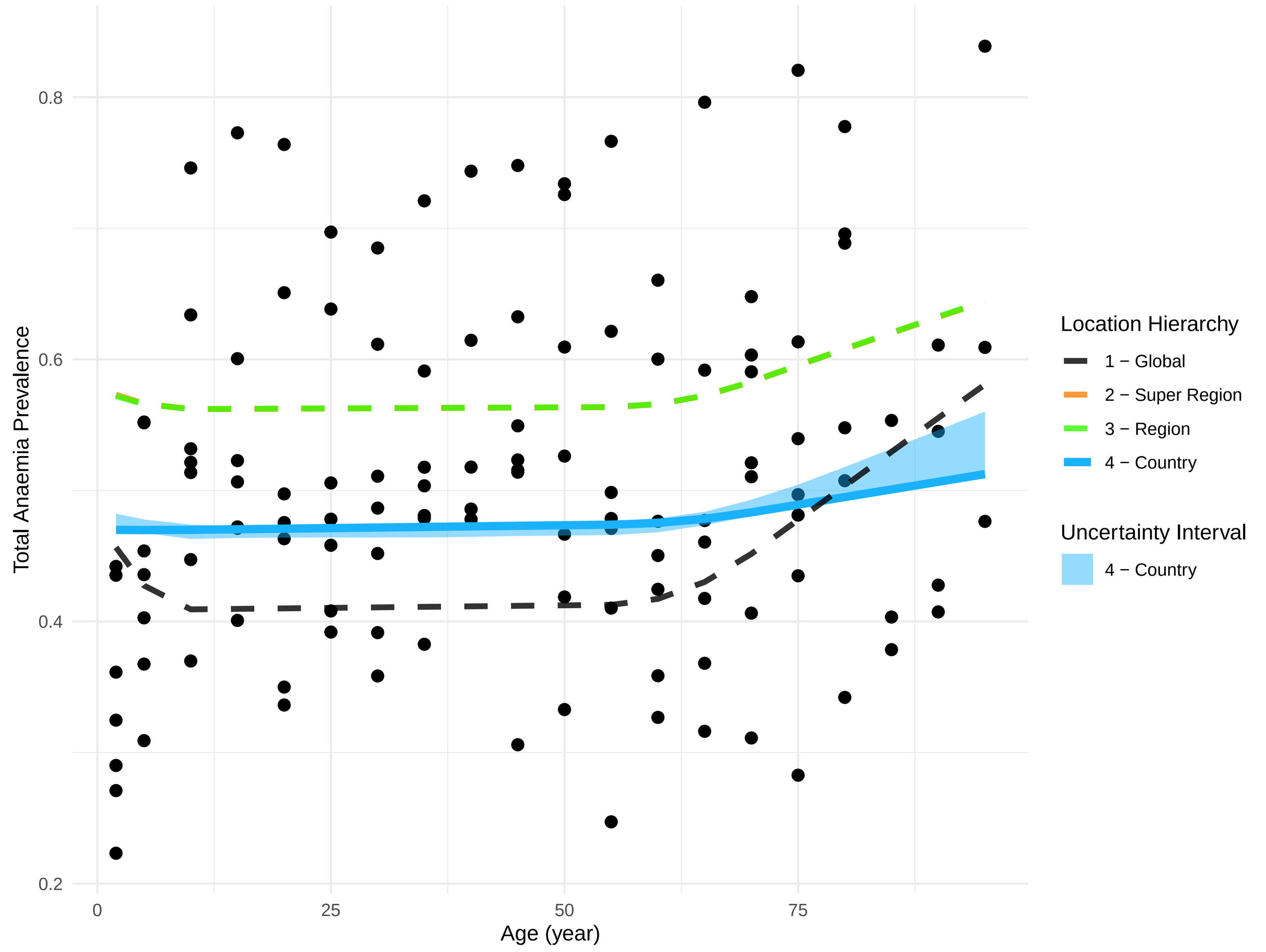

MR−BRT Age/Sex Fits − Non−pregnant Females 2+ Years Old − Total Anaemia Prevalence − Azad Jammu & Kashmir
WHO−2001 Adjustment Method

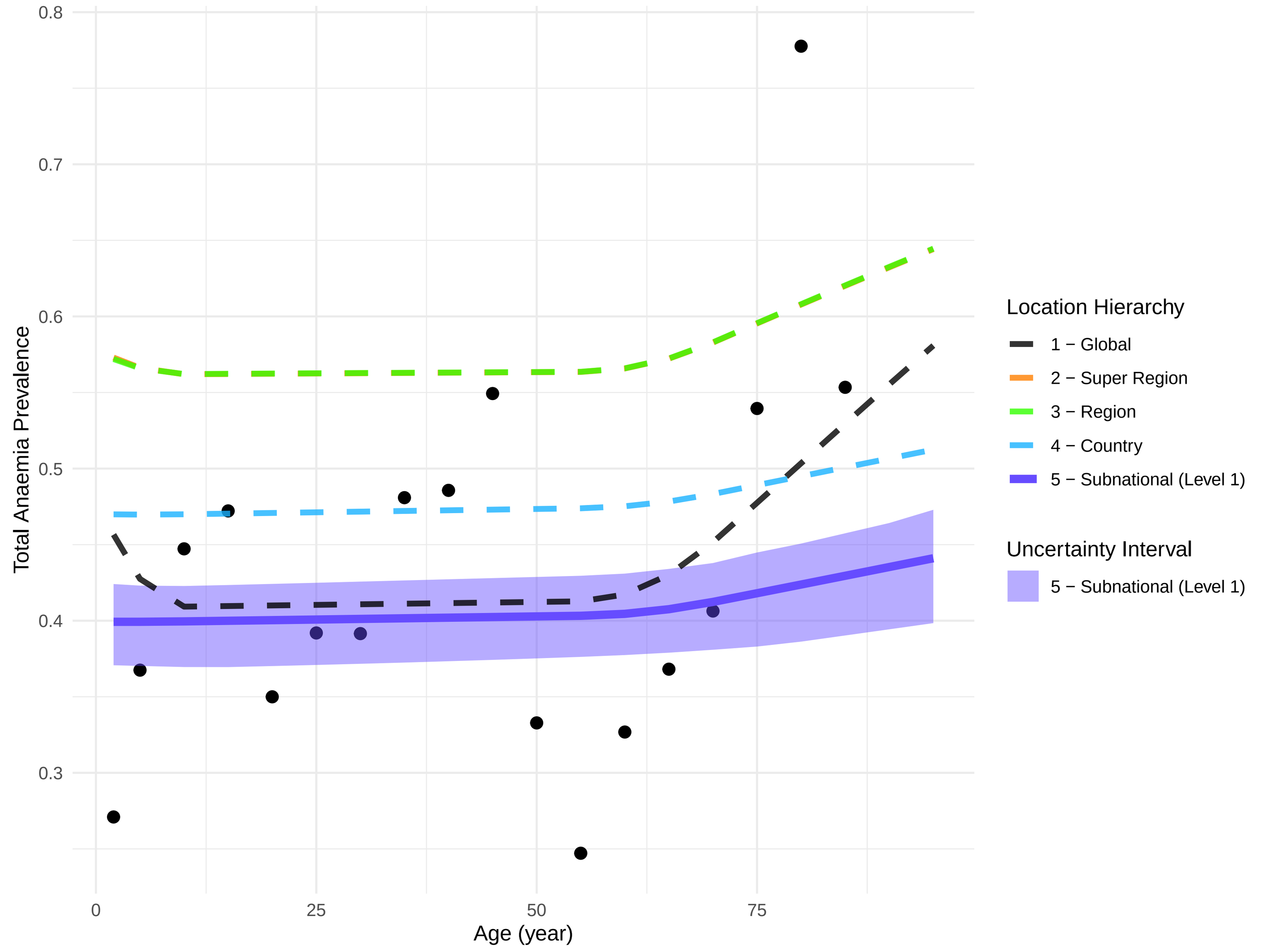

MR−BRT Age/Sex Fits − Non−pregnant Females 2+ Years Old − Total Anaemia Prevalence − Balochistan
WHO−2001 Adjustment Method

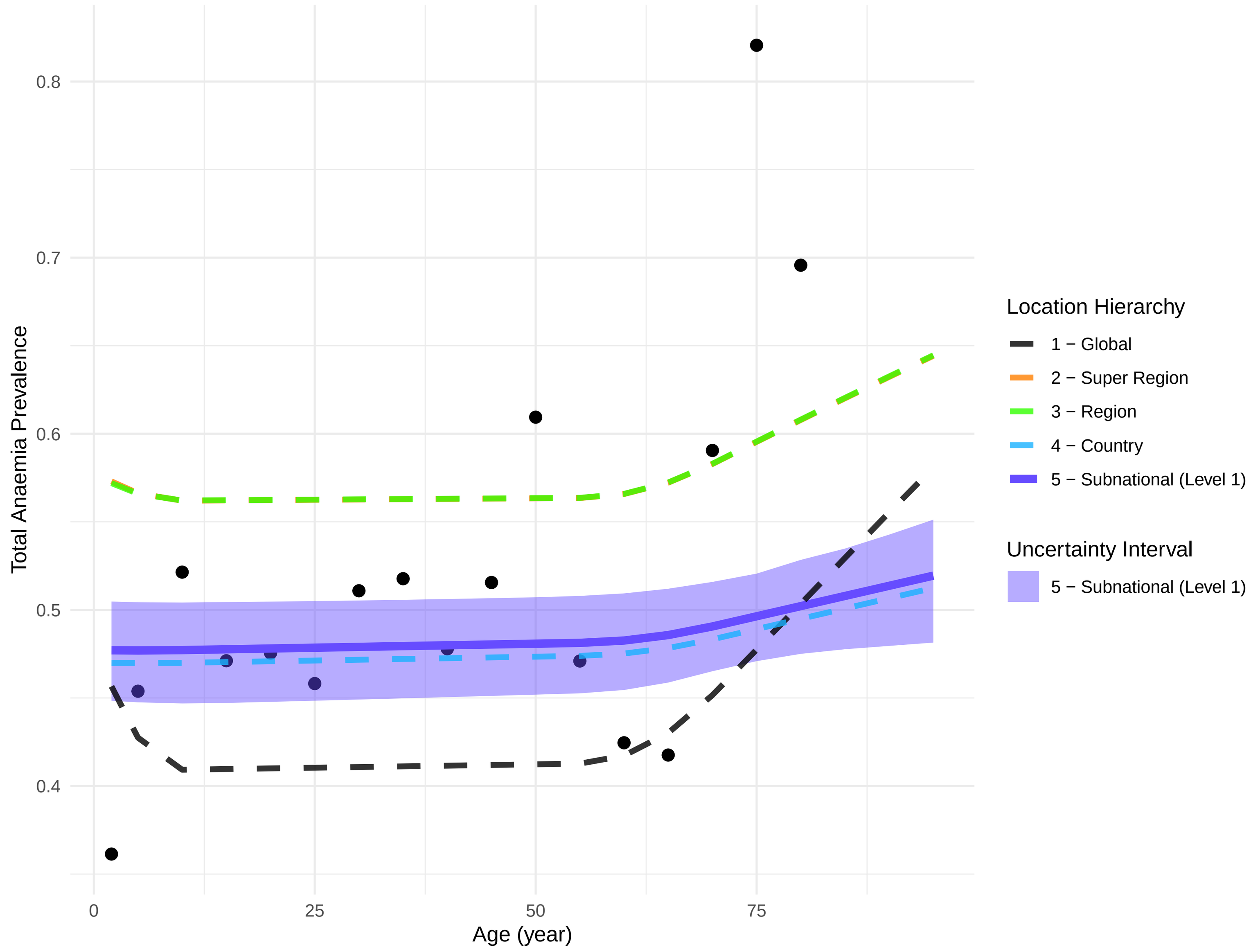

MR−BRT Age/Sex Fits − Non−pregnant Females 2+ Years Old − Total Anaemia Prevalence − Gilgit−Baltistan
WHO−2001 Adjustment Method

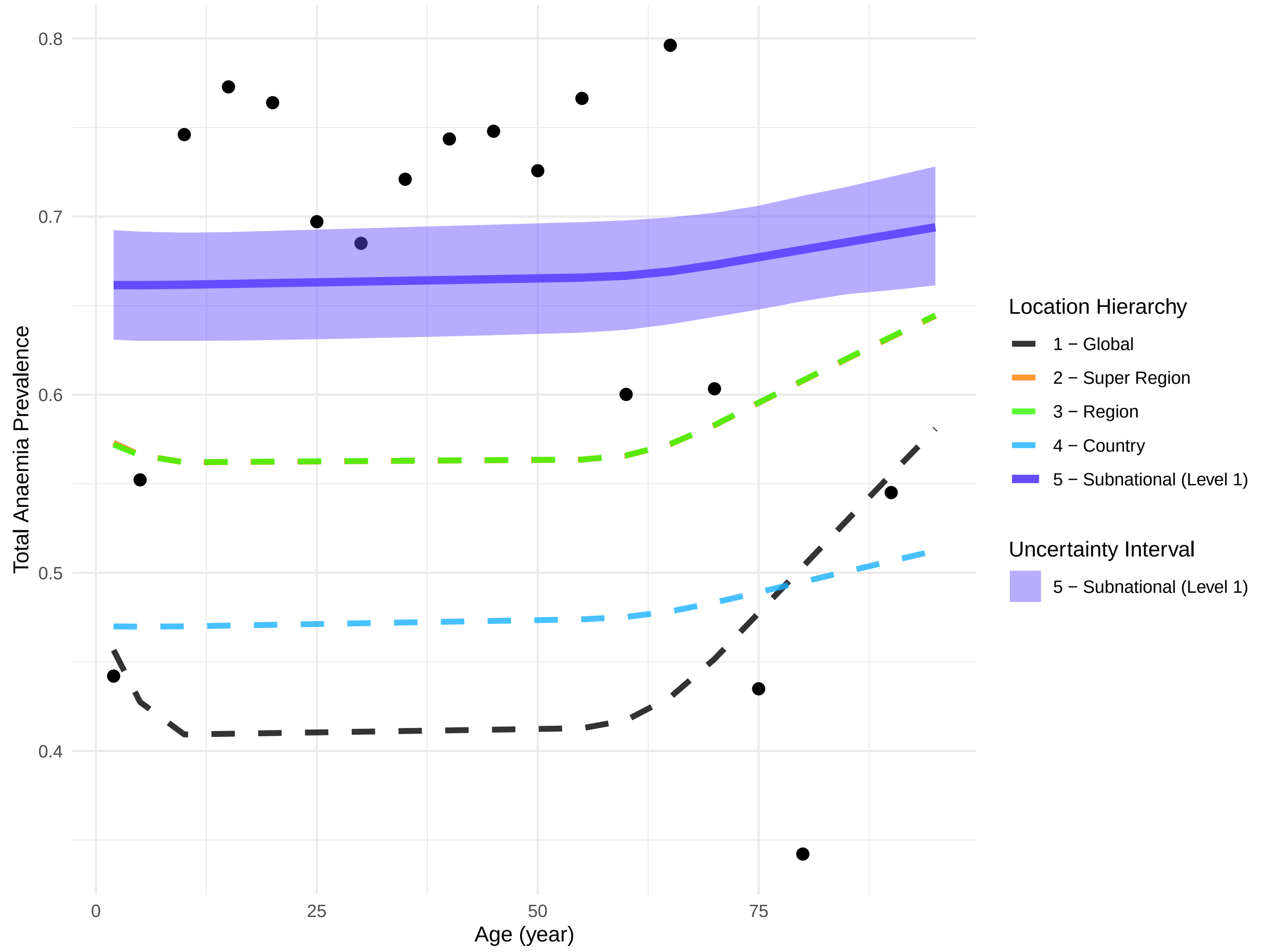

MR−BRT Age/Sex Fits − Non−pregnant Females 2+ Years Old − Total Anaemia Prevalence − Khyber Pakhtunkhwa
WHO−2001 Adjustment Method

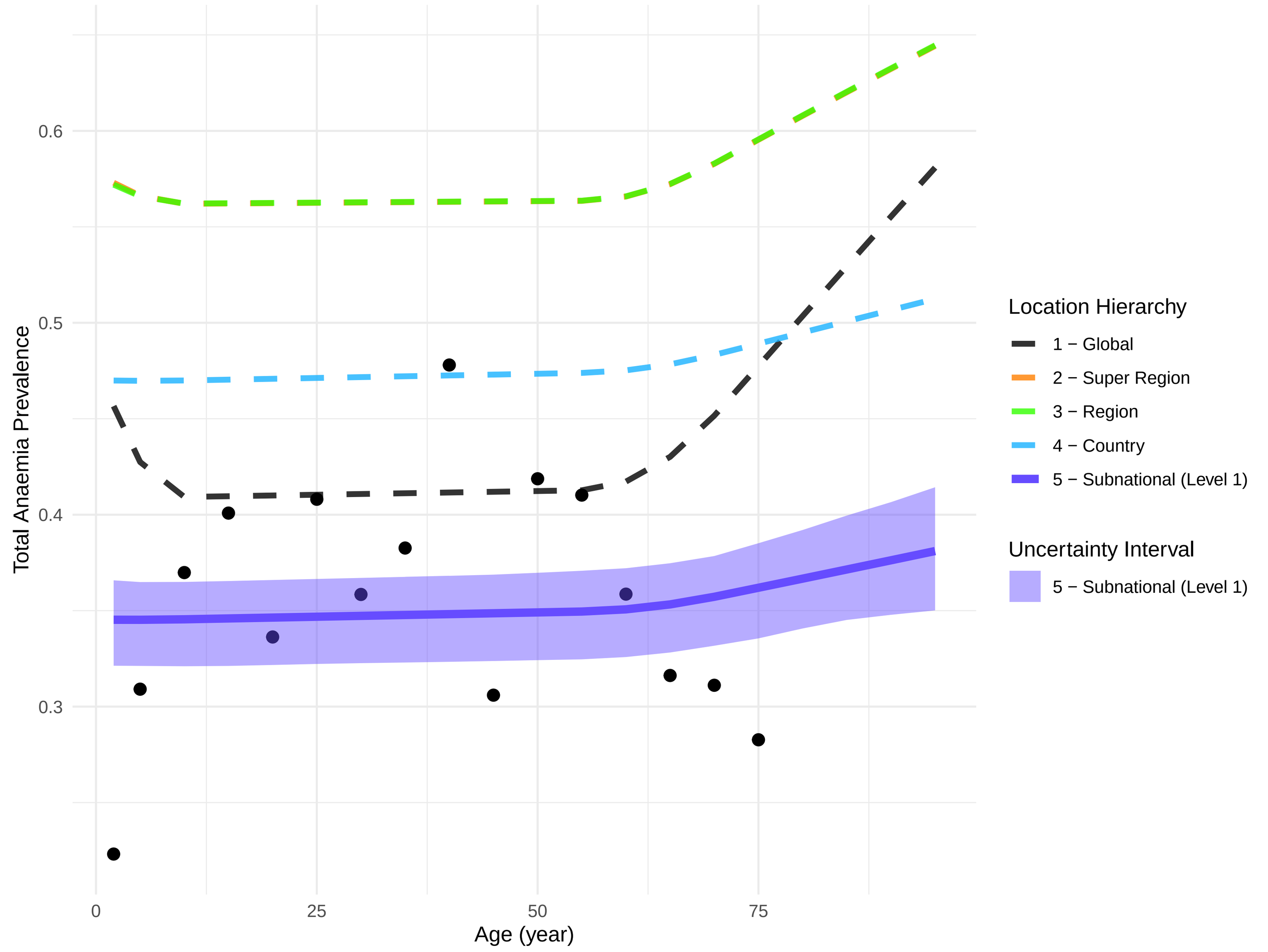

MR−BRT Age/Sex Fits − Non−pregnant Females 2+ Years Old − Total Anaemia Prevalence − Punjab
WHO−2001 Adjustment Method

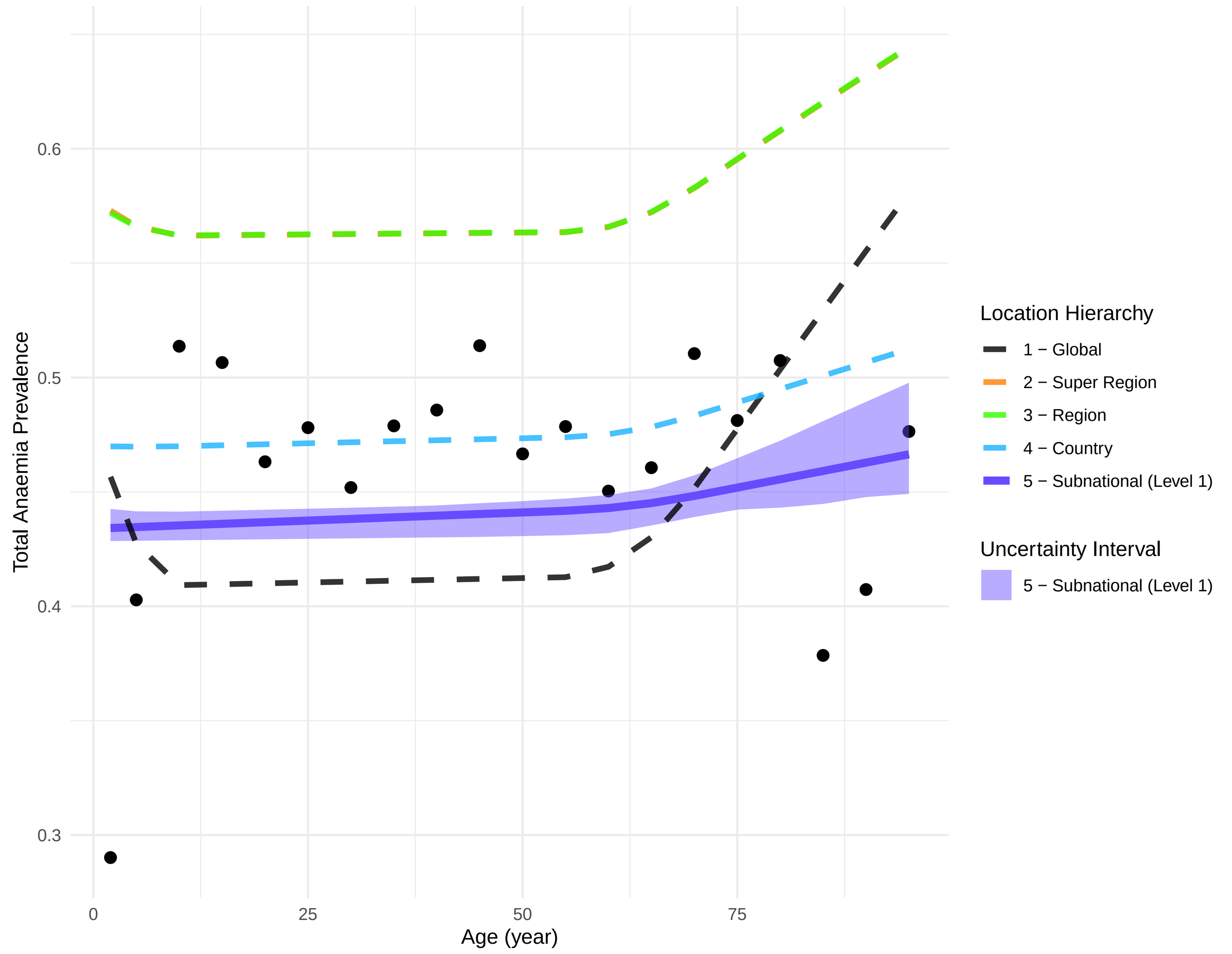

MR−BRT Age/Sex Fits − Non−pregnant Females 2+ Years Old − Total Anaemia Prevalence − Sindh
WHO−2001 Adjustment Method

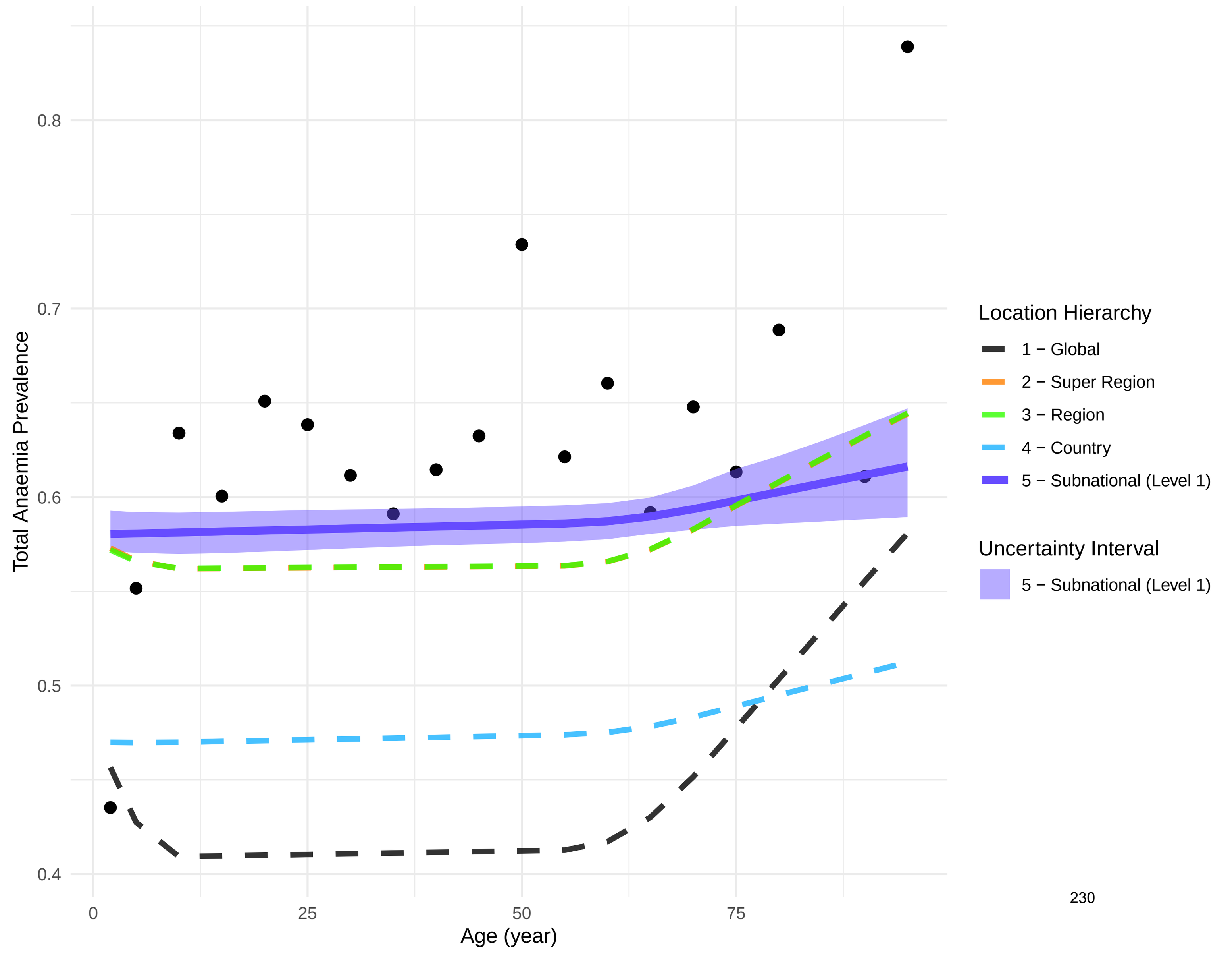

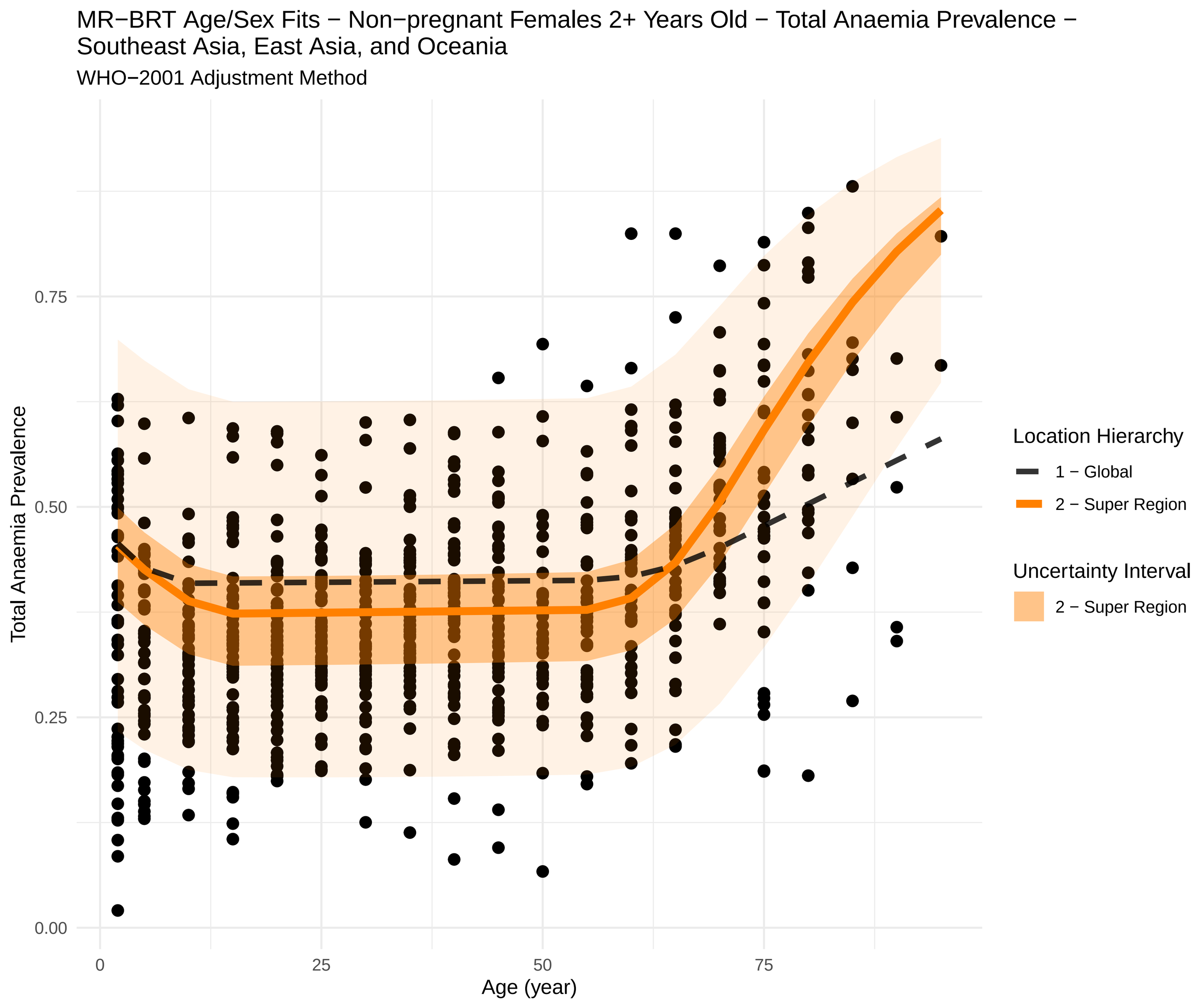
MR−BRT Age/Sex Fits − Non−pregnant Females 2+ Years Old − Total Anaemia Prevalence − Southeast Asia, East Asia, and Oceania
WHO−2001 Adjustment Method
Total Anaemia Prevalence
Age (year)
Location Hierarchy
1 − Global
2 − Super Region
Uncertainty Interval
2 − Super Region

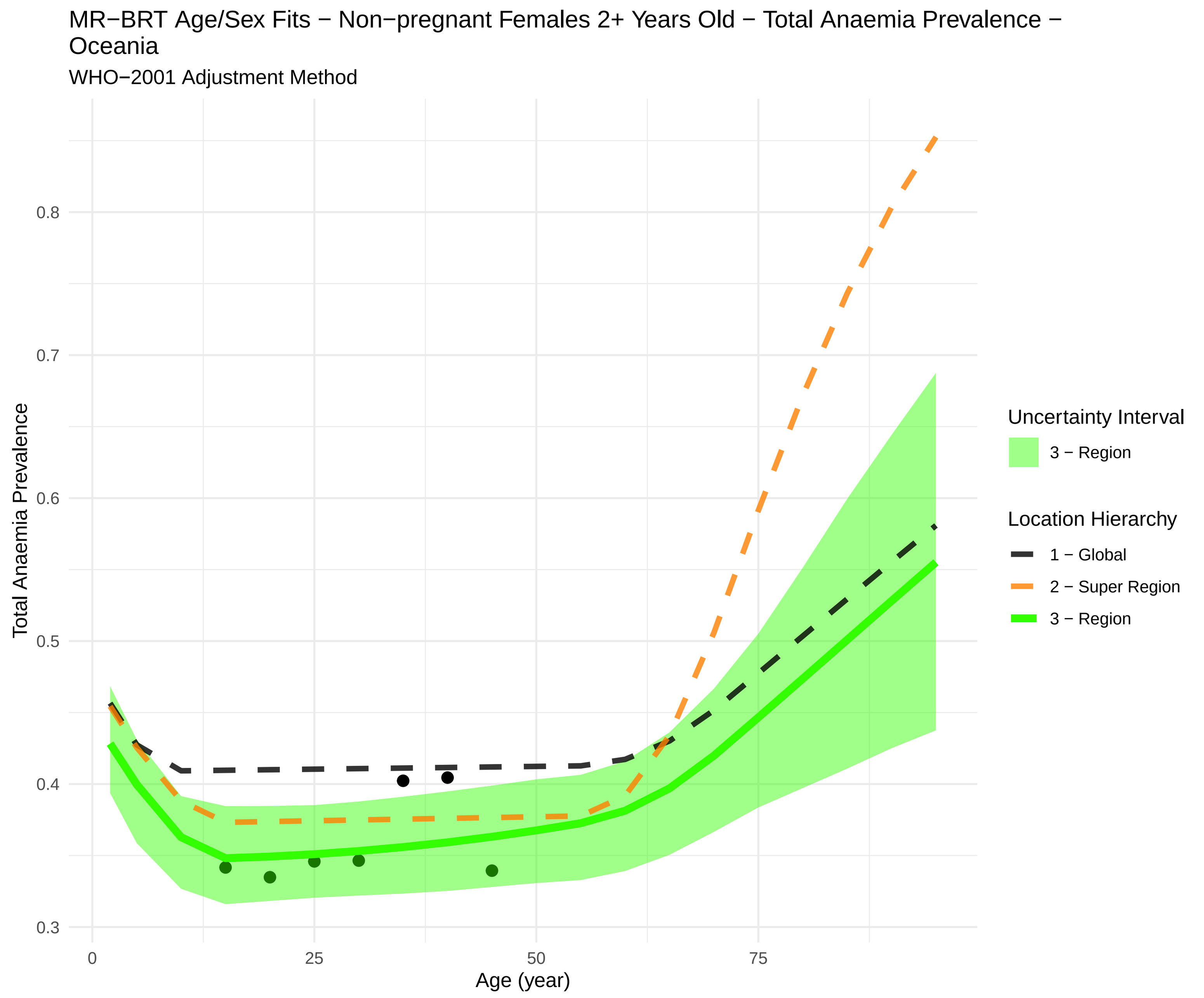
MR−BRT Age/Sex Fits − Non−pregnant Females 2+ Years Old − Total Anaemia Prevalence − Oceania
WHO−2001 Adjustment Method
Total Anaemia Prevalence
Age (year)
Uncertainty Interval
3 − Region
Location Hierarchy
1 − Global
2 − Super Region
3 − Region

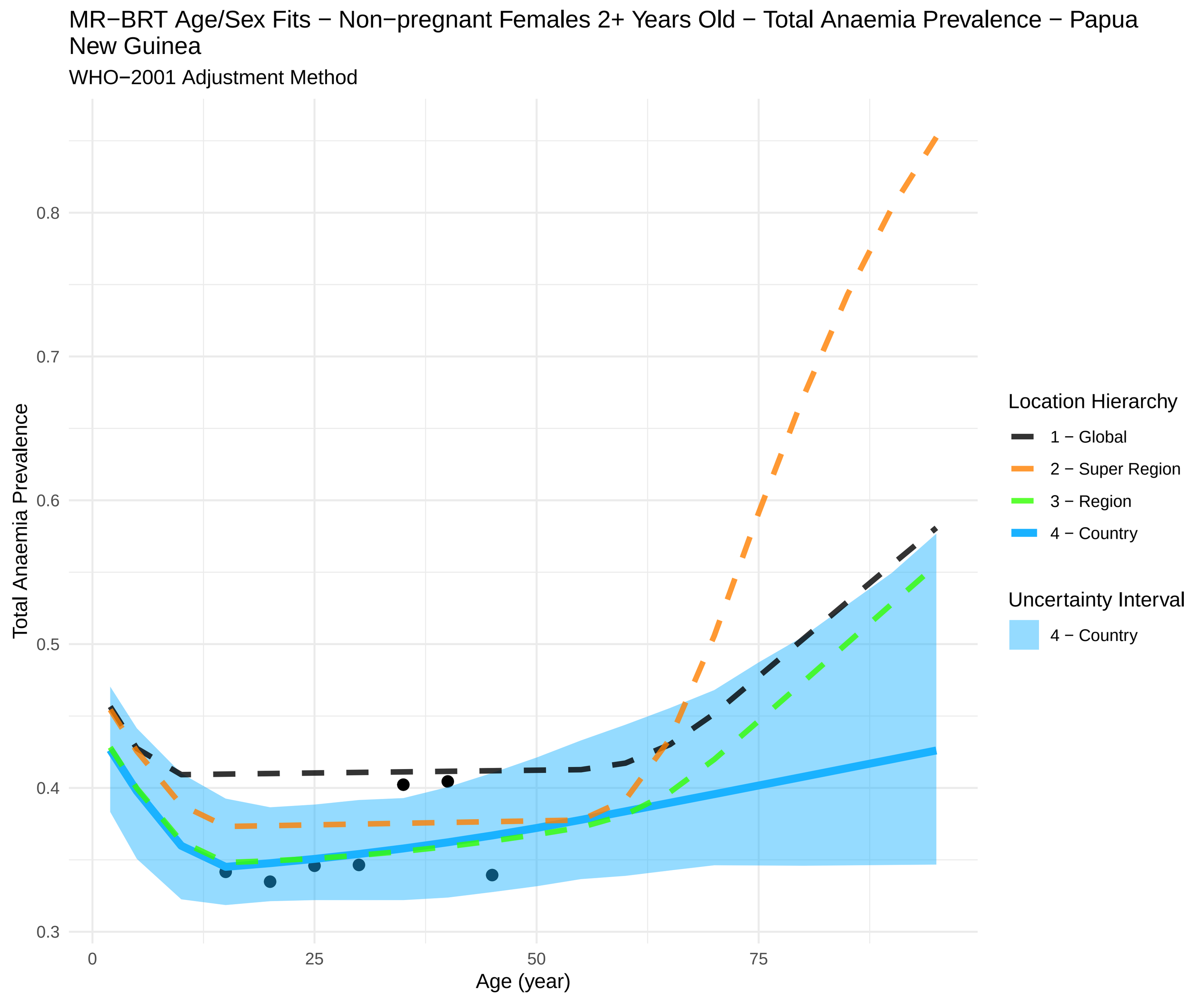
MR−BRT Age/Sex Fits − Non−pregnant Females 2+ Years Old − Total Anaemia Prevalence − Papua New Guinea
WHO−2001 Adjustment Method
Total Anaemia Prevalence
Age (year)
Location Hierarchy
1 − Global
2 − Super Region
3 − Region
4 − Country
Uncertainty Interval
4 − Country

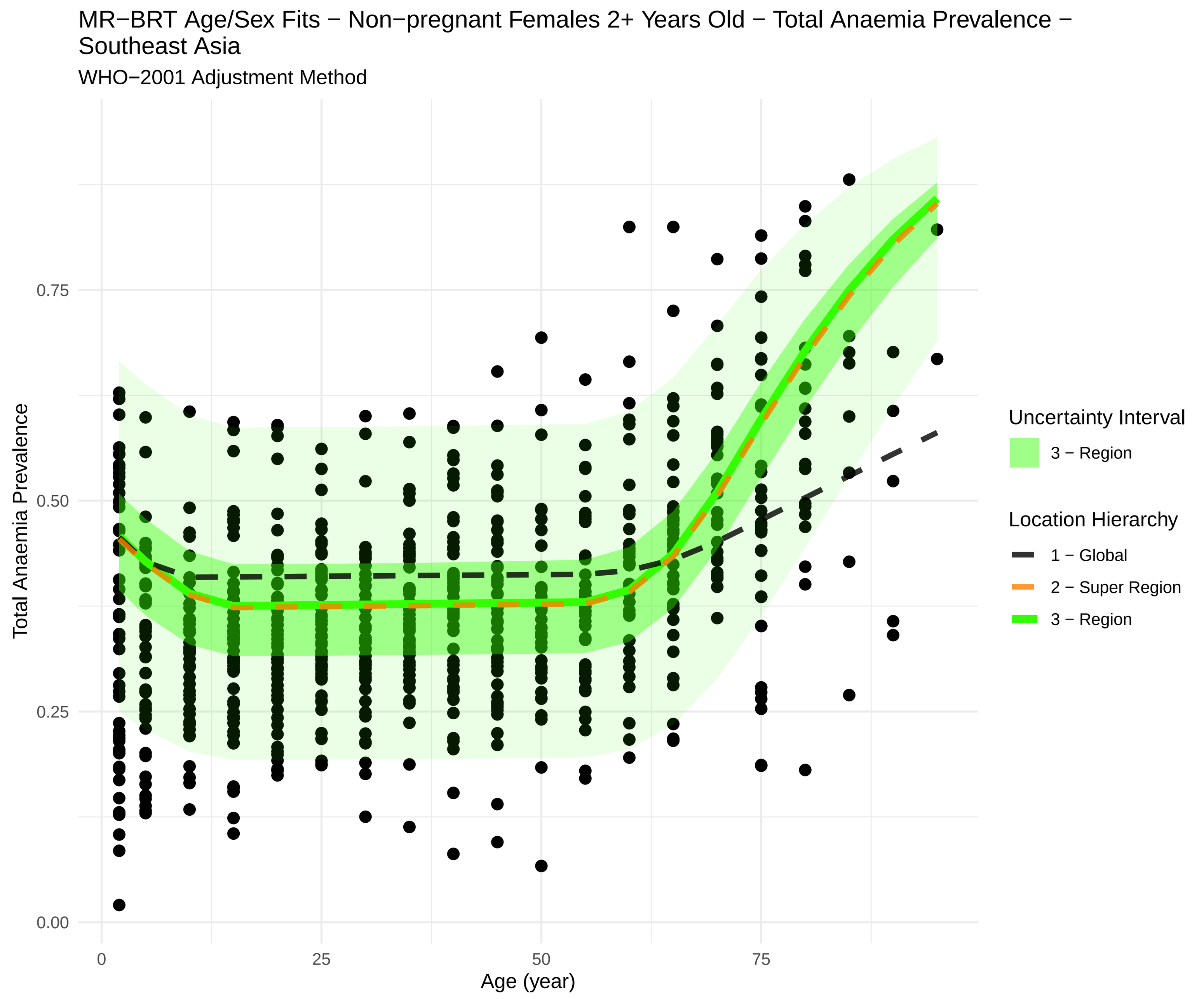
MR−BRT Age/Sex Fits − Non−pregnant Females 2+ Years Old − Total Anaemia Prevalence − Southeast Asia
WHO−2001 Adjustment Method
Total Anaemia Prevalence
Age (year)
Uncertainty Interval
3 − Region
Location Hierarchy
1 − Global
2 − Super Region
3 − Region

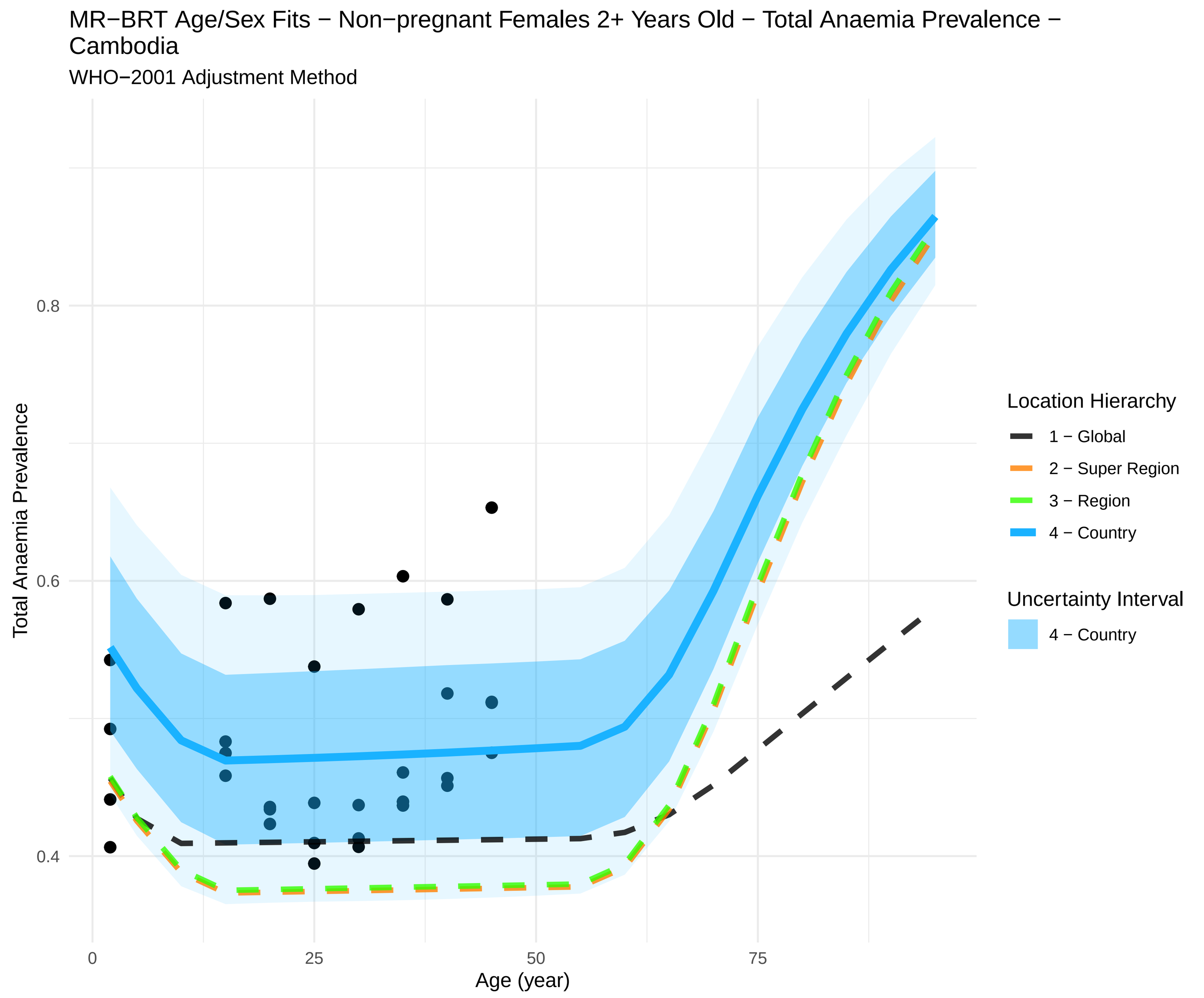
MR−BRT Age/Sex Fits − Non−pregnant Females 2+ Years Old − Total Anaemia Prevalence − Cambodia
WHO−2001 Adjustment Method
Total Anaemia Prevalence
Age (year)
Location Hierarchy
1 − Global
2 − Super Region
3 − Region
4 − Country
Uncertainty Interval
4 − Country

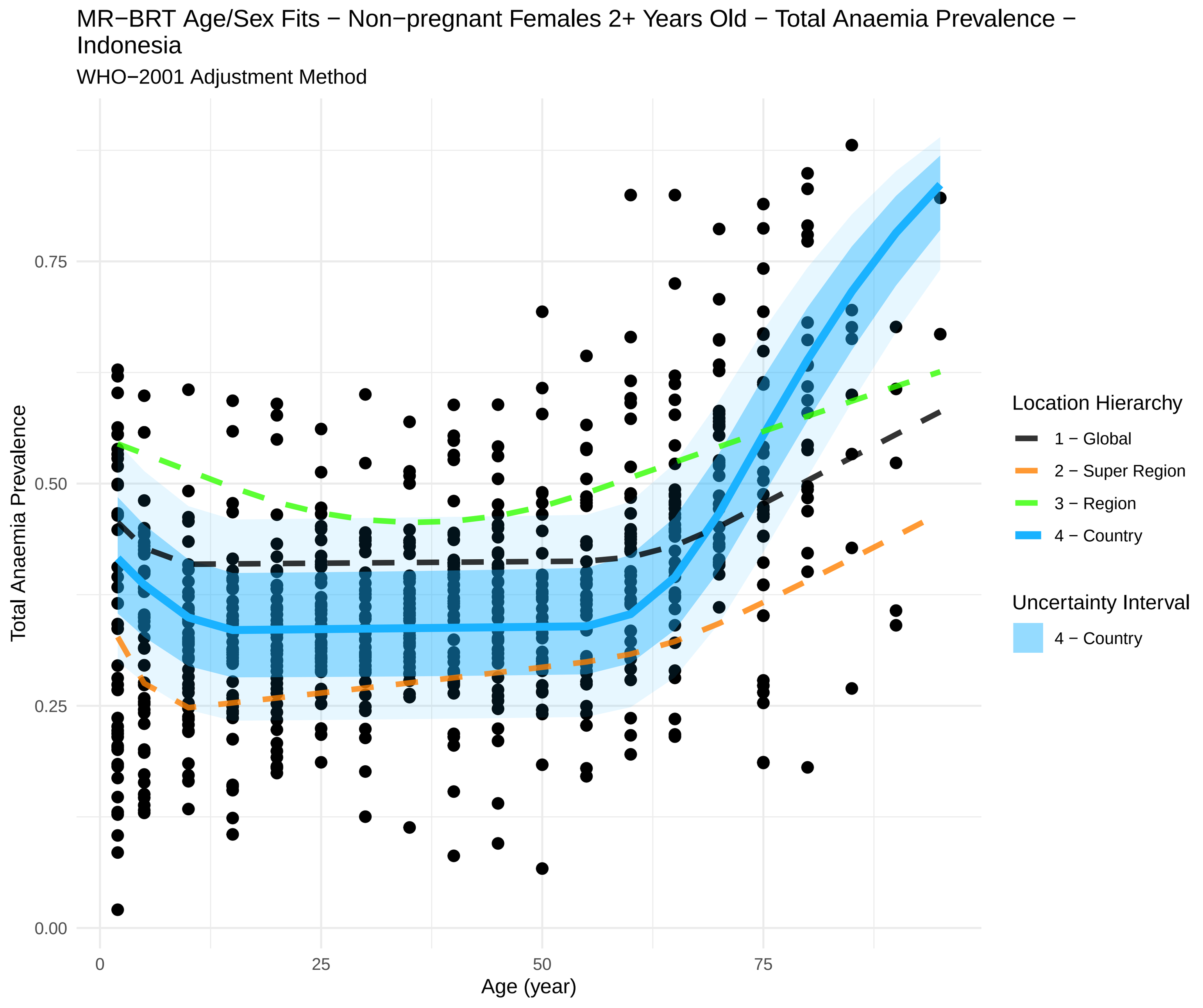
MR−BRT Age/Sex Fits − Non−pregnant Females 2+ Years Old − Total Anaemia Prevalence − Indonesia
WHO−2001 Adjustment Method
Total Anaemia Prevalence
Age (year)
Location Hierarchy
1 − Global
2 − Super Region
3 − Region
4 − Country
Uncertainty Interval
4 − Country

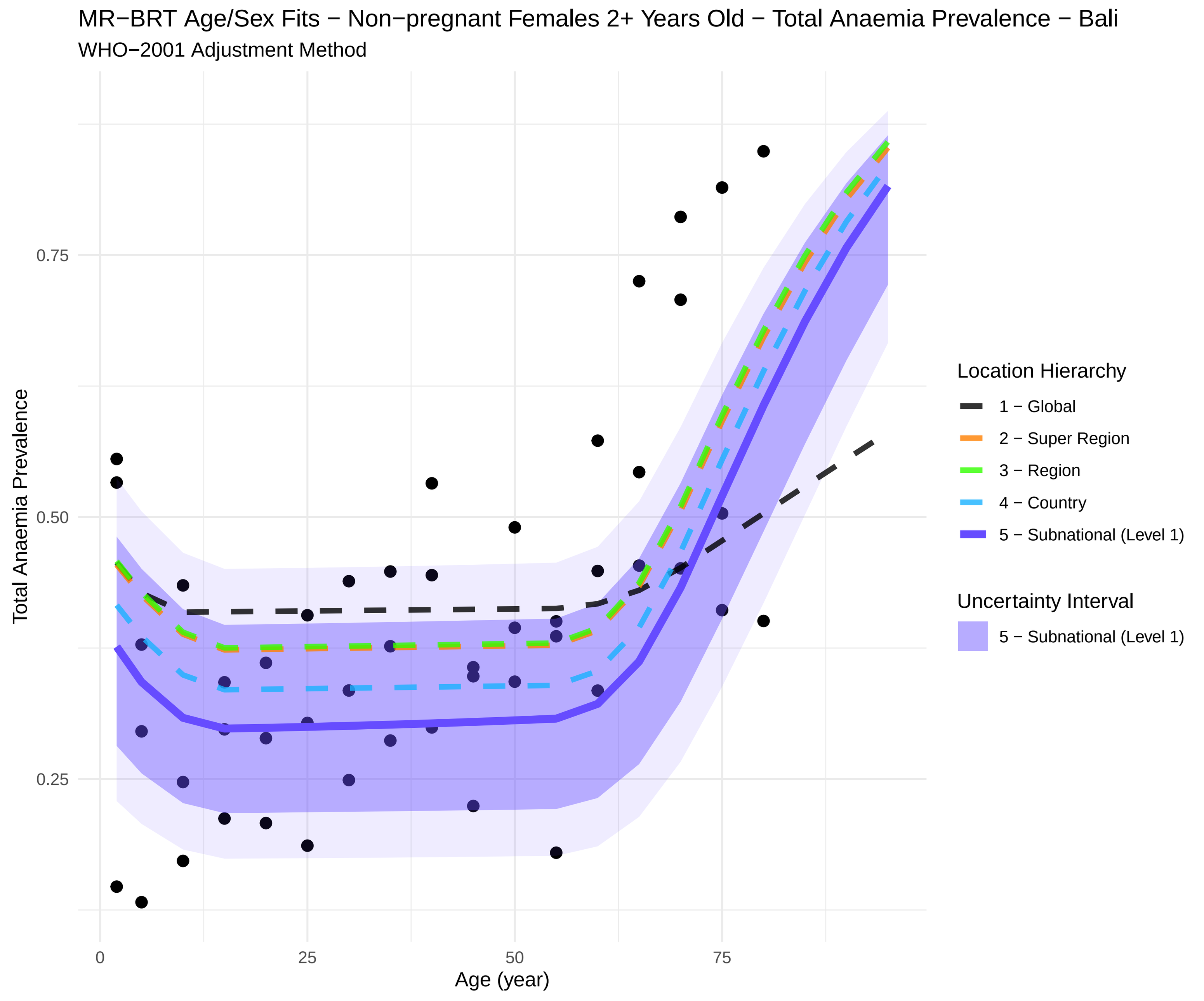
MR−BRT Age/Sex Fits − Non−pregnant Females 2+ Years Old − Total Anaemia Prevalence − Bali
WHO−2001 Adjustment Method
Total Anaemia Prevalence
Age (year)
Location Hierarchy
1 − Global
2 − Super Region
3 − Region
4 − Country
5 − Subnational (Level 1)
Uncertainty Interval
5 − Subnational (Level 1)

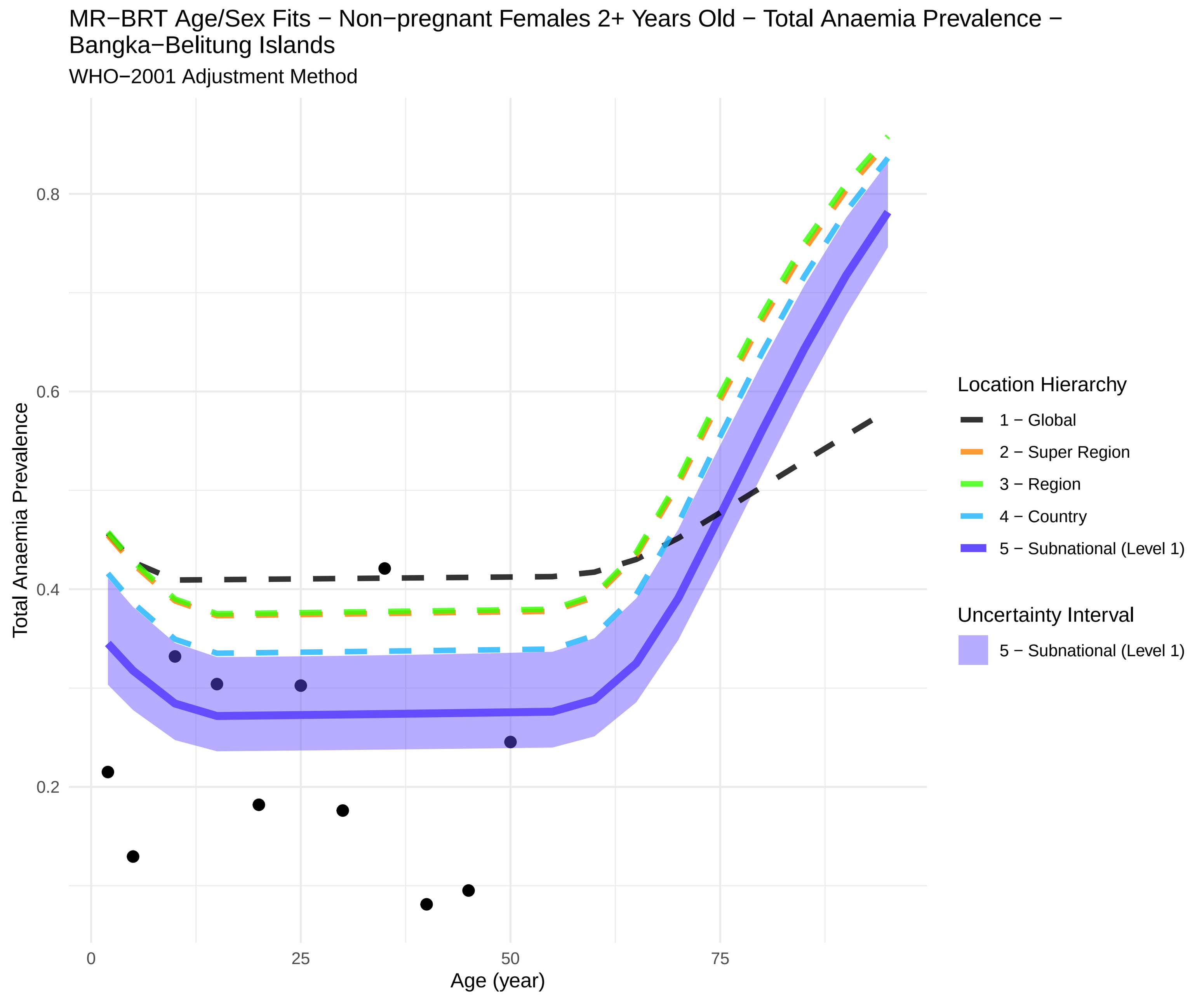
MR−BRT Age/Sex Fits − Non−pregnant Females 2+ Years Old − Total Anaemia Prevalence − Bangka−Belitung Islands
WHO−2001 Adjustment Method
Total Anaemia Prevalence
Age (year)
Location Hierarchy
1 − Global
2 − Super Region
3 − Region
4 − Country
5 − Subnational (Level 1)
Uncertainty Interval
5 − Subnational (Level 1)

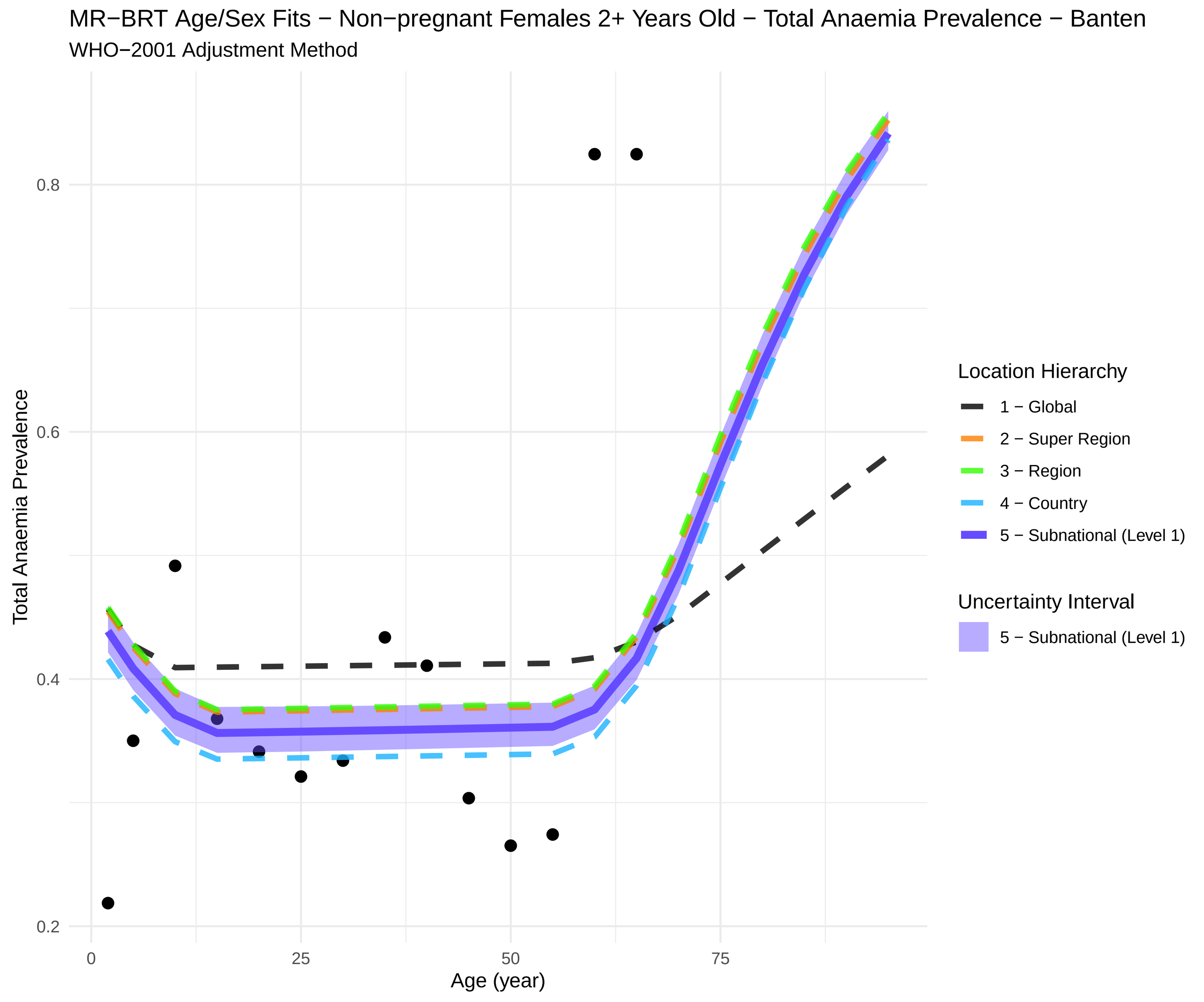
MR−BRT Age/Sex Fits − Non−pregnant Females 2+ Years Old − Total Anaemia Prevalence − Banten
WHO−2001 Adjustment Method
Total Anaemia Prevalence
Age (year)
Location Hierarchy
1 − Global
2 − Super Region
3 − Region
4 − Country
5 − Subnational (Level 1)
Uncertainty Interval
5 − Subnational (Level 1)

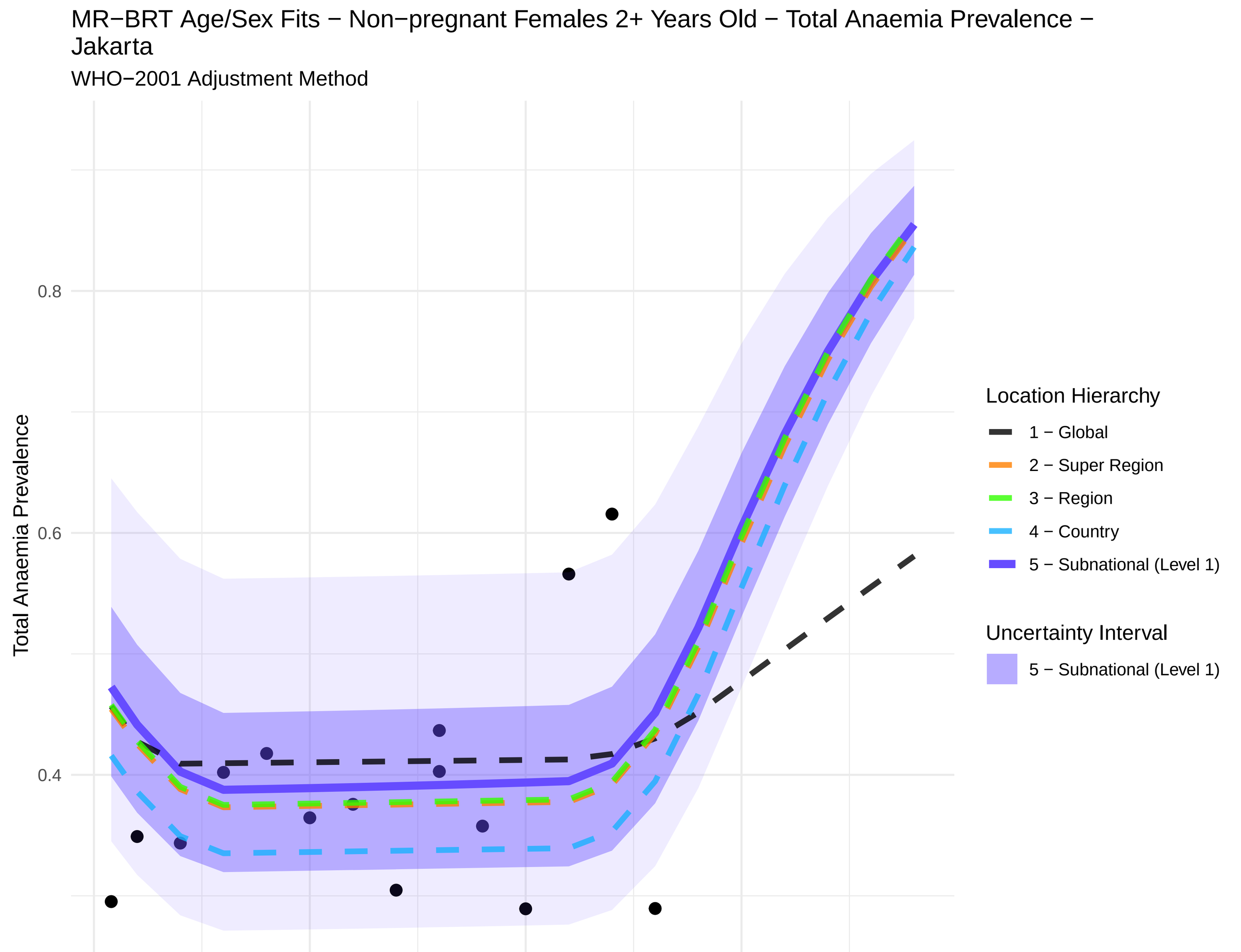
MR−BRT Age/Sex Fits − Non−pregnant Females 2+ Years Old − Total Anaemia Prevalence − Jakarta
WHO−2001 Adjustment Method
Total Anaemia Prevalence
Age (year)
Location Hierarchy
1 − Global
2 − Super Region
3 − Region
4 − Country
5 − Subnational (Level 1)
Uncertainty Interval
5 − Subnational (Level 1)

MR−BRT Age/Sex Fits − Non−pregnant Females 2+ Years Old − Total Anaemia Prevalence − West Java
WHO−2001 Adjustment Method

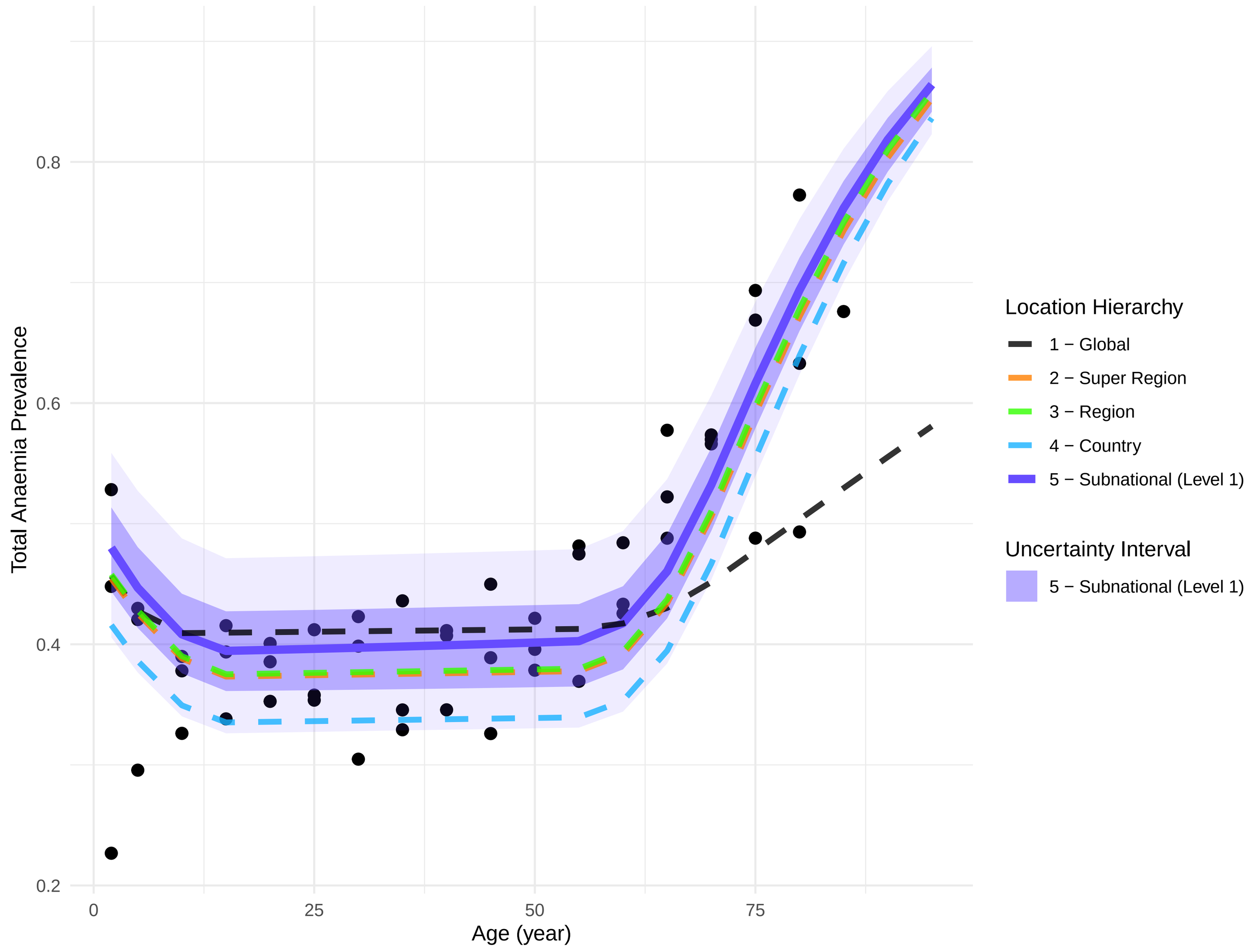

MR−BRT Age/Sex Fits − Non−pregnant Females 2+ Years Old − Total Anaemia Prevalence − Central Java
WHO−2001 Adjustment Method

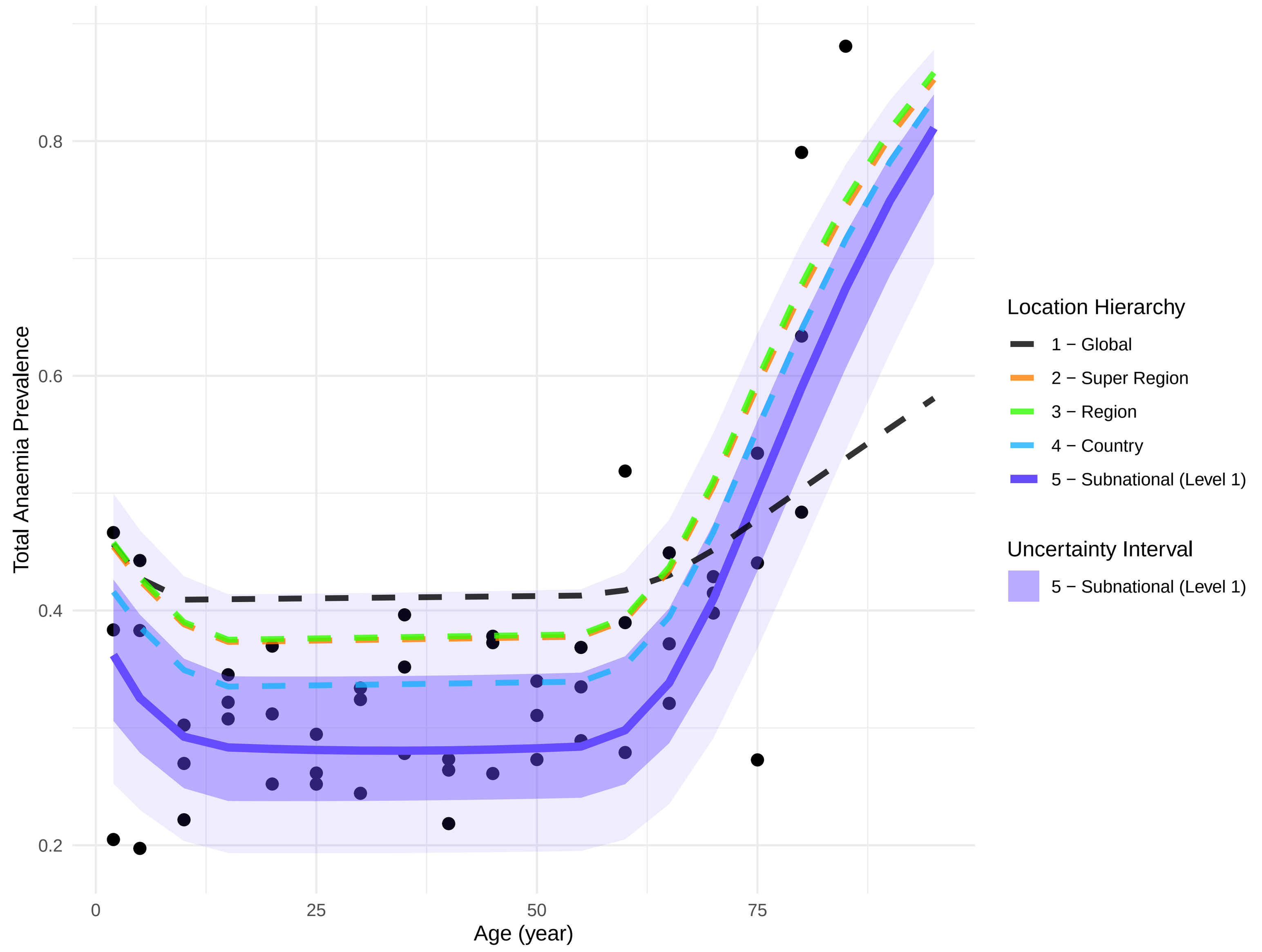

MR−BRT Age/Sex Fits − Non−pregnant Females 2+ Years Old − Total Anaemia Prevalence − East Java
WHO−2001 Adjustment Method

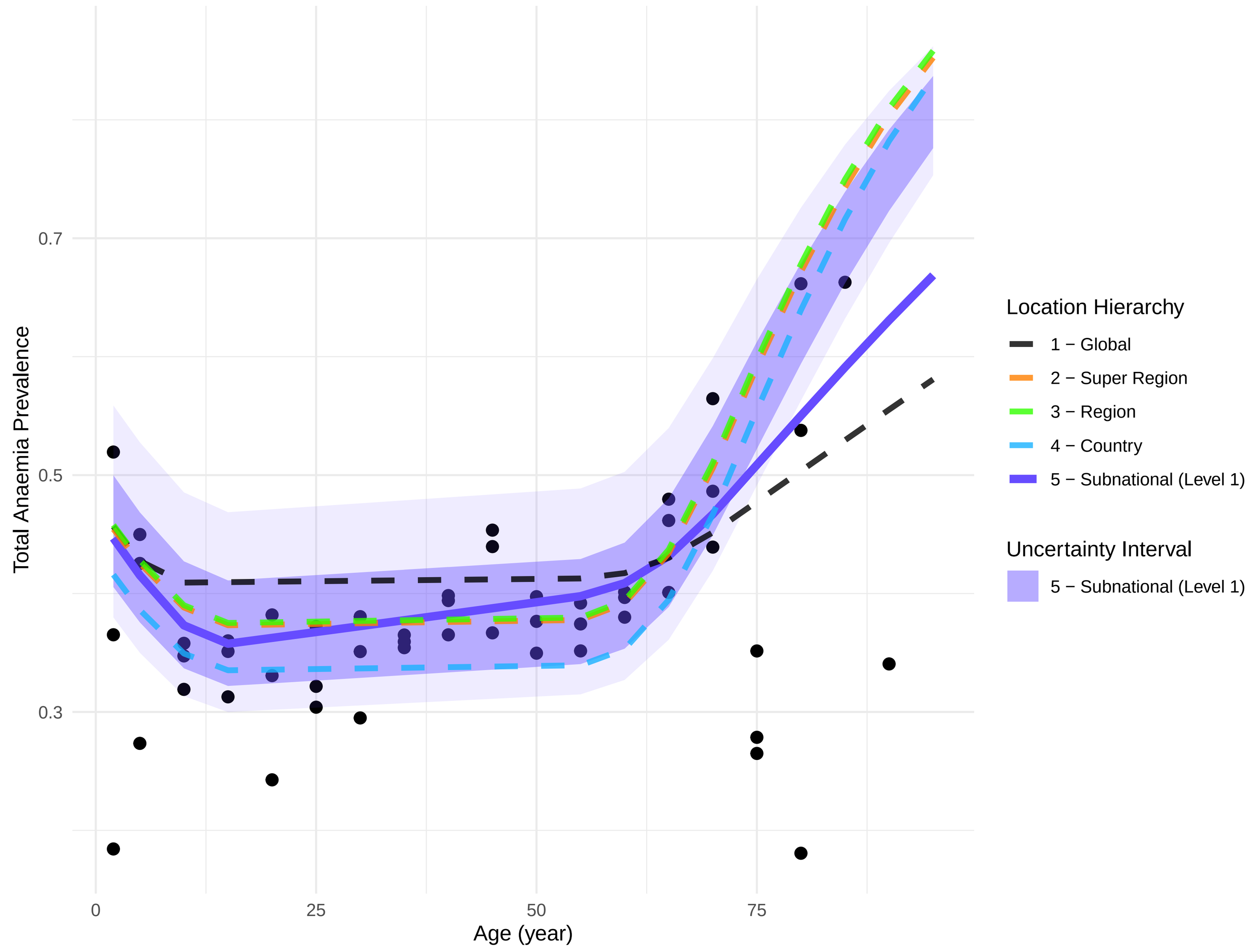

MR−BRT Age/Sex Fits − Non−pregnant Females 2+ Years Old − Total Anaemia Prevalence − South Kalimantan
WHO−2001 Adjustment Method

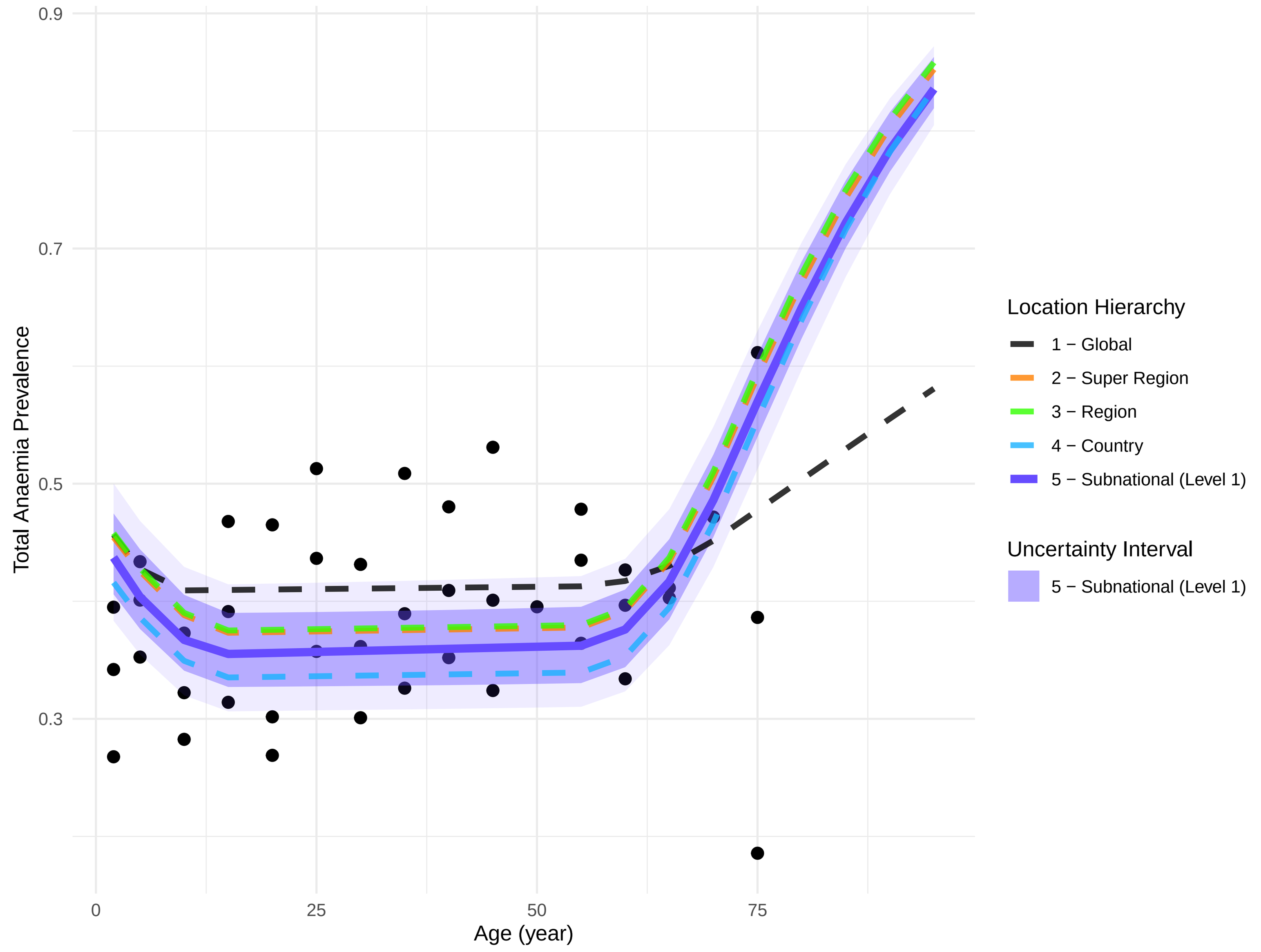

MR−BRT Age/Sex Fits − Non−pregnant Females 2+ Years Old − Total Anaemia Prevalence − Central Kalimantan
WHO−2001 Adjustment Method

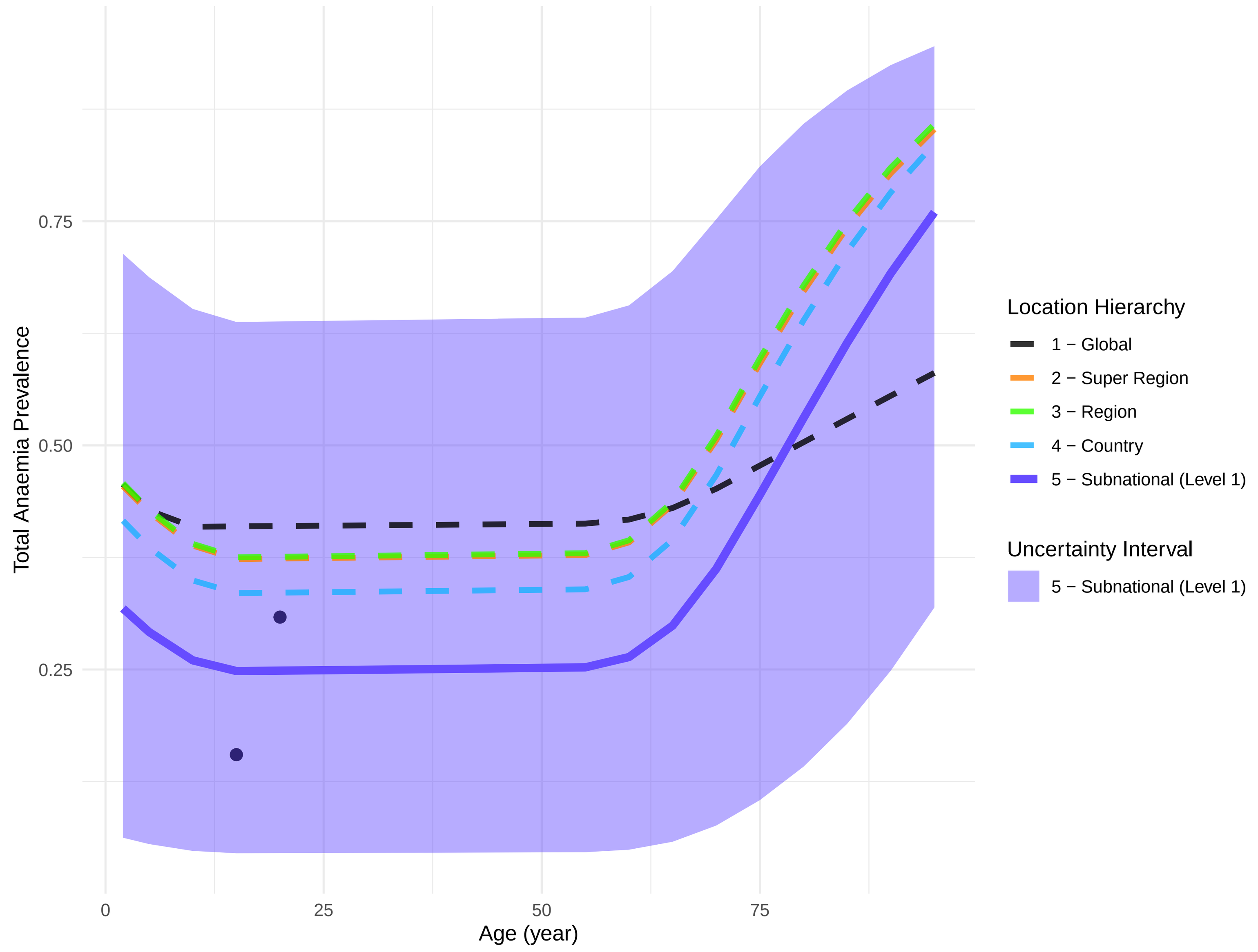

MR−BRT Age/Sex Fits − Non−pregnant Females 2+ Years Old − Total Anaemia Prevalence − East Kalimantan
WHO−2001 Adjustment Method

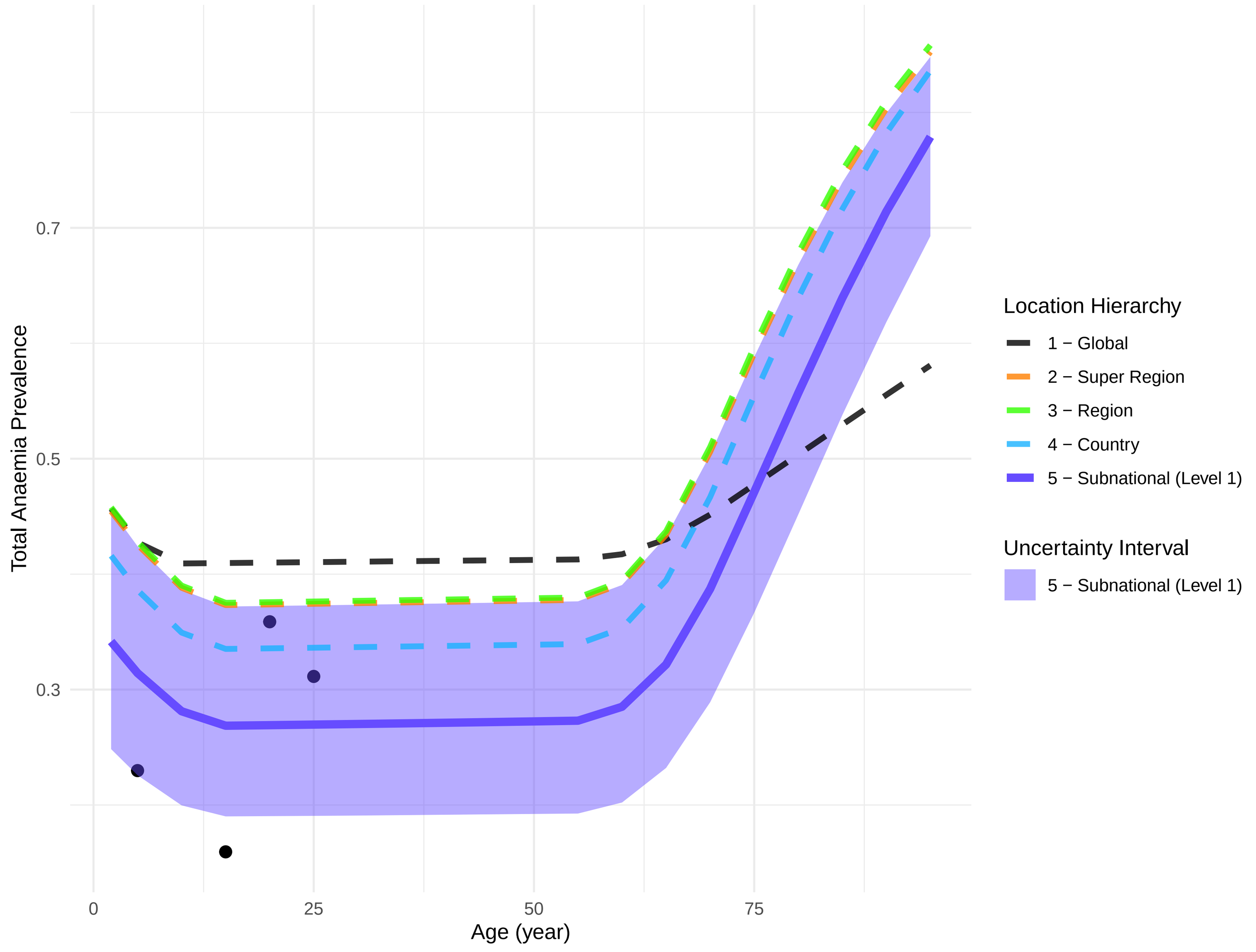

MR−BRT Age/Sex Fits − Non−pregnant Females 2+ Years Old − Total Anaemia Prevalence − Riau Islands
WHO−2001 Adjustment Method

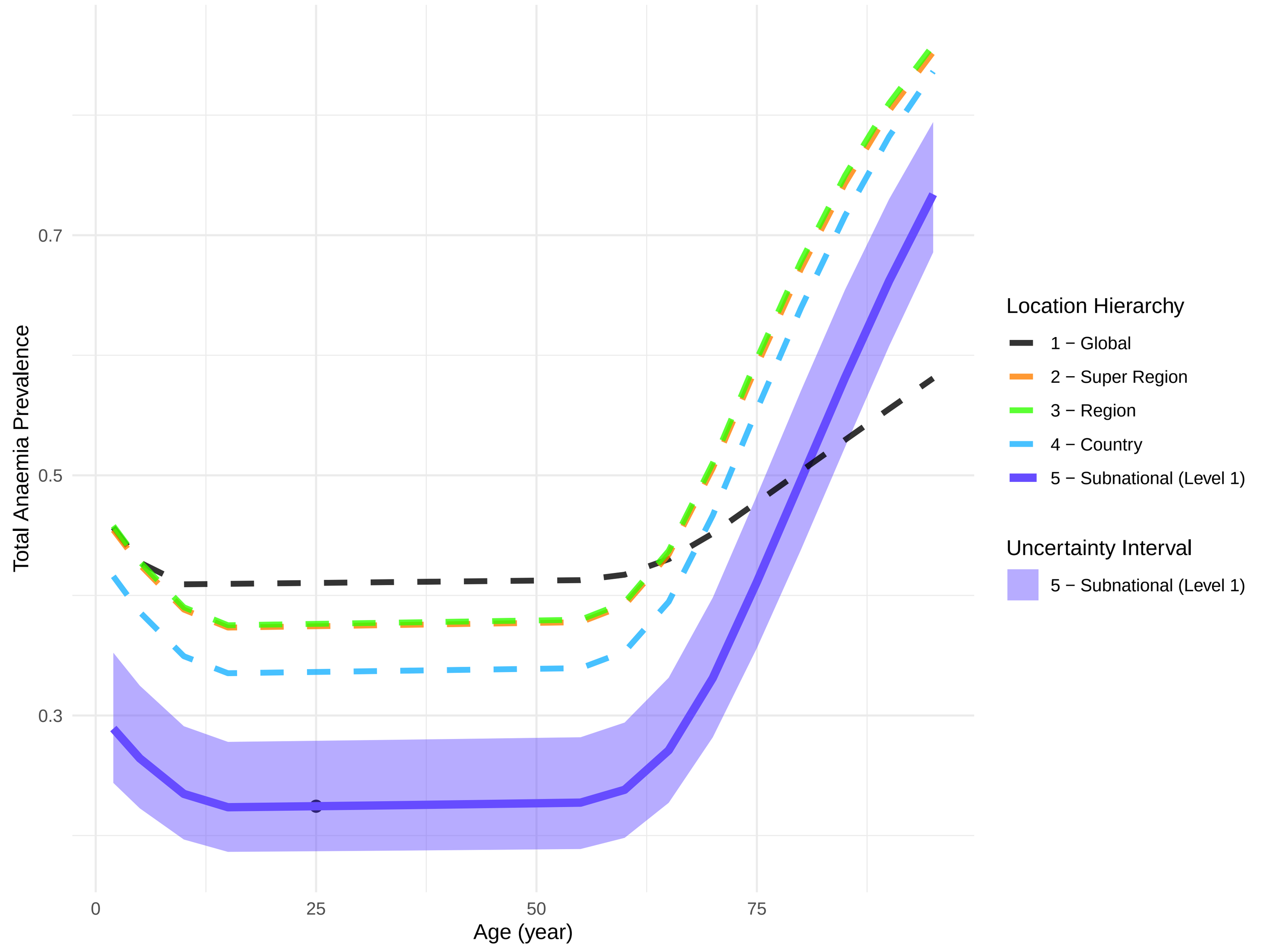

MR−BRT Age/Sex Fits − Non−pregnant Females 2+ Years Old − Total Anaemia Prevalence − Lampung
WHO−2001 Adjustment Method

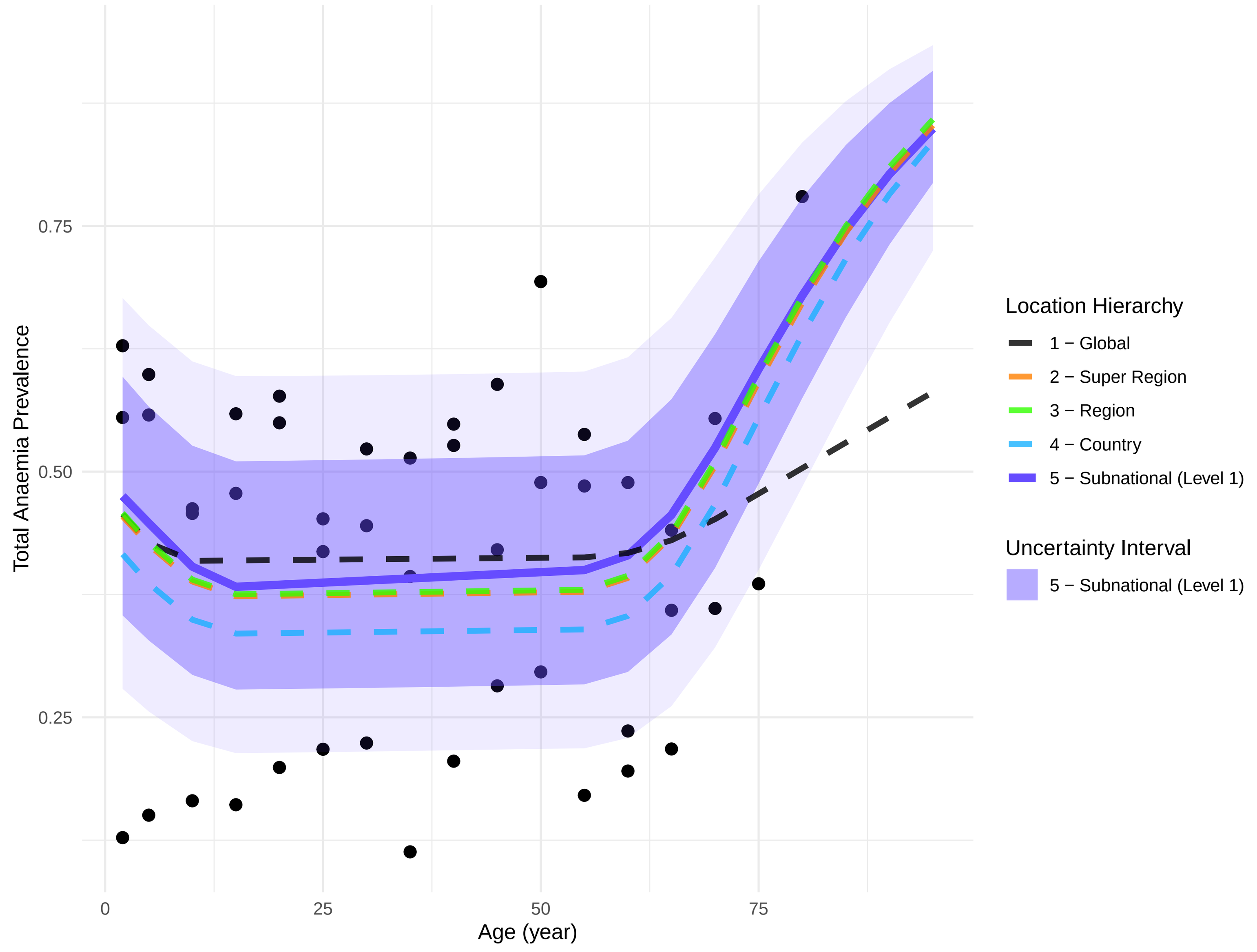

MR−BRT Age/Sex Fits − Non−pregnant Females 2+ Years Old − Total Anaemia Prevalence − West Nusa Tenggara
WHO−2001 Adjustment Method

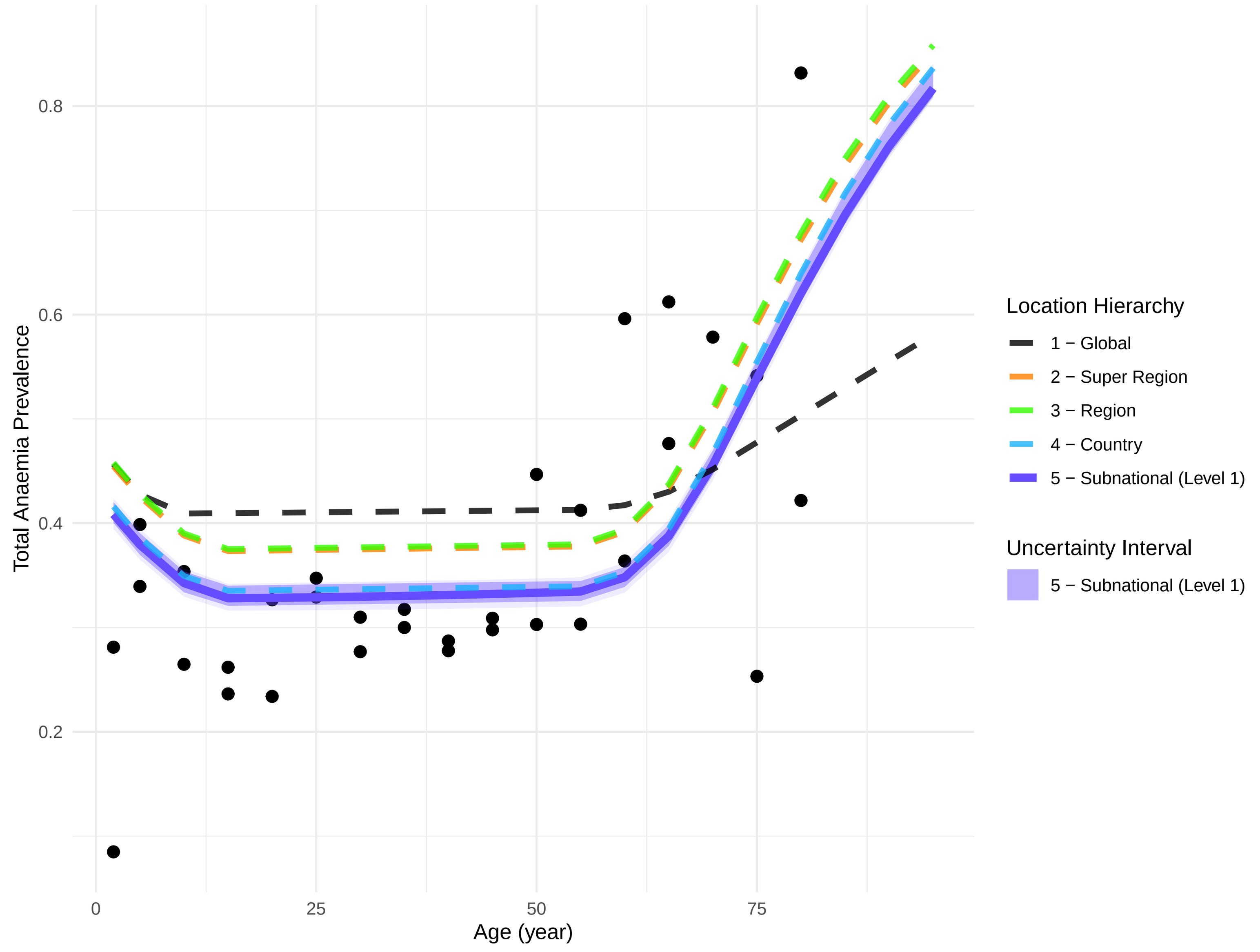

MR−BRT Age/Sex Fits − Non−pregnant Females 2+ Years Old − Total Anaemia Prevalence − Riau
WHO−2001 Adjustment Method

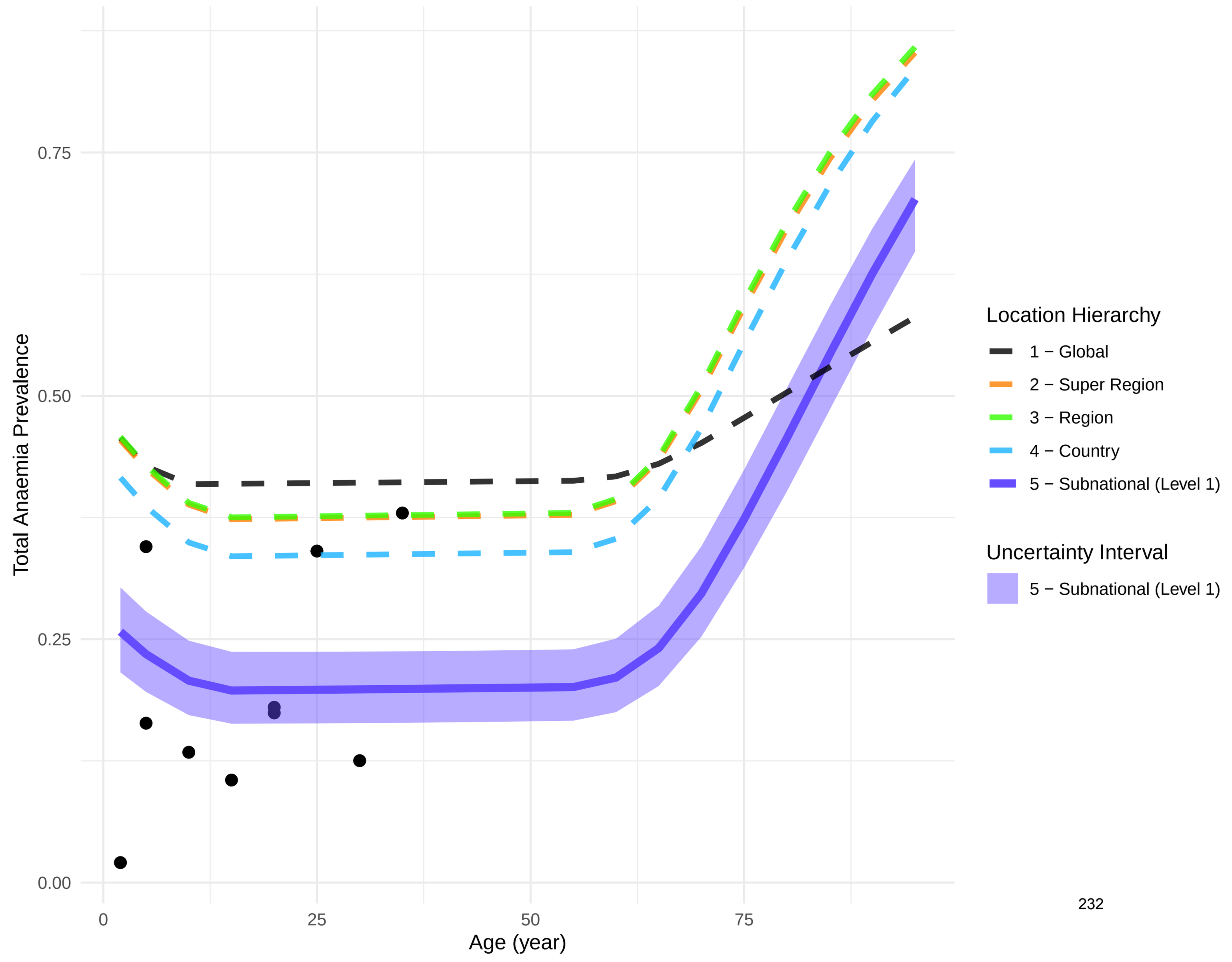

MR−BRT Age/Sex Fits − Non−pregnant Females 2+ Years Old − Total Anaemia Prevalence − West Sulawesi
WHO−2001 Adjustment Method

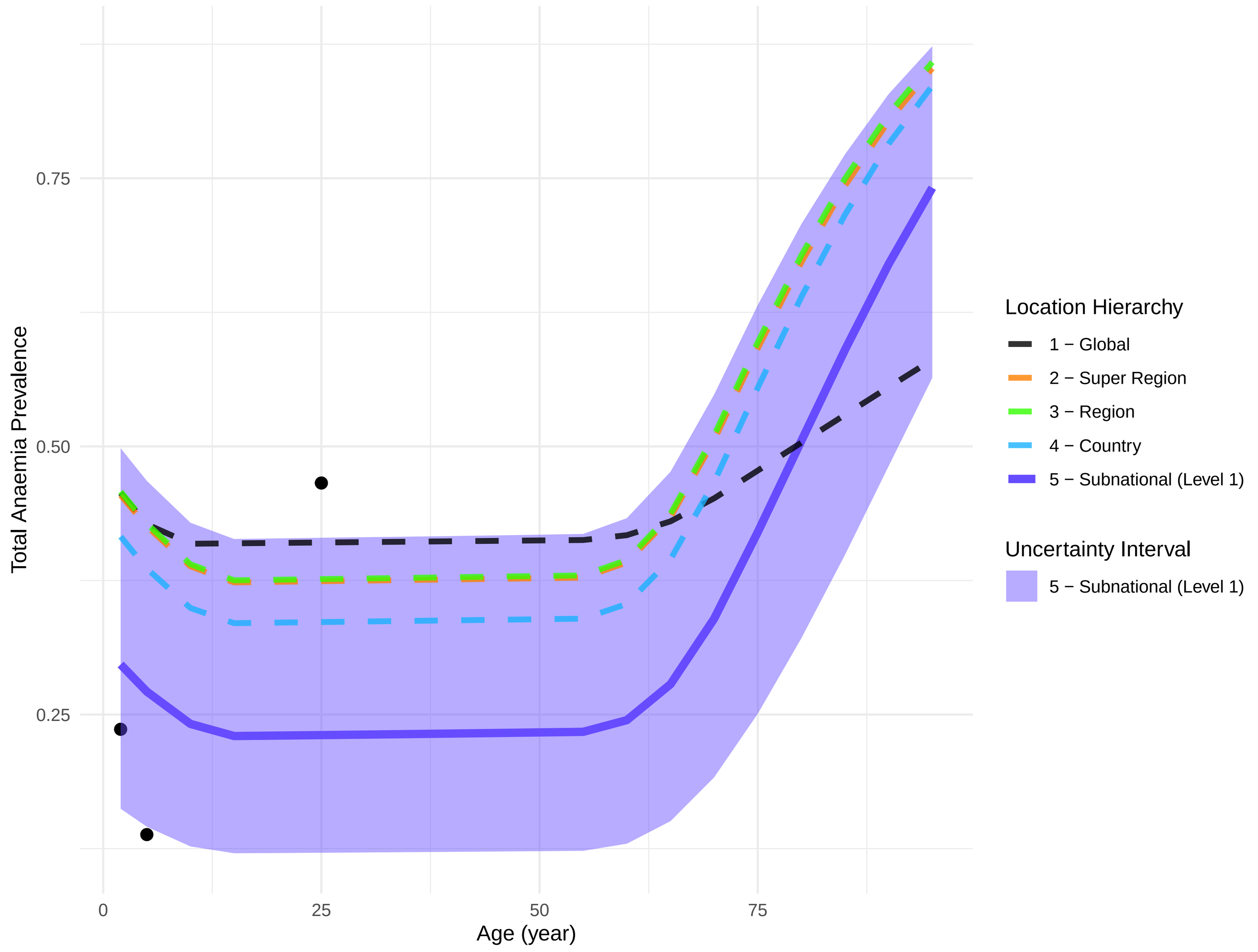

MR−BRT Age/Sex Fits − Non−pregnant Females 2+ Years Old − Total Anaemia Prevalence − South Sulawesi
WHO−2001 Adjustment Method

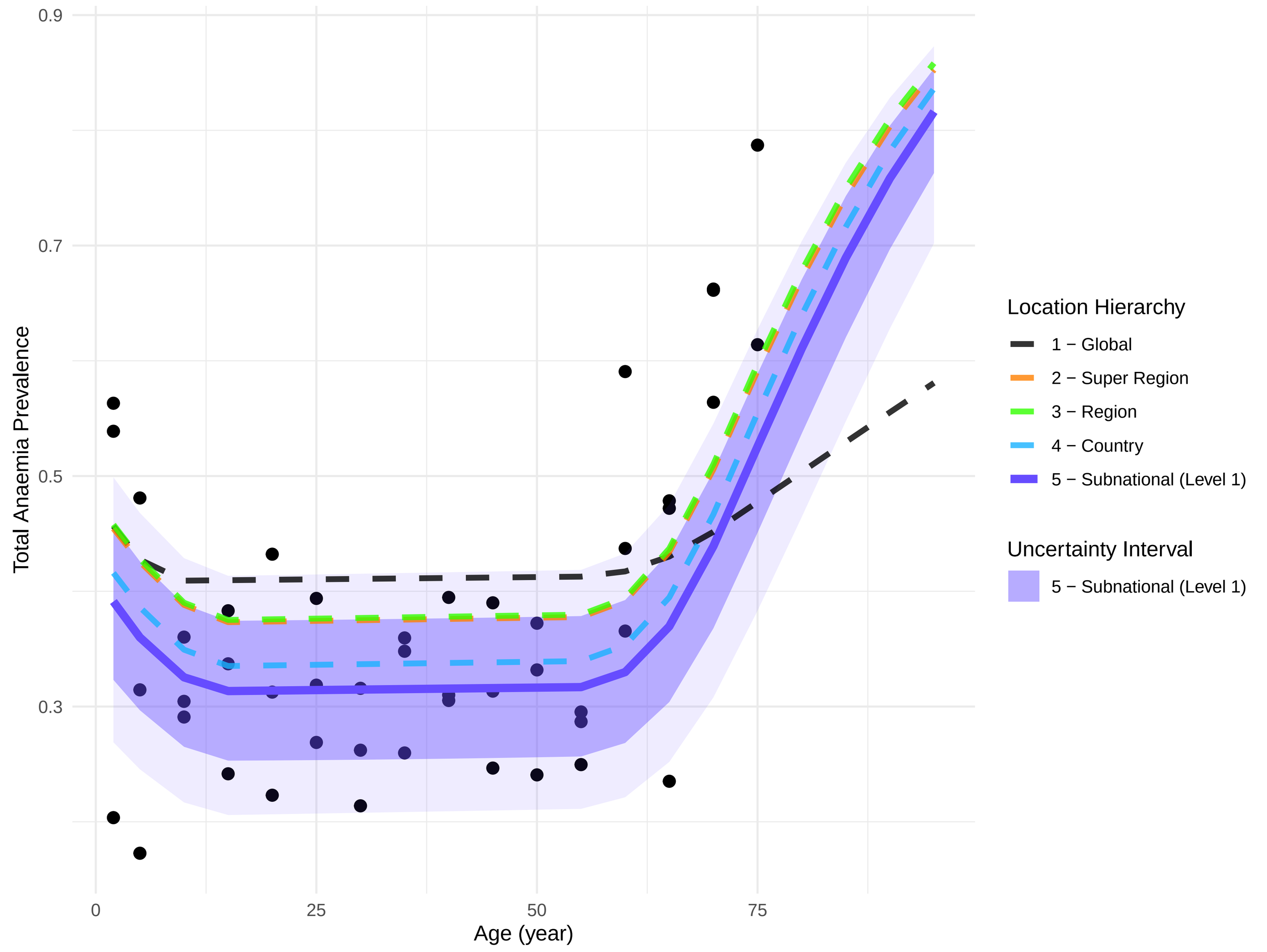

MR−BRT Age/Sex Fits − Non−pregnant Females 2+ Years Old − Total Anaemia Prevalence − West Sumatra
WHO−2001 Adjustment Method

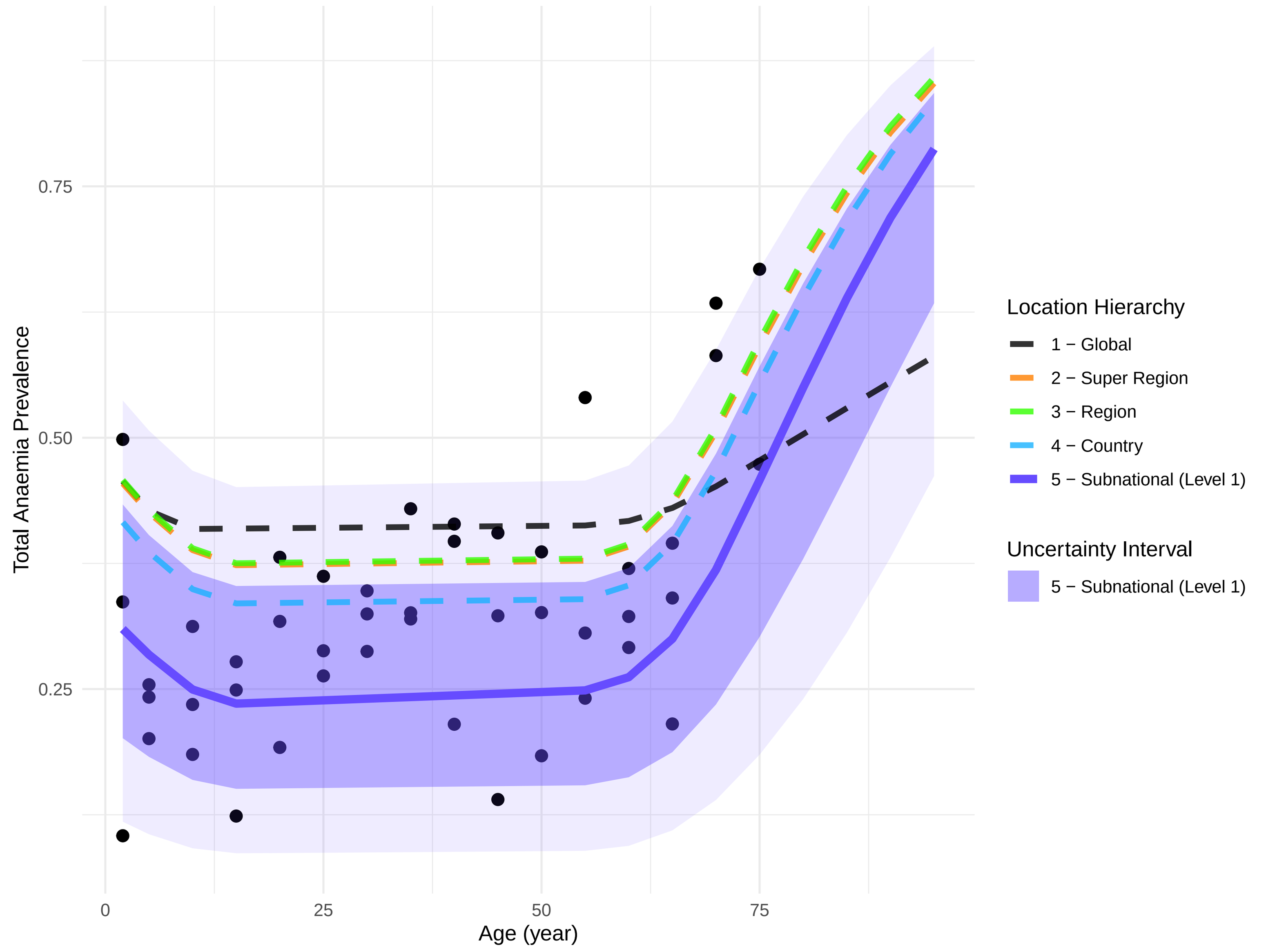

MR−BRT Age/Sex Fits − Non−pregnant Females 2+ Years Old − Total Anaemia Prevalence − South Sumatra
WHO−2001 Adjustment Method

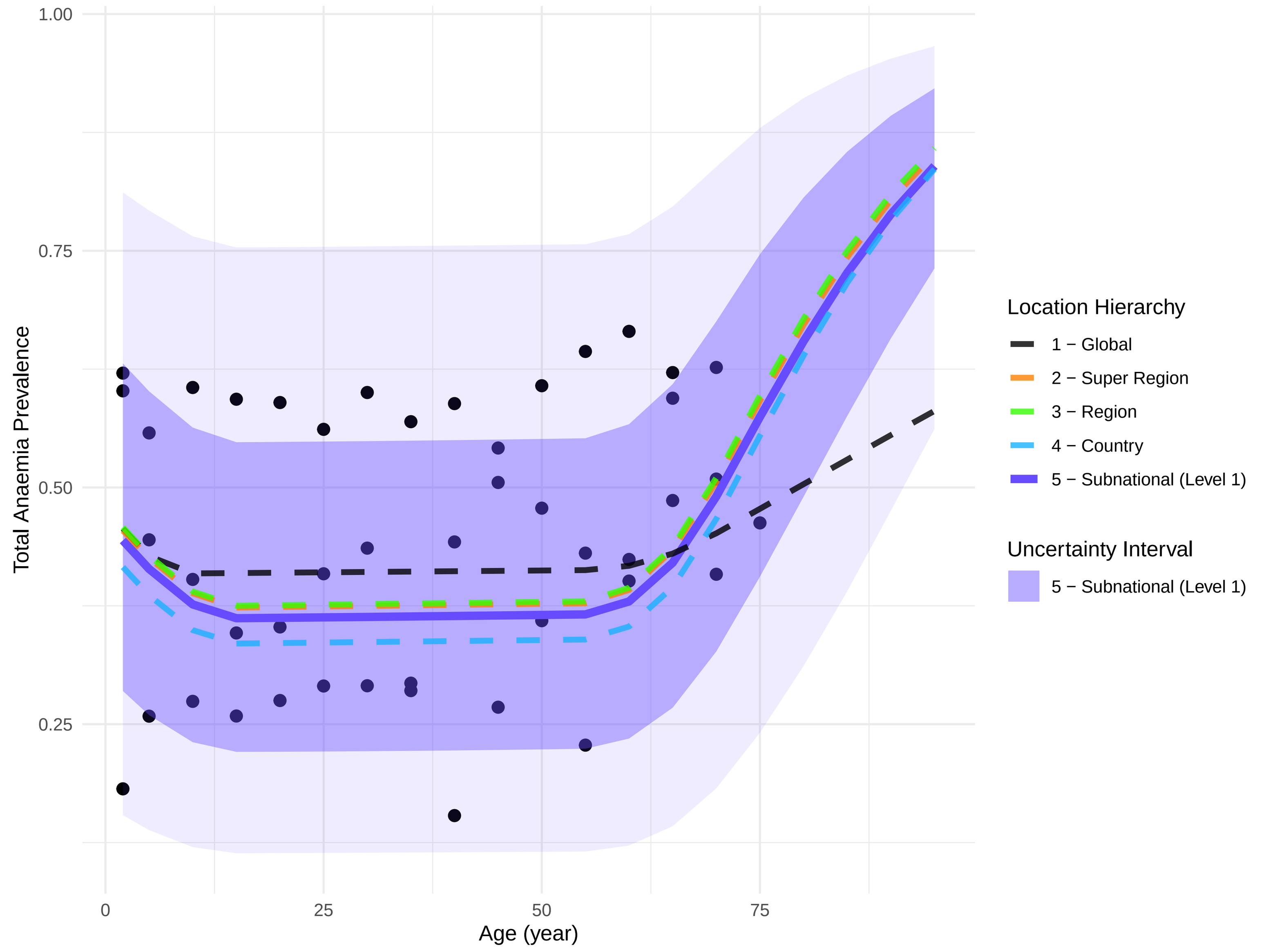

MR−BRT Age/Sex Fits − Non−pregnant Females 2+ Years Old − Total Anaemia Prevalence − North Sumatra
WHO−2001 Adjustment Method

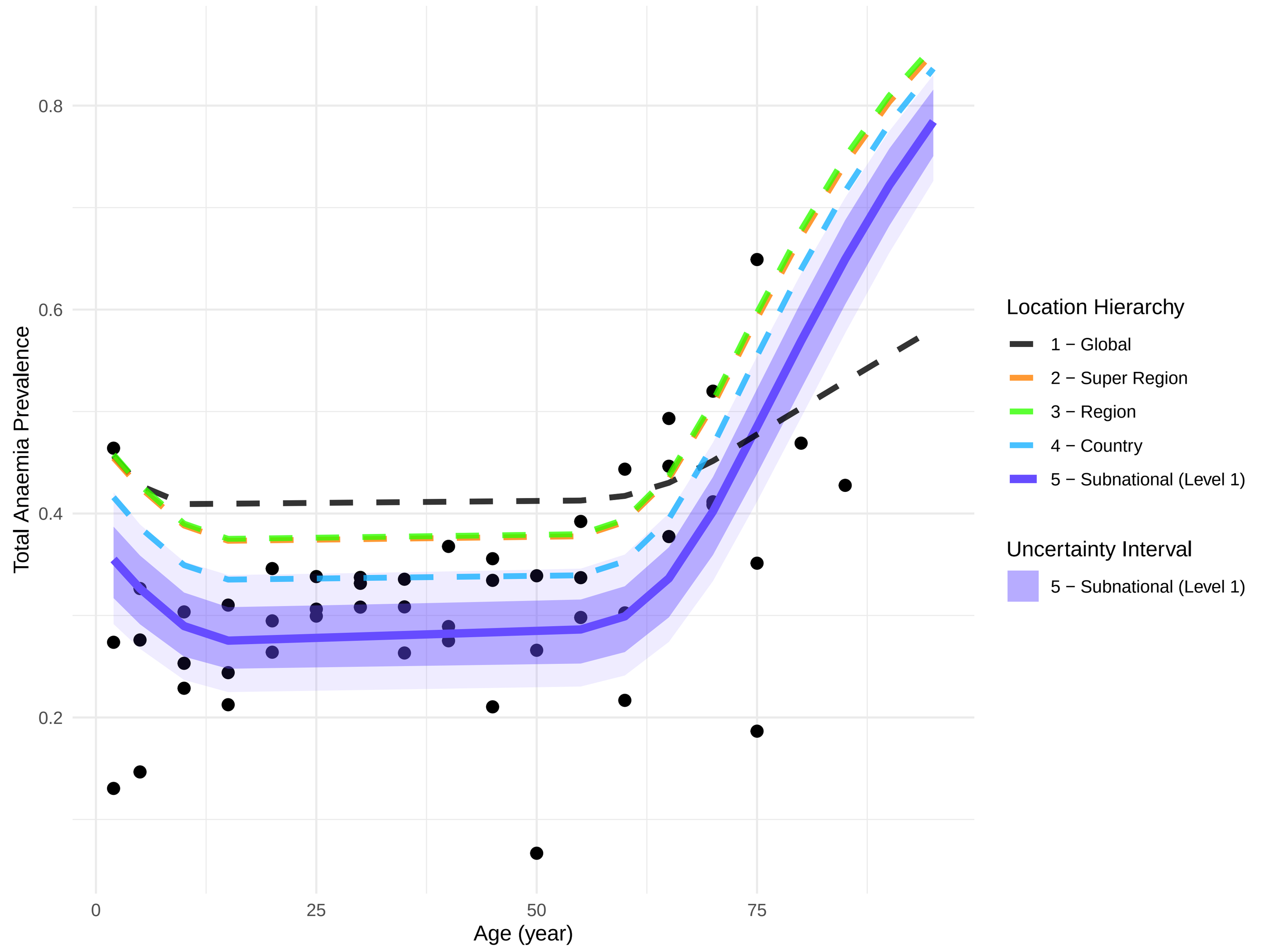

MR−BRT Age/Sex Fits − Non−pregnant Females 2+ Years Old − Total Anaemia Prevalence − Yogyakarta
WHO−2001 Adjustment Method

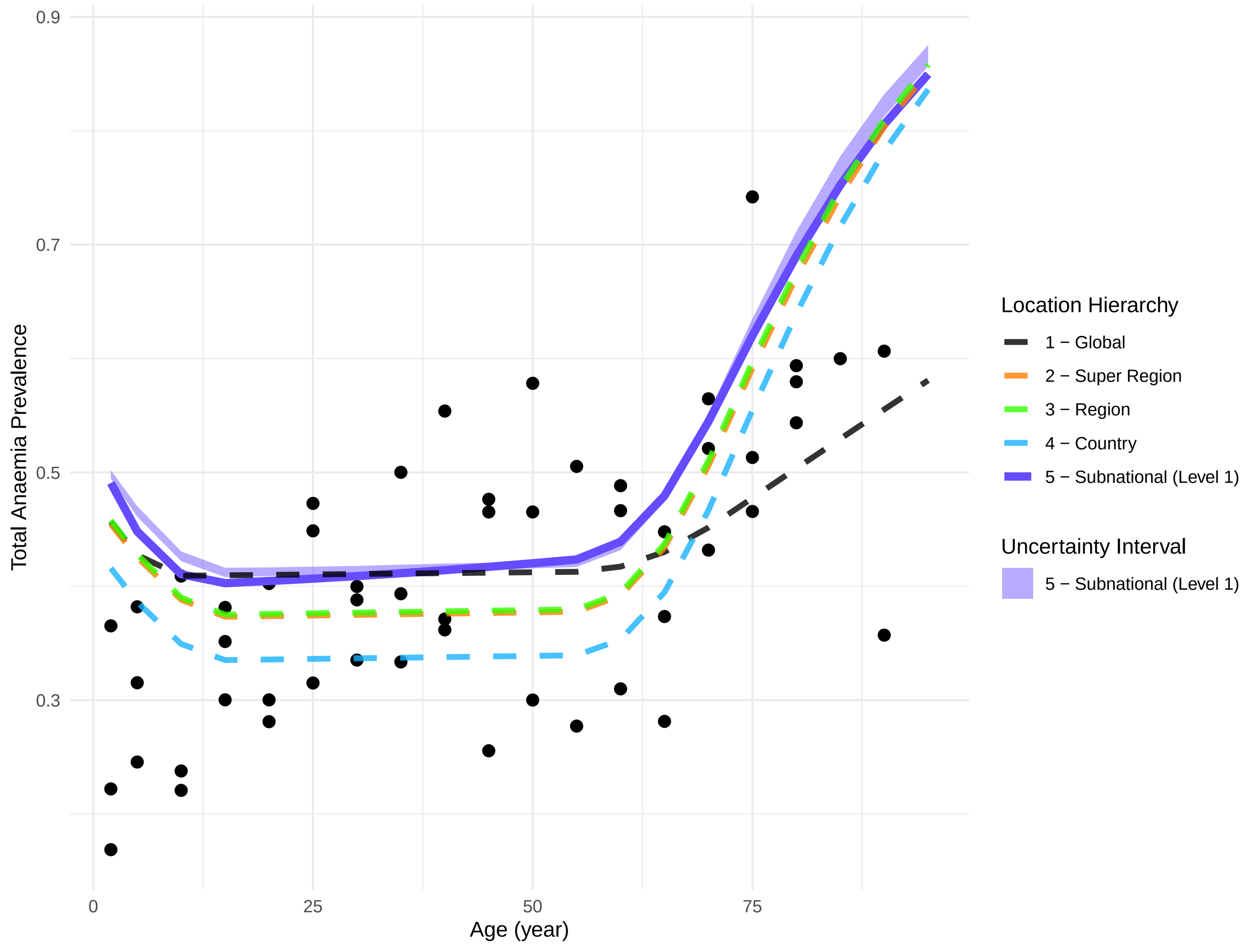

MR−BRT Age/Sex Fits − Non−pregnant Females 2+ Years Old − Total Anaemia Prevalence − Myanmar
WHO−2001 Adjustment Method

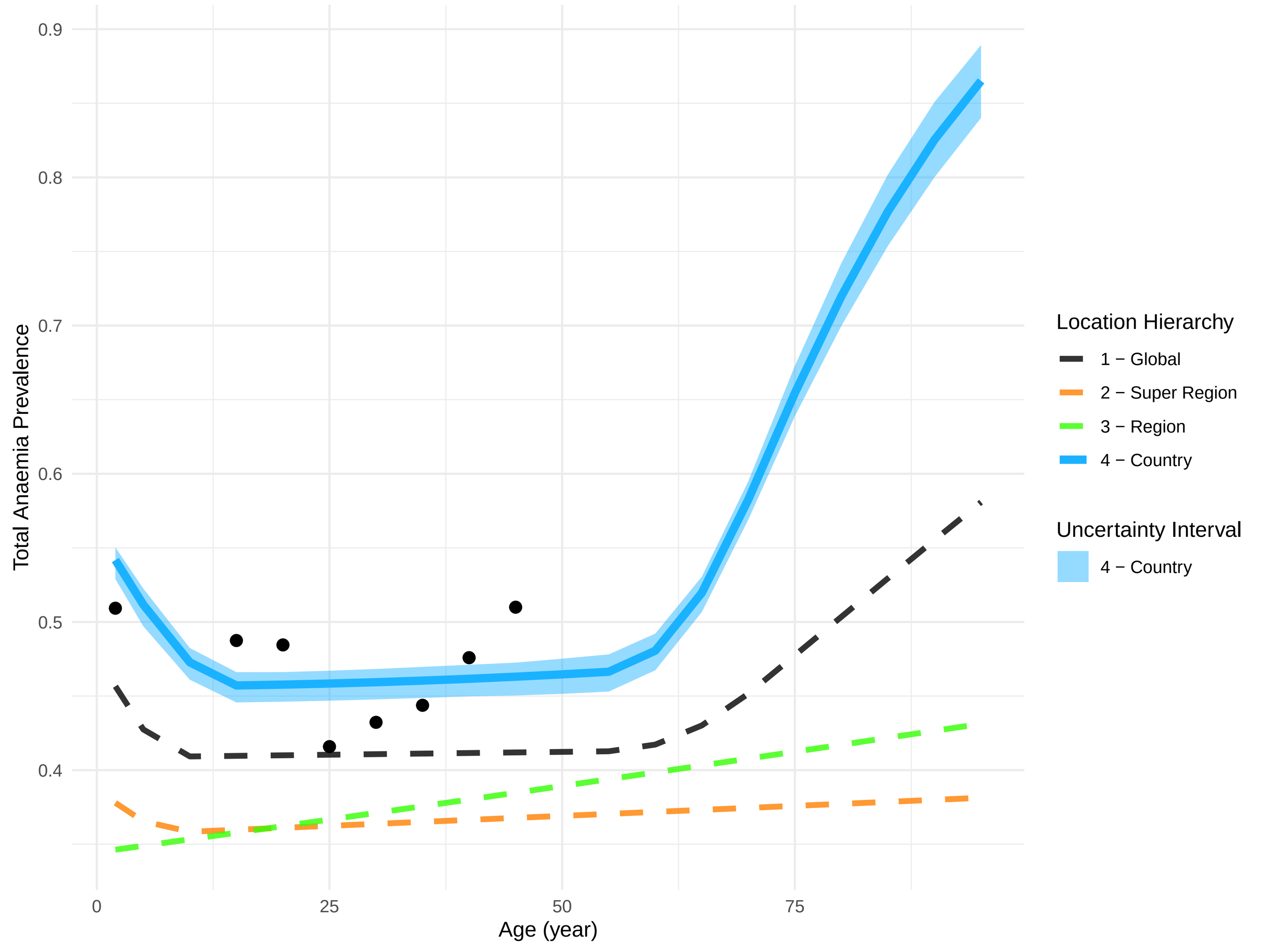

MR−BRT Age/Sex Fits − Non−pregnant Females 2+ Years Old − Total Anaemia Prevalence − Timor−Leste
WHO−2001 Adjustment Method

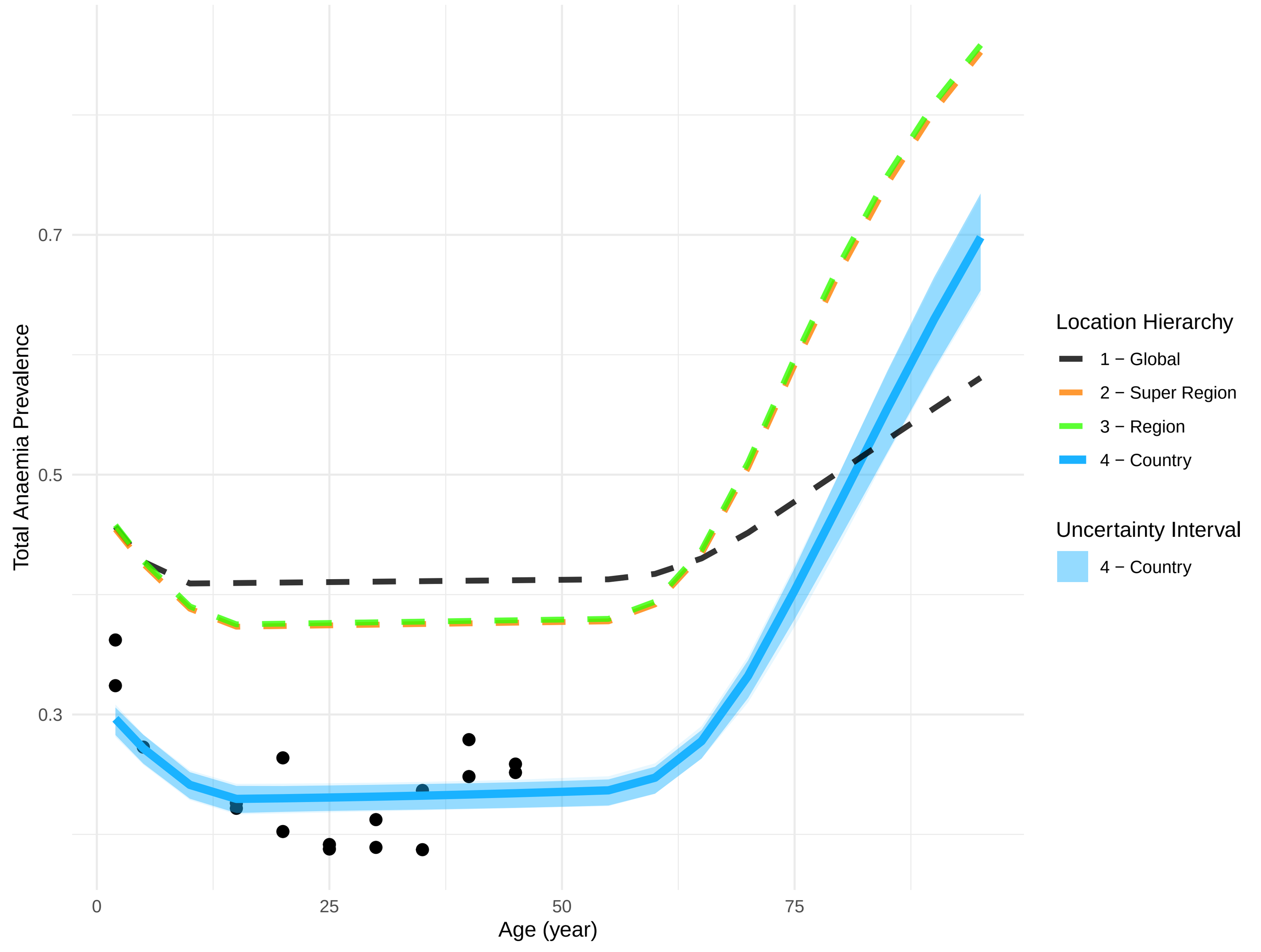

MR−BRT Age/Sex Fits − Non−pregnant Females 2+ Years Old − Total Anaemia Prevalence − Sub−Saharan Africa
WHO−2001 Adjustment Method

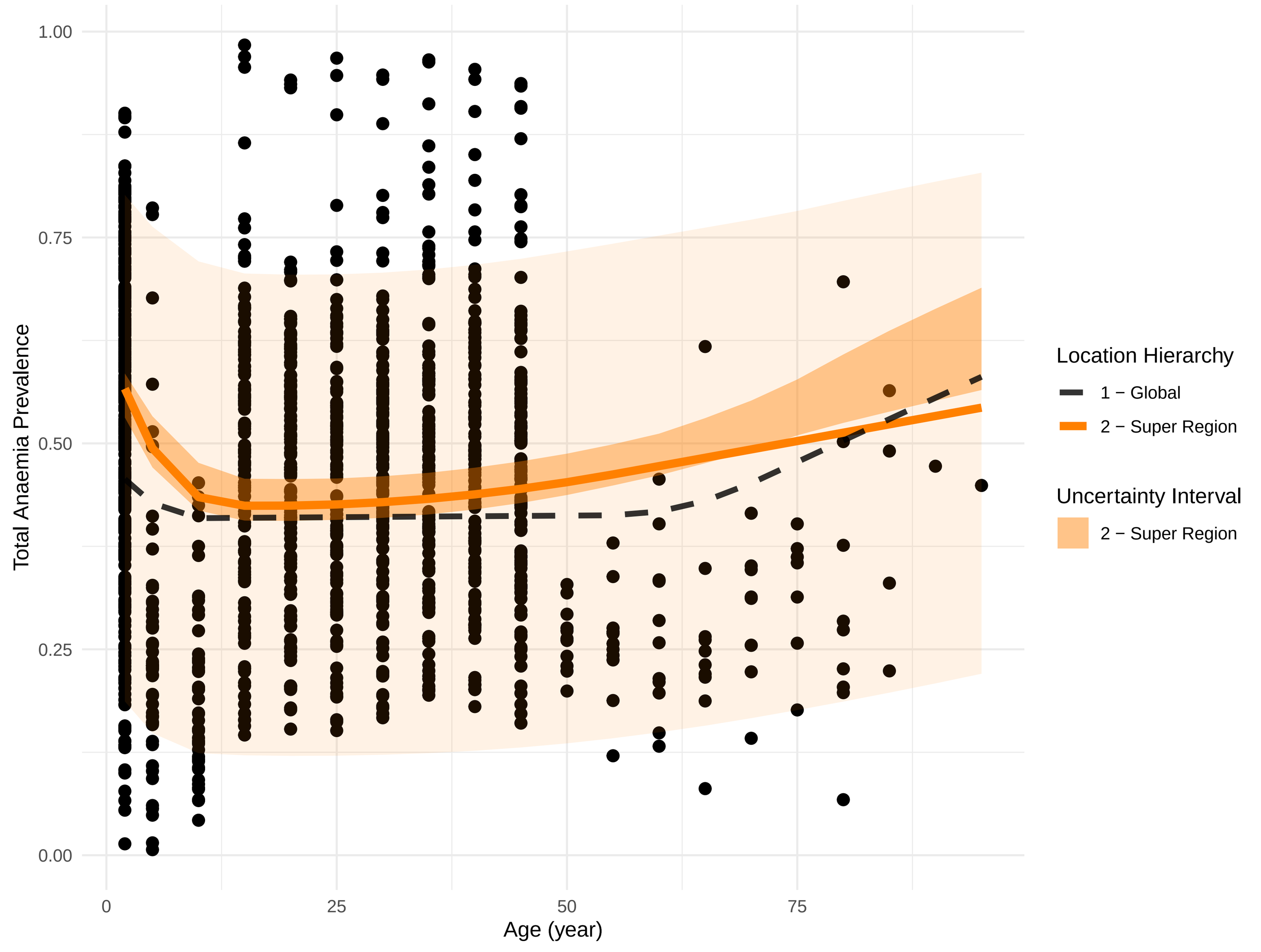

MR−BRT Age/Sex Fits − Non−pregnant Females 2+ Years Old − Total Anaemia Prevalence − Central Sub−Saharan Africa
WHO−2001 Adjustment Method

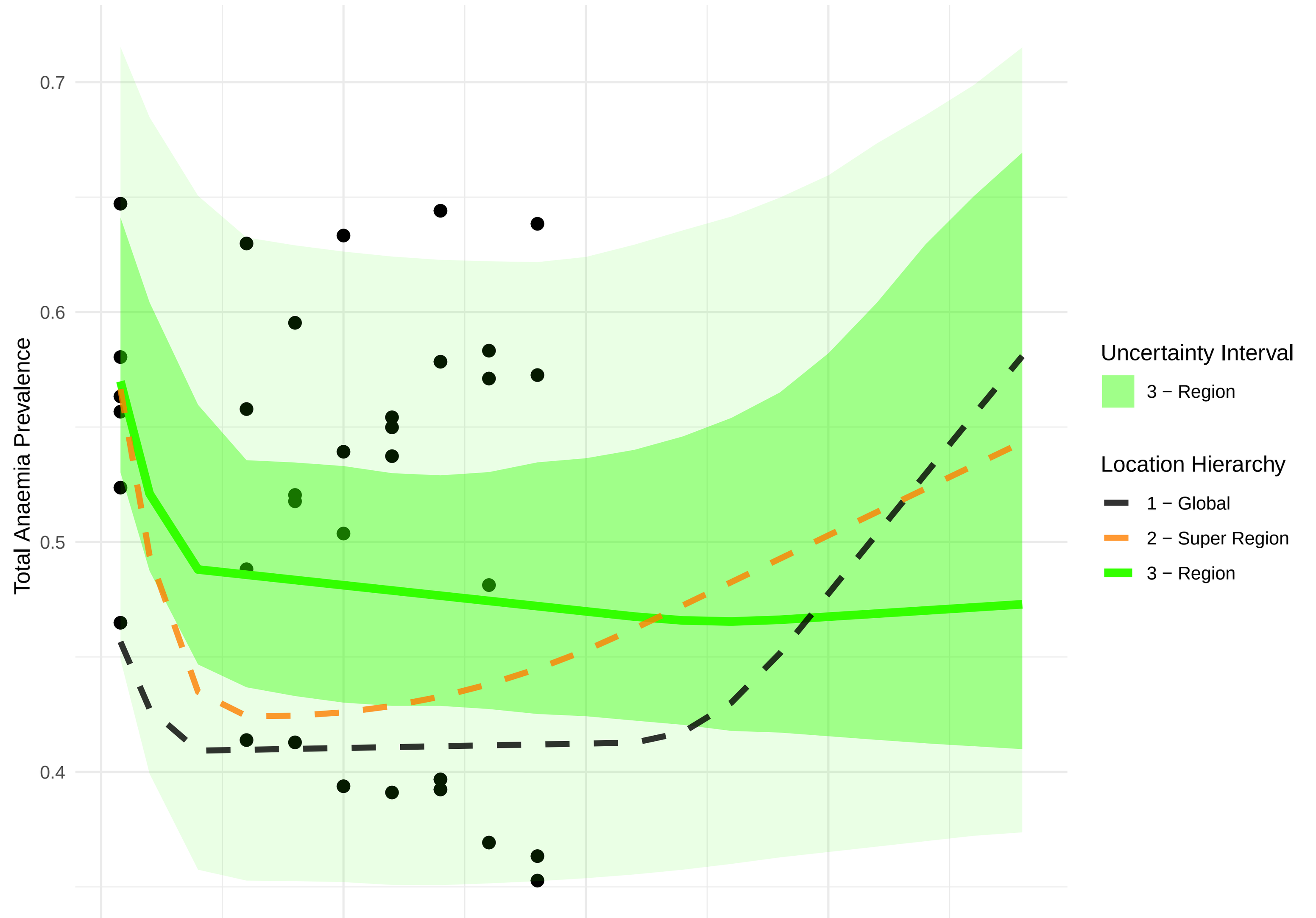

MR−BRT Age/Sex Fits − Non−pregnant Females 2+ Years Old − Total Anaemia Prevalence − Angola
WHO−2001 Adjustment Method

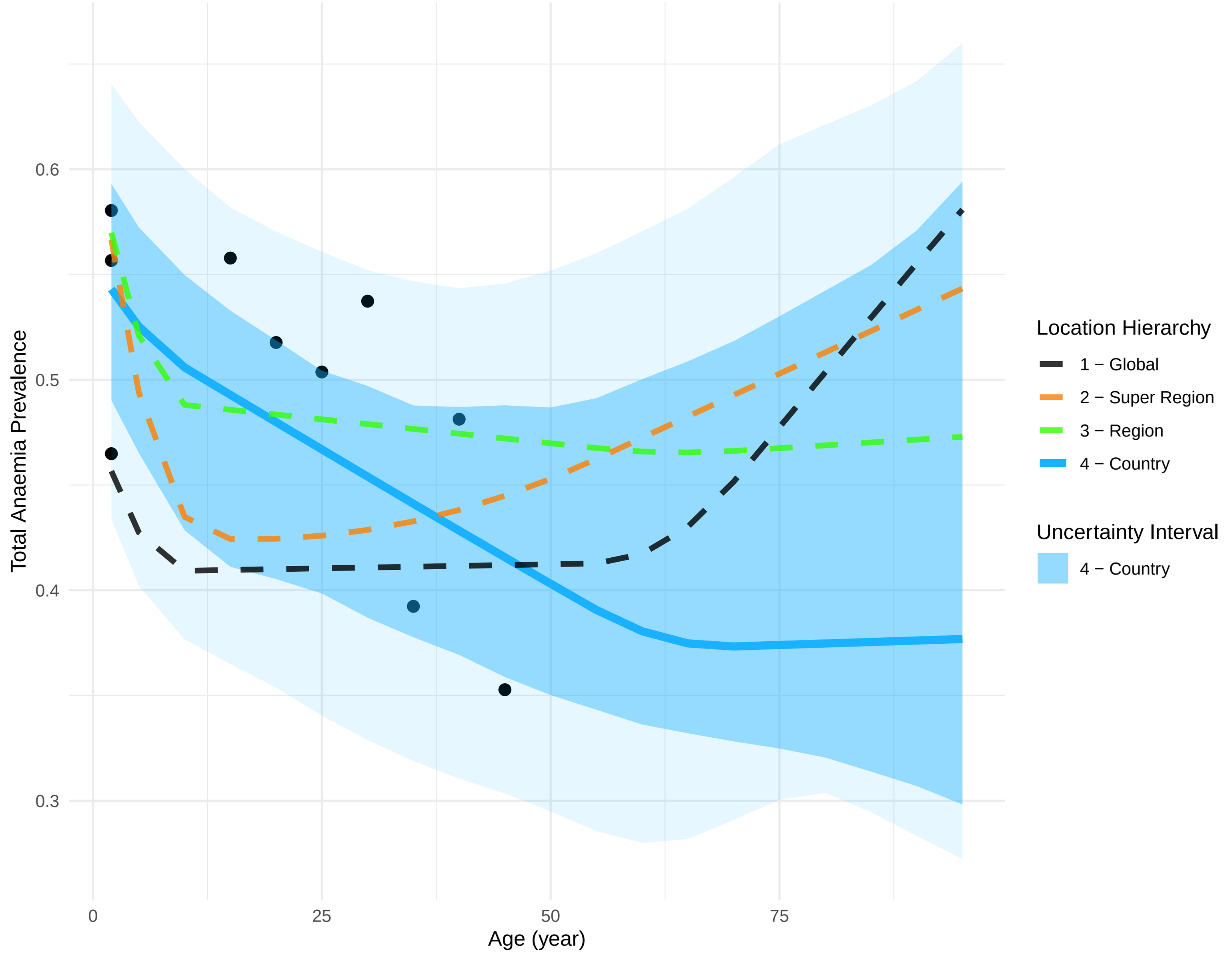

MR−BRT Age/Sex Fits − Non−pregnant Females 2+ Years Old − Total Anaemia Prevalence − Democratic Republic of the Congo
WHO−2001 Adjustment Method

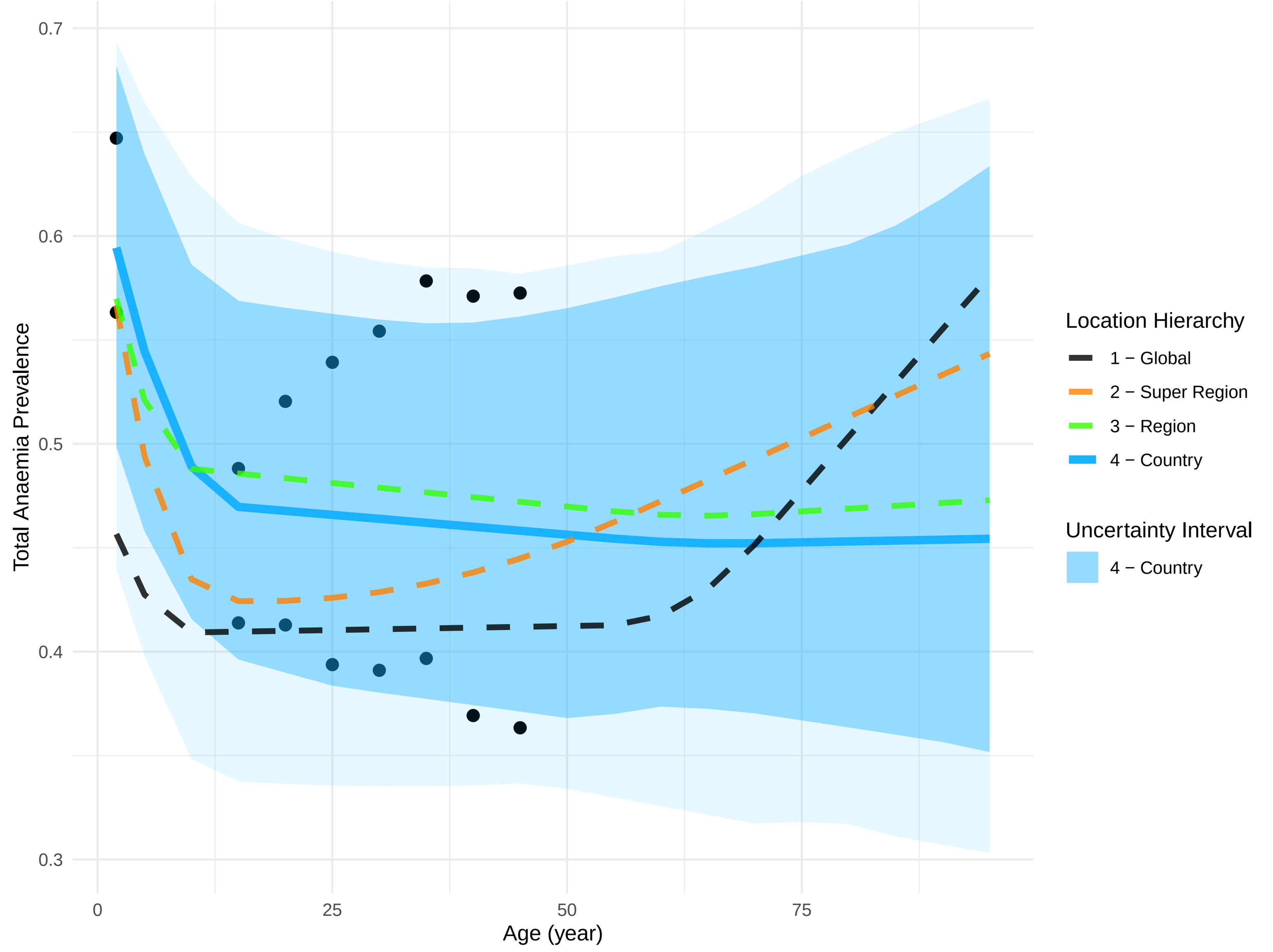

MR−BRT Age/Sex Fits − Non−pregnant Females 2+ Years Old − Total Anaemia Prevalence − Gabon
WHO−2001 Adjustment Method

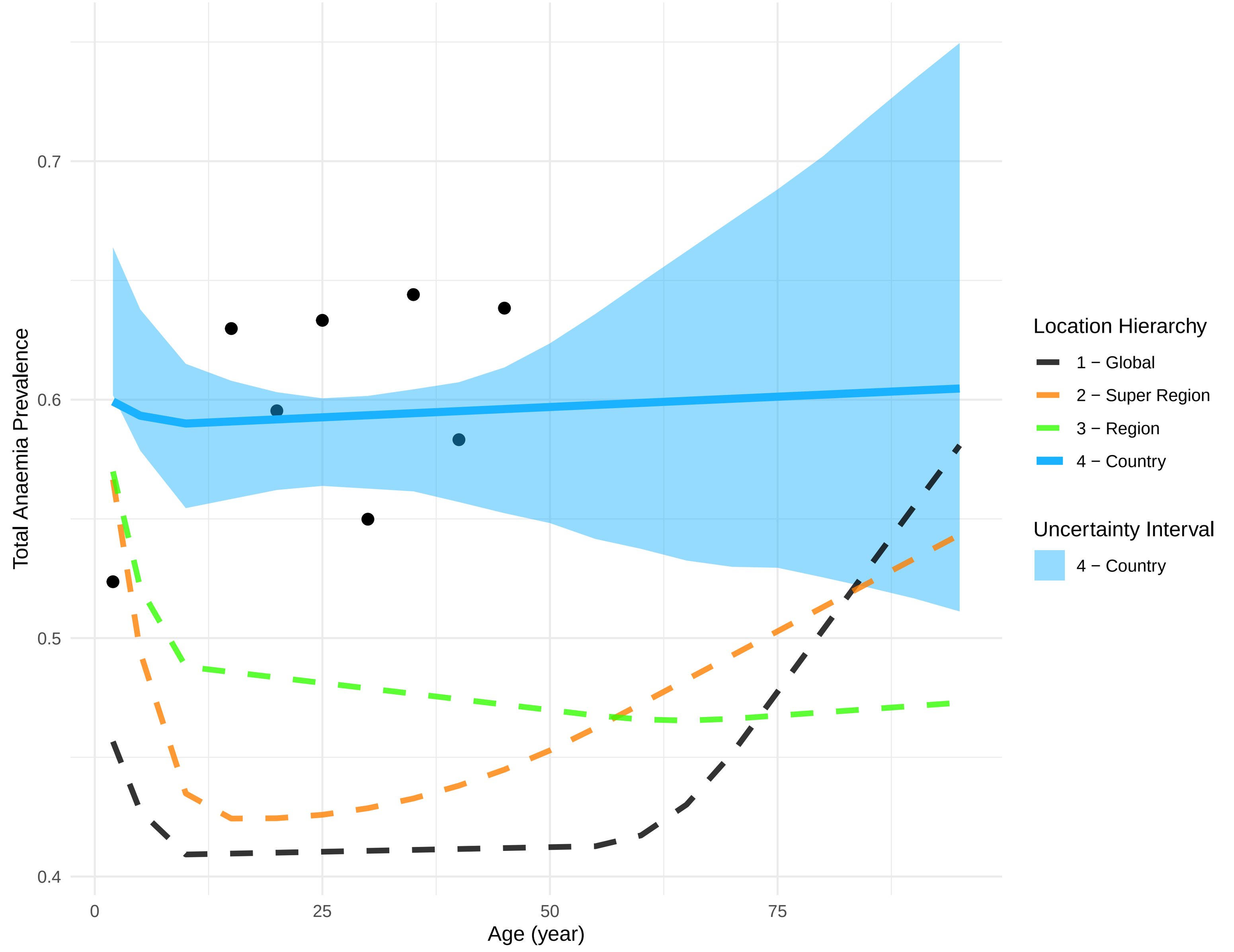

MR−BRT Age/Sex Fits − Non−pregnant Females 2+ Years Old − Total Anaemia Prevalence − Eastern Sub−Saharan Africa
WHO−2001 Adjustment Method

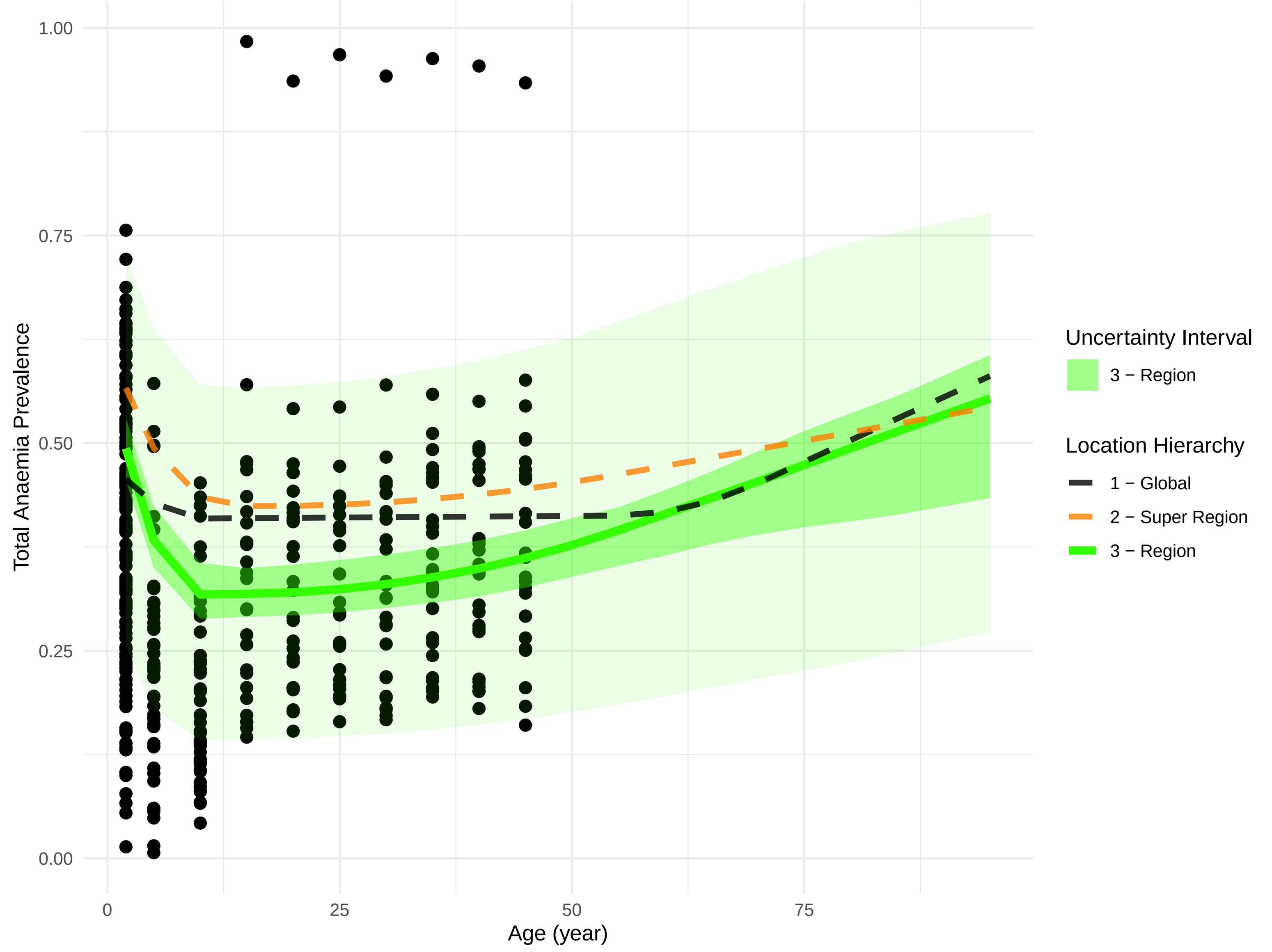

MR−BRT Age/Sex Fits − Non−pregnant Females 2+ Years Old − Total Anaemia Prevalence − Burundi
WHO−2001 Adjustment Method

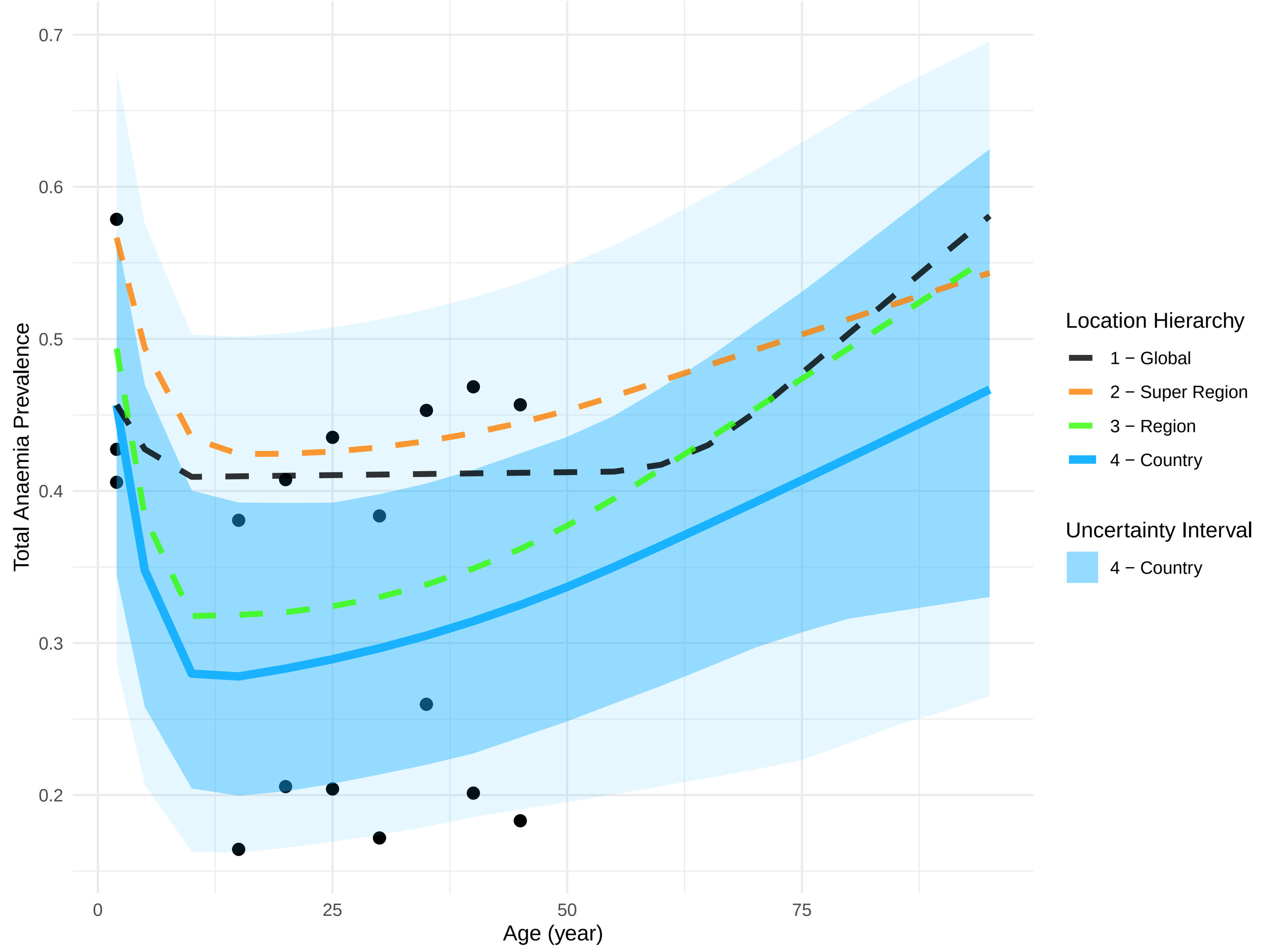

MR−BRT Age/Sex Fits − Non−pregnant Females 2+ Years Old − Total Anaemia Prevalence − Ethiopia
WHO−2001 Adjustment Method

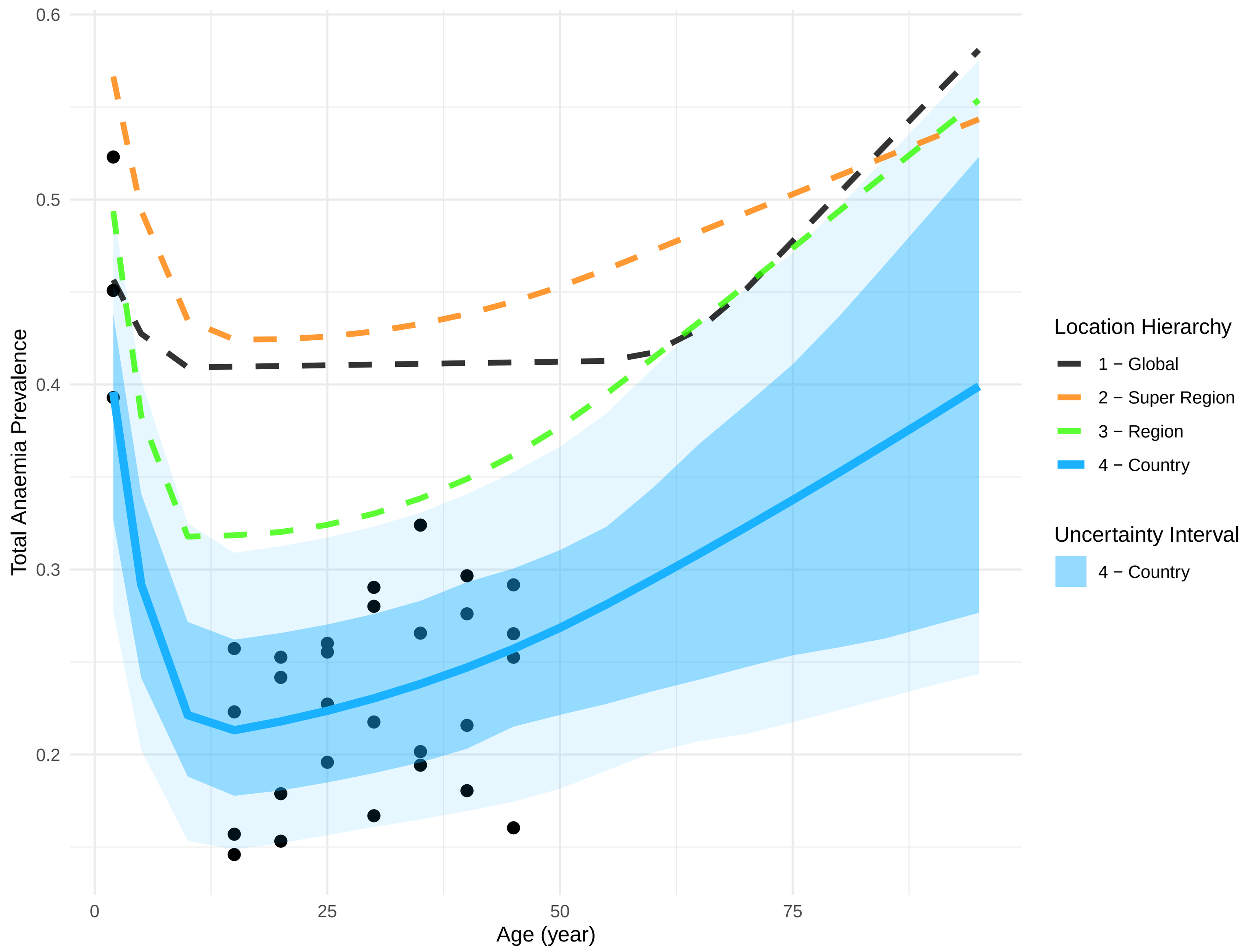

MR−BRT Age/Sex Fits − Non−pregnant Females 2+ Years Old − Total Anaemia Prevalence − Kenya
WHO−2001 Adjustment Method

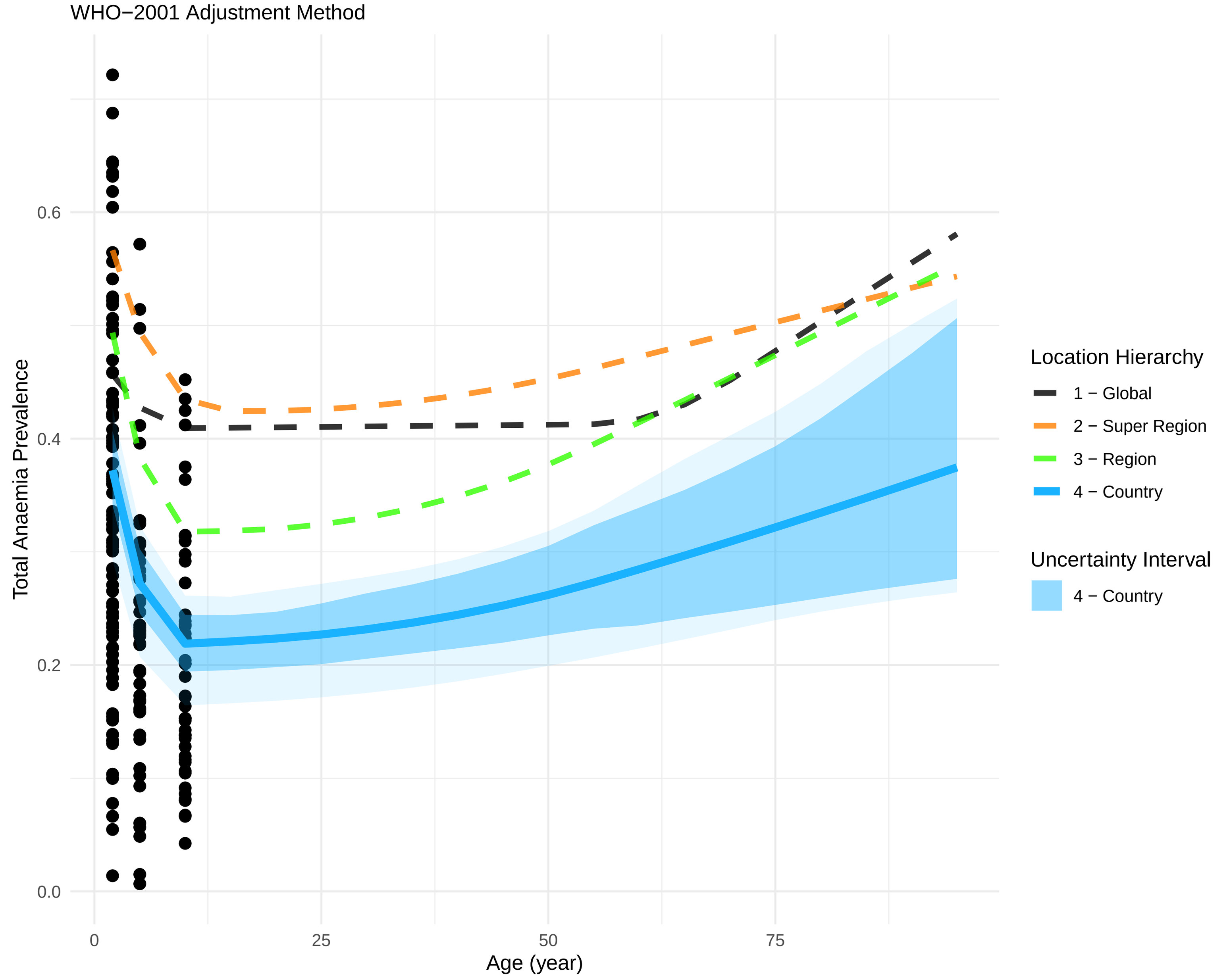

MR−BRT Age/Sex Fits − Non−pregnant Females 2+ Years Old − Total Anaemia Prevalence − Baringo
WHO−2001 Adjustment Method

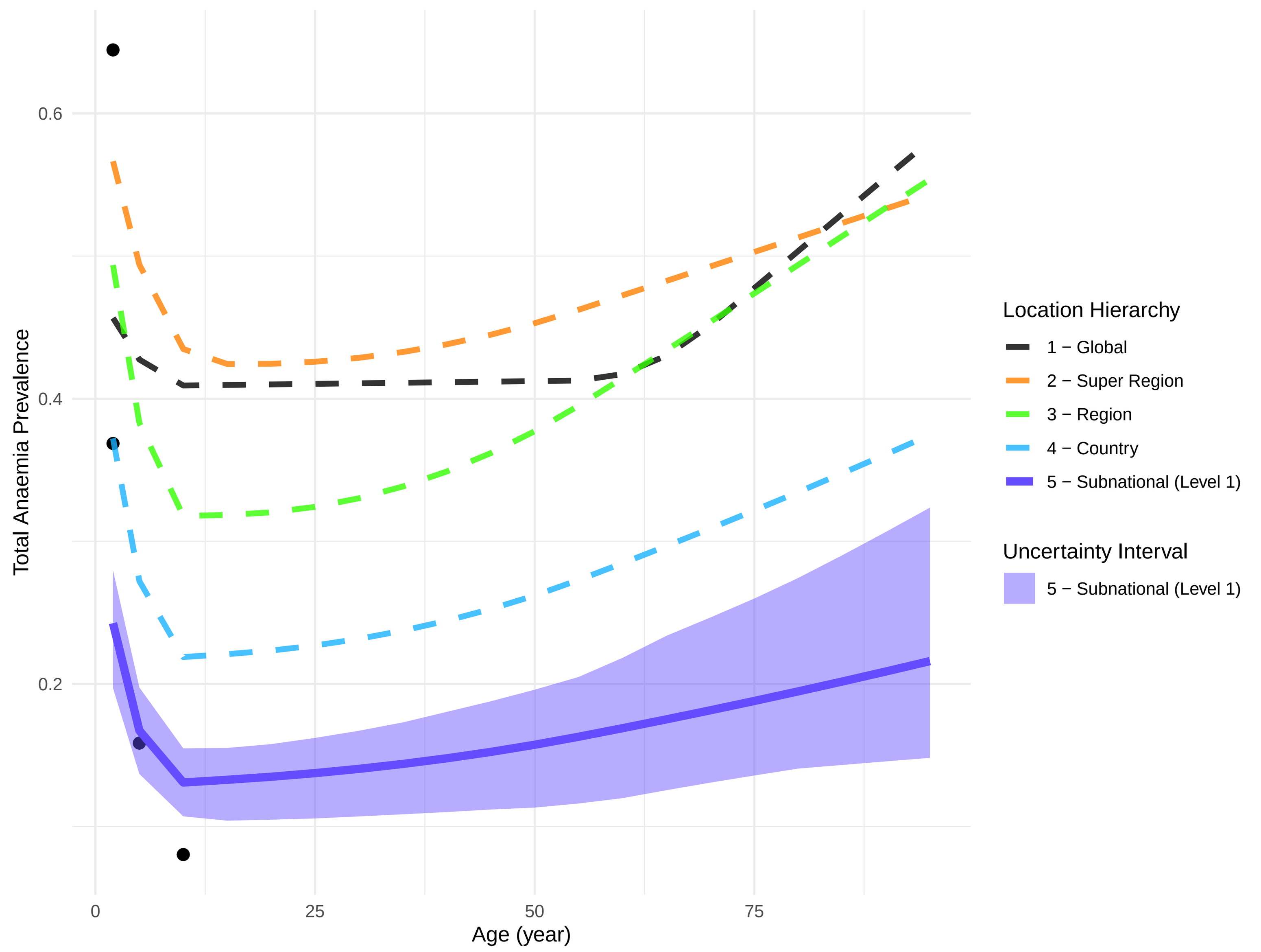

MR−BRT Age/Sex Fits − Non−pregnant Females 2+ Years Old − Total Anaemia Prevalence − Bomet
WHO−2001 Adjustment Method

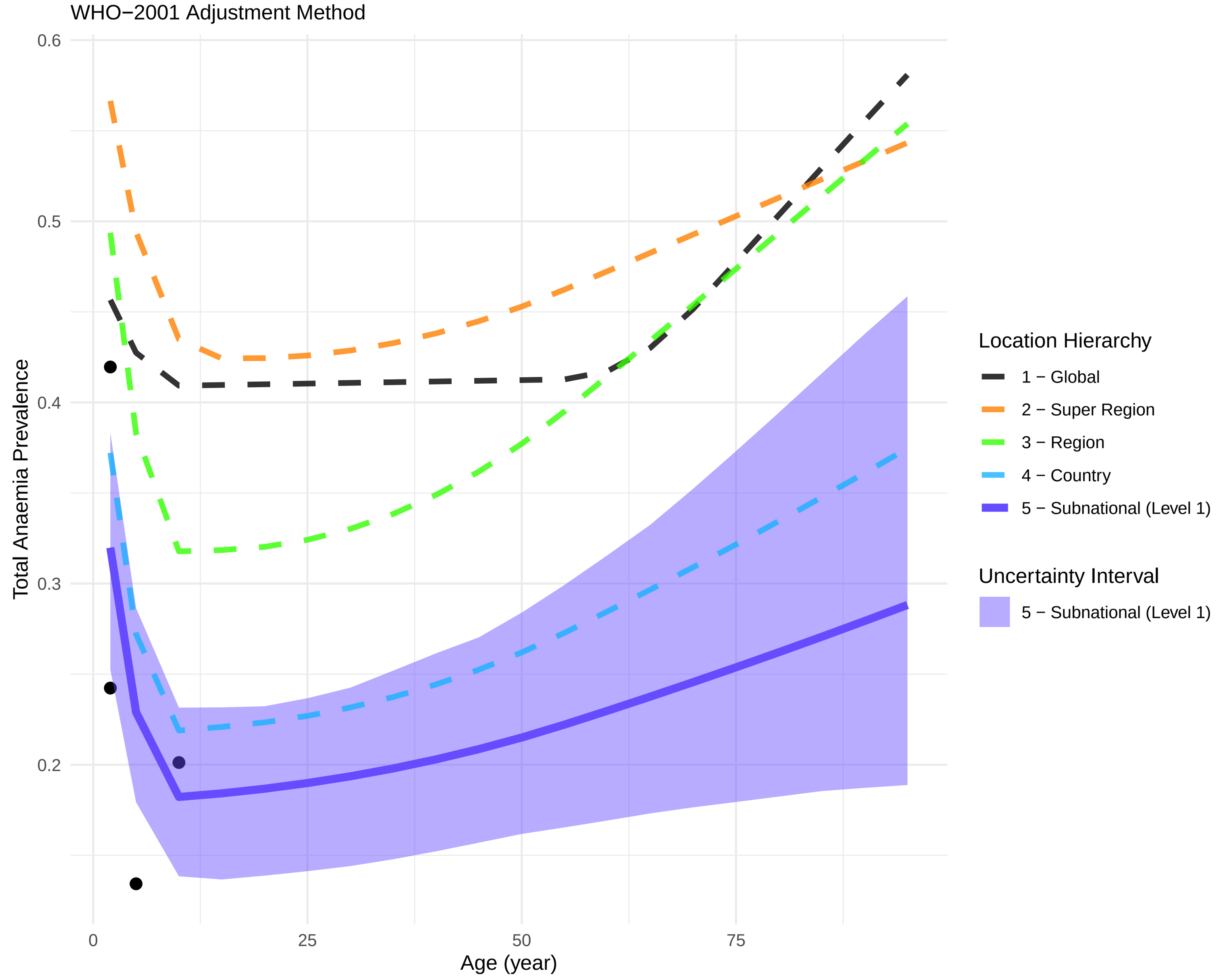

MR−BRT Age/Sex Fits − Non−pregnant Females 2+ Years Old − Total Anaemia Prevalence − Bungoma
WHO−2001 Adjustment Method

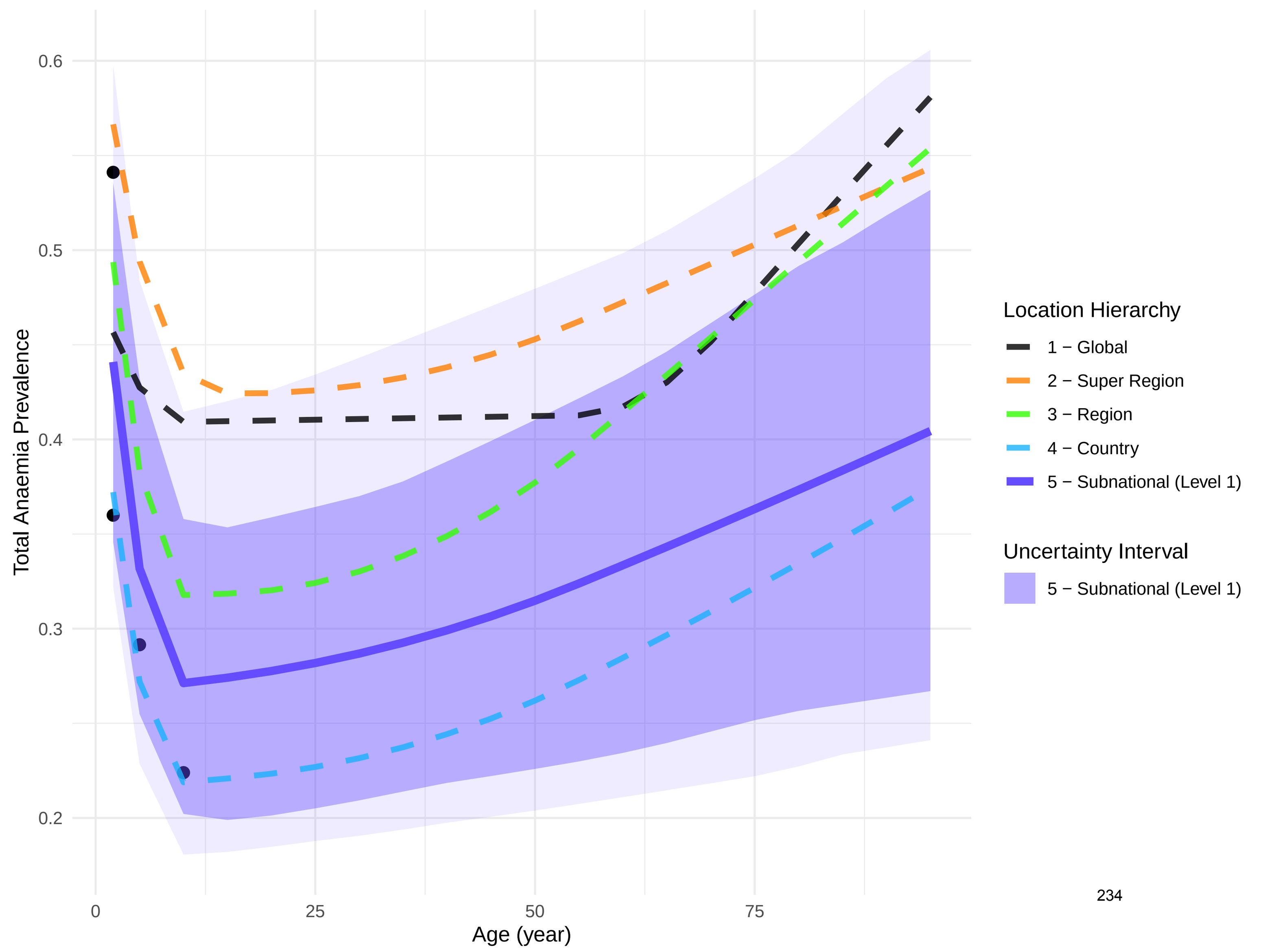

MR−BRT Age/Sex Fits − Non−pregnant Females 2+ Years Old − Total Anaemia Prevalence − Busia
WHO−2001 Adjustment Method

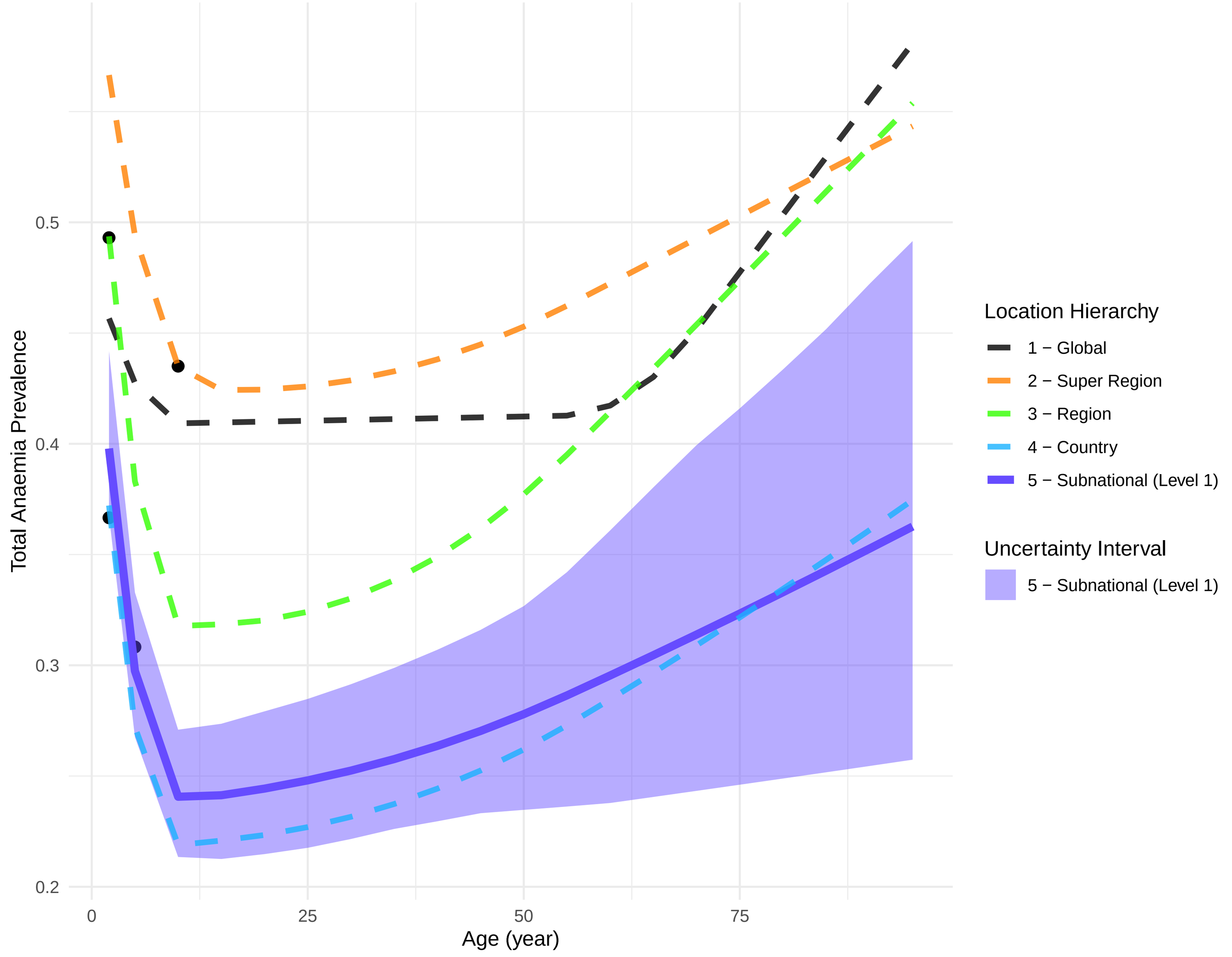

MR−BRT Age/Sex Fits − Non−pregnant Females 2+ Years Old − Total Anaemia Prevalence − Elgeyo−Marakwet
WHO−2001 Adjustment Method

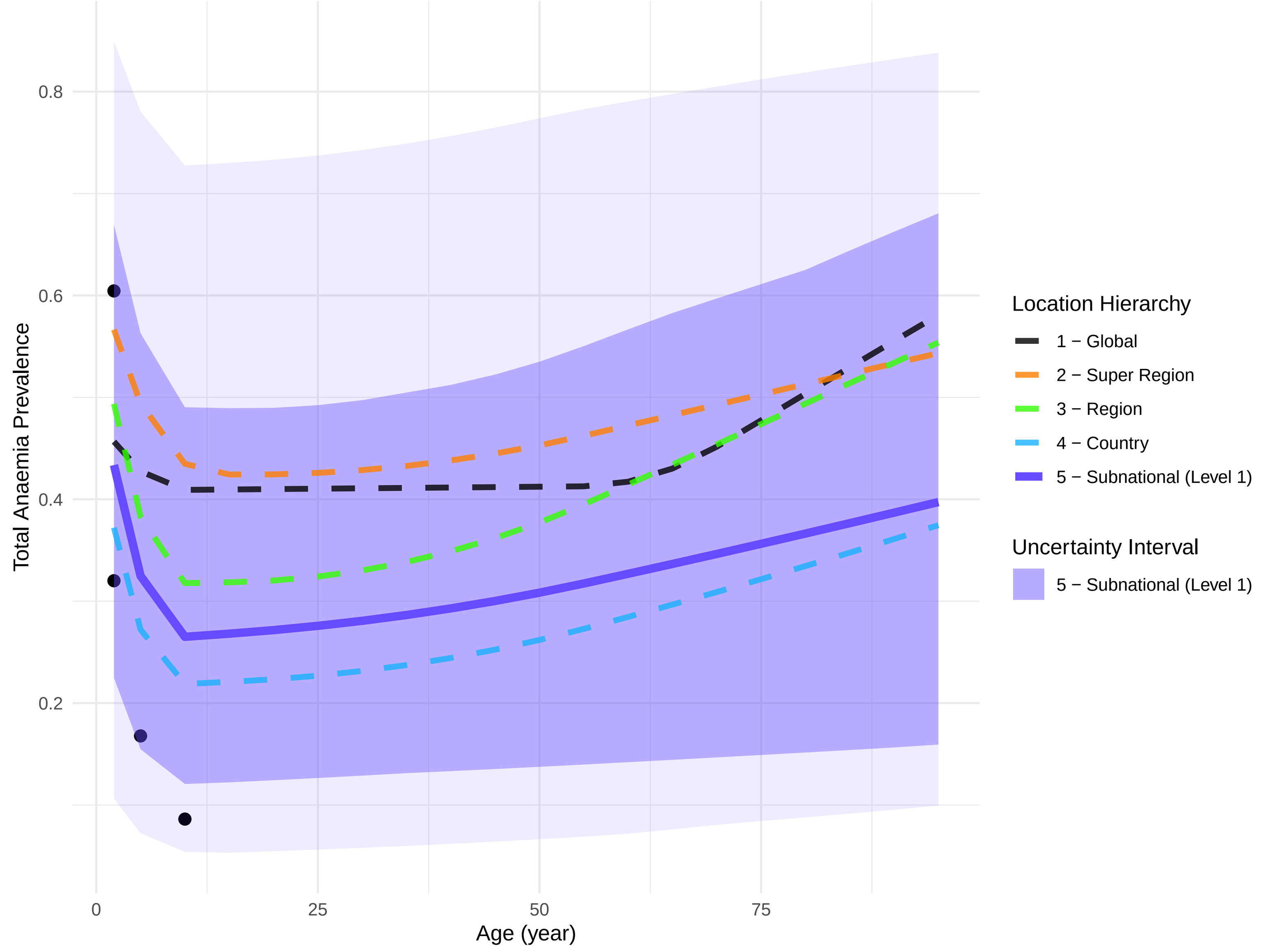

MR−BRT Age/Sex Fits − Non−pregnant Females 2+ Years Old − Total Anaemia Prevalence − Embu
WHO−2001 Adjustment Method

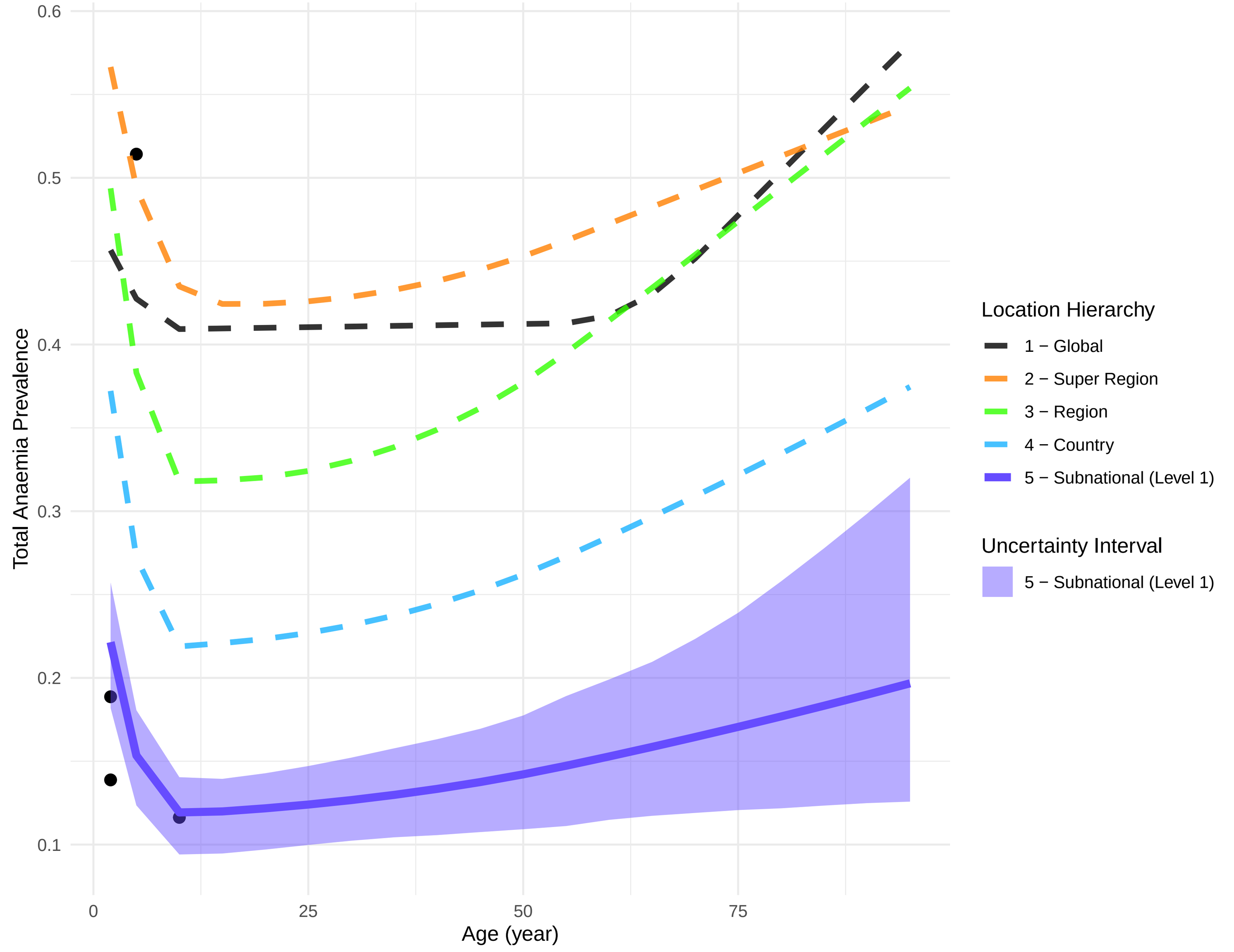

MR−BRT Age/Sex Fits − Non−pregnant Females 2+ Years Old − Total Anaemia Prevalence − Garissa
WHO−2001 Adjustment Method

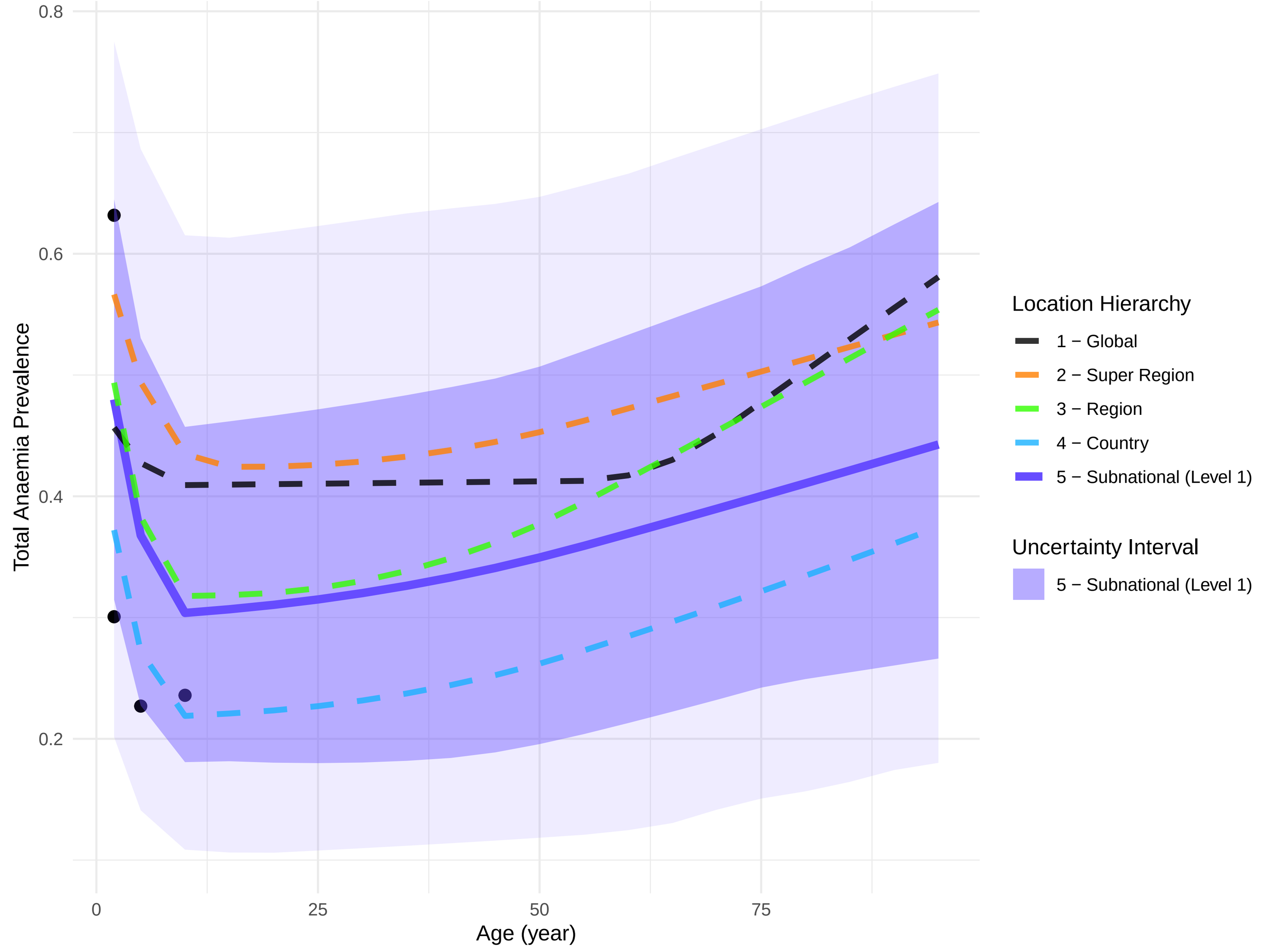

MR−BRT Age/Sex Fits − Non−pregnant Females 2+ Years Old − Total Anaemia Prevalence − Homa Bay
WHO−2001 Adjustment Method

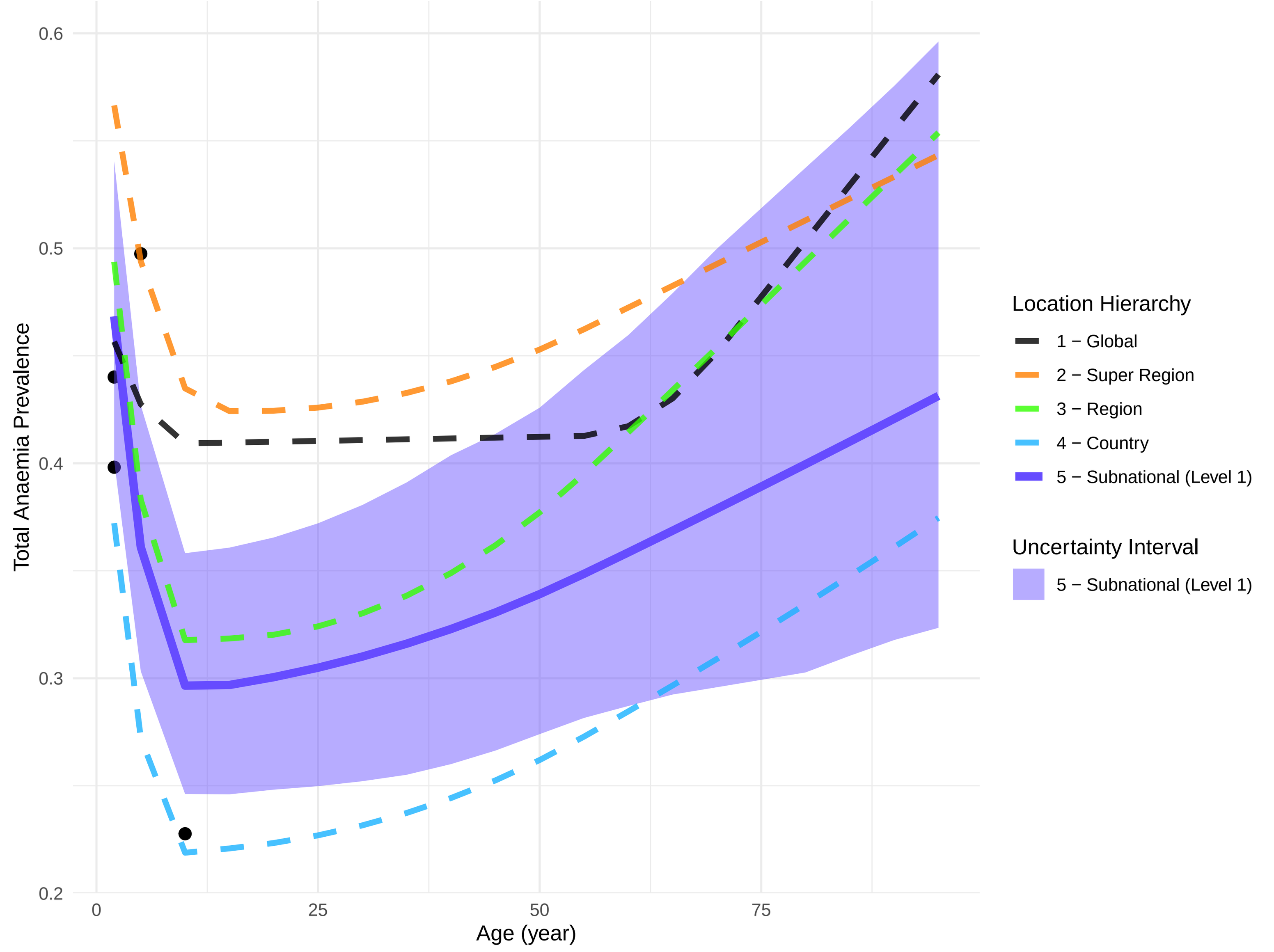

MR−BRT Age/Sex Fits − Non−pregnant Females 2+ Years Old − Total Anaemia Prevalence − Isiolo
WHO−2001 Adjustment Method

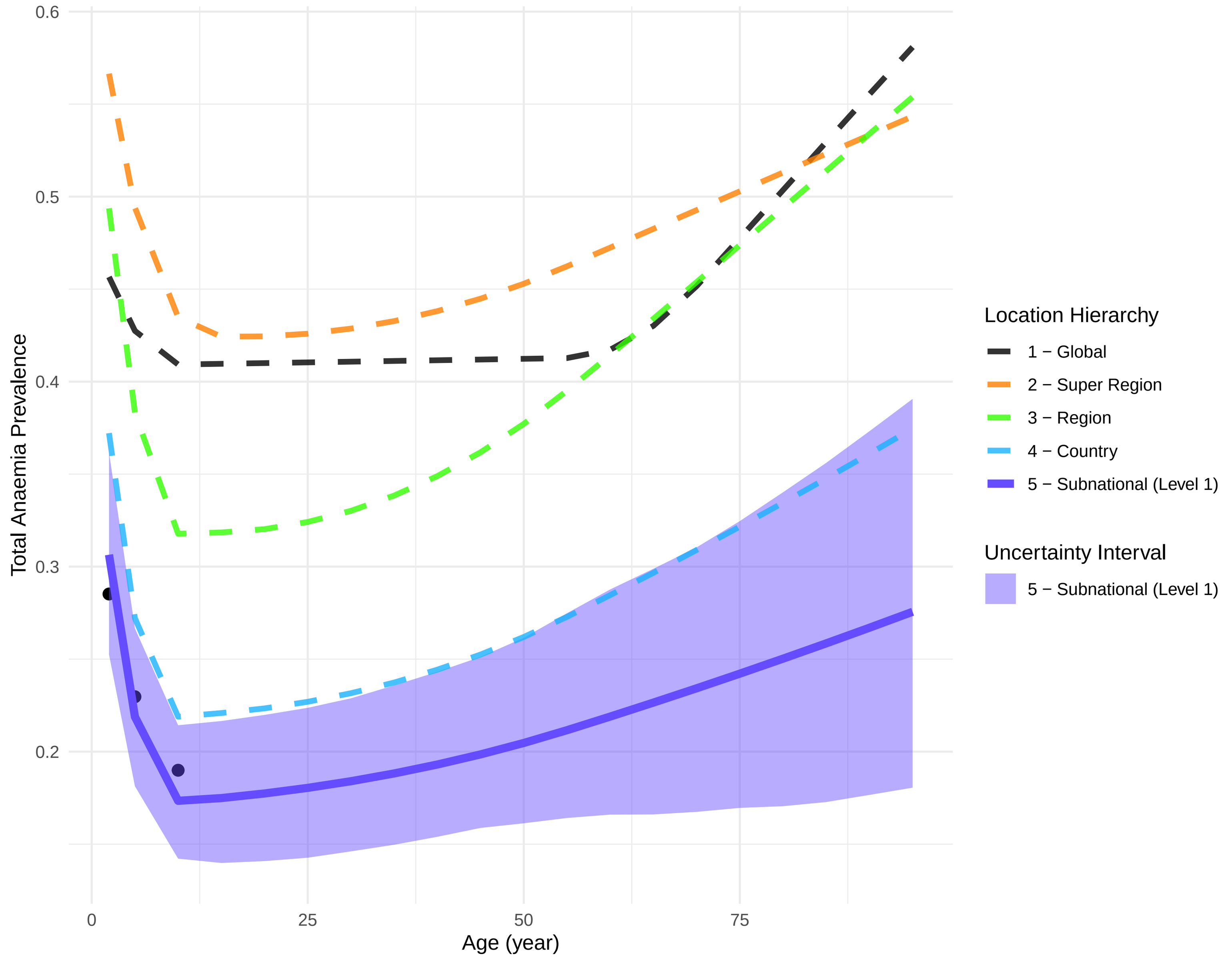

MR−BRT Age/Sex Fits − Non−pregnant Females 2+ Years Old − Total Anaemia Prevalence − Kajiado
WHO−2001 Adjustment Method

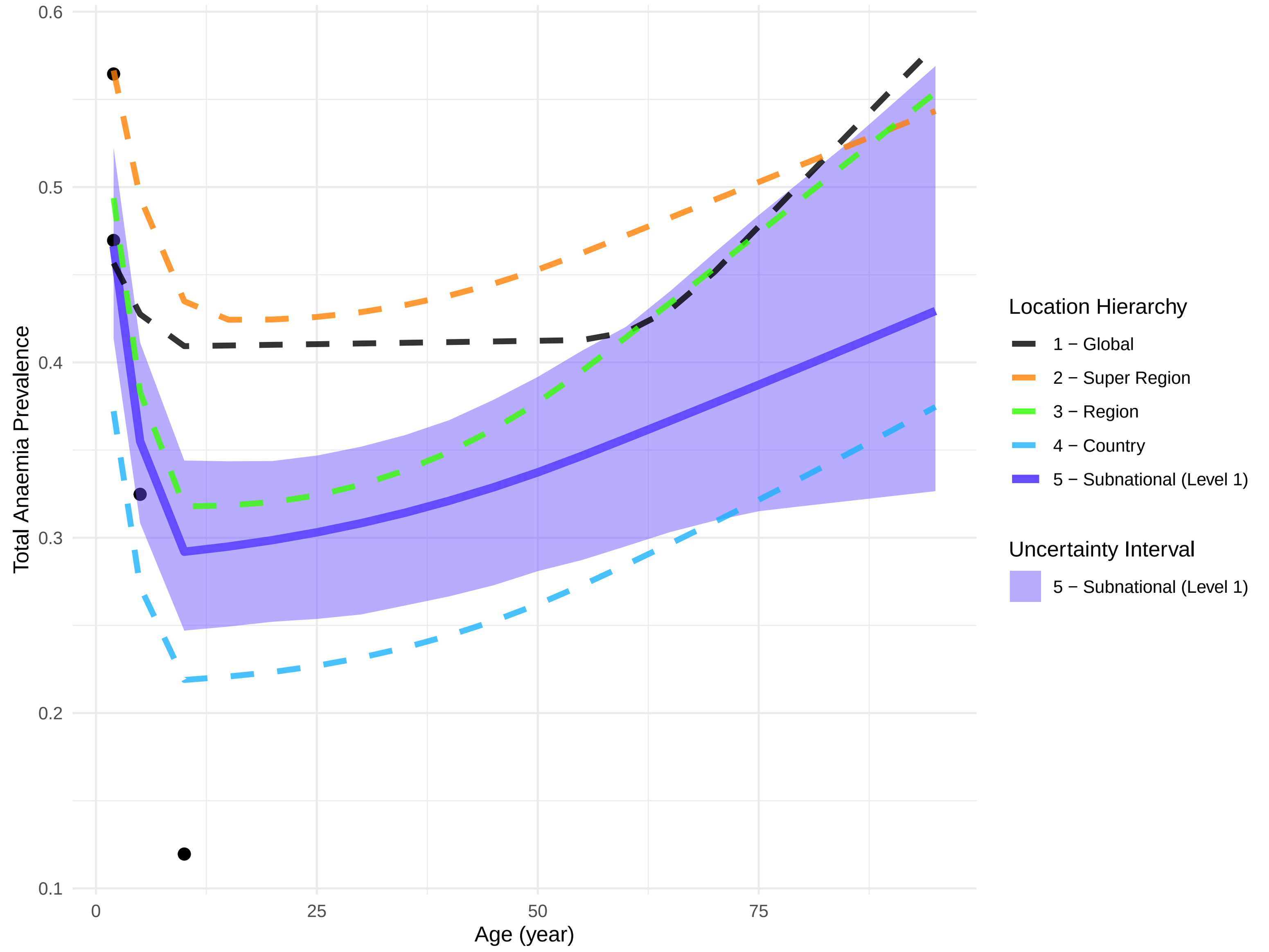

MR−BRT Age/Sex Fits − Non−pregnant Females 2+ Years Old − Total Anaemia Prevalence − Kakamega
WHO−2001 Adjustment Method

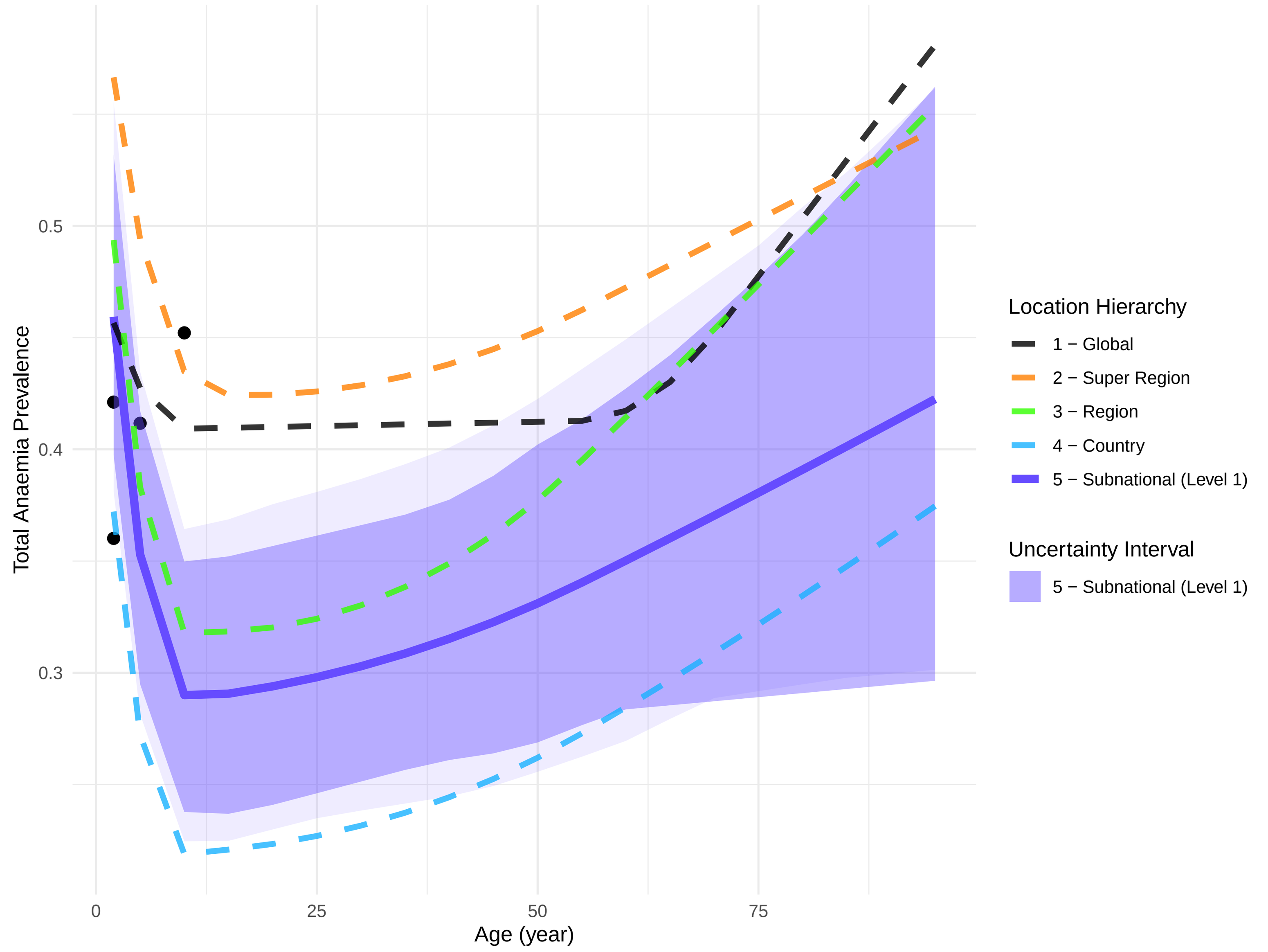

MR−BRT Age/Sex Fits − Non−pregnant Females 2+ Years Old − Total Anaemia Prevalence − Kericho
WHO−2001 Adjustment Method

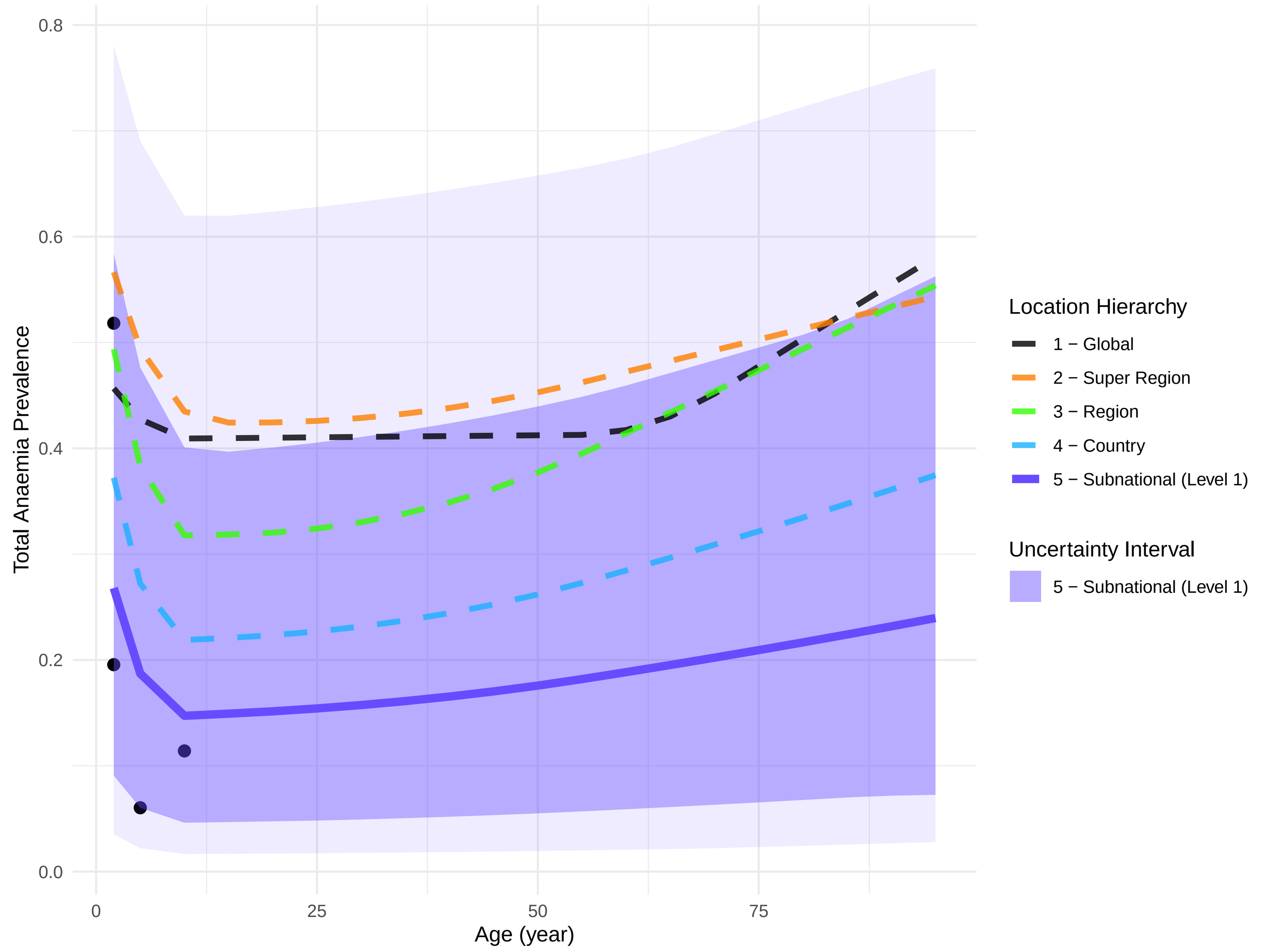

MR−BRT Age/Sex Fits − Non−pregnant Females 2+ Years Old − Total Anaemia Prevalence − Kiambu
WHO−2001 Adjustment Method

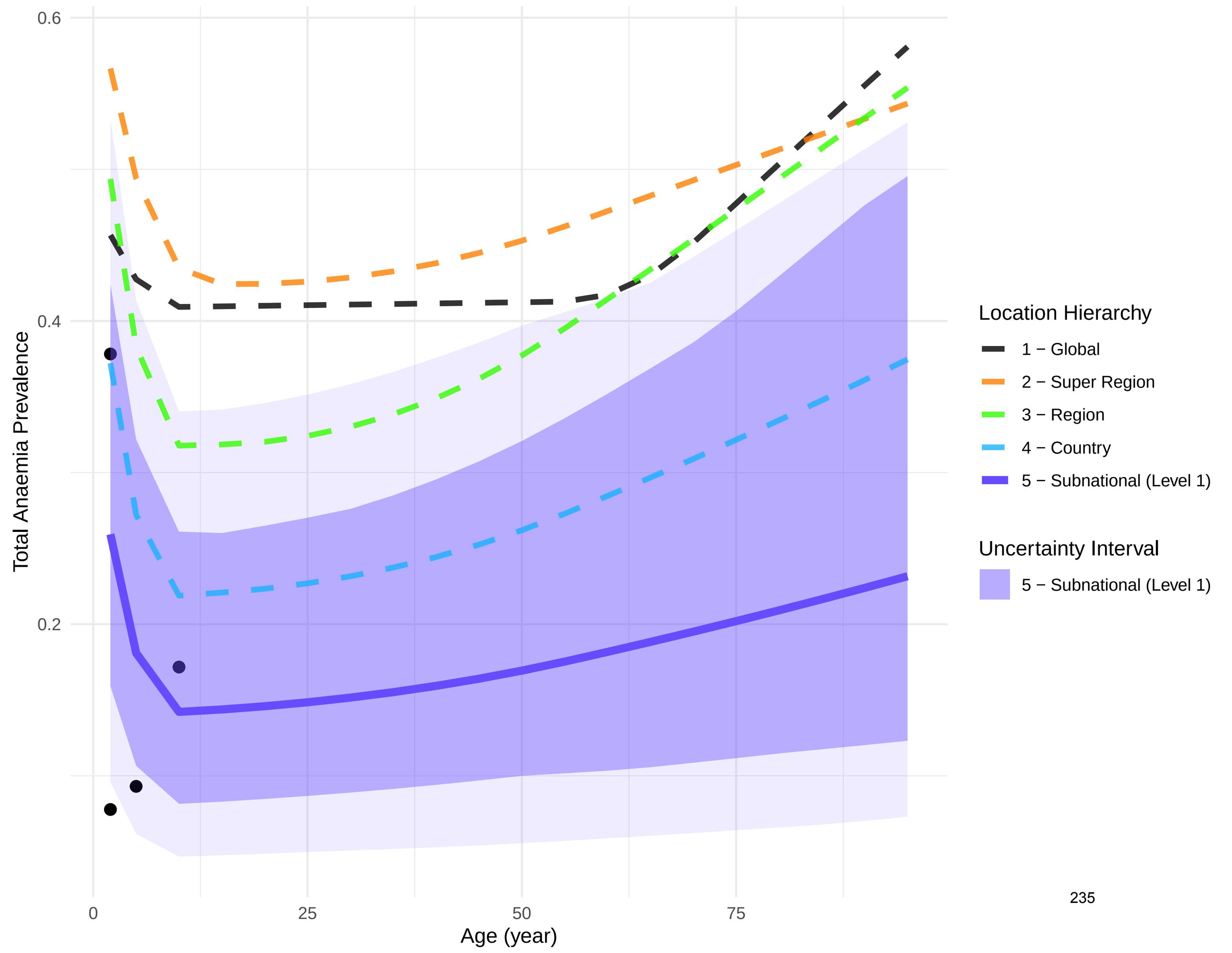

MR−BRT Age/Sex Fits − Non−pregnant Females 2+ Years Old − Total Anaemia Prevalence − Kilifi
WHO−2001 Adjustment Method

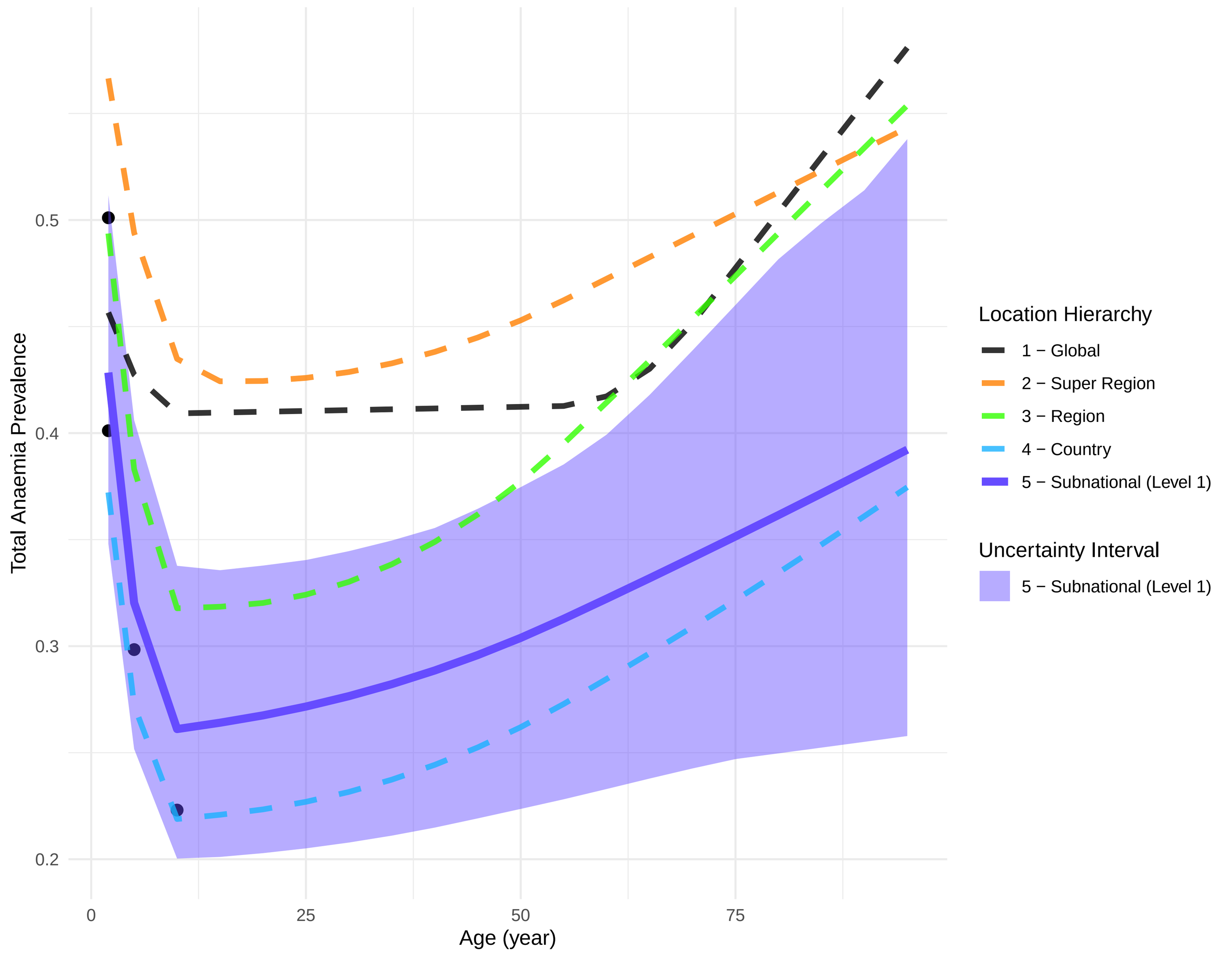

MR−BRT Age/Sex Fits − Non−pregnant Females 2+ Years Old − Total Anaemia Prevalence − Kirinyaga
WHO−2001 Adjustment Method

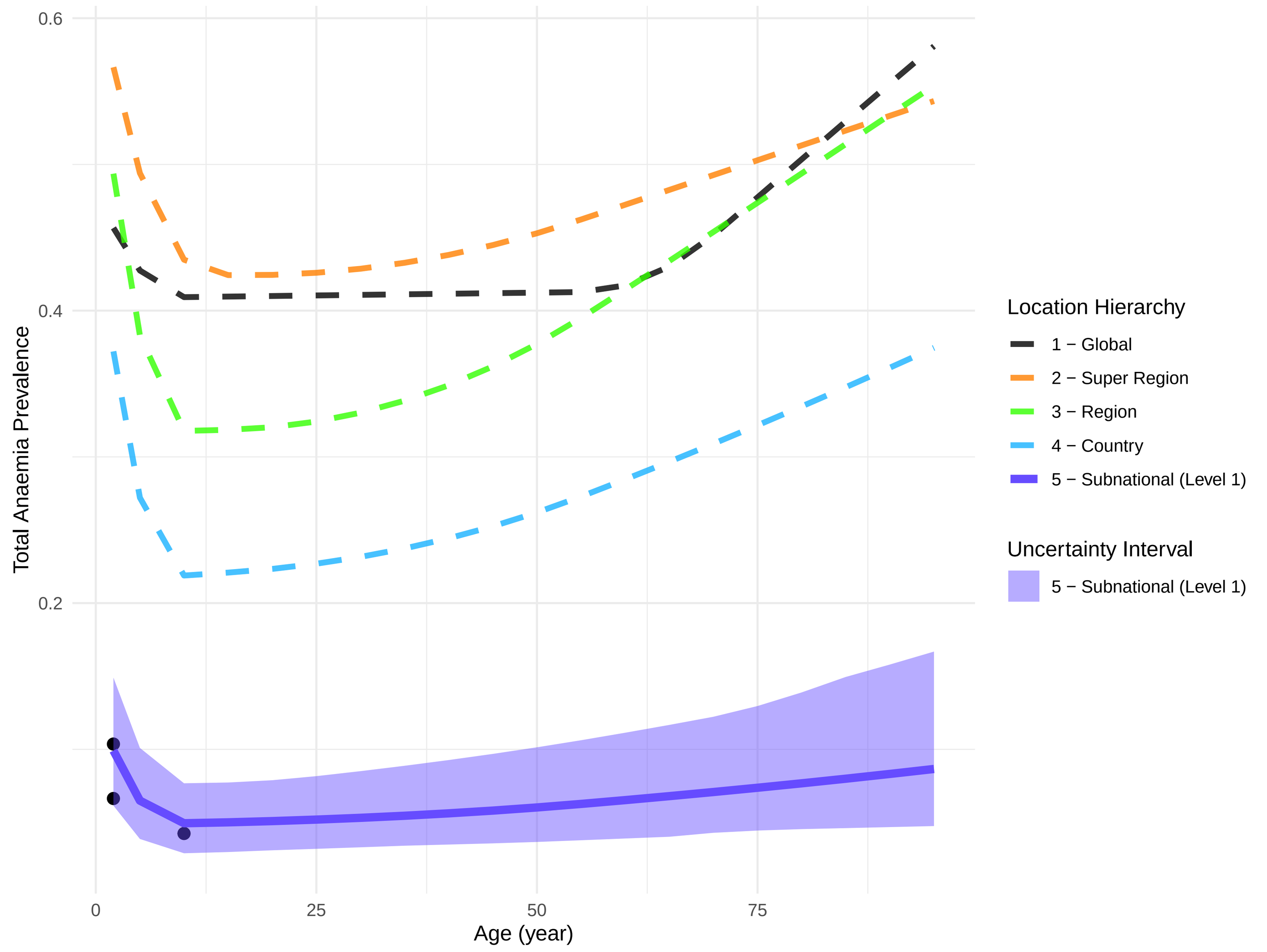

MR−BRT Age/Sex Fits − Non−pregnant Females 2+ Years Old − Total Anaemia Prevalence − Kisii
WHO−2001 Adjustment Method

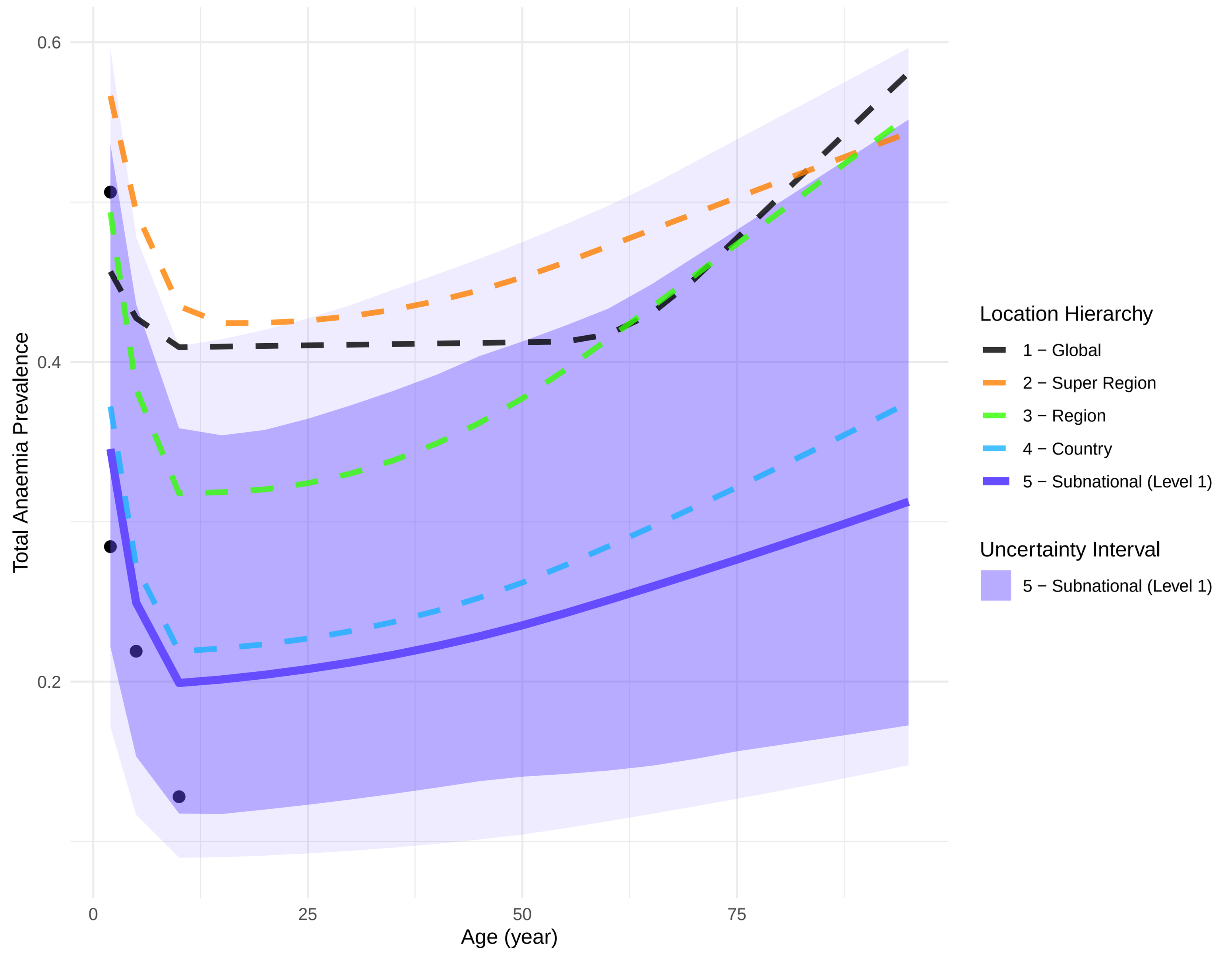

MR−BRT Age/Sex Fits − Non−pregnant Females 2+ Years Old − Total Anaemia Prevalence − Kisumu
WHO−2001 Adjustment Method

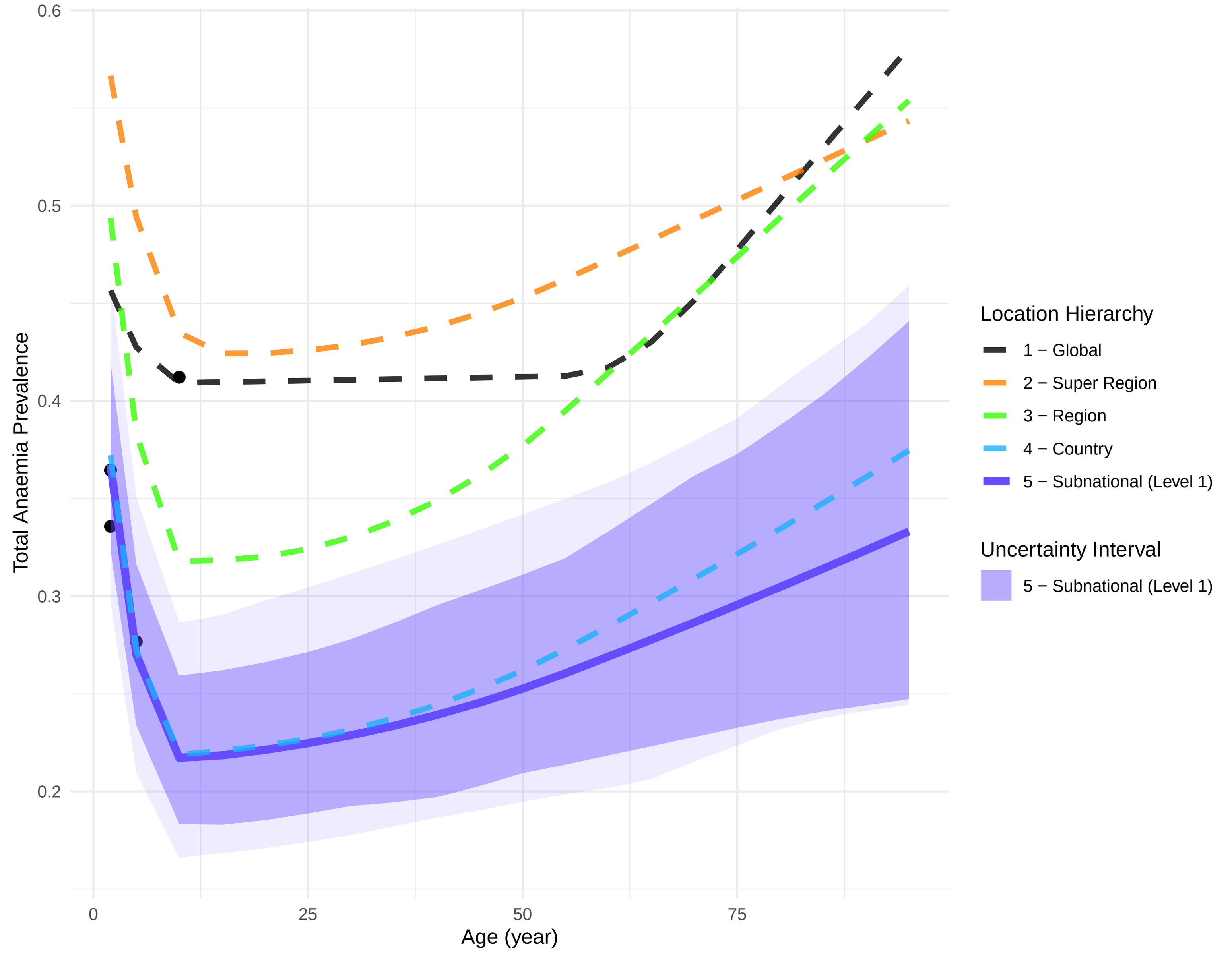

MR−BRT Age/Sex Fits − Non−pregnant Females 2+ Years Old − Total Anaemia Prevalence − Kitui
WHO−2001 Adjustment Method

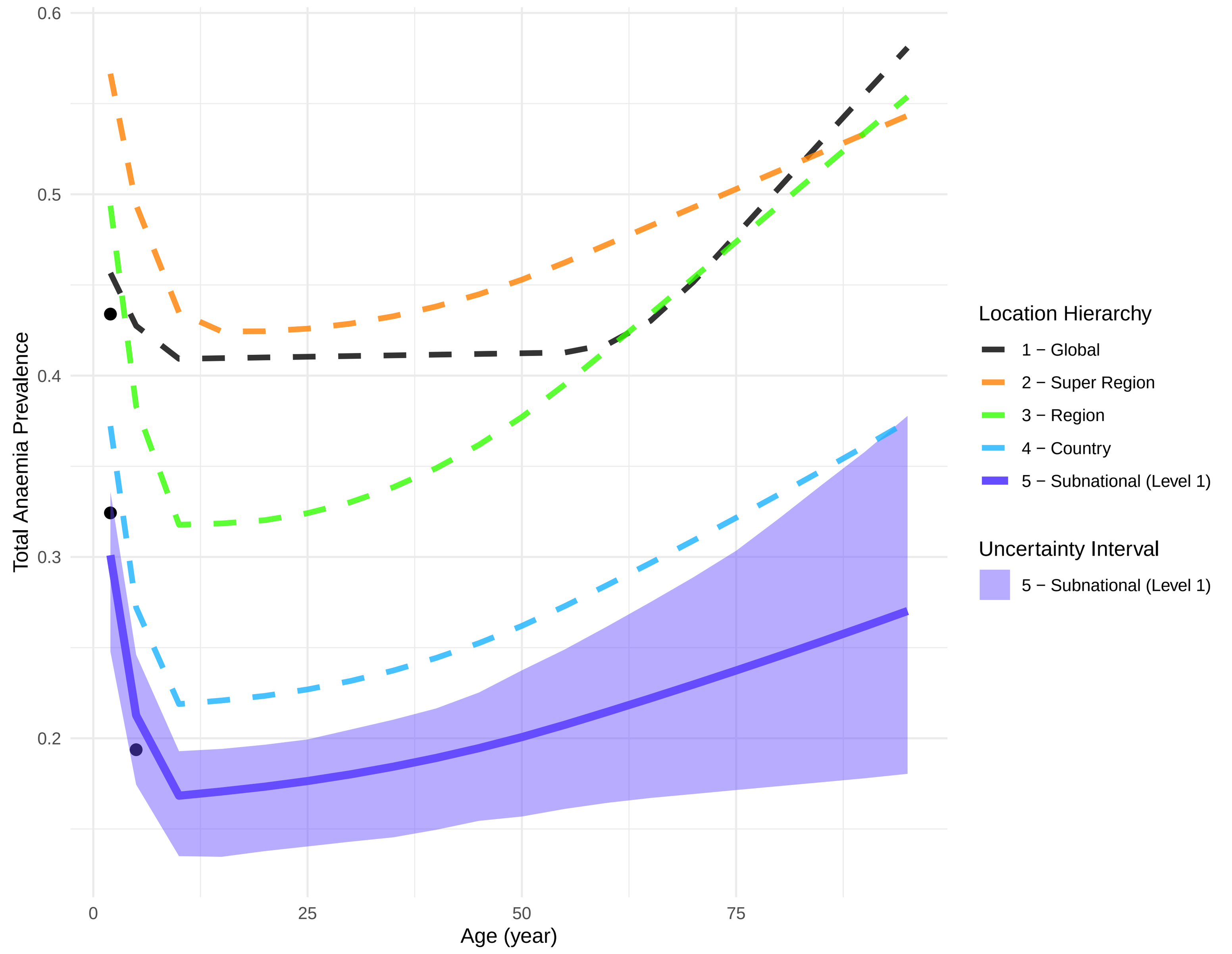

MR−BRT Age/Sex Fits − Non−pregnant Females 2+ Years Old − Total Anaemia Prevalence − Kwale
WHO−2001 Adjustment Method

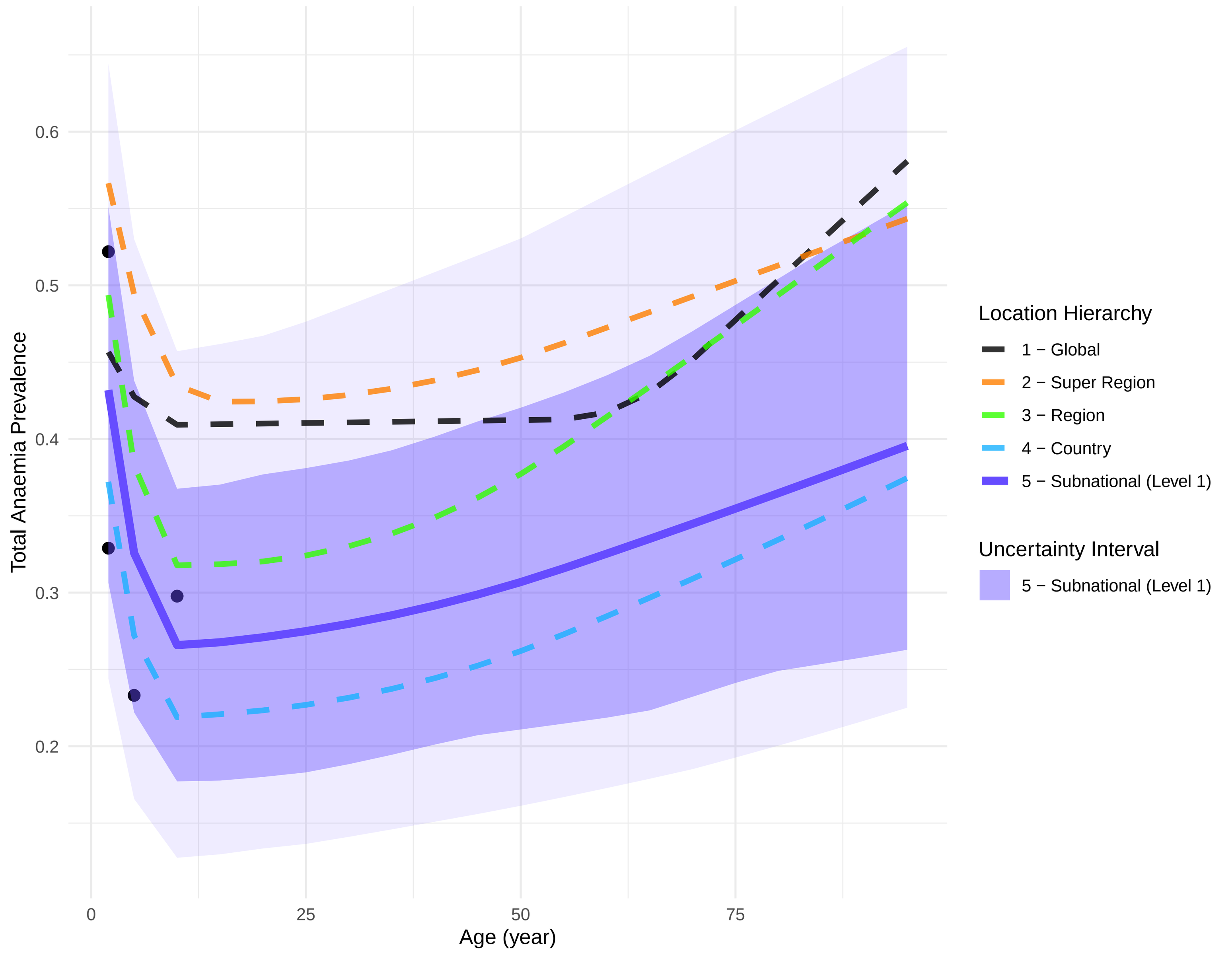

MR−BRT Age/Sex Fits − Non−pregnant Females 2+ Years Old − Total Anaemia Prevalence − Laikipia
WHO−2001 Adjustment Method

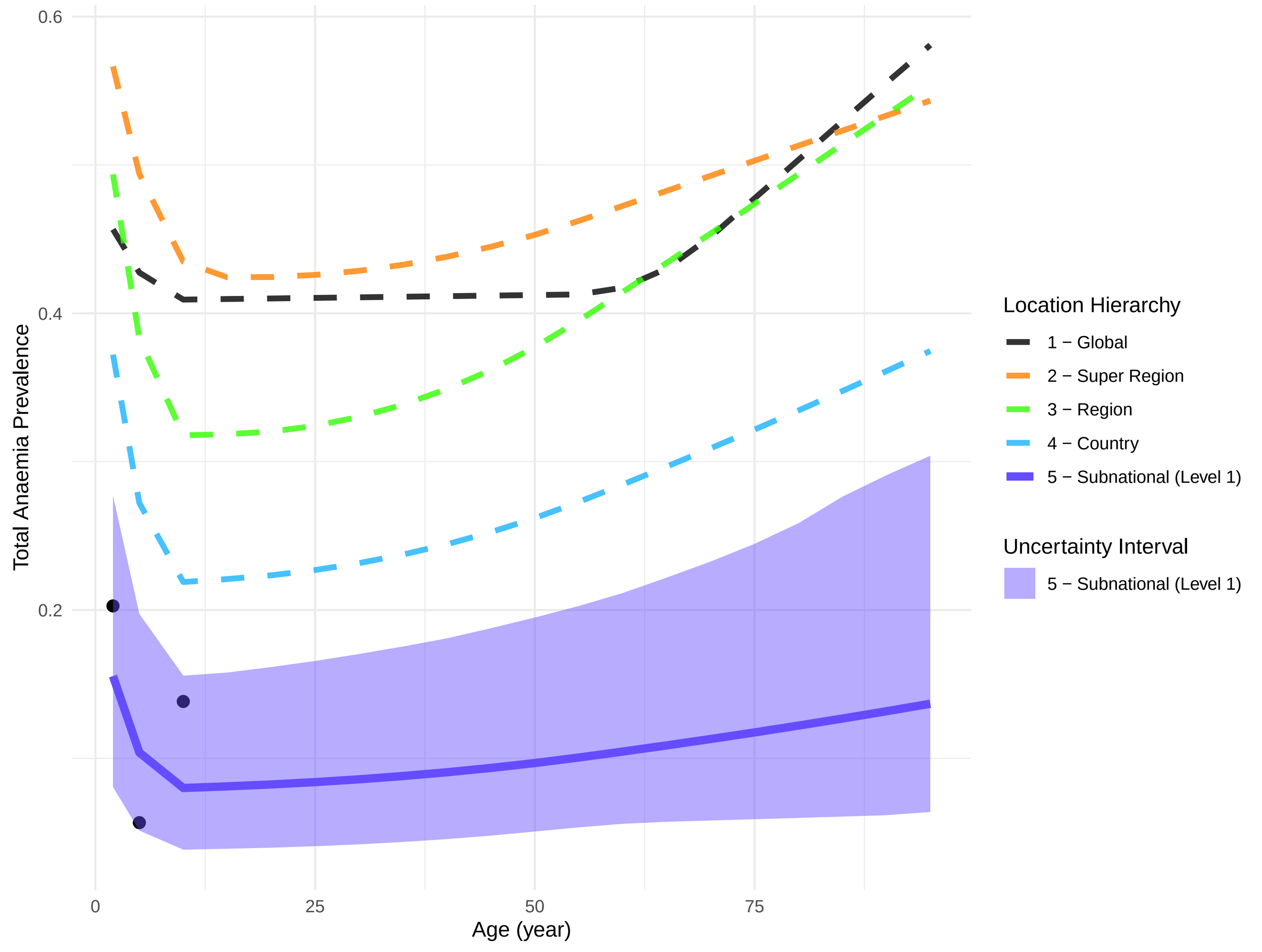

MR−BRT Age/Sex Fits − Non−pregnant Females 2+ Years Old − Total Anaemia Prevalence − Lamu
WHO−2001 Adjustment Method

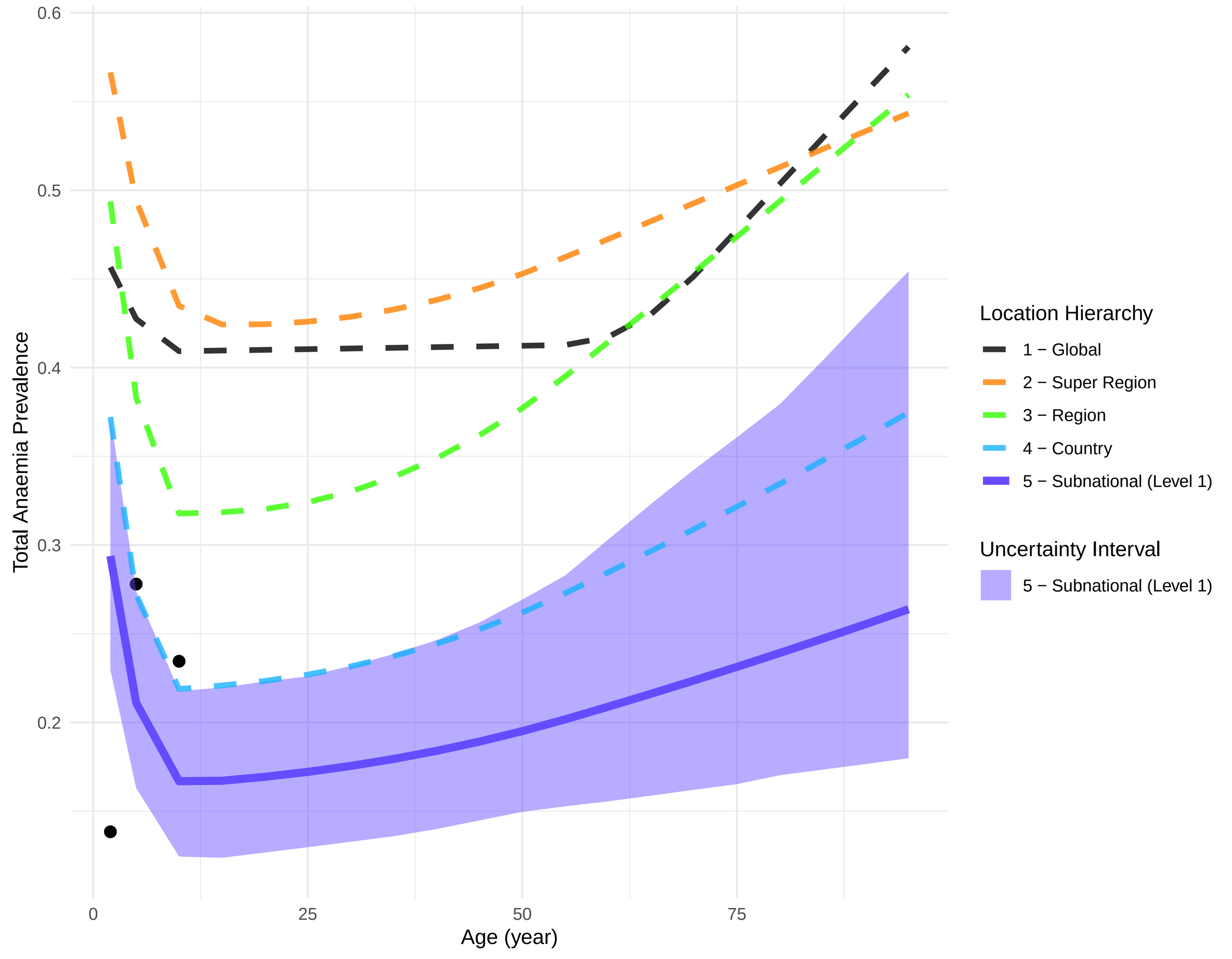

MR−BRT Age/Sex Fits − Non−pregnant Females 2+ Years Old − Total Anaemia Prevalence − Machakos
WHO−2001 Adjustment Method

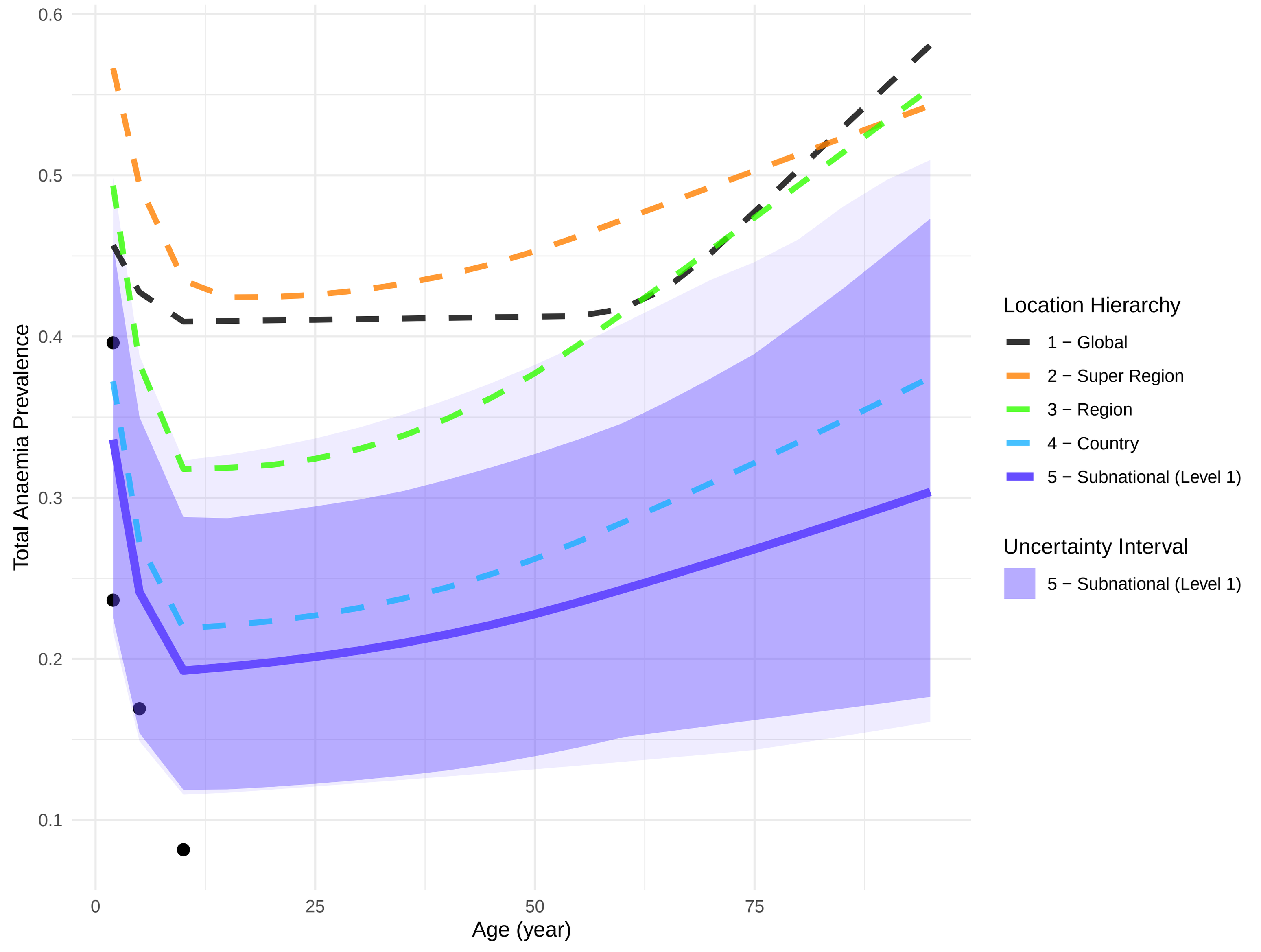

MR−BRT Age/Sex Fits − Non−pregnant Females 2+ Years Old − Total Anaemia Prevalence − Makueni
WHO−2001 Adjustment Method

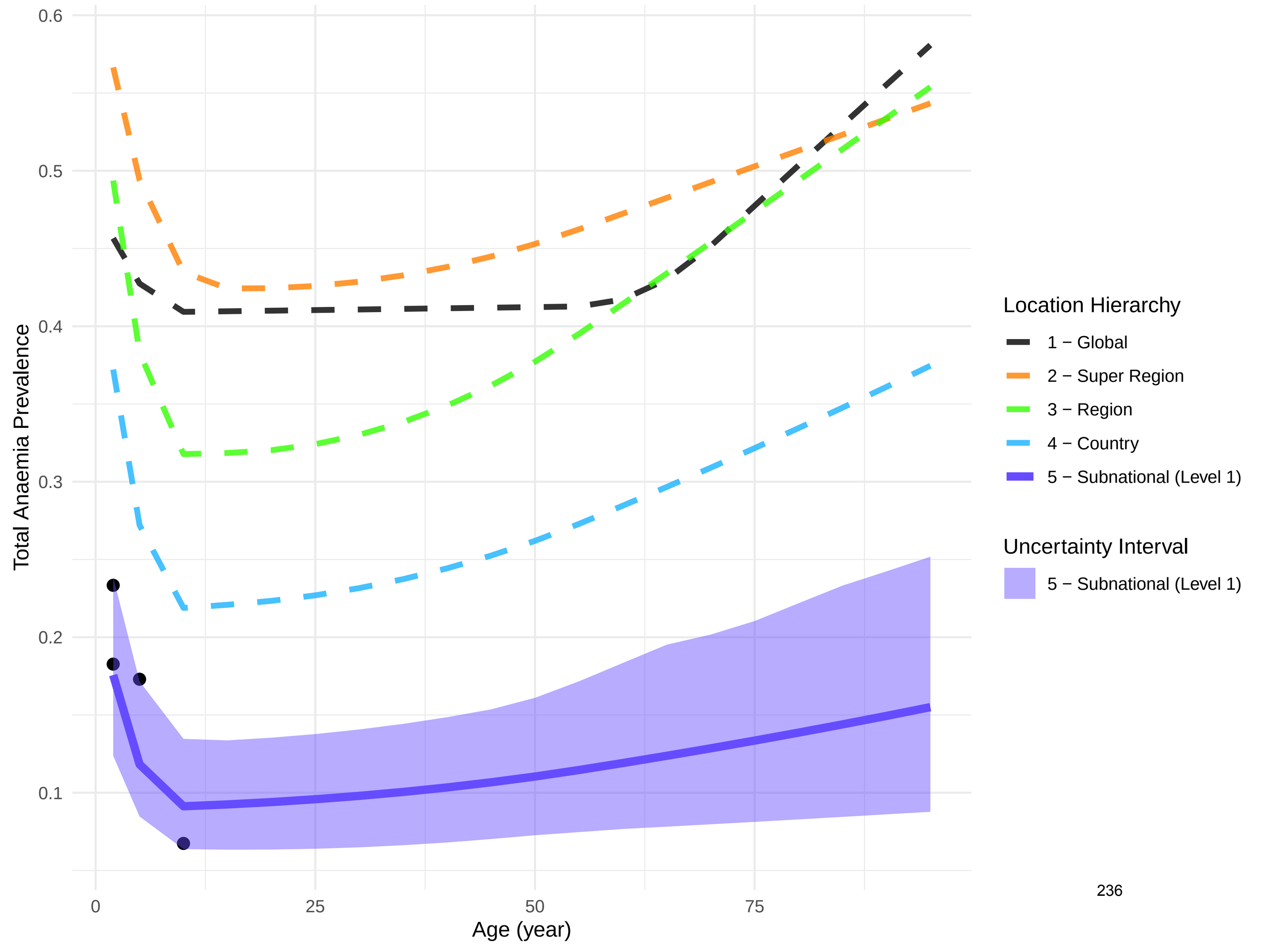

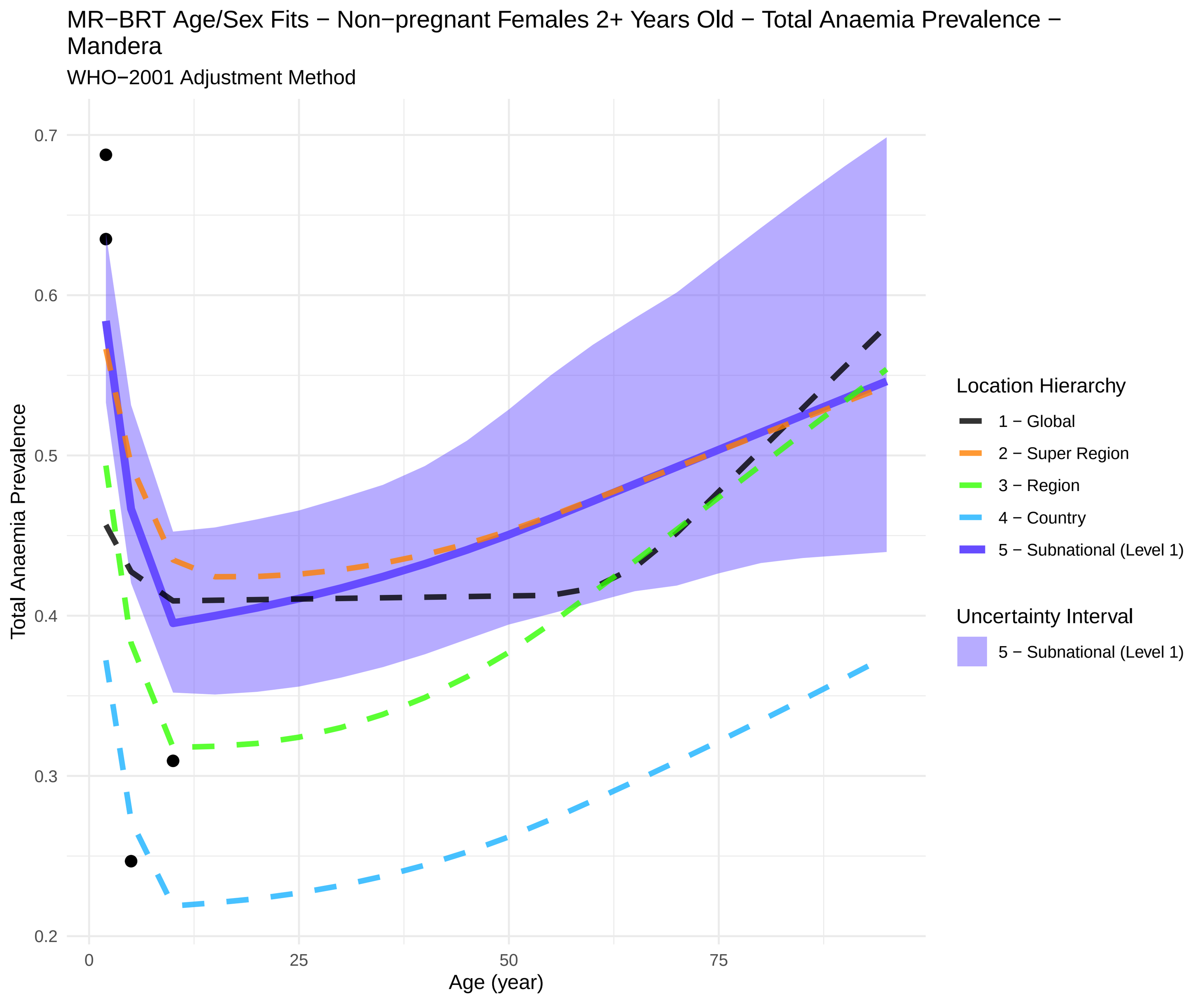
MR−BRT Age/Sex Fits − Non−pregnant Females 2+ Years Old − Total Anaemia Prevalence − Mandera
WHO−2001 Adjustment Method
Total Anaemia Prevalence
Age (year)
Location Hierarchy
1 − Global
2 − Super Region
3 − Region
4 − Country
5 − Subnational (Level 1)
Uncertainty Interval
5 − Subnational (Level 1)

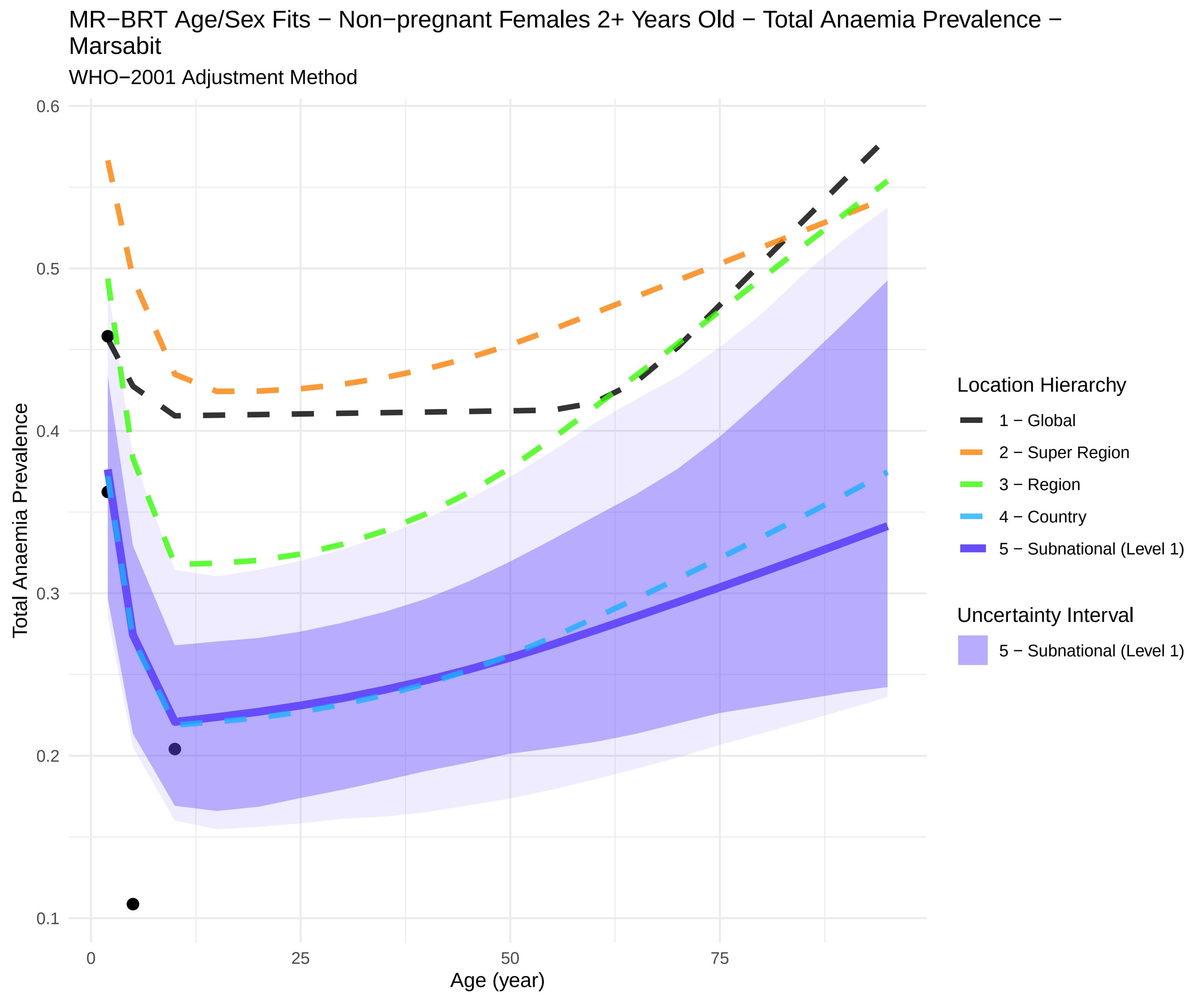
MR−BRT Age/Sex Fits − Non−pregnant Females 2+ Years Old − Total Anaemia Prevalence − Marsabit
WHO−2001 Adjustment Method
Total Anaemia Prevalence
Age (year)
Location Hierarchy
1 − Global
2 − Super Region
3 − Region
4 − Country
5 − Subnational (Level 1)
Uncertainty Interval
5 − Subnational (Level 1)

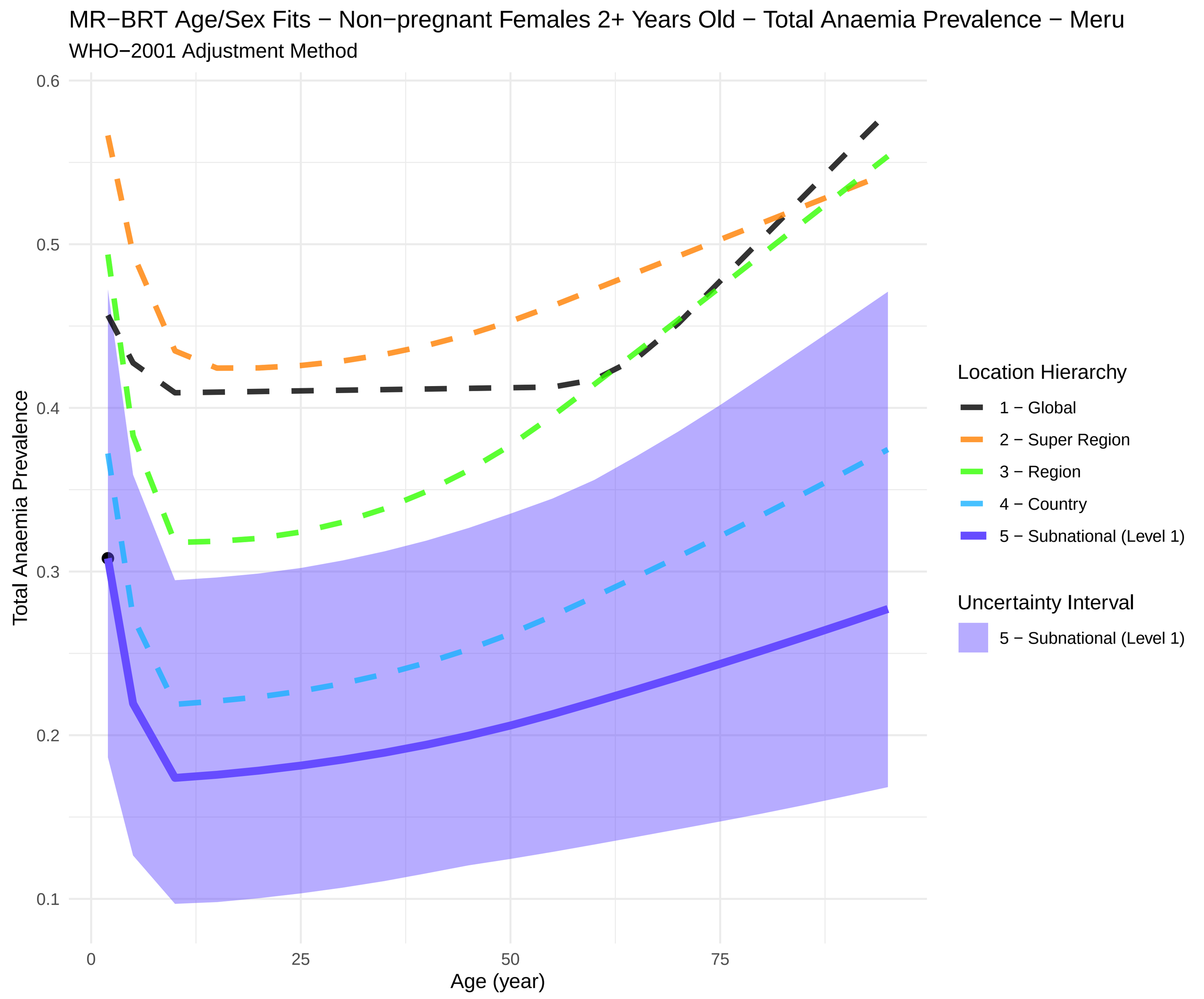
MR−BRT Age/Sex Fits − Non−pregnant Females 2+ Years Old − Total Anaemia Prevalence − Meru
WHO−2001 Adjustment Method
Total Anaemia Prevalence
Age (year)
Location Hierarchy
1 − Global
2 − Super Region
3 − Region
4 − Country
5 − Subnational (Level 1)
Uncertainty Interval
5 − Subnational (Level 1)

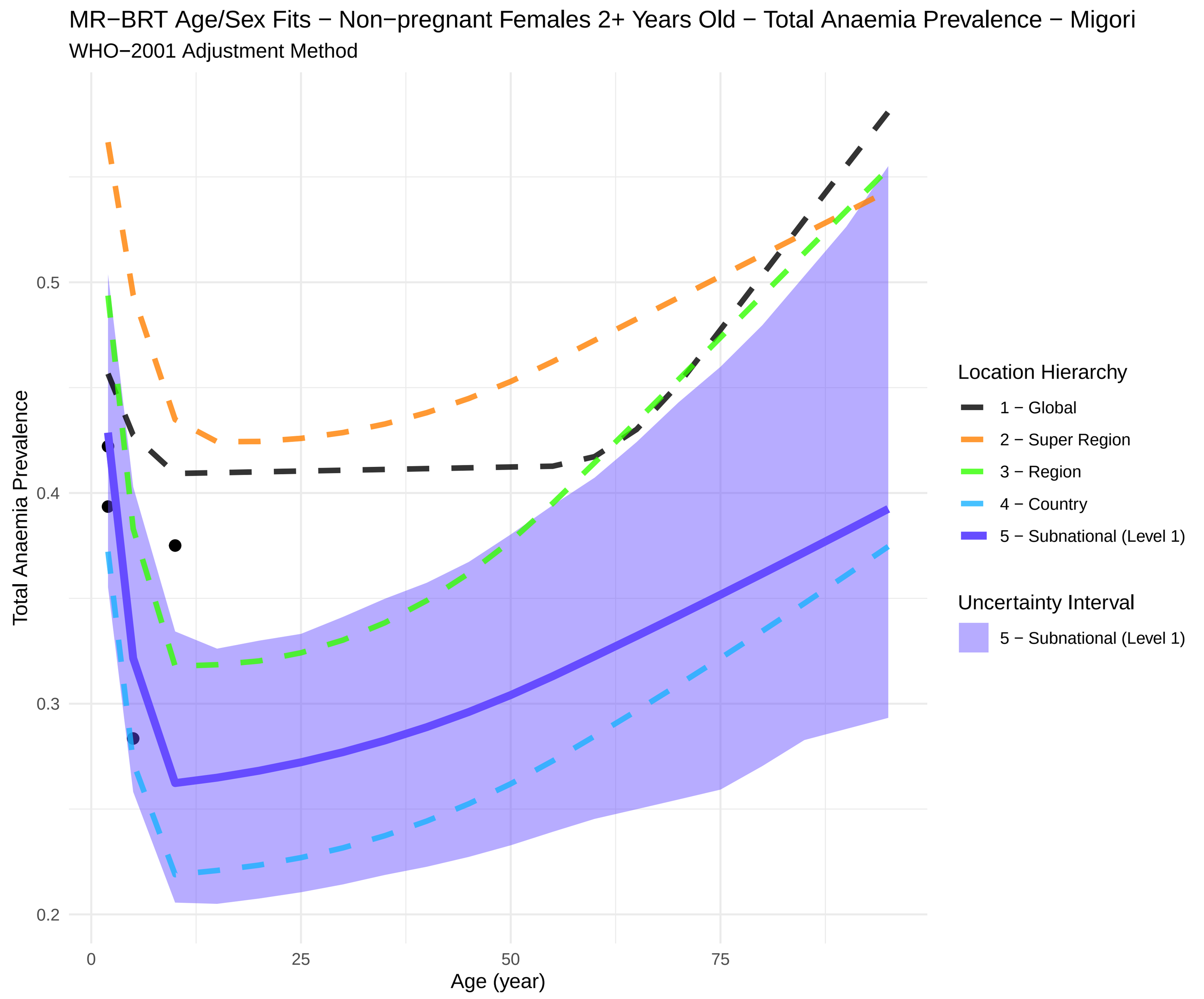
MR−BRT Age/Sex Fits − Non−pregnant Females 2+ Years Old − Total Anaemia Prevalence − Migori
WHO−2001 Adjustment Method
Total Anaemia Prevalence
Age (year)
Location Hierarchy
1 − Global
2 − Super Region
3 − Region
4 − Country
5 − Subnational (Level 1)
Uncertainty Interval
5 − Subnational (Level 1)

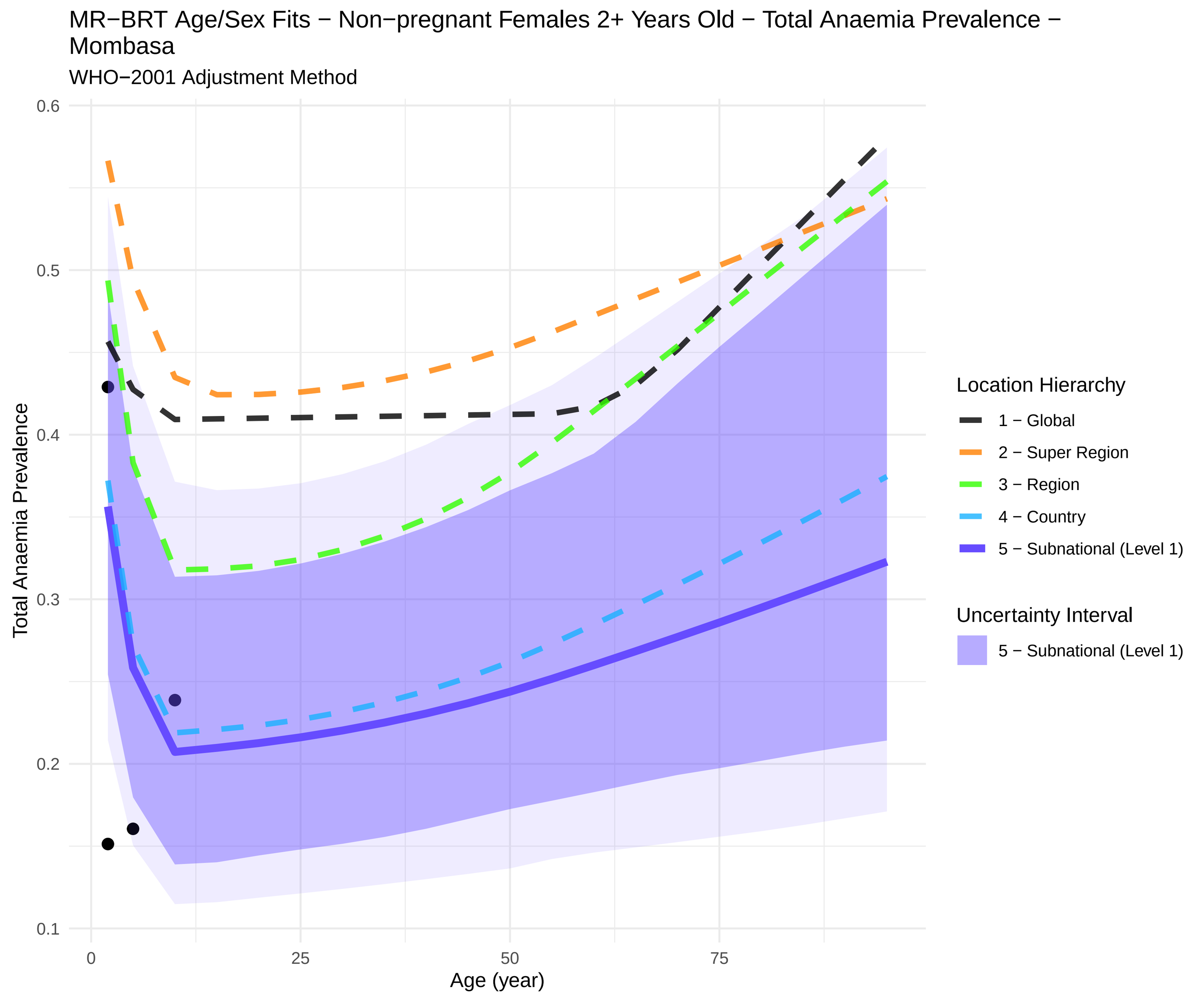
MR−BRT Age/Sex Fits − Non−pregnant Females 2+ Years Old − Total Anaemia Prevalence − Mombasa
WHO−2001 Adjustment Method
Total Anaemia Prevalence
Age (year)
Location Hierarchy
1 − Global
2 − Super Region
3 − Region
4 − Country
5 − Subnational (Level 1)
Uncertainty Interval
5 − Subnational (Level 1)

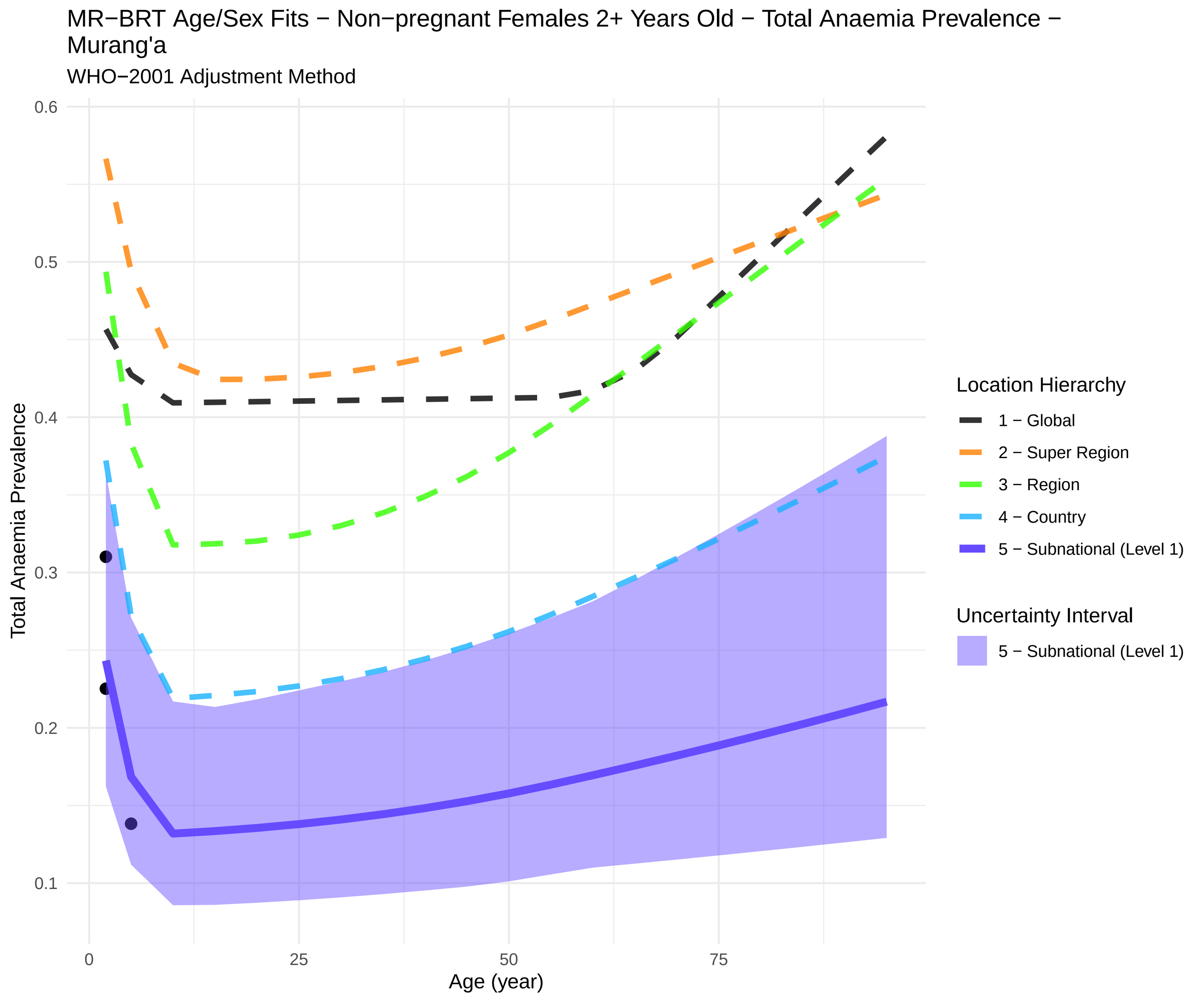
MR−BRT Age/Sex Fits − Non−pregnant Females 2+ Years Old − Total Anaemia Prevalence − Murang'a
WHO−2001 Adjustment Method
Total Anaemia Prevalence
Age (year)
Location Hierarchy
1 − Global
2 − Super Region
3 − Region
4 − Country
5 − Subnational (Level 1)
Uncertainty Interval
5 − Subnational (Level 1)

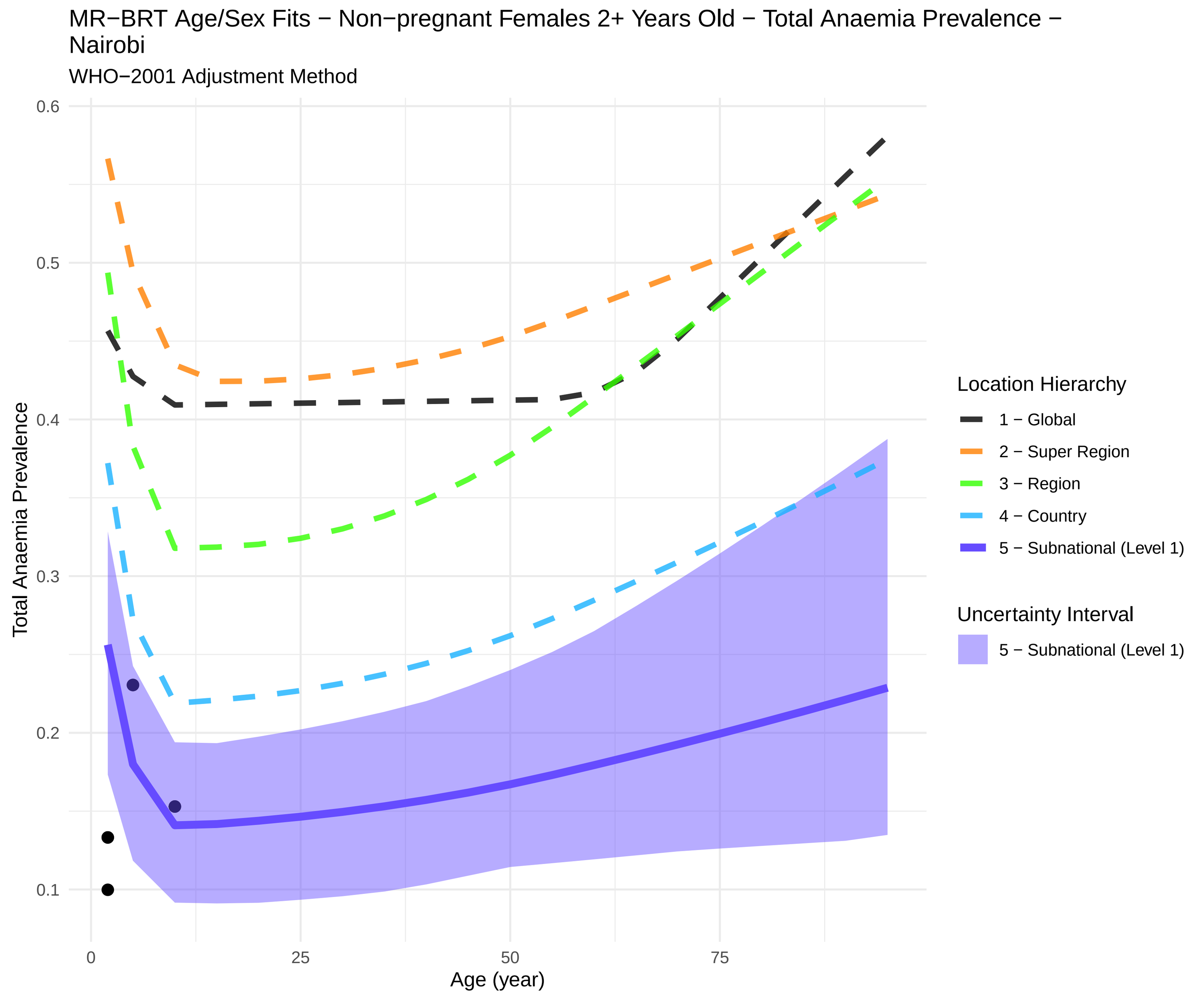
MR−BRT Age/Sex Fits − Non−pregnant Females 2+ Years Old − Total Anaemia Prevalence − Nairobi
WHO−2001 Adjustment Method
Total Anaemia Prevalence
Age (year)
Location Hierarchy
1 − Global
2 − Super Region
3 − Region
4 − Country
5 − Subnational (Level 1)
Uncertainty Interval
5 − Subnational (Level 1)

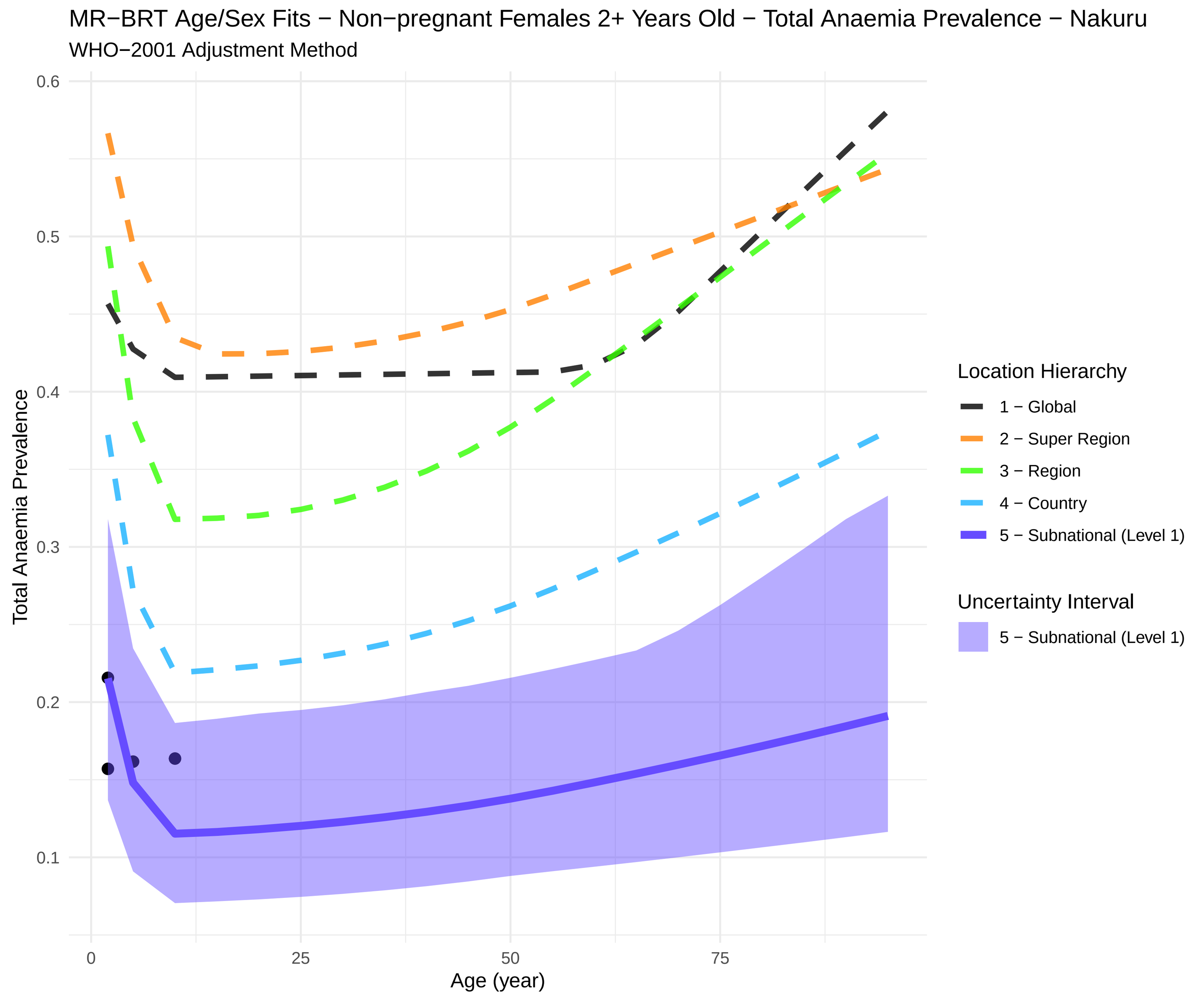
MR−BRT Age/Sex Fits − Non−pregnant Females 2+ Years Old − Total Anaemia Prevalence − Nakuru
WHO−2001 Adjustment Method
Total Anaemia Prevalence
Age (year)
Location Hierarchy
1 − Global
2 − Super Region
3 − Region
4 − Country
5 − Subnational (Level 1)
Uncertainty Interval
5 − Subnational (Level 1)

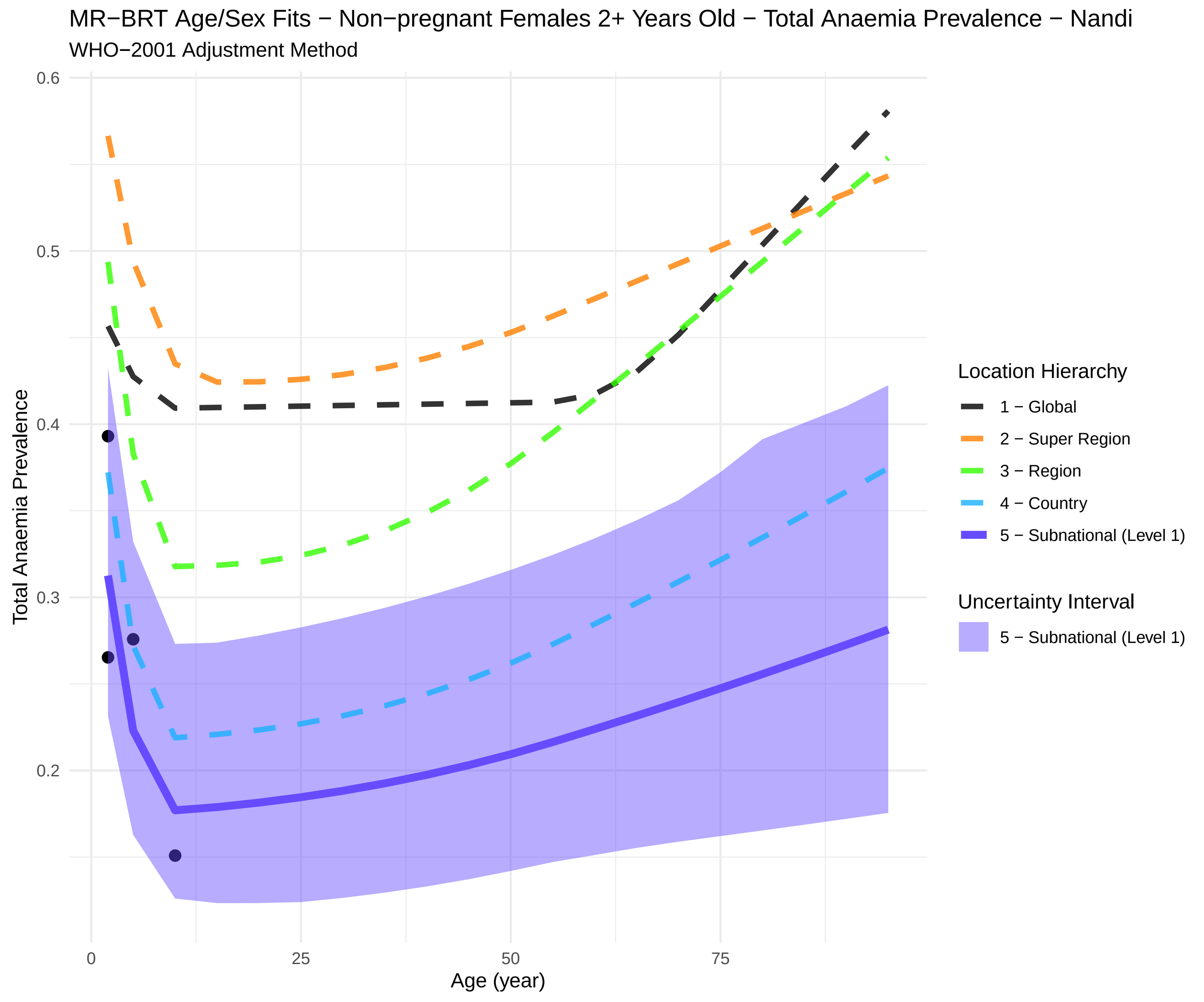
MR−BRT Age/Sex Fits − Non−pregnant Females 2+ Years Old − Total Anaemia Prevalence − Nandi
WHO−2001 Adjustment Method
Total Anaemia Prevalence
Age (year)
Location Hierarchy
1 − Global
2 − Super Region
3 − Region
4 − Country
5 − Subnational (Level 1)
Uncertainty Interval
5 − Subnational (Level 1)

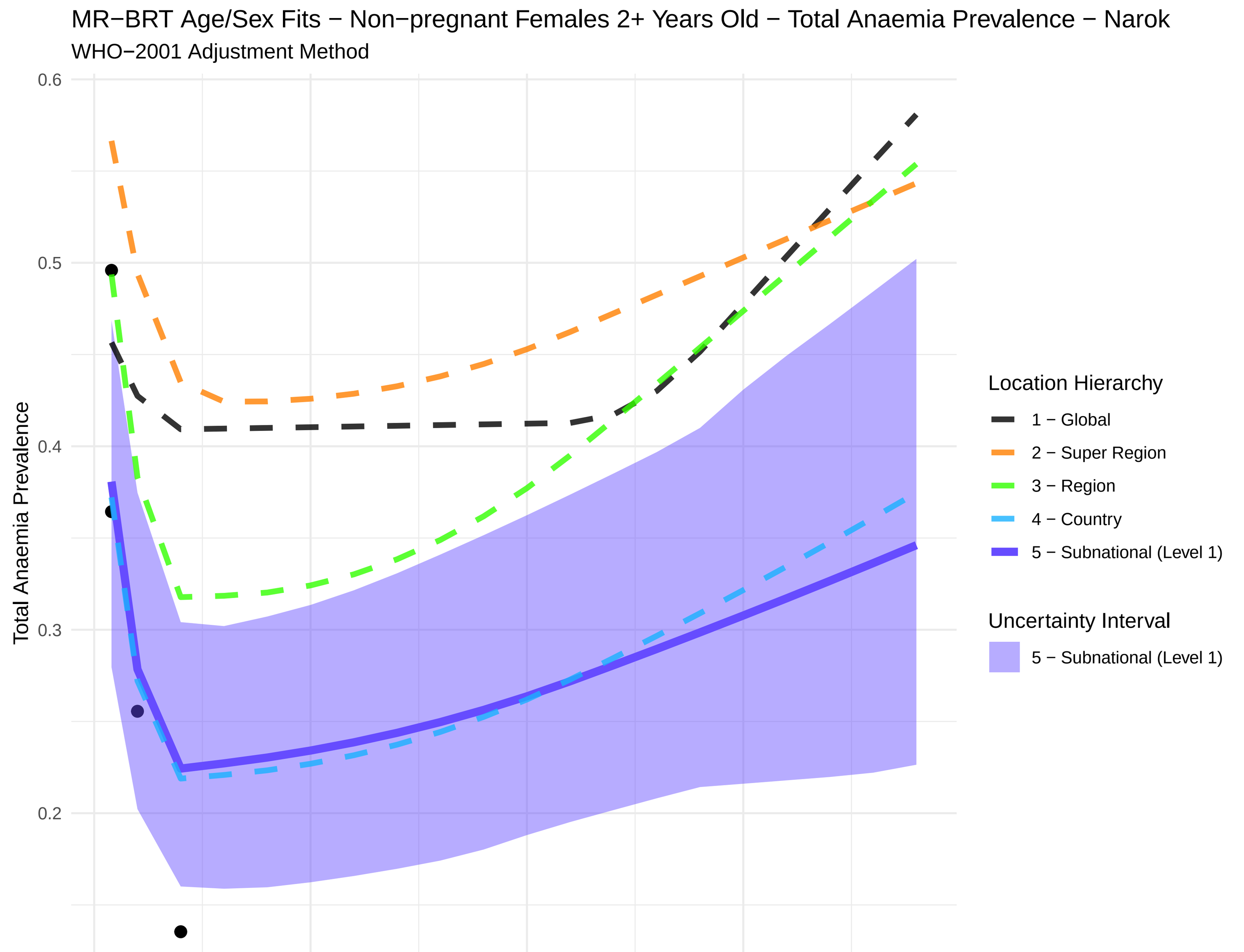
MR−BRT Age/Sex Fits − Non−pregnant Females 2+ Years Old − Total Anaemia Prevalence − Narok
WHO−2001 Adjustment Method
Total Anaemia Prevalence
Age (year)
Location Hierarchy
1 − Global
2 − Super Region
3 − Region
4 − Country
5 − Subnational (Level 1)
Uncertainty Interval
5 − Subnational (Level 1)

MR−BRT Age/Sex Fits − Non−pregnant Females 2+ Years Old − Total Anaemia Prevalence − Nyamira
WHO−2001 Adjustment Method

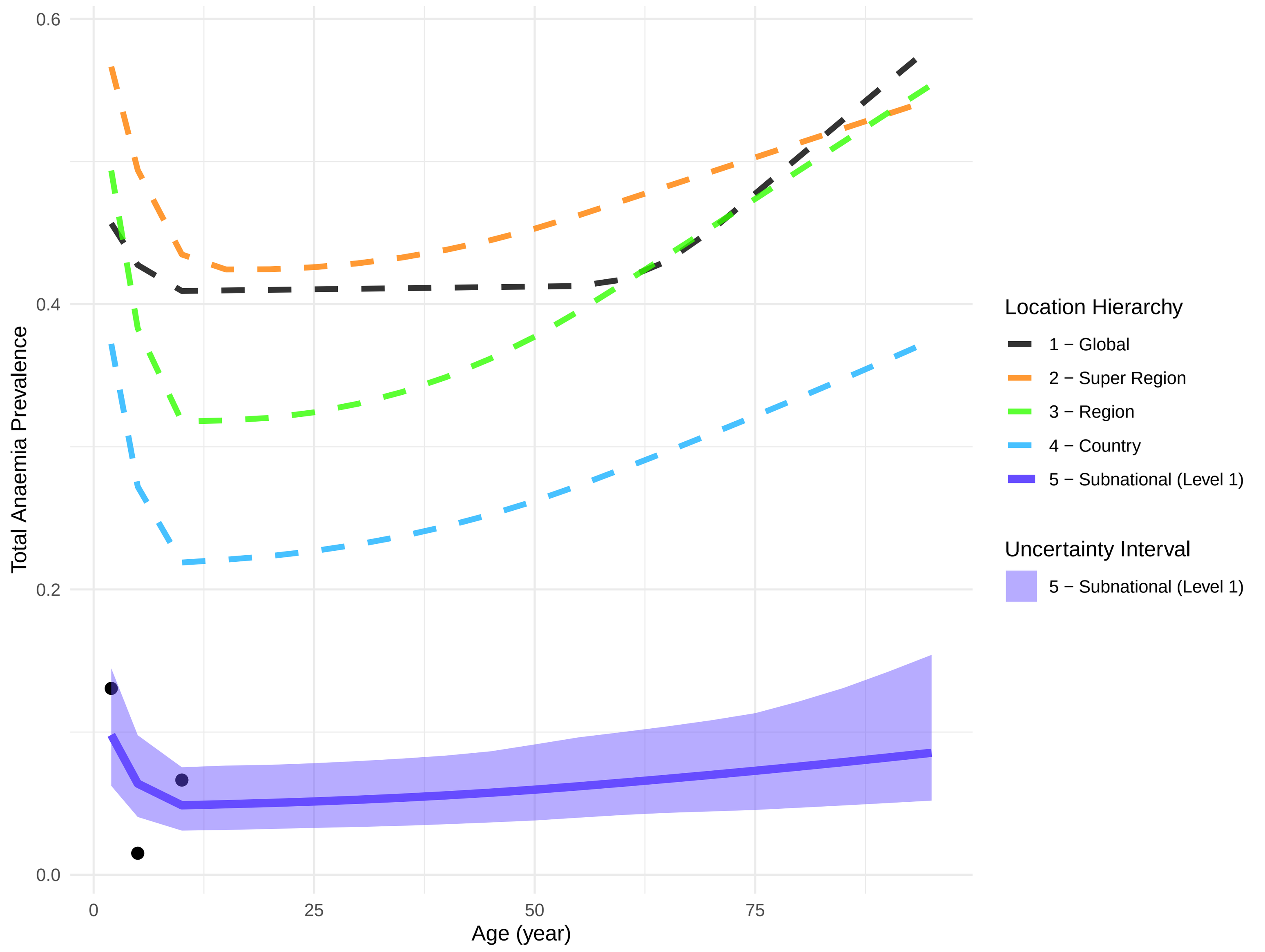

MR−BRT Age/Sex Fits − Non−pregnant Females 2+ Years Old − Total Anaemia Prevalence − Nyandarua
WHO−2001 Adjustment Method

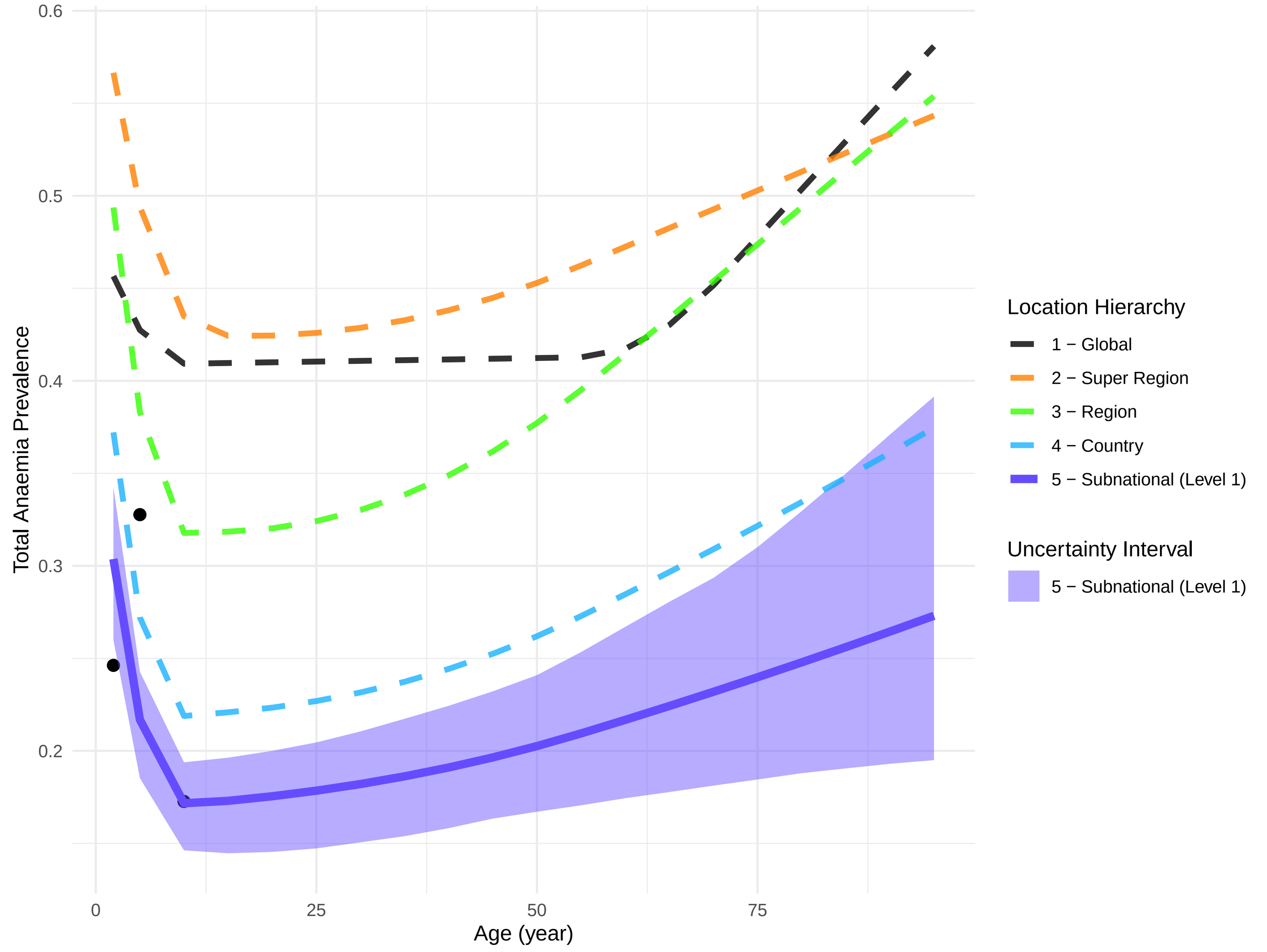

MR−BRT Age/Sex Fits − Non−pregnant Females 2+ Years Old − Total Anaemia Prevalence − Nyeri
WHO−2001 Adjustment Method

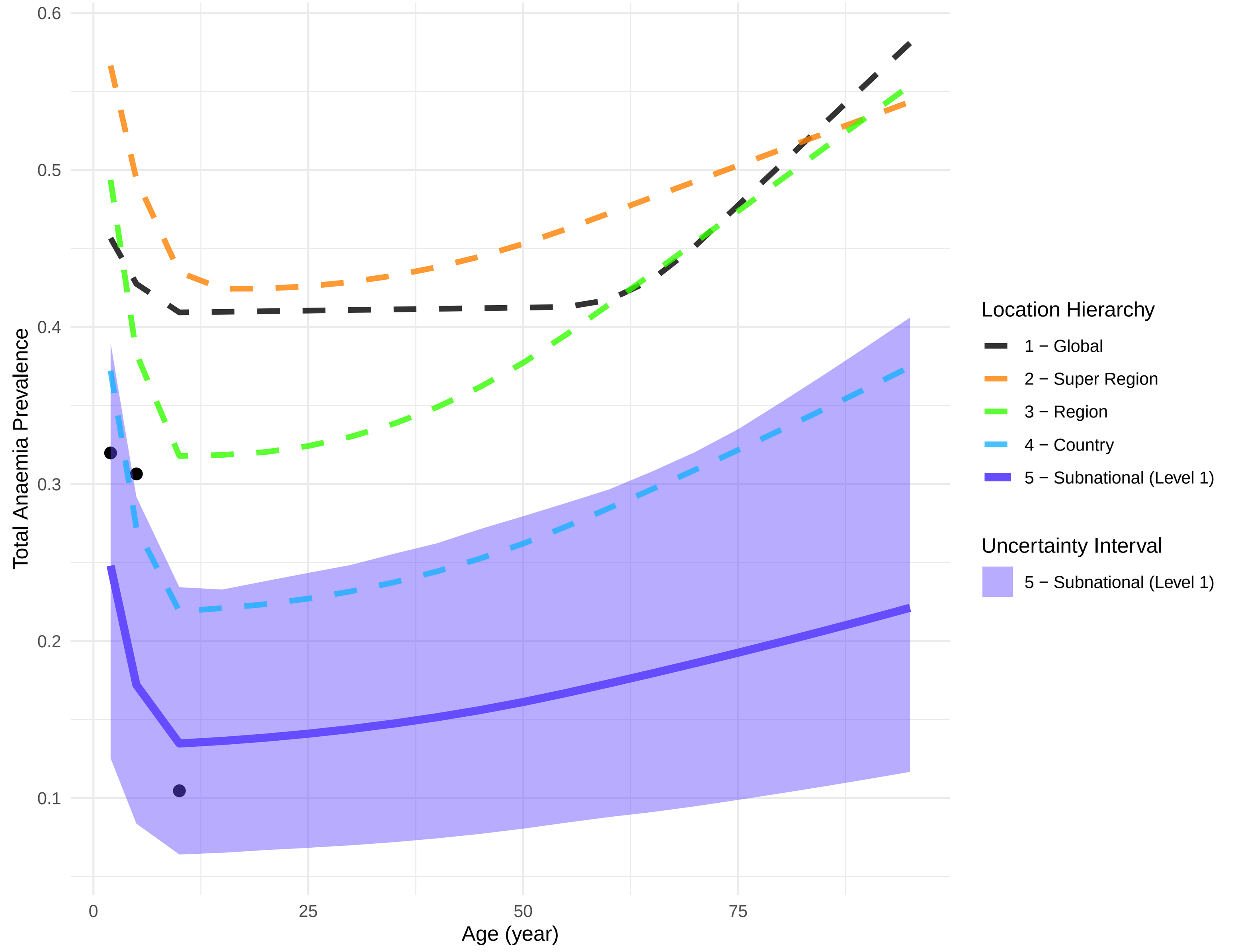

MR−BRT Age/Sex Fits − Non−pregnant Females 2+ Years Old − Total Anaemia Prevalence − Samburu
WHO−2001 Adjustment Method

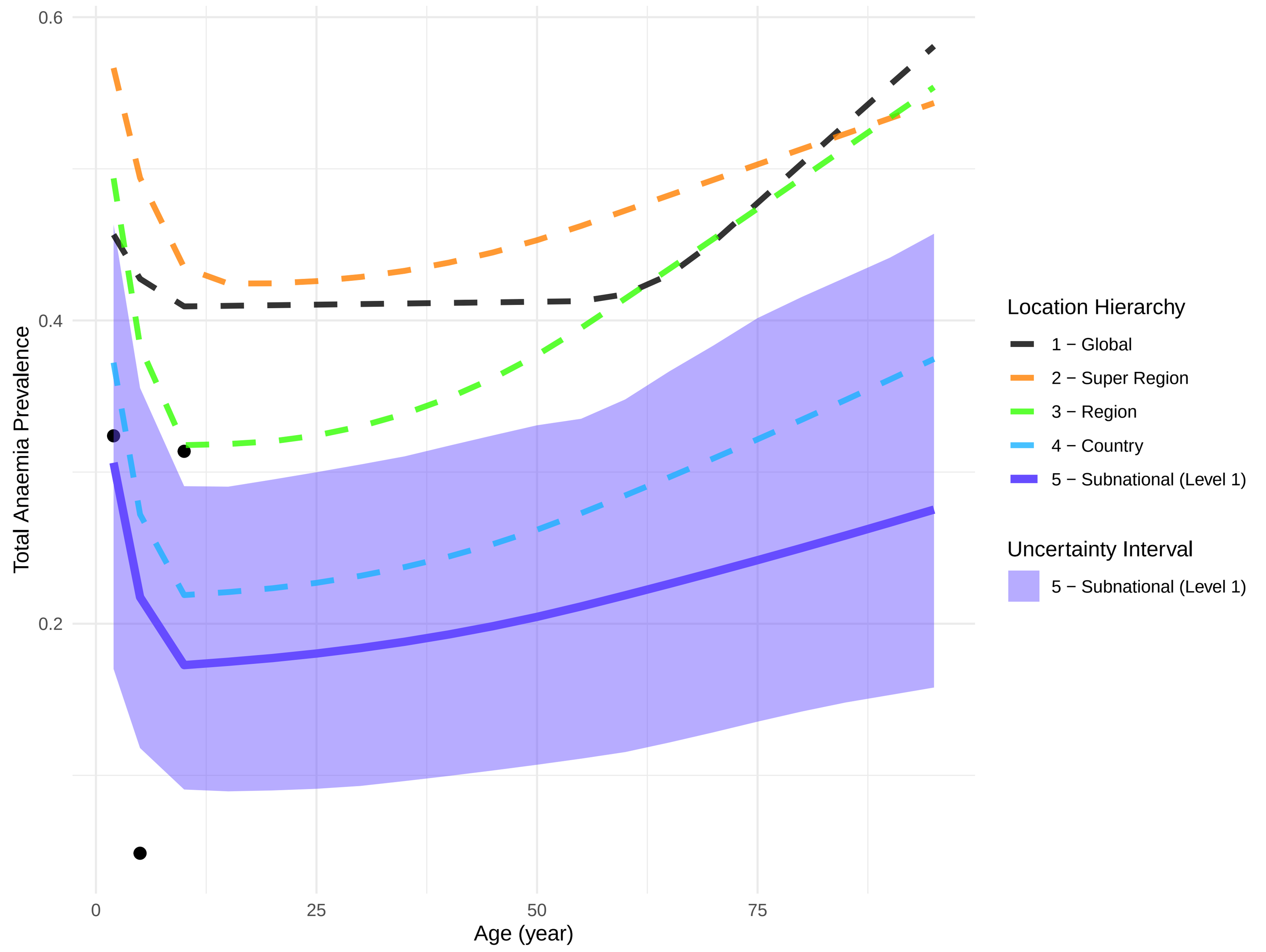

MR−BRT Age/Sex Fits − Non−pregnant Females 2+ Years Old − Total Anaemia Prevalence − Siaya
WHO−2001 Adjustment Method

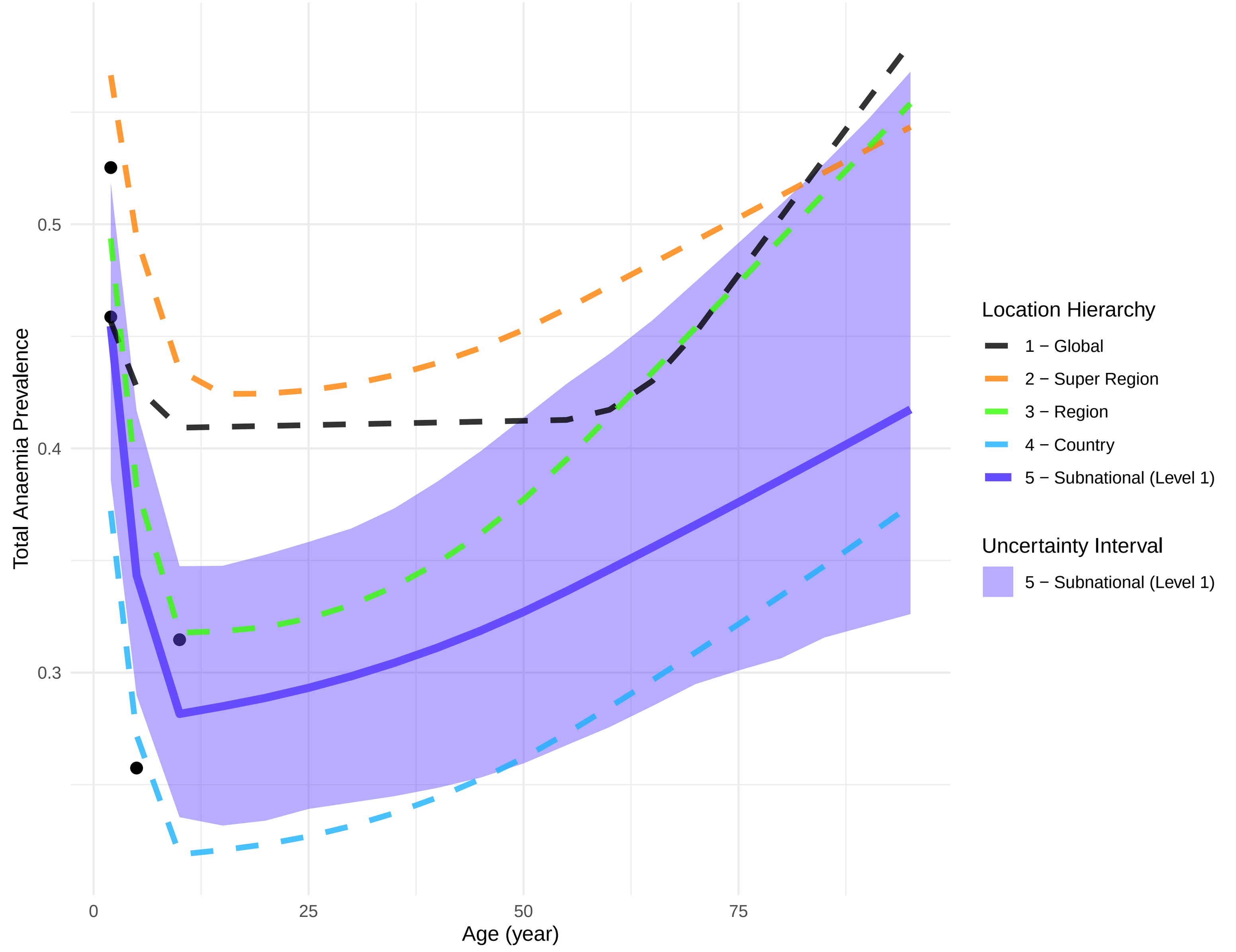

MR−BRT Age/Sex Fits − Non−pregnant Females 2+ Years Old − Total Anaemia Prevalence − Taita Taveta
WHO−2001 Adjustment Method

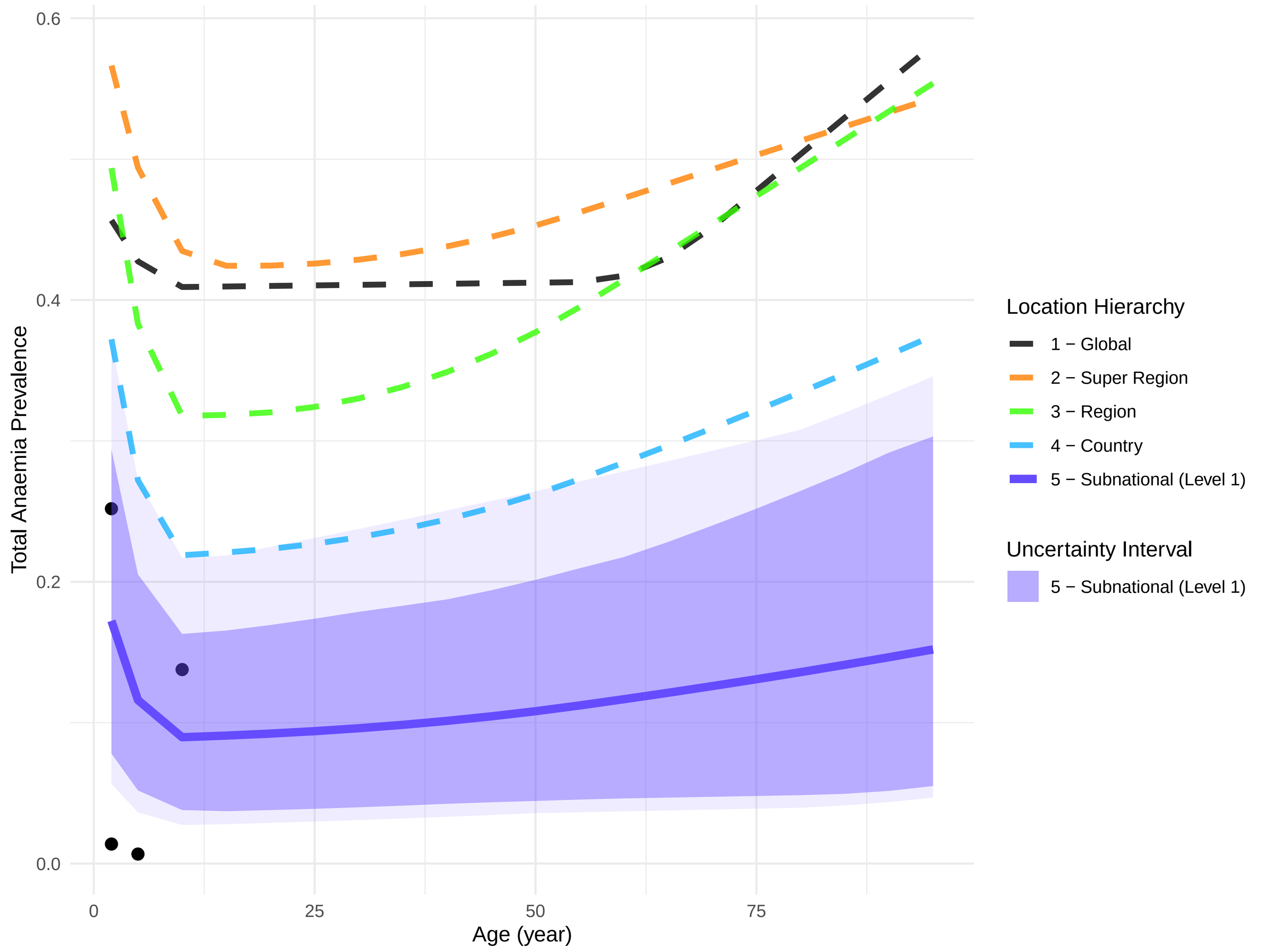

MR−BRT Age/Sex Fits − Non−pregnant Females 2+ Years Old − Total Anaemia Prevalence − Tana River
WHO−2001 Adjustment Method

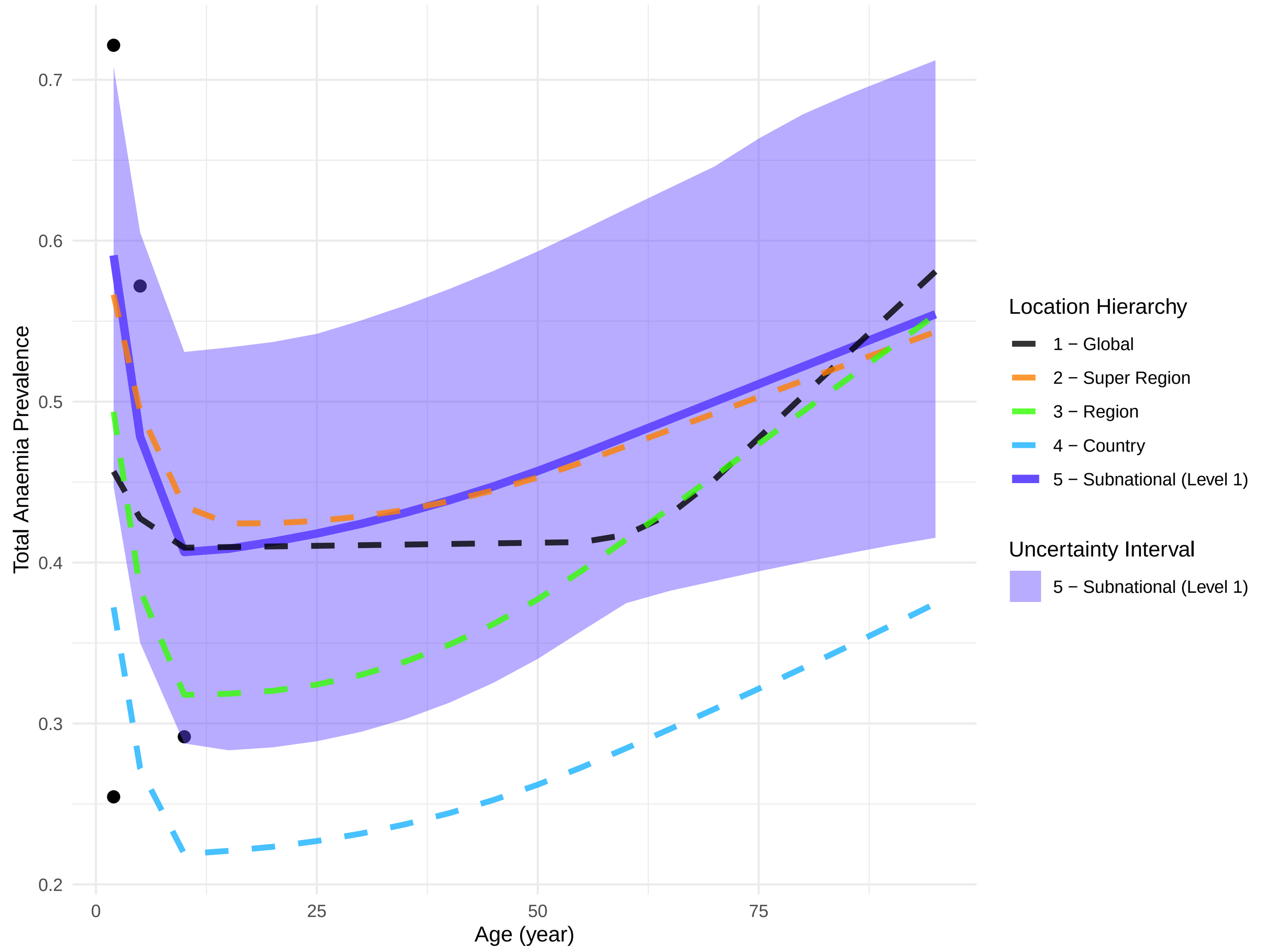

MR−BRT Age/Sex Fits − Non−pregnant Females 2+ Years Old − Total Anaemia Prevalence − Tharaka Nithi
WHO−2001 Adjustment Method

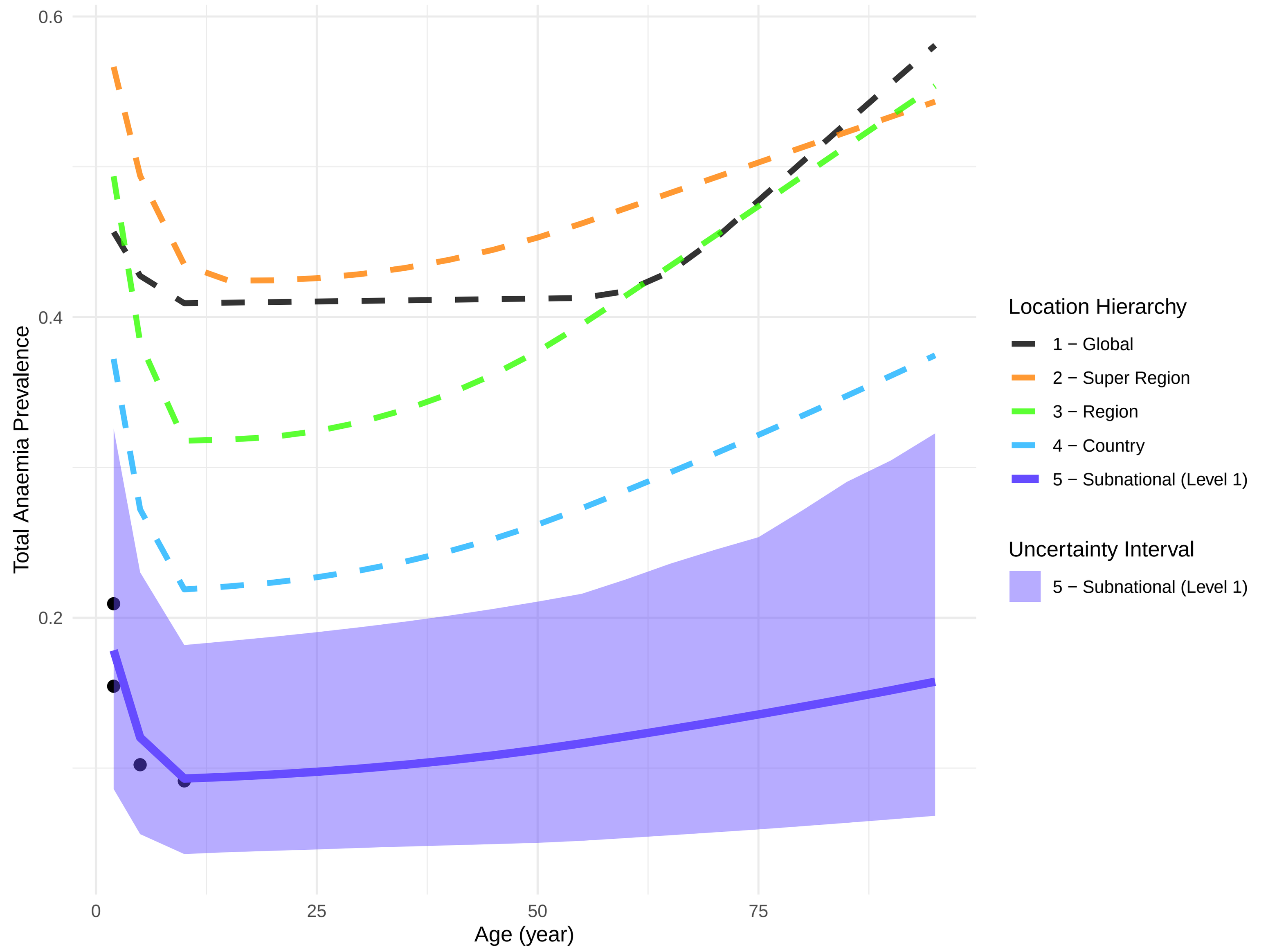

MR−BRT Age/Sex Fits − Non−pregnant Females 2+ Years Old − Total Anaemia Prevalence − Trans Nzoia
WHO−2001 Adjustment Method

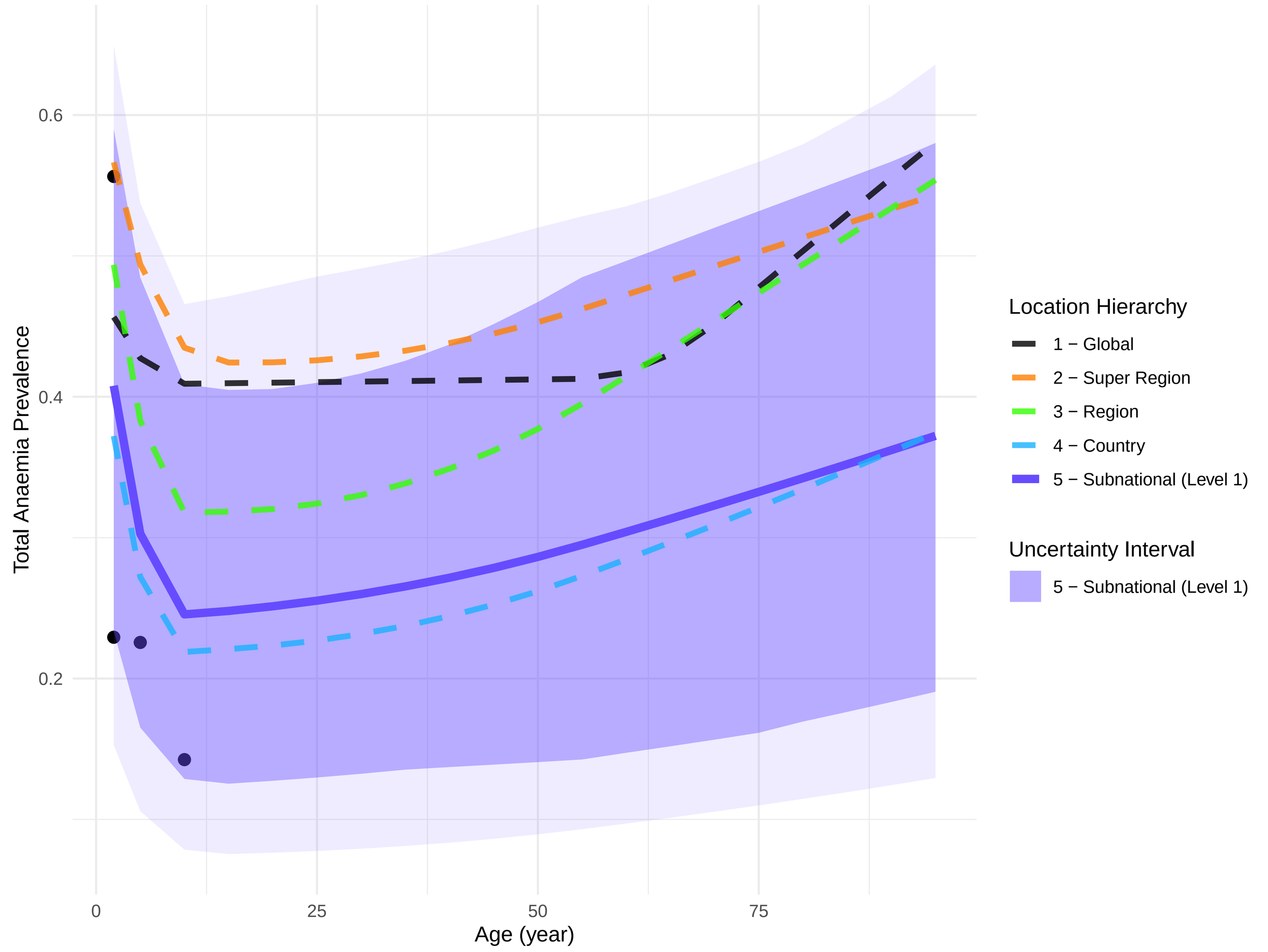

MR−BRT Age/Sex Fits − Non−pregnant Females 2+ Years Old − Total Anaemia Prevalence − Turkana
WHO−2001 Adjustment Method

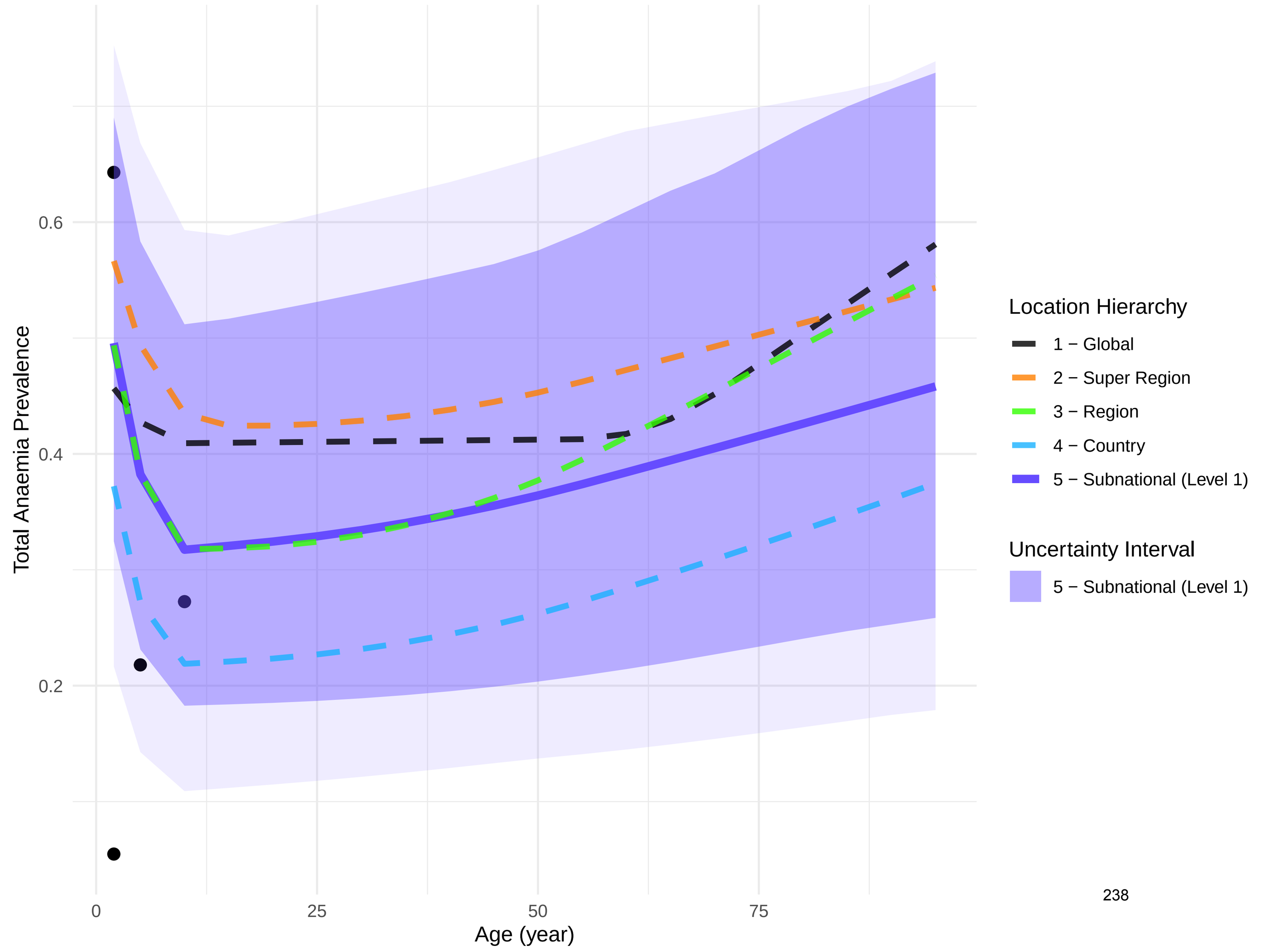

MR−BRT Age/Sex Fits − Non−pregnant Females 2+ Years Old − Total Anaemia Prevalence − Uasin Gishu
WHO−2001 Adjustment Method

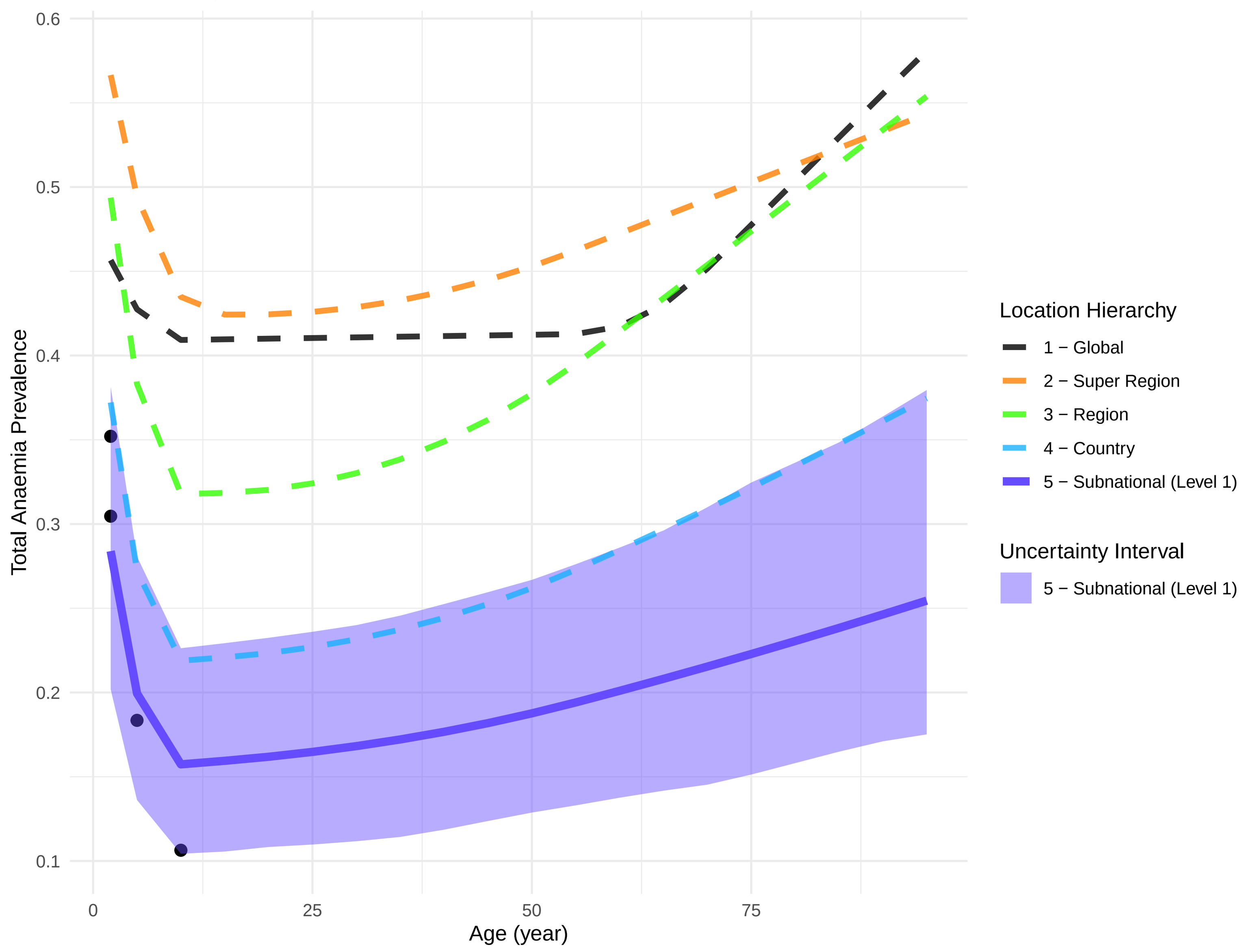

MR−BRT Age/Sex Fits − Non−pregnant Females 2+ Years Old − Total Anaemia Prevalence − Vihiga
WHO−2001 Adjustment Method

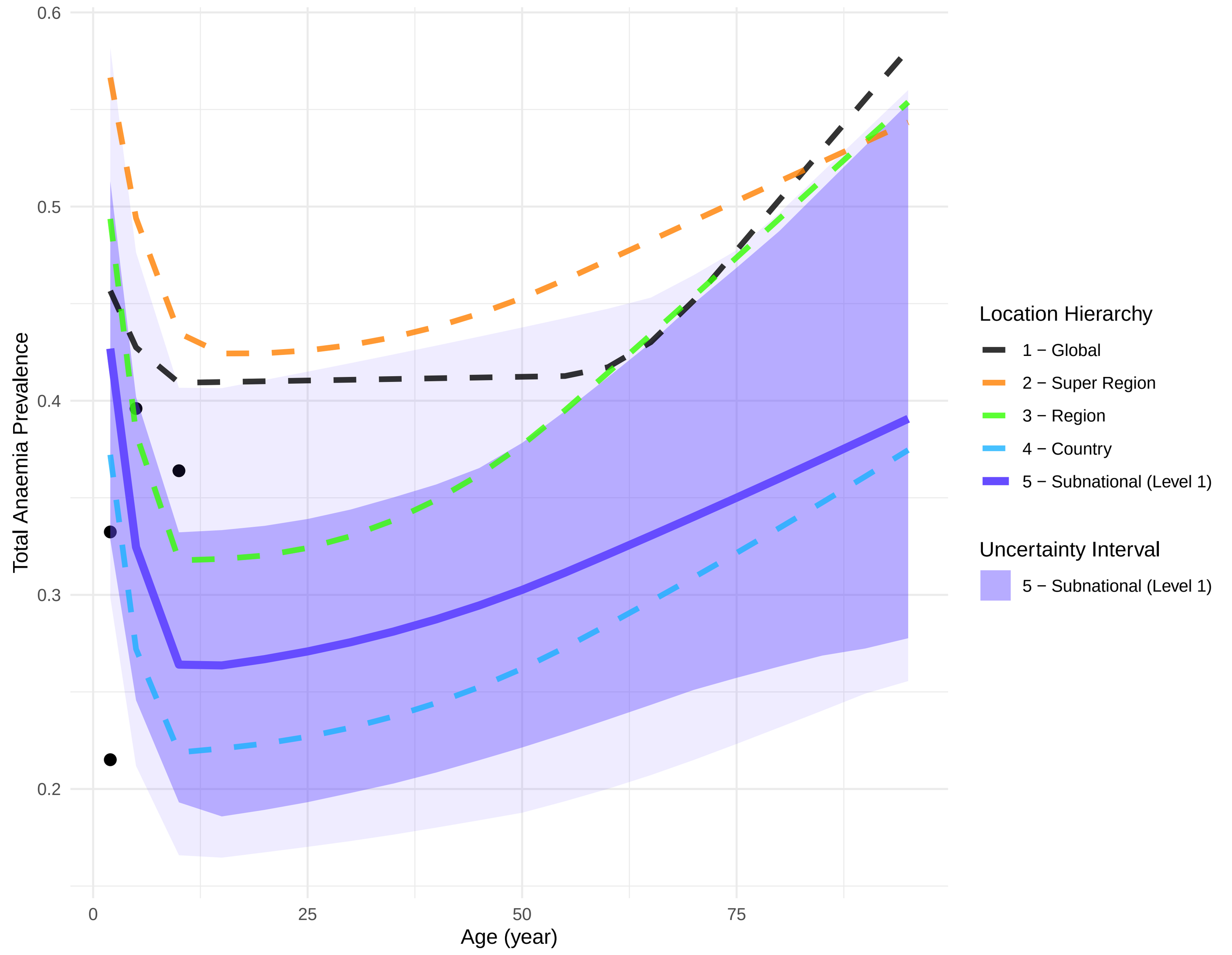

MR−BRT Age/Sex Fits − Non−pregnant Females 2+ Years Old − Total Anaemia Prevalence − Wajir
WHO−2001 Adjustment Method

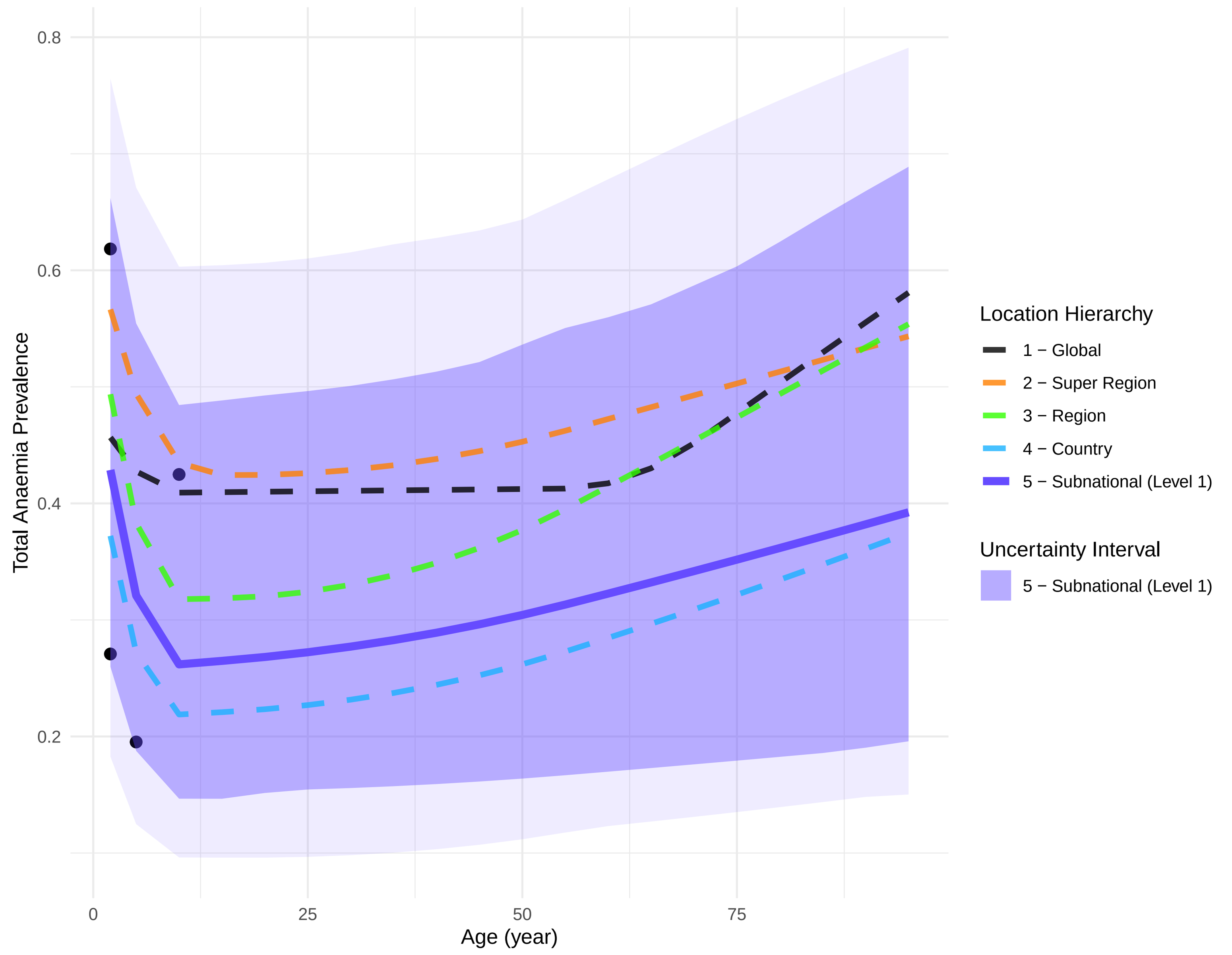

MR−BRT Age/Sex Fits − Non−pregnant Females 2+ Years Old − Total Anaemia Prevalence − West Pokot
WHO−2001 Adjustment Method

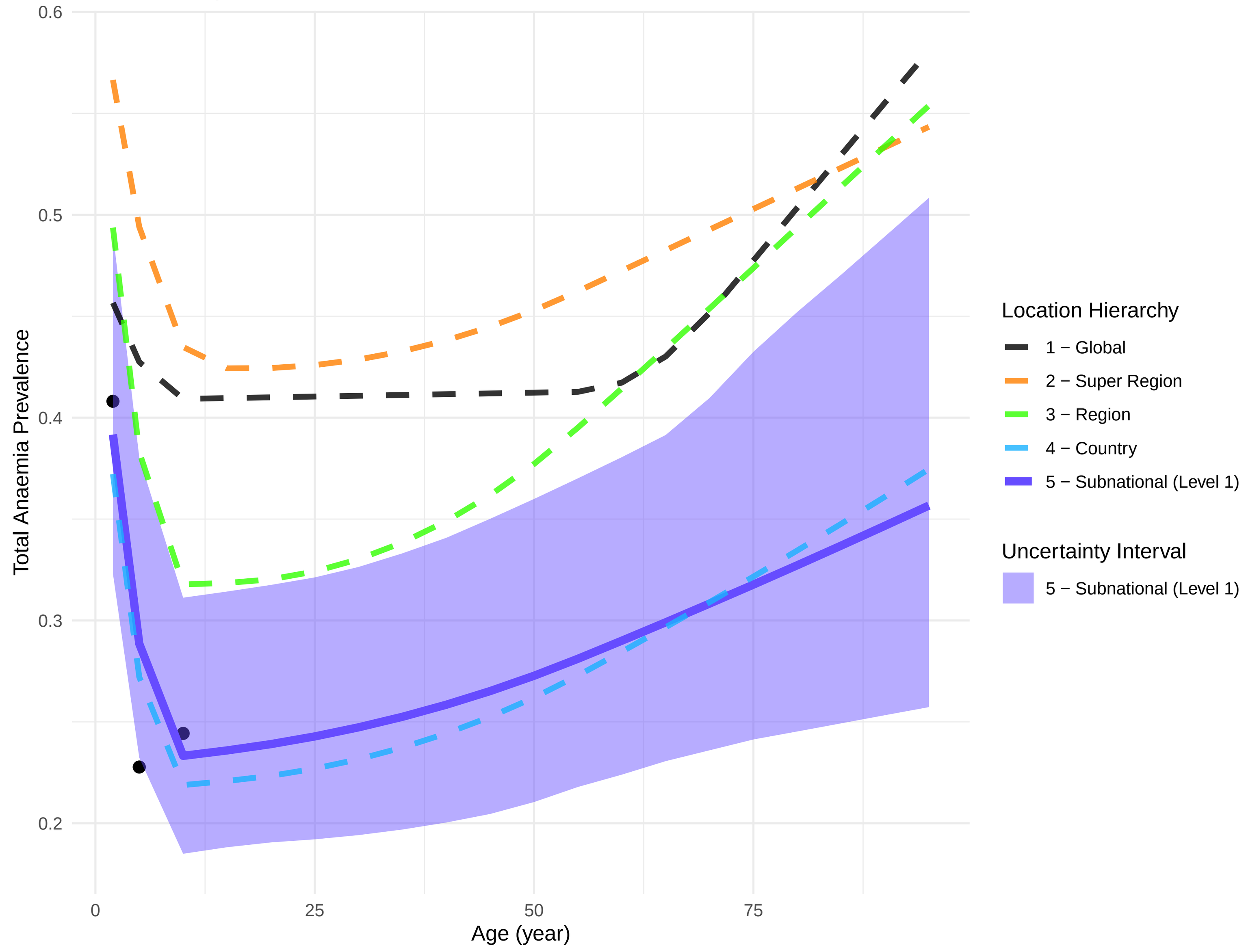

MR−BRT Age/Sex Fits − Non−pregnant Females 2+ Years Old − Total Anaemia Prevalence − Madagascar
WHO−2001 Adjustment Method

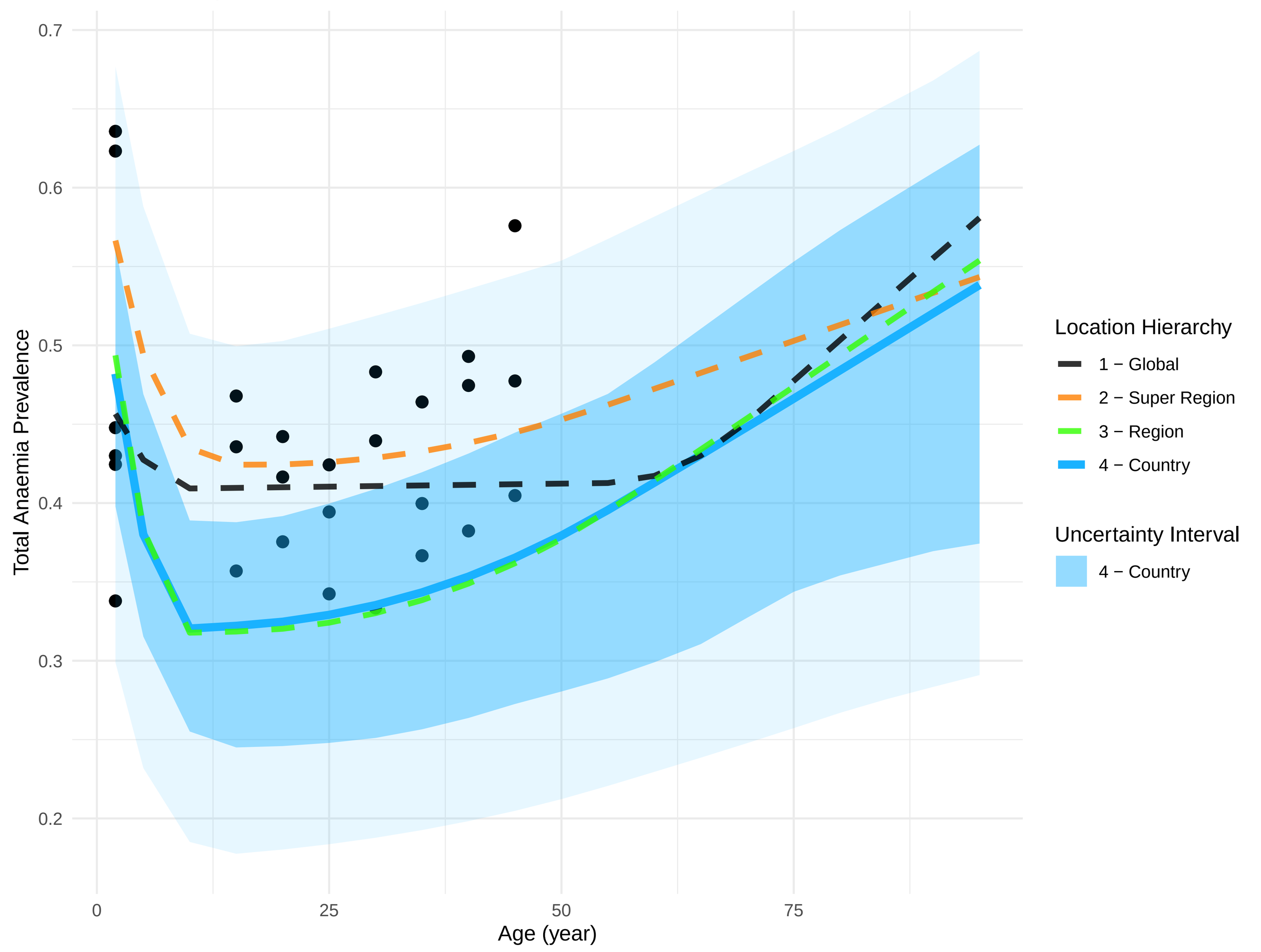

MR−BRT Age/Sex Fits − Non−pregnant Females 2+ Years Old − Total Anaemia Prevalence − Malawi
WHO−2001 Adjustment Method

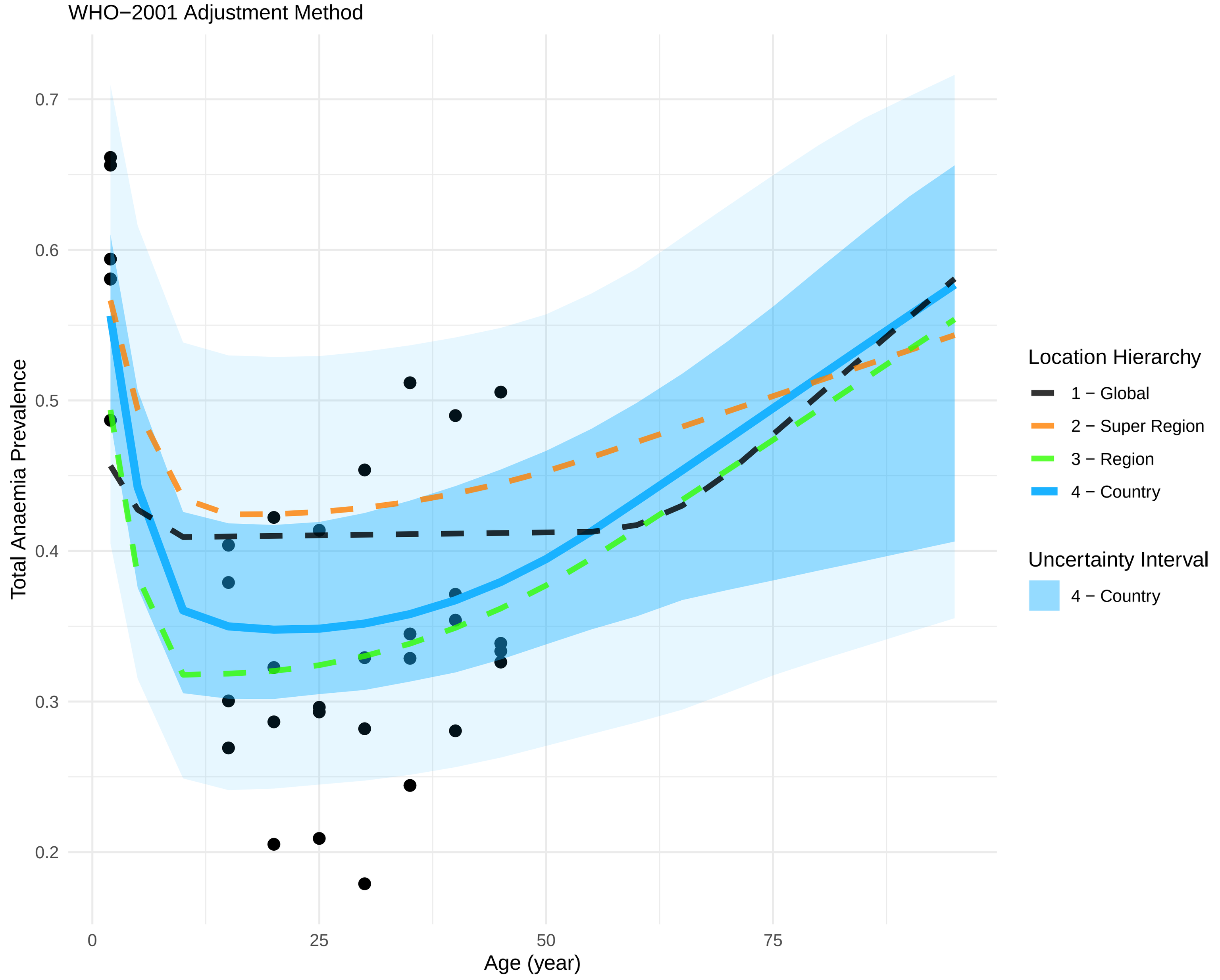

MR−BRT Age/Sex Fits − Non−pregnant Females 2+ Years Old − Total Anaemia Prevalence − Mozambique
WHO−2001 Adjustment Method

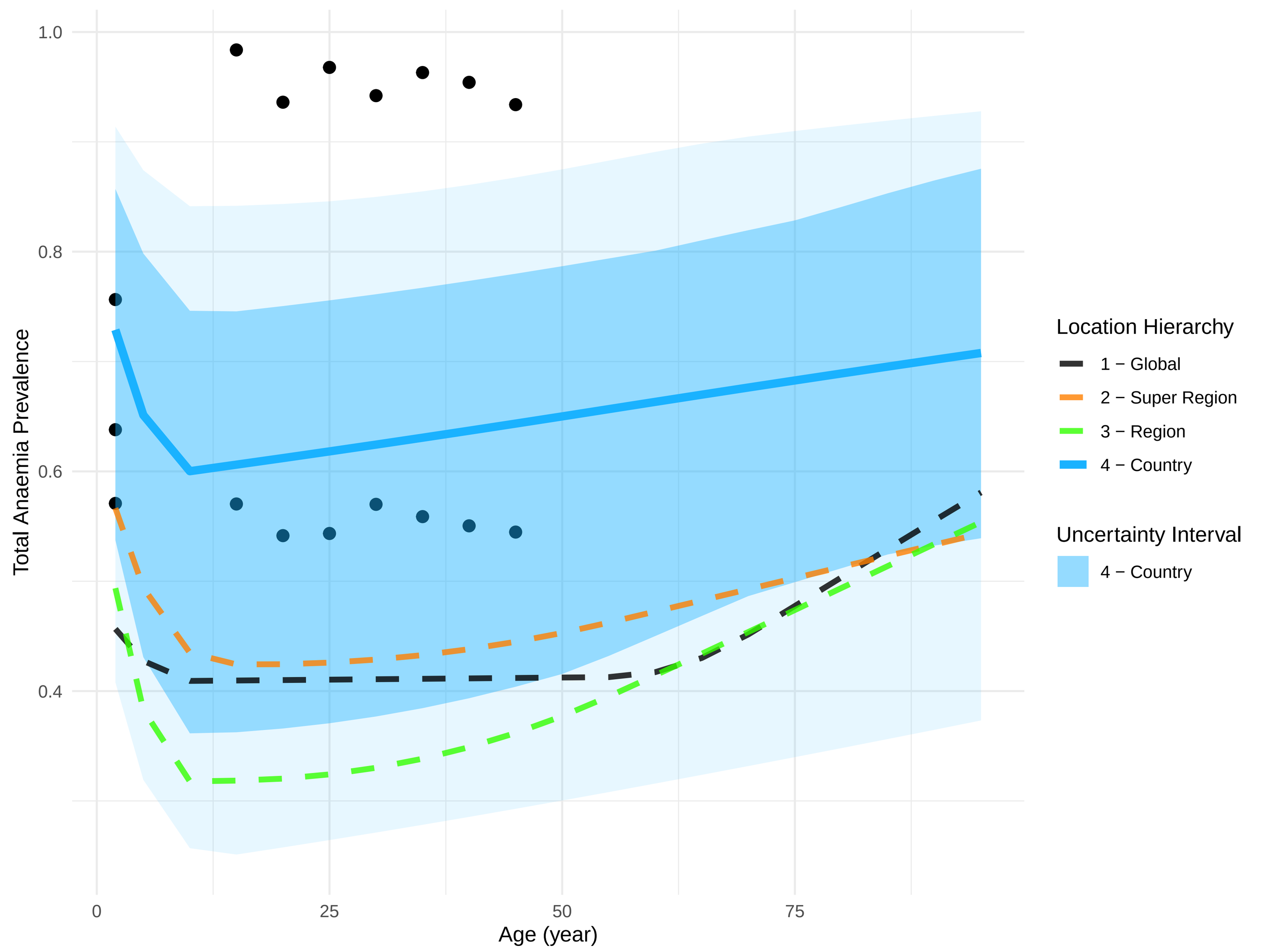

MR−BRT Age/Sex Fits − Non−pregnant Females 2+ Years Old − Total Anaemia Prevalence − Rwanda
WHO−2001 Adjustment Method

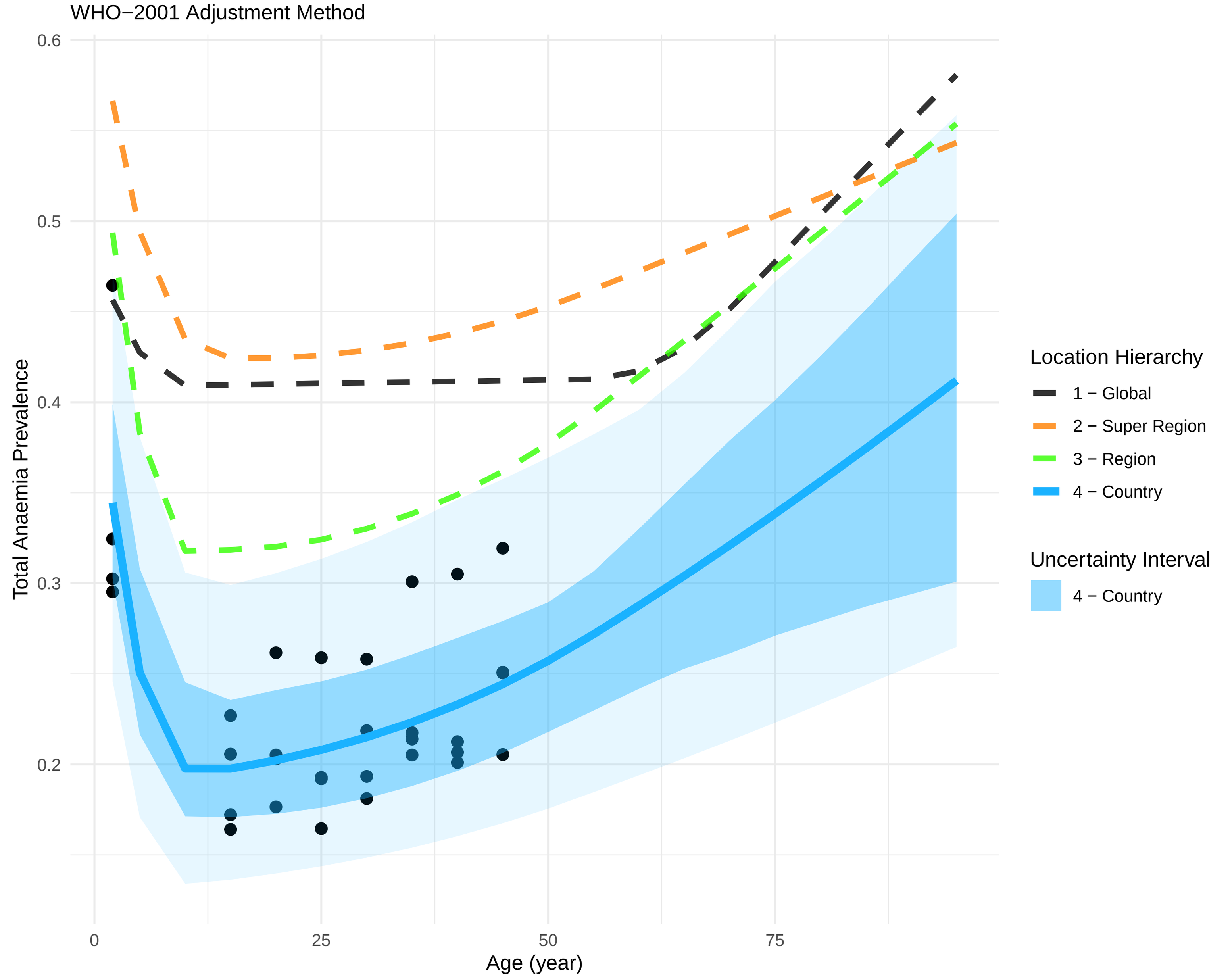

MR−BRT Age/Sex Fits − Non−pregnant Females 2+ Years Old − Total Anaemia Prevalence − Uganda
WHO−2001 Adjustment Method

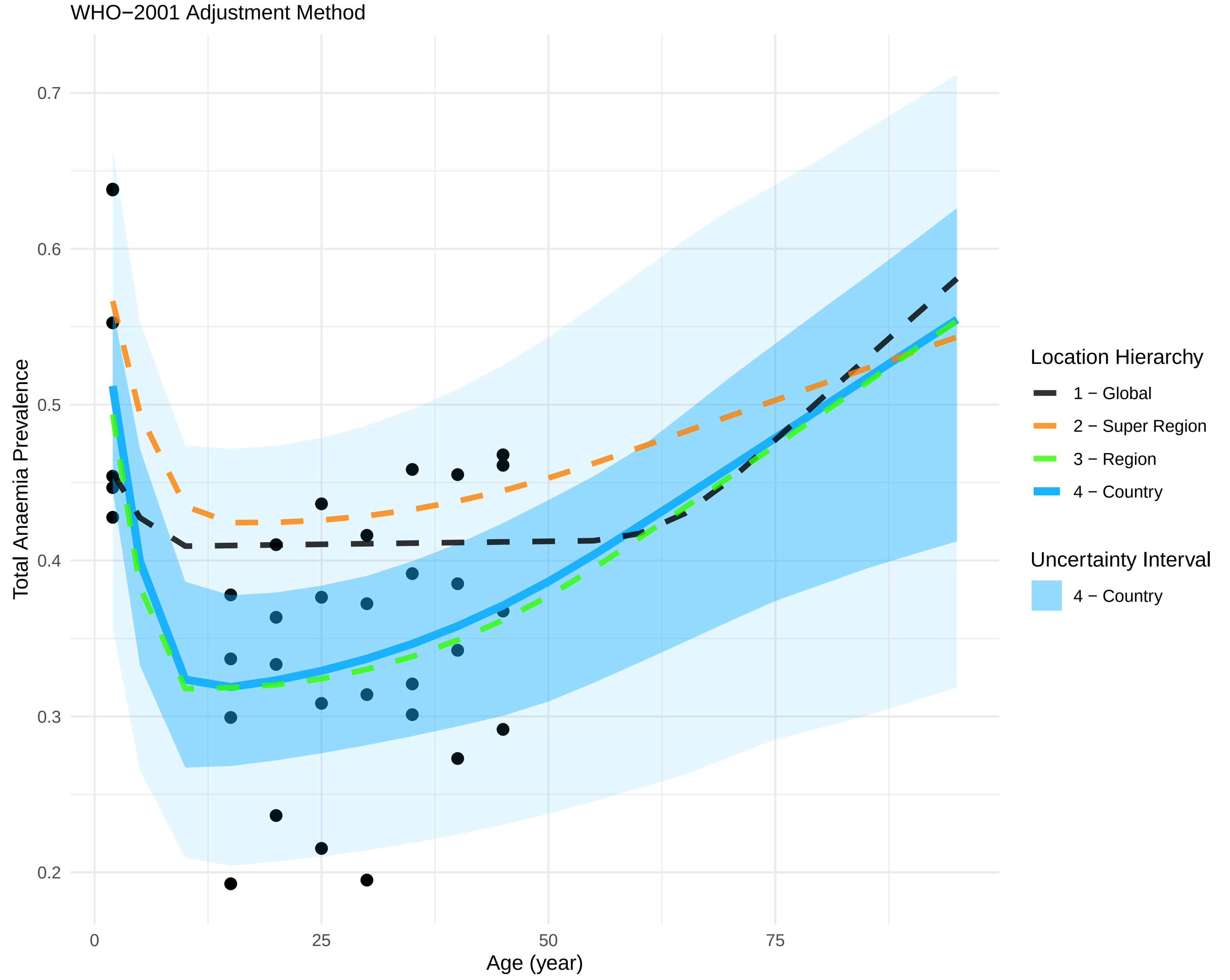

MR−BRT Age/Sex Fits − Non−pregnant Females 2+ Years Old − Total Anaemia Prevalence − United Republic of Tanzania
WHO−2001 Adjustment Method

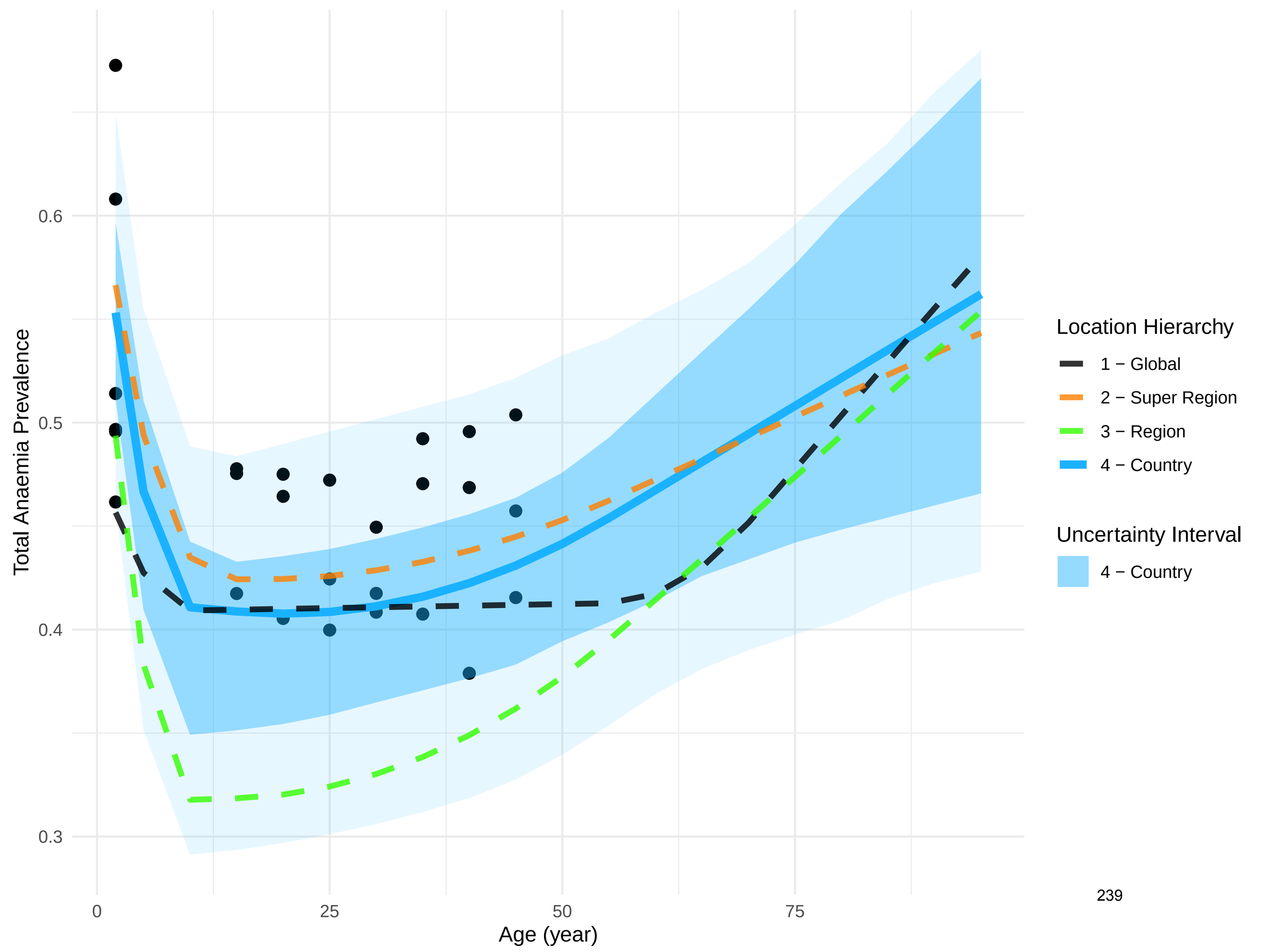

MR−BRT Age/Sex Fits − Non−pregnant Females 2+ Years Old − Total Anaemia Prevalence − Zambia
WHO−2001 Adjustment Method

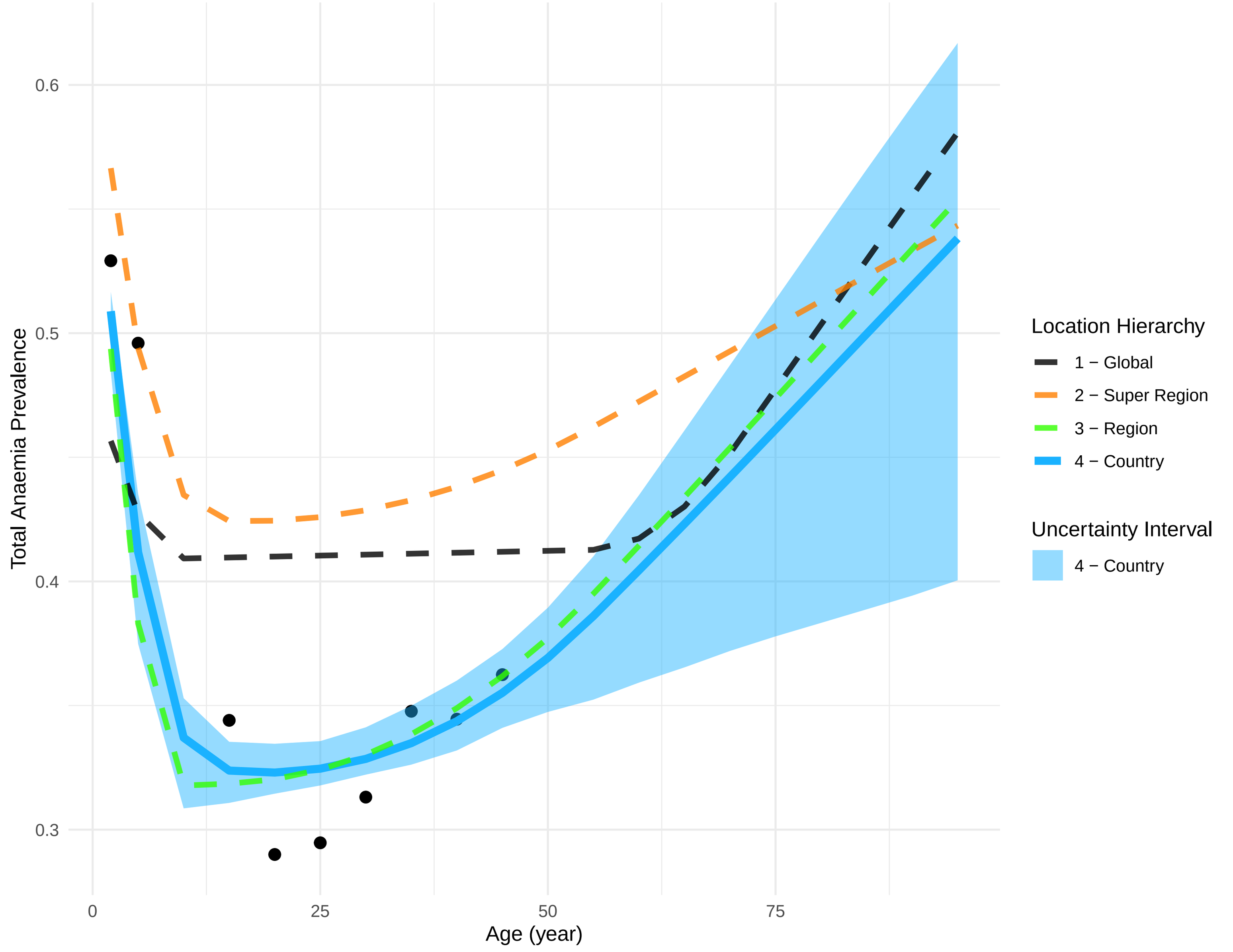

MR−BRT Age/Sex Fits − Non−pregnant Females 2+ Years Old − Total Anaemia Prevalence − Southern Sub−Saharan Africa
WHO−2001 Adjustment Method

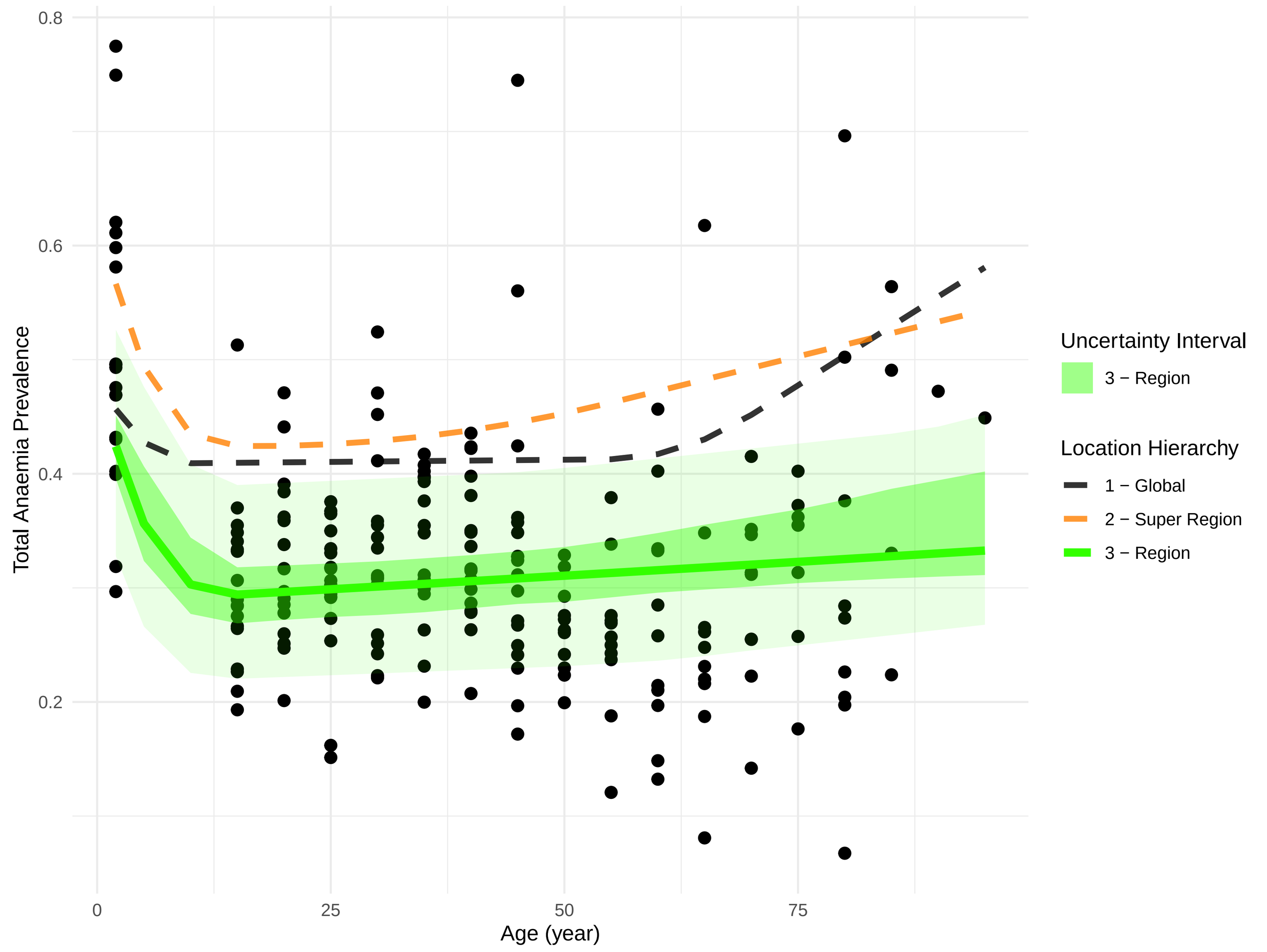

MR−BRT Age/Sex Fits − Non−pregnant Females 2+ Years Old − Total Anaemia Prevalence − Eswatini
WHO−2001 Adjustment Method

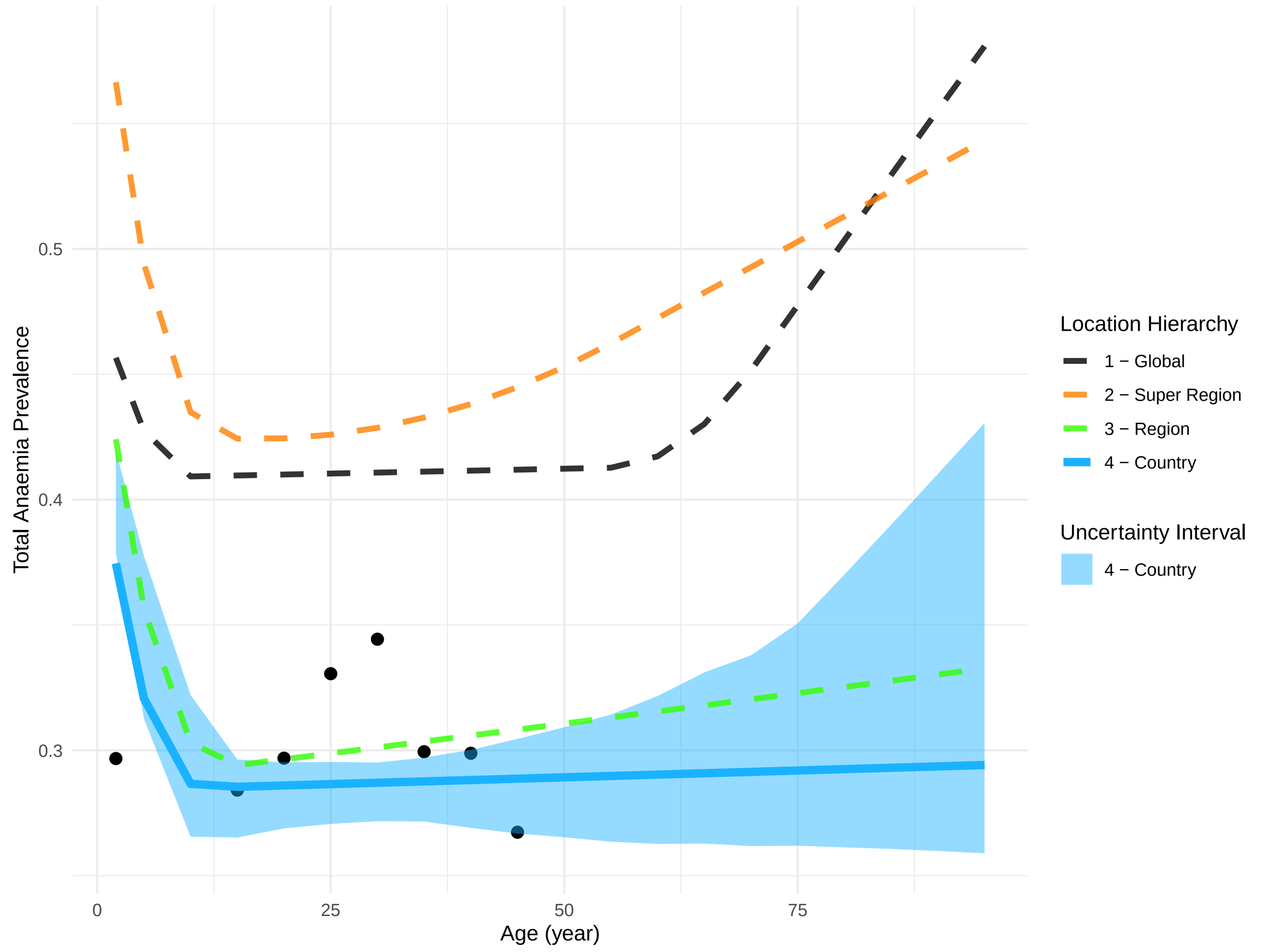

MR−BRT Age/Sex Fits − Non−pregnant Females 2+ Years Old − Total Anaemia Prevalence − Lesotho
WHO−2001 Adjustment Method

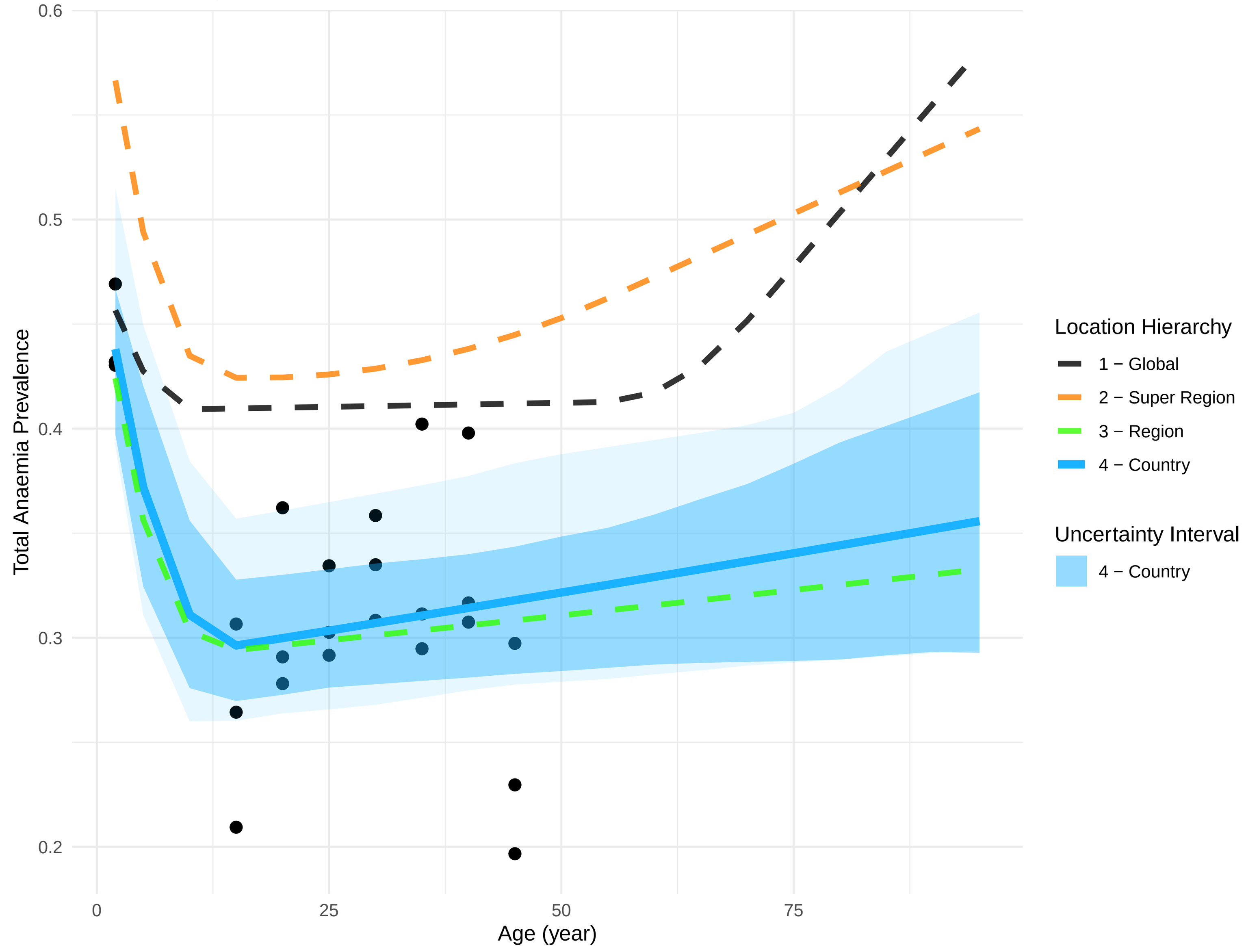

MR−BRT Age/Sex Fits − Non−pregnant Females 2+ Years Old − Total Anaemia Prevalence − Namibia
WHO−2001 Adjustment Method

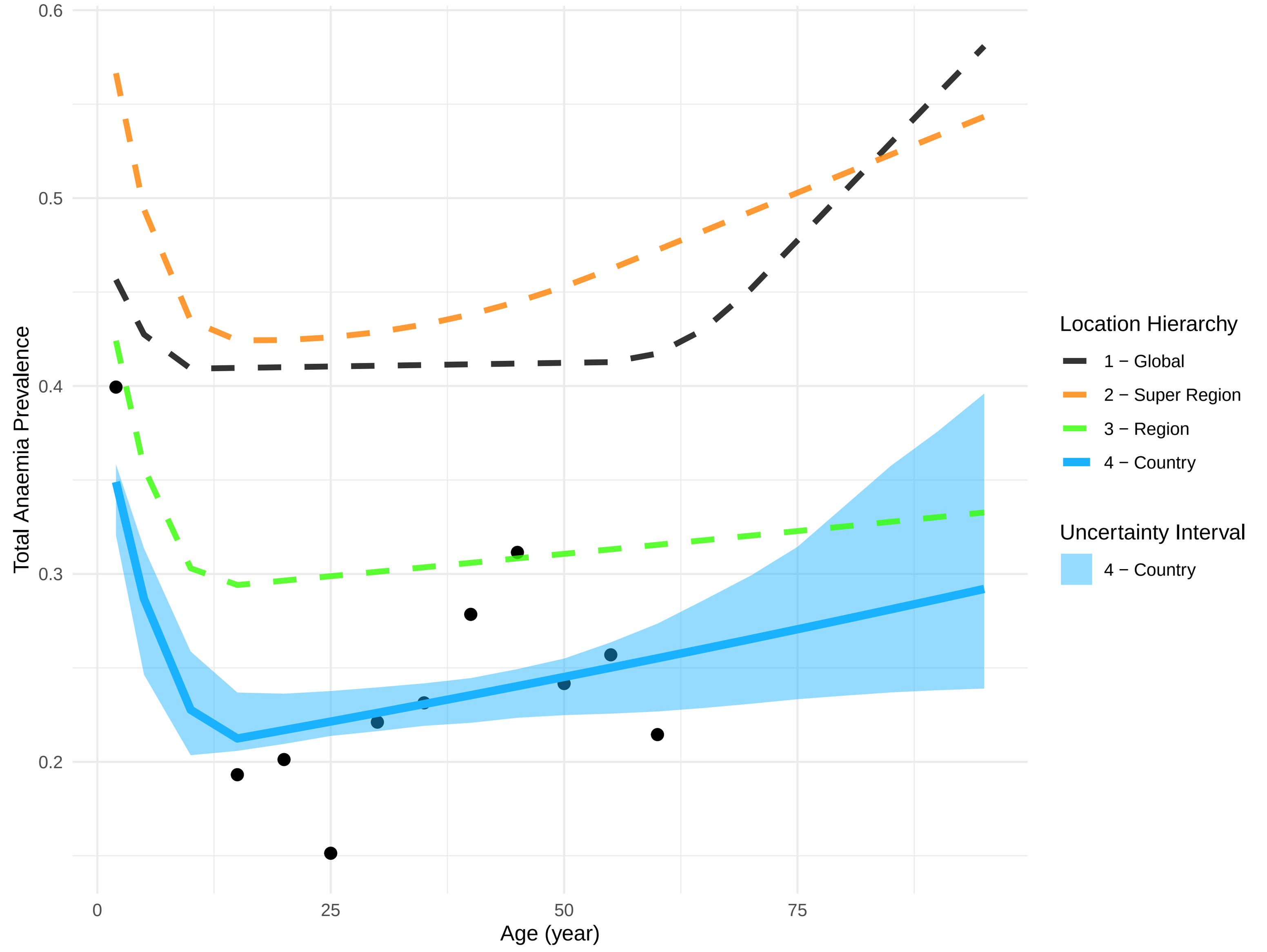

MR−BRT Age/Sex Fits − Non−pregnant Females 2+ Years Old − Total Anaemia Prevalence − South Africa
WHO−2001 Adjustment Method

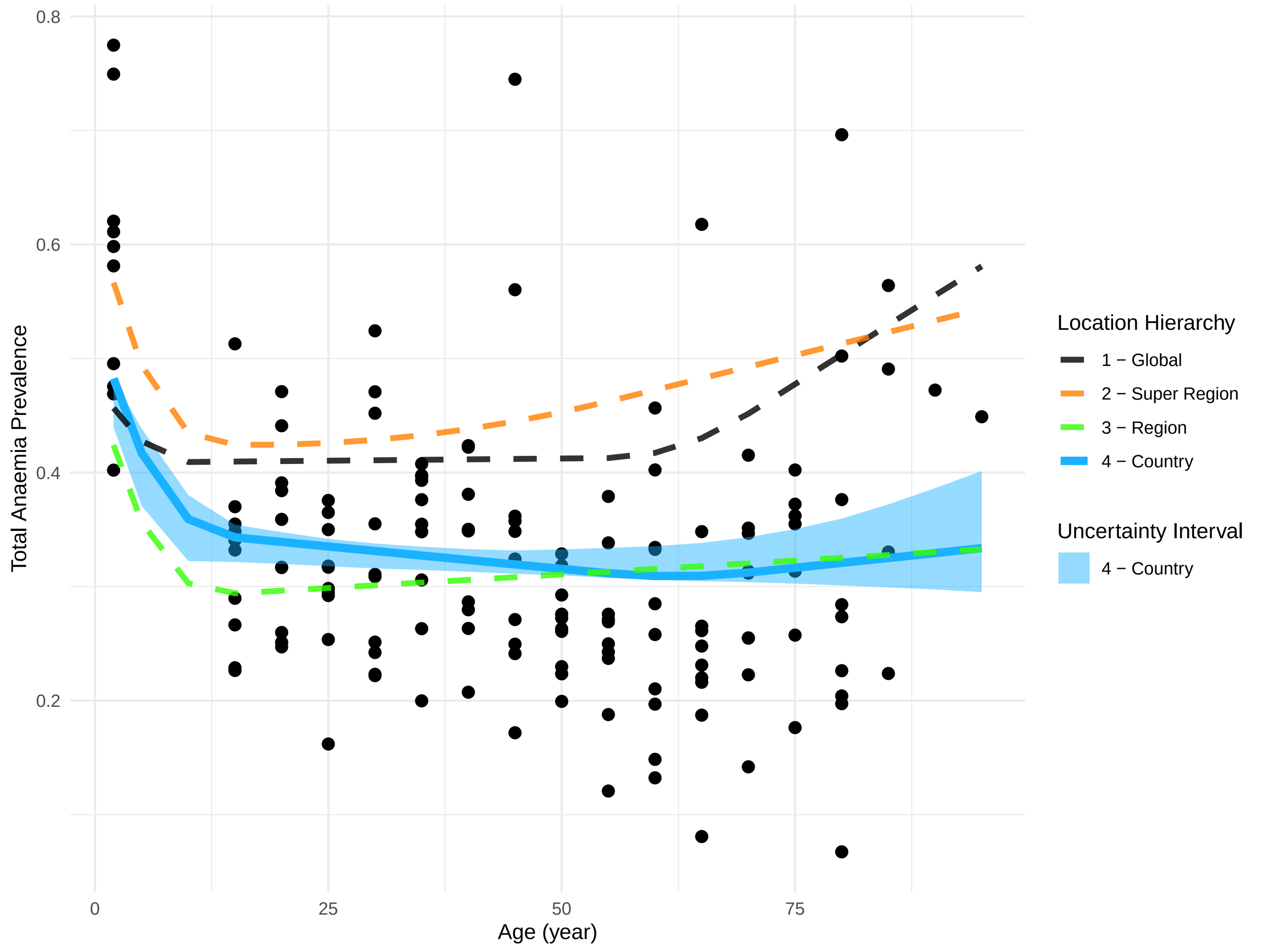

MR−BRT Age/Sex Fits − Non−pregnant Females 2+ Years Old − Total Anaemia Prevalence − Eastern Cape
WHO−2001 Adjustment Method

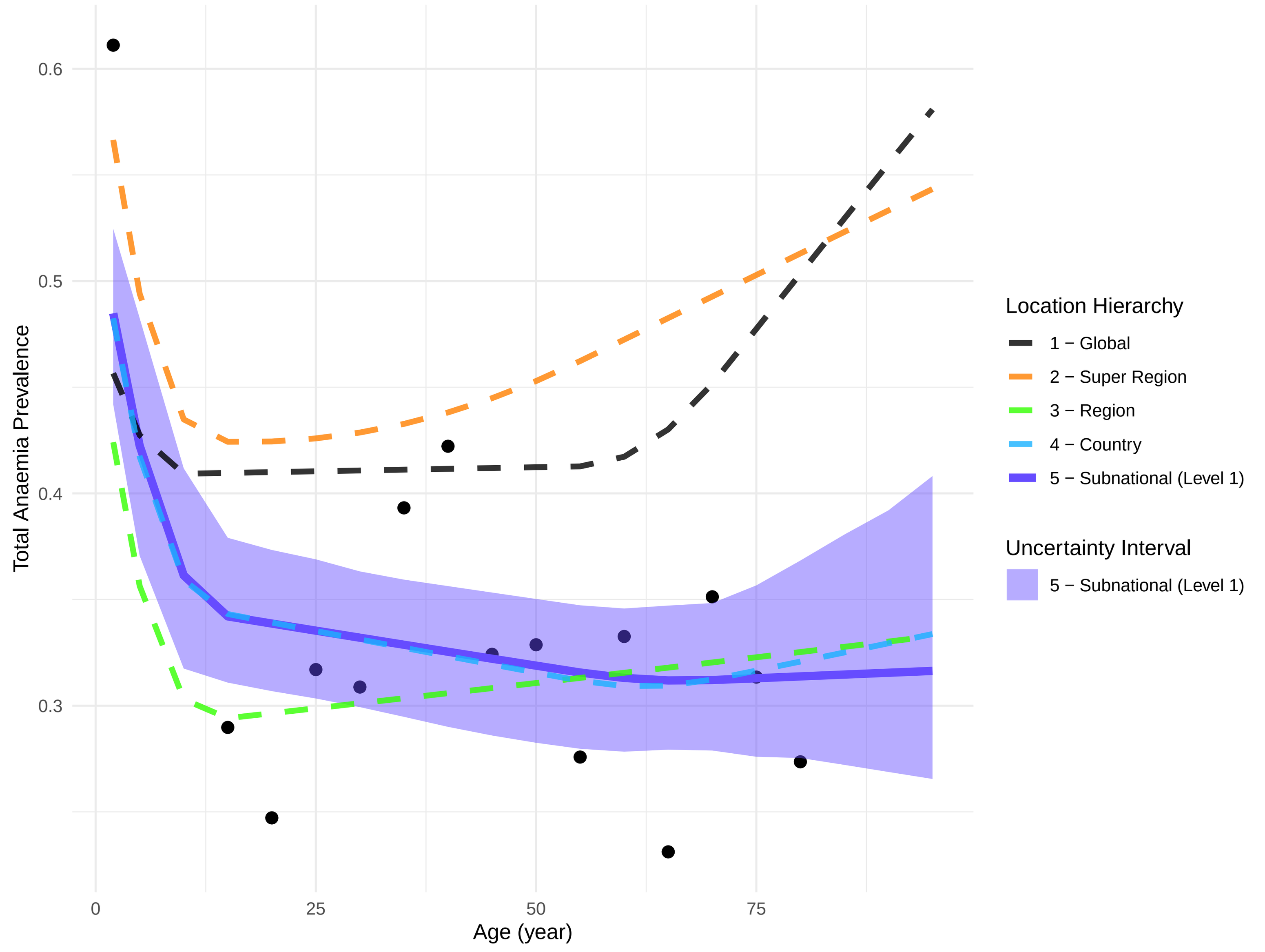

MR−BRT Age/Sex Fits − Non−pregnant Females 2+ Years Old − Total Anaemia Prevalence − Free State
WHO−2001 Adjustment Method

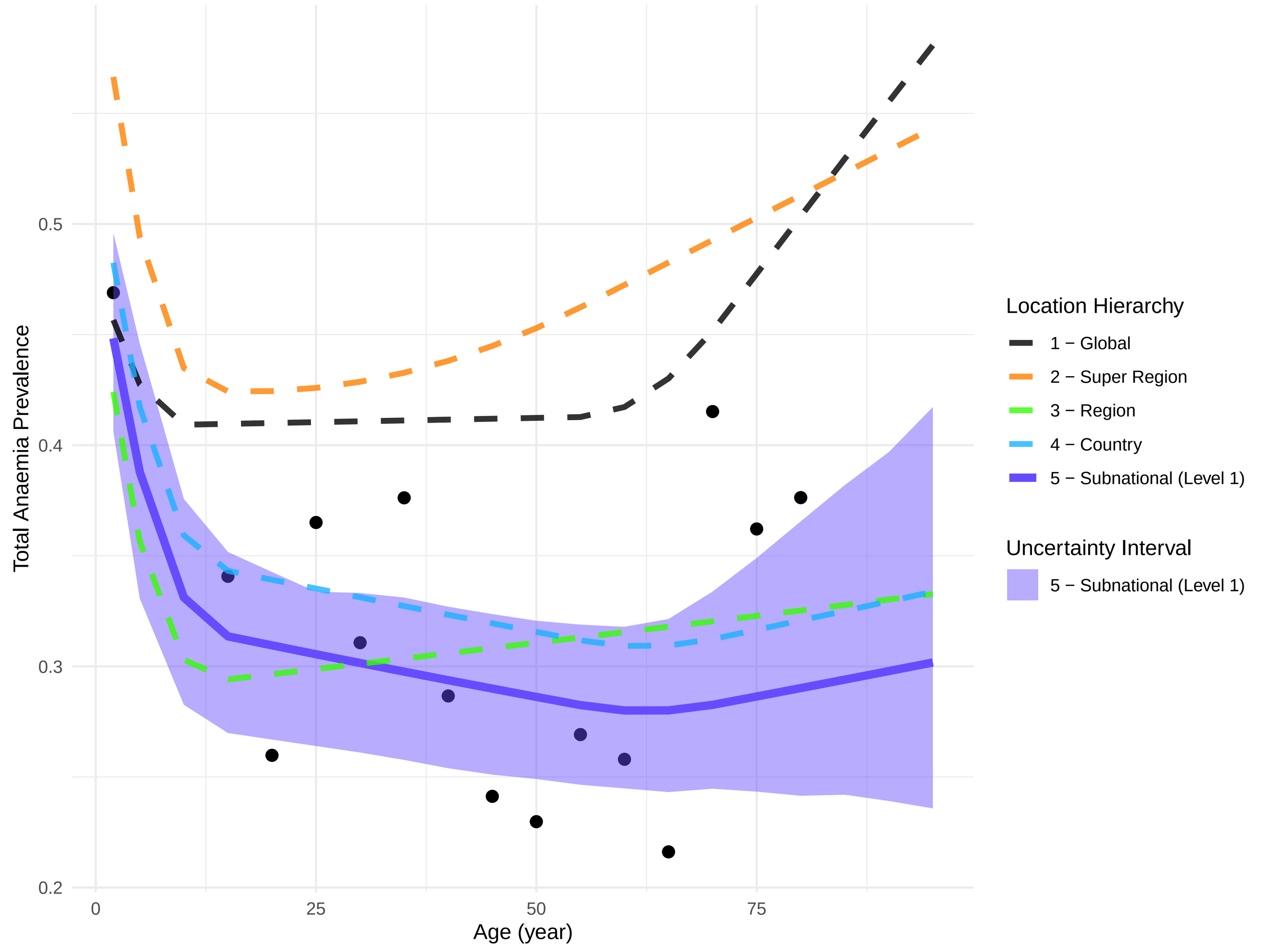

MR−BRT Age/Sex Fits − Non−pregnant Females 2+ Years Old − Total Anaemia Prevalence − Gauteng
WHO−2001 Adjustment Method

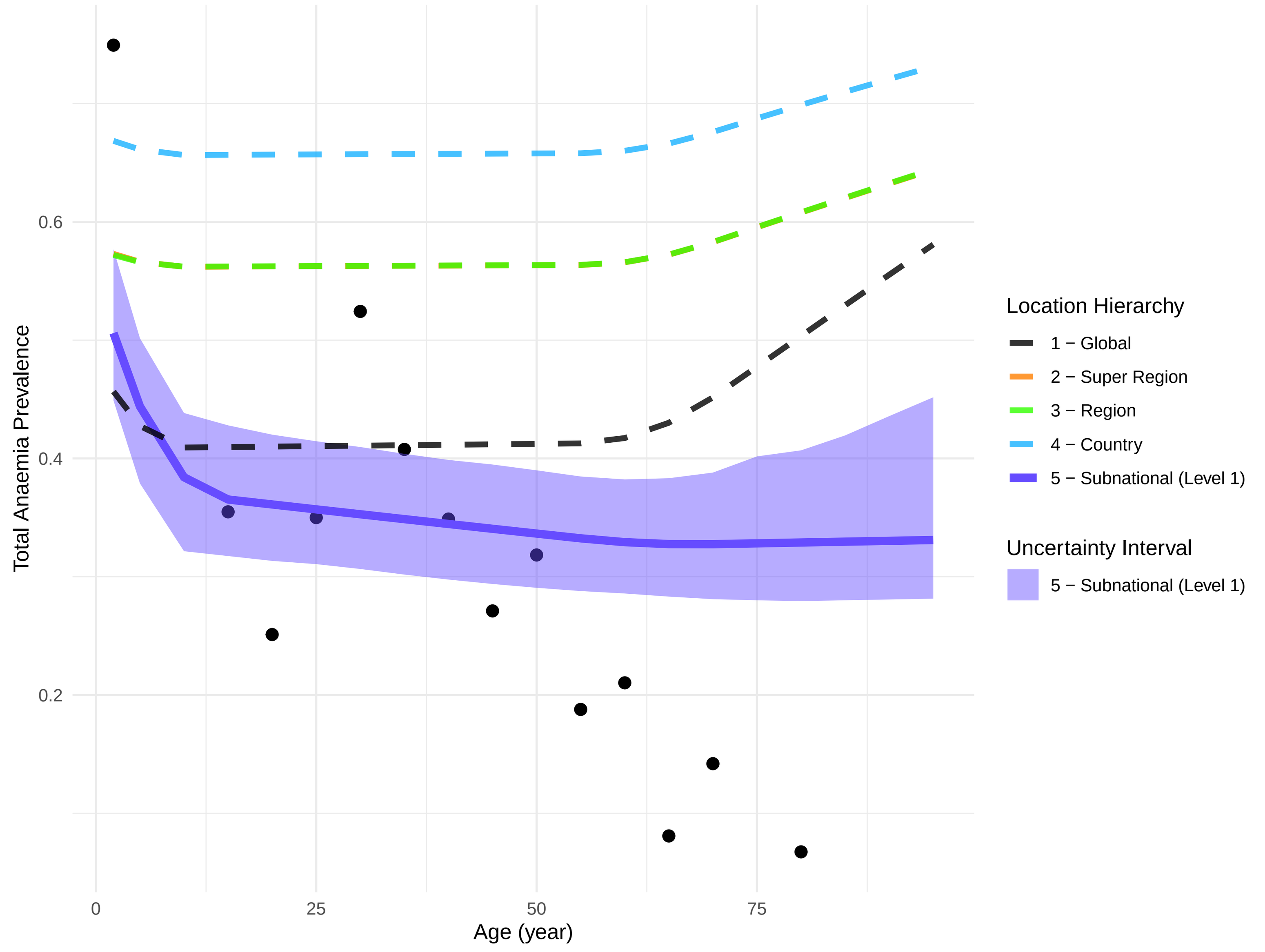

MR−BRT Age/Sex Fits − Non−pregnant Females 2+ Years Old − Total Anaemia Prevalence − KwaZulu−Natal
WHO−2001 Adjustment Method

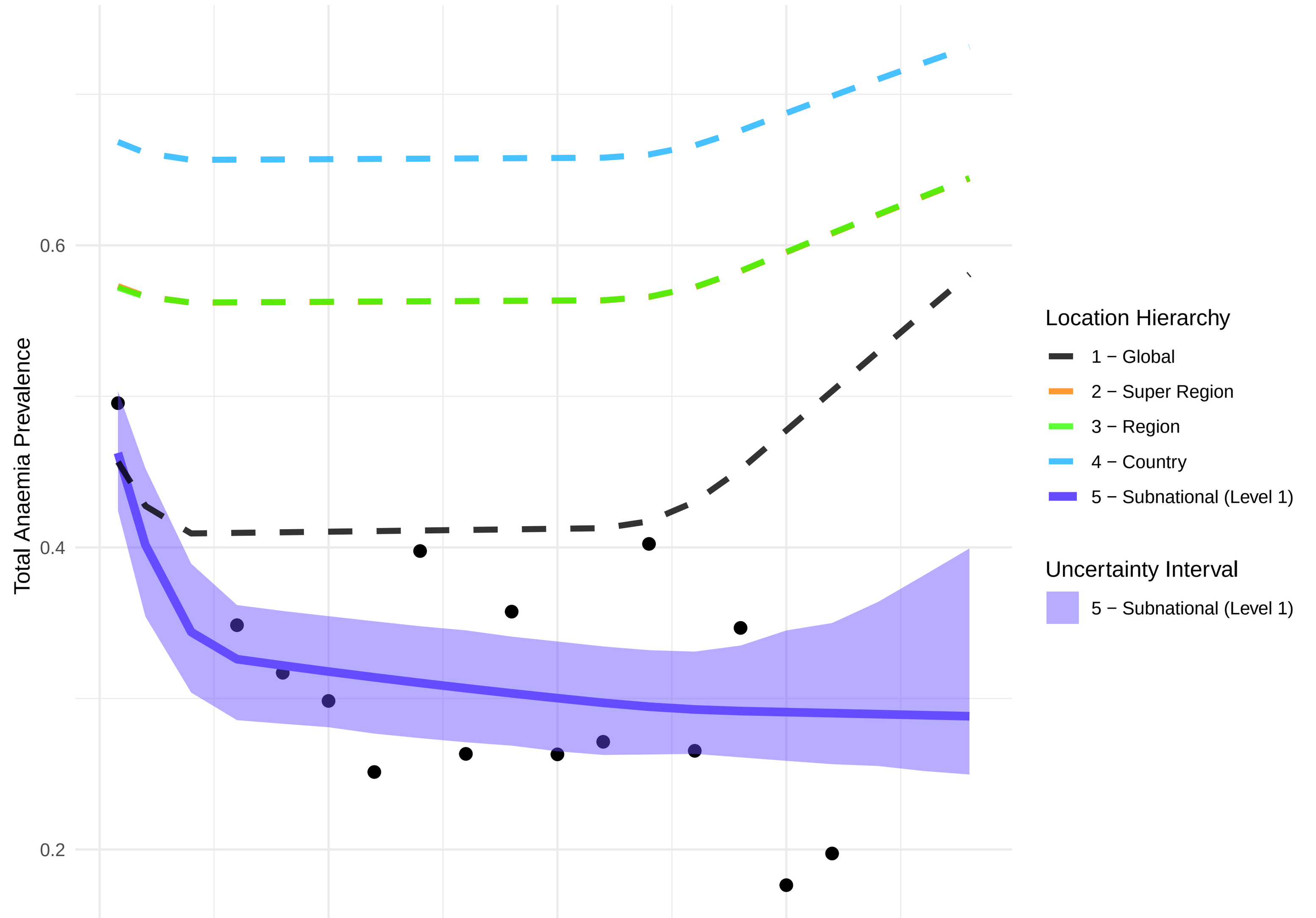

MR−BRT Age/Sex Fits − Non−pregnant Females 2+ Years Old − Total Anaemia Prevalence − Limpopo
WHO−2001 Adjustment Method

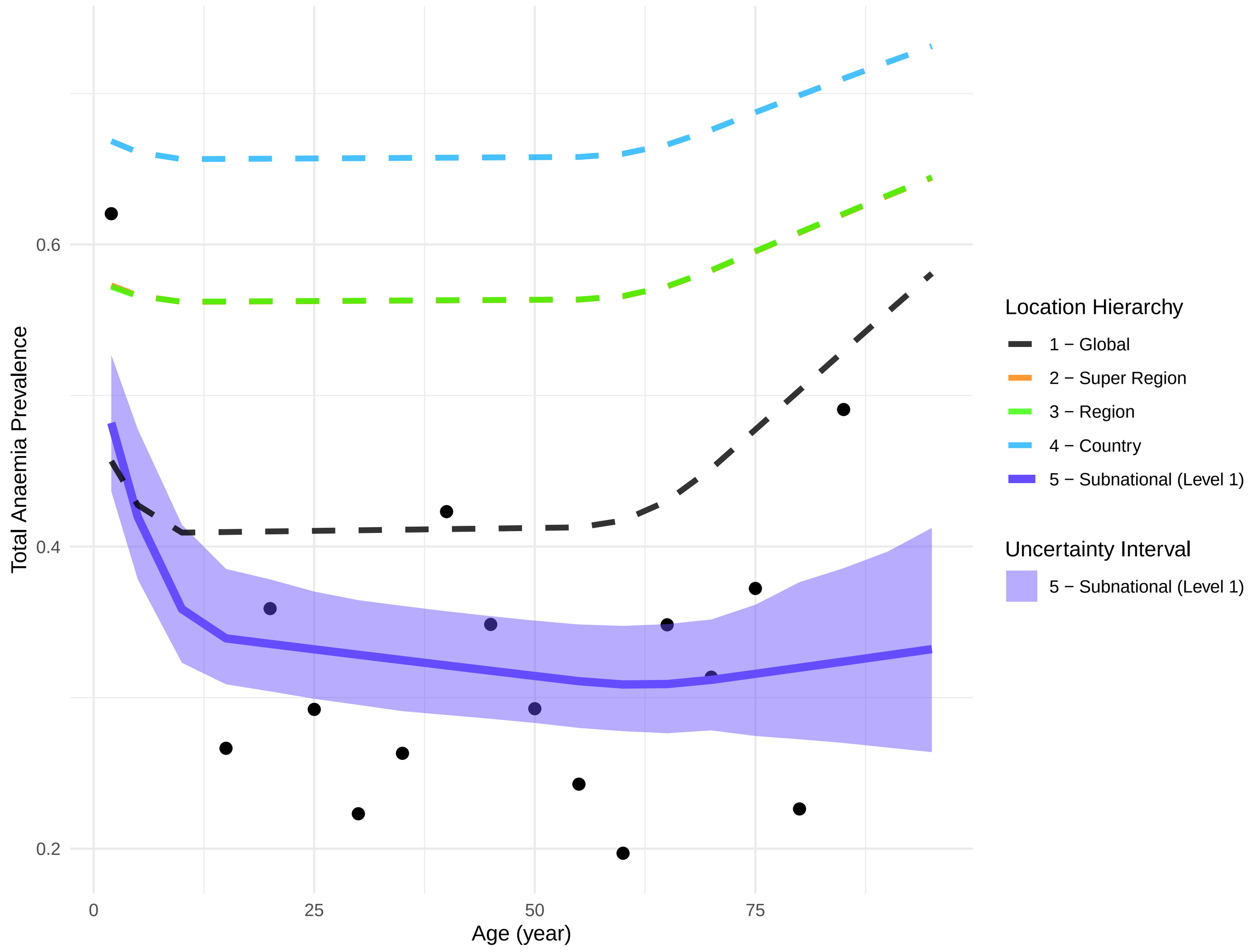

MR−BRT Age/Sex Fits − Non−pregnant Females 2+ Years Old − Total Anaemia Prevalence − Mpumalanga
WHO−2001 Adjustment Method

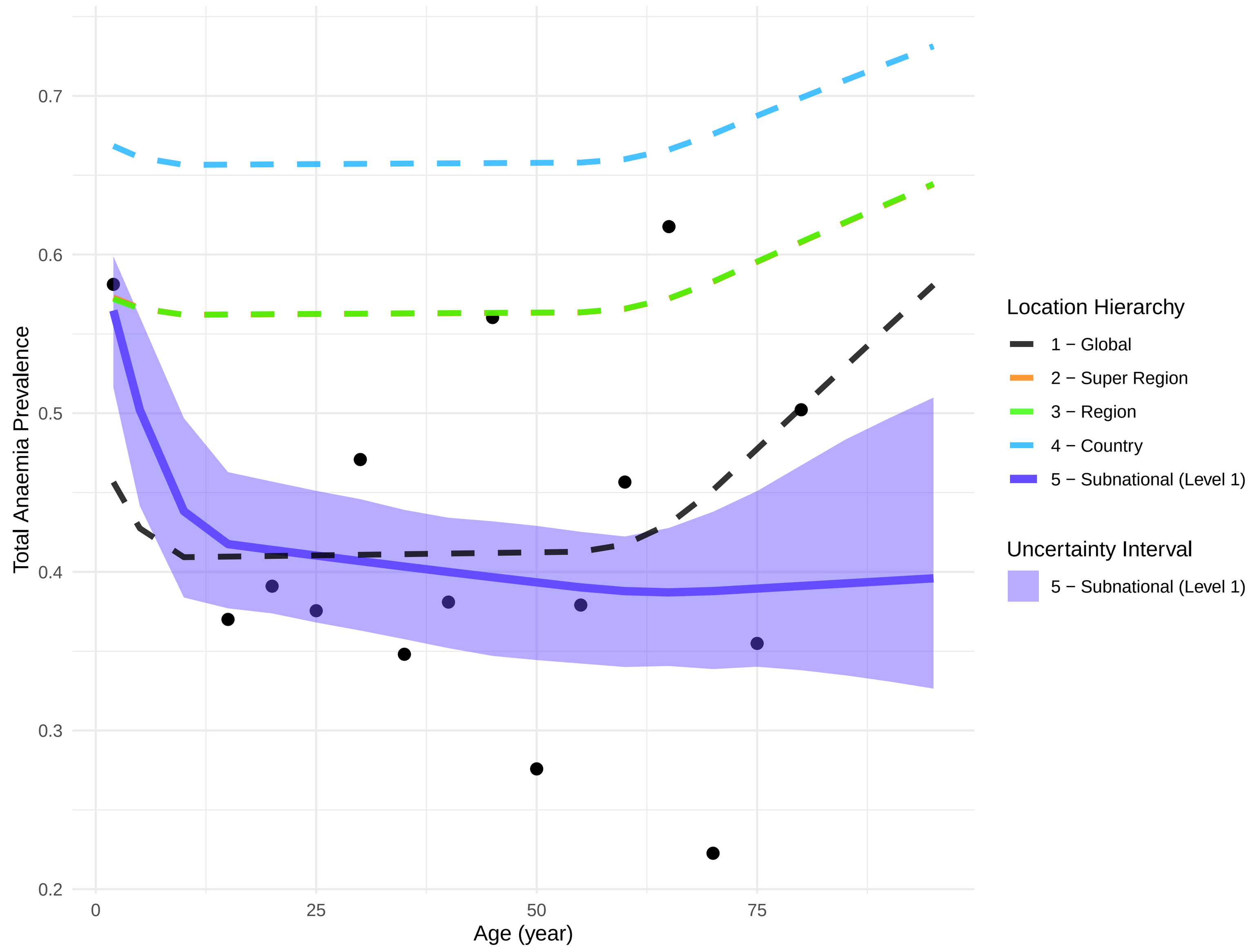

MR−BRT Age/Sex Fits − Non−pregnant Females 2+ Years Old − Total Anaemia Prevalence − North−West
WHO−2001 Adjustment Method

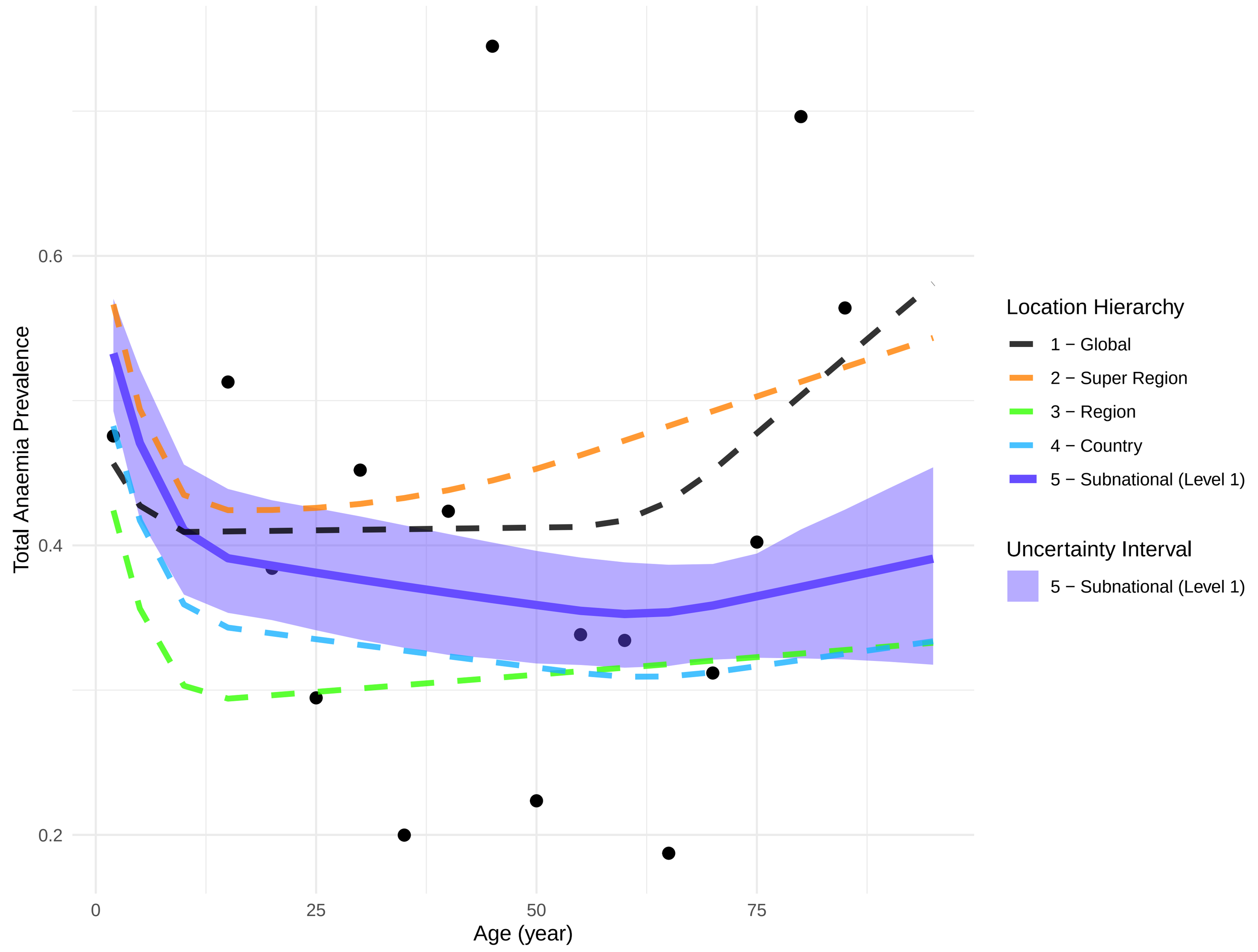

MR−BRT Age/Sex Fits − Non−pregnant Females 2+ Years Old − Total Anaemia Prevalence − Northern Cape
WHO−2001 Adjustment Method

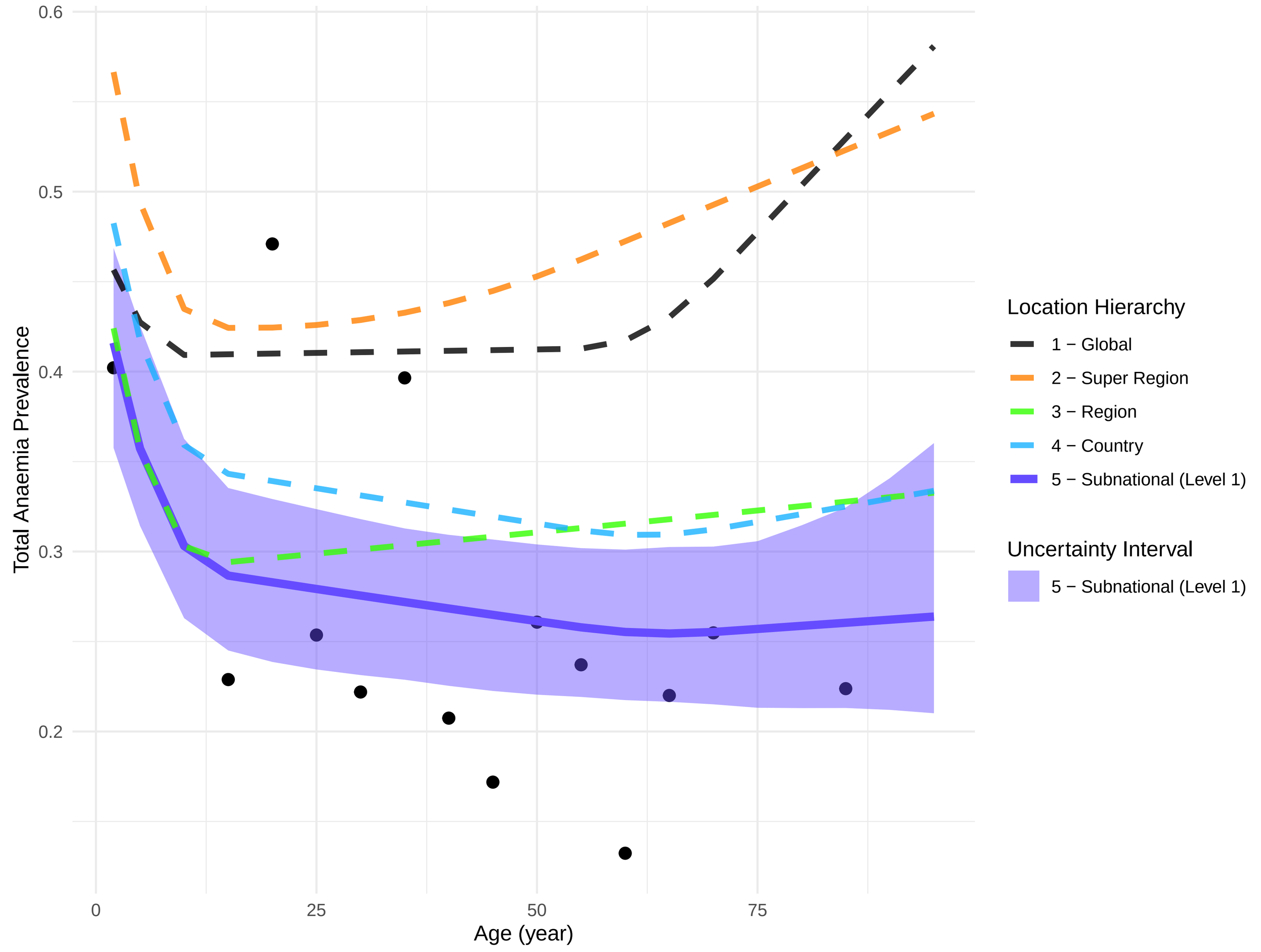

MR−BRT Age/Sex Fits − Non−pregnant Females 2+ Years Old − Total Anaemia Prevalence − Western Cape
WHO−2001 Adjustment Method

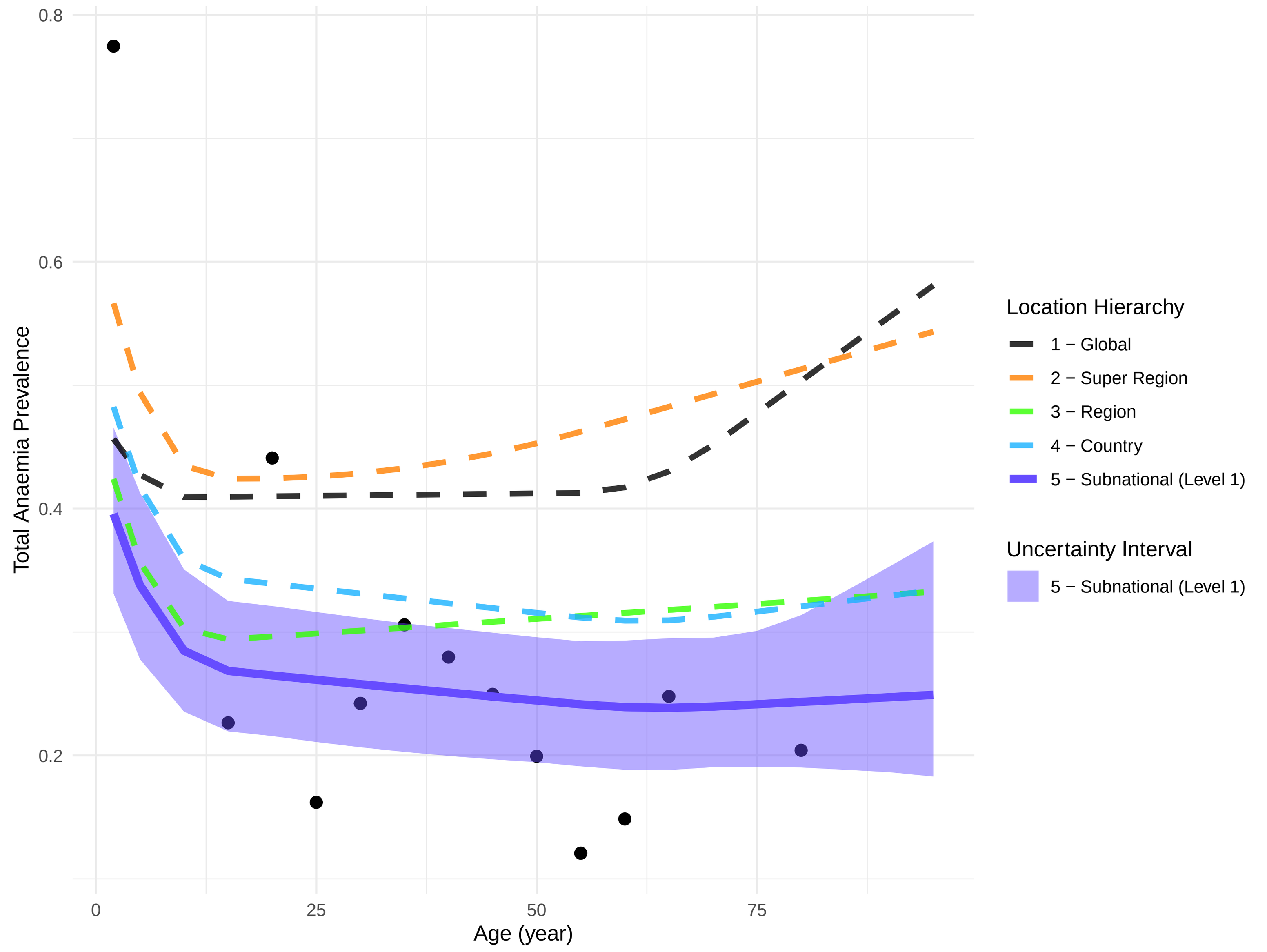

MR−BRT Age/Sex Fits − Non−pregnant Females 2+ Years Old − Total Anaemia Prevalence − Zimbabwe
WHO−2001 Adjustment Method

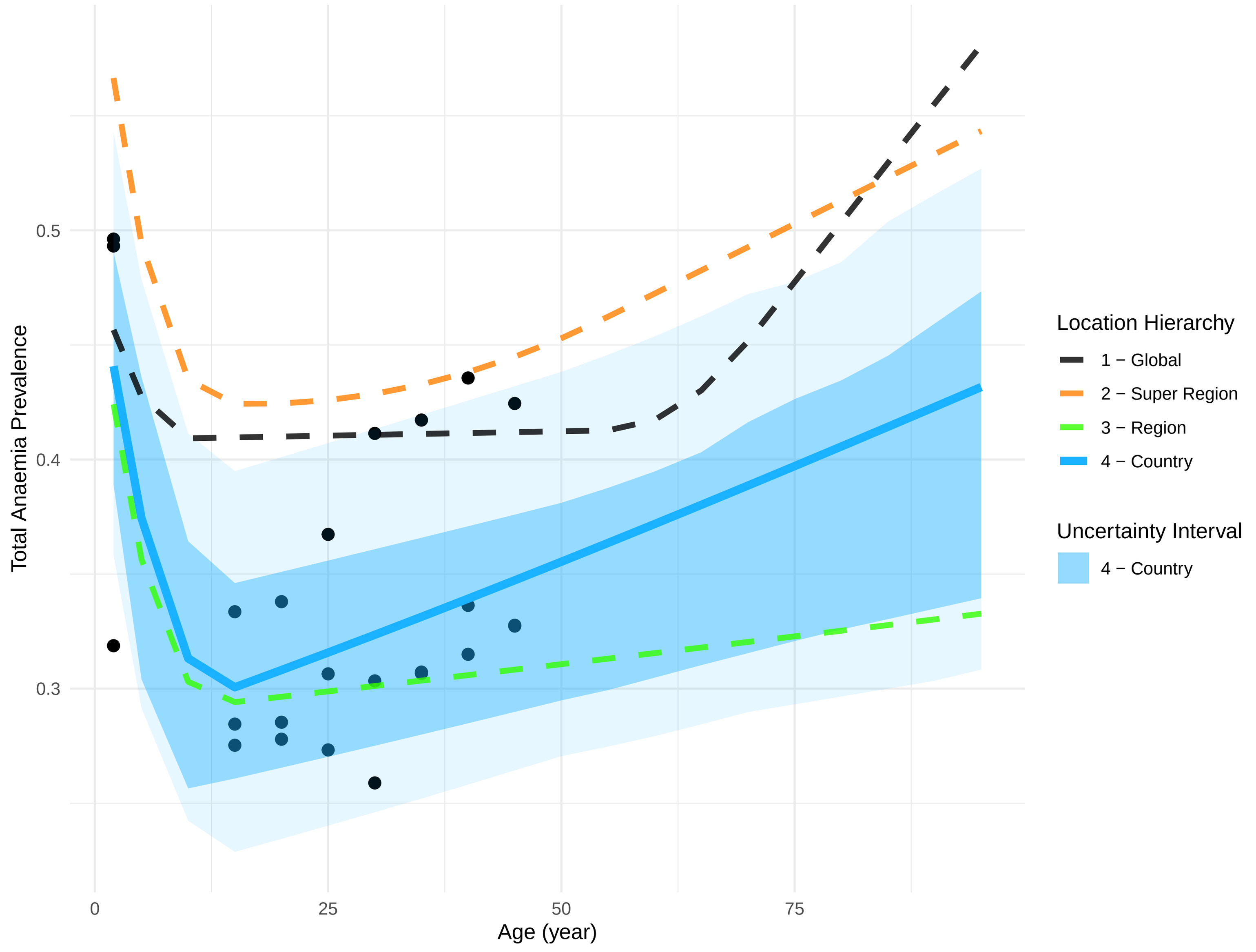

MR−BRT Age/Sex Fits − Non−pregnant Females 2+ Years Old − Total Anaemia Prevalence − Western Sub−Saharan Africa
WHO−2001 Adjustment Method

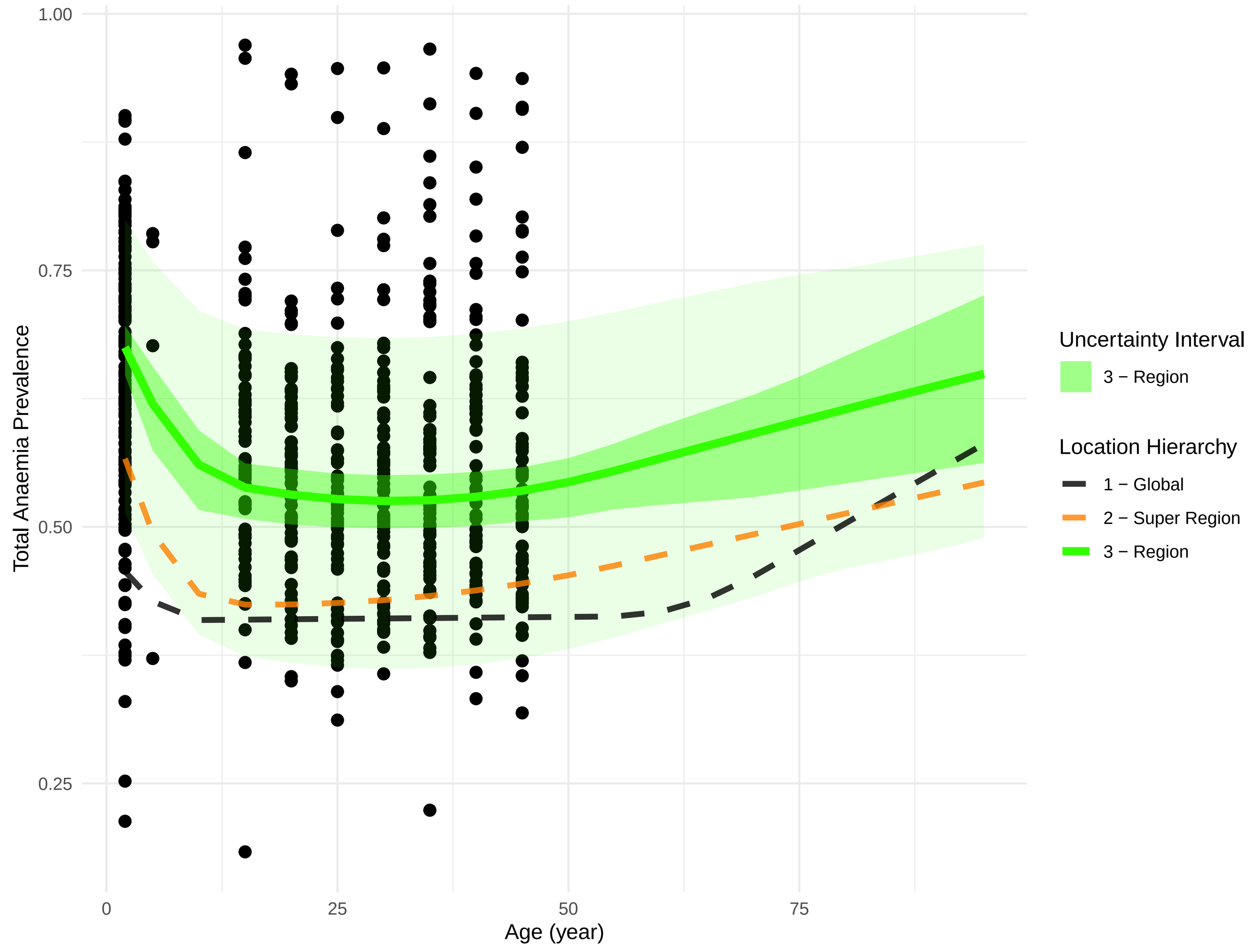

MR−BRT Age/Sex Fits − Non−pregnant Females 2+ Years Old − Total Anaemia Prevalence − Benin
WHO−2001 Adjustment Method

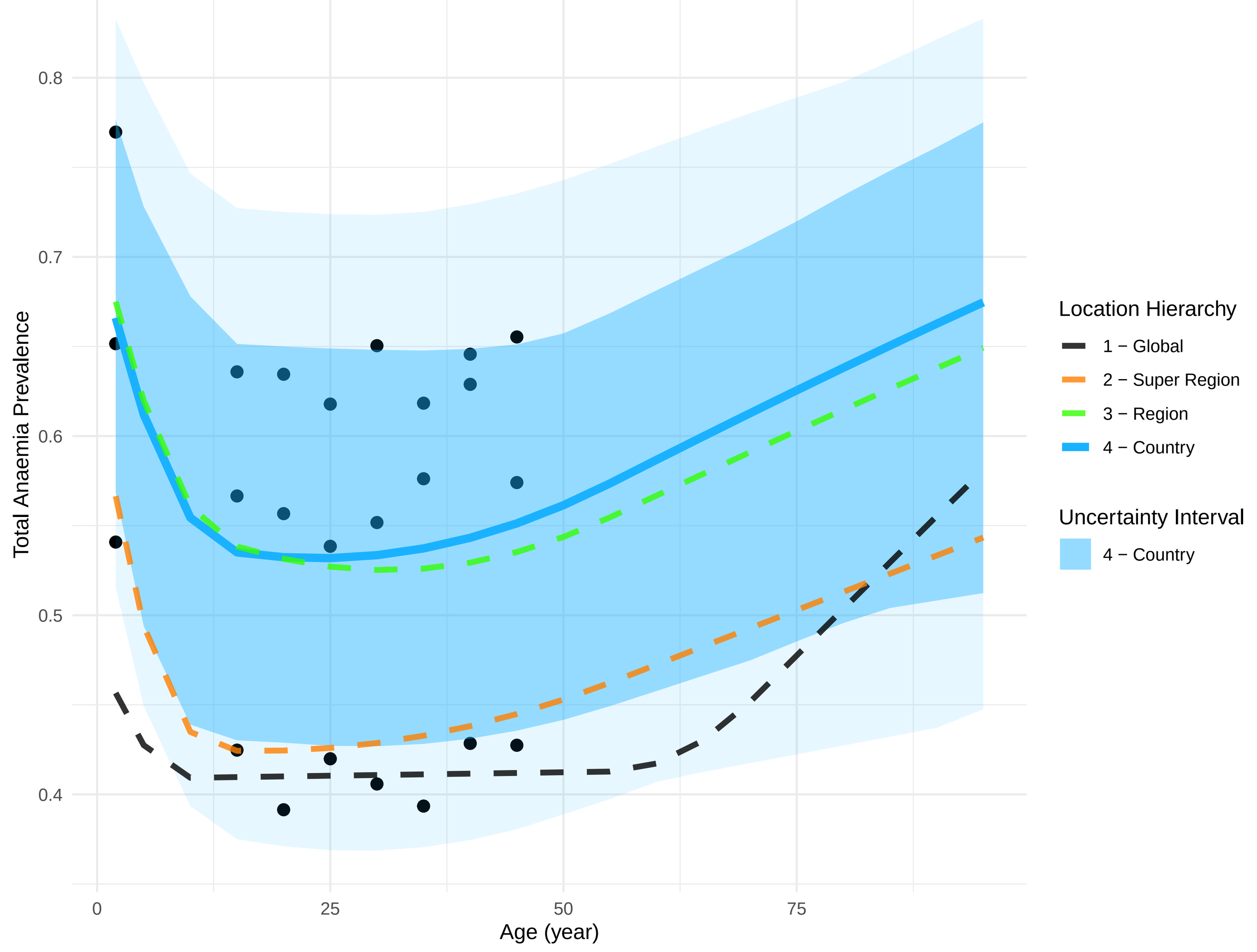

MR−BRT Age/Sex Fits − Non−pregnant Females 2+ Years Old − Total Anaemia Prevalence − Burkina Faso
WHO−2001 Adjustment Method

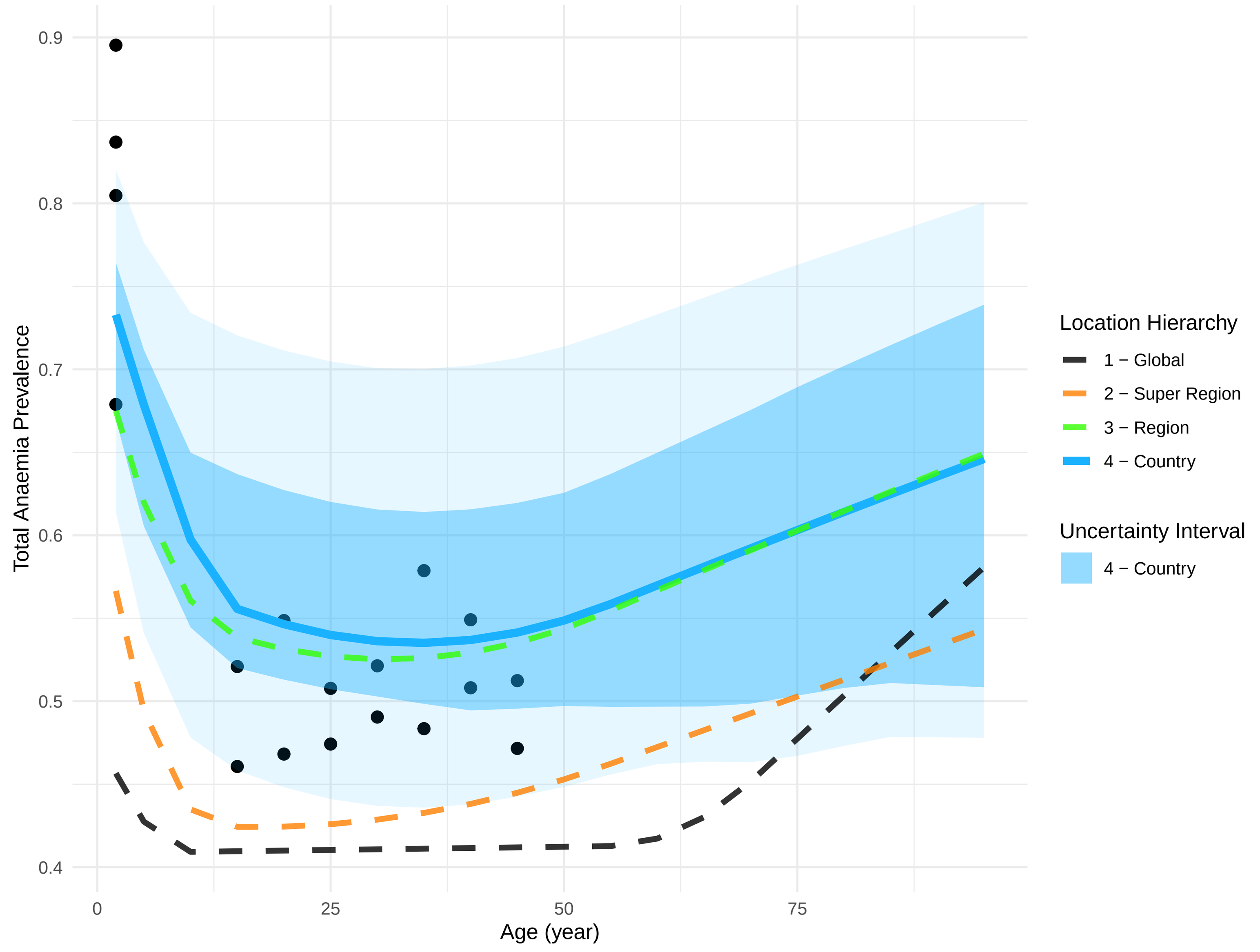

MR−BRT Age/Sex Fits − Non−pregnant Females 2+ Years Old − Total Anaemia Prevalence − Cameroon
WHO−2001 Adjustment Method

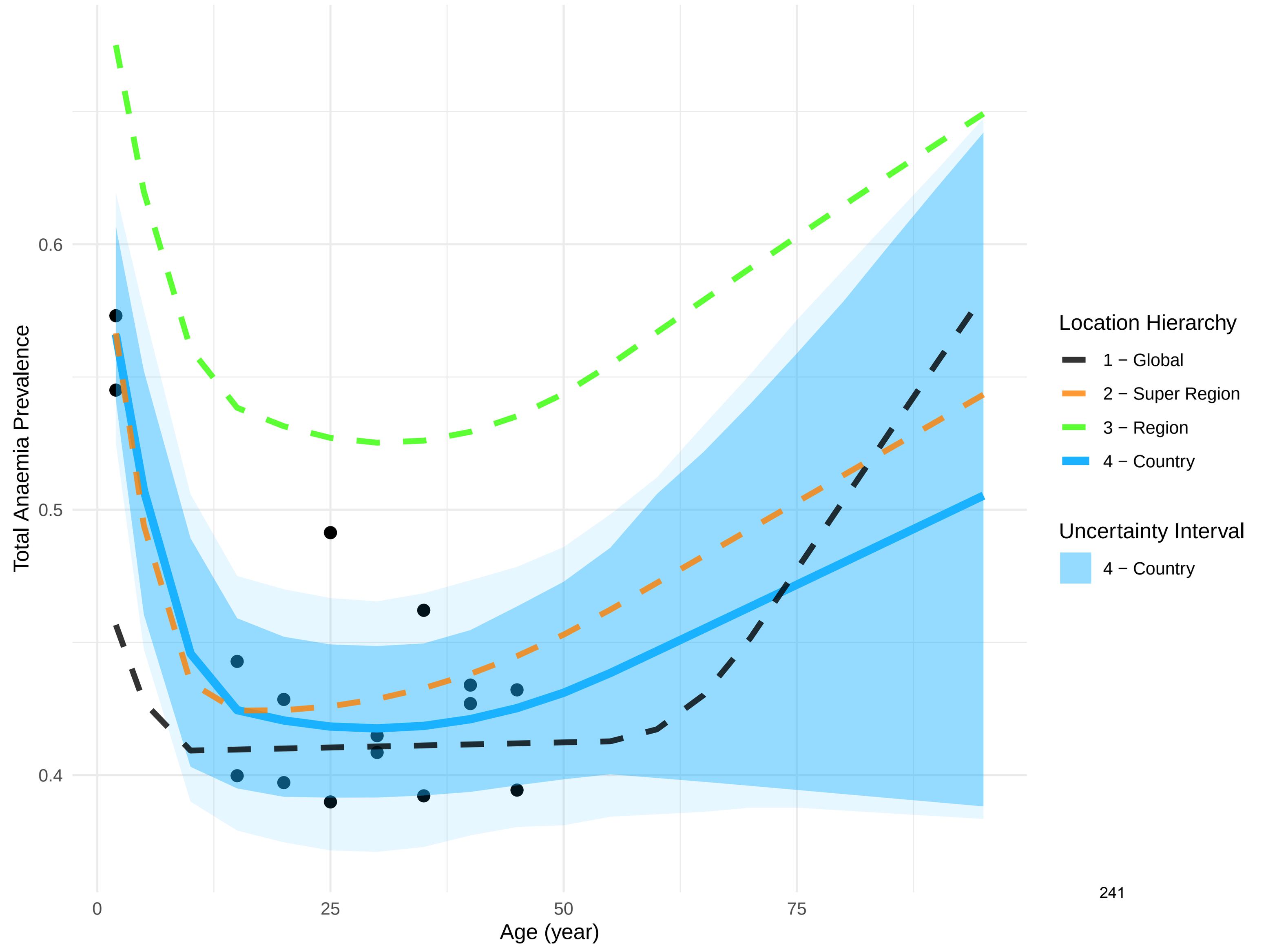

MR−BRT Age/Sex Fits − Non−pregnant Females 2+ Years Old − Total Anaemia Prevalence − Côte d'Ivoire
WHO−2001 Adjustment Method

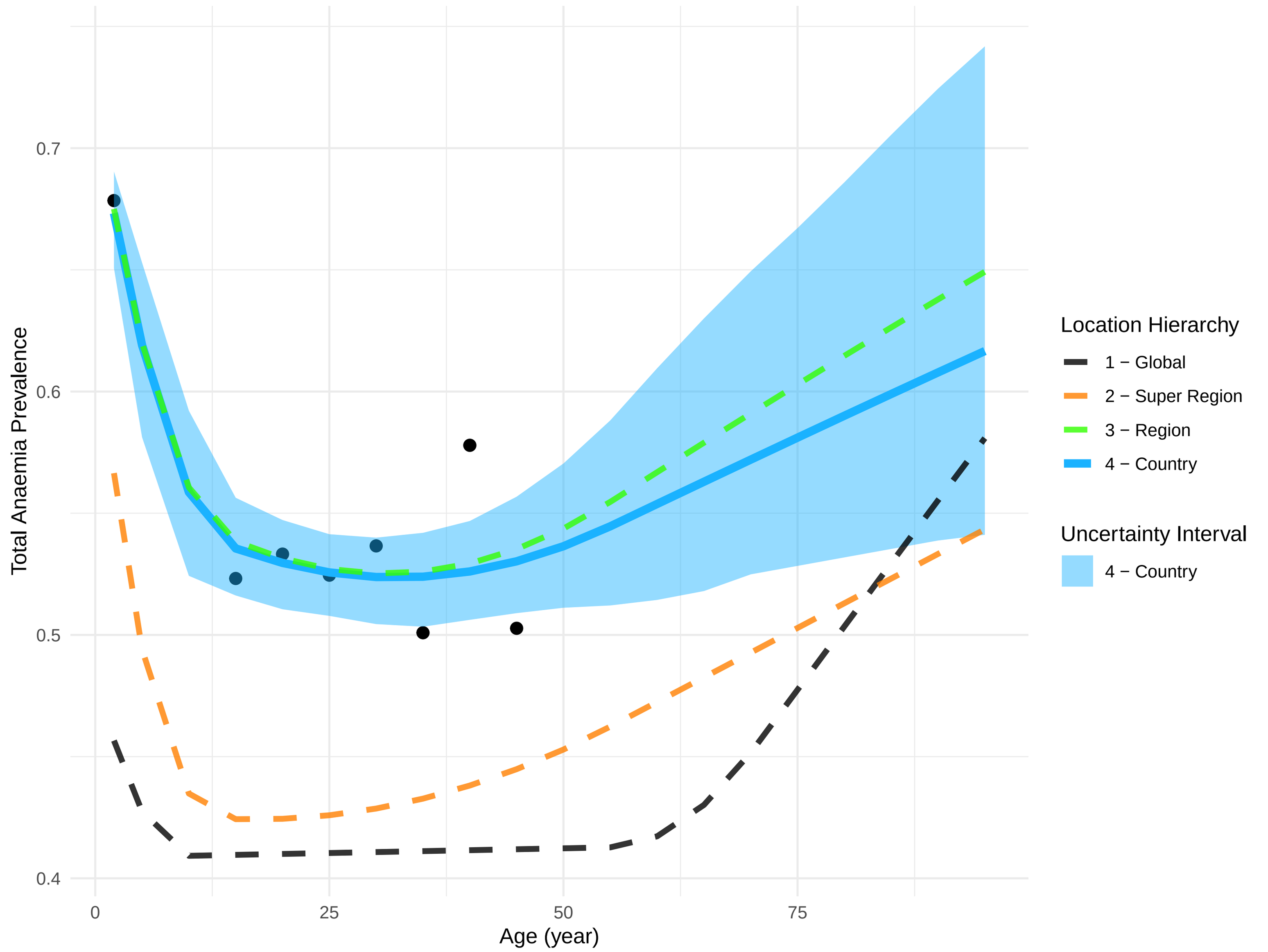

MR−BRT Age/Sex Fits − Non−pregnant Females 2+ Years Old − Total Anaemia Prevalence − Gambia
WHO−2001 Adjustment Method

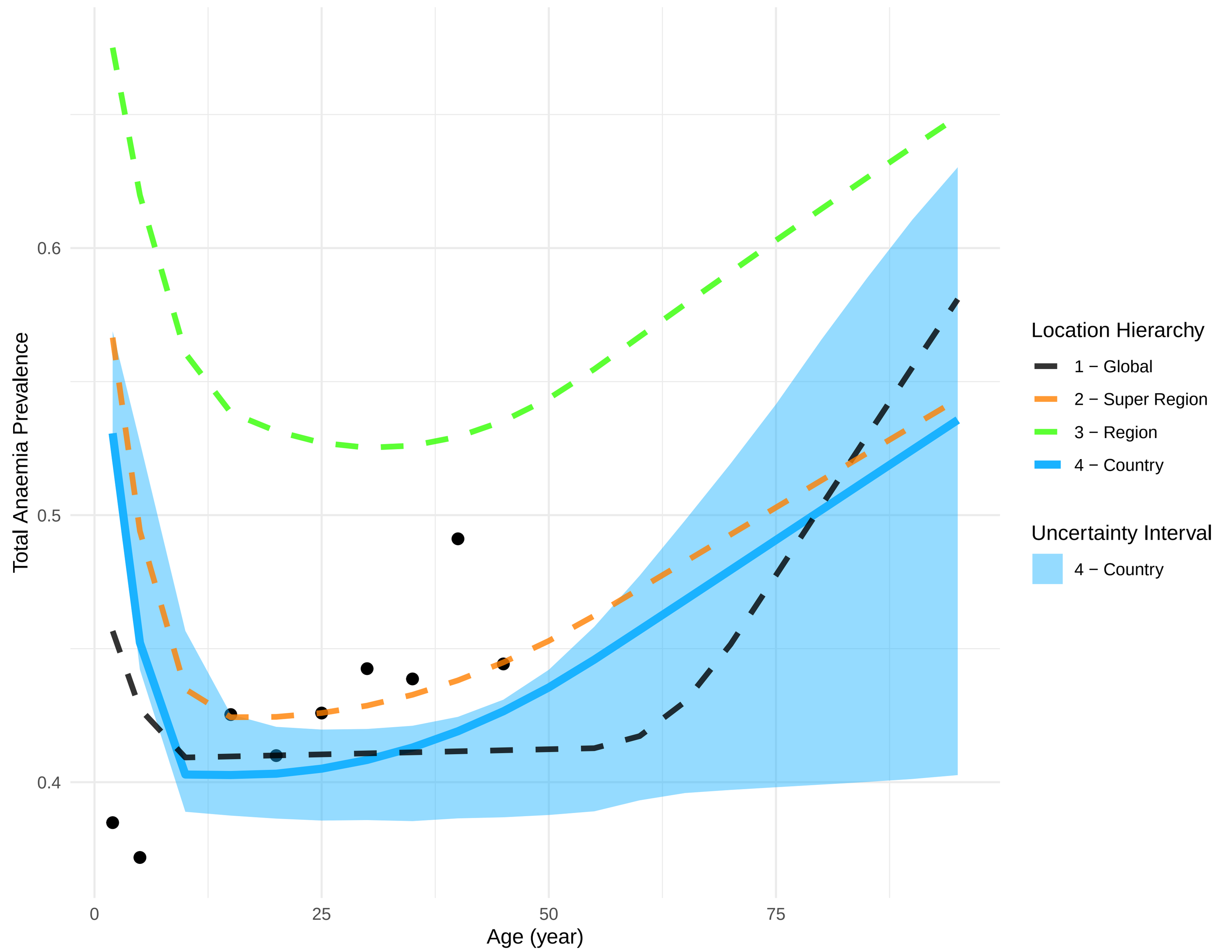

MR−BRT Age/Sex Fits − Non−pregnant Females 2+ Years Old − Total Anaemia Prevalence − Ghana
WHO−2001 Adjustment Method

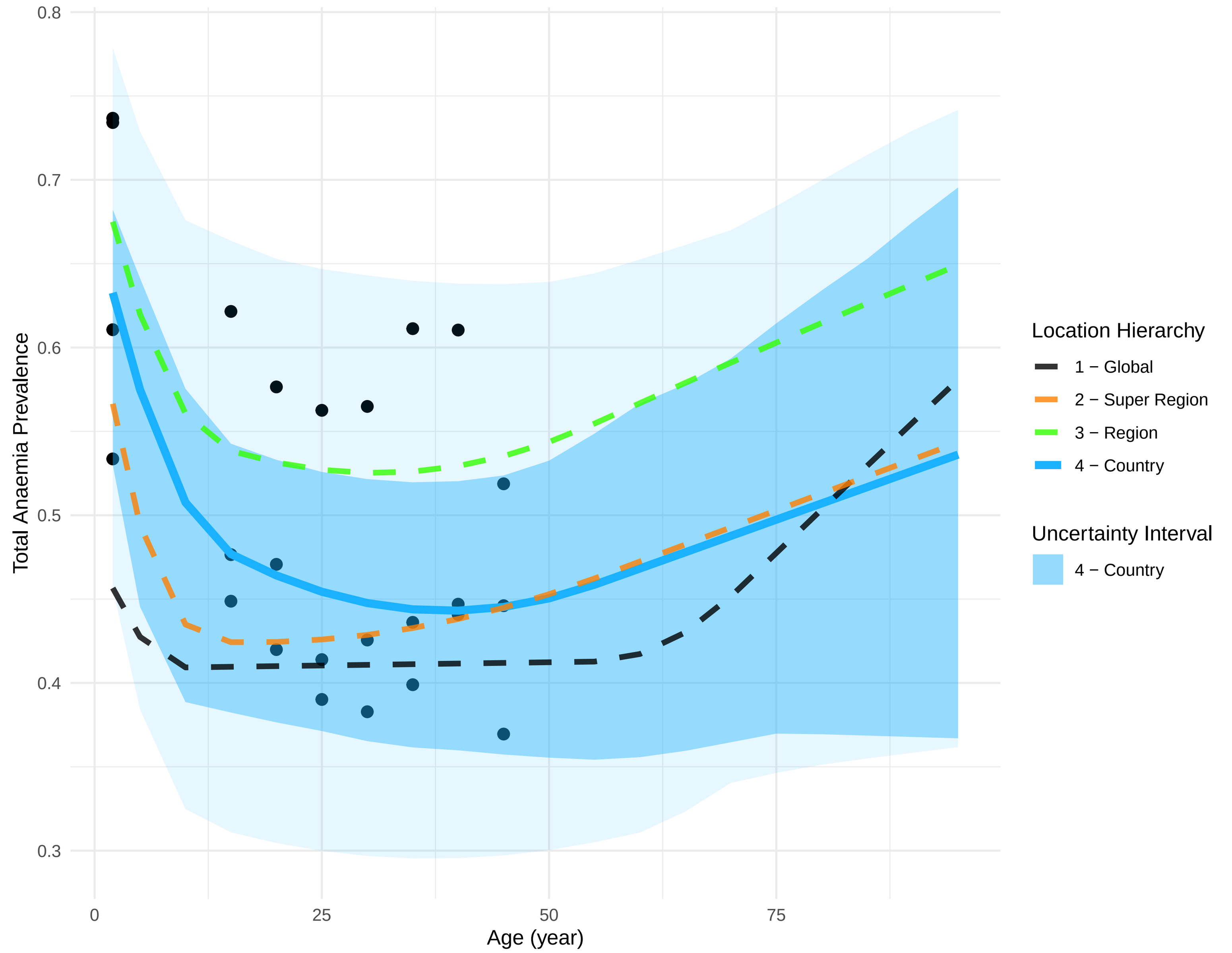

MR−BRT Age/Sex Fits − Non−pregnant Females 2+ Years Old − Total Anaemia Prevalence − Guinea
WHO−2001 Adjustment Method

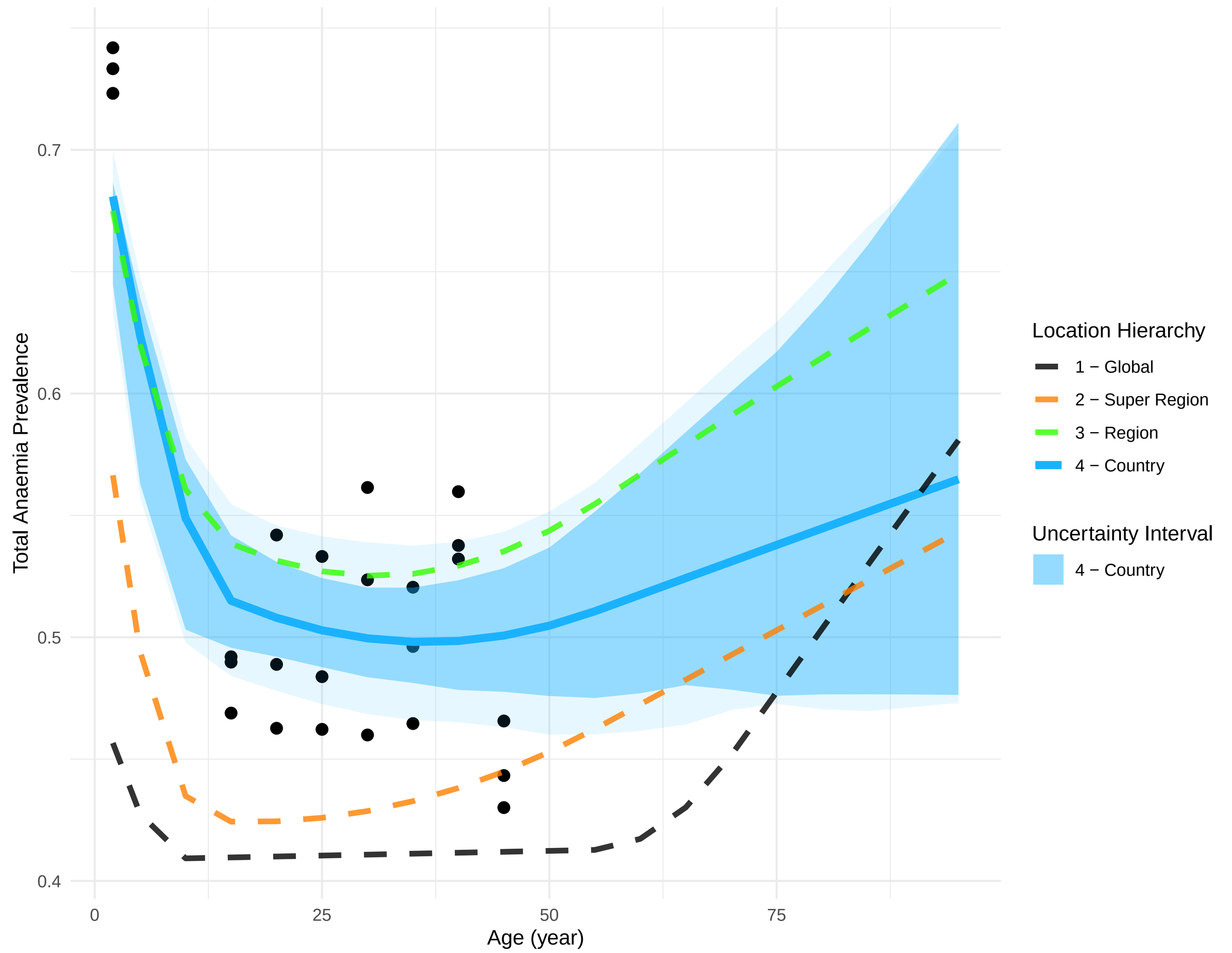

MR−BRT Age/Sex Fits − Non−pregnant Females 2+ Years Old − Total Anaemia Prevalence − Liberia
WHO−2001 Adjustment Method

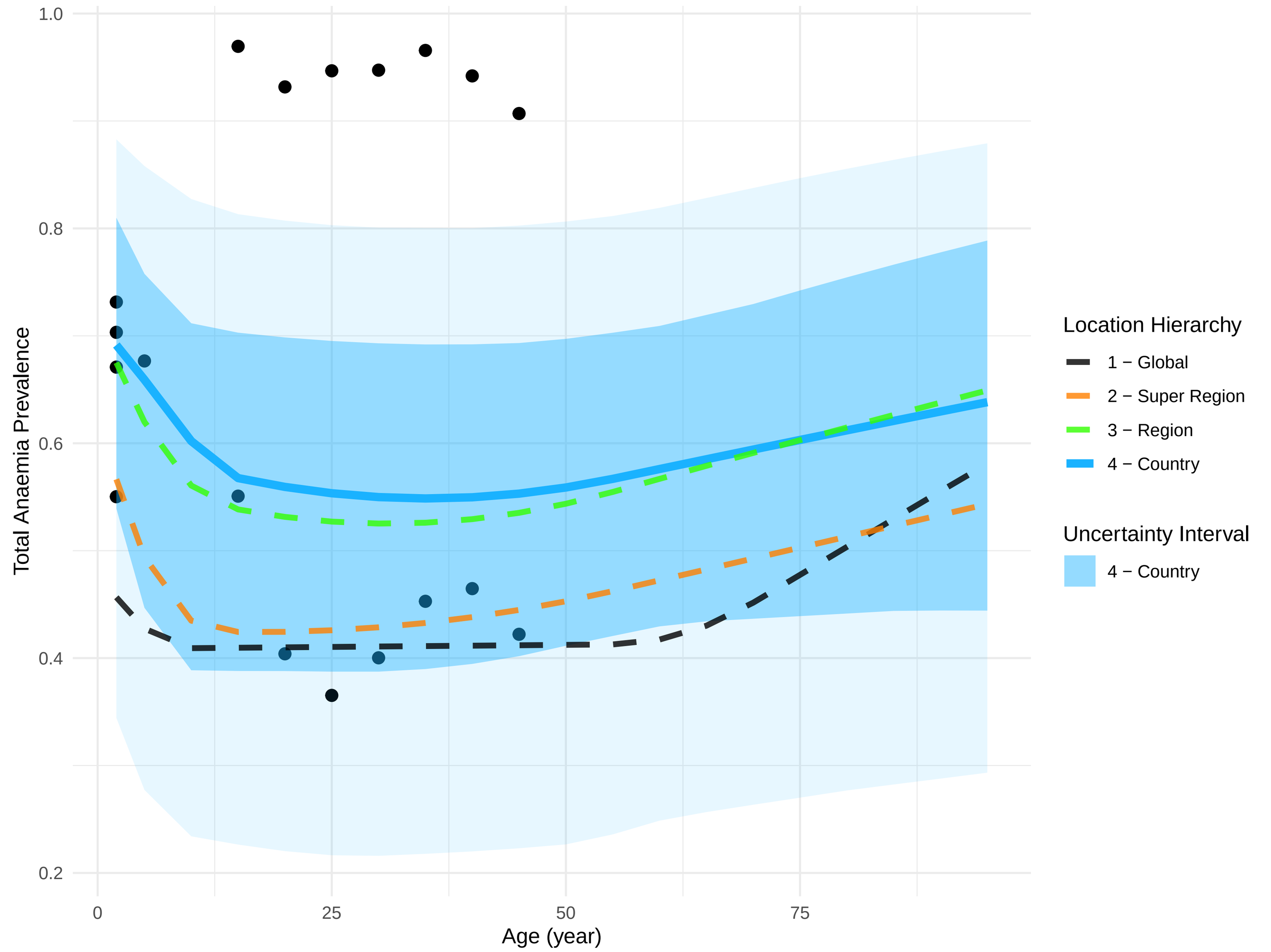

MR−BRT Age/Sex Fits − Non−pregnant Females 2+ Years Old − Total Anaemia Prevalence − Mali
WHO−2001 Adjustment Method

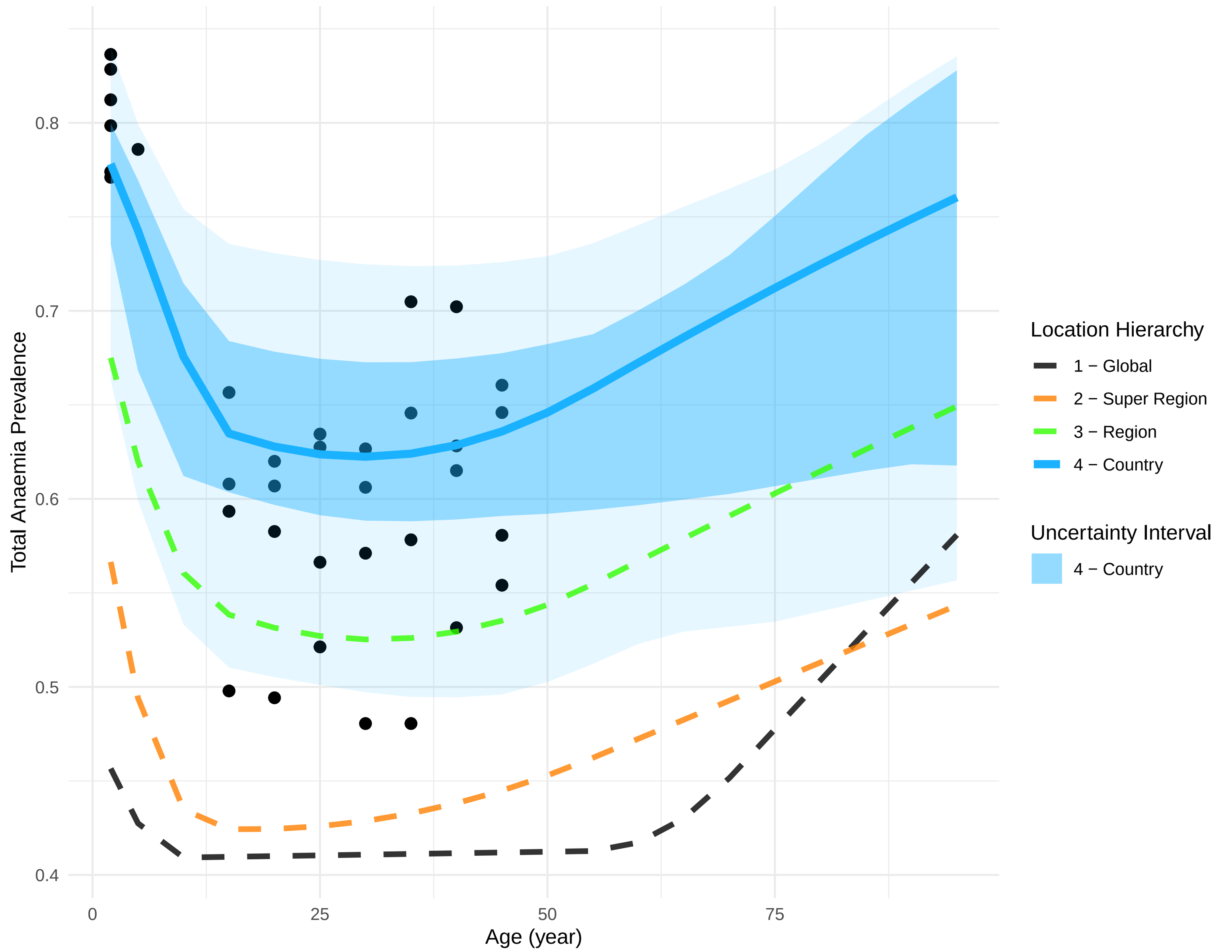

MR−BRT Age/Sex Fits − Non−pregnant Females 2+ Years Old − Total Anaemia Prevalence − Niger
WHO−2001 Adjustment Method

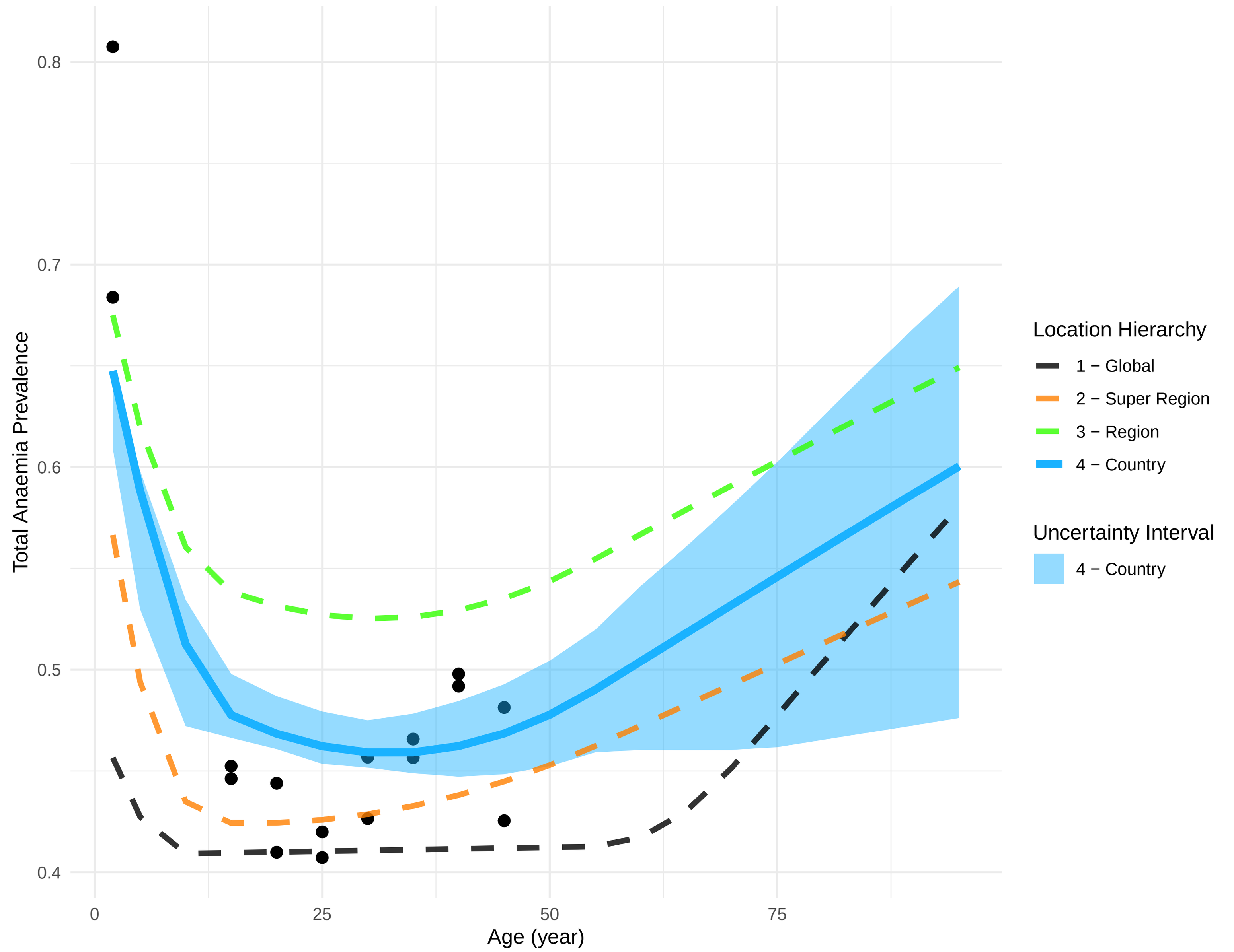

MR−BRT Age/Sex Fits − Non−pregnant Females 2+ Years Old − Total Anaemia Prevalence − Nigeria
WHO−2001 Adjustment Method

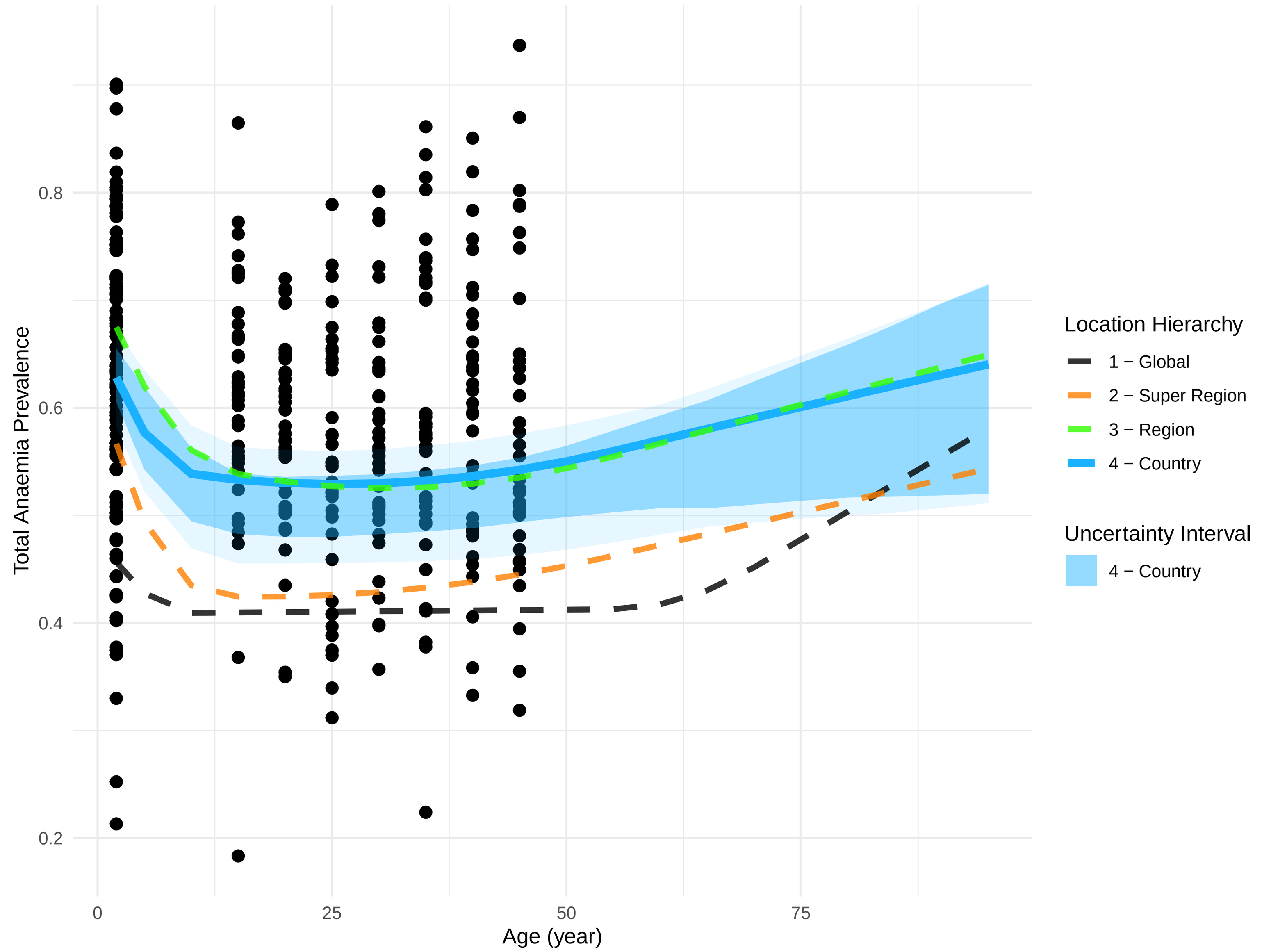

MR−BRT Age/Sex Fits − Non−pregnant Females 2+ Years Old − Total Anaemia Prevalence − Abia
WHO−2001 Adjustment Method

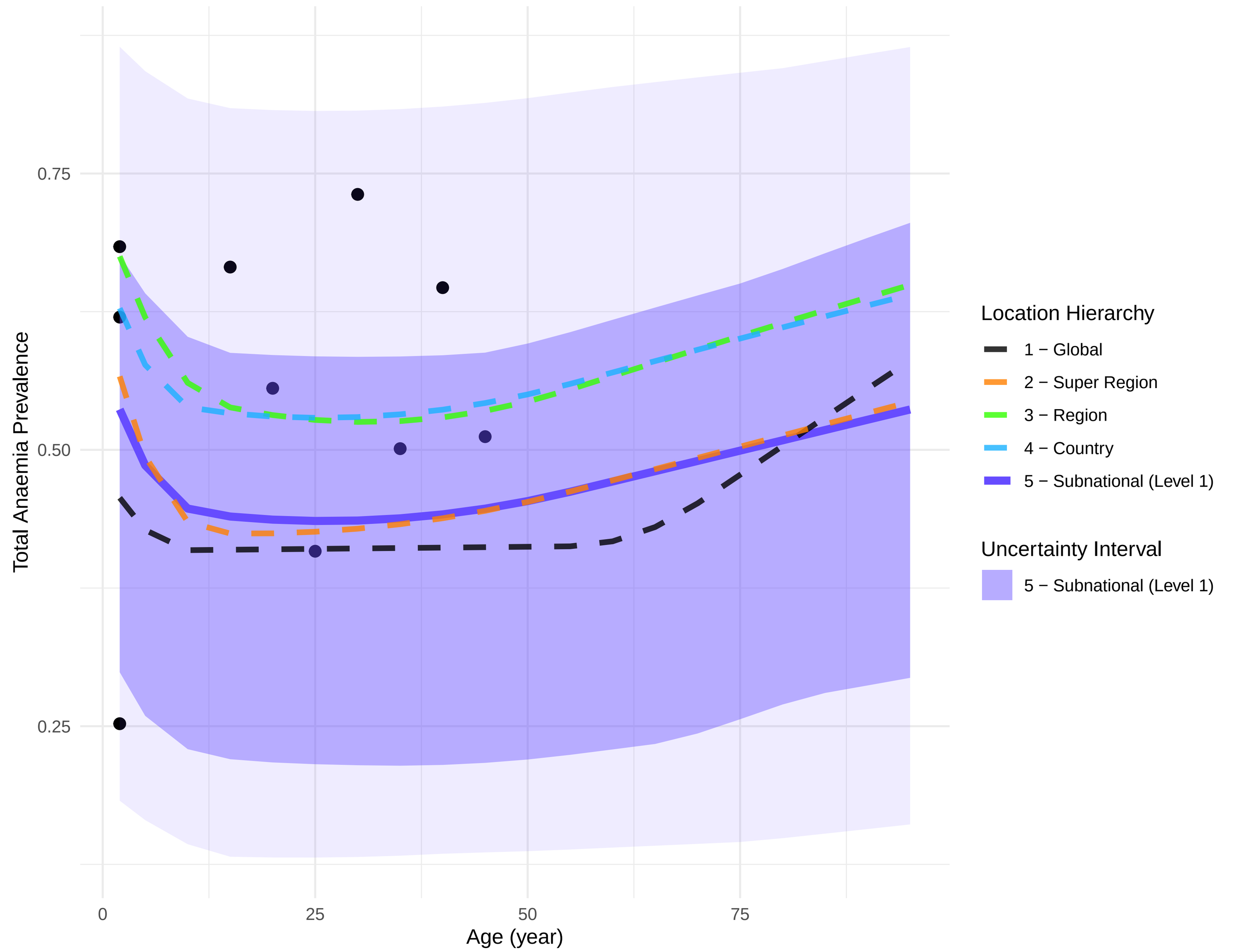

MR−BRT Age/Sex Fits − Non−pregnant Females 2+ Years Old − Total Anaemia Prevalence − Adamawa
WHO−2001 Adjustment Method

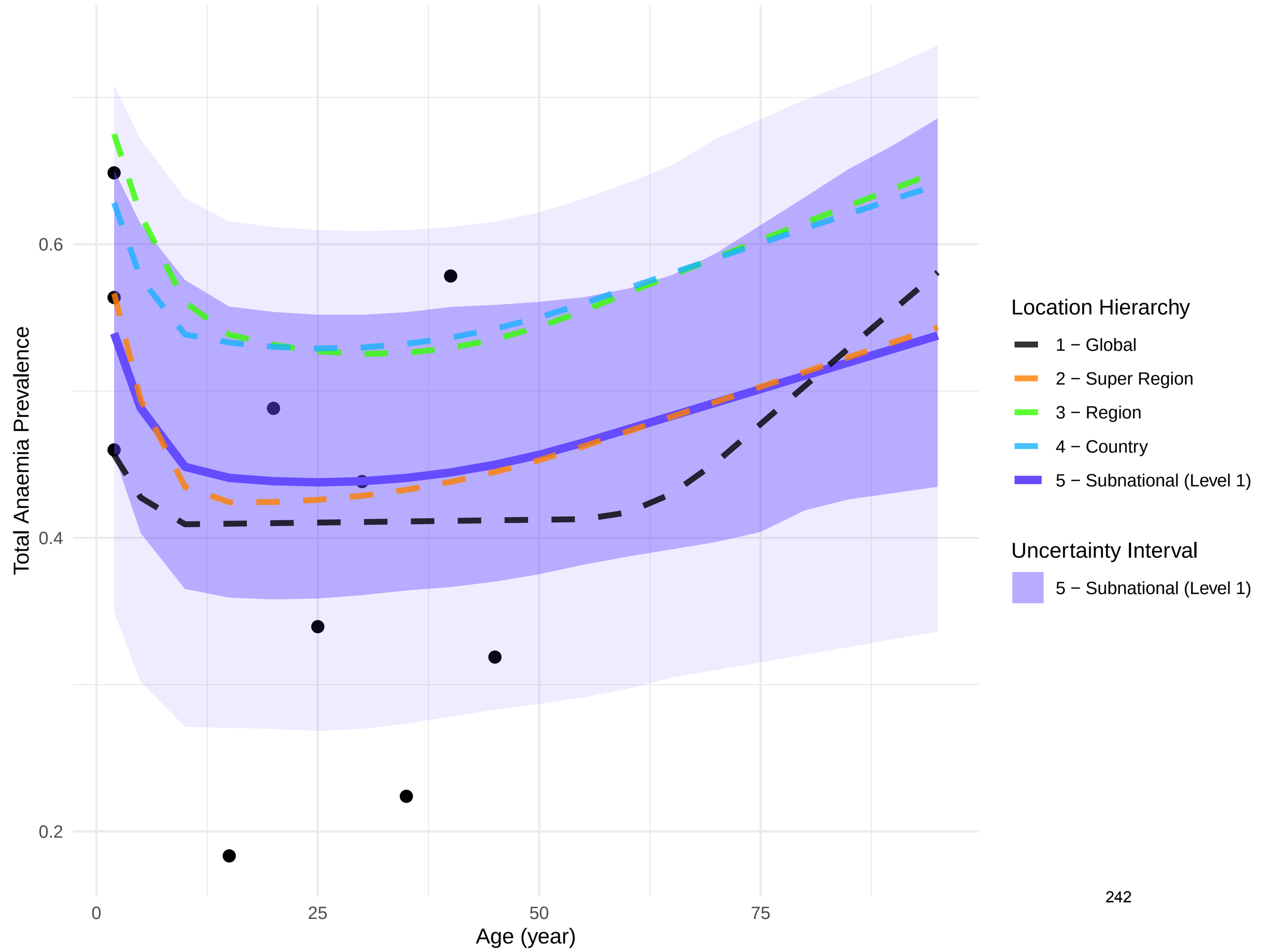

MR−BRT Age/Sex Fits − Non−pregnant Females 2+ Years Old − Total Anaemia Prevalence − Akwa Ibom
WHO−2001 Adjustment Method

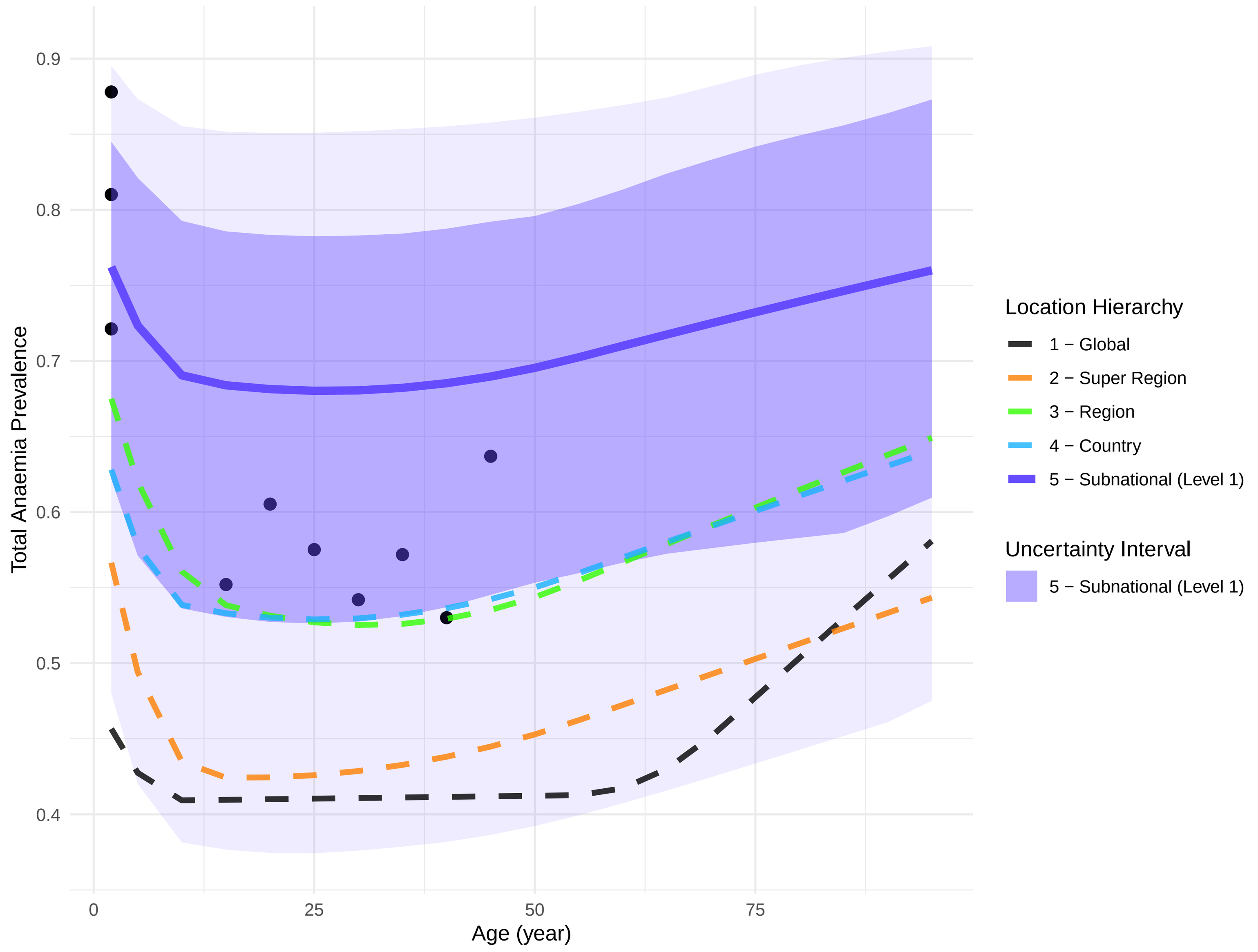

MR−BRT Age/Sex Fits − Non−pregnant Females 2+ Years Old − Total Anaemia Prevalence − Anambra
WHO−2001 Adjustment Method

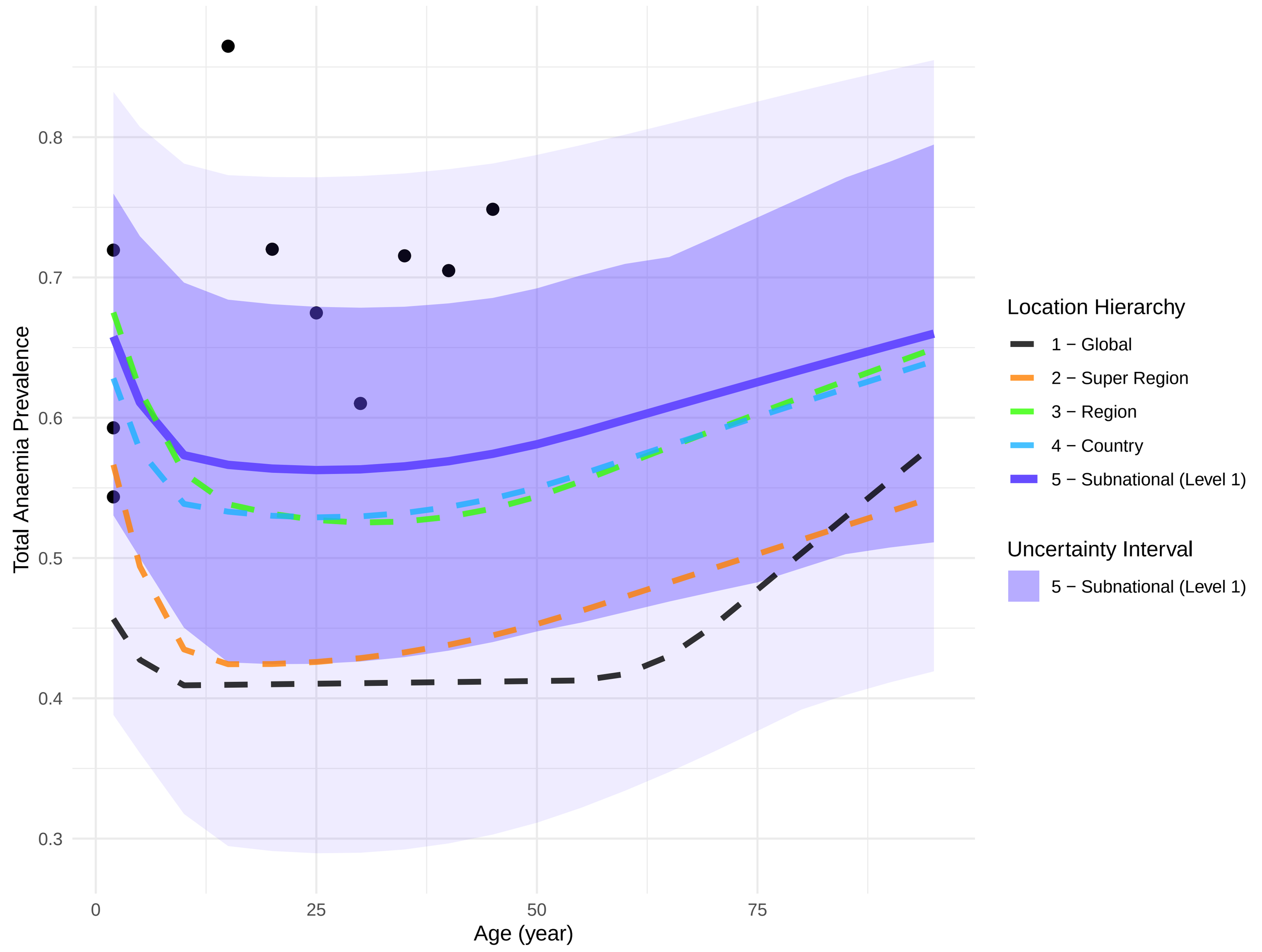

MR−BRT Age/Sex Fits − Non−pregnant Females 2+ Years Old − Total Anaemia Prevalence − Bauchi
WHO−2001 Adjustment Method

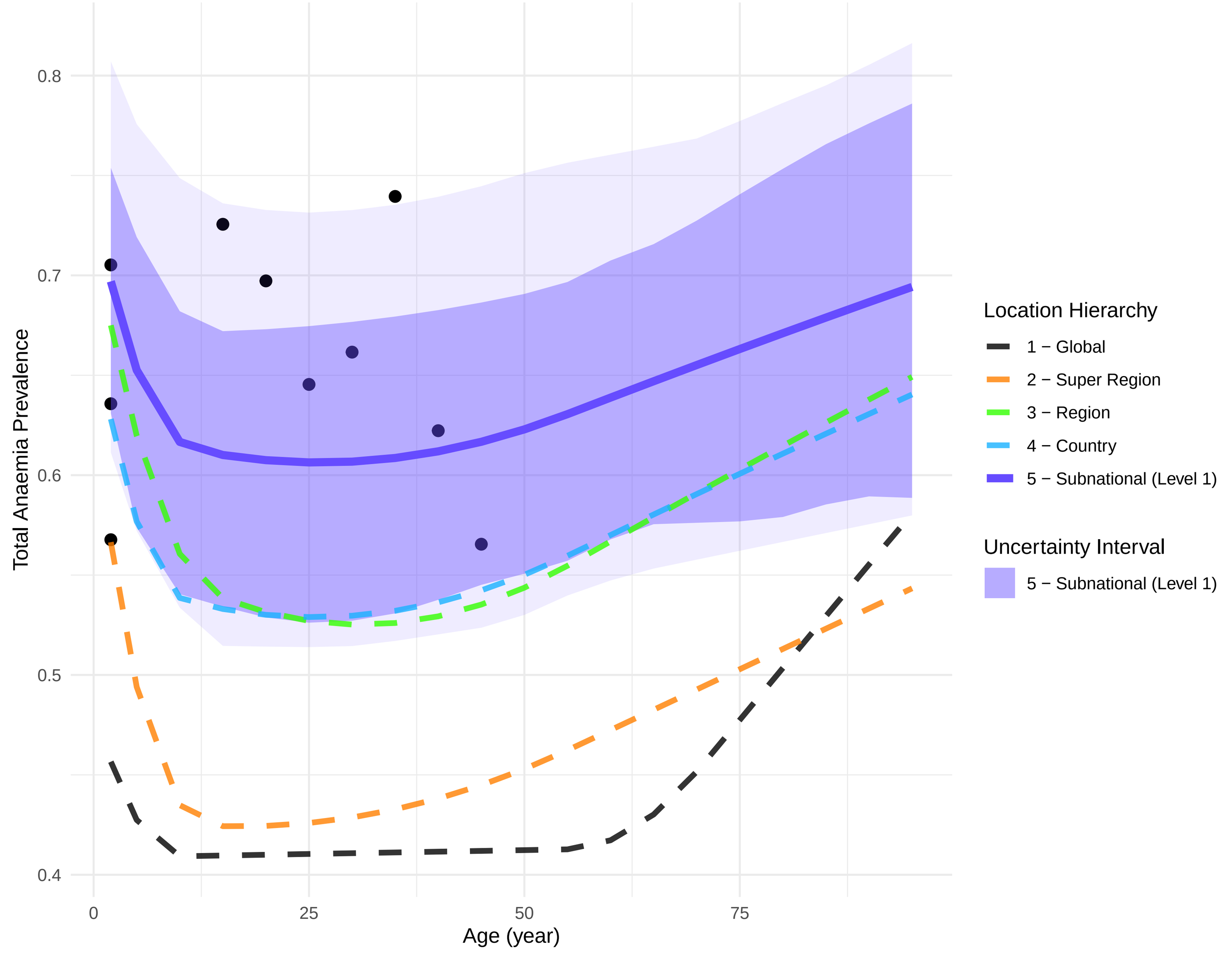

MR−BRT Age/Sex Fits − Non−pregnant Females 2+ Years Old − Total Anaemia Prevalence − Bayelsa
WHO−2001 Adjustment Method

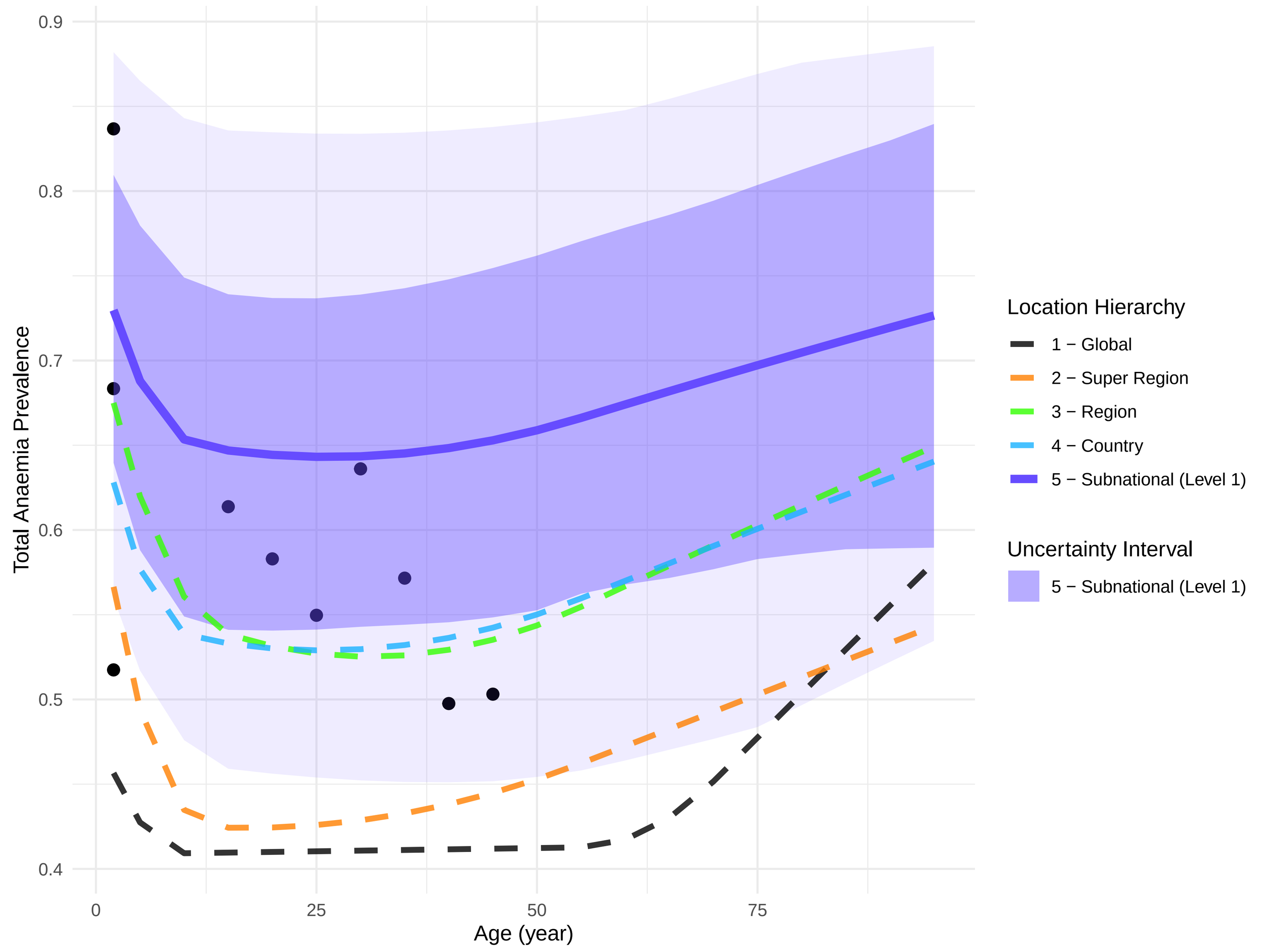

MR−BRT Age/Sex Fits − Non−pregnant Females 2+ Years Old − Total Anaemia Prevalence − Benue
WHO−2001 Adjustment Method

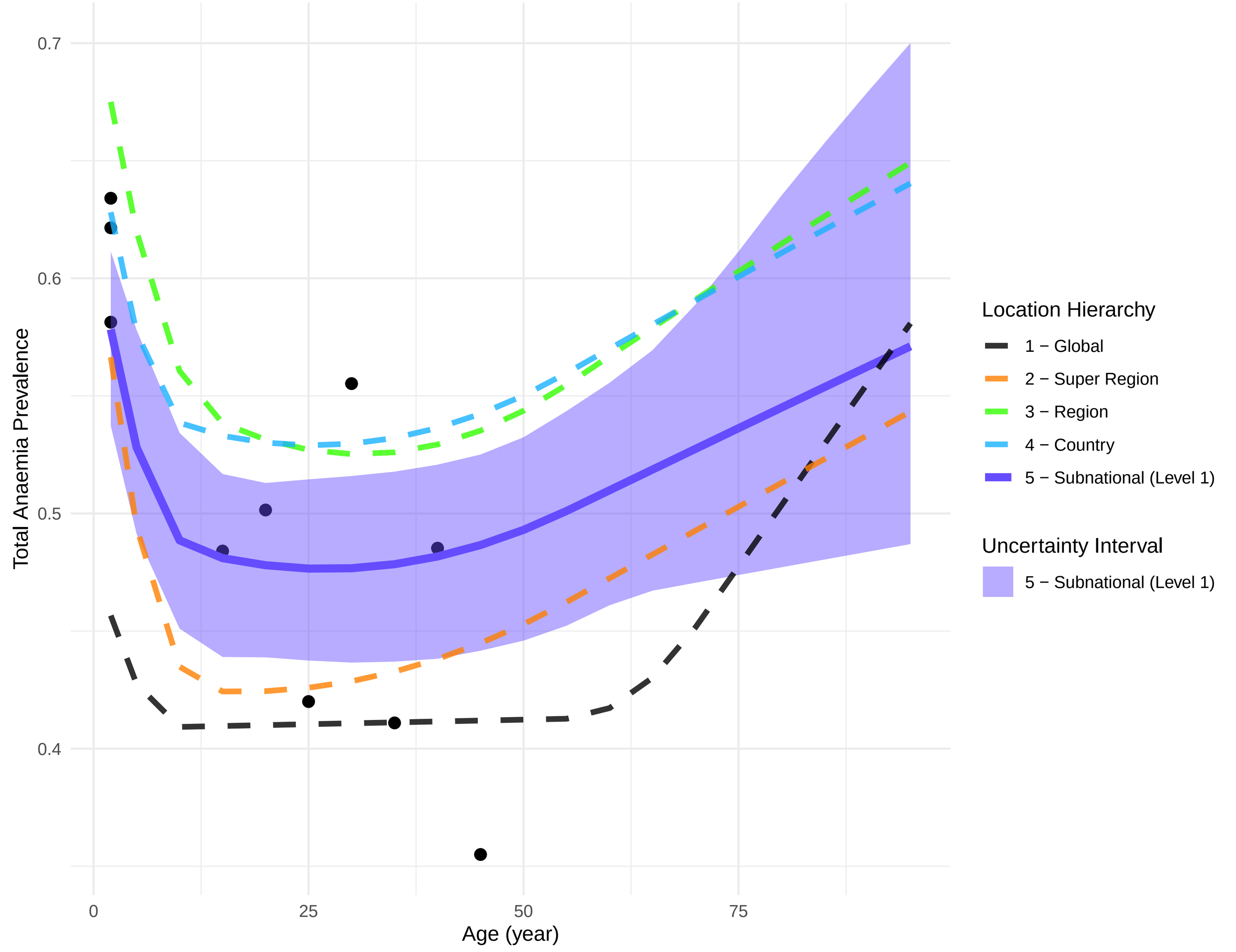

MR−BRT Age/Sex Fits − Non−pregnant Females 2+ Years Old − Total Anaemia Prevalence − Borno
WHO−2001 Adjustment Method

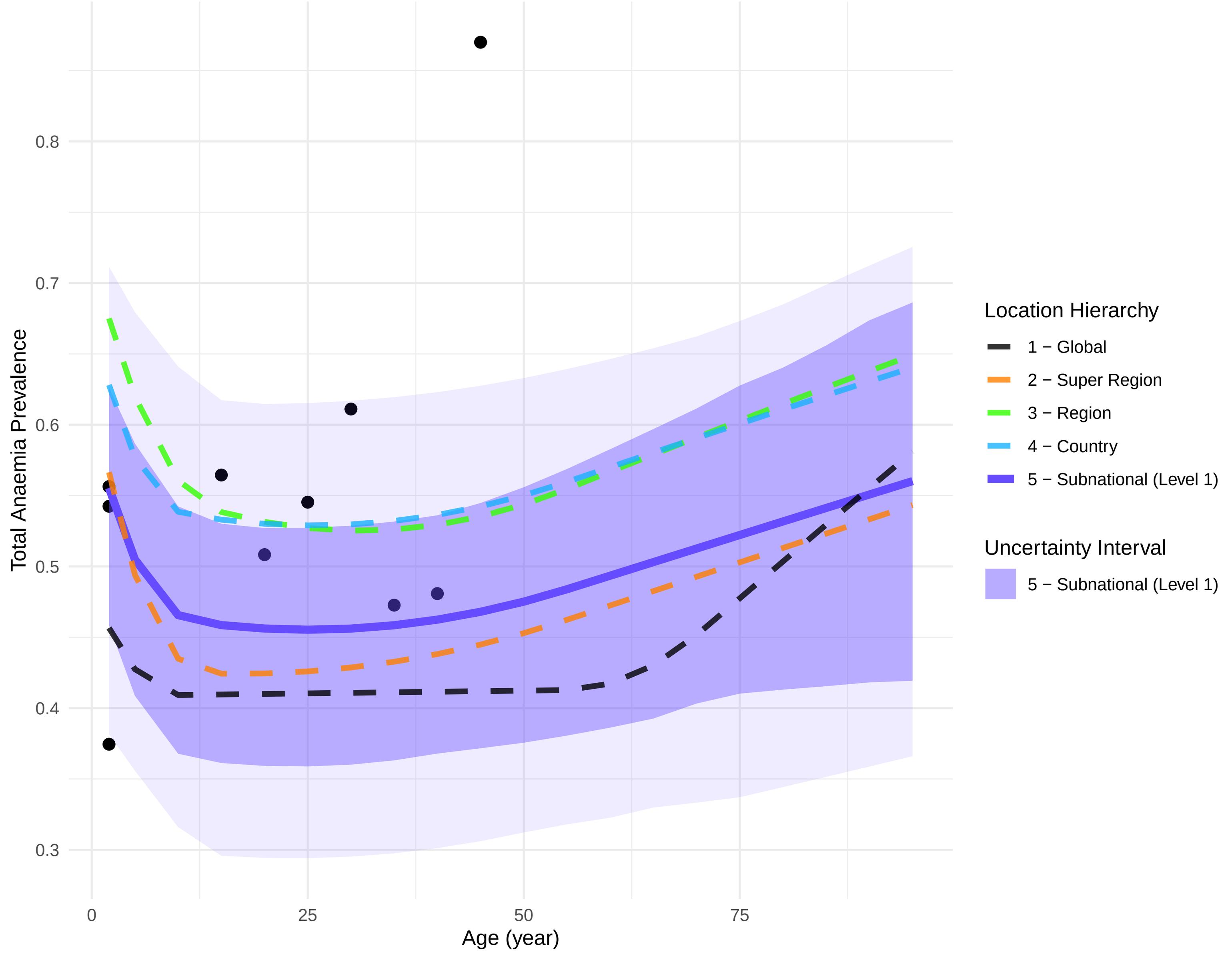

MR−BRT Age/Sex Fits − Non−pregnant Females 2+ Years Old − Total Anaemia Prevalence − Cross River
WHO−2001 Adjustment Method

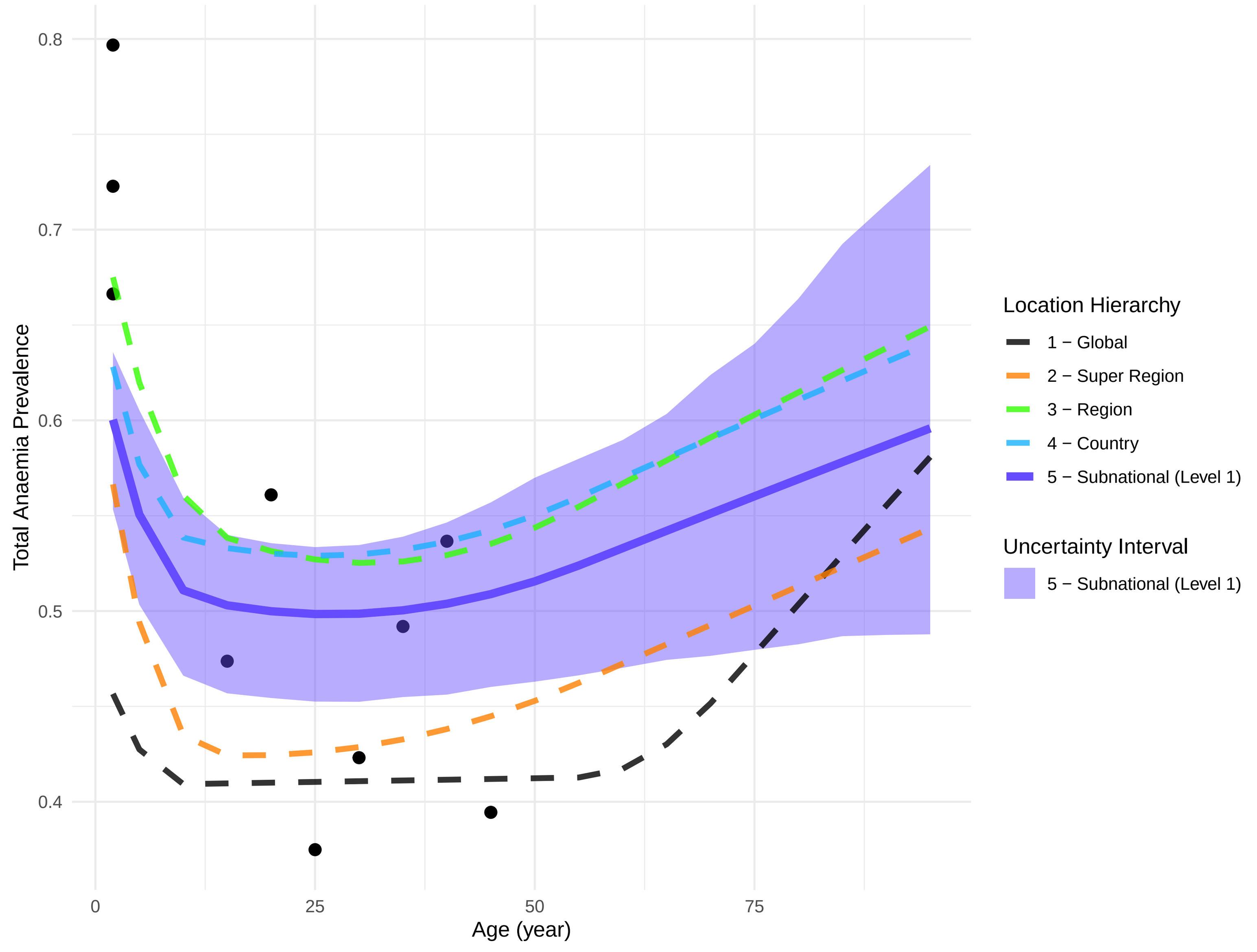

MR−BRT Age/Sex Fits − Non−pregnant Females 2+ Years Old − Total Anaemia Prevalence − Delta
WHO−2001 Adjustment Method

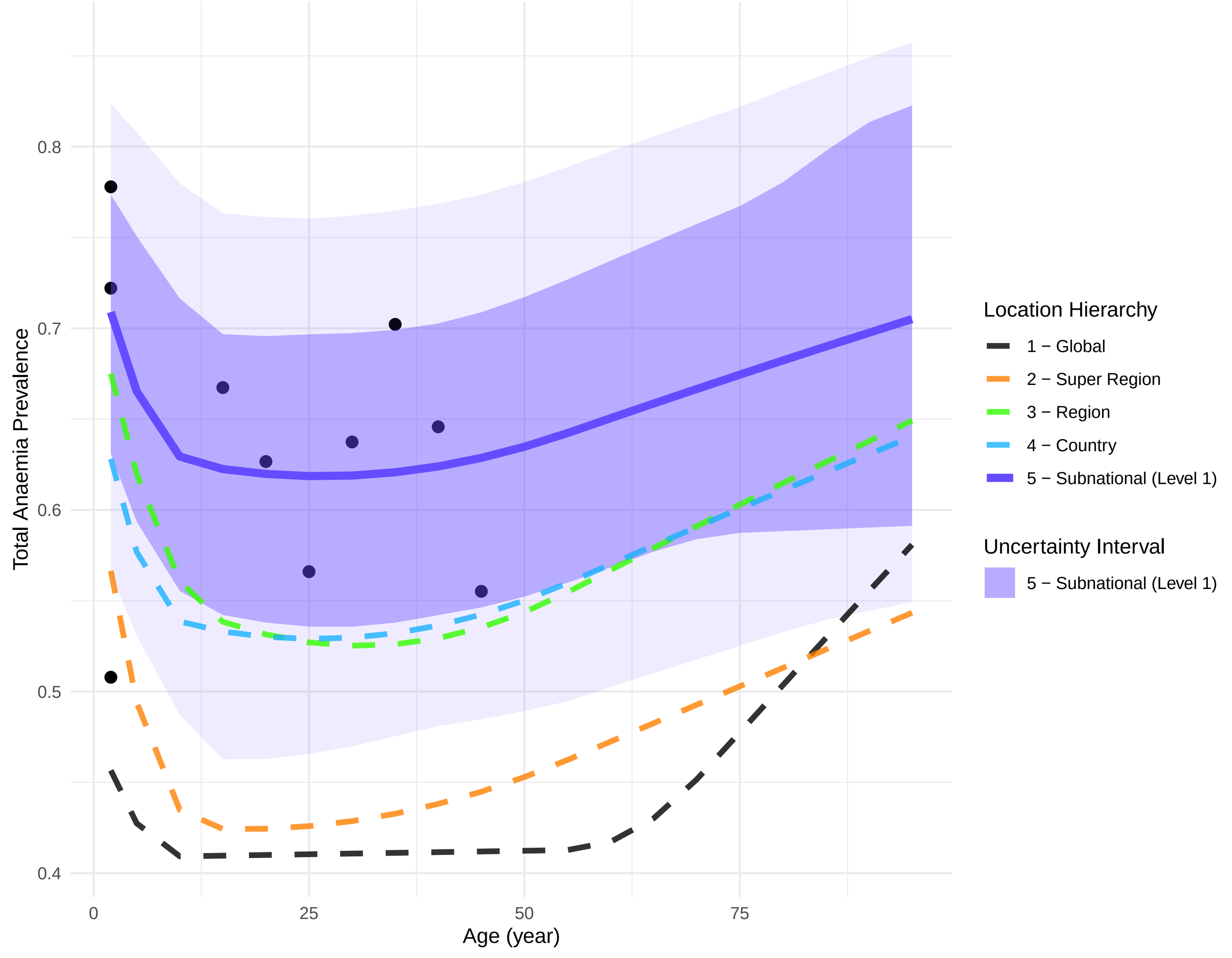

MR−BRT Age/Sex Fits − Non−pregnant Females 2+ Years Old − Total Anaemia Prevalence − Ebonyi
WHO−2001 Adjustment Method

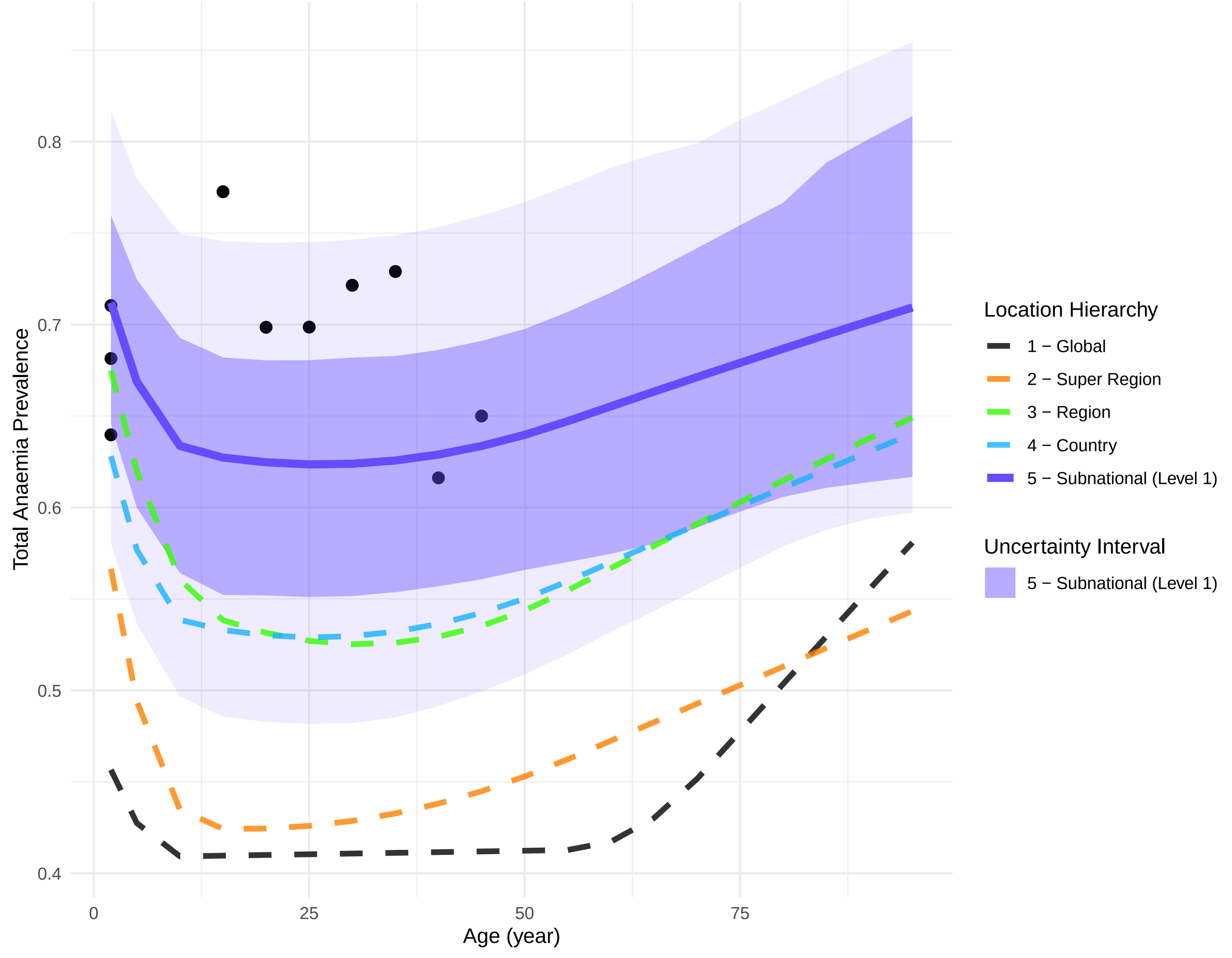

MR−BRT Age/Sex Fits − Non−pregnant Females 2+ Years Old − Total Anaemia Prevalence − Edo
WHO−2001 Adjustment Method

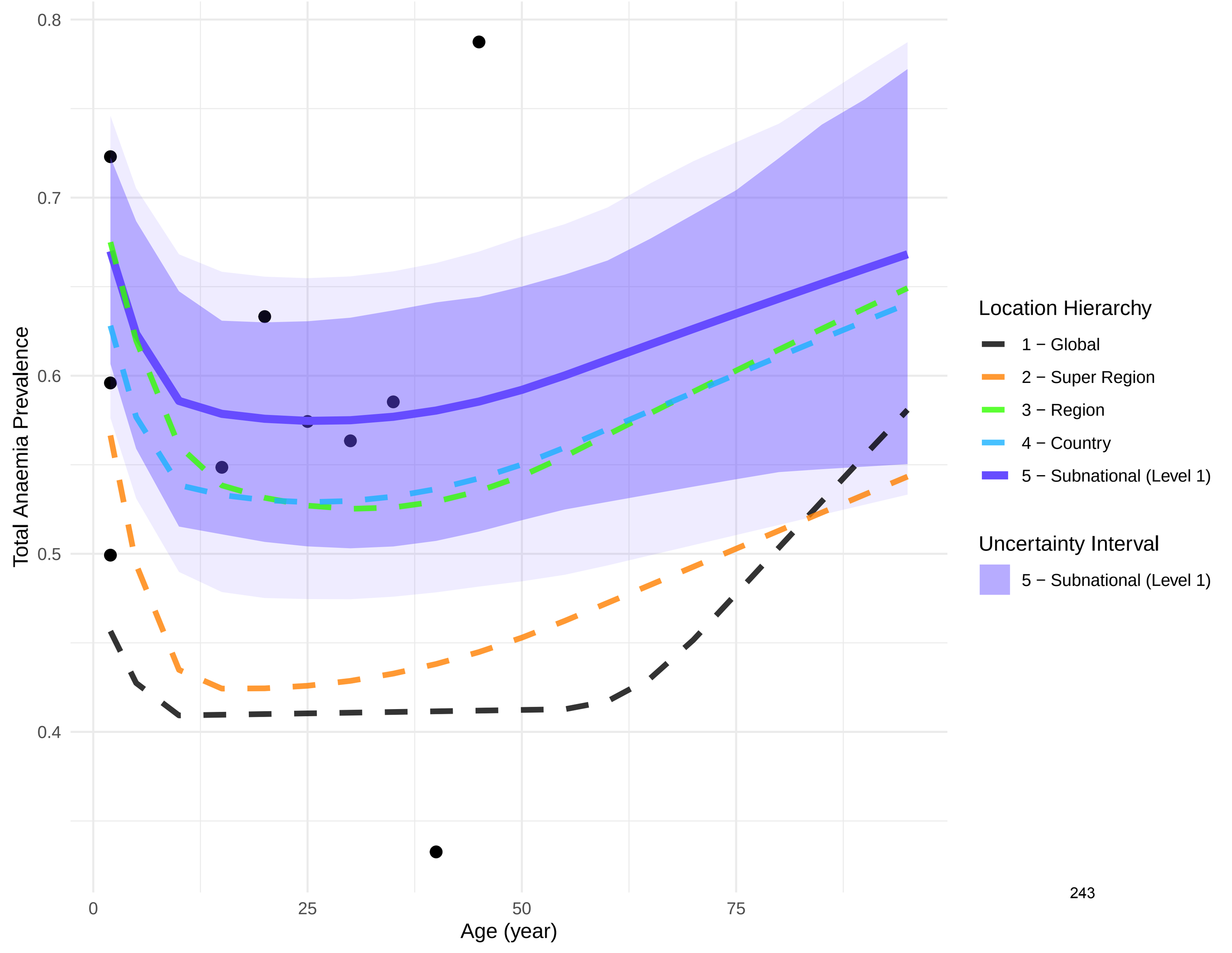

MR−BRT Age/Sex Fits − Non−pregnant Females 2+ Years Old − Total Anaemia Prevalence − Ekiti
WHO−2001 Adjustment Method

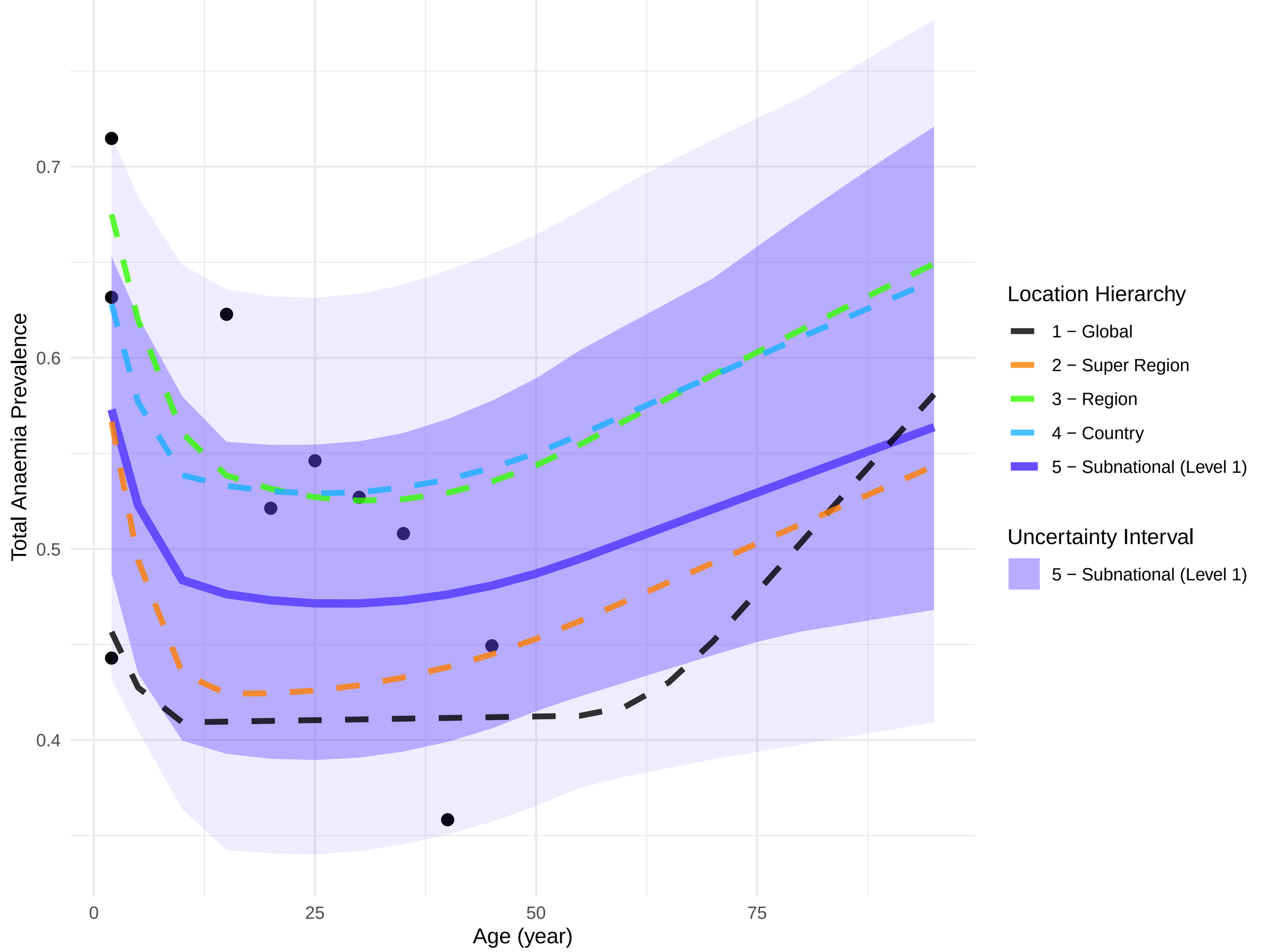

MR−BRT Age/Sex Fits − Non−pregnant Females 2+ Years Old − Total Anaemia Prevalence − Enugu
WHO−2001 Adjustment Method

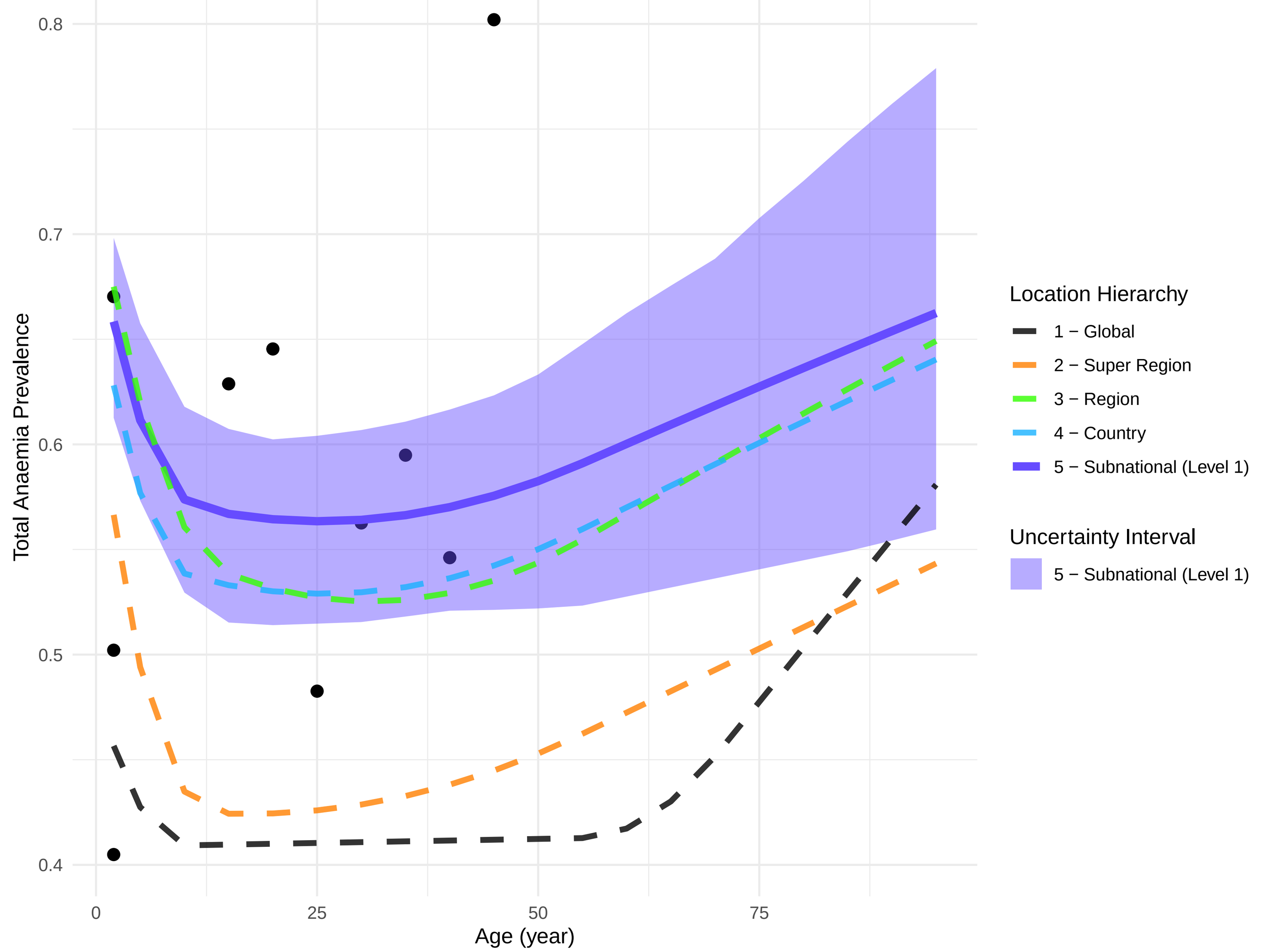

MR−BRT Age/Sex Fits − Non−pregnant Females 2+ Years Old − Total Anaemia Prevalence − FCT (Abuja)
WHO−2001 Adjustment Method

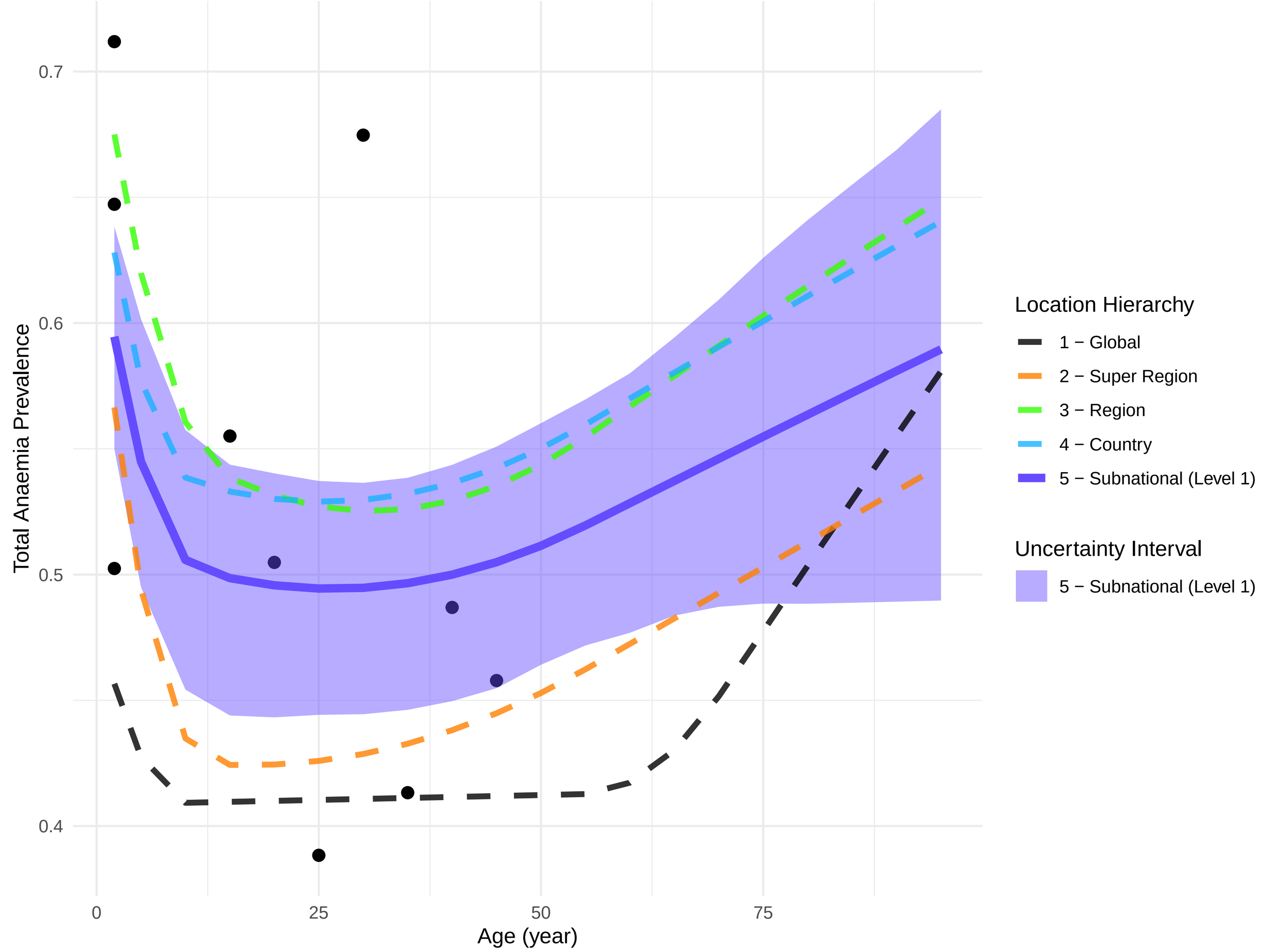

MR−BRT Age/Sex Fits − Non−pregnant Females 2+ Years Old − Total Anaemia Prevalence − Gombe
WHO−2001 Adjustment Method

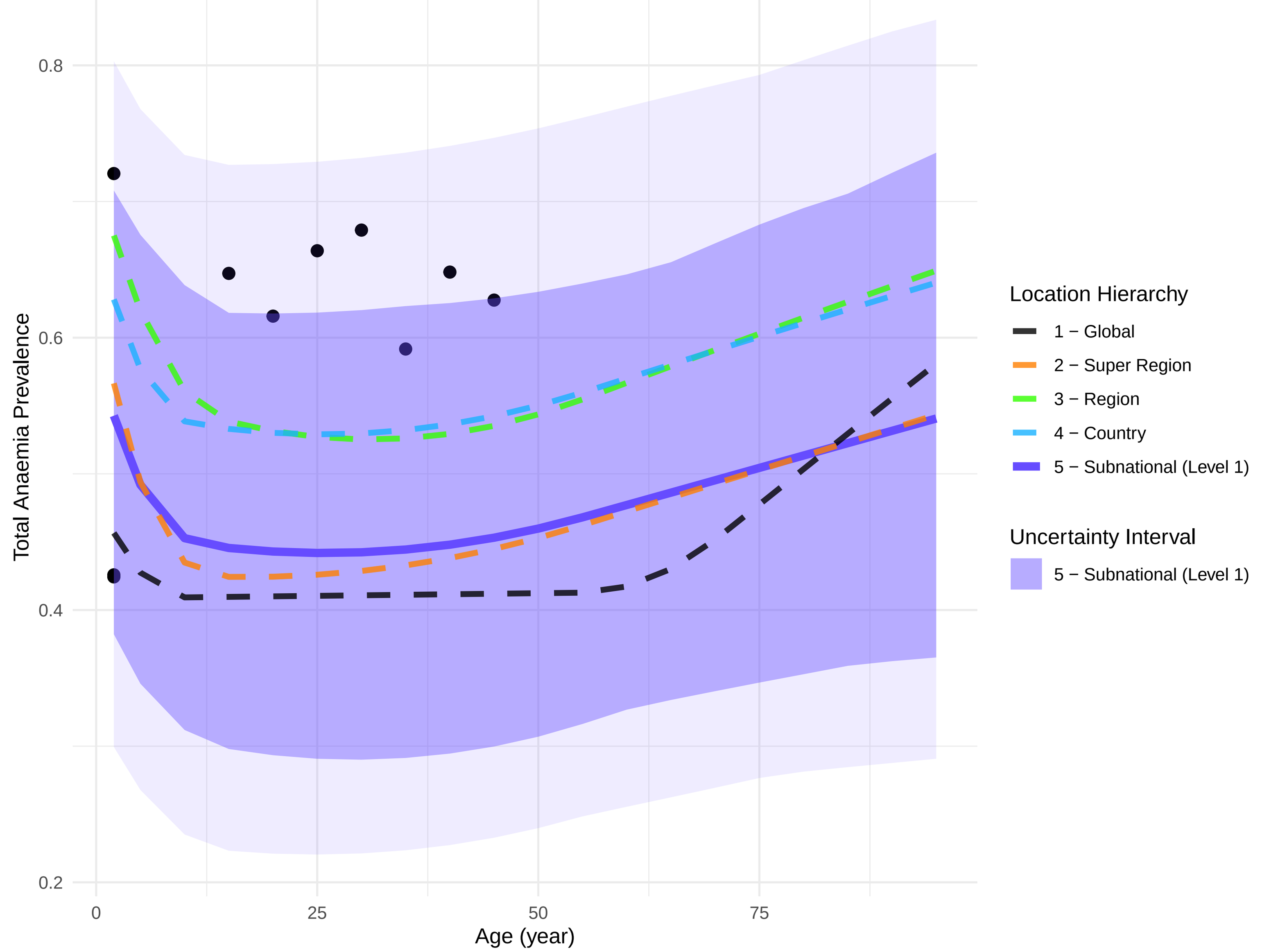

MR−BRT Age/Sex Fits − Non−pregnant Females 2+ Years Old − Total Anaemia Prevalence − Imo
WHO−2001 Adjustment Method

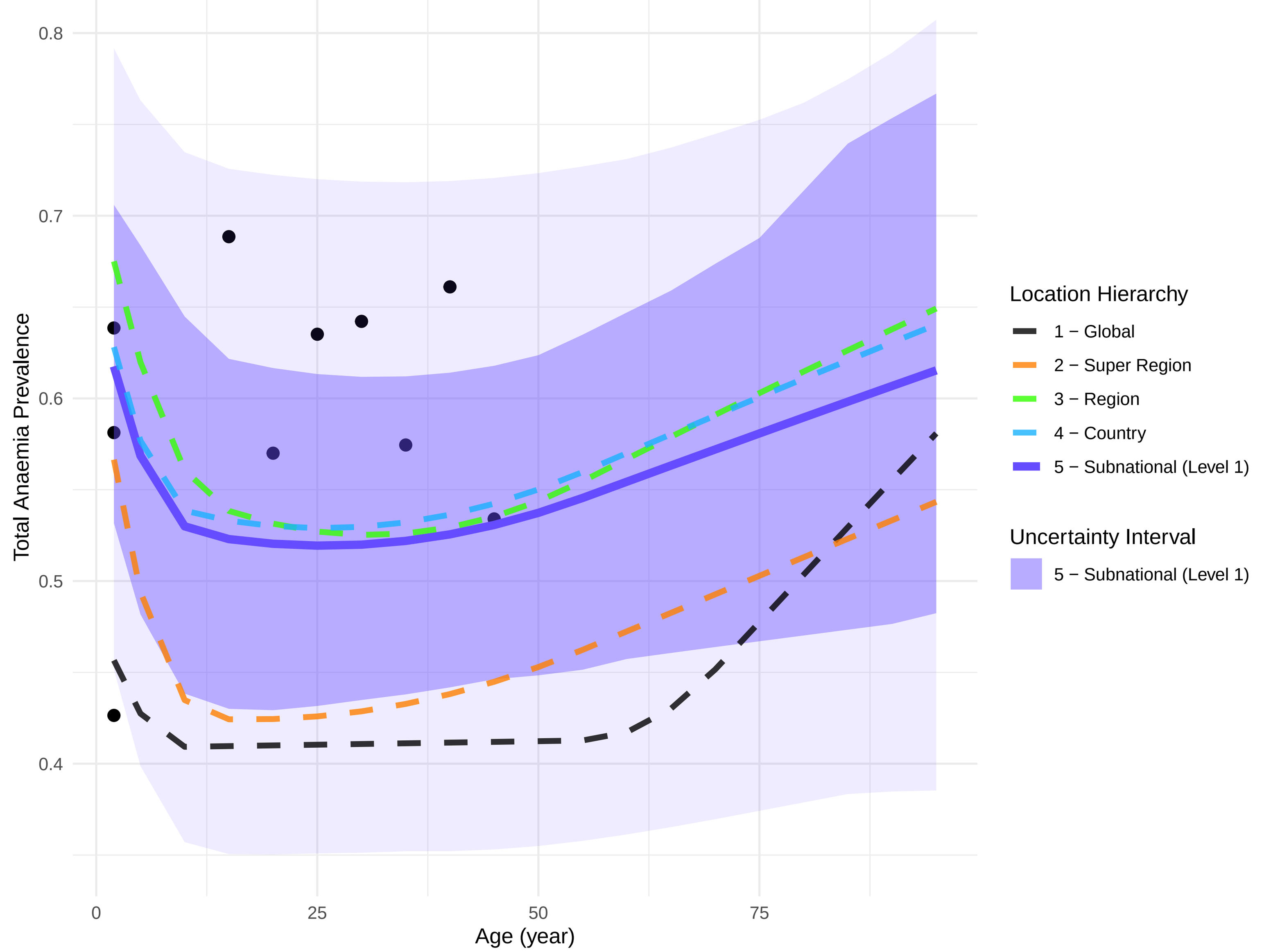

MR−BRT Age/Sex Fits − Non−pregnant Females 2+ Years Old − Total Anaemia Prevalence − Jigawa
WHO−2001 Adjustment Method

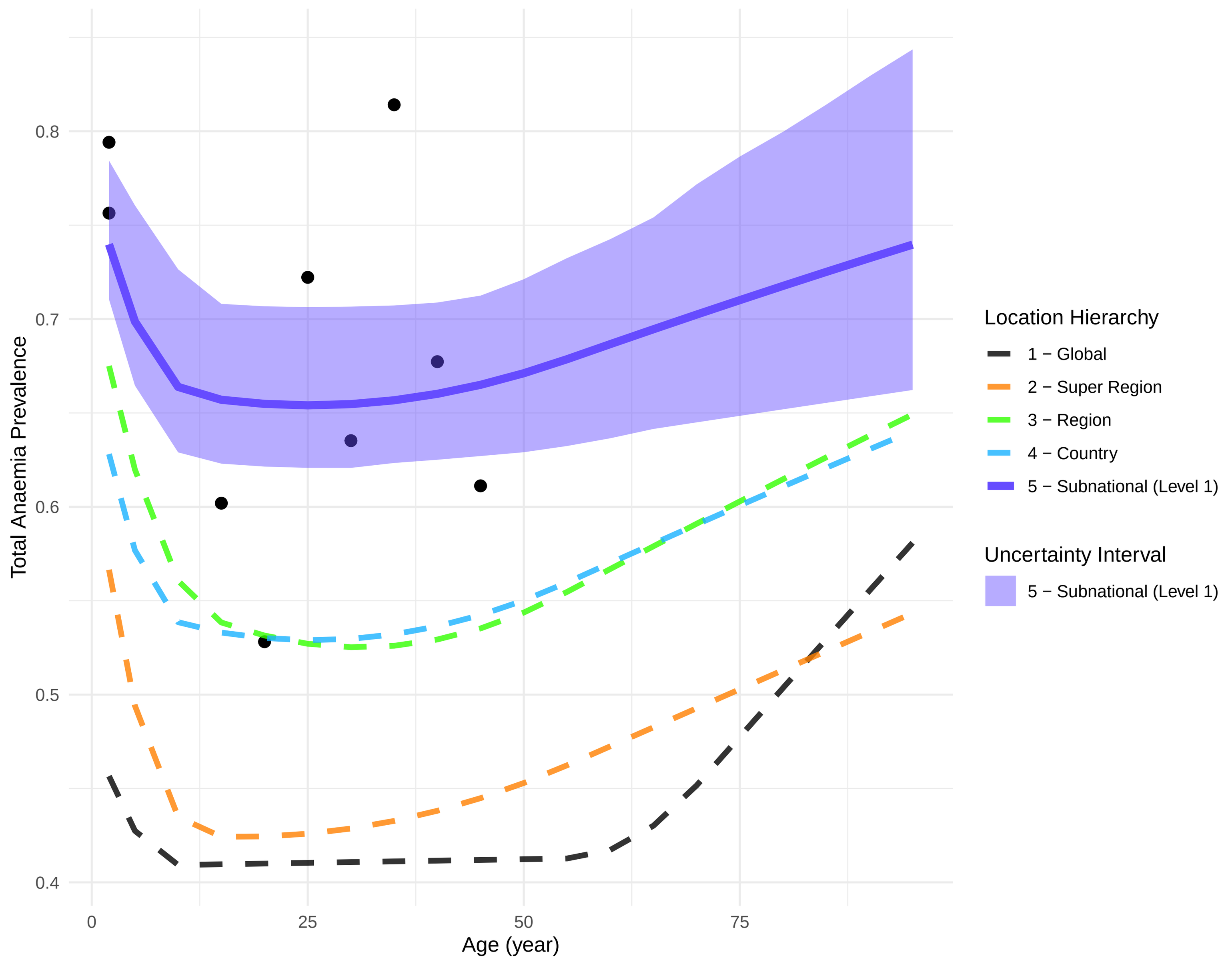

MR−BRT Age/Sex Fits − Non−pregnant Females 2+ Years Old − Total Anaemia Prevalence − Kaduna
WHO−2001 Adjustment Method

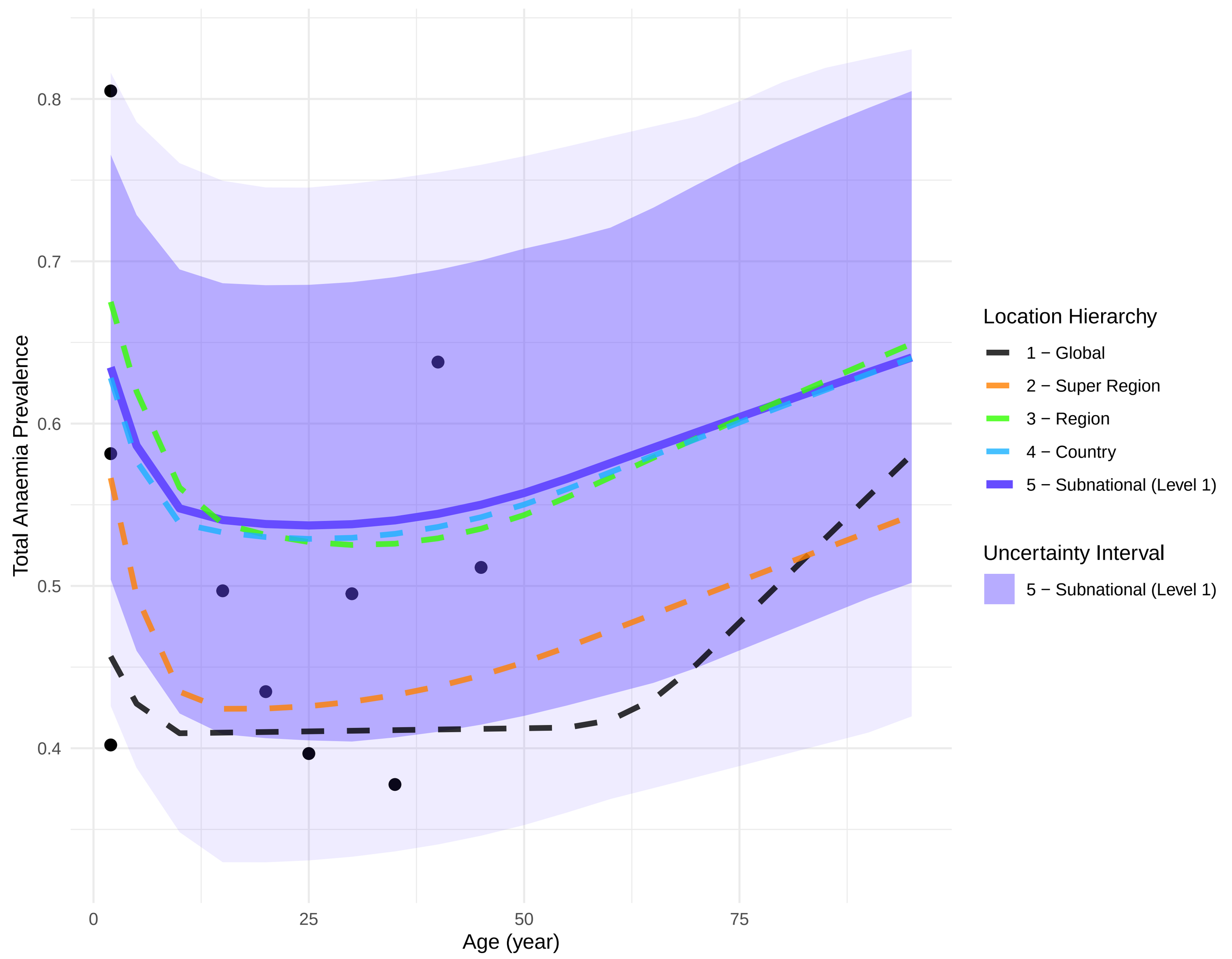

MR−BRT Age/Sex Fits − Non−pregnant Females 2+ Years Old − Total Anaemia Prevalence − Kano
WHO−2001 Adjustment Method

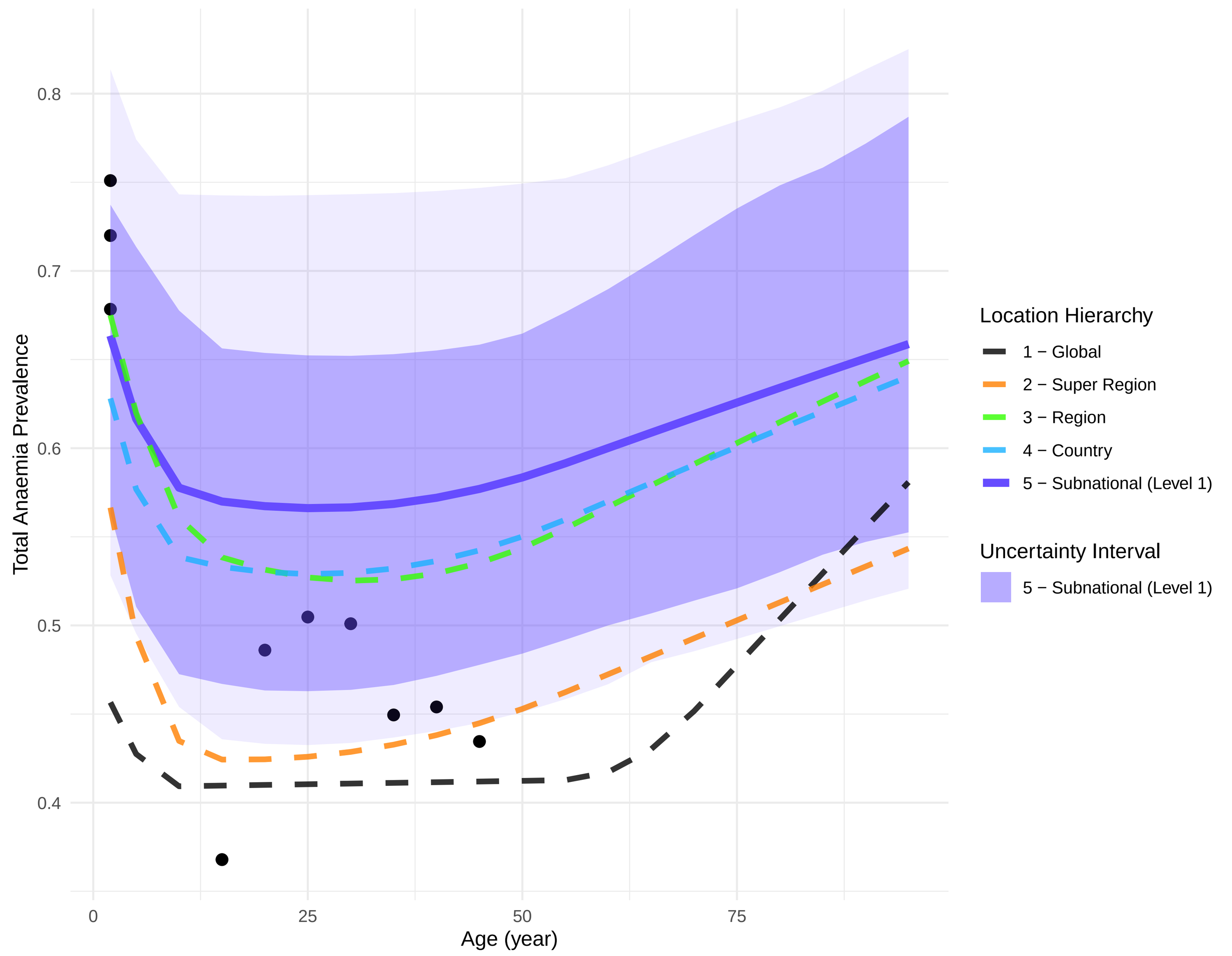

MR−BRT Age/Sex Fits − Non−pregnant Females 2+ Years Old − Total Anaemia Prevalence − Katsina
WHO−2001 Adjustment Method

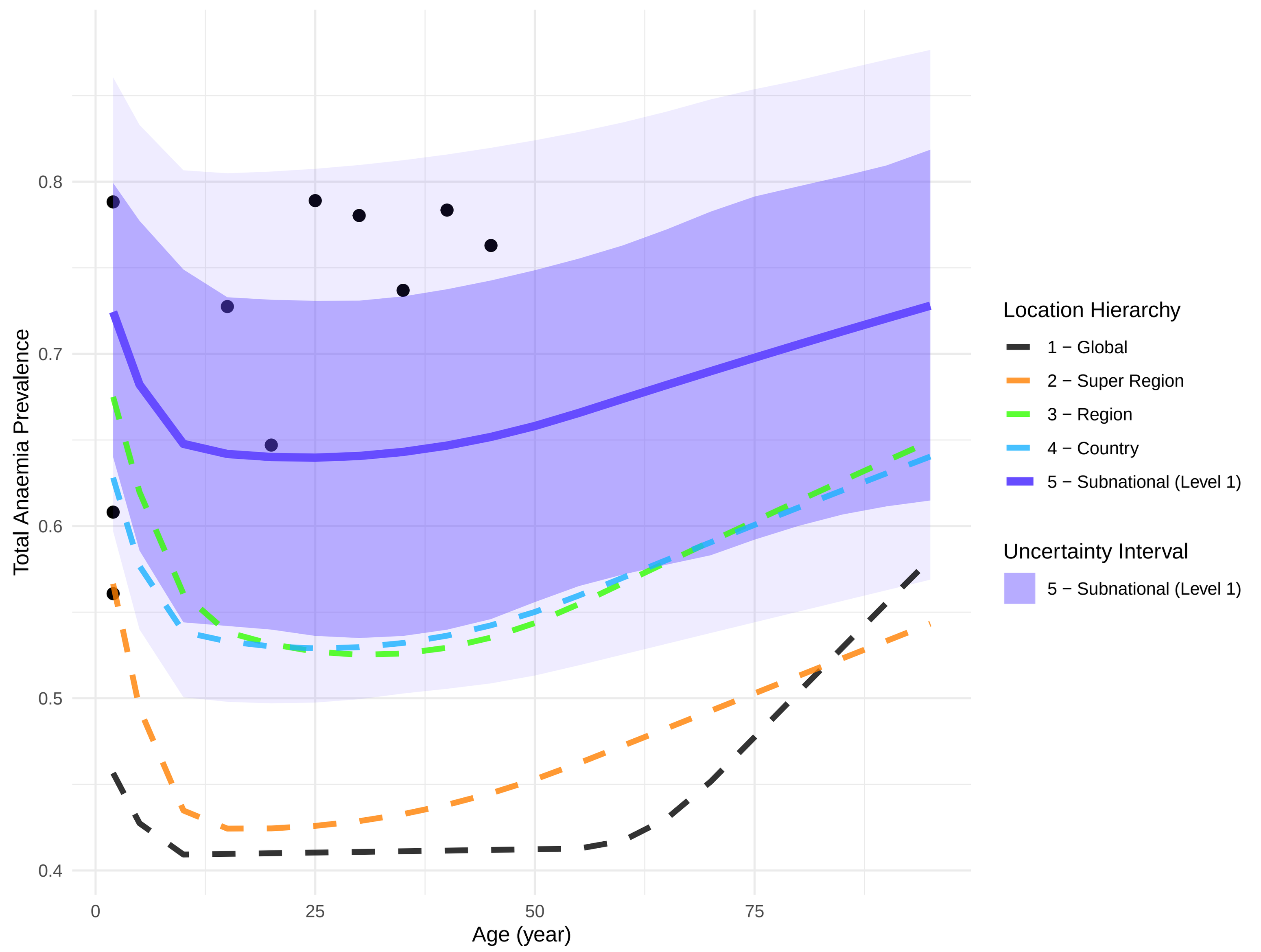

MR−BRT Age/Sex Fits − Non−pregnant Females 2+ Years Old − Total Anaemia Prevalence − Kebbi
WHO−2001 Adjustment Method

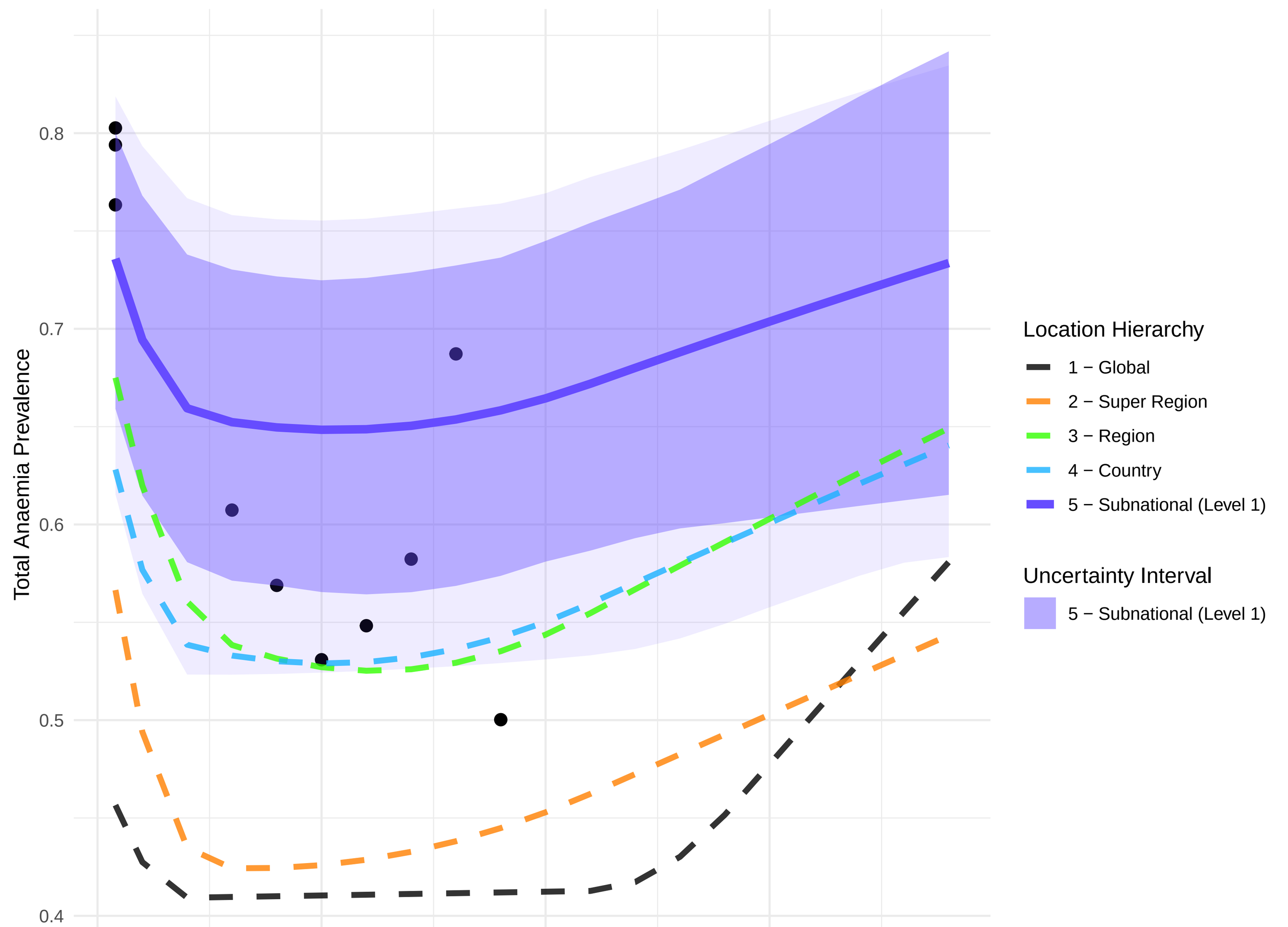

MR−BRT Age/Sex Fits − Non−pregnant Females 2+ Years Old − Total Anaemia Prevalence − Kogi
WHO−2001 Adjustment Method

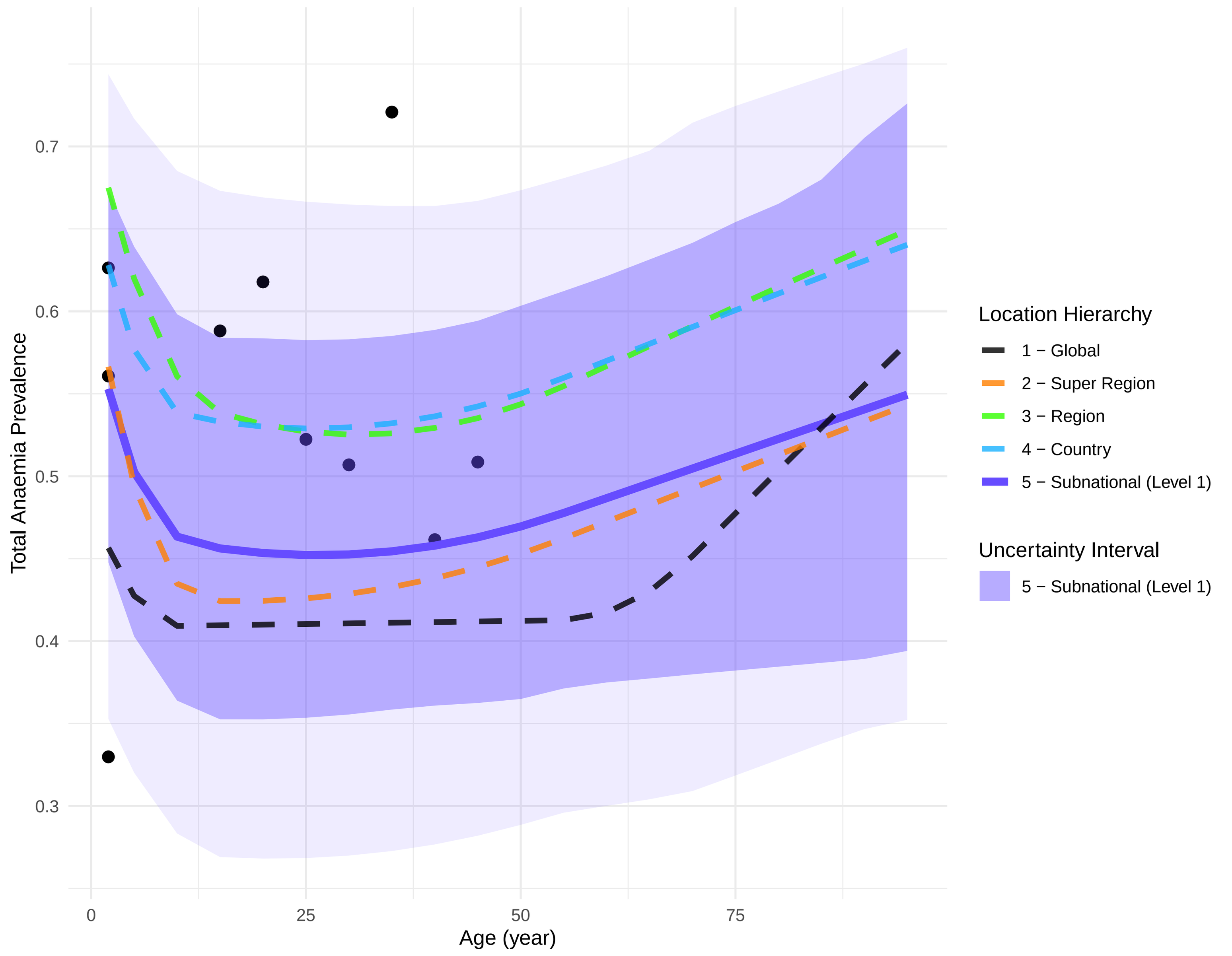

MR−BRT Age/Sex Fits − Non−pregnant Females 2+ Years Old − Total Anaemia Prevalence − Kwara
WHO−2001 Adjustment Method

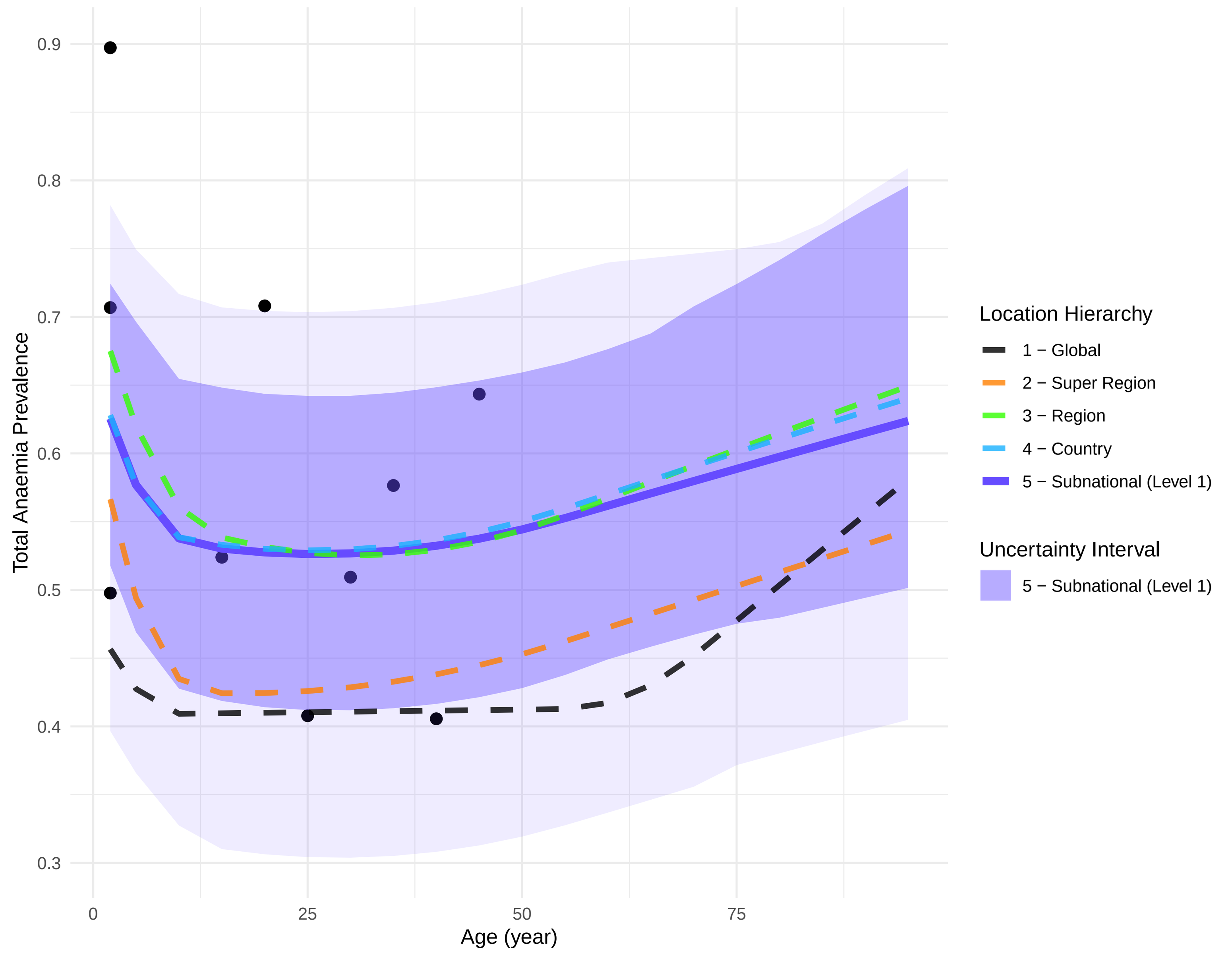

MR−BRT Age/Sex Fits − Non−pregnant Females 2+ Years Old − Total Anaemia Prevalence − Lagos
WHO−2001 Adjustment Method

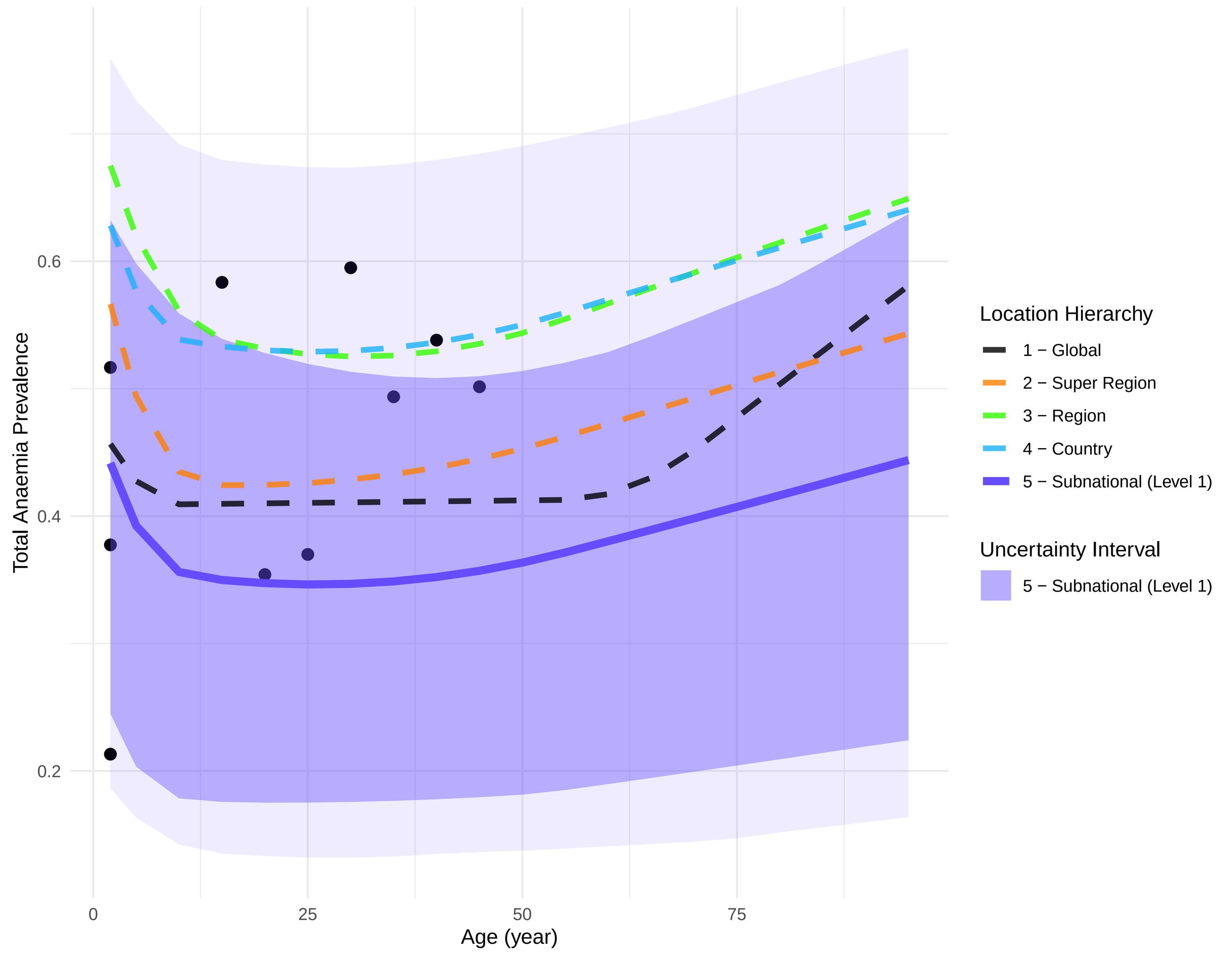

MR−BRT Age/Sex Fits − Non−pregnant Females 2+ Years Old − Total Anaemia Prevalence − Nasarawa
WHO−2001 Adjustment Method

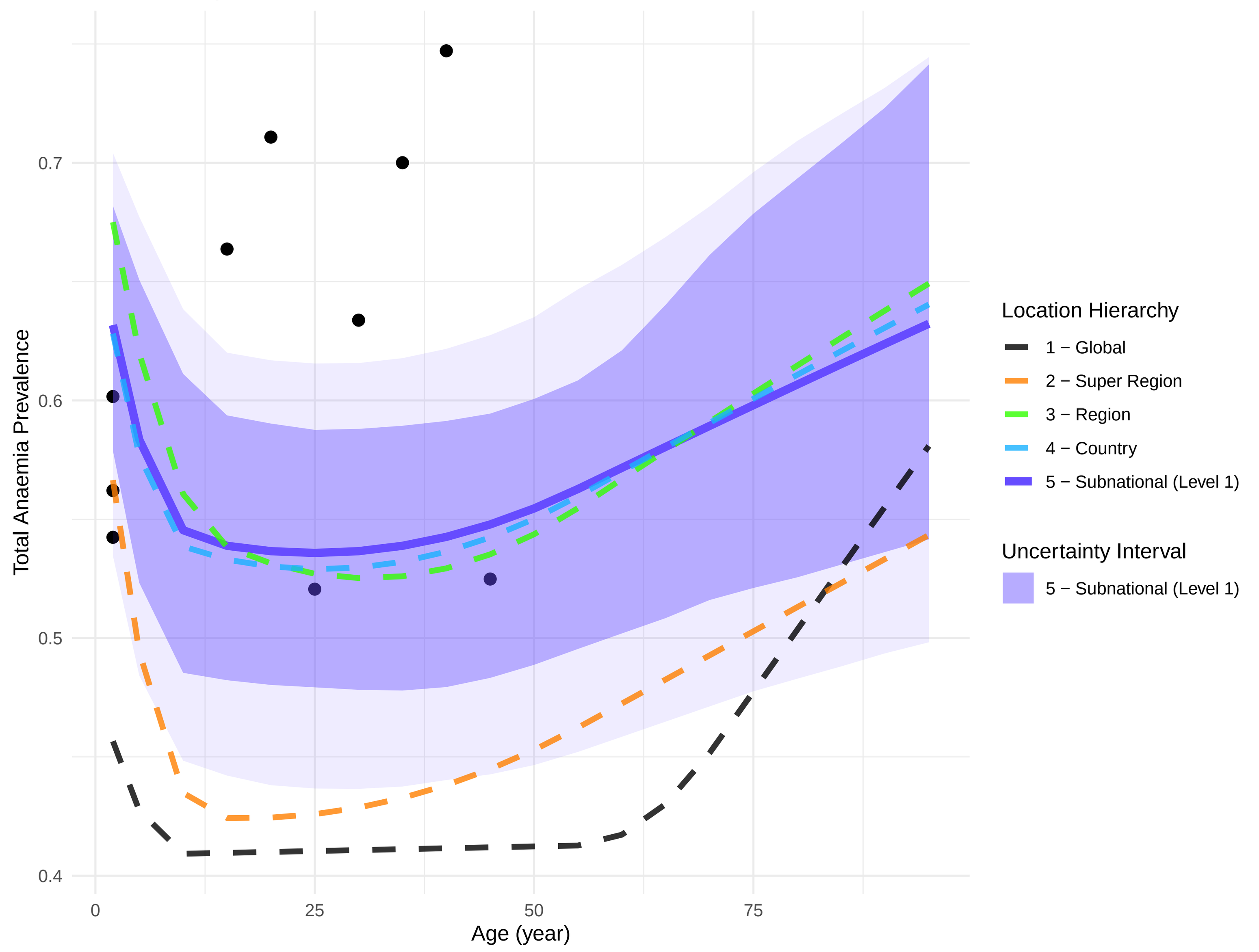

MR−BRT Age/Sex Fits − Non−pregnant Females 2+ Years Old − Total Anaemia Prevalence − Niger
WHO−2001 Adjustment Method

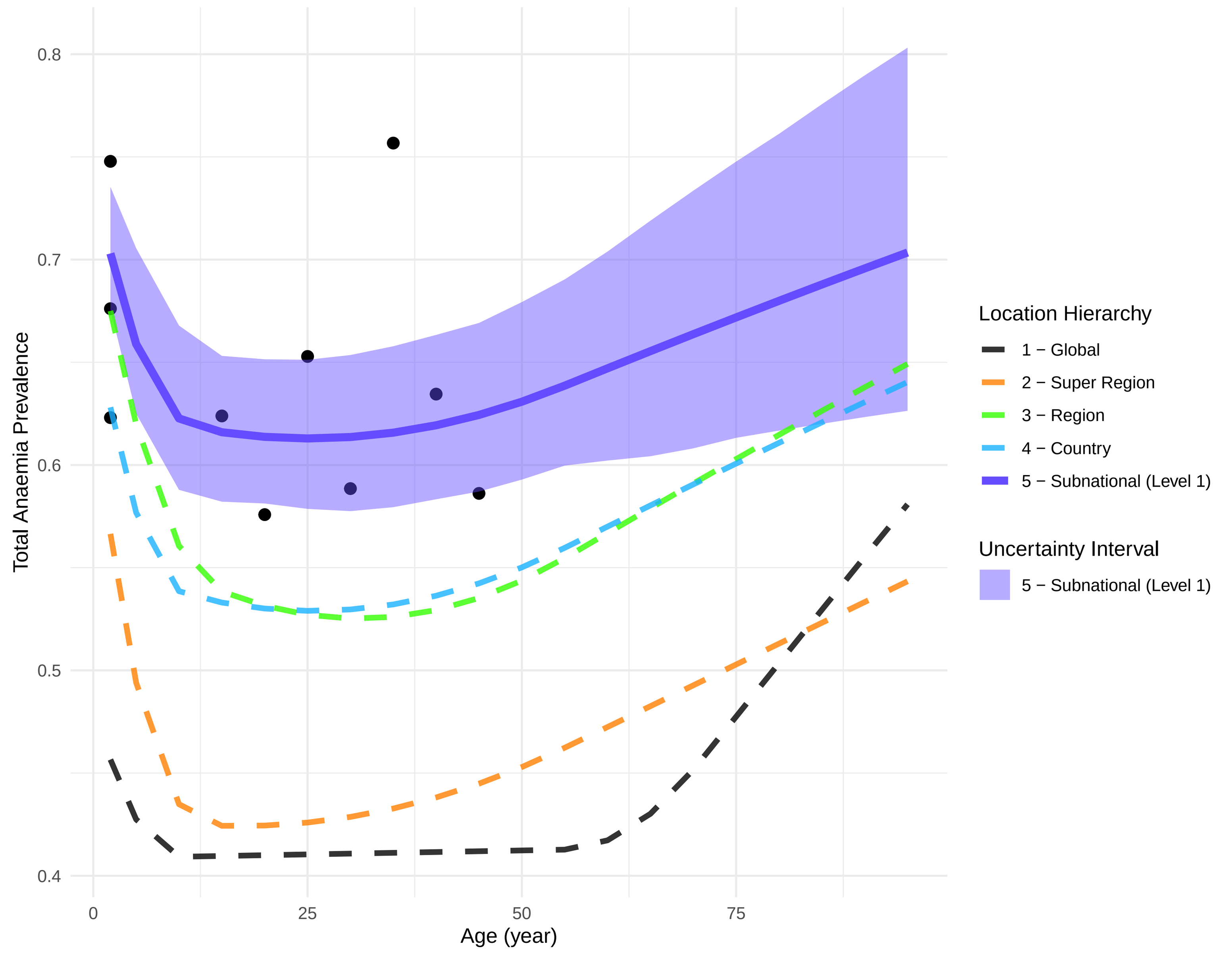

MR−BRT Age/Sex Fits − Non−pregnant Females 2+ Years Old − Total Anaemia Prevalence − Ogun
WHO−2001 Adjustment Method

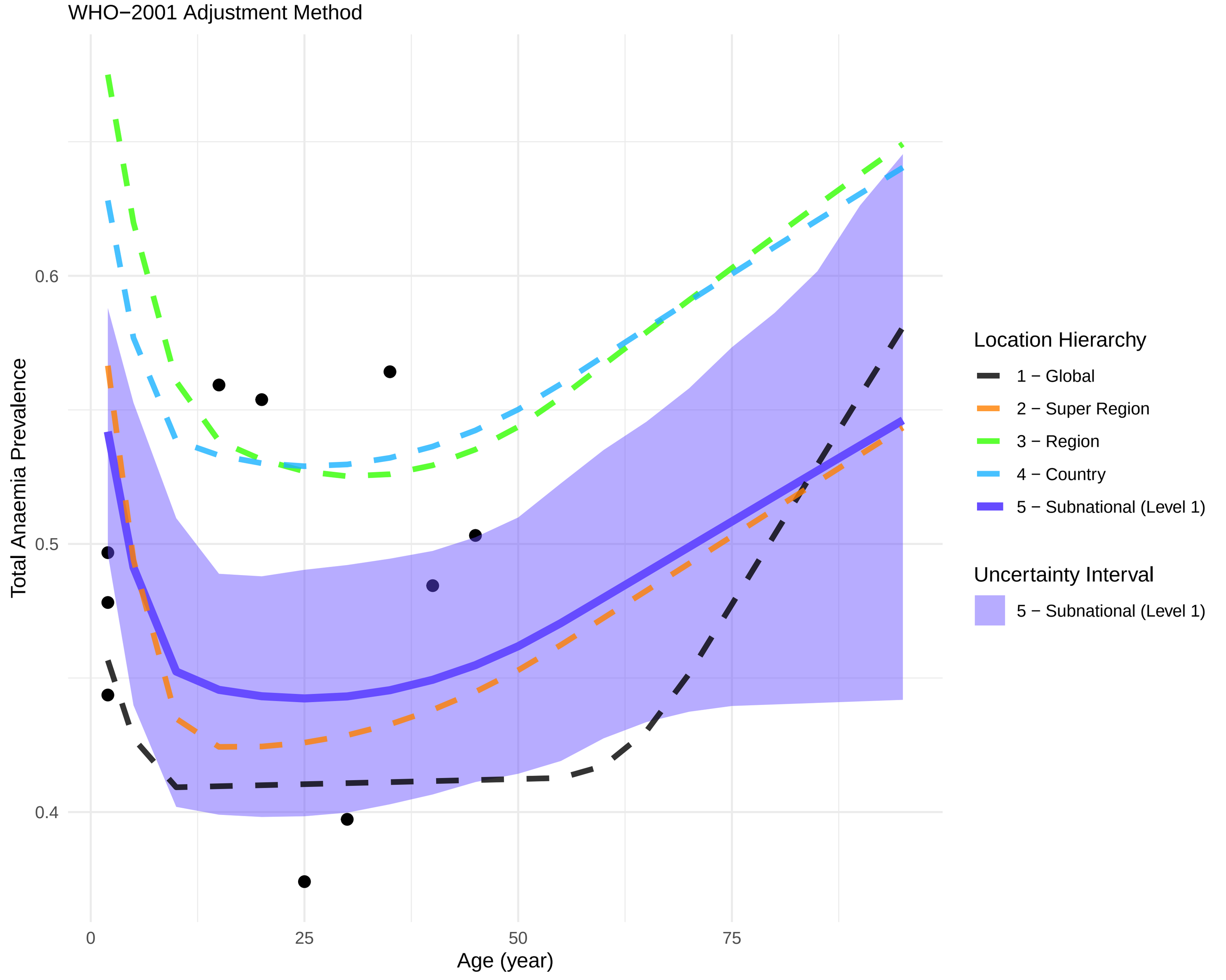

MR−BRT Age/Sex Fits − Non−pregnant Females 2+ Years Old − Total Anaemia Prevalence − Ondo
WHO−2001 Adjustment Method

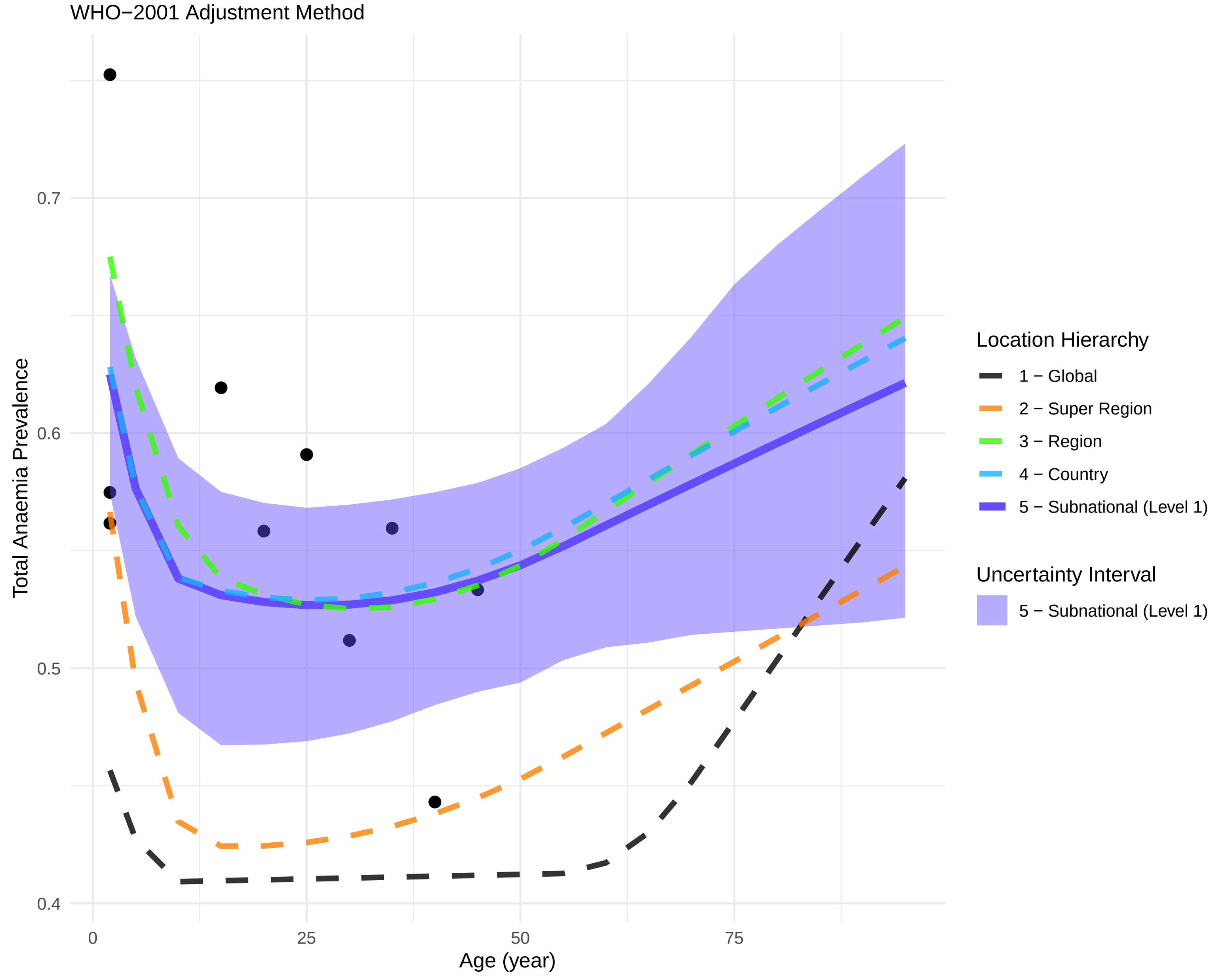

MR−BRT Age/Sex Fits − Non−pregnant Females 2+ Years Old − Total Anaemia Prevalence − Osun
WHO−2001 Adjustment Method

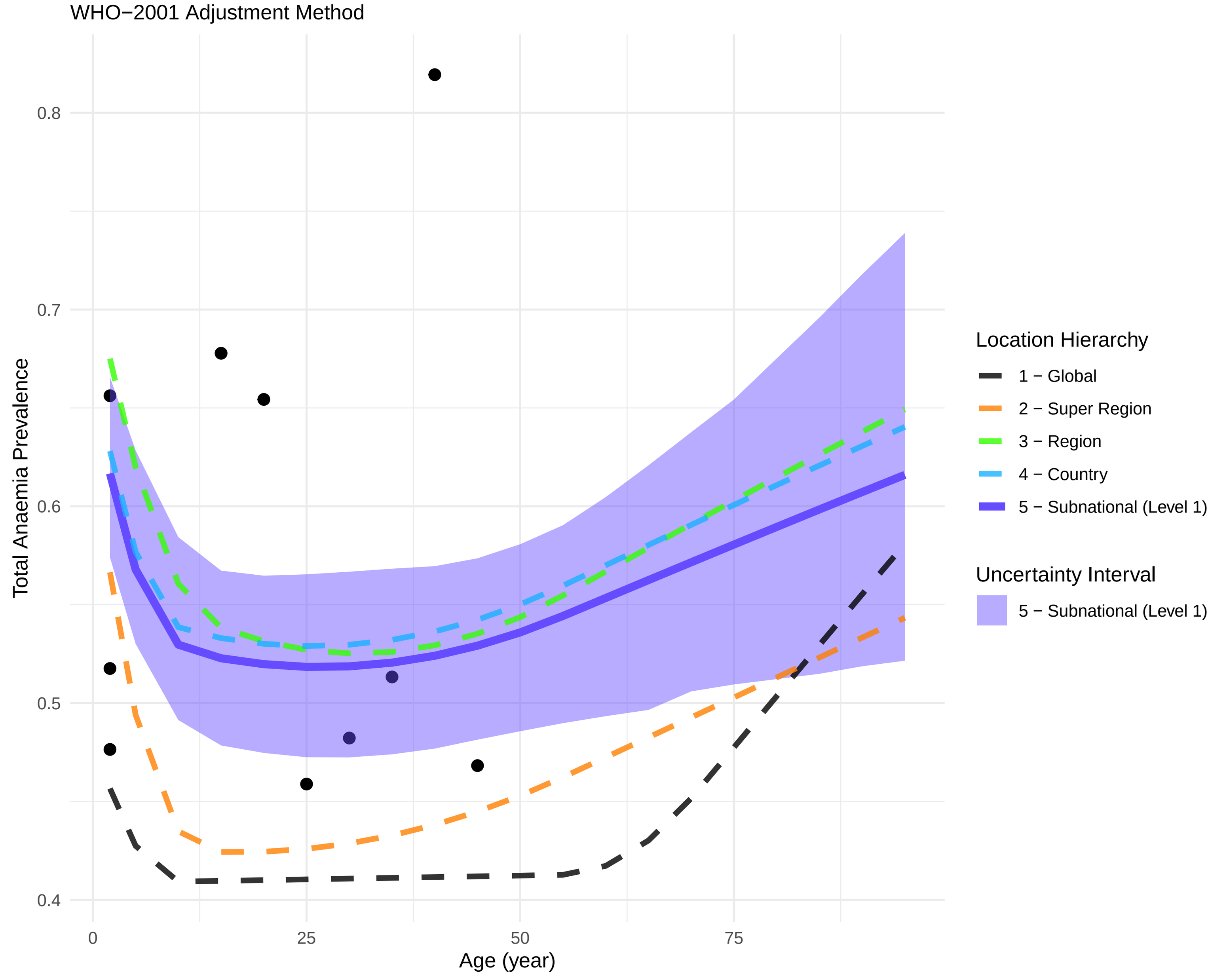

MR−BRT Age/Sex Fits − Non−pregnant Females 2+ Years Old − Total Anaemia Prevalence − Oyo
WHO−2001 Adjustment Method

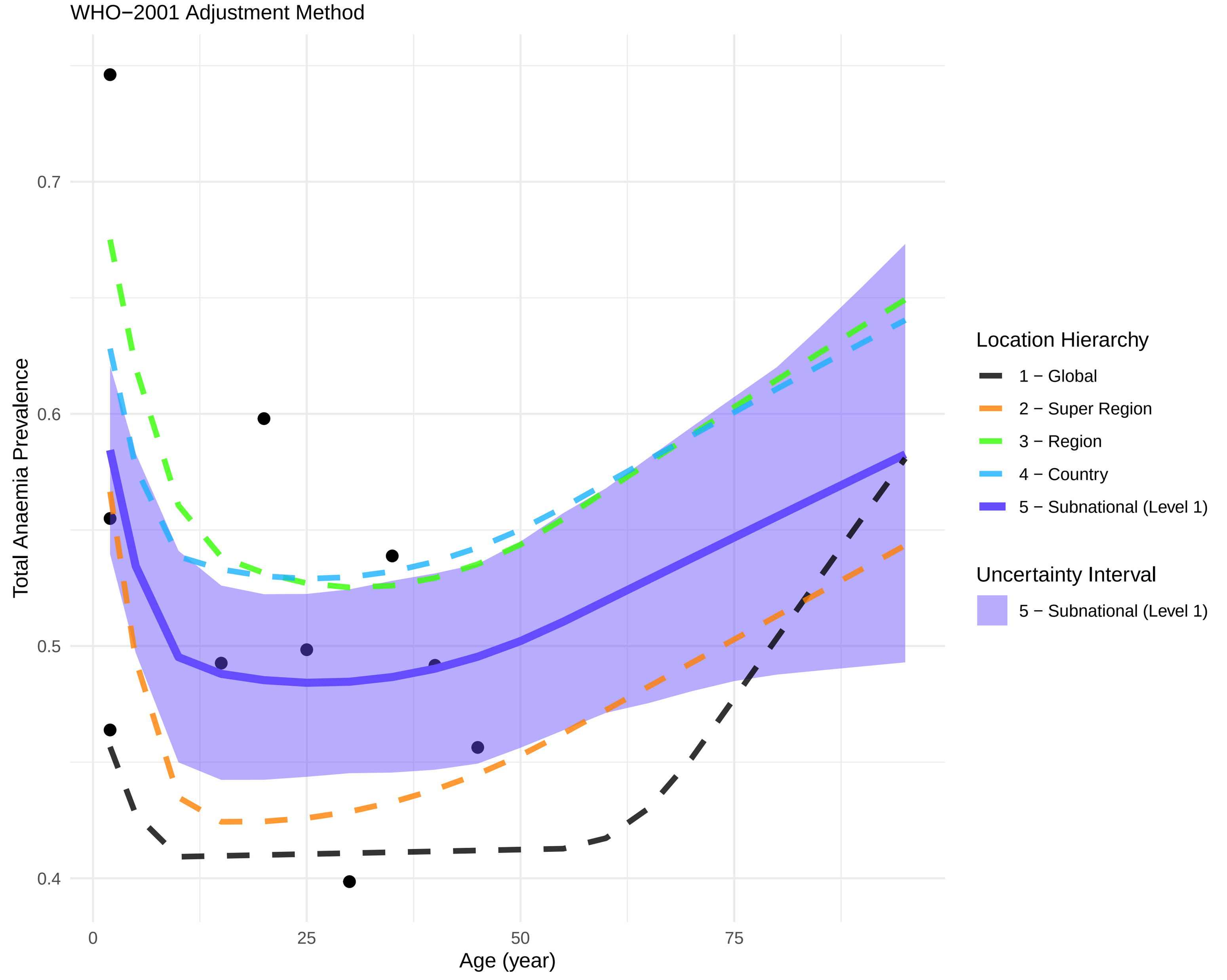

MR−BRT Age/Sex Fits − Non−pregnant Females 2+ Years Old − Total Anaemia Prevalence − Plateau
WHO−2001 Adjustment Method

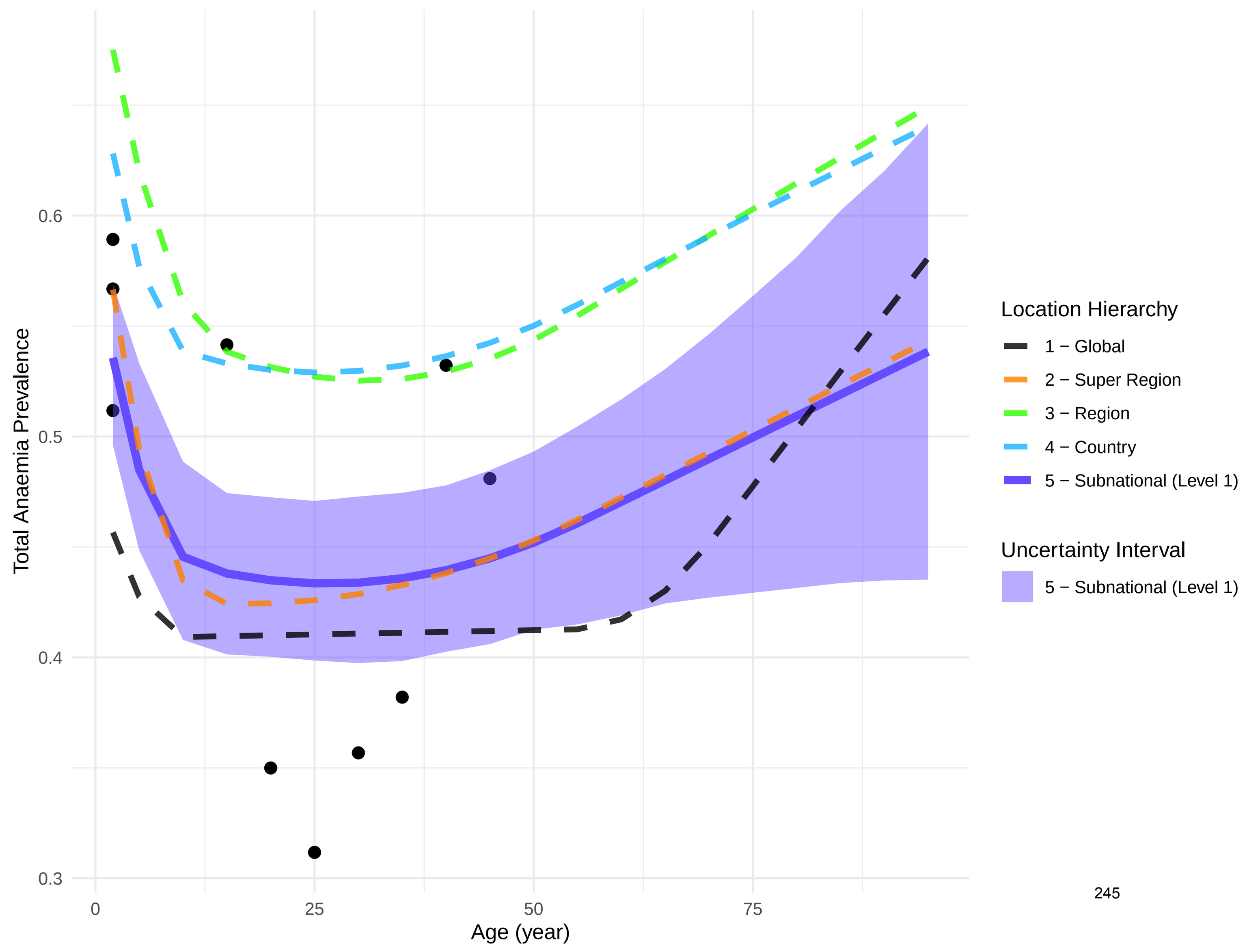

MR−BRT Age/Sex Fits − Non−pregnant Females 2+ Years Old − Total Anaemia Prevalence − Rivers
WHO−2001 Adjustment Method

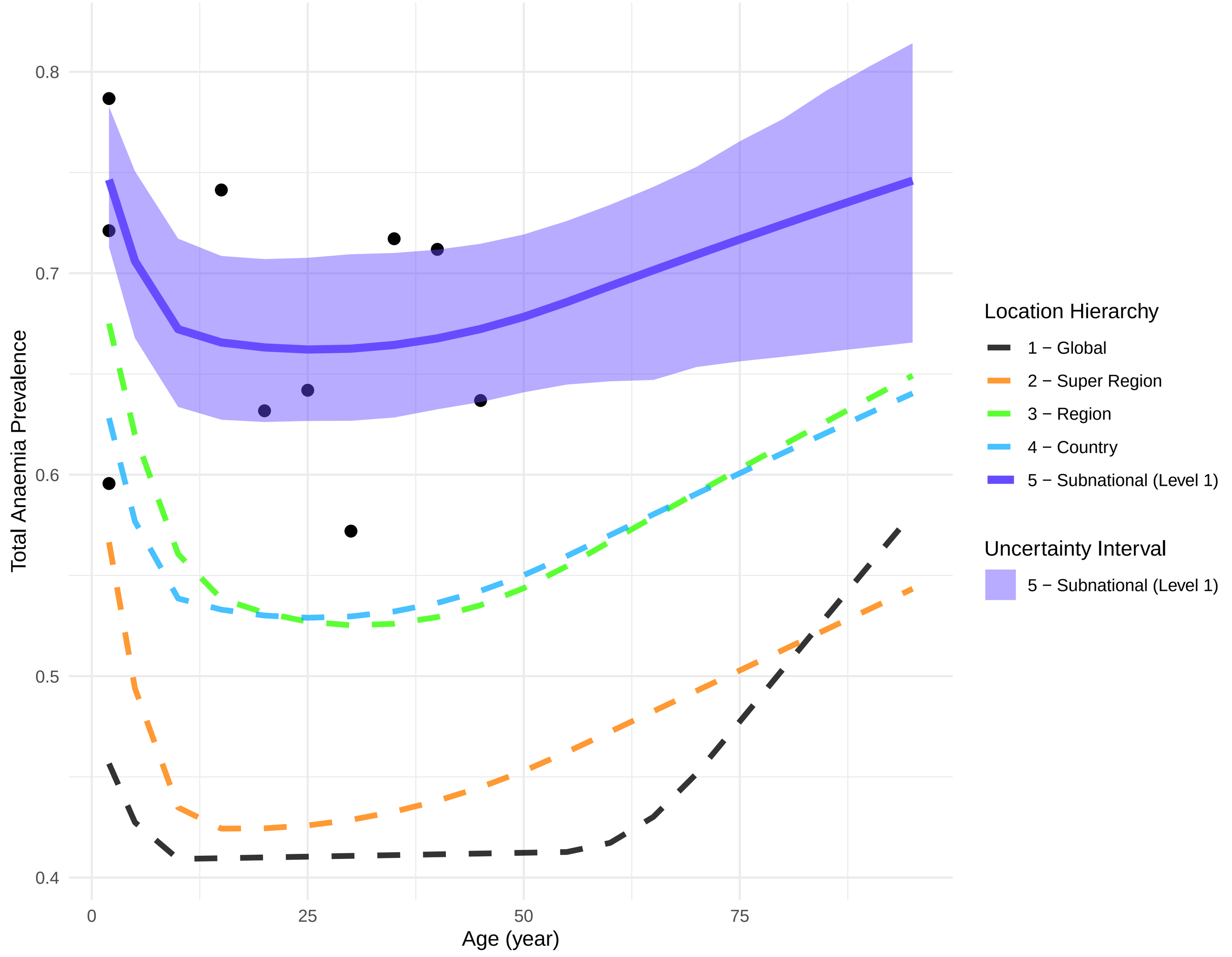

MR−BRT Age/Sex Fits − Non−pregnant Females 2+ Years Old − Total Anaemia Prevalence − Sokoto
WHO−2001 Adjustment Method

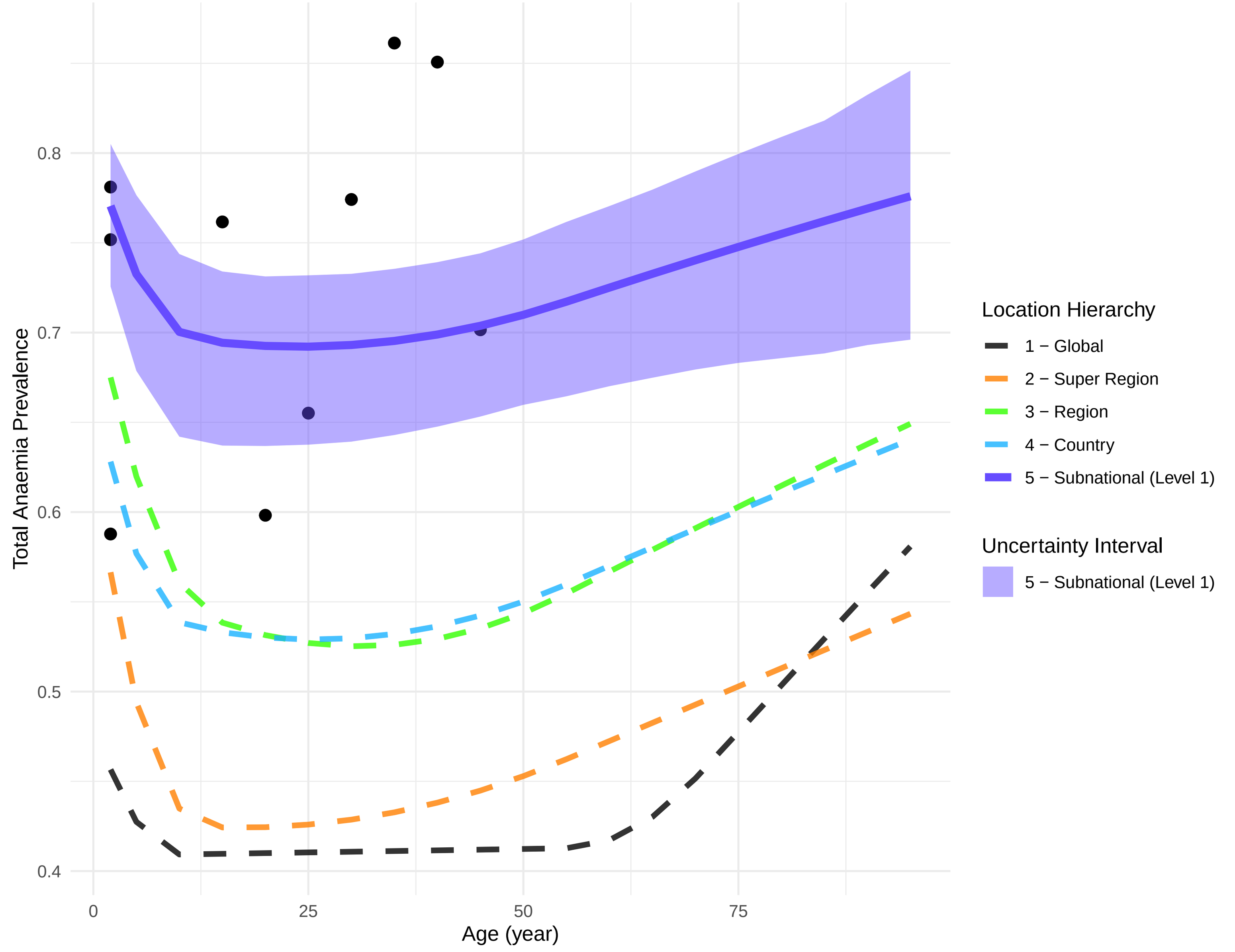

MR−BRT Age/Sex Fits − Non−pregnant Females 2+ Years Old − Total Anaemia Prevalence − Taraba
WHO−2001 Adjustment Method

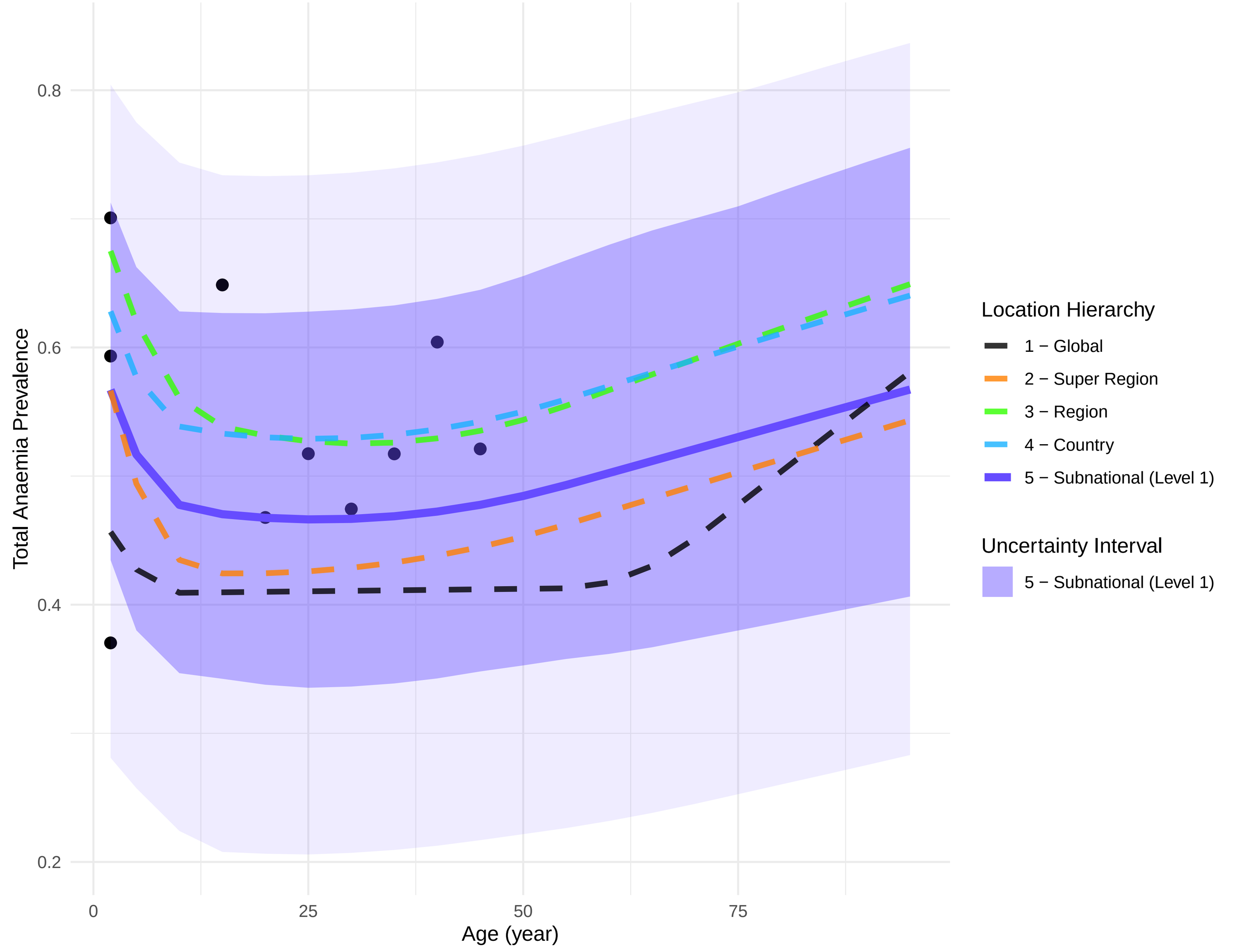

MR−BRT Age/Sex Fits − Non−pregnant Females 2+ Years Old − Total Anaemia Prevalence − Yobe
WHO−2001 Adjustment Method

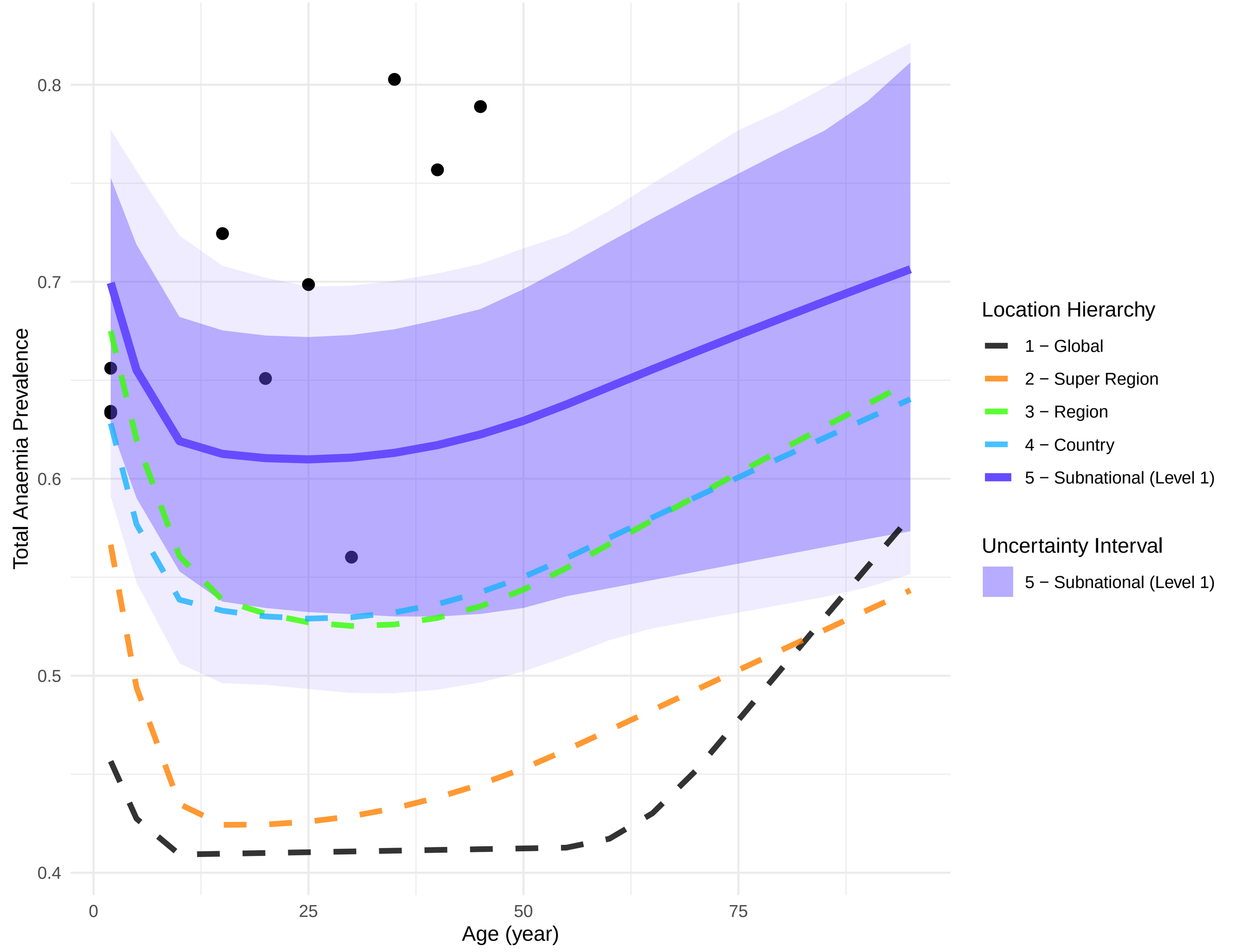

MR−BRT Age/Sex Fits − Non−pregnant Females 2+ Years Old − Total Anaemia Prevalence − Zamfara
WHO−2001 Adjustment Method

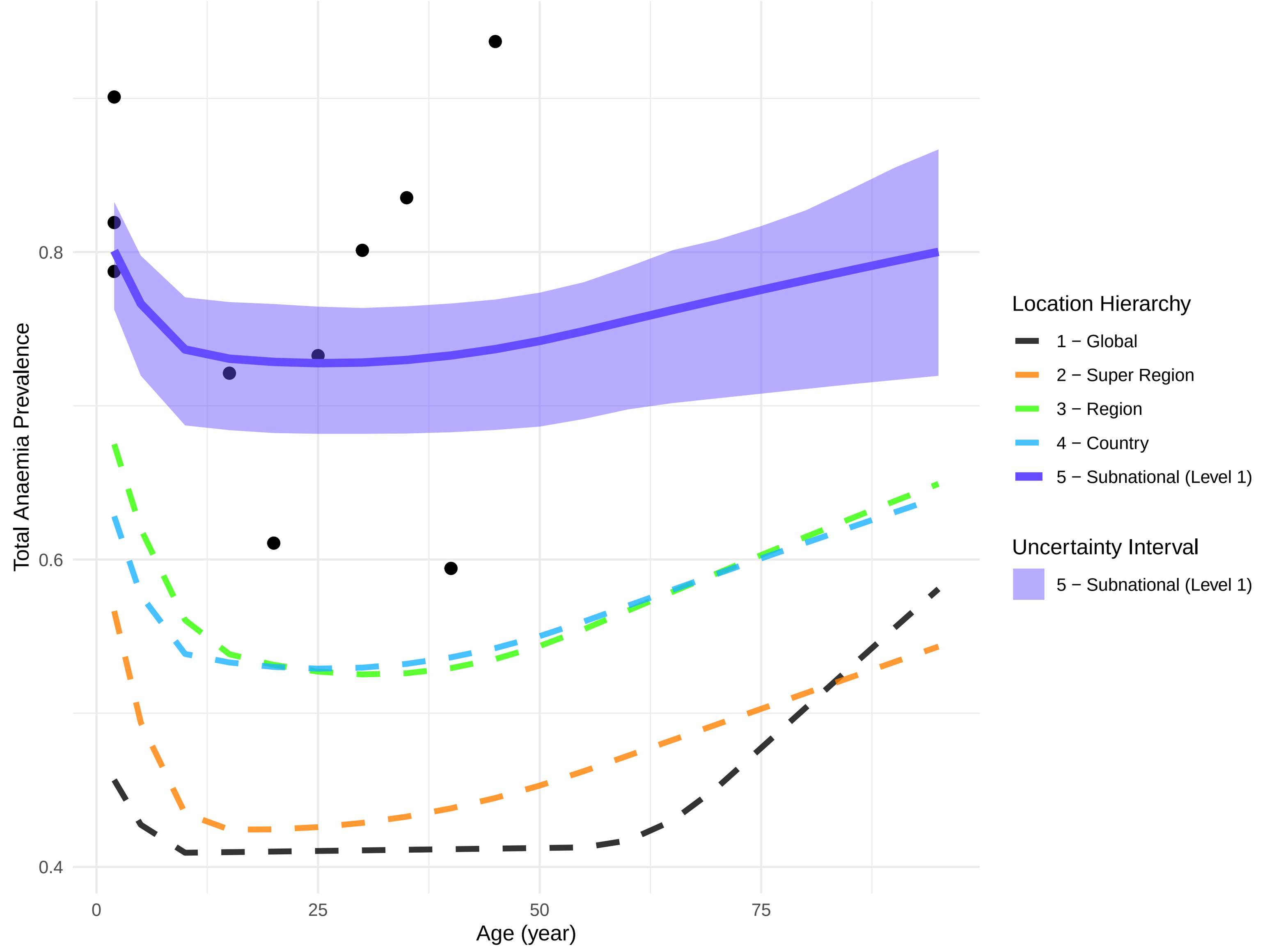

MR−BRT Age/Sex Fits − Non−pregnant Females 2+ Years Old − Total Anaemia Prevalence − Senegal
WHO−2001 Adjustment Method

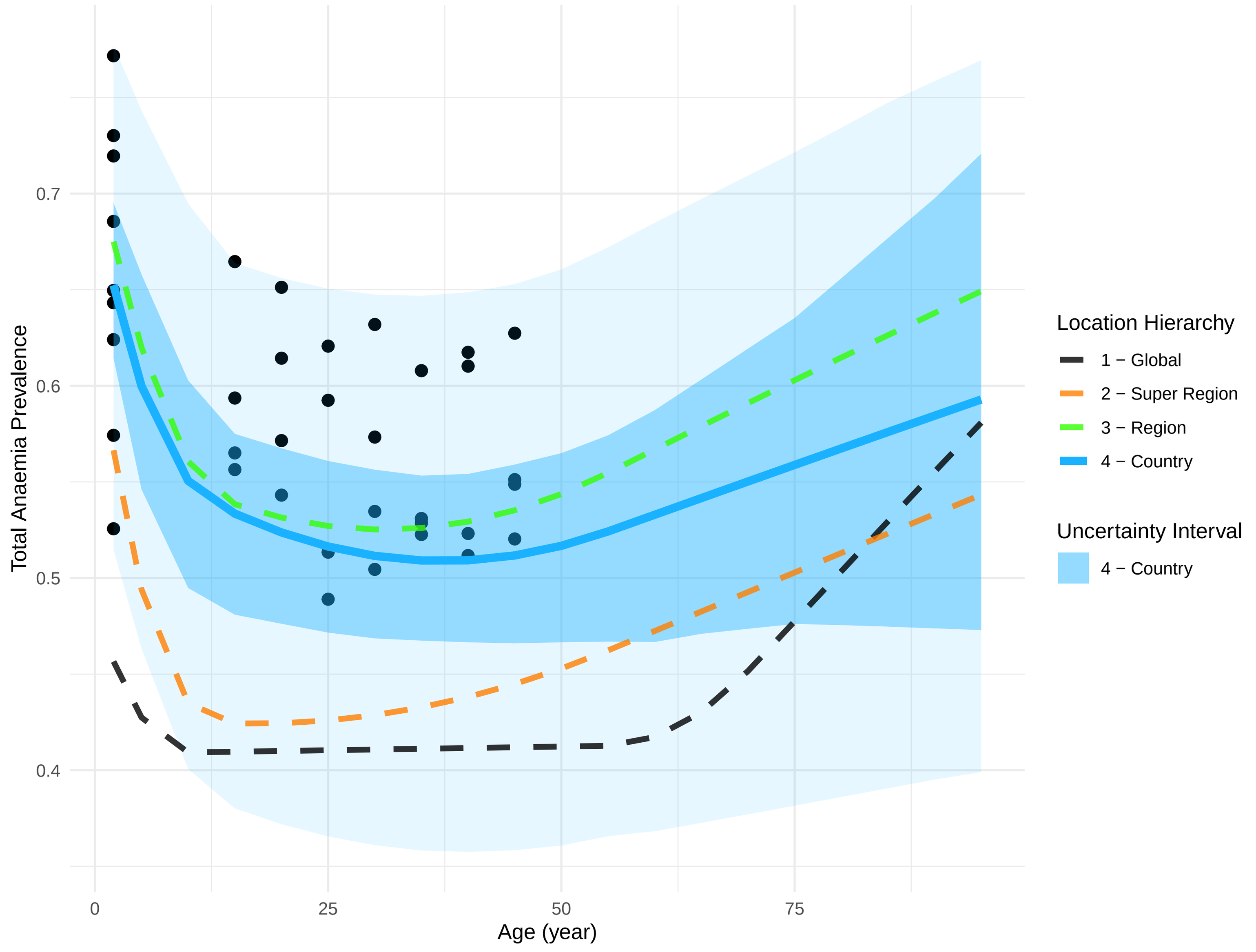

MR−BRT Age/Sex Fits − Non−pregnant Females 2+ Years Old − Total Anaemia Prevalence − Sierra Leone
WHO−2001 Adjustment Method

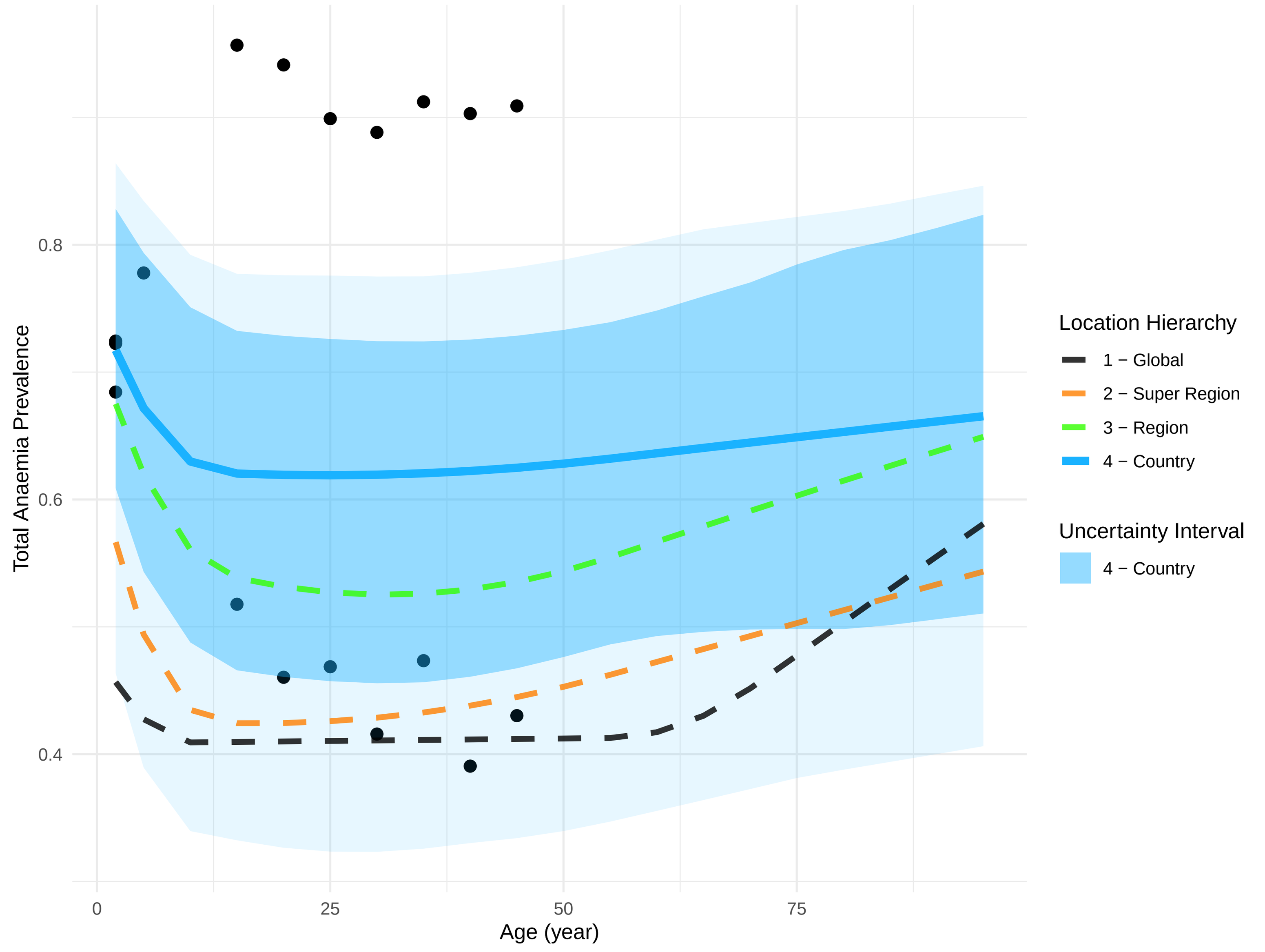

MR−BRT Age/Sex Fits − Non−pregnant Females 2+ Years Old − Total Anaemia Prevalence − Togo
WHO−2001 Adjustment Method

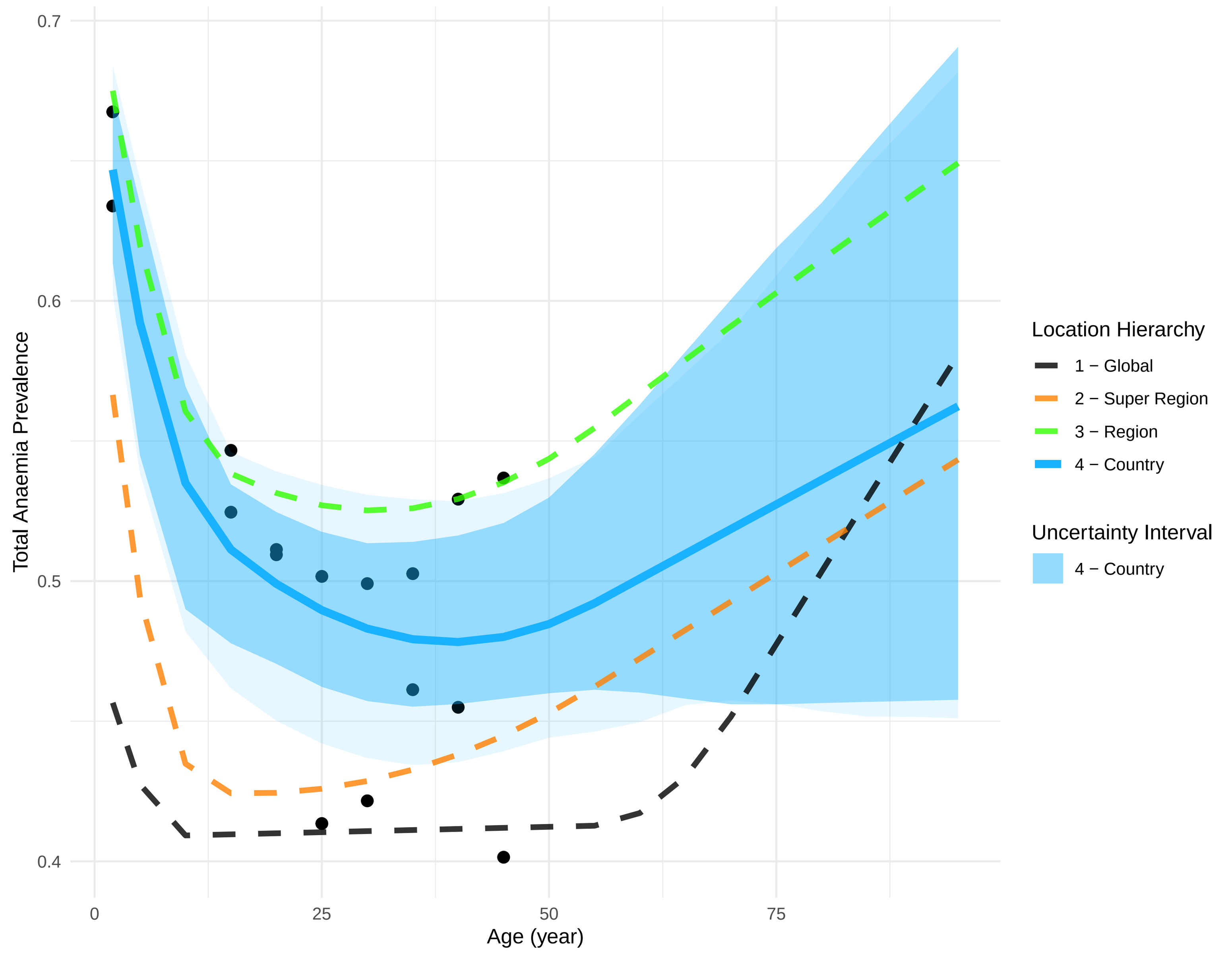

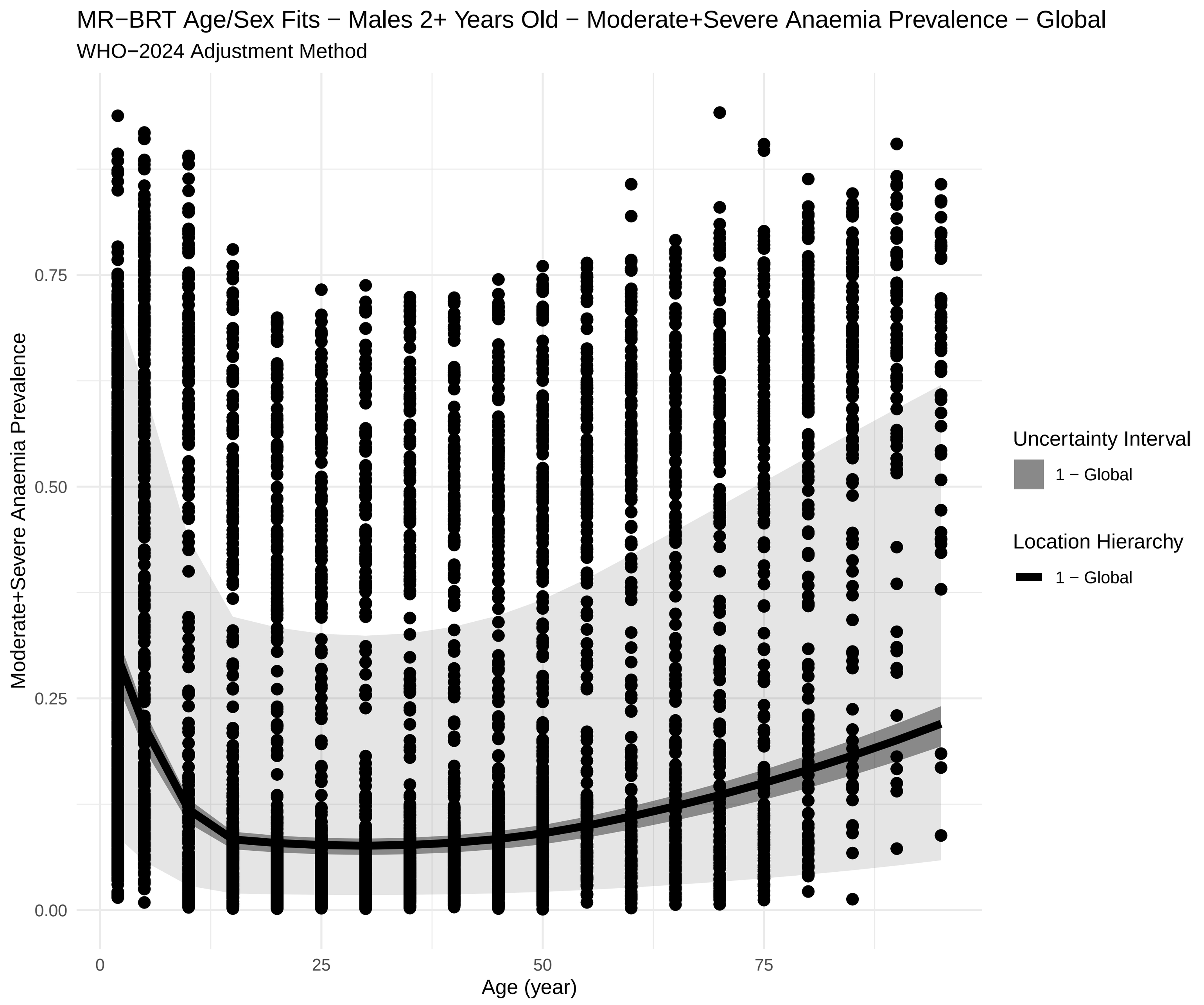
MR−BRT Age/Sex Fits − Males 2+ Years Old − Moderate+Severe Anaemia Prevalence − Global
WHO−2024 Adjustment Method
Moderate+Severe Anaemia Prevalence
Age (year)
Uncertainty Interval
1 − Global
Location Hierarchy
1 − Global

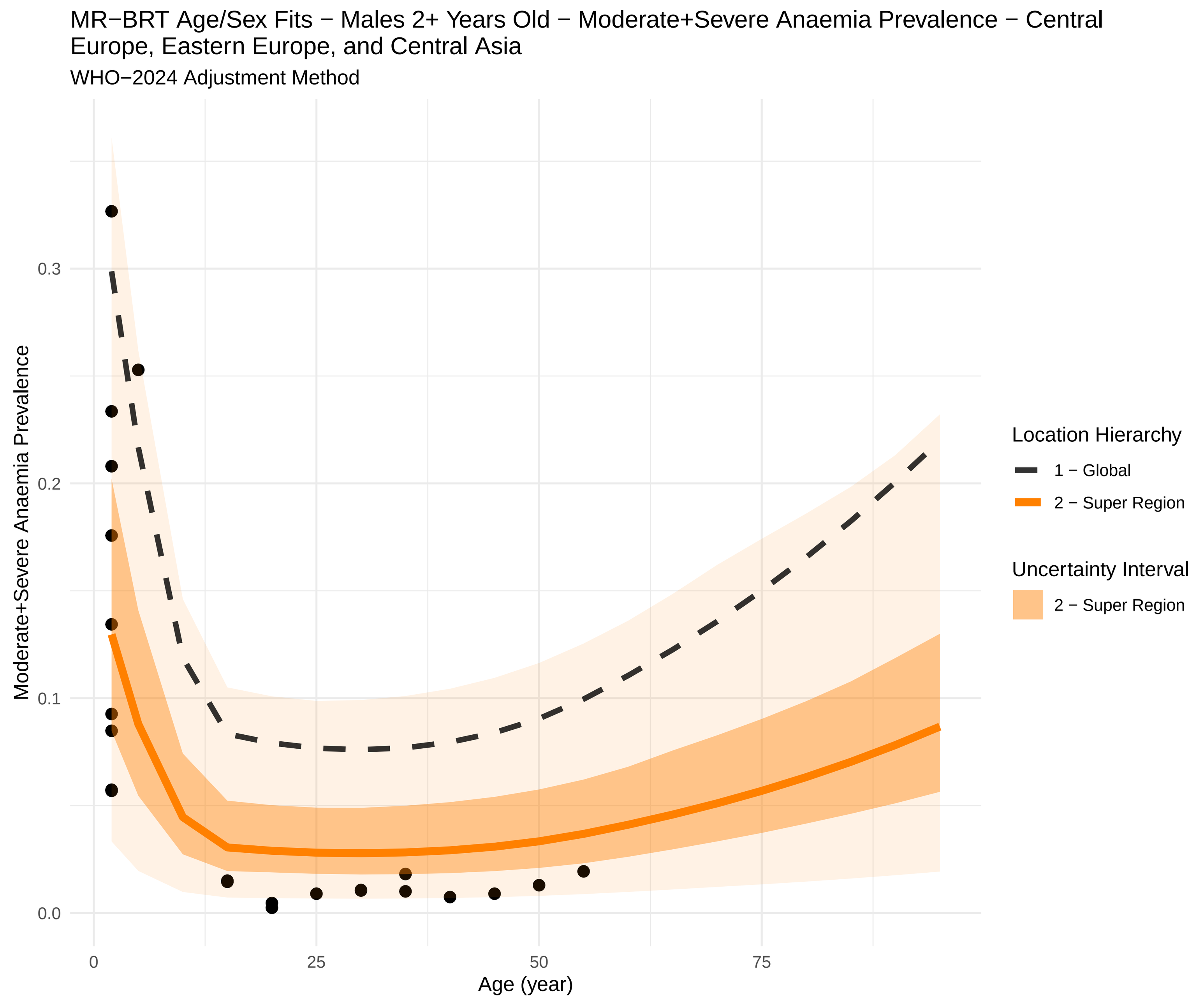
MR−BRT Age/Sex Fits − Males 2+ Years Old − Moderate+Severe Anaemia Prevalence − Central Europe, Eastern Europe, and Central Asia
WHO−2024 Adjustment Method
Moderate+Severe Anaemia Prevalence
Age (year)
Location Hierarchy
1 − Global
2 − Super Region
Uncertainty Interval
2 − Super Region

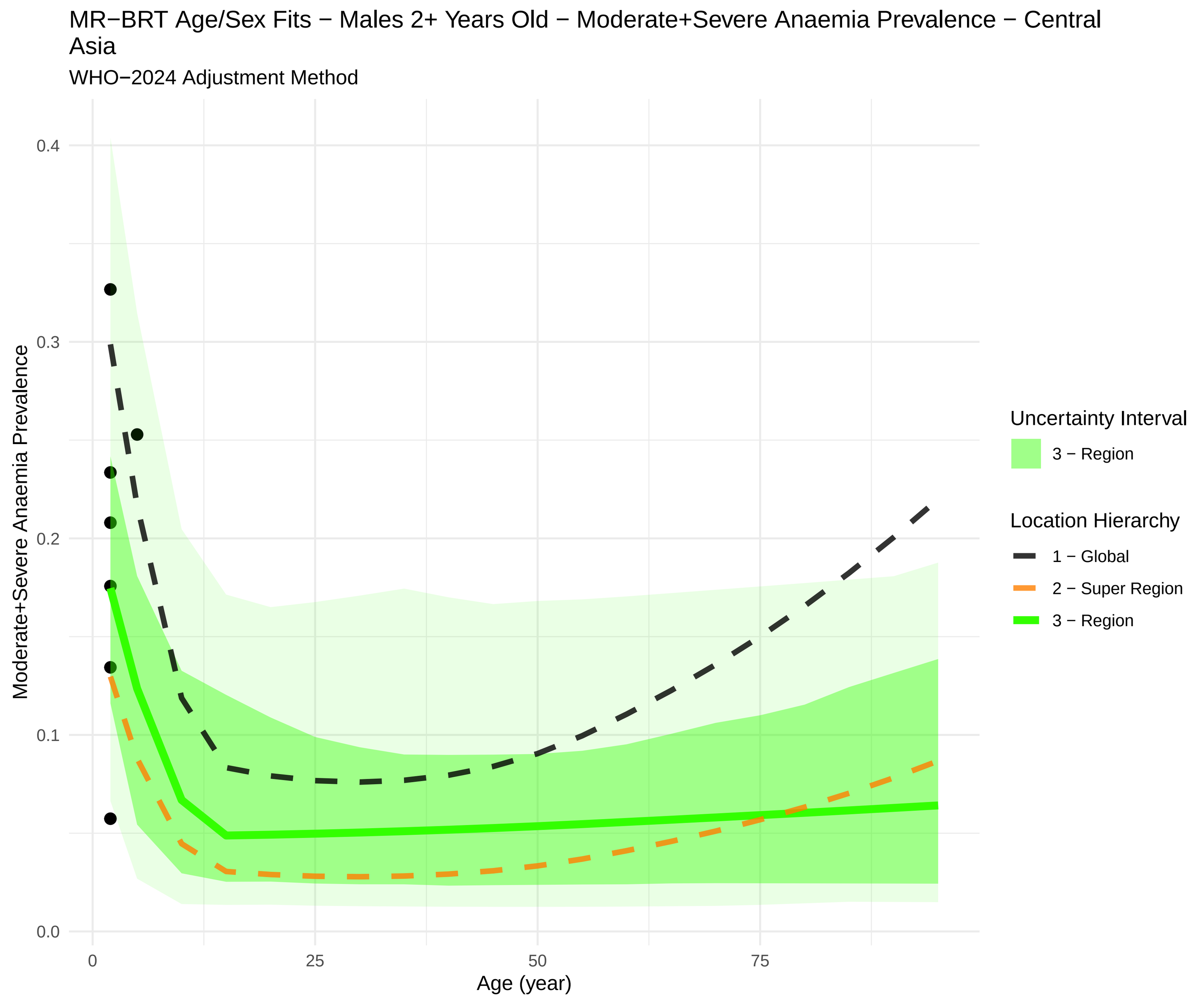
MR−BRT Age/Sex Fits − Males 2+ Years Old − Moderate+Severe Anaemia Prevalence − Central Asia
WHO−2024 Adjustment Method
Moderate+Severe Anaemia Prevalence
Age (year)
Uncertainty Interval
3 − Region
Location Hierarchy
1 − Global
2 − Super Region
3 − Region

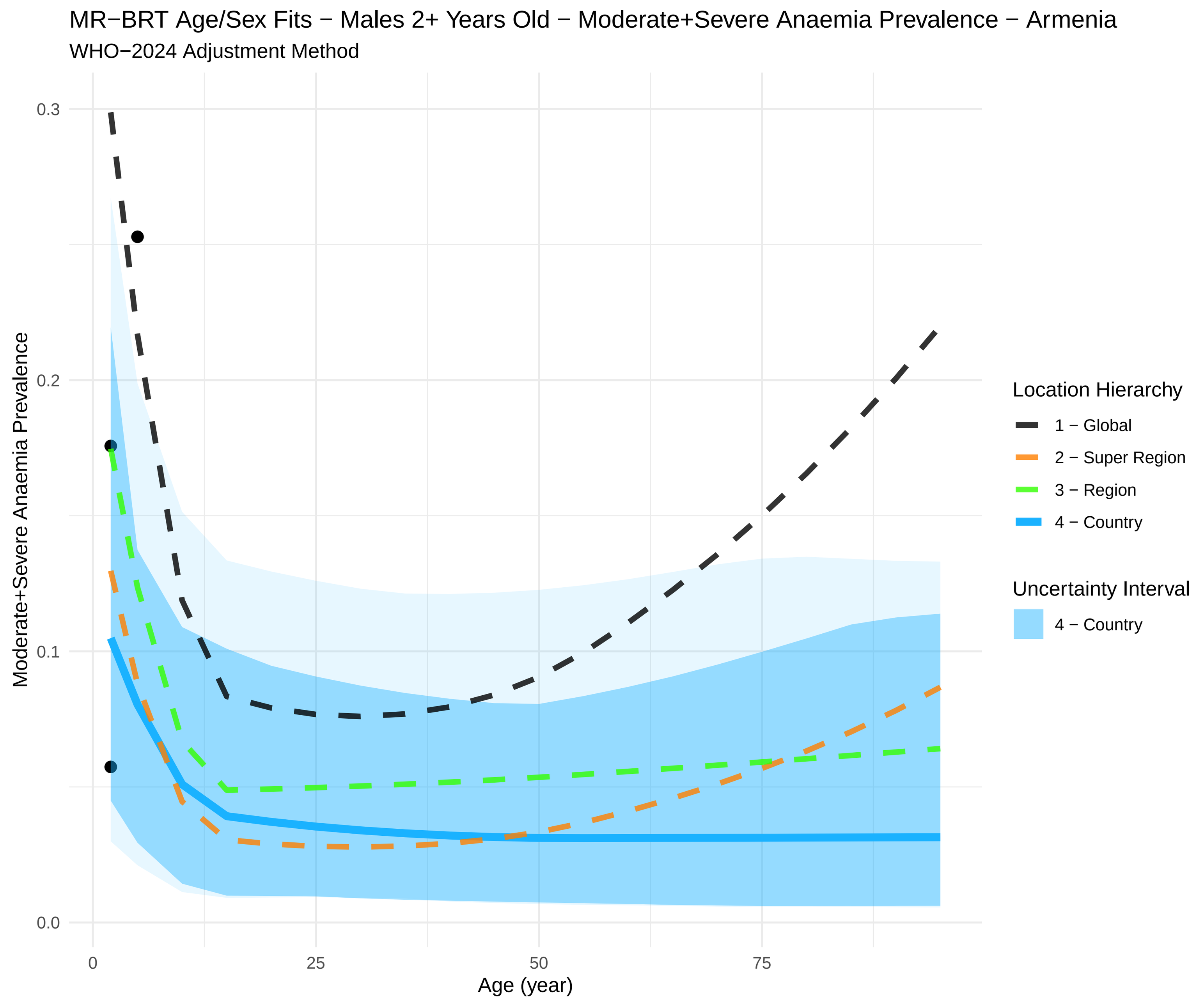
MR−BRT Age/Sex Fits − Males 2+ Years Old − Moderate+Severe Anaemia Prevalence − Armenia
WHO−2024 Adjustment Method
Moderate+Severe Anaemia Prevalence
Age (year)
Location Hierarchy
1 − Global
2 − Super Region
3 − Region
4 − Country
Uncertainty Interval
4 − Country

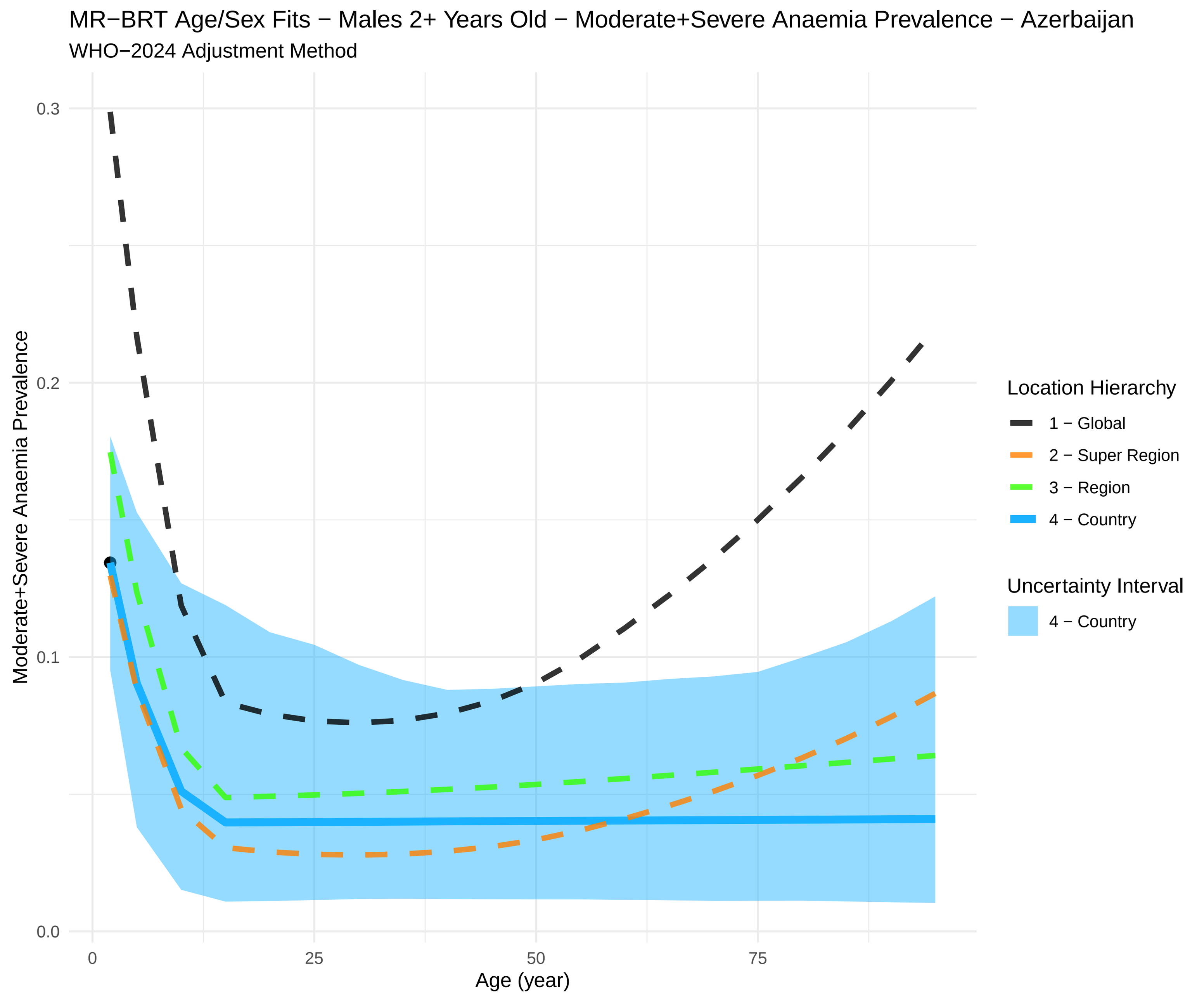
MR−BRT Age/Sex Fits − Males 2+ Years Old − Moderate+Severe Anaemia Prevalence − Azerbaijan
WHO−2024 Adjustment Method
Moderate+Severe Anaemia Prevalence
Age (year)
Location Hierarchy
1 − Global
2 − Super Region
3 − Region
4 − Country
Uncertainty Interval
4 − Country

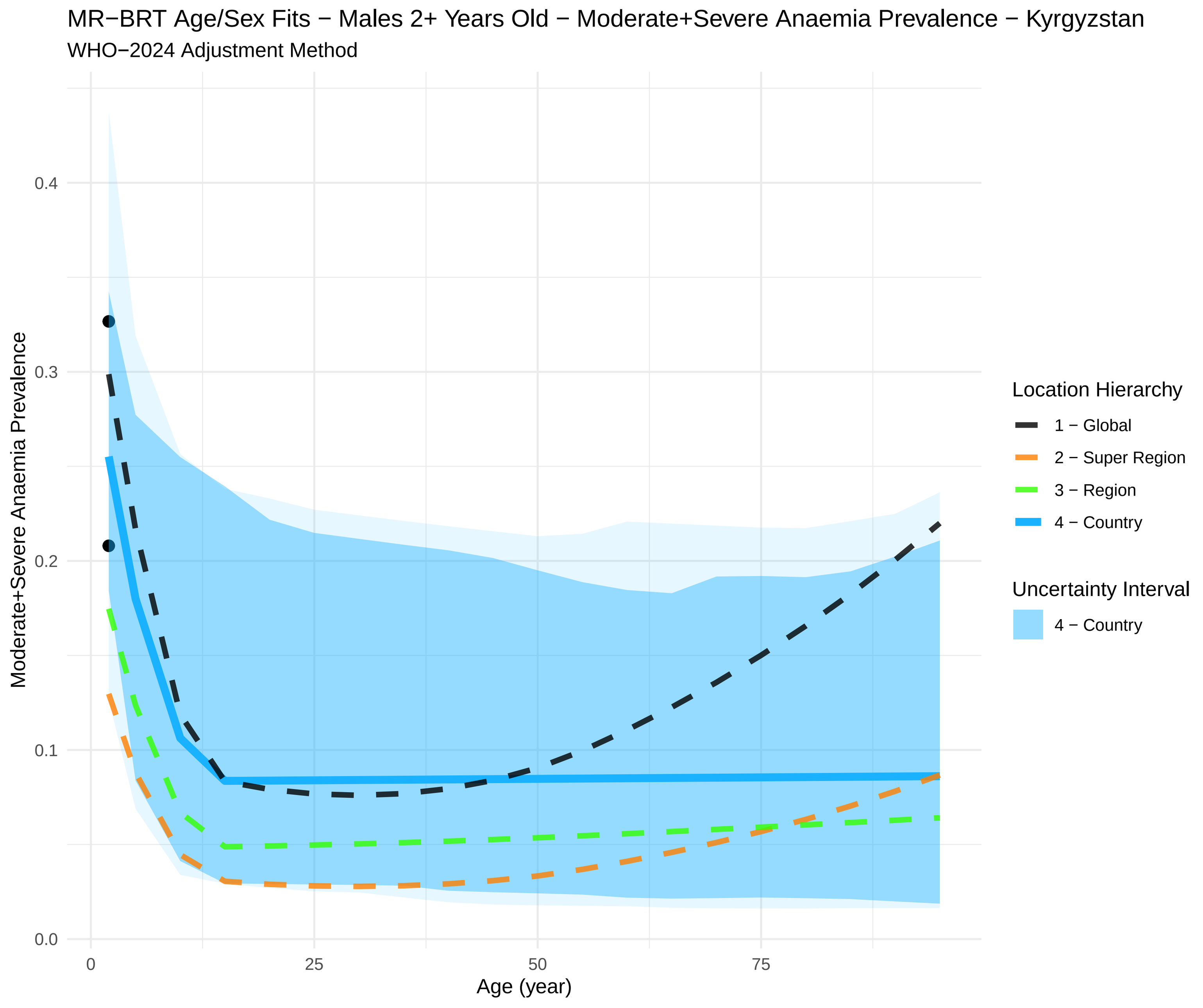
MR−BRT Age/Sex Fits − Males 2+ Years Old − Moderate+Severe Anaemia Prevalence − Kyrgyzstan
WHO−2024 Adjustment Method
Moderate+Severe Anaemia Prevalence
Age (year)
Location Hierarchy
1 − Global
2 − Super Region
3 − Region
4 − Country
Uncertainty Interval
4 − Country

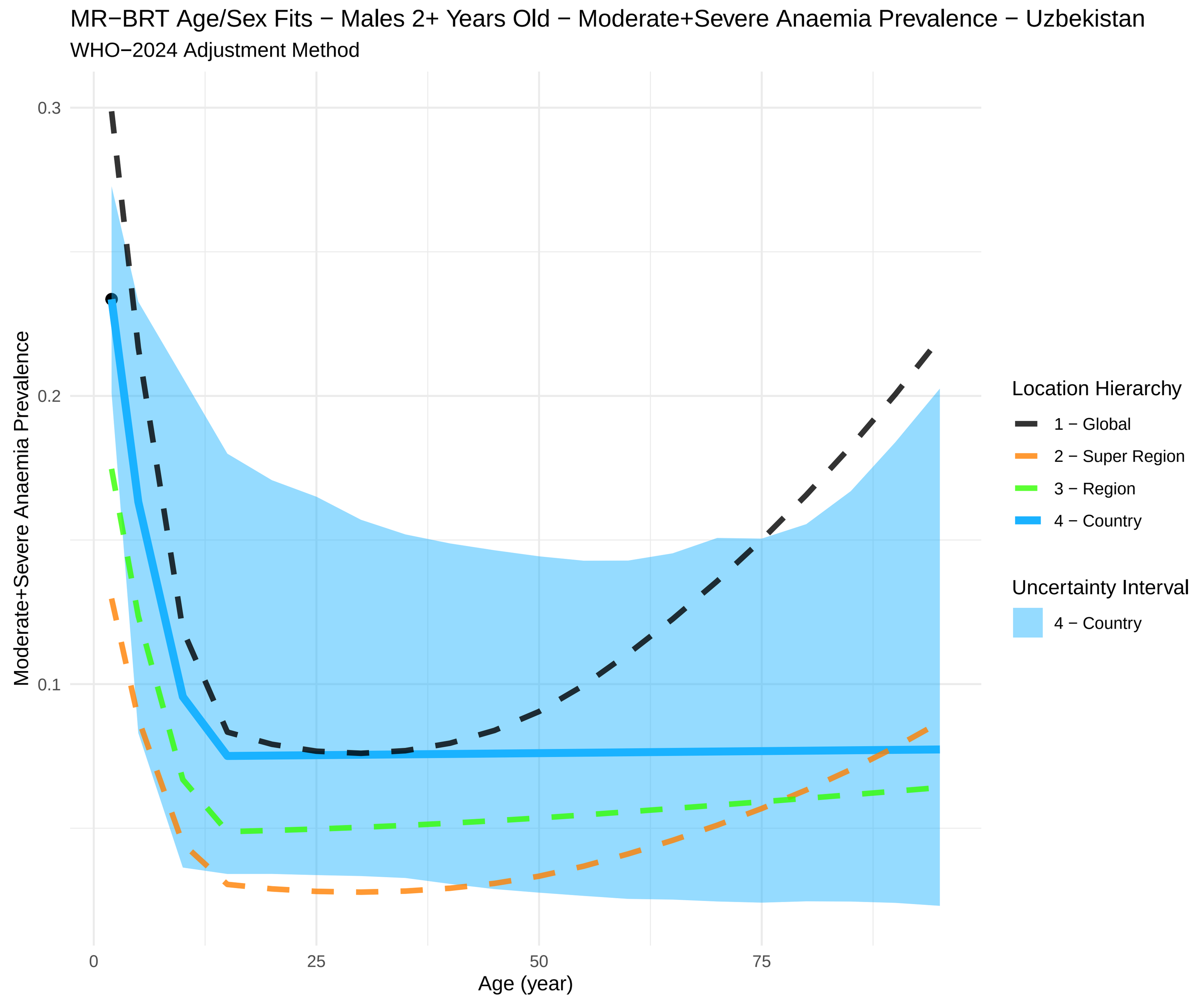
MR−BRT Age/Sex Fits − Males 2+ Years Old − Moderate+Severe Anaemia Prevalence − Uzbekistan
WHO−2024 Adjustment Method
Moderate+Severe Anaemia Prevalence
Age (year)
Location Hierarchy
1 − Global
2 − Super Region
3 − Region
4 − Country
Uncertainty Interval
4 − Country

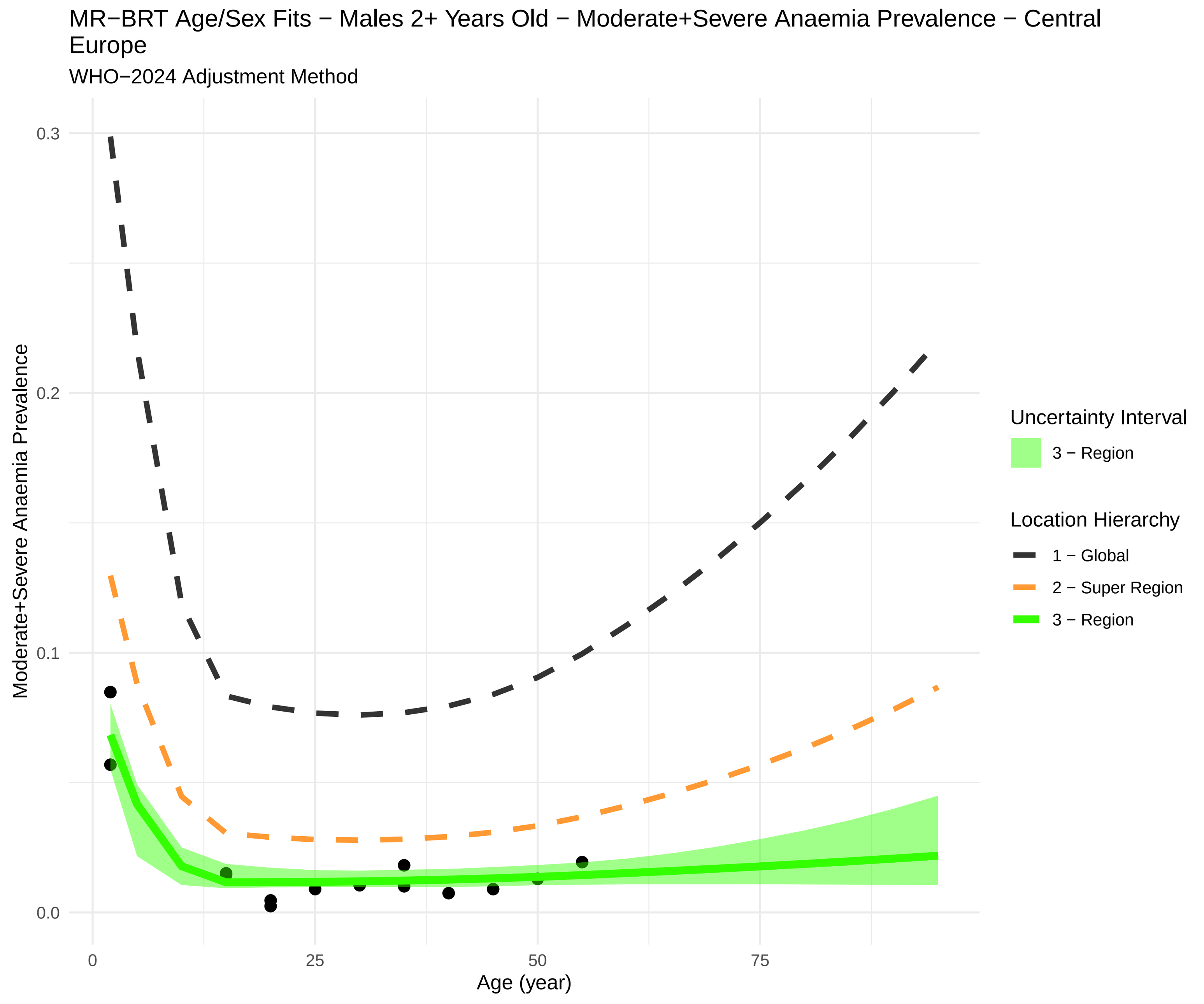
MR−BRT Age/Sex Fits − Males 2+ Years Old − Moderate+Severe Anaemia Prevalence − Central Europe
WHO−2024 Adjustment Method
Moderate+Severe Anaemia Prevalence
Age (year)
Uncertainty Interval
3 − Region
Location Hierarchy
1 − Global
2 − Super Region
3 − Region

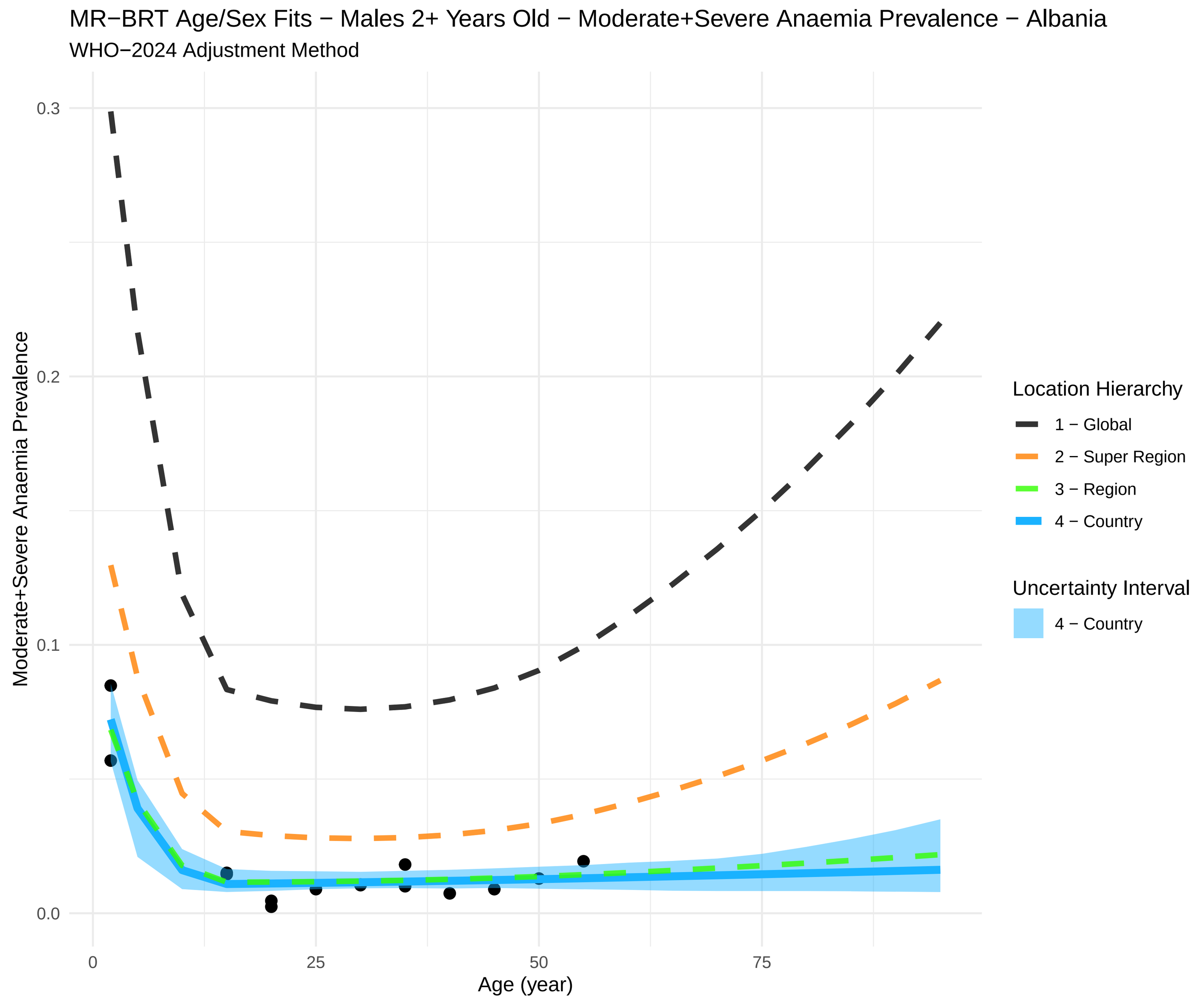
MR−BRT Age/Sex Fits − Males 2+ Years Old − Moderate+Severe Anaemia Prevalence − Albania
WHO−2024 Adjustment Method
Moderate+Severe Anaemia Prevalence
Age (year)
Location Hierarchy
1 − Global
2 − Super Region
3 − Region
4 − Country
Uncertainty Interval
4 − Country

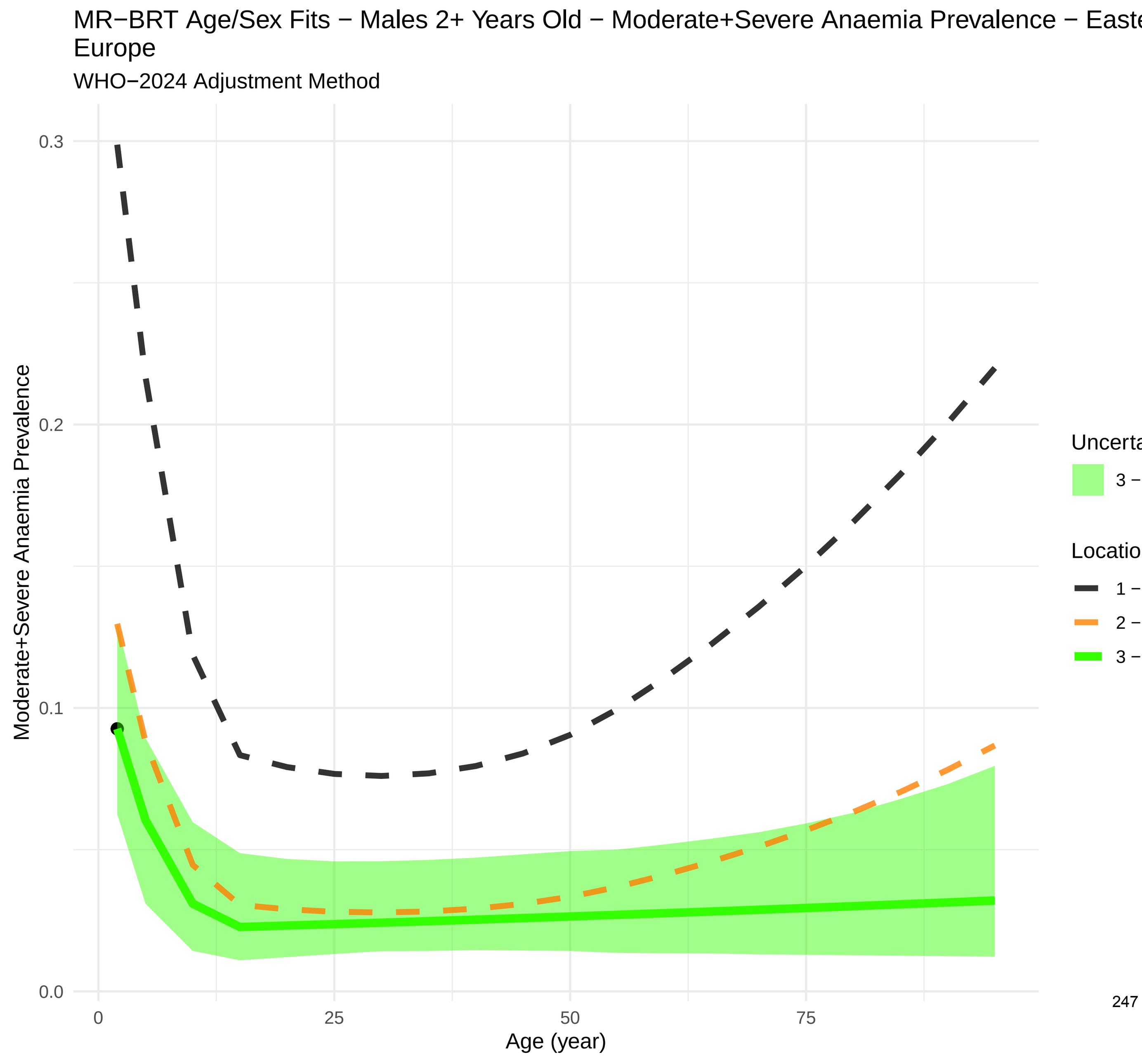
MR−BRT Age/Sex Fits − Males 2+ Years Old − Moderate+Severe Anaemia Prevalence − Eastern Europe
WHO−2024 Adjustment Method
Moderate+Severe Anaemia Prevalence
Age (year)
Uncertainty Interval
3 − Region
Location Hierarchy
1 − Global
2 − Super Region
3 − Region

MR−BRT Age/Sex Fits − Males 2+ Years Old − Moderate+Severe Anaemia Prevalence − Republic of Moldova
WHO−2024 Adjustment Method

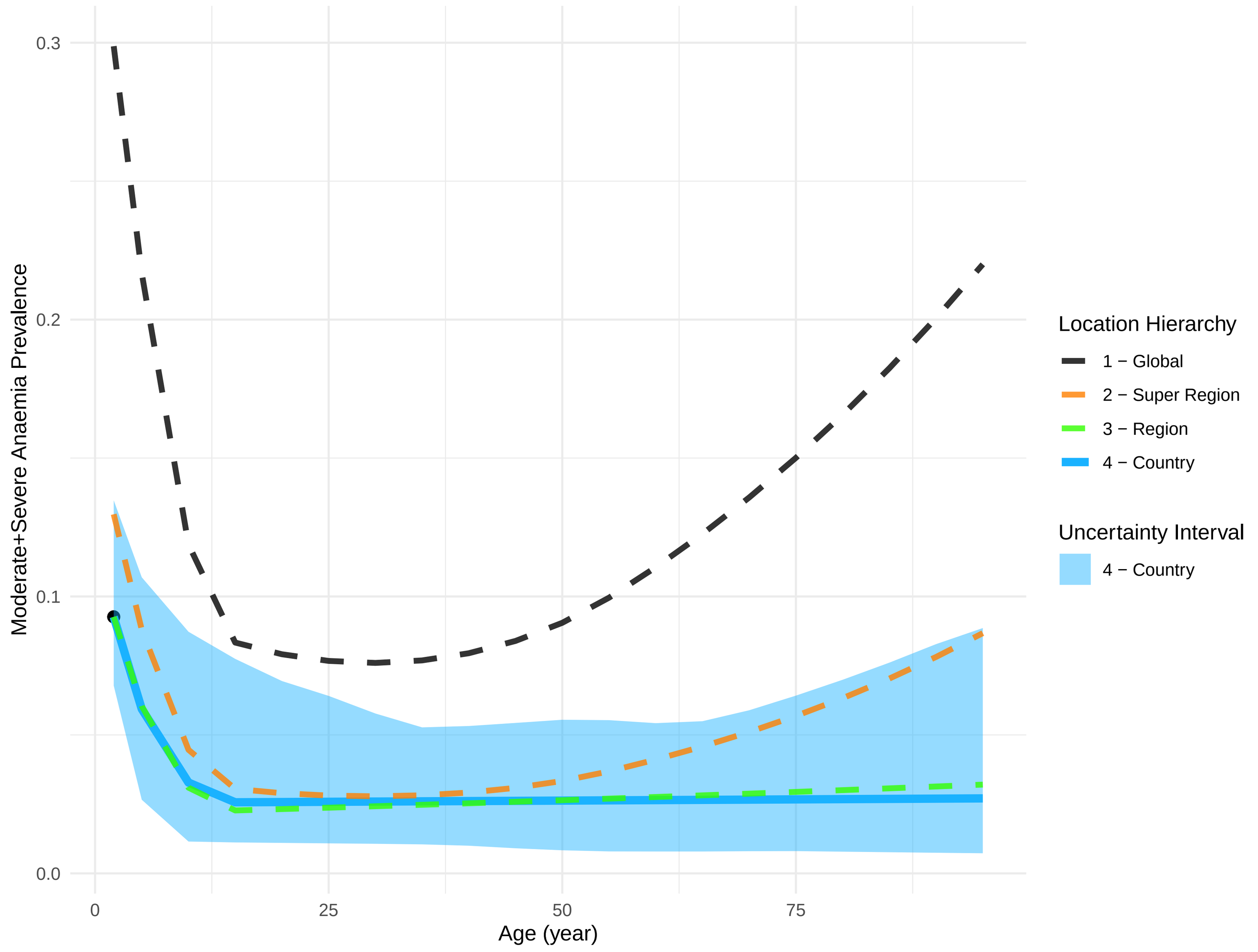

MR−BRT Age/Sex Fits − Males 2+ Years Old − Moderate+Severe Anaemia Prevalence − High−income
WHO−2024 Adjustment Method

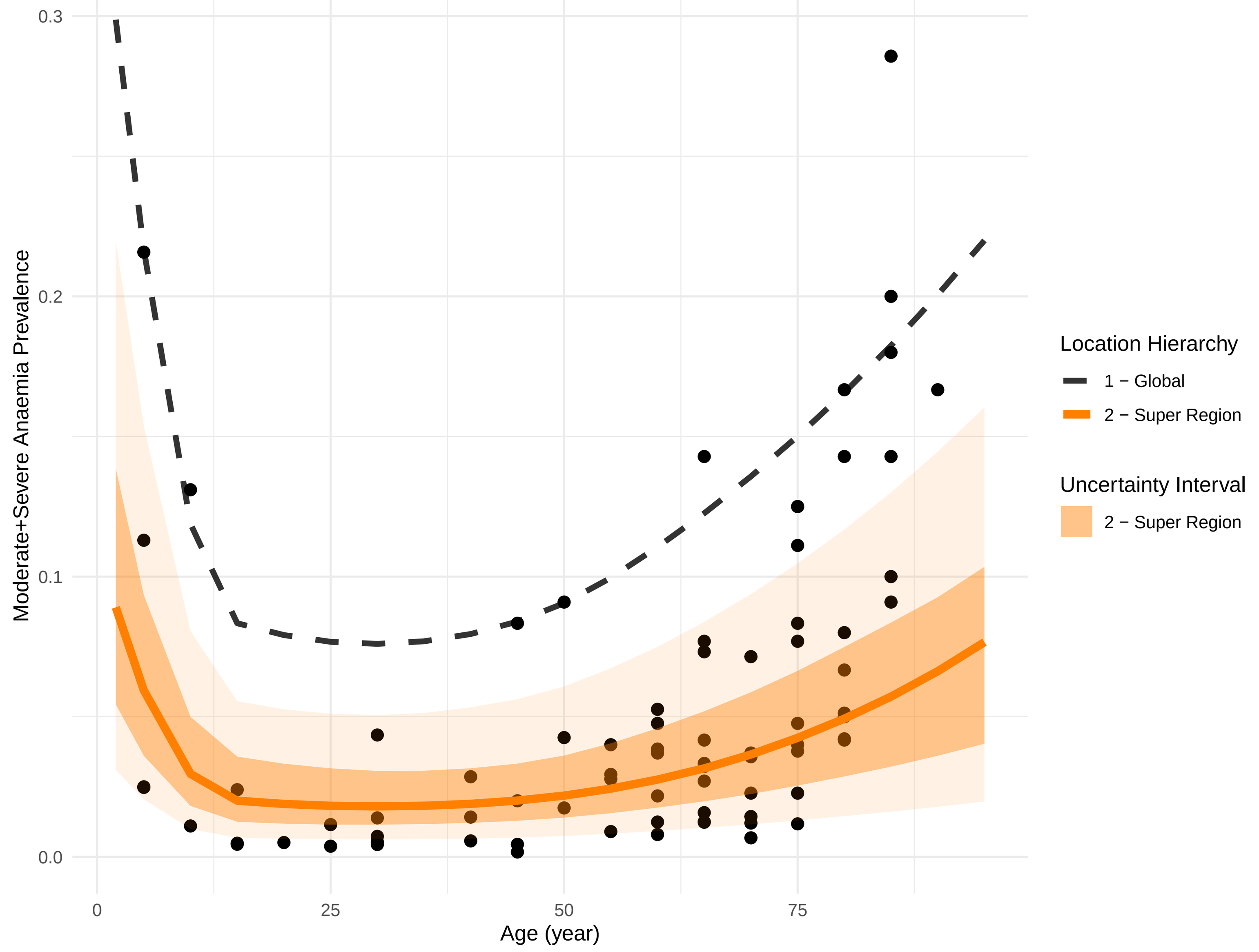

MR−BRT Age/Sex Fits − Males 2+ Years Old − Moderate+Severe Anaemia Prevalence − Western Europe
WHO−2024 Adjustment Method

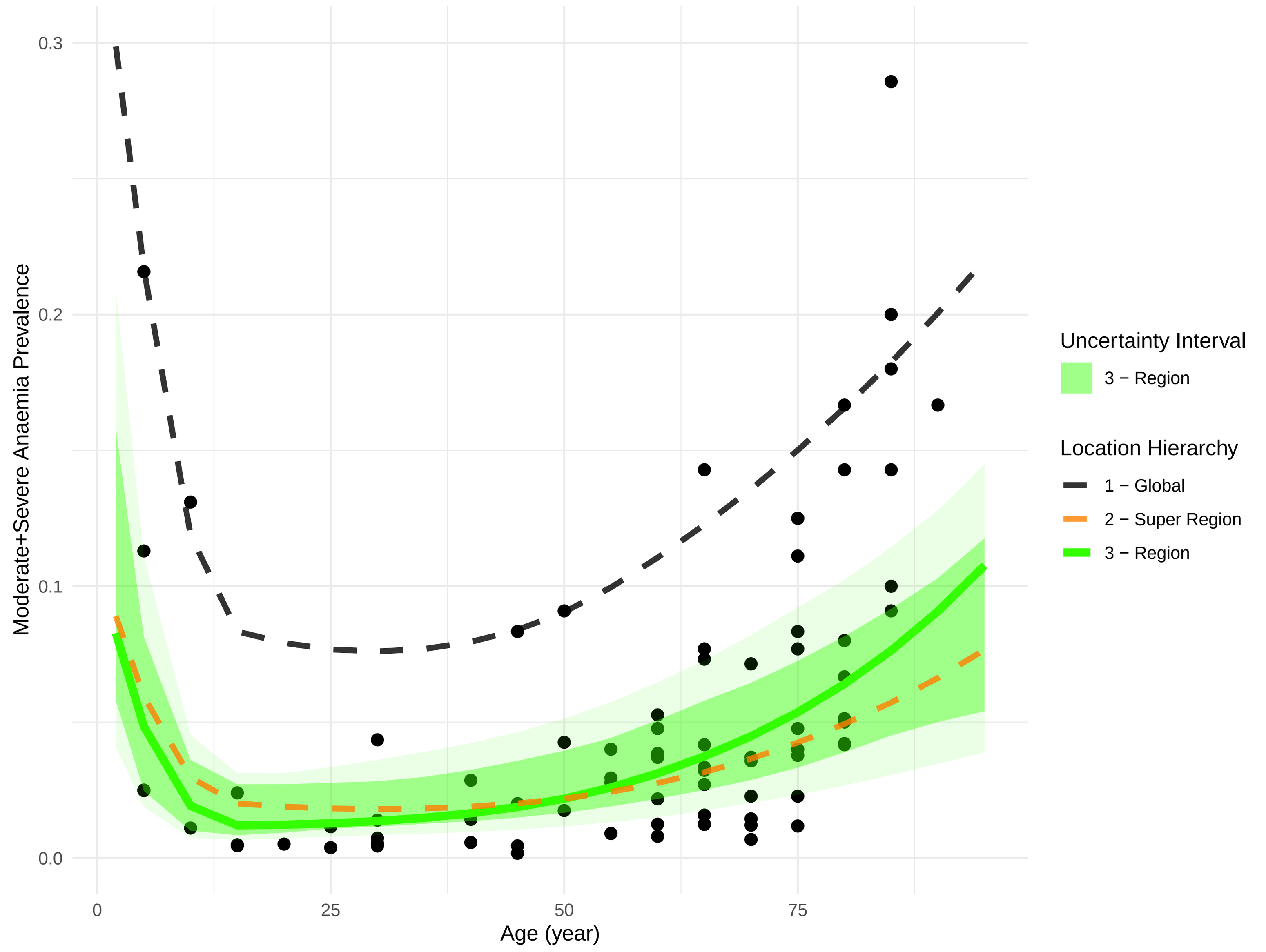

MR−BRT Age/Sex Fits − Males 2+ Years Old − Moderate+Severe Anaemia Prevalence − United Kingdom
WHO−2024 Adjustment Method

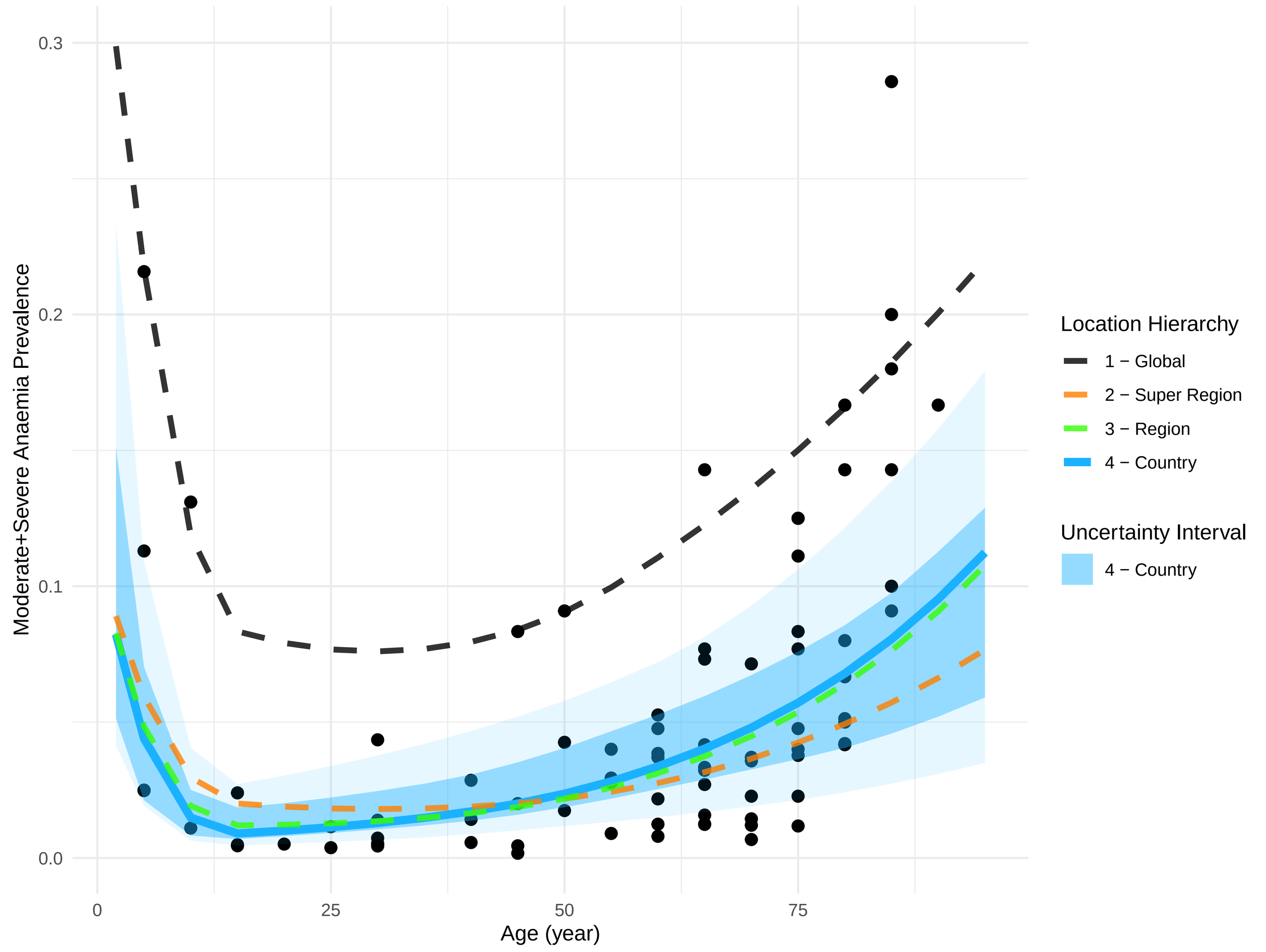

MR−BRT Age/Sex Fits − Males 2+ Years Old − Moderate+Severe Anaemia Prevalence − England
WHO−2024 Adjustment Method

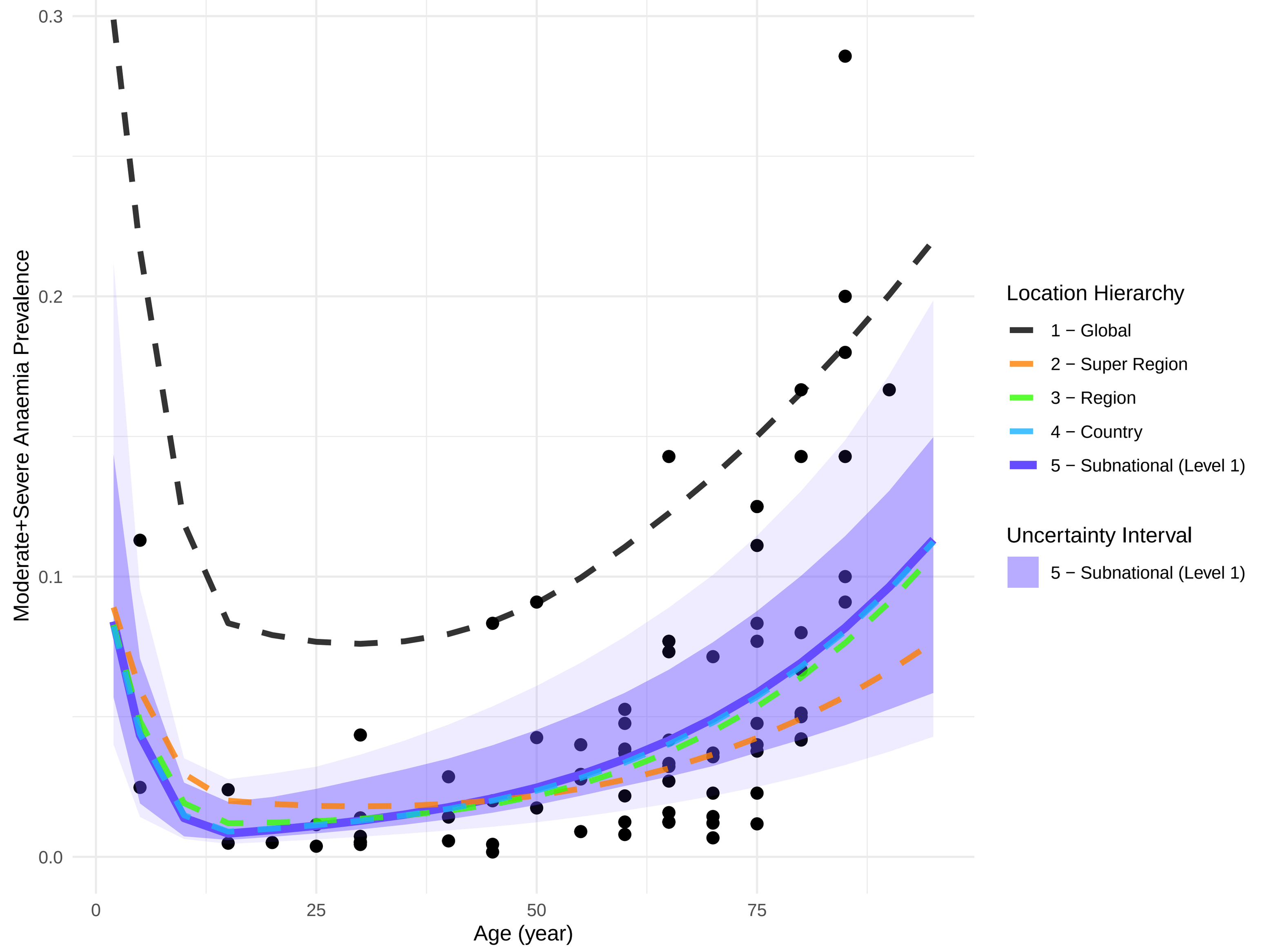

MR−BRT Age/Sex Fits − Males 2+ Years Old − Moderate+Severe Anaemia Prevalence − East of England
WHO−2024 Adjustment Method

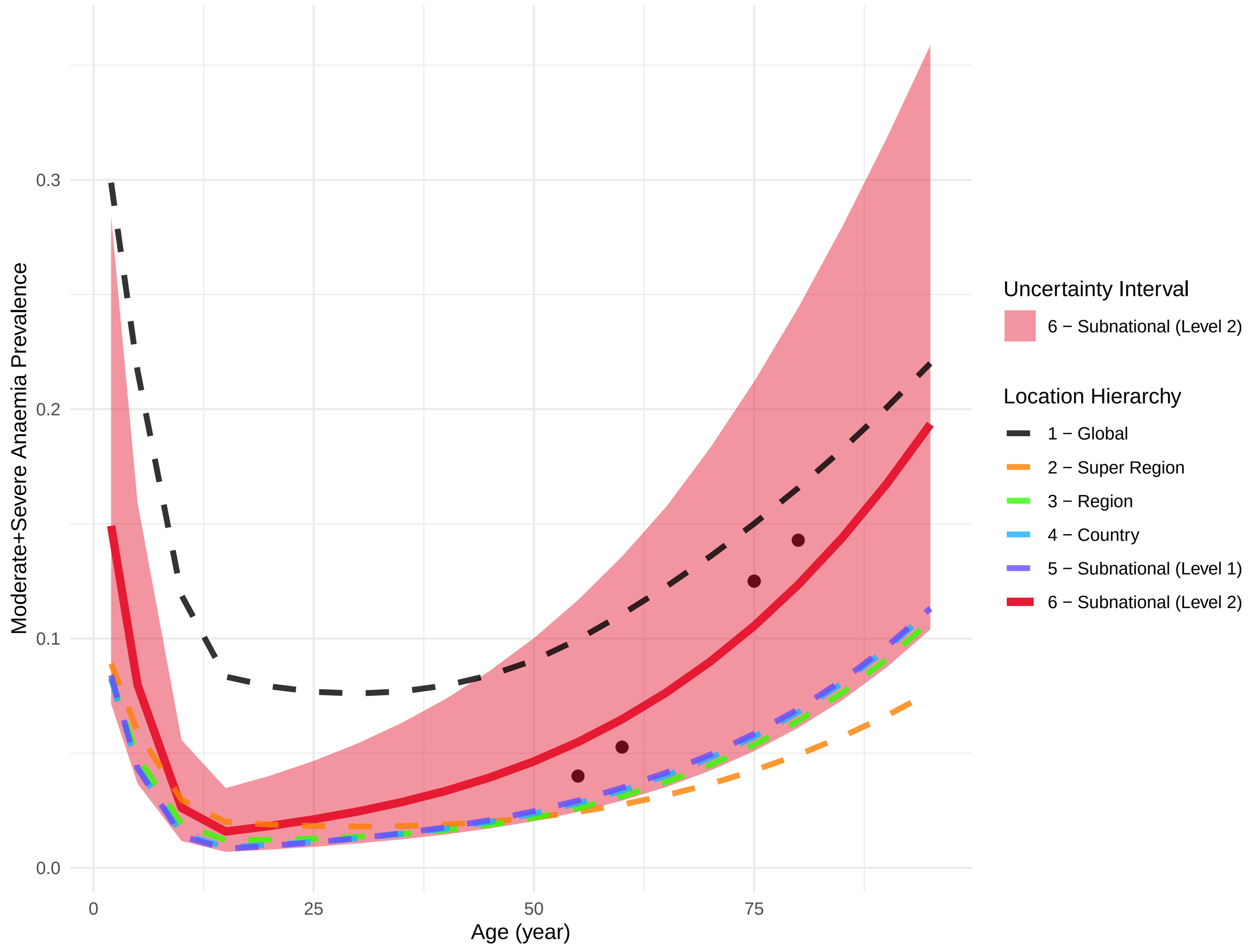

MR−BRT Age/Sex Fits − Males 2+ Years Old − Moderate+Severe Anaemia Prevalence − Greater London
WHO−2024 Adjustment Method

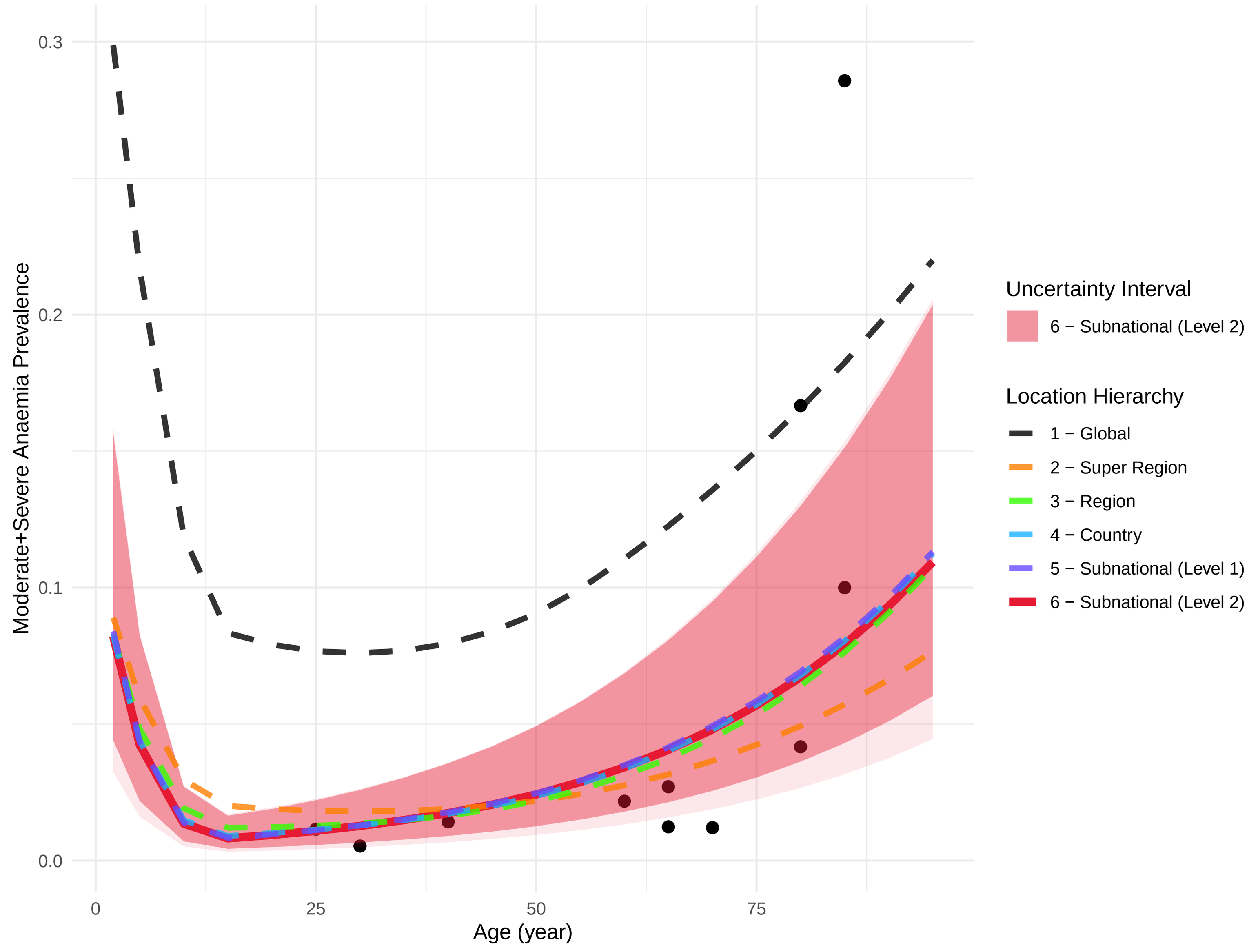

MR−BRT Age/Sex Fits − Males 2+ Years Old − Moderate+Severe Anaemia Prevalence − North West England
WHO−2024 Adjustment Method

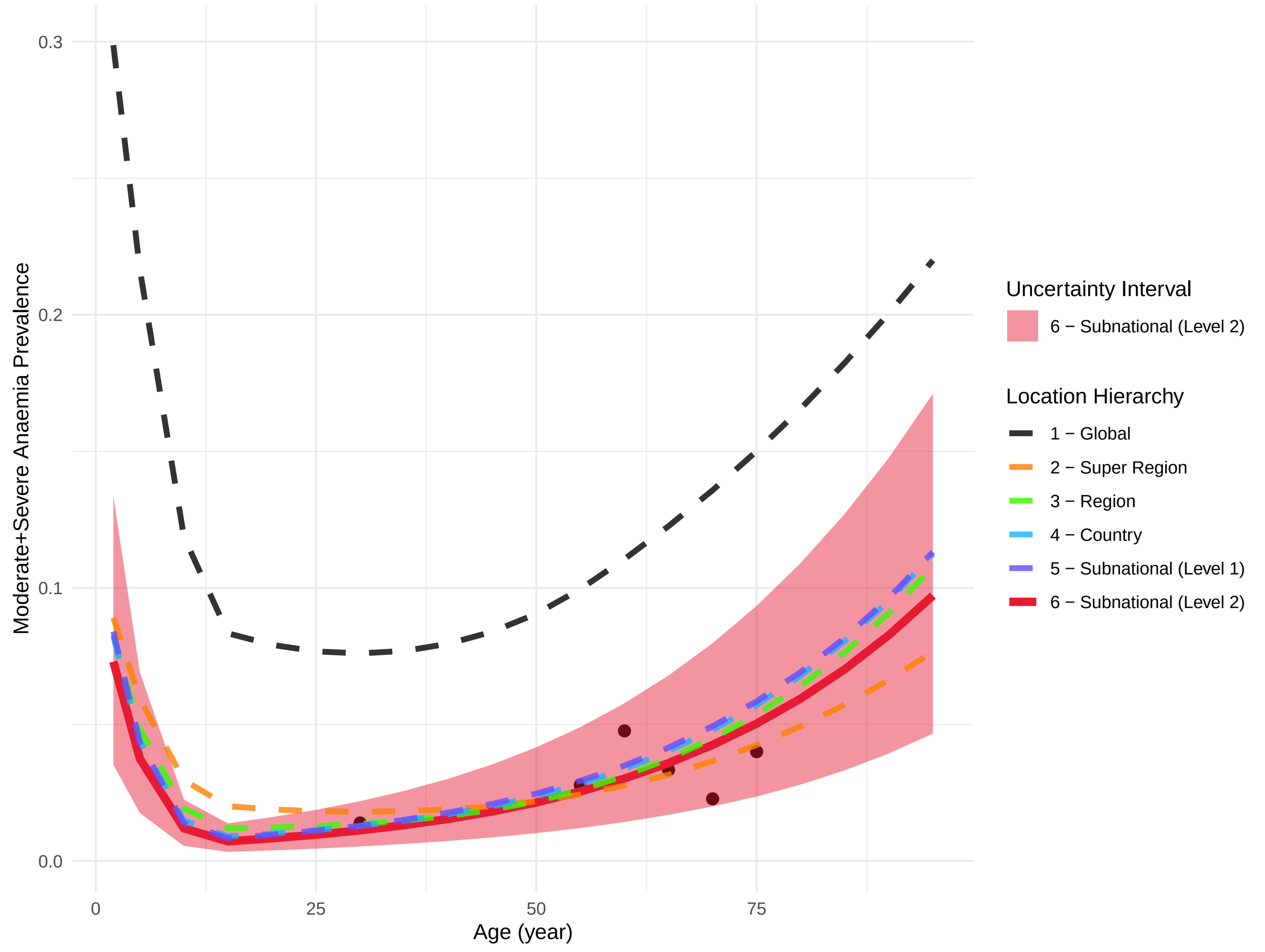

MR−BRT Age/Sex Fits − Males 2+ Years Old − Moderate+Severe Anaemia Prevalence − South East England
WHO−2024 Adjustment Method

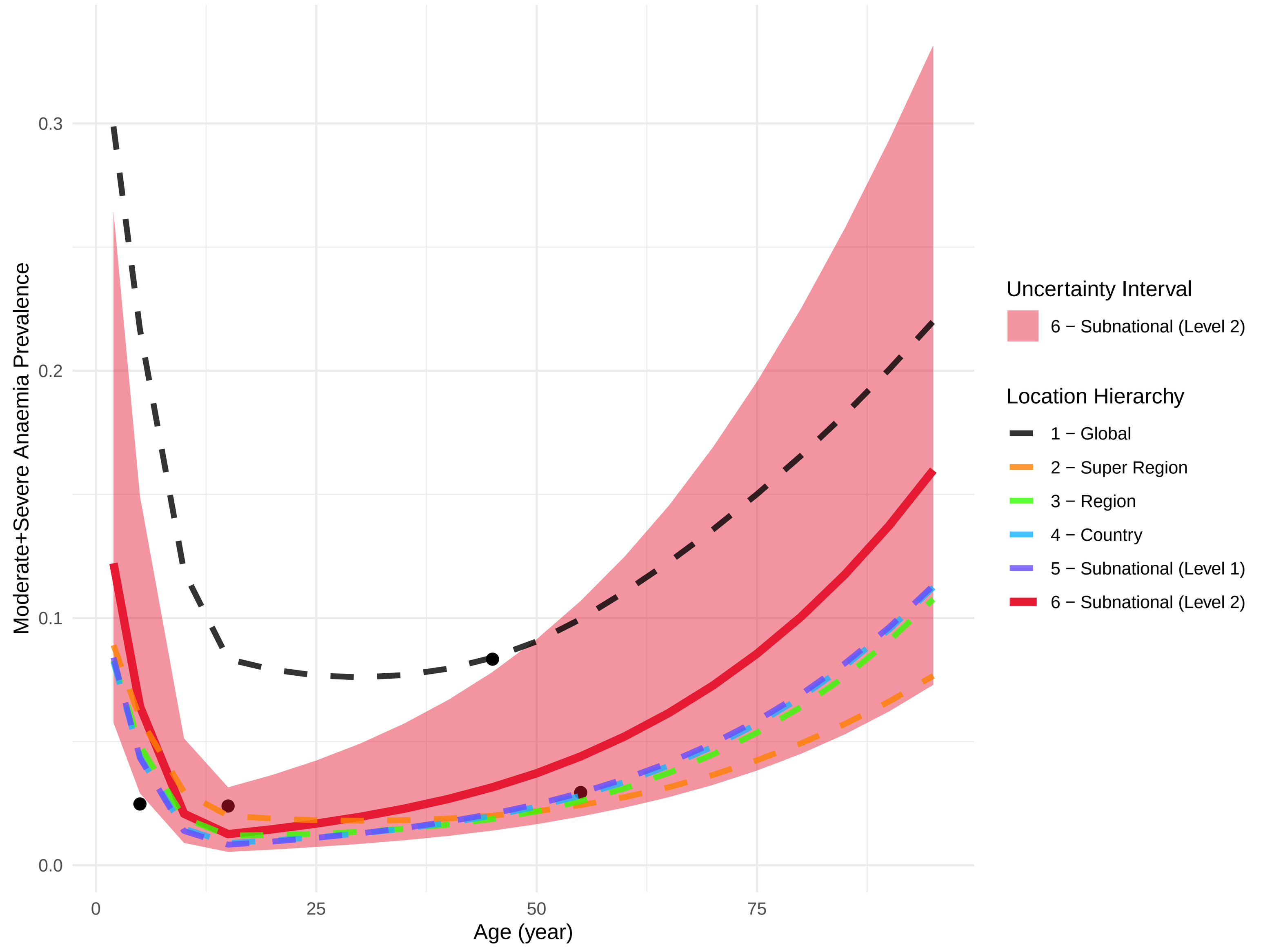

MR−BRT Age/Sex Fits − Males 2+ Years Old − Moderate+Severe Anaemia Prevalence − South West England
WHO−2024 Adjustment Method

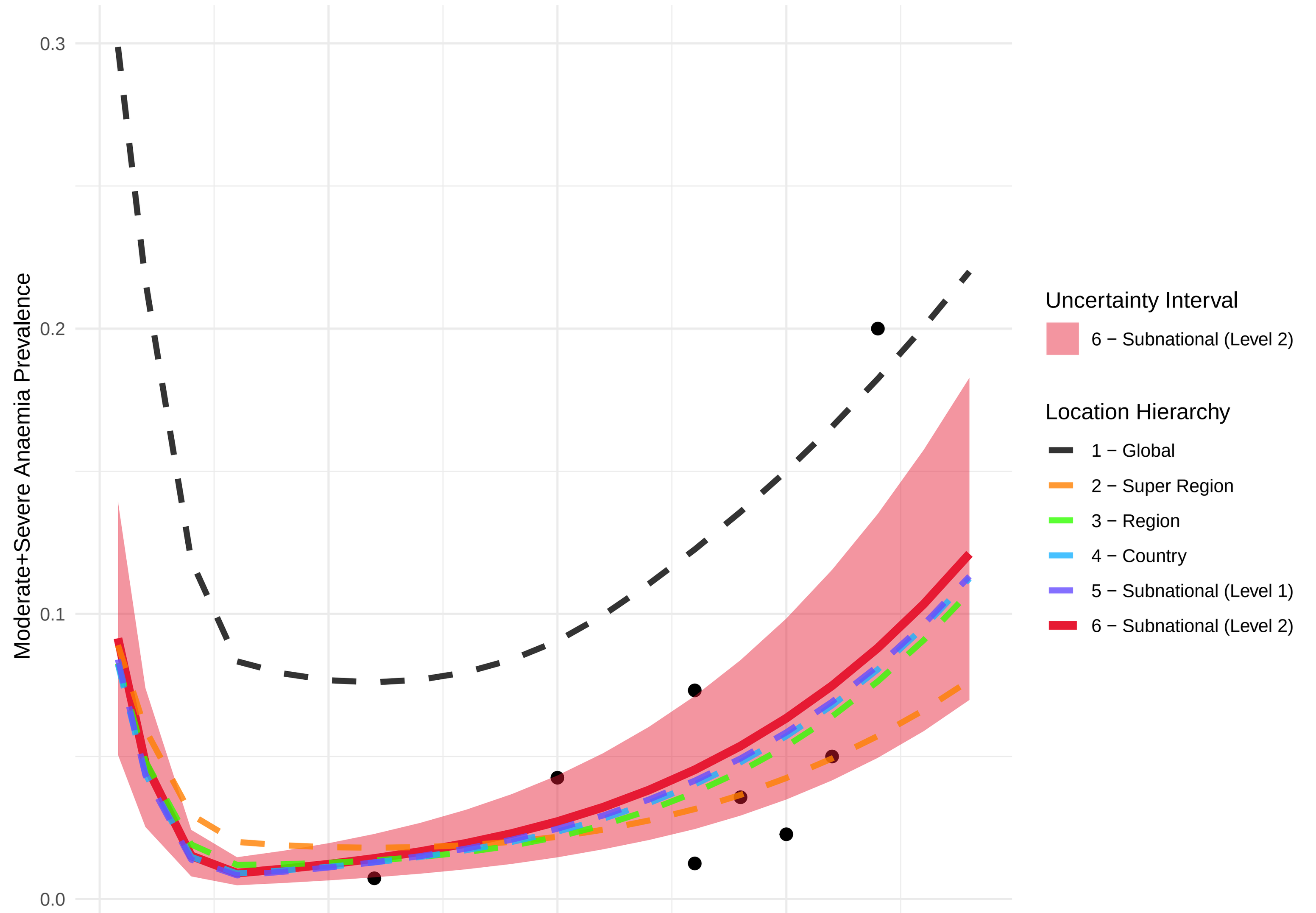

MR–BRT Age/Sex Fits – Males 2+ Years Old – Moderate+Severe Anaemia Prevalence – West Midlands
WHO–2024 Adjustment Method

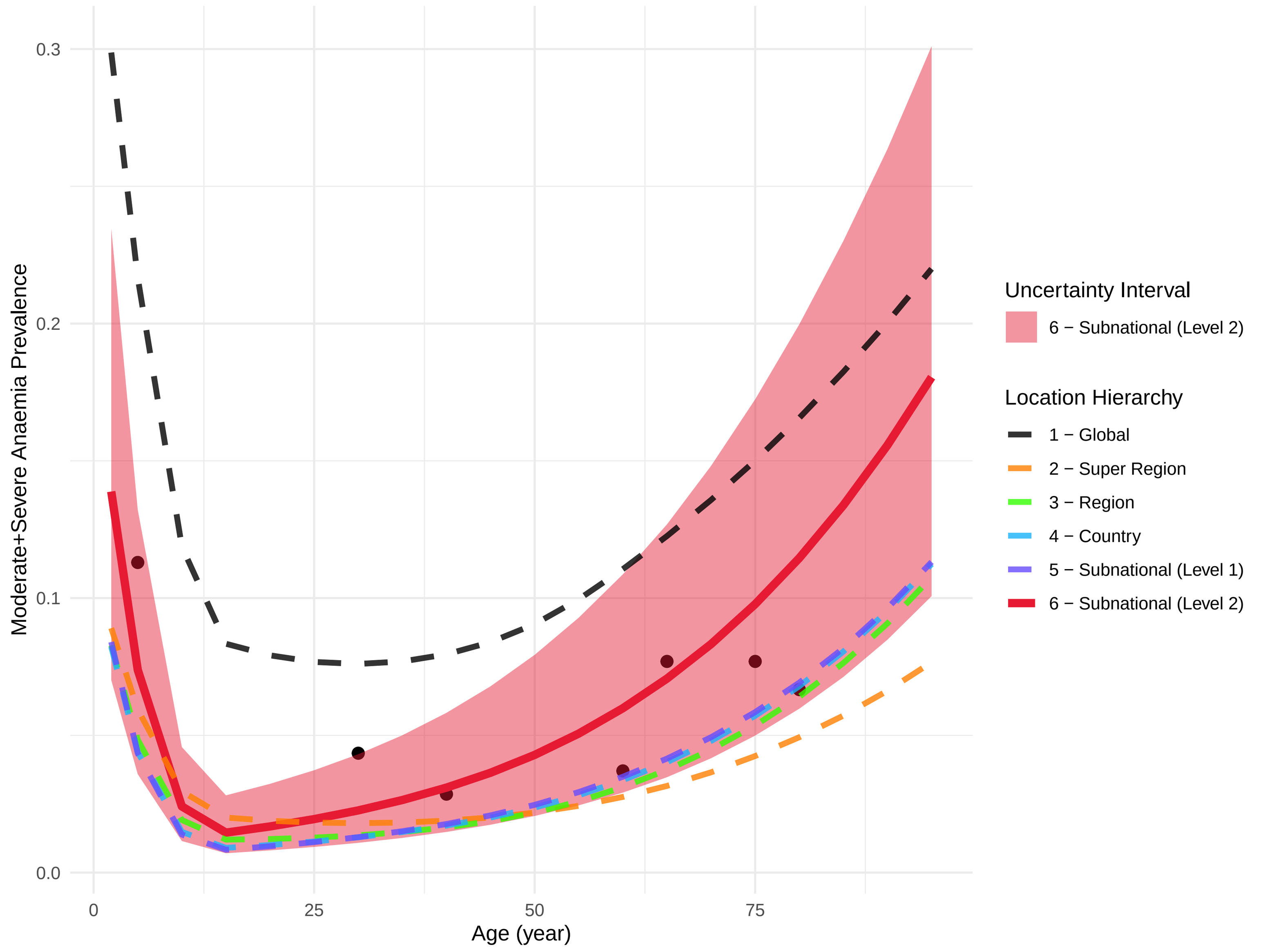

MR–BRT Age/Sex Fits – Males 2+ Years Old – Moderate+Severe Anaemia Prevalence – Yorkshire and the Humber
WHO–2024 Adjustment Method

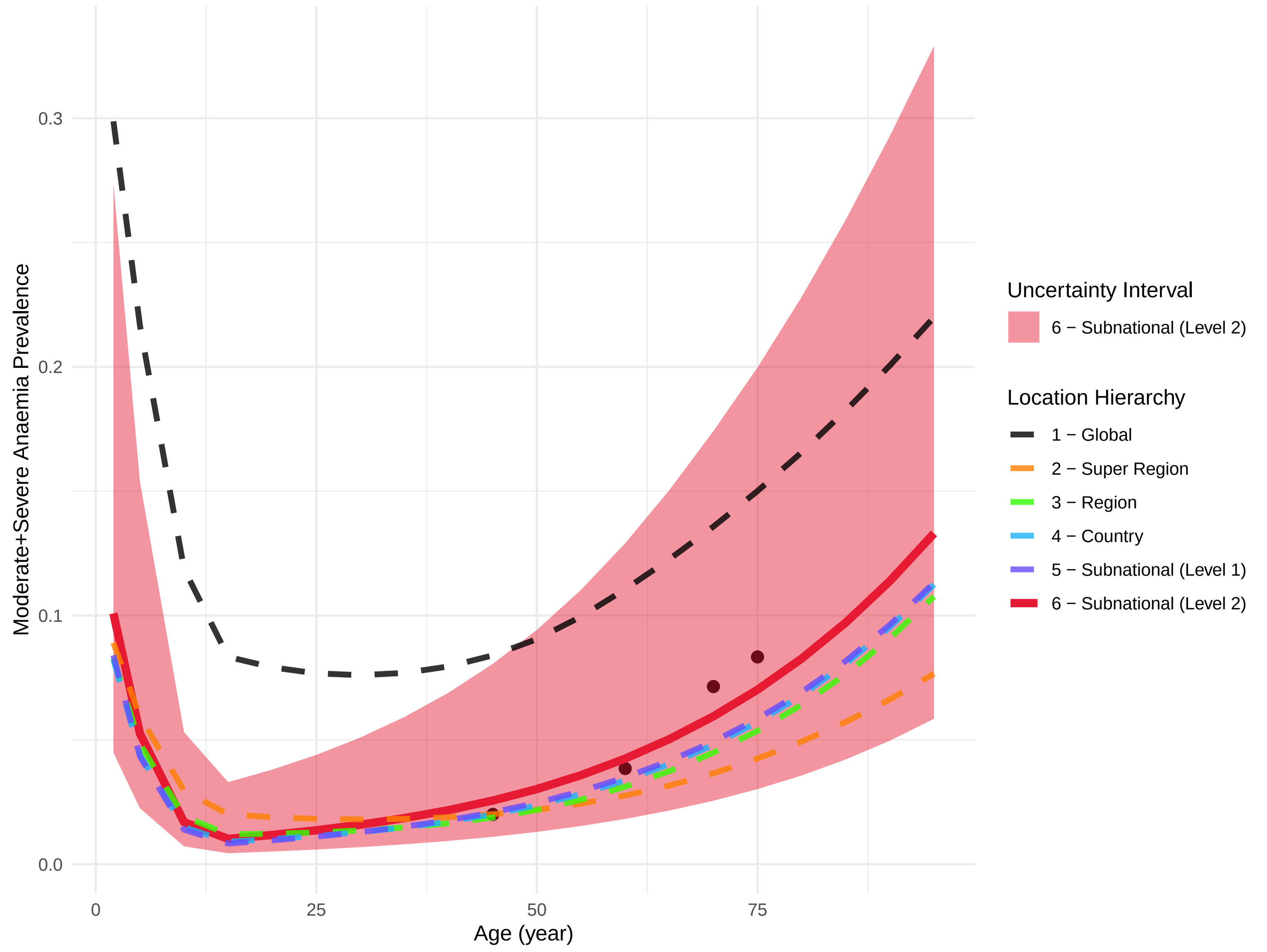

MR–BRT Age/Sex Fits – Males 2+ Years Old – Moderate+Severe Anaemia Prevalence – Scotland
WHO–2024 Adjustment Method

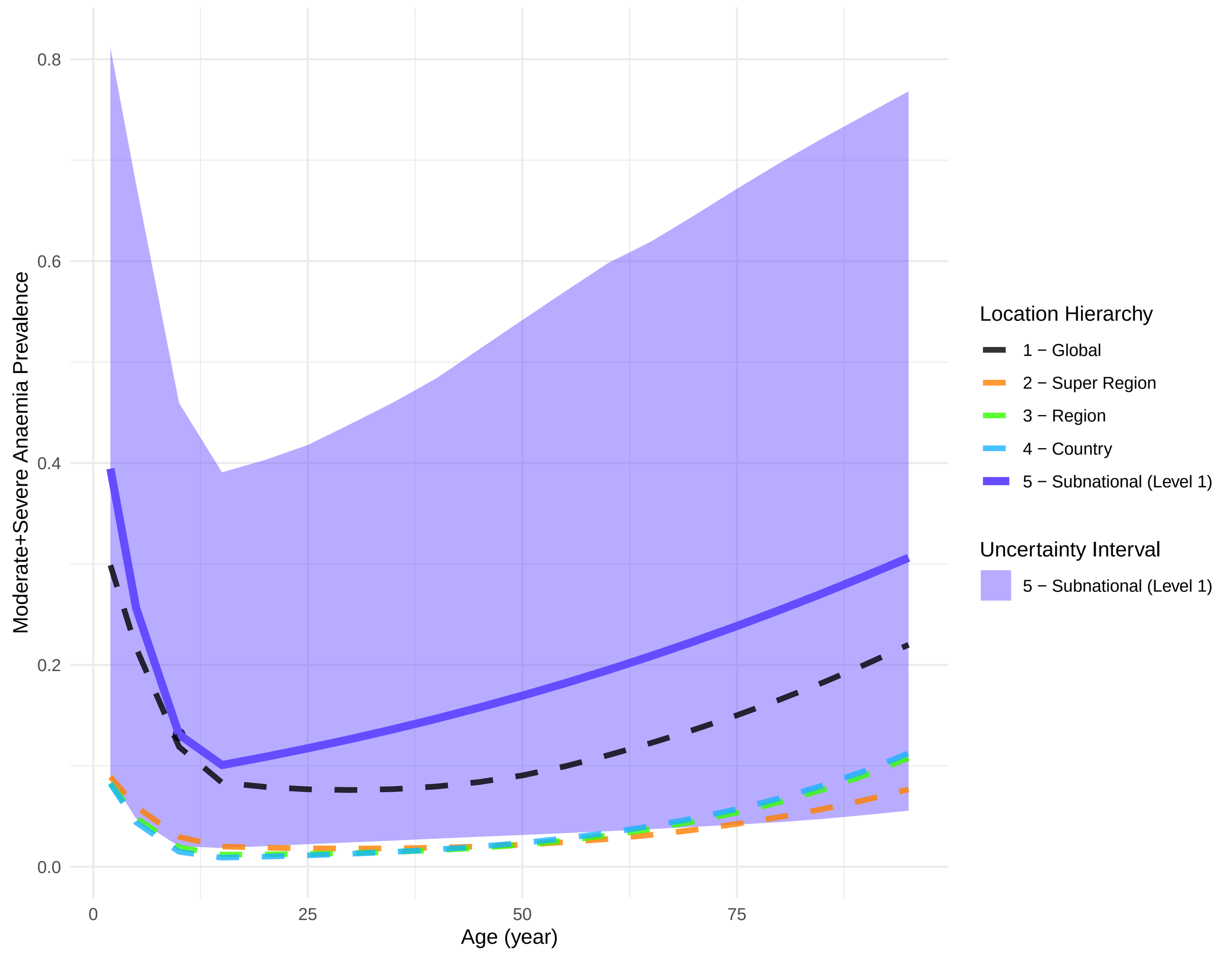

MR–BRT Age/Sex Fits – Males 2+ Years Old – Moderate+Severe Anaemia Prevalence – Wales
WHO–2024 Adjustment Method

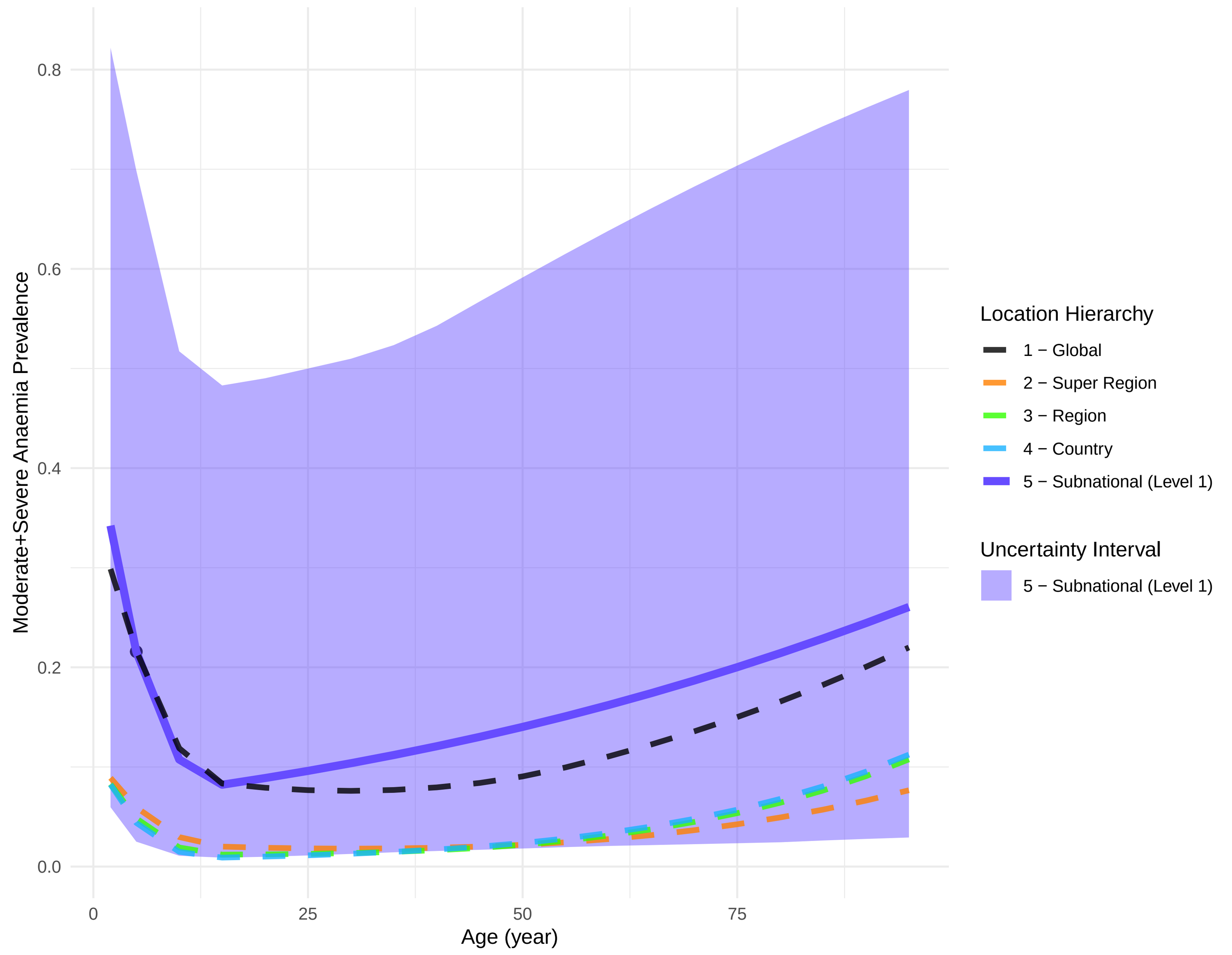

MR–BRT Age/Sex Fits – Males 2+ Years Old – Moderate+Severe Anaemia Prevalence – Latin America and Caribbean
WHO–2024 Adjustment Method

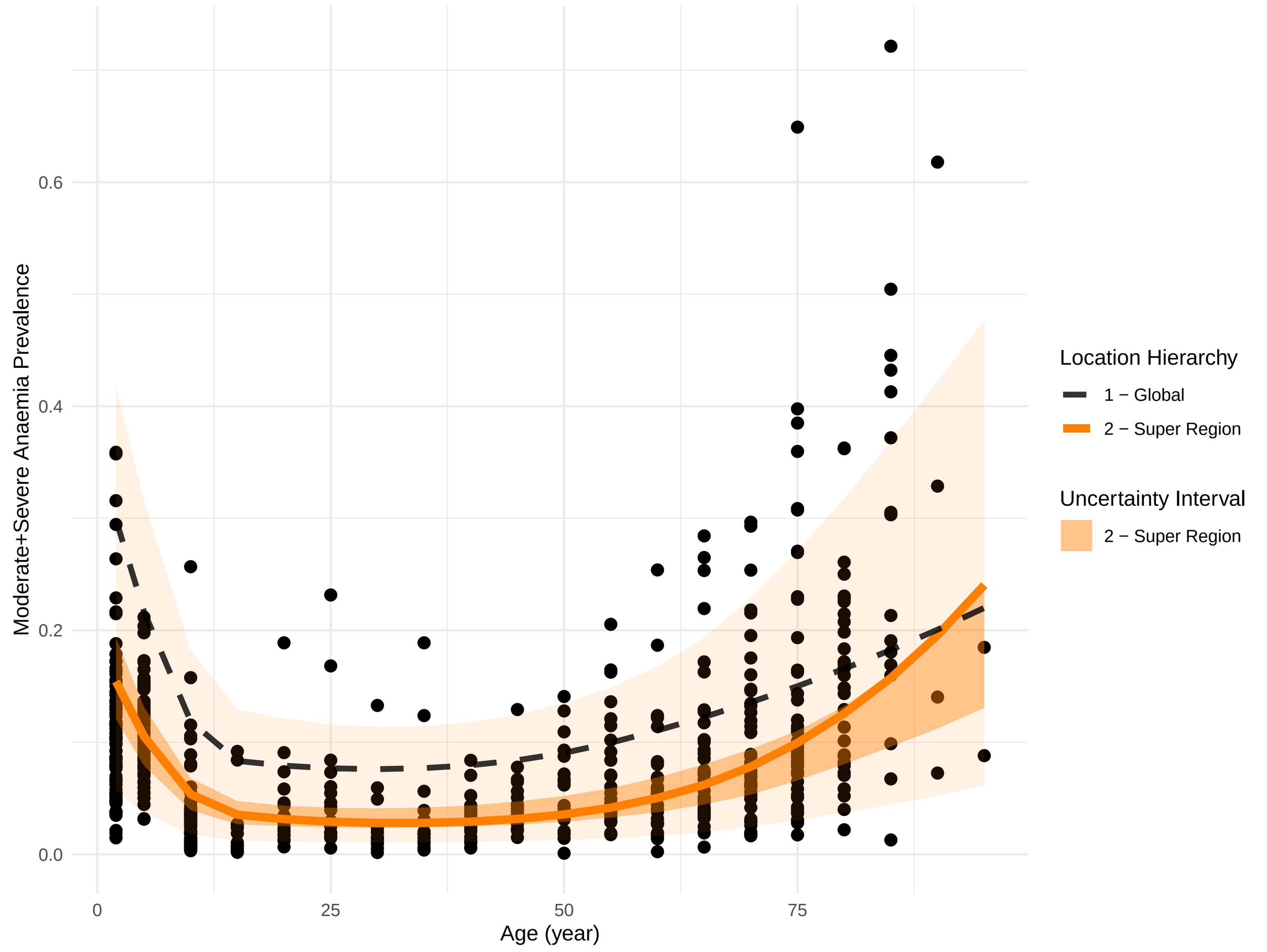

MR–BRT Age/Sex Fits – Males 2+ Years Old – Moderate+Severe Anaemia Prevalence – Andean Latin America
WHO–2024 Adjustment Method

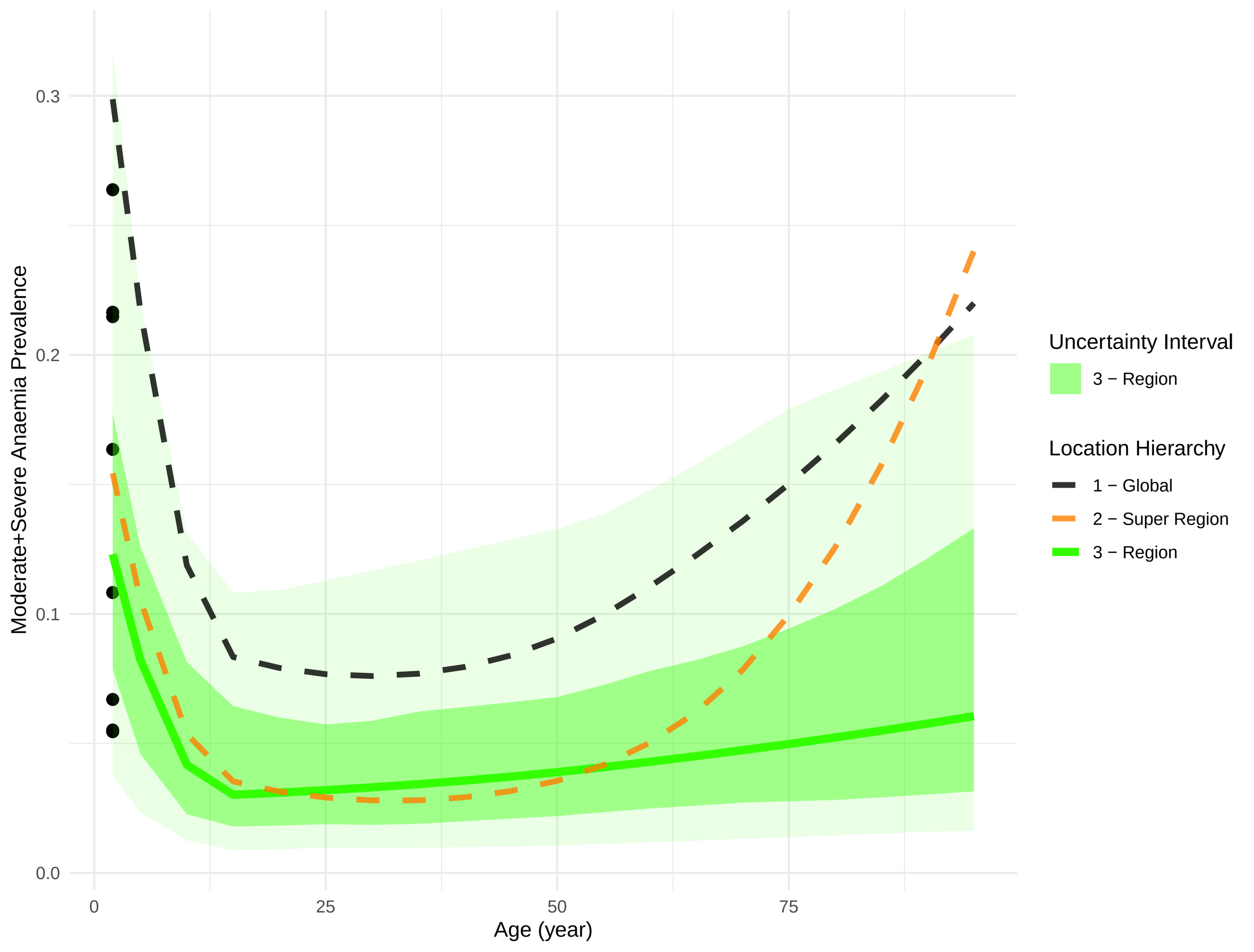

MR–BRT Age/Sex Fits – Males 2+ Years Old – Moderate+Severe Anaemia Prevalence – Bolivia (Plurinational State of)
WHO–2024 Adjustment Method

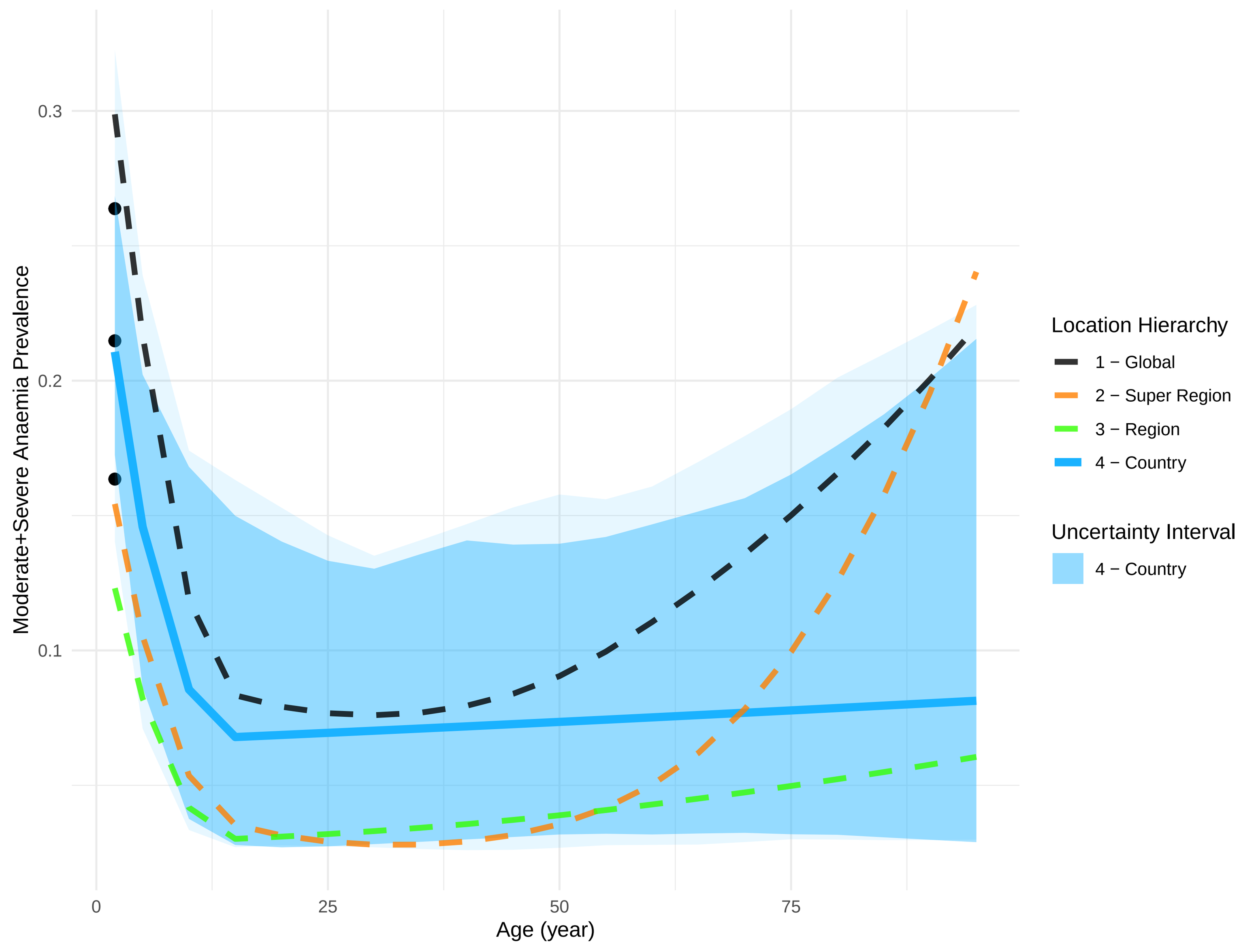

MR–BRT Age/Sex Fits – Males 2+ Years Old – Moderate+Severe Anaemia Prevalence – Peru
WHO–2024 Adjustment Method

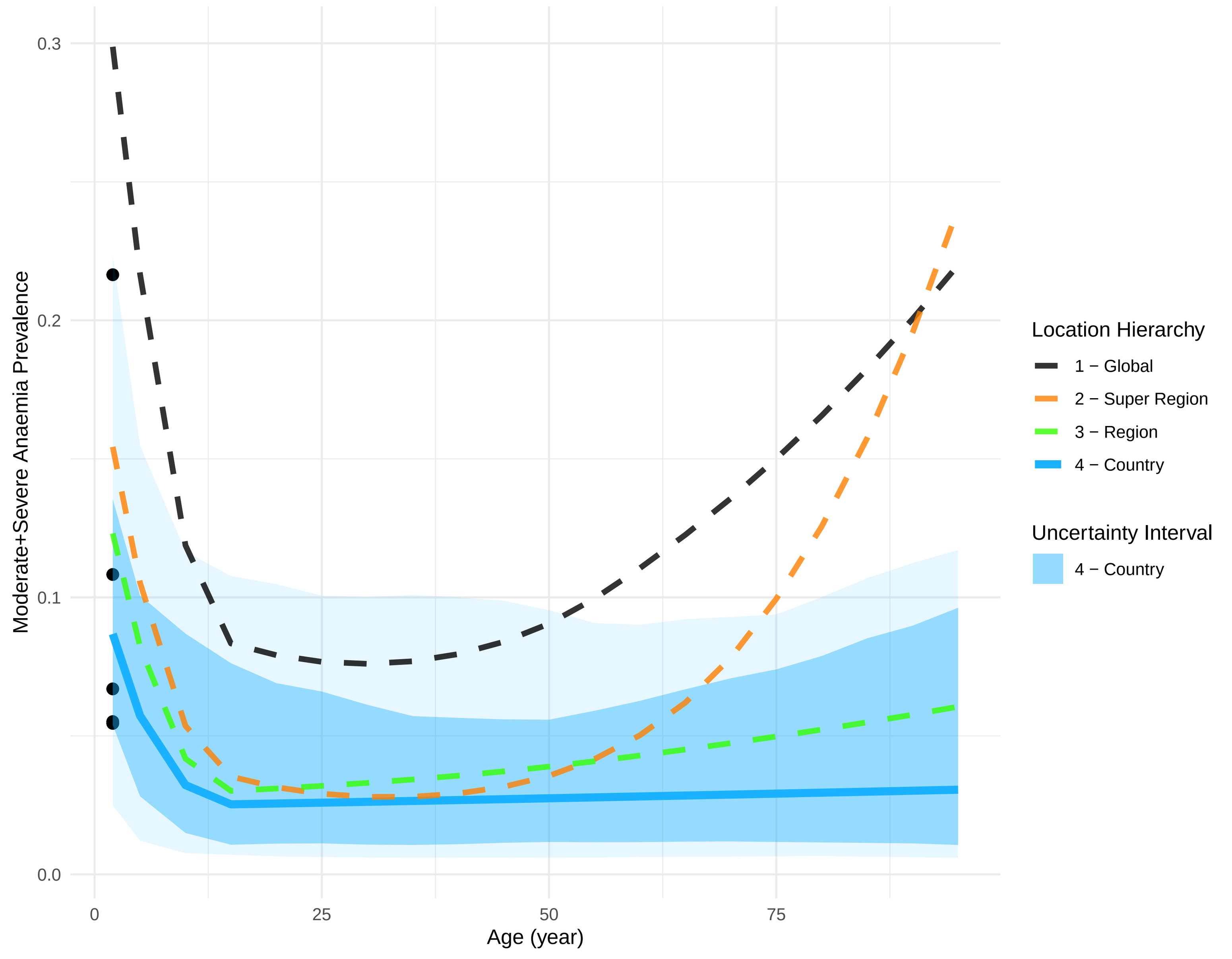

MR–BRT Age/Sex Fits – Males 2+ Years Old – Moderate+Severe Anaemia Prevalence – Caribbean
WHO–2024 Adjustment Method

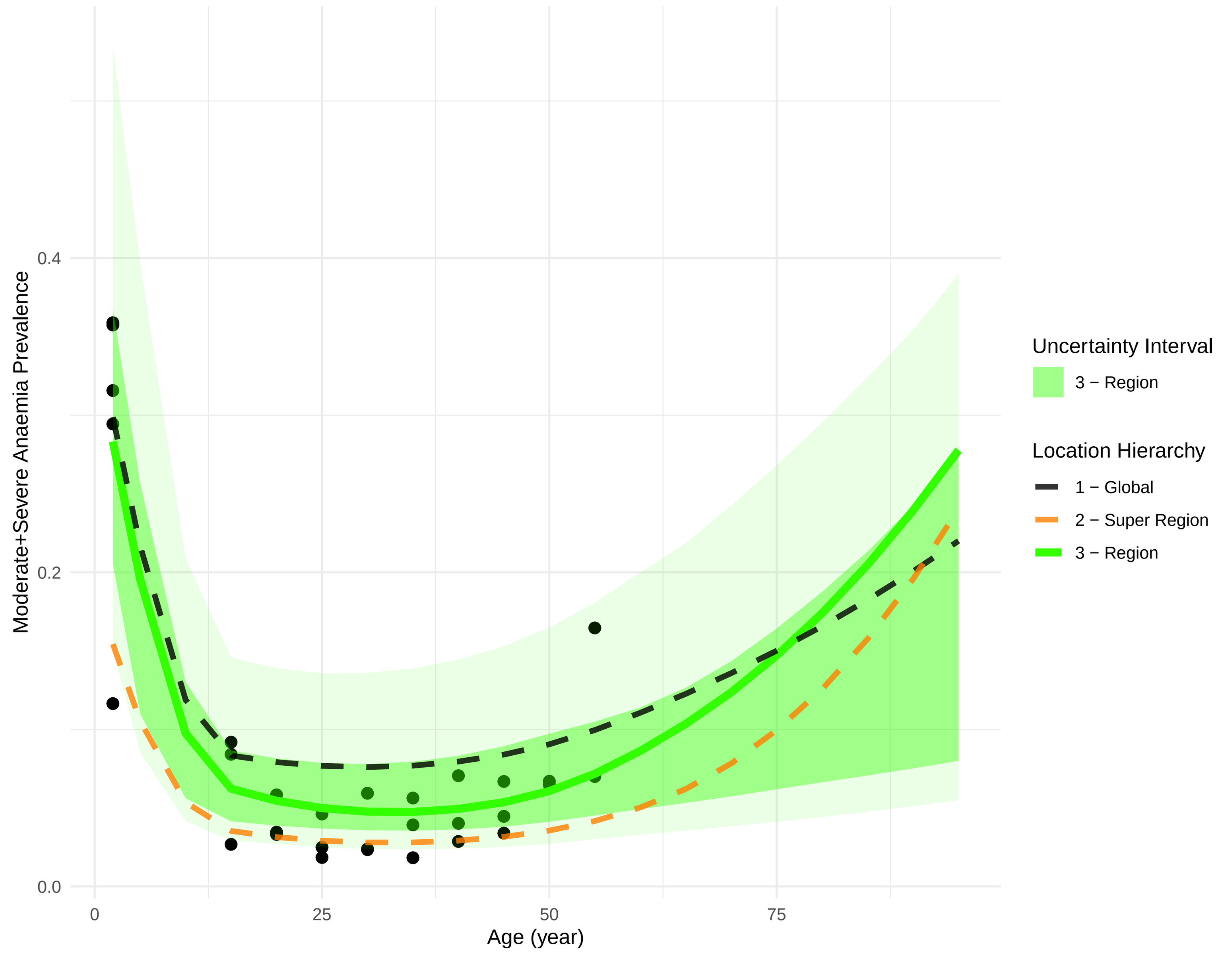

MR–BRT Age/Sex Fits – Males 2+ Years Old – Moderate+Severe Anaemia Prevalence – Guyana
WHO–2024 Adjustment Method

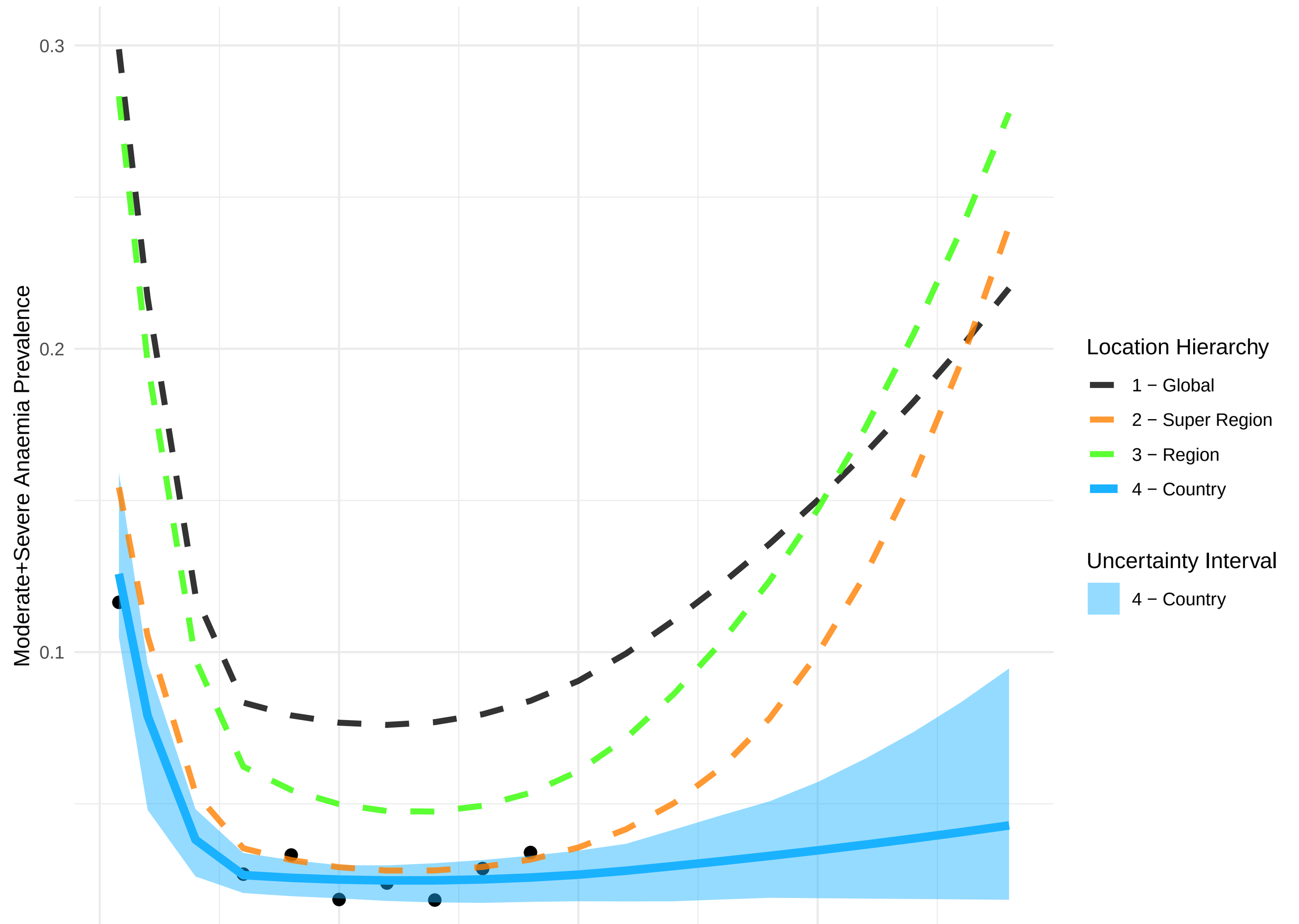

MR−BRT Age/Sex Fits − Males 2+ Years Old − Moderate+Severe Anaemia Prevalence − Haiti
WHO−2024 Adjustment Method

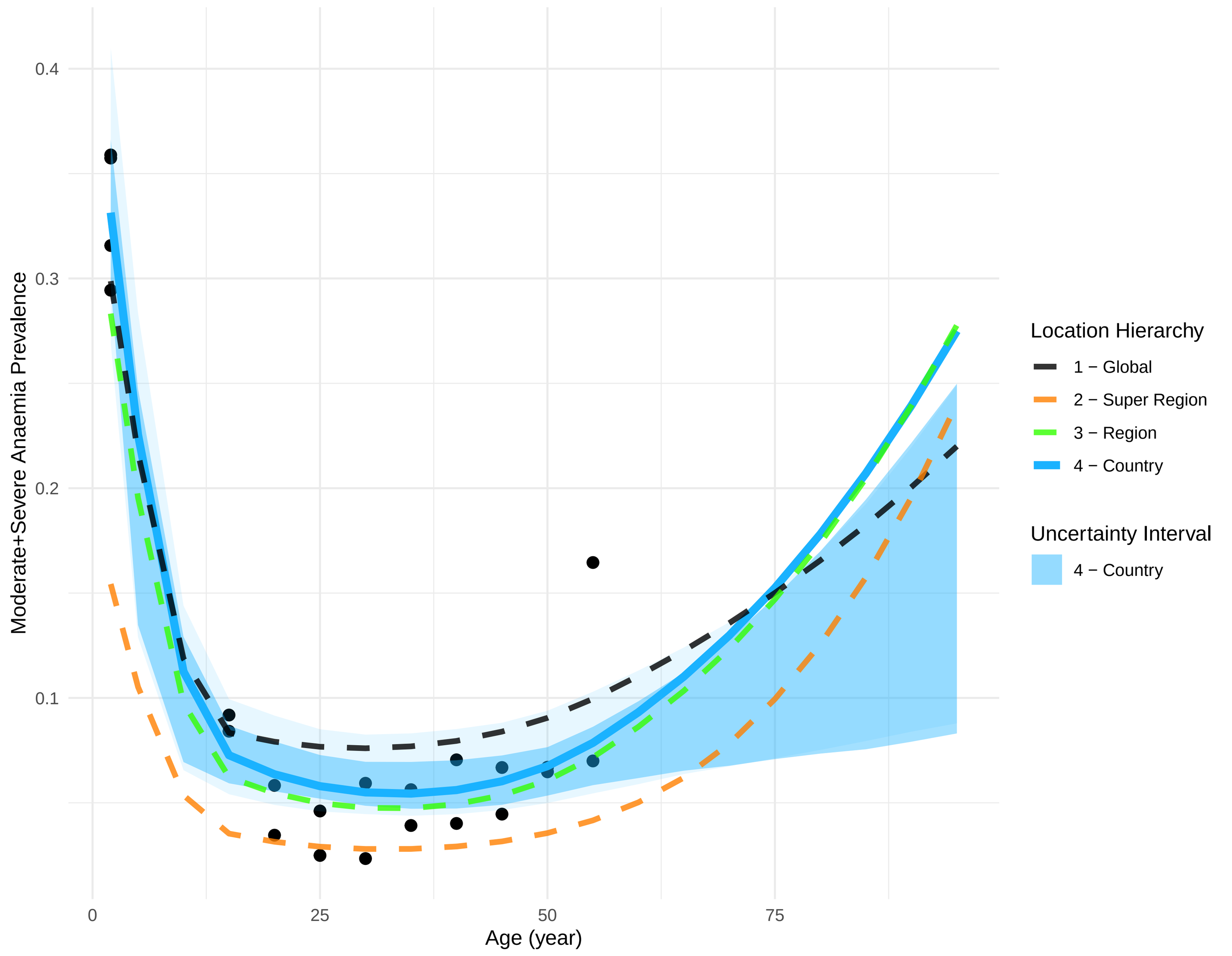

MR−BRT Age/Sex Fits − Males 2+ Years Old − Moderate+Severe Anaemia Prevalence − Central Latin America
WHO−2024 Adjustment Method

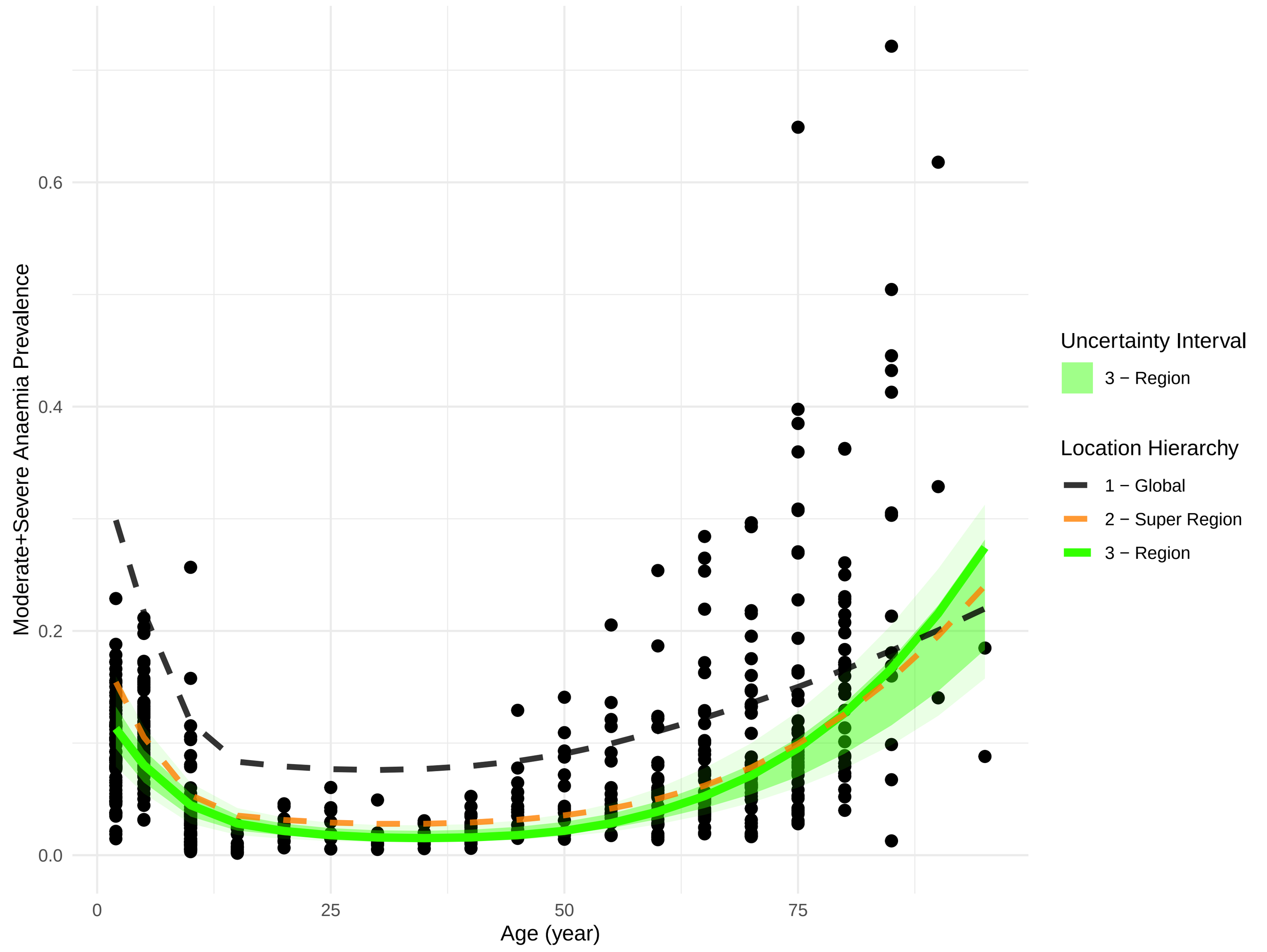

MR−BRT Age/Sex Fits − Males 2+ Years Old − Moderate+Severe Anaemia Prevalence − Guatemala
WHO−2024 Adjustment Method

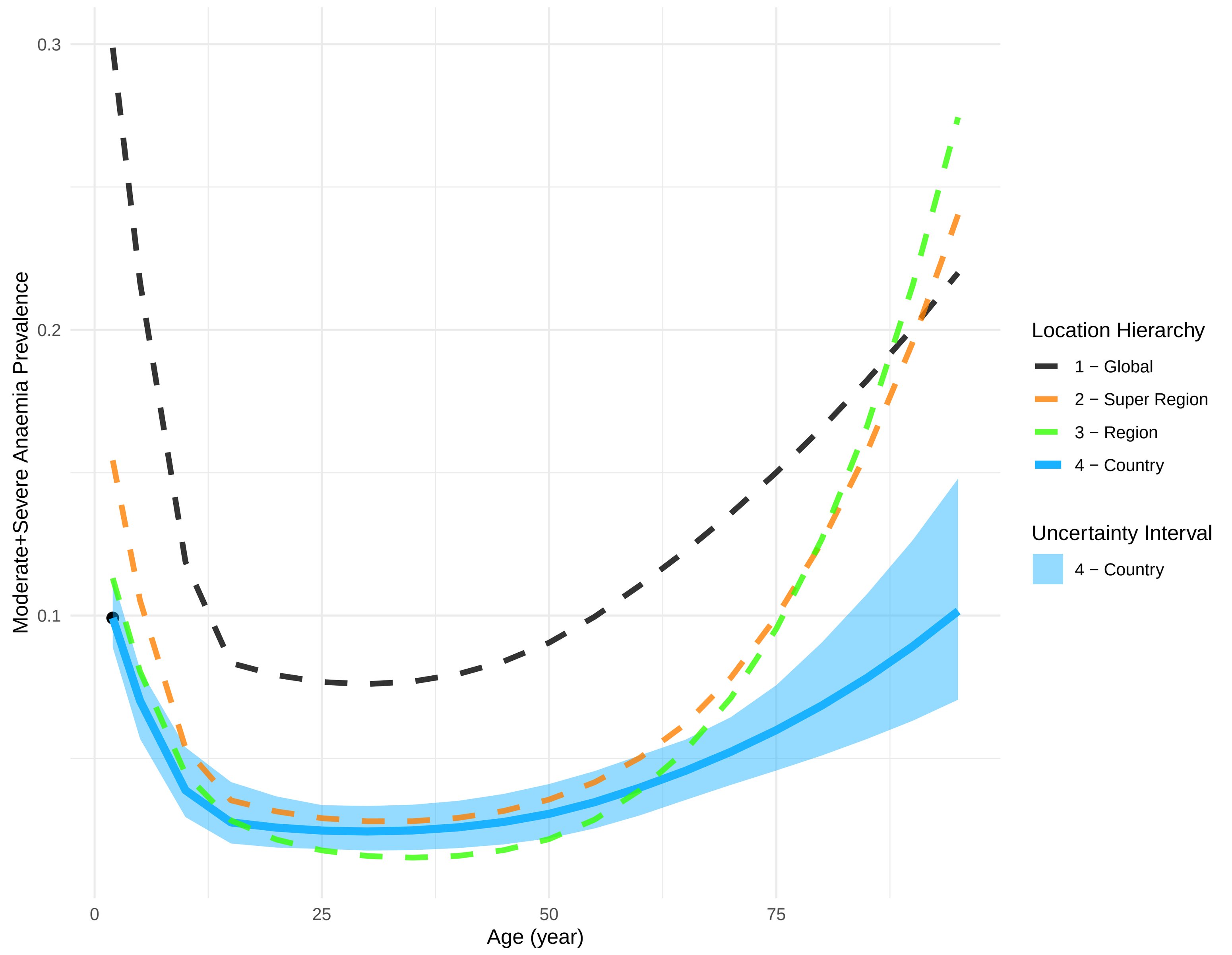

MR−BRT Age/Sex Fits − Males 2+ Years Old − Moderate+Severe Anaemia Prevalence − Honduras
WHO−2024 Adjustment Method

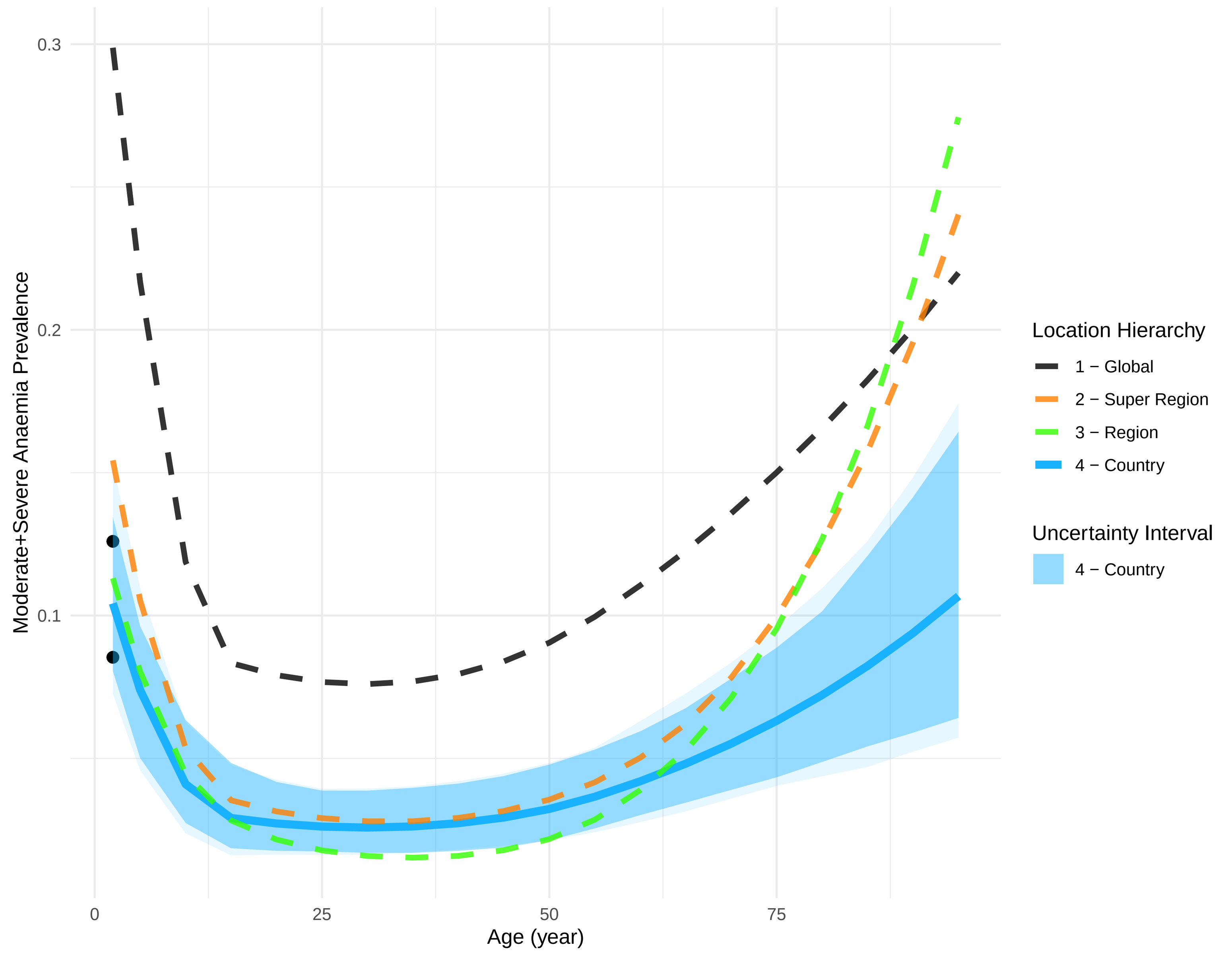

MR−BRT Age/Sex Fits − Males 2+ Years Old − Moderate+Severe Anaemia Prevalence − Mexico
WHO−2024 Adjustment Method

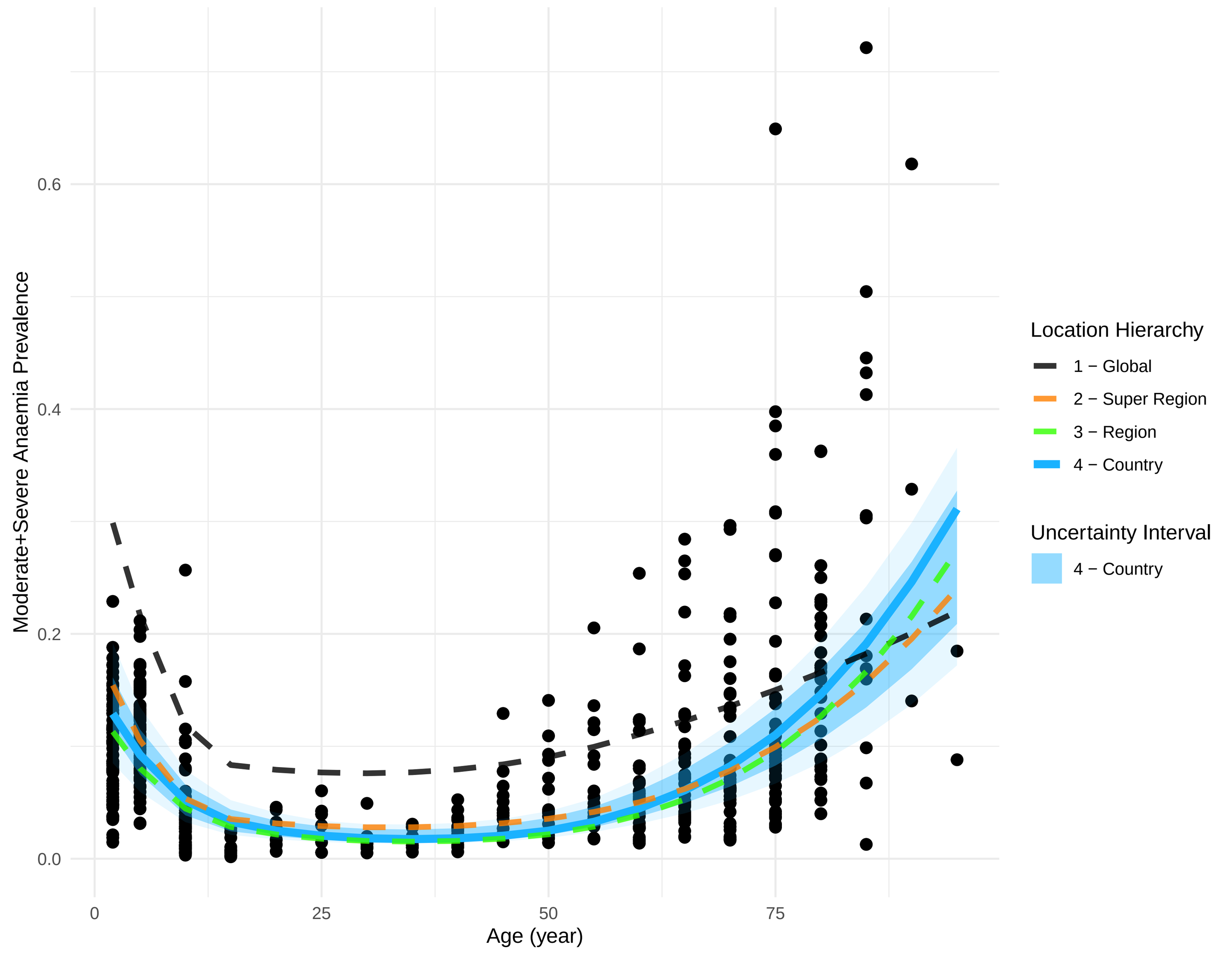

MR−BRT Age/Sex Fits − Males 2+ Years Old − Moderate+Severe Anaemia Prevalence − Aguascalientes
WHO−2024 Adjustment Method

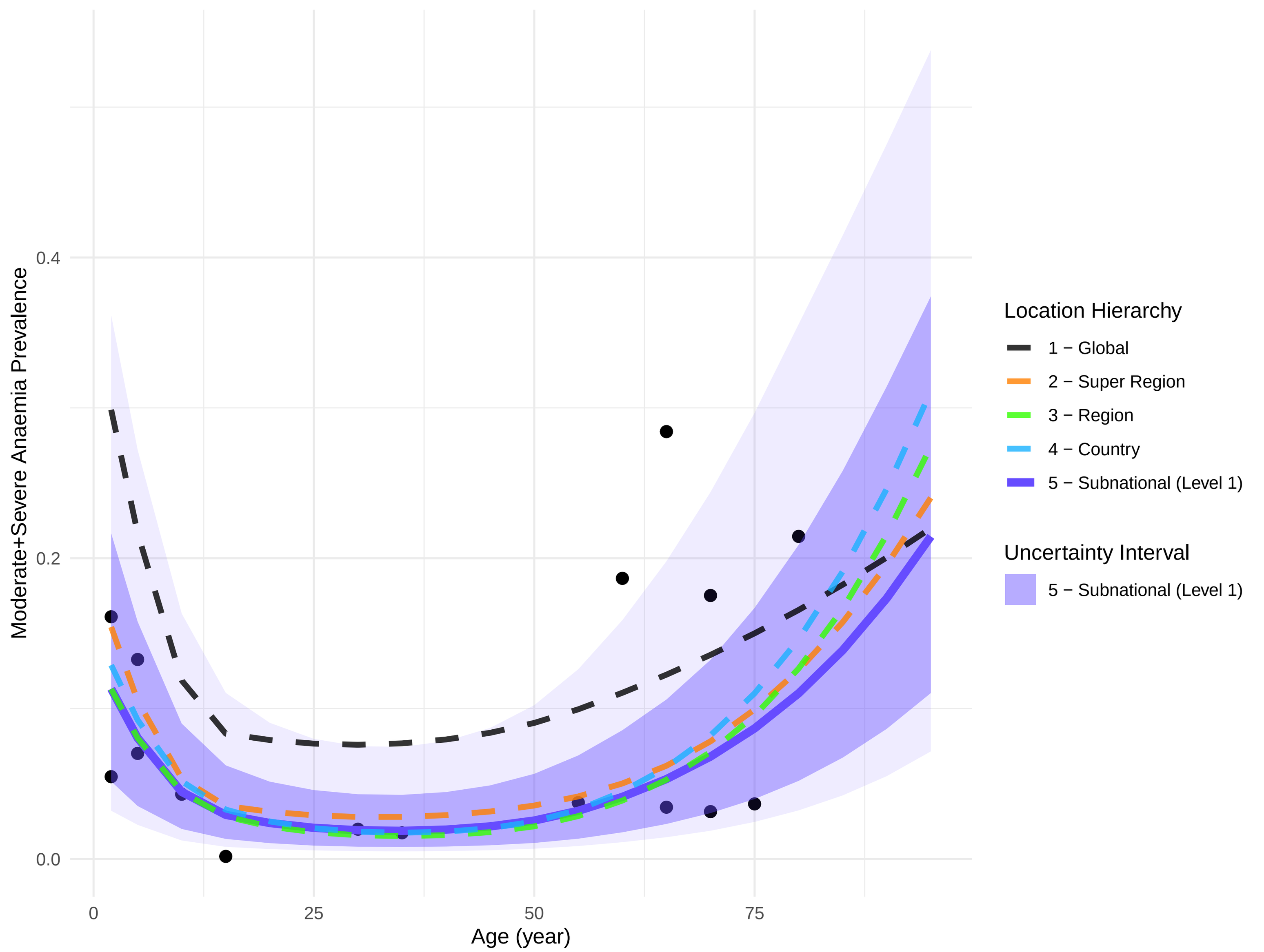

MR−BRT Age/Sex Fits − Males 2+ Years Old − Moderate+Severe Anaemia Prevalence − Baja California
WHO−2024 Adjustment Method

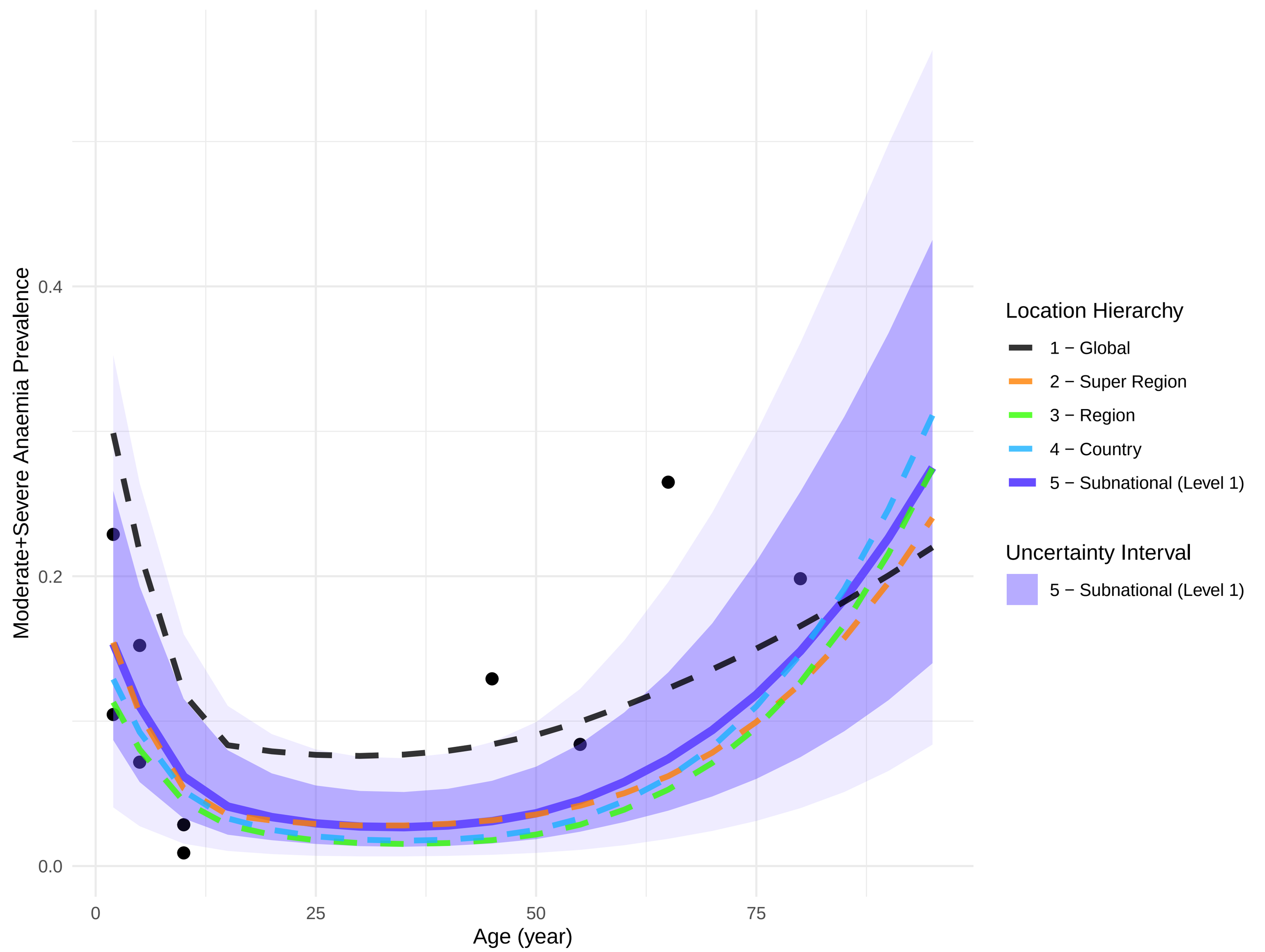

MR−BRT Age/Sex Fits − Males 2+ Years Old − Moderate+Severe Anaemia Prevalence − Baja California Sur
WHO−2024 Adjustment Method

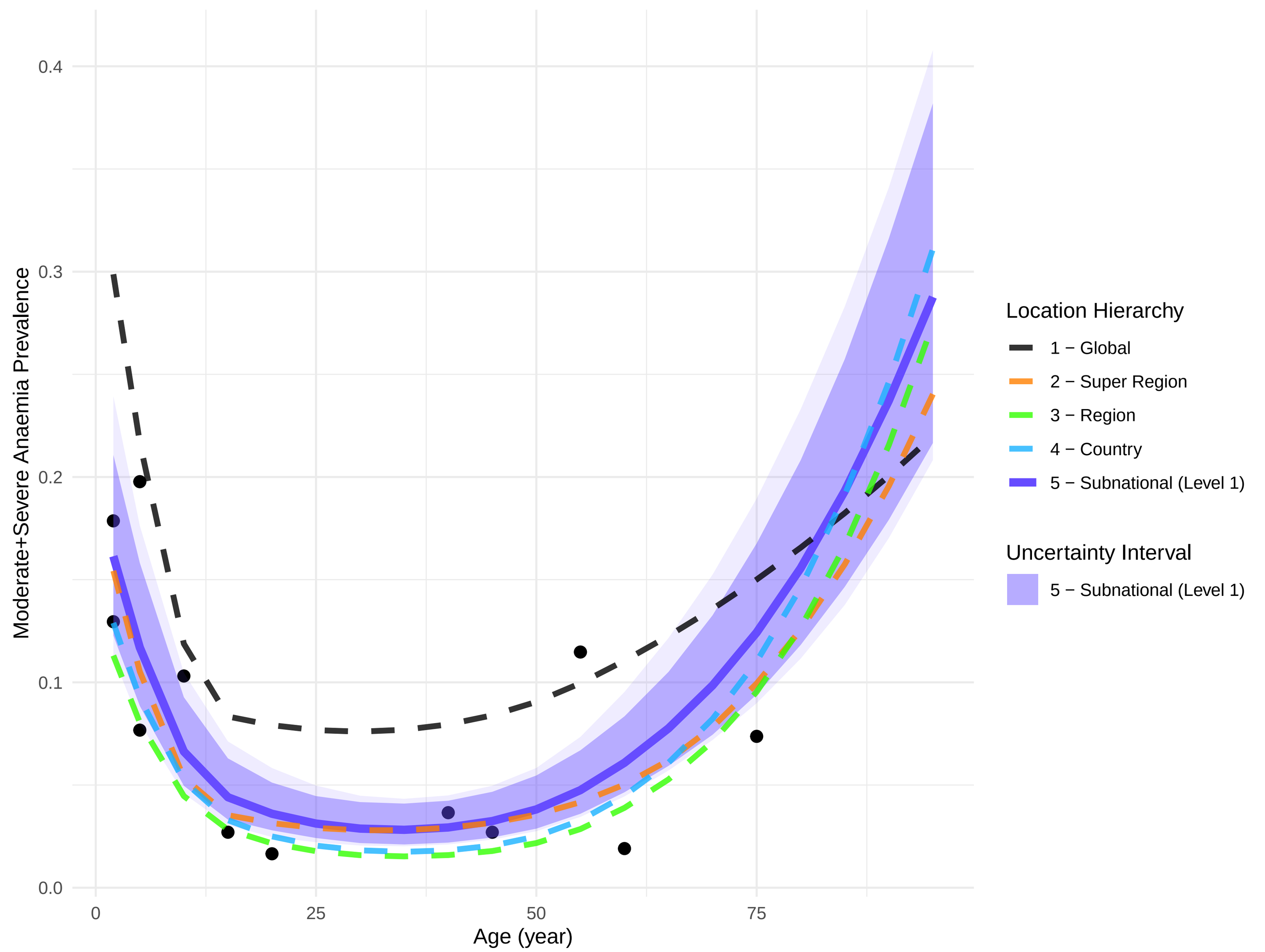

MR−BRT Age/Sex Fits − Males 2+ Years Old − Moderate+Severe Anaemia Prevalence − Campeche
WHO−2024 Adjustment Method

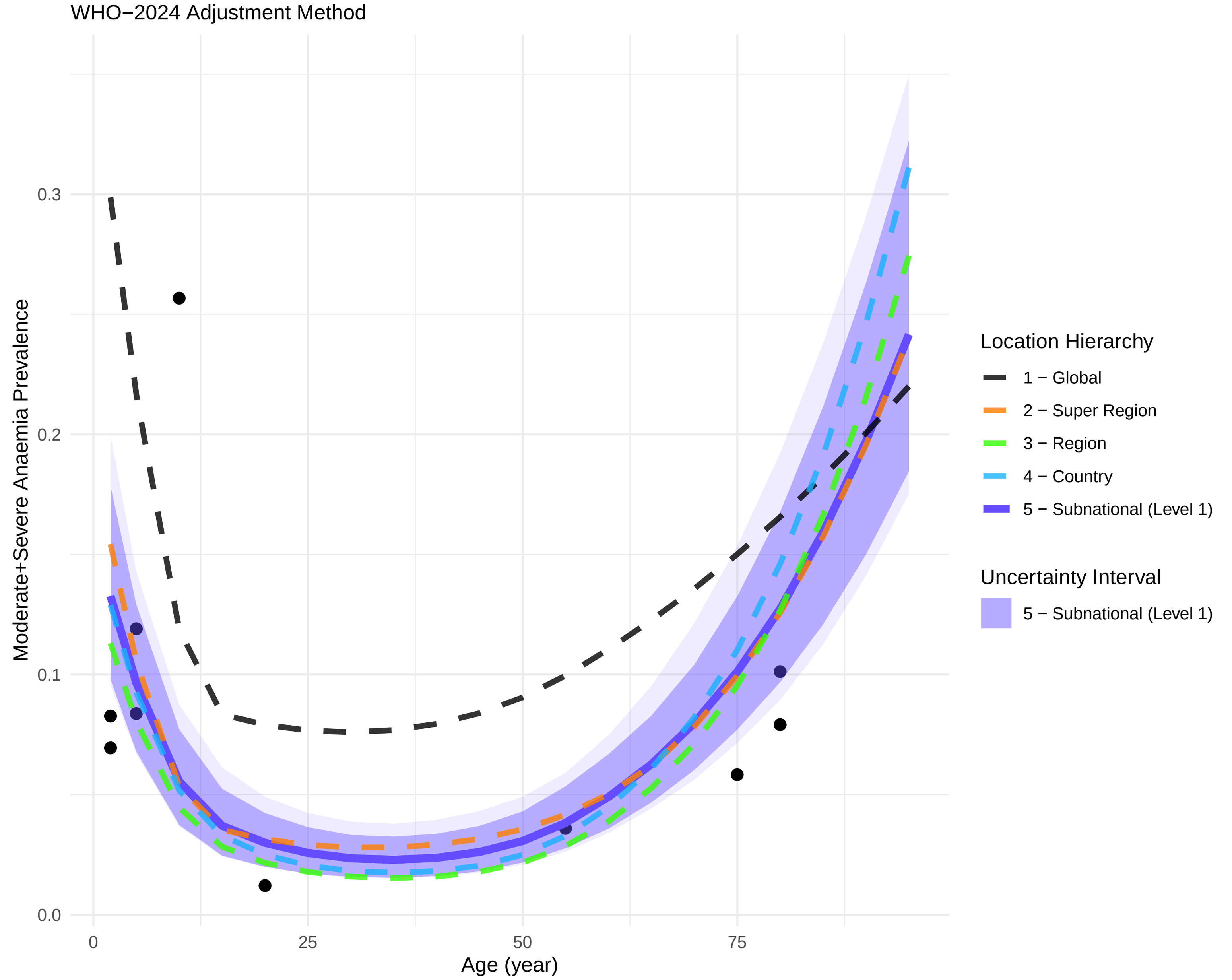

MR−BRT Age/Sex Fits − Males 2+ Years Old − Moderate+Severe Anaemia Prevalence − Chiapas
WHO−2024 Adjustment Method

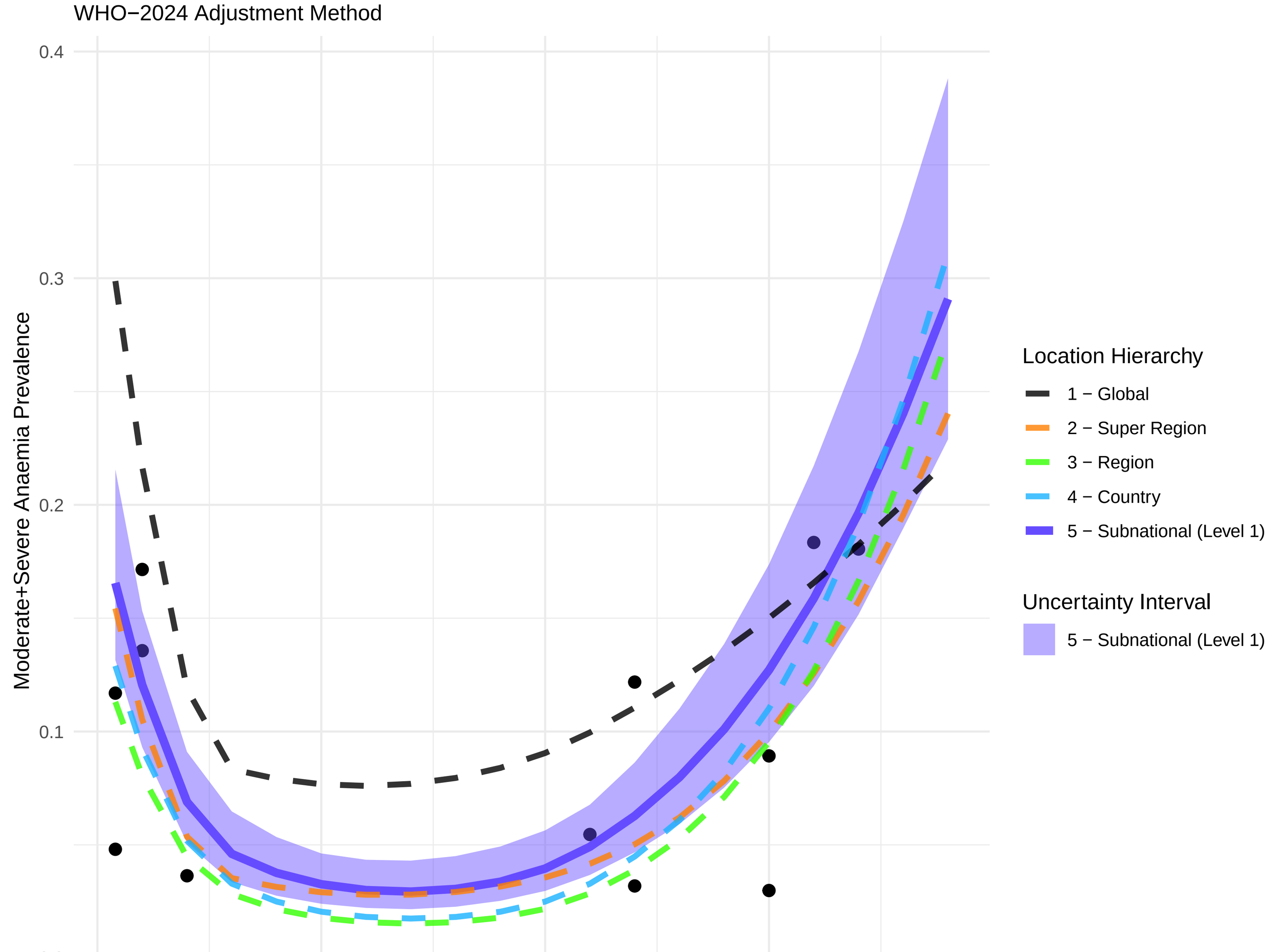

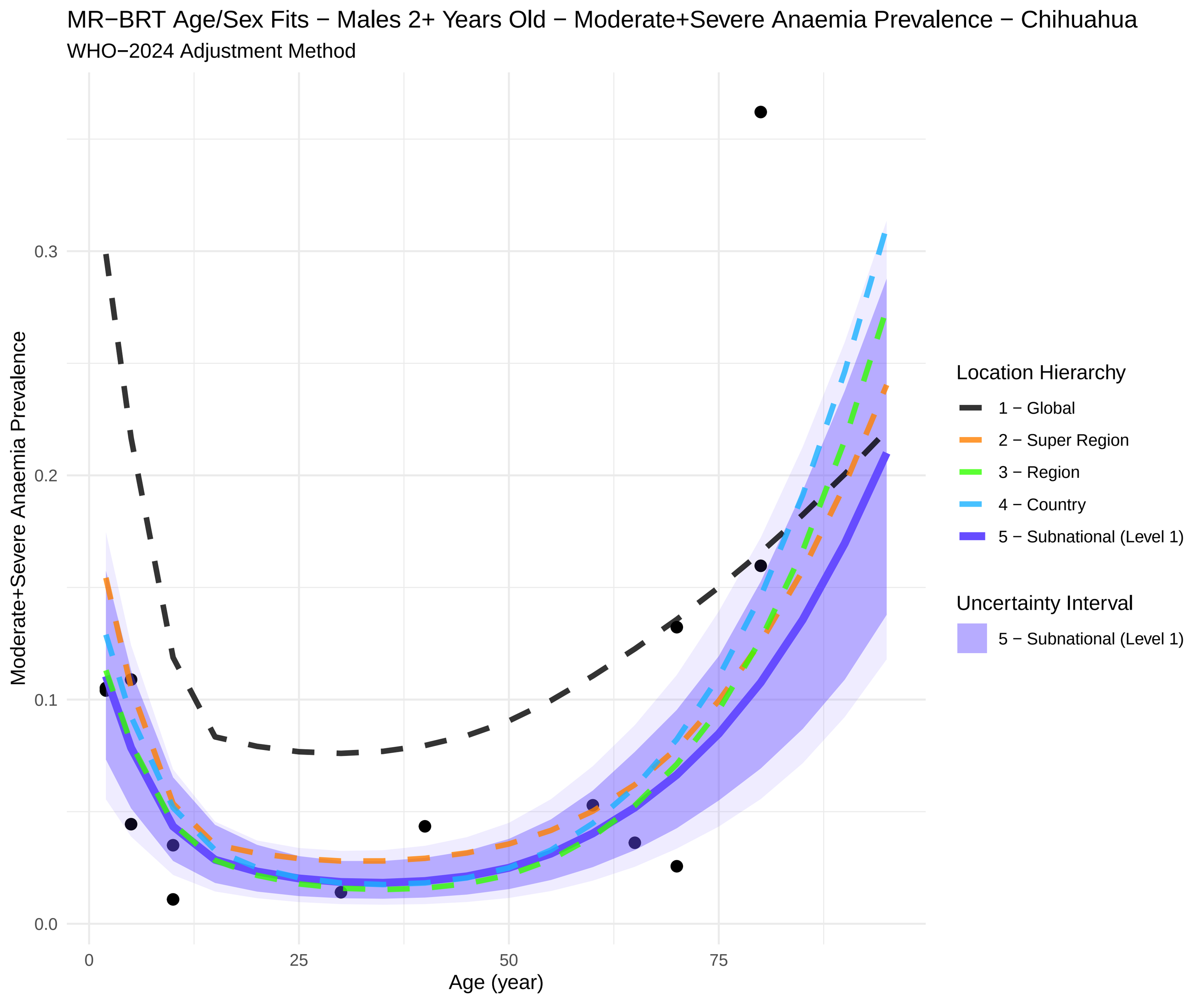
MR−BRT Age/Sex Fits − Males 2+ Years Old − Moderate+Severe Anaemia Prevalence − Chihuahua
WHO−2024 Adjustment Method
Moderate+Severe Anaemia Prevalence
Age (year)
Location Hierarchy
1 − Global
2 − Super Region
3 − Region
4 − Country
5 − Subnational (Level 1)
Uncertainty Interval
5 − Subnational (Level 1)

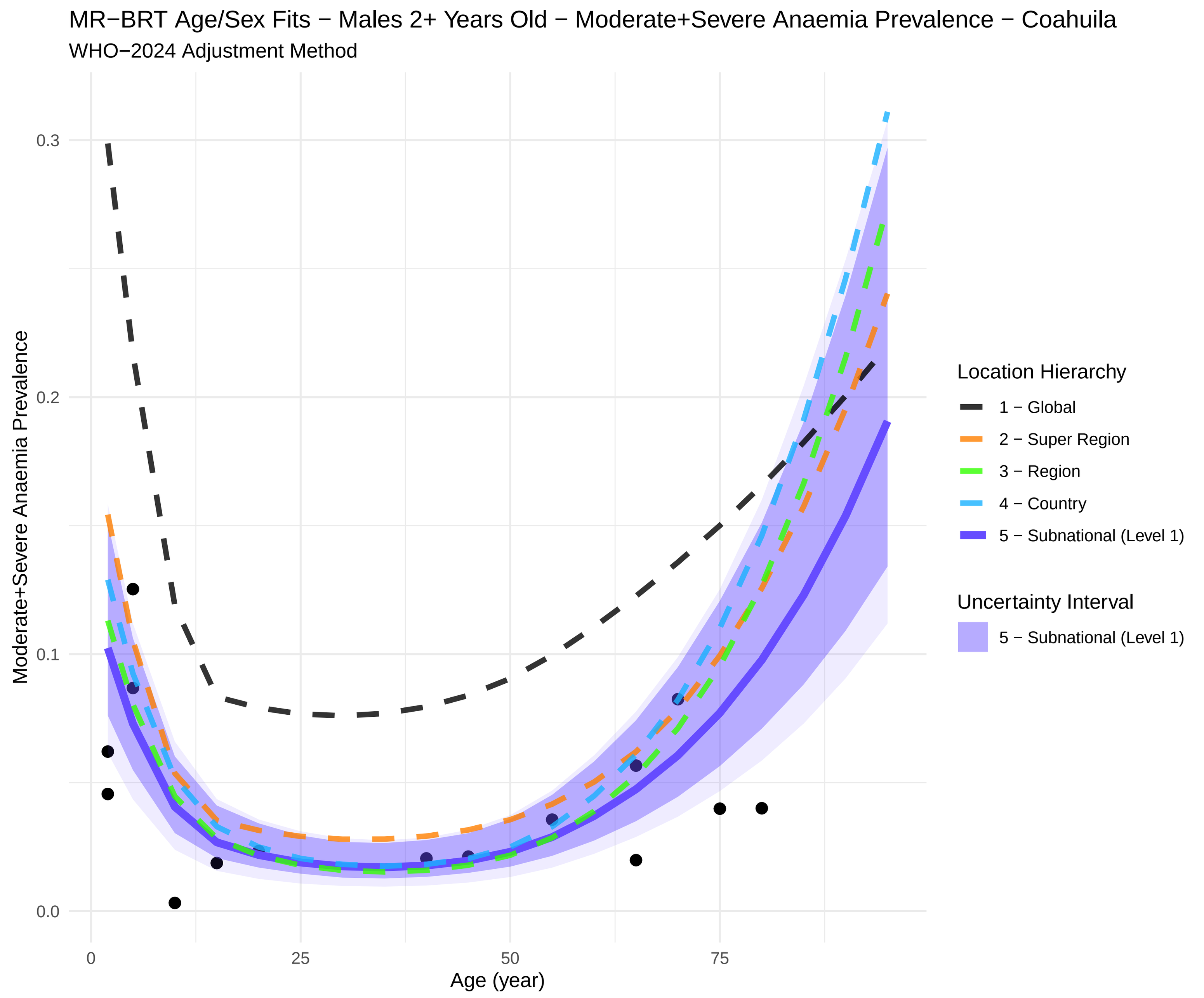
MR−BRT Age/Sex Fits − Males 2+ Years Old − Moderate+Severe Anaemia Prevalence − Coahuila
WHO−2024 Adjustment Method
Moderate+Severe Anaemia Prevalence
Age (year)
Location Hierarchy
1 − Global
2 − Super Region
3 − Region
4 − Country
5 − Subnational (Level 1)
Uncertainty Interval
5 − Subnational (Level 1)

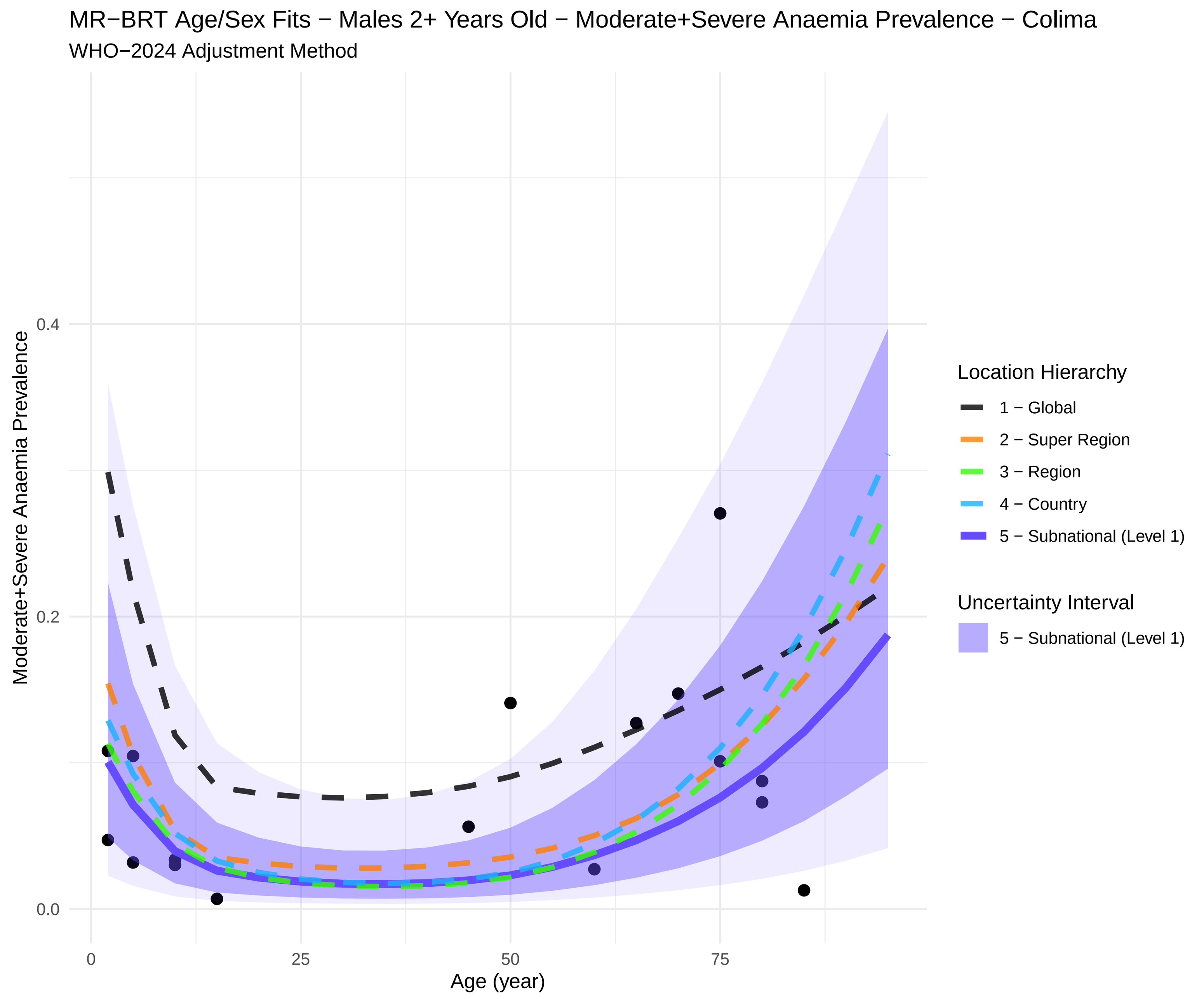
MR−BRT Age/Sex Fits − Males 2+ Years Old − Moderate+Severe Anaemia Prevalence − Colima
WHO−2024 Adjustment Method
Moderate+Severe Anaemia Prevalence
Age (year)
Location Hierarchy
1 − Global
2 − Super Region
3 − Region
4 − Country
5 − Subnational (Level 1)
Uncertainty Interval
5 − Subnational (Level 1)

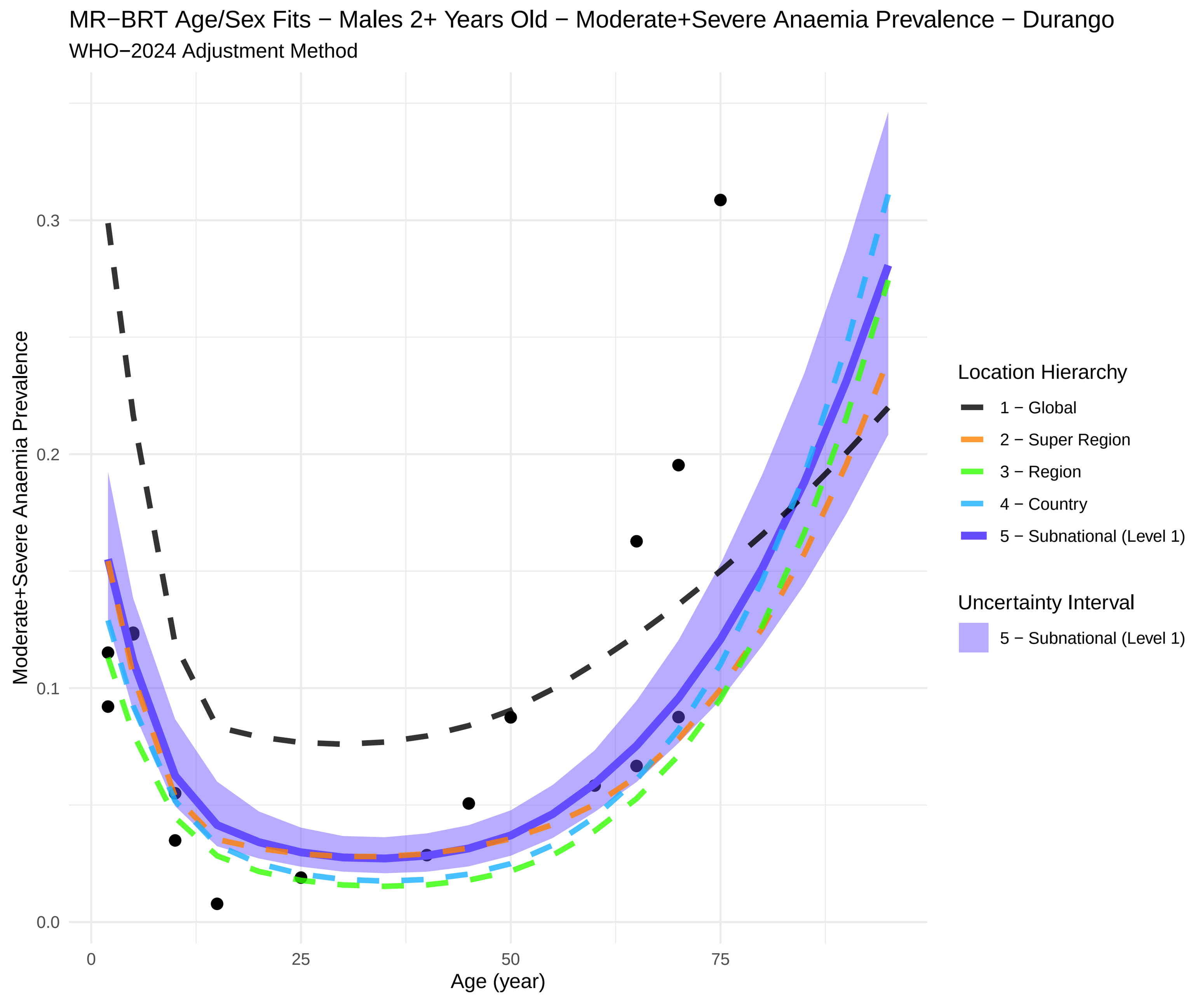
MR−BRT Age/Sex Fits − Males 2+ Years Old − Moderate+Severe Anaemia Prevalence − Durango
WHO−2024 Adjustment Method
Moderate+Severe Anaemia Prevalence
Age (year)
Location Hierarchy
1 − Global
2 − Super Region
3 − Region
4 − Country
5 − Subnational (Level 1)
Uncertainty Interval
5 − Subnational (Level 1)

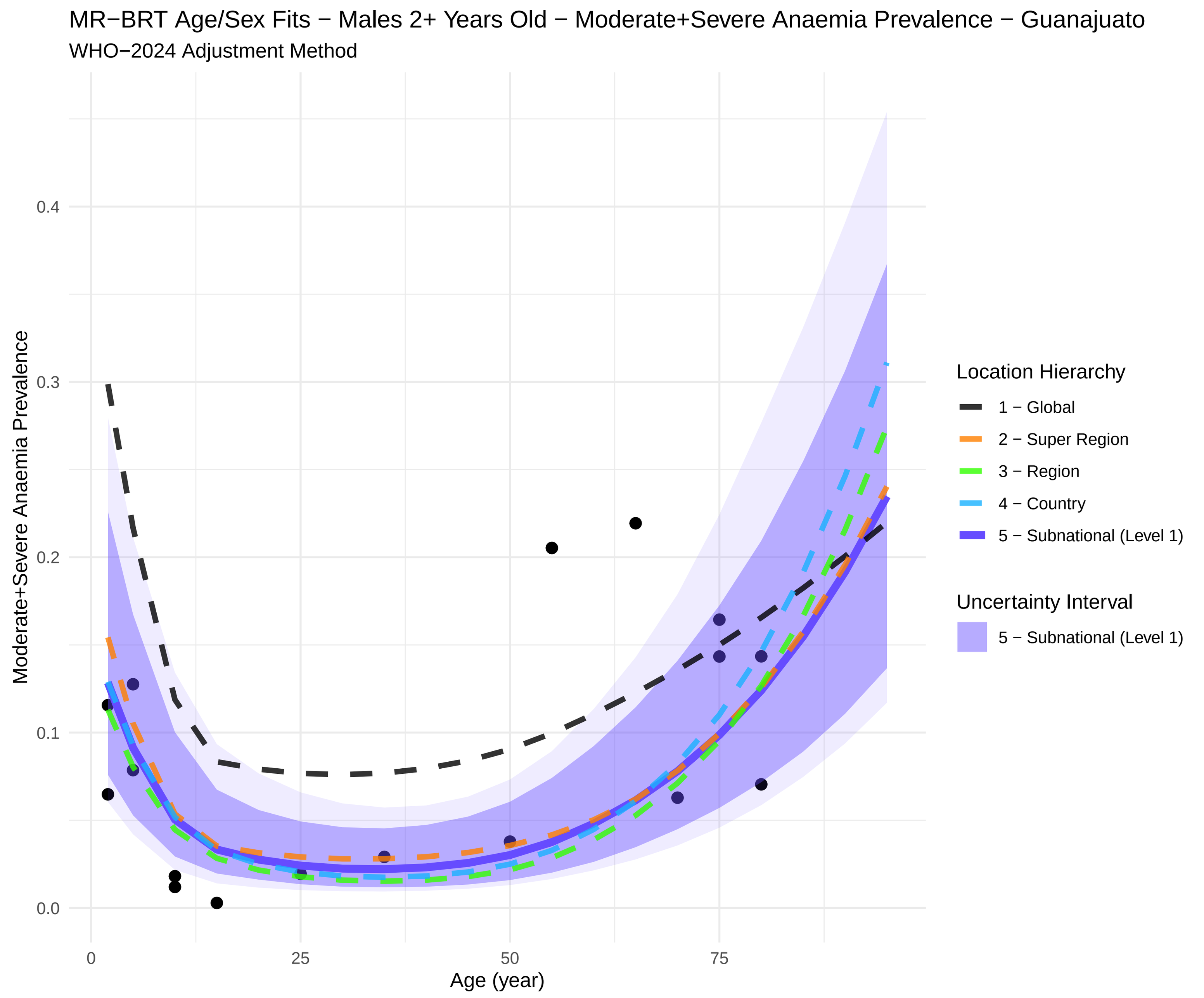
MR−BRT Age/Sex Fits − Males 2+ Years Old − Moderate+Severe Anaemia Prevalence − Guanajuato
WHO−2024 Adjustment Method
Moderate+Severe Anaemia Prevalence
Age (year)
Location Hierarchy
1 − Global
2 − Super Region
3 − Region
4 − Country
5 − Subnational (Level 1)
Uncertainty Interval
5 − Subnational (Level 1)

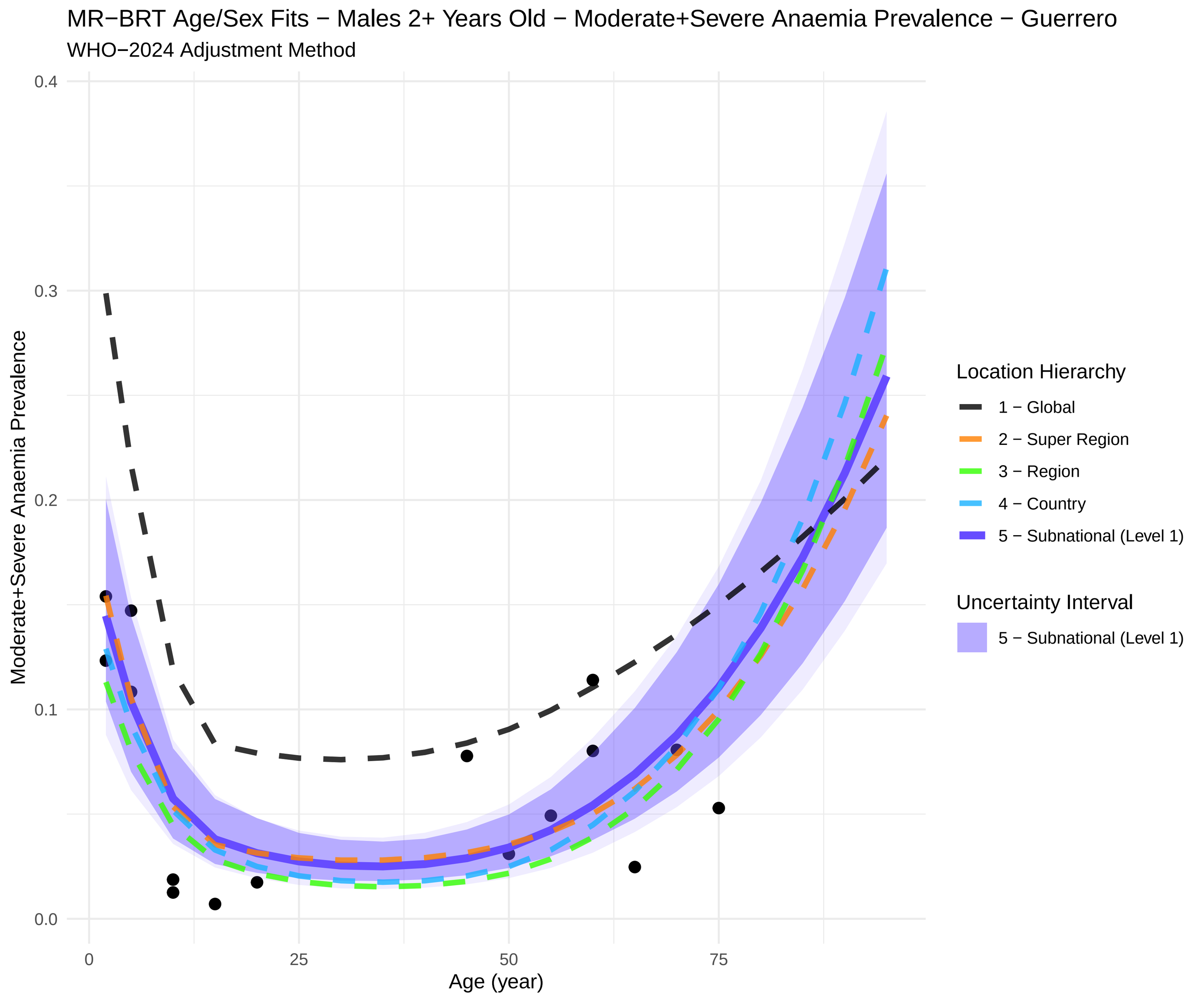
MR−BRT Age/Sex Fits − Males 2+ Years Old − Moderate+Severe Anaemia Prevalence − Guerrero
WHO−2024 Adjustment Method
Moderate+Severe Anaemia Prevalence
Age (year)
Location Hierarchy
1 − Global
2 − Super Region
3 − Region
4 − Country
5 − Subnational (Level 1)
Uncertainty Interval
5 − Subnational (Level 1)

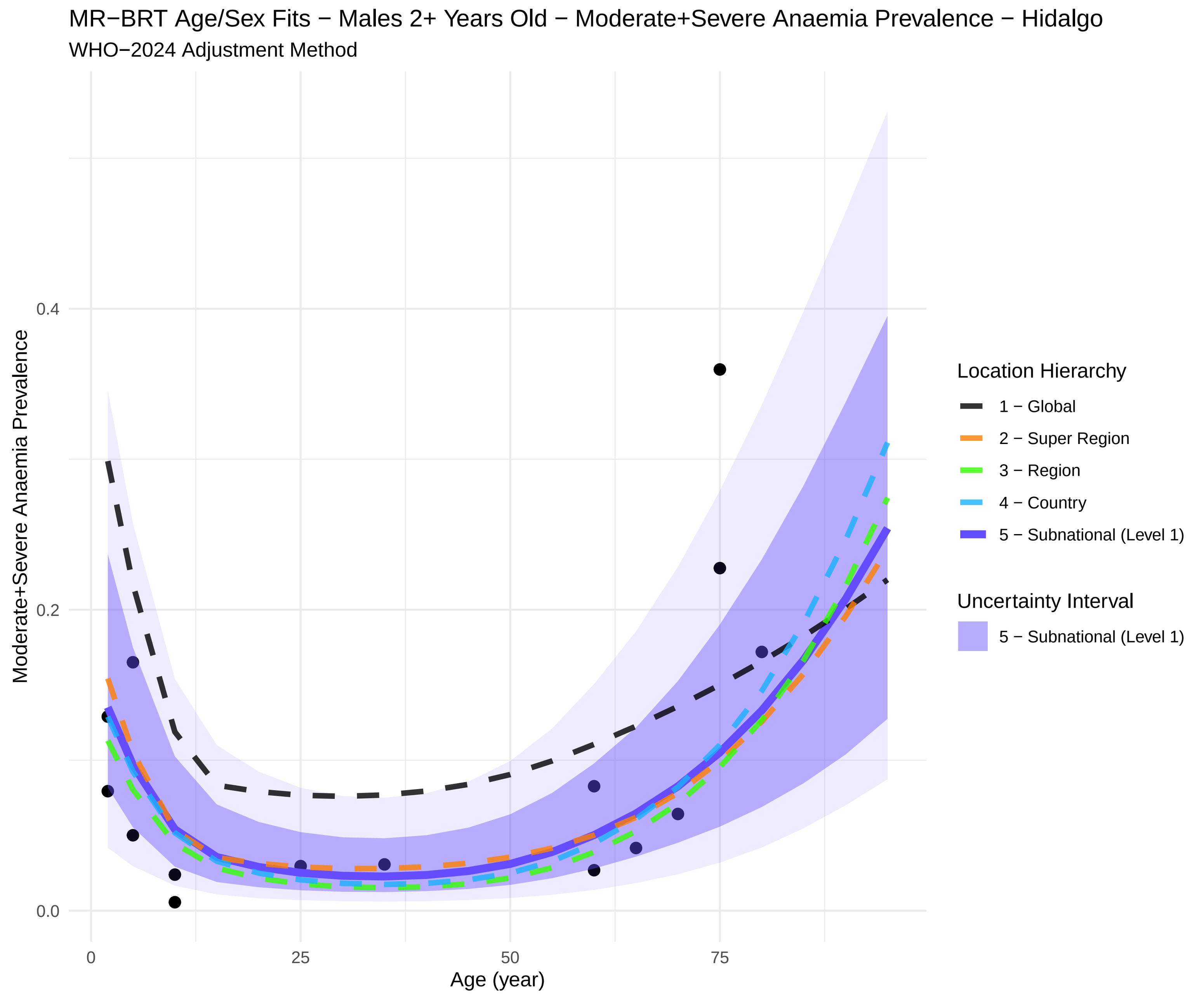
MR−BRT Age/Sex Fits − Males 2+ Years Old − Moderate+Severe Anaemia Prevalence − Hidalgo
WHO−2024 Adjustment Method
Moderate+Severe Anaemia Prevalence
Age (year)
Location Hierarchy
1 − Global
2 − Super Region
3 − Region
4 − Country
5 − Subnational (Level 1)
Uncertainty Interval
5 − Subnational (Level 1)

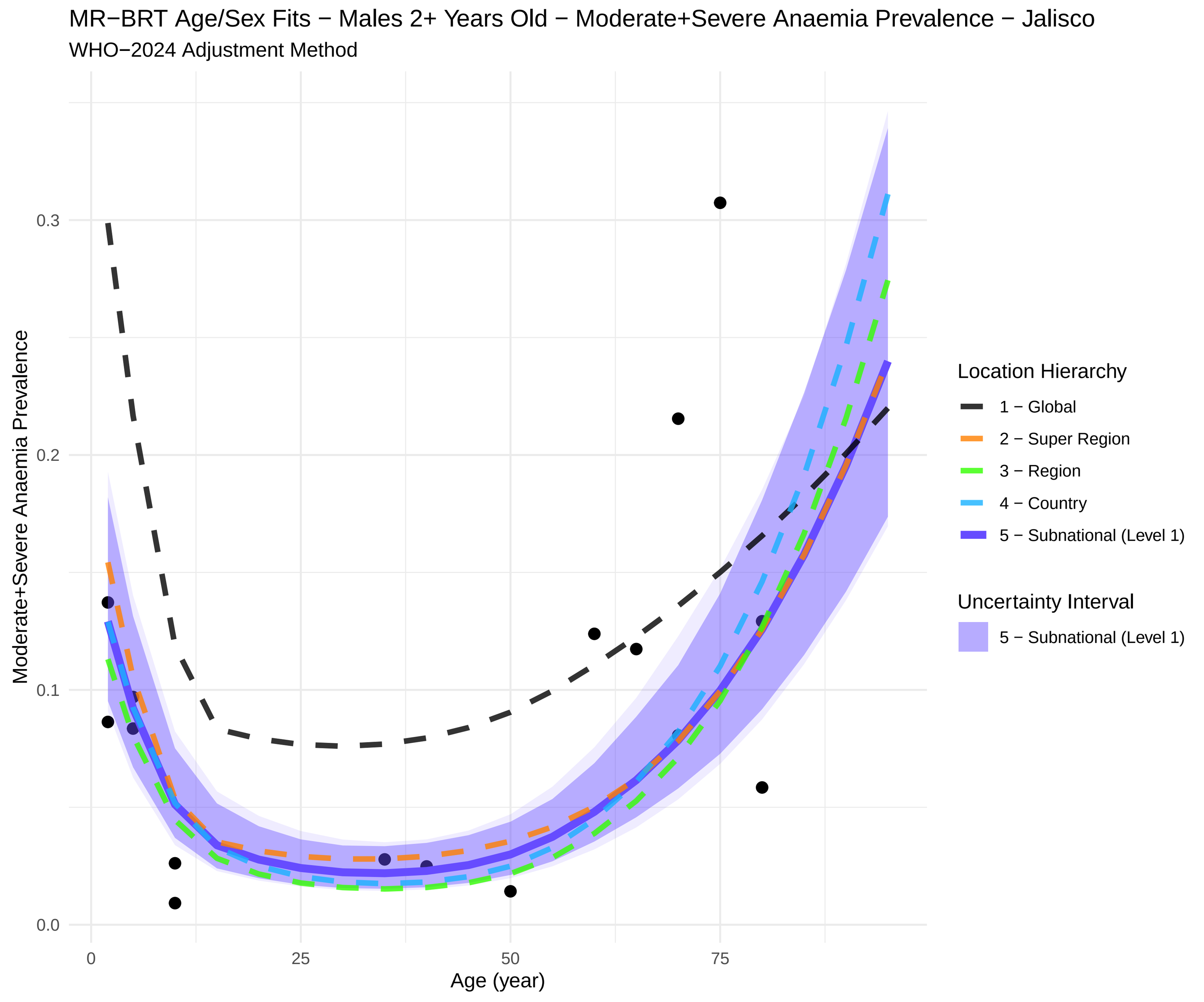
MR−BRT Age/Sex Fits − Males 2+ Years Old − Moderate+Severe Anaemia Prevalence − Jalisco
WHO−2024 Adjustment Method
Moderate+Severe Anaemia Prevalence
Age (year)
Location Hierarchy
1 − Global
2 − Super Region
3 − Region
4 − Country
5 − Subnational (Level 1)
Uncertainty Interval
5 − Subnational (Level 1)

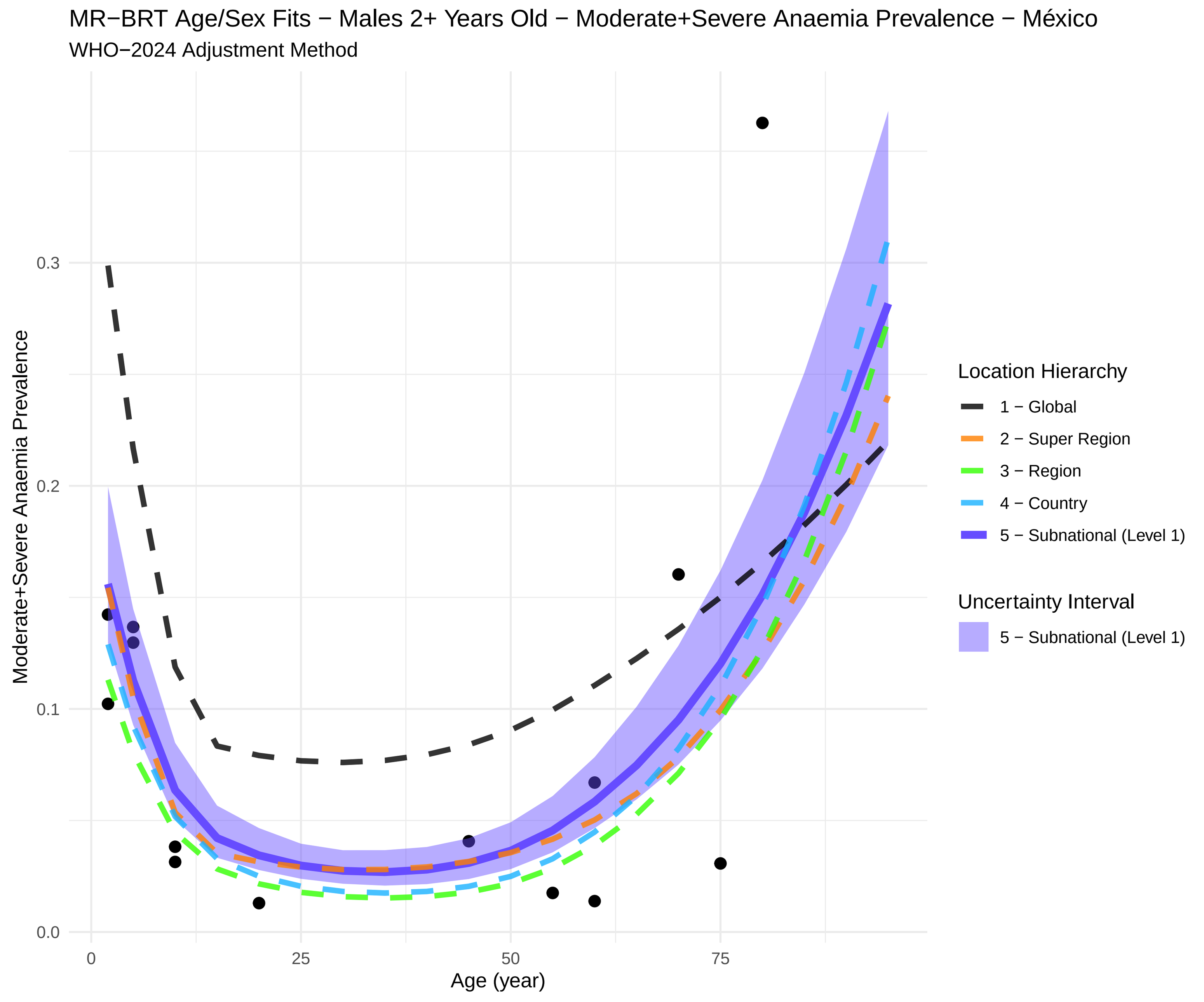
MR−BRT Age/Sex Fits − Males 2+ Years Old − Moderate+Severe Anaemia Prevalence − México
WHO−2024 Adjustment Method
Moderate+Severe Anaemia Prevalence
Age (year)
Location Hierarchy
1 − Global
2 − Super Region
3 − Region
4 − Country
5 − Subnational (Level 1)
Uncertainty Interval
5 − Subnational (Level 1)

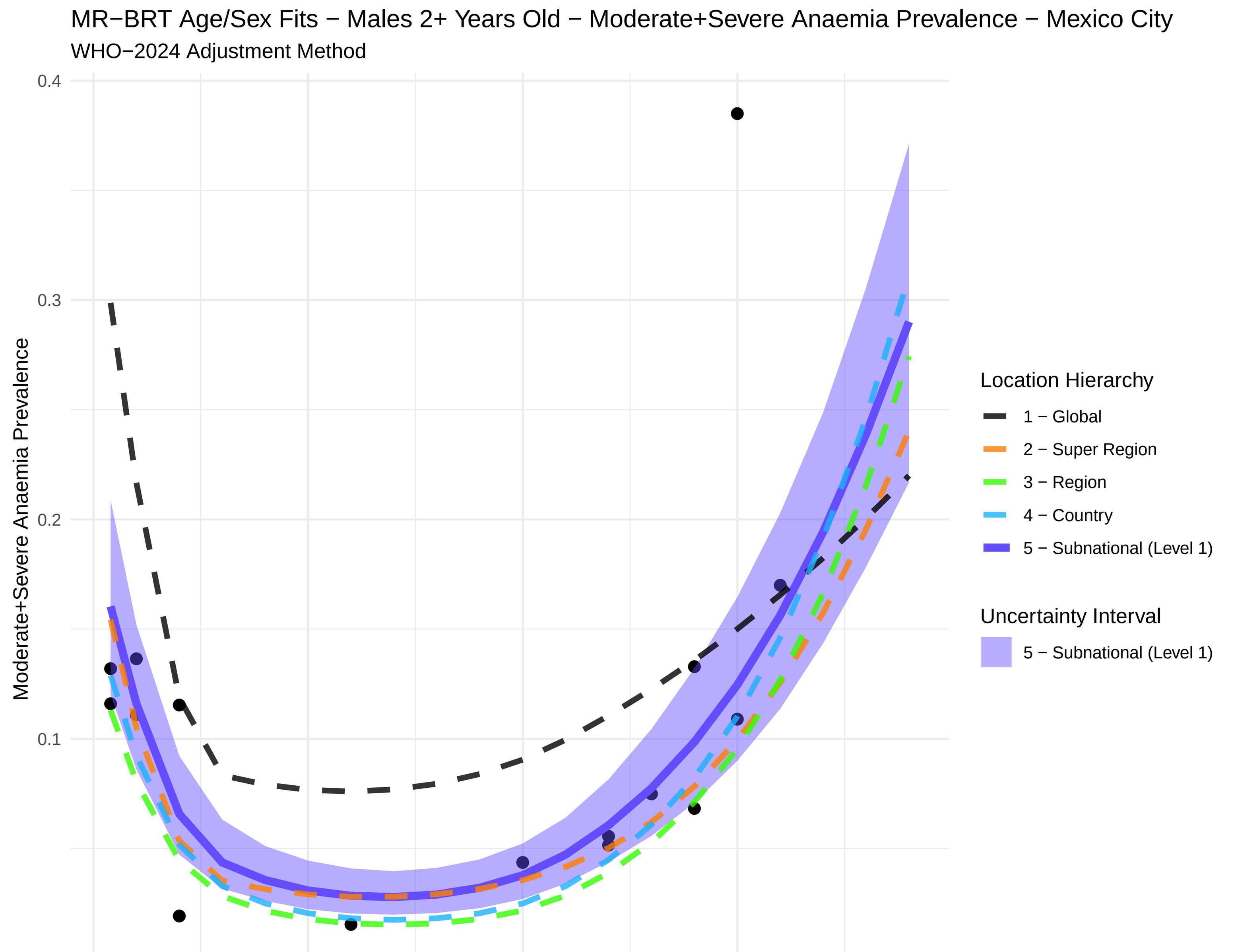
MR−BRT Age/Sex Fits − Males 2+ Years Old − Moderate+Severe Anaemia Prevalence − Mexico City
WHO−2024 Adjustment Method
Moderate+Severe Anaemia Prevalence
Age (year)
Location Hierarchy
1 − Global
2 − Super Region
3 − Region
4 − Country
5 − Subnational (Level 1)
Uncertainty Interval
5 − Subnational (Level 1)

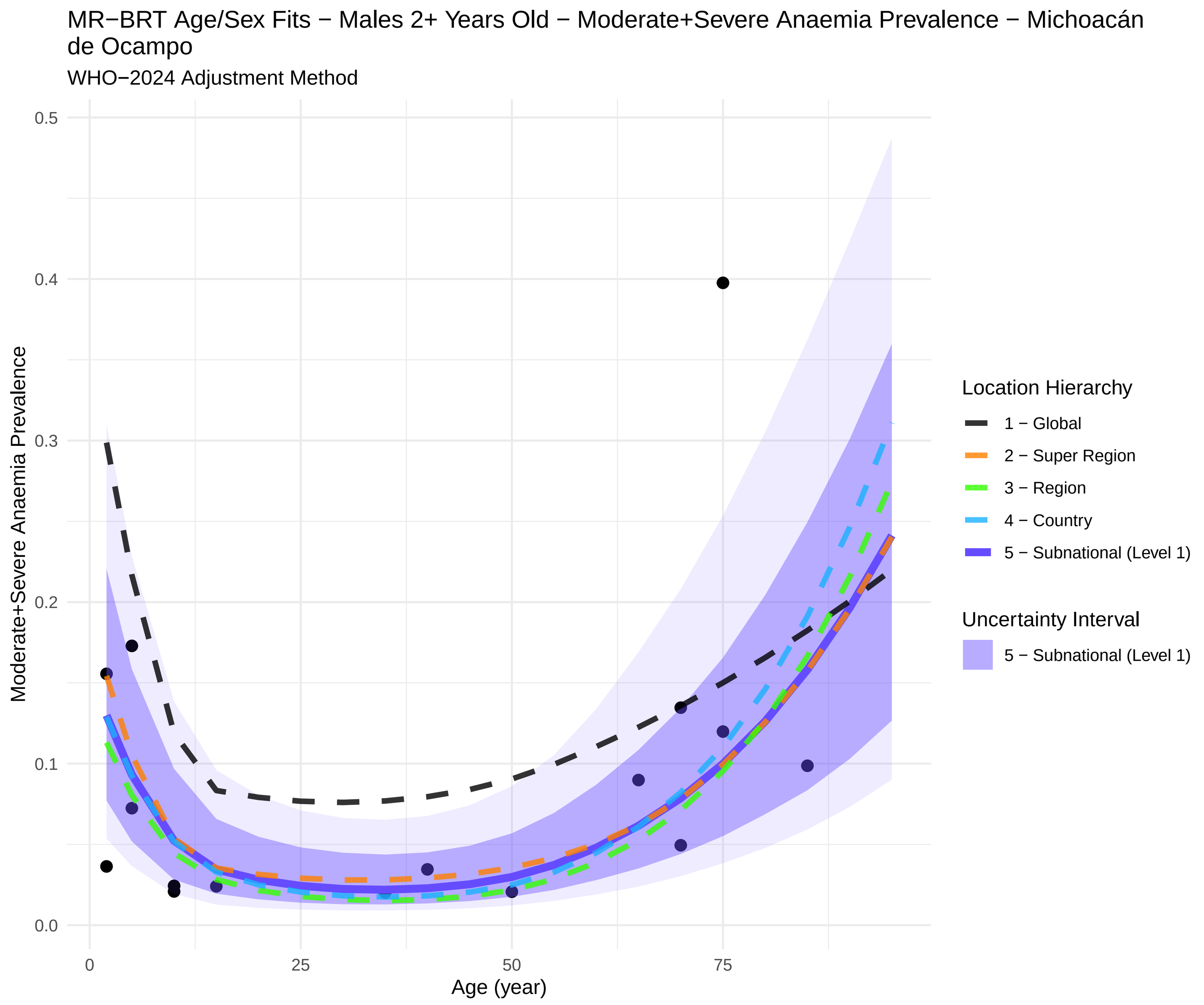
MR−BRT Age/Sex Fits − Males 2+ Years Old − Moderate+Severe Anaemia Prevalence − Michoacán de Ocampo
WHO−2024 Adjustment Method
Moderate+Severe Anaemia Prevalence
Age (year)
Location Hierarchy
1 − Global
2 − Super Region
3 − Region
4 − Country
5 − Subnational (Level 1)
Uncertainty Interval
5 − Subnational (Level 1)

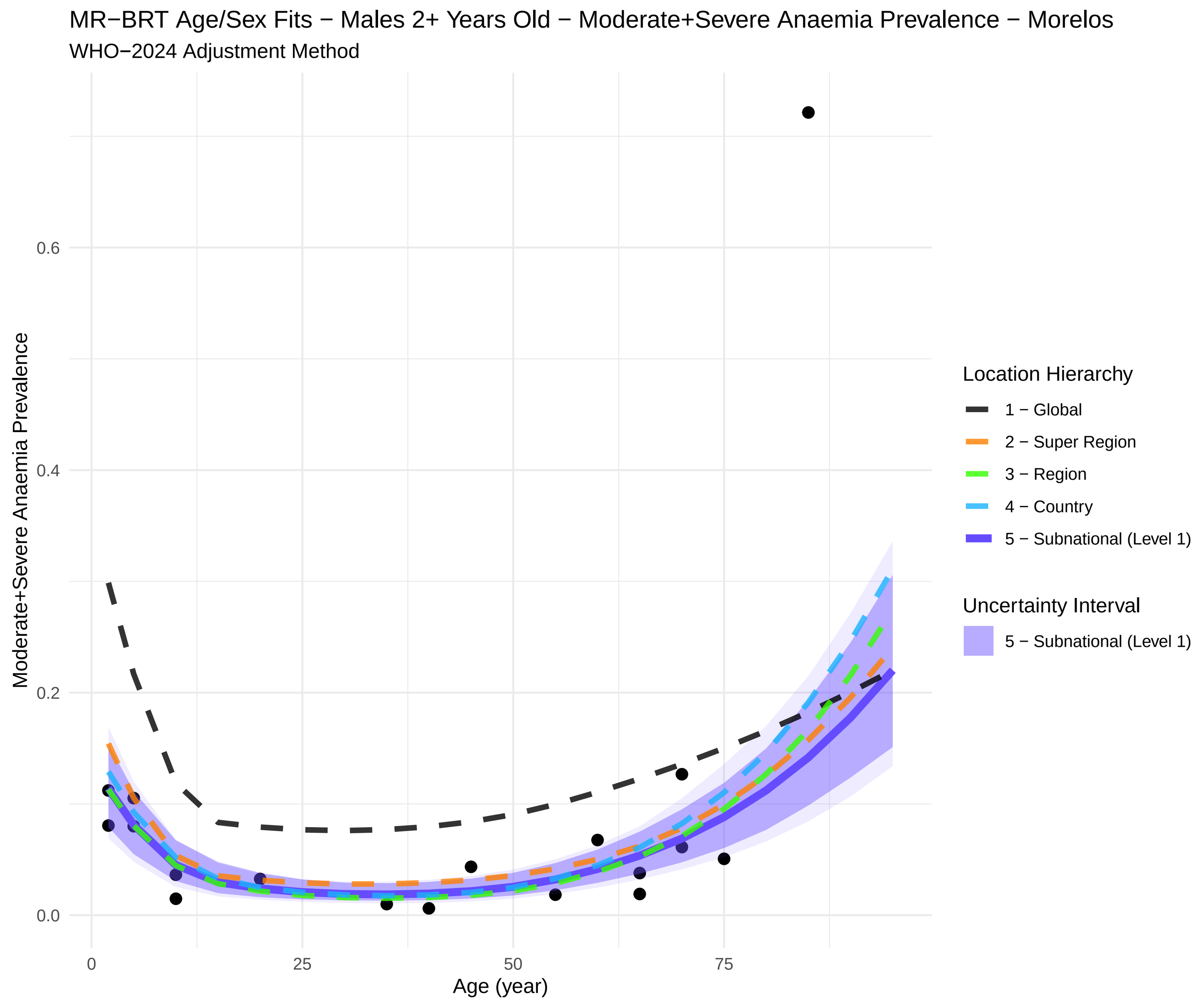
MR−BRT Age/Sex Fits − Males 2+ Years Old − Moderate+Severe Anaemia Prevalence − Morelos
WHO−2024 Adjustment Method
Moderate+Severe Anaemia Prevalence
Age (year)
Location Hierarchy
1 − Global
2 − Super Region
3 − Region
4 − Country
5 − Subnational (Level 1)
Uncertainty Interval
5 − Subnational (Level 1)

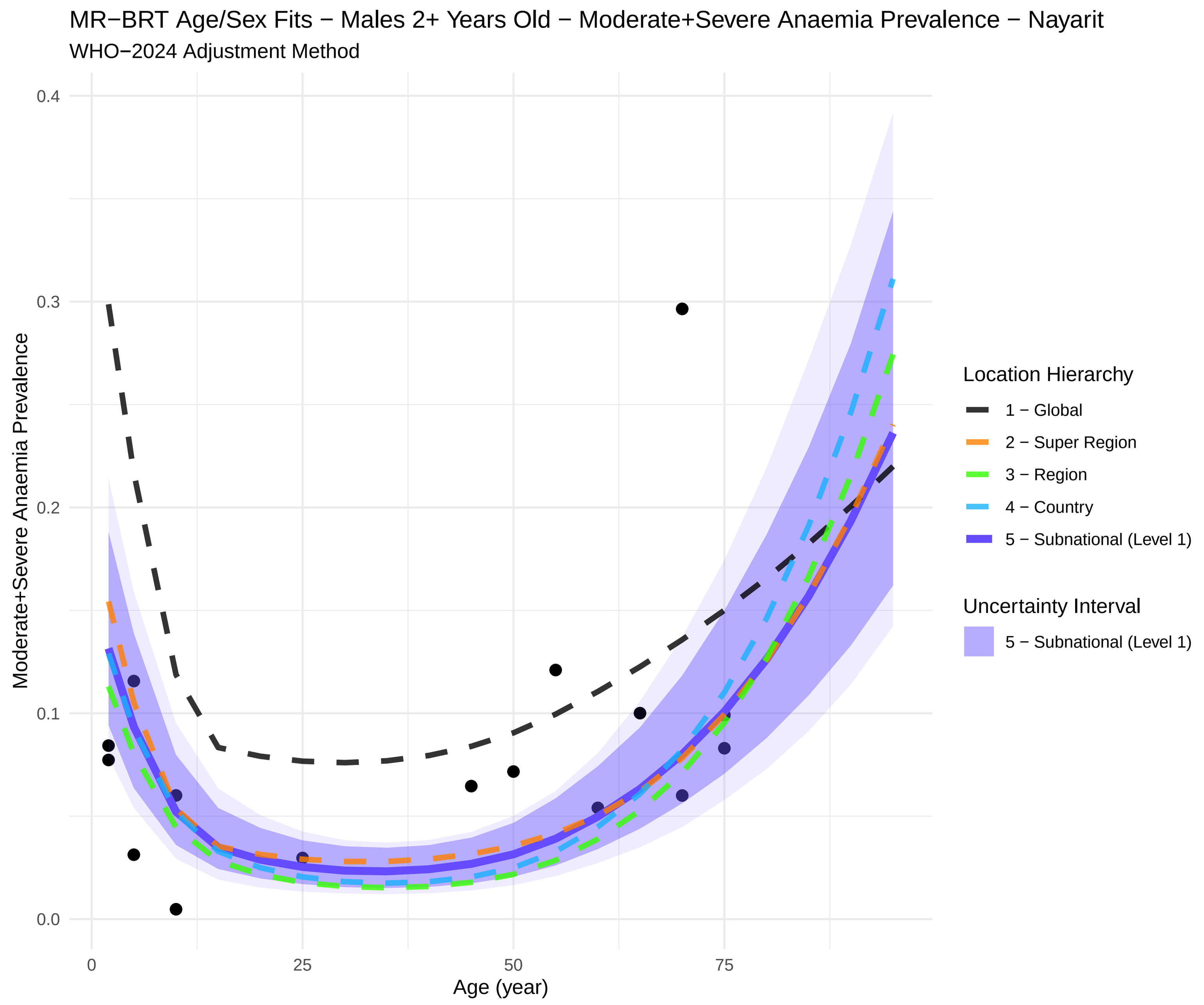
MR−BRT Age/Sex Fits − Males 2+ Years Old − Moderate+Severe Anaemia Prevalence − Nayarit
WHO−2024 Adjustment Method
Moderate+Severe Anaemia Prevalence
Age (year)
Location Hierarchy
1 − Global
2 − Super Region
3 − Region
4 − Country
5 − Subnational (Level 1)
Uncertainty Interval
5 − Subnational (Level 1)

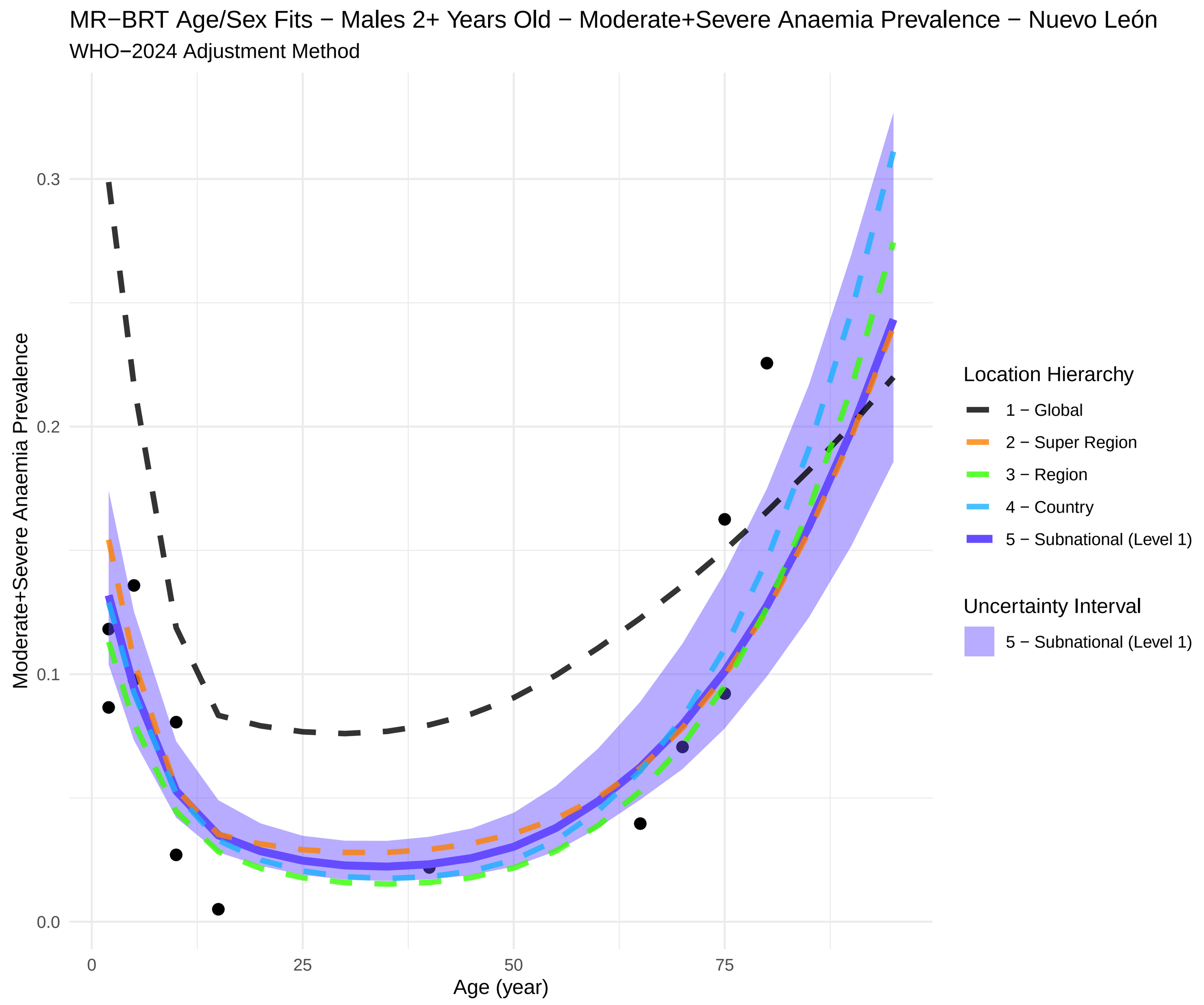
MR−BRT Age/Sex Fits − Males 2+ Years Old − Moderate+Severe Anaemia Prevalence − Nuevo León
WHO−2024 Adjustment Method
Moderate+Severe Anaemia Prevalence
Age (year)
Location Hierarchy
1 − Global
2 − Super Region
3 − Region
4 − Country
5 − Subnational (Level 1)
Uncertainty Interval
5 − Subnational (Level 1)

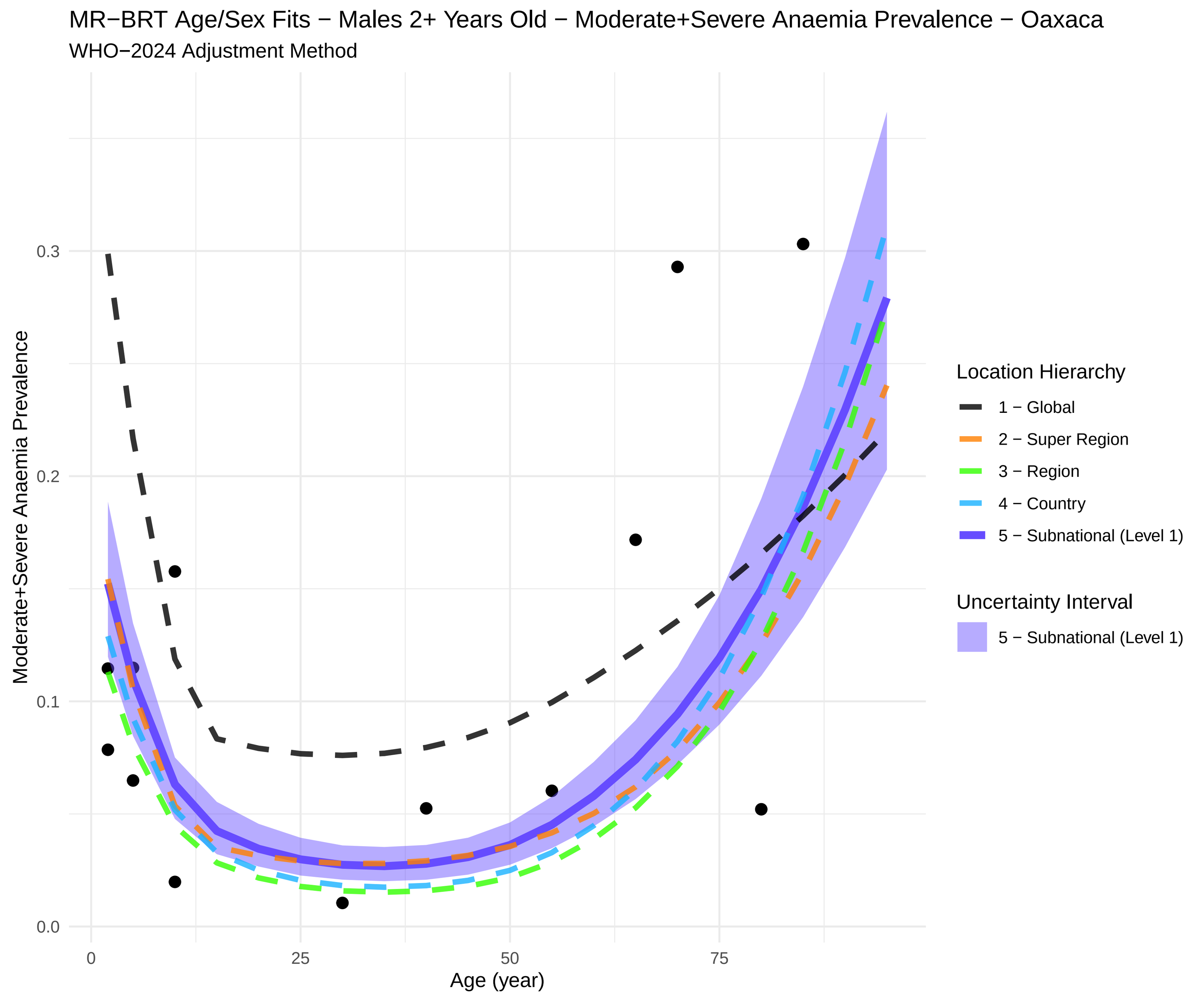
MR−BRT Age/Sex Fits − Males 2+ Years Old − Moderate+Severe Anaemia Prevalence − Oaxaca
WHO−2024 Adjustment Method
Moderate+Severe Anaemia Prevalence
Age (year)
Location Hierarchy
1 − Global
2 − Super Region
3 − Region
4 − Country
5 − Subnational (Level 1)
Uncertainty Interval
5 − Subnational (Level 1)

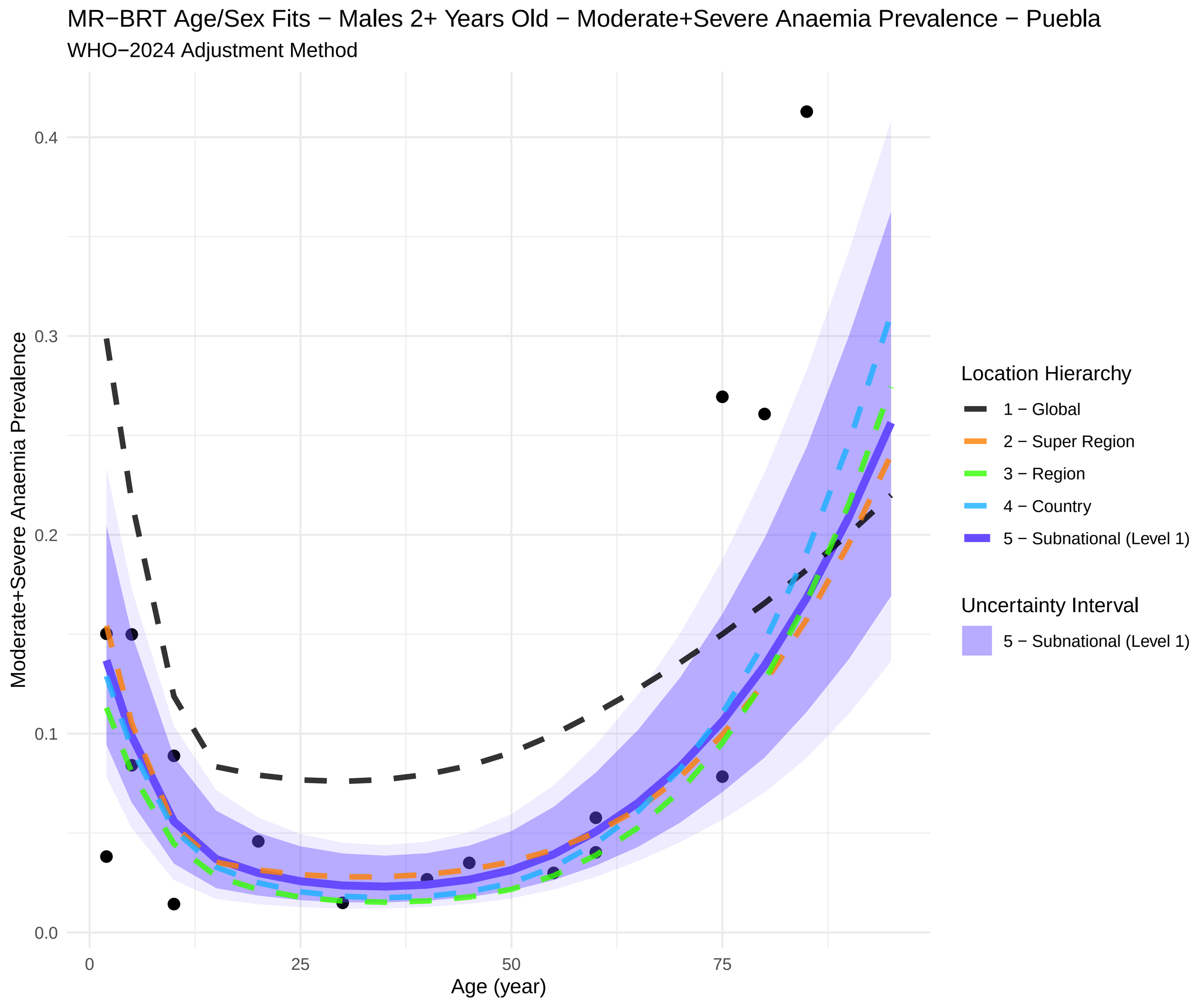
MR−BRT Age/Sex Fits − Males 2+ Years Old − Moderate+Severe Anaemia Prevalence − Puebla
WHO−2024 Adjustment Method
Moderate+Severe Anaemia Prevalence
Age (year)
Location Hierarchy
1 − Global
2 − Super Region
3 − Region
4 − Country
5 − Subnational (Level 1)
Uncertainty Interval
5 − Subnational (Level 1)

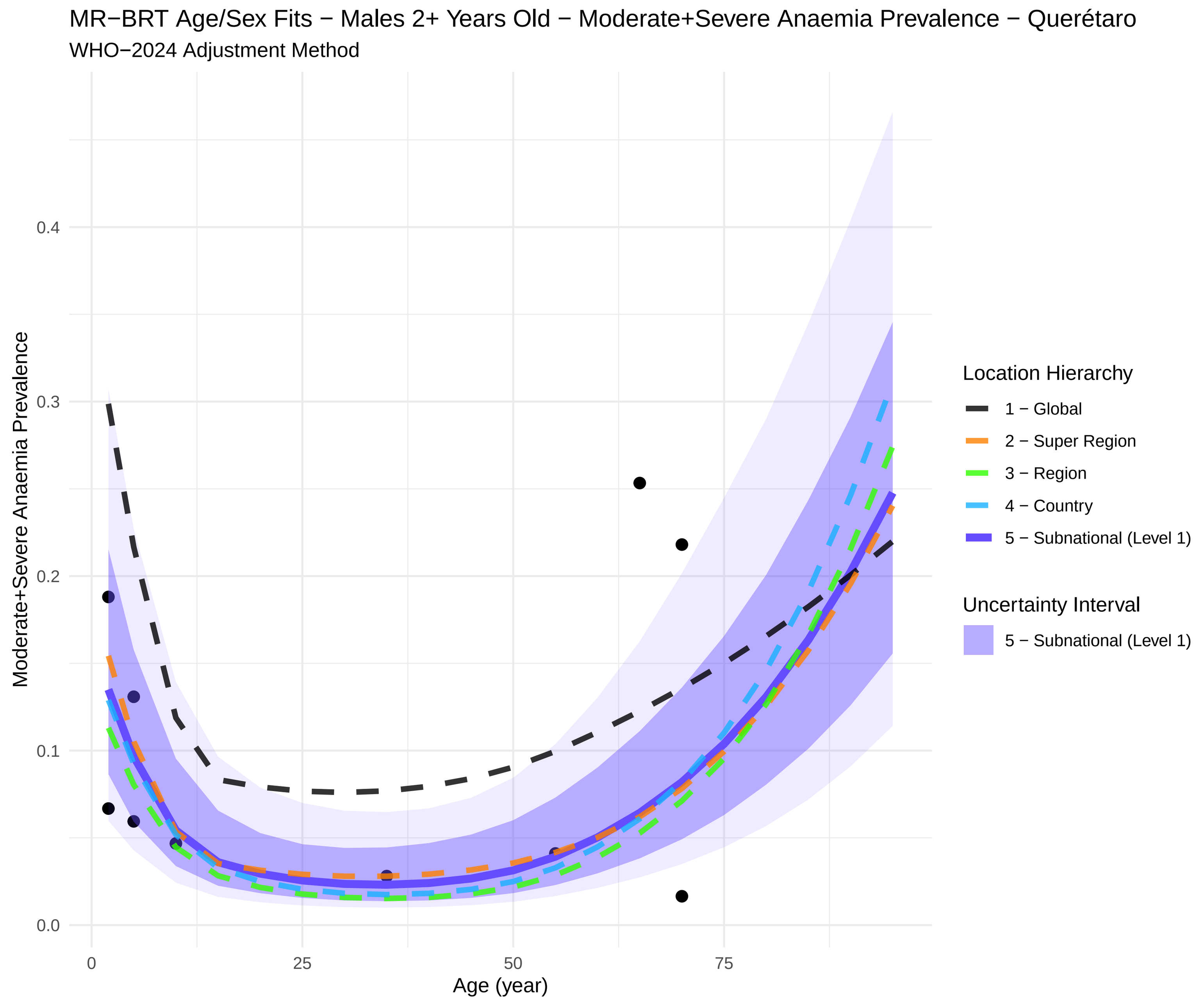
MR−BRT Age/Sex Fits − Males 2+ Years Old − Moderate+Severe Anaemia Prevalence − Querétaro
WHO−2024 Adjustment Method
Moderate+Severe Anaemia Prevalence
Age (year)
Location Hierarchy
1 − Global
2 − Super Region
3 − Region
4 − Country
5 − Subnational (Level 1)
Uncertainty Interval
5 − Subnational (Level 1)

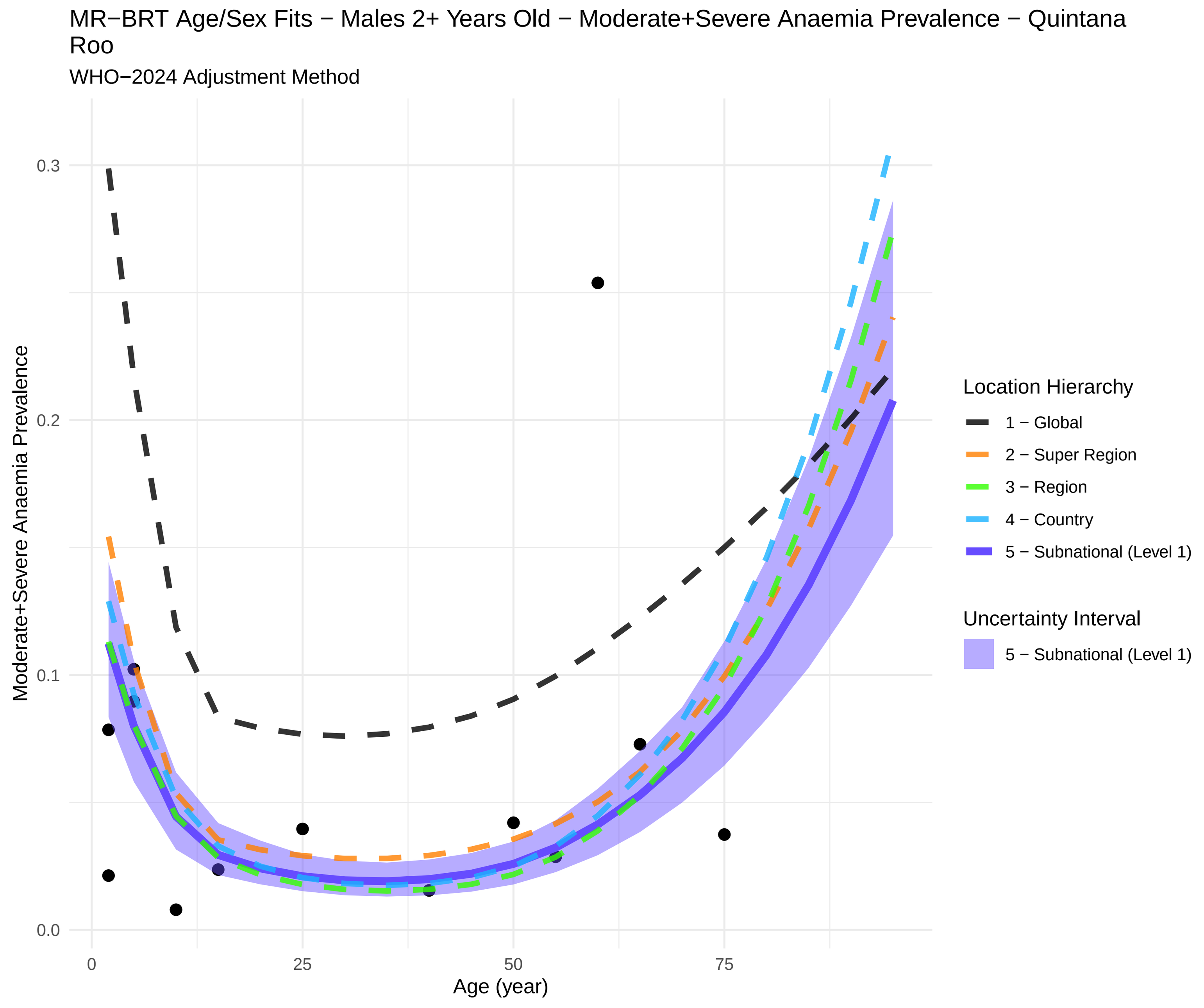
MR−BRT Age/Sex Fits − Males 2+ Years Old − Moderate+Severe Anaemia Prevalence − Quintana Roo
WHO−2024 Adjustment Method
Moderate+Severe Anaemia Prevalence
Age (year)
Location Hierarchy
1 − Global
2 − Super Region
3 − Region
4 − Country
5 − Subnational (Level 1)
Uncertainty Interval
5 − Subnational (Level 1)

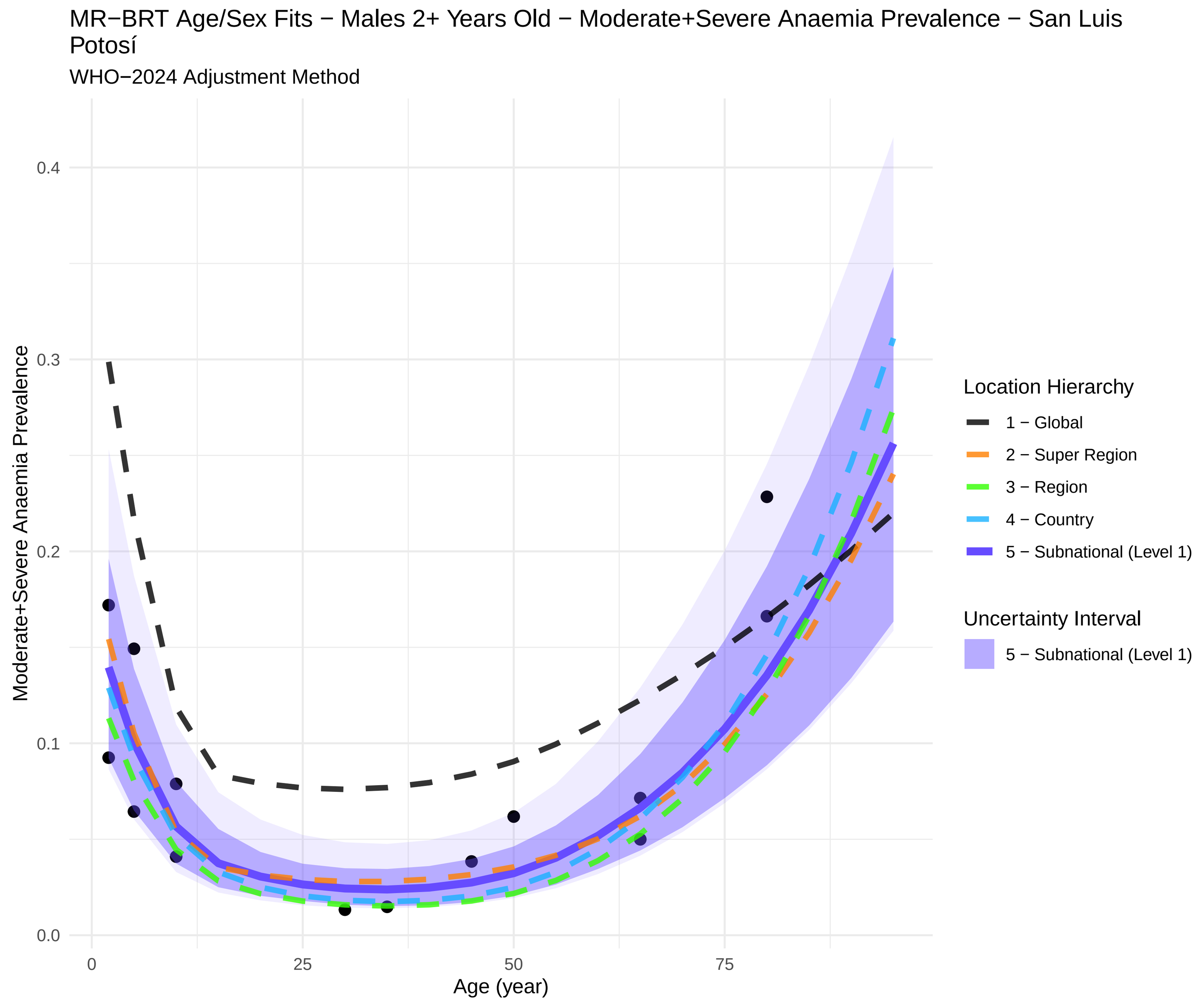
MR−BRT Age/Sex Fits − Males 2+ Years Old − Moderate+Severe Anaemia Prevalence − San Luis Potosí
WHO−2024 Adjustment Method
Moderate+Severe Anaemia Prevalence
Age (year)
Location Hierarchy
1 − Global
2 − Super Region
3 − Region
4 − Country
5 − Subnational (Level 1)
Uncertainty Interval
5 − Subnational (Level 1)

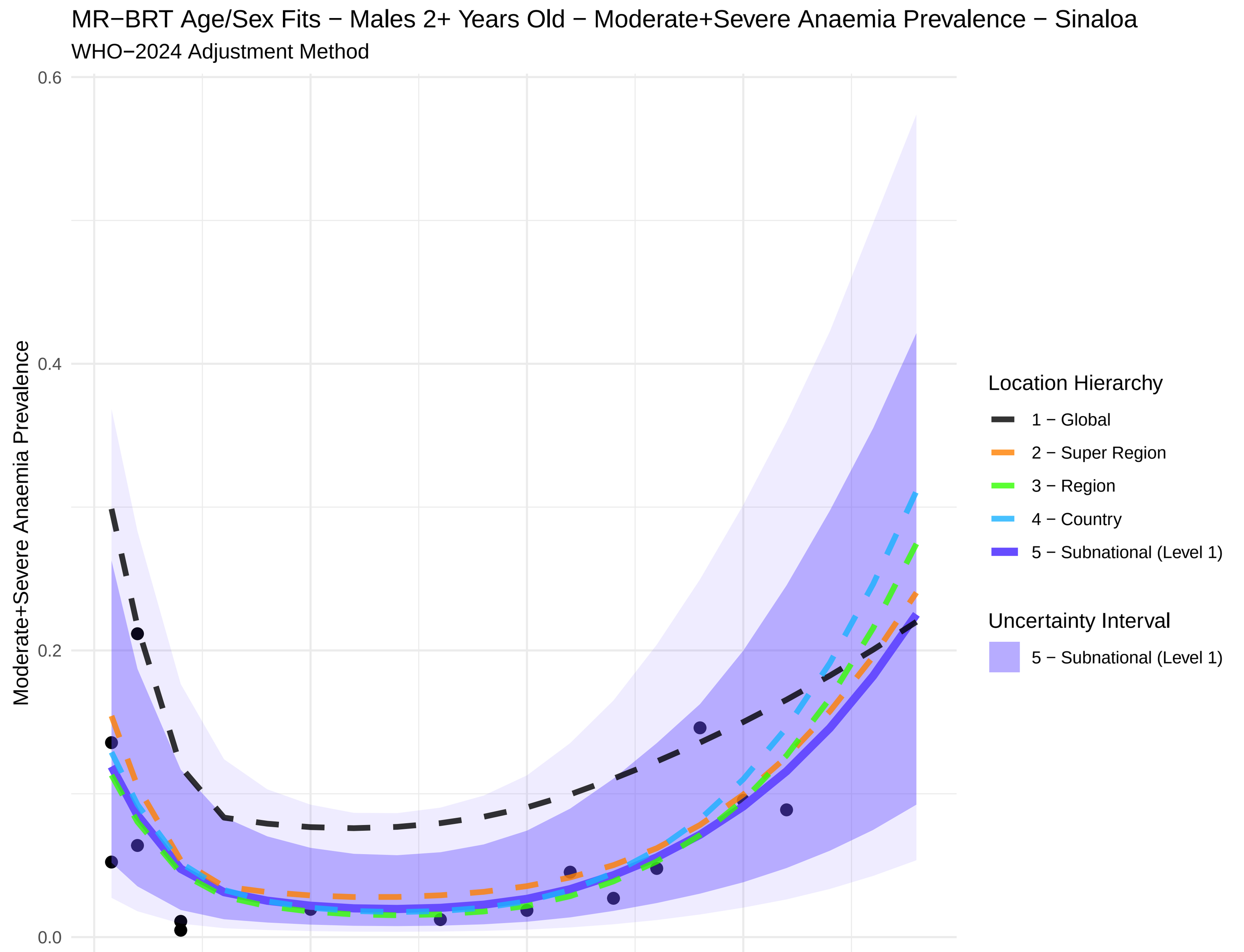
MR−BRT Age/Sex Fits − Males 2+ Years Old − Moderate+Severe Anaemia Prevalence − Sinaloa
WHO−2024 Adjustment Method
Moderate+Severe Anaemia Prevalence
Age (year)
Location Hierarchy
1 − Global
2 − Super Region
3 − Region
4 − Country
5 − Subnational (Level 1)
Uncertainty Interval
5 − Subnational (Level 1)

MR−BRT Age/Sex Fits − Males 2+ Years Old − Moderate+Severe Anaemia Prevalence − Sonora
WHO−2024 Adjustment Method

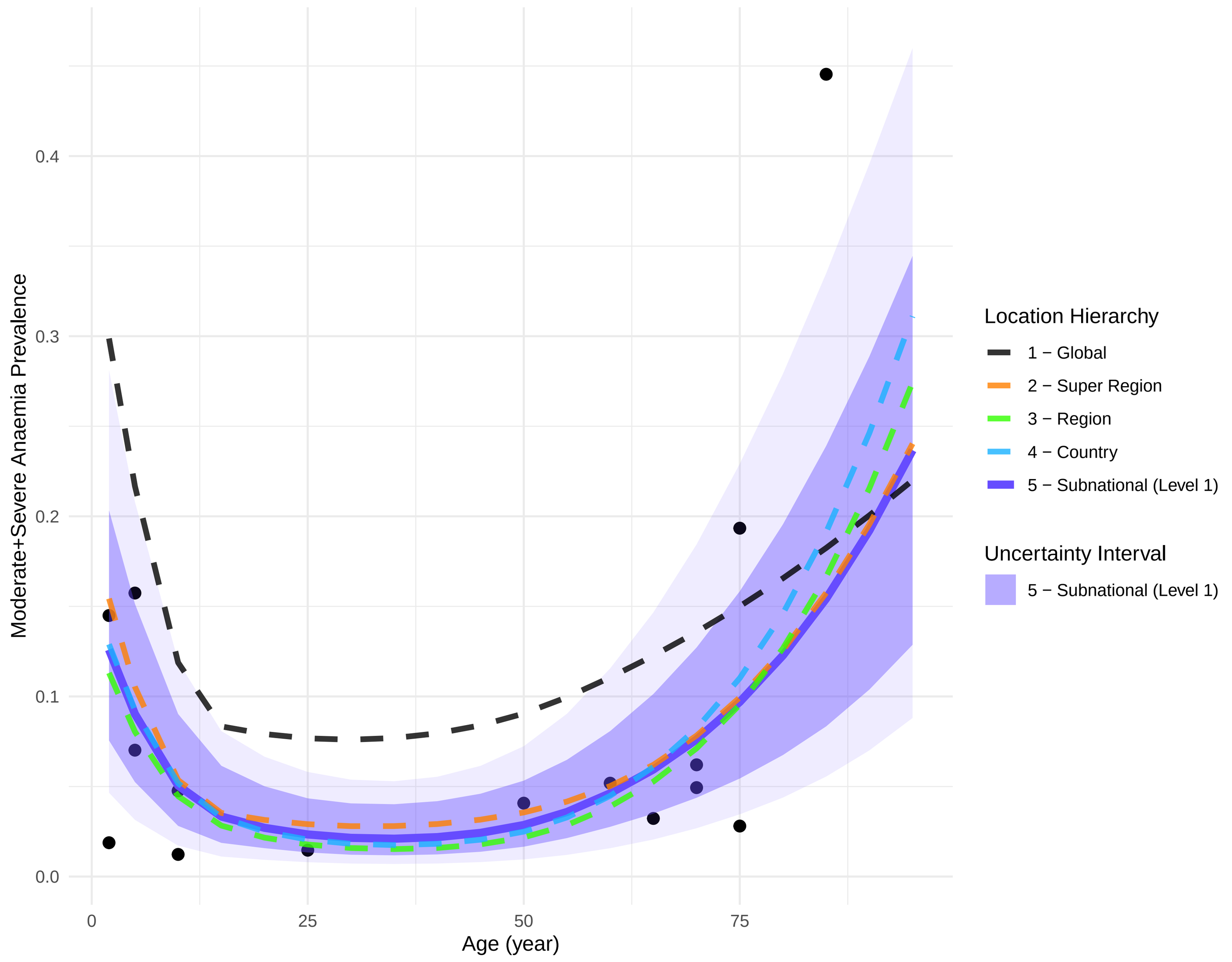

MR−BRT Age/Sex Fits − Males 2+ Years Old − Moderate+Severe Anaemia Prevalence − Tabasco
WHO−2024 Adjustment Method

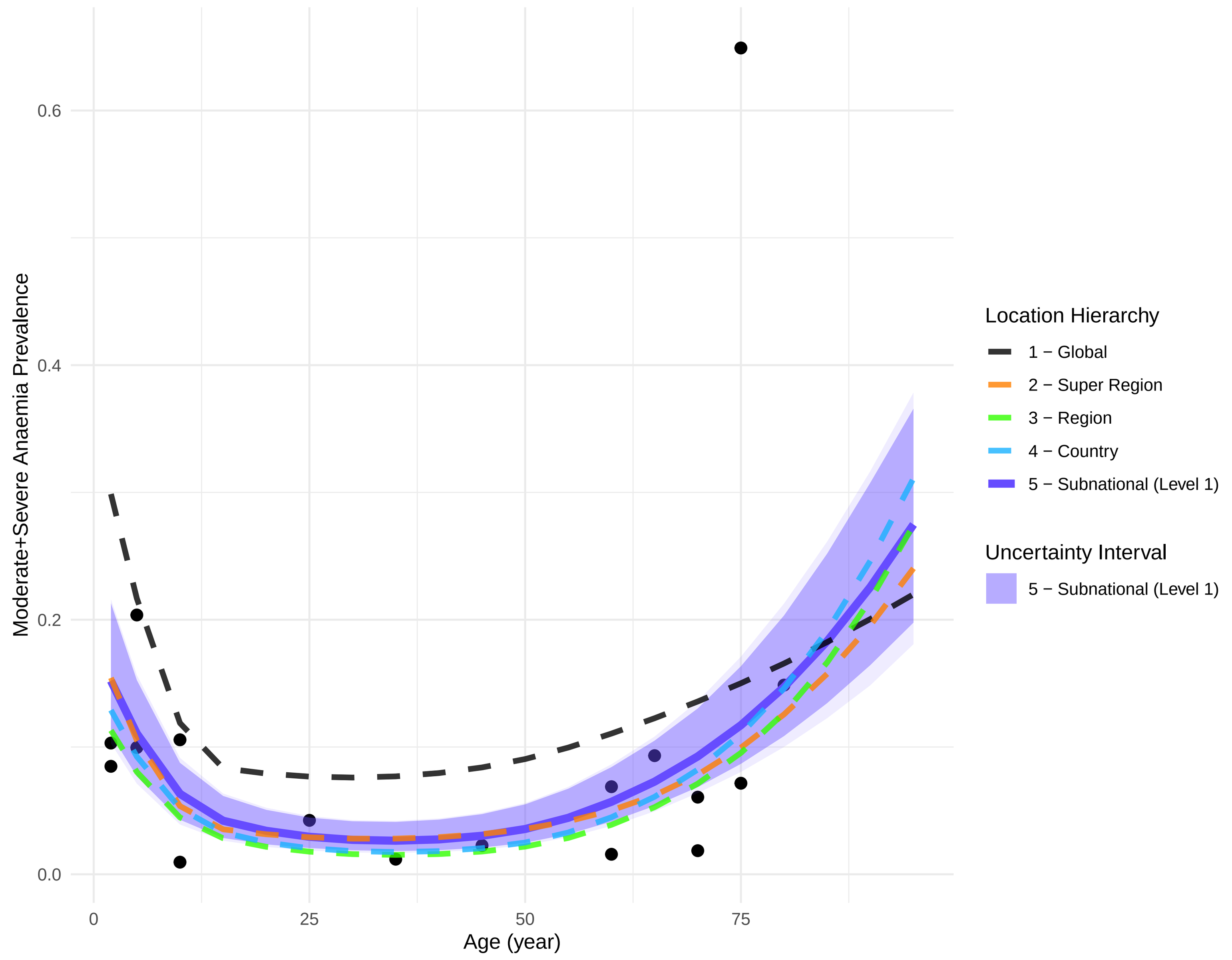

MR−BRT Age/Sex Fits − Males 2+ Years Old − Moderate+Severe Anaemia Prevalence − Tamaulipas
WHO−2024 Adjustment Method

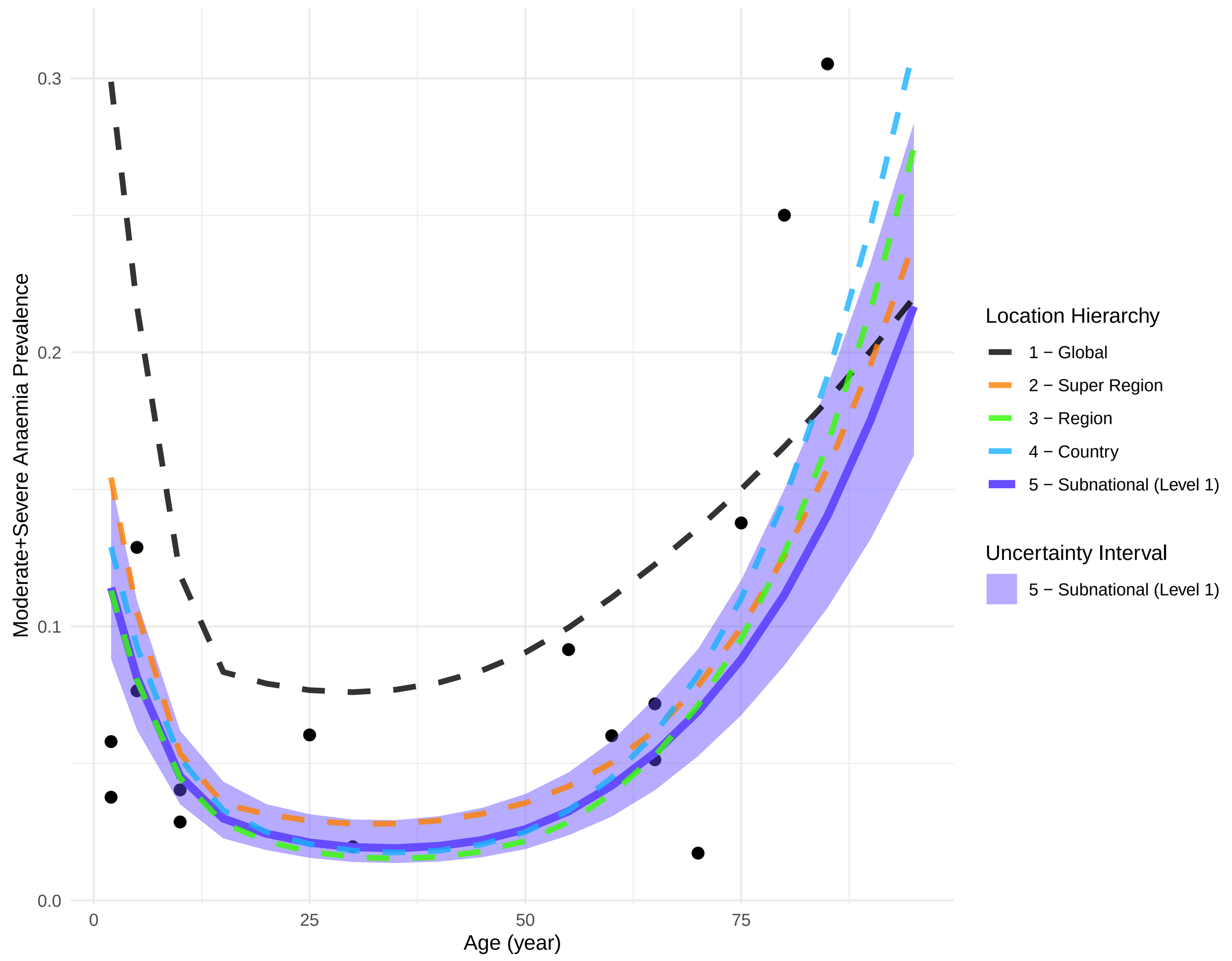

MR−BRT Age/Sex Fits − Males 2+ Years Old − Moderate+Severe Anaemia Prevalence − Tlaxcala
WHO−2024 Adjustment Method

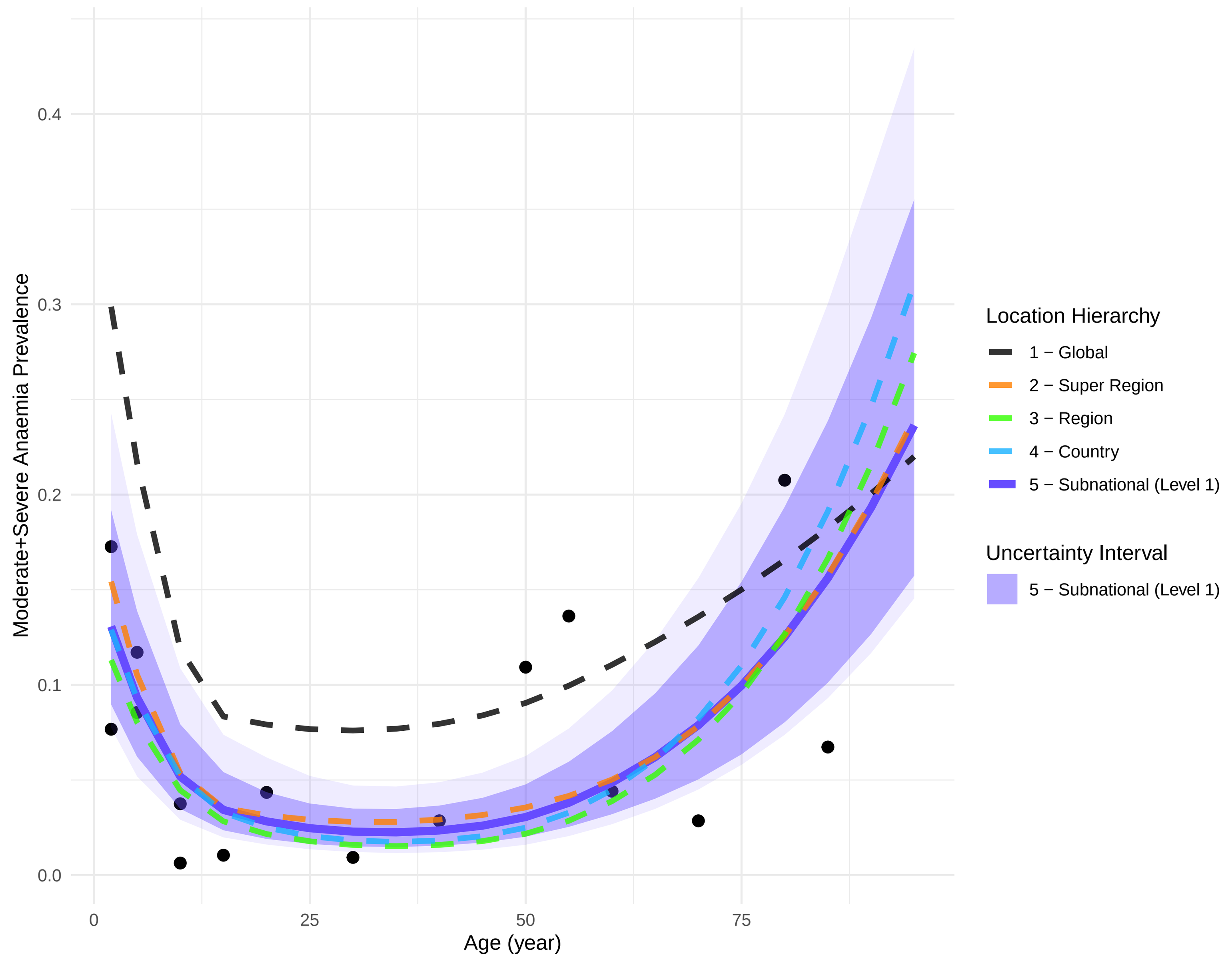

MR−BRT Age/Sex Fits − Males 2+ Years Old − Moderate+Severe Anaemia Prevalence − Veracruz de Ignacio de la Llave
WHO−2024 Adjustment Method

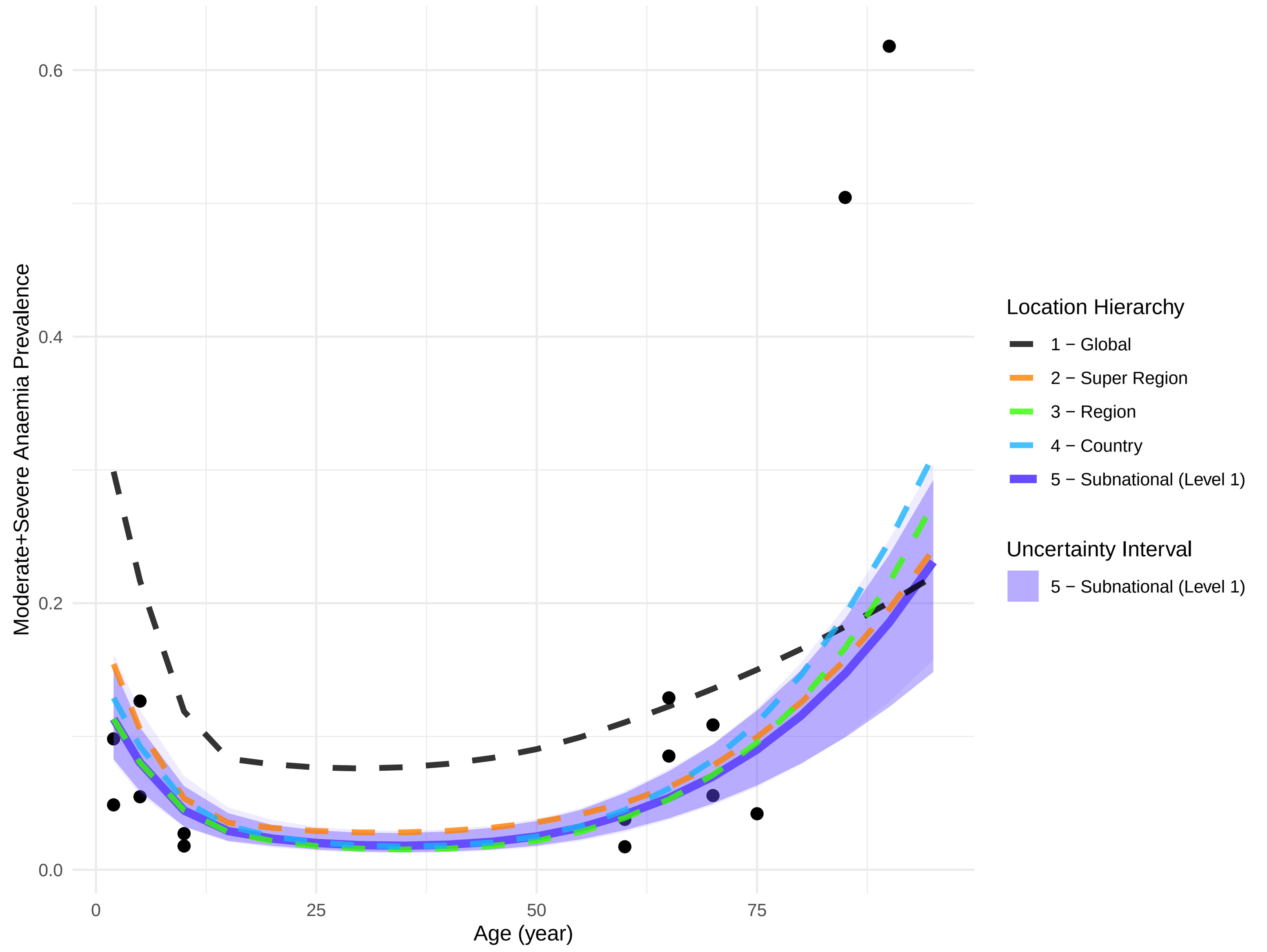

MR−BRT Age/Sex Fits − Males 2+ Years Old − Moderate+Severe Anaemia Prevalence − Yucatán
WHO−2024 Adjustment Method

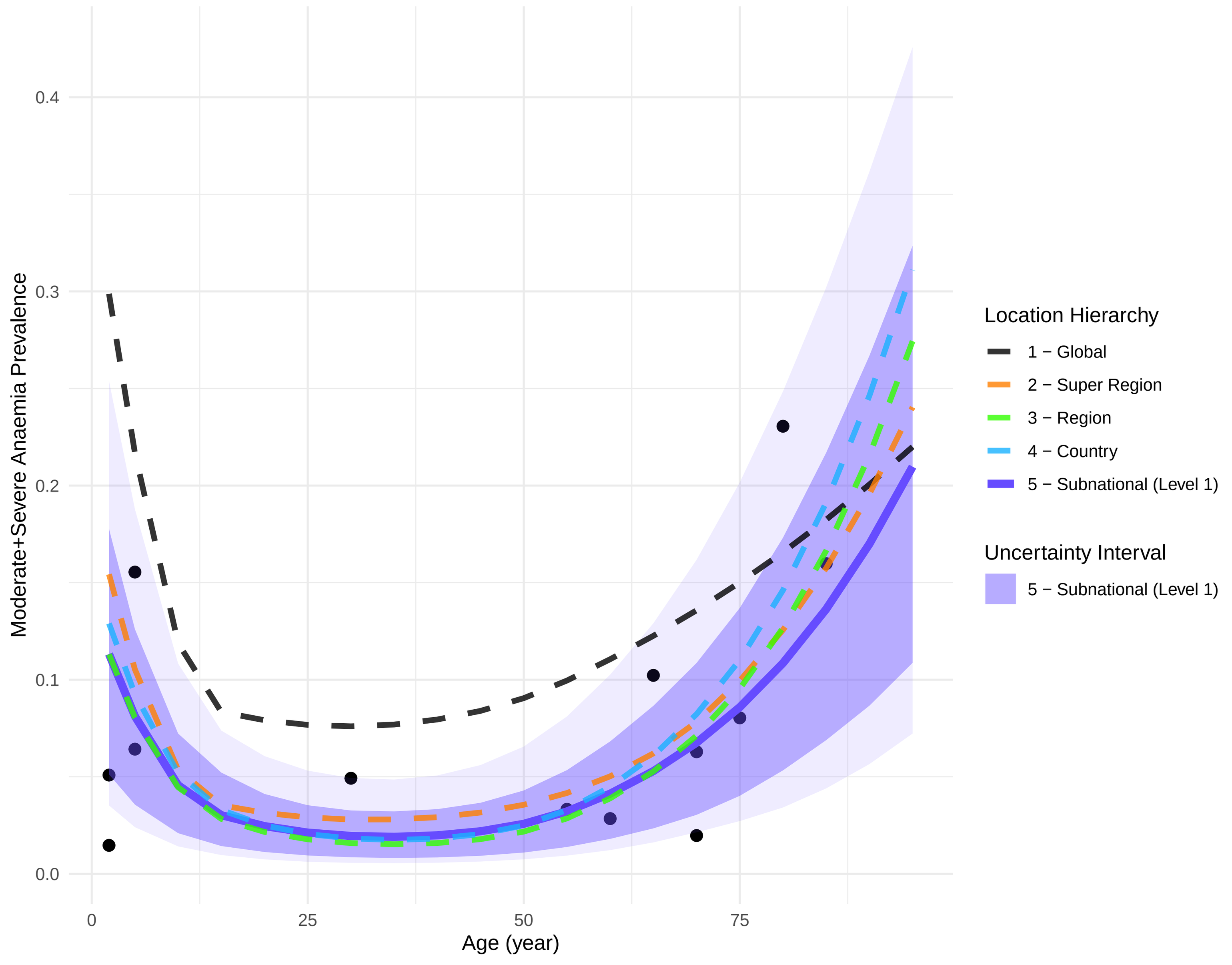

MR−BRT Age/Sex Fits − Males 2+ Years Old − Moderate+Severe Anaemia Prevalence − Zacatecas
WHO−2024 Adjustment Method

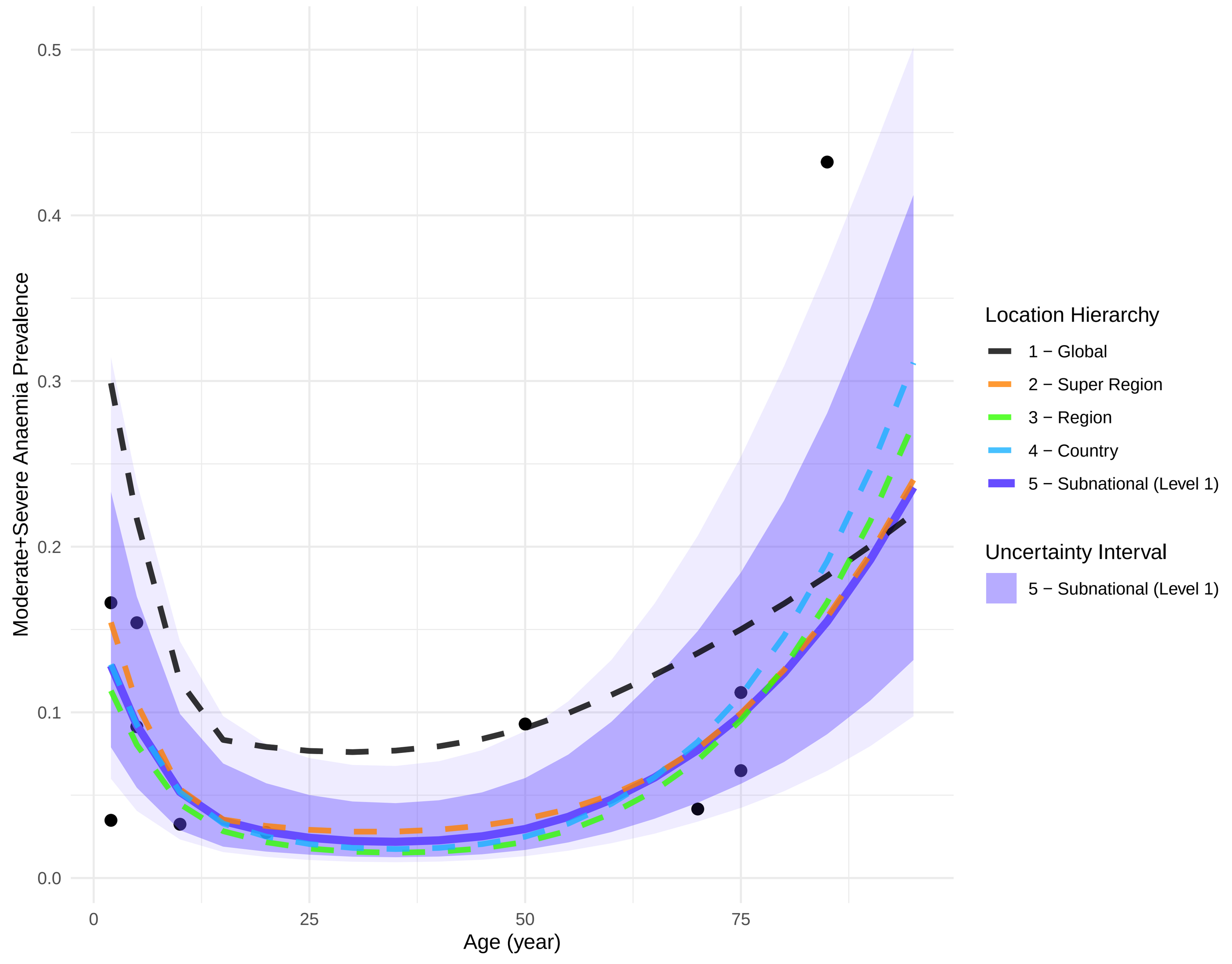

MR−BRT Age/Sex Fits − Males 2+ Years Old − Moderate+Severe Anaemia Prevalence − Tropical Latin America
WHO−2024 Adjustment Method

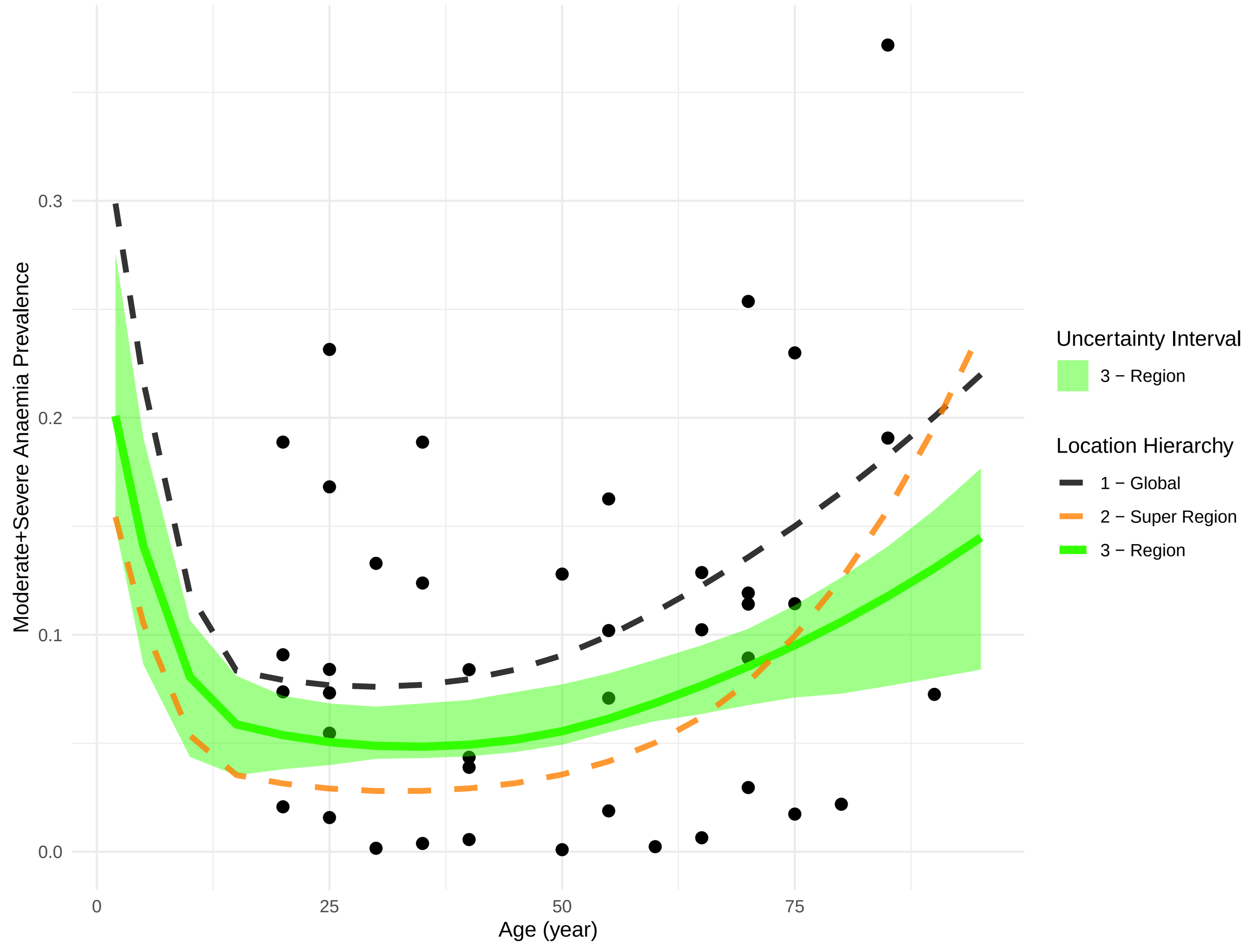

MR−BRT Age/Sex Fits − Males 2+ Years Old − Moderate+Severe Anaemia Prevalence − Brazil
WHO−2024 Adjustment Method

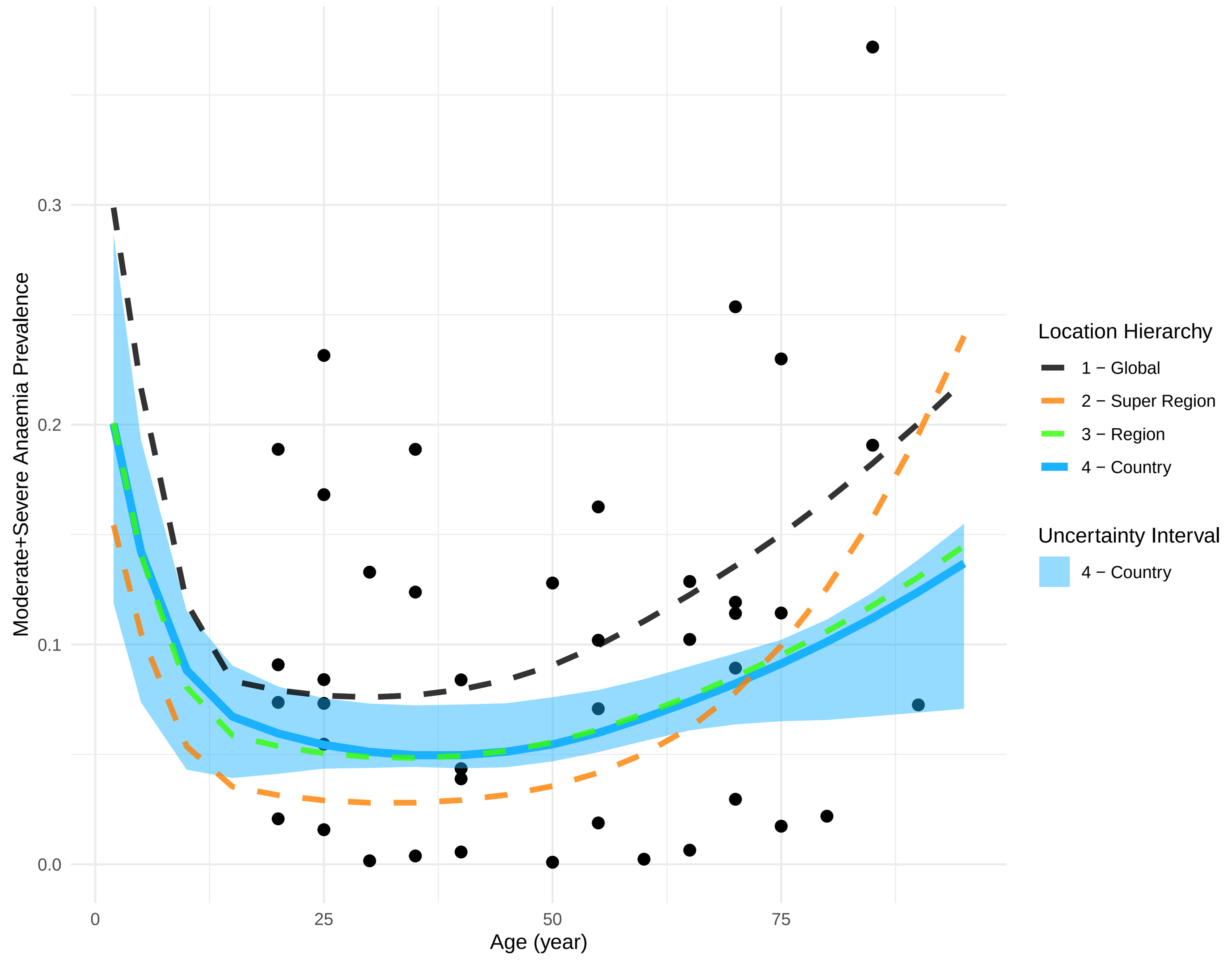

MR−BRT Age/Sex Fits − Males 2+ Years Old − Moderate+Severe Anaemia Prevalence − Acre
WHO−2024 Adjustment Method

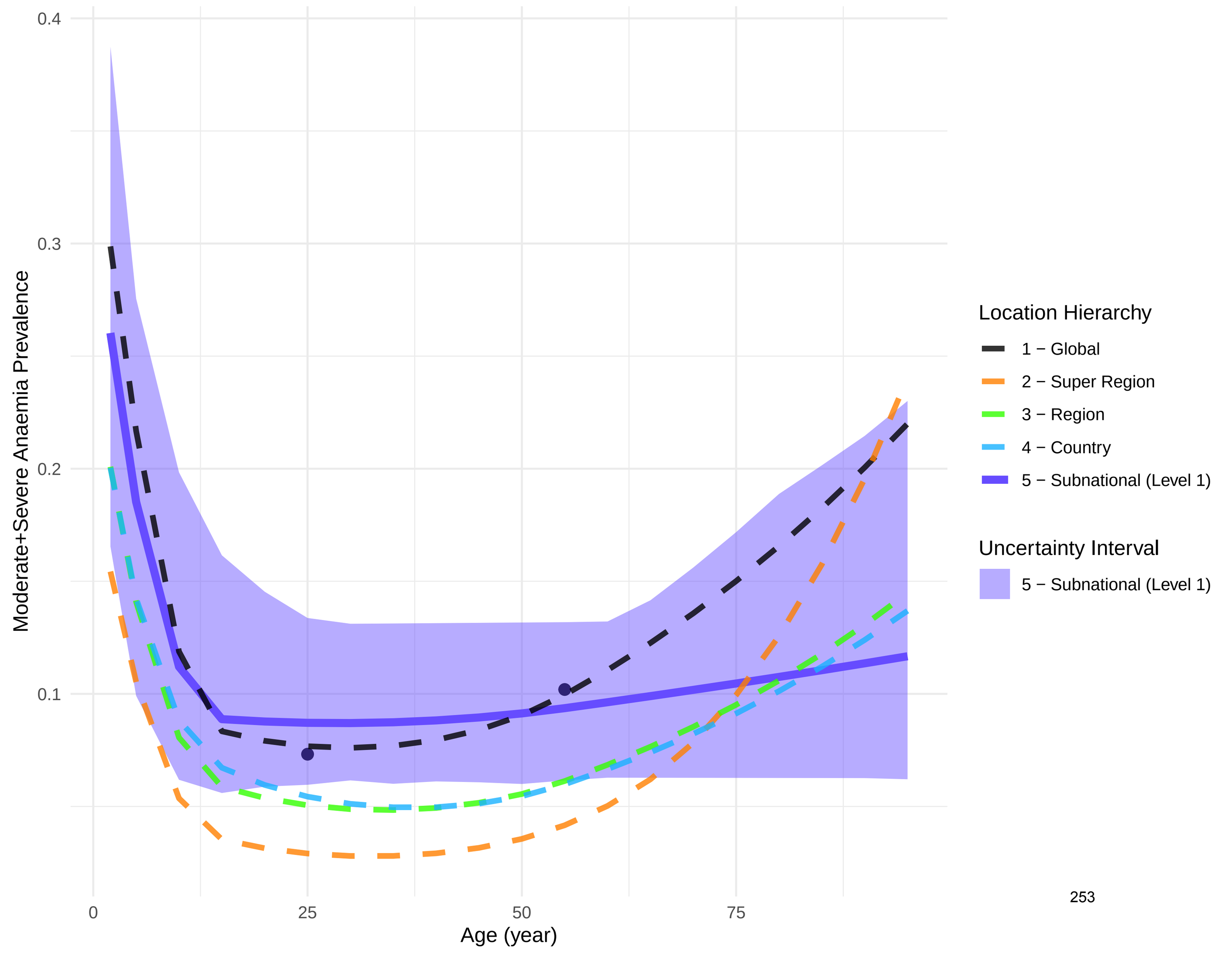

MR−BRT Age/Sex Fits − Males 2+ Years Old − Moderate+Severe Anaemia Prevalence − Alagoas
WHO−2024 Adjustment Method

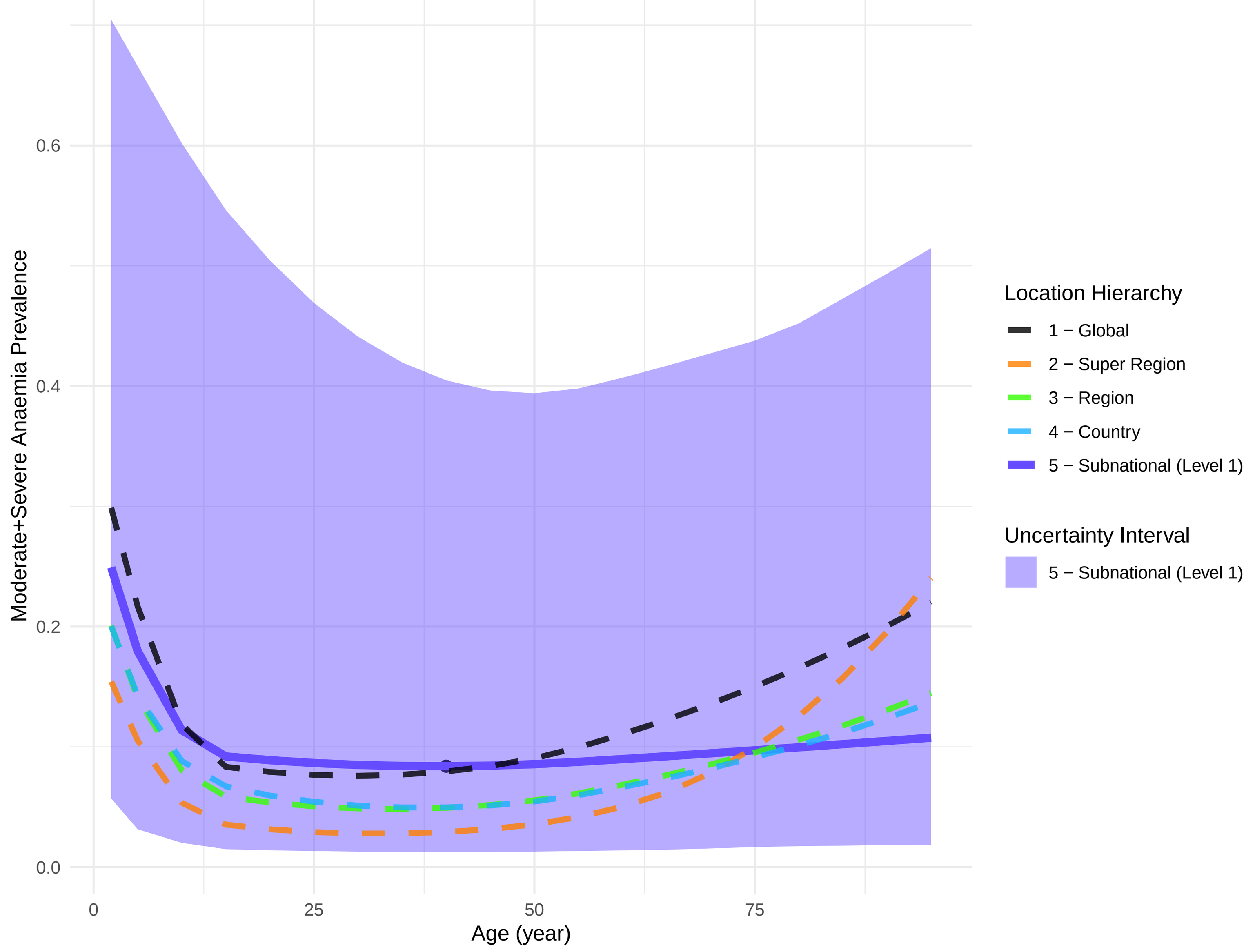

MR−BRT Age/Sex Fits − Males 2+ Years Old − Moderate+Severe Anaemia Prevalence − Amapá
WHO−2024 Adjustment Method

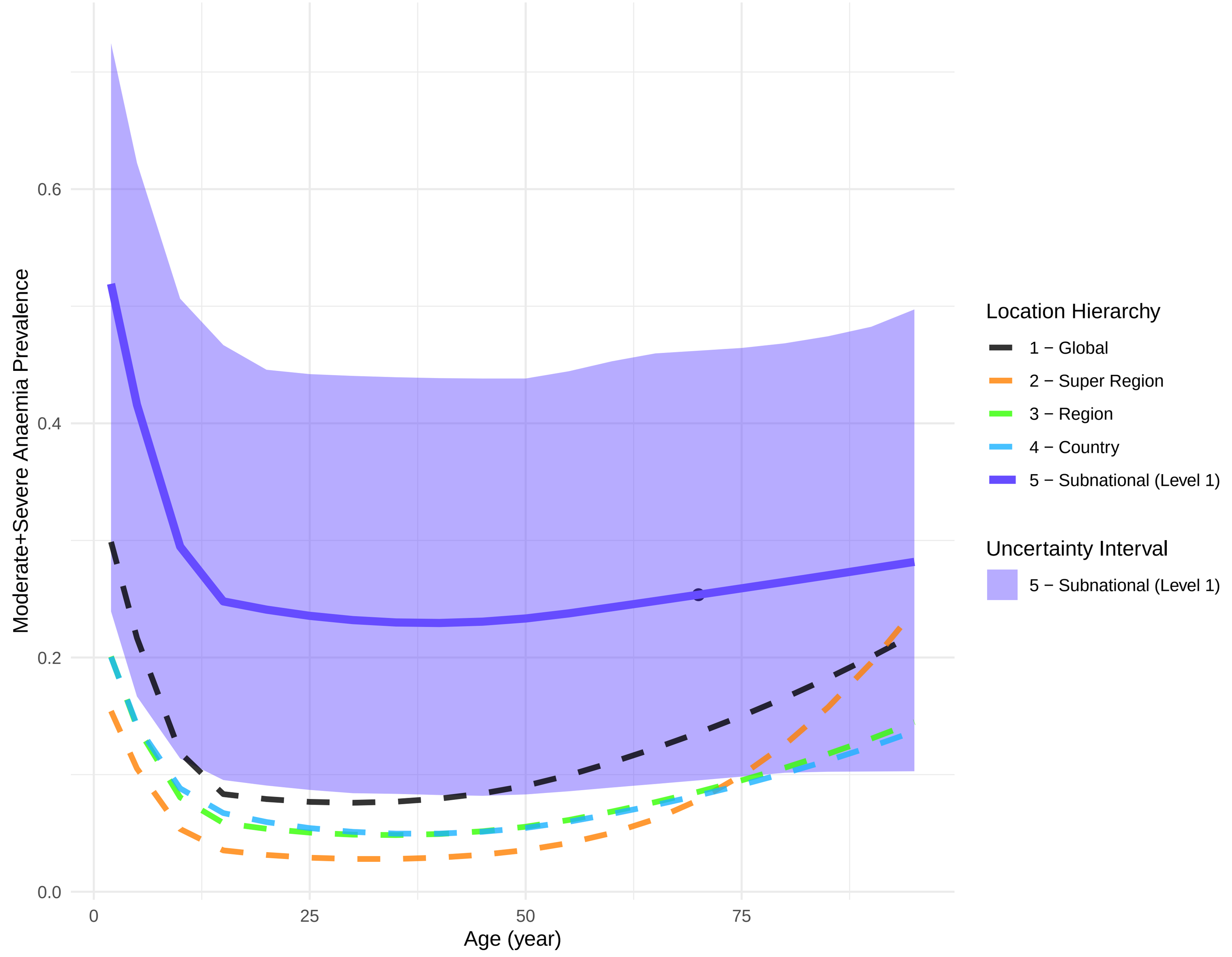

MR−BRT Age/Sex Fits − Males 2+ Years Old − Moderate+Severe Anaemia Prevalence − Amazonas
WHO−2024 Adjustment Method

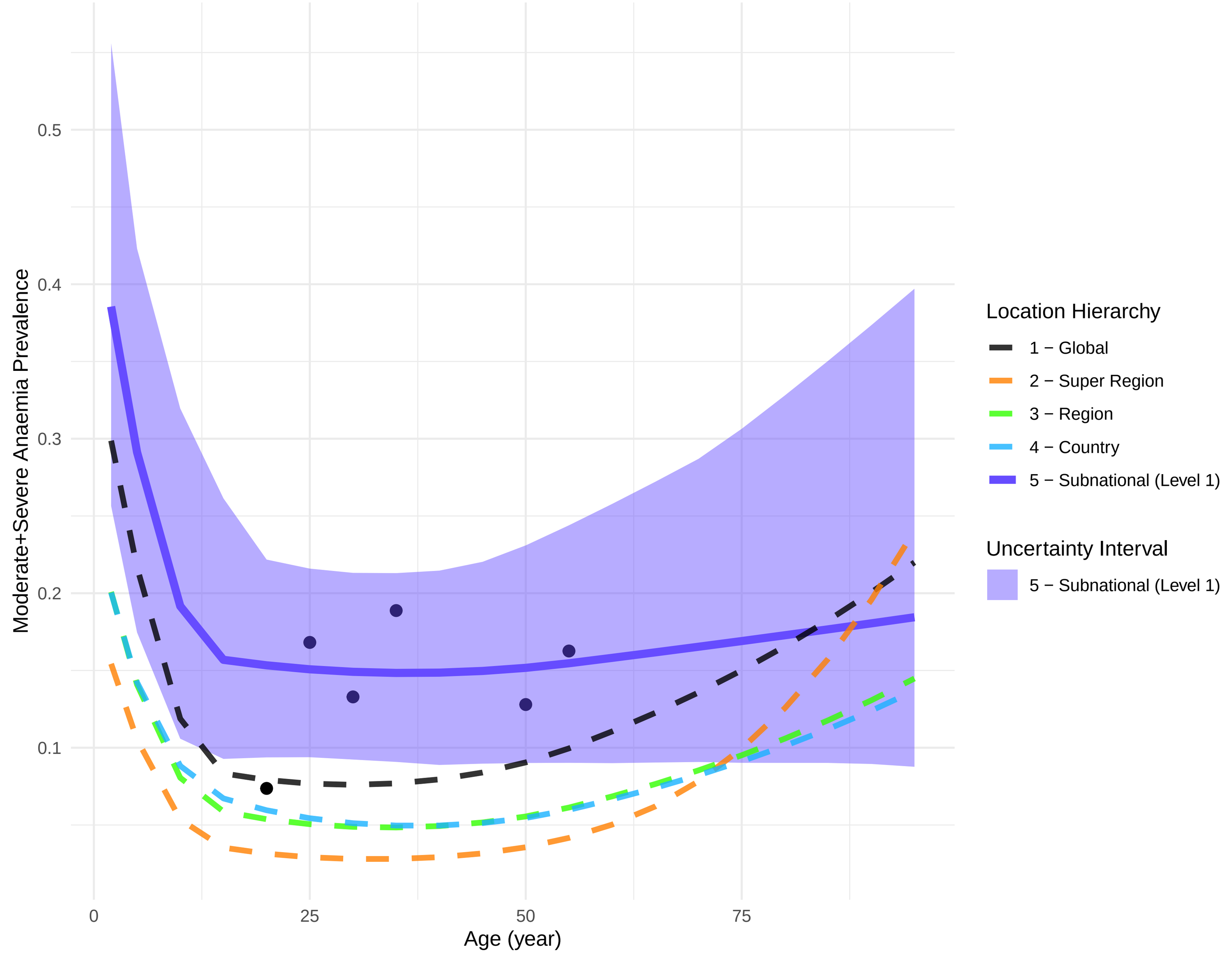

MR−BRT Age/Sex Fits − Males 2+ Years Old − Moderate+Severe Anaemia Prevalence − Maranhão
WHO−2024 Adjustment Method

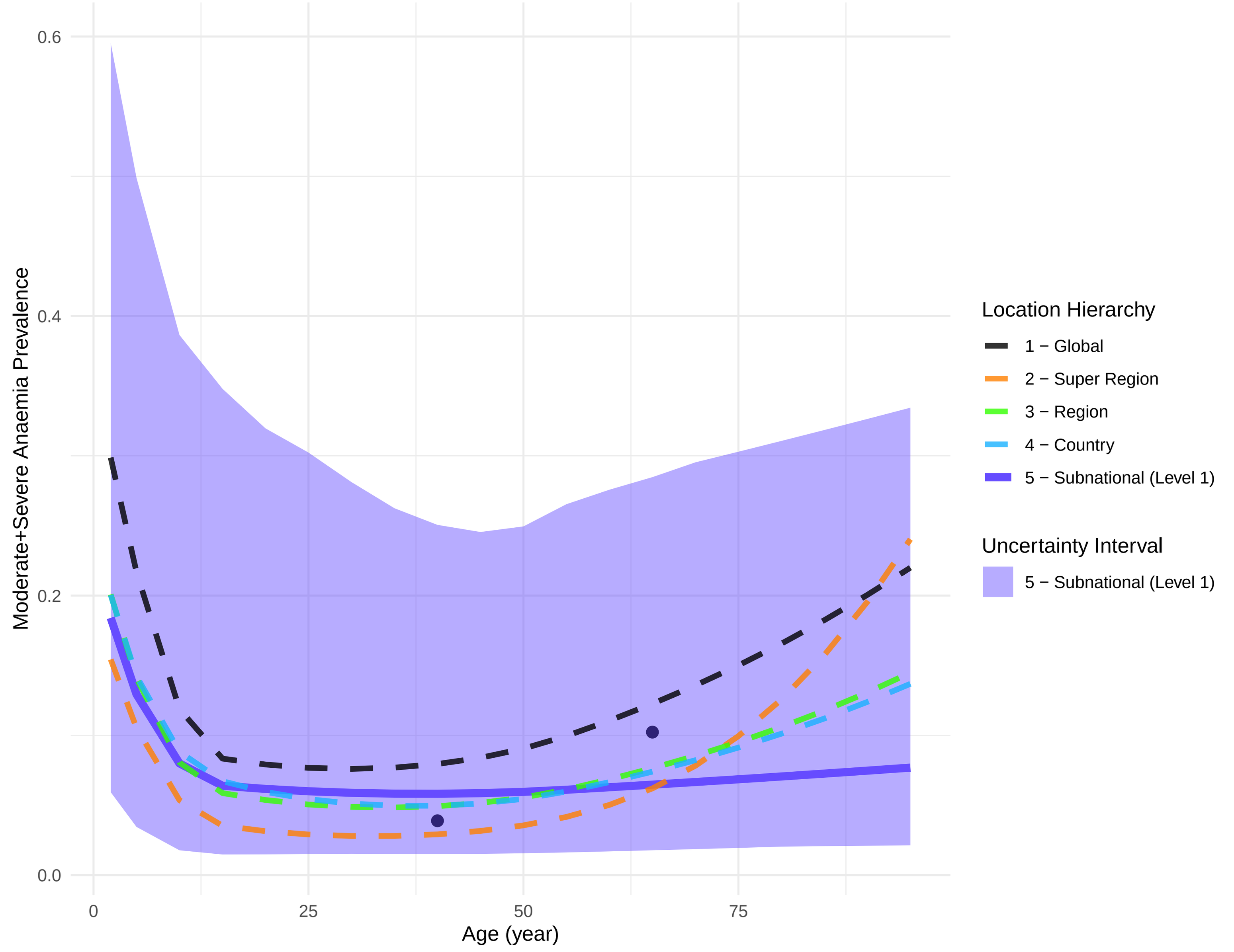

MR−BRT Age/Sex Fits − Males 2+ Years Old − Moderate+Severe Anaemia Prevalence − Minas Gerais
WHO−2024 Adjustment Method

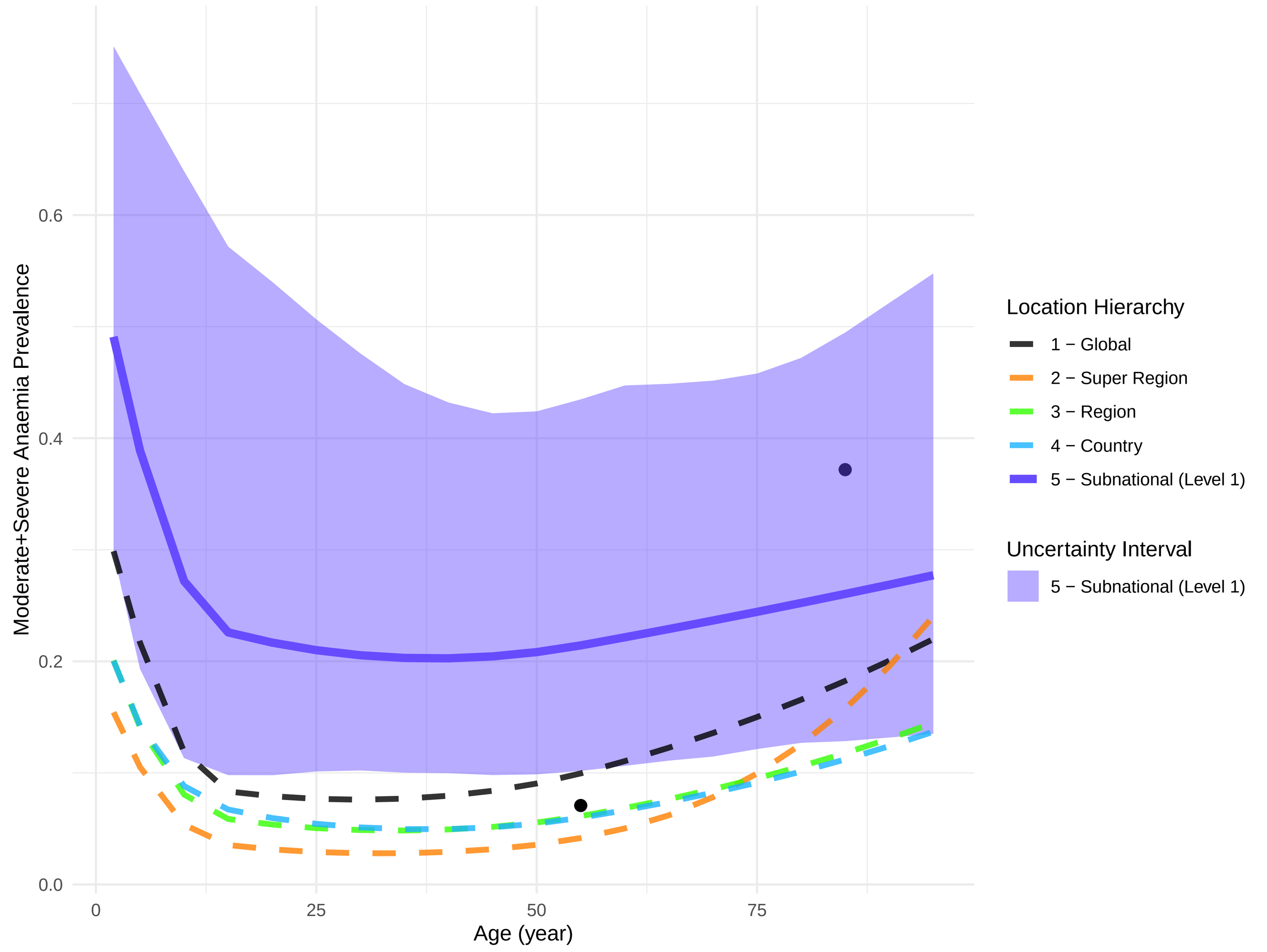

MR−BRT Age/Sex Fits − Males 2+ Years Old − Moderate+Severe Anaemia Prevalence − Paraíba
WHO−2024 Adjustment Method

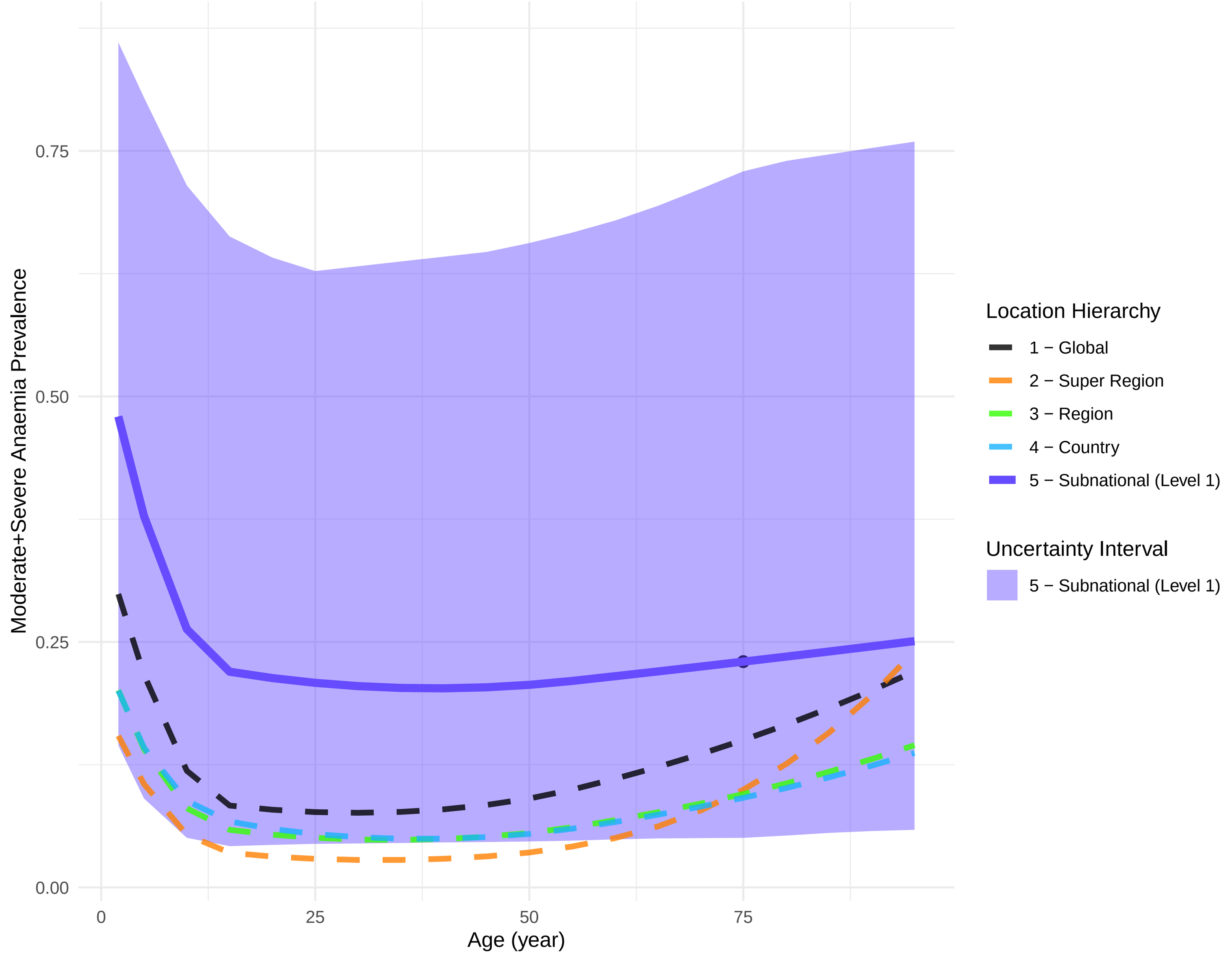

MR−BRT Age/Sex Fits − Males 2+ Years Old − Moderate+Severe Anaemia Prevalence − Paraná
WHO−2024 Adjustment Method

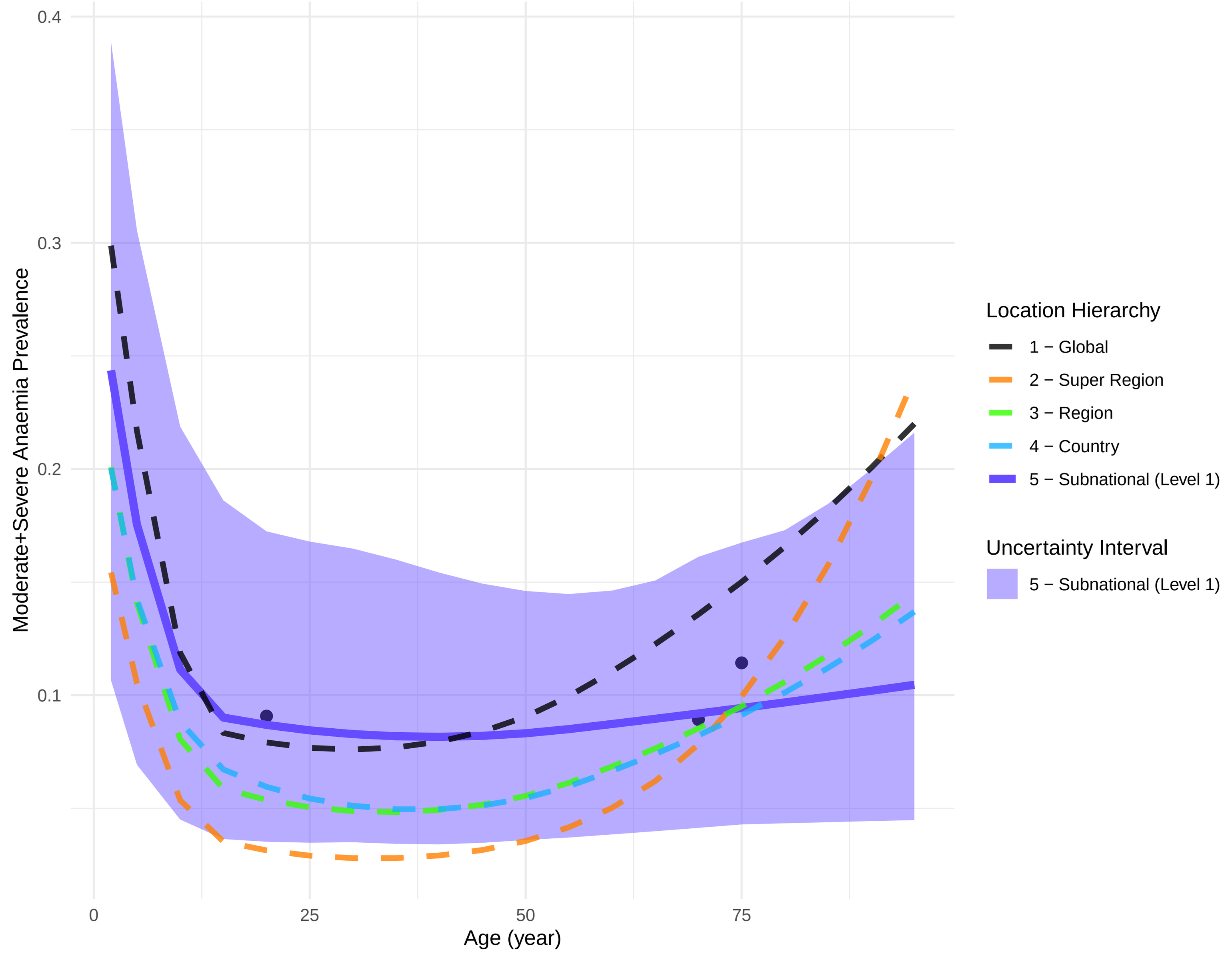

MR−BRT Age/Sex Fits − Males 2+ Years Old − Moderate+Severe Anaemia Prevalence − Piaui
WHO−2024 Adjustment Method

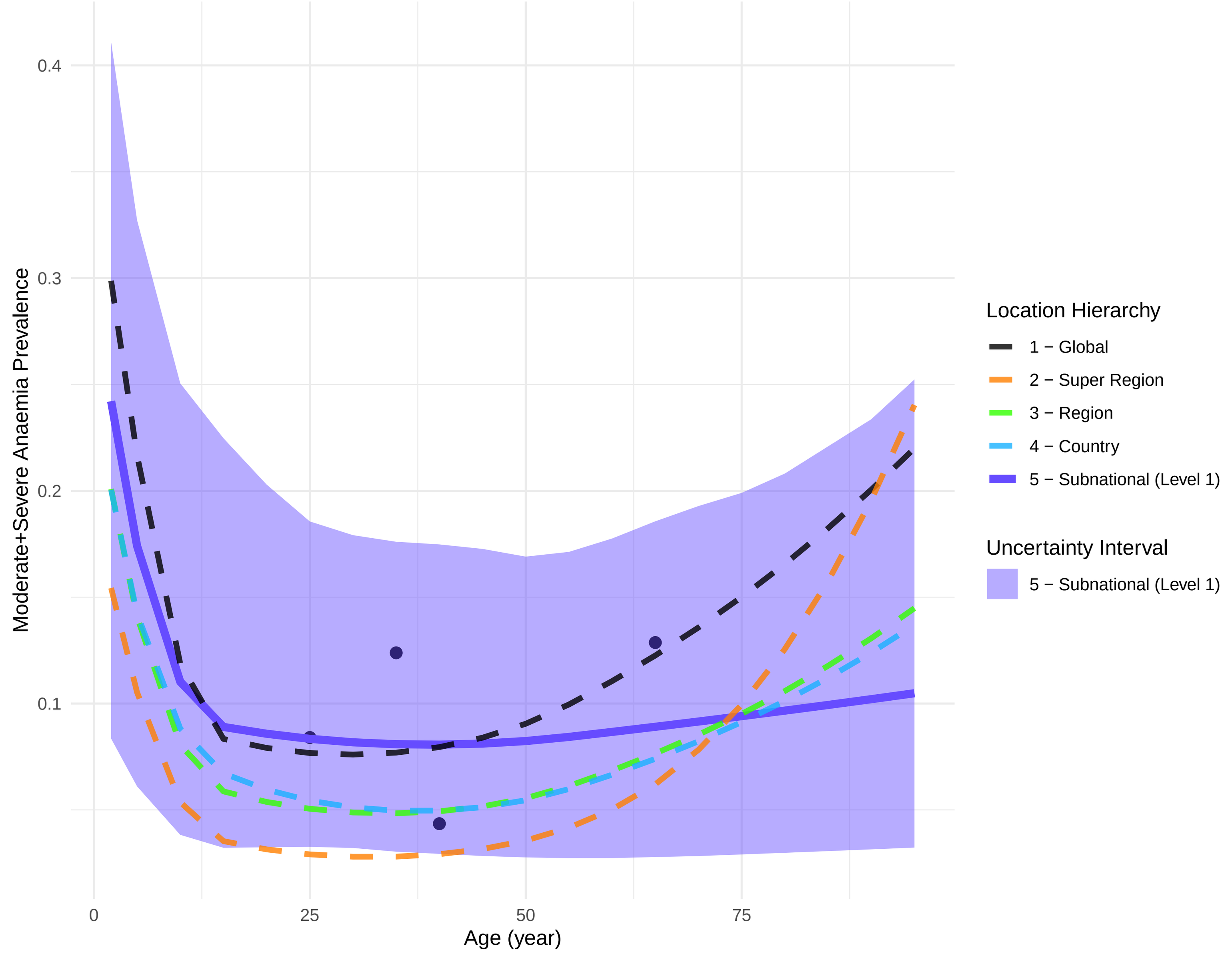

MR−BRT Age/Sex Fits − Males 2+ Years Old − Moderate+Severe Anaemia Prevalence − Rio de Janeiro
WHO−2024 Adjustment Method

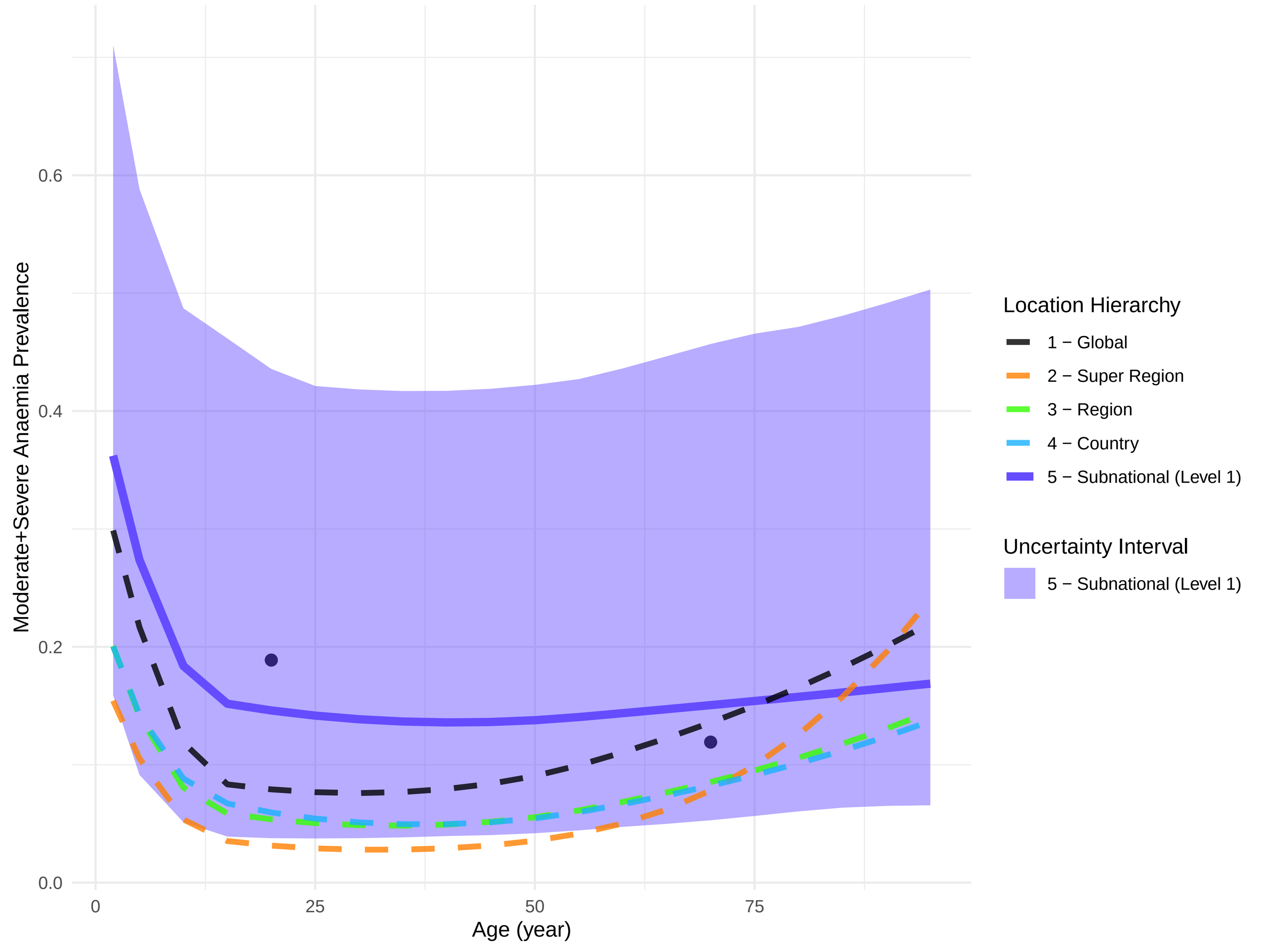

MR−BRT Age/Sex Fits − Males 2+ Years Old − Moderate+Severe Anaemia Prevalence − Rondônia
WHO−2024 Adjustment Method

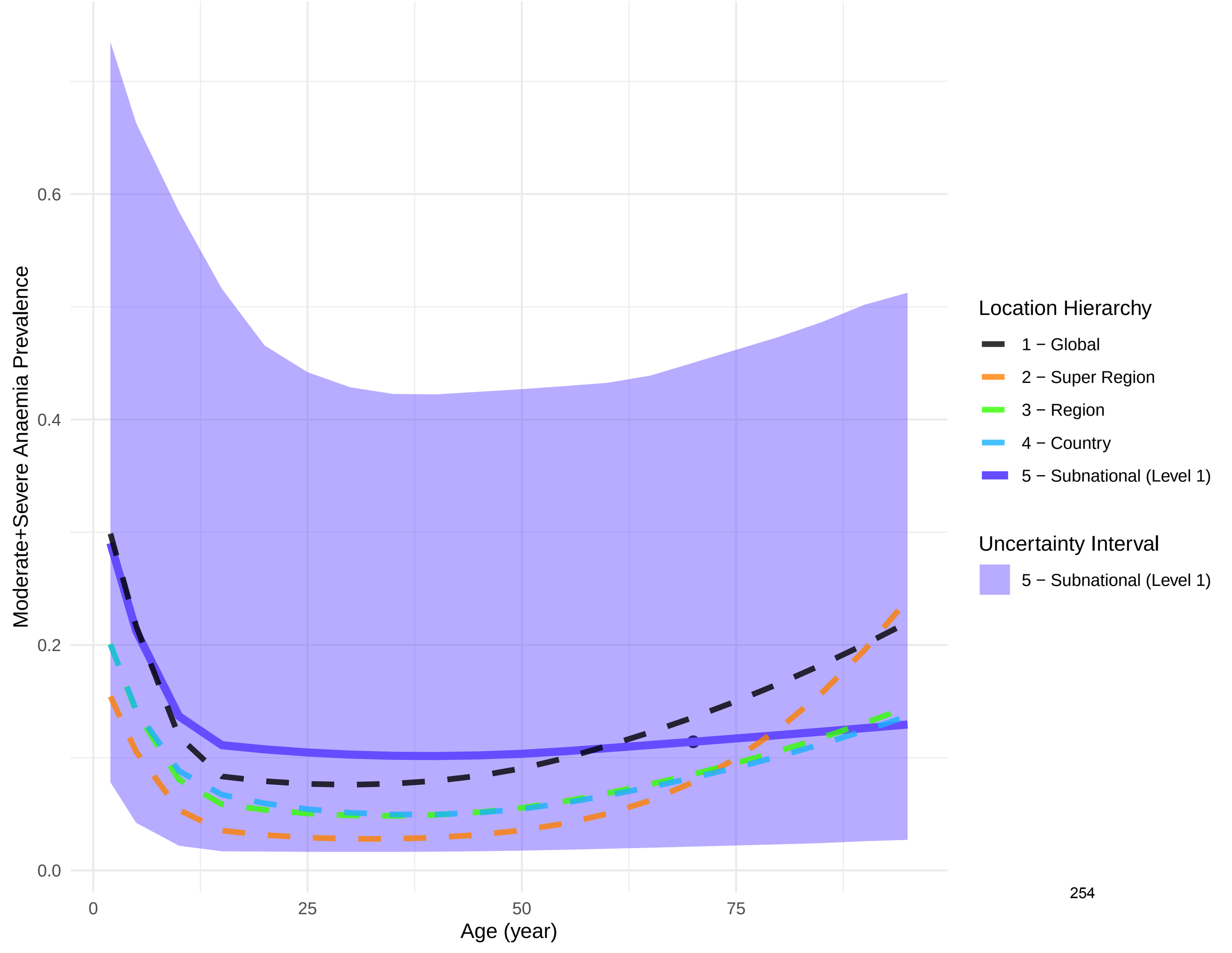

MR−BRT Age/Sex Fits − Males 2+ Years Old − Moderate+Severe Anaemia Prevalence − Roraima
WHO−2024 Adjustment Method

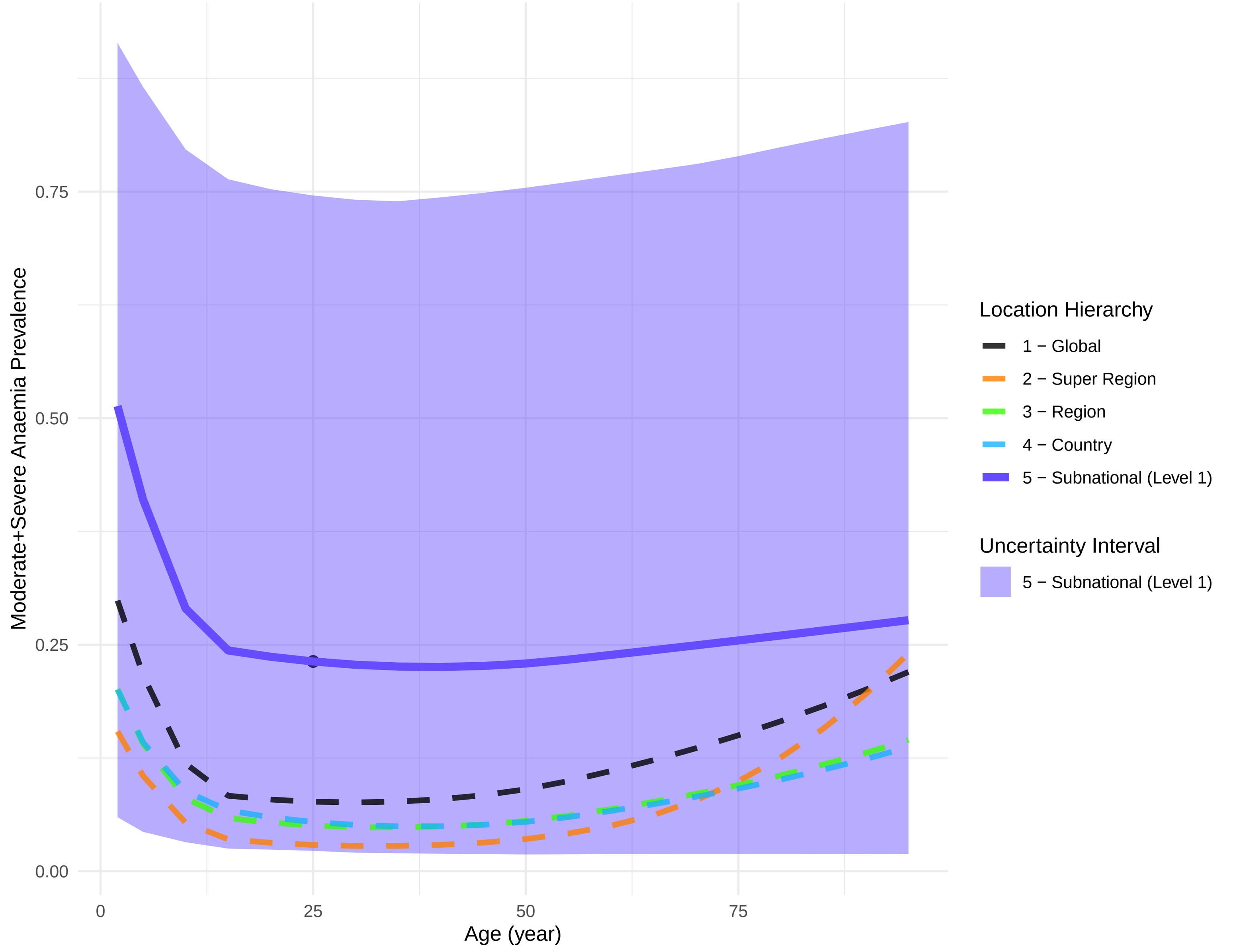

MR−BRT Age/Sex Fits − Males 2+ Years Old − Moderate+Severe Anaemia Prevalence − São Paulo
WHO−2024 Adjustment Method

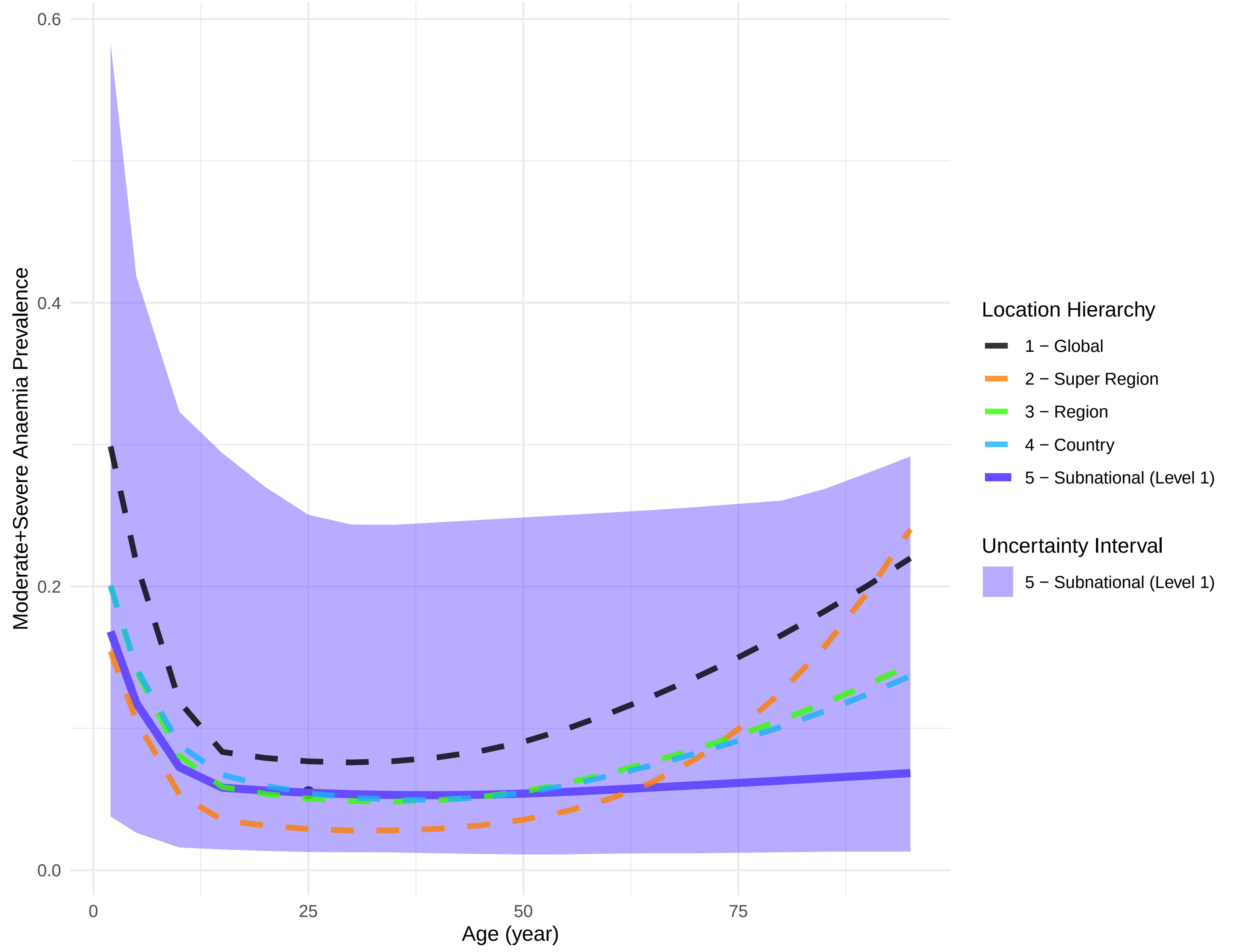

MR−BRT Age/Sex Fits − Males 2+ Years Old − Moderate+Severe Anaemia Prevalence − North Africa and Middle East
WHO−2024 Adjustment Method

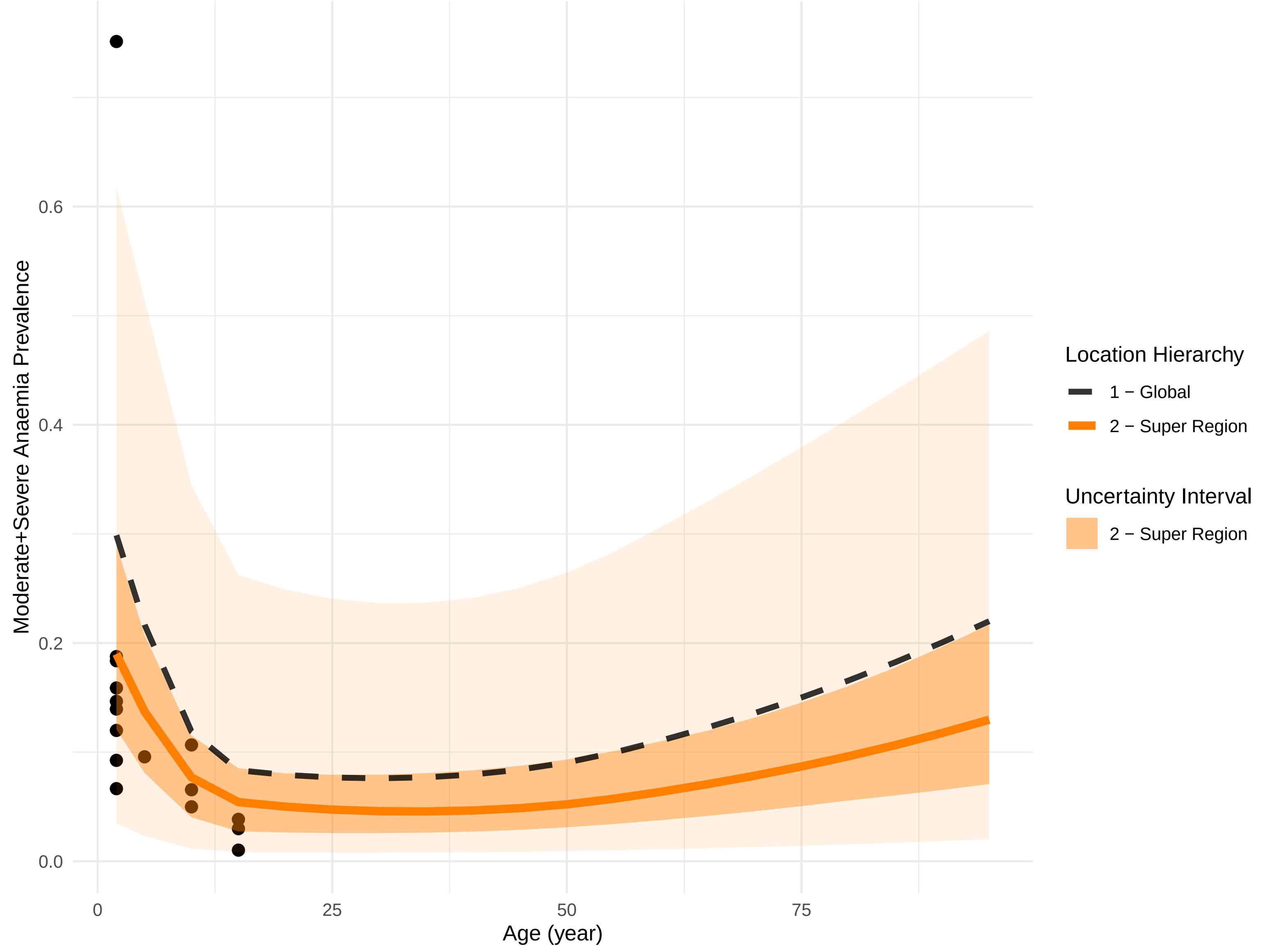

MR−BRT Age/Sex Fits − Males 2+ Years Old − Moderate+Severe Anaemia Prevalence − North Africa and Middle East
WHO−2024 Adjustment Method

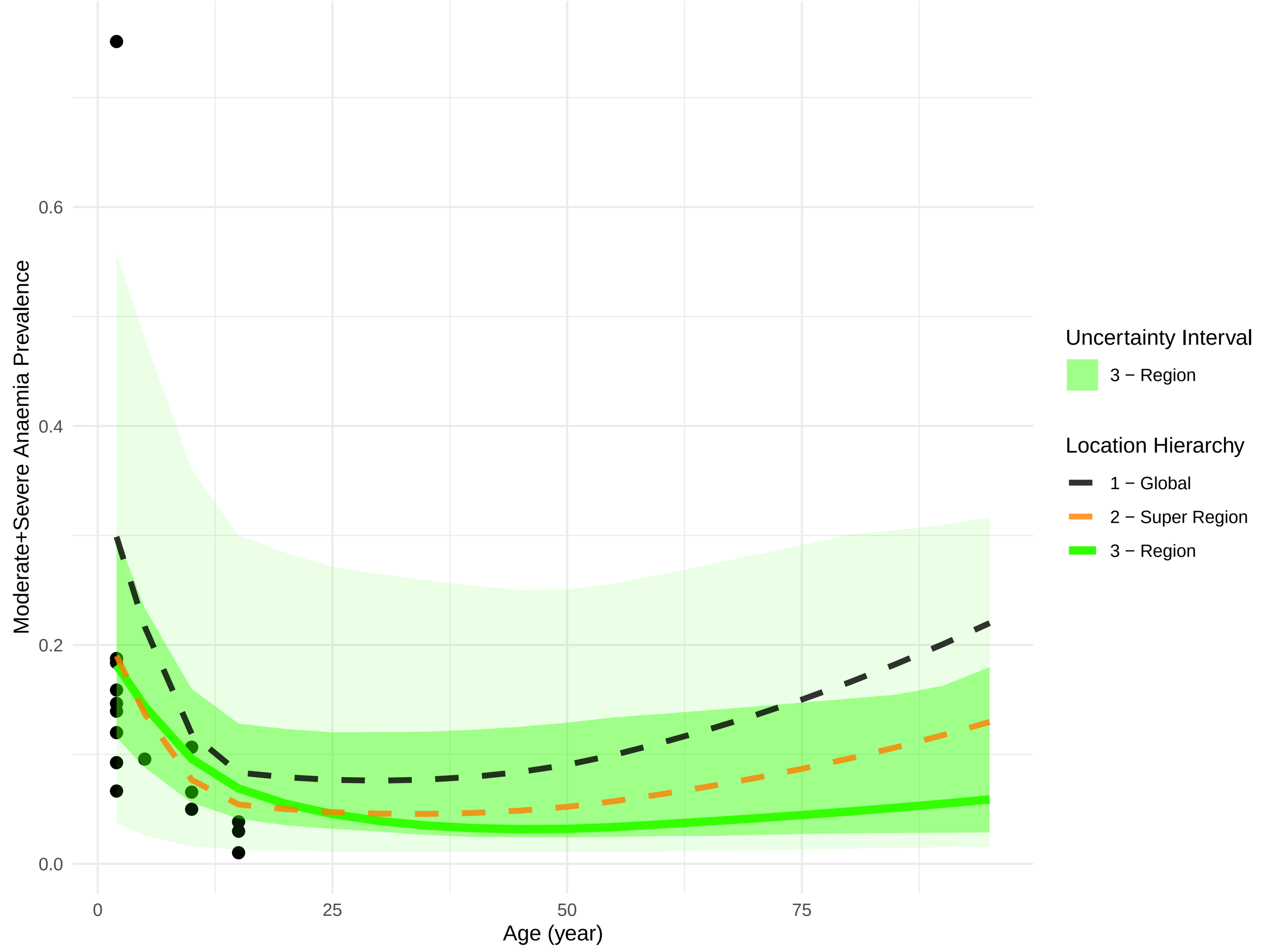

MR−BRT Age/Sex Fits − Males 2+ Years Old − Moderate+Severe Anaemia Prevalence − Egypt
WHO−2024 Adjustment Method

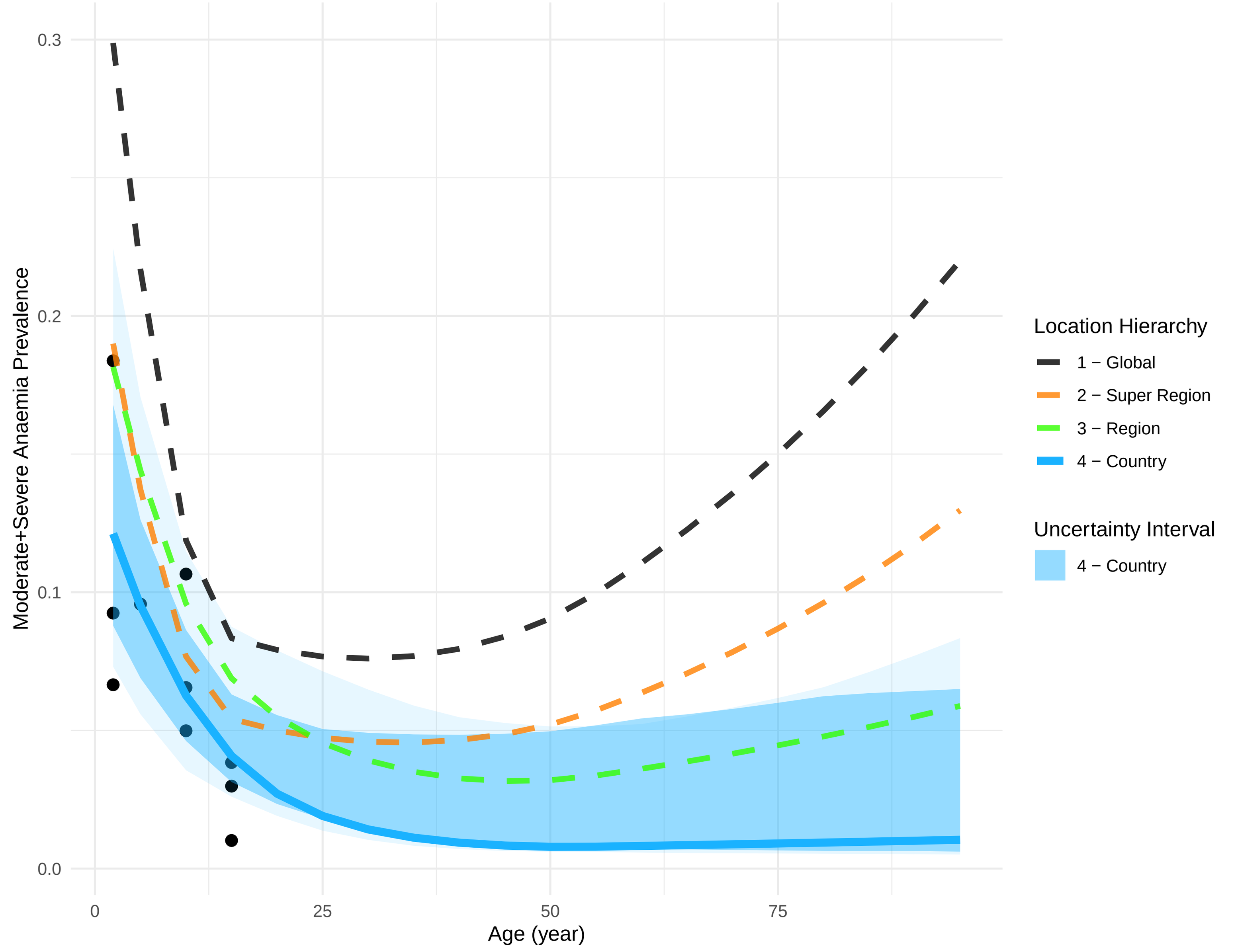

MR−BRT Age/Sex Fits − Males 2+ Years Old − Moderate+Severe Anaemia Prevalence − Jordan
WHO−2024 Adjustment Method

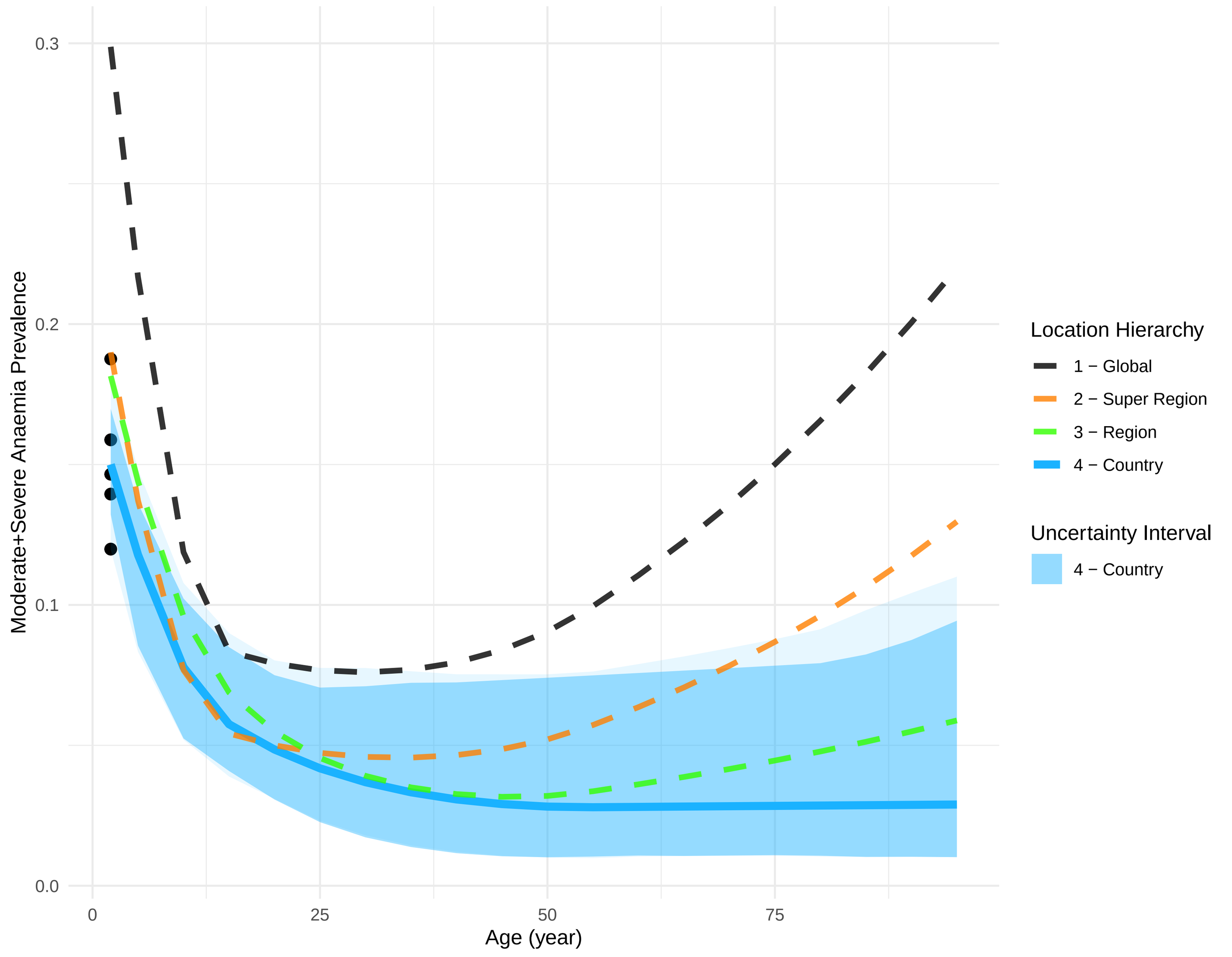

MR−BRT Age/Sex Fits − Males 2+ Years Old − Moderate+Severe Anaemia Prevalence − Yemen
WHO−2024 Adjustment Method

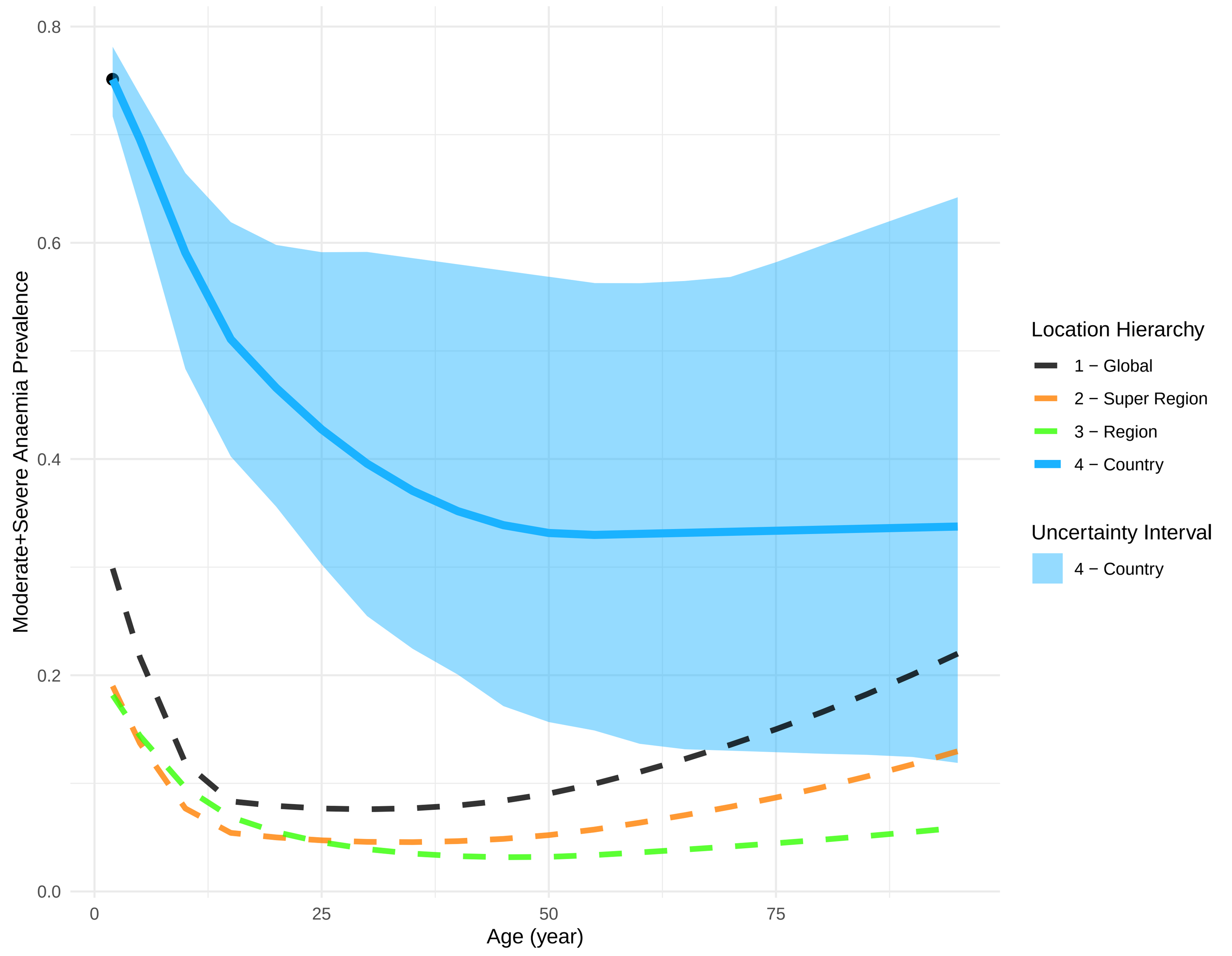

MR−BRT Age/Sex Fits − Males 2+ Years Old − Moderate+Severe Anaemia Prevalence − South Asia
WHO−2024 Adjustment Method

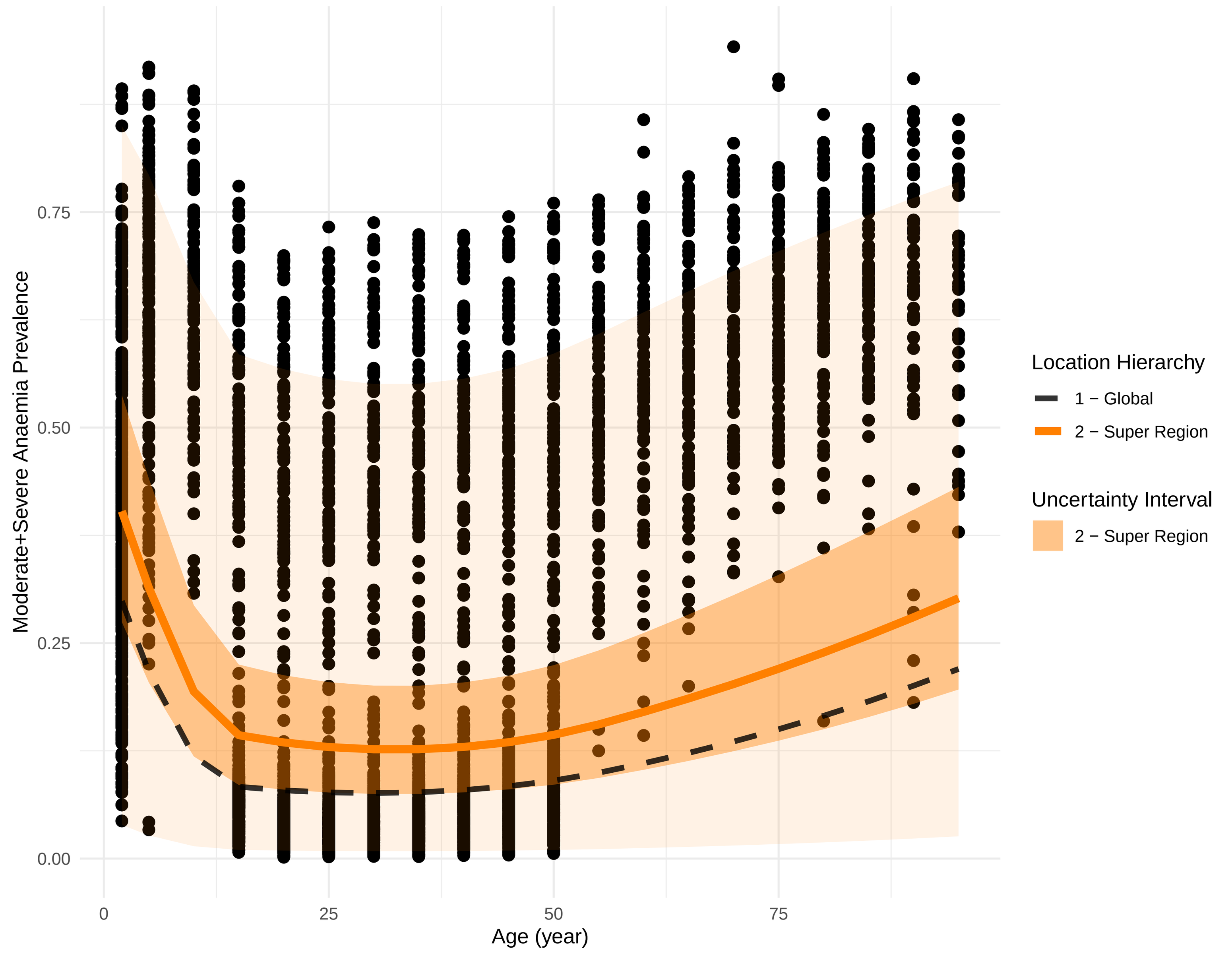

MR−BRT Age/Sex Fits − Males 2+ Years Old − Moderate+Severe Anaemia Prevalence − South Asia
WHO−2024 Adjustment Method

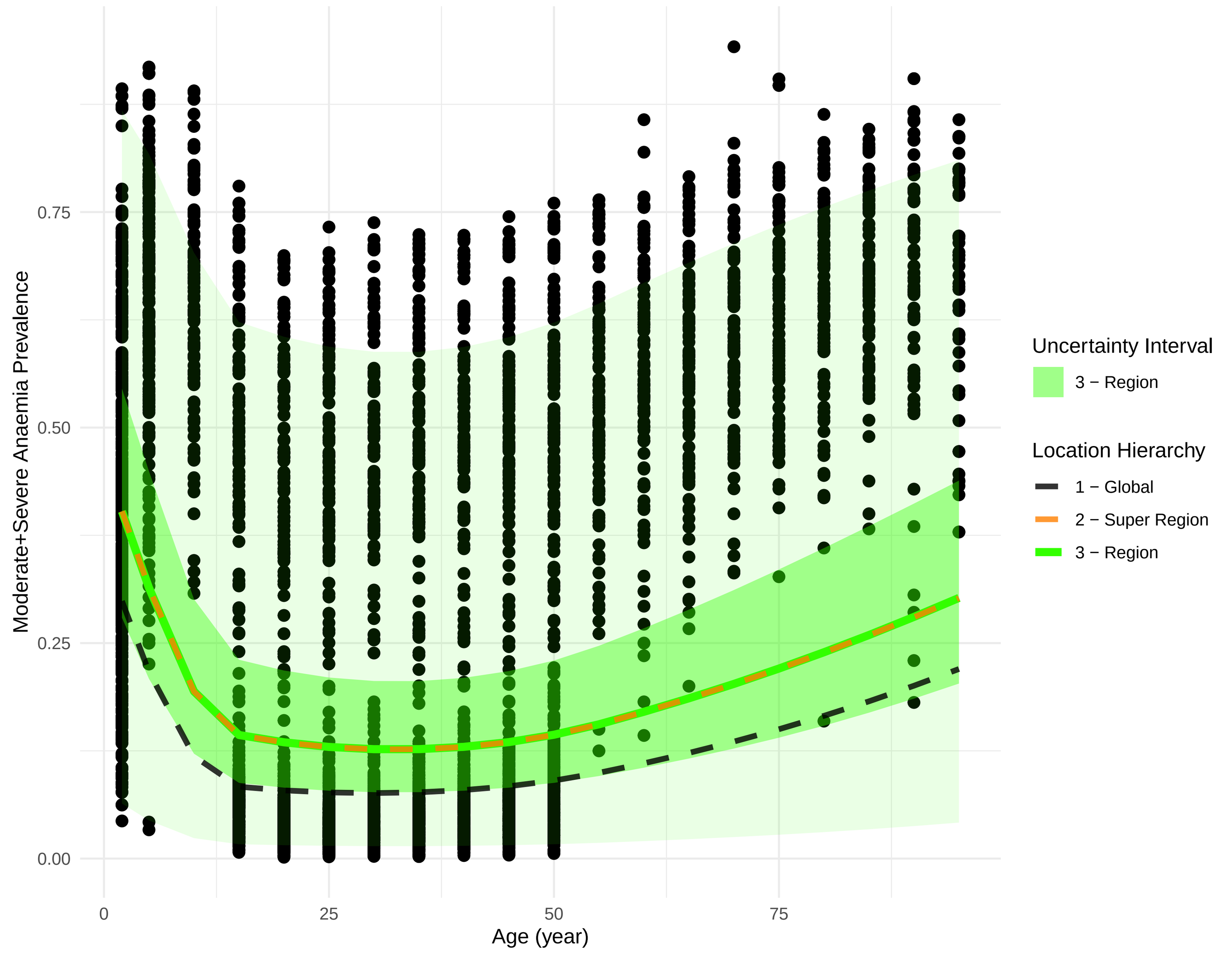

MR−BRT Age/Sex Fits − Males 2+ Years Old − Moderate+Severe Anaemia Prevalence − Bangladesh
WHO−2024 Adjustment Method

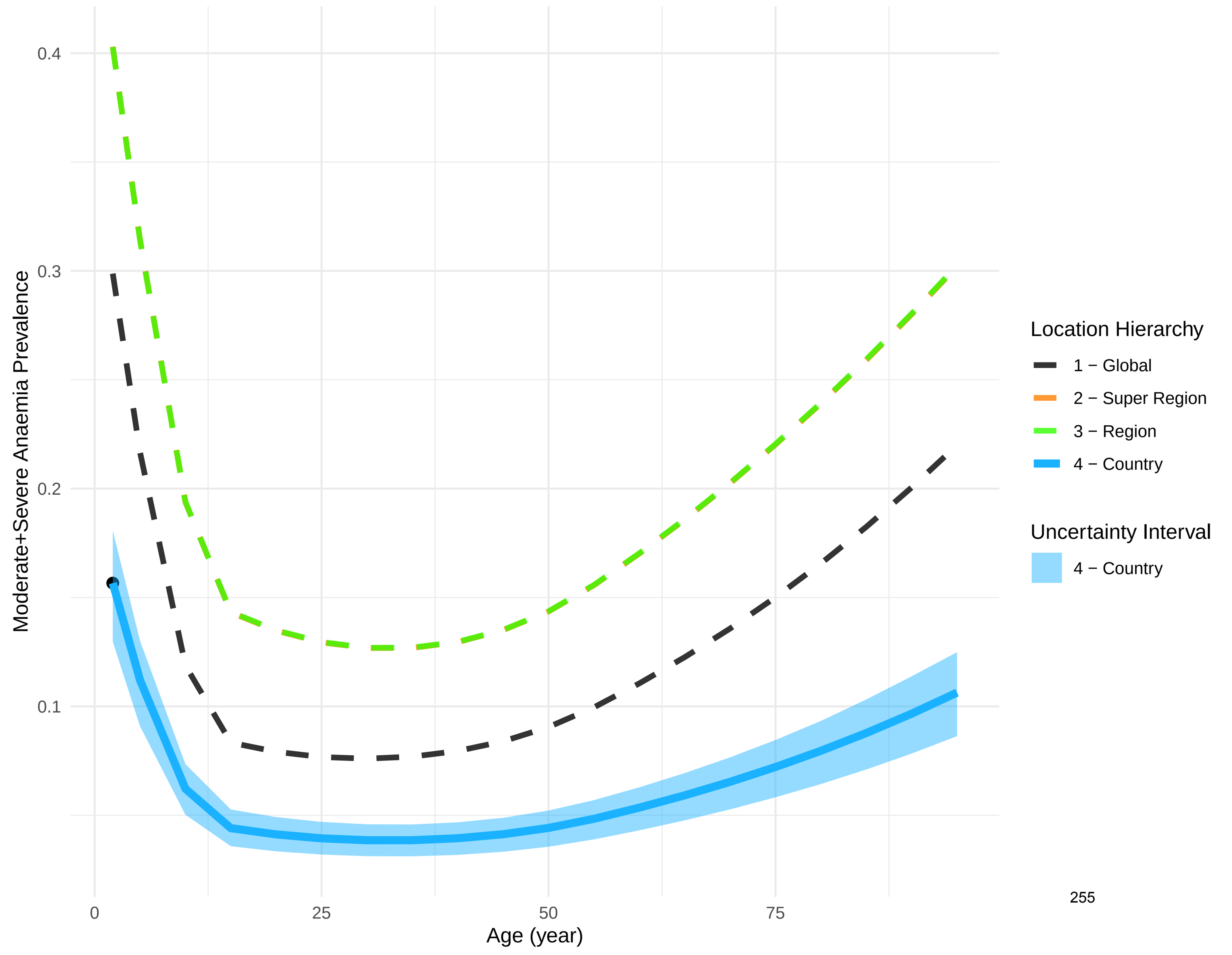

MR−BRT Age/Sex Fits – Males 2+ Years Old – Moderate+Severe Anaemia Prevalence – India
WHO−2024 Adjustment Method

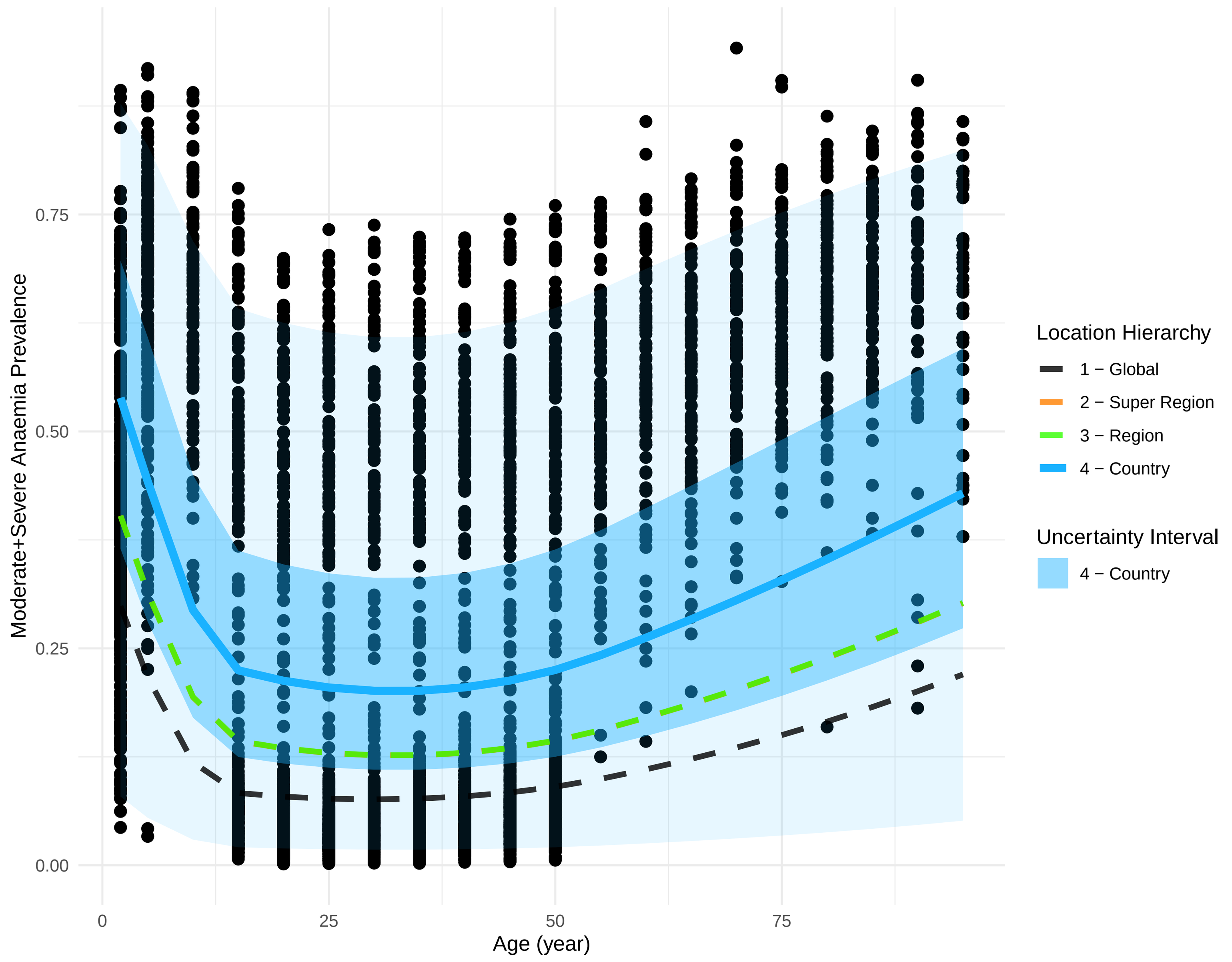

MR−BRT Age/Sex Fits – Males 2+ Years Old – Moderate+Severe Anaemia Prevalence – Andhra Pradesh
WHO−2024 Adjustment Method

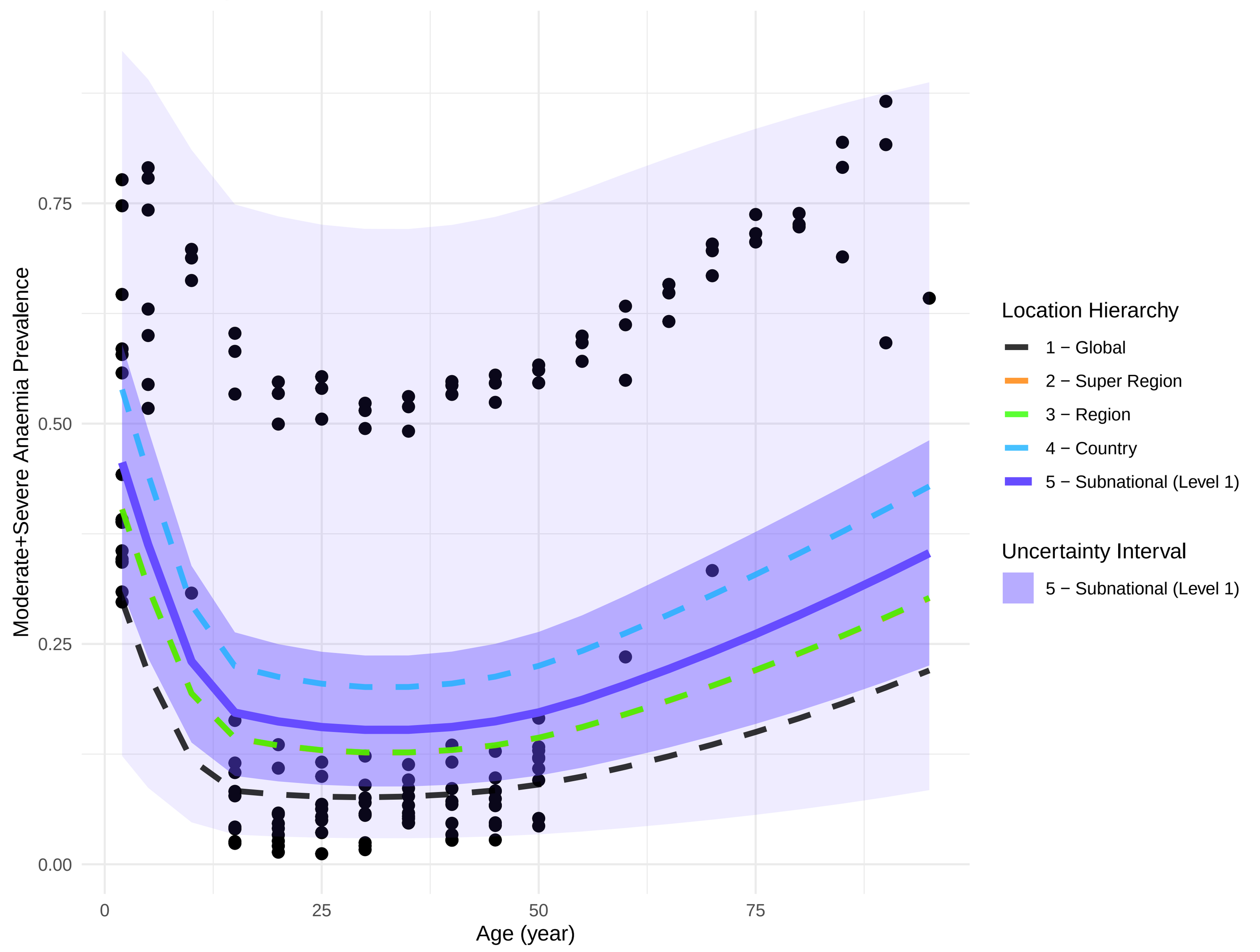

MR−BRT Age/Sex Fits – Males 2+ Years Old – Moderate+Severe Anaemia Prevalence – Andhra Pradesh, Rural
WHO−2024 Adjustment Method

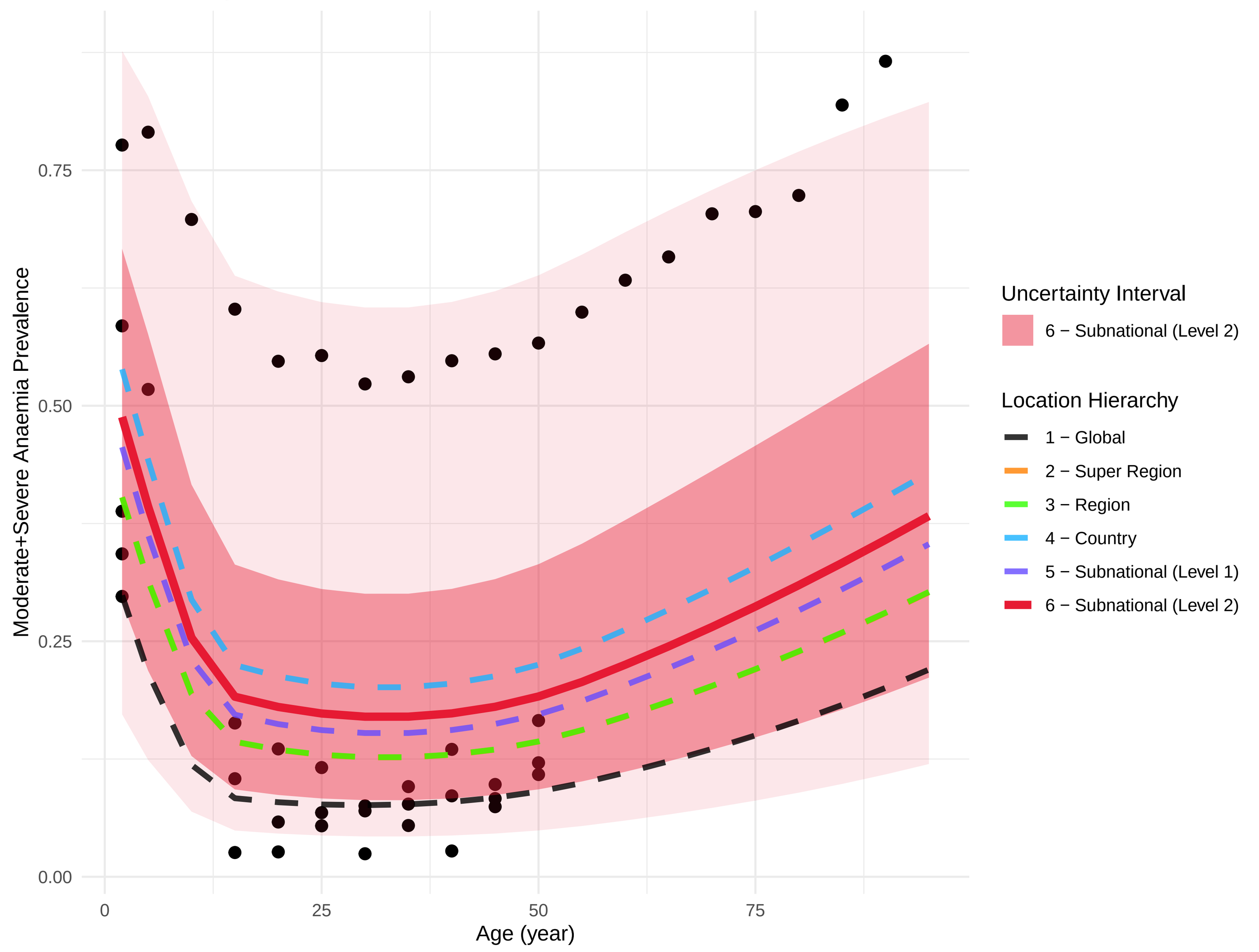

MR−BRT Age/Sex Fits – Males 2+ Years Old – Moderate+Severe Anaemia Prevalence – Andhra Pradesh, Urban
WHO−2024 Adjustment Method

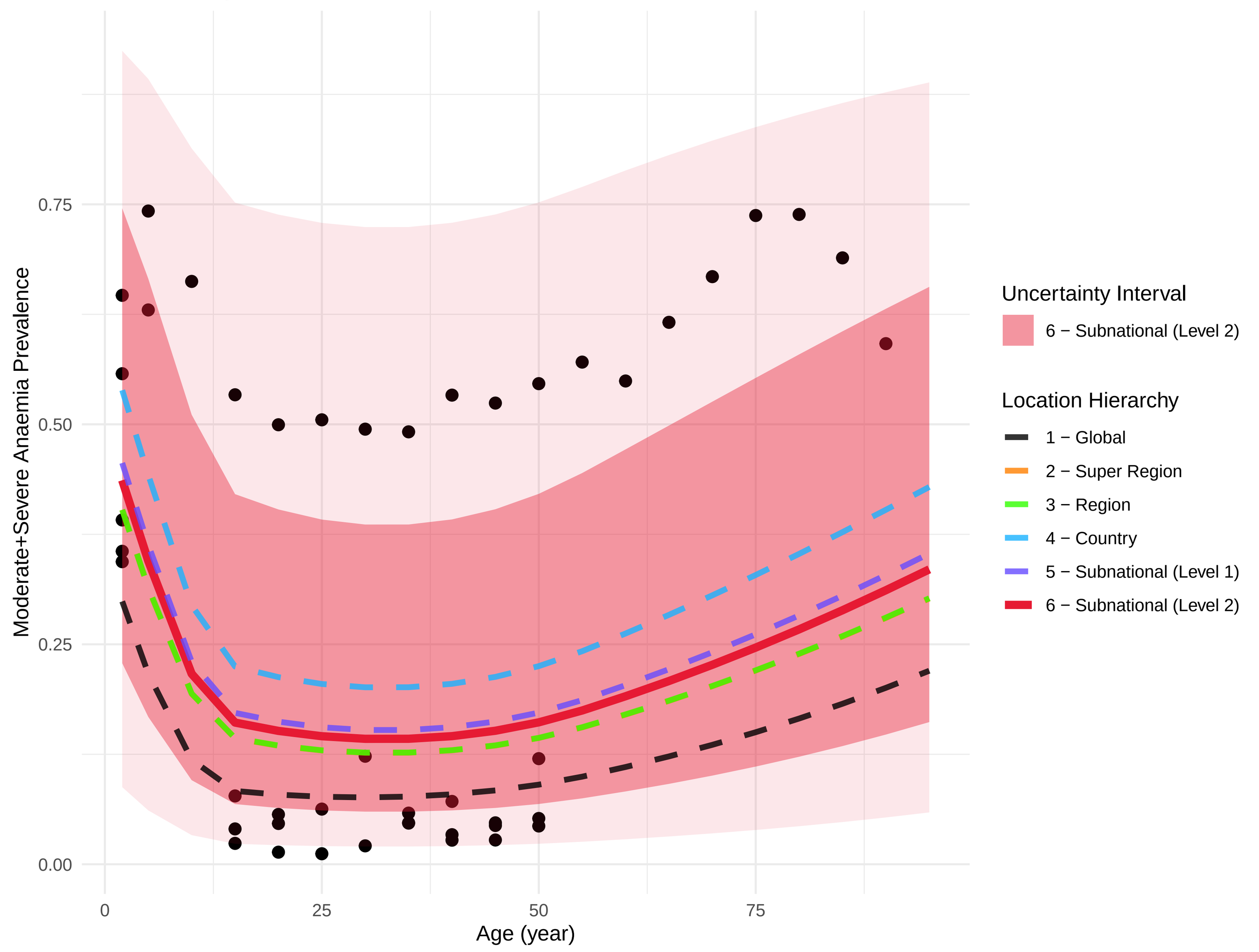

MR−BRT Age/Sex Fits – Males 2+ Years Old – Moderate+Severe Anaemia Prevalence – Arunachal Pradesh
WHO−2024 Adjustment Method

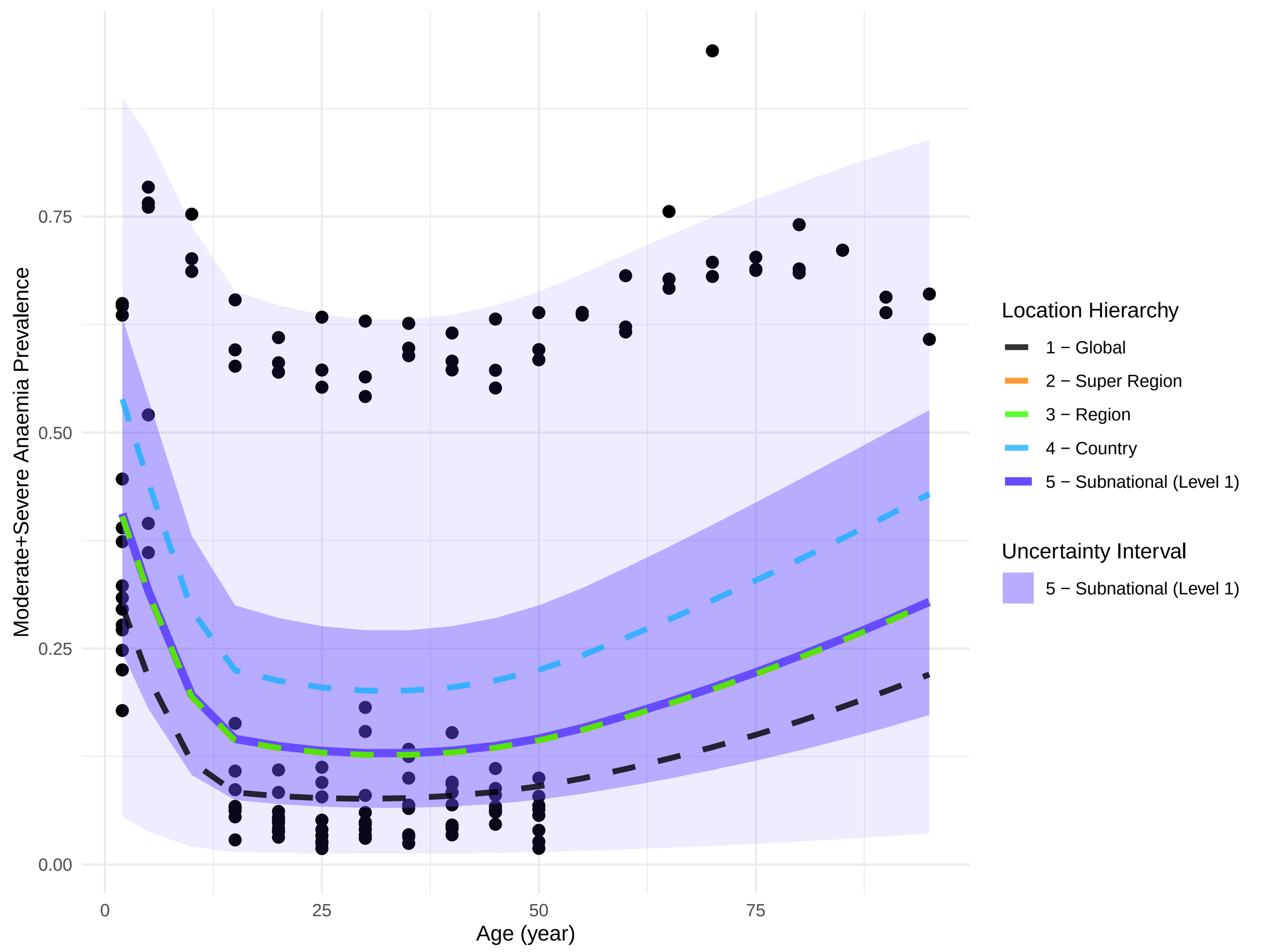

MR−BRT Age/Sex Fits – Males 2+ Years Old – Moderate+Severe Anaemia Prevalence – Arunachal Pradesh, Rural
WHO−2024 Adjustment Method

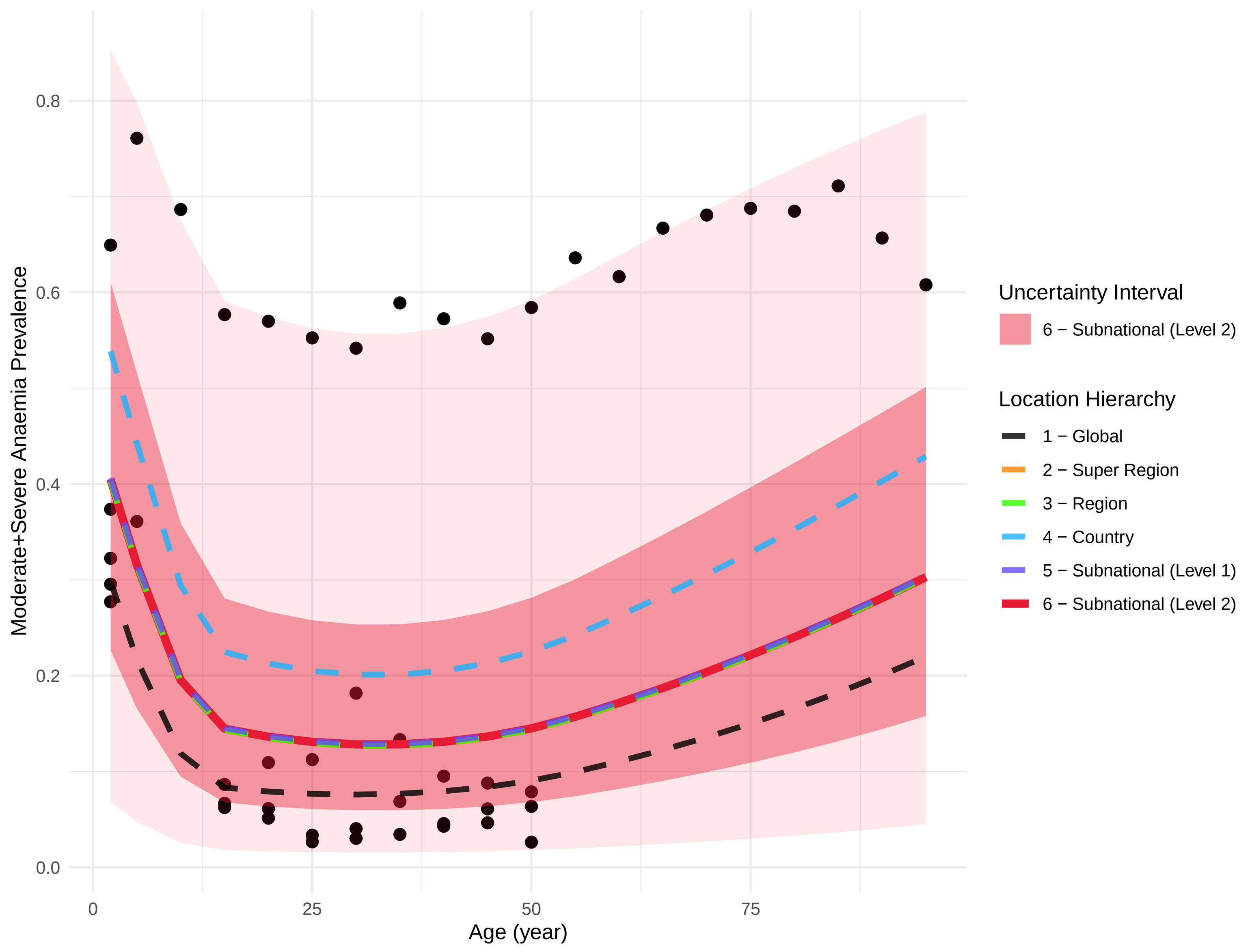

MR−BRT Age/Sex Fits – Males 2+ Years Old – Moderate+Severe Anaemia Prevalence – Arunachal Pradesh, Urban
WHO−2024 Adjustment Method

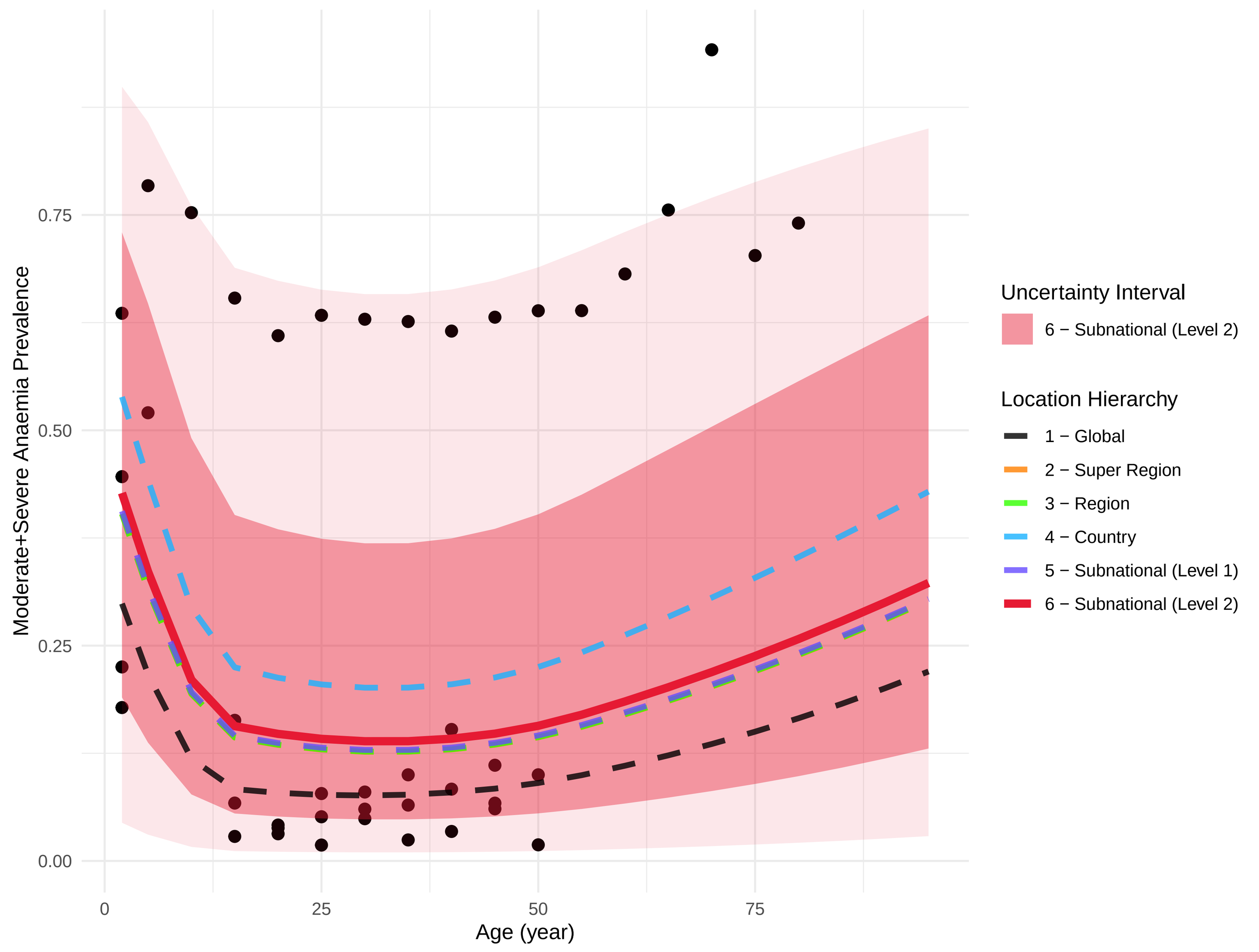

MR−BRT Age/Sex Fits – Males 2+ Years Old – Moderate+Severe Anaemia Prevalence – Assam
WHO−2024 Adjustment Method

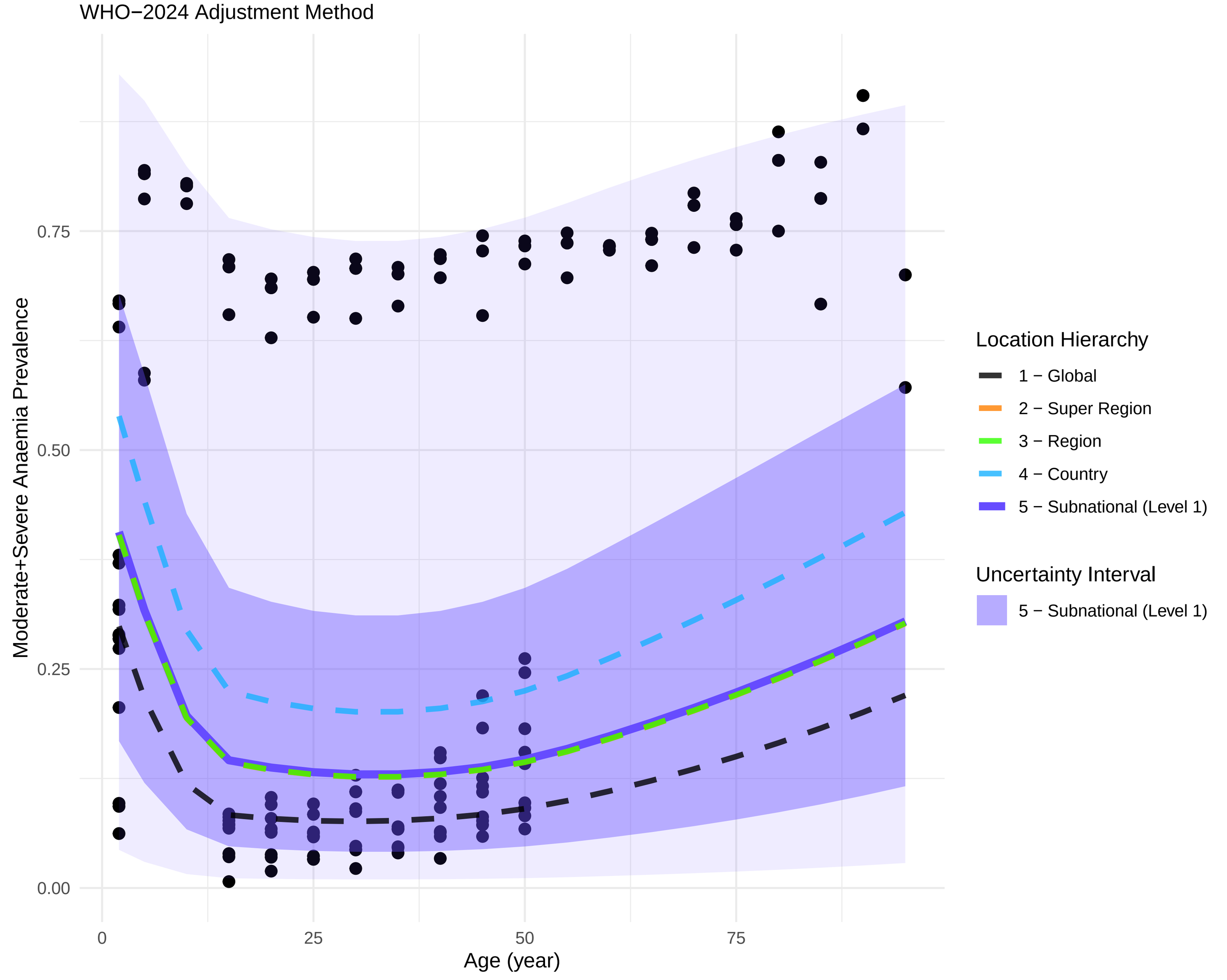

MR−BRT Age/Sex Fits – Males 2+ Years Old – Moderate+Severe Anaemia Prevalence – Assam, Rural
WHO−2024 Adjustment Method

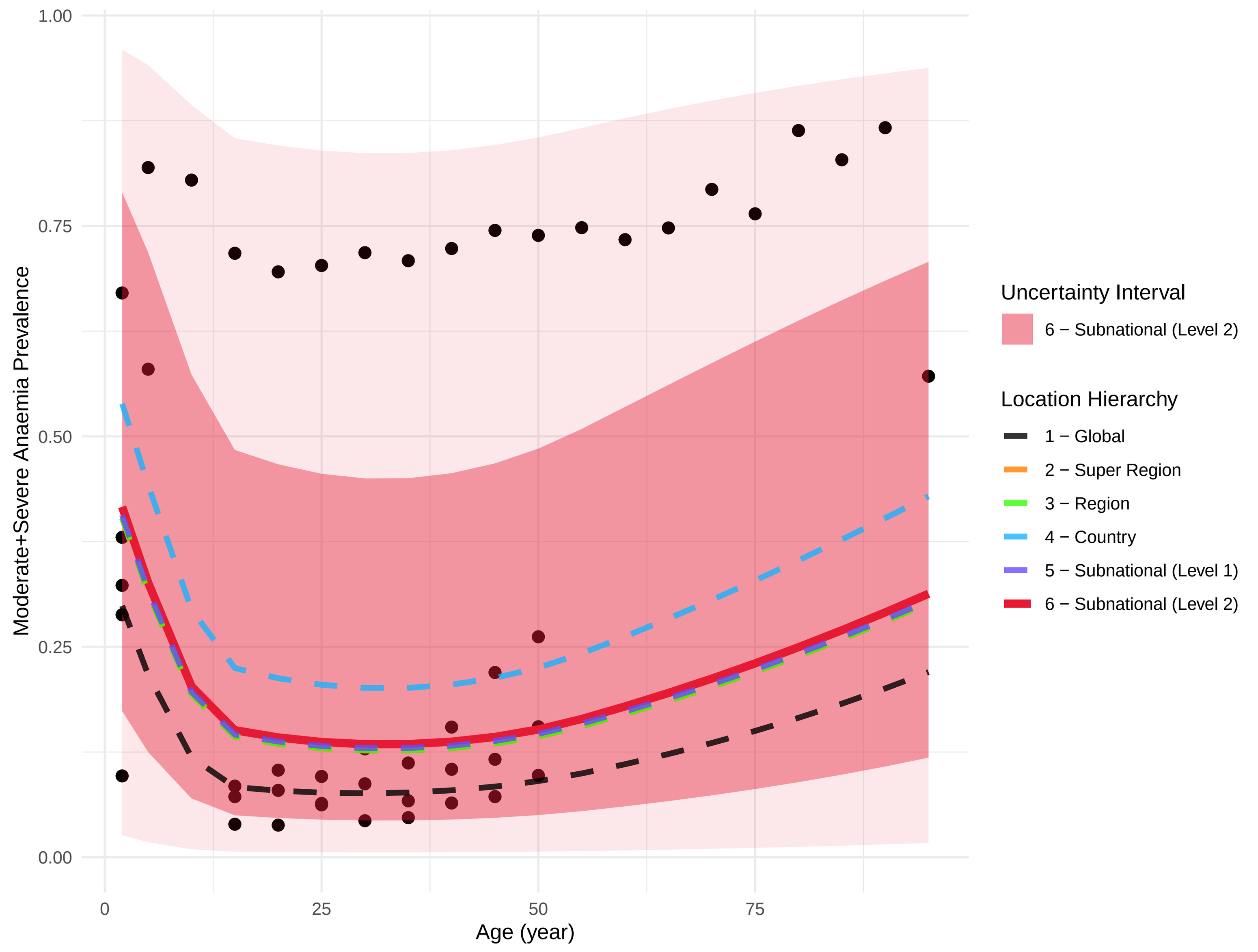

MR−BRT Age/Sex Fits – Males 2+ Years Old – Moderate+Severe Anaemia Prevalence – Assam, Urban
WHO−2024 Adjustment Method

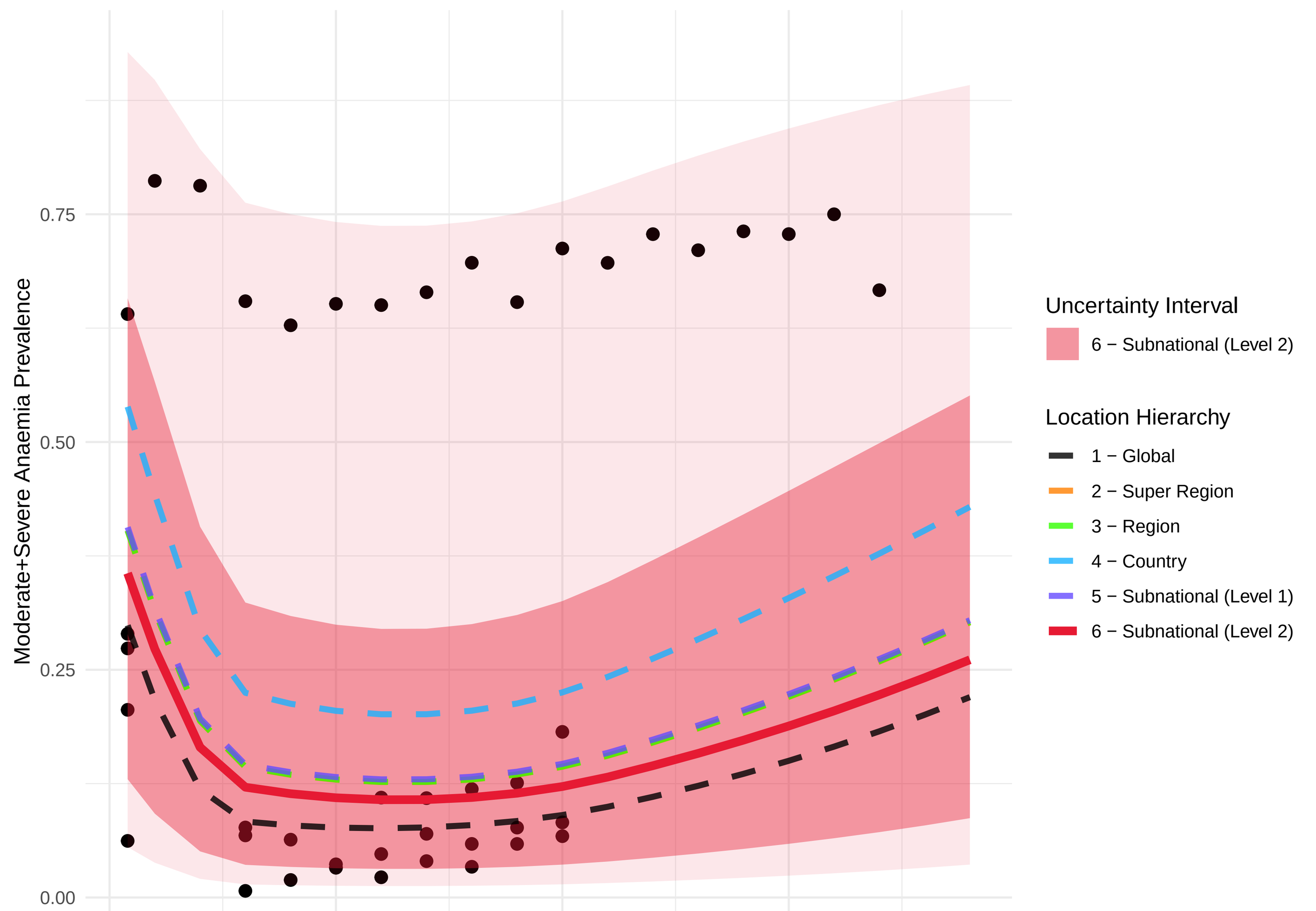

MR−BRT Age/Sex Fits − Males 2+ Years Old − Moderate+Severe Anaemia Prevalence − Bihar
WHO−2024 Adjustment Method

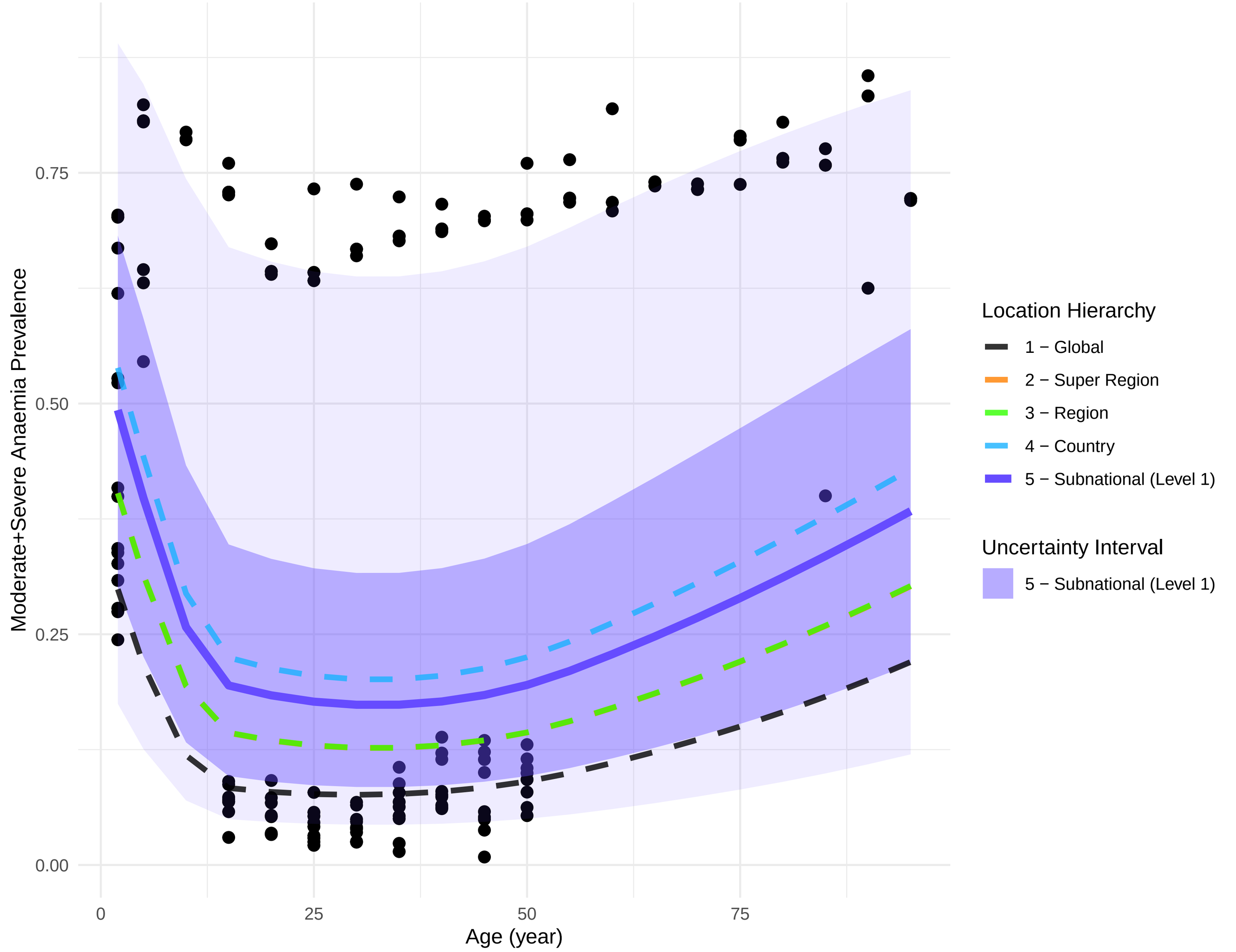

MR−BRT Age/Sex Fits − Males 2+ Years Old − Moderate+Severe Anaemia Prevalence − Bihar, Rural
WHO−2024 Adjustment Method

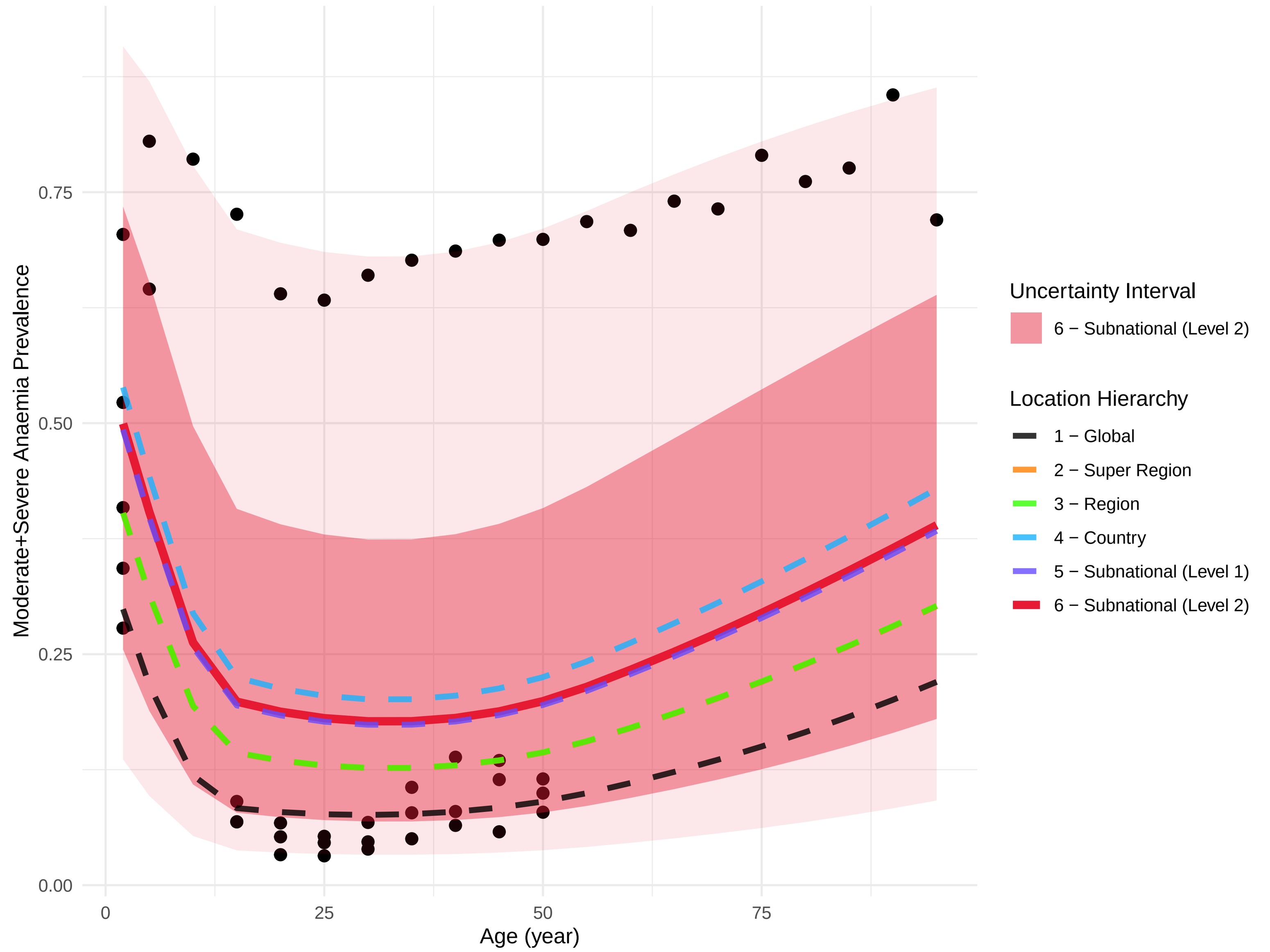

MR−BRT Age/Sex Fits − Males 2+ Years Old − Moderate+Severe Anaemia Prevalence − Bihar, Urban
WHO−2024 Adjustment Method

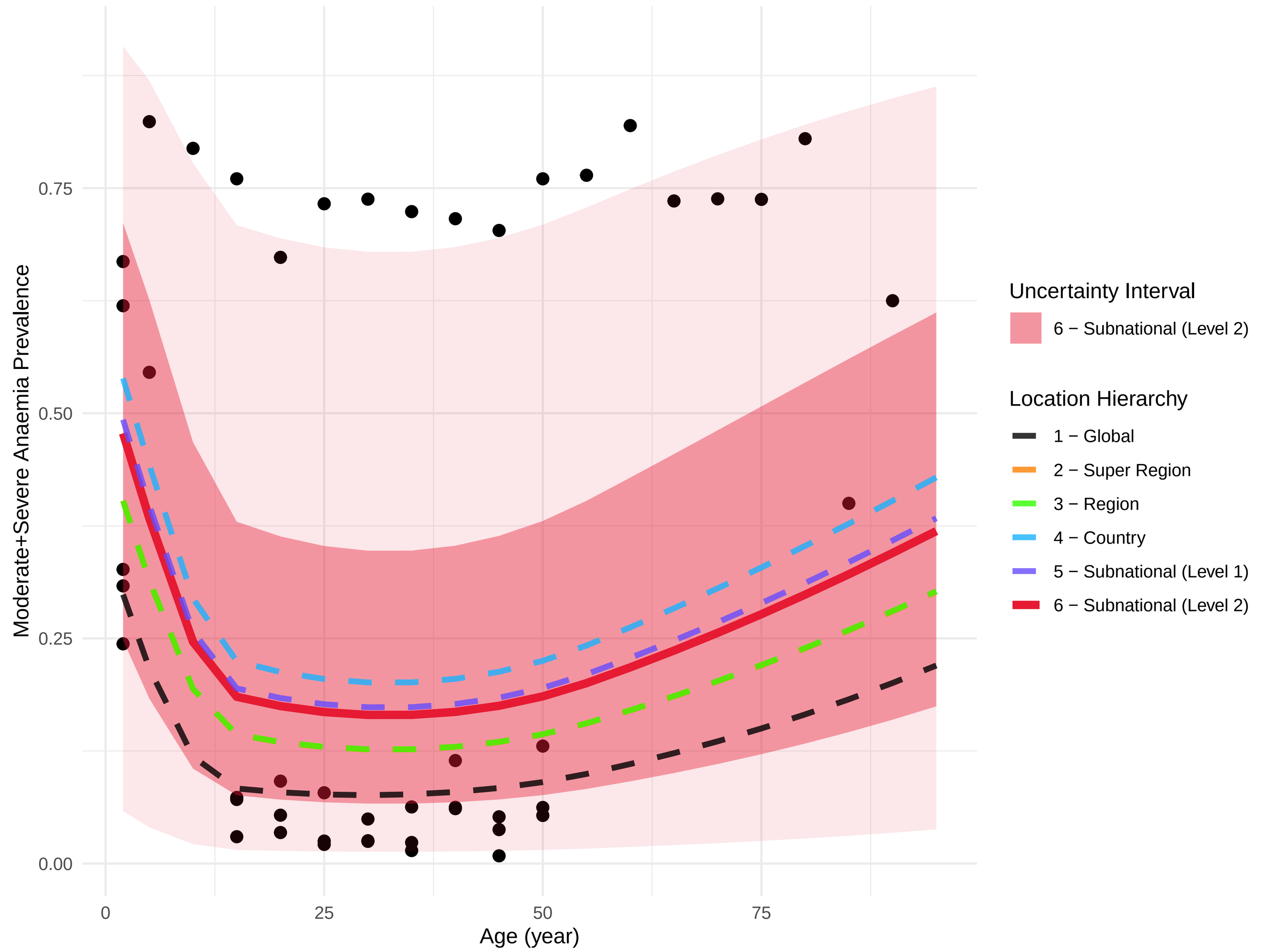

MR−BRT Age/Sex Fits − Males 2+ Years Old − Moderate+Severe Anaemia Prevalence − Chhattisgarh
WHO−2024 Adjustment Method

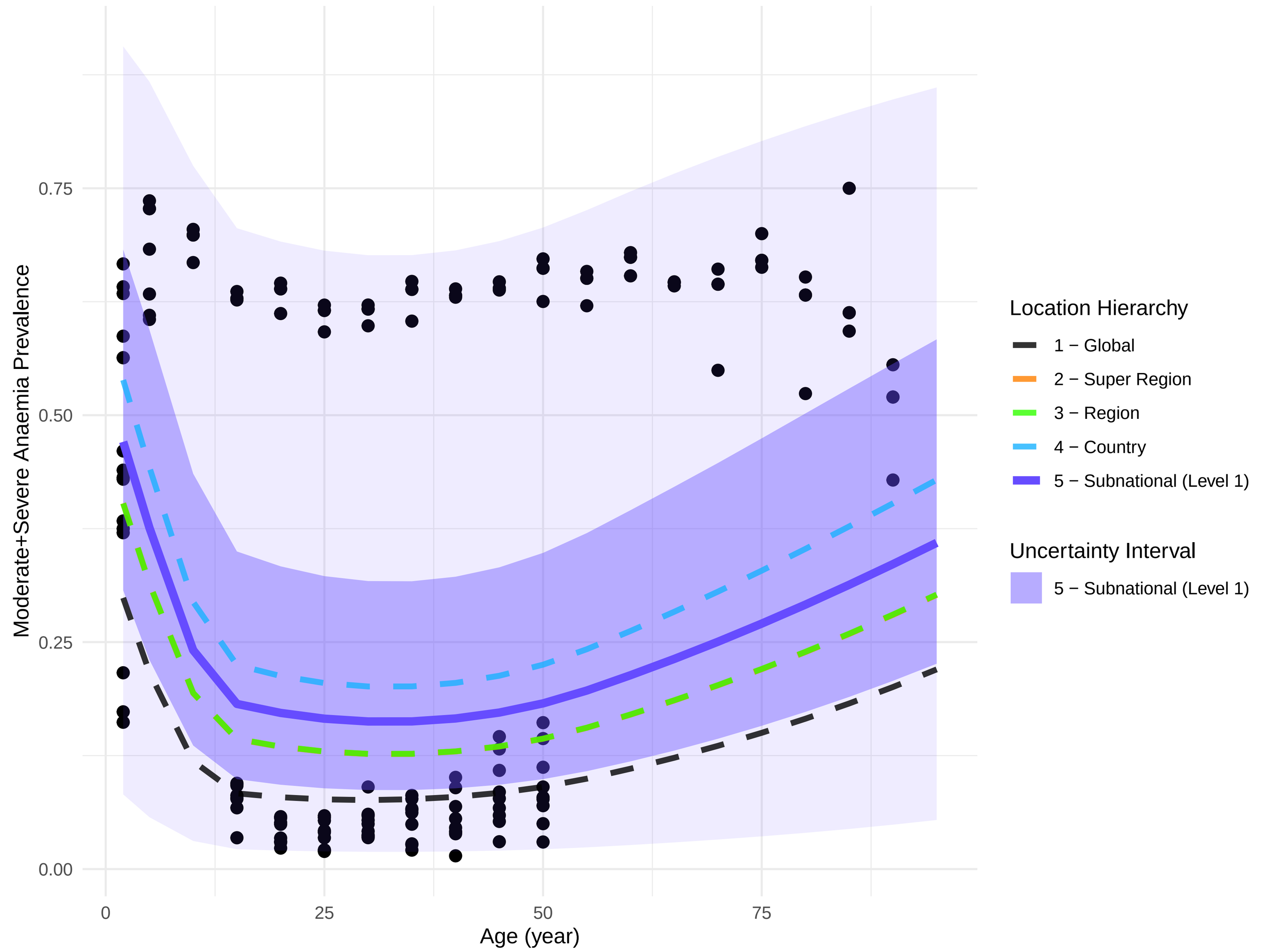

MR−BRT Age/Sex Fits − Males 2+ Years Old − Moderate+Severe Anaemia Prevalence − Chhattisgarh, Rural
WHO−2024 Adjustment Method

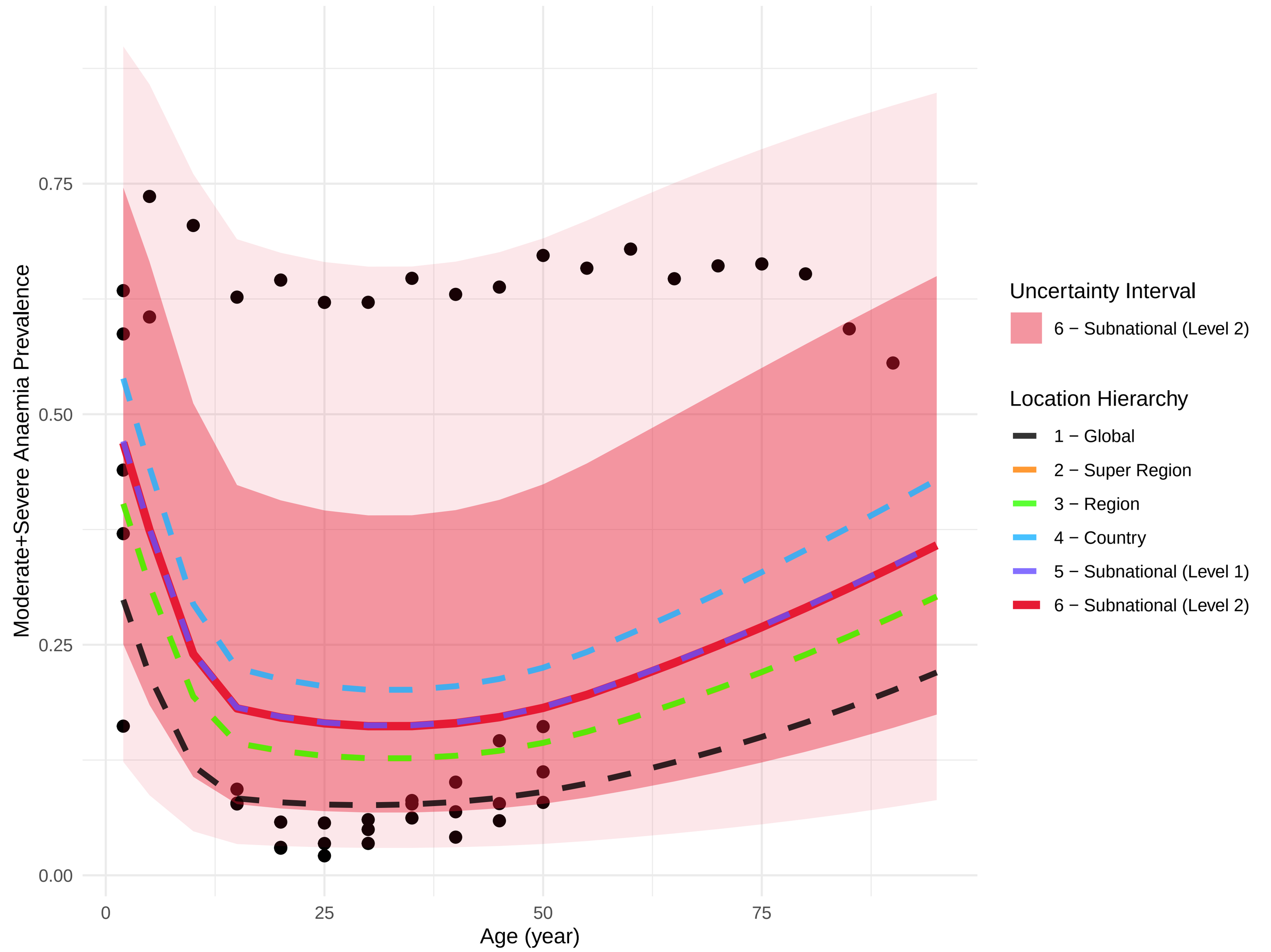

MR−BRT Age/Sex Fits − Males 2+ Years Old − Moderate+Severe Anaemia Prevalence − Chhattisgarh, Urban
WHO−2024 Adjustment Method

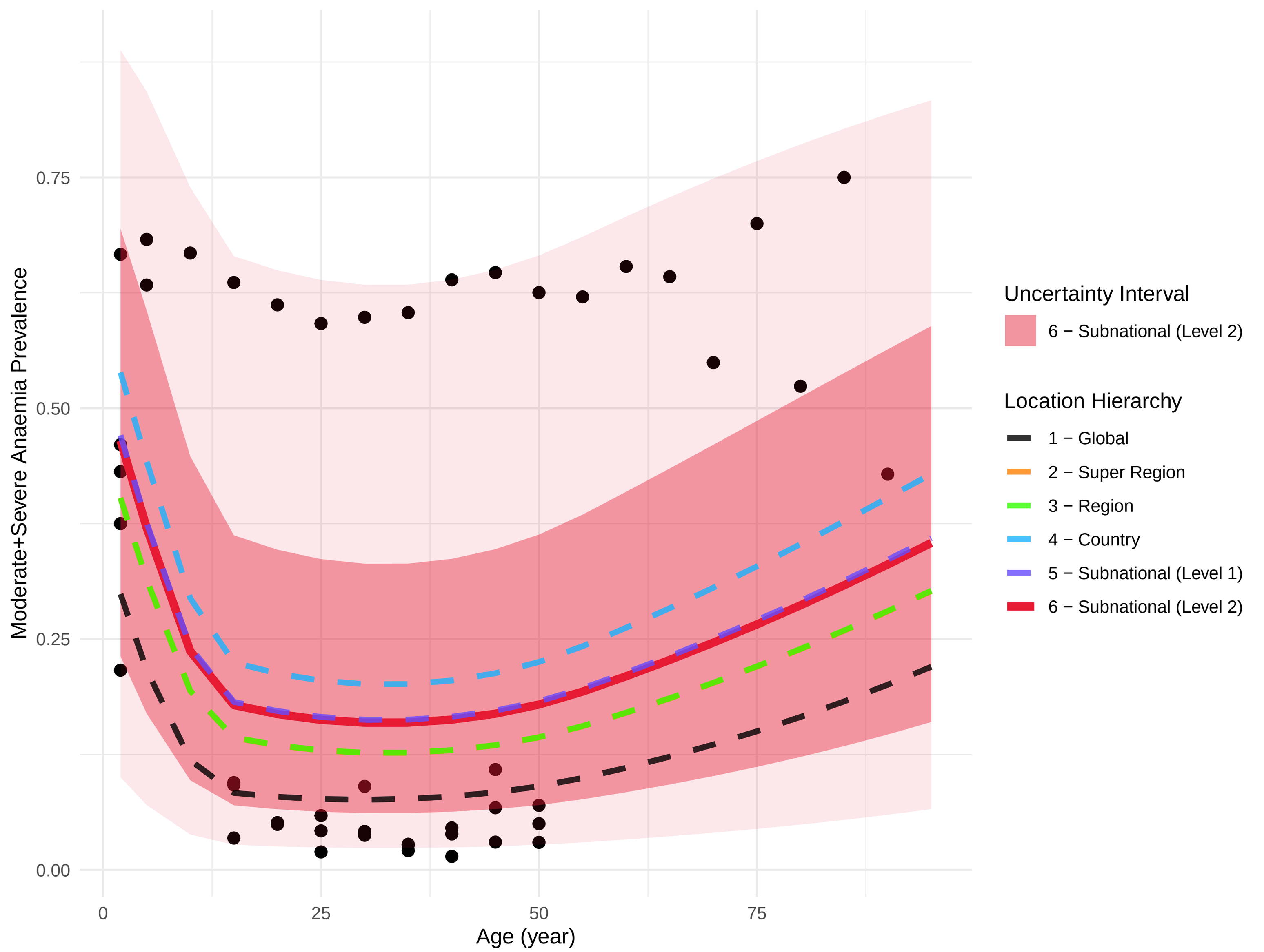

MR−BRT Age/Sex Fits − Males 2+ Years Old − Moderate+Severe Anaemia Prevalence − Delhi
WHO−2024 Adjustment Method

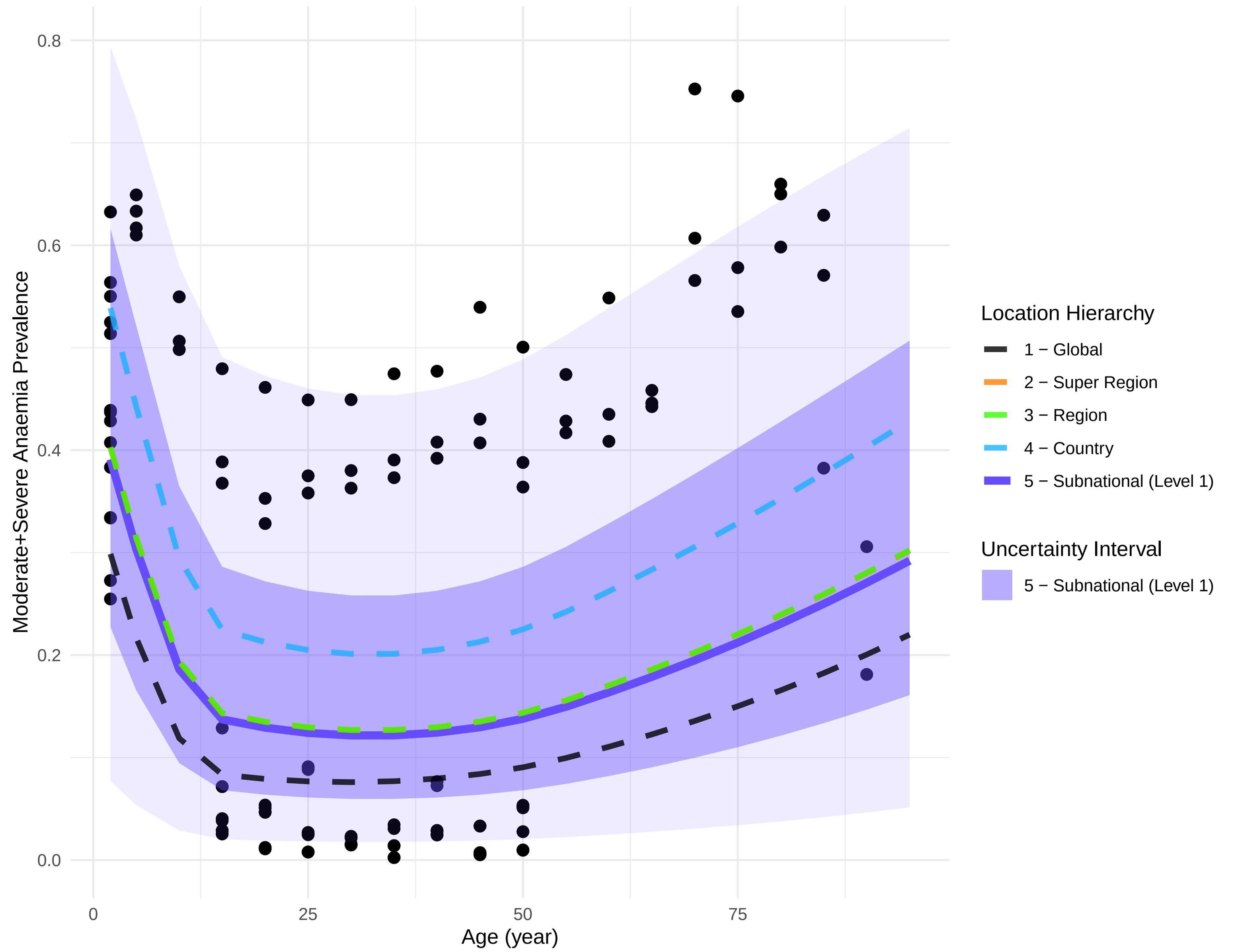

MR−BRT Age/Sex Fits − Males 2+ Years Old − Moderate+Severe Anaemia Prevalence − Delhi, Rural
WHO−2024 Adjustment Method

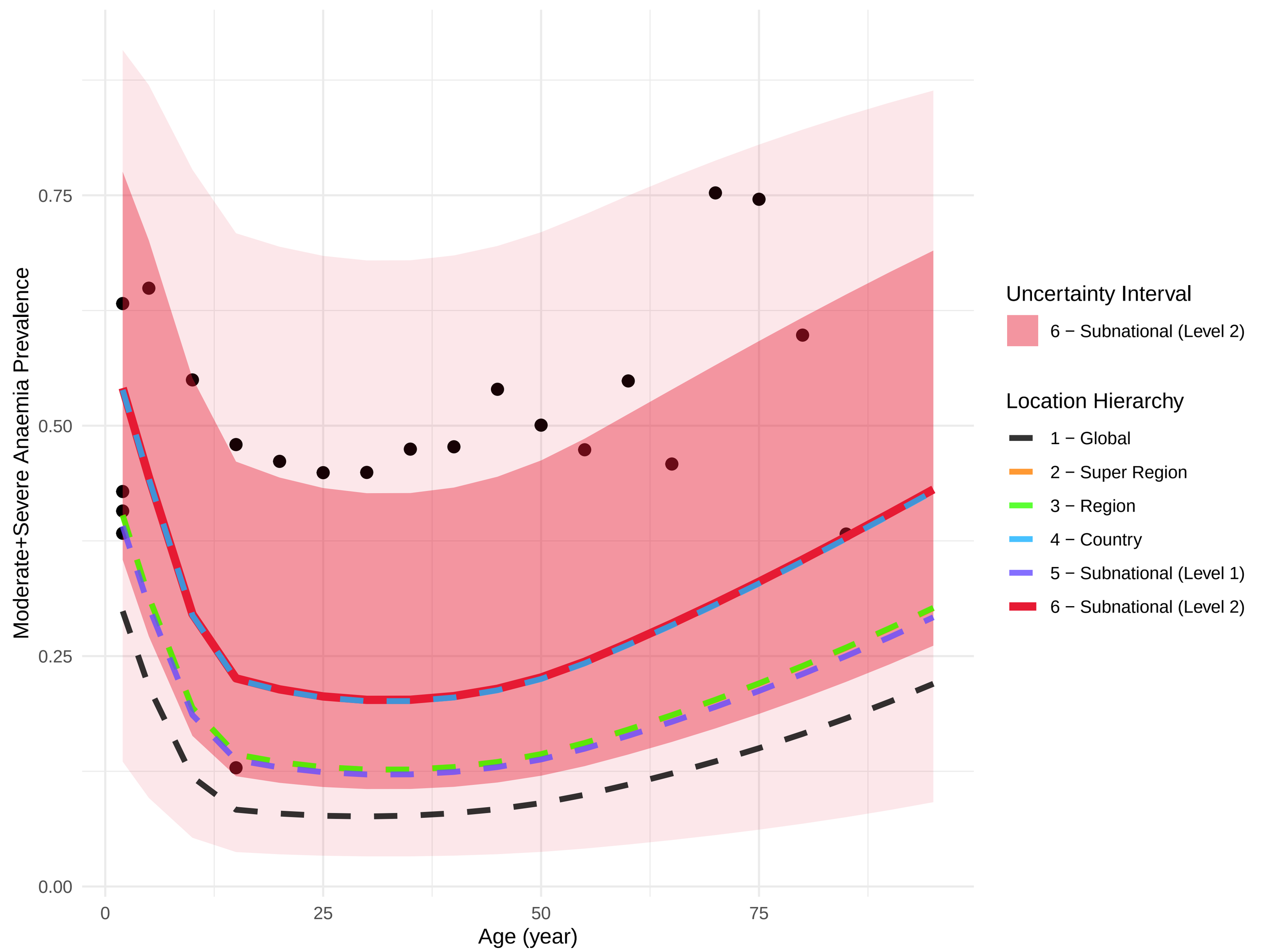

MR−BRT Age/Sex Fits − Males 2+ Years Old − Moderate+Severe Anaemia Prevalence − Delhi, Urban
WHO−2024 Adjustment Method

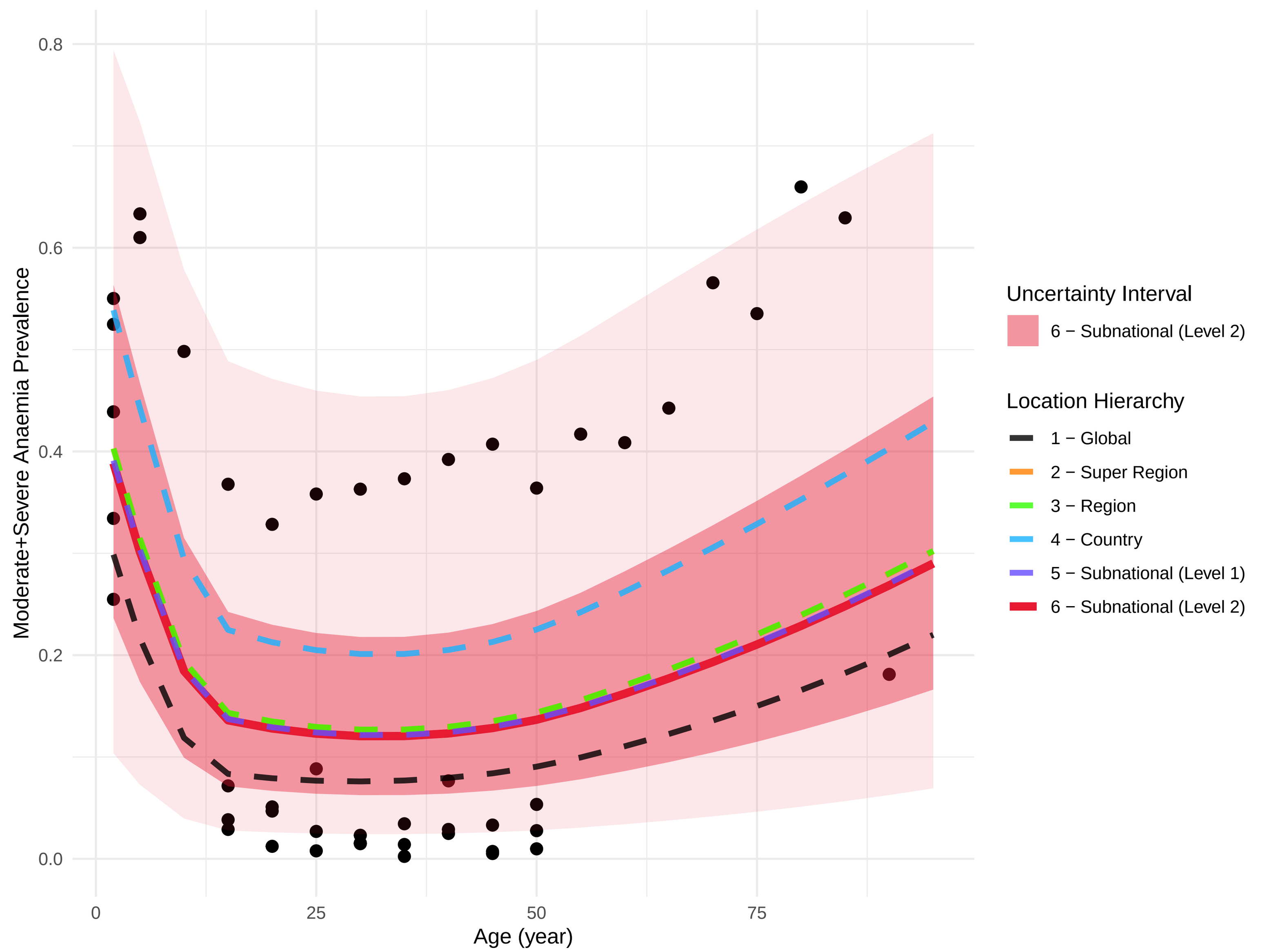

MR−BRT Age/Sex Fits − Males 2+ Years Old − Moderate+Severe Anaemia Prevalence − Goa
WHO−2024 Adjustment Method

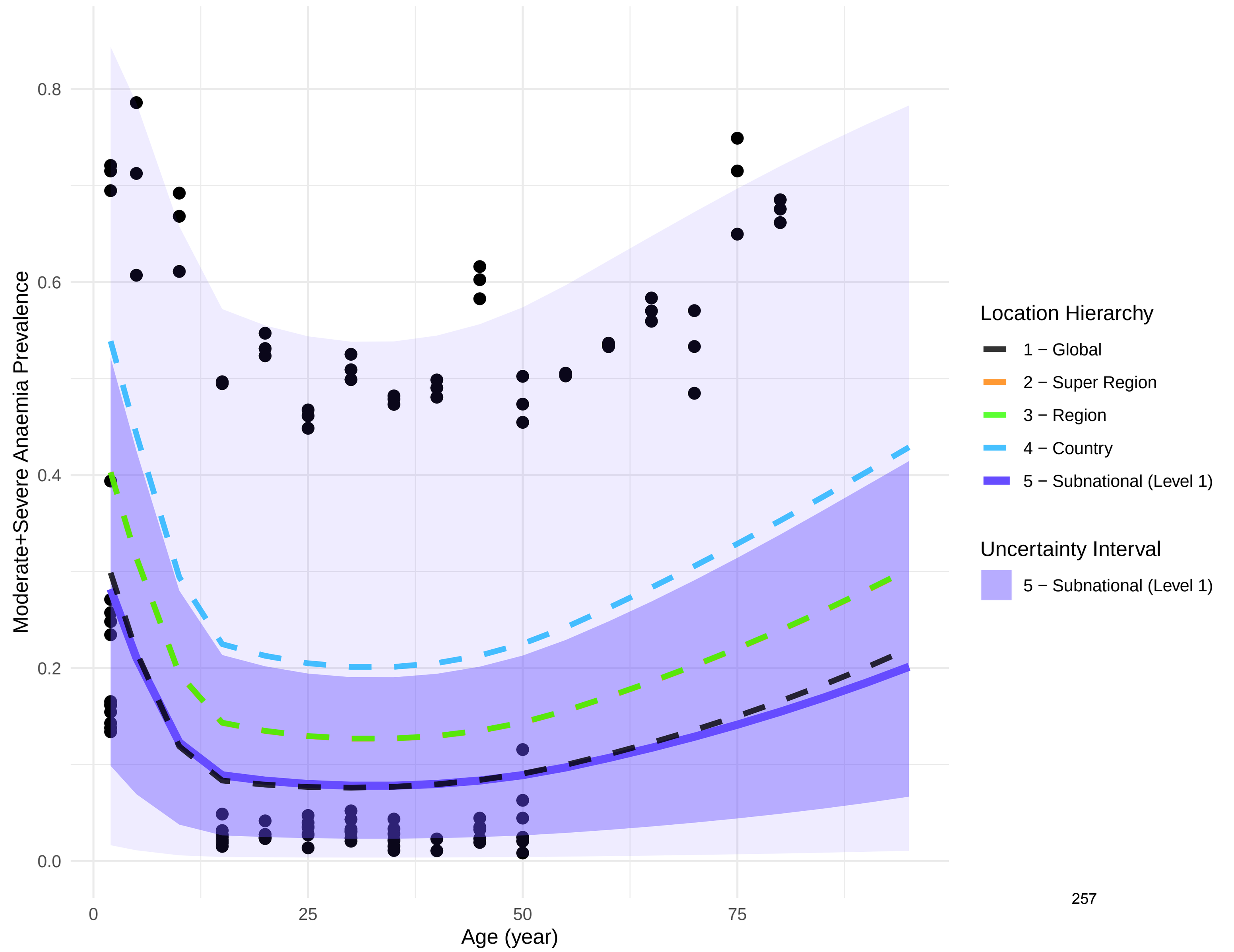

MR−BRT Age/Sex Fits − Males 2+ Years Old − Moderate+Severe Anaemia Prevalence − Goa, Rural
WHO−2024 Adjustment Method

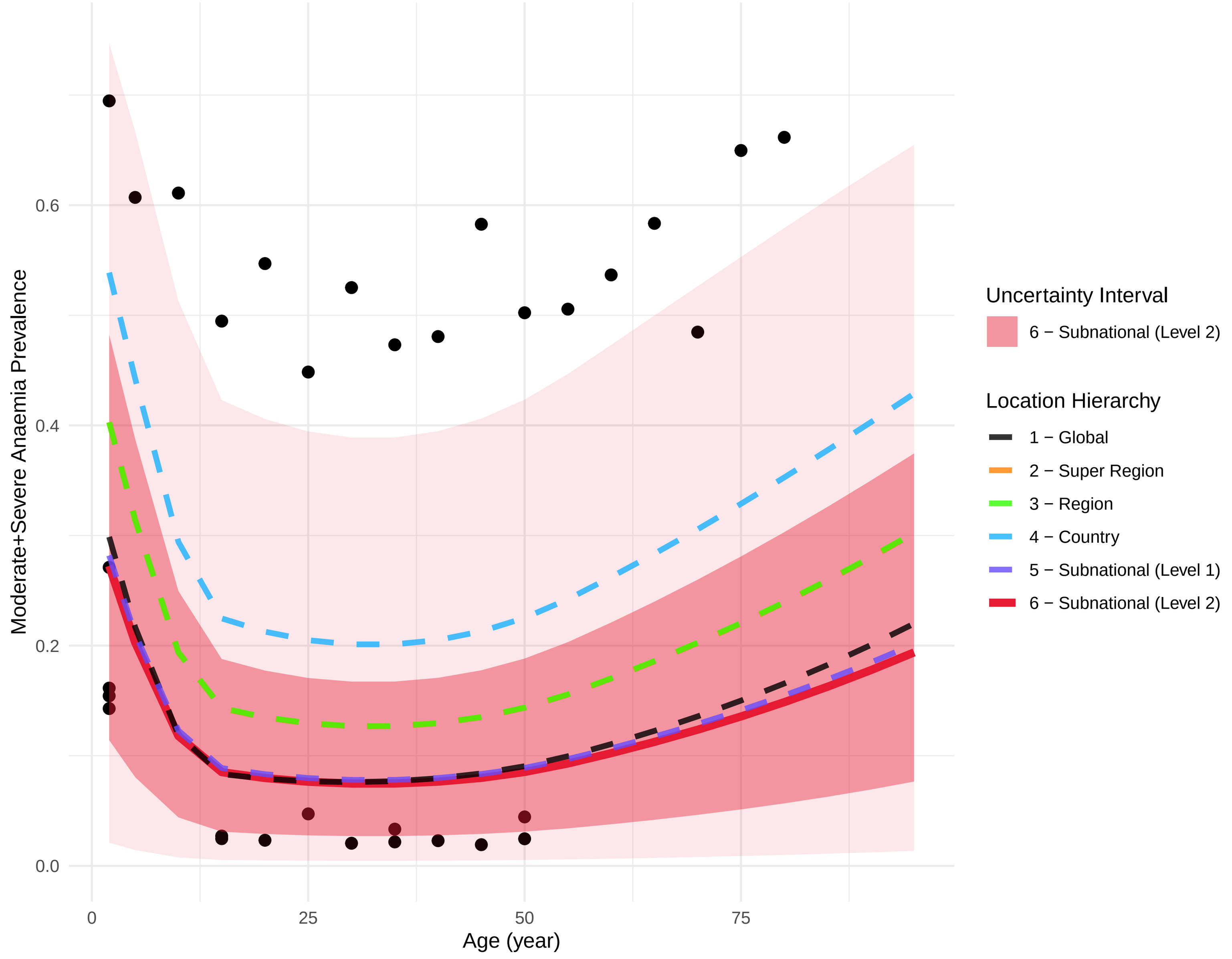

MR−BRT Age/Sex Fits − Males 2+ Years Old − Moderate+Severe Anaemia Prevalence − Goa, Urban
WHO−2024 Adjustment Method

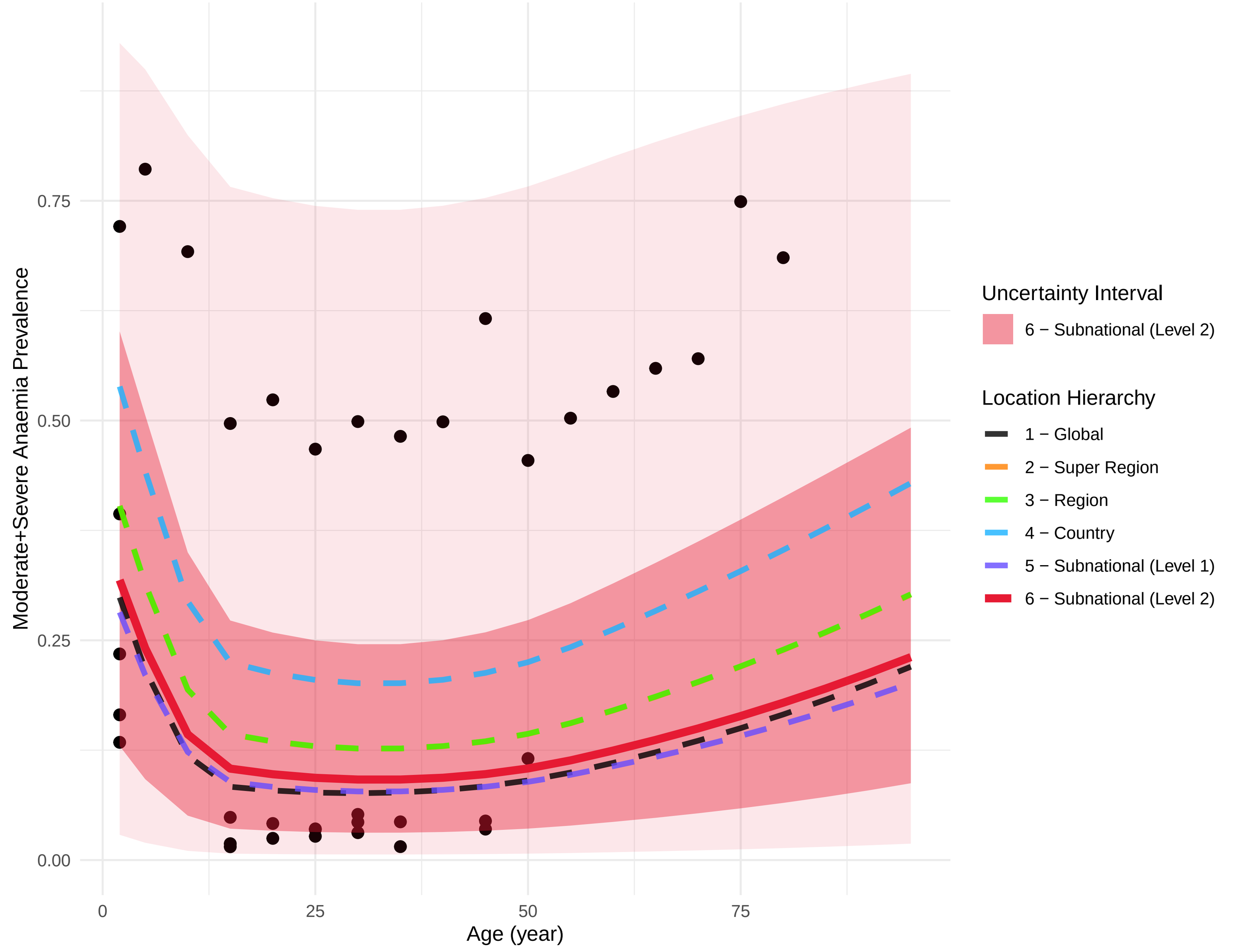

MR−BRT Age/Sex Fits − Males 2+ Years Old − Moderate+Severe Anaemia Prevalence − Gujarat
WHO−2024 Adjustment Method

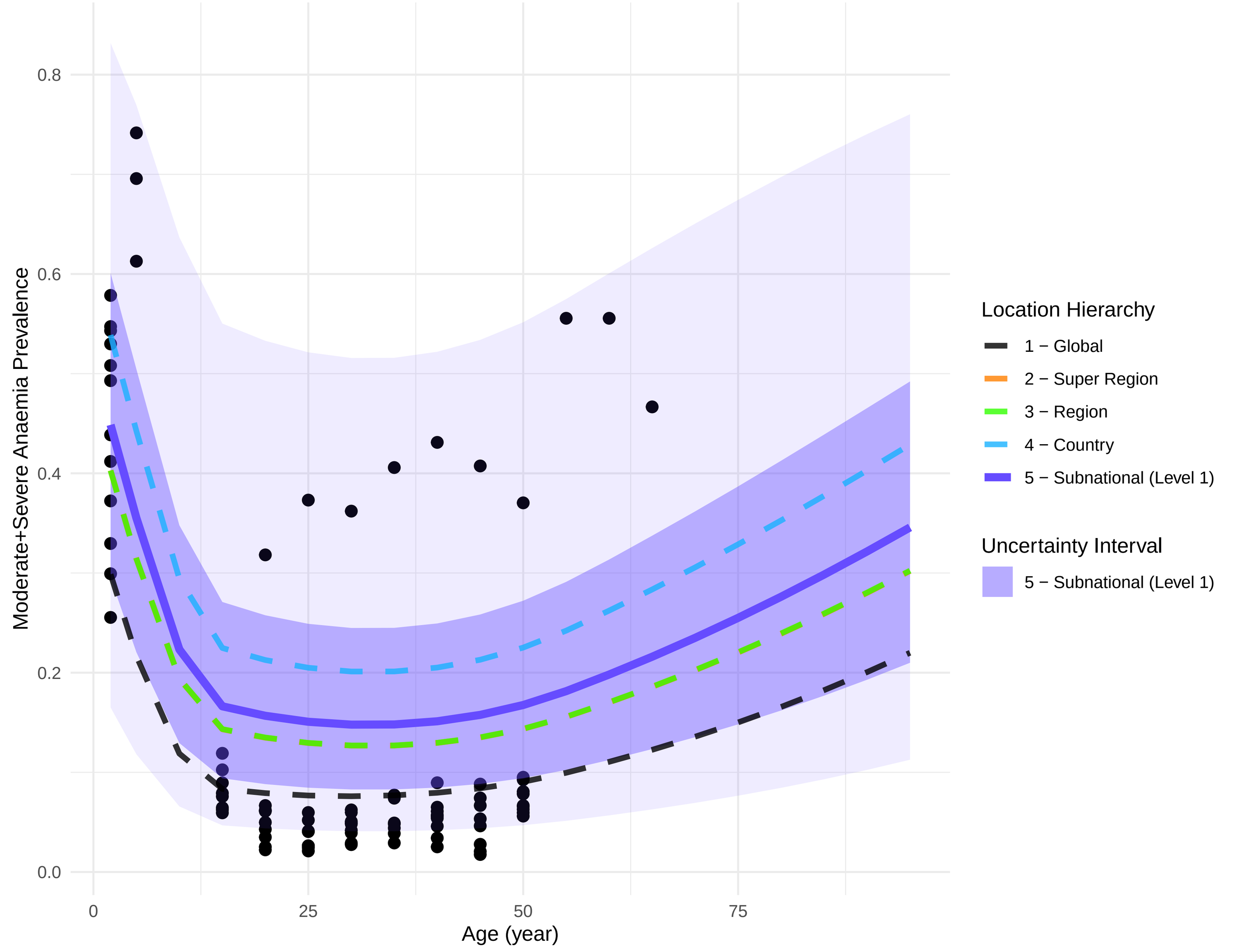

MR−BRT Age/Sex Fits − Males 2+ Years Old − Moderate+Severe Anaemia Prevalence − Gujarat, Rural
WHO−2024 Adjustment Method

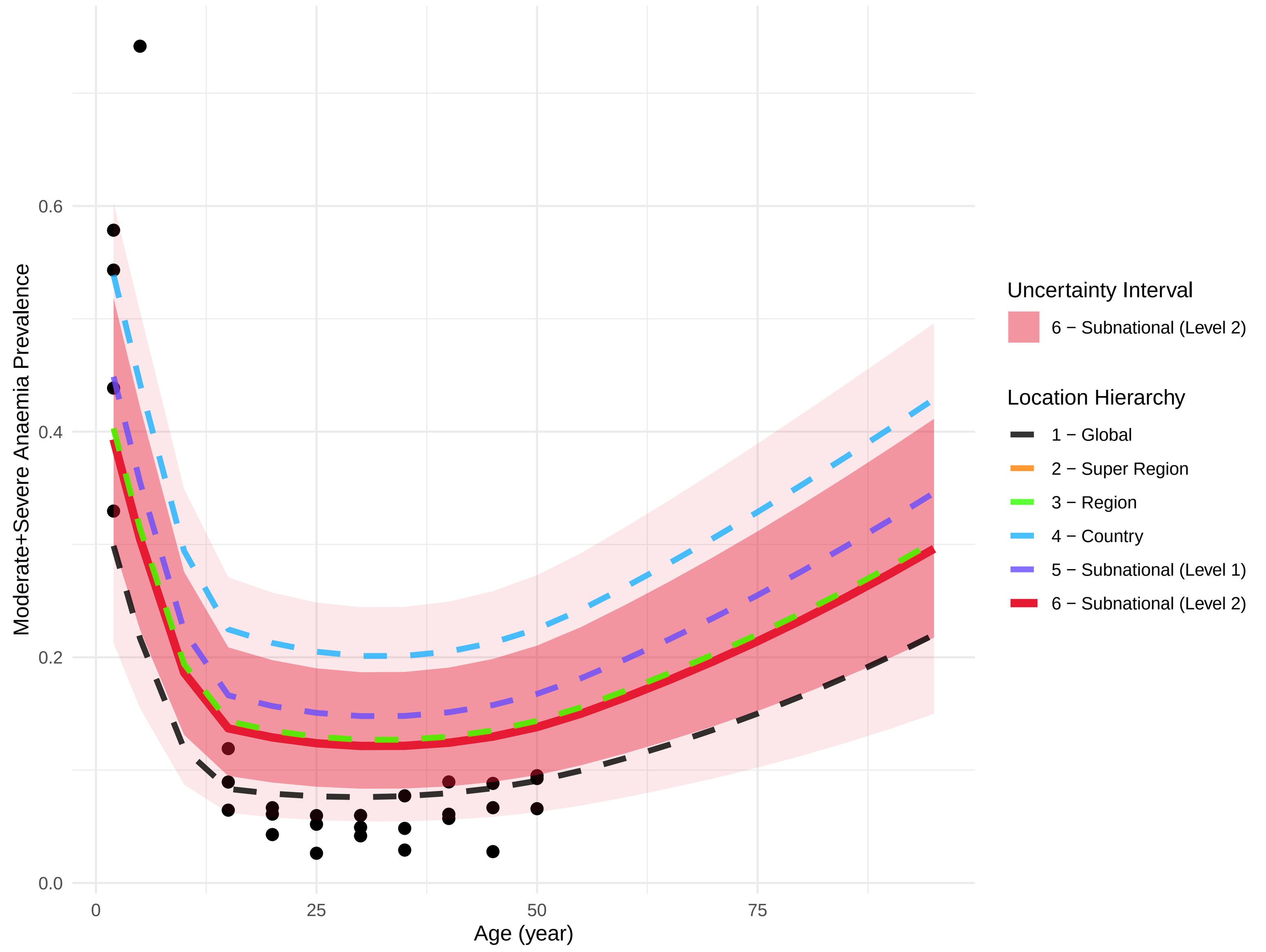

MR−BRT Age/Sex Fits − Males 2+ Years Old − Moderate+Severe Anaemia Prevalence − Gujarat, Urban
WHO−2024 Adjustment Method

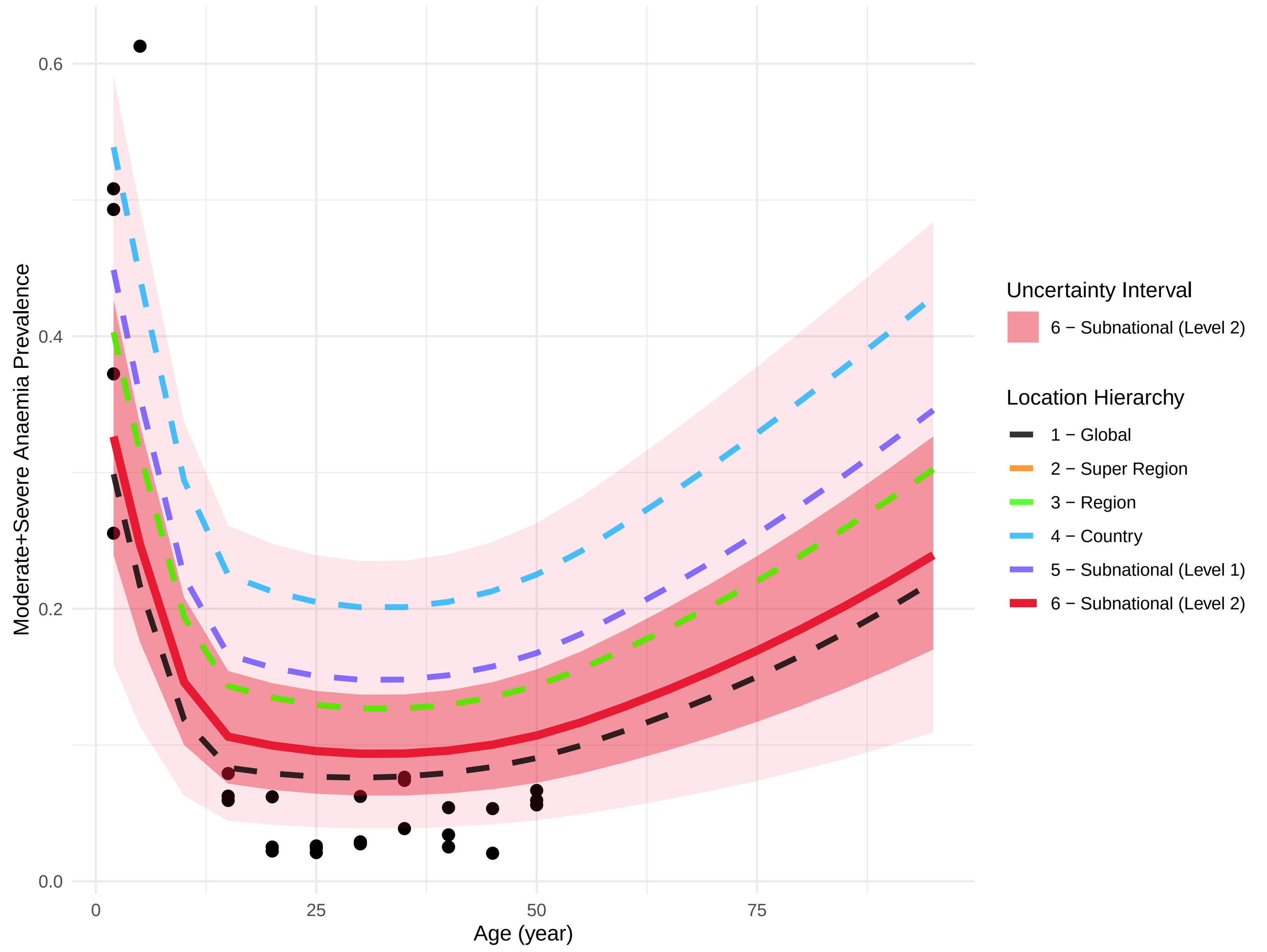

MR−BRT Age/Sex Fits − Males 2+ Years Old − Moderate+Severe Anaemia Prevalence − Haryana
WHO−2024 Adjustment Method

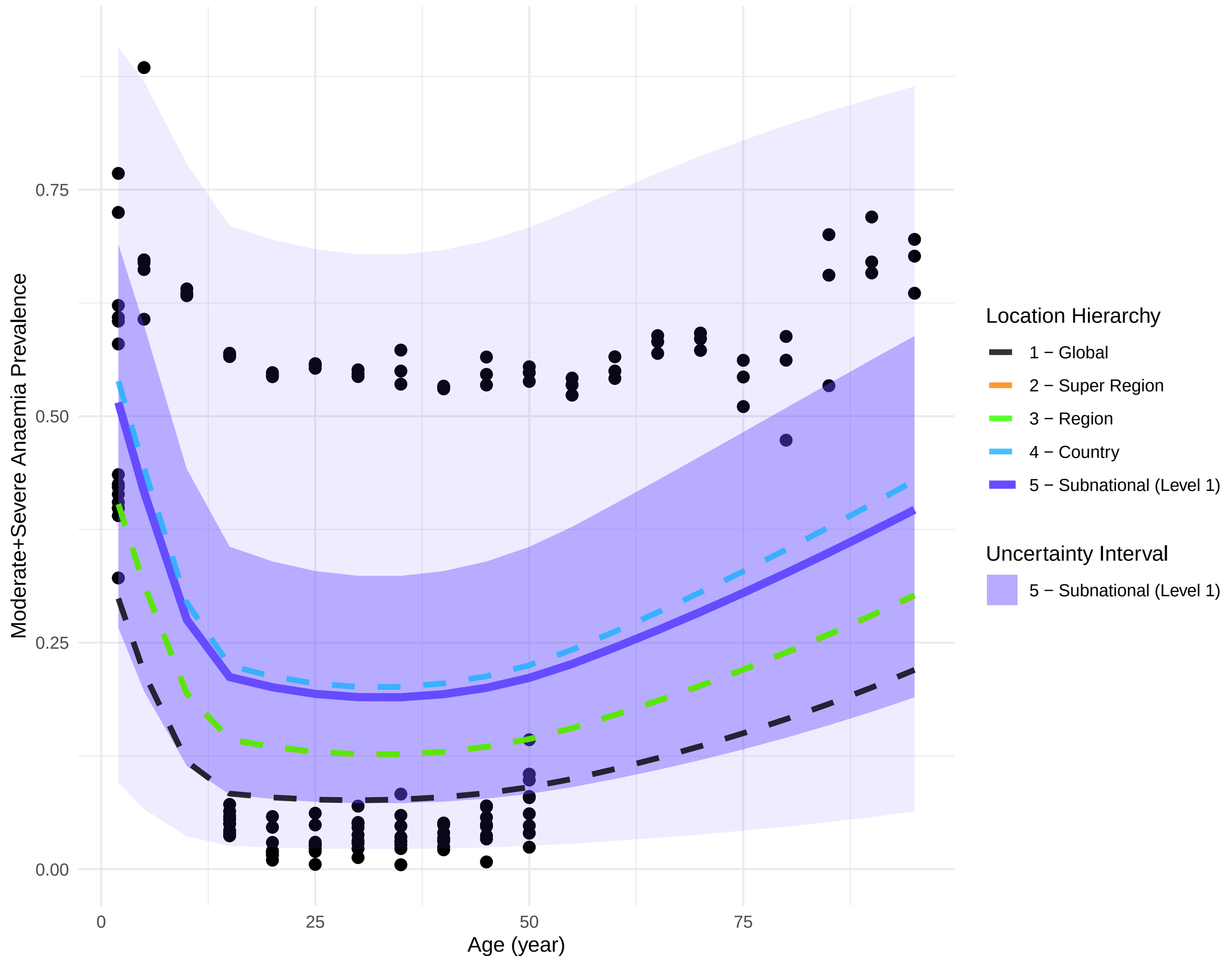

MR−BRT Age/Sex Fits − Males 2+ Years Old − Moderate+Severe Anaemia Prevalence − Haryana, Rural
WHO−2024 Adjustment Method

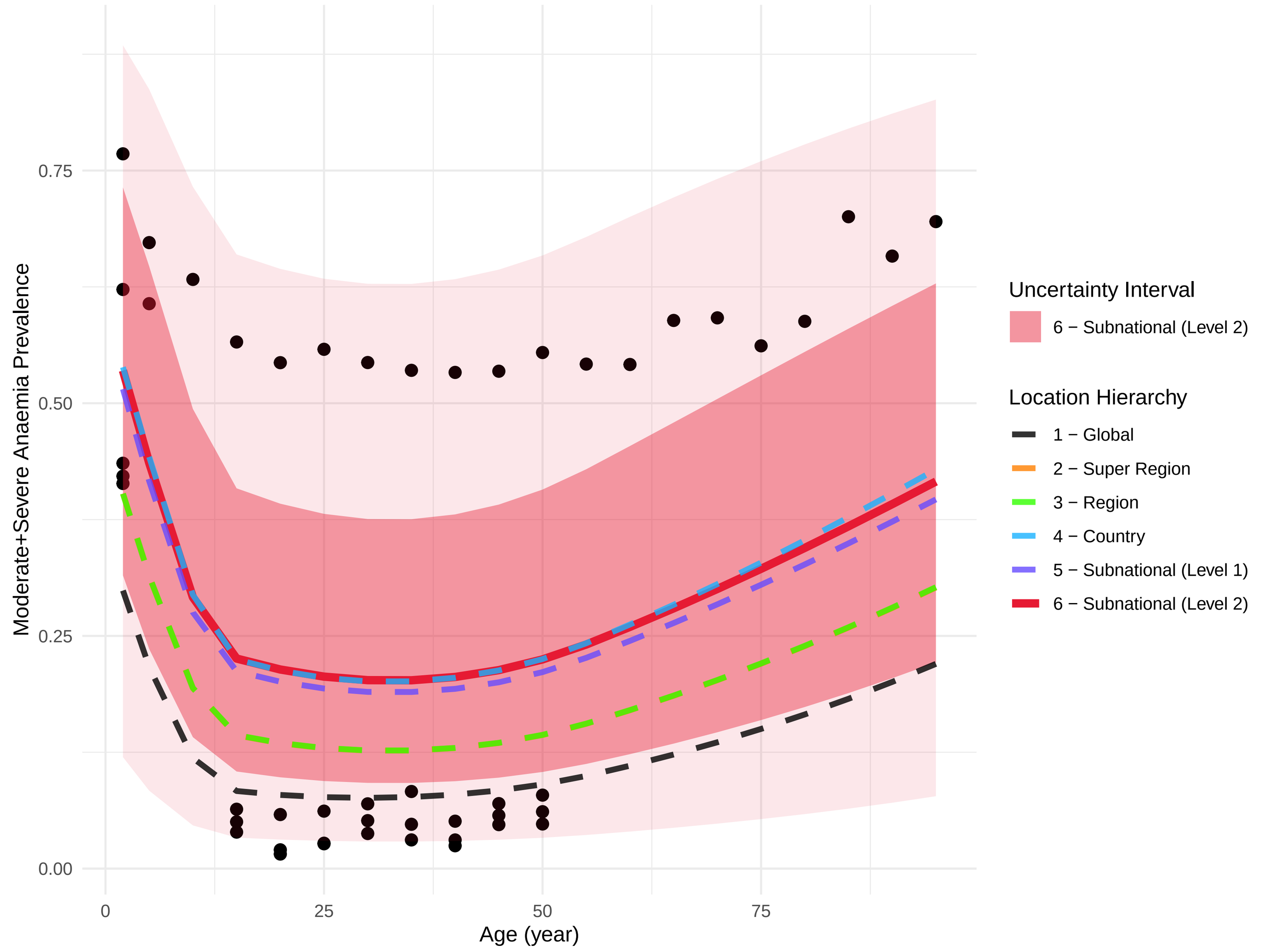

MR−BRT Age/Sex Fits − Males 2+ Years Old − Moderate+Severe Anaemia Prevalence − Haryana, Urban
WHO−2024 Adjustment Method

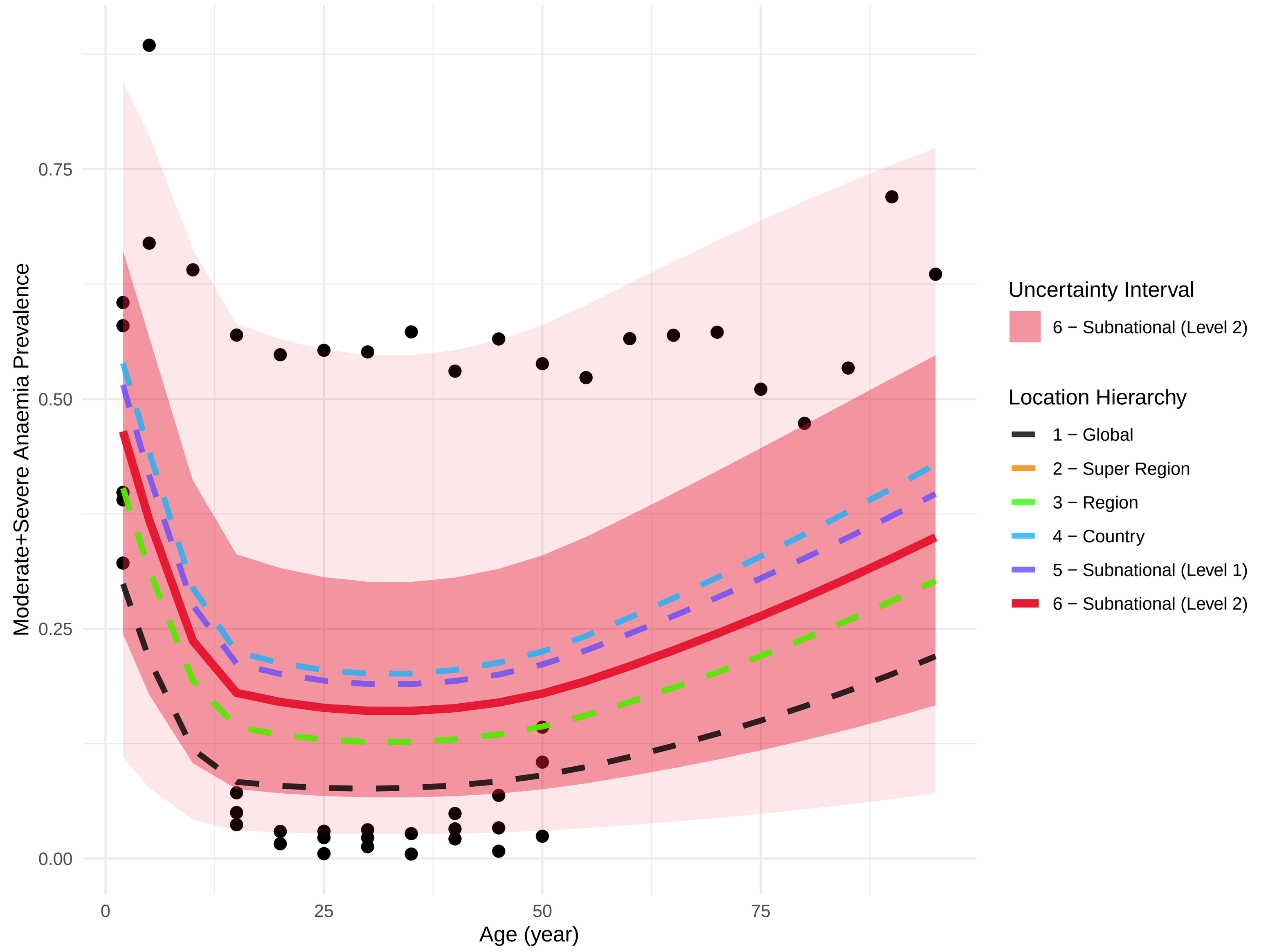

MR−BRT Age/Sex Fits − Males 2+ Years Old − Moderate+Severe Anaemia Prevalence − Himachal Pradesh
WHO−2024 Adjustment Method

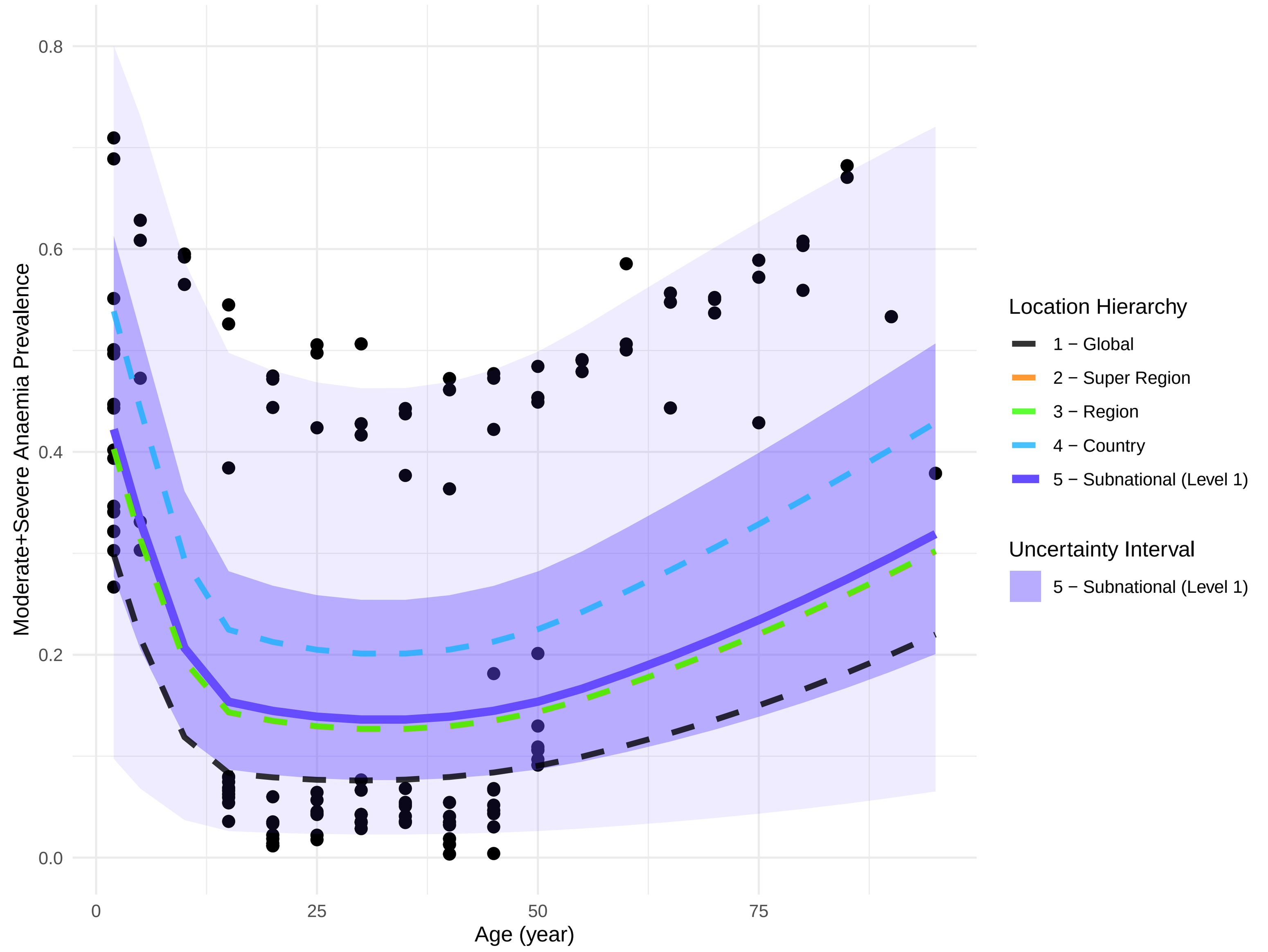

MR−BRT Age/Sex Fits − Males 2+ Years Old − Moderate+Severe Anaemia Prevalence − Himachal Pradesh, Rural
WHO−2024 Adjustment Method

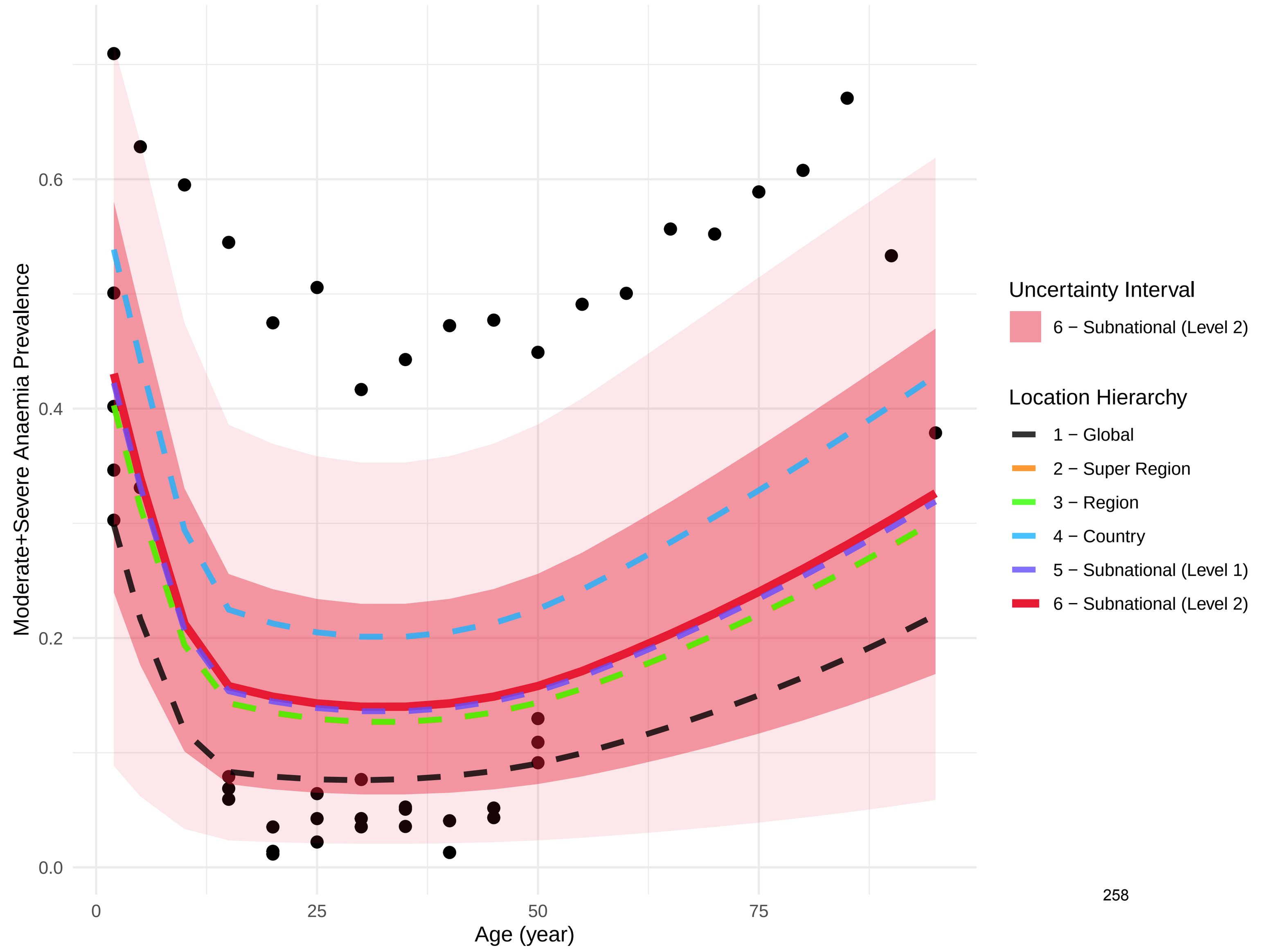

MR−BRT Age/Sex Fits − Males 2+ Years Old − Moderate+Severe Anaemia Prevalence − Himachal Pradesh, Urban
WHO−2024 Adjustment Method

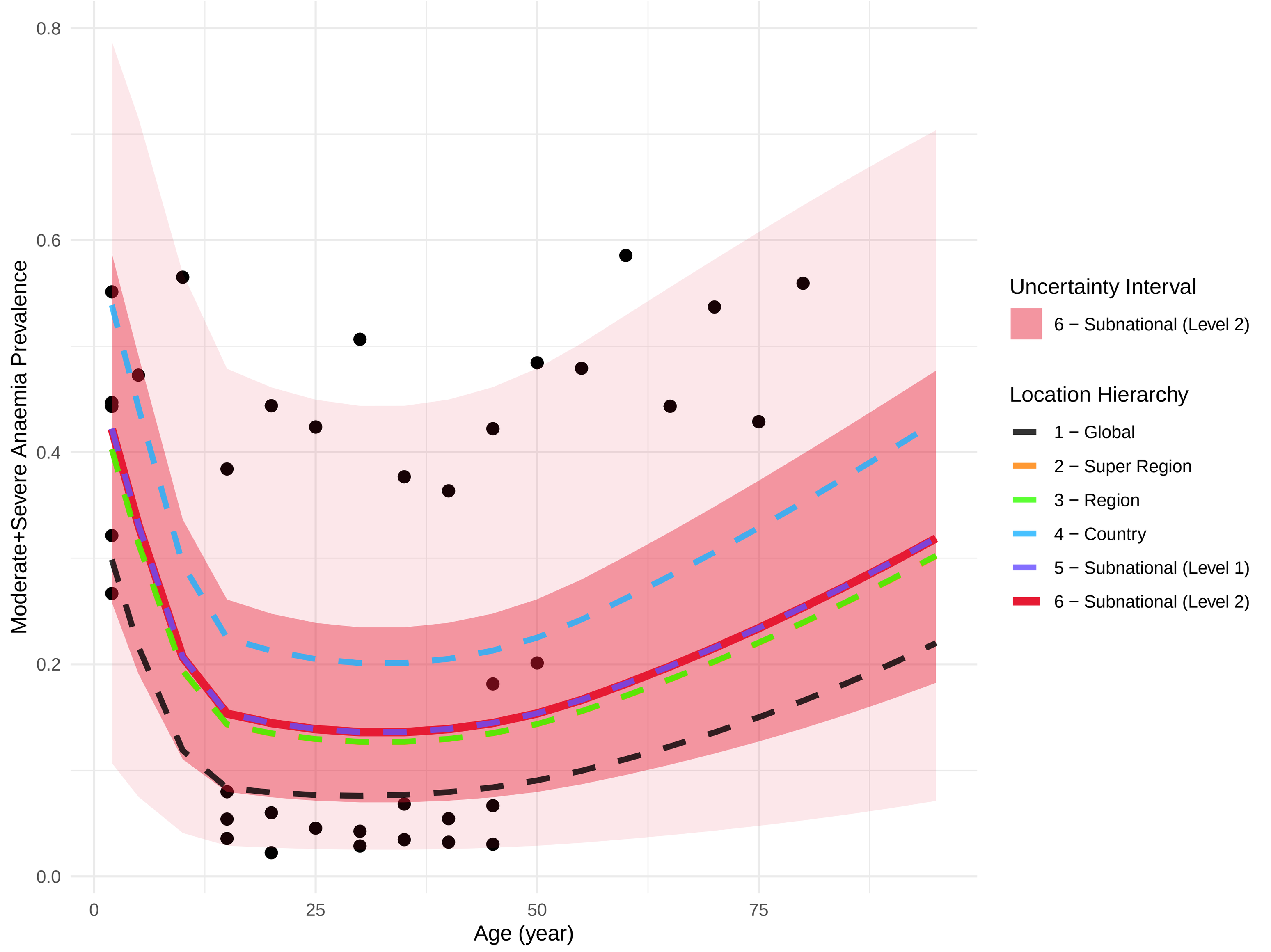

MR−BRT Age/Sex Fits − Males 2+ Years Old − Moderate+Severe Anaemia Prevalence − Jammu & Kashmir and Ladakh
WHO−2024 Adjustment Method

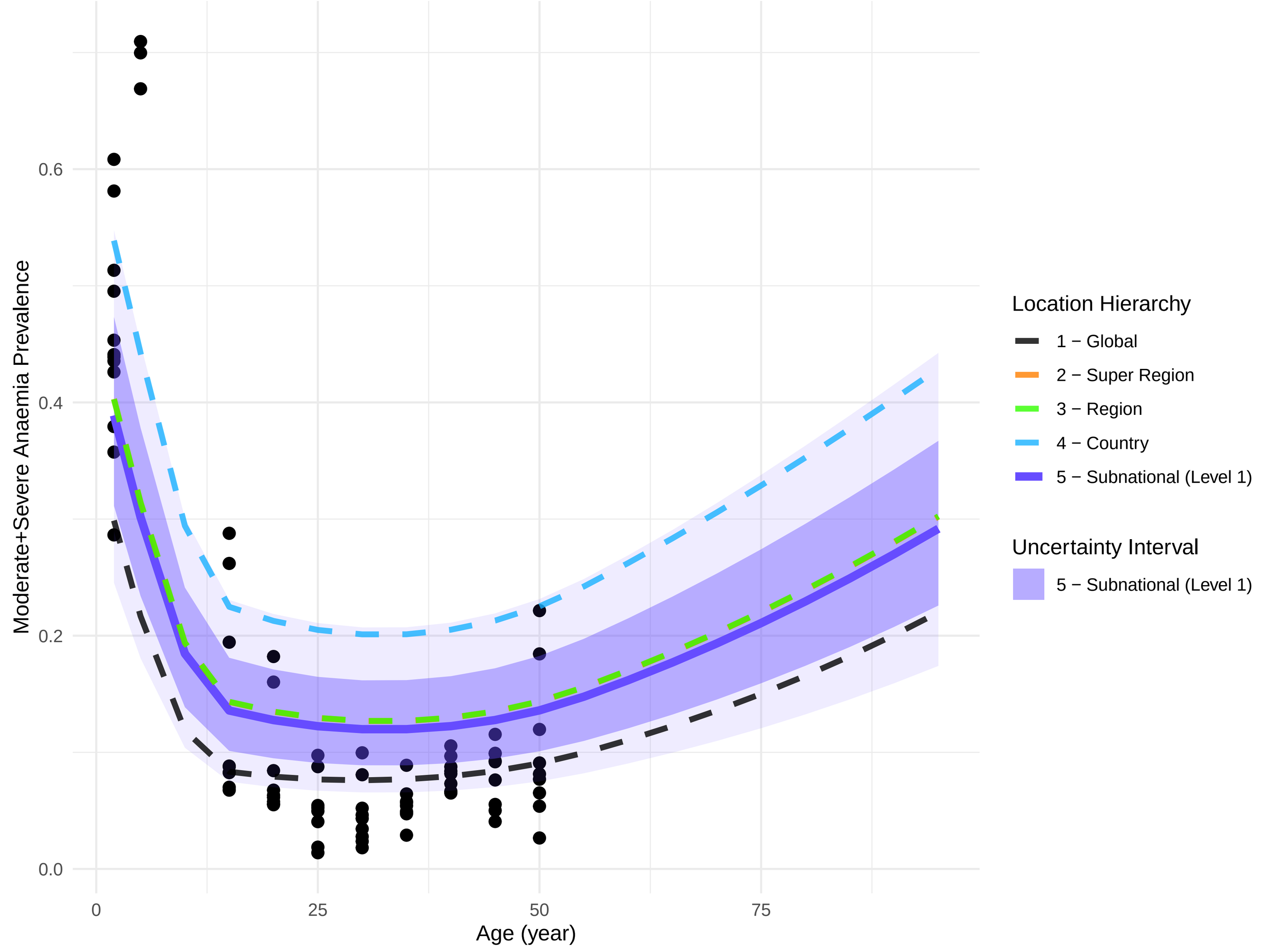

MR−BRT Age/Sex Fits − Males 2+ Years Old − Moderate+Severe Anaemia Prevalence − Jammu & Kashmir and Ladakh, Rural
WHO−2024 Adjustment Method

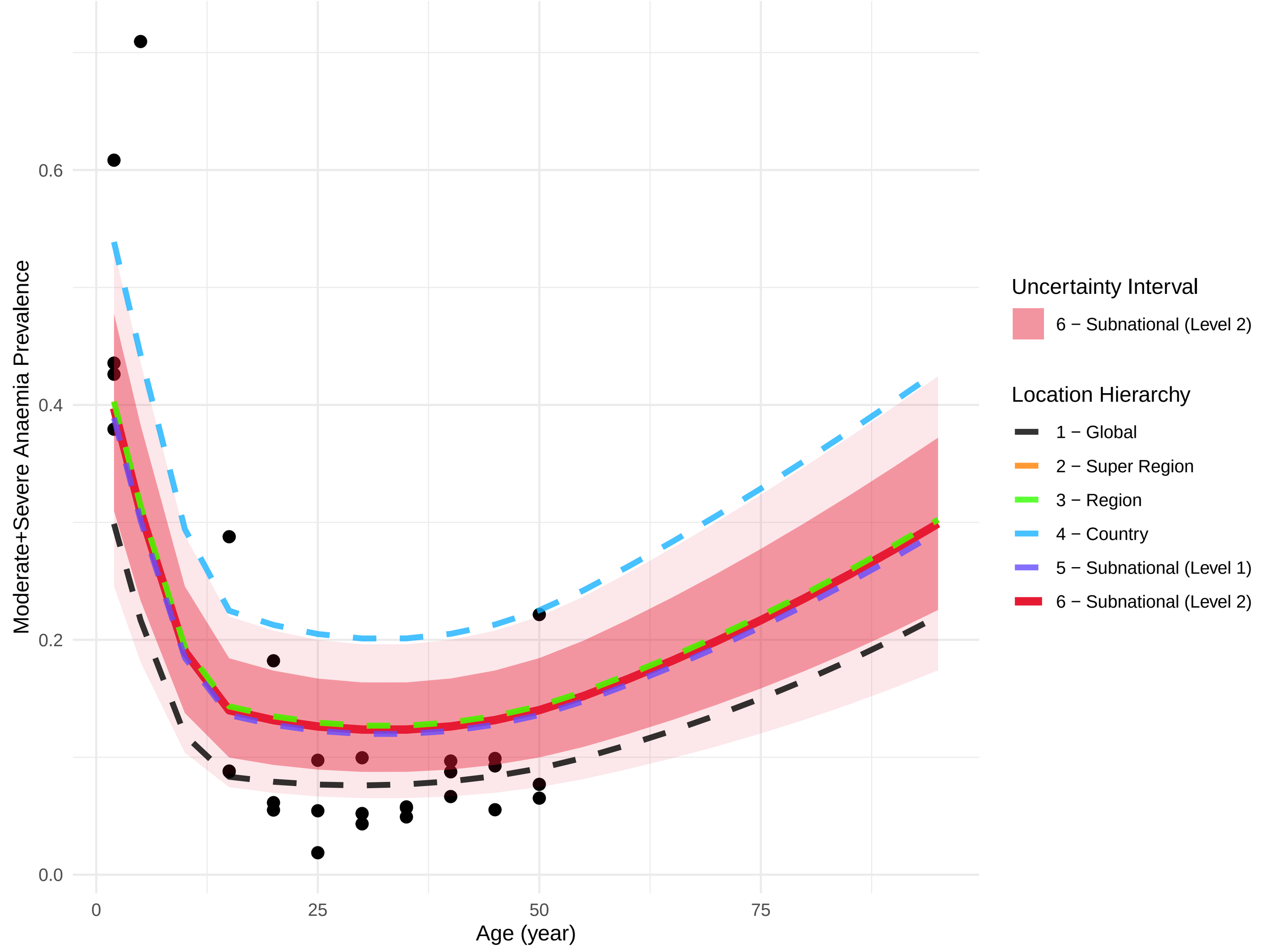

MR−BRT Age/Sex Fits − Males 2+ Years Old − Moderate+Severe Anaemia Prevalence − Jammu & Kashmir and Ladakh, Urban
WHO−2024 Adjustment Method

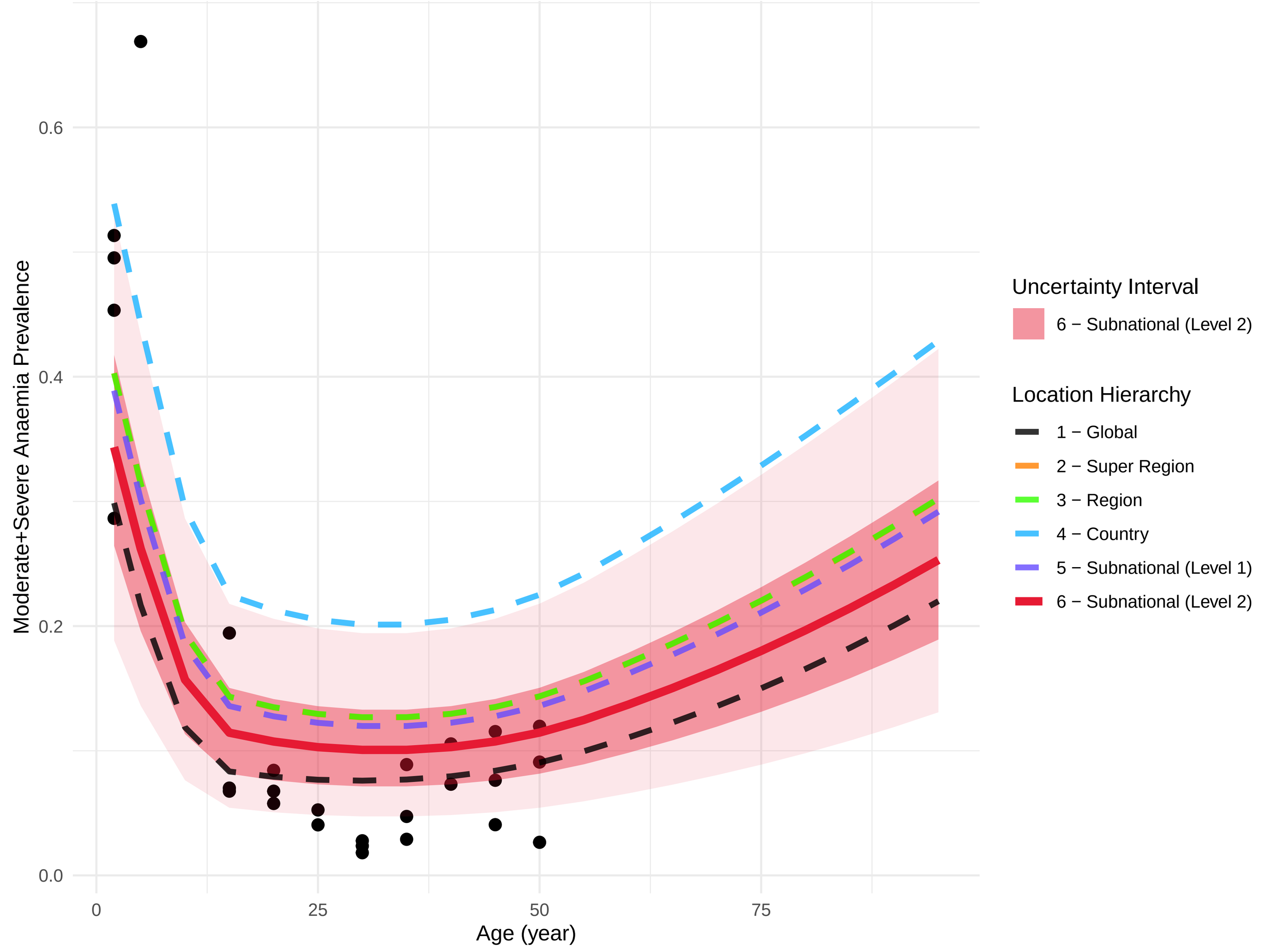

MR−BRT Age/Sex Fits − Males 2+ Years Old − Moderate+Severe Anaemia Prevalence − Jharkhand
WHO−2024 Adjustment Method

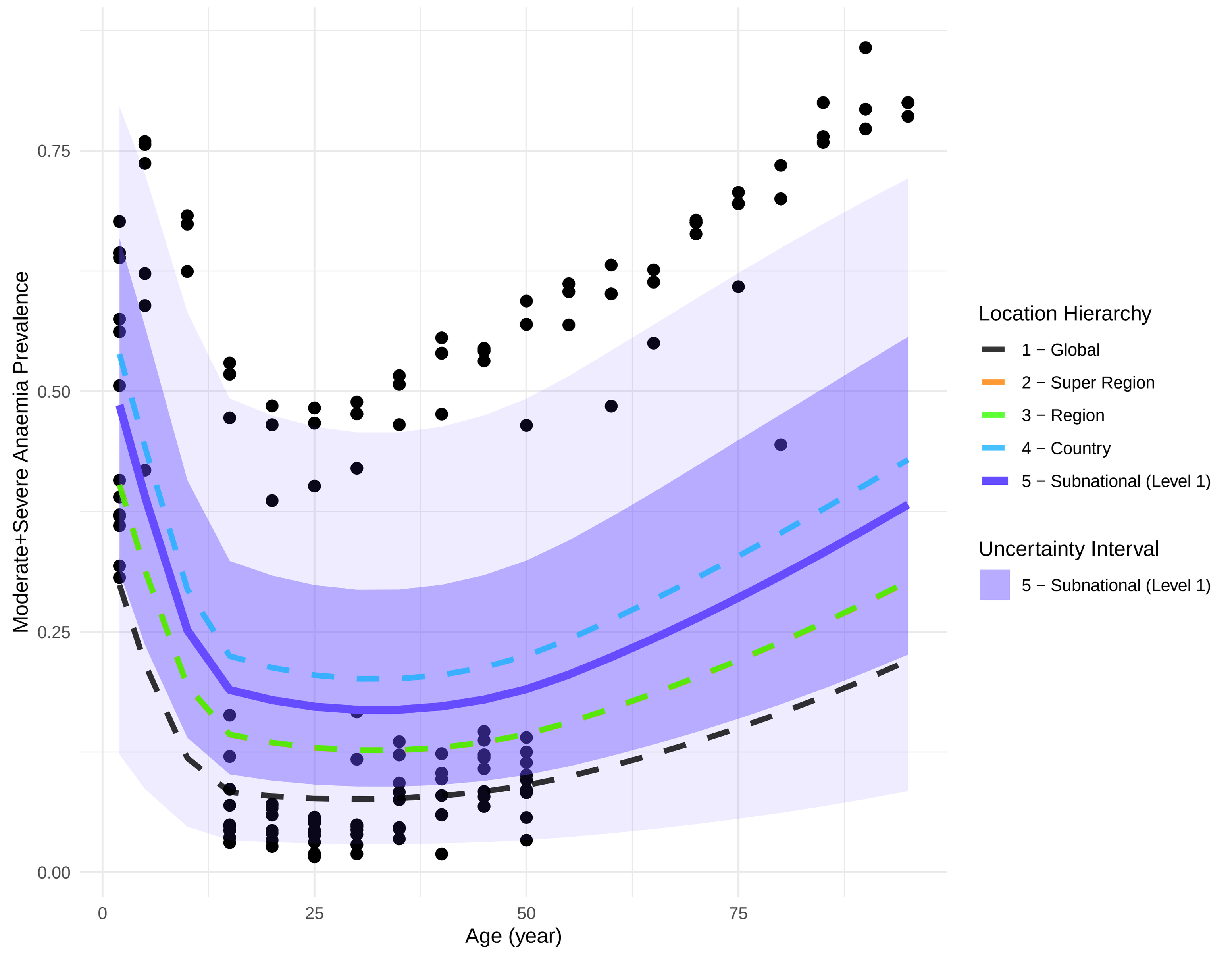

MR−BRT Age/Sex Fits − Males 2+ Years Old − Moderate+Severe Anaemia Prevalence − Jharkhand, Rural
WHO−2024 Adjustment Method

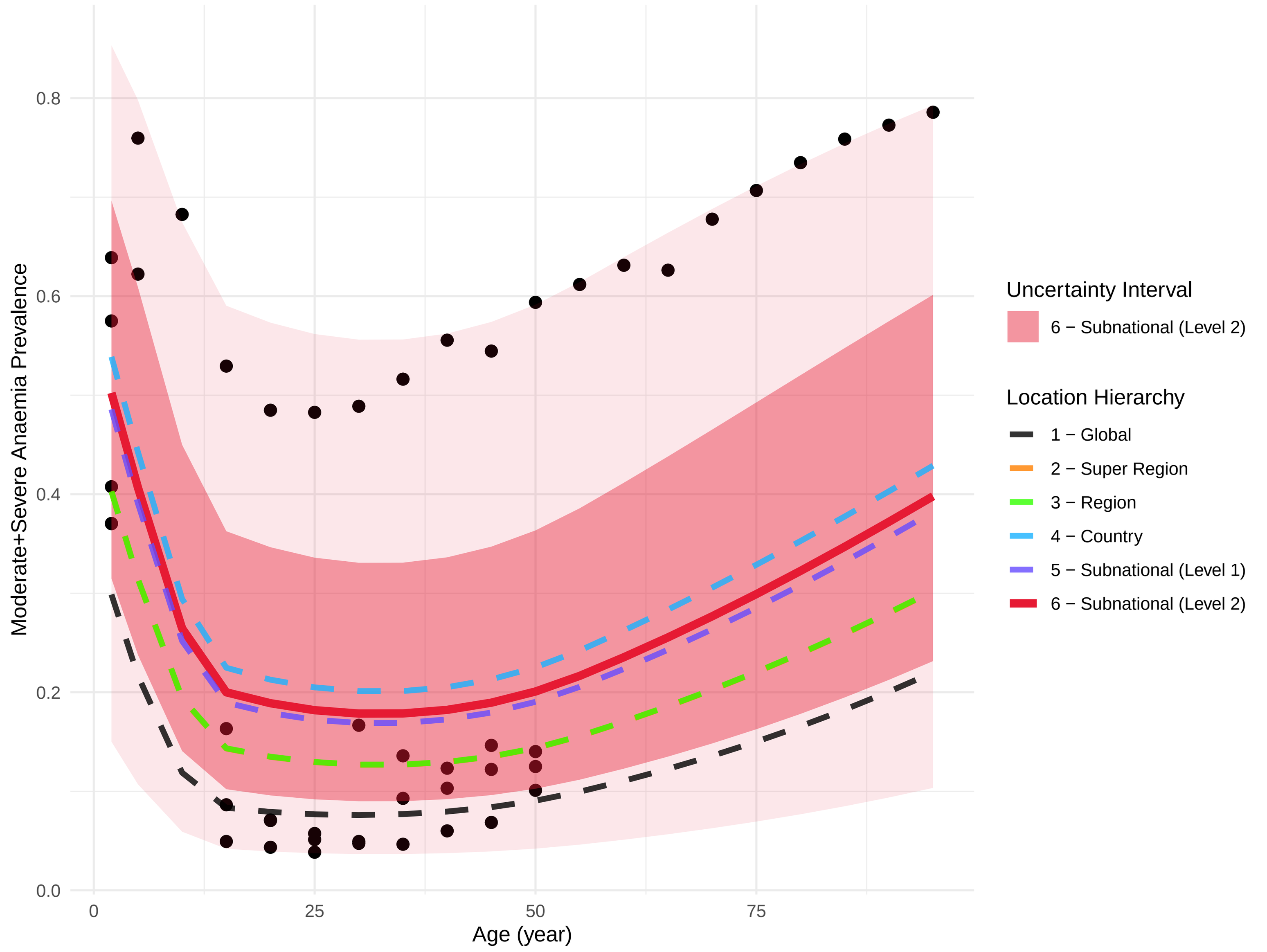

MR−BRT Age/Sex Fits − Males 2+ Years Old − Moderate+Severe Anaemia Prevalence − Jharkhand, Urban
WHO−2024 Adjustment Method

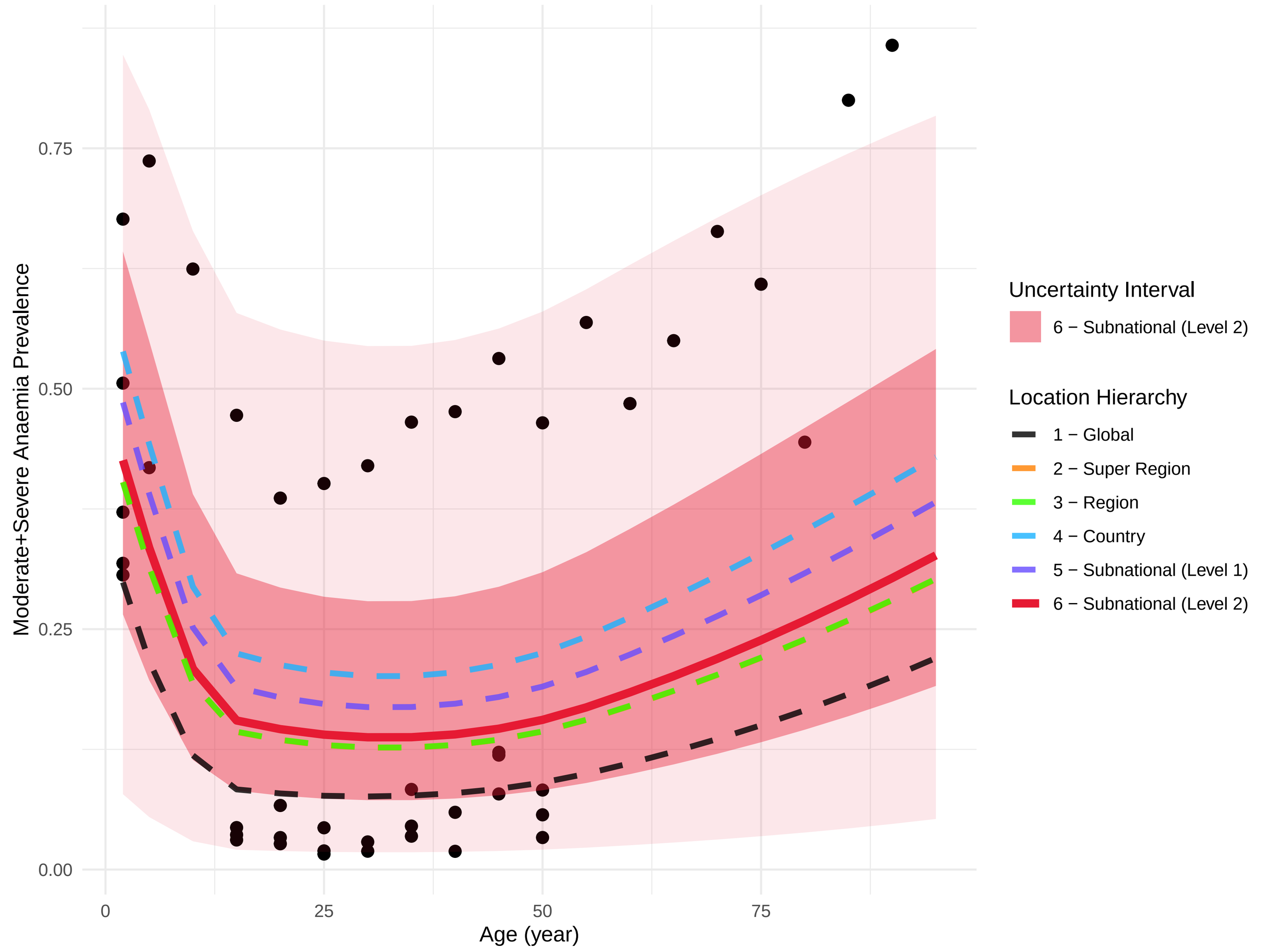

MR−BRT Age/Sex Fits − Males 2+ Years Old − Moderate+Severe Anaemia Prevalence − Karnataka
WHO−2024 Adjustment Method

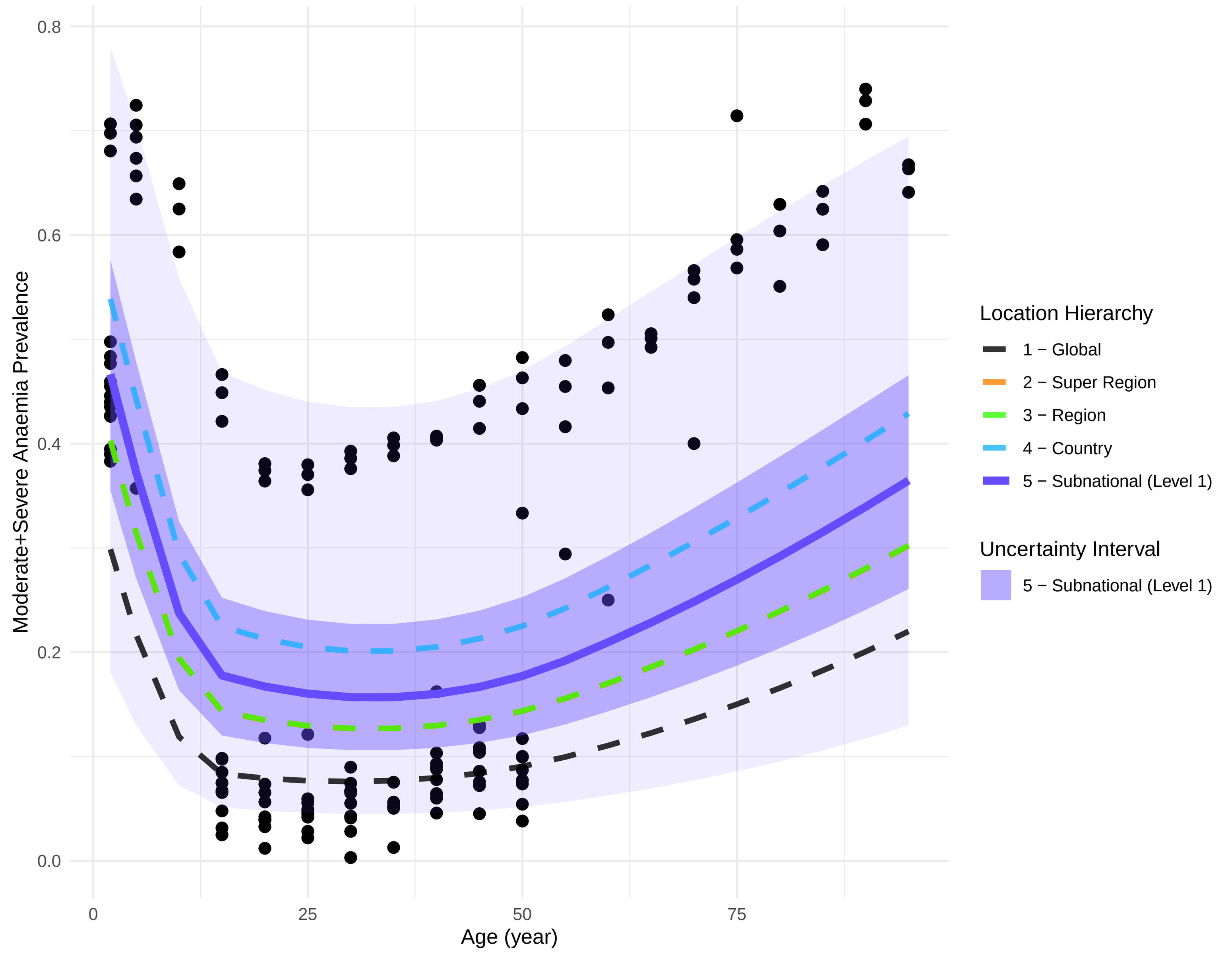

MR−BRT Age/Sex Fits − Males 2+ Years Old − Moderate+Severe Anaemia Prevalence − Karnataka, Rural
WHO−2024 Adjustment Method

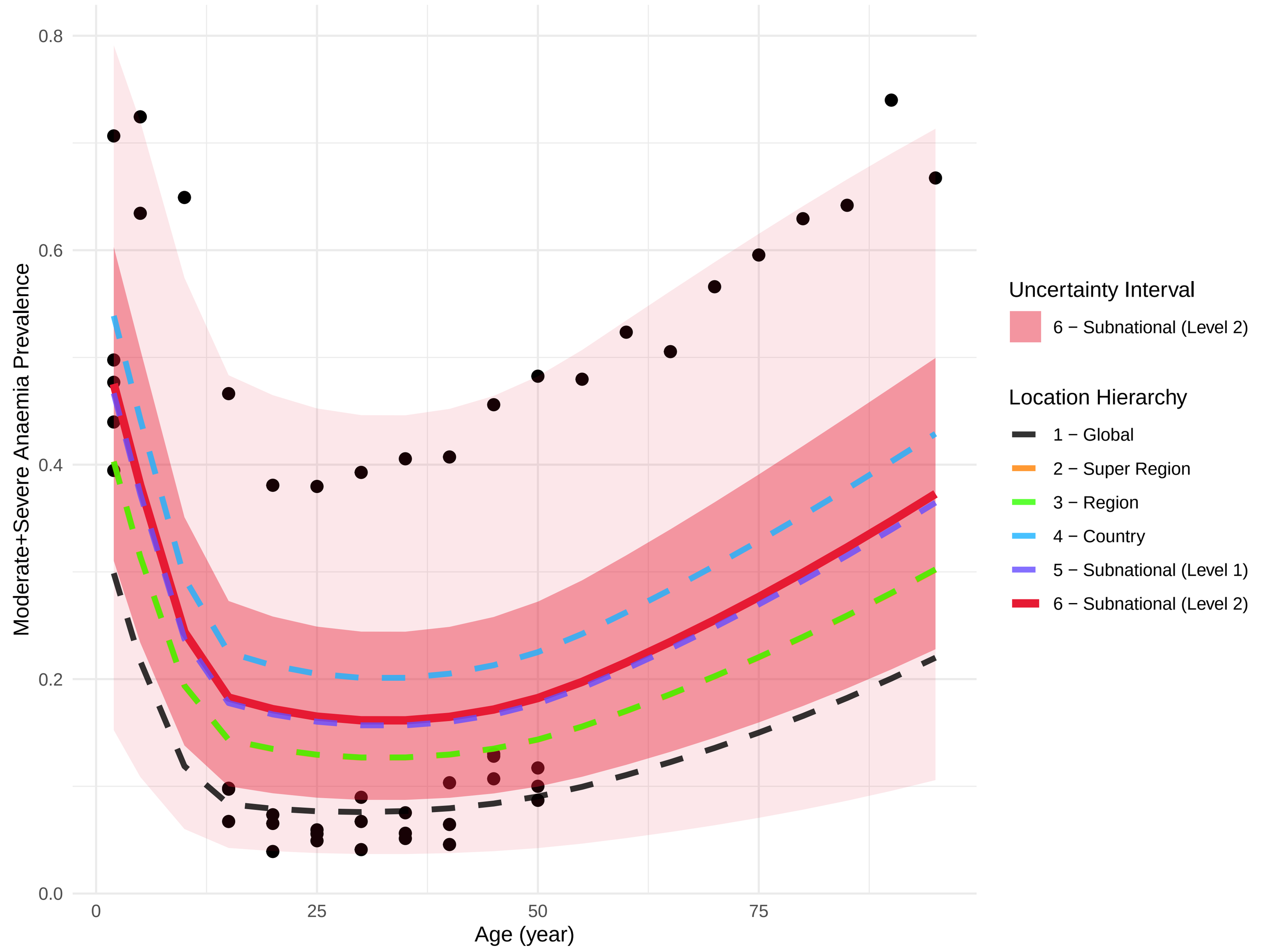

MR−BRT Age/Sex Fits − Males 2+ Years Old − Moderate+Severe Anaemia Prevalence − Karnataka, Urban
WHO−2024 Adjustment Method

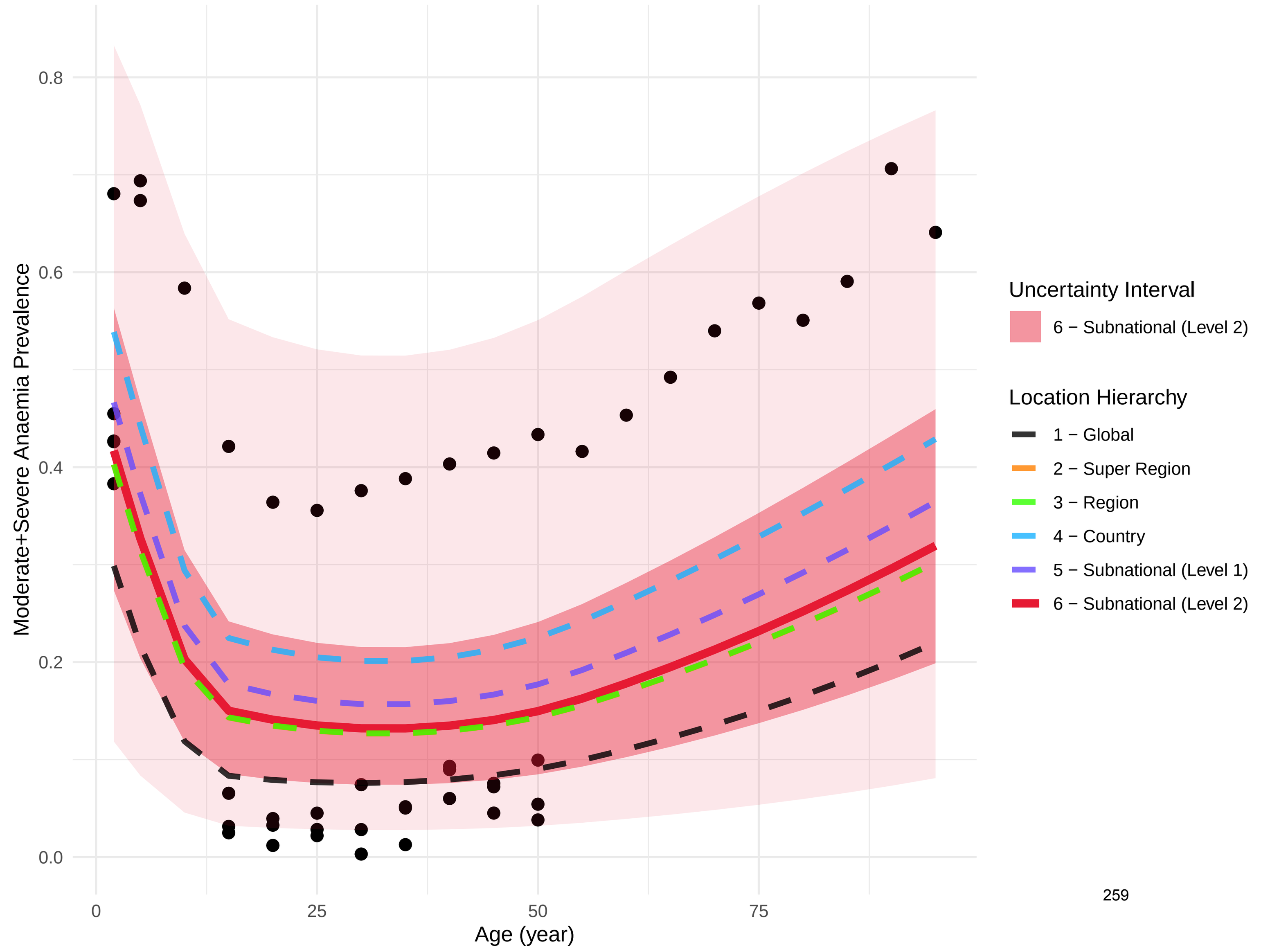

MR−BRT Age/Sex Fits − Males 2+ Years Old − Moderate+Severe Anaemia Prevalence − Kerala
WHO−2024 Adjustment Method

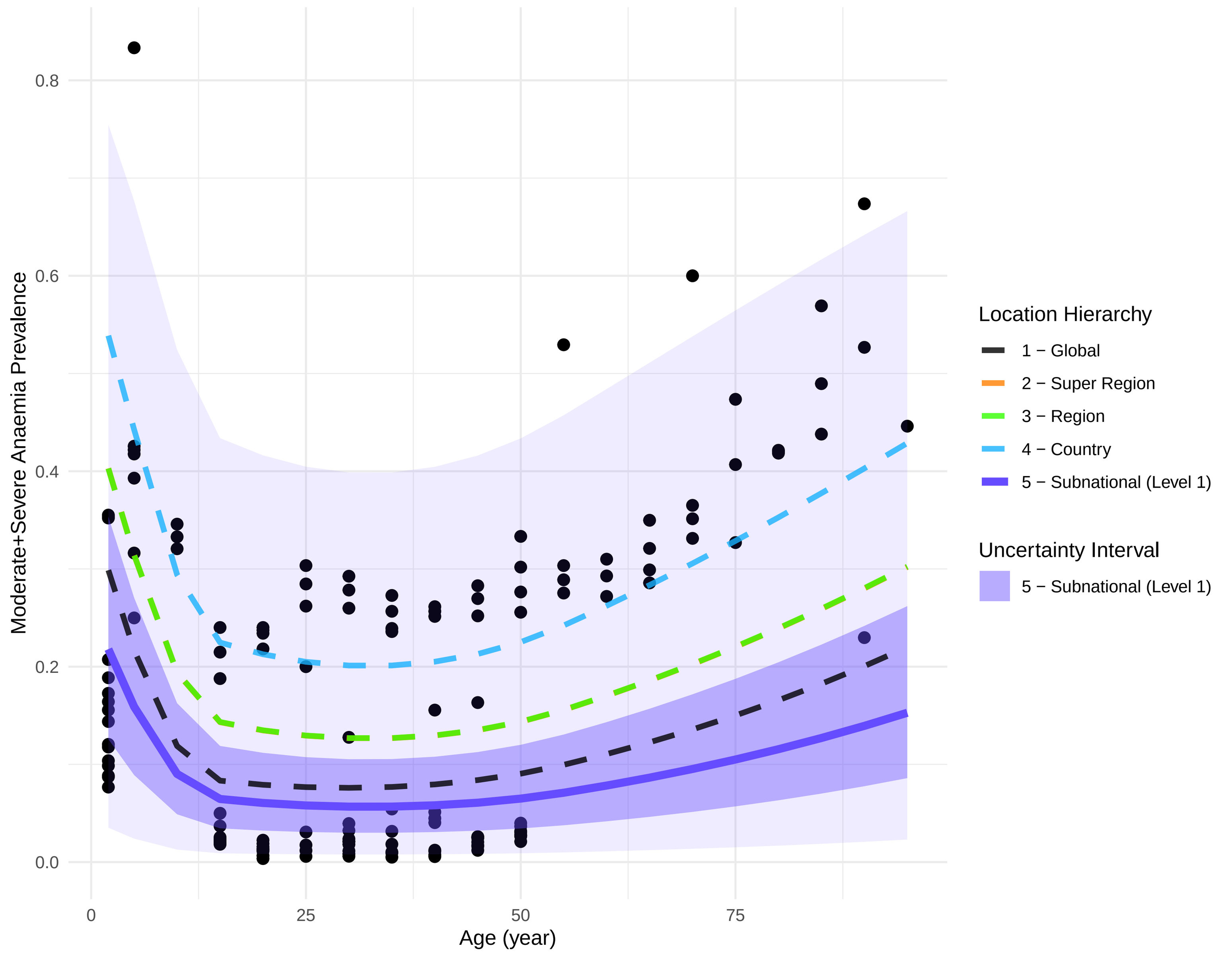

MR−BRT Age/Sex Fits − Males 2+ Years Old − Moderate+Severe Anaemia Prevalence − Kerala, Rural
WHO−2024 Adjustment Method

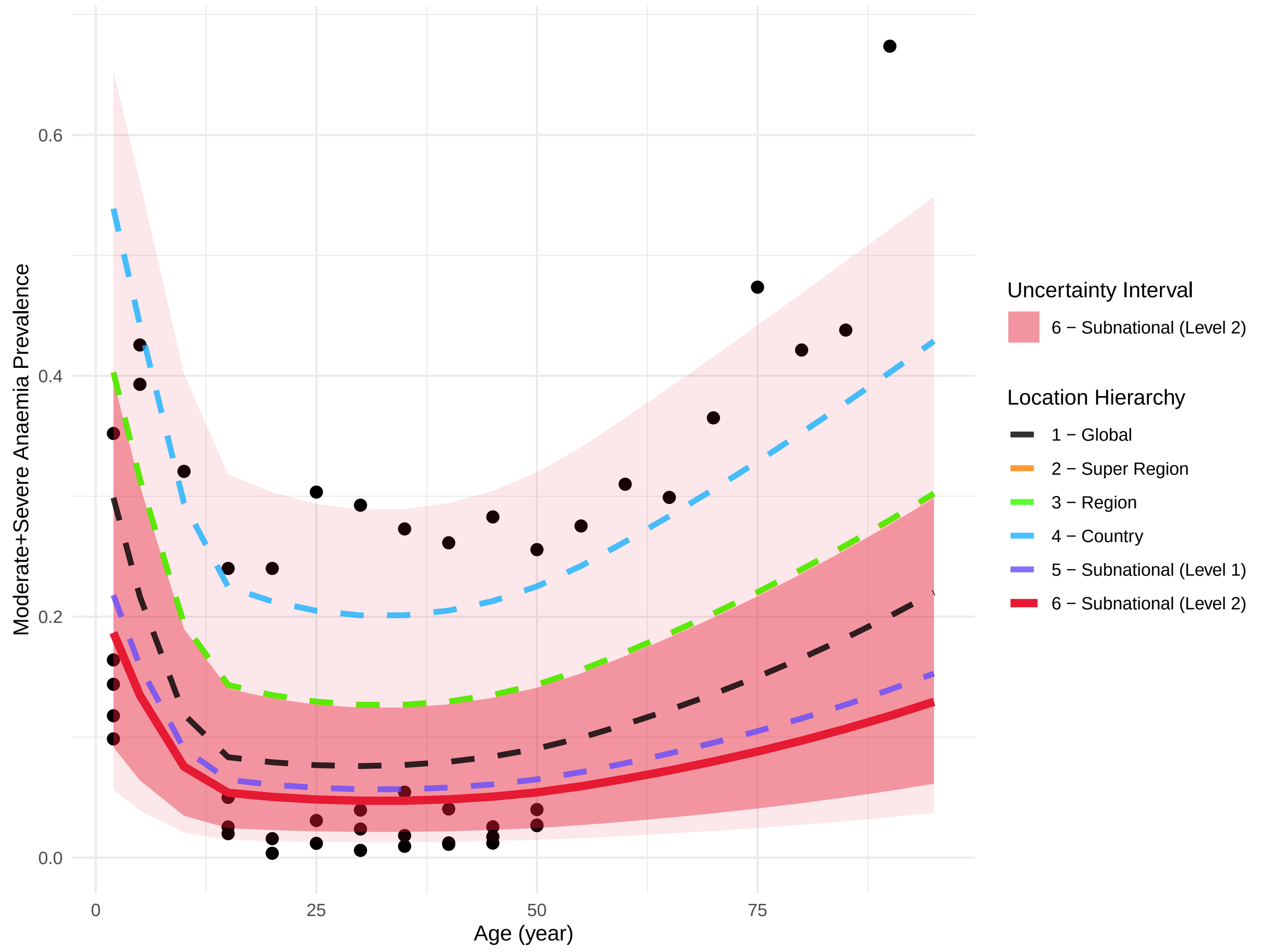

MR−BRT Age/Sex Fits − Males 2+ Years Old − Moderate+Severe Anaemia Prevalence − Kerala, Urban
WHO−2024 Adjustment Method

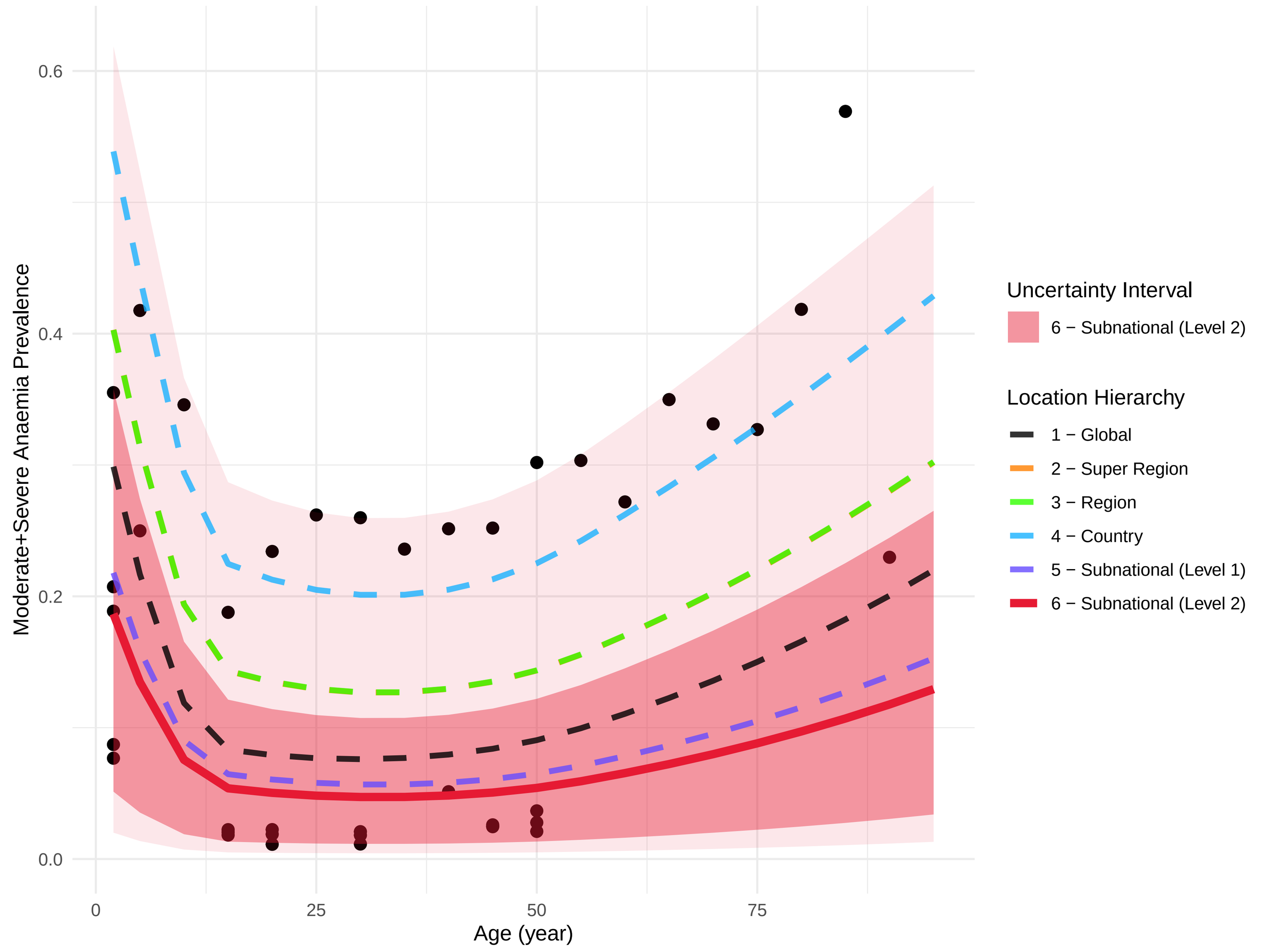

MR−BRT Age/Sex Fits − Males 2+ Years Old − Moderate+Severe Anaemia Prevalence − Madhya Pradesh
WHO−2024 Adjustment Method

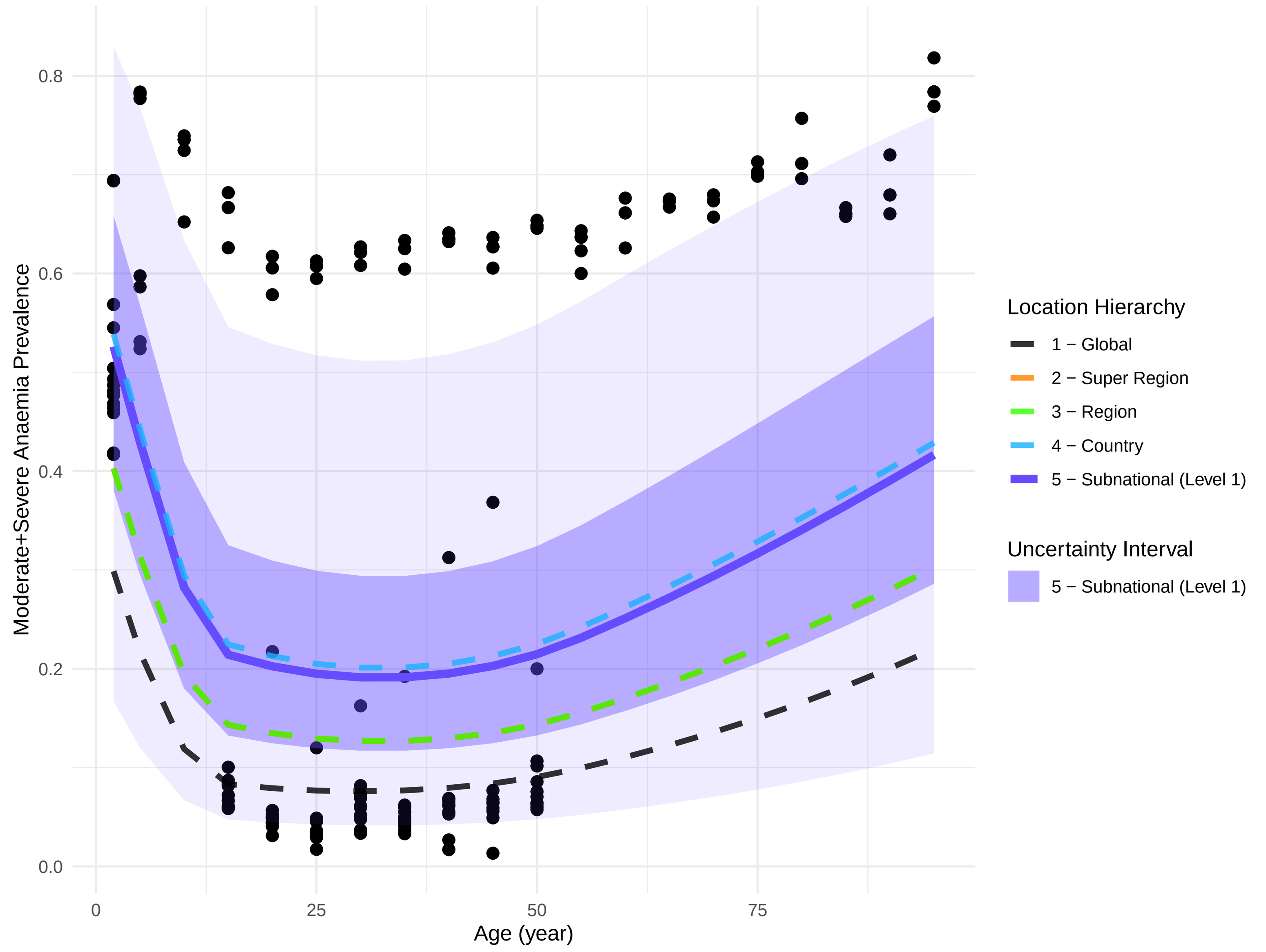

MR−BRT Age/Sex Fits − Males 2+ Years Old − Moderate+Severe Anaemia Prevalence − Madhya Pradesh, Rural
WHO−2024 Adjustment Method

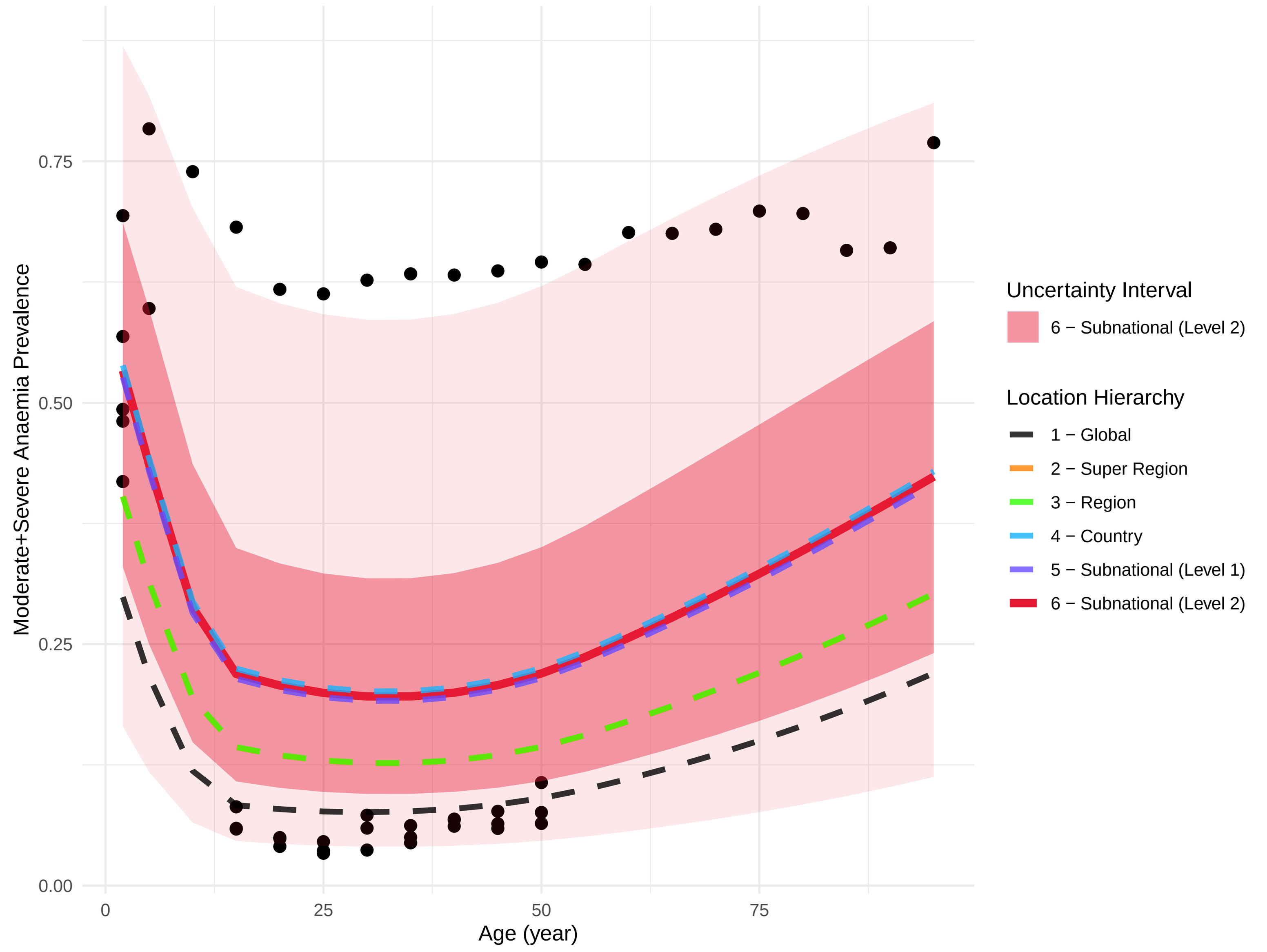

MR−BRT Age/Sex Fits − Males 2+ Years Old − Moderate+Severe Anaemia Prevalence − Madhya Pradesh, Urban
WHO−2024 Adjustment Method

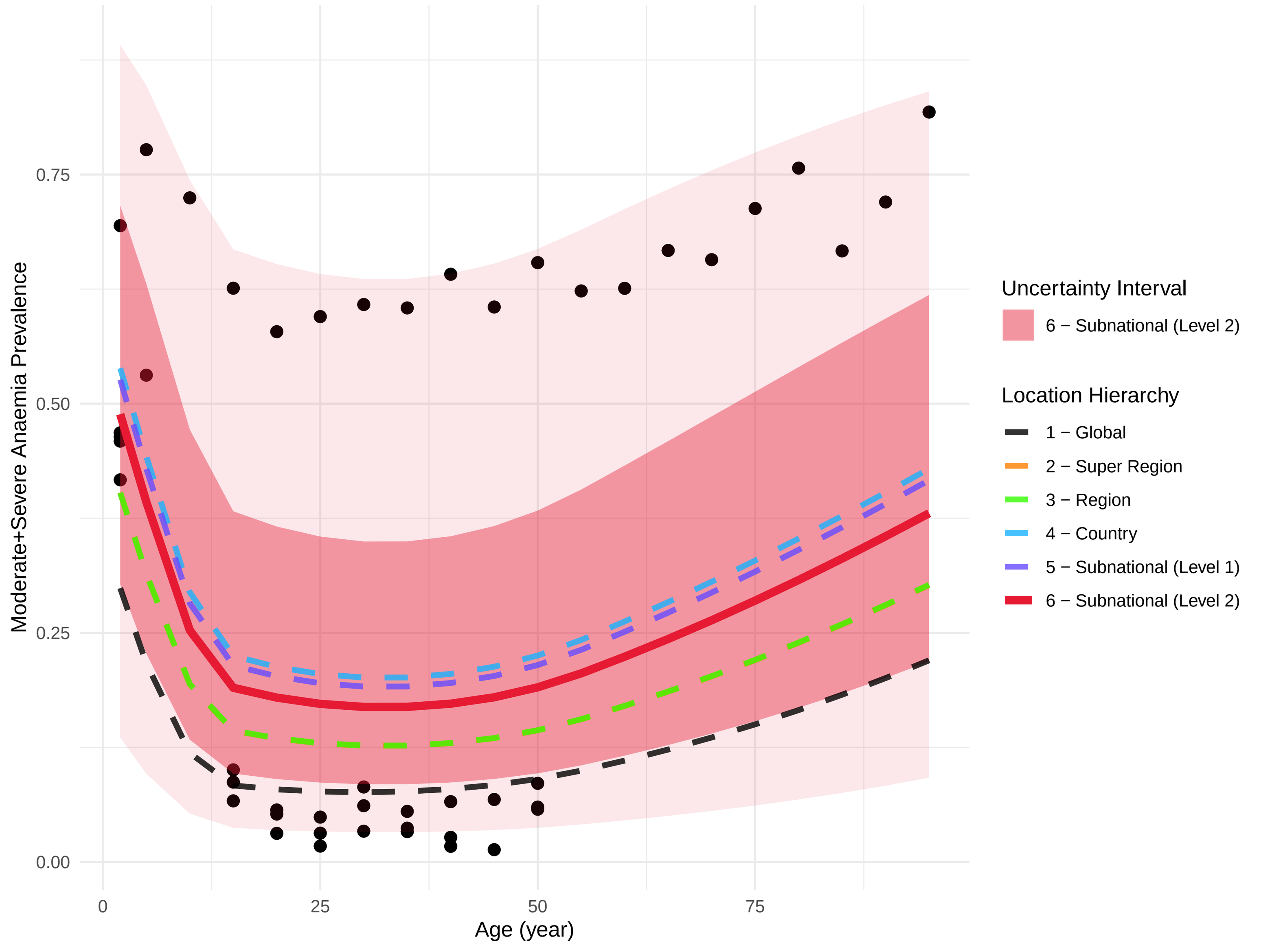

MR−BRT Age/Sex Fits − Males 2+ Years Old − Moderate+Severe Anaemia Prevalence − Maharashtra
WHO−2024 Adjustment Method

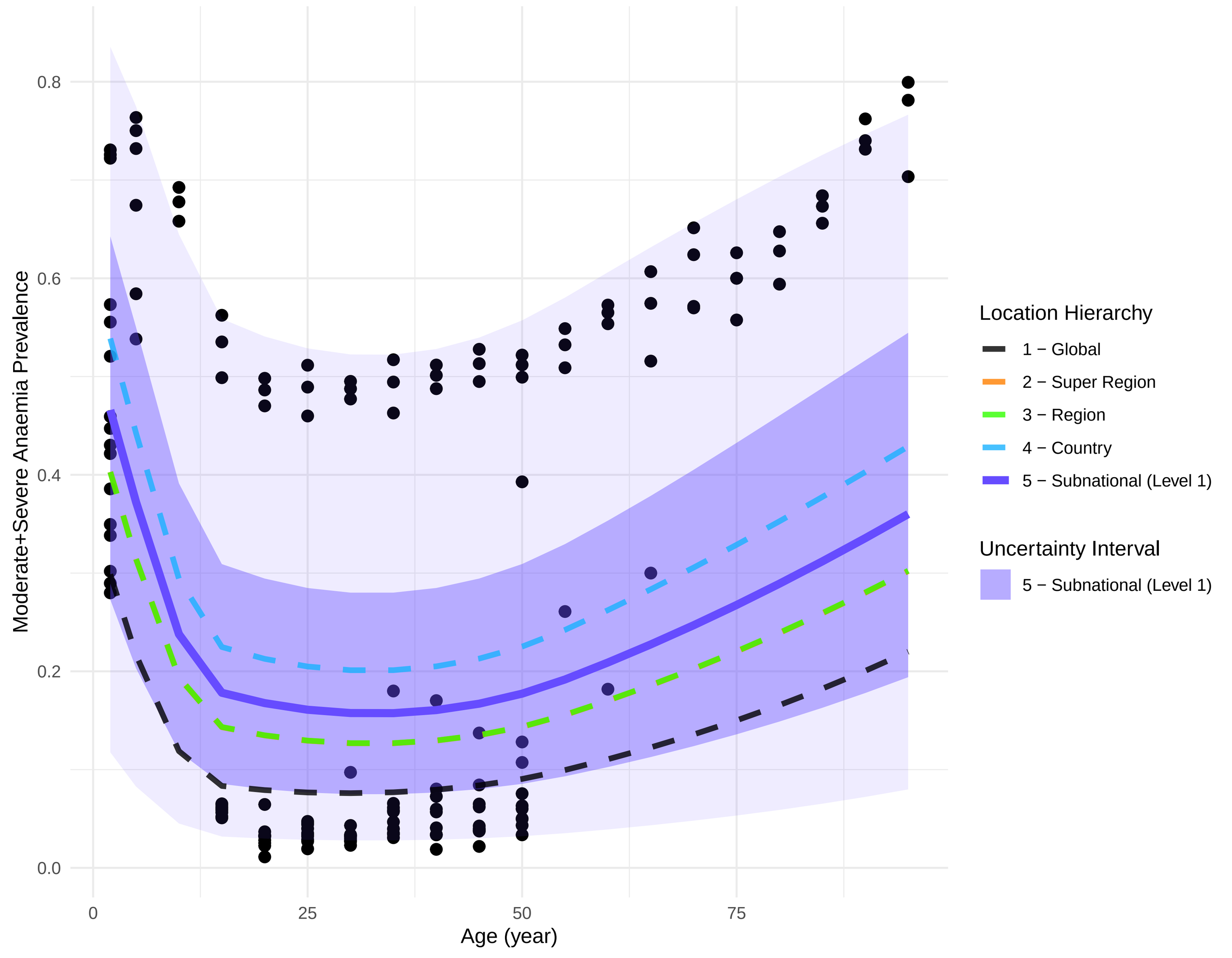

MR−BRT Age/Sex Fits − Males 2+ Years Old − Moderate+Severe Anaemia Prevalence − Maharashtra, Rural
WHO−2024 Adjustment Method

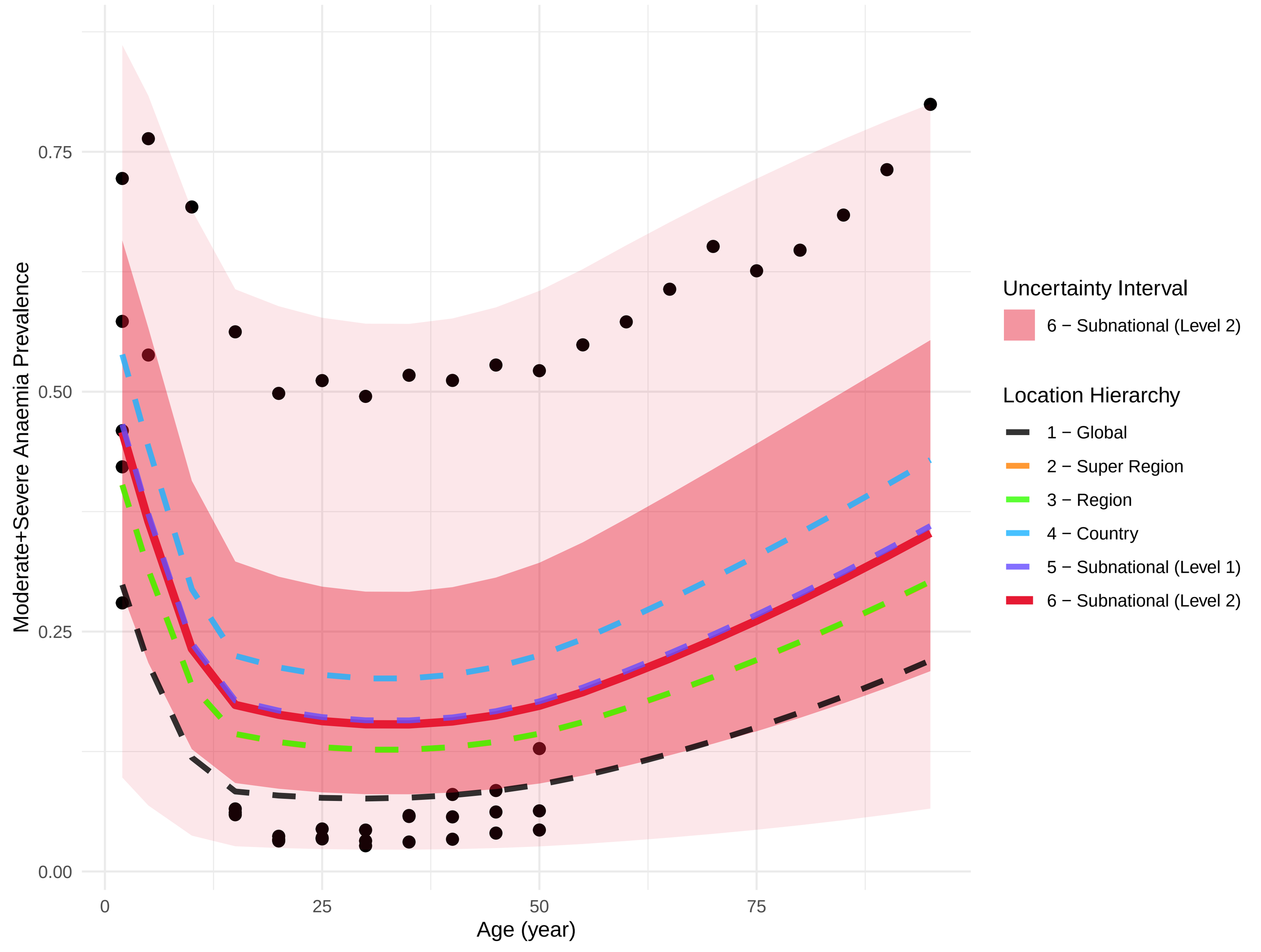

MR−BRT Age/Sex Fits − Males 2+ Years Old − Moderate+Severe Anaemia Prevalence − Maharashtra, Urban
WHO−2024 Adjustment Method

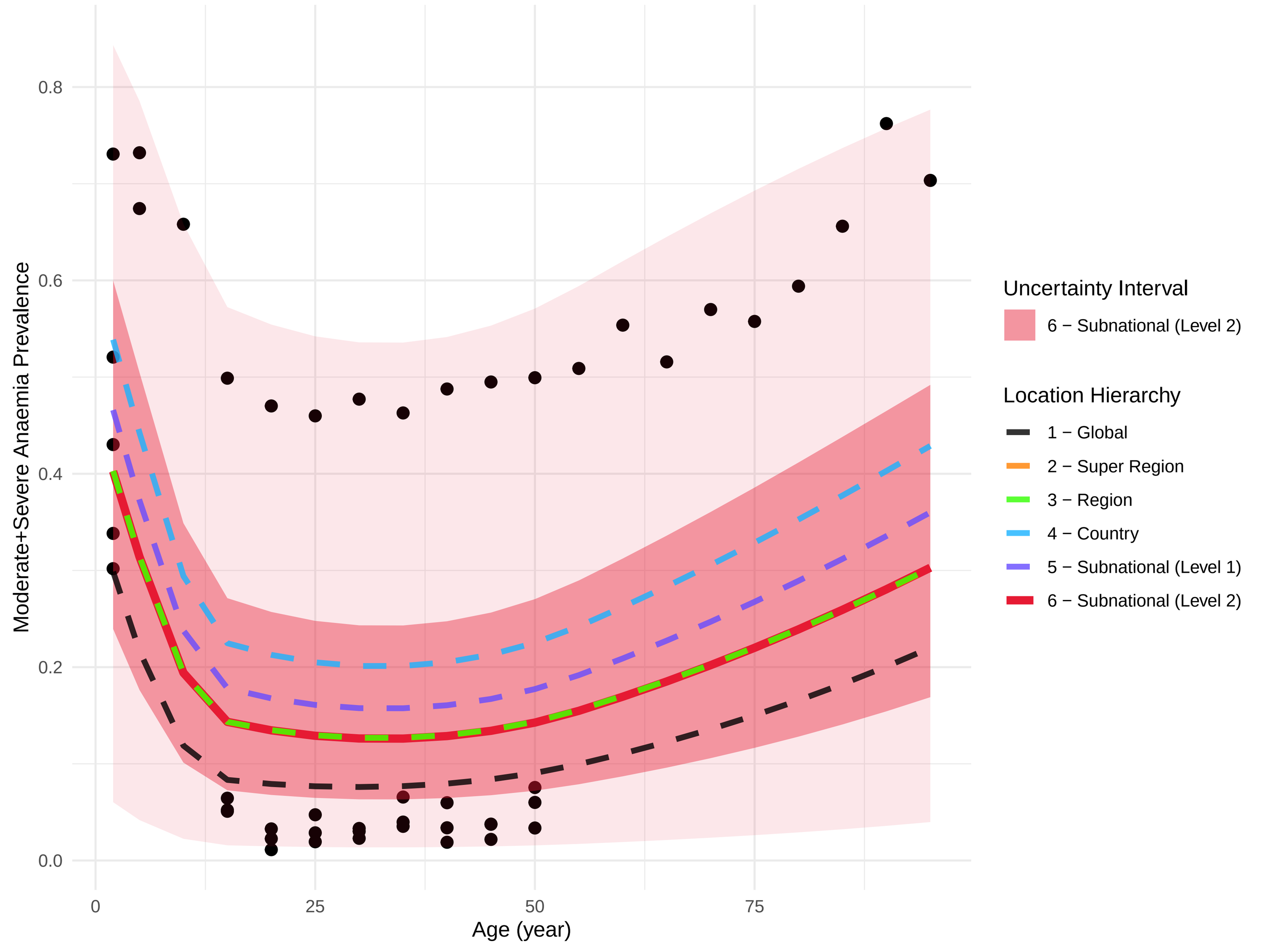

MR−BRT Age/Sex Fits − Males 2+ Years Old − Moderate+Severe Anaemia Prevalence − Manipur
WHO−2024 Adjustment Method

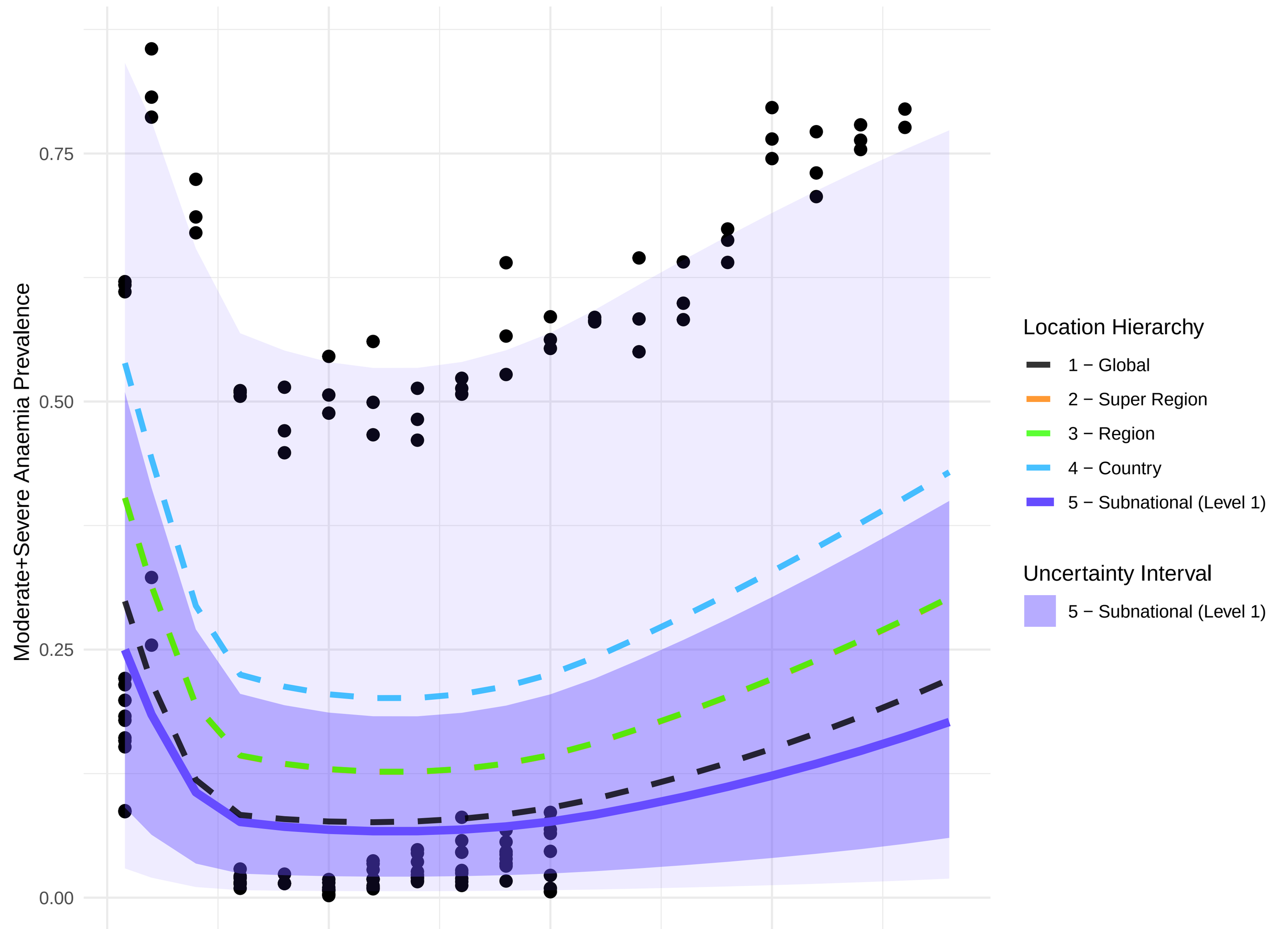

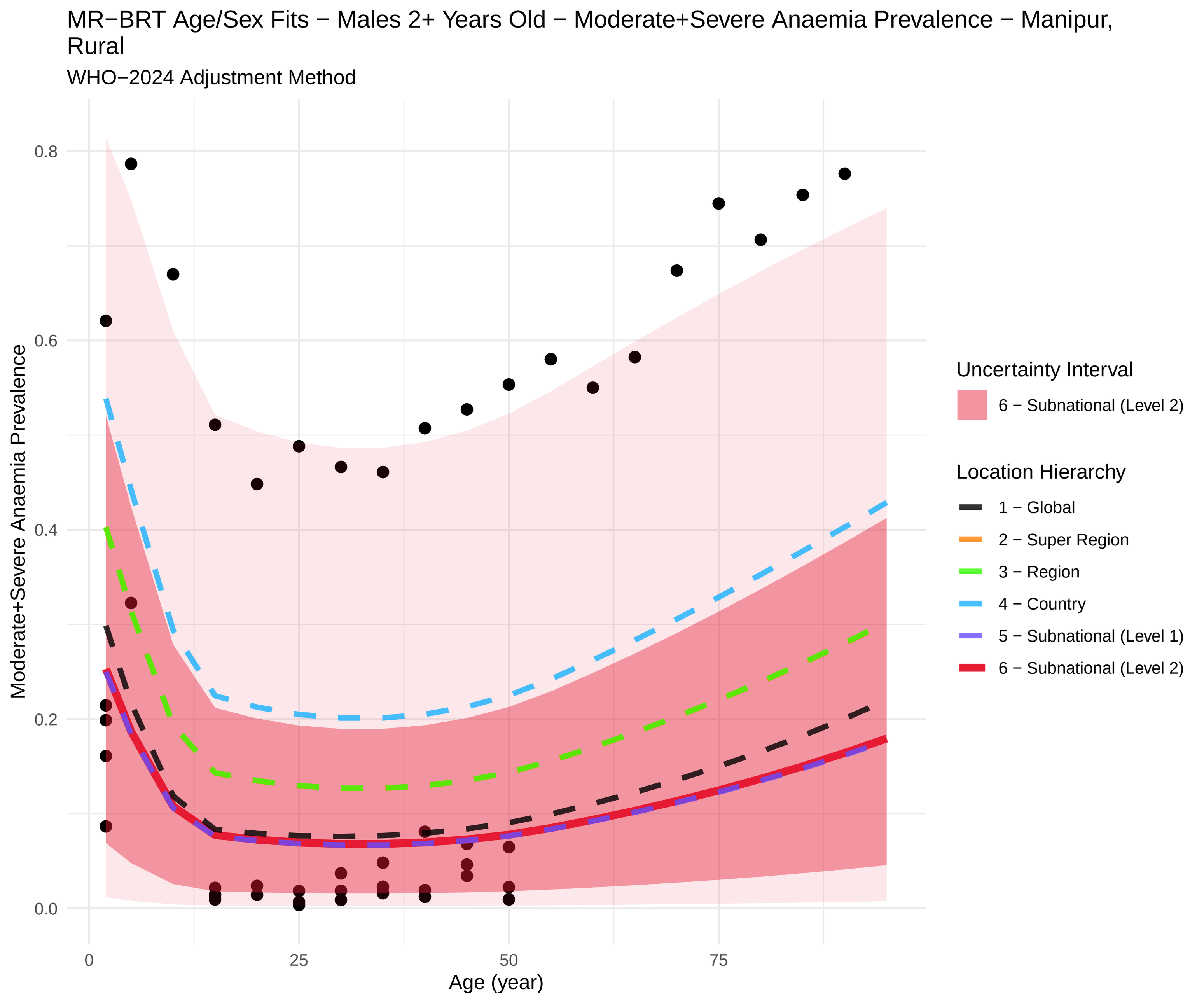
MR−BRT Age/Sex Fits − Males 2+ Years Old − Moderate+Severe Anaemia Prevalence − Manipur, Rural
WHO−2024 Adjustment Method
Moderate+Severe Anaemia Prevalence
Age (year)
Uncertainty Interval
6 − Subnational (Level 2)
Location Hierarchy
1 − Global
2 − Super Region
3 − Region
4 − Country
5 − Subnational (Level 1)
6 − Subnational (Level 2)

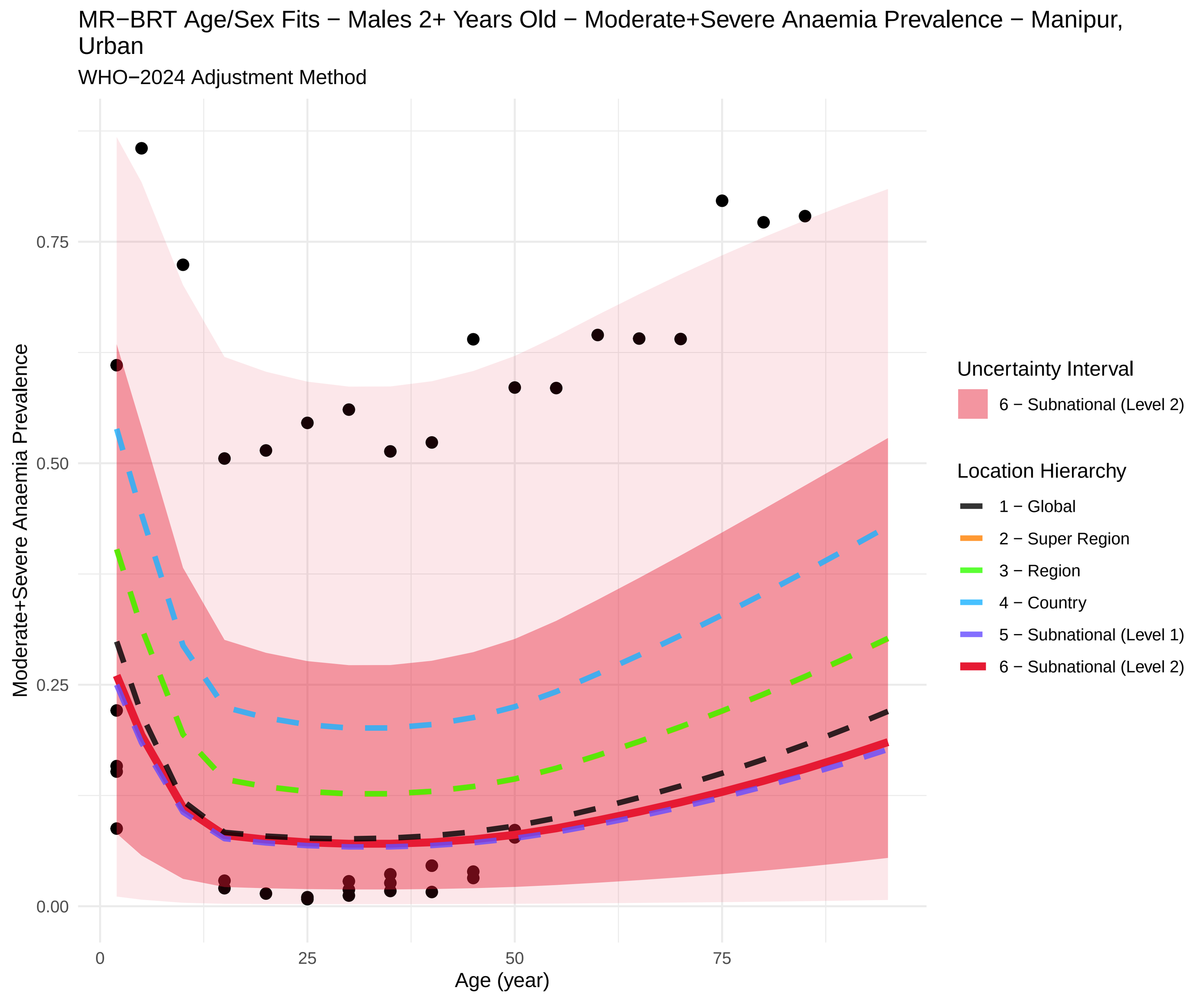
MR−BRT Age/Sex Fits − Males 2+ Years Old − Moderate+Severe Anaemia Prevalence − Manipur, Urban
WHO−2024 Adjustment Method
Moderate+Severe Anaemia Prevalence
Age (year)
Uncertainty Interval
6 − Subnational (Level 2)
Location Hierarchy
1 − Global
2 − Super Region
3 − Region
4 − Country
5 − Subnational (Level 1)
6 − Subnational (Level 2)

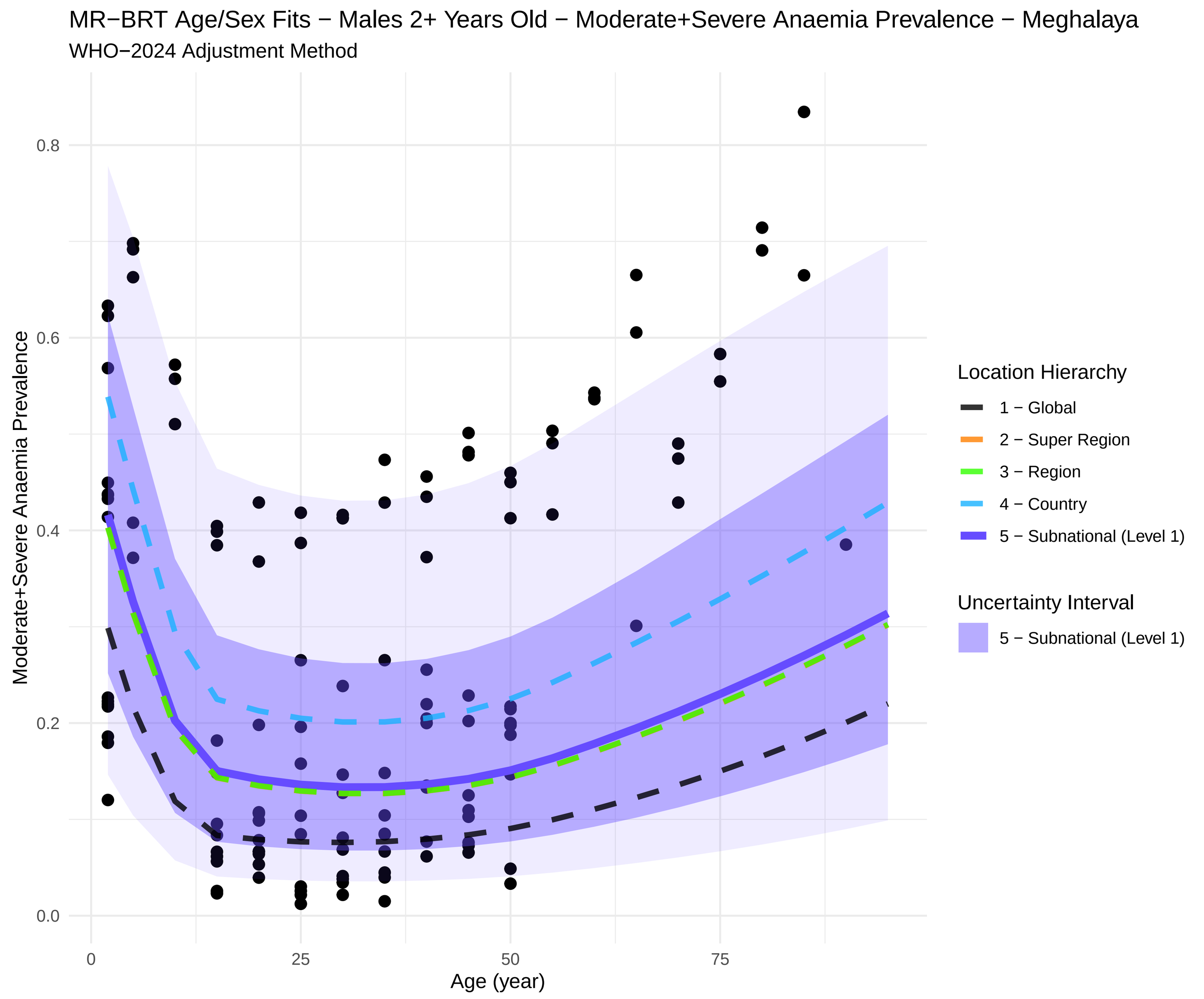
MR−BRT Age/Sex Fits − Males 2+ Years Old − Moderate+Severe Anaemia Prevalence − Meghalaya
WHO−2024 Adjustment Method
Moderate+Severe Anaemia Prevalence
Age (year)
Location Hierarchy
1 − Global
2 − Super Region
3 − Region
4 − Country
5 − Subnational (Level 1)
Uncertainty Interval
5 − Subnational (Level 1)

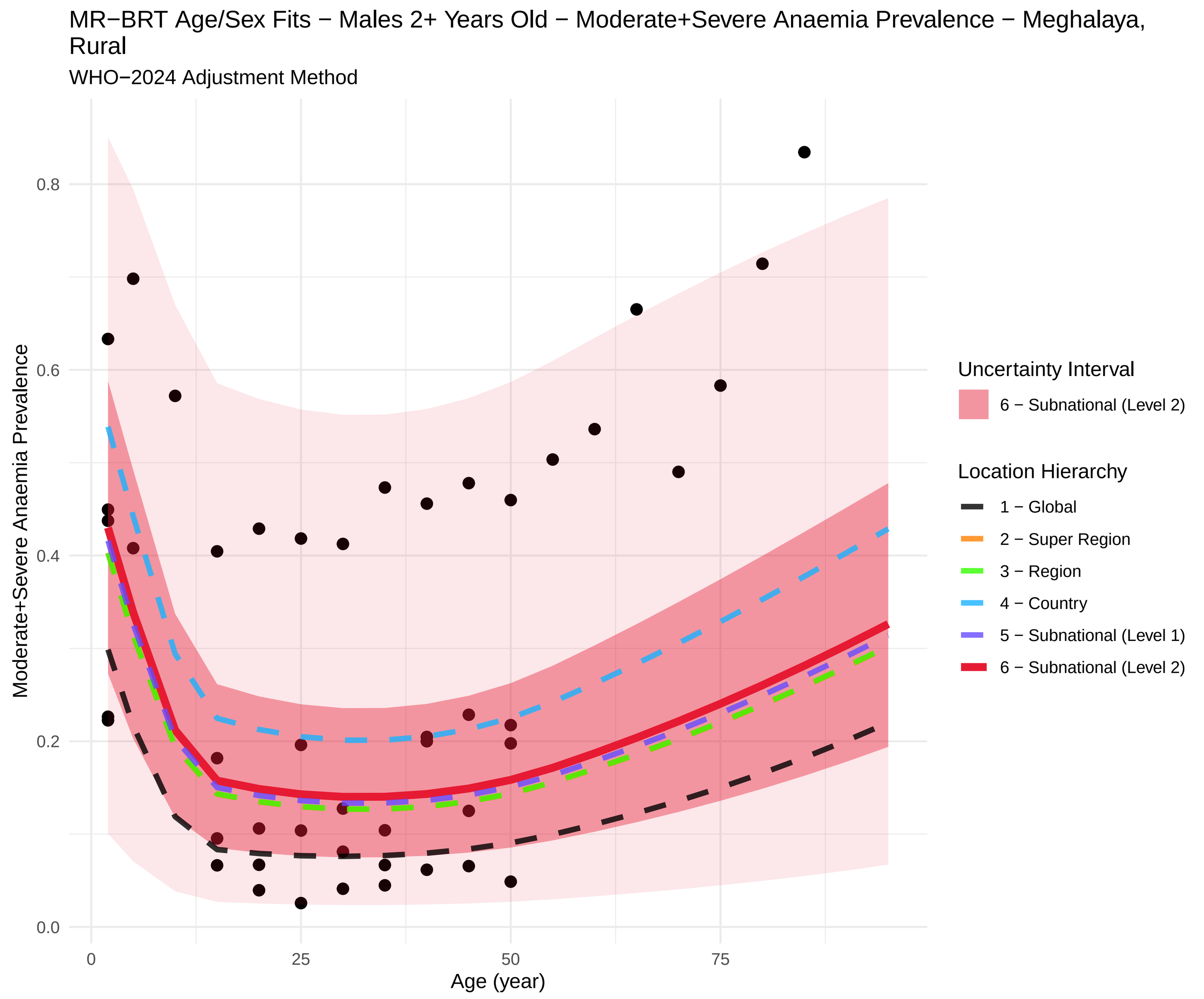
MR−BRT Age/Sex Fits − Males 2+ Years Old − Moderate+Severe Anaemia Prevalence − Meghalaya, Rural
WHO−2024 Adjustment Method
Moderate+Severe Anaemia Prevalence
Age (year)
Uncertainty Interval
6 − Subnational (Level 2)
Location Hierarchy
1 − Global
2 − Super Region
3 − Region
4 − Country
5 − Subnational (Level 1)
6 − Subnational (Level 2)

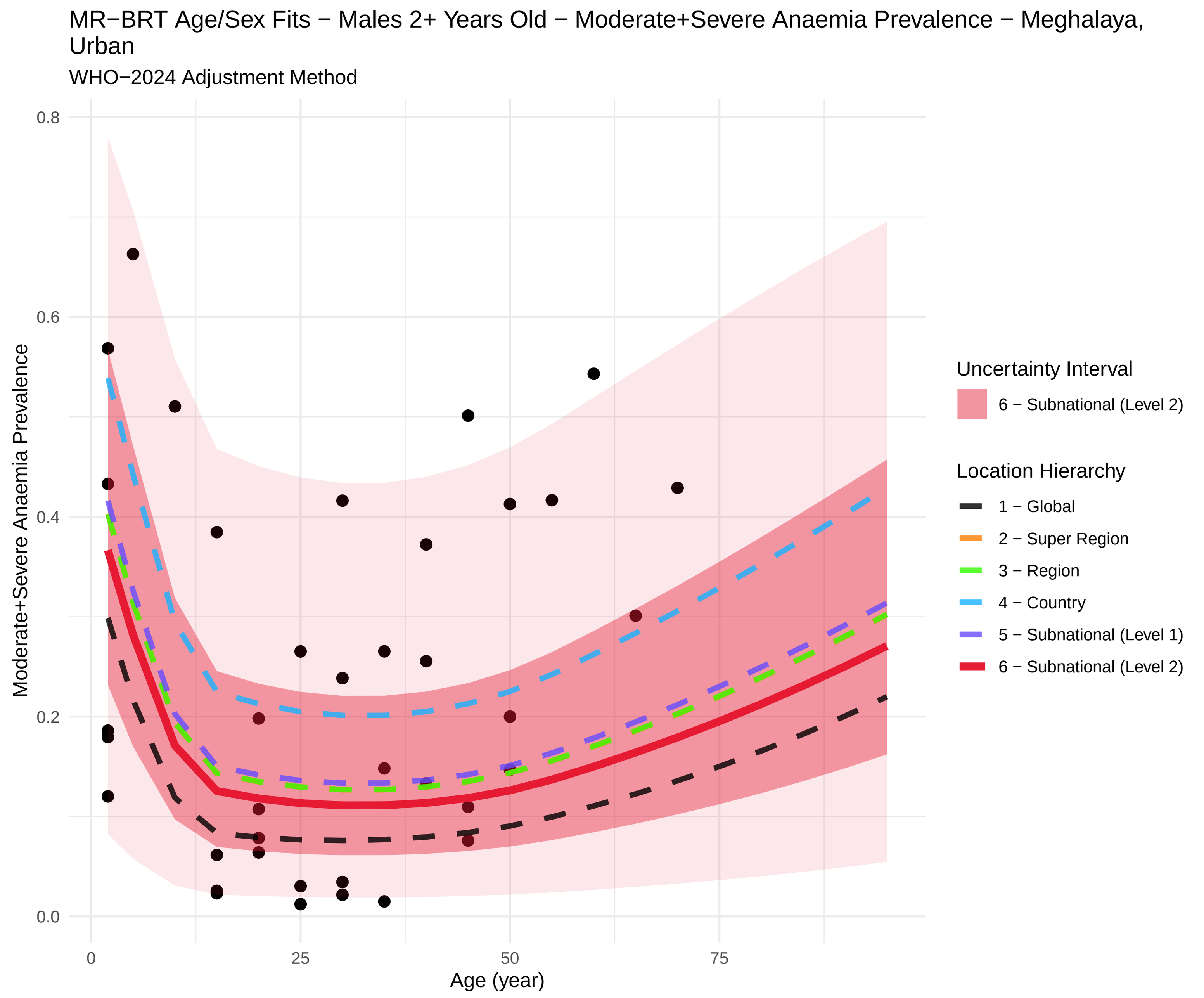
MR−BRT Age/Sex Fits − Males 2+ Years Old − Moderate+Severe Anaemia Prevalence − Meghalaya, Urban
WHO−2024 Adjustment Method
Moderate+Severe Anaemia Prevalence
Age (year)
Uncertainty Interval
6 − Subnational (Level 2)
Location Hierarchy
1 − Global
2 − Super Region
3 − Region
4 − Country
5 − Subnational (Level 1)
6 − Subnational (Level 2)

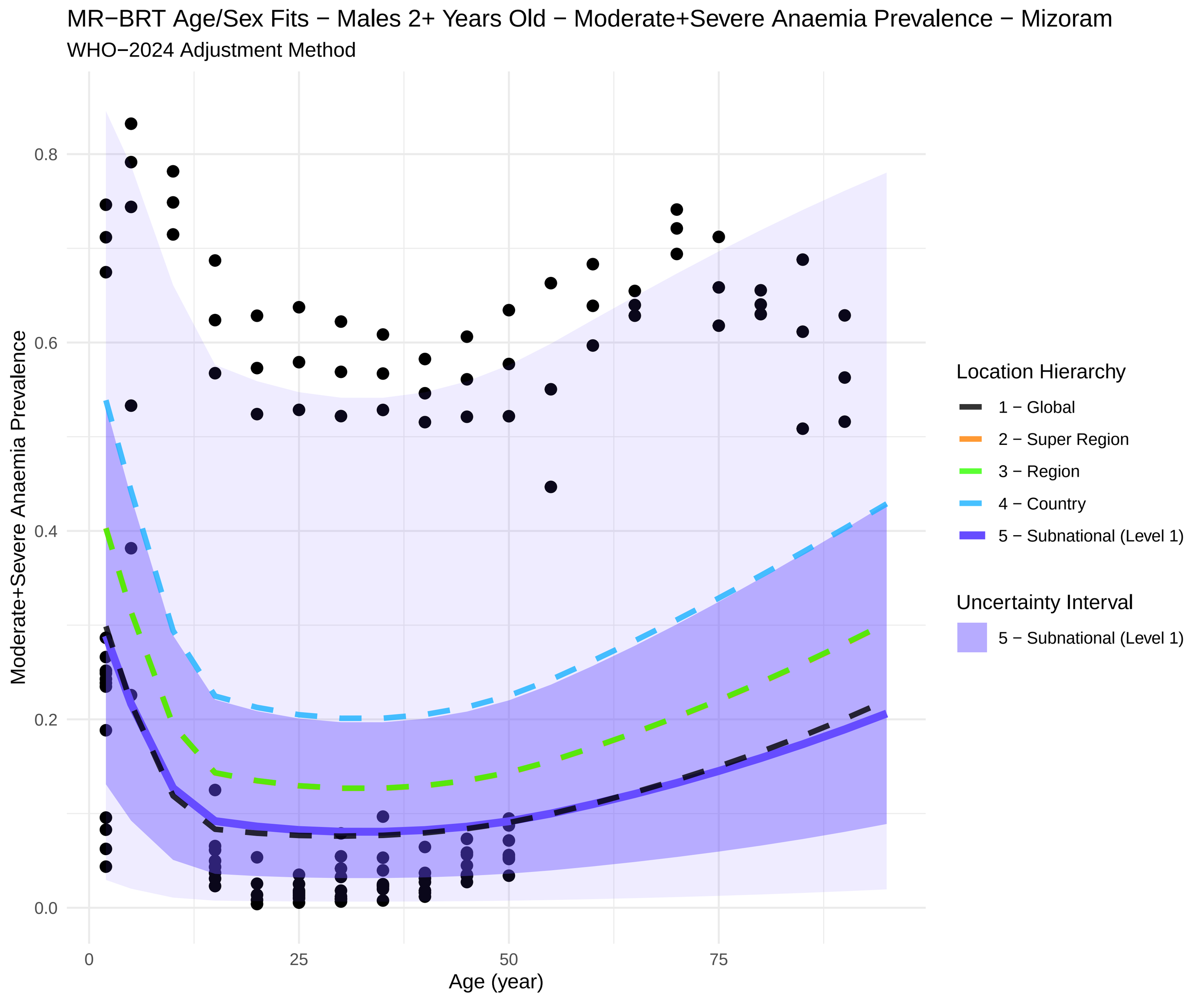
MR−BRT Age/Sex Fits − Males 2+ Years Old − Moderate+Severe Anaemia Prevalence − Mizoram
WHO−2024 Adjustment Method
Moderate+Severe Anaemia Prevalence
Age (year)
Location Hierarchy
1 − Global
2 − Super Region
3 − Region
4 − Country
5 − Subnational (Level 1)
Uncertainty Interval
5 − Subnational (Level 1)

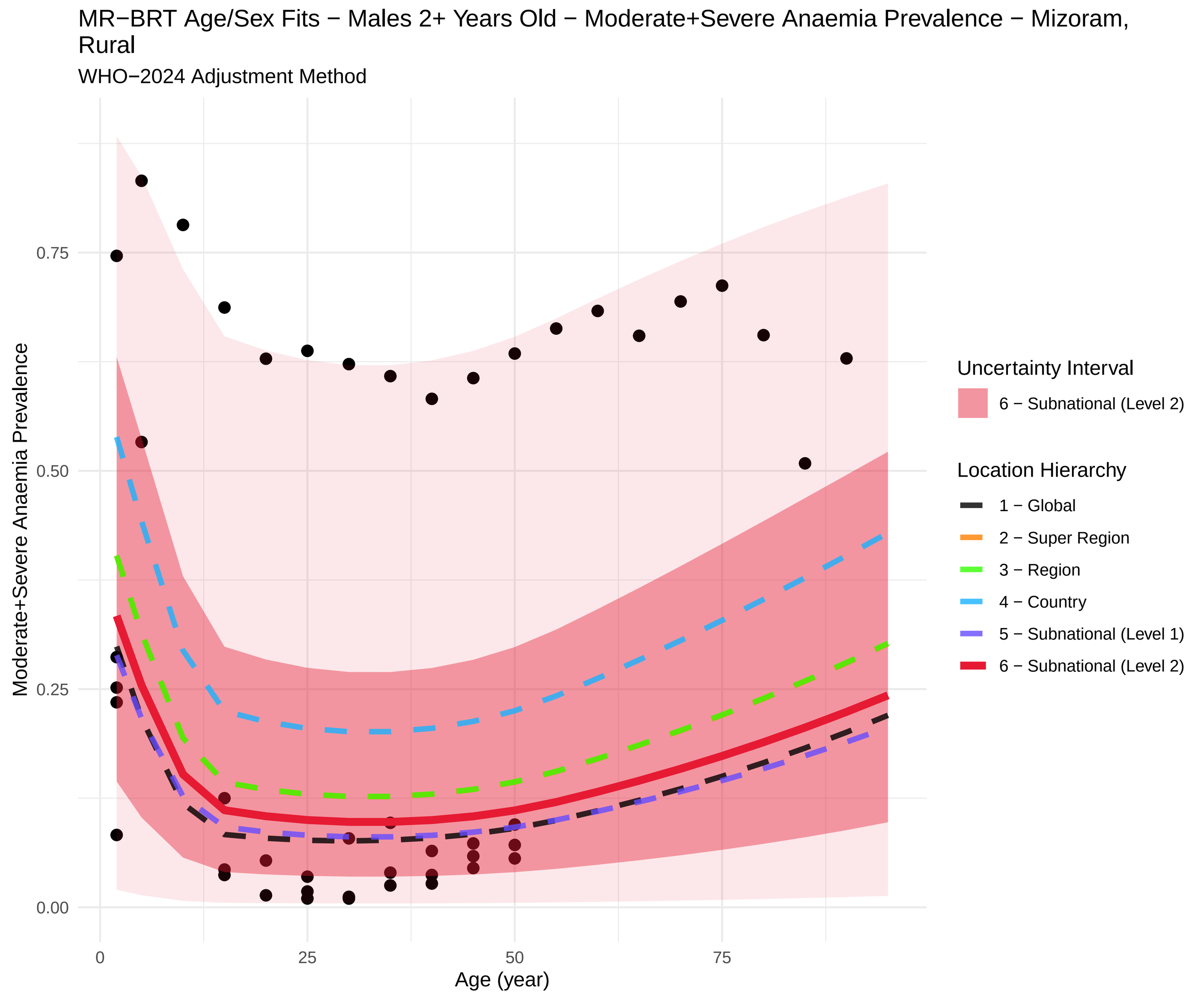
MR−BRT Age/Sex Fits − Males 2+ Years Old − Moderate+Severe Anaemia Prevalence − Mizoram, Rural
WHO−2024 Adjustment Method
Moderate+Severe Anaemia Prevalence
Age (year)
Uncertainty Interval
6 − Subnational (Level 2)
Location Hierarchy
1 − Global
2 − Super Region
3 − Region
4 − Country
5 − Subnational (Level 1)
6 − Subnational (Level 2)

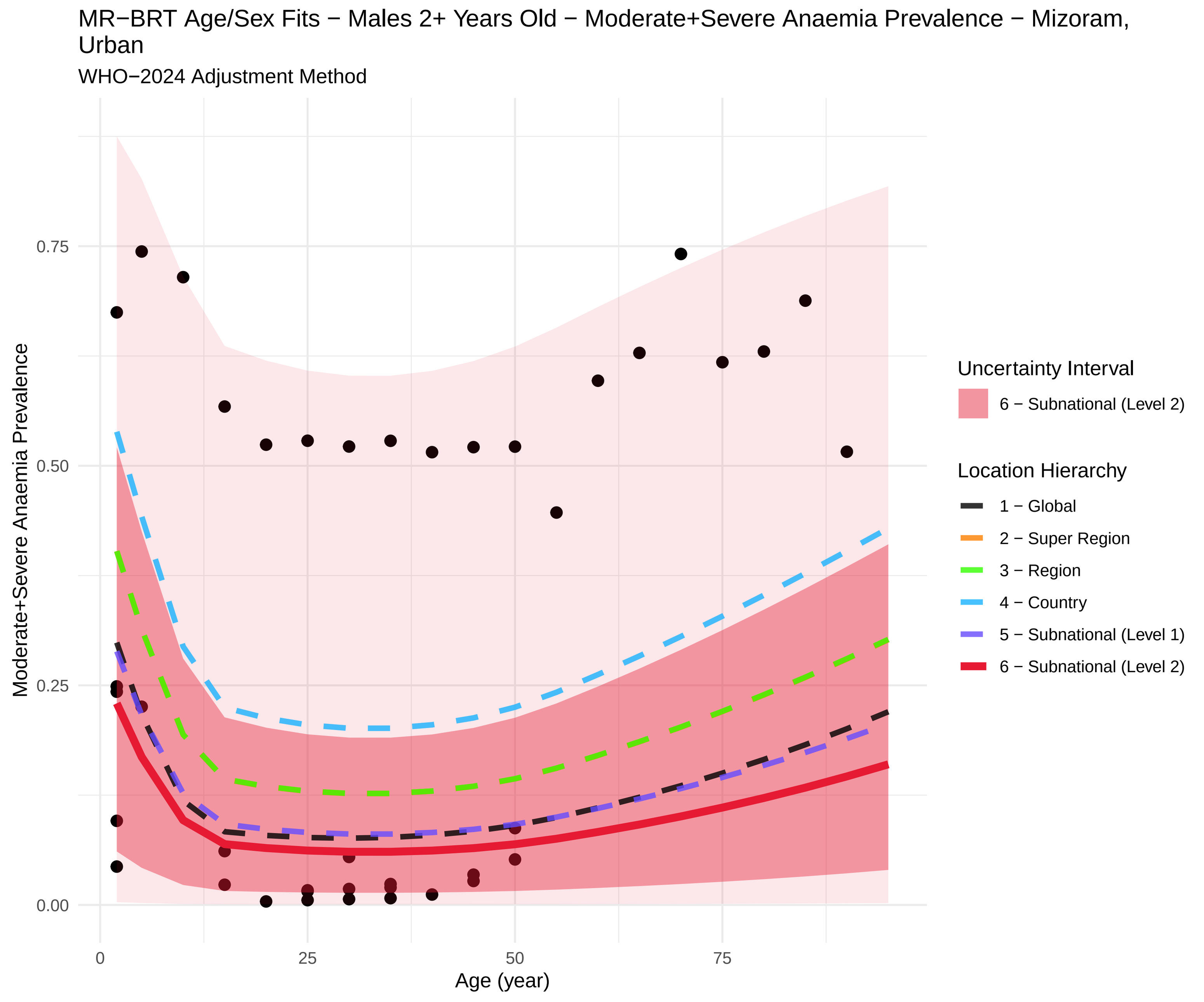
MR−BRT Age/Sex Fits − Males 2+ Years Old − Moderate+Severe Anaemia Prevalence − Mizoram, Urban
WHO−2024 Adjustment Method
Moderate+Severe Anaemia Prevalence
Age (year)
Uncertainty Interval
6 − Subnational (Level 2)
Location Hierarchy
1 − Global
2 − Super Region
3 − Region
4 − Country
5 − Subnational (Level 1)
6 − Subnational (Level 2)

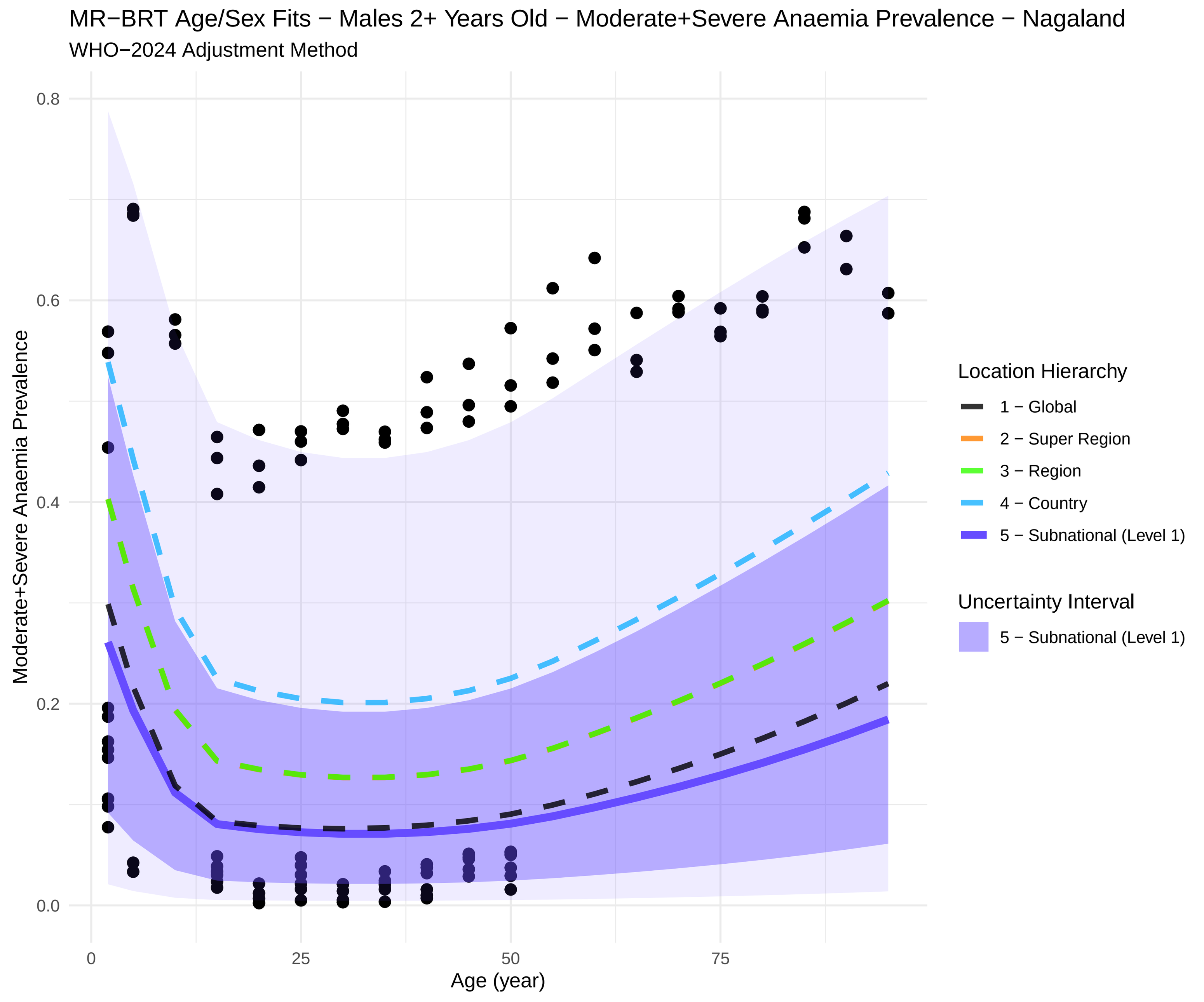
MR−BRT Age/Sex Fits − Males 2+ Years Old − Moderate+Severe Anaemia Prevalence − Nagaland
WHO−2024 Adjustment Method
Moderate+Severe Anaemia Prevalence
Age (year)
Location Hierarchy
1 − Global
2 − Super Region
3 − Region
4 − Country
5 − Subnational (Level 1)
Uncertainty Interval
5 − Subnational (Level 1)

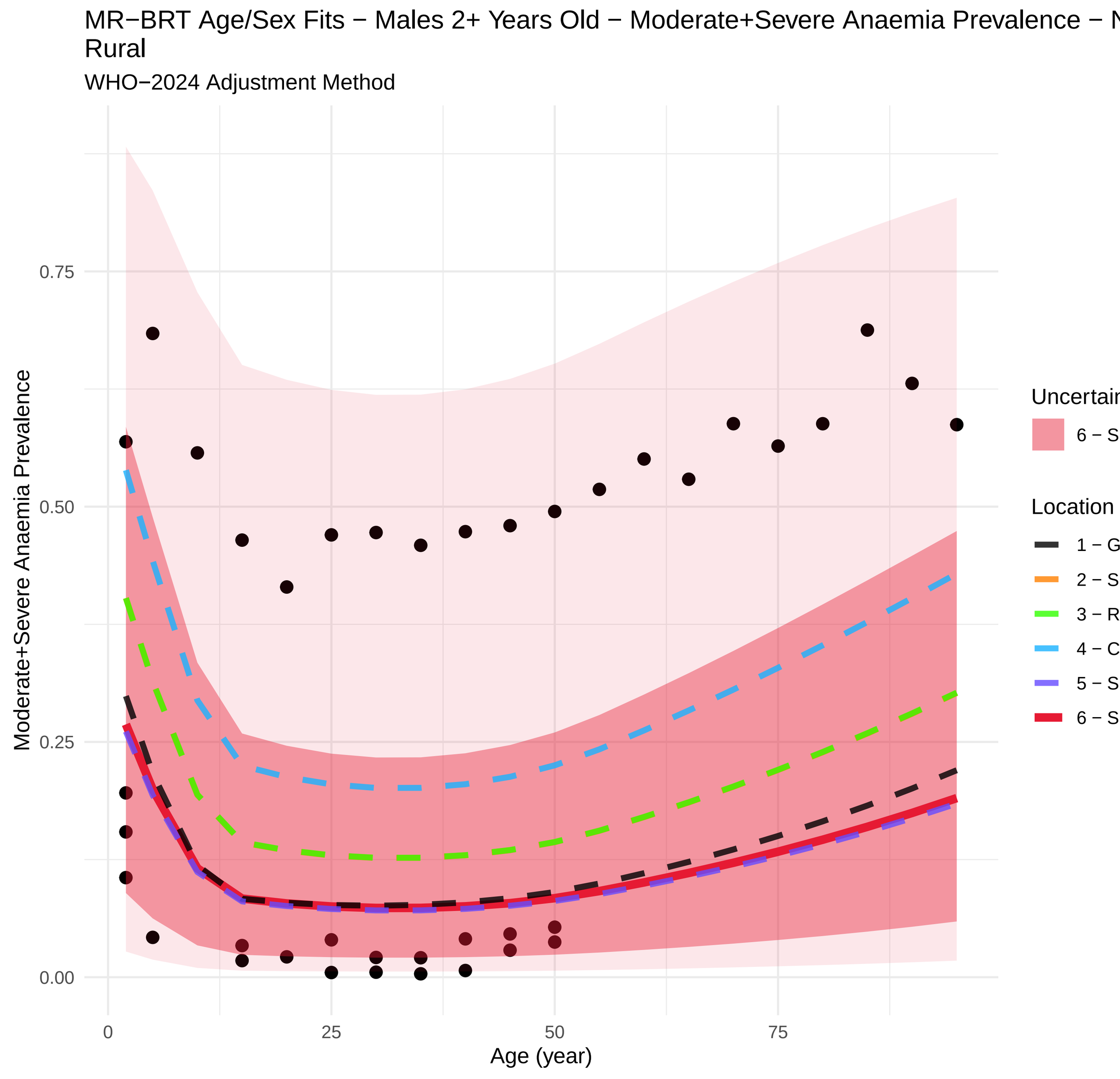
MR−BRT Age/Sex Fits − Males 2+ Years Old − Moderate+Severe Anaemia Prevalence − Nagaland, Rural
WHO−2024 Adjustment Method
Moderate+Severe Anaemia Prevalence
Age (year)
Uncertainty Interval
6 − Subnational (Level 2)
Location Hierarchy
1 − Global
2 − Super Region
3 − Region
4 − Country
5 − Subnational (Level 1)
6 − Subnational (Level 2)

MR−BRT Age/Sex Fits − Males 2+ Years Old − Moderate+Severe Anaemia Prevalence − Nagaland, Urban
WHO−2024 Adjustment Method

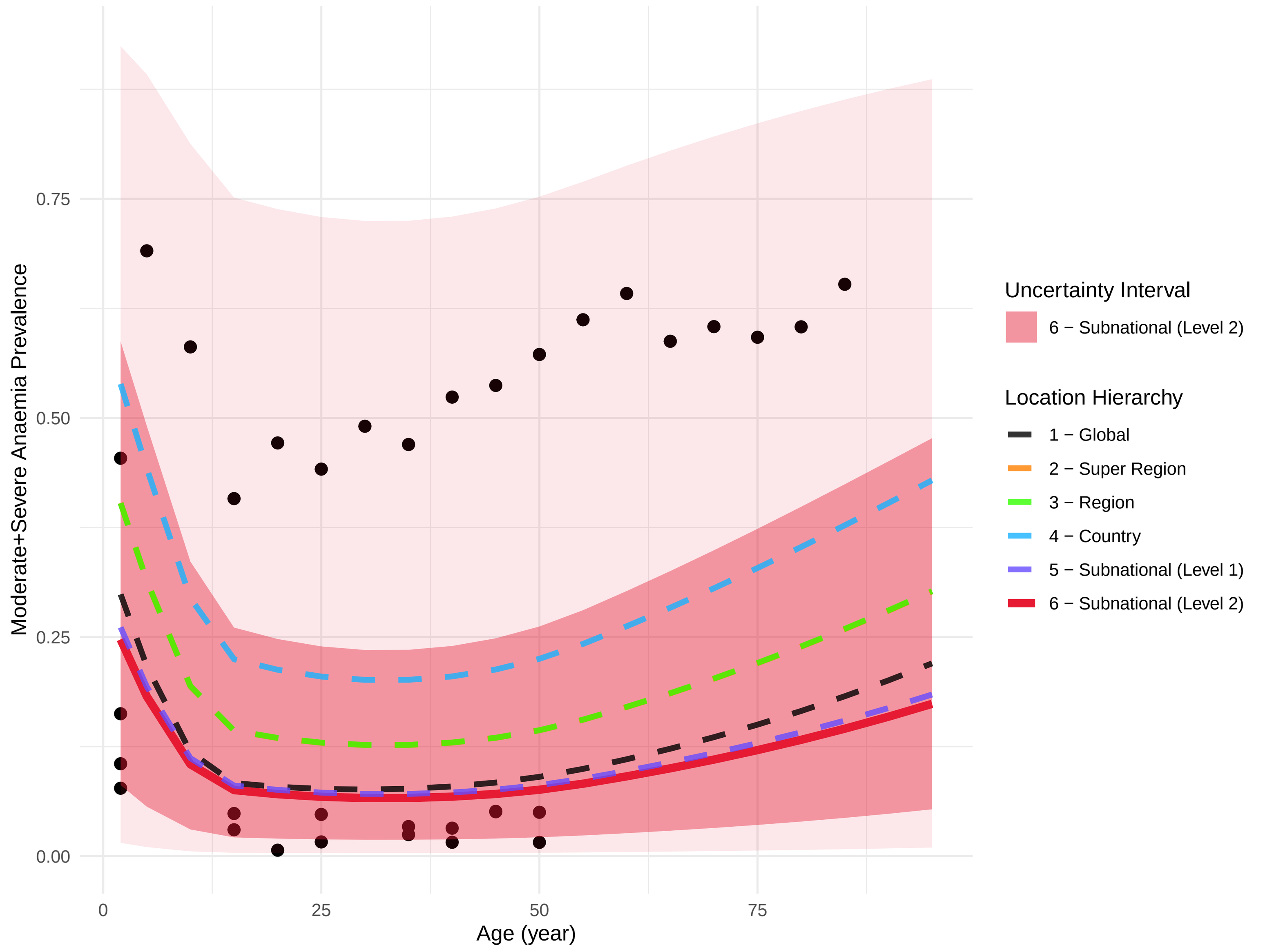

MR−BRT Age/Sex Fits − Males 2+ Years Old − Moderate+Severe Anaemia Prevalence − Odisha
WHO−2024 Adjustment Method

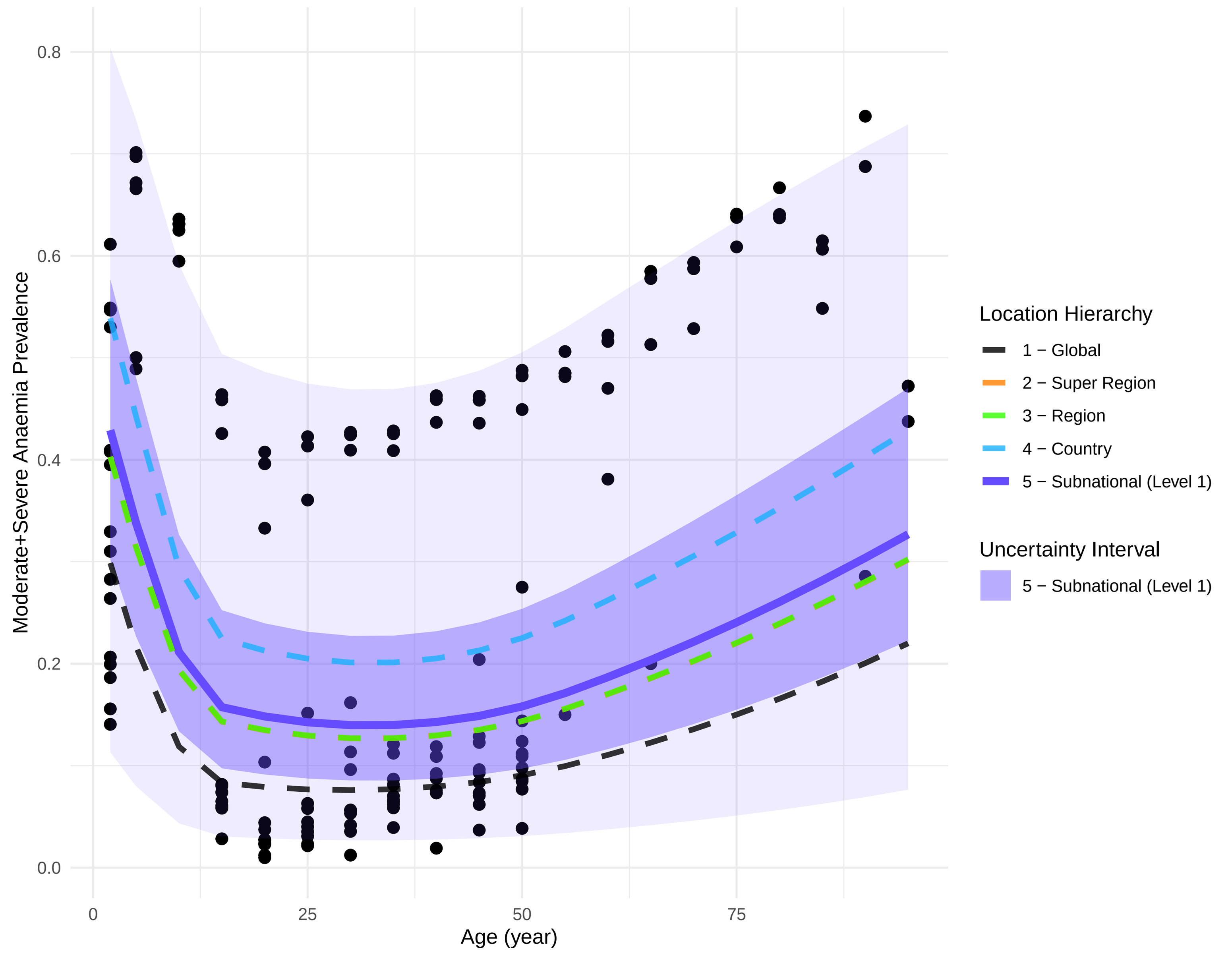

MR−BRT Age/Sex Fits − Males 2+ Years Old − Moderate+Severe Anaemia Prevalence − Odisha, Rural
WHO−2024 Adjustment Method

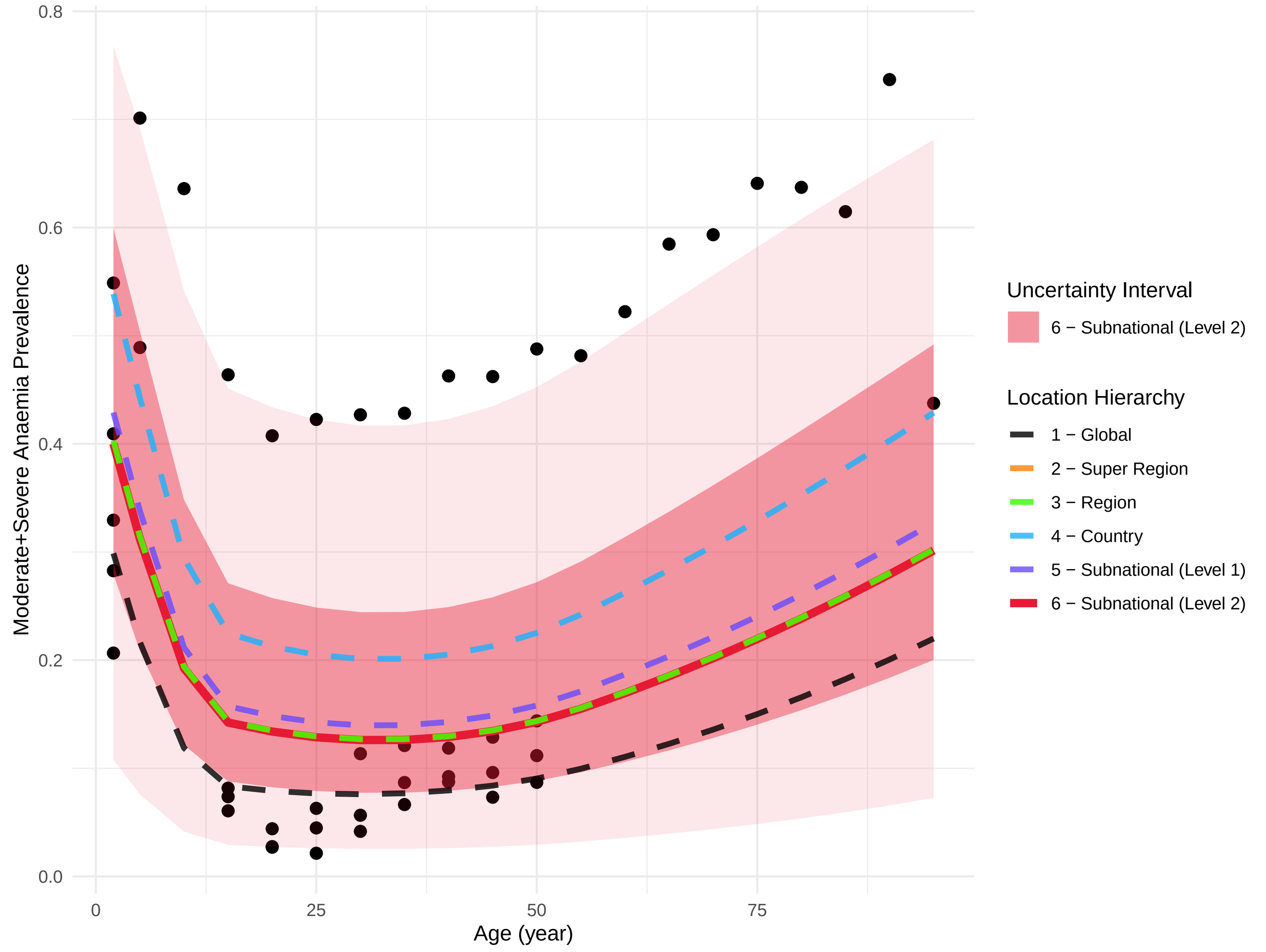

MR−BRT Age/Sex Fits − Males 2+ Years Old − Moderate+Severe Anaemia Prevalence − Odisha, Urban
WHO−2024 Adjustment Method

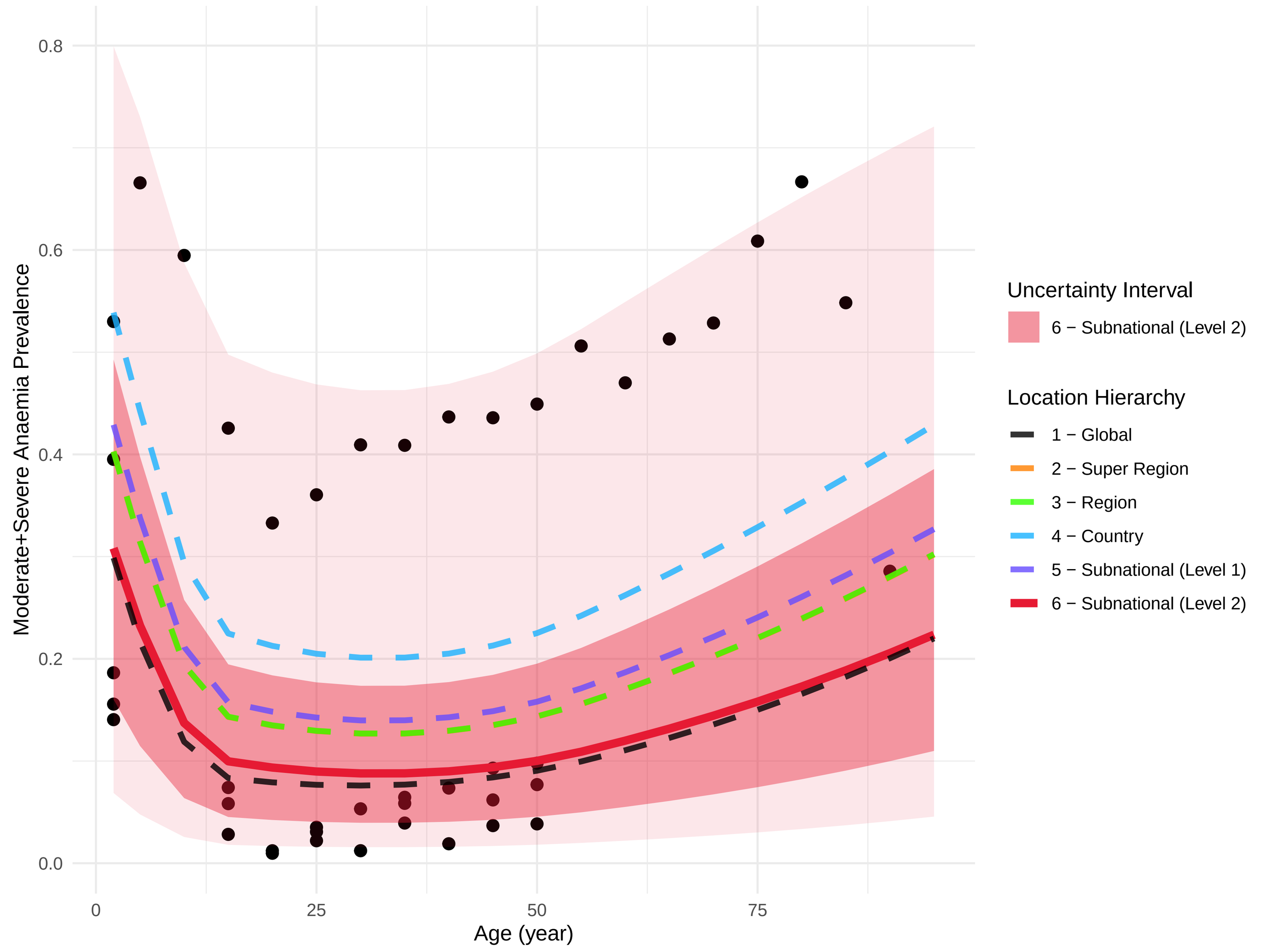

MR−BRT Age/Sex Fits − Males 2+ Years Old − Moderate+Severe Anaemia Prevalence − Other Union Territories
WHO−2024 Adjustment Method

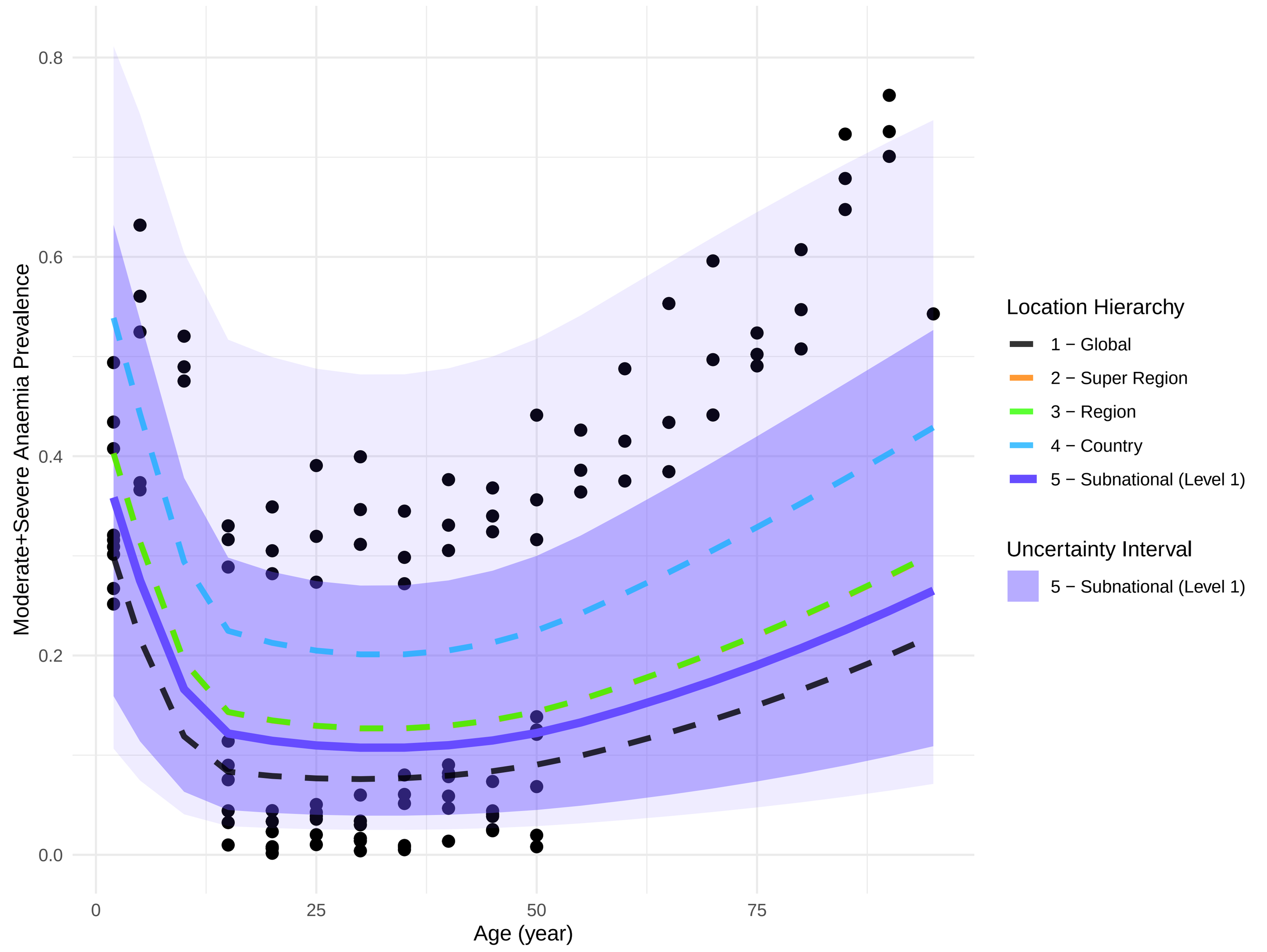

MR−BRT Age/Sex Fits − Males 2+ Years Old − Moderate+Severe Anaemia Prevalence − Other Union Territories, Rural
WHO−2024 Adjustment Method

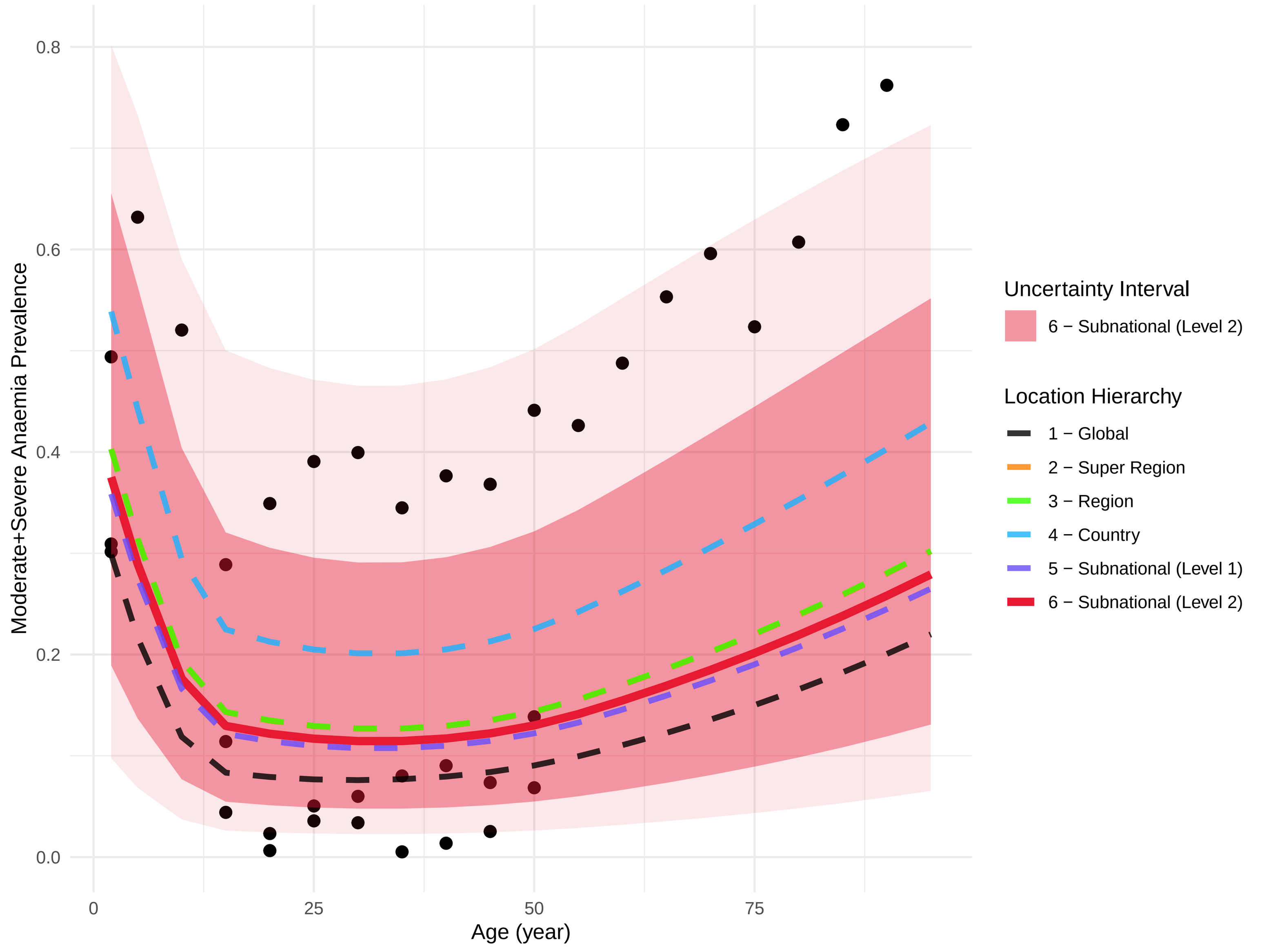

MR−BRT Age/Sex Fits − Males 2+ Years Old − Moderate+Severe Anaemia Prevalence − Other Union Territories, Urban
WHO−2024 Adjustment Method

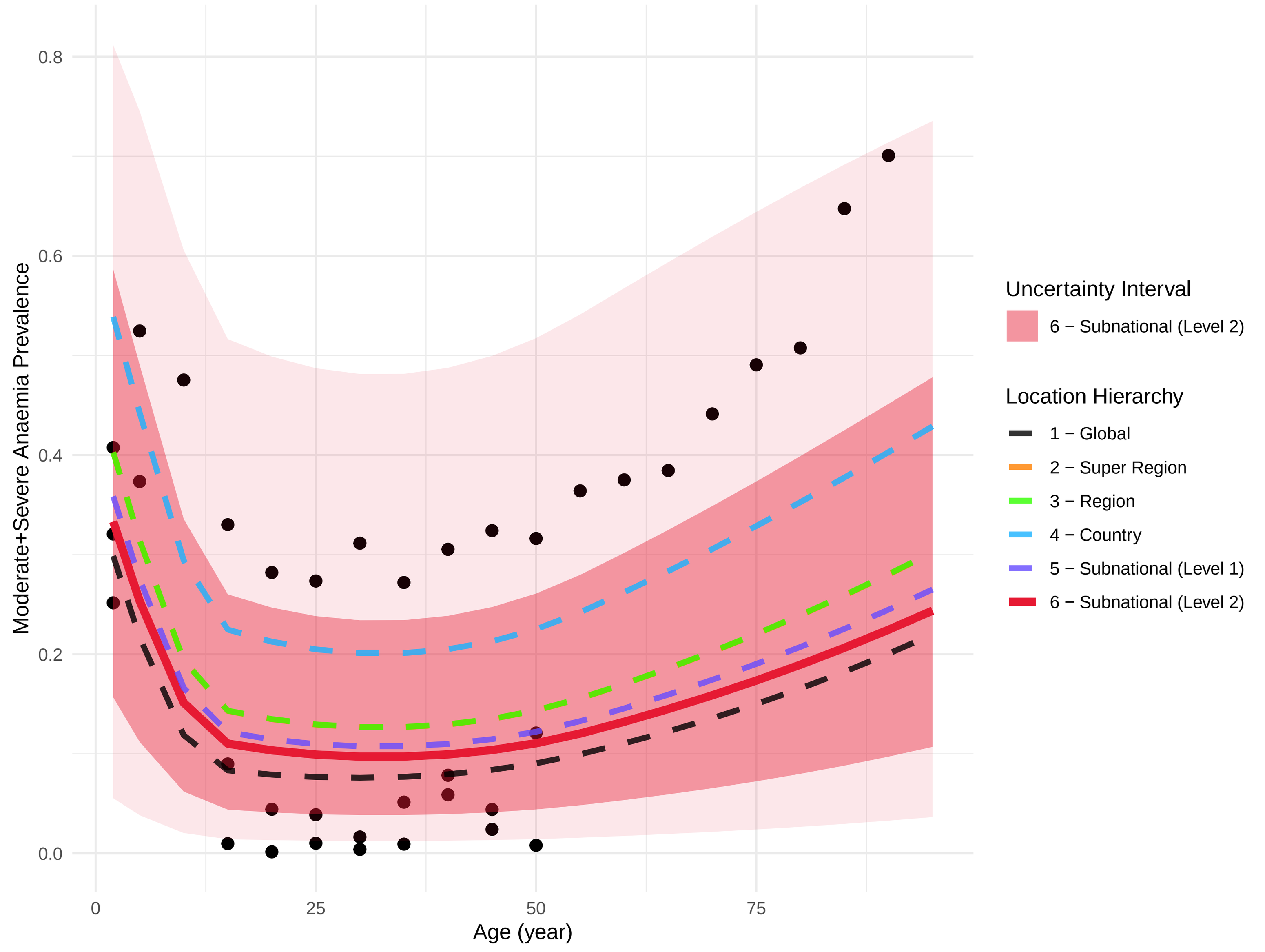

MR−BRT Age/Sex Fits − Males 2+ Years Old − Moderate+Severe Anaemia Prevalence − Punjab
WHO−2024 Adjustment Method

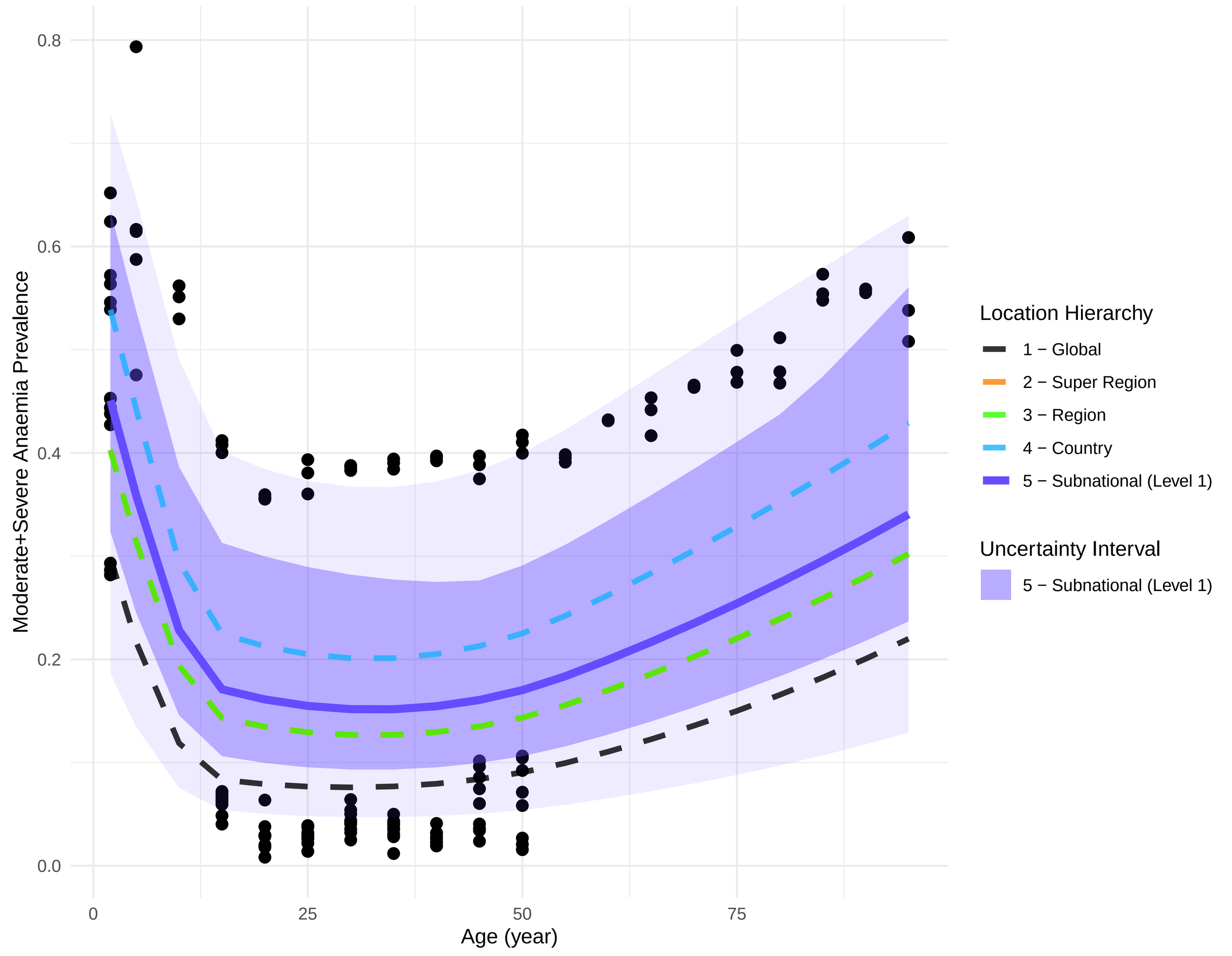

MR−BRT Age/Sex Fits − Males 2+ Years Old − Moderate+Severe Anaemia Prevalence − Punjab, Rural
WHO−2024 Adjustment Method

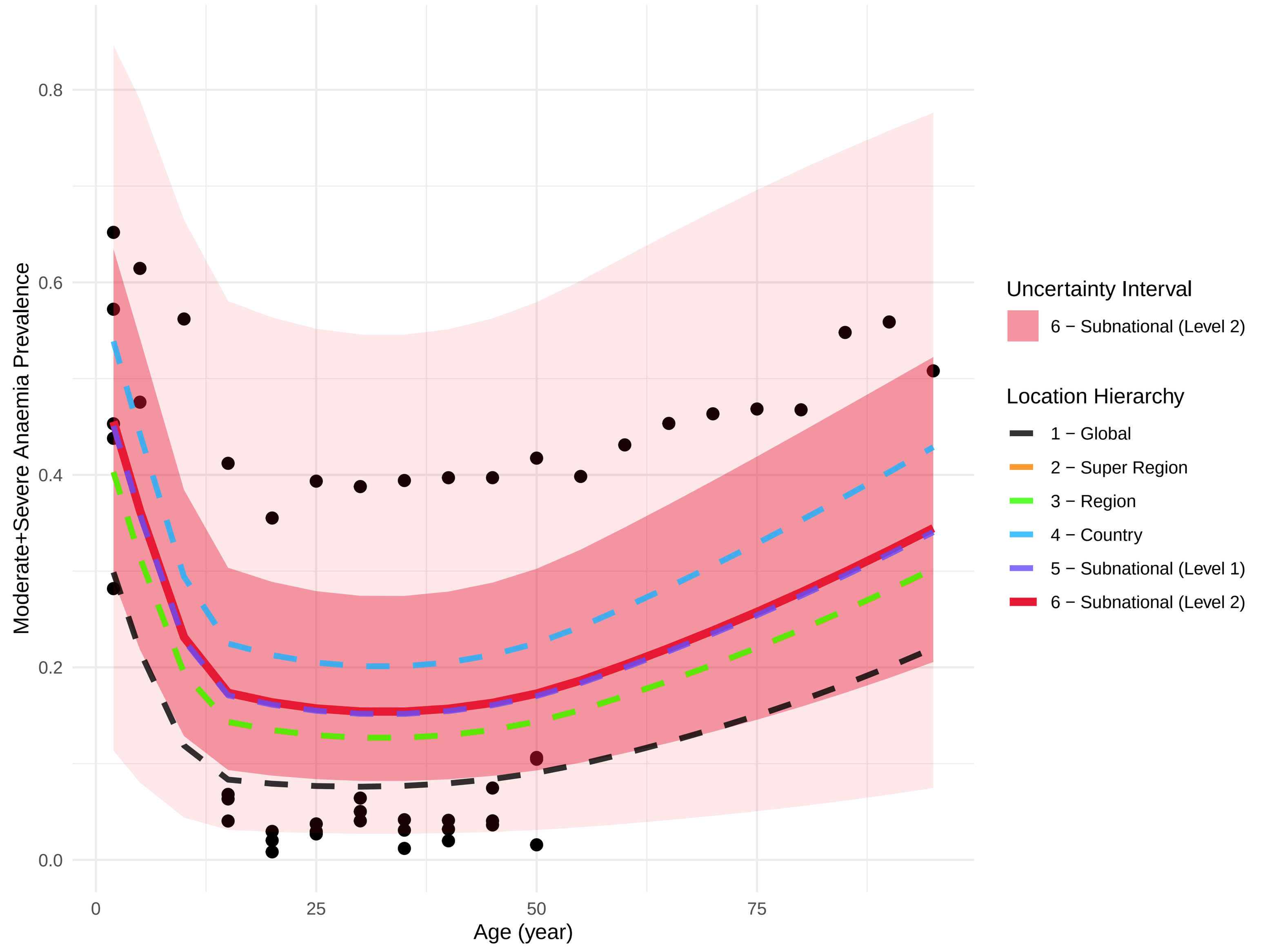

MR−BRT Age/Sex Fits − Males 2+ Years Old − Moderate+Severe Anaemia Prevalence − Punjab, Urban
WHO−2024 Adjustment Method

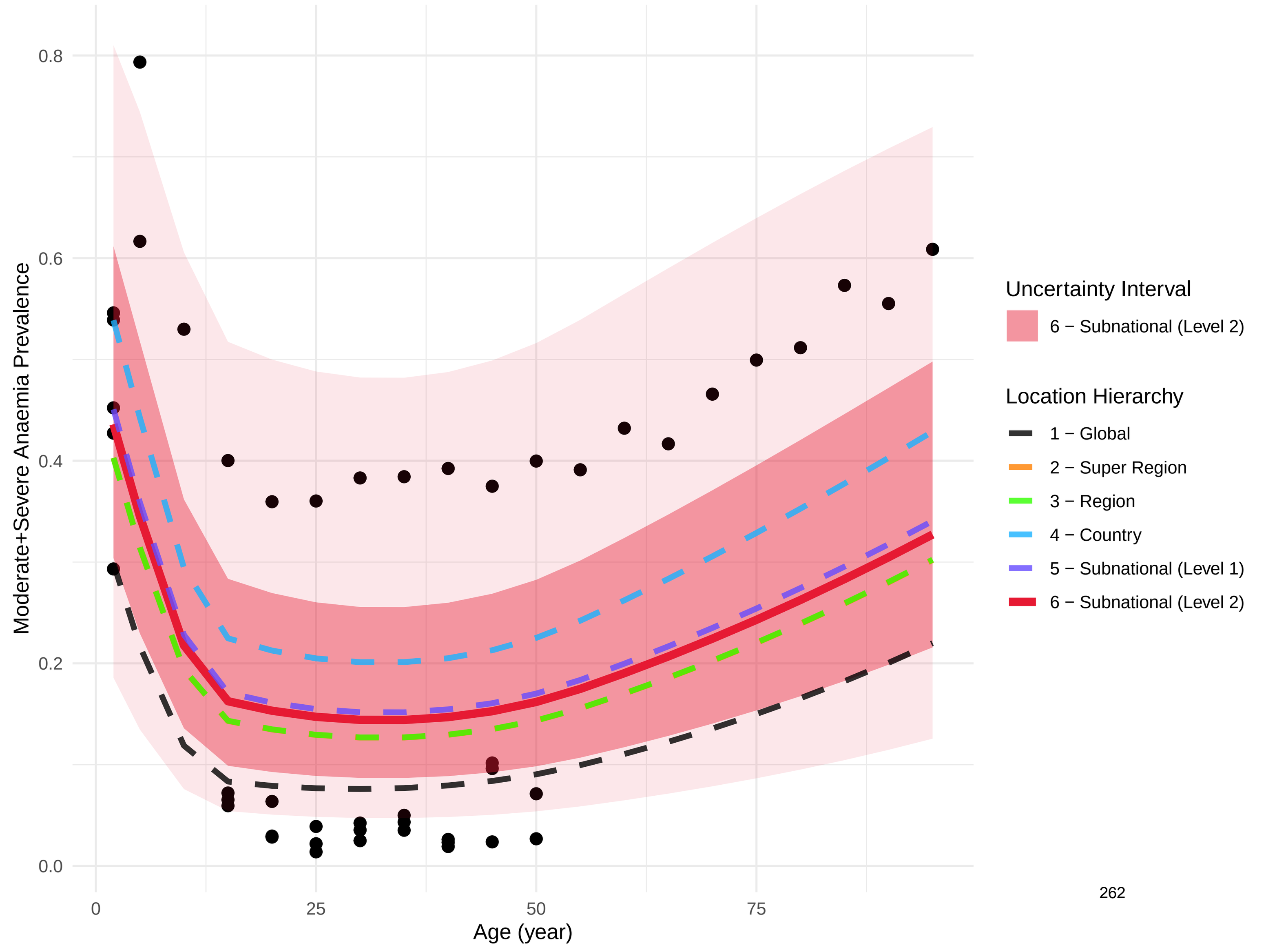

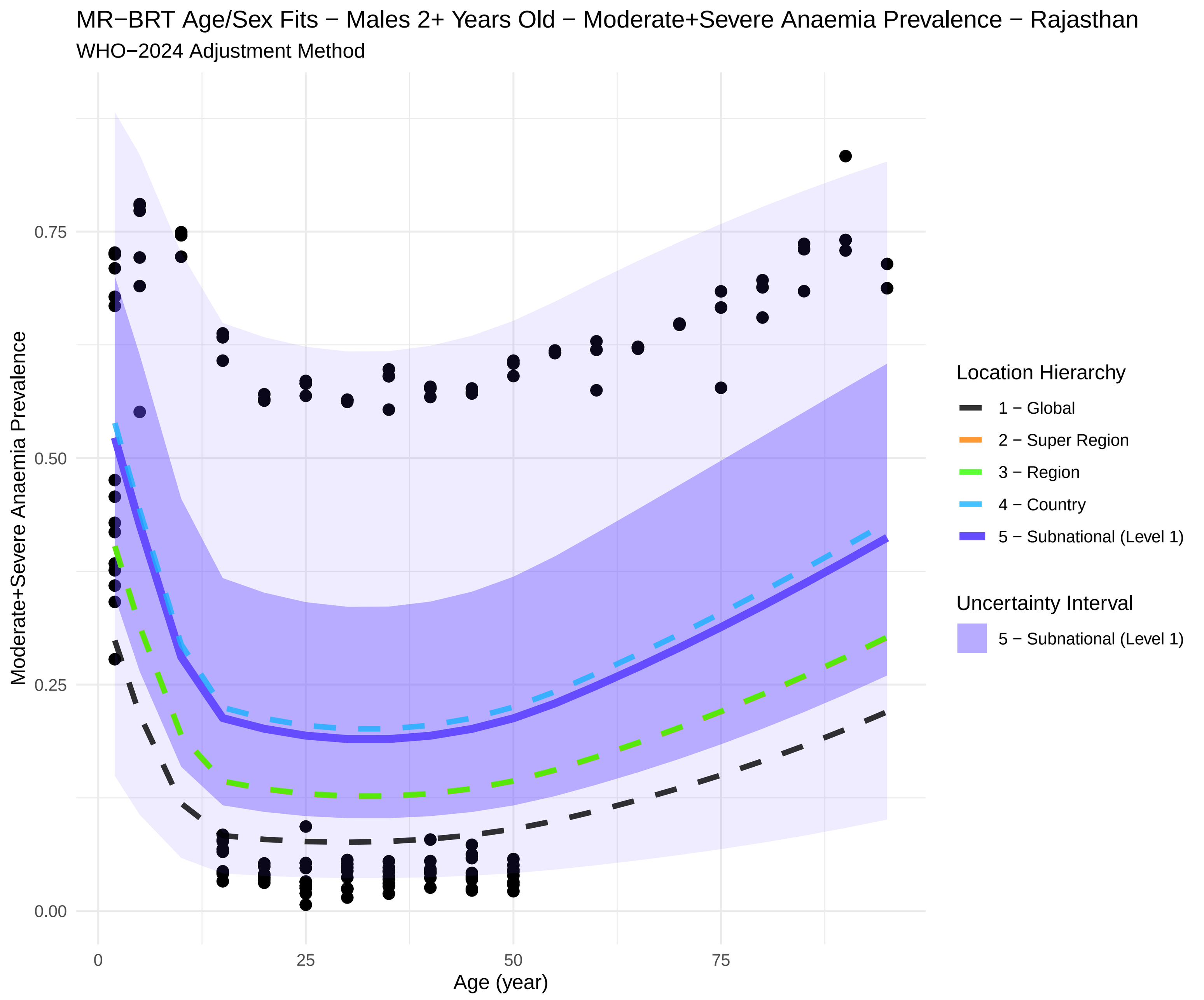
MR−BRT Age/Sex Fits − Males 2+ Years Old − Moderate+Severe Anaemia Prevalence − Rajasthan
WHO−2024 Adjustment Method
Moderate+Severe Anaemia Prevalence
Age (year)
Location Hierarchy
1 − Global
2 − Super Region
3 − Region
4 − Country
5 − Subnational (Level 1)
Uncertainty Interval
5 − Subnational (Level 1)

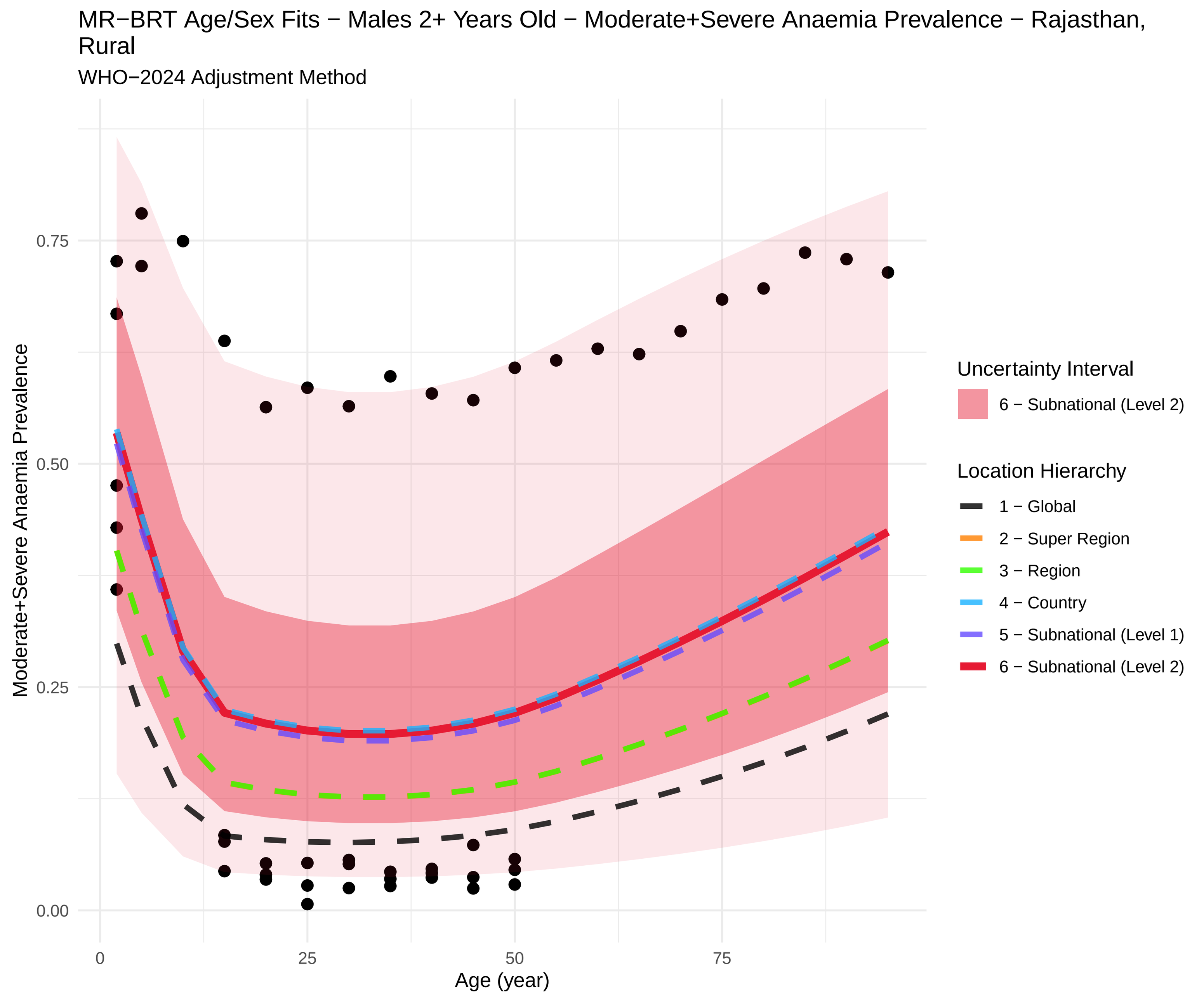
MR−BRT Age/Sex Fits − Males 2+ Years Old − Moderate+Severe Anaemia Prevalence − Rajasthan, Rural
WHO−2024 Adjustment Method
Moderate+Severe Anaemia Prevalence
Age (year)
Uncertainty Interval
6 − Subnational (Level 2)
Location Hierarchy
1 − Global
2 − Super Region
3 − Region
4 − Country
5 − Subnational (Level 1)
6 − Subnational (Level 2)

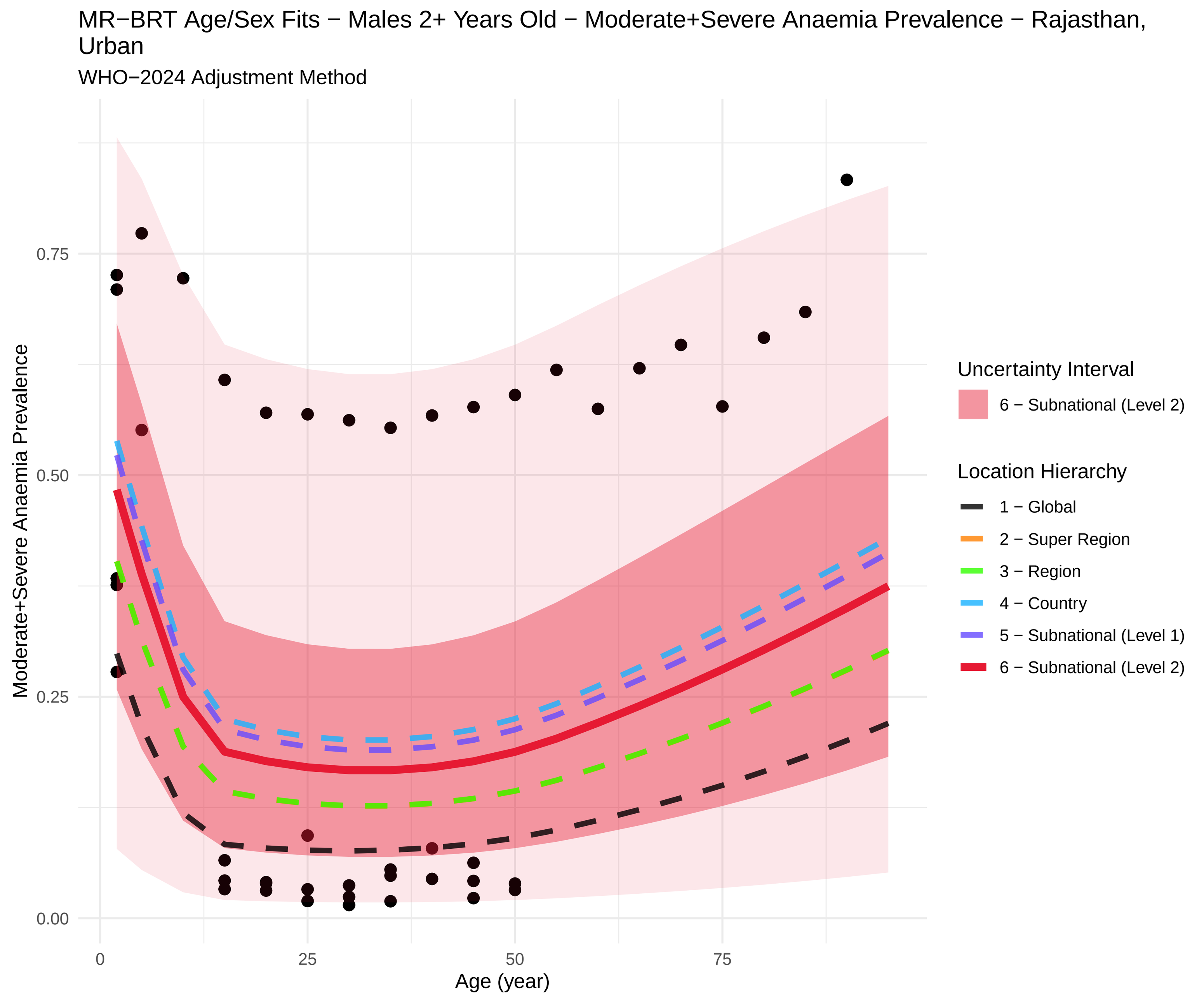
MR−BRT Age/Sex Fits − Males 2+ Years Old − Moderate+Severe Anaemia Prevalence − Rajasthan, Urban
WHO−2024 Adjustment Method
Moderate+Severe Anaemia Prevalence
Age (year)
Uncertainty Interval
6 − Subnational (Level 2)
Location Hierarchy
1 − Global
2 − Super Region
3 − Region
4 − Country
5 − Subnational (Level 1)
6 − Subnational (Level 2)

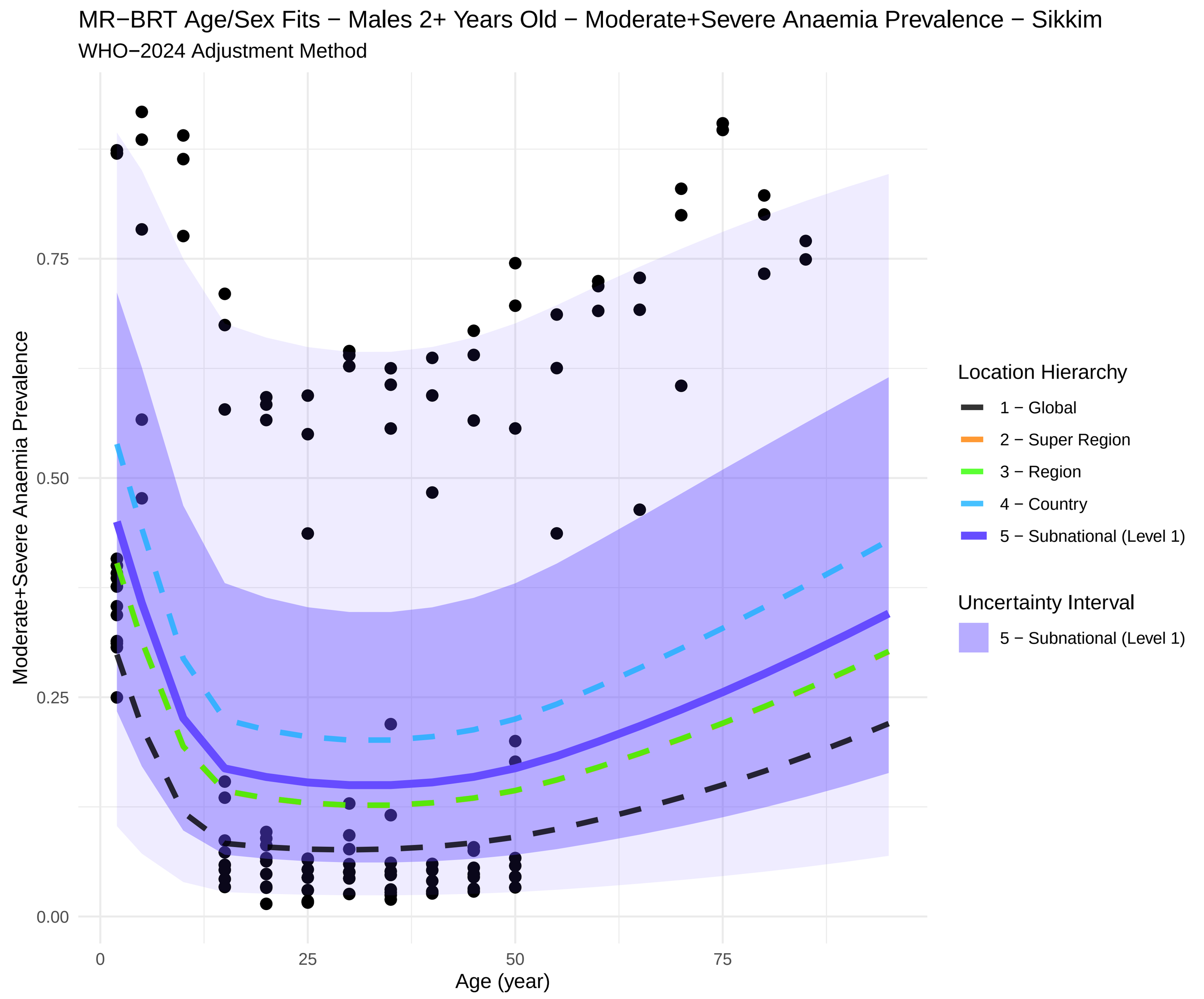
MR−BRT Age/Sex Fits − Males 2+ Years Old − Moderate+Severe Anaemia Prevalence − Sikkim
WHO−2024 Adjustment Method
Moderate+Severe Anaemia Prevalence
Age (year)
Location Hierarchy
1 − Global
2 − Super Region
3 − Region
4 − Country
5 − Subnational (Level 1)
Uncertainty Interval
5 − Subnational (Level 1)

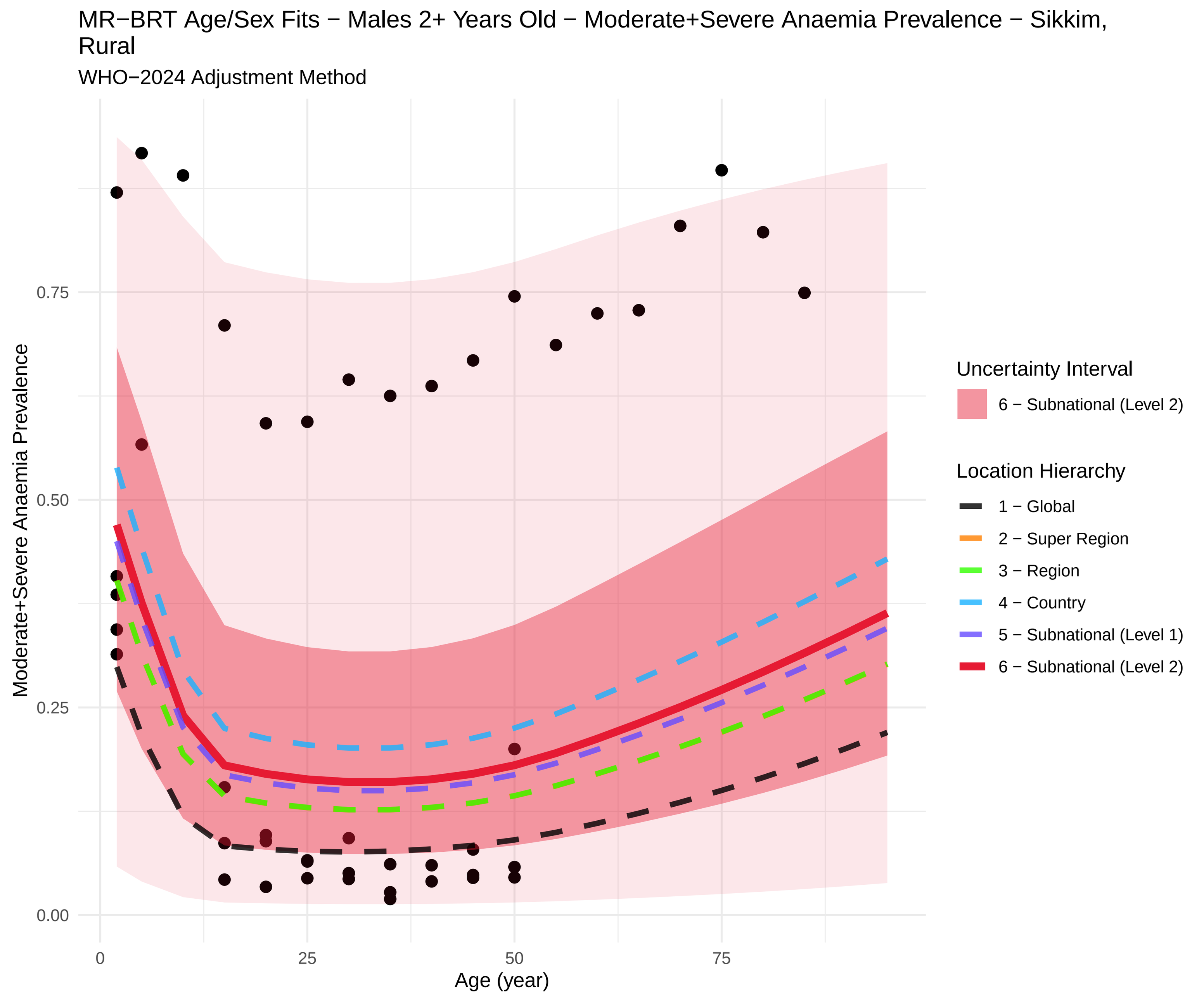
MR−BRT Age/Sex Fits − Males 2+ Years Old − Moderate+Severe Anaemia Prevalence − Sikkim, Rural
WHO−2024 Adjustment Method
Moderate+Severe Anaemia Prevalence
Age (year)
Uncertainty Interval
6 − Subnational (Level 2)
Location Hierarchy
1 − Global
2 − Super Region
3 − Region
4 − Country
5 − Subnational (Level 1)
6 − Subnational (Level 2)

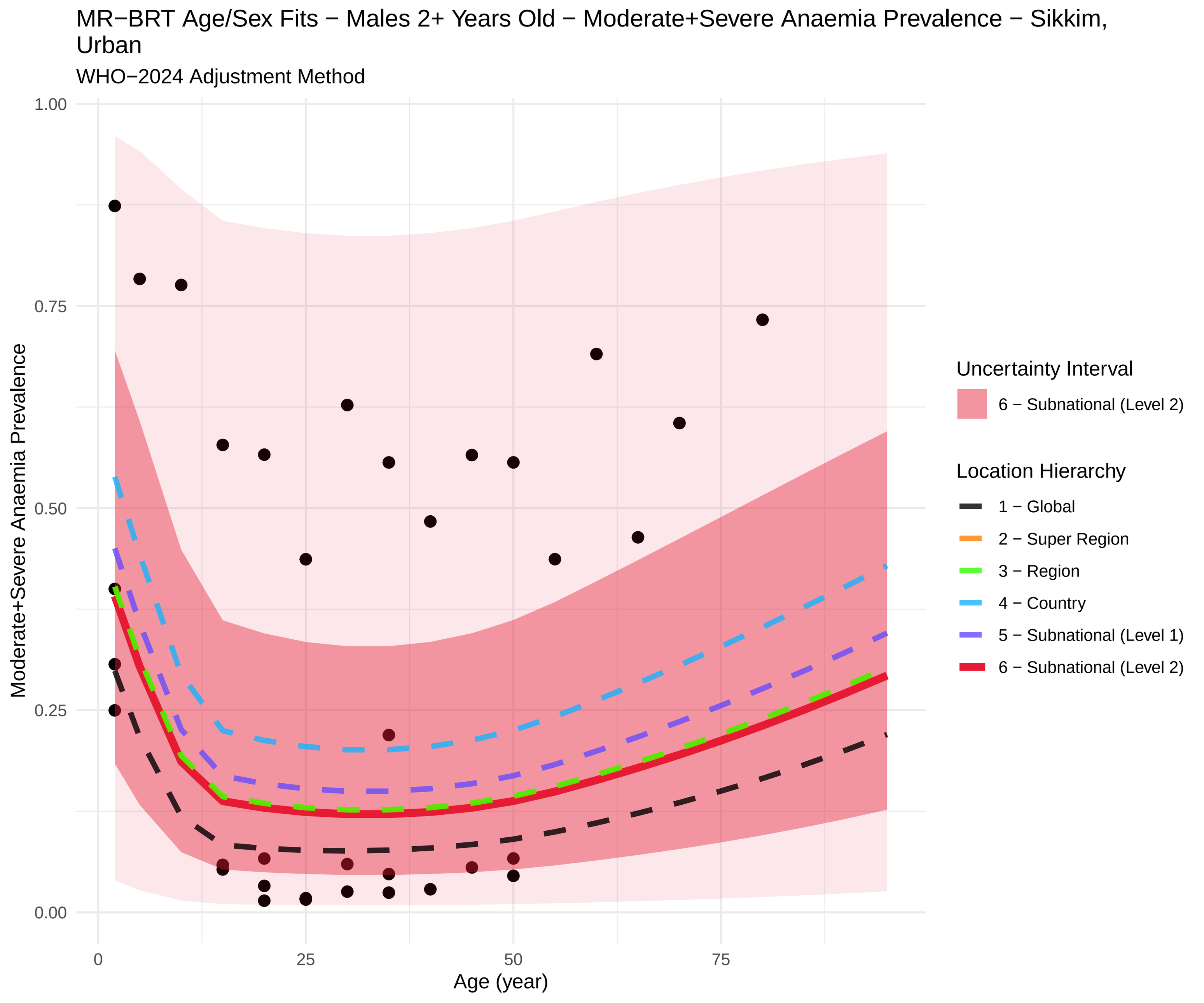
MR−BRT Age/Sex Fits − Males 2+ Years Old − Moderate+Severe Anaemia Prevalence − Sikkim, Urban
WHO−2024 Adjustment Method
Moderate+Severe Anaemia Prevalence
Age (year)
Uncertainty Interval
6 − Subnational (Level 2)
Location Hierarchy
1 − Global
2 − Super Region
3 − Region
4 − Country
5 − Subnational (Level 1)
6 − Subnational (Level 2)

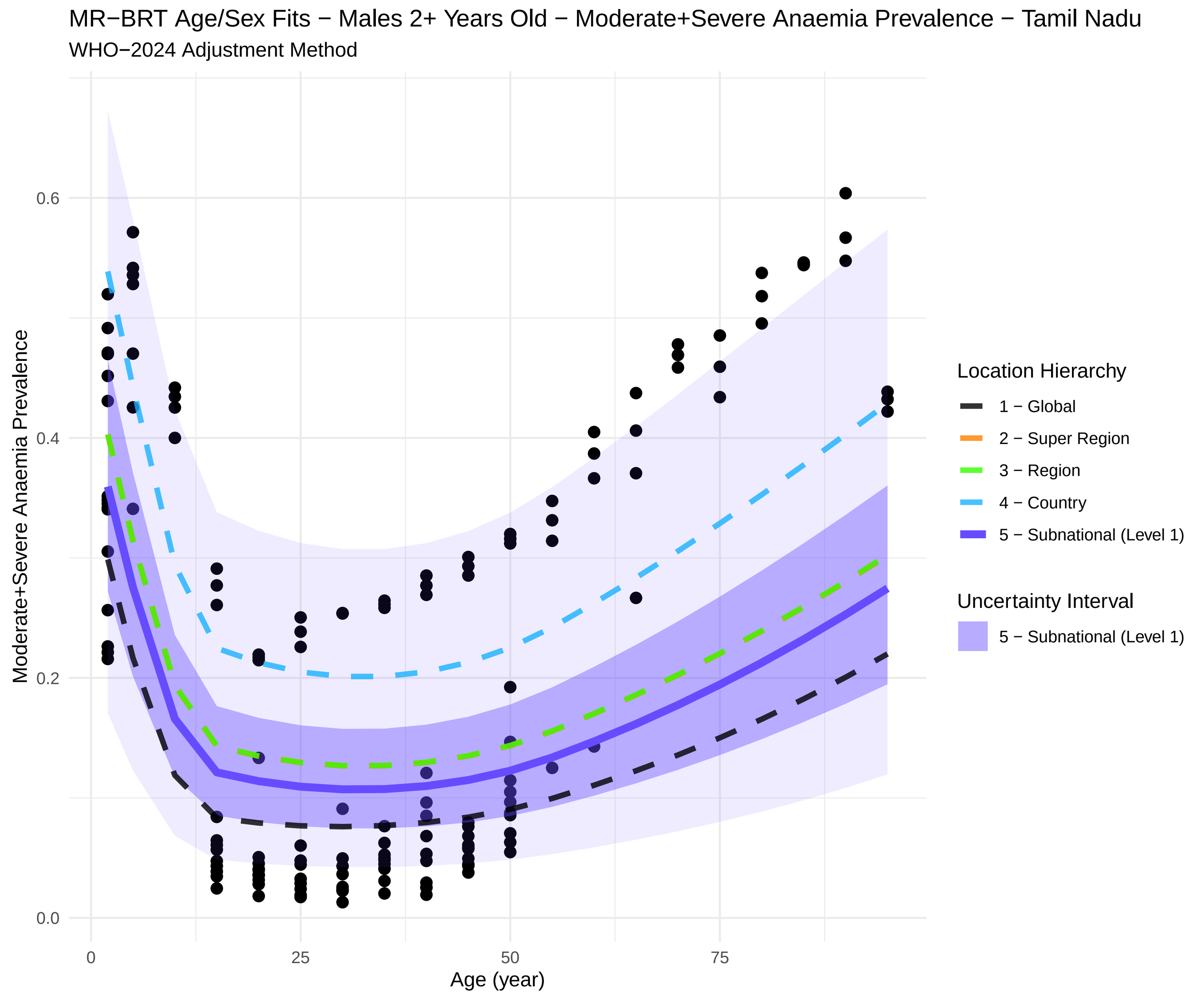
MR−BRT Age/Sex Fits − Males 2+ Years Old − Moderate+Severe Anaemia Prevalence − Tamil Nadu
WHO−2024 Adjustment Method
Moderate+Severe Anaemia Prevalence
Age (year)
Location Hierarchy
1 − Global
2 − Super Region
3 − Region
4 − Country
5 − Subnational (Level 1)
Uncertainty Interval
5 − Subnational (Level 1)

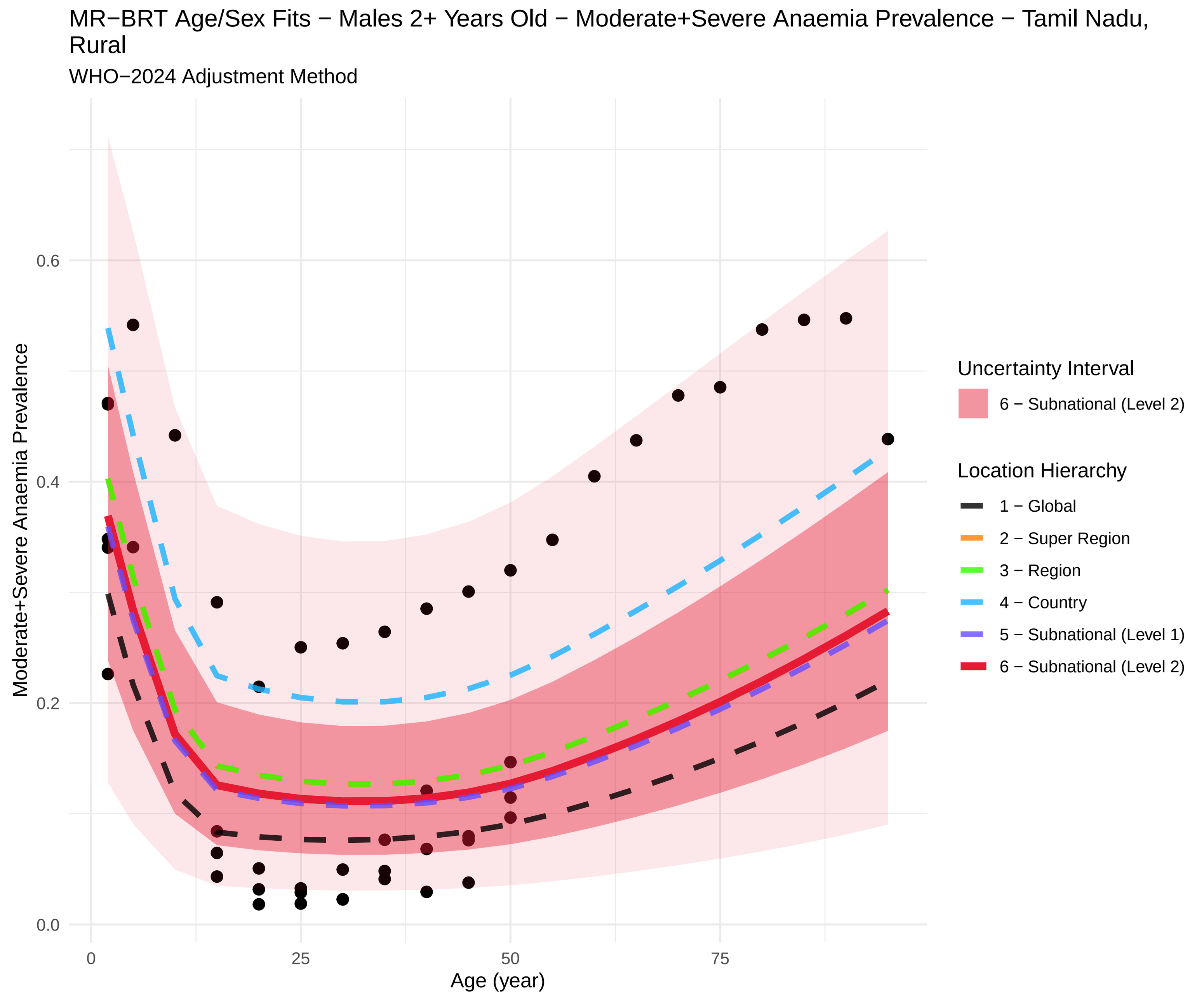
MR−BRT Age/Sex Fits − Males 2+ Years Old − Moderate+Severe Anaemia Prevalence − Tamil Nadu, Rural
WHO−2024 Adjustment Method
Moderate+Severe Anaemia Prevalence
Age (year)
Uncertainty Interval
6 − Subnational (Level 2)
Location Hierarchy
1 − Global
2 − Super Region
3 − Region
4 − Country
5 − Subnational (Level 1)
6 − Subnational (Level 2)

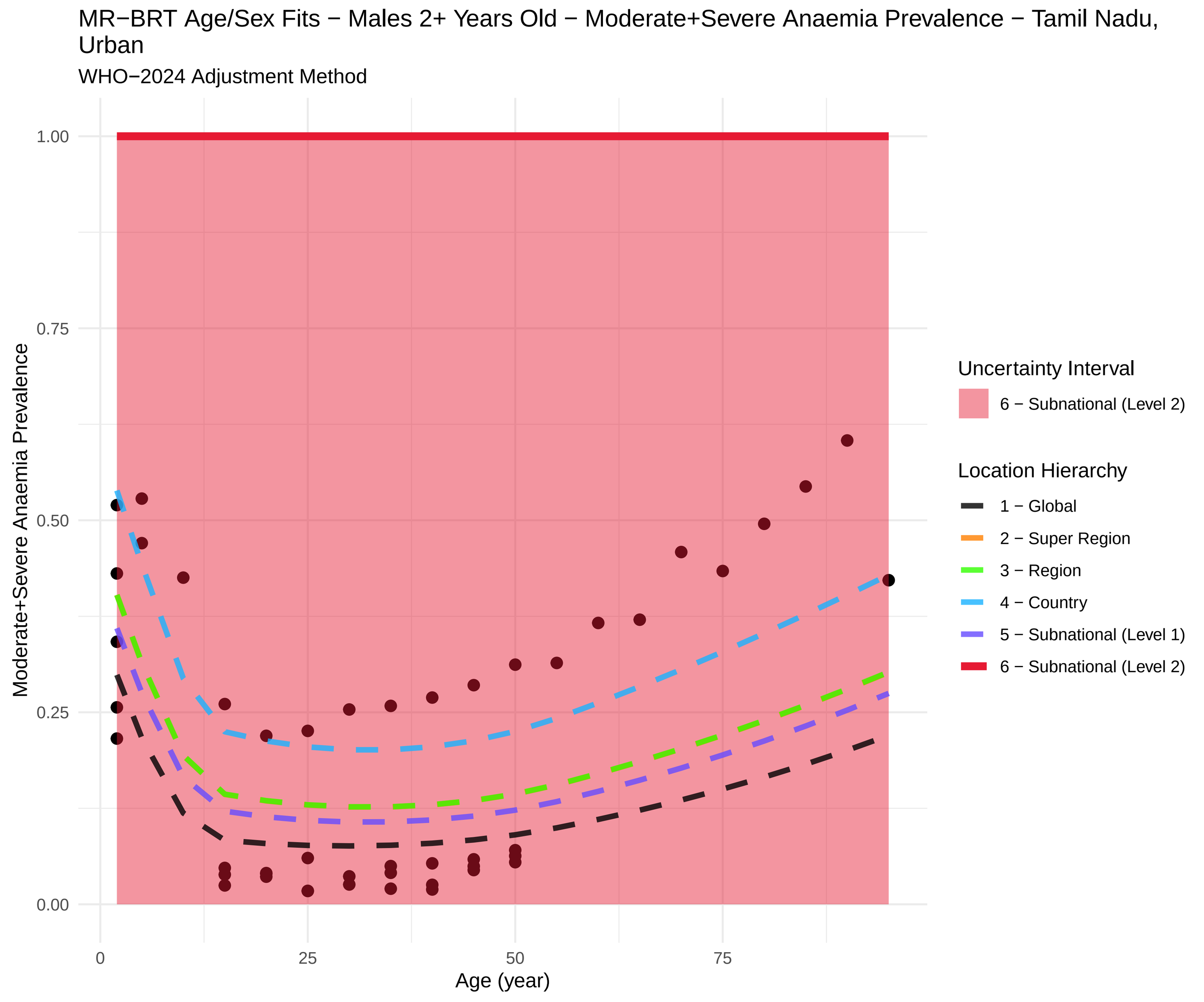
MR−BRT Age/Sex Fits − Males 2+ Years Old − Moderate+Severe Anaemia Prevalence − Tamil Nadu, Urban
WHO−2024 Adjustment Method
Moderate+Severe Anaemia Prevalence
Age (year)
Uncertainty Interval
6 − Subnational (Level 2)
Location Hierarchy
1 − Global
2 − Super Region
3 − Region
4 − Country
5 − Subnational (Level 1)
6 − Subnational (Level 2)

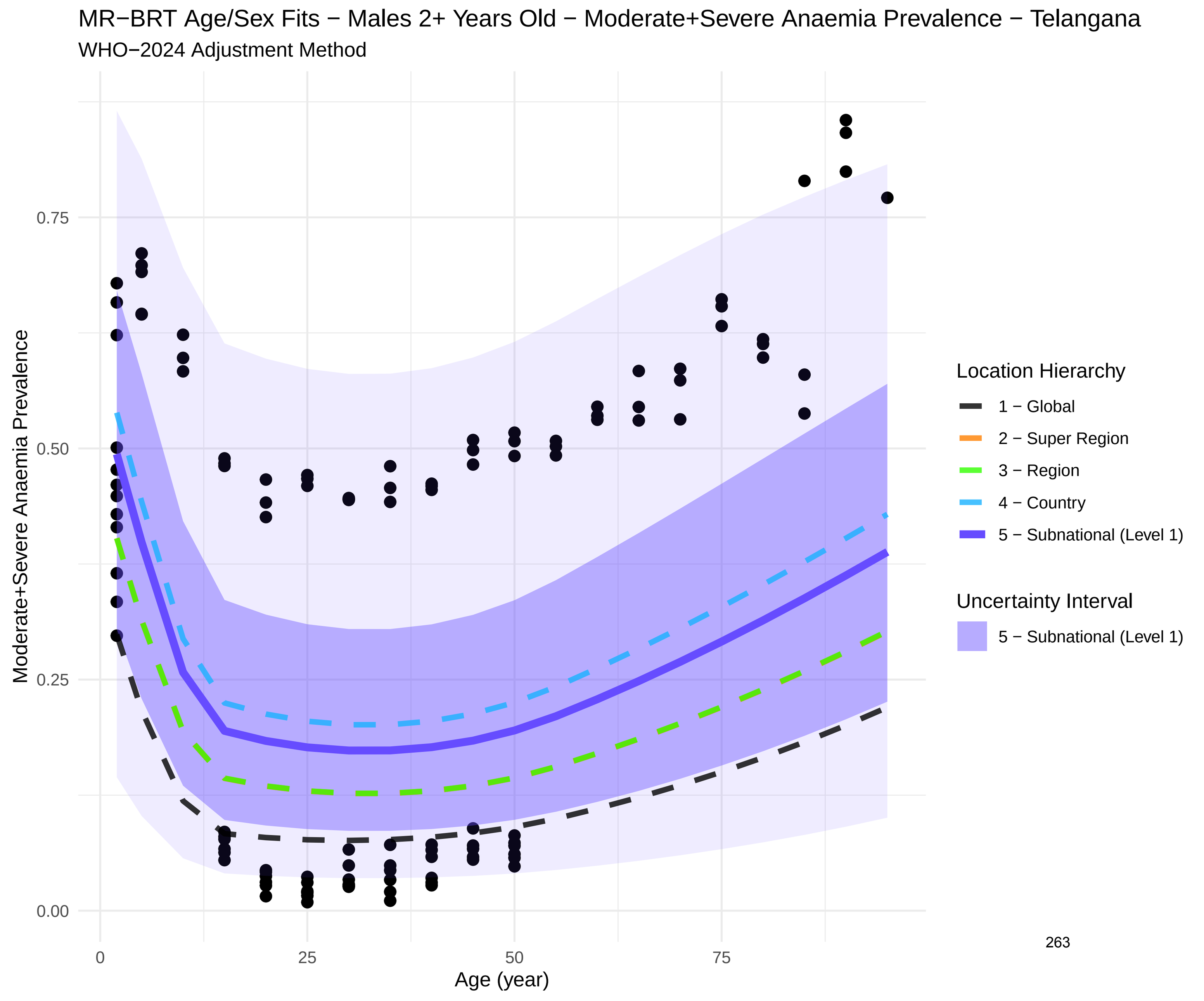
MR−BRT Age/Sex Fits − Males 2+ Years Old − Moderate+Severe Anaemia Prevalence − Telangana
WHO−2024 Adjustment Method
Moderate+Severe Anaemia Prevalence
Age (year)
Location Hierarchy
1 − Global
2 − Super Region
3 − Region
4 − Country
5 − Subnational (Level 1)
Uncertainty Interval
5 − Subnational (Level 1)

MR−BRT Age/Sex Fits − Males 2+ Years Old − Moderate+Severe Anaemia Prevalence − Telangana, Rural
WHO−2024 Adjustment Method

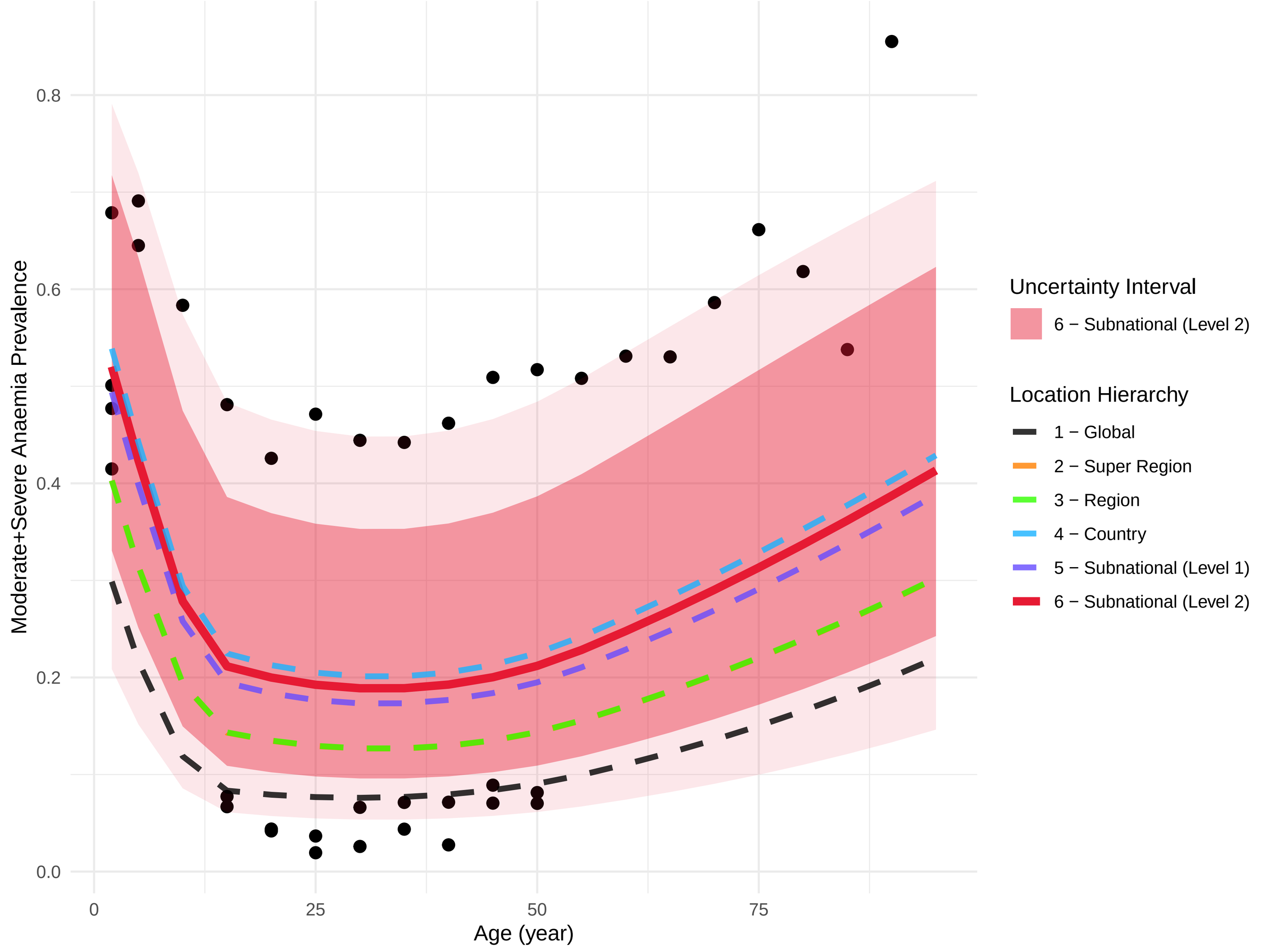

MR−BRT Age/Sex Fits − Males 2+ Years Old − Moderate+Severe Anaemia Prevalence − Telangana, Urban
WHO−2024 Adjustment Method

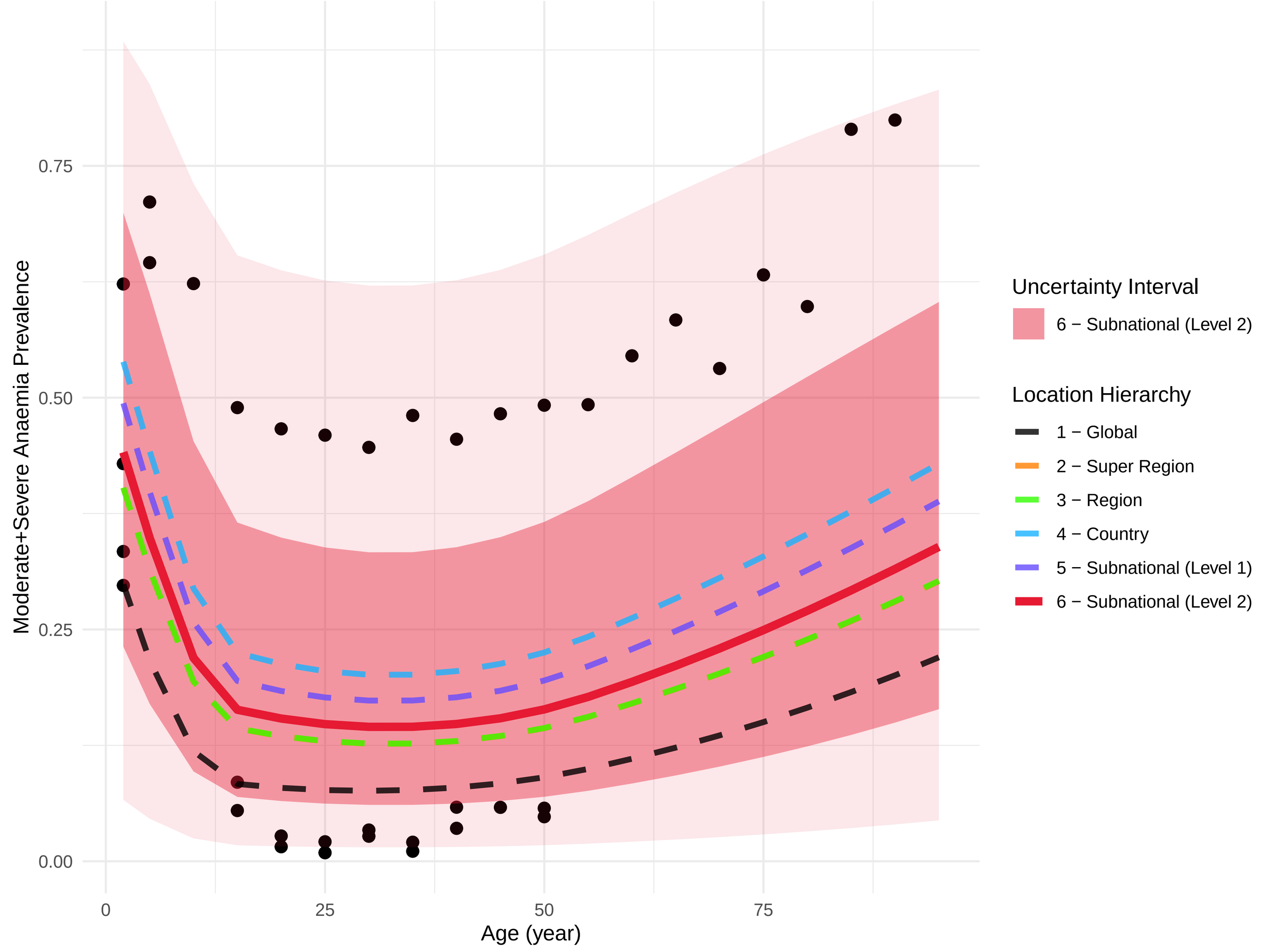

MR−BRT Age/Sex Fits − Males 2+ Years Old − Moderate+Severe Anaemia Prevalence − Tripura
WHO−2024 Adjustment Method

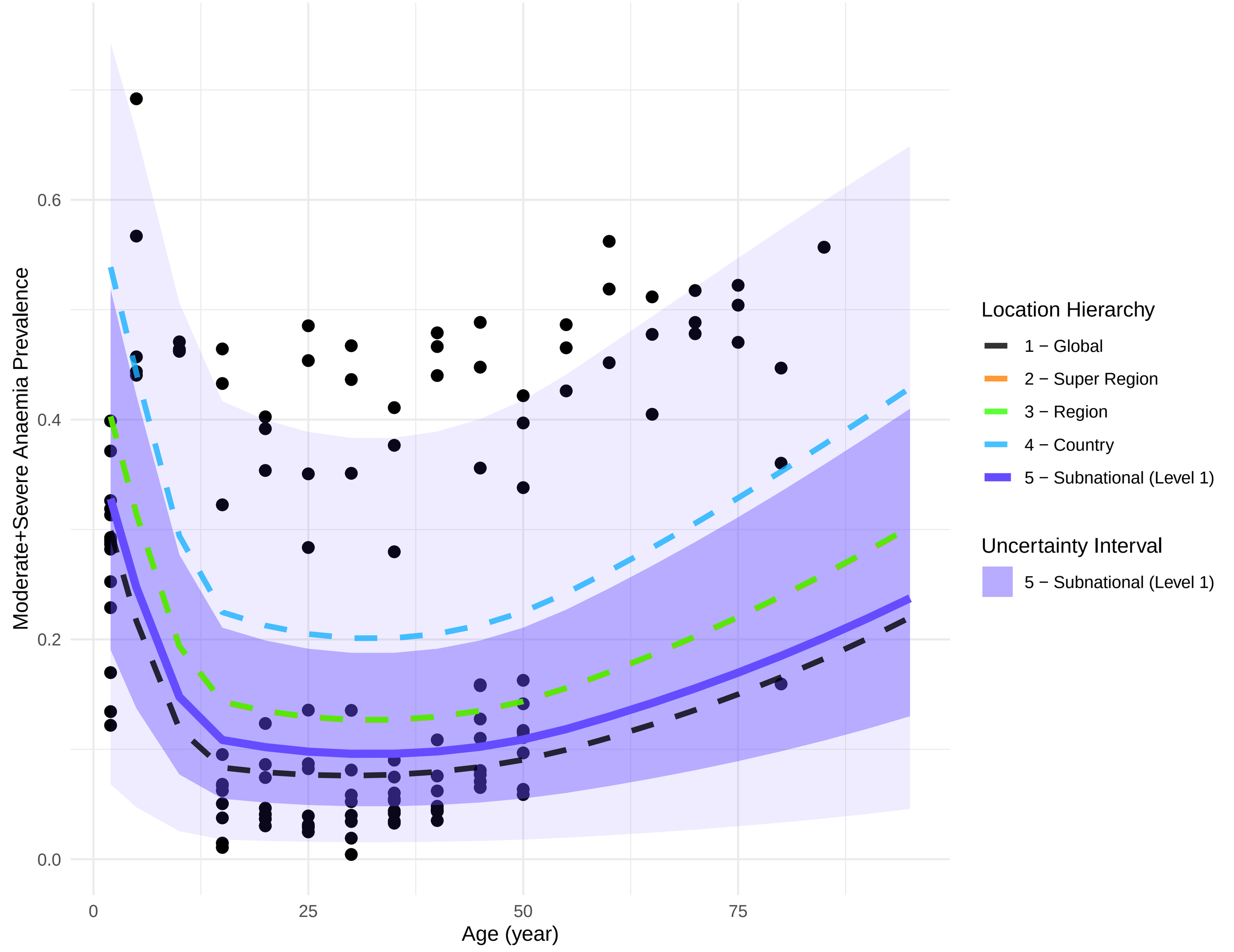

MR−BRT Age/Sex Fits − Males 2+ Years Old − Moderate+Severe Anaemia Prevalence − Tripura, Rural
WHO−2024 Adjustment Method

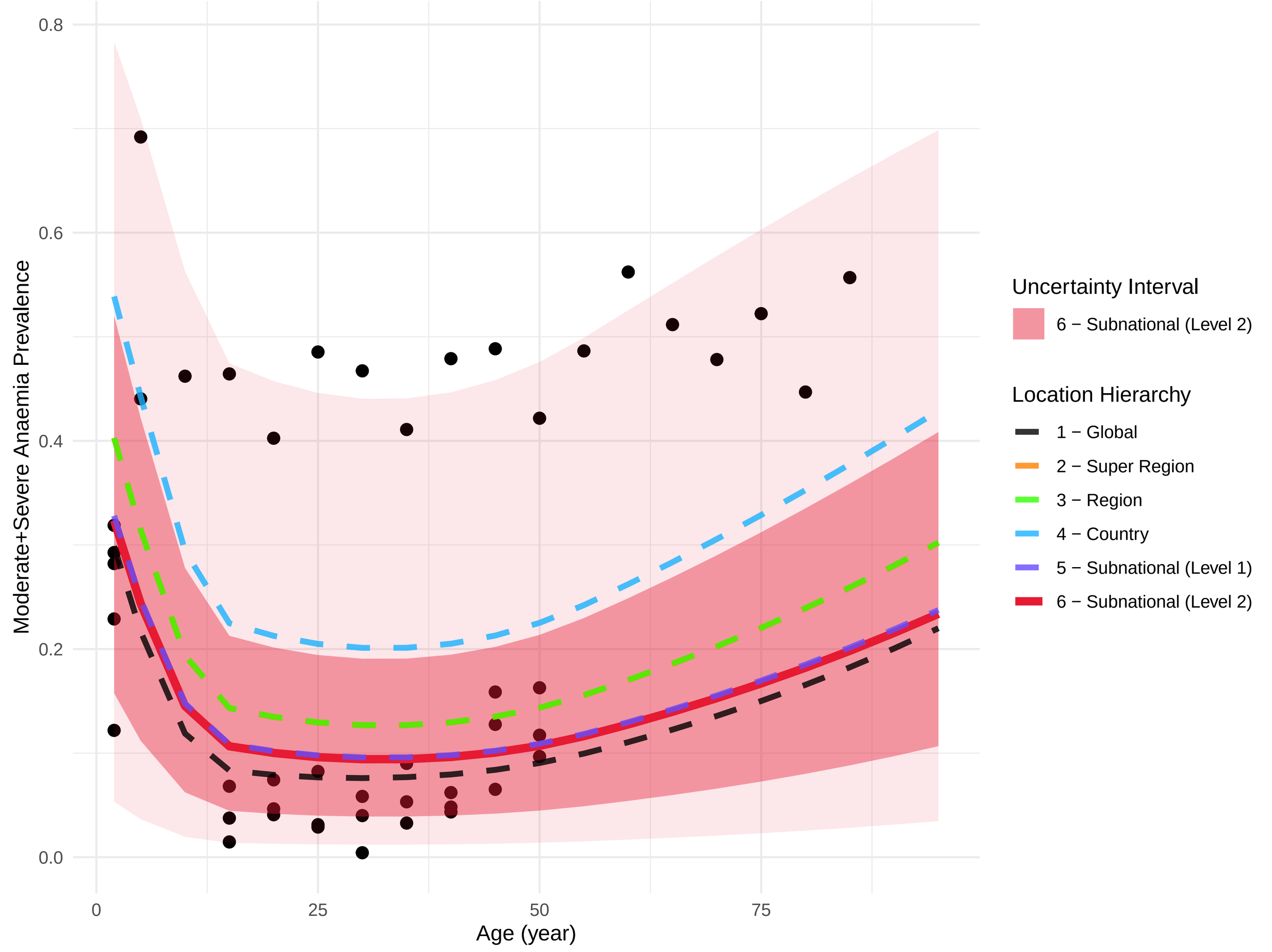

MR−BRT Age/Sex Fits − Males 2+ Years Old − Moderate+Severe Anaemia Prevalence − Tripura, Urban
WHO−2024 Adjustment Method

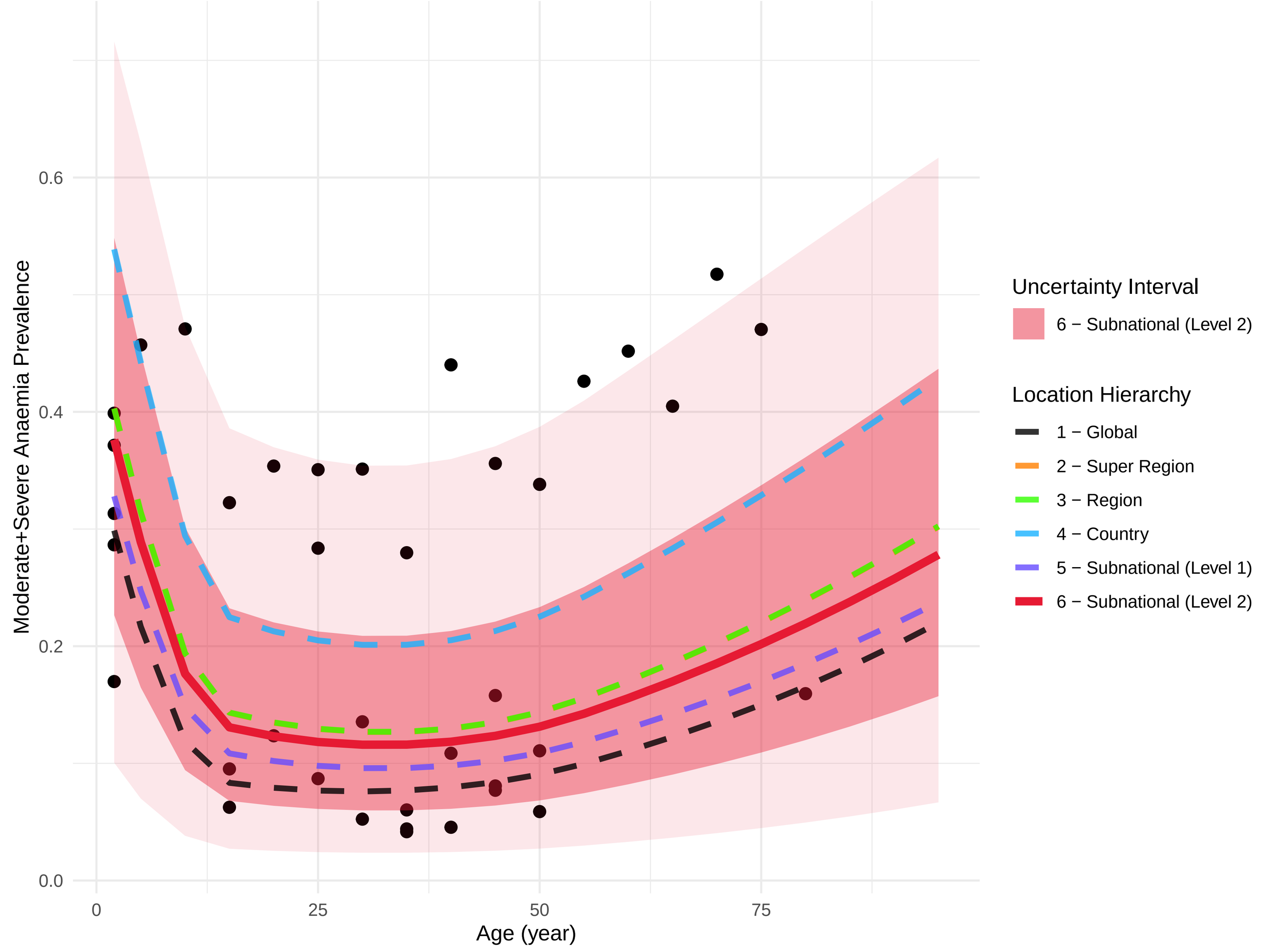

MR−BRT Age/Sex Fits − Males 2+ Years Old − Moderate+Severe Anaemia Prevalence − Uttar Pradesh
WHO−2024 Adjustment Method

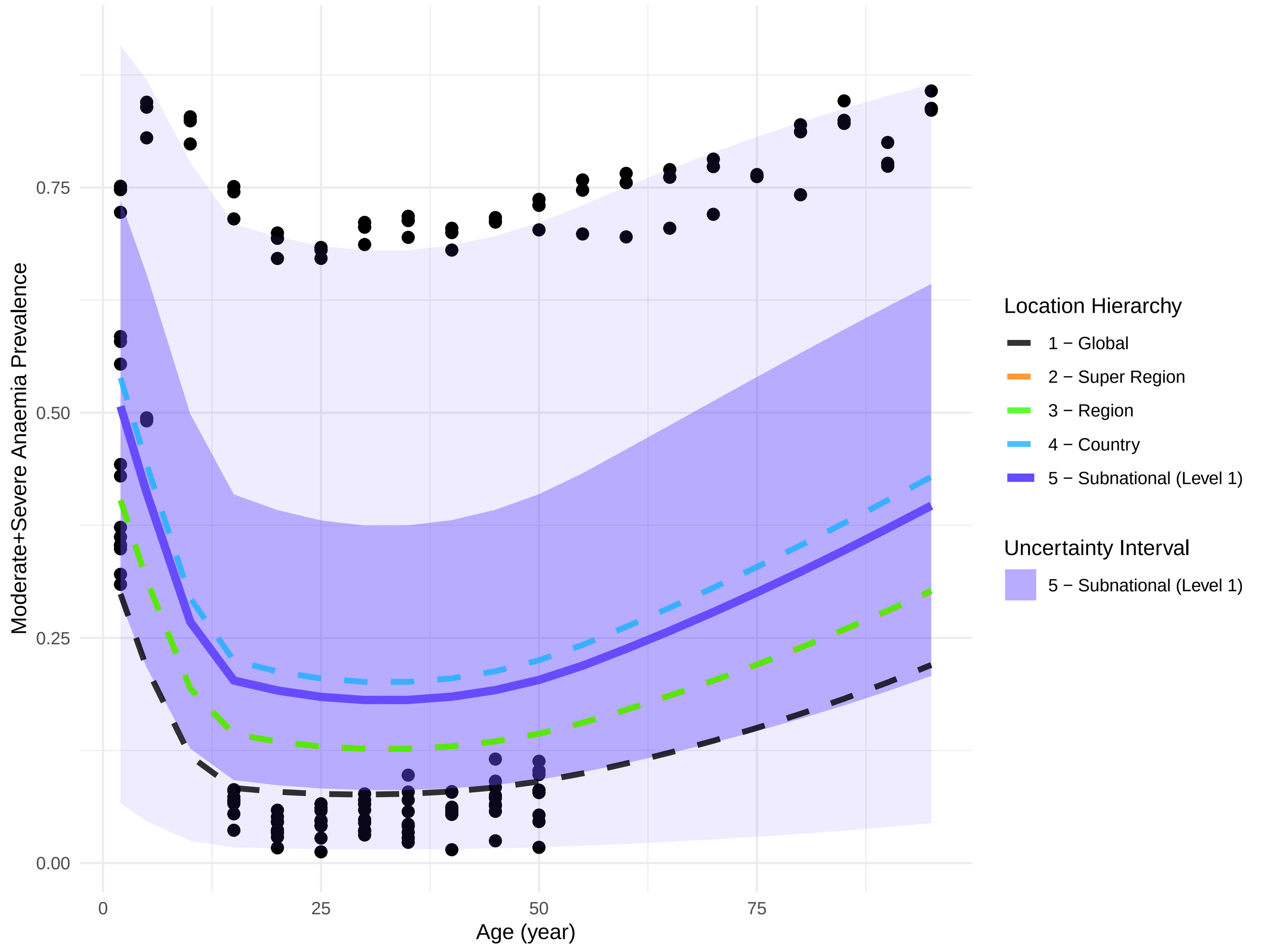

MR−BRT Age/Sex Fits − Males 2+ Years Old − Moderate+Severe Anaemia Prevalence − Uttar Pradesh, Rural
WHO−2024 Adjustment Method

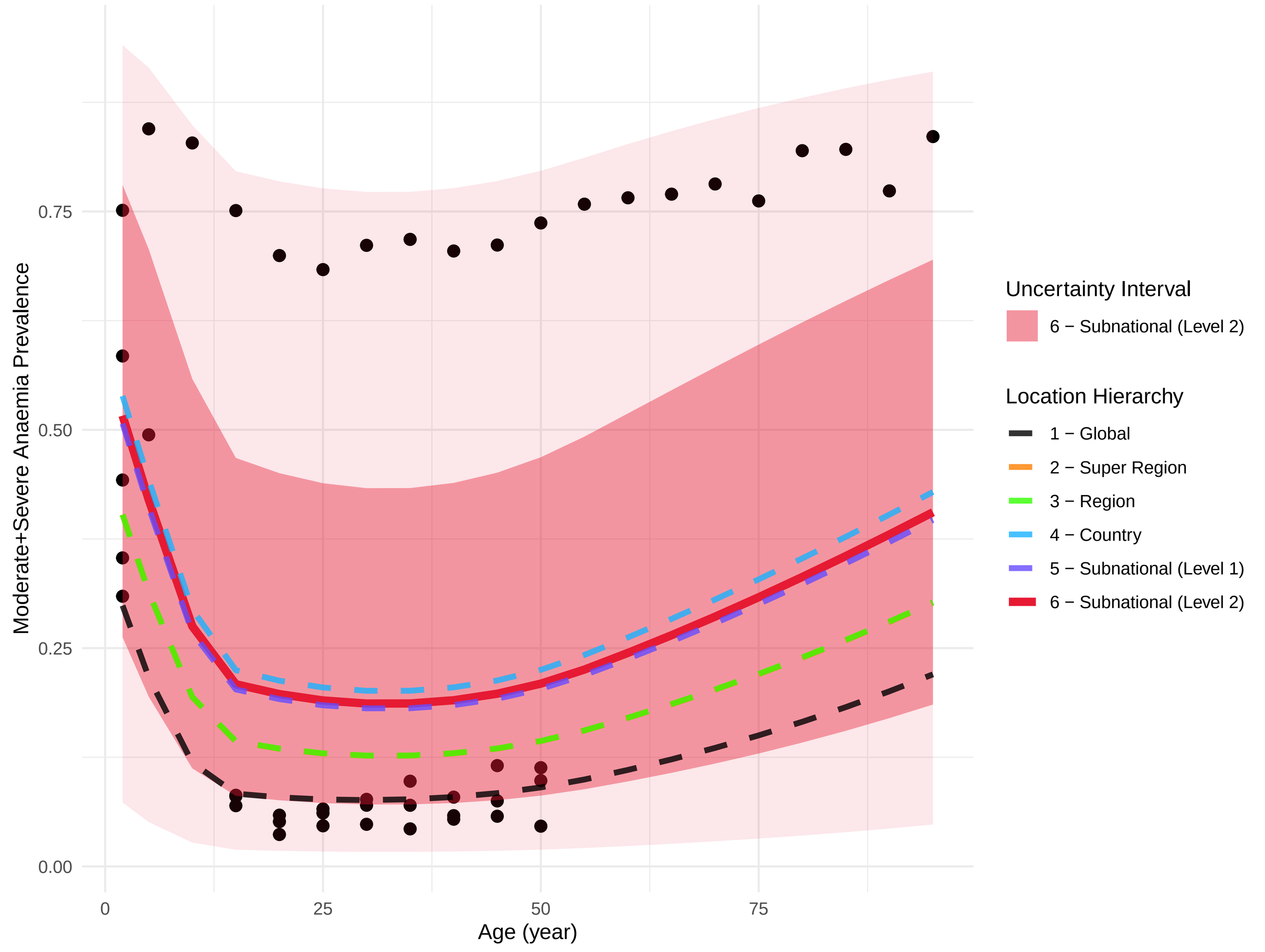

MR−BRT Age/Sex Fits − Males 2+ Years Old − Moderate+Severe Anaemia Prevalence − Uttar Pradesh, Urban
WHO−2024 Adjustment Method

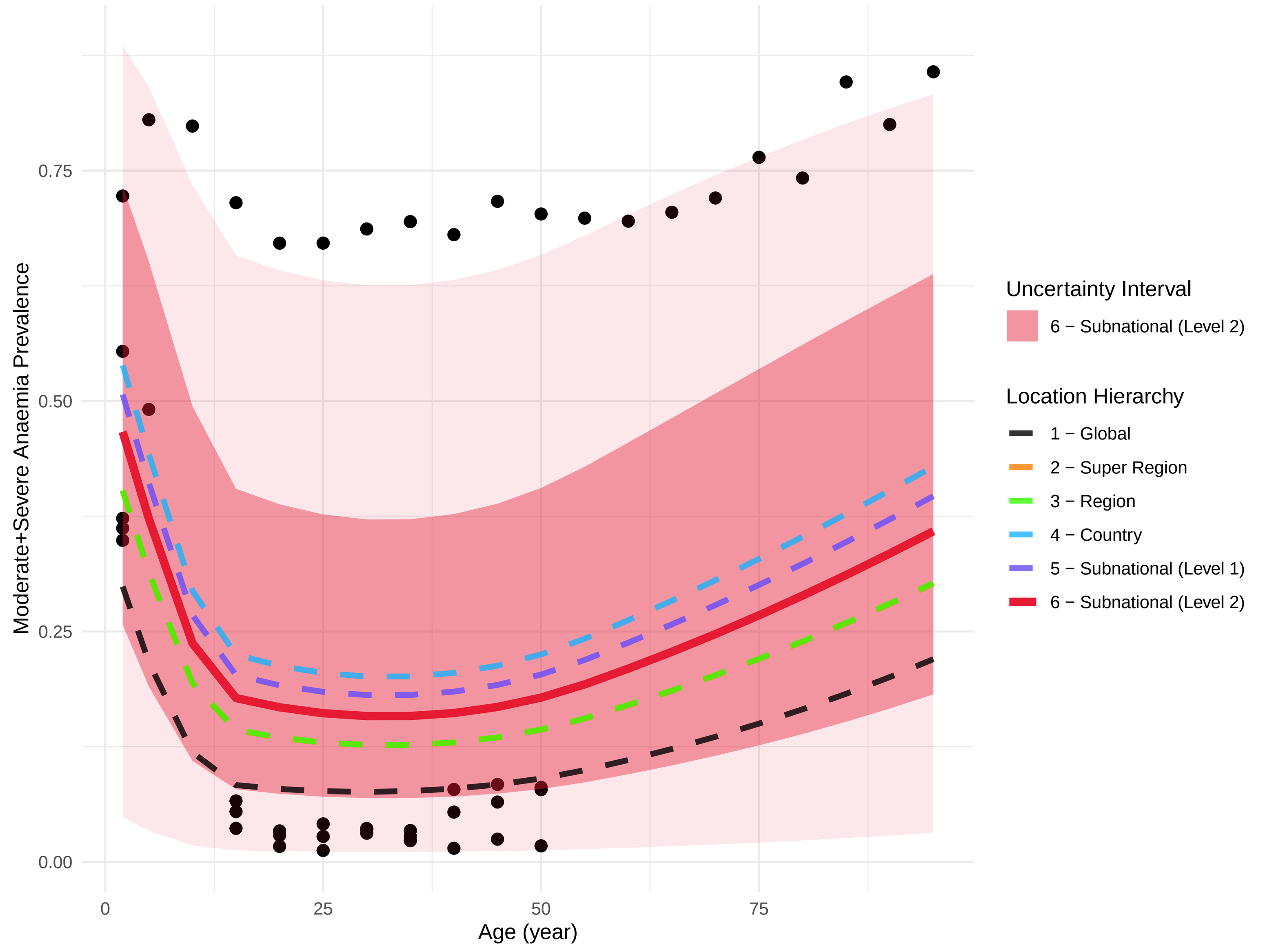

MR−BRT Age/Sex Fits − Males 2+ Years Old − Moderate+Severe Anaemia Prevalence − Uttarakhand
WHO−2024 Adjustment Method

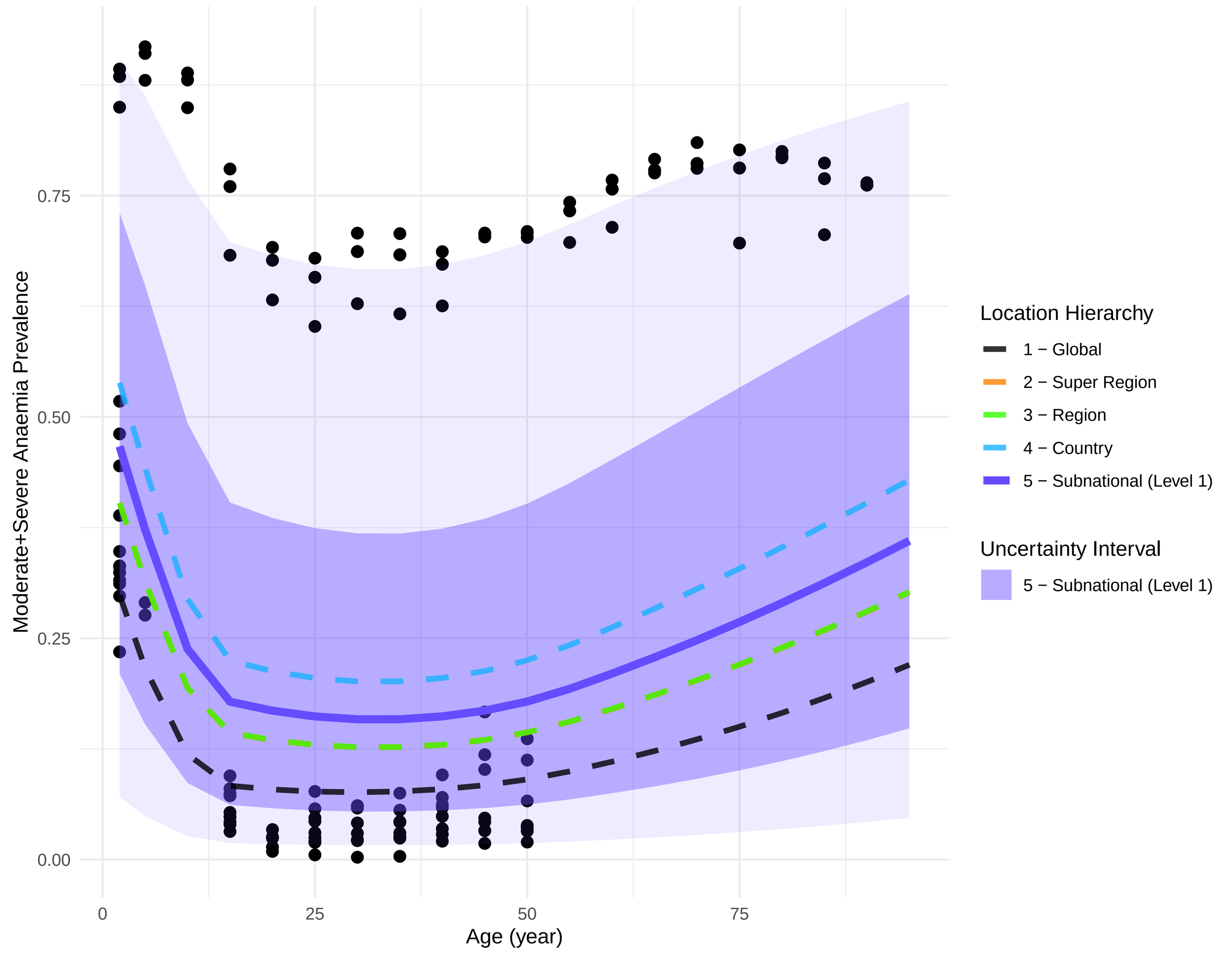

MR−BRT Age/Sex Fits − Males 2+ Years Old − Moderate+Severe Anaemia Prevalence − Uttarakhand, Rural
WHO−2024 Adjustment Method

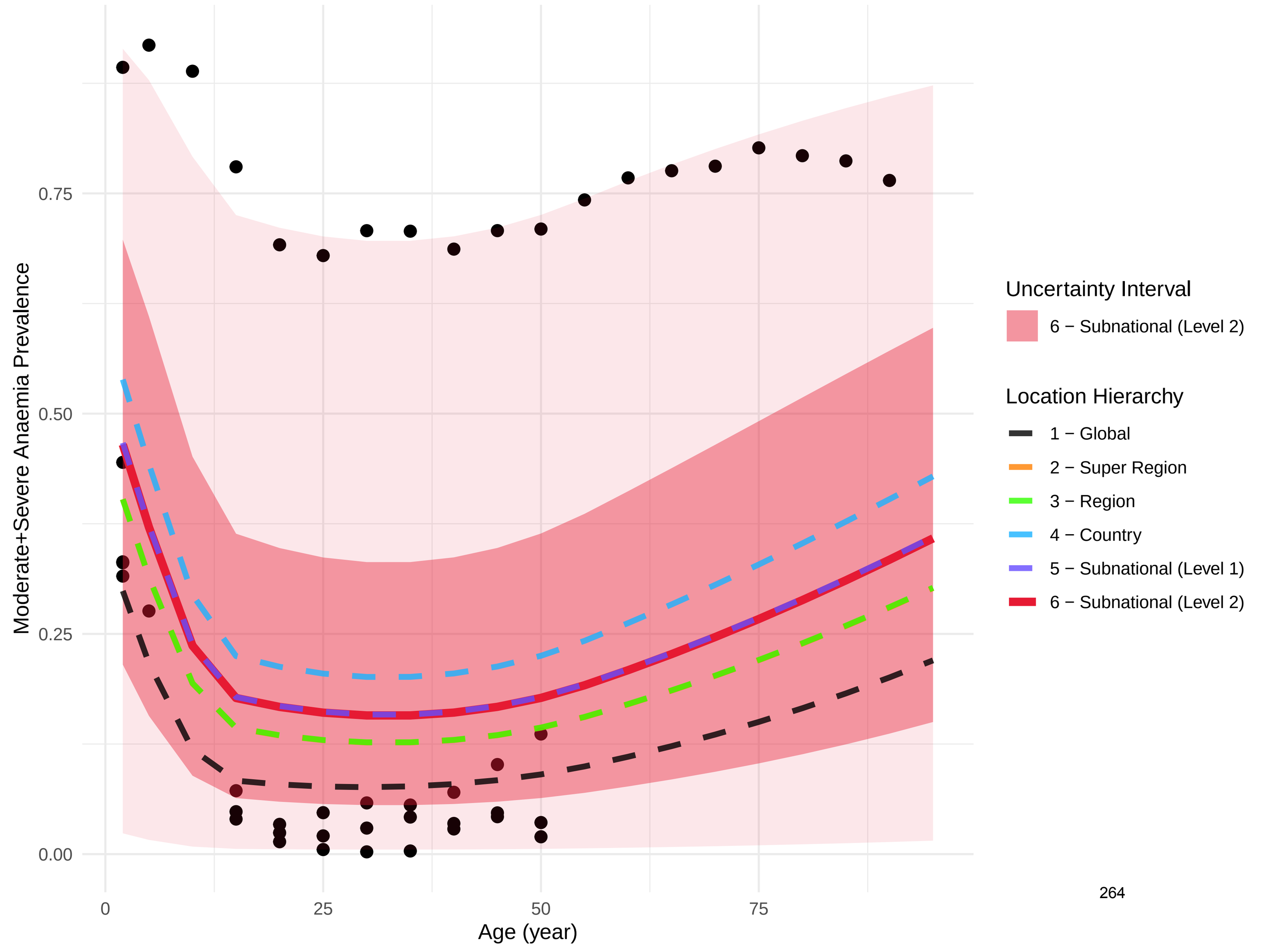

MR−BRT Age/Sex Fits − Males 2+ Years Old − Moderate+Severe Anaemia Prevalence − Uttarakhand, Urban
WHO−2024 Adjustment Method

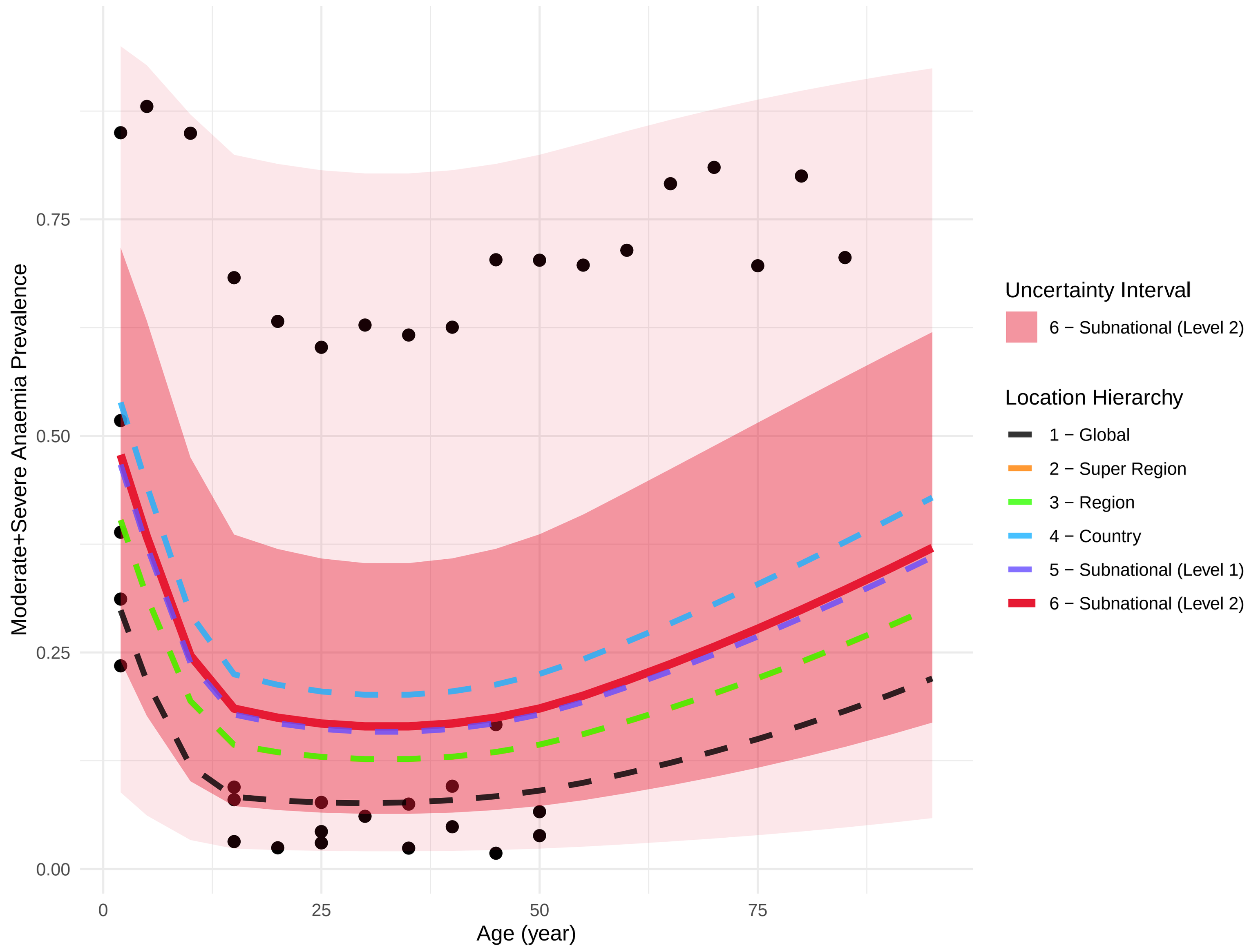

MR−BRT Age/Sex Fits − Males 2+ Years Old − Moderate+Severe Anaemia Prevalence − West Bengal
WHO−2024 Adjustment Method

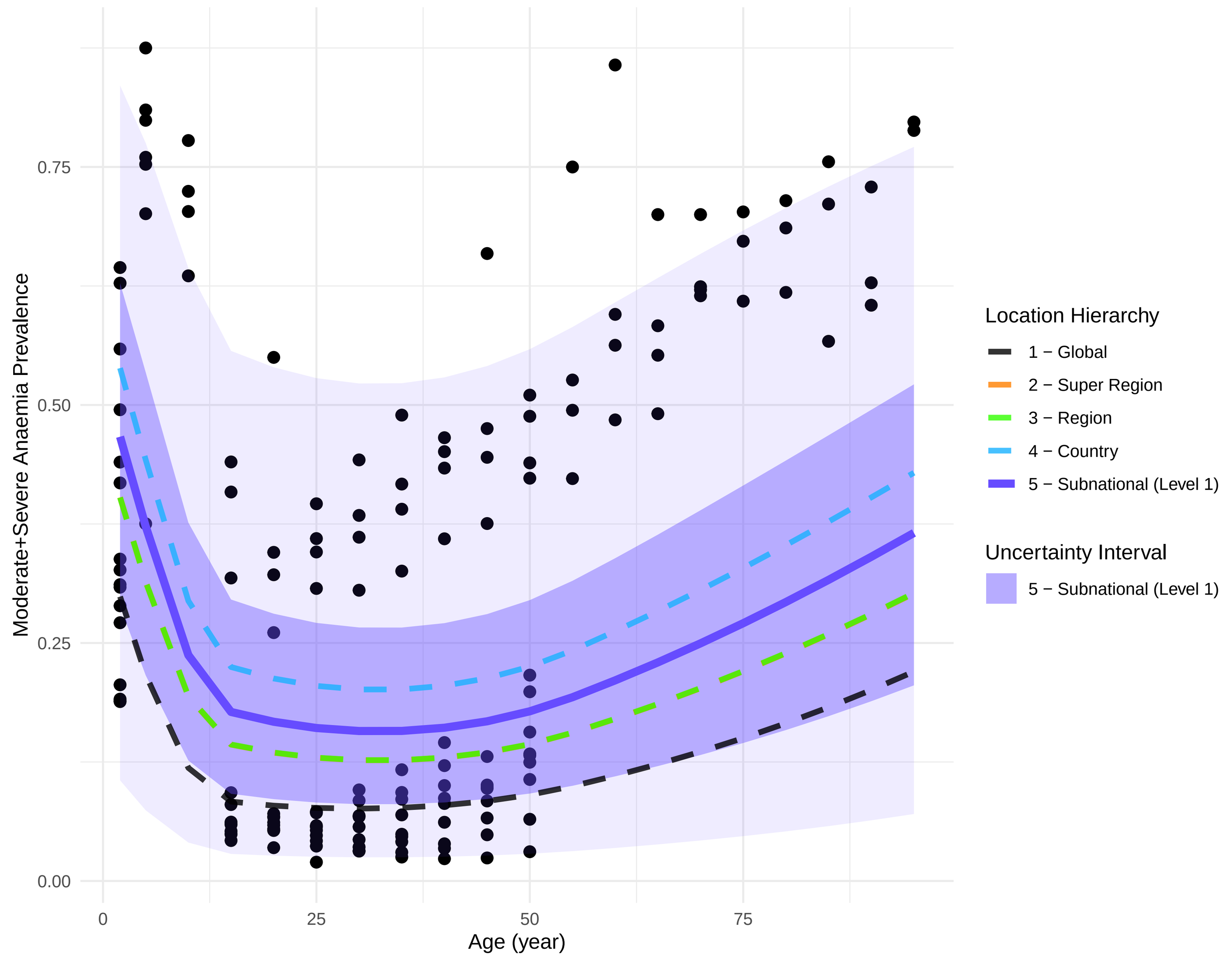

MR−BRT Age/Sex Fits − Males 2+ Years Old − Moderate+Severe Anaemia Prevalence − West Bengal, Rural
WHO−2024 Adjustment Method

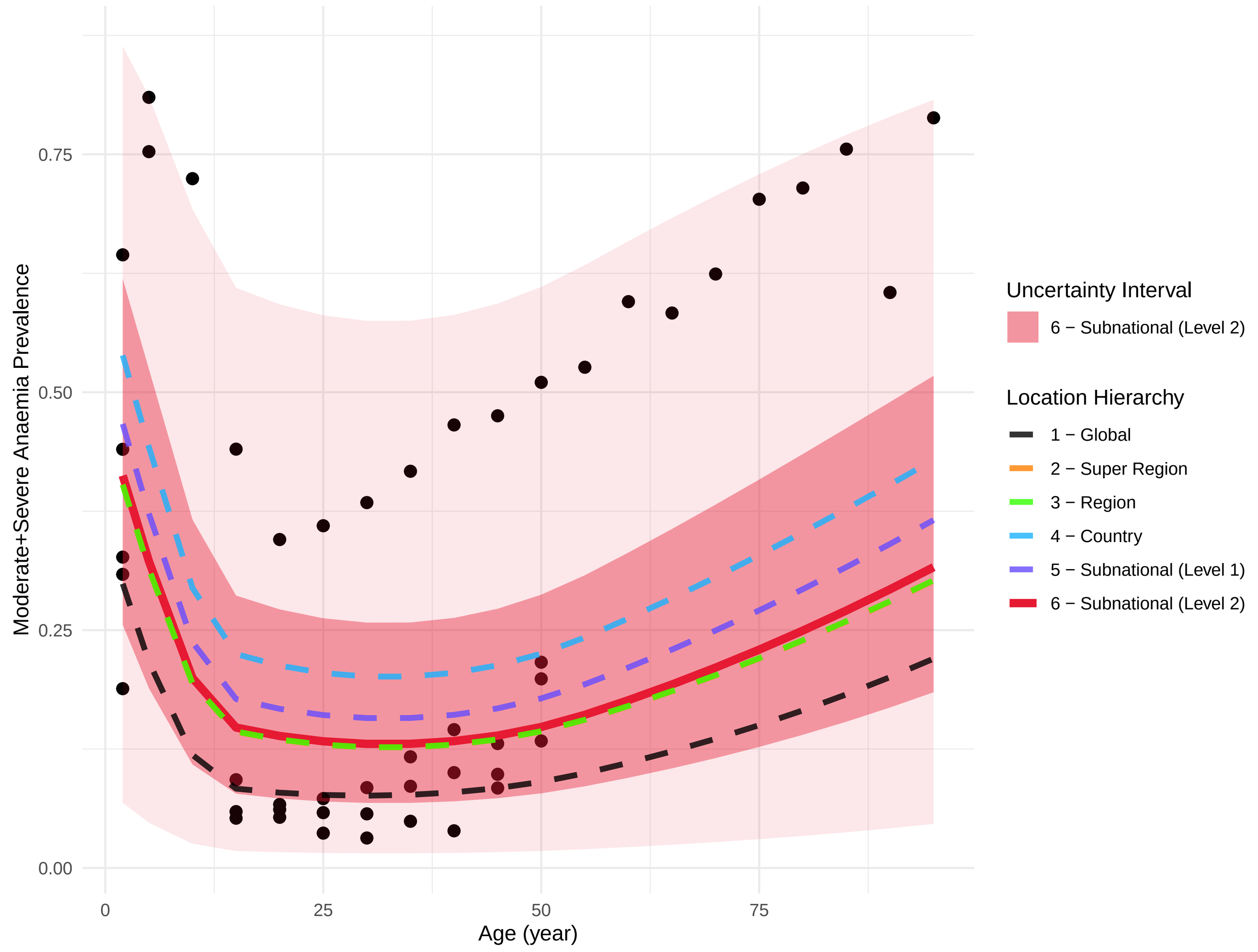

MR−BRT Age/Sex Fits − Males 2+ Years Old − Moderate+Severe Anaemia Prevalence − West Bengal, Urban
WHO−2024 Adjustment Method

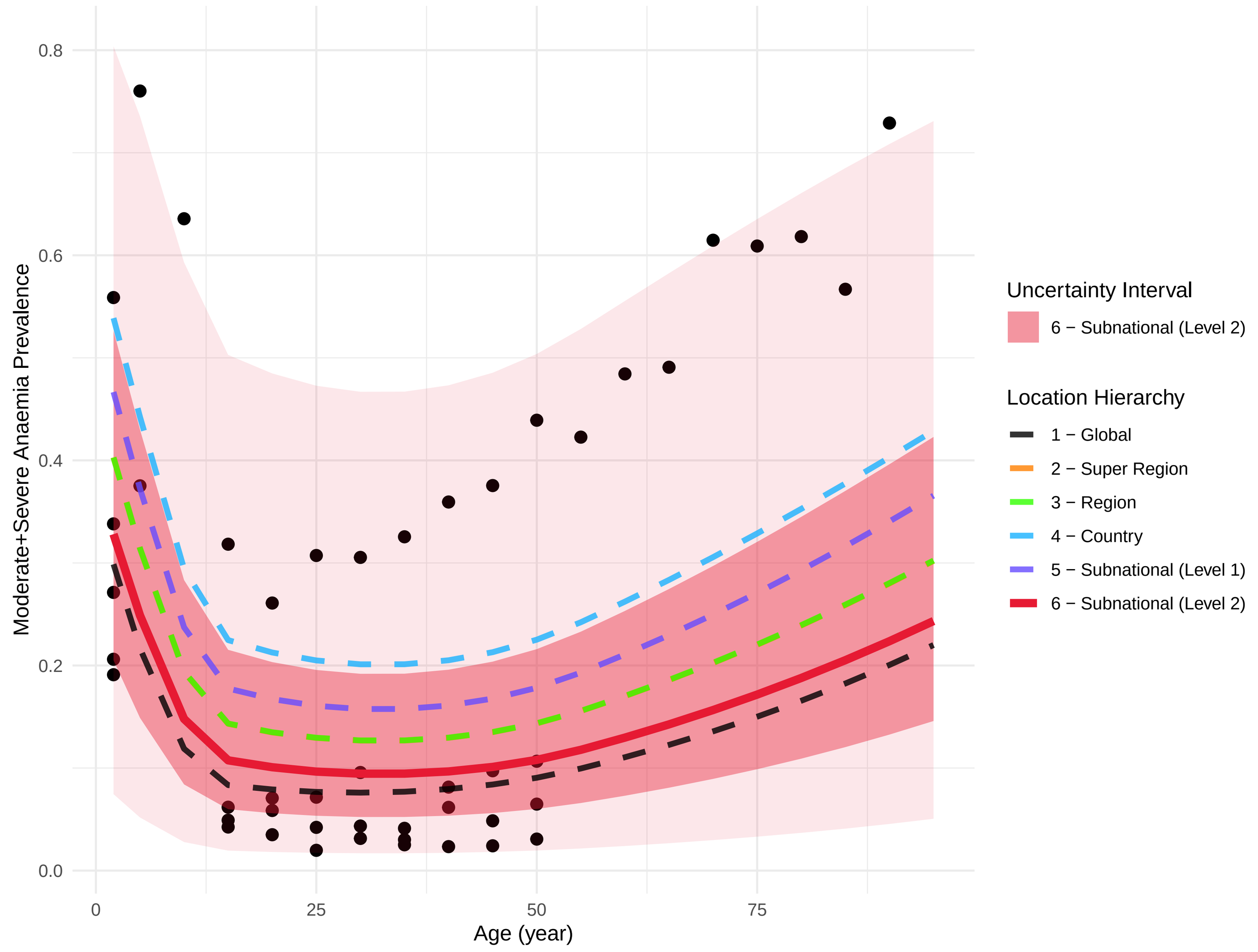

MR−BRT Age/Sex Fits − Males 2+ Years Old − Moderate+Severe Anaemia Prevalence − Nepal
WHO−2024 Adjustment Method

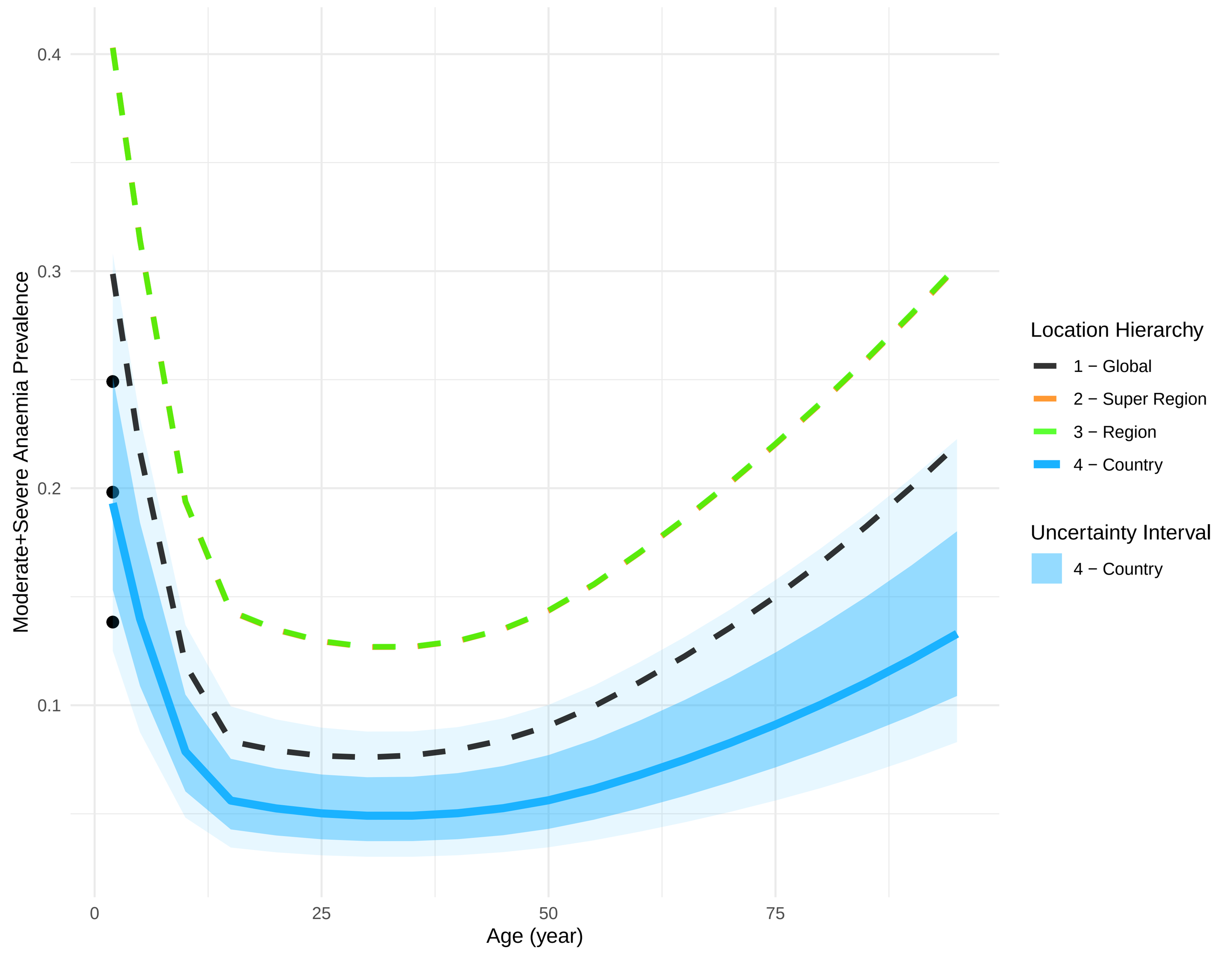

MR−BRT Age/Sex Fits − Males 2+ Years Old − Moderate+Severe Anaemia Prevalence − Southeast Asia, East Asia, and Oceania
WHO−2024 Adjustment Method

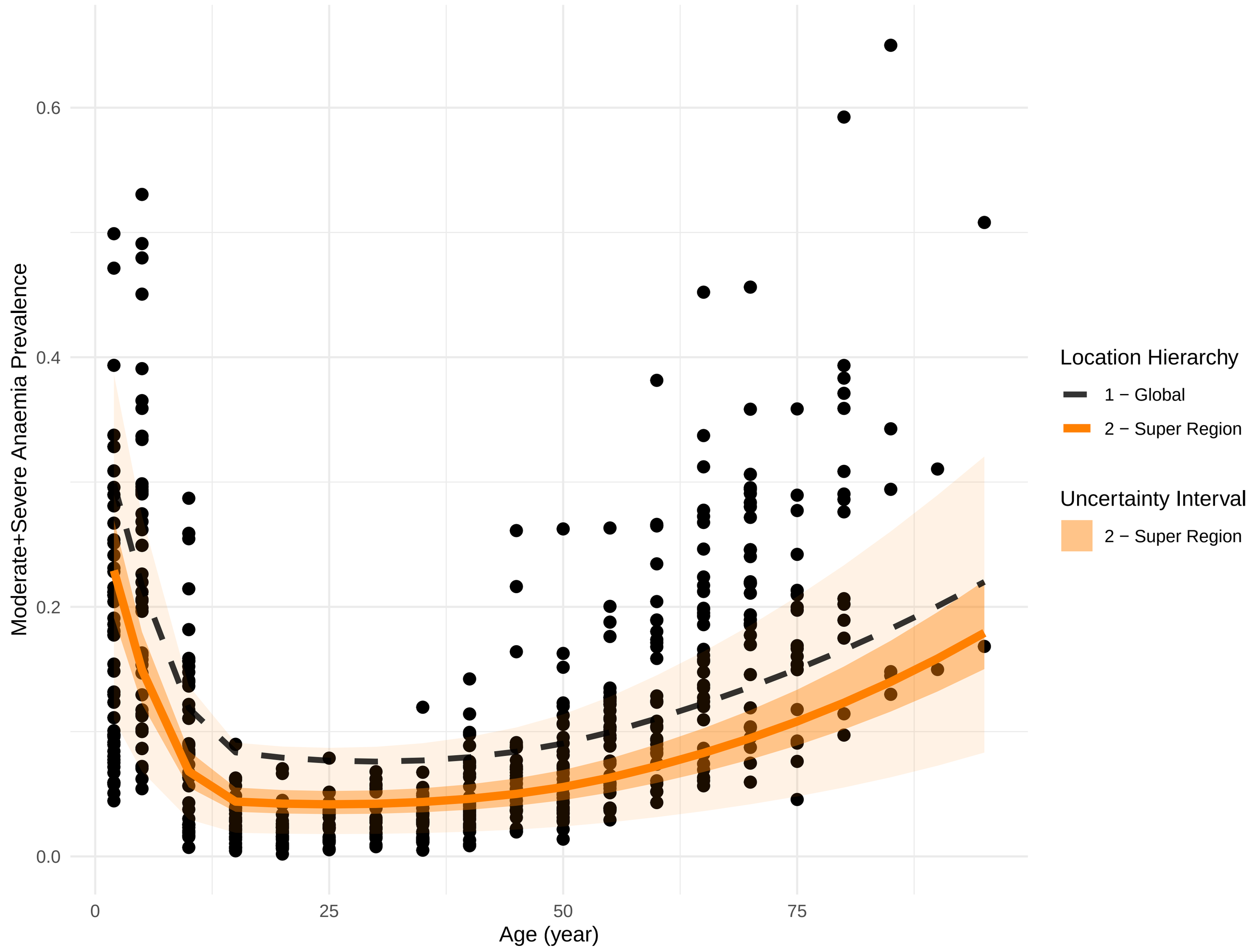

MR−BRT Age/Sex Fits − Males 2+ Years Old − Moderate+Severe Anaemia Prevalence − Southeast Asia
WHO−2024 Adjustment Method

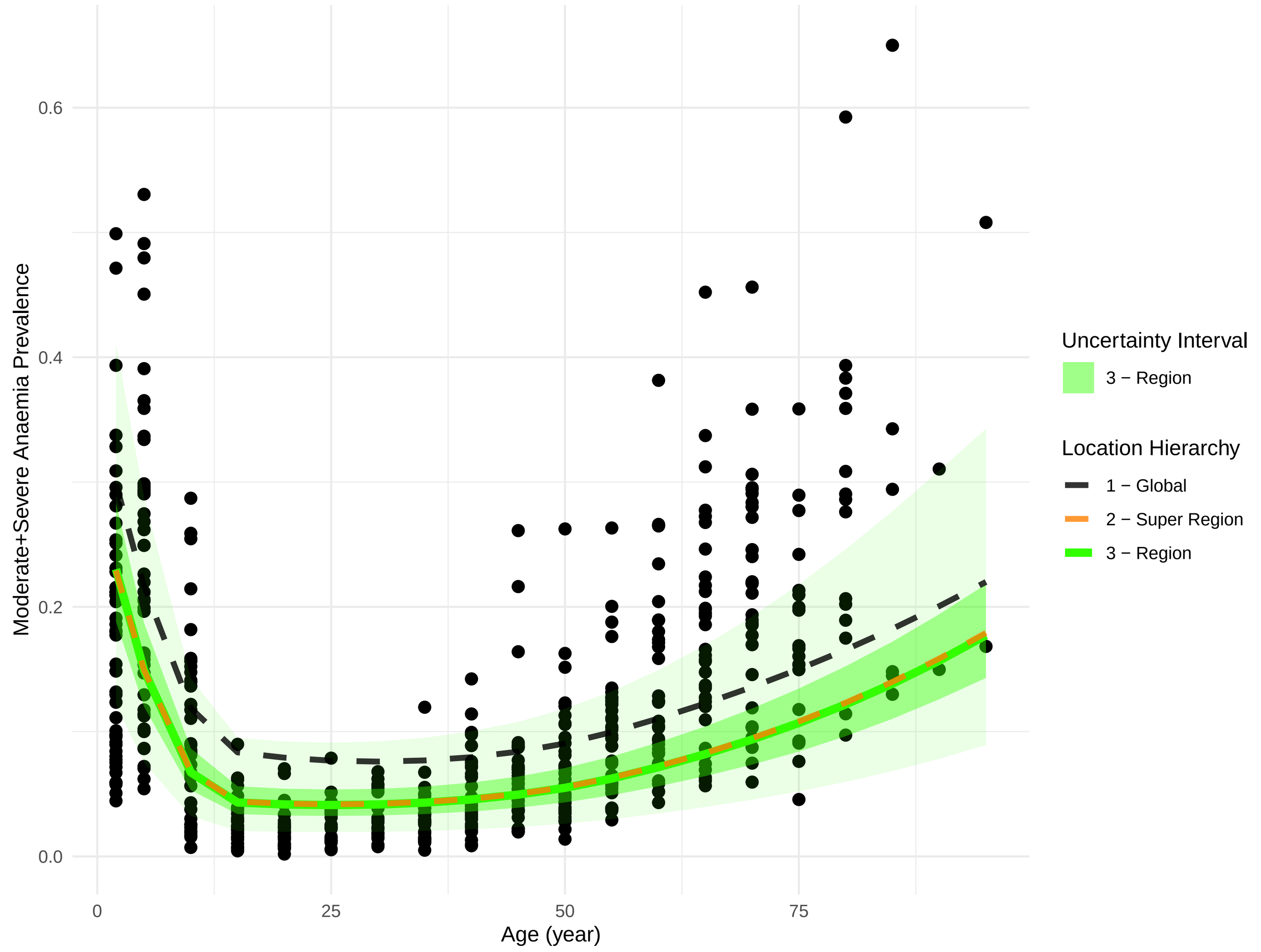

MR−BRT Age/Sex Fits − Males 2+ Years Old − Moderate+Severe Anaemia Prevalence − Cambodia
WHO−2024 Adjustment Method

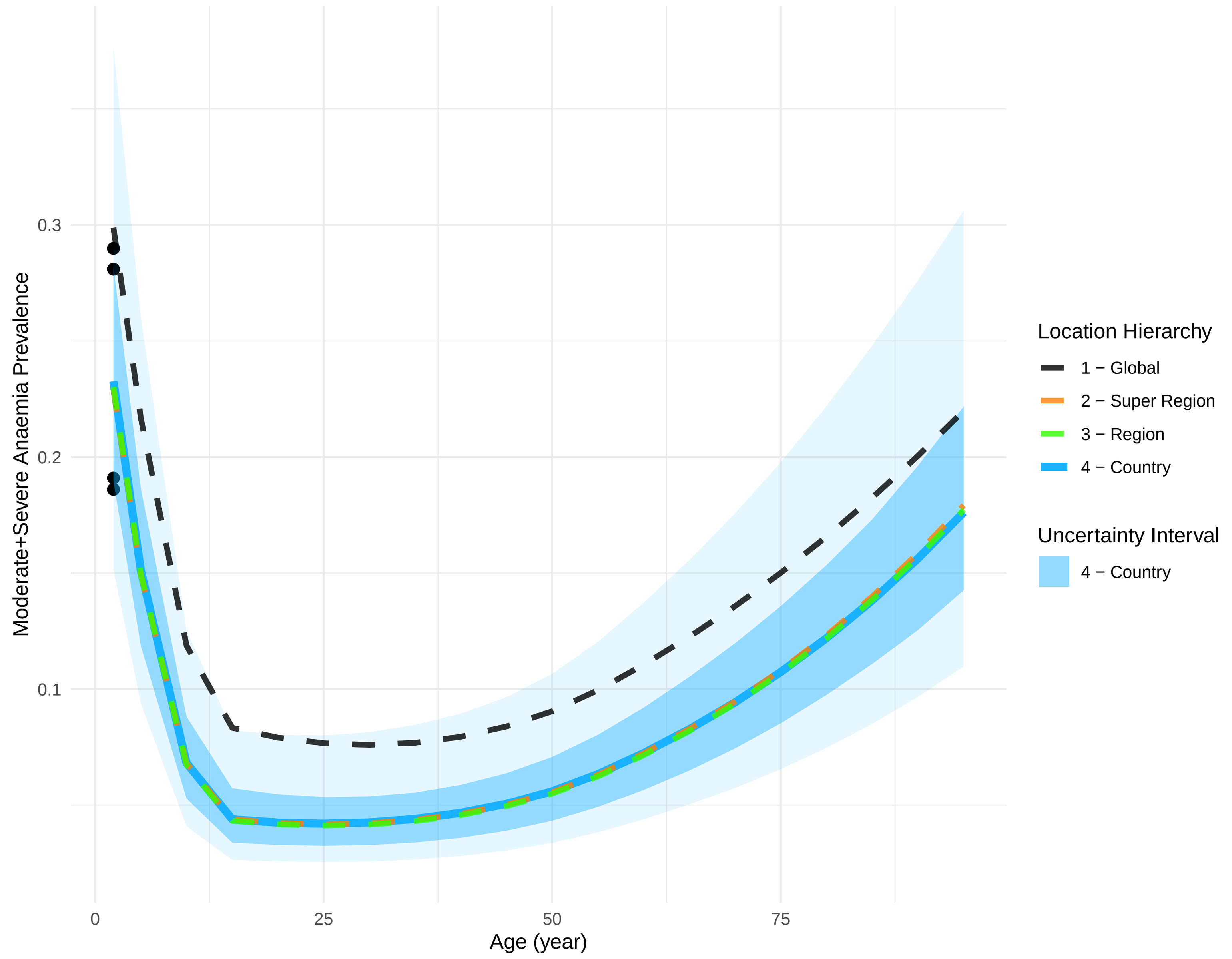

MR−BRT Age/Sex Fits − Males 2+ Years Old − Moderate+Severe Anaemia Prevalence − Indonesia
WHO−2024 Adjustment Method

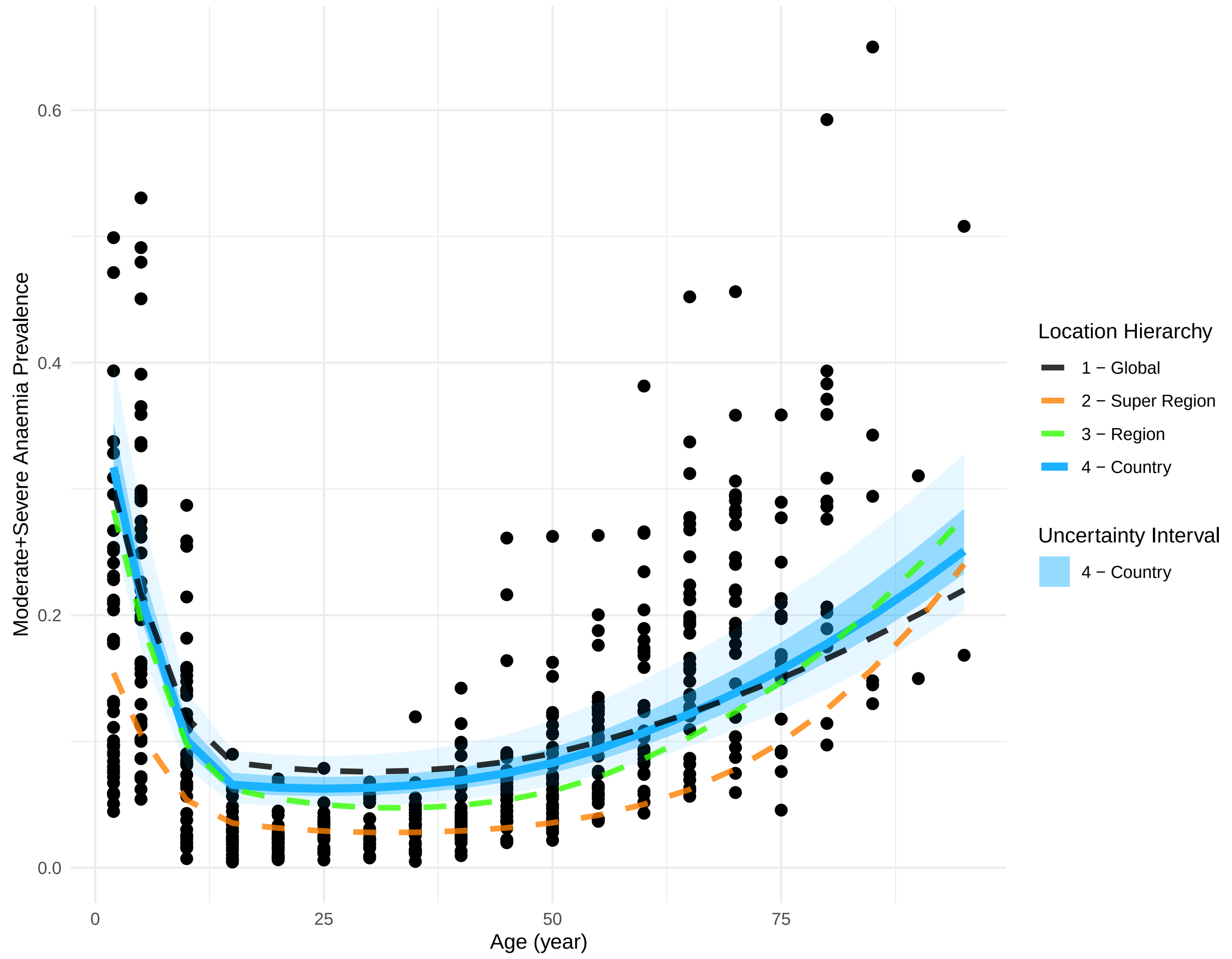

MR−BRT Age/Sex Fits − Males 2+ Years Old − Moderate+Severe Anaemia Prevalence − Bali
WHO−2024 Adjustment Method

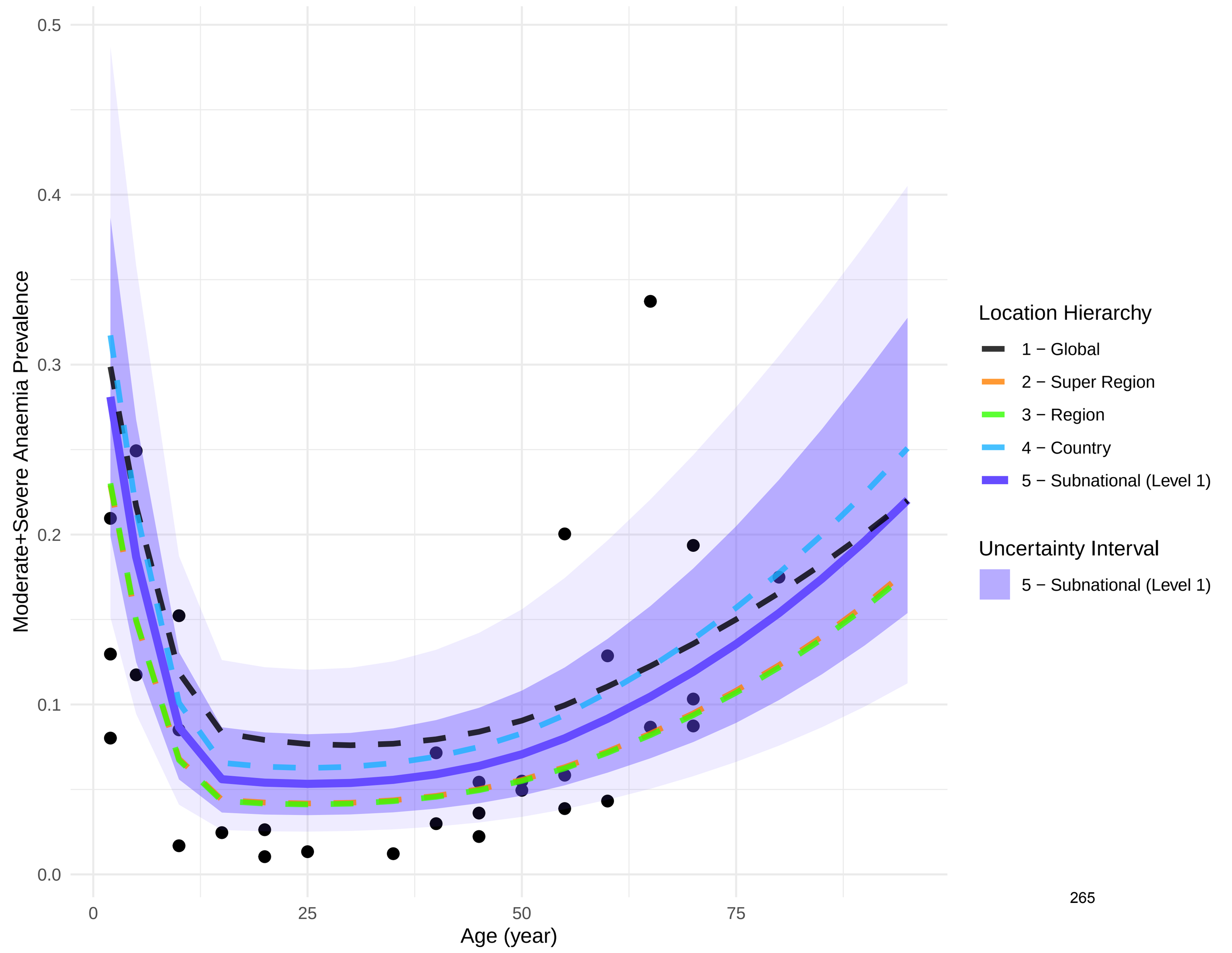

MR−BRT Age/Sex Fits − Males 2+ Years Old − Moderate+Severe Anaemia Prevalence − Bangka−Belitung Islands
WHO−2024 Adjustment Method

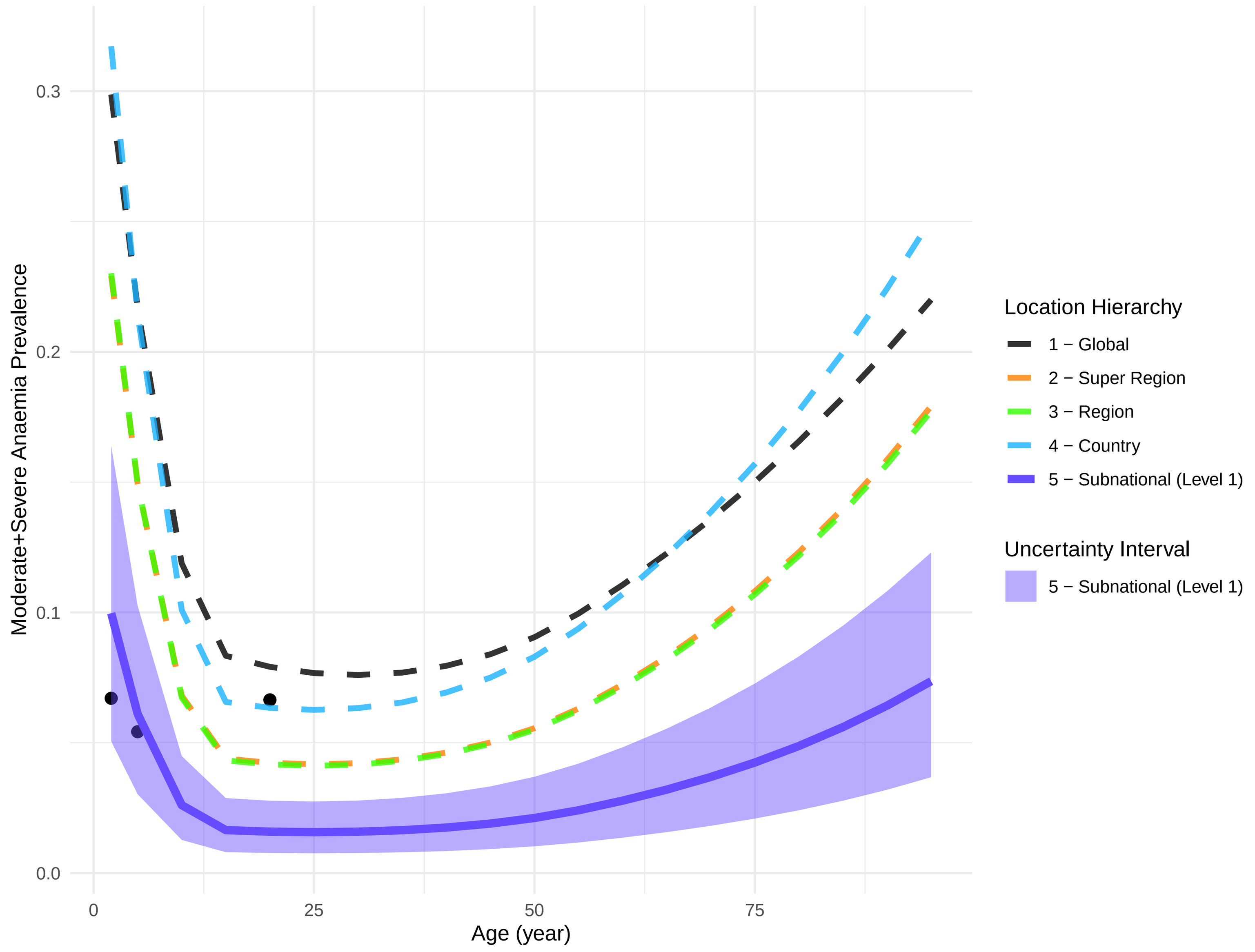

MR−BRT Age/Sex Fits − Males 2+ Years Old − Moderate+Severe Anaemia Prevalence − Banten
WHO−2024 Adjustment Method

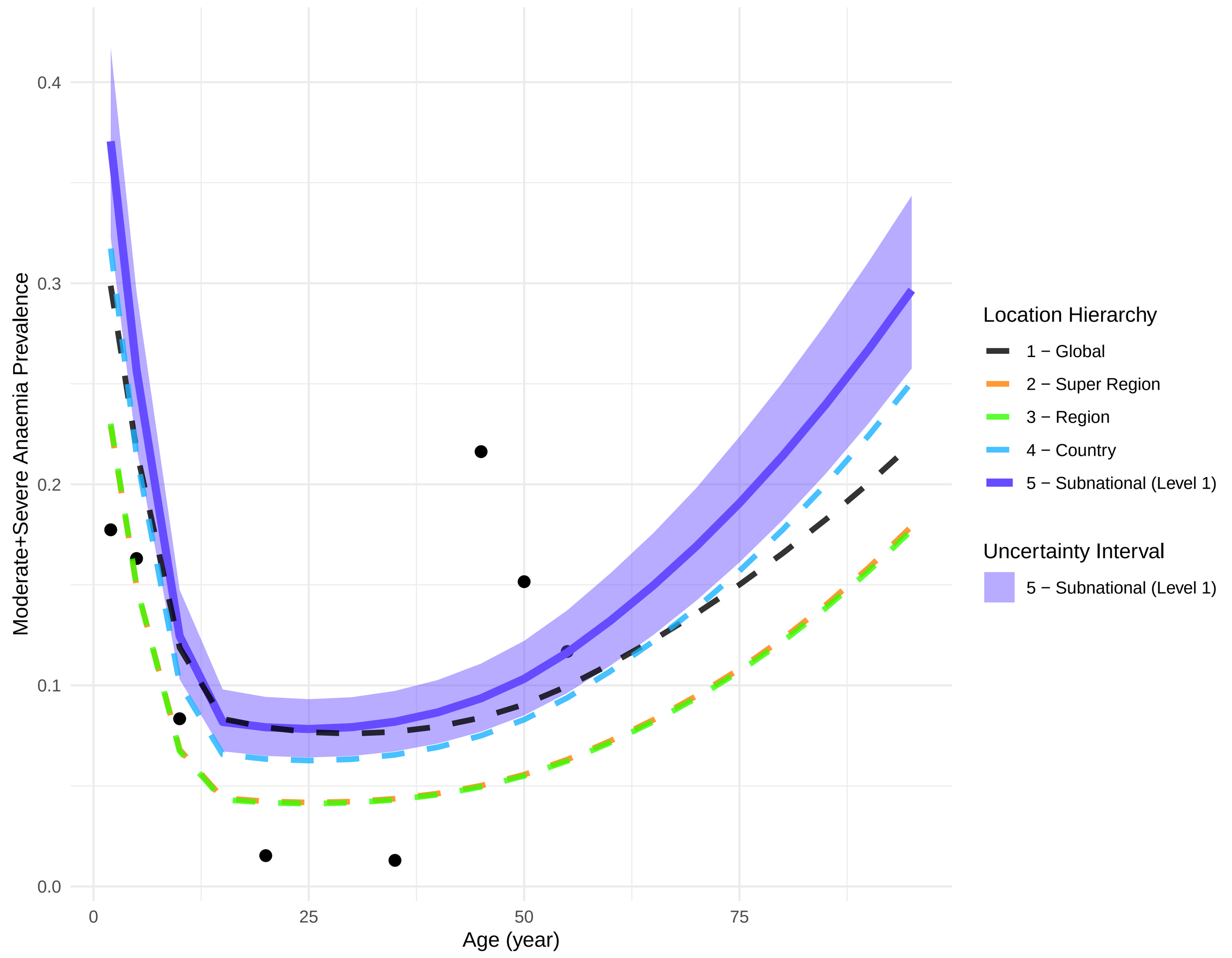

MR−BRT Age/Sex Fits − Males 2+ Years Old − Moderate+Severe Anaemia Prevalence − Jakarta
WHO−2024 Adjustment Method

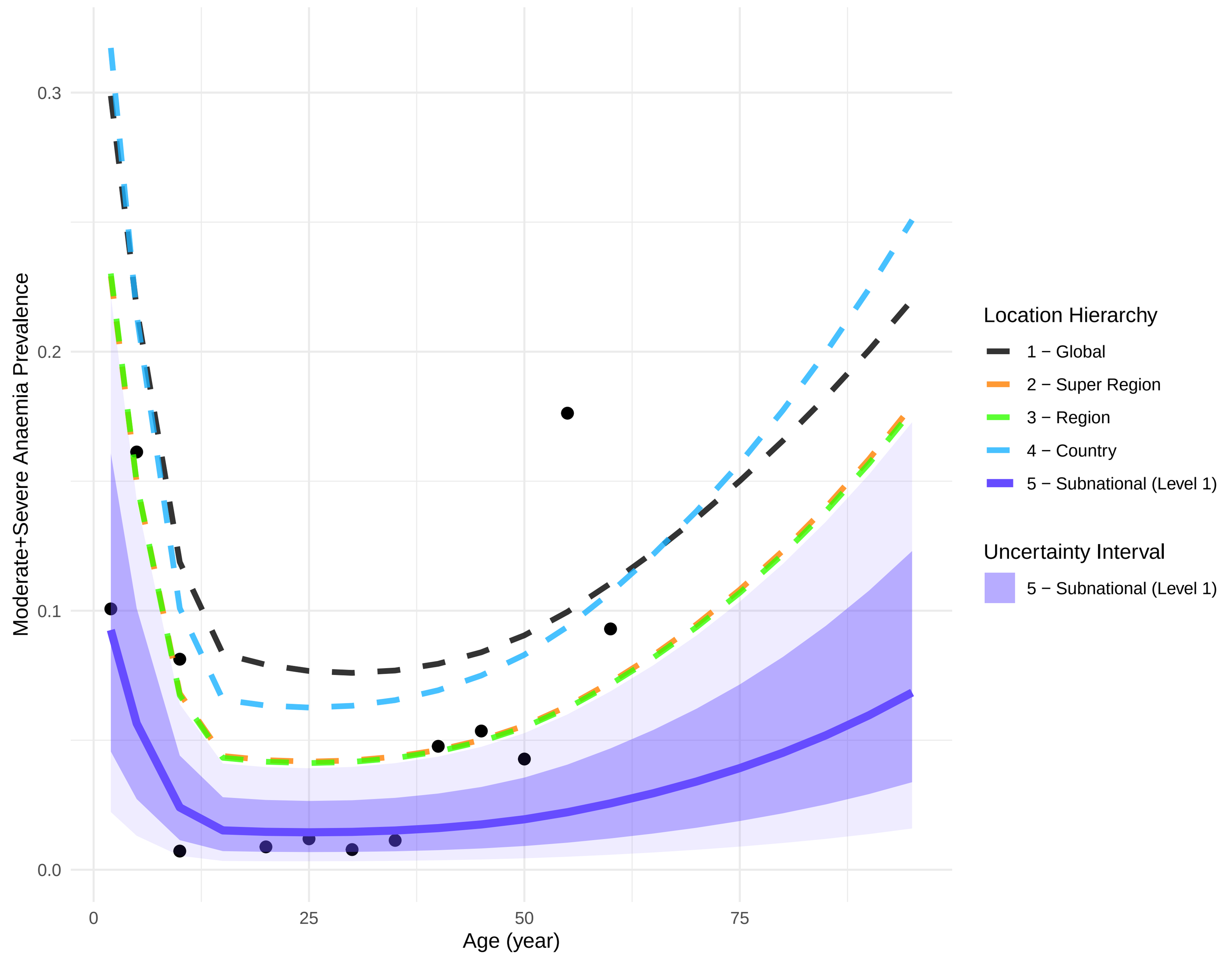

MR−BRT Age/Sex Fits − Males 2+ Years Old − Moderate+Severe Anaemia Prevalence − West Java
WHO−2024 Adjustment Method

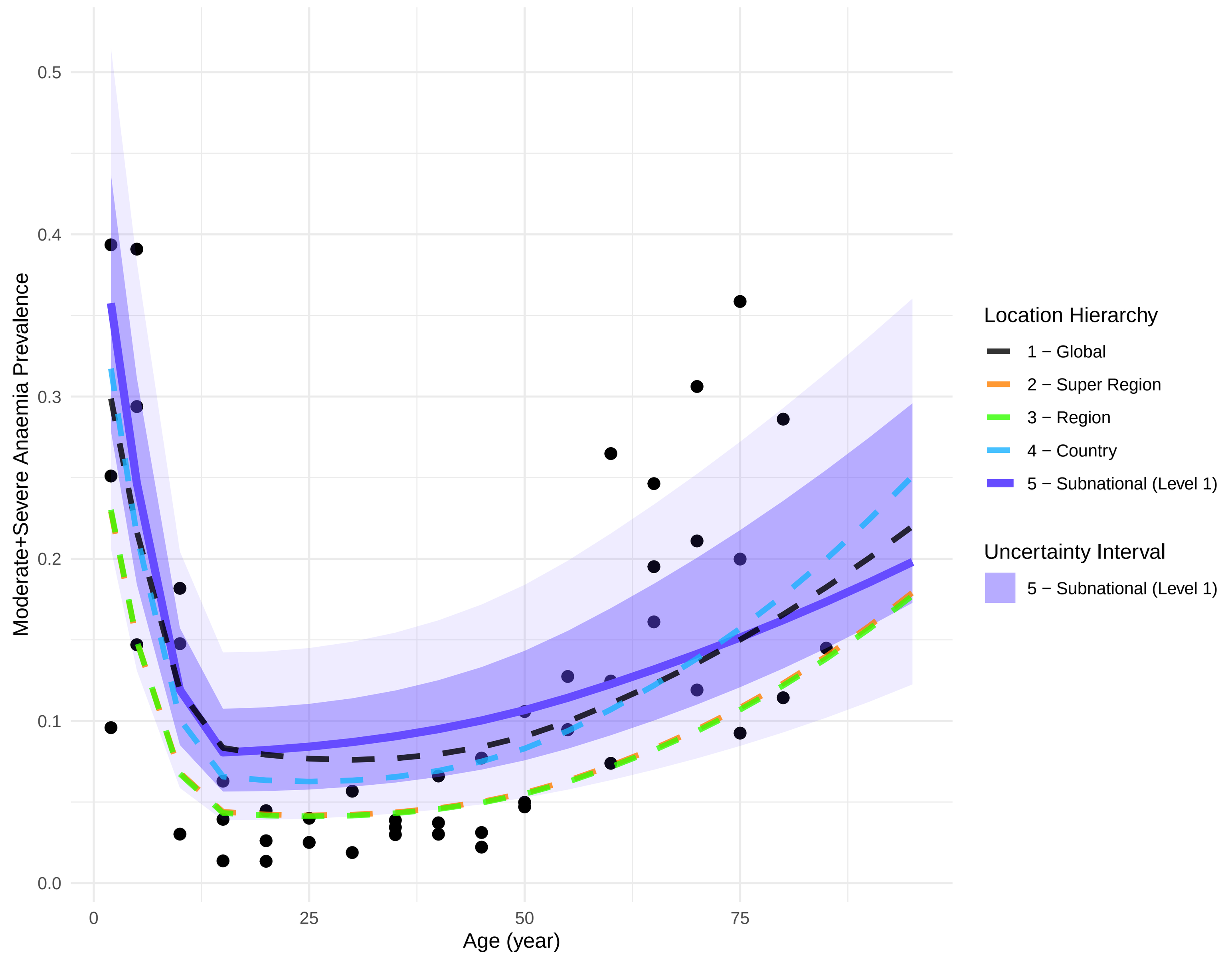

MR−BRT Age/Sex Fits − Males 2+ Years Old − Moderate+Severe Anaemia Prevalence − Central Java
WHO−2024 Adjustment Method

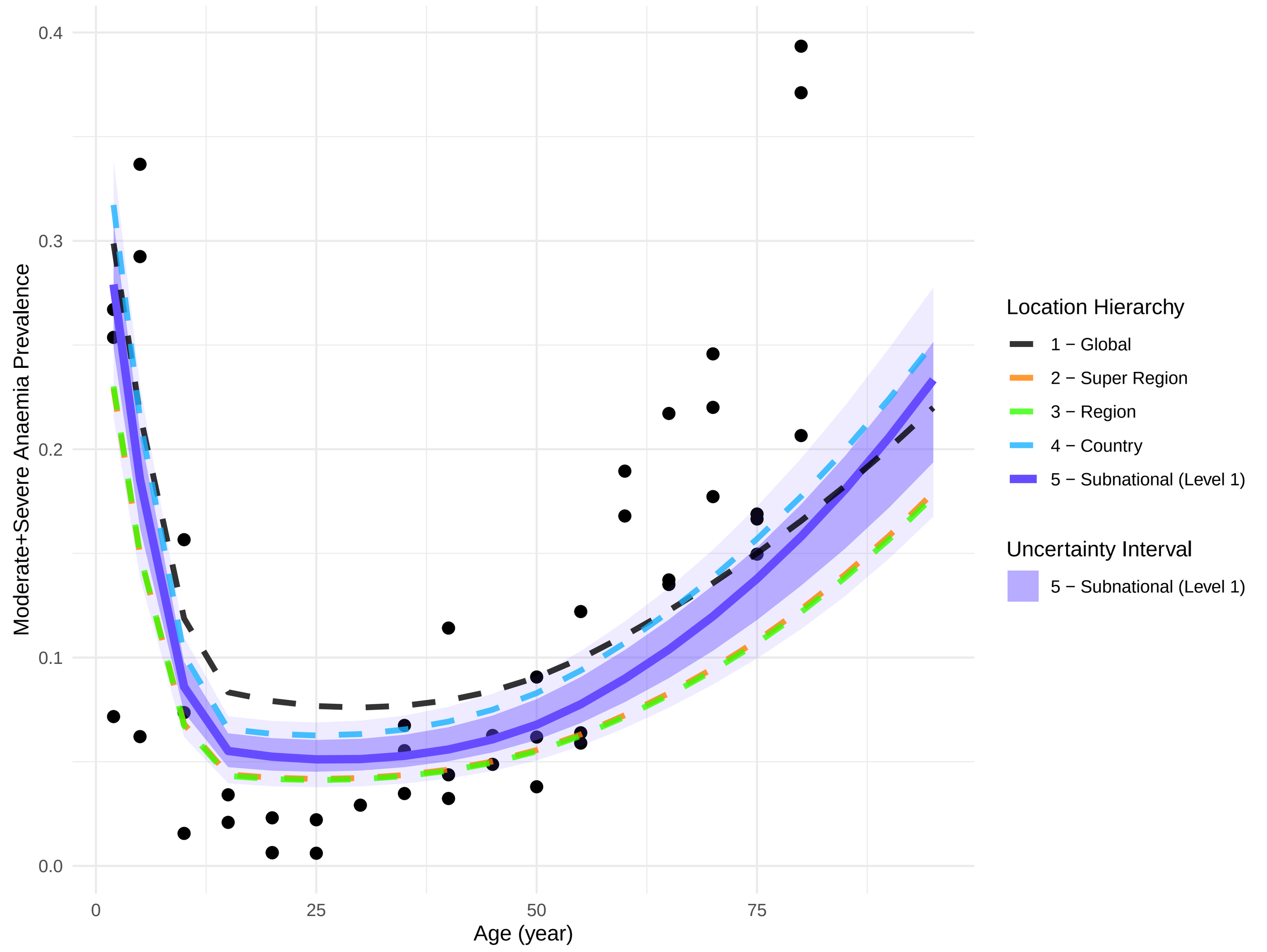

MR−BRT Age/Sex Fits − Males 2+ Years Old − Moderate+Severe Anaemia Prevalence − East Java
WHO−2024 Adjustment Method

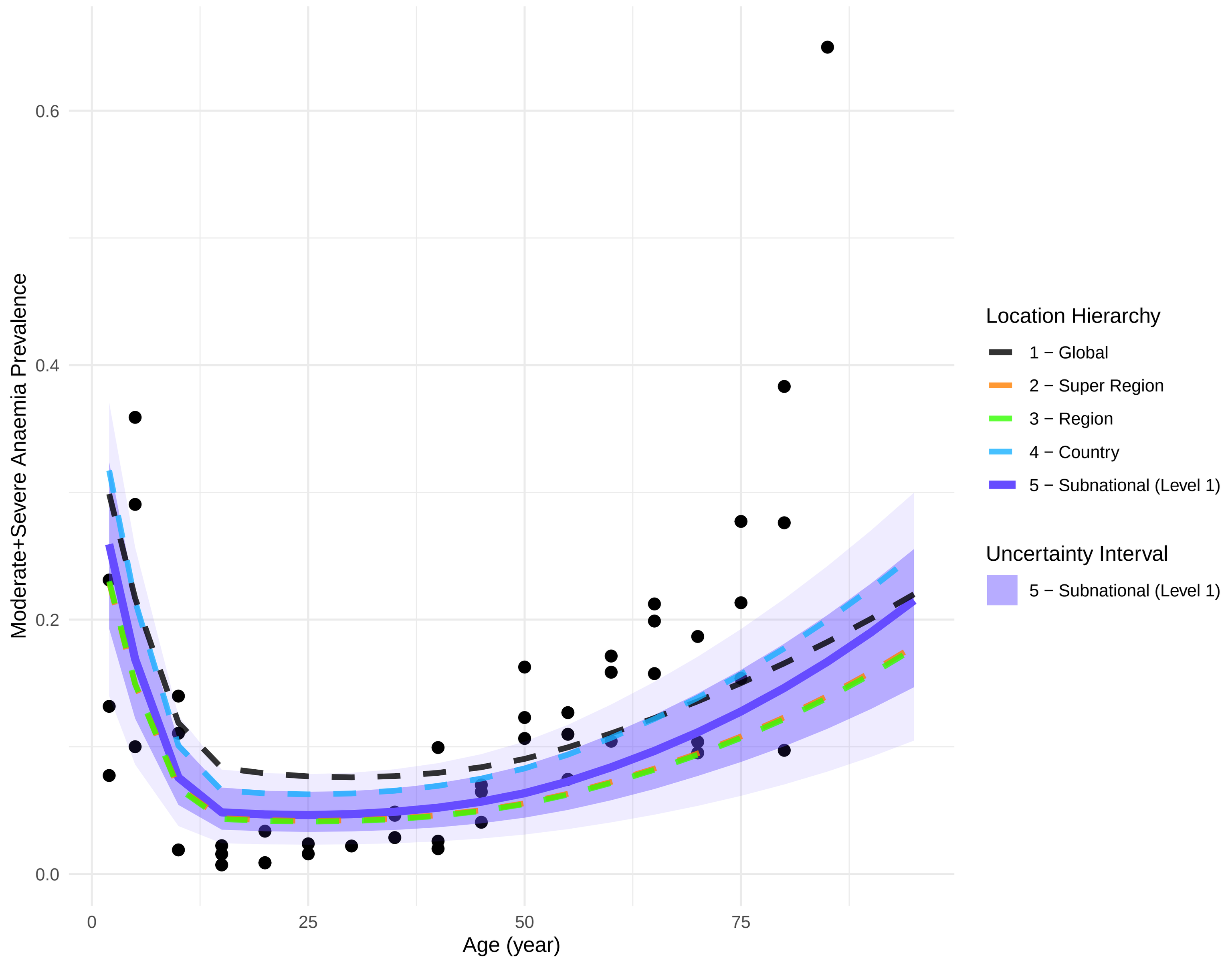

MR−BRT Age/Sex Fits − Males 2+ Years Old − Moderate+Severe Anaemia Prevalence − South Kalimantan
WHO−2024 Adjustment Method

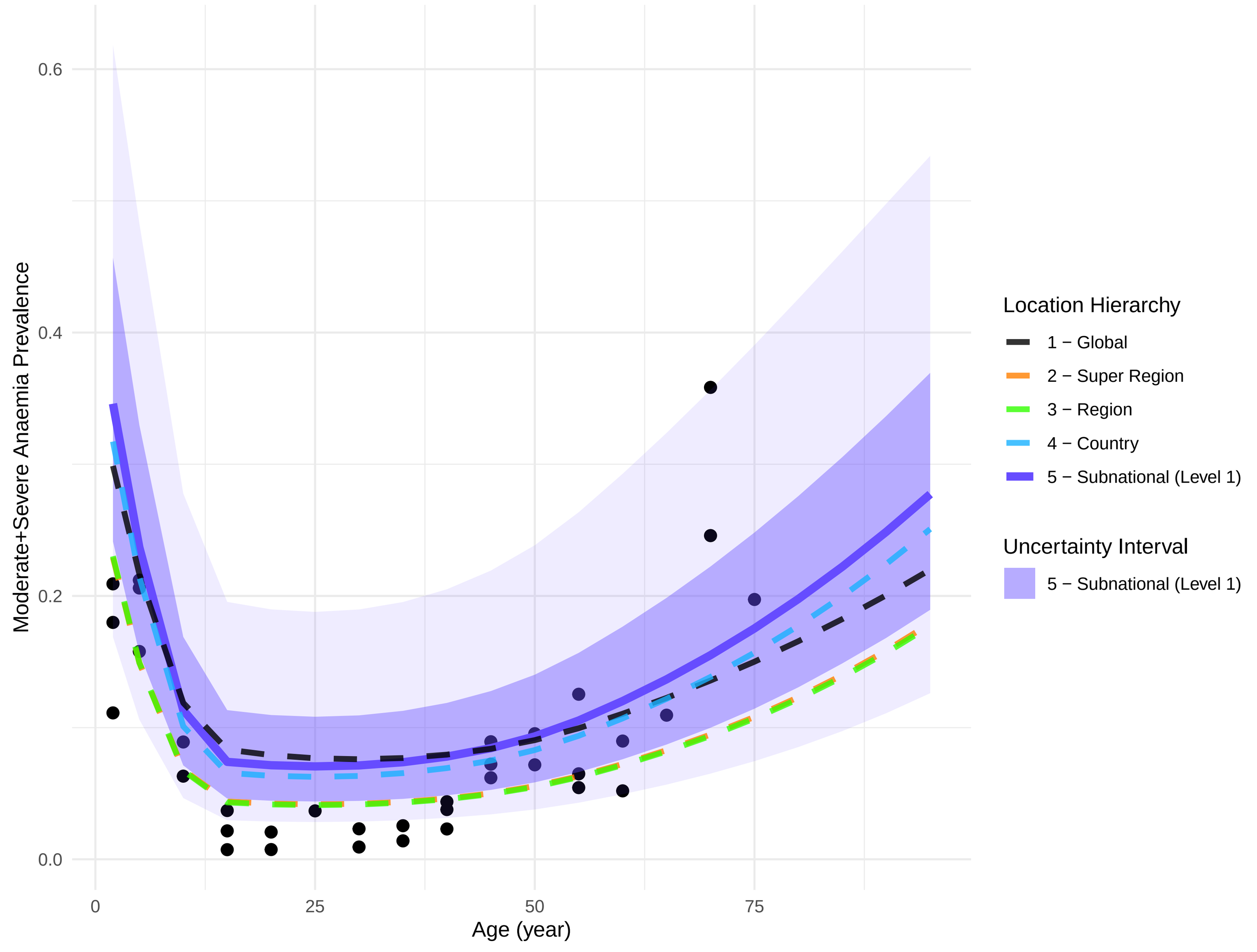

MR−BRT Age/Sex Fits − Males 2+ Years Old − Moderate+Severe Anaemia Prevalence − East Kalimantan
WHO−2024 Adjustment Method

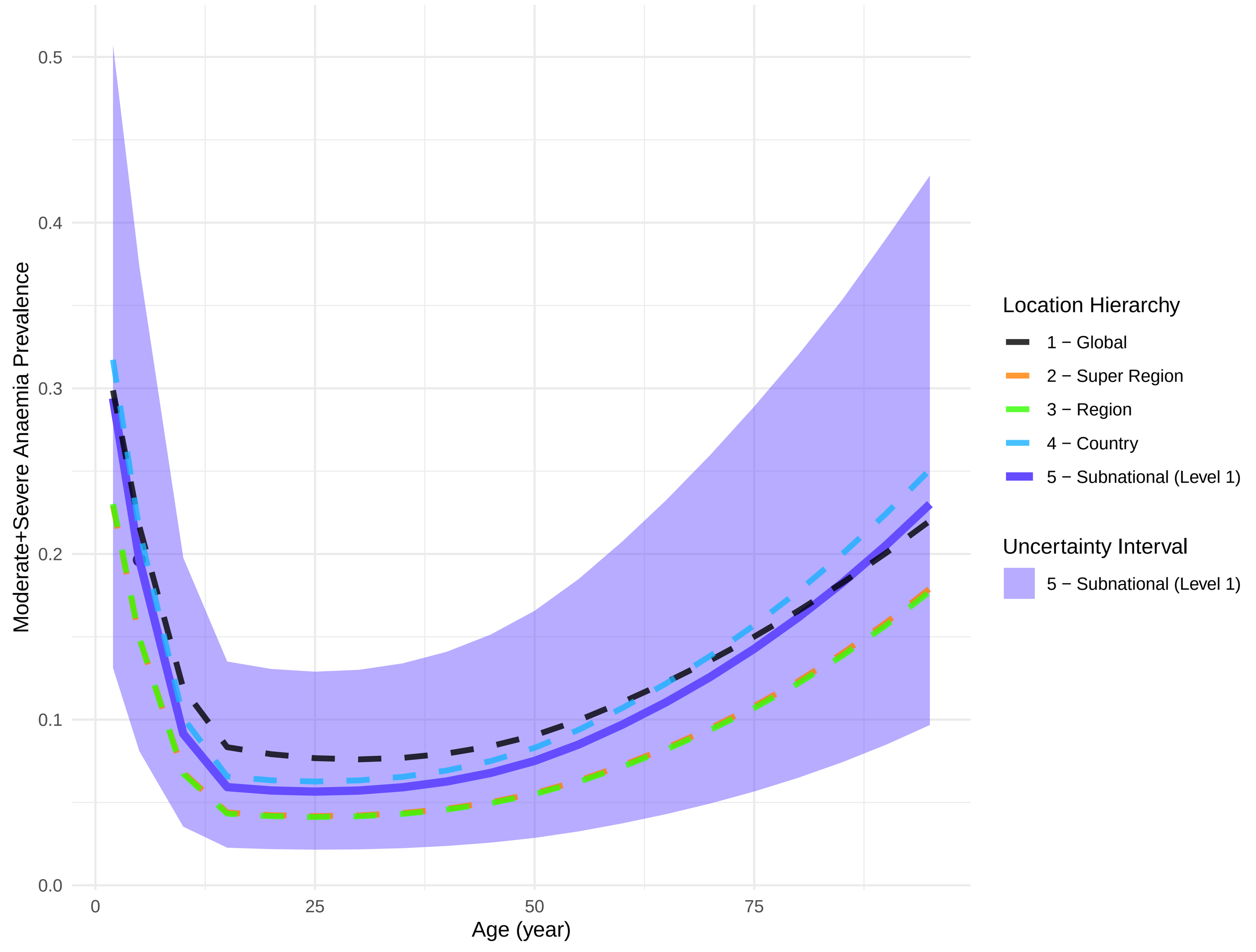

MR−BRT Age/Sex Fits − Males 2+ Years Old − Moderate+Severe Anaemia Prevalence − Lampung
WHO−2024 Adjustment Method

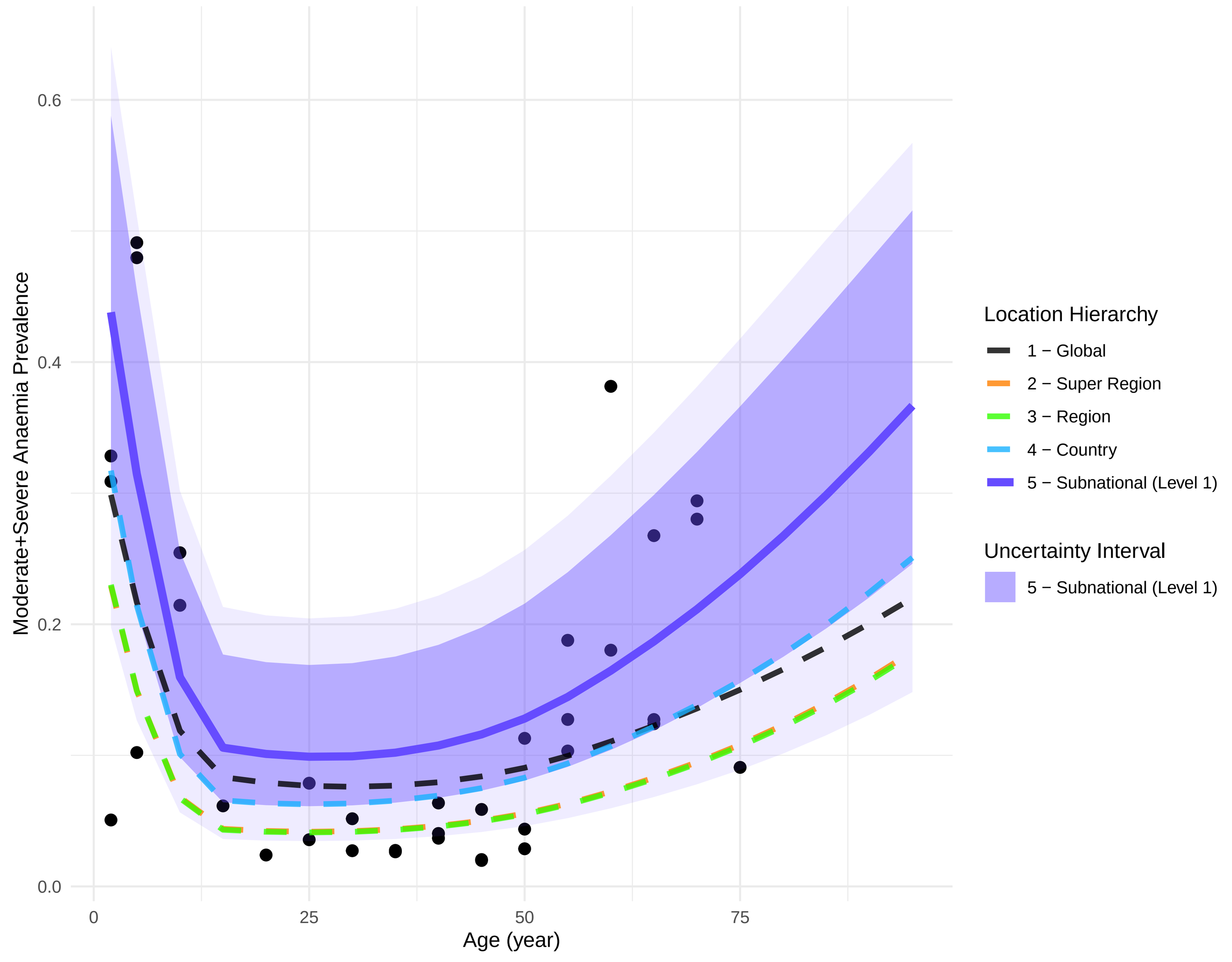

MR−BRT Age/Sex Fits − Males 2+ Years Old − Moderate+Severe Anaemia Prevalence − West Nusa Tenggara
WHO−2024 Adjustment Method

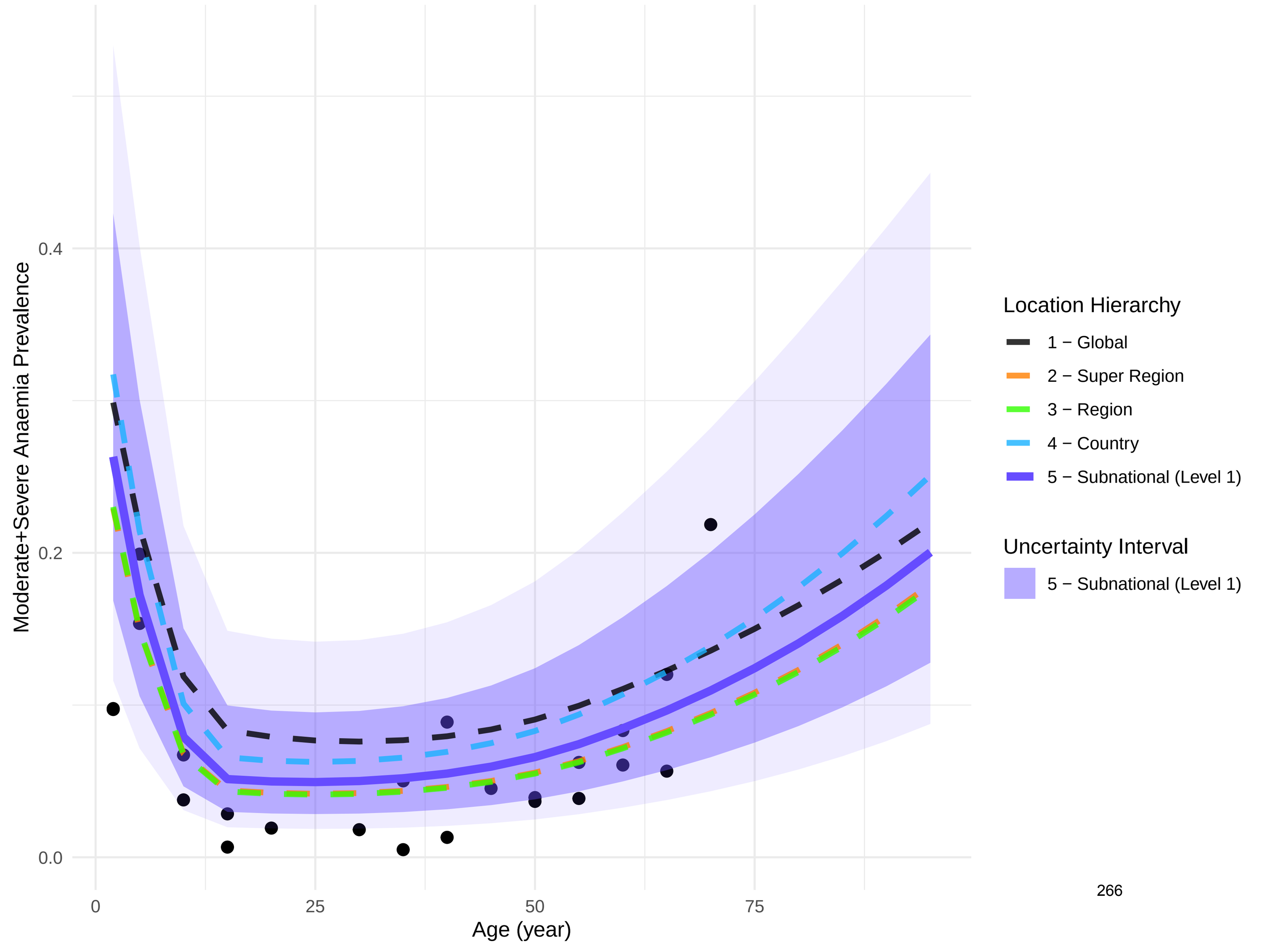

MR−BRT Age/Sex Fits − Males 2+ Years Old − Moderate+Severe Anaemia Prevalence − Riau
WHO−2024 Adjustment Method

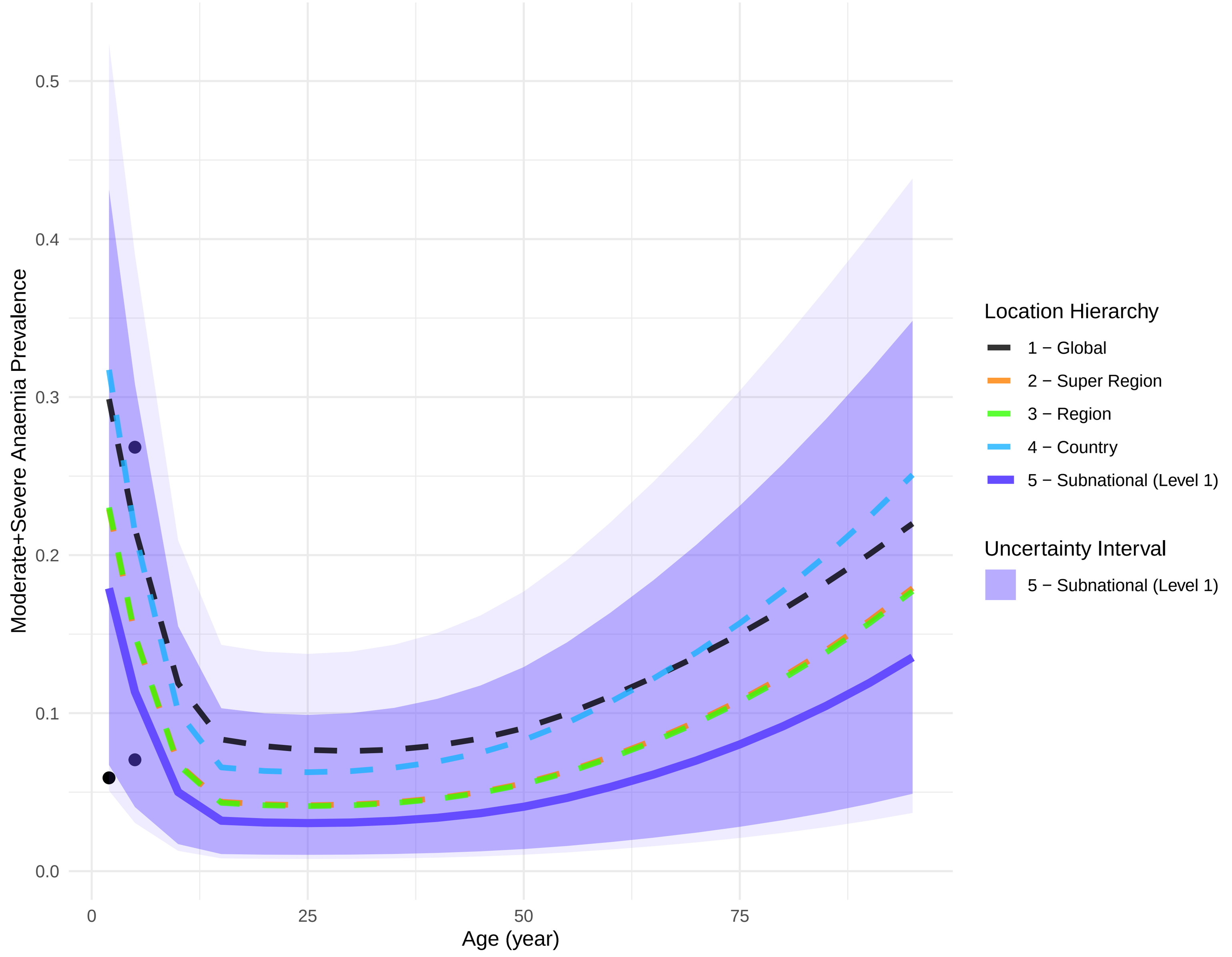

MR−BRT Age/Sex Fits − Males 2+ Years Old − Moderate+Severe Anaemia Prevalence − West Sulawesi
WHO−2024 Adjustment Method

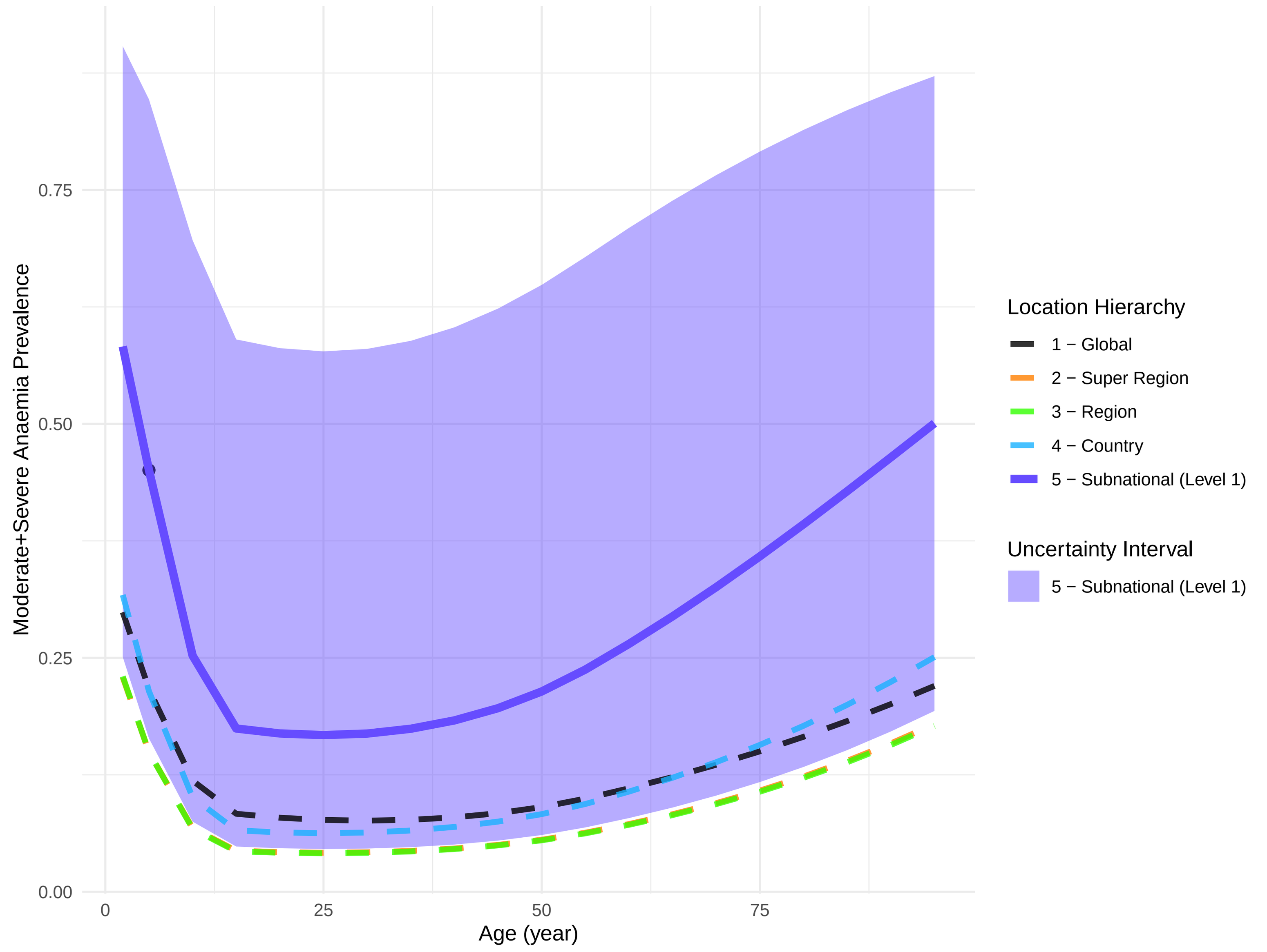

MR−BRT Age/Sex Fits − Males 2+ Years Old − Moderate+Severe Anaemia Prevalence − South Sulawesi
WHO−2024 Adjustment Method

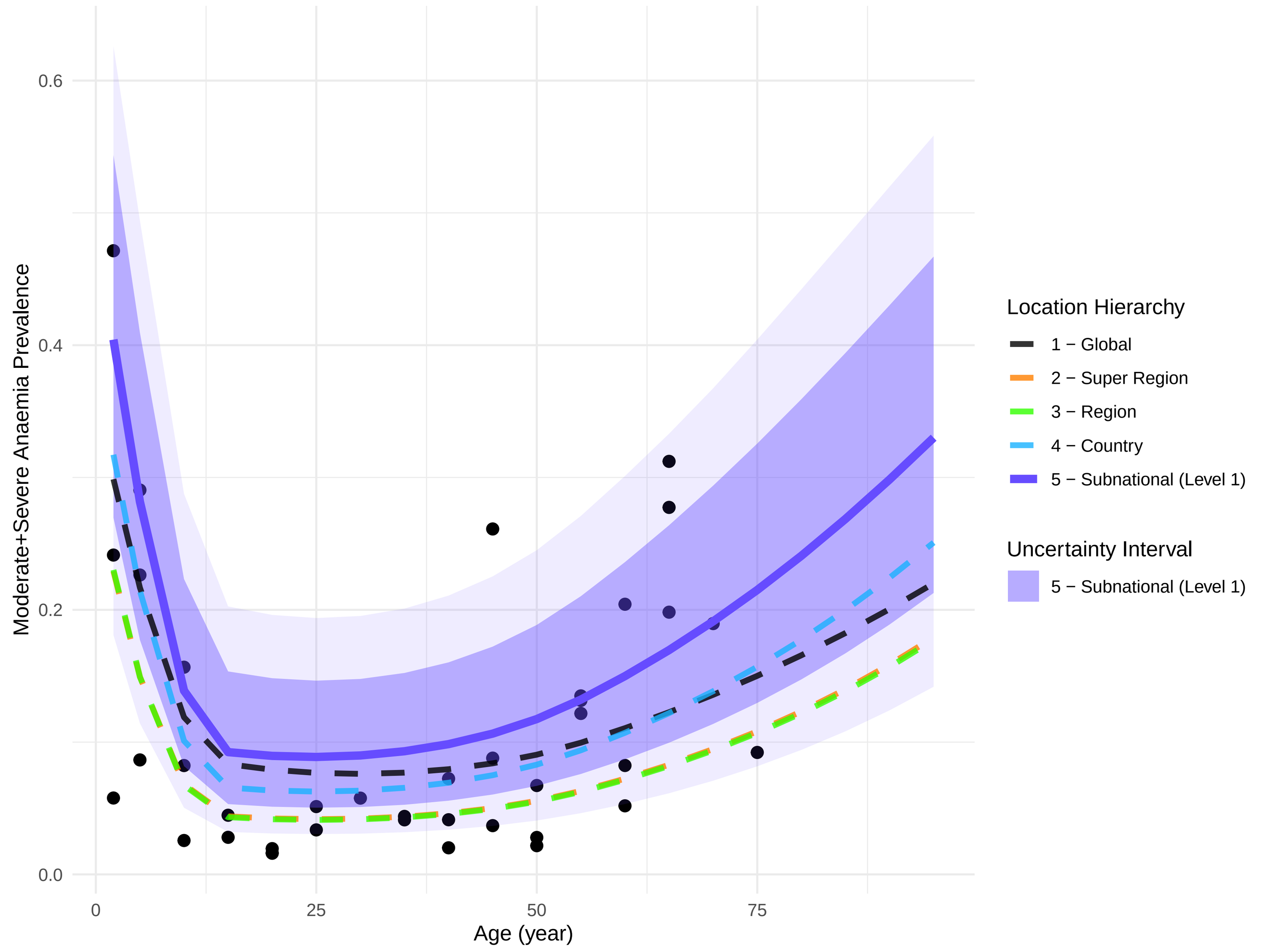

MR−BRT Age/Sex Fits − Males 2+ Years Old − Moderate+Severe Anaemia Prevalence − West Sumatra
WHO−2024 Adjustment Method

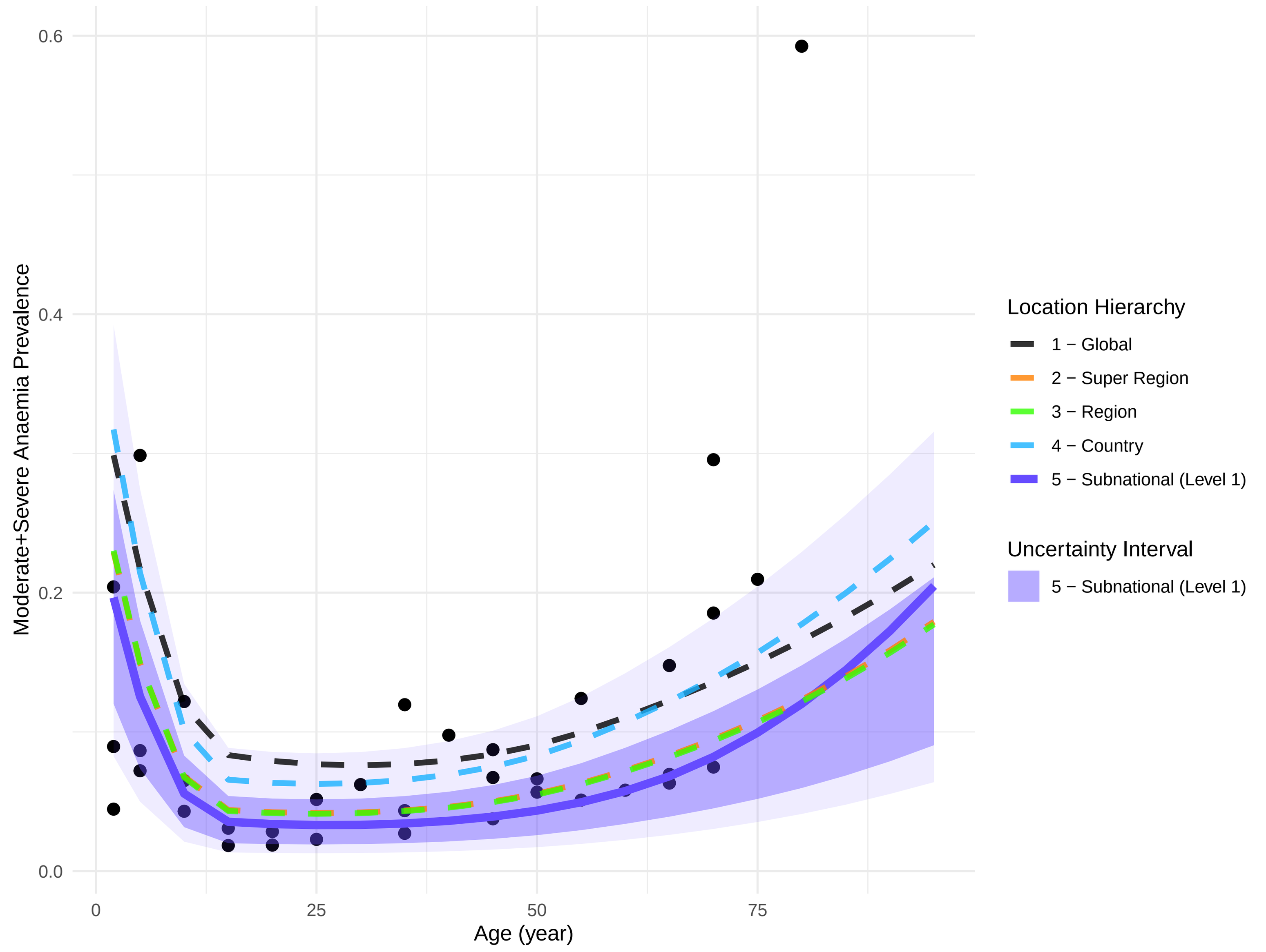

MR−BRT Age/Sex Fits − Males 2+ Years Old − Moderate+Severe Anaemia Prevalence − South Sumatra
WHO−2024 Adjustment Method

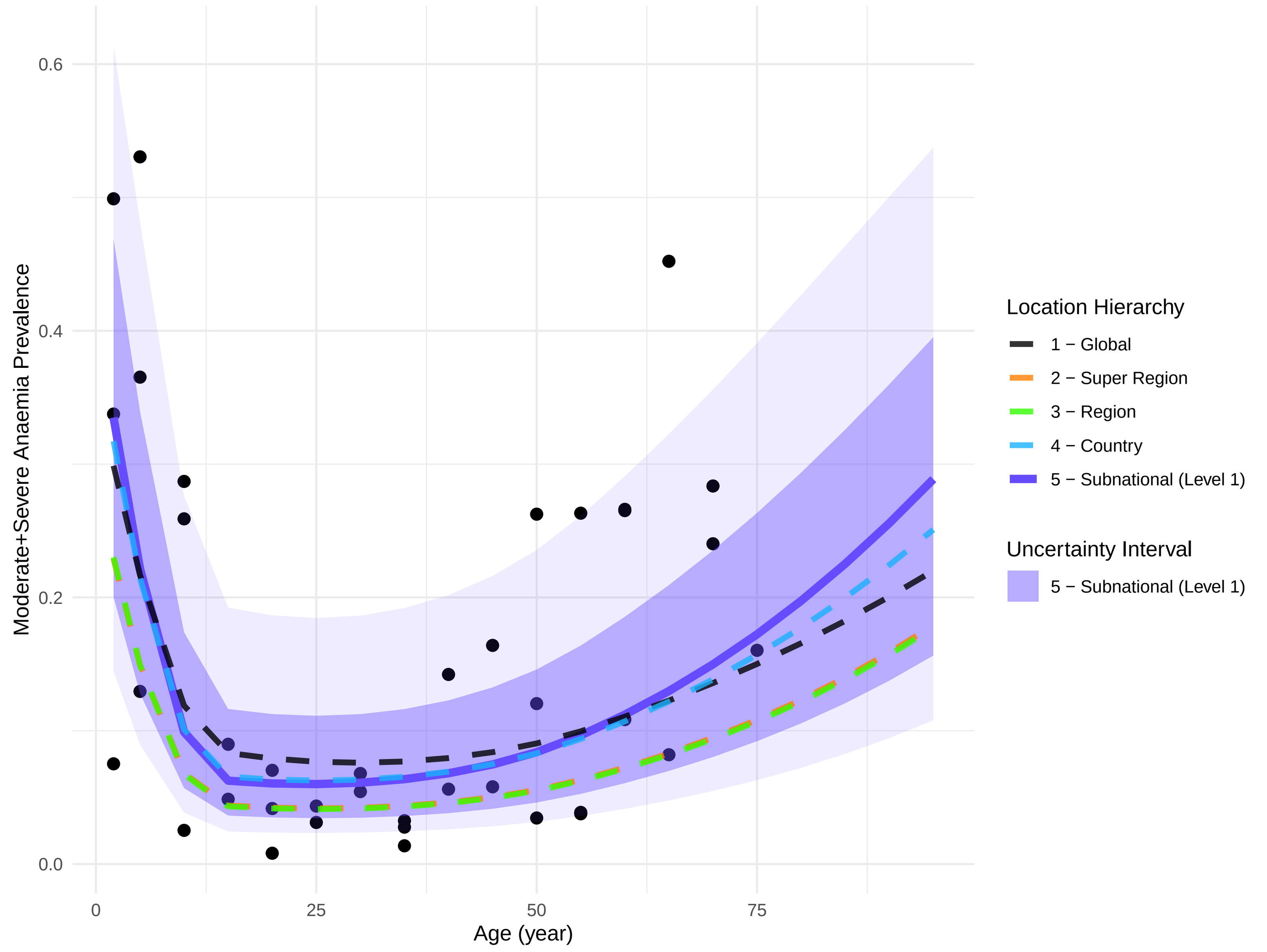

MR−BRT Age/Sex Fits − Males 2+ Years Old − Moderate+Severe Anaemia Prevalence − North Sumatra
WHO−2024 Adjustment Method

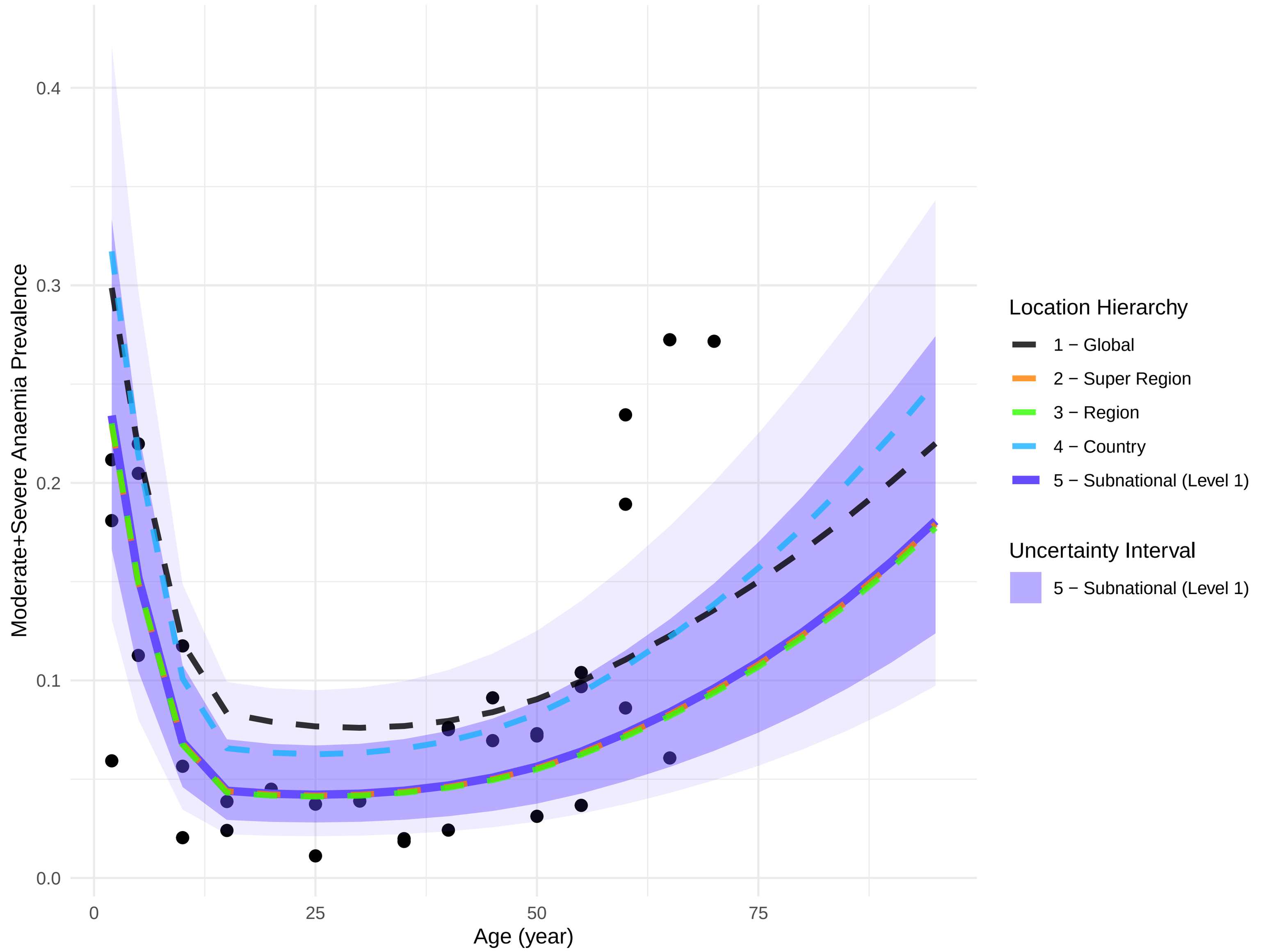

MR−BRT Age/Sex Fits − Males 2+ Years Old − Moderate+Severe Anaemia Prevalence − Yogyakarta
WHO−2024 Adjustment Method

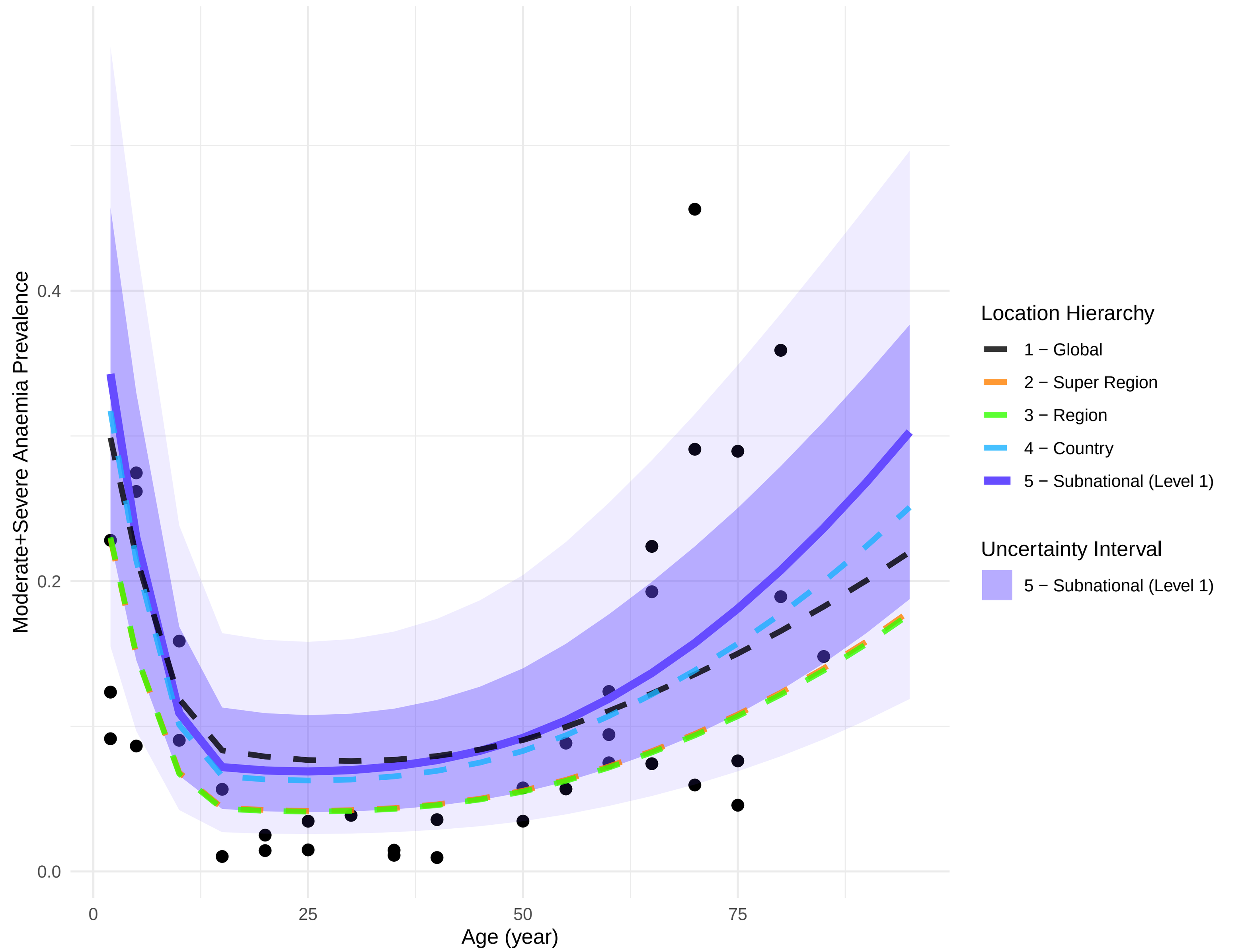

MR−BRT Age/Sex Fits − Males 2+ Years Old − Moderate+Severe Anaemia Prevalence − Myanmar
WHO−2024 Adjustment Method

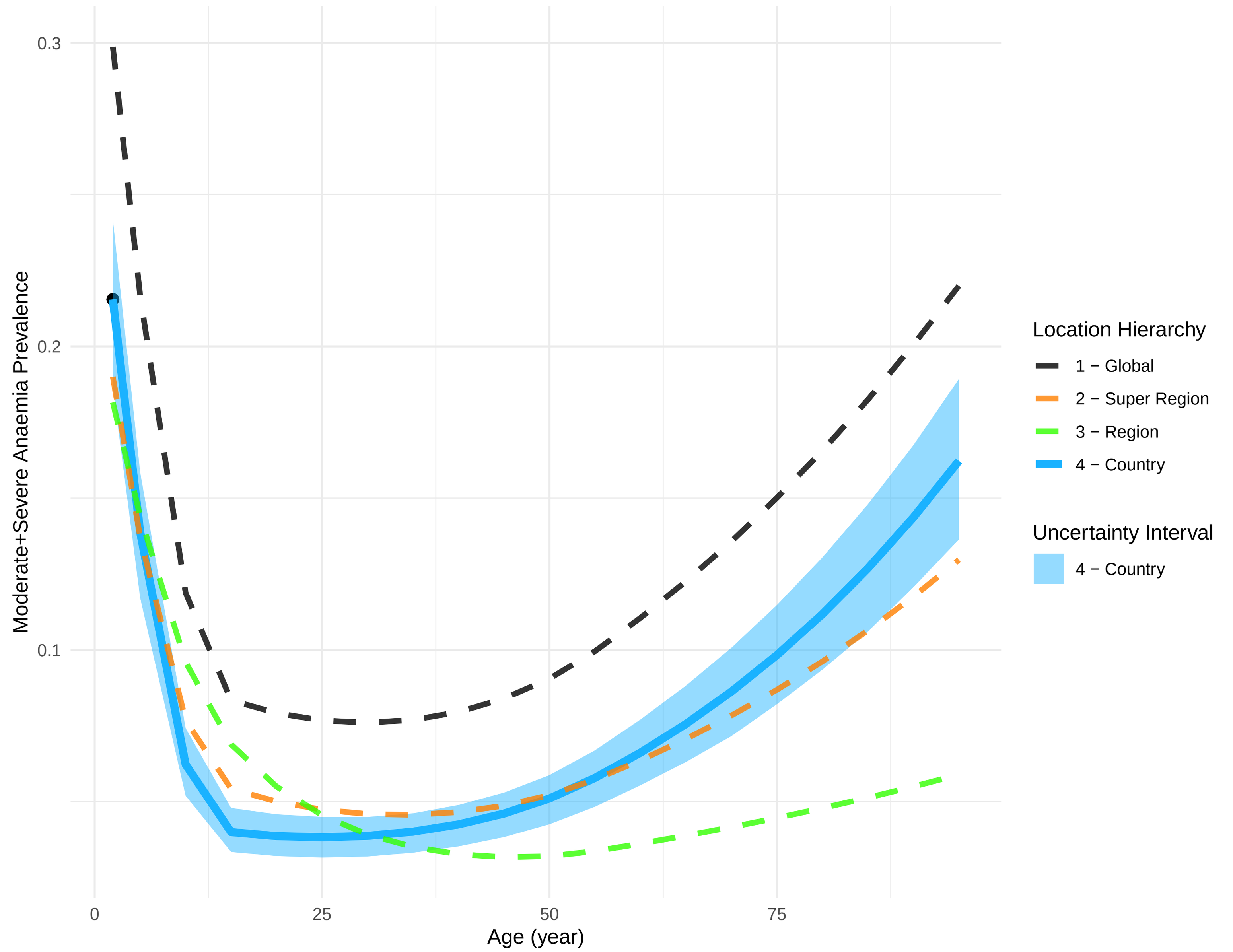

MR−BRT Age/Sex Fits − Males 2+ Years Old − Moderate+Severe Anaemia Prevalence − Timor−Leste
WHO−2024 Adjustment Method

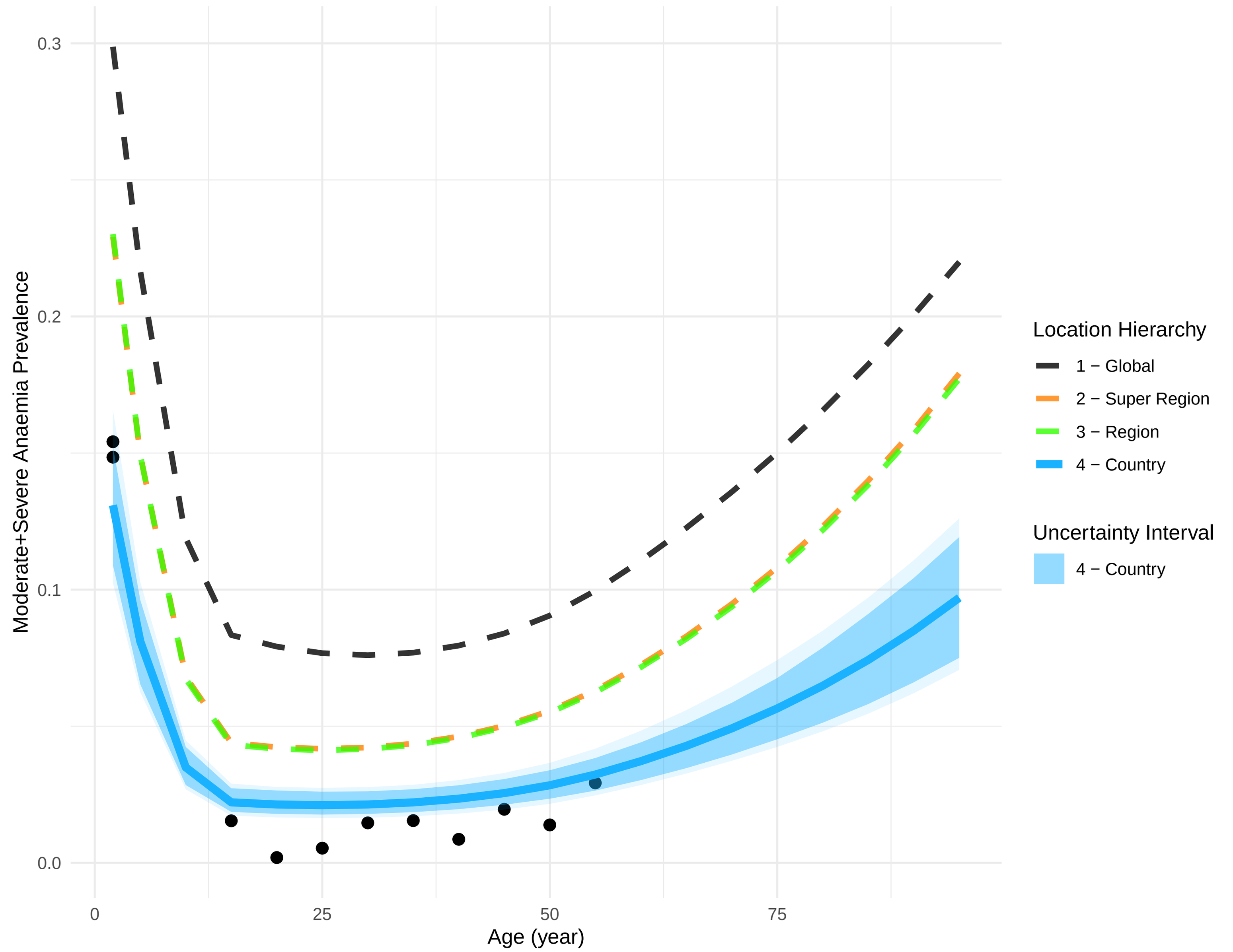

MR−BRT Age/Sex Fits − Males 2+ Years Old − Moderate+Severe Anaemia Prevalence − Sub−Saharan Africa
WHO−2024 Adjustment Method

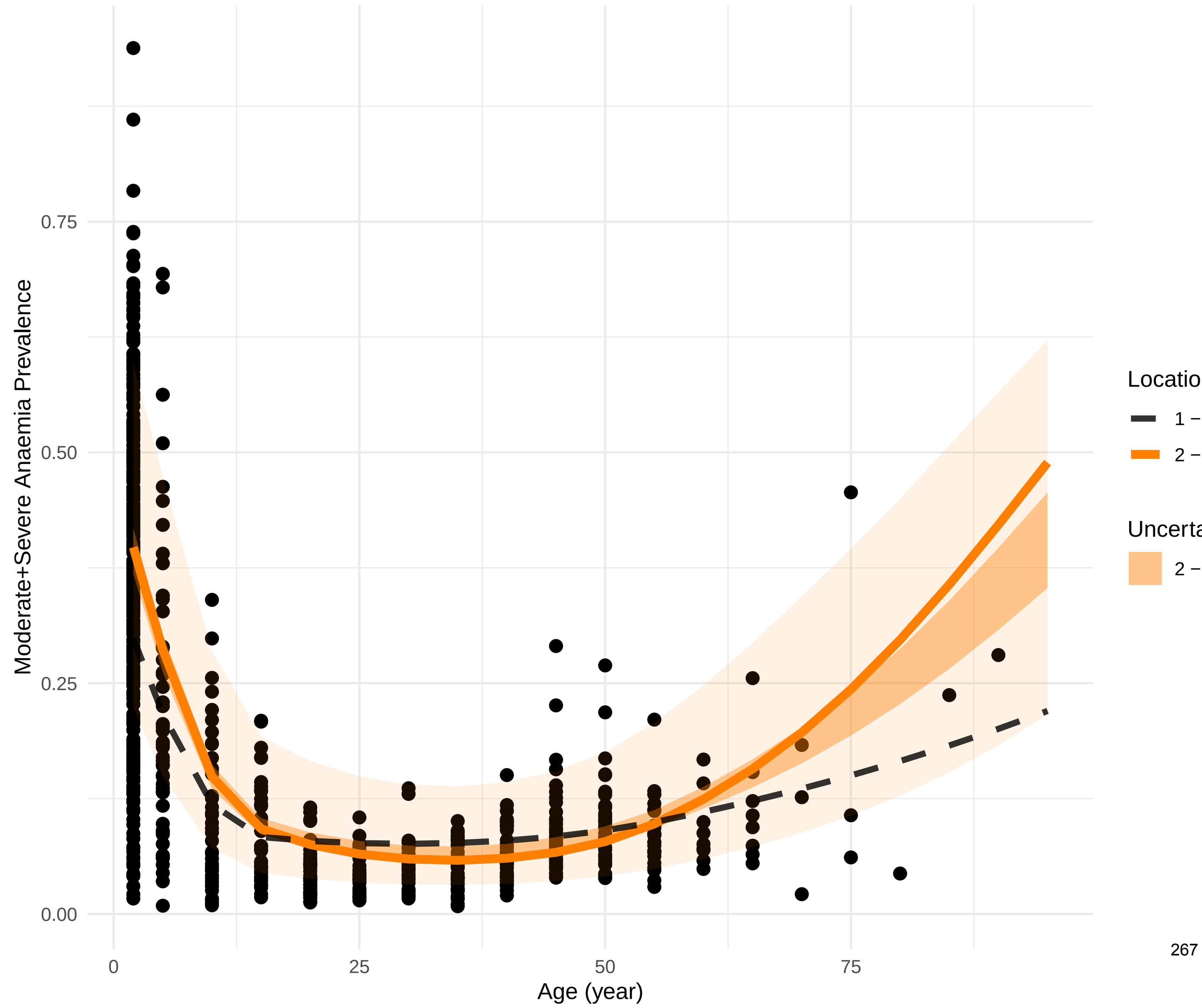

MR−BRT Age/Sex Fits − Males 2+ Years Old − Moderate+Severe Anaemia Prevalence − Central Sub−Saharan Africa
WHO−2024 Adjustment Method

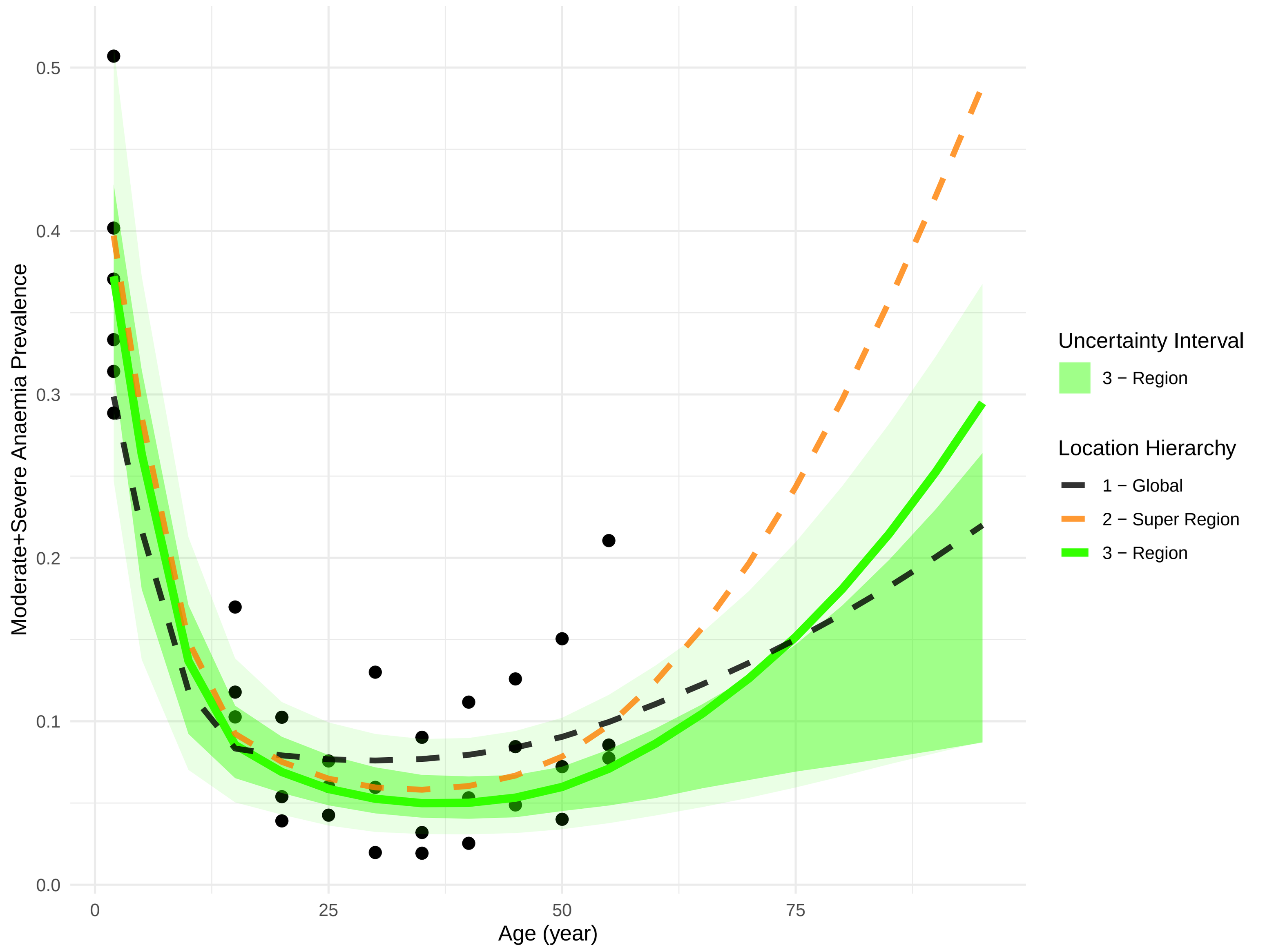

MR−BRT Age/Sex Fits − Males 2+ Years Old − Moderate+Severe Anaemia Prevalence − Angola
WHO−2024 Adjustment Method

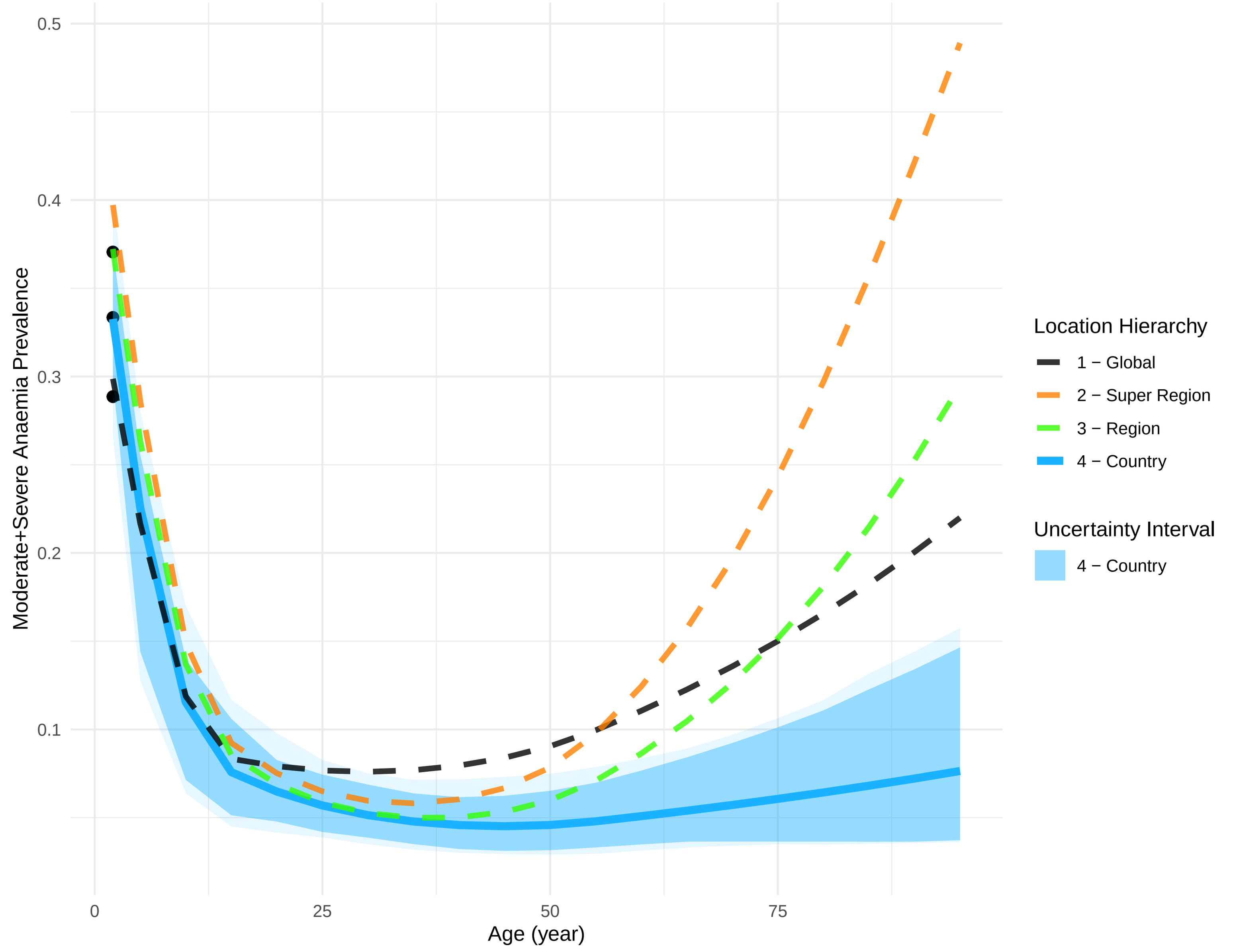

MR−BRT Age/Sex Fits − Males 2+ Years Old − Moderate+Severe Anaemia Prevalence − Democratic Republic of the Congo
WHO−2024 Adjustment Method

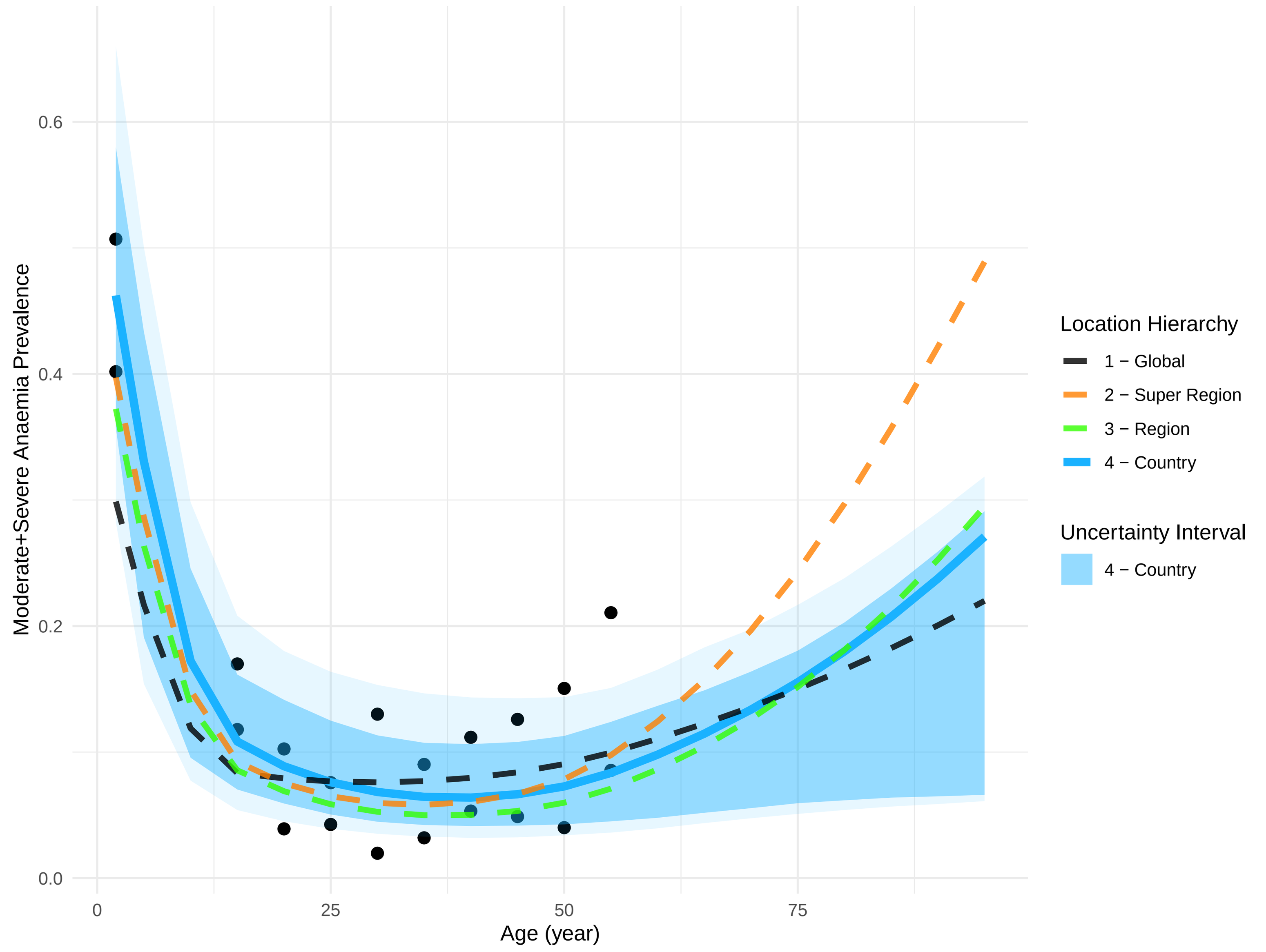

MR−BRT Age/Sex Fits − Males 2+ Years Old − Moderate+Severe Anaemia Prevalence − Gabon
WHO−2024 Adjustment Method

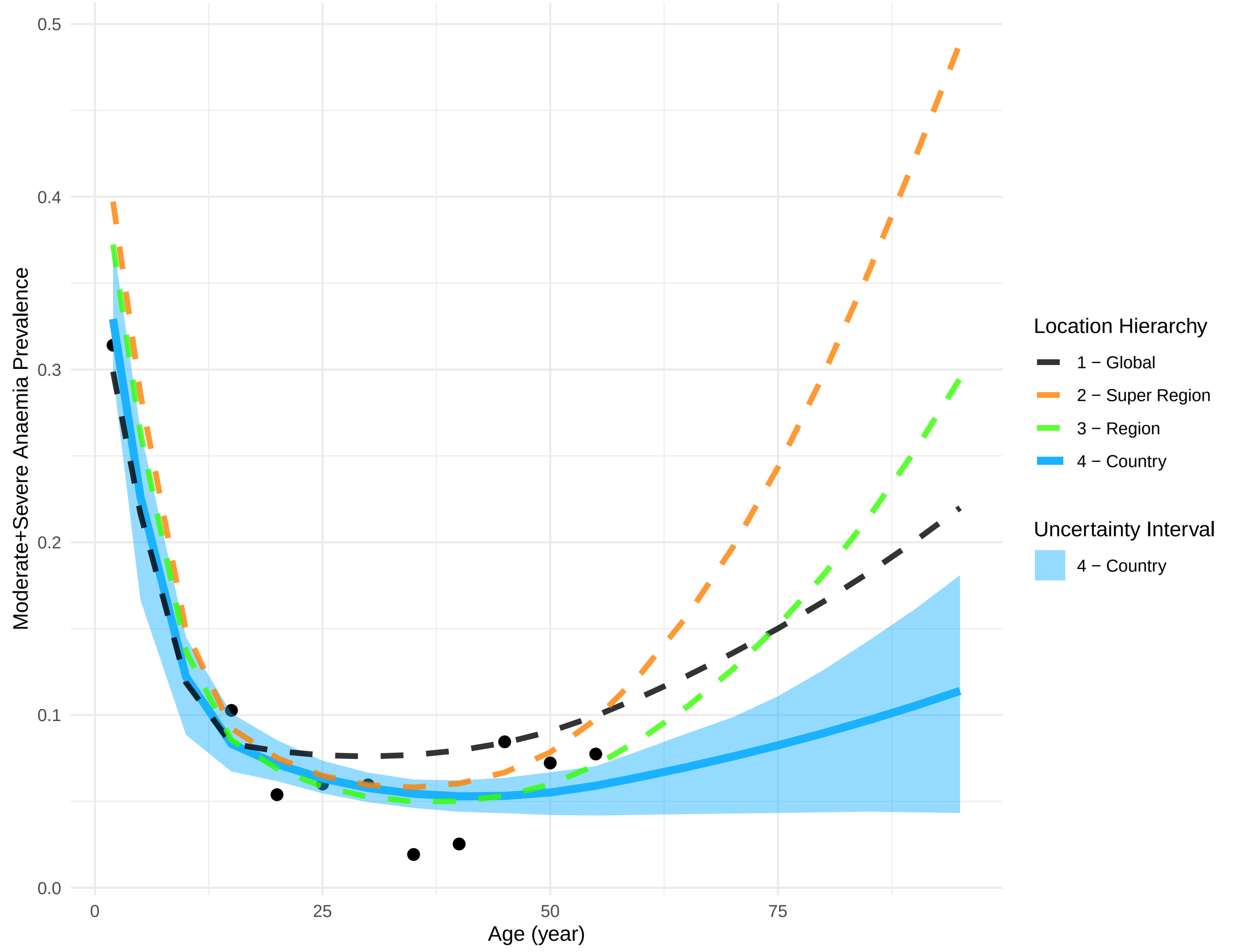

MR−BRT Age/Sex Fits − Males 2+ Years Old − Moderate+Severe Anaemia Prevalence − Eastern Sub−Saharan Africa
WHO−2024 Adjustment Method

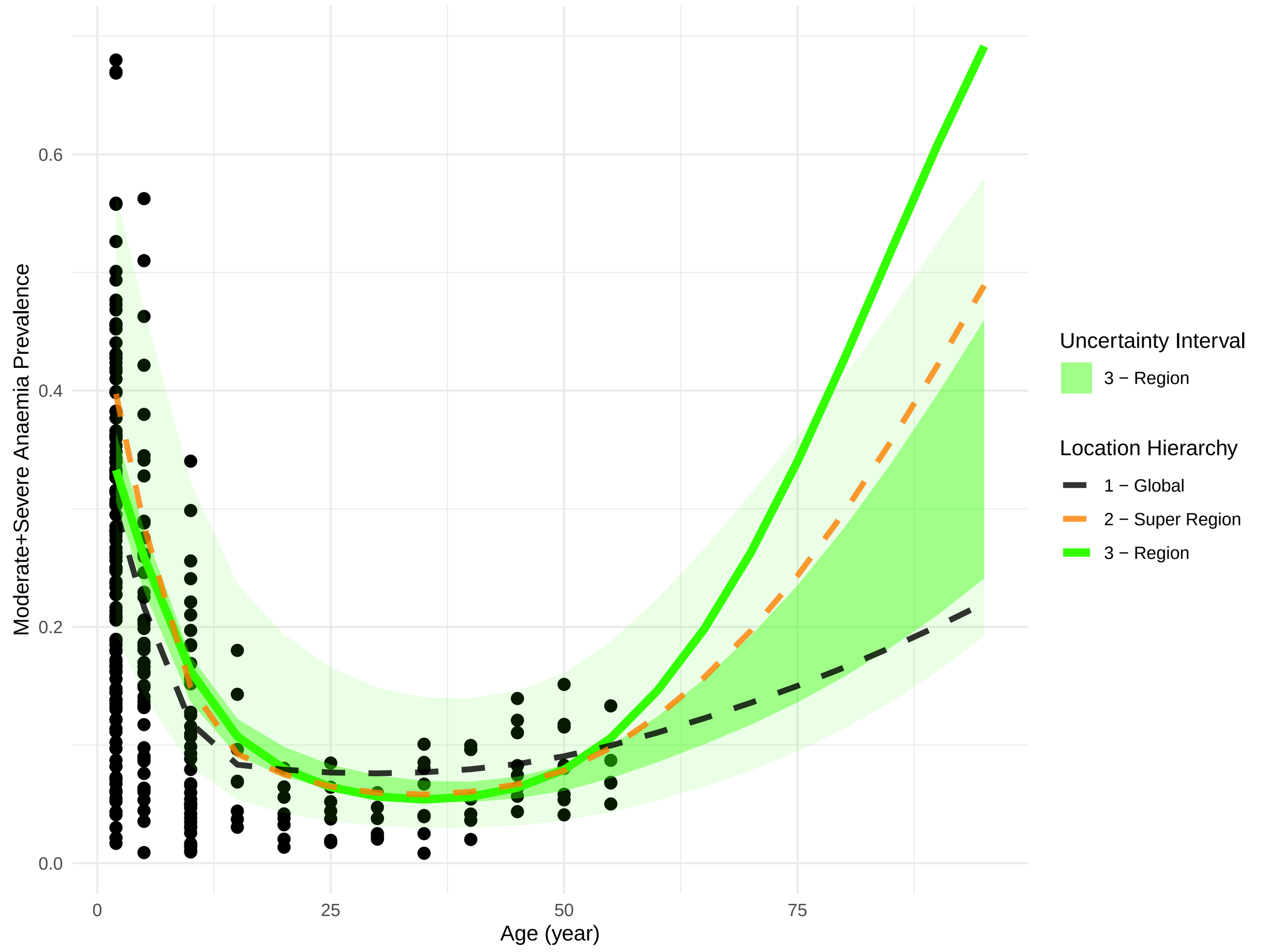

MR−BRT Age/Sex Fits − Males 2+ Years Old − Moderate+Severe Anaemia Prevalence − Burundi
WHO−2024 Adjustment Method

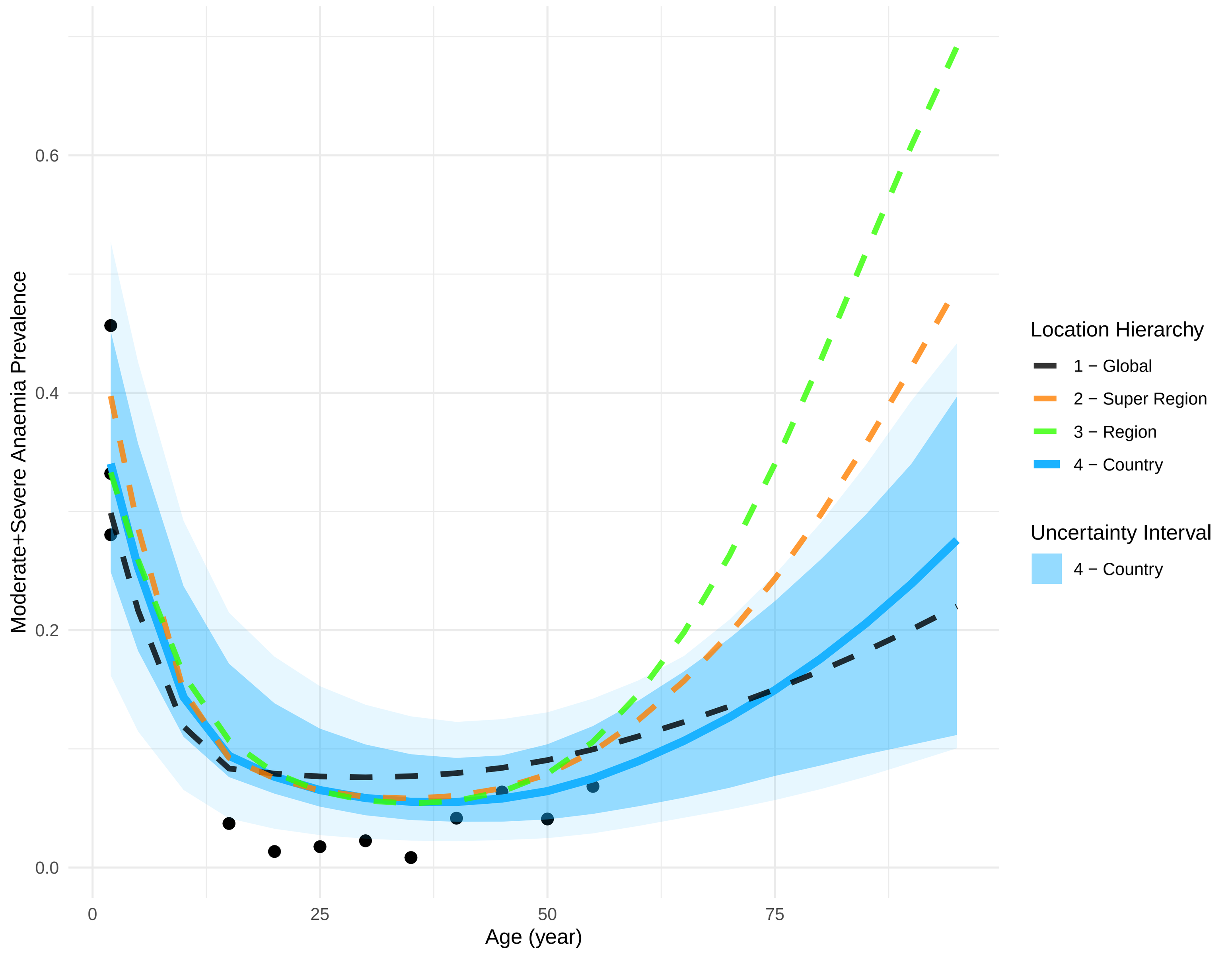

MR−BRT Age/Sex Fits − Males 2+ Years Old − Moderate+Severe Anaemia Prevalence − Ethiopia
WHO−2024 Adjustment Method

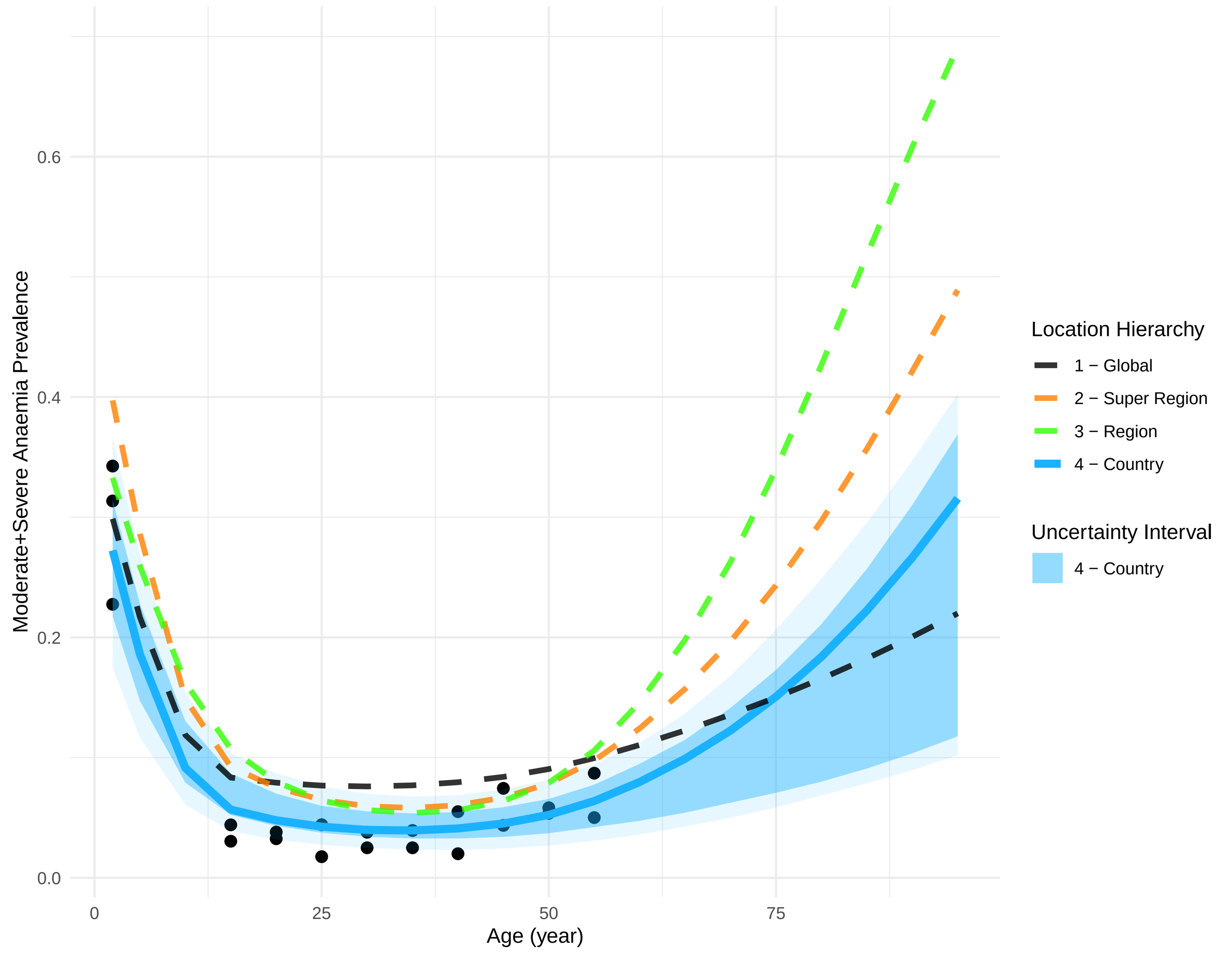

MR−BRT Age/Sex Fits − Males 2+ Years Old − Moderate+Severe Anaemia Prevalence − Kenya
WHO−2024 Adjustment Method

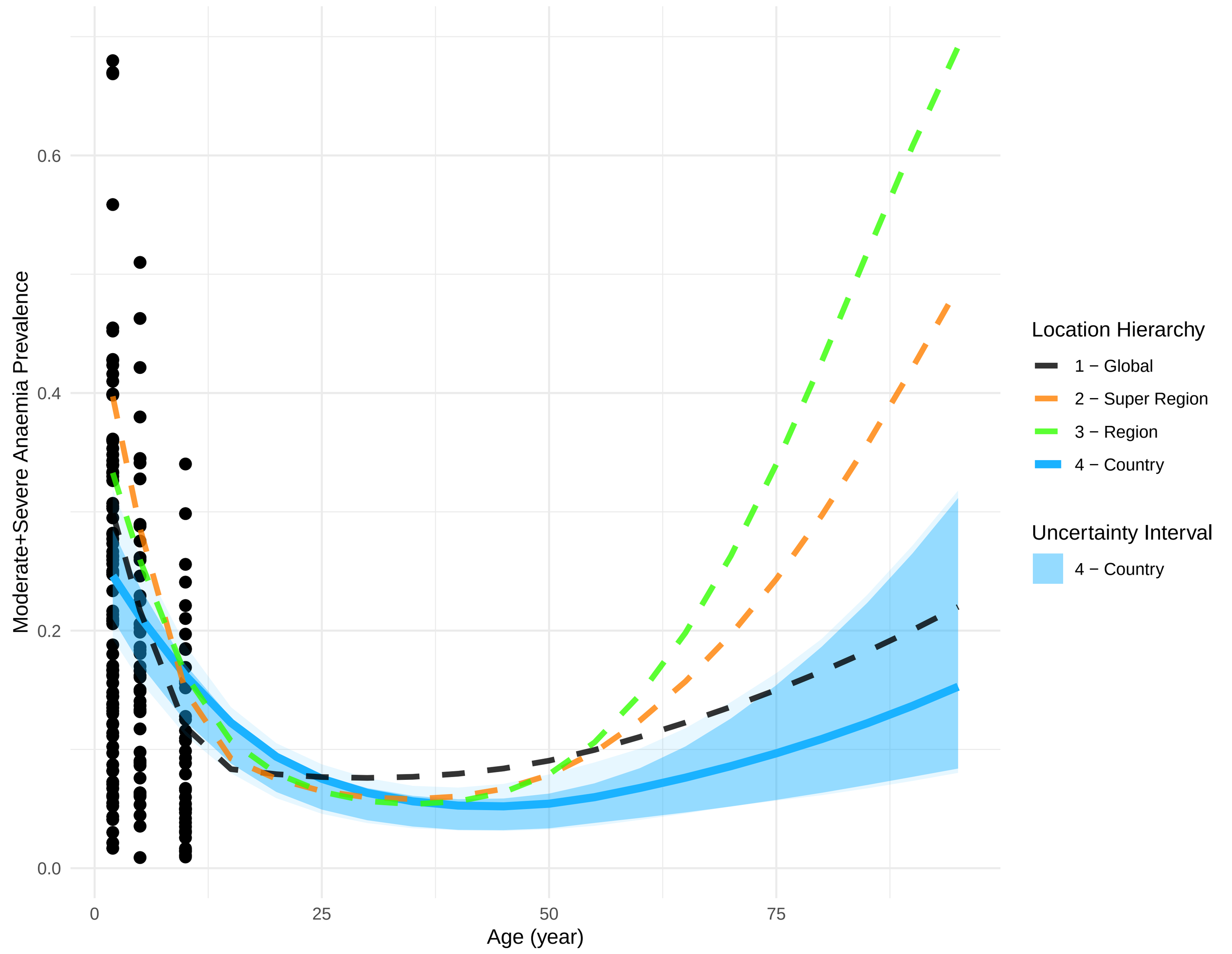

MR−BRT Age/Sex Fits − Males 2+ Years Old − Moderate+Severe Anaemia Prevalence − Baringo
WHO−2024 Adjustment Method

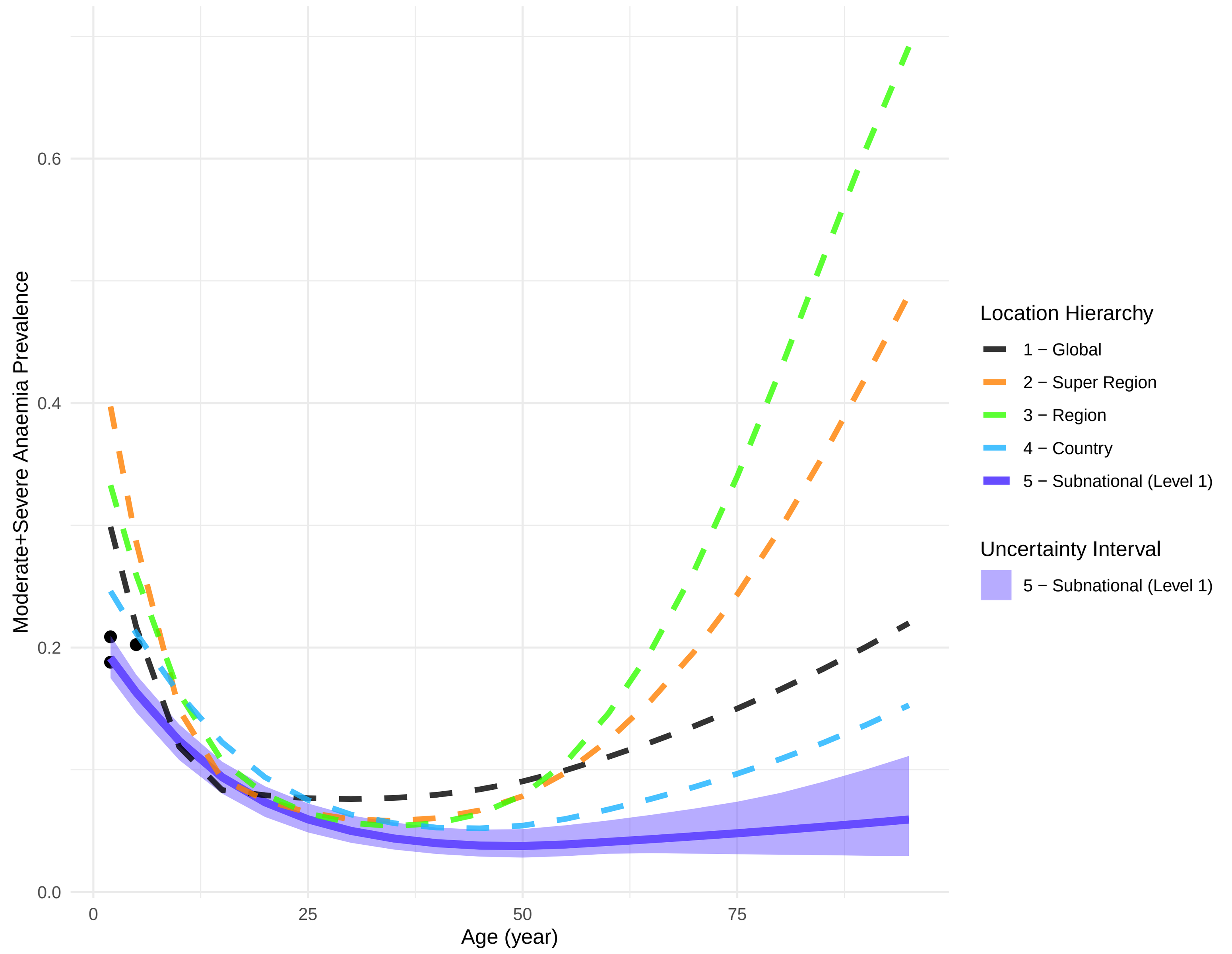

MR−BRT Age/Sex Fits − Males 2+ Years Old − Moderate+Severe Anaemia Prevalence − Bomet
WHO−2024 Adjustment Method

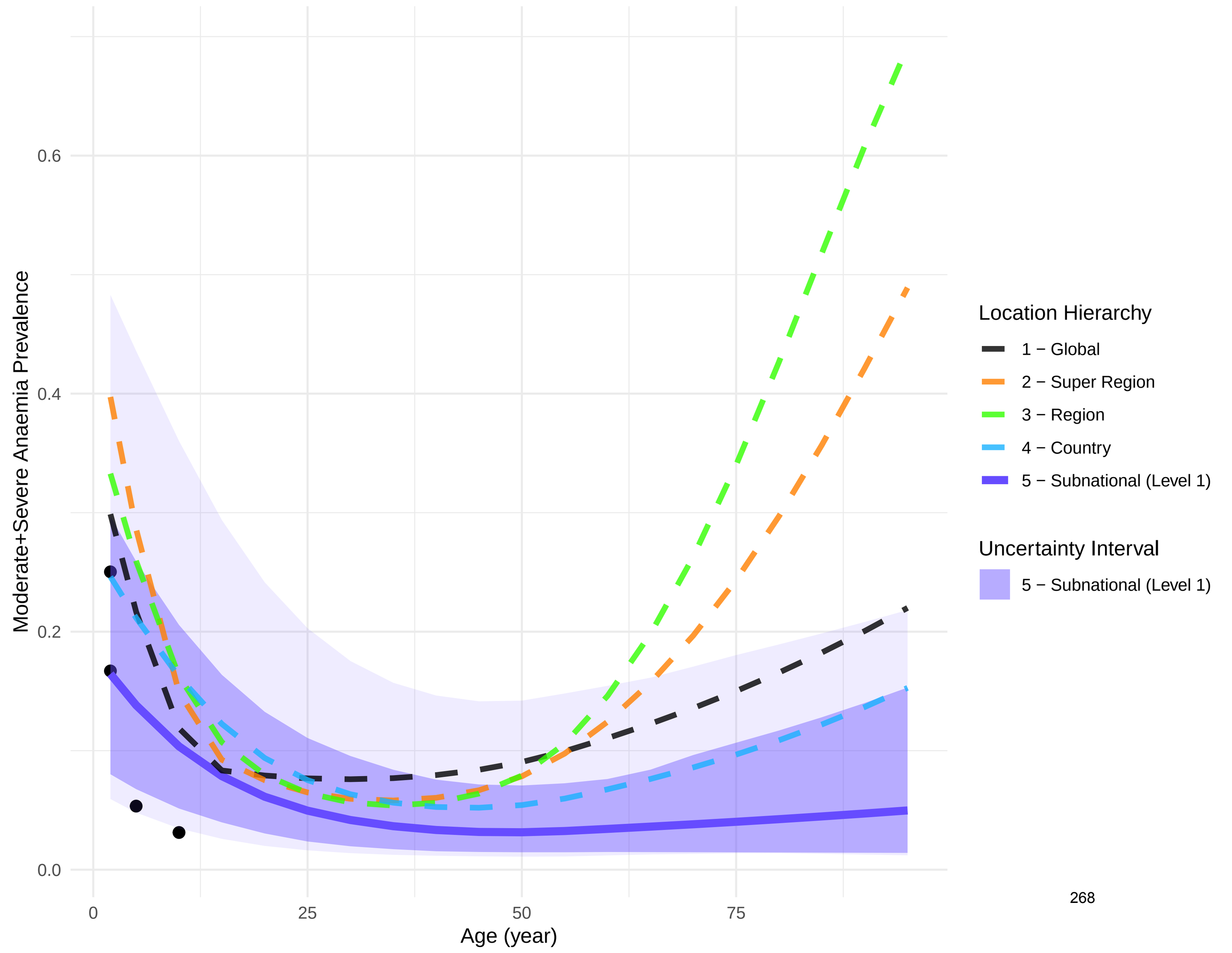

MR−BRT Age/Sex Fits − Males 2+ Years Old − Moderate+Severe Anaemia Prevalence − Bungoma
WHO−2024 Adjustment Method

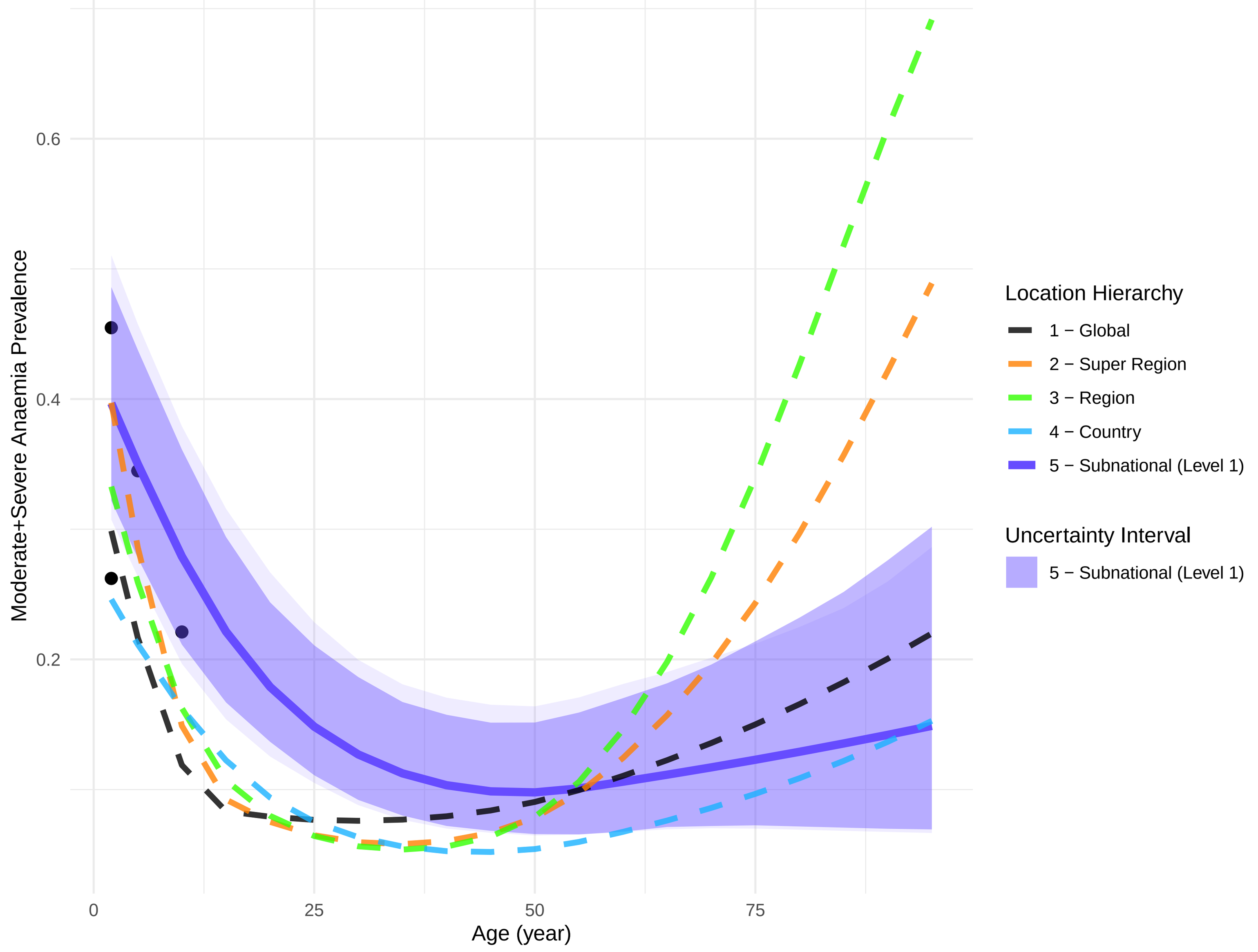

MR−BRT Age/Sex Fits − Males 2+ Years Old − Moderate+Severe Anaemia Prevalence − Busia
WHO−2024 Adjustment Method

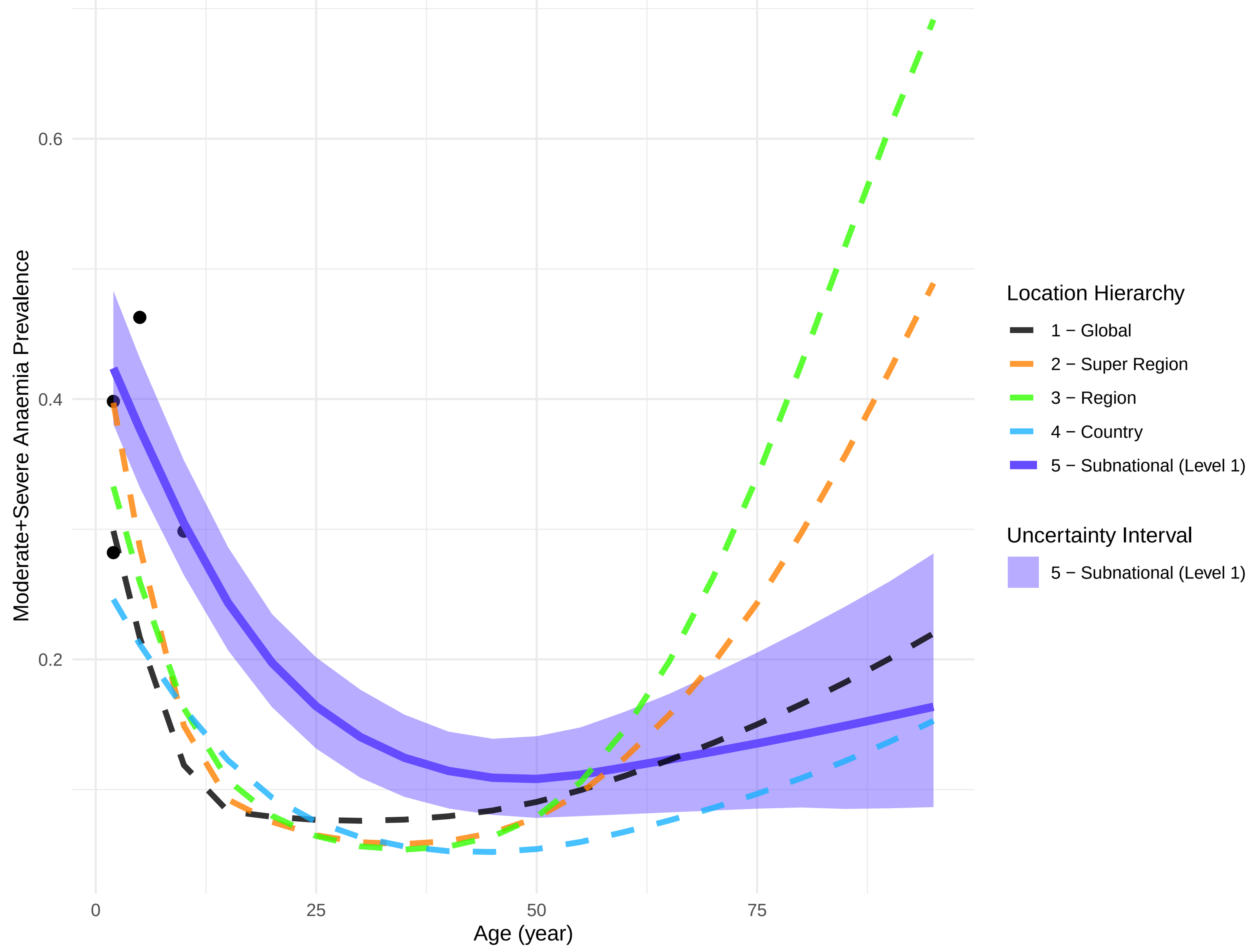

MR−BRT Age/Sex Fits − Males 2+ Years Old − Moderate+Severe Anaemia Prevalence − Elgeyo−Marakwet
WHO−2024 Adjustment Method

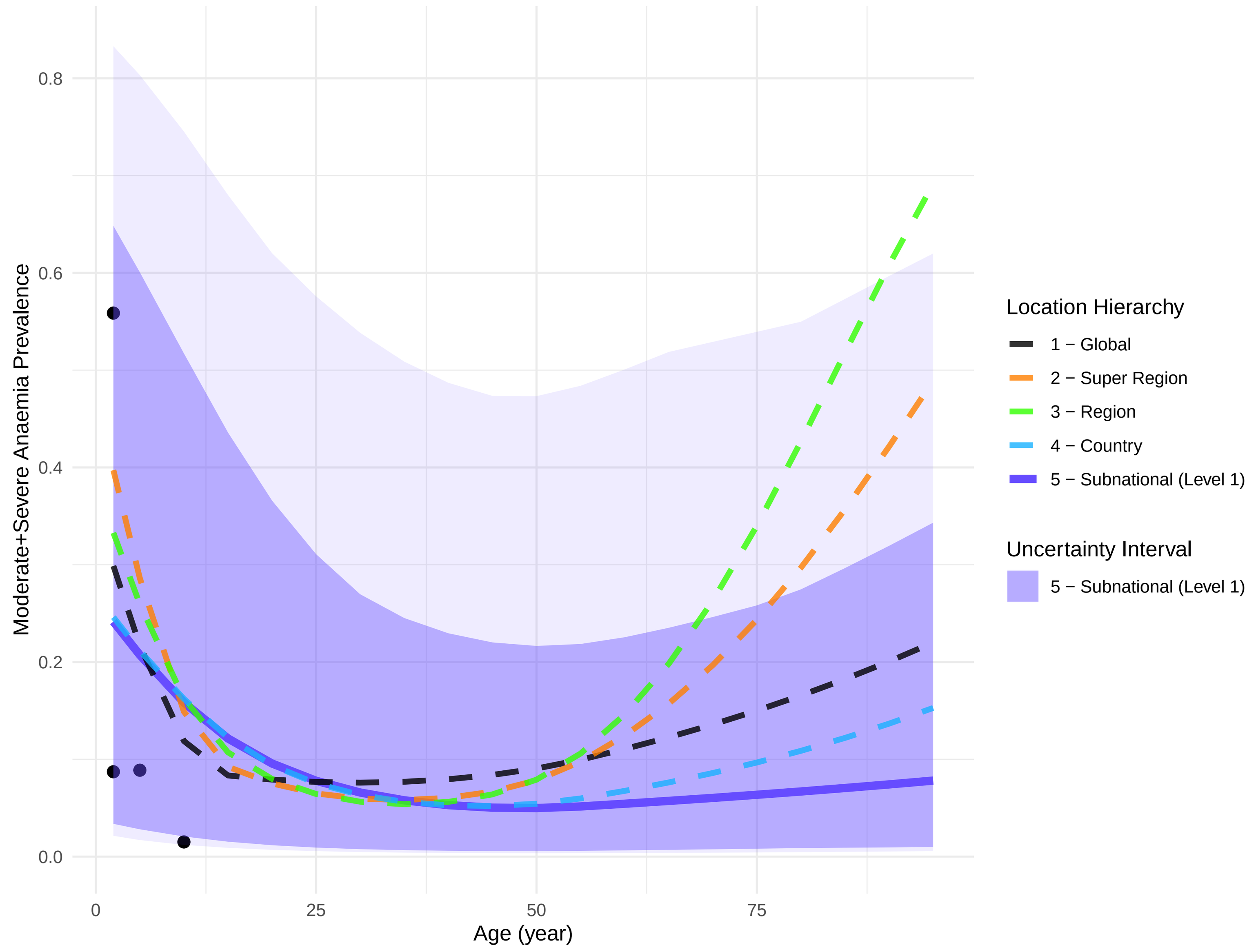

MR−BRT Age/Sex Fits − Males 2+ Years Old − Moderate+Severe Anaemia Prevalence − Embu
WHO−2024 Adjustment Method

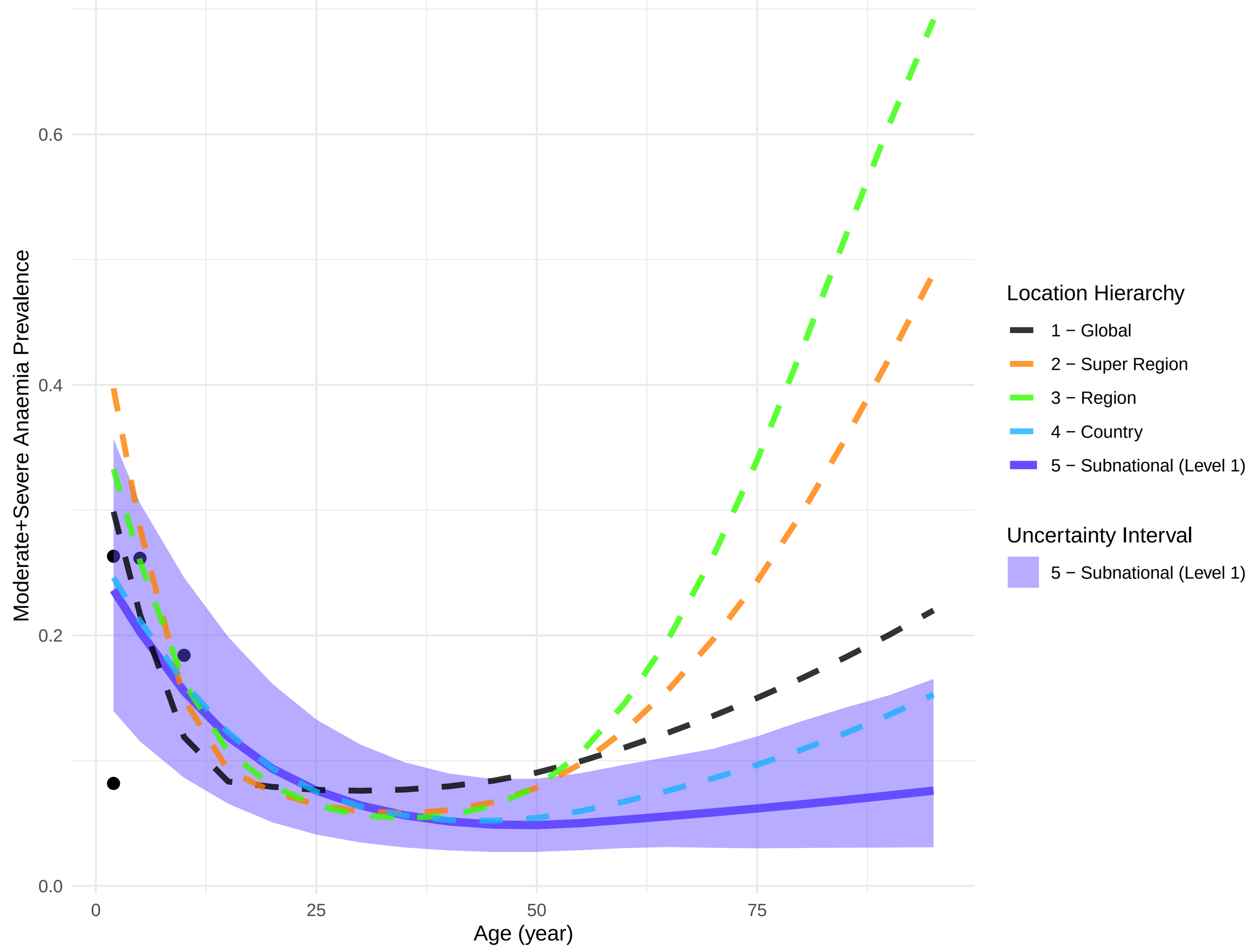

MR−BRT Age/Sex Fits − Males 2+ Years Old − Moderate+Severe Anaemia Prevalence − Garissa
WHO−2024 Adjustment Method

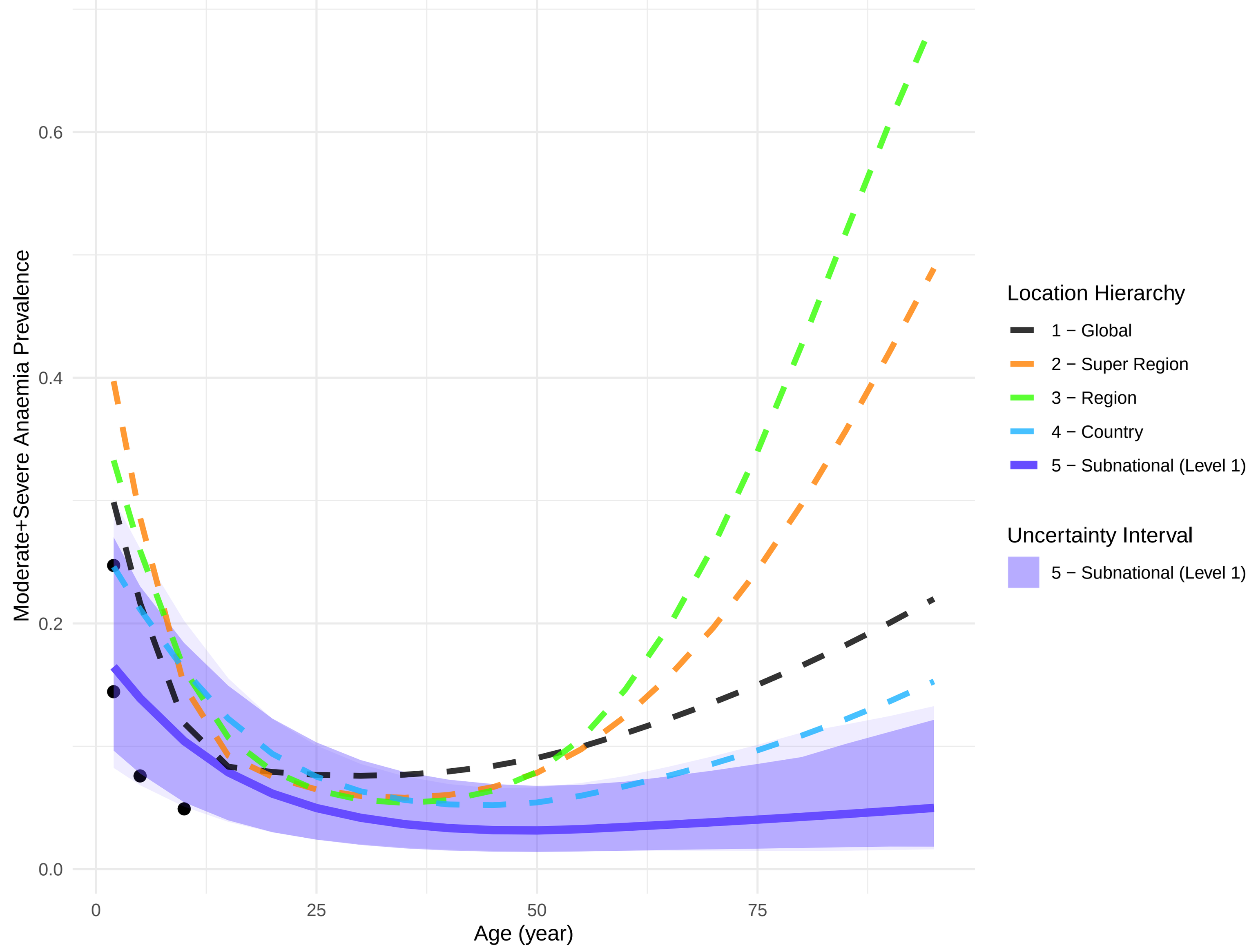

MR−BRT Age/Sex Fits − Males 2+ Years Old − Moderate+Severe Anaemia Prevalence − Homa Bay
WHO−2024 Adjustment Method

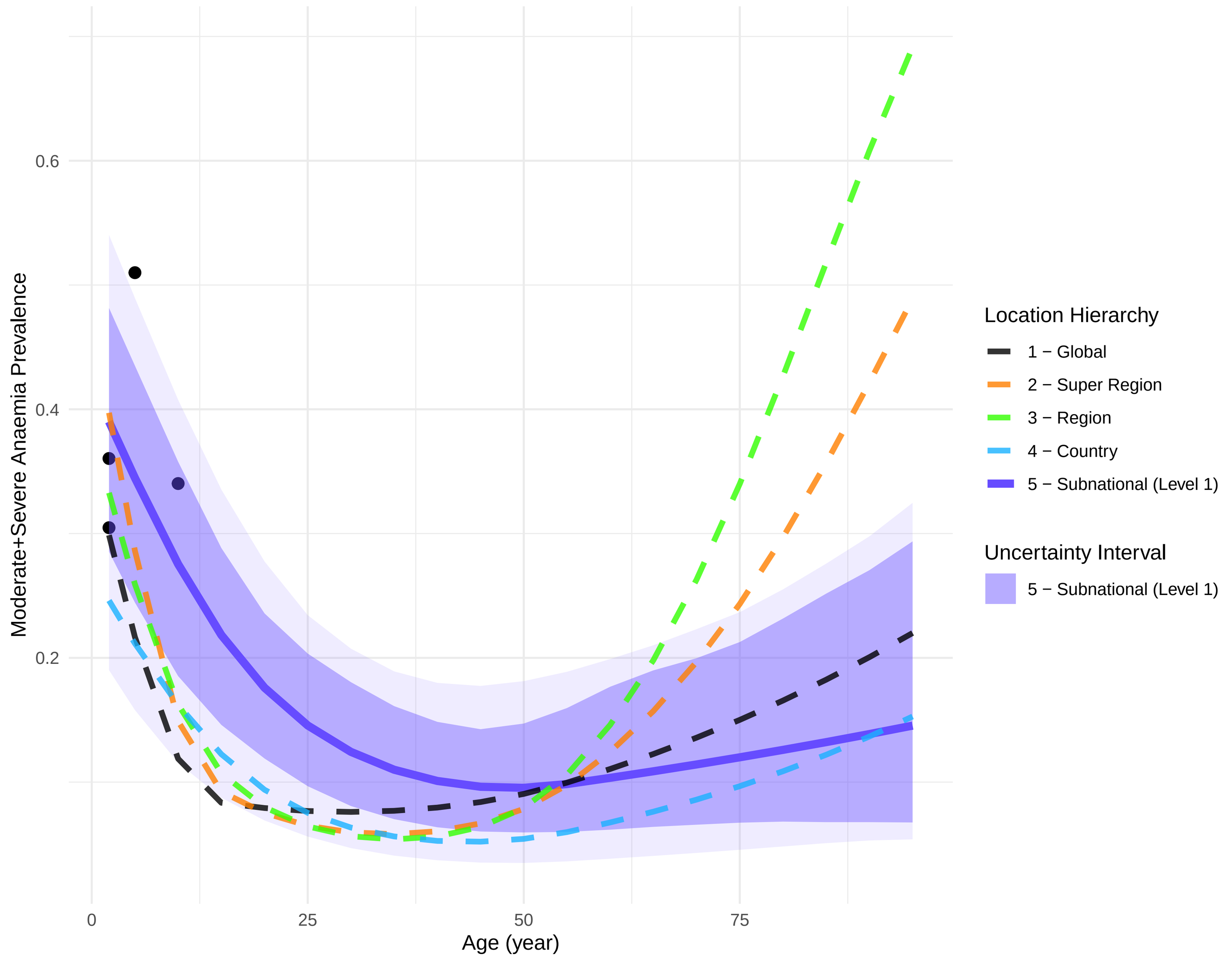

MR−BRT Age/Sex Fits − Males 2+ Years Old − Moderate+Severe Anaemia Prevalence − Isiolo
WHO−2024 Adjustment Method

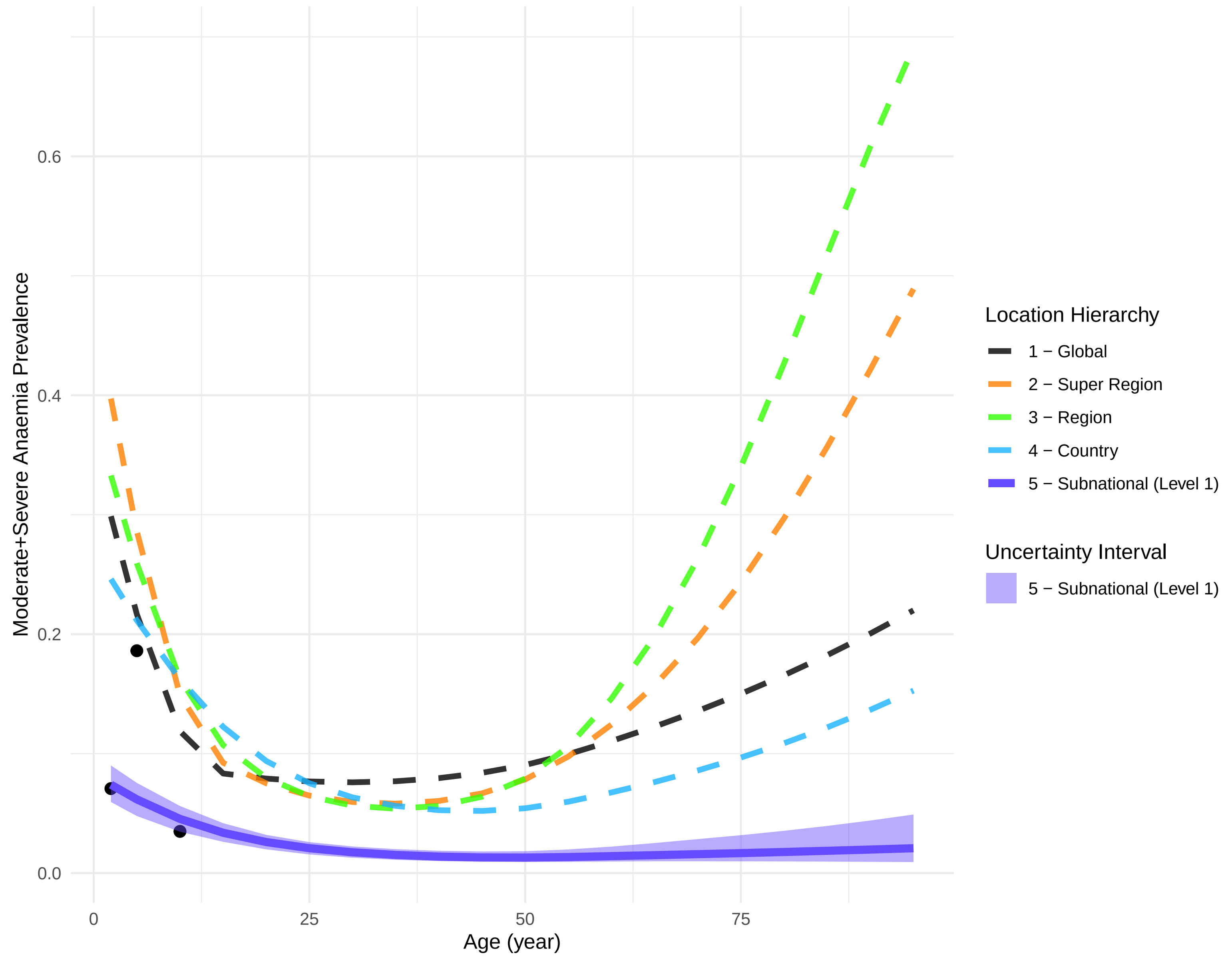

MR−BRT Age/Sex Fits − Males 2+ Years Old − Moderate+Severe Anaemia Prevalence − Kajiado
WHO−2024 Adjustment Method

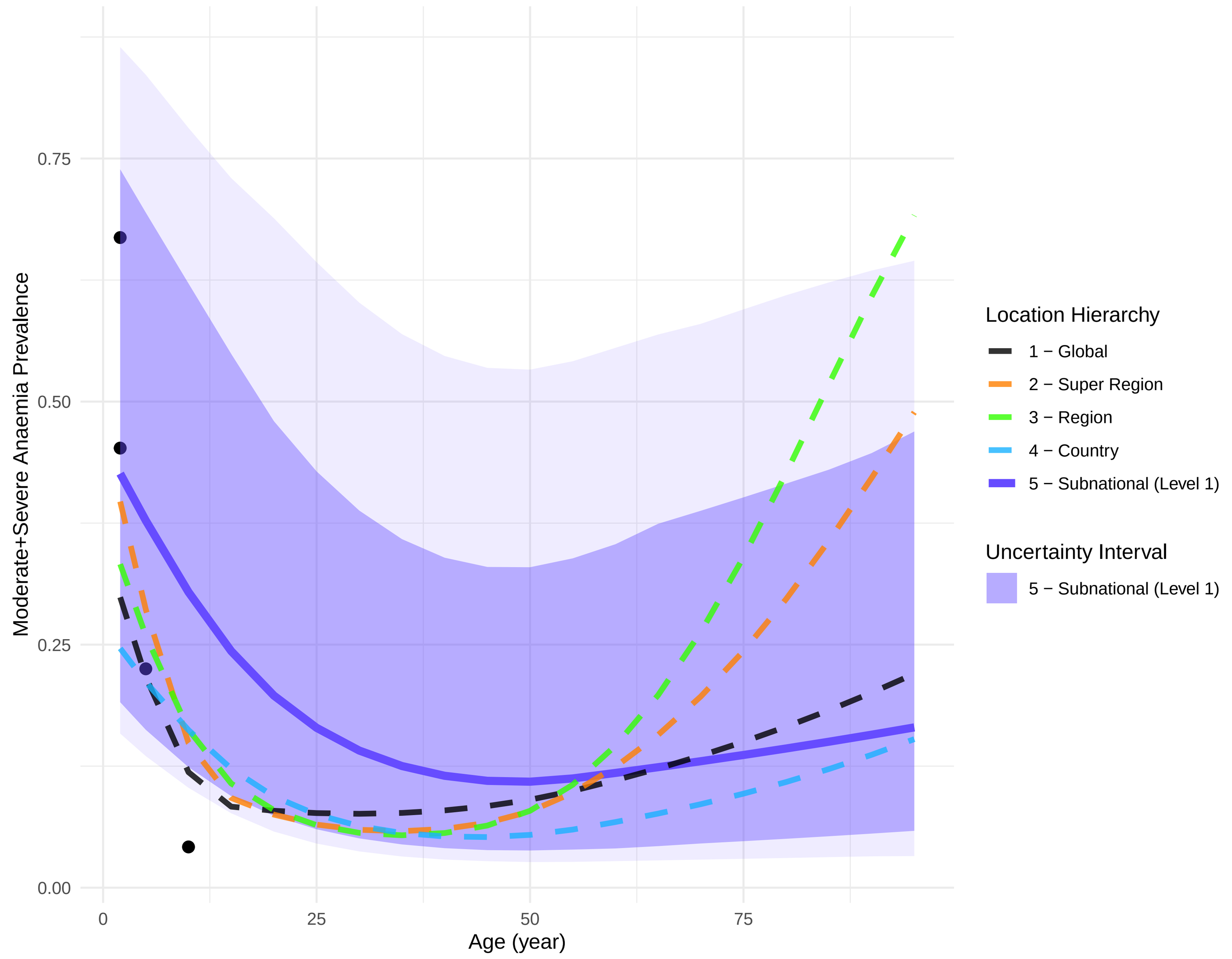

MR−BRT Age/Sex Fits − Males 2+ Years Old − Moderate+Severe Anaemia Prevalence − Kakamega
WHO−2024 Adjustment Method

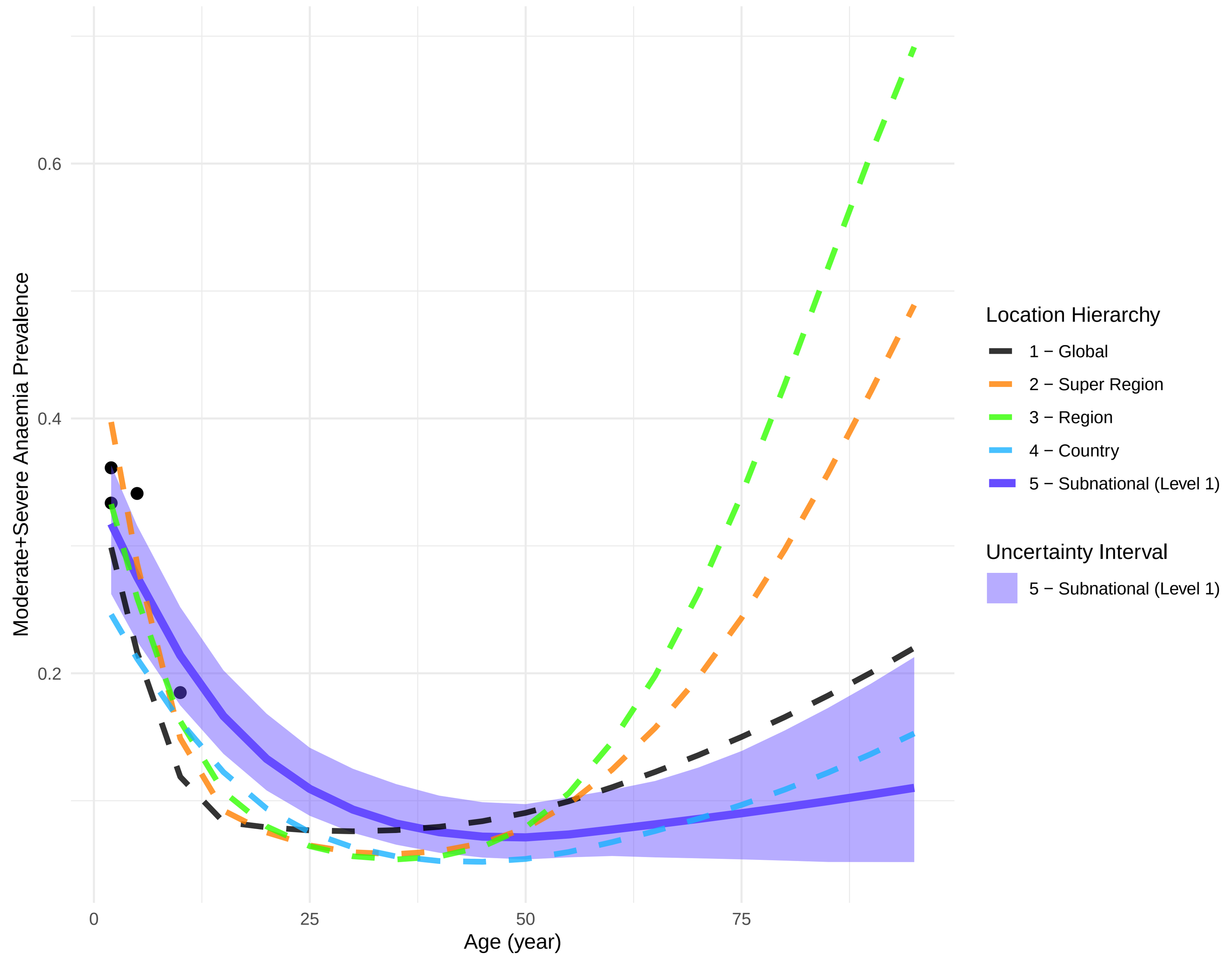

MR−BRT Age/Sex Fits − Males 2+ Years Old − Moderate+Severe Anaemia Prevalence − Kericho
WHO−2024 Adjustment Method

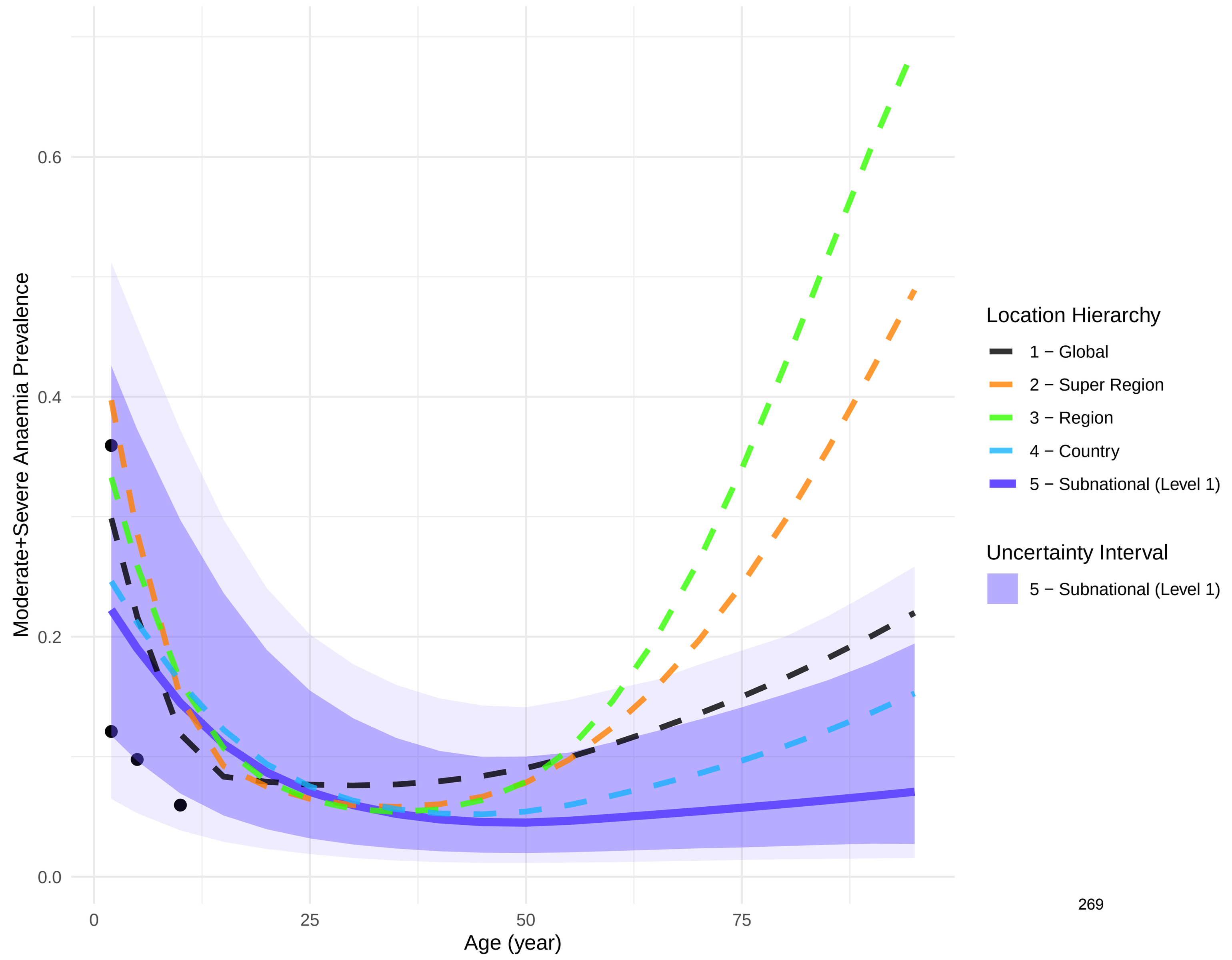

MR−BRT Age/Sex Fits − Males 2+ Years Old − Moderate+Severe Anaemia Prevalence − Kiambu
WHO−2024 Adjustment Method

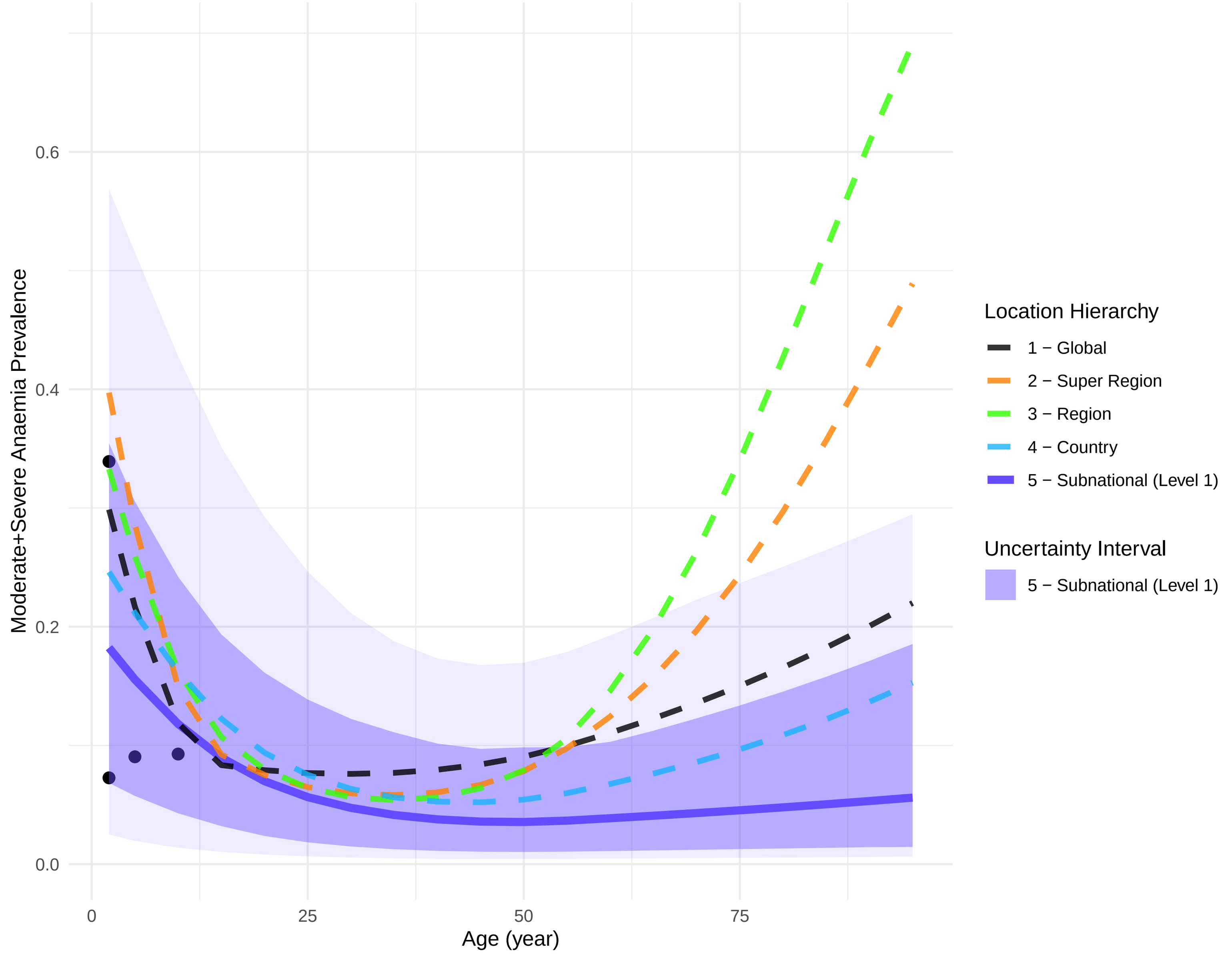

MR−BRT Age/Sex Fits − Males 2+ Years Old − Moderate+Severe Anaemia Prevalence − Kilifi
WHO−2024 Adjustment Method

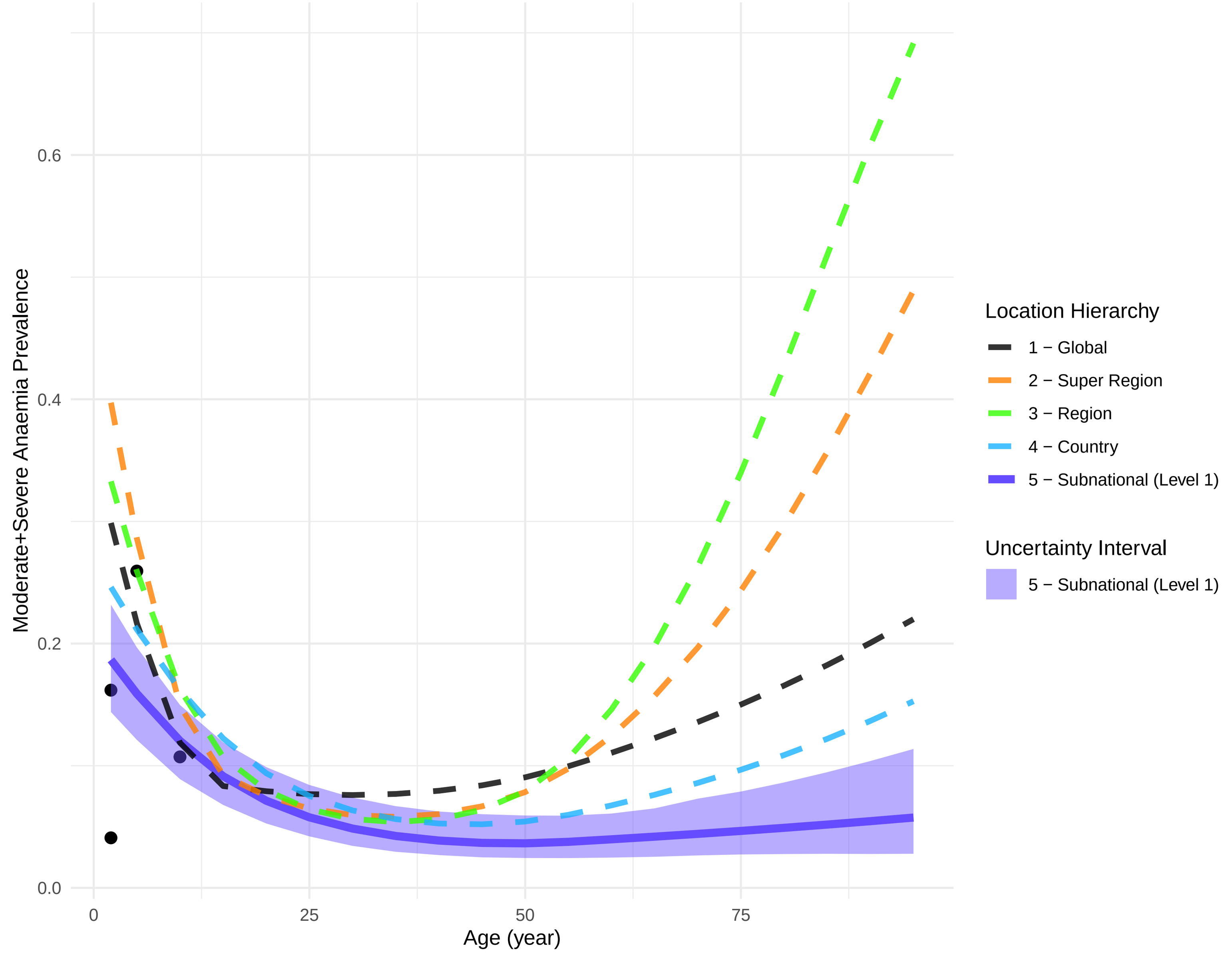

MR−BRT Age/Sex Fits − Males 2+ Years Old − Moderate+Severe Anaemia Prevalence − Kirinyaga
WHO−2024 Adjustment Method

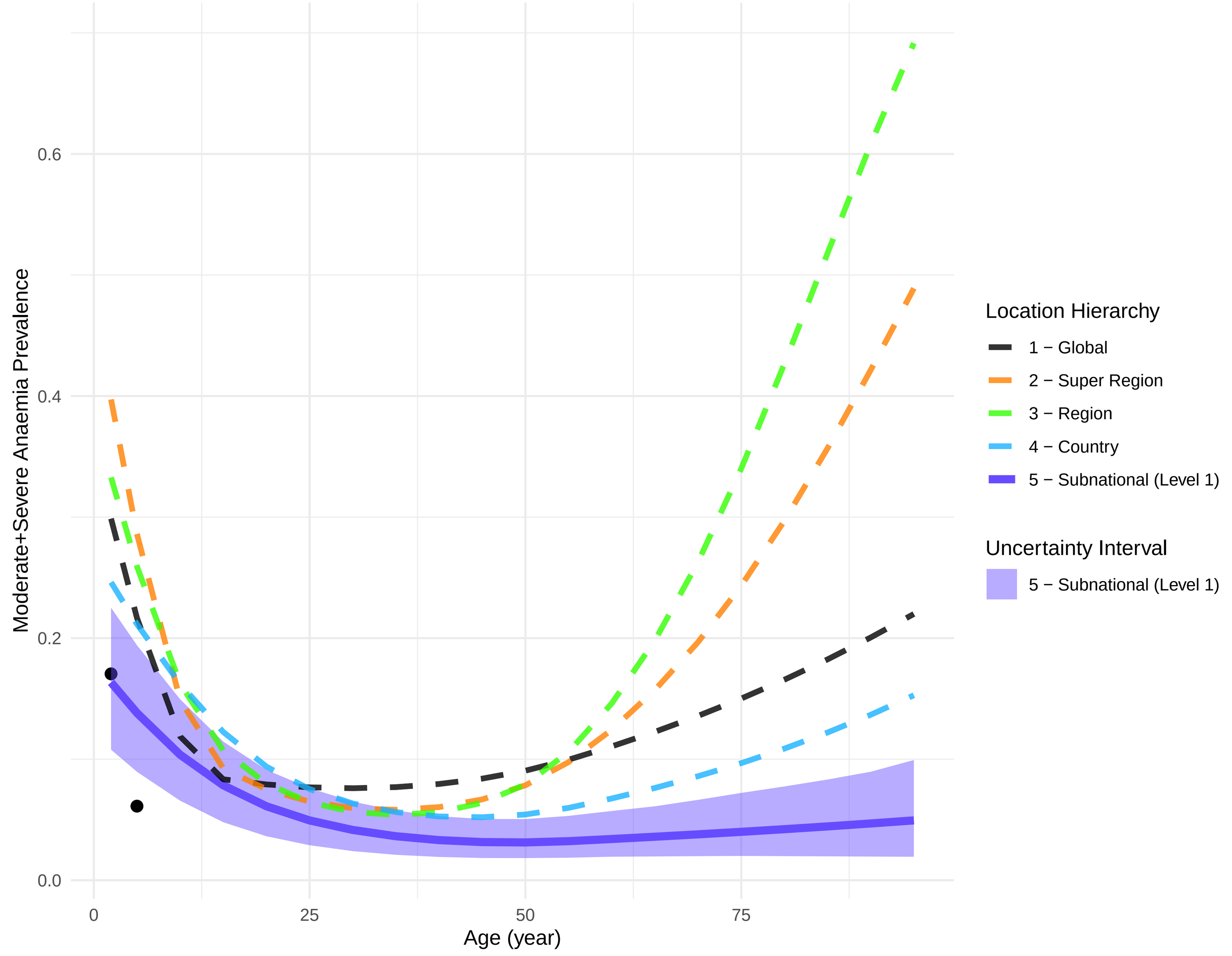

MR−BRT Age/Sex Fits − Males 2+ Years Old − Moderate+Severe Anaemia Prevalence − Kisii
WHO−2024 Adjustment Method

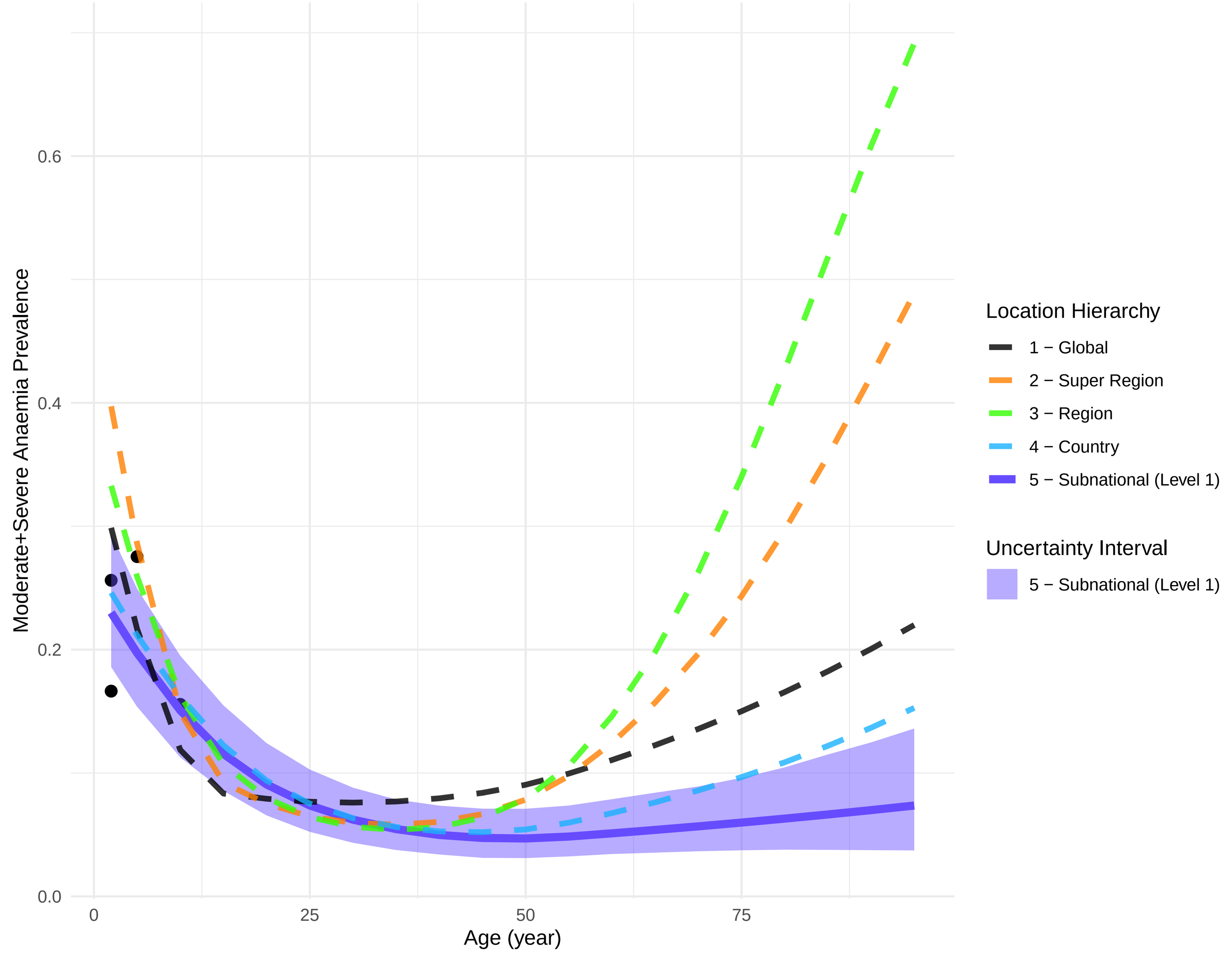

MR−BRT Age/Sex Fits − Males 2+ Years Old − Moderate+Severe Anaemia Prevalence − Kisumu
WHO−2024 Adjustment Method

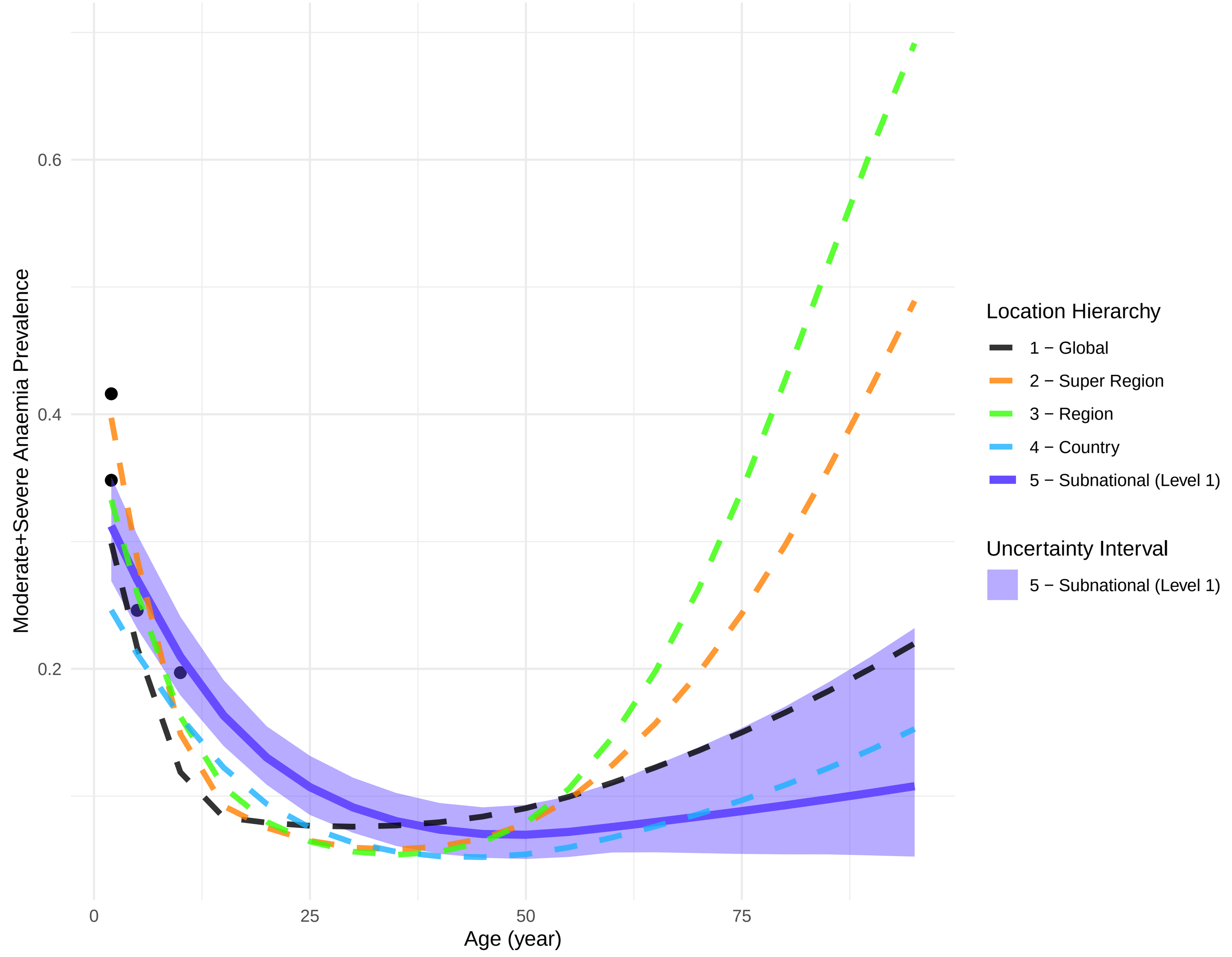

MR−BRT Age/Sex Fits − Males 2+ Years Old − Moderate+Severe Anaemia Prevalence − Kitui
WHO−2024 Adjustment Method

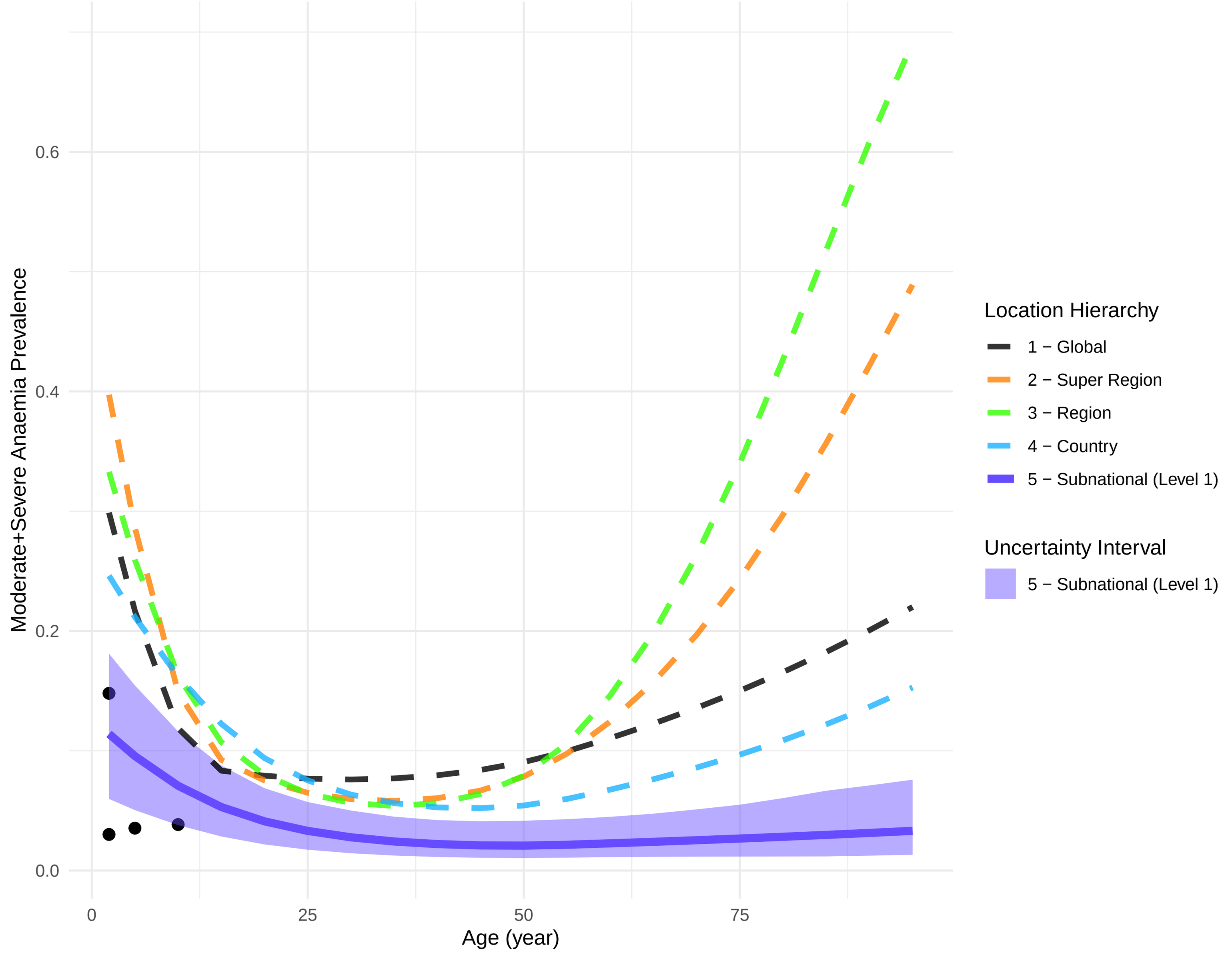

MR−BRT Age/Sex Fits − Males 2+ Years Old − Moderate+Severe Anaemia Prevalence − Kwale
WHO−2024 Adjustment Method

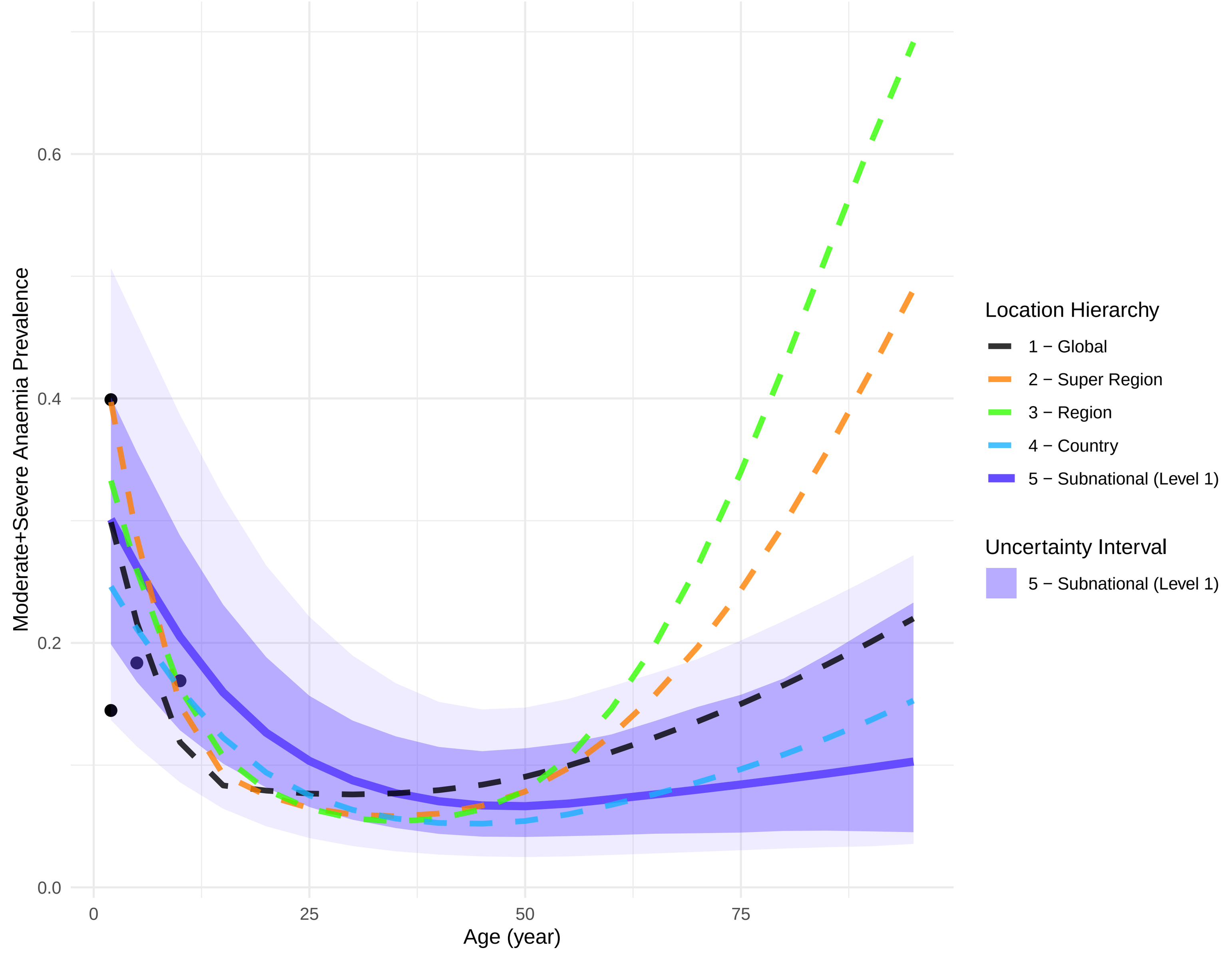

MR−BRT Age/Sex Fits − Males 2+ Years Old − Moderate+Severe Anaemia Prevalence − Laikipia
WHO−2024 Adjustment Method

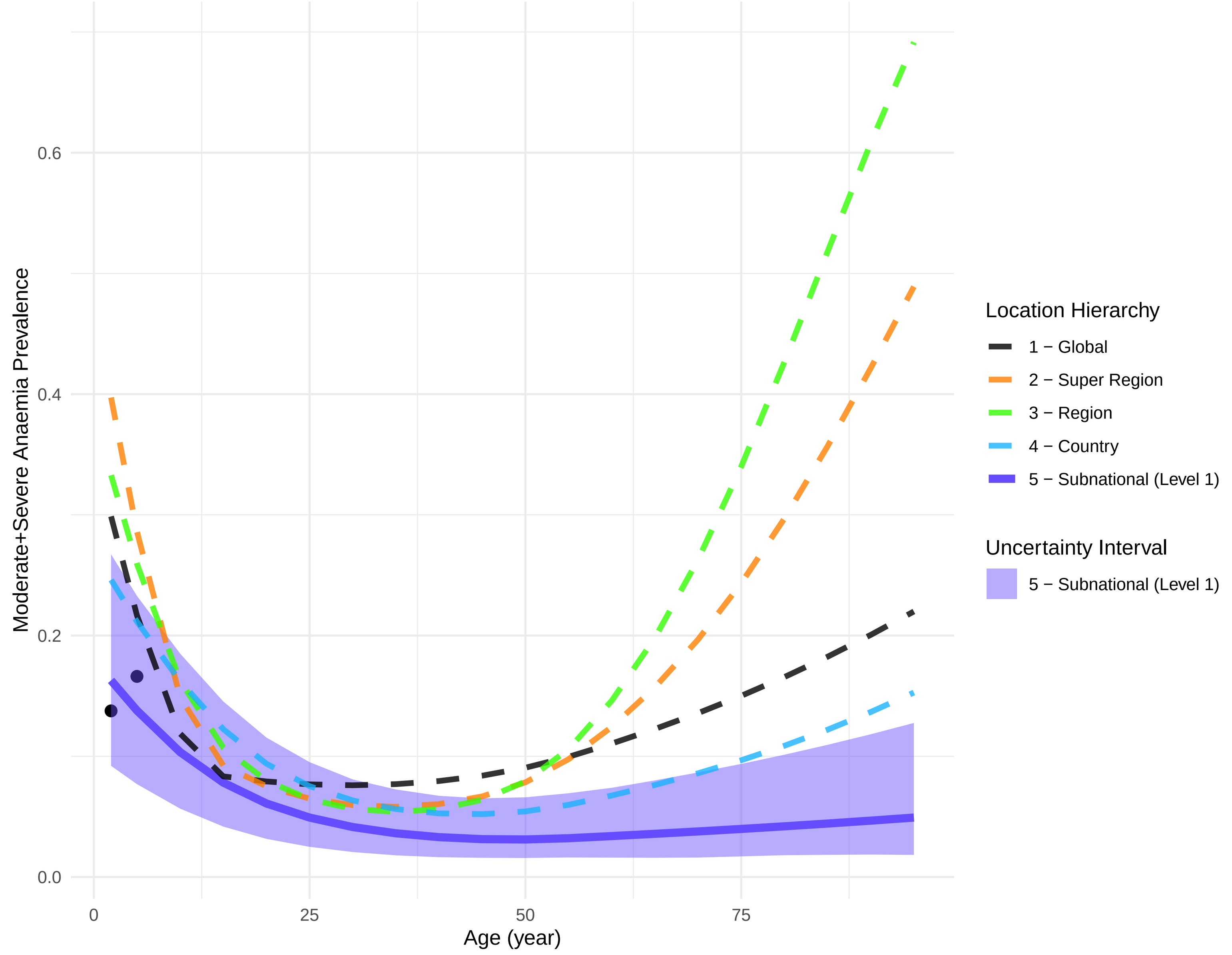

MR−BRT Age/Sex Fits − Males 2+ Years Old − Moderate+Severe Anaemia Prevalence − Lamu
WHO−2024 Adjustment Method

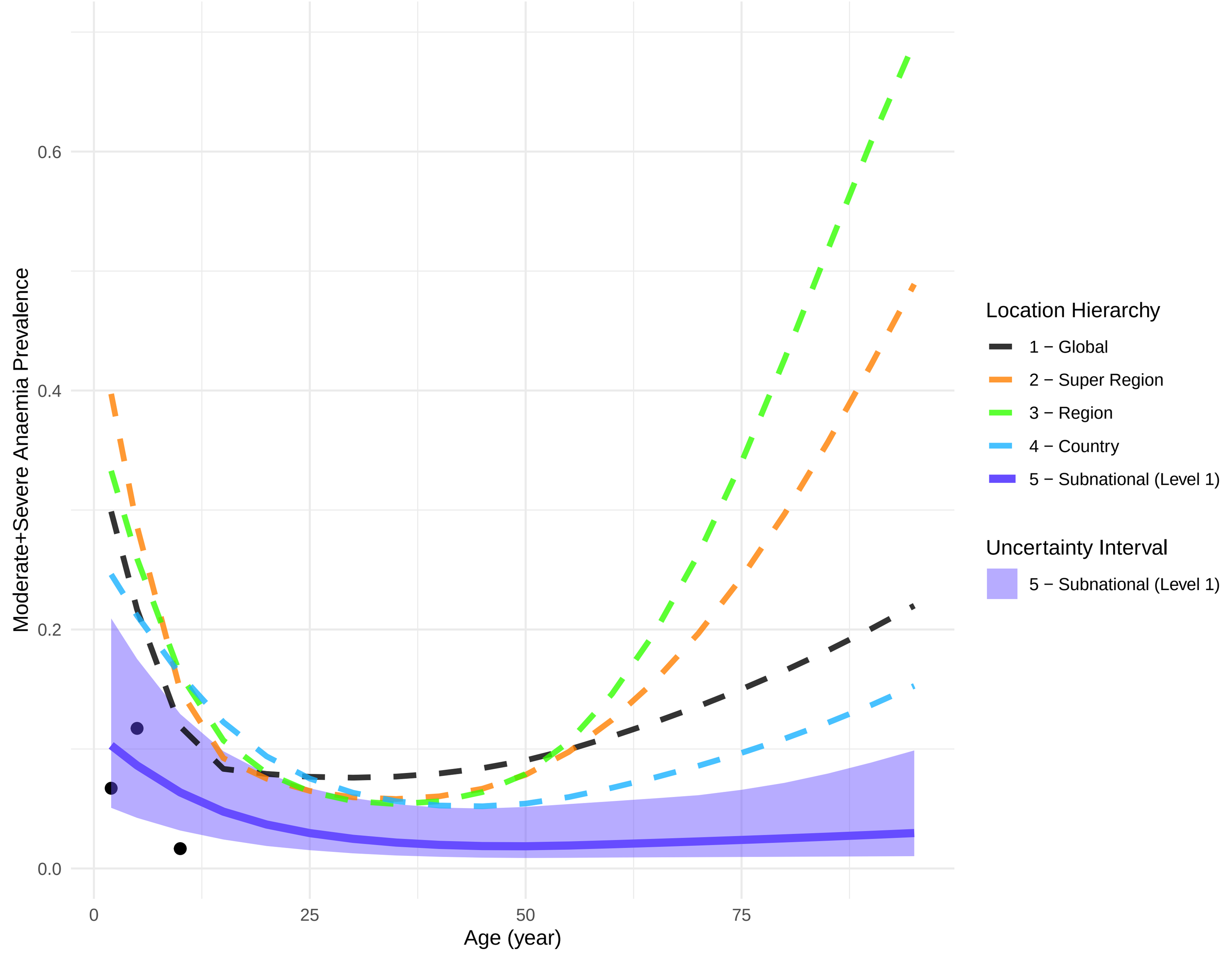

MR−BRT Age/Sex Fits − Males 2+ Years Old − Moderate+Severe Anaemia Prevalence − Machakos
WHO−2024 Adjustment Method

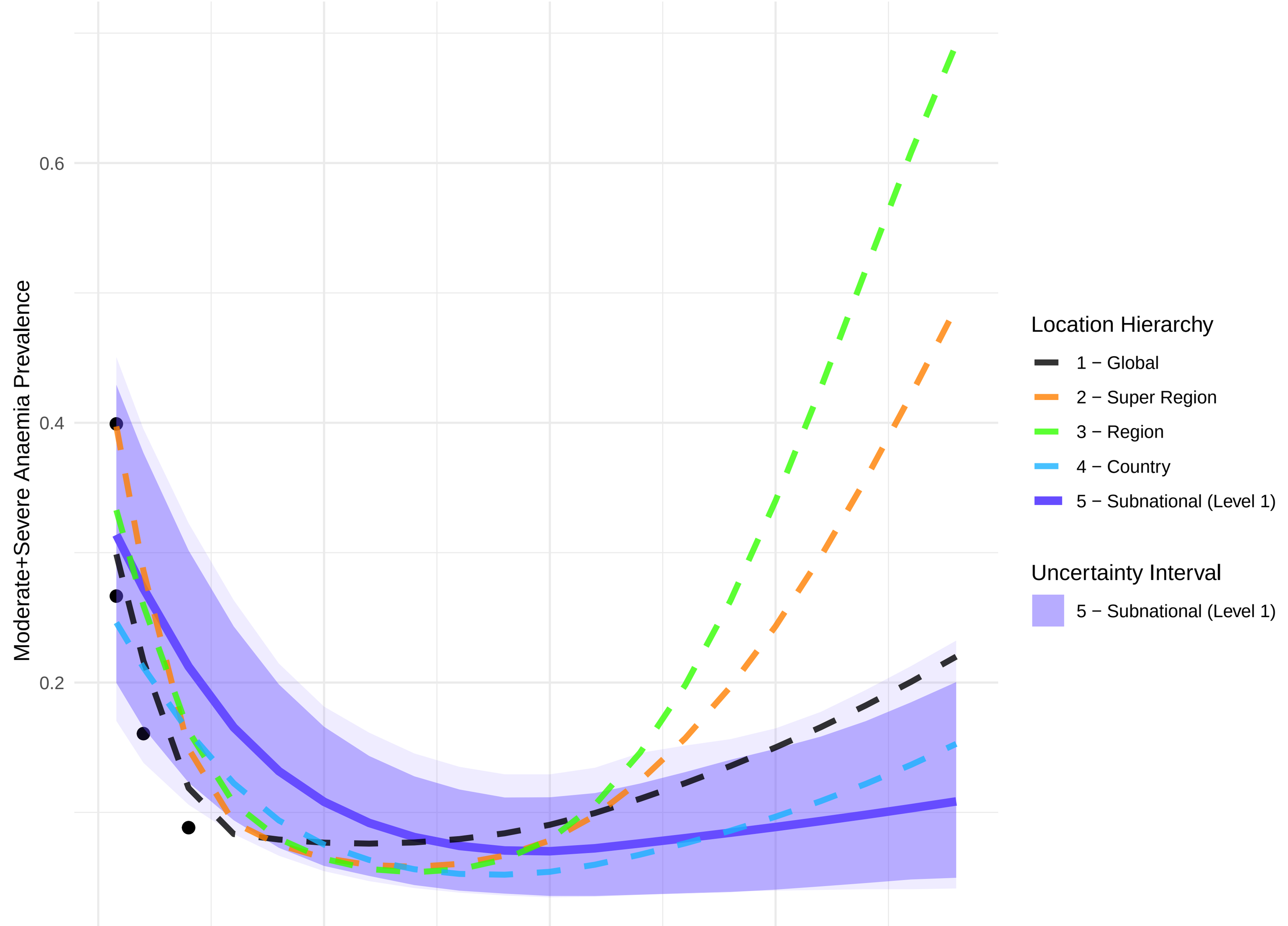

MR−BRT Age/Sex Fits − Males 2+ Years Old − Moderate+Severe Anaemia Prevalence − Makueni
WHO−2024 Adjustment Method

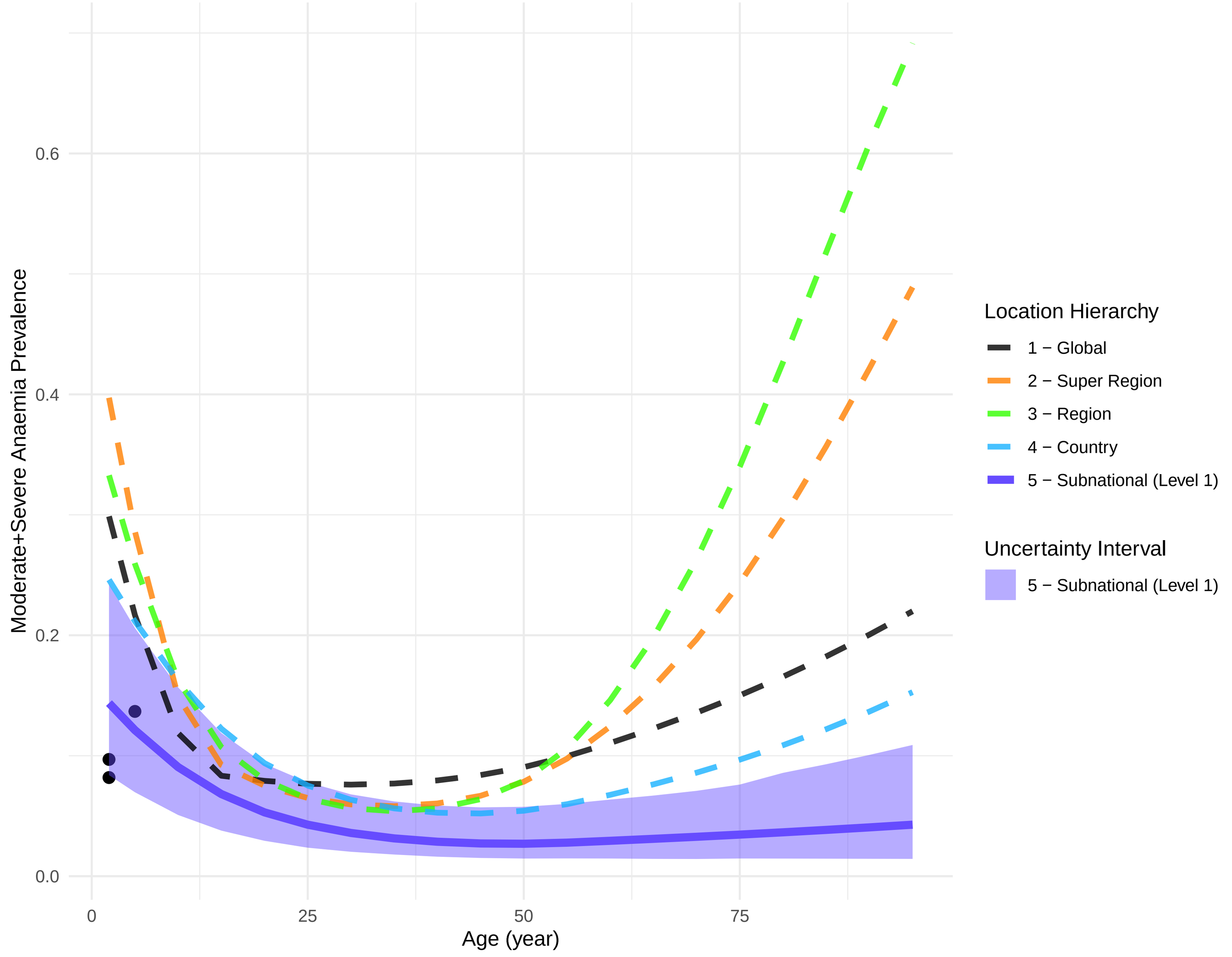

MR−BRT Age/Sex Fits − Males 2+ Years Old − Moderate+Severe Anaemia Prevalence − Mandera
WHO−2024 Adjustment Method

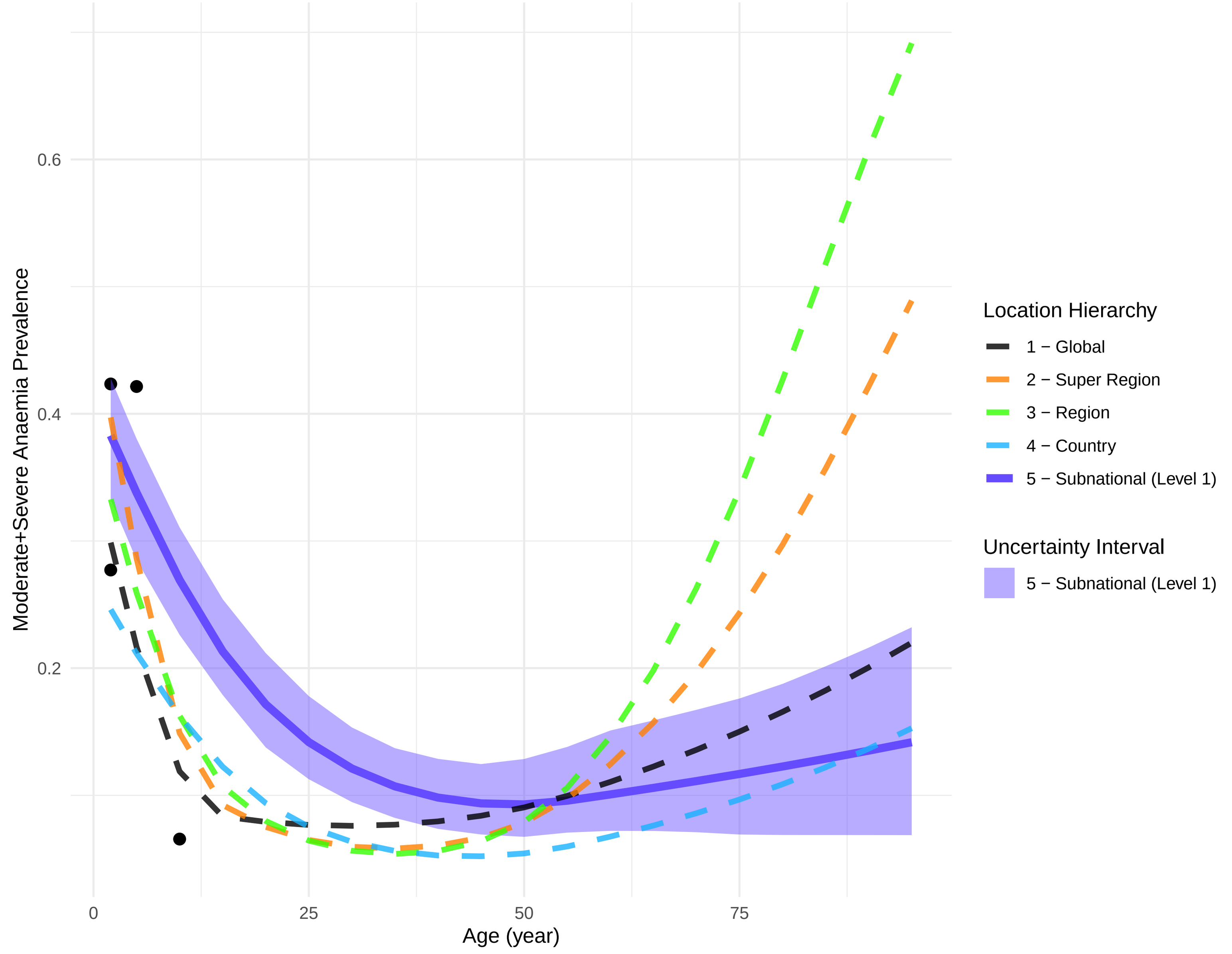

MR−BRT Age/Sex Fits − Males 2+ Years Old − Moderate+Severe Anaemia Prevalence − Marsabit
WHO−2024 Adjustment Method

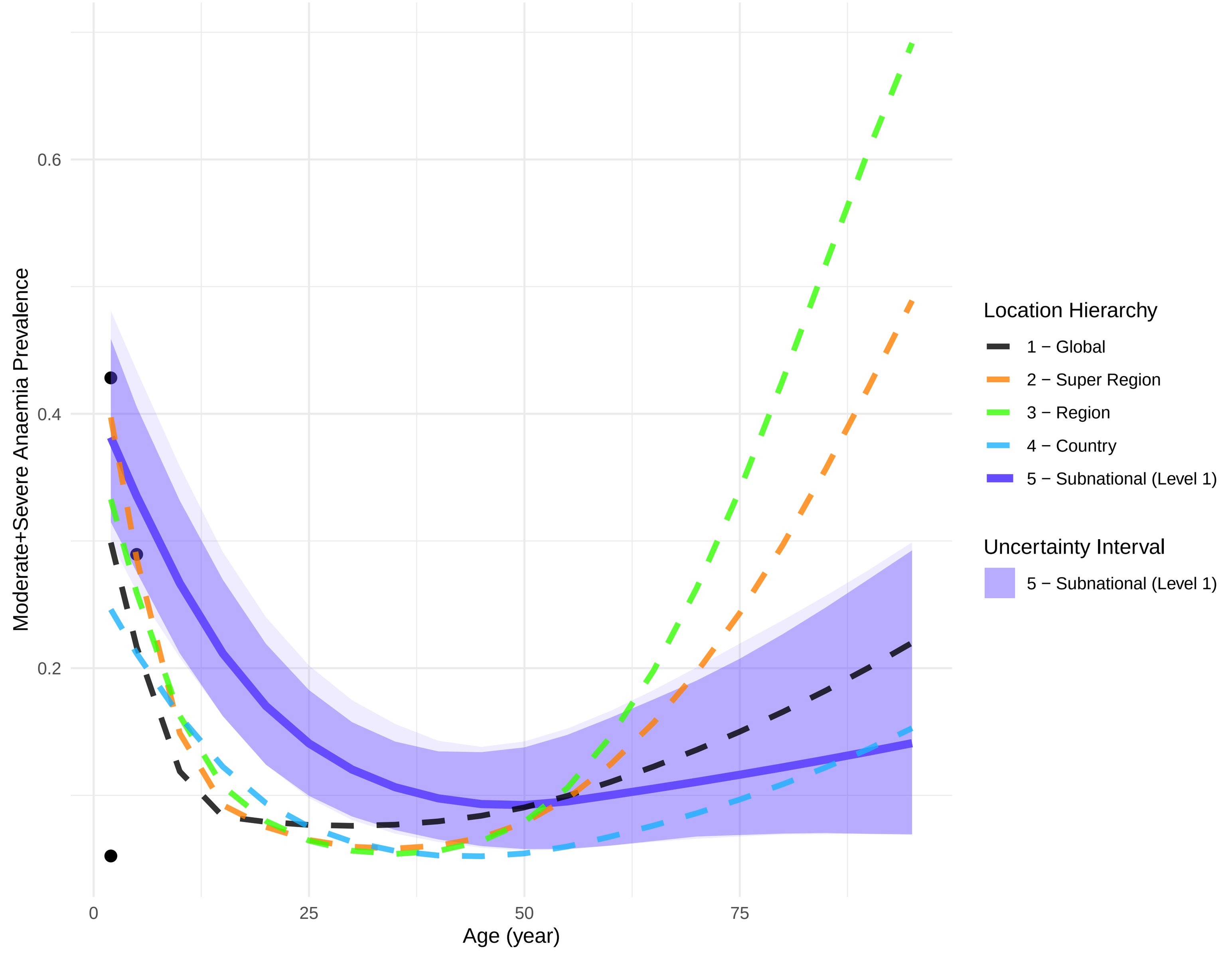

MR−BRT Age/Sex Fits − Males 2+ Years Old − Moderate+Severe Anaemia Prevalence − Meru
WHO−2024 Adjustment Method

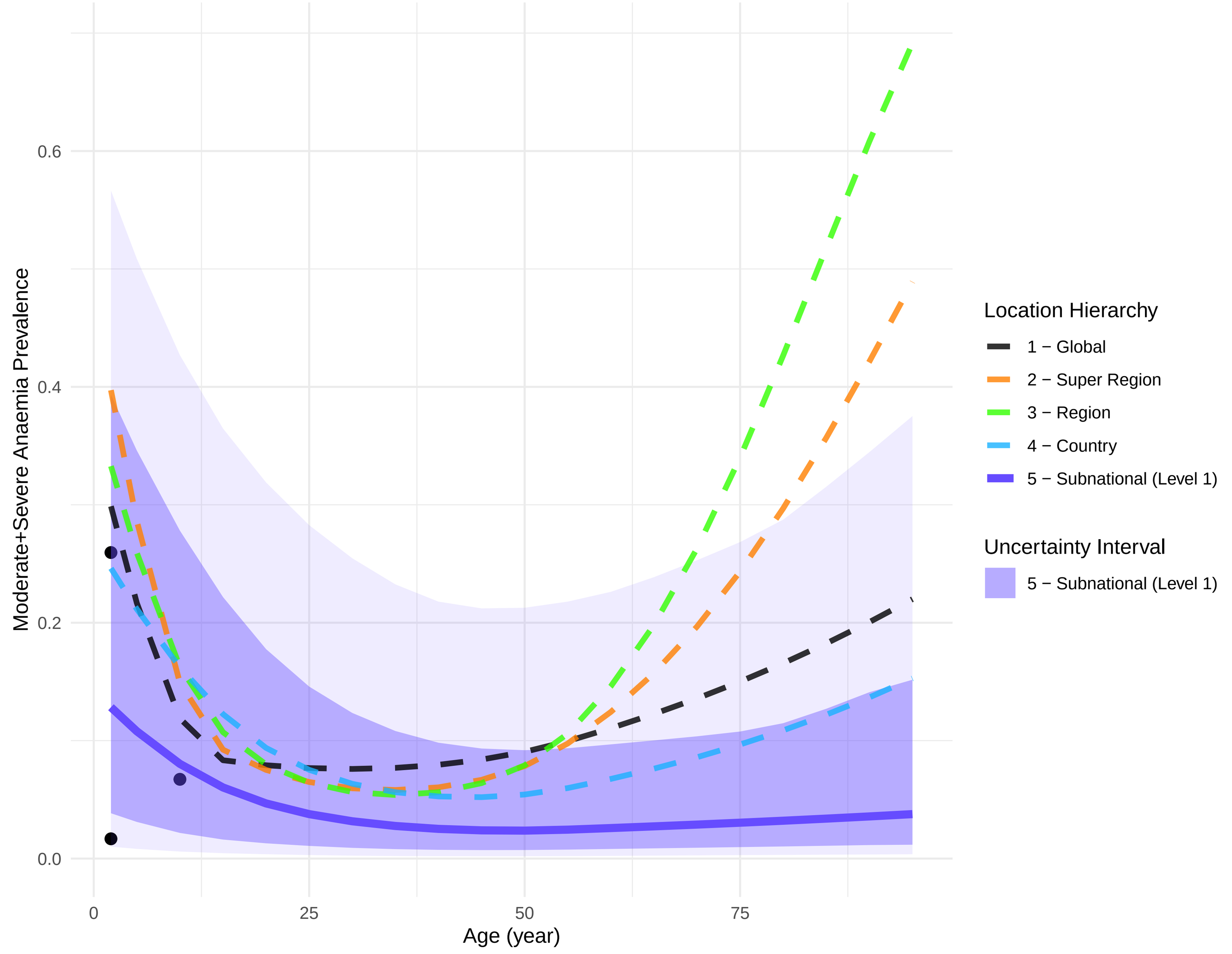

MR−BRT Age/Sex Fits − Males 2+ Years Old − Moderate+Severe Anaemia Prevalence − Migori
WHO−2024 Adjustment Method

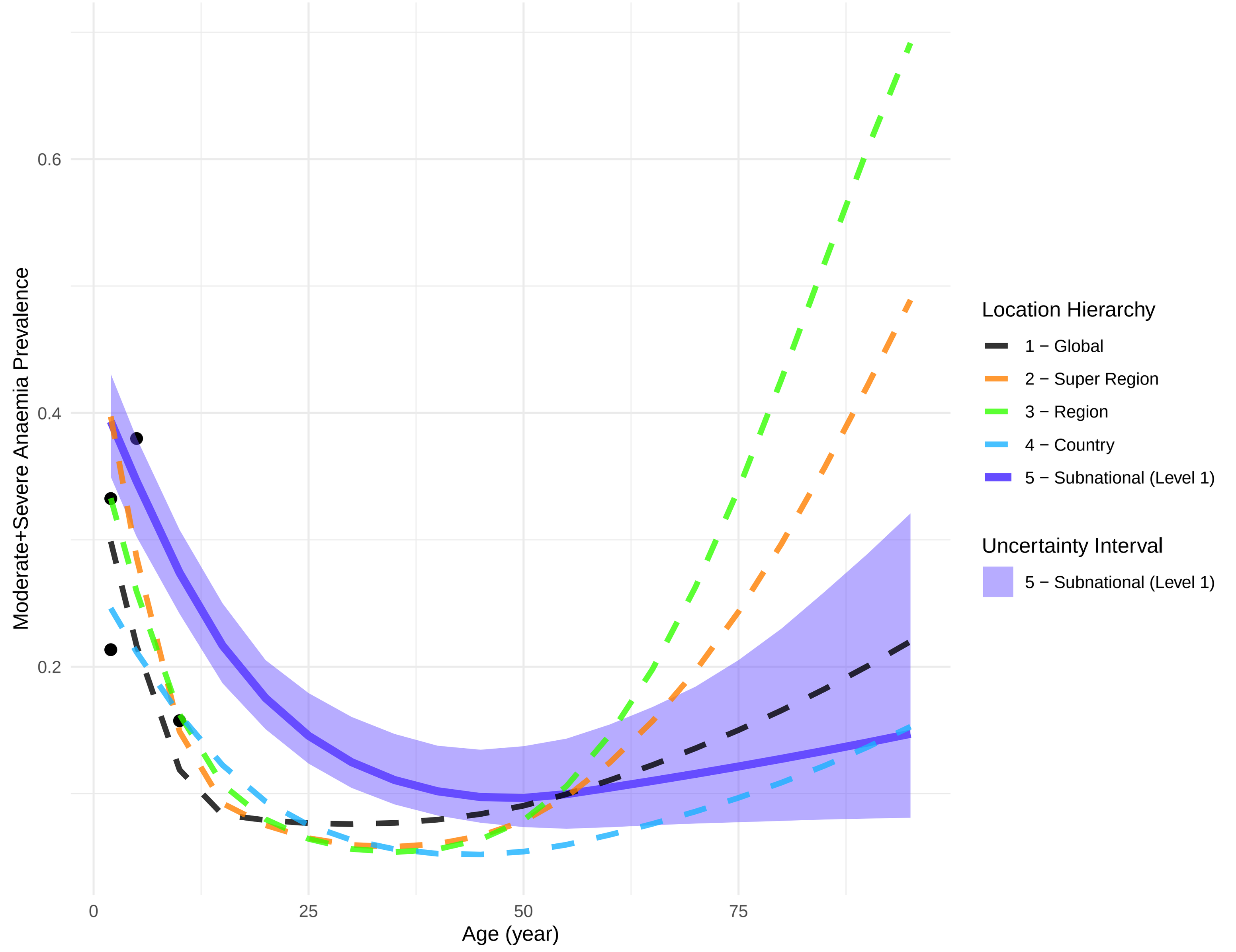

MR−BRT Age/Sex Fits − Males 2+ Years Old − Moderate+Severe Anaemia Prevalence − Mombasa
WHO−2024 Adjustment Method

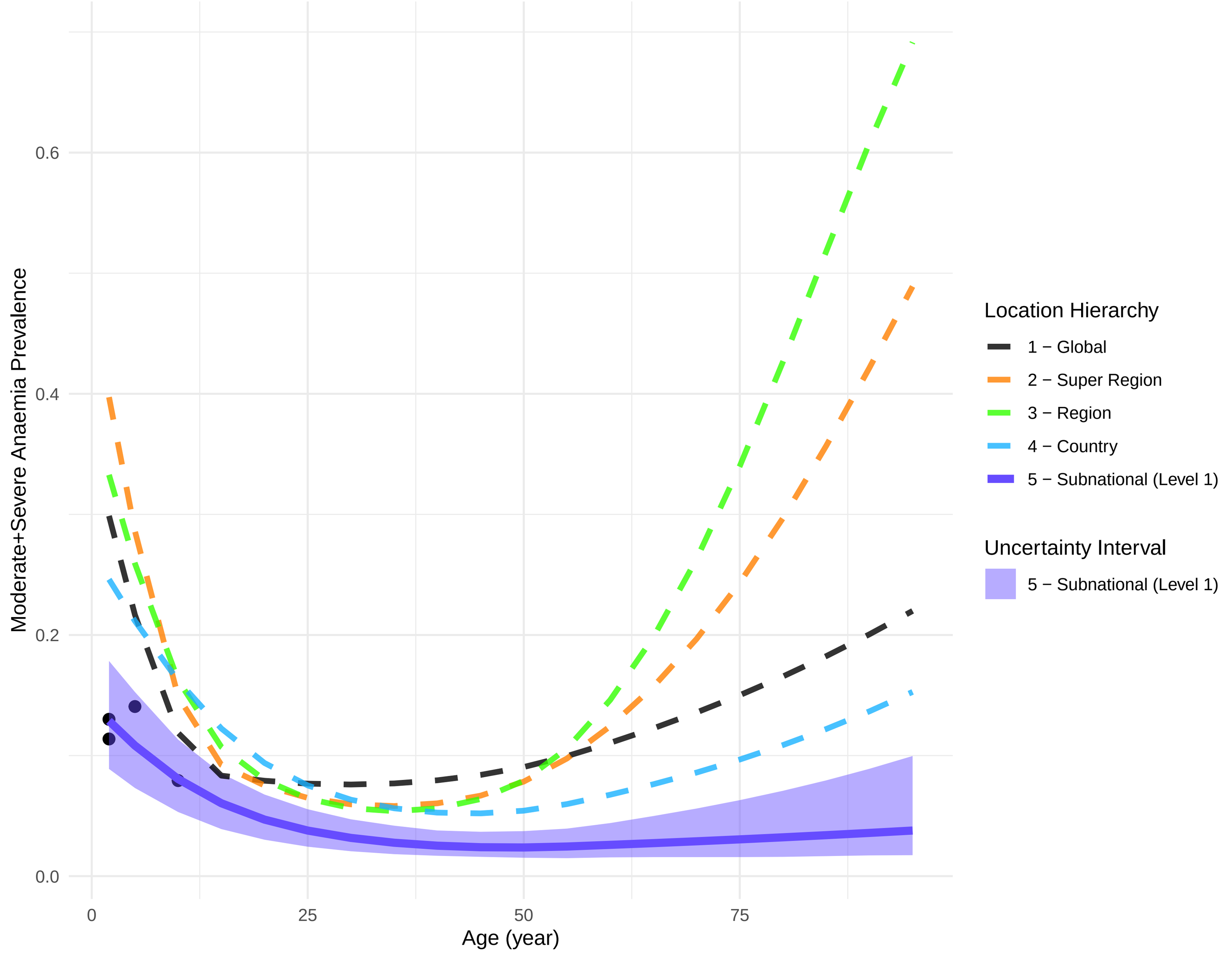

MR−BRT Age/Sex Fits − Males 2+ Years Old − Moderate+Severe Anaemia Prevalence − Murang'a
WHO−2024 Adjustment Method

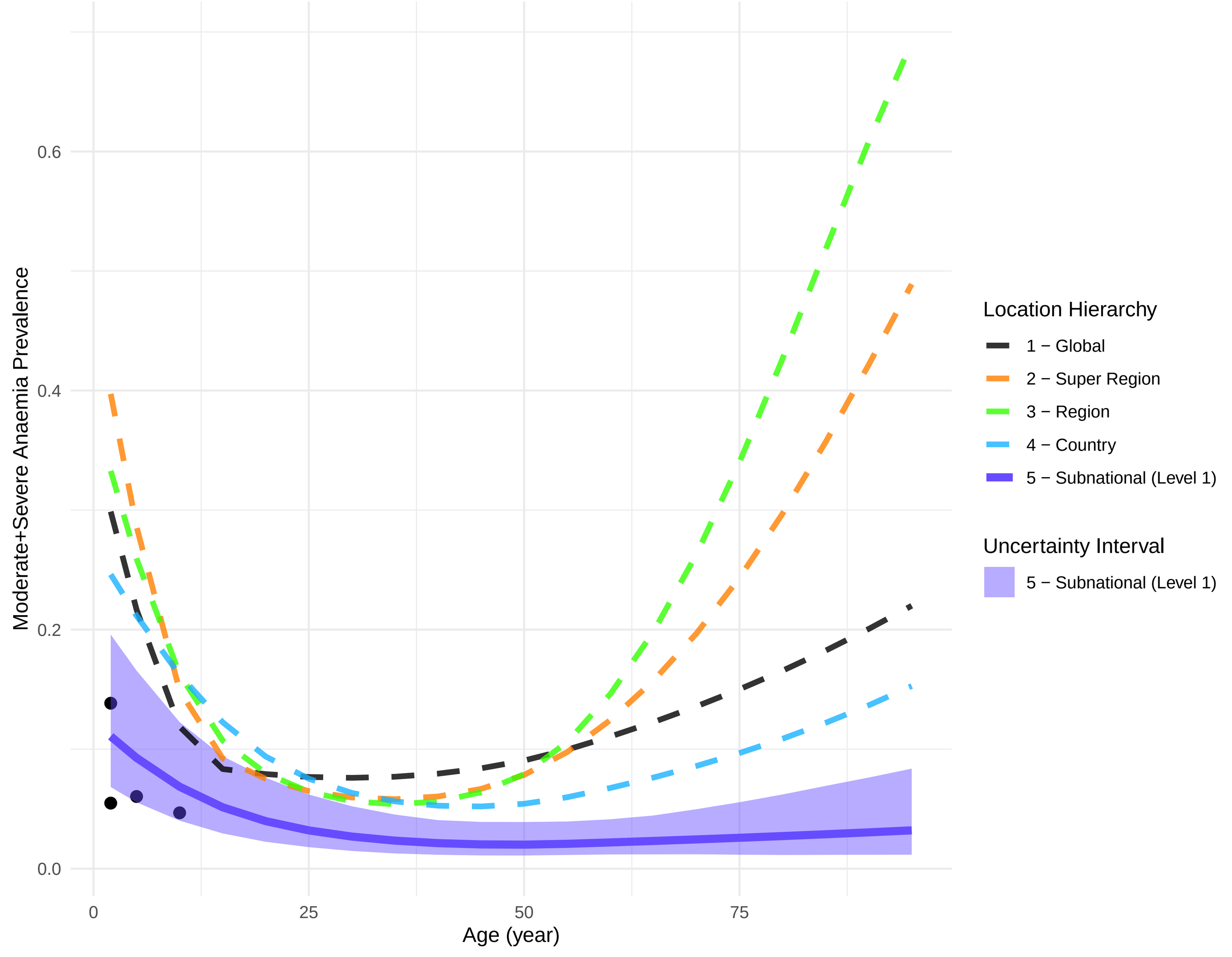

MR−BRT Age/Sex Fits − Males 2+ Years Old − Moderate+Severe Anaemia Prevalence − Nairobi
WHO−2024 Adjustment Method

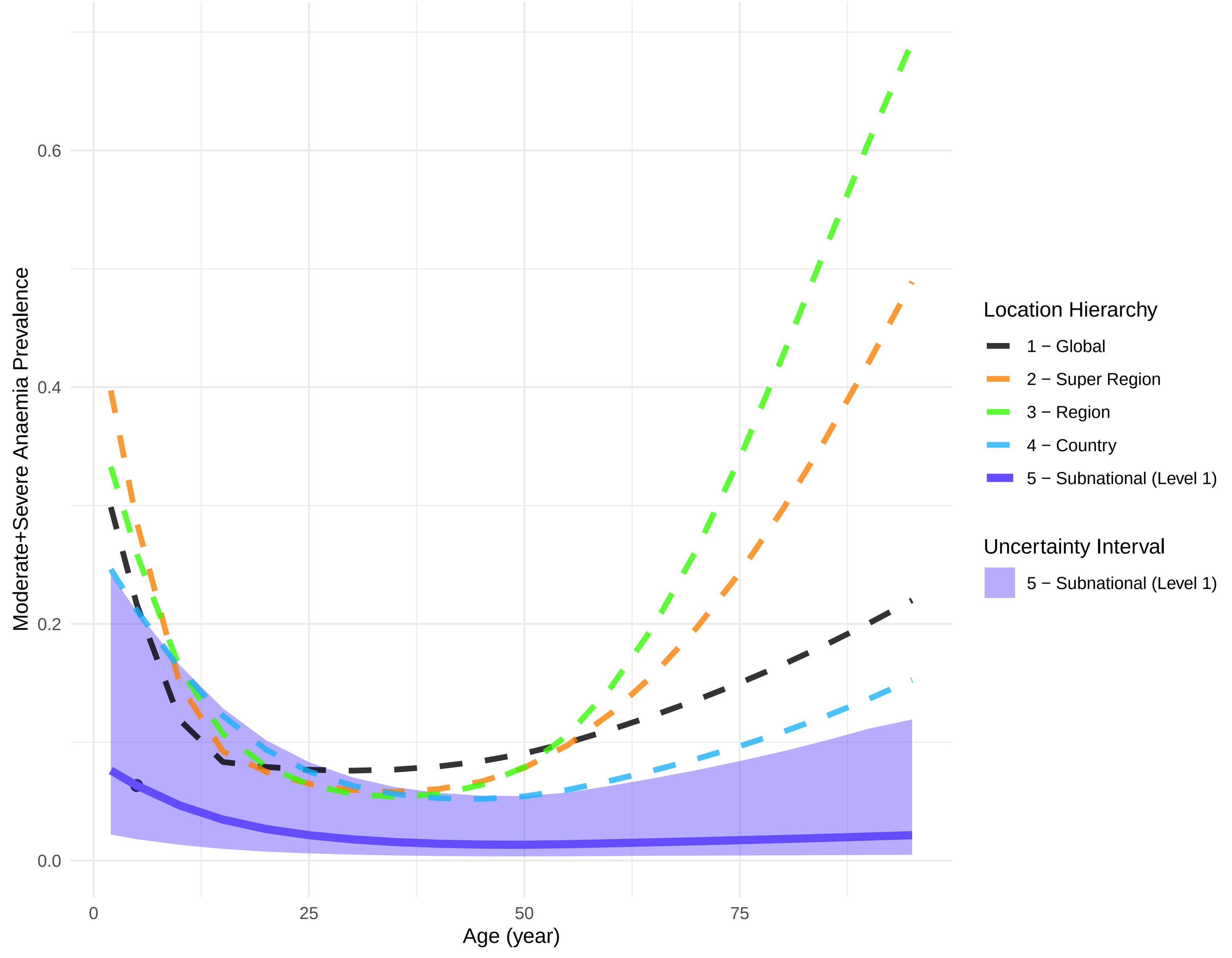

MR−BRT Age/Sex Fits − Males 2+ Years Old − Moderate+Severe Anaemia Prevalence − Nakuru
WHO−2024 Adjustment Method

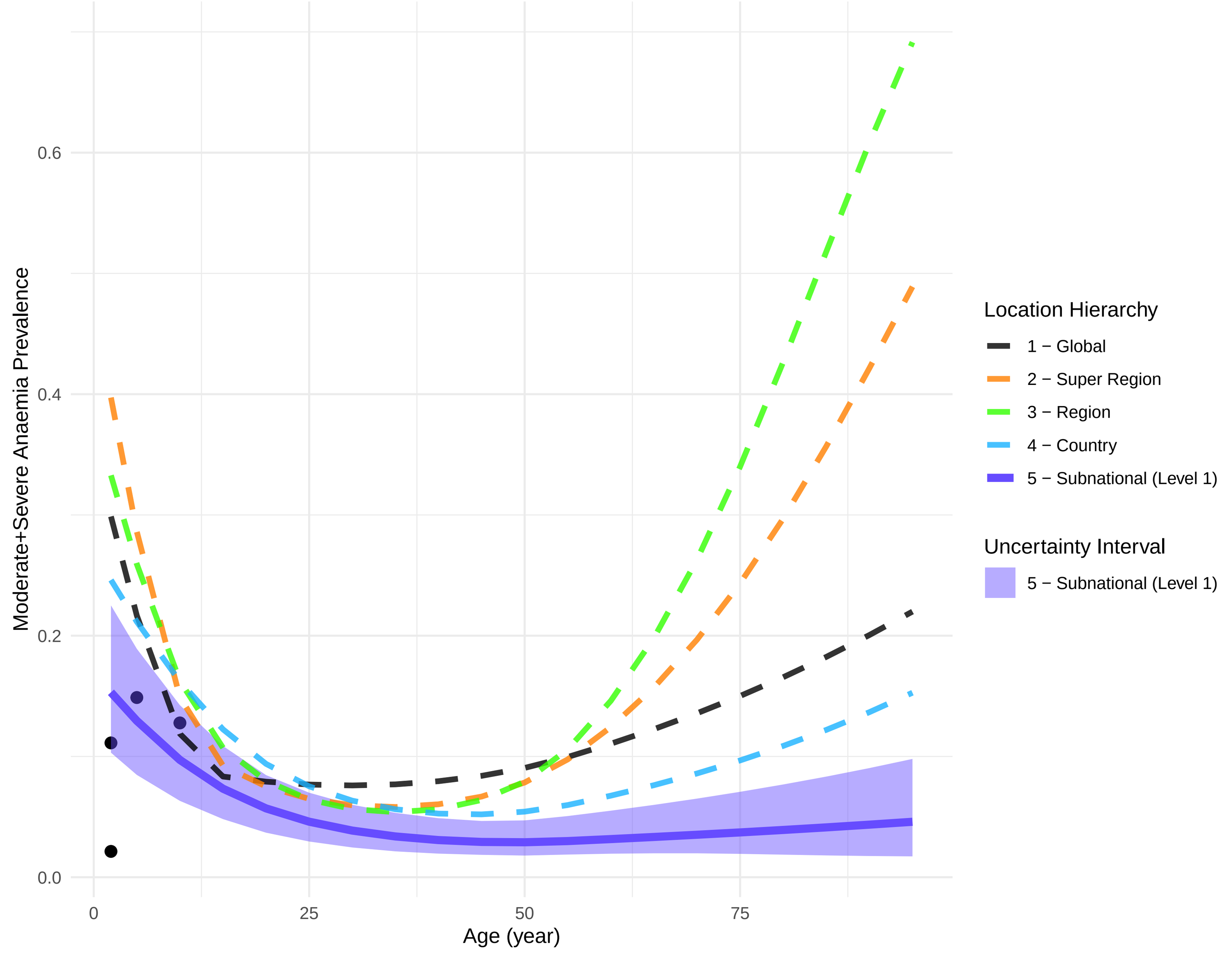

MR−BRT Age/Sex Fits − Males 2+ Years Old − Moderate+Severe Anaemia Prevalence − Nandi
WHO−2024 Adjustment Method

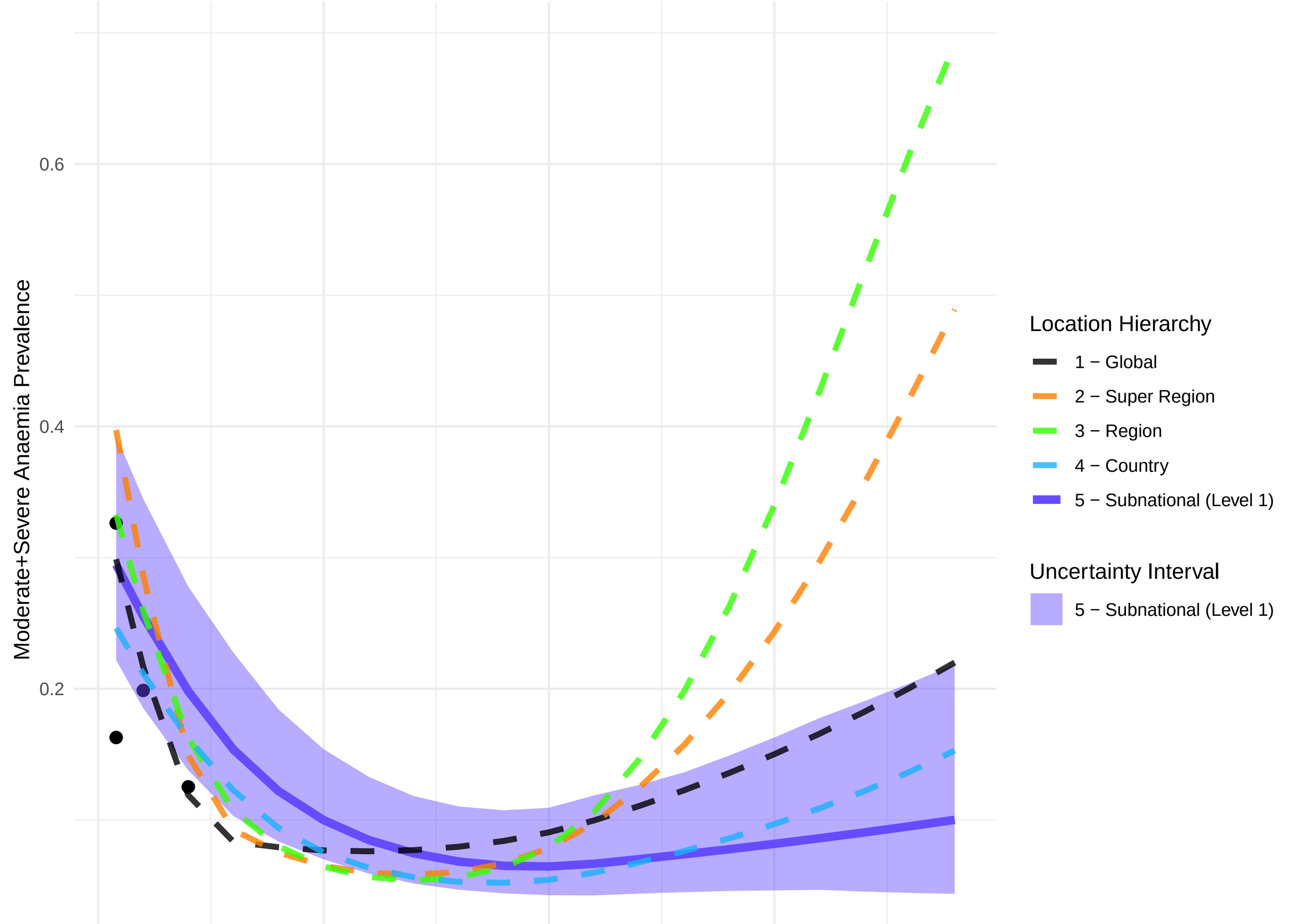

MR−BRT Age/Sex Fits − Males 2+ Years Old − Moderate+Severe Anaemia Prevalence − Narok
WHO−2024 Adjustment Method

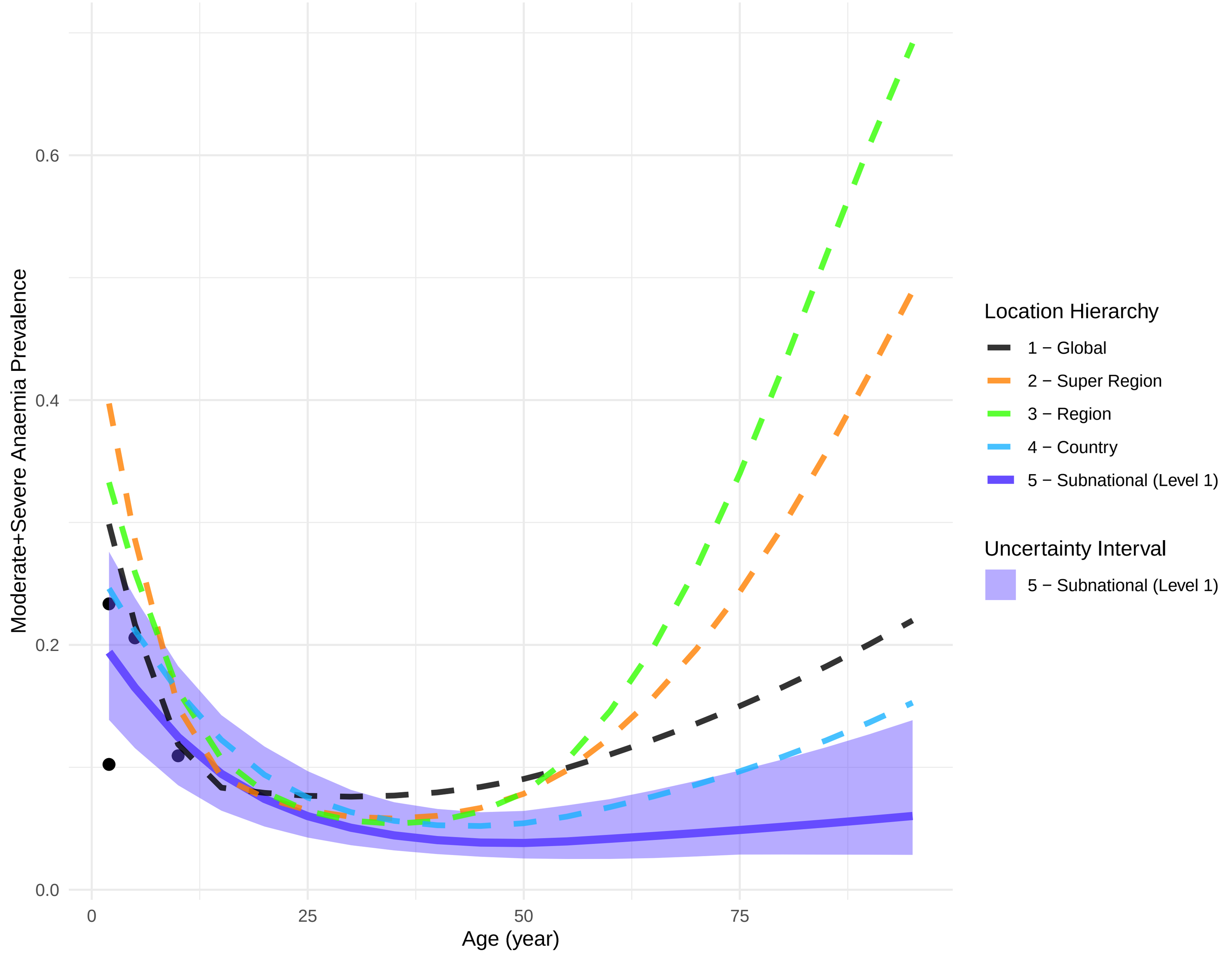

MR−BRT Age/Sex Fits − Males 2+ Years Old − Moderate+Severe Anaemia Prevalence − Nyamira
WHO−2024 Adjustment Method

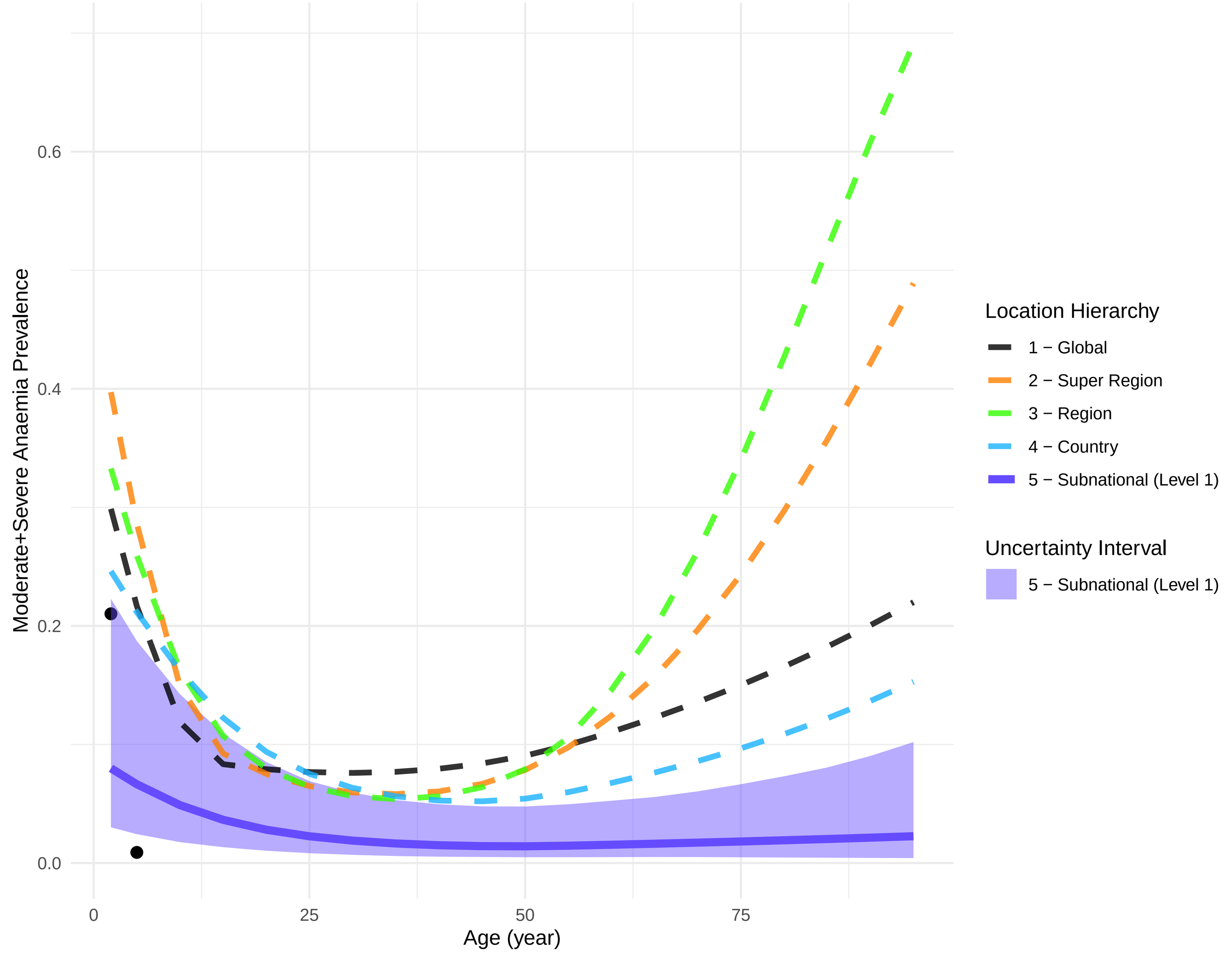

MR−BRT Age/Sex Fits − Males 2+ Years Old − Moderate+Severe Anaemia Prevalence − Nyandarua
WHO−2024 Adjustment Method

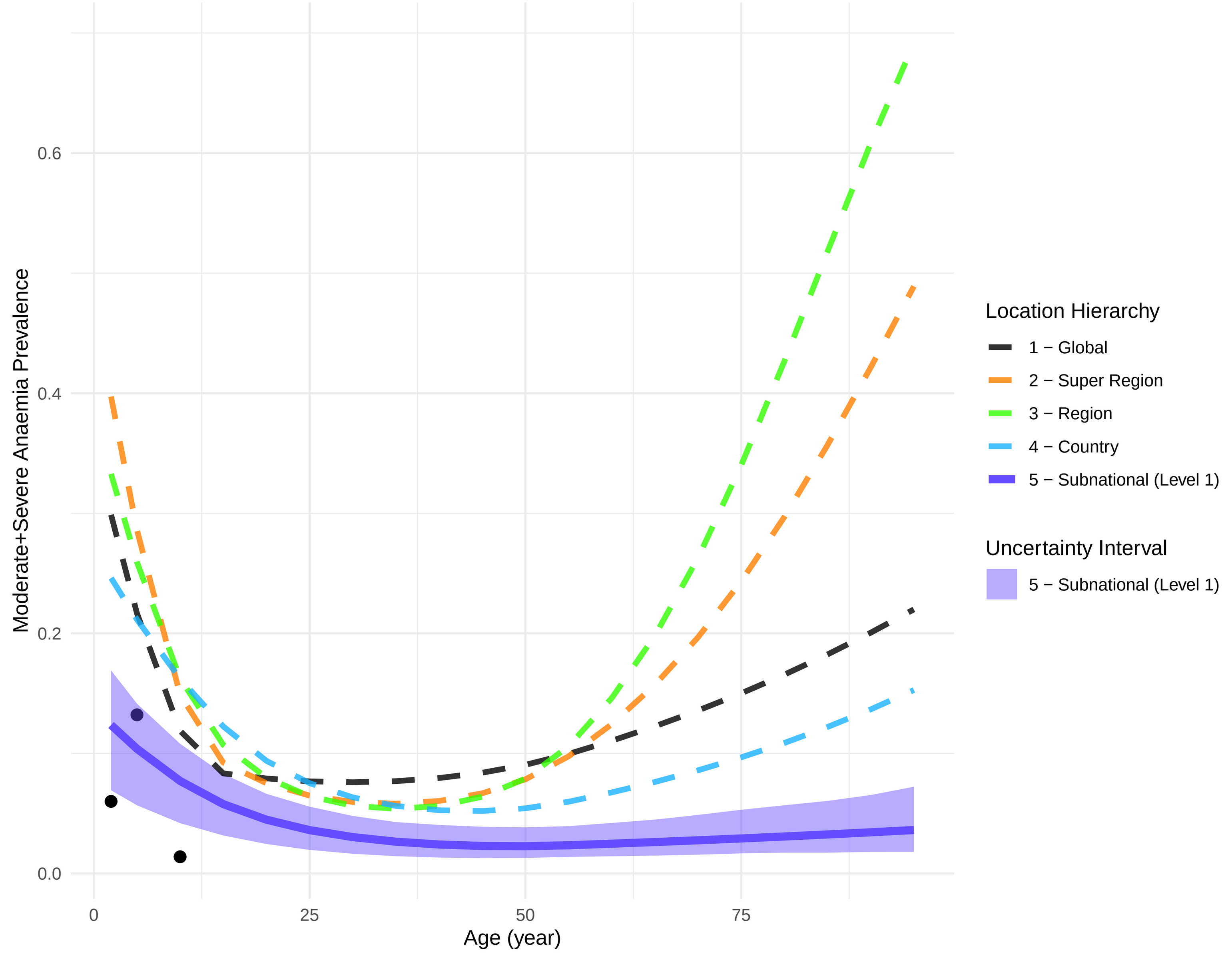

MR−BRT Age/Sex Fits − Males 2+ Years Old − Moderate+Severe Anaemia Prevalence − Nyeri
WHO−2024 Adjustment Method

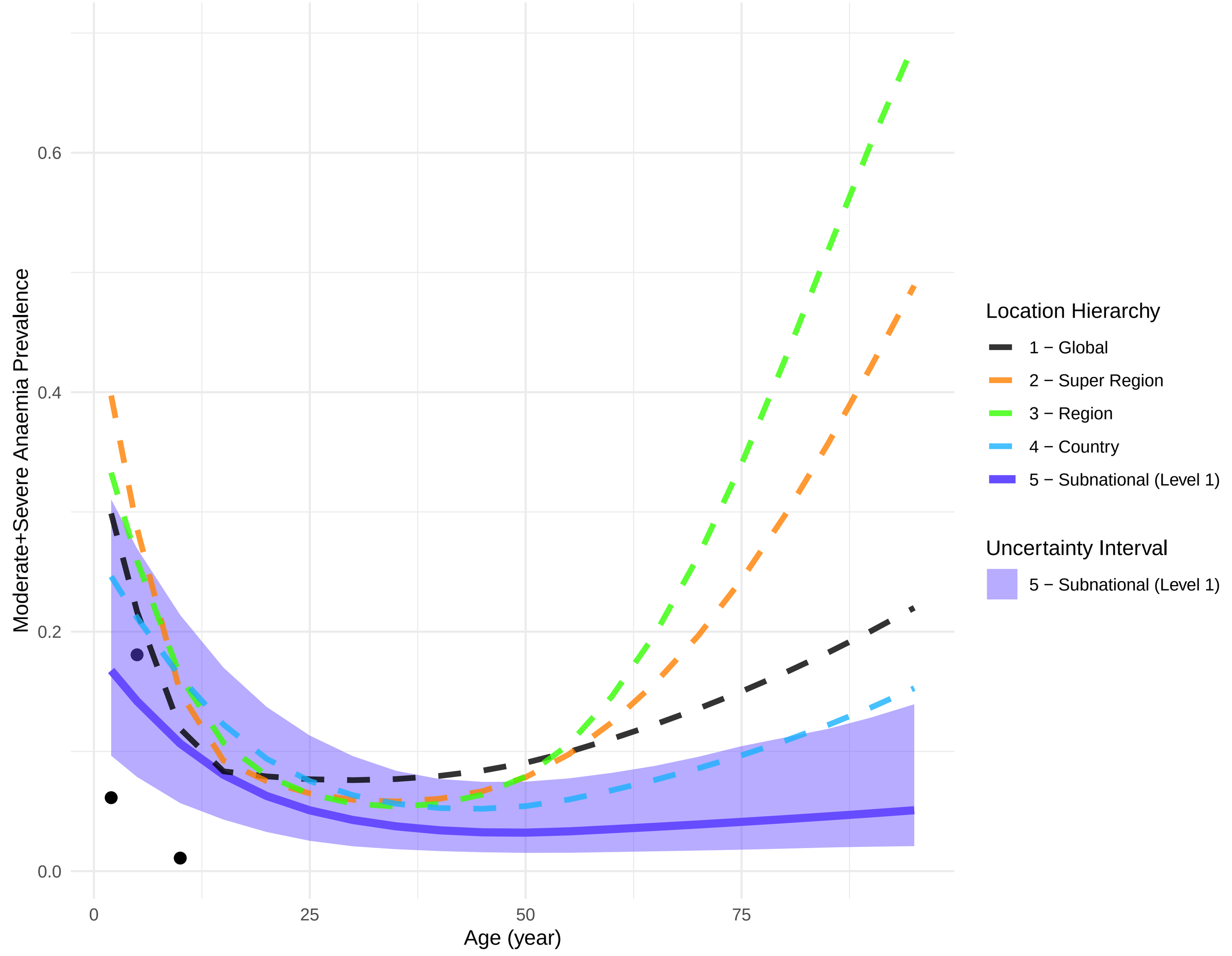

MR−BRT Age/Sex Fits − Males 2+ Years Old − Moderate+Severe Anaemia Prevalence − Samburu
WHO−2024 Adjustment Method

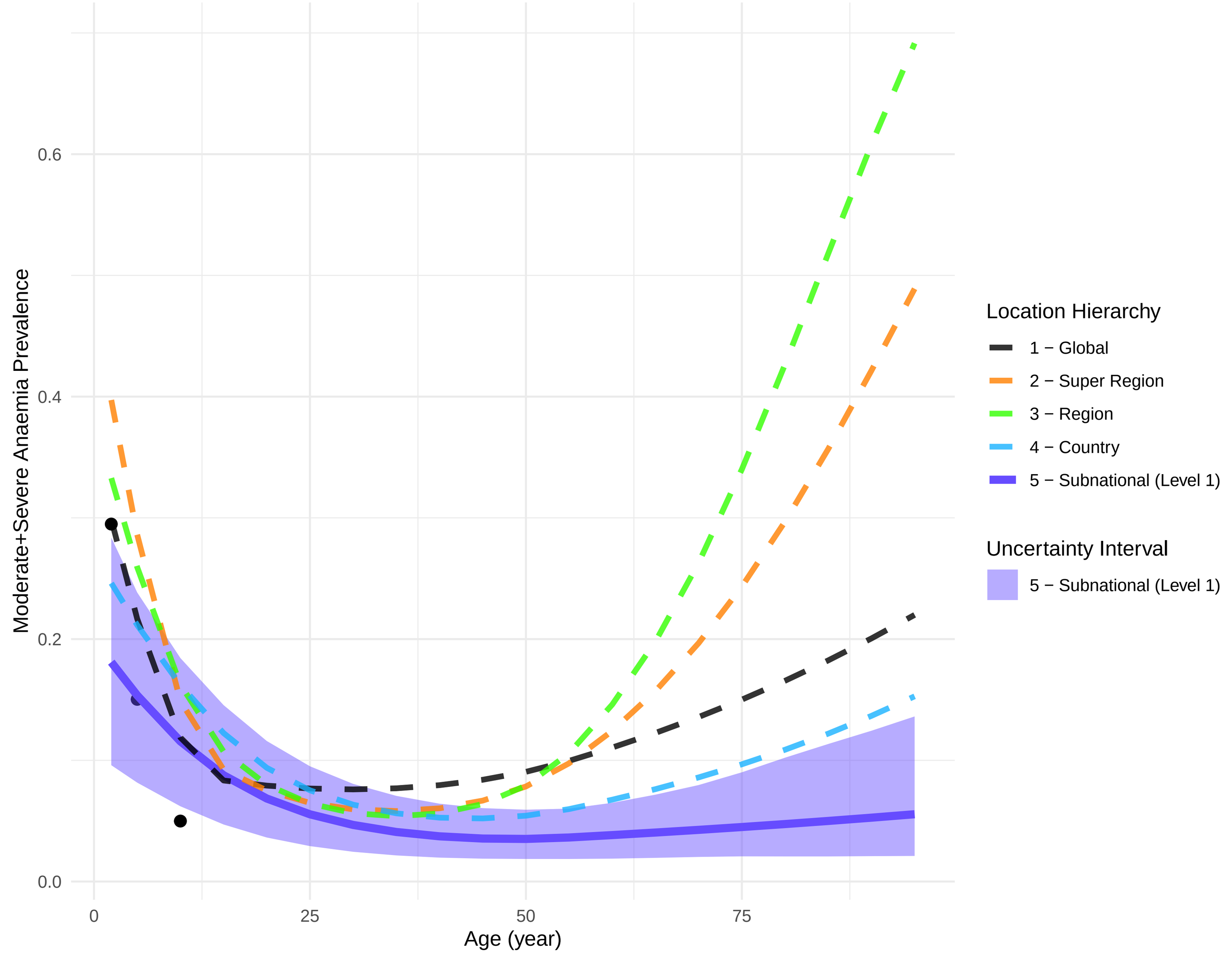

MR−BRT Age/Sex Fits − Males 2+ Years Old − Moderate+Severe Anaemia Prevalence − Siaya
WHO−2024 Adjustment Method

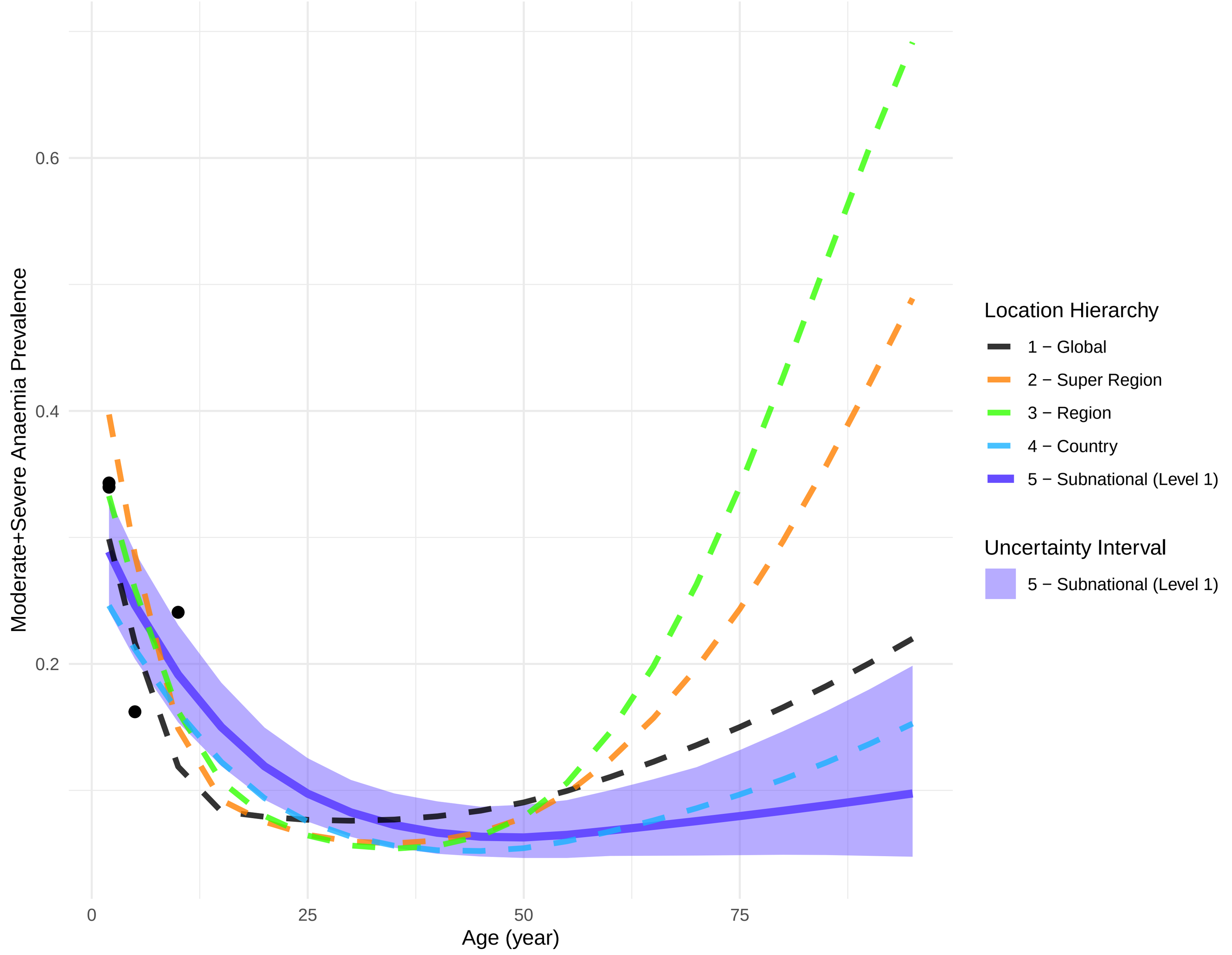

MR−BRT Age/Sex Fits − Males 2+ Years Old − Moderate+Severe Anaemia Prevalence − Taita Taveta
WHO−2024 Adjustment Method

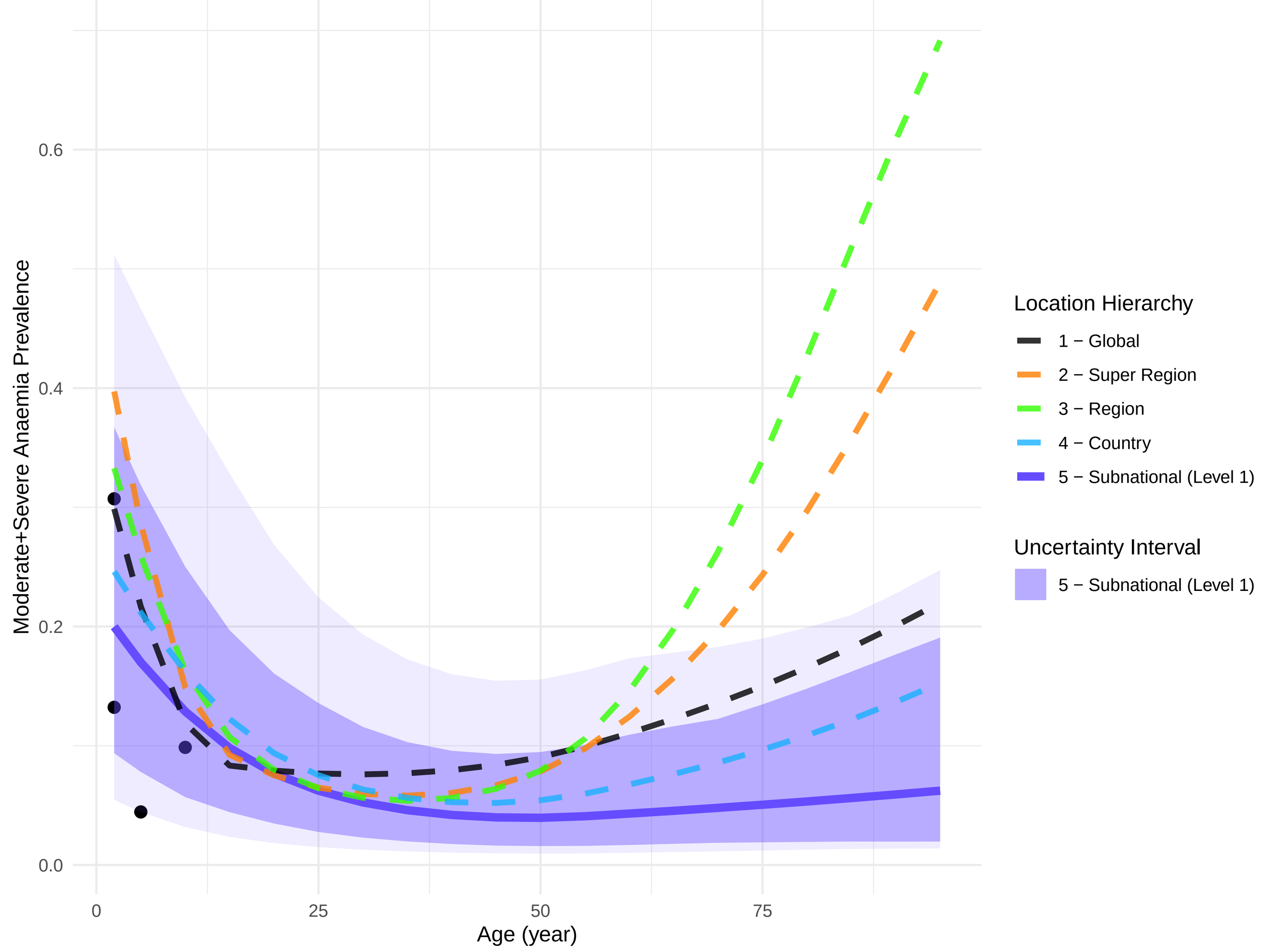

MR−BRT Age/Sex Fits − Males 2+ Years Old − Moderate+Severe Anaemia Prevalence − Tana River
WHO−2024 Adjustment Method

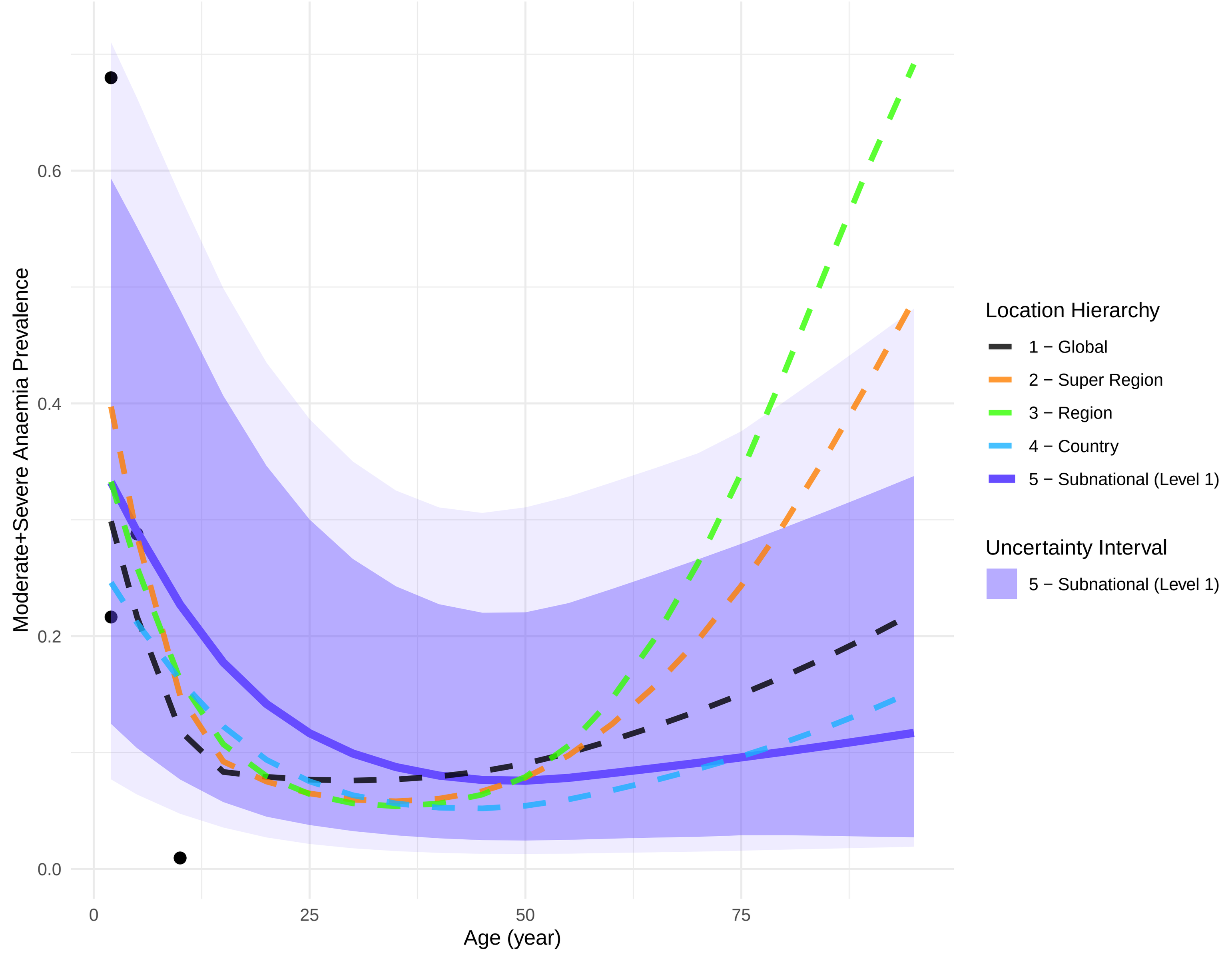

MR−BRT Age/Sex Fits − Males 2+ Years Old − Moderate+Severe Anaemia Prevalence − Tharaka Nithi
WHO−2024 Adjustment Method

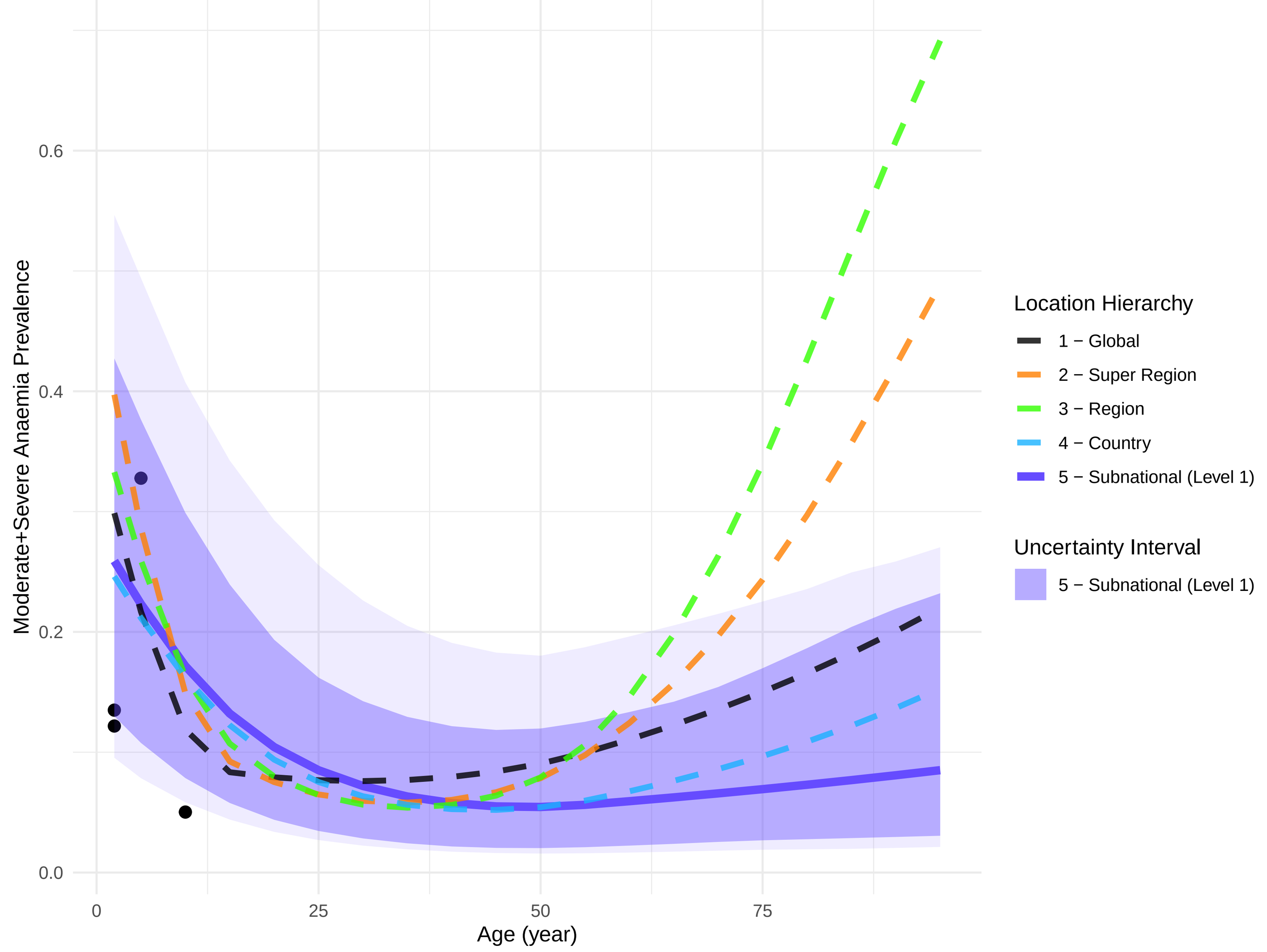

MR−BRT Age/Sex Fits − Males 2+ Years Old − Moderate+Severe Anaemia Prevalence − Trans Nzoia
WHO−2024 Adjustment Method

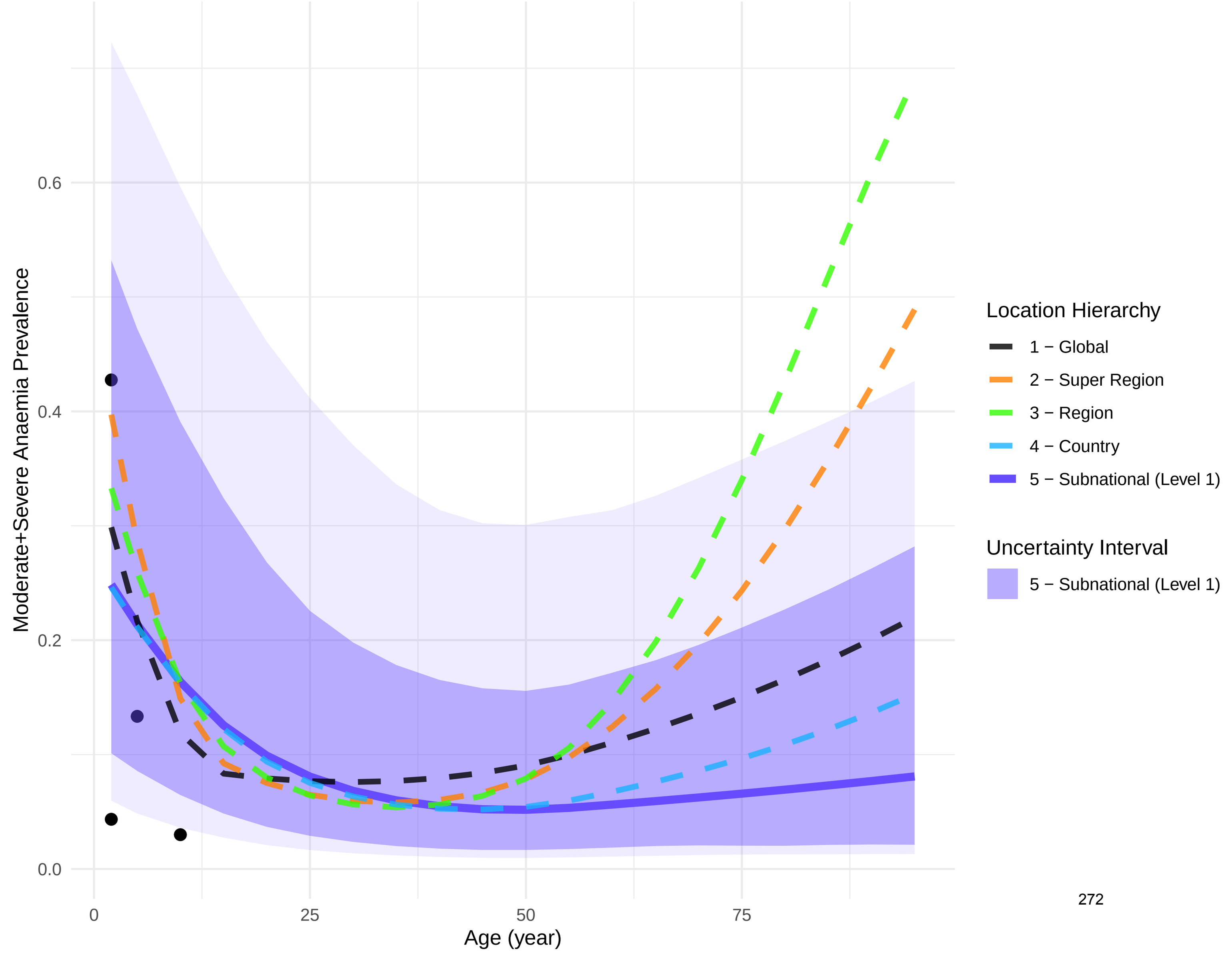

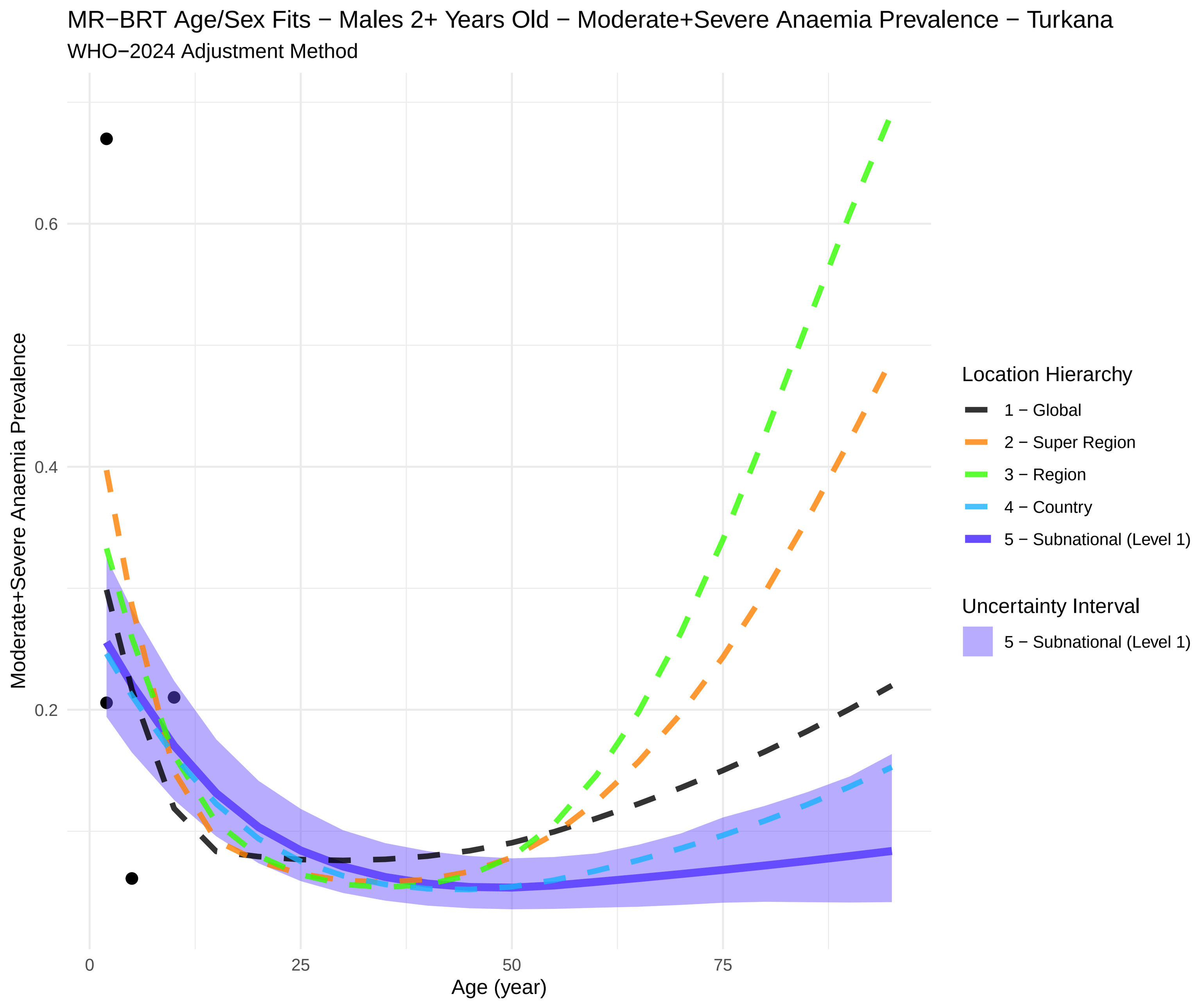
MR−BRT Age/Sex Fits − Males 2+ Years Old − Moderate+Severe Anaemia Prevalence − Turkana
WHO−2024 Adjustment Method
Moderate+Severe Anaemia Prevalence
Age (year)
Location Hierarchy
1 − Global
2 − Super Region
3 − Region
4 − Country
5 − Subnational (Level 1)
Uncertainty Interval
5 − Subnational (Level 1)

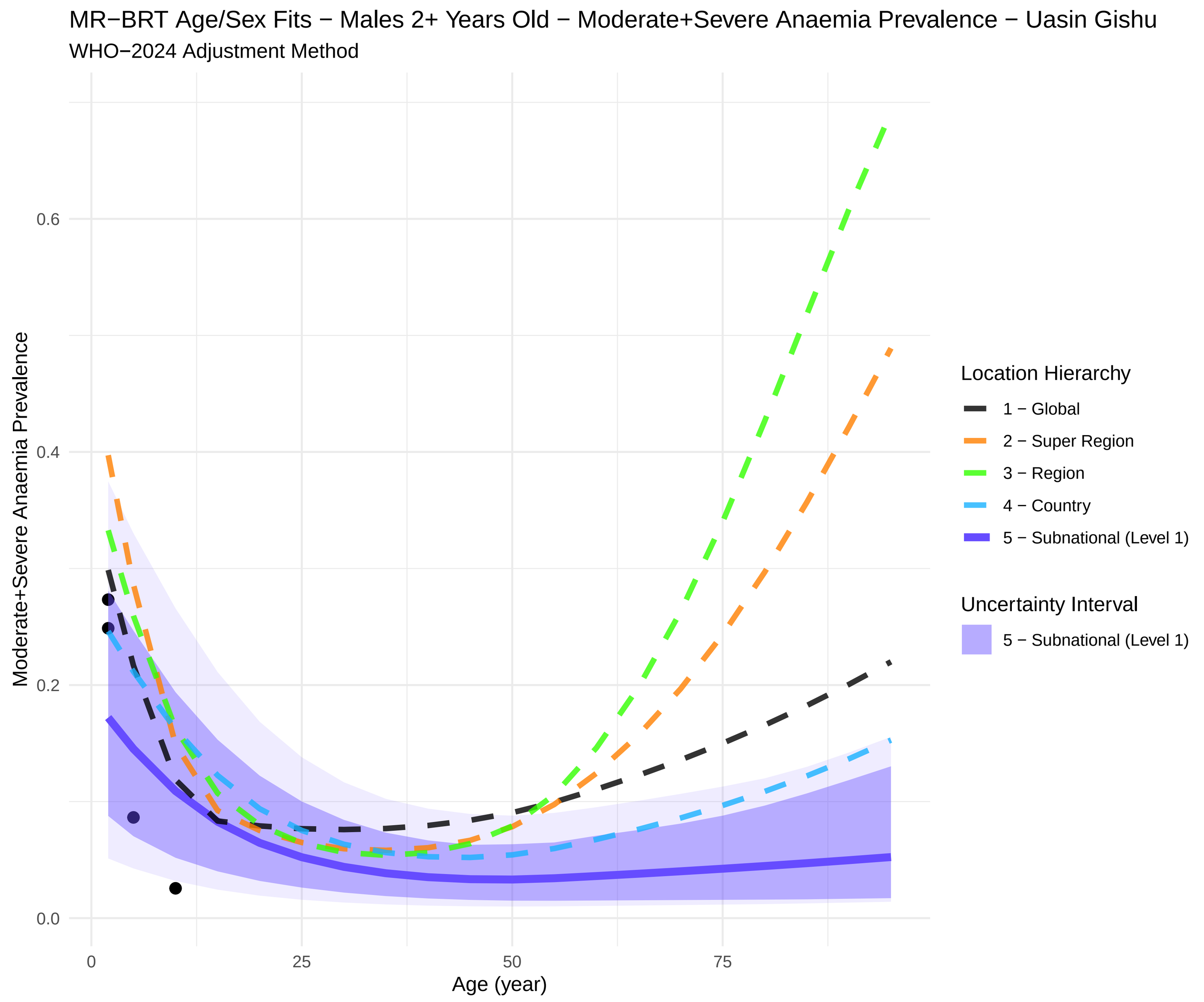
MR−BRT Age/Sex Fits − Males 2+ Years Old − Moderate+Severe Anaemia Prevalence − Uasin Gishu
WHO−2024 Adjustment Method
Moderate+Severe Anaemia Prevalence
Age (year)
Location Hierarchy
1 − Global
2 − Super Region
3 − Region
4 − Country
5 − Subnational (Level 1)
Uncertainty Interval
5 − Subnational (Level 1)

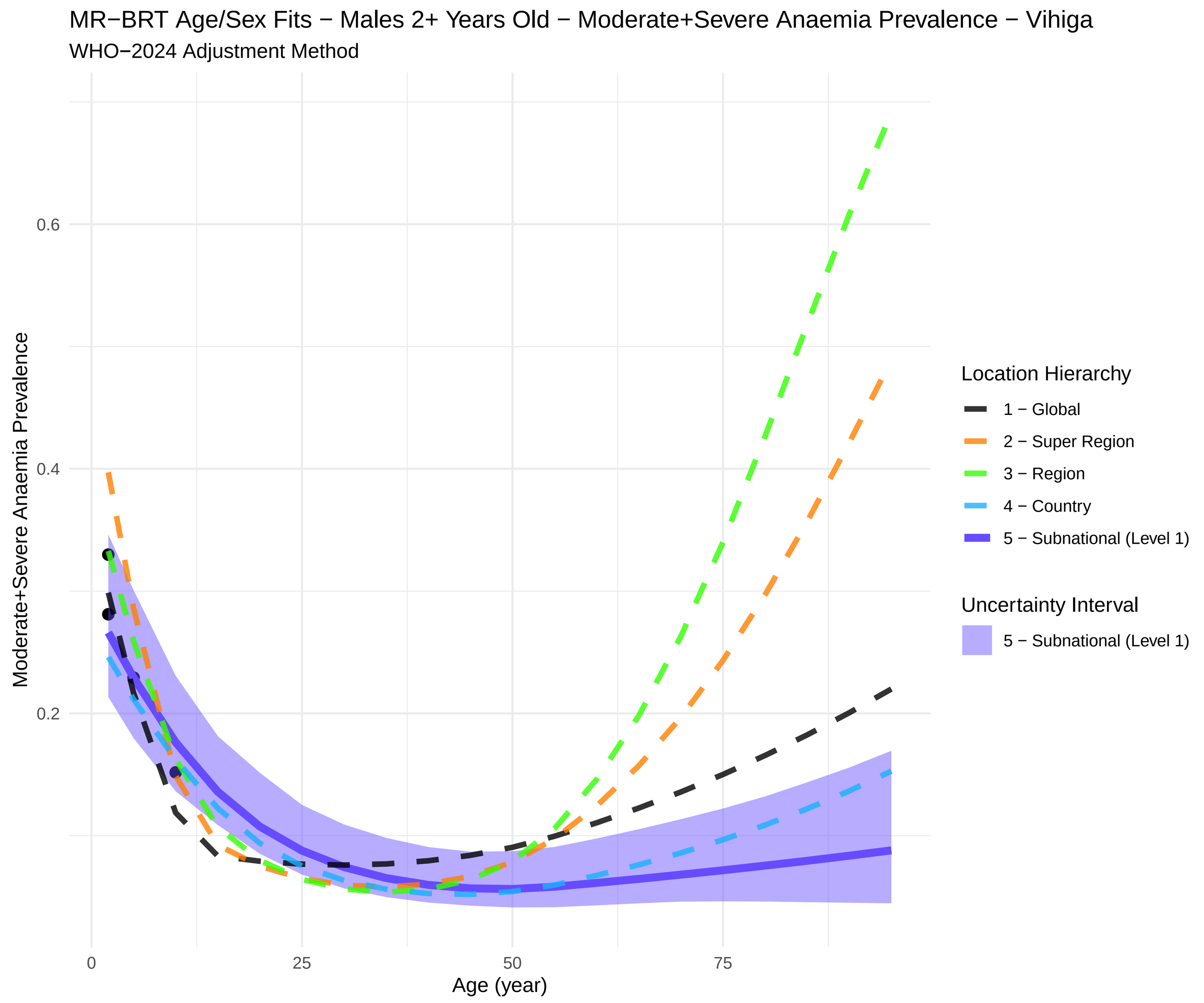
MR−BRT Age/Sex Fits − Males 2+ Years Old − Moderate+Severe Anaemia Prevalence − Vihiga
WHO−2024 Adjustment Method
Moderate+Severe Anaemia Prevalence
Age (year)
Location Hierarchy
1 − Global
2 − Super Region
3 − Region
4 − Country
5 − Subnational (Level 1)
Uncertainty Interval
5 − Subnational (Level 1)

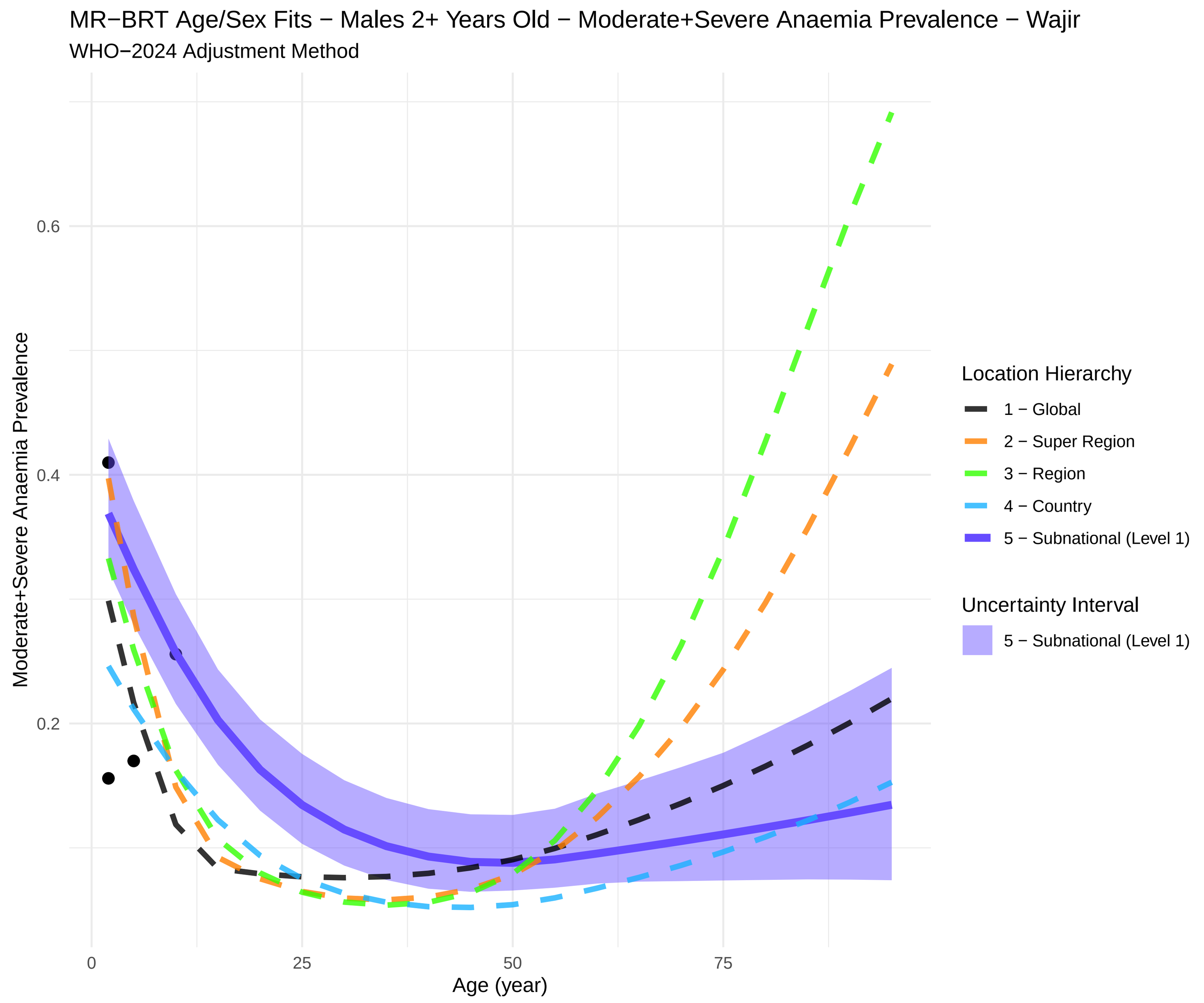
MR−BRT Age/Sex Fits − Males 2+ Years Old − Moderate+Severe Anaemia Prevalence − Wajir
WHO−2024 Adjustment Method
Moderate+Severe Anaemia Prevalence
Age (year)
Location Hierarchy
1 − Global
2 − Super Region
3 − Region
4 − Country
5 − Subnational (Level 1)
Uncertainty Interval
5 − Subnational (Level 1)

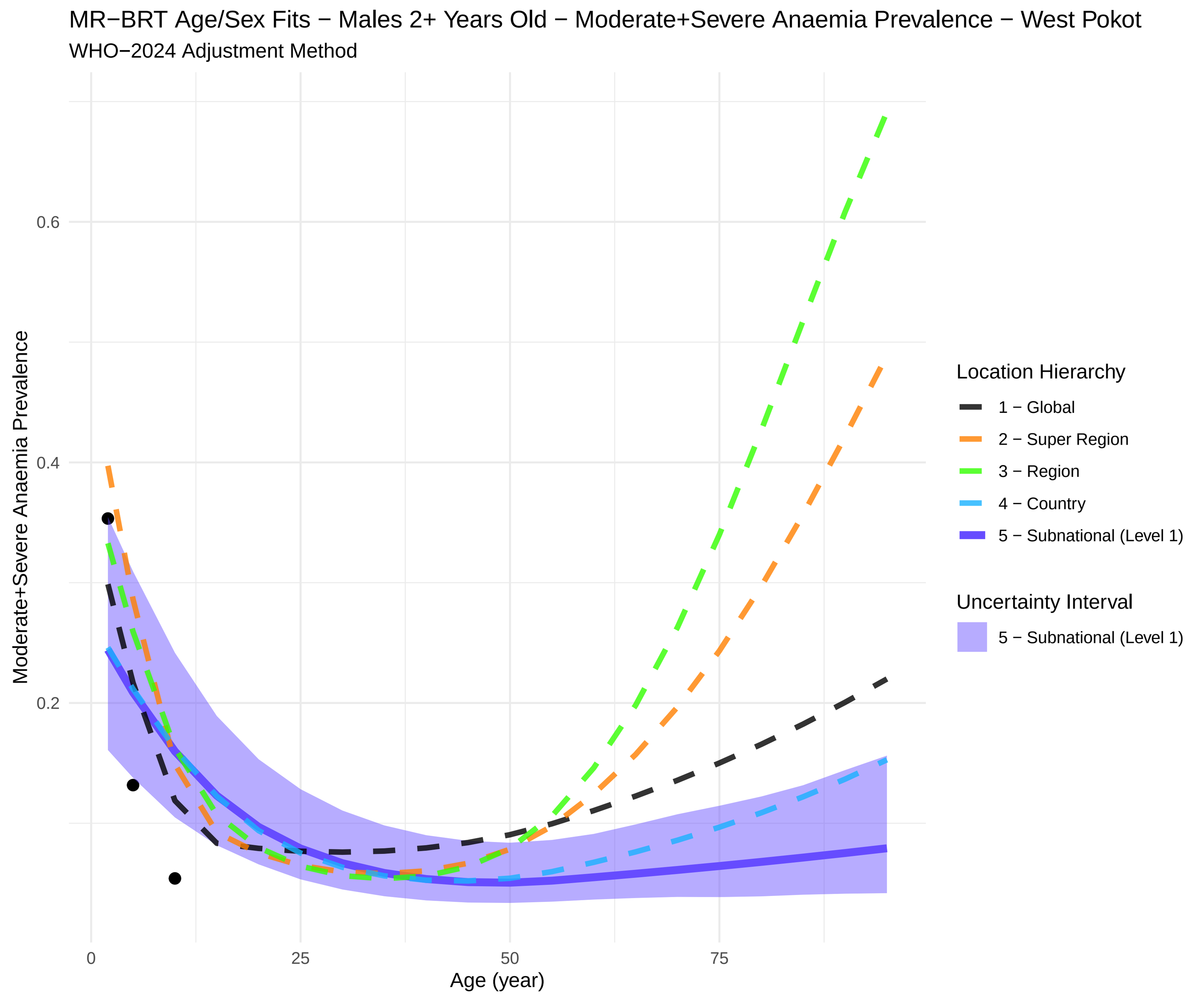
MR−BRT Age/Sex Fits − Males 2+ Years Old − Moderate+Severe Anaemia Prevalence − West Pokot
WHO−2024 Adjustment Method
Moderate+Severe Anaemia Prevalence
Age (year)
Location Hierarchy
1 − Global
2 − Super Region
3 − Region
4 − Country
5 − Subnational (Level 1)
Uncertainty Interval
5 − Subnational (Level 1)

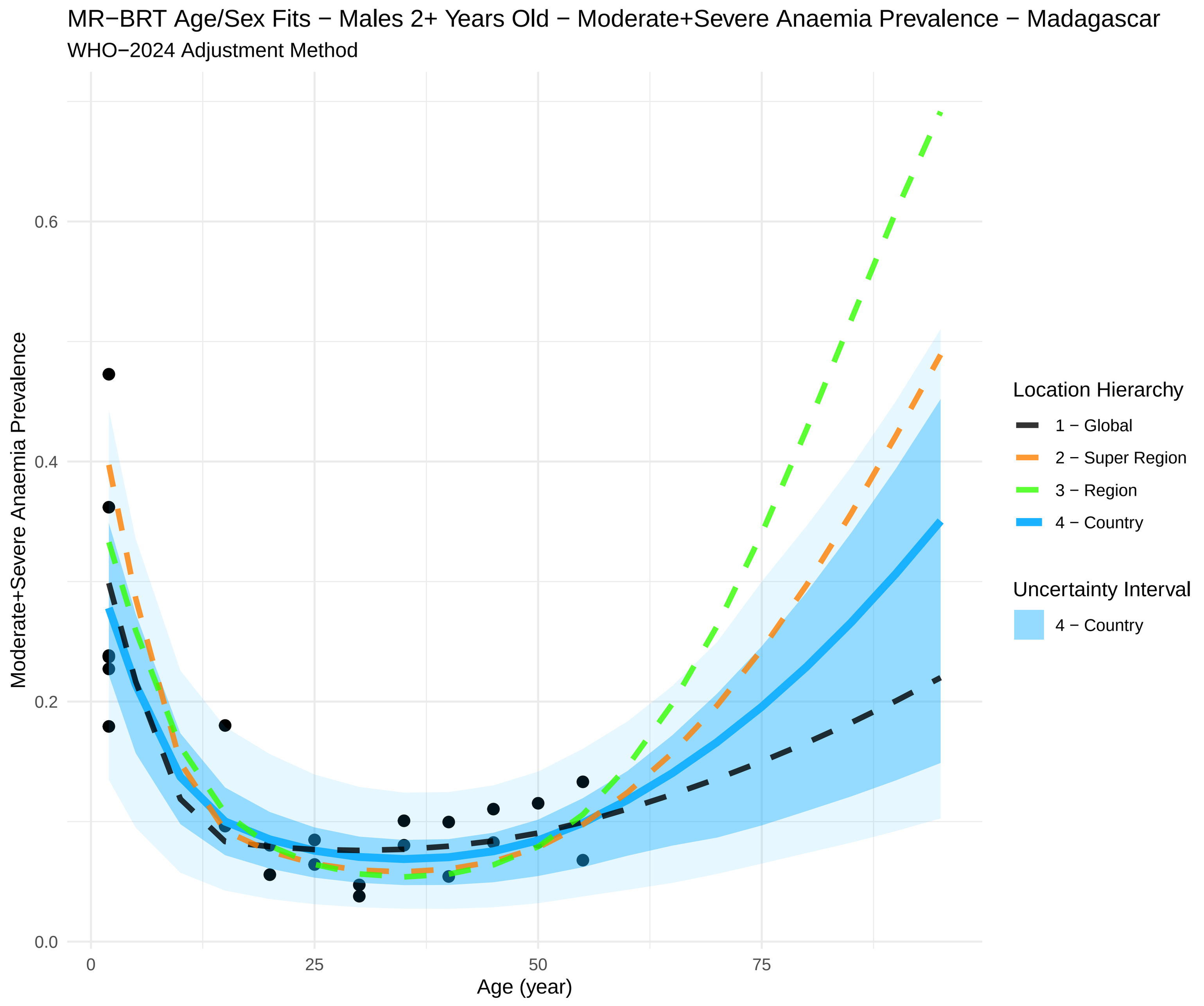
MR−BRT Age/Sex Fits − Males 2+ Years Old − Moderate+Severe Anaemia Prevalence − Madagascar
WHO−2024 Adjustment Method
Moderate+Severe Anaemia Prevalence
Age (year)
Location Hierarchy
1 − Global
2 − Super Region
3 − Region
4 − Country
Uncertainty Interval
4 − Country

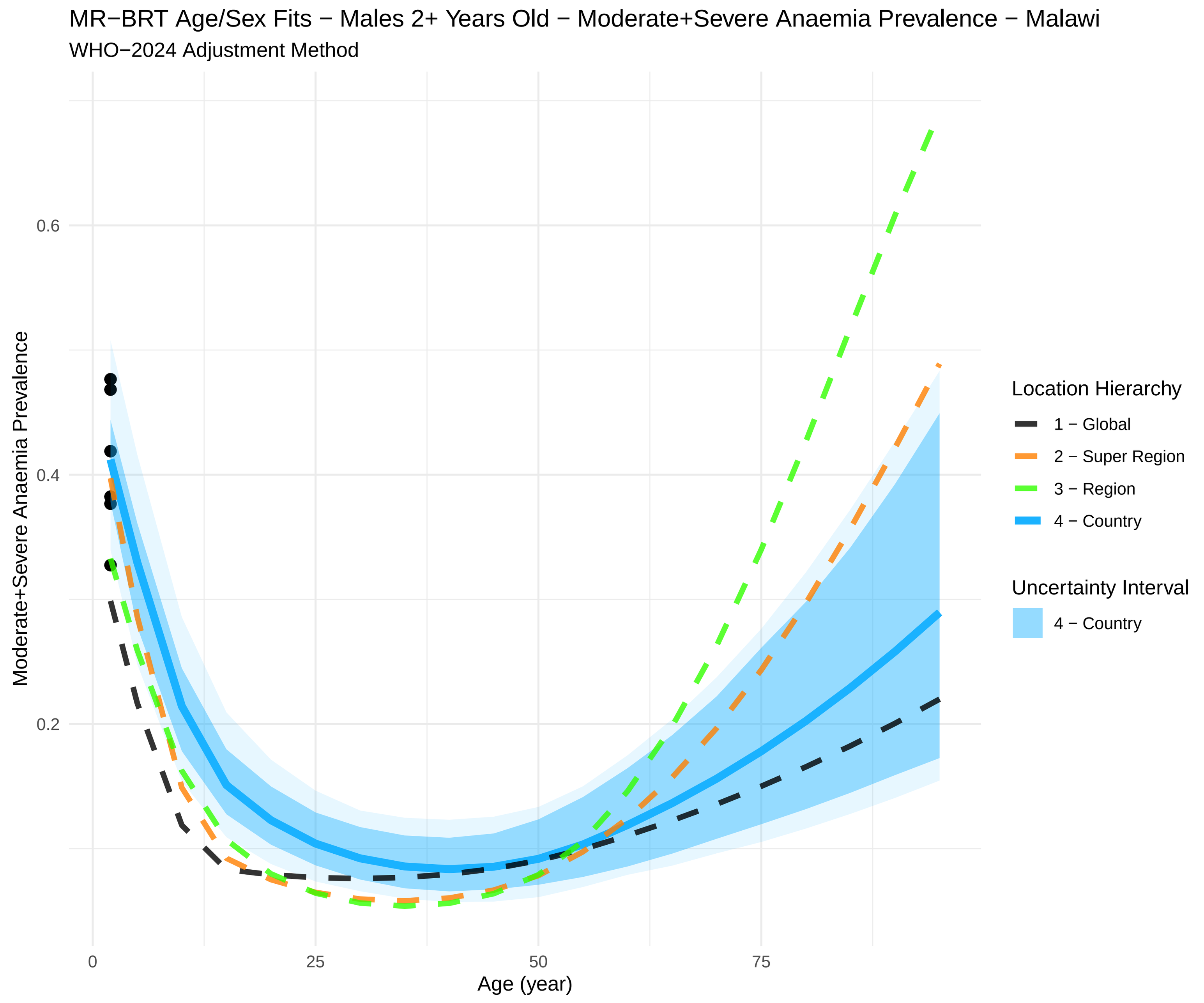
MR−BRT Age/Sex Fits − Males 2+ Years Old − Moderate+Severe Anaemia Prevalence − Malawi
WHO−2024 Adjustment Method
Moderate+Severe Anaemia Prevalence
Age (year)
Location Hierarchy
1 − Global
2 − Super Region
3 − Region
4 − Country
Uncertainty Interval
4 − Country

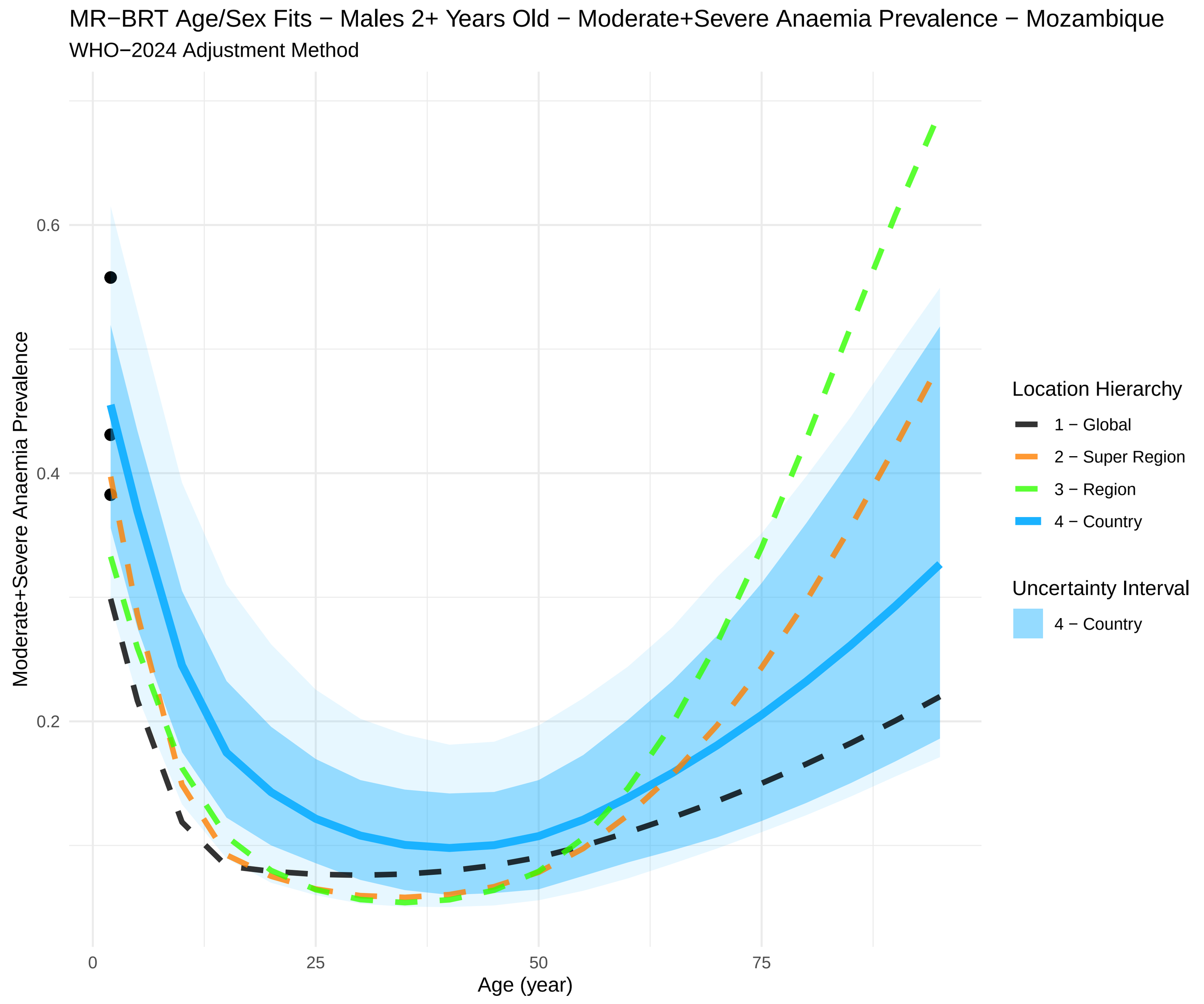
MR−BRT Age/Sex Fits − Males 2+ Years Old − Moderate+Severe Anaemia Prevalence − Mozambique
WHO−2024 Adjustment Method
Moderate+Severe Anaemia Prevalence
Age (year)
Location Hierarchy
1 − Global
2 − Super Region
3 − Region
4 − Country
Uncertainty Interval
4 − Country

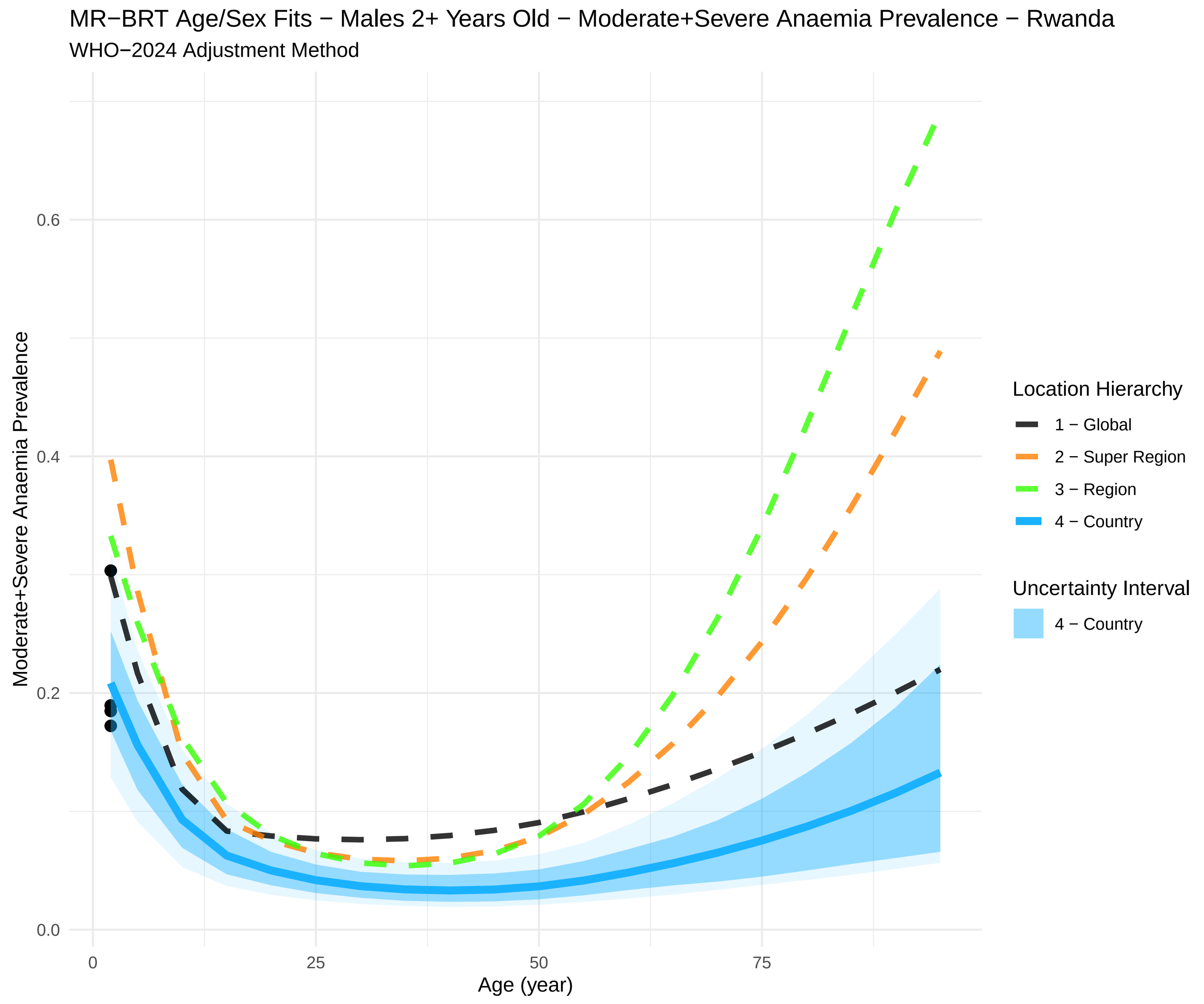
MR−BRT Age/Sex Fits − Males 2+ Years Old − Moderate+Severe Anaemia Prevalence − Rwanda
WHO−2024 Adjustment Method
Moderate+Severe Anaemia Prevalence
Age (year)
Location Hierarchy
1 − Global
2 − Super Region
3 − Region
4 − Country
Uncertainty Interval
4 − Country

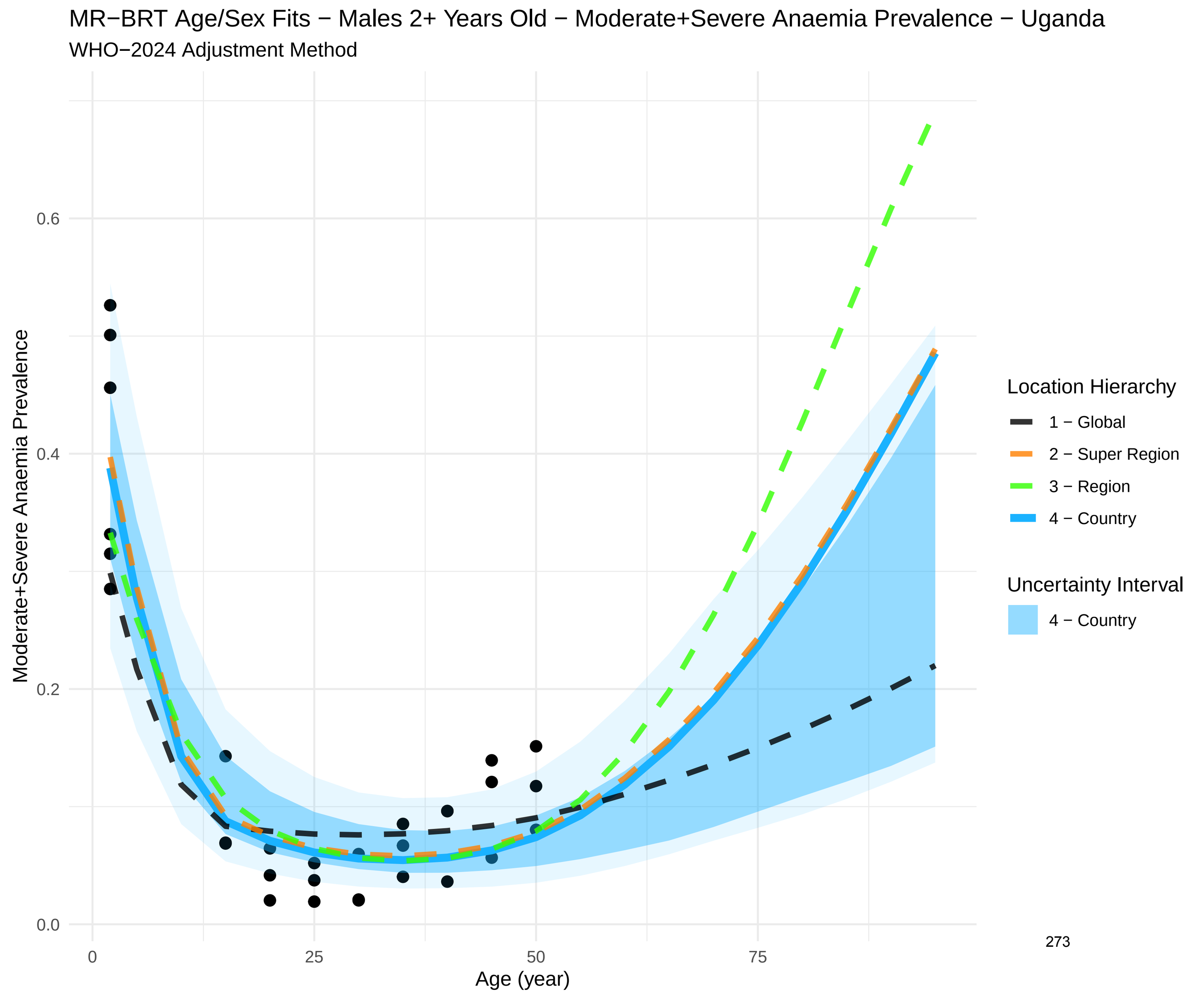
MR−BRT Age/Sex Fits − Males 2+ Years Old − Moderate+Severe Anaemia Prevalence − Uganda
WHO−2024 Adjustment Method
Moderate+Severe Anaemia Prevalence
Age (year)
Location Hierarchy
1 − Global
2 − Super Region
3 − Region
4 − Country
Uncertainty Interval
4 − Country

MR−BRT Age/Sex Fits − Males 2+ Years Old − Moderate+Severe Anaemia Prevalence − United Republic of Tanzania
WHO−2024 Adjustment Method

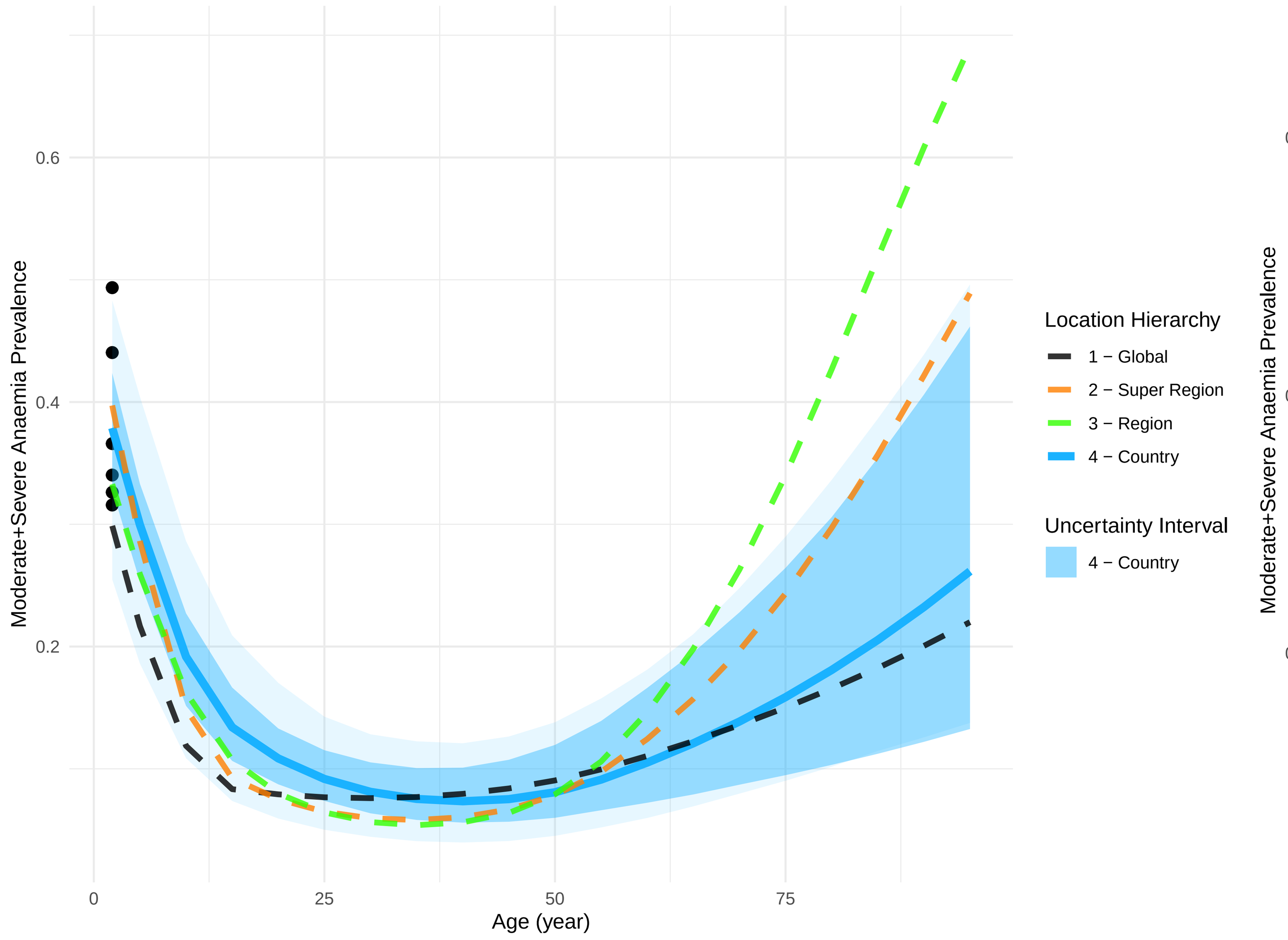

MR−BRT Age/Sex Fits − Males 2+ Years Old − Moderate+Severe Anaemia Prevalence − Zambia
WHO−2024 Adjustment Method

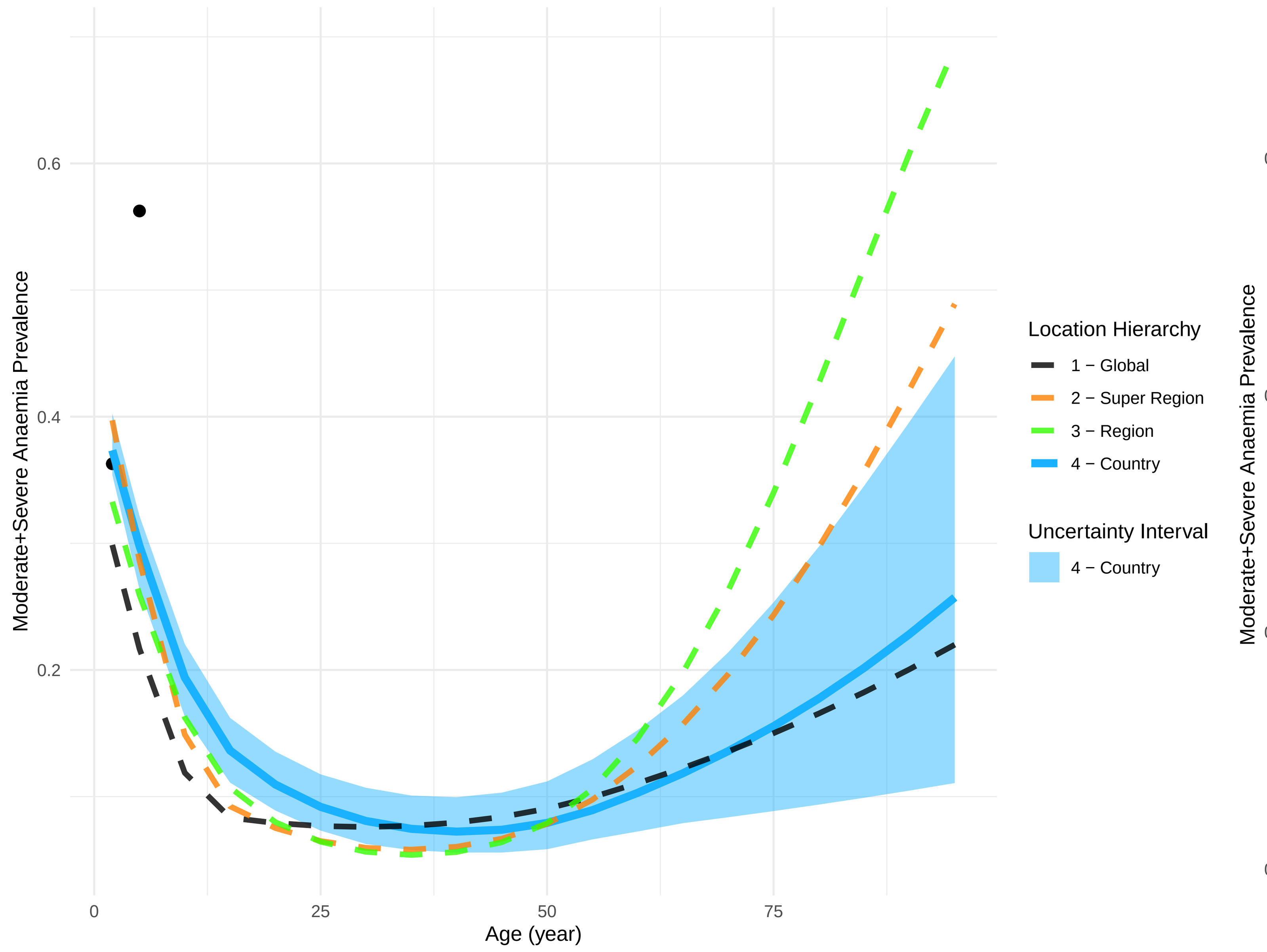

MR−BRT Age/Sex Fits − Males 2+ Years Old − Moderate+Severe Anaemia Prevalence − Southern Sub−Saharan Africa
WHO−2024 Adjustment Method

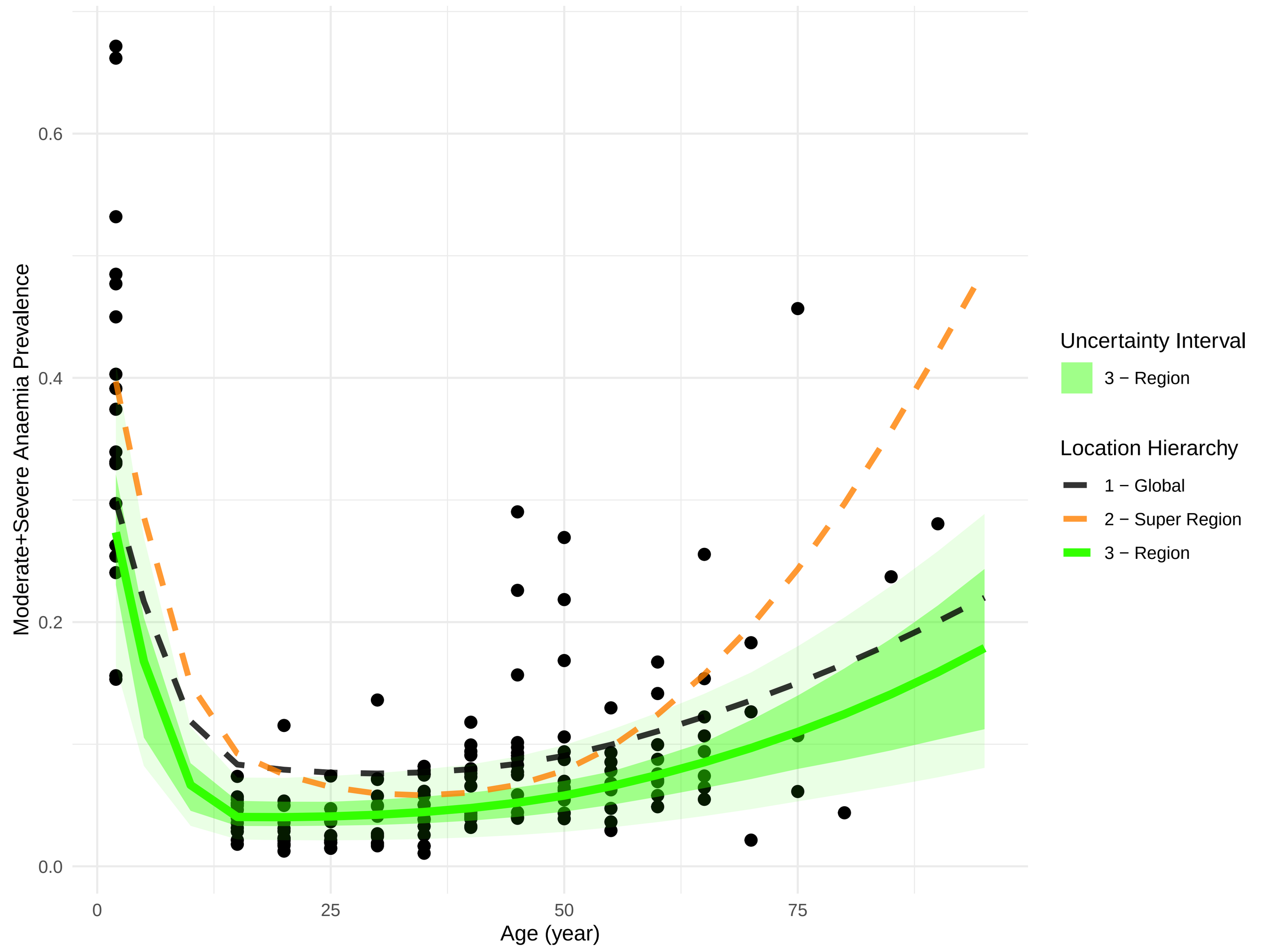

MR−BRT Age/Sex Fits − Males 2+ Years Old − Moderate+Severe Anaemia Prevalence − Eswatini
WHO−2024 Adjustment Method

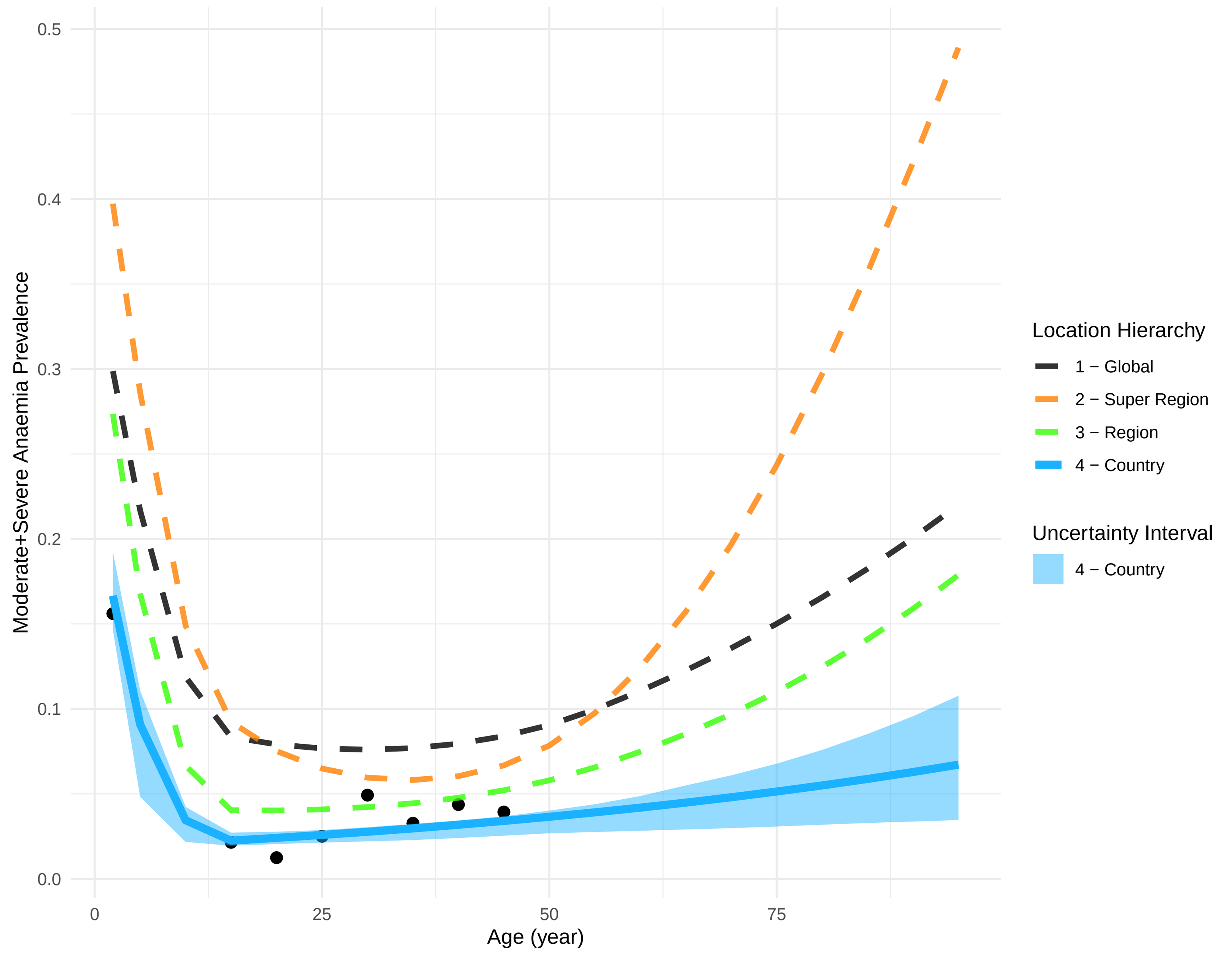

MR−BRT Age/Sex Fits − Males 2+ Years Old − Moderate+Severe Anaemia Prevalence − Lesotho
WHO−2024 Adjustment Method

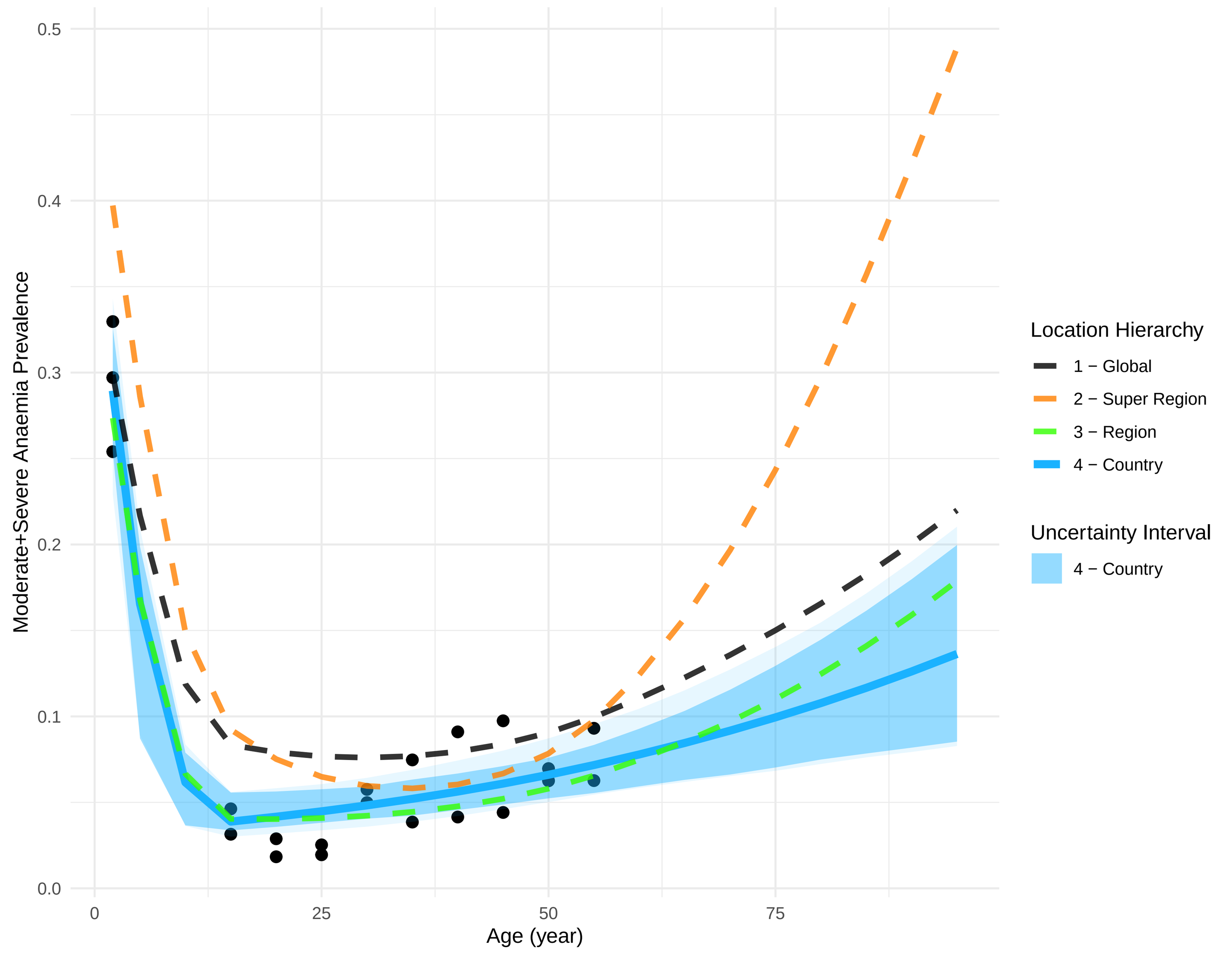

MR−BRT Age/Sex Fits − Males 2+ Years Old − Moderate+Severe Anaemia Prevalence − Namibia
WHO−2024 Adjustment Method

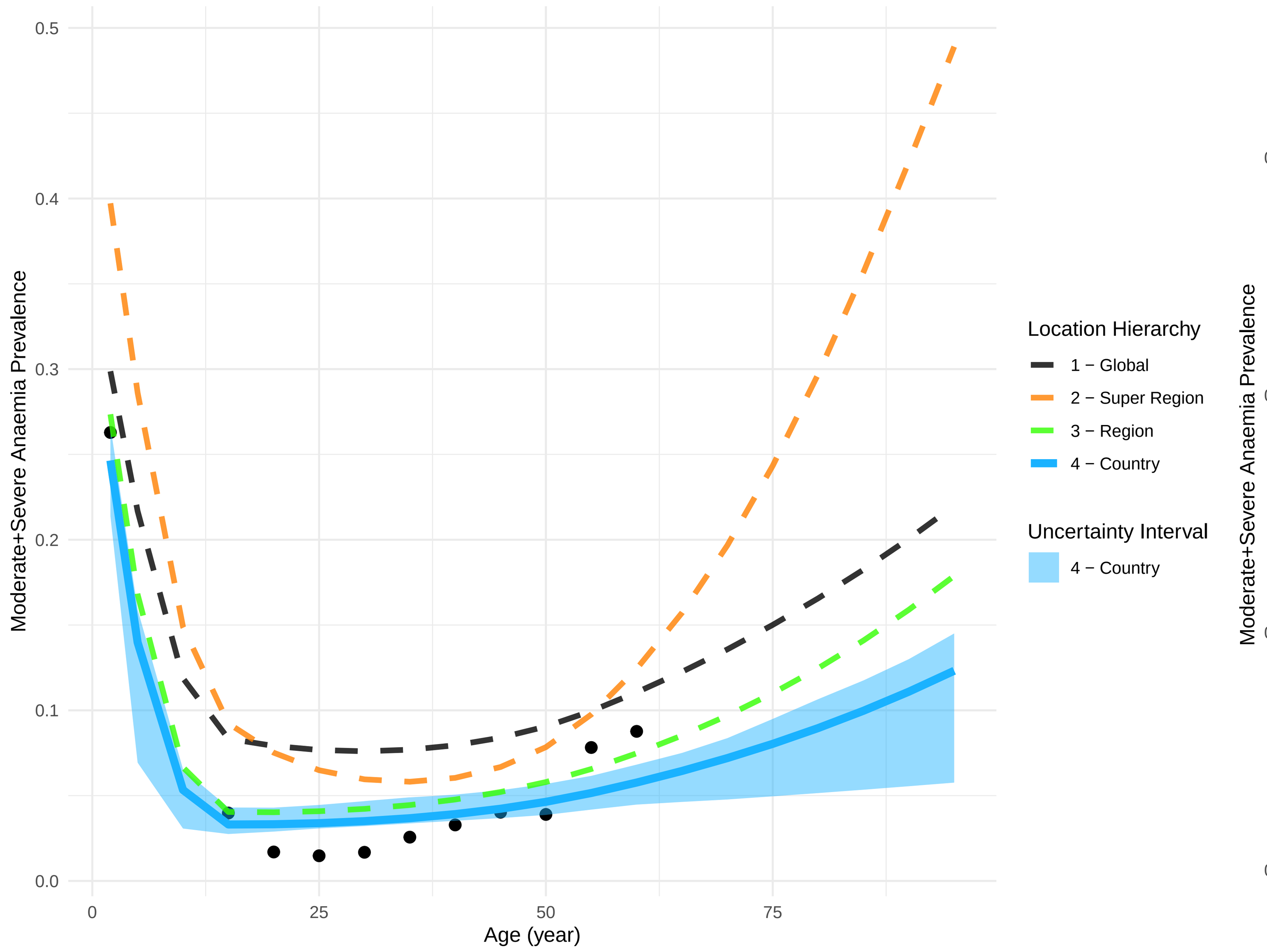

MR−BRT Age/Sex Fits − Males 2+ Years Old − Moderate+Severe Anaemia Prevalence − South Africa
WHO−2024 Adjustment Method

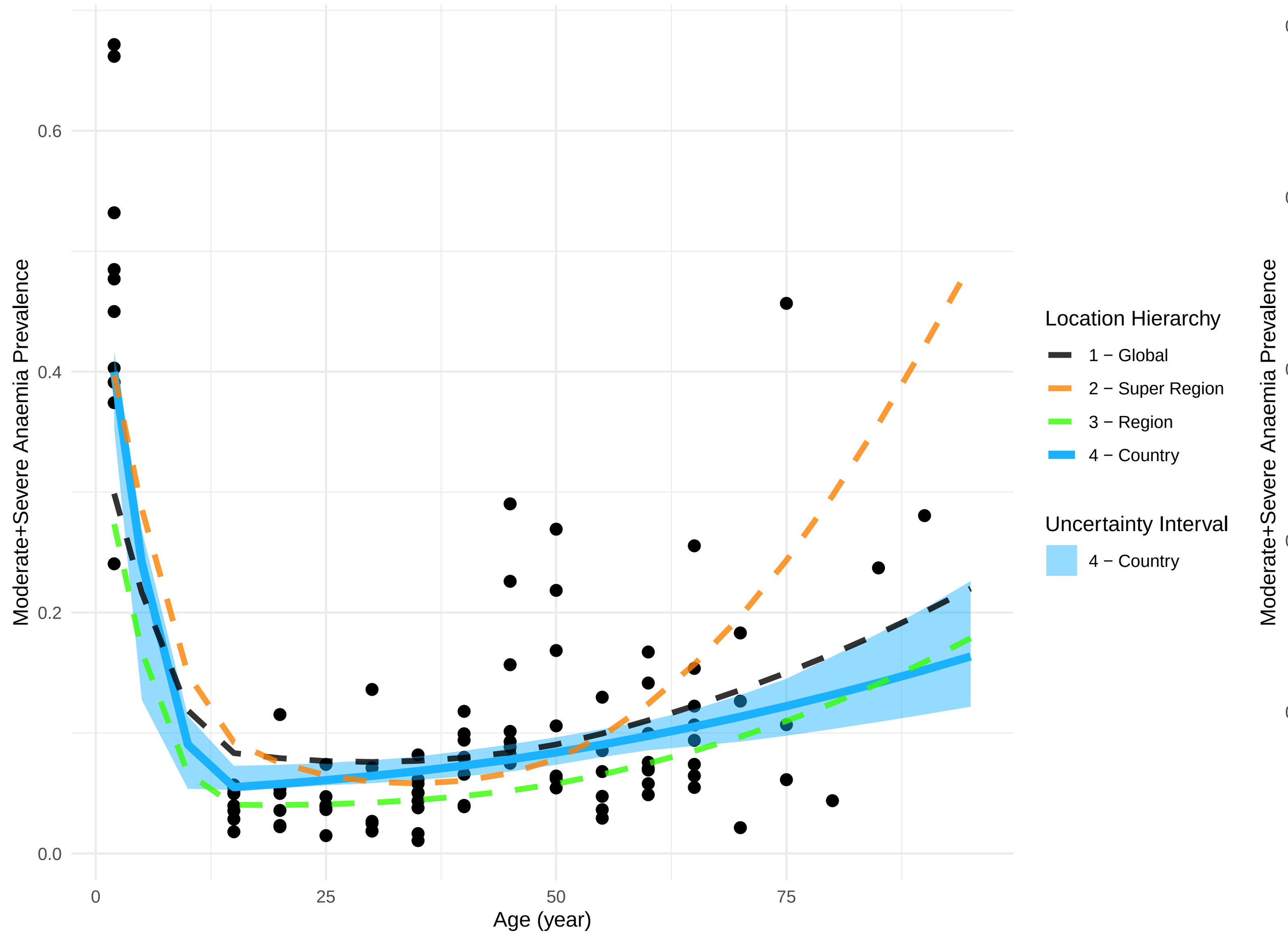

MR−BRT Age/Sex Fits − Males 2+ Years Old − Moderate+Severe Anaemia Prevalence − Eastern Cape
WHO−2024 Adjustment Method

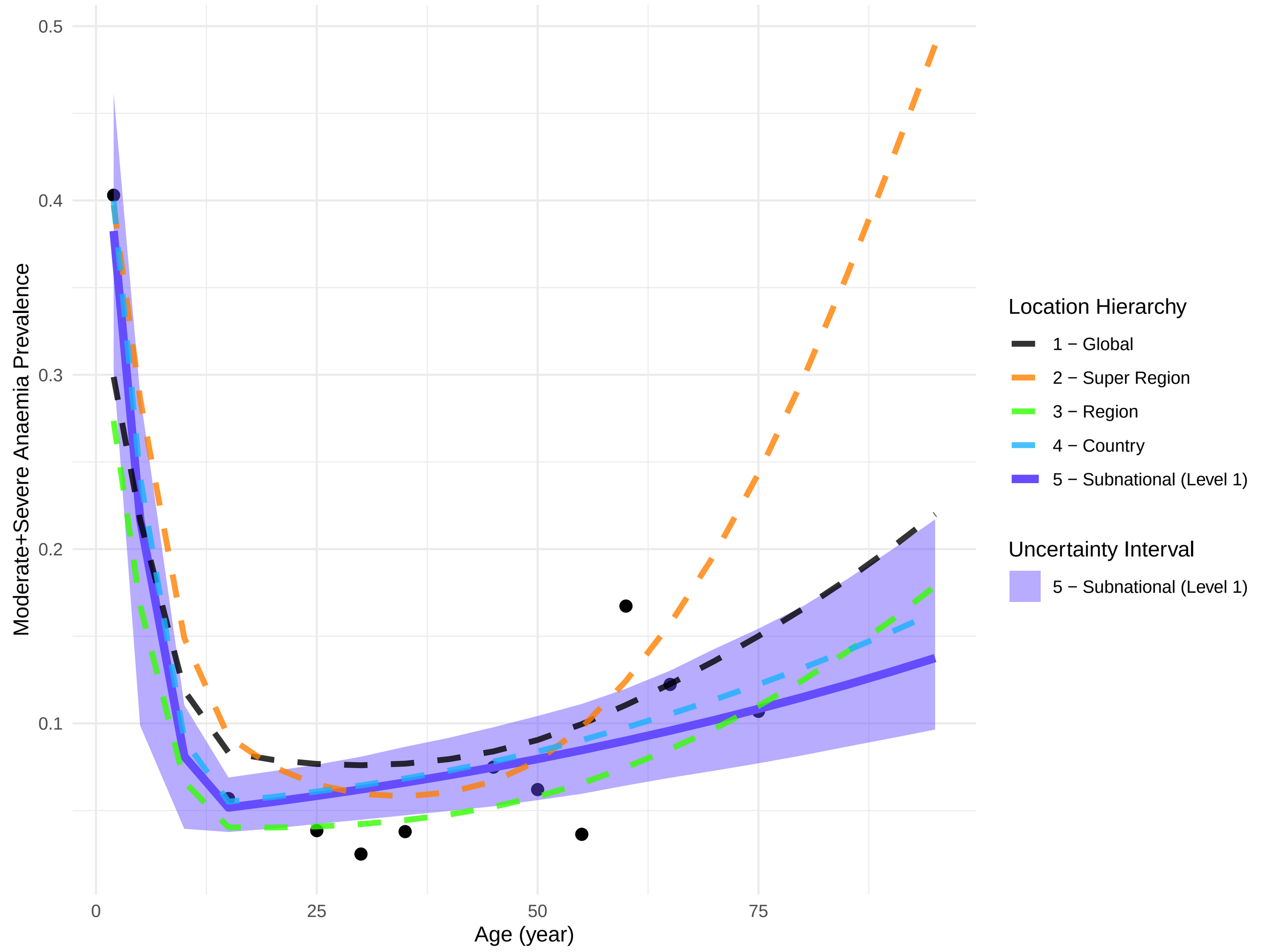

MR−BRT Age/Sex Fits − Males 2+ Years Old − Moderate+Severe Anaemia Prevalence − Free State
WHO−2024 Adjustment Method

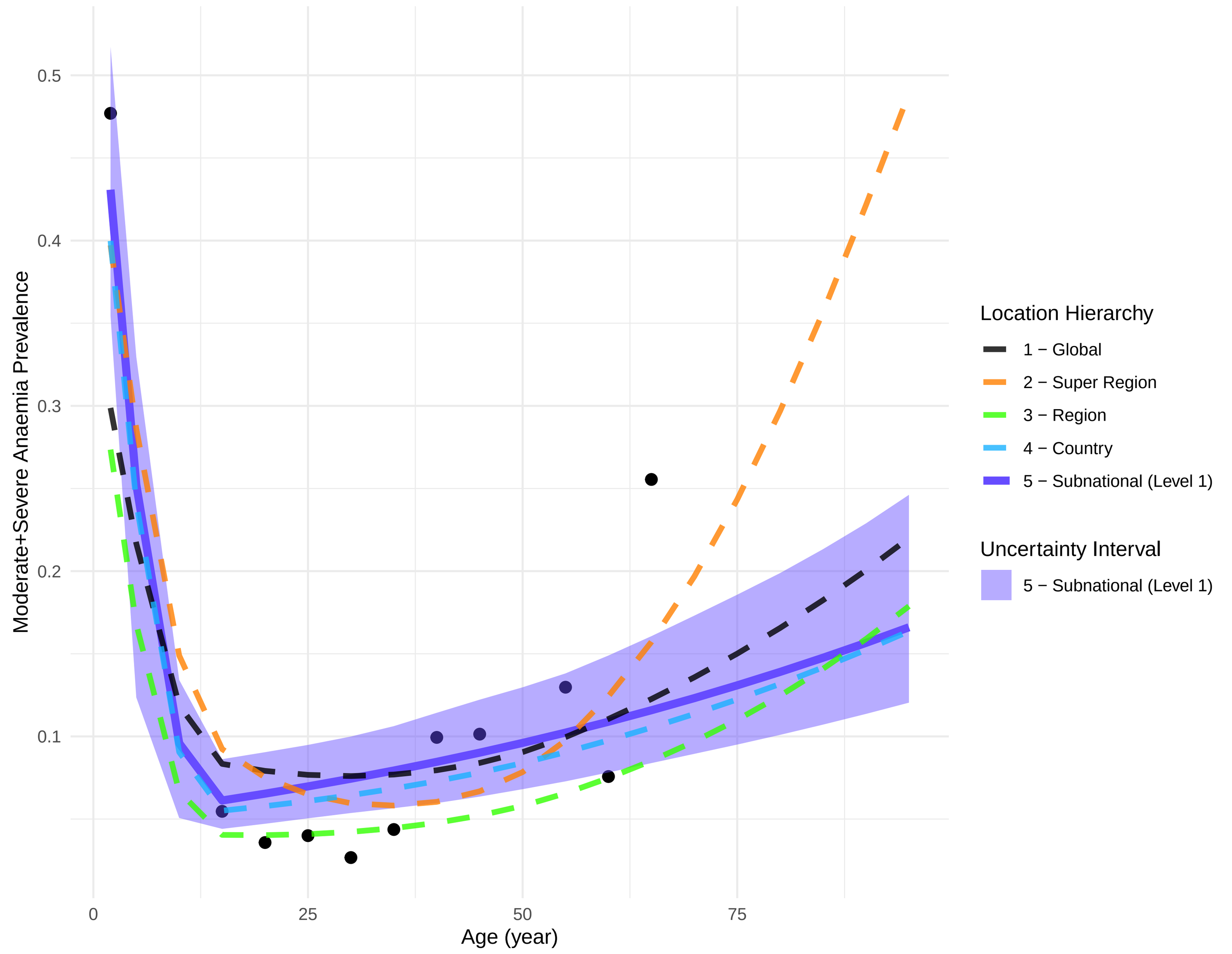

MR−BRT Age/Sex Fits − Males 2+ Years Old − Moderate+Severe Anaemia Prevalence − Gauteng
WHO−2024 Adjustment Method

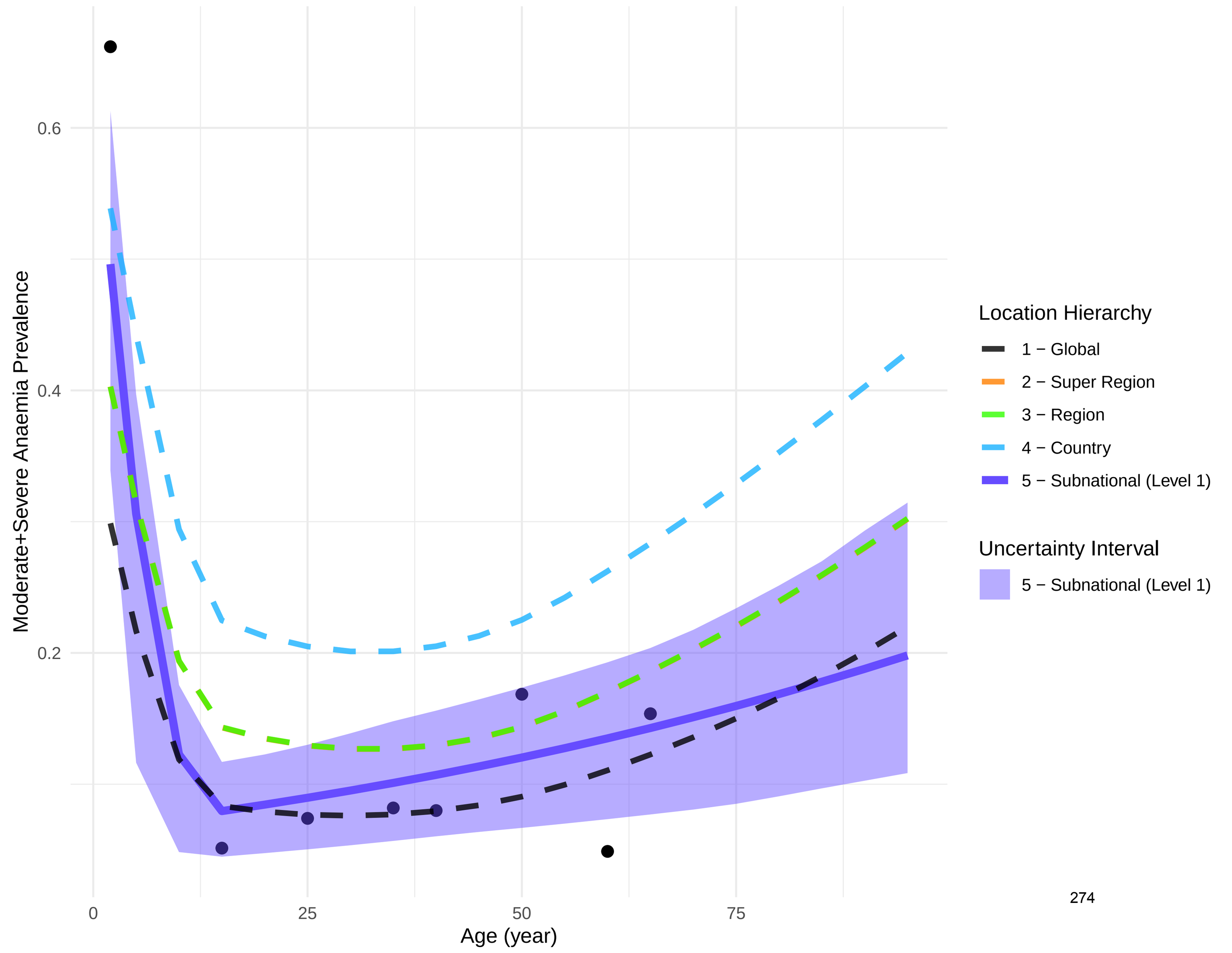

MR−BRT Age/Sex Fits − Males 2+ Years Old − Moderate+Severe Anaemia Prevalence − KwaZulu−Natal
WHO−2024 Adjustment Method

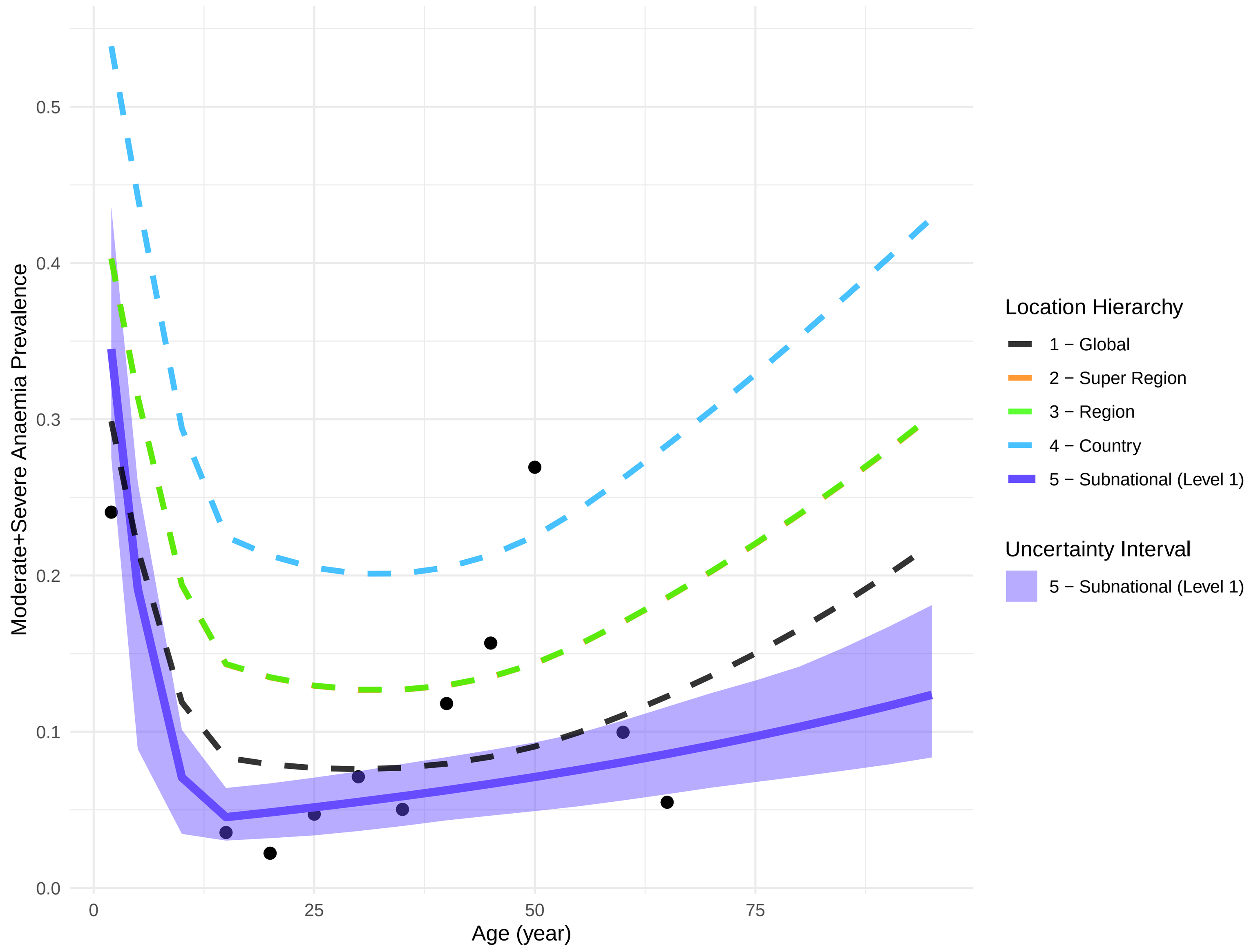

MR−BRT Age/Sex Fits − Males 2+ Years Old − Moderate+Severe Anaemia Prevalence − Limpopo
WHO−2024 Adjustment Method

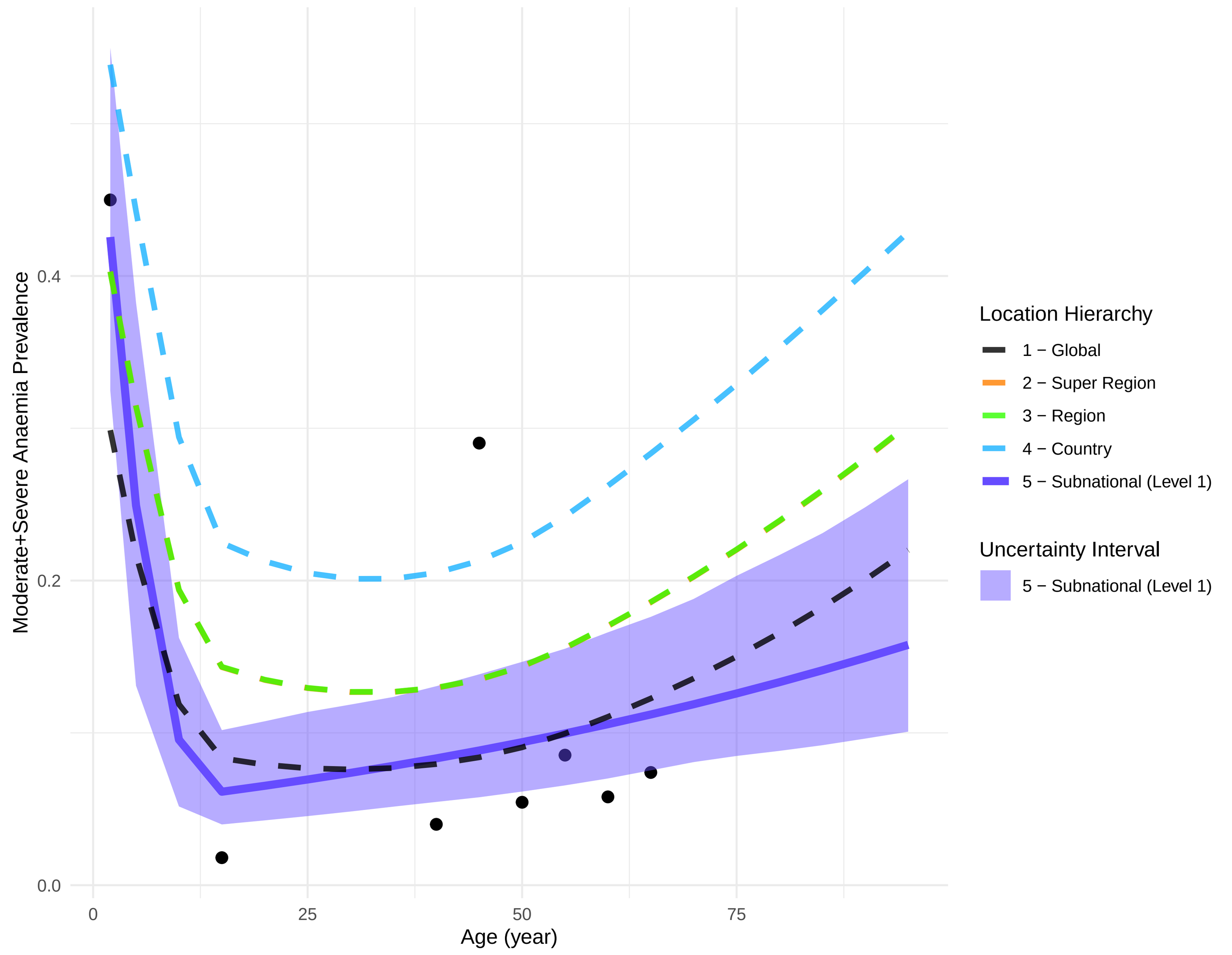

MR−BRT Age/Sex Fits − Males 2+ Years Old − Moderate+Severe Anaemia Prevalence − Mpumalanga
WHO−2024 Adjustment Method

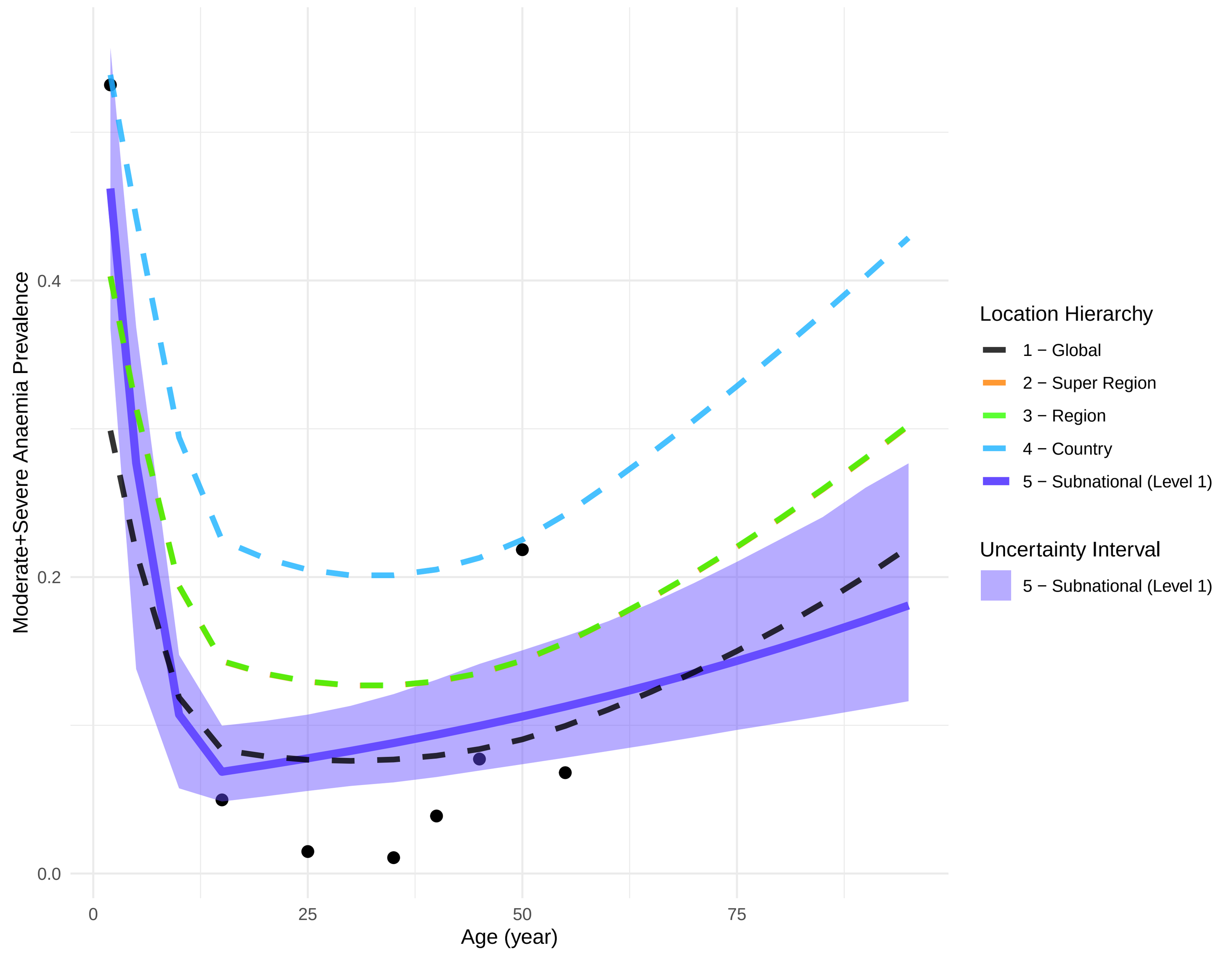

MR−BRT Age/Sex Fits − Males 2+ Years Old − Moderate+Severe Anaemia Prevalence − North−West
WHO−2024 Adjustment Method

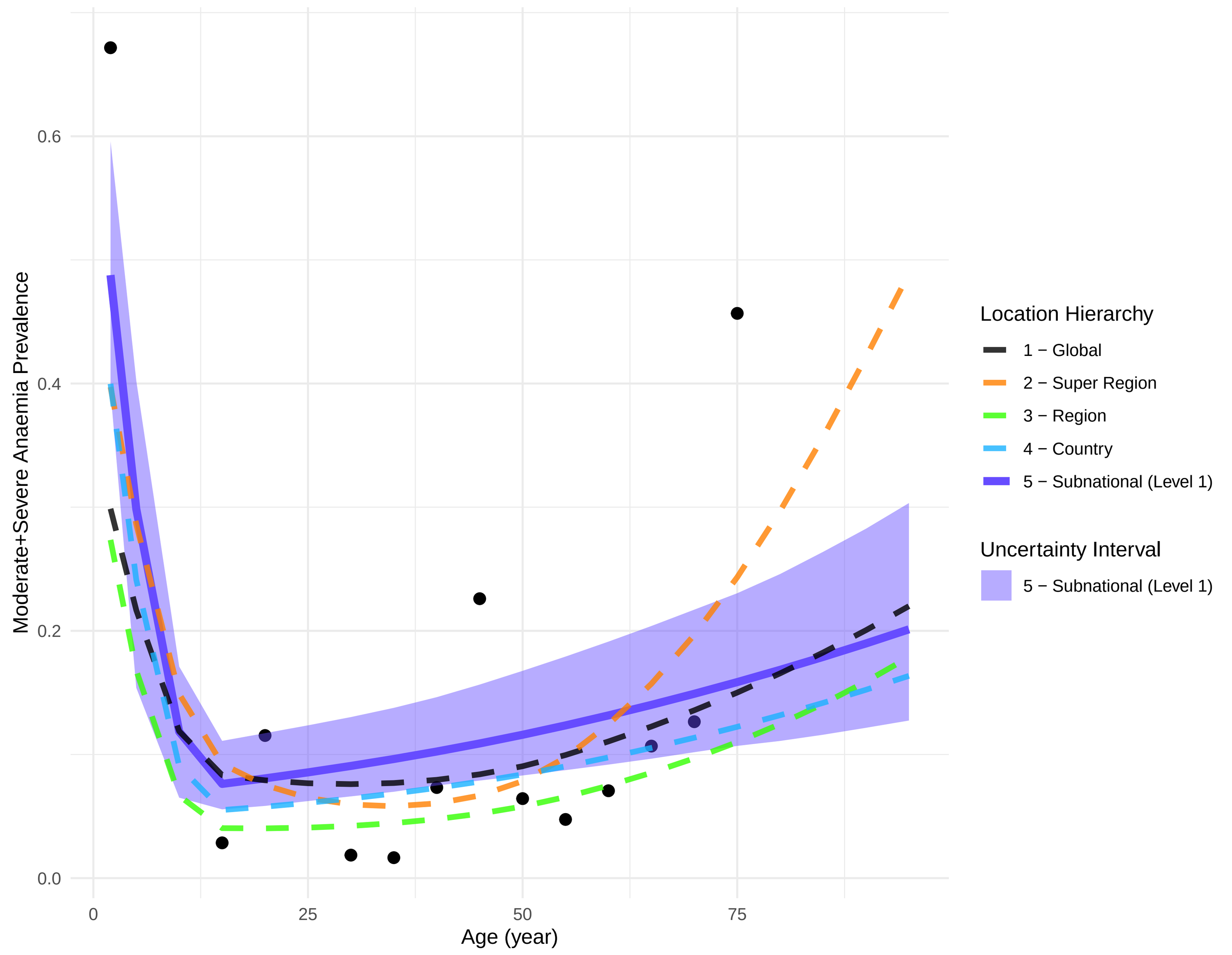

MR−BRT Age/Sex Fits − Males 2+ Years Old − Moderate+Severe Anaemia Prevalence − Northern Cape
WHO−2024 Adjustment Method

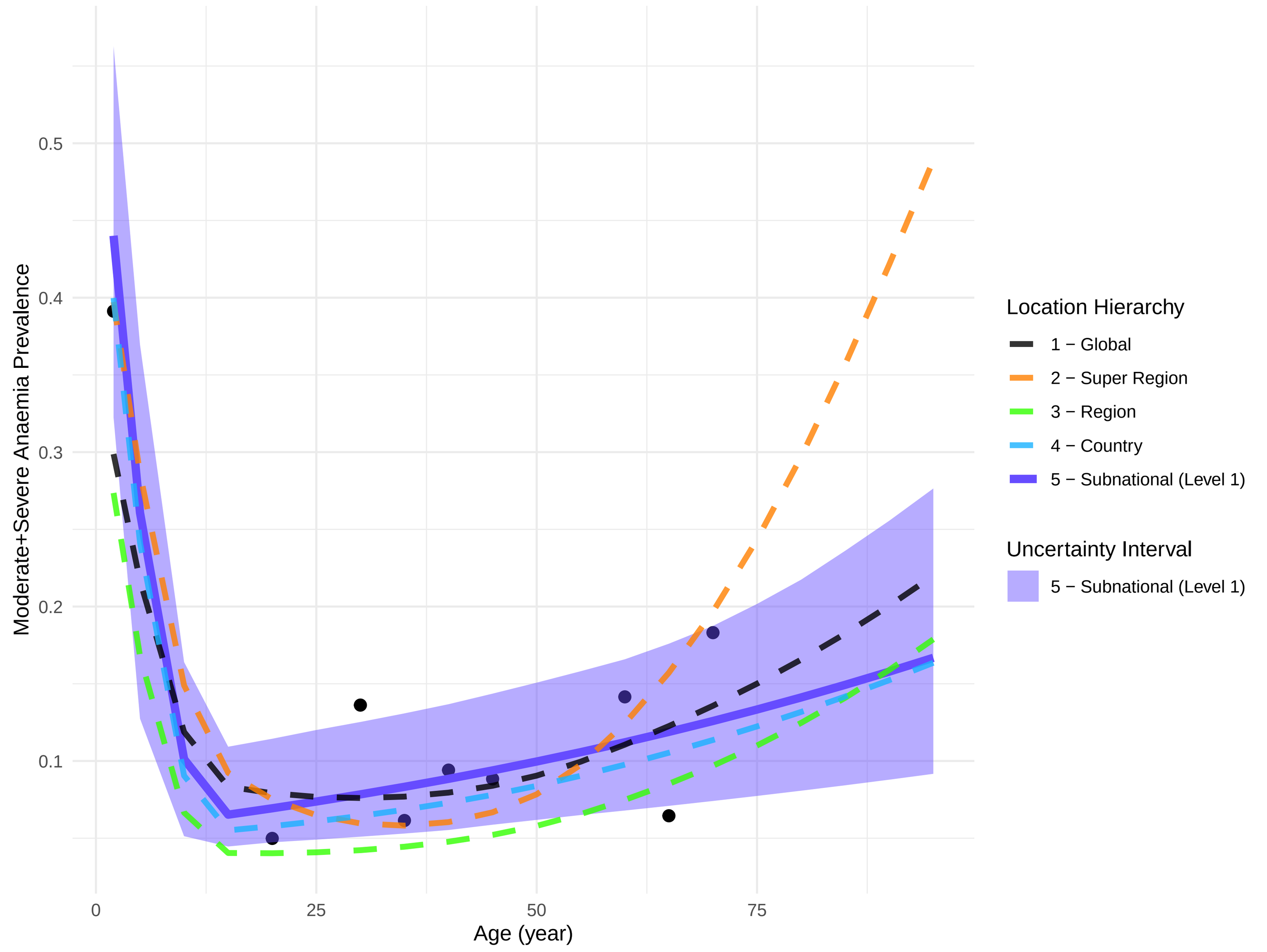

MR−BRT Age/Sex Fits − Males 2+ Years Old − Moderate+Severe Anaemia Prevalence − Western Cape
WHO−2024 Adjustment Method

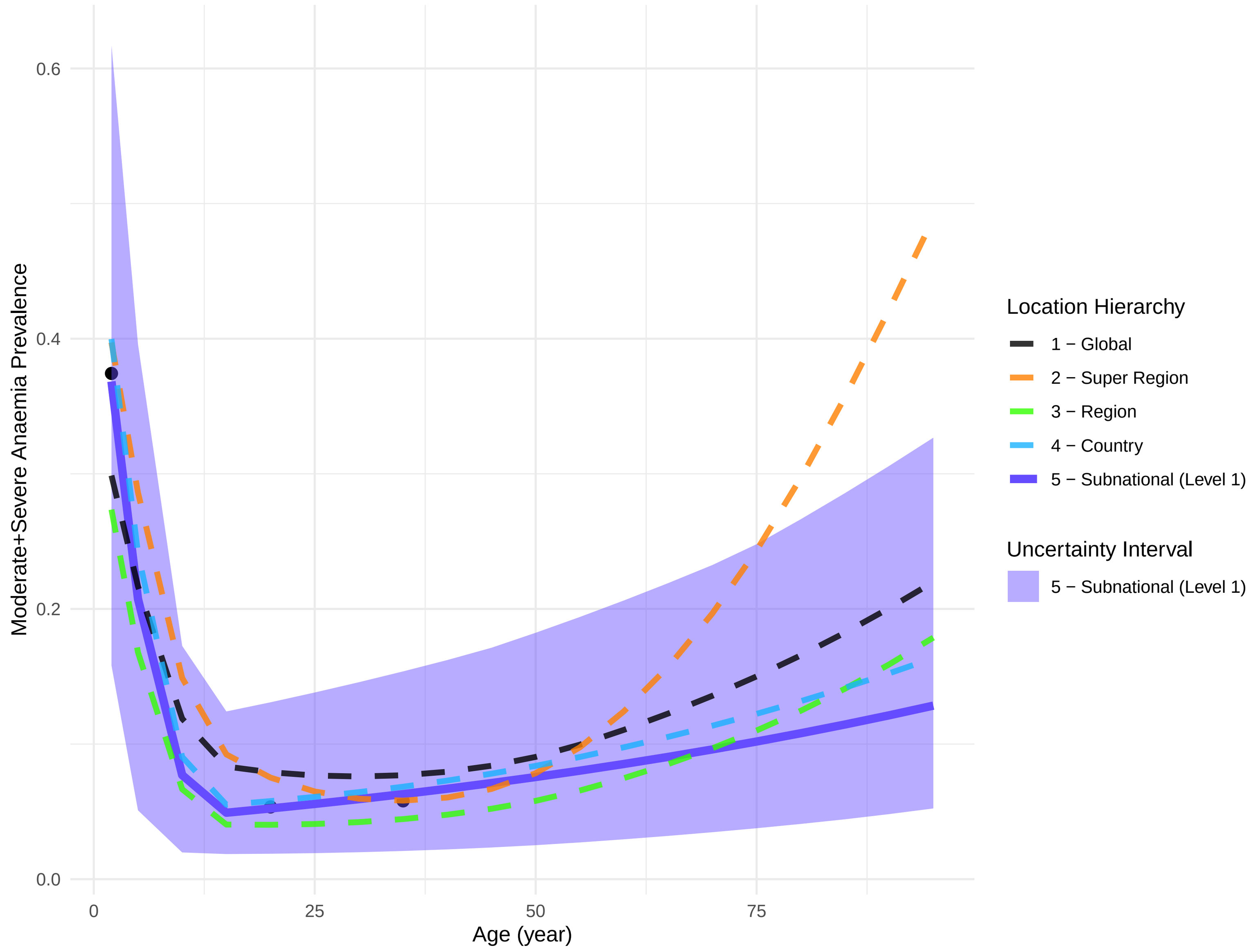

MR−BRT Age/Sex Fits − Males 2+ Years Old − Moderate+Severe Anaemia Prevalence − Zimbabwe
WHO−2024 Adjustment Method

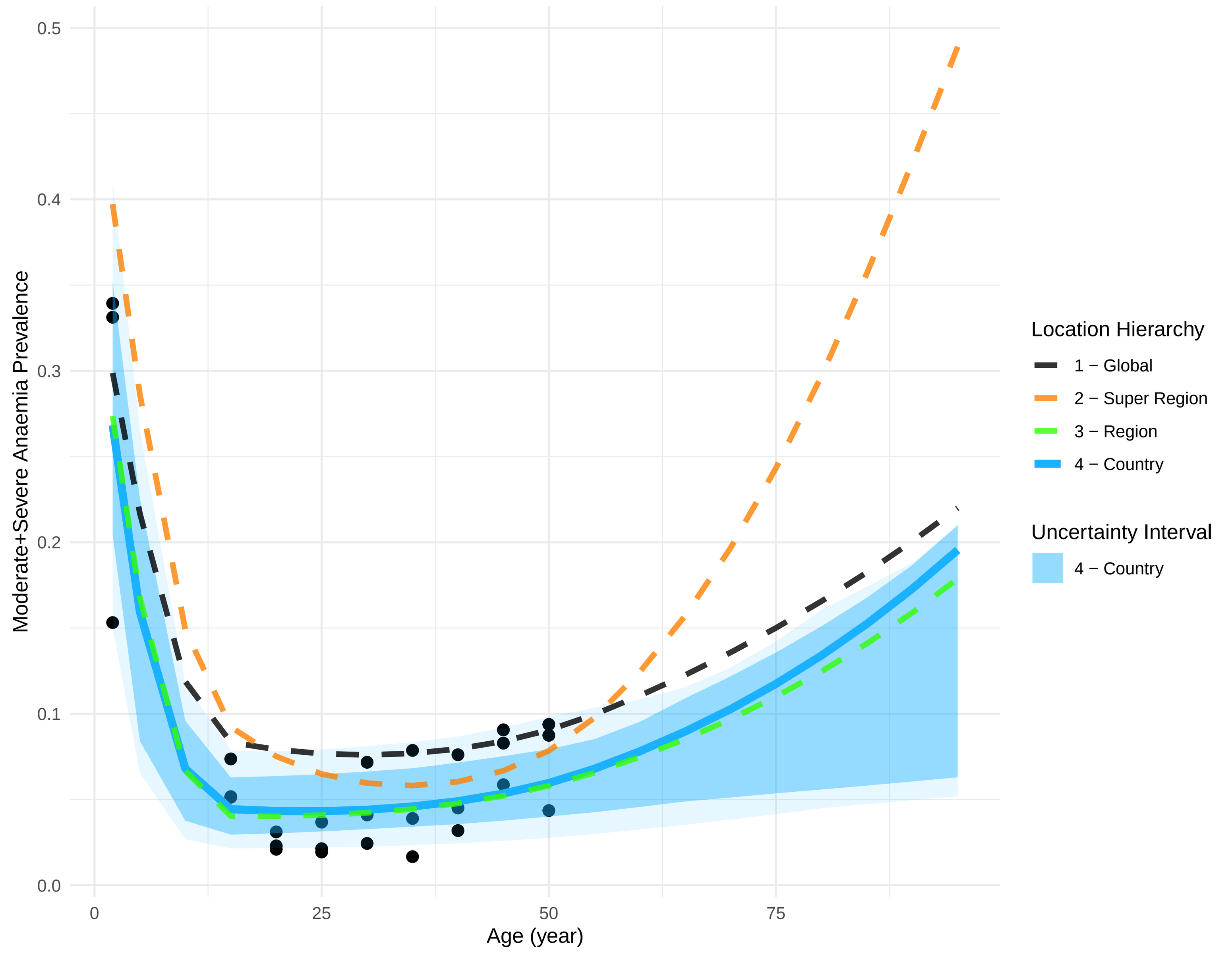

MR−BRT Age/Sex Fits − Males 2+ Years Old − Moderate+Severe Anaemia Prevalence − Western Sub−Saharan Africa
WHO−2024 Adjustment Method

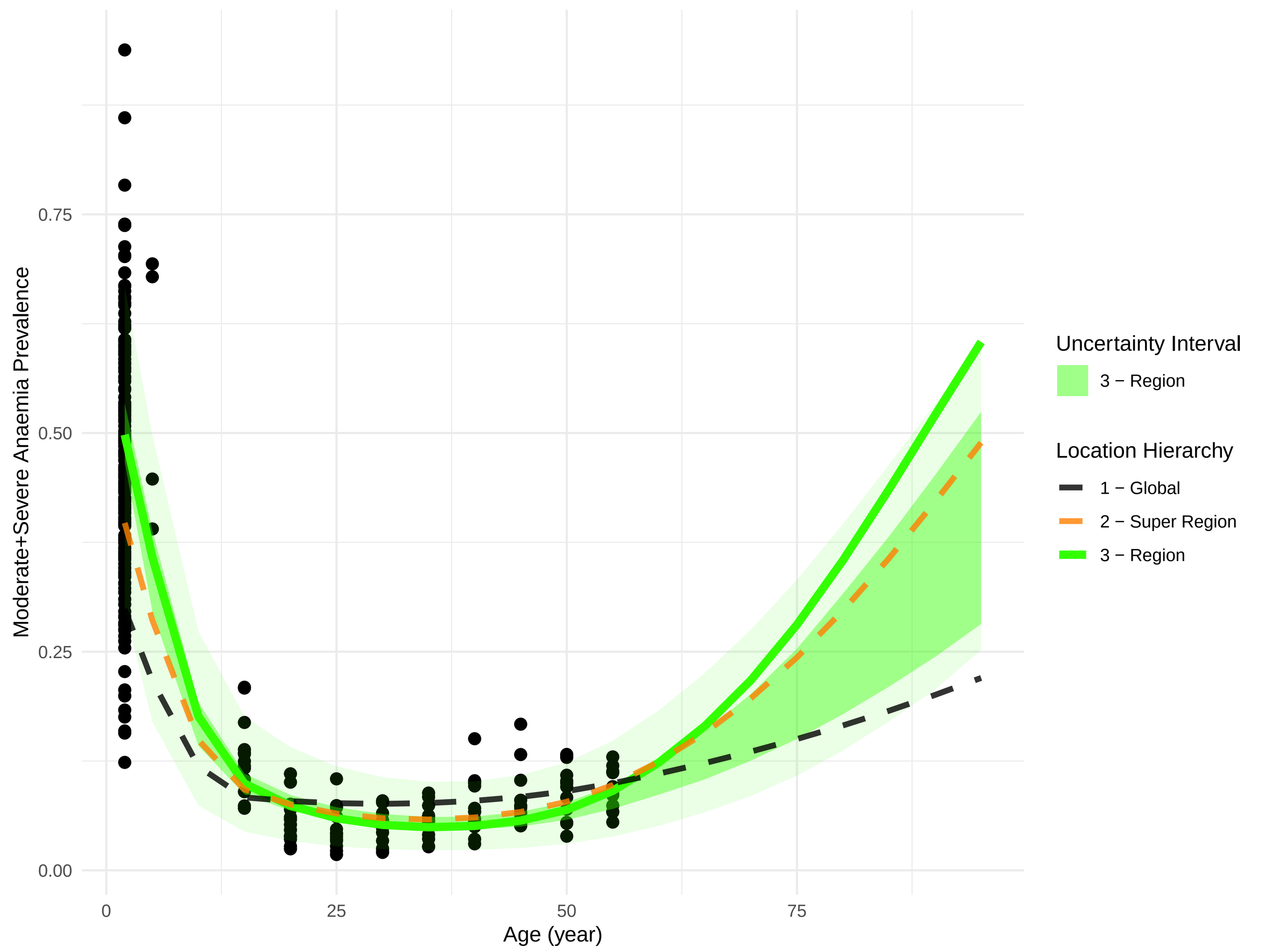

MR−BRT Age/Sex Fits − Males 2+ Years Old − Moderate+Severe Anaemia Prevalence − Benin
WHO−2024 Adjustment Method

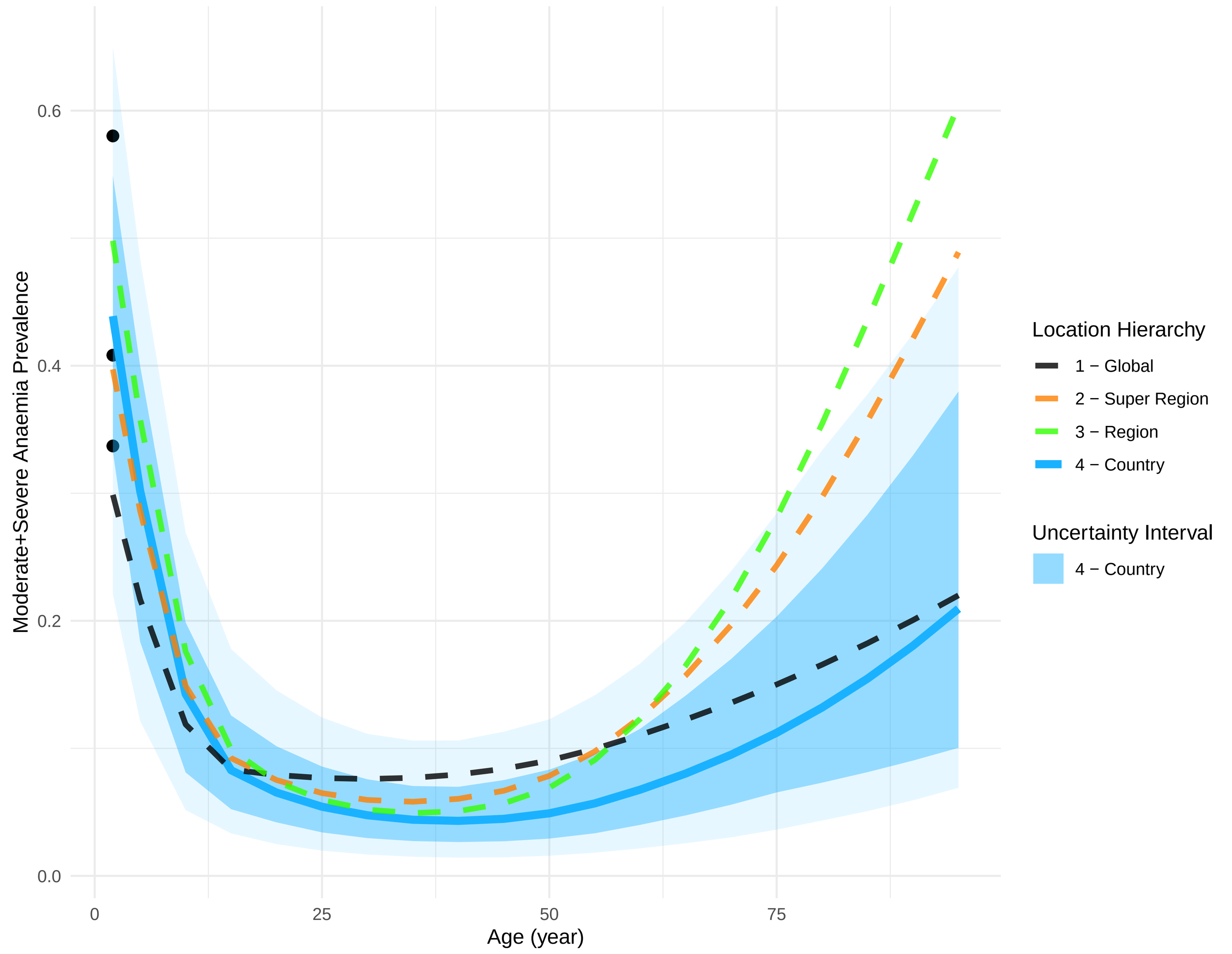

MR−BRT Age/Sex Fits − Males 2+ Years Old − Moderate+Severe Anaemia Prevalence − Burkina Faso
WHO−2024 Adjustment Method

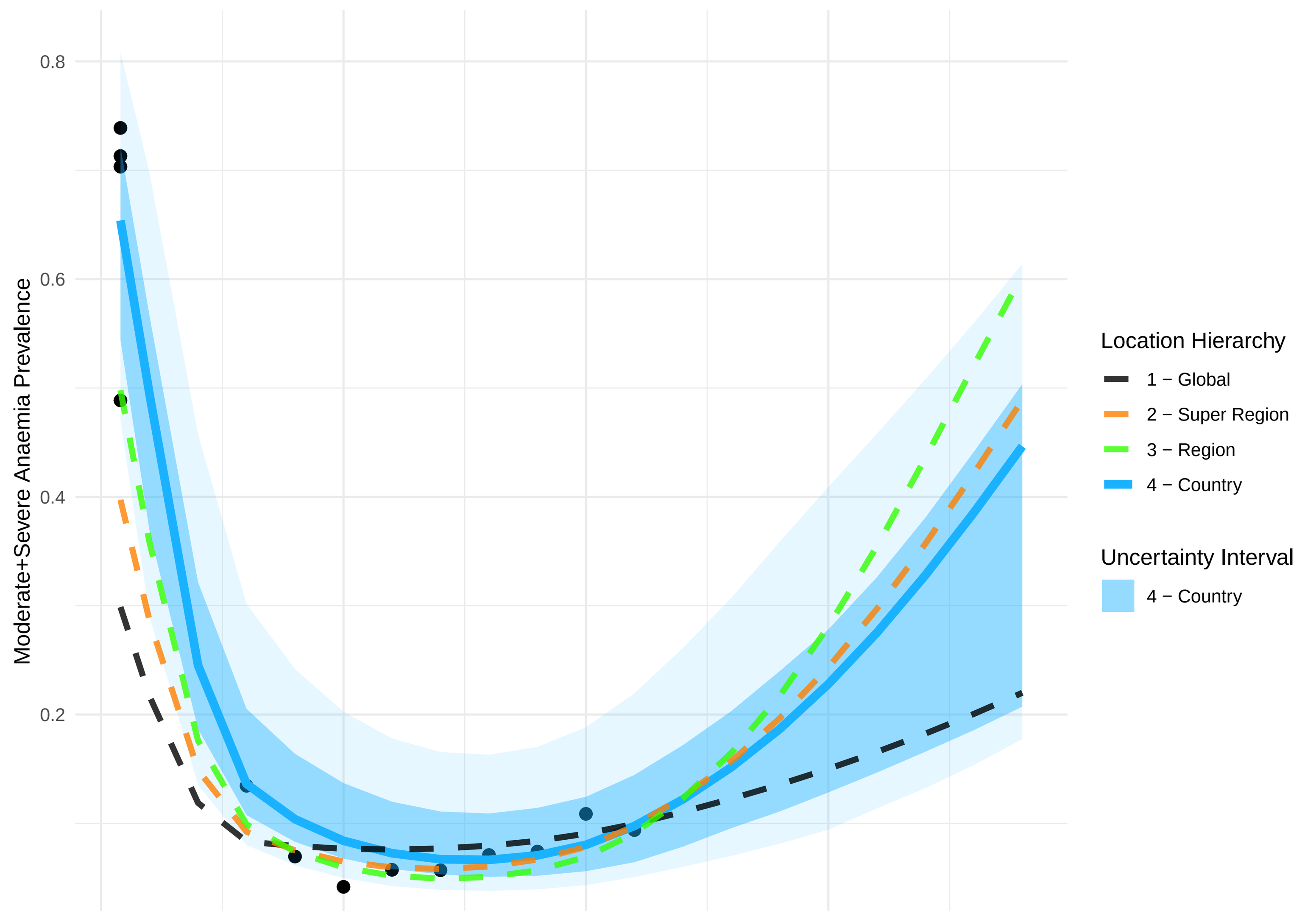

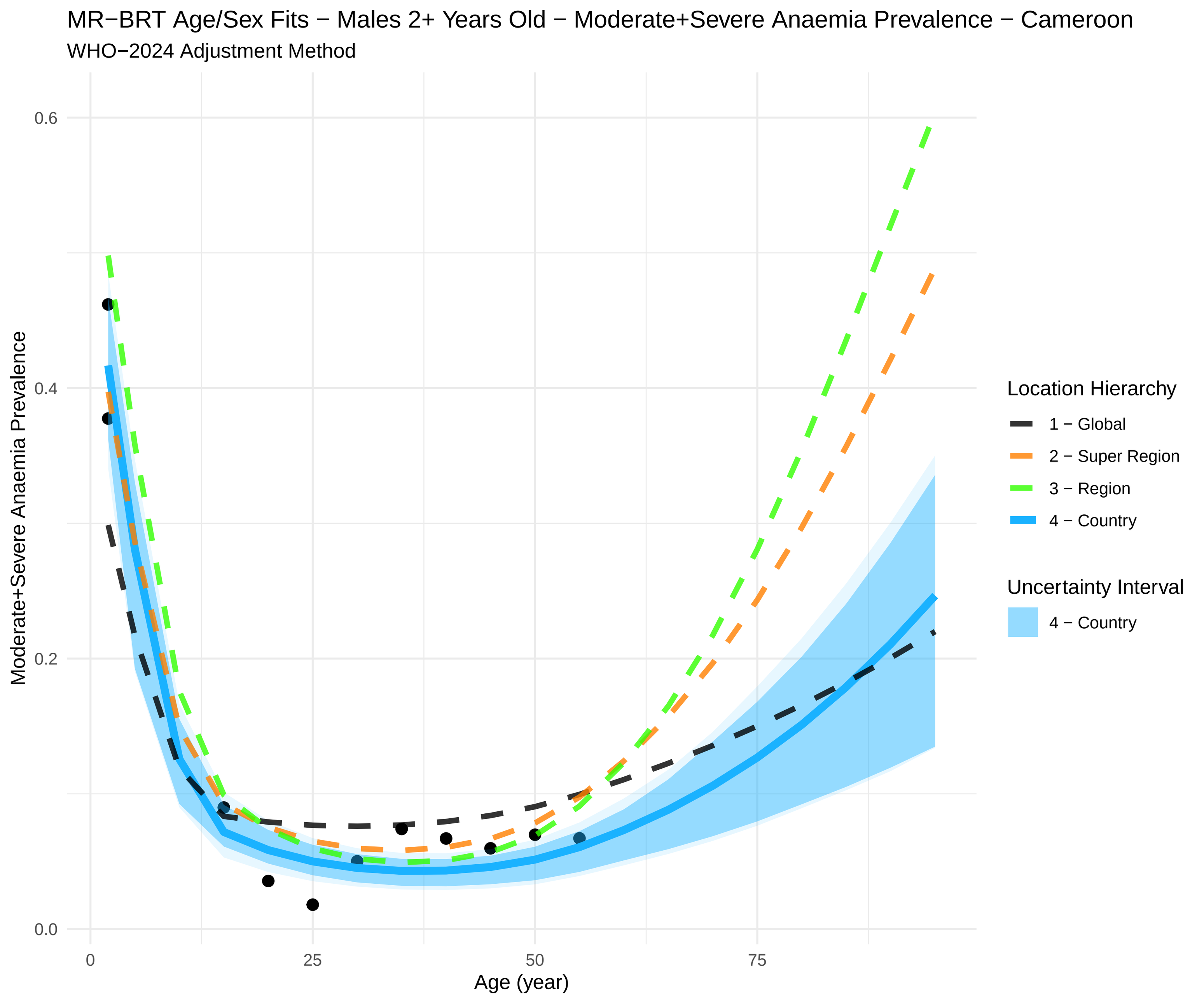
MR−BRT Age/Sex Fits − Males 2+ Years Old − Moderate+Severe Anaemia Prevalence − Cameroon
WHO−2024 Adjustment Method
Moderate+Severe Anaemia Prevalence
Age (year)
Location Hierarchy
1 − Global
2 − Super Region
3 − Region
4 − Country
Uncertainty Interval
4 − Country

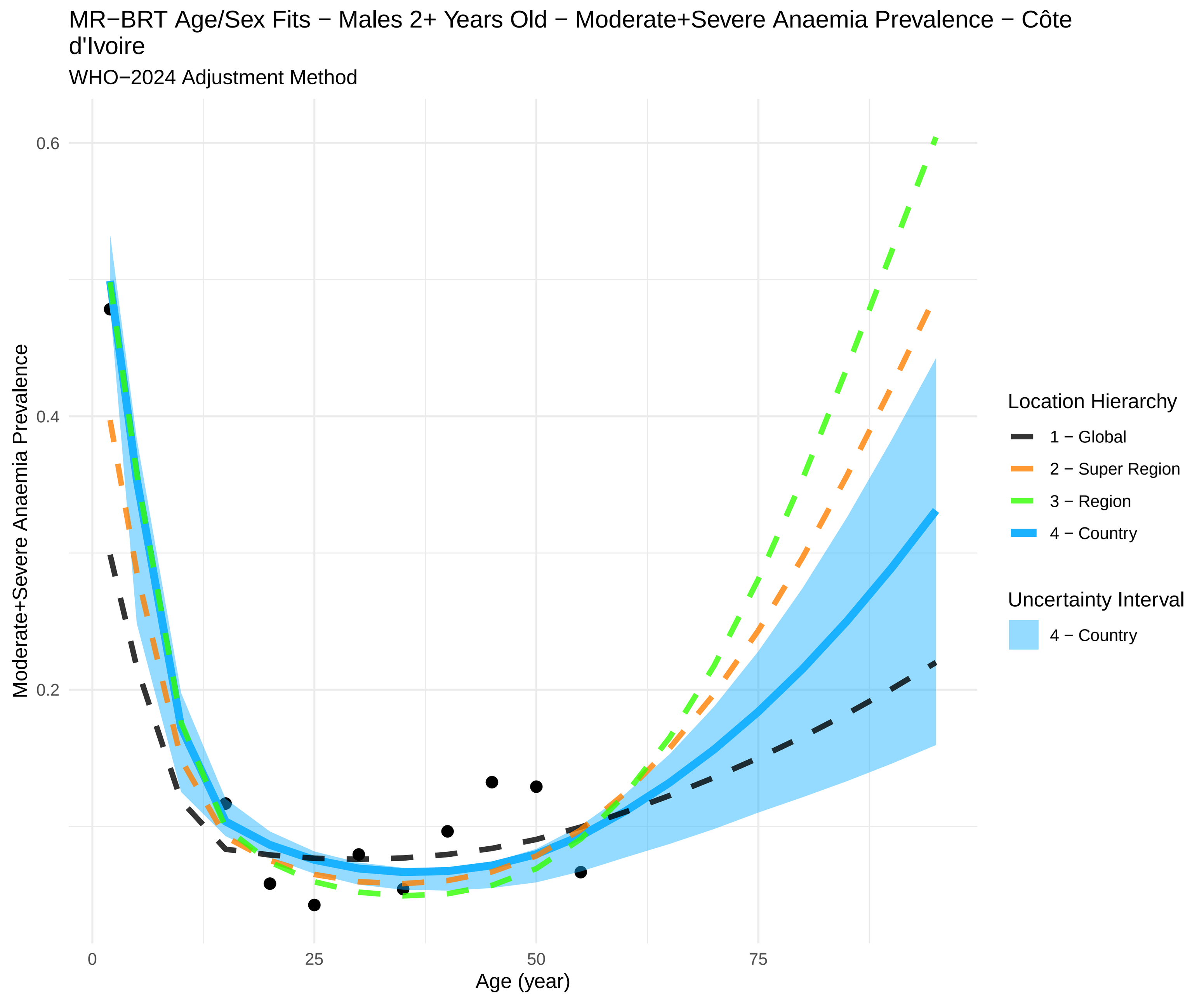
MR−BRT Age/Sex Fits − Males 2+ Years Old − Moderate+Severe Anaemia Prevalence − Côte d'Ivoire
WHO−2024 Adjustment Method
Moderate+Severe Anaemia Prevalence
Age (year)
Location Hierarchy
1 − Global
2 − Super Region
3 − Region
4 − Country
Uncertainty Interval
4 − Country

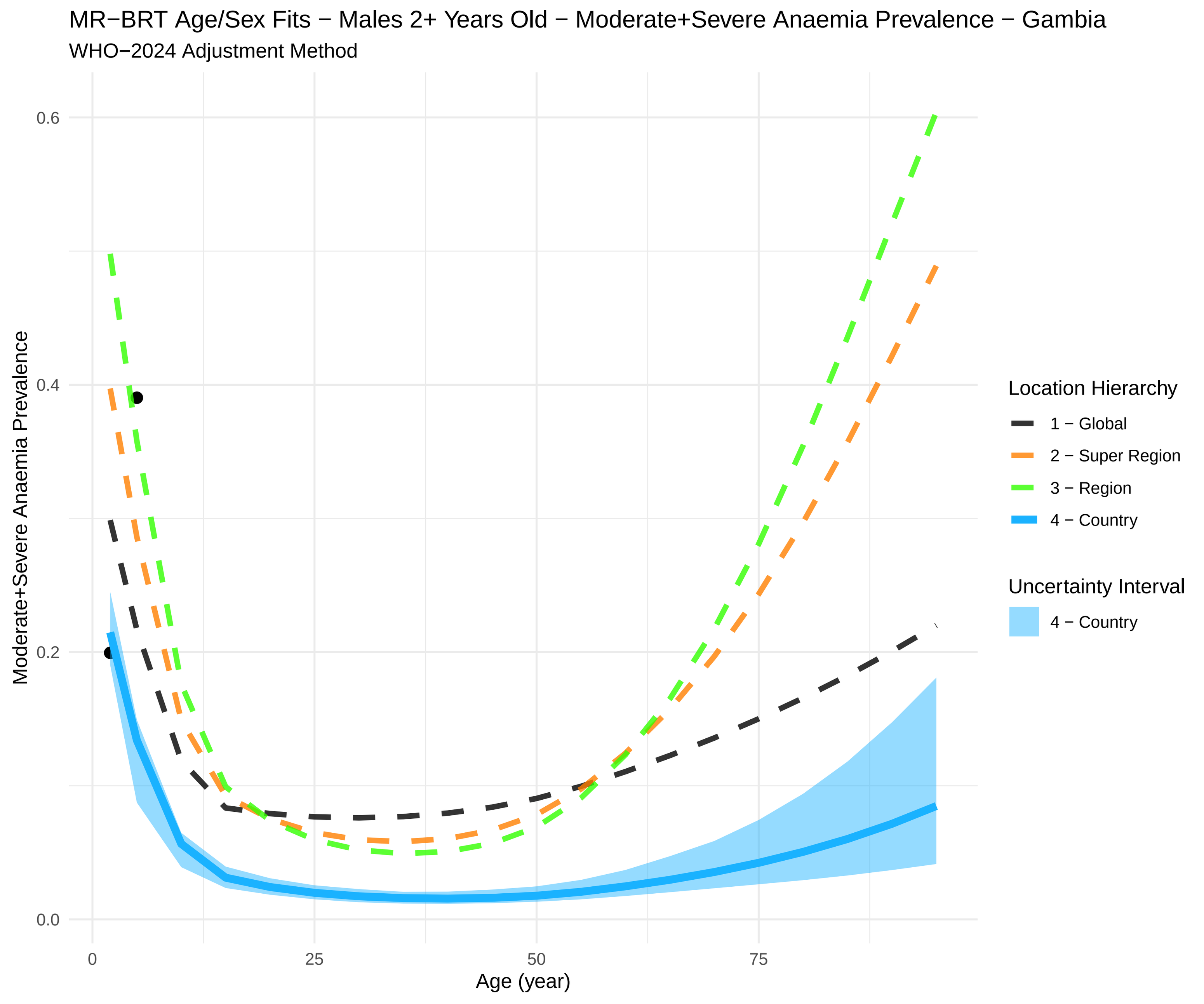
MR−BRT Age/Sex Fits − Males 2+ Years Old − Moderate+Severe Anaemia Prevalence − Gambia
WHO−2024 Adjustment Method
Moderate+Severe Anaemia Prevalence
Age (year)
Location Hierarchy
1 − Global
2 − Super Region
3 − Region
4 − Country
Uncertainty Interval
4 − Country

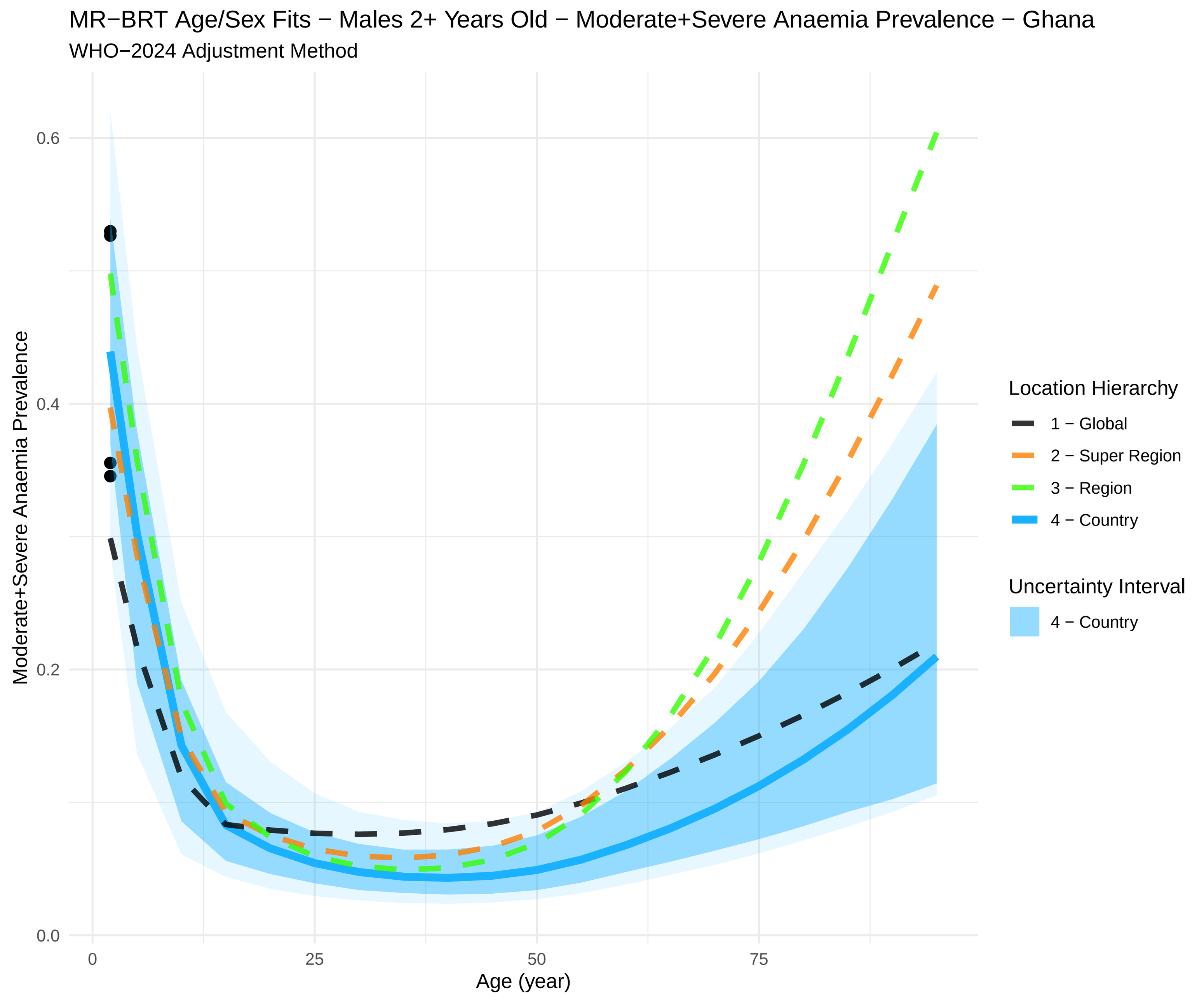
MR−BRT Age/Sex Fits − Males 2+ Years Old − Moderate+Severe Anaemia Prevalence − Ghana
WHO−2024 Adjustment Method
Moderate+Severe Anaemia Prevalence
Age (year)
Location Hierarchy
1 − Global
2 − Super Region
3 − Region
4 − Country
Uncertainty Interval
4 − Country

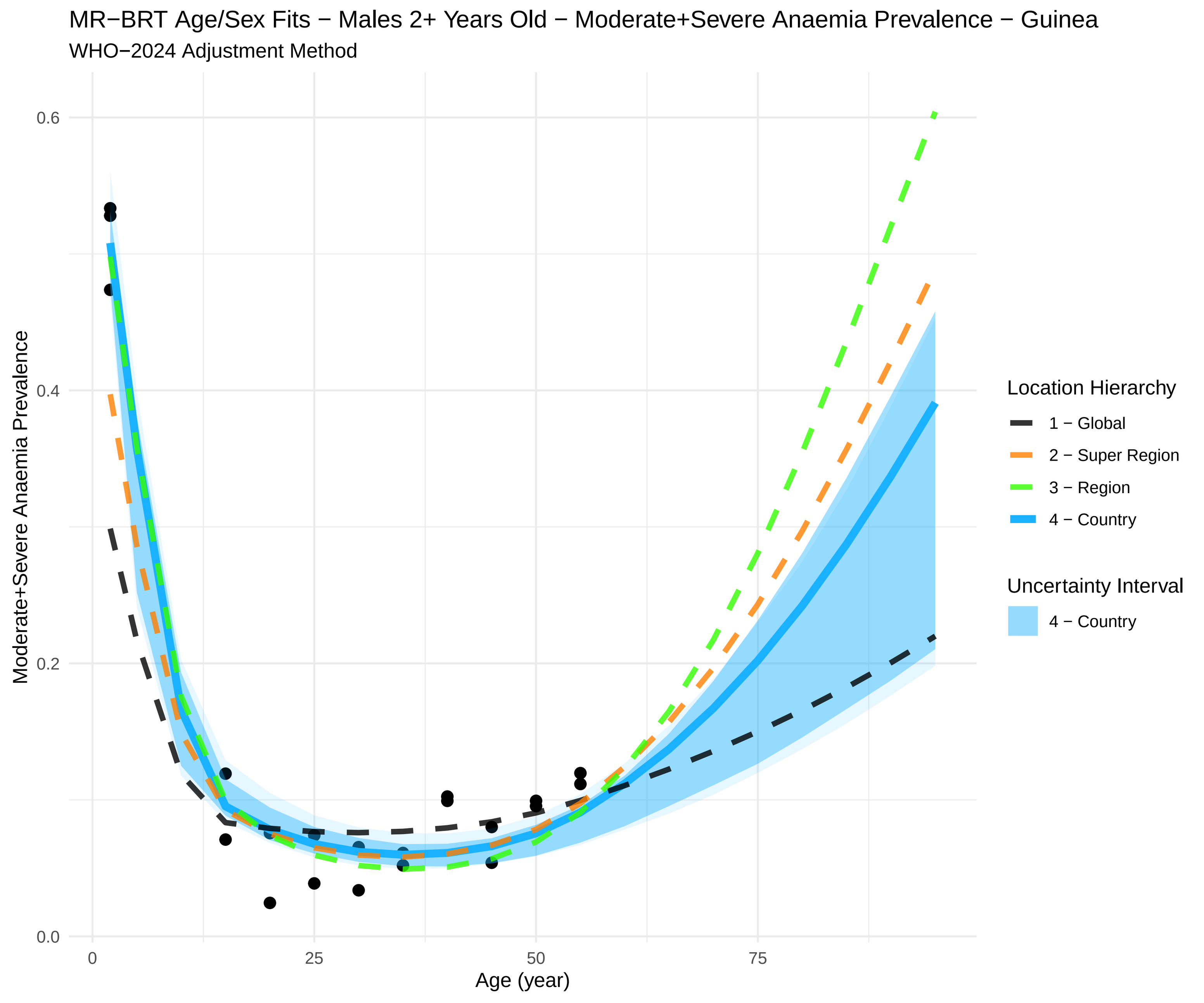
MR−BRT Age/Sex Fits − Males 2+ Years Old − Moderate+Severe Anaemia Prevalence − Guinea
WHO−2024 Adjustment Method
Moderate+Severe Anaemia Prevalence
Age (year)
Location Hierarchy
1 − Global
2 − Super Region
3 − Region
4 − Country
Uncertainty Interval
4 − Country

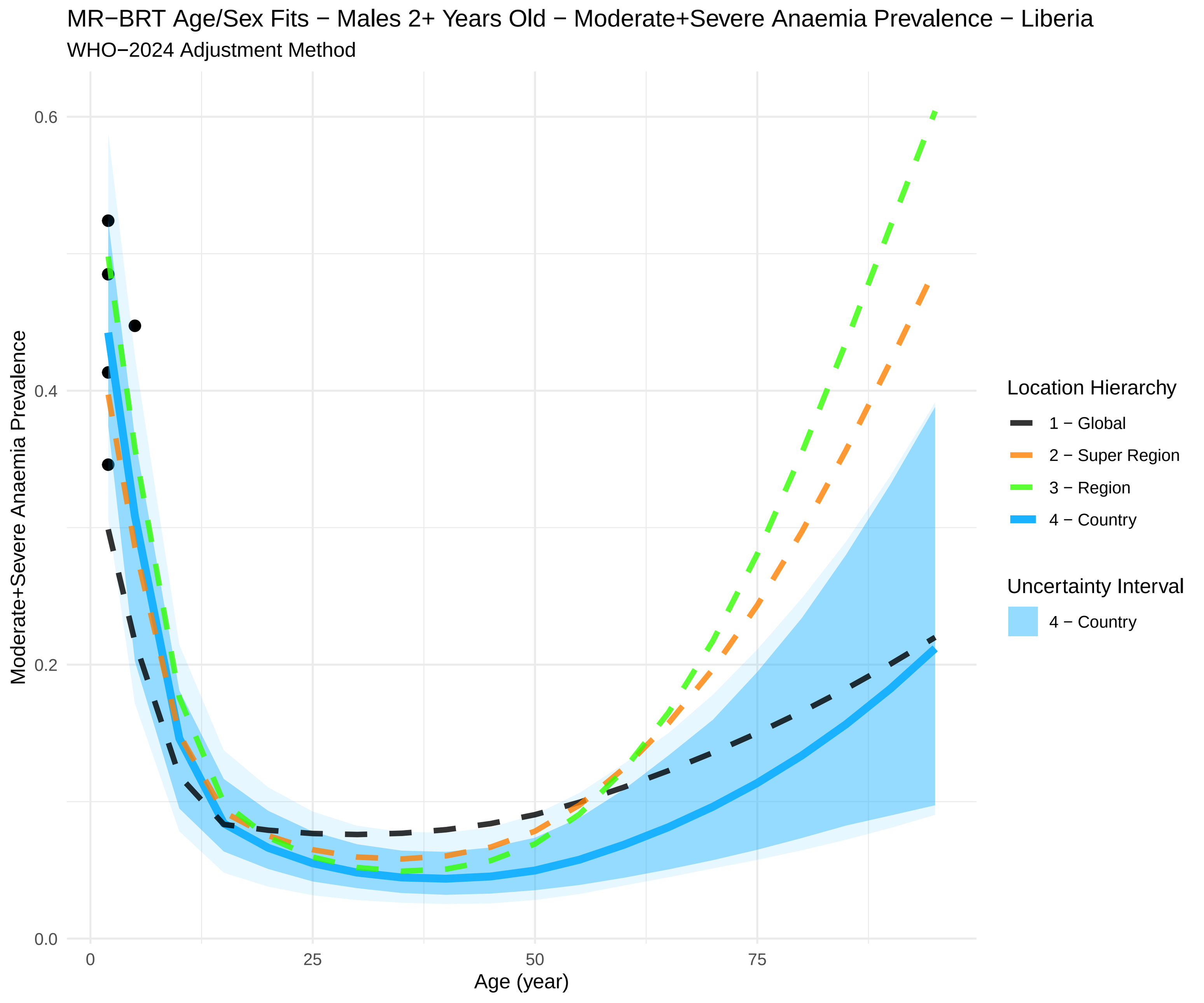
MR−BRT Age/Sex Fits − Males 2+ Years Old − Moderate+Severe Anaemia Prevalence − Liberia
WHO−2024 Adjustment Method
Moderate+Severe Anaemia Prevalence
Age (year)
Location Hierarchy
1 − Global
2 − Super Region
3 − Region
4 − Country
Uncertainty Interval
4 − Country

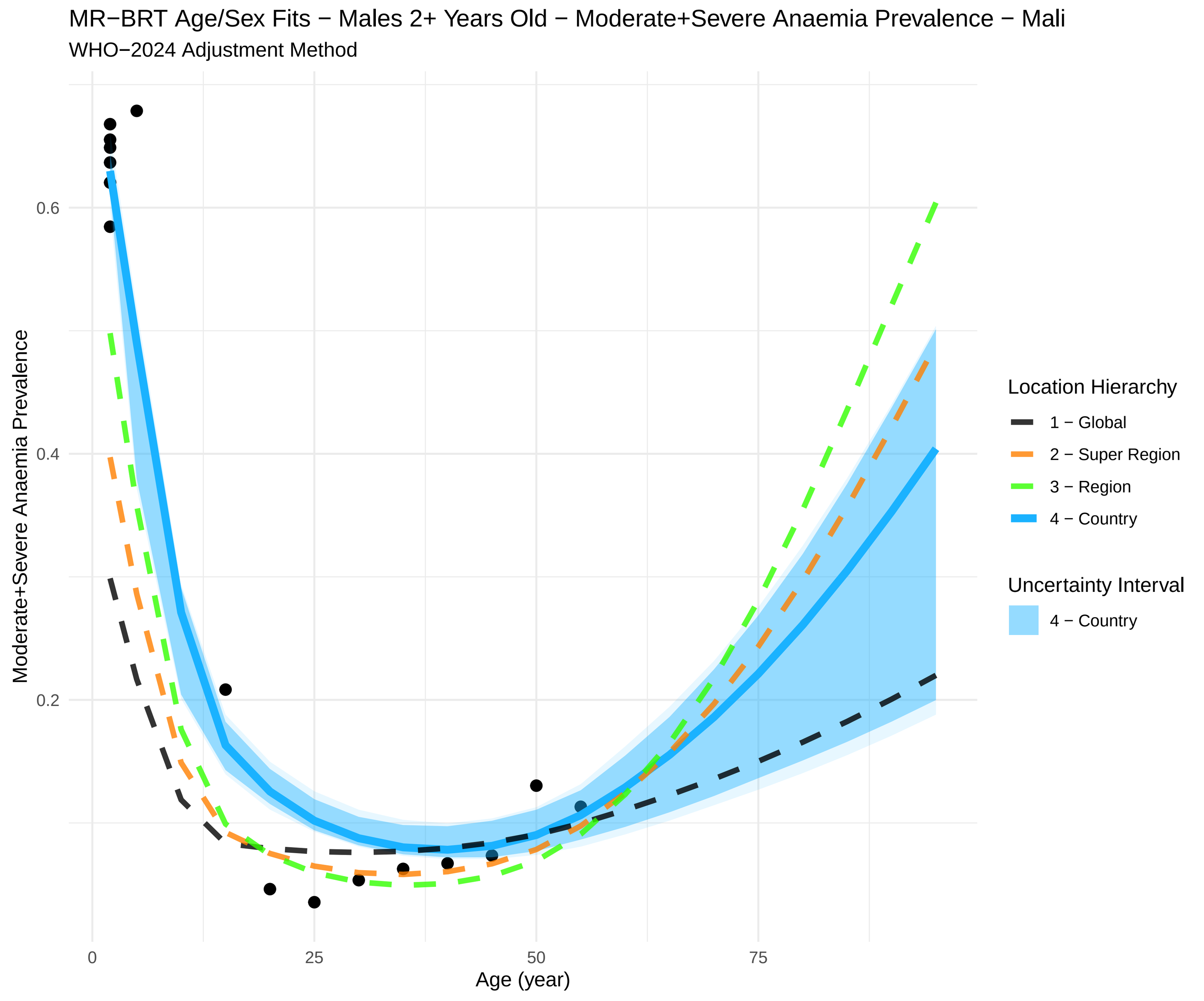
MR−BRT Age/Sex Fits − Males 2+ Years Old − Moderate+Severe Anaemia Prevalence − Mali
WHO−2024 Adjustment Method
Moderate+Severe Anaemia Prevalence
Age (year)
Location Hierarchy
1 − Global
2 − Super Region
3 − Region
4 − Country
Uncertainty Interval
4 − Country

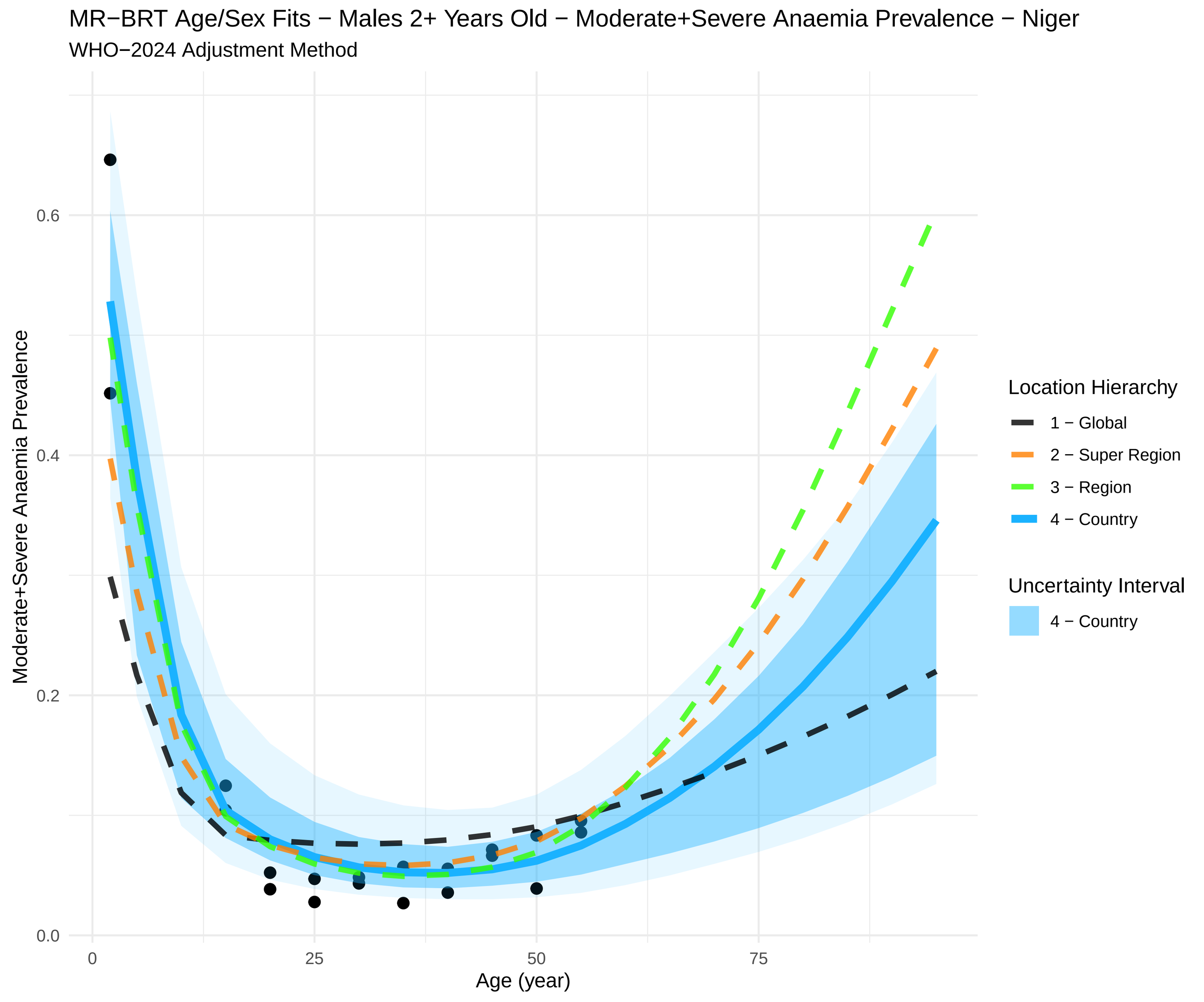
MR−BRT Age/Sex Fits − Males 2+ Years Old − Moderate+Severe Anaemia Prevalence − Niger
WHO−2024 Adjustment Method
Moderate+Severe Anaemia Prevalence
Age (year)
Location Hierarchy
1 − Global
2 − Super Region
3 − Region
4 − Country
Uncertainty Interval
4 − Country

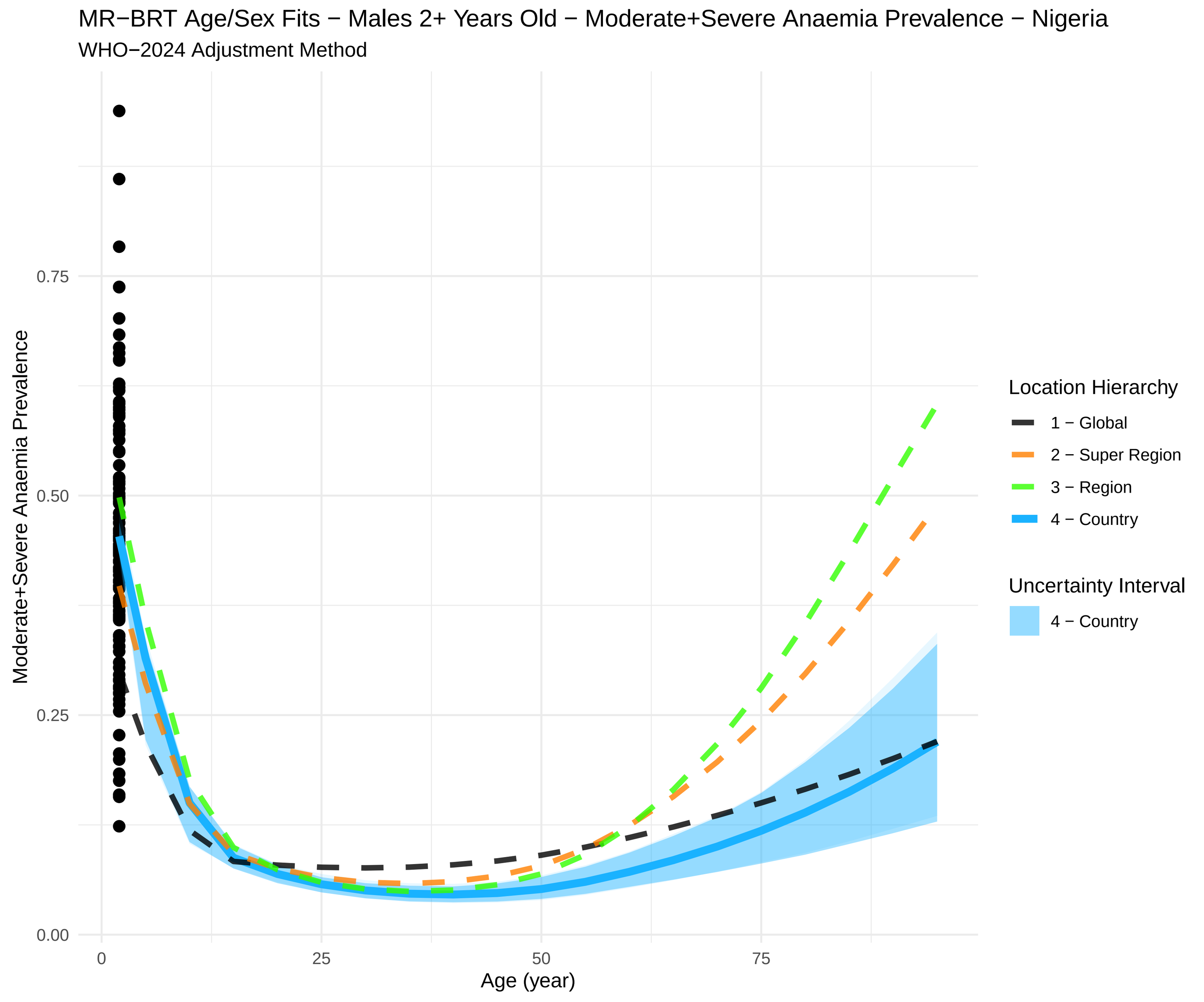
MR−BRT Age/Sex Fits − Males 2+ Years Old − Moderate+Severe Anaemia Prevalence − Nigeria
WHO−2024 Adjustment Method
Moderate+Severe Anaemia Prevalence
Age (year)
Location Hierarchy
1 − Global
2 − Super Region
3 − Region
4 − Country
Uncertainty Interval
4 − Country

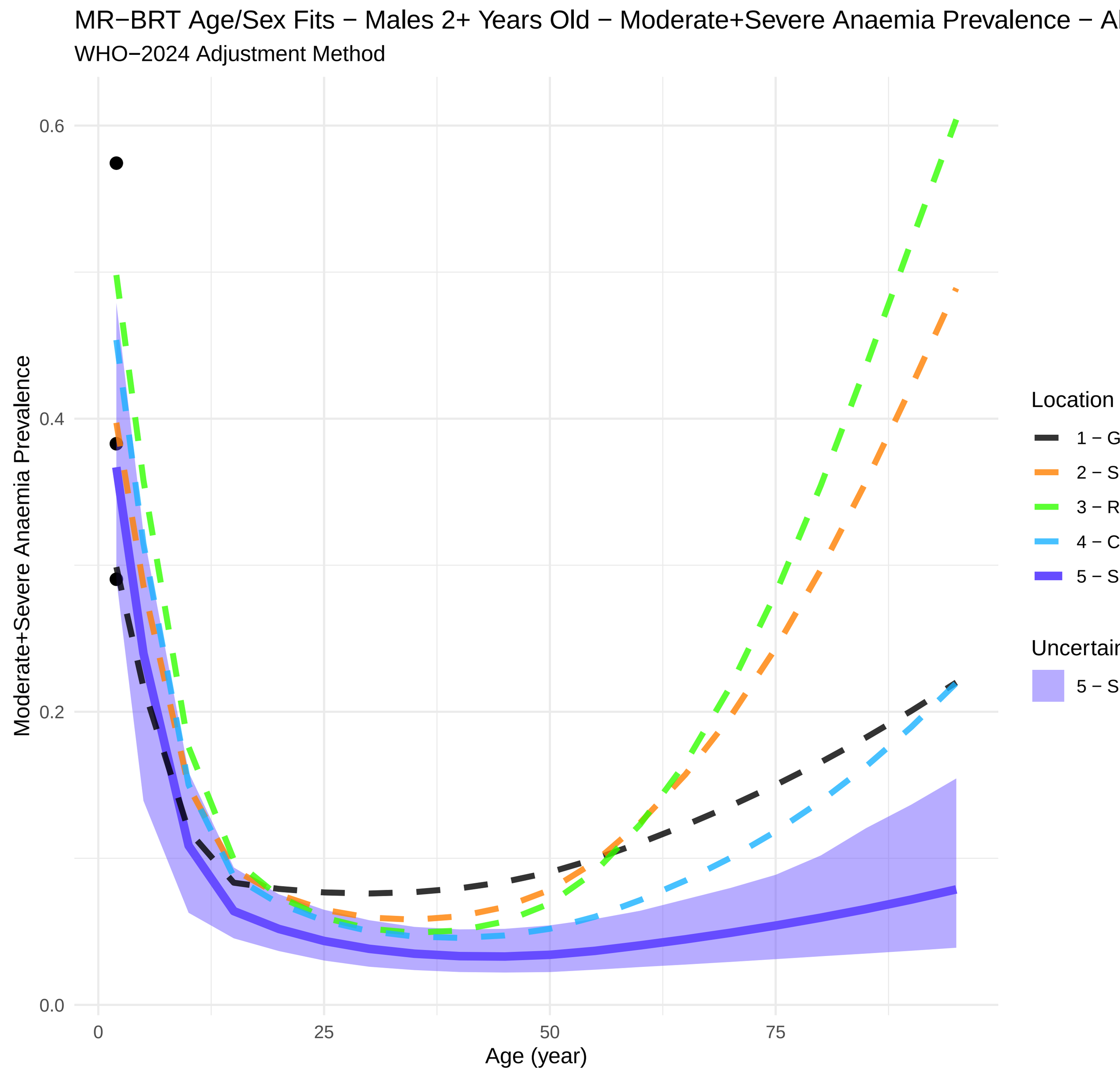
MR−BRT Age/Sex Fits − Males 2+ Years Old − Moderate+Severe Anaemia Prevalence − Abia
WHO−2024 Adjustment Method
Moderate+Severe Anaemia Prevalence
Age (year)
Location Hierarchy
1 − Global
2 − Super Region
3 − Region
4 − Country
5 − Subnational (Level 1)
Uncertainty Interval
5 − Subnational (Level 1)

MR−BRT Age/Sex Fits − Males 2+ Years Old − Moderate+Severe Anaemia Prevalence − Adamawa
WHO−2024 Adjustment Method

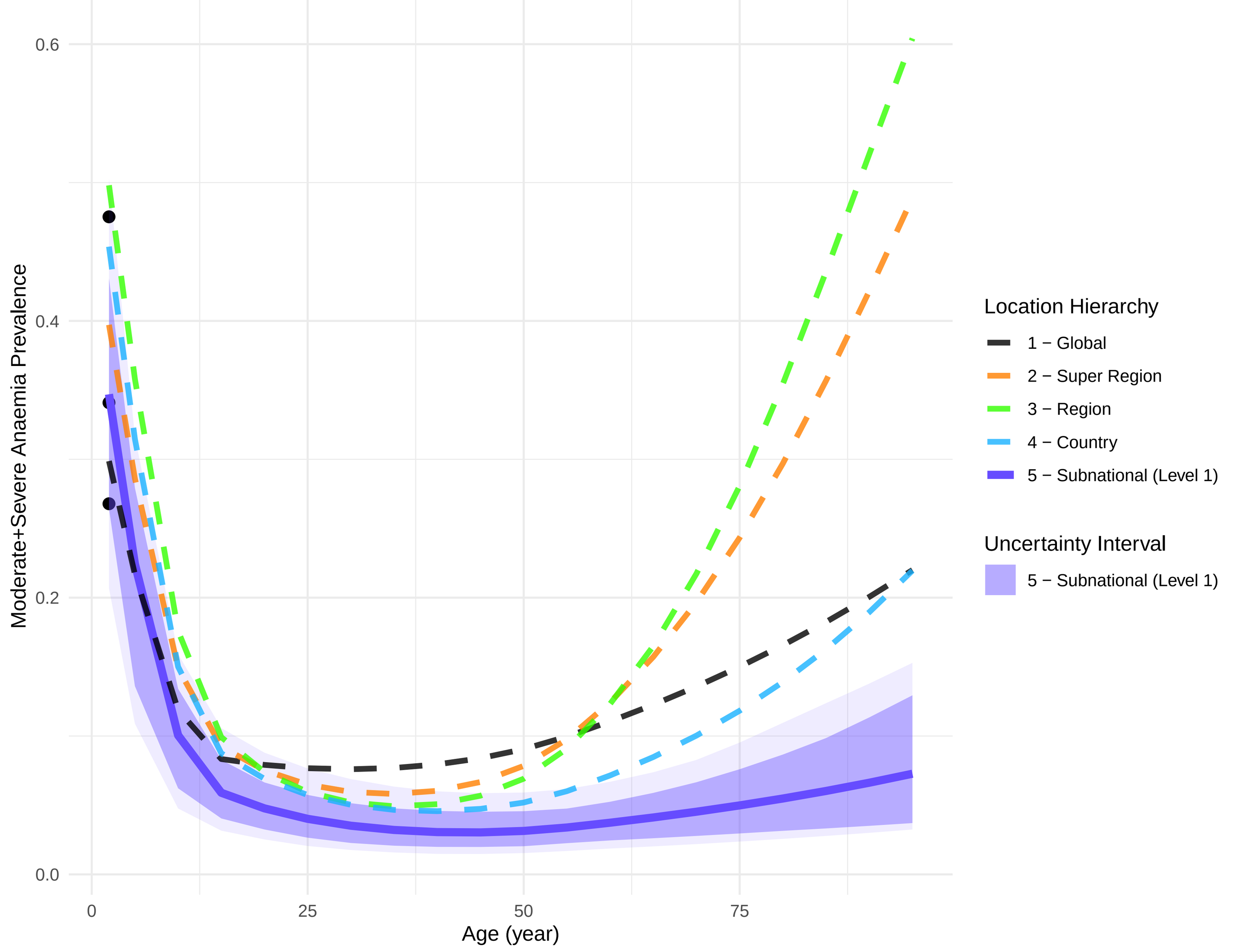

MR−BRT Age/Sex Fits − Males 2+ Years Old − Moderate+Severe Anaemia Prevalence − Akwa Ibom
WHO−2024 Adjustment Method

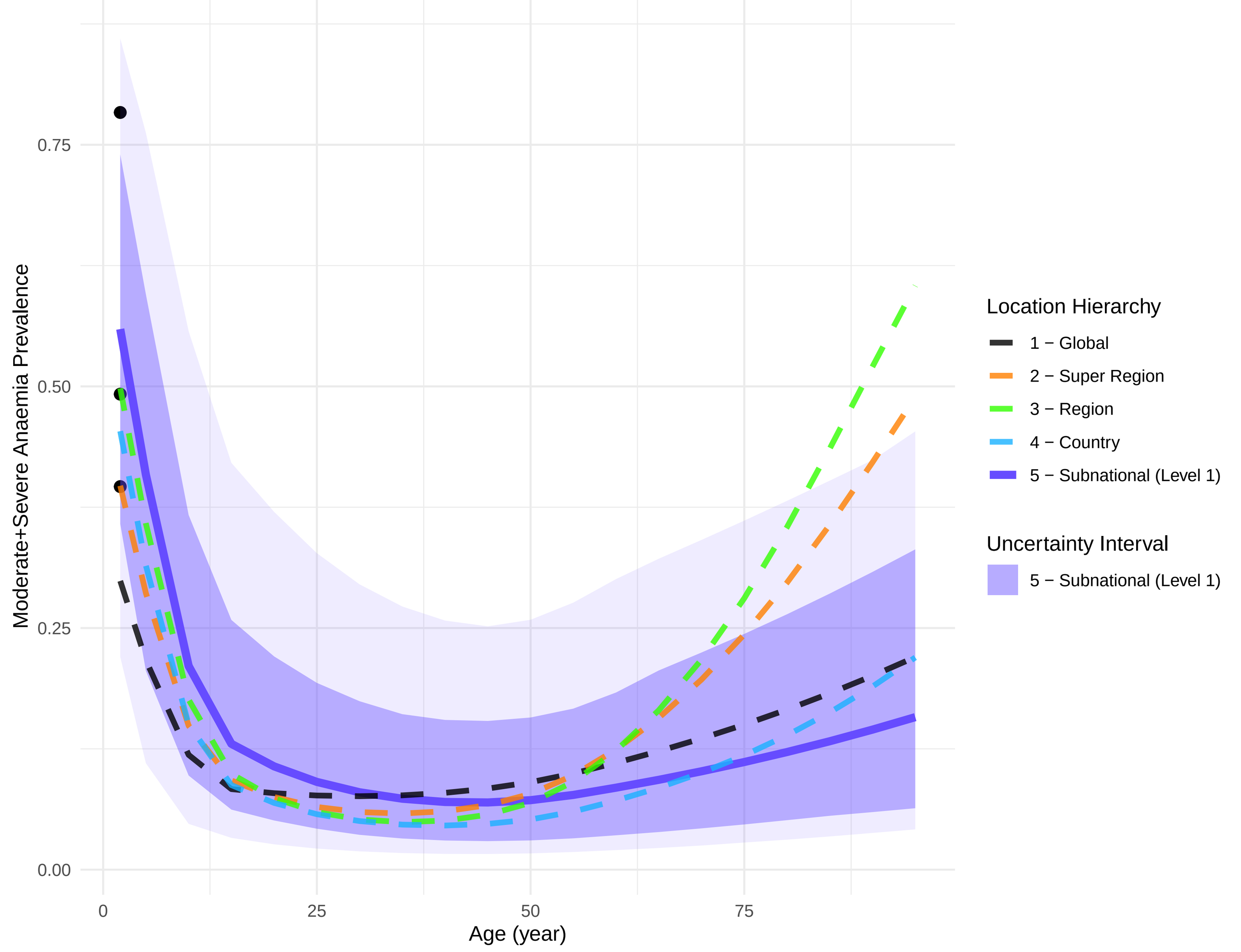

MR−BRT Age/Sex Fits − Males 2+ Years Old − Moderate+Severe Anaemia Prevalence − Anambra
WHO−2024 Adjustment Method

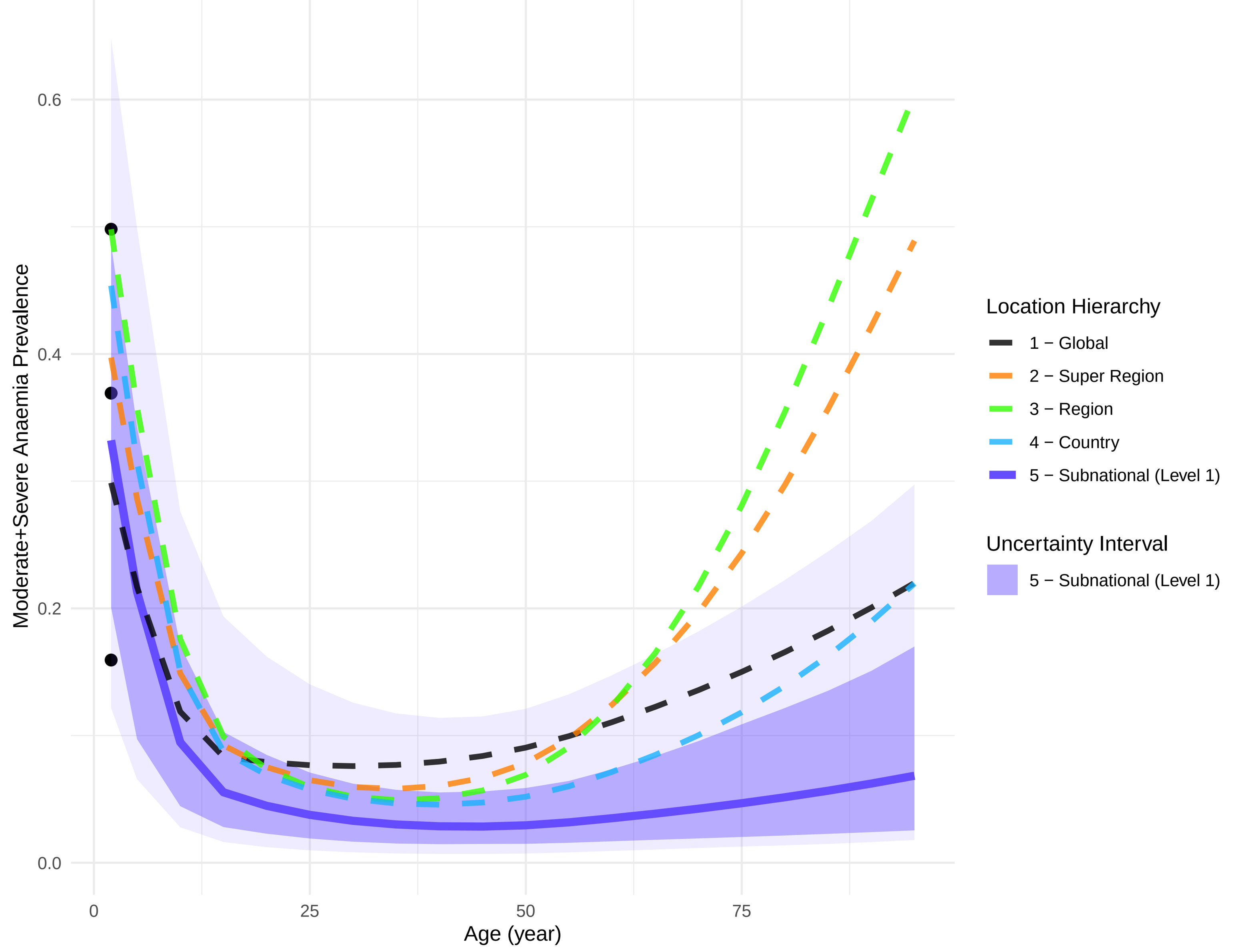

MR−BRT Age/Sex Fits − Males 2+ Years Old − Moderate+Severe Anaemia Prevalence − Bauchi
WHO−2024 Adjustment Method

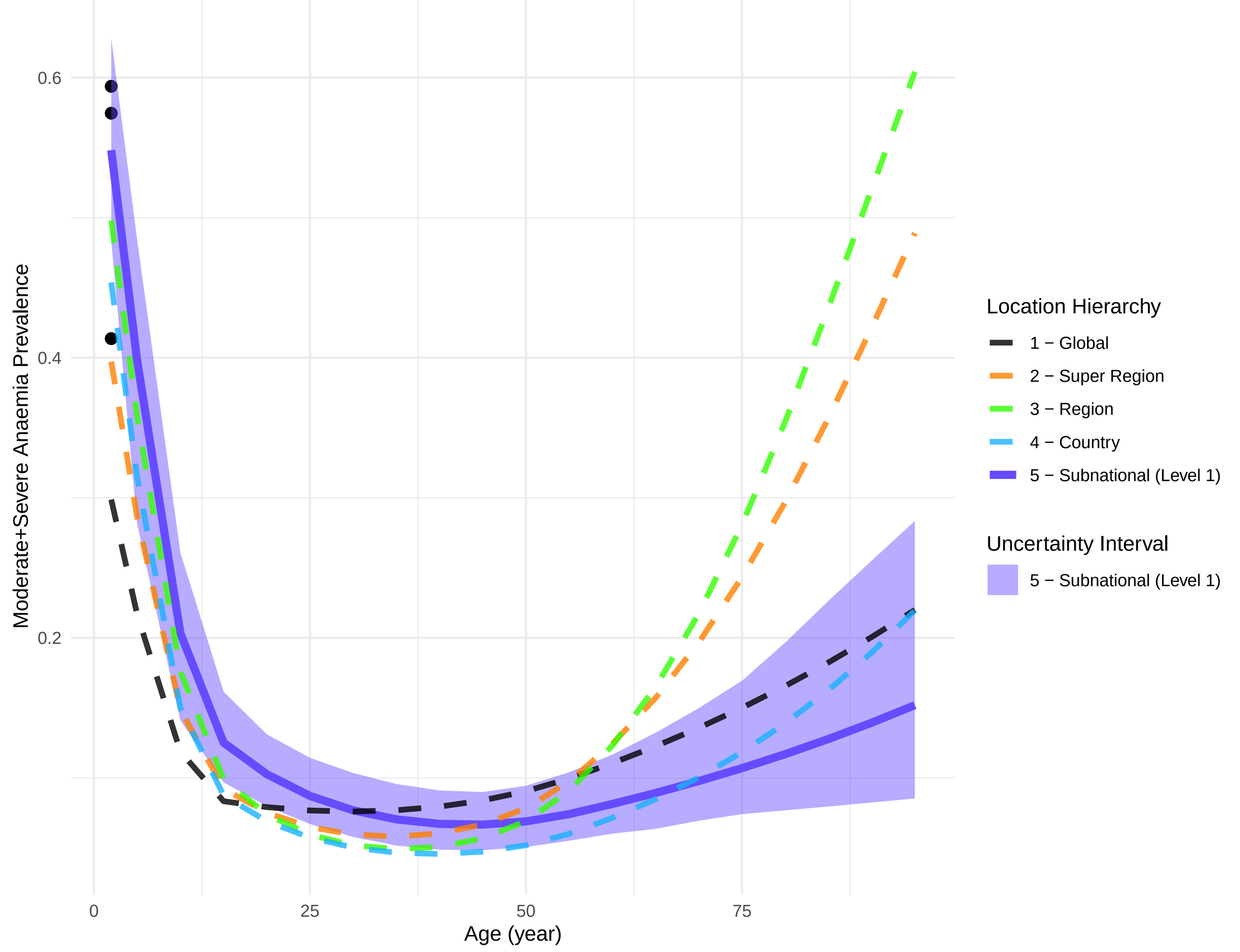

MR−BRT Age/Sex Fits − Males 2+ Years Old − Moderate+Severe Anaemia Prevalence − Bayelsa
WHO−2024 Adjustment Method

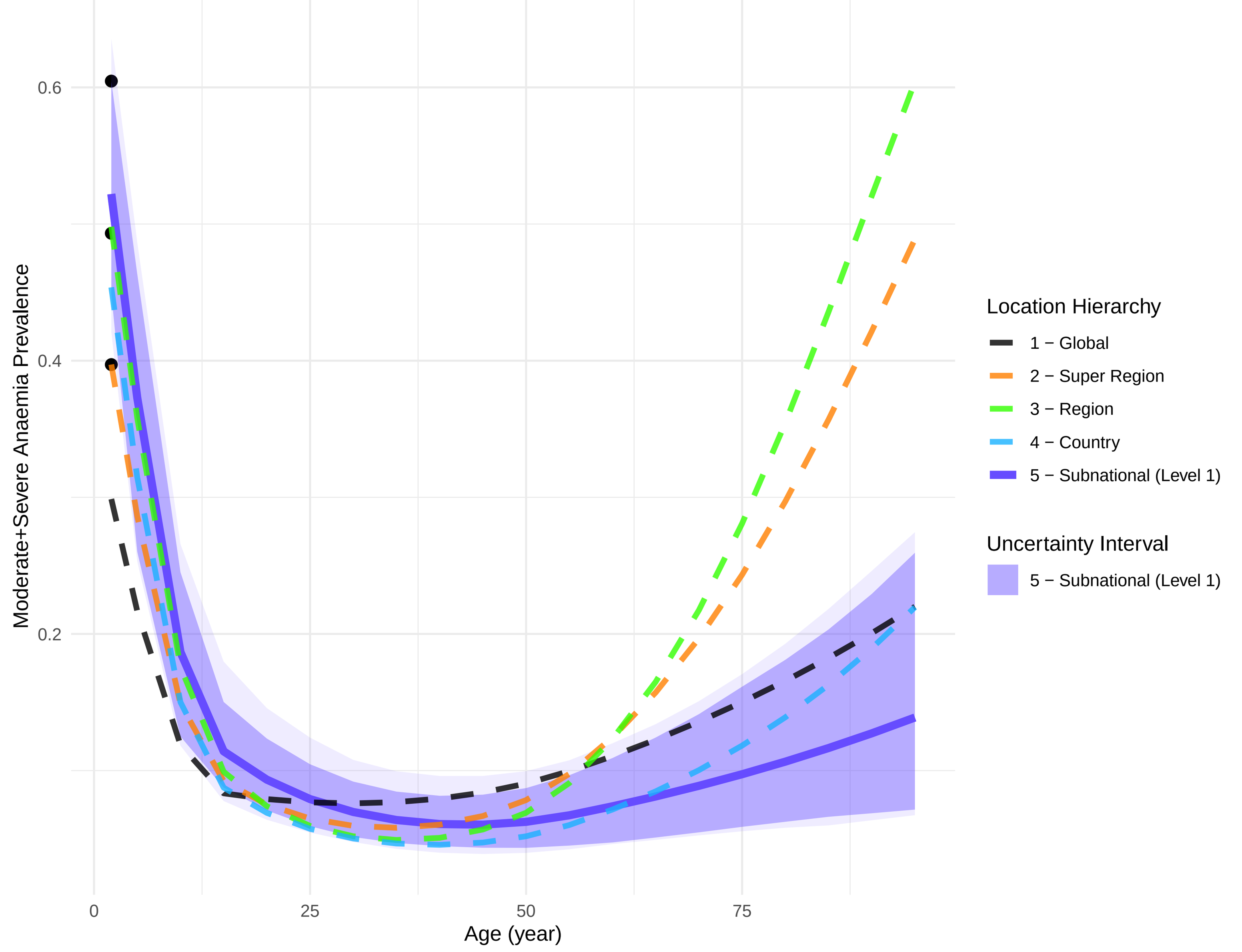

MR−BRT Age/Sex Fits − Males 2+ Years Old − Moderate+Severe Anaemia Prevalence − Benue
WHO−2024 Adjustment Method

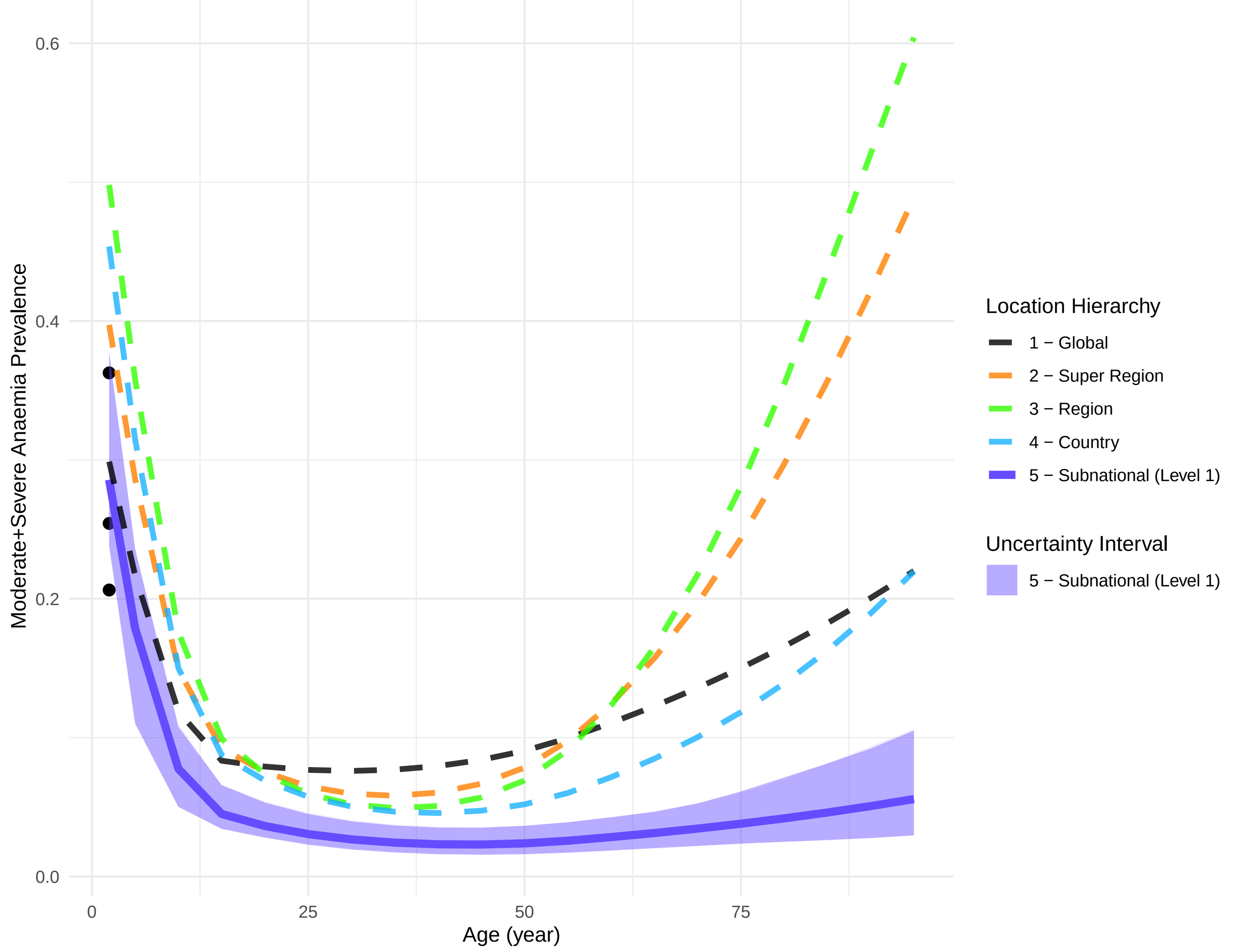

MR−BRT Age/Sex Fits − Males 2+ Years Old − Moderate+Severe Anaemia Prevalence − Borno
WHO−2024 Adjustment Method

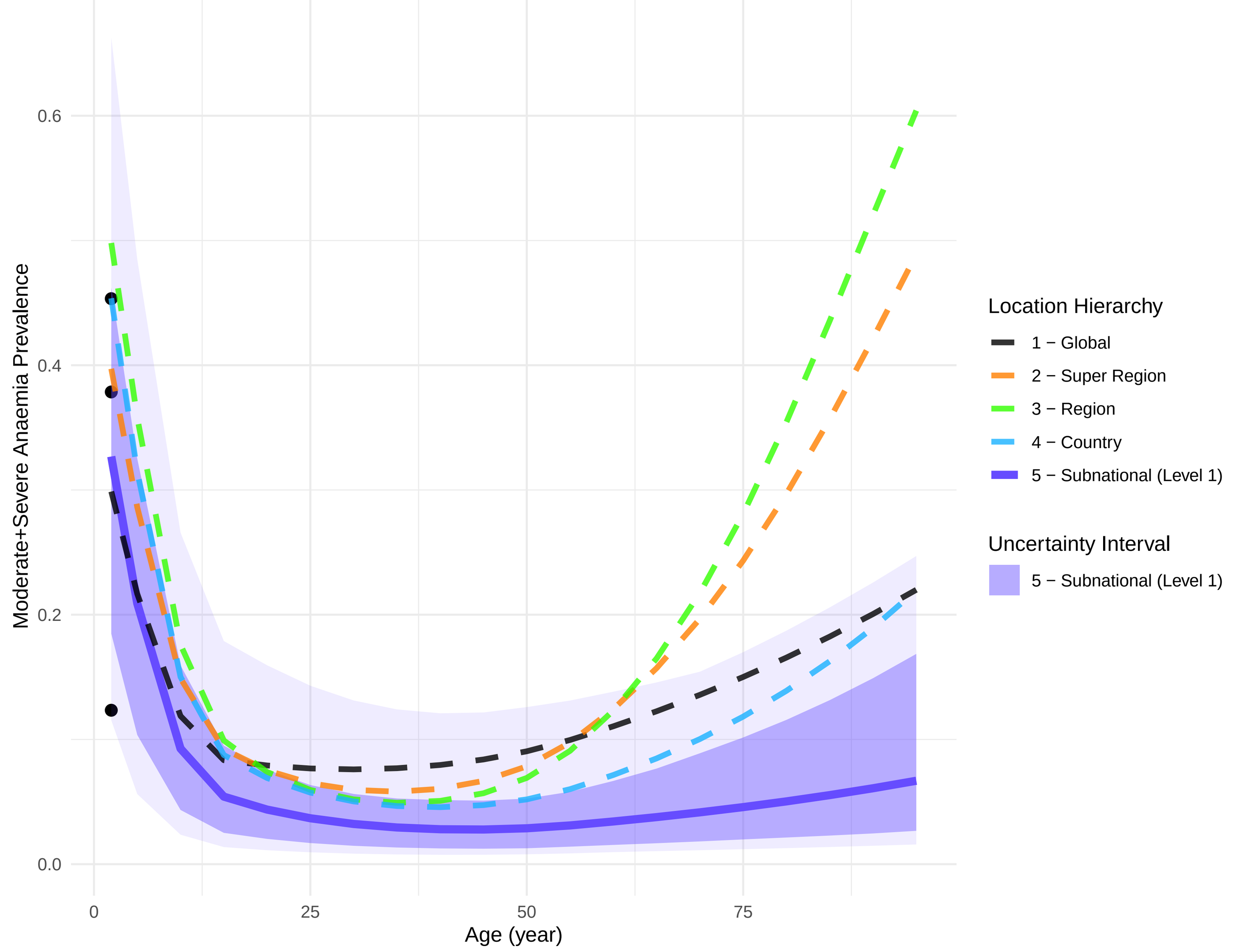

MR−BRT Age/Sex Fits − Males 2+ Years Old − Moderate+Severe Anaemia Prevalence − Cross River
WHO−2024 Adjustment Method

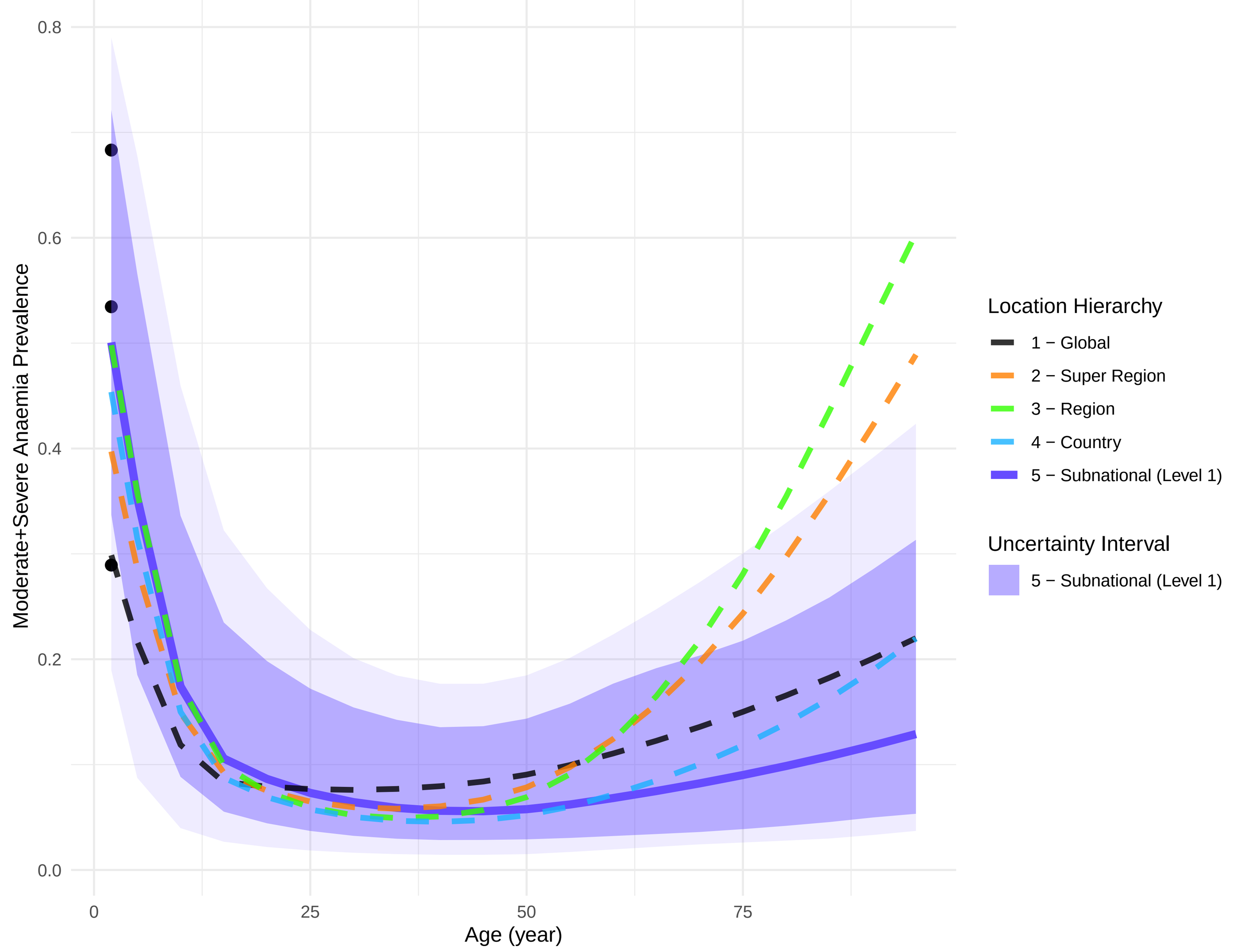

MR−BRT Age/Sex Fits − Males 2+ Years Old − Moderate+Severe Anaemia Prevalence − Delta
WHO−2024 Adjustment Method

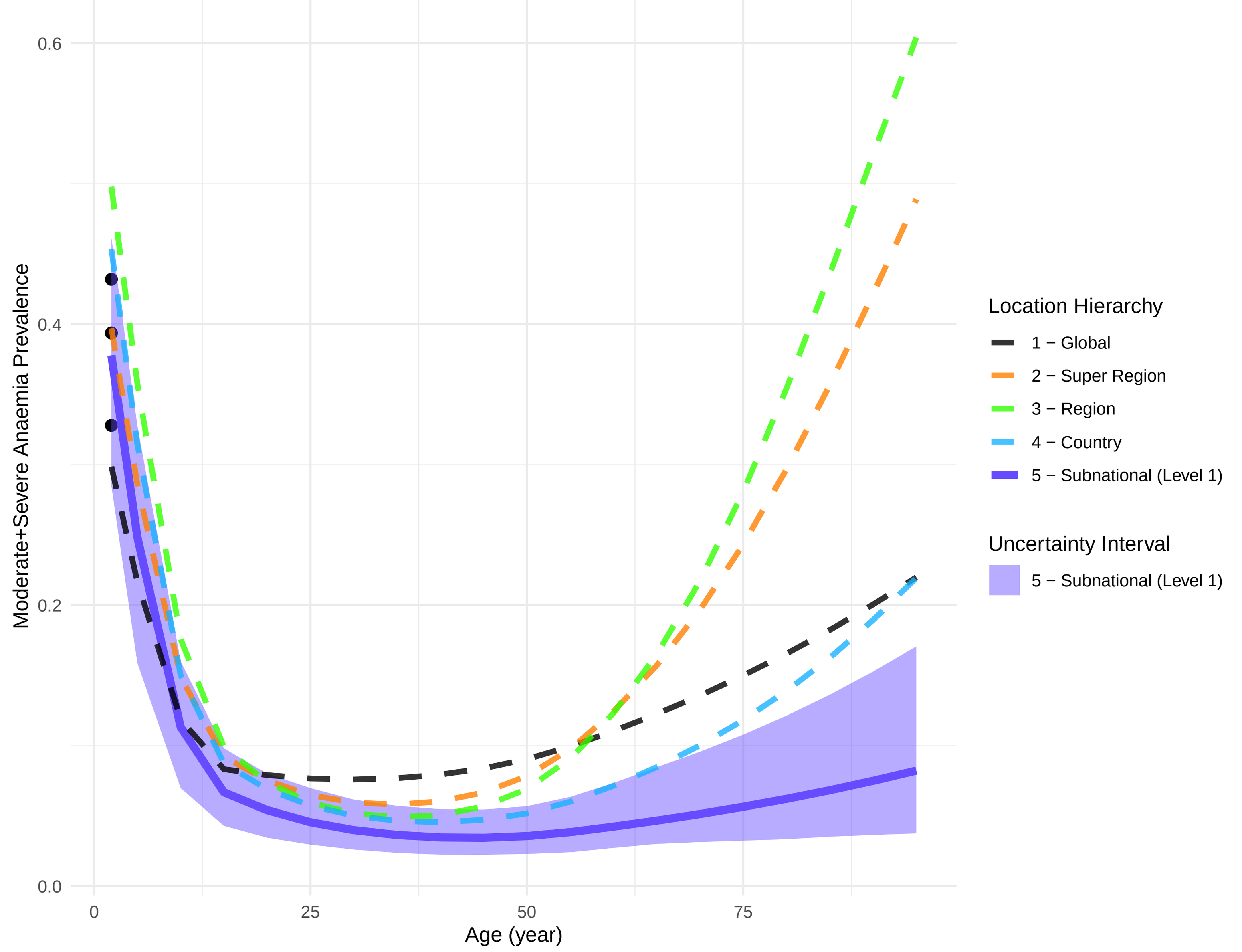

MR−BRT Age/Sex Fits − Males 2+ Years Old − Moderate+Severe Anaemia Prevalence − Ebonyi
WHO−2024 Adjustment Method

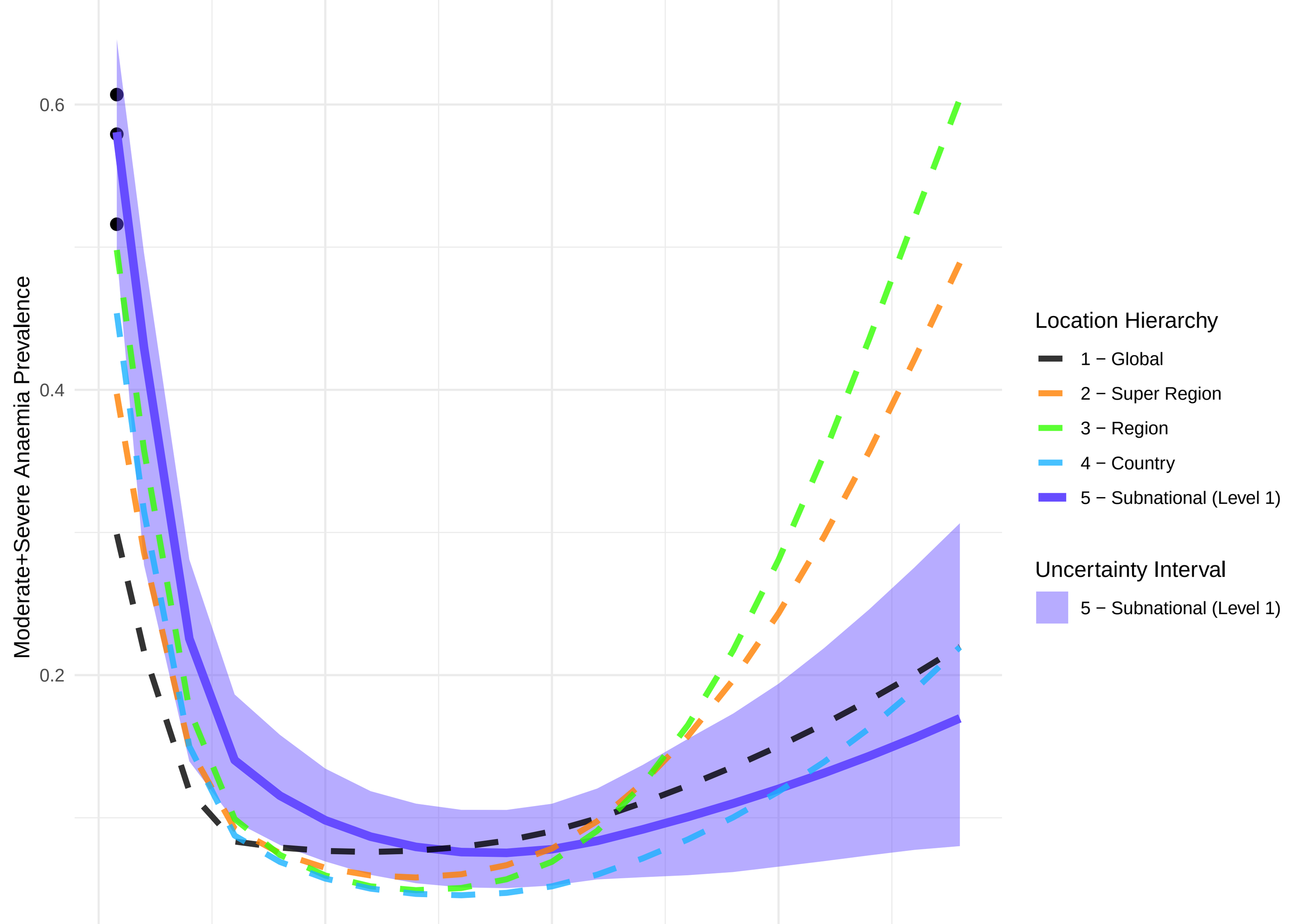

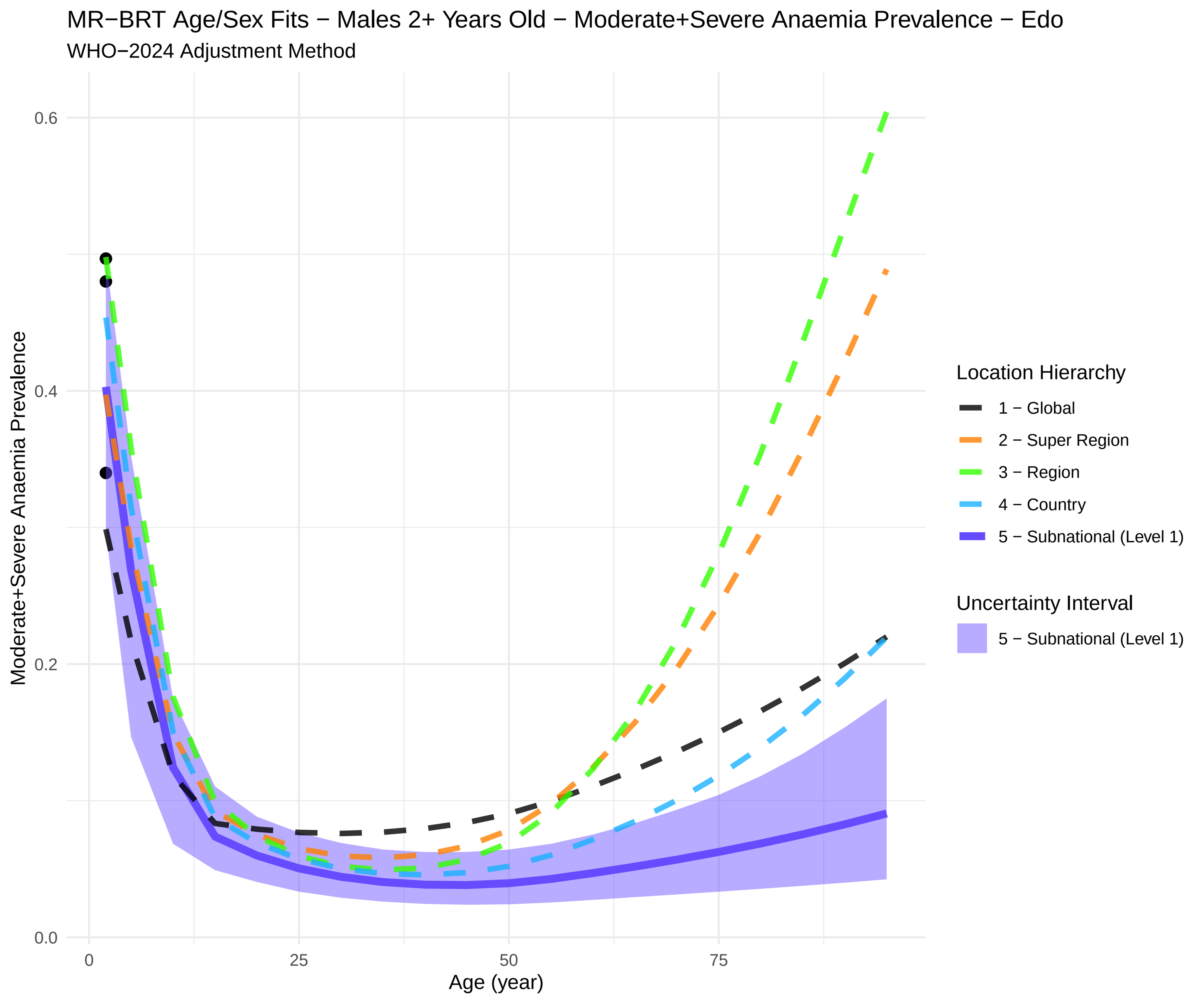
MR−BRT Age/Sex Fits − Males 2+ Years Old − Moderate+Severe Anaemia Prevalence − Edo
WHO−2024 Adjustment Method
Moderate+Severe Anaemia Prevalence
Age (year)
Location Hierarchy
1 − Global
2 − Super Region
3 − Region
4 − Country
5 − Subnational (Level 1)
Uncertainty Interval
5 − Subnational (Level 1)

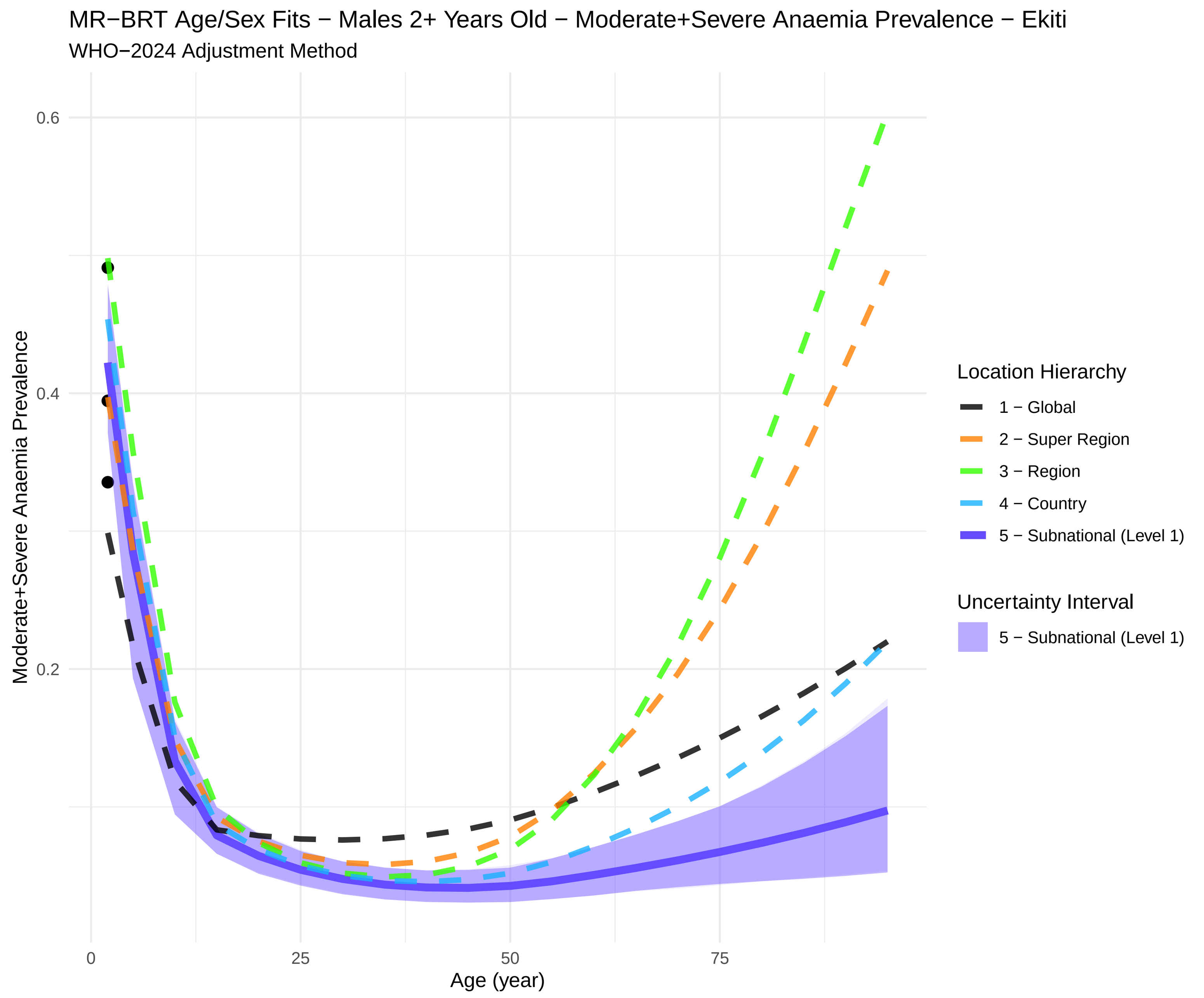
MR−BRT Age/Sex Fits − Males 2+ Years Old − Moderate+Severe Anaemia Prevalence − Ekiti
WHO−2024 Adjustment Method
Moderate+Severe Anaemia Prevalence
Age (year)
Location Hierarchy
1 − Global
2 − Super Region
3 − Region
4 − Country
5 − Subnational (Level 1)
Uncertainty Interval
5 − Subnational (Level 1)

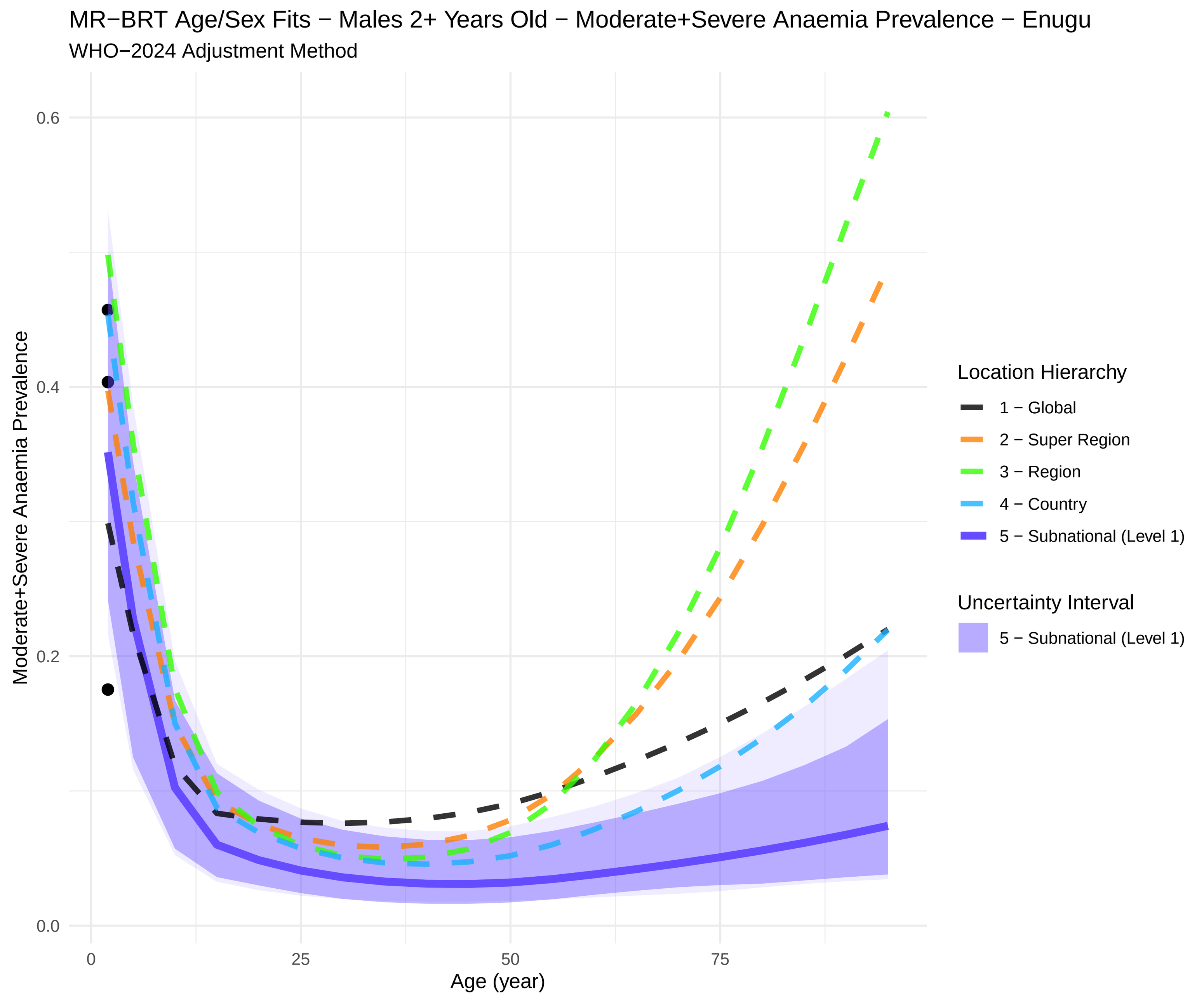
MR−BRT Age/Sex Fits − Males 2+ Years Old − Moderate+Severe Anaemia Prevalence − Enugu
WHO−2024 Adjustment Method
Moderate+Severe Anaemia Prevalence
Age (year)
Location Hierarchy
1 − Global
2 − Super Region
3 − Region
4 − Country
5 − Subnational (Level 1)
Uncertainty Interval
5 − Subnational (Level 1)

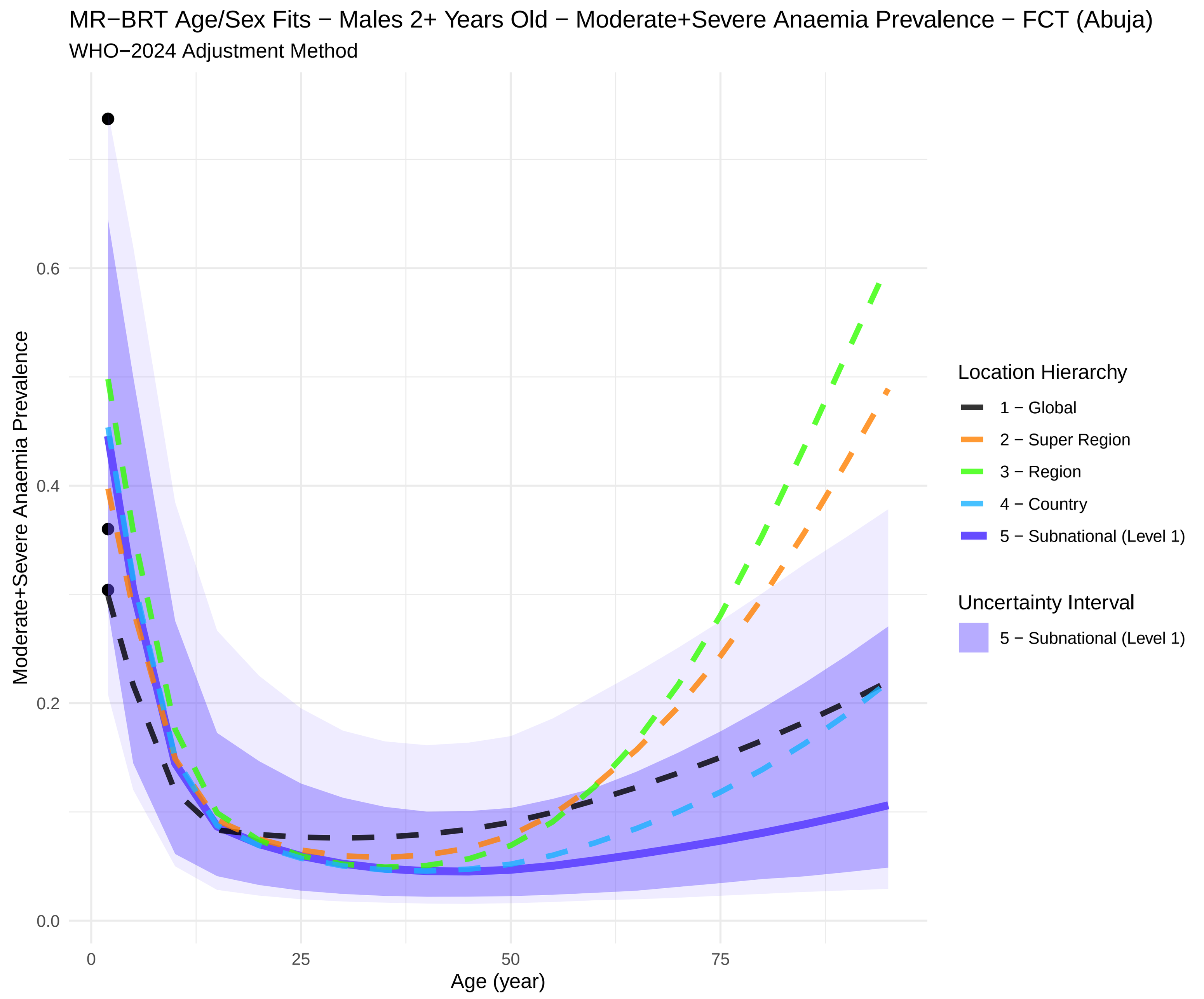
MR−BRT Age/Sex Fits − Males 2+ Years Old − Moderate+Severe Anaemia Prevalence − FCT (Abuja)
WHO−2024 Adjustment Method
Moderate+Severe Anaemia Prevalence
Age (year)
Location Hierarchy
1 − Global
2 − Super Region
3 − Region
4 − Country
5 − Subnational (Level 1)
Uncertainty Interval
5 − Subnational (Level 1)

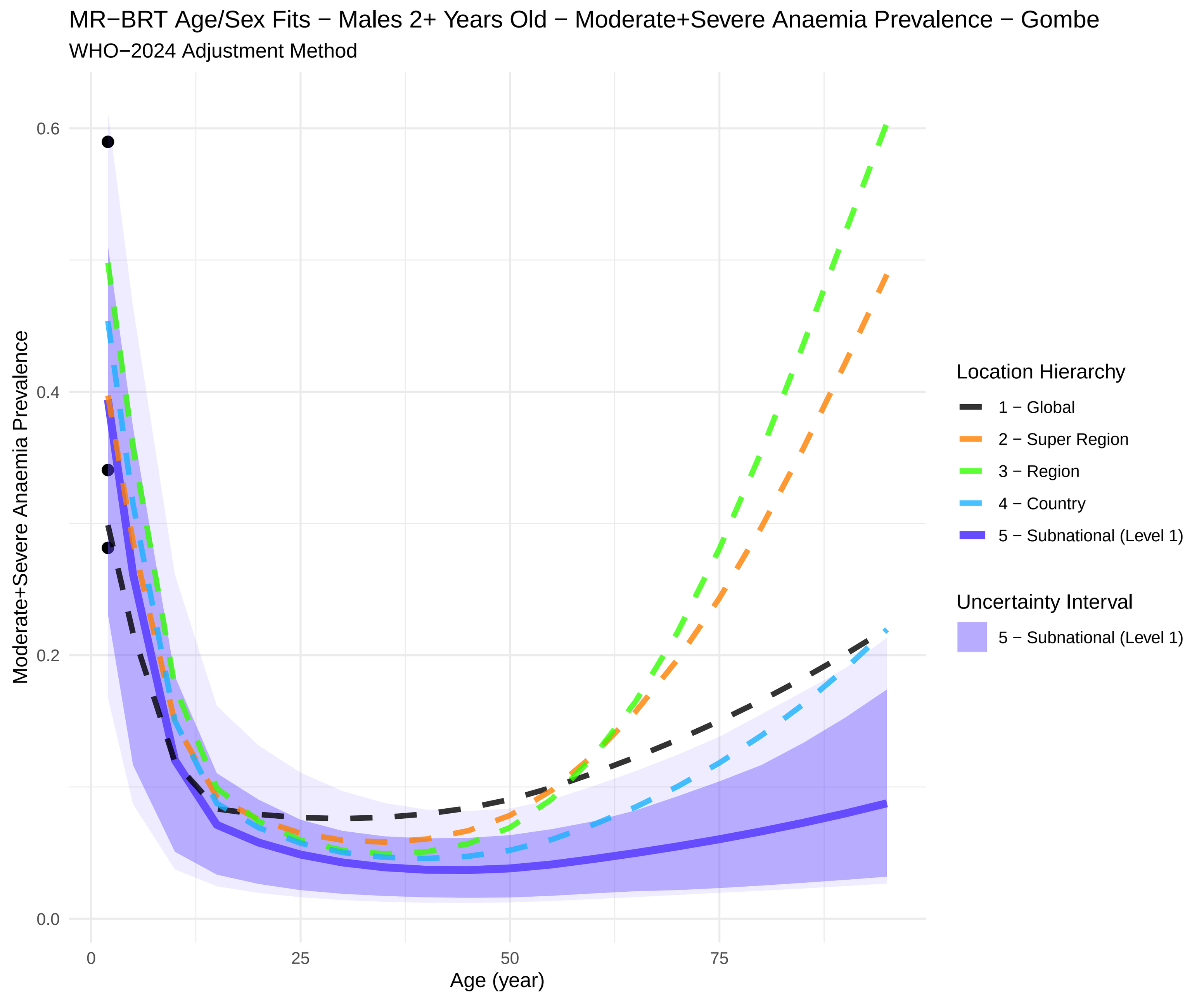
MR−BRT Age/Sex Fits − Males 2+ Years Old − Moderate+Severe Anaemia Prevalence − Gombe
WHO−2024 Adjustment Method
Moderate+Severe Anaemia Prevalence
Age (year)
Location Hierarchy
1 − Global
2 − Super Region
3 − Region
4 − Country
5 − Subnational (Level 1)
Uncertainty Interval
5 − Subnational (Level 1)

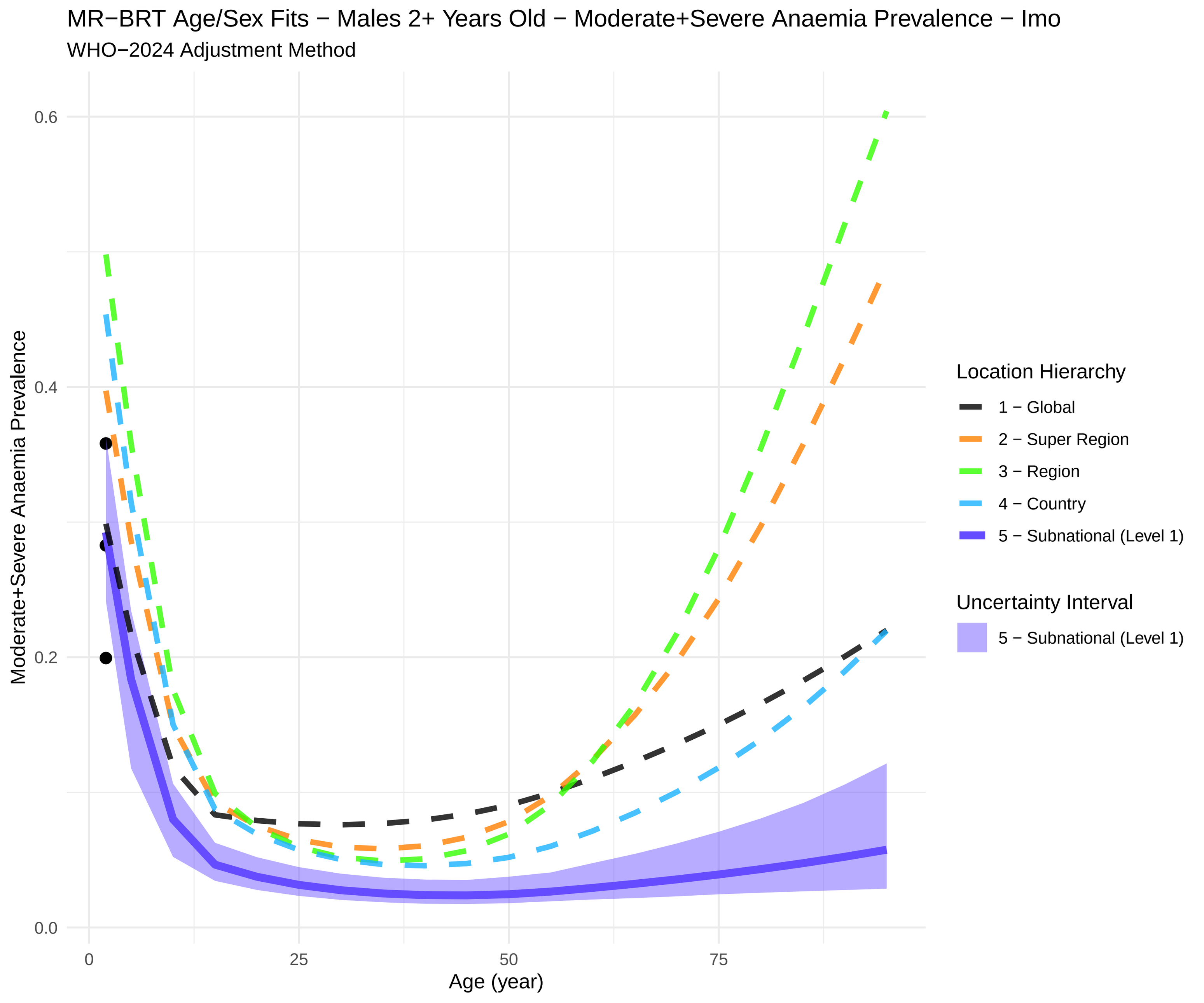
MR−BRT Age/Sex Fits − Males 2+ Years Old − Moderate+Severe Anaemia Prevalence − Imo
WHO−2024 Adjustment Method
Moderate+Severe Anaemia Prevalence
Age (year)
Location Hierarchy
1 − Global
2 − Super Region
3 − Region
4 − Country
5 − Subnational (Level 1)
Uncertainty Interval
5 − Subnational (Level 1)

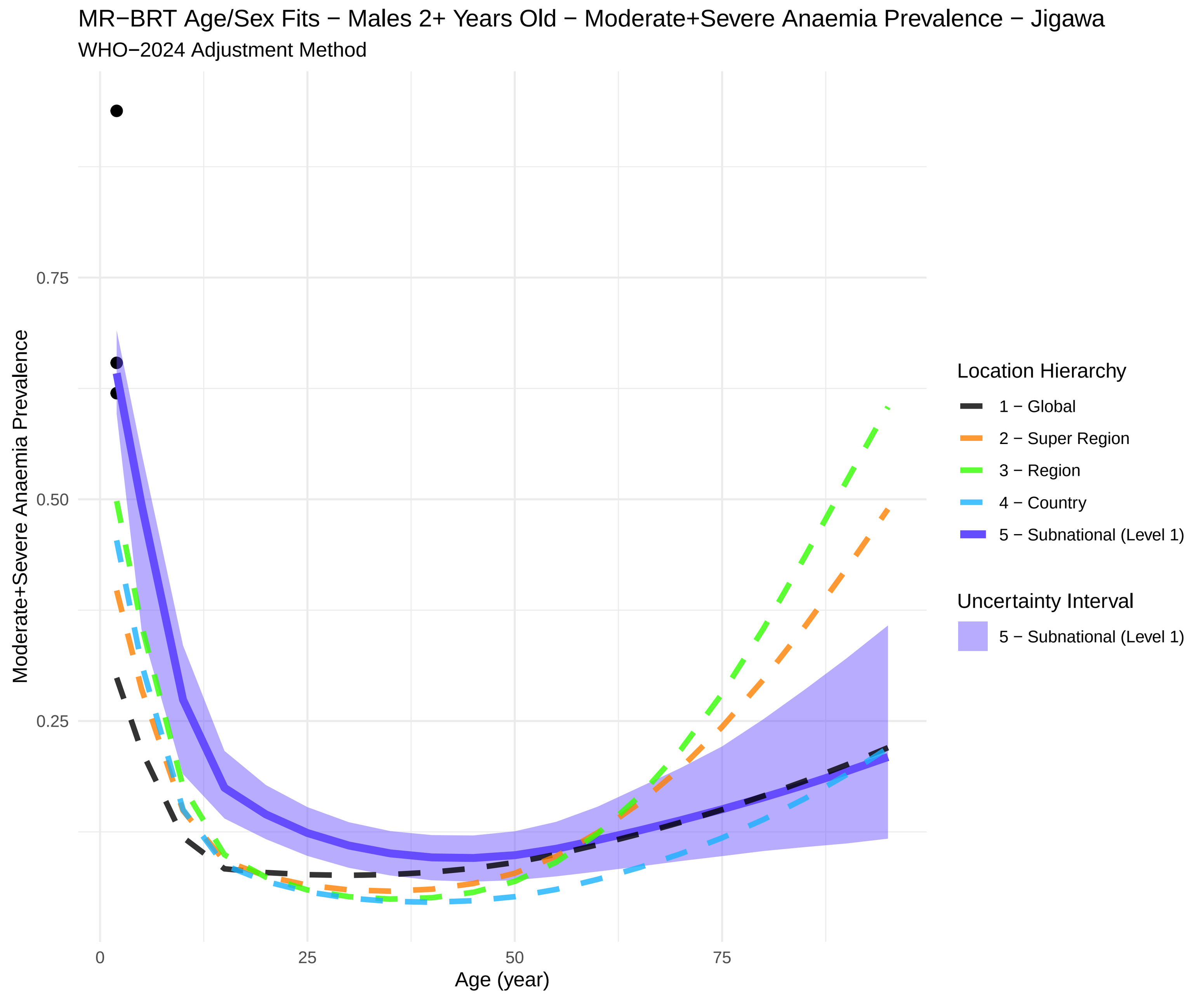
MR−BRT Age/Sex Fits − Males 2+ Years Old − Moderate+Severe Anaemia Prevalence − Jigawa
WHO−2024 Adjustment Method
Moderate+Severe Anaemia Prevalence
Age (year)
Location Hierarchy
1 − Global
2 − Super Region
3 − Region
4 − Country
5 − Subnational (Level 1)
Uncertainty Interval
5 − Subnational (Level 1)

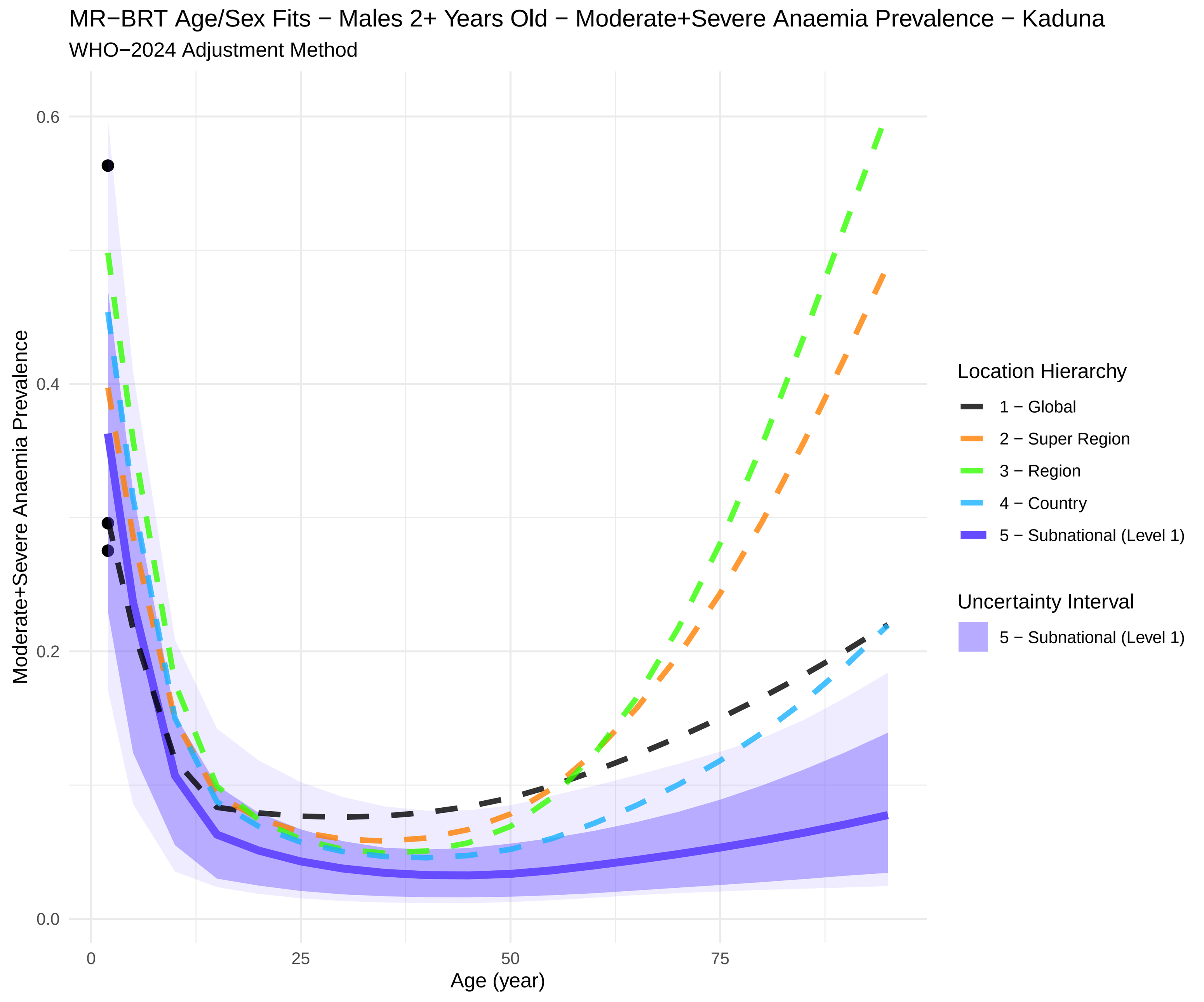
MR−BRT Age/Sex Fits − Males 2+ Years Old − Moderate+Severe Anaemia Prevalence − Kaduna
WHO−2024 Adjustment Method
Moderate+Severe Anaemia Prevalence
Age (year)
Location Hierarchy
1 − Global
2 − Super Region
3 − Region
4 − Country
5 − Subnational (Level 1)
Uncertainty Interval
5 − Subnational (Level 1)

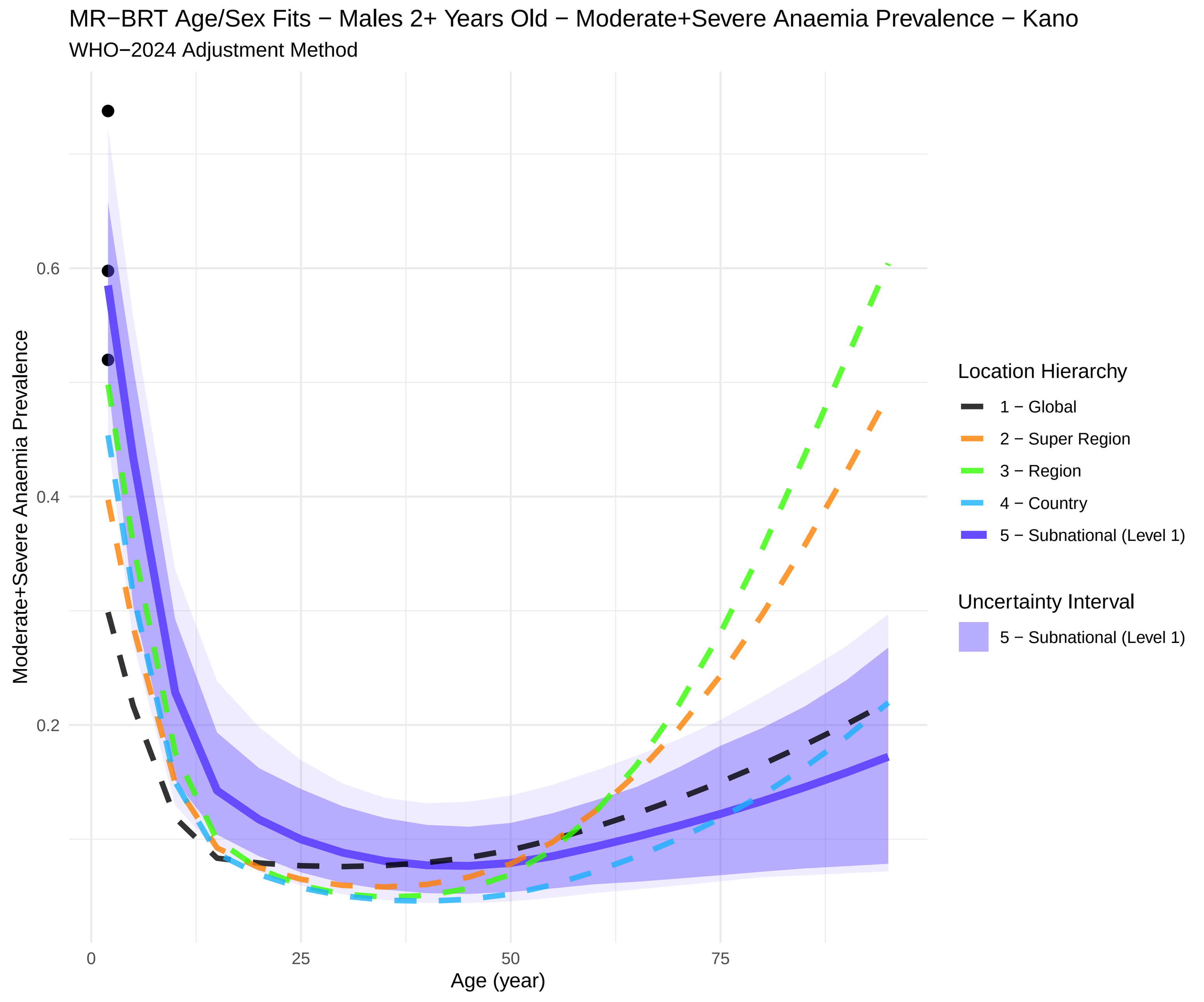
MR−BRT Age/Sex Fits − Males 2+ Years Old − Moderate+Severe Anaemia Prevalence − Kano
WHO−2024 Adjustment Method
Moderate+Severe Anaemia Prevalence
Age (year)
Location Hierarchy
1 − Global
2 − Super Region
3 − Region
4 − Country
5 − Subnational (Level 1)
Uncertainty Interval
5 − Subnational (Level 1)

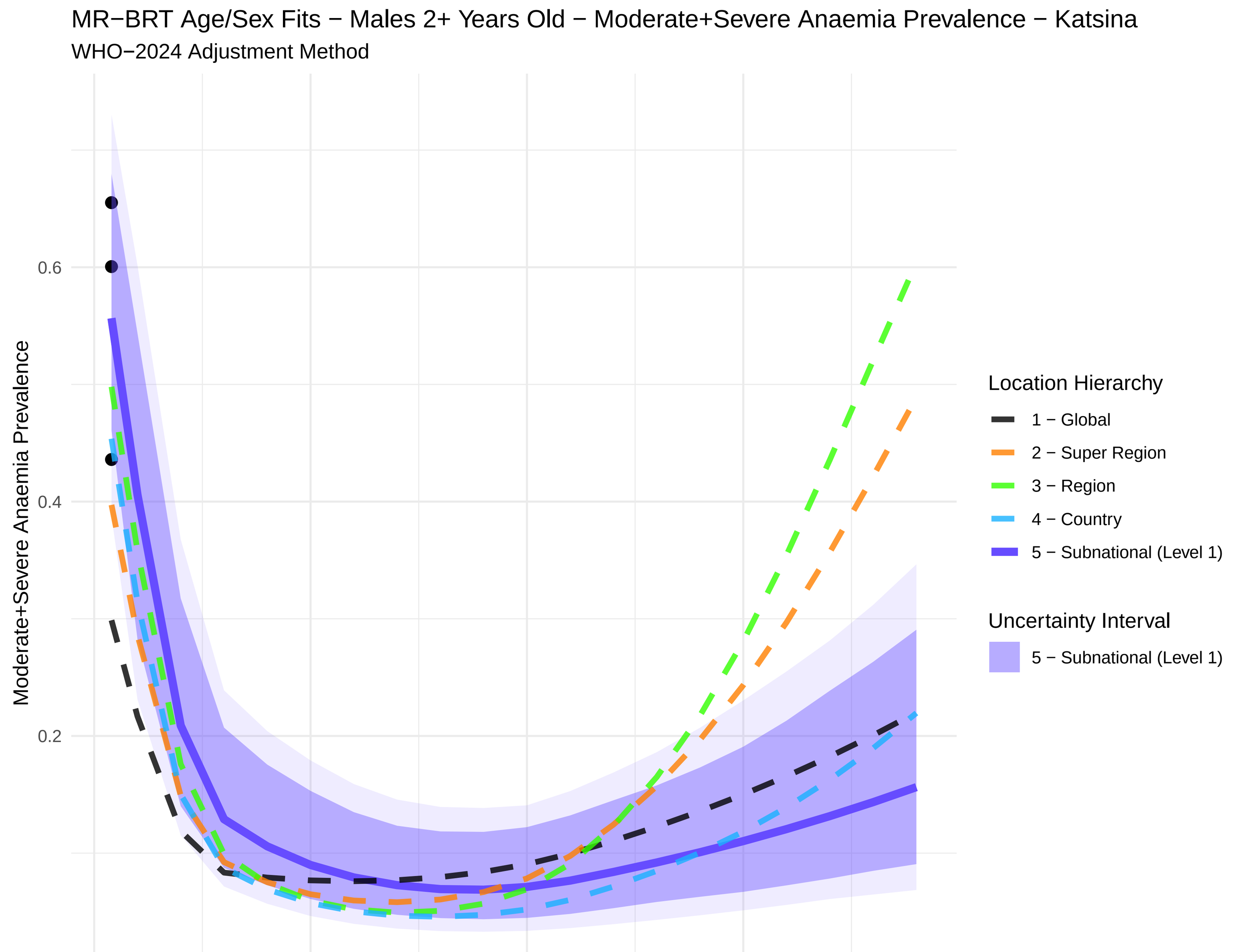
MR−BRT Age/Sex Fits − Males 2+ Years Old − Moderate+Severe Anaemia Prevalence − Katsina
WHO−2024 Adjustment Method
Moderate+Severe Anaemia Prevalence
Age (year)
Location Hierarchy
1 − Global
2 − Super Region
3 − Region
4 − Country
5 − Subnational (Level 1)
Uncertainty Interval
5 − Subnational (Level 1)

MR−BRT Age/Sex Fits − Males 2+ Years Old − Moderate+Severe Anaemia Prevalence − Kebbi
WHO−2024 Adjustment Method

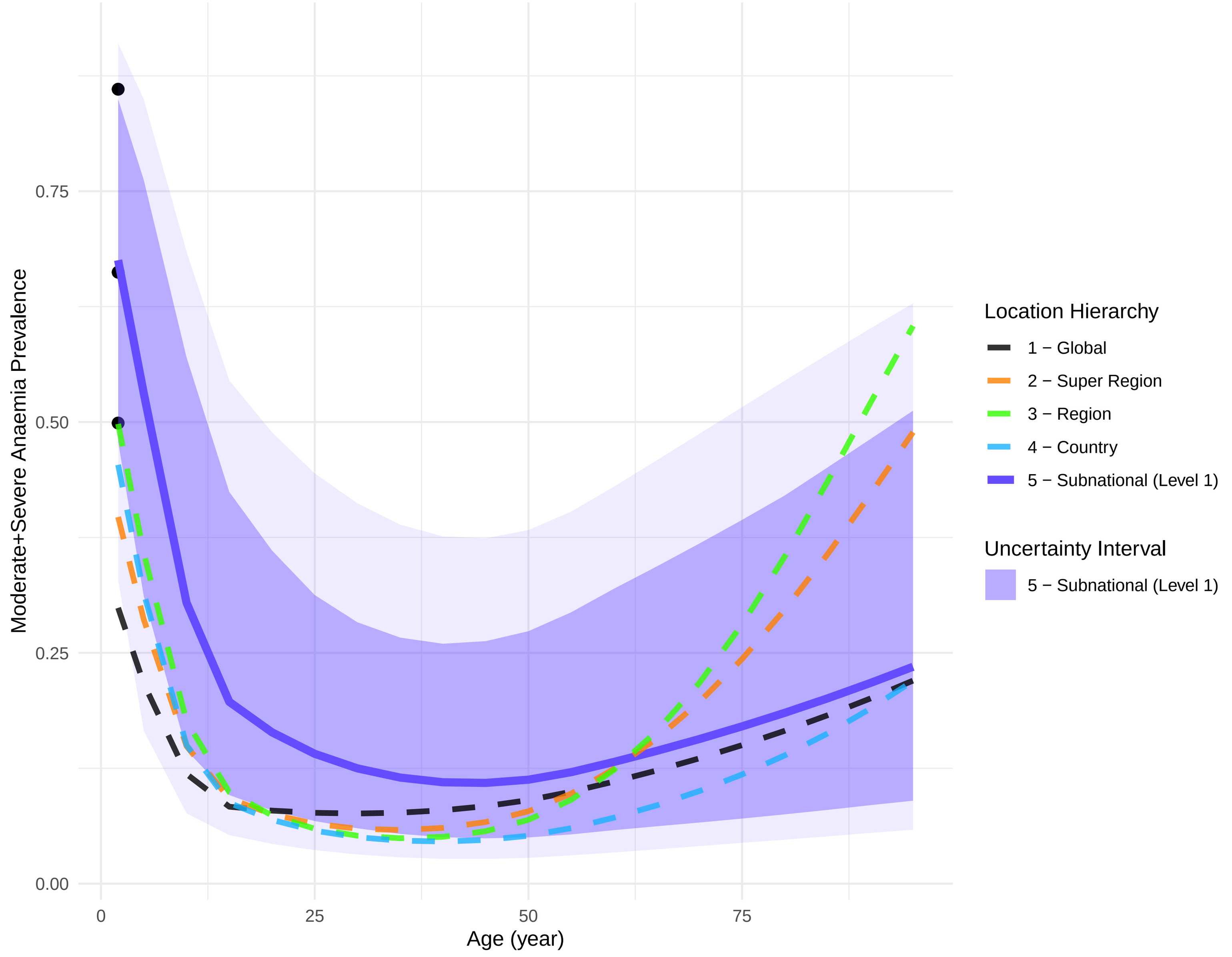

MR−BRT Age/Sex Fits − Males 2+ Years Old − Moderate+Severe Anaemia Prevalence − Kogi
WHO−2024 Adjustment Method

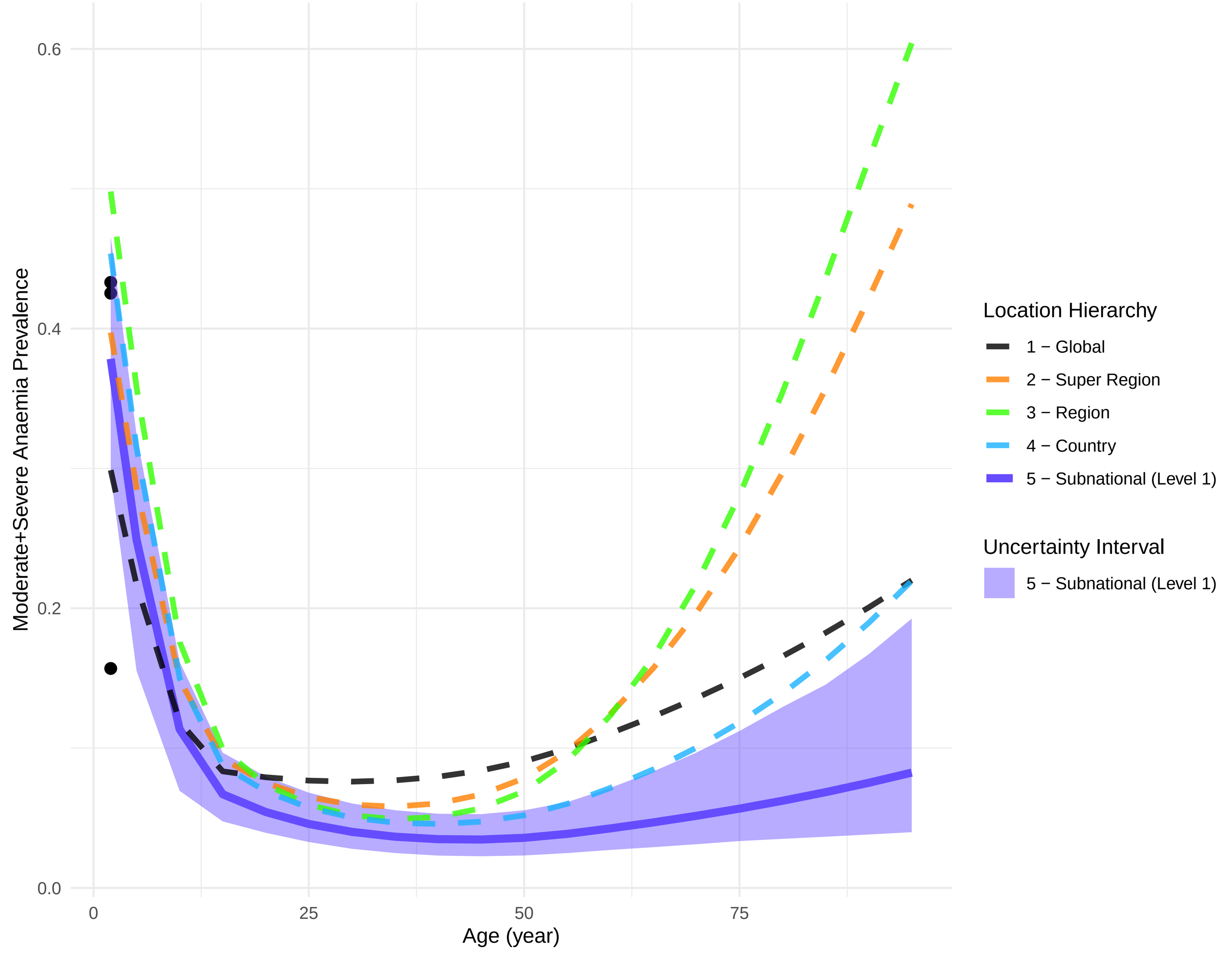

MR−BRT Age/Sex Fits − Males 2+ Years Old − Moderate+Severe Anaemia Prevalence − Kwara
WHO−2024 Adjustment Method

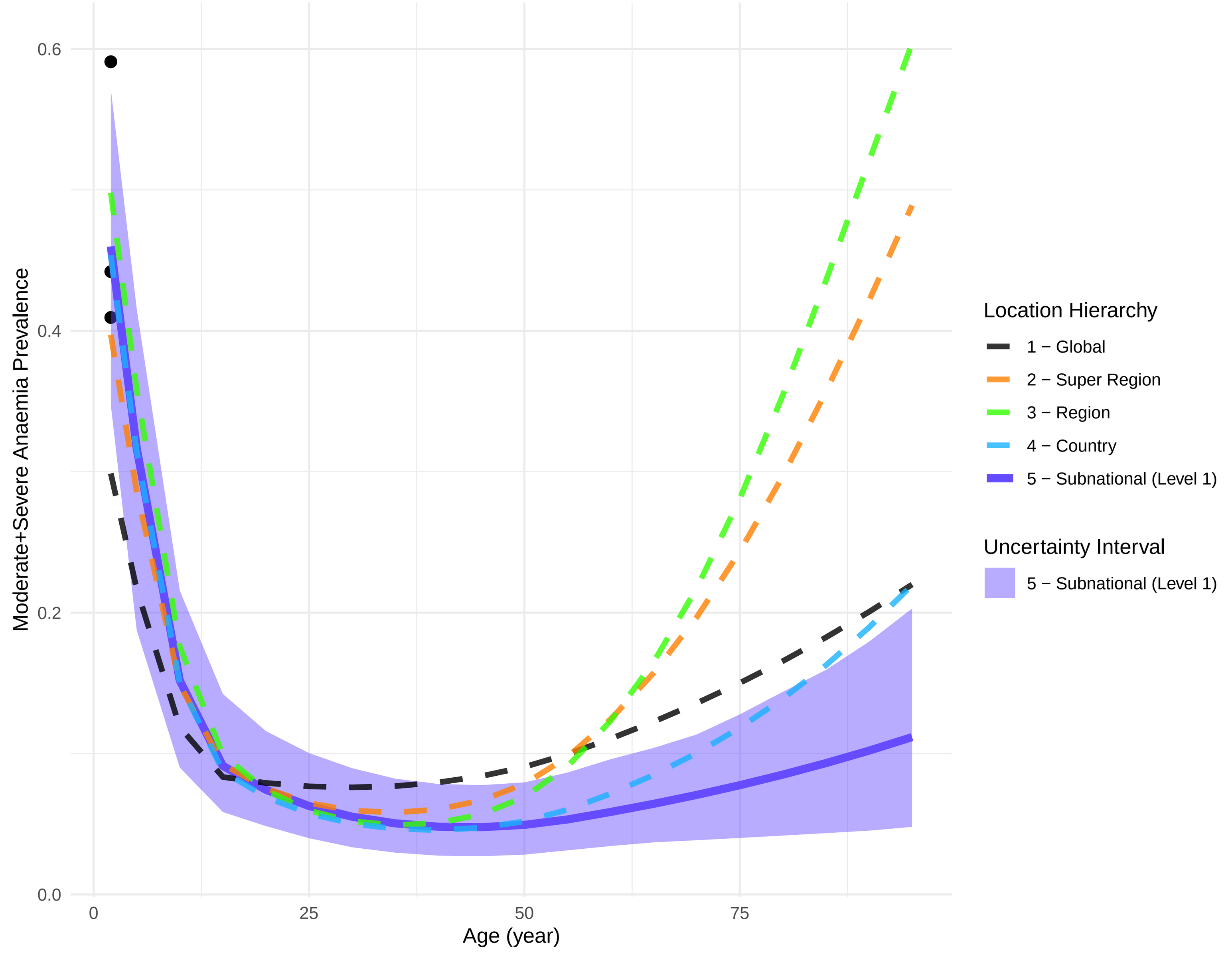

MR−BRT Age/Sex Fits − Males 2+ Years Old − Moderate+Severe Anaemia Prevalence − Lagos
WHO−2024 Adjustment Method

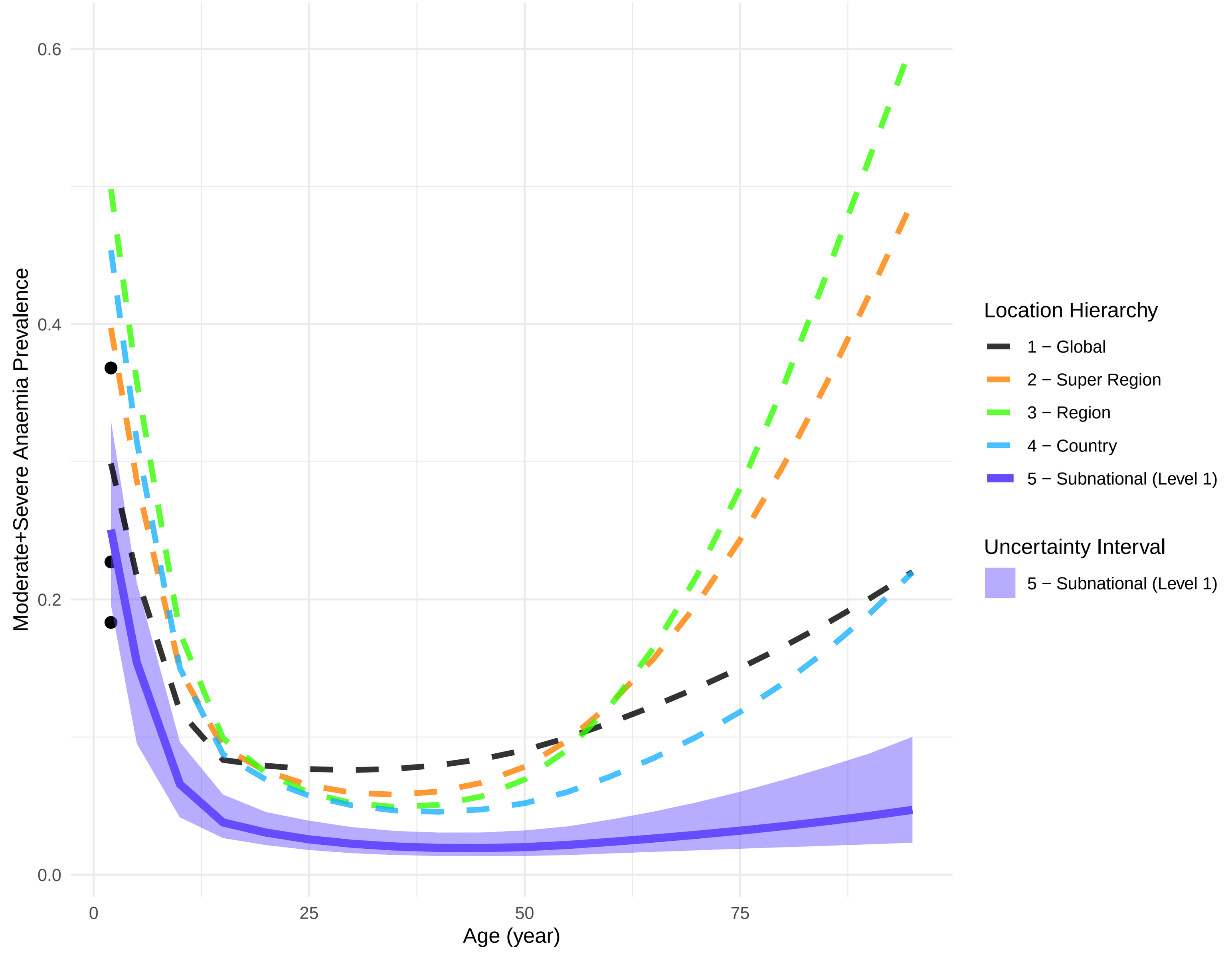

MR−BRT Age/Sex Fits − Males 2+ Years Old − Moderate+Severe Anaemia Prevalence − Nasarawa
WHO−2024 Adjustment Method

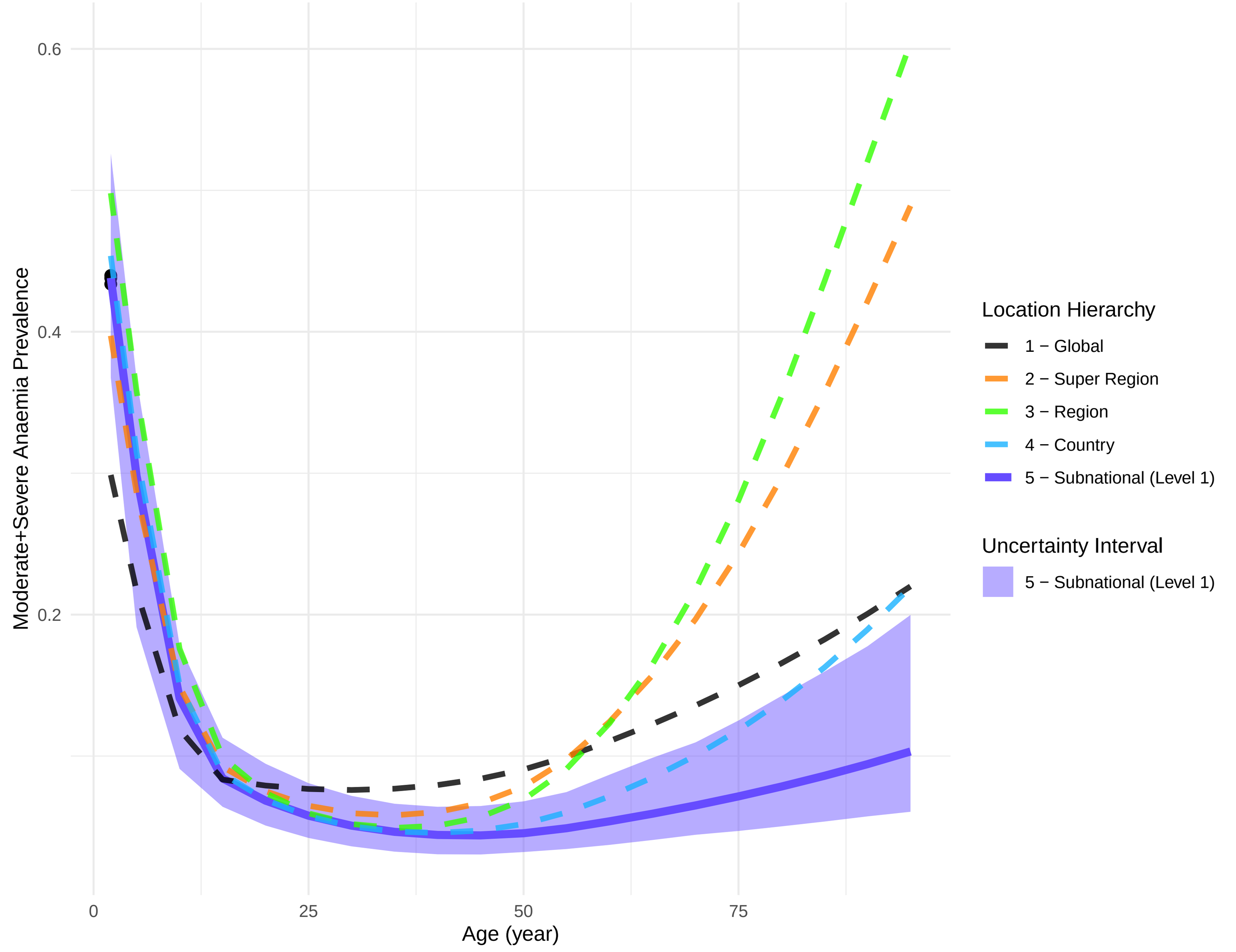

MR−BRT Age/Sex Fits − Males 2+ Years Old − Moderate+Severe Anaemia Prevalence − Niger
WHO−2024 Adjustment Method

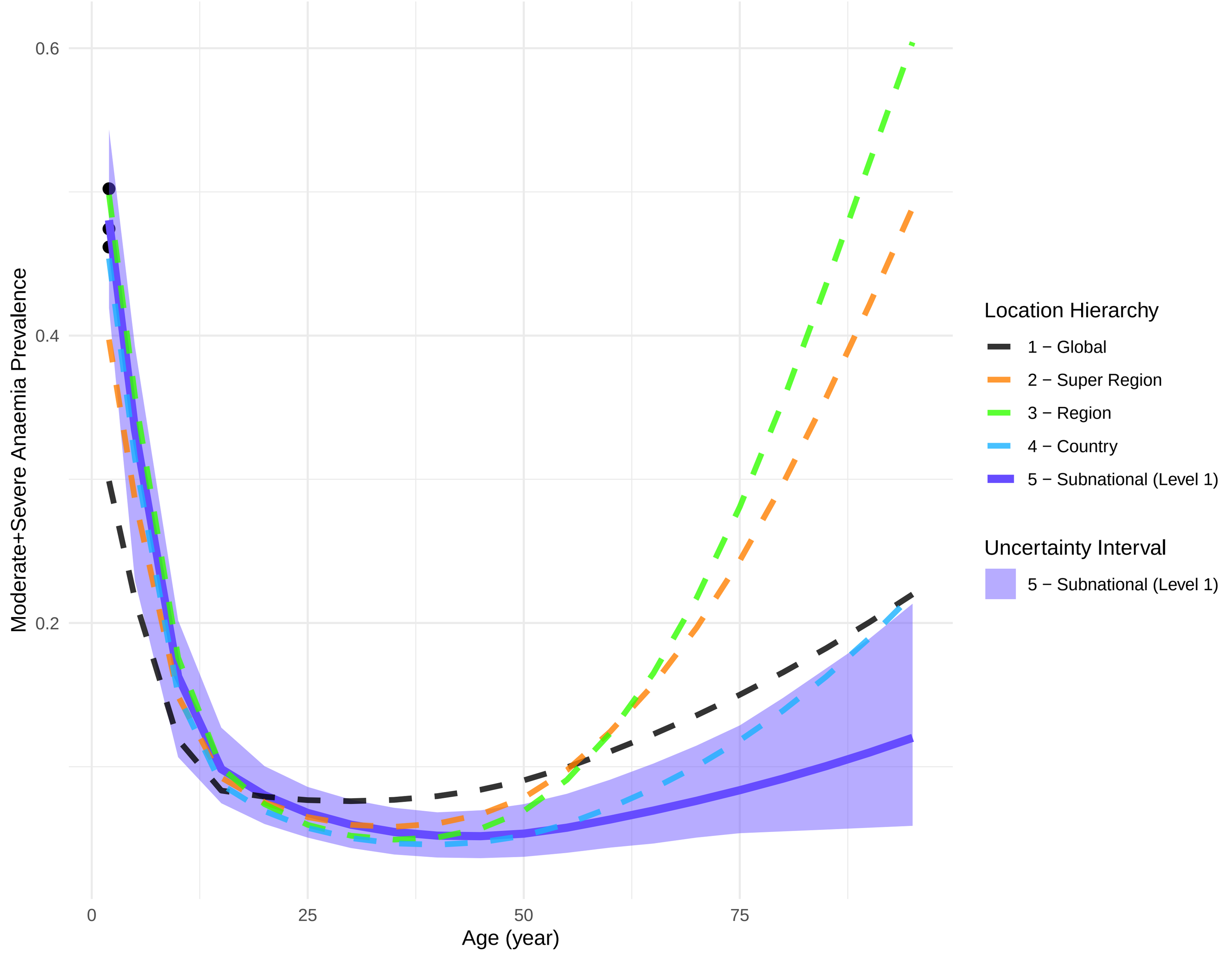

MR−BRT Age/Sex Fits − Males 2+ Years Old − Moderate+Severe Anaemia Prevalence − Ogun
WHO−2024 Adjustment Method

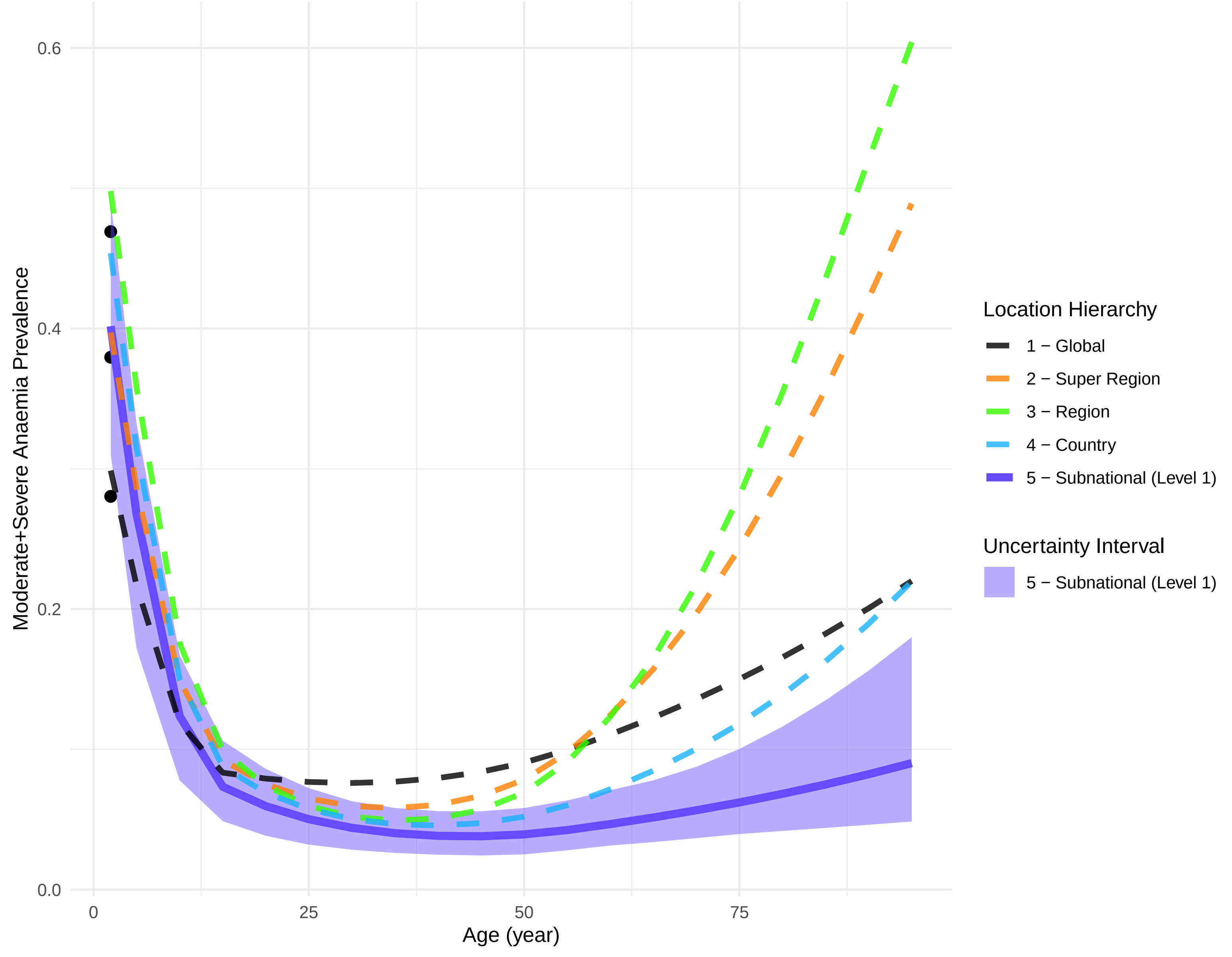

MR−BRT Age/Sex Fits − Males 2+ Years Old − Moderate+Severe Anaemia Prevalence − Ondo
WHO−2024 Adjustment Method

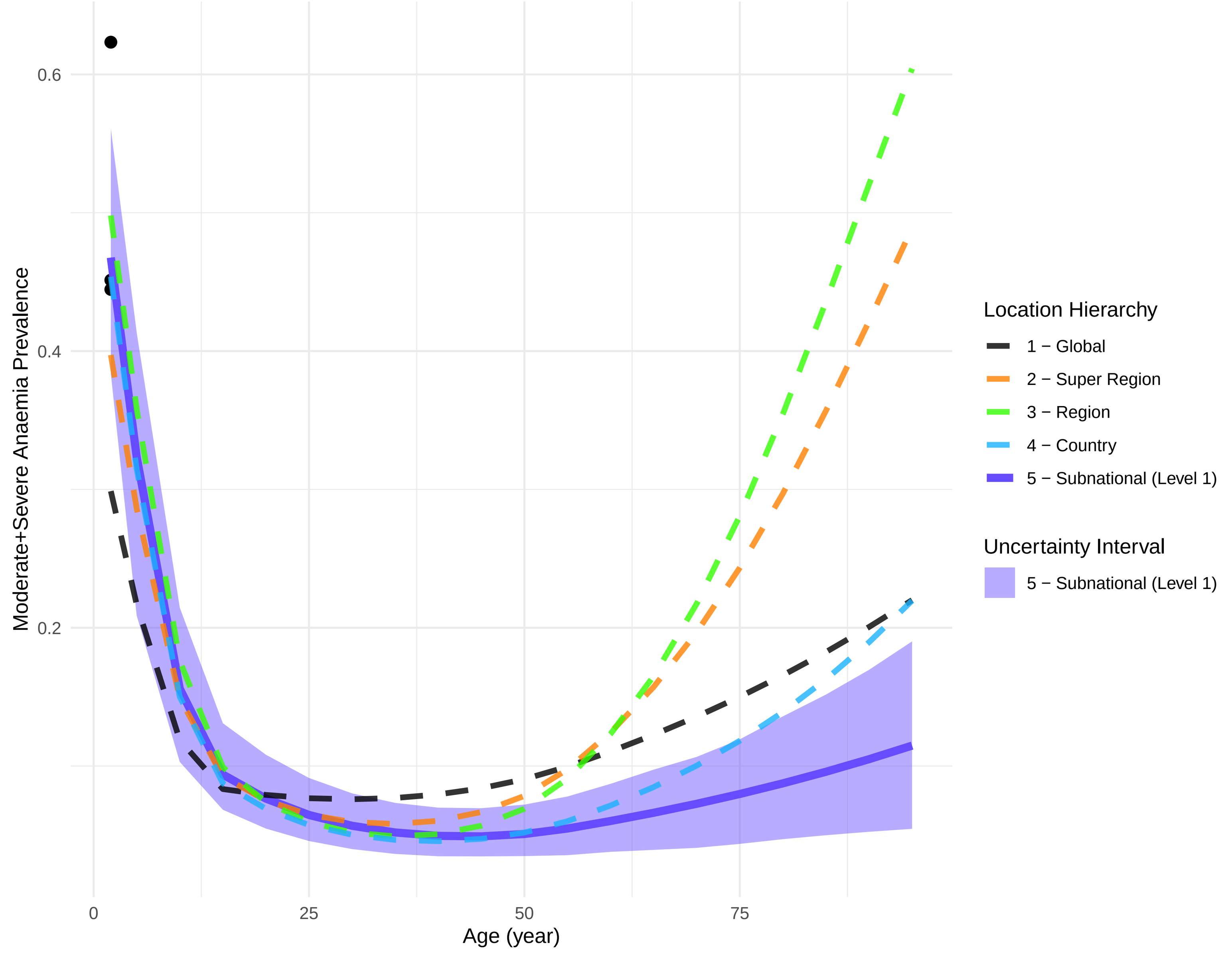

MR−BRT Age/Sex Fits − Males 2+ Years Old − Moderate+Severe Anaemia Prevalence − Osun
WHO−2024 Adjustment Method

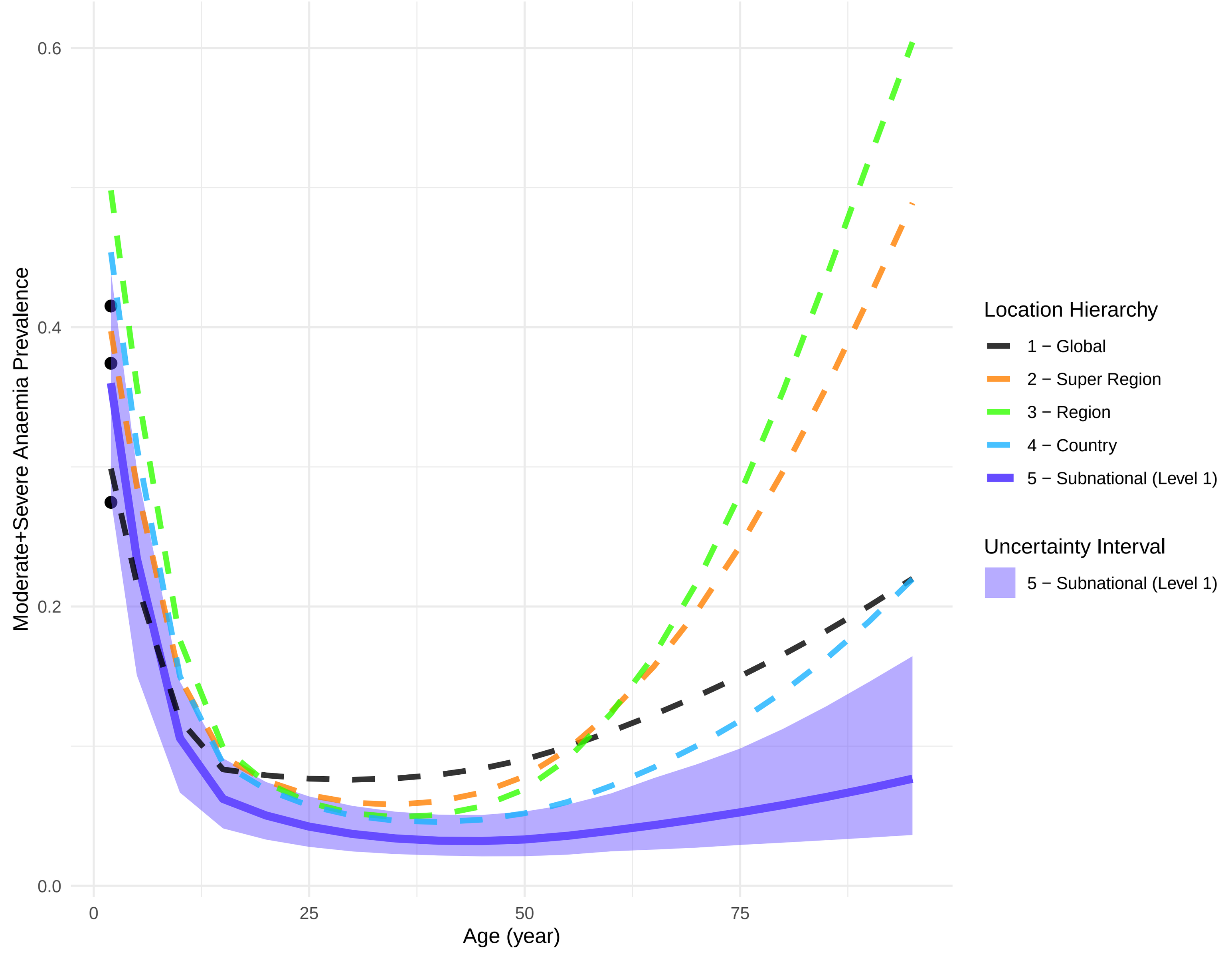

MR−BRT Age/Sex Fits − Males 2+ Years Old − Moderate+Severe Anaemia Prevalence − Oyo
WHO−2024 Adjustment Method

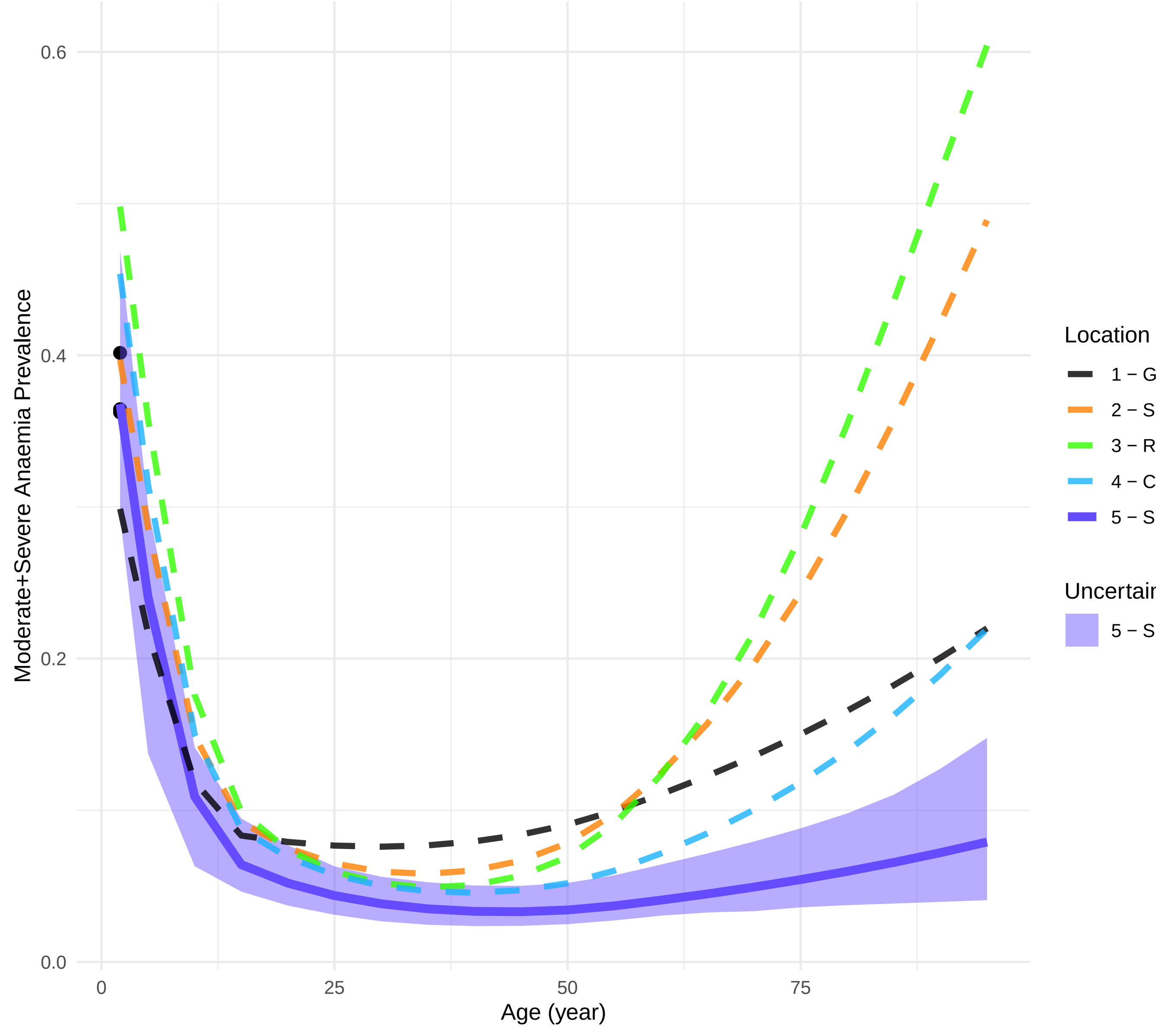

MR−BRT Age/Sex Fits − Males 2+ Years Old − Moderate+Severe Anaemia Prevalence − Plateau
WHO−2024 Adjustment Method

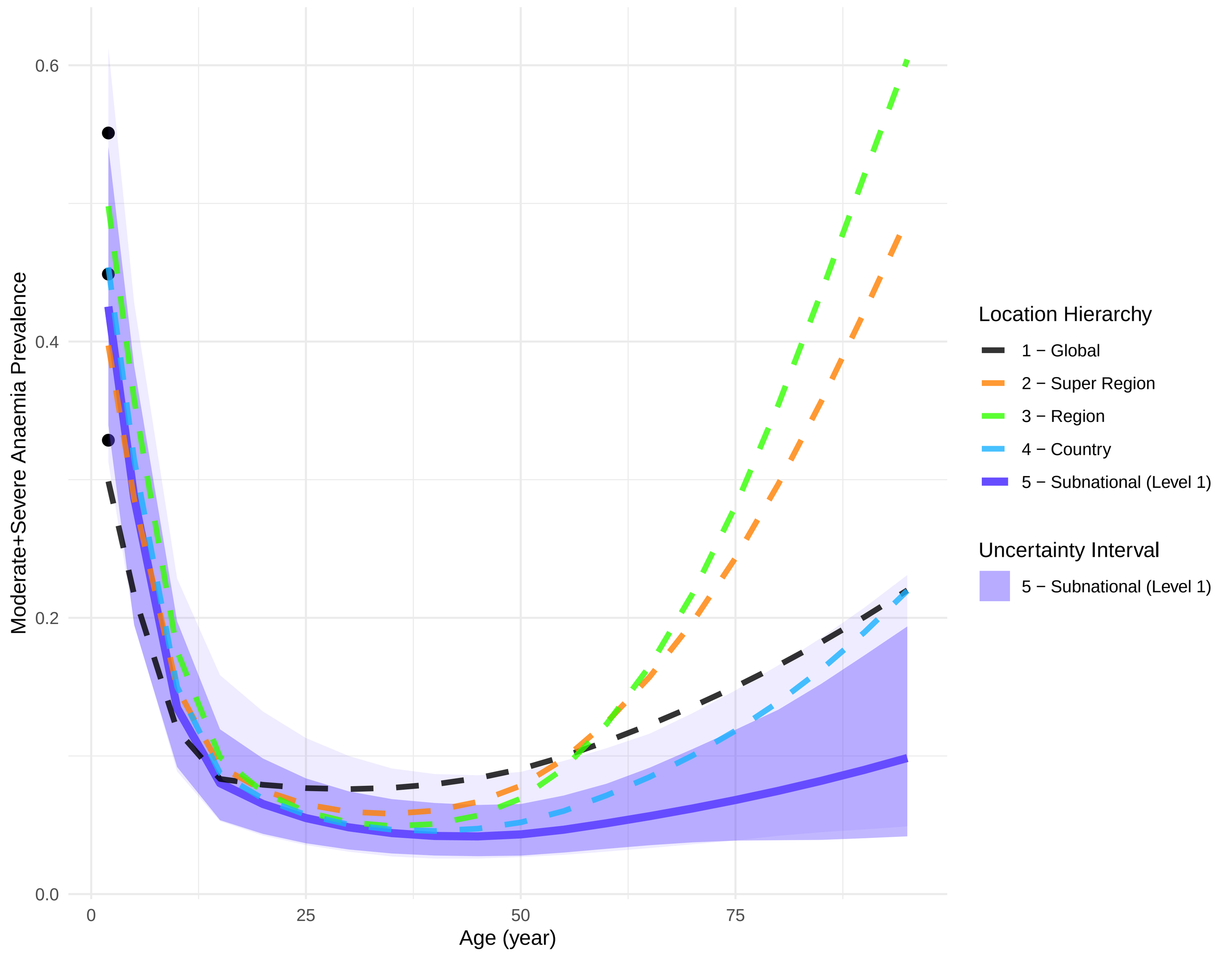

MR−BRT Age/Sex Fits − Males 2+ Years Old − Moderate+Severe Anaemia Prevalence − Rivers
WHO−2024 Adjustment Method

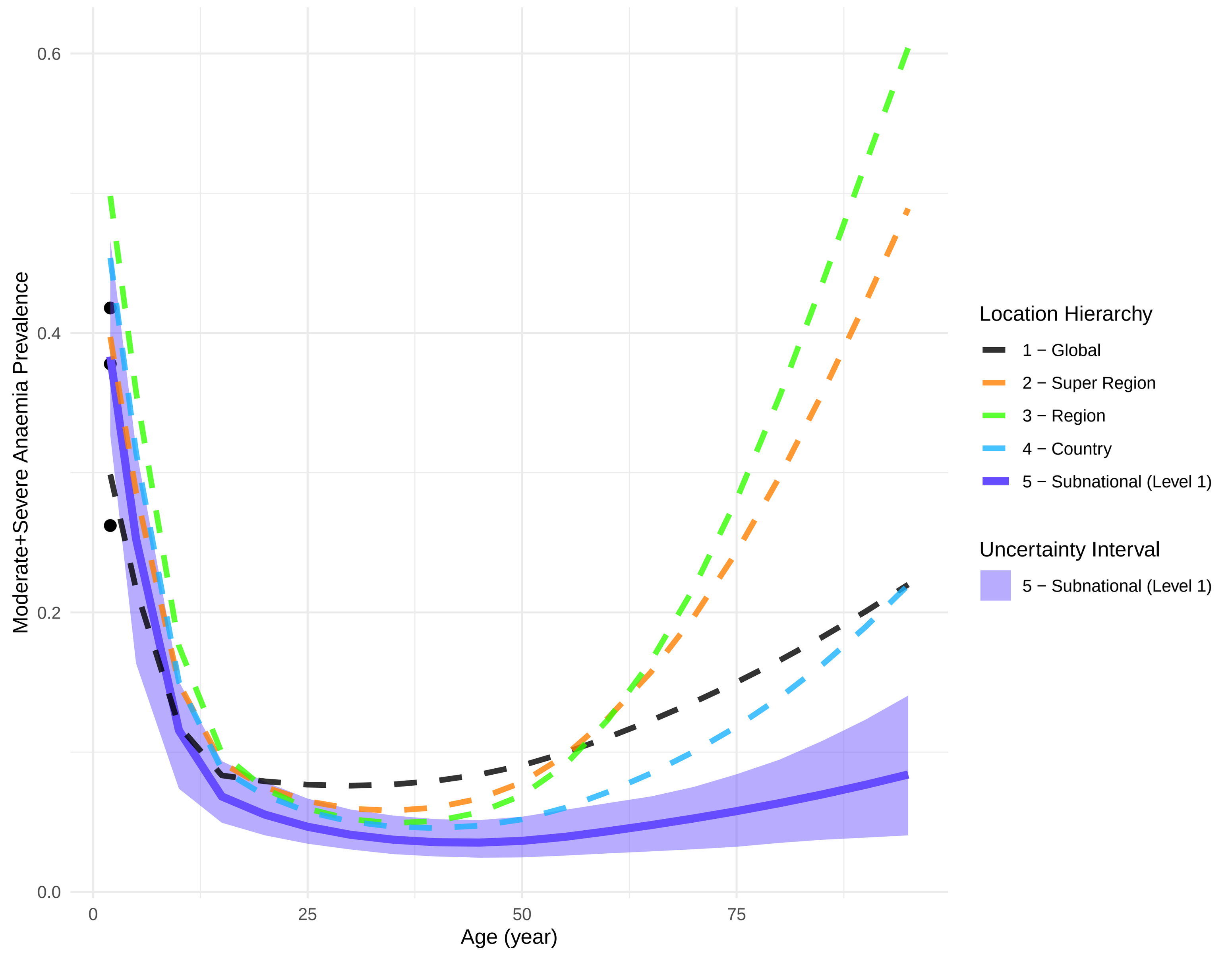

MR−BRT Age/Sex Fits − Males 2+ Years Old − Moderate+Severe Anaemia Prevalence − Sokoto
WHO−2024 Adjustment Method

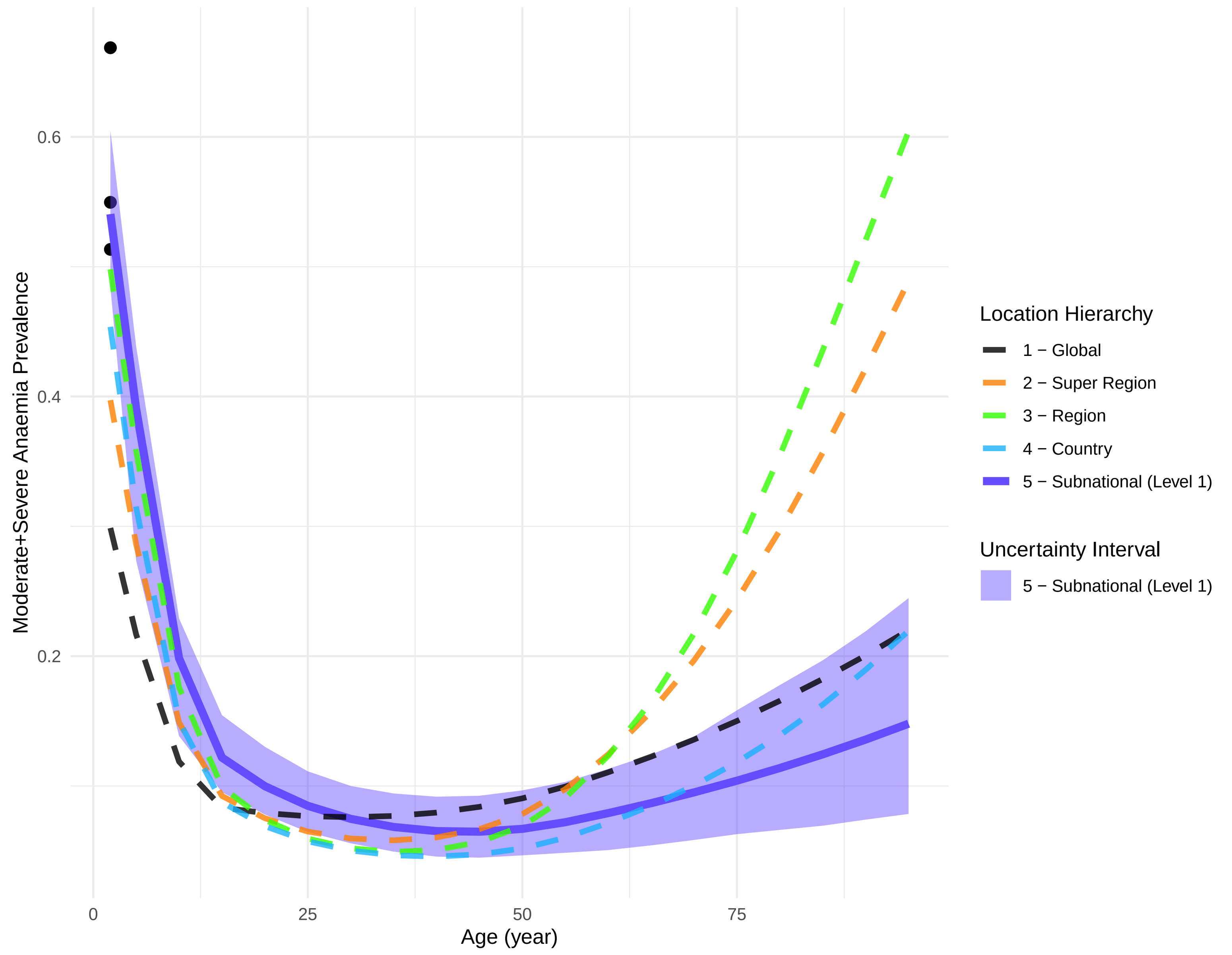

MR−BRT Age/Sex Fits − Males 2+ Years Old − Moderate+Severe Anaemia Prevalence − Taraba
WHO−2024 Adjustment Method

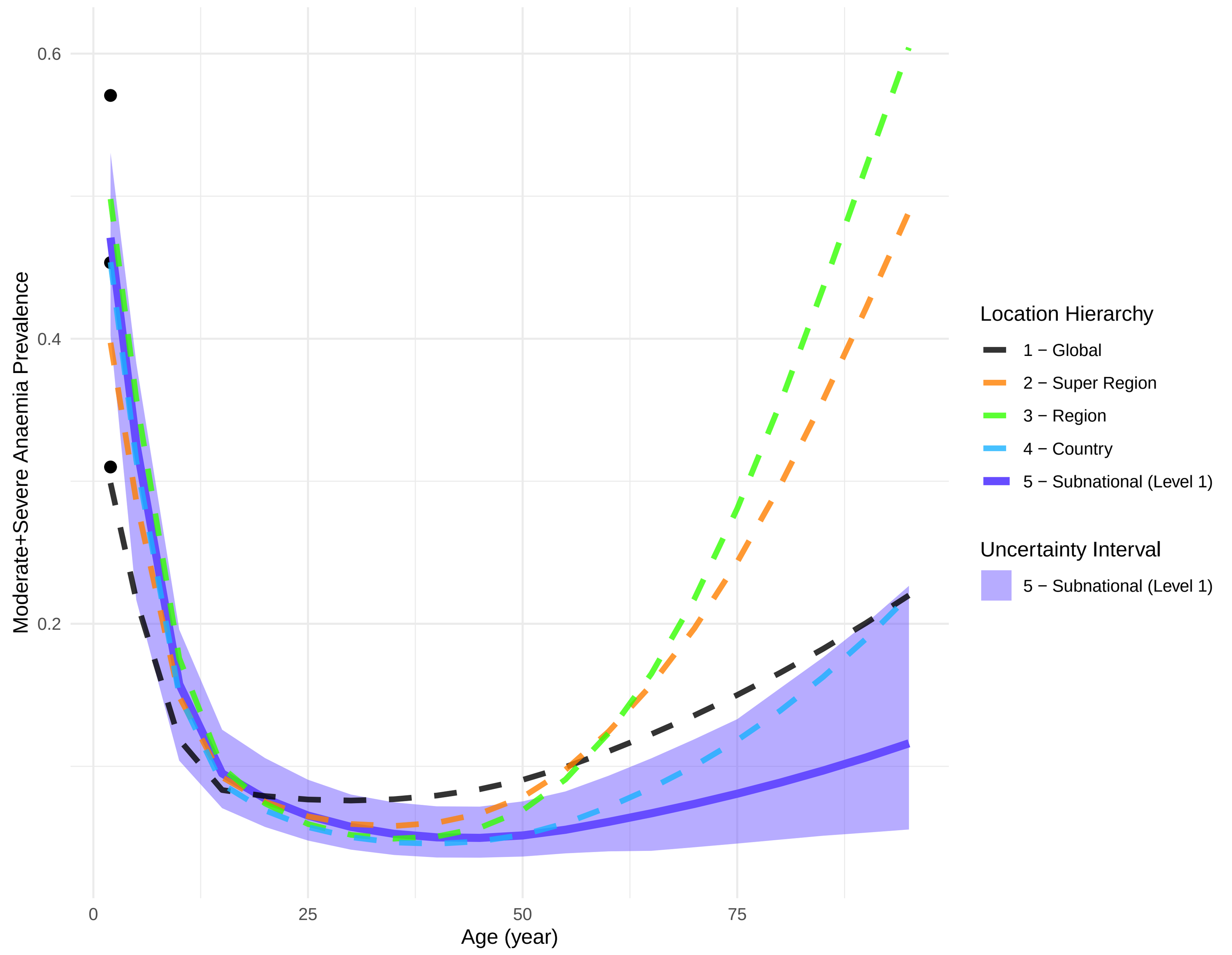

MR−BRT Age/Sex Fits − Males 2+ Years Old − Moderate+Severe Anaemia Prevalence − Yobe
WHO−2024 Adjustment Method

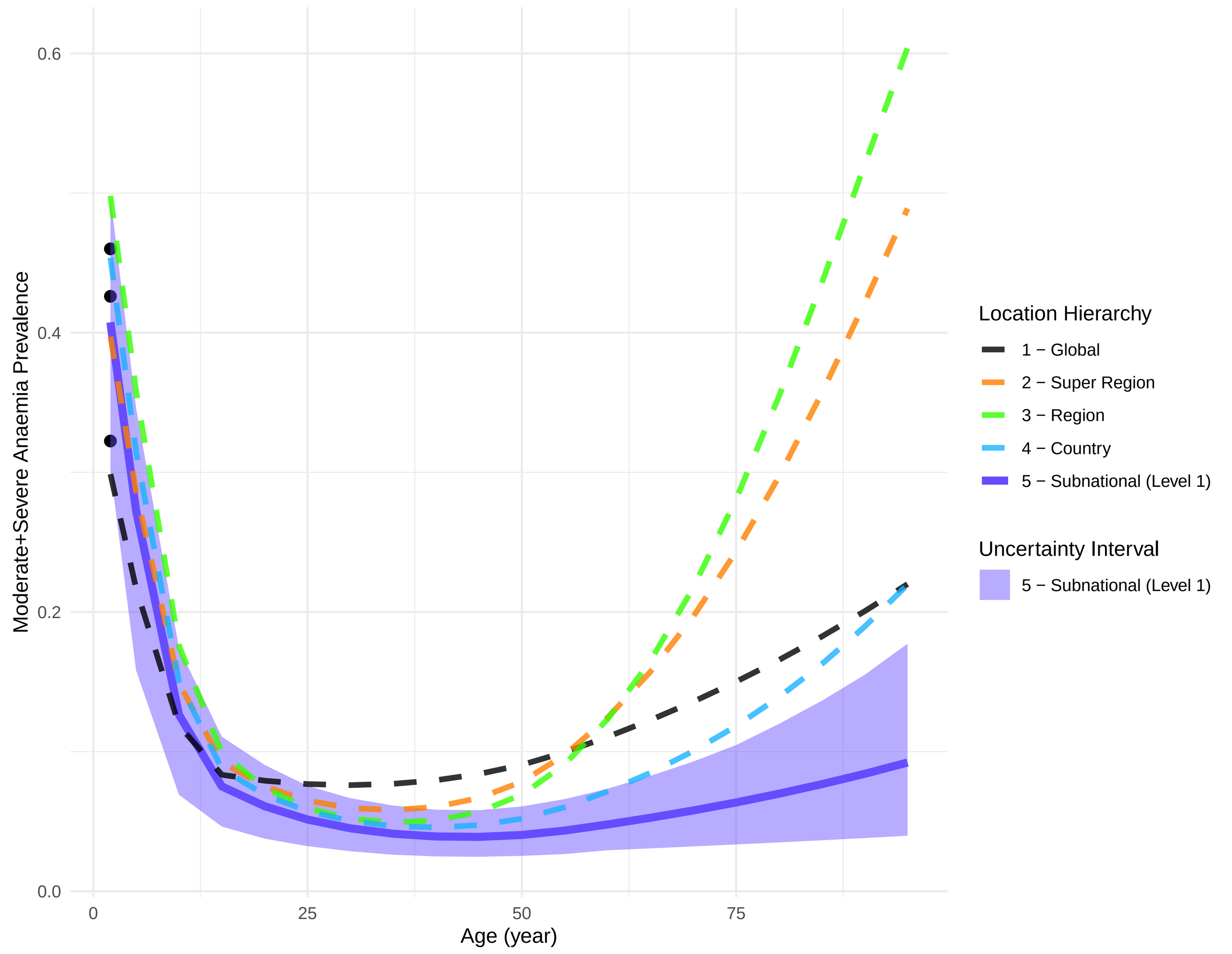

MR−BRT Age/Sex Fits − Males 2+ Years Old − Moderate+Severe Anaemia Prevalence − Zamfara
WHO−2024 Adjustment Method

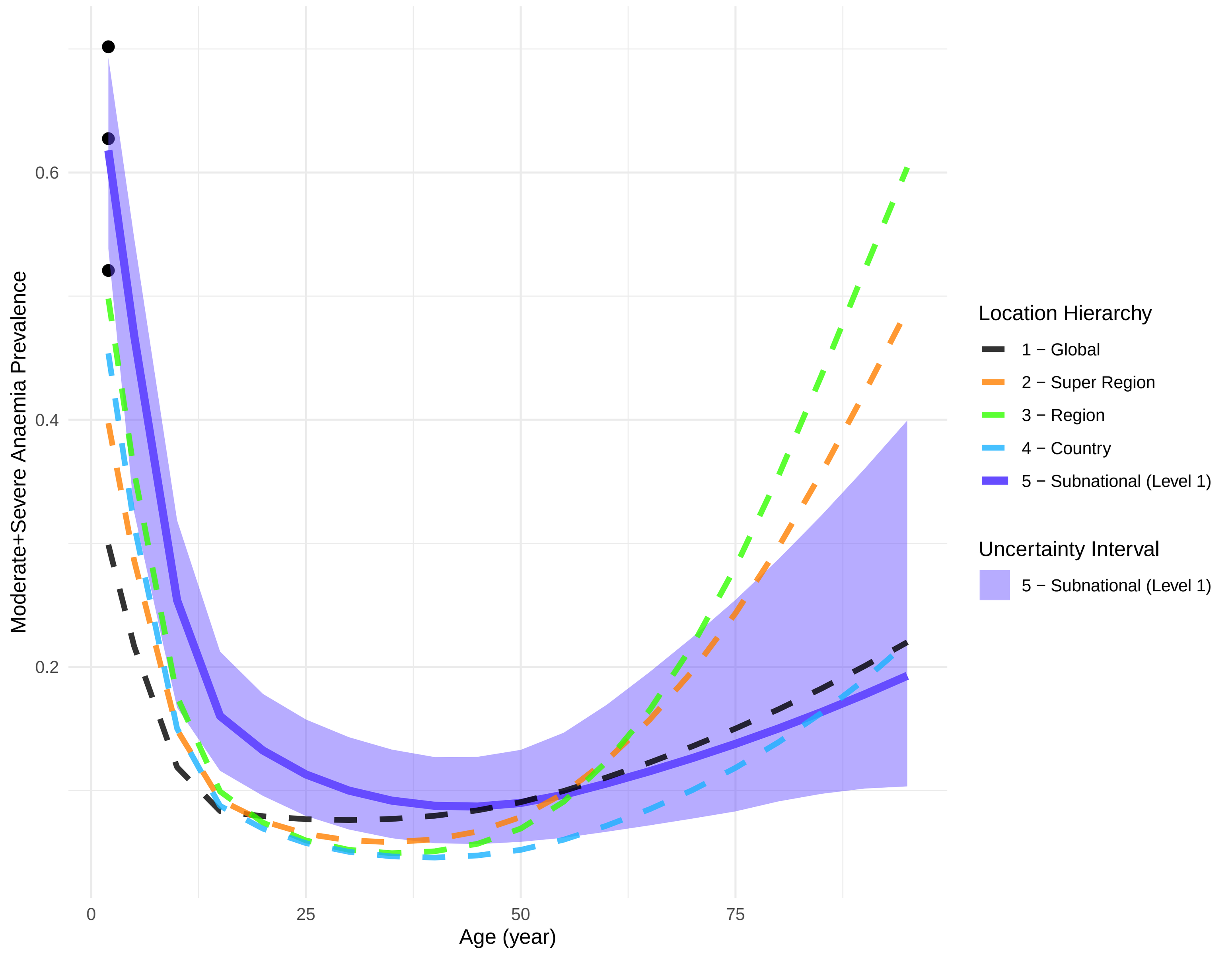

MR−BRT Age/Sex Fits − Males 2+ Years Old − Moderate+Severe Anaemia Prevalence − Senegal
WHO−2024 Adjustment Method

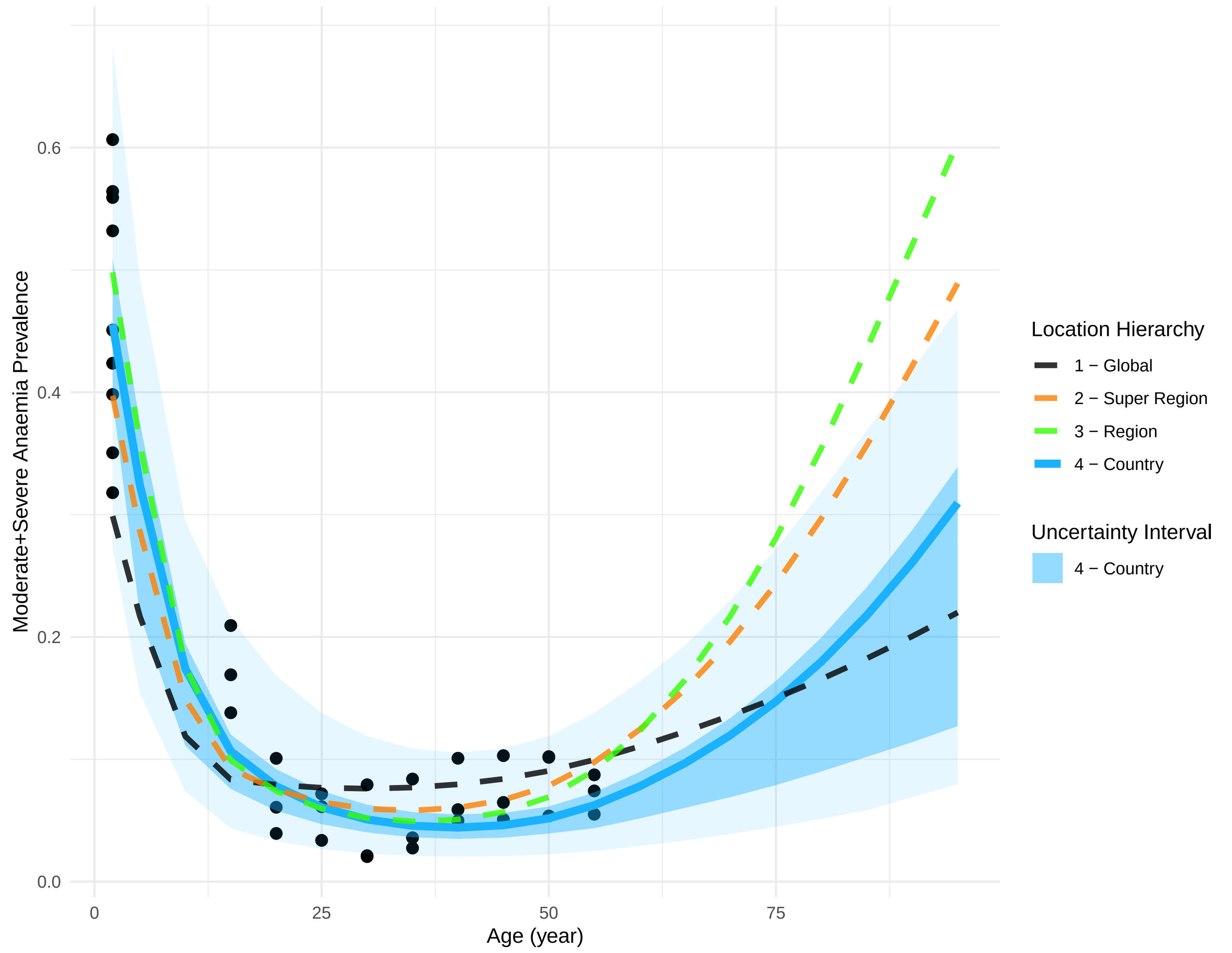

MR−BRT Age/Sex Fits − Males 2+ Years Old − Moderate+Severe Anaemia Prevalence − Sierra Leone
WHO−2024 Adjustment Method

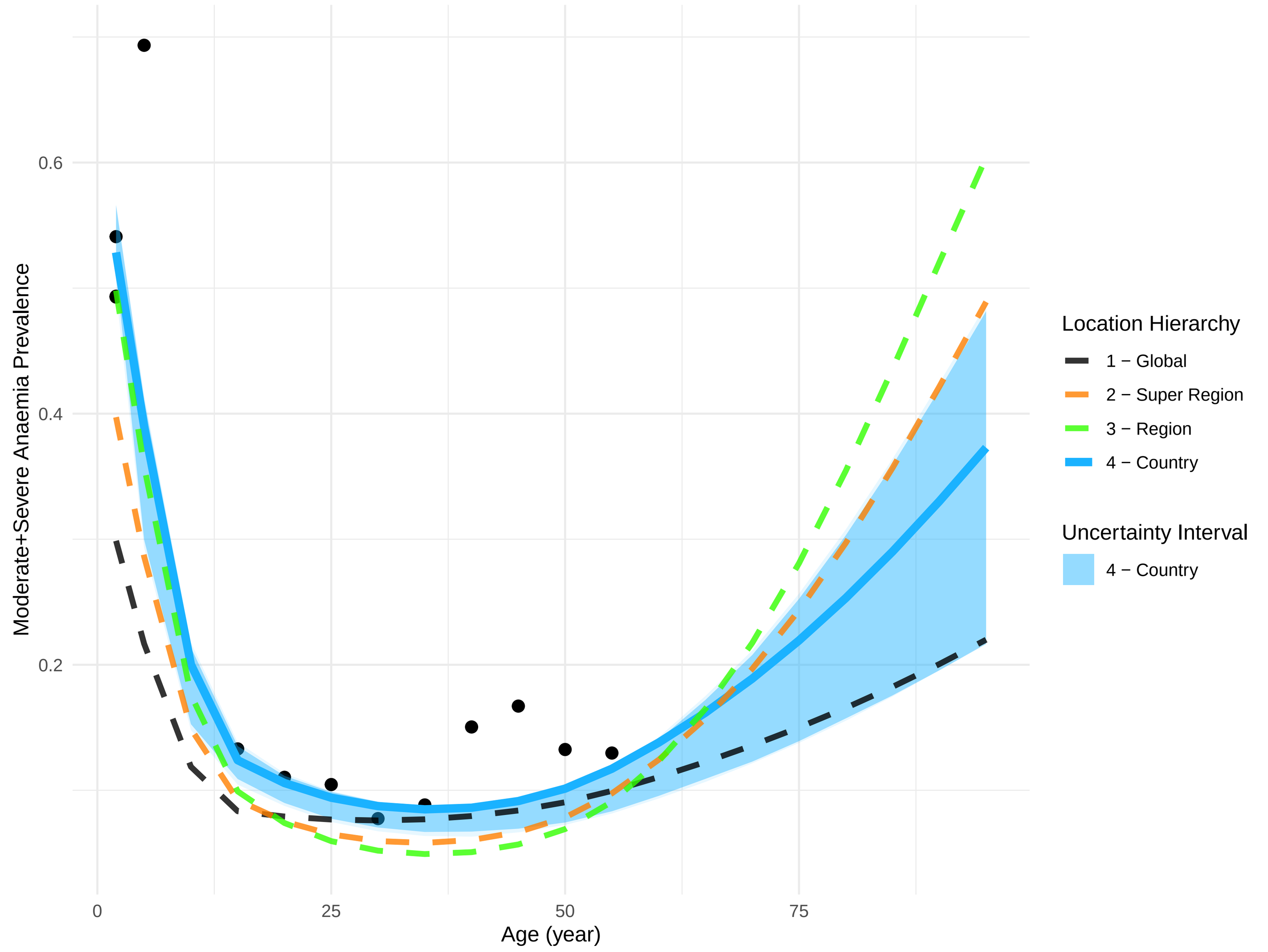

MR−BRT Age/Sex Fits − Males 2+ Years Old − Moderate+Severe Anaemia Prevalence − Togo
WHO−2024 Adjustment Method

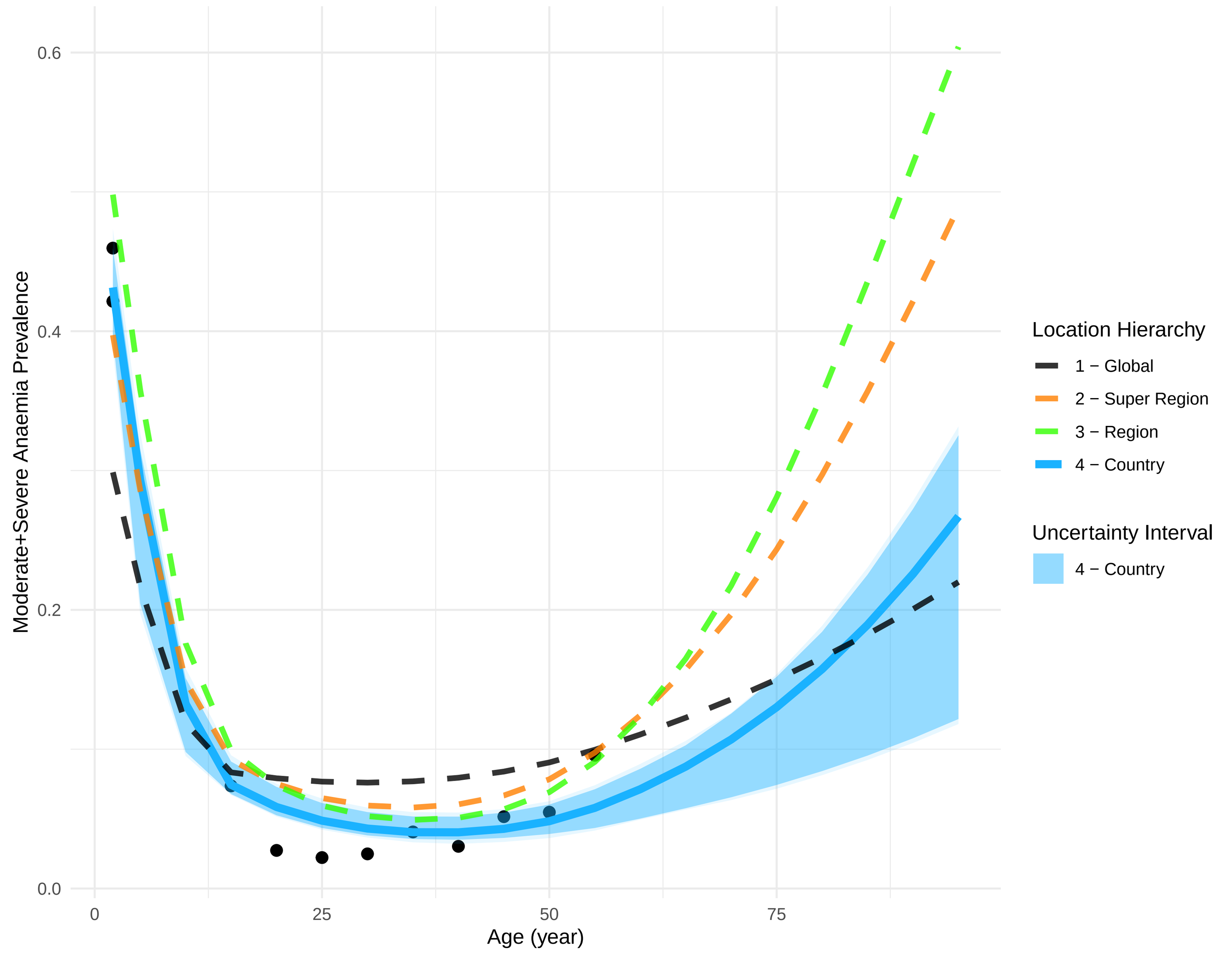

MR−BRT Age/Sex Fits − Non−pregnant Females 2+ Years Old − Moderate+Severe Anaemia Prevalence − Global
WHO−2024 Adjustment Method

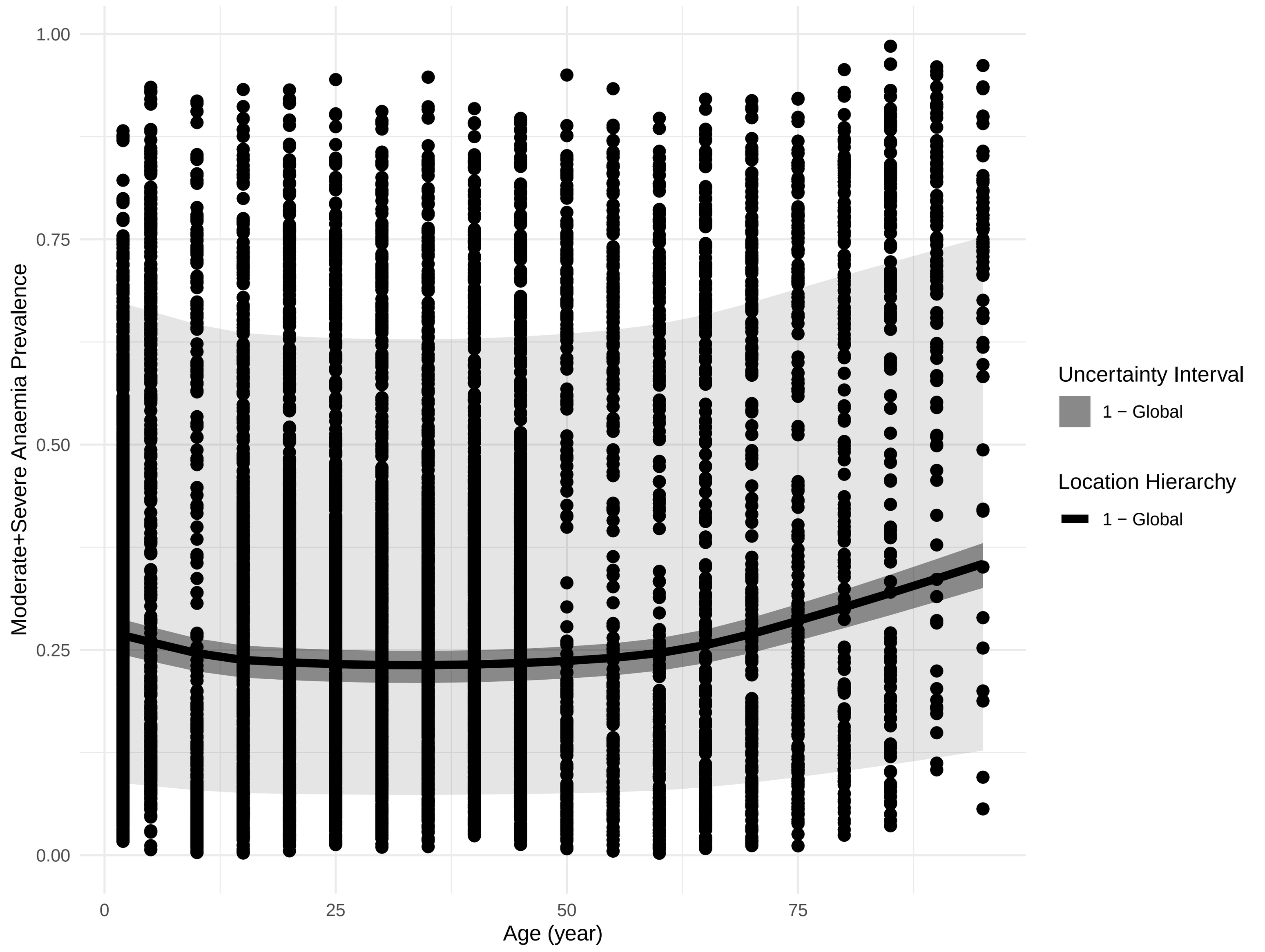

MR−BRT Age/Sex Fits − Non−pregnant Females 2+ Years Old − Moderate+Severe Anaemia Prevalence − Central Europe, Eastern Europe, and Central Asia
WHO−2024 Adjustment Method

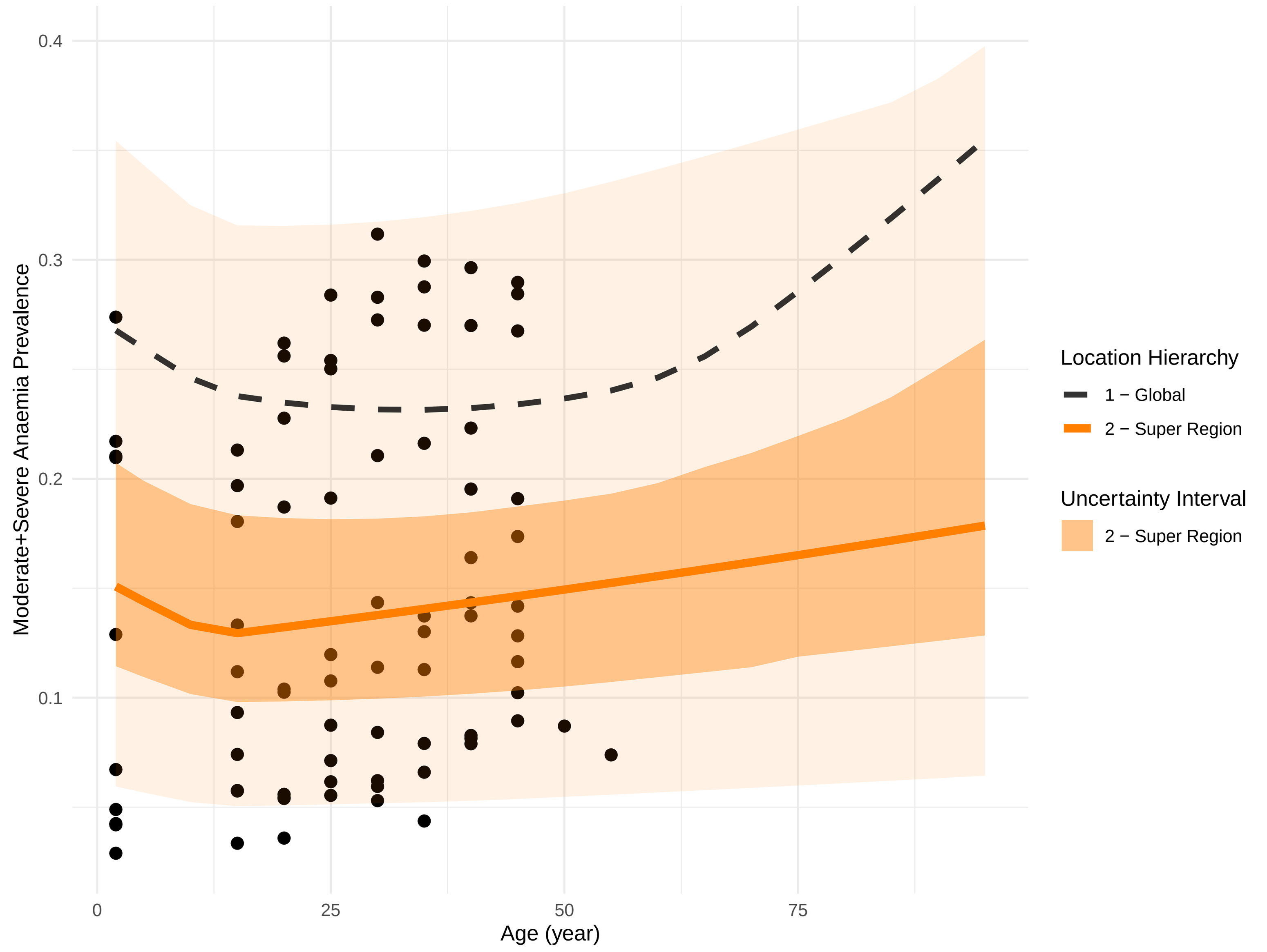

MR−BRT Age/Sex Fits − Non−pregnant Females 2+ Years Old − Moderate+Severe Anaemia Prevalence − Central Asia
WHO−2024 Adjustment Method

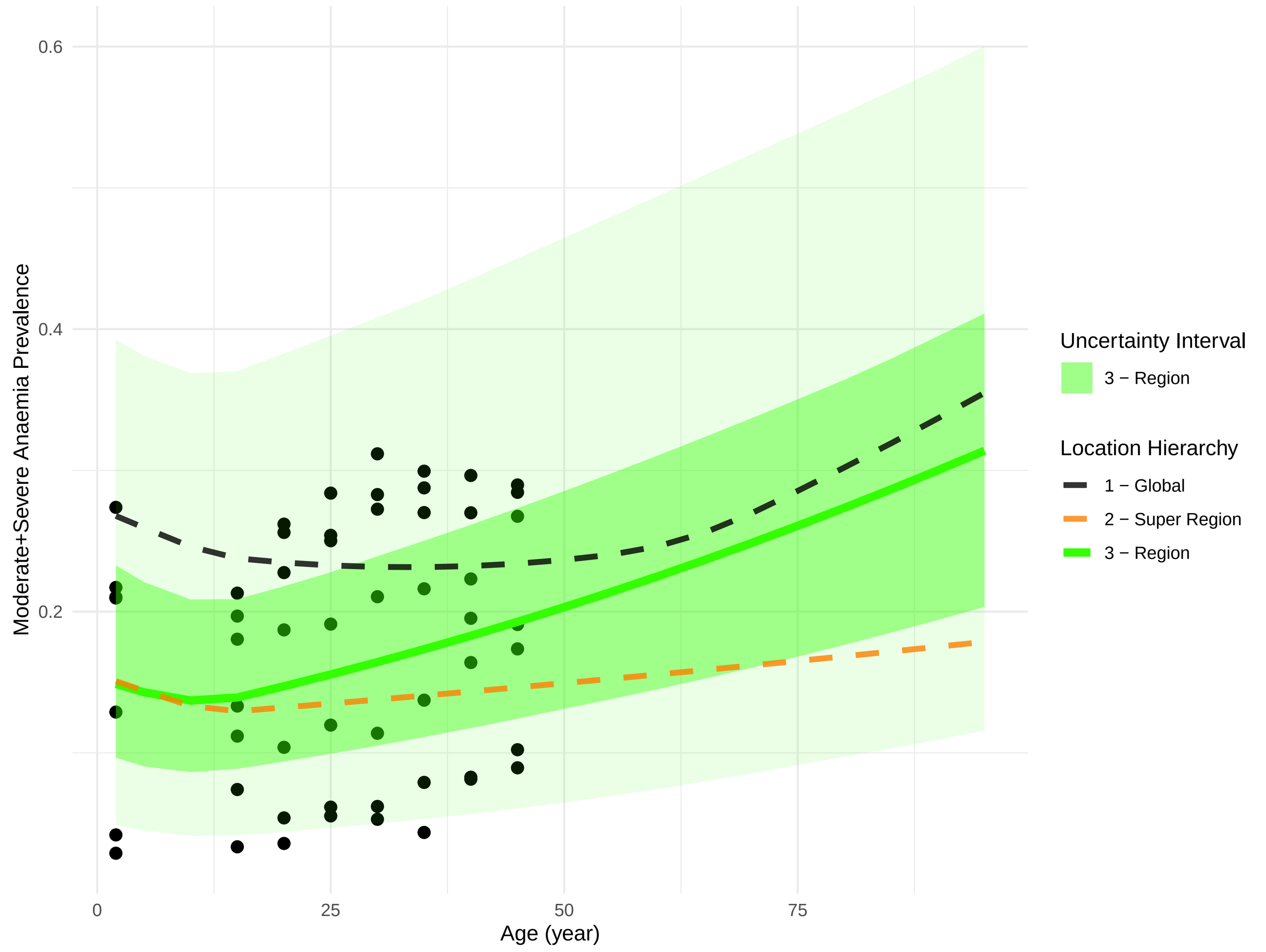

MR−BRT Age/Sex Fits − Non−pregnant Females 2+ Years Old − Moderate+Severe Anaemia Prevalence − Armenia
WHO−2024 Adjustment Method

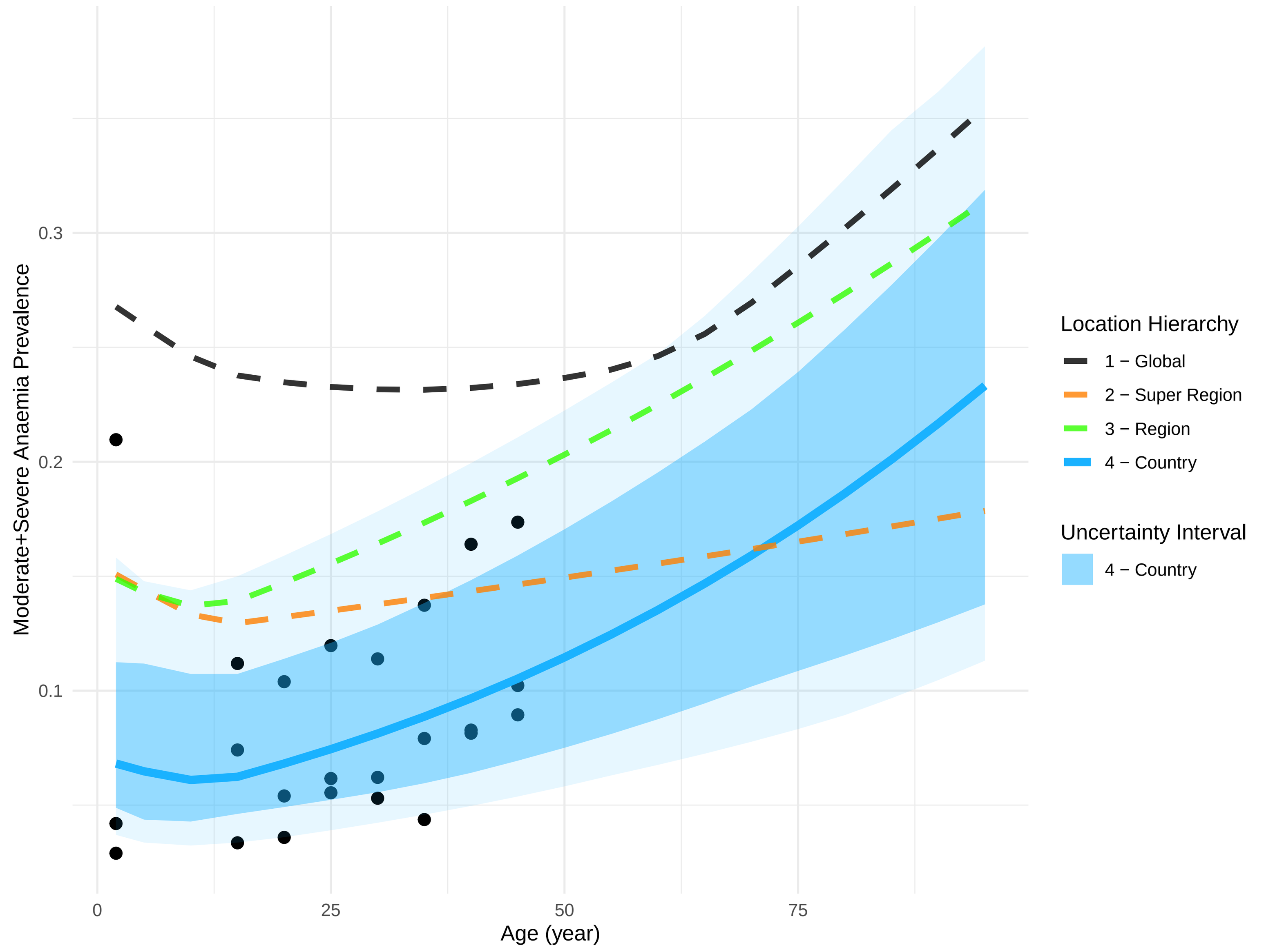

MR−BRT Age/Sex Fits − Non−pregnant Females 2+ Years Old − Moderate+Severe Anaemia Prevalence − Azerbaijan
WHO−2024 Adjustment Method

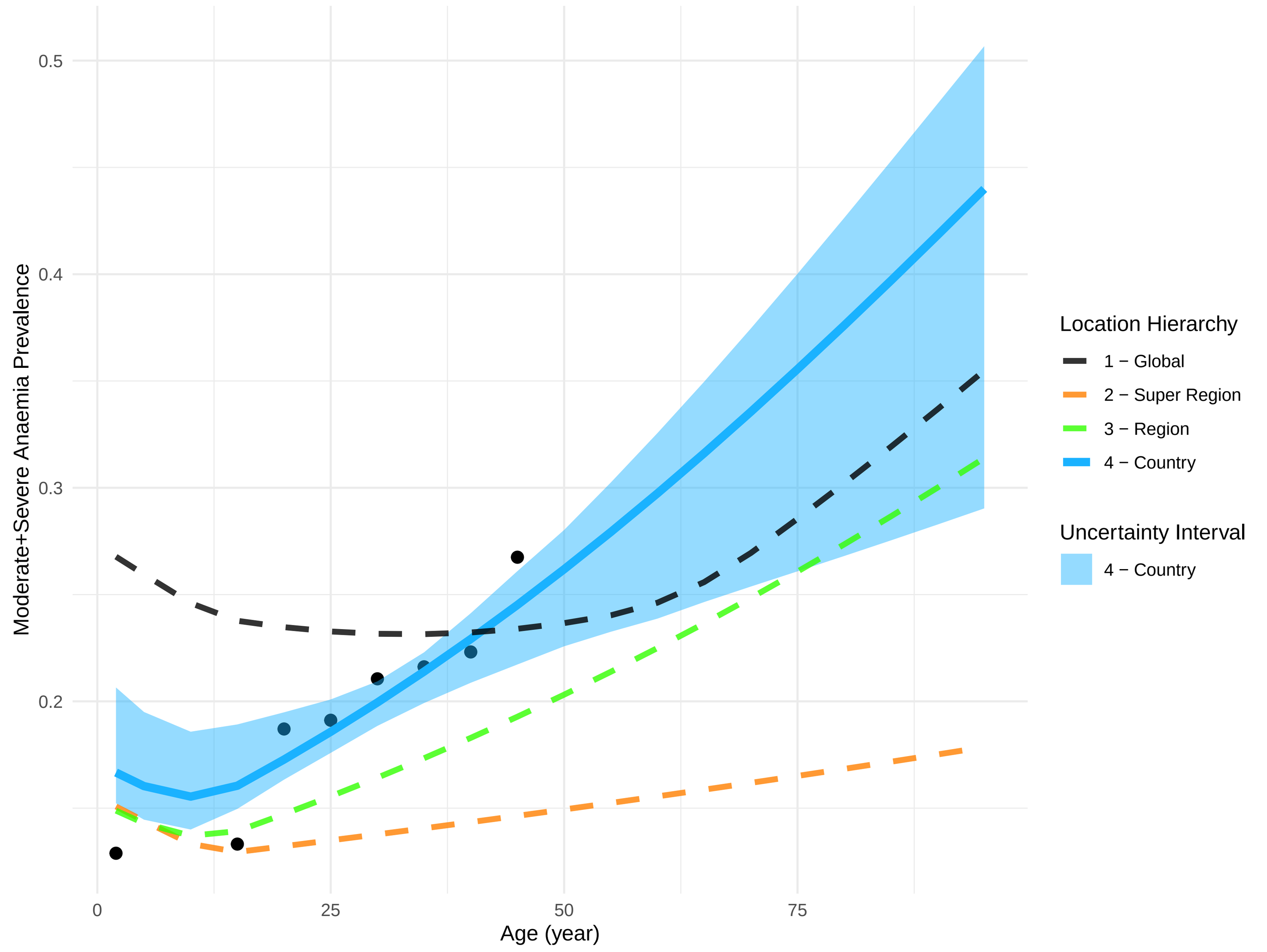

MR−BRT Age/Sex Fits − Non−pregnant Females 2+ Years Old − Moderate+Severe Anaemia Prevalence − Kyrgyzstan
WHO−2024 Adjustment Method

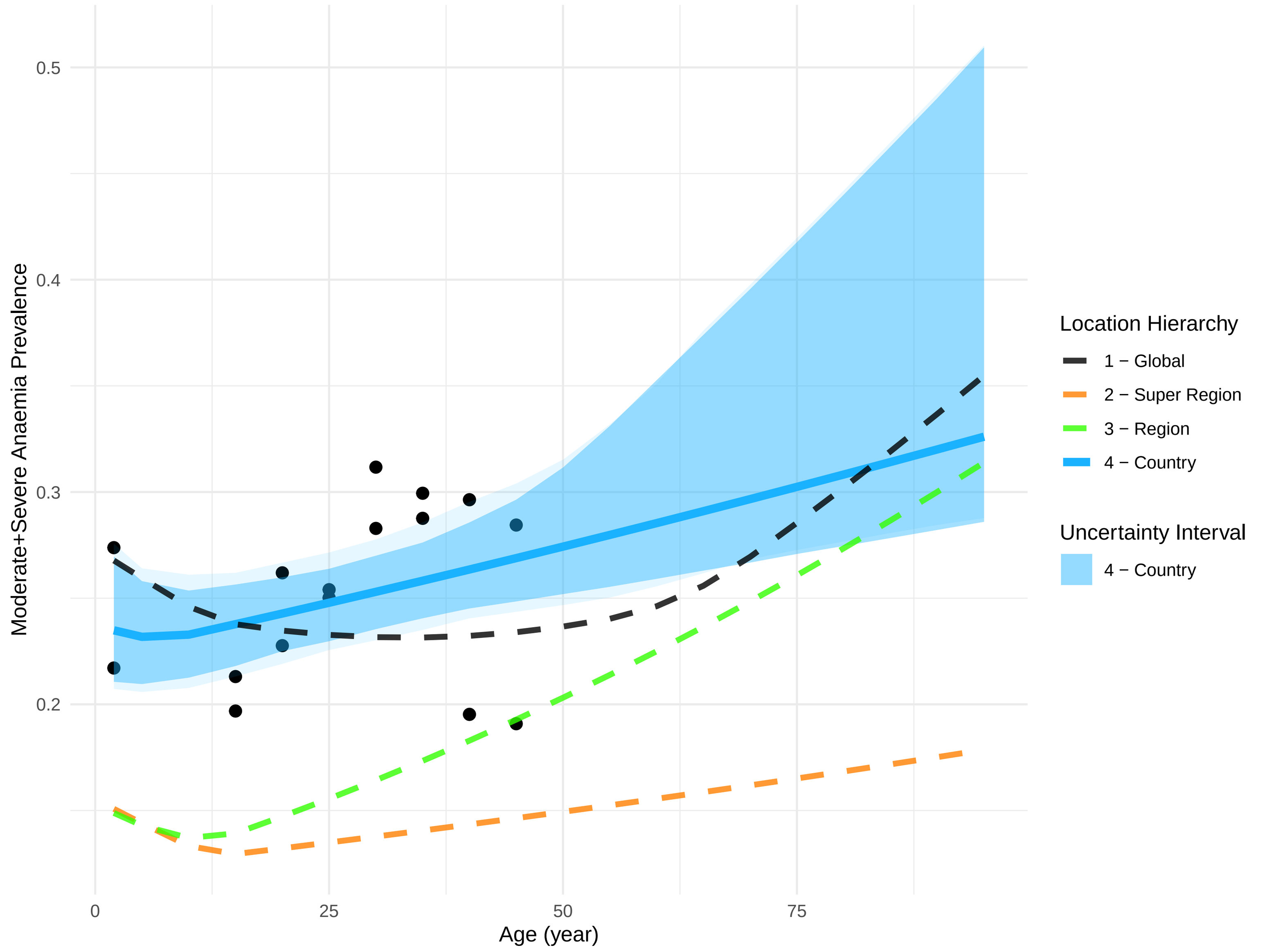

MR−BRT Age/Sex Fits − Non−pregnant Females 2+ Years Old − Moderate+Severe Anaemia Prevalence − Tajikistan
WHO−2024 Adjustment Method

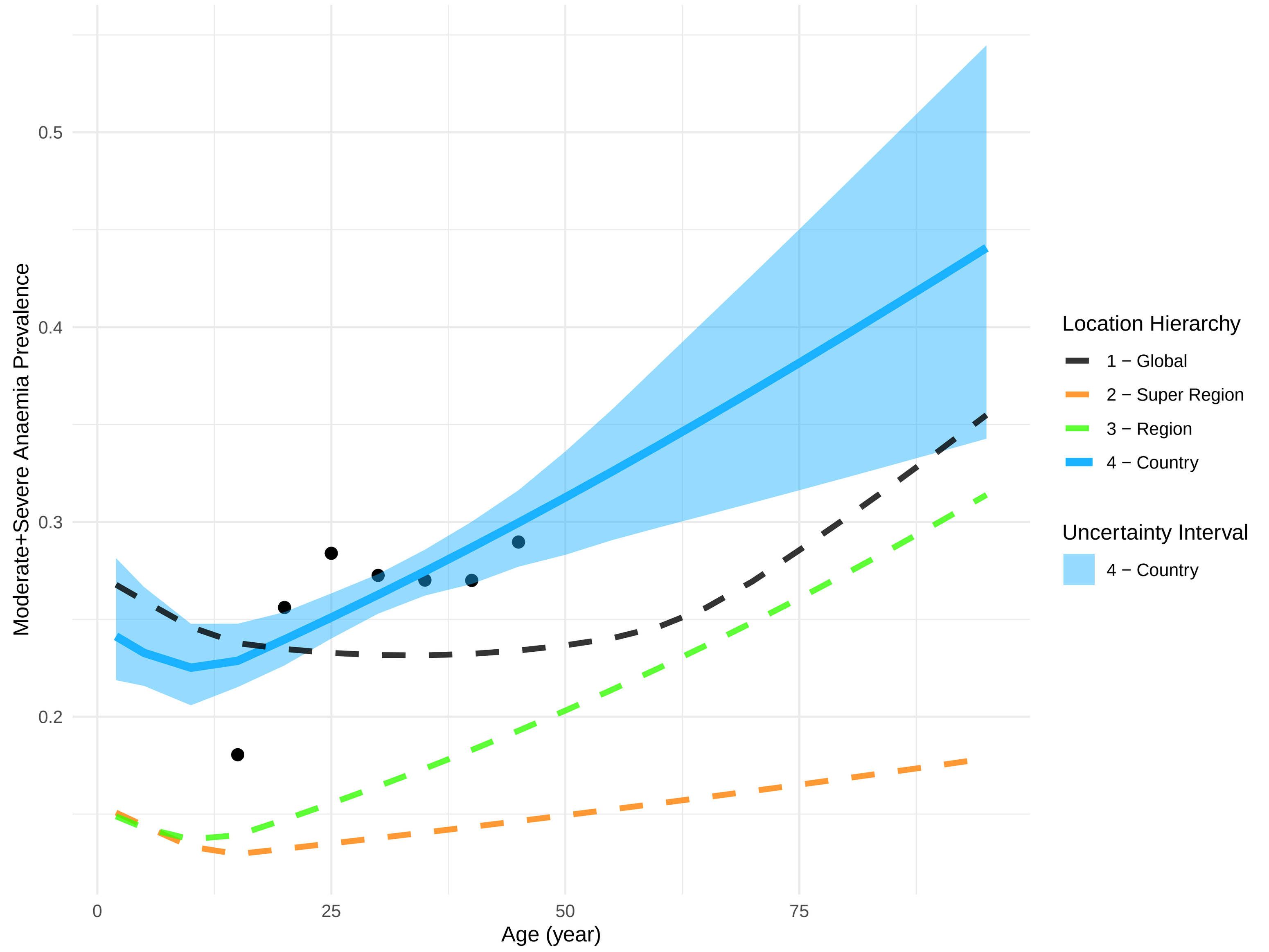

MR−BRT Age/Sex Fits − Non−pregnant Females 2+ Years Old − Moderate+Severe Anaemia Prevalence − Uzbekistan
WHO−2024 Adjustment Method

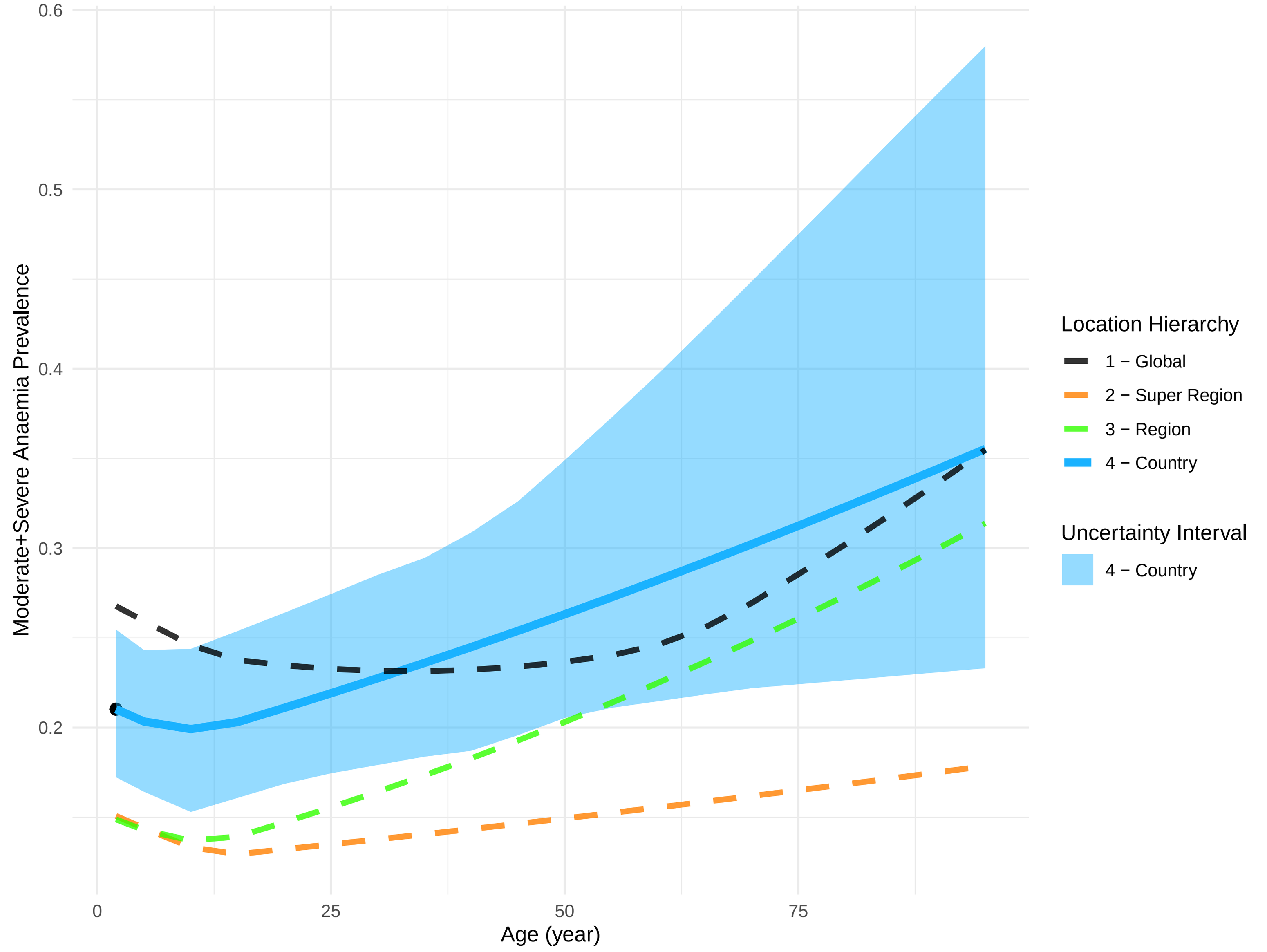

MR−BRT Age/Sex Fits − Non−pregnant Females 2+ Years Old − Moderate+Severe Anaemia Prevalence − Central Europe
WHO−2024 Adjustment Method

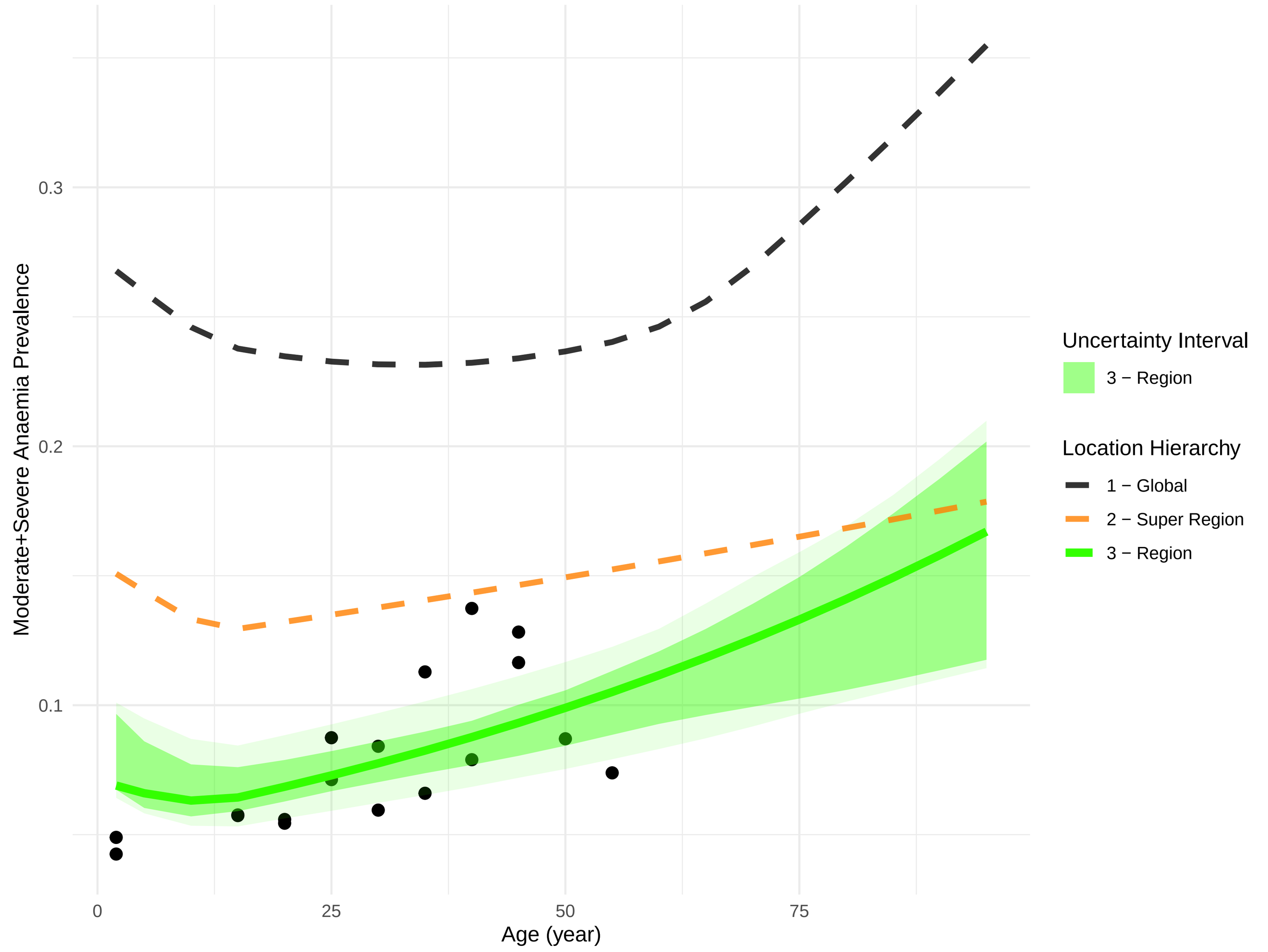

MR−BRT Age/Sex Fits − Non−pregnant Females 2+ Years Old − Moderate+Severe Anaemia Prevalence − Albania
WHO−2024 Adjustment Method

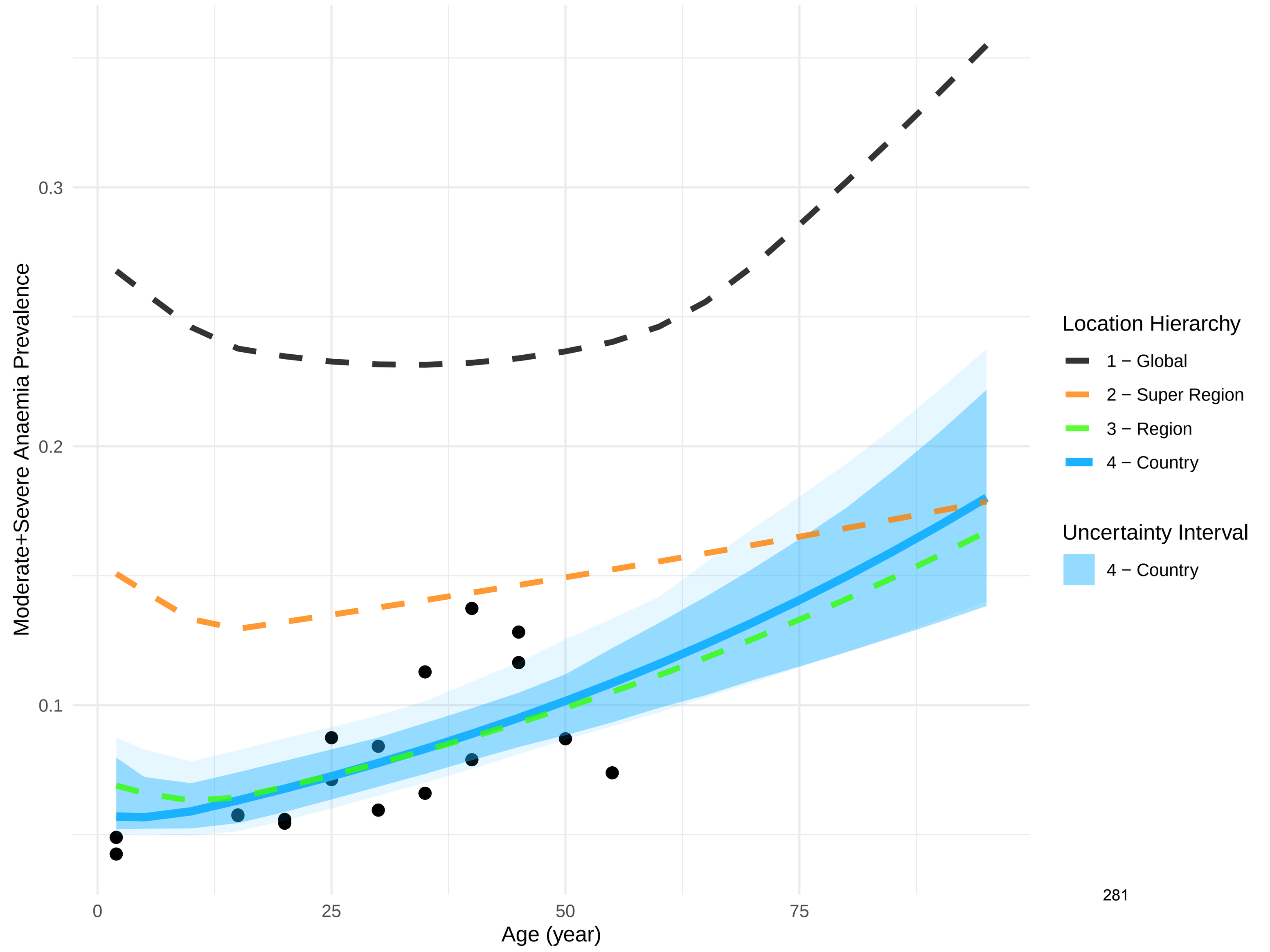

MR−BRT Age/Sex Fits − Non−pregnant Females 2+ Years Old − Moderate+Severe Anaemia Prevalence − Eastern Europe
WHO−2024 Adjustment Method

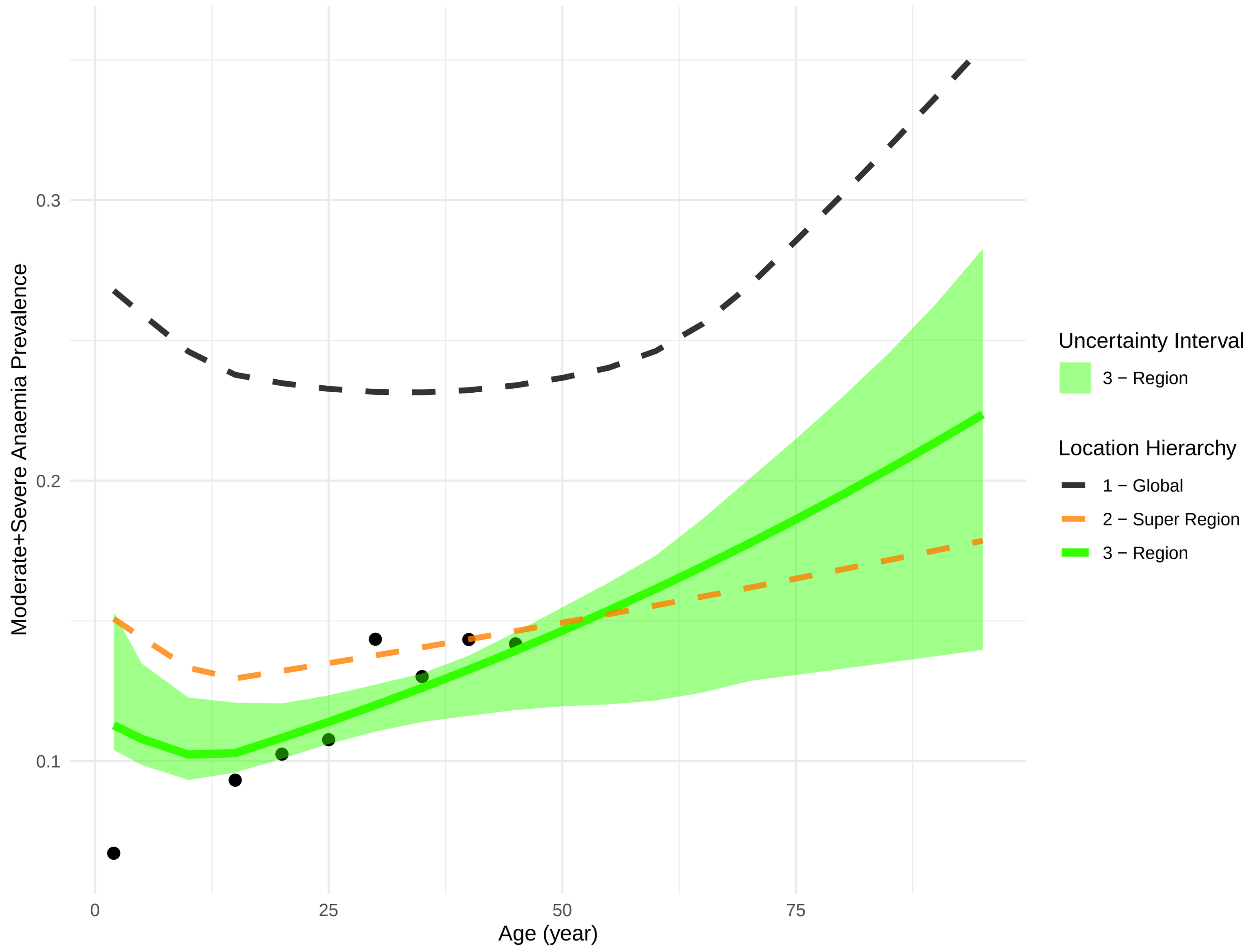

MR−BRT Age/Sex Fits − Non−pregnant Females 2+ Years Old − Moderate+Severe Anaemia Prevalence − Republic of Moldova
WHO−2024 Adjustment Method

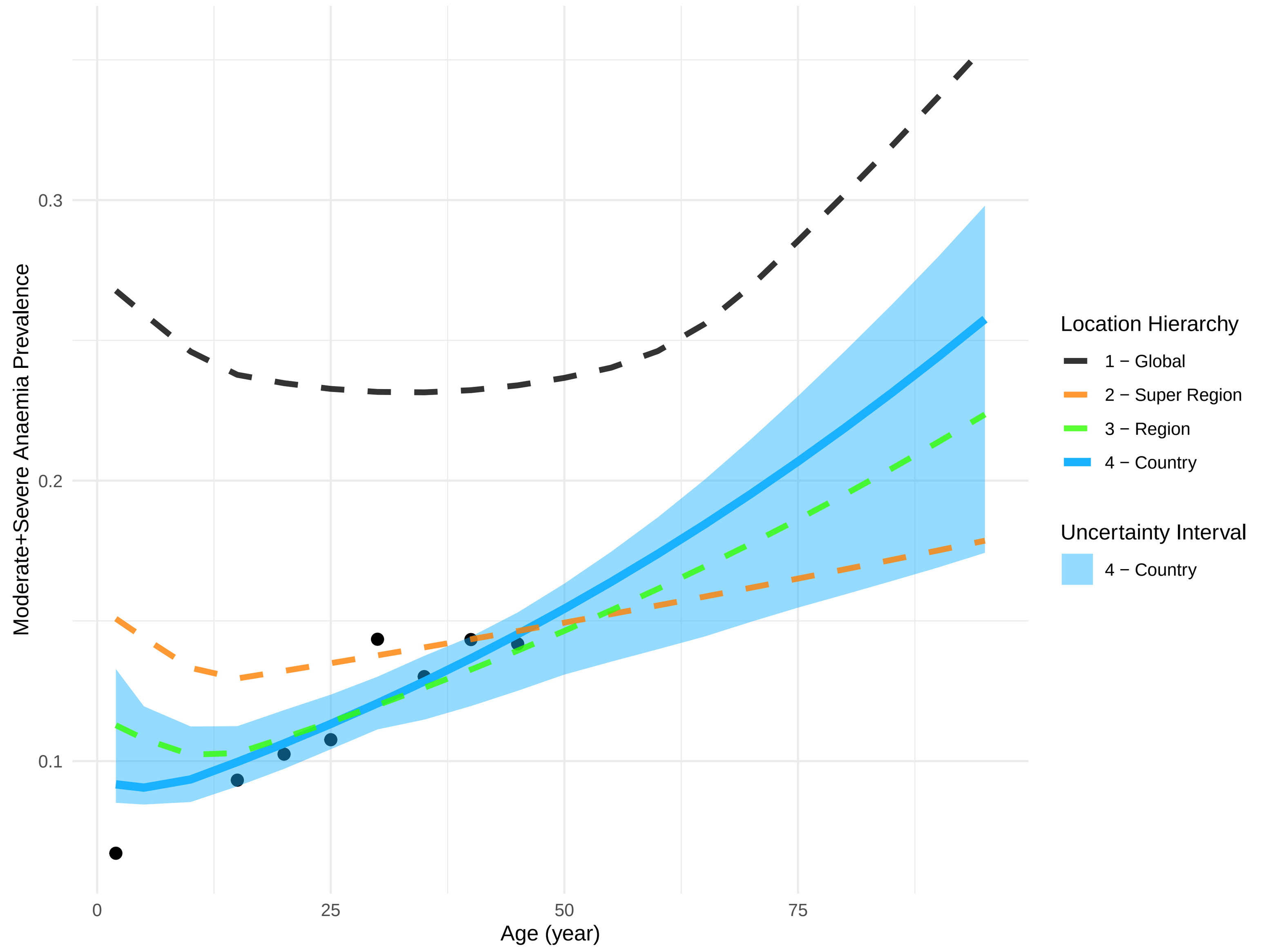

MR−BRT Age/Sex Fits − Non−pregnant Females 2+ Years Old − Moderate+Severe Anaemia Prevalence − High−income
WHO−2024 Adjustment Method

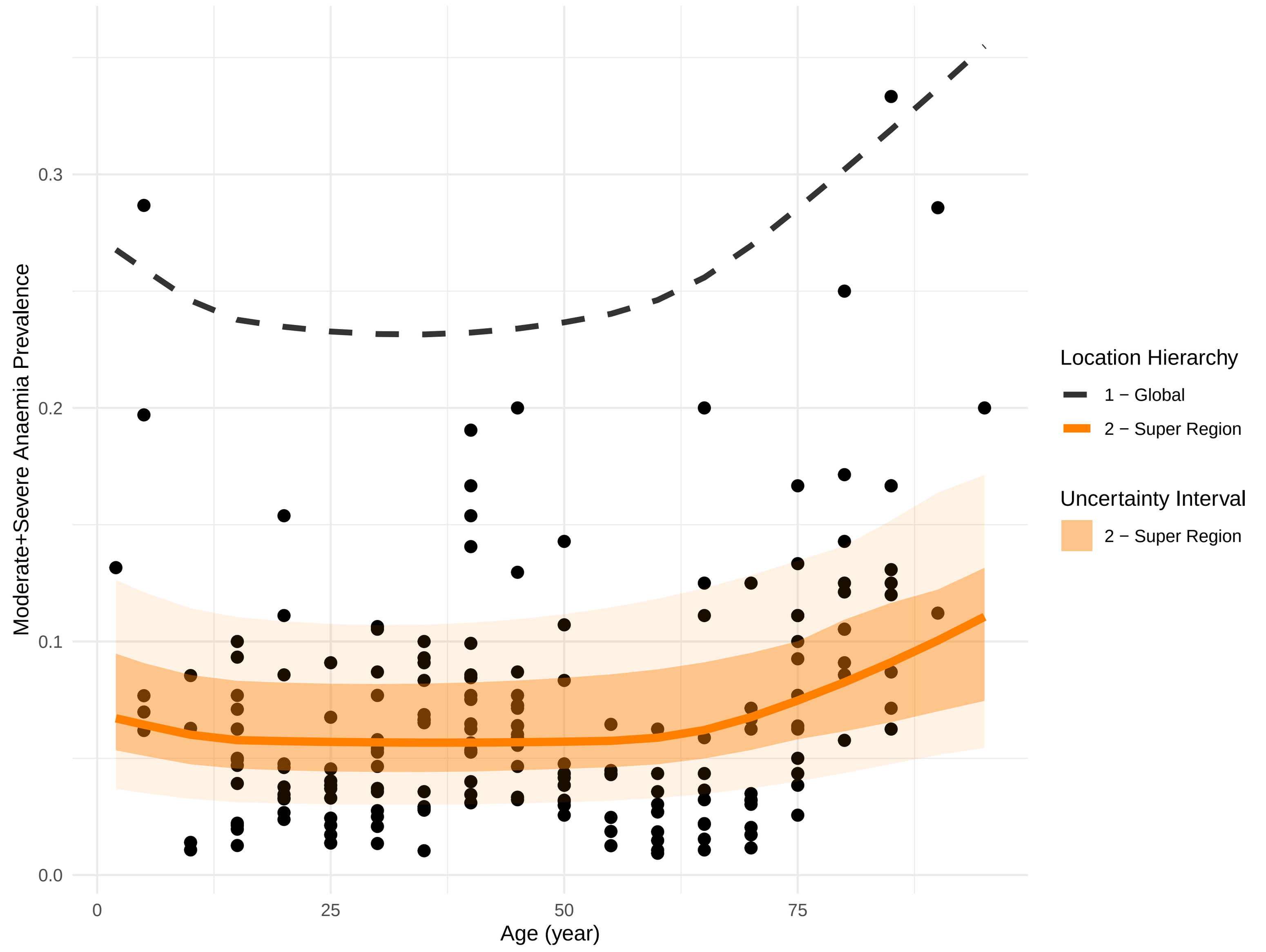

MR−BRT Age/Sex Fits − Non−pregnant Females 2+ Years Old − Moderate+Severe Anaemia Prevalence − Western Europe
WHO−2024 Adjustment Method

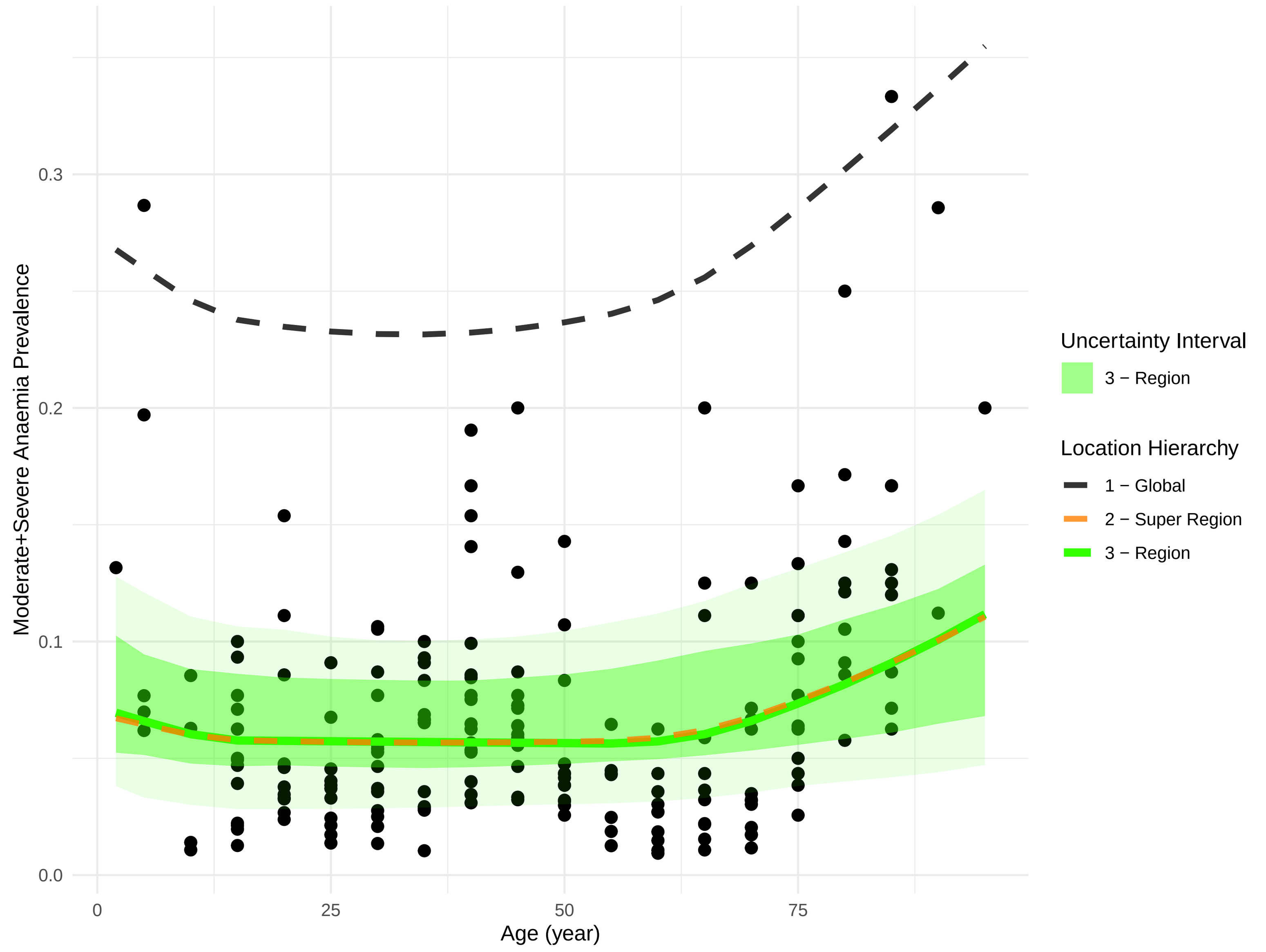

MR−BRT Age/Sex Fits − Non−pregnant Females 2+ Years Old − Moderate+Severe Anaemia Prevalence − United Kingdom
WHO−2024 Adjustment Method

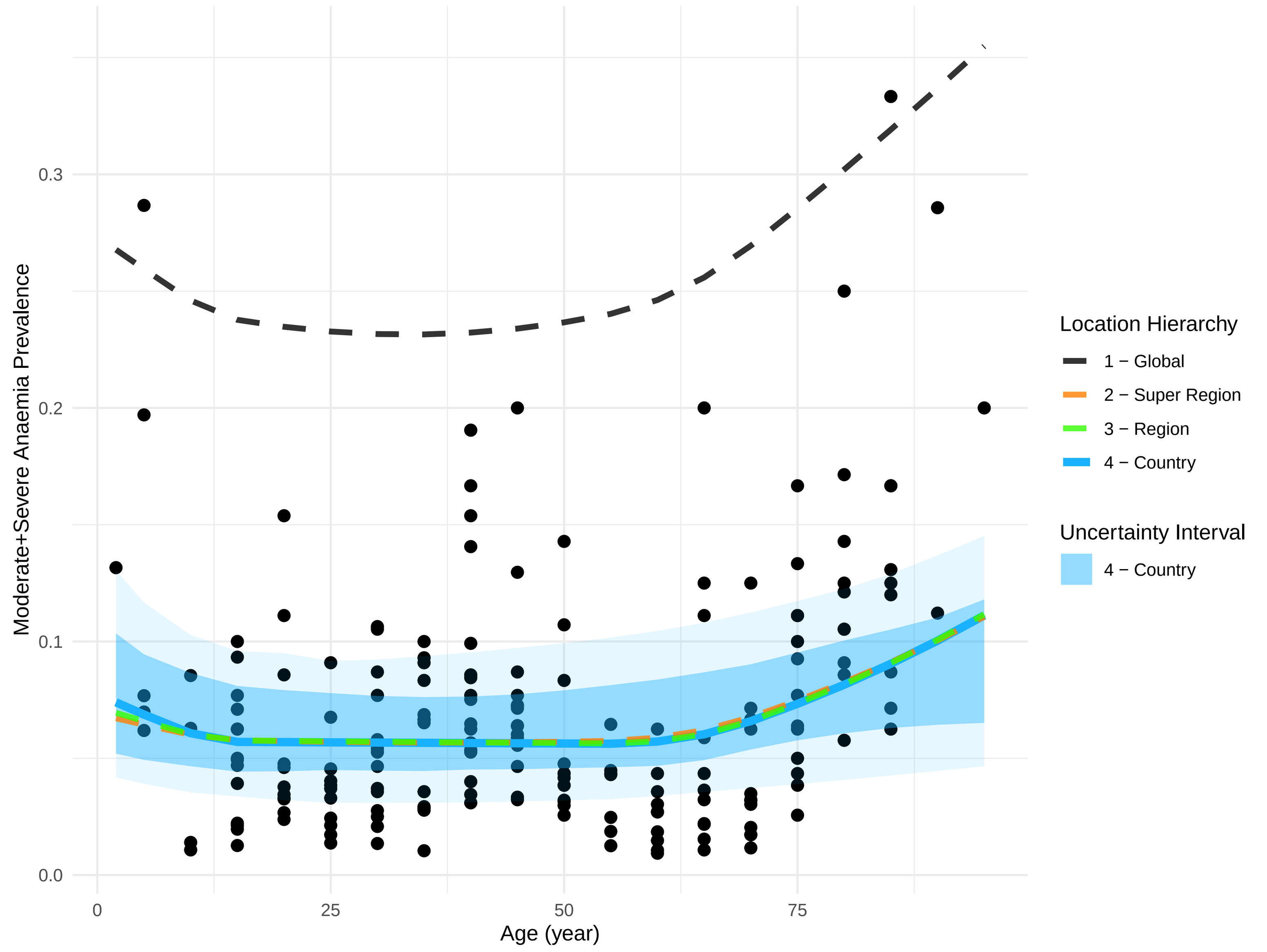

MR−BRT Age/Sex Fits − Non−pregnant Females 2+ Years Old − Moderate+Severe Anaemia Prevalence − England
WHO−2024 Adjustment Method

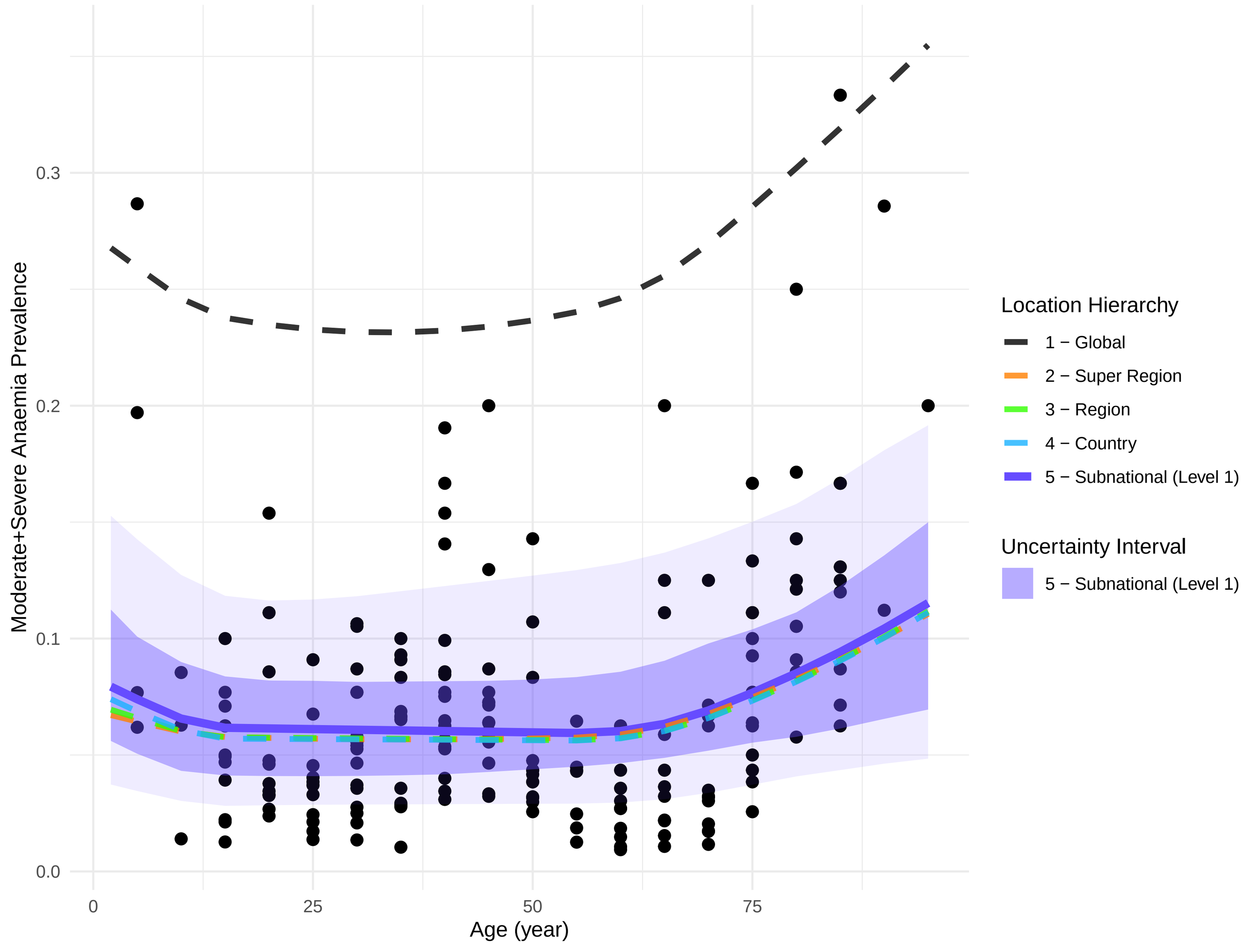

MR−BRT Age/Sex Fits − Non−pregnant Females 2+ Years Old − Moderate+Severe Anaemia Prevalence − East Midlands
WHO−2024 Adjustment Method

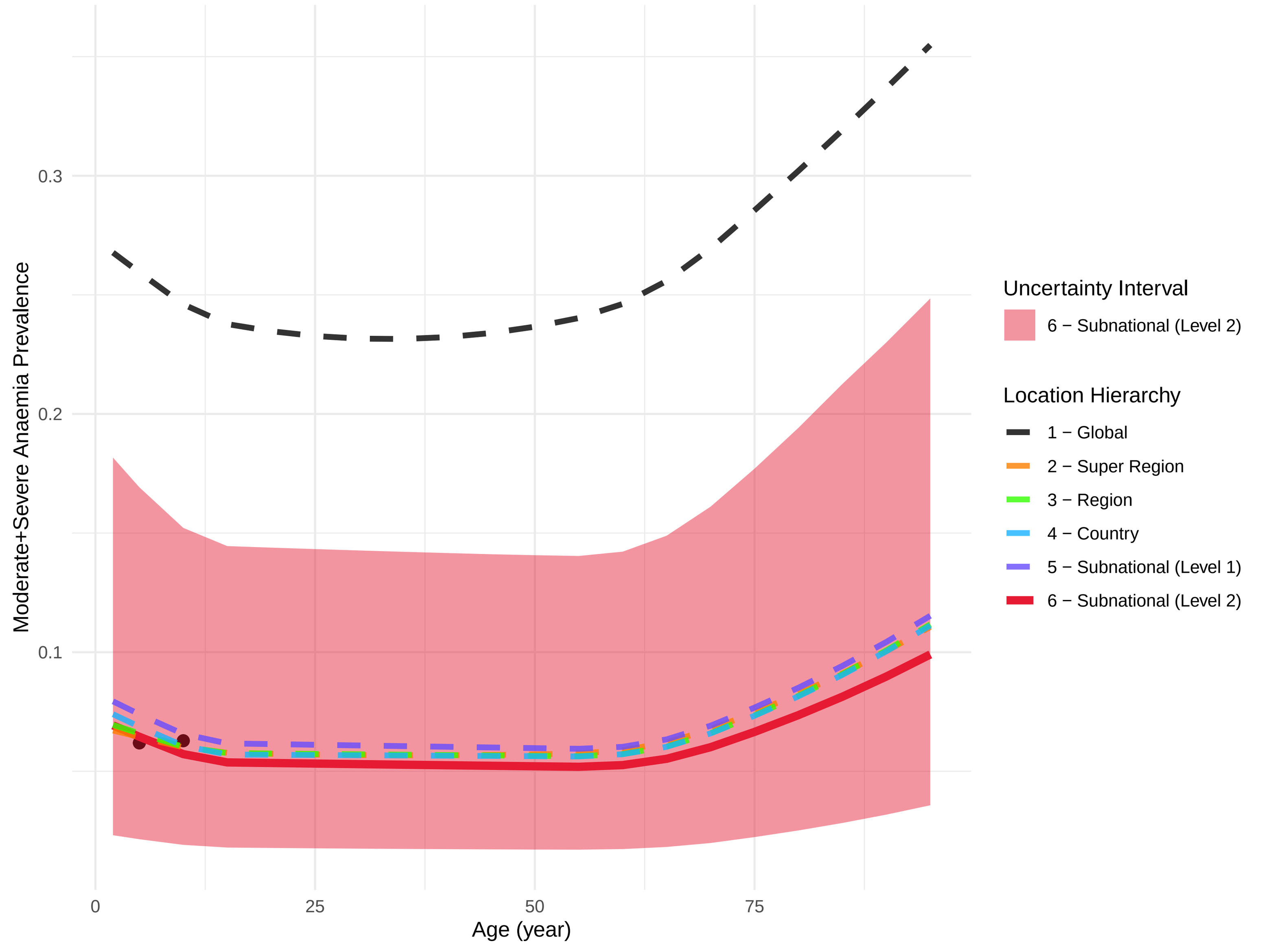

MR−BRT Age/Sex Fits − Non−pregnant Females 2+ Years Old − Moderate+Severe Anaemia Prevalence − East of England
WHO−2024 Adjustment Method

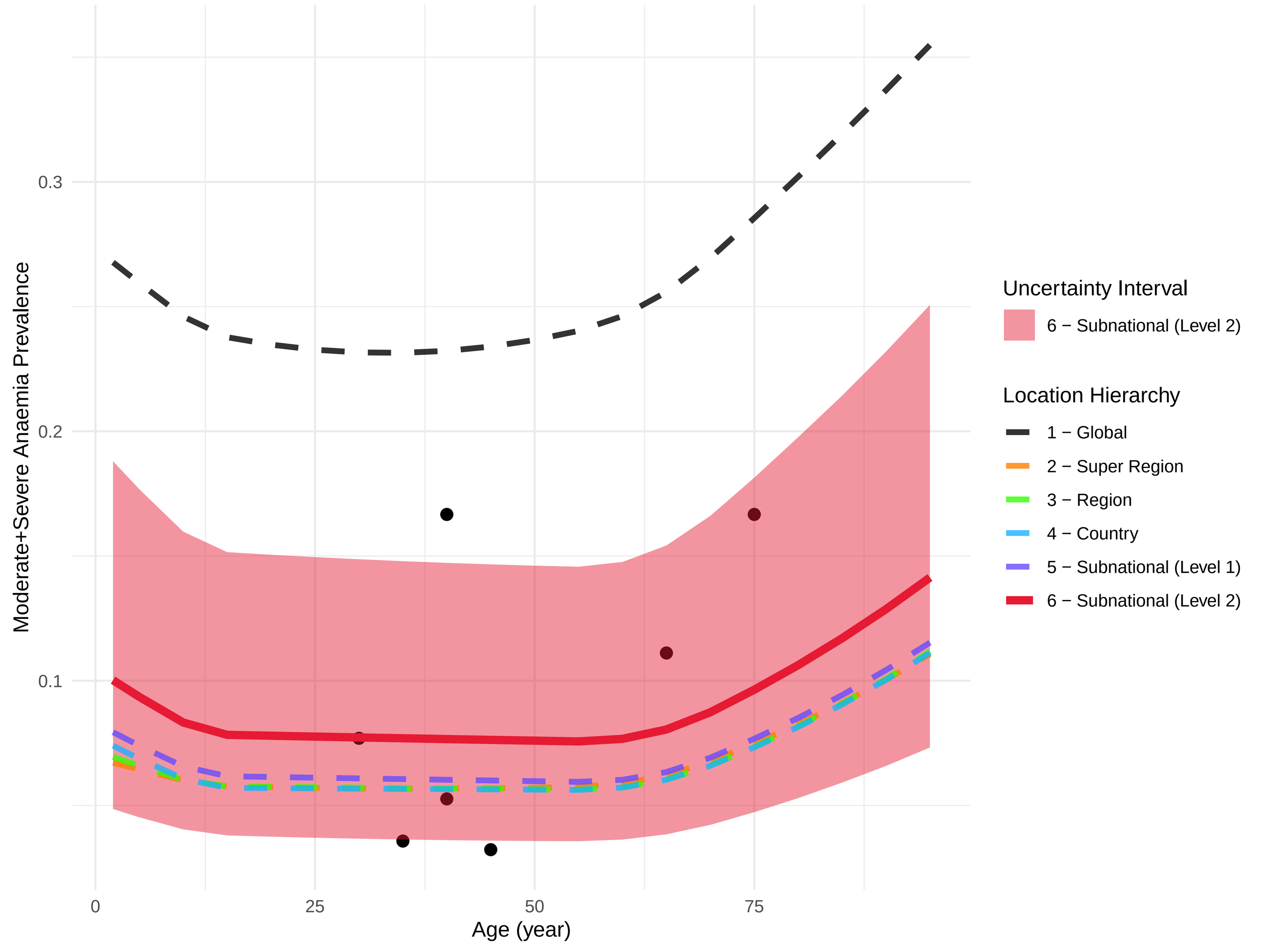

MR−BRT Age/Sex Fits − Non−pregnant Females 2+ Years Old − Moderate+Severe Anaemia Prevalence − Greater London
WHO−2024 Adjustment Method

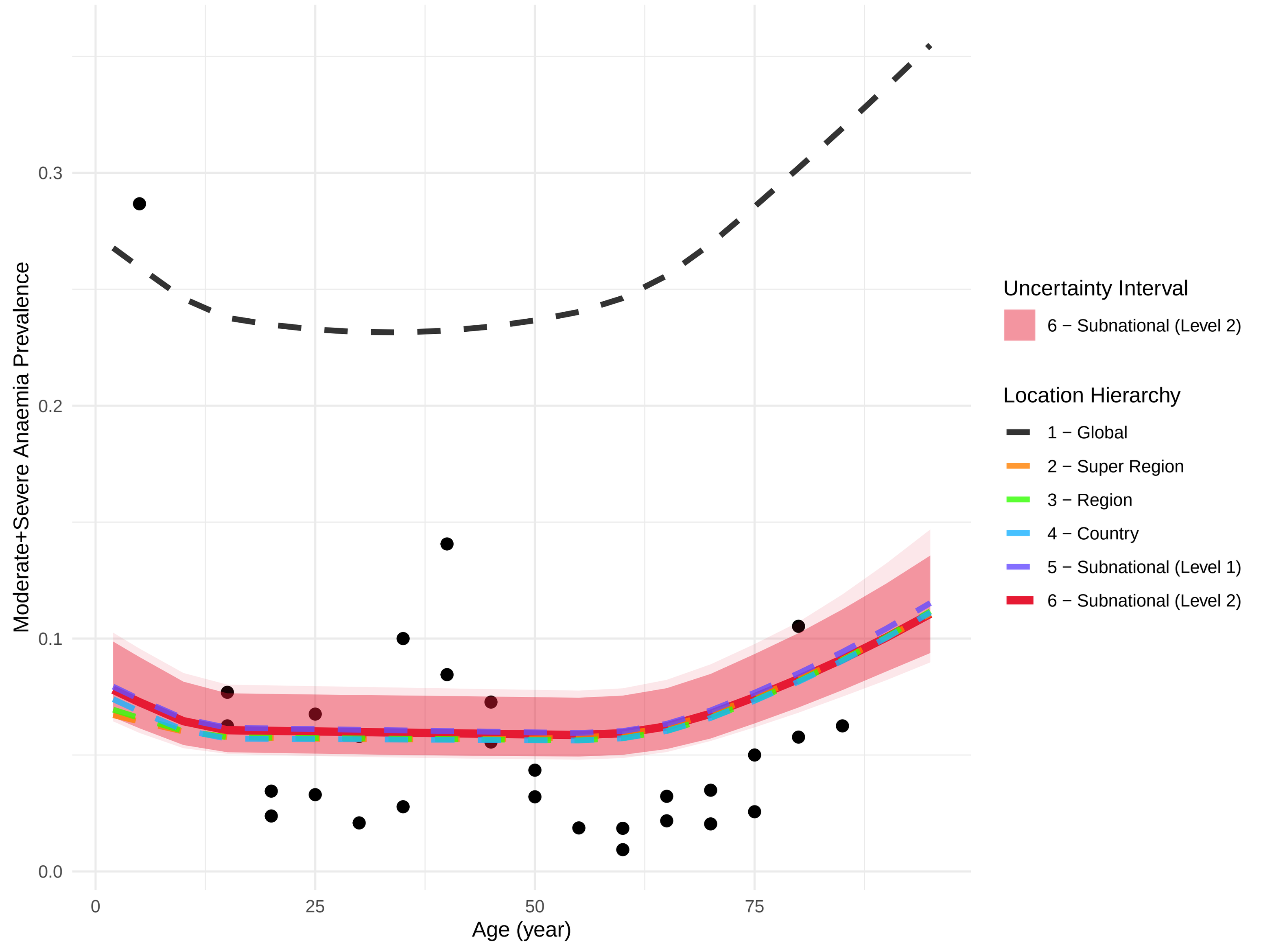

MR−BRT Age/Sex Fits − Non−pregnant Females 2+ Years Old − Moderate+Severe Anaemia Prevalence − North East England
WHO−2024 Adjustment Method

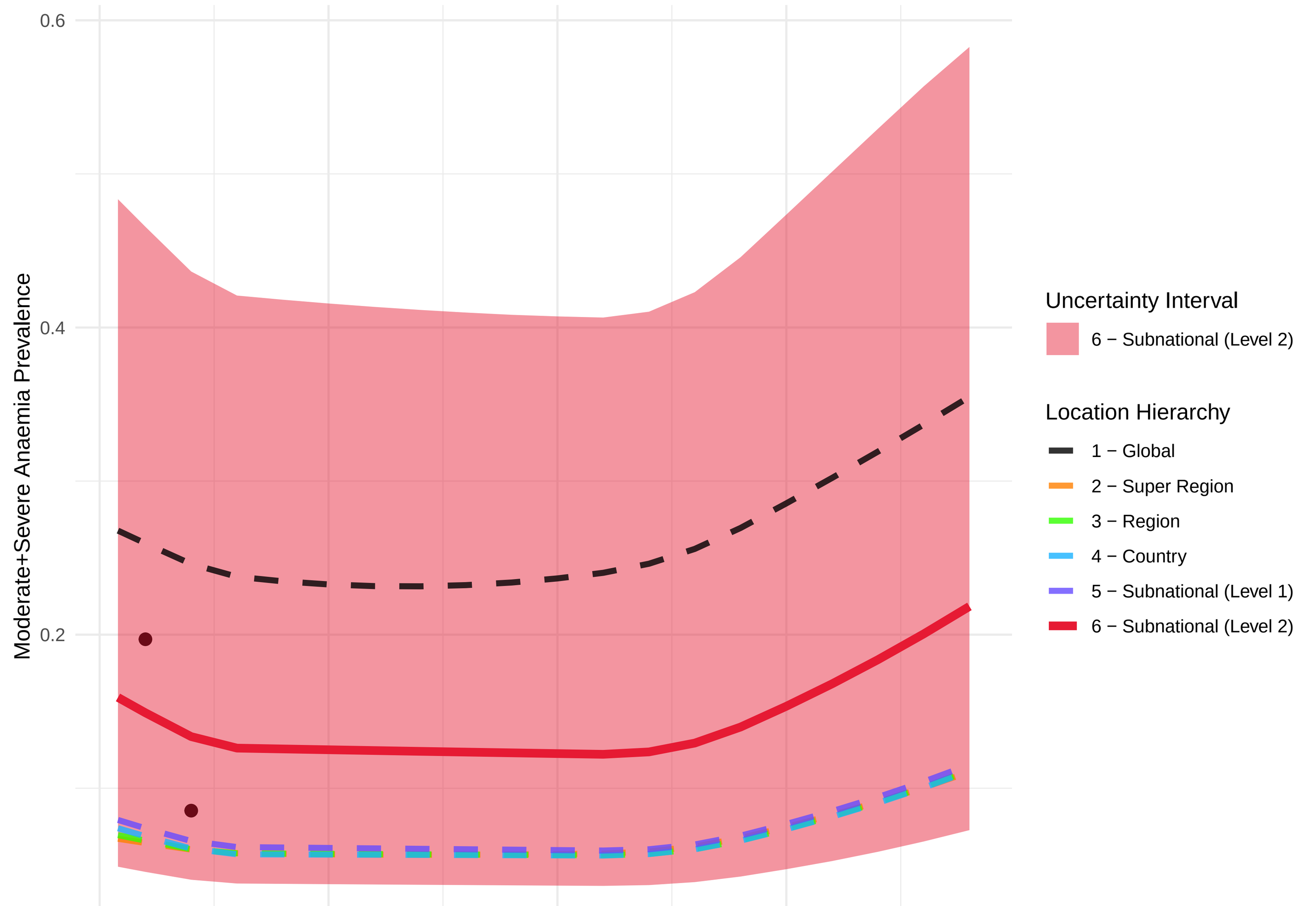

MR−BRT Age/Sex Fits − Non−pregnant Females 2+ Years Old − Moderate+Severe Anaemia Prevalence − North West England
WHO−2024 Adjustment Method

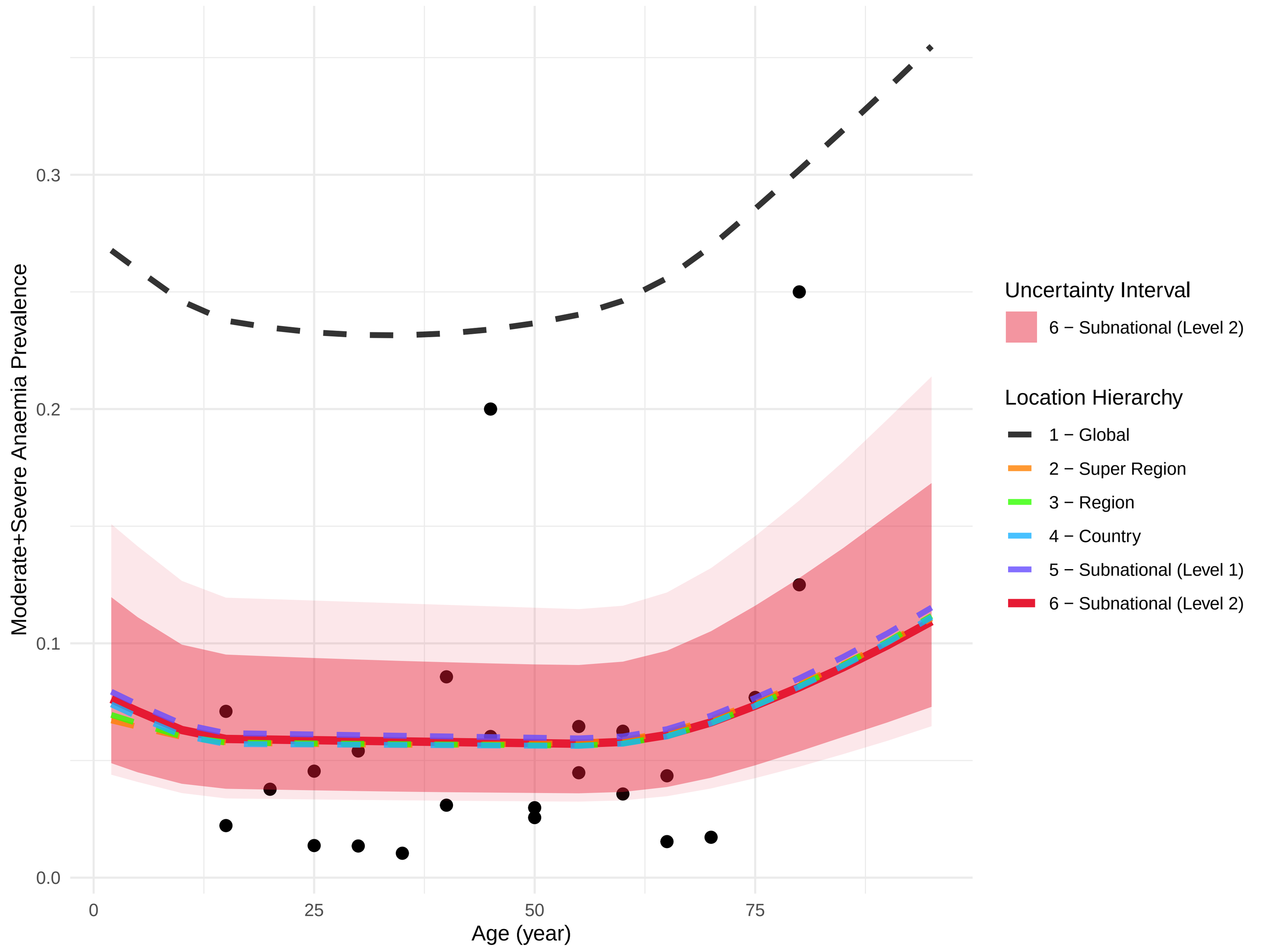

MR−BRT Age/Sex Fits − Non−pregnant Females 2+ Years Old − Moderate+Severe Anaemia Prevalence − South East England
WHO−2024 Adjustment Method

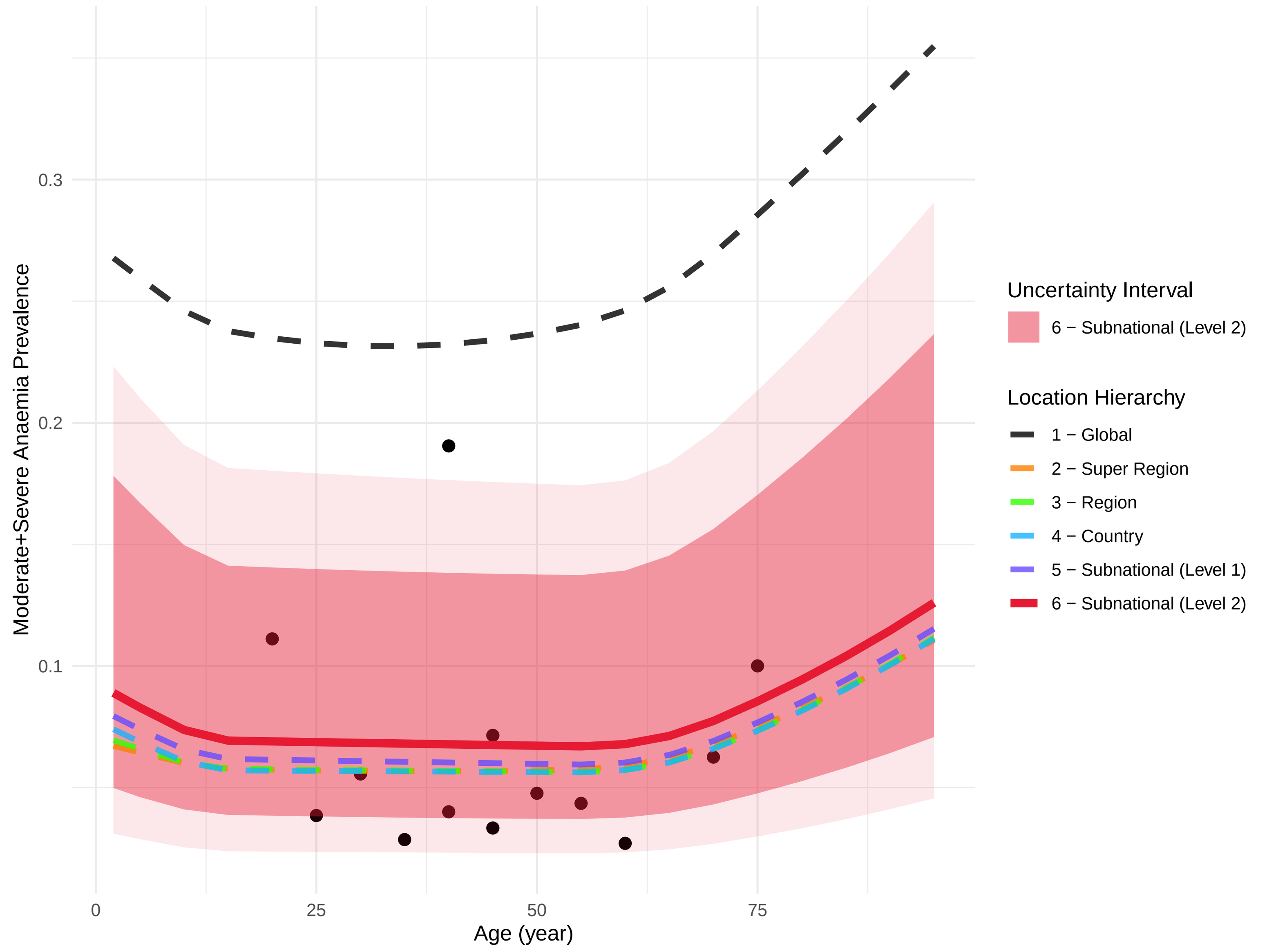

MR−BRT Age/Sex Fits − Non−pregnant Females 2+ Years Old − Moderate+Severe Anaemia Prevalence − South West England
WHO−2024 Adjustment Method

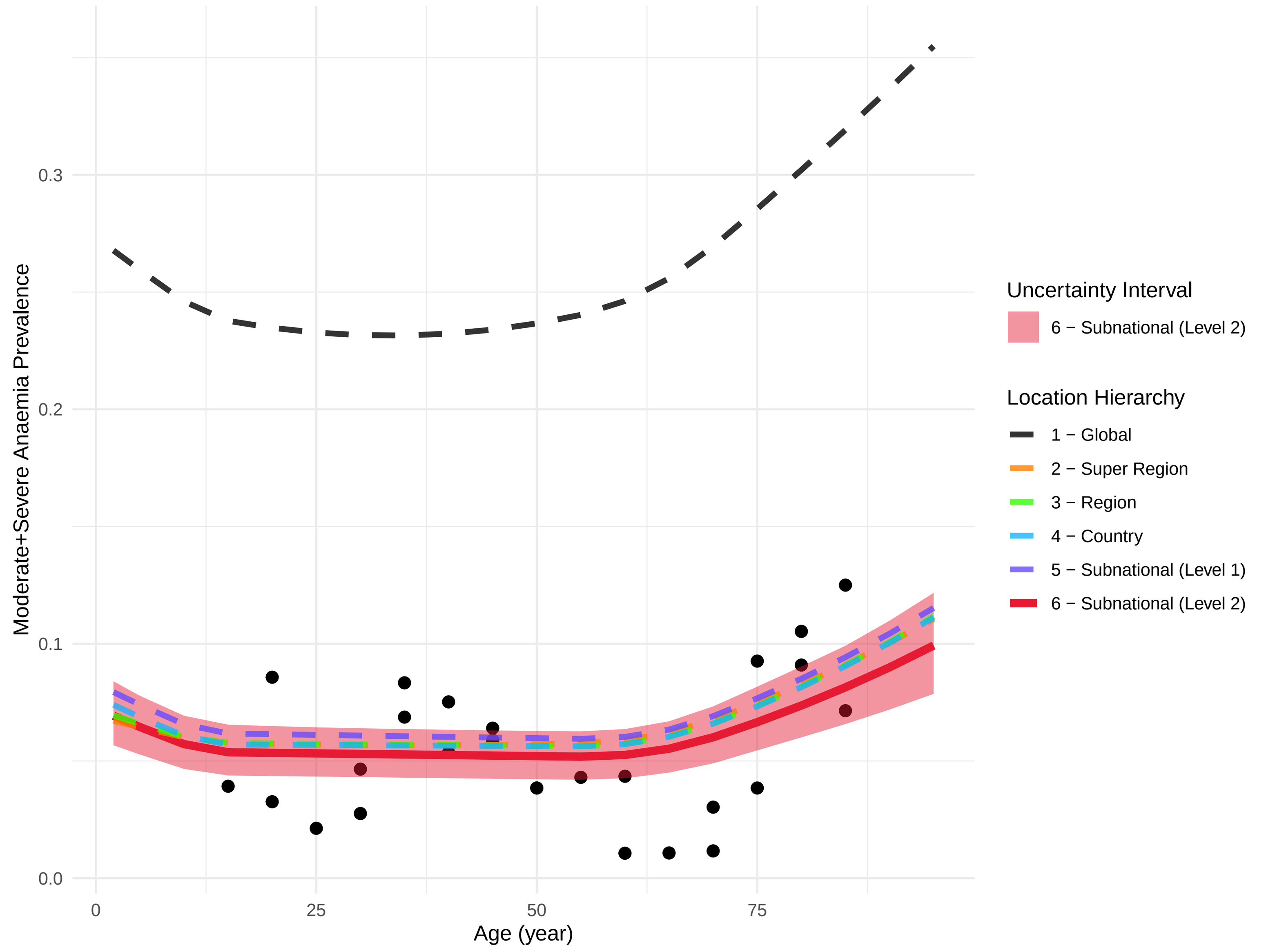

MR−BRT Age/Sex Fits − Non−pregnant Females 2+ Years Old − Moderate+Severe Anaemia Prevalence − West Midlands
WHO−2024 Adjustment Method

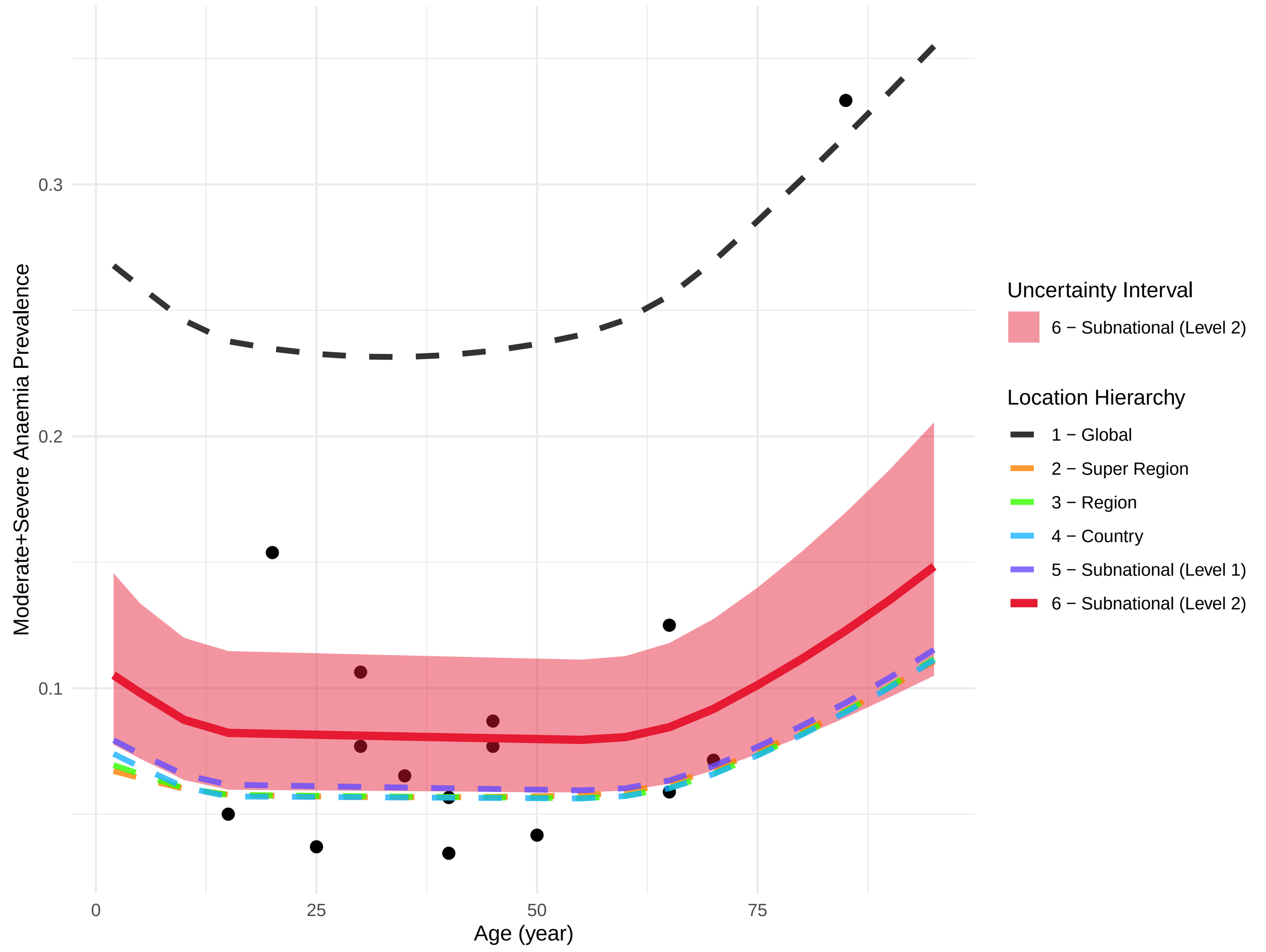

MR−BRT Age/Sex Fits − Non−pregnant Females 2+ Years Old − Moderate+Severe Anaemia Prevalence − Yorkshire and the Humber
WHO−2024 Adjustment Method

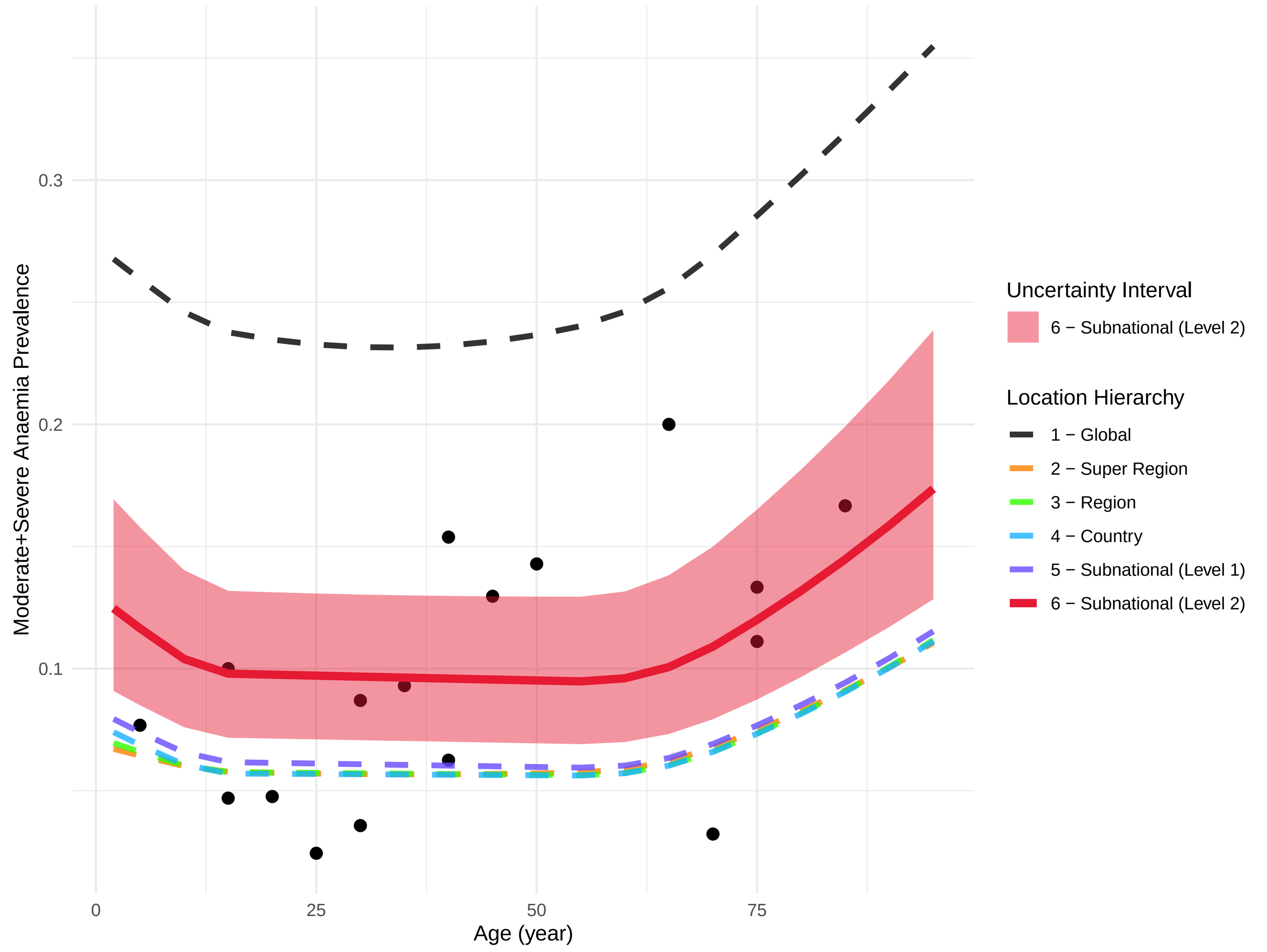

MR−BRT Age/Sex Fits − Non−pregnant Females 2+ Years Old − Moderate+Severe Anaemia Prevalence − Scotland
WHO−2024 Adjustment Method

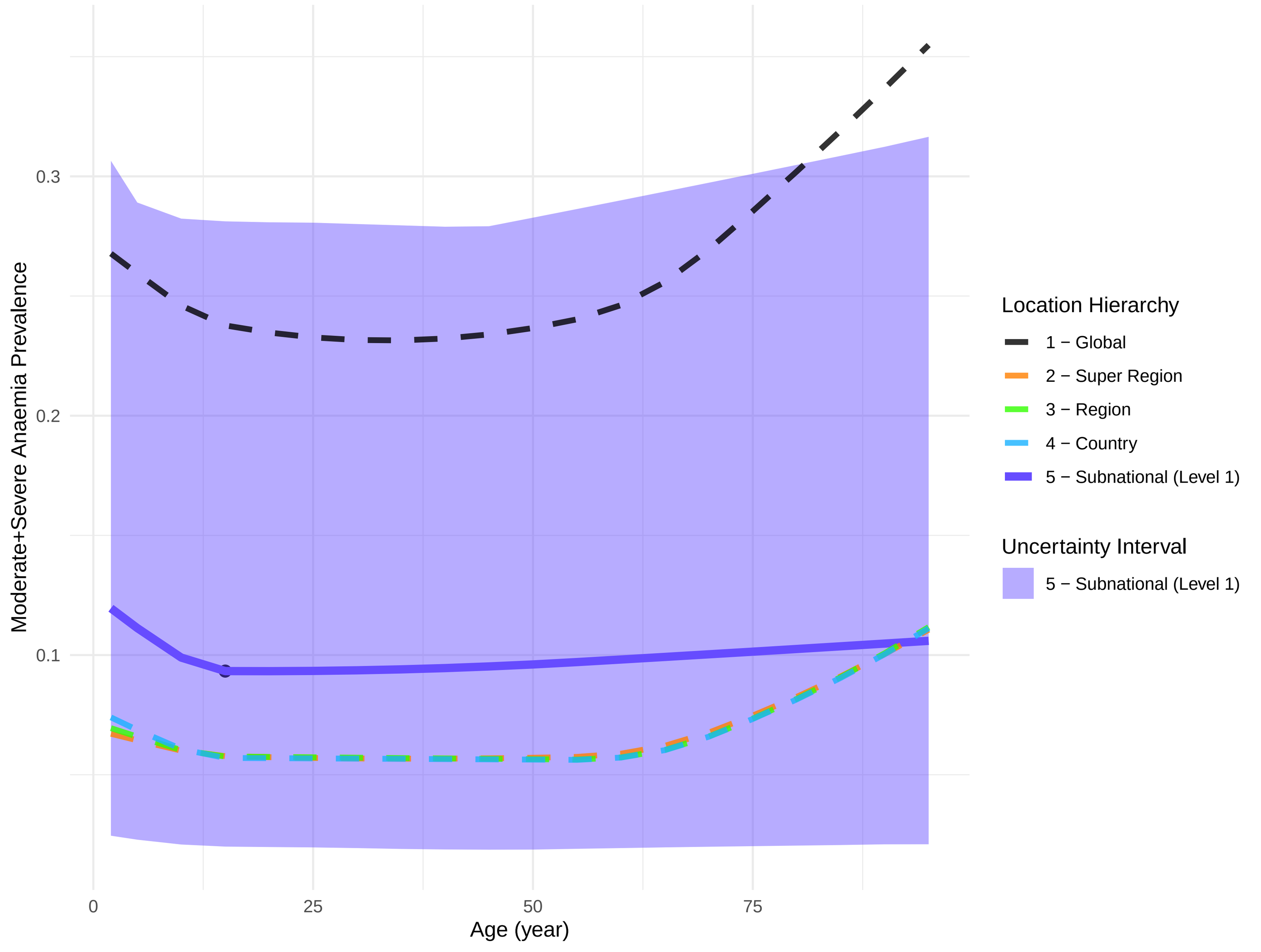

MR−BRT Age/Sex Fits − Non−pregnant Females 2+ Years Old − Moderate+Severe Anaemia Prevalence − Latin America and Caribbean
WHO−2024 Adjustment Method

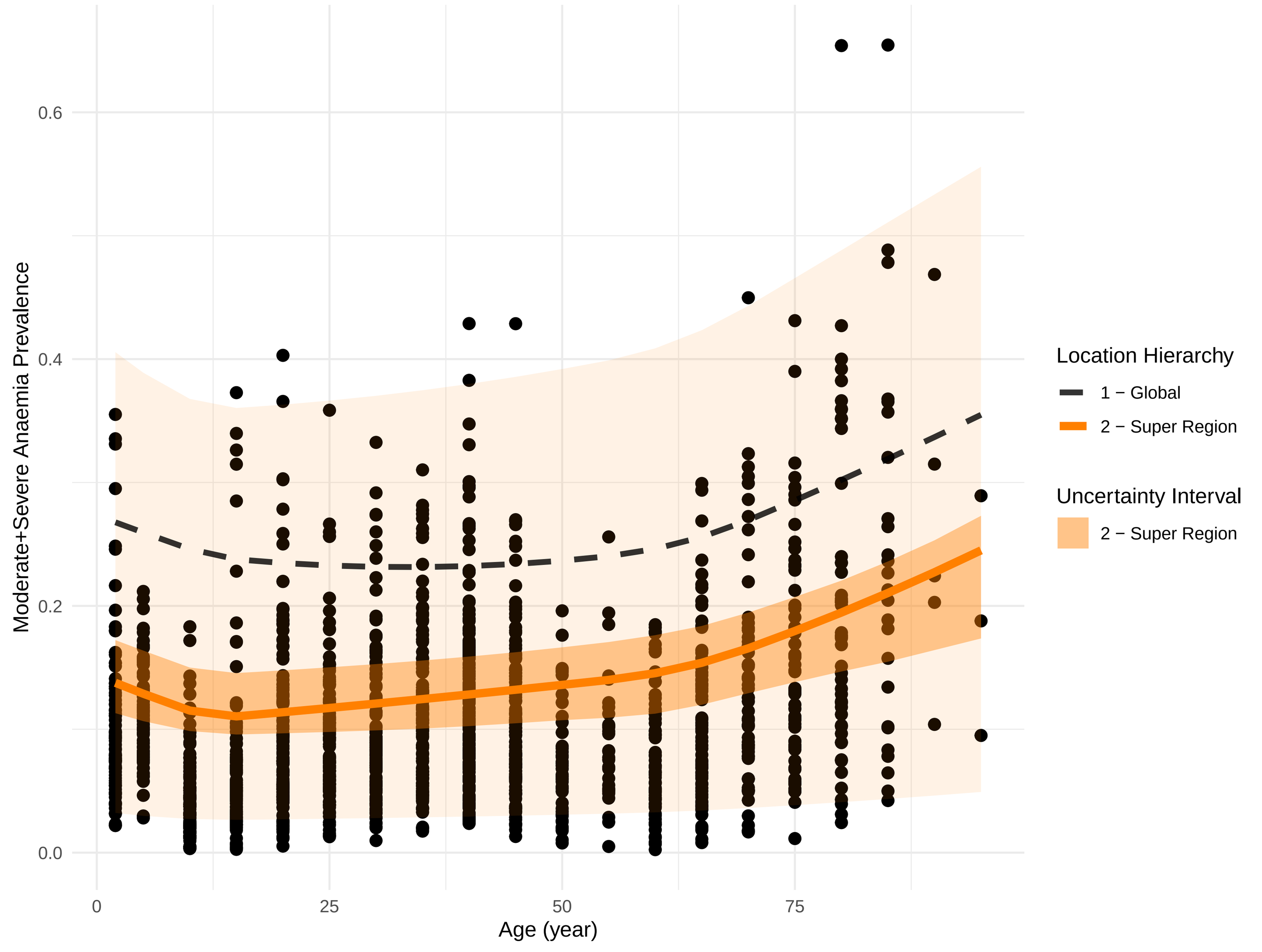

MR−BRT Age/Sex Fits − Non−pregnant Females 2+ Years Old − Moderate+Severe Anaemia Prevalence − Andean Latin America
WHO−2024 Adjustment Method

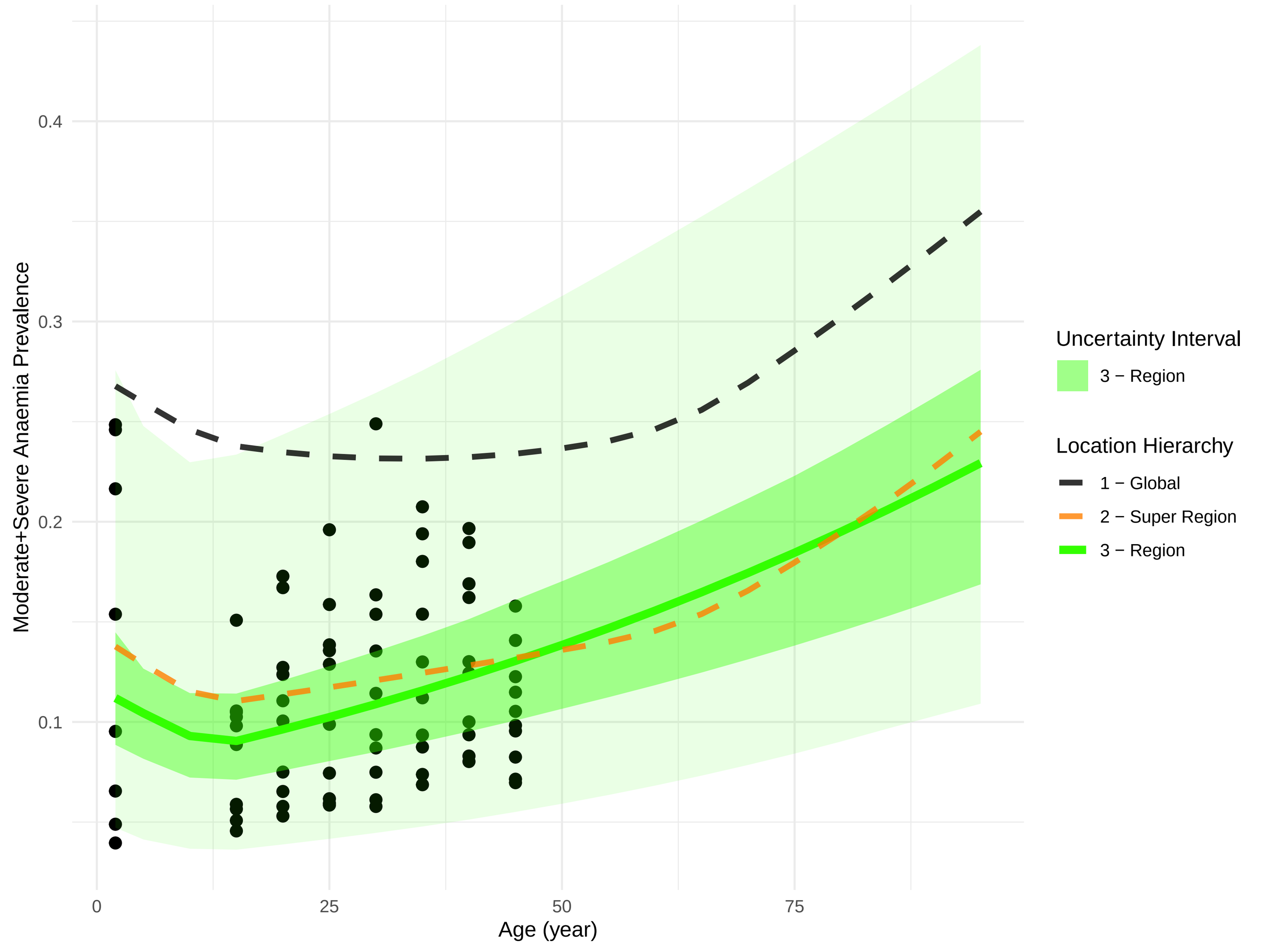

MR−BRT Age/Sex Fits − Non−pregnant Females 2+ Years Old − Moderate+Severe Anaemia Prevalence − Bolivia (Plurinational State of)
WHO−2024 Adjustment Method

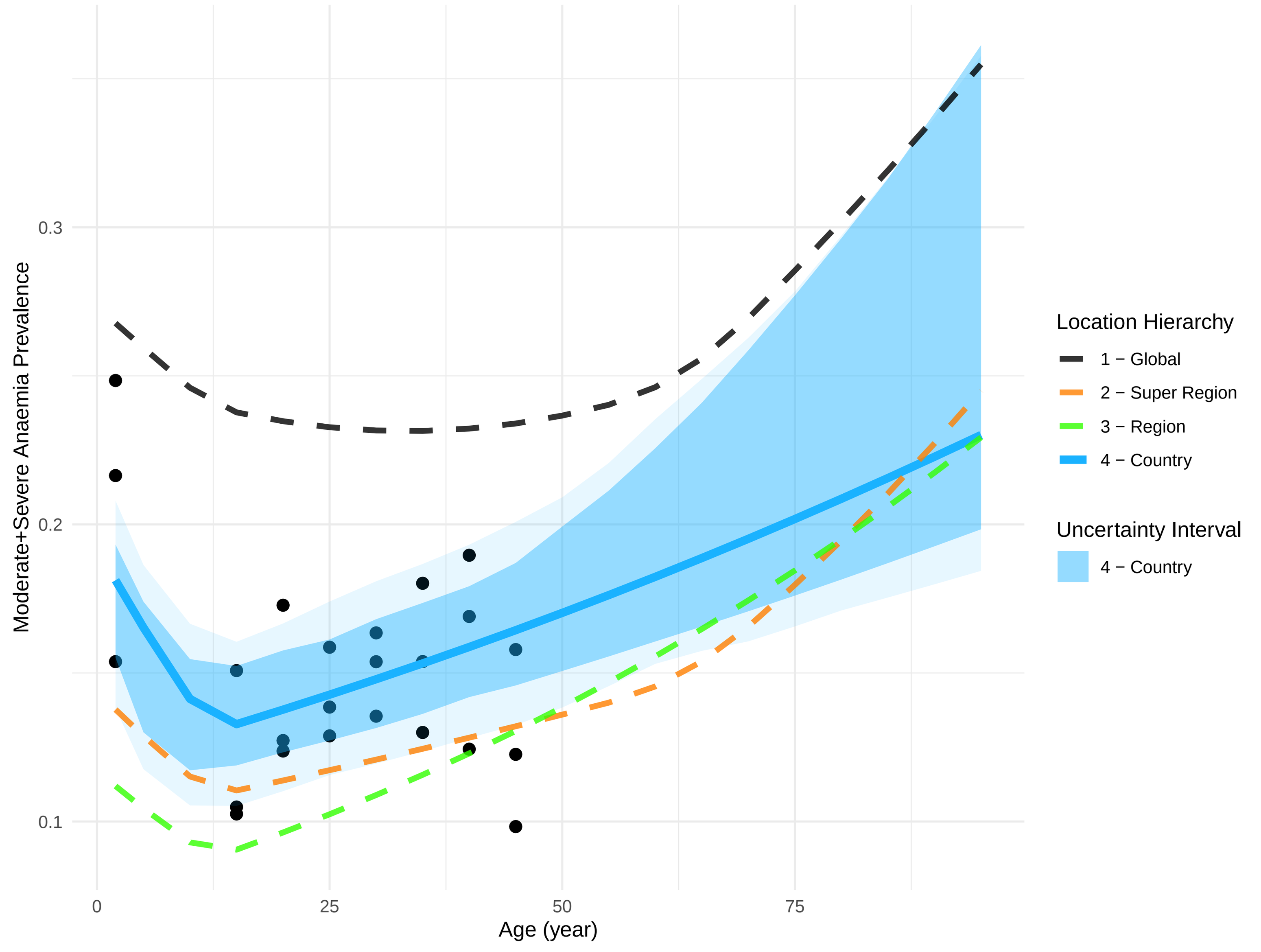

MR−BRT Age/Sex Fits − Non−pregnant Females 2+ Years Old − Moderate+Severe Anaemia Prevalence − Peru
WHO−2024 Adjustment Method

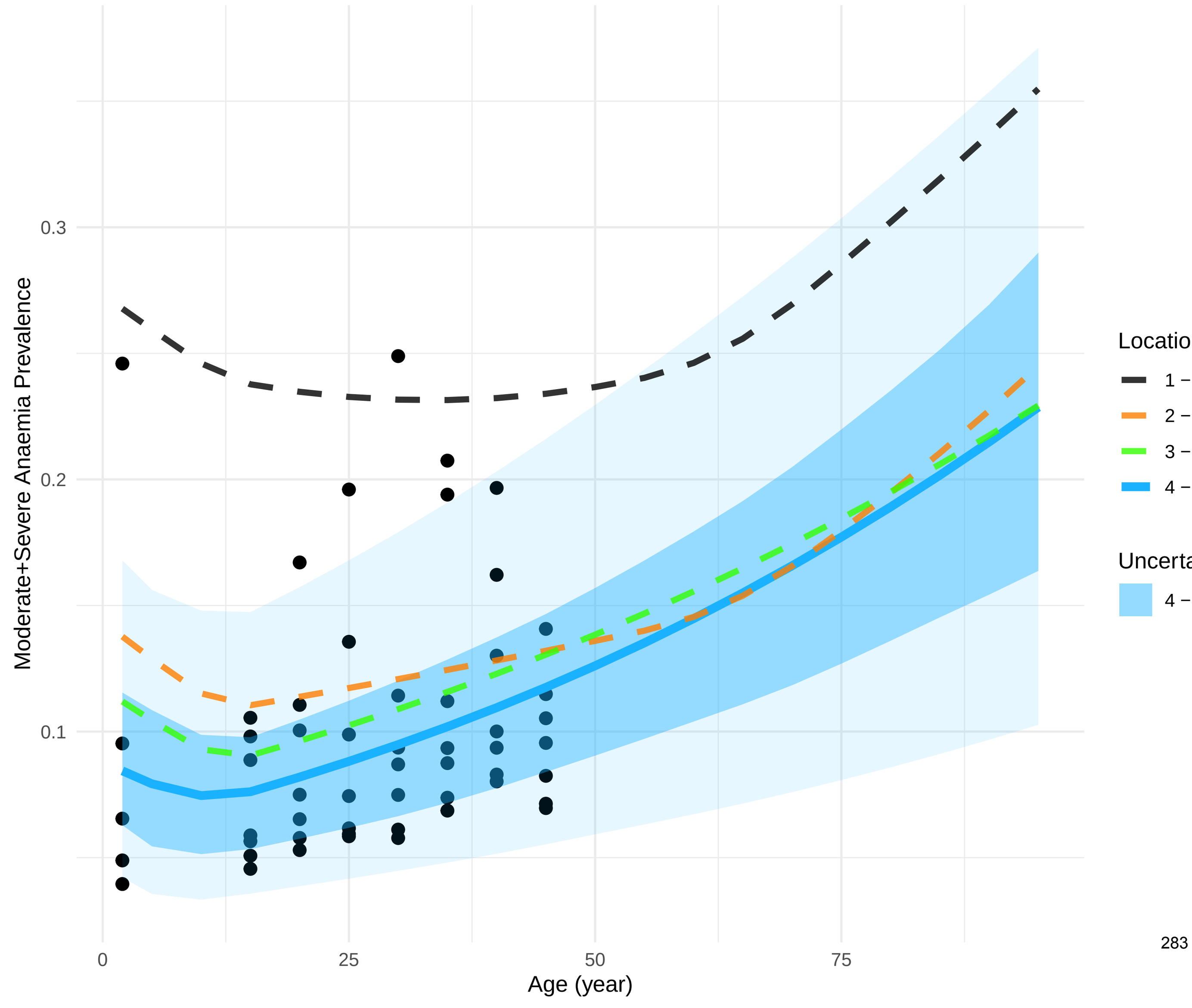

MR−BRT Age/Sex Fits − Non−pregnant Females 2+ Years Old − Moderate+Severe Anaemia Prevalence − Caribbean
WHO−2024 Adjustment Method

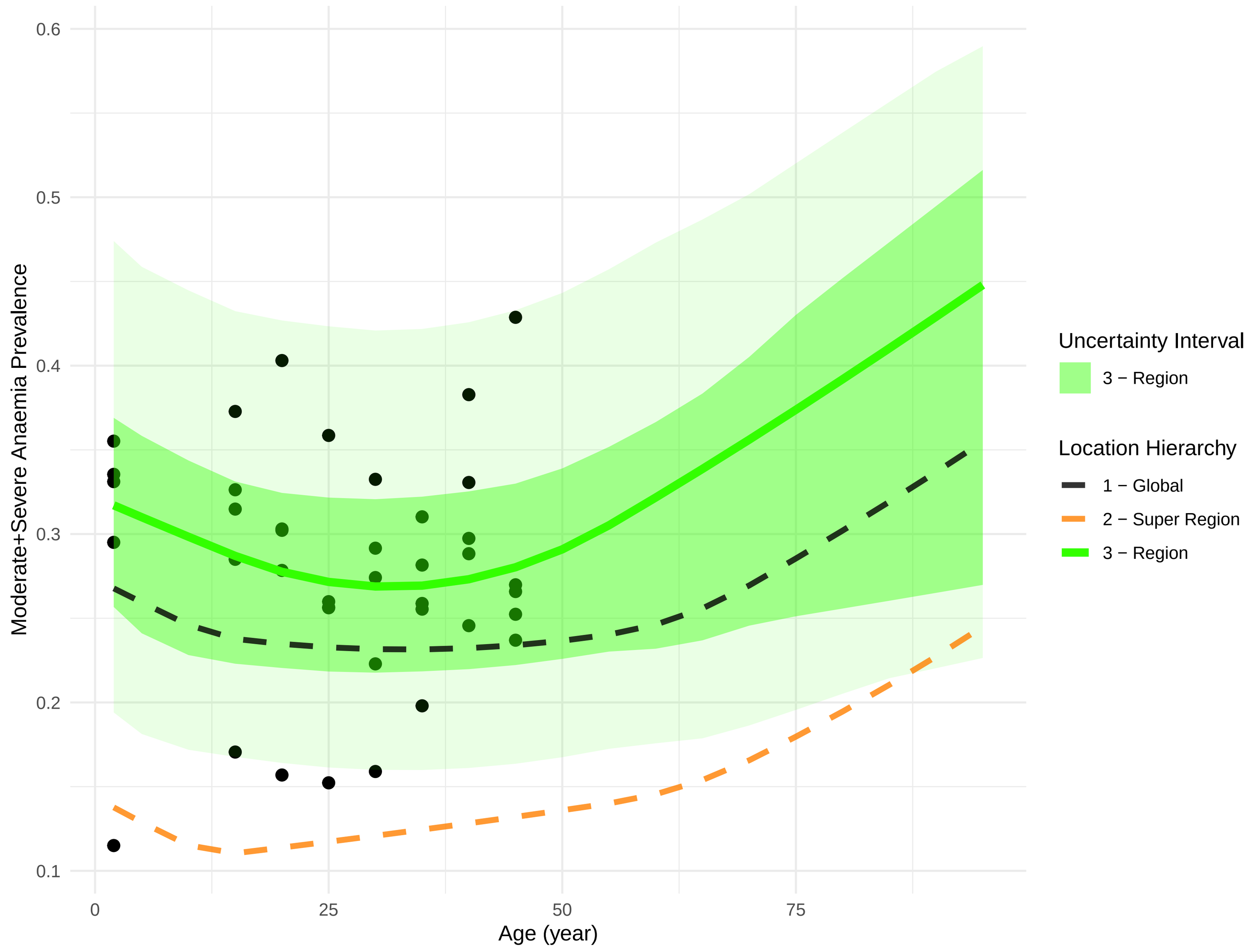

MR−BRT Age/Sex Fits − Non−pregnant Females 2+ Years Old − Moderate+Severe Anaemia Prevalence − Guyana
WHO−2024 Adjustment Method

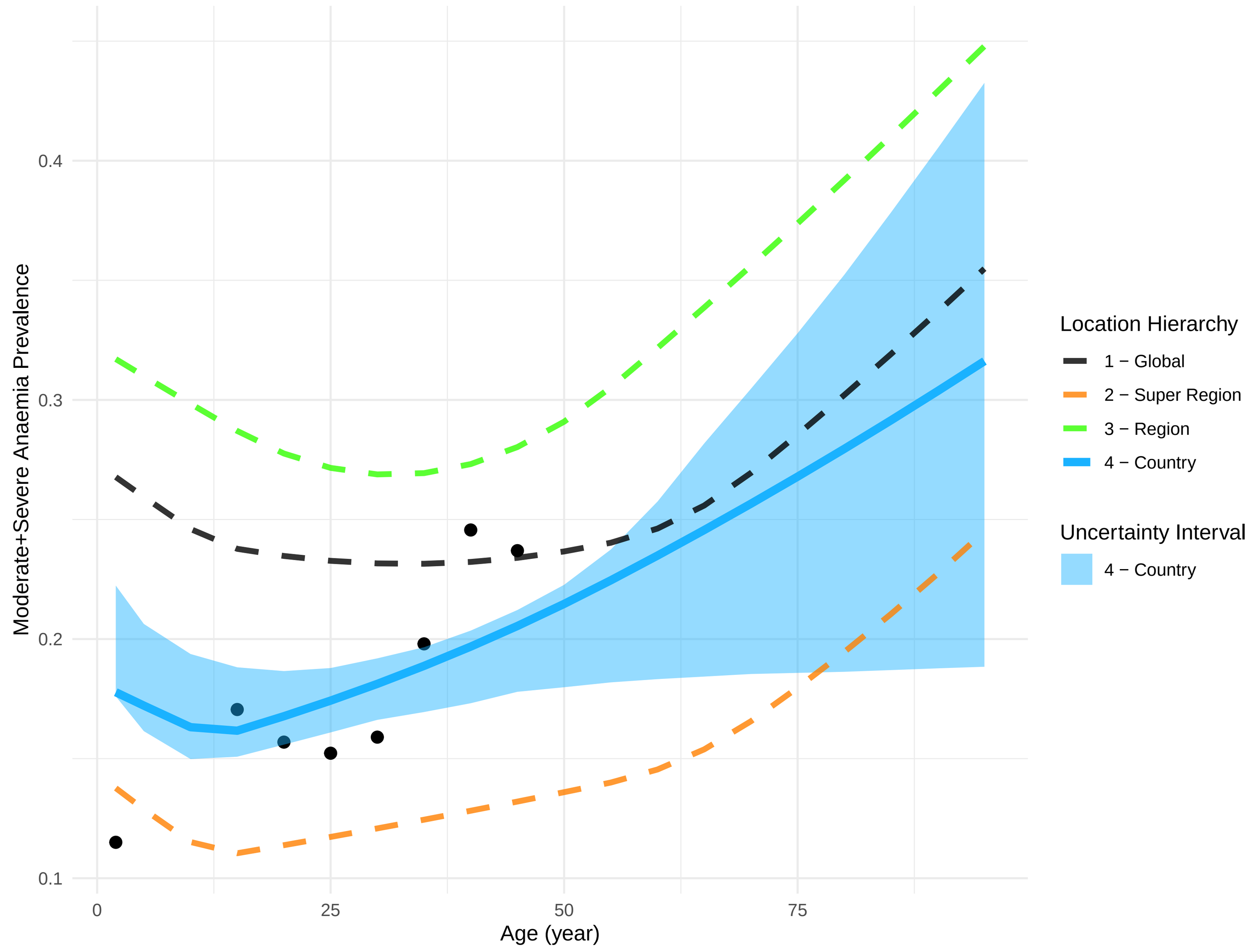

MR−BRT Age/Sex Fits − Non−pregnant Females 2+ Years Old − Moderate+Severe Anaemia Prevalence − Haiti
WHO−2024 Adjustment Method

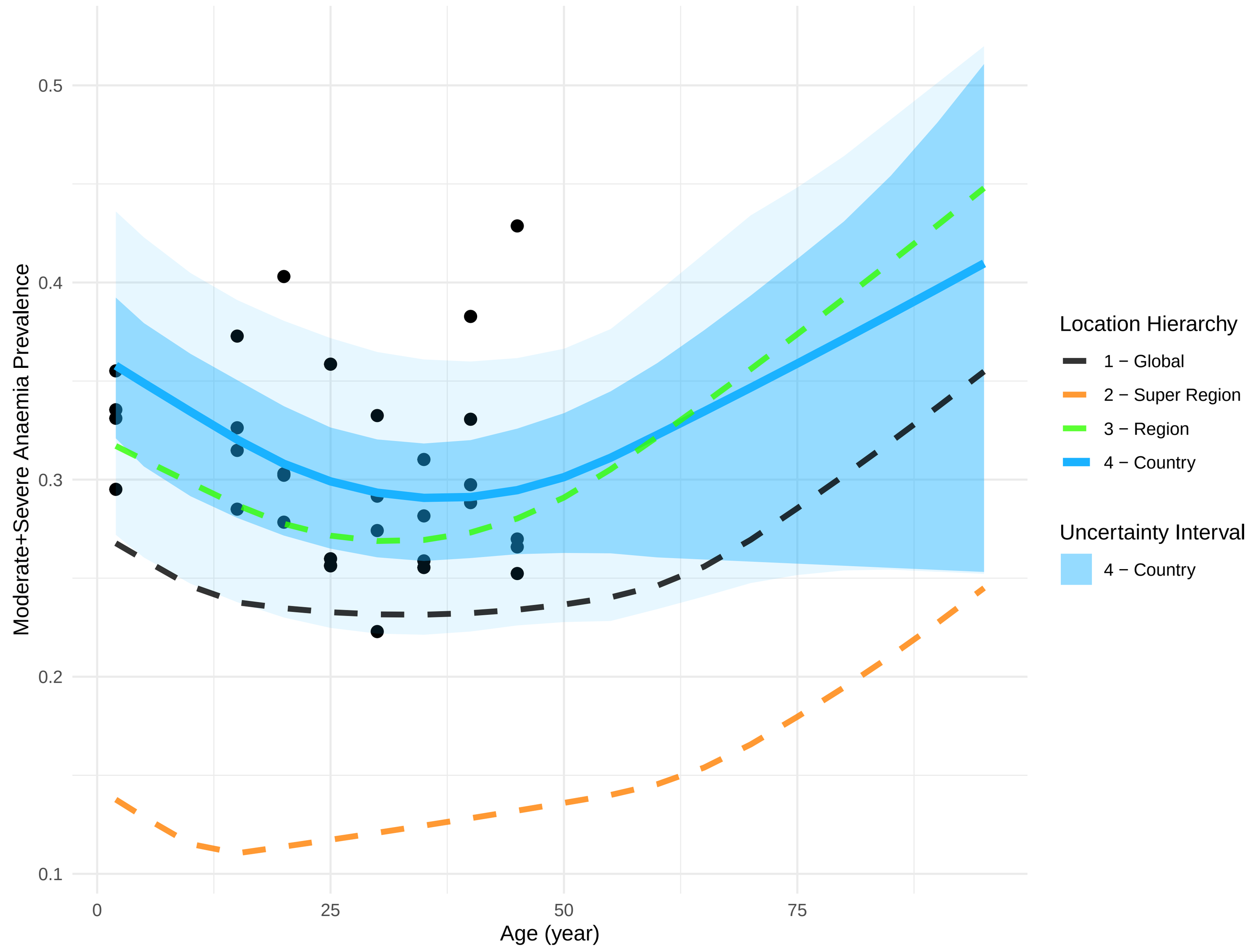

MR−BRT Age/Sex Fits − Non−pregnant Females 2+ Years Old − Moderate+Severe Anaemia Prevalence − Central Latin America
WHO−2024 Adjustment Method

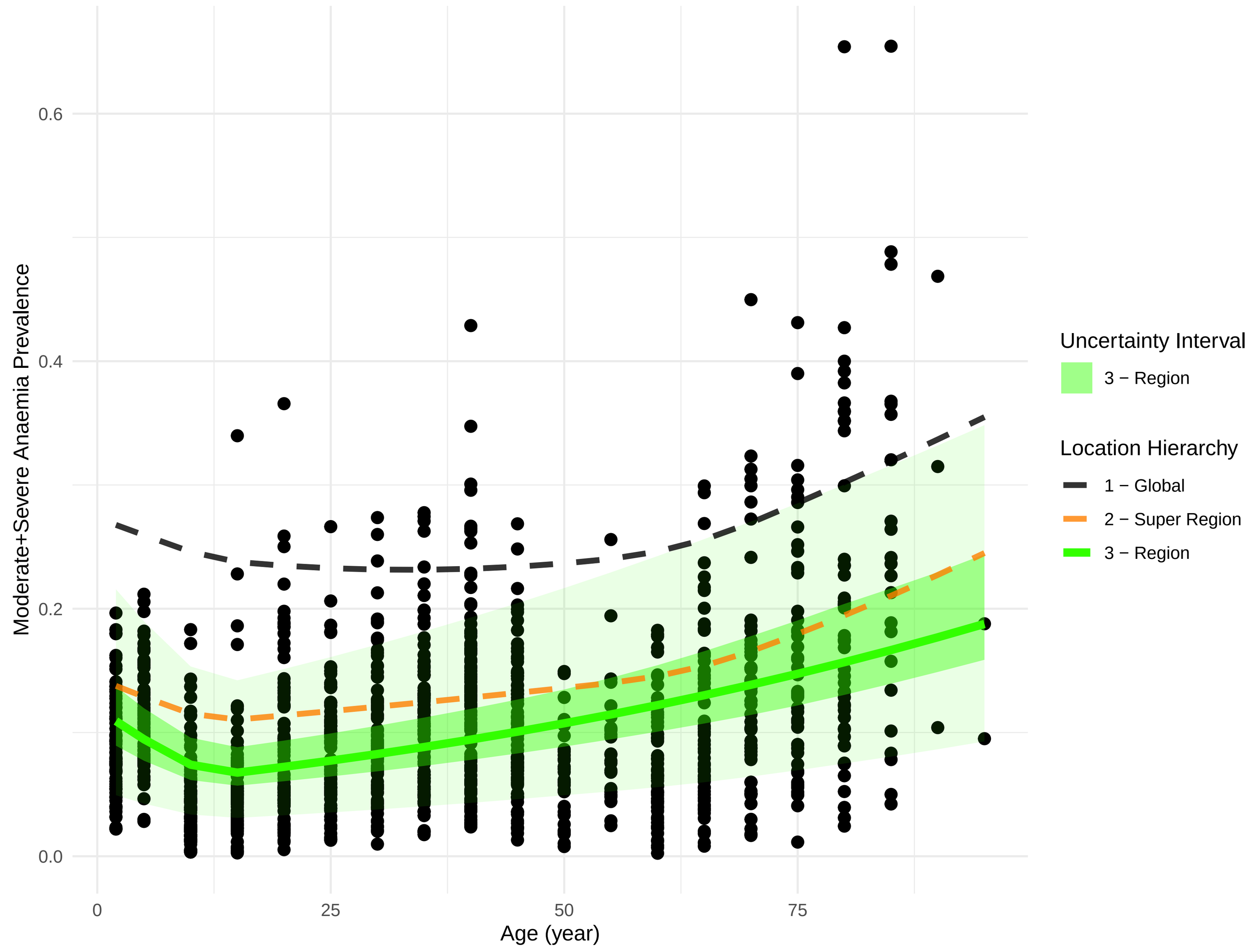

MR−BRT Age/Sex Fits − Non−pregnant Females 2+ Years Old − Moderate+Severe Anaemia Prevalence − El Salvador
WHO−2024 Adjustment Method

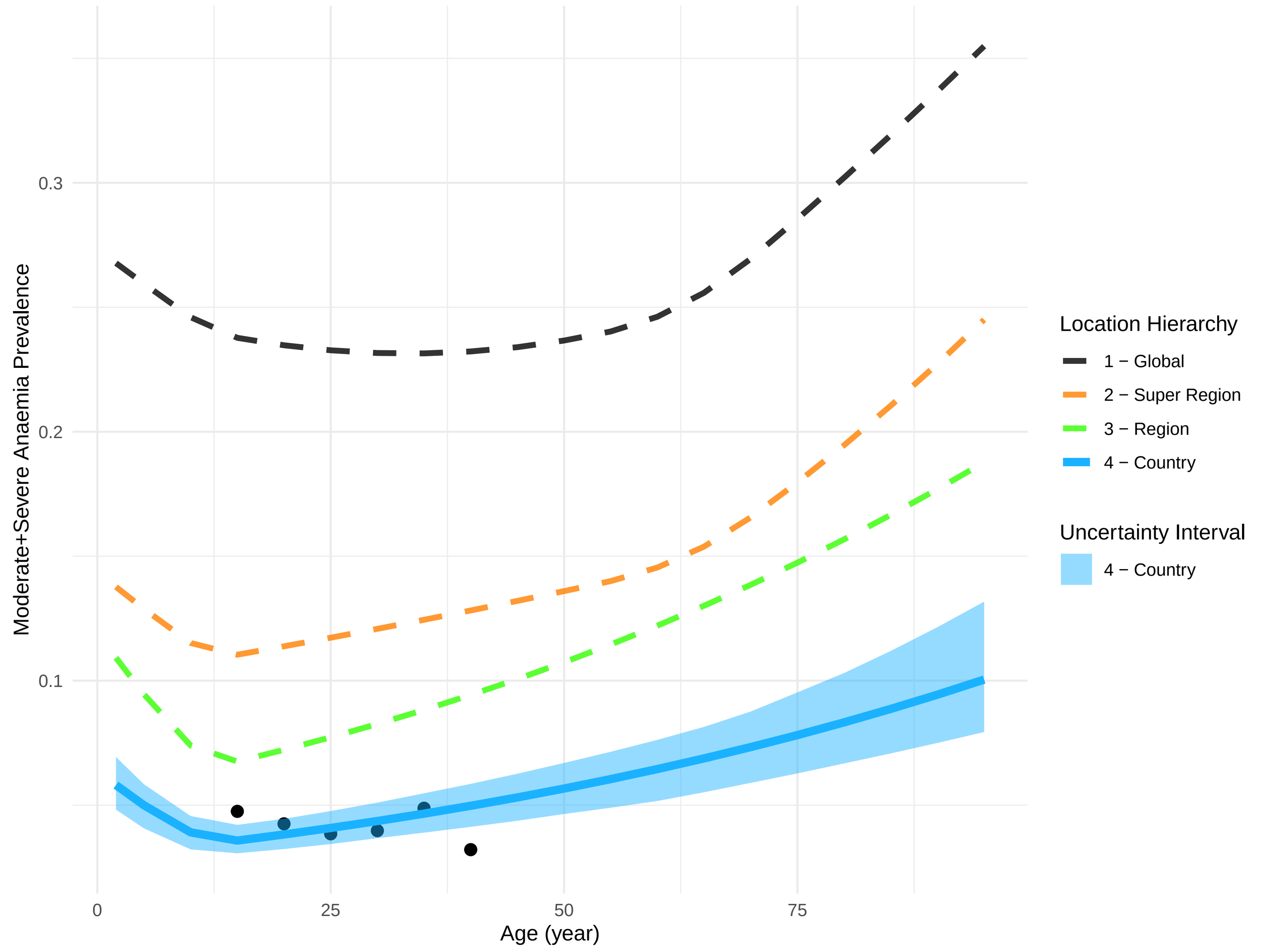

MR−BRT Age/Sex Fits − Non−pregnant Females 2+ Years Old − Moderate+Severe Anaemia Prevalence − Guatemala
WHO−2024 Adjustment Method

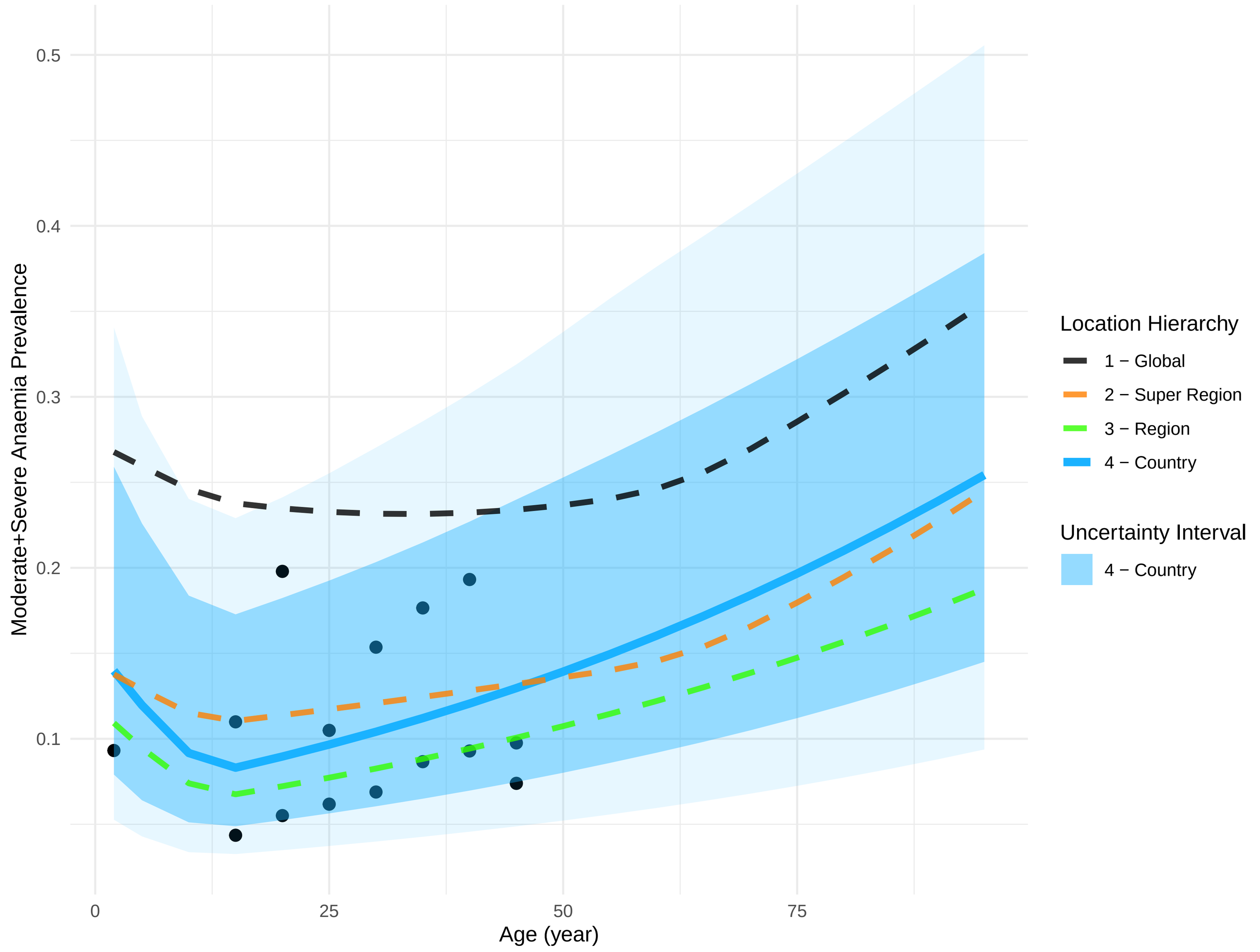

MR−BRT Age/Sex Fits − Non−pregnant Females 2+ Years Old − Moderate+Severe Anaemia Prevalence − Honduras
WHO−2024 Adjustment Method

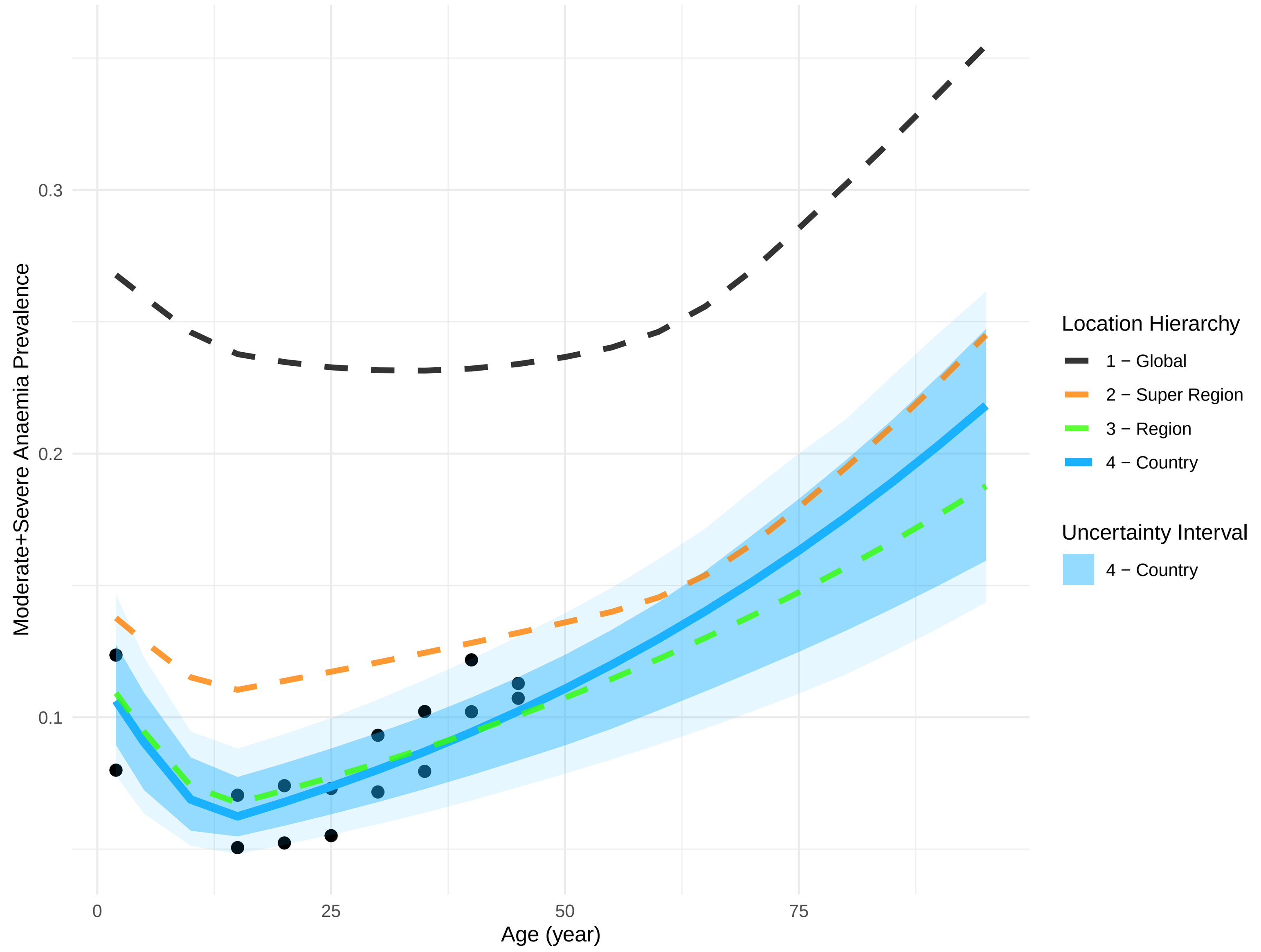

MR−BRT Age/Sex Fits − Non−pregnant Females 2+ Years Old − Moderate+Severe Anaemia Prevalence − Mexico
WHO−2024 Adjustment Method

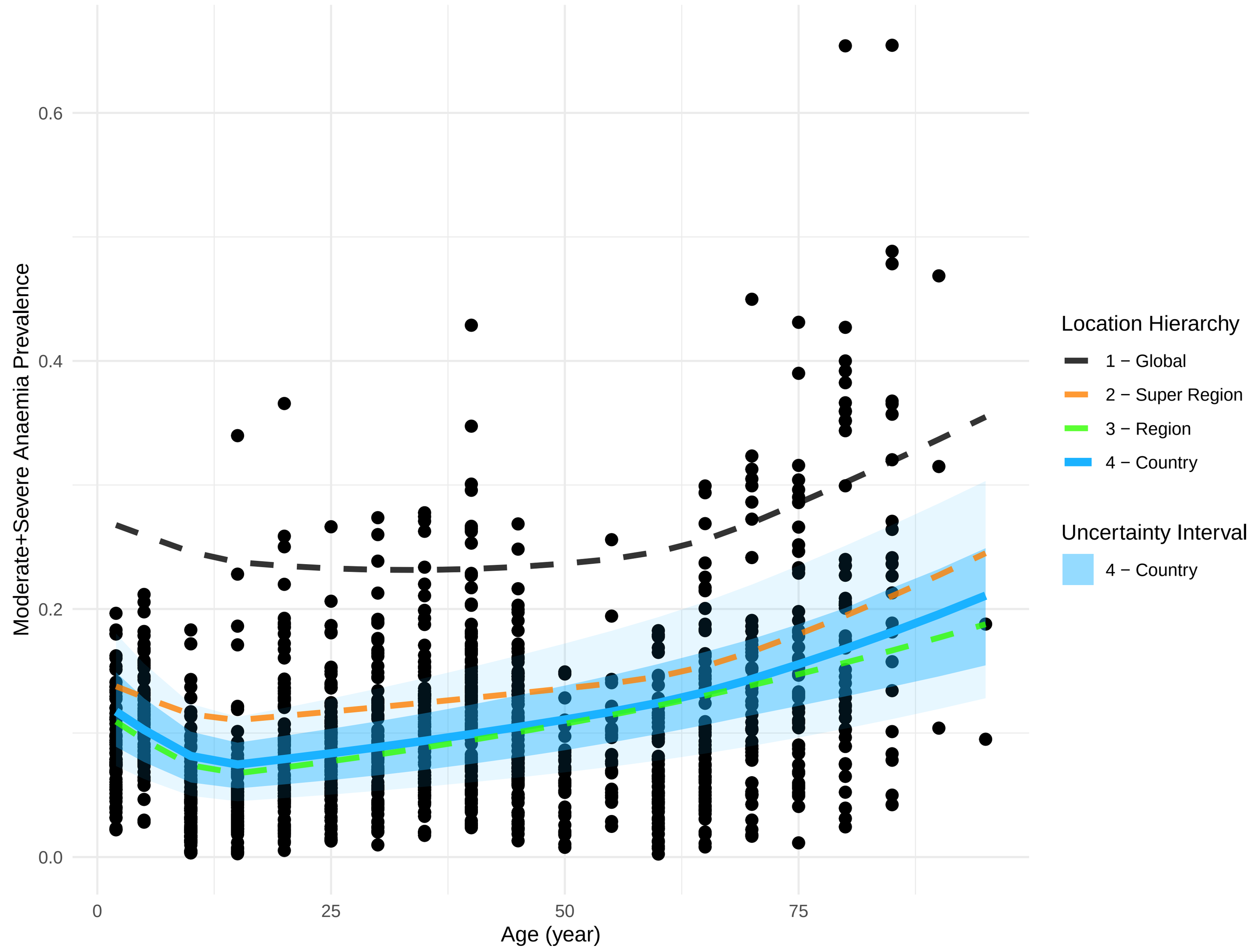

MR−BRT Age/Sex Fits − Non−pregnant Females 2+ Years Old − Moderate+Severe Anaemia Prevalence − Aguascalientes
WHO−2024 Adjustment Method

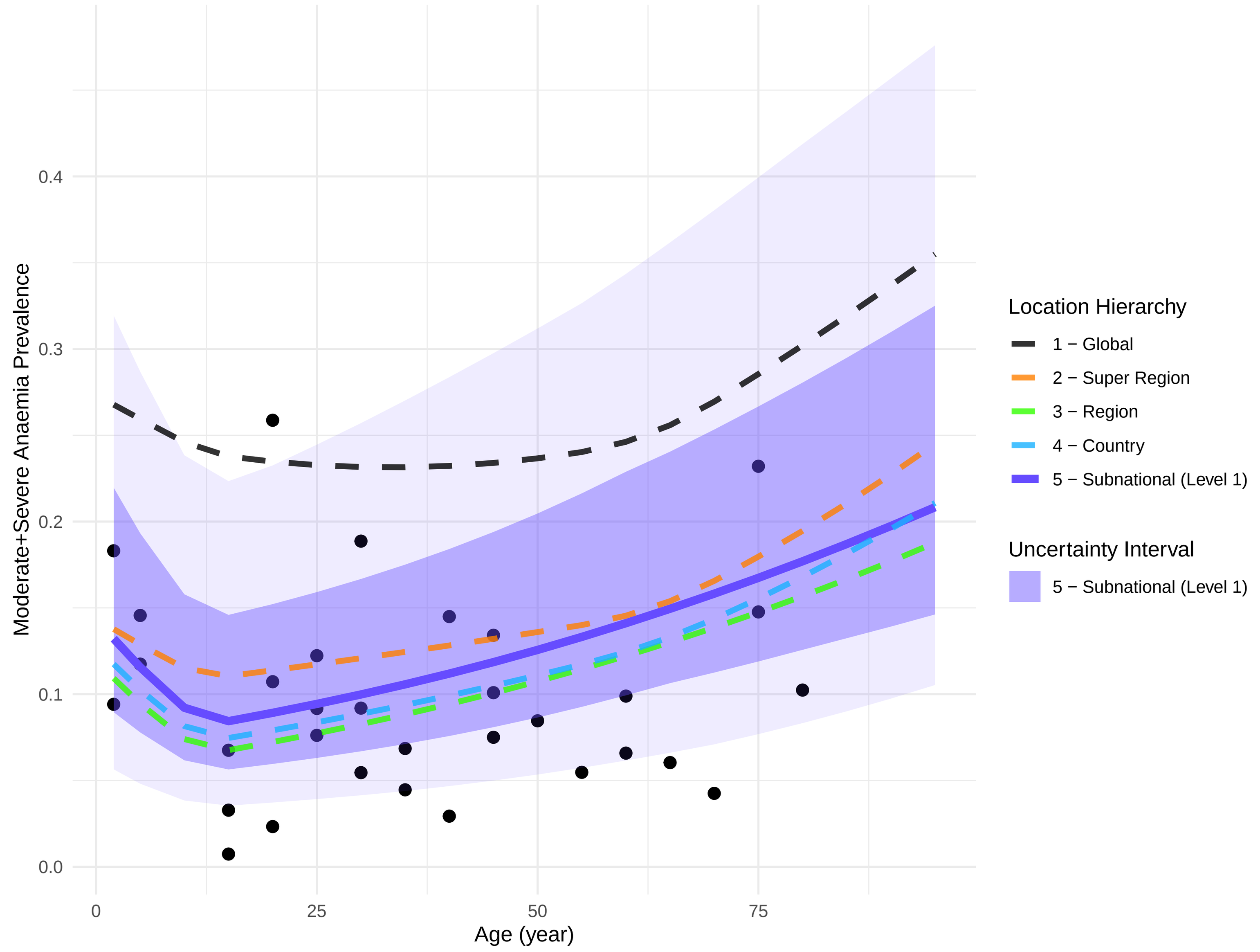

MR−BRT Age/Sex Fits − Non−pregnant Females 2+ Years Old − Moderate+Severe Anaemia Prevalence − Baja California
WHO−2024 Adjustment Method

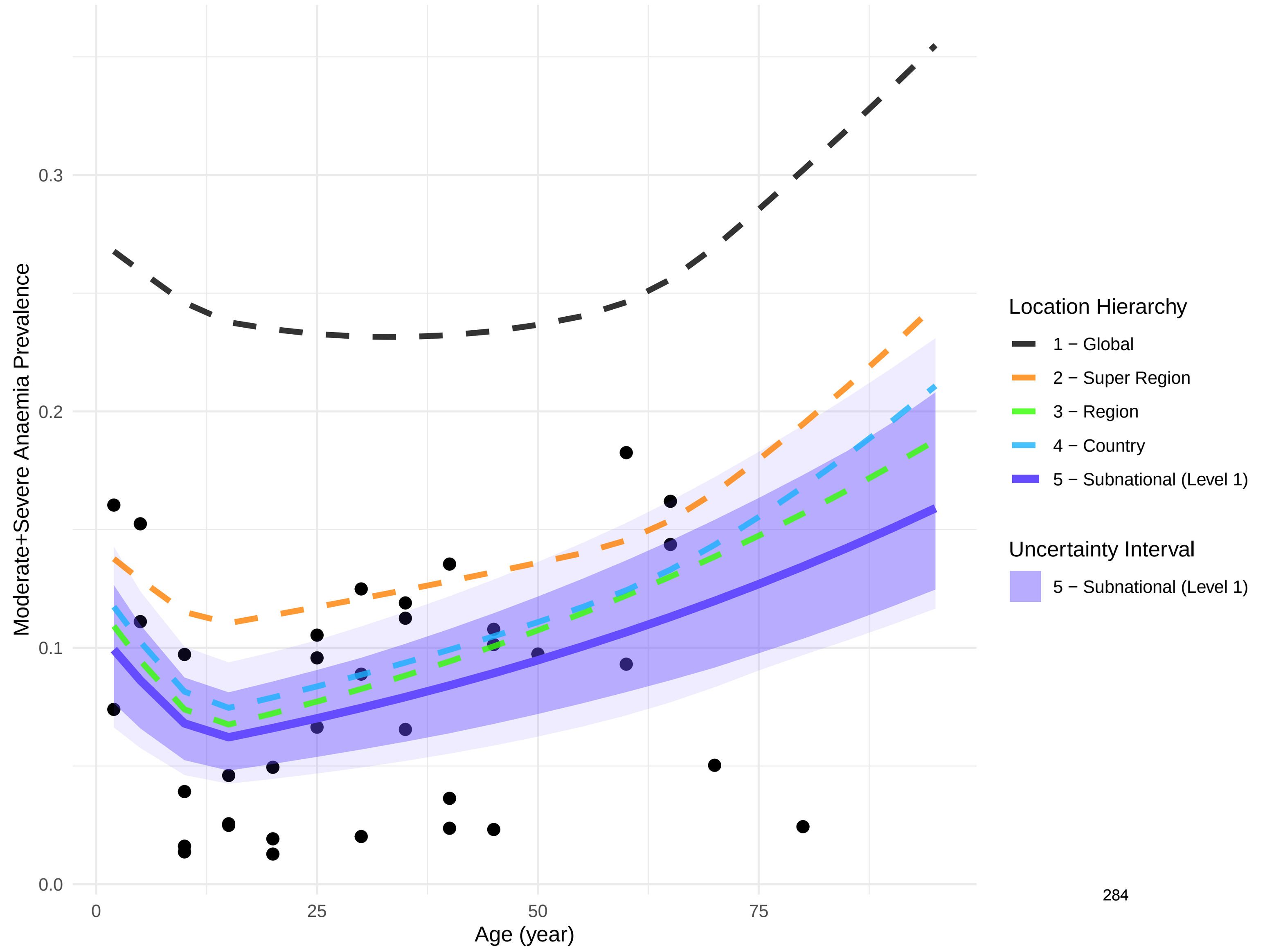

MR−BRT Age/Sex Fits − Non−pregnant Females 2+ Years Old − Moderate+Severe Anaemia Prevalence − Baja California Sur
WHO−2024 Adjustment Method

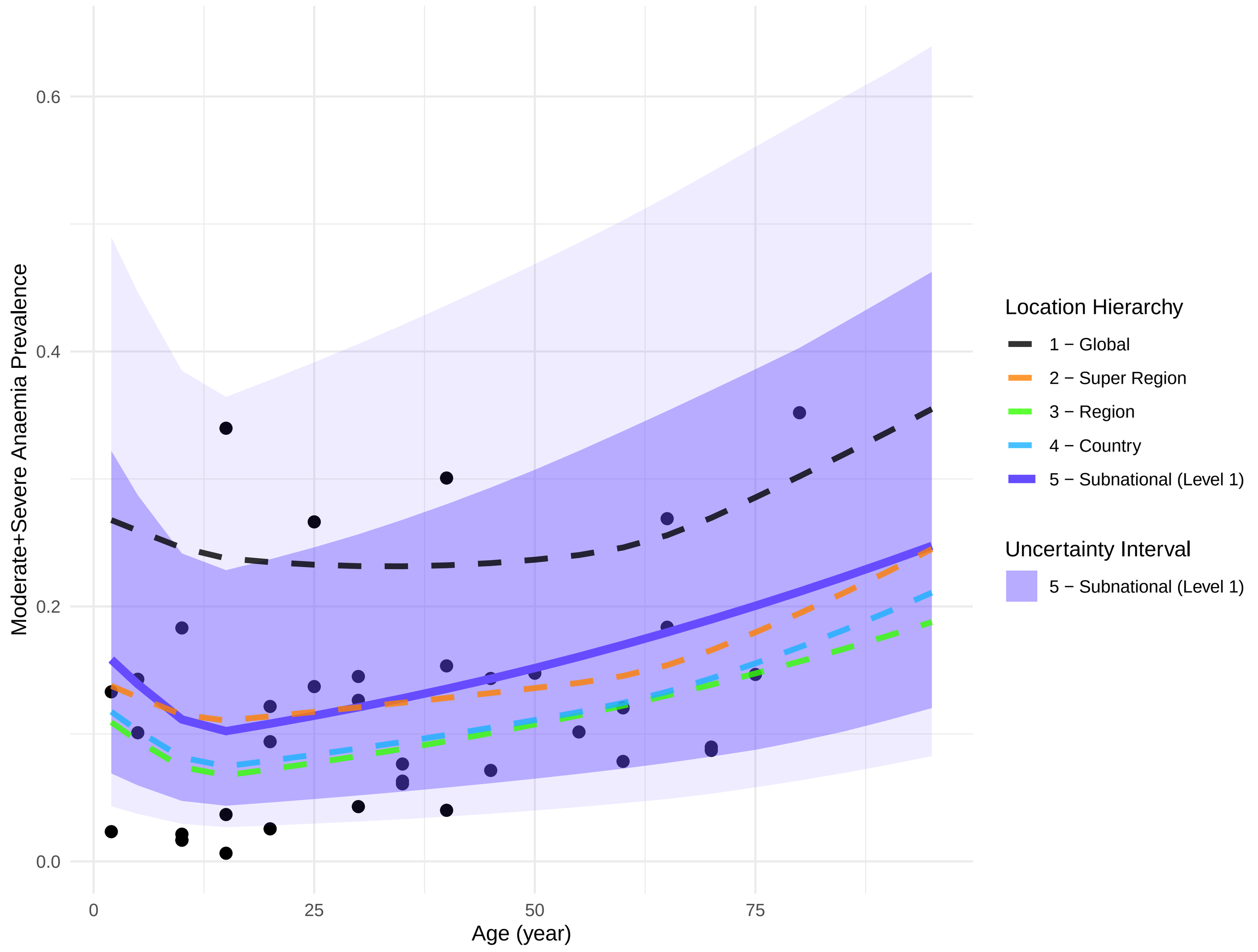

MR−BRT Age/Sex Fits − Non−pregnant Females 2+ Years Old − Moderate+Severe Anaemia Prevalence − Campeche
WHO−2024 Adjustment Method

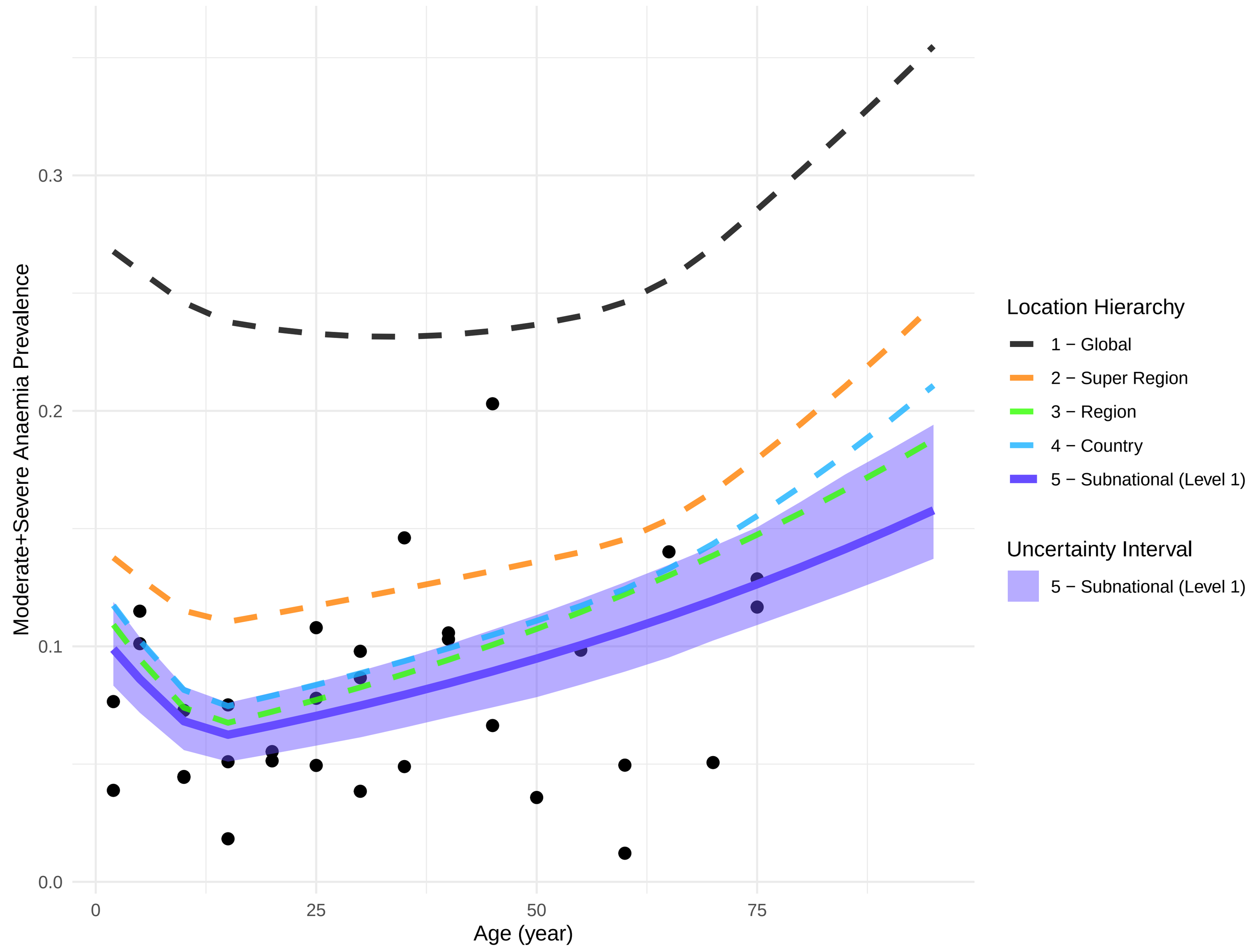

MR−BRT Age/Sex Fits − Non−pregnant Females 2+ Years Old − Moderate+Severe Anaemia Prevalence − Chiapas
WHO−2024 Adjustment Method

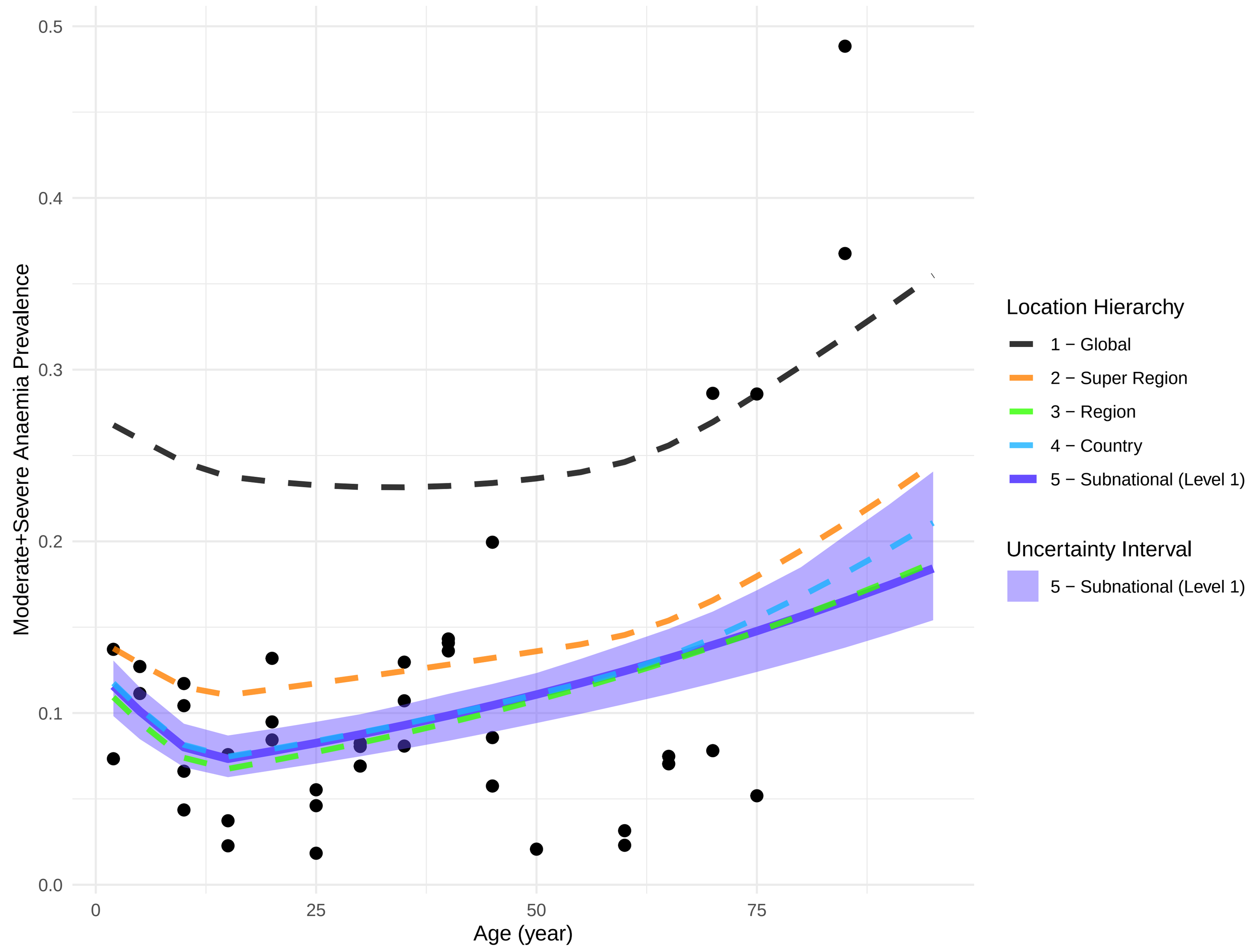

MR−BRT Age/Sex Fits − Non−pregnant Females 2+ Years Old − Moderate+Severe Anaemia Prevalence − Chihuahua
WHO−2024 Adjustment Method

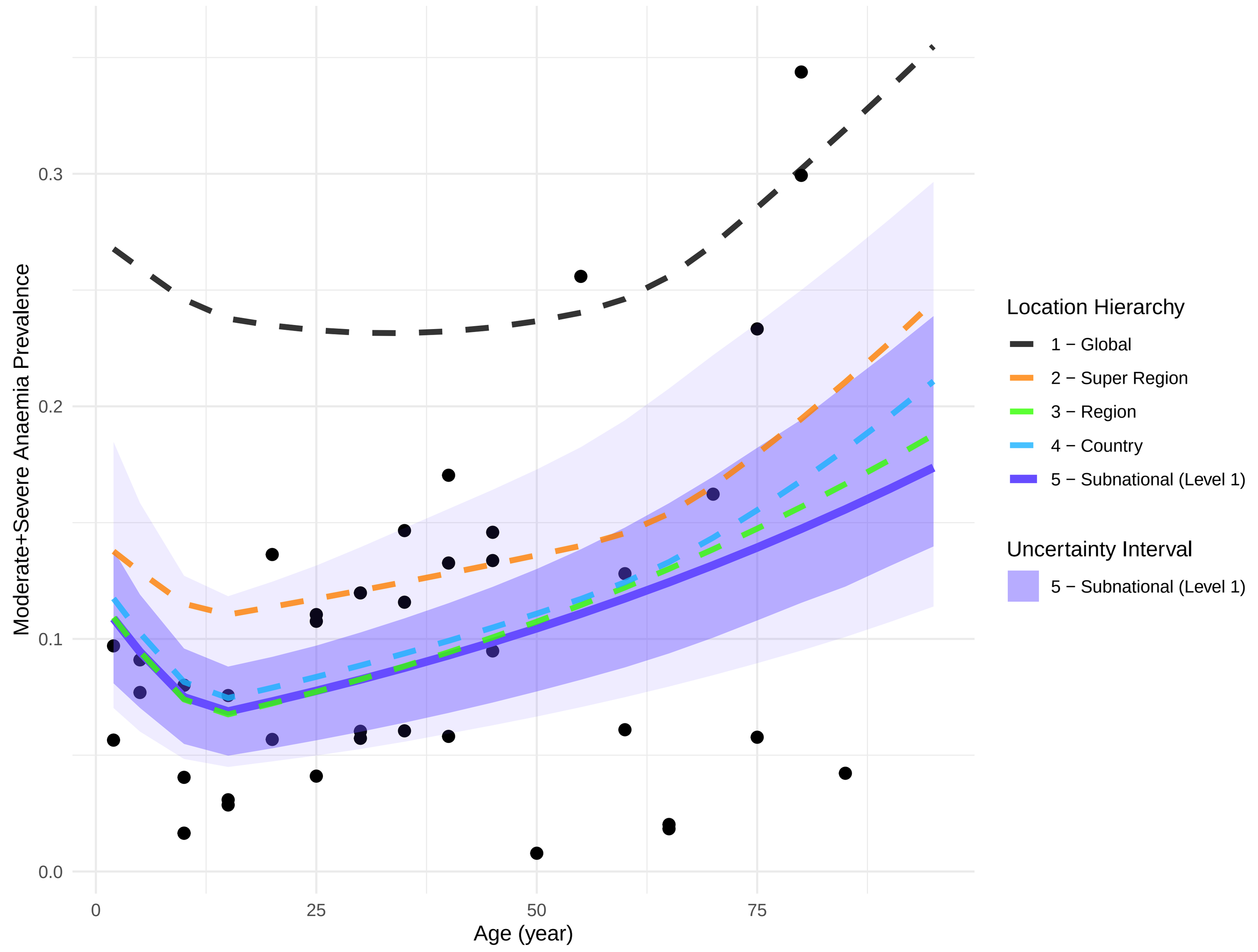

MR−BRT Age/Sex Fits − Non−pregnant Females 2+ Years Old − Moderate+Severe Anaemia Prevalence − Coahuila
WHO−2024 Adjustment Method

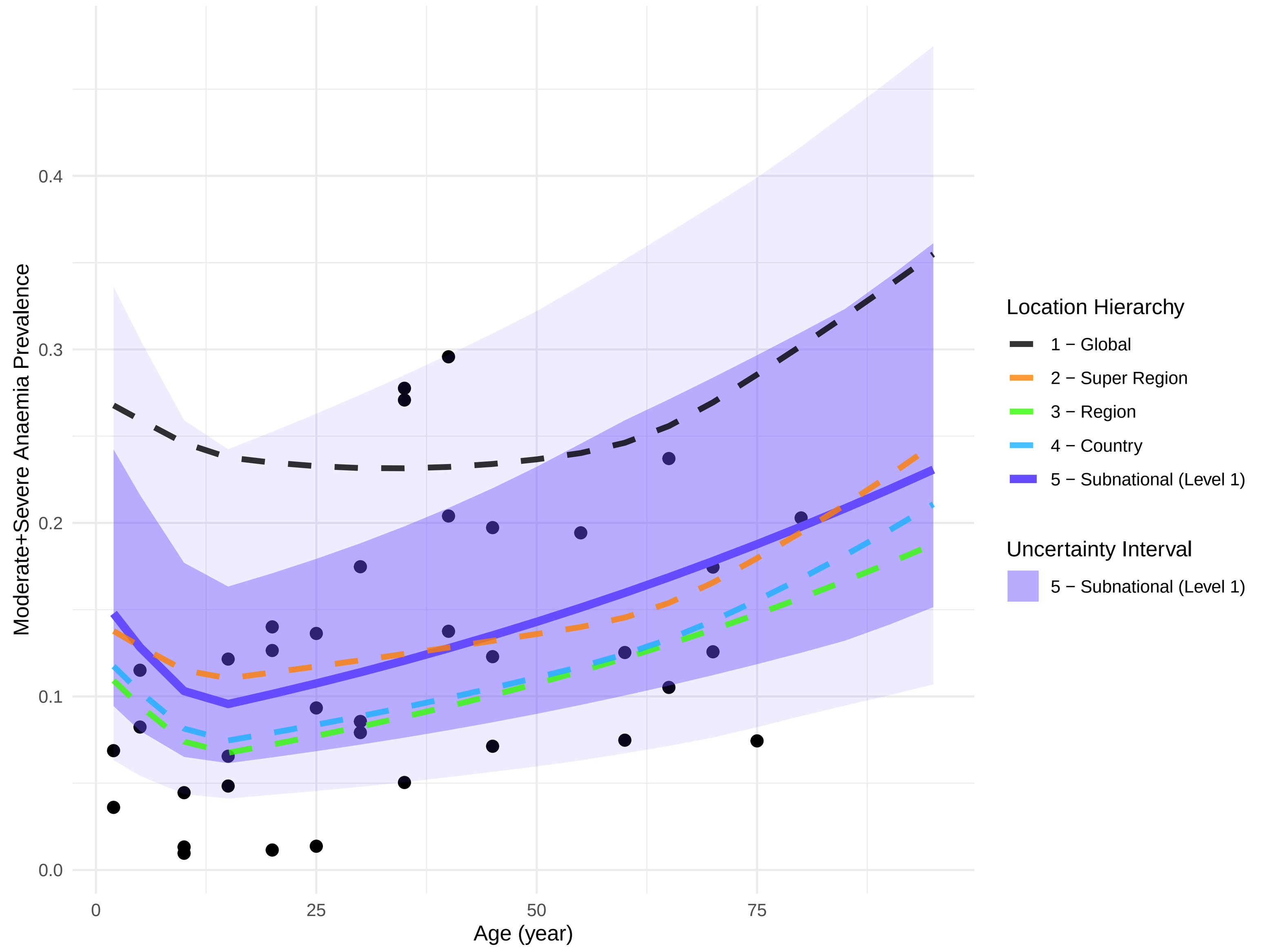

MR−BRT Age/Sex Fits − Non−pregnant Females 2+ Years Old − Moderate+Severe Anaemia Prevalence − Colima
WHO−2024 Adjustment Method

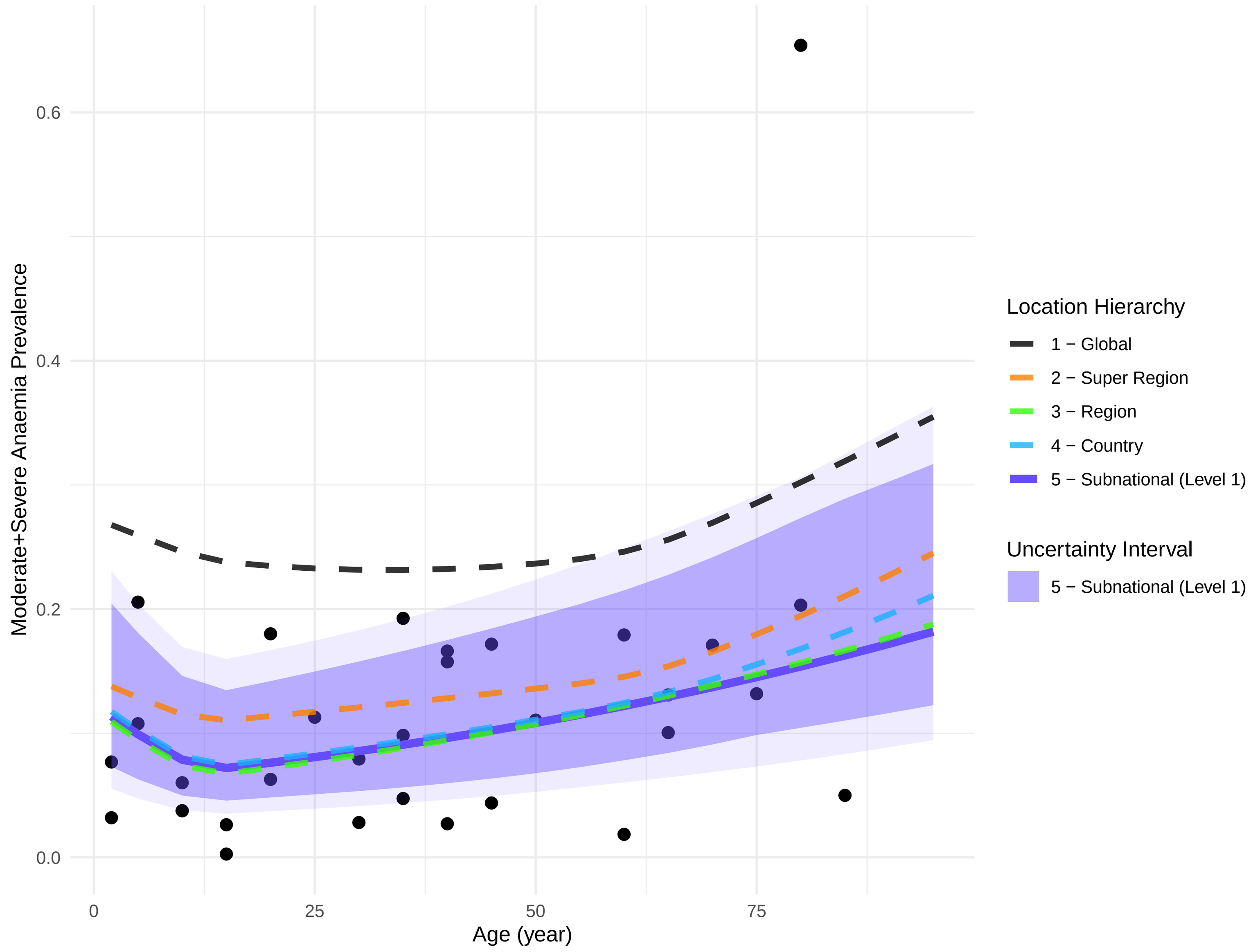

MR−BRT Age/Sex Fits − Non−pregnant Females 2+ Years Old − Moderate+Severe Anaemia Prevalence − Durango
WHO−2024 Adjustment Method

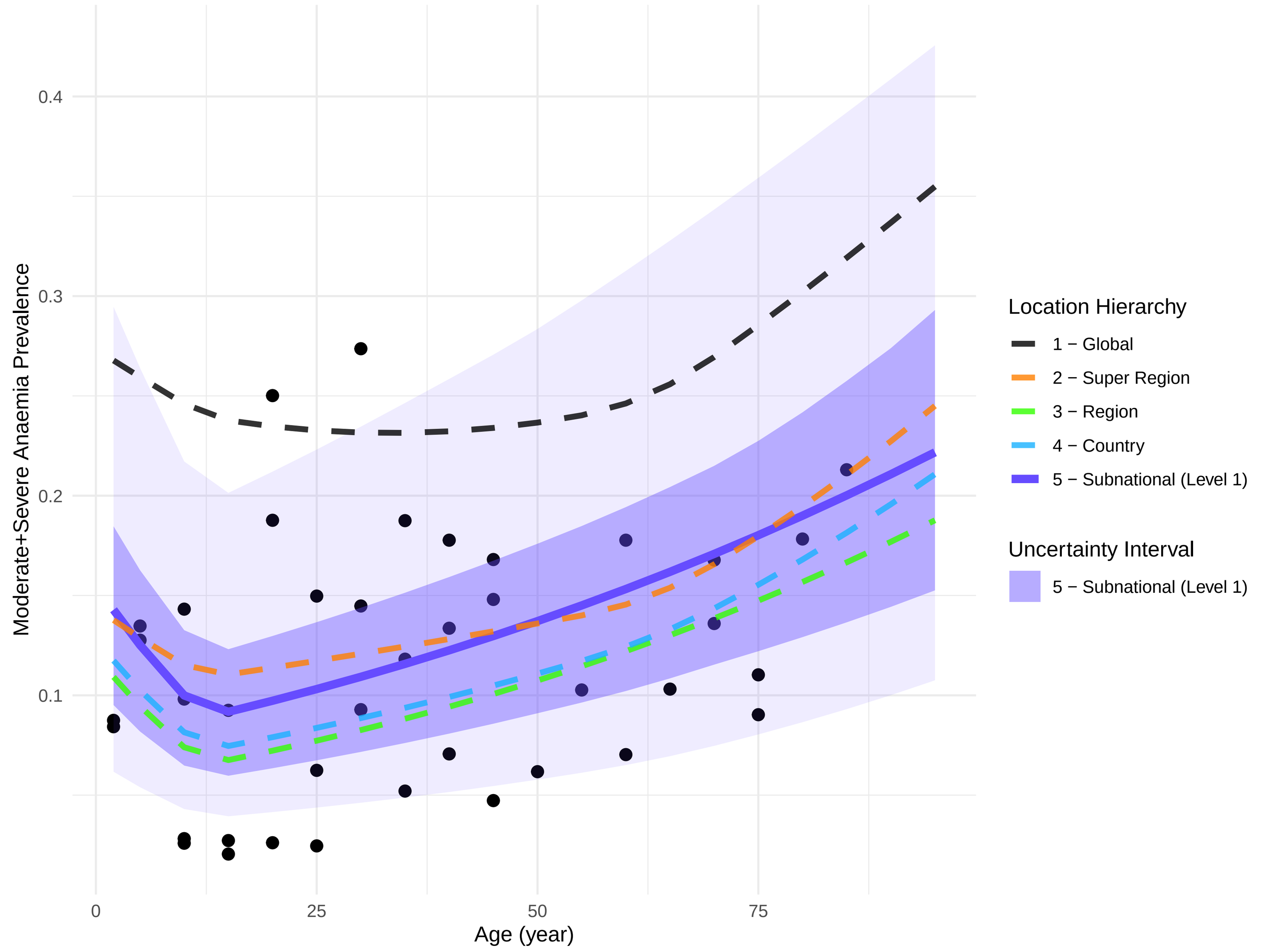

MR−BRT Age/Sex Fits − Non−pregnant Females 2+ Years Old − Moderate+Severe Anaemia Prevalence − Guanajuato
WHO−2024 Adjustment Method

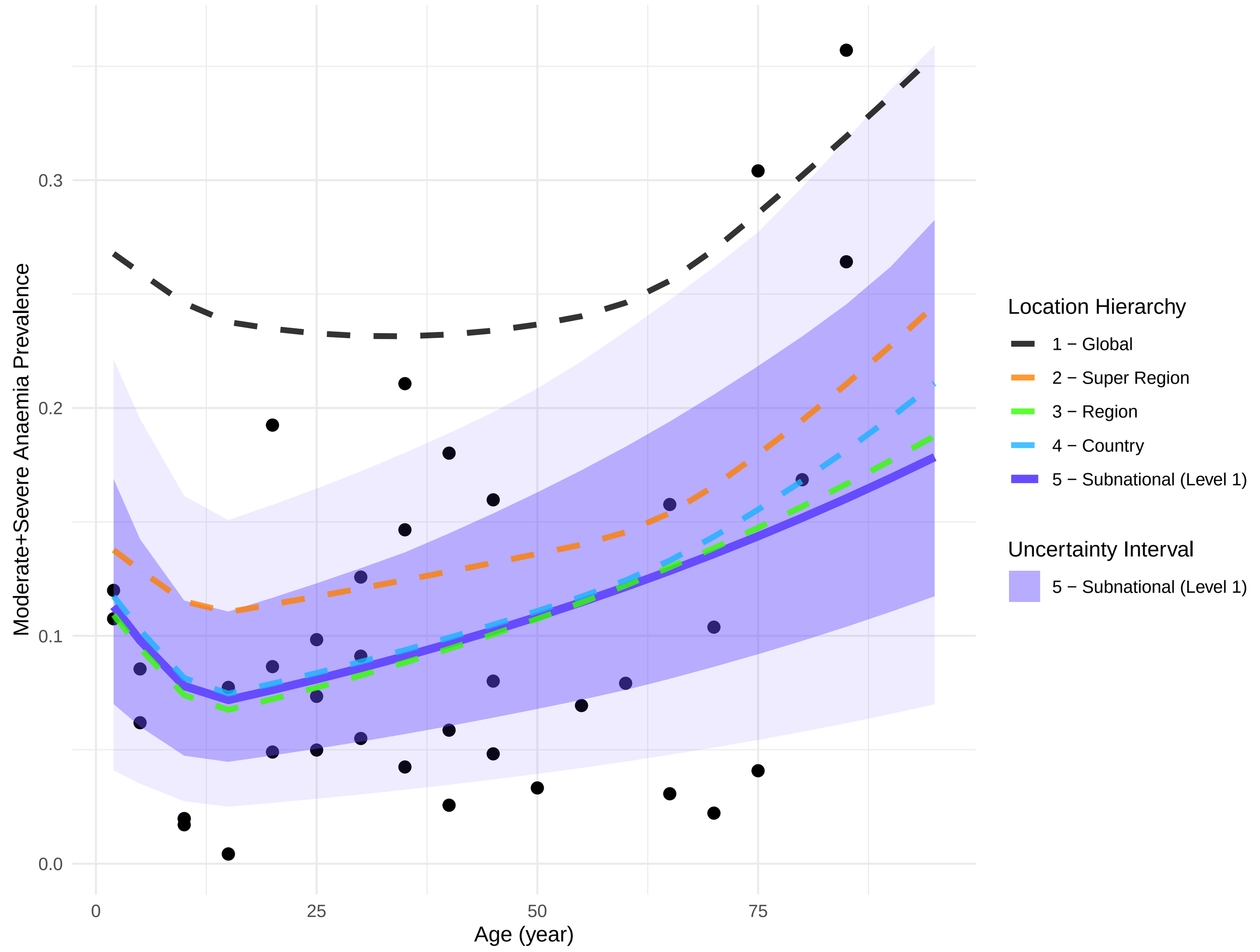

MR−BRT Age/Sex Fits − Non−pregnant Females 2+ Years Old − Moderate+Severe Anaemia Prevalence − Guerrero
WHO−2024 Adjustment Method

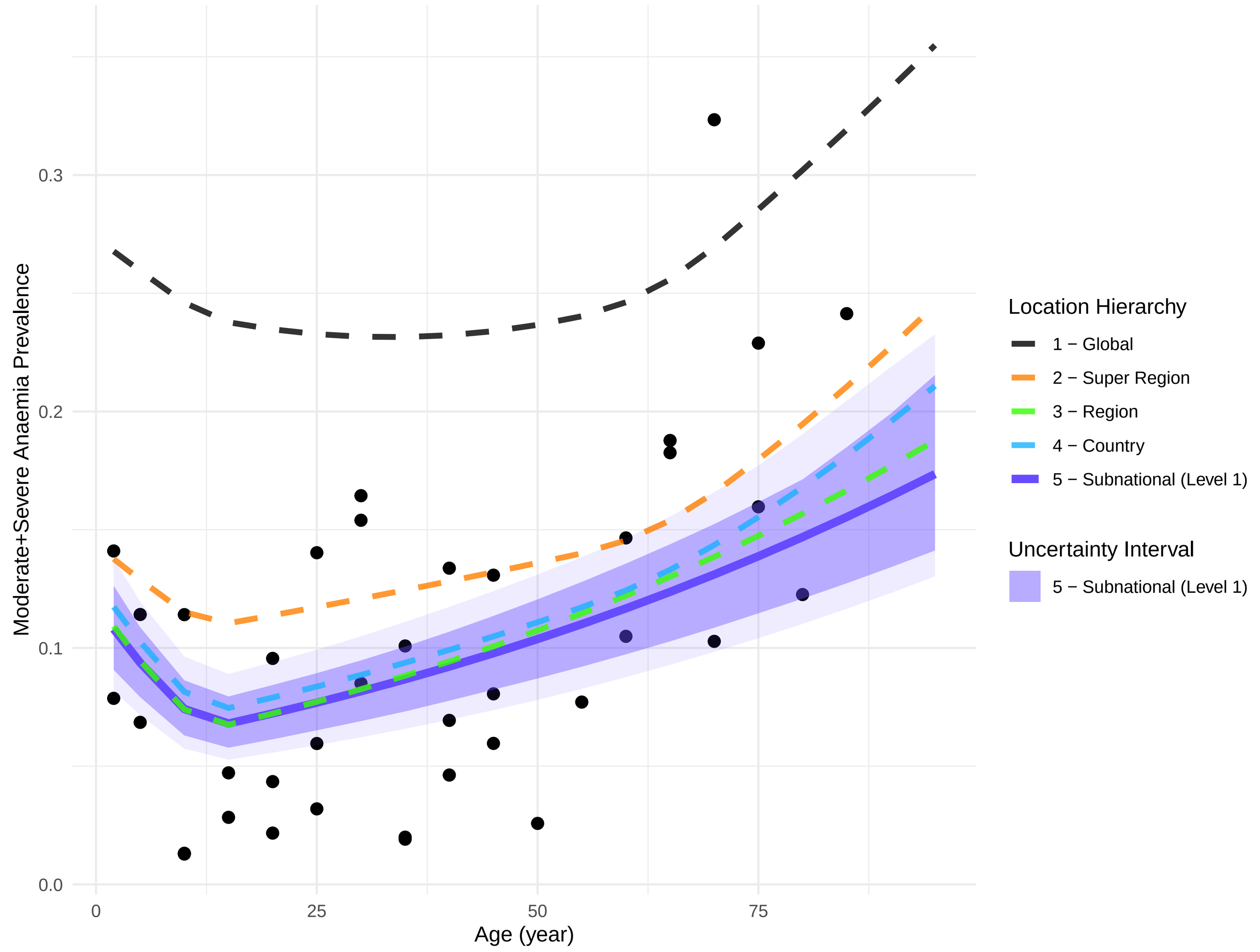

MR−BRT Age/Sex Fits − Non−pregnant Females 2+ Years Old − Moderate+Severe Anaemia Prevalence − Hidalgo
WHO−2024 Adjustment Method

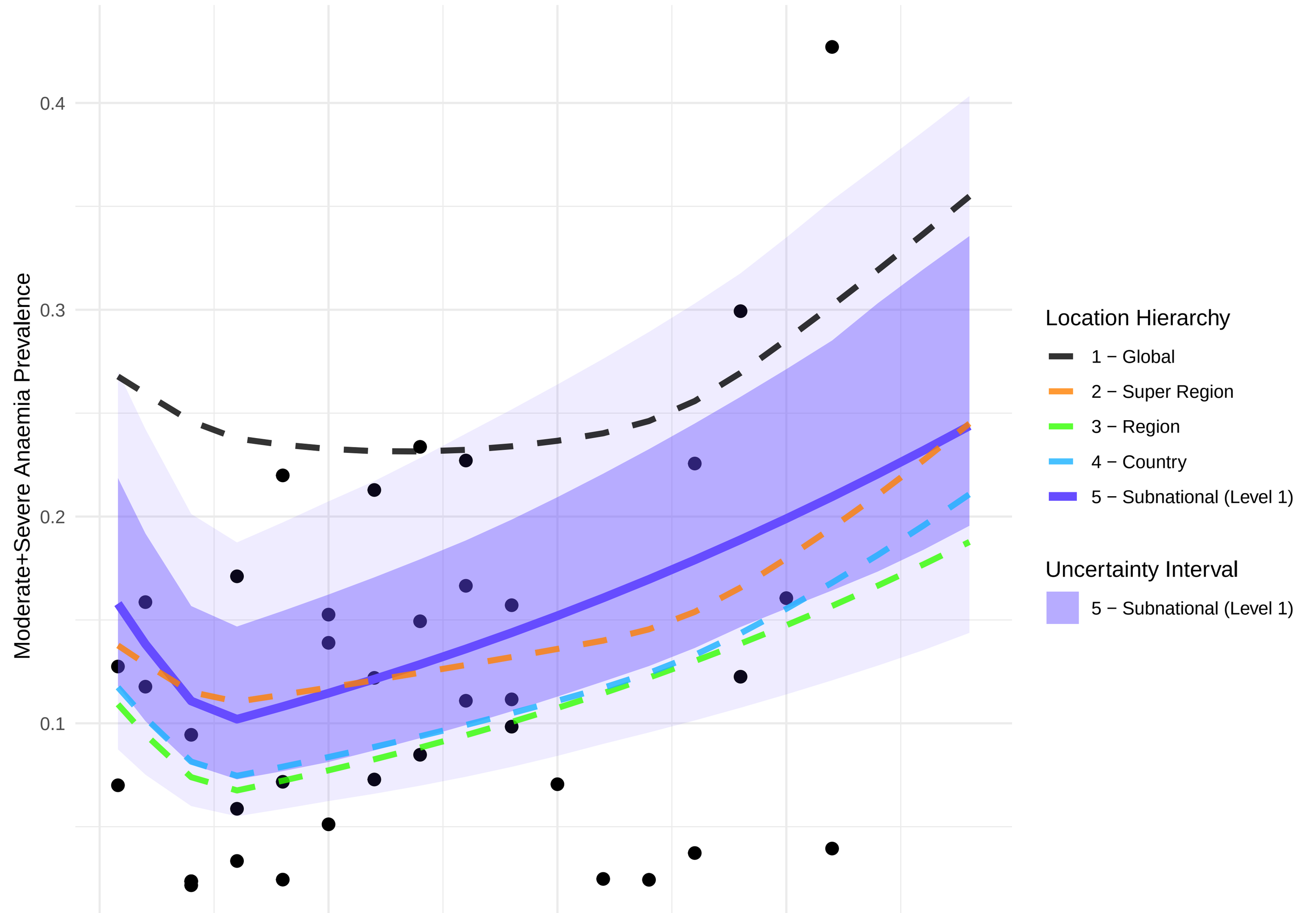

MR−BRT Age/Sex Fits − Non−pregnant Females 2+ Years Old − Moderate+Severe Anaemia Prevalence − Jalisco
WHO−2024 Adjustment Method

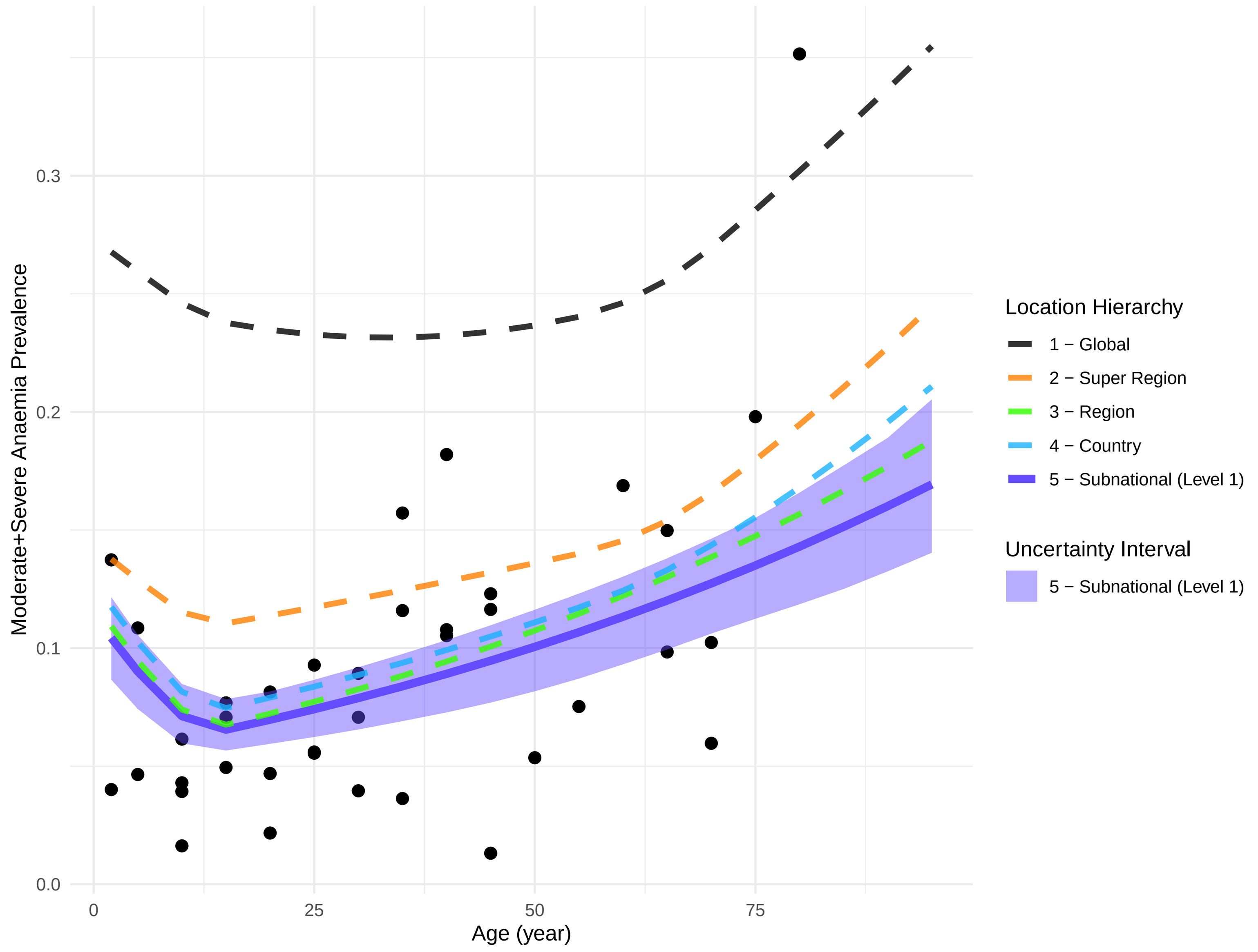

MR−BRT Age/Sex Fits − Non−pregnant Females 2+ Years Old − Moderate+Severe Anaemia Prevalence − México
WHO−2024 Adjustment Method

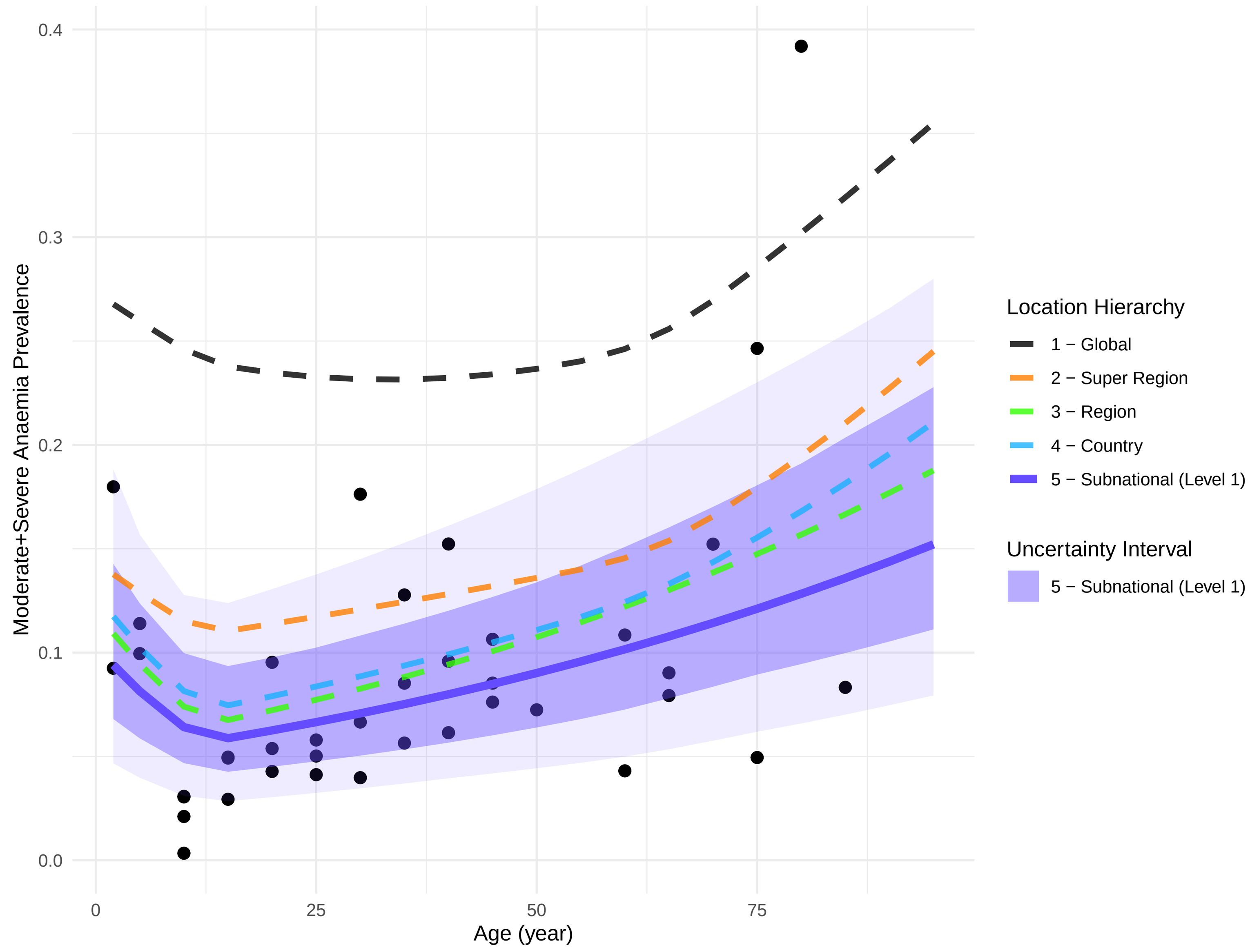

MR−BRT Age/Sex Fits − Non−pregnant Females 2+ Years Old − Moderate+Severe Anaemia Prevalence − Mexico City
WHO−2024 Adjustment Method

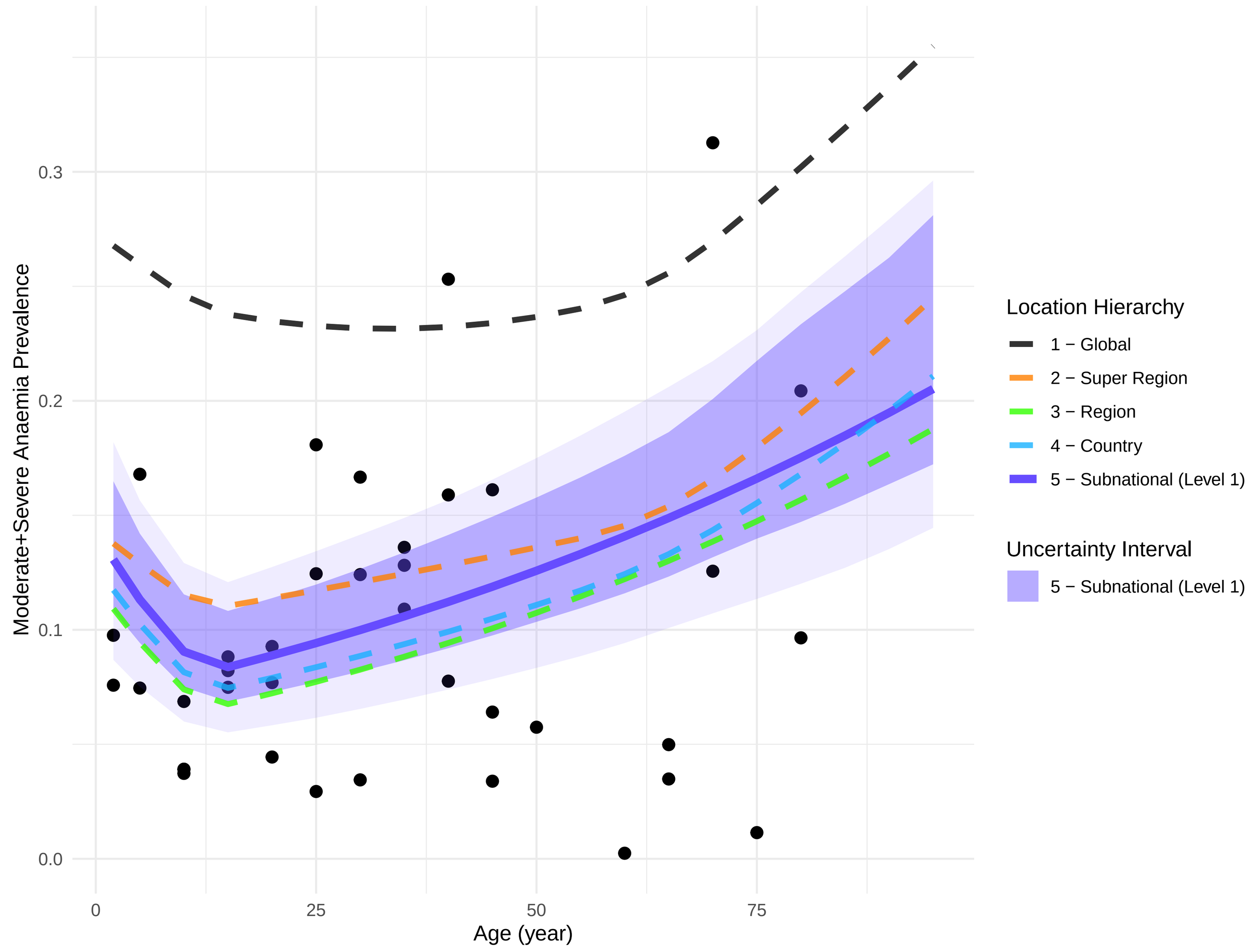

MR−BRT Age/Sex Fits − Non−pregnant Females 2+ Years Old − Moderate+Severe Anaemia Prevalence − Michoacán de Ocampo
WHO−2024 Adjustment Method

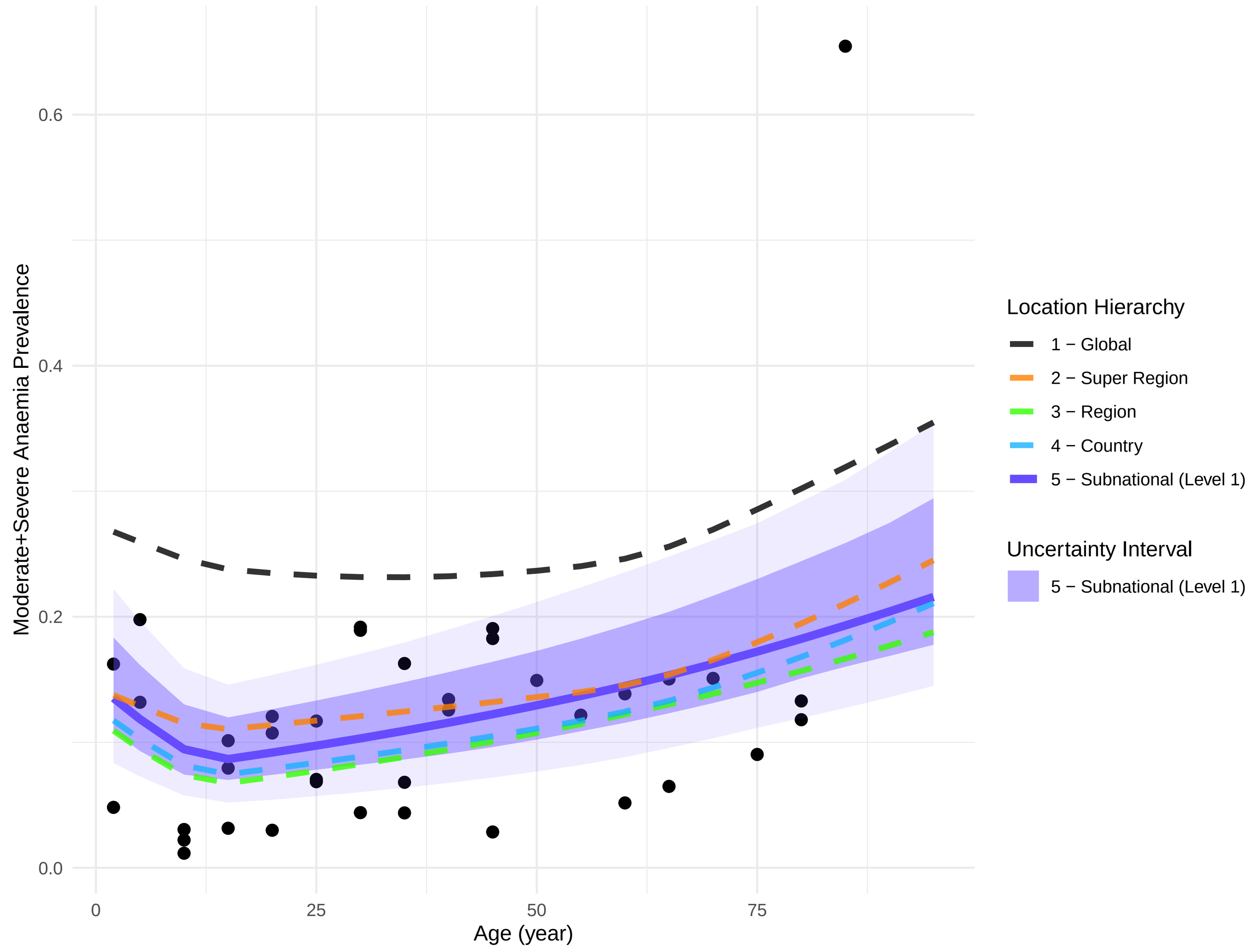

MR−BRT Age/Sex Fits − Non−pregnant Females 2+ Years Old − Moderate+Severe Anaemia Prevalence − Morelos
WHO−2024 Adjustment Method

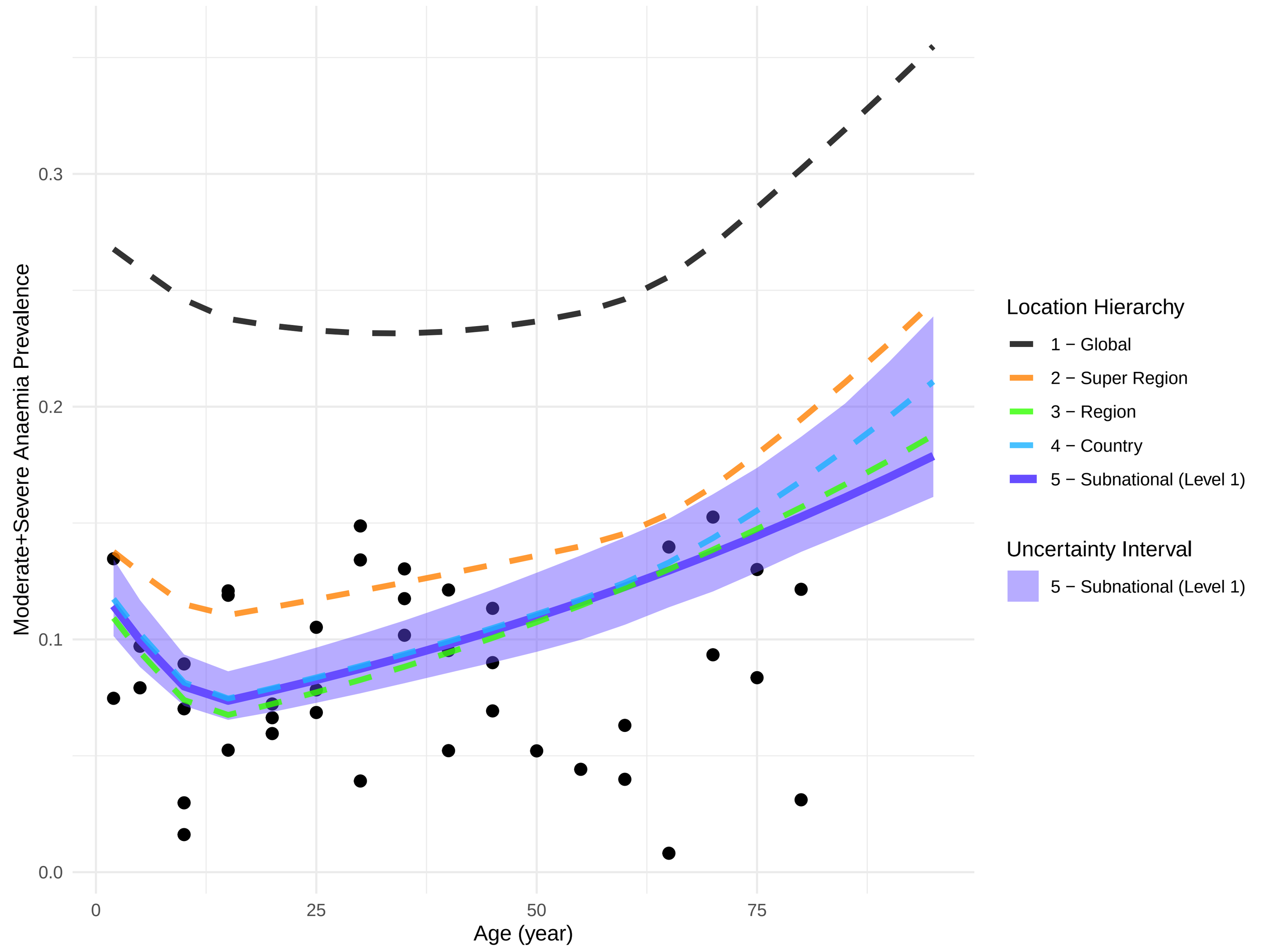

MR−BRT Age/Sex Fits − Non−pregnant Females 2+ Years Old − Moderate+Severe Anaemia Prevalence − Nayarit
WHO−2024 Adjustment Method

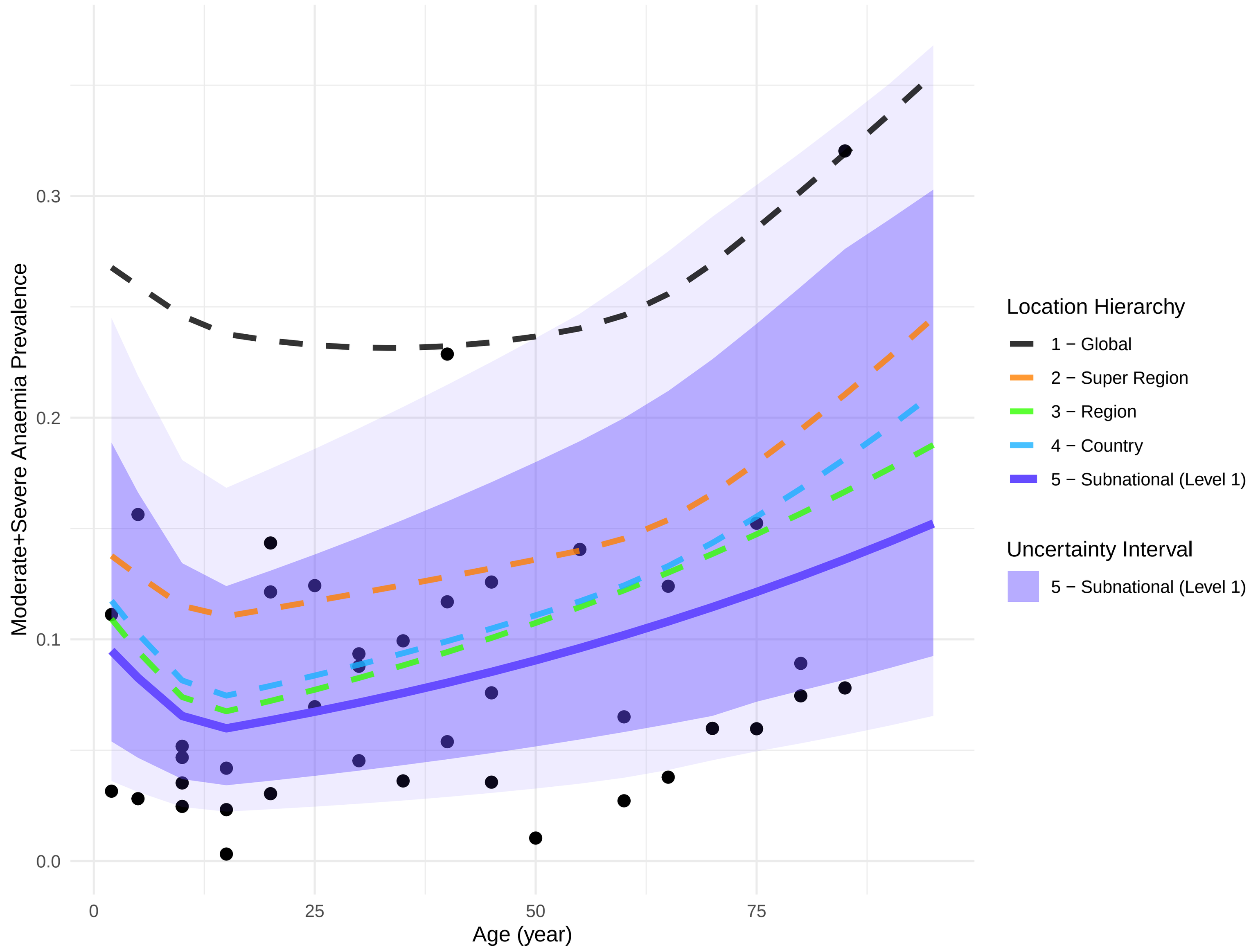

MR−BRT Age/Sex Fits − Non−pregnant Females 2+ Years Old − Moderate+Severe Anaemia Prevalence − Nuevo León
WHO−2024 Adjustment Method

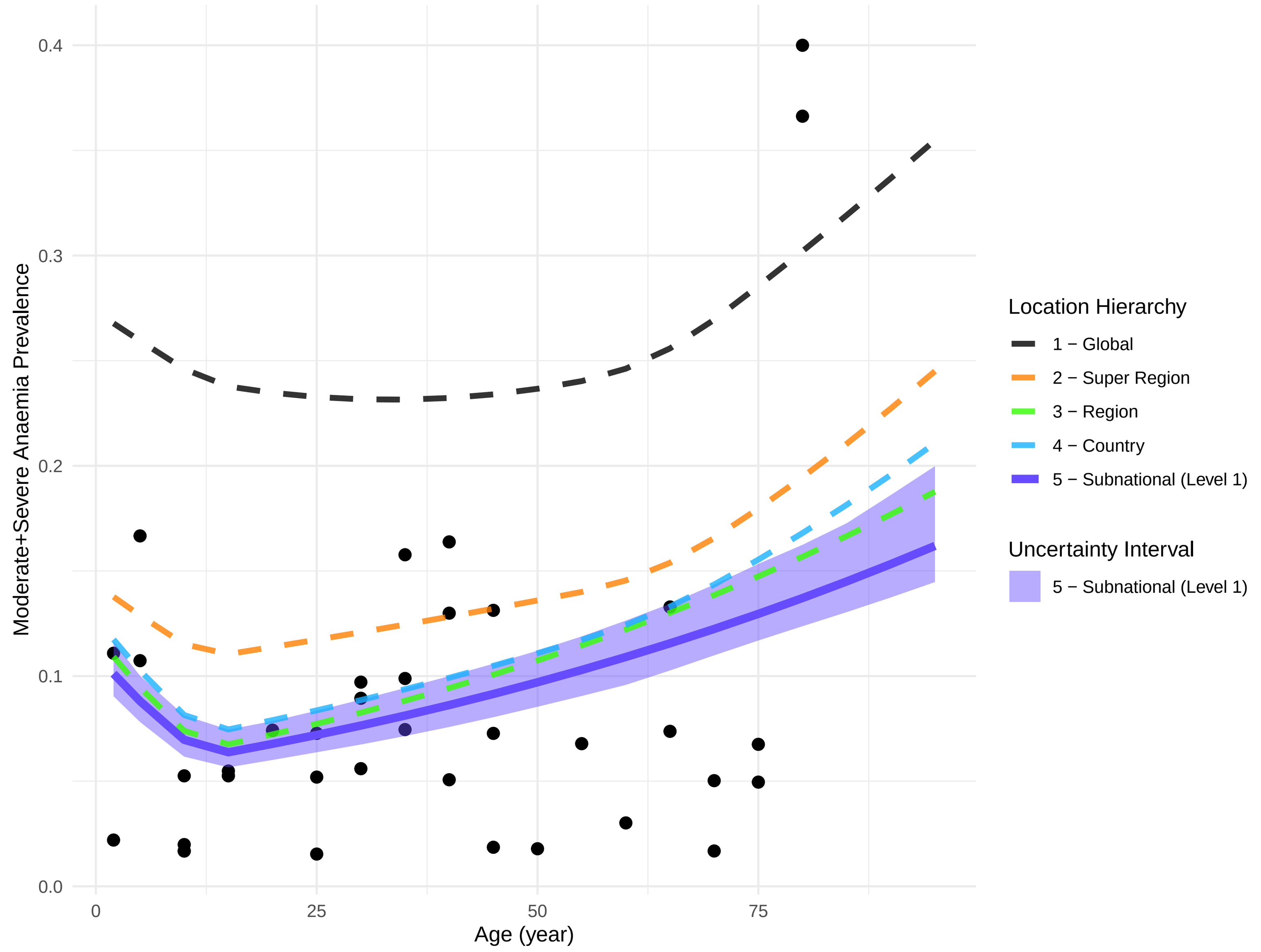

MR−BRT Age/Sex Fits − Non−pregnant Females 2+ Years Old − Moderate+Severe Anaemia Prevalence − Oaxaca
WHO−2024 Adjustment Method

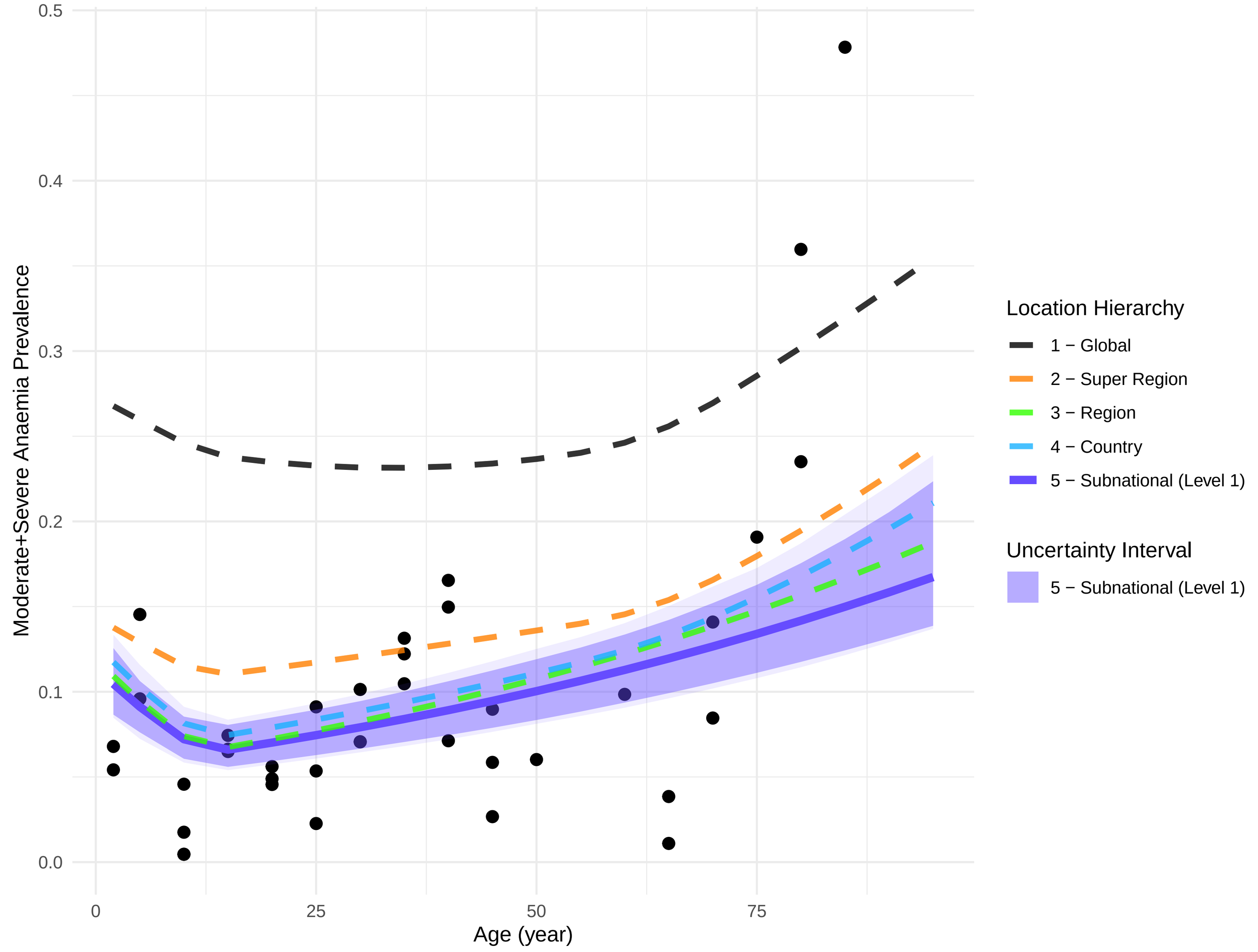

MR−BRT Age/Sex Fits − Non−pregnant Females 2+ Years Old − Moderate+Severe Anaemia Prevalence − Puebla
WHO−2024 Adjustment Method

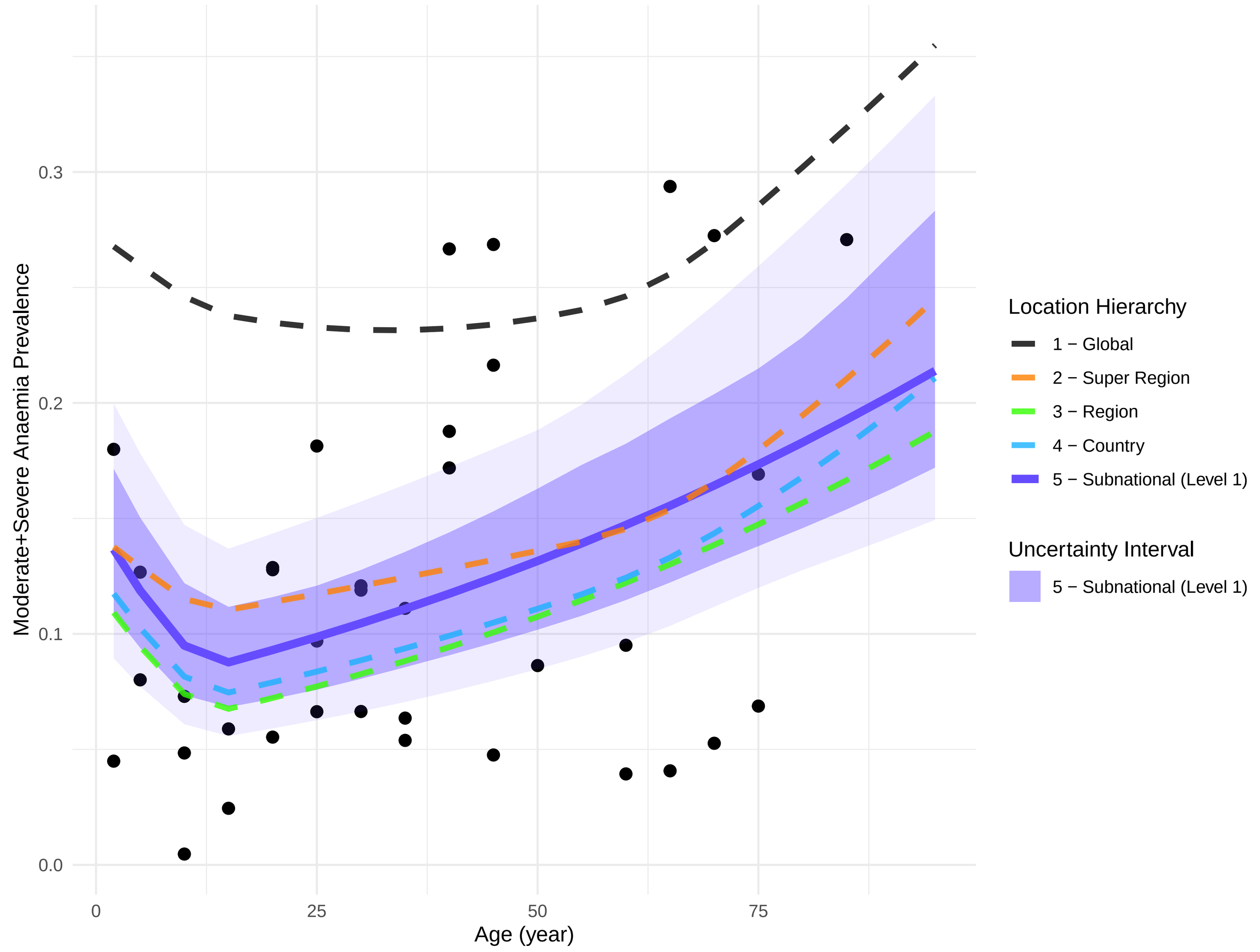

MR−BRT Age/Sex Fits − Non−pregnant Females 2+ Years Old − Moderate+Severe Anaemia Prevalence − Querétaro
WHO−2024 Adjustment Method

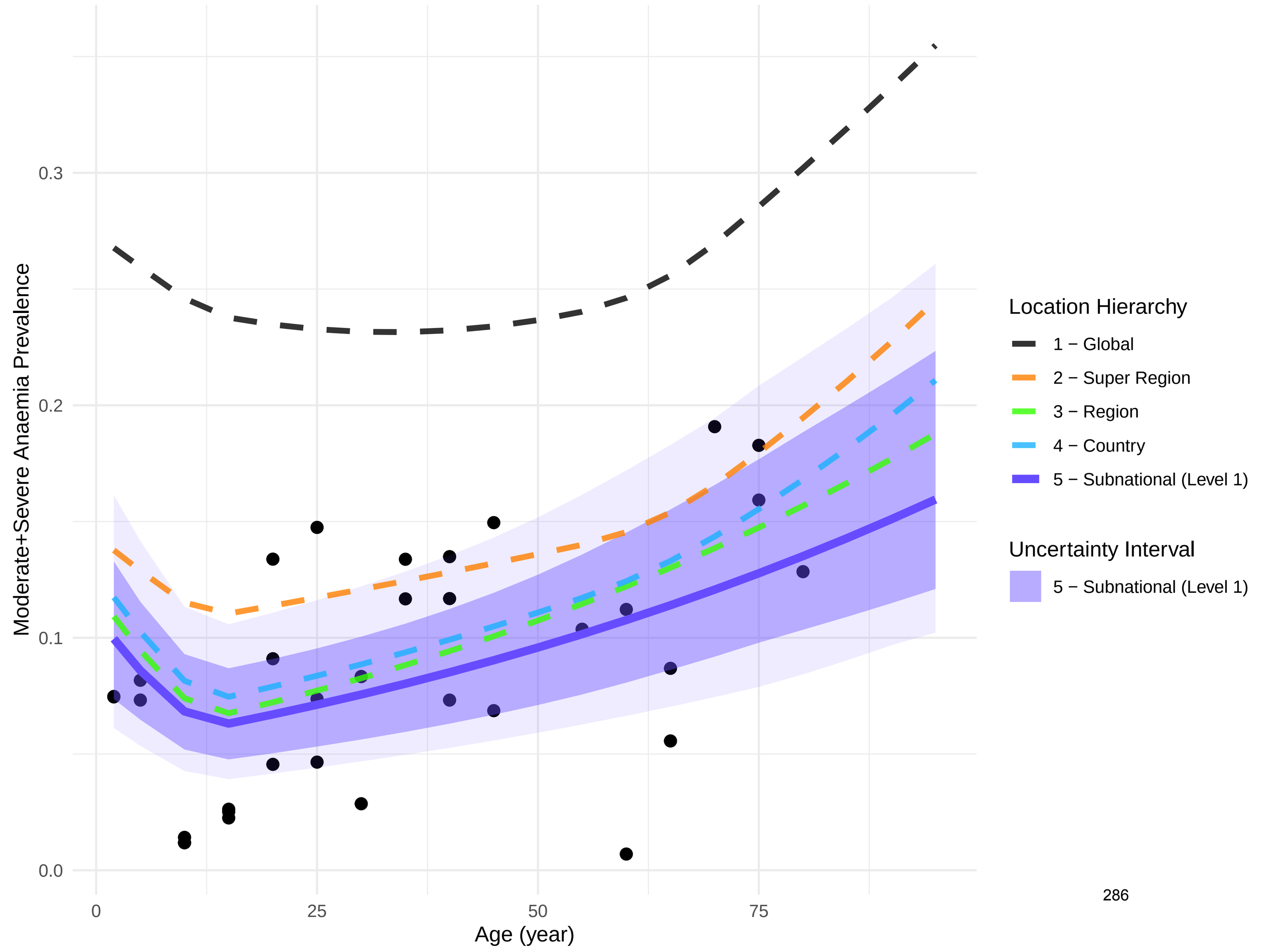

MR−BRT Age/Sex Fits − Non−pregnant Females 2+ Years Old − Moderate+Severe Anaemia Prevalence − Quintana Roo
WHO−2024 Adjustment Method

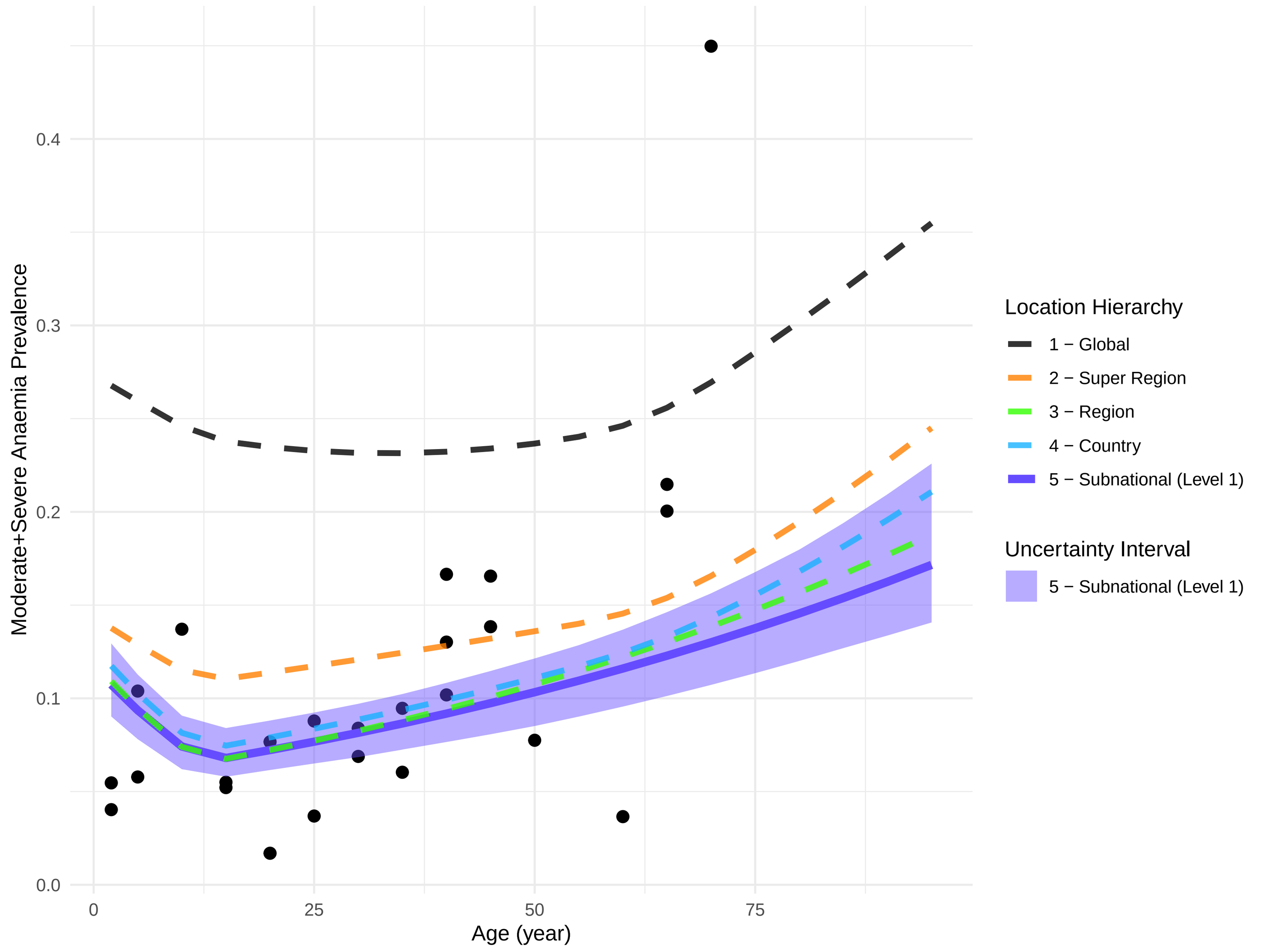

MR−BRT Age/Sex Fits − Non−pregnant Females 2+ Years Old − Moderate+Severe Anaemia Prevalence − San Luis Potosí
WHO−2024 Adjustment Method

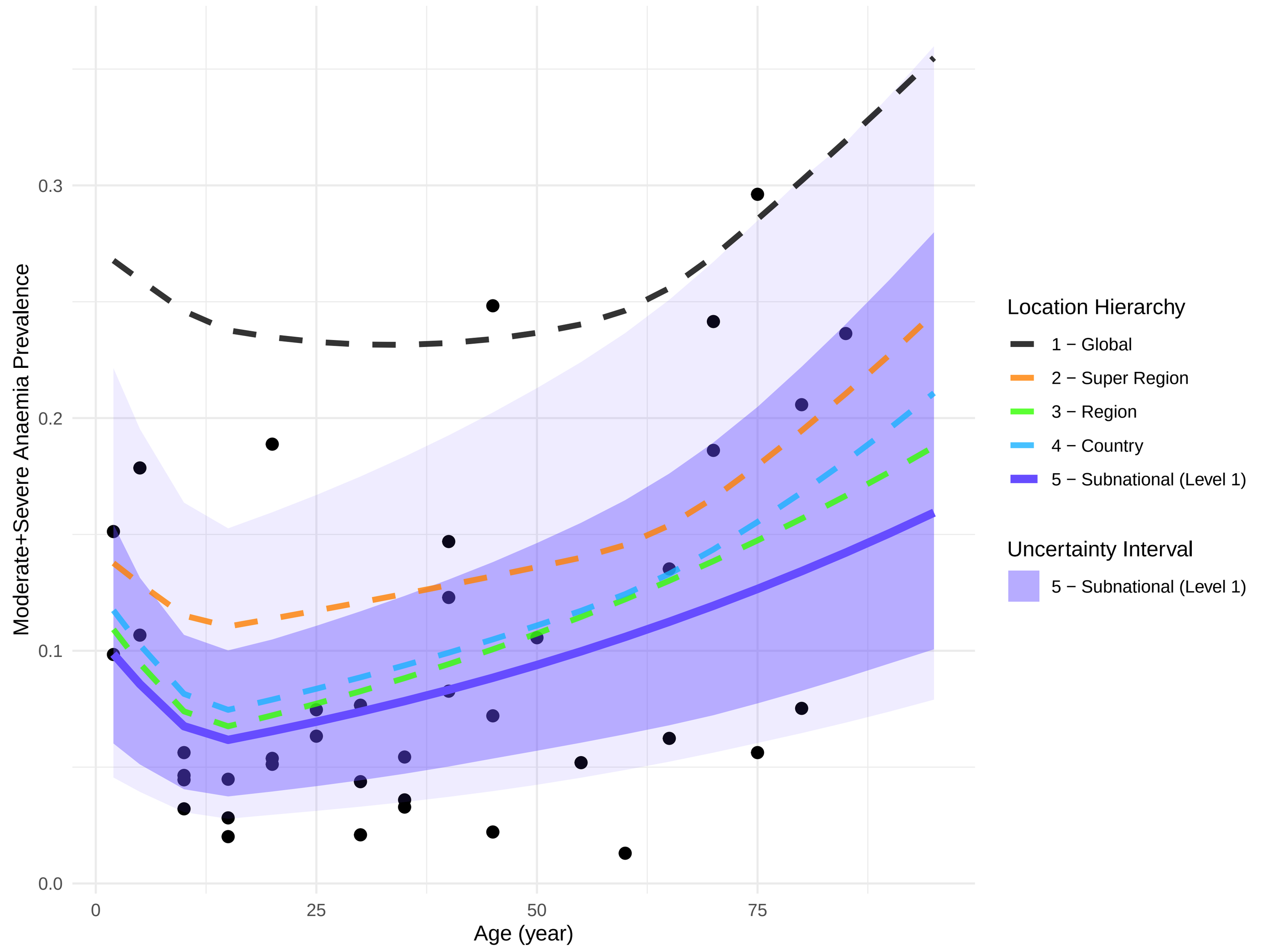

MR−BRT Age/Sex Fits − Non−pregnant Females 2+ Years Old − Moderate+Severe Anaemia Prevalence − Sinaloa
WHO−2024 Adjustment Method

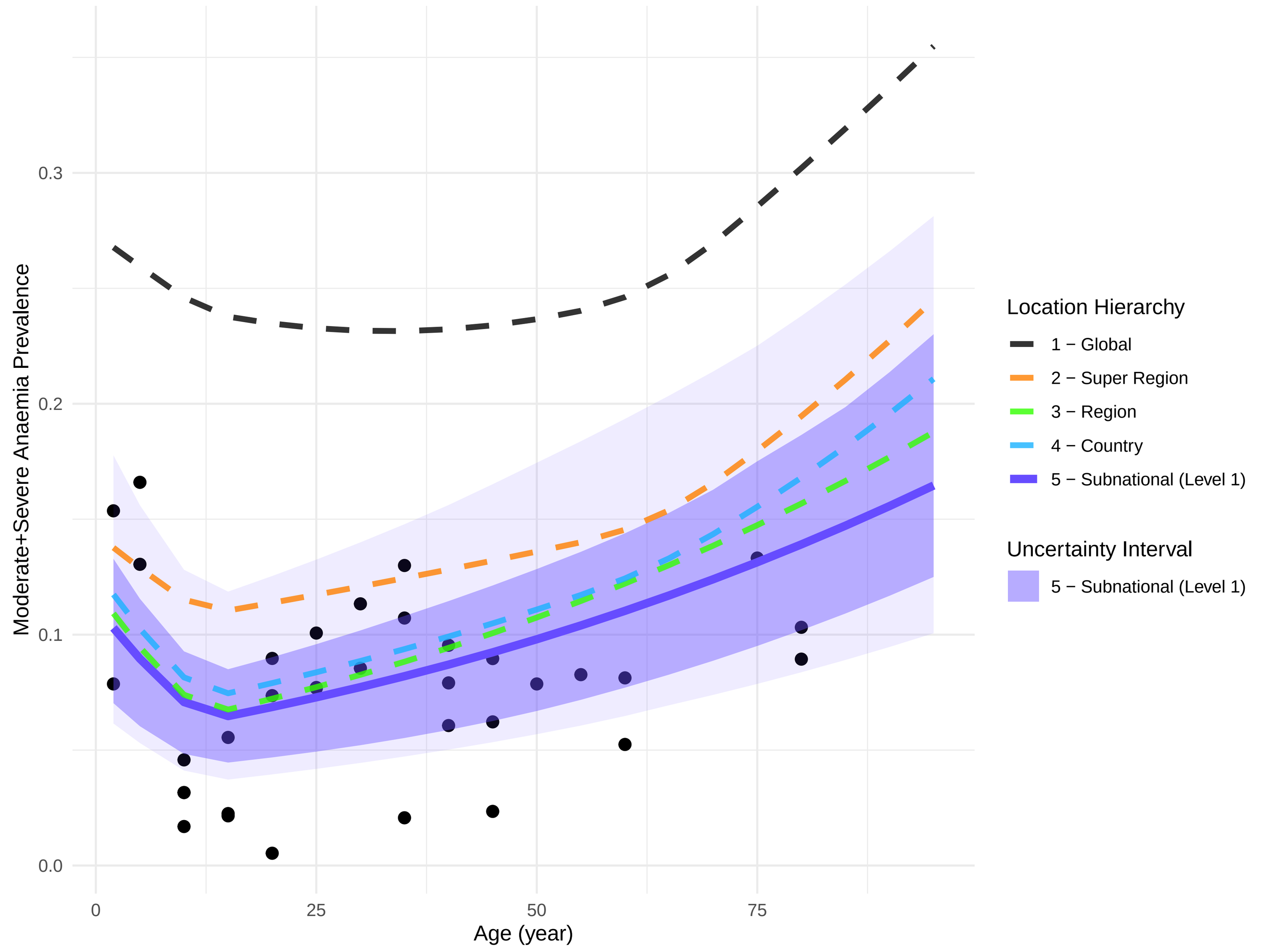

MR−BRT Age/Sex Fits − Non−pregnant Females 2+ Years Old − Moderate+Severe Anaemia Prevalence − Sonora
WHO−2024 Adjustment Method

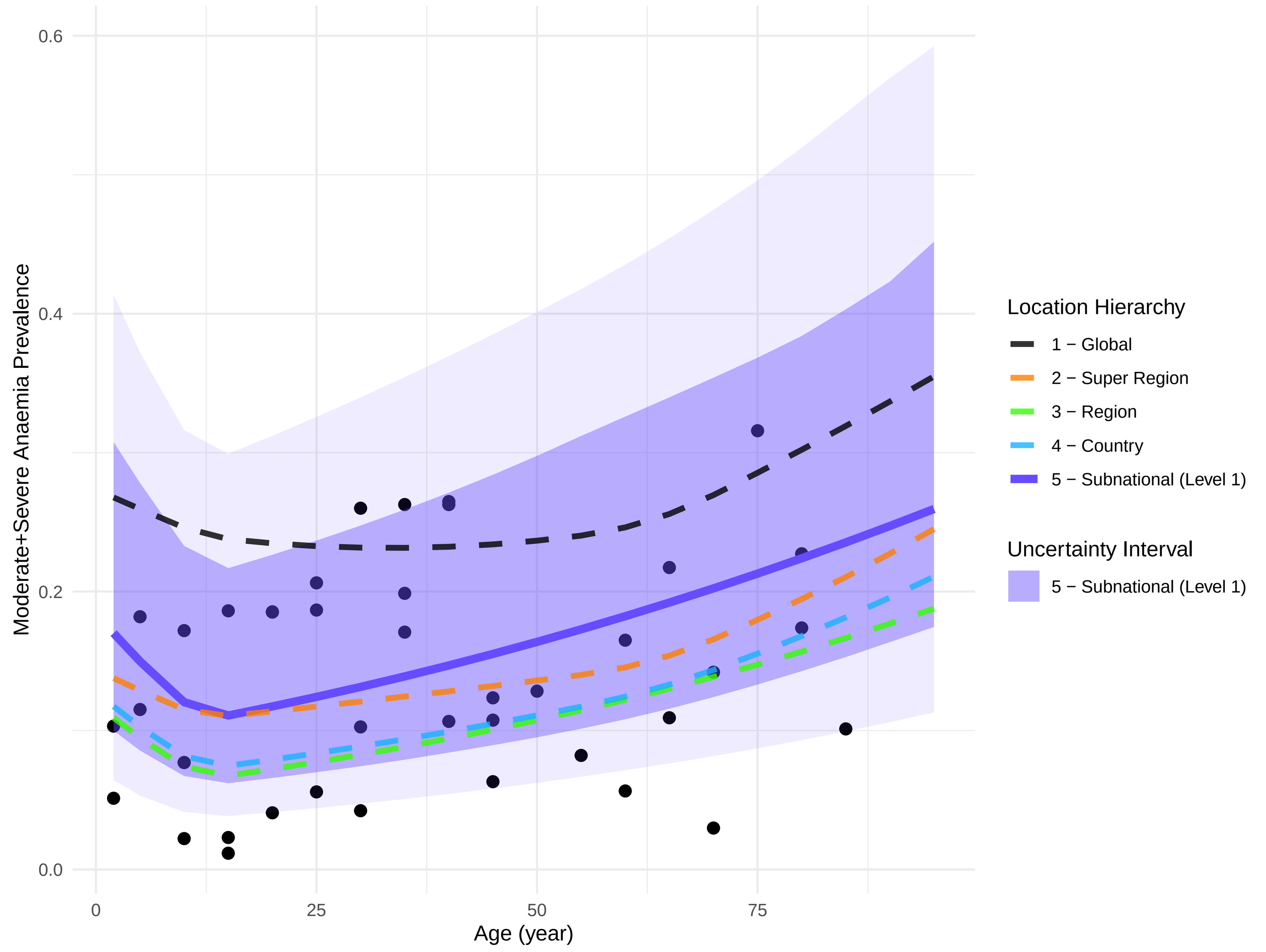

MR−BRT Age/Sex Fits − Non−pregnant Females 2+ Years Old − Moderate+Severe Anaemia Prevalence − Tabasco
WHO−2024 Adjustment Method

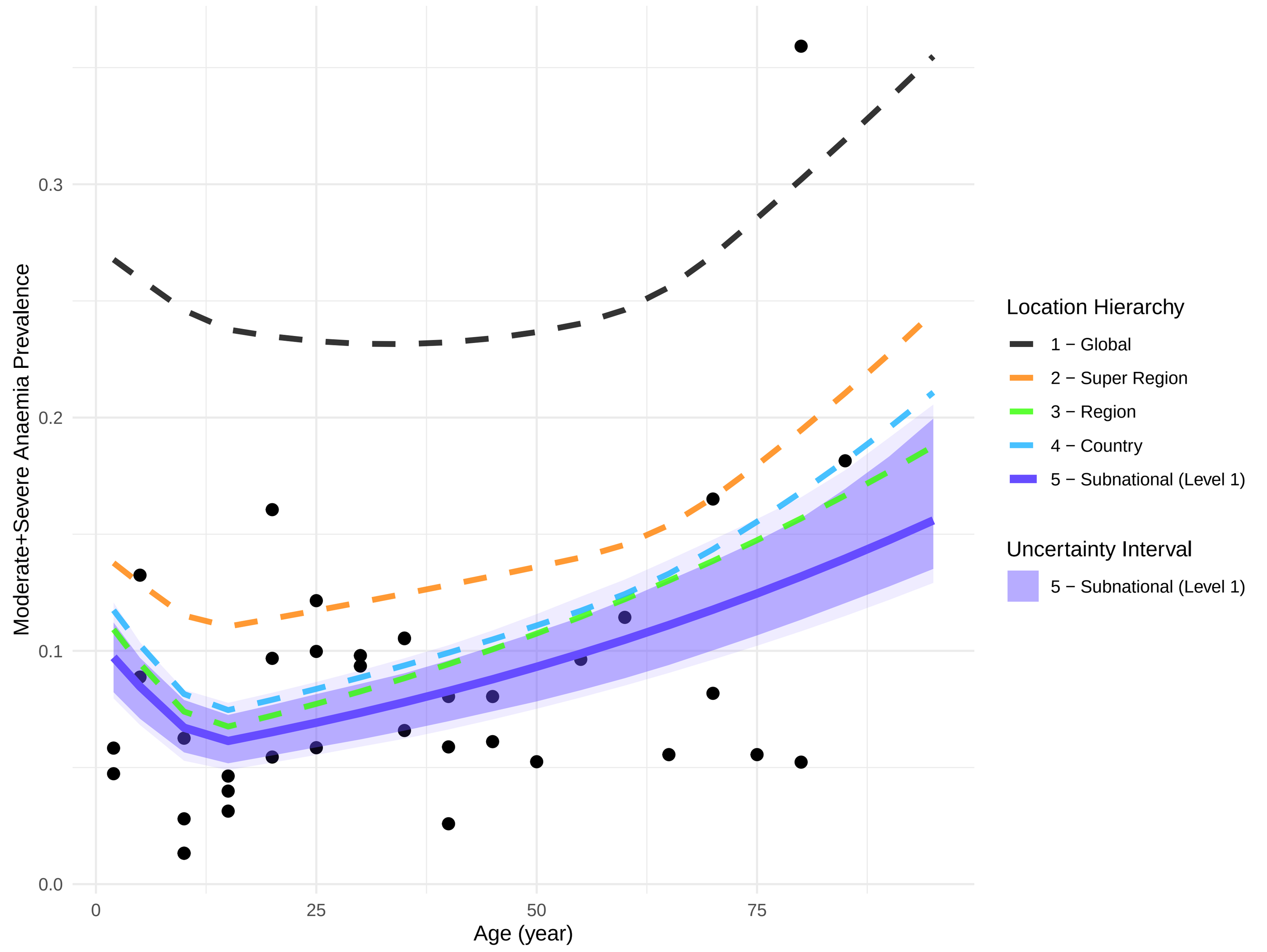

MR−BRT Age/Sex Fits − Non−pregnant Females 2+ Years Old − Moderate+Severe Anaemia Prevalence − Tamaulipas
WHO−2024 Adjustment Method

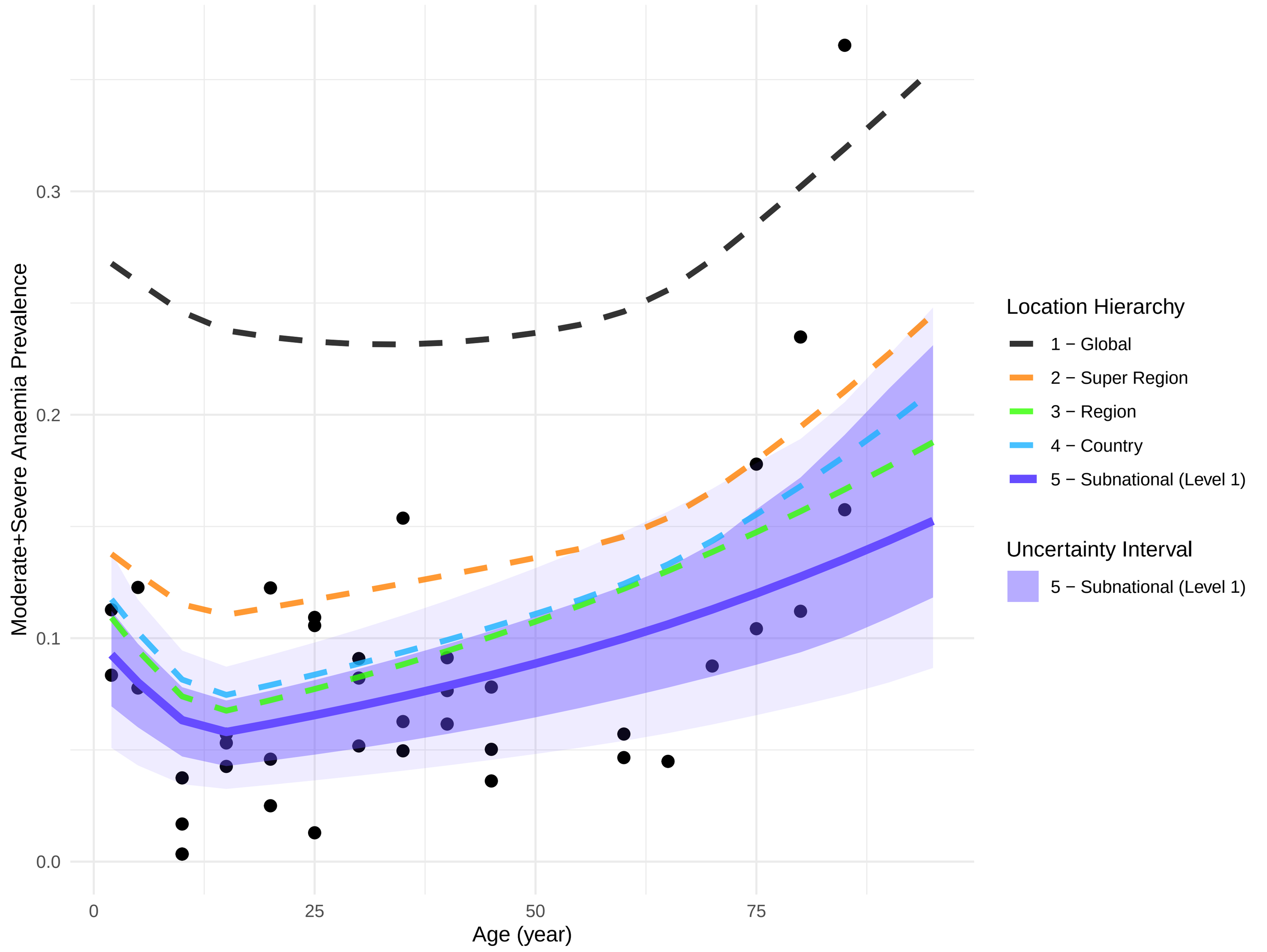

MR−BRT Age/Sex Fits − Non−pregnant Females 2+ Years Old − Moderate+Severe Anaemia Prevalence − Tlaxcala
WHO−2024 Adjustment Method

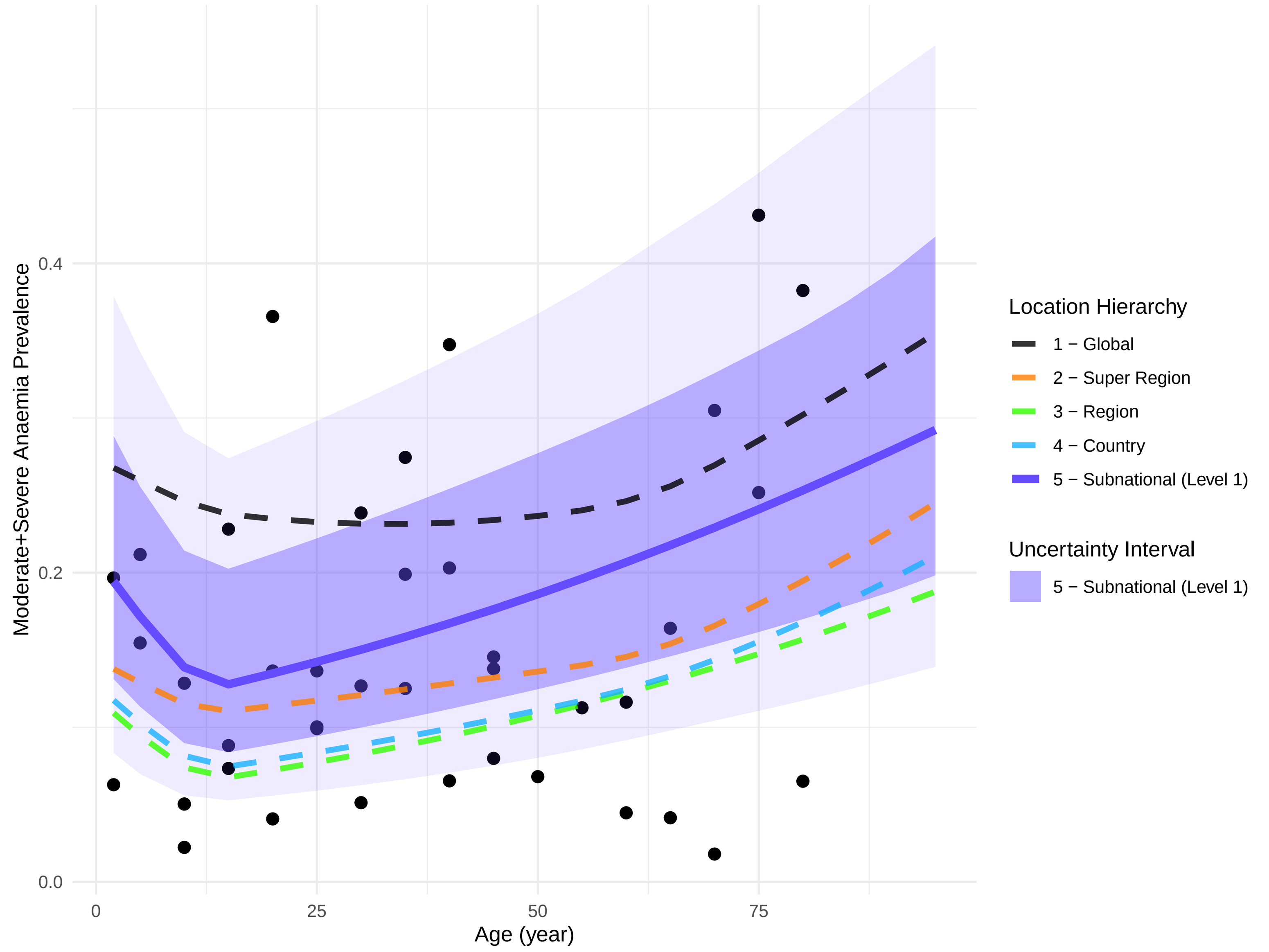

MR−BRT Age/Sex Fits − Non−pregnant Females 2+ Years Old − Moderate+Severe Anaemia Prevalence − Veracruz de Ignacio de la Llave
WHO−2024 Adjustment Method

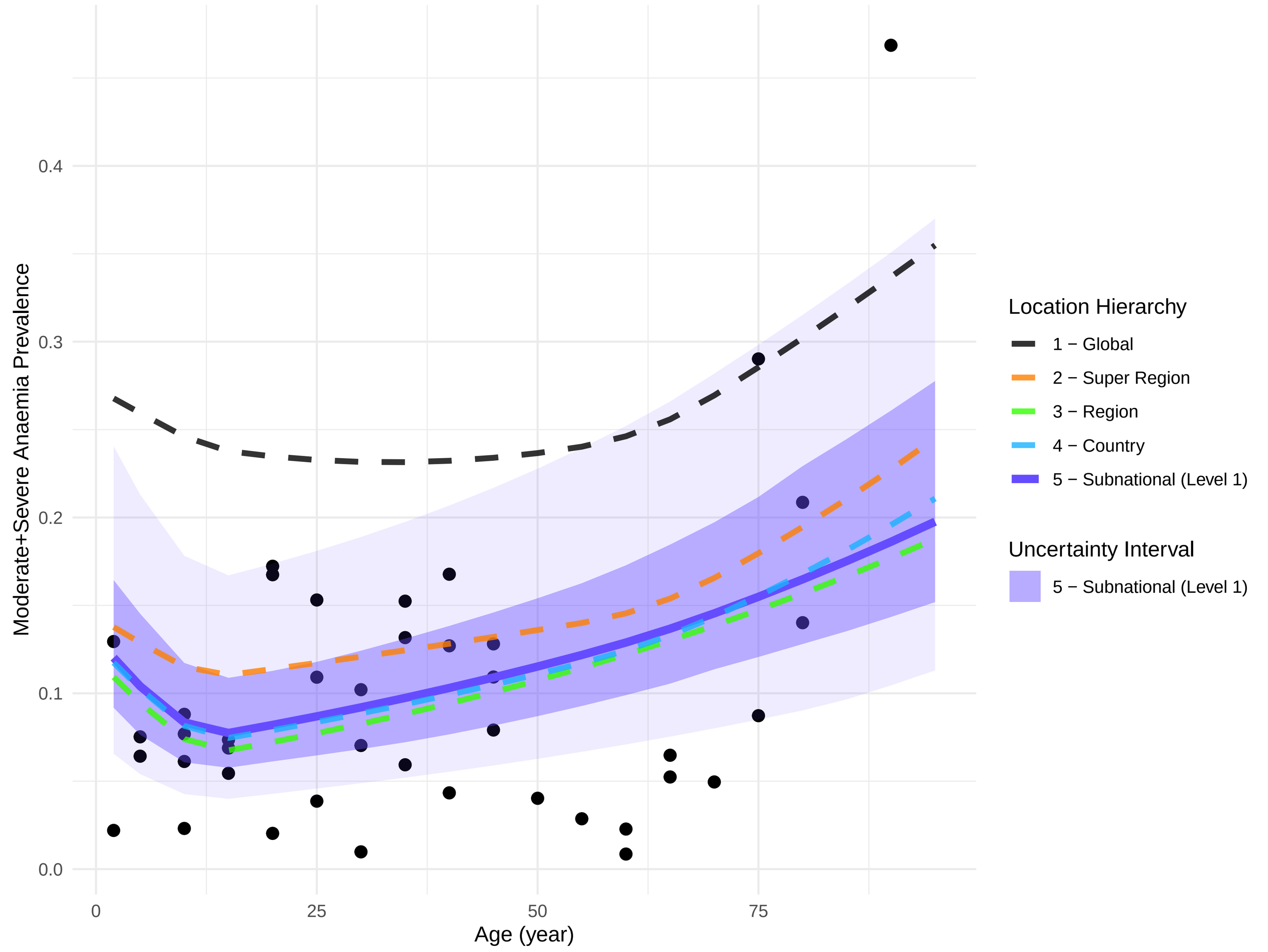

MR−BRT Age/Sex Fits − Non−pregnant Females 2+ Years Old − Moderate+Severe Anaemia Prevalence − Yucatán
WHO−2024 Adjustment Method

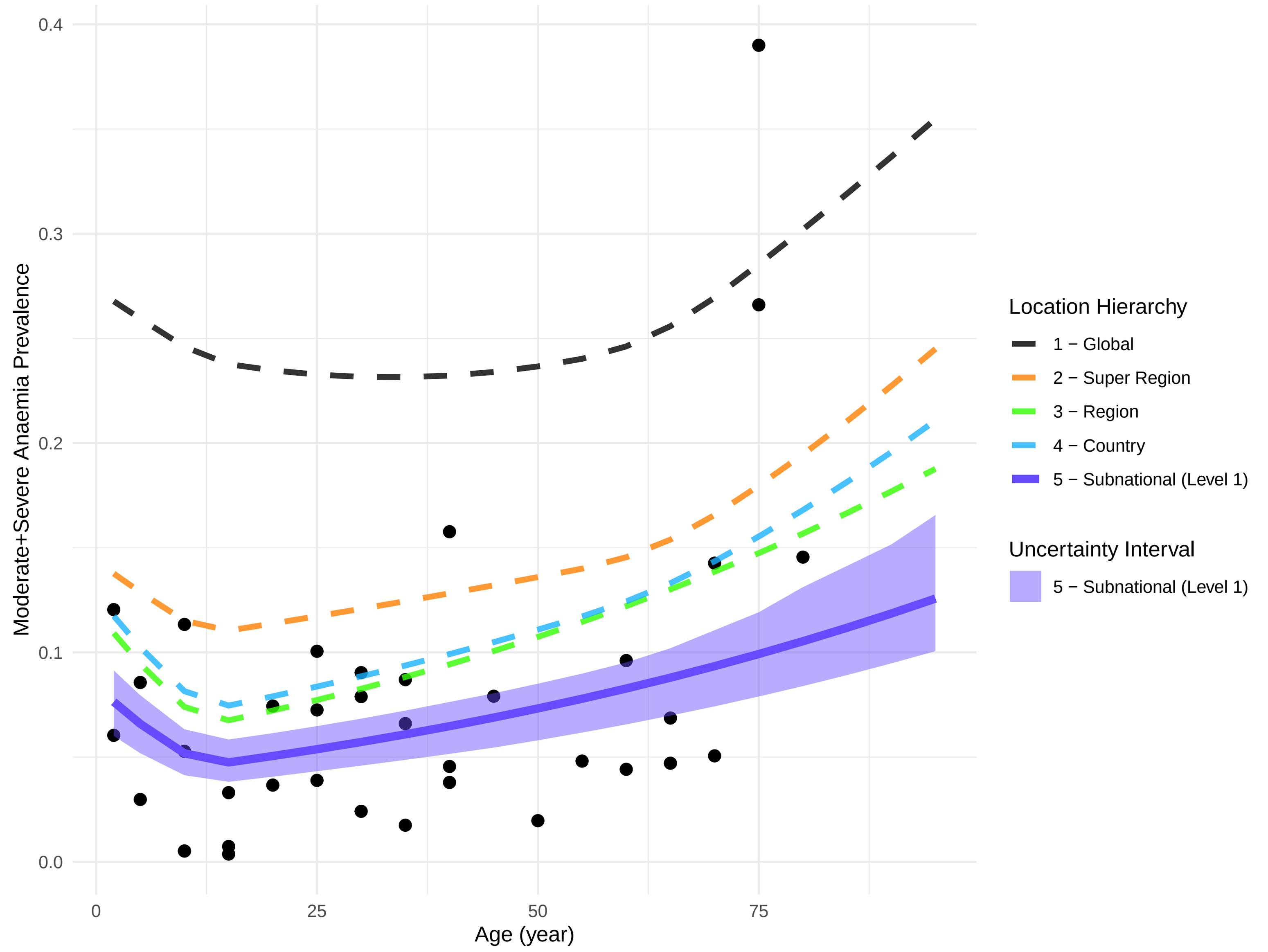

MR−BRT Age/Sex Fits − Non−pregnant Females 2+ Years Old − Moderate+Severe Anaemia Prevalence − Zacatecas
WHO−2024 Adjustment Method

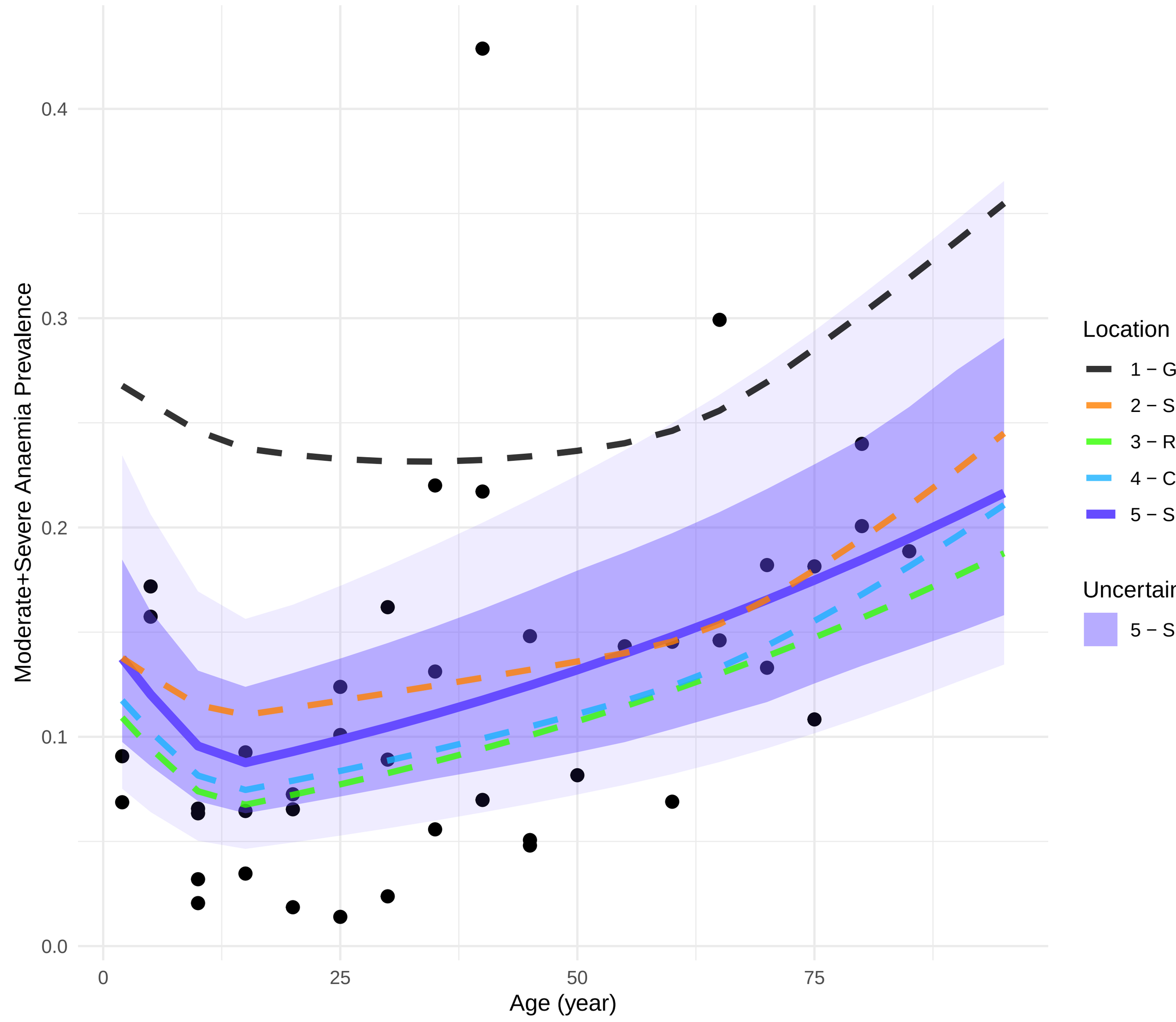

MR−BRT Age/Sex Fits − Non−pregnant Females 2+ Years Old − Moderate+Severe Anaemia Prevalence − Tropical Latin America
WHO−2024 Adjustment Method

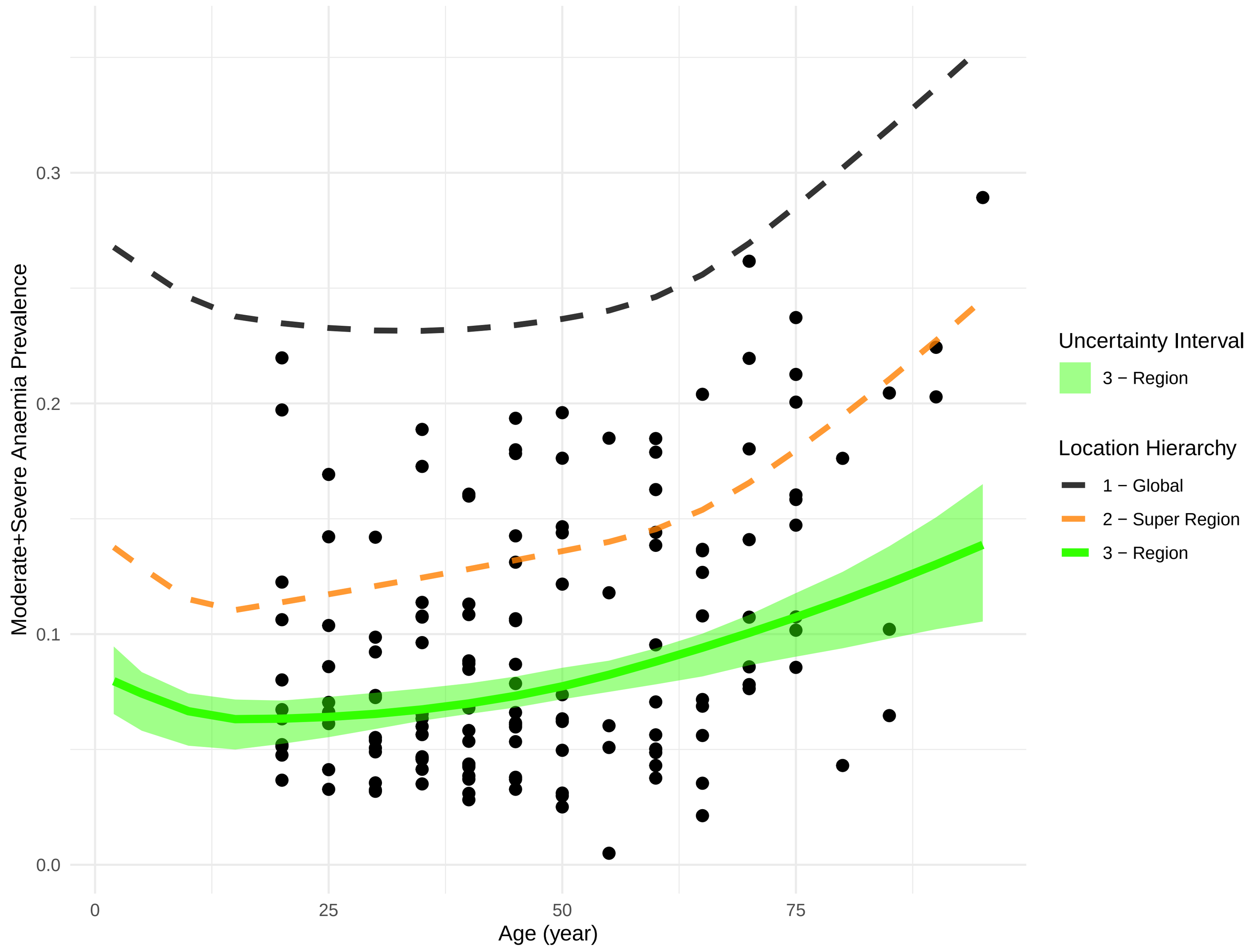

MR−BRT Age/Sex Fits − Non−pregnant Females 2+ Years Old − Moderate+Severe Anaemia Prevalence − Brazil
WHO−2024 Adjustment Method

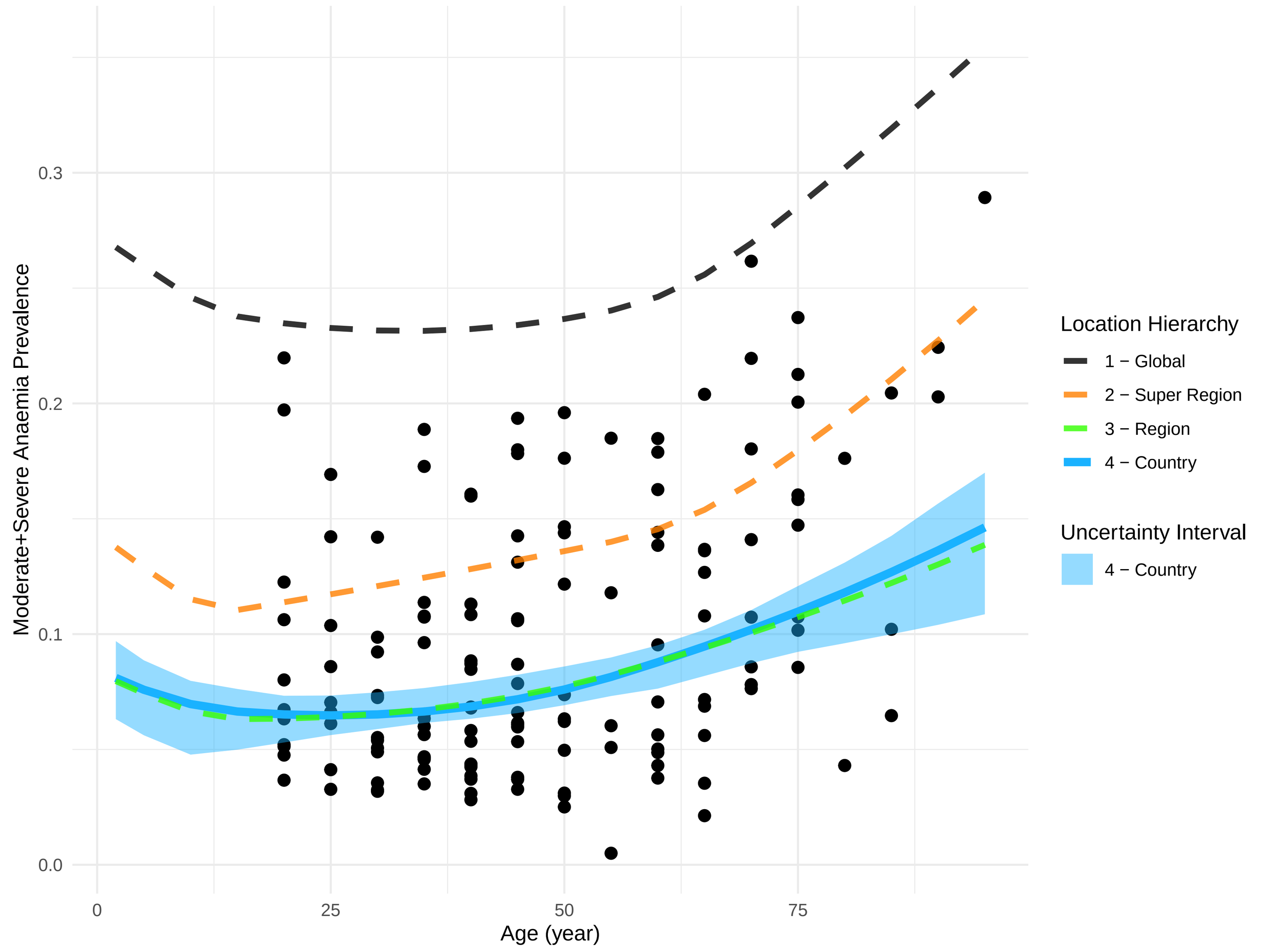

MR−BRT Age/Sex Fits − Non−pregnant Females 2+ Years Old − Moderate+Severe Anaemia Prevalence − Acre
WHO−2024 Adjustment Method

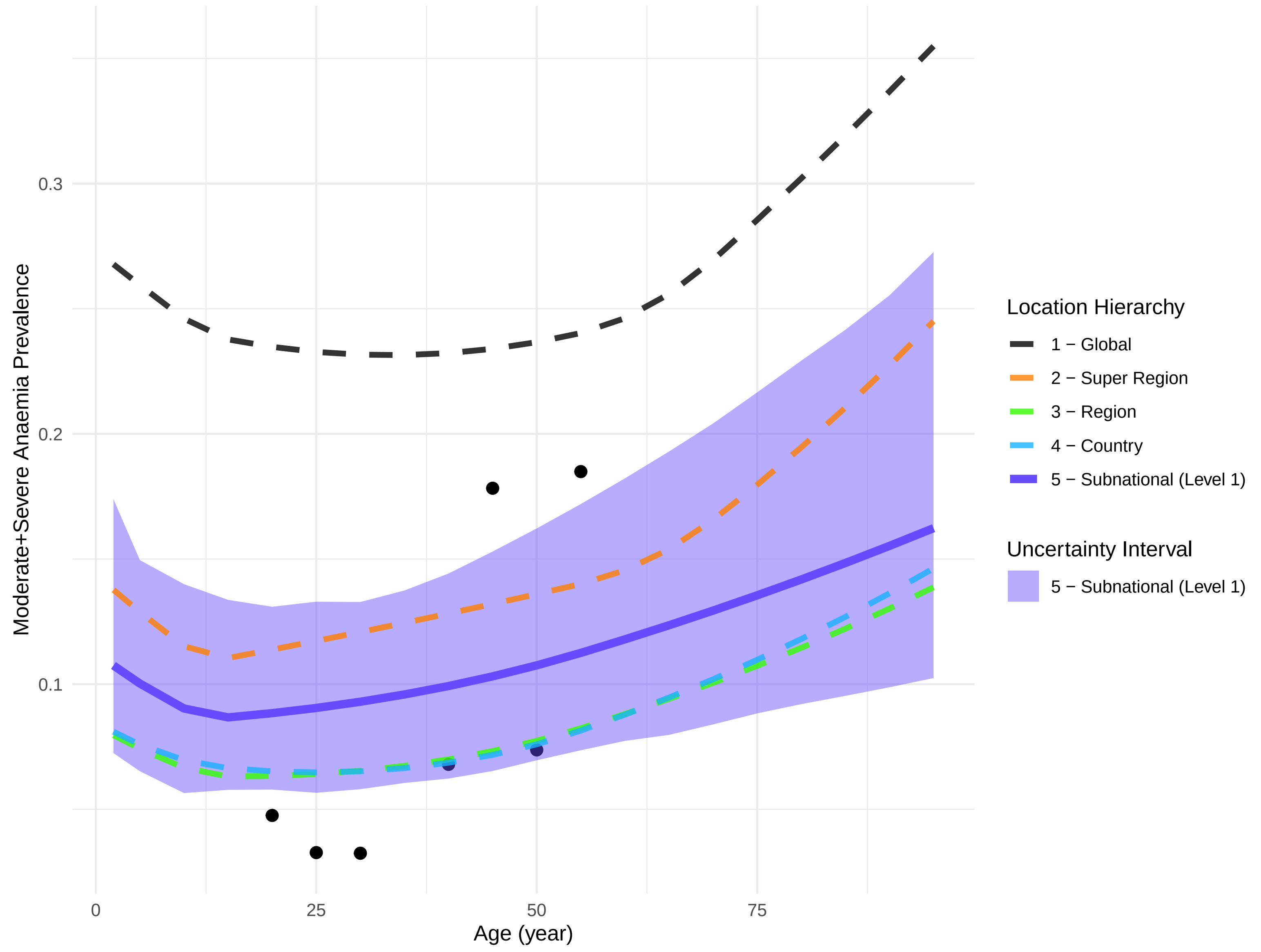

MR−BRT Age/Sex Fits − Non−pregnant Females 2+ Years Old − Moderate+Severe Anaemia Prevalence − Alagoas
WHO−2024 Adjustment Method

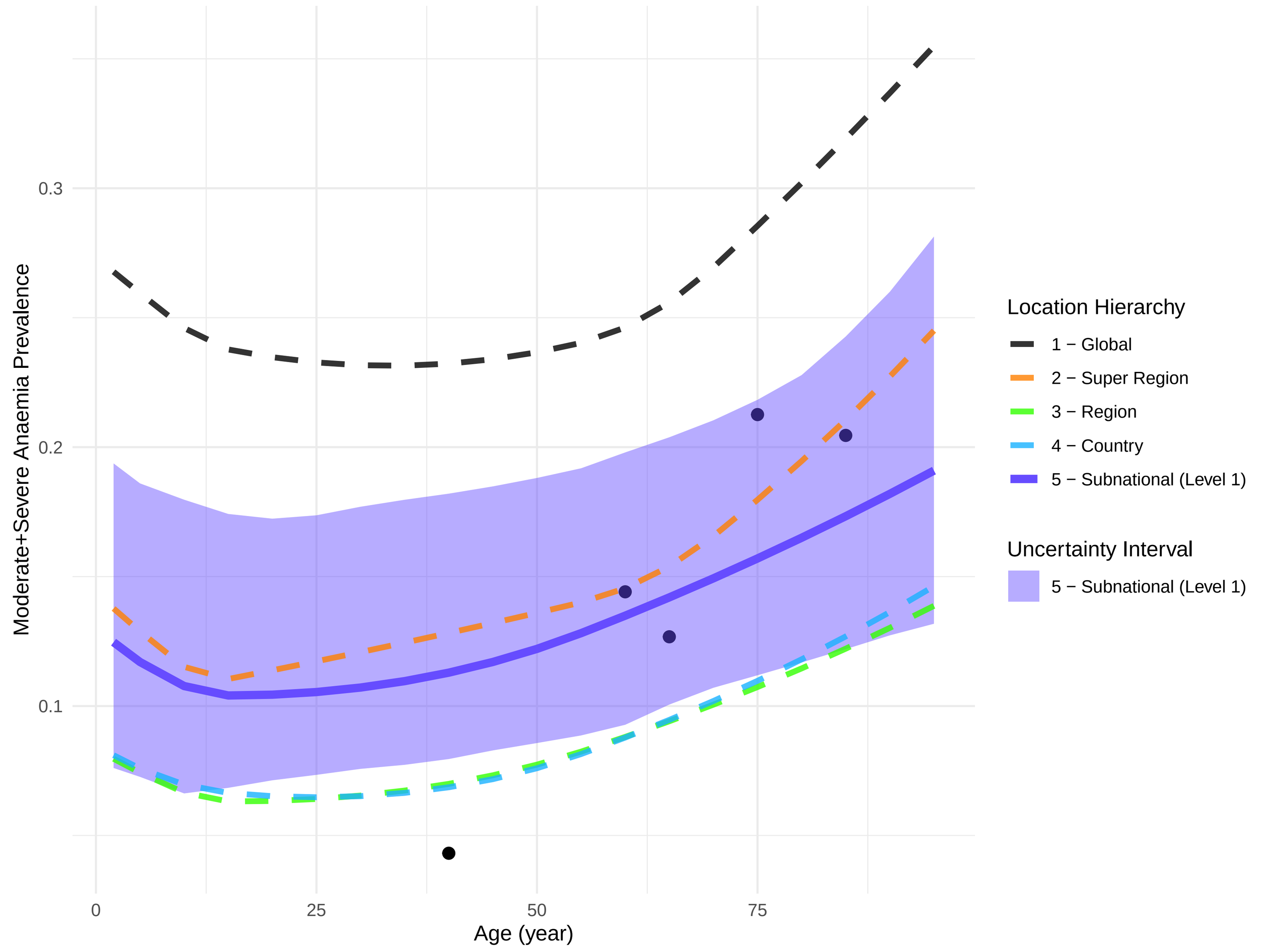

MR−BRT Age/Sex Fits − Non−pregnant Females 2+ Years Old − Moderate+Severe Anaemia Prevalence − Amapá
WHO−2024 Adjustment Method

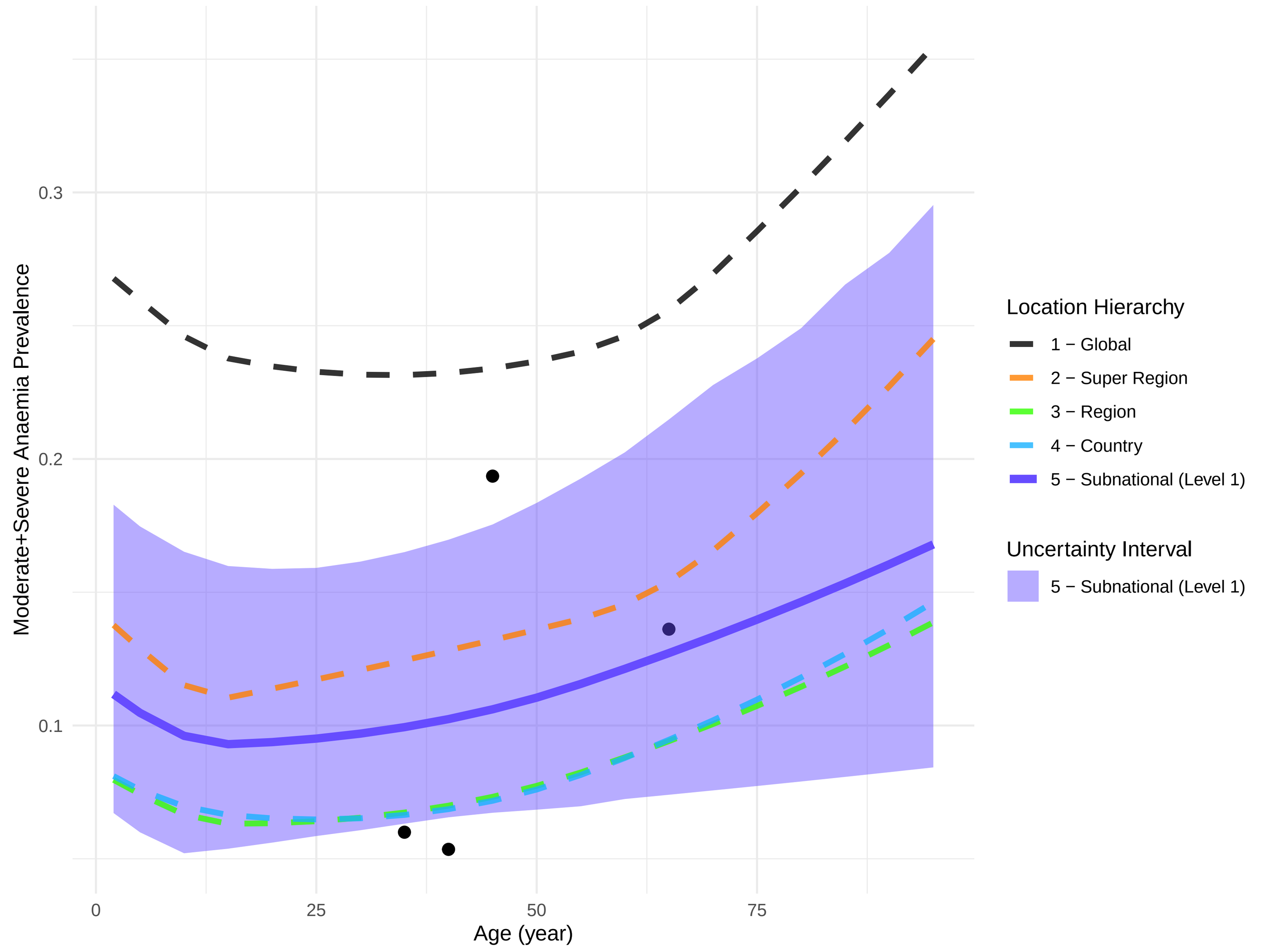

MR−BRT Age/Sex Fits − Non−pregnant Females 2+ Years Old − Moderate+Severe Anaemia Prevalence − Amazonas
WHO−2024 Adjustment Method

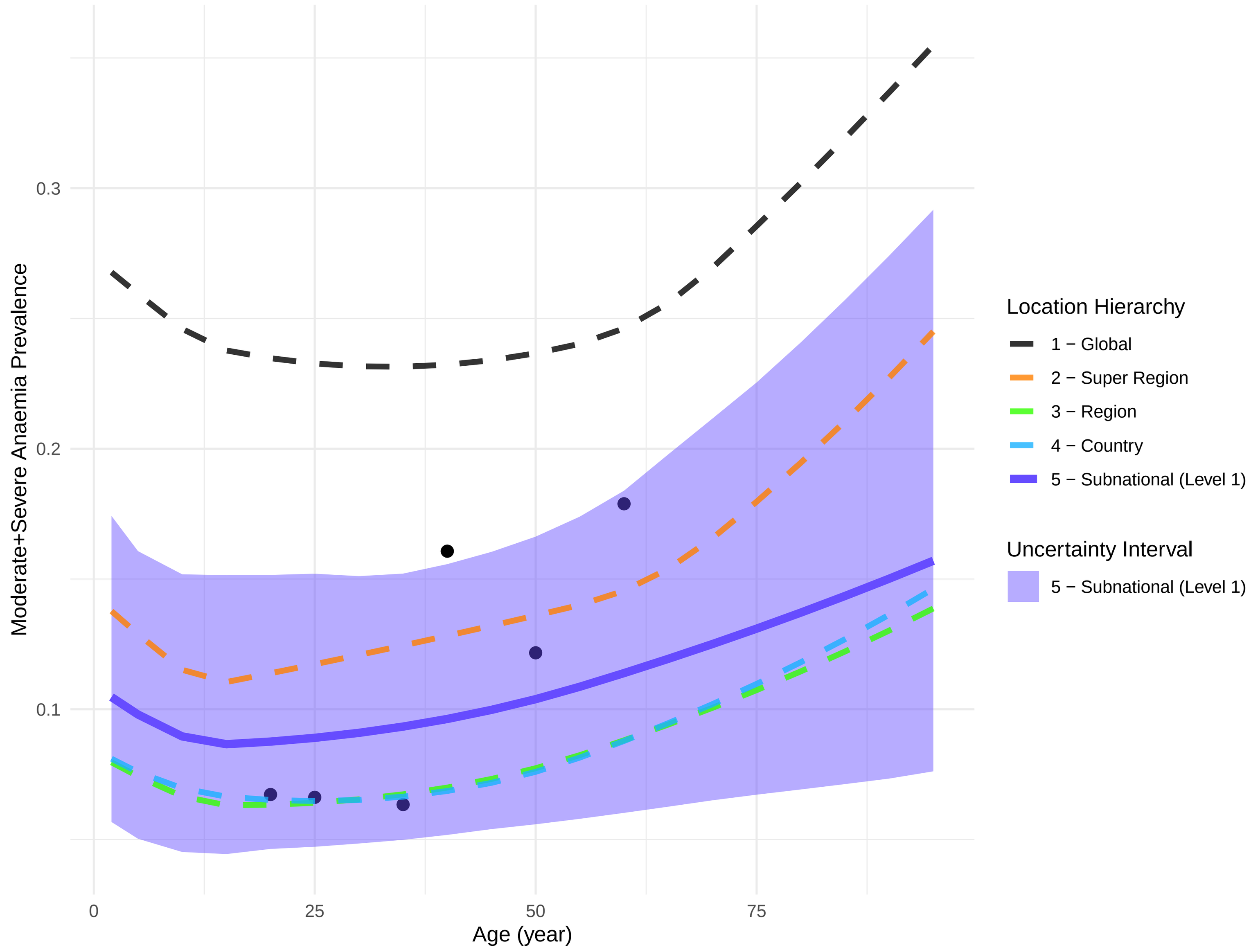

MR−BRT Age/Sex Fits − Non−pregnant Females 2+ Years Old − Moderate+Severe Anaemia Prevalence − Bahia
WHO−2024 Adjustment Method

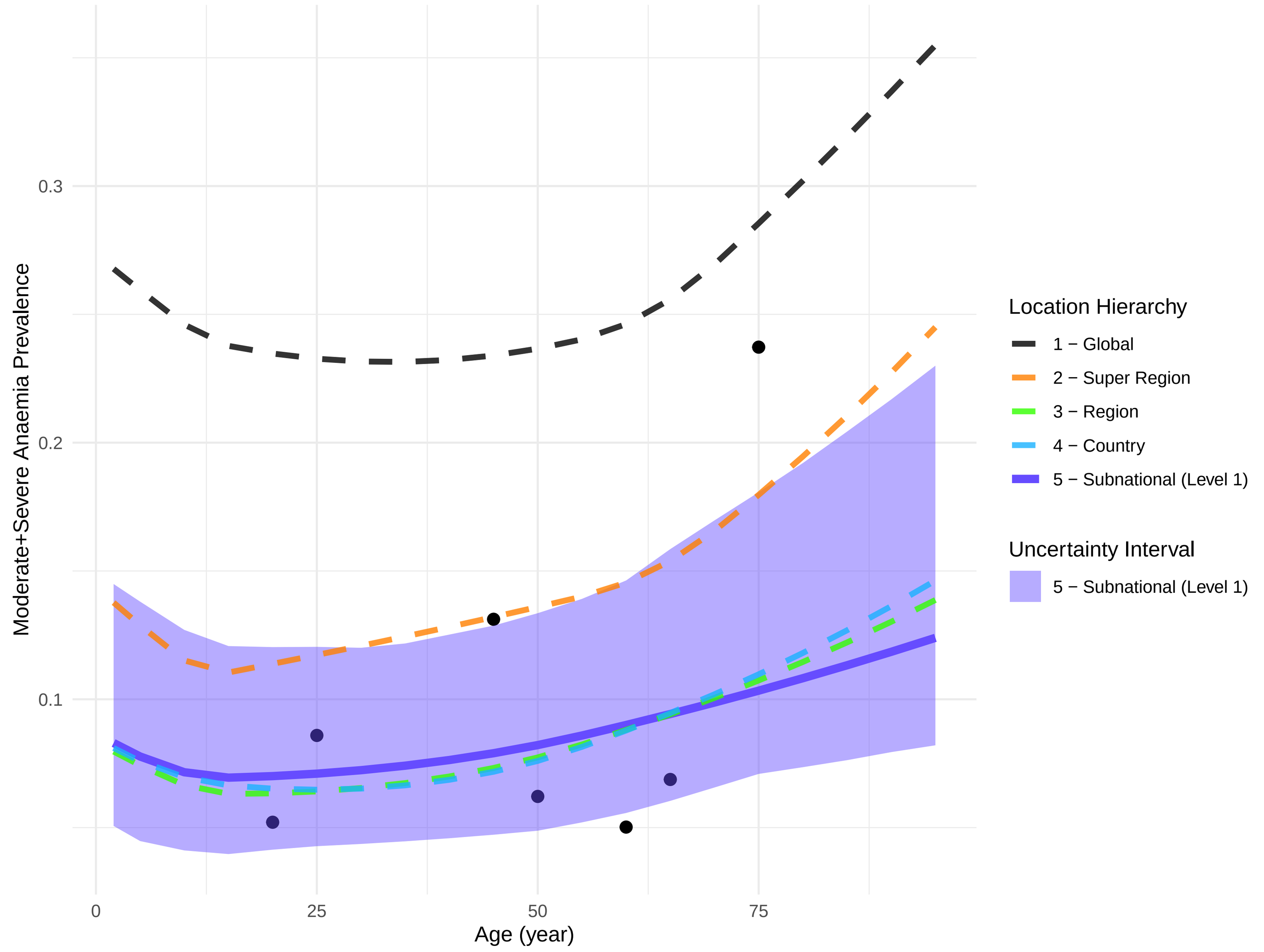

MR−BRT Age/Sex Fits − Non−pregnant Females 2+ Years Old − Moderate+Severe Anaemia Prevalence − Ceará
WHO−2024 Adjustment Method

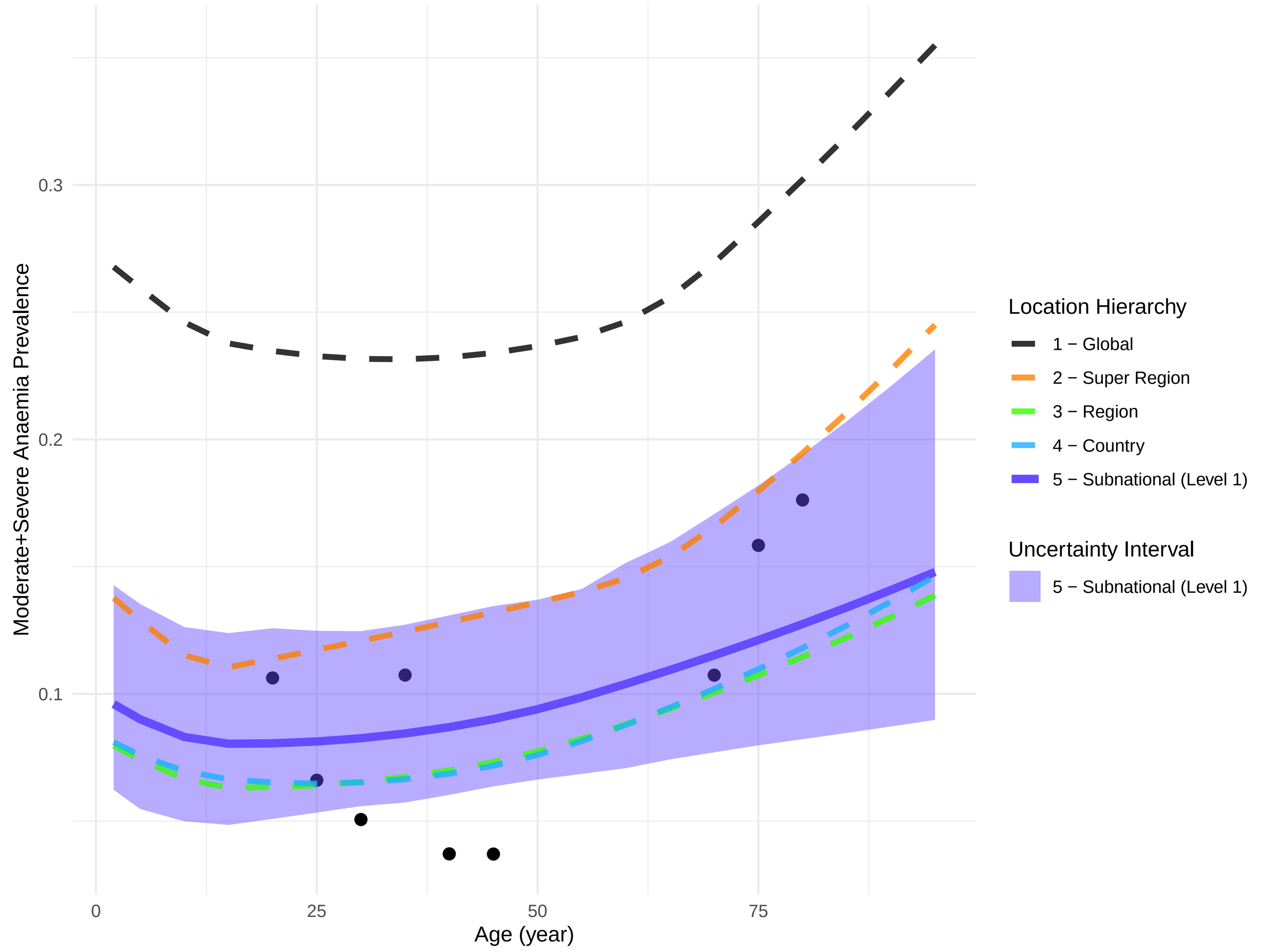

MR−BRT Age/Sex Fits − Non−pregnant Females 2+ Years Old − Moderate+Severe Anaemia Prevalence − Distrito Federal
WHO−2024 Adjustment Method

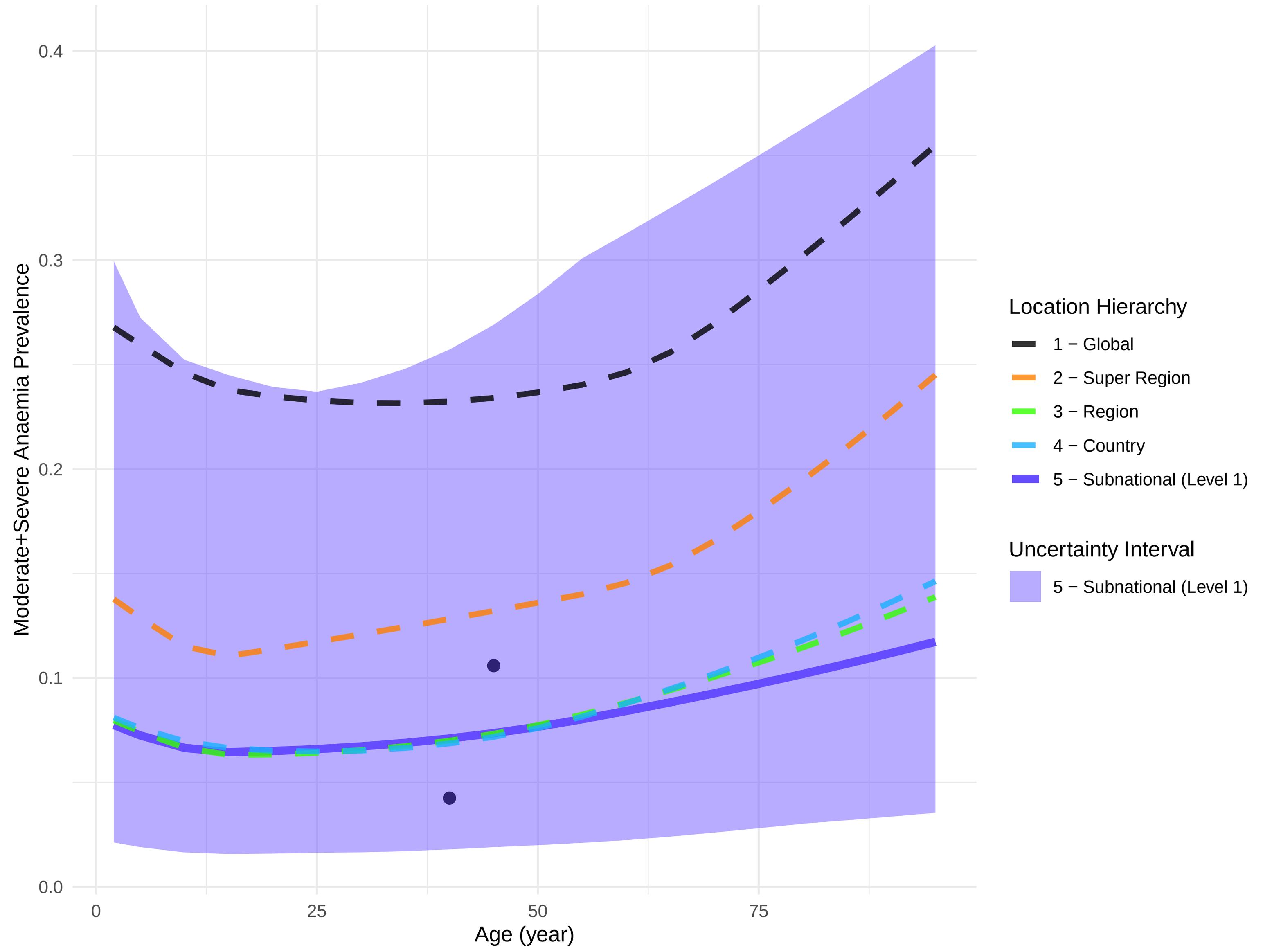

MR−BRT Age/Sex Fits − Non−pregnant Females 2+ Years Old − Moderate+Severe Anaemia Prevalence − Espírito Santo
WHO−2024 Adjustment Method

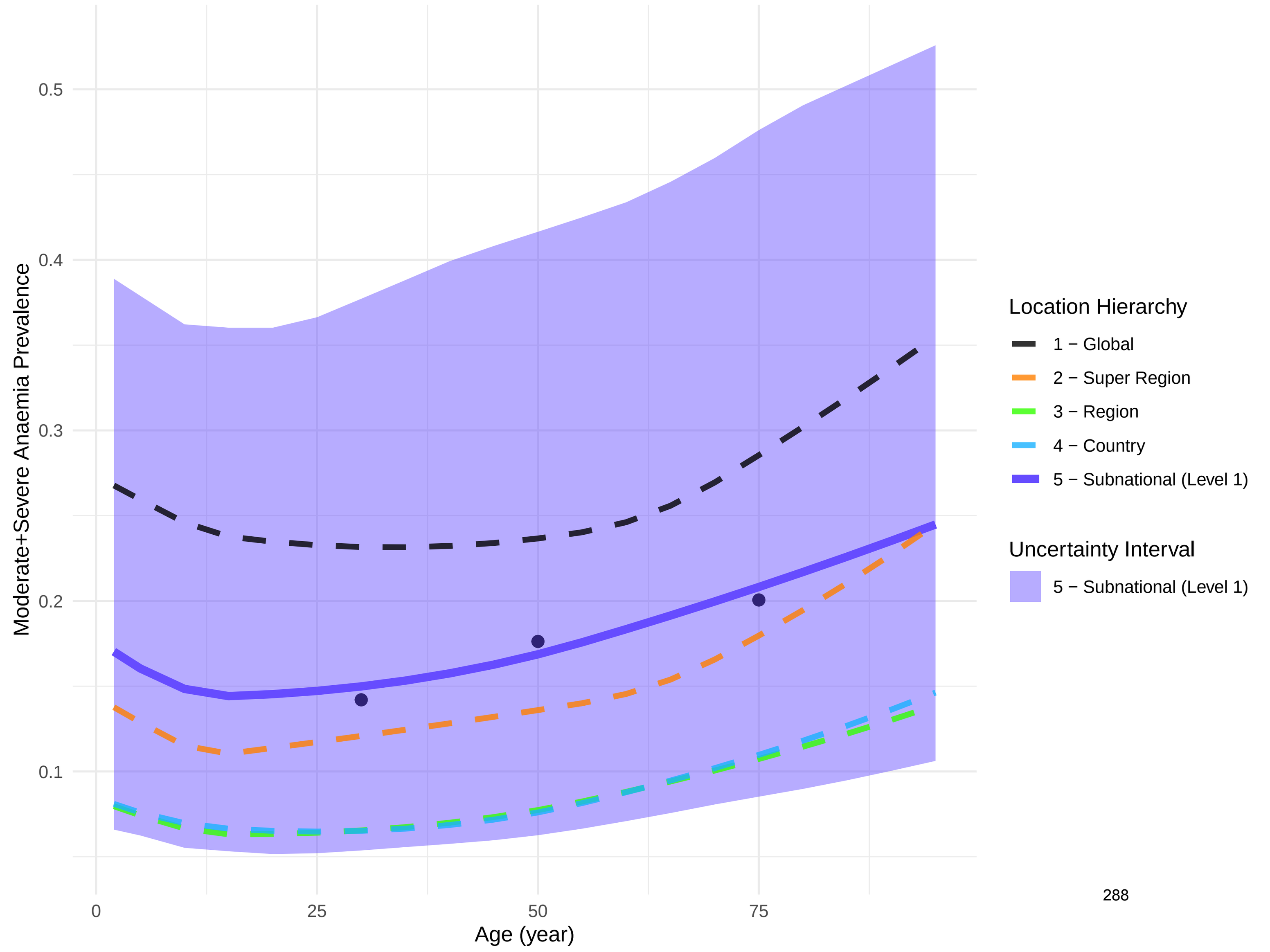

MR−BRT Age/Sex Fits − Non−pregnant Females 2+ Years Old − Moderate+Severe Anaemia Prevalence − Goiás
WHO−2024 Adjustment Method

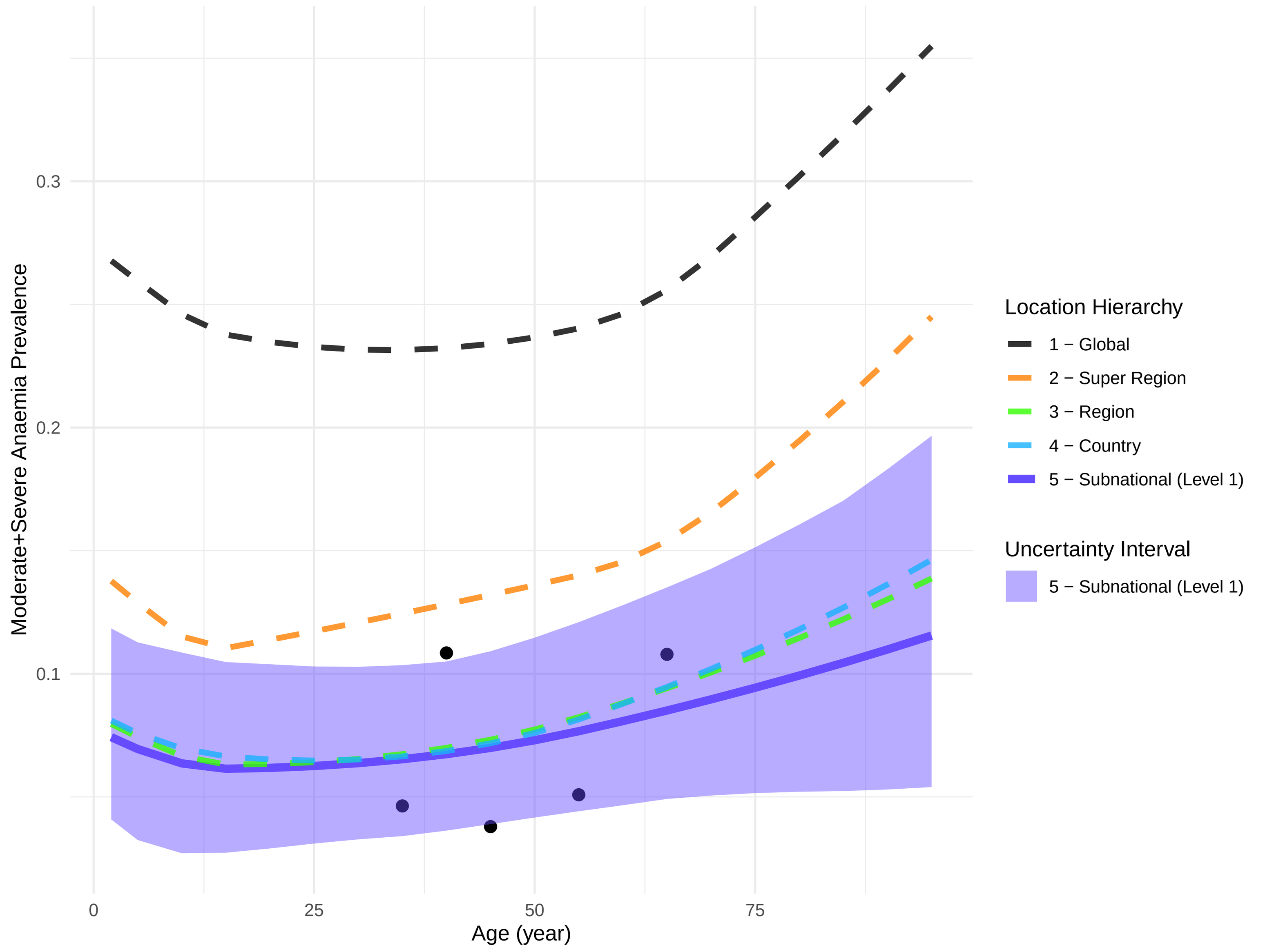

MR−BRT Age/Sex Fits − Non−pregnant Females 2+ Years Old − Moderate+Severe Anaemia Prevalence − Maranhão
WHO−2024 Adjustment Method

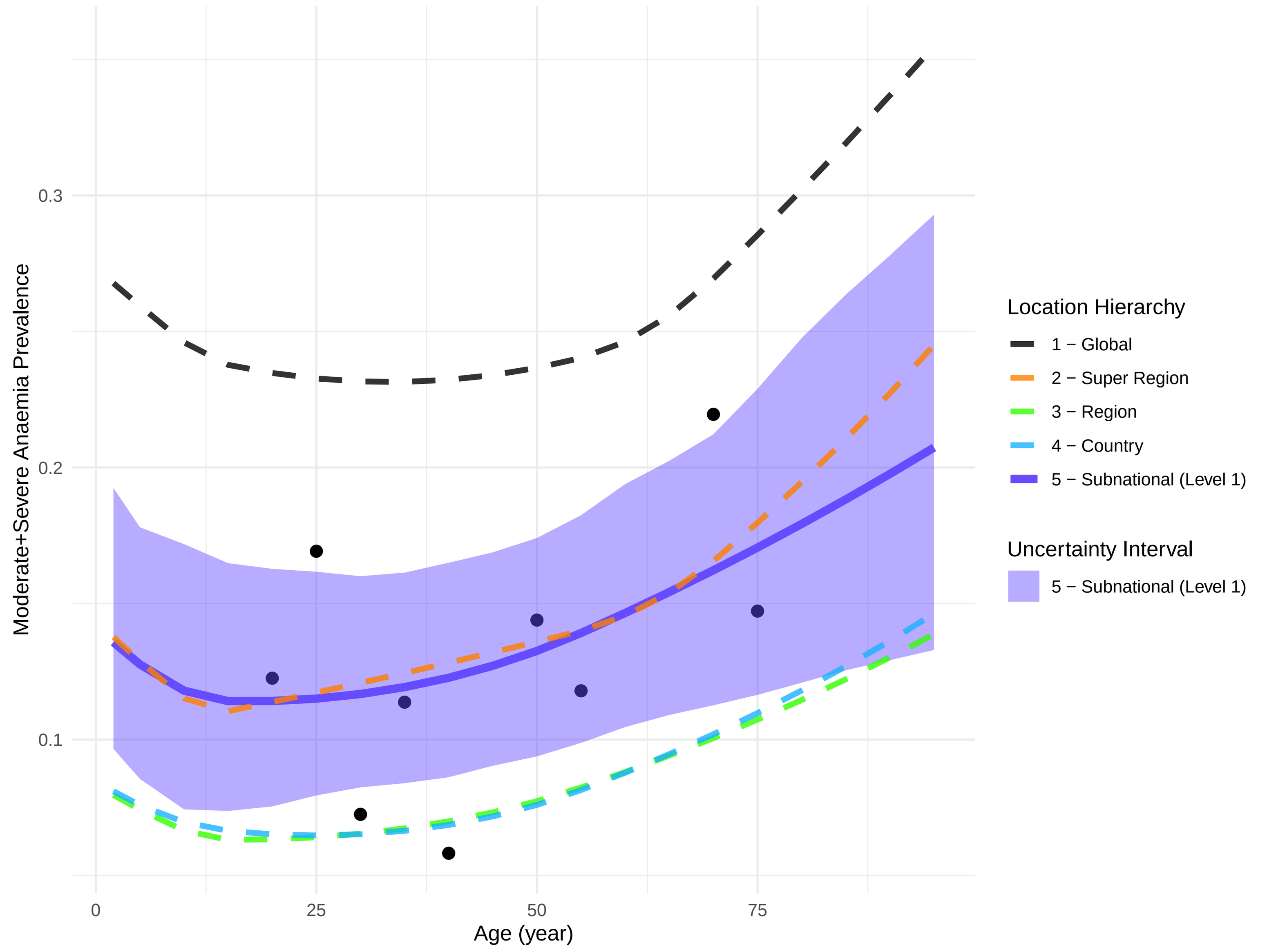

MR−BRT Age/Sex Fits − Non−pregnant Females 2+ Years Old − Moderate+Severe Anaemia Prevalence − Mato Grosso
WHO−2024 Adjustment Method

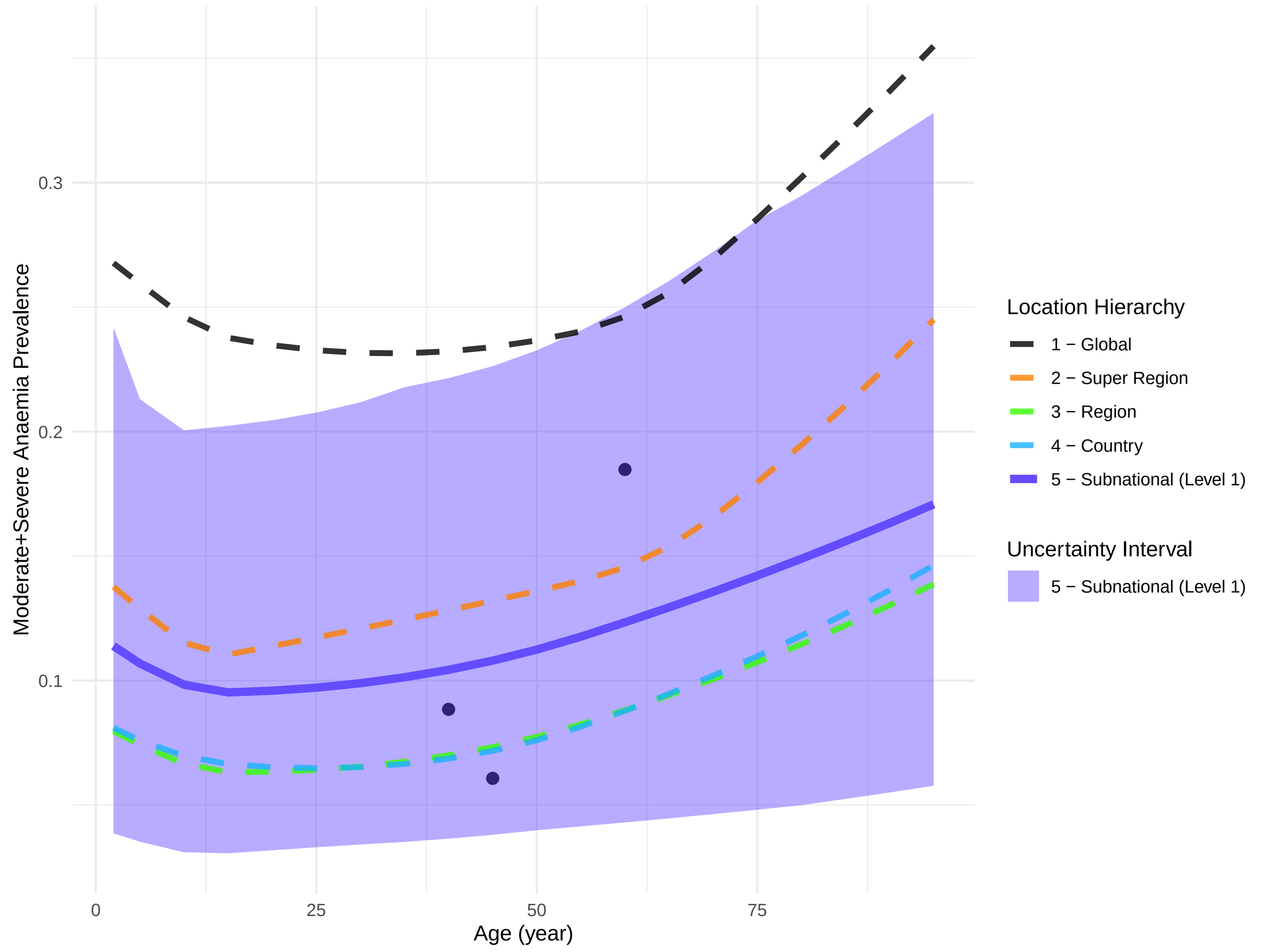

MR−BRT Age/Sex Fits − Non−pregnant Females 2+ Years Old − Moderate+Severe Anaemia Prevalence − Mato Grosso do Sul
WHO−2024 Adjustment Method

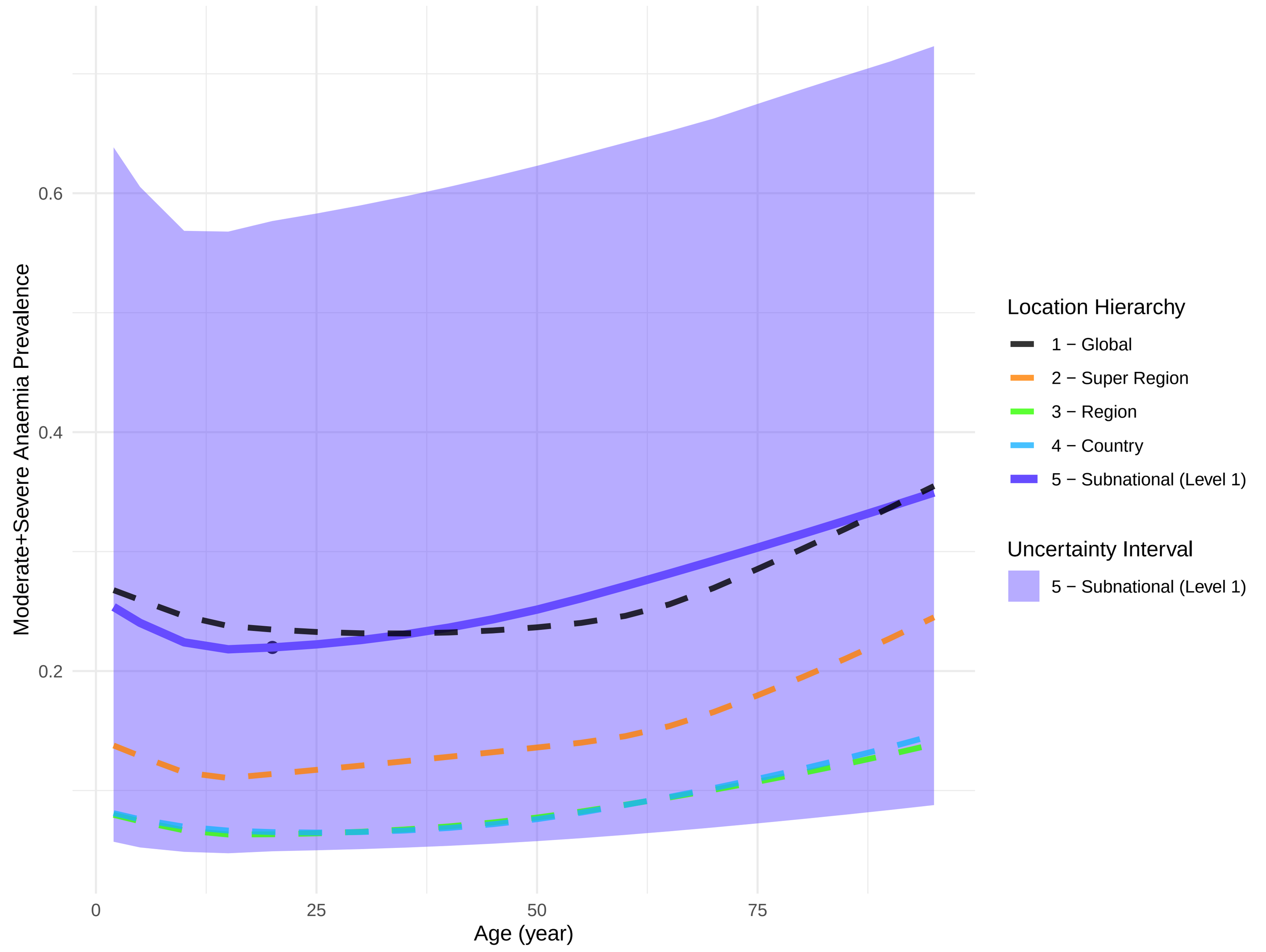

MR−BRT Age/Sex Fits − Non−pregnant Females 2+ Years Old − Moderate+Severe Anaemia Prevalence − Minas Gerais
WHO−2024 Adjustment Method

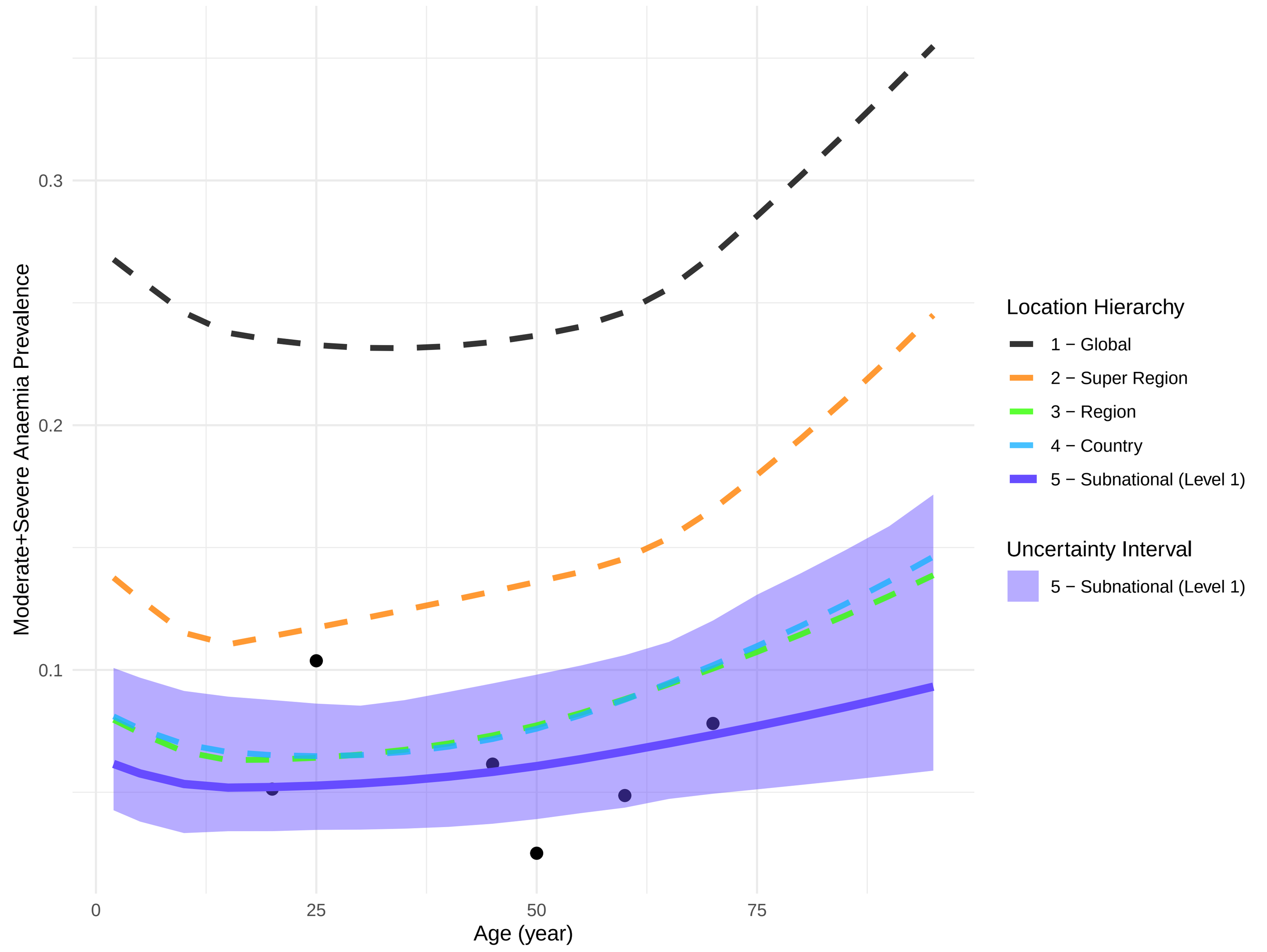

MR−BRT Age/Sex Fits − Non−pregnant Females 2+ Years Old − Moderate+Severe Anaemia Prevalence − Pará
WHO−2024 Adjustment Method

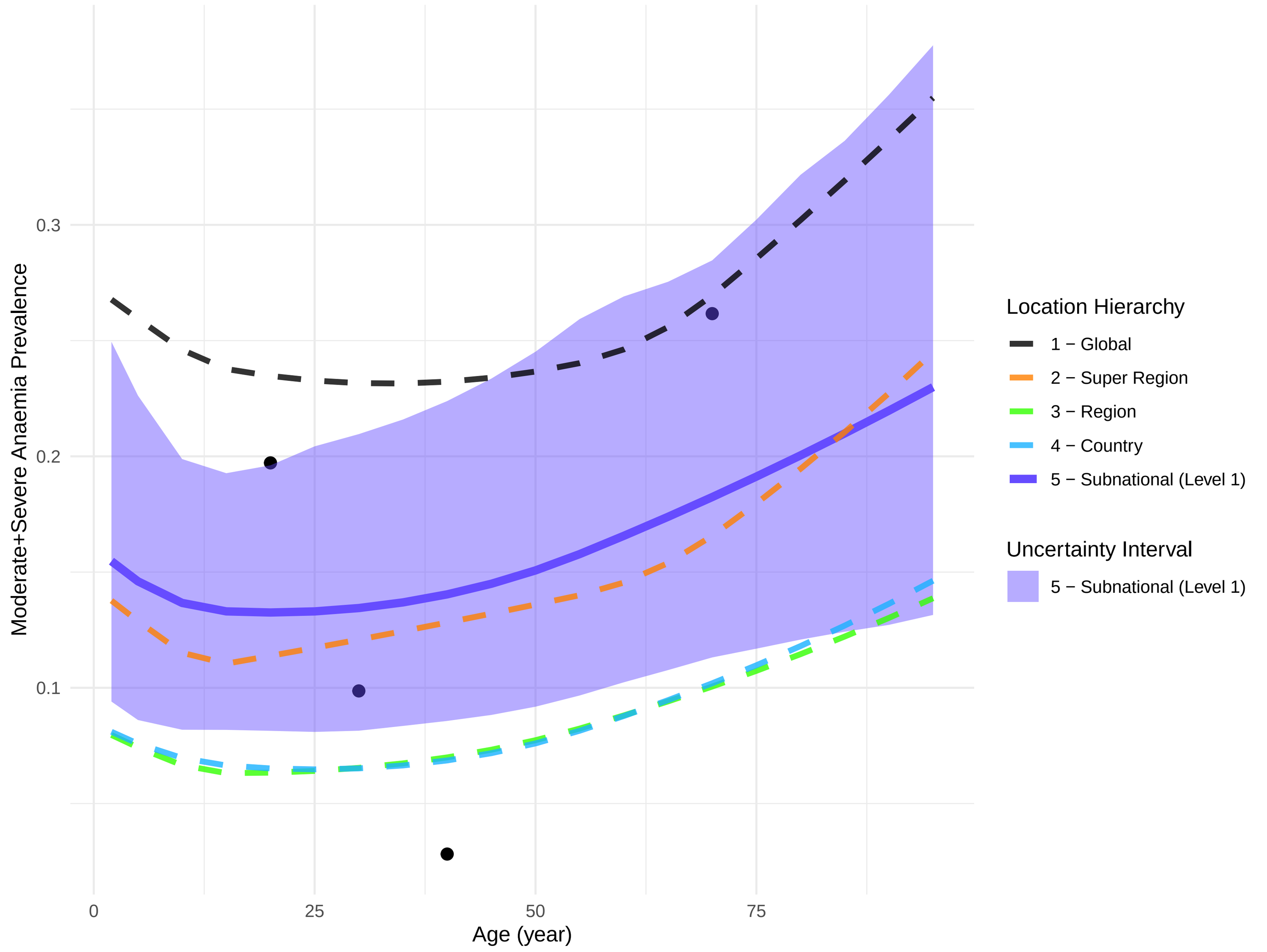

MR−BRT Age/Sex Fits − Non−pregnant Females 2+ Years Old − Moderate+Severe Anaemia Prevalence − Paraíba
WHO−2024 Adjustment Method

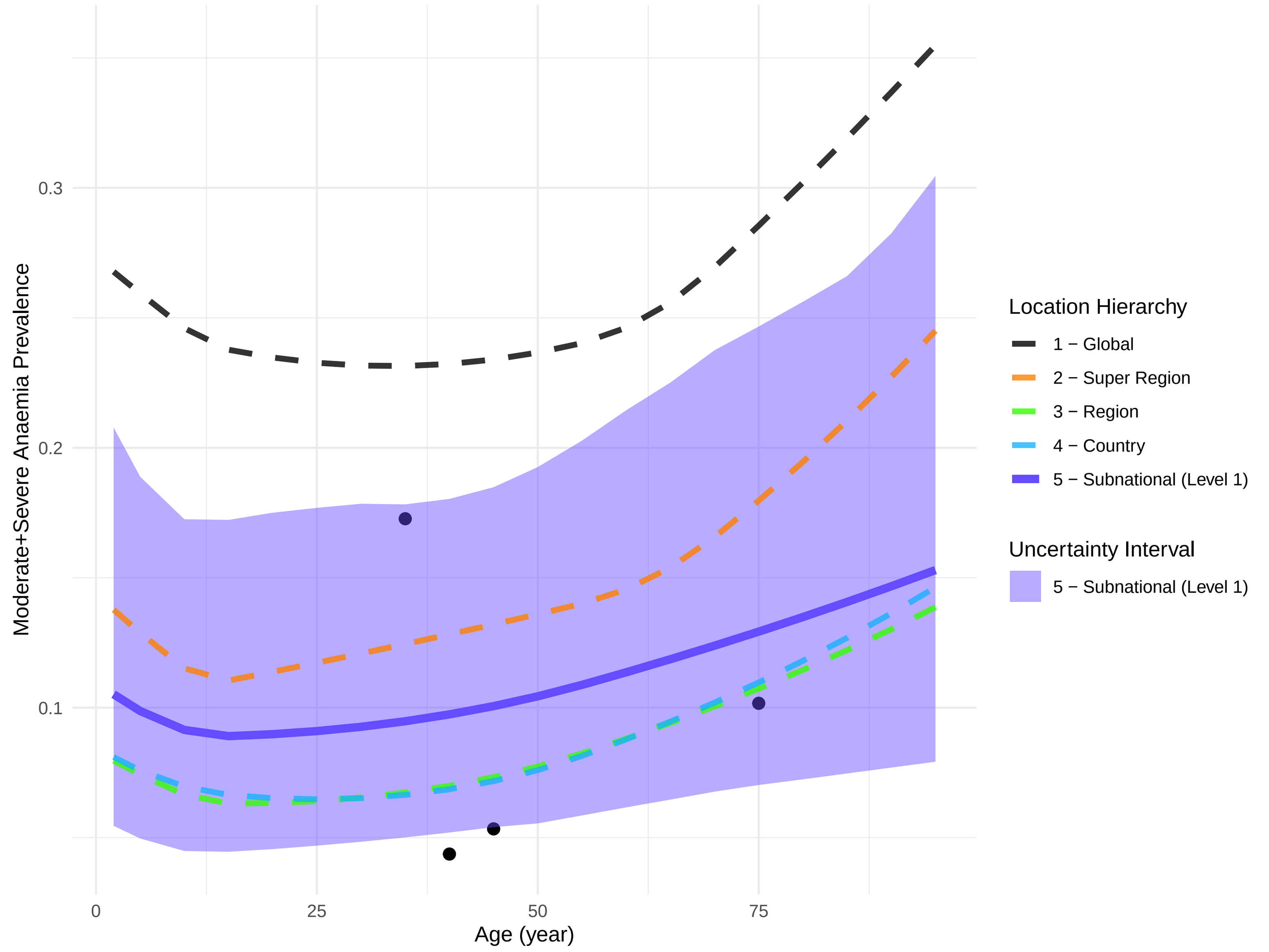

MR−BRT Age/Sex Fits − Non−pregnant Females 2+ Years Old − Moderate+Severe Anaemia Prevalence − Paraná
WHO−2024 Adjustment Method

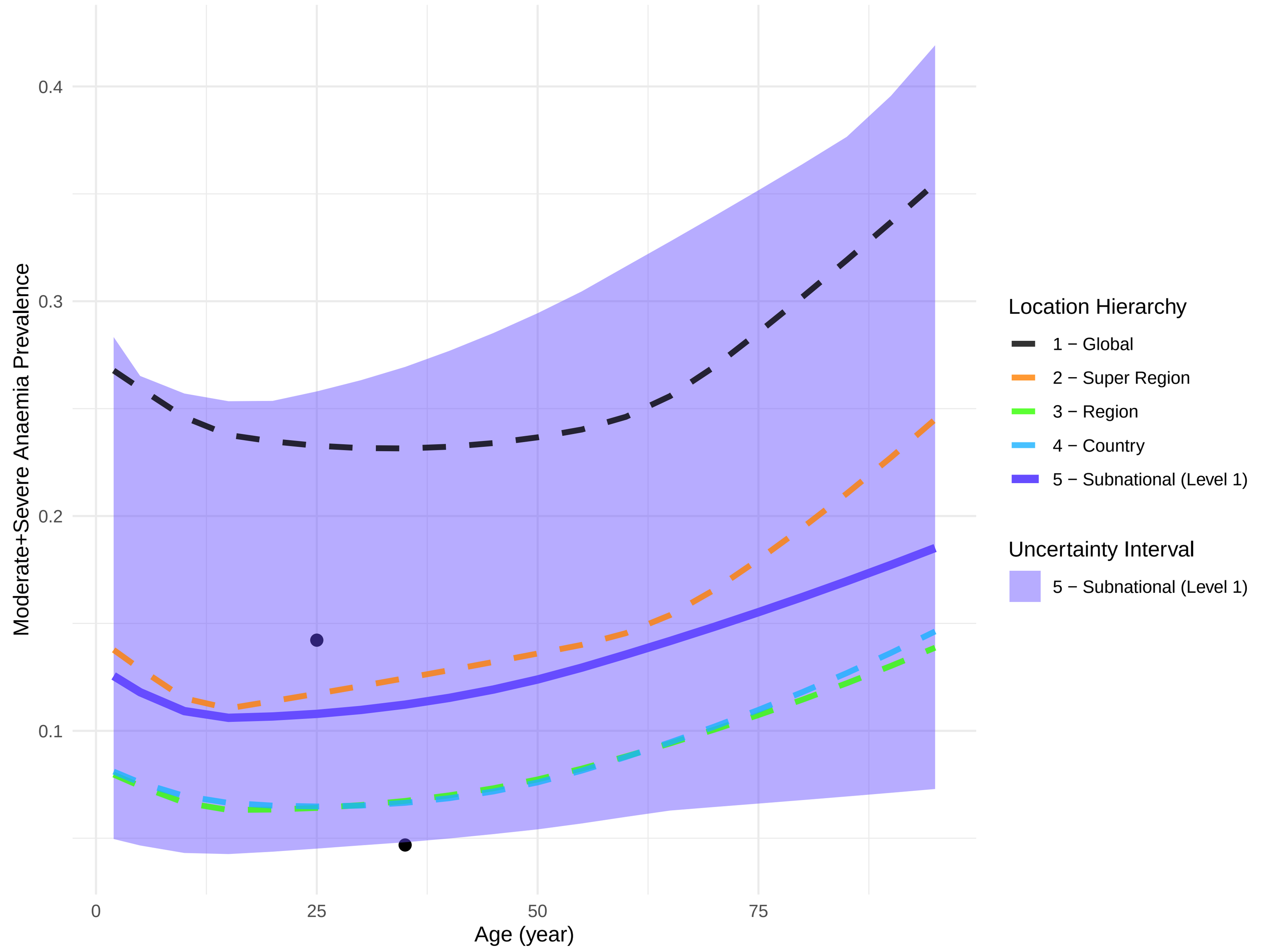

MR−BRT Age/Sex Fits − Non−pregnant Females 2+ Years Old − Moderate+Severe Anaemia Prevalence − Pernambuco
WHO−2024 Adjustment Method

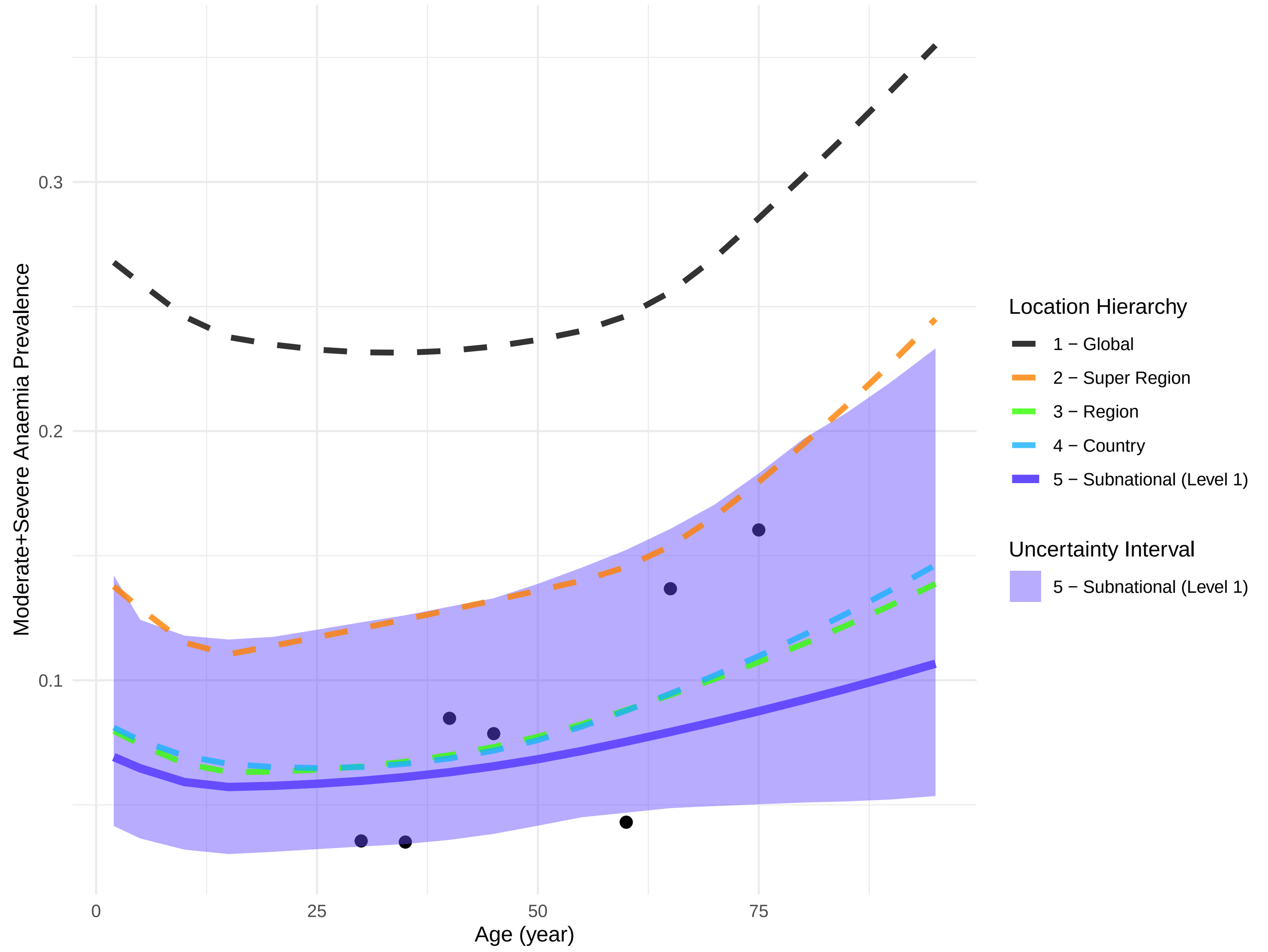

MR−BRT Age/Sex Fits − Non−pregnant Females 2+ Years Old − Moderate+Severe Anaemia Prevalence − Piauí
WHO−2024 Adjustment Method

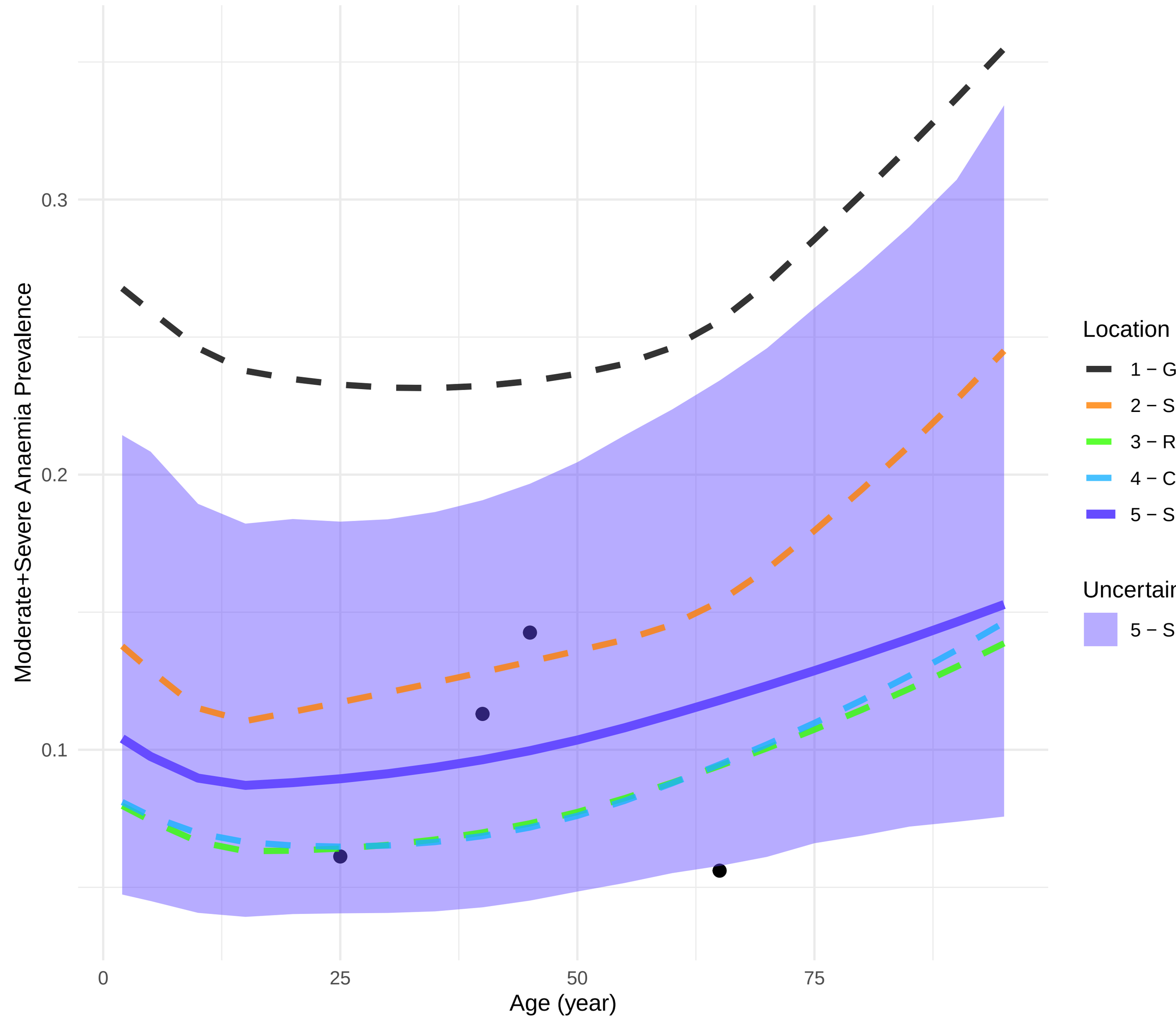

MR−BRT Age/Sex Fits − Non−pregnant Females 2+ Years Old − Moderate+Severe Anaemia Prevalence − Rio de Janeiro
WHO−2024 Adjustment Method

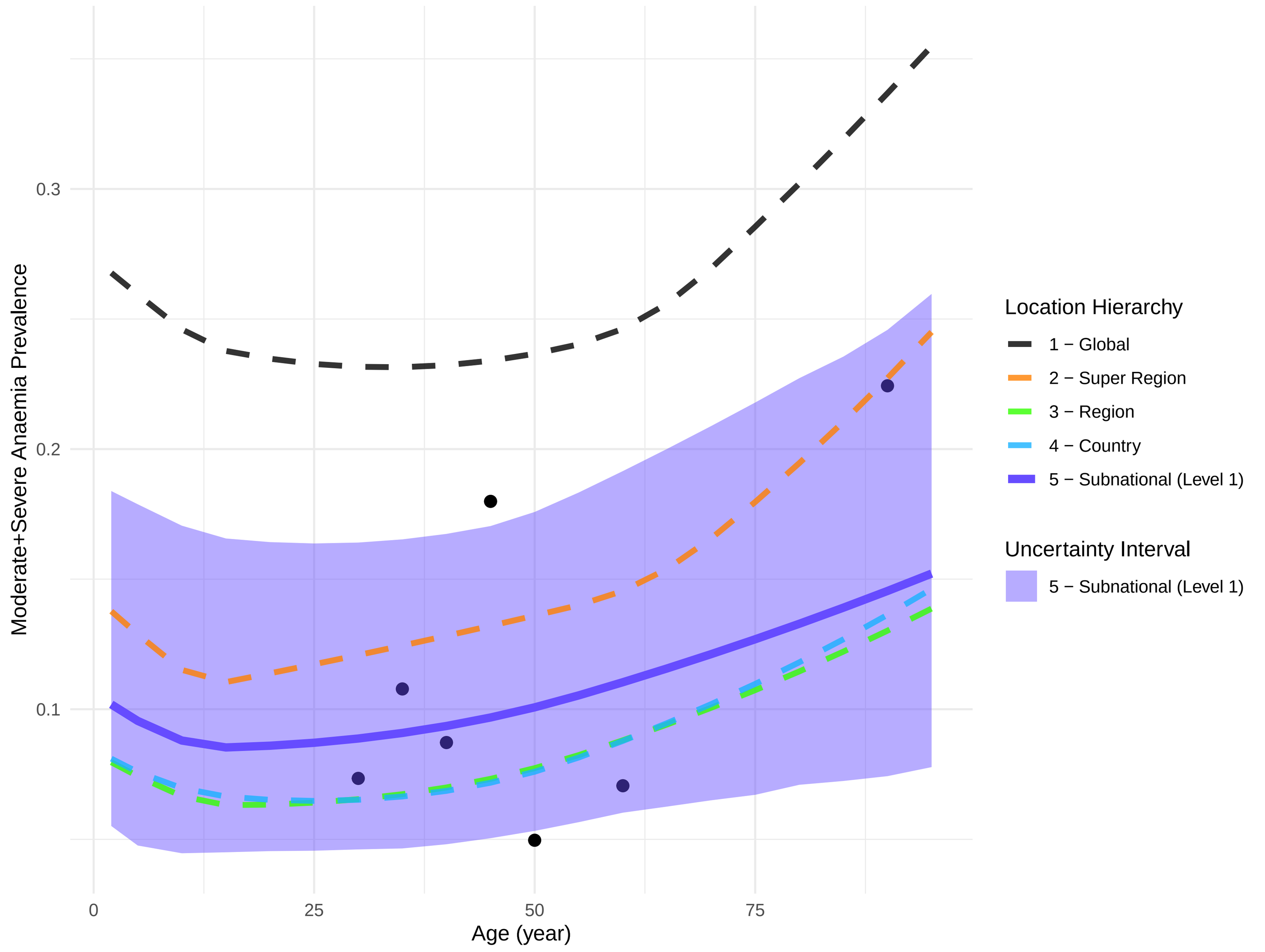

MR−BRT Age/Sex Fits − Non−pregnant Females 2+ Years Old − Moderate+Severe Anaemia Prevalence − Rio Grande do Norte
WHO−2024 Adjustment Method

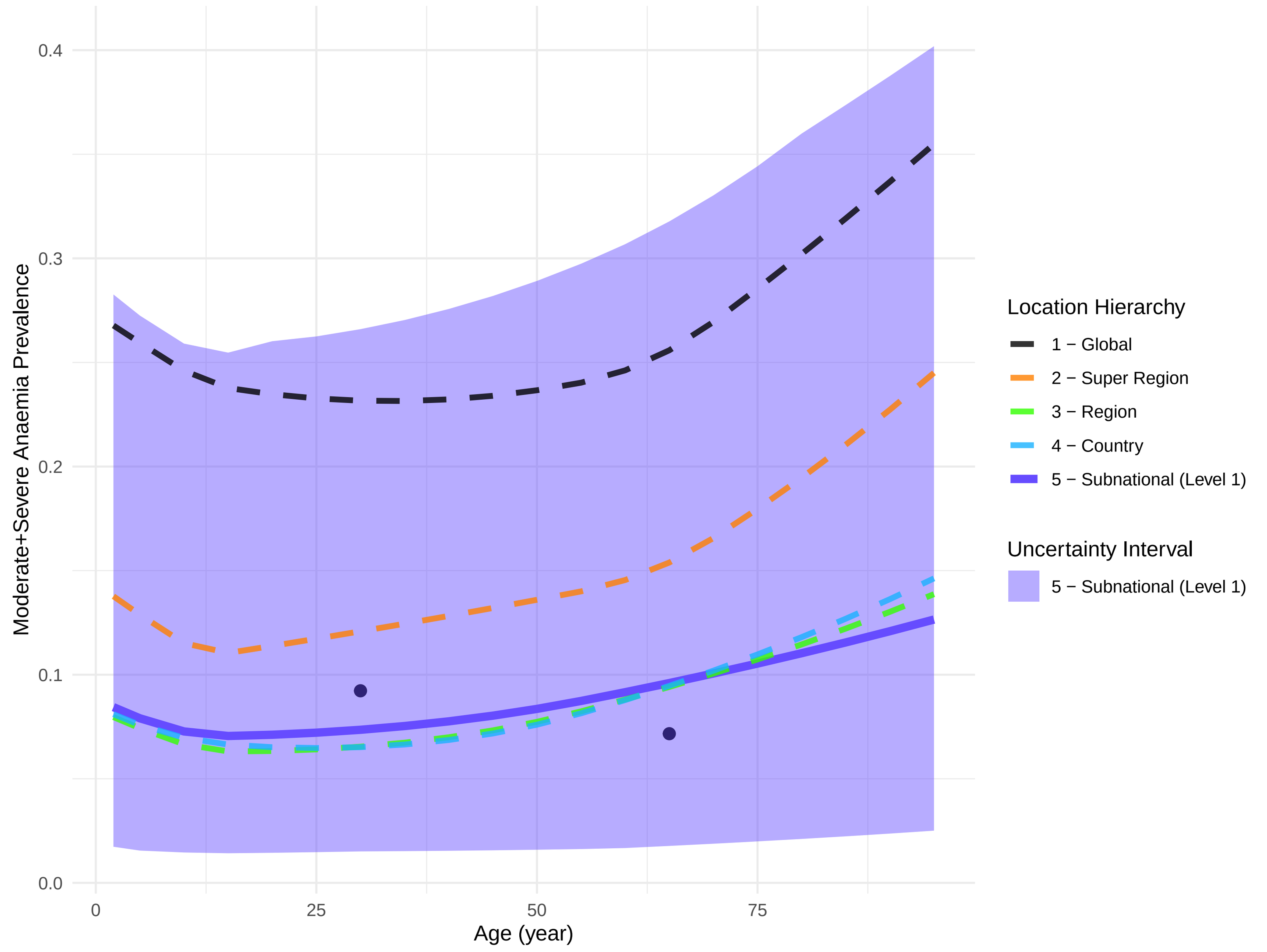

MR−BRT Age/Sex Fits − Non−pregnant Females 2+ Years Old − Moderate+Severe Anaemia Prevalence − Rio Grande do Sul
WHO−2024 Adjustment Method

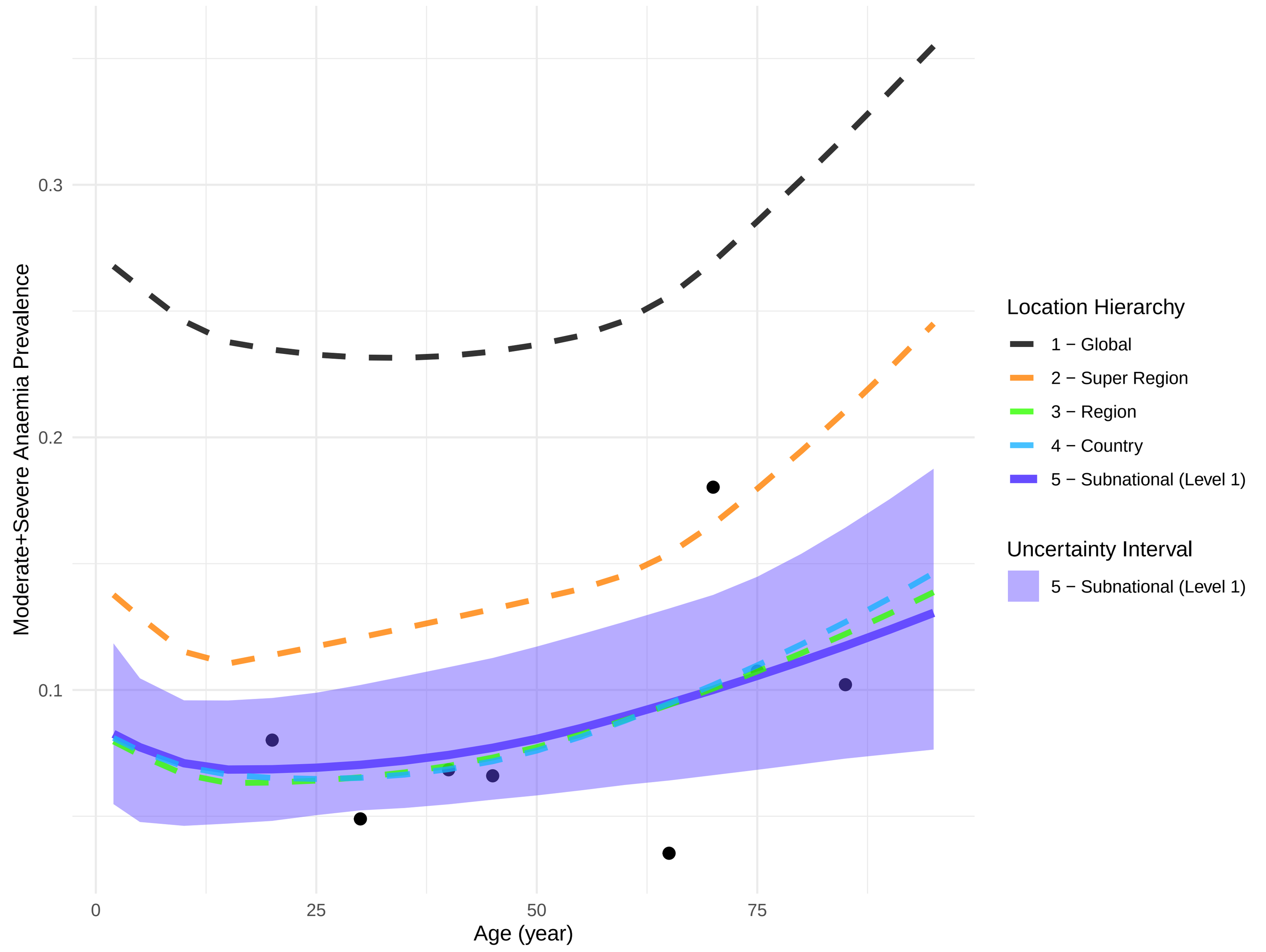

MR−BRT Age/Sex Fits − Non−pregnant Females 2+ Years Old − Moderate+Severe Anaemia Prevalence − Rondônia
WHO−2024 Adjustment Method

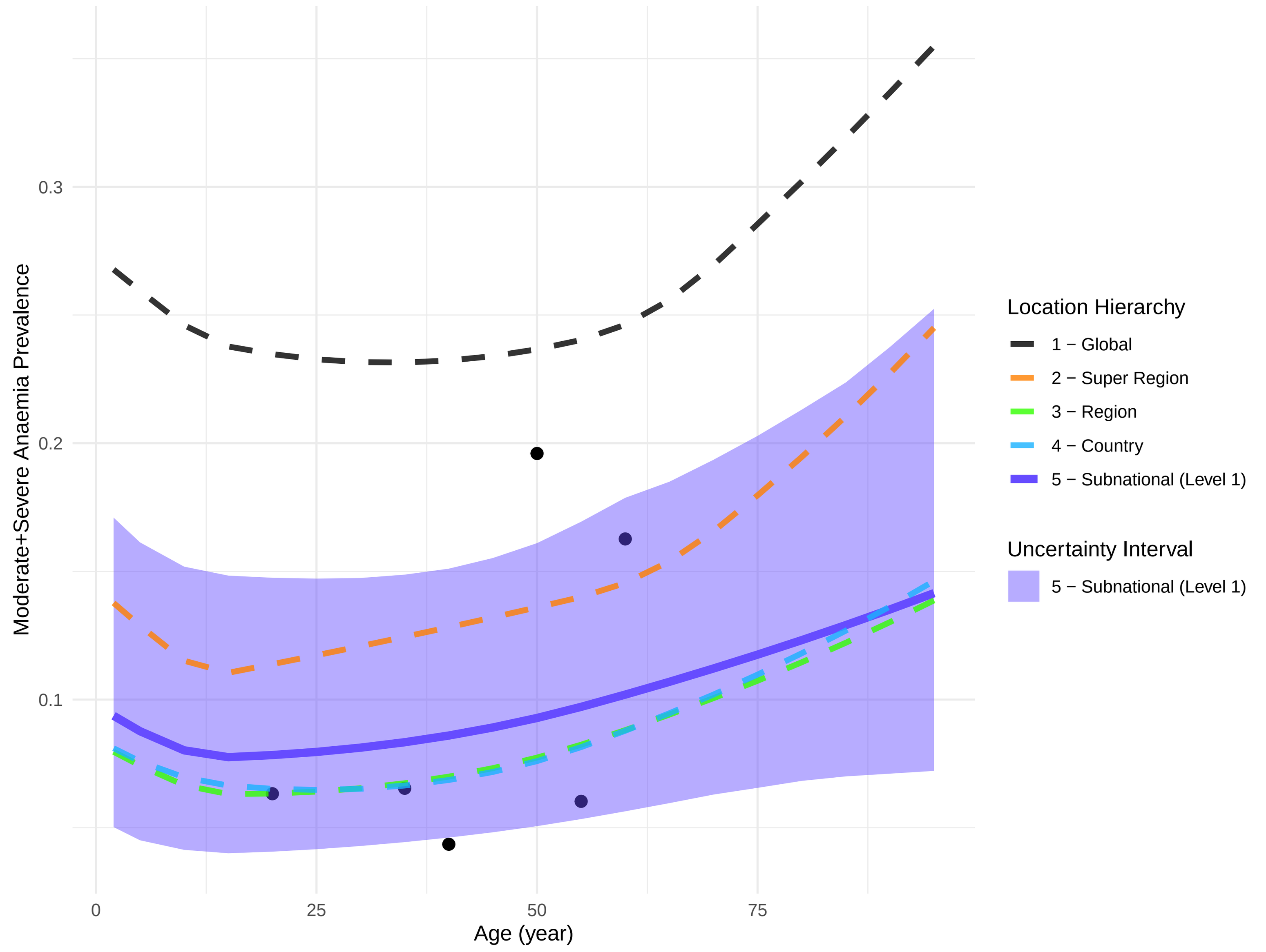

MR−BRT Age/Sex Fits − Non−pregnant Females 2+ Years Old − Moderate+Severe Anaemia Prevalence − Roraima
WHO−2024 Adjustment Method

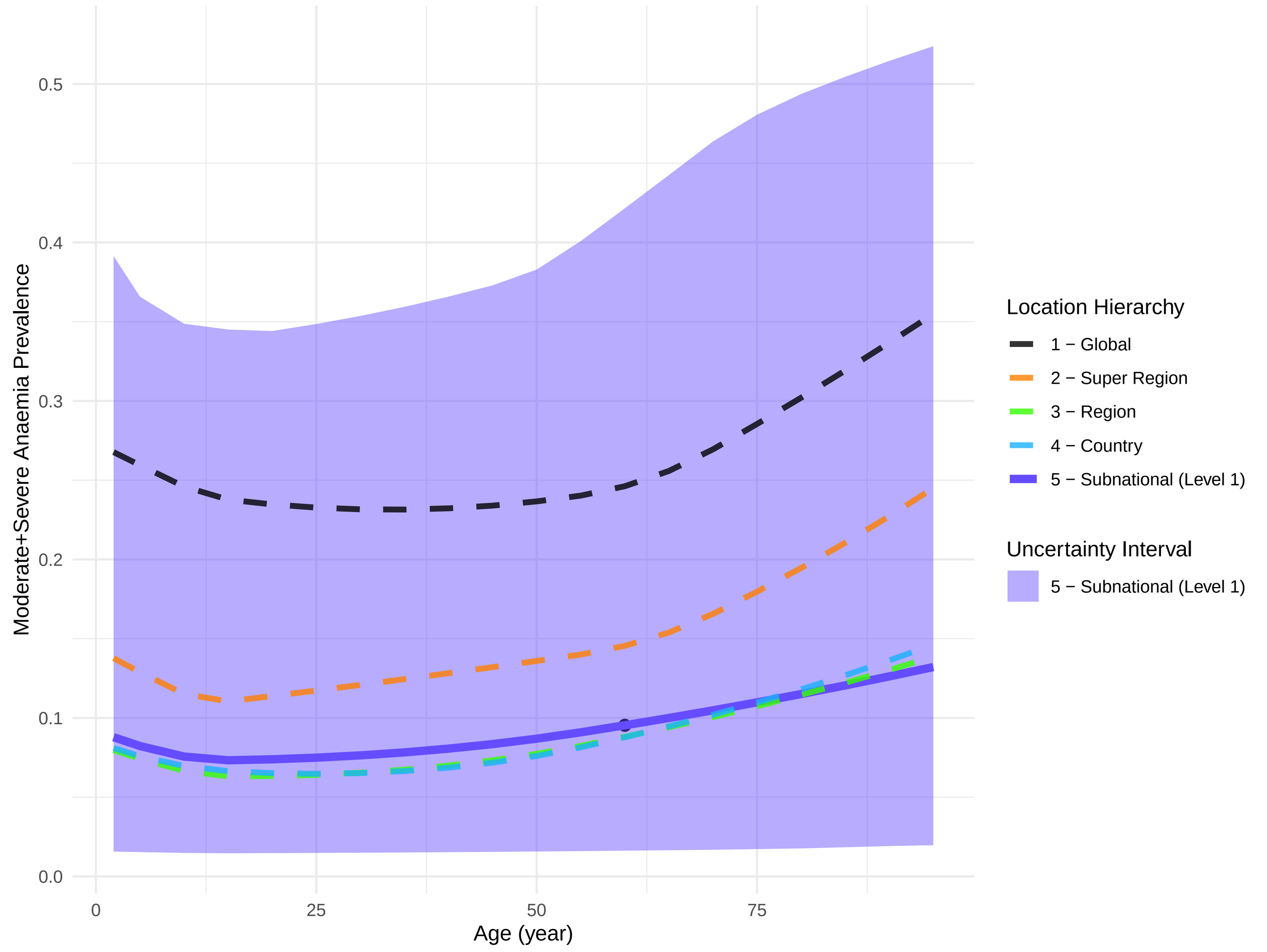

MR−BRT Age/Sex Fits − Non−pregnant Females 2+ Years Old − Moderate+Severe Anaemia Prevalence − Santa Catarina
WHO−2024 Adjustment Method

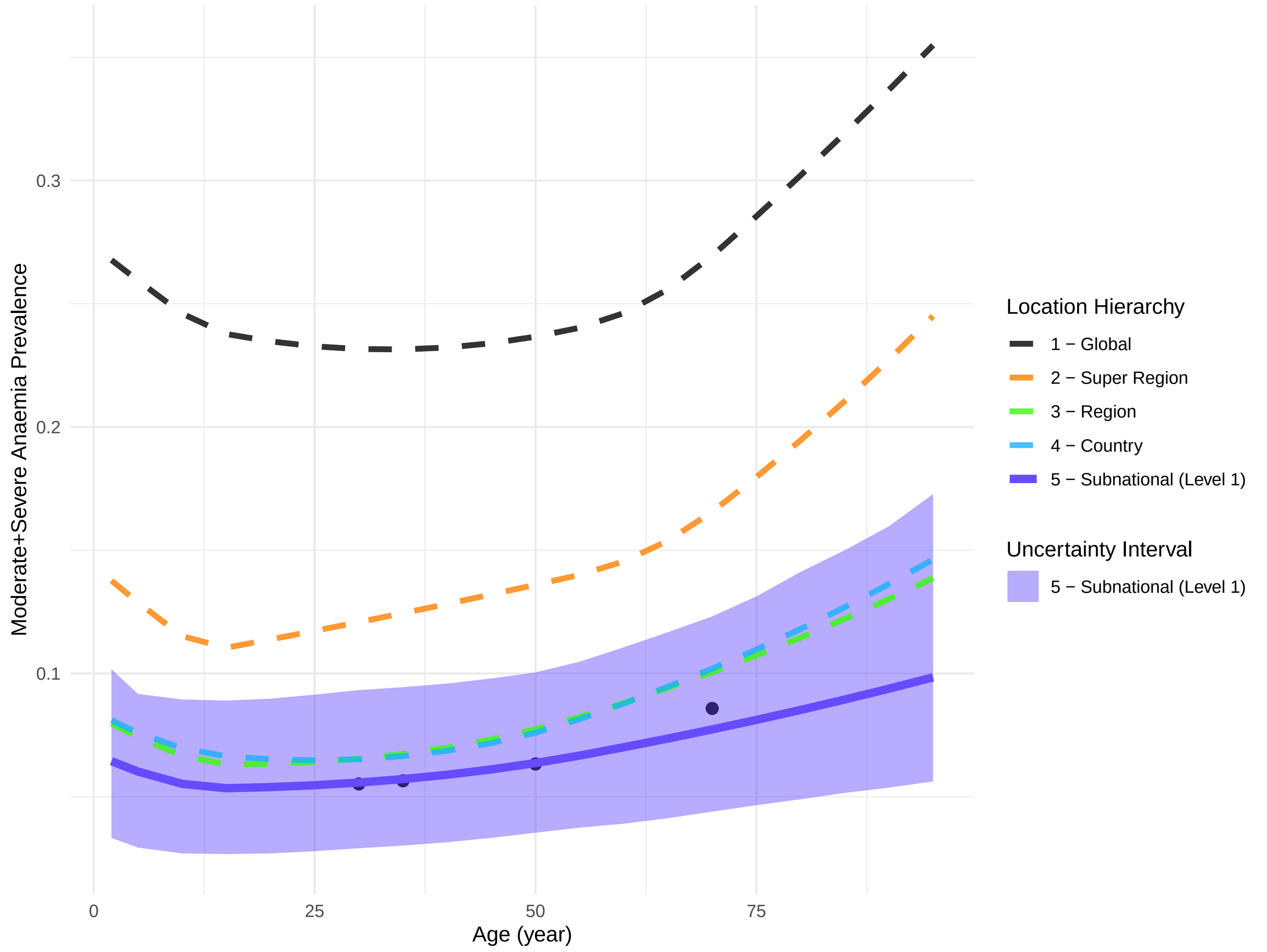

MR−BRT Age/Sex Fits − Non−pregnant Females 2+ Years Old − Moderate+Severe Anaemia Prevalence − São Paulo
WHO−2024 Adjustment Method

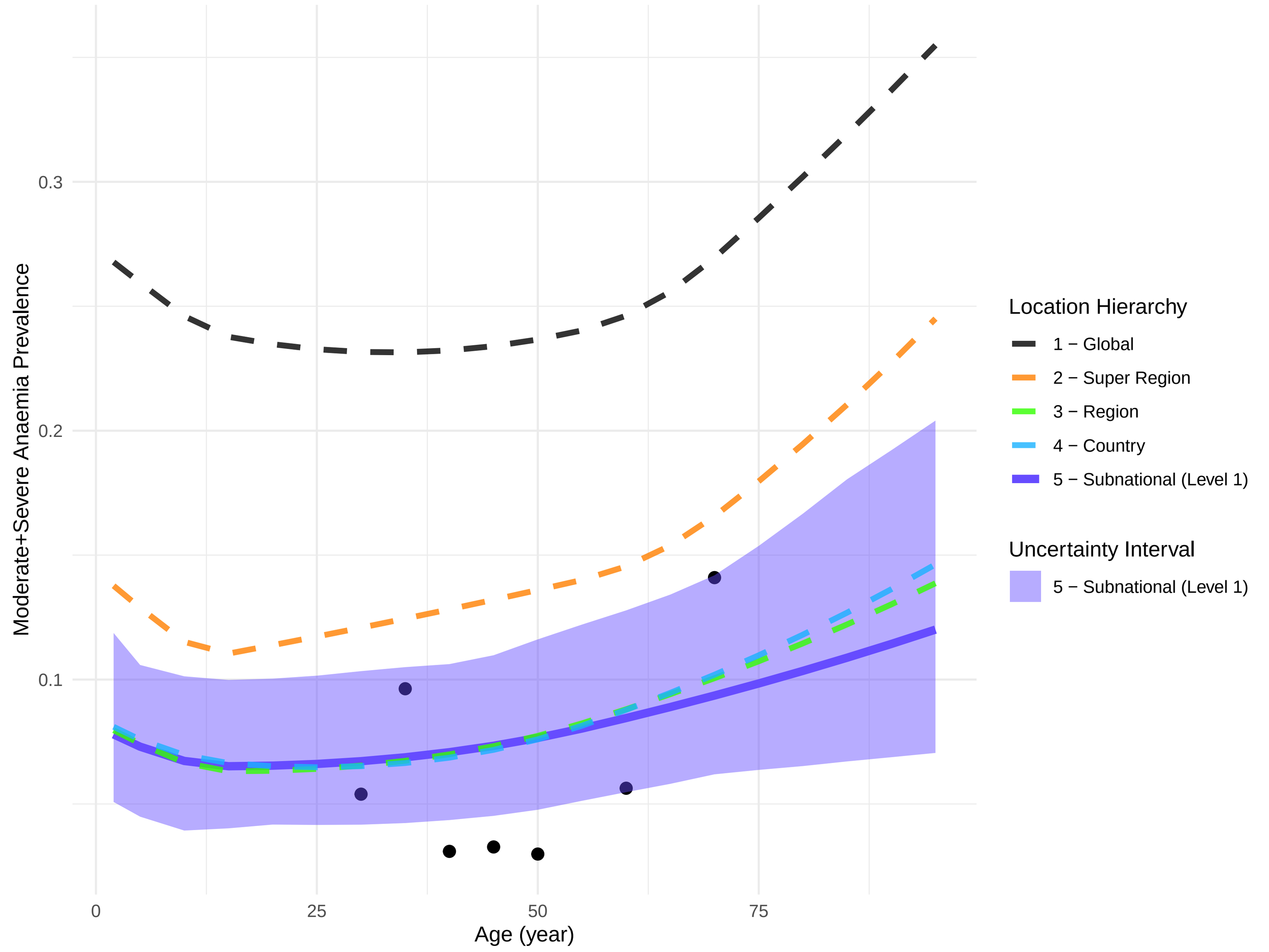

MR−BRT Age/Sex Fits − Non−pregnant Females 2+ Years Old − Moderate+Severe Anaemia Prevalence − Sergipe
WHO−2024 Adjustment Method

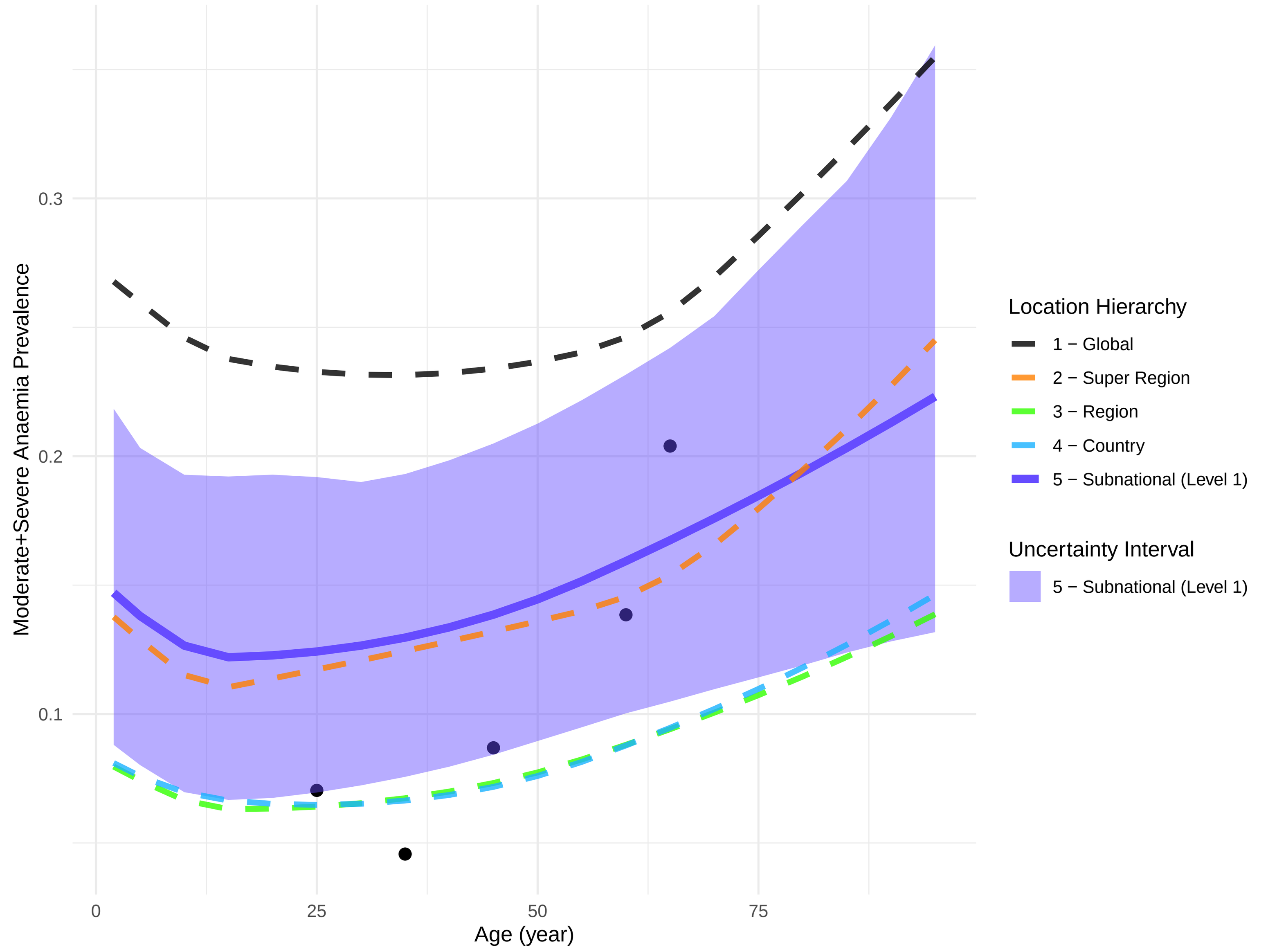

MR−BRT Age/Sex Fits − Non−pregnant Females 2+ Years Old − Moderate+Severe Anaemia Prevalence − Tocantins
WHO−2024 Adjustment Method

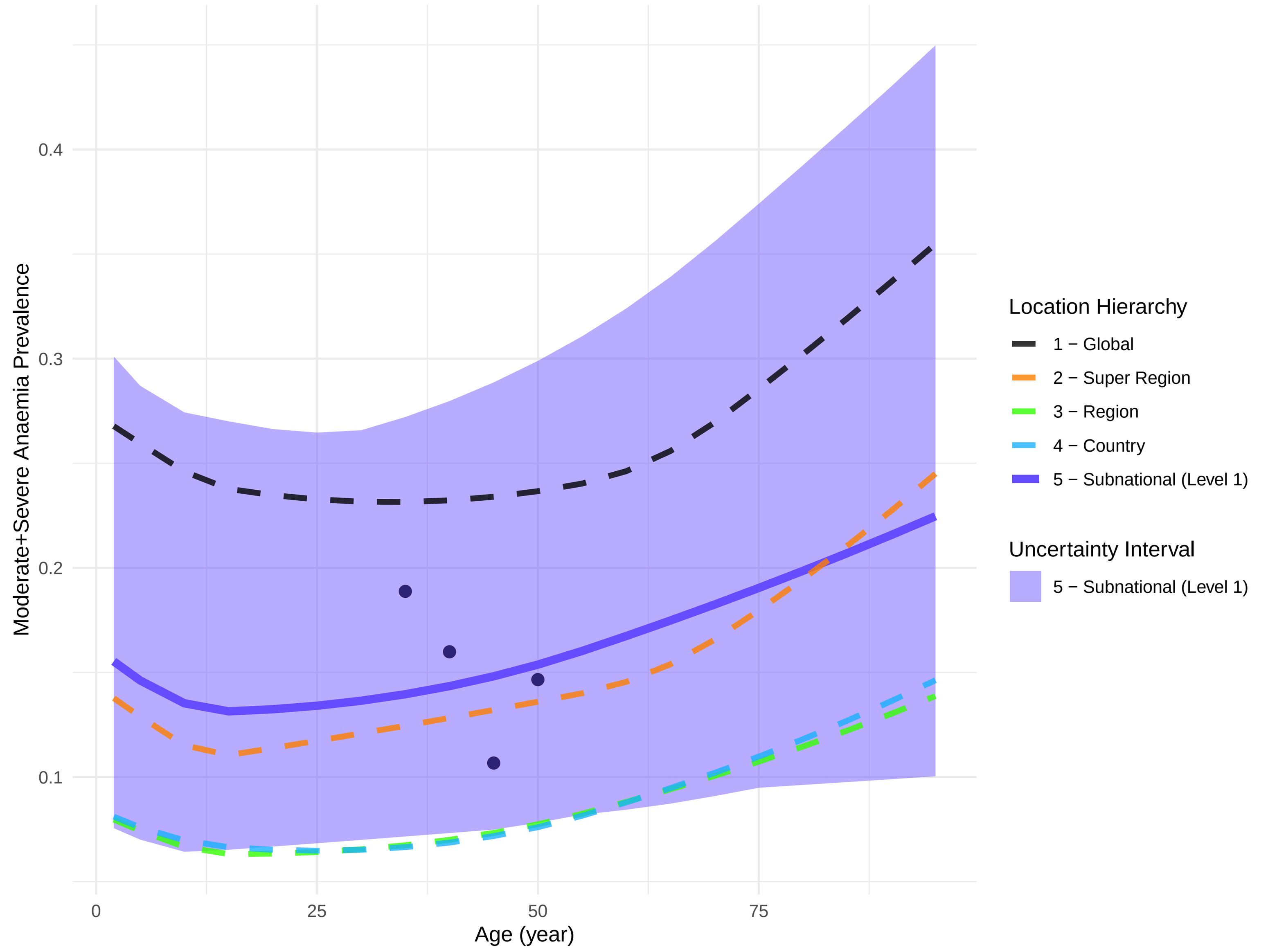

MR−BRT Age/Sex Fits − Non−pregnant Females 2+ Years Old − Moderate+Severe Anaemia Prevalence − North Africa and Middle East
WHO−2024 Adjustment Method

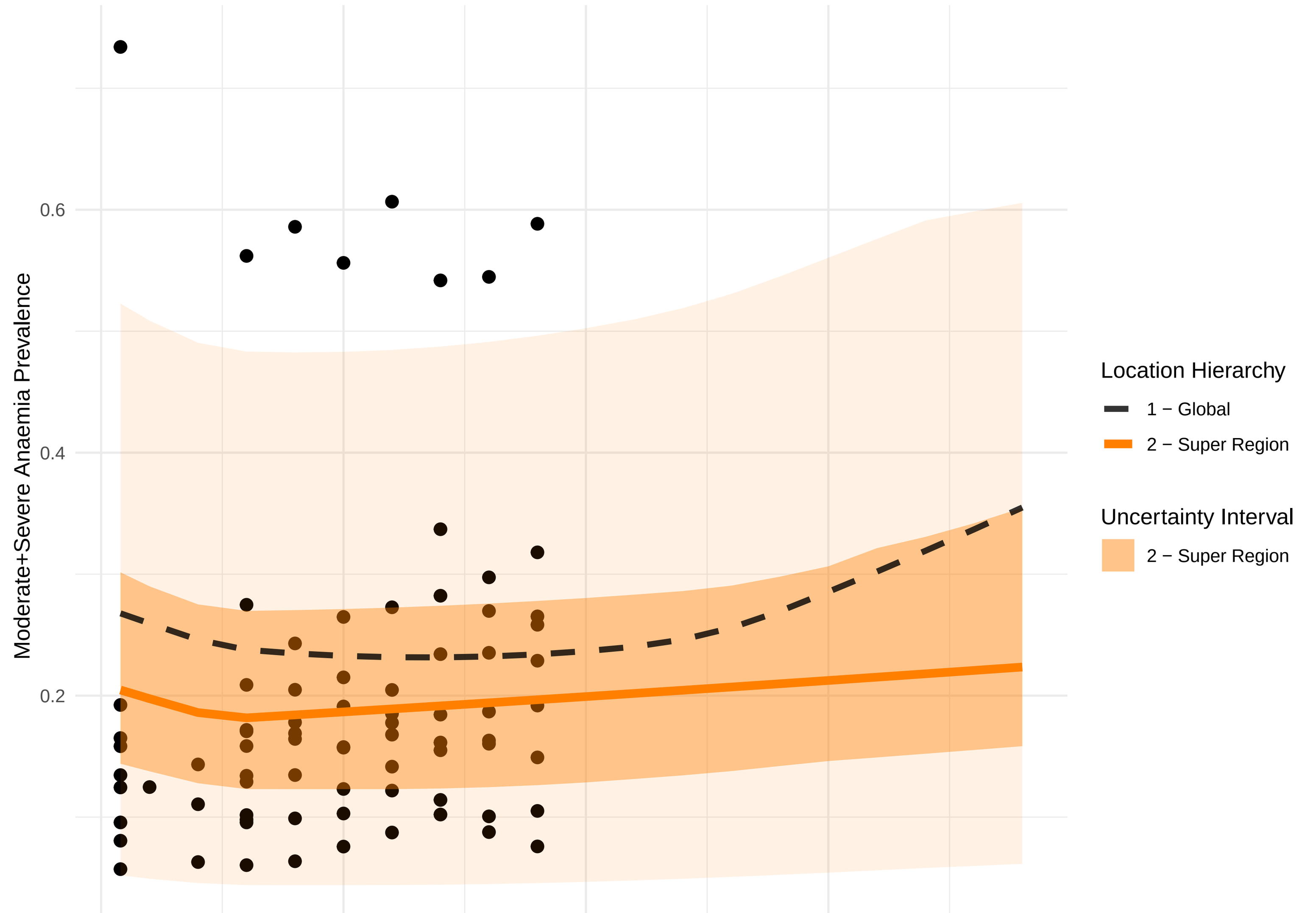

MR−BRT Age/Sex Fits − Non-pregnant Females 2+ Years Old − Moderate+Severe Anaemia Prevalence − North Africa and Middle East
WHO−2024 Adjustment Method

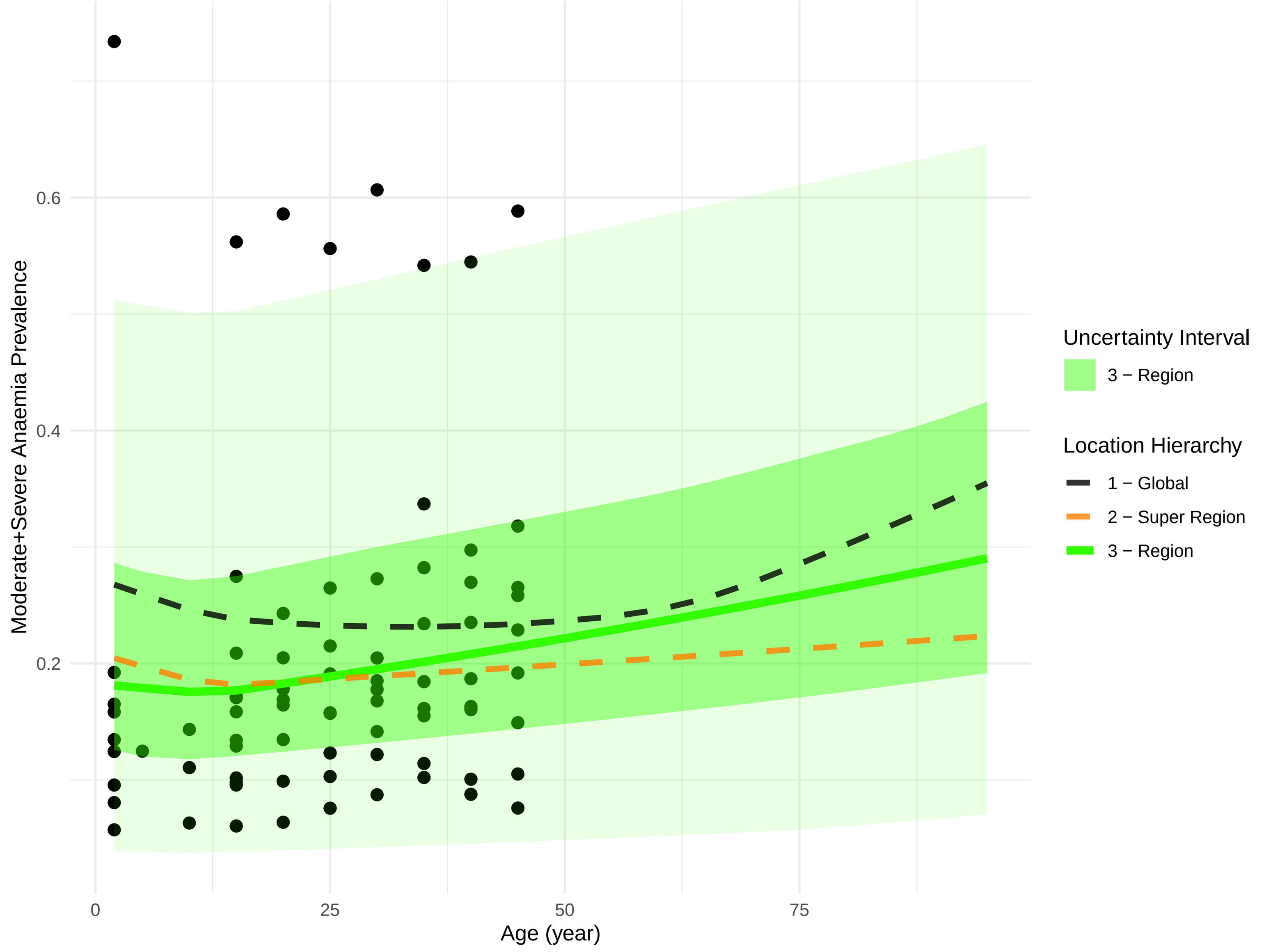

MR−BRT Age/Sex Fits − Non-pregnant Females 2+ Years Old − Moderate+Severe Anaemia Prevalence − Egypt
WHO−2024 Adjustment Method

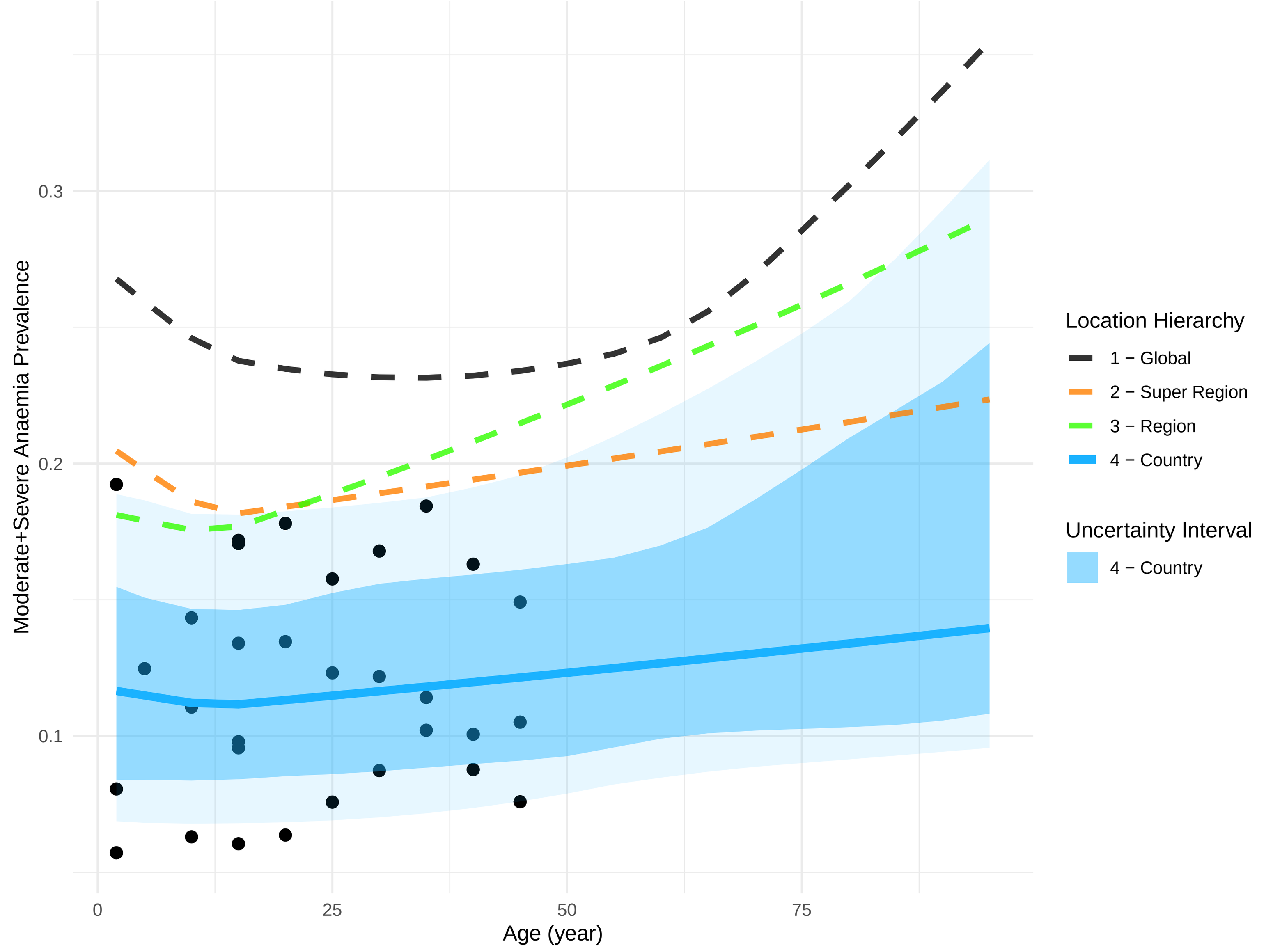

MR−BRT Age/Sex Fits − Non-pregnant Females 2+ Years Old − Moderate+Severe Anaemia Prevalence − Jordan
WHO−2024 Adjustment Method

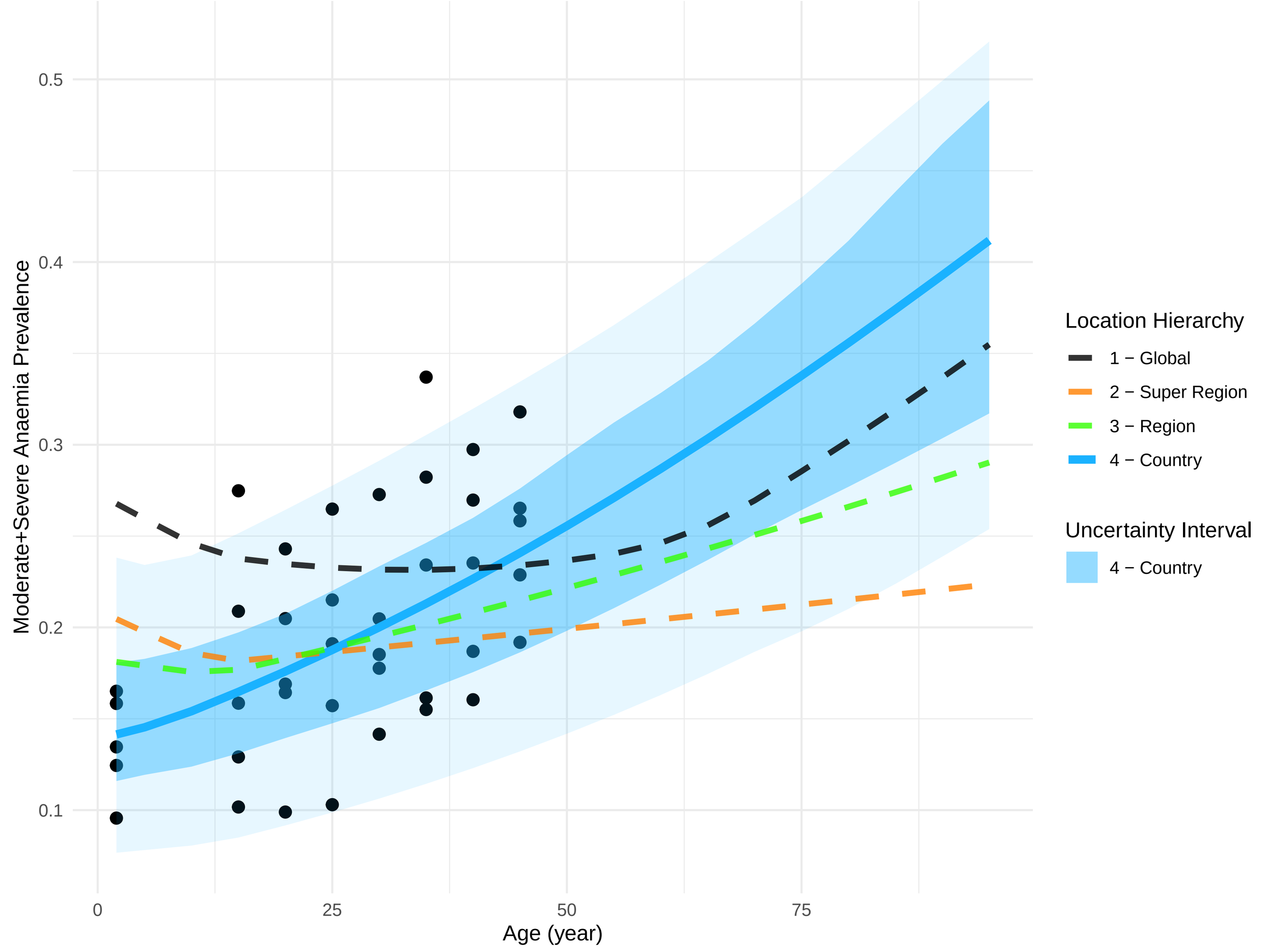

MR−BRT Age/Sex Fits − Non-pregnant Females 2+ Years Old − Moderate+Severe Anaemia Prevalence − Yemen
WHO−2024 Adjustment Method

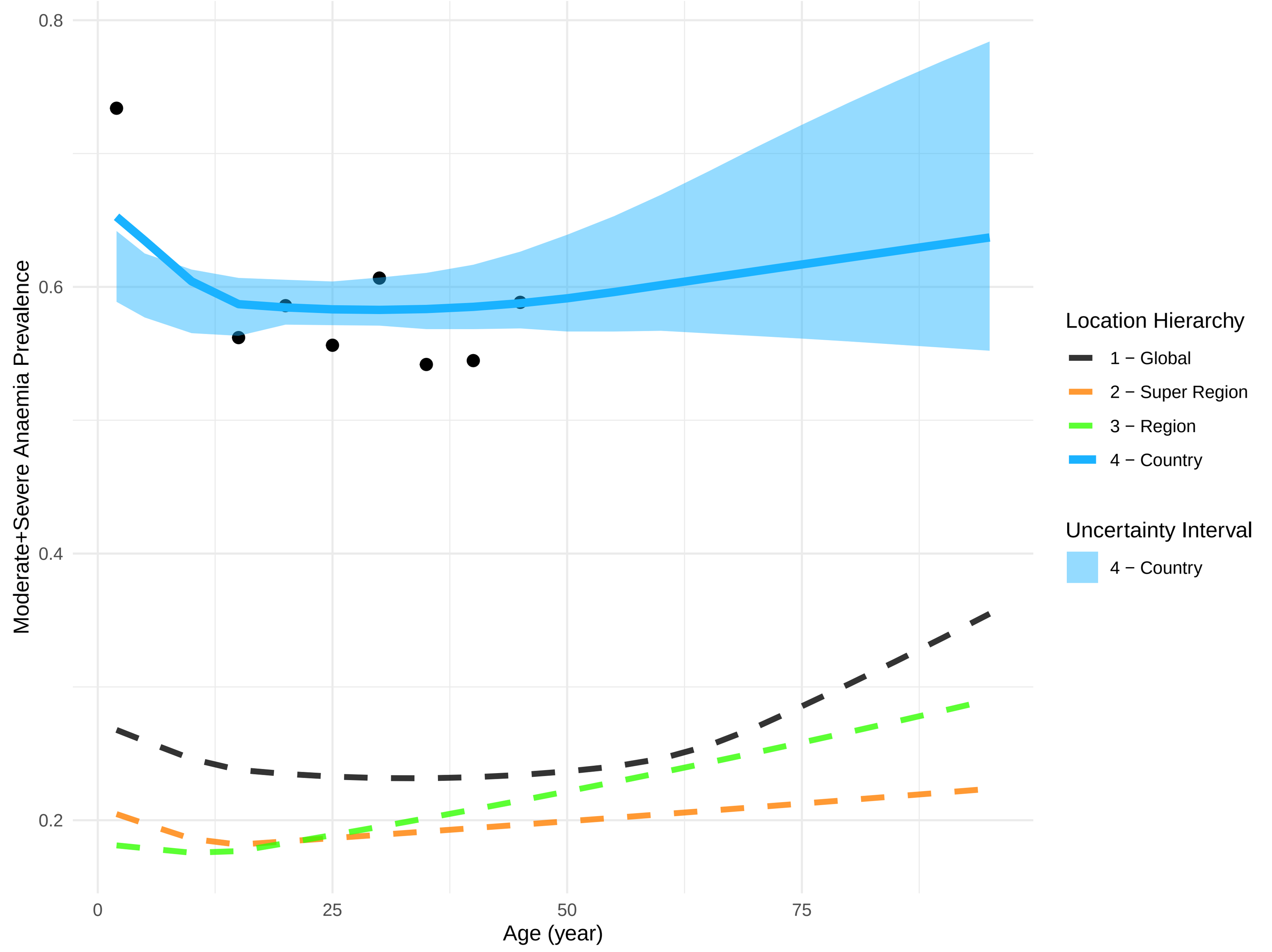

MR−BRT Age/Sex Fits − Non-pregnant Females 2+ Years Old − Moderate+Severe Anaemia Prevalence − South Asia
WHO−2024 Adjustment Method

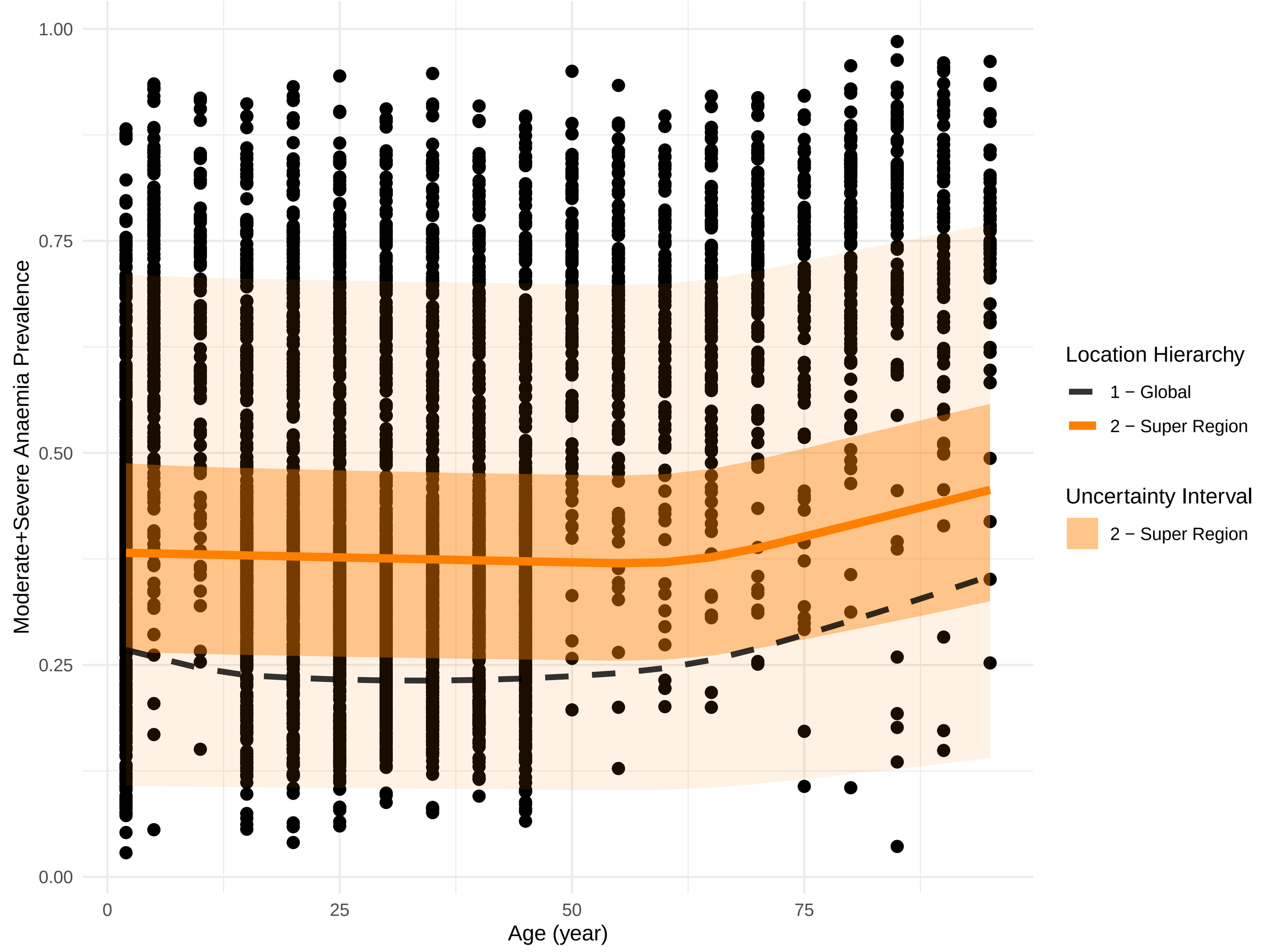

MR−BRT Age/Sex Fits − Non-pregnant Females 2+ Years Old − Moderate+Severe Anaemia Prevalence − South Asia
WHO−2024 Adjustment Method

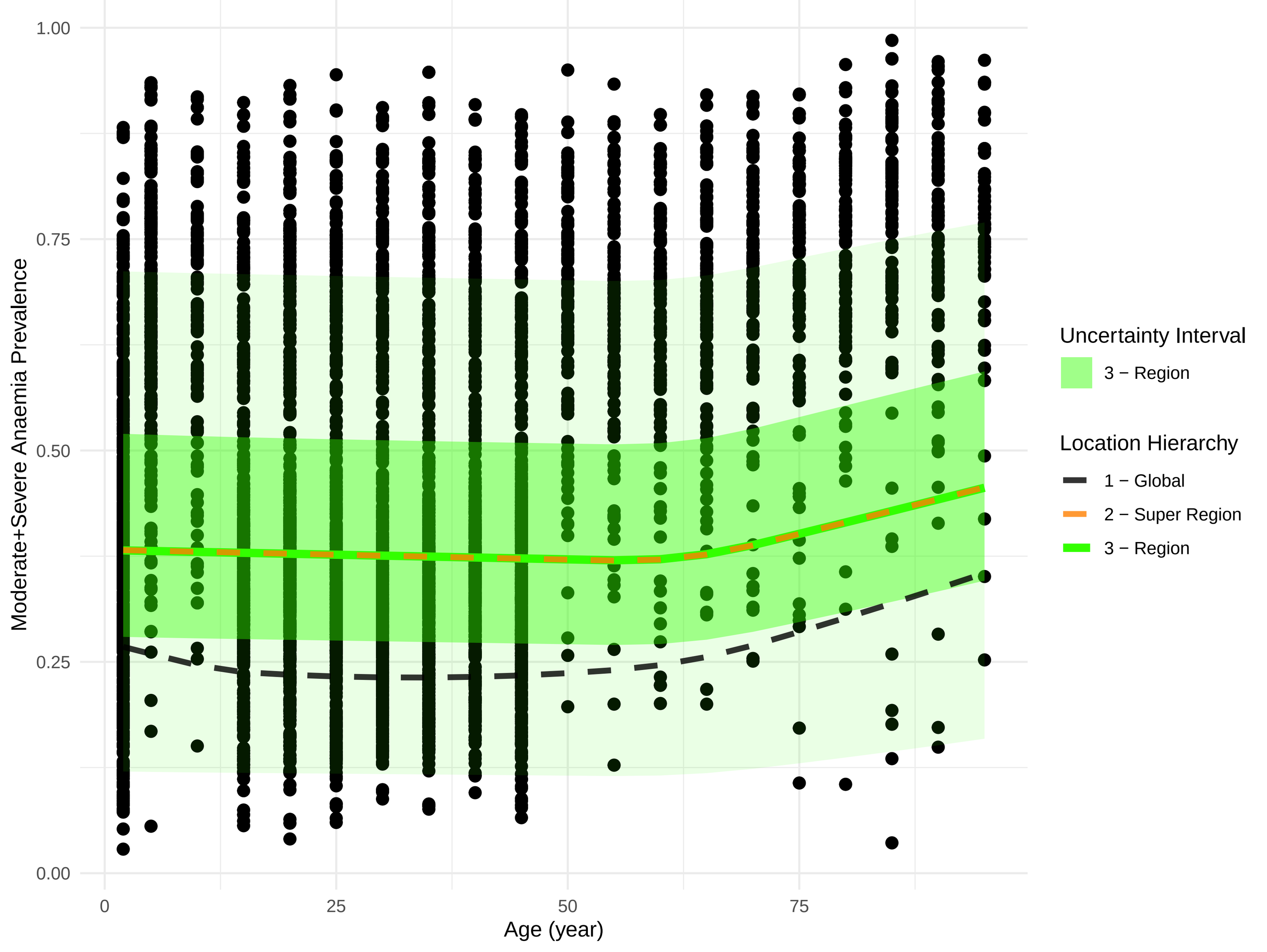

MR−BRT Age/Sex Fits − Non-pregnant Females 2+ Years Old − Moderate+Severe Anaemia Prevalence − Bangladesh
WHO−2024 Adjustment Method

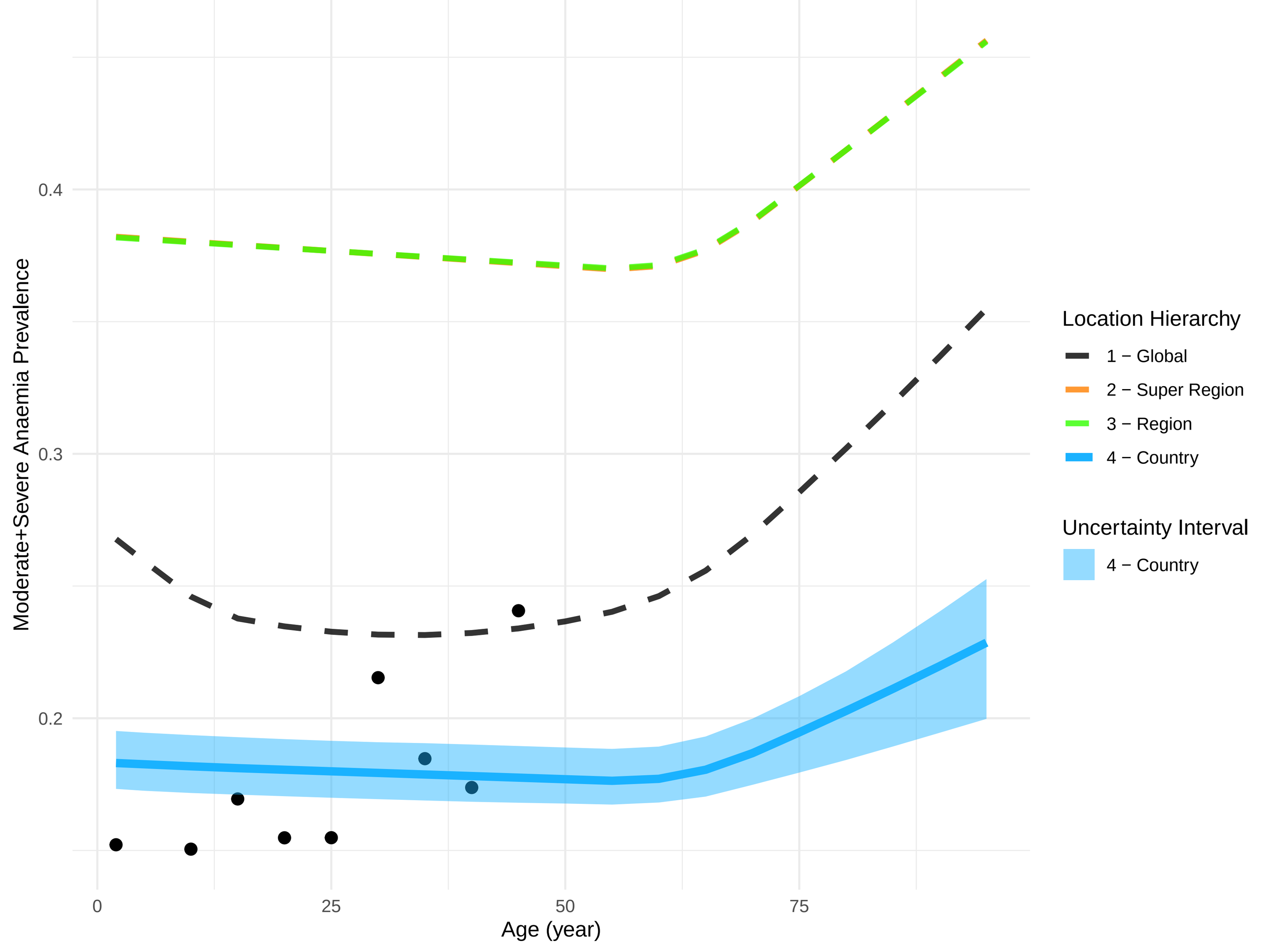

MR−BRT Age/Sex Fits − Non-pregnant Females 2+ Years Old − Moderate+Severe Anaemia Prevalence − India
WHO−2024 Adjustment Method

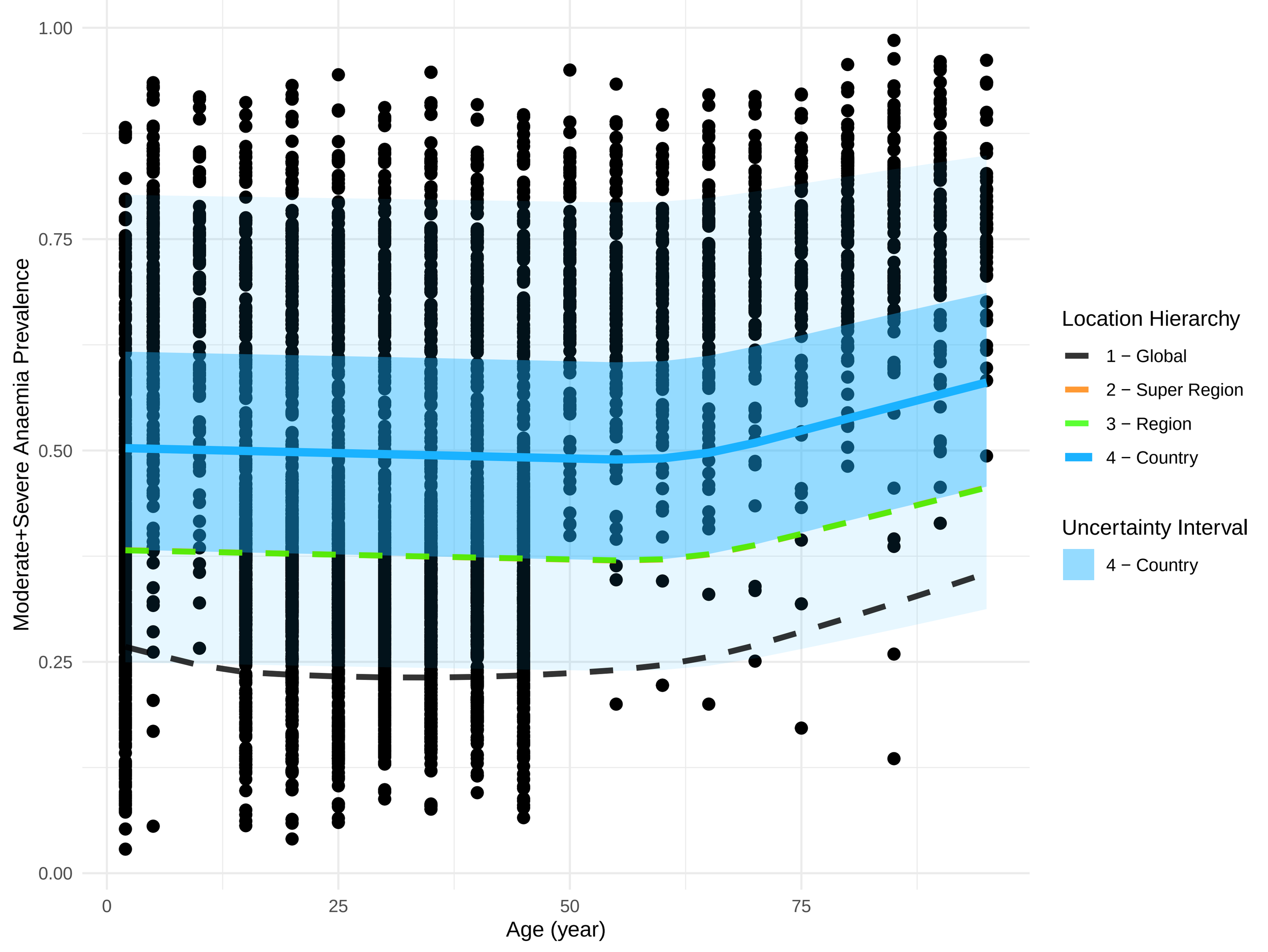

MR−BRT Age/Sex Fits − Non-pregnant Females 2+ Years Old − Moderate+Severe Anaemia Prevalence − Andhra Pradesh
WHO−2024 Adjustment Method

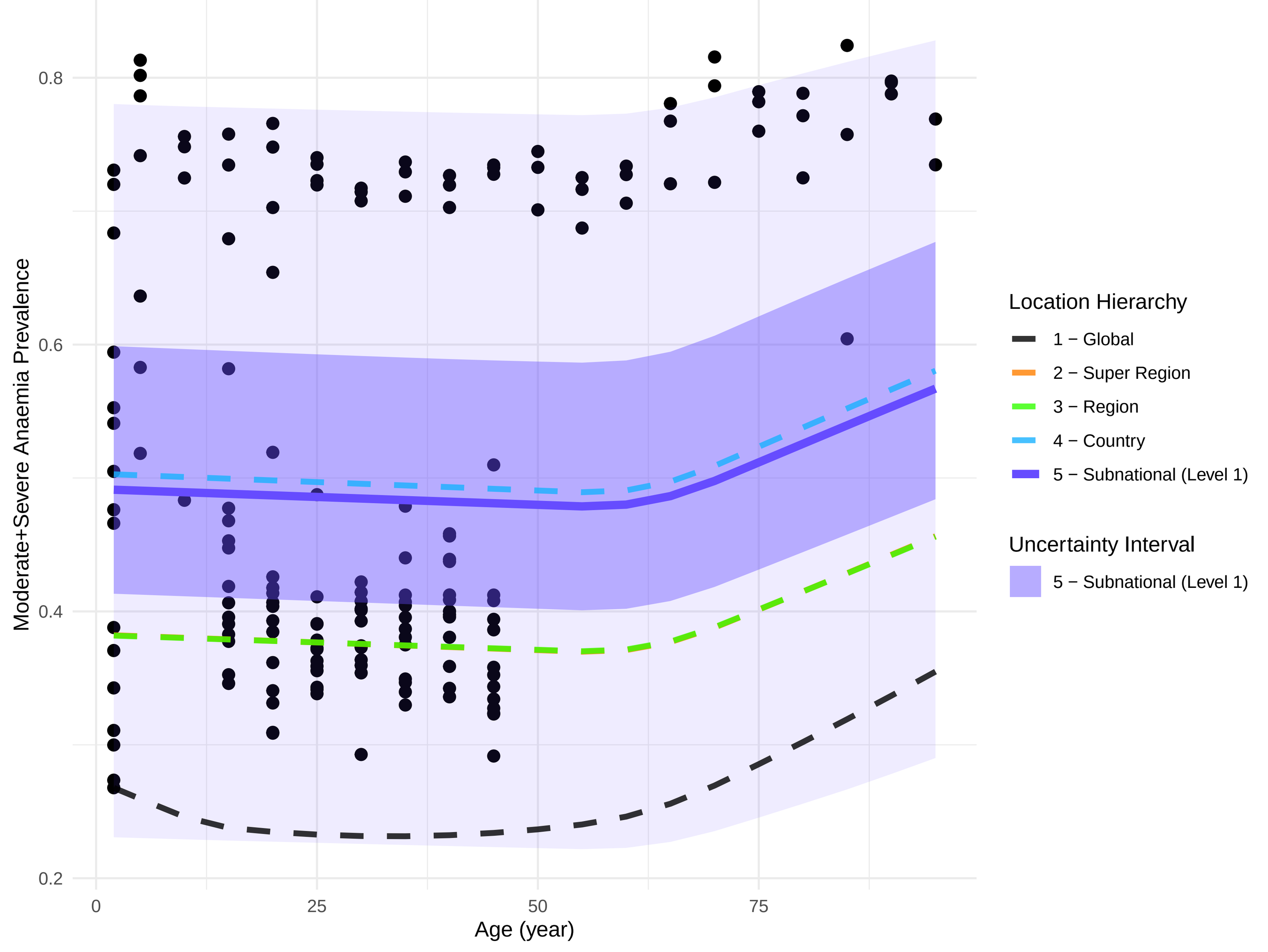

MR−BRT Age/Sex Fits − Non-pregnant Females 2+ Years Old − Moderate+Severe Anaemia Prevalence − Andhra Pradesh, Rural
WHO−2024 Adjustment Method

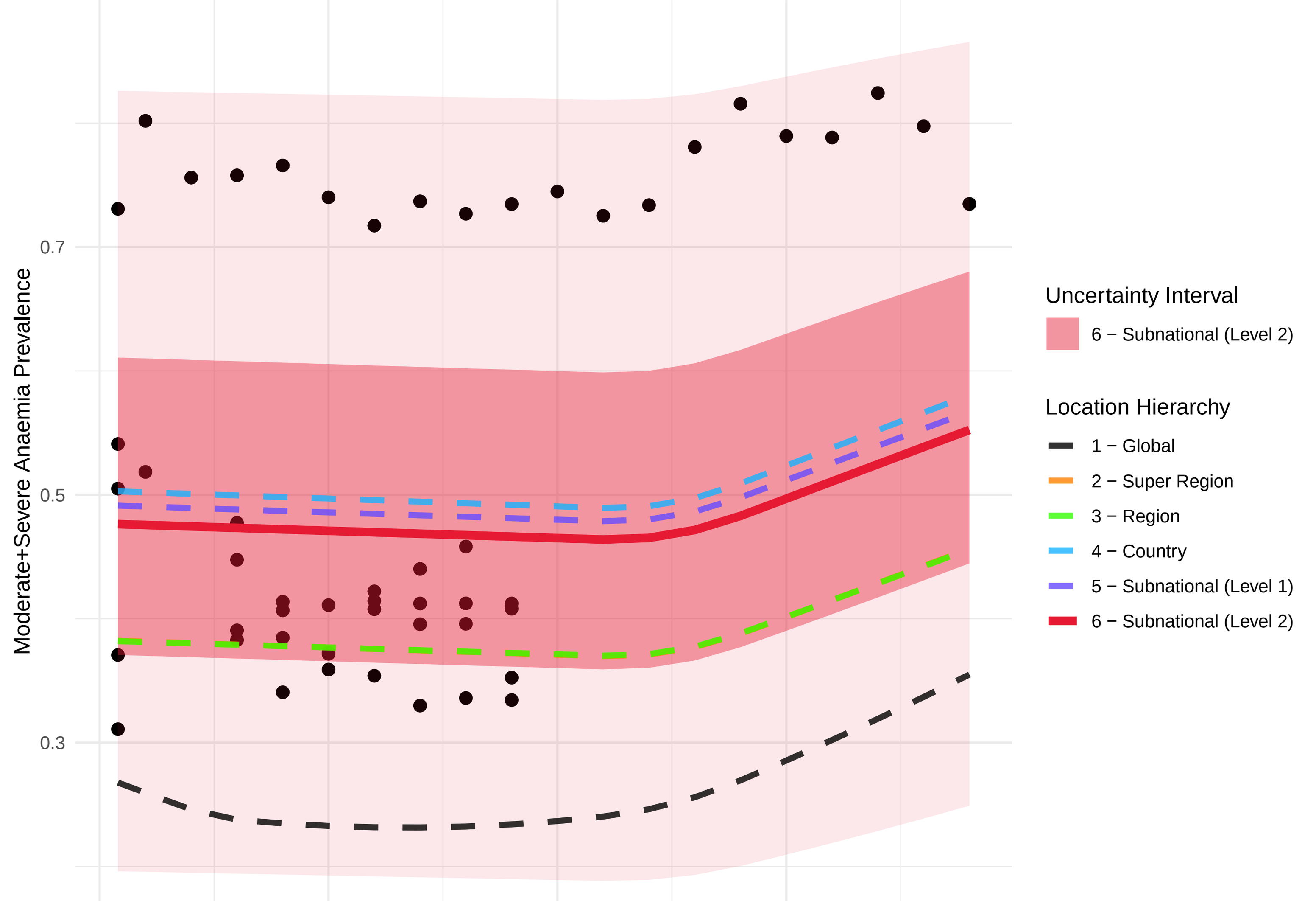

MR−BRT Age/Sex Fits − Non−pregnant Females 2+ Years Old − Moderate+Severe Anaemia Prevalence − Andhra Pradesh, Urban
WHO−2024 Adjustment Method

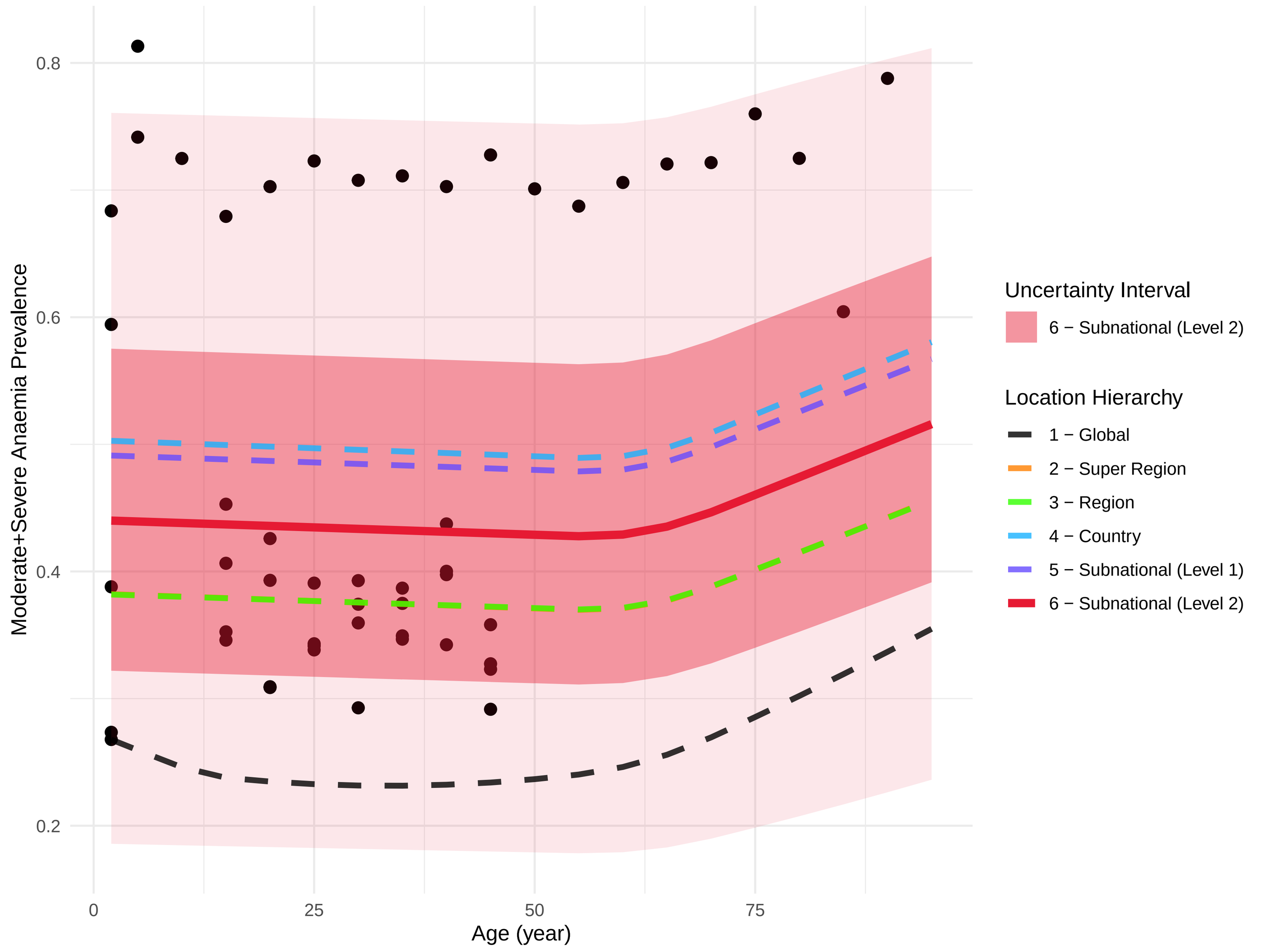

MR−BRT Age/Sex Fits − Non−pregnant Females 2+ Years Old − Moderate+Severe Anaemia Prevalence − Arunachal Pradesh
WHO−2024 Adjustment Method

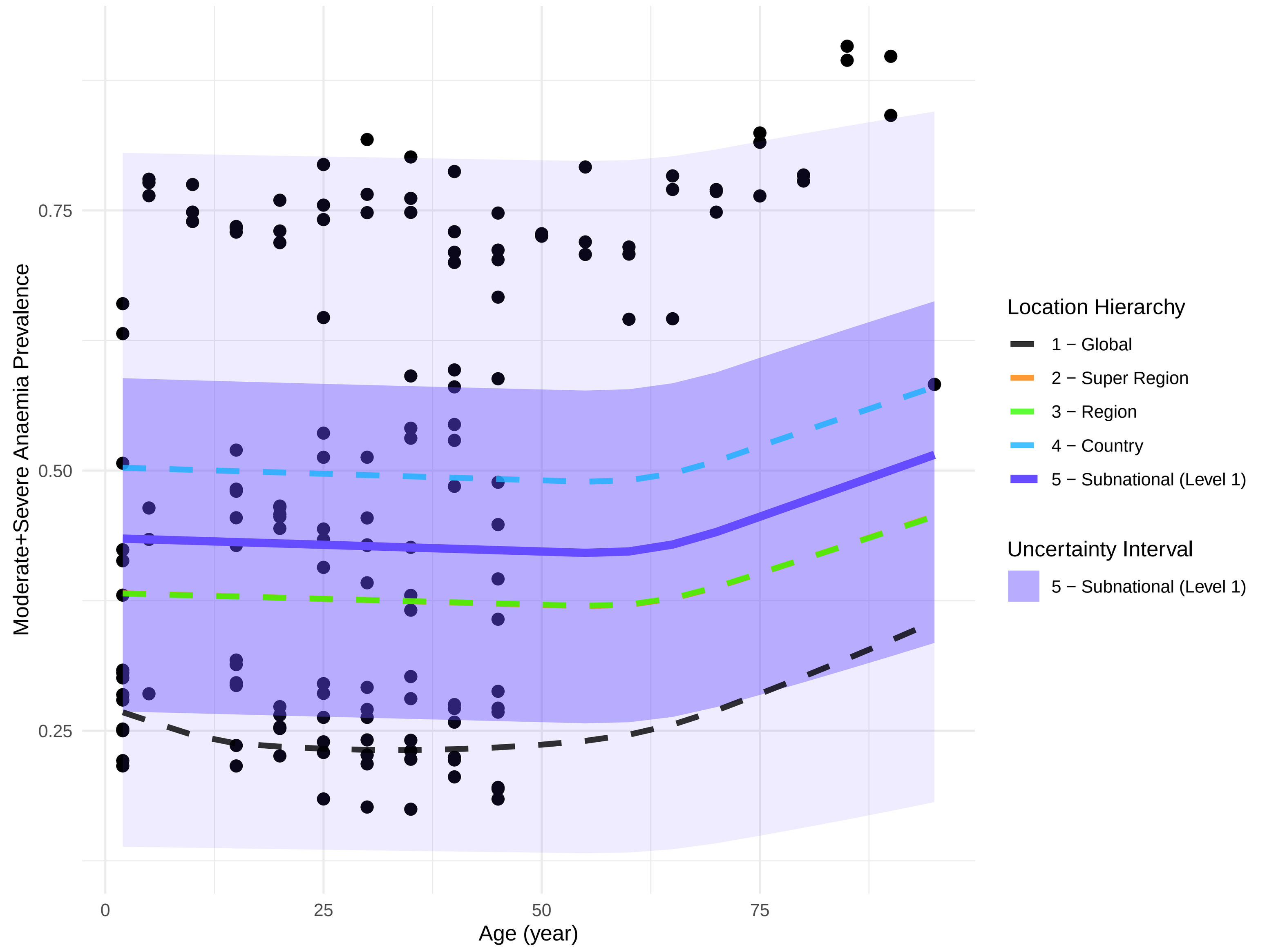

MR−BRT Age/Sex Fits − Non−pregnant Females 2+ Years Old − Moderate+Severe Anaemia Prevalence − Arunachal Pradesh, Rural
WHO−2024 Adjustment Method

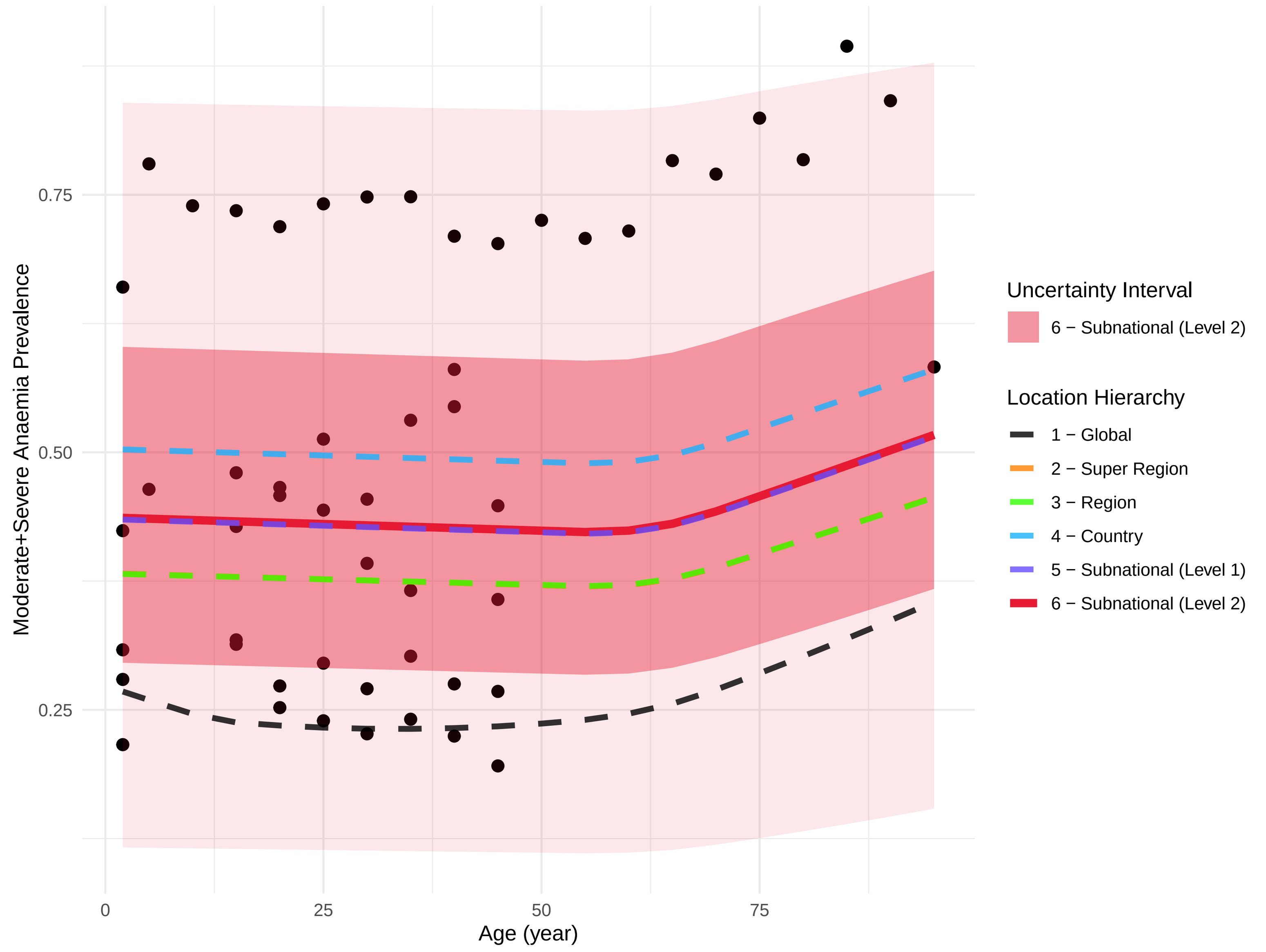

MR−BRT Age/Sex Fits − Non−pregnant Females 2+ Years Old − Moderate+Severe Anaemia Prevalence − Arunachal Pradesh, Urban
WHO−2024 Adjustment Method

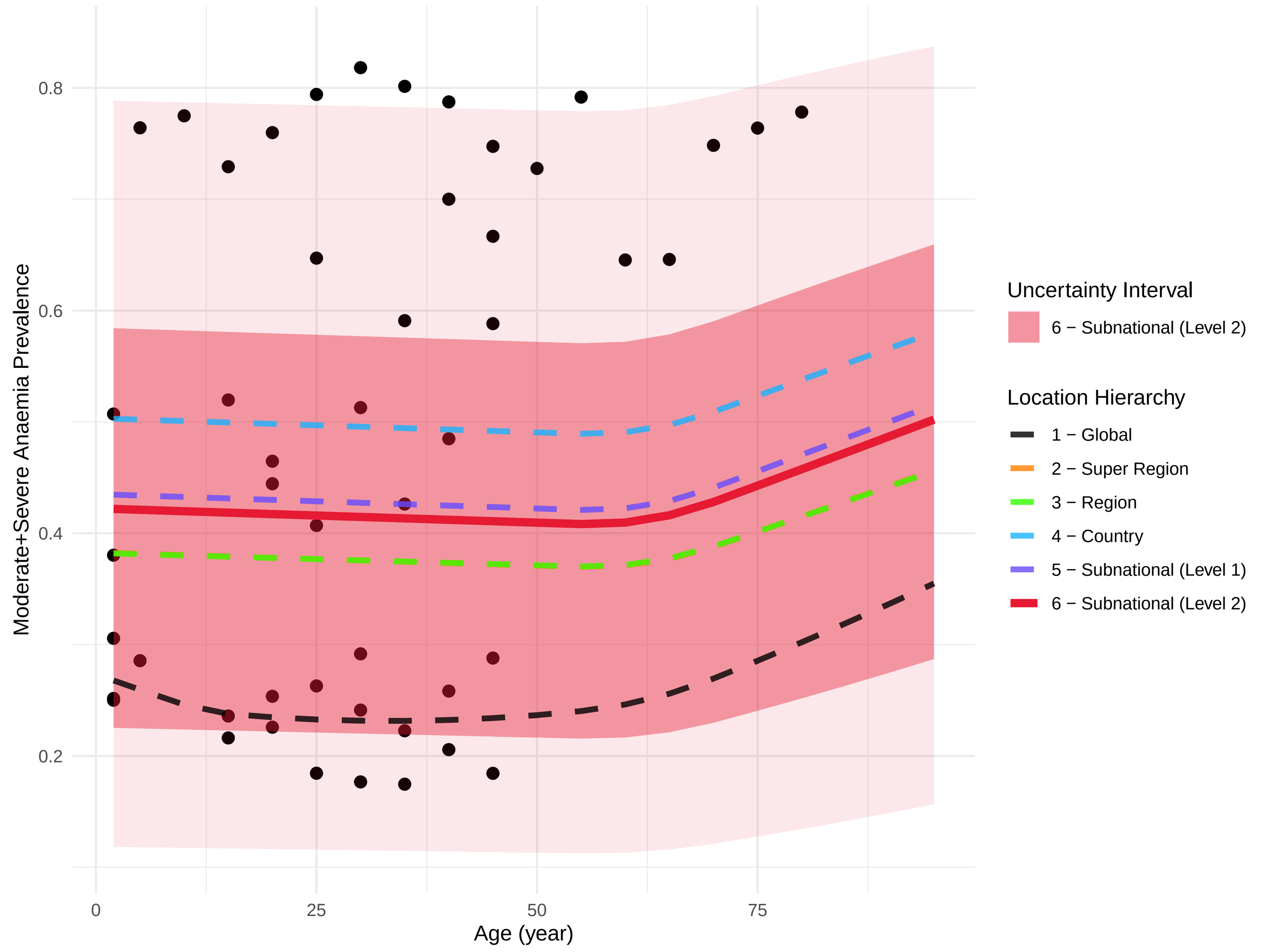

MR−BRT Age/Sex Fits − Non−pregnant Females 2+ Years Old − Moderate+Severe Anaemia Prevalence − Assam
WHO−2024 Adjustment Method

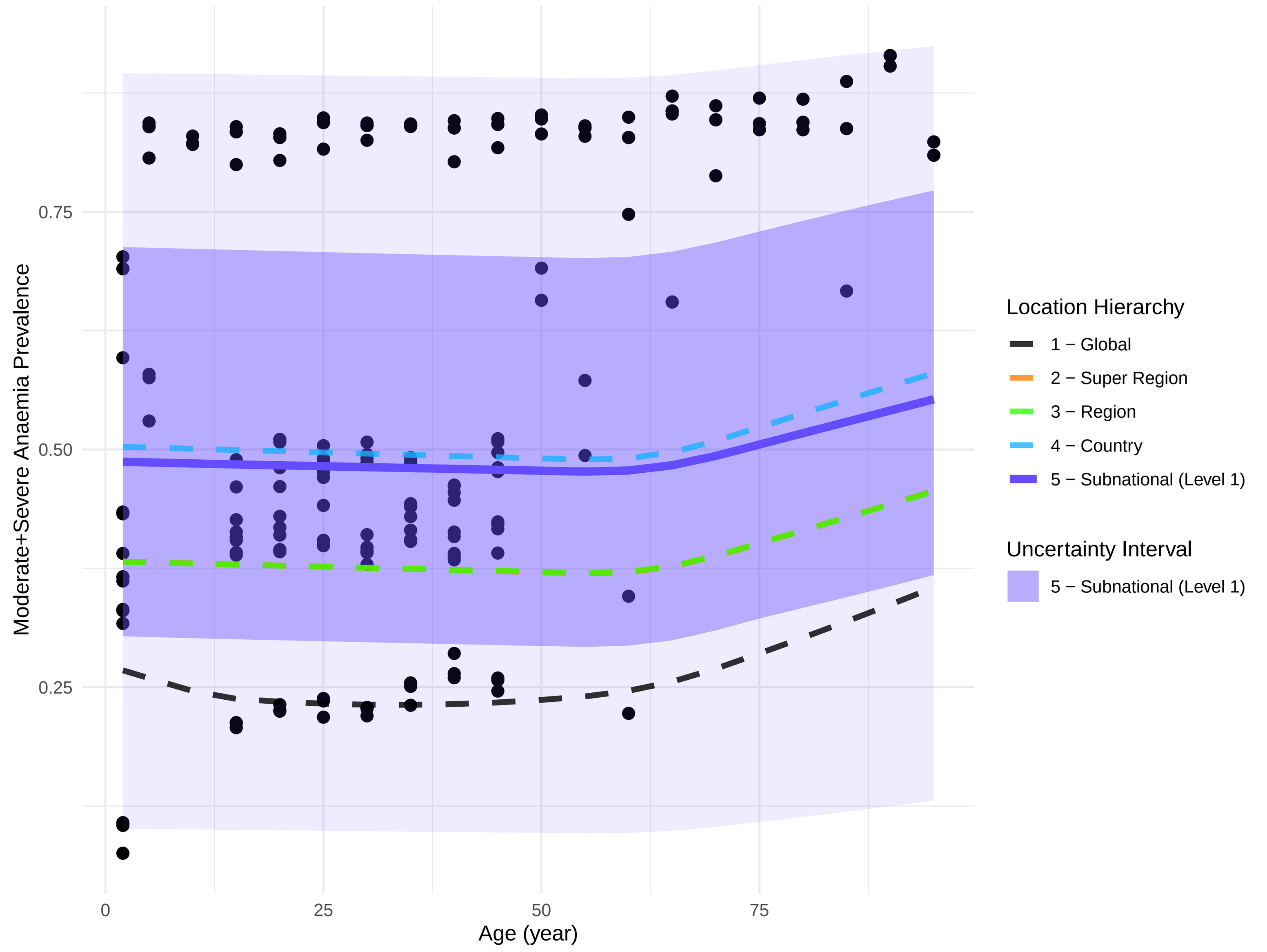

MR−BRT Age/Sex Fits − Non−pregnant Females 2+ Years Old − Moderate+Severe Anaemia Prevalence − Assam, Rural
WHO−2024 Adjustment Method

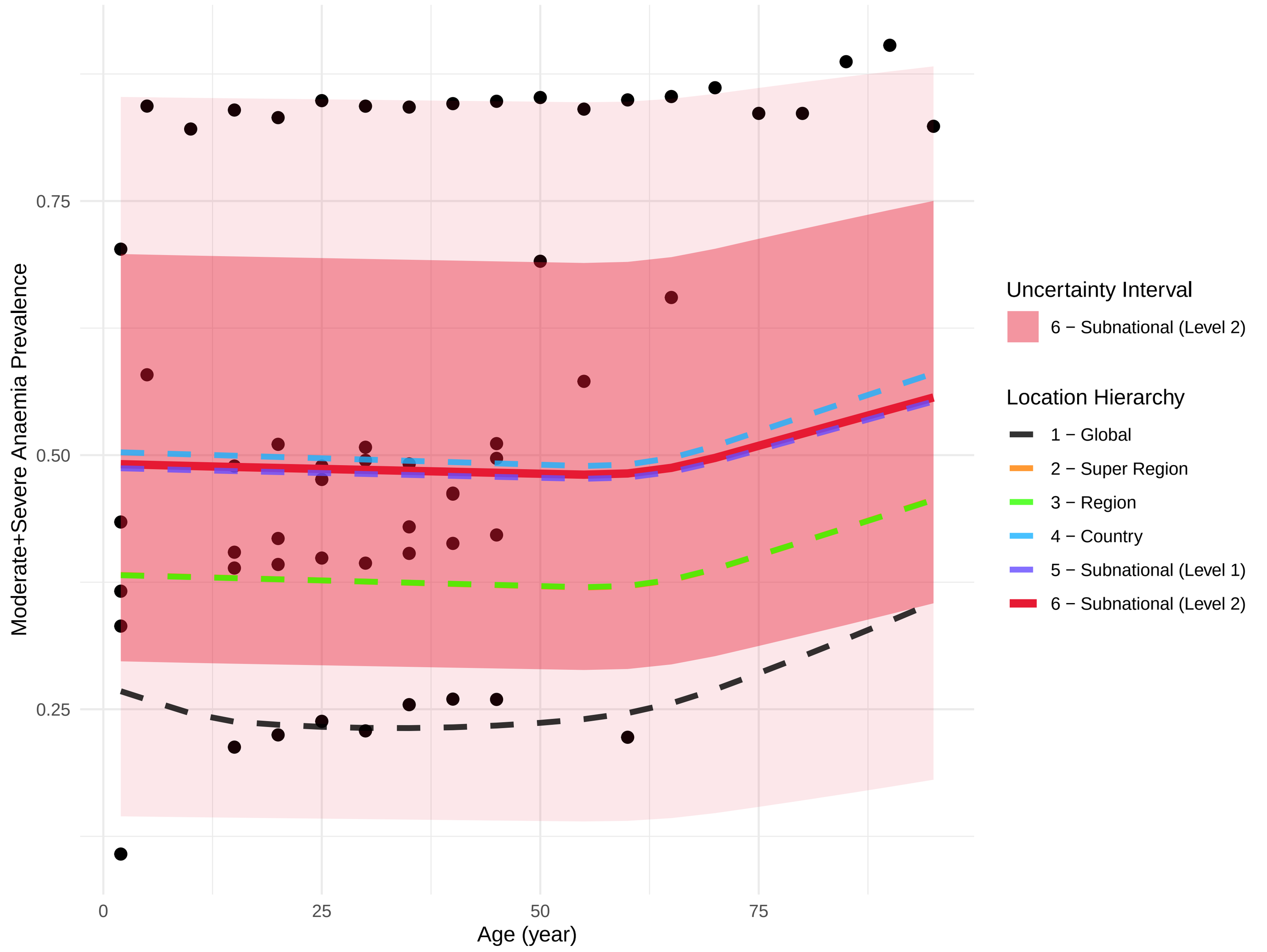

MR−BRT Age/Sex Fits − Non−pregnant Females 2+ Years Old − Moderate+Severe Anaemia Prevalence − Assam, Urban
WHO−2024 Adjustment Method

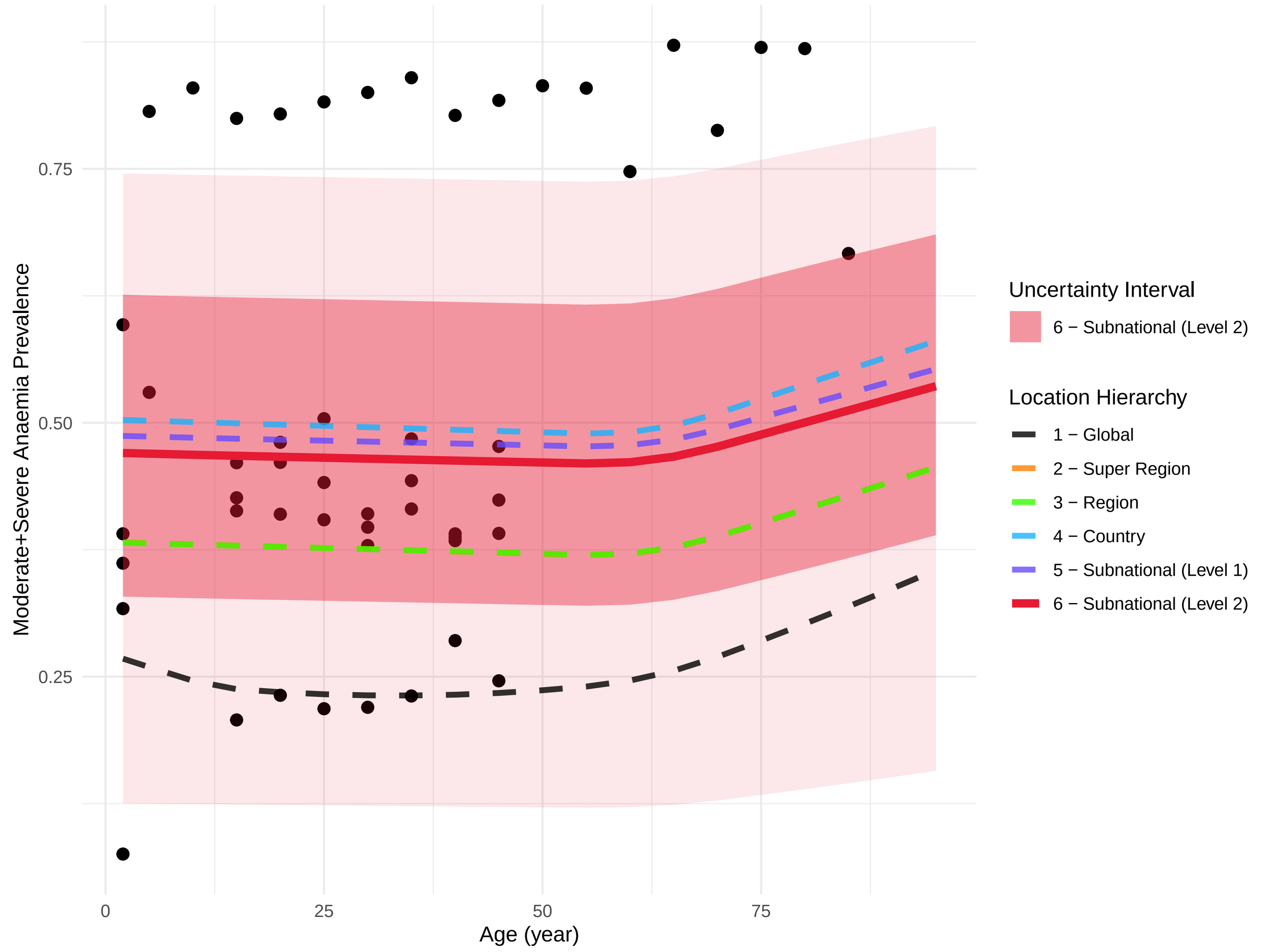

MR−BRT Age/Sex Fits − Non−pregnant Females 2+ Years Old − Moderate+Severe Anaemia Prevalence − Bihar
WHO−2024 Adjustment Method

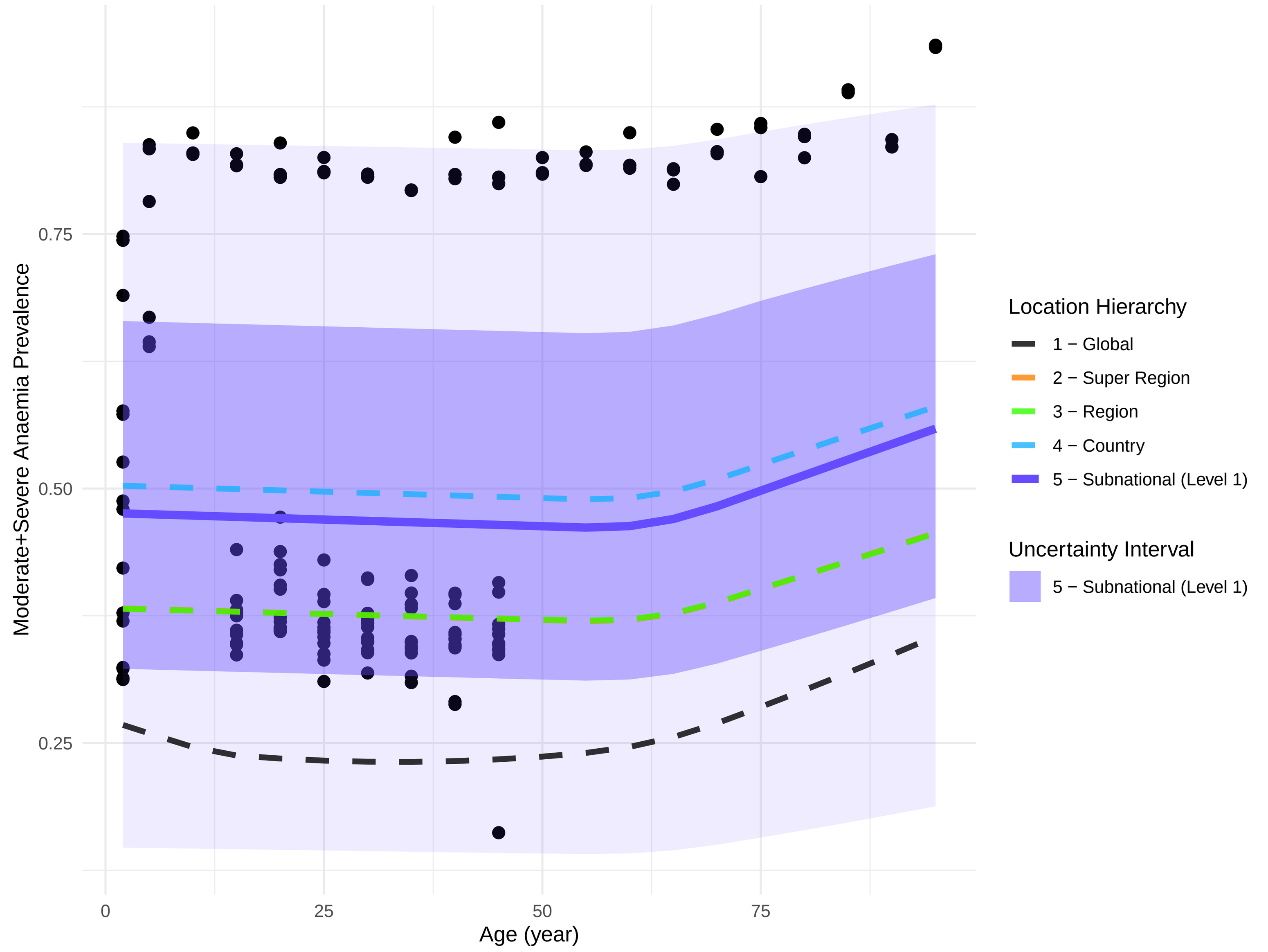

MR−BRT Age/Sex Fits − Non−pregnant Females 2+ Years Old − Moderate+Severe Anaemia Prevalence − Bihar, Rural
WHO−2024 Adjustment Method

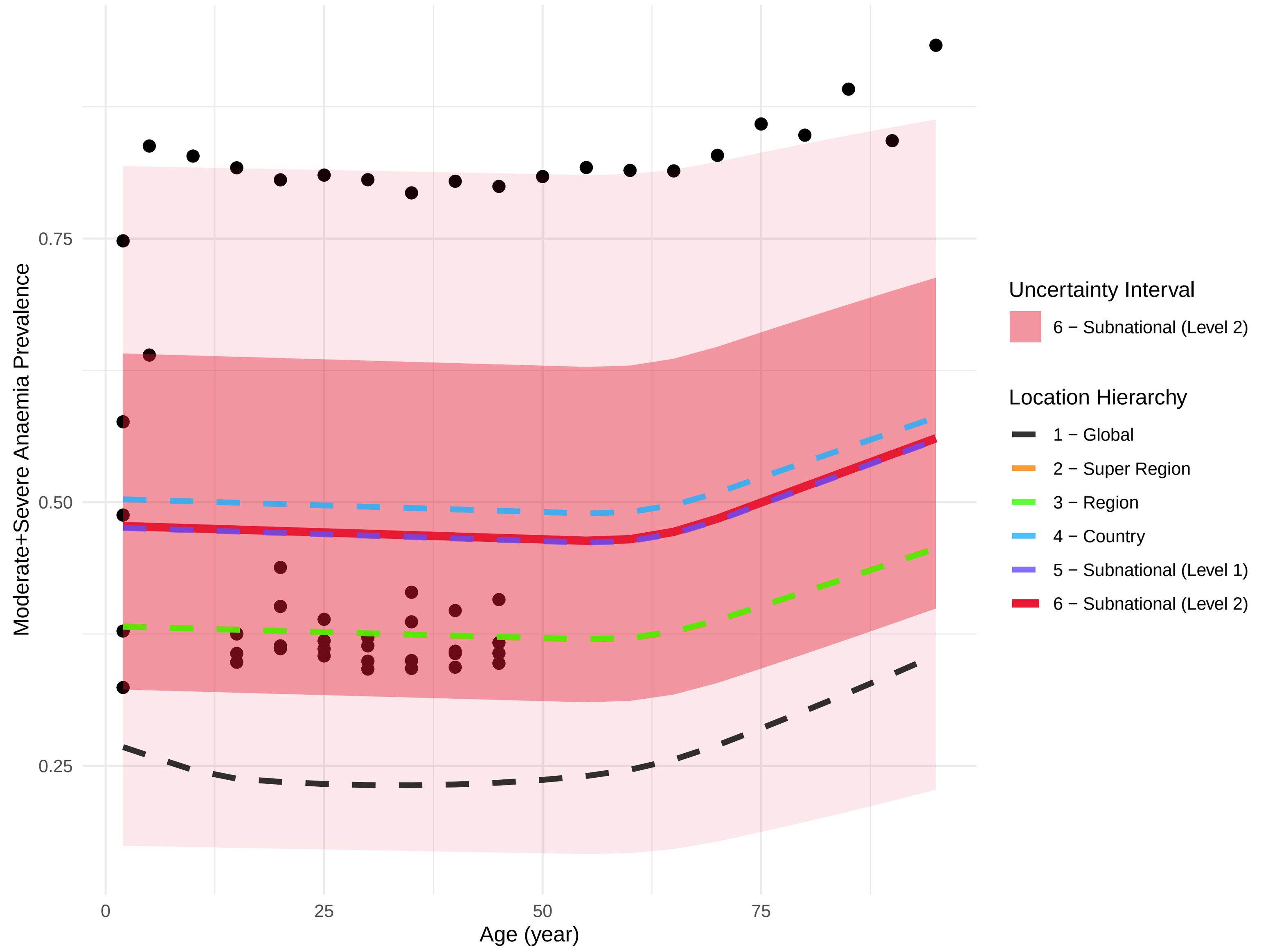

MR−BRT Age/Sex Fits − Non−pregnant Females 2+ Years Old − Moderate+Severe Anaemia Prevalence − Bihar, Urban
WHO−2024 Adjustment Method

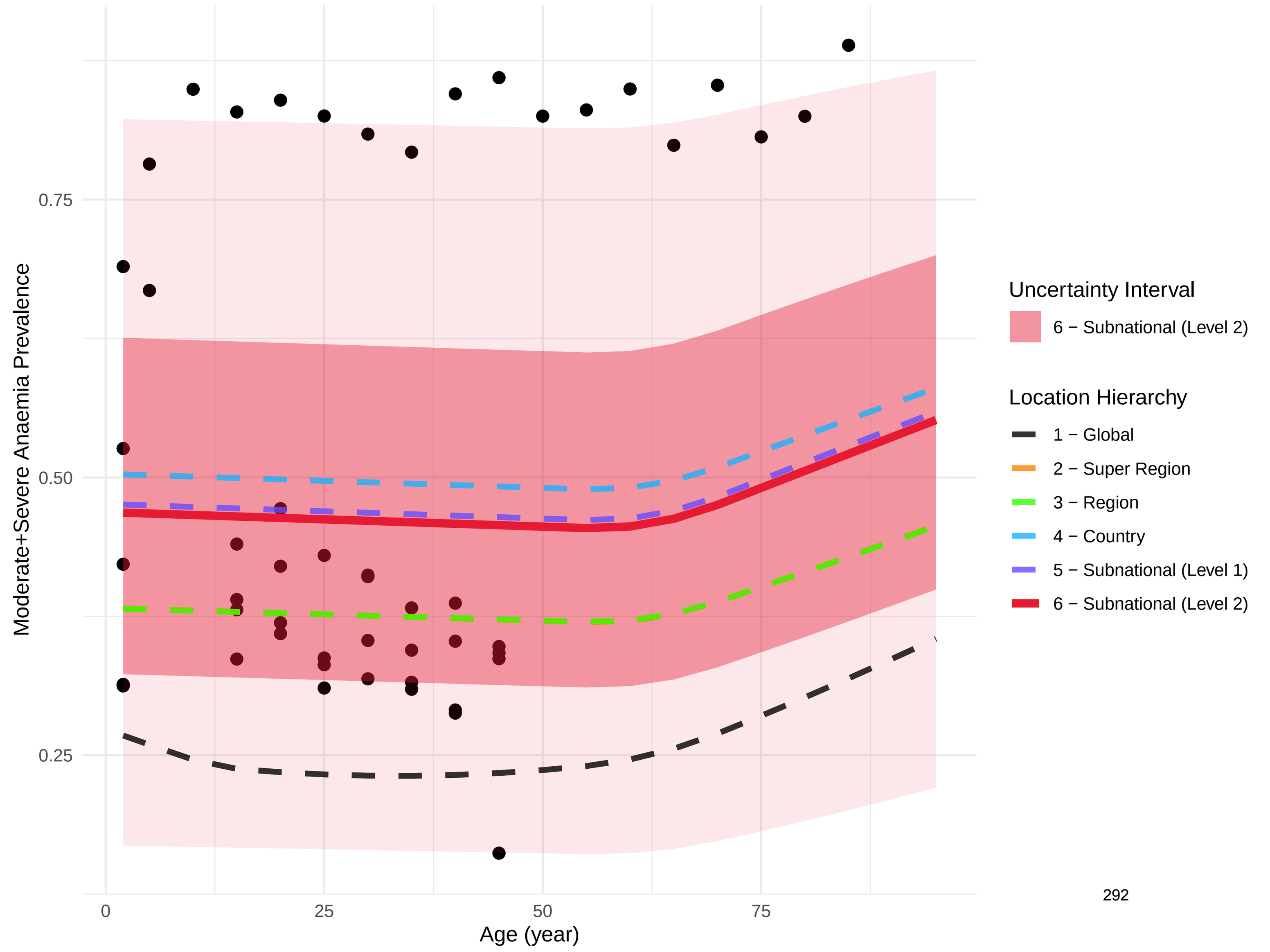

MR−BRT Age/Sex Fits − Non−pregnant Females 2+ Years Old − Moderate+Severe Anaemia Prevalence − Chhattisgarh
WHO−2024 Adjustment Method

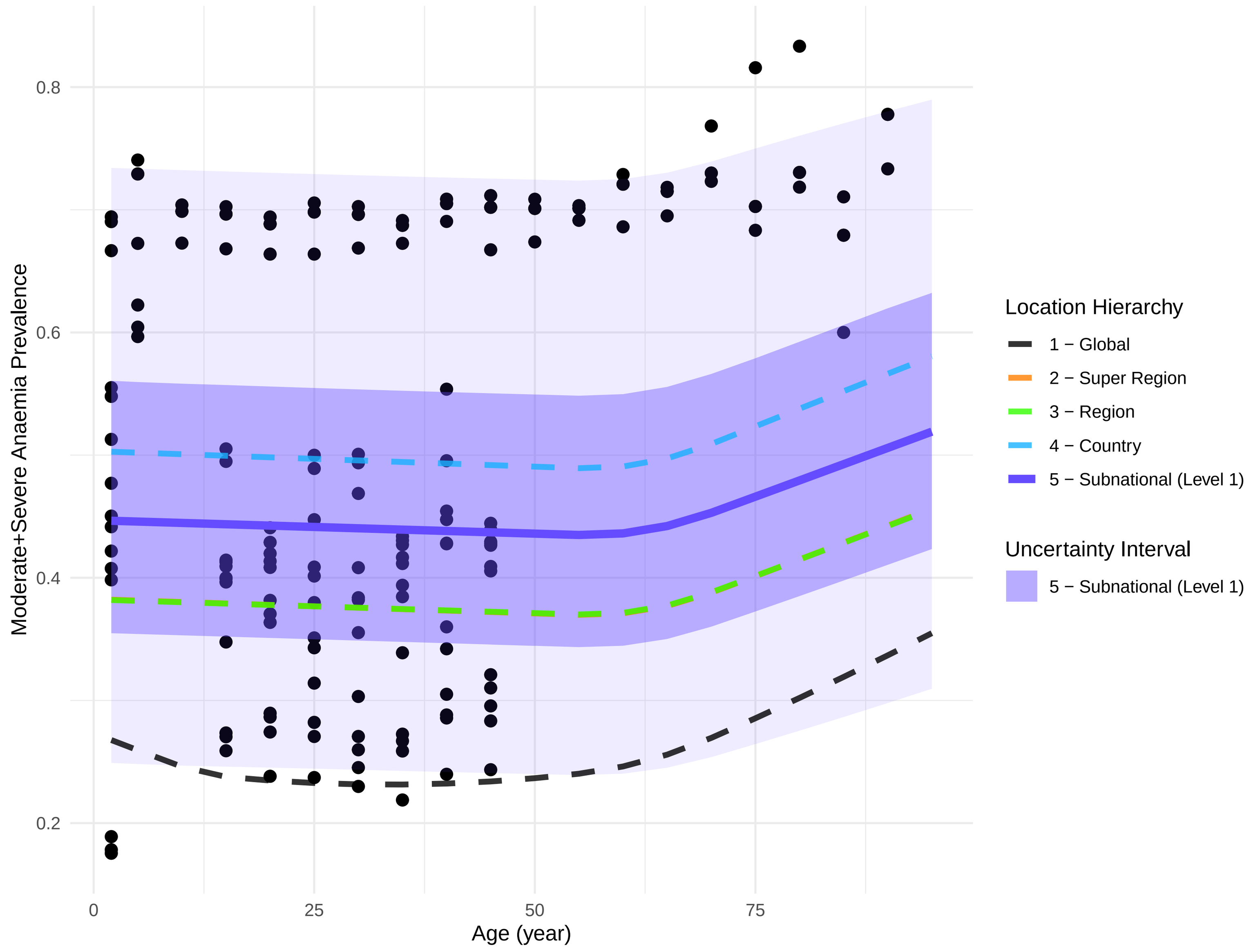

MR−BRT Age/Sex Fits − Non−pregnant Females 2+ Years Old − Moderate+Severe Anaemia Prevalence − Chhattisgarh, Rural
WHO−2024 Adjustment Method

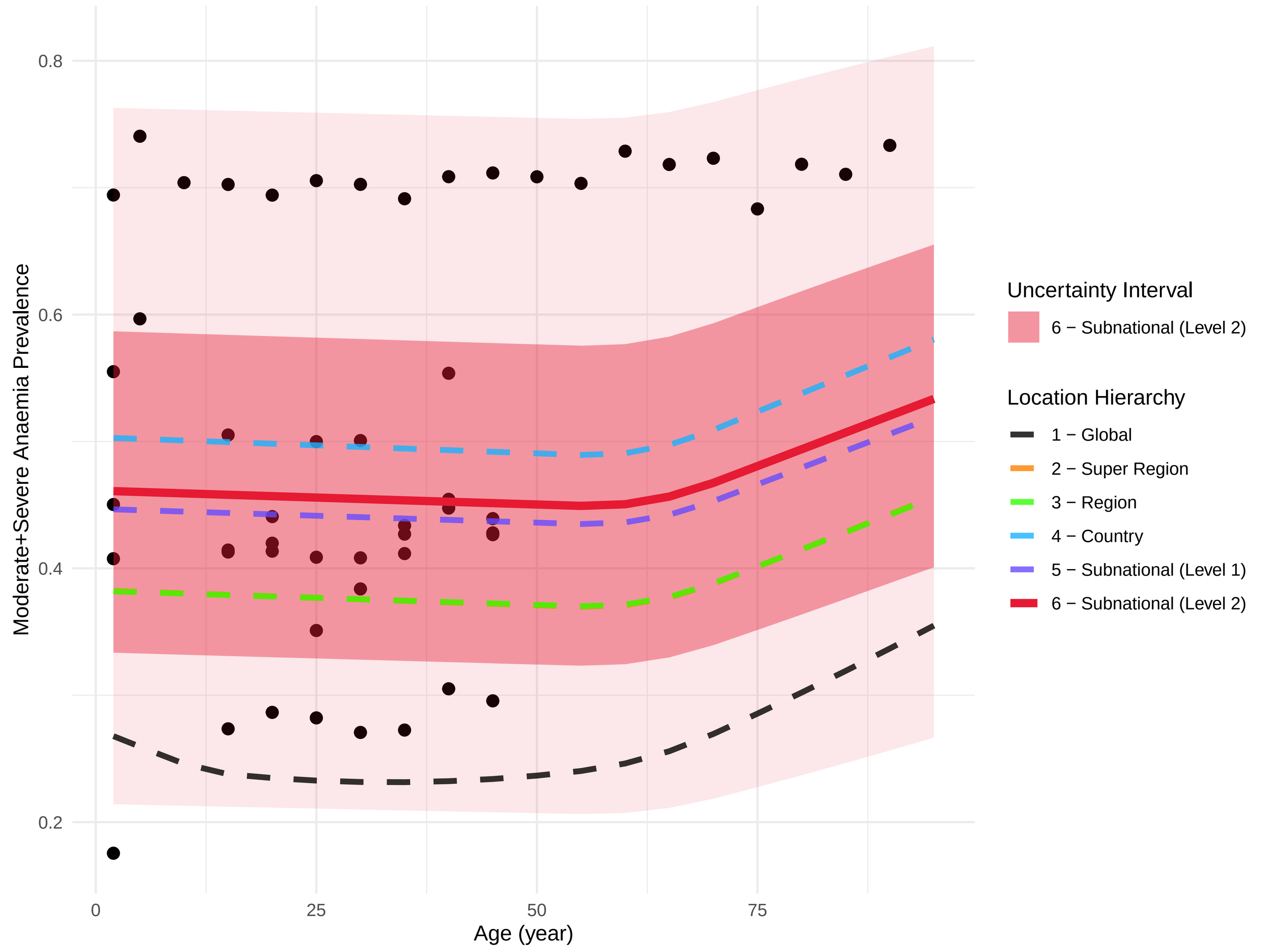

MR−BRT Age/Sex Fits − Non−pregnant Females 2+ Years Old − Moderate+Severe Anaemia Prevalence − Chhattisgarh, Urban
WHO−2024 Adjustment Method

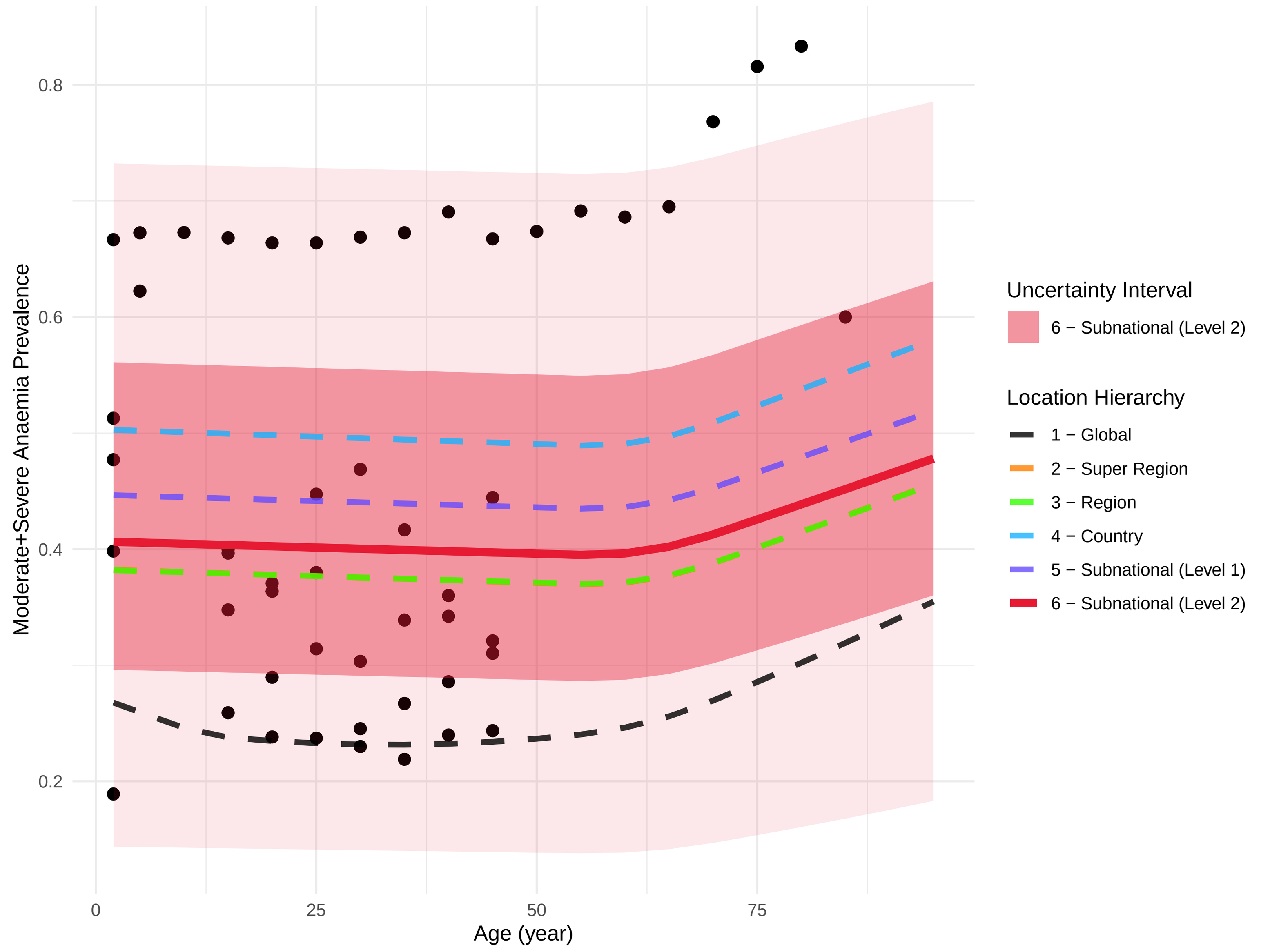

MR−BRT Age/Sex Fits − Non−pregnant Females 2+ Years Old − Moderate+Severe Anaemia Prevalence − Delhi
WHO−2024 Adjustment Method

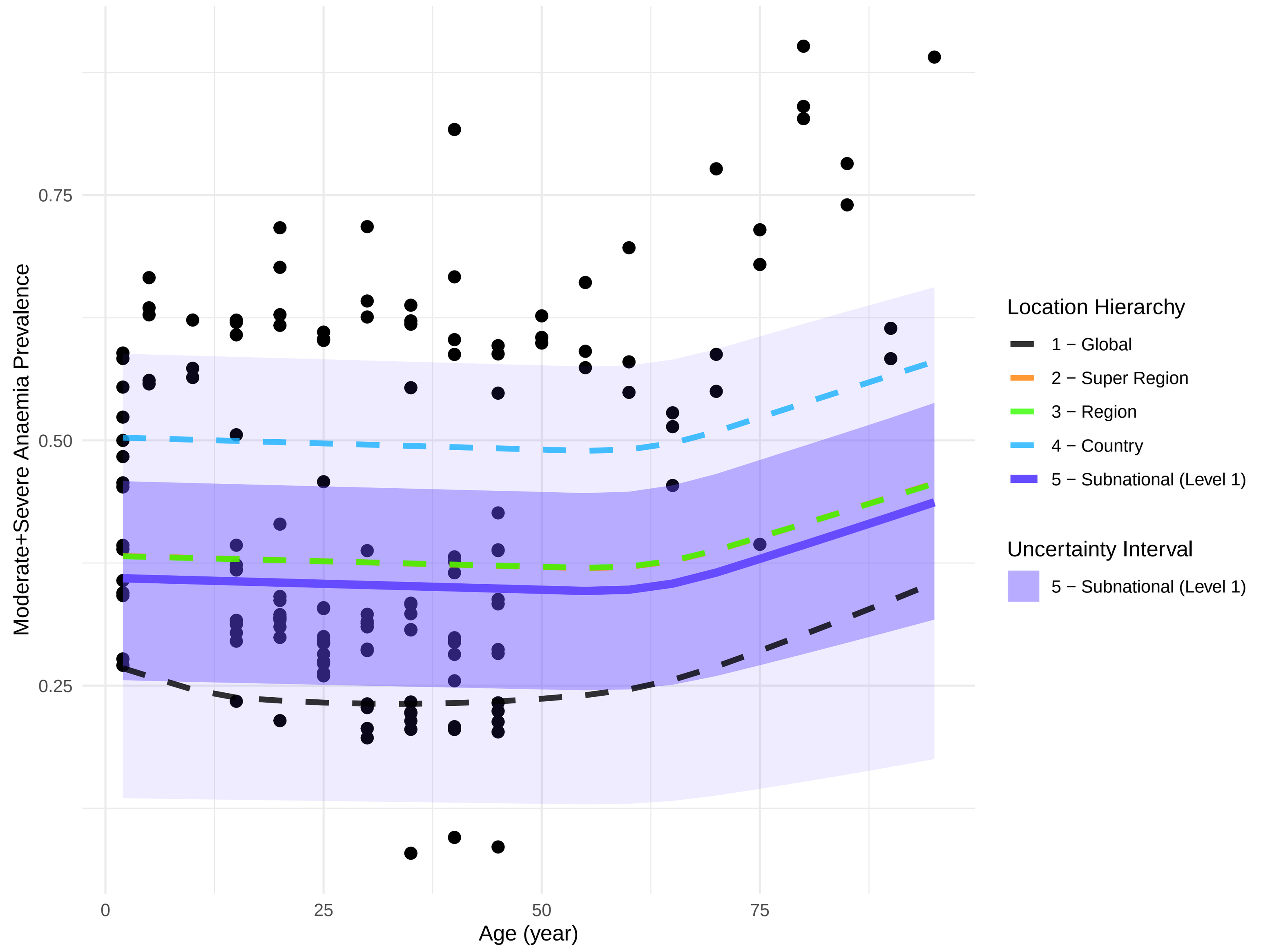

MR−BRT Age/Sex Fits − Non−pregnant Females 2+ Years Old − Moderate+Severe Anaemia Prevalence − Delhi, Rural
WHO−2024 Adjustment Method

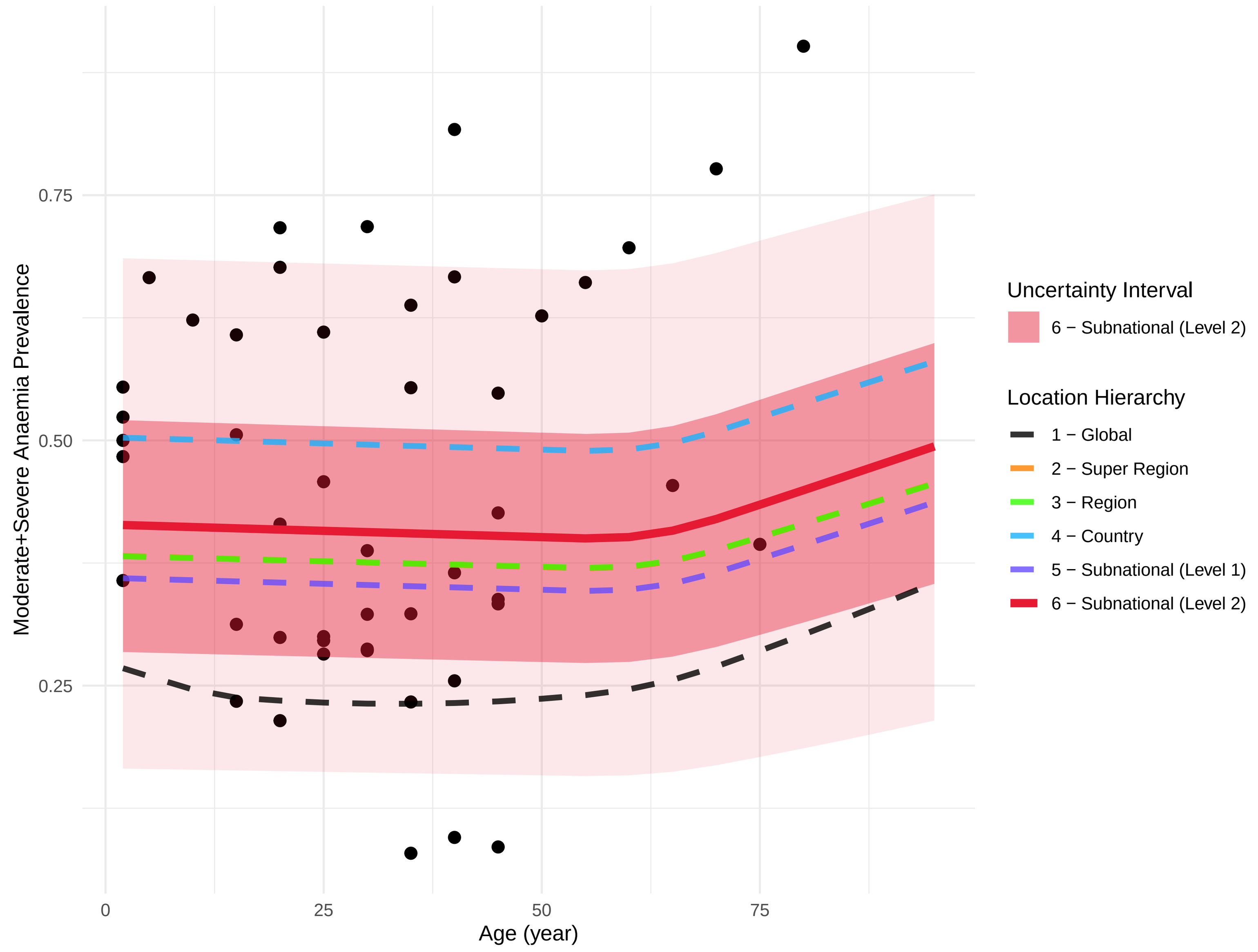

MR−BRT Age/Sex Fits − Non−pregnant Females 2+ Years Old − Moderate+Severe Anaemia Prevalence − Delhi, Urban
WHO−2024 Adjustment Method

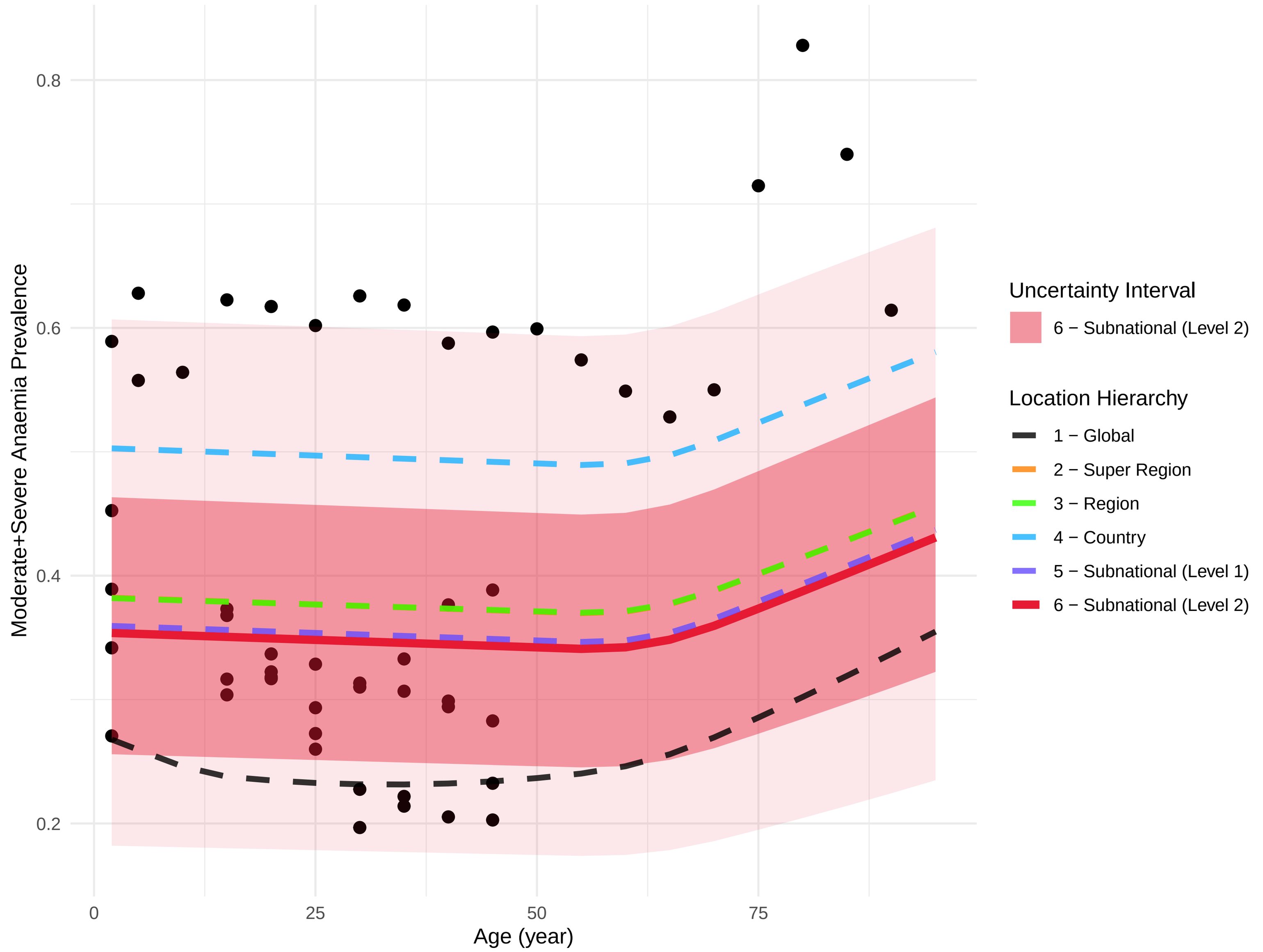

MR−BRT Age/Sex Fits − Non−pregnant Females 2+ Years Old − Moderate+Severe Anaemia Prevalence − Goa
WHO−2024 Adjustment Method

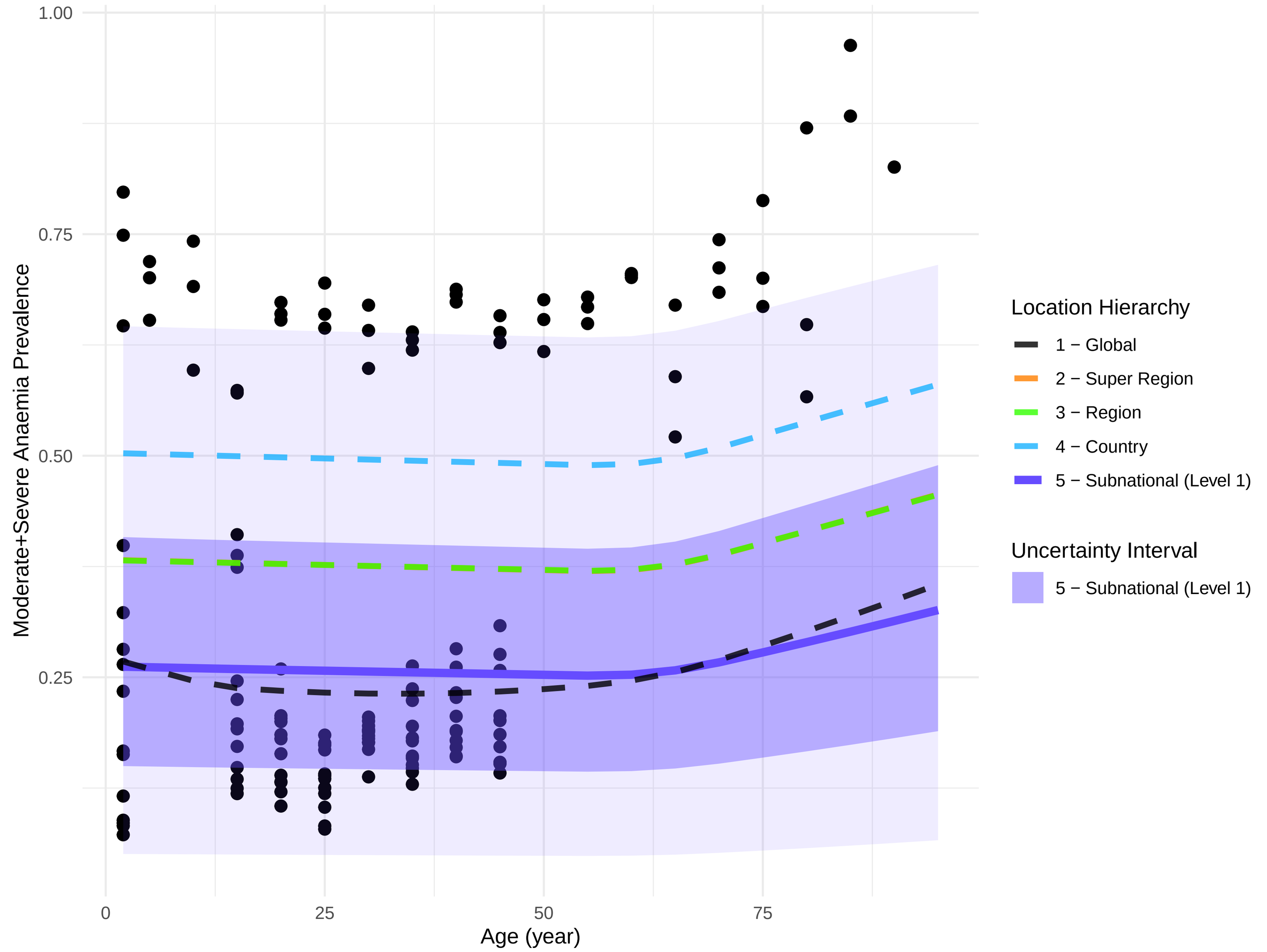

MR−BRT Age/Sex Fits − Non−pregnant Females 2+ Years Old − Moderate+Severe Anaemia Prevalence − Goa, Rural
WHO−2024 Adjustment Method

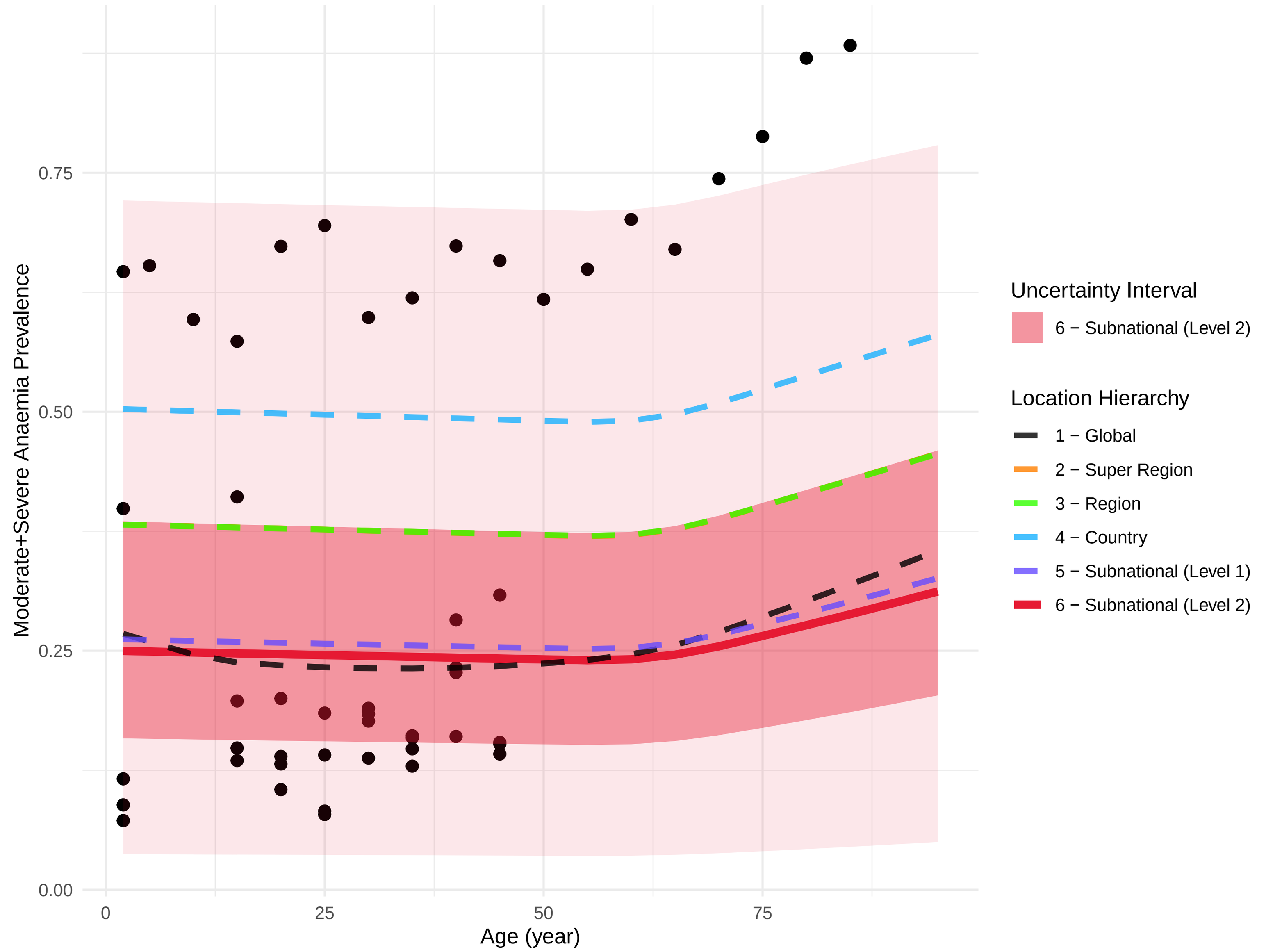

MR−BRT Age/Sex Fits − Non−pregnant Females 2+ Years Old − Moderate+Severe Anaemia Prevalence − Goa, Urban
WHO−2024 Adjustment Method

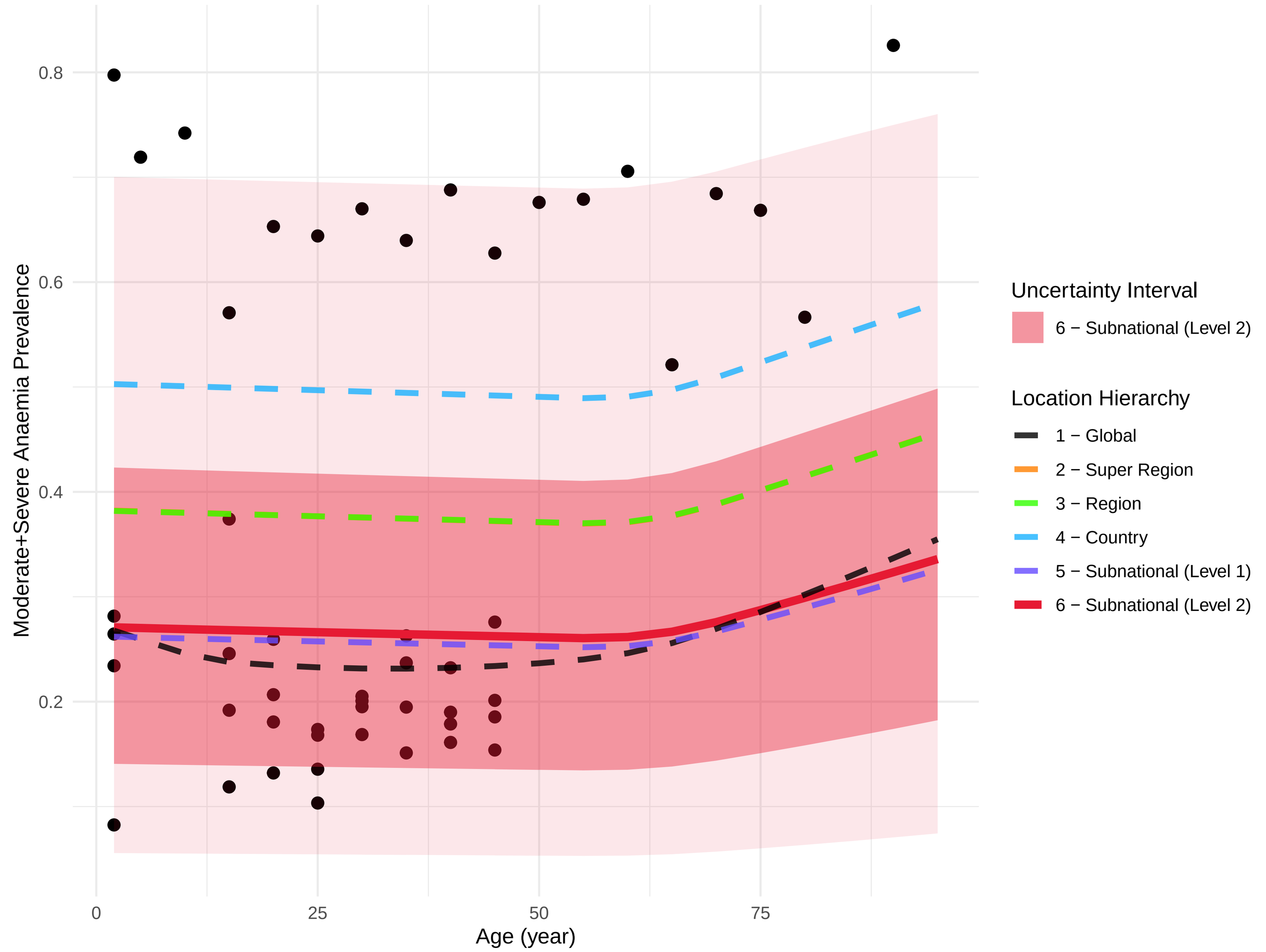

MR−BRT Age/Sex Fits − Non−pregnant Females 2+ Years Old − Moderate+Severe Anaemia Prevalence − Gujarat
WHO−2024 Adjustment Method

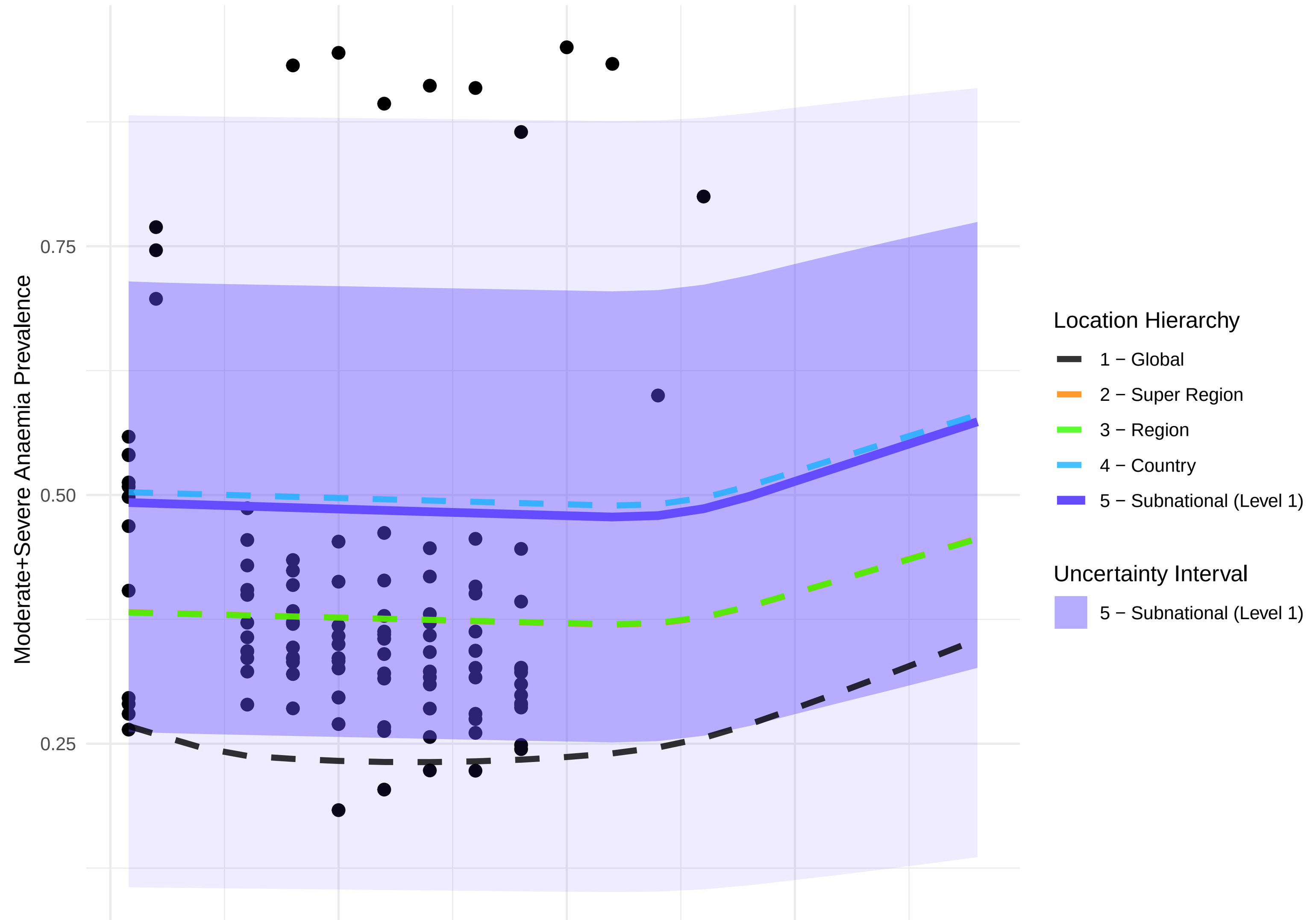

MR−BRT Age/Sex Fits − Non−pregnant Females 2+ Years Old − Moderate+Severe Anaemia Prevalence − Gujarat, Rural
WHO−2024 Adjustment Method

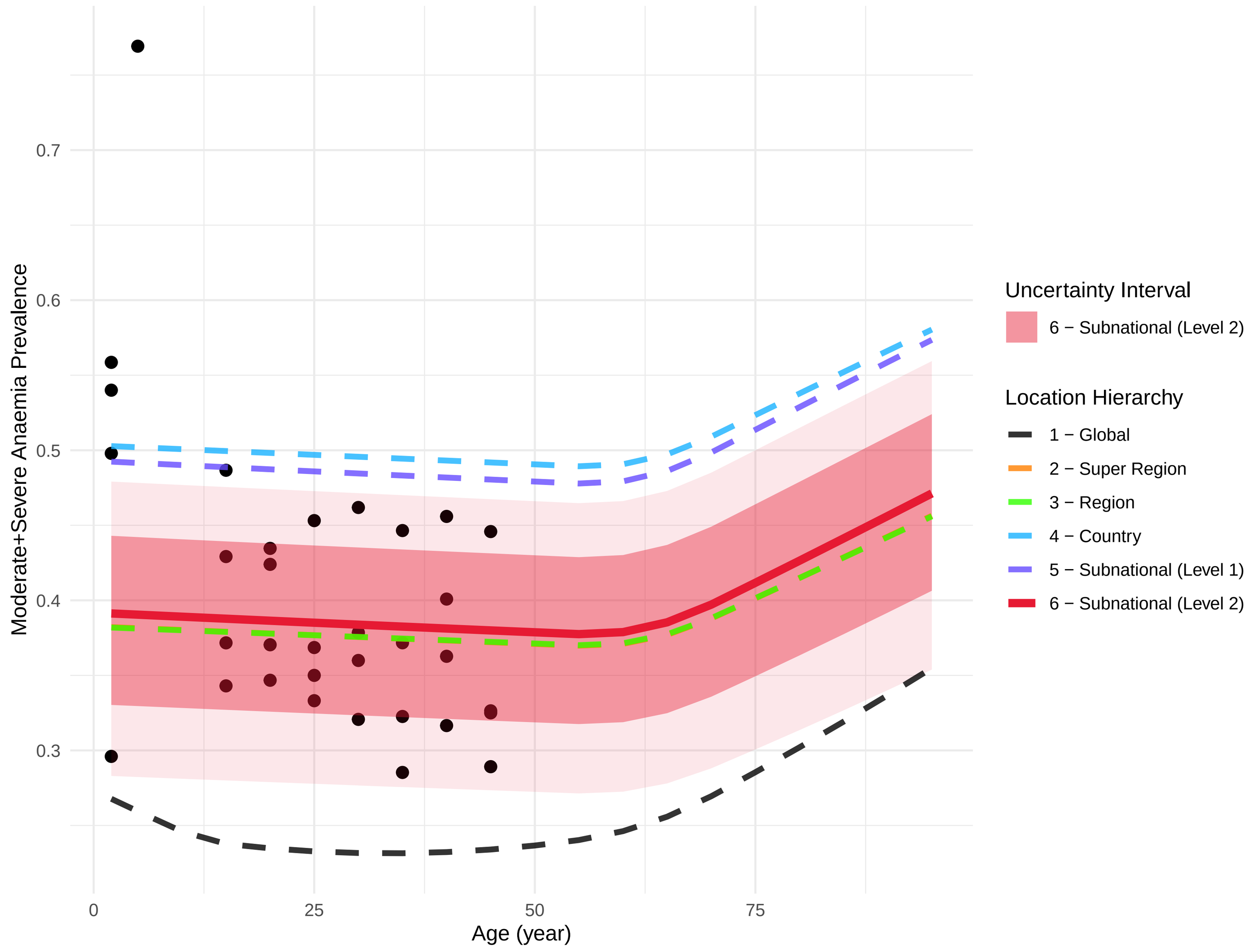

MR−BRT Age/Sex Fits − Non−pregnant Females 2+ Years Old − Moderate+Severe Anaemia Prevalence − Gujarat, Urban
WHO−2024 Adjustment Method

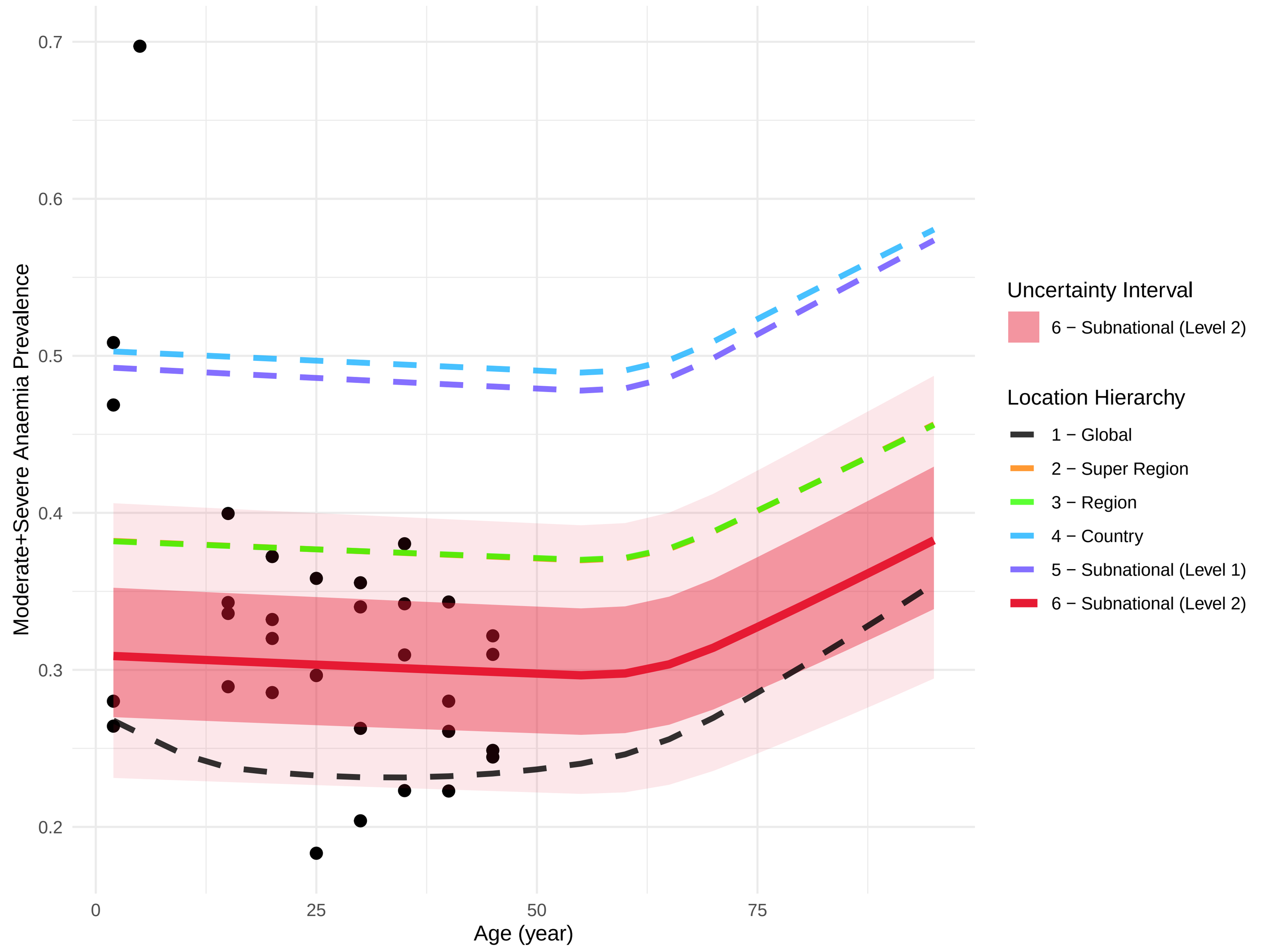

MR−BRT Age/Sex Fits − Non−pregnant Females 2+ Years Old − Moderate+Severe Anaemia Prevalence − Haryana
WHO−2024 Adjustment Method

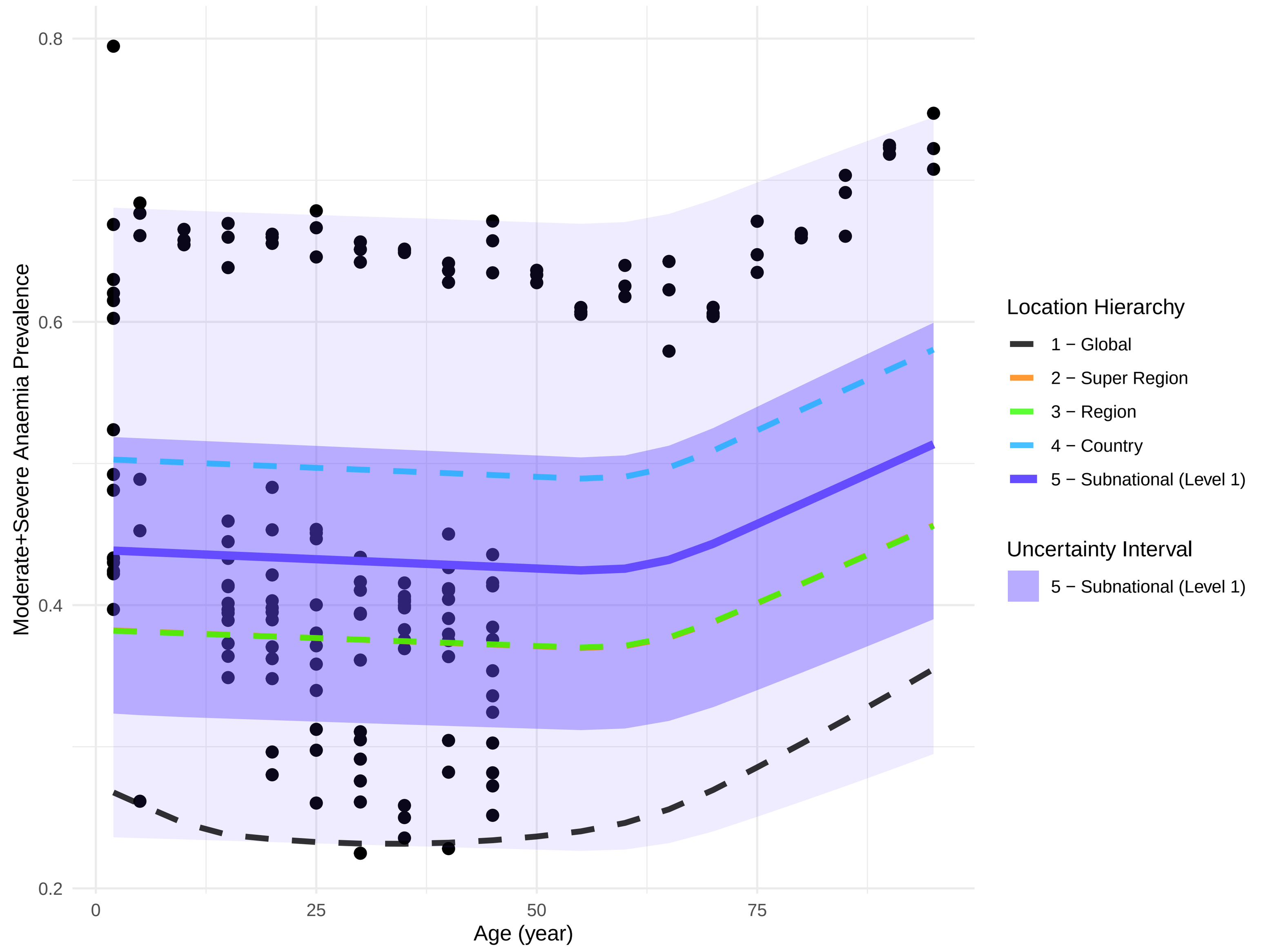

MR−BRT Age/Sex Fits − Non−pregnant Females 2+ Years Old − Moderate+Severe Anaemia Prevalence − Haryana, Rural
WHO−2024 Adjustment Method

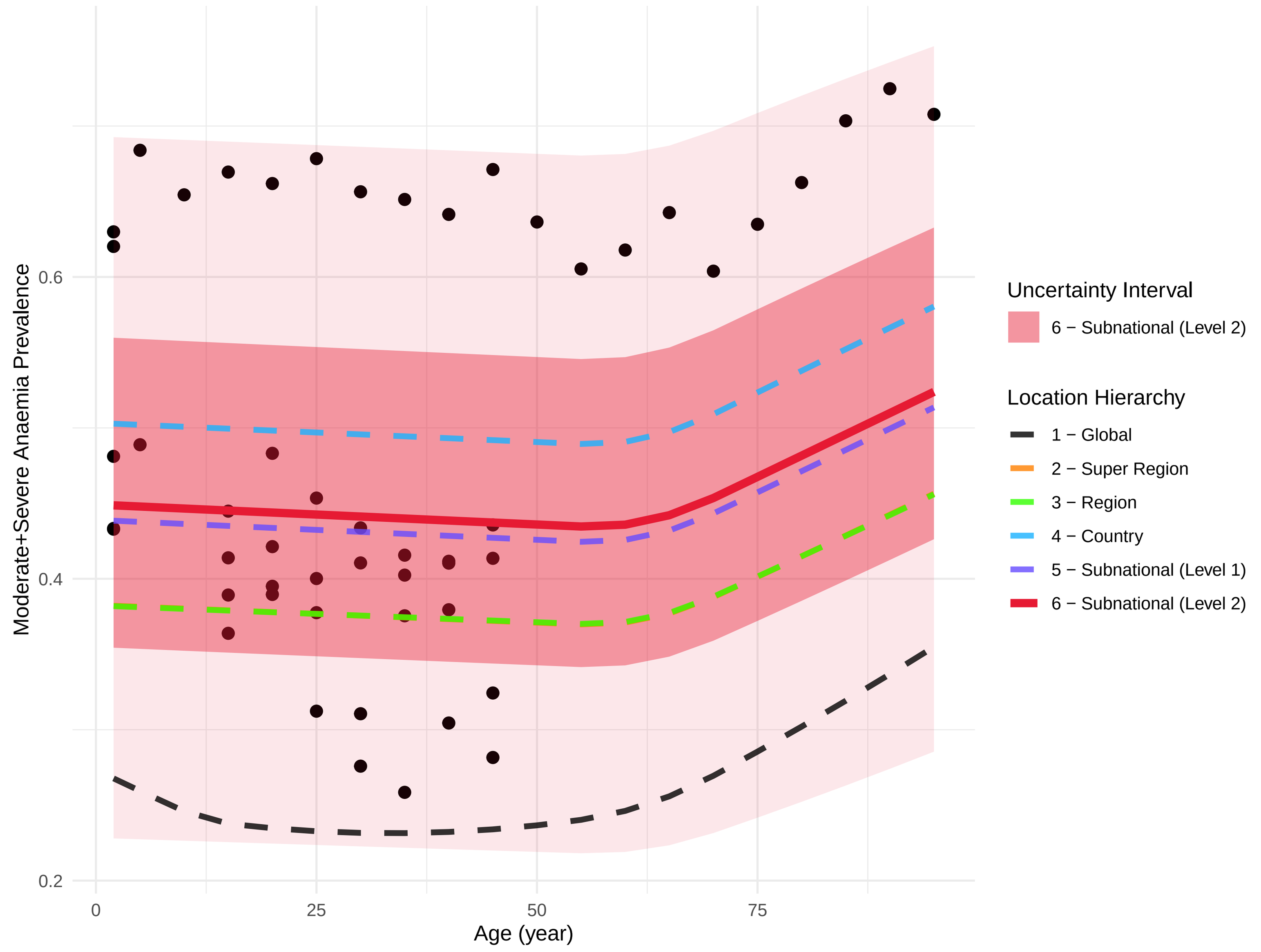

MR−BRT Age/Sex Fits − Non−pregnant Females 2+ Years Old − Moderate+Severe Anaemia Prevalence − Haryana, Urban
WHO−2024 Adjustment Method

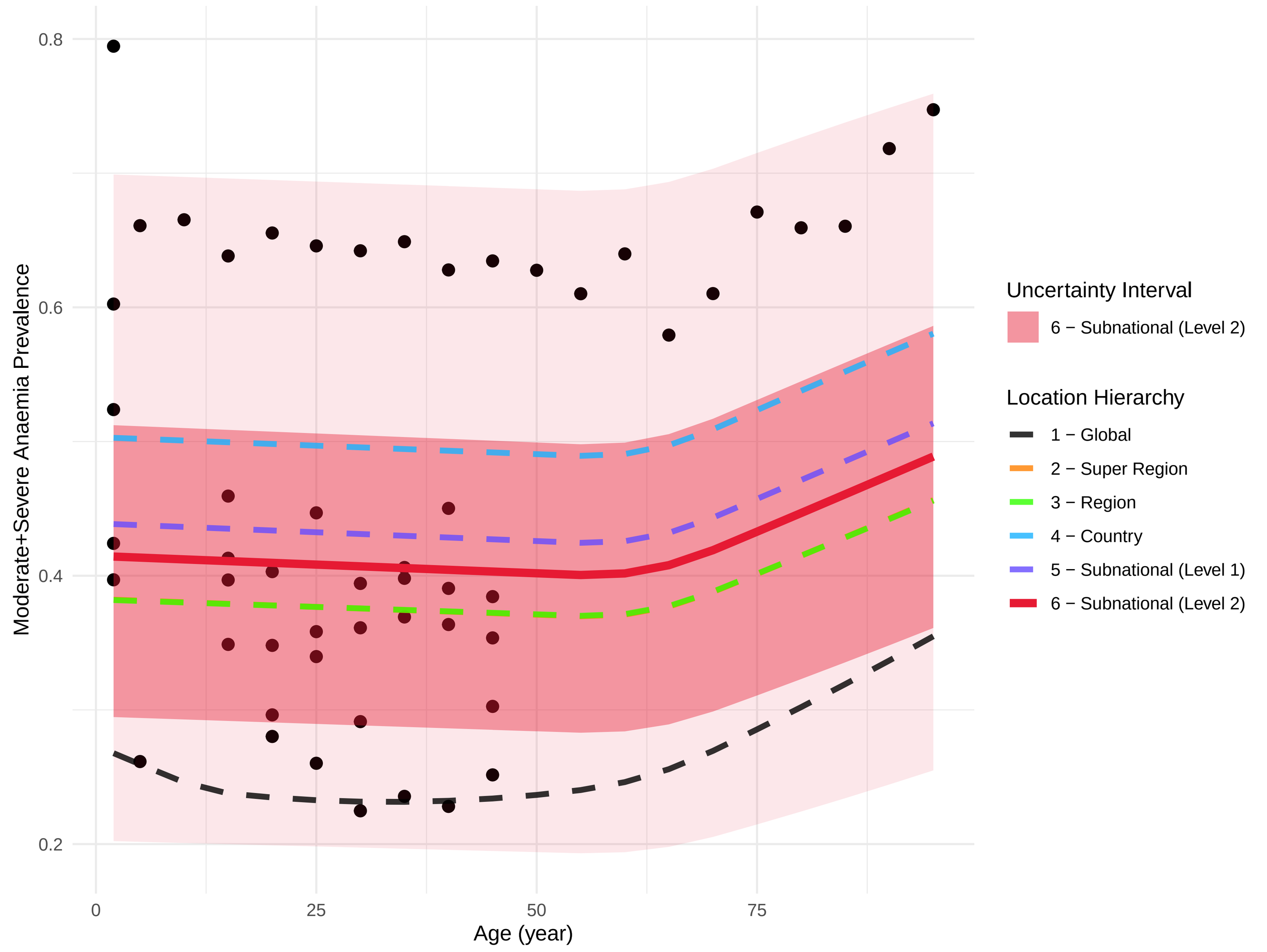

MR−BRT Age/Sex Fits − Non−pregnant Females 2+ Years Old − Moderate+Severe Anaemia Prevalence − Himachal Pradesh
WHO−2024 Adjustment Method

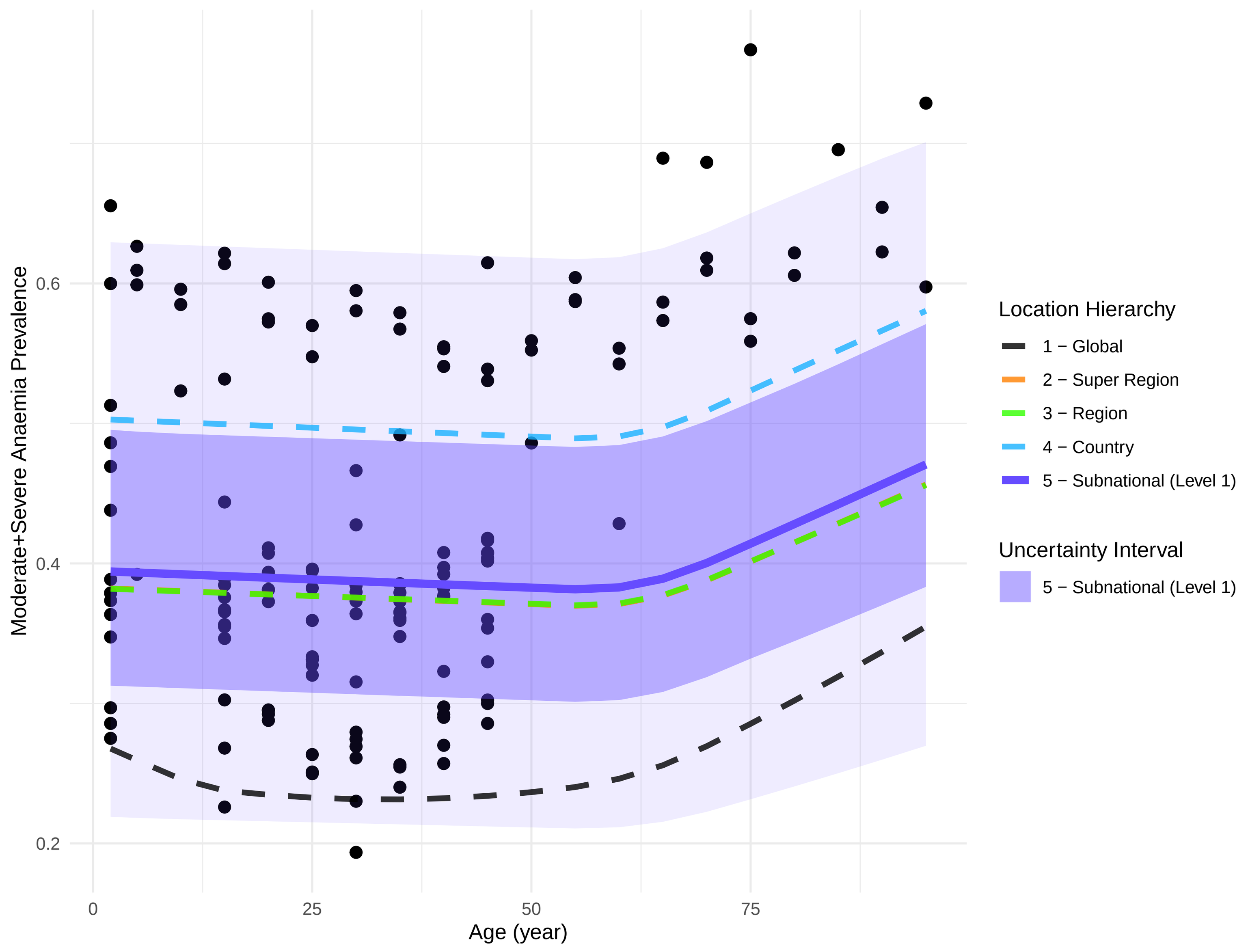

MR−BRT Age/Sex Fits − Non−pregnant Females 2+ Years Old − Moderate+Severe Anaemia Prevalence − Himachal Pradesh, Rural
WHO−2024 Adjustment Method

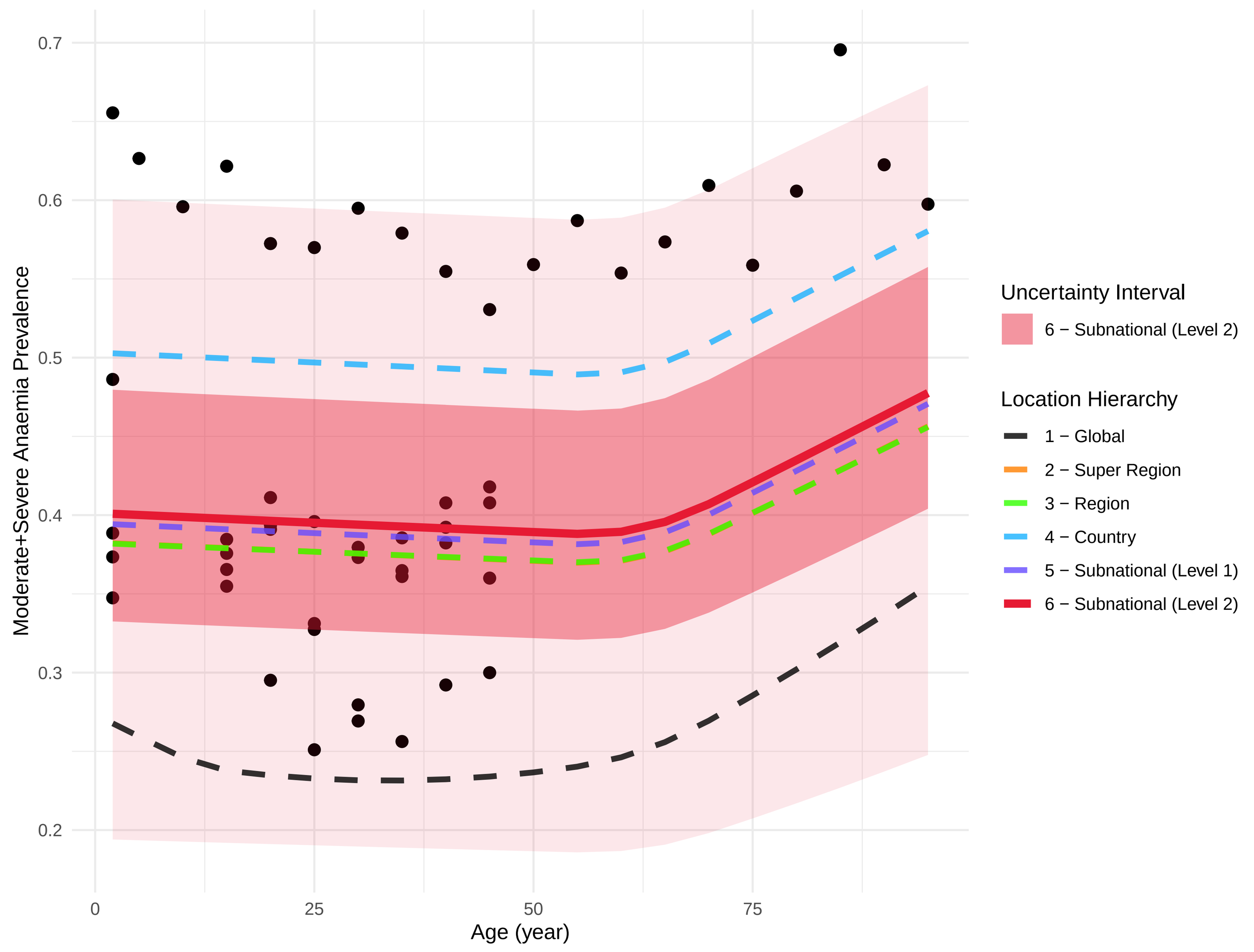

MR−BRT Age/Sex Fits − Non−pregnant Females 2+ Years Old − Moderate+Severe Anaemia Prevalence − Himachal Pradesh, Urban
WHO−2024 Adjustment Method

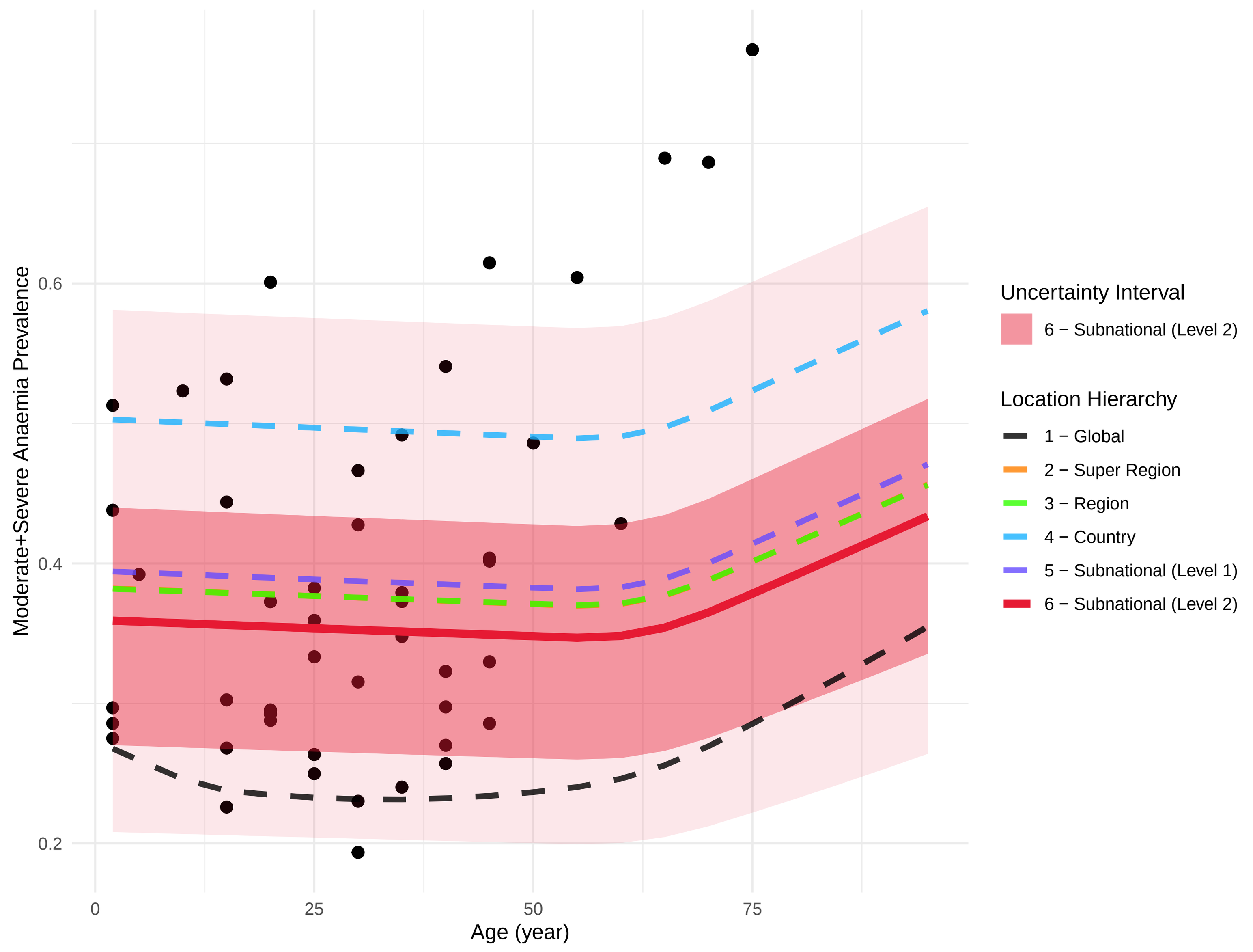

MR−BRT Age/Sex Fits − Non−pregnant Females 2+ Years Old − Moderate+Severe Anaemia Prevalence − Jammu & Kashmir and Ladakh
WHO−2024 Adjustment Method

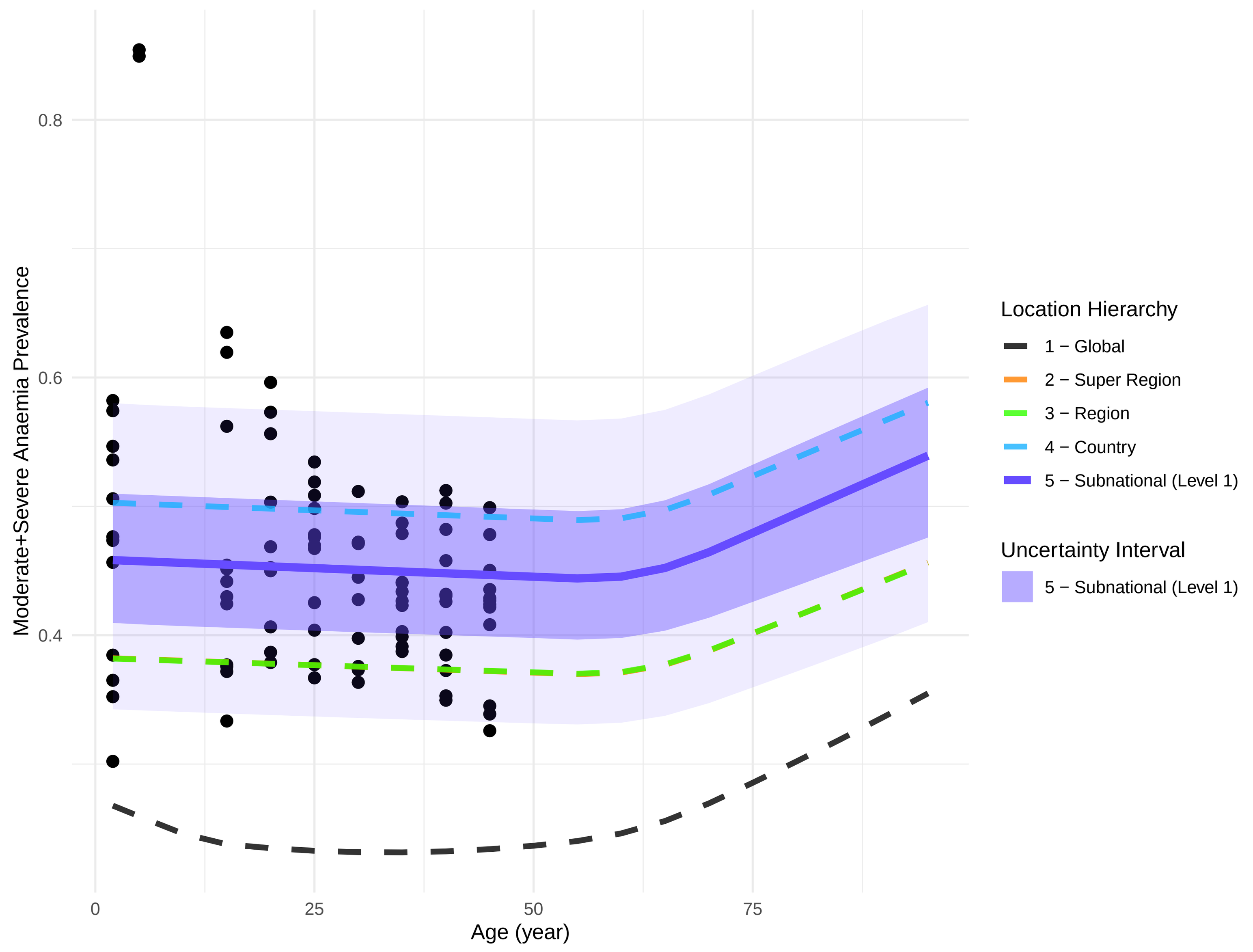

MR−BRT Age/Sex Fits − Non−pregnant Females 2+ Years Old − Moderate+Severe Anaemia Prevalence − Jammu & Kashmir and Ladakh, Rural
WHO−2024 Adjustment Method

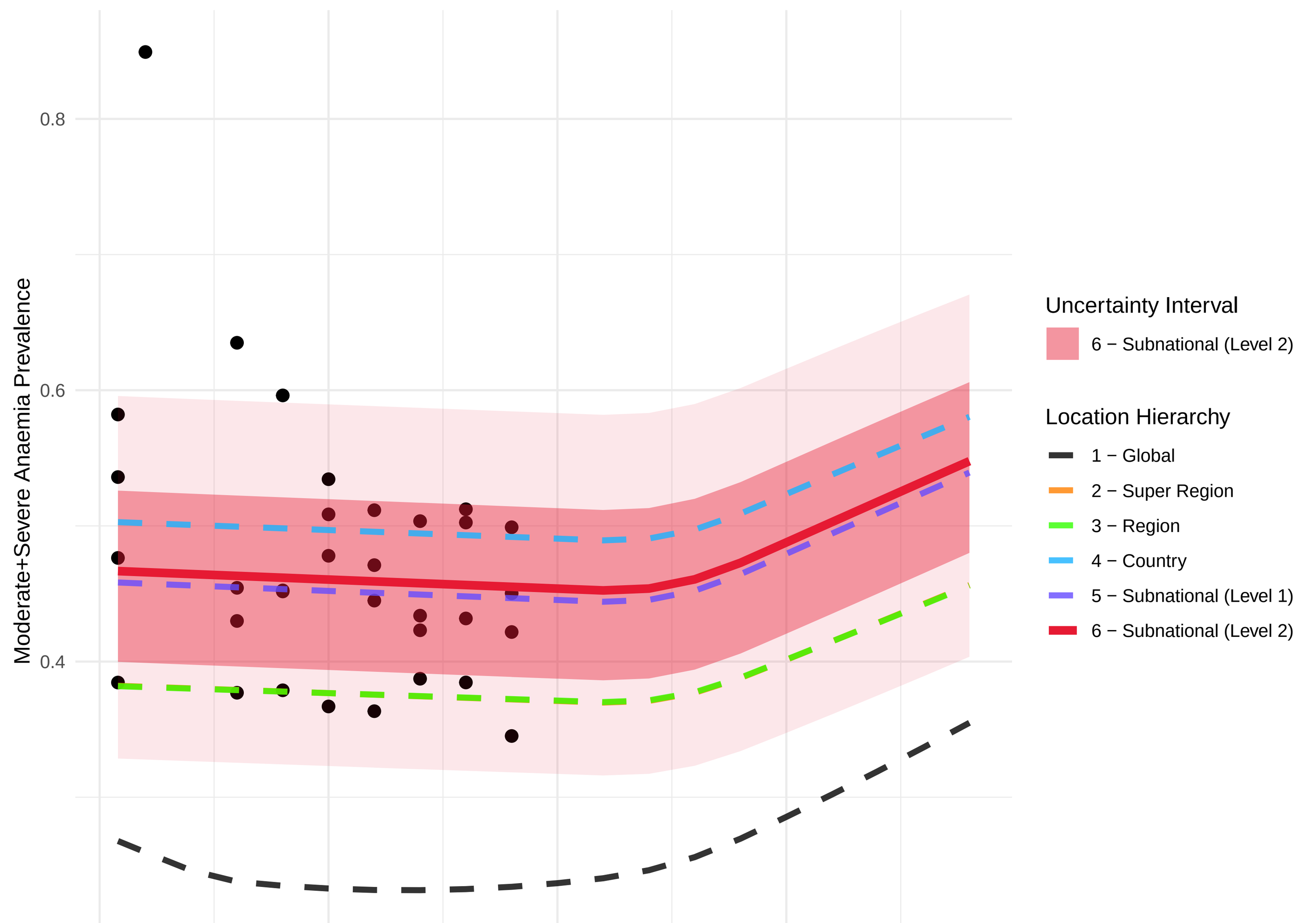

MR−BRT Age/Sex Fits − Non−pregnant Females 2+ Years Old − Moderate+Severe Anaemia Prevalence − Jammu & Kashmir and Ladakh, Urban
WHO−2024 Adjustment Method

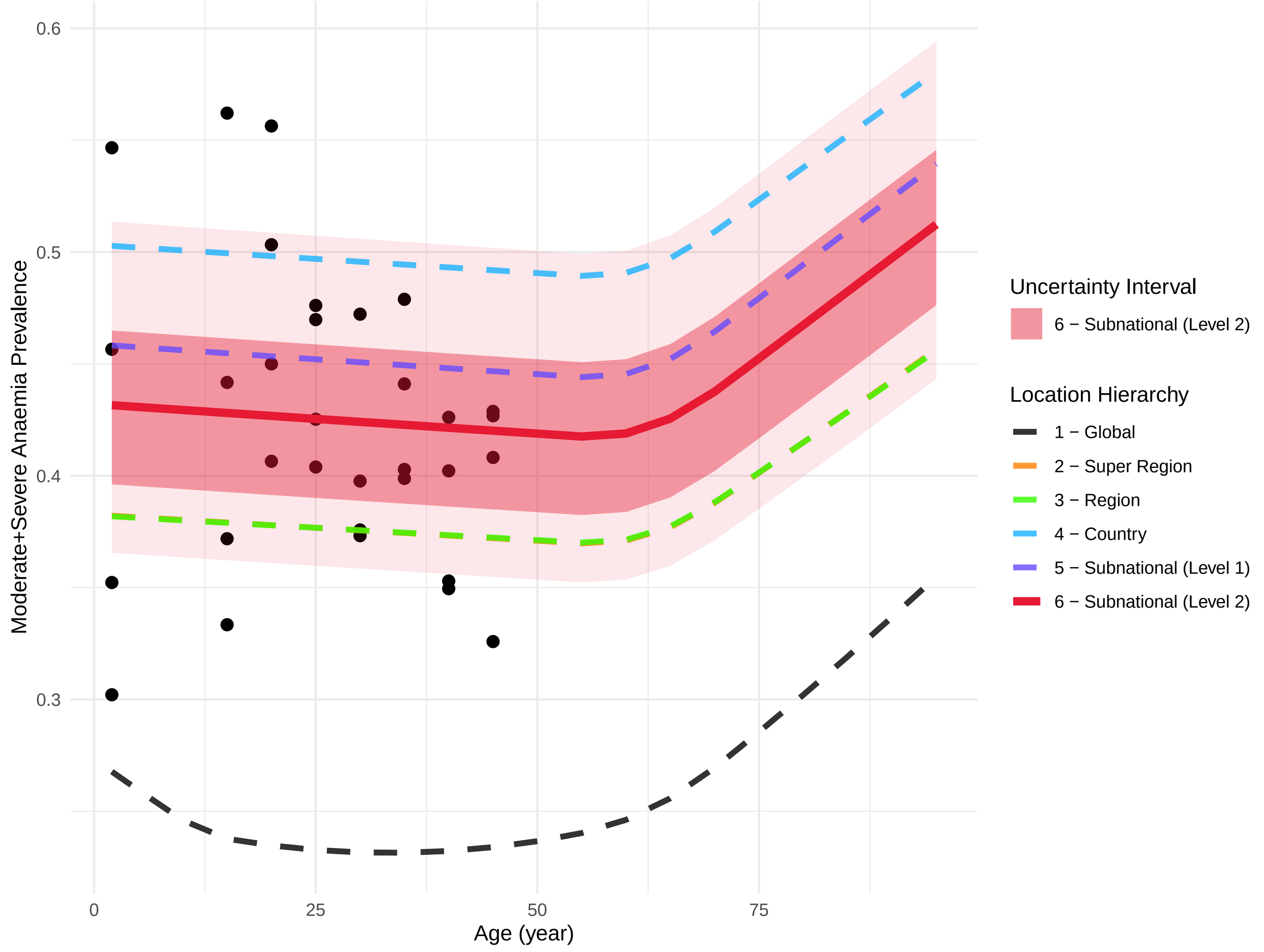

MR−BRT Age/Sex Fits − Non−pregnant Females 2+ Years Old − Moderate+Severe Anaemia Prevalence − Jharkhand
WHO−2024 Adjustment Method

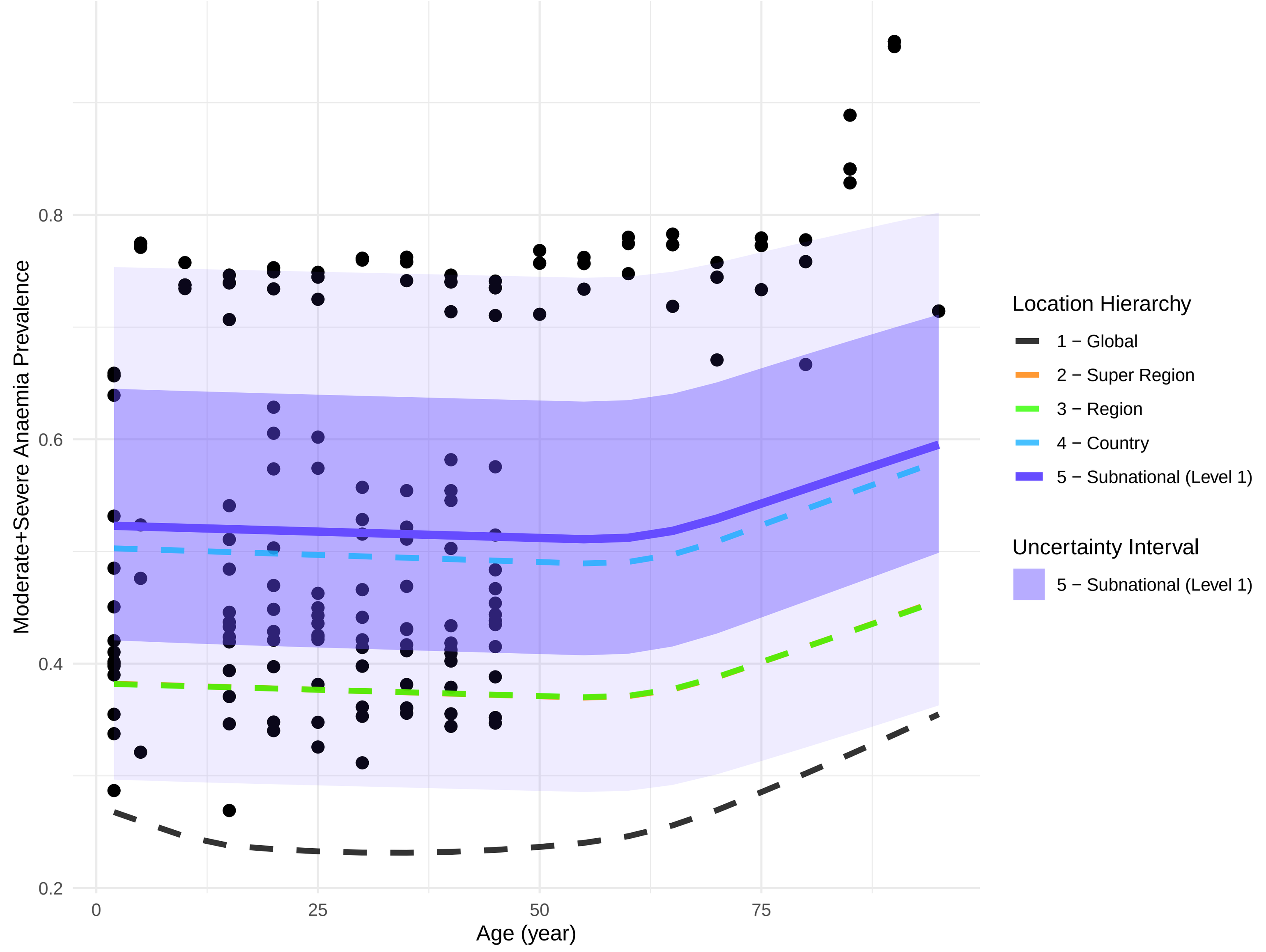

MR−BRT Age/Sex Fits − Non−pregnant Females 2+ Years Old − Moderate+Severe Anaemia Prevalence − Jharkhand, Rural
WHO−2024 Adjustment Method

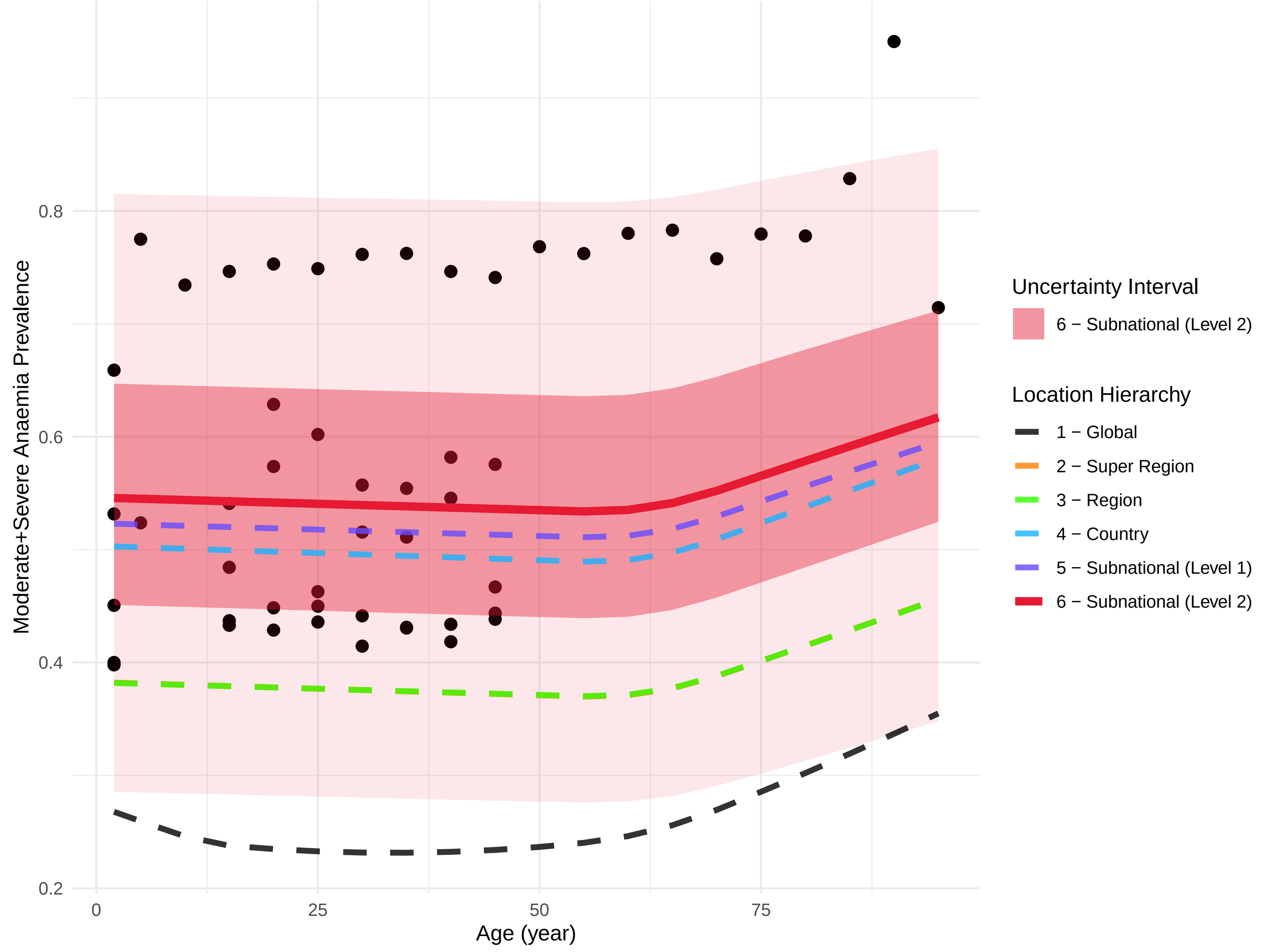

MR−BRT Age/Sex Fits − Non−pregnant Females 2+ Years Old − Moderate+Severe Anaemia Prevalence − Jharkhand, Urban
WHO−2024 Adjustment Method

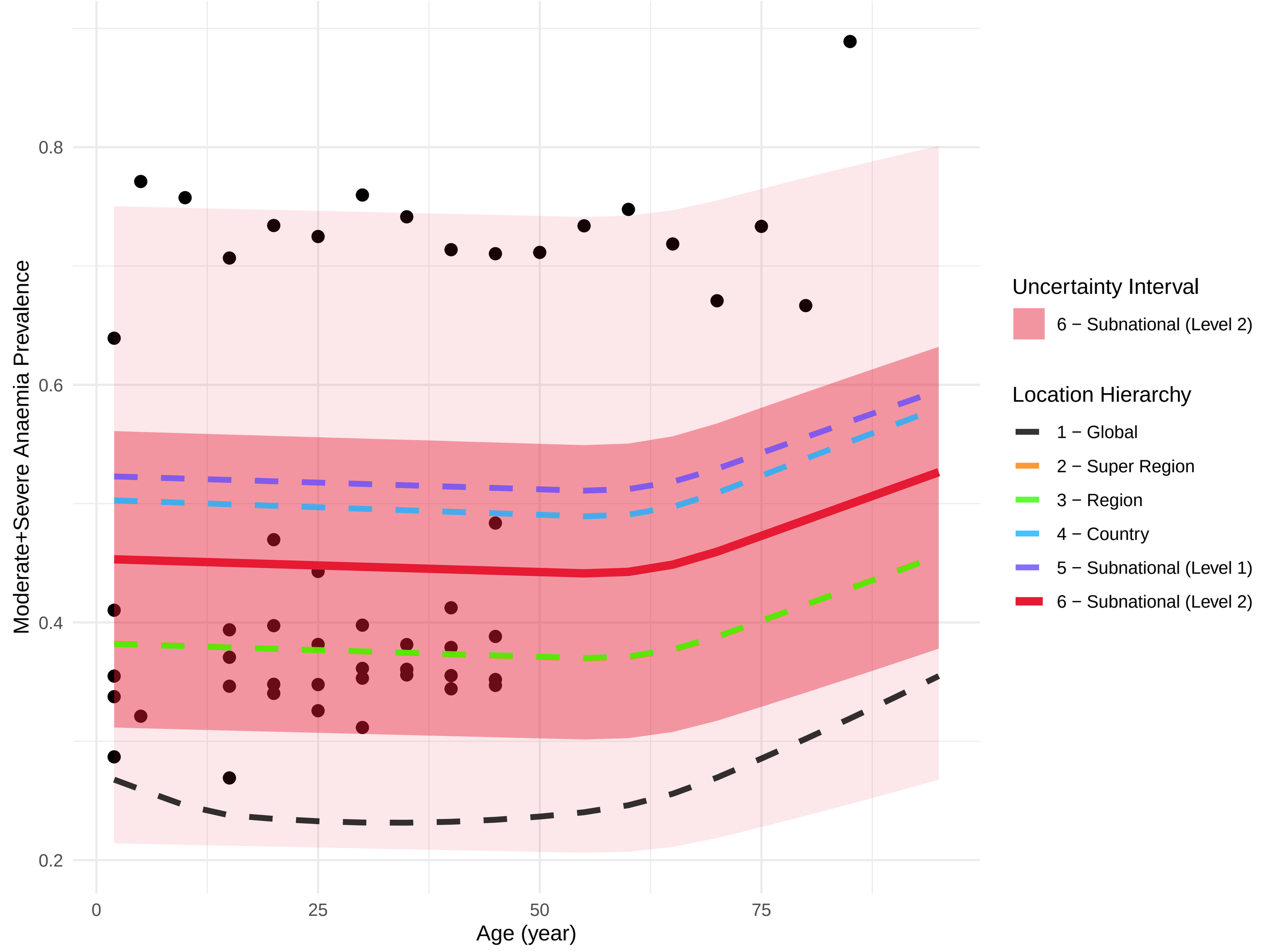

MR−BRT Age/Sex Fits − Non−pregnant Females 2+ Years Old − Moderate+Severe Anaemia Prevalence − Karnataka
WHO−2024 Adjustment Method

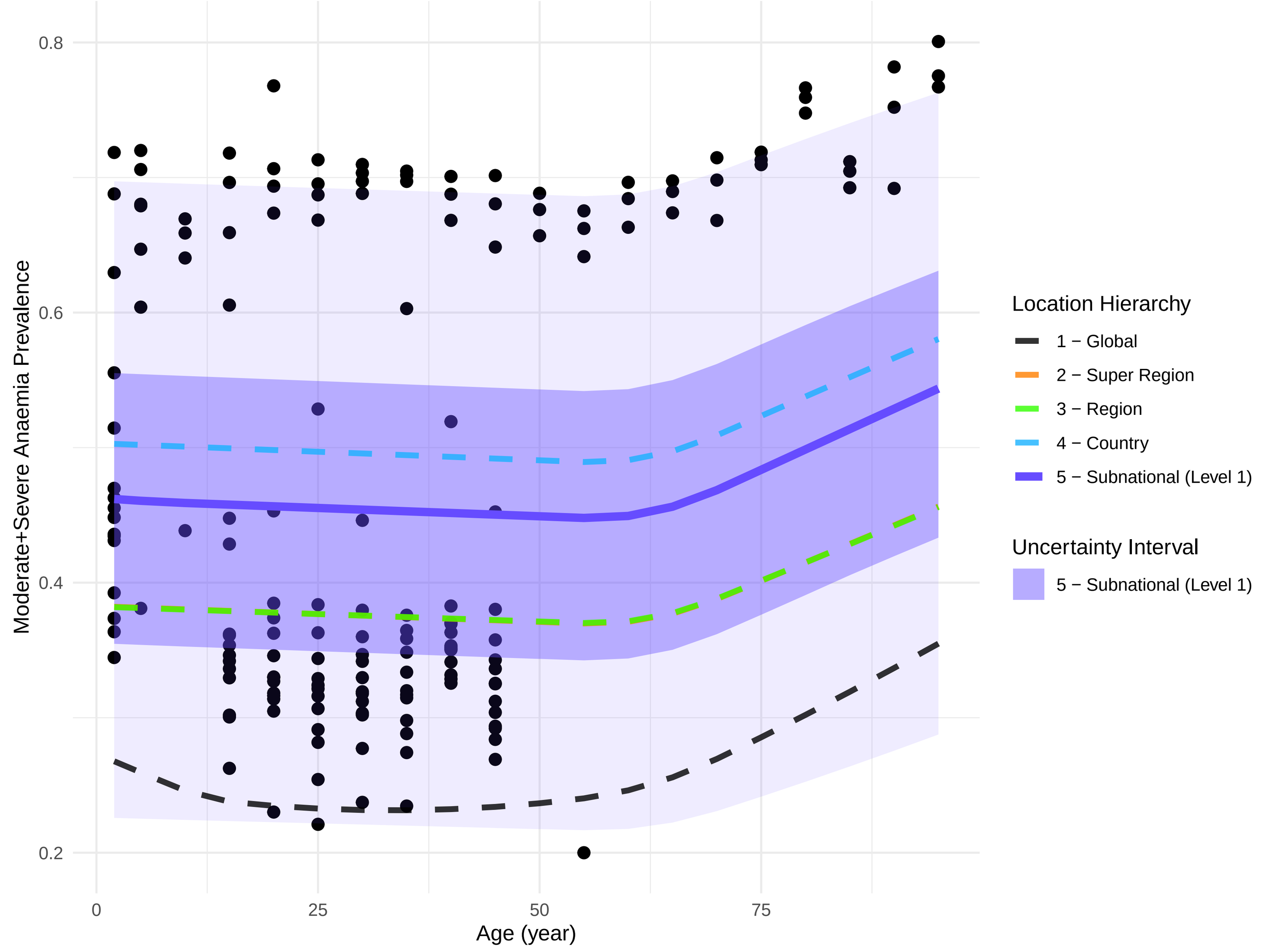

MR−BRT Age/Sex Fits − Non−pregnant Females 2+ Years Old − Moderate+Severe Anaemia Prevalence − Karnataka, Rural
WHO−2024 Adjustment Method

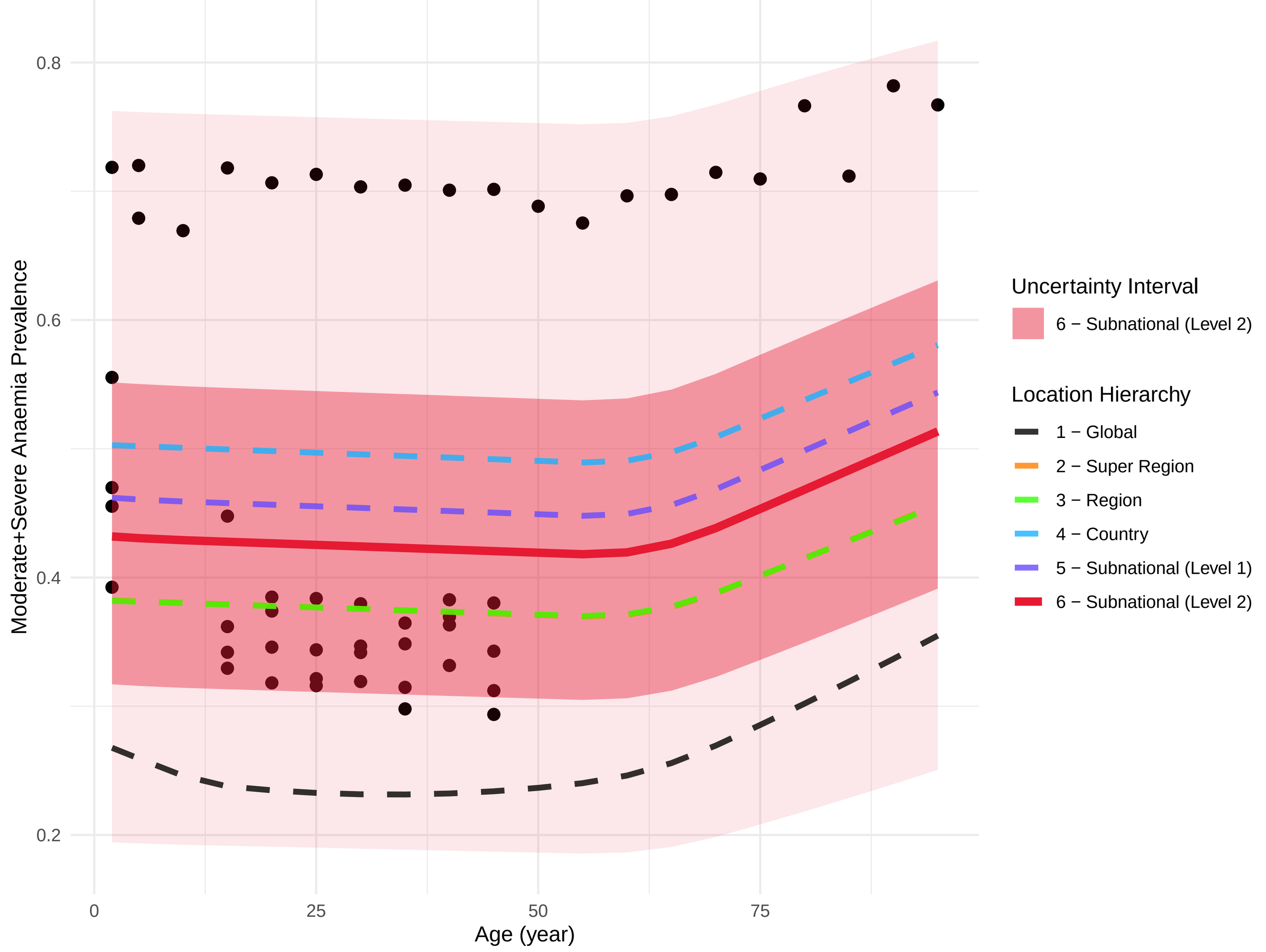

MR−BRT Age/Sex Fits − Non−pregnant Females 2+ Years Old − Moderate+Severe Anaemia Prevalence − Karnataka, Urban
WHO−2024 Adjustment Method

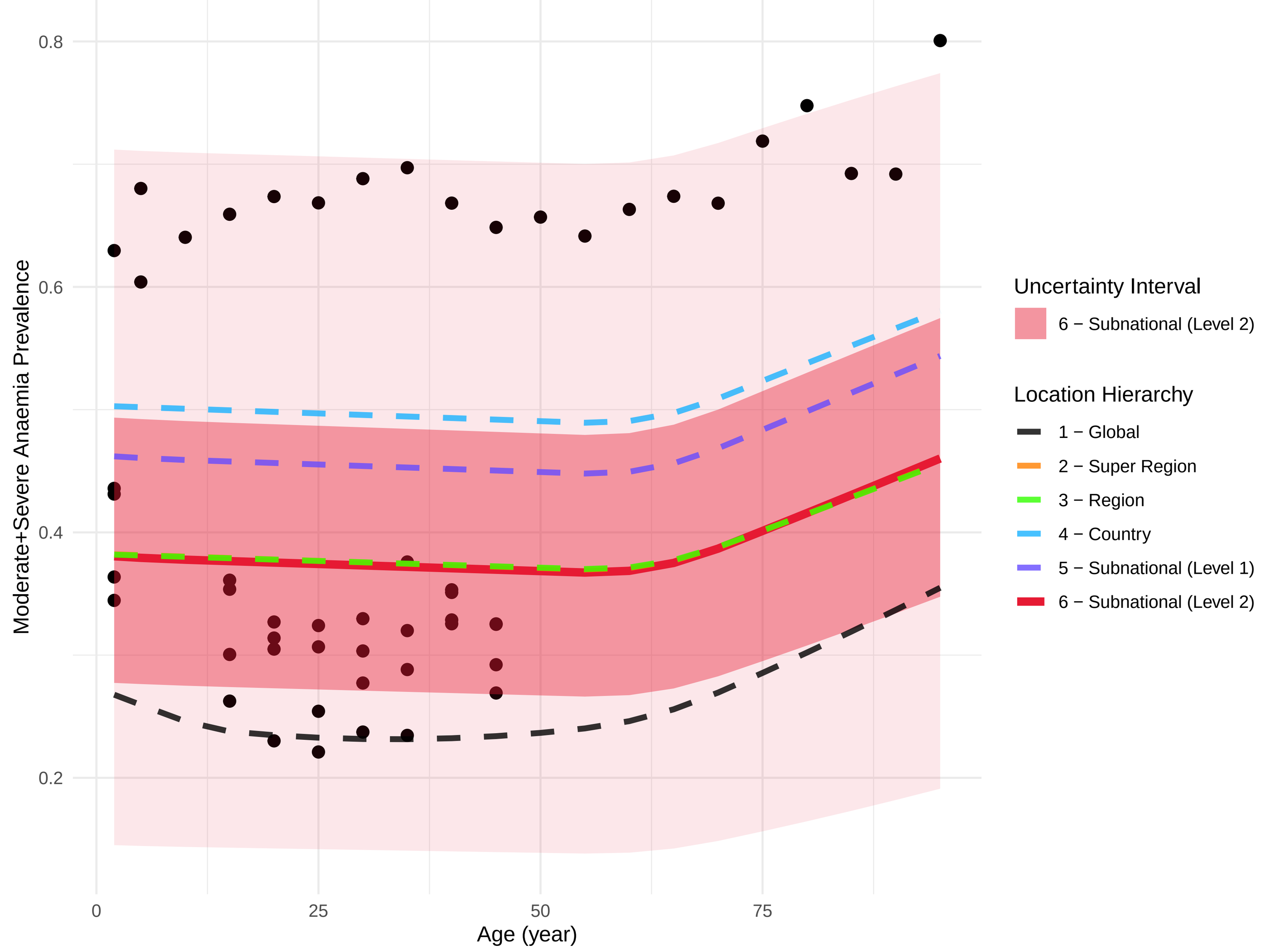

MR−BRT Age/Sex Fits − Non−pregnant Females 2+ Years Old − Moderate+Severe Anaemia Prevalence − Kerala
WHO−2024 Adjustment Method

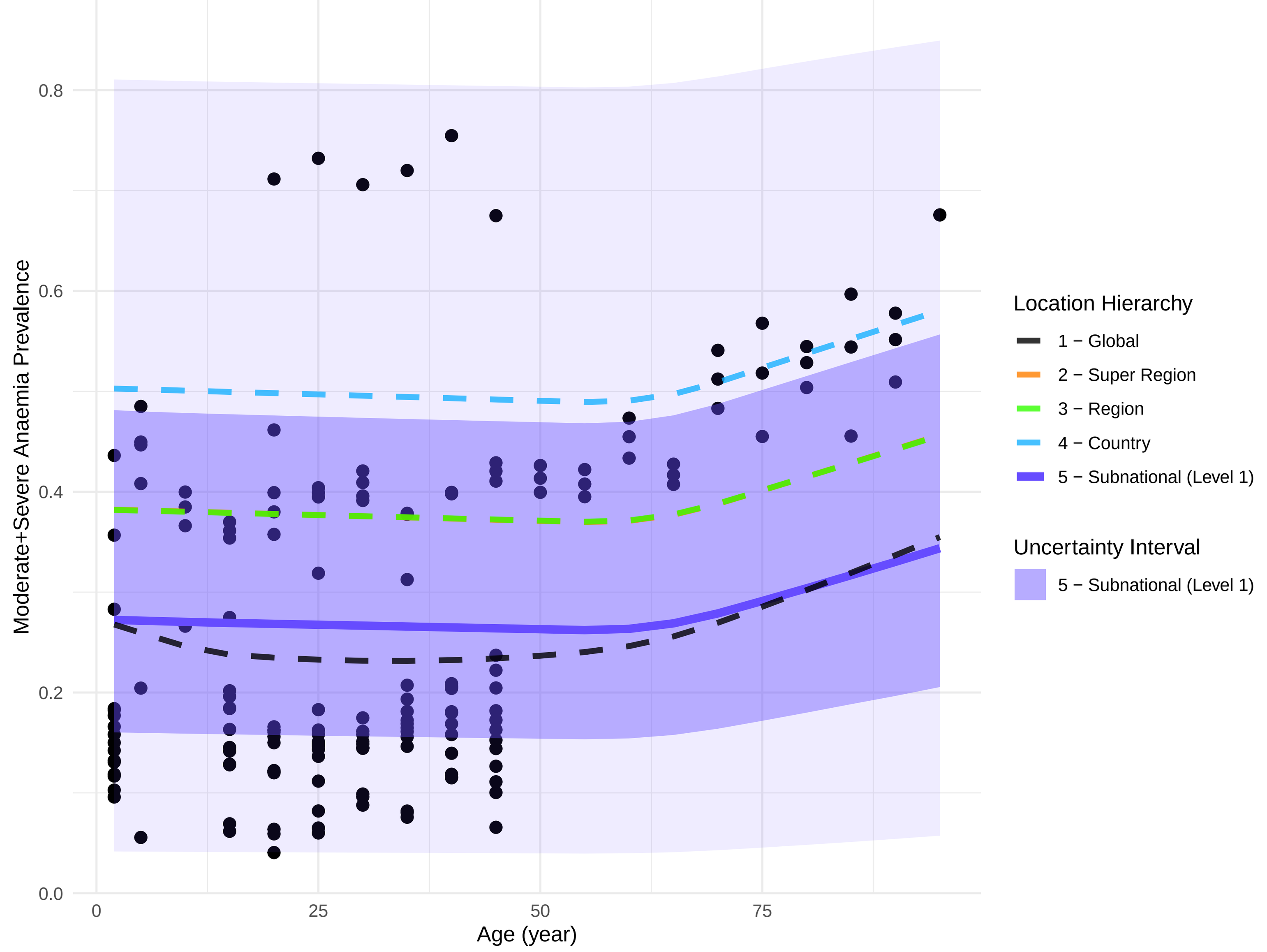

MR−BRT Age/Sex Fits − Non−pregnant Females 2+ Years Old − Moderate+Severe Anaemia Prevalence − Kerala, Rural
WHO−2024 Adjustment Method

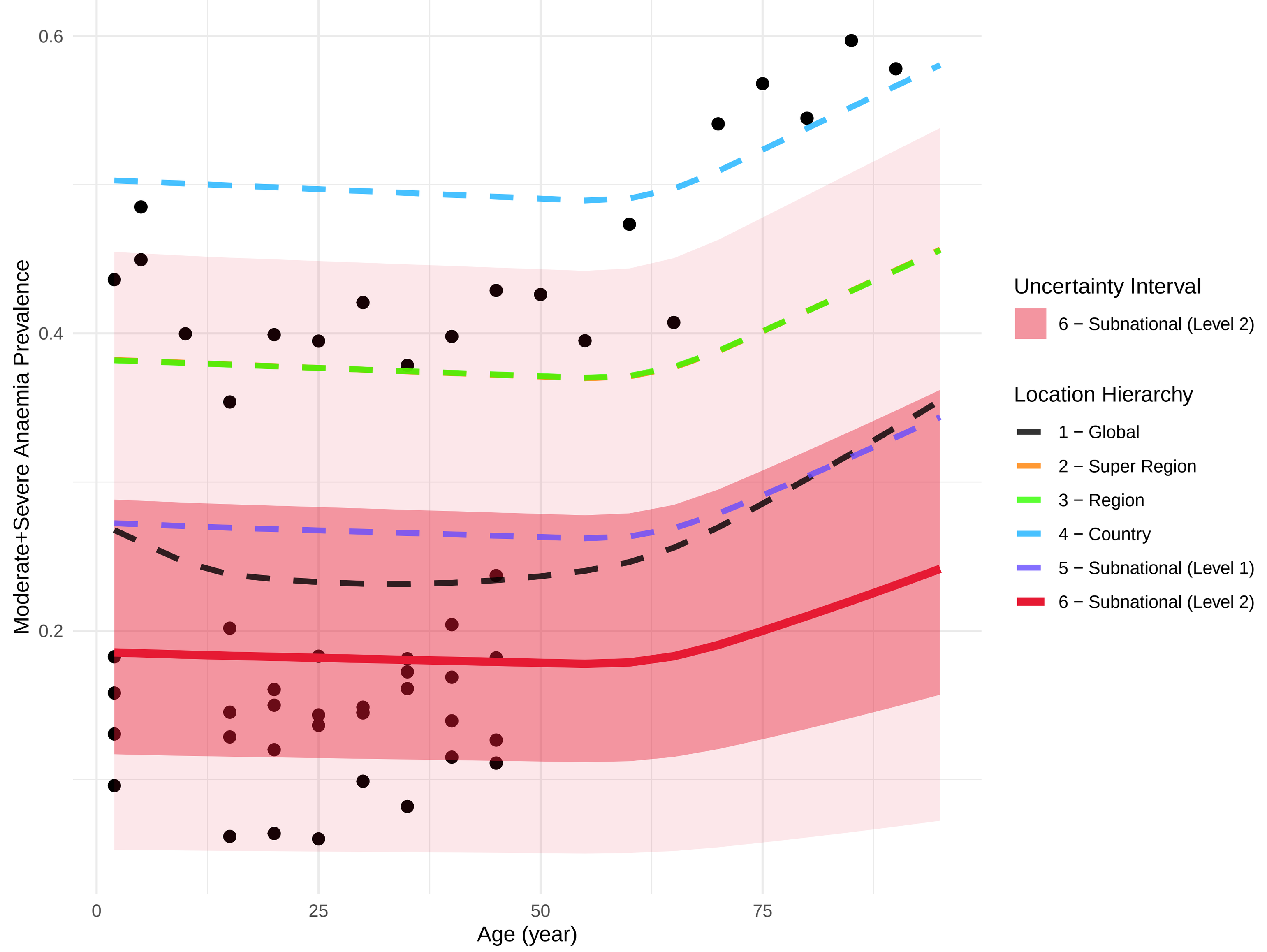

MR−BRT Age/Sex Fits − Non−pregnant Females 2+ Years Old − Moderate+Severe Anaemia Prevalence − Kerala, Urban
WHO−2024 Adjustment Method

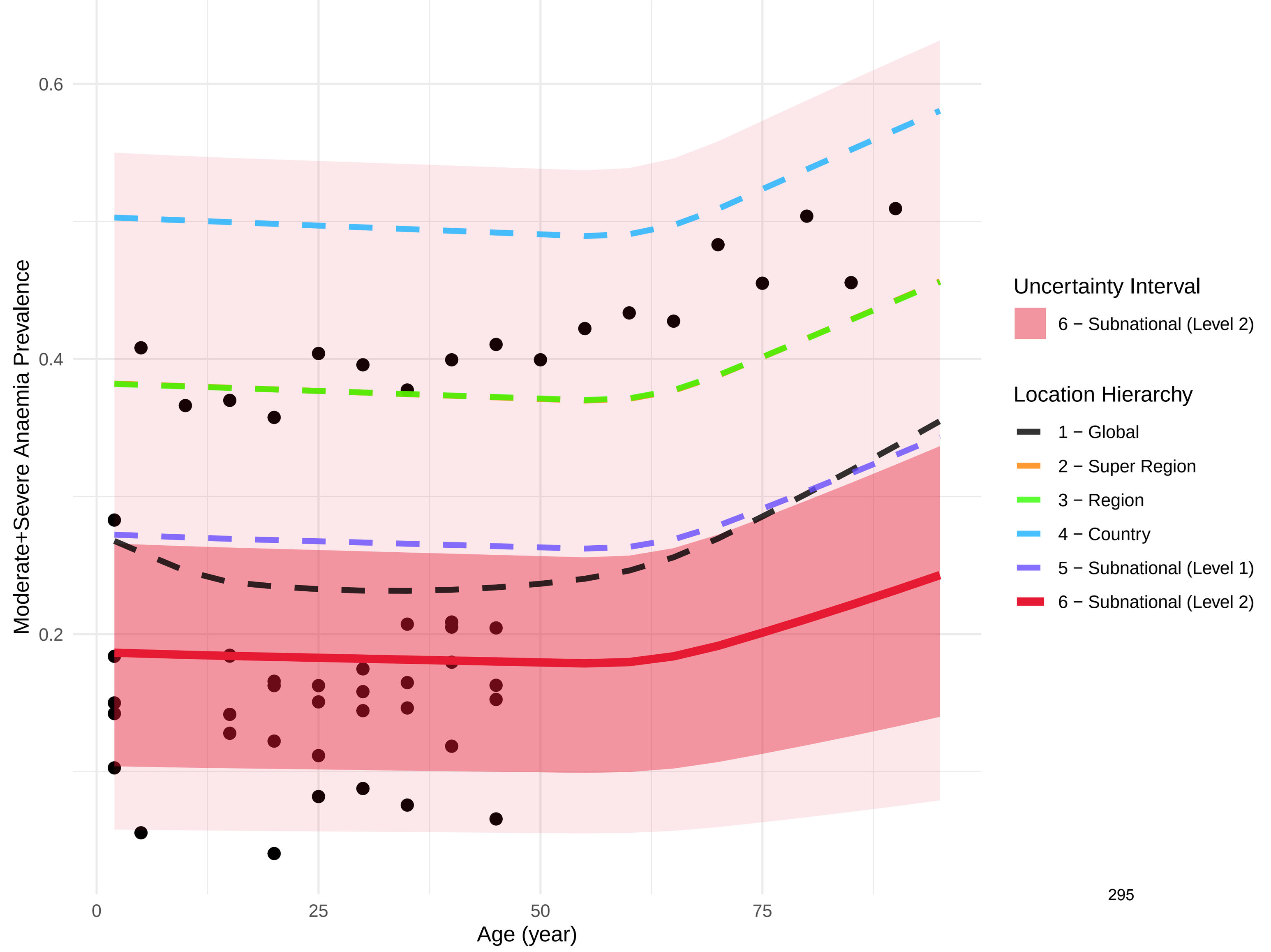

MR−BRT Age/Sex Fits − Non−pregnant Females 2+ Years Old − Moderate+Severe Anaemia Prevalence − Madhya Pradesh
WHO−2024 Adjustment Method

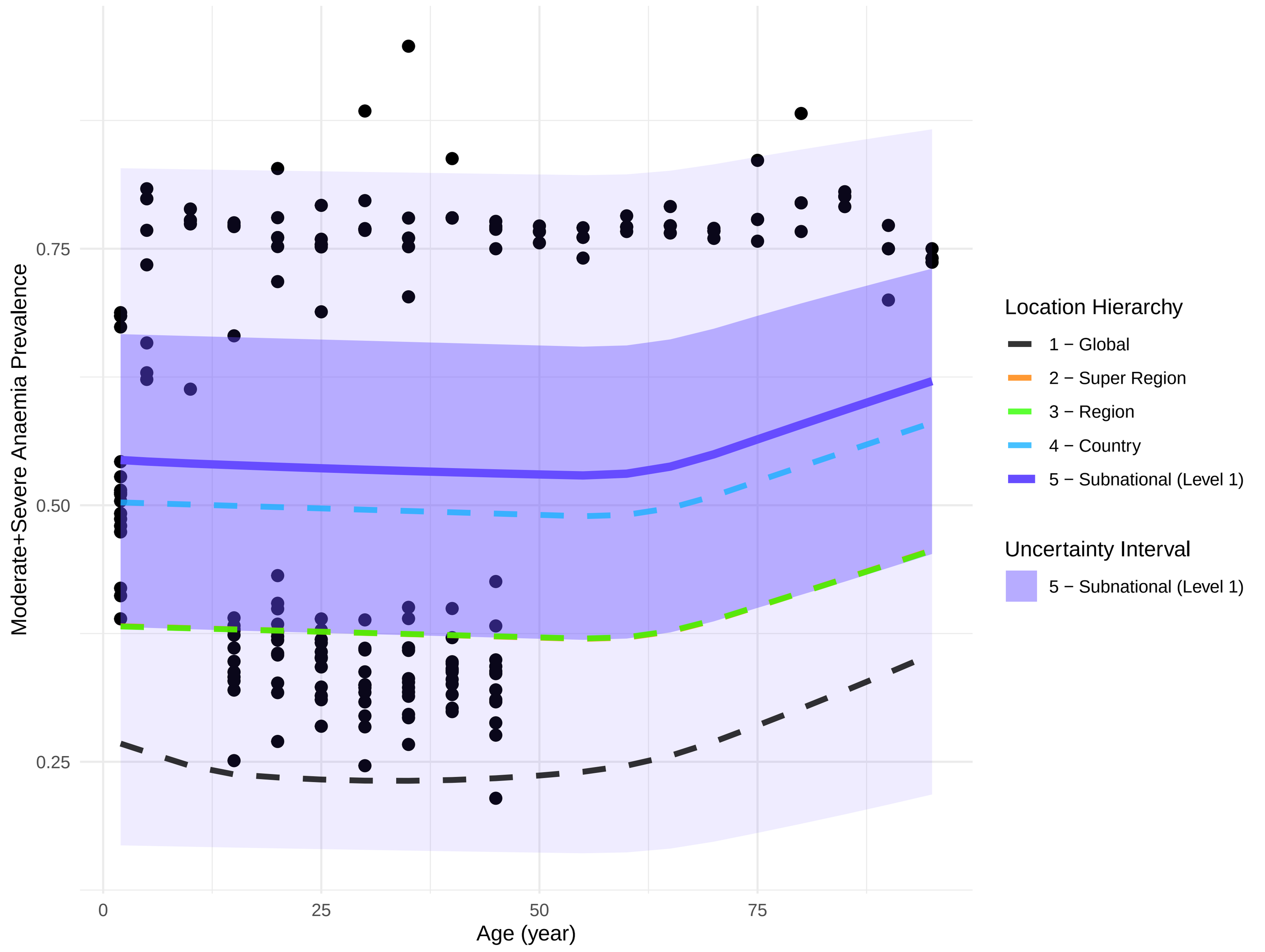

MR−BRT Age/Sex Fits − Non−pregnant Females 2+ Years Old − Moderate+Severe Anaemia Prevalence − Madhya Pradesh, Rural
WHO−2024 Adjustment Method

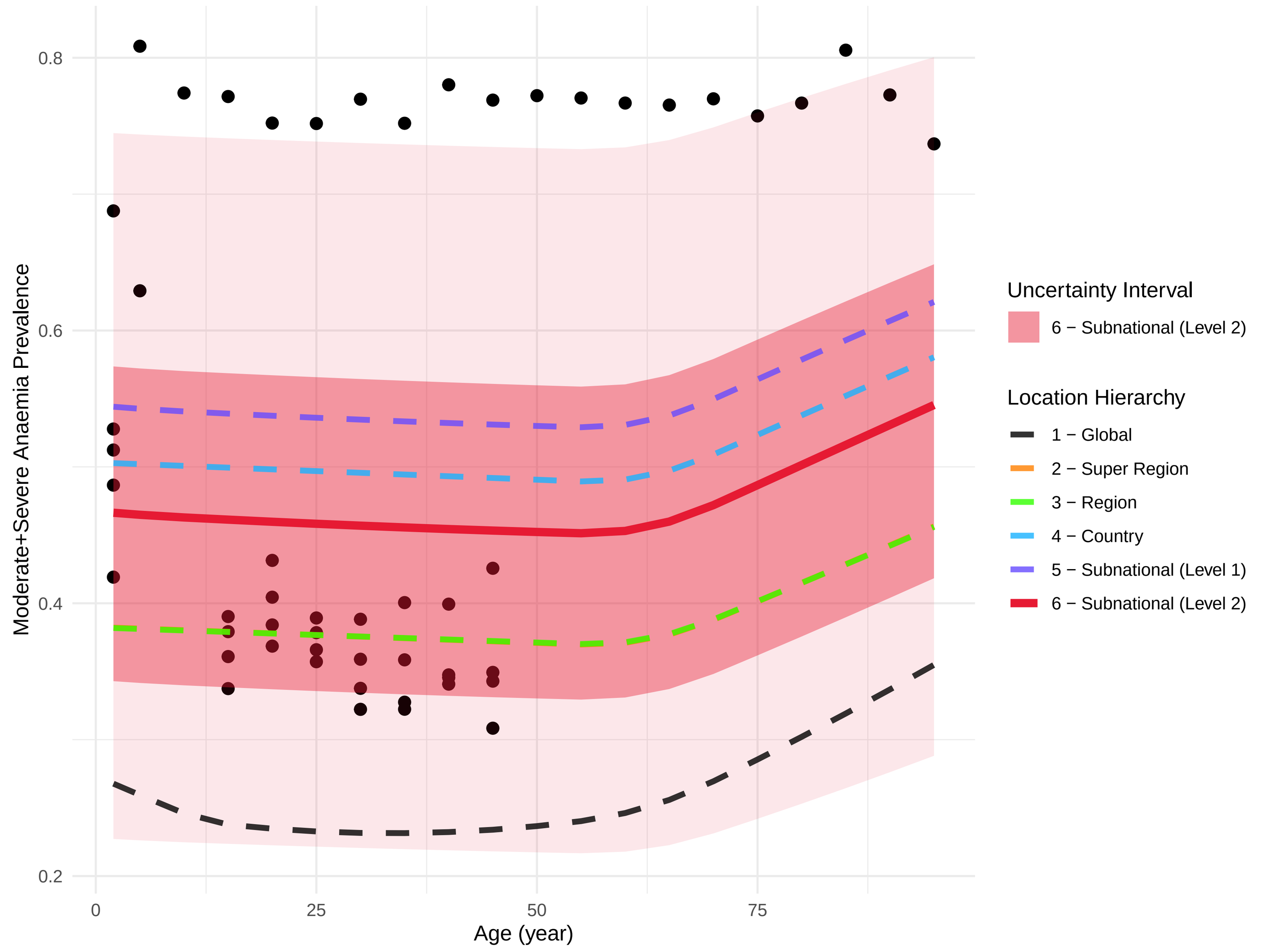

MR−BRT Age/Sex Fits − Non−pregnant Females 2+ Years Old − Moderate+Severe Anaemia Prevalence − Madhya Pradesh, Urban
WHO−2024 Adjustment Method

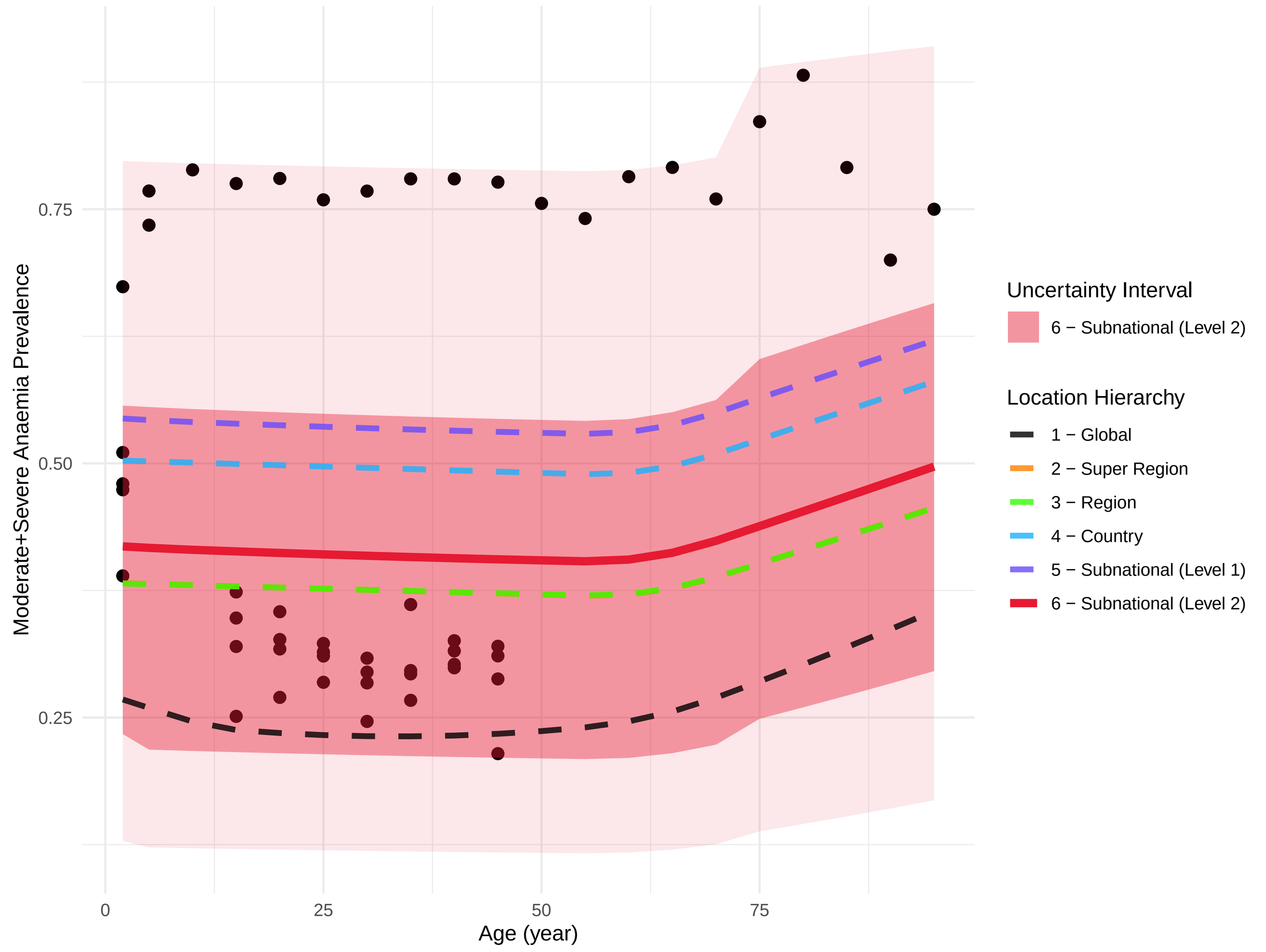

MR−BRT Age/Sex Fits − Non−pregnant Females 2+ Years Old − Moderate+Severe Anaemia Prevalence − Maharashtra
WHO−2024 Adjustment Method

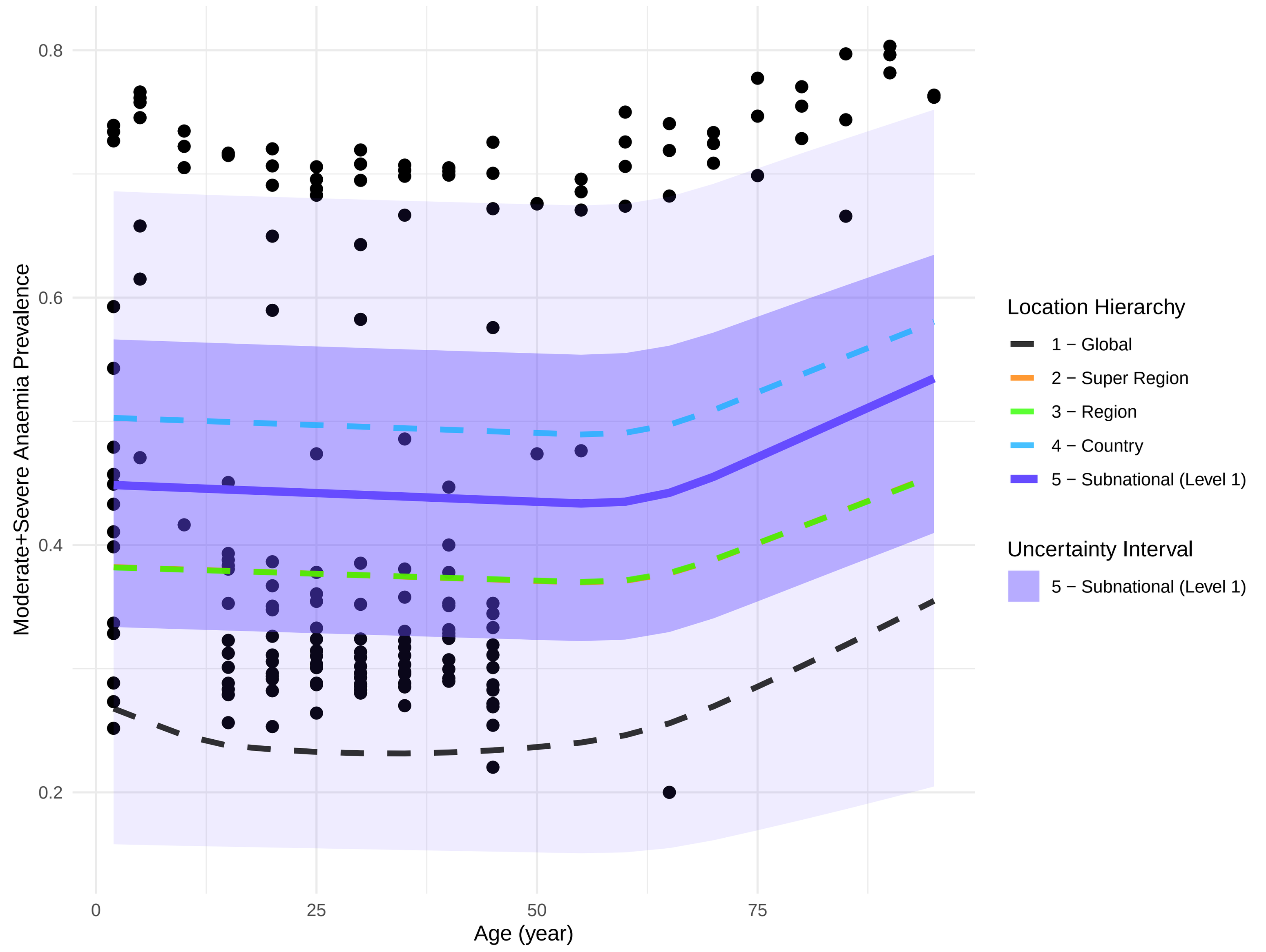

MR−BRT Age/Sex Fits − Non−pregnant Females 2+ Years Old − Moderate+Severe Anaemia Prevalence − Maharashtra, Rural
WHO−2024 Adjustment Method

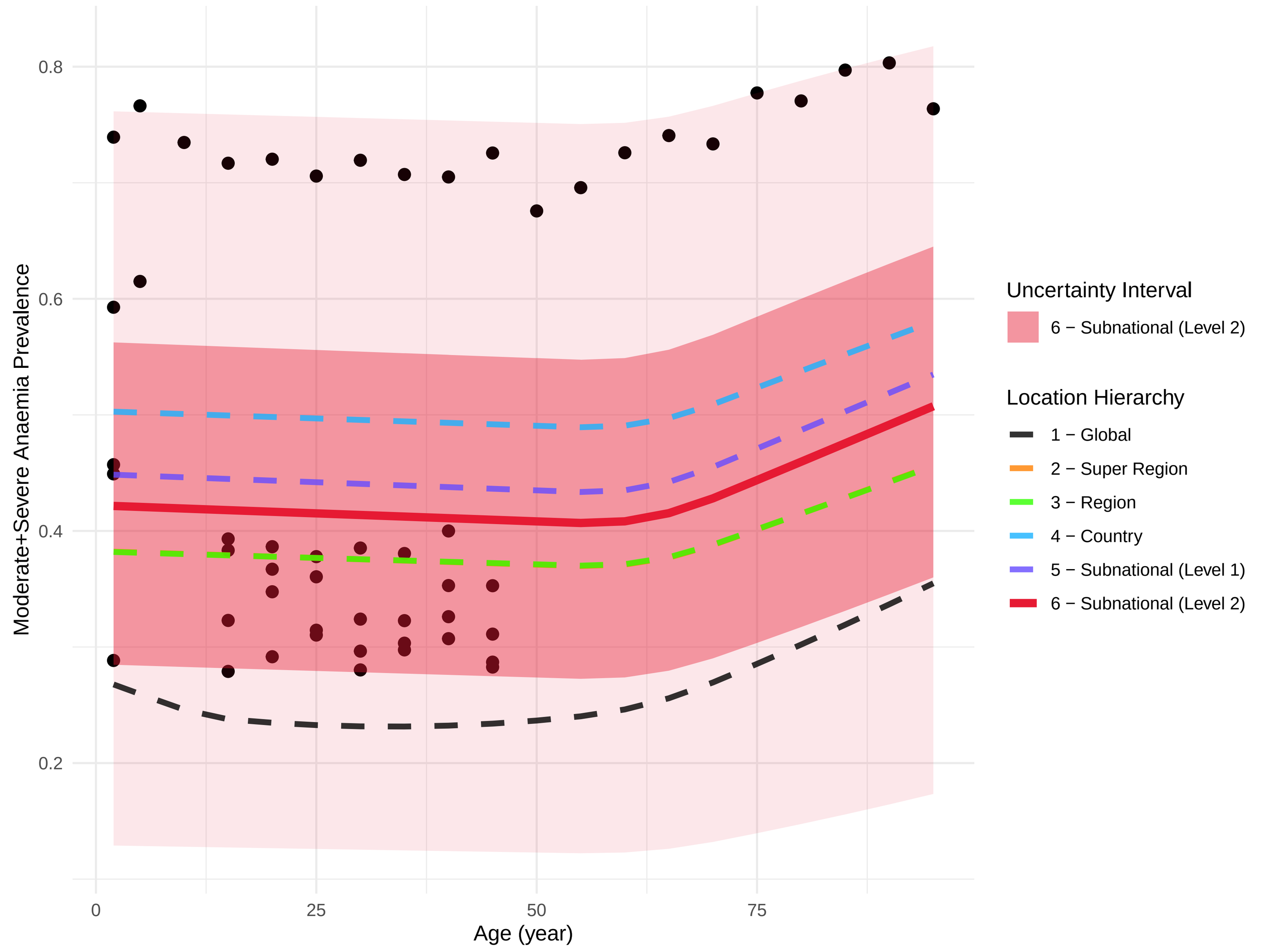

MR−BRT Age/Sex Fits − Non−pregnant Females 2+ Years Old − Moderate+Severe Anaemia Prevalence − Maharashtra, Urban
WHO−2024 Adjustment Method

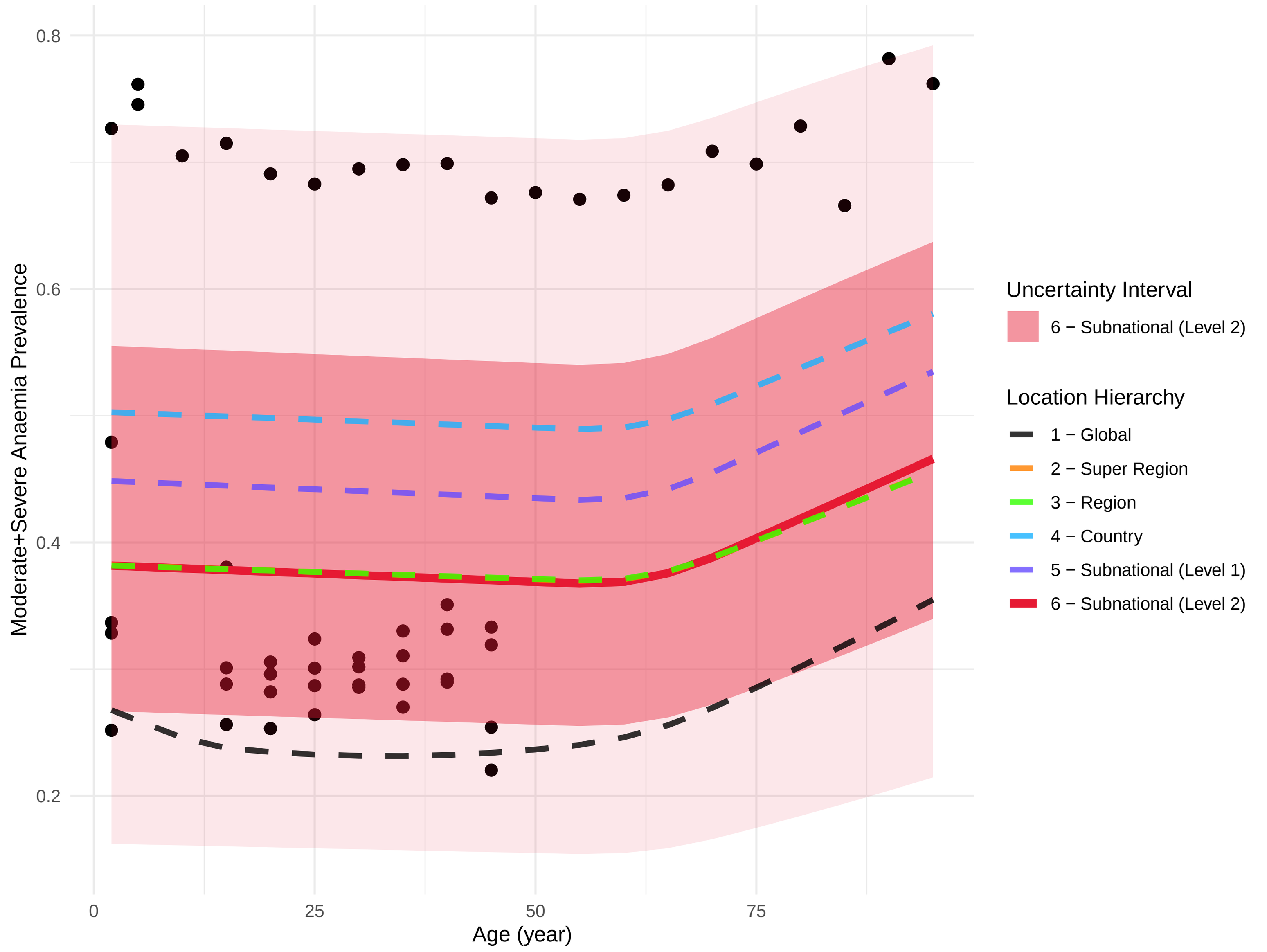

MR−BRT Age/Sex Fits − Non−pregnant Females 2+ Years Old − Moderate+Severe Anaemia Prevalence − Manipur
WHO−2024 Adjustment Method

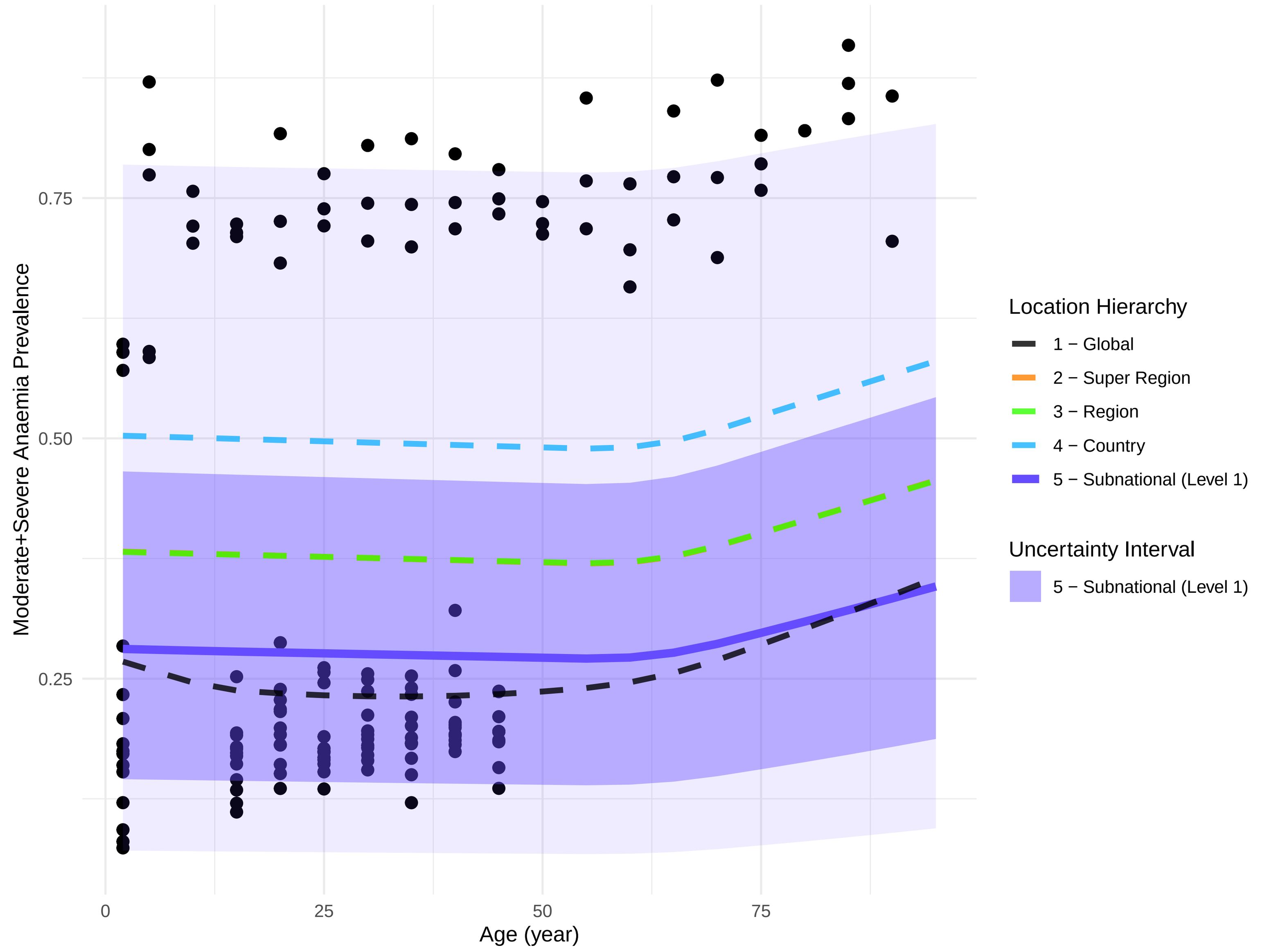

MR−BRT Age/Sex Fits − Non−pregnant Females 2+ Years Old − Moderate+Severe Anaemia Prevalence − Manipur, Rural
WHO−2024 Adjustment Method

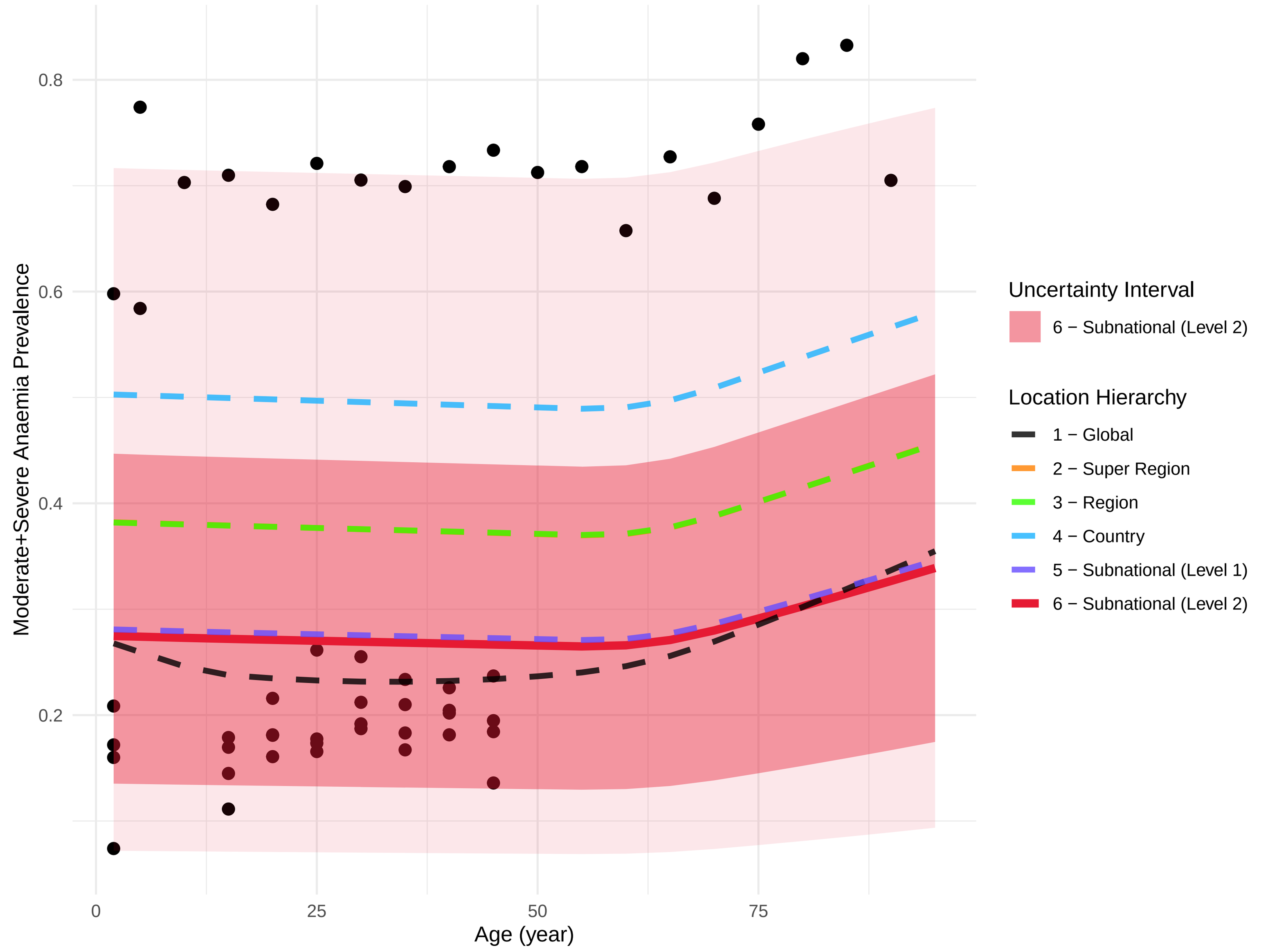

MR−BRT Age/Sex Fits − Non−pregnant Females 2+ Years Old − Moderate+Severe Anaemia Prevalence − Manipur, Urban
WHO−2024 Adjustment Method

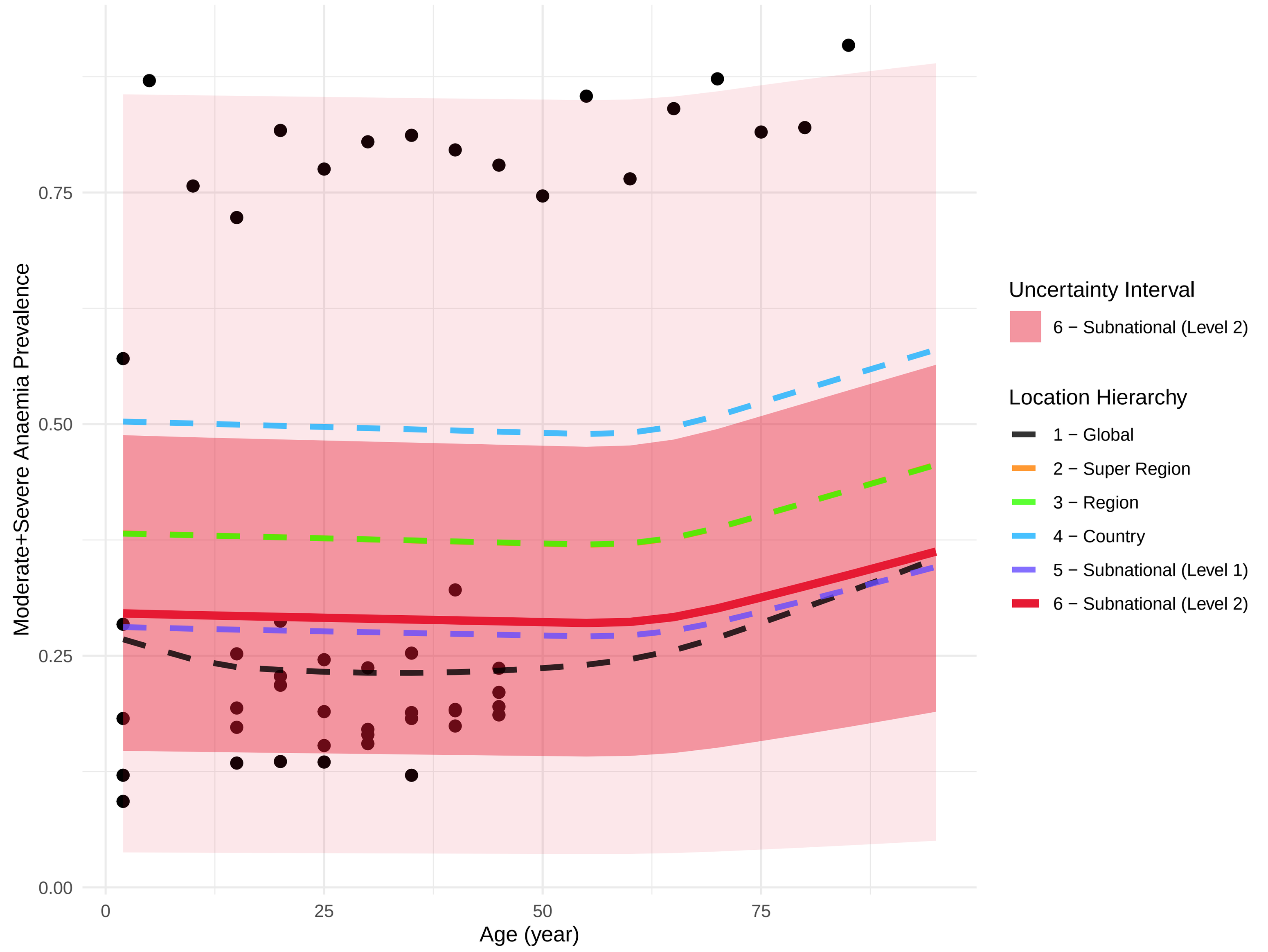

MR−BRT Age/Sex Fits − Non−pregnant Females 2+ Years Old − Moderate+Severe Anaemia Prevalence − Meghalaya
WHO−2024 Adjustment Method

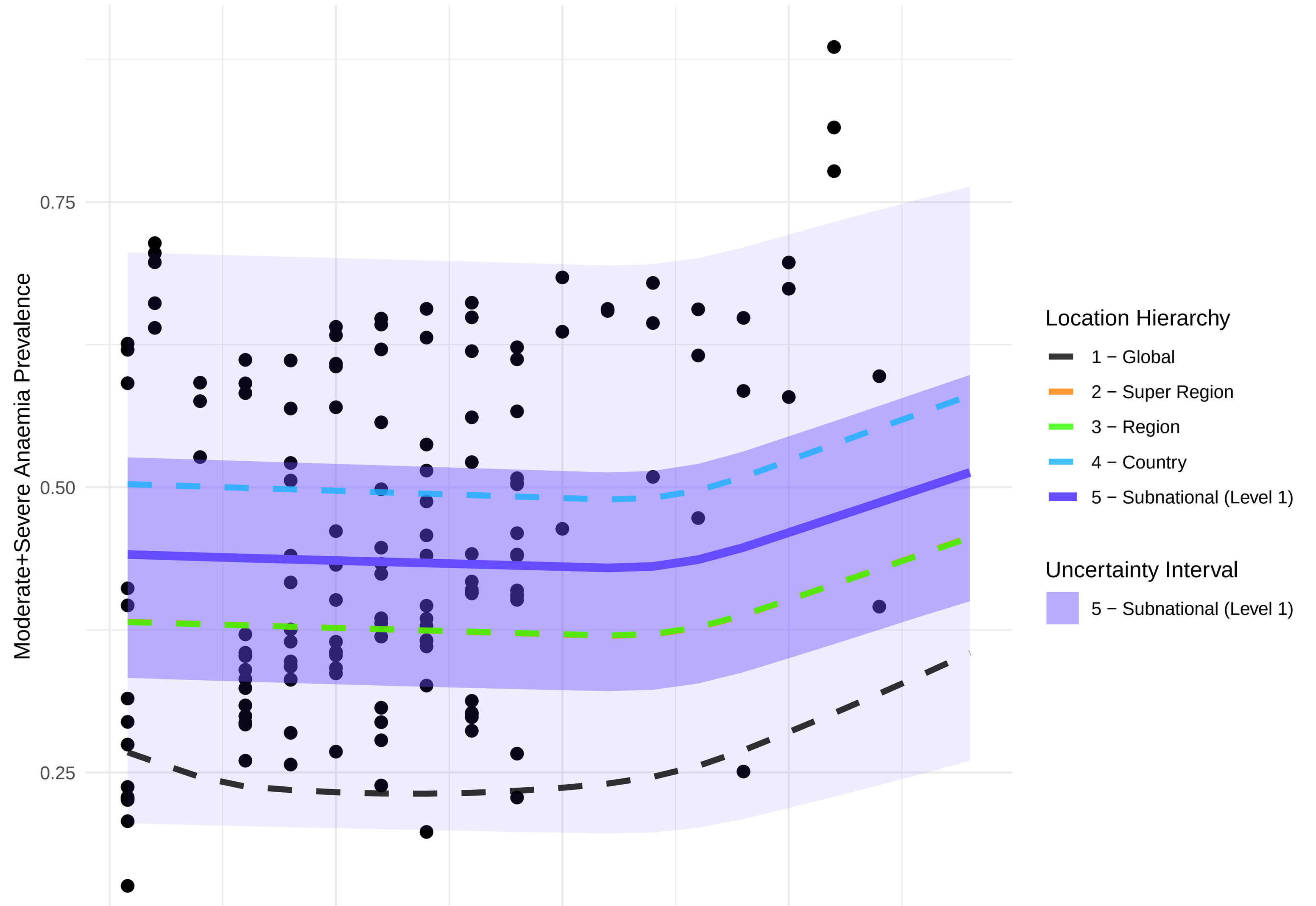

MR−BRT Age/Sex Fits − Non−pregnant Females 2+ Years Old − Moderate+Severe Anaemia Prevalence − Meghalaya, Rural
WHO−2024 Adjustment Method

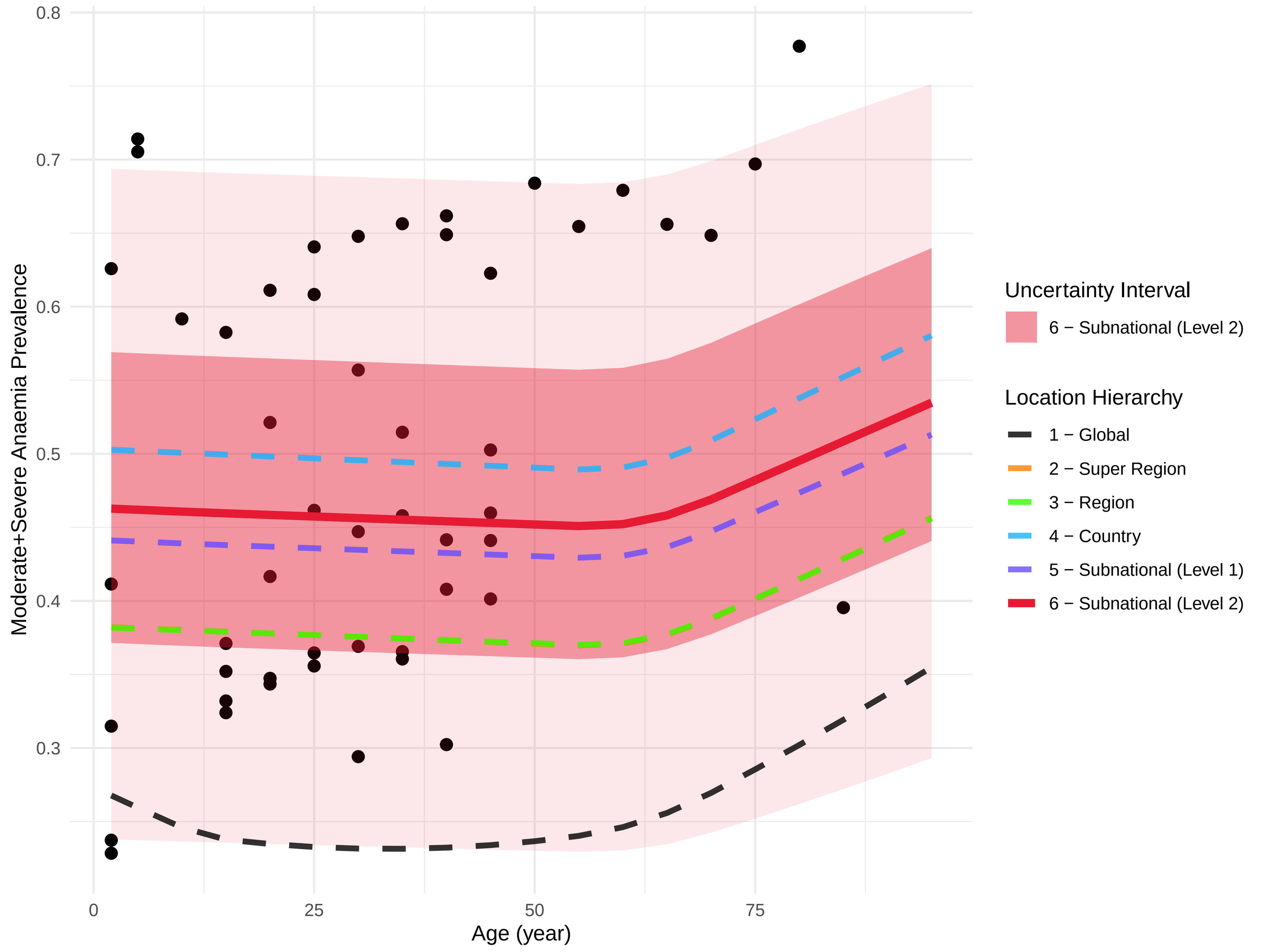

MR−BRT Age/Sex Fits − Non−pregnant Females 2+ Years Old − Moderate+Severe Anaemia Prevalence − Meghalaya, Urban
WHO−2024 Adjustment Method

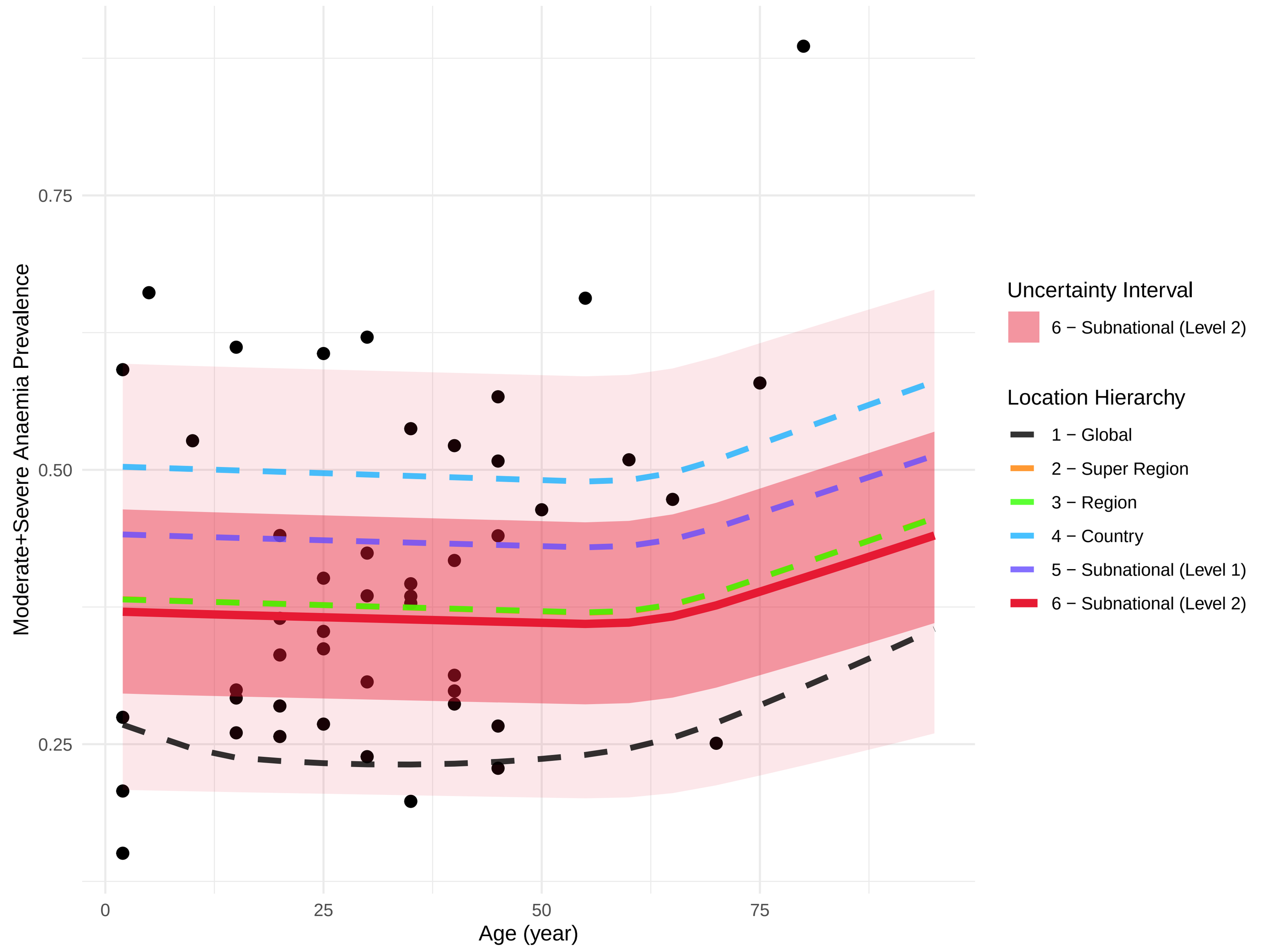

MR−BRT Age/Sex Fits − Non−pregnant Females 2+ Years Old − Moderate+Severe Anaemia Prevalence − Mizoram
WHO−2024 Adjustment Method

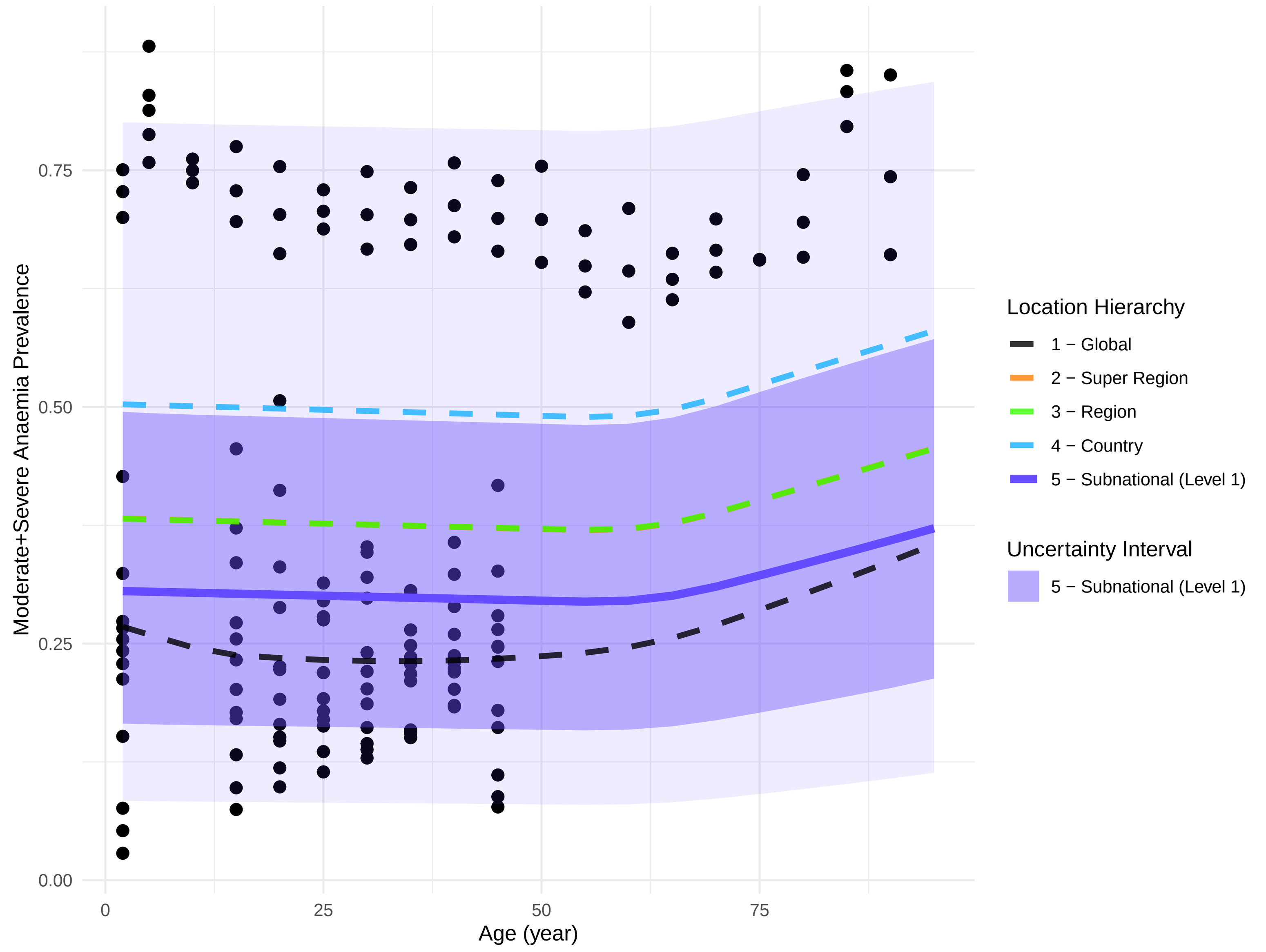

MR−BRT Age/Sex Fits − Non−pregnant Females 2+ Years Old − Moderate+Severe Anaemia Prevalence − Mizoram, Rural
WHO−2024 Adjustment Method

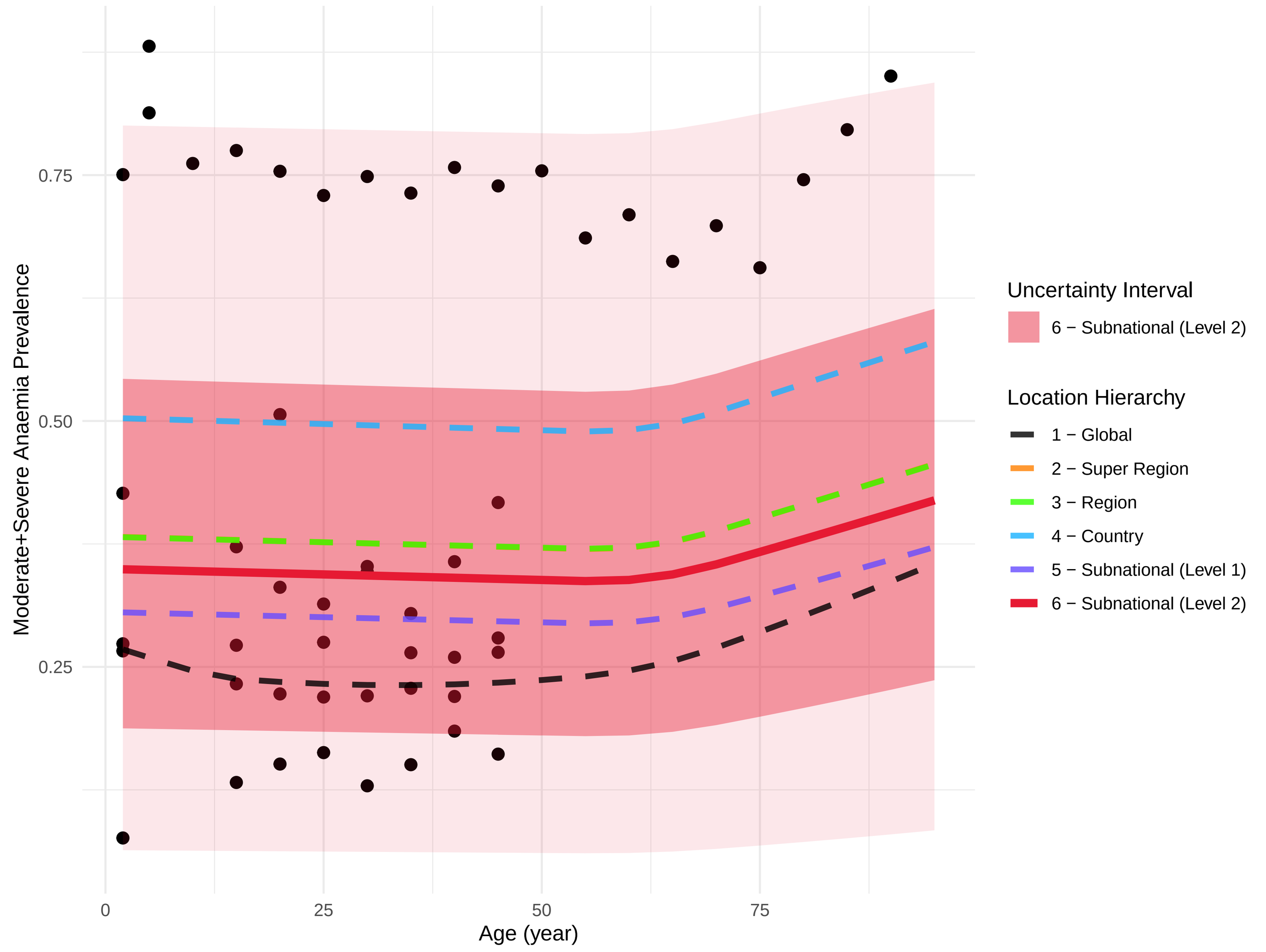

MR−BRT Age/Sex Fits − Non−pregnant Females 2+ Years Old − Moderate+Severe Anaemia Prevalence − Mizoram, Urban
WHO−2024 Adjustment Method

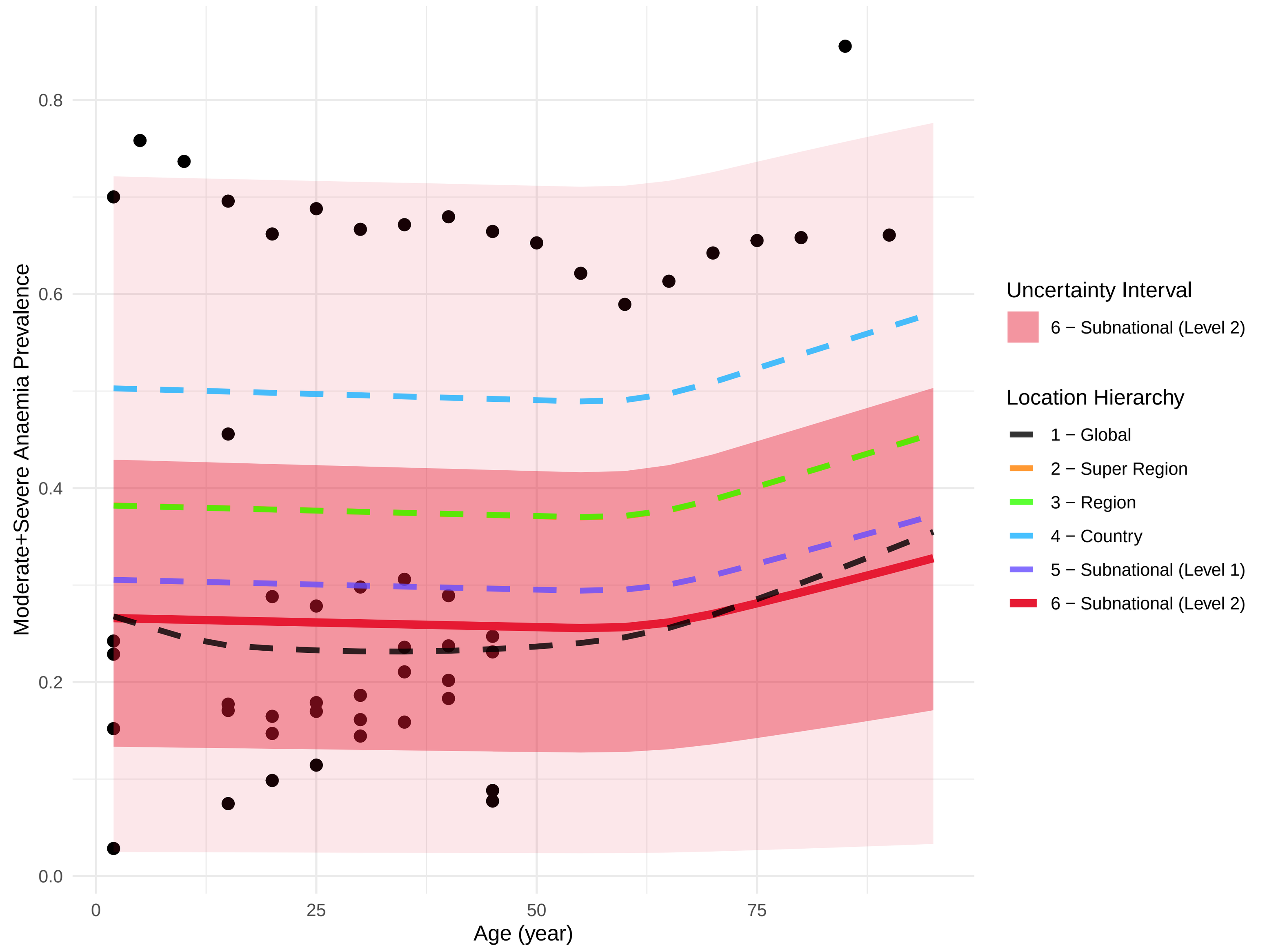

MR−BRT Age/Sex Fits − Non−pregnant Females 2+ Years Old − Moderate+Severe Anaemia Prevalence − Nagaland
WHO−2024 Adjustment Method

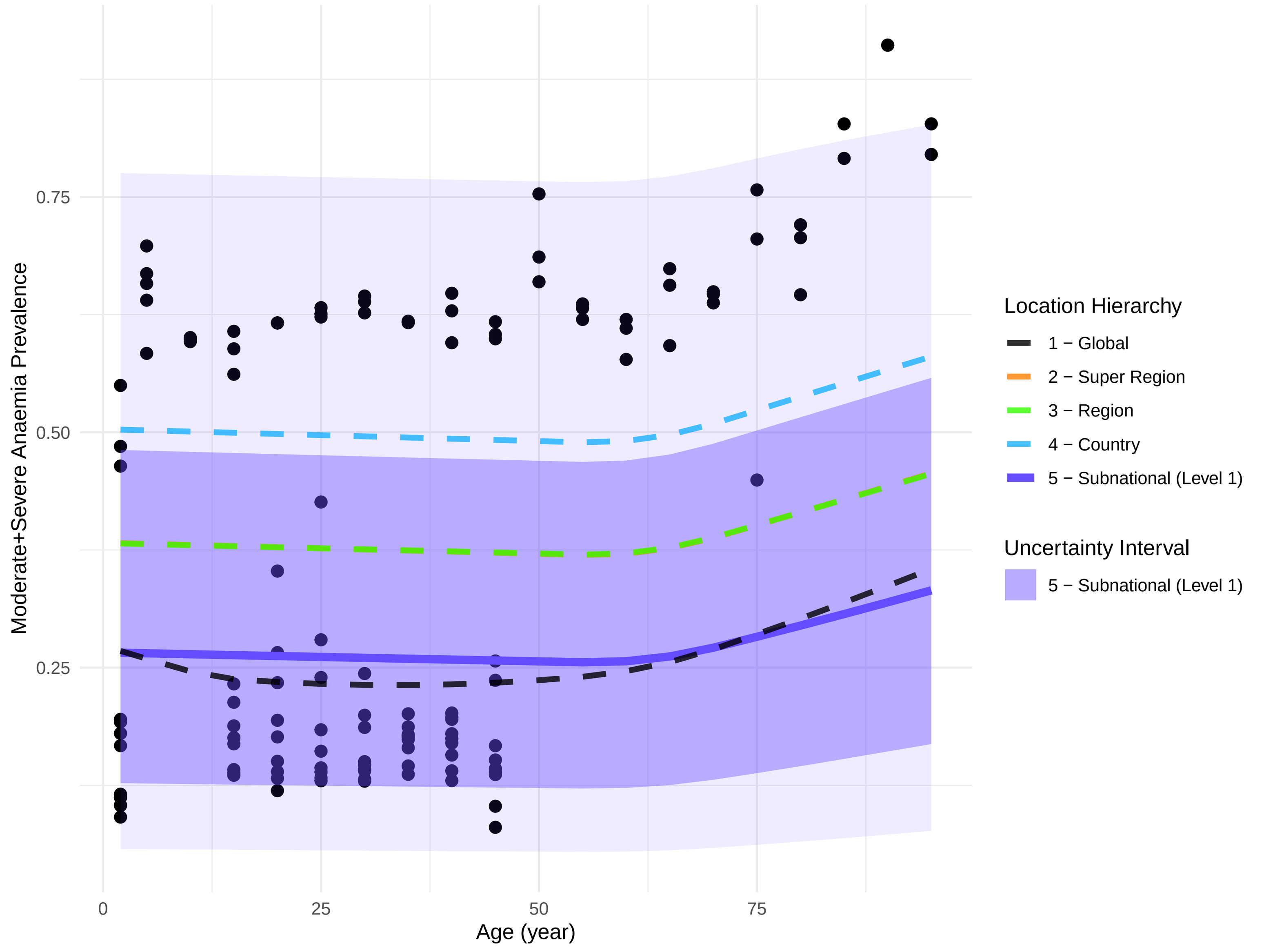

MR−BRT Age/Sex Fits − Non−pregnant Females 2+ Years Old − Moderate+Severe Anaemia Prevalence − Nagaland, Rural
WHO−2024 Adjustment Method

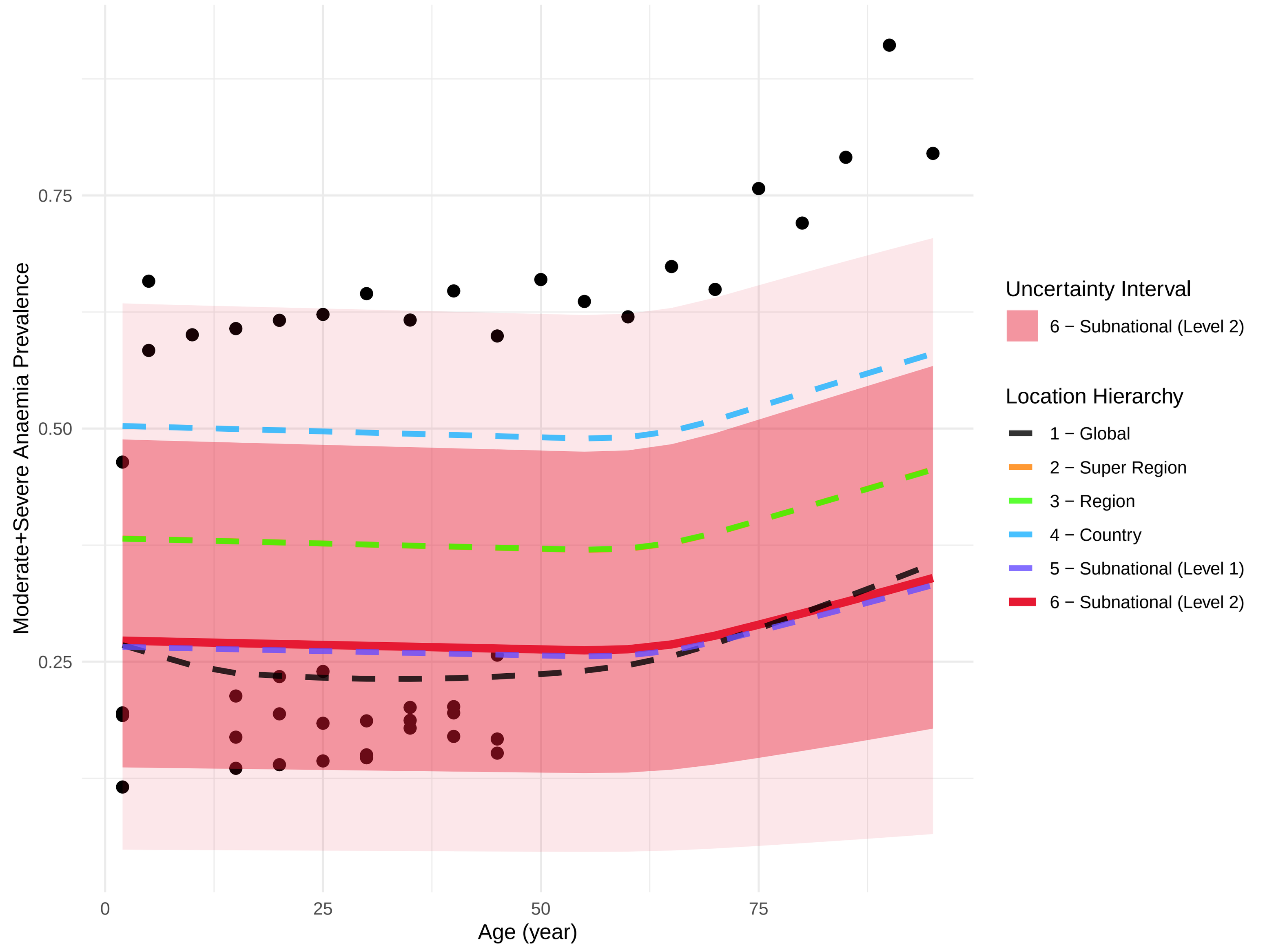

MR−BRT Age/Sex Fits − Non−pregnant Females 2+ Years Old − Moderate+Severe Anaemia Prevalence − Nagaland, Urban
WHO−2024 Adjustment Method

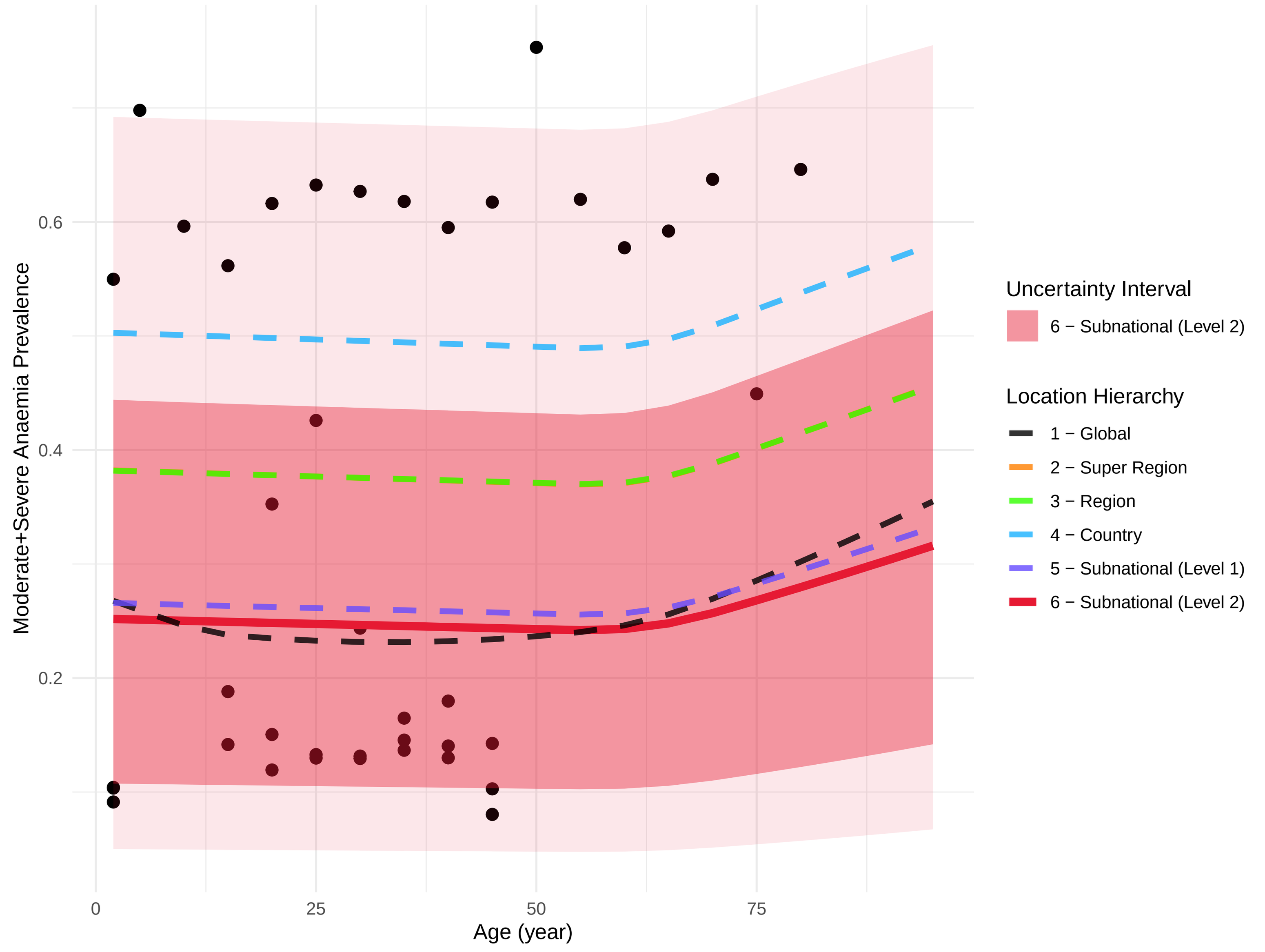

MR−BRT Age/Sex Fits − Non−pregnant Females 2+ Years Old − Moderate+Severe Anaemia Prevalence − Odisha
WHO−2024 Adjustment Method

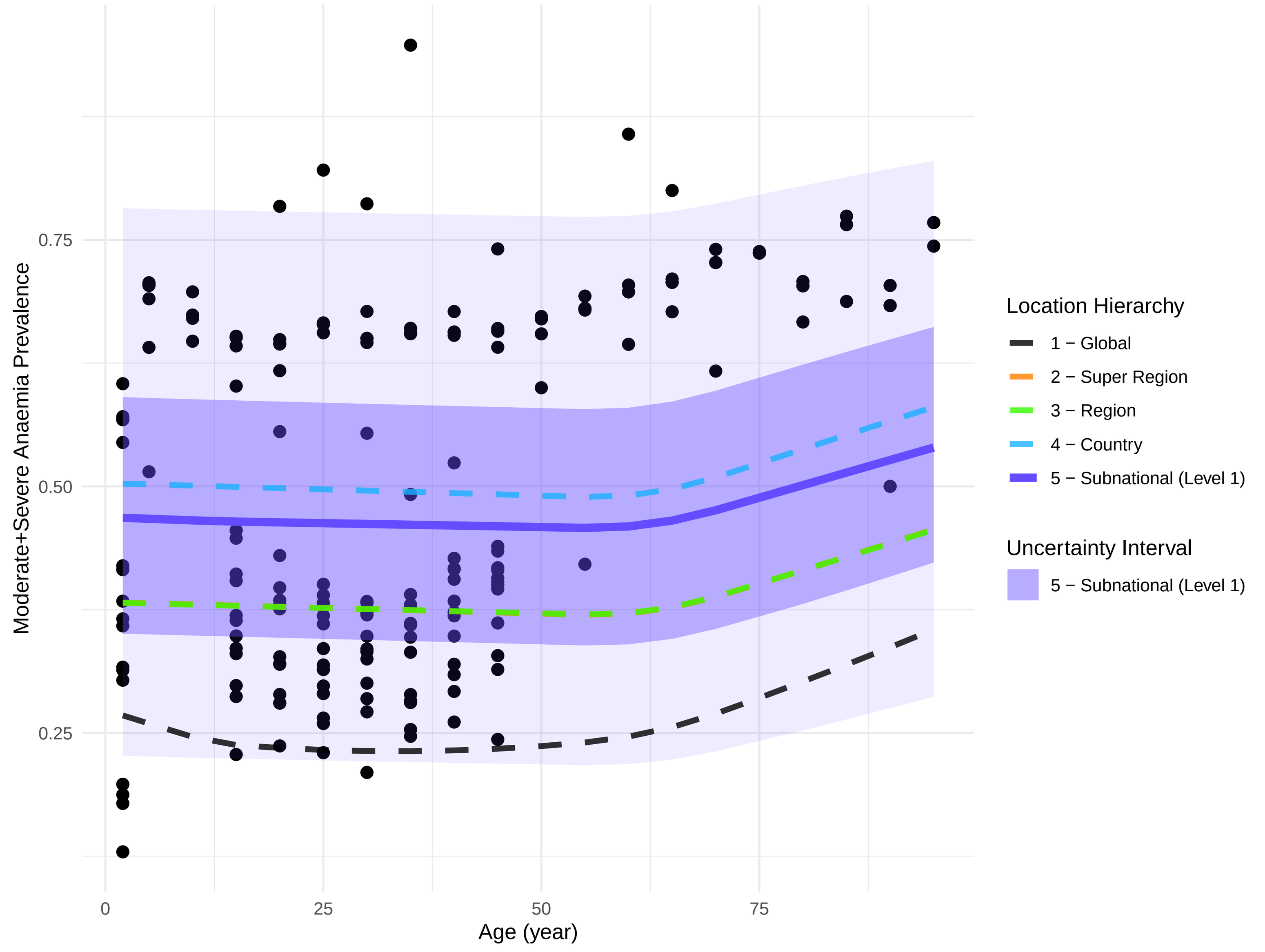

MR−BRT Age/Sex Fits − Non−pregnant Females 2+ Years Old − Moderate+Severe Anaemia Prevalence − Odisha, Rural
WHO−2024 Adjustment Method

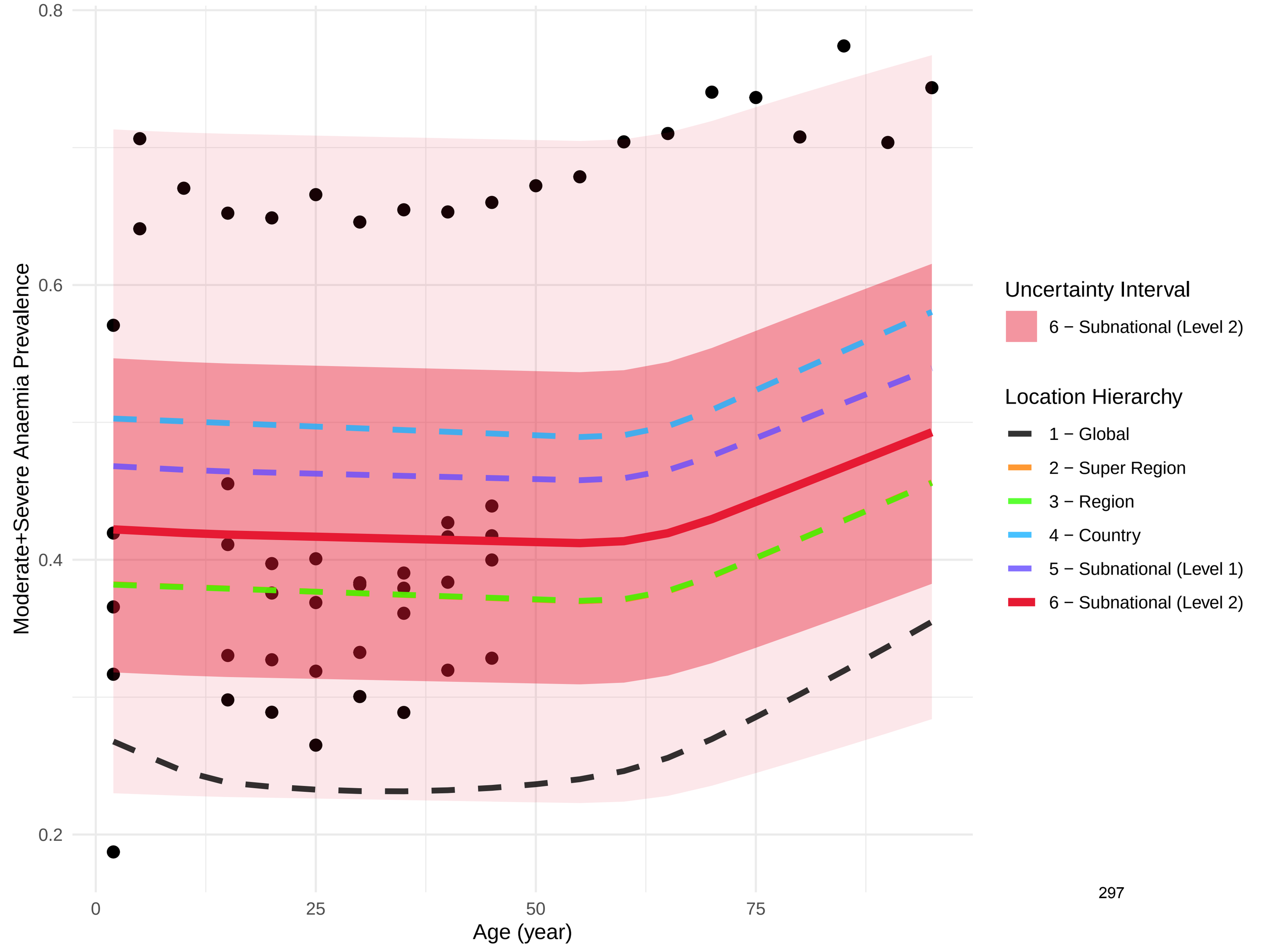

MR−BRT Age/Sex Fits − Non−pregnant Females 2+ Years Old − Moderate+Severe Anaemia Prevalence − Odisha, Urban
WHO−2024 Adjustment Method

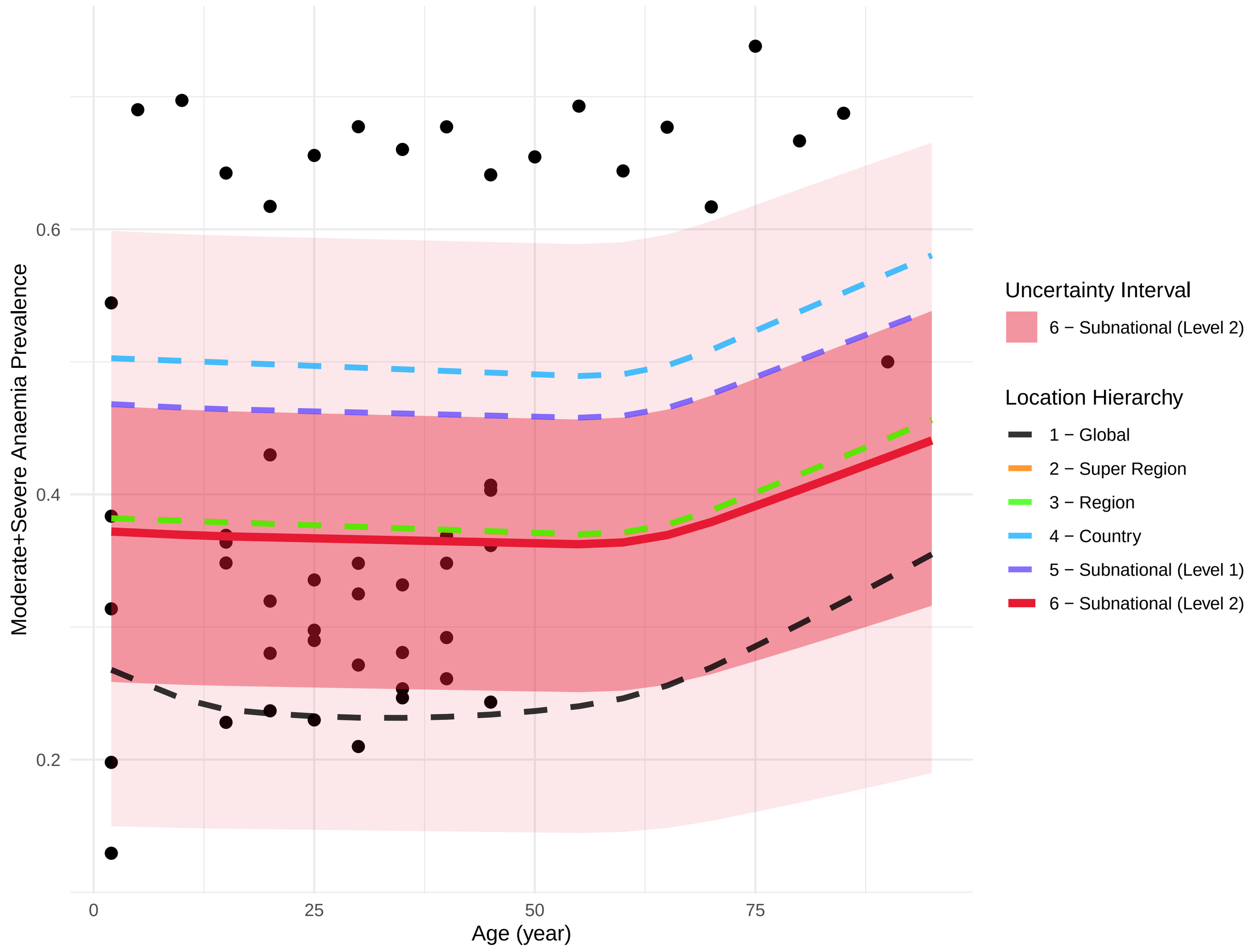

MR−BRT Age/Sex Fits − Non−pregnant Females 2+ Years Old − Moderate+Severe Anaemia Prevalence − Other Union Territories
WHO−2024 Adjustment Method

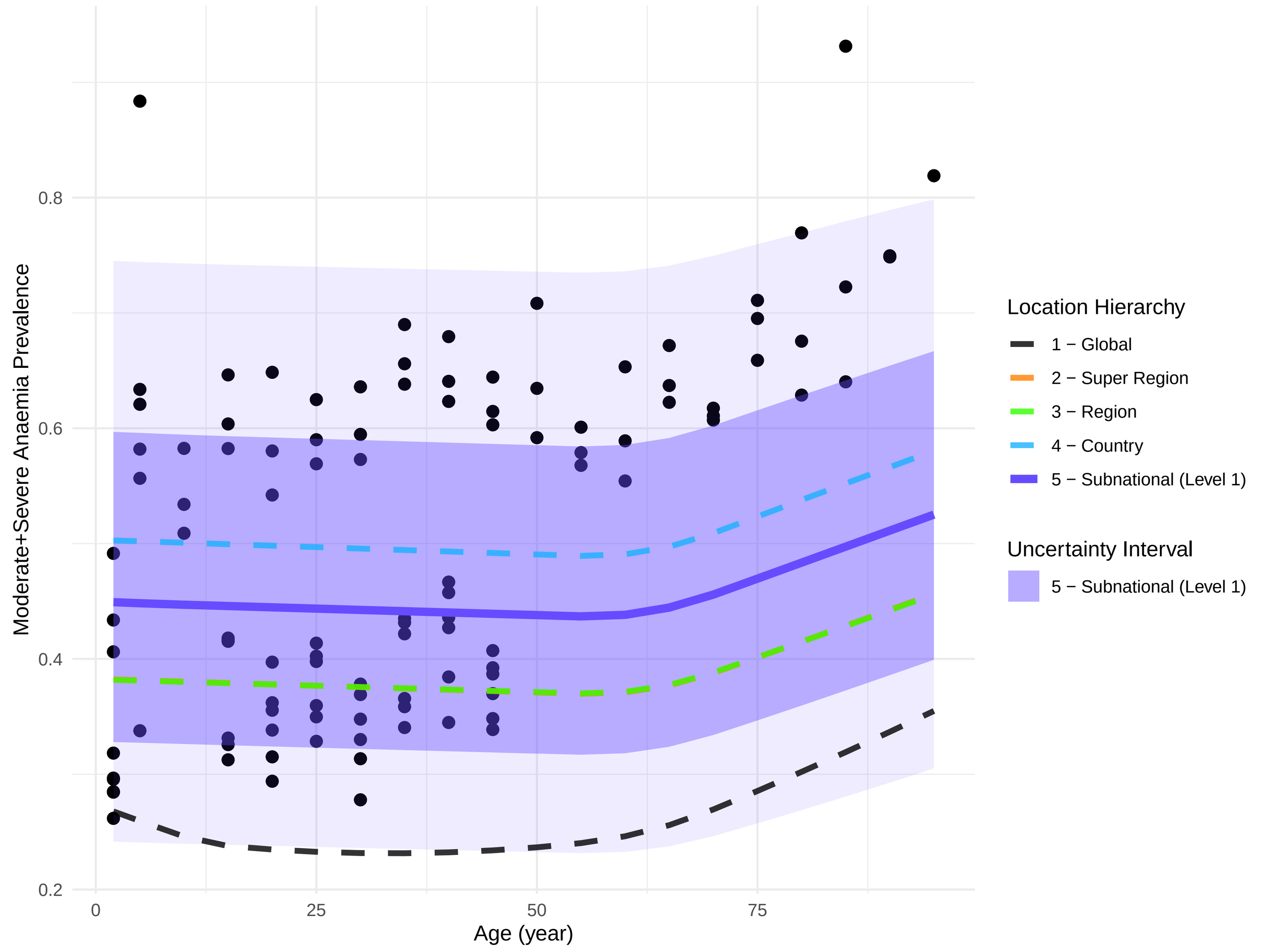

MR−BRT Age/Sex Fits − Non−pregnant Females 2+ Years Old − Moderate+Severe Anaemia Prevalence − Other Union Territories, Rural
WHO−2024 Adjustment Method

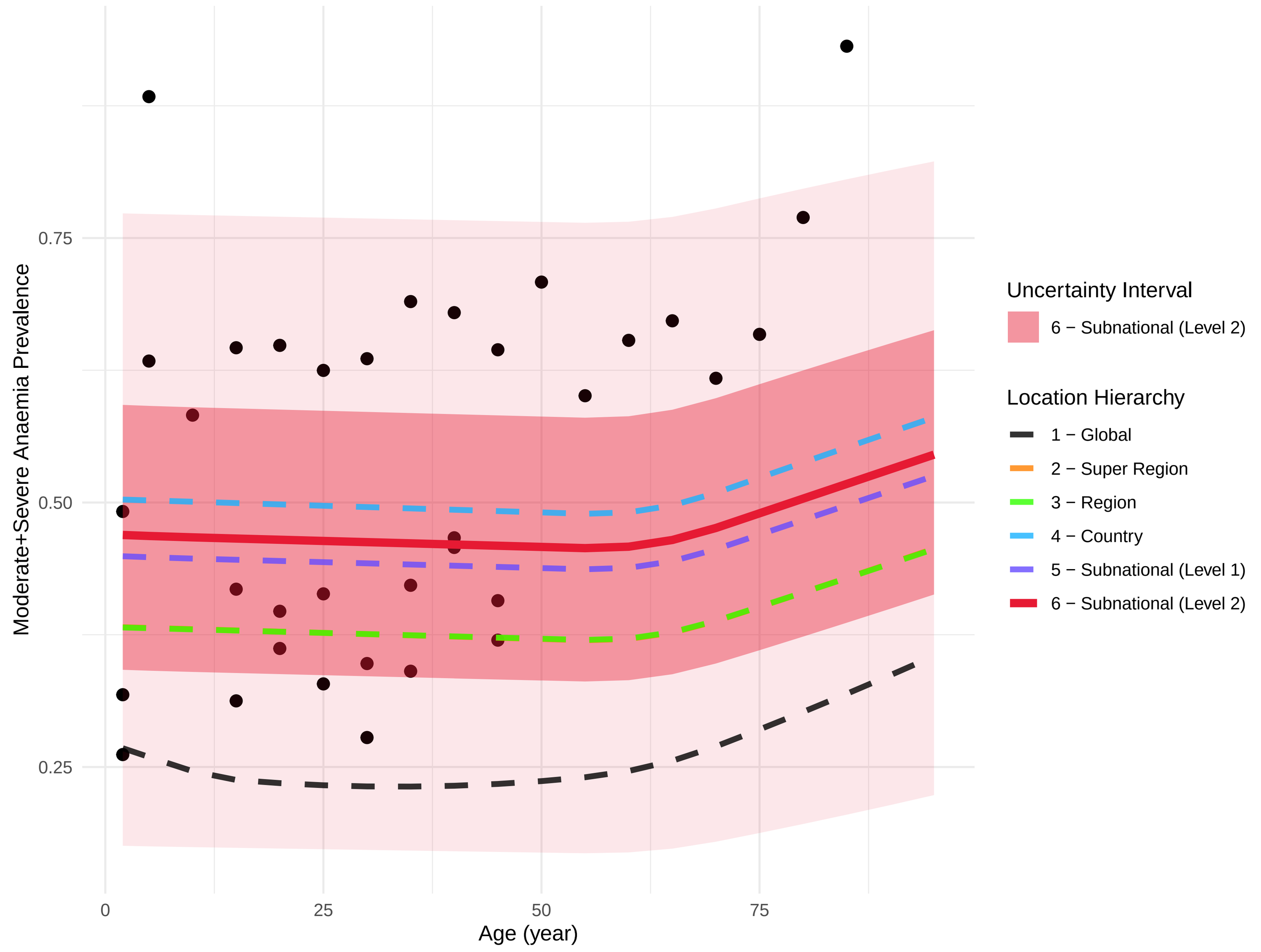

MR−BRT Age/Sex Fits − Non−pregnant Females 2+ Years Old − Moderate+Severe Anaemia Prevalence − Other Union Territories, Urban
WHO−2024 Adjustment Method

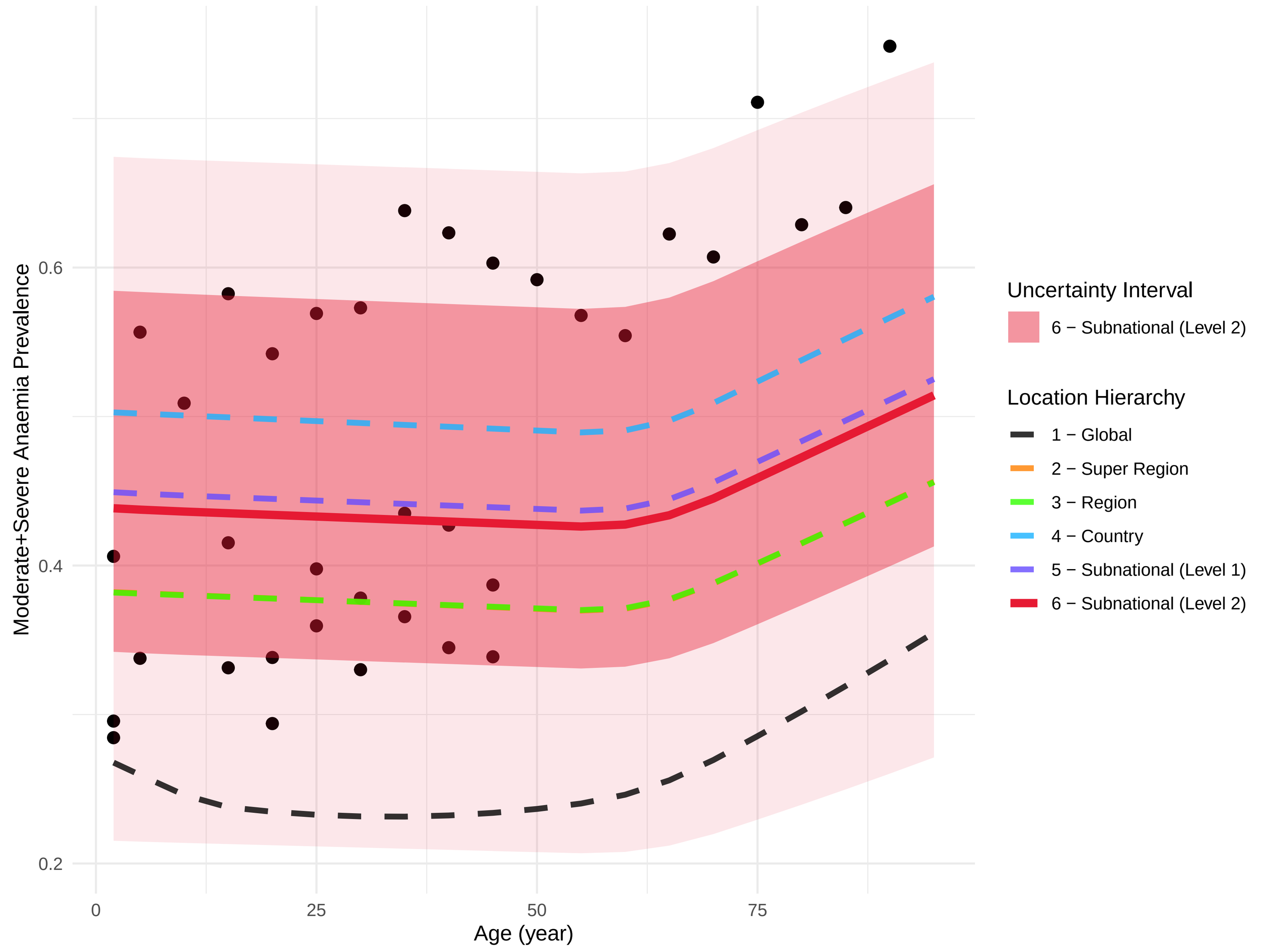

MR−BRT Age/Sex Fits − Non−pregnant Females 2+ Years Old − Moderate+Severe Anaemia Prevalence − Punjab
WHO−2024 Adjustment Method

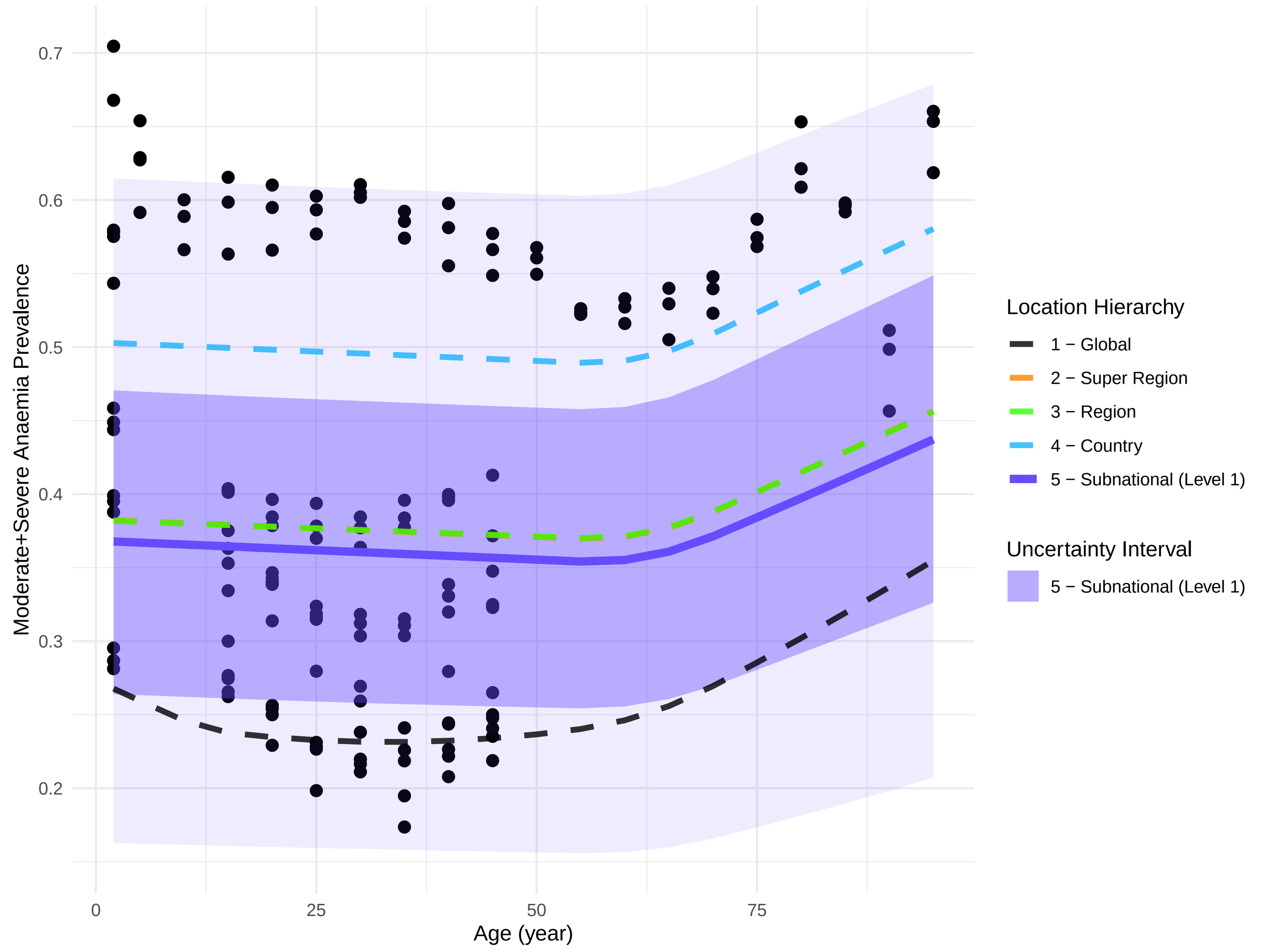

MR−BRT Age/Sex Fits − Non−pregnant Females 2+ Years Old − Moderate+Severe Anaemia Prevalence − Punjab, Rural
WHO−2024 Adjustment Method

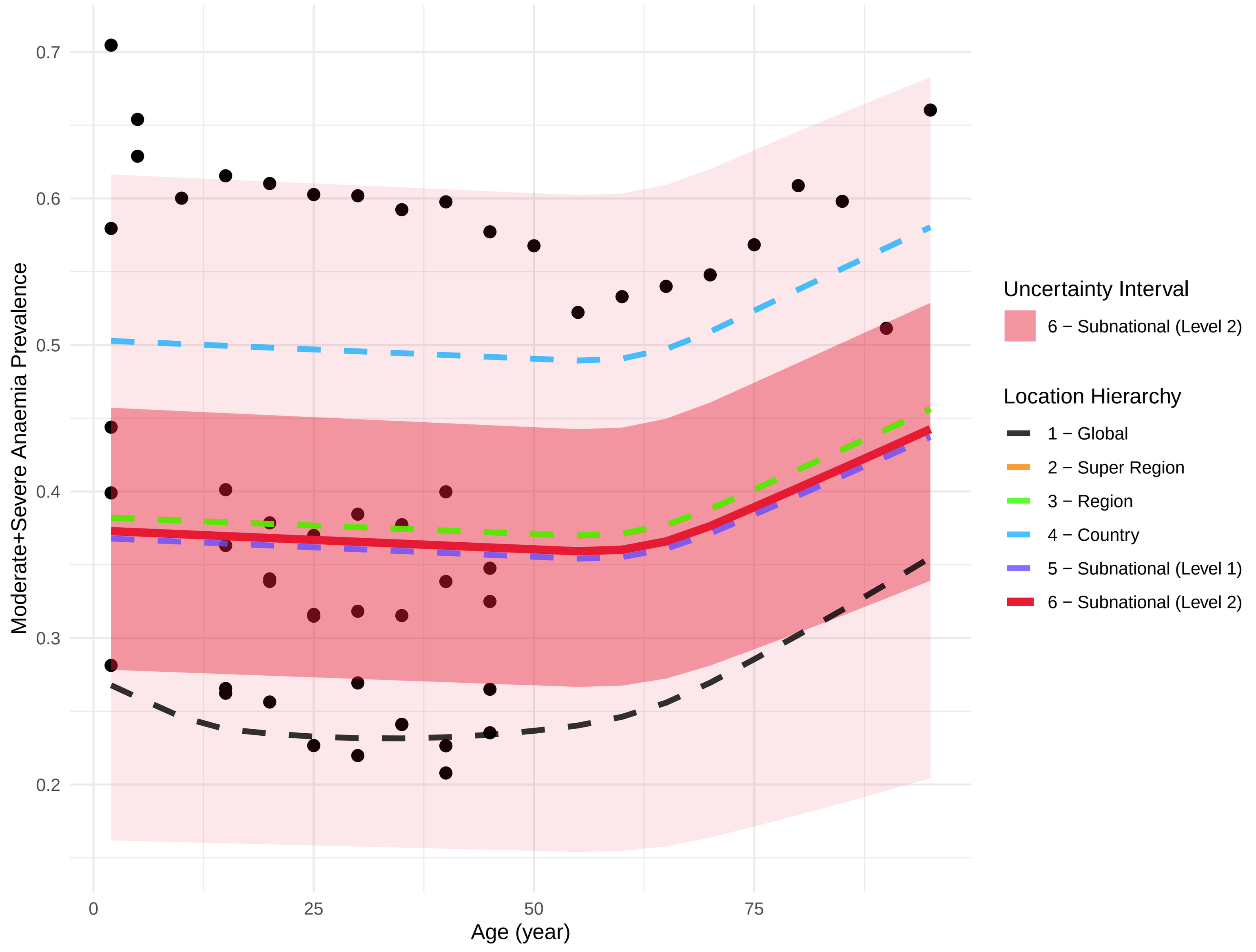

MR−BRT Age/Sex Fits − Non−pregnant Females 2+ Years Old − Moderate+Severe Anaemia Prevalence − Punjab, Urban
WHO−2024 Adjustment Method

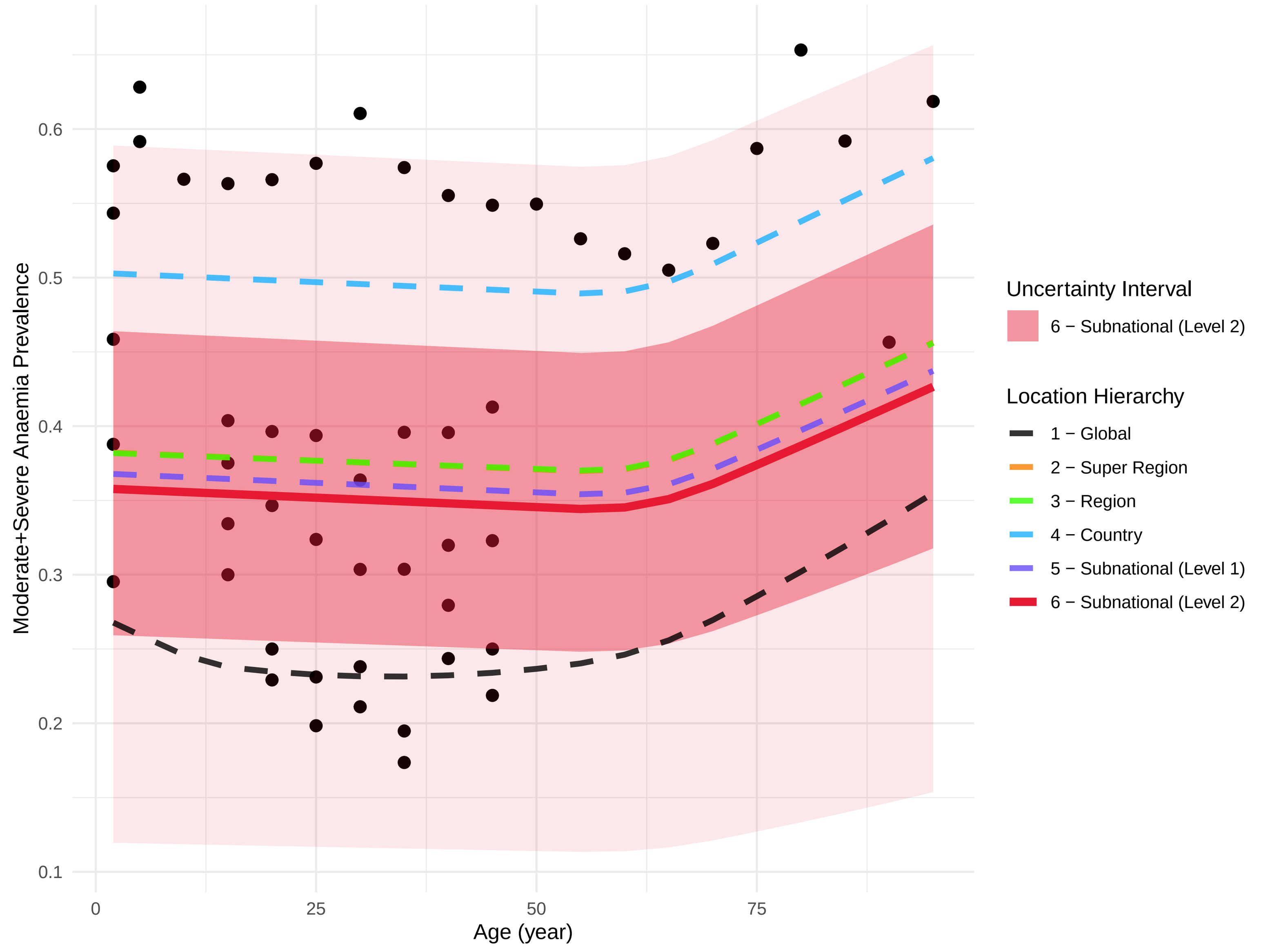

MR−BRT Age/Sex Fits − Non−pregnant Females 2+ Years Old − Moderate+Severe Anaemia Prevalence − Rajasthan
WHO−2024 Adjustment Method

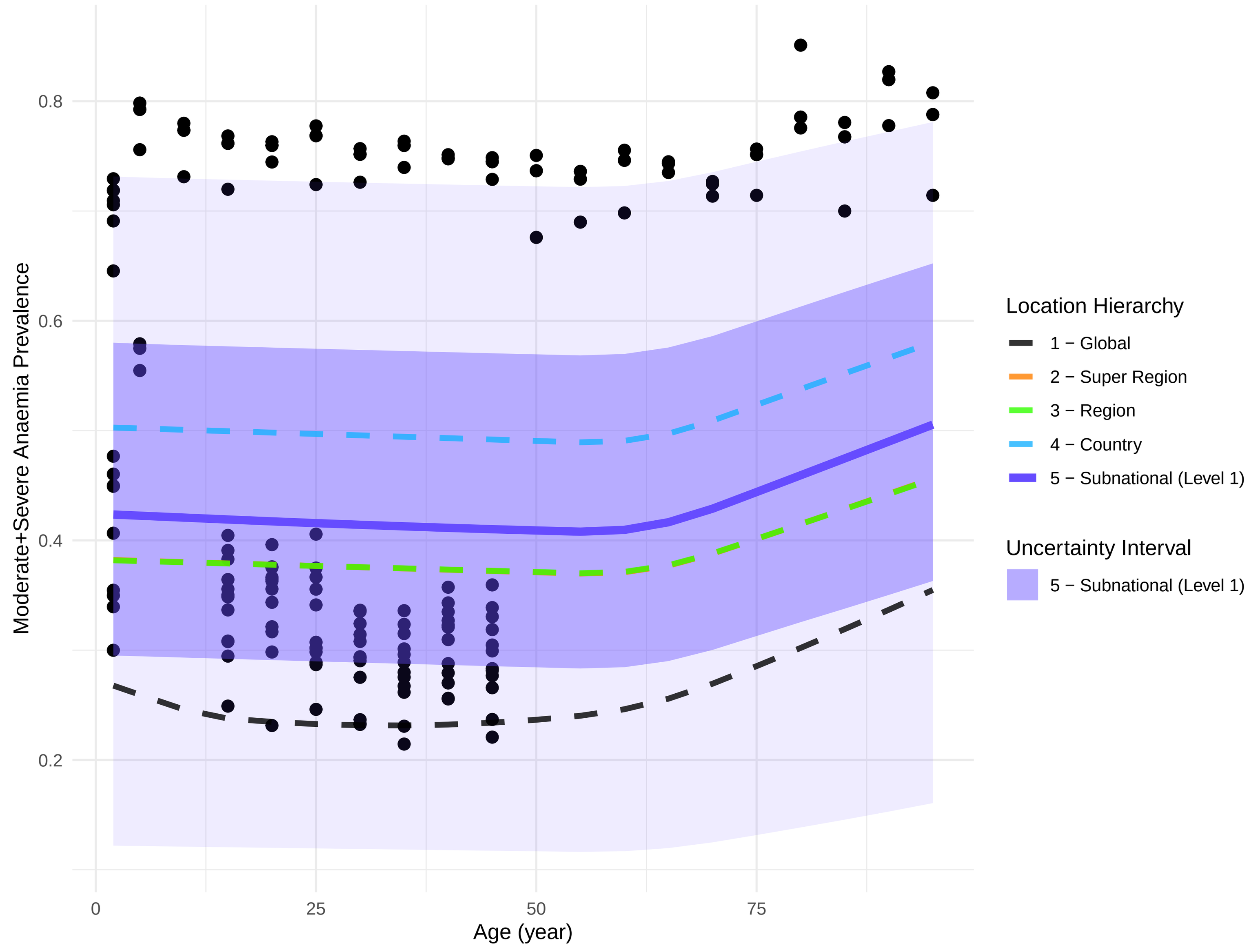

MR−BRT Age/Sex Fits − Non−pregnant Females 2+ Years Old − Moderate+Severe Anaemia Prevalence − Rajasthan, Rural
WHO−2024 Adjustment Method

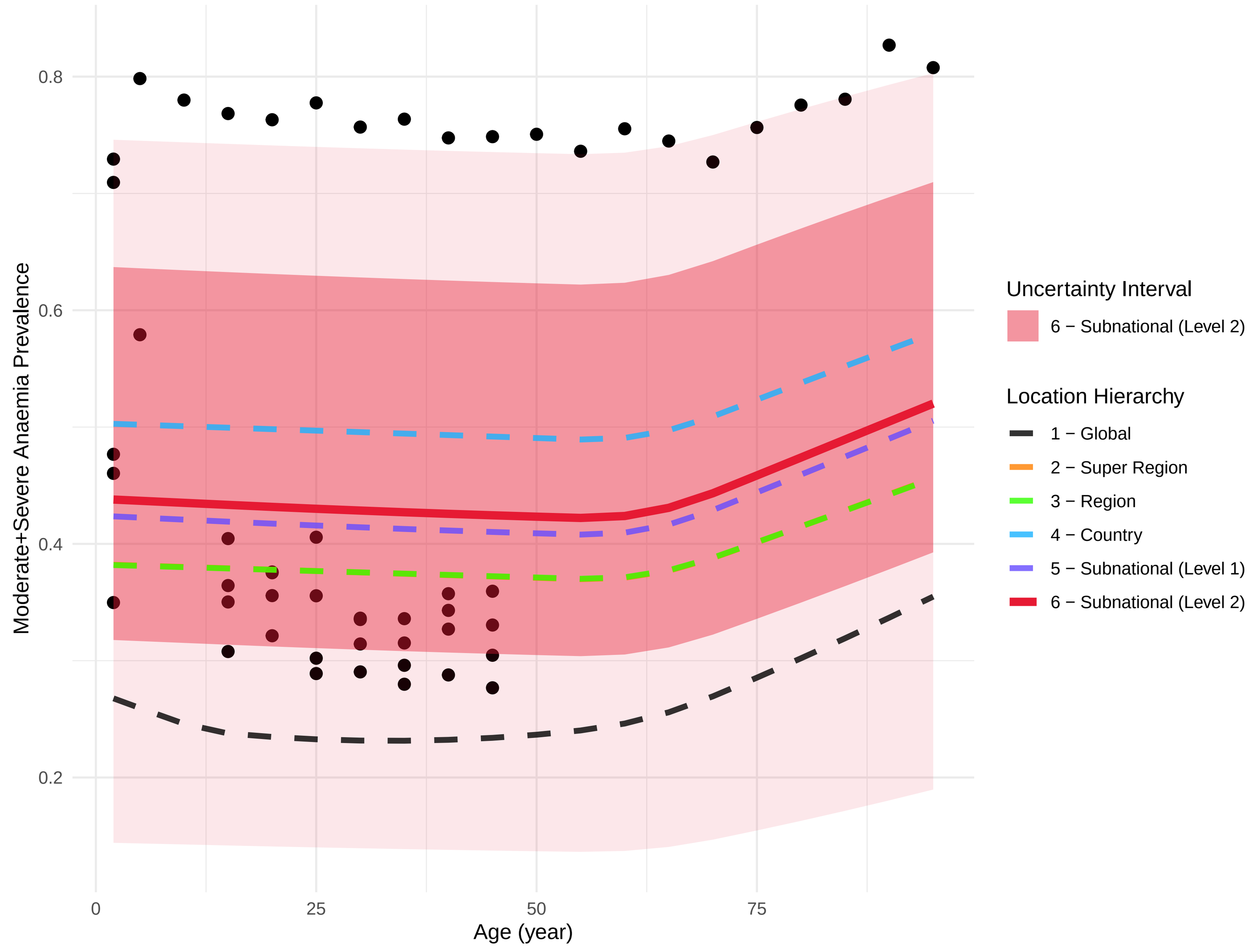

MR−BRT Age/Sex Fits − Non−pregnant Females 2+ Years Old − Moderate+Severe Anaemia Prevalence − Rajasthan, Urban
WHO−2024 Adjustment Method

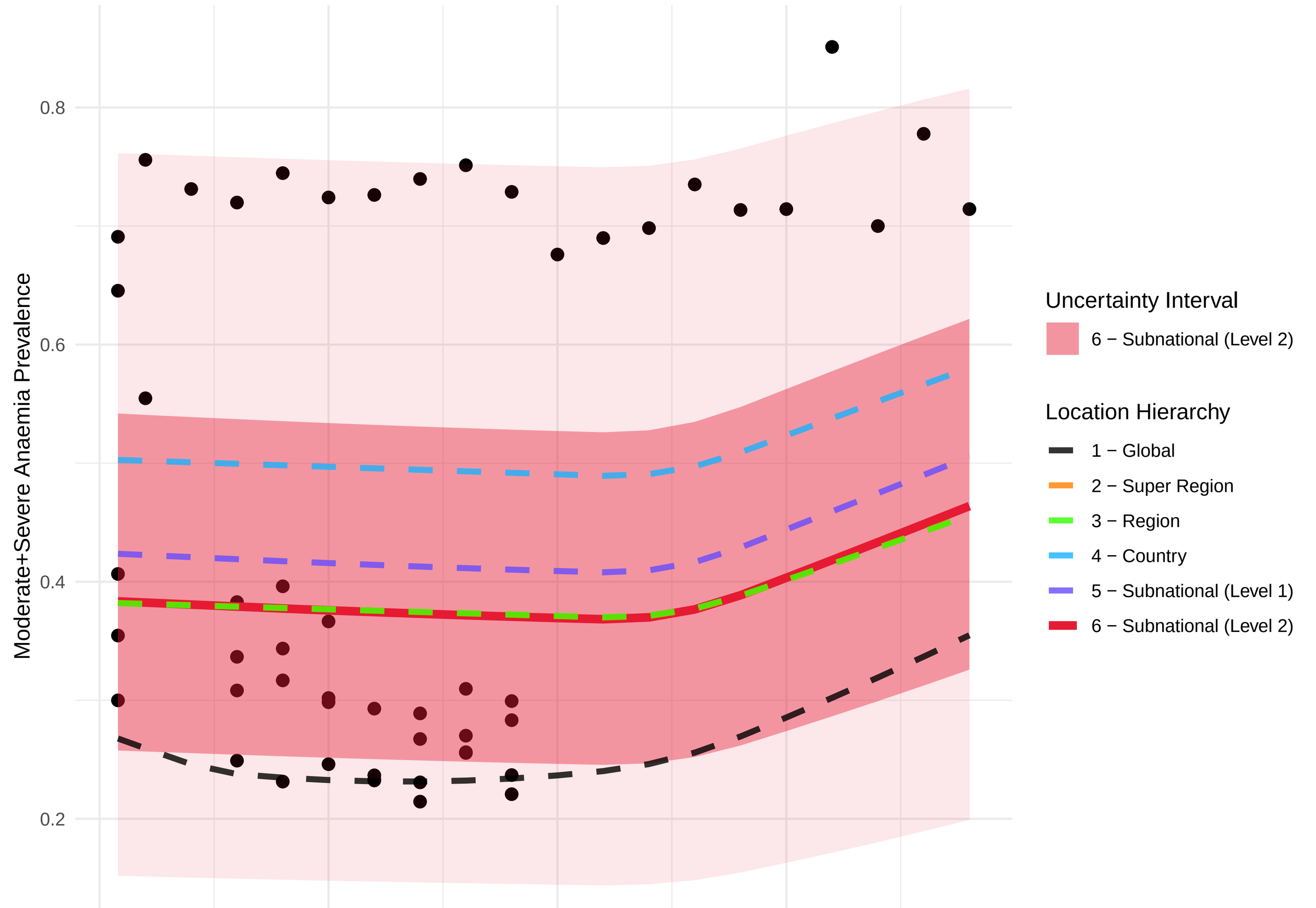

MR−BRT Age/Sex Fits − Non−pregnant Females 2+ Years Old − Moderate+Severe Anaemia Prevalence − Sikkim
WHO−2024 Adjustment Method

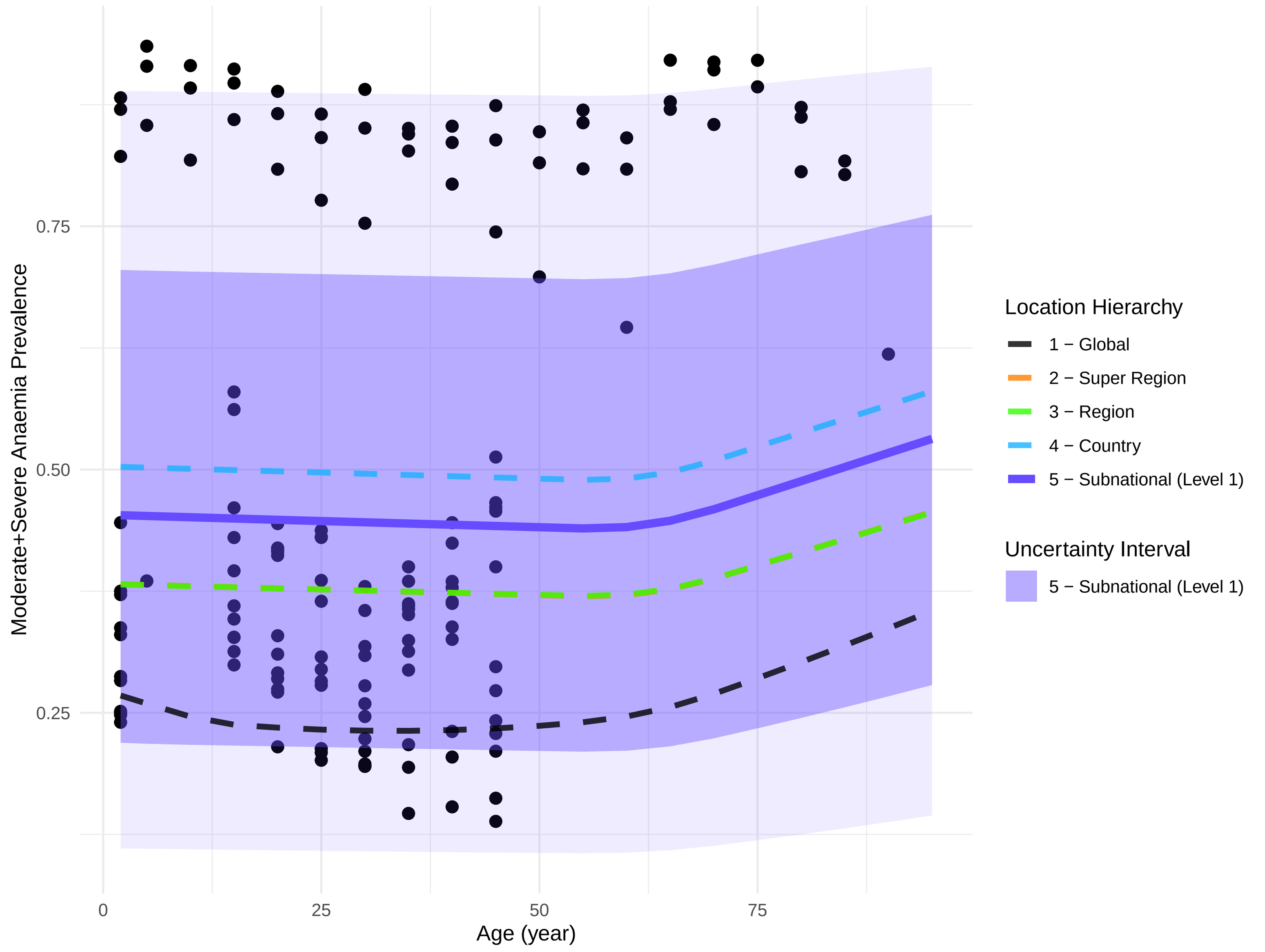

MR−BRT Age/Sex Fits − Non−pregnant Females 2+ Years Old − Moderate+Severe Anaemia Prevalence − Sikkim, Rural
WHO−2024 Adjustment Method

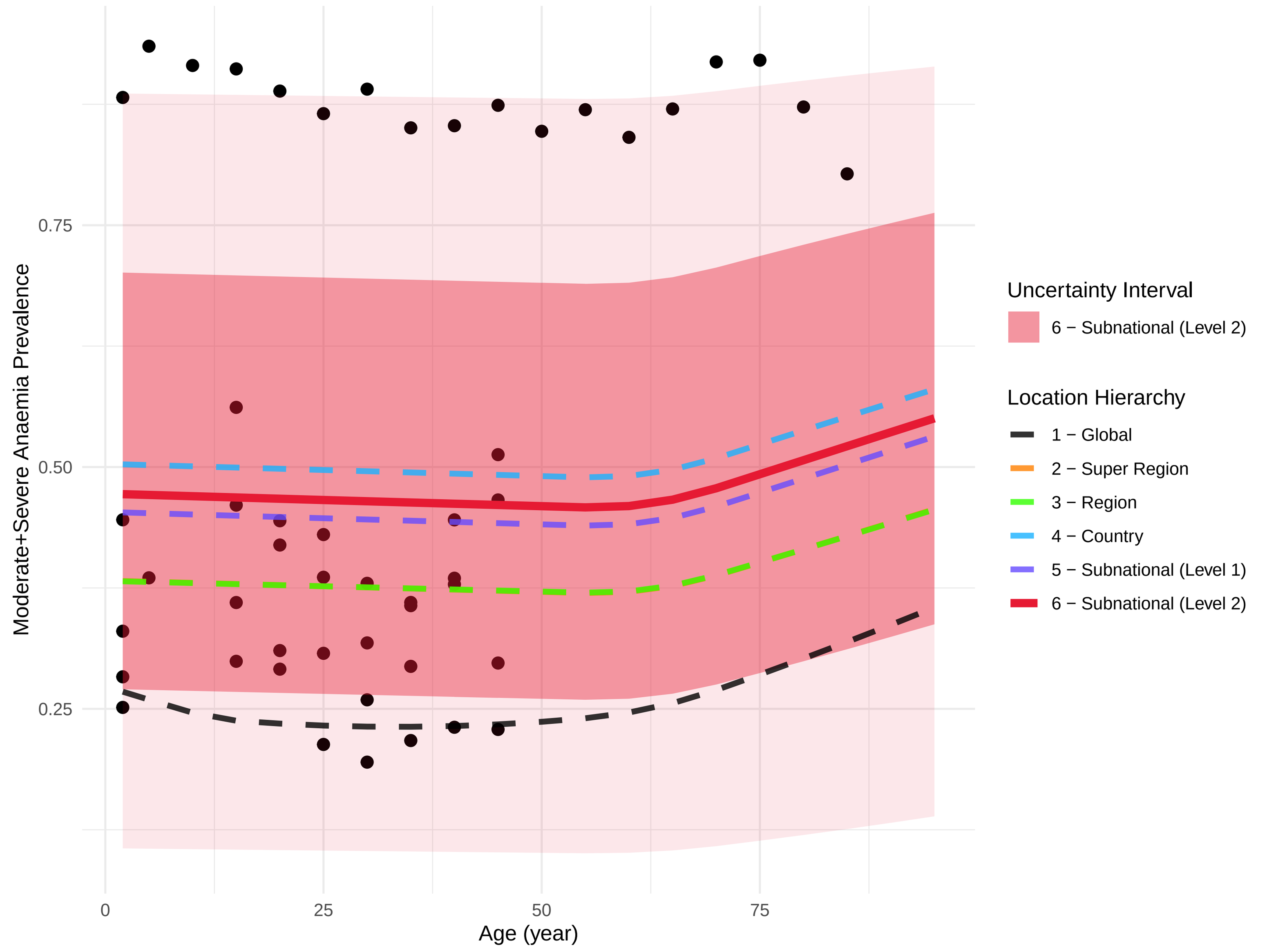

MR−BRT Age/Sex Fits − Non−pregnant Females 2+ Years Old − Moderate+Severe Anaemia Prevalence − Sikkim, Urban
WHO−2024 Adjustment Method

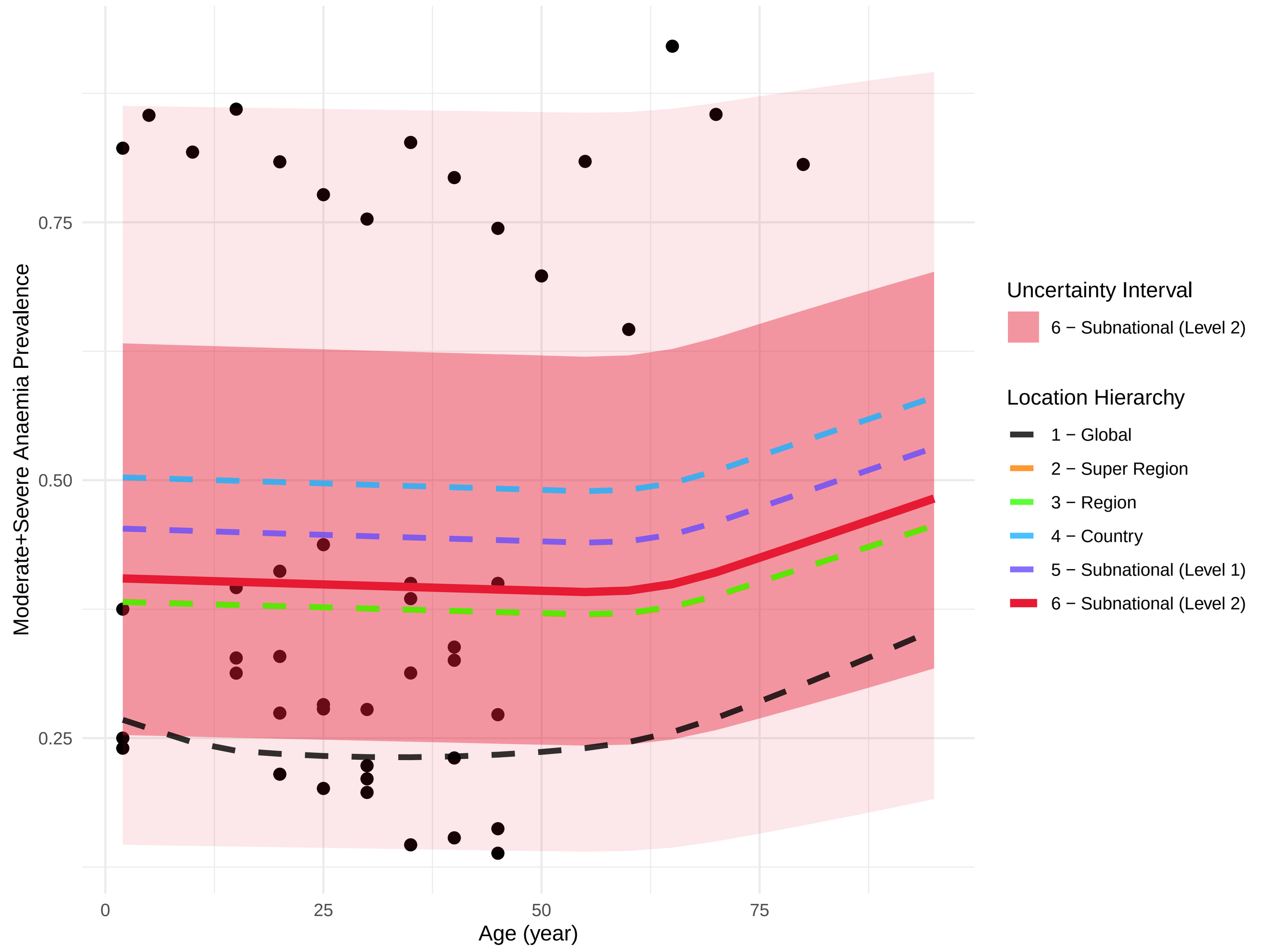

MR−BRT Age/Sex Fits − Non−pregnant Females 2+ Years Old − Moderate+Severe Anaemia Prevalence − Tamil Nadu
WHO−2024 Adjustment Method

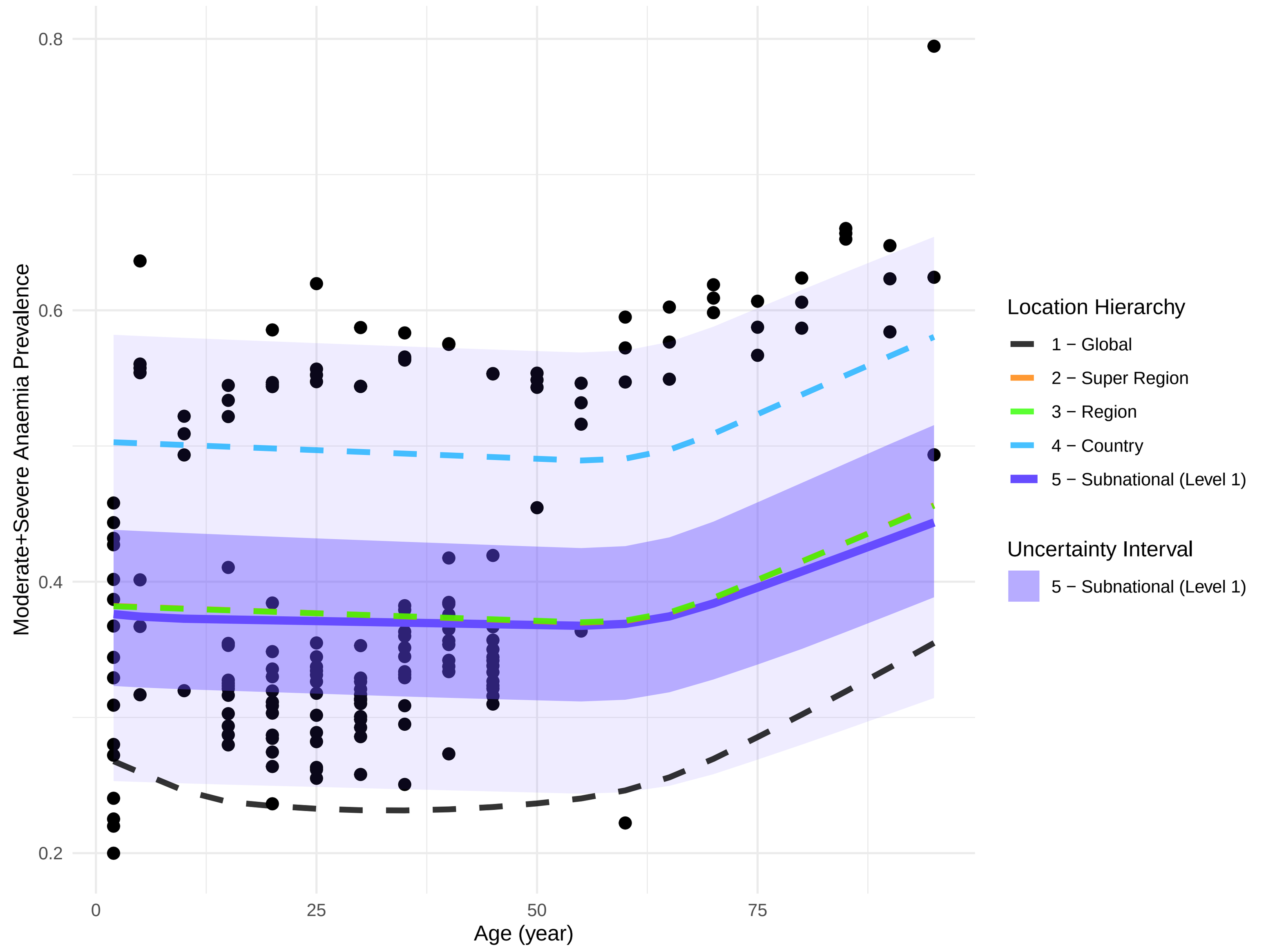

MR−BRT Age/Sex Fits − Non−pregnant Females 2+ Years Old − Moderate+Severe Anaemia Prevalence − Tamil Nadu, Rural
WHO−2024 Adjustment Method

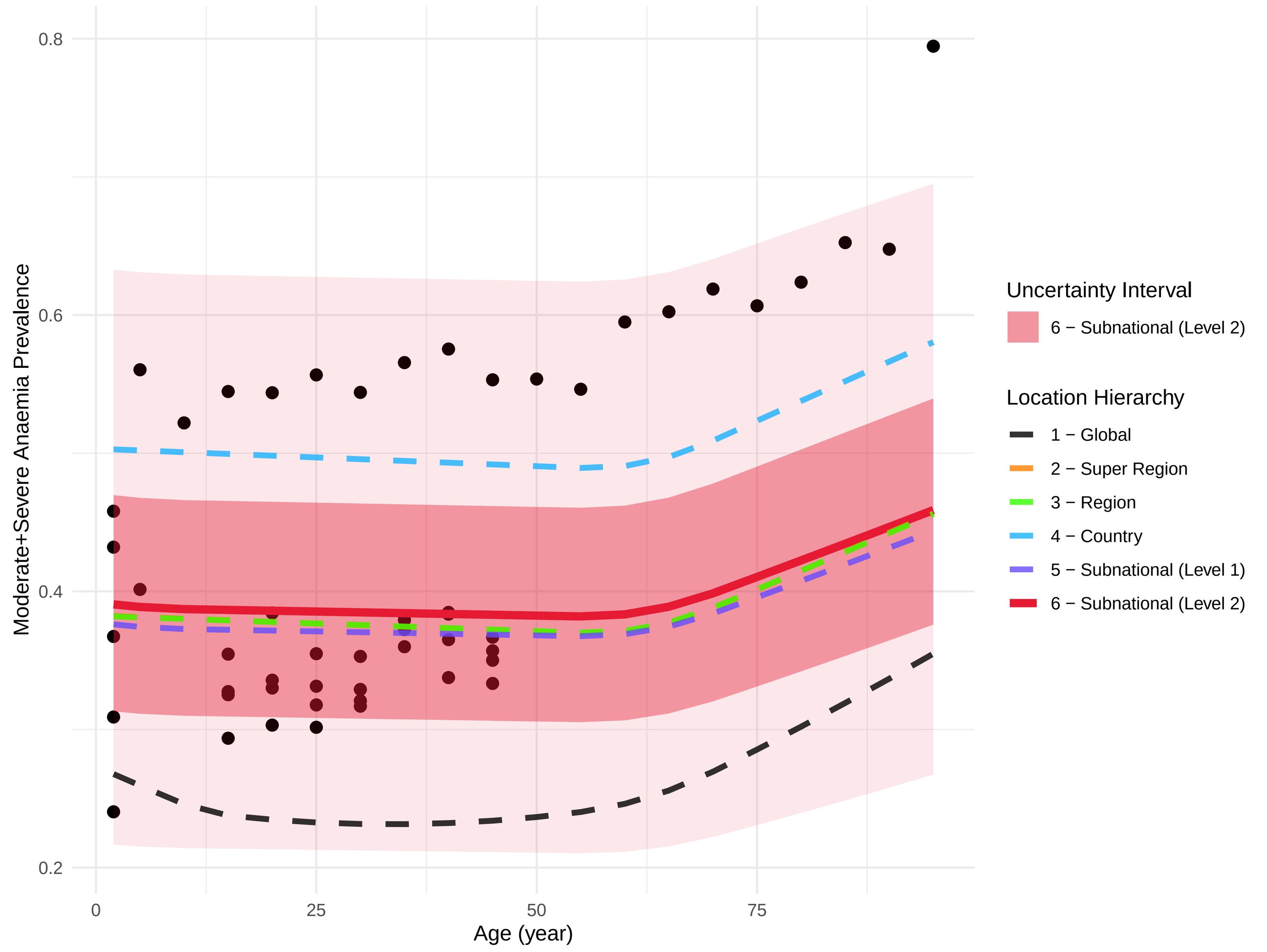

MR−BRT Age/Sex Fits − Non−pregnant Females 2+ Years Old − Moderate+Severe Anaemia Prevalence − Tamil Nadu, Urban
WHO−2024 Adjustment Method

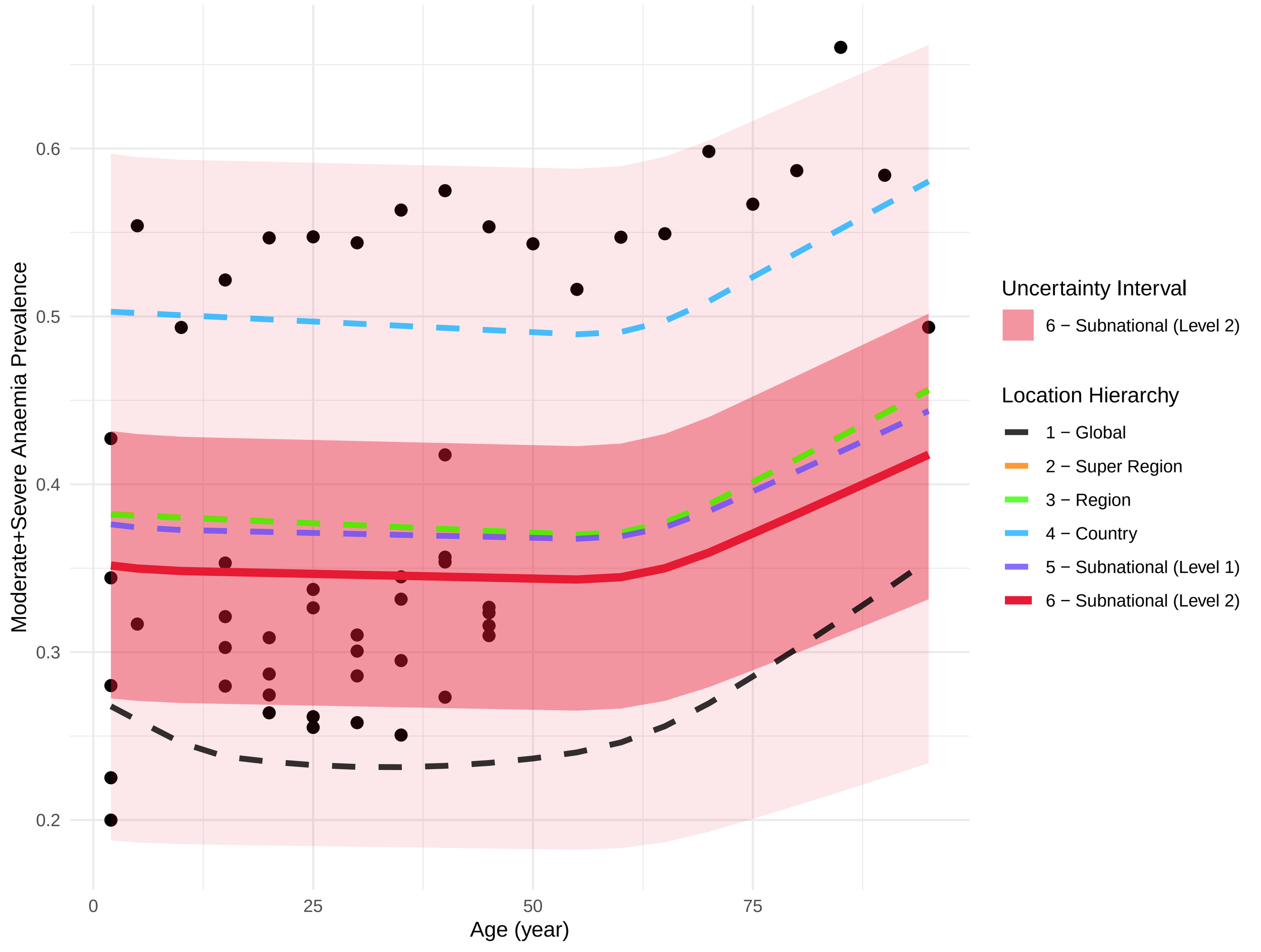

MR−BRT Age/Sex Fits − Non−pregnant Females 2+ Years Old − Moderate+Severe Anaemia Prevalence − Telangana
WHO−2024 Adjustment Method

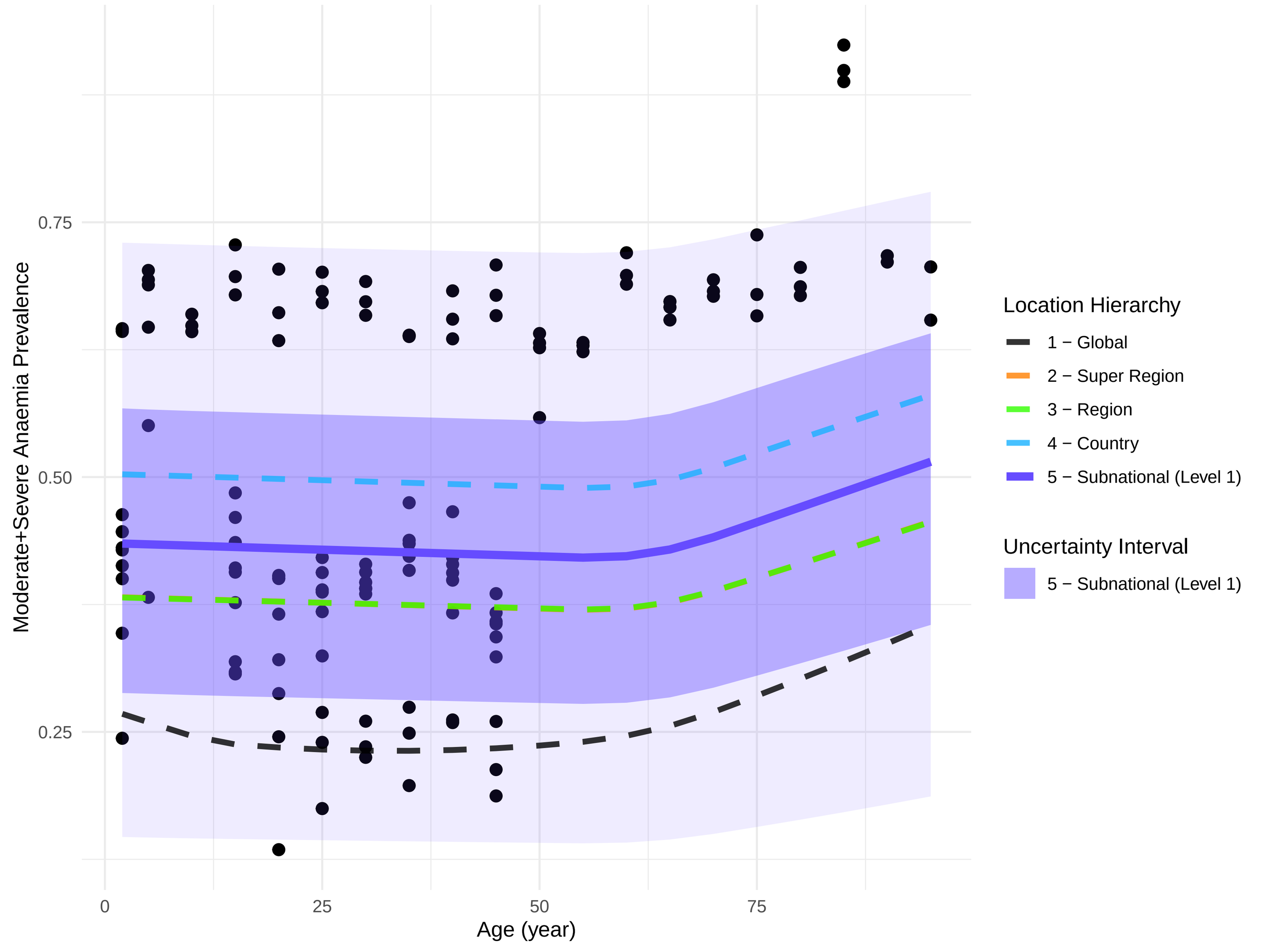

MR−BRT Age/Sex Fits − Non−pregnant Females 2+ Years Old − Moderate+Severe Anaemia Prevalence − Telangana, Rural
WHO−2024 Adjustment Method

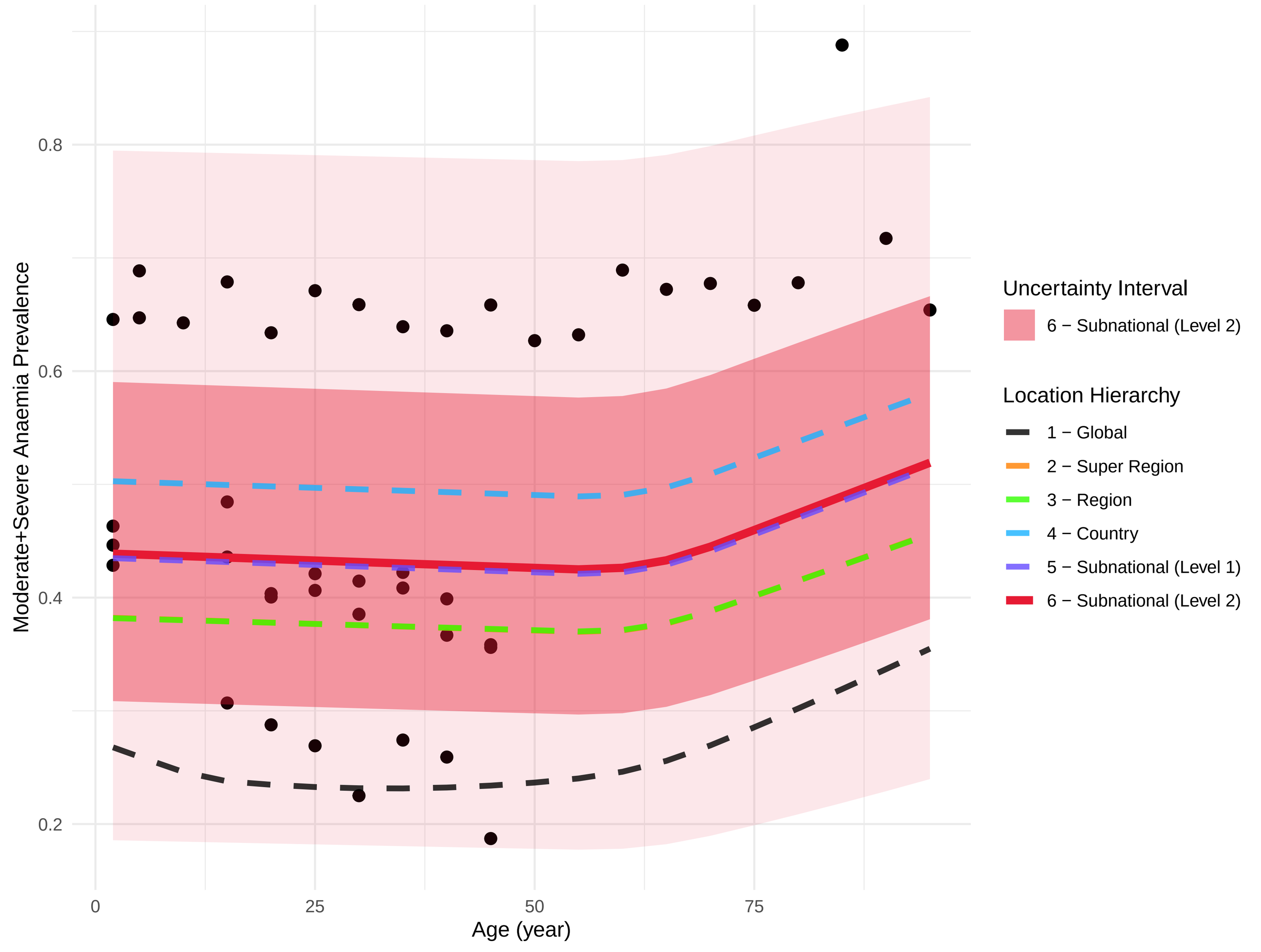

MR−BRT Age/Sex Fits − Non−pregnant Females 2+ Years Old − Moderate+Severe Anaemia Prevalence − Telangana, Urban
WHO−2024 Adjustment Method

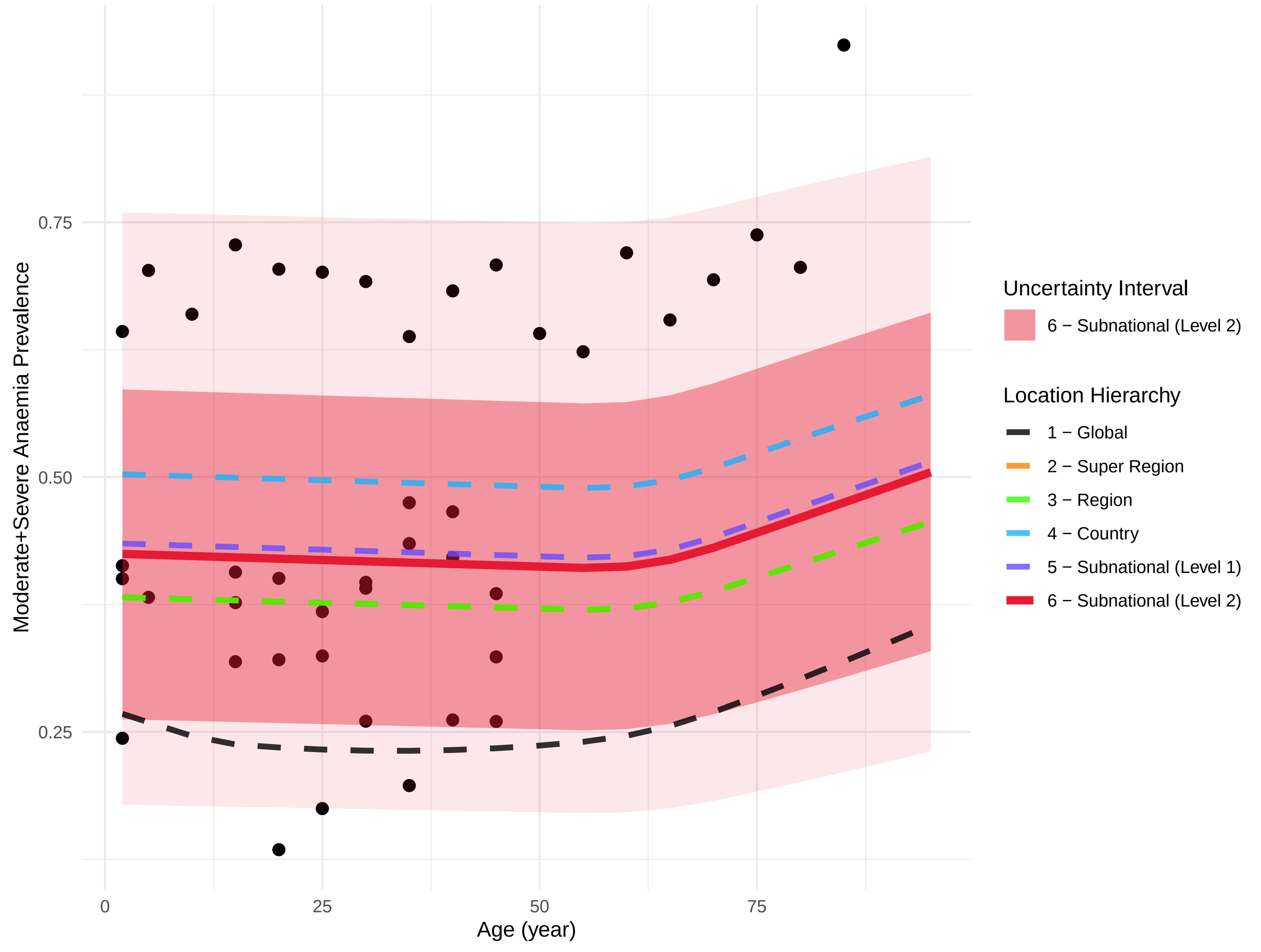

MR−BRT Age/Sex Fits − Non−pregnant Females 2+ Years Old − Moderate+Severe Anaemia Prevalence − Tripura
WHO−2024 Adjustment Method

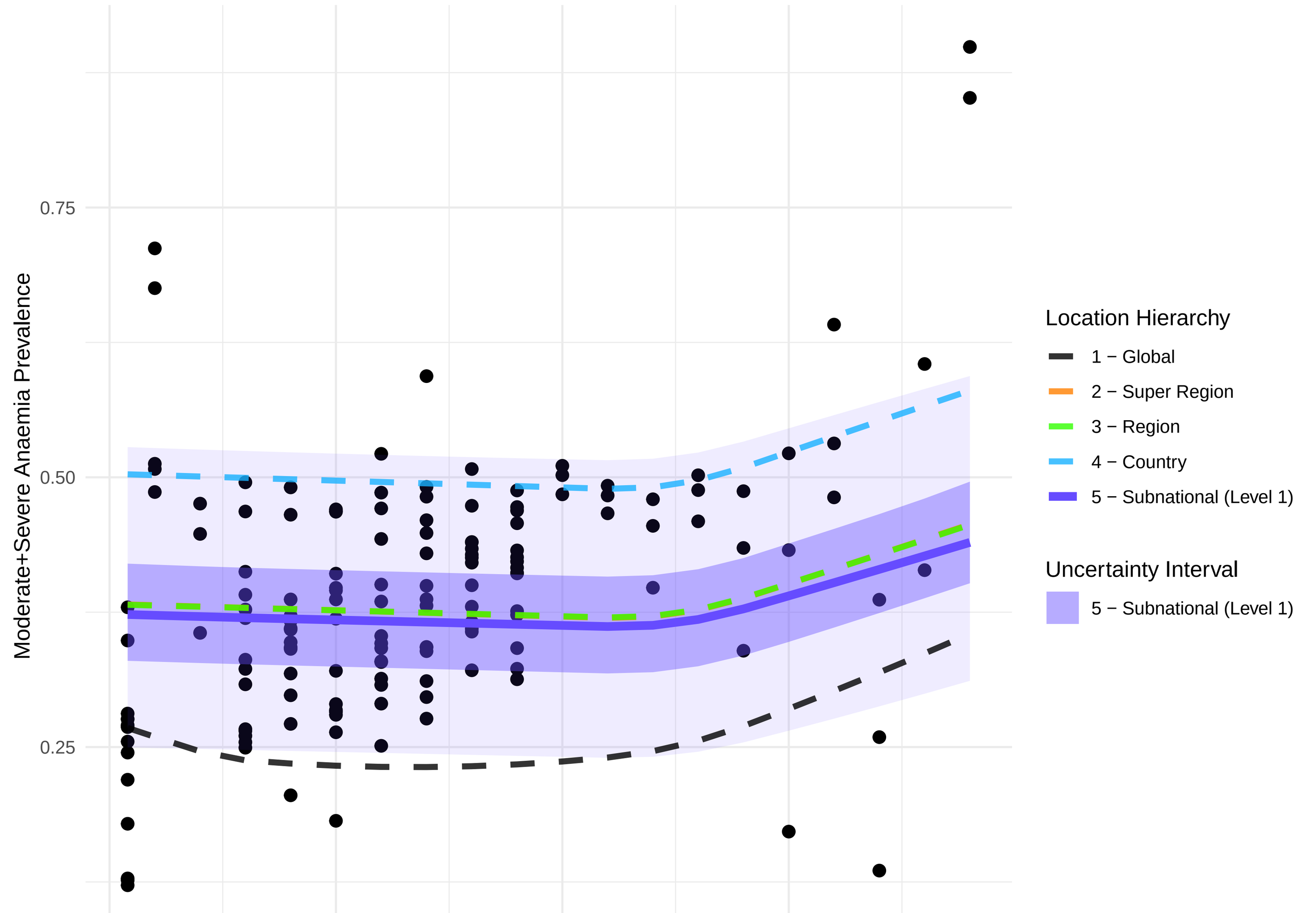

MR−BRT Age/Sex Fits − Non−pregnant Females 2+ Years Old − Moderate+Severe Anaemia Prevalence − Tripura, Rural
WHO−2024 Adjustment Method

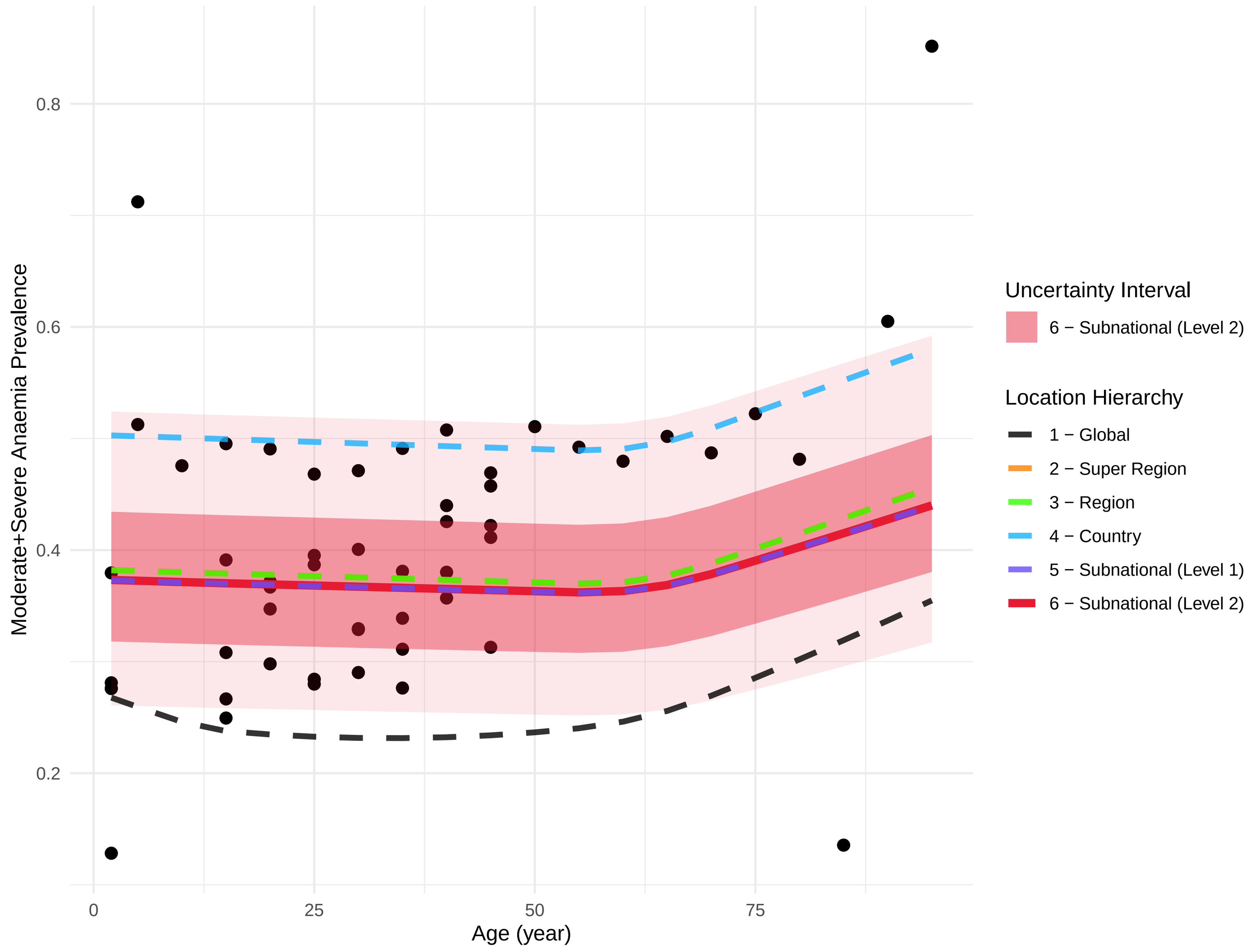

MR−BRT Age/Sex Fits − Non−pregnant Females 2+ Years Old − Moderate+Severe Anaemia Prevalence − Tripura, Urban
WHO−2024 Adjustment Method

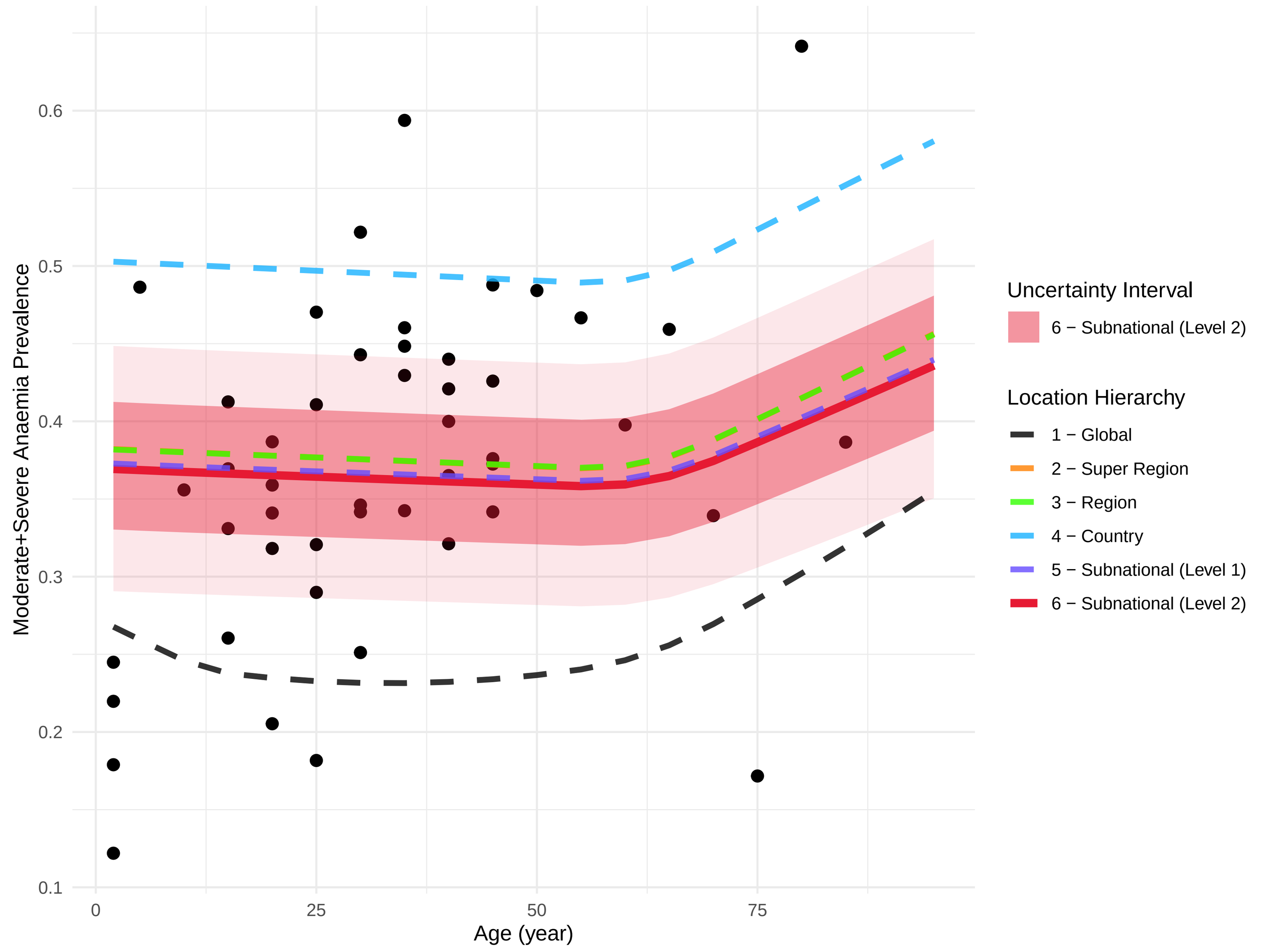

MR−BRT Age/Sex Fits − Non−pregnant Females 2+ Years Old − Moderate+Severe Anaemia Prevalence − Uttar Pradesh
WHO−2024 Adjustment Method

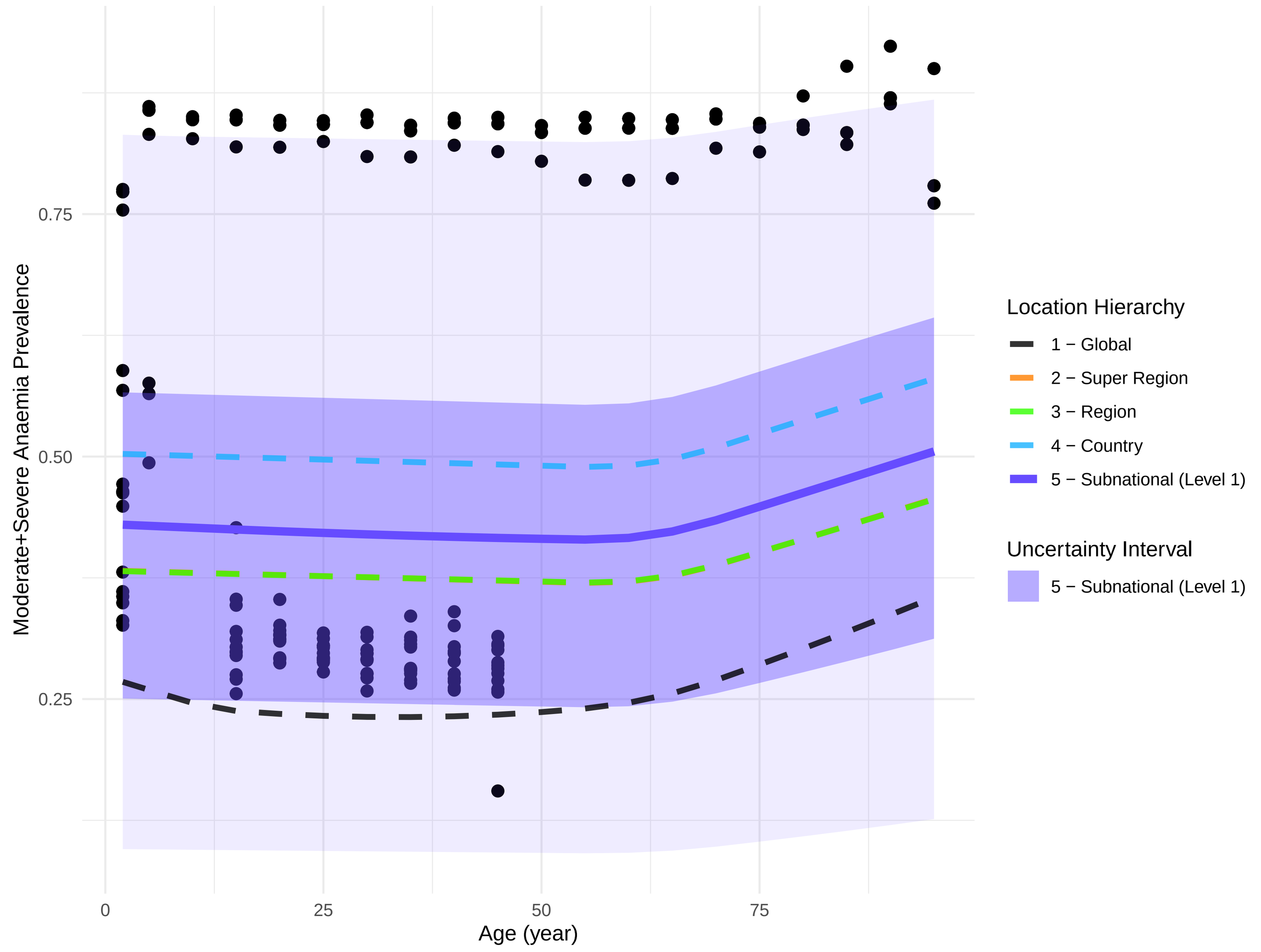

MR−BRT Age/Sex Fits − Non−pregnant Females 2+ Years Old − Moderate+Severe Anaemia Prevalence − Uttar Pradesh, Rural
WHO−2024 Adjustment Method

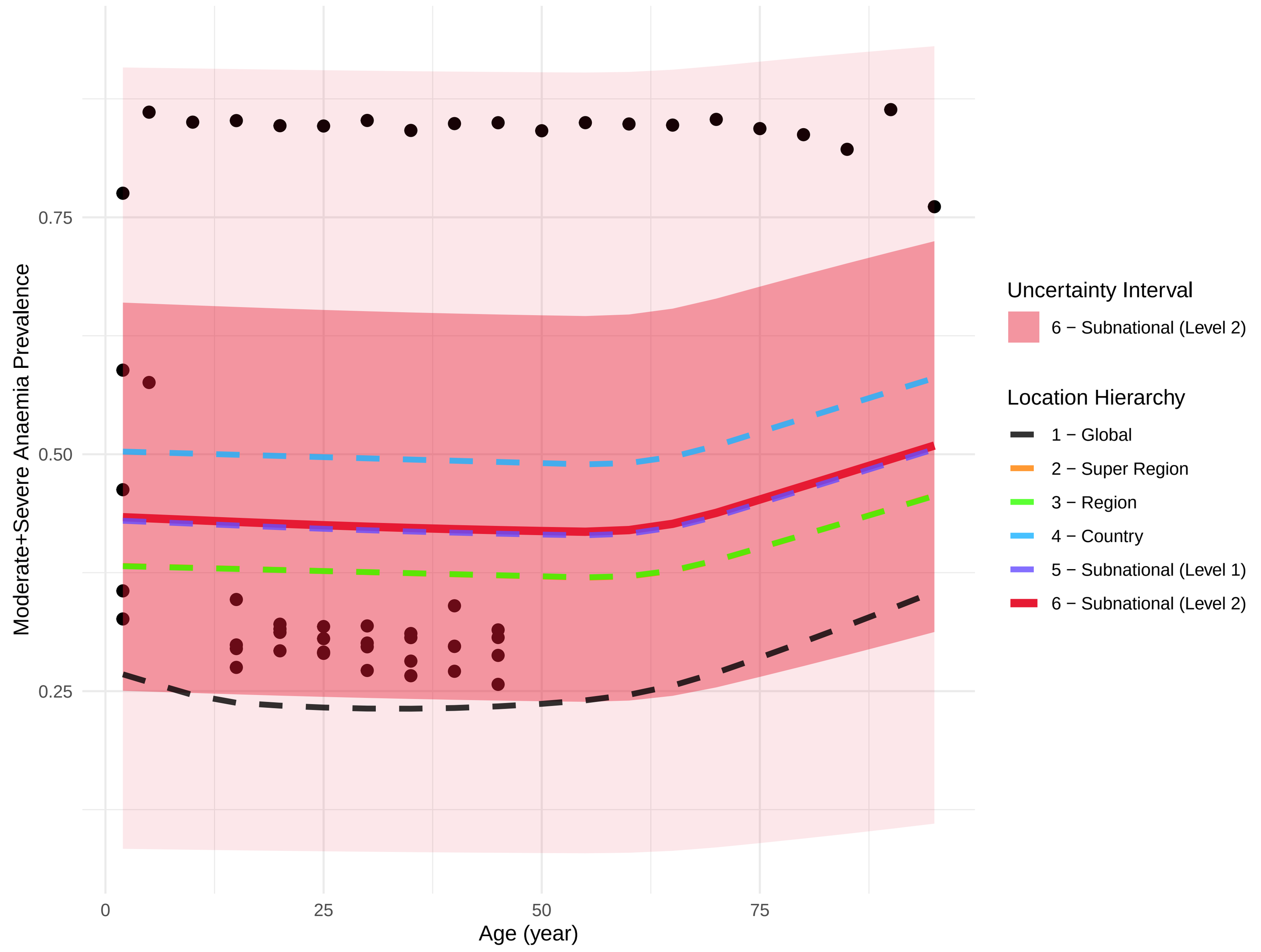

MR−BRT Age/Sex Fits − Non−pregnant Females 2+ Years Old − Moderate+Severe Anaemia Prevalence − Uttar Pradesh, Urban
WHO−2024 Adjustment Method

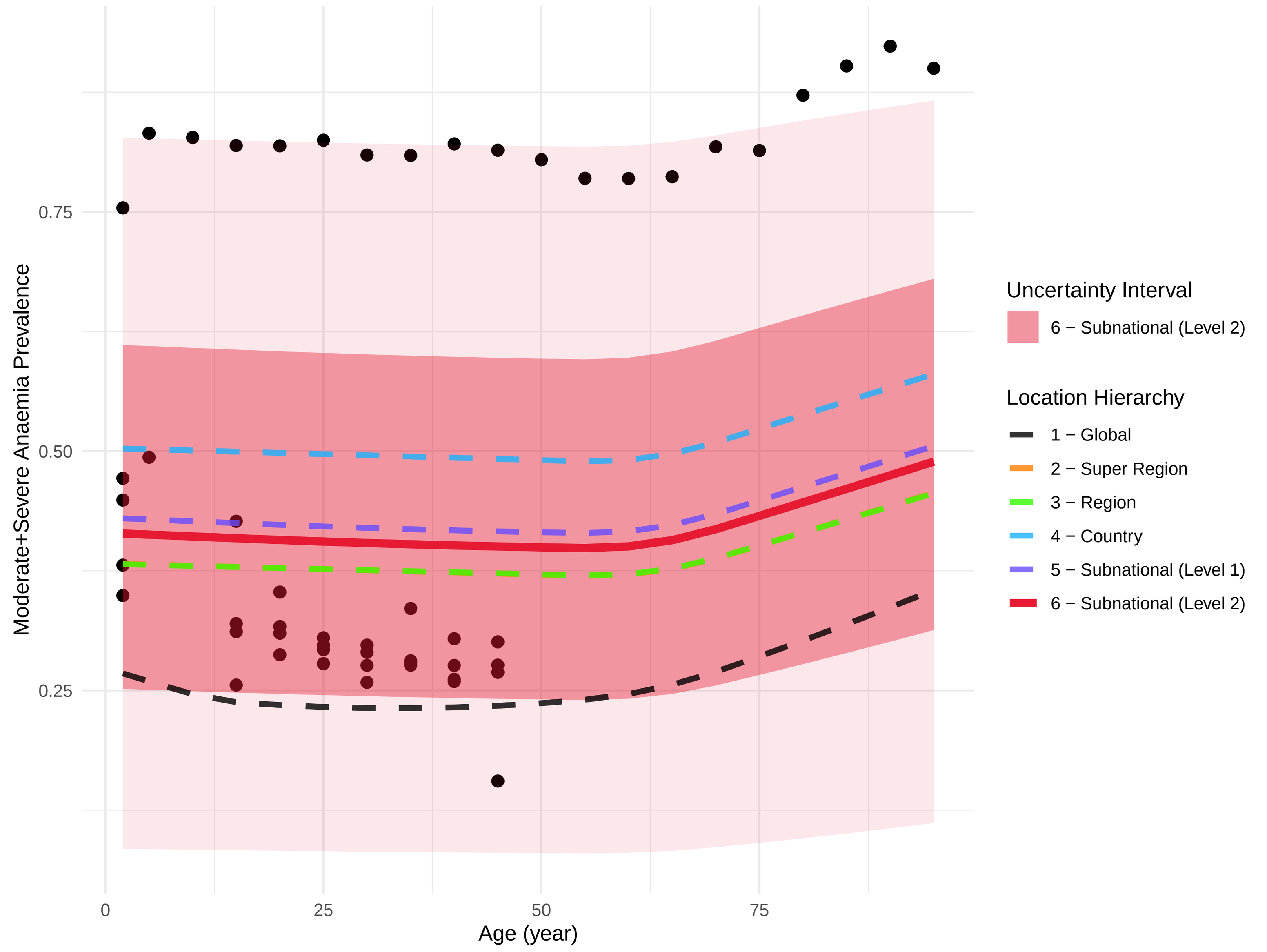

MR−BRT Age/Sex Fits − Non−pregnant Females 2+ Years Old − Moderate+Severe Anaemia Prevalence − Uttarakhand
WHO−2024 Adjustment Method

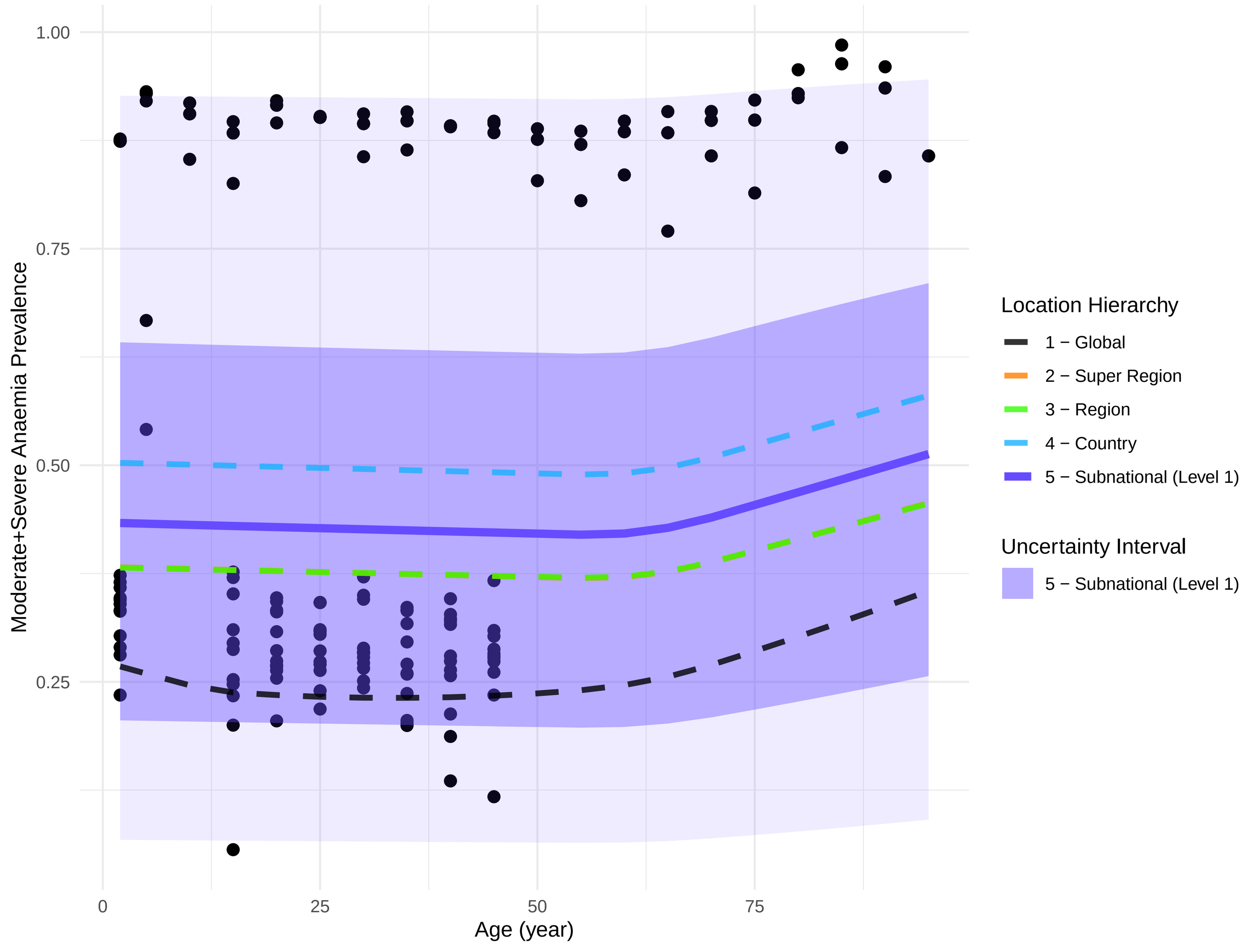

MR−BRT Age/Sex Fits − Non−pregnant Females 2+ Years Old − Moderate+Severe Anaemia Prevalence − Uttarakhand, Rural
WHO−2024 Adjustment Method

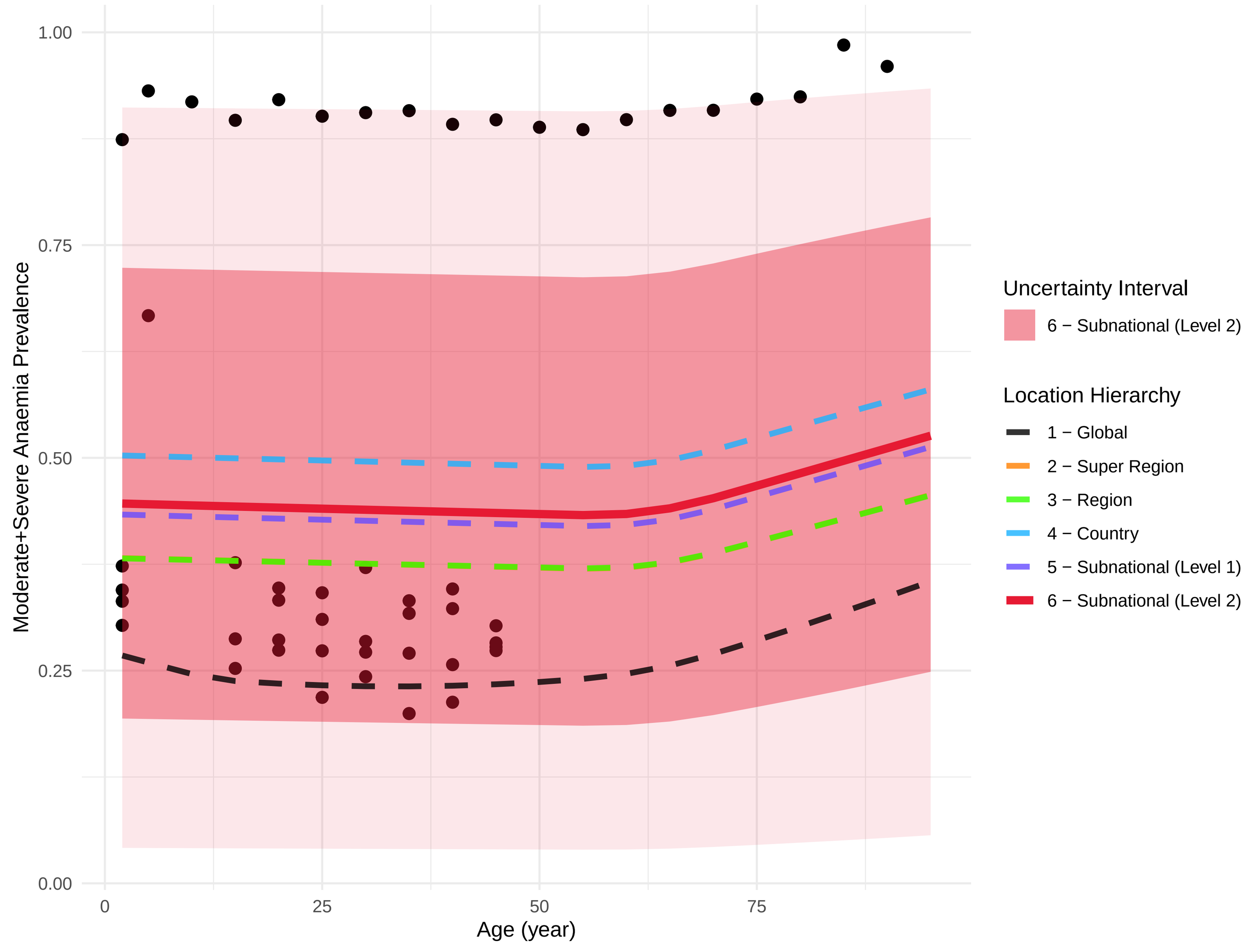

MR−BRT Age/Sex Fits − Non−pregnant Females 2+ Years Old − Moderate+Severe Anaemia Prevalence − Uttarakhand, Urban
WHO−2024 Adjustment Method

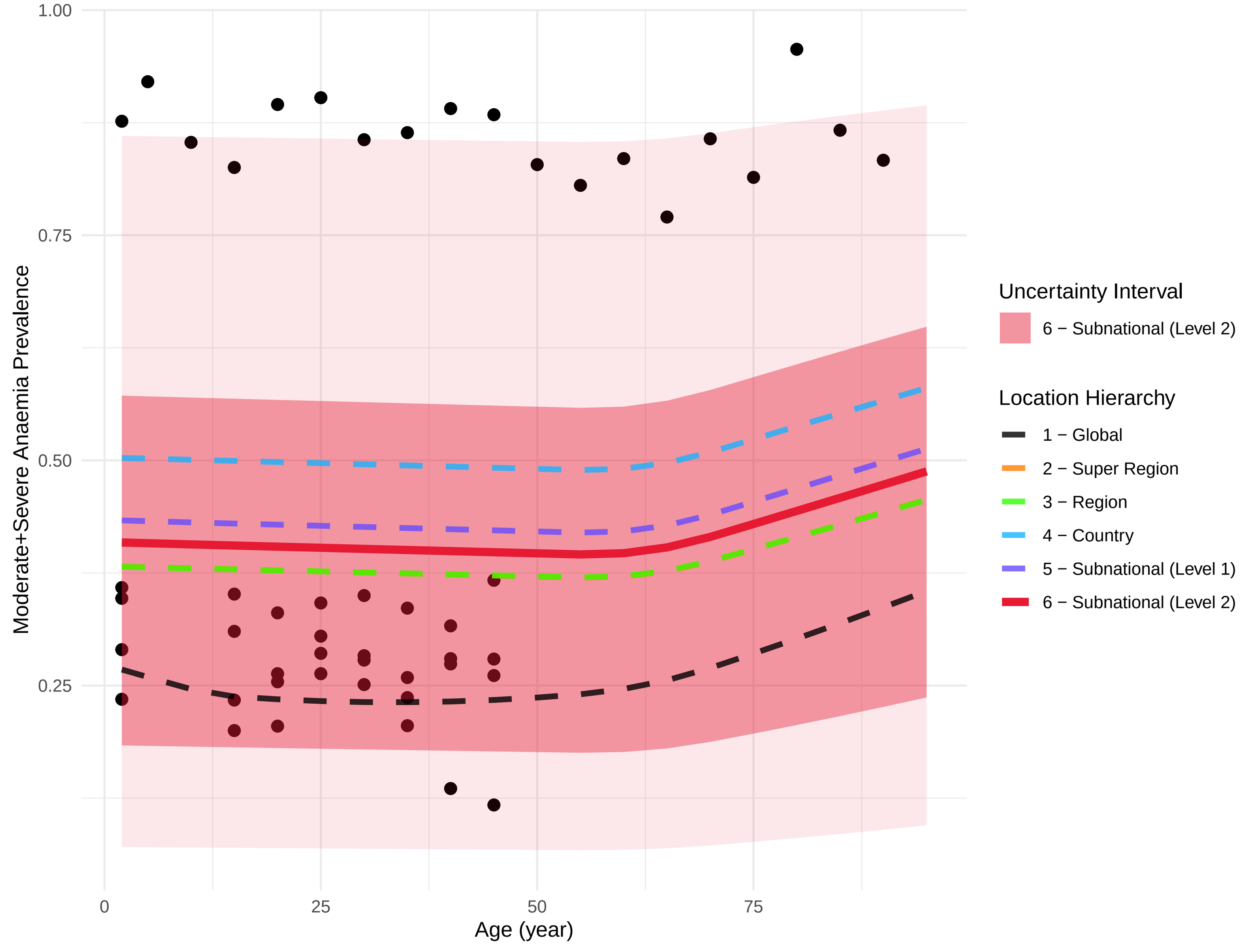

MR−BRT Age/Sex Fits − Non−pregnant Females 2+ Years Old − Moderate+Severe Anaemia Prevalence − West Bengal
WHO−2024 Adjustment Method

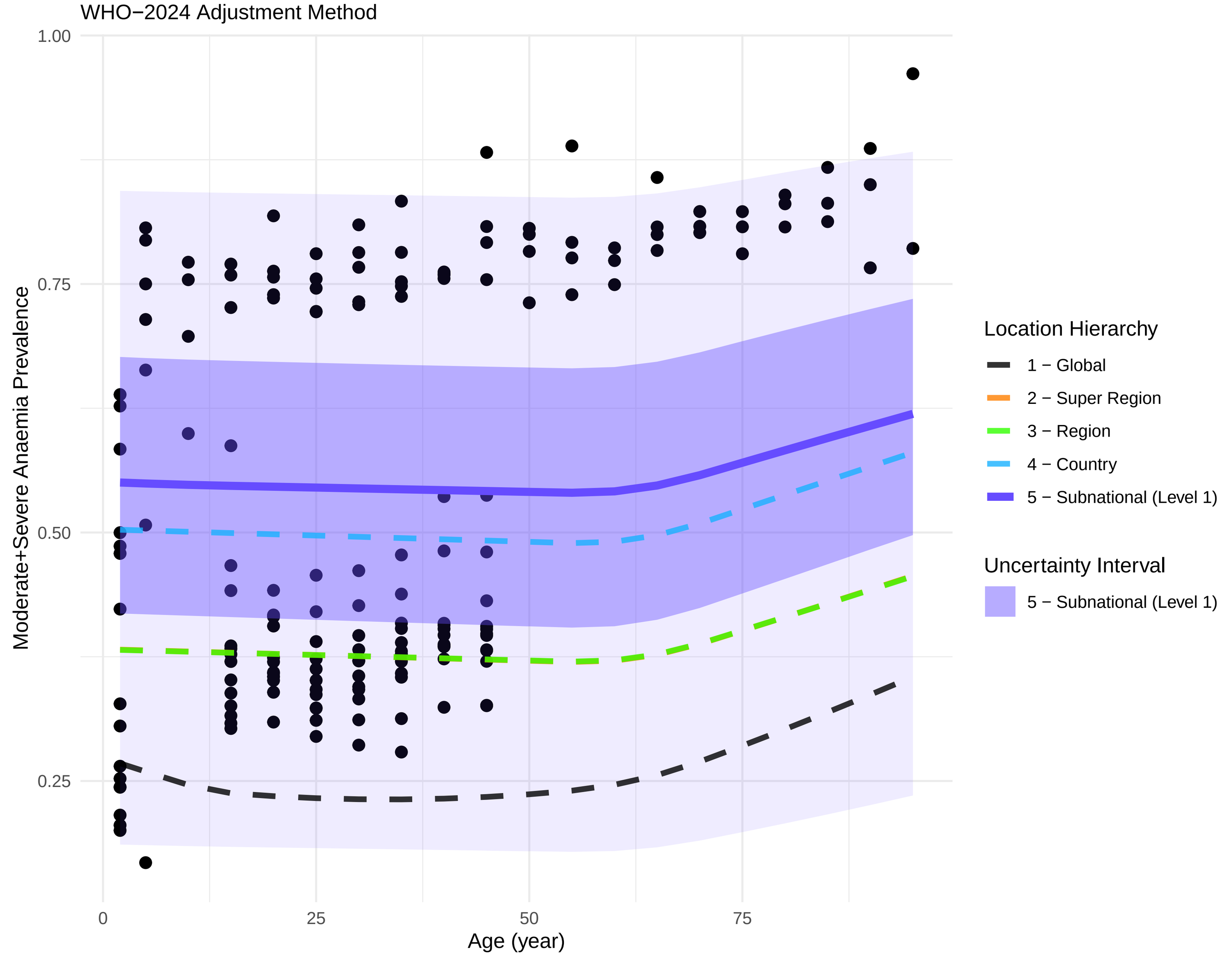

MR−BRT Age/Sex Fits − Non−pregnant Females 2+ Years Old − Moderate+Severe Anaemia Prevalence − West Bengal, Rural
WHO−2024 Adjustment Method

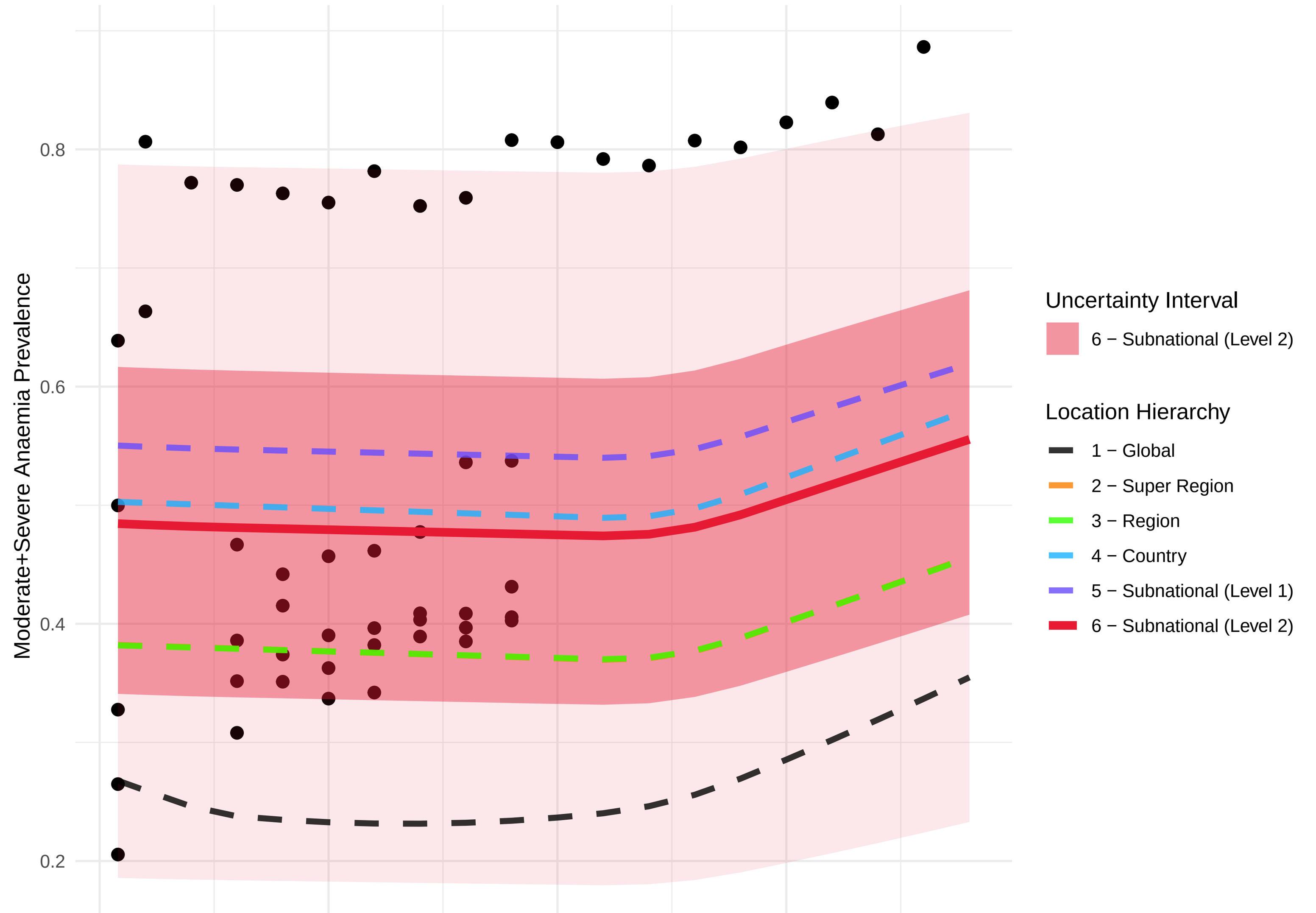

MR−BRT Age/Sex Fits − Non−pregnant Females 2+ Years Old − Moderate+Severe Anaemia Prevalence − West Bengal, Urban
WHO−2024 Adjustment Method

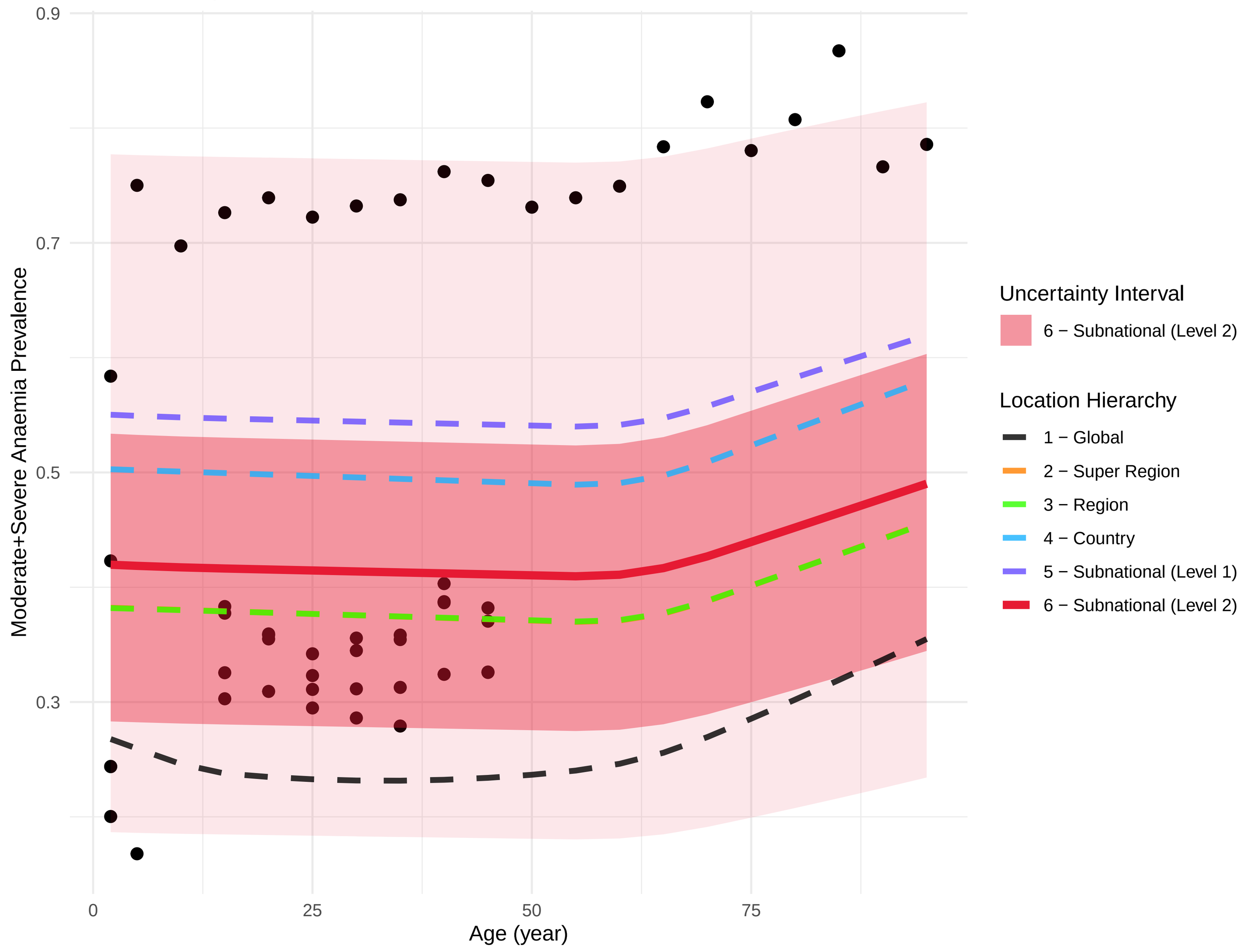

MR−BRT Age/Sex Fits − Non−pregnant Females 2+ Years Old − Moderate+Severe Anaemia Prevalence − Nepal
WHO−2024 Adjustment Method

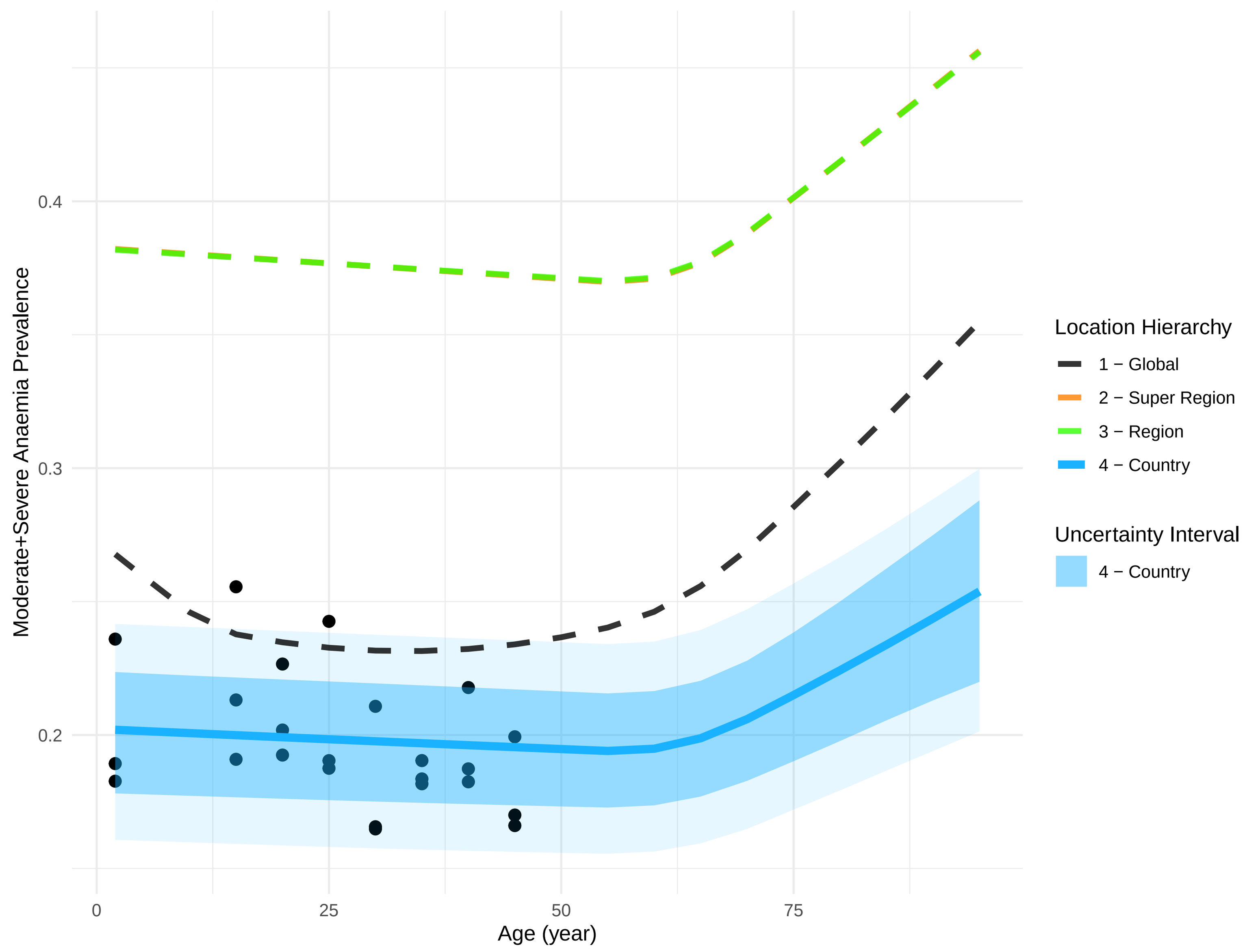

MR−BRT Age/Sex Fits − Non−pregnant Females 2+ Years Old − Moderate+Severe Anaemia Prevalence − Pakistan
WHO−2024 Adjustment Method

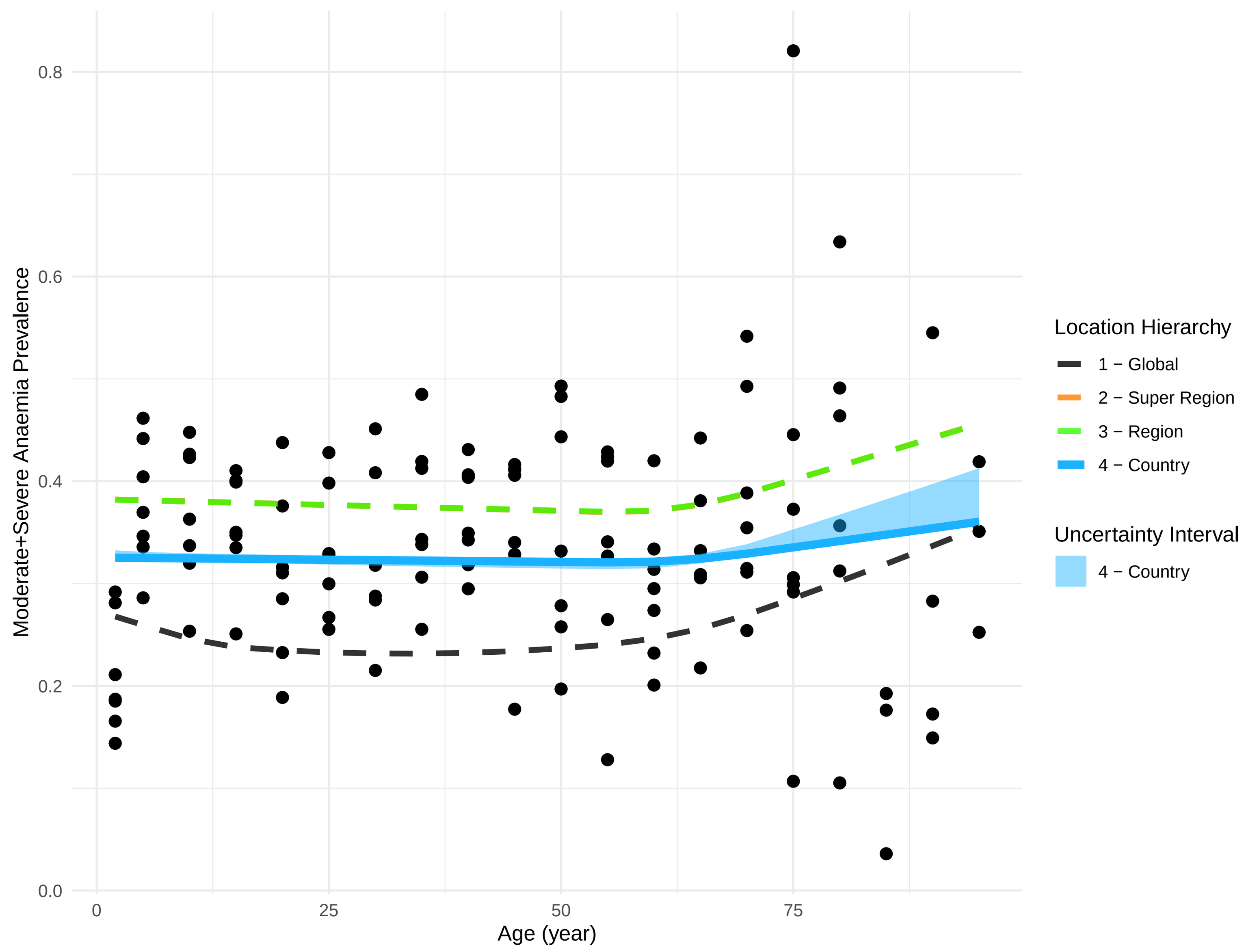

MR−BRT Age/Sex Fits − Non−pregnant Females 2+ Years Old − Moderate+Severe Anaemia Prevalence − Azad Jammu & Kashmir
WHO−2024 Adjustment Method

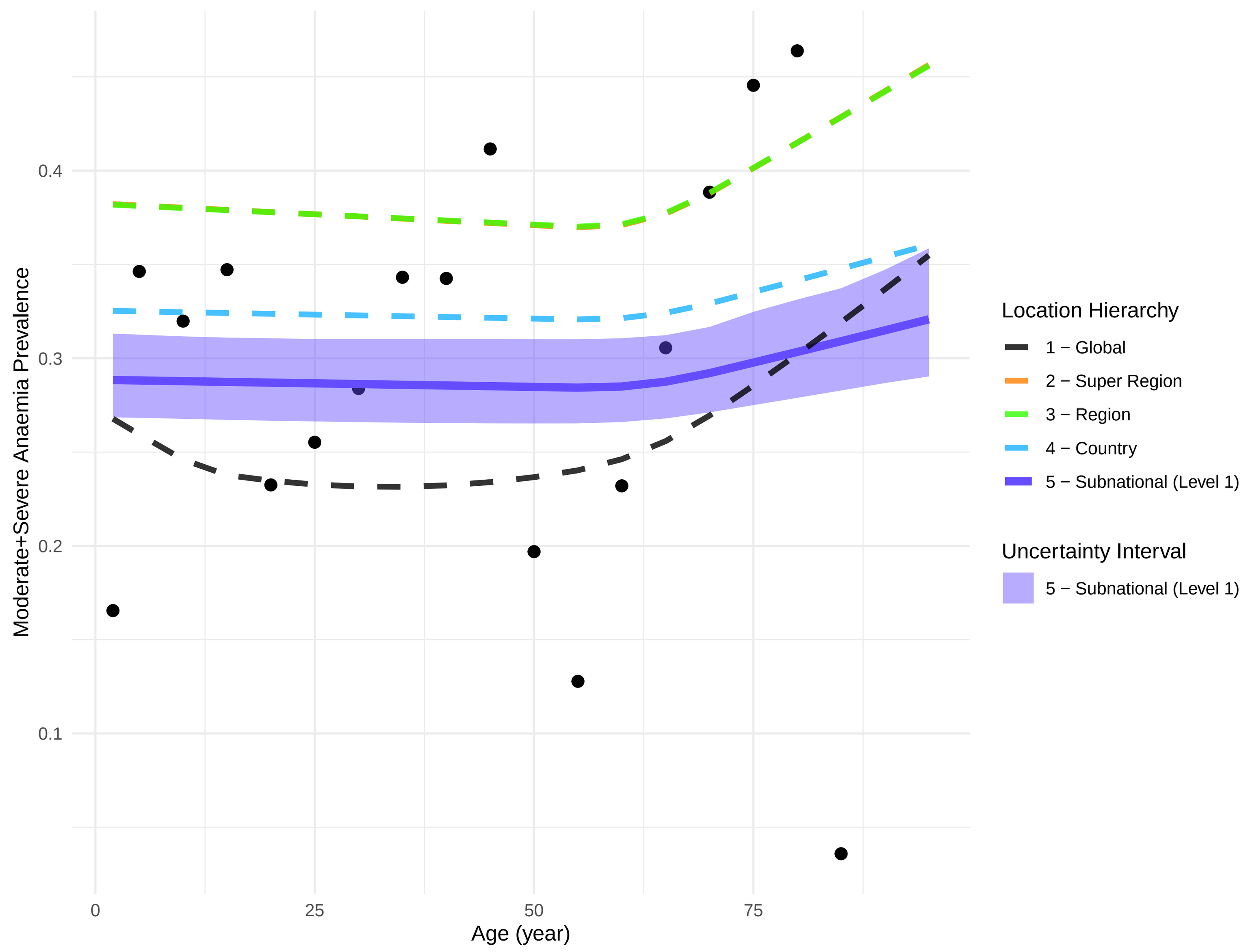

MR−BRT Age/Sex Fits − Non−pregnant Females 2+ Years Old − Moderate+Severe Anaemia Prevalence − Balochistan
WHO−2024 Adjustment Method

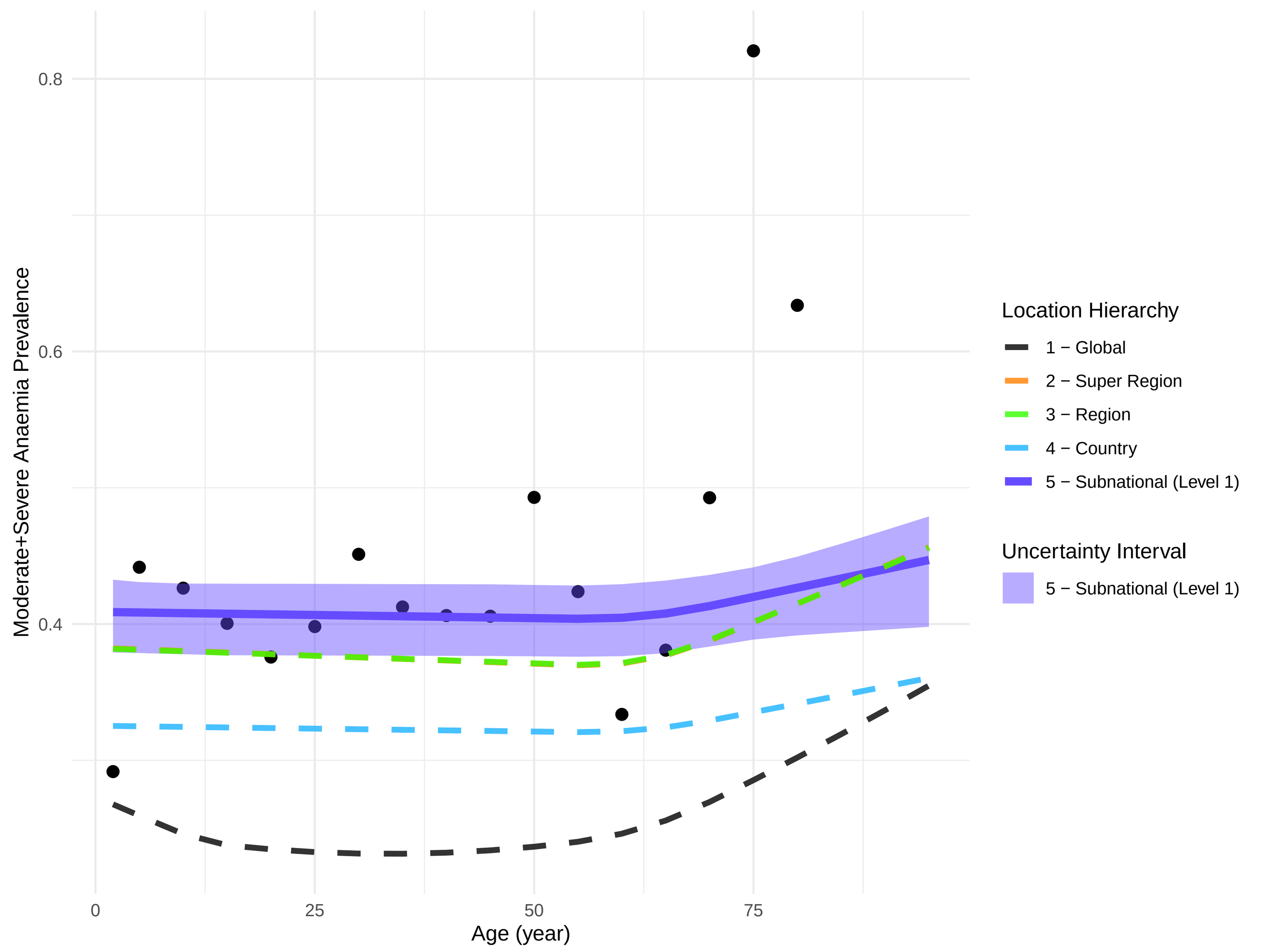

MR−BRT Age/Sex Fits − Non−pregnant Females 2+ Years Old − Moderate+Severe Anaemia Prevalence − Gilgit−Baltistan
WHO−2024 Adjustment Method

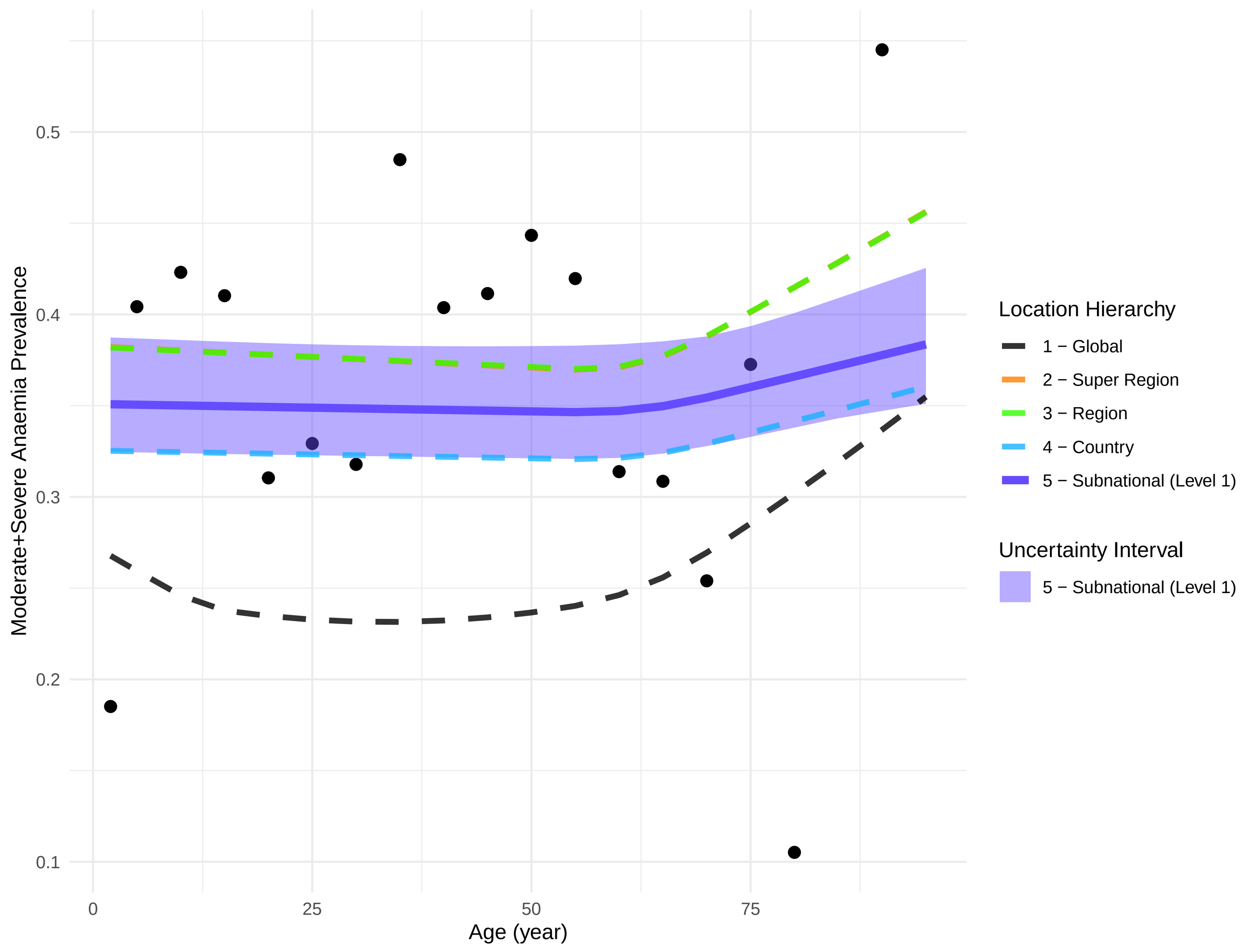

MR−BRT Age/Sex Fits − Non−pregnant Females 2+ Years Old − Moderate+Severe Anaemia Prevalence − Khyber Pakhtunkhwa
WHO−2024 Adjustment Method

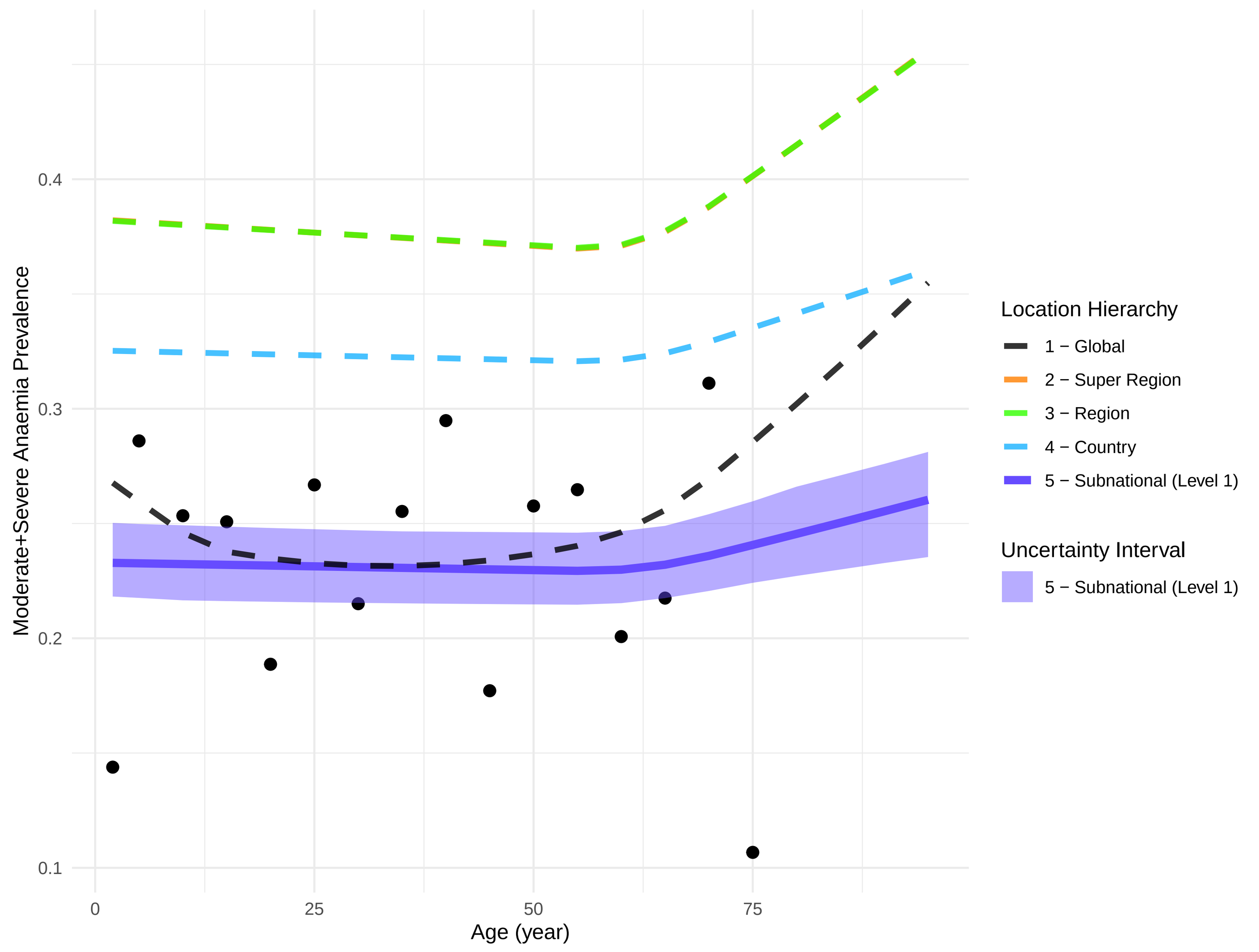

MR−BRT Age/Sex Fits − Non−pregnant Females 2+ Years Old − Moderate+Severe Anaemia Prevalence − Punjab
WHO−2024 Adjustment Method

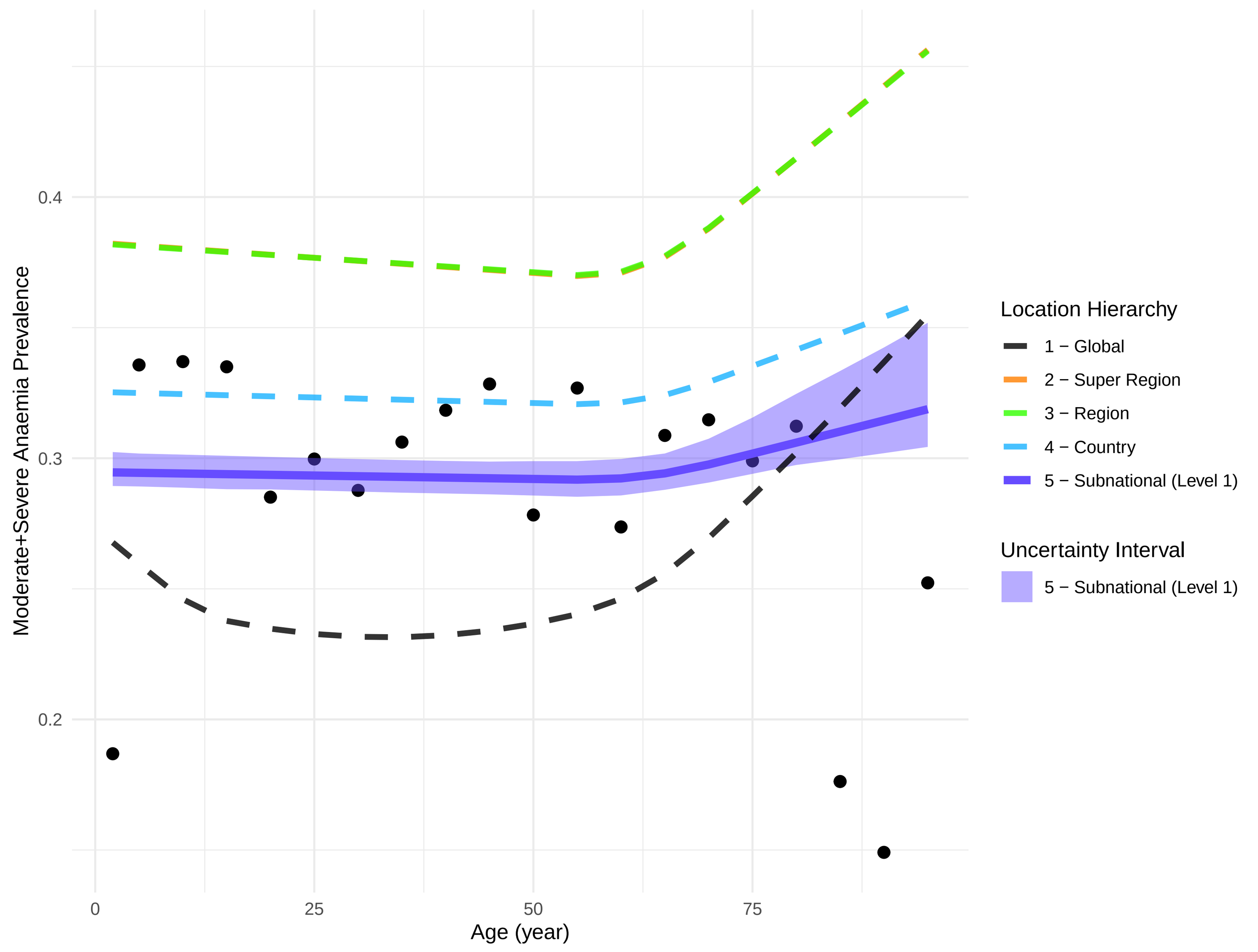

MR−BRT Age/Sex Fits − Non−pregnant Females 2+ Years Old − Moderate+Severe Anaemia Prevalence − Sindh
WHO−2024 Adjustment Method

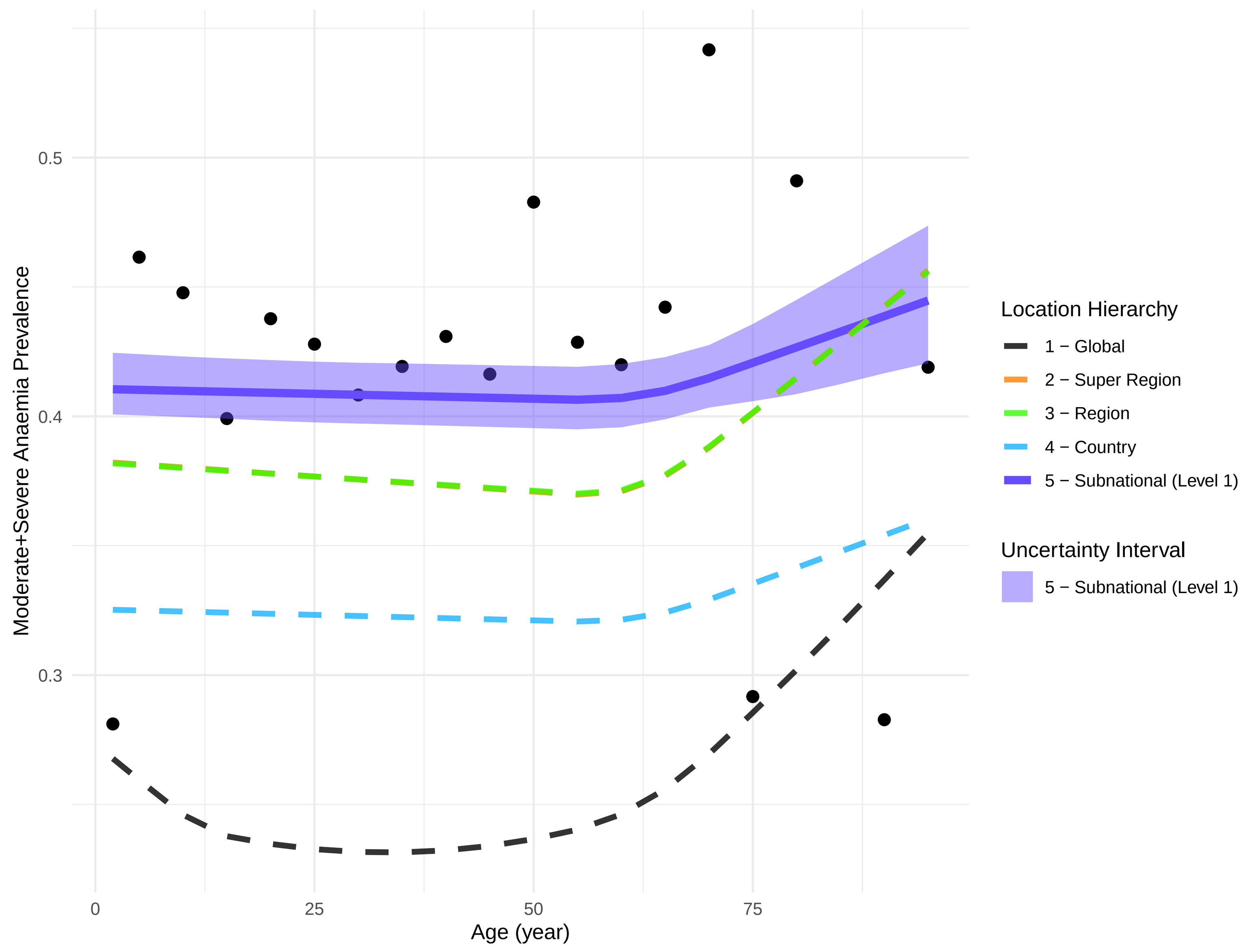

MR−BRT Age/Sex Fits − Non−pregnant Females 2+ Years Old − Moderate+Severe Anaemia Prevalence − Southeast Asia, East Asia, and Oceania
WHO−2024 Adjustment Method

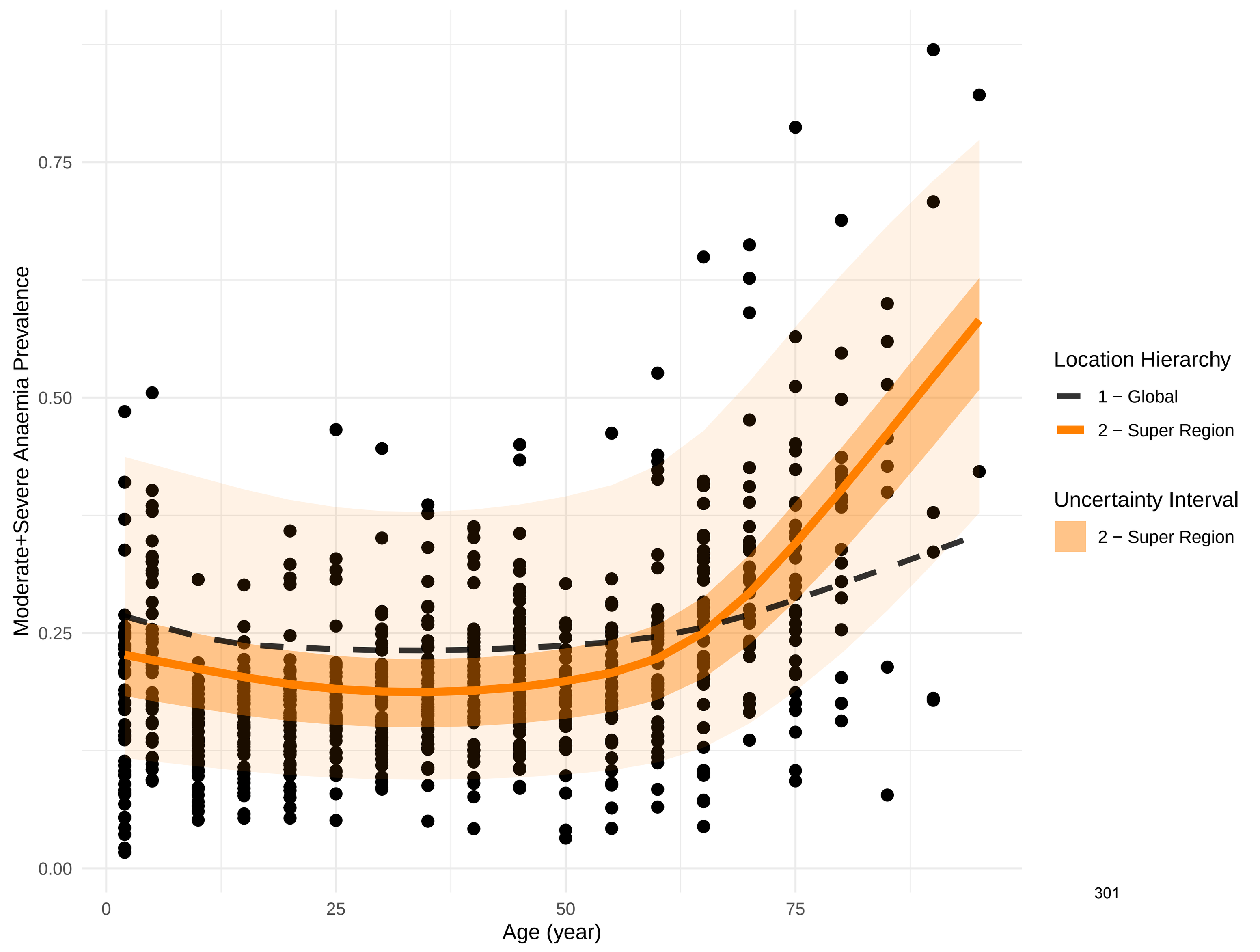

MR−BRT Age/Sex Fits − Non−pregnant Females 2+ Years Old − Moderate+Severe Anaemia Prevalence − Oceania
WHO−2024 Adjustment Method

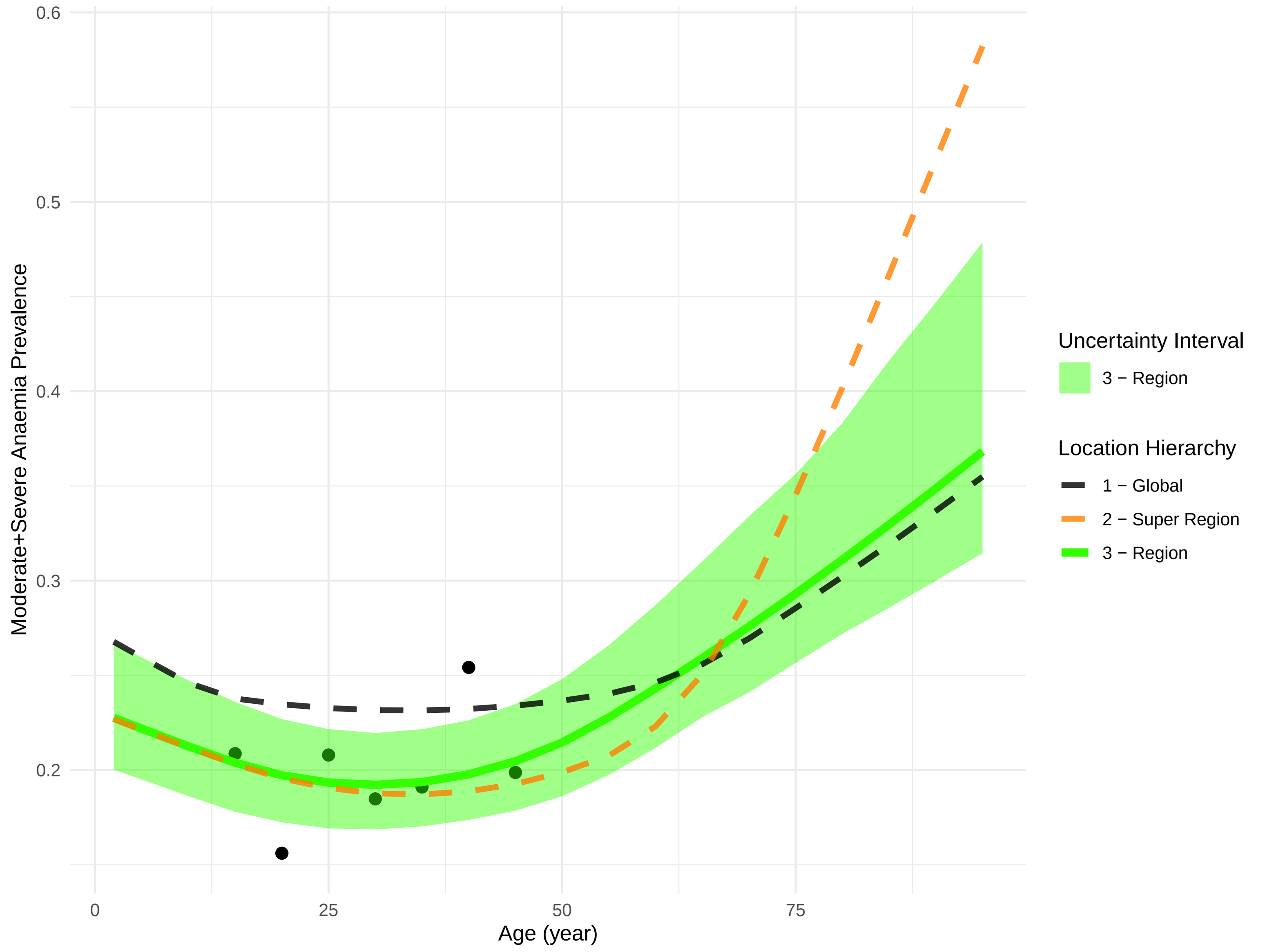

MR−BRT Age/Sex Fits − Non−pregnant Females 2+ Years Old − Moderate+Severe Anaemia Prevalence − Papua New Guinea
WHO−2024 Adjustment Method

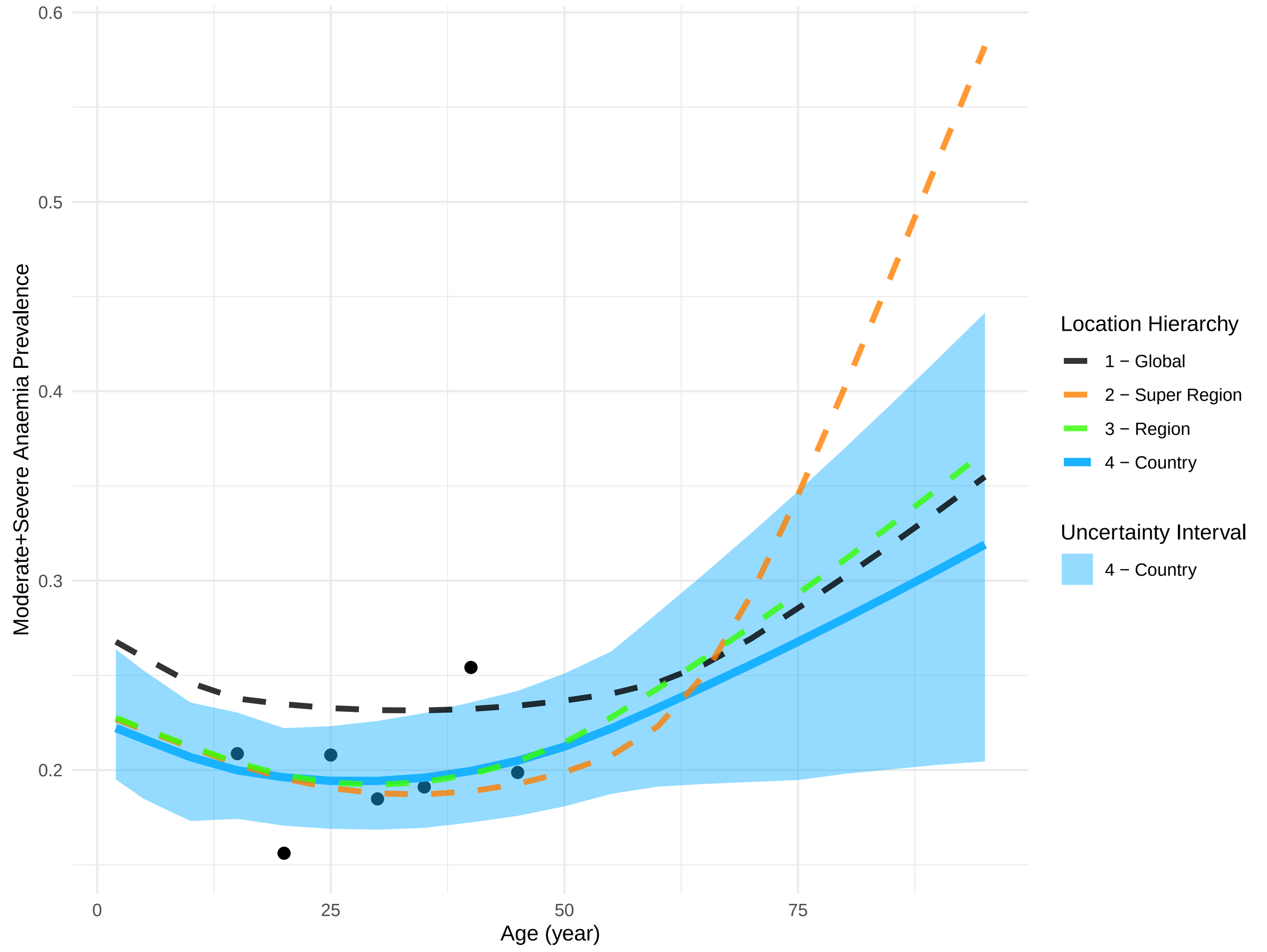

MR−BRT Age/Sex Fits − Non−pregnant Females 2+ Years Old − Moderate+Severe Anaemia Prevalence − Southeast Asia
WHO−2024 Adjustment Method

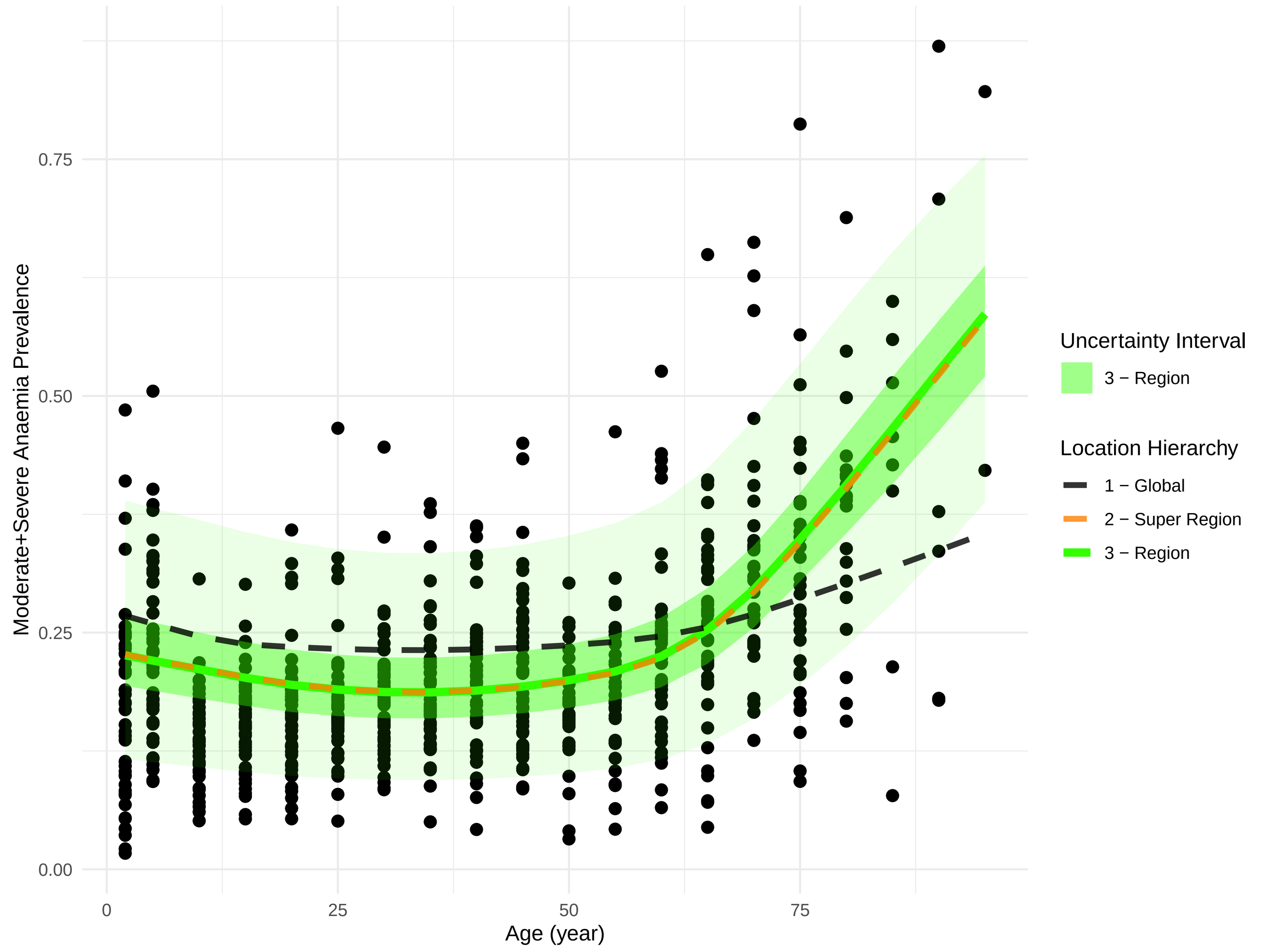

MR−BRT Age/Sex Fits − Non−pregnant Females 2+ Years Old − Moderate+Severe Anaemia Prevalence − Cambodia
WHO−2024 Adjustment Method

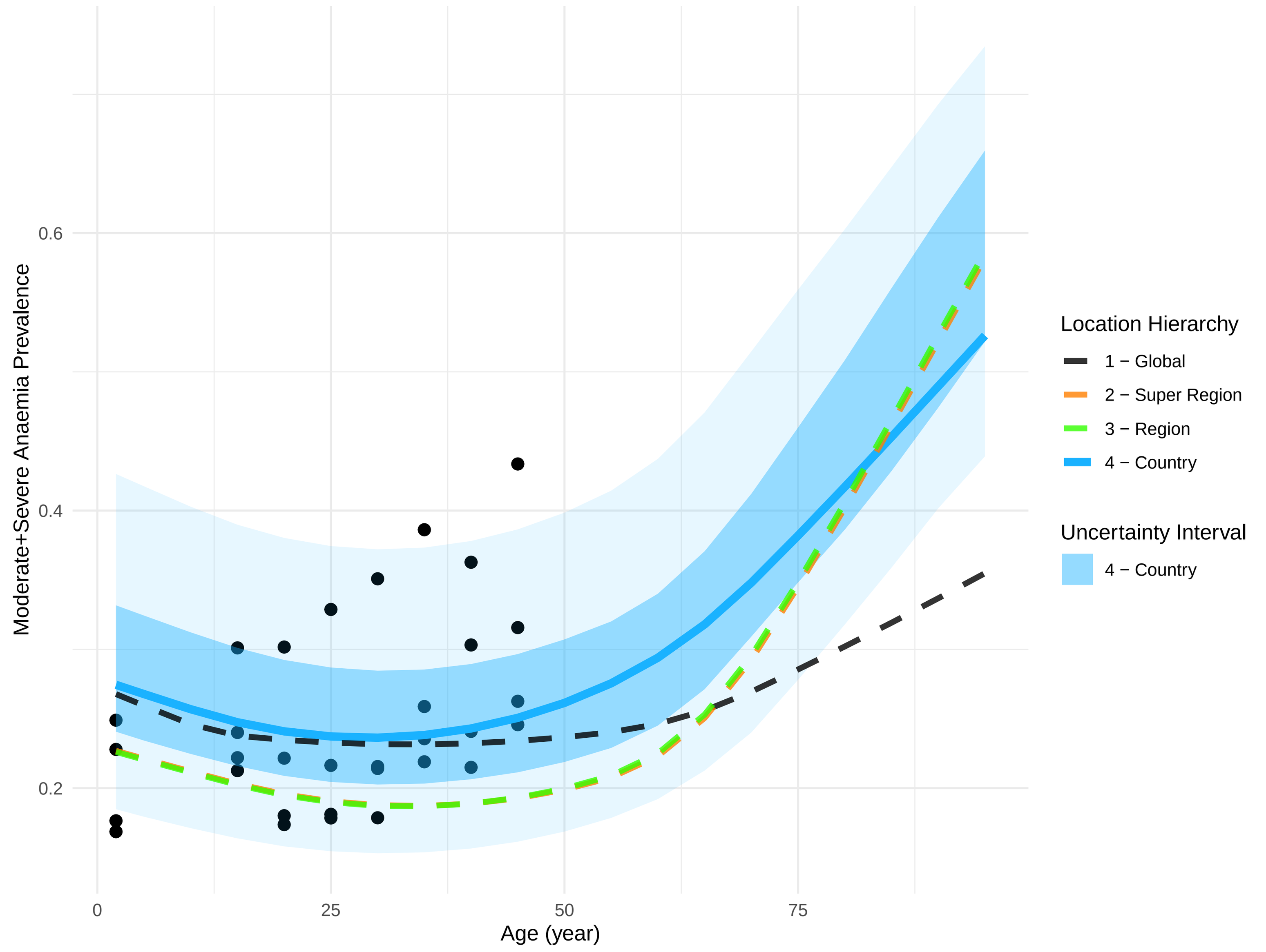

MR−BRT Age/Sex Fits − Non−pregnant Females 2+ Years Old − Moderate+Severe Anaemia Prevalence − Indonesia
WHO−2024 Adjustment Method

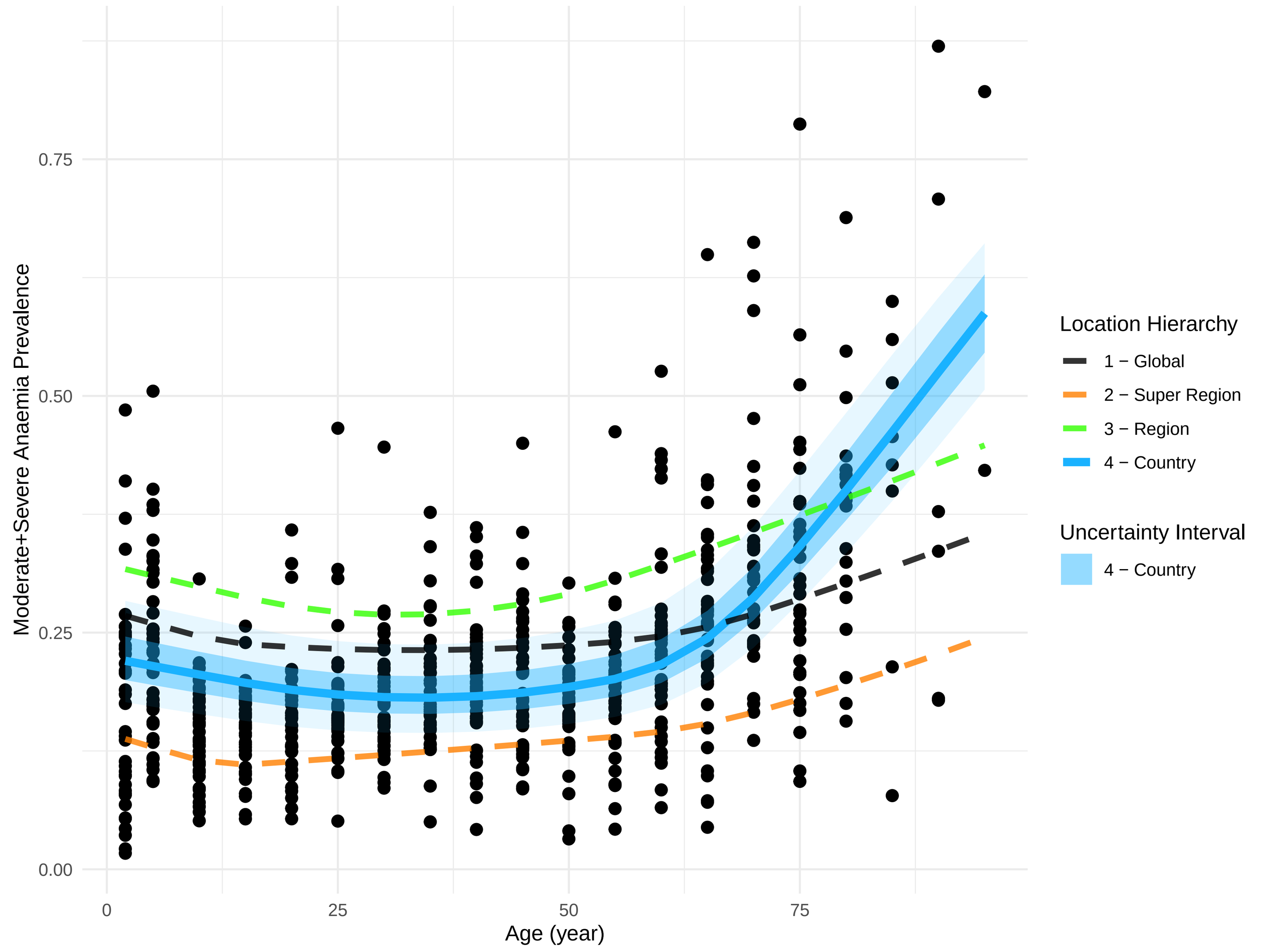

MR−BRT Age/Sex Fits − Non−pregnant Females 2+ Years Old − Moderate+Severe Anaemia Prevalence − Bali
WHO−2024 Adjustment Method

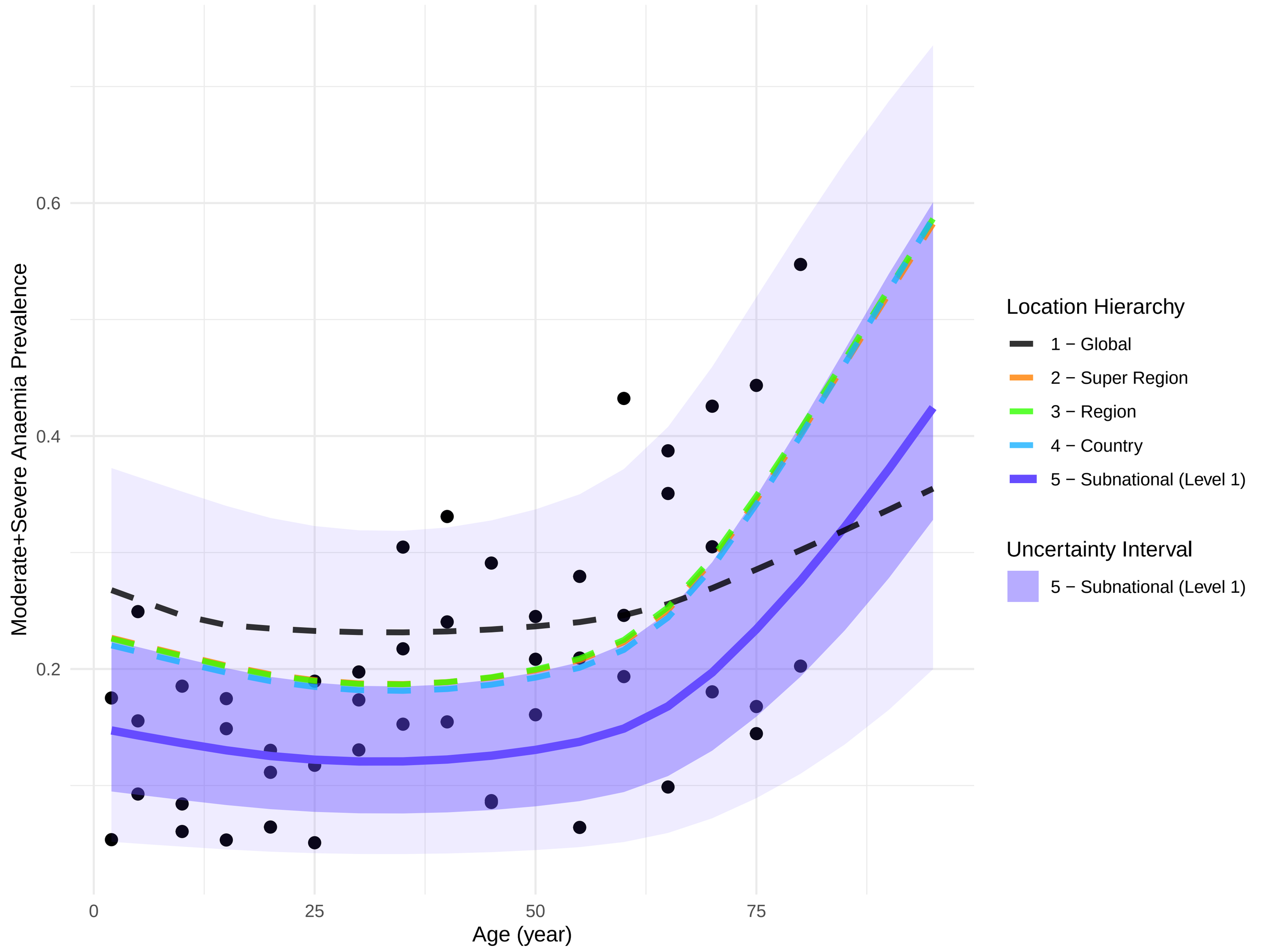

MR−BRT Age/Sex Fits − Non−pregnant Females 2+ Years Old − Moderate+Severe Anaemia Prevalence − Bangka−Belitung Islands
WHO−2024 Adjustment Method

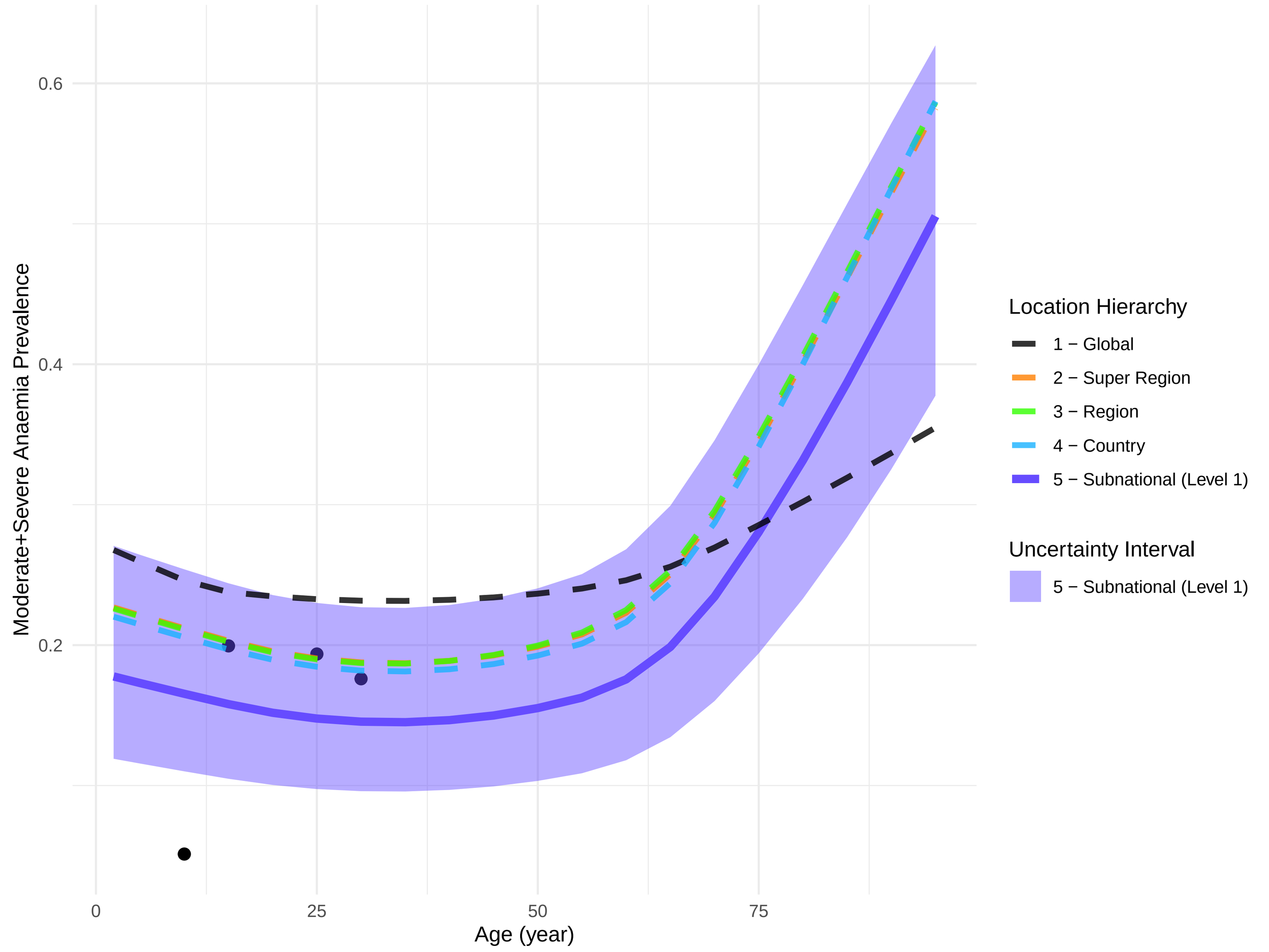

MR−BRT Age/Sex Fits − Non−pregnant Females 2+ Years Old − Moderate+Severe Anaemia Prevalence − Banten
WHO−2024 Adjustment Method

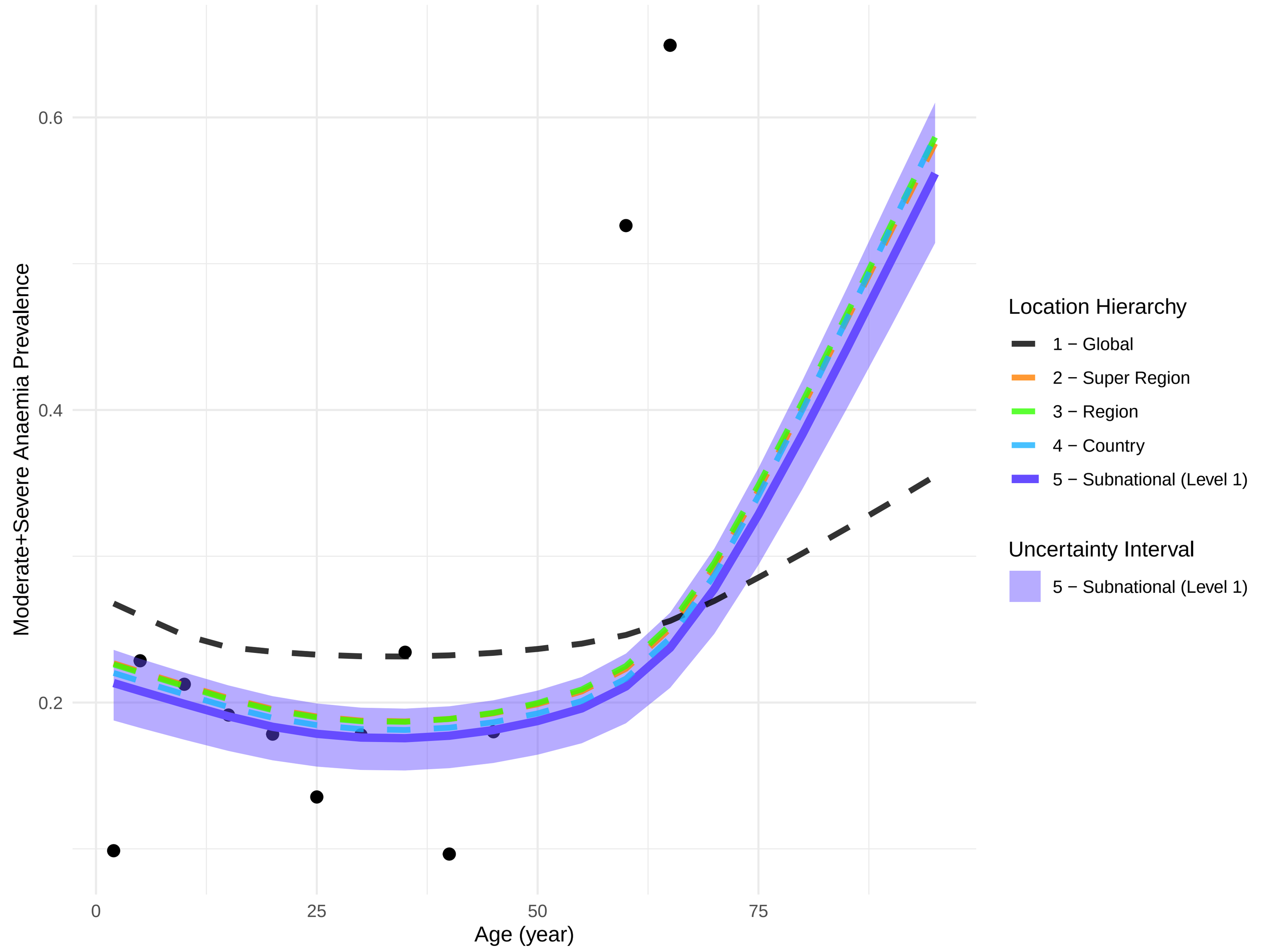

MR−BRT Age/Sex Fits − Non−pregnant Females 2+ Years Old − Moderate+Severe Anaemia Prevalence − Jakarta
WHO−2024 Adjustment Method

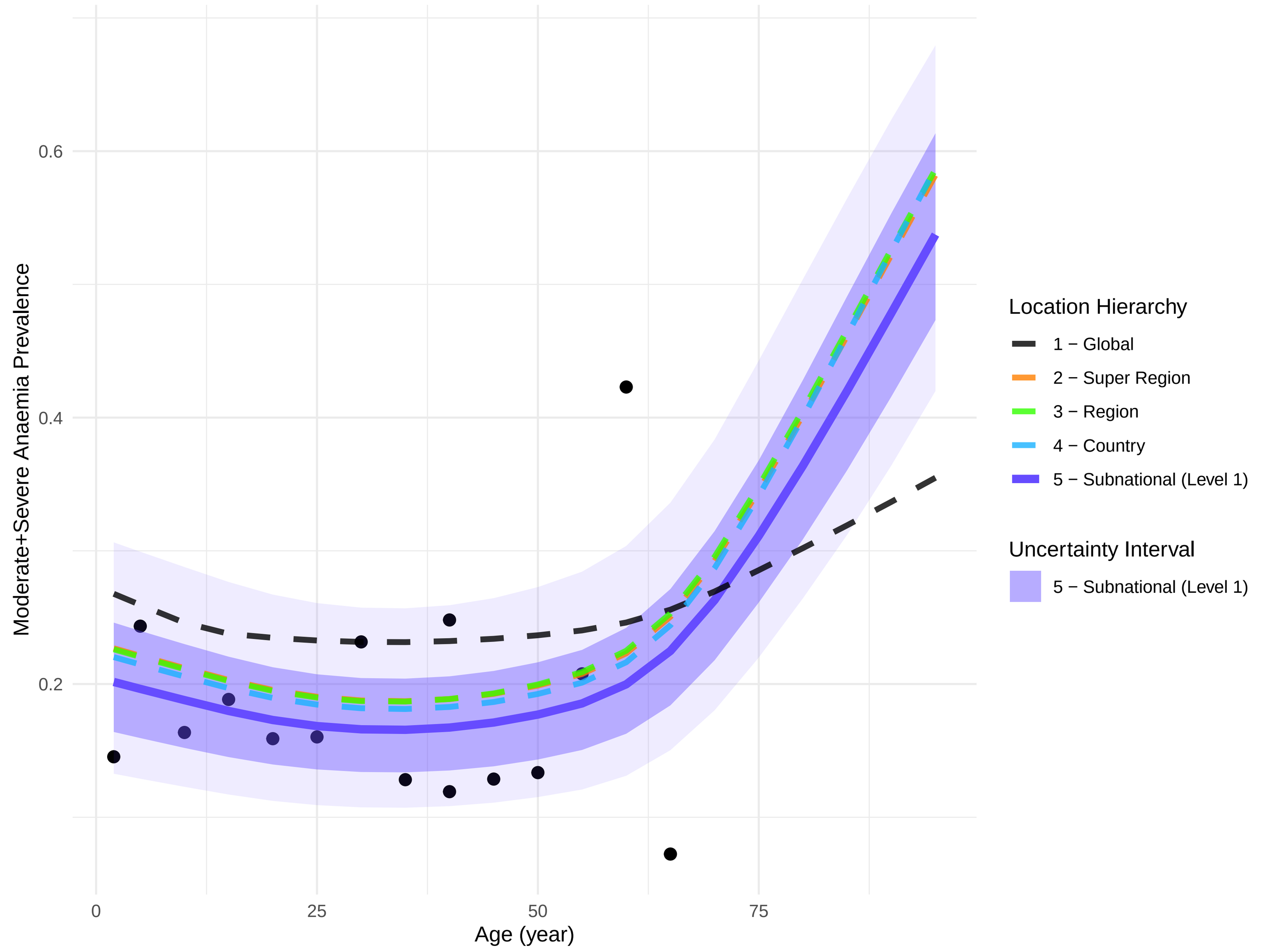

MR−BRT Age/Sex Fits − Non−pregnant Females 2+ Years Old − Moderate+Severe Anaemia Prevalence − West Java
WHO−2024 Adjustment Method

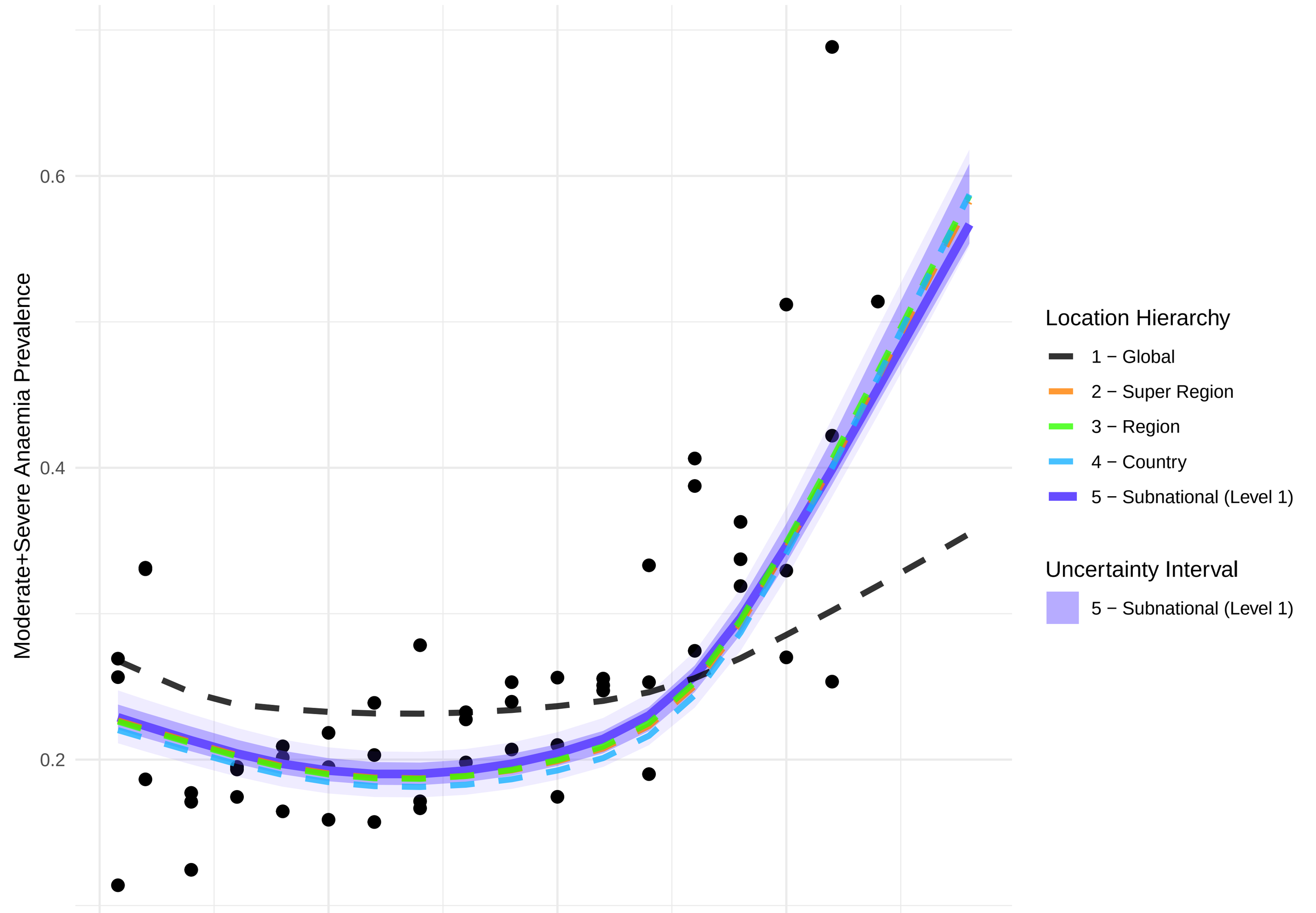

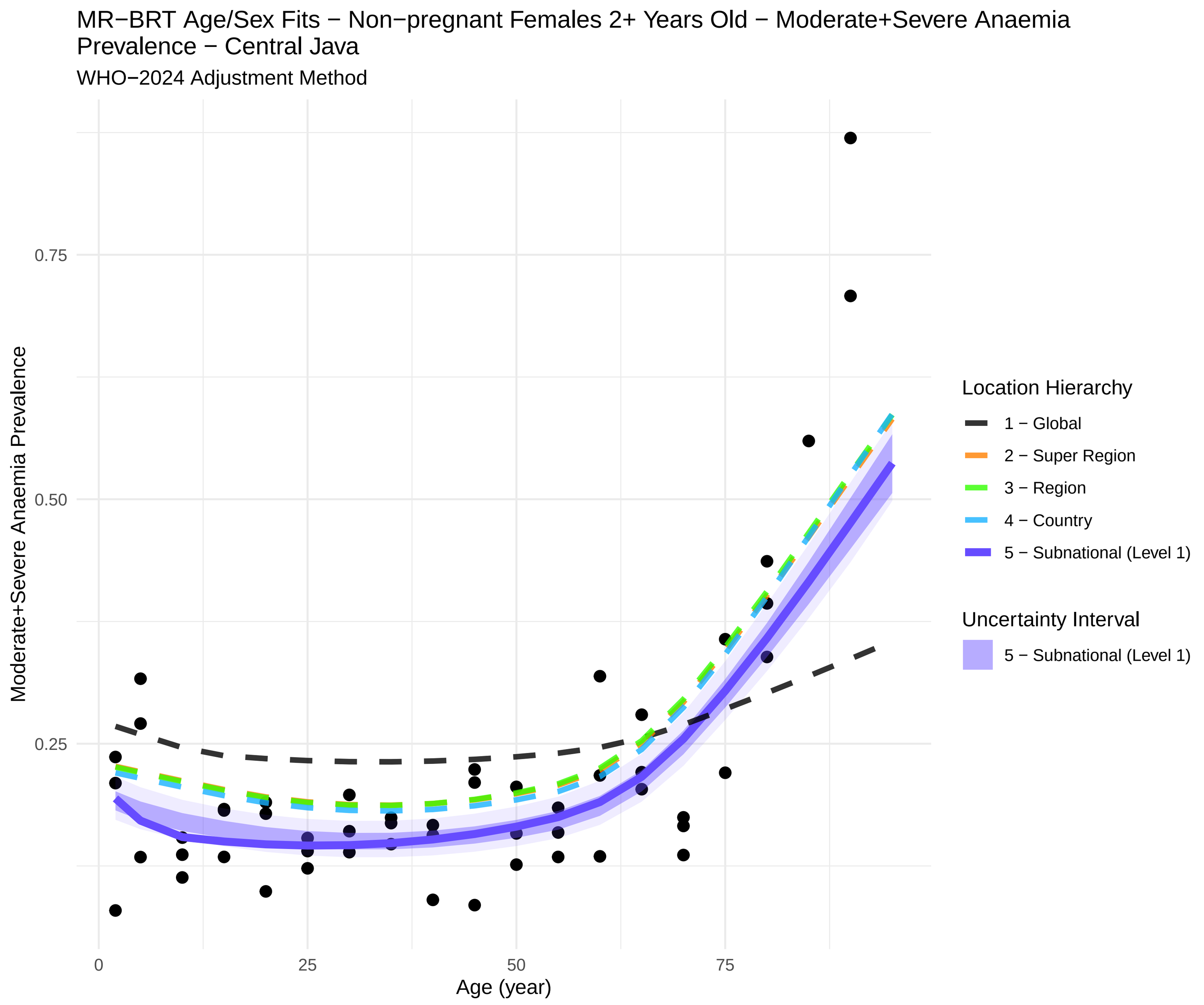
MR−BRT Age/Sex Fits − Non−pregnant Females 2+ Years Old − Moderate+Severe Anaemia
Prevalence − Central Java
WHO−2024 Adjustment Method
Moderate+Severe Anaemia Prevalence
Age (year)
Location Hierarchy
1 − Global
2 − Super Region
3 − Region
4 − Country
5 − Subnational (Level 1)
Uncertainty Interval
5 − Subnational (Level 1)

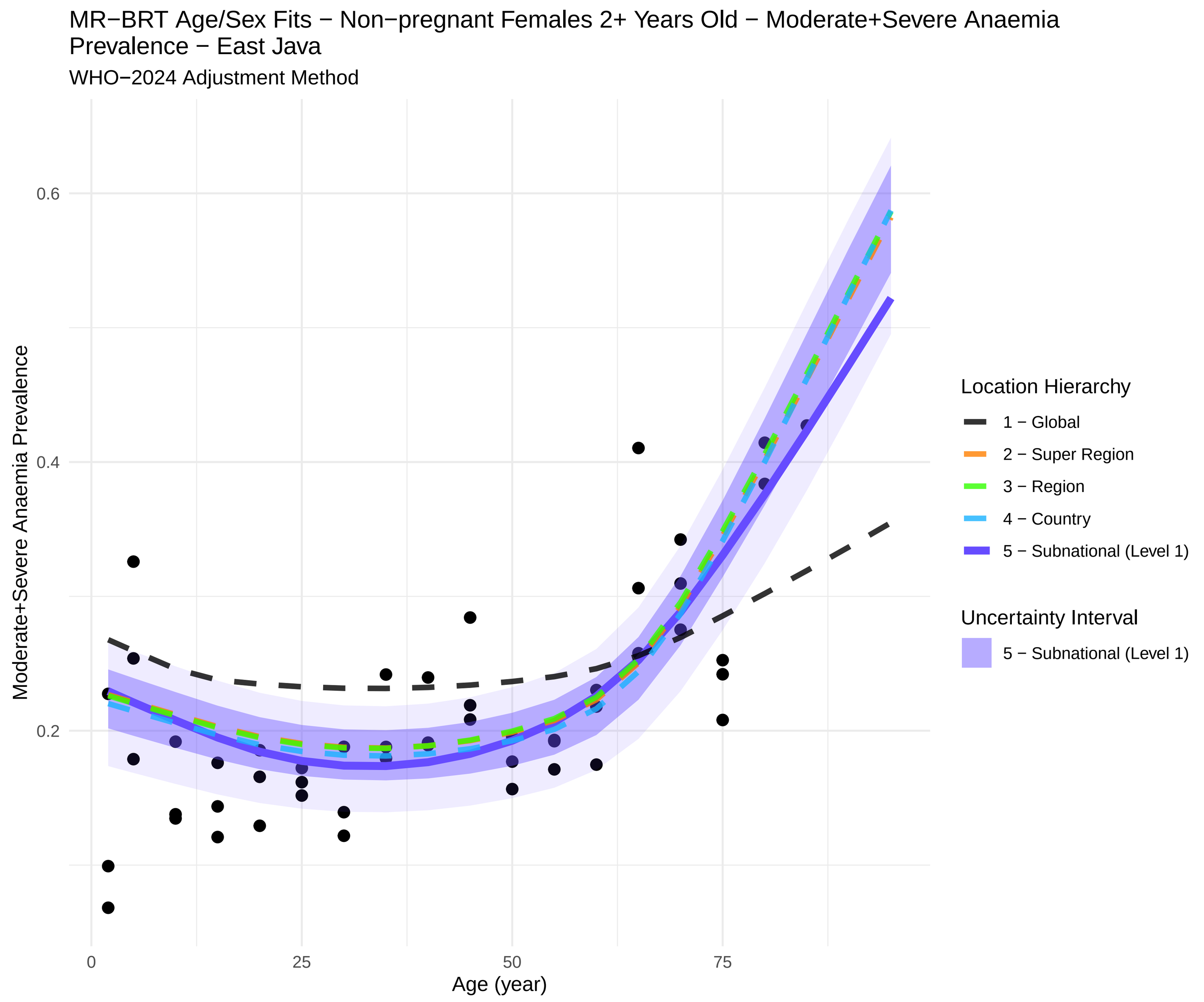
MR−BRT Age/Sex Fits − Non−pregnant Females 2+ Years Old − Moderate+Severe Anaemia
Prevalence − East Java
WHO−2024 Adjustment Method
Moderate+Severe Anaemia Prevalence
Age (year)
Location Hierarchy
1 − Global
2 − Super Region
3 − Region
4 − Country
5 − Subnational (Level 1)
Uncertainty Interval
5 − Subnational (Level 1)

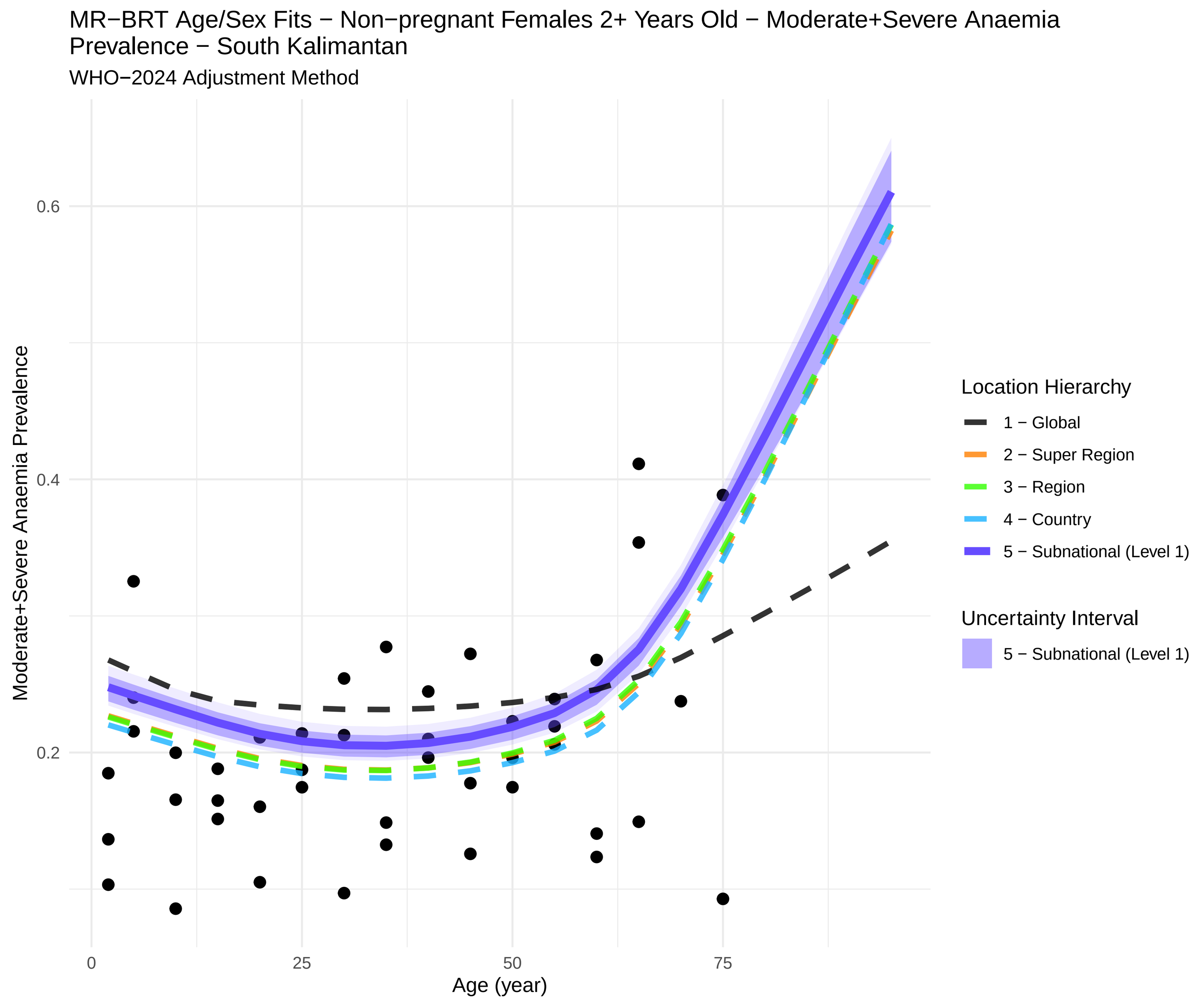
MR−BRT Age/Sex Fits − Non−pregnant Females 2+ Years Old − Moderate+Severe Anaemia
Prevalence − South Kalimantan
WHO−2024 Adjustment Method
Moderate+Severe Anaemia Prevalence
Age (year)
Location Hierarchy
1 − Global
2 − Super Region
3 − Region
4 − Country
5 − Subnational (Level 1)
Uncertainty Interval
5 − Subnational (Level 1)

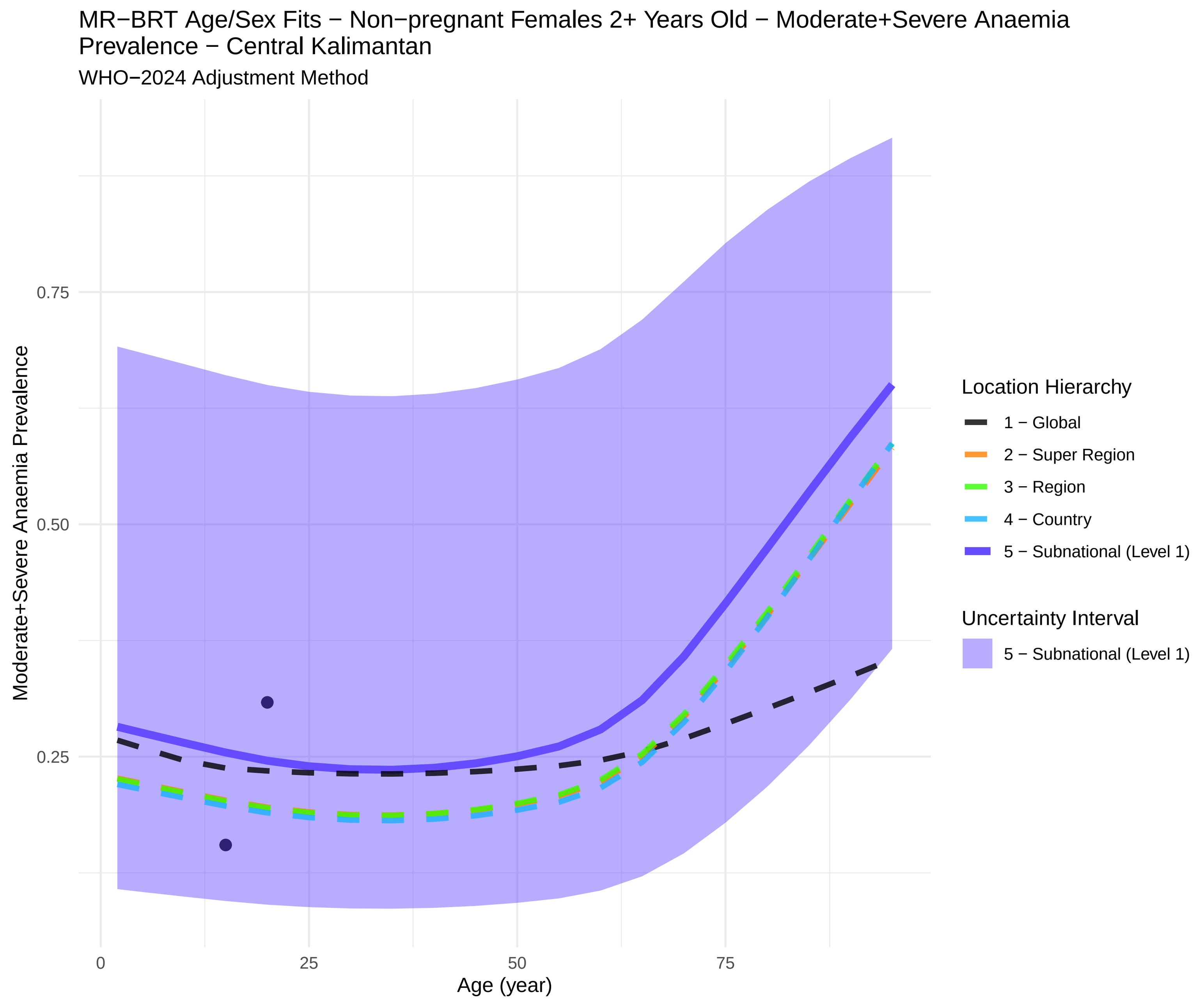
MR−BRT Age/Sex Fits − Non−pregnant Females 2+ Years Old − Moderate+Severe Anaemia
Prevalence − Central Kalimantan
WHO−2024 Adjustment Method
Moderate+Severe Anaemia Prevalence
Age (year)
Location Hierarchy
1 − Global
2 − Super Region
3 − Region
4 − Country
5 − Subnational (Level 1)
Uncertainty Interval
5 − Subnational (Level 1)

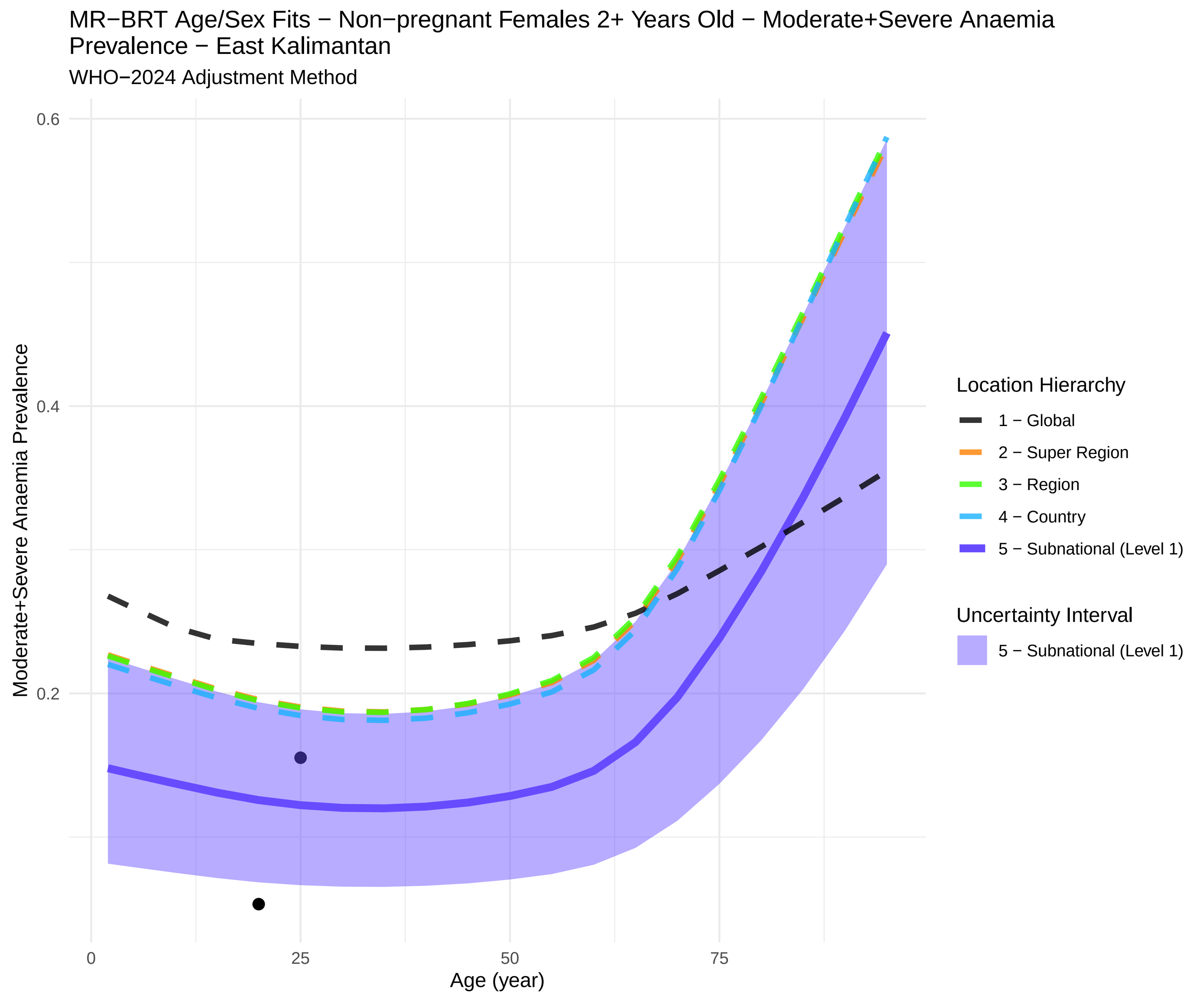
MR−BRT Age/Sex Fits − Non−pregnant Females 2+ Years Old − Moderate+Severe Anaemia
Prevalence − East Kalimantan
WHO−2024 Adjustment Method
Moderate+Severe Anaemia Prevalence
Age (year)
Location Hierarchy
1 − Global
2 − Super Region
3 − Region
4 − Country
5 − Subnational (Level 1)
Uncertainty Interval
5 − Subnational (Level 1)

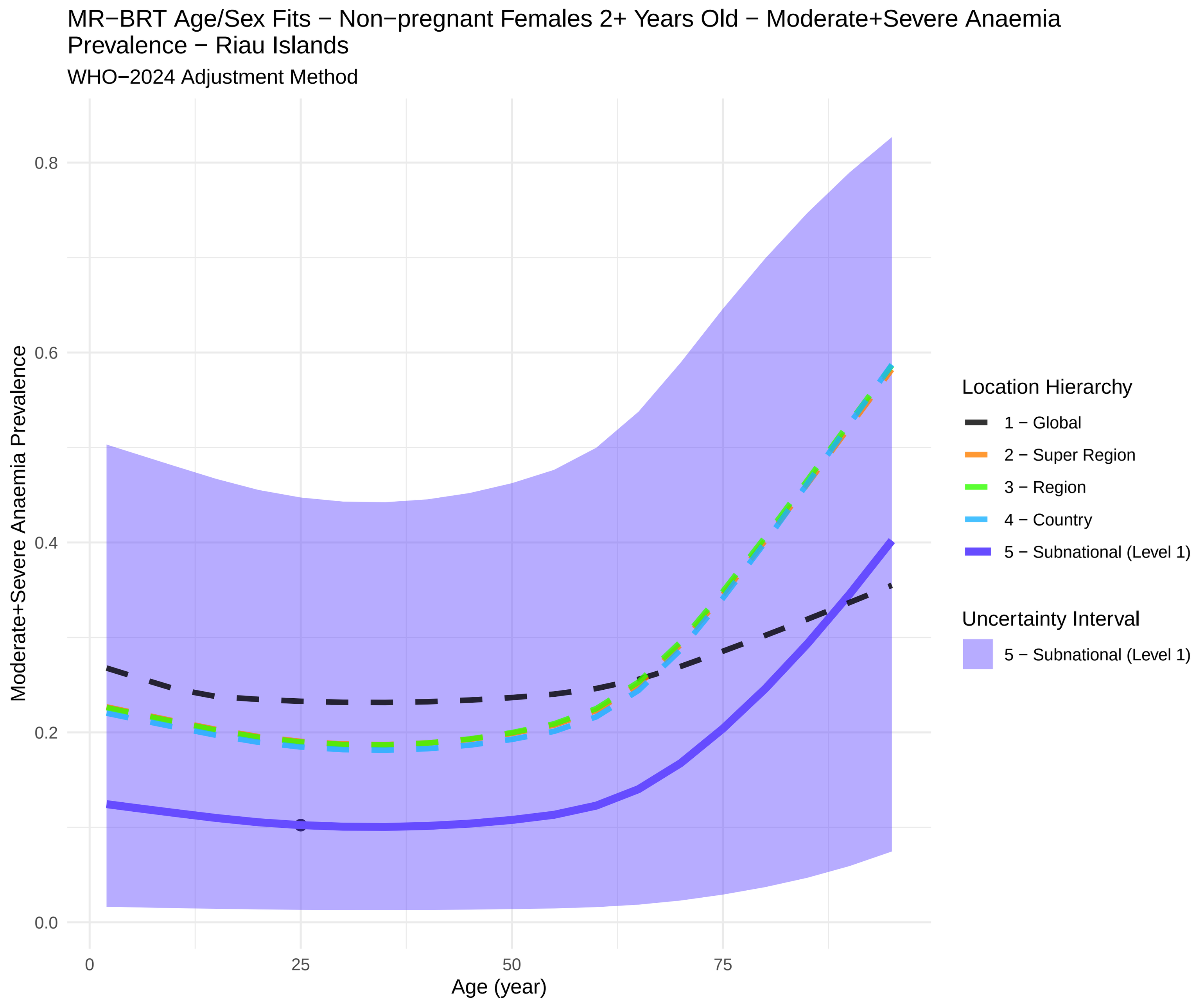
MR−BRT Age/Sex Fits − Non−pregnant Females 2+ Years Old − Moderate+Severe Anaemia
Prevalence − Riau Islands
WHO−2024 Adjustment Method
Moderate+Severe Anaemia Prevalence
Age (year)
Location Hierarchy
1 − Global
2 − Super Region
3 − Region
4 − Country
5 − Subnational (Level 1)
Uncertainty Interval
5 − Subnational (Level 1)

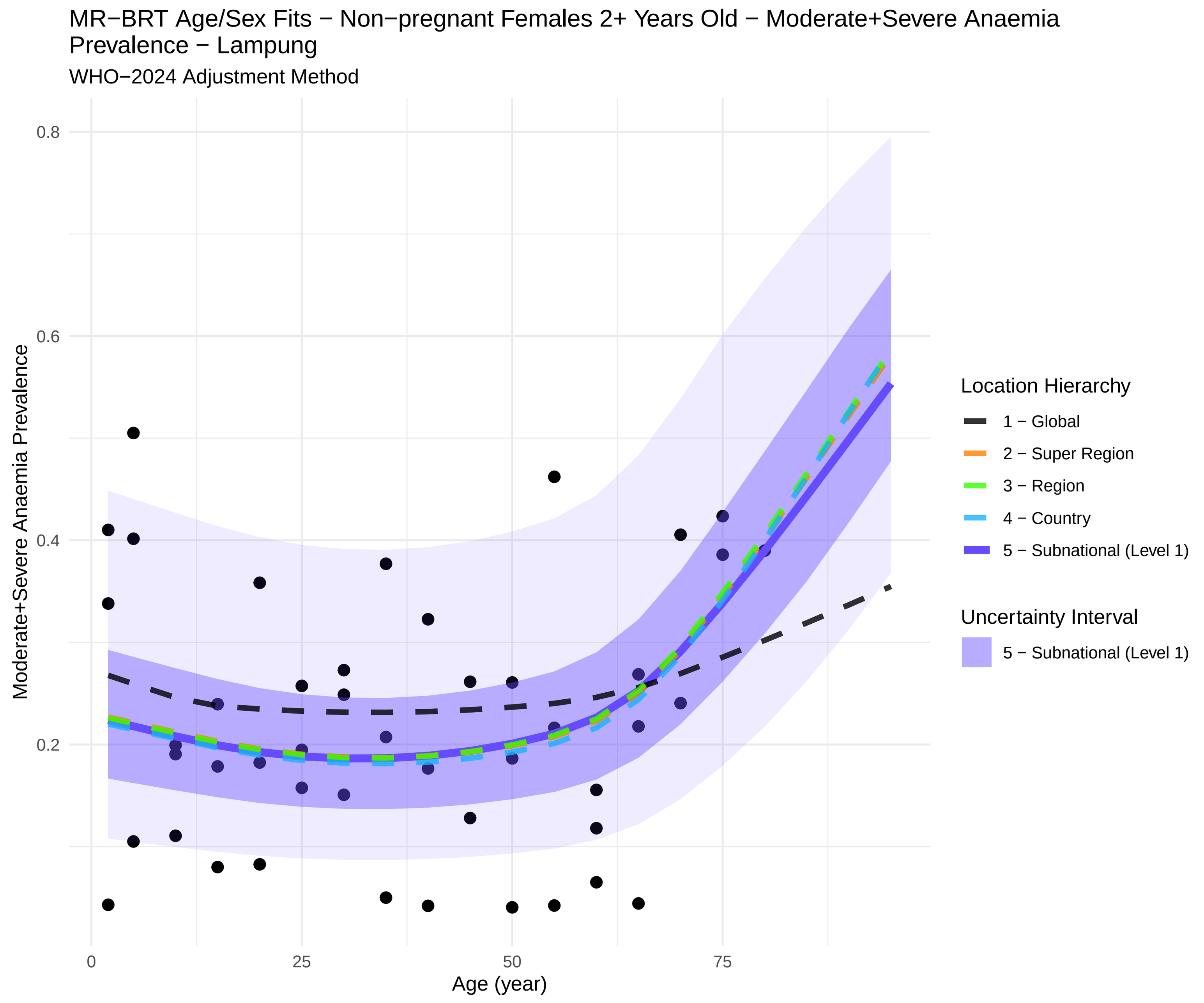
MR−BRT Age/Sex Fits − Non−pregnant Females 2+ Years Old − Moderate+Severe Anaemia
Prevalence − Lampung
WHO−2024 Adjustment Method
Moderate+Severe Anaemia Prevalence
Age (year)
Location Hierarchy
1 − Global
2 − Super Region
3 − Region
4 − Country
5 − Subnational (Level 1)
Uncertainty Interval
5 − Subnational (Level 1)

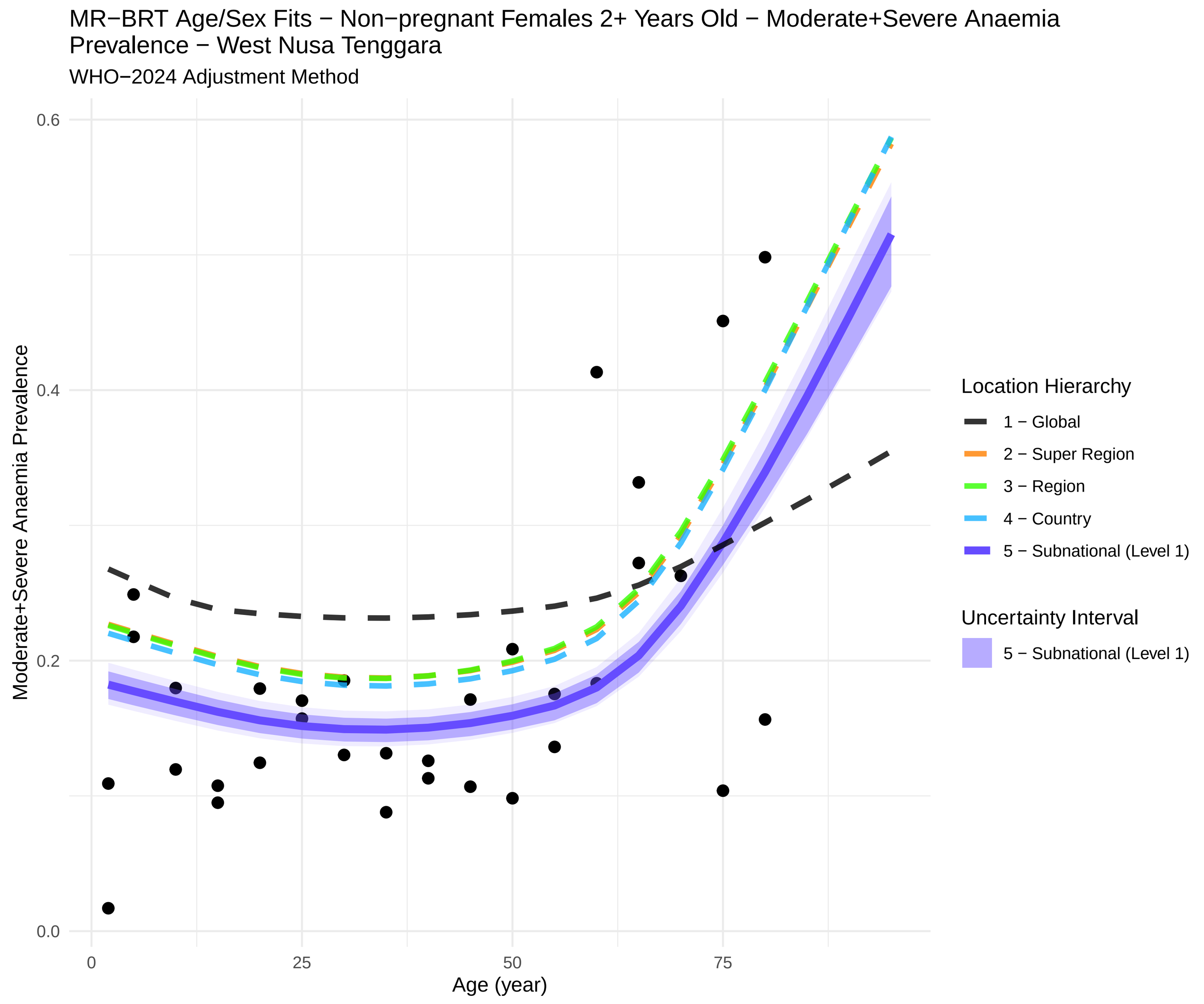
MR−BRT Age/Sex Fits − Non−pregnant Females 2+ Years Old − Moderate+Severe Anaemia
Prevalence − West Nusa Tenggara
WHO−2024 Adjustment Method
Moderate+Severe Anaemia Prevalence
Age (year)
Location Hierarchy
1 − Global
2 − Super Region
3 − Region
4 − Country
5 − Subnational (Level 1)
Uncertainty Interval
5 − Subnational (Level 1)

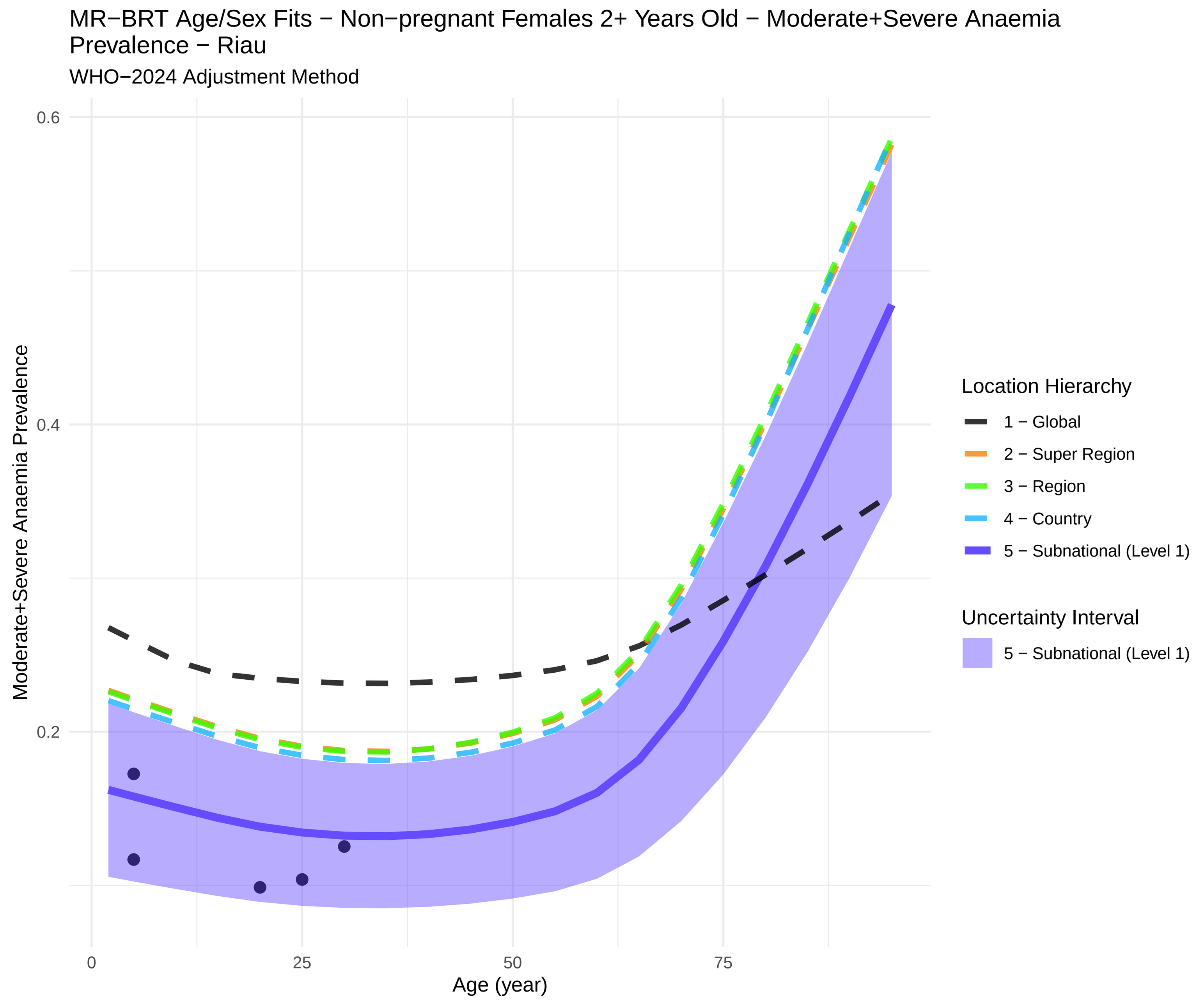
MR−BRT Age/Sex Fits − Non−pregnant Females 2+ Years Old − Moderate+Severe Anaemia
Prevalence − Riau
WHO−2024 Adjustment Method
Moderate+Severe Anaemia Prevalence
Age (year)
Location Hierarchy
1 − Global
2 − Super Region
3 − Region
4 − Country
5 − Subnational (Level 1)
Uncertainty Interval
5 − Subnational (Level 1)

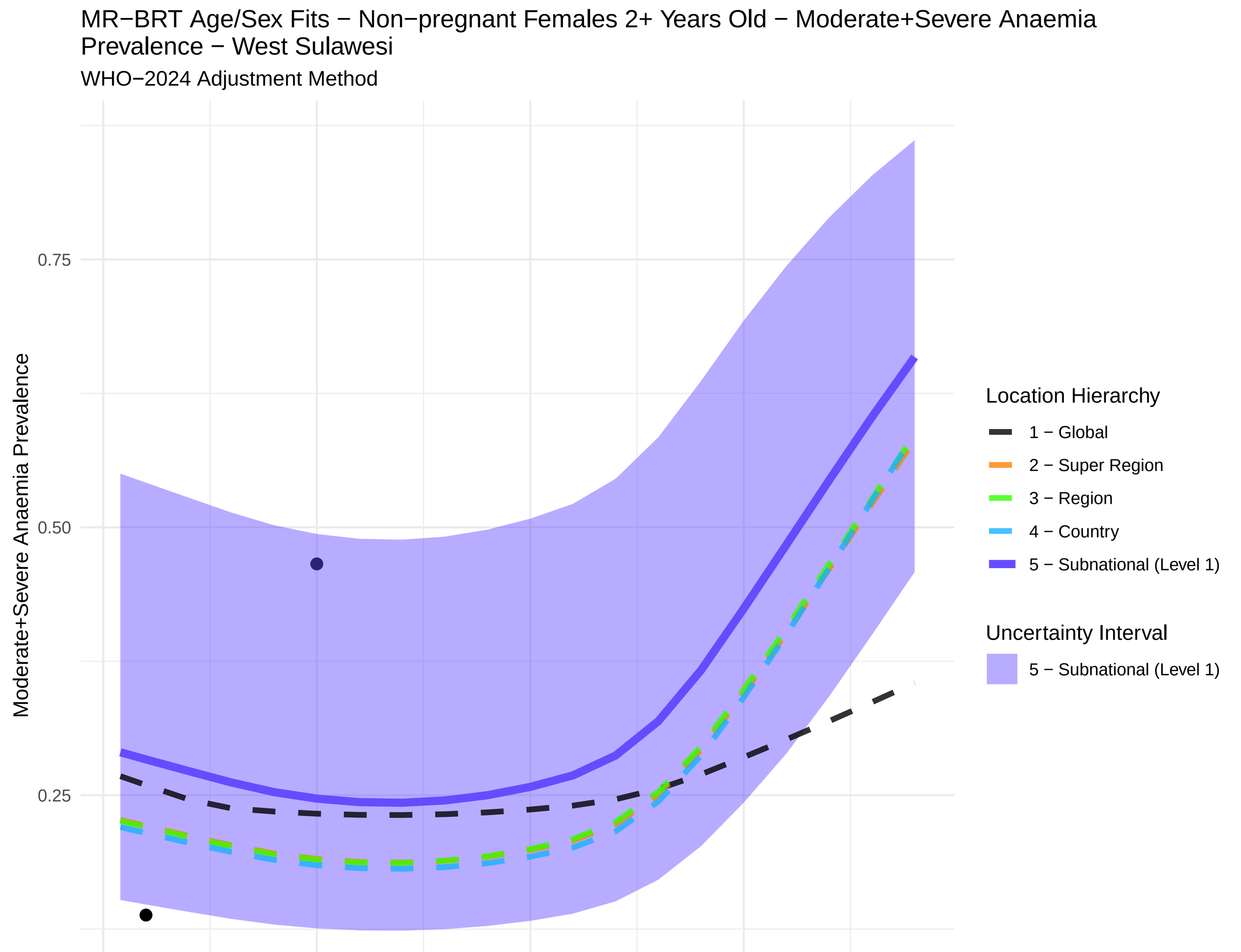
MR−BRT Age/Sex Fits − Non−pregnant Females 2+ Years Old − Moderate+Severe Anaemia
Prevalence − West Sulawesi
WHO−2024 Adjustment Method
Moderate+Severe Anaemia Prevalence
Age (year)
Location Hierarchy
1 − Global
2 − Super Region
3 − Region
4 − Country
5 − Subnational (Level 1)
Uncertainty Interval
5 − Subnational (Level 1)

MR−BRT Age/Sex Fits − Non−pregnant Females 2+ Years Old − Moderate+Severe Anaemia Prevalence − South Sulawesi
WHO−2024 Adjustment Method

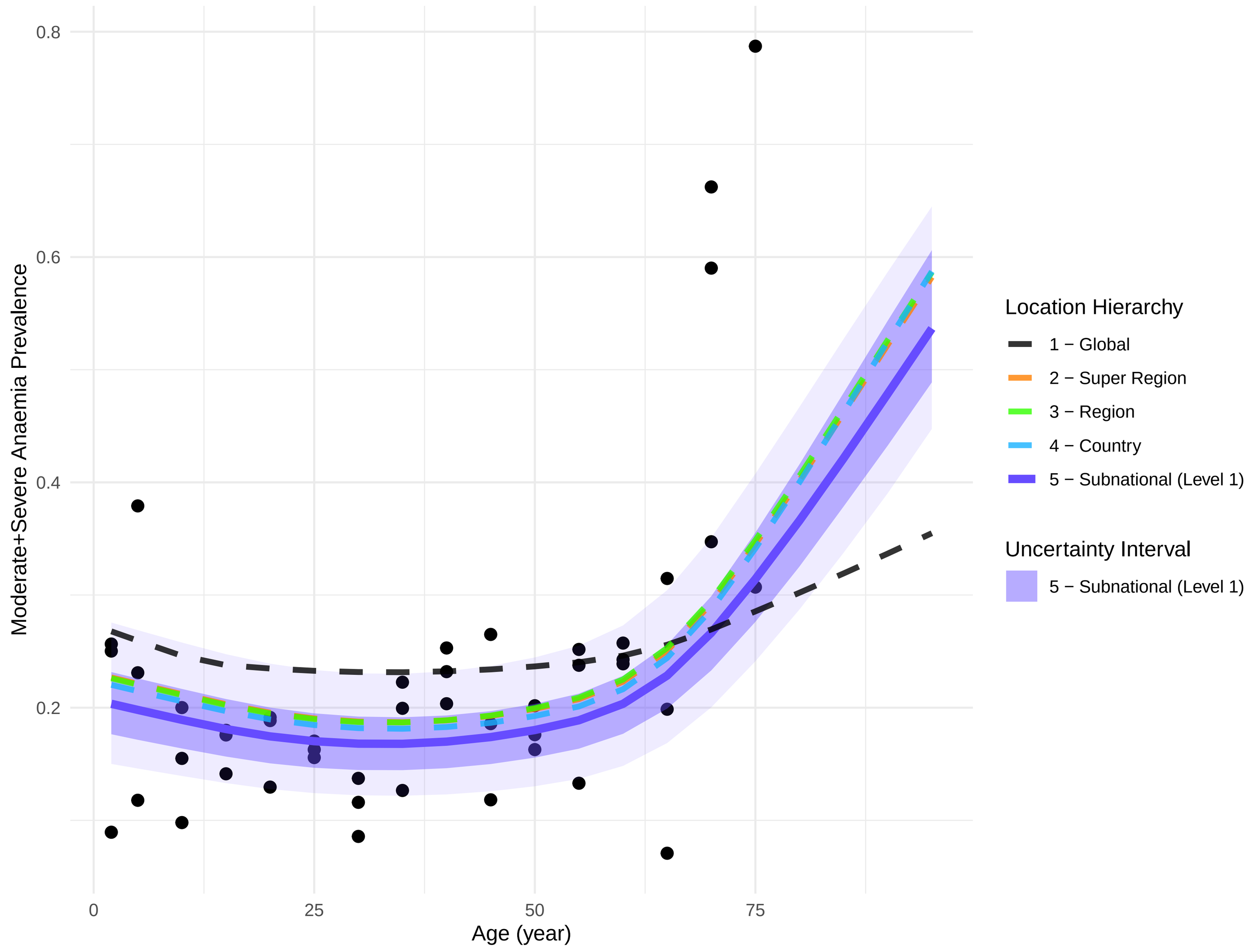

MR−BRT Age/Sex Fits − Non−pregnant Females 2+ Years Old − Moderate+Severe Anaemia Prevalence − West Sumatra
WHO−2024 Adjustment Method

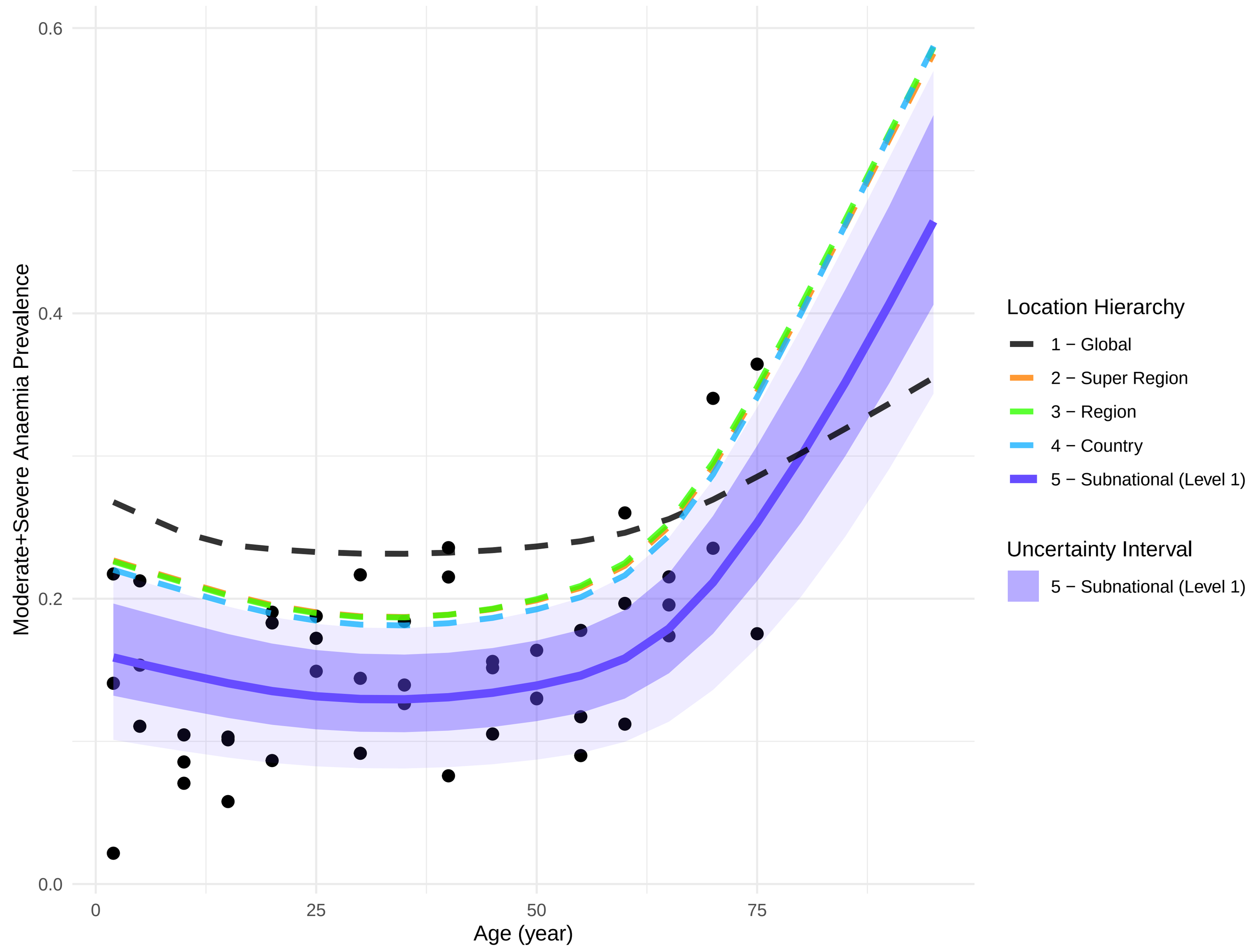

MR−BRT Age/Sex Fits − Non−pregnant Females 2+ Years Old − Moderate+Severe Anaemia Prevalence − South Sumatra
WHO−2024 Adjustment Method

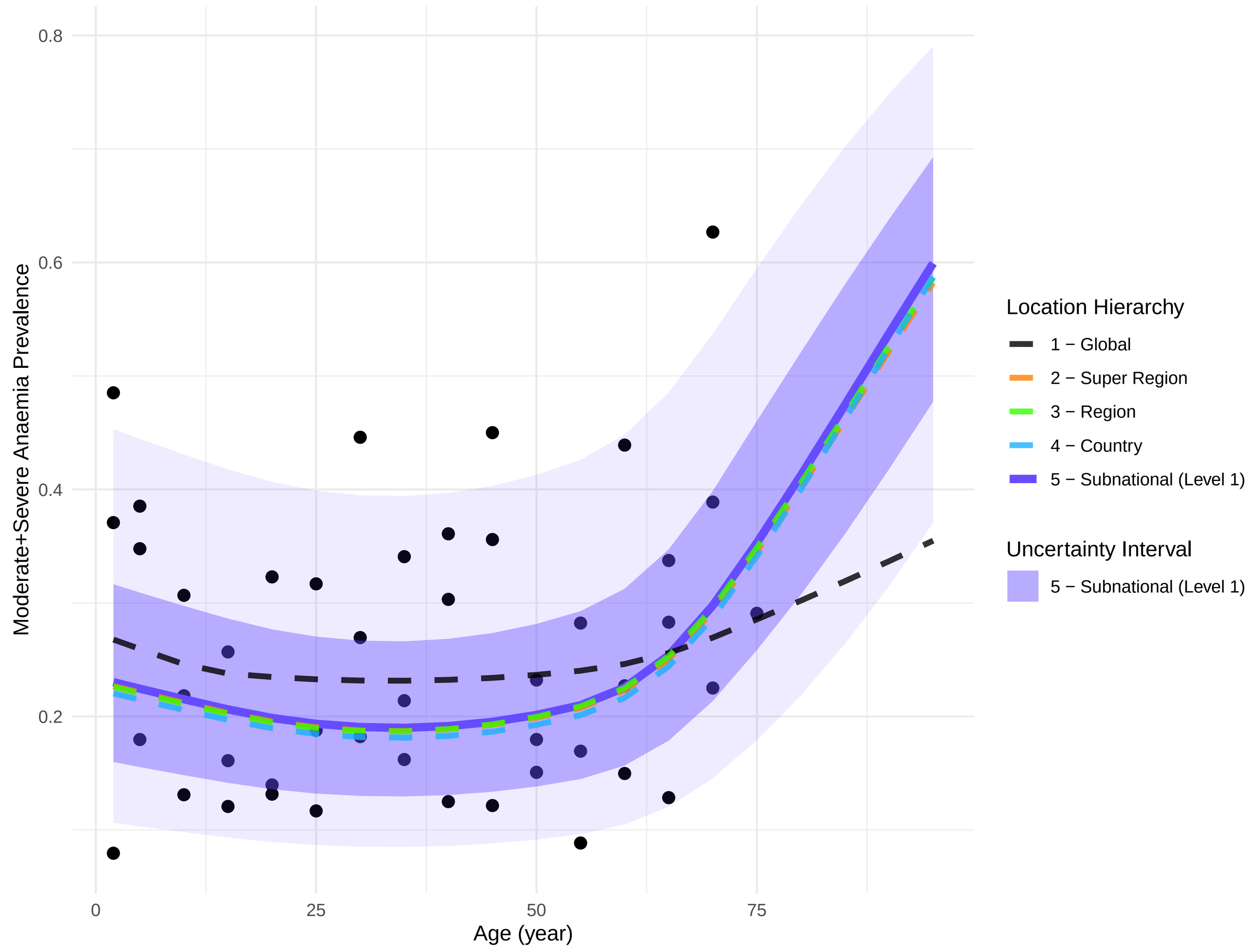

MR−BRT Age/Sex Fits − Non−pregnant Females 2+ Years Old − Moderate+Severe Anaemia Prevalence − North Sumatra
WHO−2024 Adjustment Method

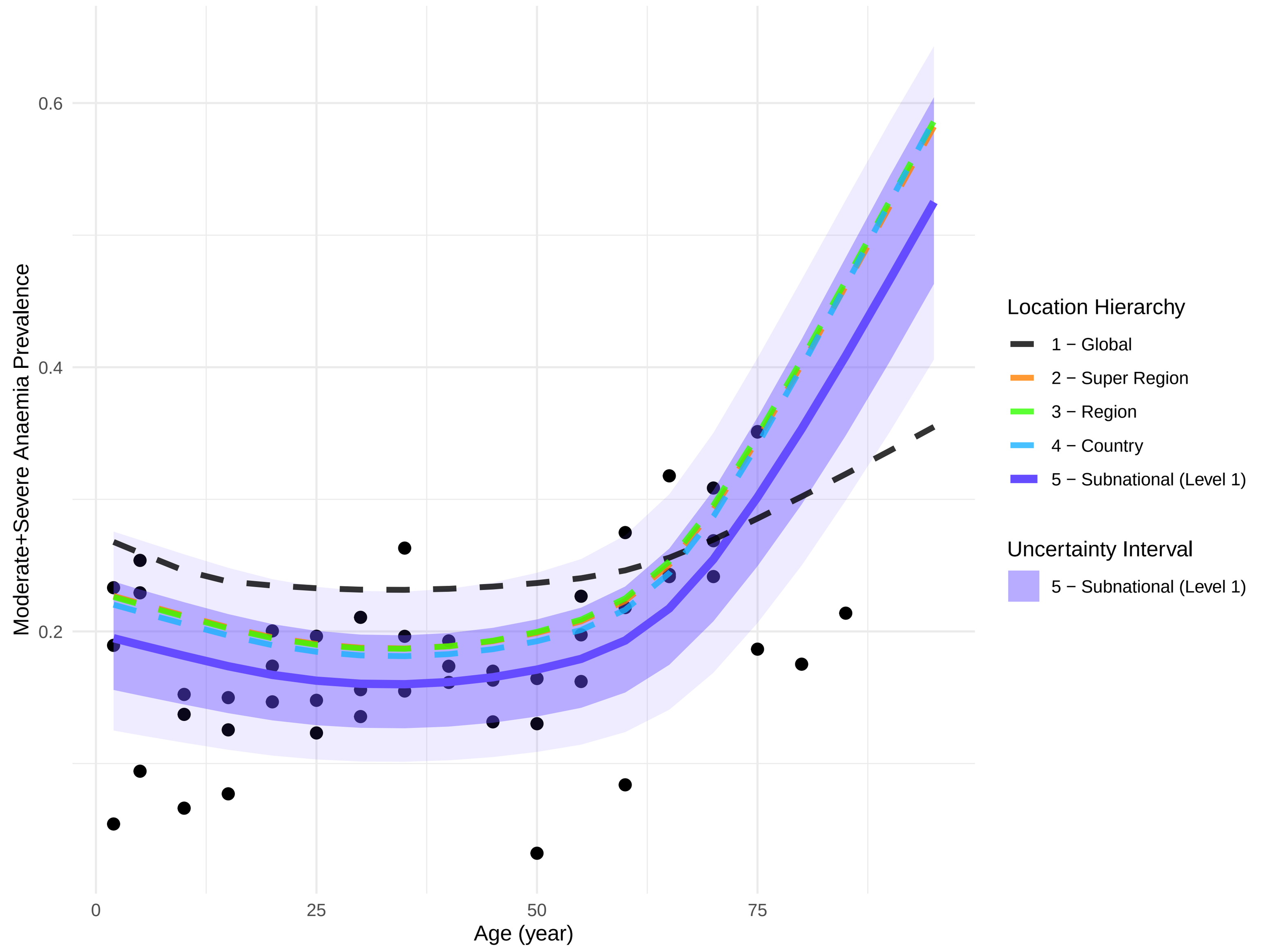

MR−BRT Age/Sex Fits − Non−pregnant Females 2+ Years Old − Moderate+Severe Anaemia Prevalence − Yogyakarta
WHO−2024 Adjustment Method

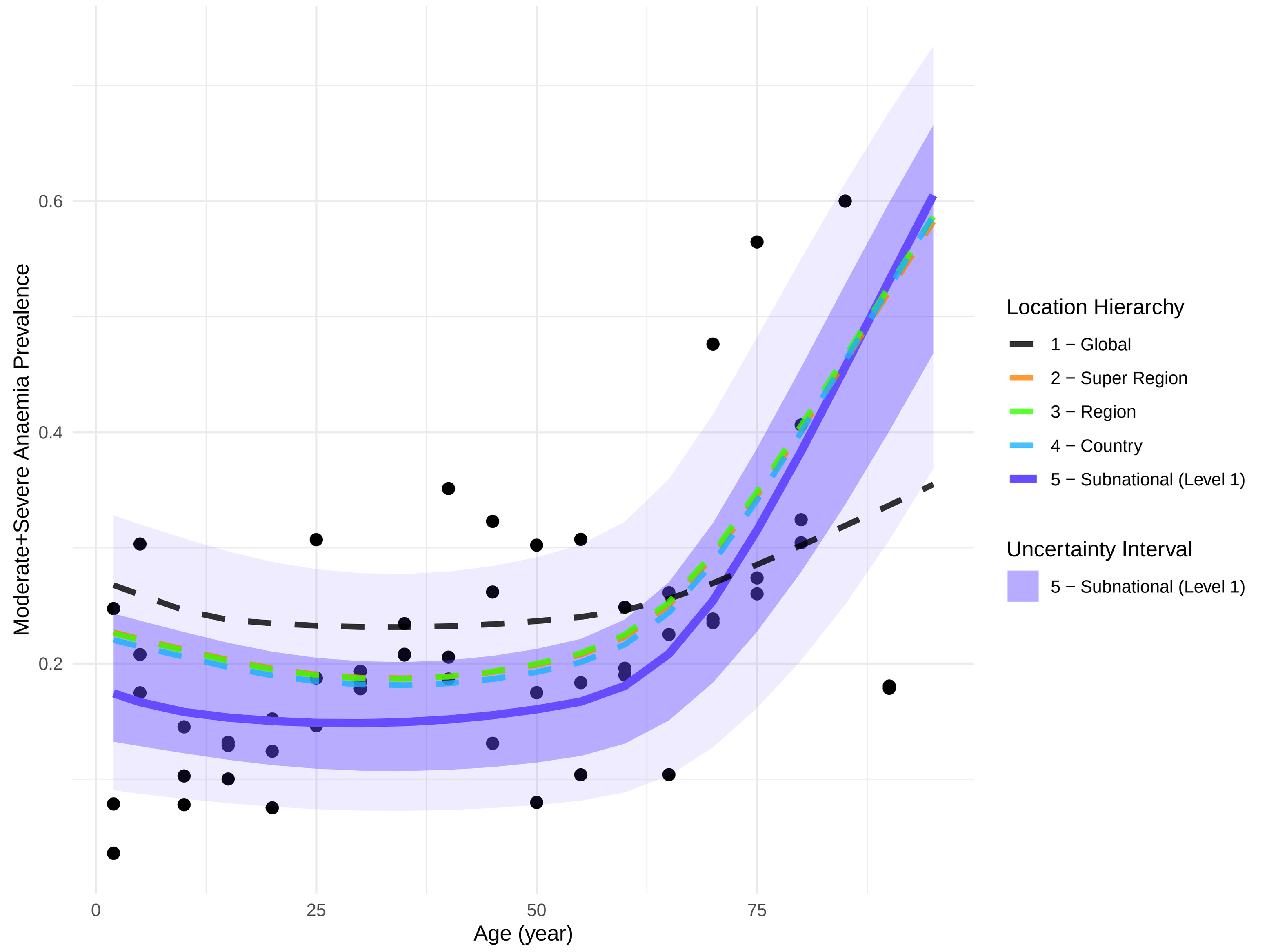

MR−BRT Age/Sex Fits − Non−pregnant Females 2+ Years Old − Moderate+Severe Anaemia Prevalence − Myanmar
WHO−2024 Adjustment Method

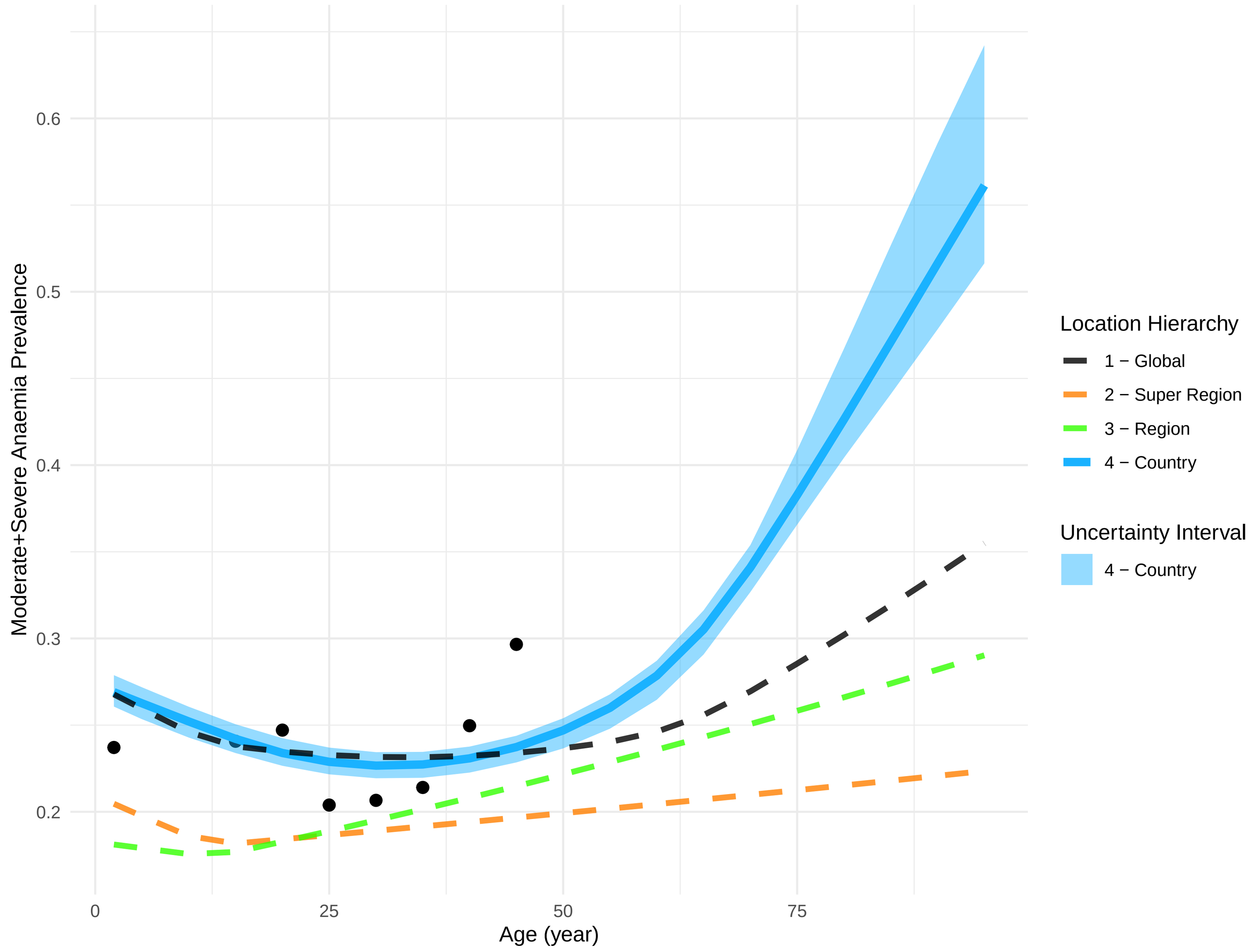

MR−BRT Age/Sex Fits − Non−pregnant Females 2+ Years Old − Moderate+Severe Anaemia Prevalence − Timor−Leste
WHO−2024 Adjustment Method

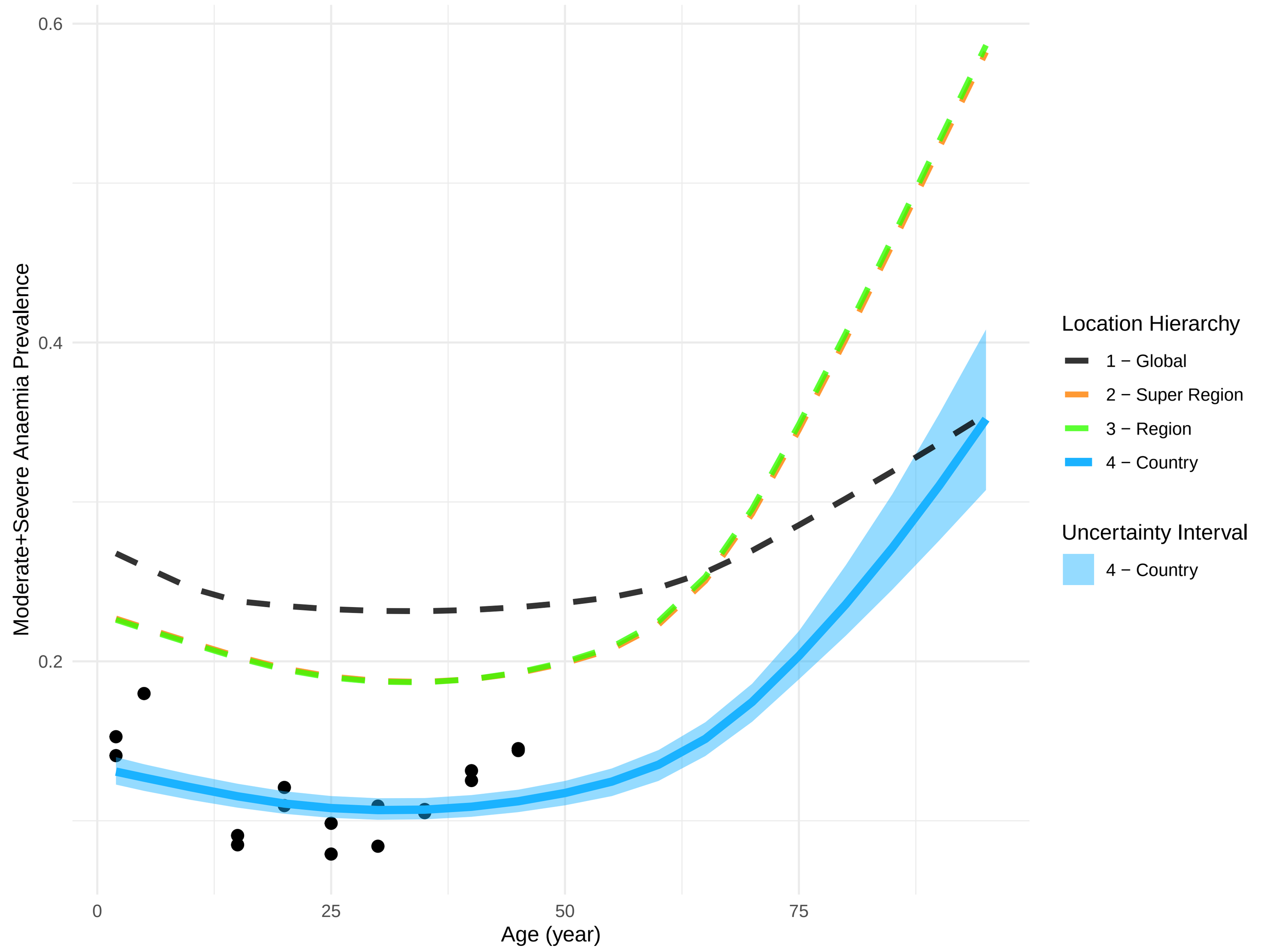

MR−BRT Age/Sex Fits − Non−pregnant Females 2+ Years Old − Moderate+Severe Anaemia Prevalence − Sub−Saharan Africa
WHO−2024 Adjustment Method

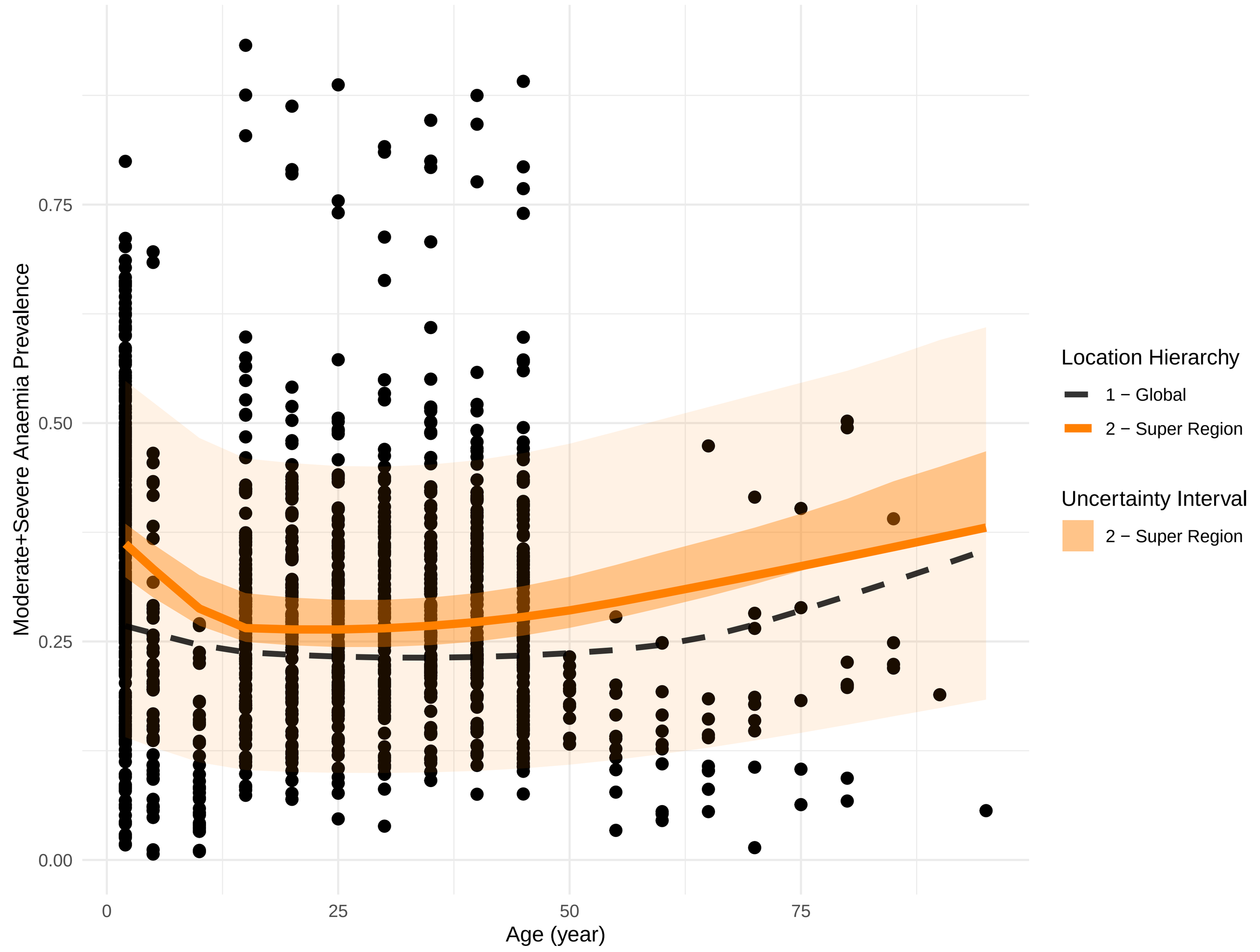

MR−BRT Age/Sex Fits − Non−pregnant Females 2+ Years Old − Moderate+Severe Anaemia Prevalence − Central Sub−Saharan Africa
WHO−2024 Adjustment Method

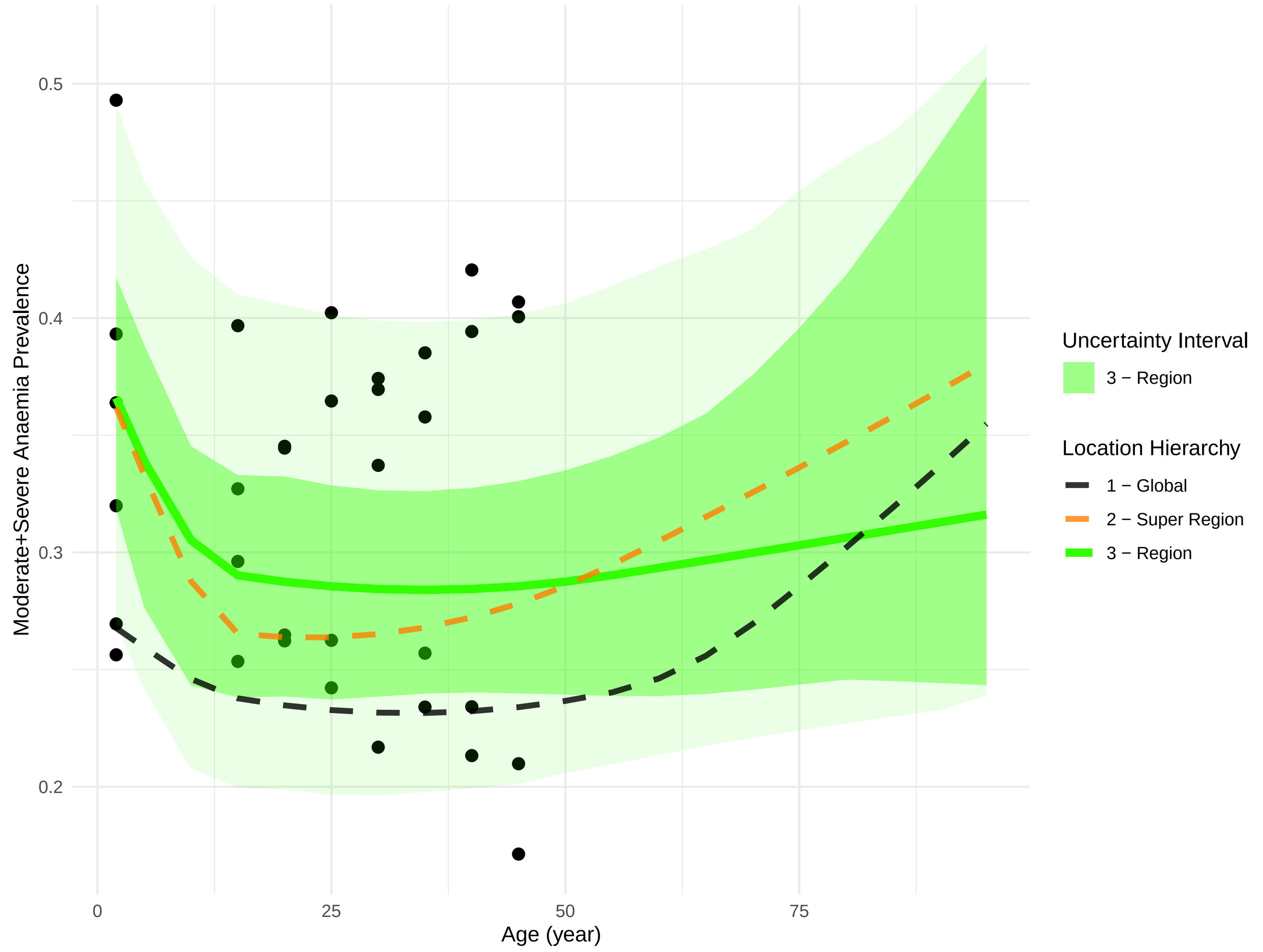

MR−BRT Age/Sex Fits − Non−pregnant Females 2+ Years Old − Moderate+Severe Anaemia Prevalence − Angola
WHO−2024 Adjustment Method

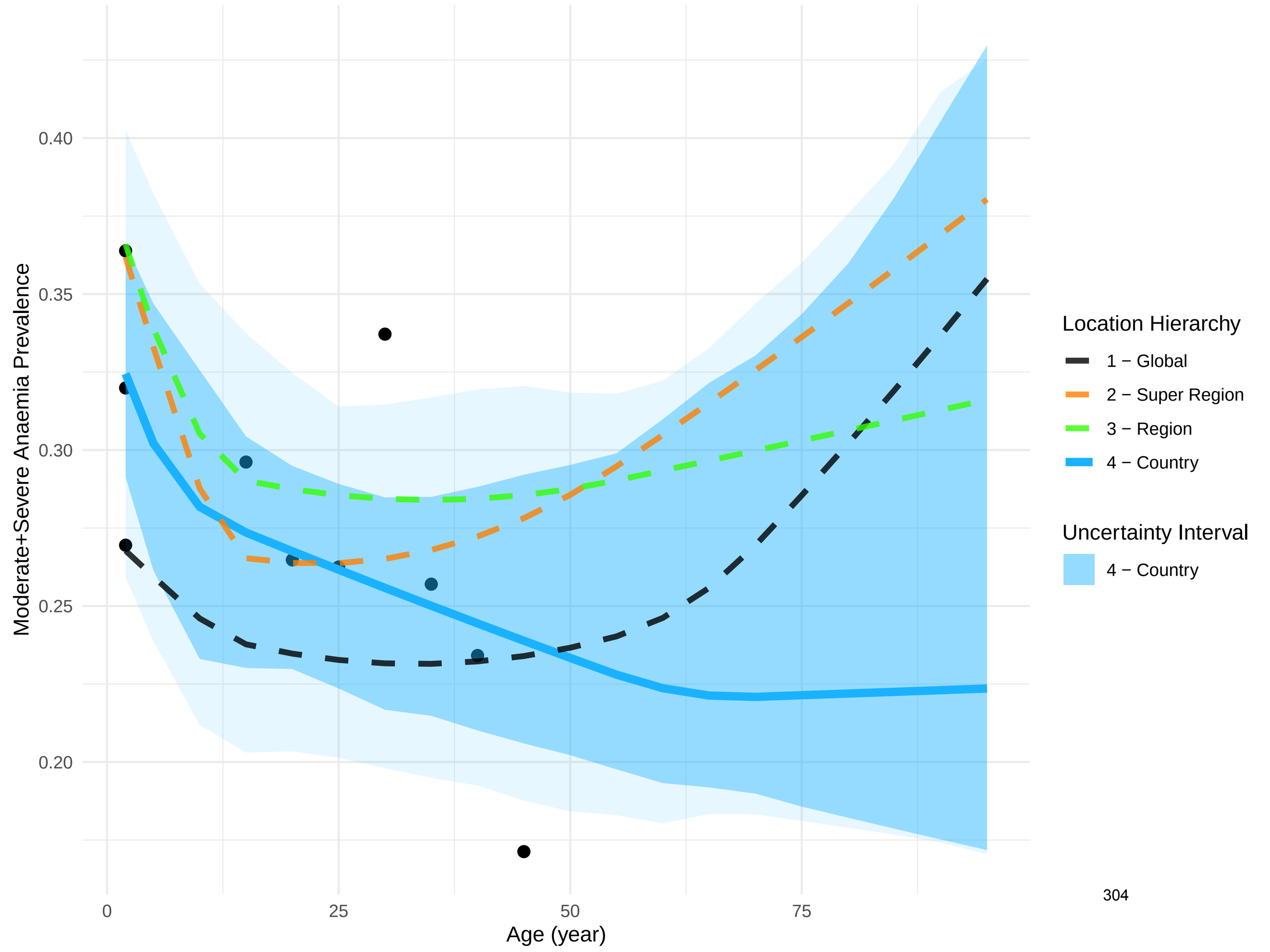

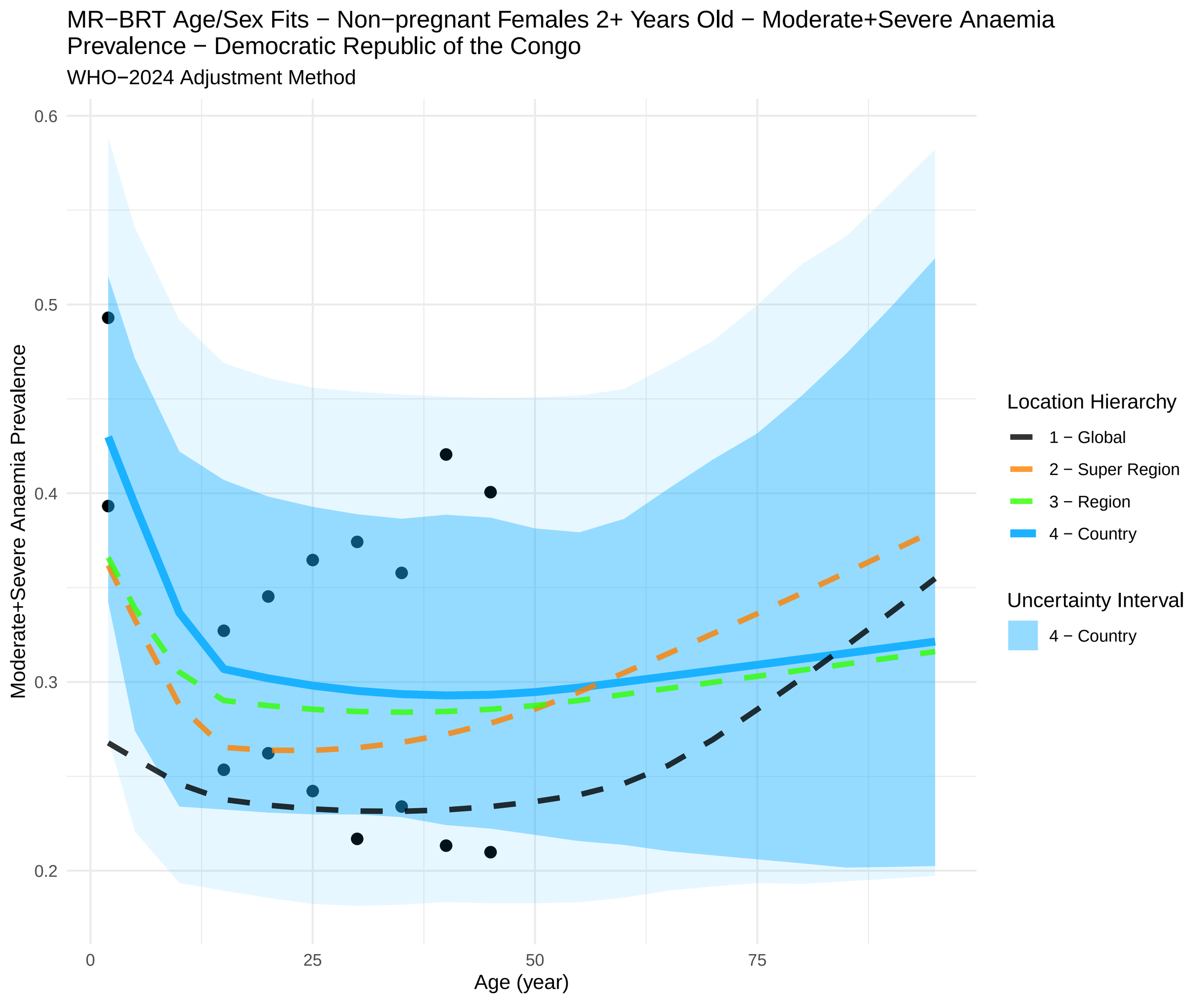
MR−BRT Age/Sex Fits − Non−pregnant Females 2+ Years Old − Moderate+Severe Anaemia Prevalence − Democratic Republic of the Congo
WHO−2024 Adjustment Method
Moderate+Severe Anaemia Prevalence
Age (year)
Location Hierarchy
1 − Global
2 − Super Region
3 − Region
4 − Country
Uncertainty Interval
4 − Country

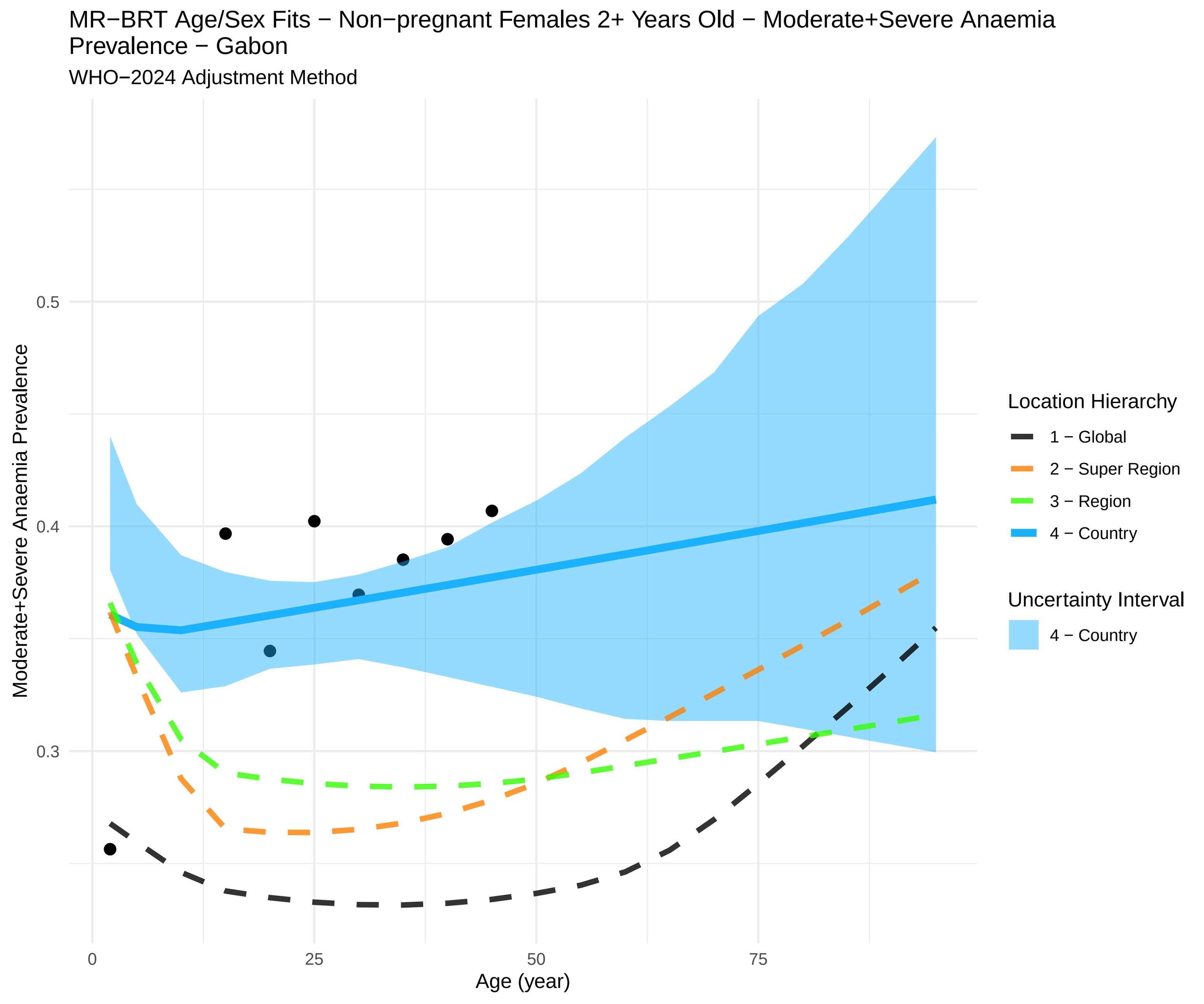
MR−BRT Age/Sex Fits − Non−pregnant Females 2+ Years Old − Moderate+Severe Anaemia Prevalence − Gabon
WHO−2024 Adjustment Method
Moderate+Severe Anaemia Prevalence
Age (year)
Location Hierarchy
1 − Global
2 − Super Region
3 − Region
4 − Country
Uncertainty Interval
4 − Country

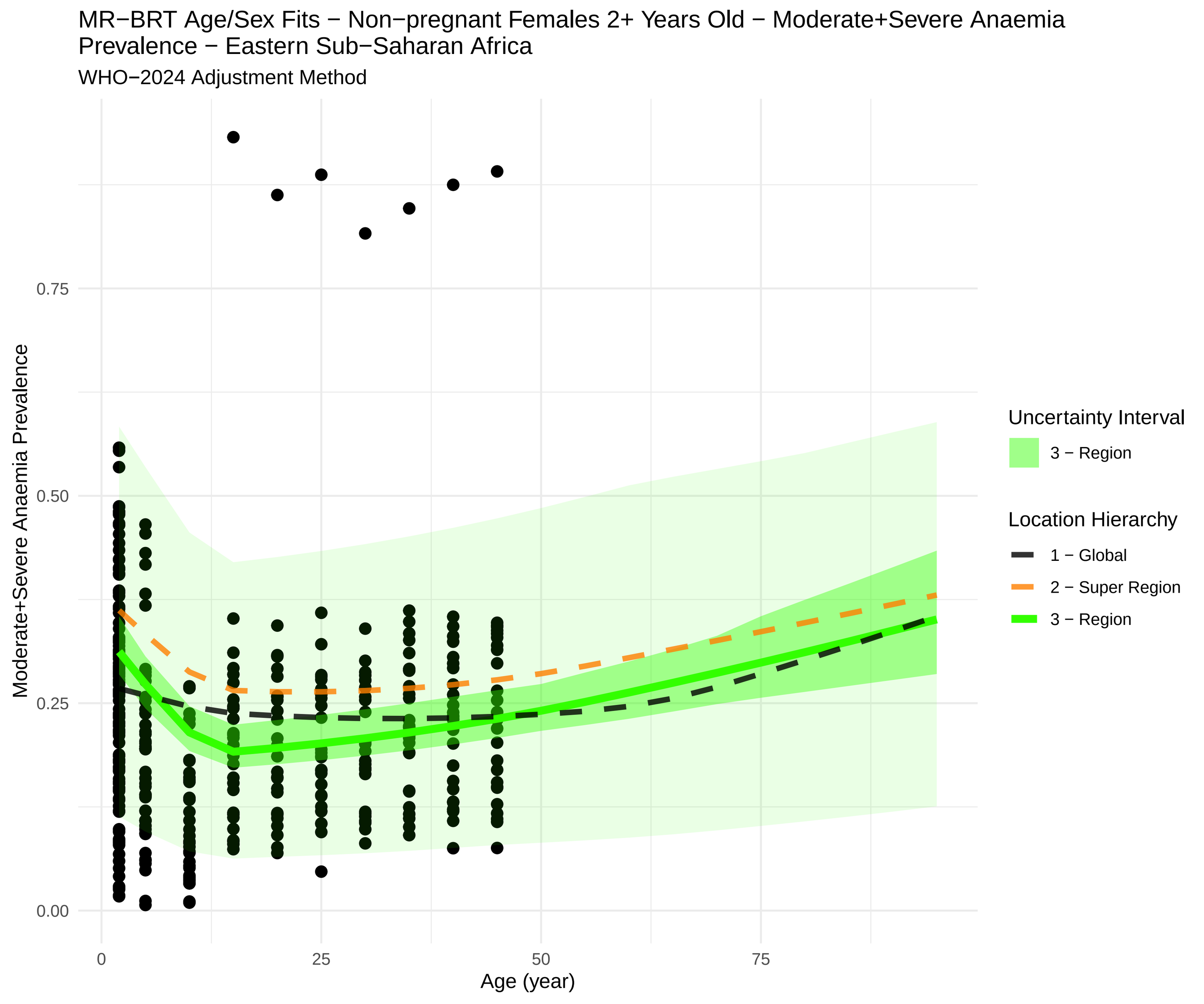
MR−BRT Age/Sex Fits − Non−pregnant Females 2+ Years Old − Moderate+Severe Anaemia Prevalence − Eastern Sub−Saharan Africa
WHO−2024 Adjustment Method
Moderate+Severe Anaemia Prevalence
Age (year)
Uncertainty Interval
3 − Region
Location Hierarchy
1 − Global
2 − Super Region
3 − Region

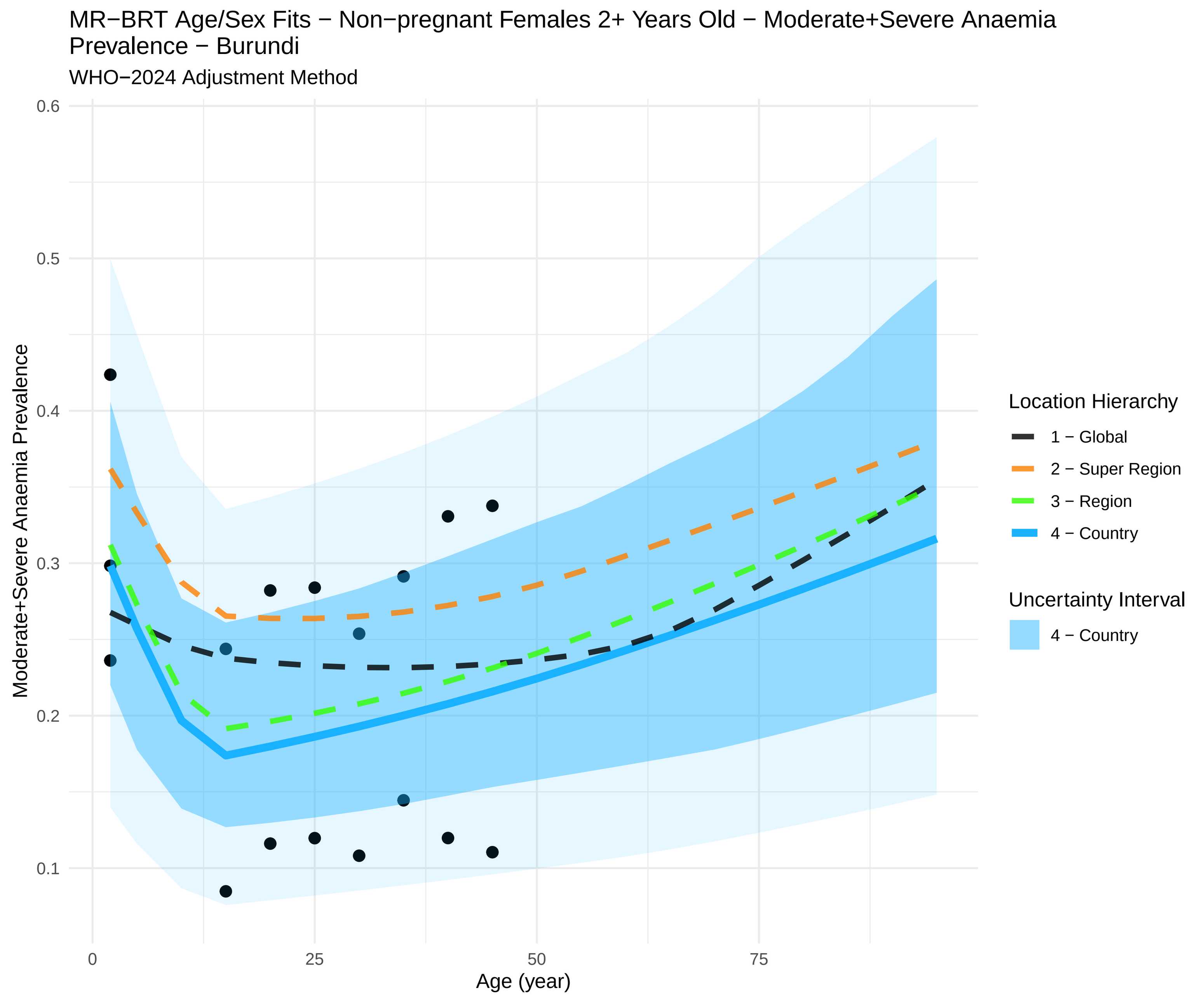
MR−BRT Age/Sex Fits − Non−pregnant Females 2+ Years Old − Moderate+Severe Anaemia Prevalence − Burundi
WHO−2024 Adjustment Method
Moderate+Severe Anaemia Prevalence
Age (year)
Location Hierarchy
1 − Global
2 − Super Region
3 − Region
4 − Country
Uncertainty Interval
4 − Country

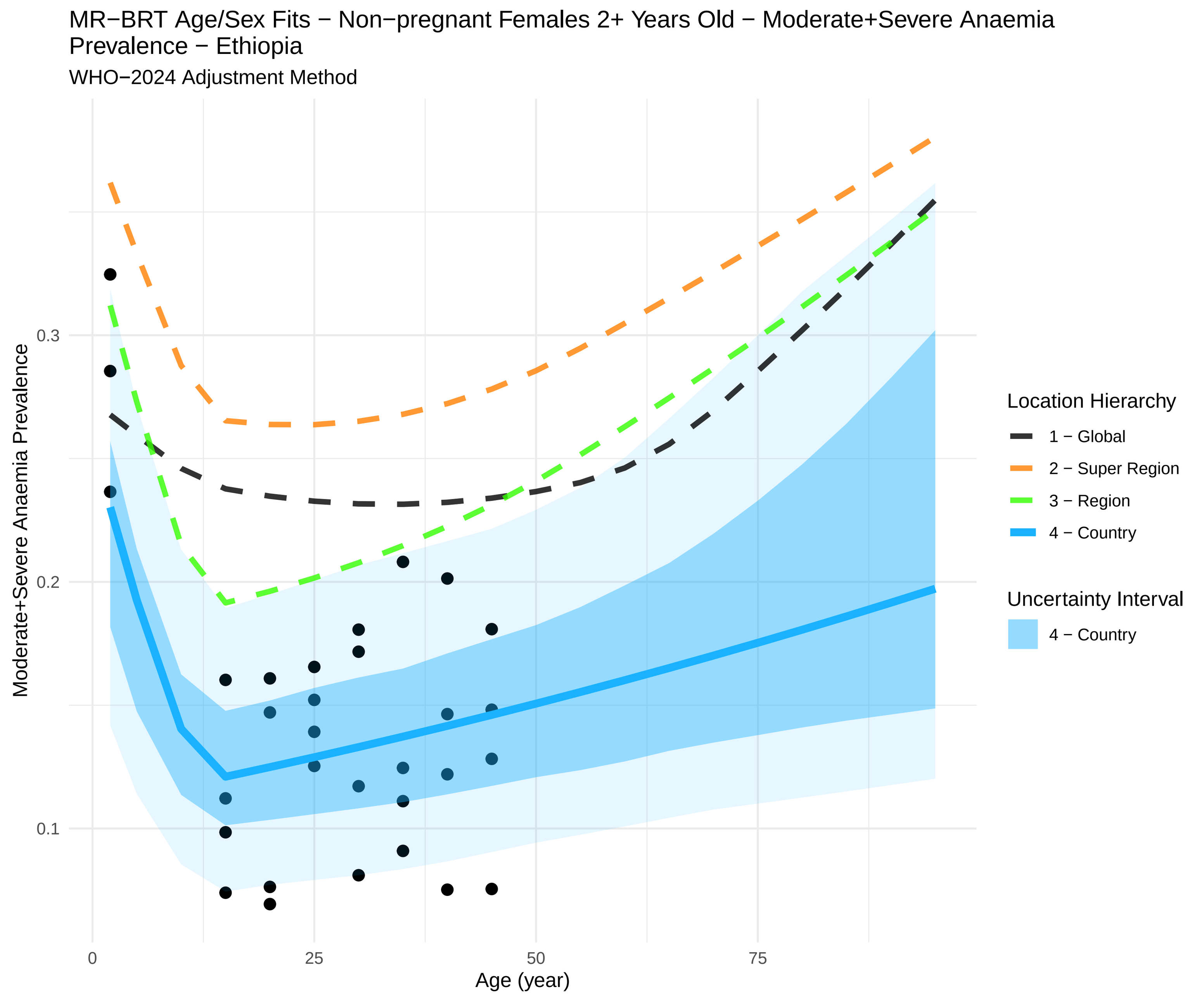
MR−BRT Age/Sex Fits − Non−pregnant Females 2+ Years Old − Moderate+Severe Anaemia Prevalence − Ethiopia
WHO−2024 Adjustment Method
Moderate+Severe Anaemia Prevalence
Age (year)
Location Hierarchy
1 − Global
2 − Super Region
3 − Region
4 − Country
Uncertainty Interval
4 − Country

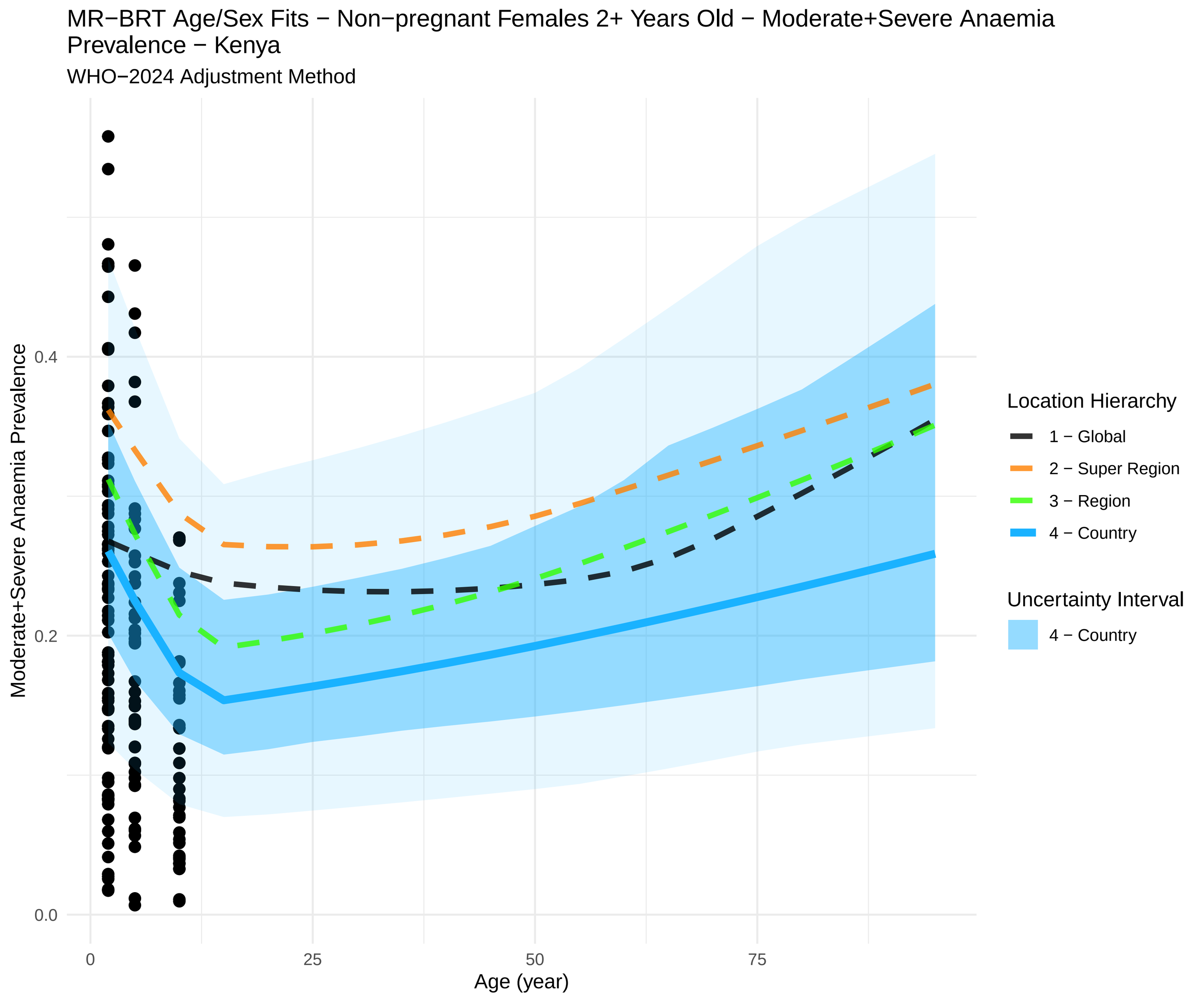
MR−BRT Age/Sex Fits − Non−pregnant Females 2+ Years Old − Moderate+Severe Anaemia Prevalence − Kenya
WHO−2024 Adjustment Method
Moderate+Severe Anaemia Prevalence
Age (year)
Location Hierarchy
1 − Global
2 − Super Region
3 − Region
4 − Country
Uncertainty Interval
4 − Country

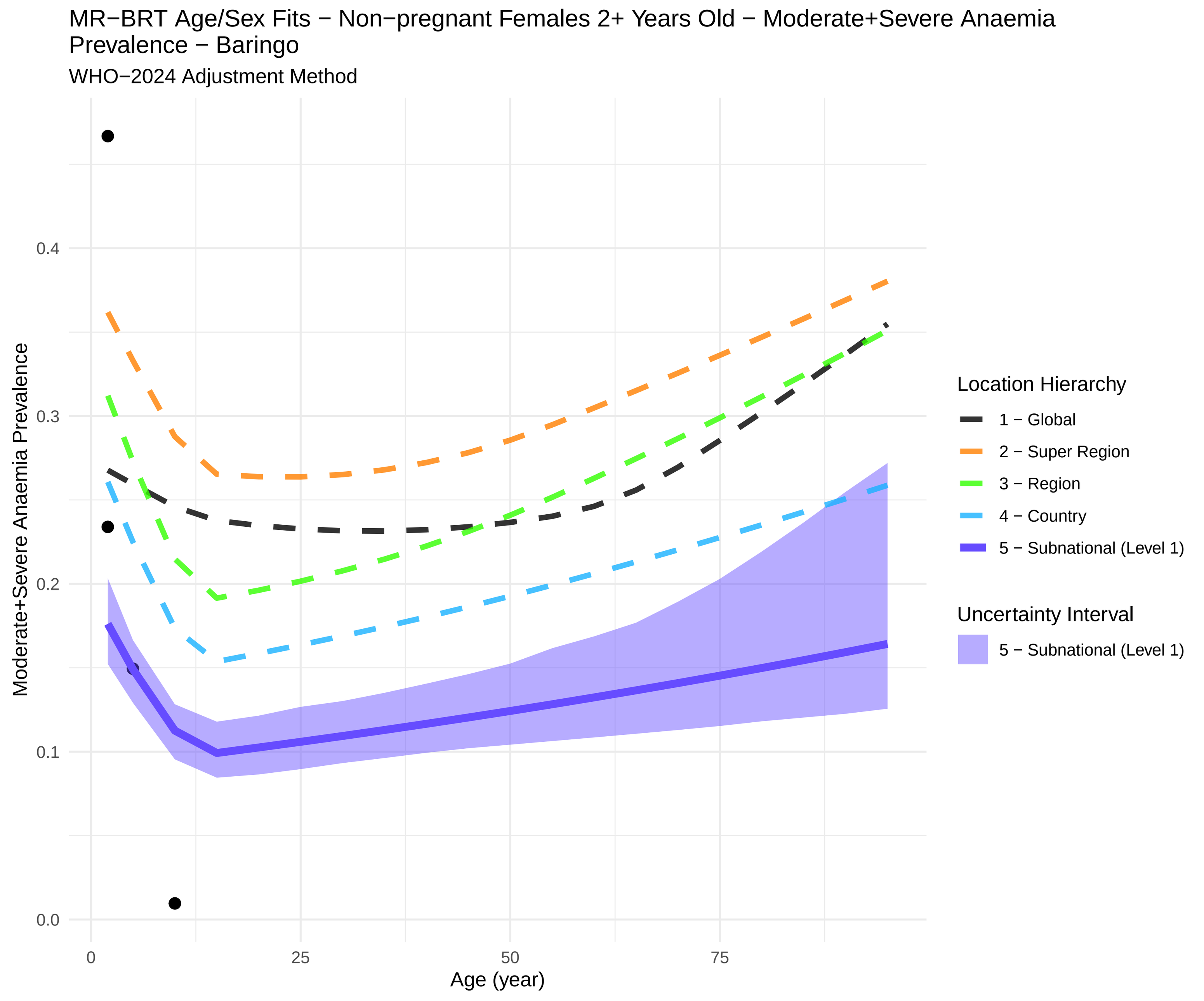
MR−BRT Age/Sex Fits − Non−pregnant Females 2+ Years Old − Moderate+Severe Anaemia Prevalence − Baringo
WHO−2024 Adjustment Method
Moderate+Severe Anaemia Prevalence
Age (year)
Location Hierarchy
1 − Global
2 − Super Region
3 − Region
4 − Country
5 − Subnational (Level 1)
Uncertainty Interval
5 − Subnational (Level 1)

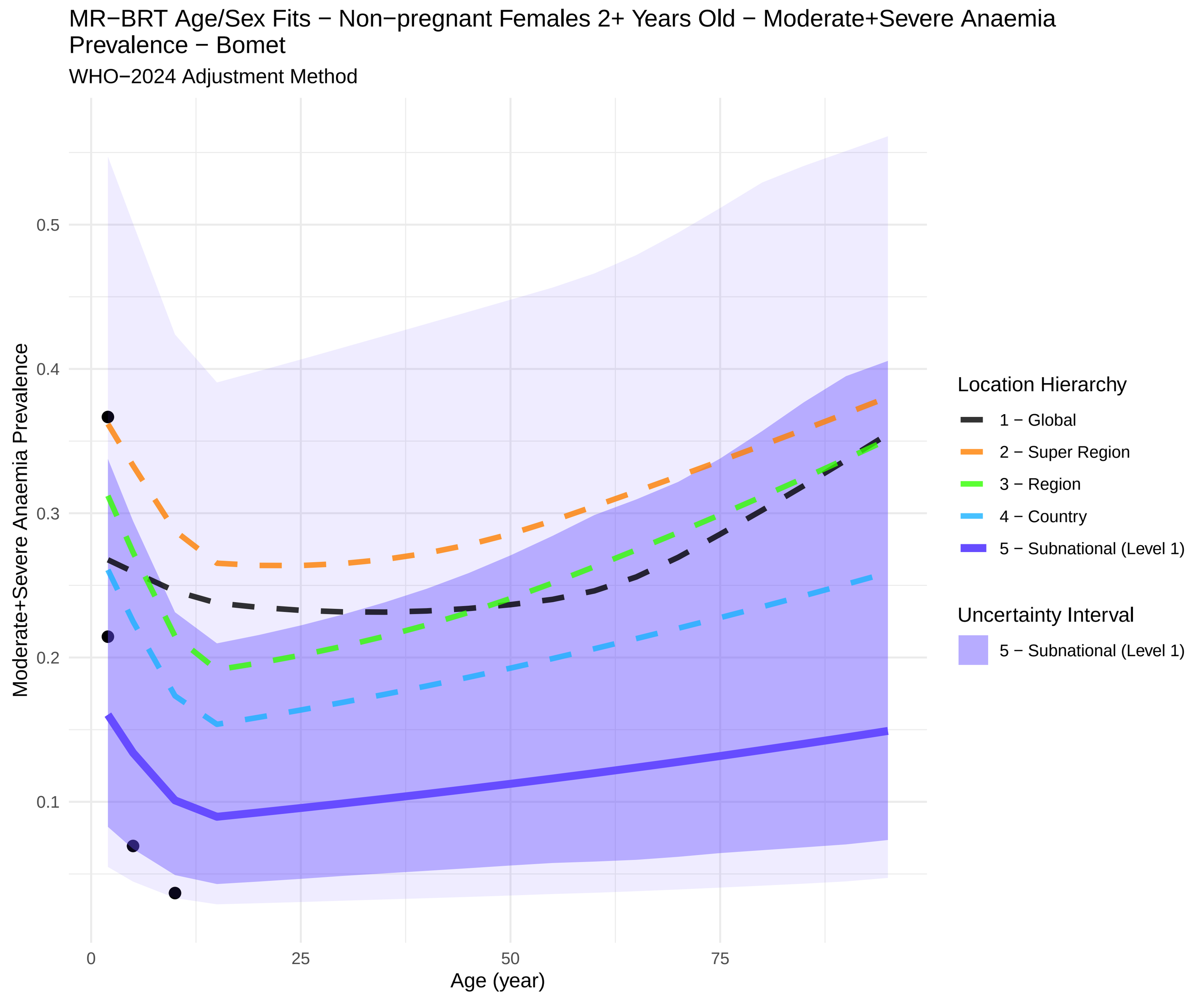
MR−BRT Age/Sex Fits − Non−pregnant Females 2+ Years Old − Moderate+Severe Anaemia Prevalence − Bomet
WHO−2024 Adjustment Method
Moderate+Severe Anaemia Prevalence
Age (year)
Location Hierarchy
1 − Global
2 − Super Region
3 − Region
4 − Country
5 − Subnational (Level 1)
Uncertainty Interval
5 − Subnational (Level 1)

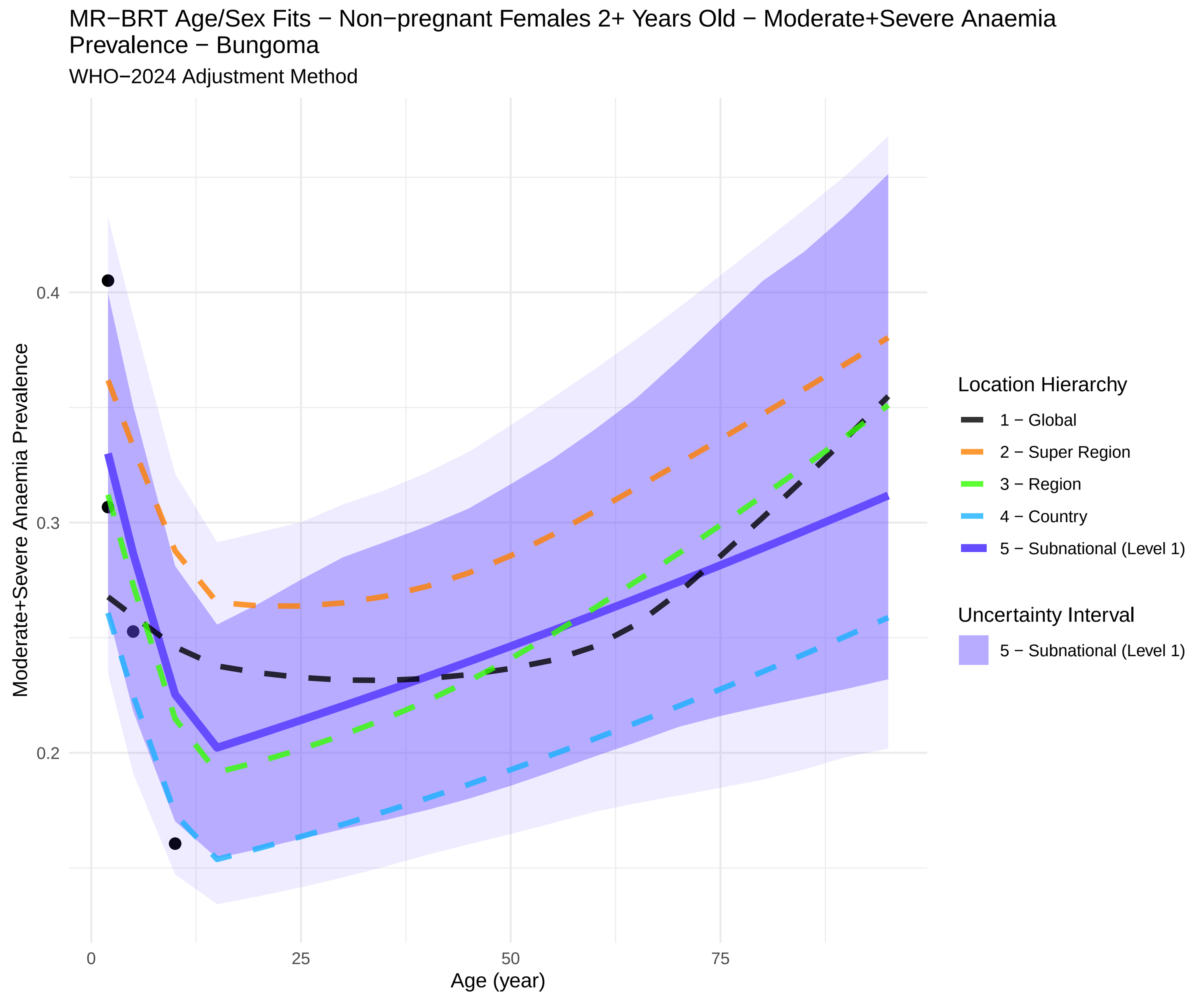
MR−BRT Age/Sex Fits − Non−pregnant Females 2+ Years Old − Moderate+Severe Anaemia Prevalence − Bungoma
WHO−2024 Adjustment Method
Moderate+Severe Anaemia Prevalence
Age (year)
Location Hierarchy
1 − Global
2 − Super Region
3 − Region
4 − Country
5 − Subnational (Level 1)
Uncertainty Interval
5 − Subnational (Level 1)

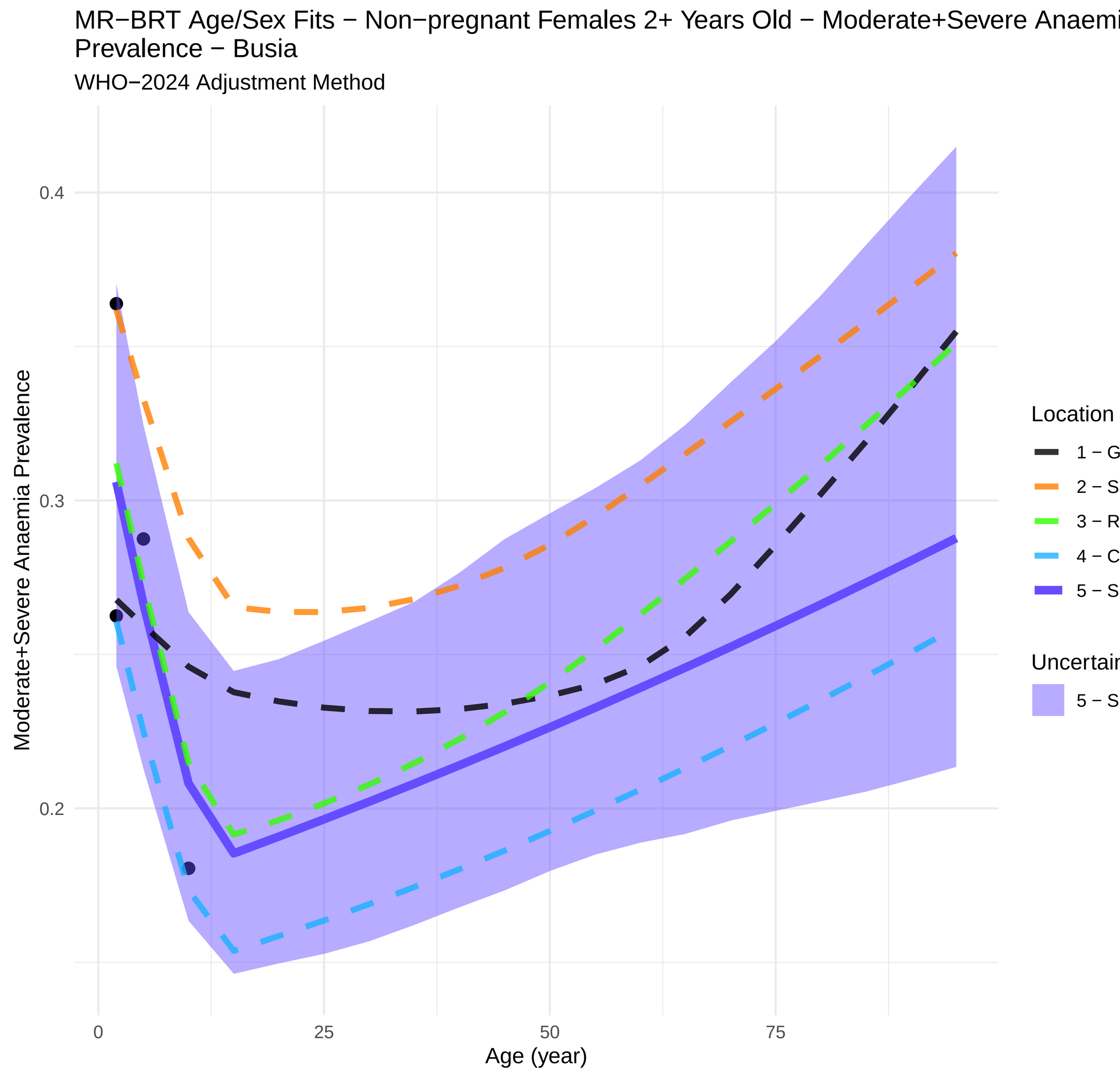
MR−BRT Age/Sex Fits − Non−pregnant Females 2+ Years Old − Moderate+Severe Anaemia Prevalence − Busia
WHO−2024 Adjustment Method
Moderate+Severe Anaemia Prevalence
Age (year)
Location Hierarchy
1 − Global
2 − Super Region
3 − Region
4 − Country
5 − Subnational (Level 1)
Uncertainty Interval
5 − Subnational (Level 1)

MR−BRT Age/Sex Fits − Non−pregnant Females 2+ Years Old − Moderate+Severe Anaemia Prevalence − Elgeyo−Marakwet
WHO−2024 Adjustment Method

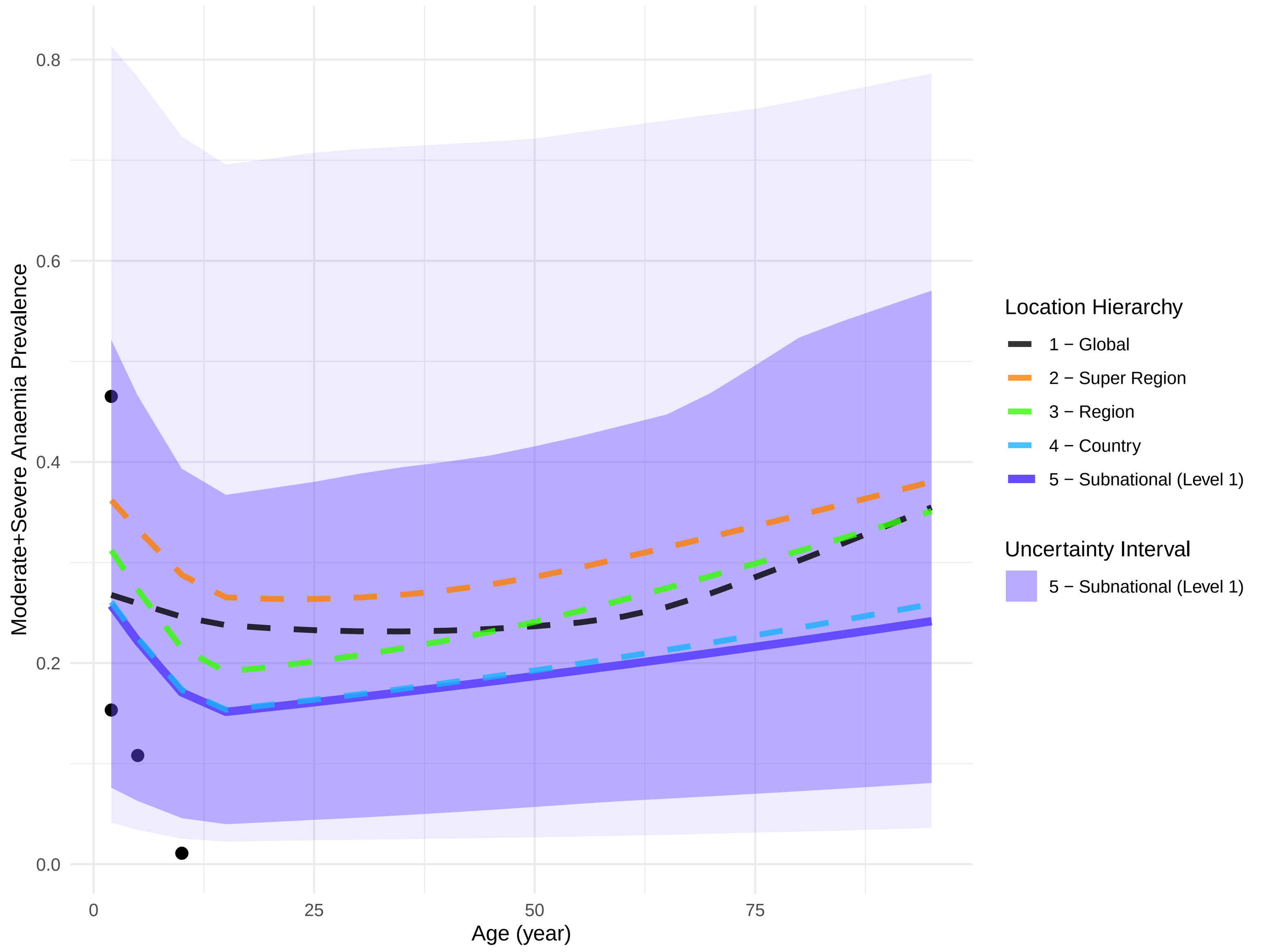

MR−BRT Age/Sex Fits − Non−pregnant Females 2+ Years Old − Moderate+Severe Anaemia Prevalence − Embu
WHO−2024 Adjustment Method

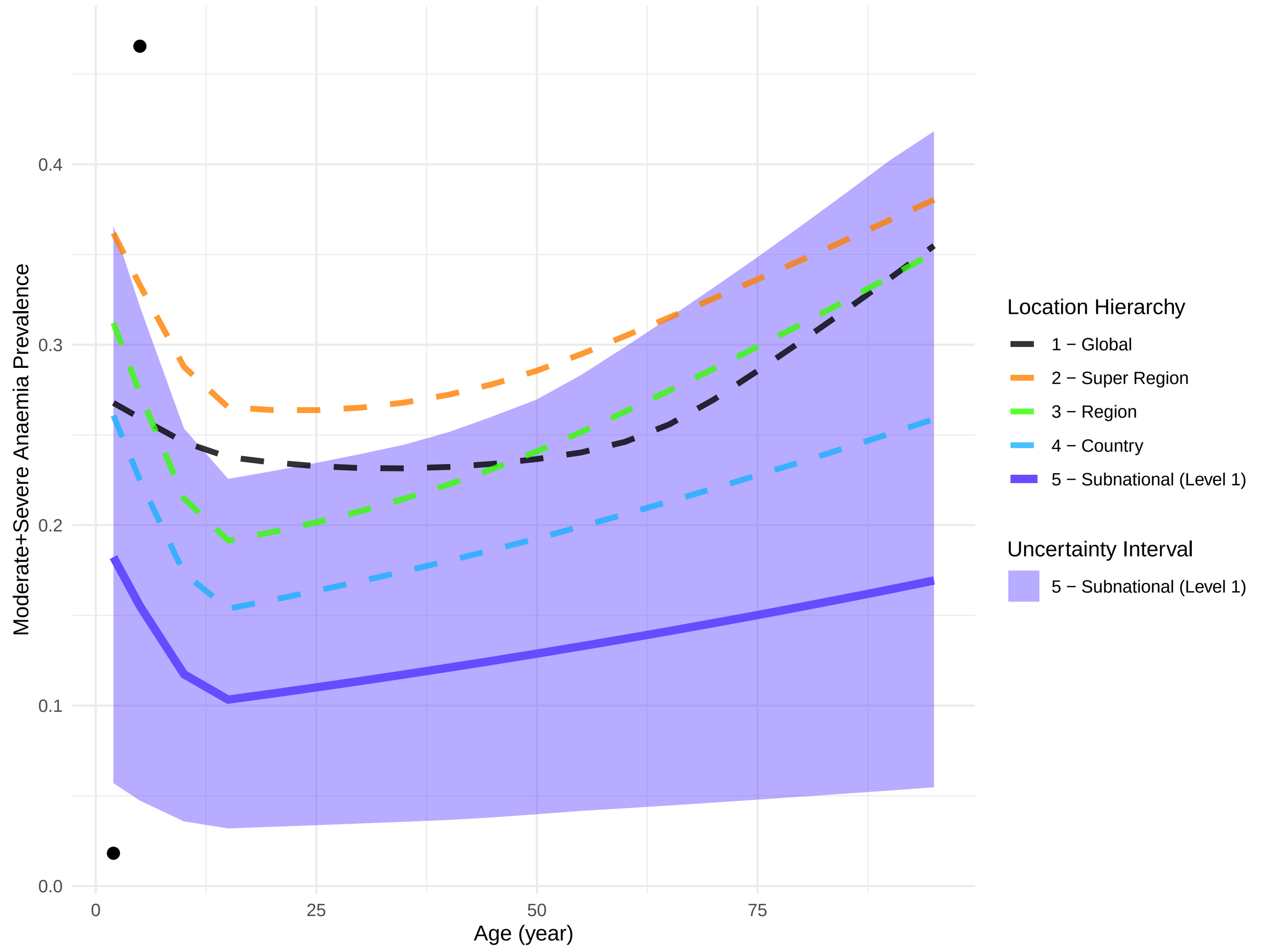

MR−BRT Age/Sex Fits − Non−pregnant Females 2+ Years Old − Moderate+Severe Anaemia Prevalence − Garissa
WHO−2024 Adjustment Method

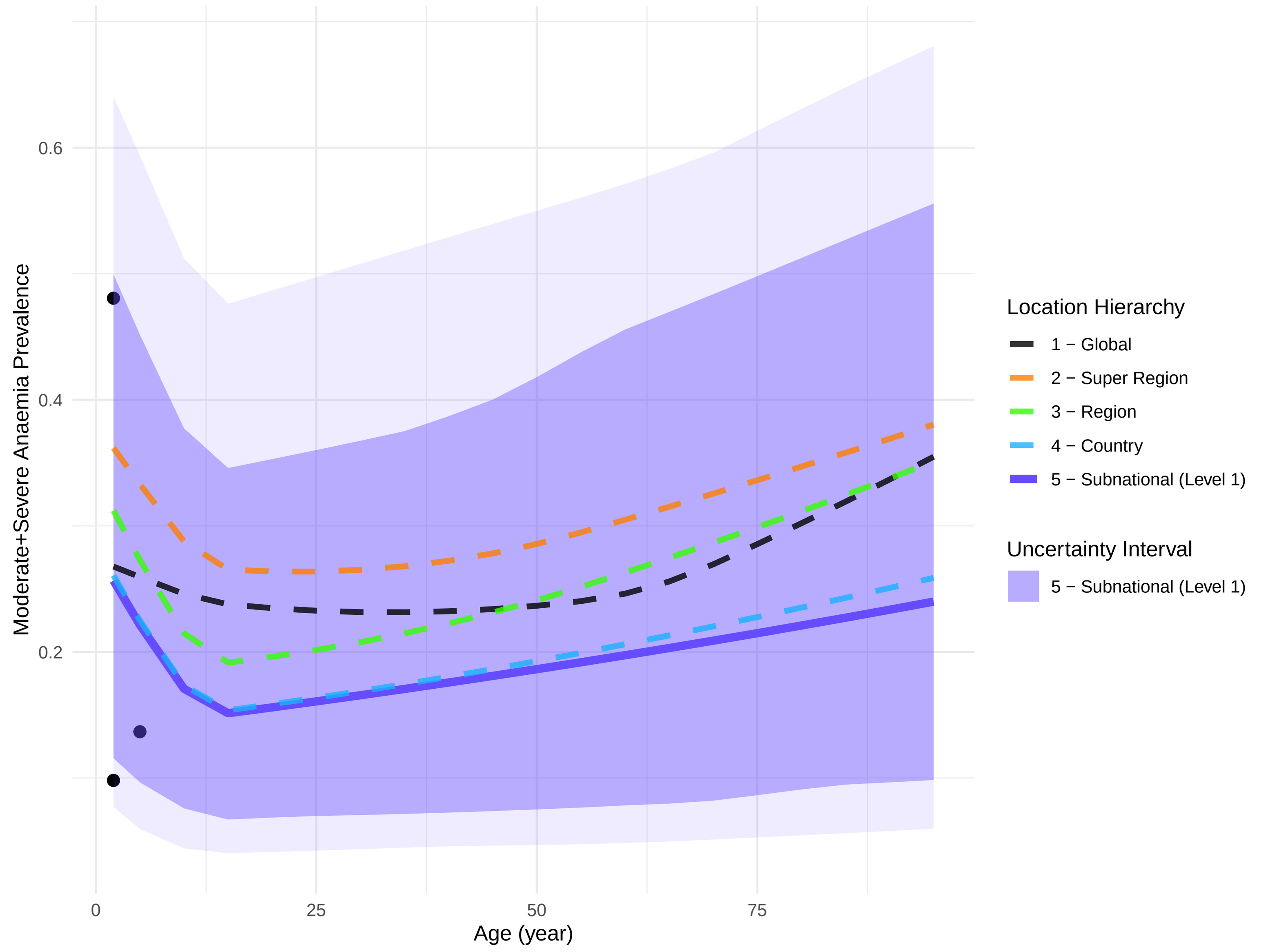

MR−BRT Age/Sex Fits − Non−pregnant Females 2+ Years Old − Moderate+Severe Anaemia Prevalence − Homa Bay
WHO−2024 Adjustment Method

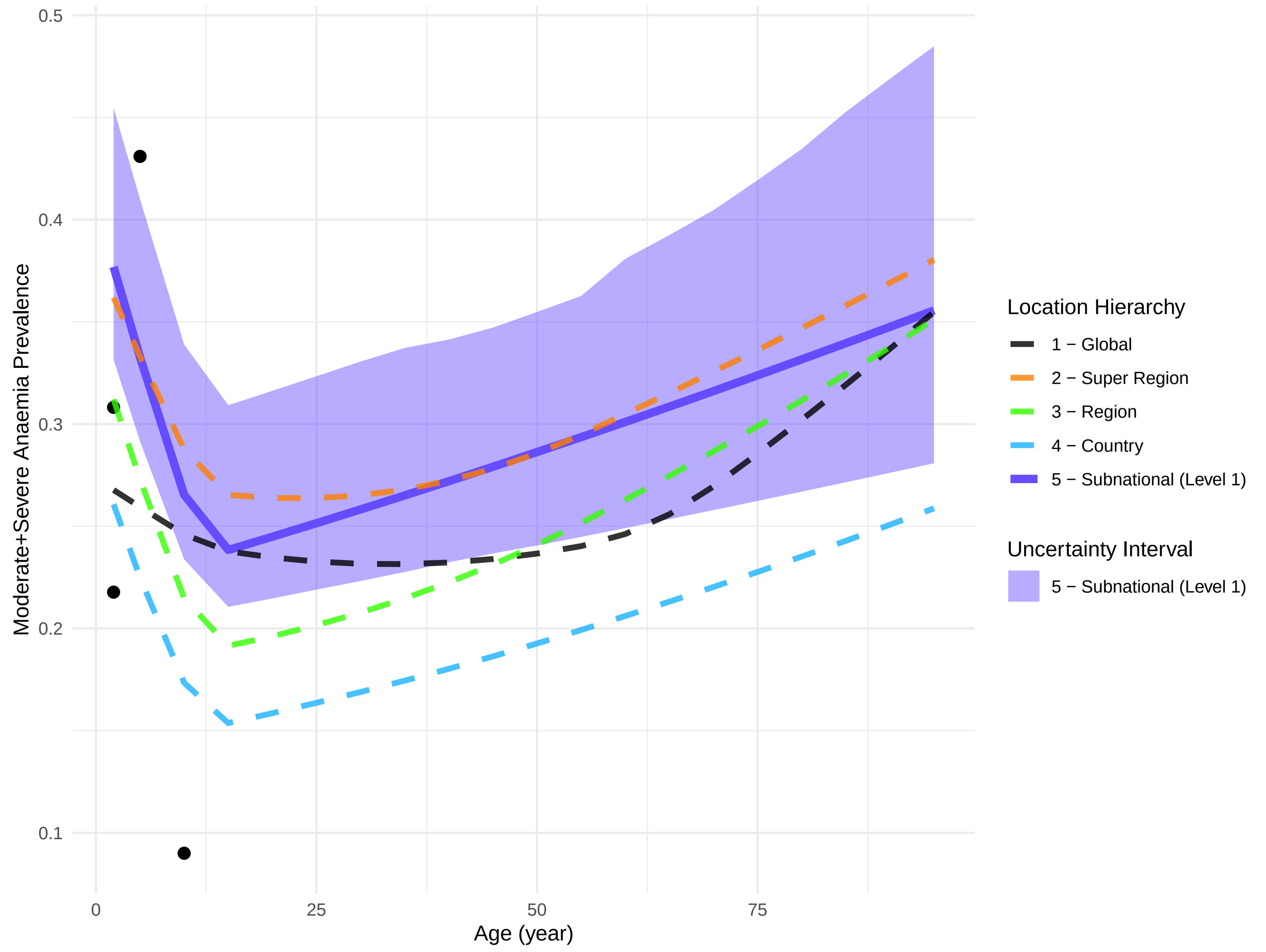

MR−BRT Age/Sex Fits − Non−pregnant Females 2+ Years Old − Moderate+Severe Anaemia Prevalence − Isiolo
WHO−2024 Adjustment Method

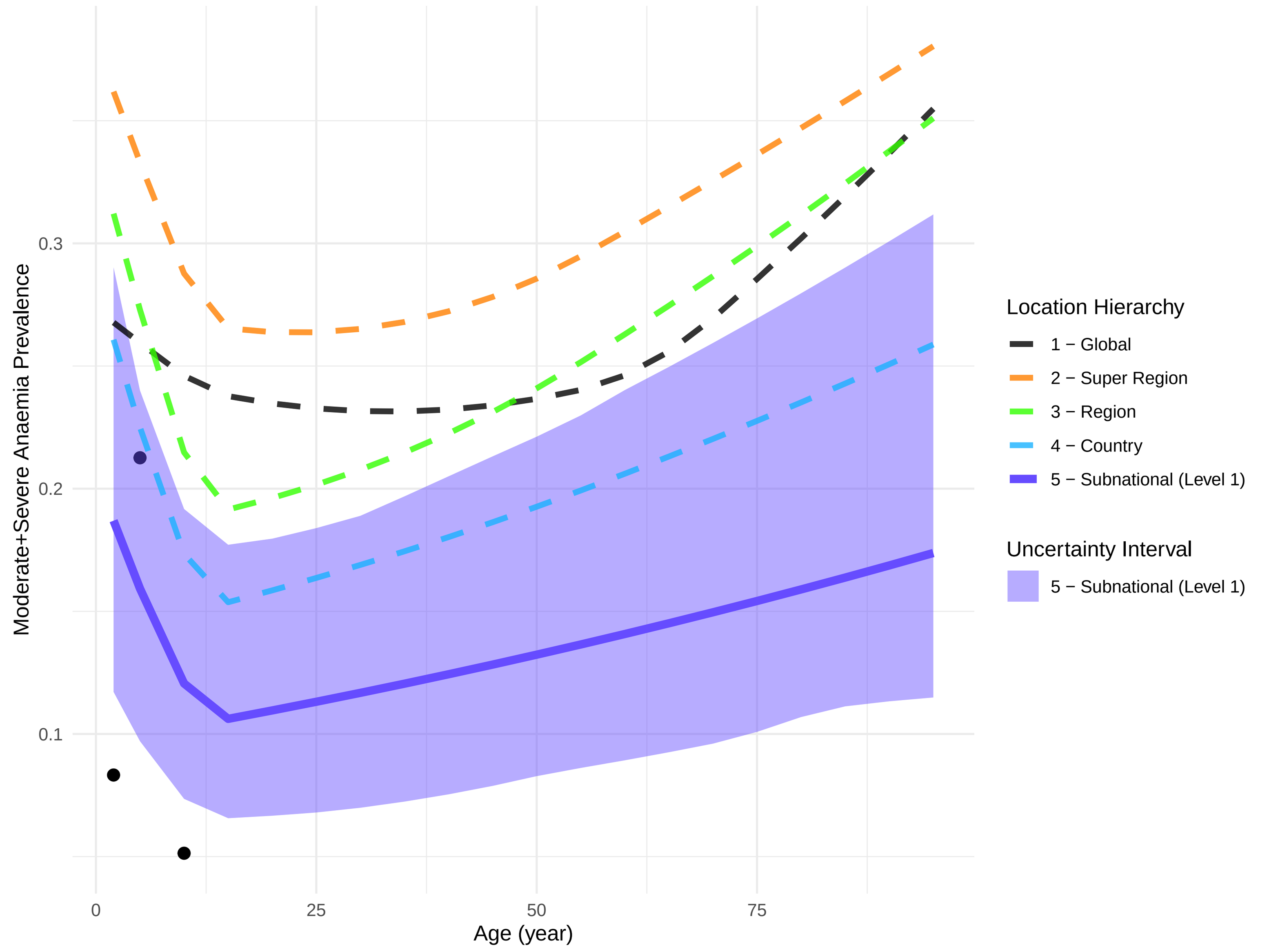

MR−BRT Age/Sex Fits − Non−pregnant Females 2+ Years Old − Moderate+Severe Anaemia Prevalence − Kajiado
WHO−2024 Adjustment Method

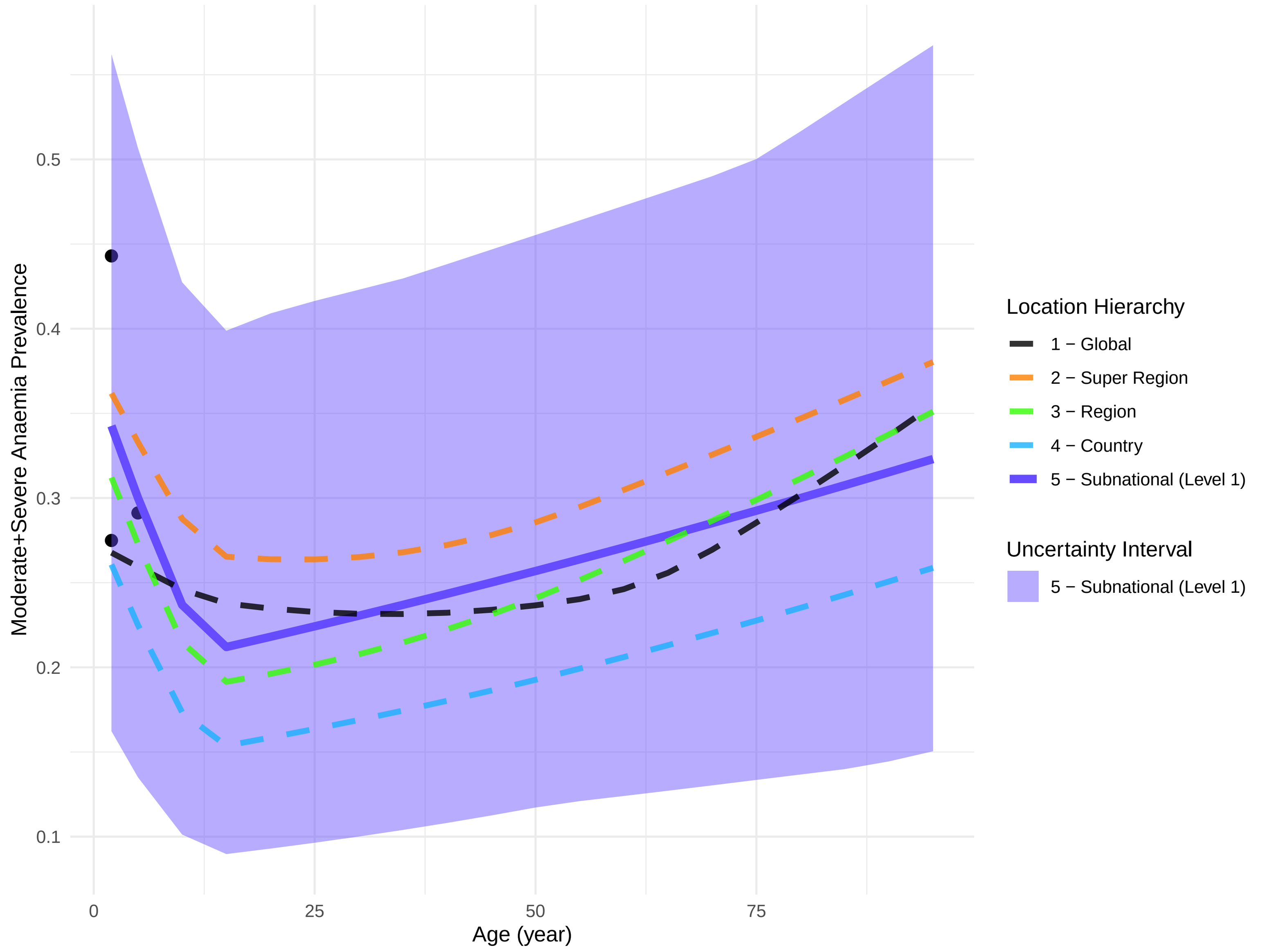

MR−BRT Age/Sex Fits − Non−pregnant Females 2+ Years Old − Moderate+Severe Anaemia Prevalence − Kakamega
WHO−2024 Adjustment Method

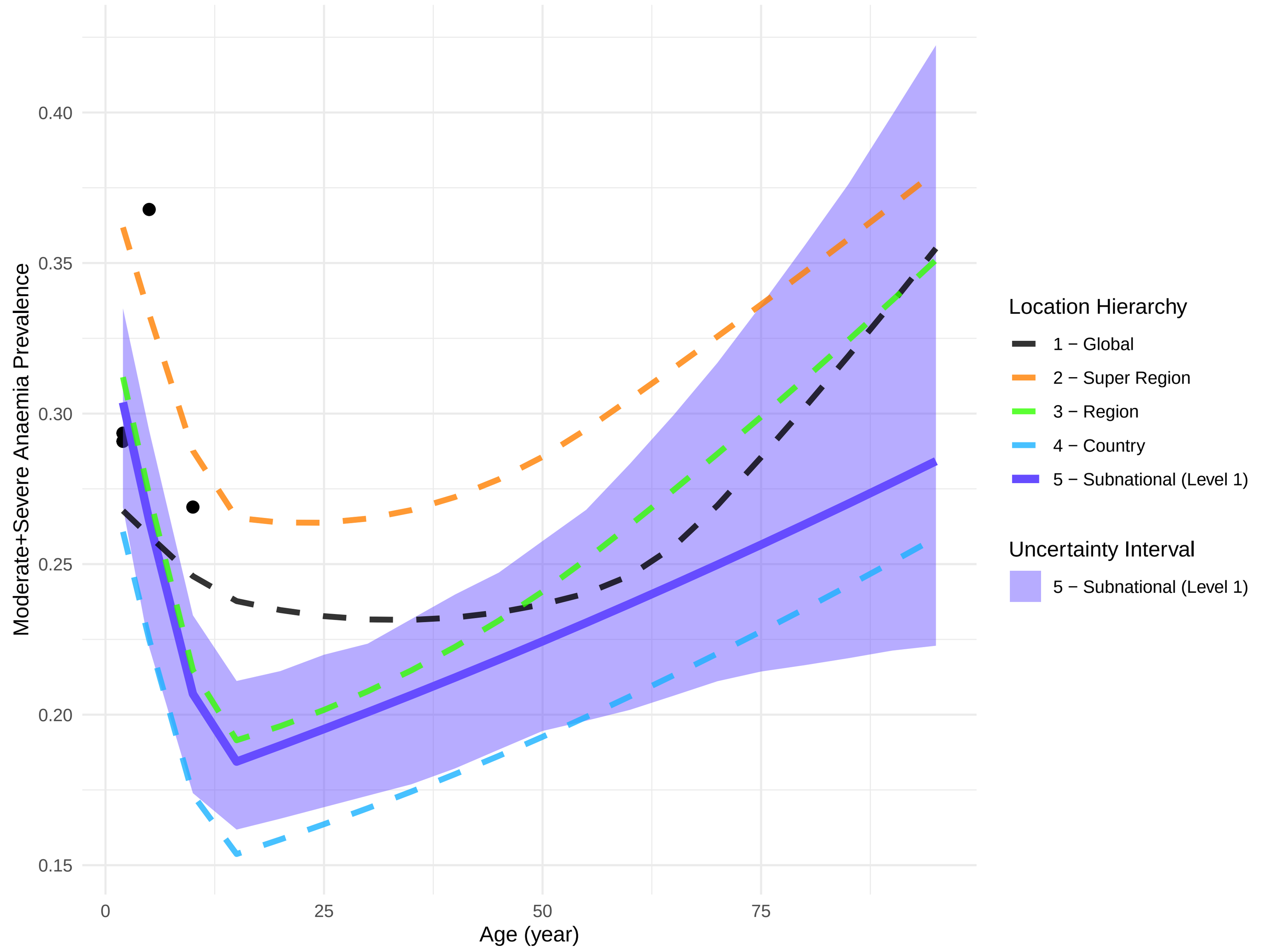

MR−BRT Age/Sex Fits − Non−pregnant Females 2+ Years Old − Moderate+Severe Anaemia Prevalence − Kericho
WHO−2024 Adjustment Method

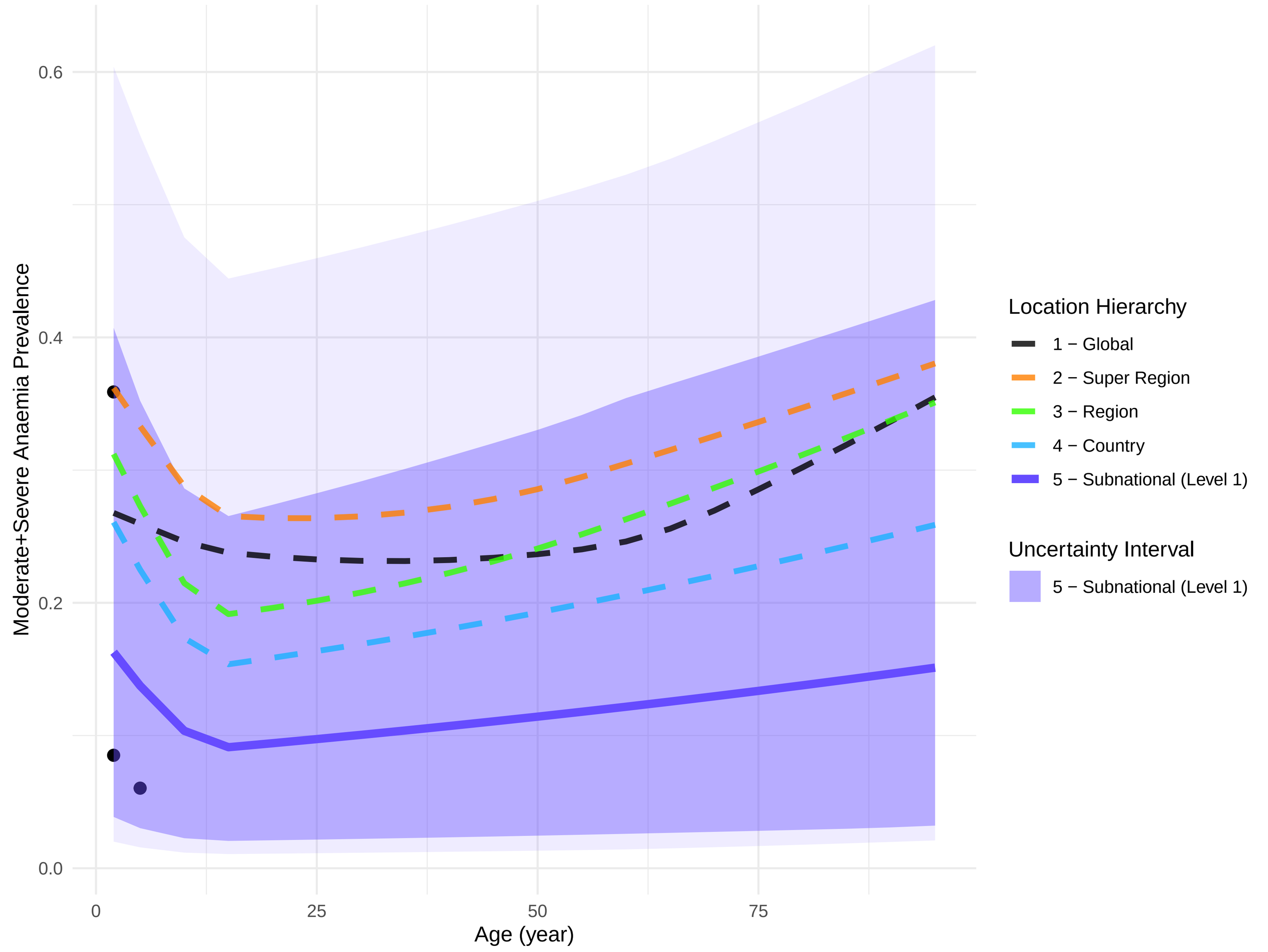

MR−BRT Age/Sex Fits − Non−pregnant Females 2+ Years Old − Moderate+Severe Anaemia Prevalence − Kiambu
WHO−2024 Adjustment Method

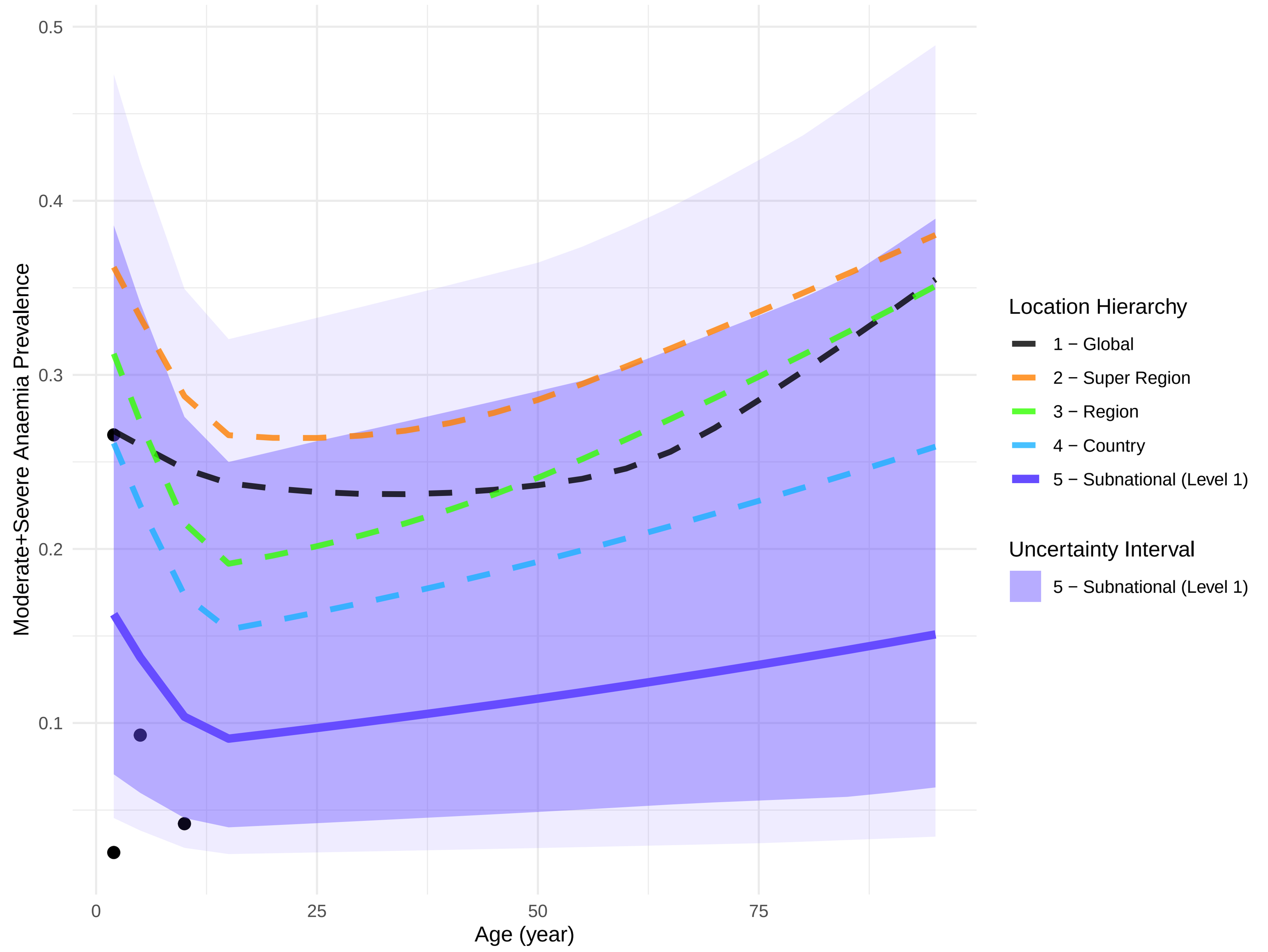

MR−BRT Age/Sex Fits − Non−pregnant Females 2+ Years Old − Moderate+Severe Anaemia Prevalence − Kilifi
WHO−2024 Adjustment Method

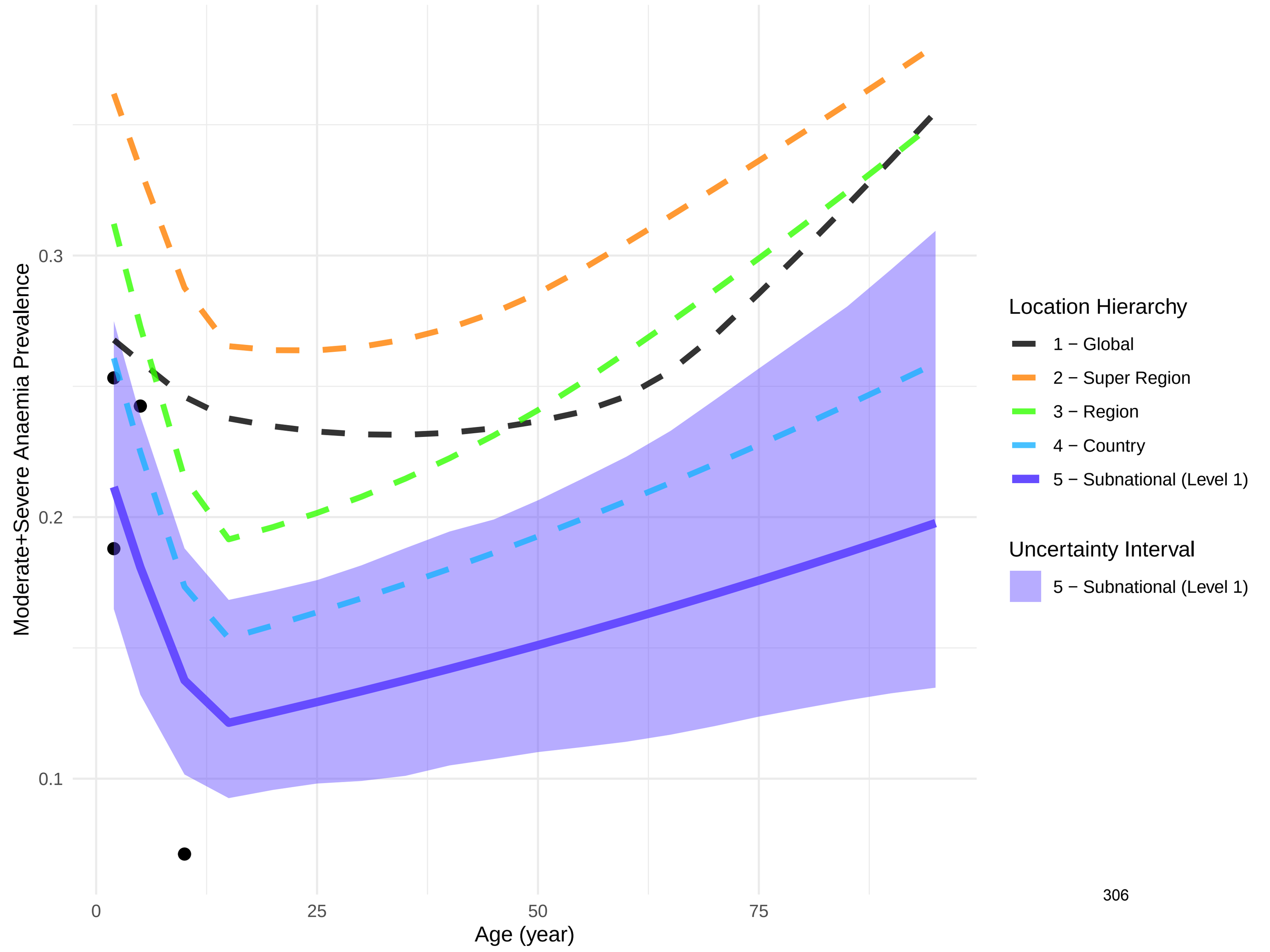

MR−BRT Age/Sex Fits − Non−pregnant Females 2+ Years Old − Moderate+Severe Anaemia Prevalence − Kirinyaga
WHO−2024 Adjustment Method

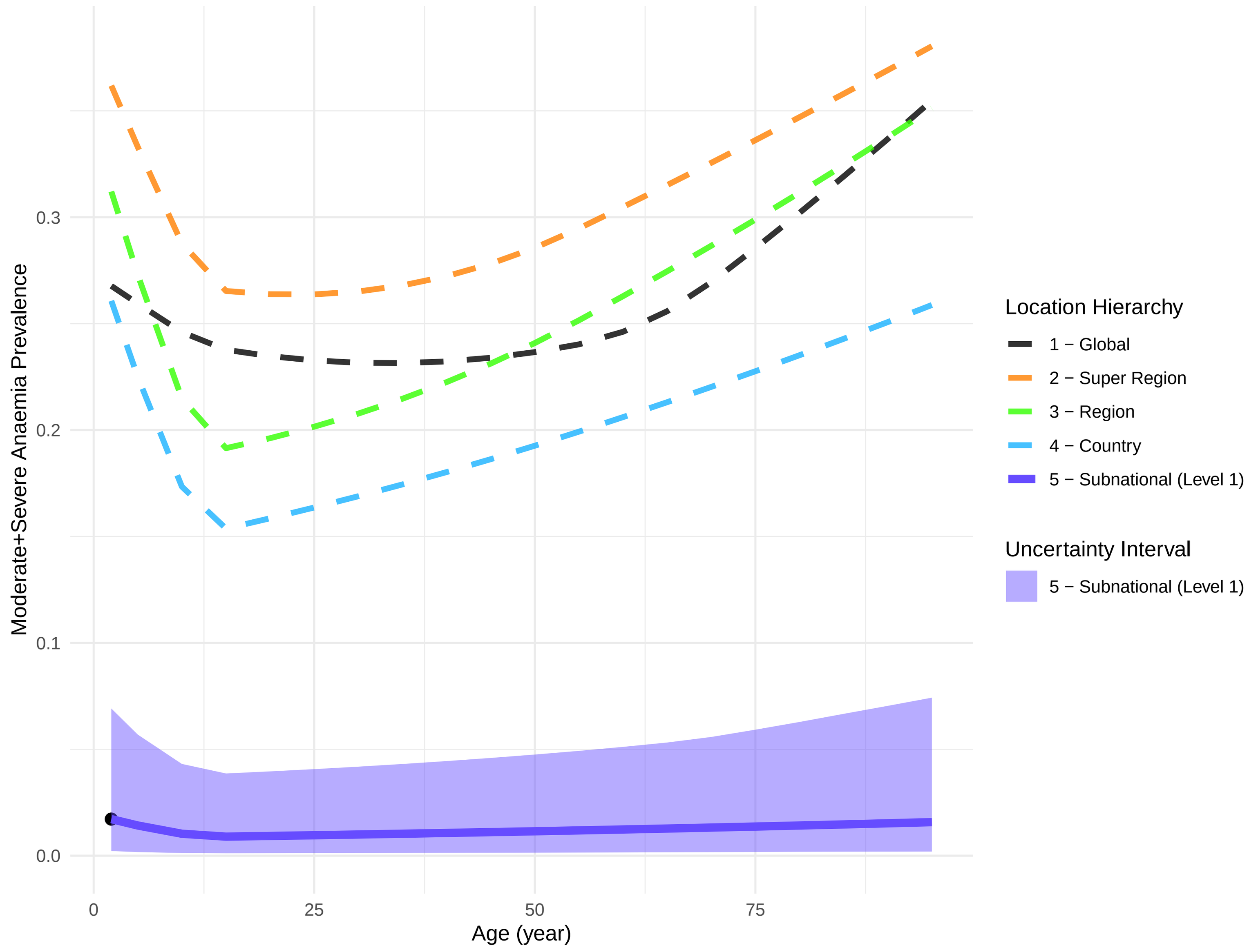

MR−BRT Age/Sex Fits − Non−pregnant Females 2+ Years Old − Moderate+Severe Anaemia Prevalence − Kisii
WHO−2024 Adjustment Method

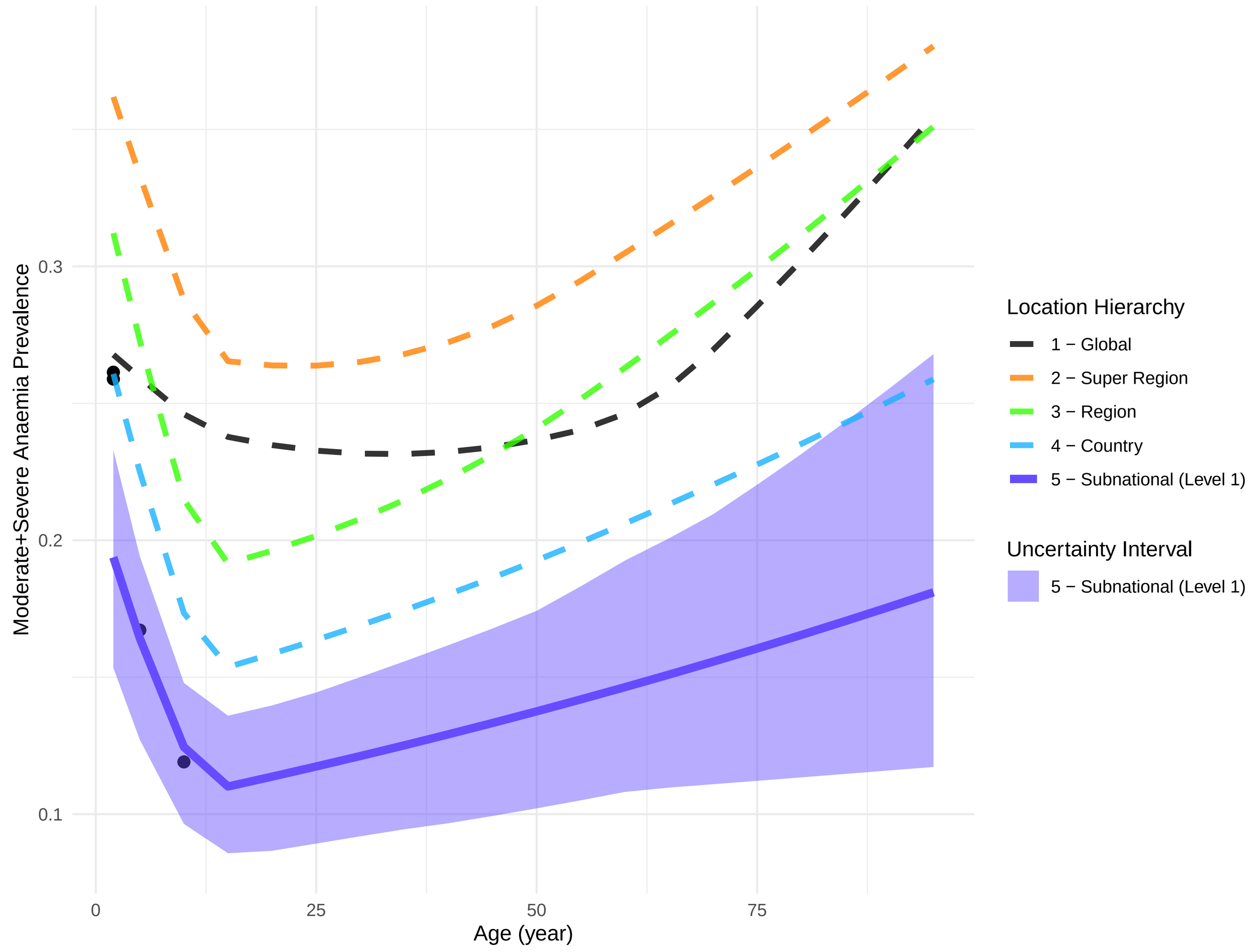

MR−BRT Age/Sex Fits − Non−pregnant Females 2+ Years Old − Moderate+Severe Anaemia Prevalence − Kisumu
WHO−2024 Adjustment Method

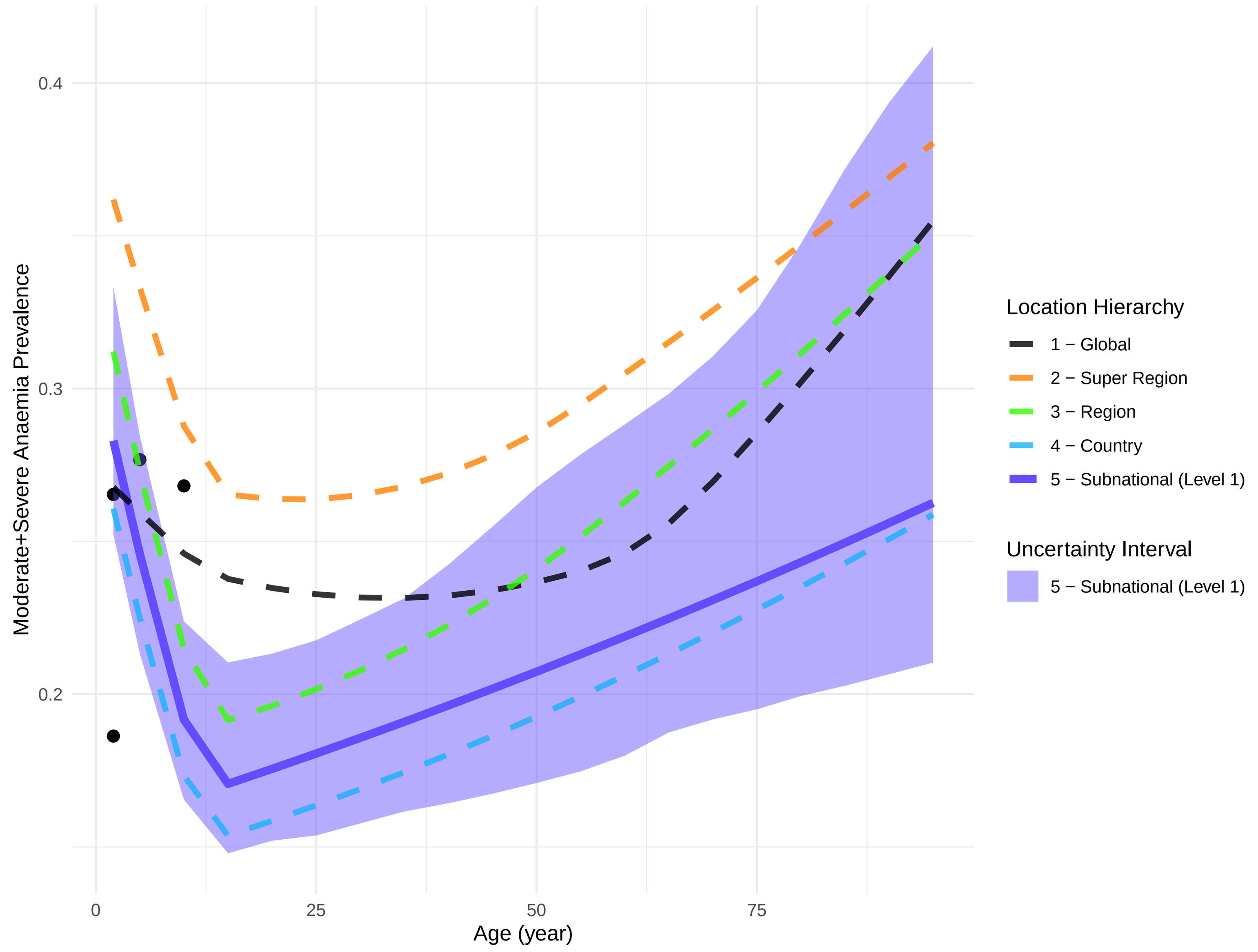

MR−BRT Age/Sex Fits − Non−pregnant Females 2+ Years Old − Moderate+Severe Anaemia Prevalence − Kitui
WHO−2024 Adjustment Method

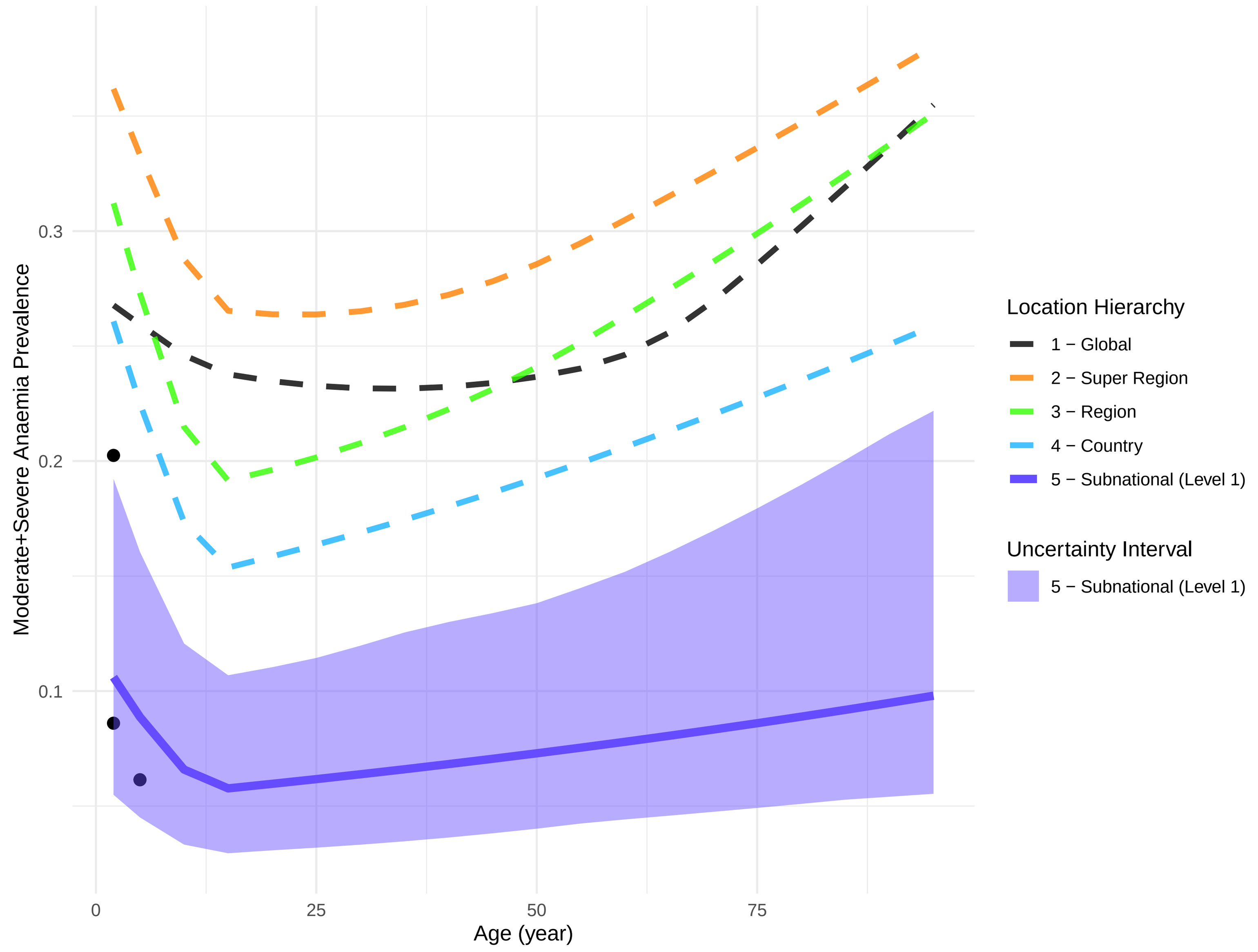

MR−BRT Age/Sex Fits − Non−pregnant Females 2+ Years Old − Moderate+Severe Anaemia Prevalence − Kwale
WHO−2024 Adjustment Method

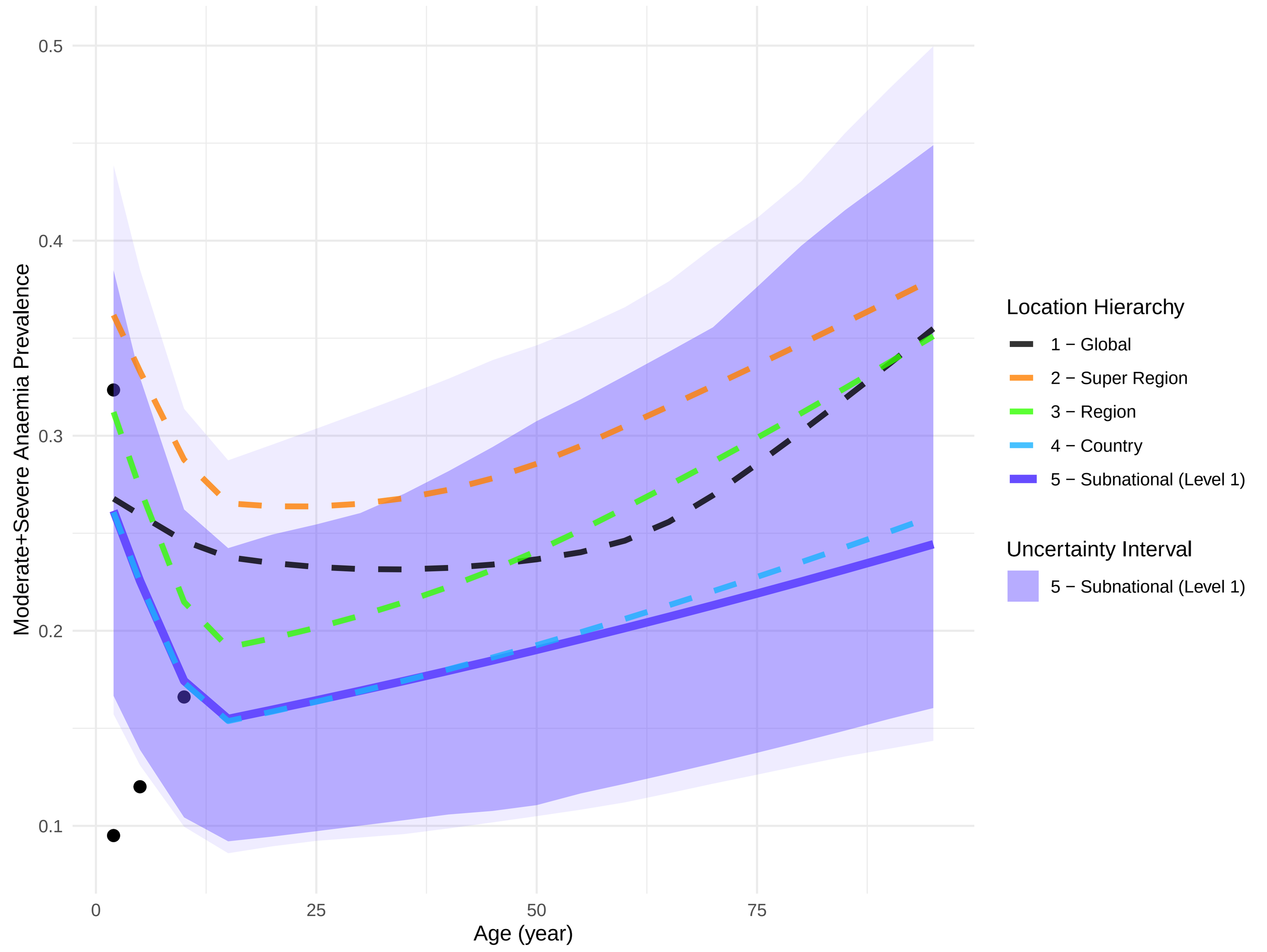

MR−BRT Age/Sex Fits − Non−pregnant Females 2+ Years Old − Moderate+Severe Anaemia Prevalence − Laikipia
WHO−2024 Adjustment Method

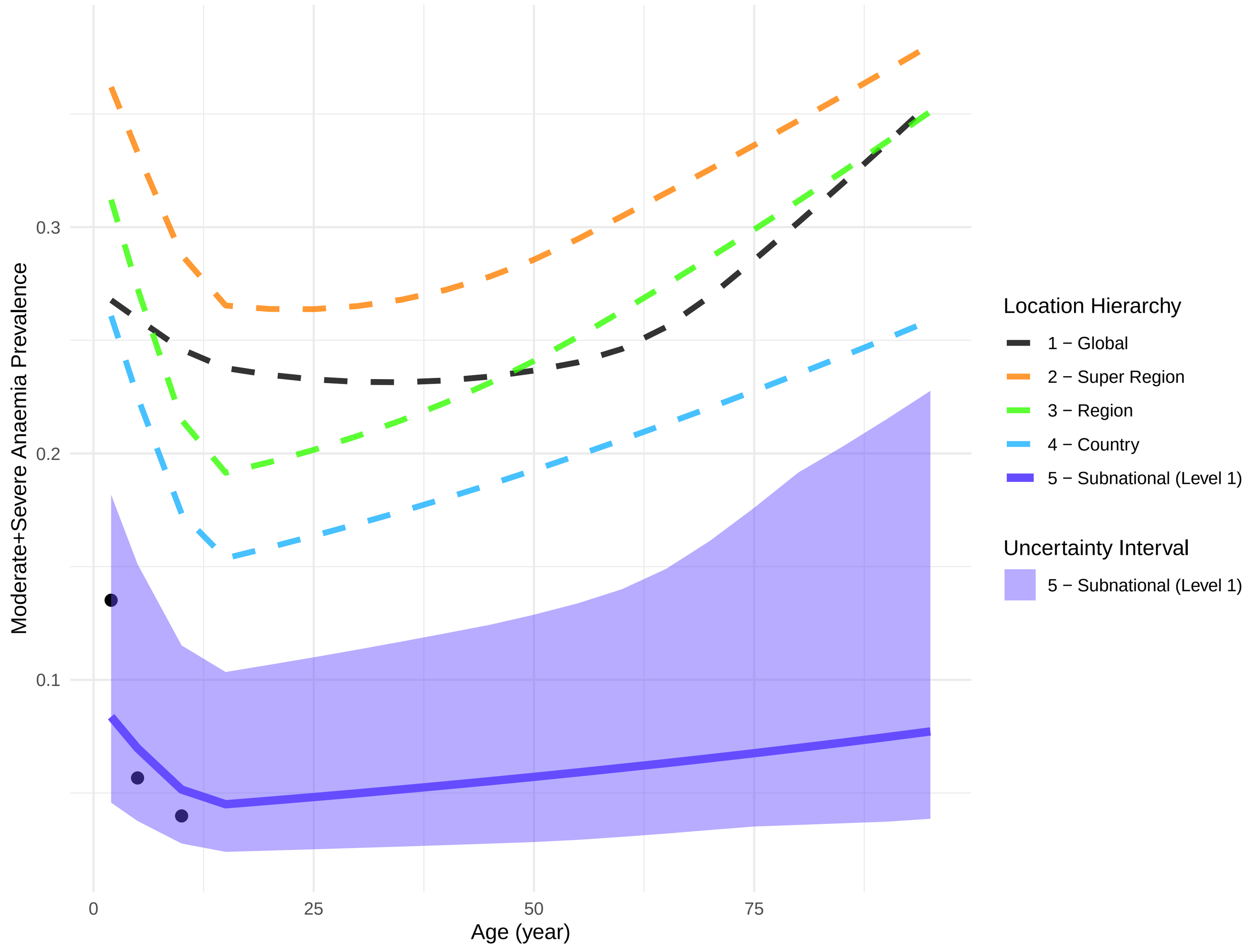

MR−BRT Age/Sex Fits − Non−pregnant Females 2+ Years Old − Moderate+Severe Anaemia Prevalence − Lamu
WHO−2024 Adjustment Method

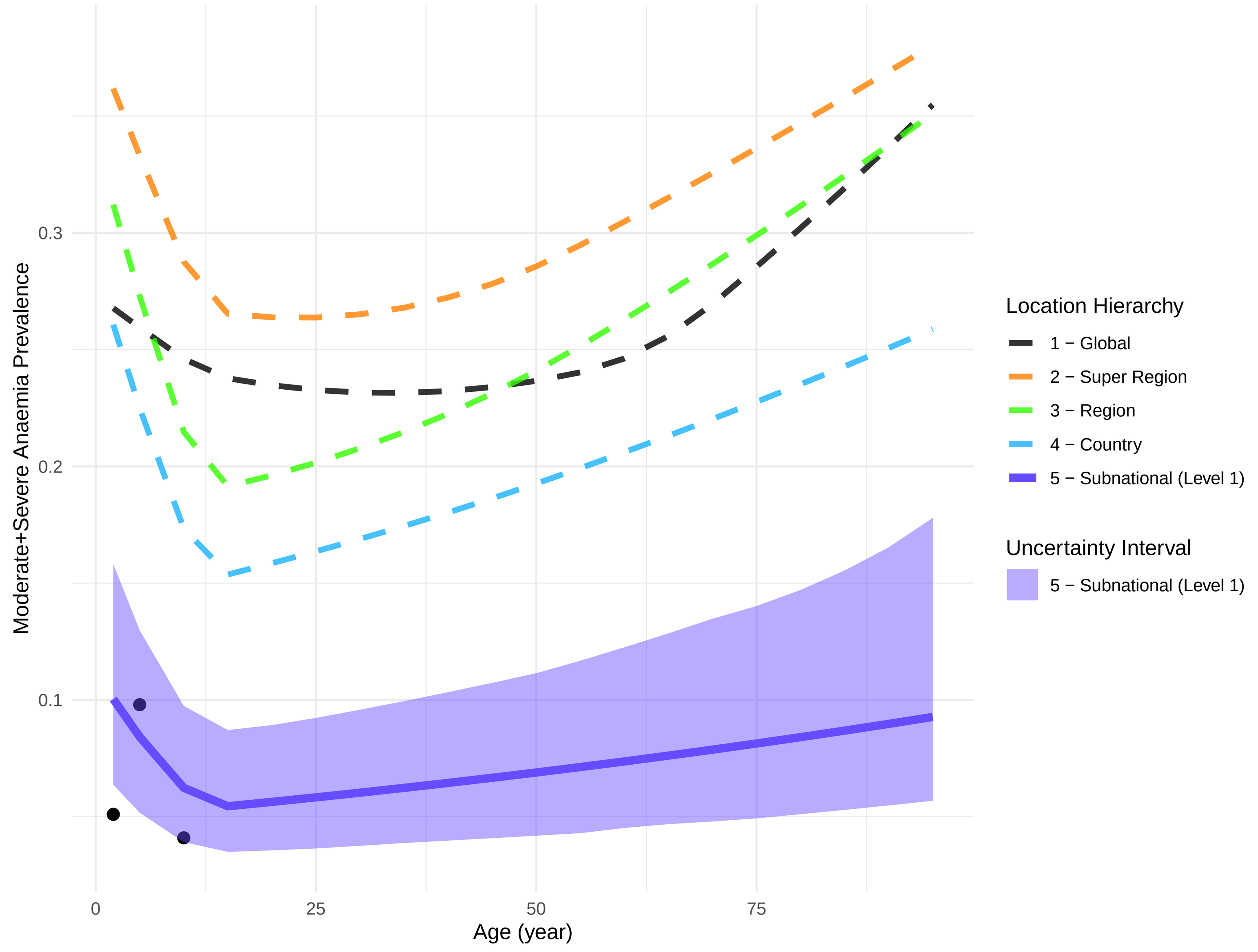

MR−BRT Age/Sex Fits − Non−pregnant Females 2+ Years Old − Moderate+Severe Anaemia Prevalence − Machakos
WHO−2024 Adjustment Method

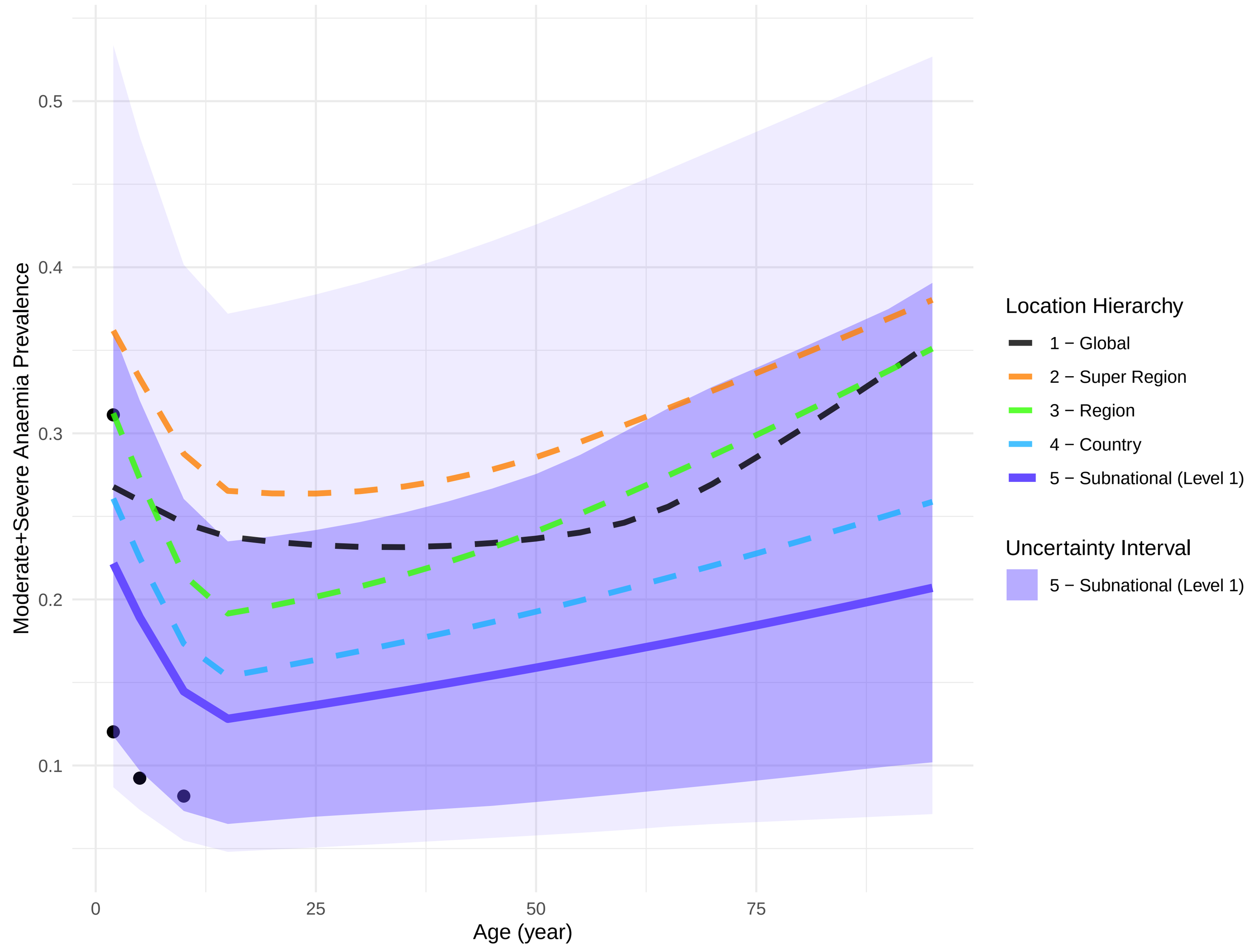

MR−BRT Age/Sex Fits − Non−pregnant Females 2+ Years Old − Moderate+Severe Anaemia Prevalence − Makueni
WHO−2024 Adjustment Method

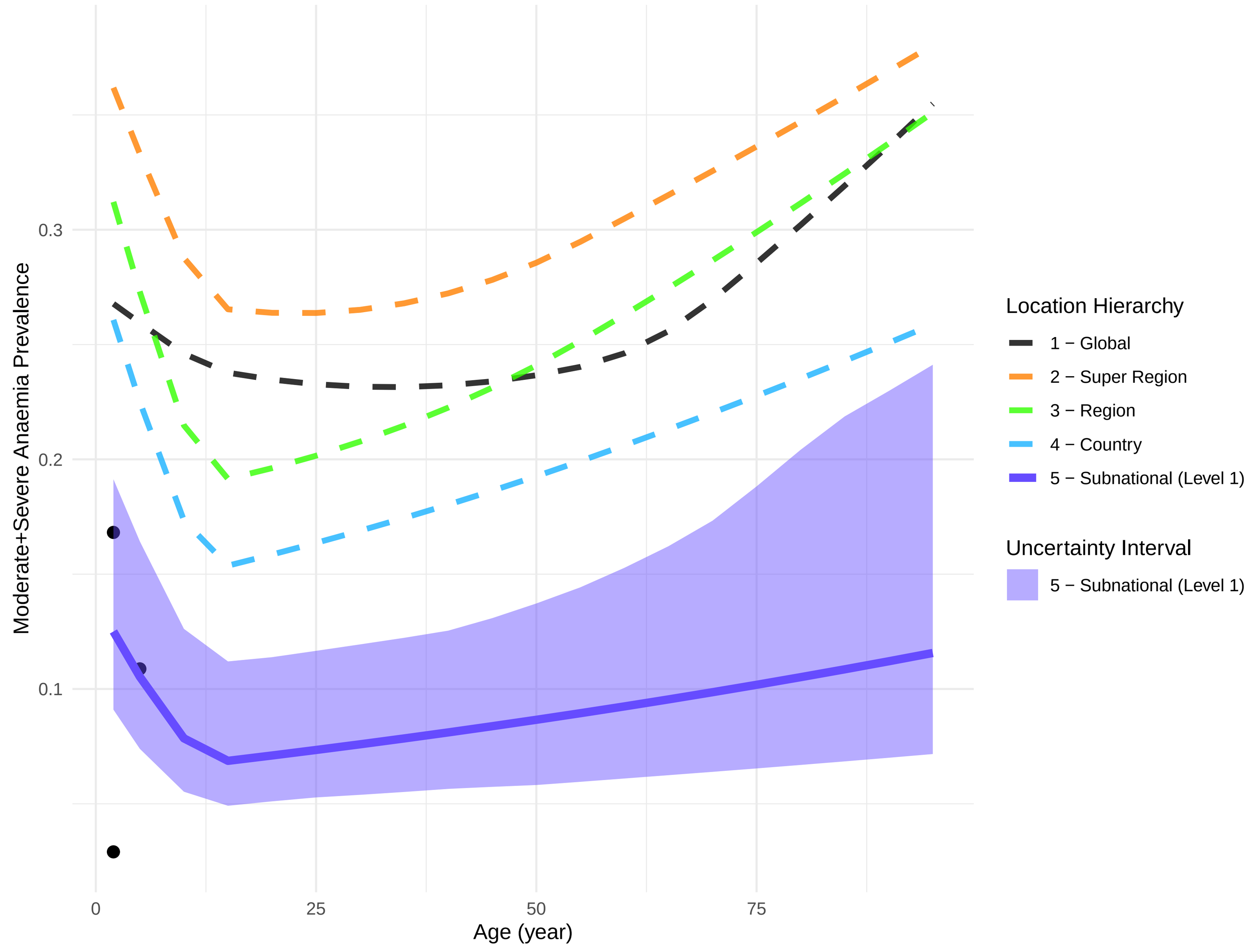

MR−BRT Age/Sex Fits − Non−pregnant Females 2+ Years Old − Moderate+Severe Anaemia Prevalence − Mandera
WHO−2024 Adjustment Method

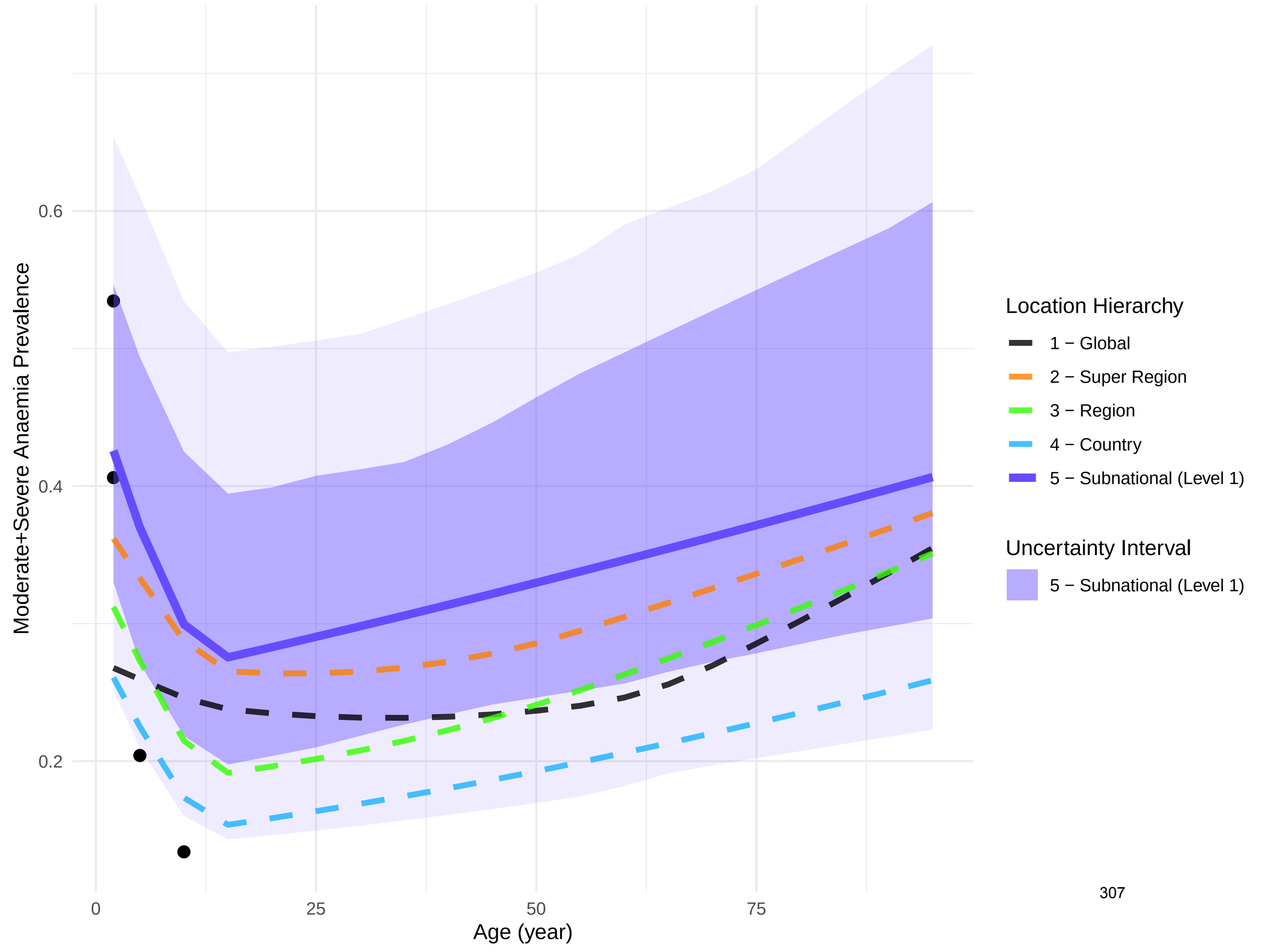

MR−BRT Age/Sex Fits − Non−pregnant Females 2+ Years Old − Moderate+Severe Anaemia Prevalence − Marsabit
WHO−2024 Adjustment Method

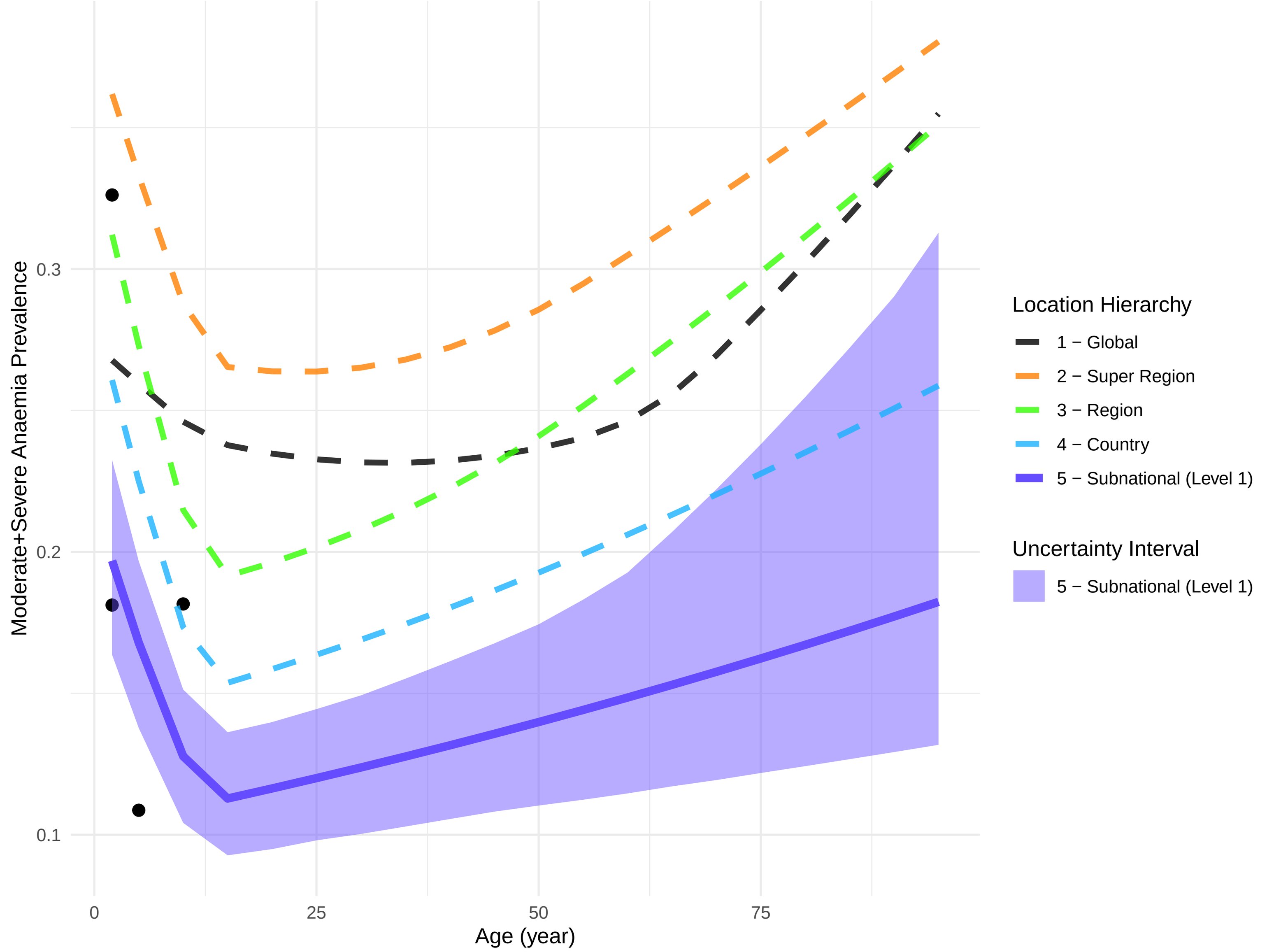

MR−BRT Age/Sex Fits − Non−pregnant Females 2+ Years Old − Moderate+Severe Anaemia Prevalence − Meru
WHO−2024 Adjustment Method

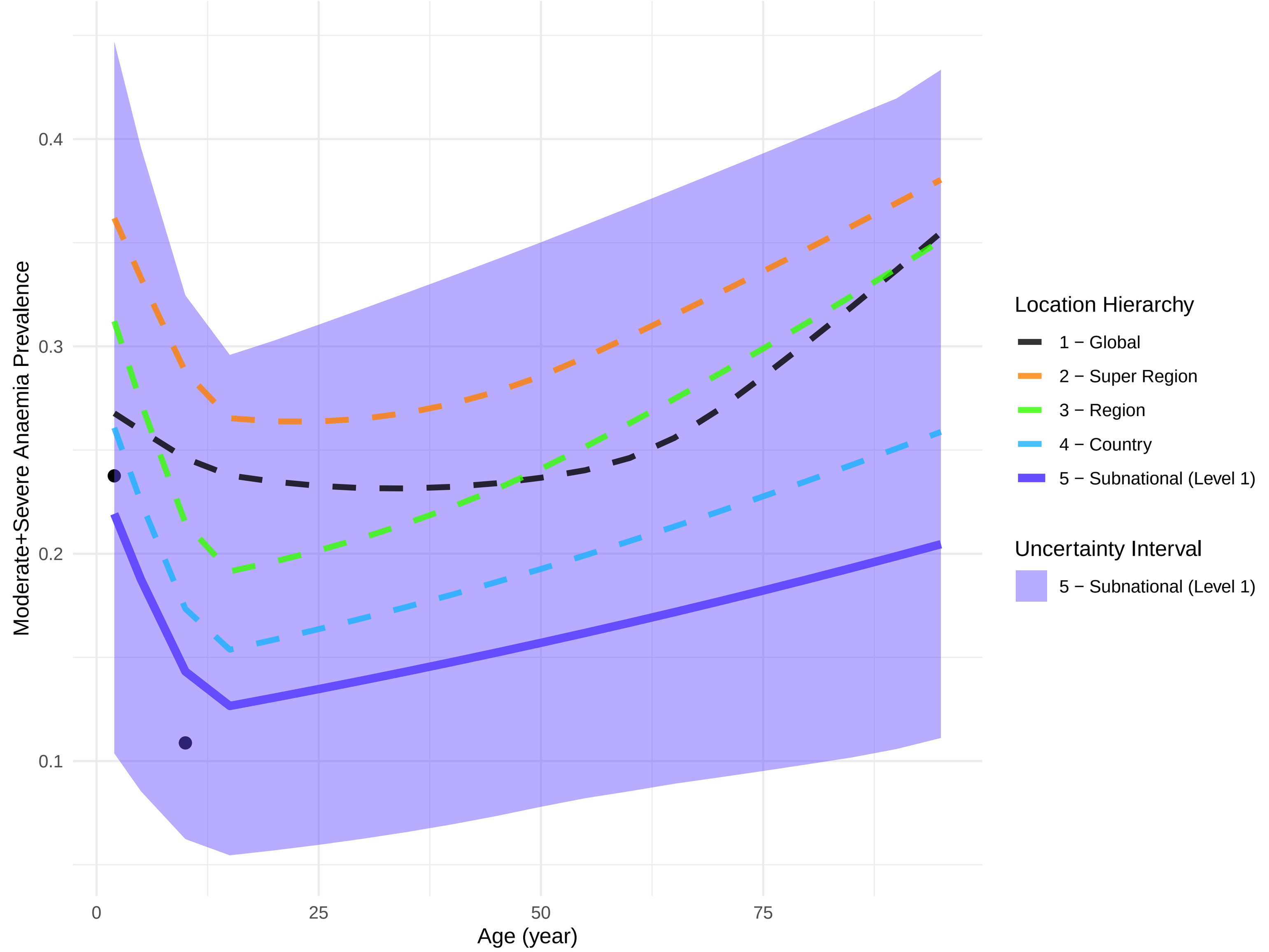

MR−BRT Age/Sex Fits − Non−pregnant Females 2+ Years Old − Moderate+Severe Anaemia Prevalence − Migori
WHO−2024 Adjustment Method

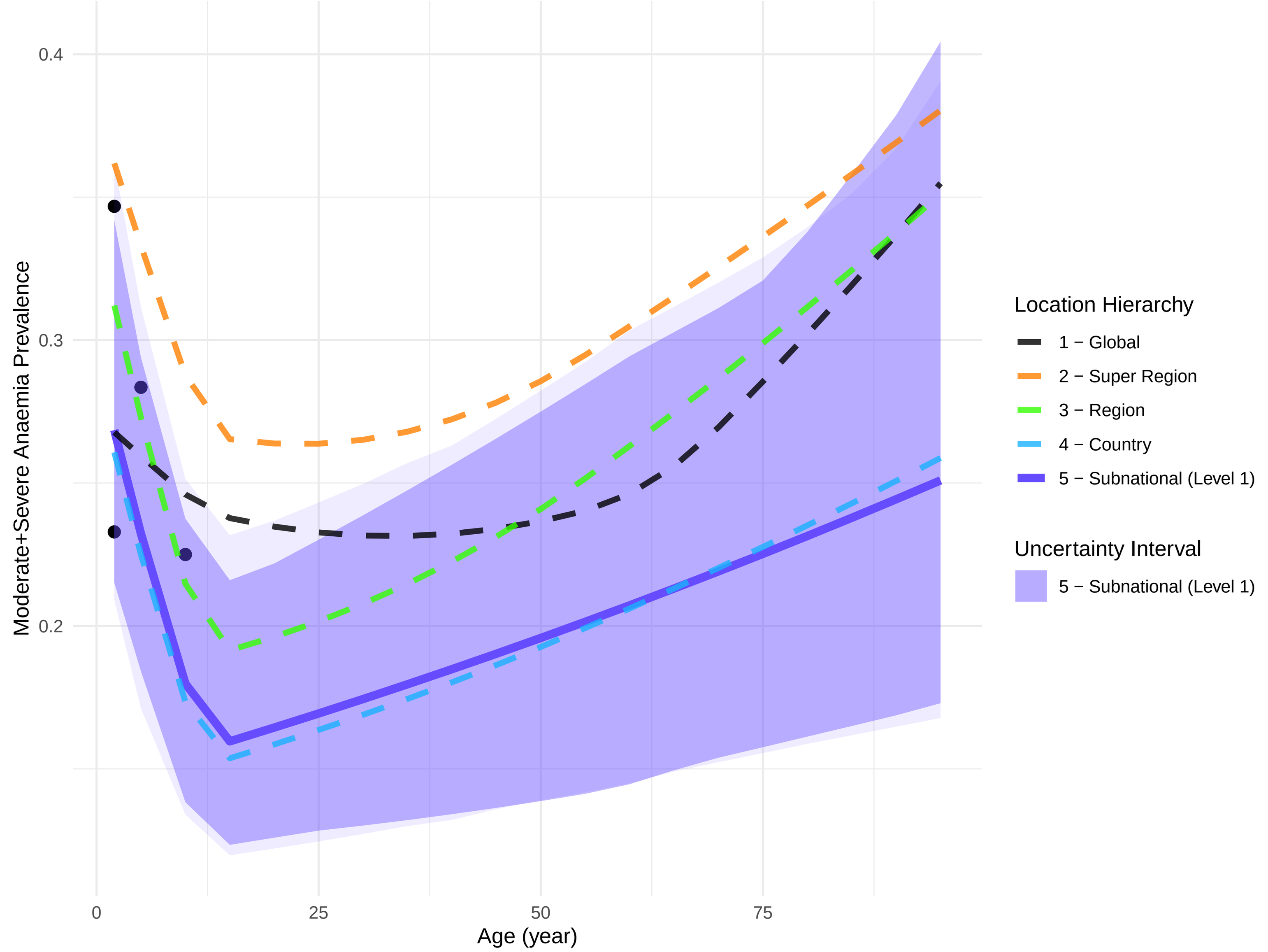

MR−BRT Age/Sex Fits − Non−pregnant Females 2+ Years Old − Moderate+Severe Anaemia Prevalence − Mombasa
WHO−2024 Adjustment Method

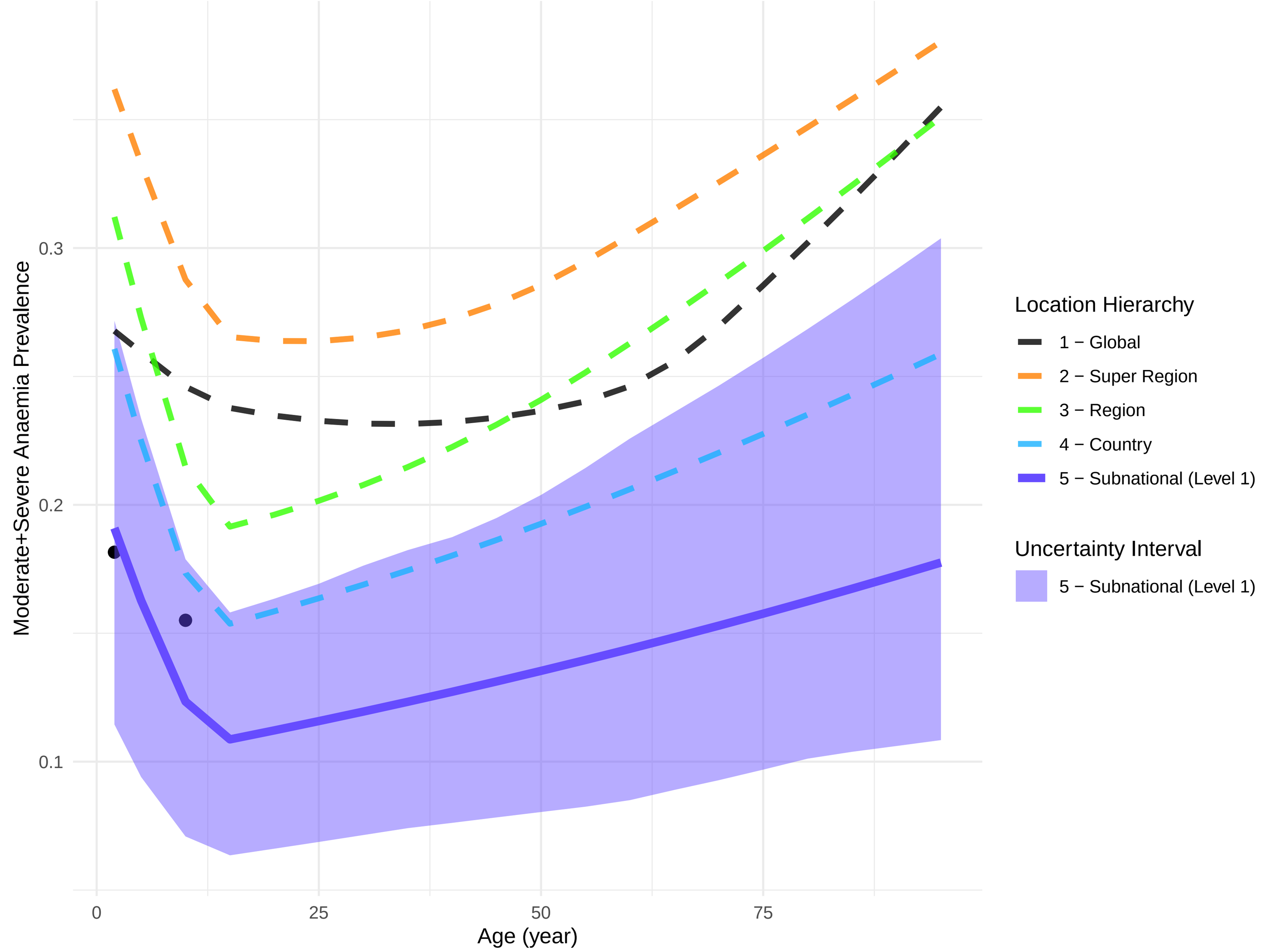

MR−BRT Age/Sex Fits − Non−pregnant Females 2+ Years Old − Moderate+Severe Anaemia Prevalence − Murang'a
WHO−2024 Adjustment Method

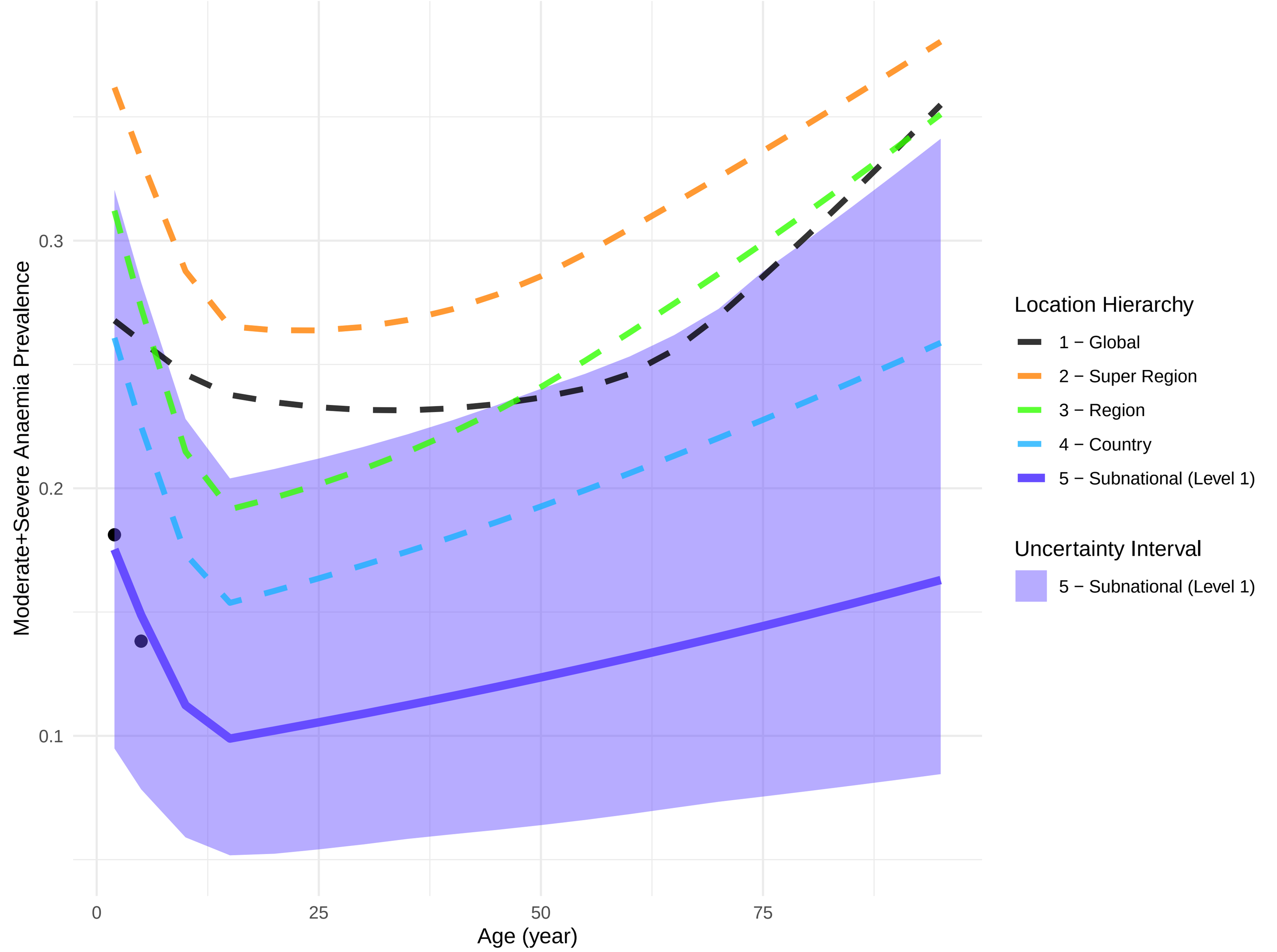

MR−BRT Age/Sex Fits − Non−pregnant Females 2+ Years Old − Moderate+Severe Anaemia Prevalence − Nairobi
WHO−2024 Adjustment Method

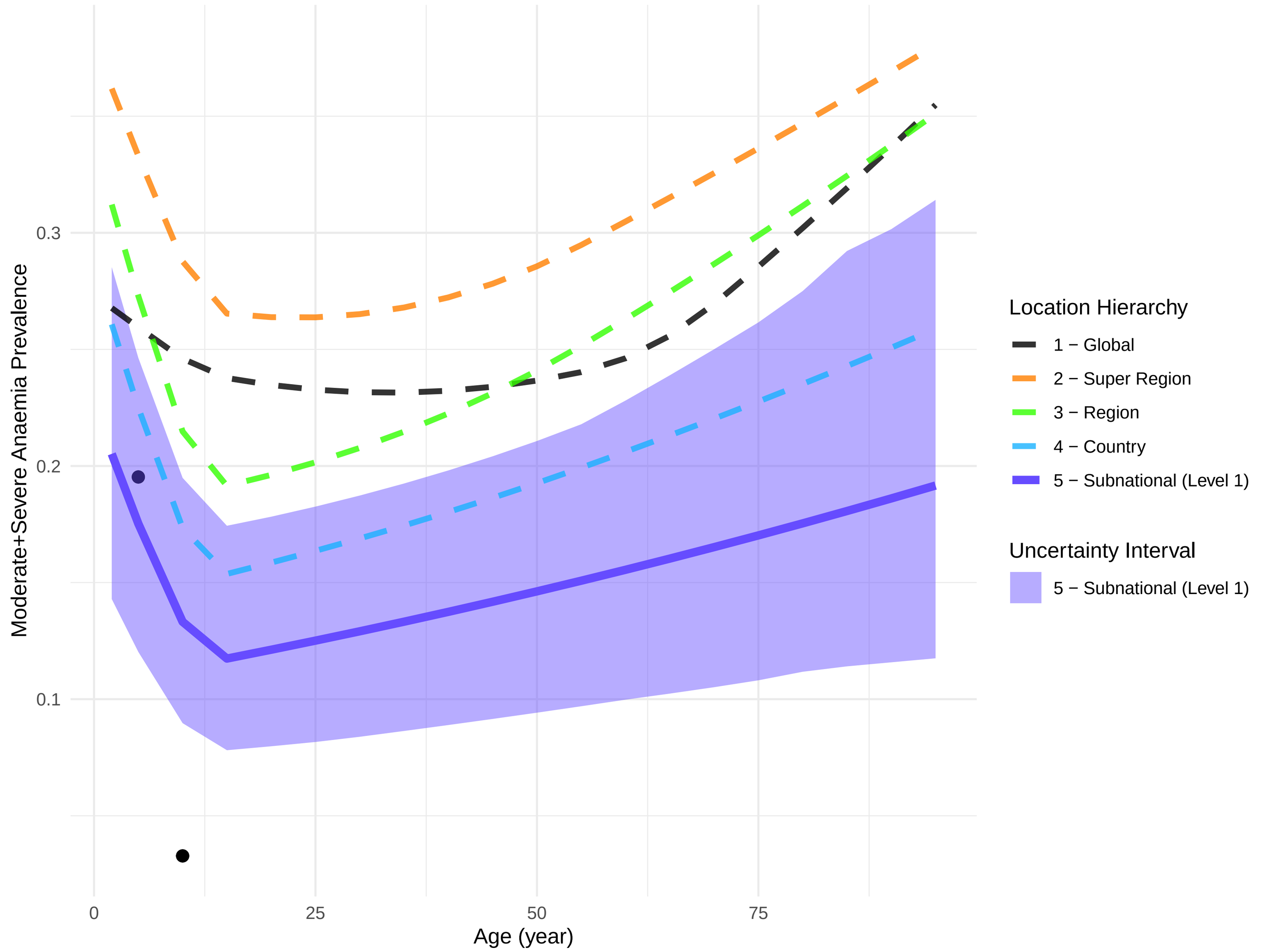

MR−BRT Age/Sex Fits − Non−pregnant Females 2+ Years Old − Moderate+Severe Anaemia Prevalence − Nakuru
WHO−2024 Adjustment Method

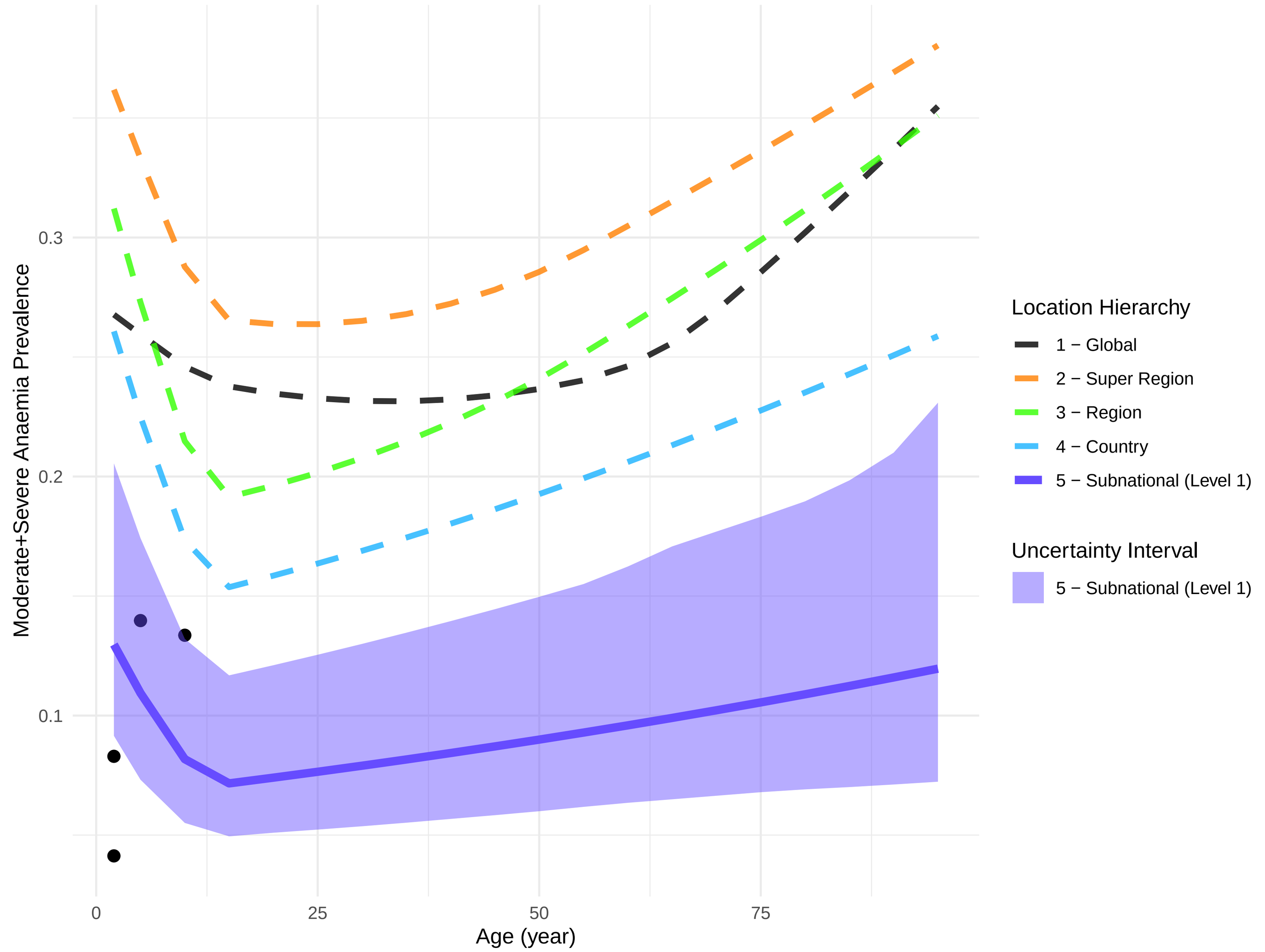

MR−BRT Age/Sex Fits − Non−pregnant Females 2+ Years Old − Moderate+Severe Anaemia Prevalence − Nandi
WHO−2024 Adjustment Method

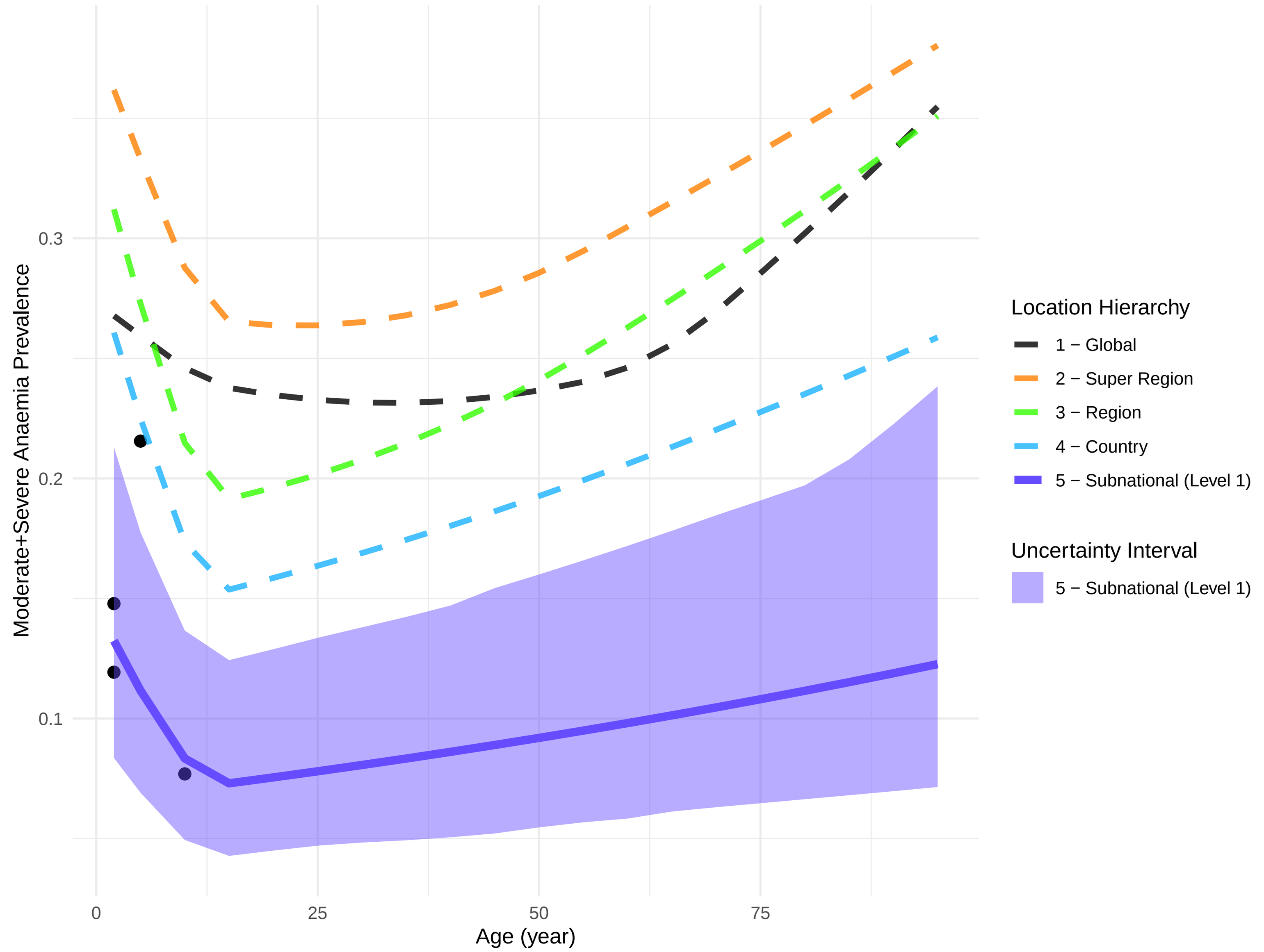

MR−BRT Age/Sex Fits − Non−pregnant Females 2+ Years Old − Moderate+Severe Anaemia Prevalence − Narok
WHO−2024 Adjustment Method

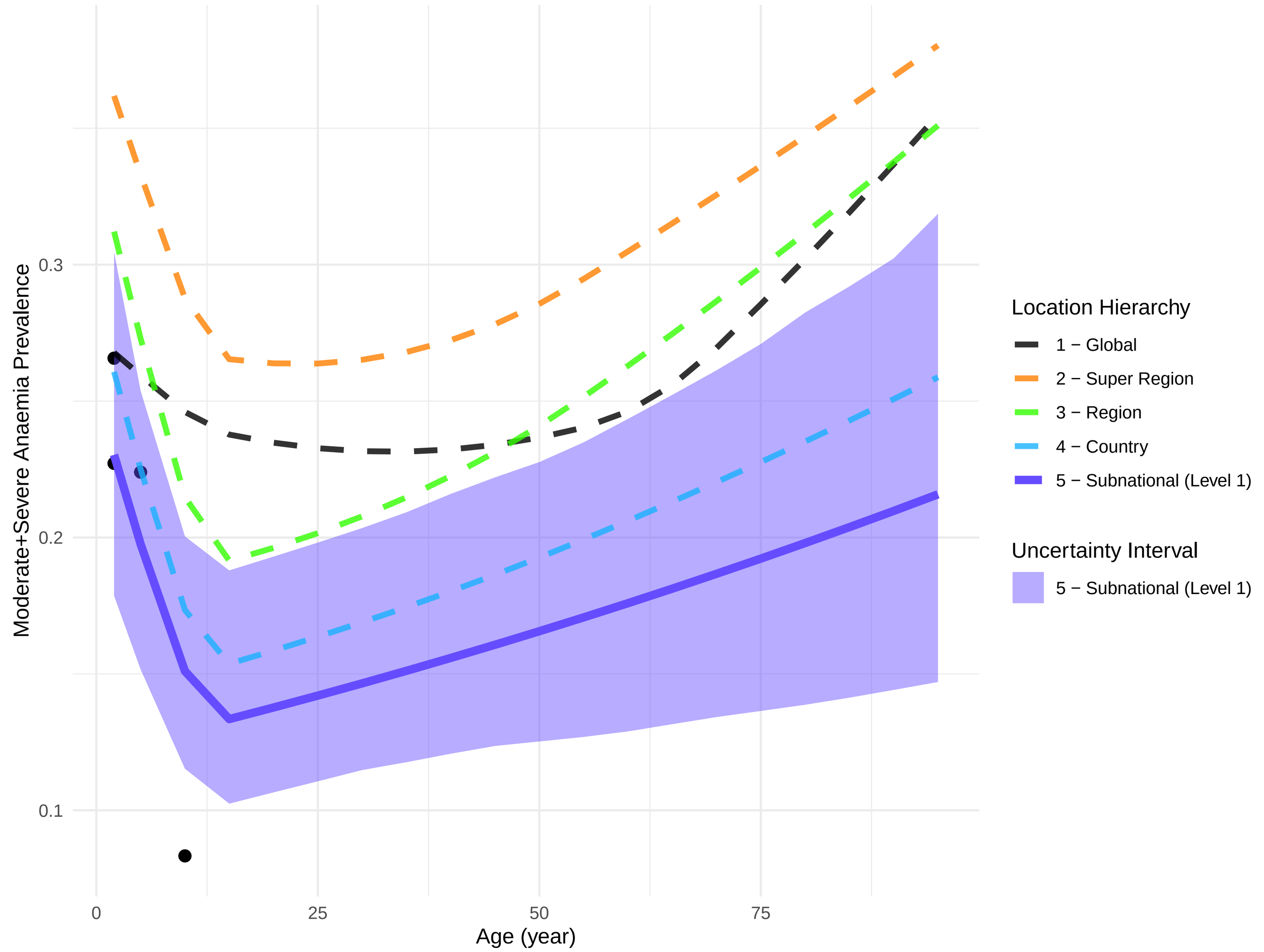

MR−BRT Age/Sex Fits − Non−pregnant Females 2+ Years Old − Moderate+Severe Anaemia Prevalence − Nyamira
WHO−2024 Adjustment Method

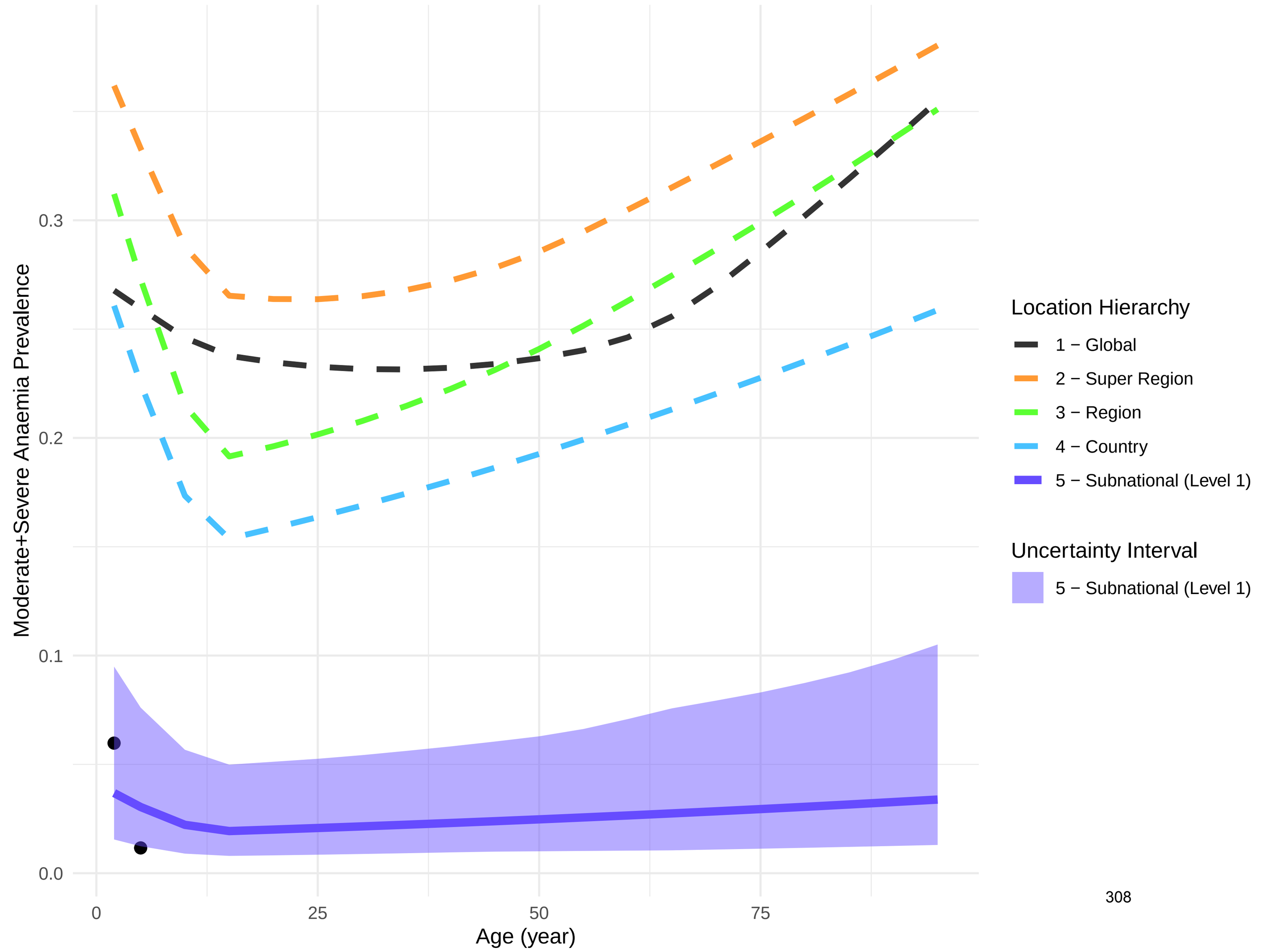

MR−BRT Age/Sex Fits − Non−pregnant Females 2+ Years Old − Moderate+Severe Anaemia Prevalence − Nyandarua
WHO−2024 Adjustment Method

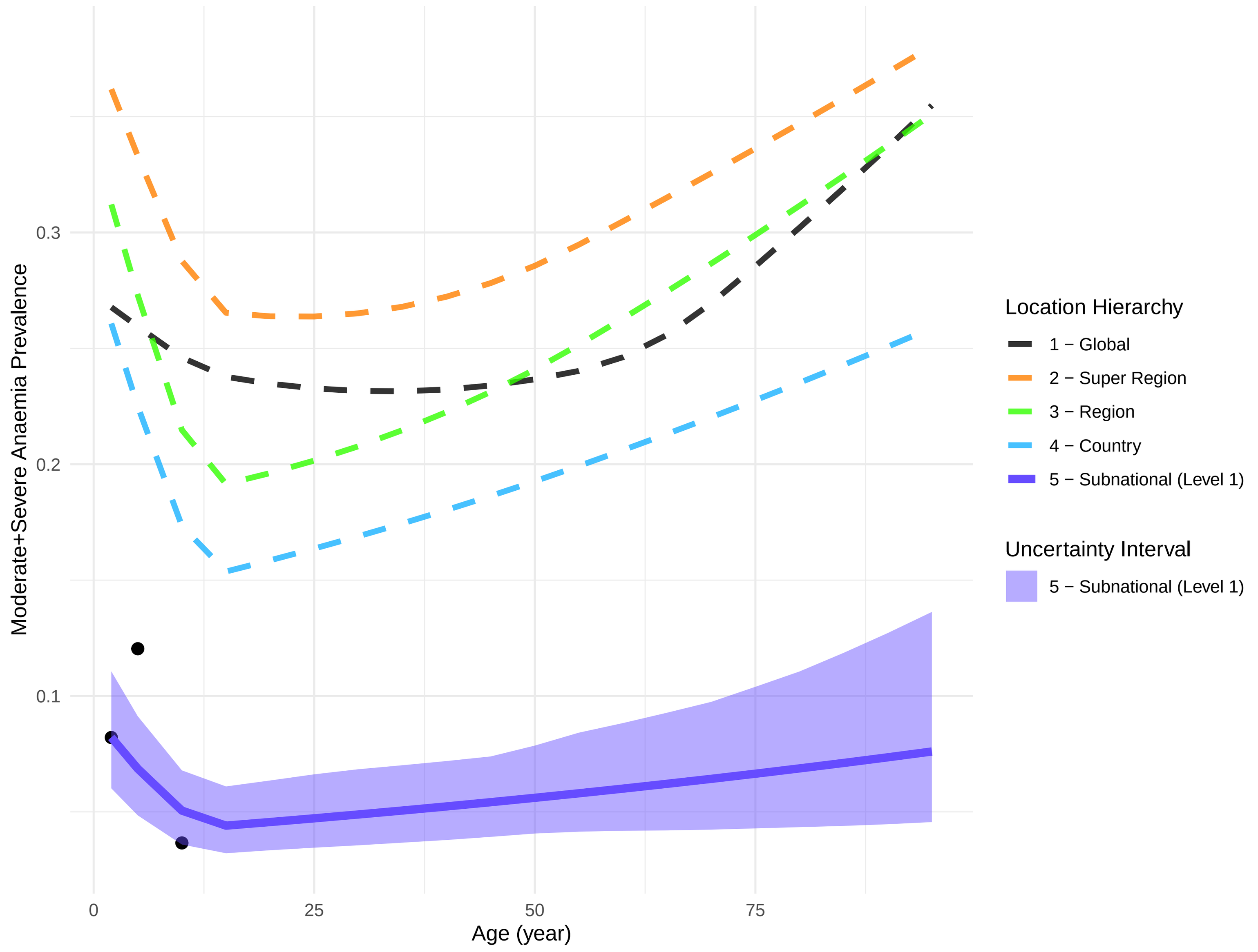

MR−BRT Age/Sex Fits − Non−pregnant Females 2+ Years Old − Moderate+Severe Anaemia Prevalence − Nyeri
WHO−2024 Adjustment Method

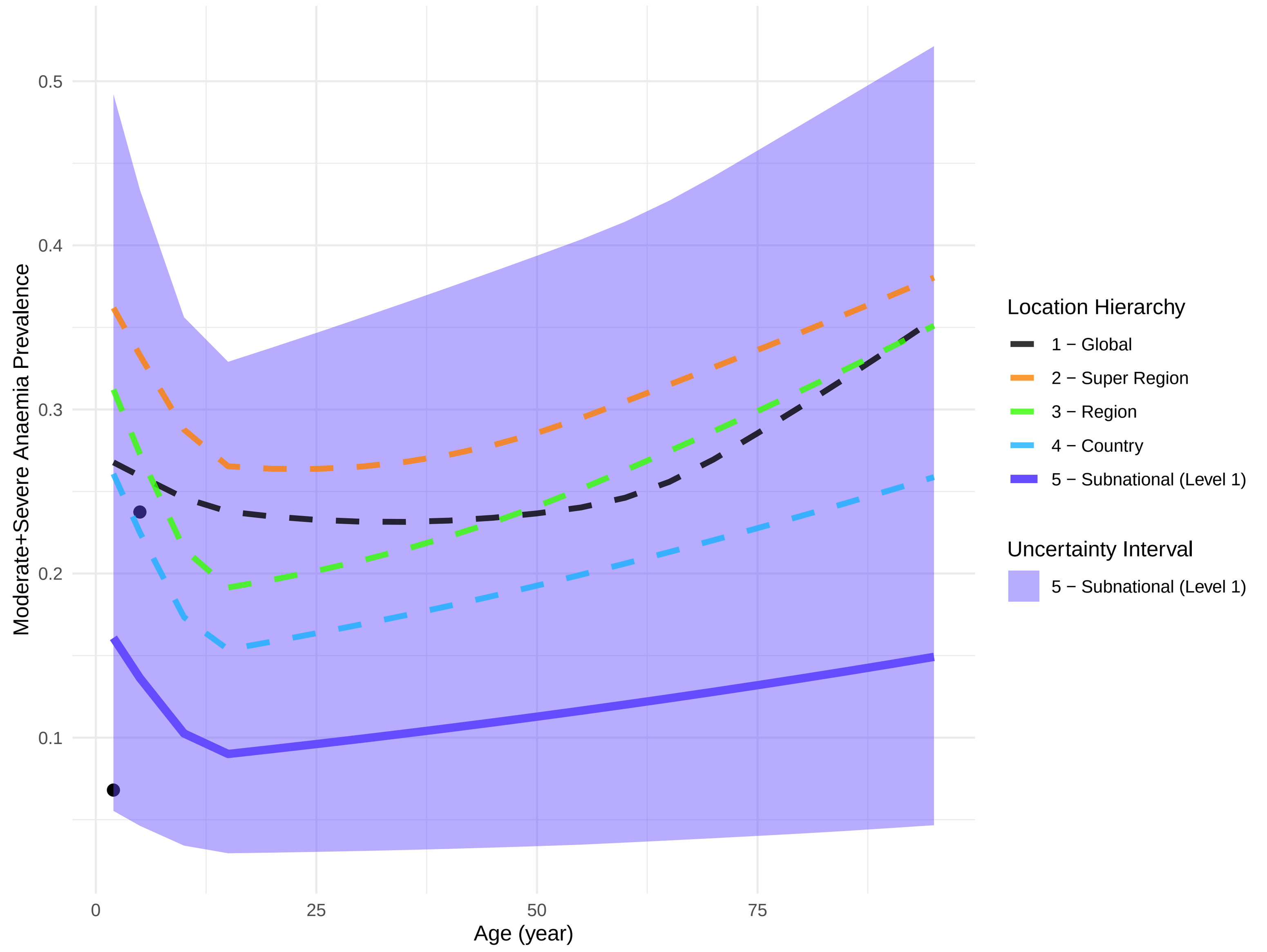

MR−BRT Age/Sex Fits − Non−pregnant Females 2+ Years Old − Moderate+Severe Anaemia Prevalence − Samburu
WHO−2024 Adjustment Method

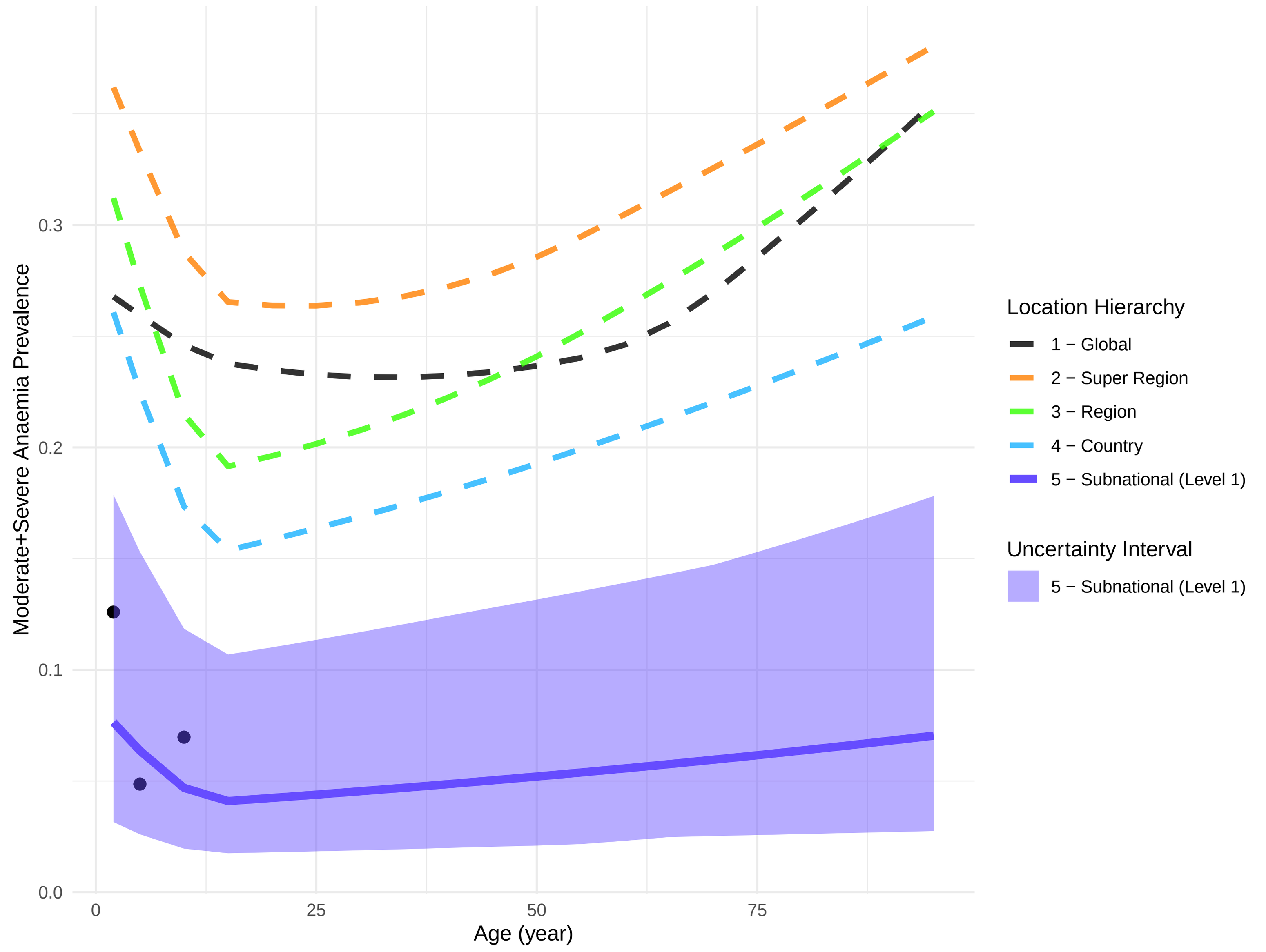

MR−BRT Age/Sex Fits − Non−pregnant Females 2+ Years Old − Moderate+Severe Anaemia Prevalence − Siaya
WHO−2024 Adjustment Method

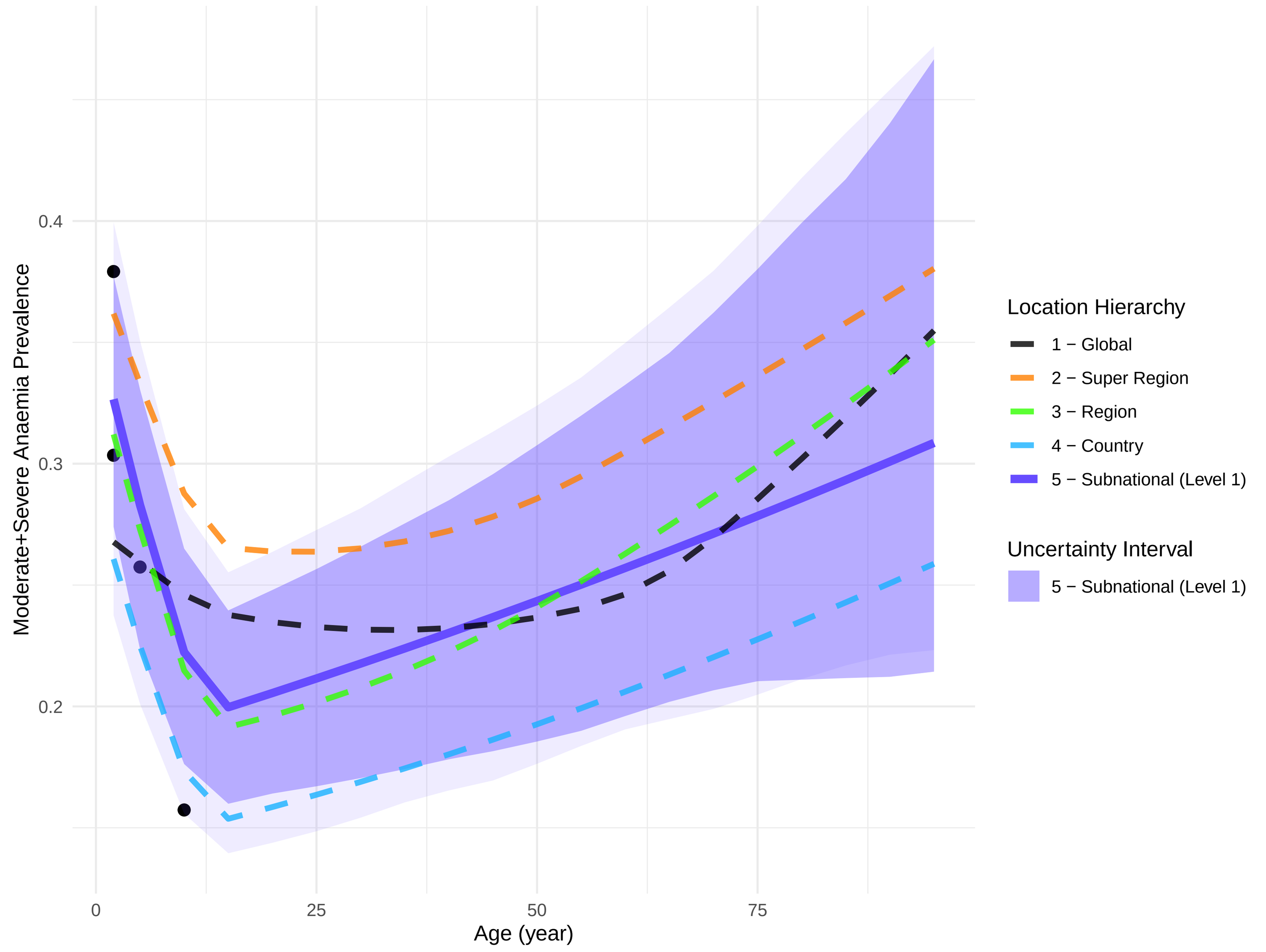

MR−BRT Age/Sex Fits − Non−pregnant Females 2+ Years Old − Moderate+Severe Anaemia Prevalence − Taita Taveta
WHO−2024 Adjustment Method

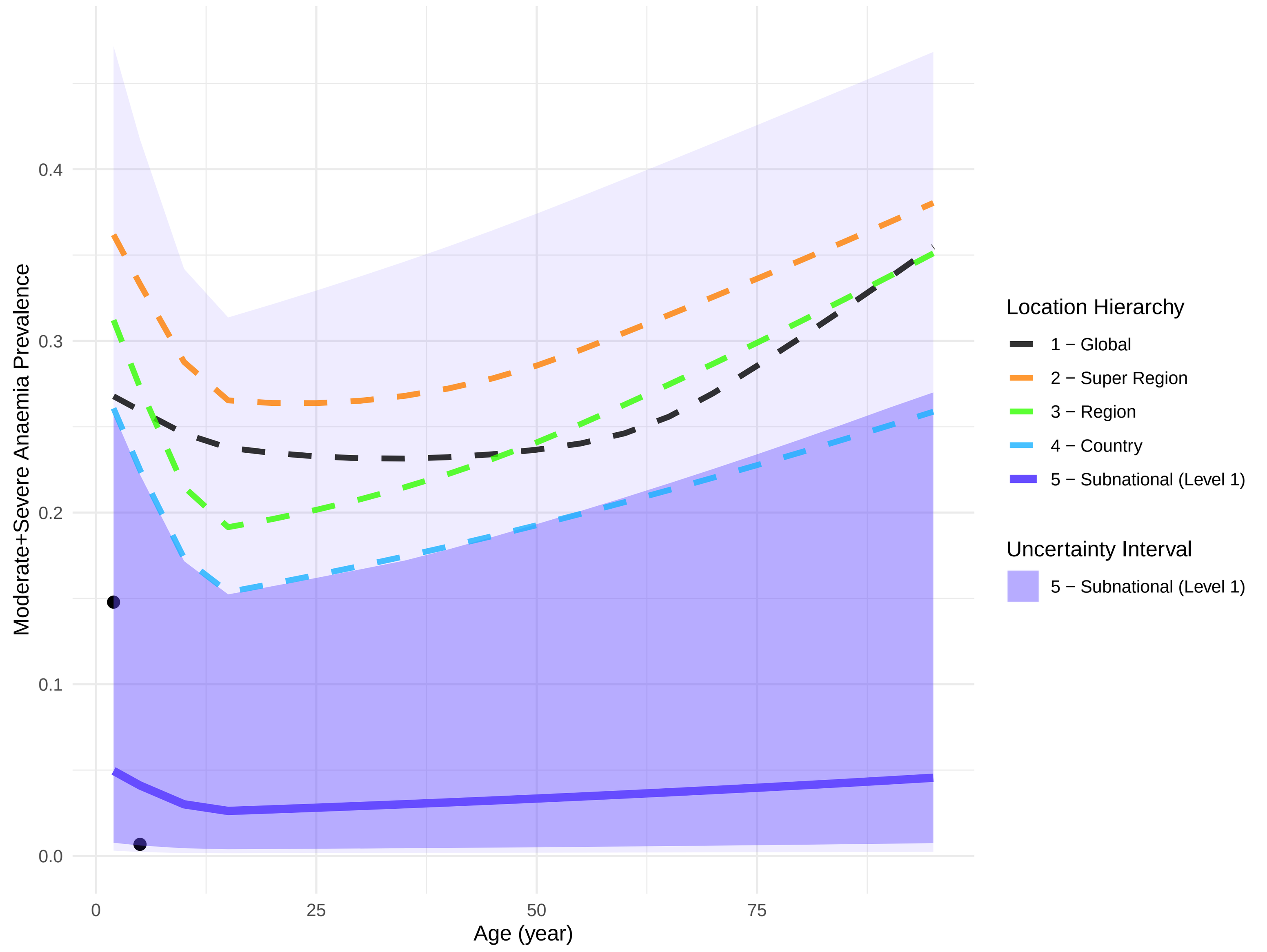

MR−BRT Age/Sex Fits − Non−pregnant Females 2+ Years Old − Moderate+Severe Anaemia Prevalence − Tana River
WHO−2024 Adjustment Method

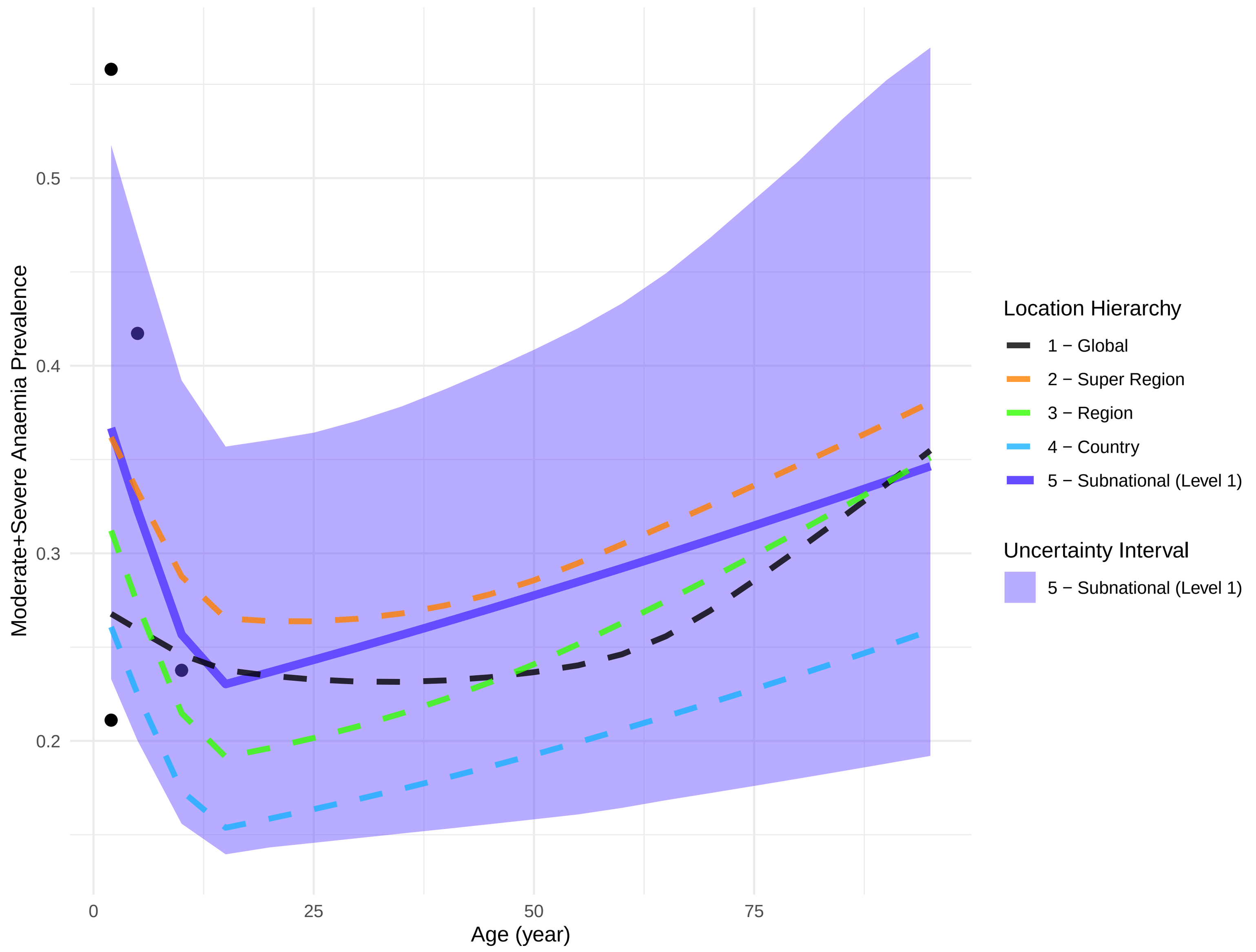

MR−BRT Age/Sex Fits − Non−pregnant Females 2+ Years Old − Moderate+Severe Anaemia Prevalence − Tharaka Nithi
WHO−2024 Adjustment Method

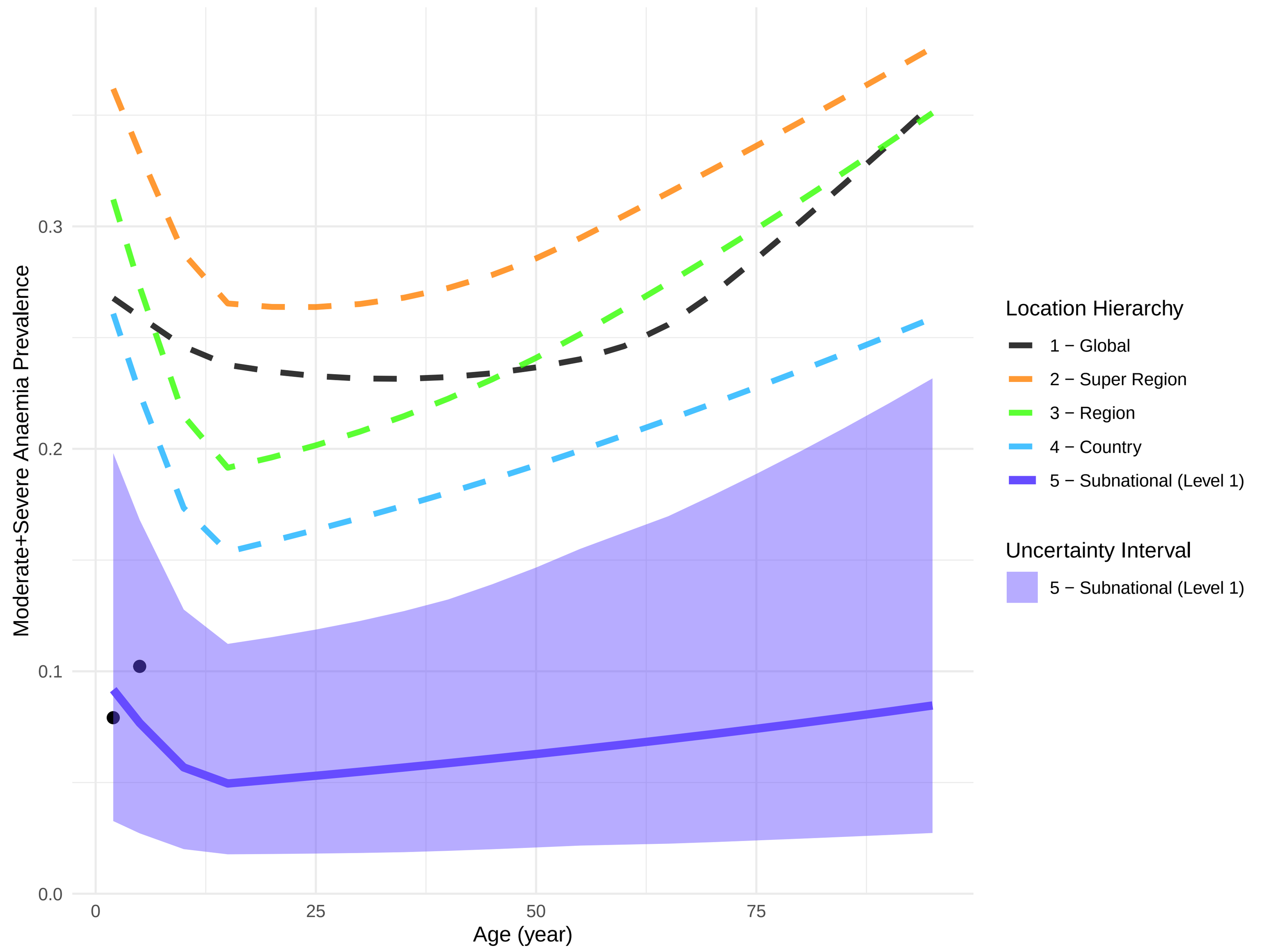

MR−BRT Age/Sex Fits − Non−pregnant Females 2+ Years Old − Moderate+Severe Anaemia Prevalence − Trans Nzoia
WHO−2024 Adjustment Method

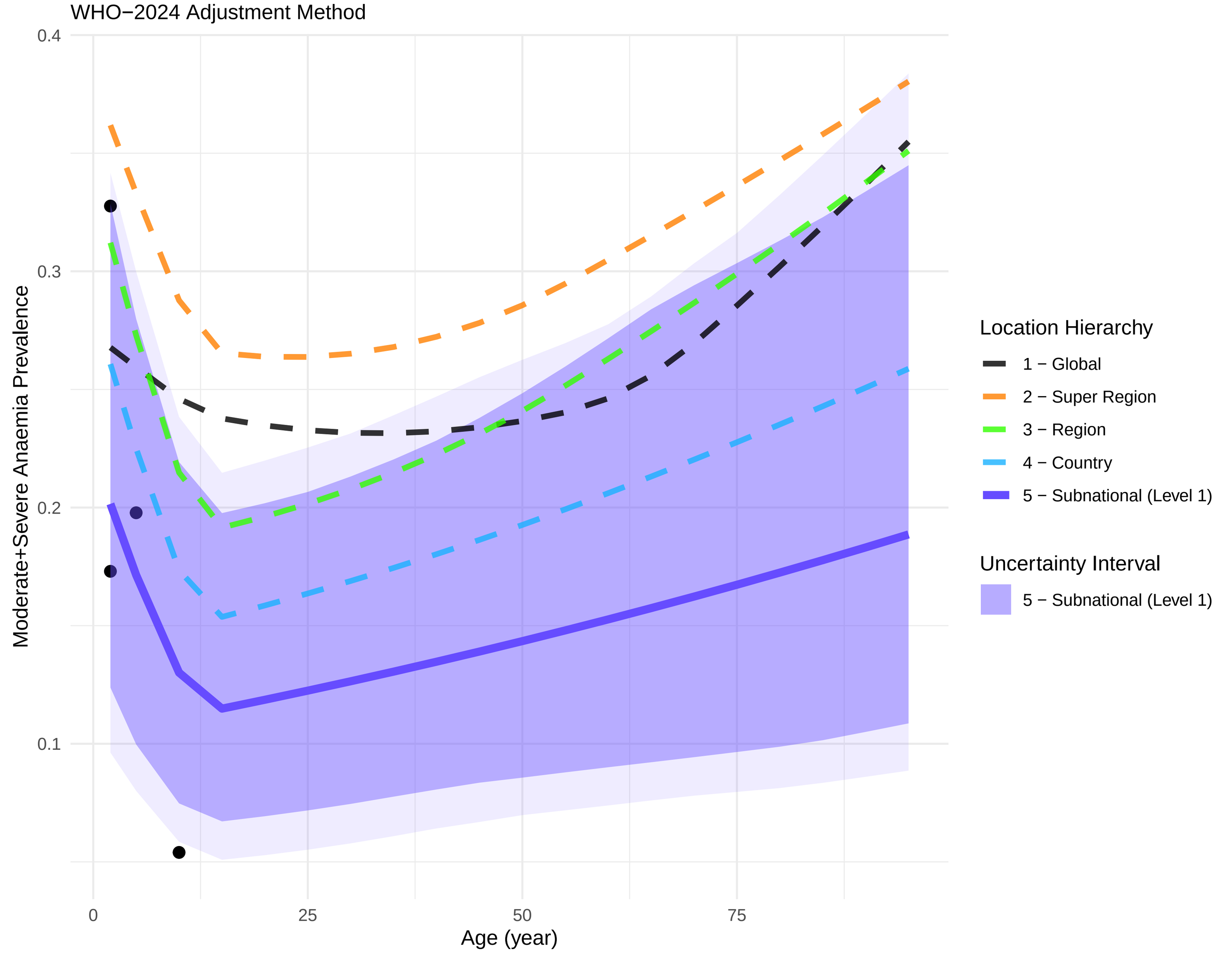

MR−BRT Age/Sex Fits − Non−pregnant Females 2+ Years Old − Moderate+Severe Anaemia Prevalence − Turkana
WHO−2024 Adjustment Method

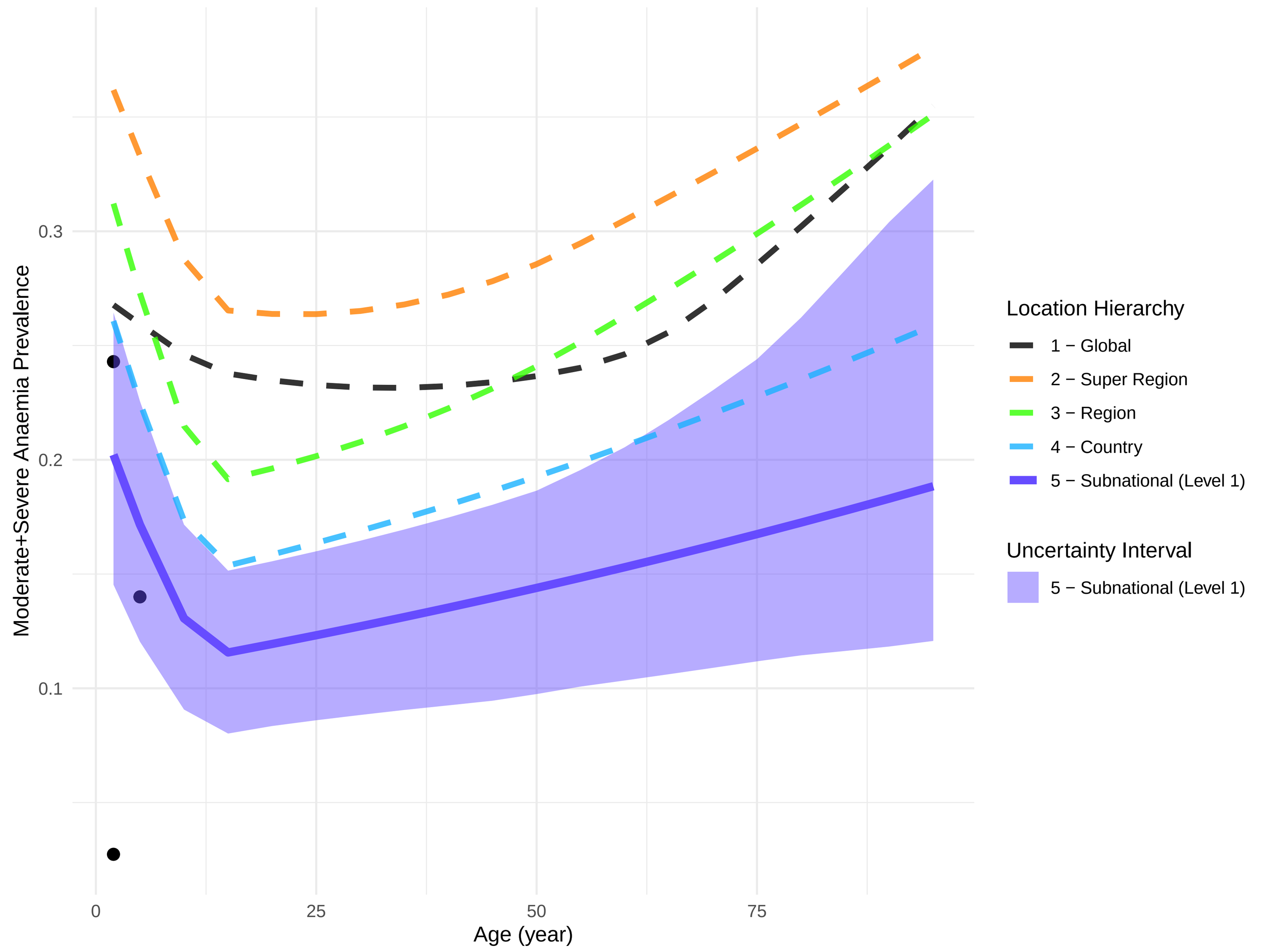

MR−BRT Age/Sex Fits − Non−pregnant Females 2+ Years Old − Moderate+Severe Anaemia Prevalence − Uasin Gishu
WHO−2024 Adjustment Method

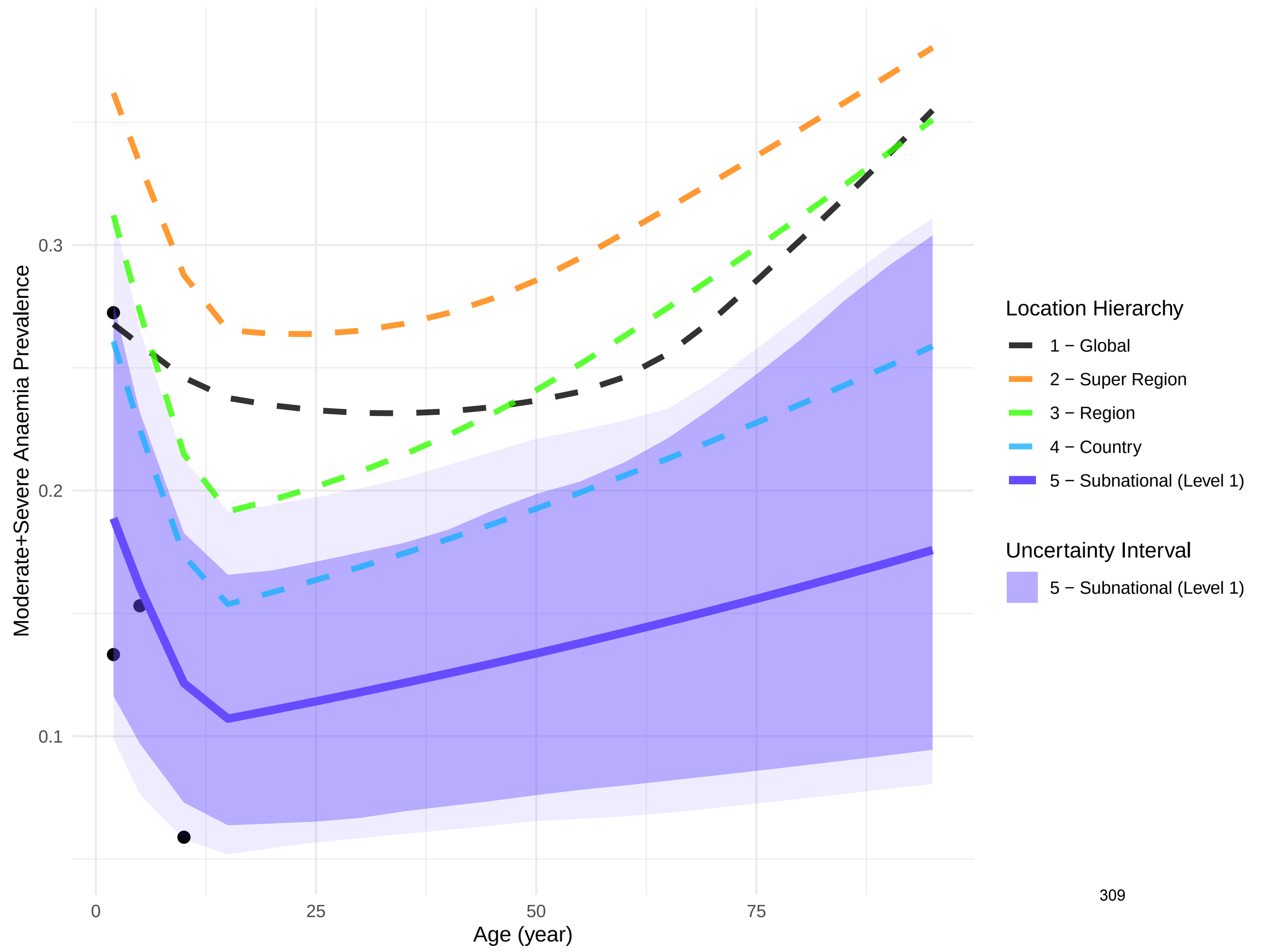

MR−BRT Age/Sex Fits − Non−pregnant Females 2+ Years Old − Moderate+Severe Anaemia Prevalence − Vihiga
WHO−2024 Adjustment Method

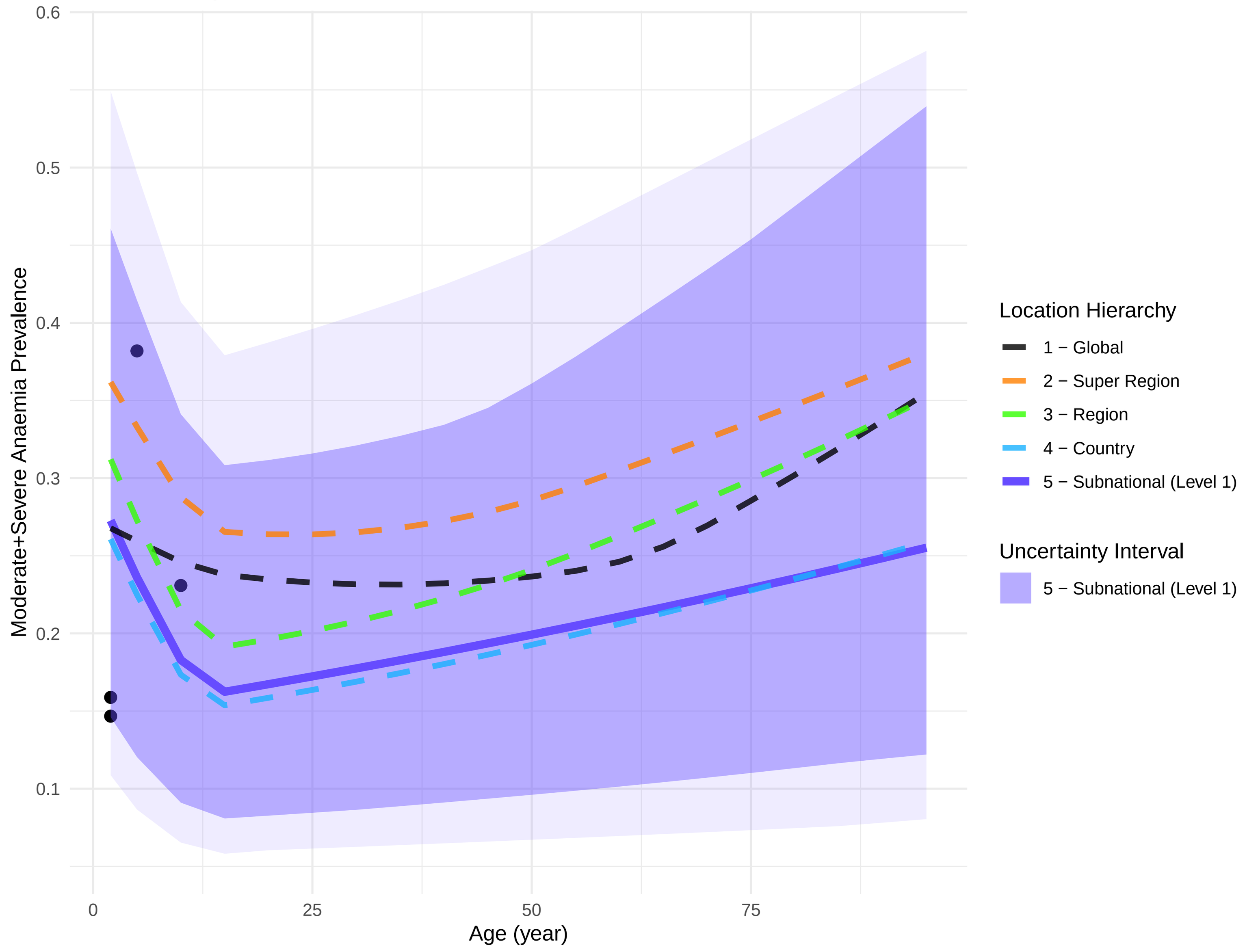

MR−BRT Age/Sex Fits − Non−pregnant Females 2+ Years Old − Moderate+Severe Anaemia Prevalence − Wajir
WHO−2024 Adjustment Method

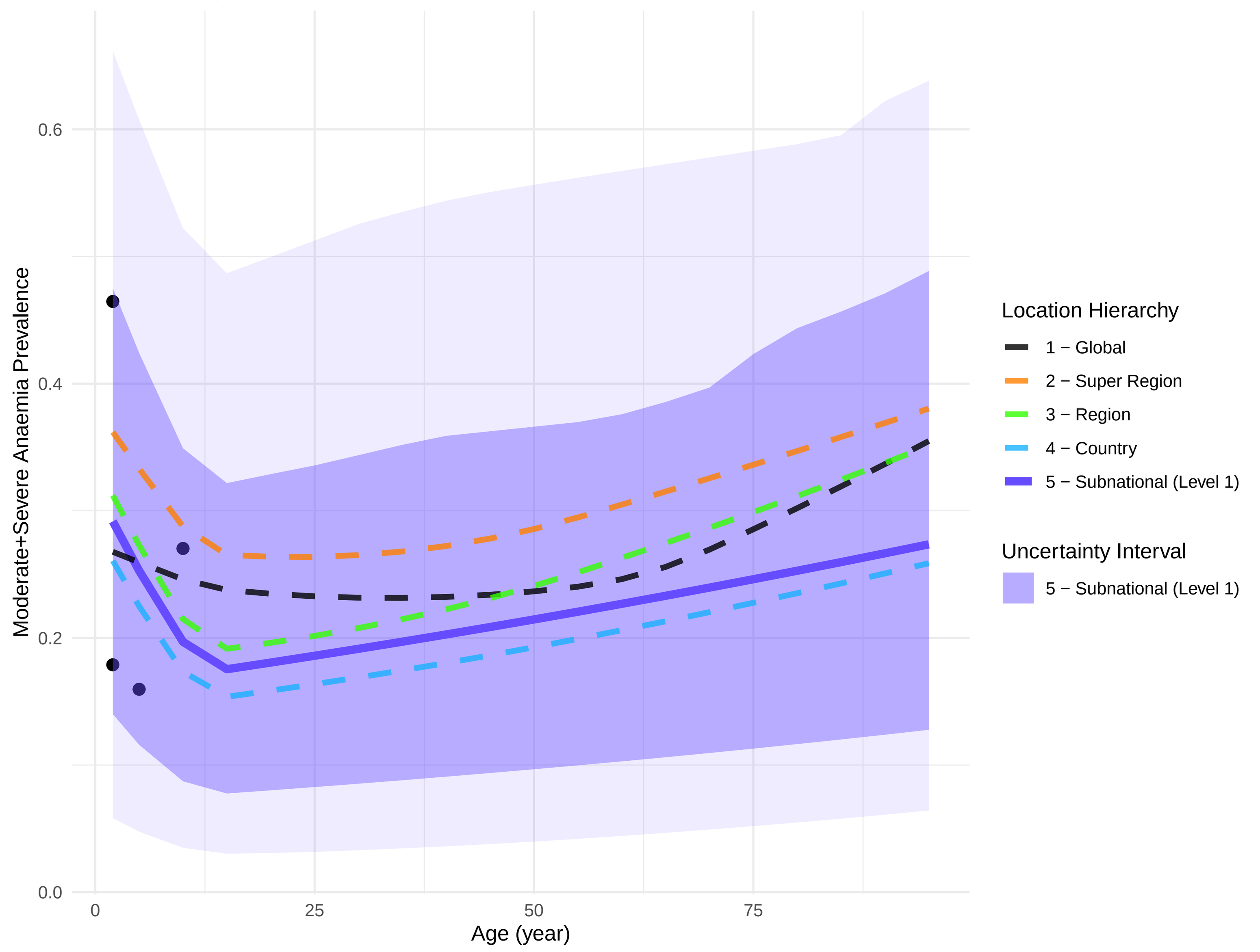

MR−BRT Age/Sex Fits − Non−pregnant Females 2+ Years Old − Moderate+Severe Anaemia Prevalence − West Pokot
WHO−2024 Adjustment Method

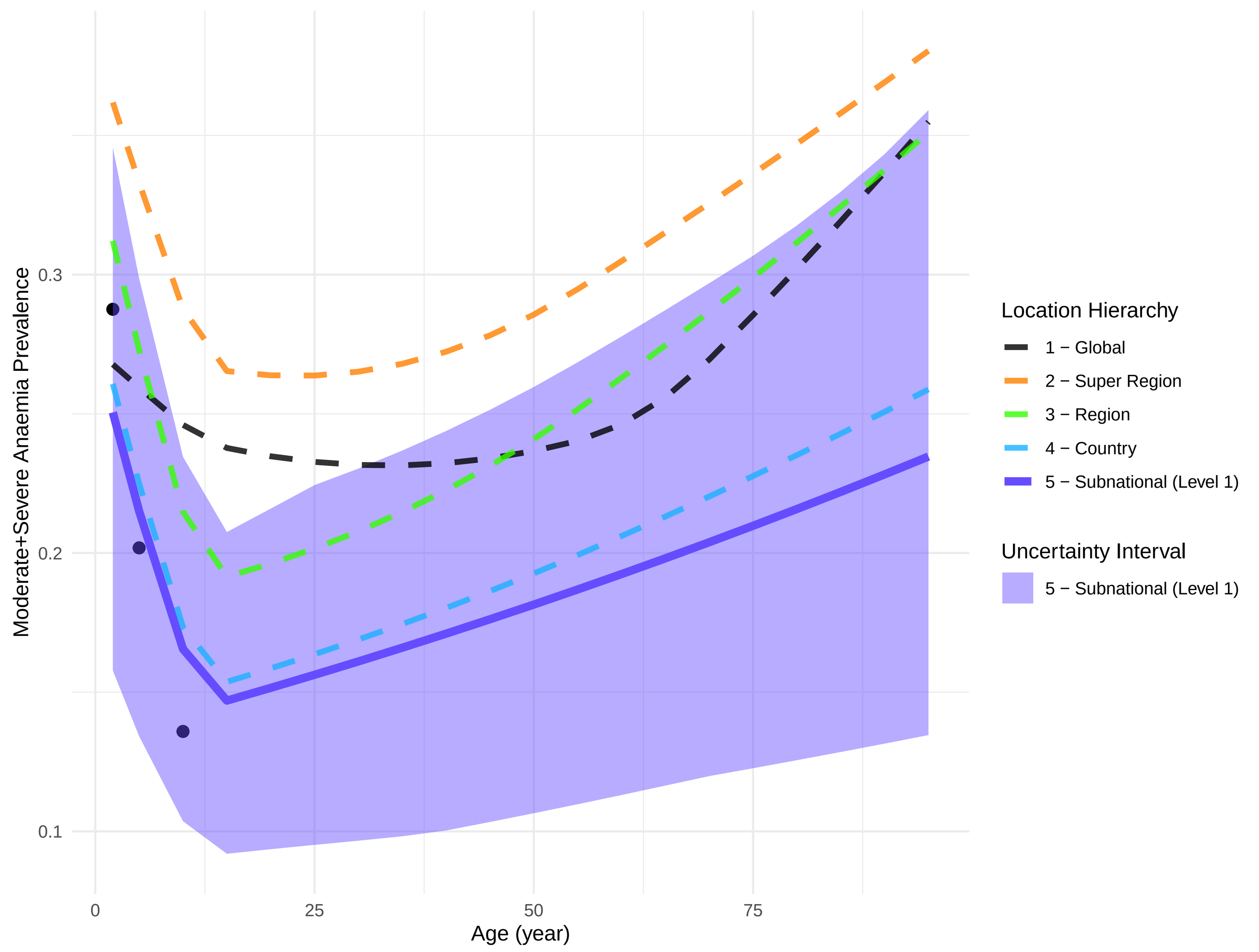

MR−BRT Age/Sex Fits − Non−pregnant Females 2+ Years Old − Moderate+Severe Anaemia Prevalence − Madagascar
WHO−2024 Adjustment Method

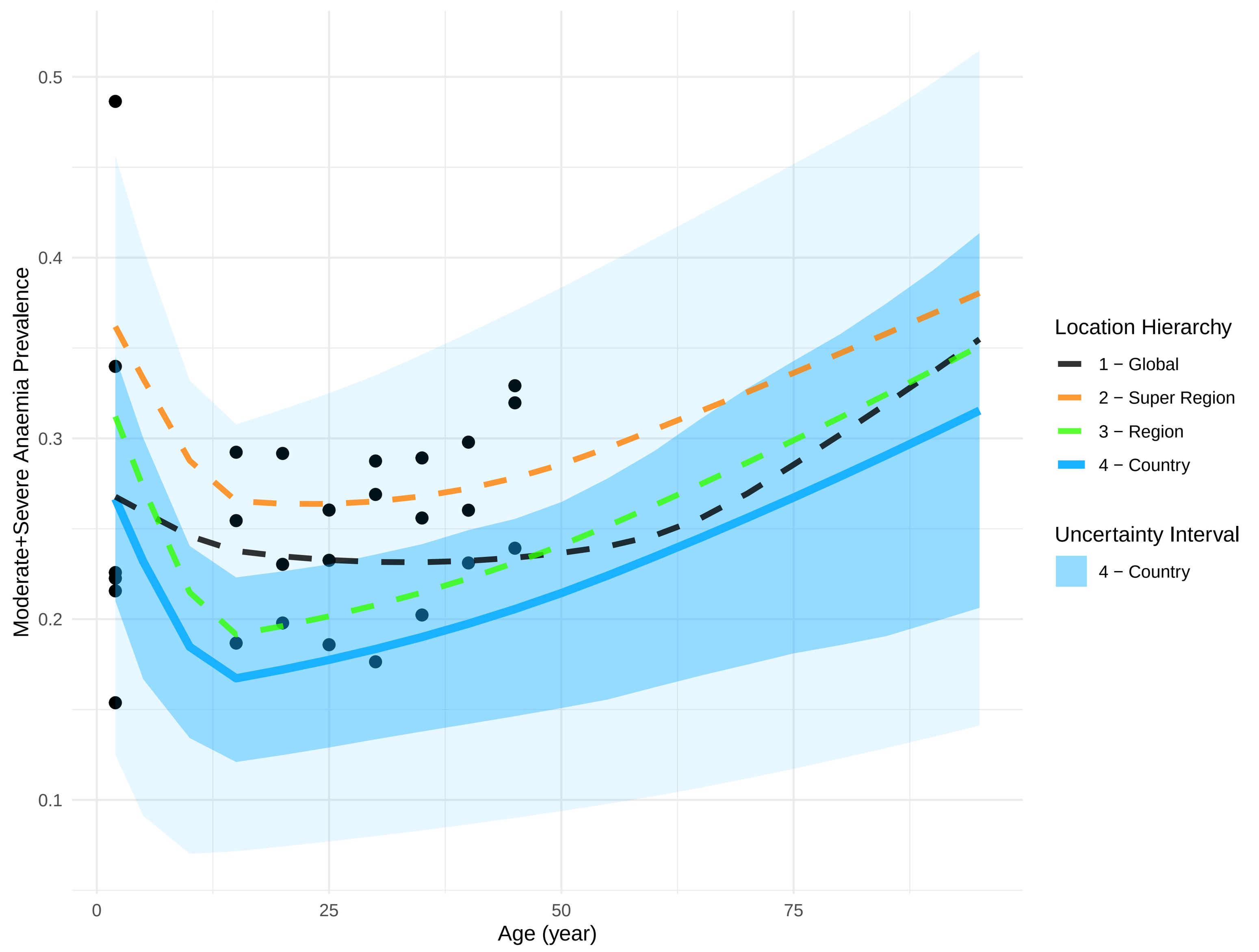

MR−BRT Age/Sex Fits − Non−pregnant Females 2+ Years Old − Moderate+Severe Anaemia Prevalence − Malawi
WHO−2024 Adjustment Method

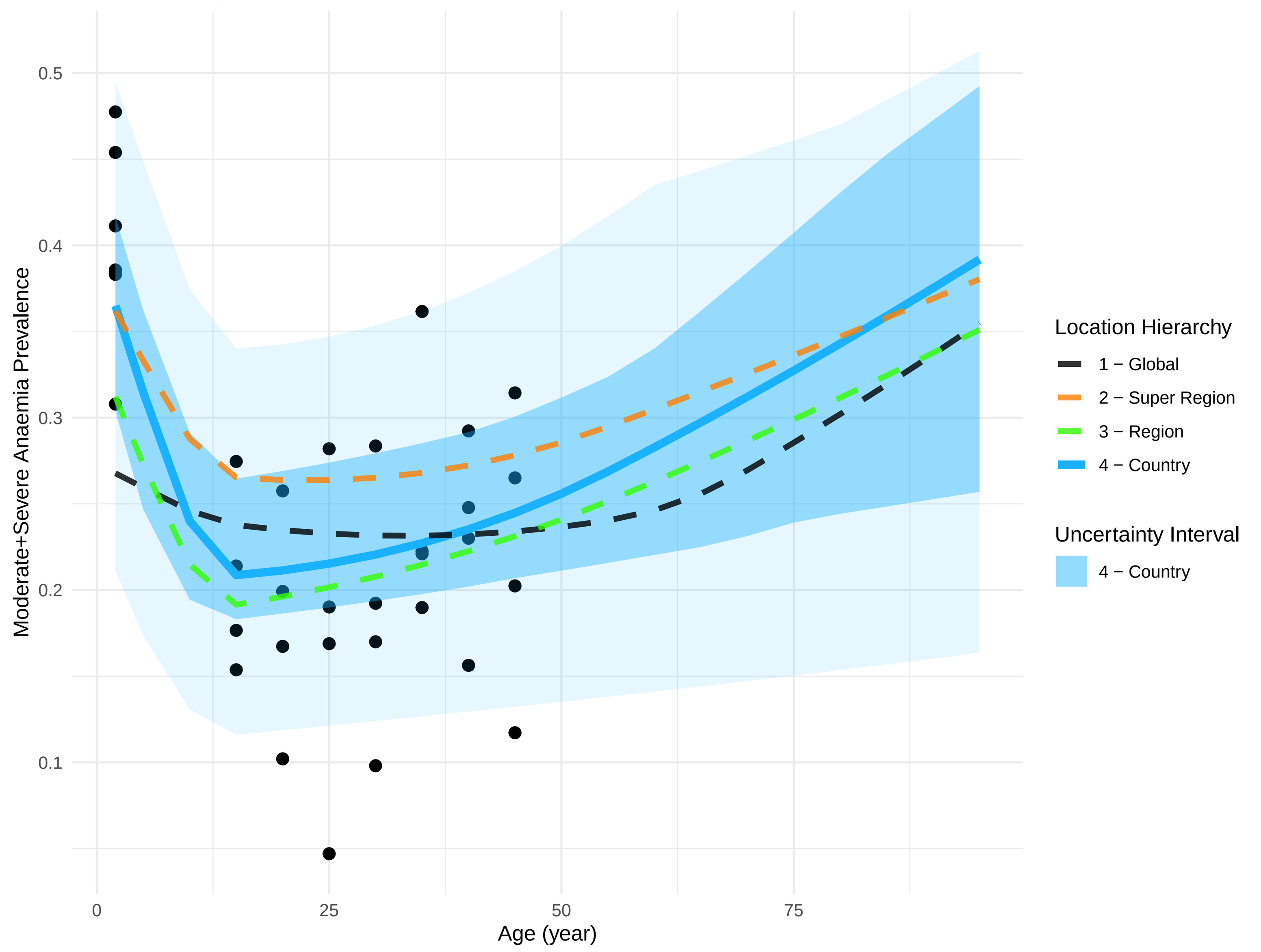

MR−BRT Age/Sex Fits − Non−pregnant Females 2+ Years Old − Moderate+Severe Anaemia Prevalence − Mozambique
WHO−2024 Adjustment Method

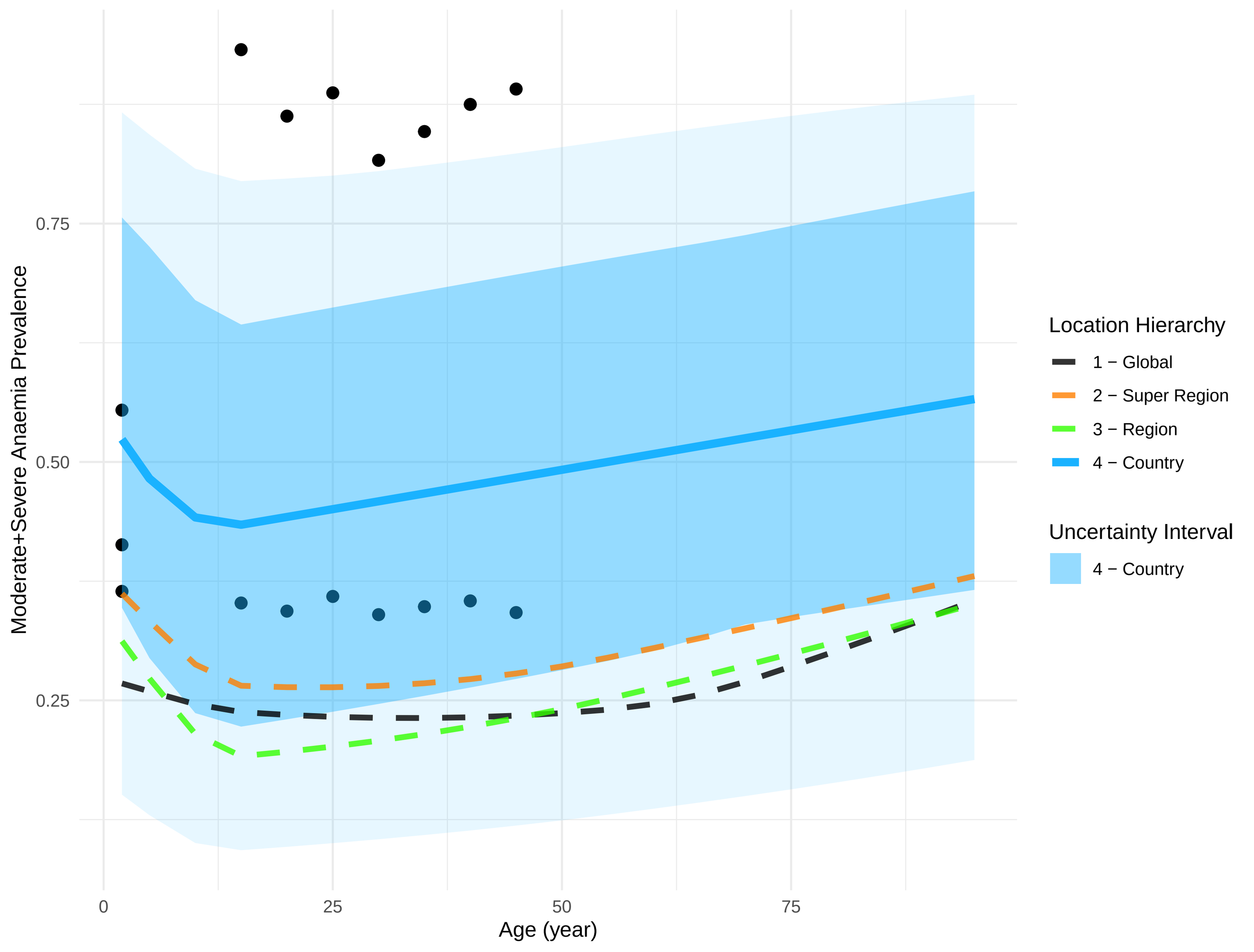

MR−BRT Age/Sex Fits − Non−pregnant Females 2+ Years Old − Moderate+Severe Anaemia Prevalence − Rwanda
WHO−2024 Adjustment Method

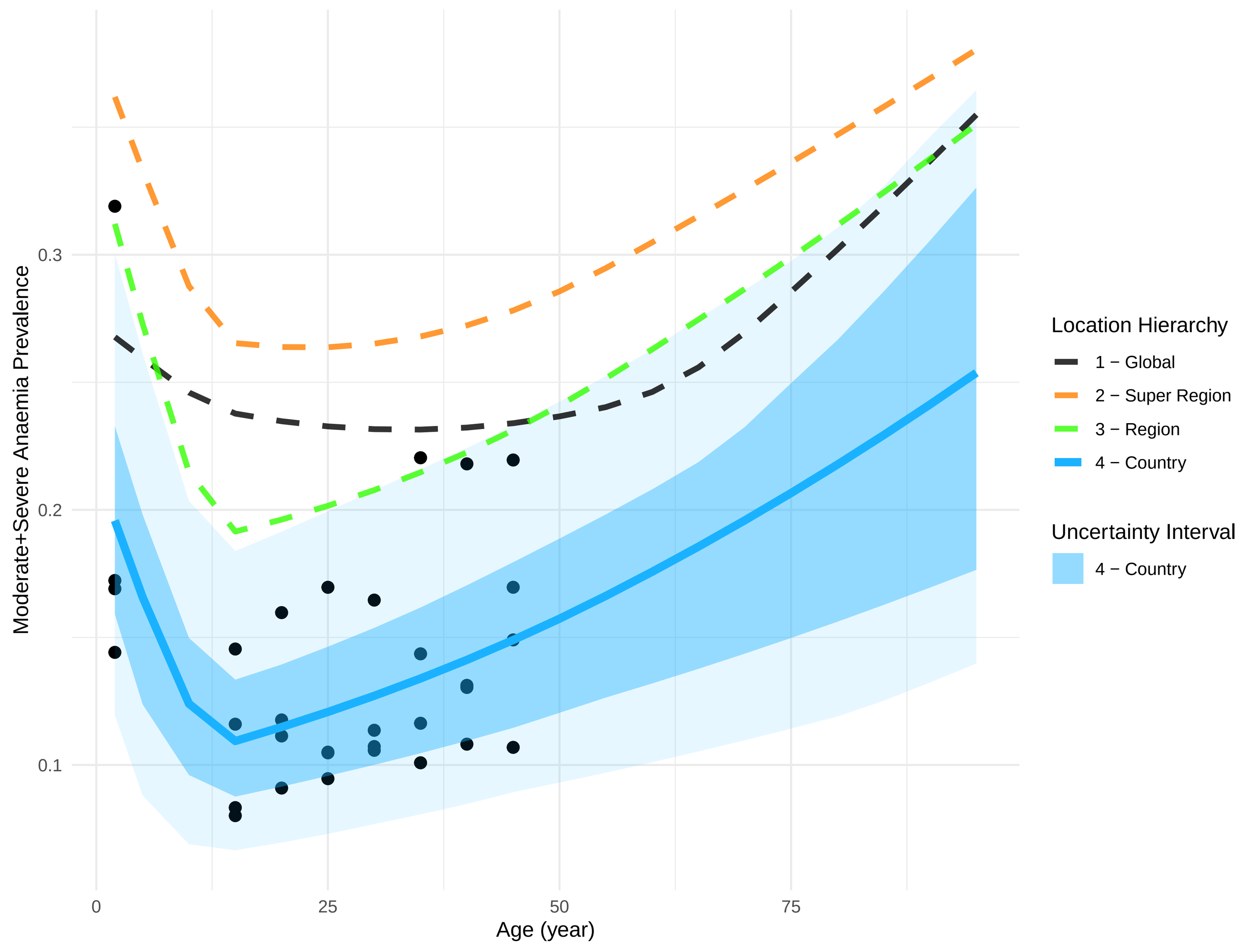

MR−BRT Age/Sex Fits − Non−pregnant Females 2+ Years Old − Moderate+Severe Anaemia Prevalence − Uganda
WHO−2024 Adjustment Method

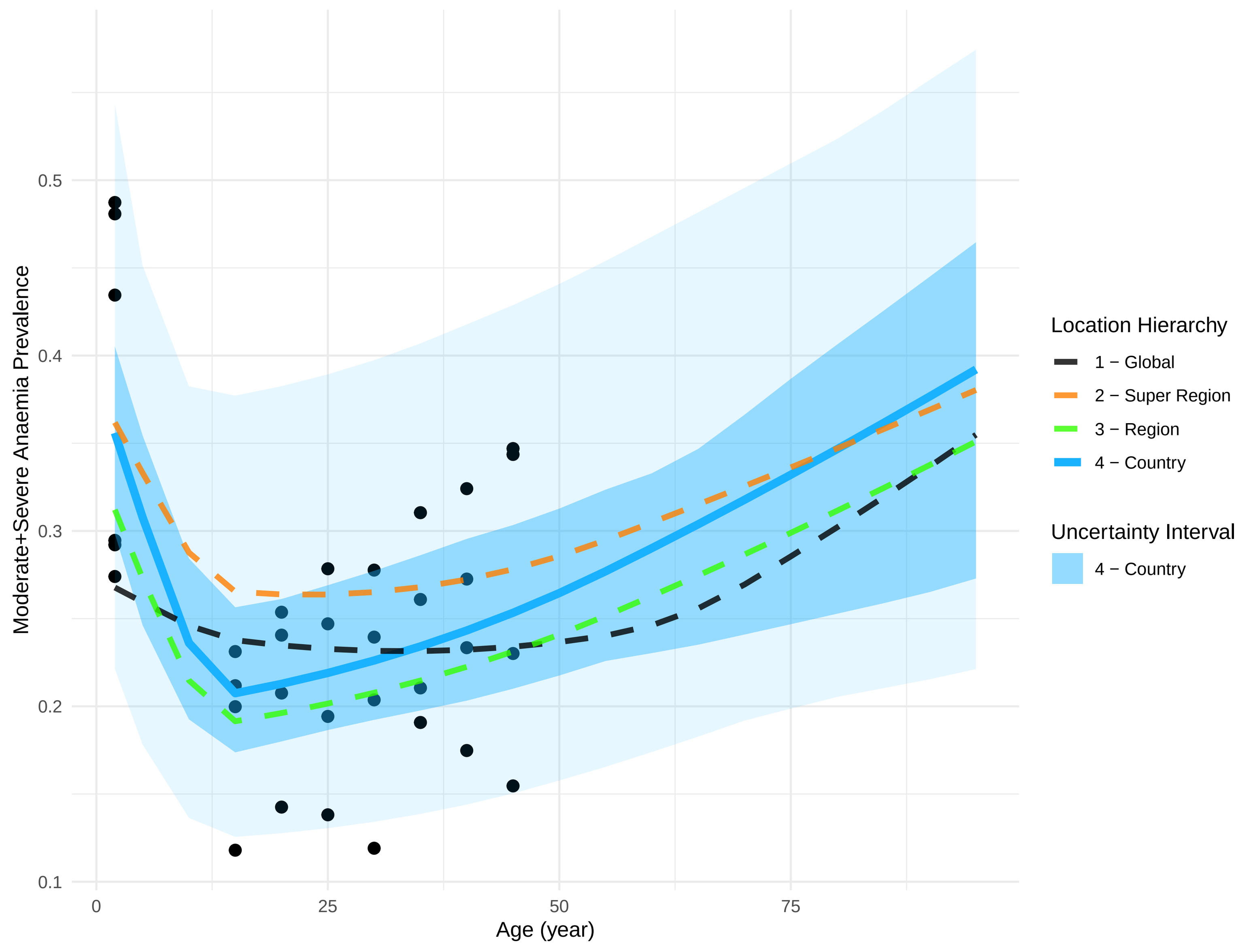

MR−BRT Age/Sex Fits − Non−pregnant Females 2+ Years Old − Moderate+Severe Anaemia Prevalence − United Republic of Tanzania
WHO−2024 Adjustment Method

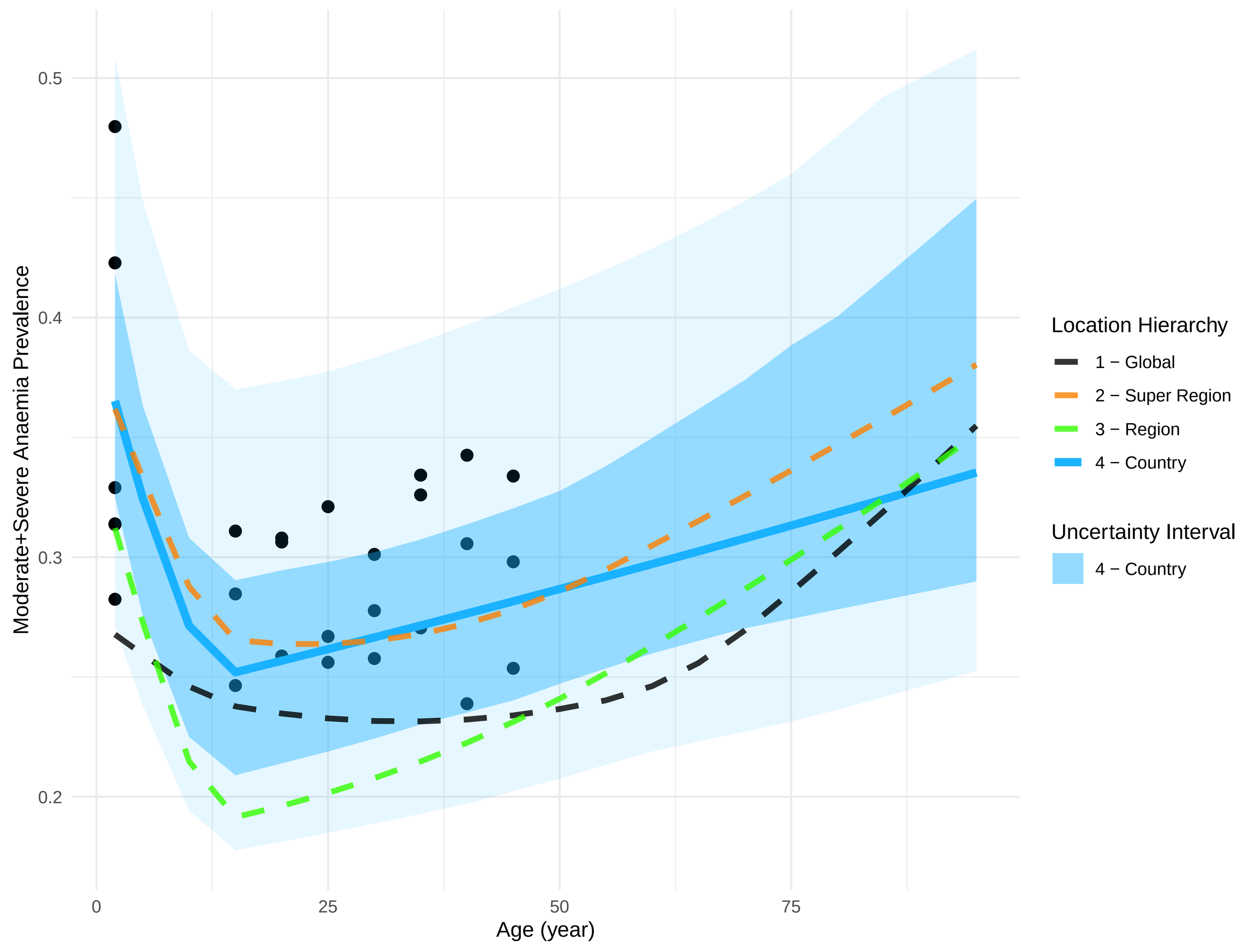

MR−BRT Age/Sex Fits − Non−pregnant Females 2+ Years Old − Moderate+Severe Anaemia Prevalence − Zambia
WHO−2024 Adjustment Method

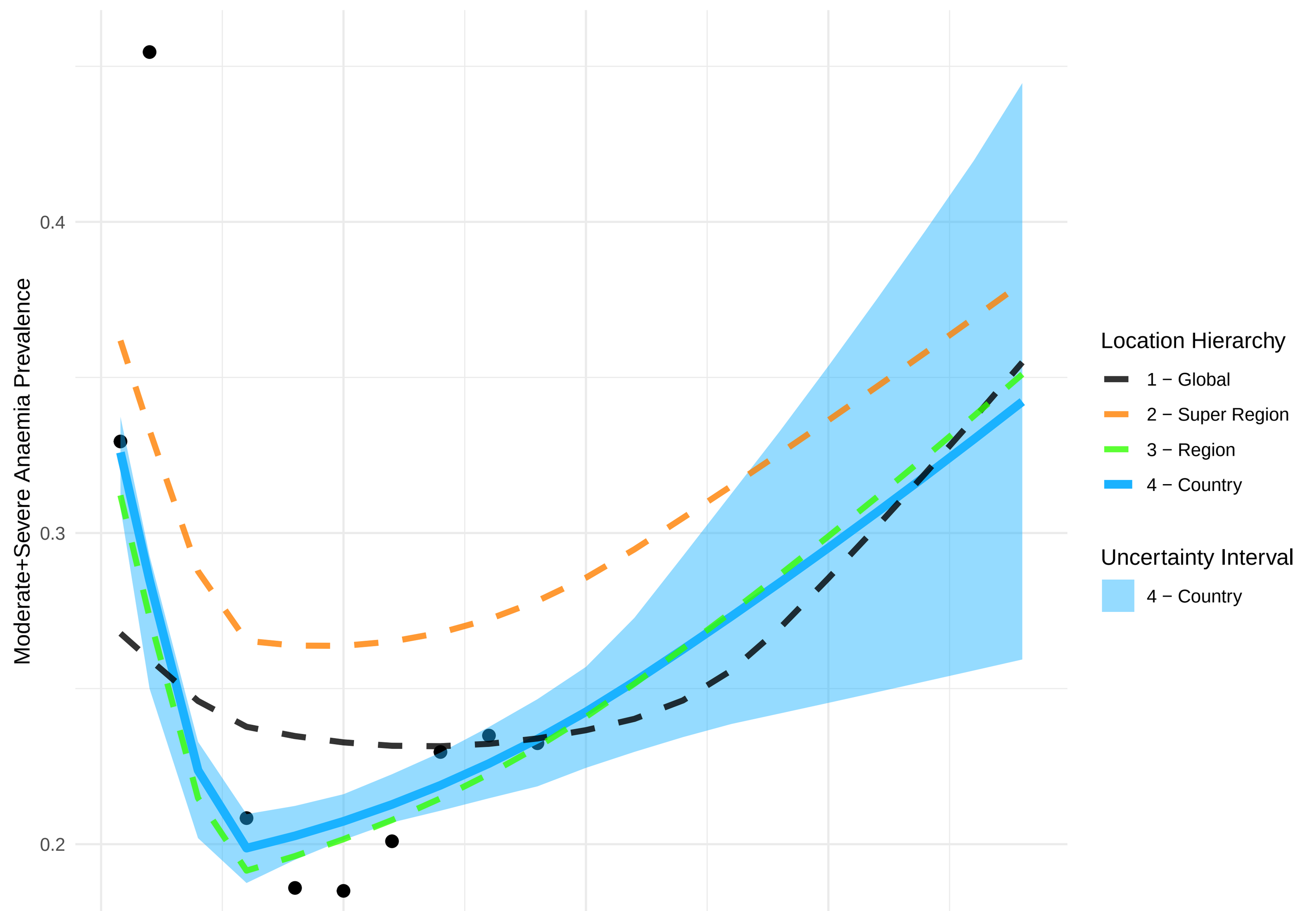

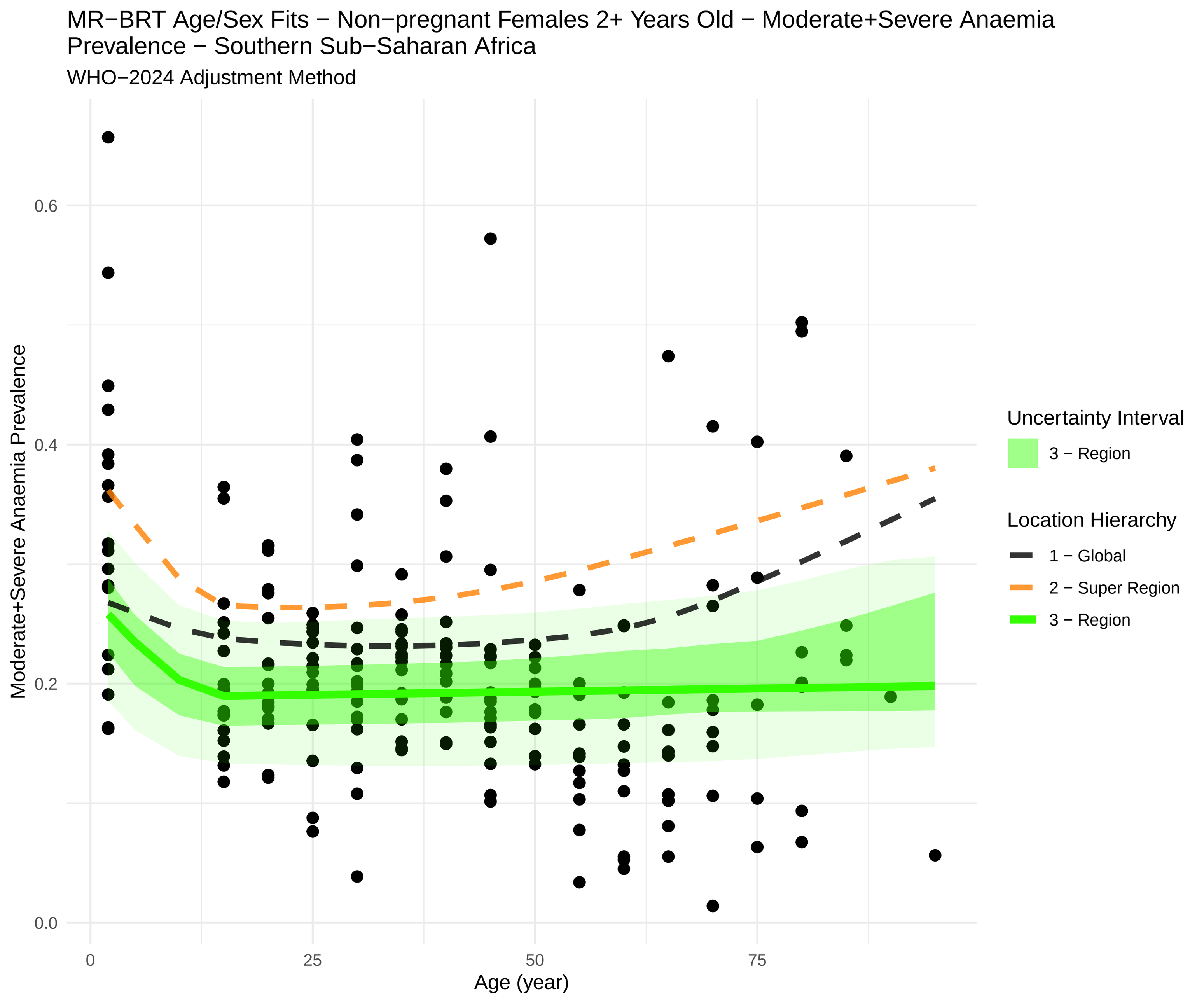
MR−BRT Age/Sex Fits − Non−pregnant Females 2+ Years Old − Moderate+Severe Anaemia Prevalence − Southern Sub−Saharan Africa
WHO−2024 Adjustment Method
Moderate+Severe Anaemia Prevalence
Age (year)
Uncertainty Interval
3 − Region
Location Hierarchy
1 − Global
2 − Super Region
3 − Region

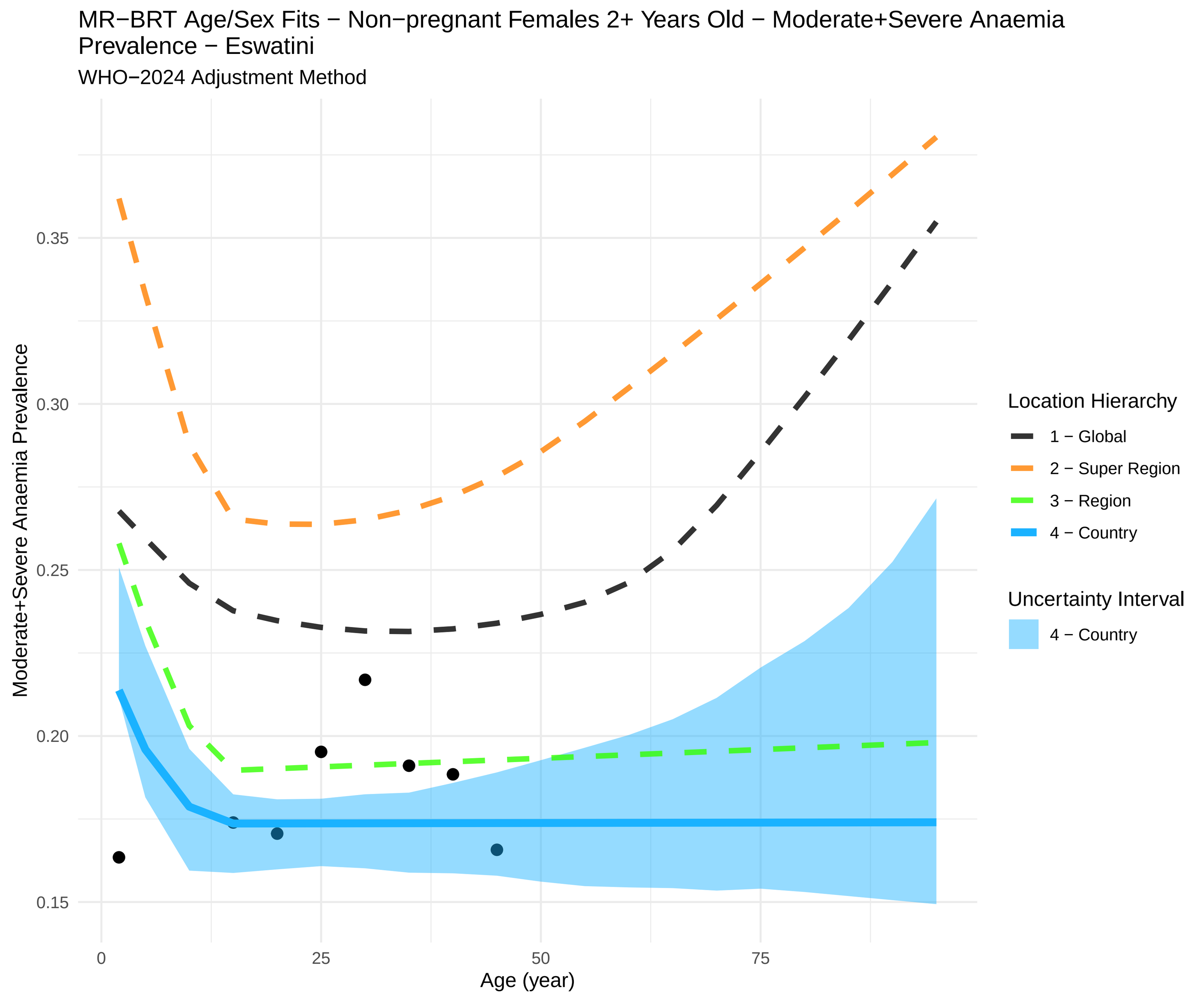
MR−BRT Age/Sex Fits − Non−pregnant Females 2+ Years Old − Moderate+Severe Anaemia Prevalence − Eswatini
WHO−2024 Adjustment Method
Moderate+Severe Anaemia Prevalence
Age (year)
Location Hierarchy
1 − Global
2 − Super Region
3 − Region
4 − Country
Uncertainty Interval
4 − Country

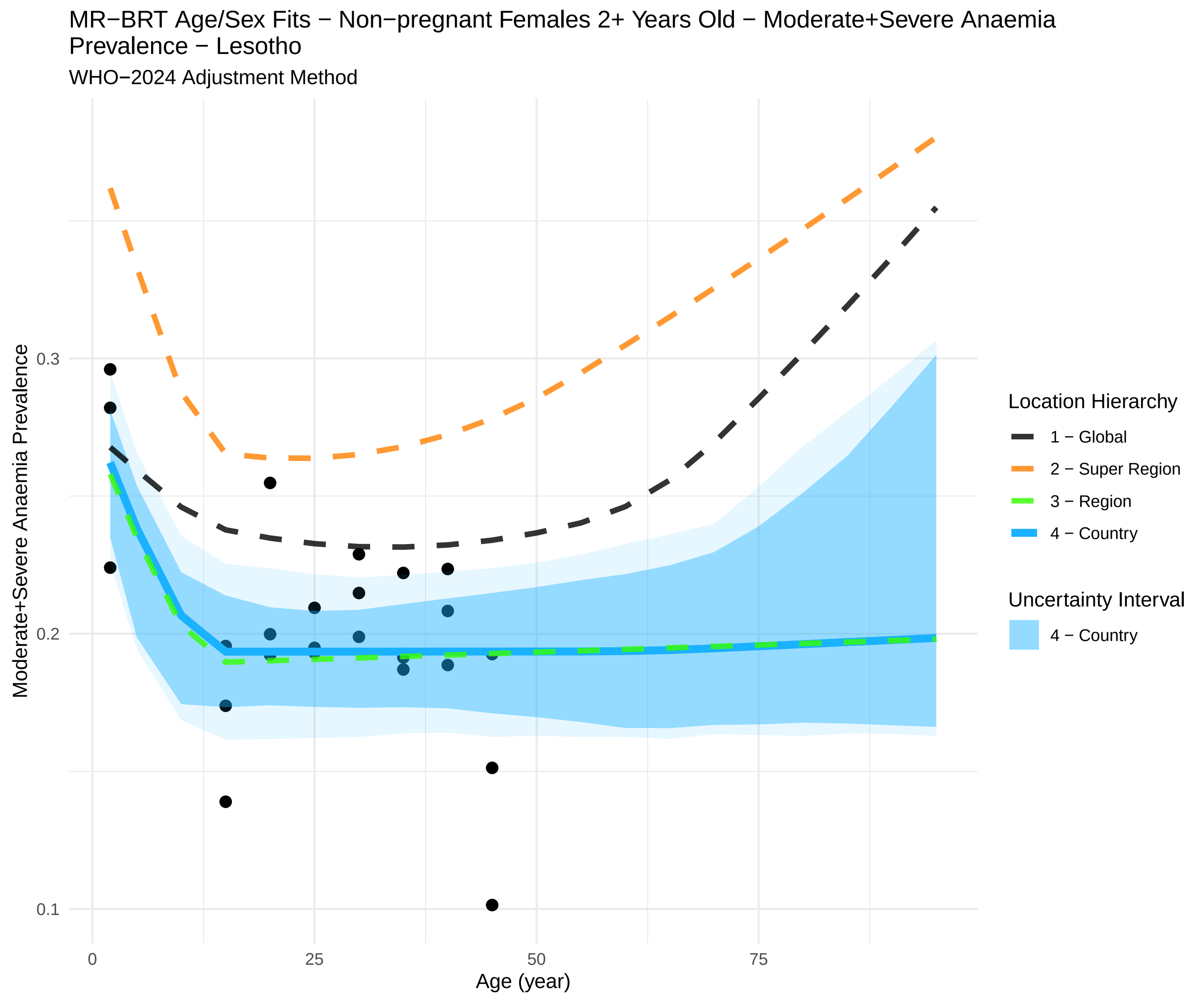
MR−BRT Age/Sex Fits − Non−pregnant Females 2+ Years Old − Moderate+Severe Anaemia Prevalence − Lesotho
WHO−2024 Adjustment Method
Moderate+Severe Anaemia Prevalence
Age (year)
Location Hierarchy
1 − Global
2 − Super Region
3 − Region
4 − Country
Uncertainty Interval
4 − Country

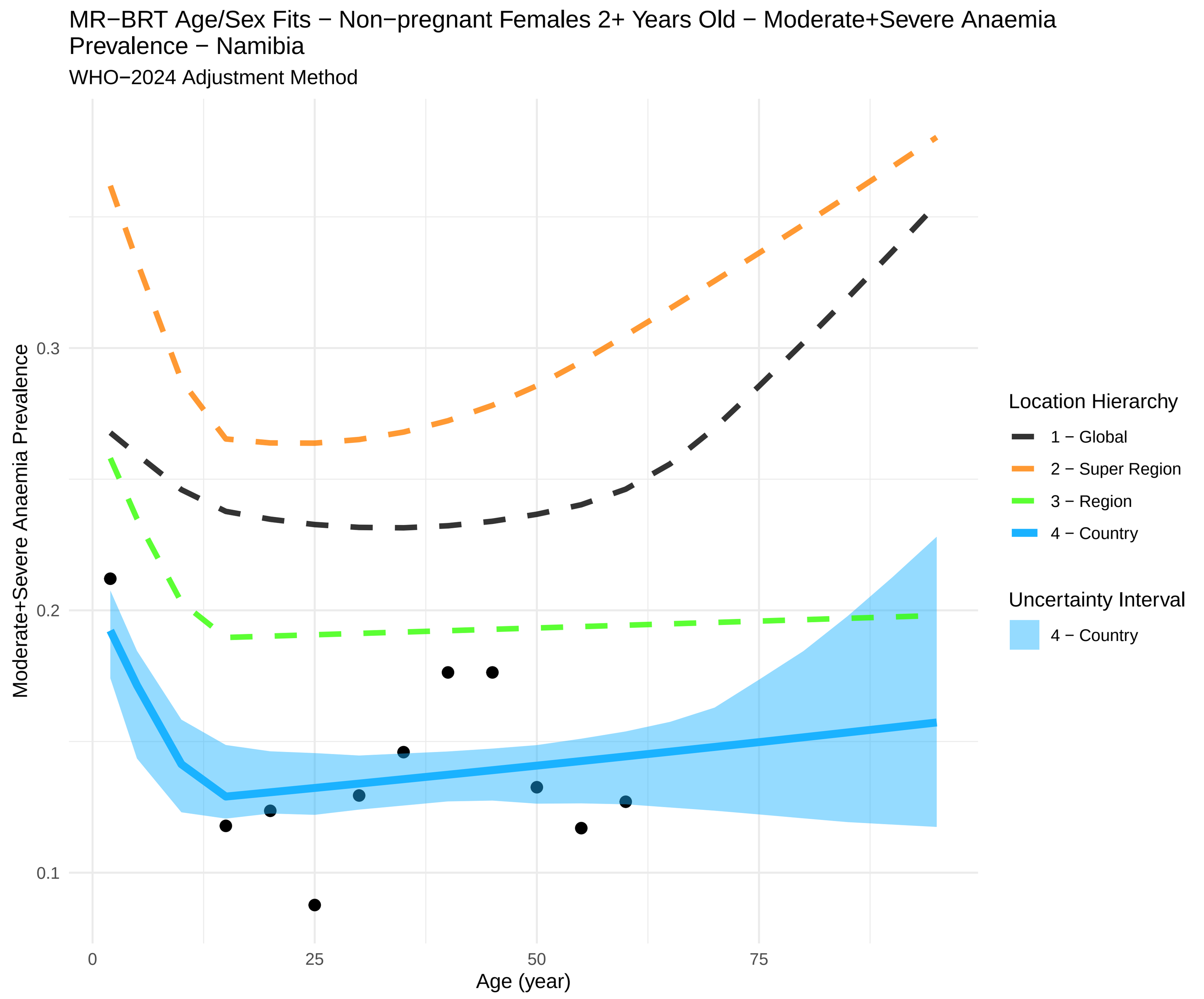
MR−BRT Age/Sex Fits − Non−pregnant Females 2+ Years Old − Moderate+Severe Anaemia Prevalence − Namibia
WHO−2024 Adjustment Method
Moderate+Severe Anaemia Prevalence
Age (year)
Location Hierarchy
1 − Global
2 − Super Region
3 − Region
4 − Country
Uncertainty Interval
4 − Country

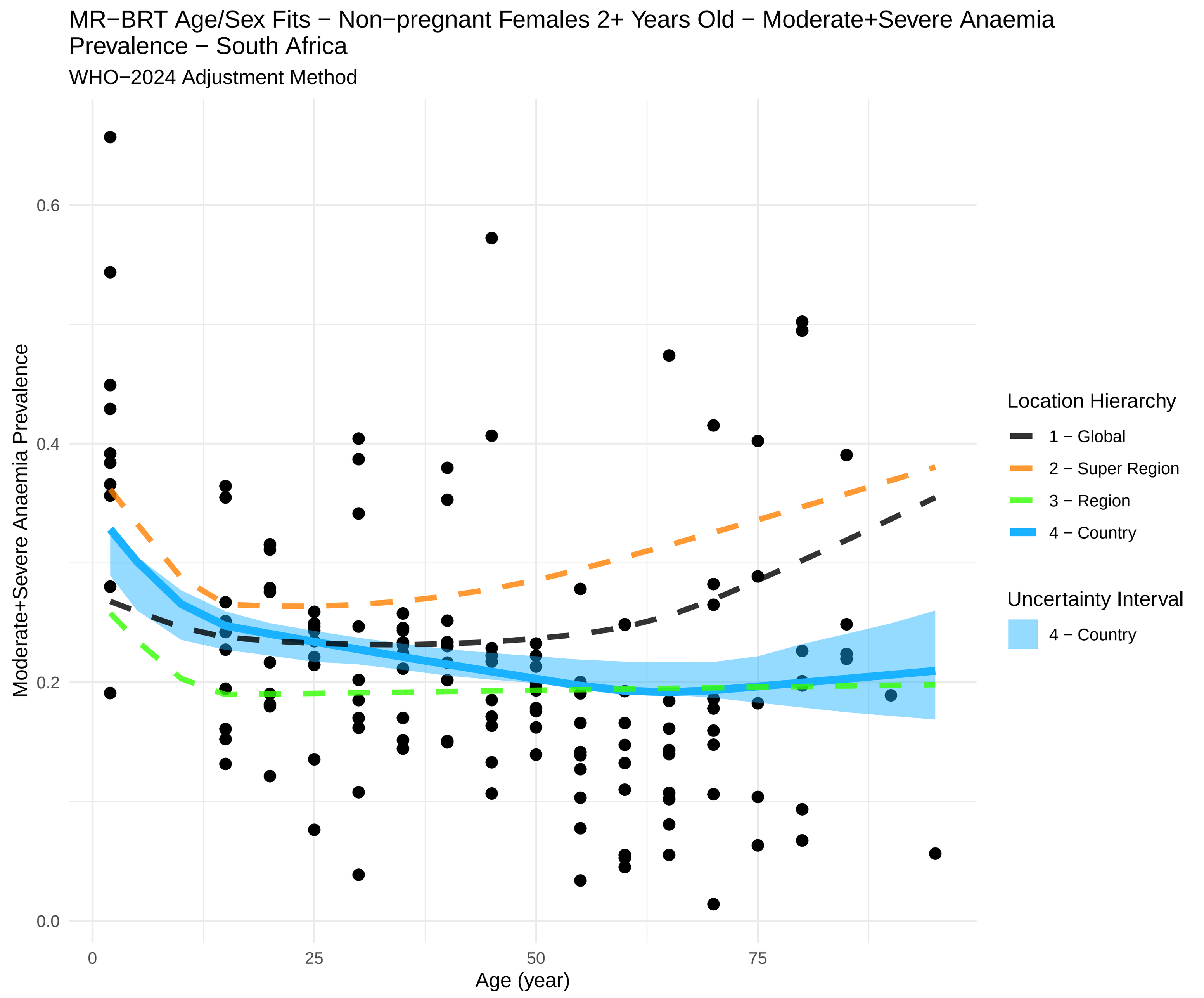
MR−BRT Age/Sex Fits − Non−pregnant Females 2+ Years Old − Moderate+Severe Anaemia Prevalence − South Africa
WHO−2024 Adjustment Method
Moderate+Severe Anaemia Prevalence
Age (year)
Location Hierarchy
1 − Global
2 − Super Region
3 − Region
4 − Country
Uncertainty Interval
4 − Country

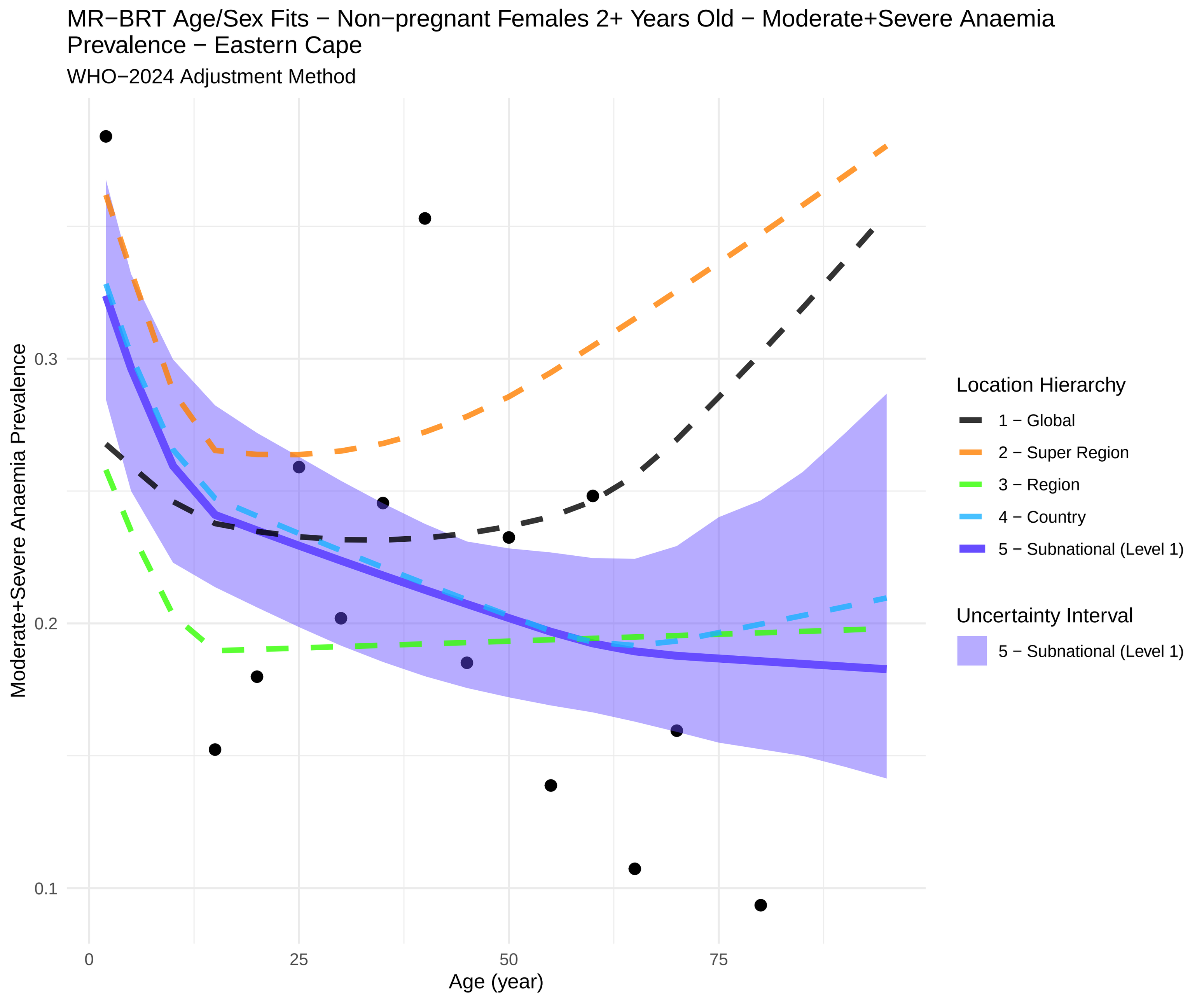
MR−BRT Age/Sex Fits − Non−pregnant Females 2+ Years Old − Moderate+Severe Anaemia Prevalence − Eastern Cape
WHO−2024 Adjustment Method
Moderate+Severe Anaemia Prevalence
Age (year)
Location Hierarchy
1 − Global
2 − Super Region
3 − Region
4 − Country
5 − Subnational (Level 1)
Uncertainty Interval
5 − Subnational (Level 1)

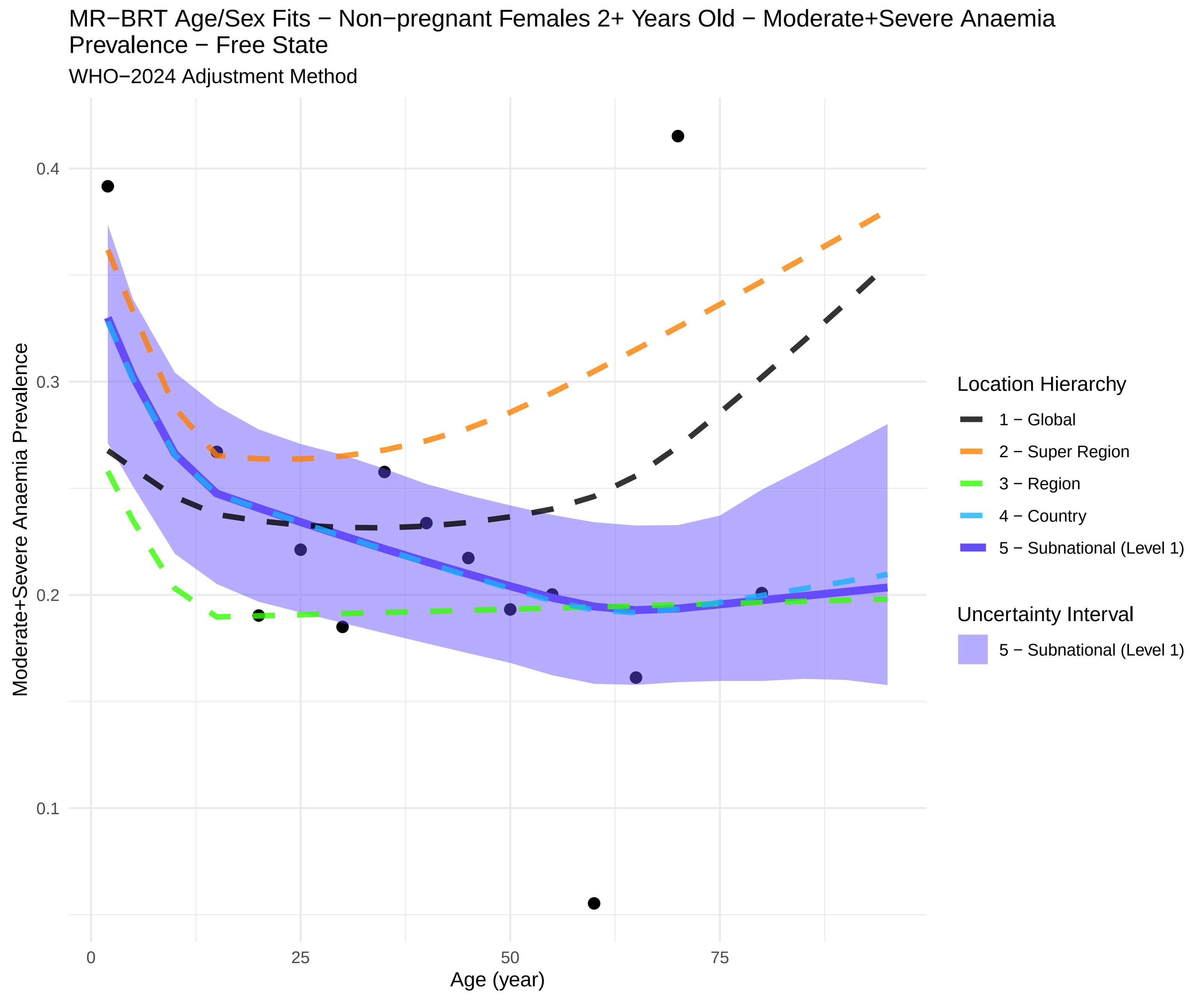
MR−BRT Age/Sex Fits − Non−pregnant Females 2+ Years Old − Moderate+Severe Anaemia Prevalence − Free State
WHO−2024 Adjustment Method
Moderate+Severe Anaemia Prevalence
Age (year)
Location Hierarchy
1 − Global
2 − Super Region
3 − Region
4 − Country
5 − Subnational (Level 1)
Uncertainty Interval
5 − Subnational (Level 1)

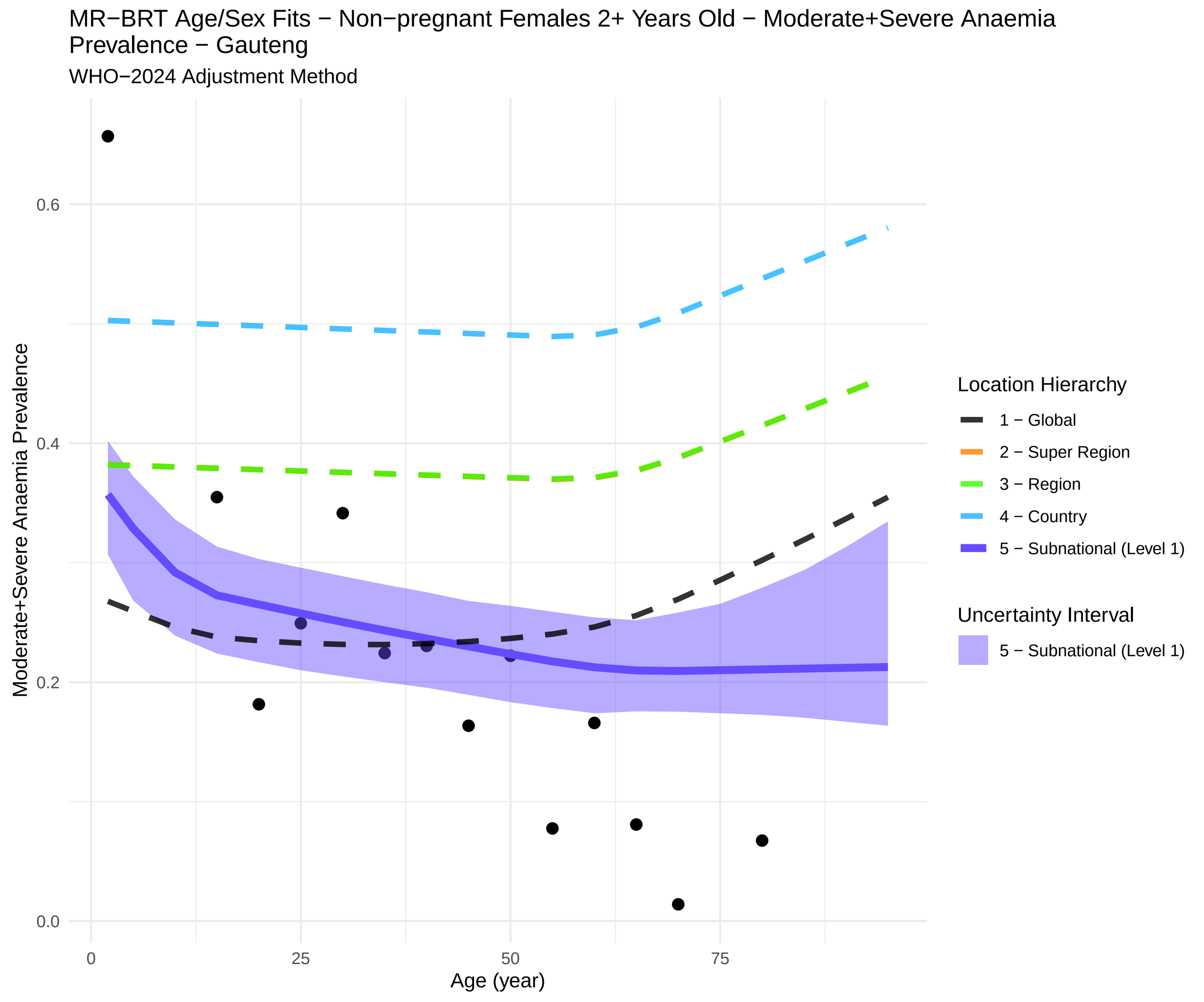
MR−BRT Age/Sex Fits − Non−pregnant Females 2+ Years Old − Moderate+Severe Anaemia Prevalence − Gauteng
WHO−2024 Adjustment Method
Moderate+Severe Anaemia Prevalence
Age (year)
Location Hierarchy
1 − Global
2 − Super Region
3 − Region
4 − Country
5 − Subnational (Level 1)
Uncertainty Interval
5 − Subnational (Level 1)

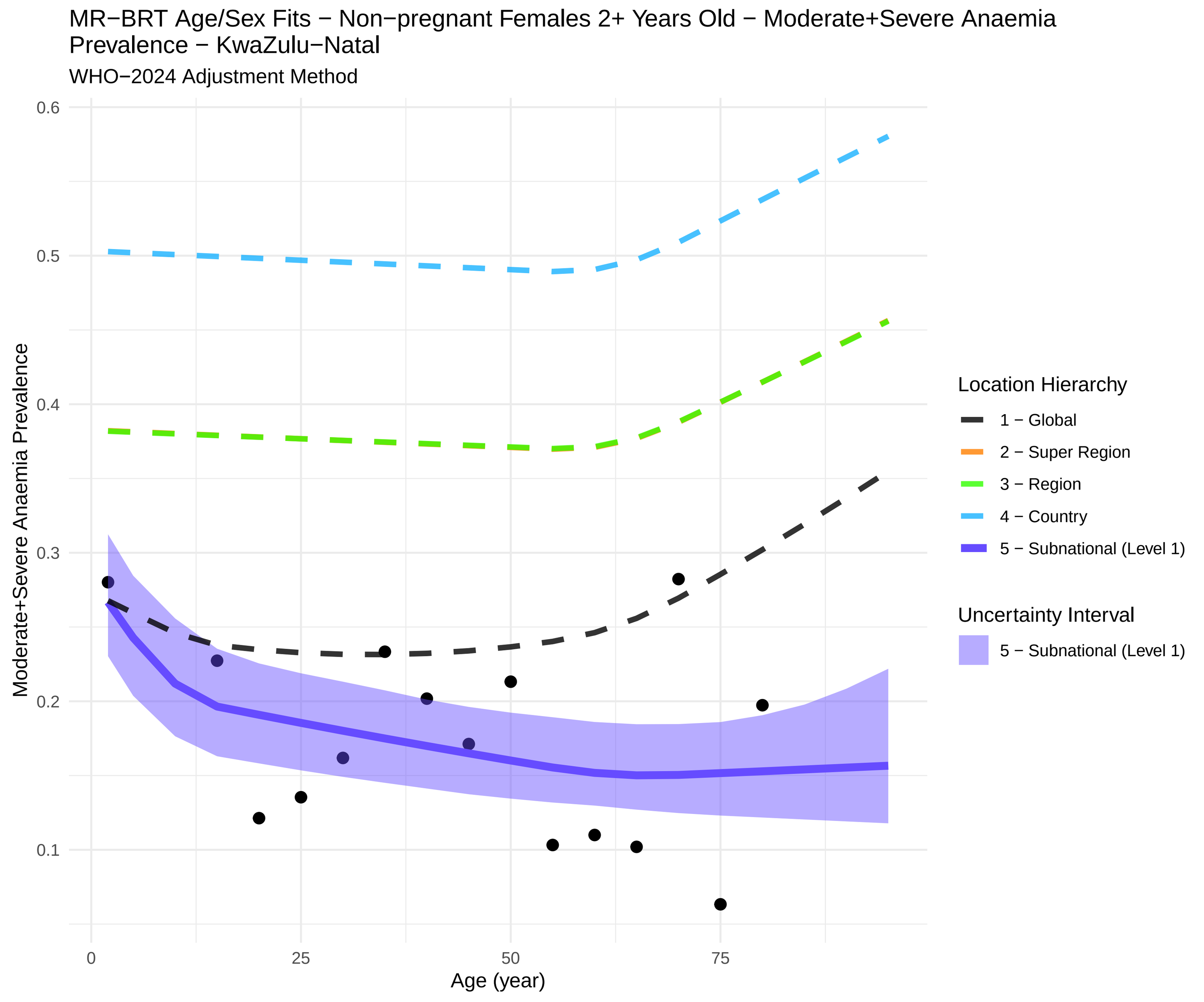
MR−BRT Age/Sex Fits − Non−pregnant Females 2+ Years Old − Moderate+Severe Anaemia Prevalence − KwaZulu−Natal
WHO−2024 Adjustment Method
Moderate+Severe Anaemia Prevalence
Age (year)
Location Hierarchy
1 − Global
2 − Super Region
3 − Region
4 − Country
5 − Subnational (Level 1)
Uncertainty Interval
5 − Subnational (Level 1)

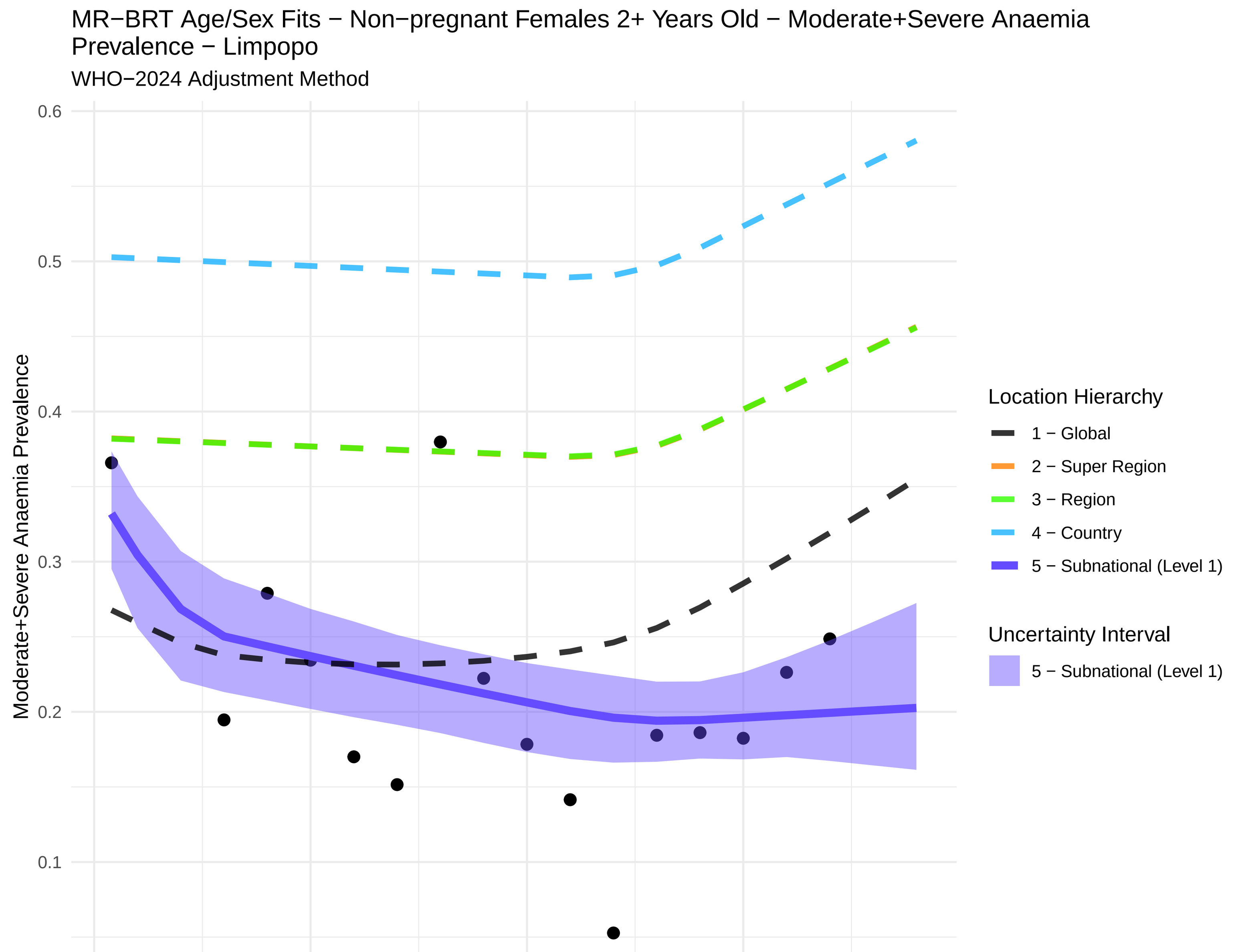
MR−BRT Age/Sex Fits − Non−pregnant Females 2+ Years Old − Moderate+Severe Anaemia Prevalence − Limpopo
WHO−2024 Adjustment Method
Moderate+Severe Anaemia Prevalence
Age (year)
Location Hierarchy
1 − Global
2 − Super Region
3 − Region
4 − Country
5 − Subnational (Level 1)
Uncertainty Interval
5 − Subnational (Level 1)

MR−BRT Age/Sex Fits − Non−pregnant Females 2+ Years Old − Moderate+Severe Anaemia Prevalence − Mpumalanga
WHO−2024 Adjustment Method

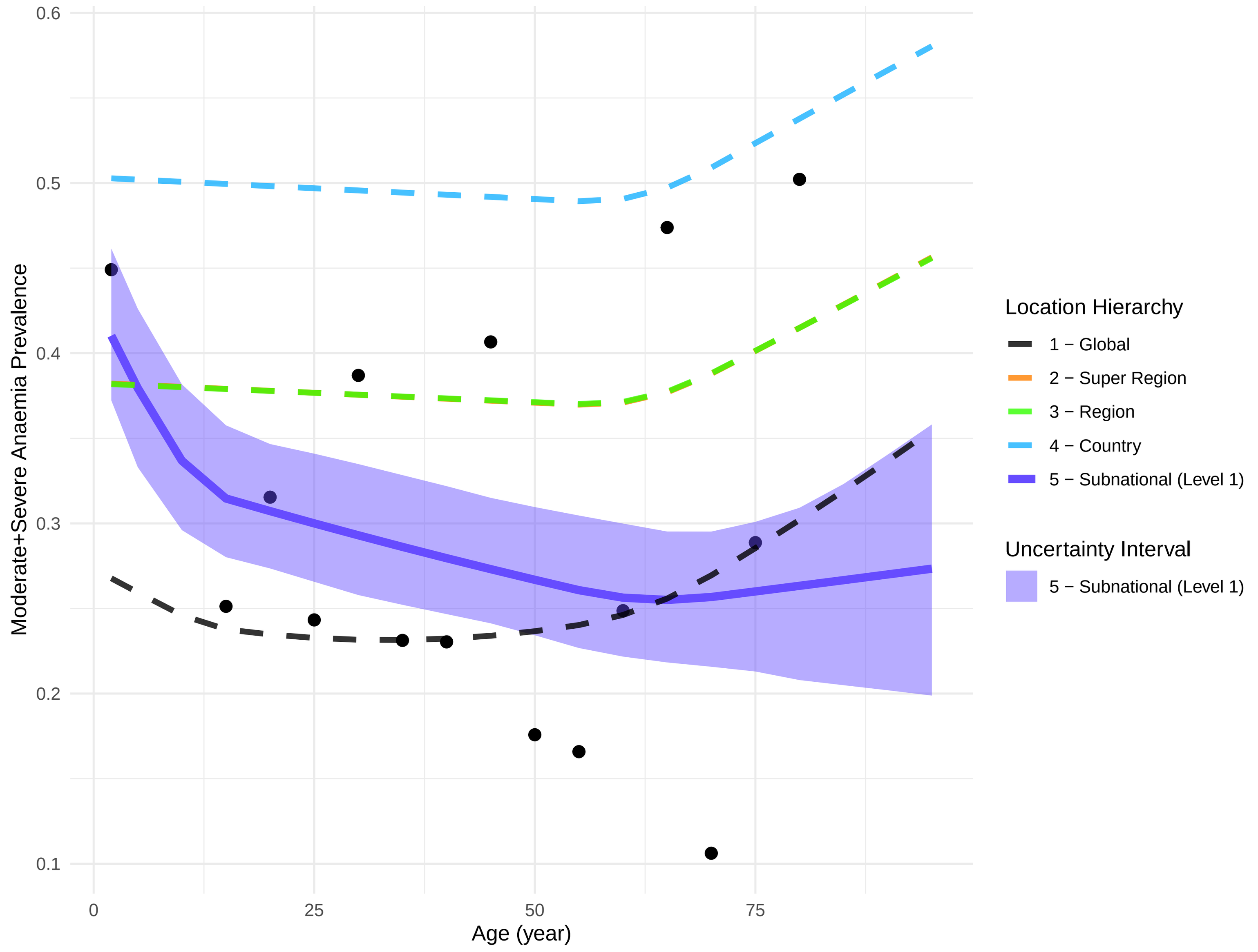

MR−BRT Age/Sex Fits − Non−pregnant Females 2+ Years Old − Moderate+Severe Anaemia Prevalence − North−West
WHO−2024 Adjustment Method

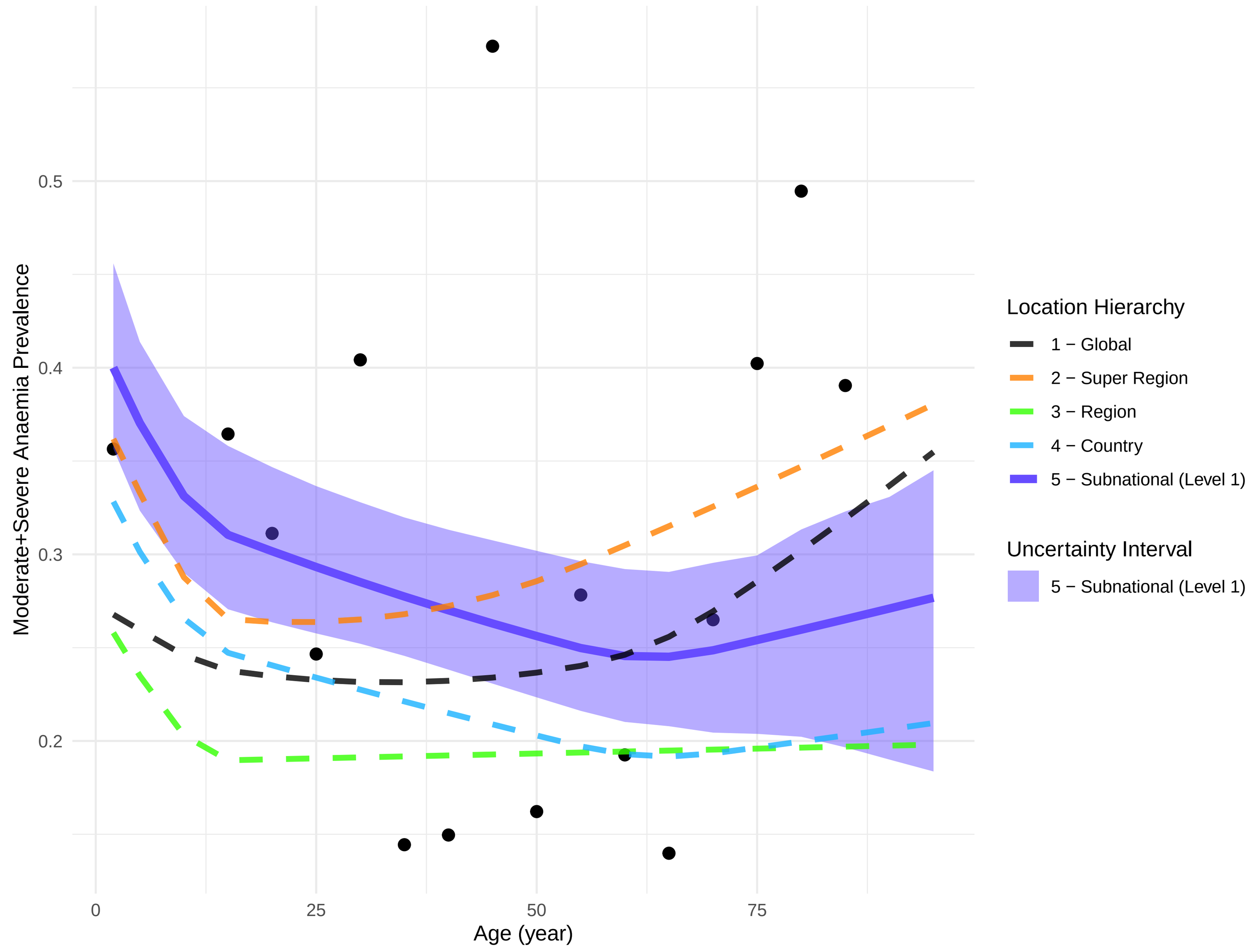

MR−BRT Age/Sex Fits − Non−pregnant Females 2+ Years Old − Moderate+Severe Anaemia Prevalence − Northern Cape
WHO−2024 Adjustment Method

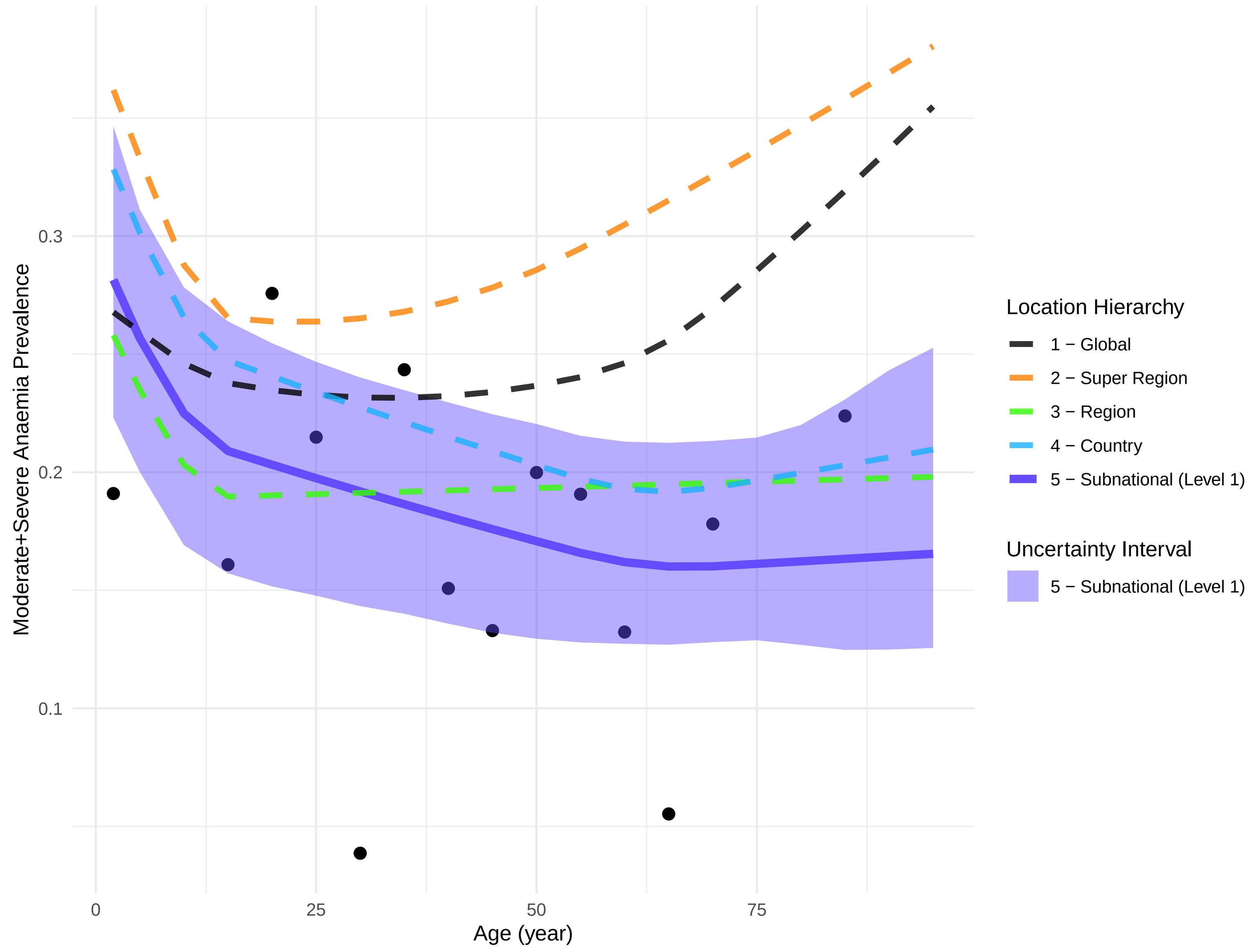

MR−BRT Age/Sex Fits − Non−pregnant Females 2+ Years Old − Moderate+Severe Anaemia Prevalence − Western Cape
WHO−2024 Adjustment Method

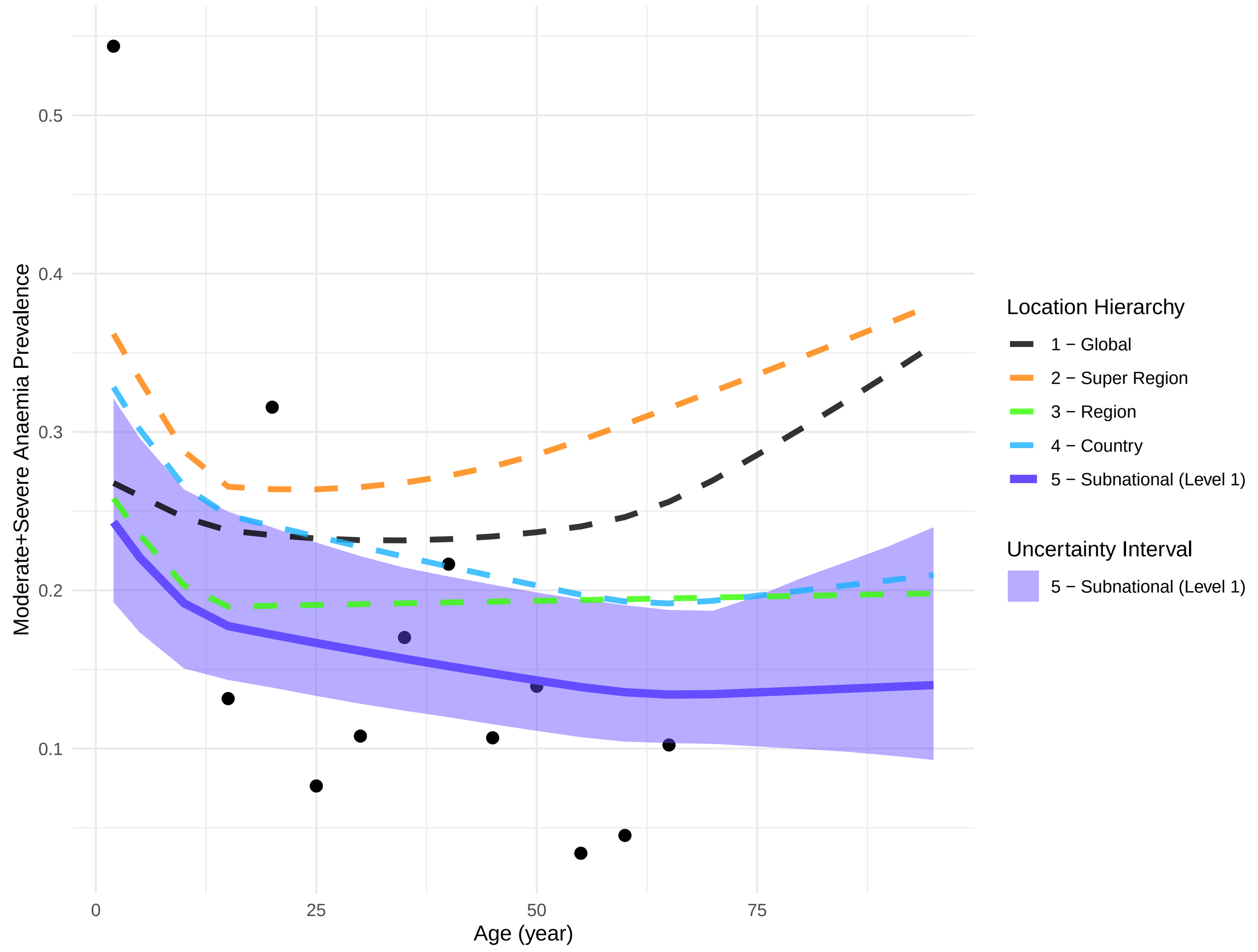

MR−BRT Age/Sex Fits − Non−pregnant Females 2+ Years Old − Moderate+Severe Anaemia Prevalence − Zimbabwe
WHO−2024 Adjustment Method

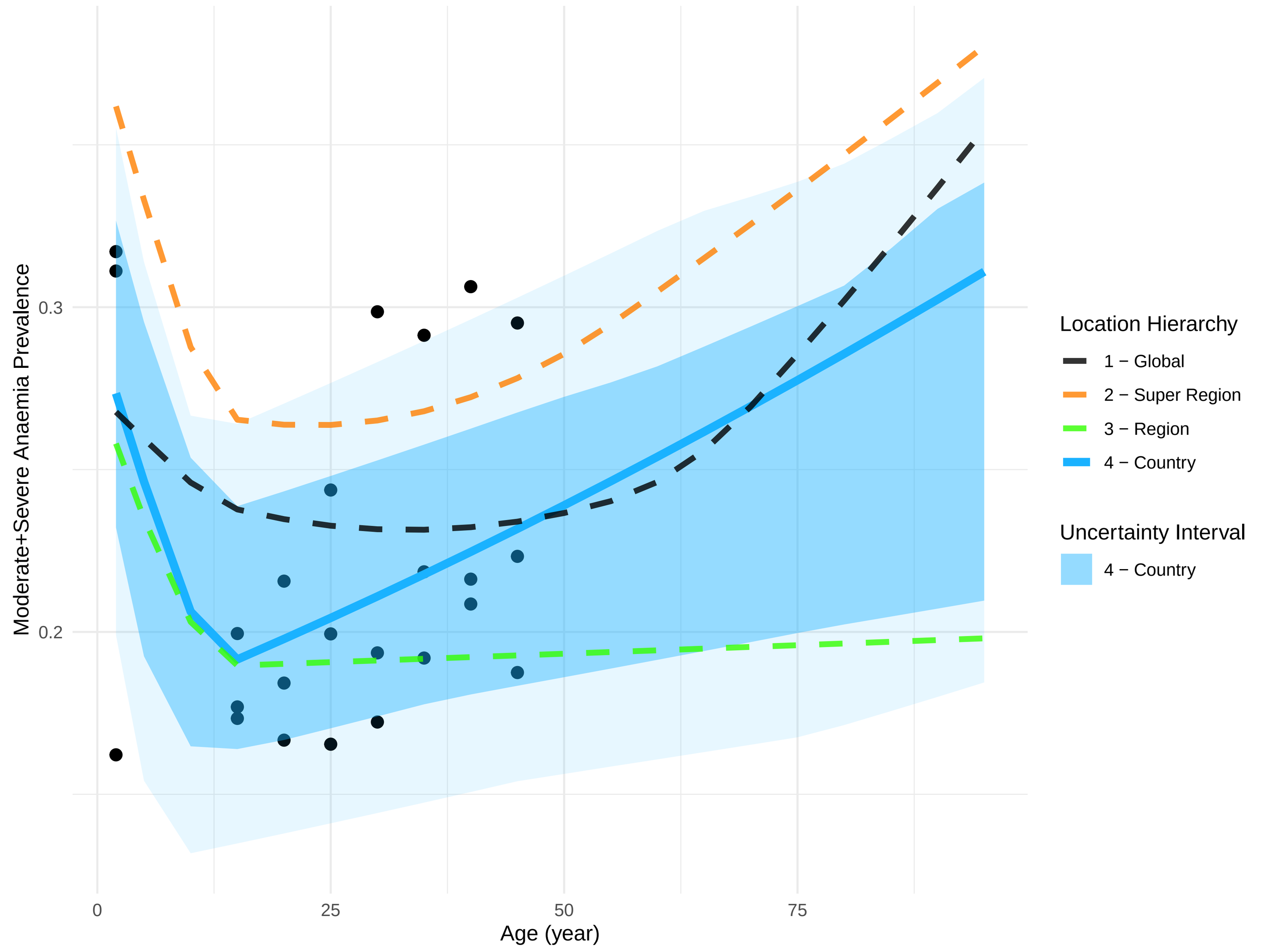

MR−BRT Age/Sex Fits − Non−pregnant Females 2+ Years Old − Moderate+Severe Anaemia Prevalence − Western Sub−Saharan Africa
WHO−2024 Adjustment Method

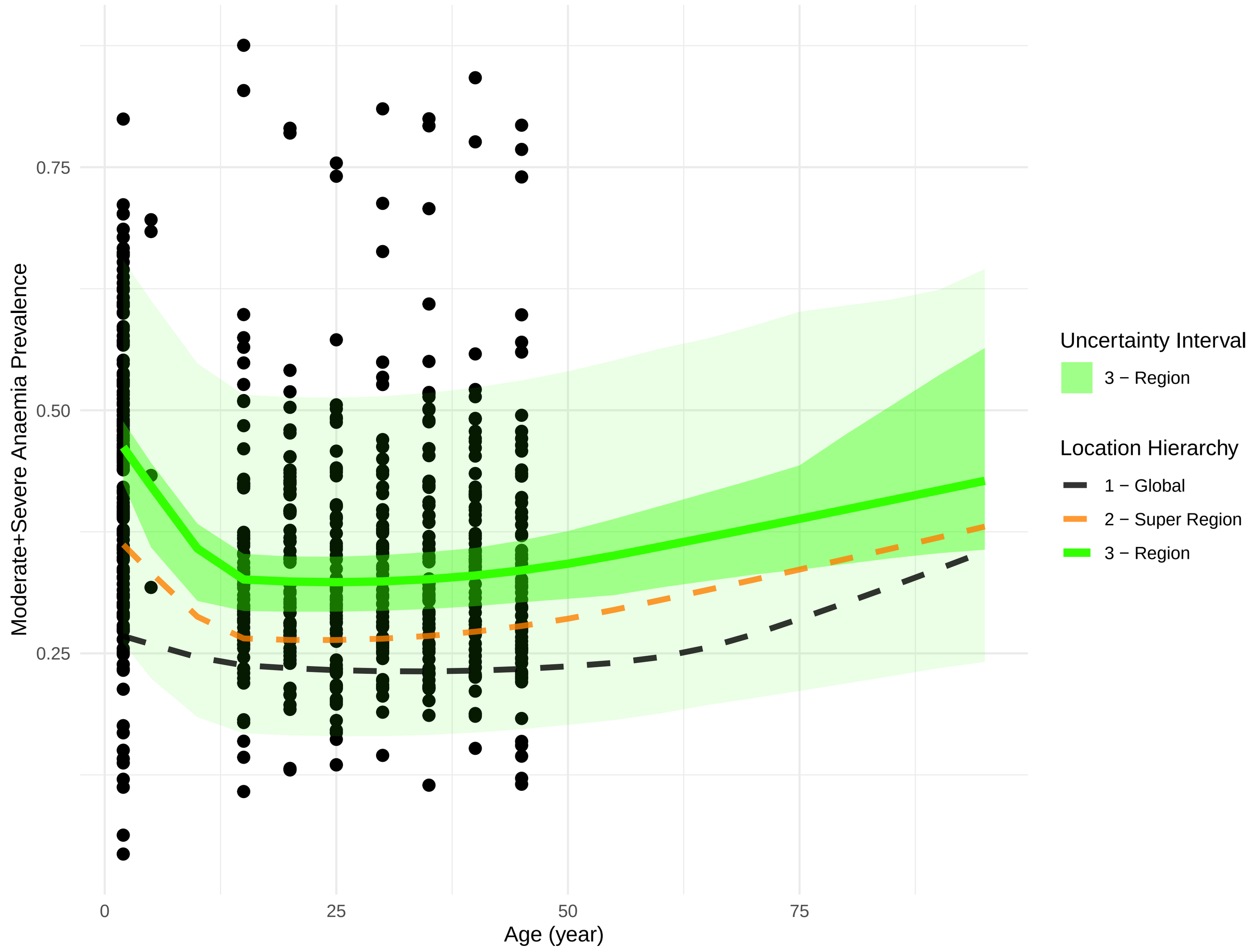

MR−BRT Age/Sex Fits − Non−pregnant Females 2+ Years Old − Moderate+Severe Anaemia Prevalence − Benin
WHO−2024 Adjustment Method

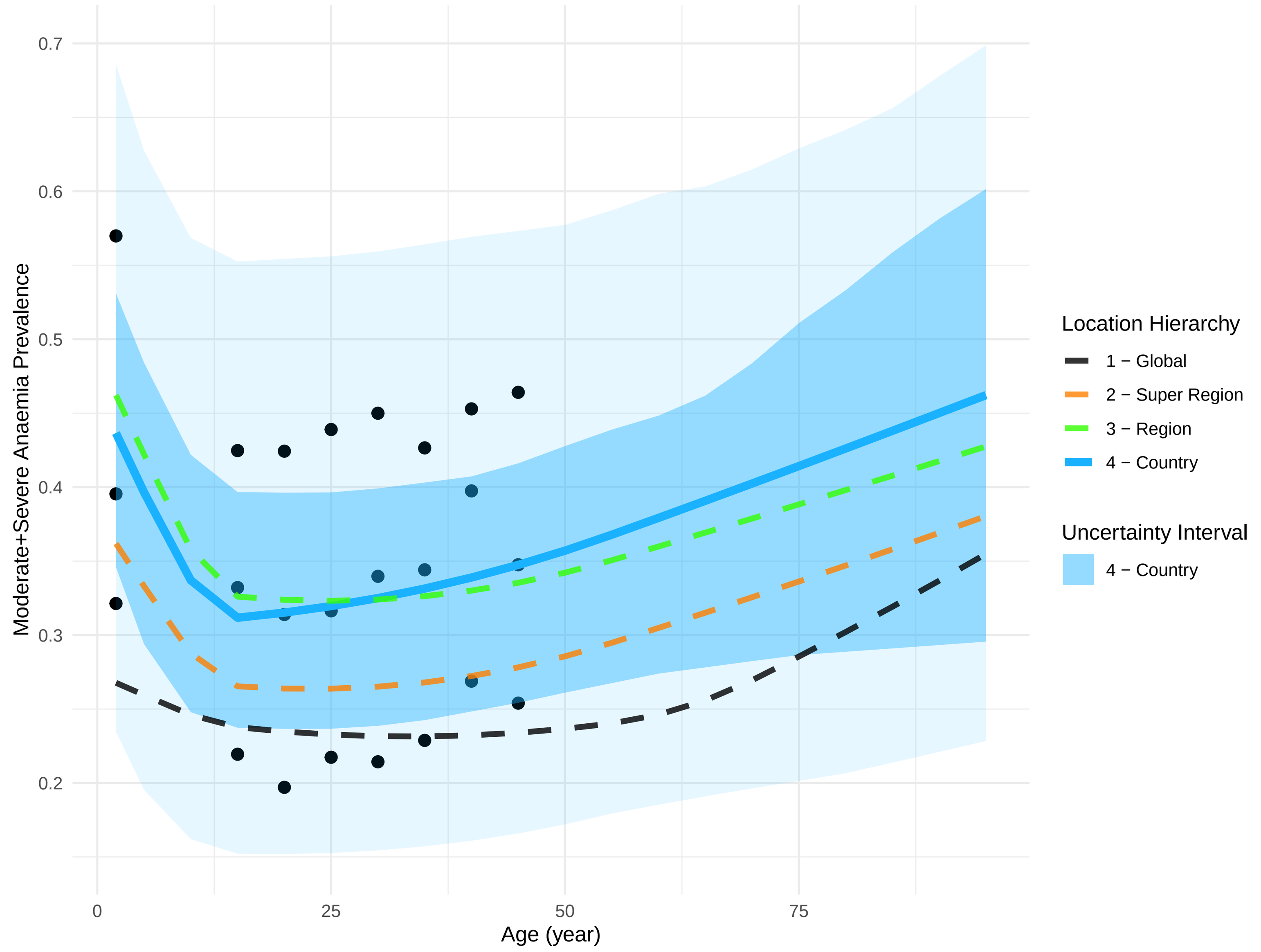

MR−BRT Age/Sex Fits − Non−pregnant Females 2+ Years Old − Moderate+Severe Anaemia Prevalence − Burkina Faso
WHO−2024 Adjustment Method

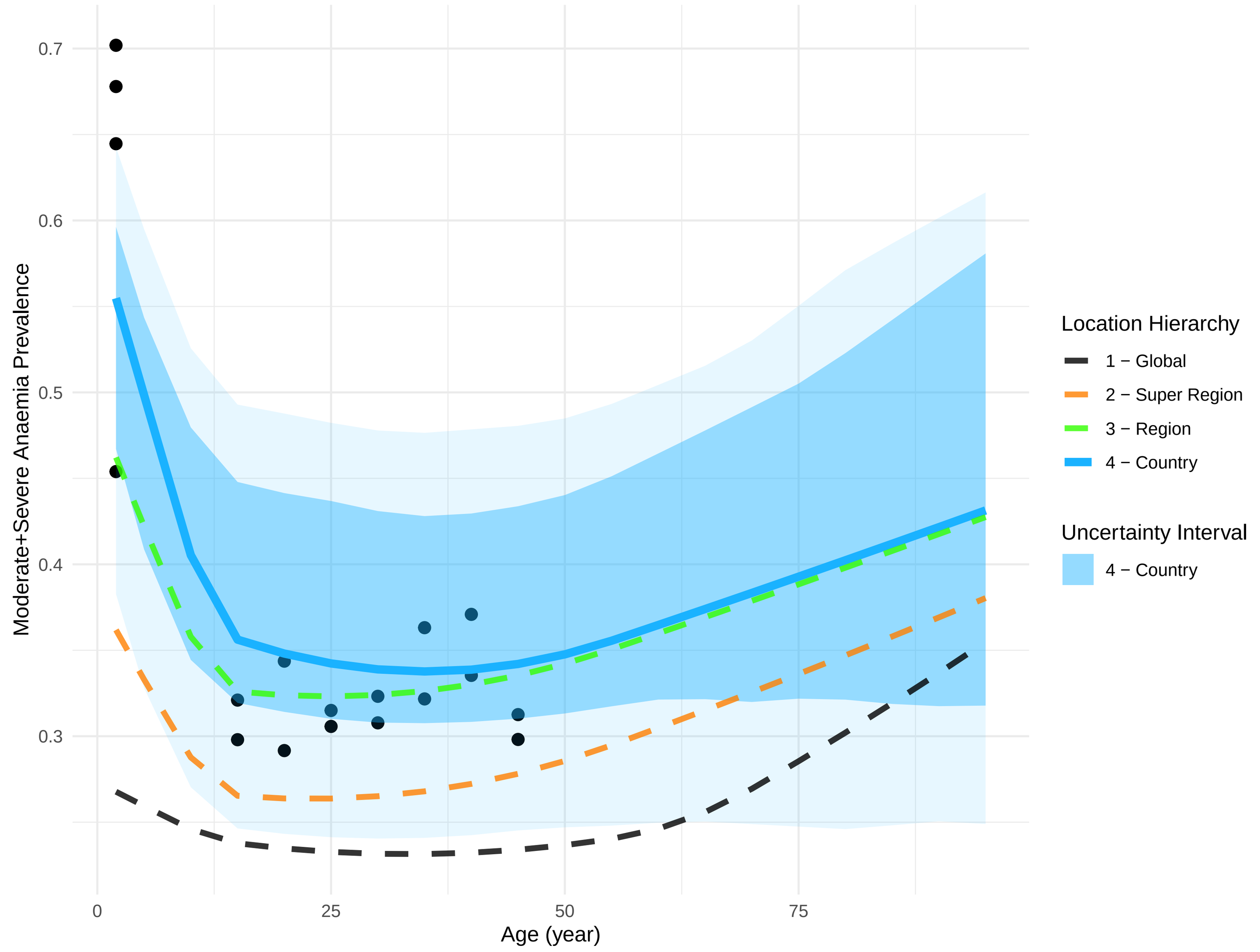

MR−BRT Age/Sex Fits − Non−pregnant Females 2+ Years Old − Moderate+Severe Anaemia Prevalence − Cameroon
WHO−2024 Adjustment Method

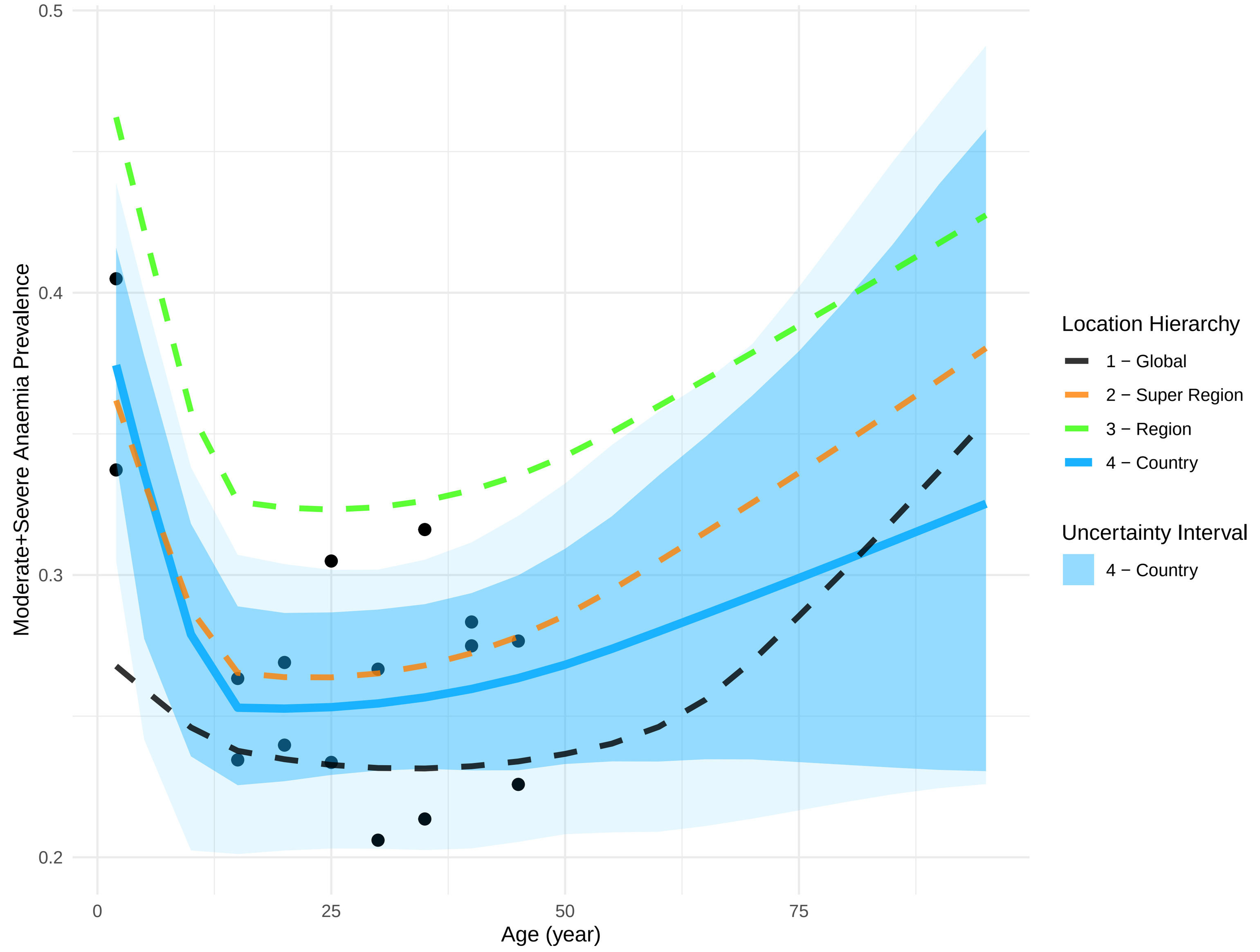

MR−BRT Age/Sex Fits − Non−pregnant Females 2+ Years Old − Moderate+Severe Anaemia Prevalence − Côte d'Ivoire
WHO−2024 Adjustment Method

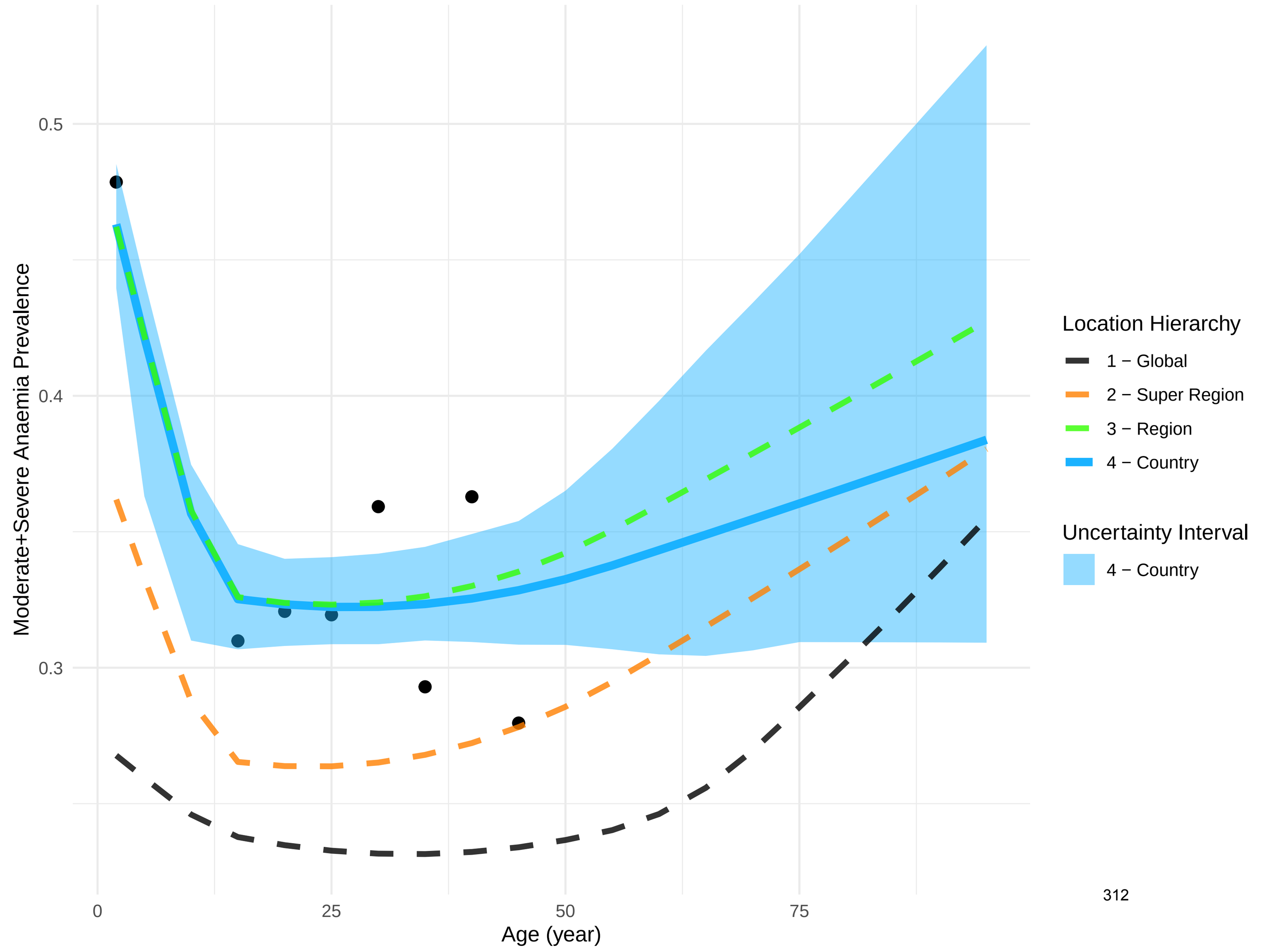

MR−BRT Age/Sex Fits − Non−pregnant Females 2+ Years Old − Moderate+Severe Anaemia Prevalence − Gambia
WHO−2024 Adjustment Method

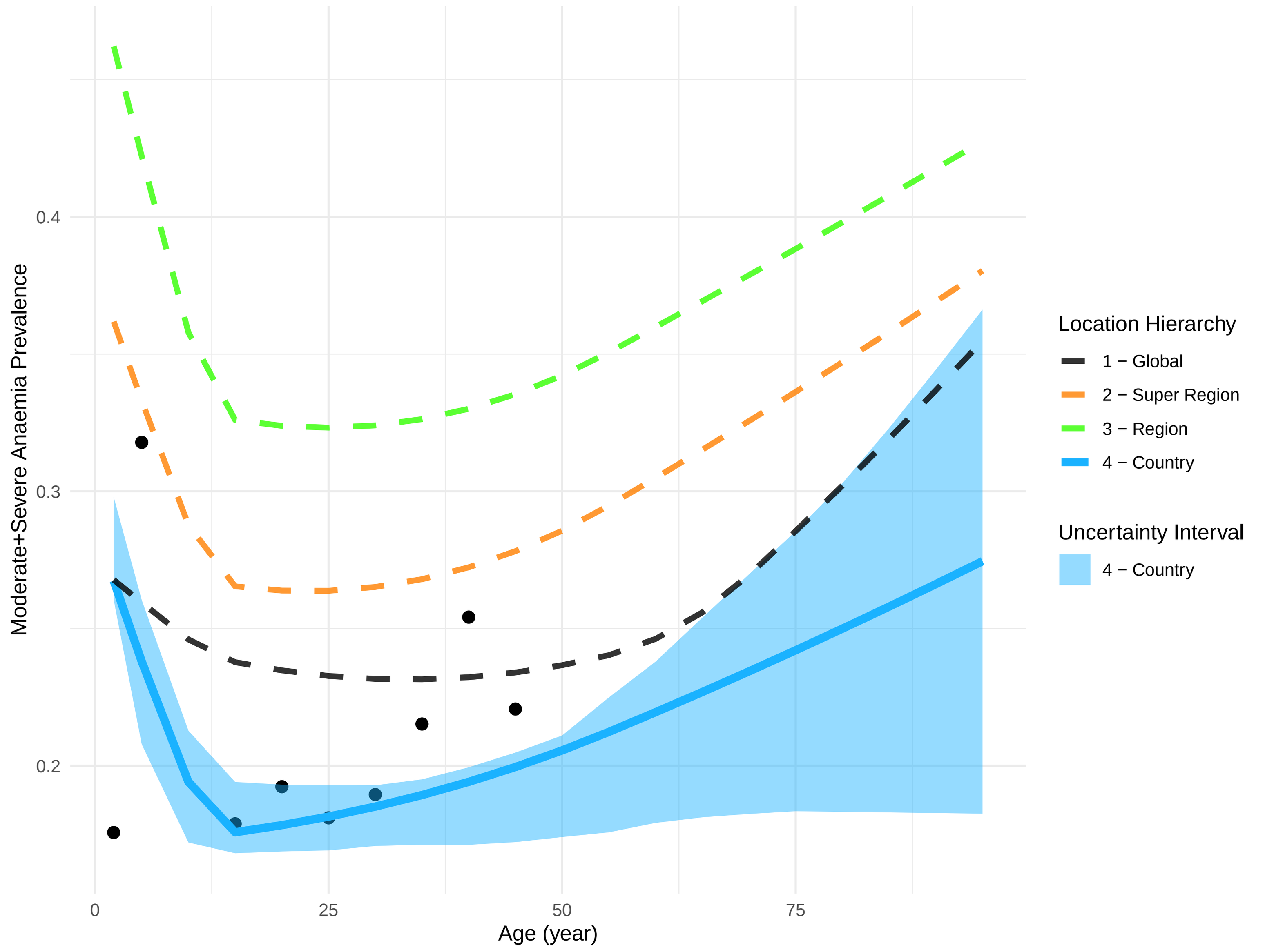

MR−BRT Age/Sex Fits − Non−pregnant Females 2+ Years Old − Moderate+Severe Anaemia Prevalence − Ghana
WHO−2024 Adjustment Method

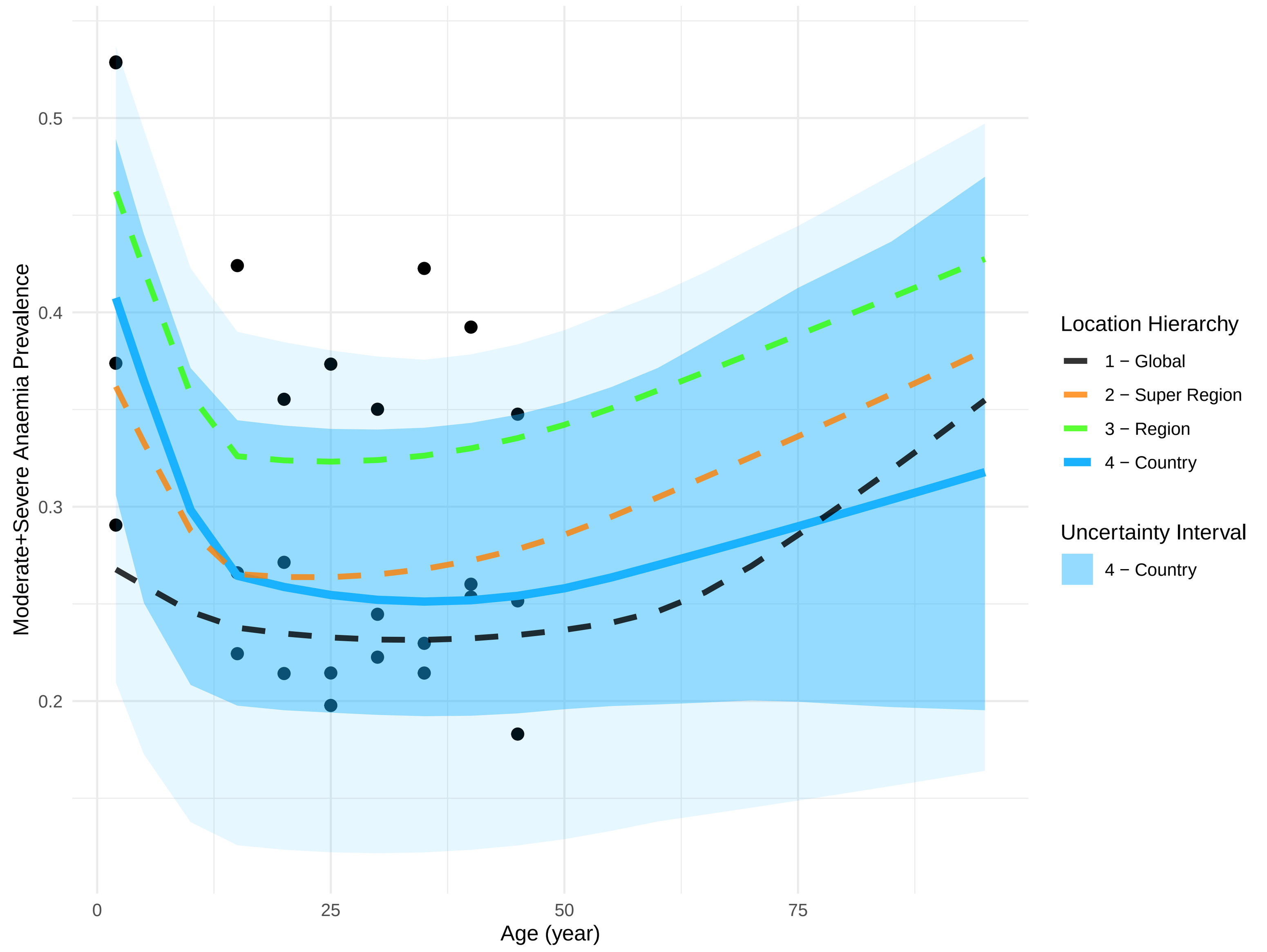

MR−BRT Age/Sex Fits − Non−pregnant Females 2+ Years Old − Moderate+Severe Anaemia Prevalence − Guinea
WHO−2024 Adjustment Method

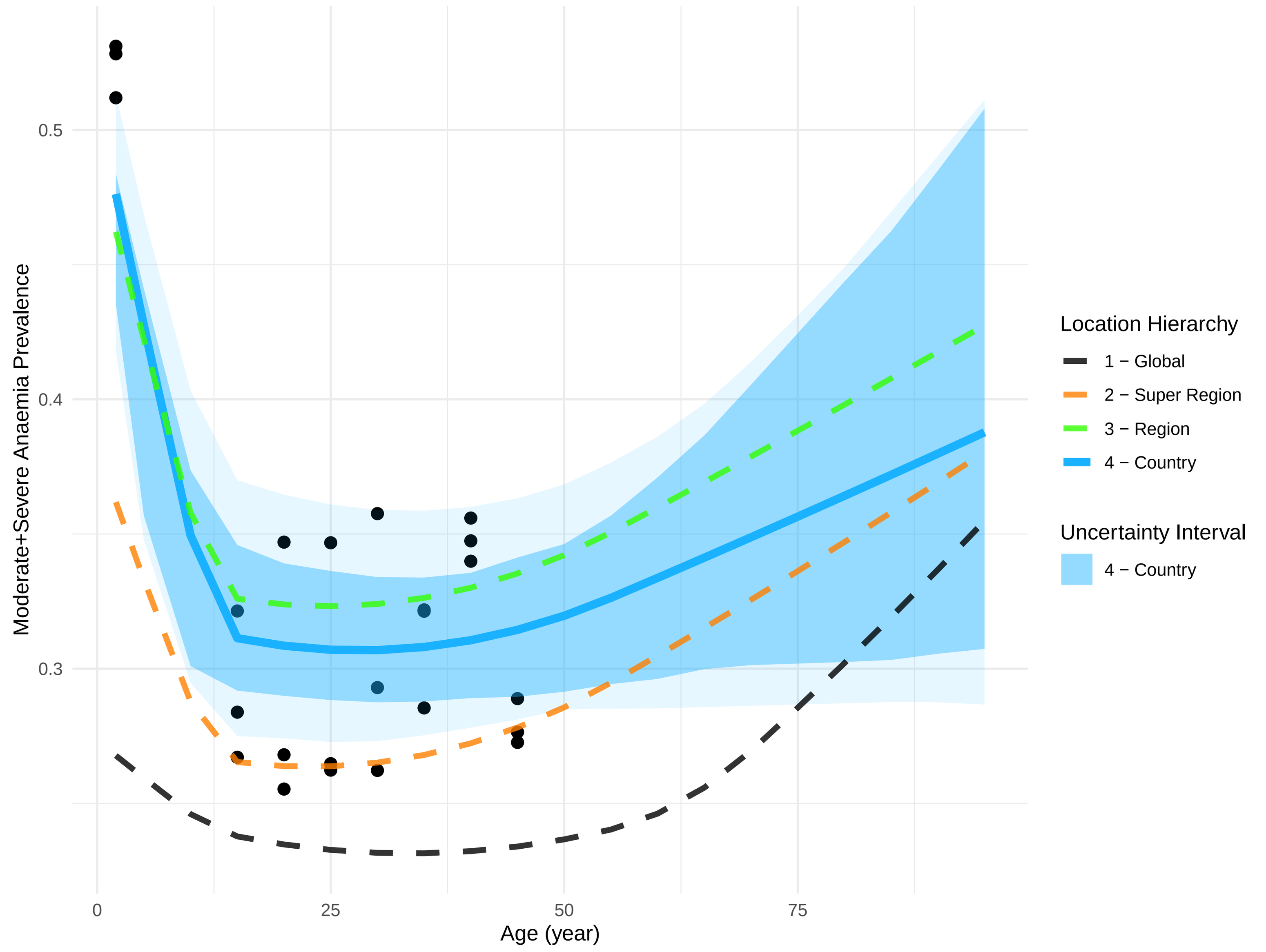

MR−BRT Age/Sex Fits − Non−pregnant Females 2+ Years Old − Moderate+Severe Anaemia Prevalence − Liberia
WHO−2024 Adjustment Method

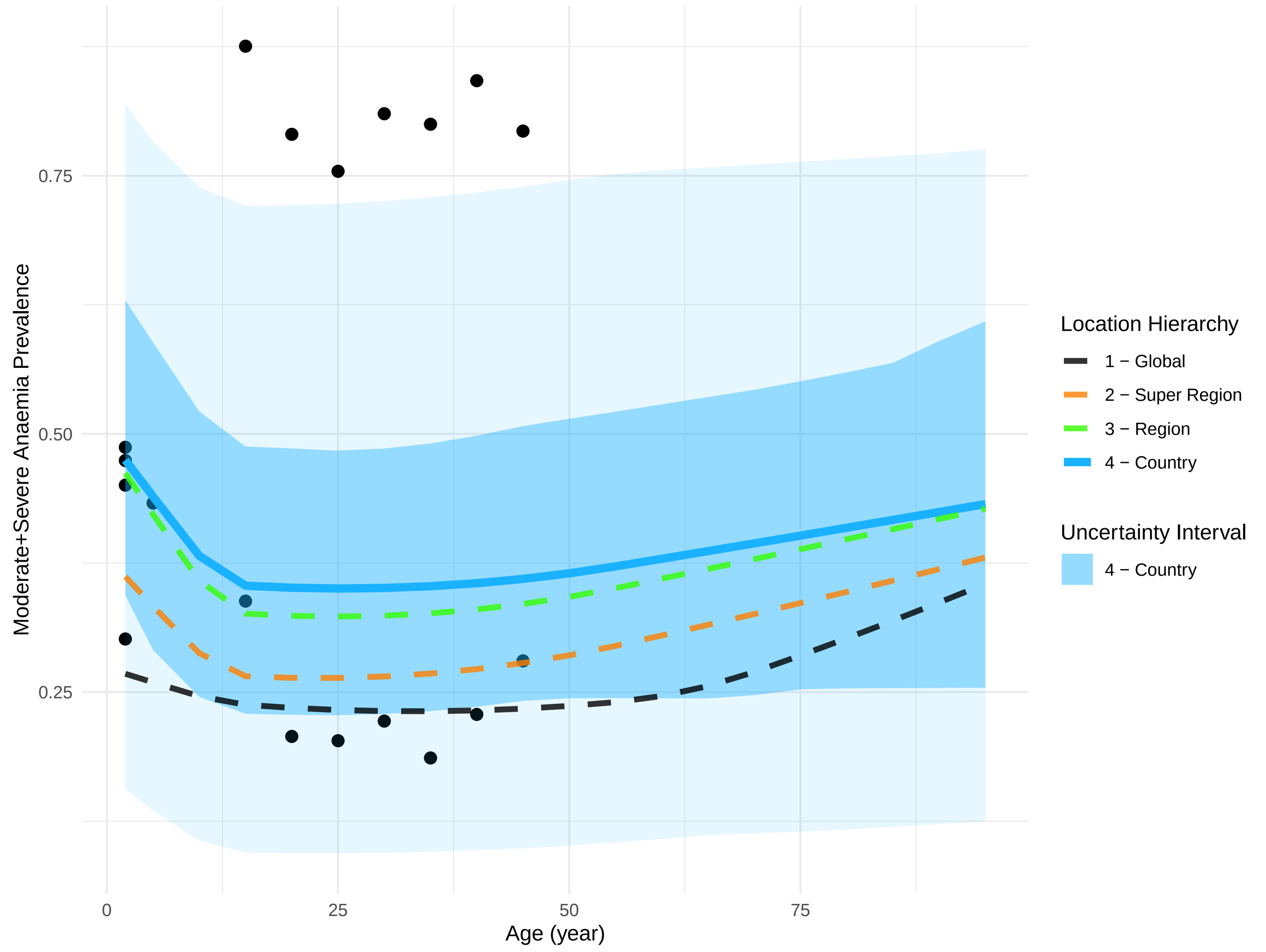

MR−BRT Age/Sex Fits − Non−pregnant Females 2+ Years Old − Moderate+Severe Anaemia Prevalence − Mali
WHO−2024 Adjustment Method

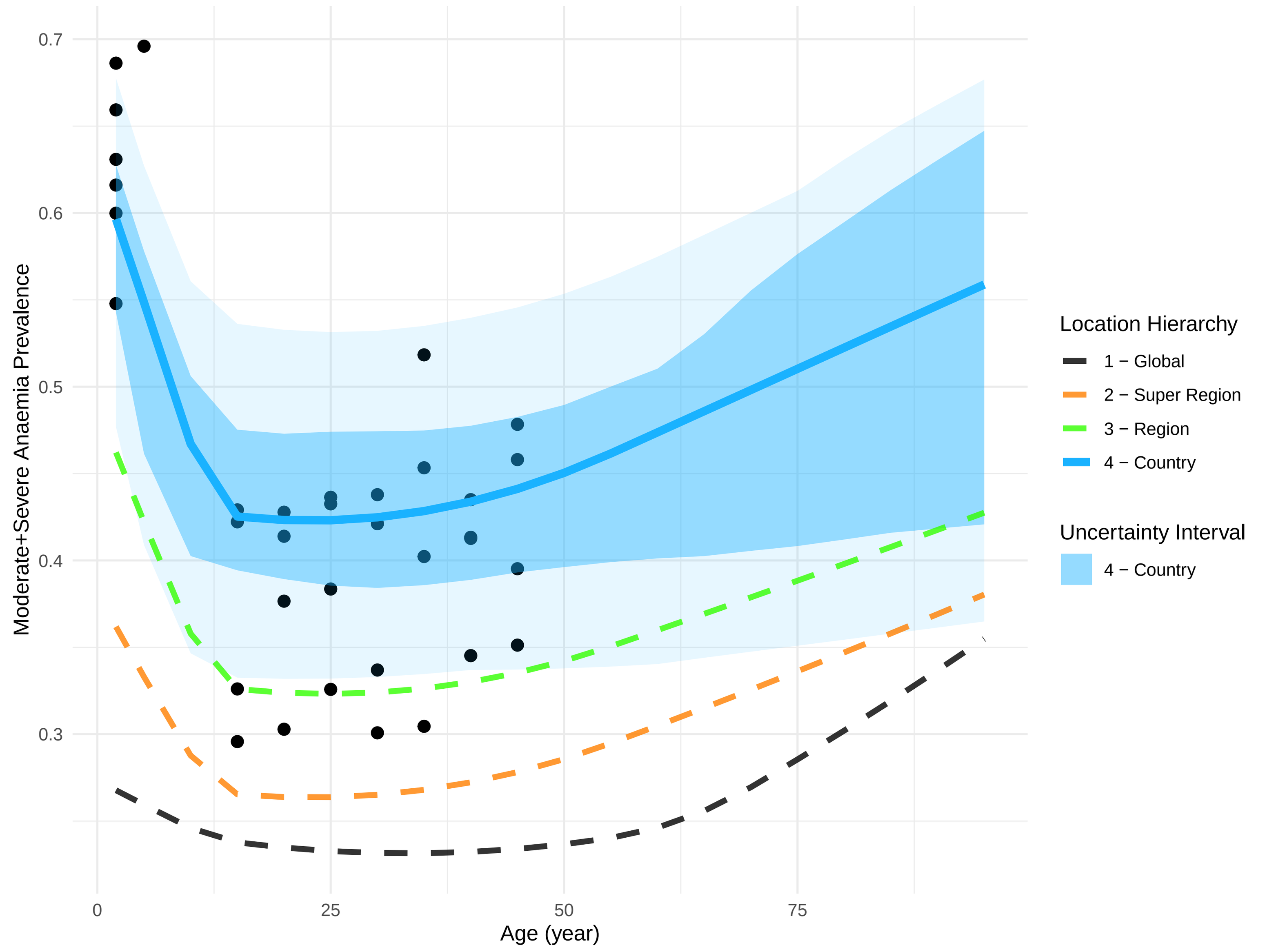

MR−BRT Age/Sex Fits − Non−pregnant Females 2+ Years Old − Moderate+Severe Anaemia Prevalence − Niger
WHO−2024 Adjustment Method

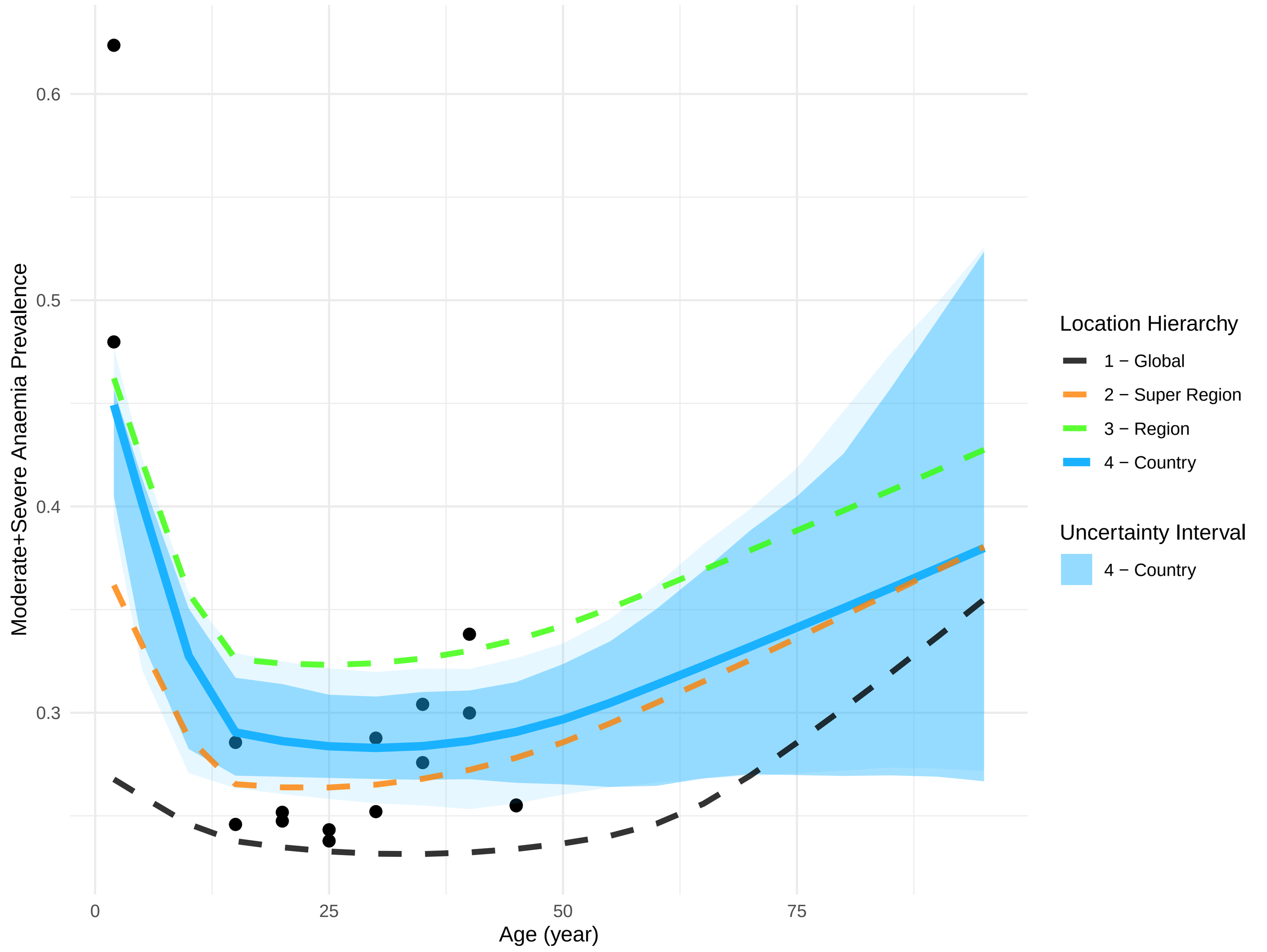

MR−BRT Age/Sex Fits − Non−pregnant Females 2+ Years Old − Moderate+Severe Anaemia Prevalence − Nigeria
WHO−2024 Adjustment Method

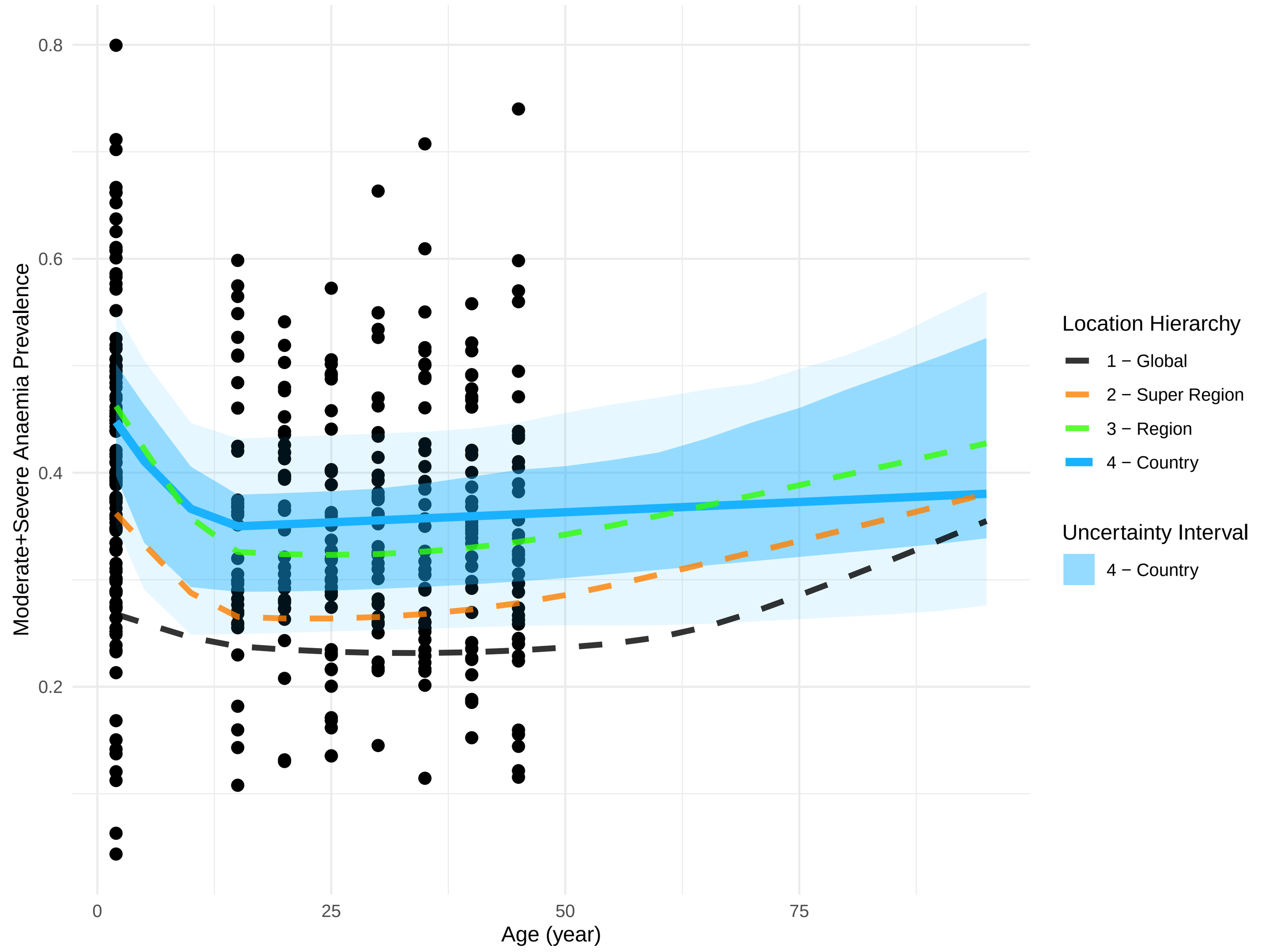

MR−BRT Age/Sex Fits − Non−pregnant Females 2+ Years Old − Moderate+Severe Anaemia Prevalence − Abia
WHO−2024 Adjustment Method

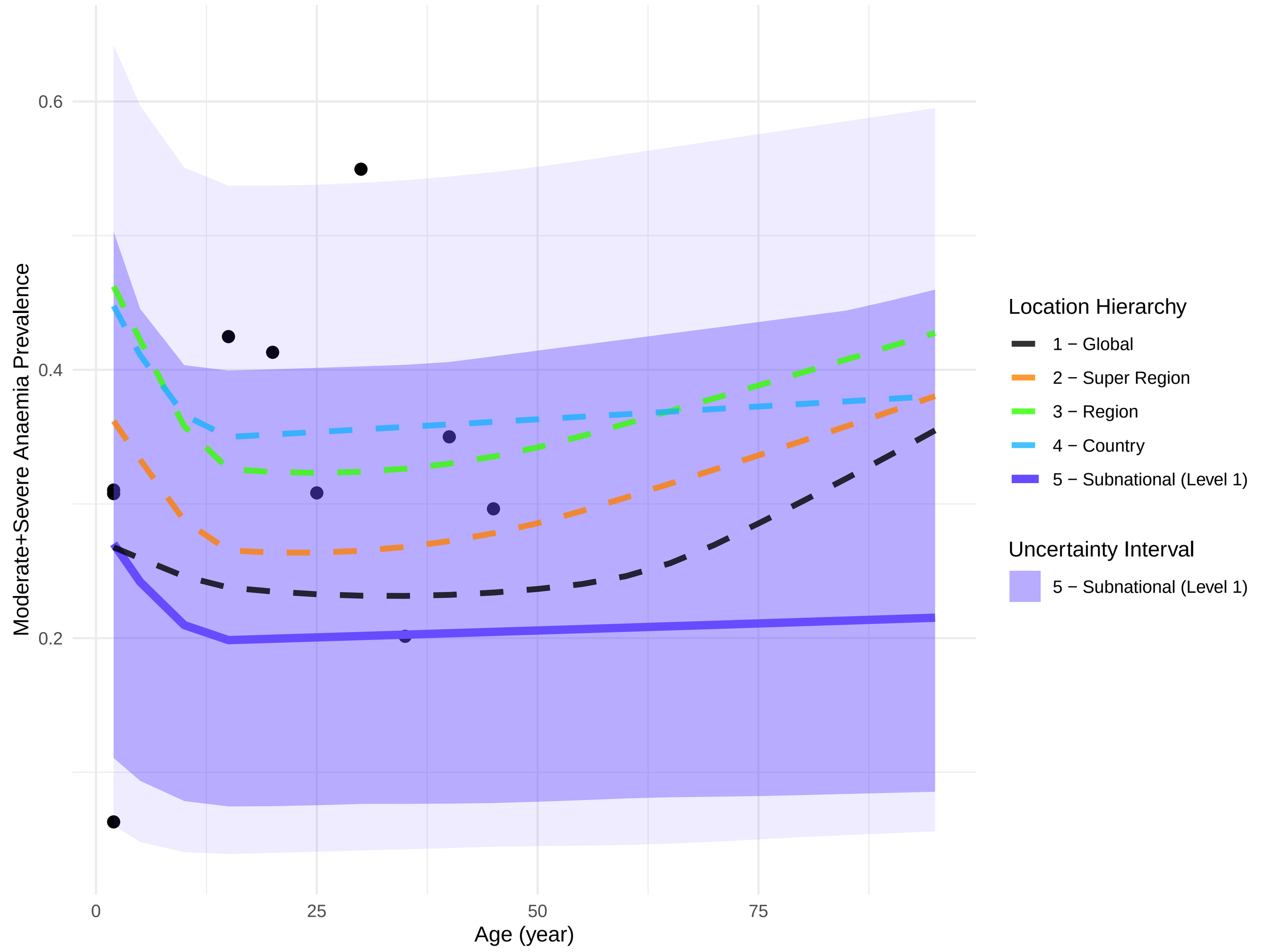

MR−BRT Age/Sex Fits − Non−pregnant Females 2+ Years Old − Moderate+Severe Anaemia Prevalence − Adamawa
WHO−2024 Adjustment Method

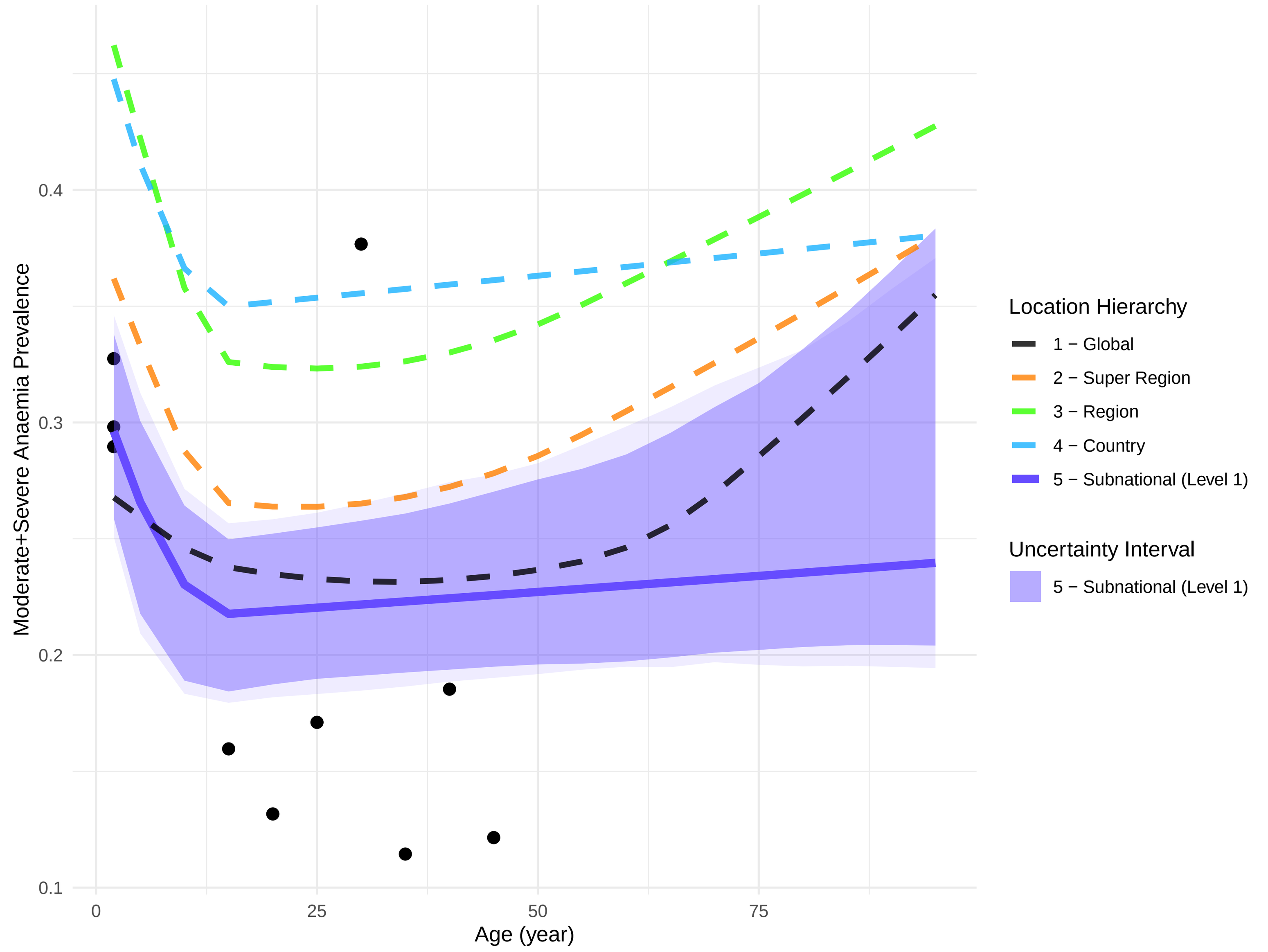

MR−BRT Age/Sex Fits − Non−pregnant Females 2+ Years Old − Moderate+Severe Anaemia Prevalence − Akwa Ibom
WHO−2024 Adjustment Method

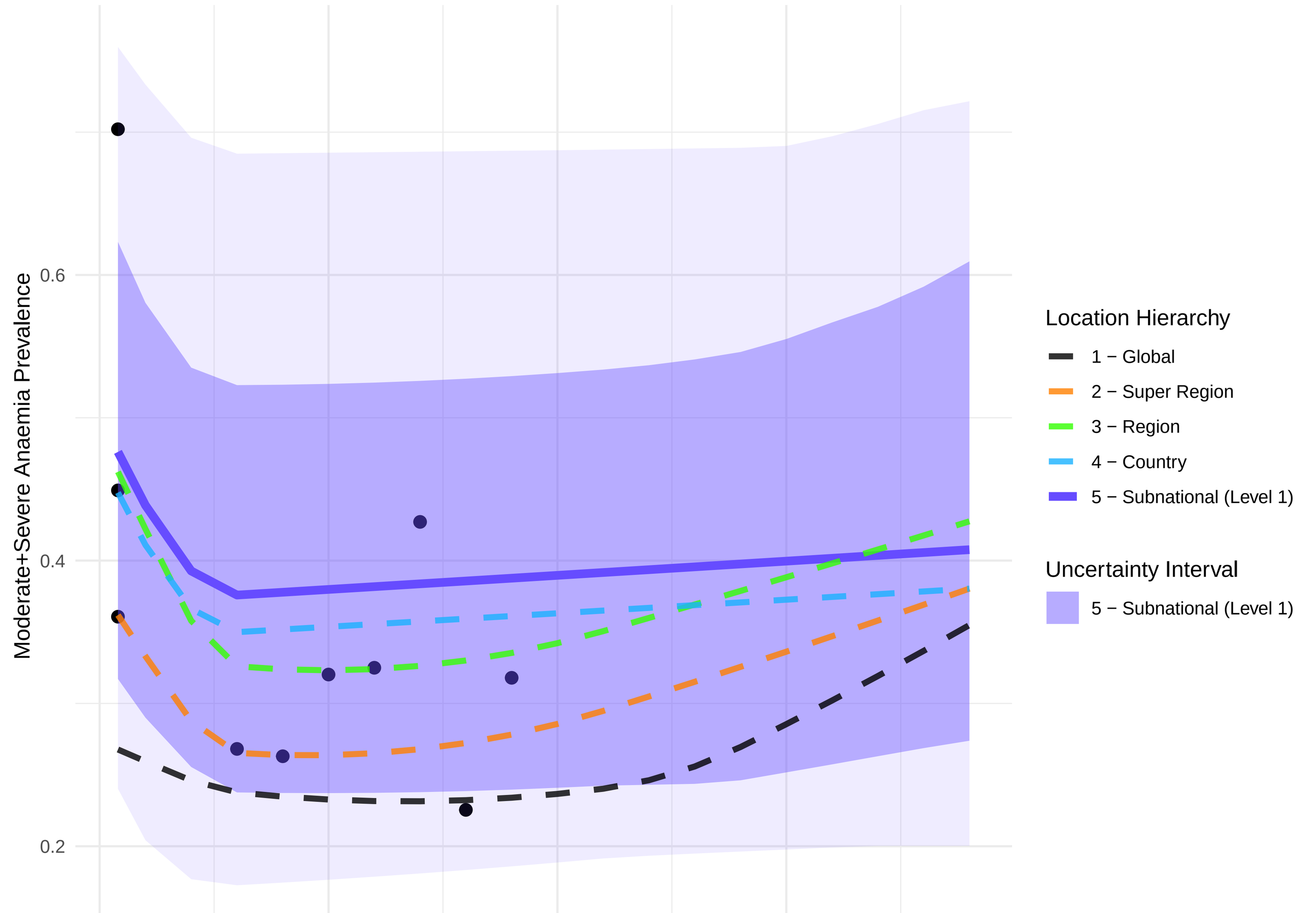

MR−BRT Age/Sex Fits − Non−pregnant Females 2+ Years Old − Moderate+Severe Anaemia Prevalence − Anambra
WHO−2024 Adjustment Method

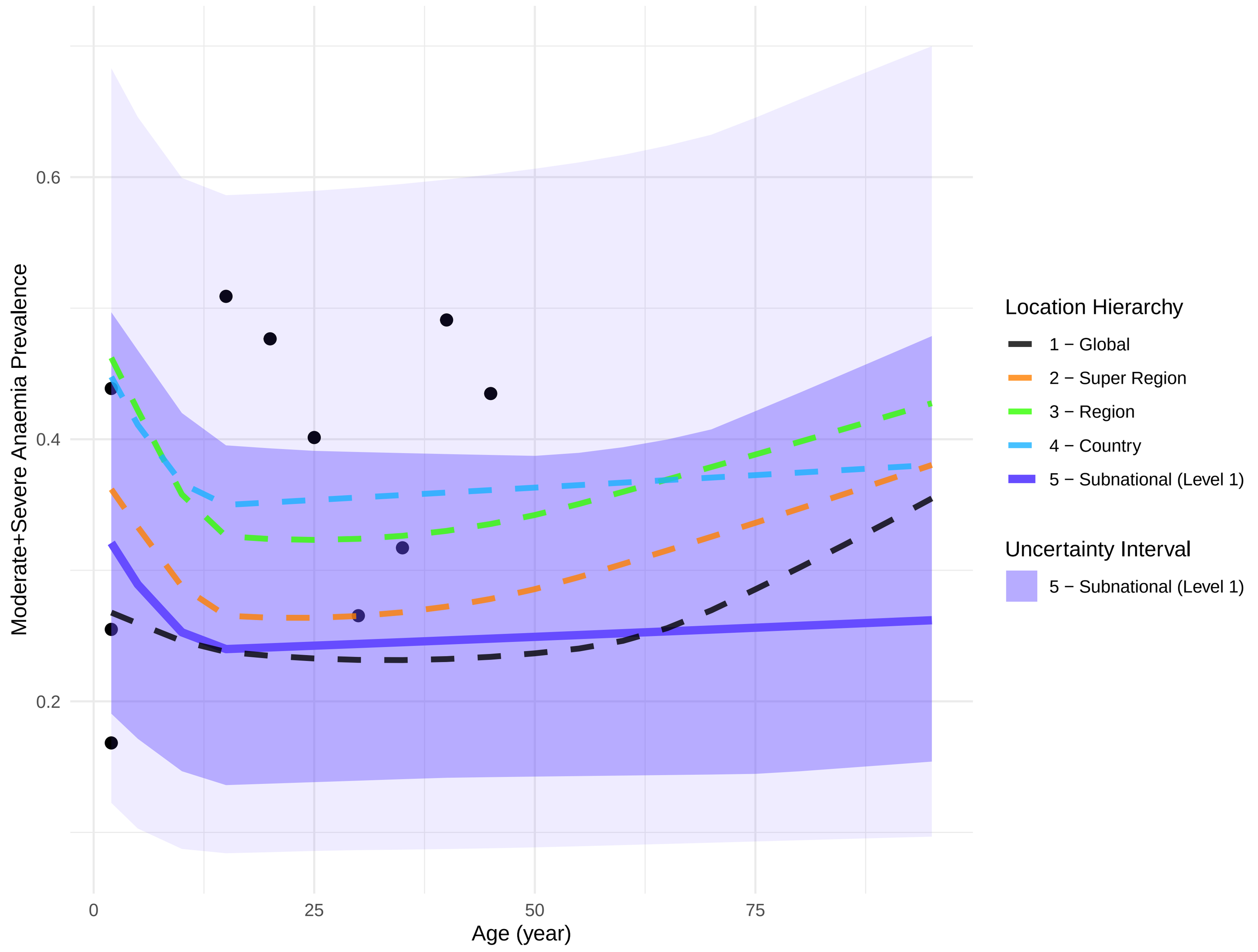

MR−BRT Age/Sex Fits − Non−pregnant Females 2+ Years Old − Moderate+Severe Anaemia Prevalence − Bauchi
WHO−2024 Adjustment Method

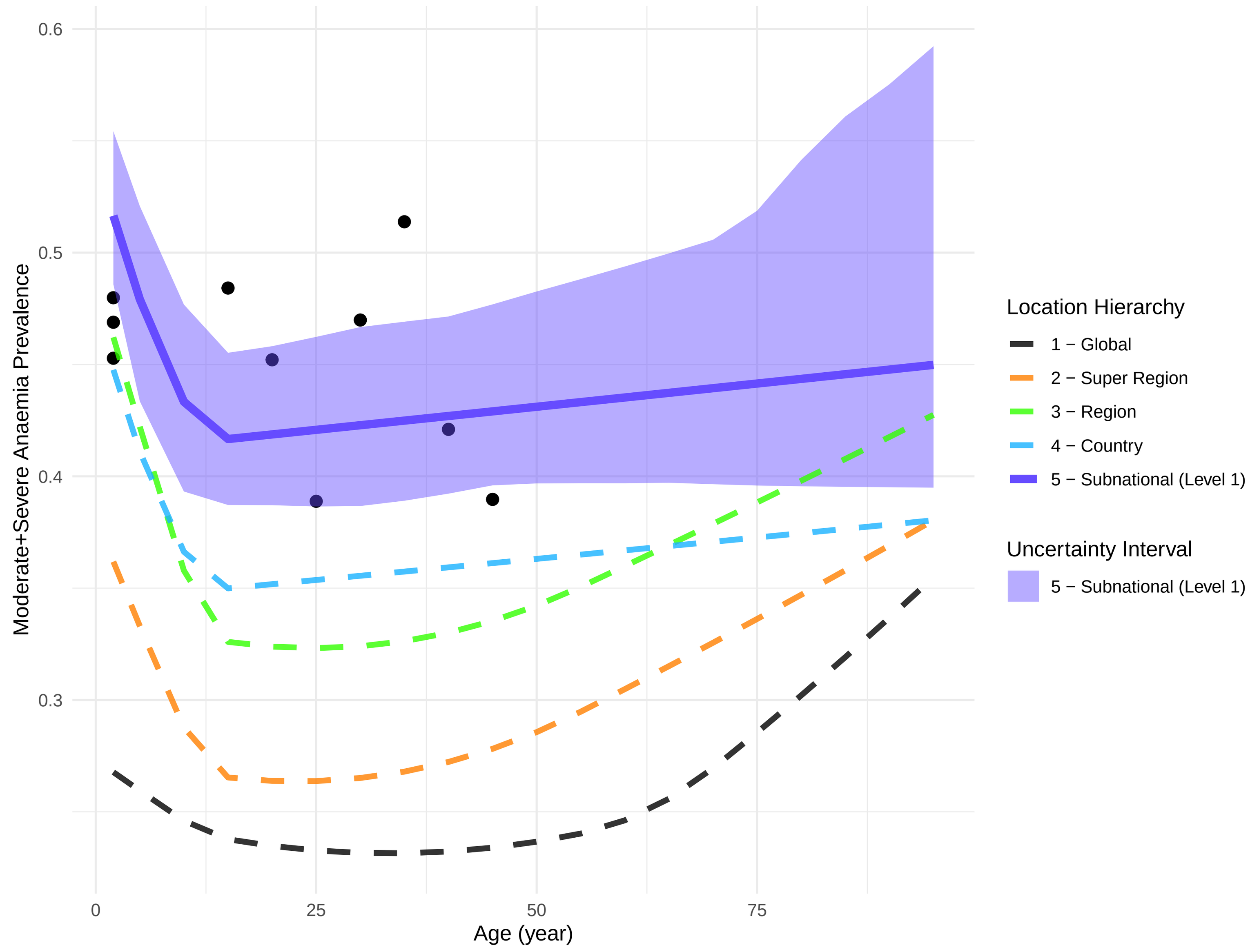

MR−BRT Age/Sex Fits − Non−pregnant Females 2+ Years Old − Moderate+Severe Anaemia Prevalence − Bayelsa
WHO−2024 Adjustment Method

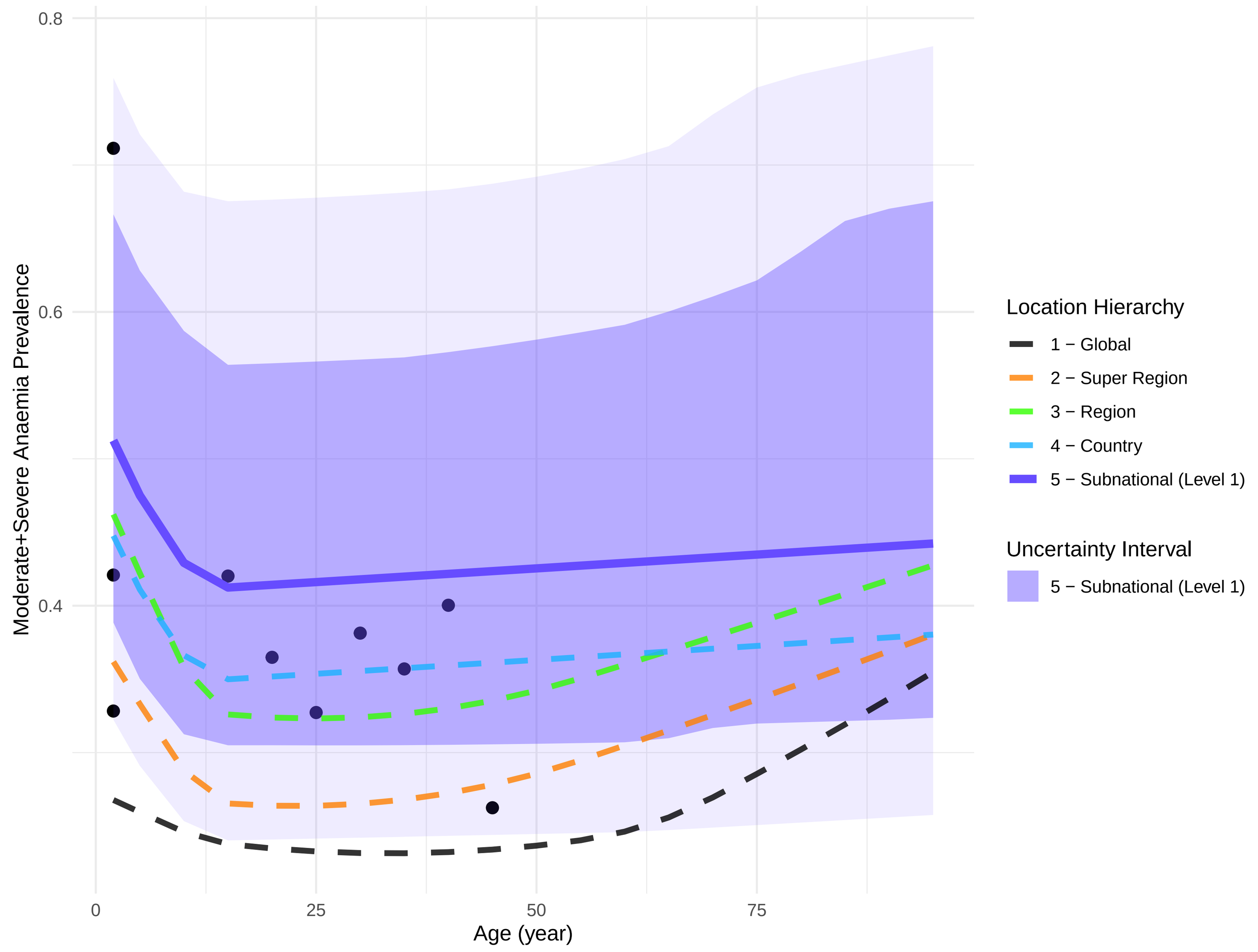

MR−BRT Age/Sex Fits − Non−pregnant Females 2+ Years Old − Moderate+Severe Anaemia Prevalence − Benue
WHO−2024 Adjustment Method

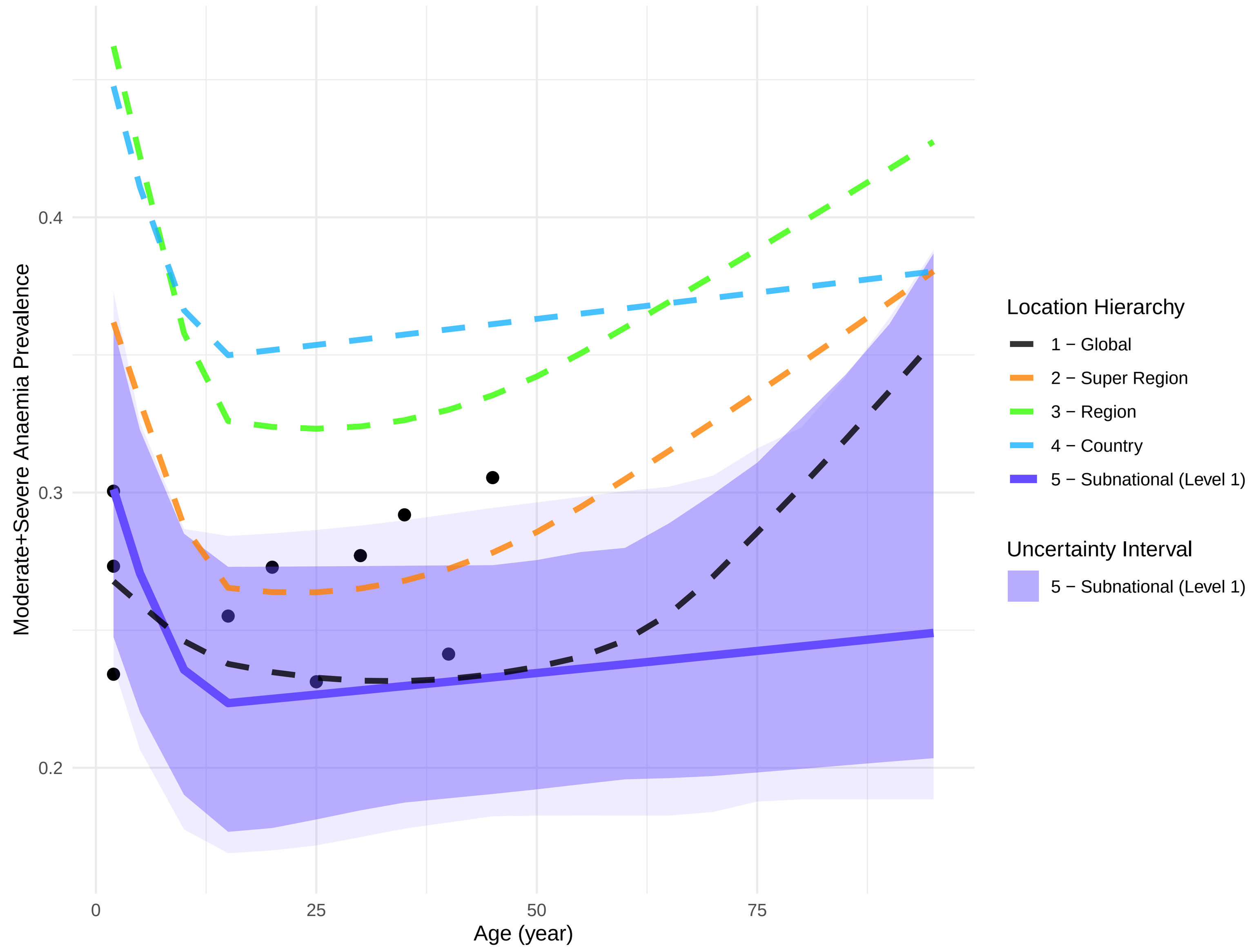

MR−BRT Age/Sex Fits − Non−pregnant Females 2+ Years Old − Moderate+Severe Anaemia Prevalence − Borno
WHO−2024 Adjustment Method

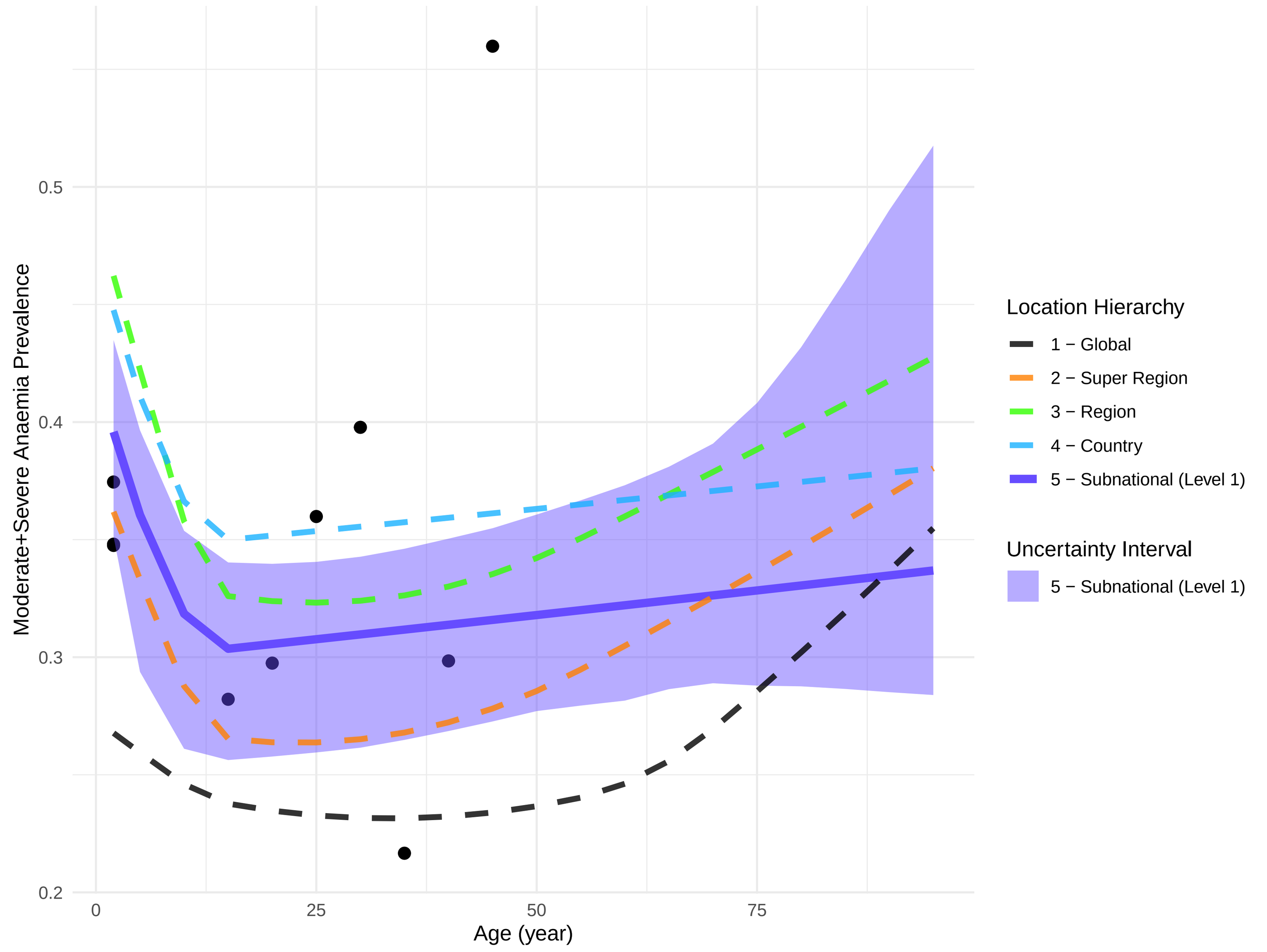

MR−BRT Age/Sex Fits − Non−pregnant Females 2+ Years Old − Moderate+Severe Anaemia Prevalence − Cross River
WHO−2024 Adjustment Method

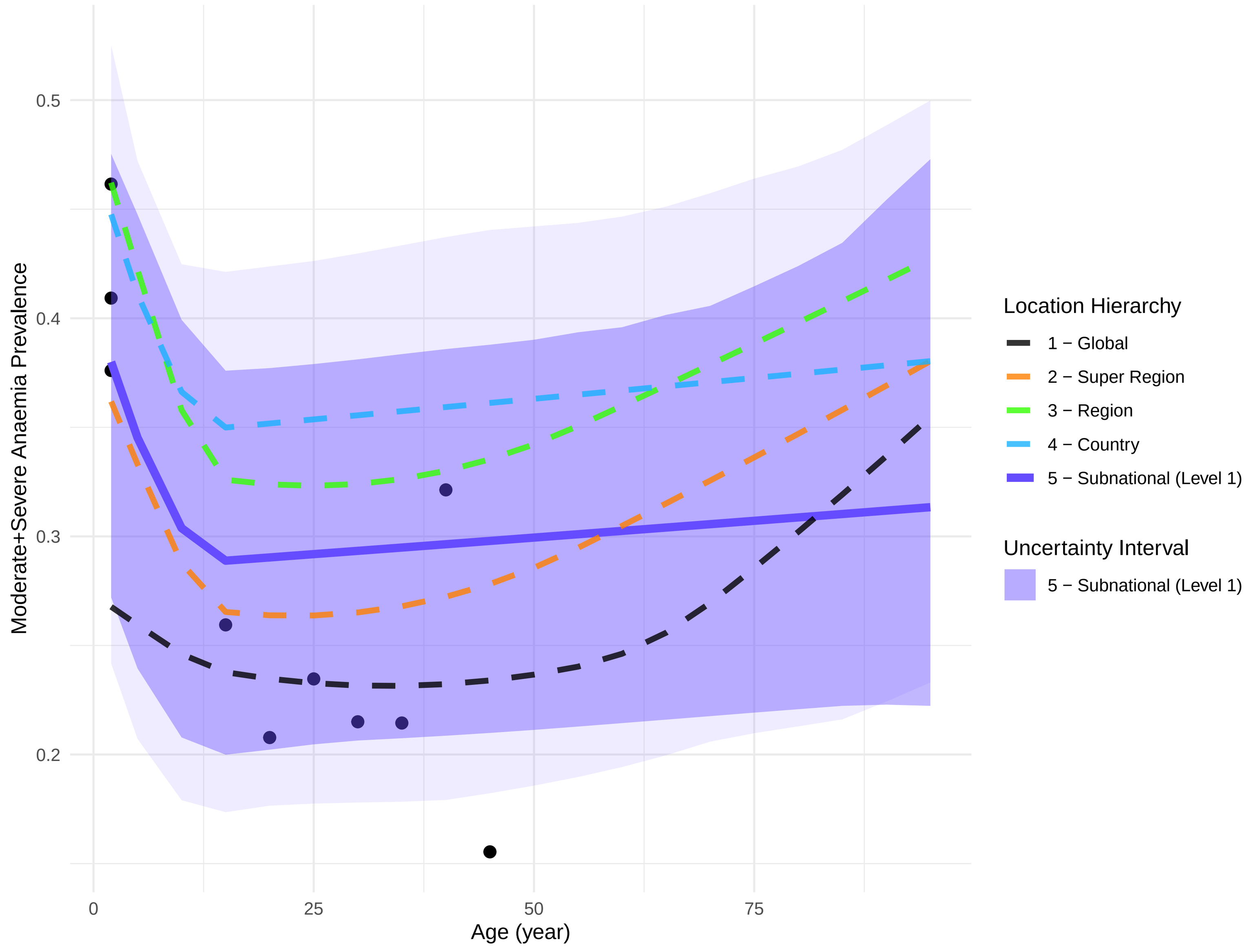

MR−BRT Age/Sex Fits − Non−pregnant Females 2+ Years Old − Moderate+Severe Anaemia Prevalence − Delta
WHO−2024 Adjustment Method

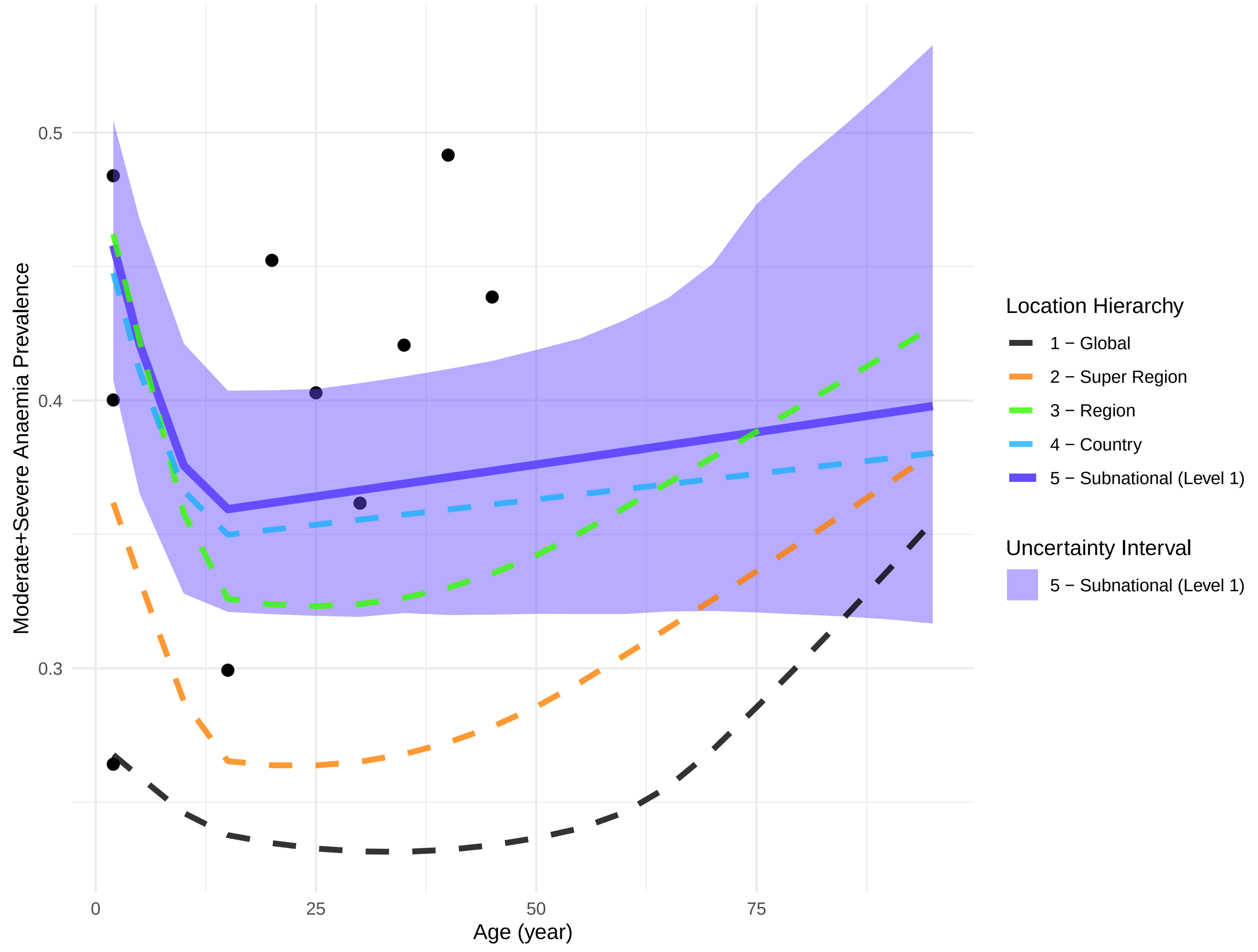

MR−BRT Age/Sex Fits − Non−pregnant Females 2+ Years Old − Moderate+Severe Anaemia Prevalence − Ebonyi
WHO−2024 Adjustment Method

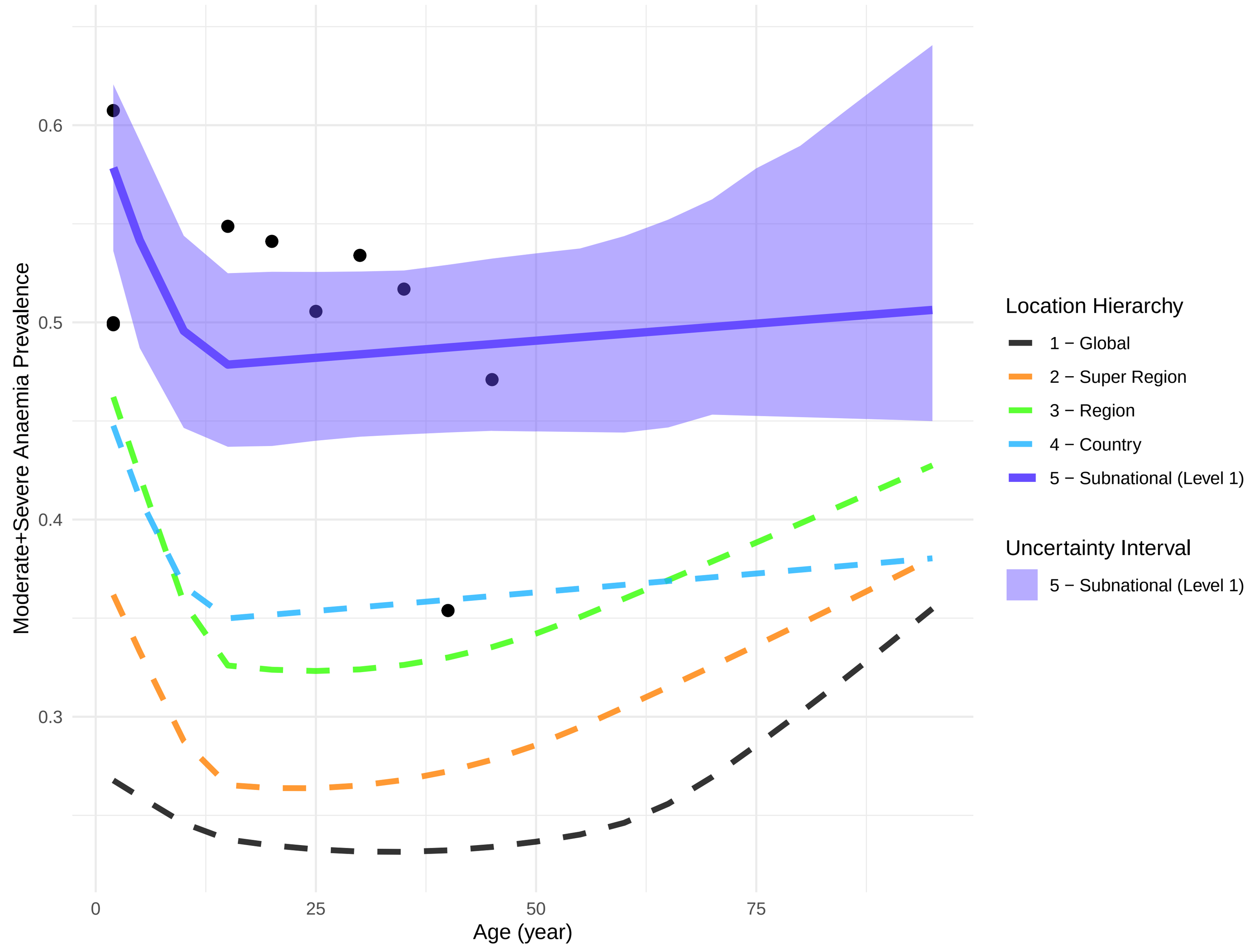

MR−BRT Age/Sex Fits − Non−pregnant Females 2+ Years Old − Moderate+Severe Anaemia Prevalence − Edo
WHO−2024 Adjustment Method

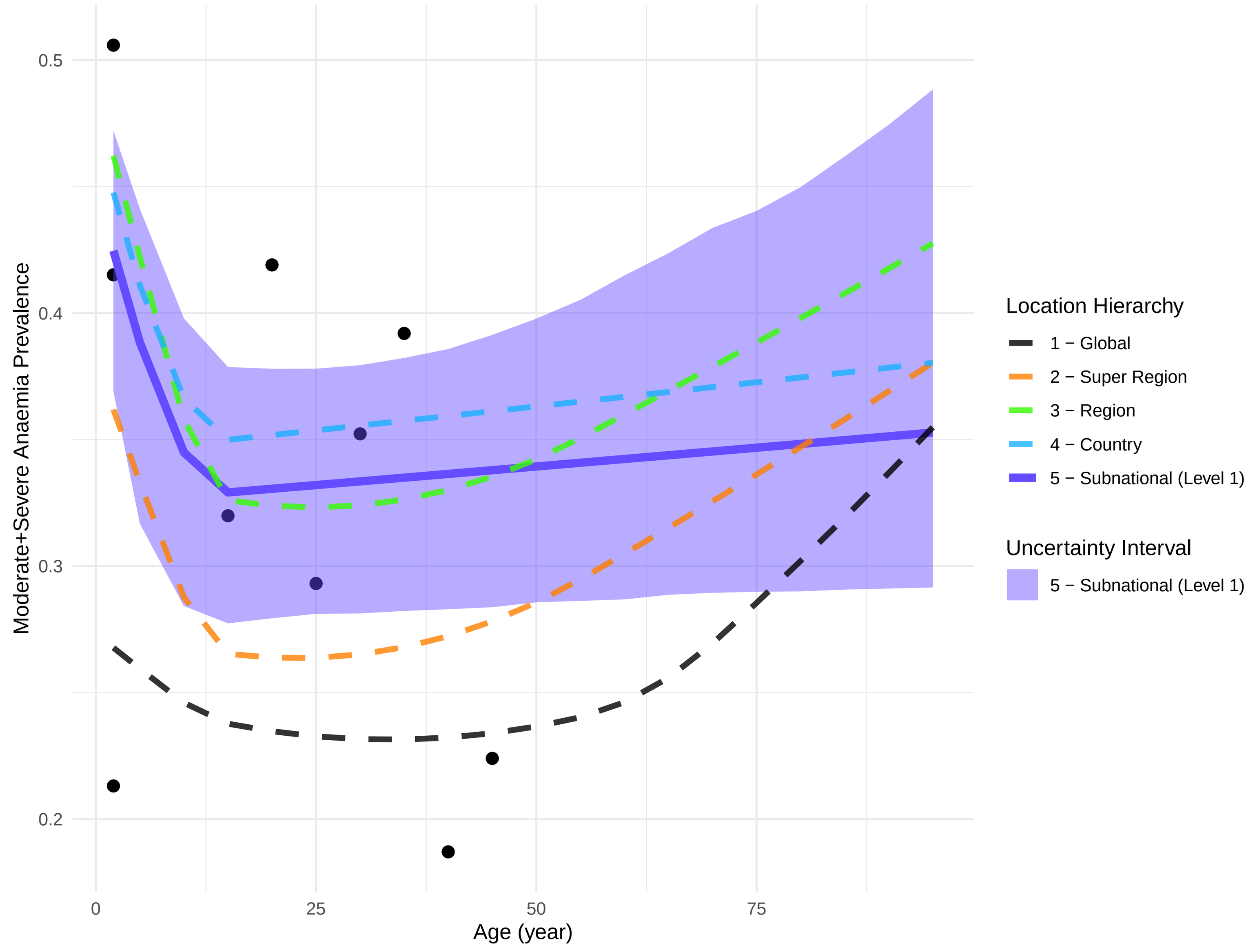

MR−BRT Age/Sex Fits − Non−pregnant Females 2+ Years Old − Moderate+Severe Anaemia Prevalence − Ekiti
WHO−2024 Adjustment Method

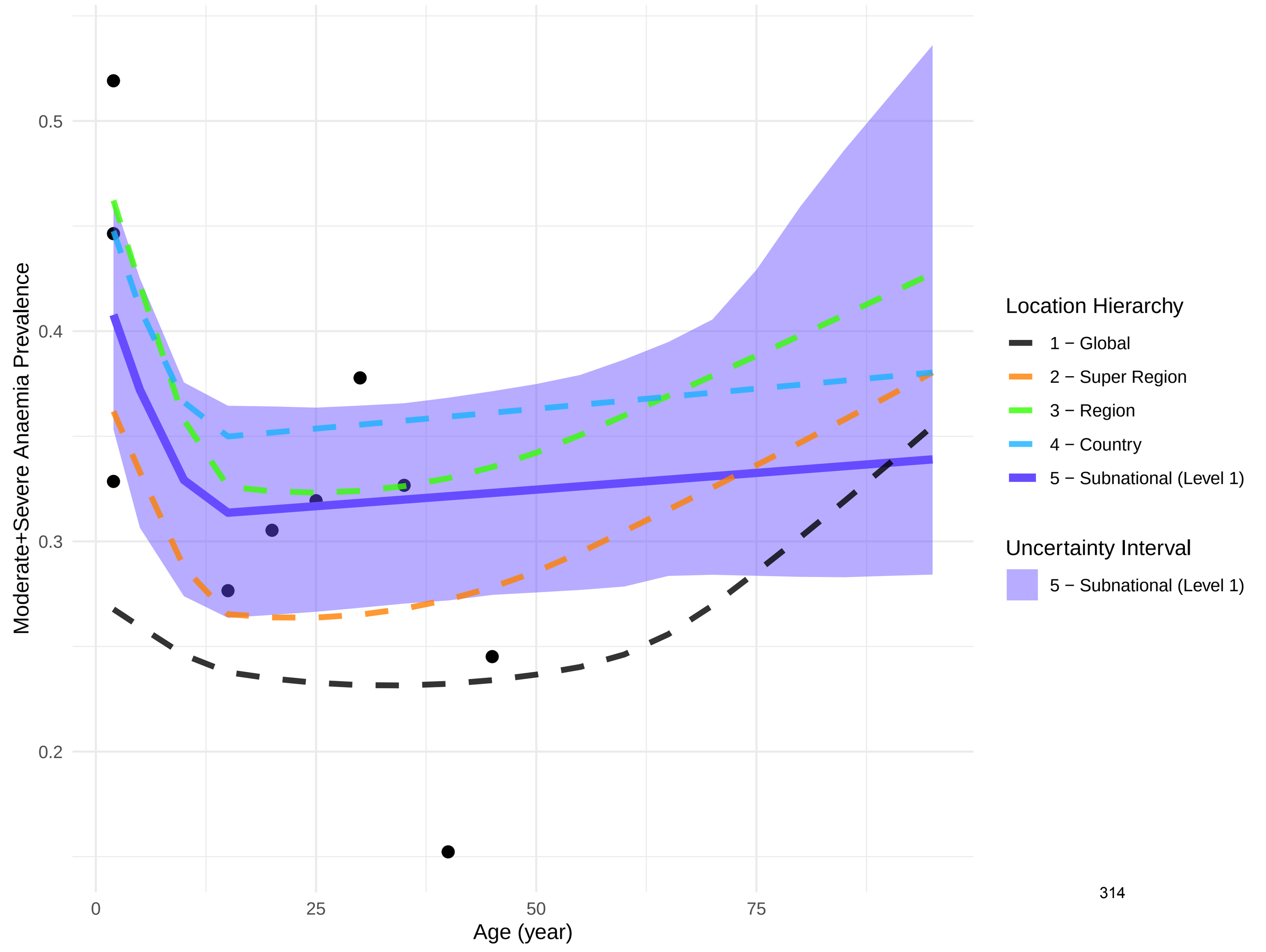

MR−BRT Age/Sex Fits − Non−pregnant Females 2+ Years Old − Moderate+Severe Anaemia Prevalence − Enugu
WHO−2024 Adjustment Method

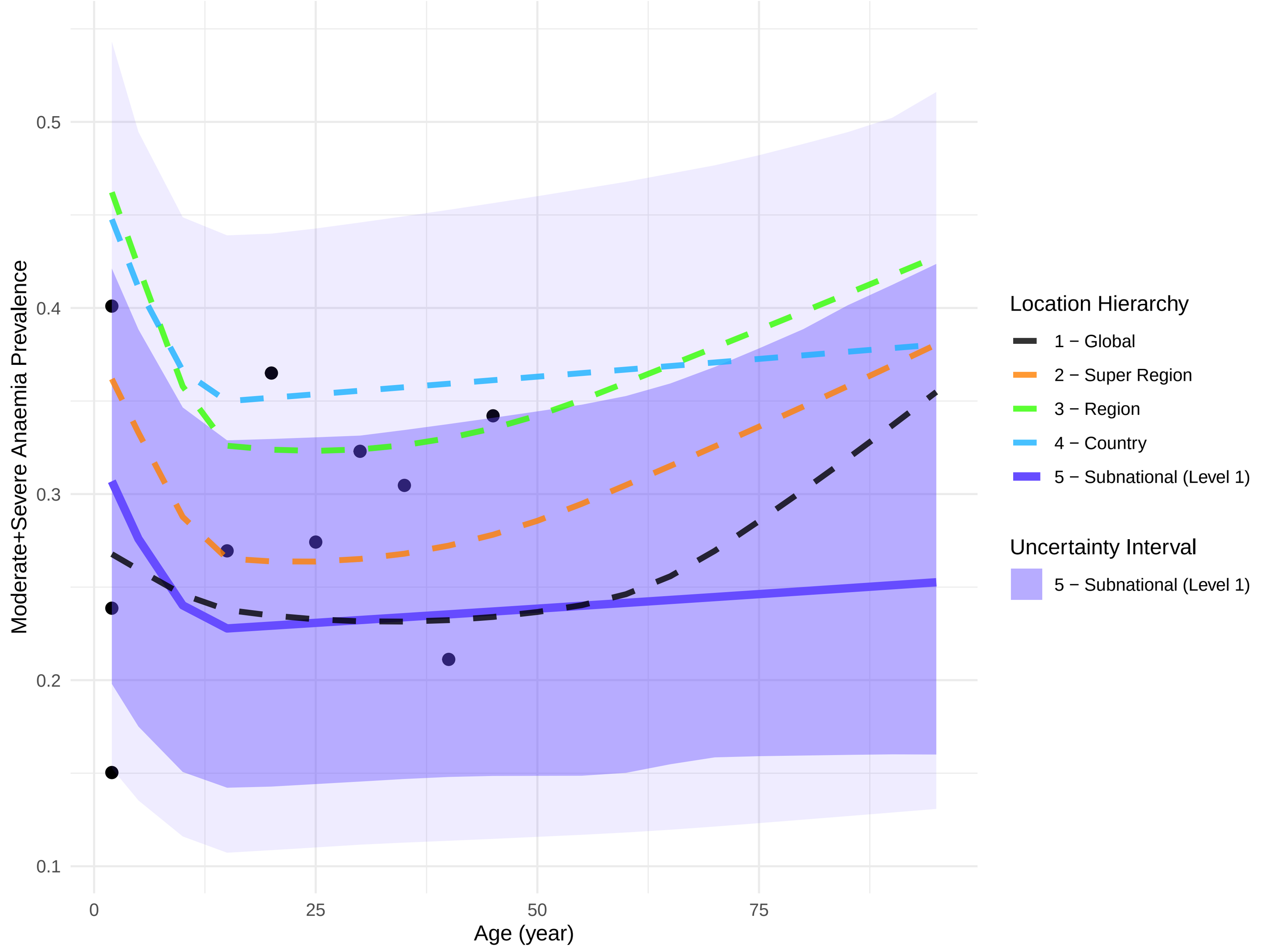

MR−BRT Age/Sex Fits − Non−pregnant Females 2+ Years Old − Moderate+Severe Anaemia Prevalence − FCT (Abuja)
WHO−2024 Adjustment Method

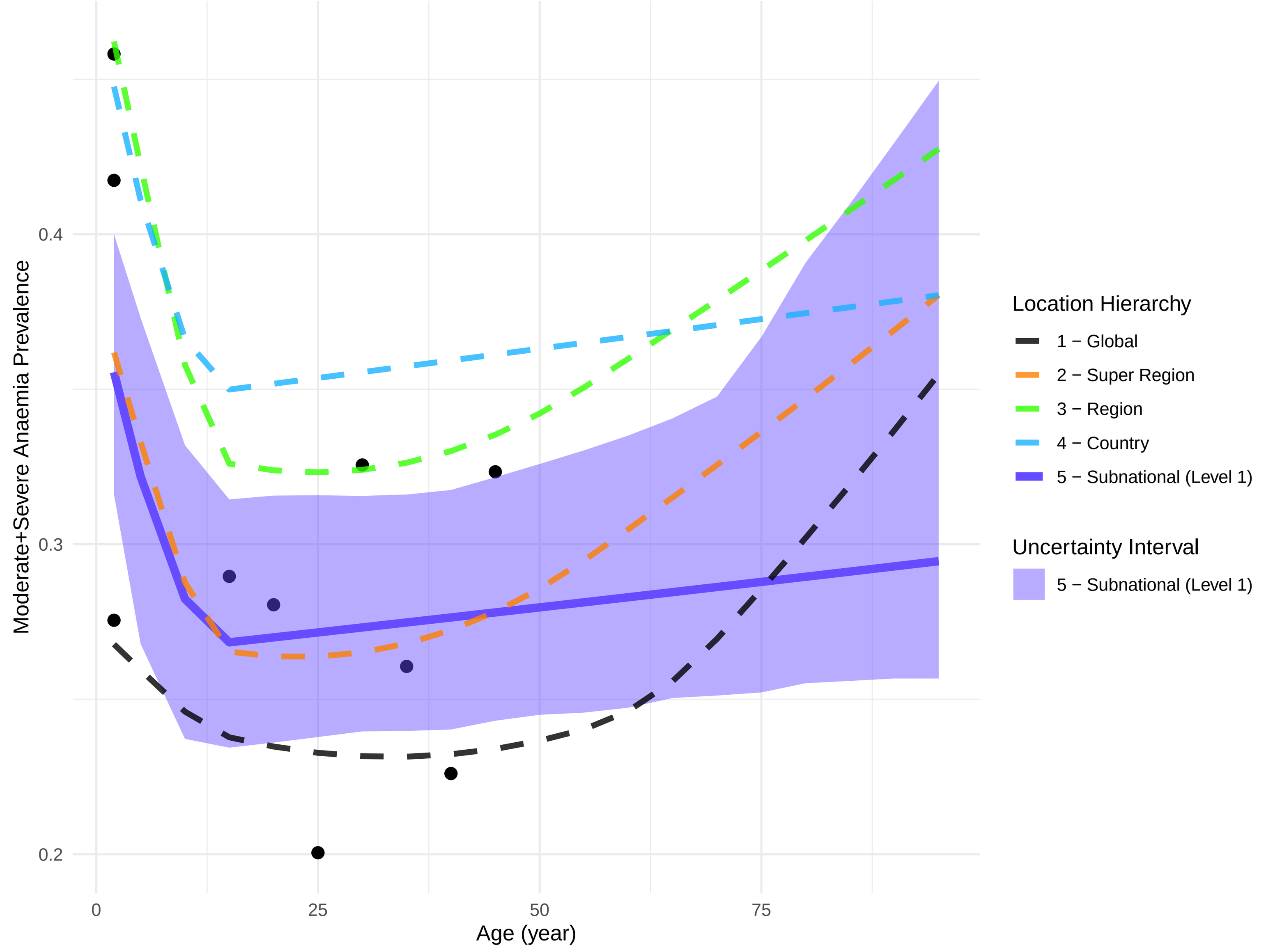

MR−BRT Age/Sex Fits − Non−pregnant Females 2+ Years Old − Moderate+Severe Anaemia Prevalence − Gombe
WHO−2024 Adjustment Method

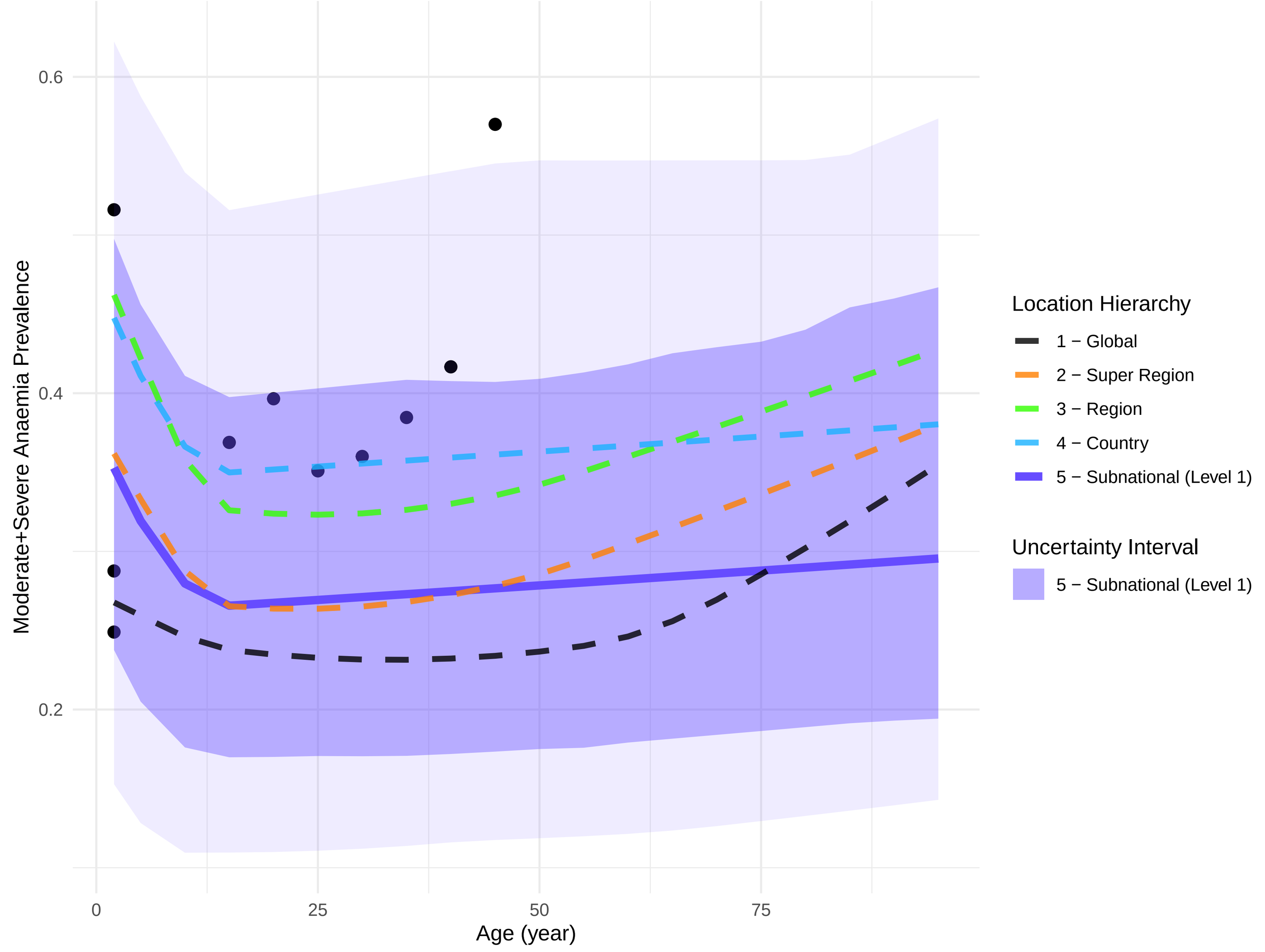

MR−BRT Age/Sex Fits − Non−pregnant Females 2+ Years Old − Moderate+Severe Anaemia Prevalence − Imo
WHO−2024 Adjustment Method

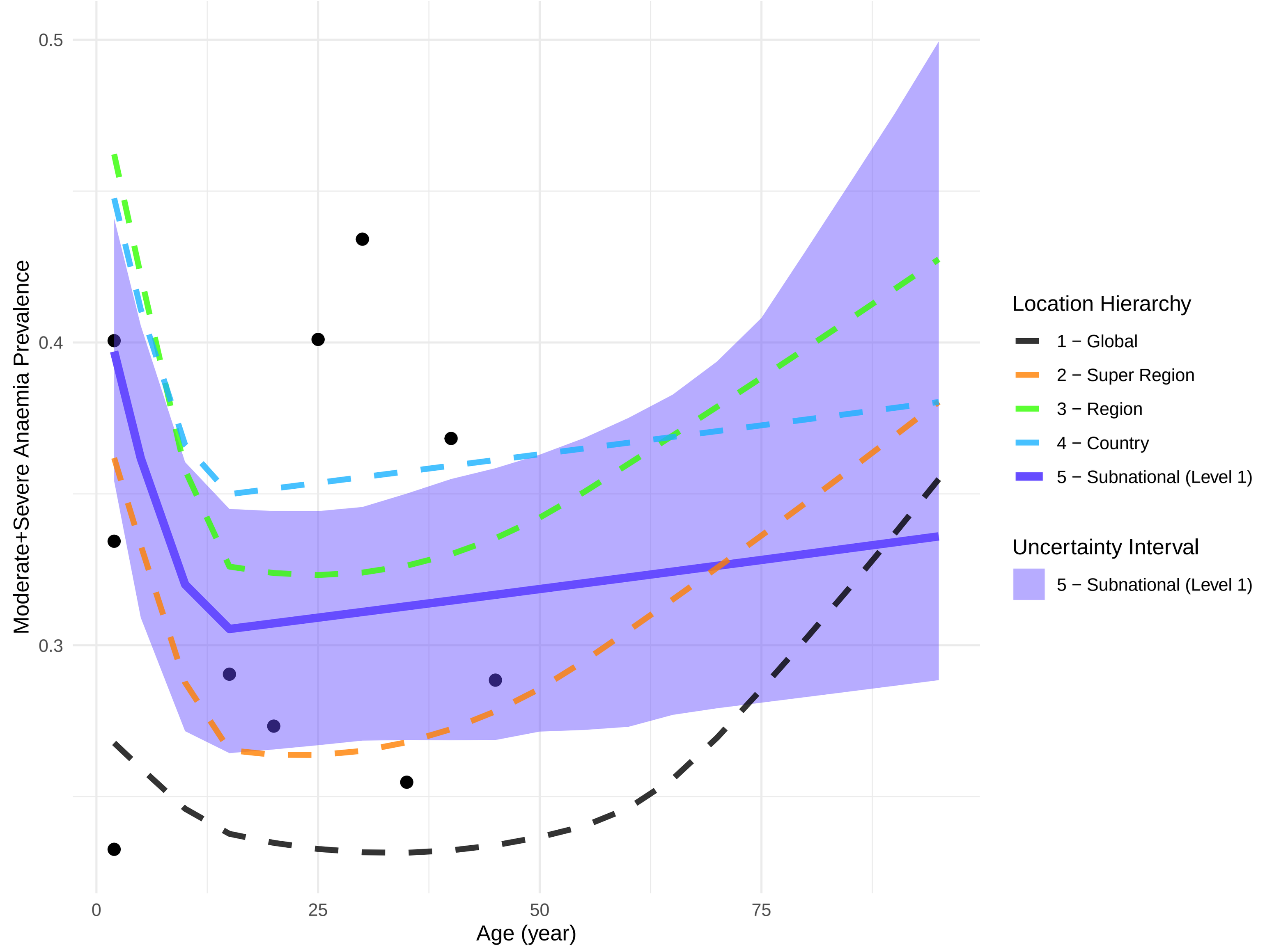

MR−BRT Age/Sex Fits − Non−pregnant Females 2+ Years Old − Moderate+Severe Anaemia Prevalence − Jigawa
WHO−2024 Adjustment Method

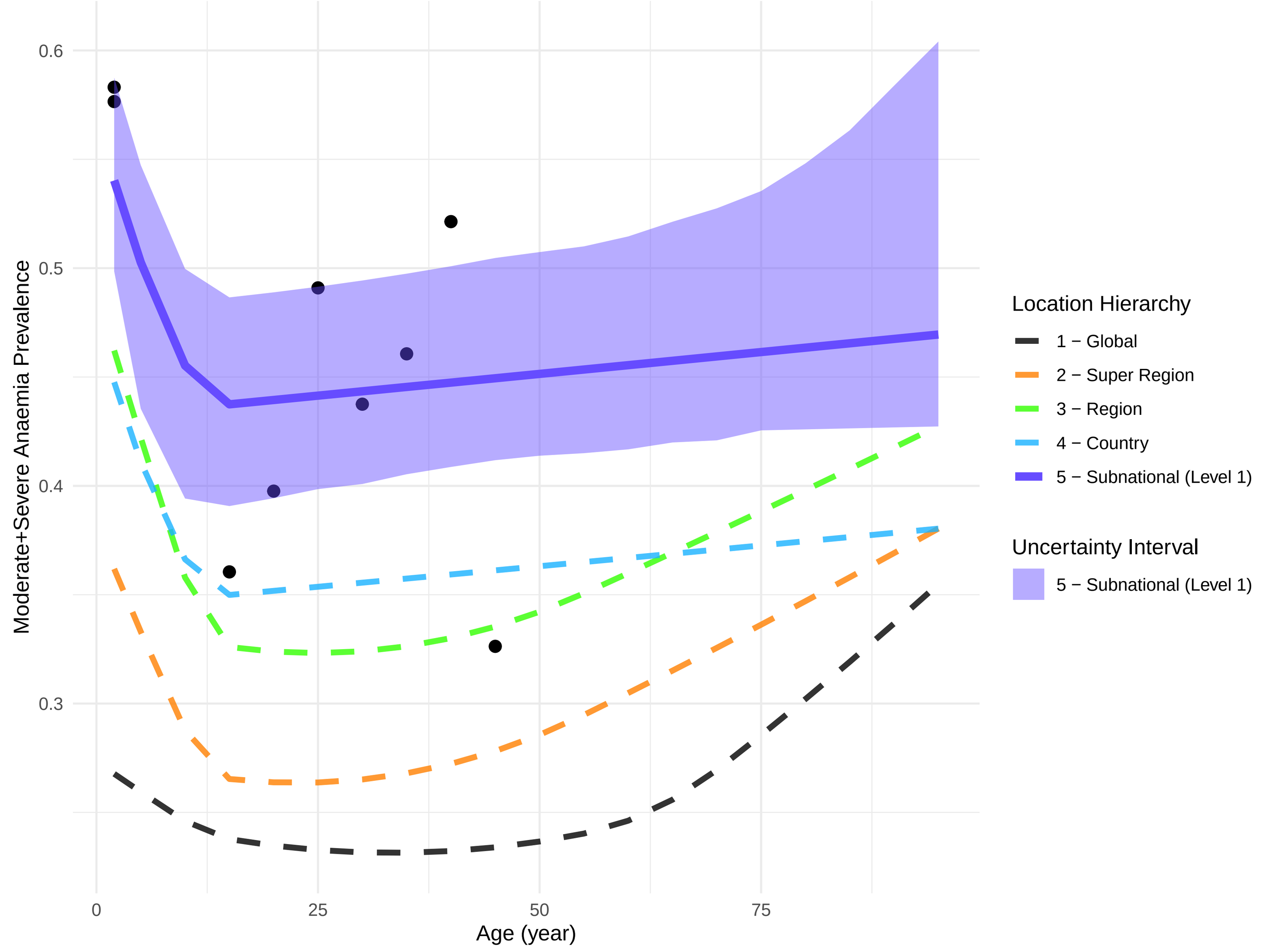

MR−BRT Age/Sex Fits − Non−pregnant Females 2+ Years Old − Moderate+Severe Anaemia Prevalence − Kaduna
WHO−2024 Adjustment Method

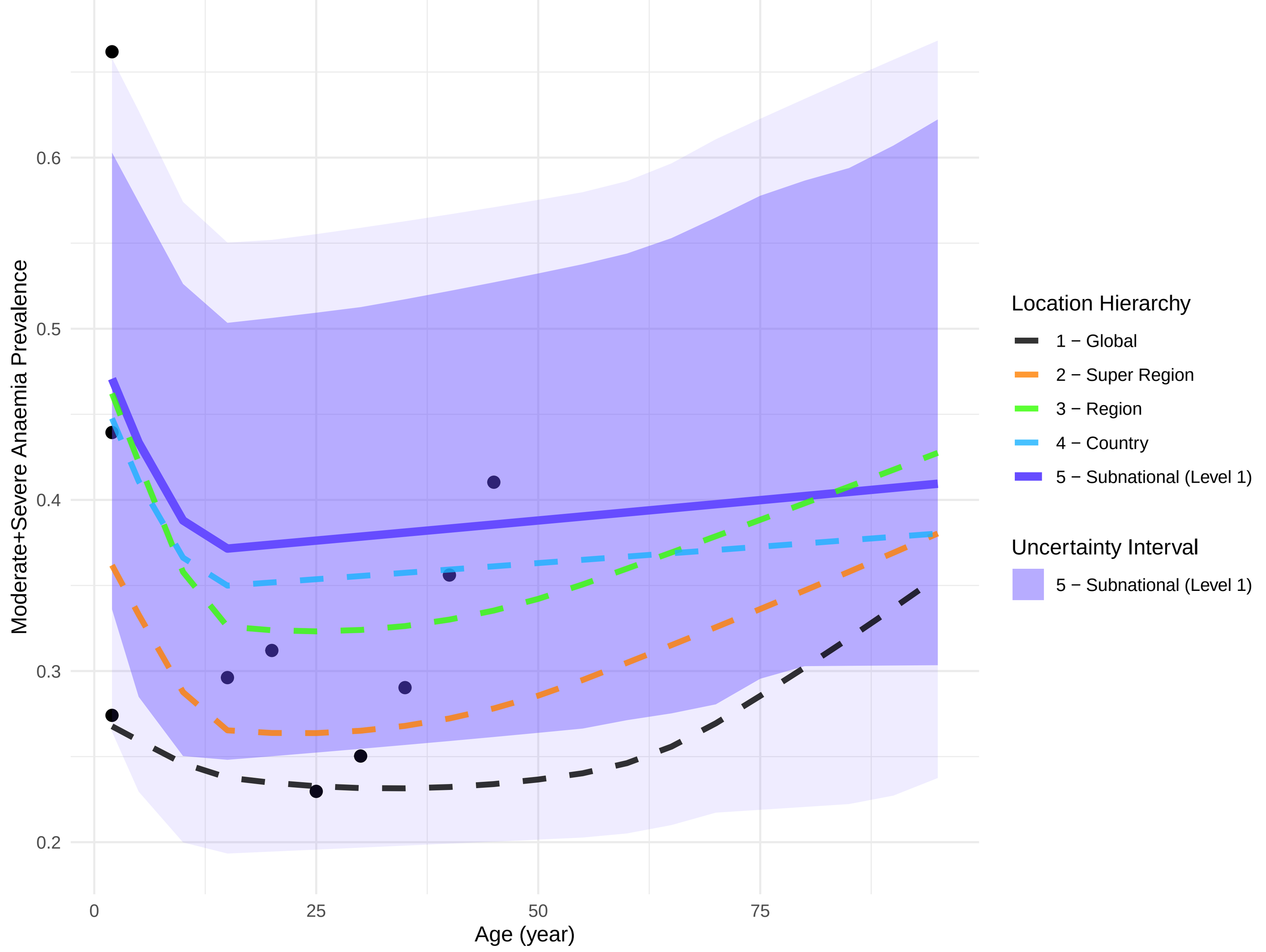

MR−BRT Age/Sex Fits − Non−pregnant Females 2+ Years Old − Moderate+Severe Anaemia Prevalence − Kano
WHO−2024 Adjustment Method

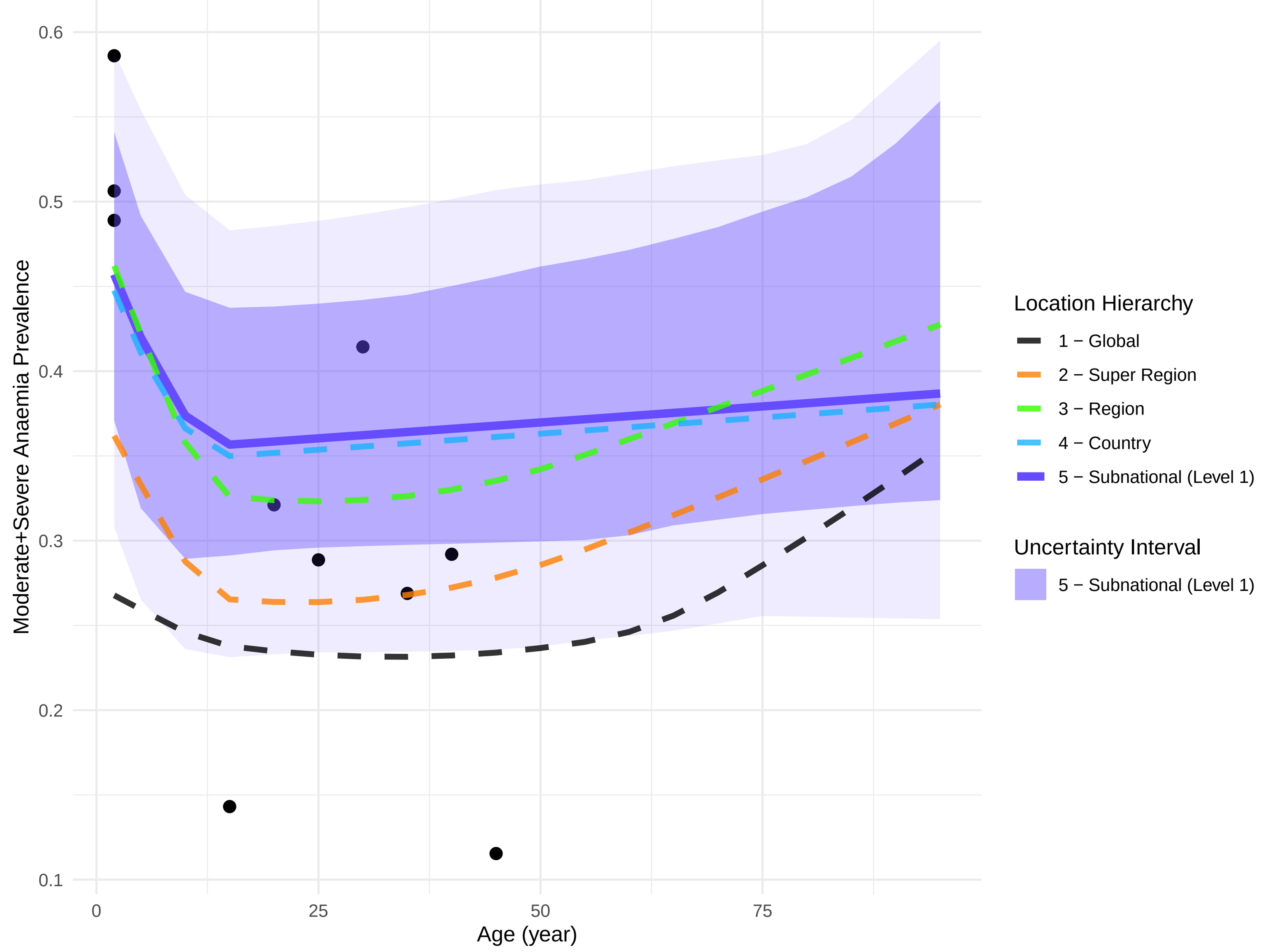

MR−BRT Age/Sex Fits − Non−pregnant Females 2+ Years Old − Moderate+Severe Anaemia Prevalence − Katsina
WHO−2024 Adjustment Method

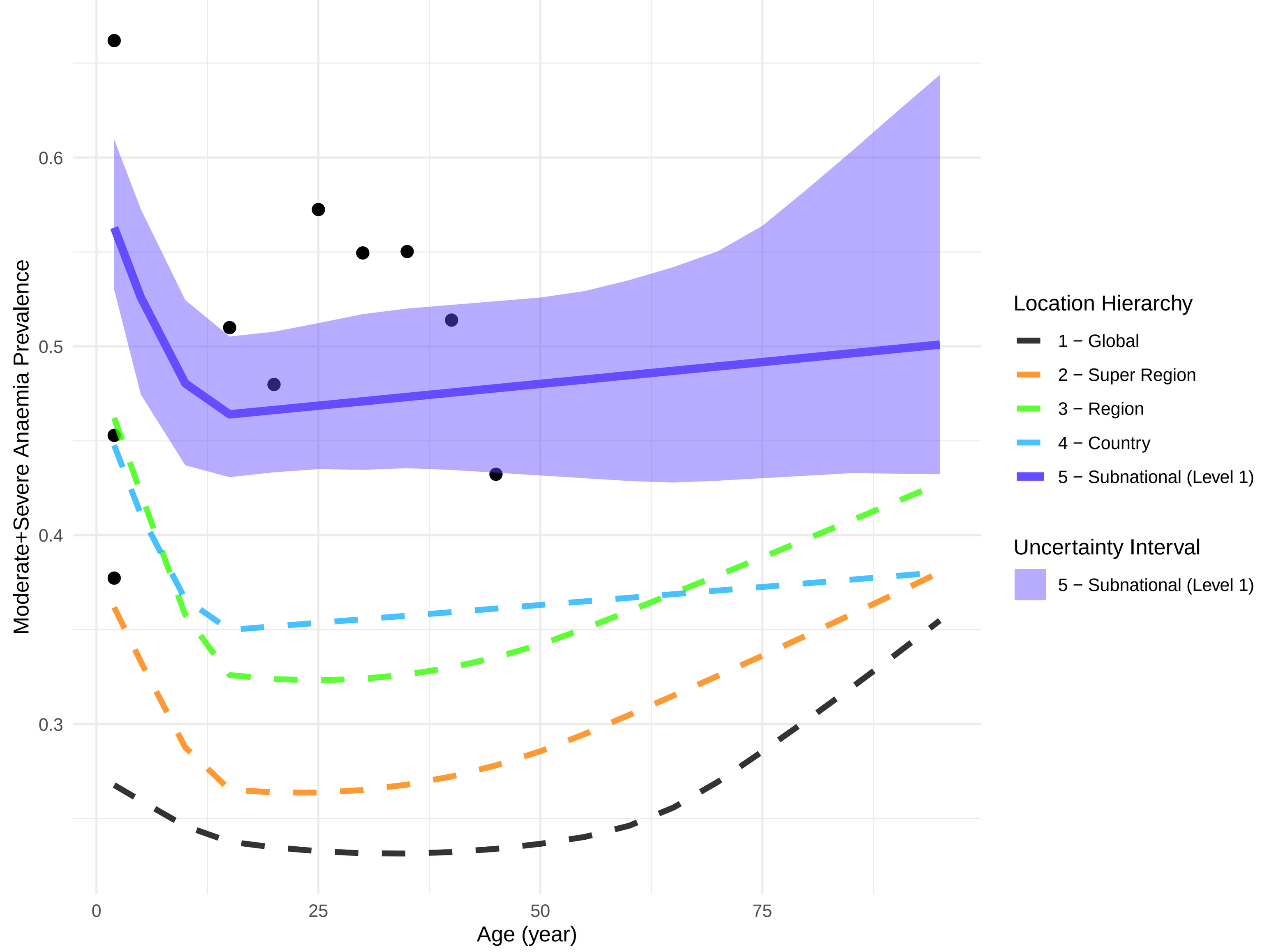

MR−BRT Age/Sex Fits − Non−pregnant Females 2+ Years Old − Moderate+Severe Anaemia Prevalence − Kebbi
WHO−2024 Adjustment Method

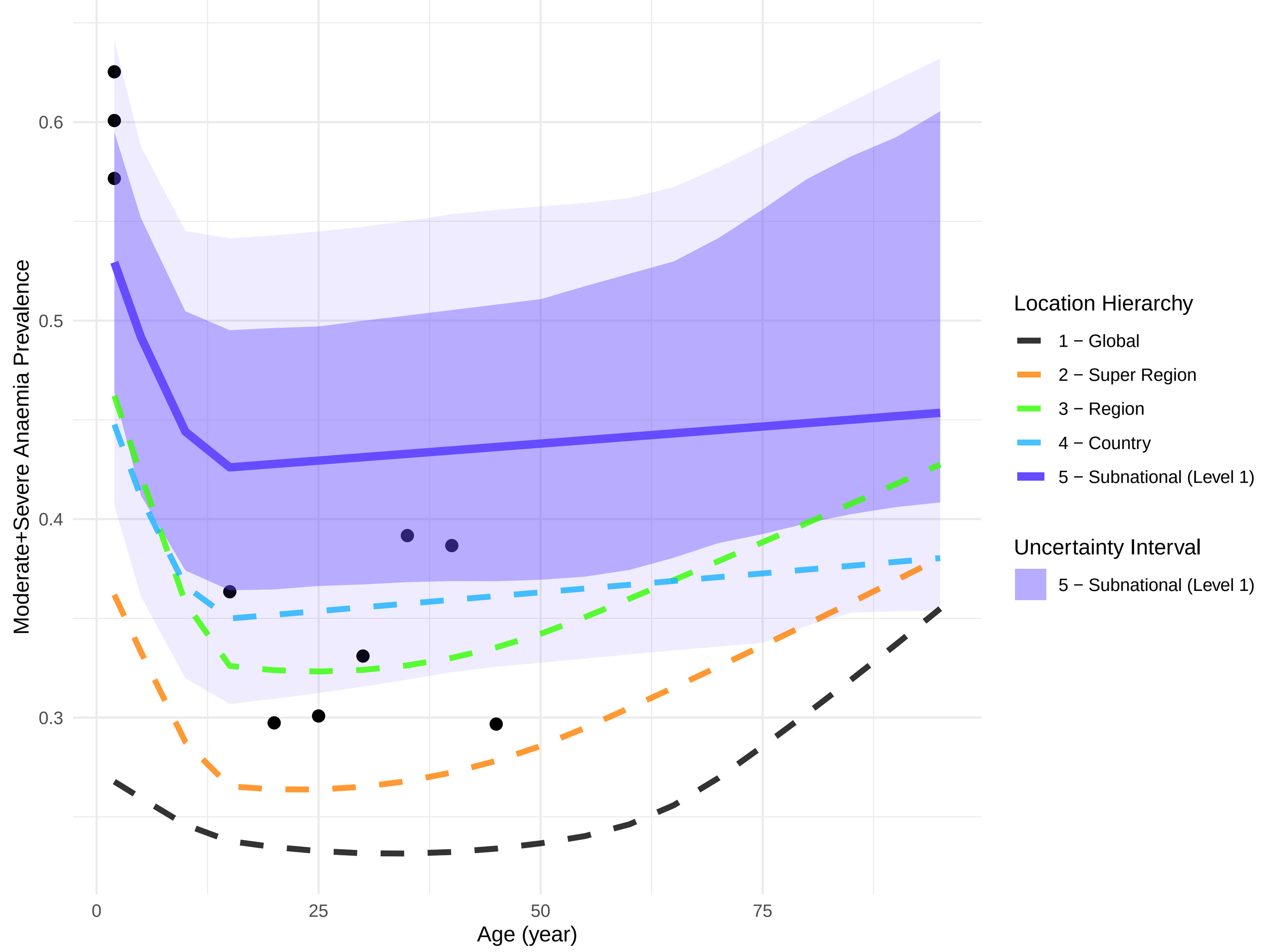

MR−BRT Age/Sex Fits − Non−pregnant Females 2+ Years Old − Moderate+Severe Anaemia Prevalence − Kogi
WHO−2024 Adjustment Method

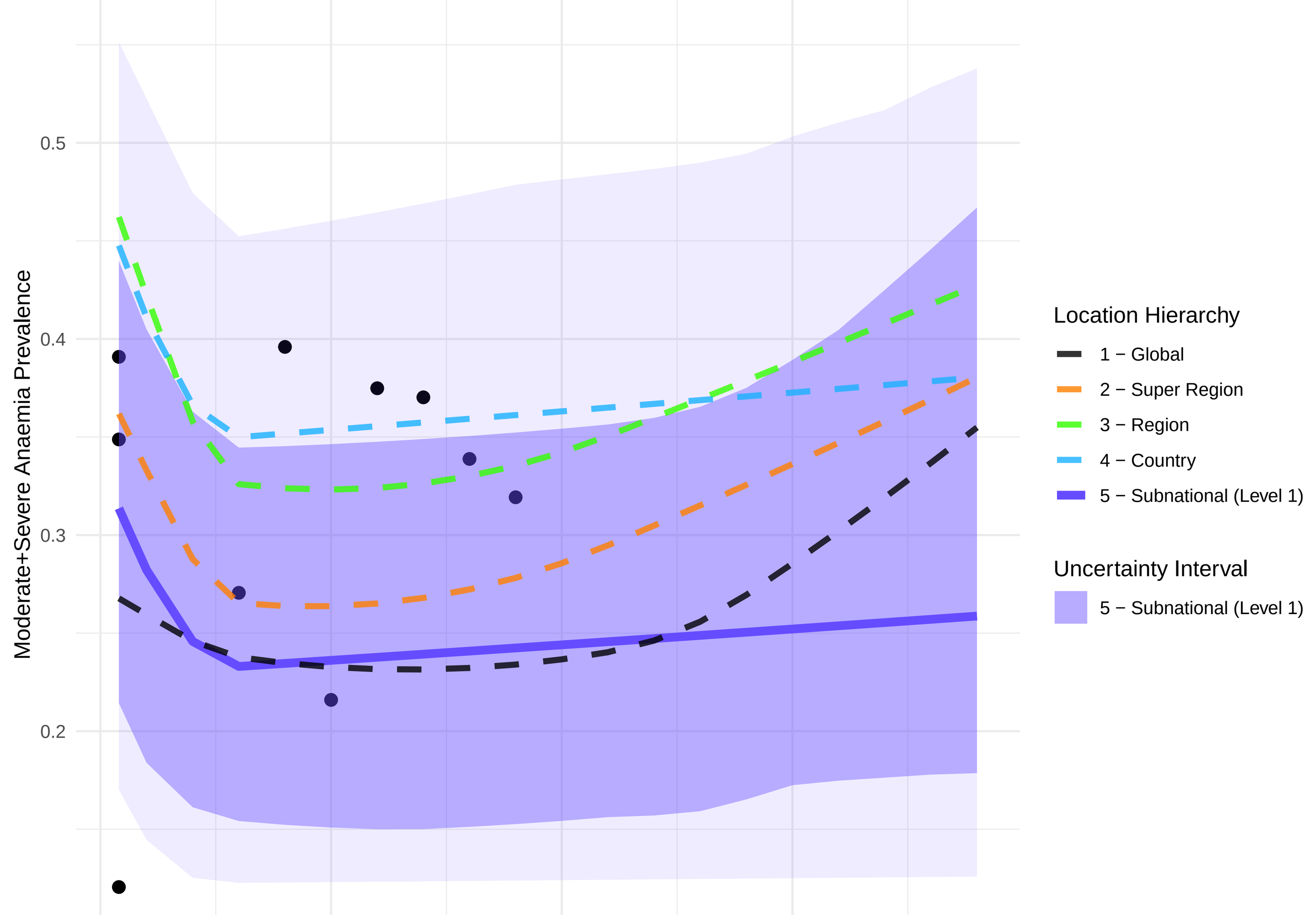

MR−BRT Age/Sex Fits − Non−pregnant Females 2+ Years Old − Moderate+Severe Anaemia Prevalence − Kwara
WHO−2024 Adjustment Method

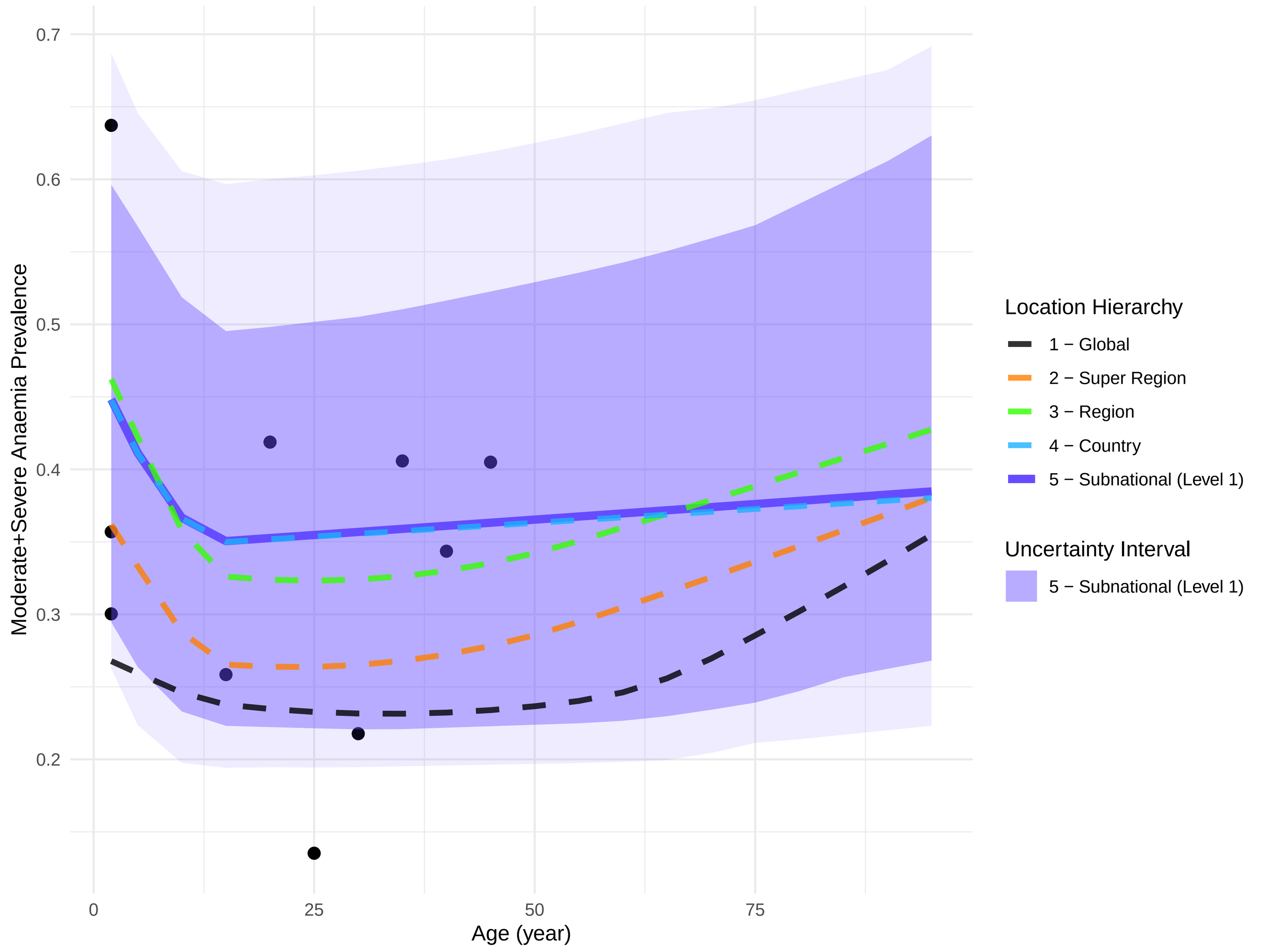

MR−BRT Age/Sex Fits − Non−pregnant Females 2+ Years Old − Moderate+Severe Anaemia Prevalence − Lagos
WHO−2024 Adjustment Method

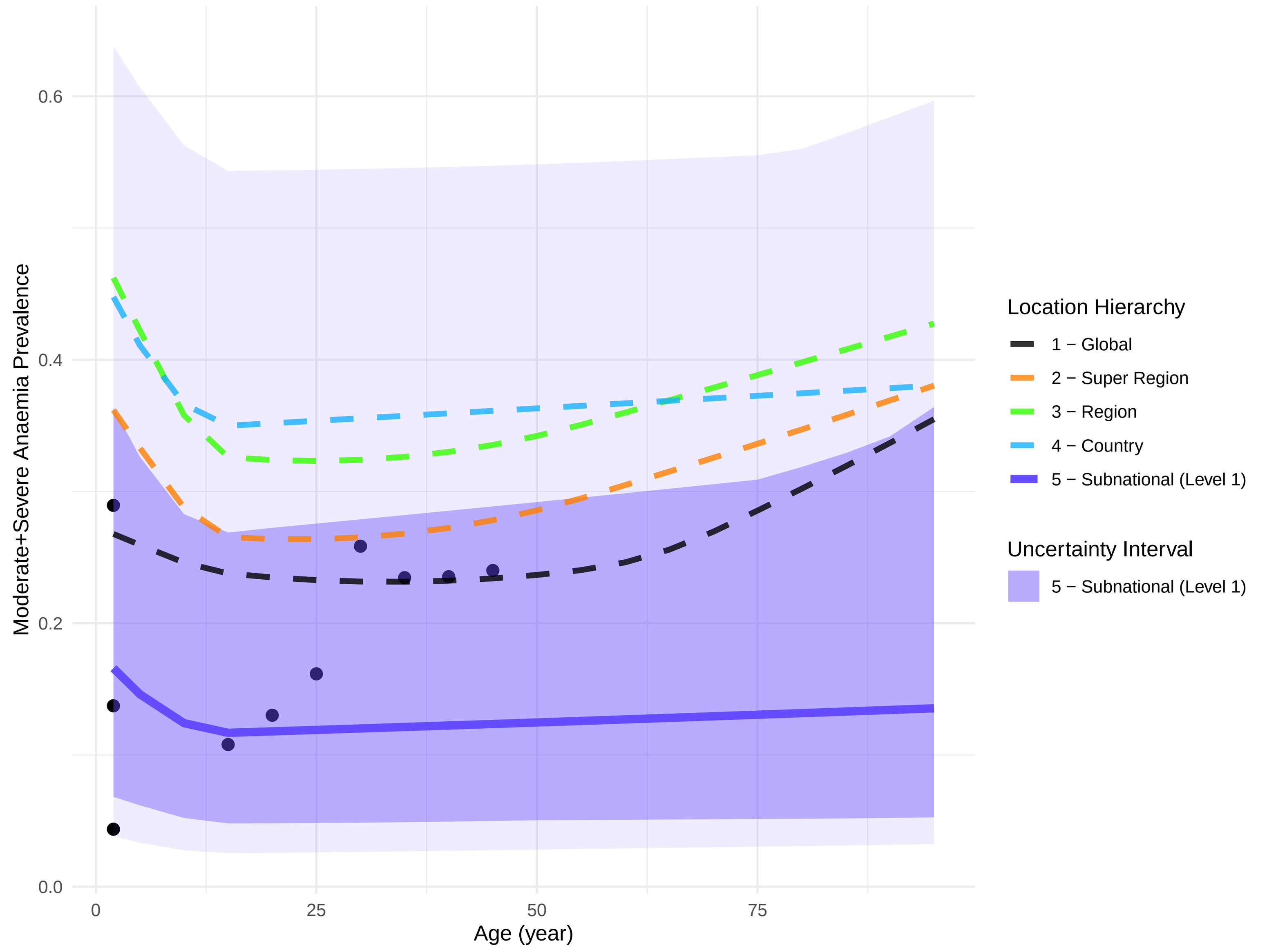

MR−BRT Age/Sex Fits − Non−pregnant Females 2+ Years Old − Moderate+Severe Anaemia Prevalence − Nasarawa
WHO−2024 Adjustment Method

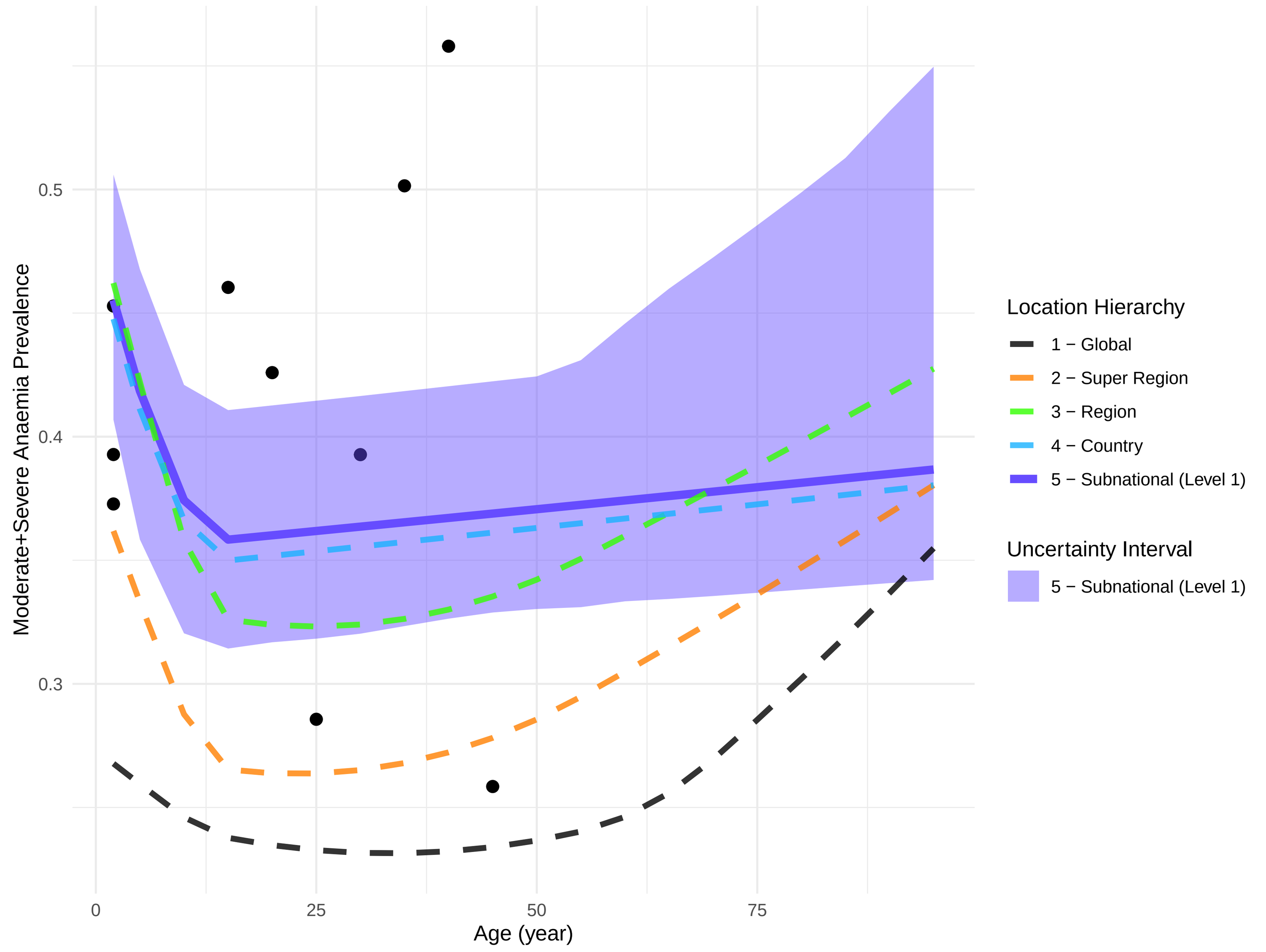

MR−BRT Age/Sex Fits − Non−pregnant Females 2+ Years Old − Moderate+Severe Anaemia Prevalence − Niger
WHO−2024 Adjustment Method

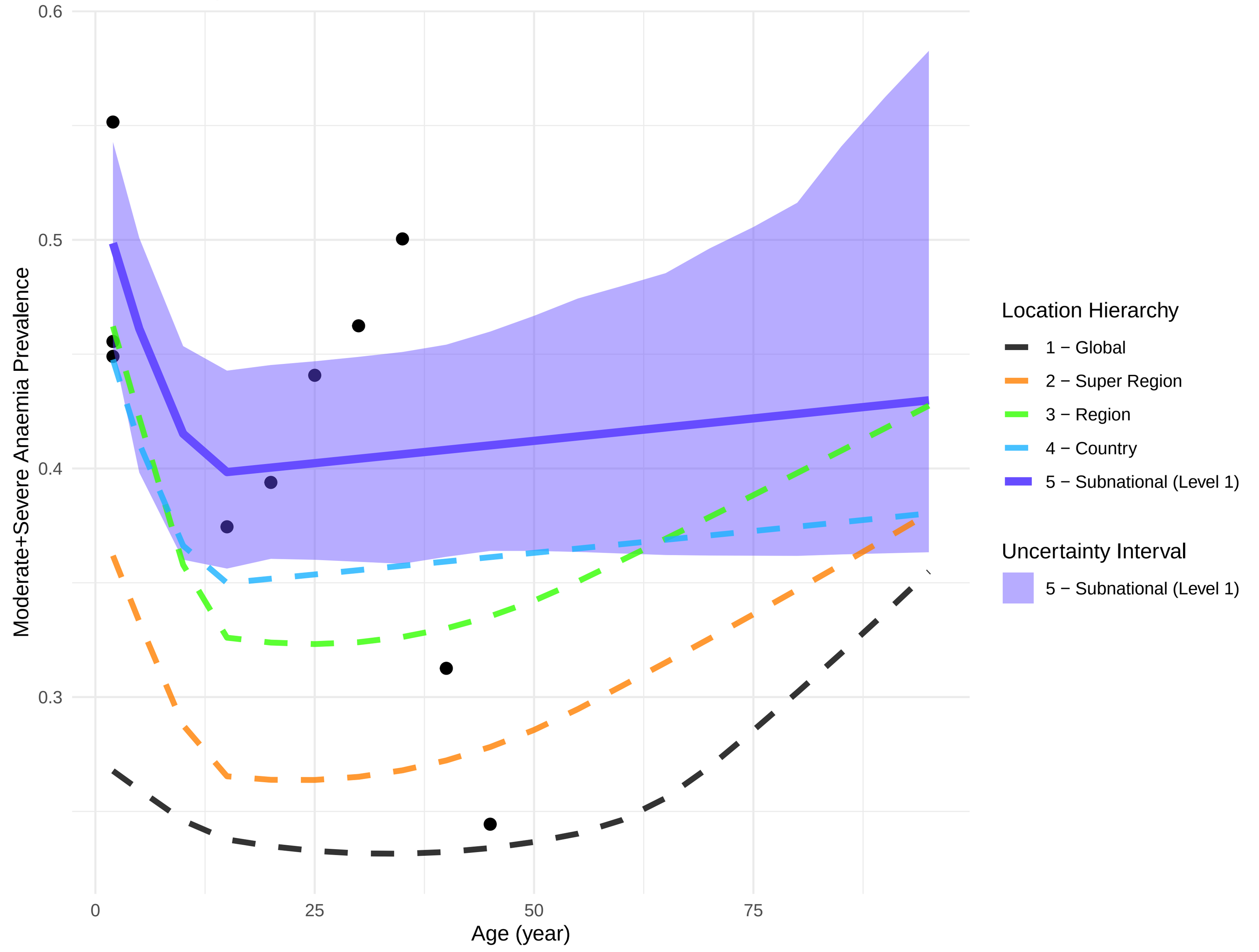

MR−BRT Age/Sex Fits − Non−pregnant Females 2+ Years Old − Moderate+Severe Anaemia Prevalence − Ogun
WHO−2024 Adjustment Method

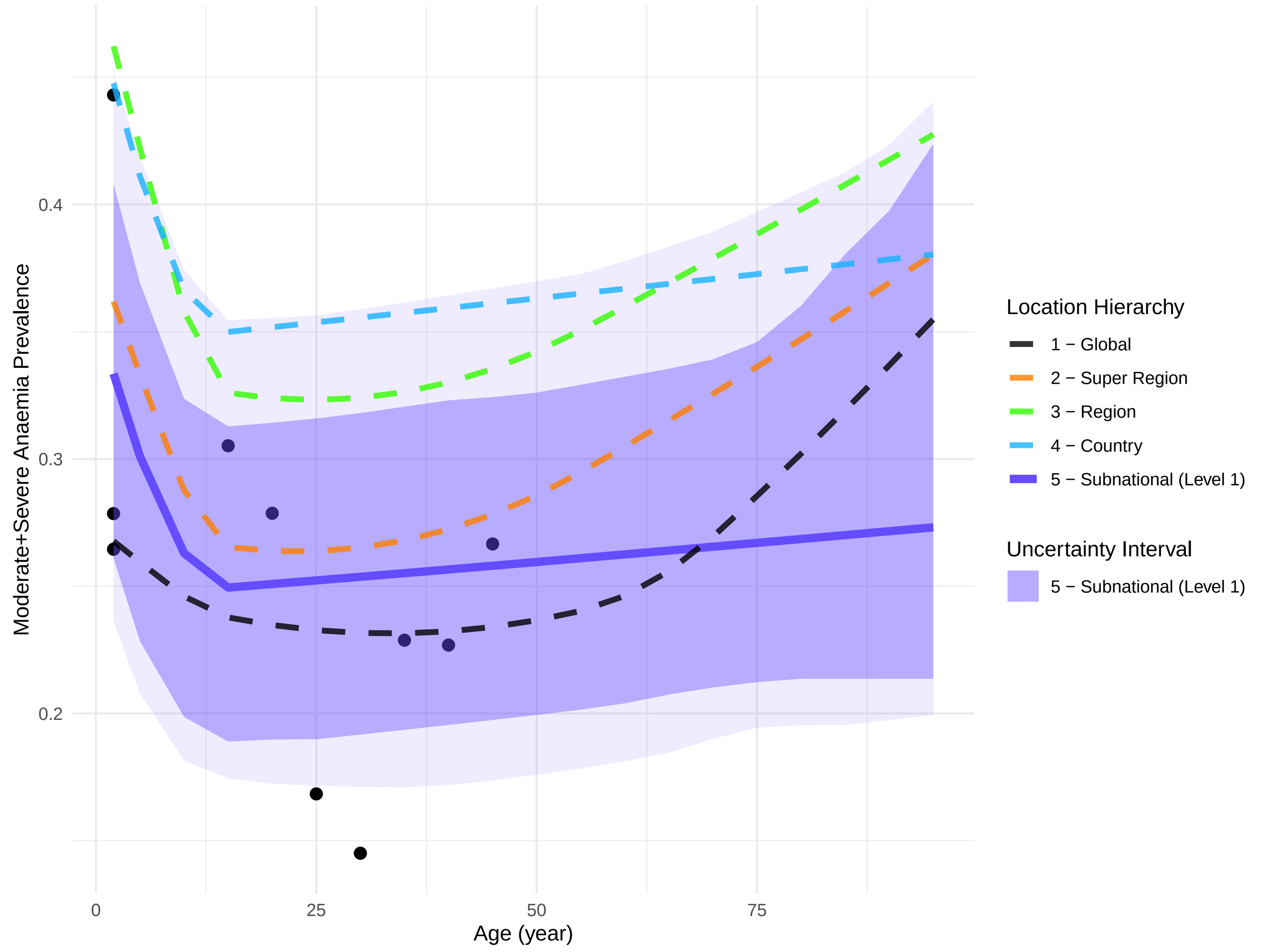

MR−BRT Age/Sex Fits − Non−pregnant Females 2+ Years Old − Moderate+Severe Anaemia Prevalence − Ondo
WHO−2024 Adjustment Method

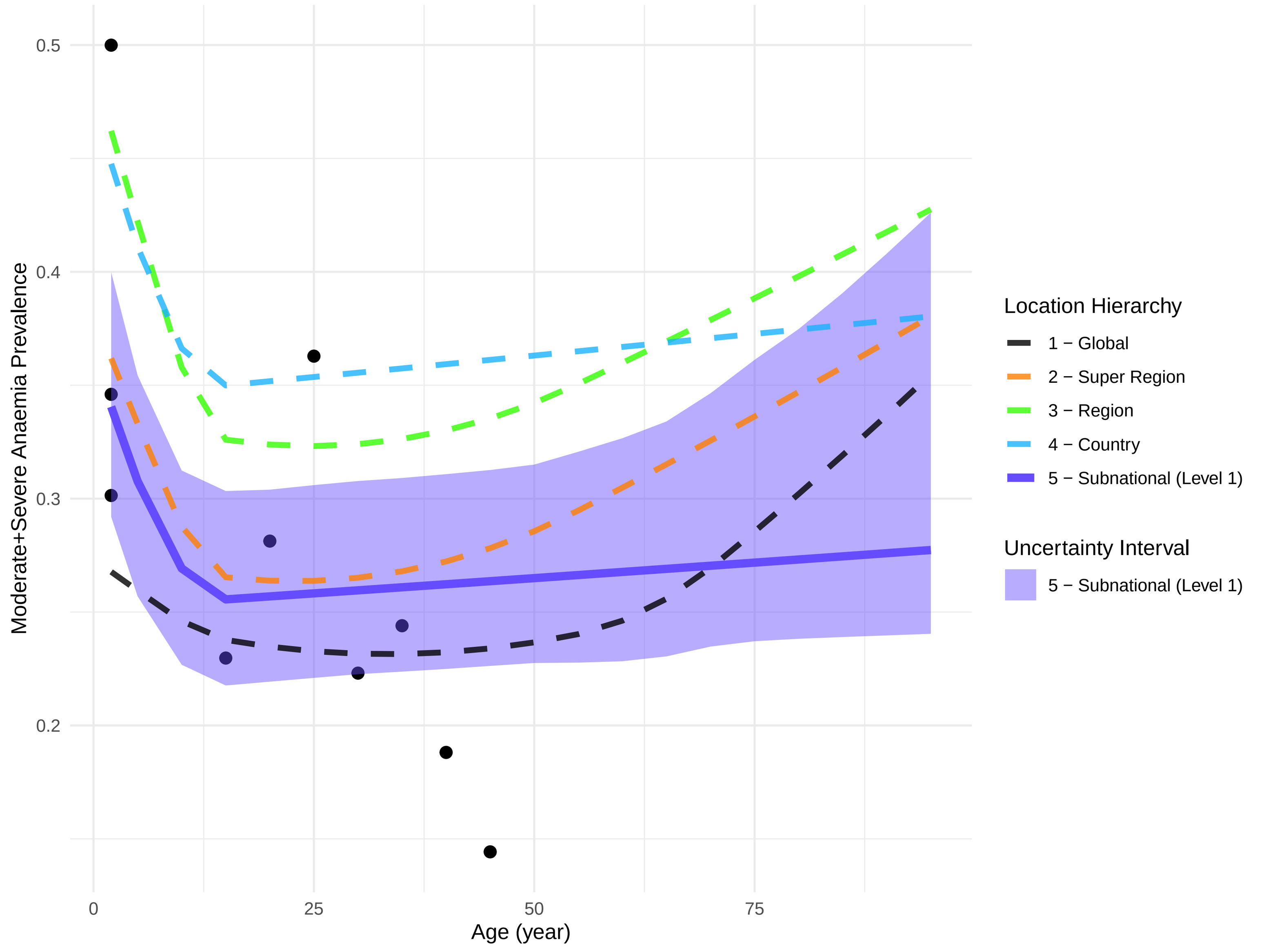

MR−BRT Age/Sex Fits − Non−pregnant Females 2+ Years Old − Moderate+Severe Anaemia Prevalence − Osun
WHO−2024 Adjustment Method

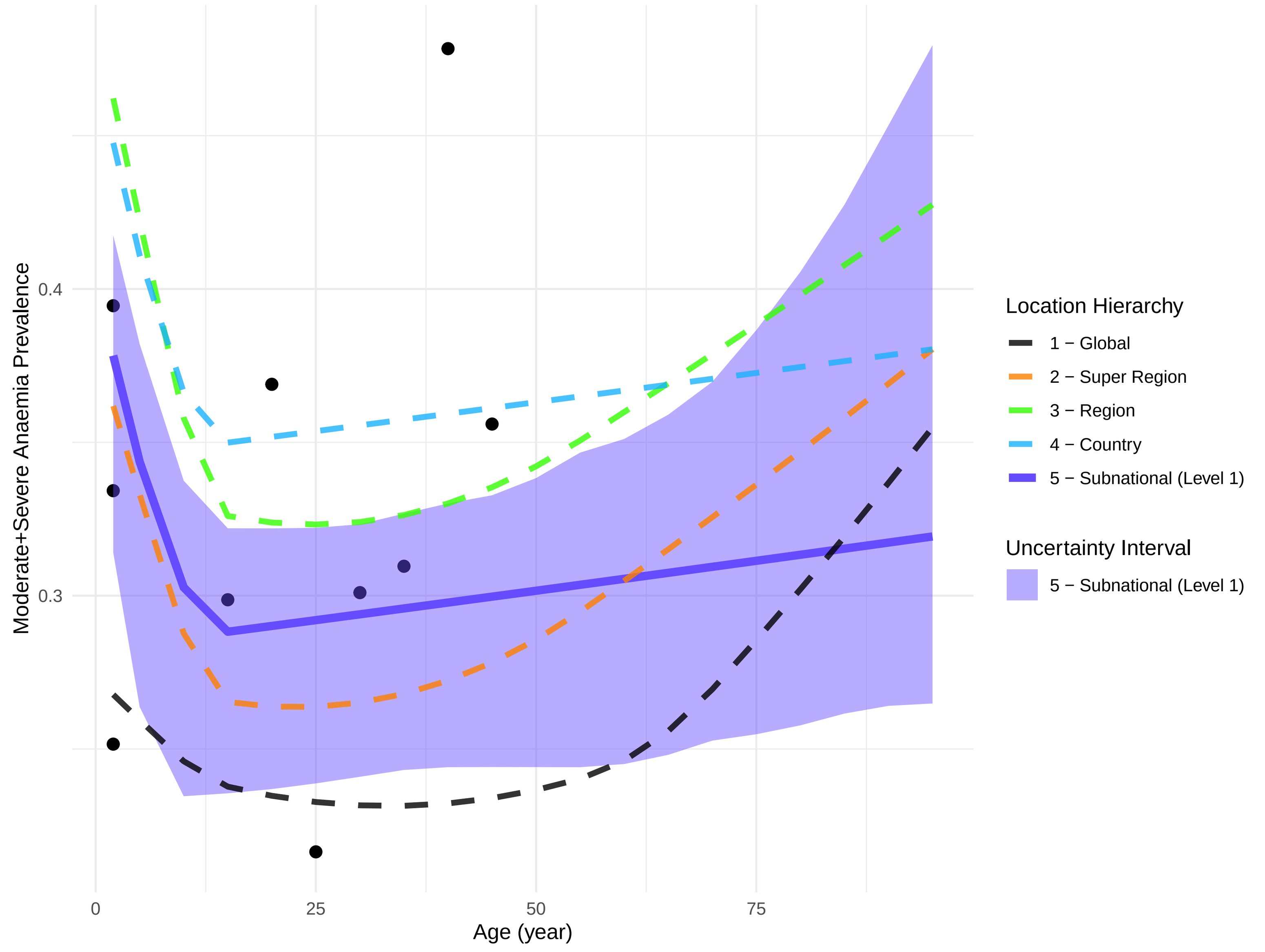

MR−BRT Age/Sex Fits − Non−pregnant Females 2+ Years Old − Moderate+Severe Anaemia Prevalence − Oyo
WHO−2024 Adjustment Method

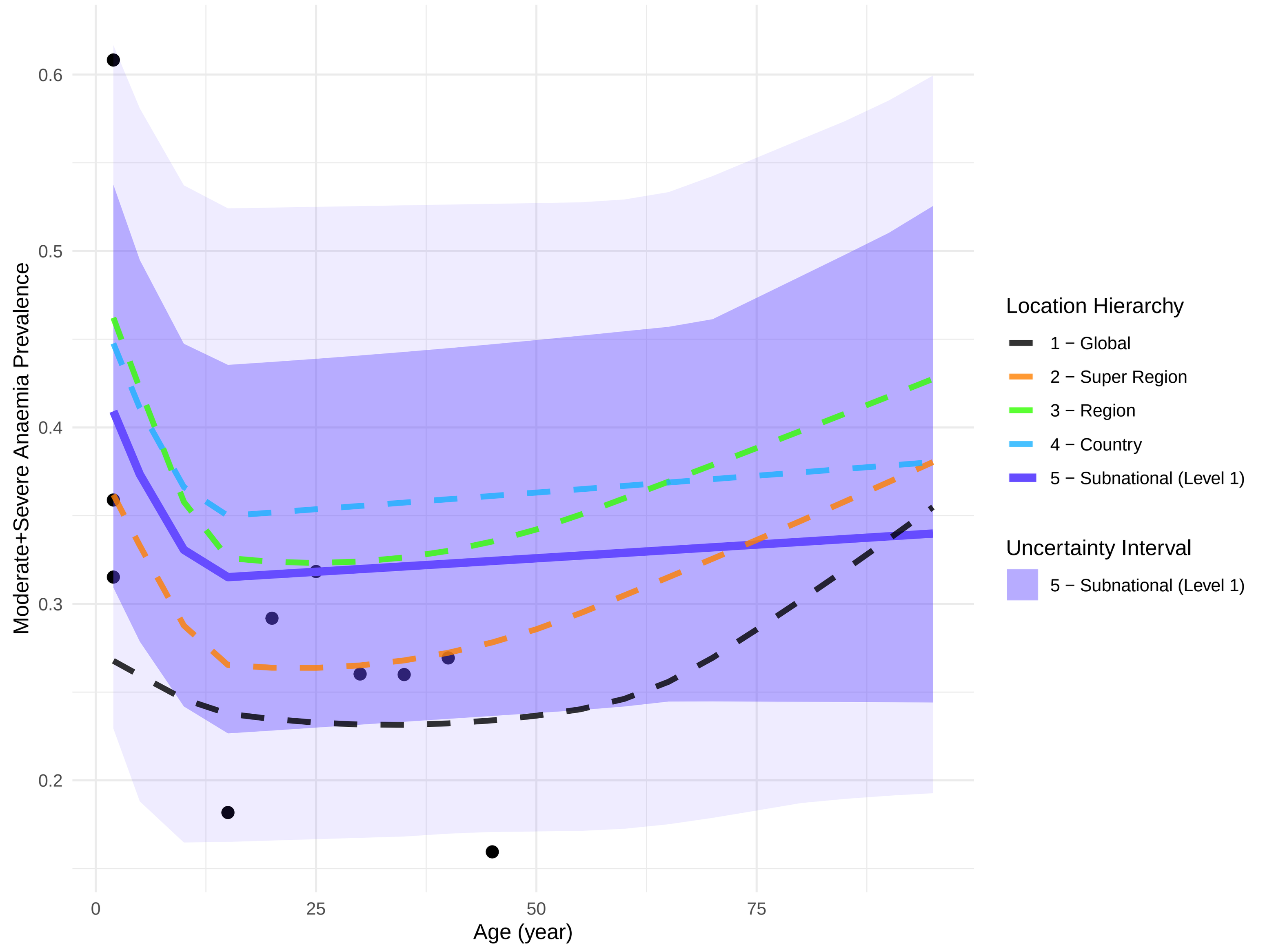

MR−BRT Age/Sex Fits − Non−pregnant Females 2+ Years Old − Moderate+Severe Anaemia Prevalence − Plateau
WHO−2024 Adjustment Method

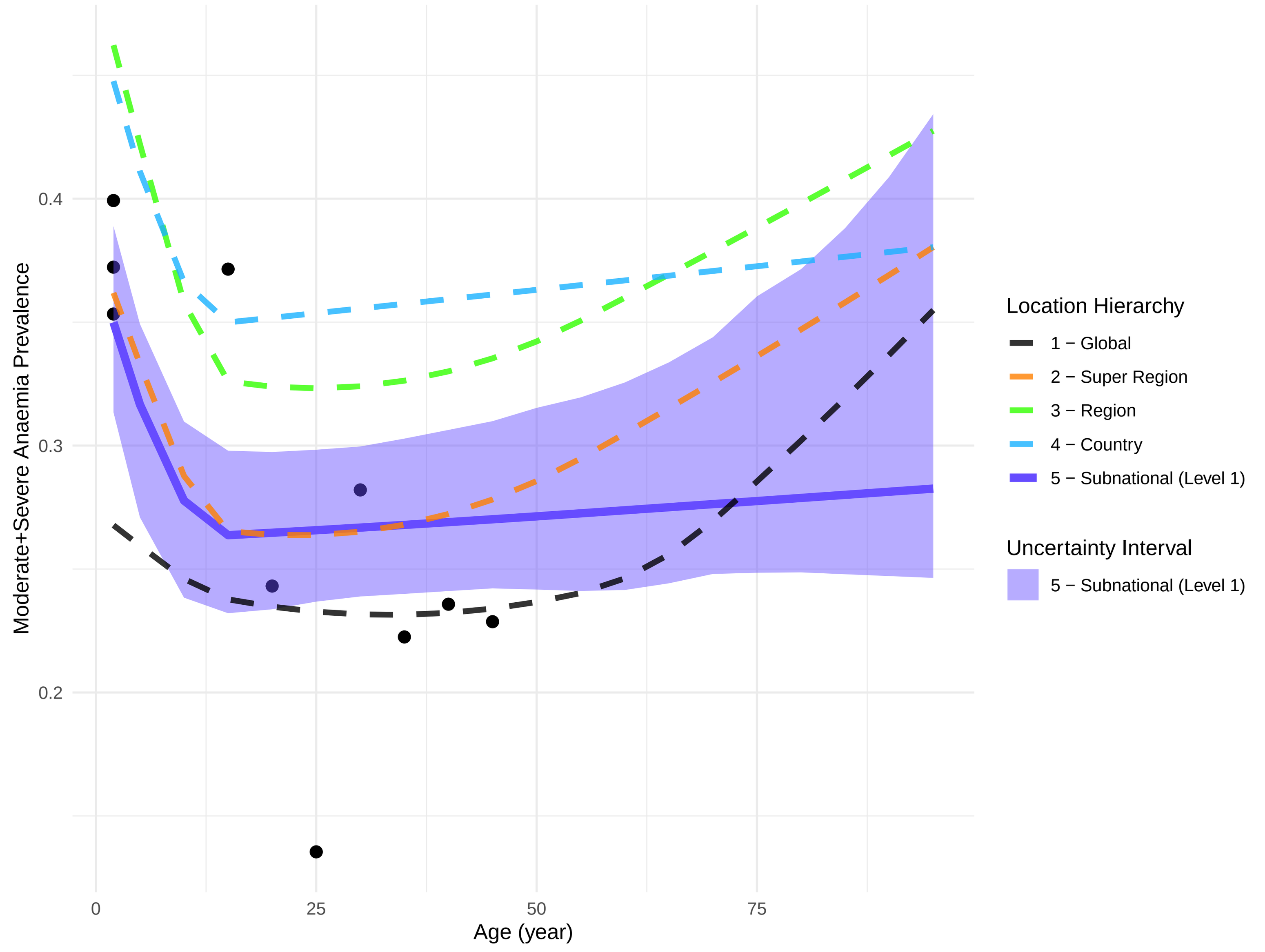

MR−BRT Age/Sex Fits − Non−pregnant Females 2+ Years Old − Moderate+Severe Anaemia Prevalence − Rivers
WHO−2024 Adjustment Method

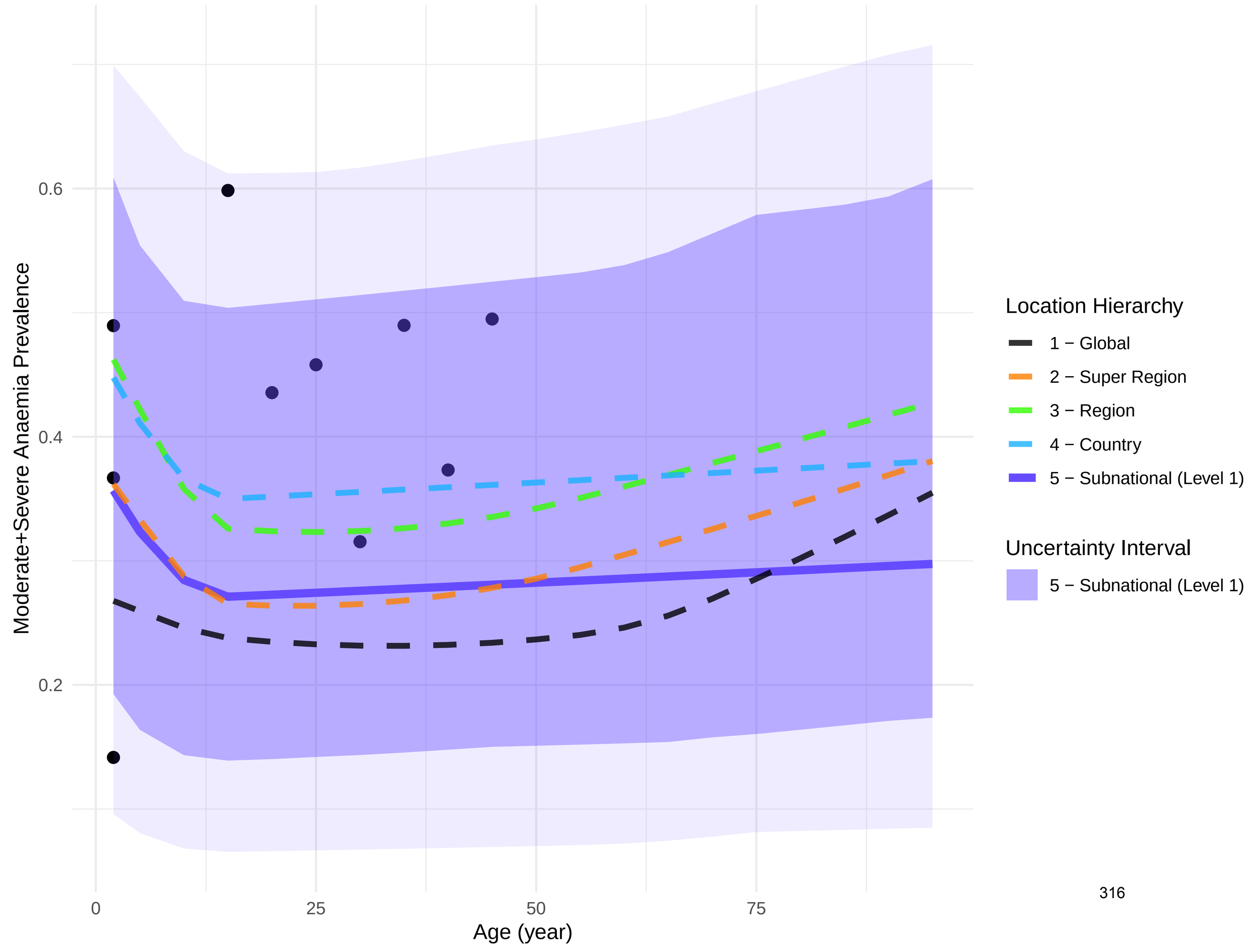

MR−BRT Age/Sex Fits − Non−pregnant Females 2+ Years Old − Moderate+Severe Anaemia Prevalence − Sokoto
WHO−2024 Adjustment Method

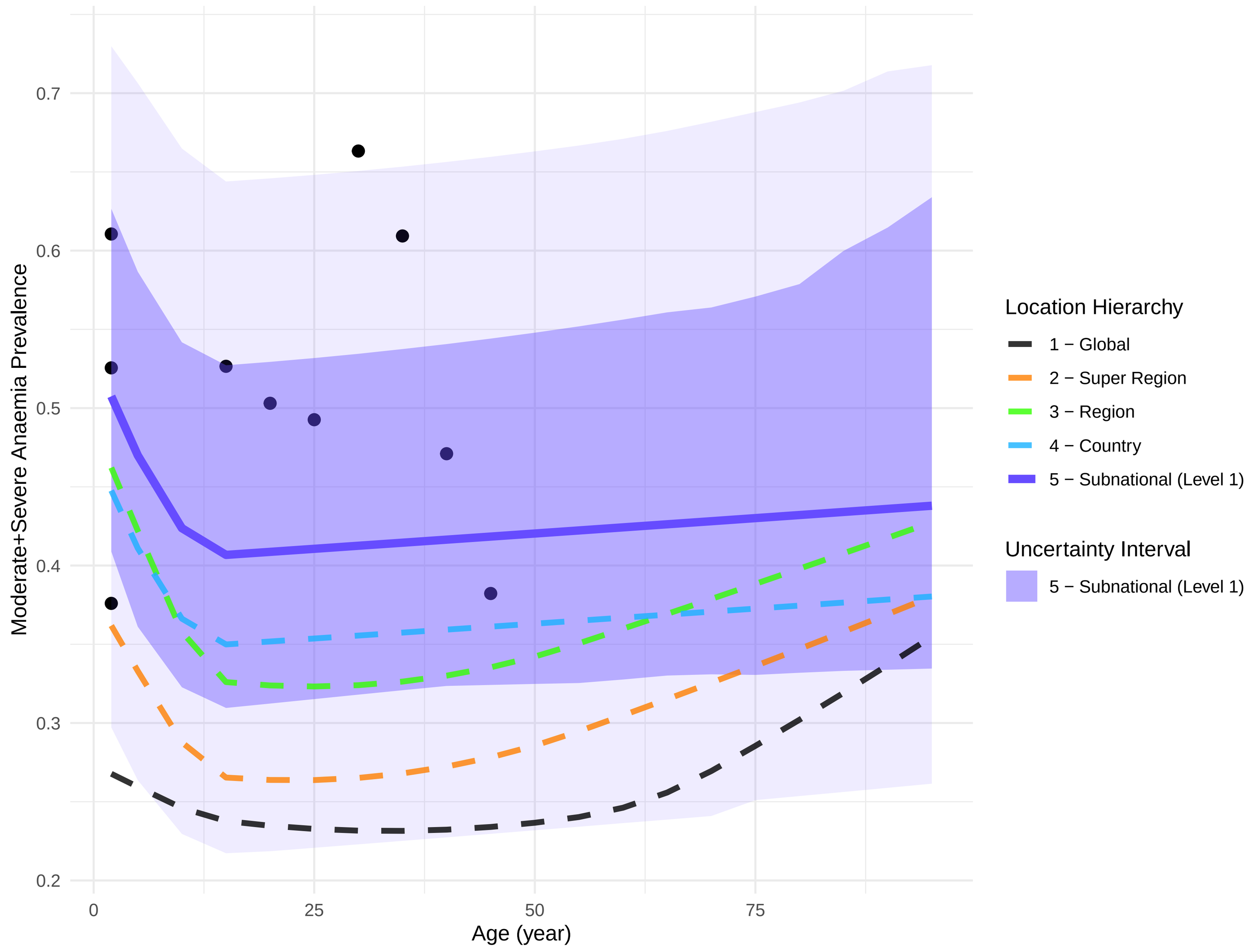

MR−BRT Age/Sex Fits − Non−pregnant Females 2+ Years Old − Moderate+Severe Anaemia Prevalence − Taraba
WHO−2024 Adjustment Method

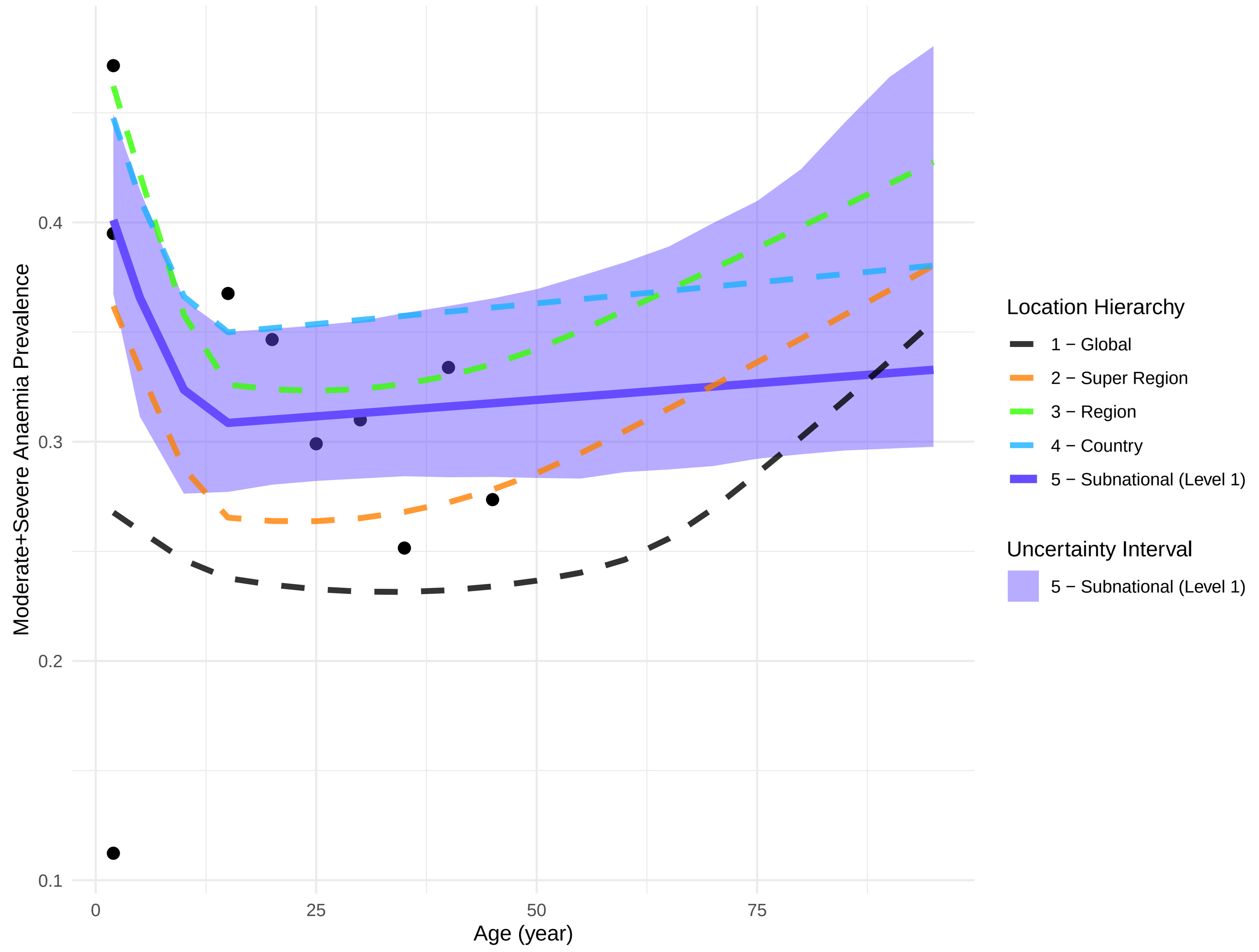

MR−BRT Age/Sex Fits − Non−pregnant Females 2+ Years Old − Moderate+Severe Anaemia Prevalence − Yobe
WHO−2024 Adjustment Method

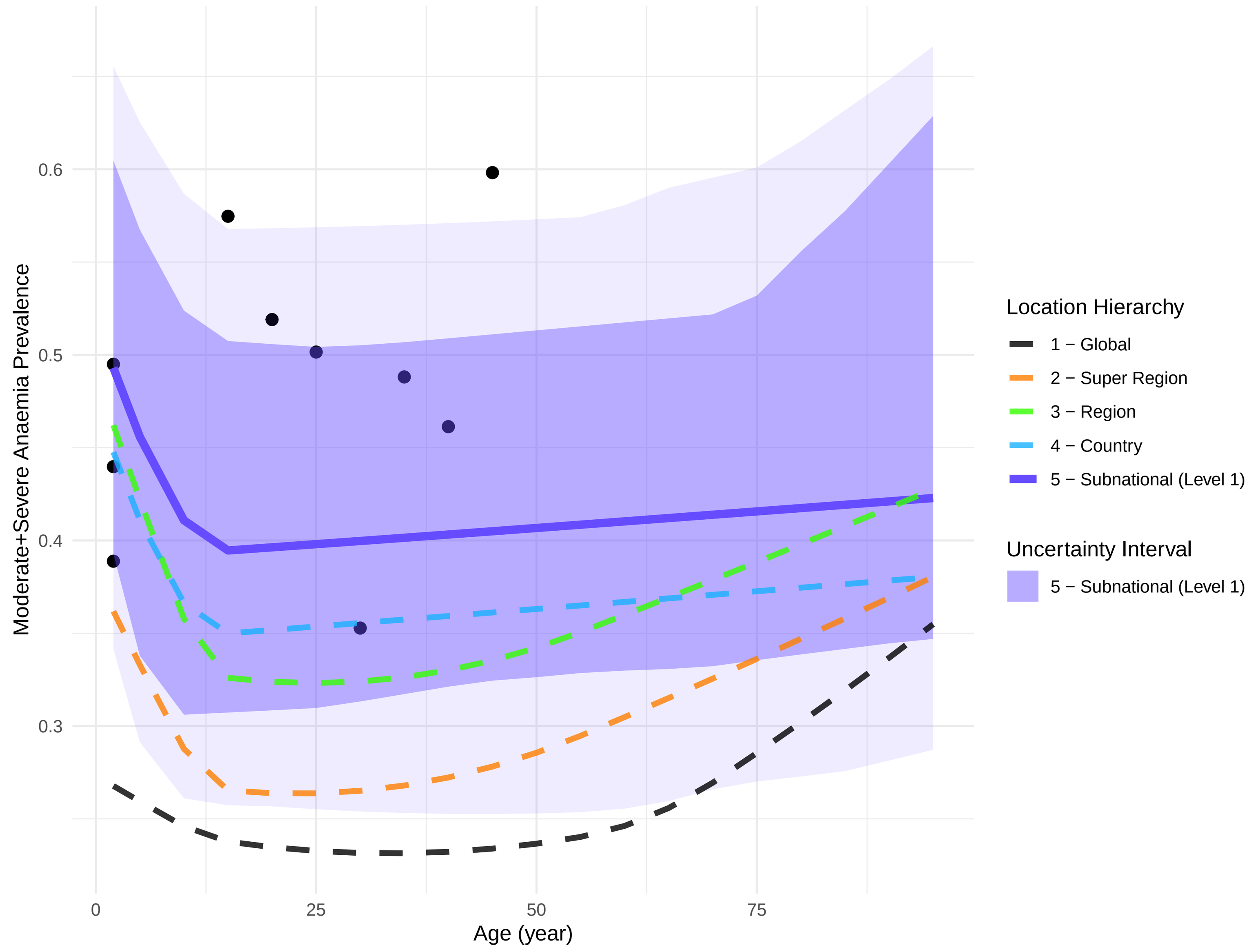

MR−BRT Age/Sex Fits − Non−pregnant Females 2+ Years Old − Moderate+Severe Anaemia Prevalence − Zamfara
WHO−2024 Adjustment Method

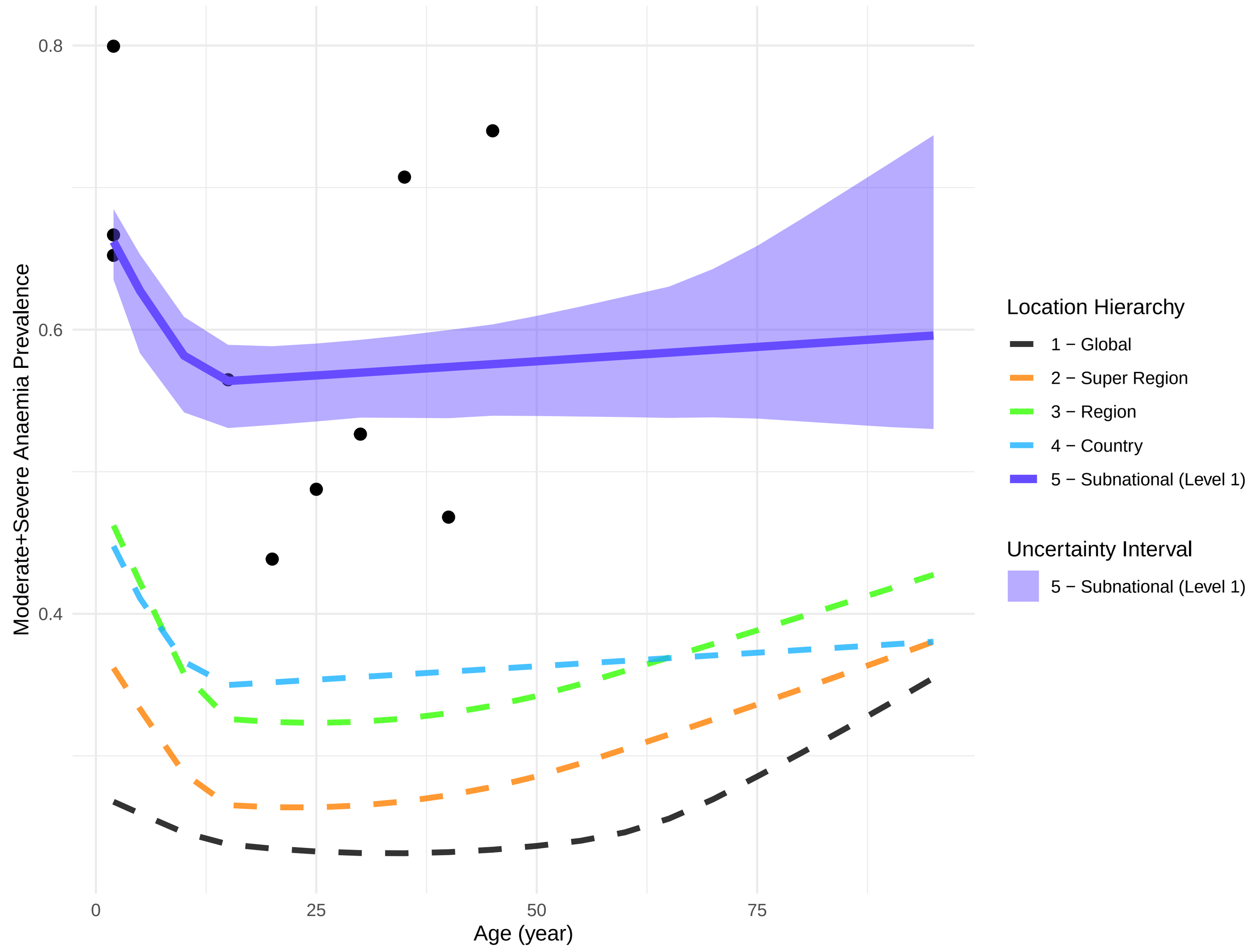

MR−BRT Age/Sex Fits − Non−pregnant Females 2+ Years Old − Moderate+Severe Anaemia Prevalence − Senegal
WHO−2024 Adjustment Method

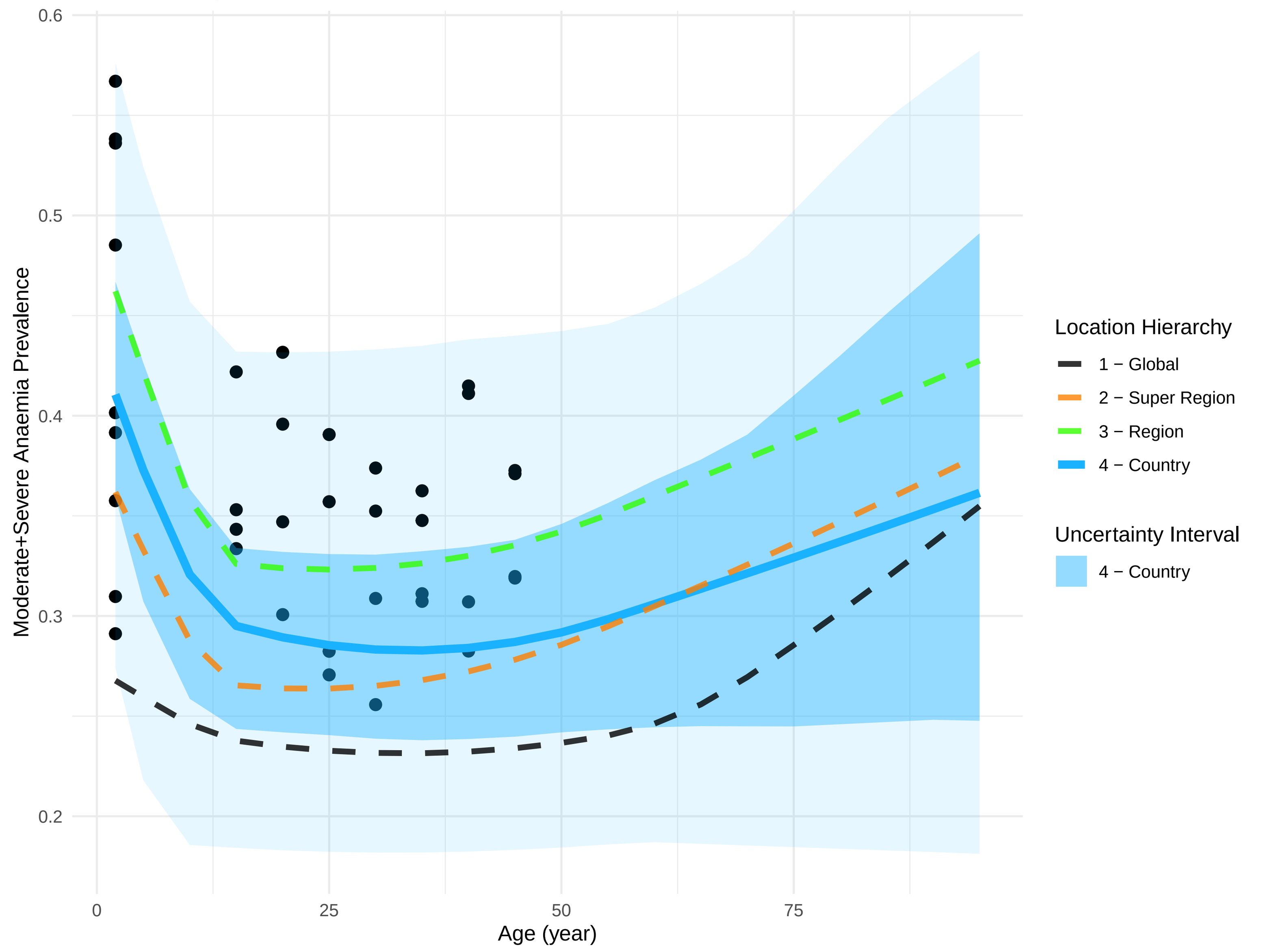

MR−BRT Age/Sex Fits − Non−pregnant Females 2+ Years Old − Moderate+Severe Anaemia Prevalence − Sierra Leone
WHO−2024 Adjustment Method

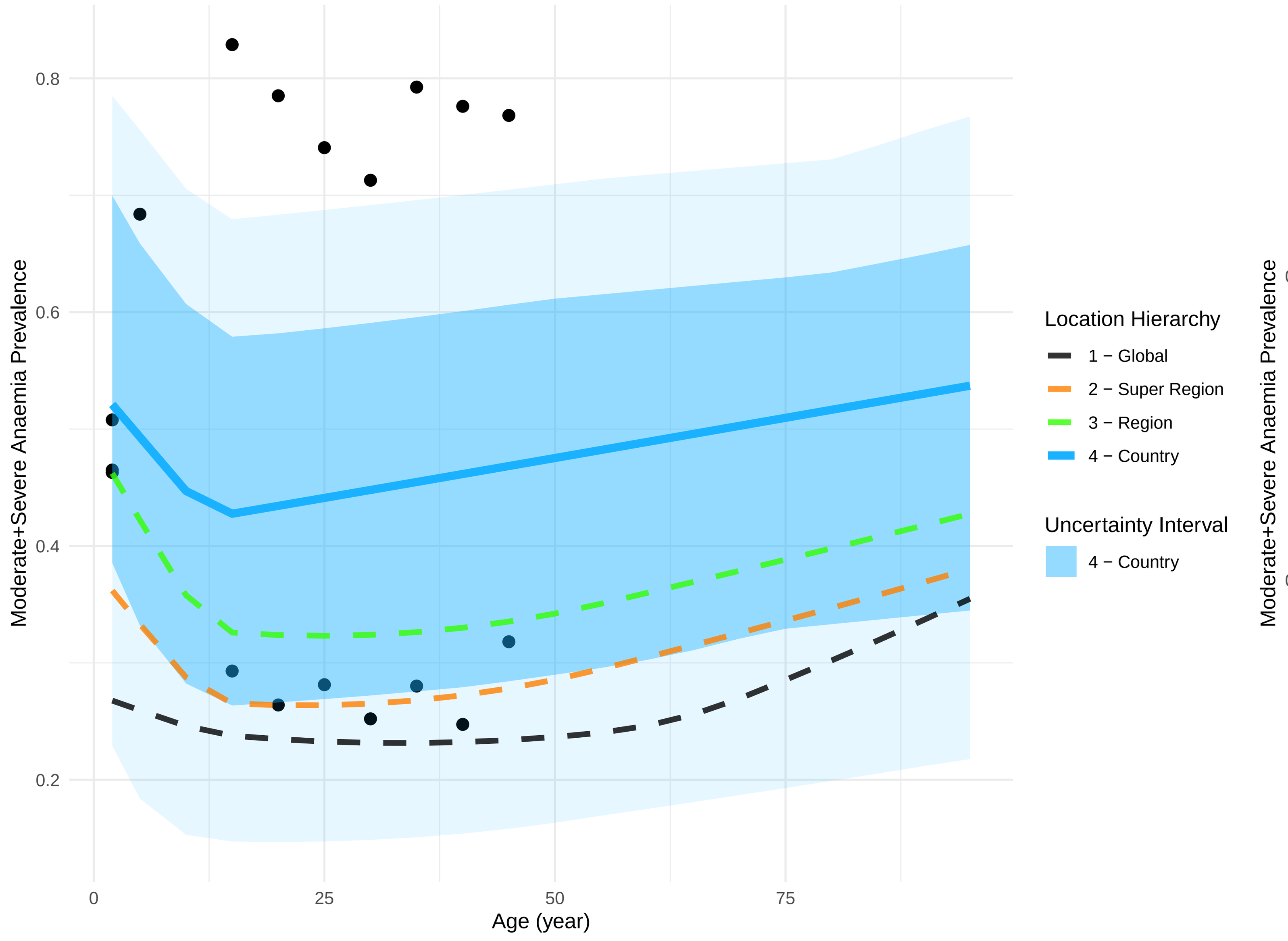

MR−BRT Age/Sex Fits − Non−pregnant Females 2+ Years Old − Moderate+Severe Anaemia Prevalence − Togo
WHO−2024 Adjustment Method

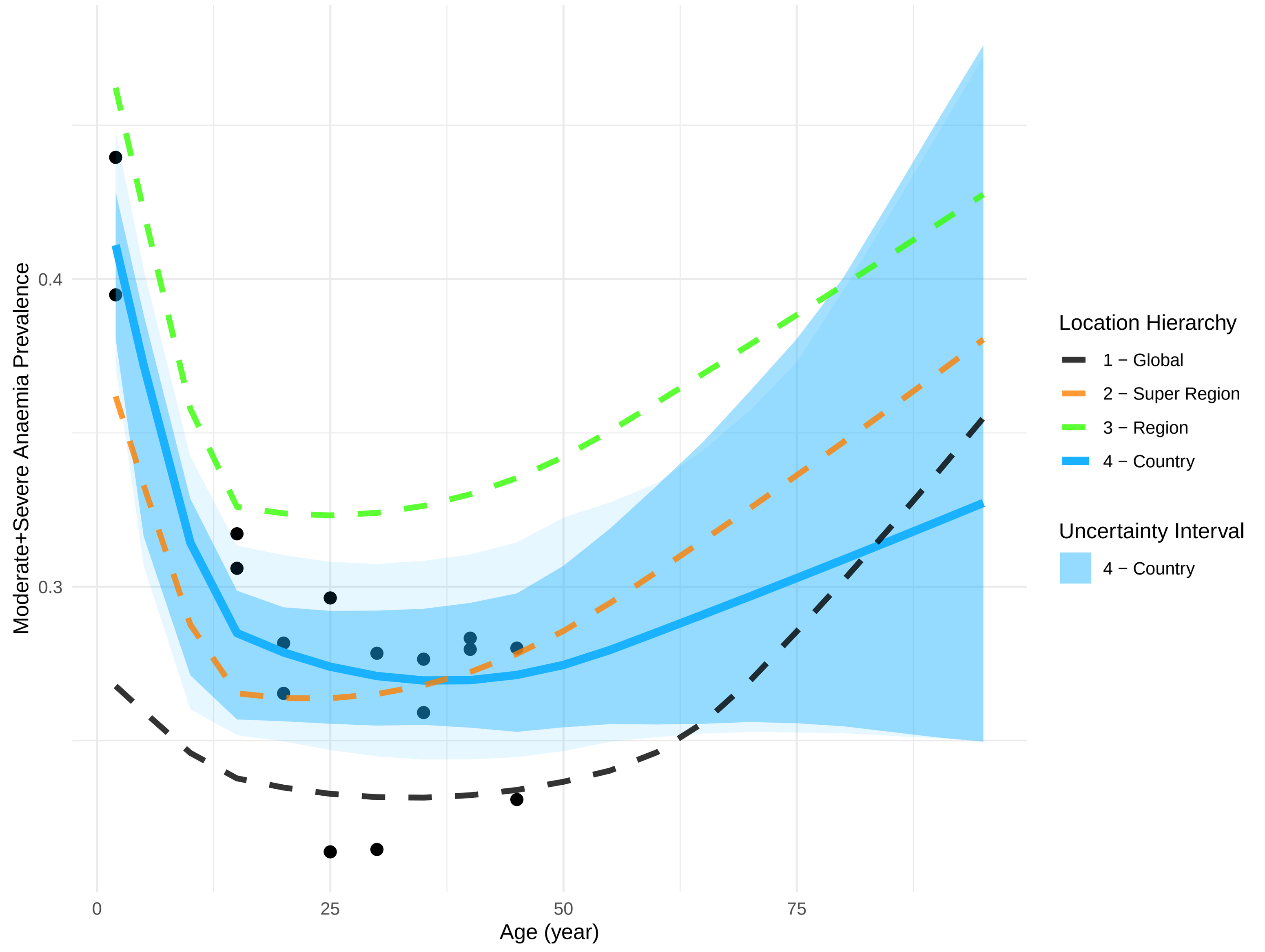

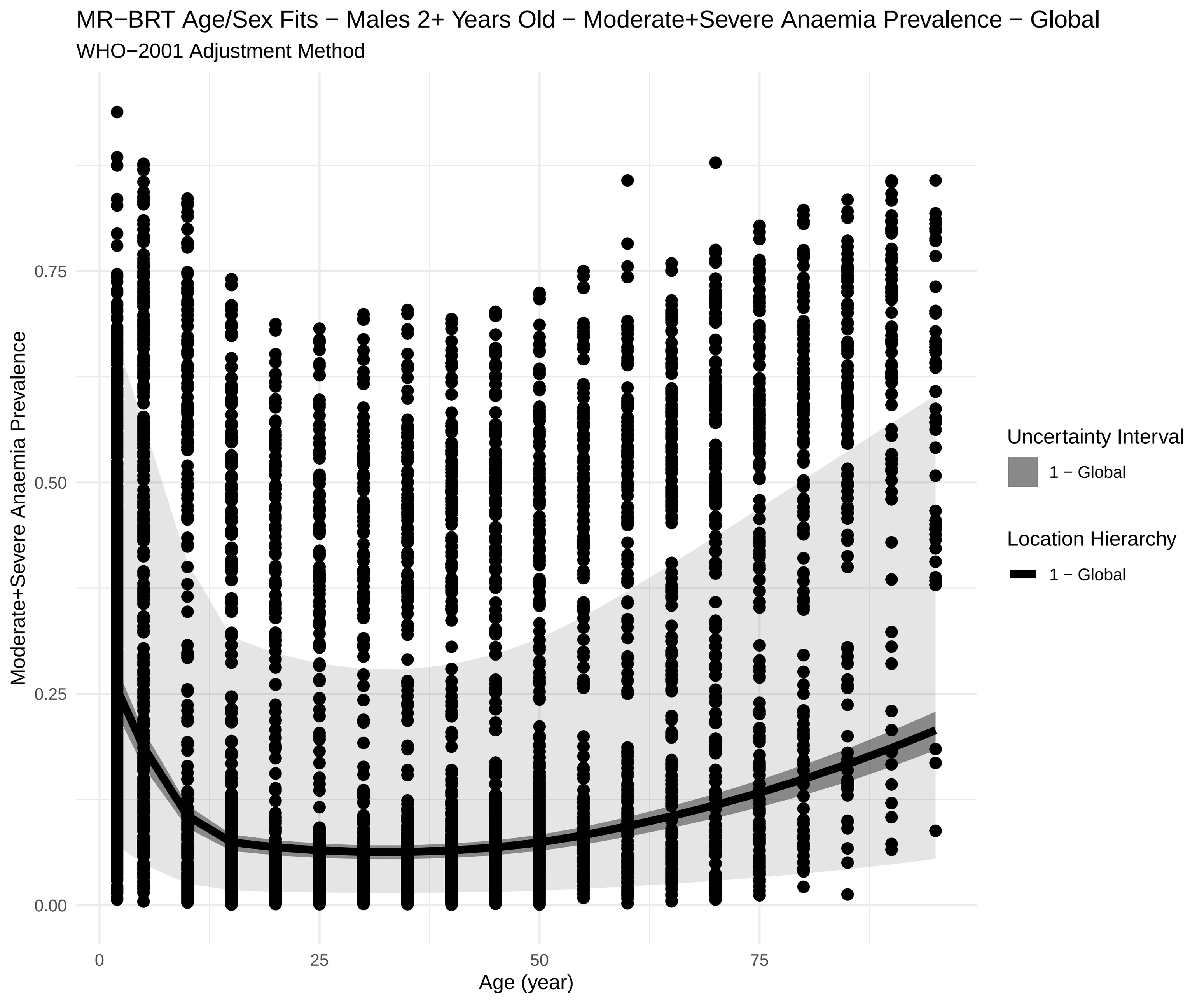
MR−BRT Age/Sex Fits − Males 2+ Years Old − Moderate+Severe Anaemia Prevalence − Global
WHO−2001 Adjustment Method
Moderate+Severe Anaemia Prevalence
Age (year)
Uncertainty Interval
1 − Global
Location Hierarchy
1 − Global

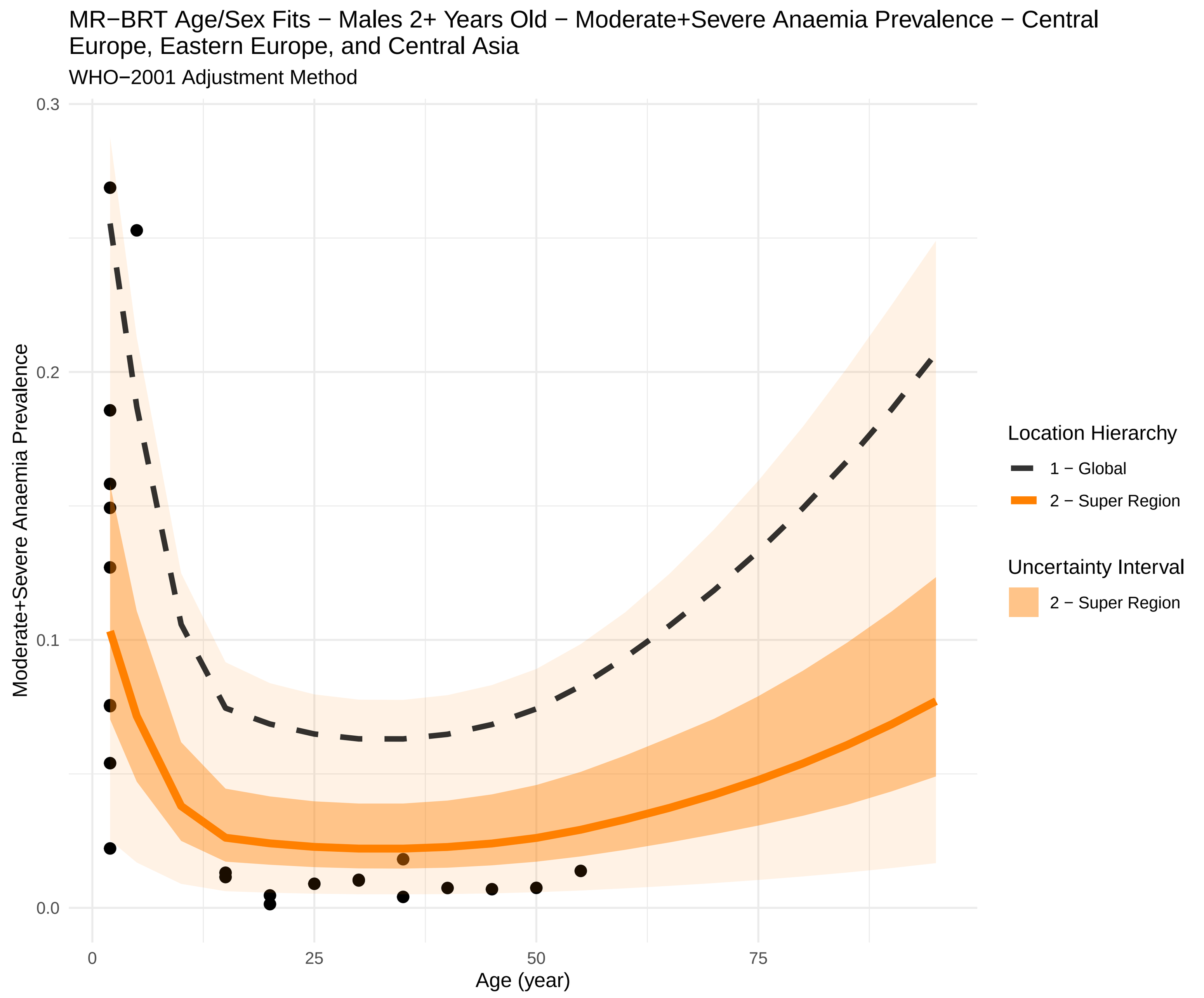
MR−BRT Age/Sex Fits − Males 2+ Years Old − Moderate+Severe Anaemia Prevalence − Central Europe, Eastern Europe, and Central Asia
WHO−2001 Adjustment Method
Moderate+Severe Anaemia Prevalence
Age (year)
Location Hierarchy
1 − Global
2 − Super Region
Uncertainty Interval
2 − Super Region

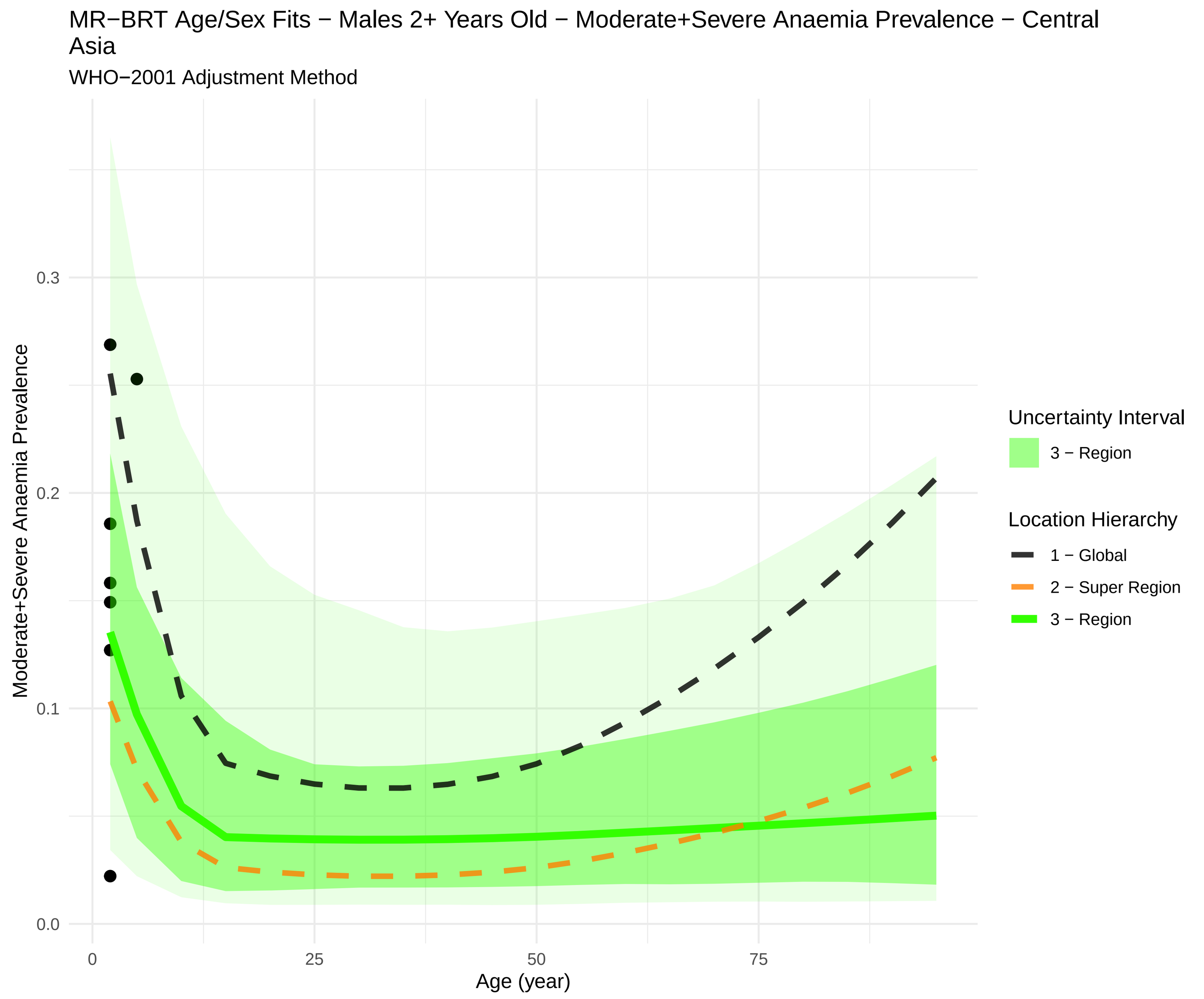
MR−BRT Age/Sex Fits − Males 2+ Years Old − Moderate+Severe Anaemia Prevalence − Central Asia
WHO−2001 Adjustment Method
Moderate+Severe Anaemia Prevalence
Age (year)
Uncertainty Interval
3 − Region
Location Hierarchy
1 − Global
2 − Super Region
3 − Region

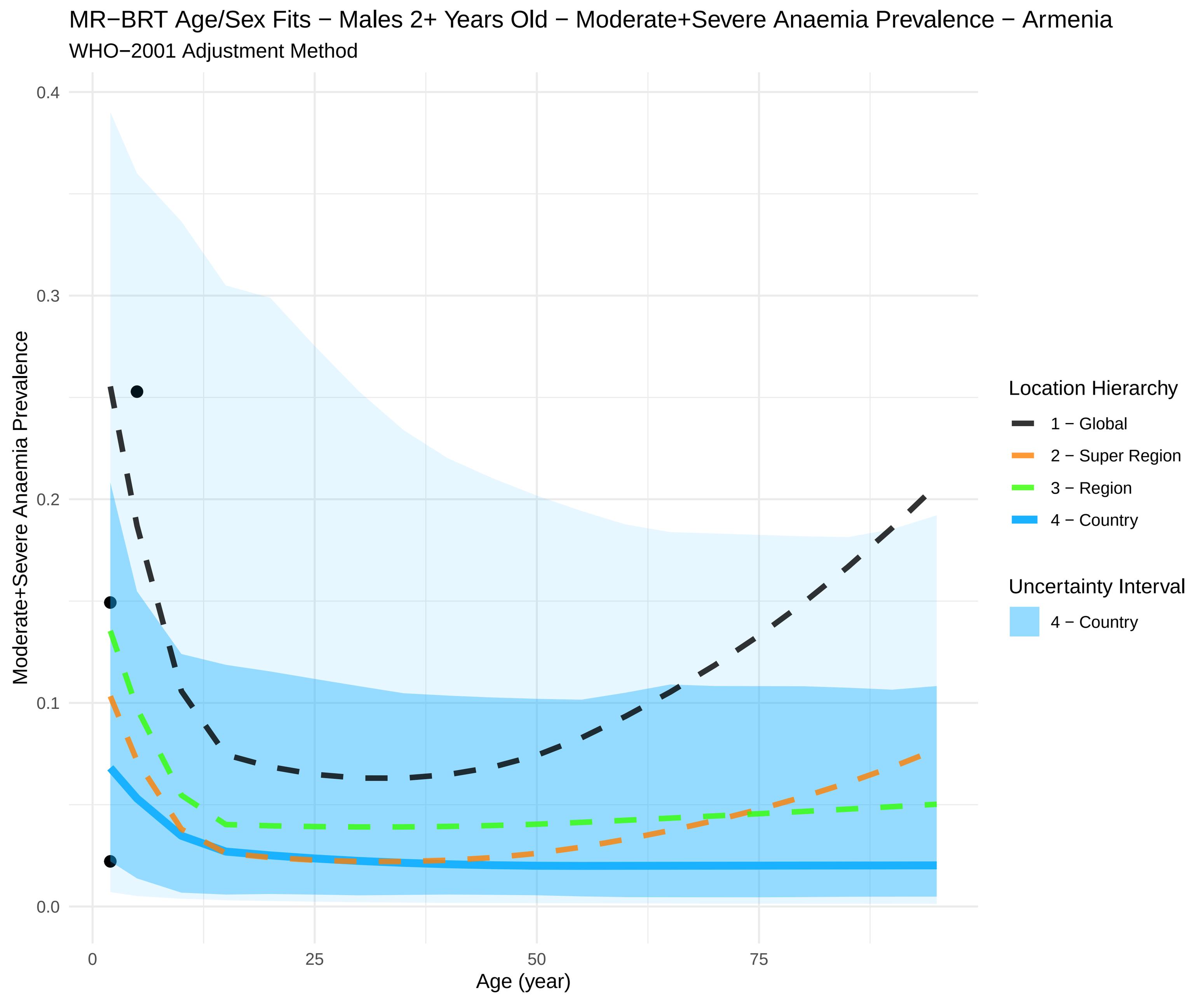
MR−BRT Age/Sex Fits − Males 2+ Years Old − Moderate+Severe Anaemia Prevalence − Armenia
WHO−2001 Adjustment Method
Moderate+Severe Anaemia Prevalence
Age (year)
Location Hierarchy
1 − Global
2 − Super Region
3 − Region
4 − Country
Uncertainty Interval
4 − Country

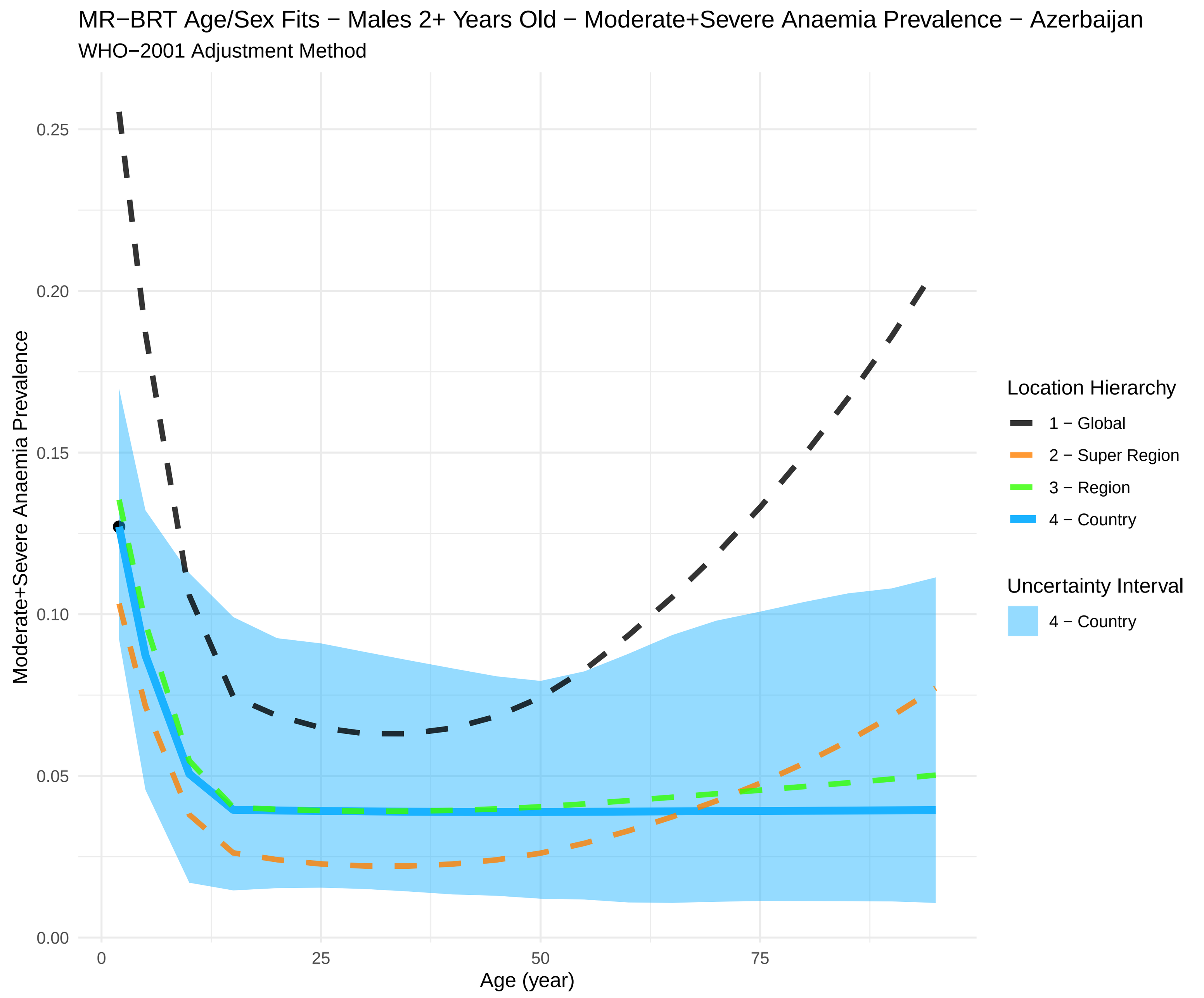
MR−BRT Age/Sex Fits − Males 2+ Years Old − Moderate+Severe Anaemia Prevalence − Azerbaijan
WHO−2001 Adjustment Method
Moderate+Severe Anaemia Prevalence
Age (year)
Location Hierarchy
1 − Global
2 − Super Region
3 − Region
4 − Country
Uncertainty Interval
4 − Country

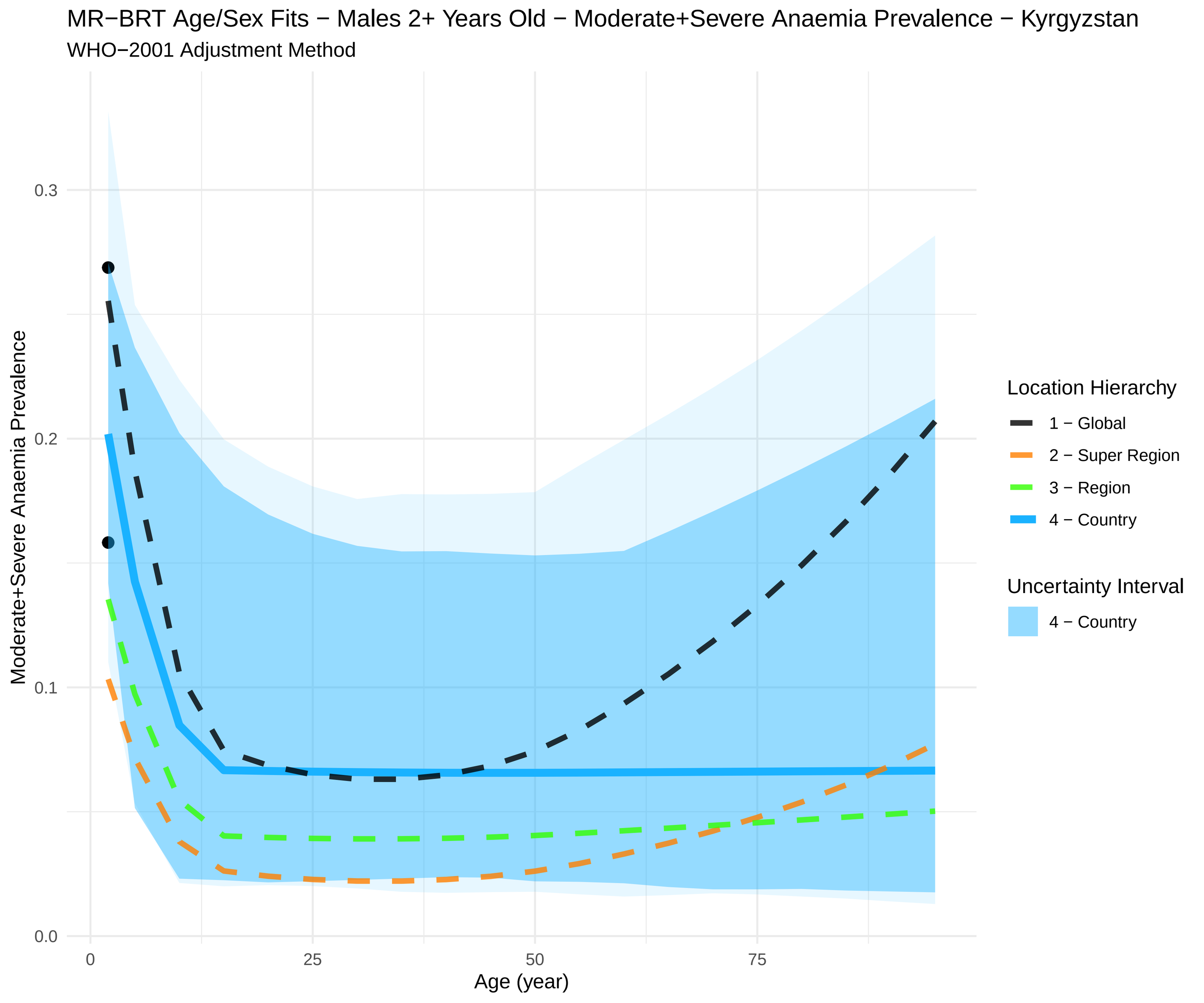
MR−BRT Age/Sex Fits − Males 2+ Years Old − Moderate+Severe Anaemia Prevalence − Kyrgyzstan
WHO−2001 Adjustment Method
Moderate+Severe Anaemia Prevalence
Age (year)
Location Hierarchy
1 − Global
2 − Super Region
3 − Region
4 − Country
Uncertainty Interval
4 − Country

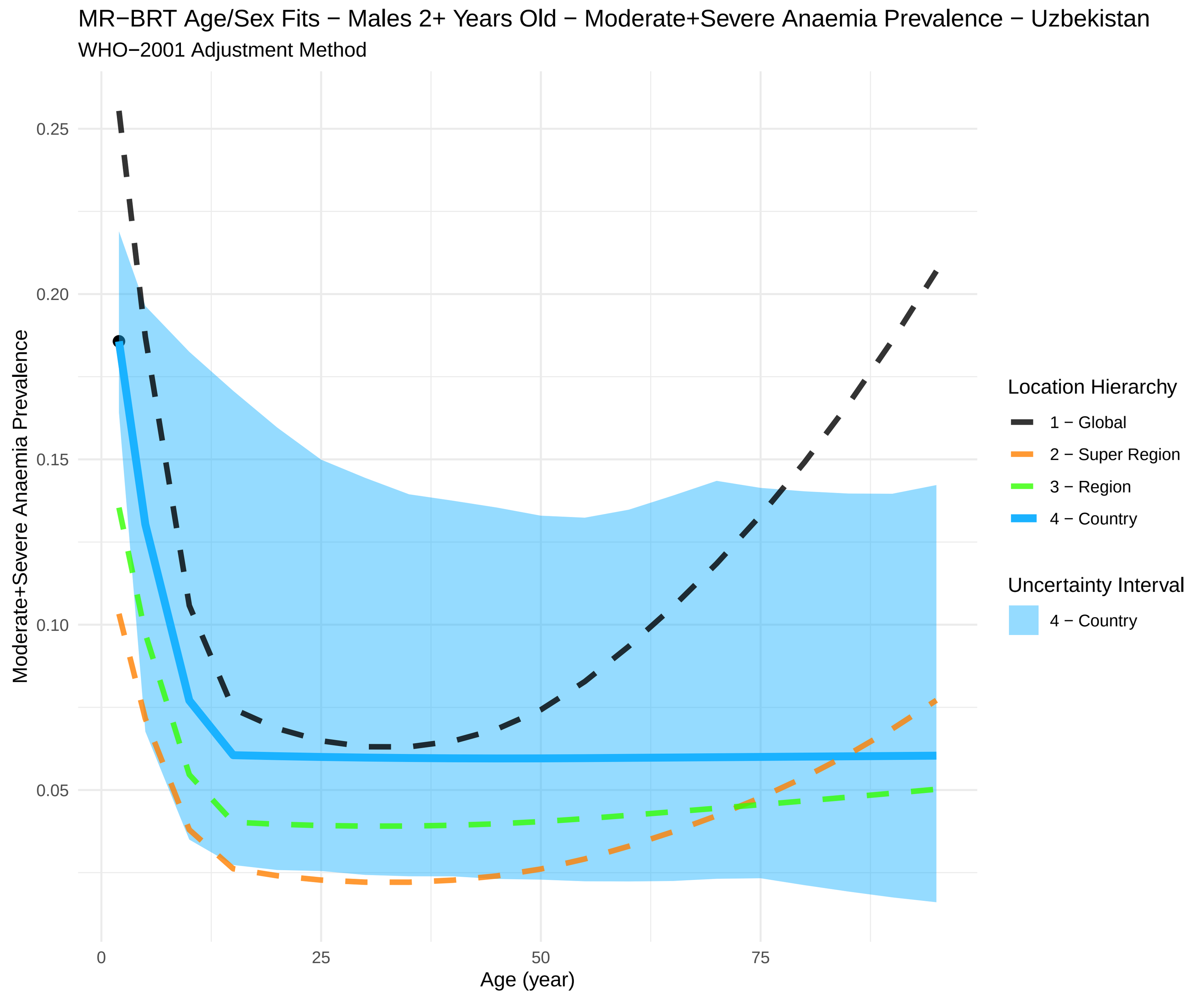
MR−BRT Age/Sex Fits − Males 2+ Years Old − Moderate+Severe Anaemia Prevalence − Uzbekistan
WHO−2001 Adjustment Method
Moderate+Severe Anaemia Prevalence
Age (year)
Location Hierarchy
1 − Global
2 − Super Region
3 − Region
4 − Country
Uncertainty Interval
4 − Country

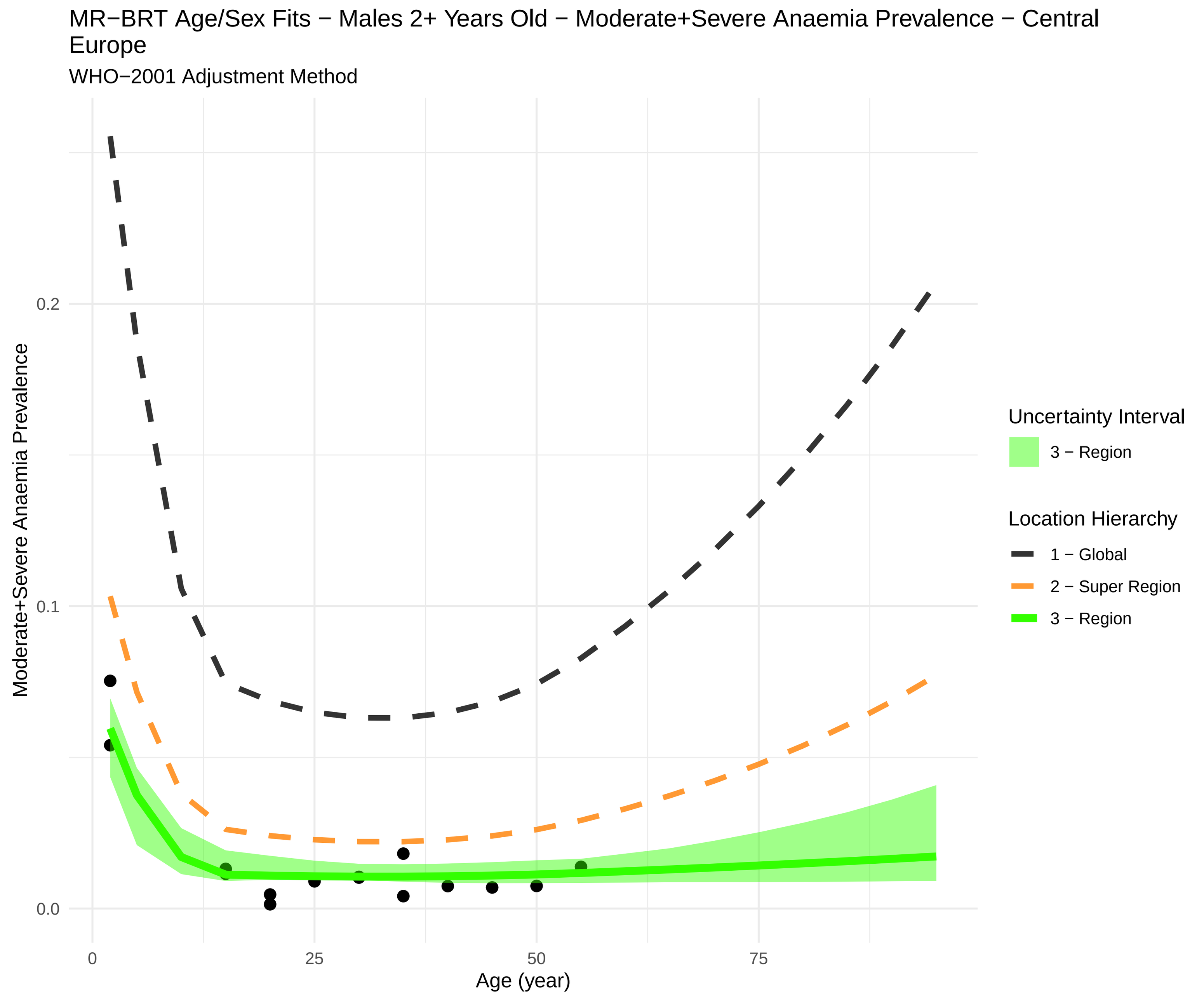
MR−BRT Age/Sex Fits − Males 2+ Years Old − Moderate+Severe Anaemia Prevalence − Central Europe
WHO−2001 Adjustment Method
Moderate+Severe Anaemia Prevalence
Age (year)
Uncertainty Interval
3 − Region
Location Hierarchy
1 − Global
2 − Super Region
3 − Region

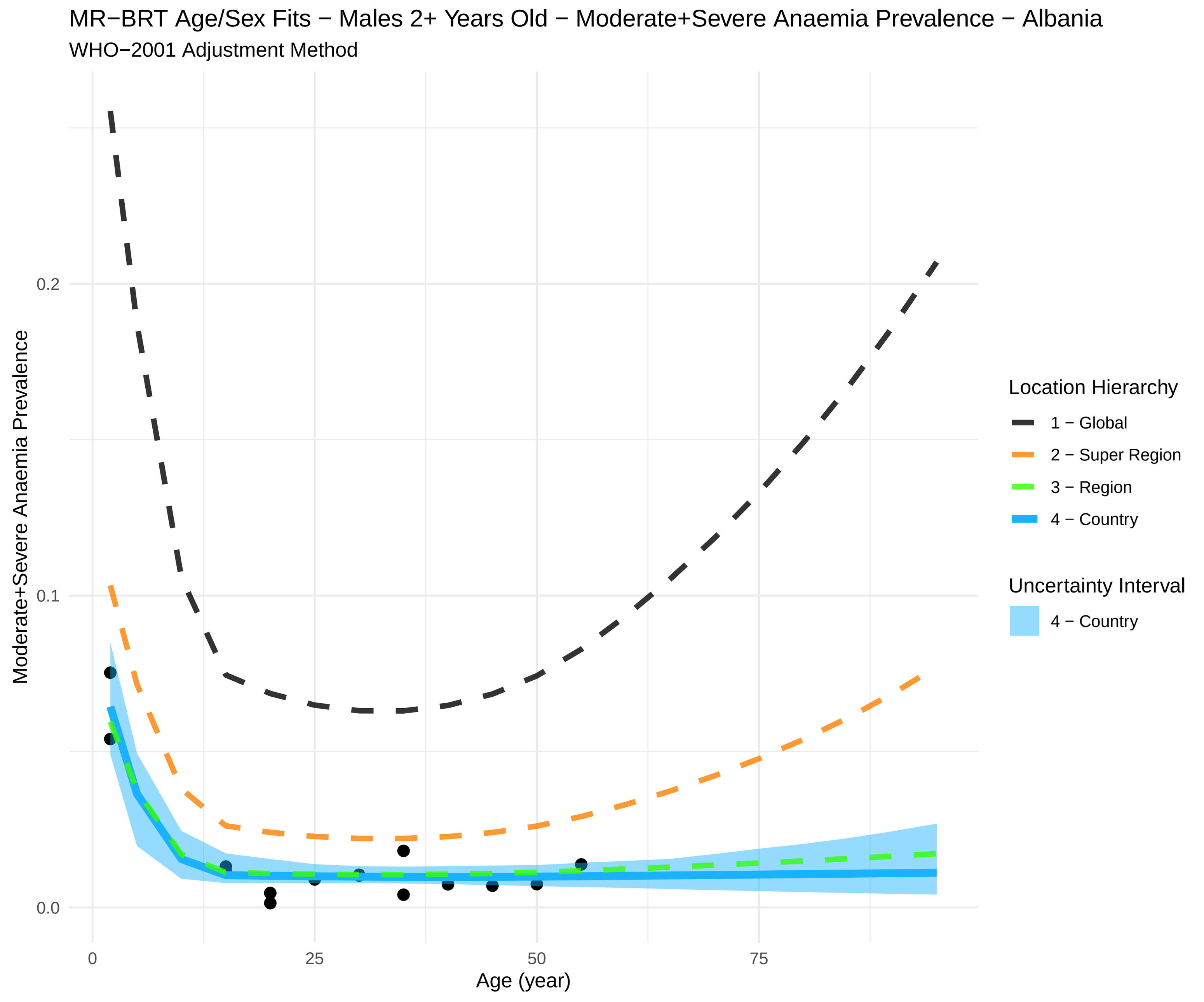
MR−BRT Age/Sex Fits − Males 2+ Years Old − Moderate+Severe Anaemia Prevalence − Albania
WHO−2001 Adjustment Method
Moderate+Severe Anaemia Prevalence
Age (year)
Location Hierarchy
1 − Global
2 − Super Region
3 − Region
4 − Country
Uncertainty Interval
4 − Country

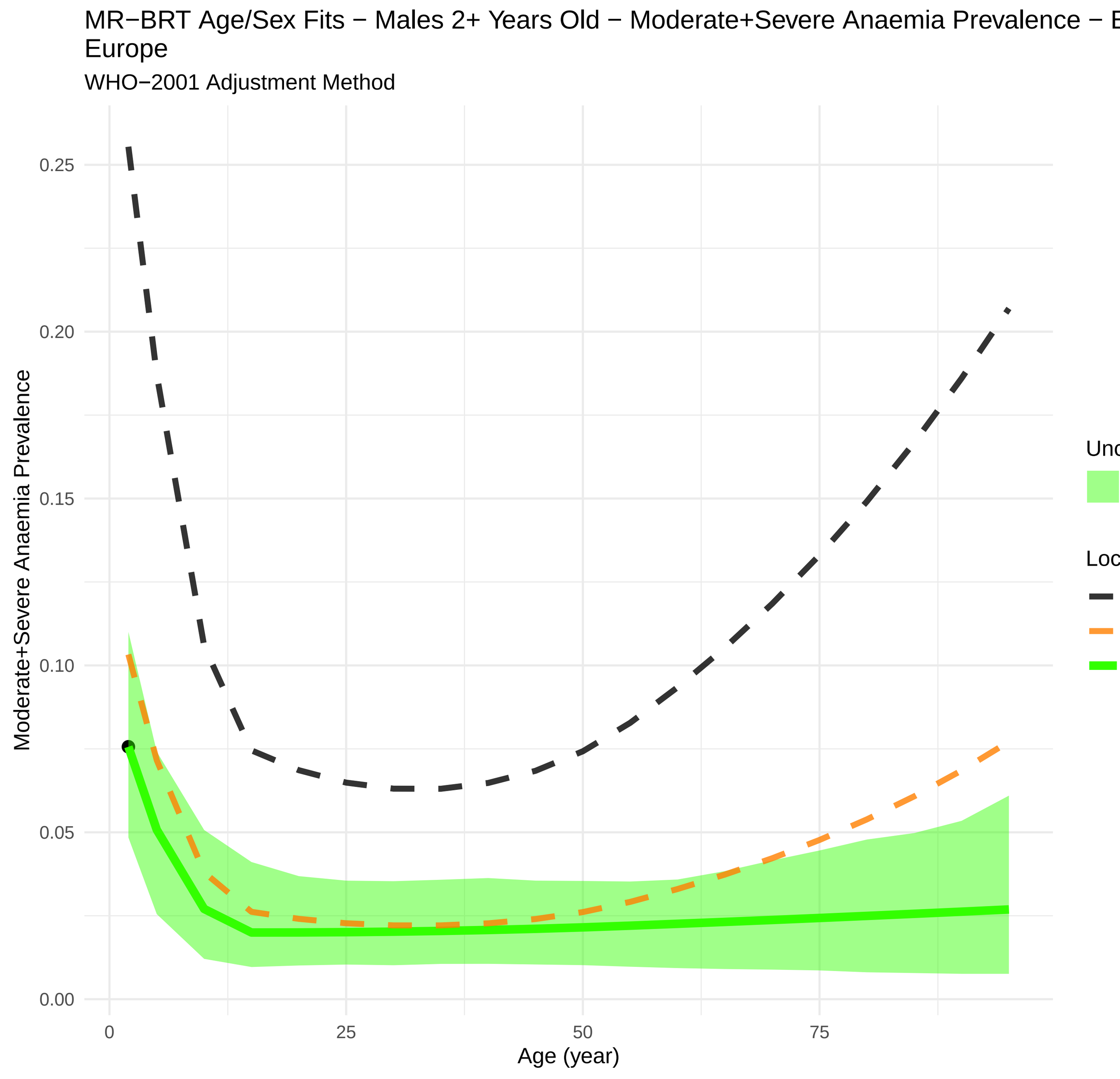
MR−BRT Age/Sex Fits − Males 2+ Years Old − Moderate+Severe Anaemia Prevalence − Eastern Europe
WHO−2001 Adjustment Method
Moderate+Severe Anaemia Prevalence
Age (year)
Uncertainty Interval
3 − Region
Location Hierarchy
1 − Global
2 − Super Region
3 − Region

MR−BRT Age/Sex Fits − Males 2+ Years Old − Moderate+Severe Anaemia Prevalence − Republic of Moldova
WHO−2001 Adjustment Method

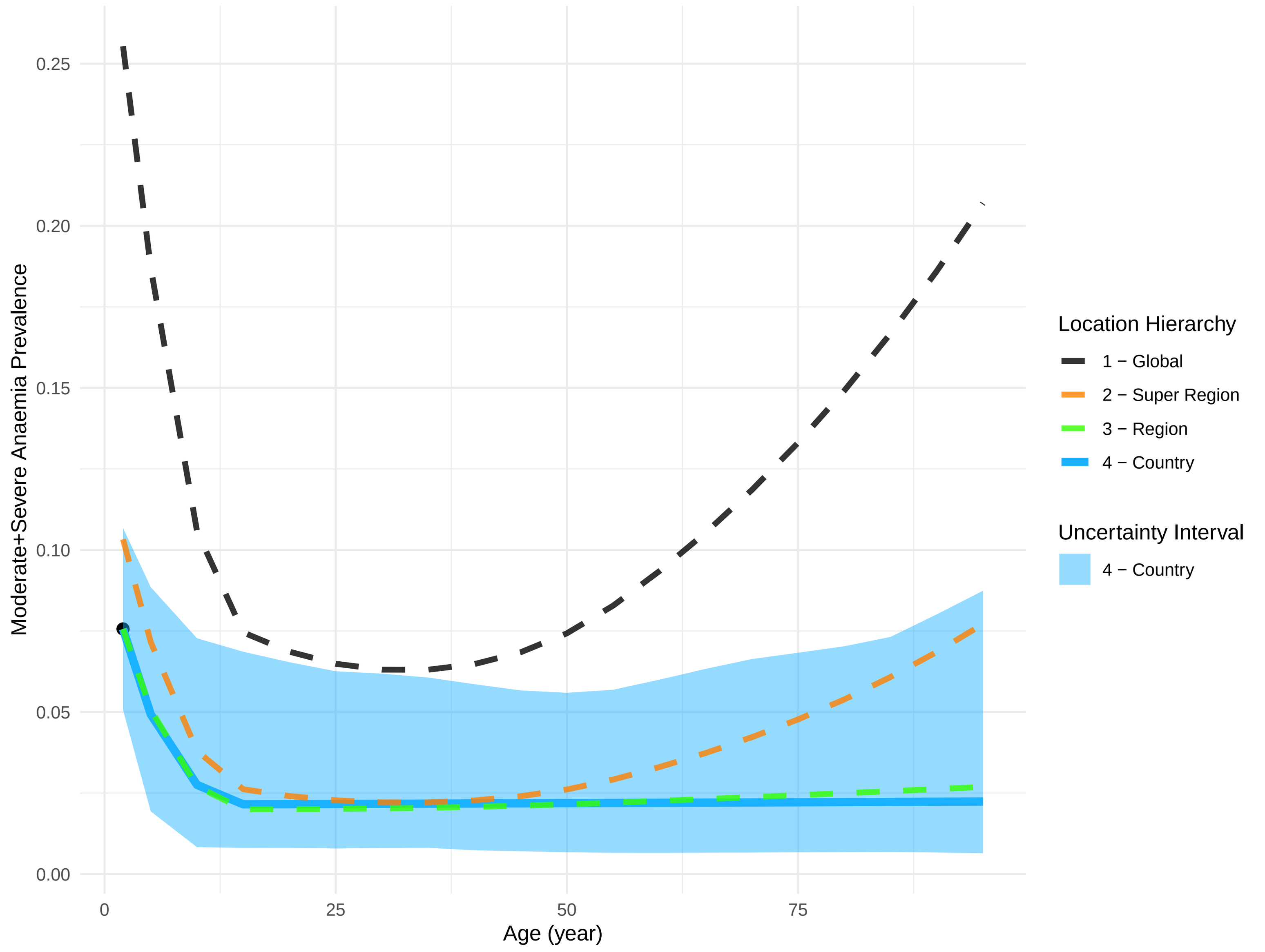

MR−BRT Age/Sex Fits − Males 2+ Years Old − Moderate+Severe Anaemia Prevalence − High−income
WHO−2001 Adjustment Method

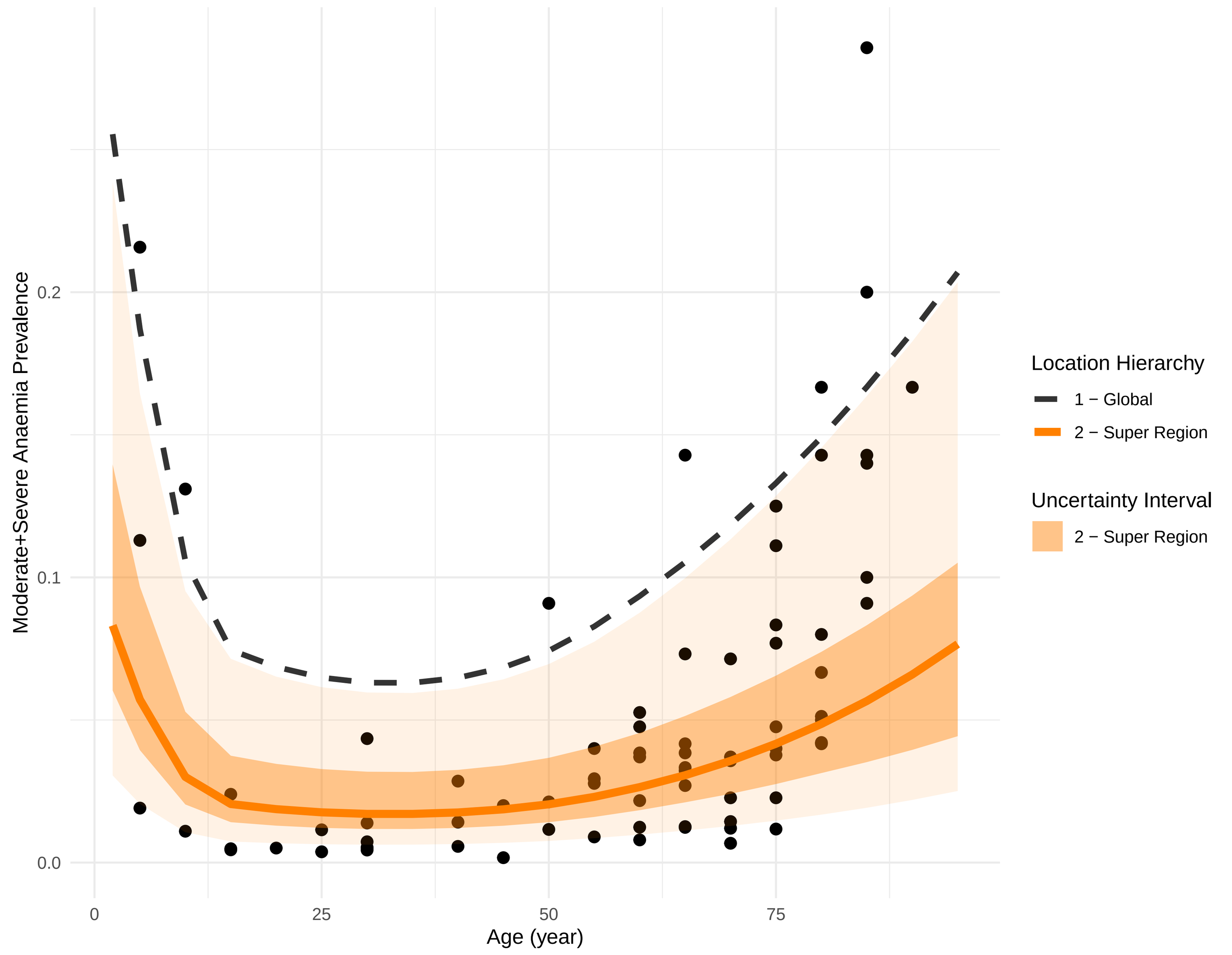

MR−BRT Age/Sex Fits − Males 2+ Years Old − Moderate+Severe Anaemia Prevalence − Western Europe
WHO−2001 Adjustment Method

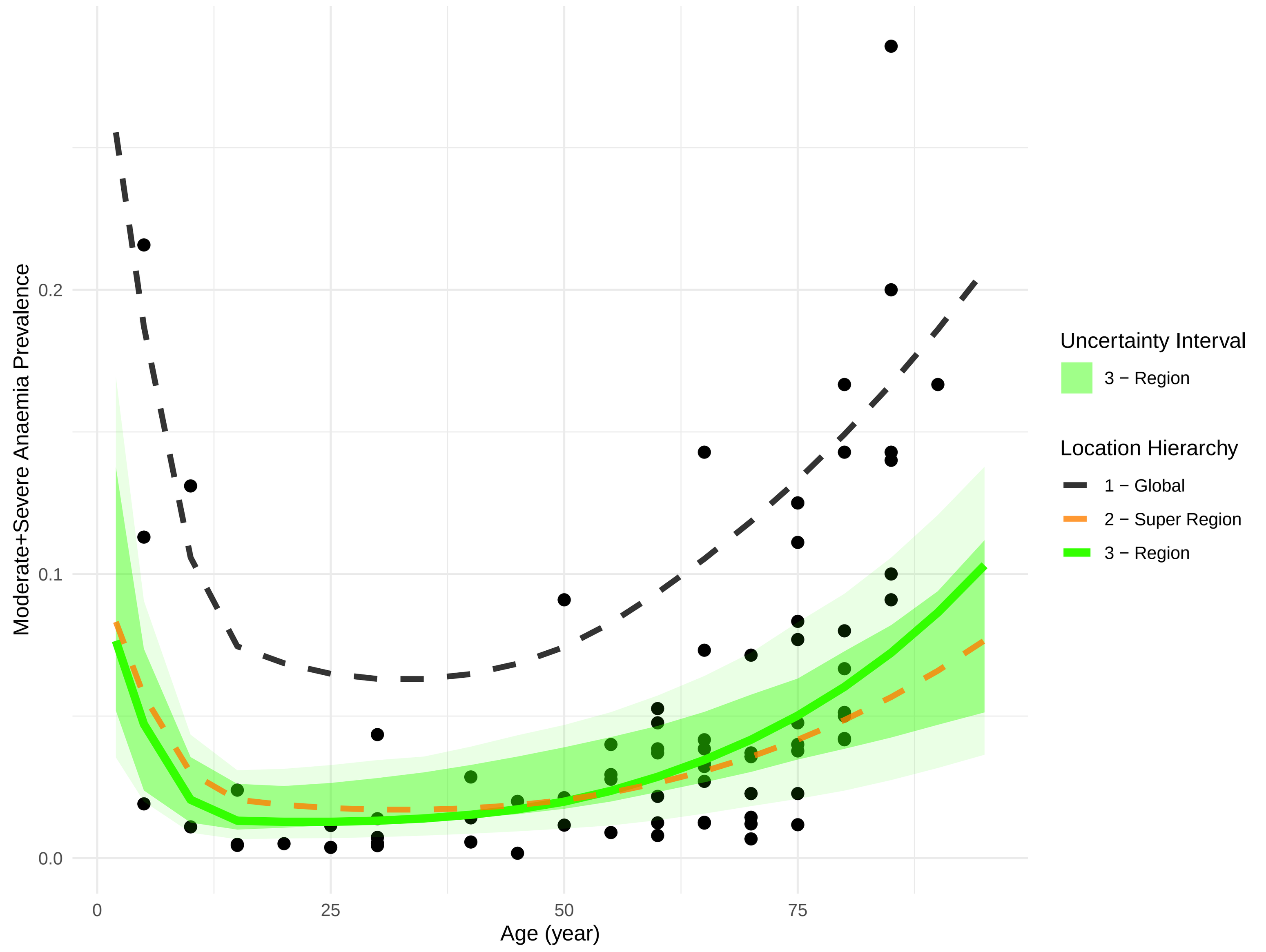

MR−BRT Age/Sex Fits − Males 2+ Years Old − Moderate+Severe Anaemia Prevalence − United Kingdom
WHO−2001 Adjustment Method

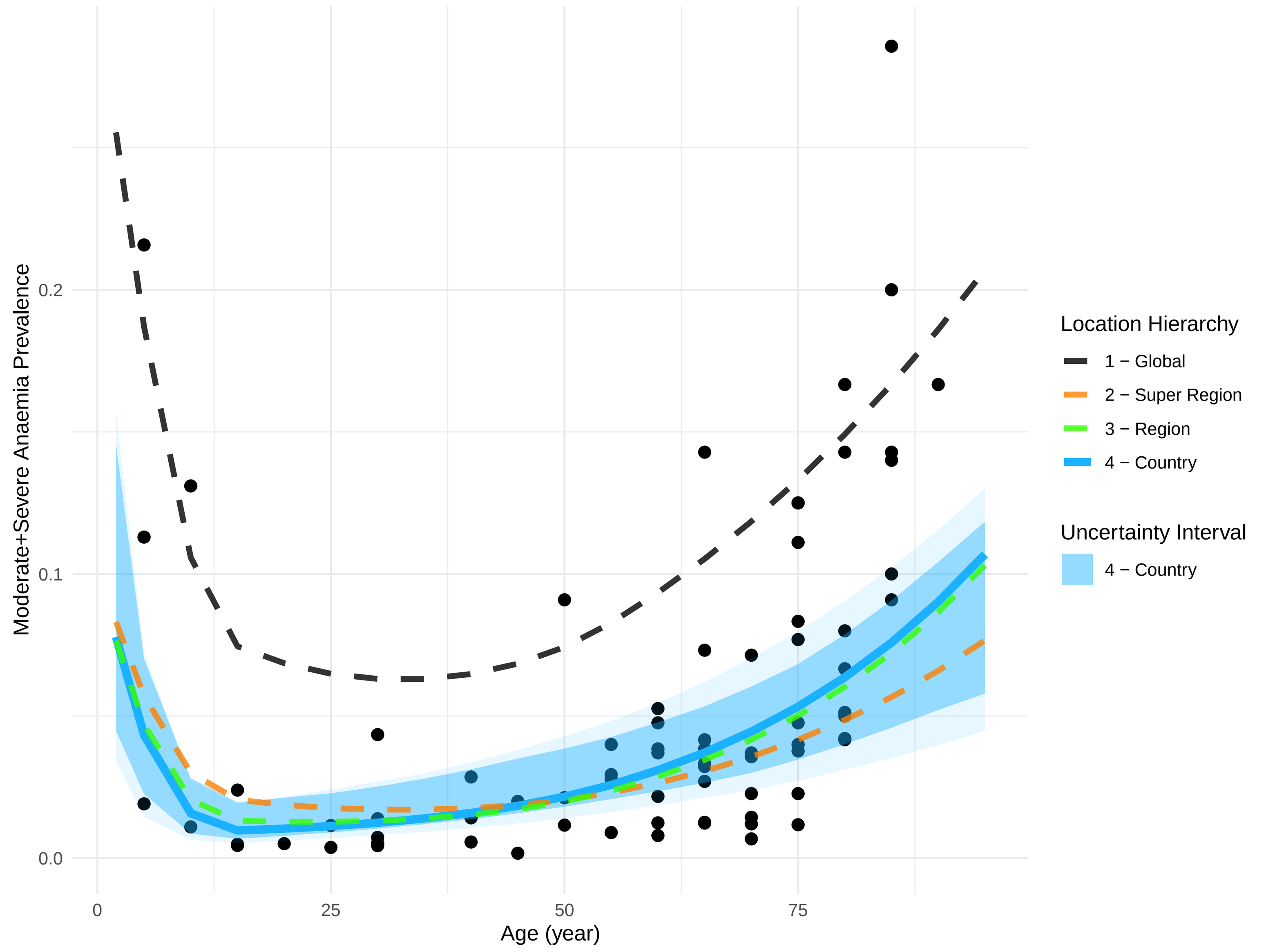

MR−BRT Age/Sex Fits − Males 2+ Years Old − Moderate+Severe Anaemia Prevalence − England
WHO−2001 Adjustment Method

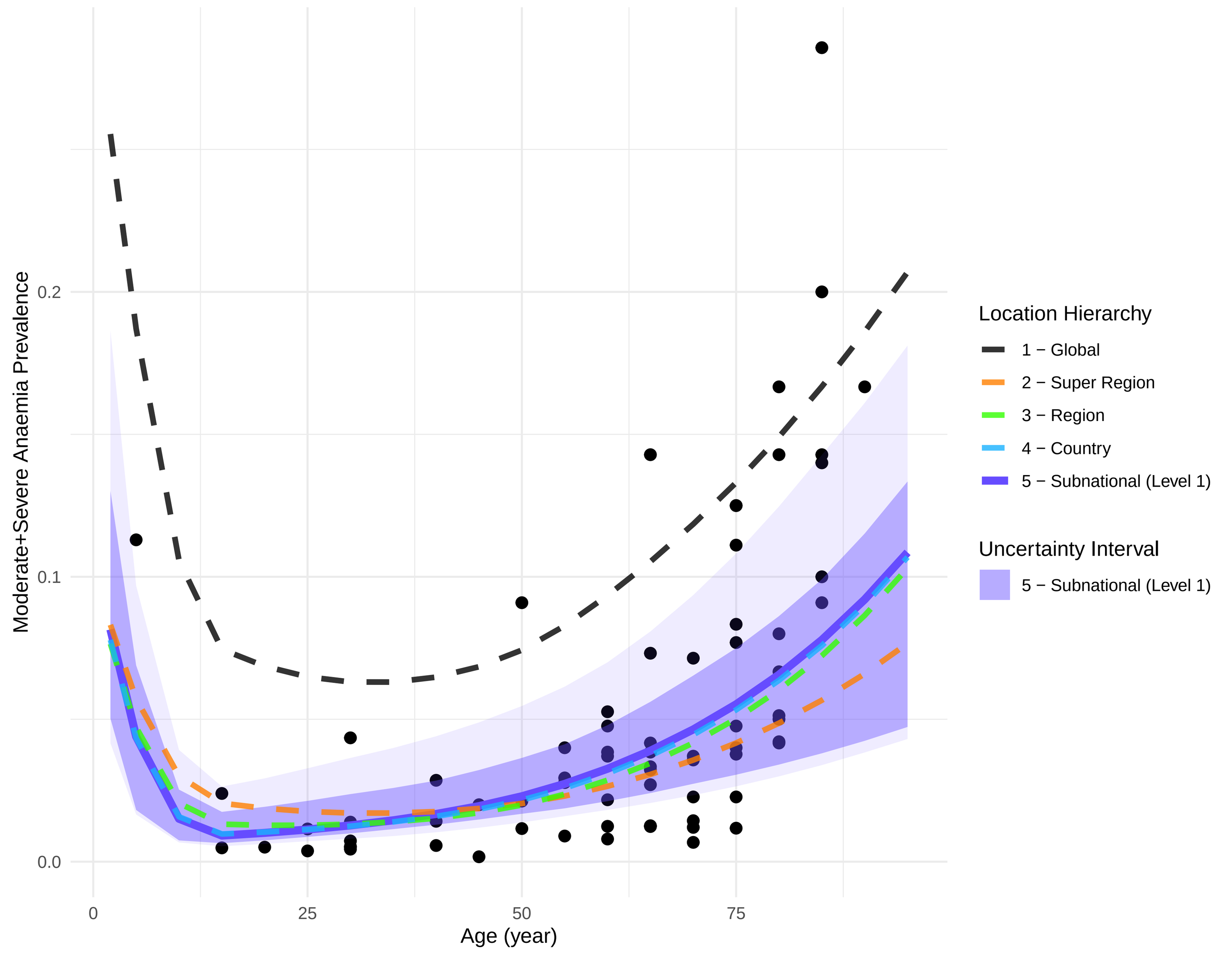

MR−BRT Age/Sex Fits − Males 2+ Years Old − Moderate+Severe Anaemia Prevalence − East of England
WHO−2001 Adjustment Method

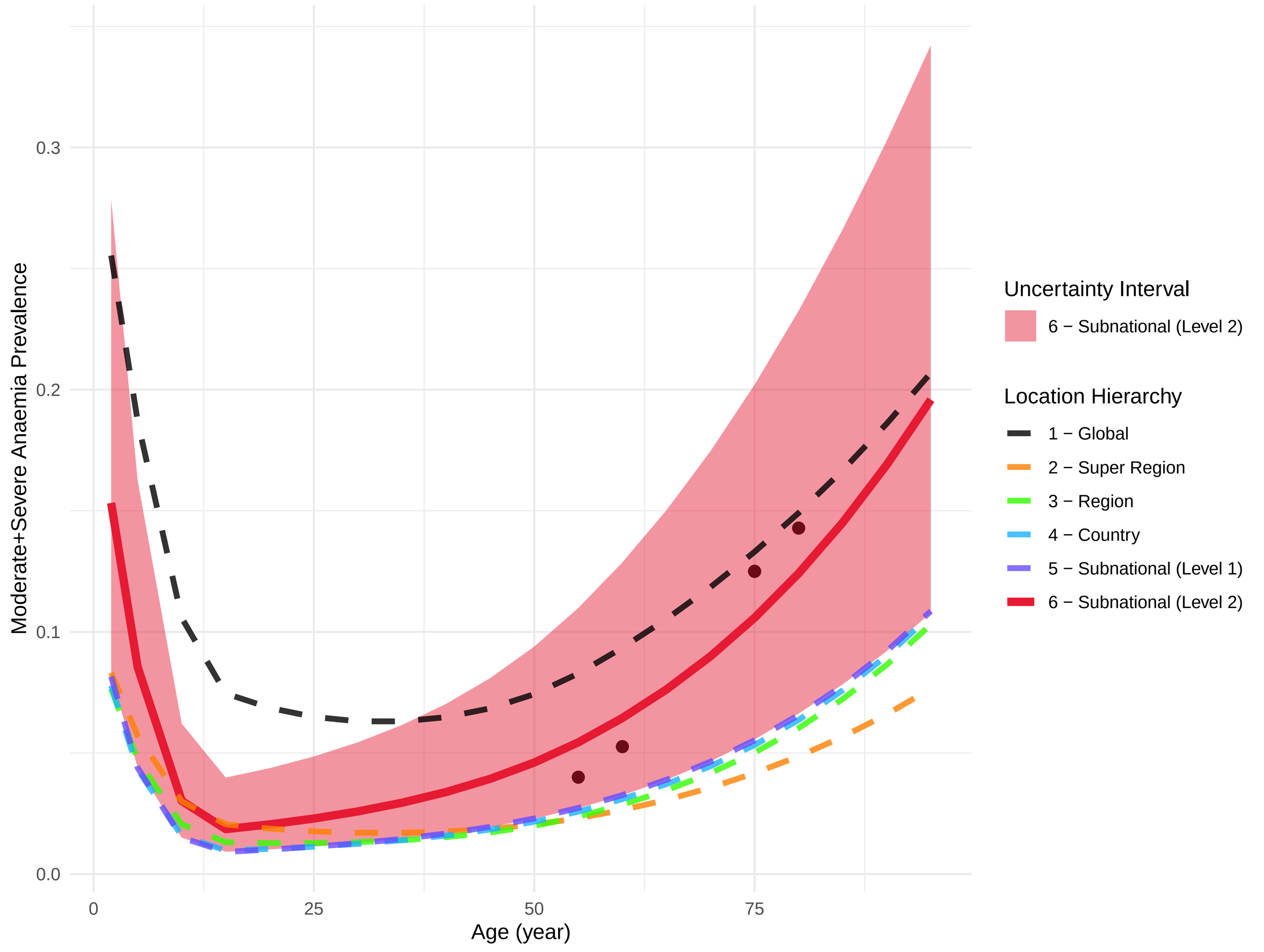

MR−BRT Age/Sex Fits − Males 2+ Years Old − Moderate+Severe Anaemia Prevalence − Greater London
WHO−2001 Adjustment Method

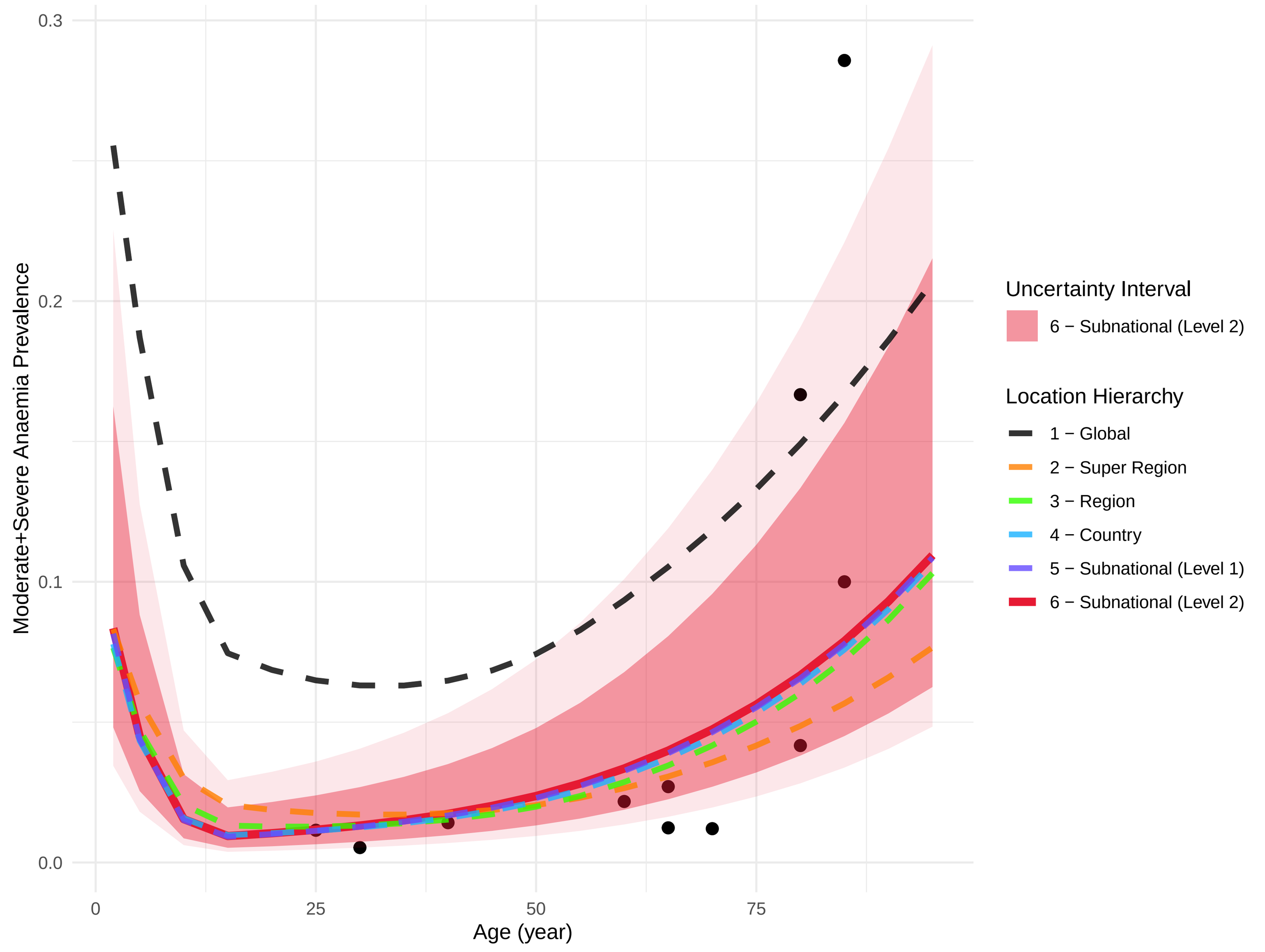

MR−BRT Age/Sex Fits − Males 2+ Years Old − Moderate+Severe Anaemia Prevalence − North West England
WHO−2001 Adjustment Method

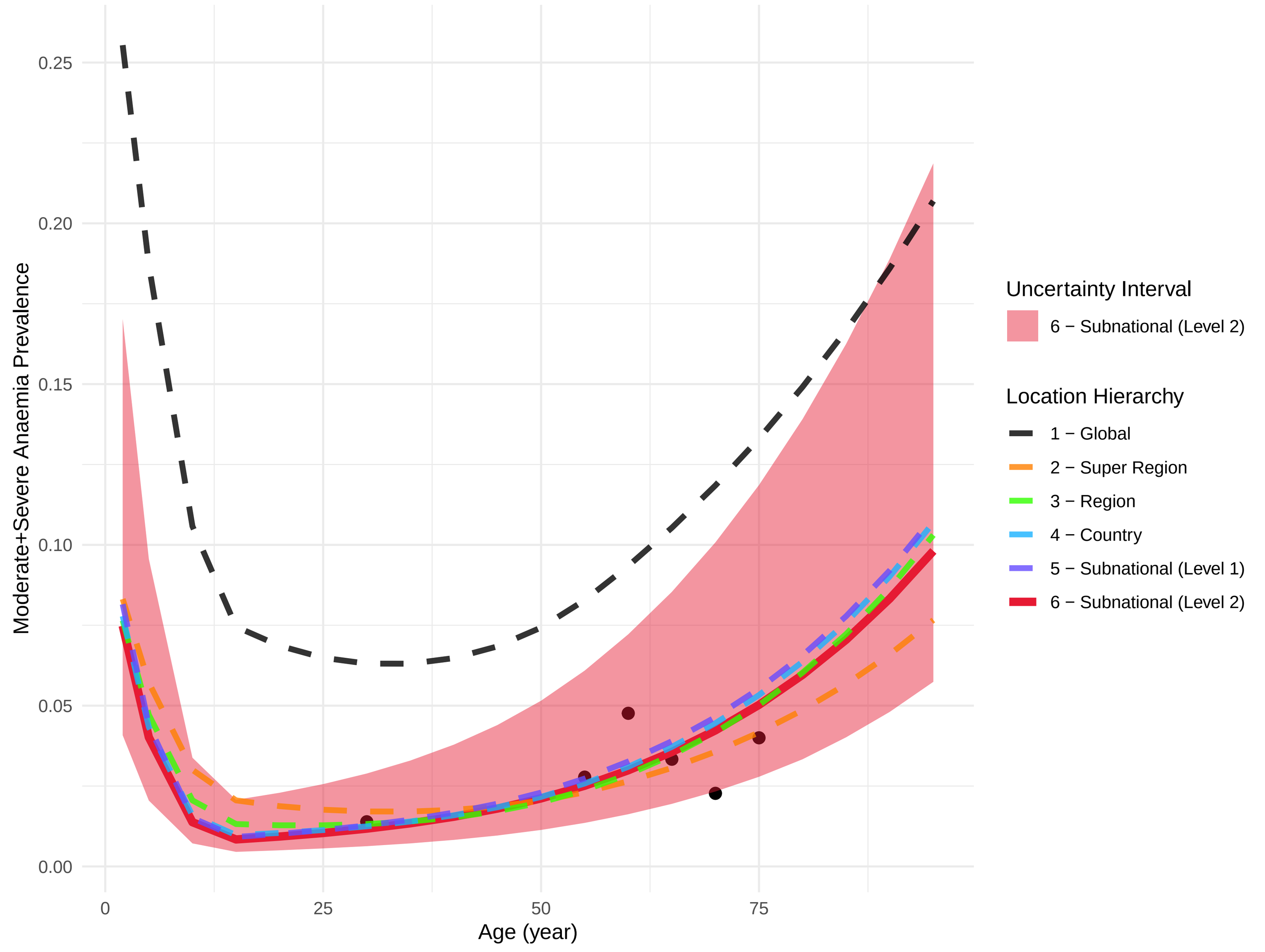

MR−BRT Age/Sex Fits − Males 2+ Years Old − Moderate+Severe Anaemia Prevalence − South East England
WHO−2001 Adjustment Method

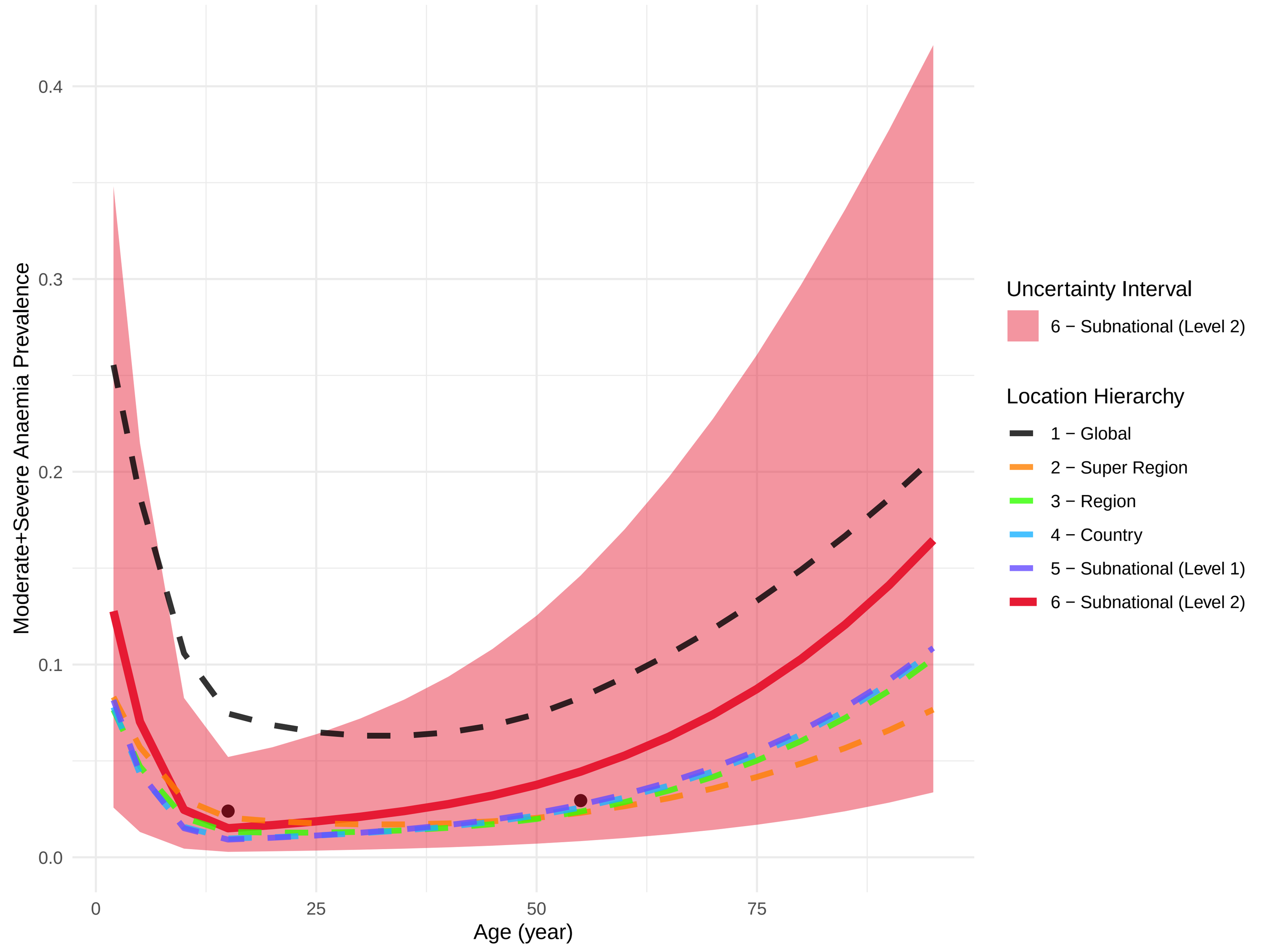

MR−BRT Age/Sex Fits − Males 2+ Years Old − Moderate+Severe Anaemia Prevalence − South West England
WHO−2001 Adjustment Method

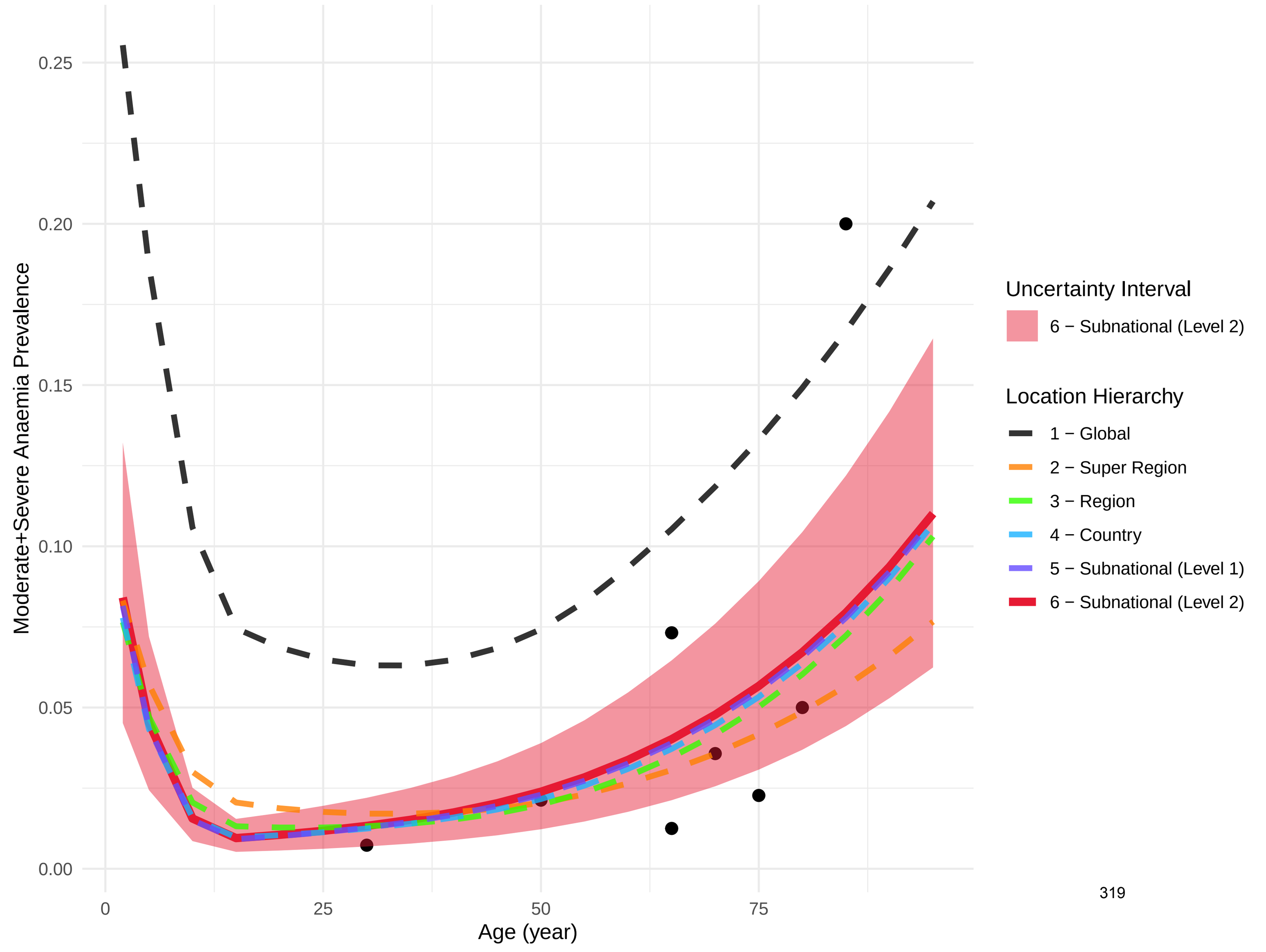

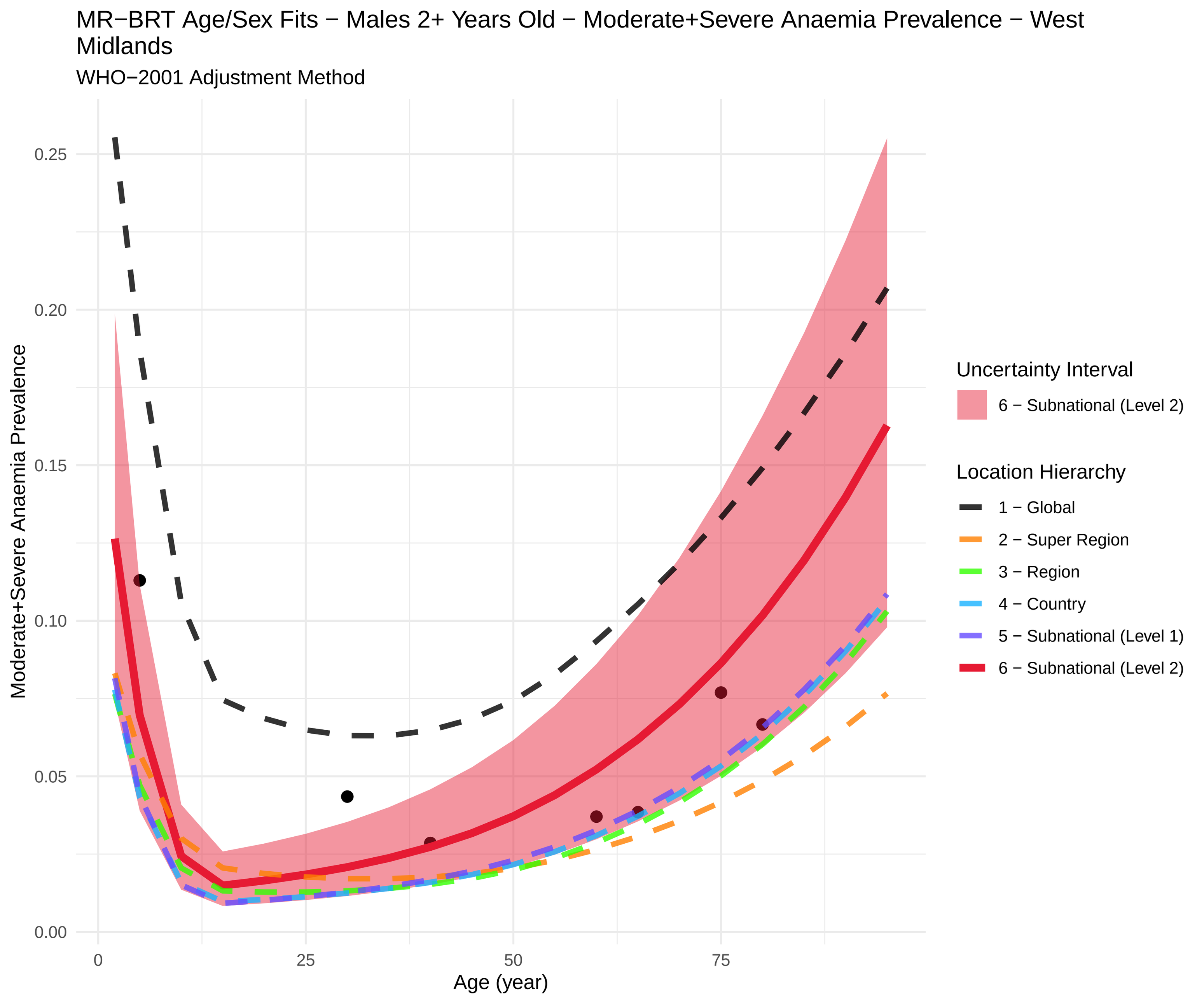
MR−BRT Age/Sex Fits − Males 2+ Years Old − Moderate+Severe Anaemia Prevalence − West Midlands
WHO−2001 Adjustment Method
Moderate+Severe Anaemia Prevalence
Age (year)
Uncertainty Interval
6 − Subnational (Level 2)
Location Hierarchy
1 − Global
2 − Super Region
3 − Region
4 − Country
5 − Subnational (Level 1)
6 − Subnational (Level 2)

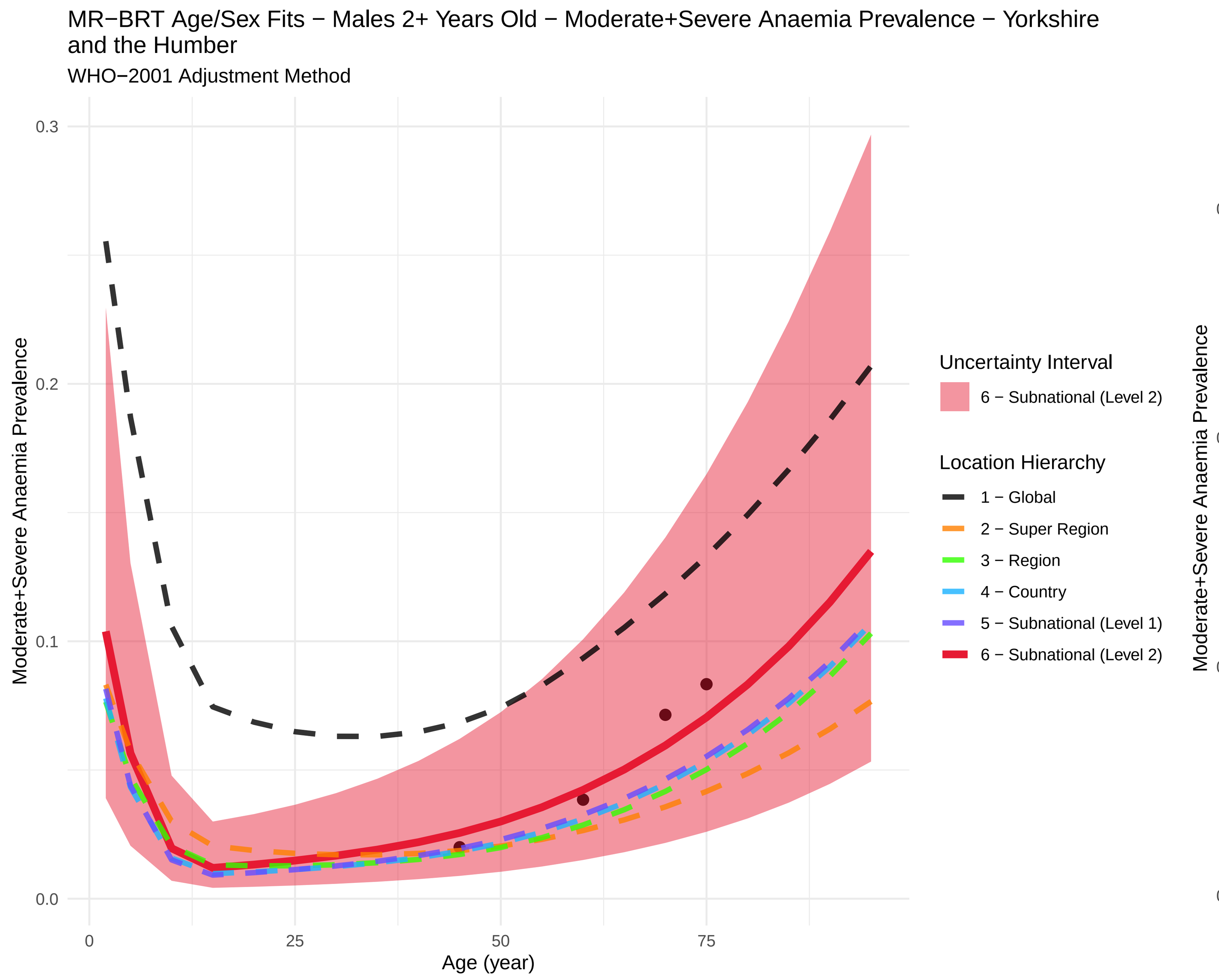
MR−BRT Age/Sex Fits − Males 2+ Years Old − Moderate+Severe Anaemia Prevalence − Yorkshire and the Humber
WHO−2001 Adjustment Method
Moderate+Severe Anaemia Prevalence
Age (year)
Uncertainty Interval
6 − Subnational (Level 2)
Location Hierarchy
1 − Global
2 − Super Region
3 − Region
4 − Country
5 − Subnational (Level 1)
6 − Subnational (Level 2)

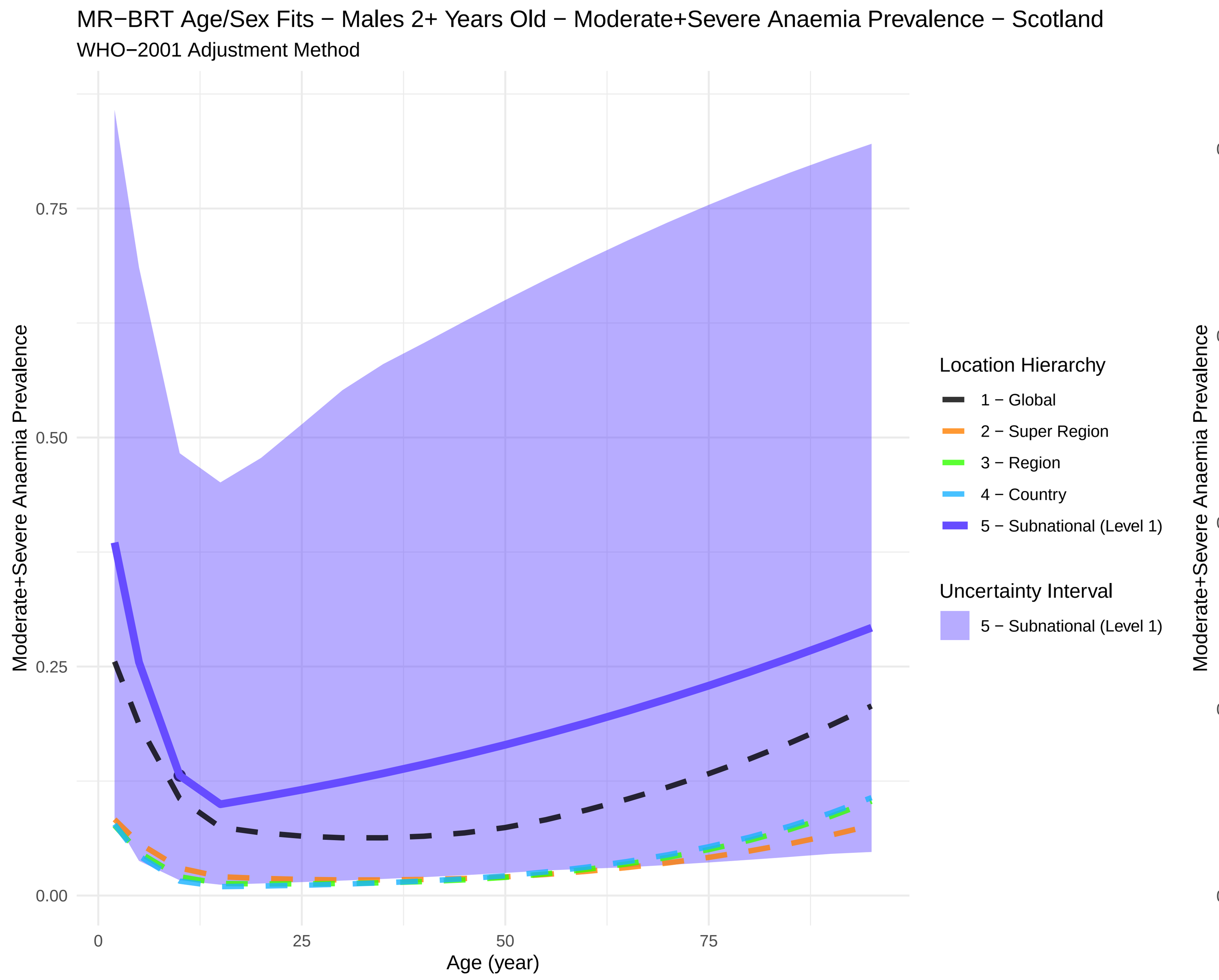
MR−BRT Age/Sex Fits − Males 2+ Years Old − Moderate+Severe Anaemia Prevalence − Scotland
WHO−2001 Adjustment Method
Moderate+Severe Anaemia Prevalence
Age (year)
Location Hierarchy
1 − Global
2 − Super Region
3 − Region
4 − Country
5 − Subnational (Level 1)
Uncertainty Interval
5 − Subnational (Level 1)

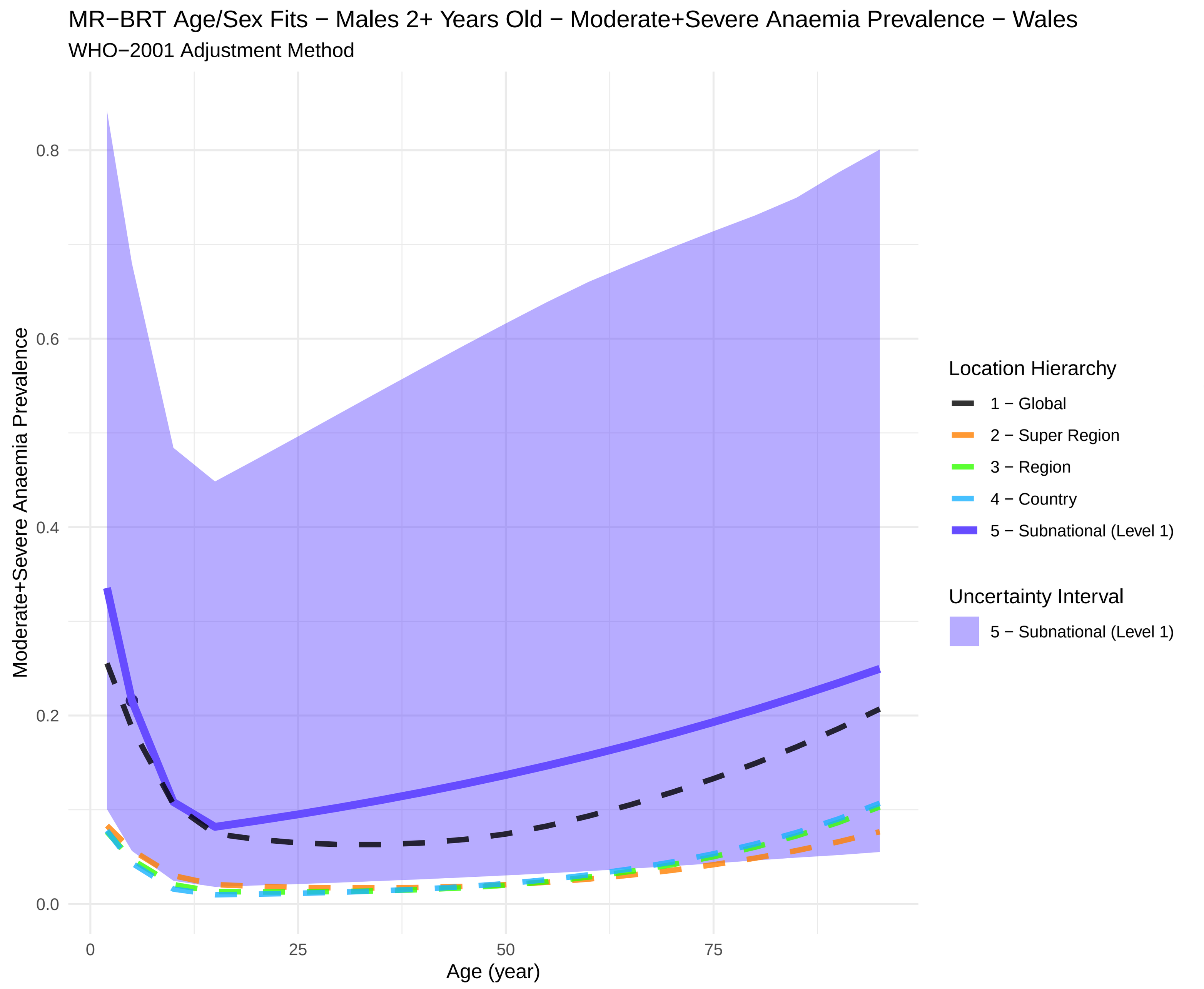
MR−BRT Age/Sex Fits − Males 2+ Years Old − Moderate+Severe Anaemia Prevalence − Wales
WHO−2001 Adjustment Method
Moderate+Severe Anaemia Prevalence
Age (year)
Location Hierarchy
1 − Global
2 − Super Region
3 − Region
4 − Country
5 − Subnational (Level 1)
Uncertainty Interval
5 − Subnational (Level 1)

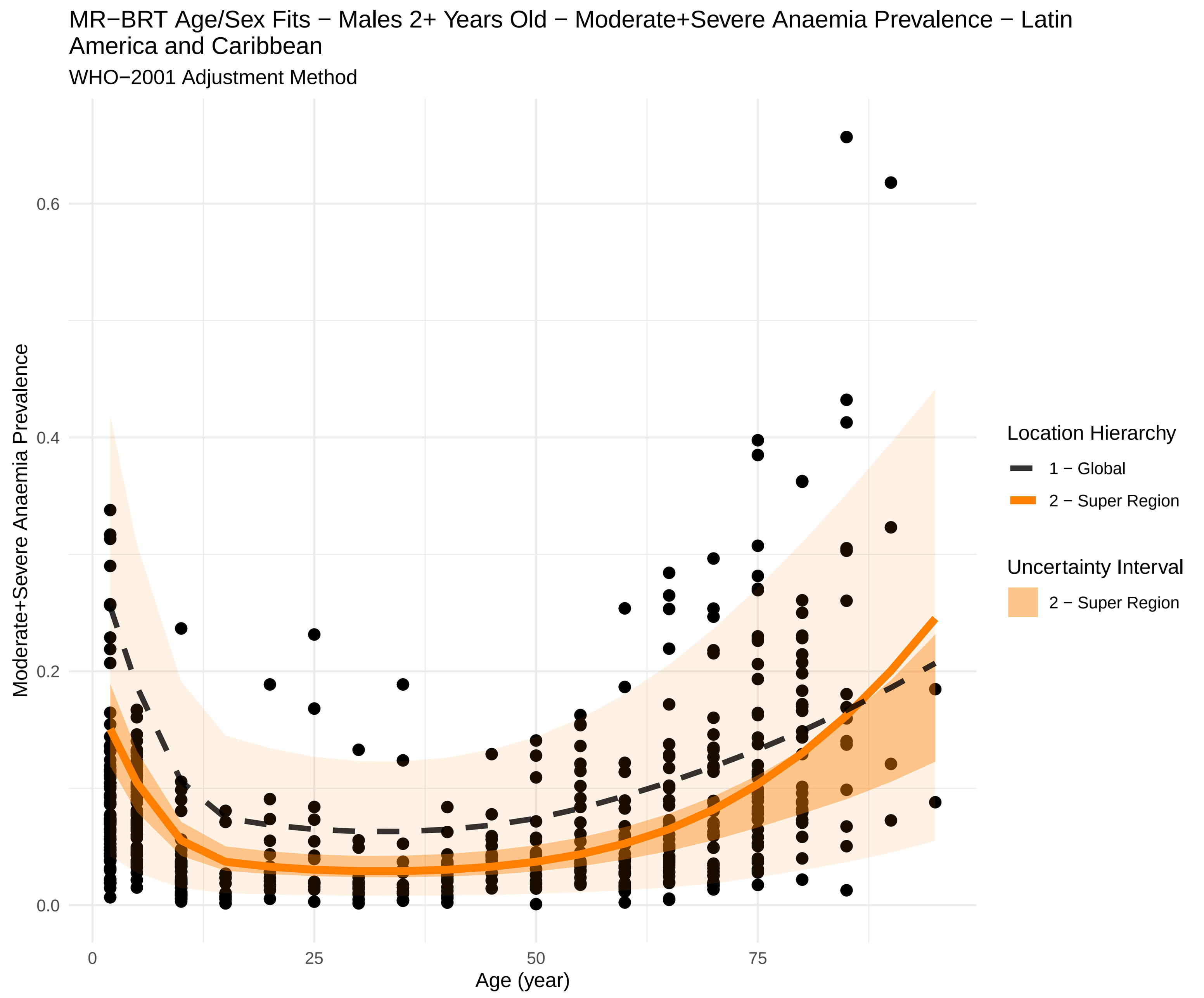
MR−BRT Age/Sex Fits − Males 2+ Years Old − Moderate+Severe Anaemia Prevalence − Latin America and Caribbean
WHO−2001 Adjustment Method
Moderate+Severe Anaemia Prevalence
Age (year)
Location Hierarchy
1 − Global
2 − Super Region
Uncertainty Interval
2 − Super Region

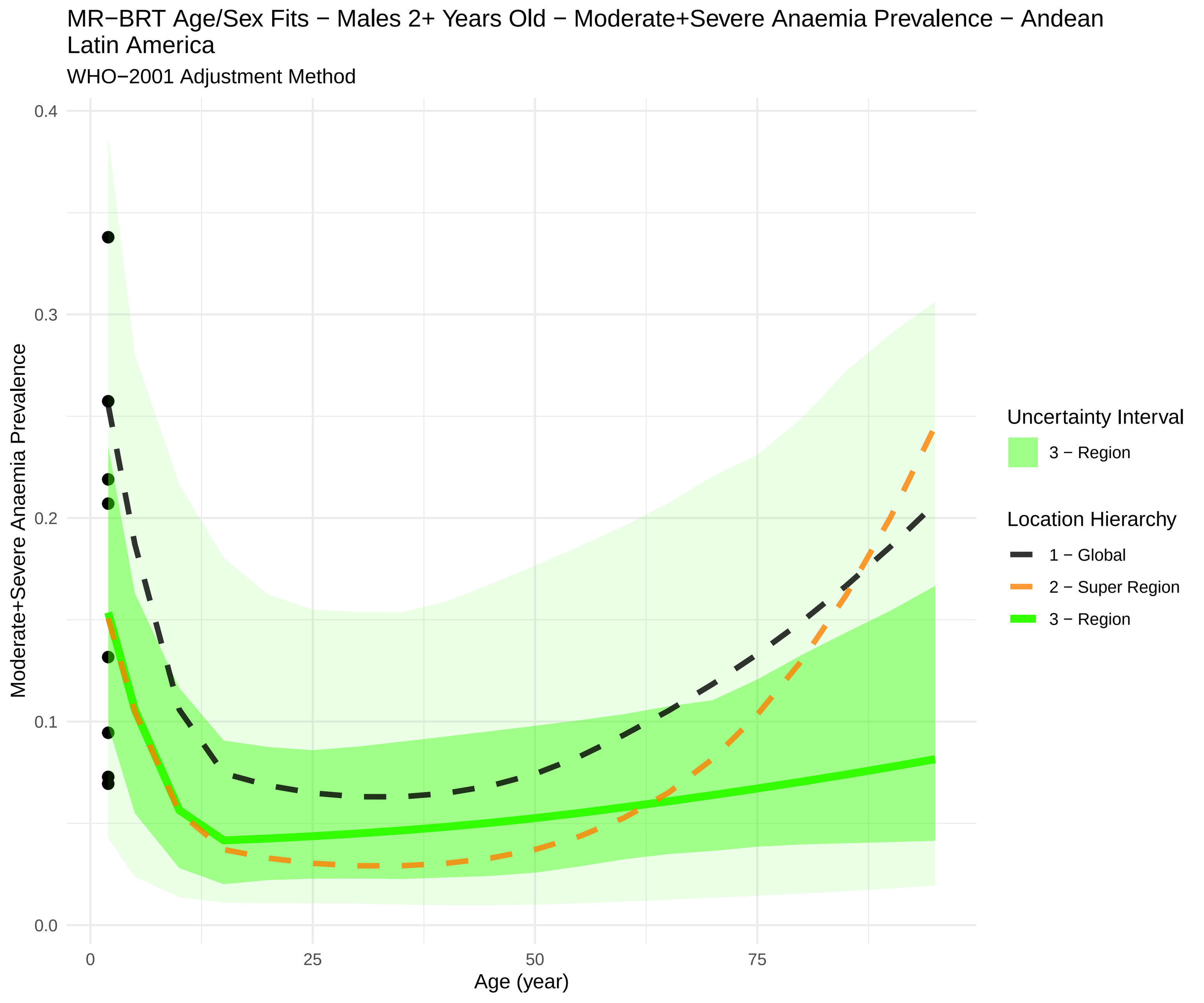
MR−BRT Age/Sex Fits − Males 2+ Years Old − Moderate+Severe Anaemia Prevalence − Andean Latin America
WHO−2001 Adjustment Method
Moderate+Severe Anaemia Prevalence
Age (year)
Uncertainty Interval
3 − Region
Location Hierarchy
1 − Global
2 − Super Region
3 − Region

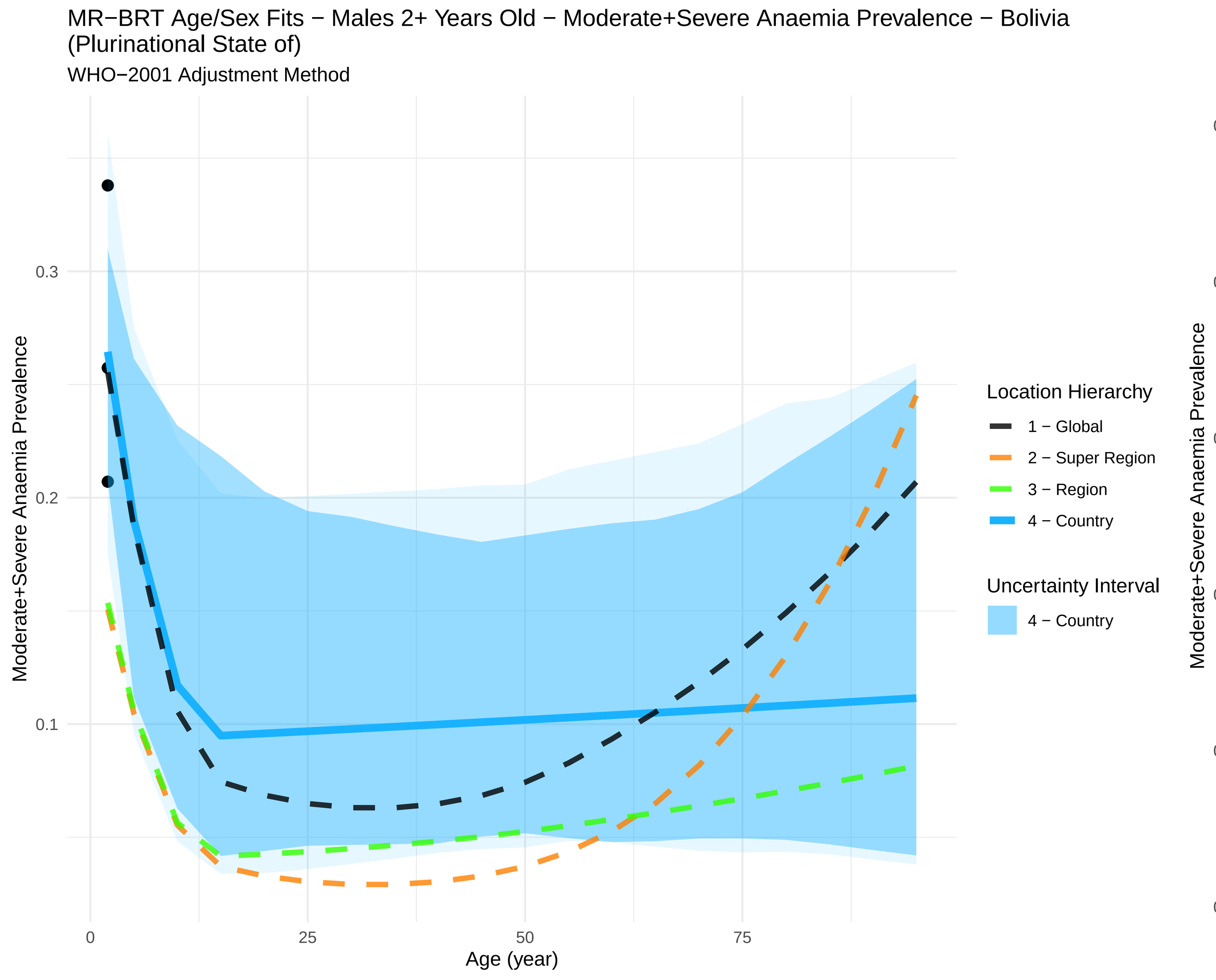
MR−BRT Age/Sex Fits − Males 2+ Years Old − Moderate+Severe Anaemia Prevalence − Bolivia (Plurinational State of)
WHO−2001 Adjustment Method
Moderate+Severe Anaemia Prevalence
Age (year)
Location Hierarchy
1 − Global
2 − Super Region
3 − Region
4 − Country
Uncertainty Interval
4 − Country

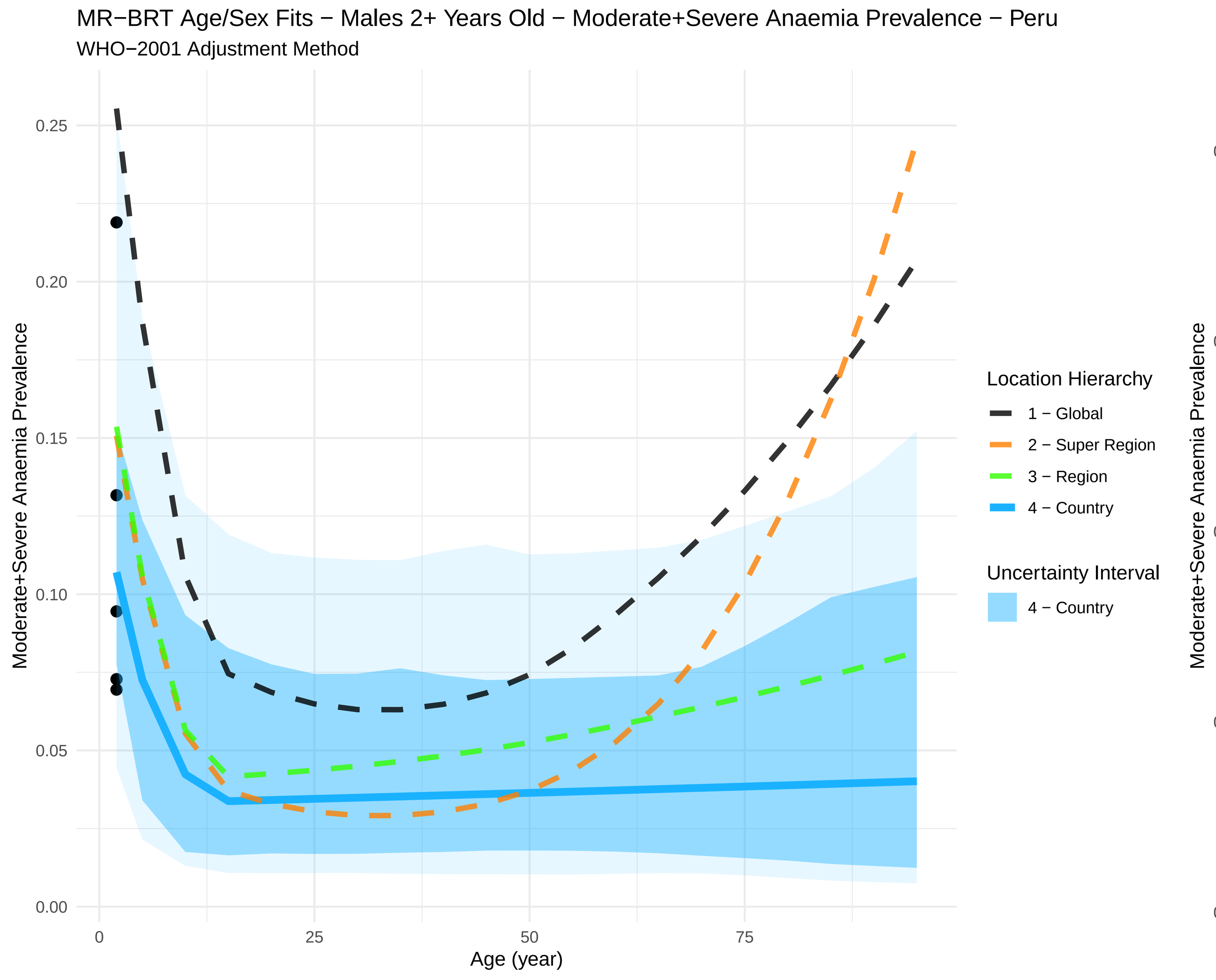
MR−BRT Age/Sex Fits − Males 2+ Years Old − Moderate+Severe Anaemia Prevalence − Peru
WHO−2001 Adjustment Method
Moderate+Severe Anaemia Prevalence
Age (year)
Location Hierarchy
1 − Global
2 − Super Region
3 − Region
4 − Country
Uncertainty Interval
4 − Country

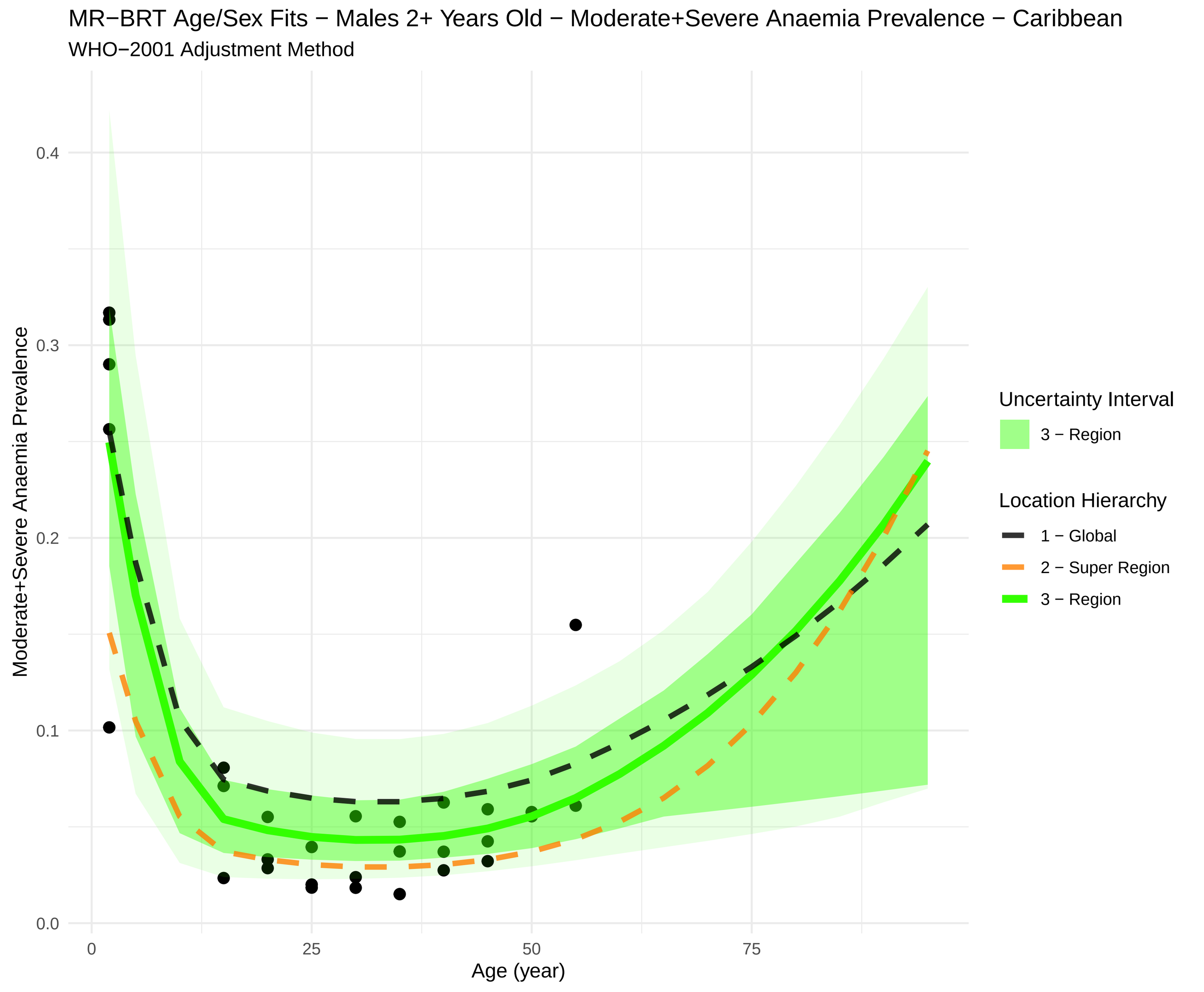
MR−BRT Age/Sex Fits − Males 2+ Years Old − Moderate+Severe Anaemia Prevalence − Caribbean
WHO−2001 Adjustment Method
Moderate+Severe Anaemia Prevalence
Age (year)
Uncertainty Interval
3 − Region
Location Hierarchy
1 − Global
2 − Super Region
3 − Region

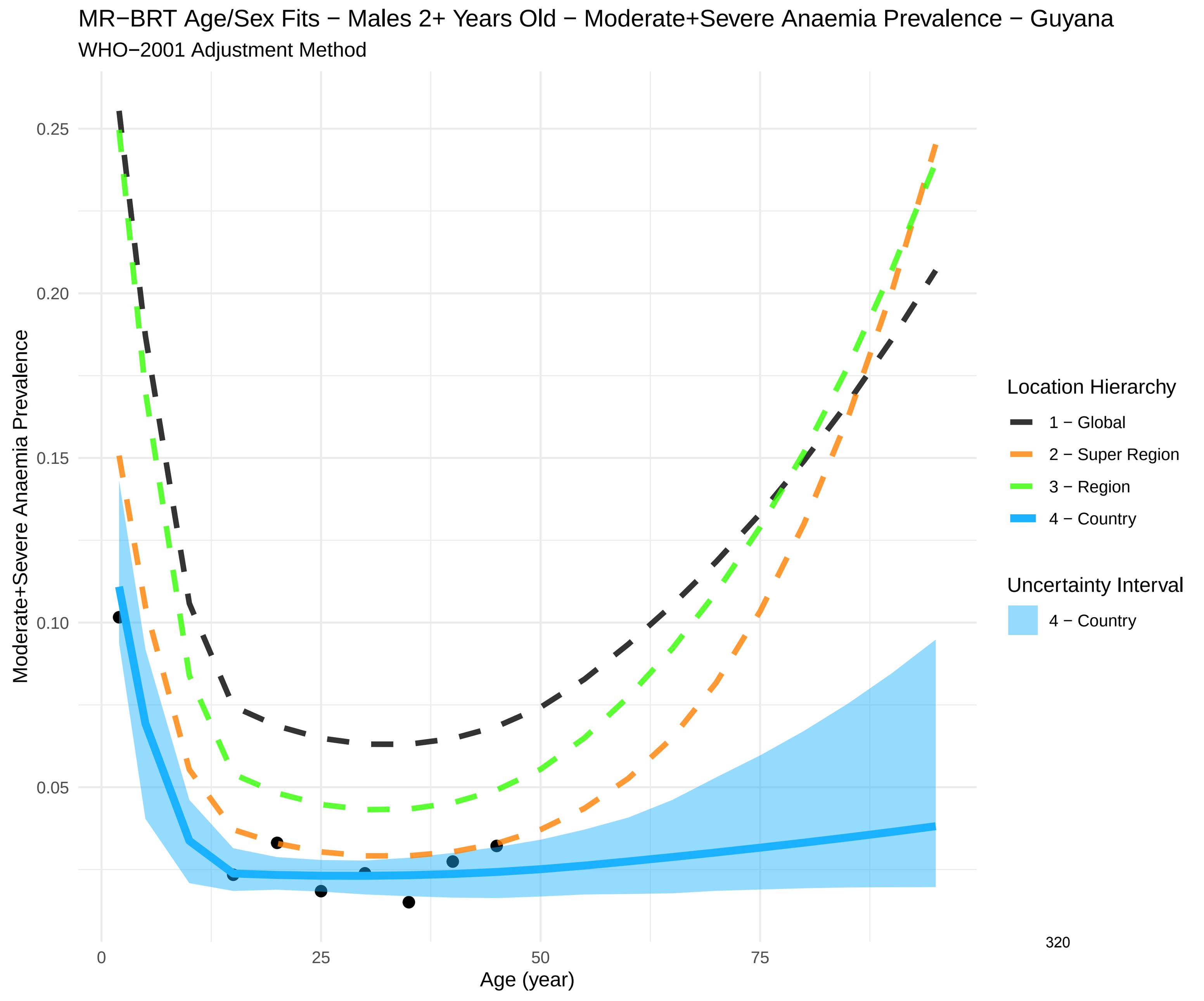
MR−BRT Age/Sex Fits − Males 2+ Years Old − Moderate+Severe Anaemia Prevalence − Guyana
WHO−2001 Adjustment Method
Moderate+Severe Anaemia Prevalence
Age (year)
Location Hierarchy
1 − Global
2 − Super Region
3 − Region
4 − Country
Uncertainty Interval
4 − Country

MR−BRT Age/Sex Fits − Males 2+ Years Old − Moderate+Severe Anaemia Prevalence − Haiti
WHO−2001 Adjustment Method

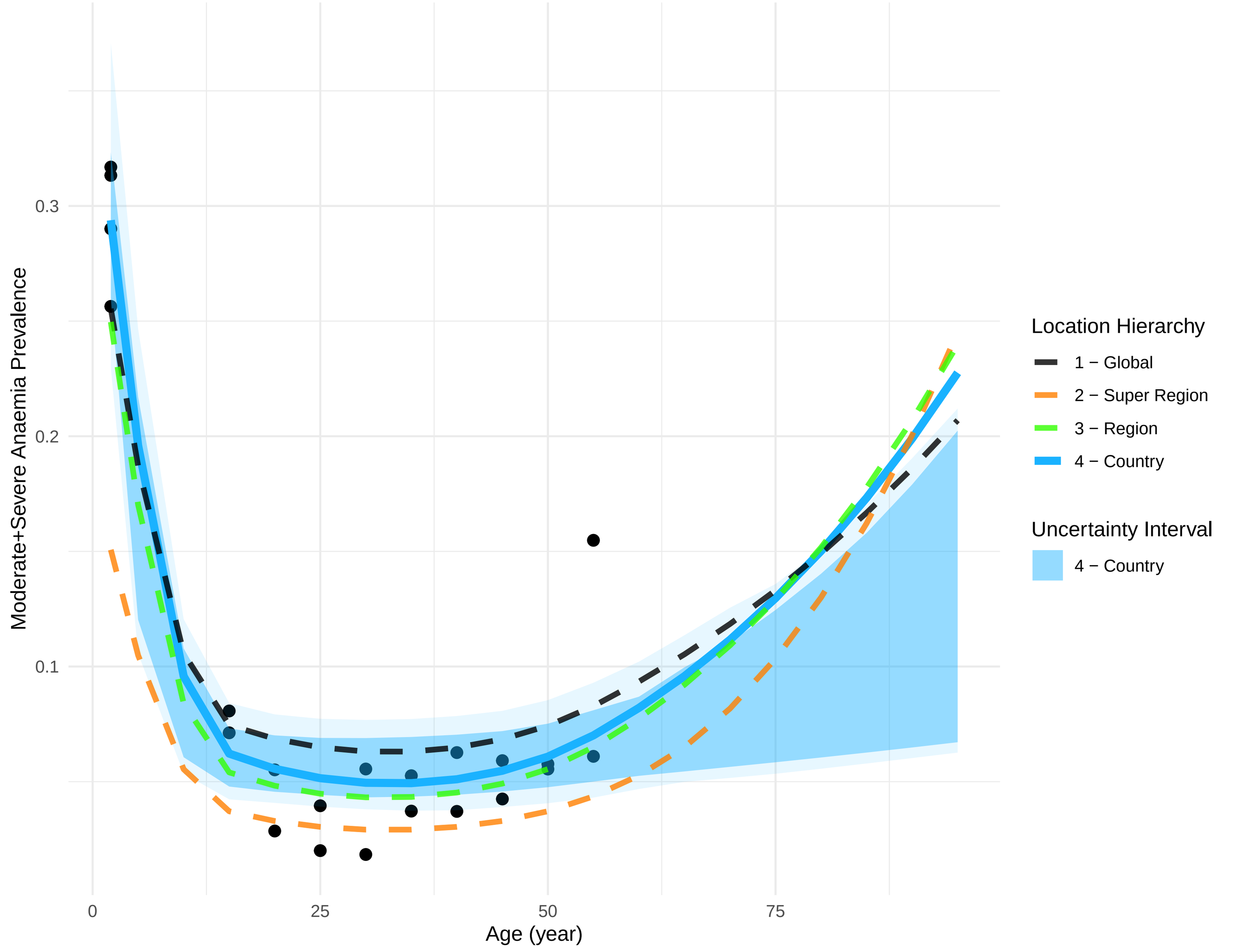

MR−BRT Age/Sex Fits − Males 2+ Years Old − Moderate+Severe Anaemia Prevalence − Central Latin America
WHO−2001 Adjustment Method

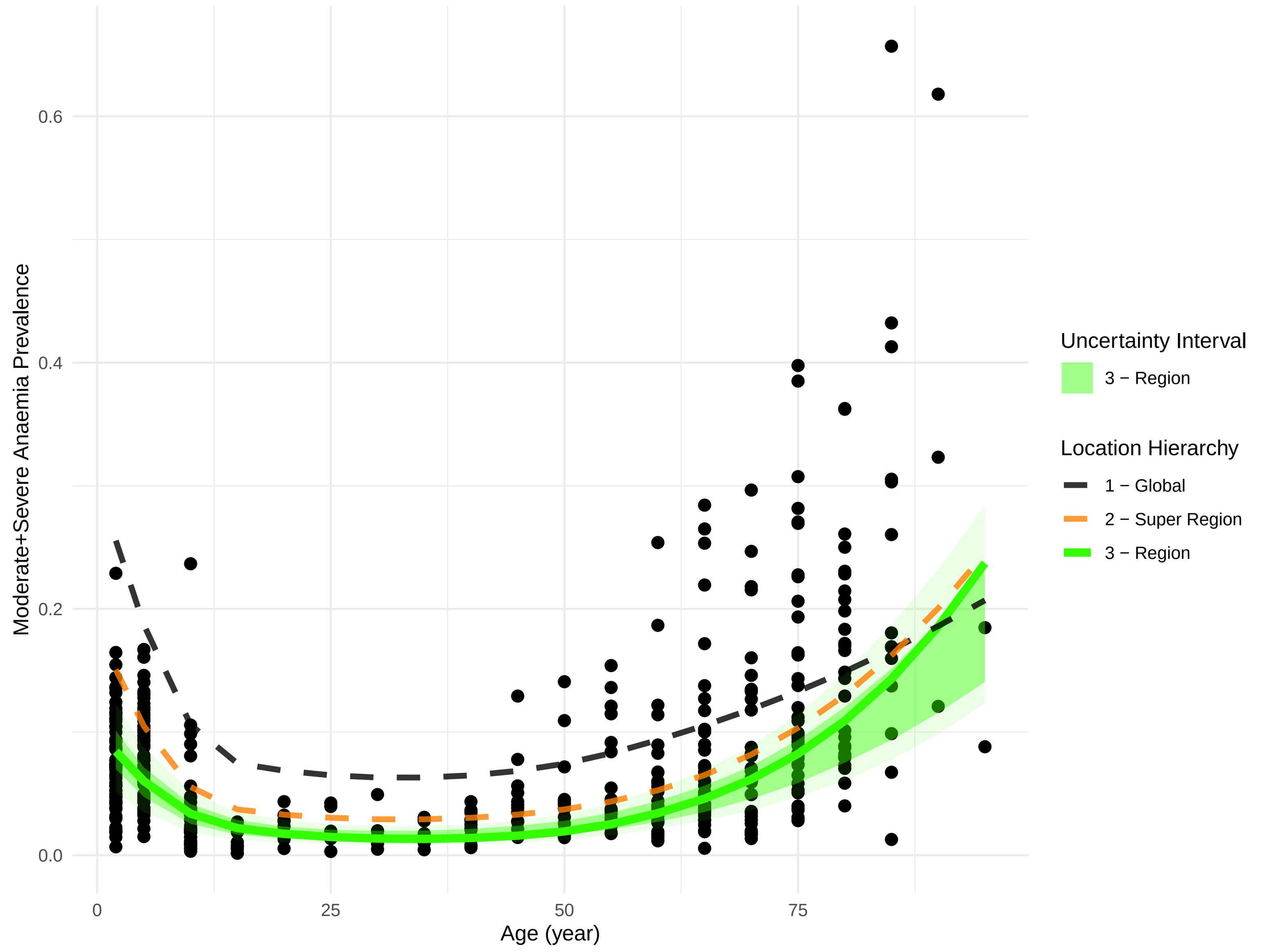

MR−BRT Age/Sex Fits − Males 2+ Years Old − Moderate+Severe Anaemia Prevalence − Guatemala
WHO−2001 Adjustment Method

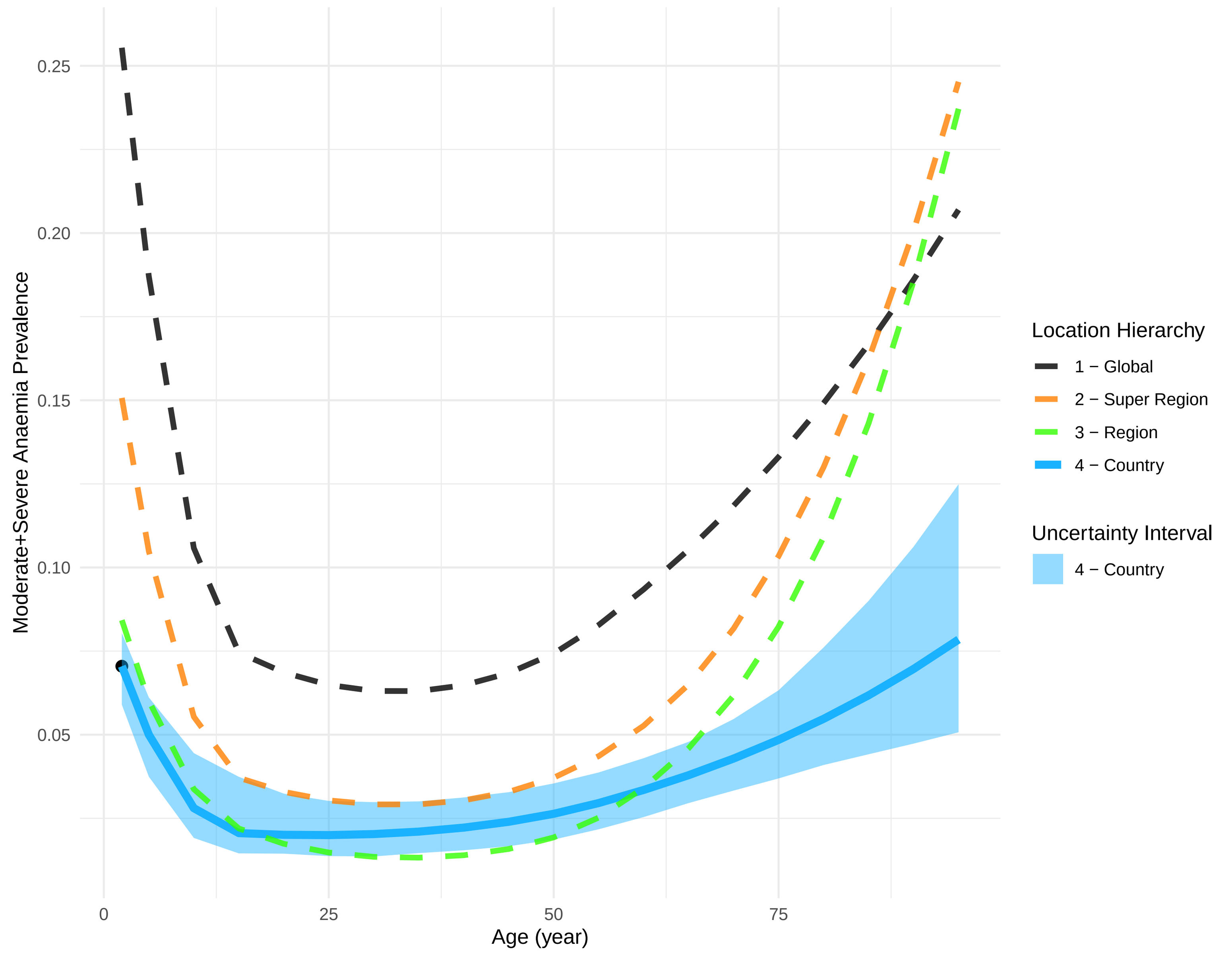

MR−BRT Age/Sex Fits − Males 2+ Years Old − Moderate+Severe Anaemia Prevalence − Honduras
WHO−2001 Adjustment Method

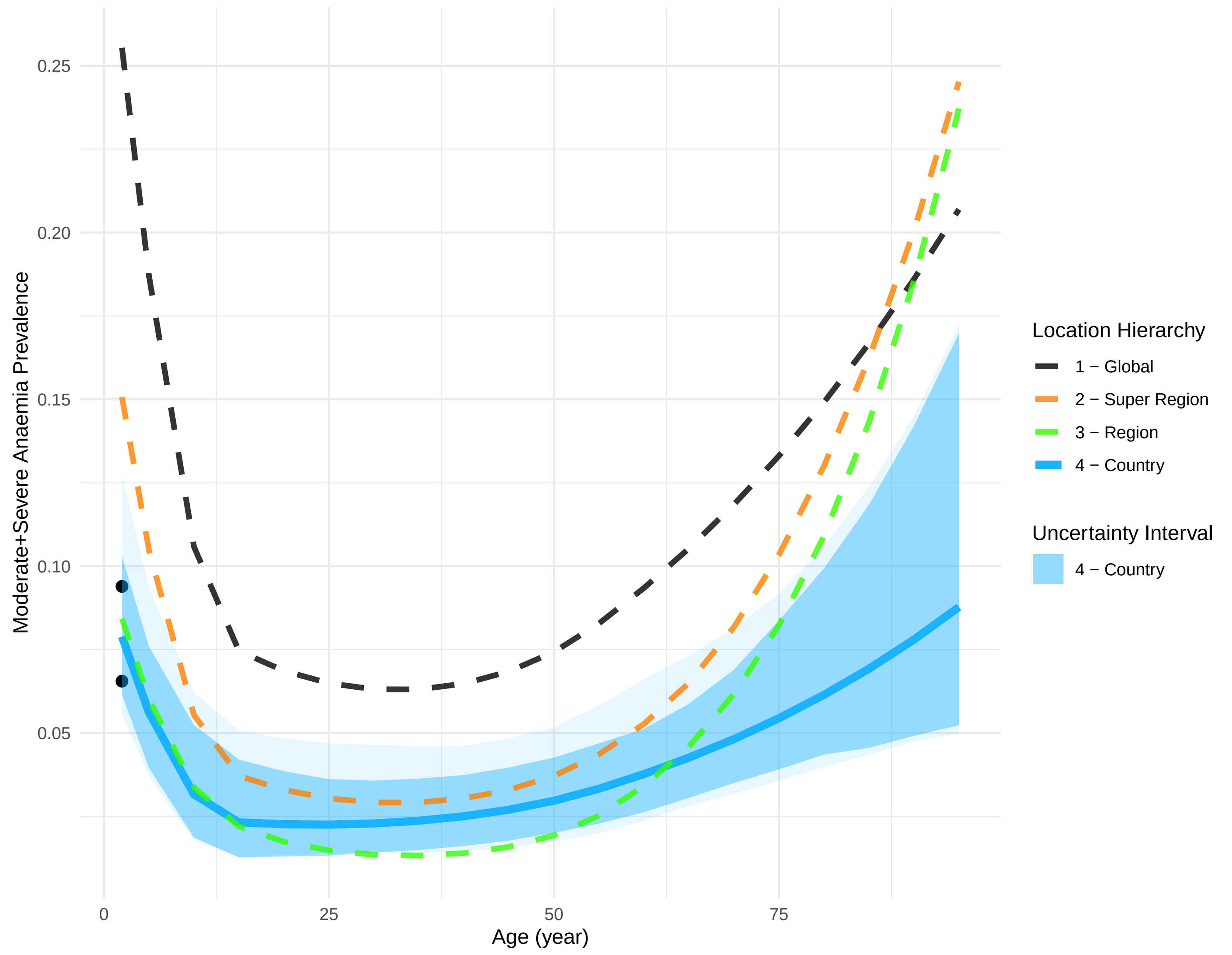

MR−BRT Age/Sex Fits − Males 2+ Years Old − Moderate+Severe Anaemia Prevalence − Mexico
WHO−2001 Adjustment Method

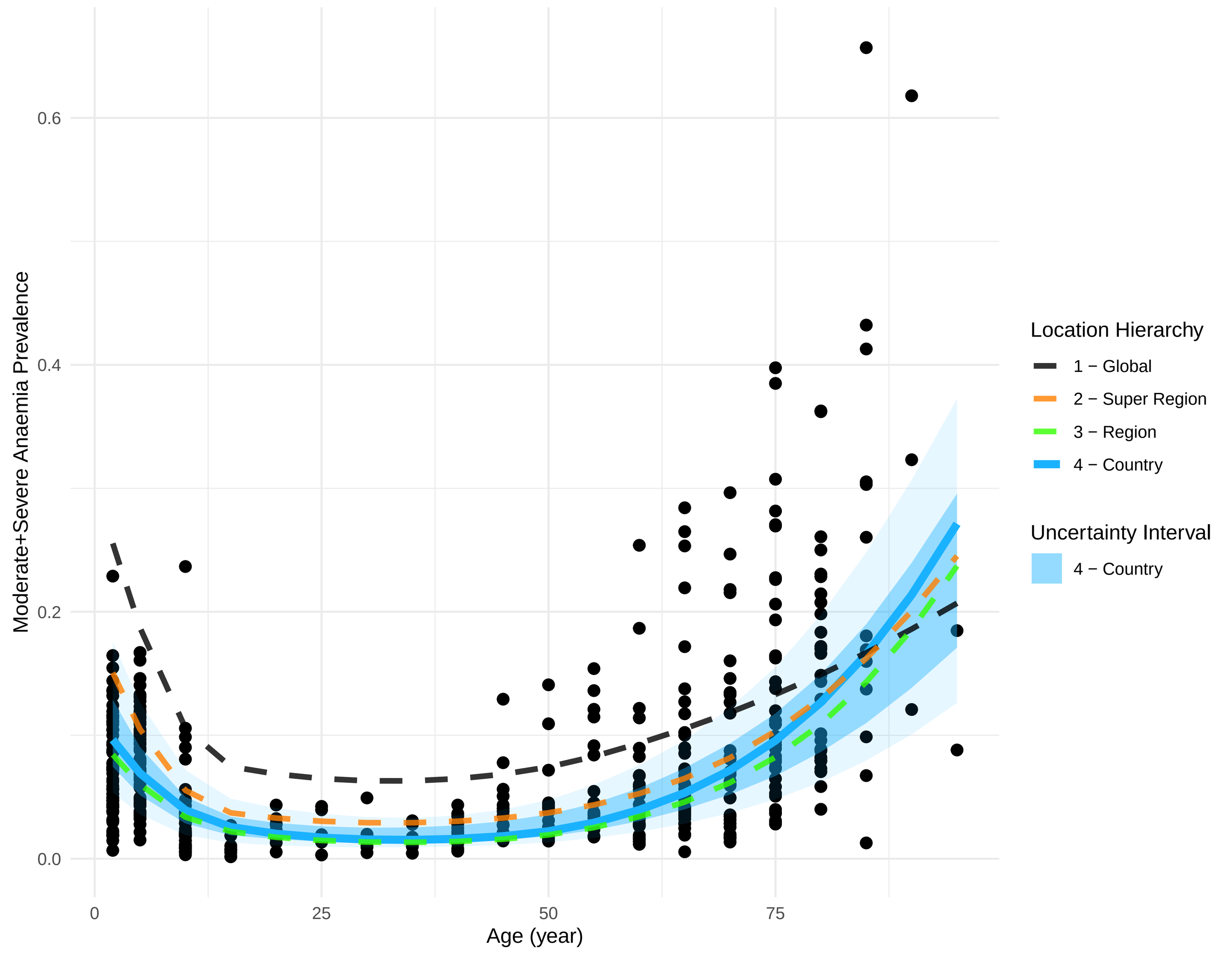

MR−BRT Age/Sex Fits − Males 2+ Years Old − Moderate+Severe Anaemia Prevalence − Aguascalientes
WHO−2001 Adjustment Method

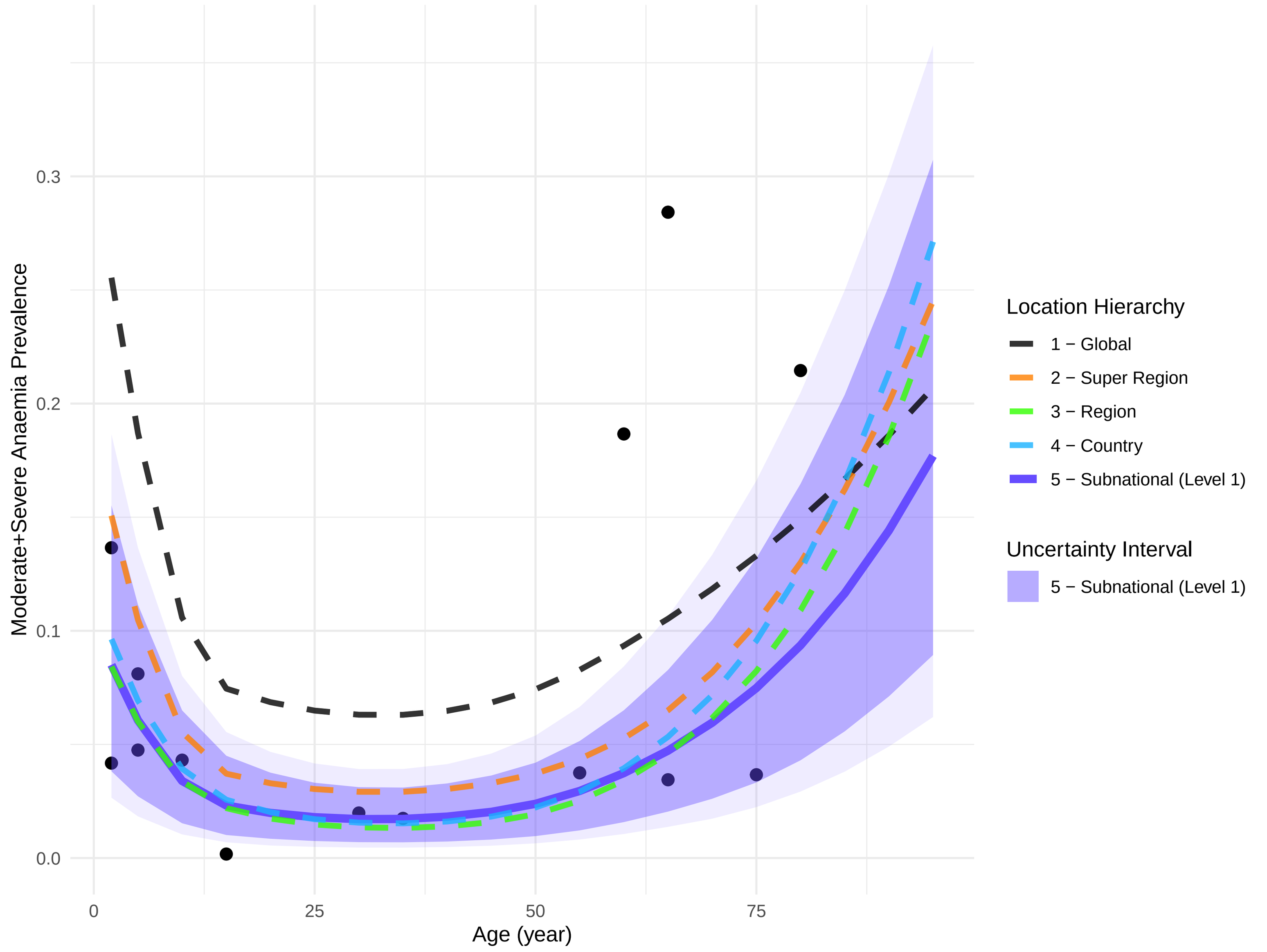

MR−BRT Age/Sex Fits − Males 2+ Years Old − Moderate+Severe Anaemia Prevalence − Baja California
WHO−2001 Adjustment Method

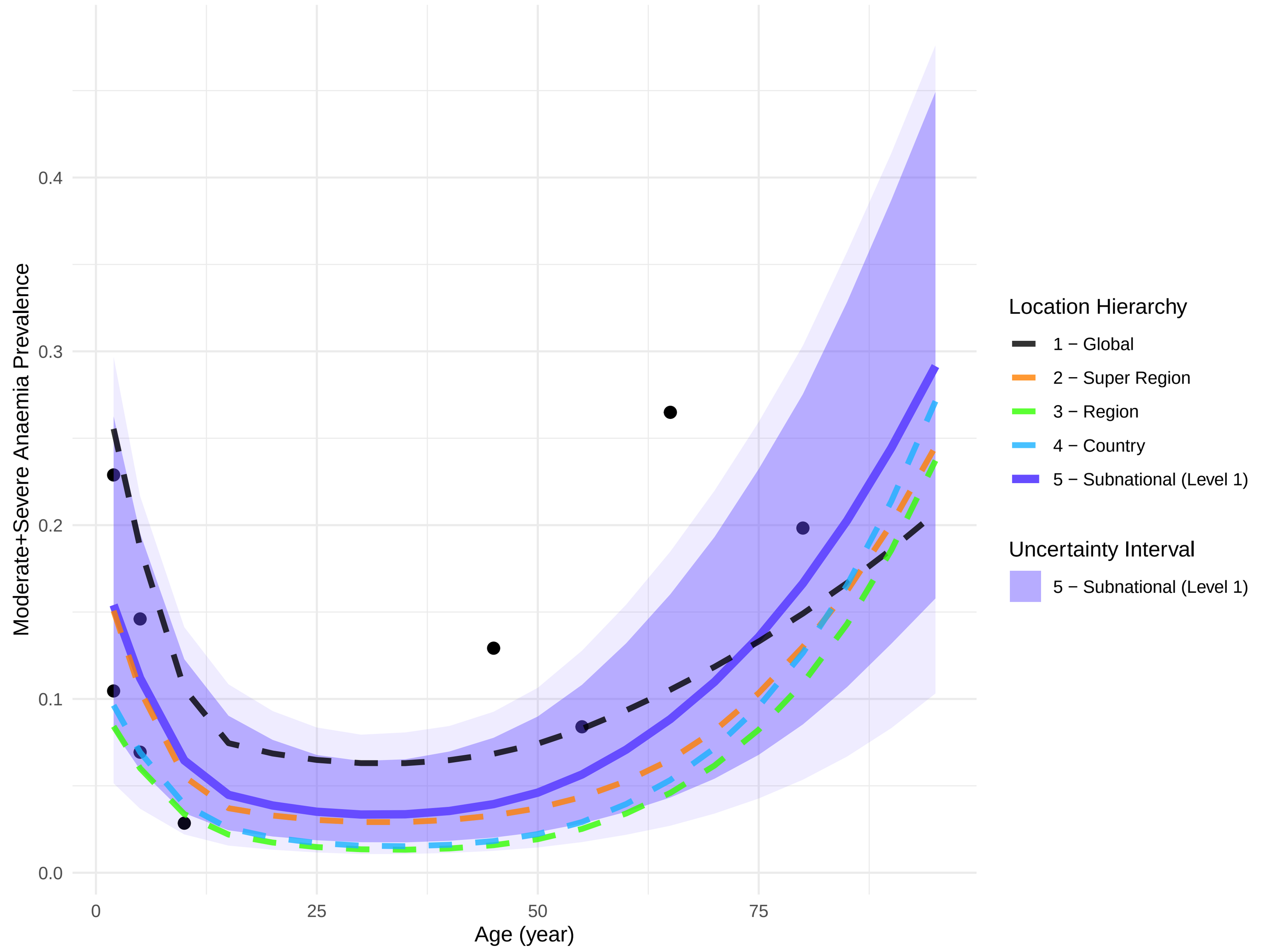

MR−BRT Age/Sex Fits − Males 2+ Years Old − Moderate+Severe Anaemia Prevalence − Baja California Sur
WHO−2001 Adjustment Method

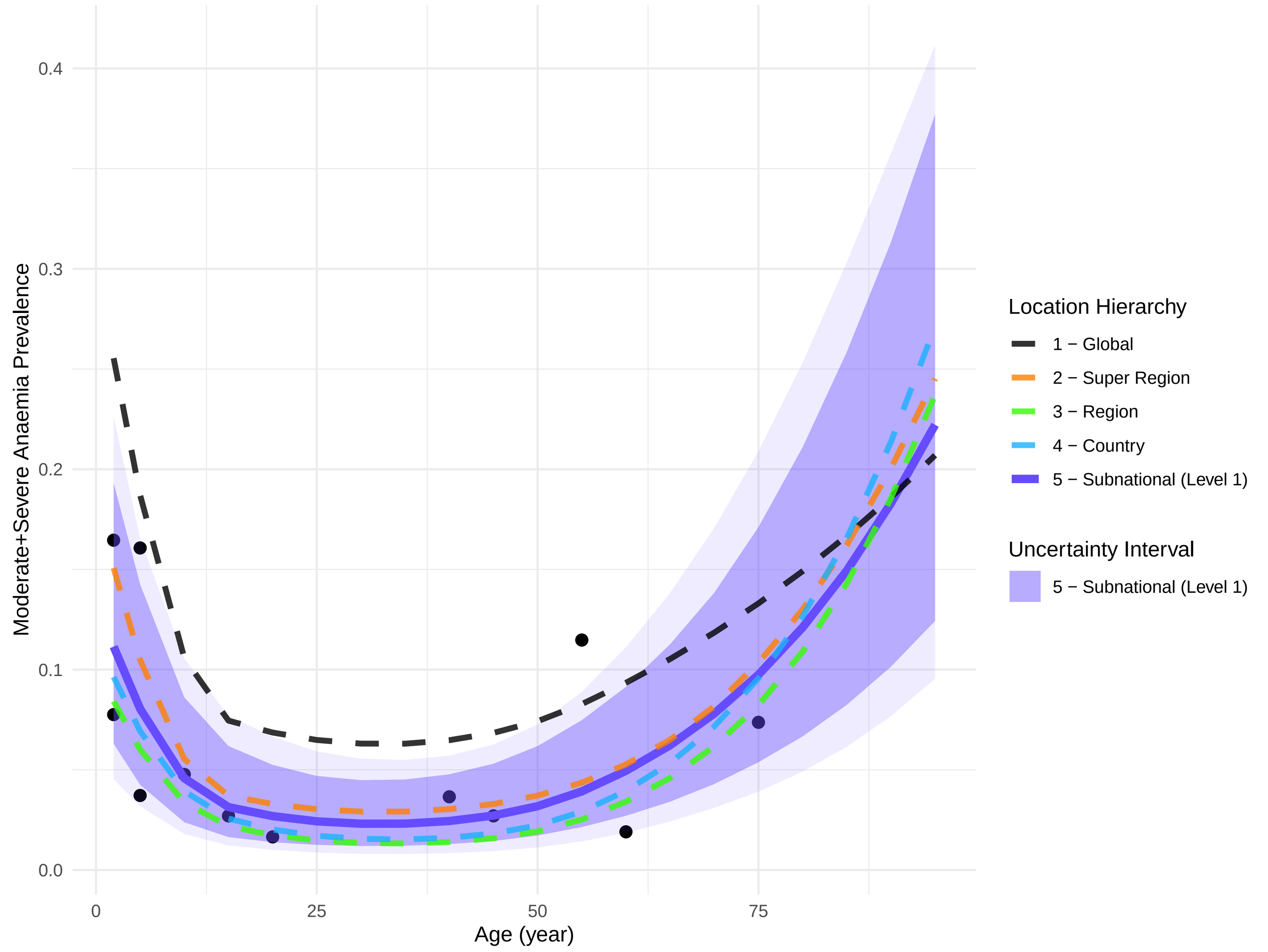

MR−BRT Age/Sex Fits − Males 2+ Years Old − Moderate+Severe Anaemia Prevalence − Campeche
WHO−2001 Adjustment Method

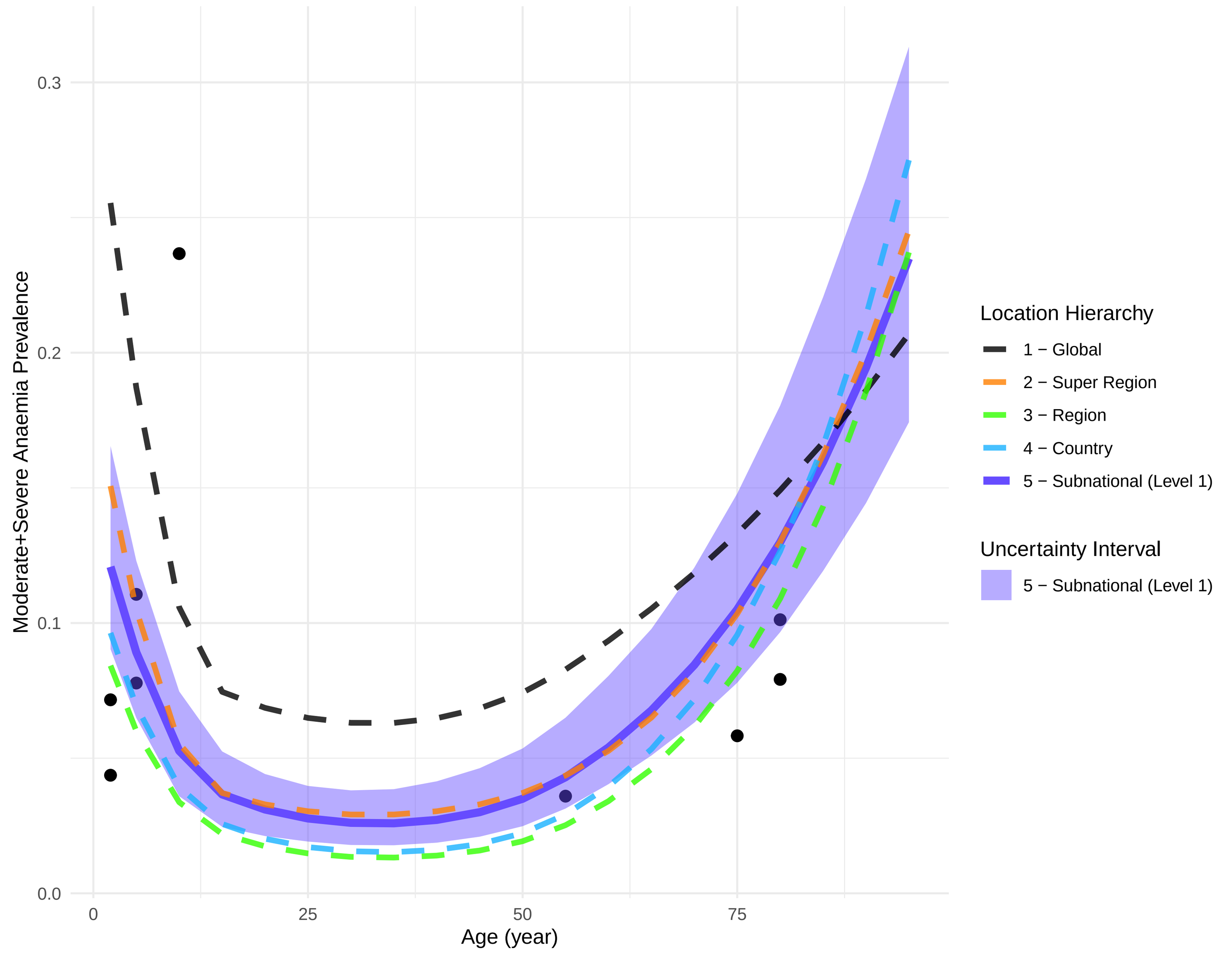

MR−BRT Age/Sex Fits − Males 2+ Years Old − Moderate+Severe Anaemia Prevalence − Chiapas
WHO−2001 Adjustment Method

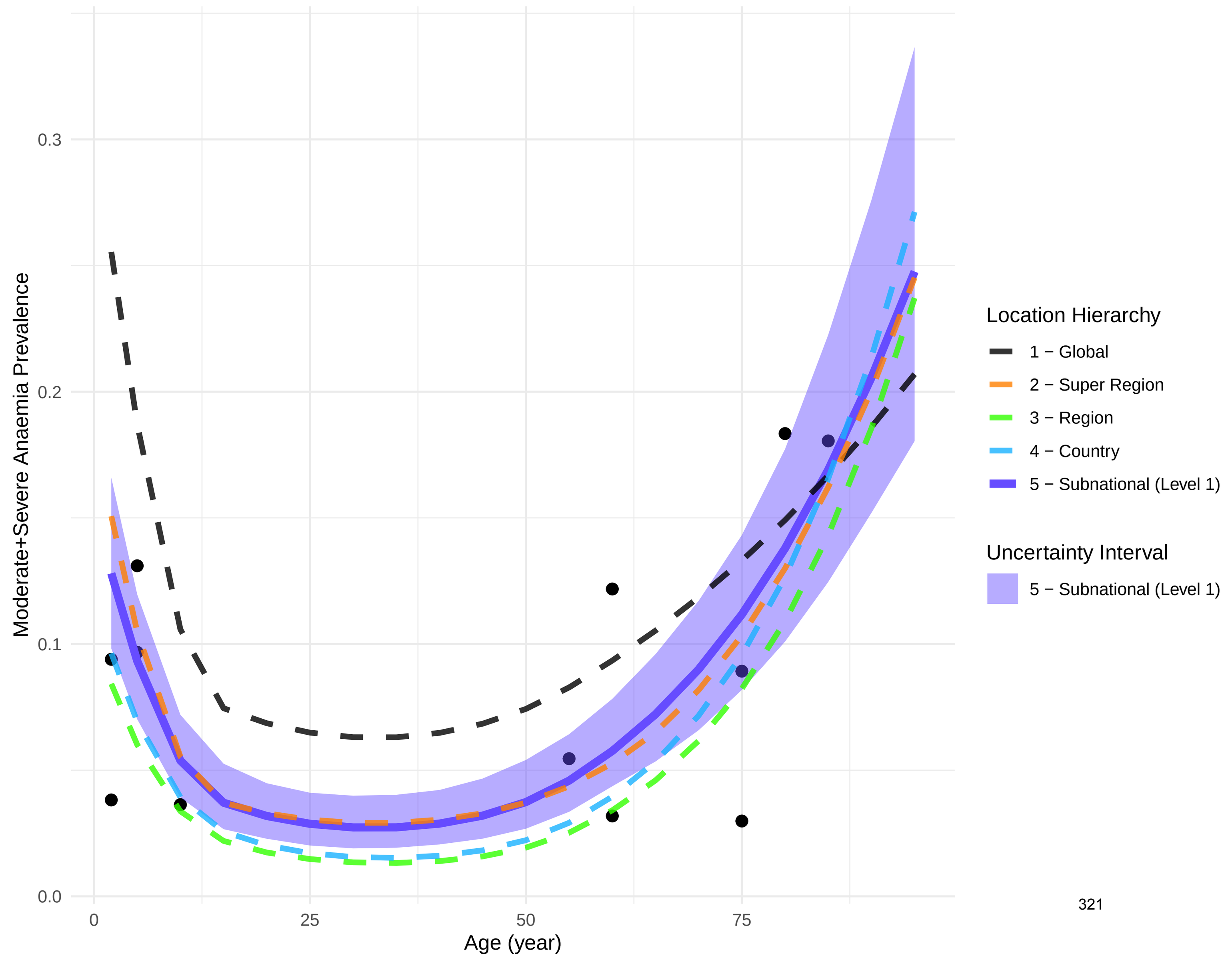

MR−BRT Age/Sex Fits − Males 2+ Years Old − Moderate+Severe Anaemia Prevalence − Chihuahua
WHO−2001 Adjustment Method

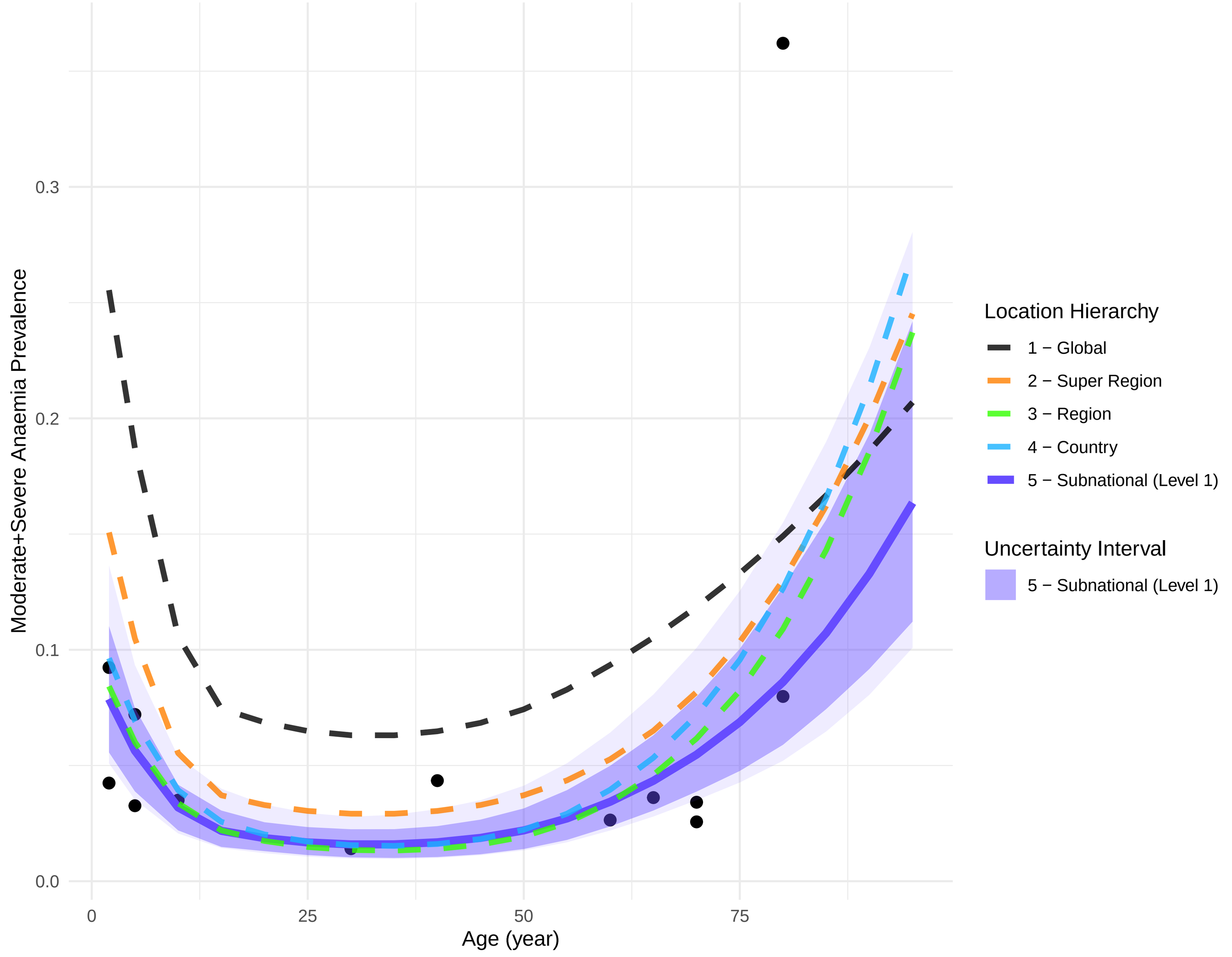

MR−BRT Age/Sex Fits − Males 2+ Years Old − Moderate+Severe Anaemia Prevalence − Coahuila
WHO−2001 Adjustment Method

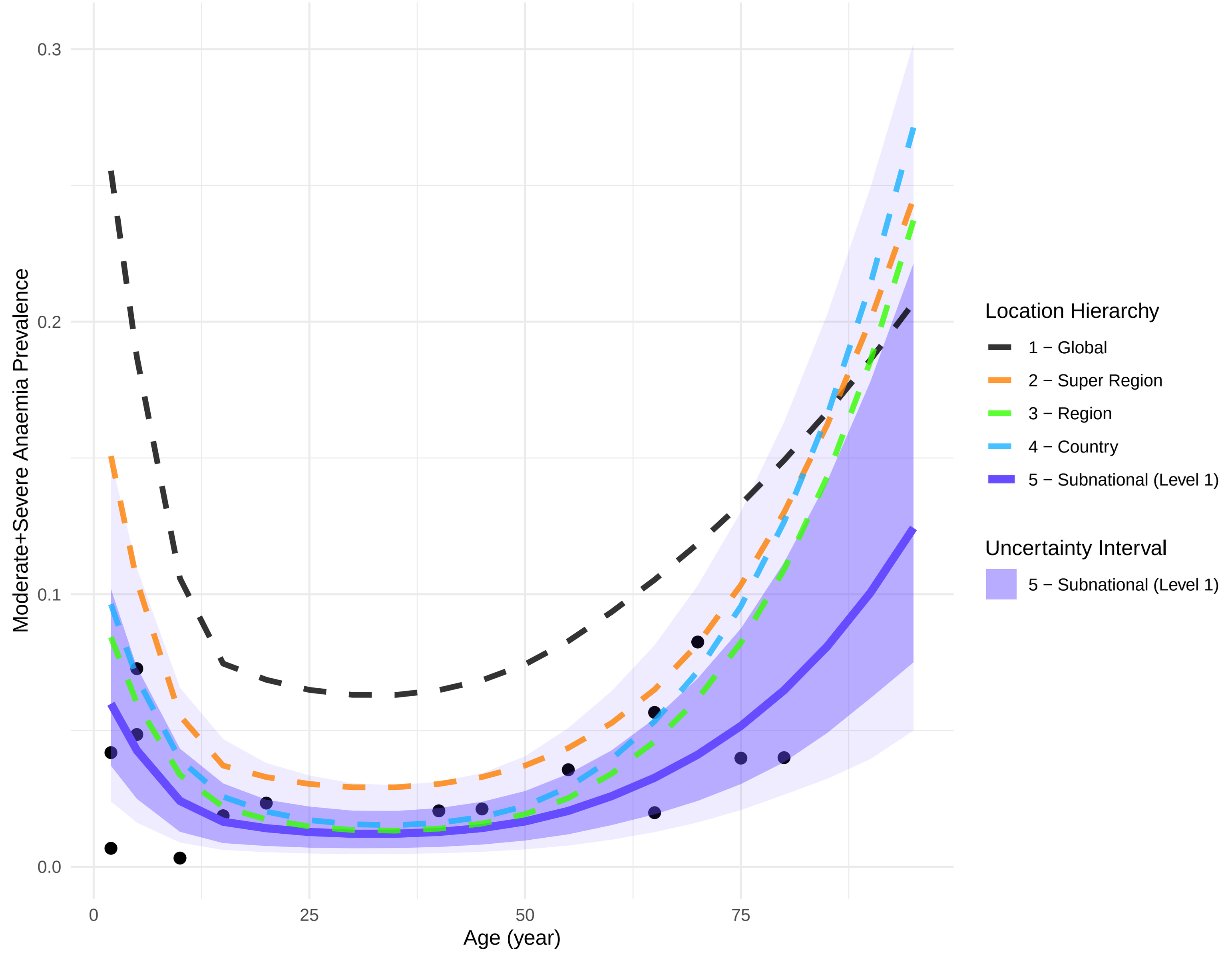

MR−BRT Age/Sex Fits − Males 2+ Years Old − Moderate+Severe Anaemia Prevalence − Colima
WHO−2001 Adjustment Method

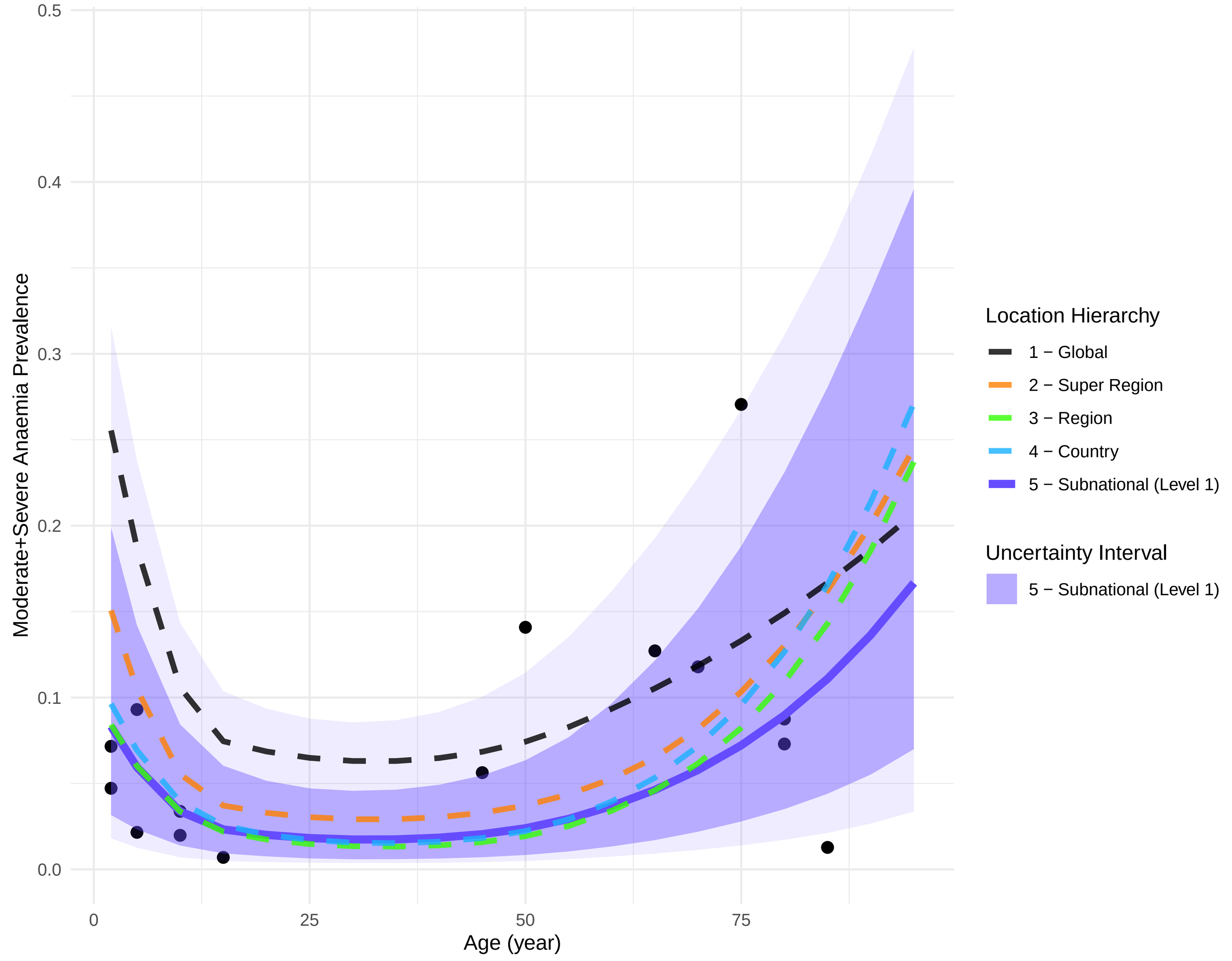

MR−BRT Age/Sex Fits − Males 2+ Years Old − Moderate+Severe Anaemia Prevalence − Durango
WHO−2001 Adjustment Method

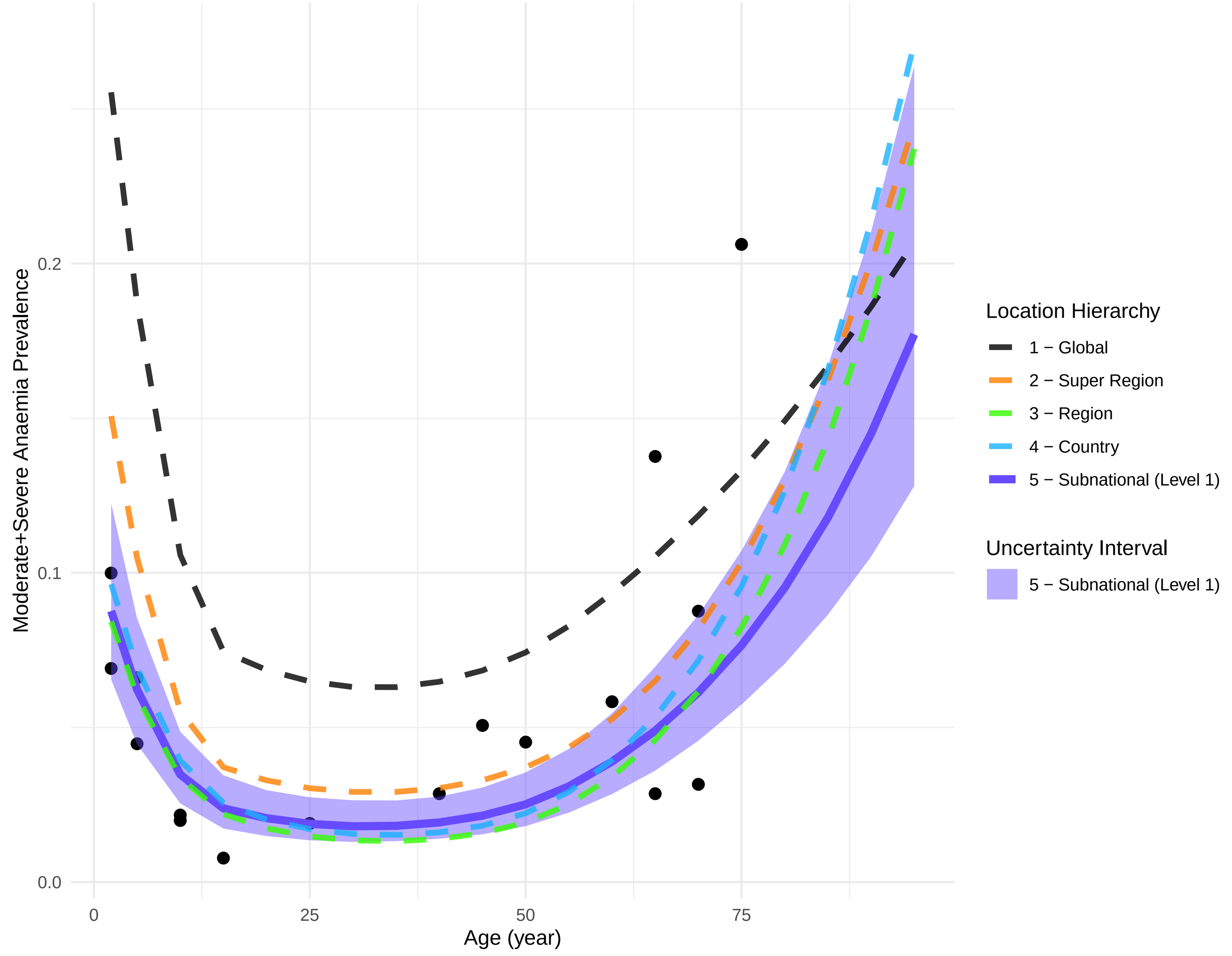

MR−BRT Age/Sex Fits − Males 2+ Years Old − Moderate+Severe Anaemia Prevalence − Guanajuato
WHO−2001 Adjustment Method

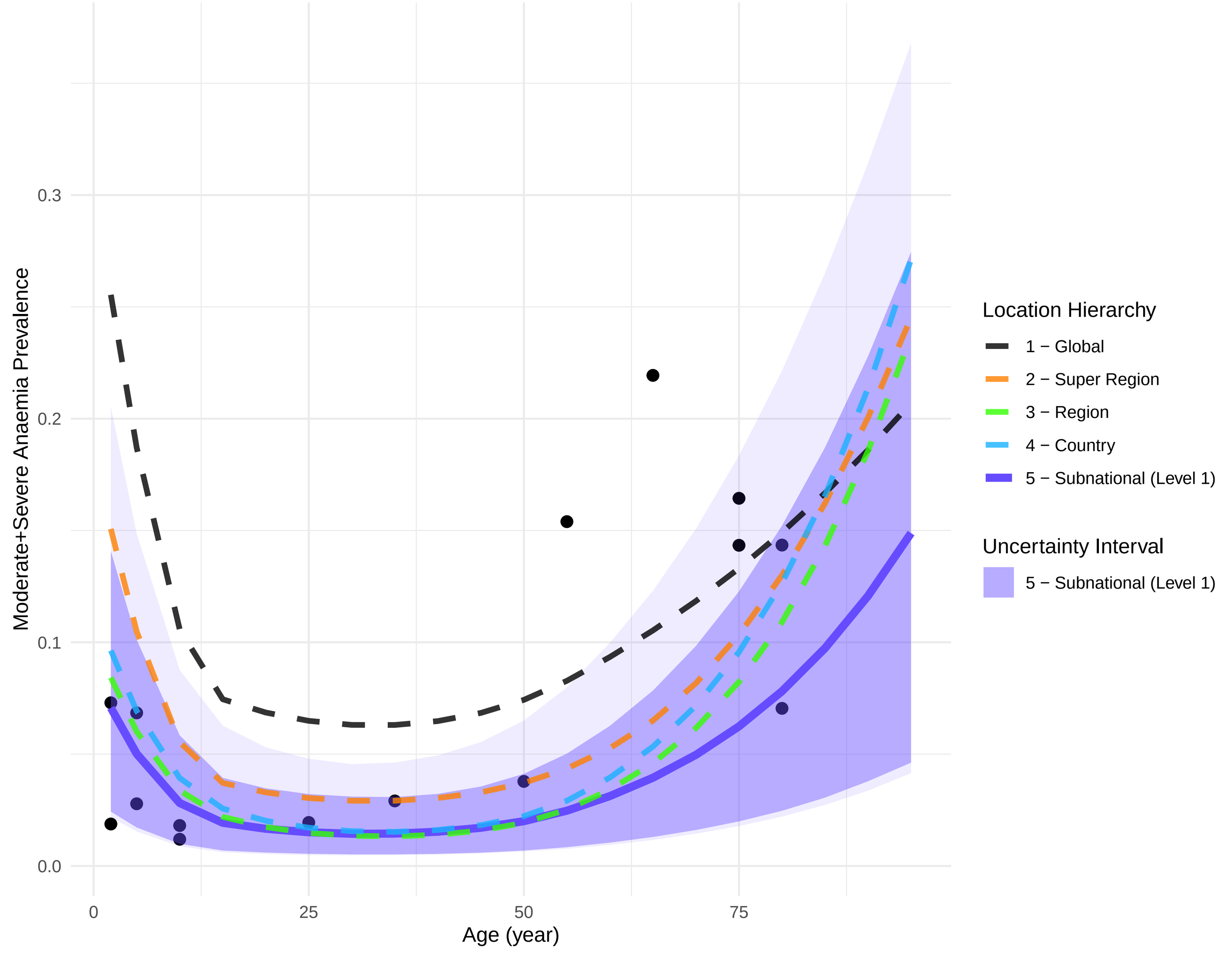

MR−BRT Age/Sex Fits − Males 2+ Years Old − Moderate+Severe Anaemia Prevalence − Guerrero
WHO−2001 Adjustment Method

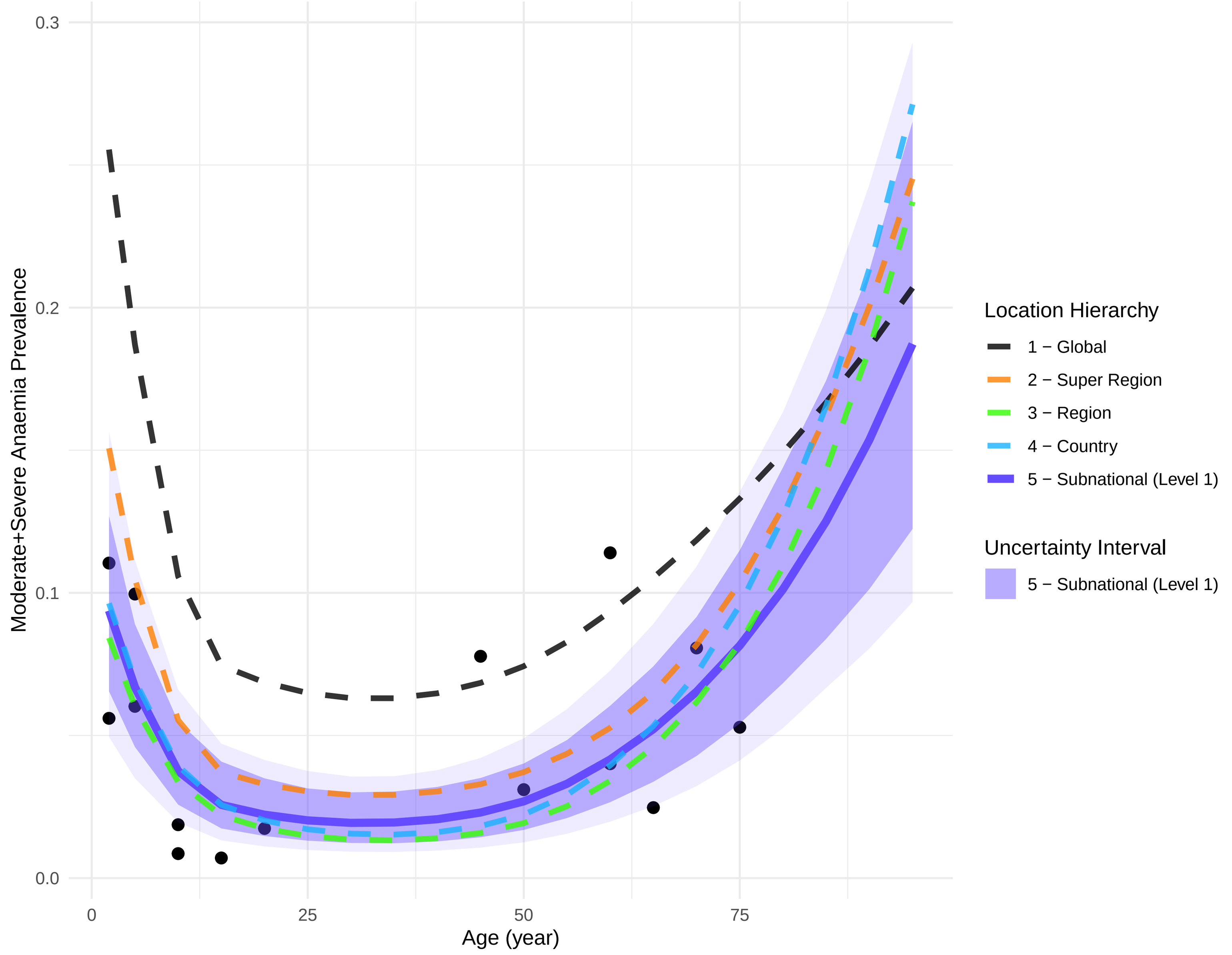

MR−BRT Age/Sex Fits − Males 2+ Years Old − Moderate+Severe Anaemia Prevalence − Hidalgo
WHO−2001 Adjustment Method

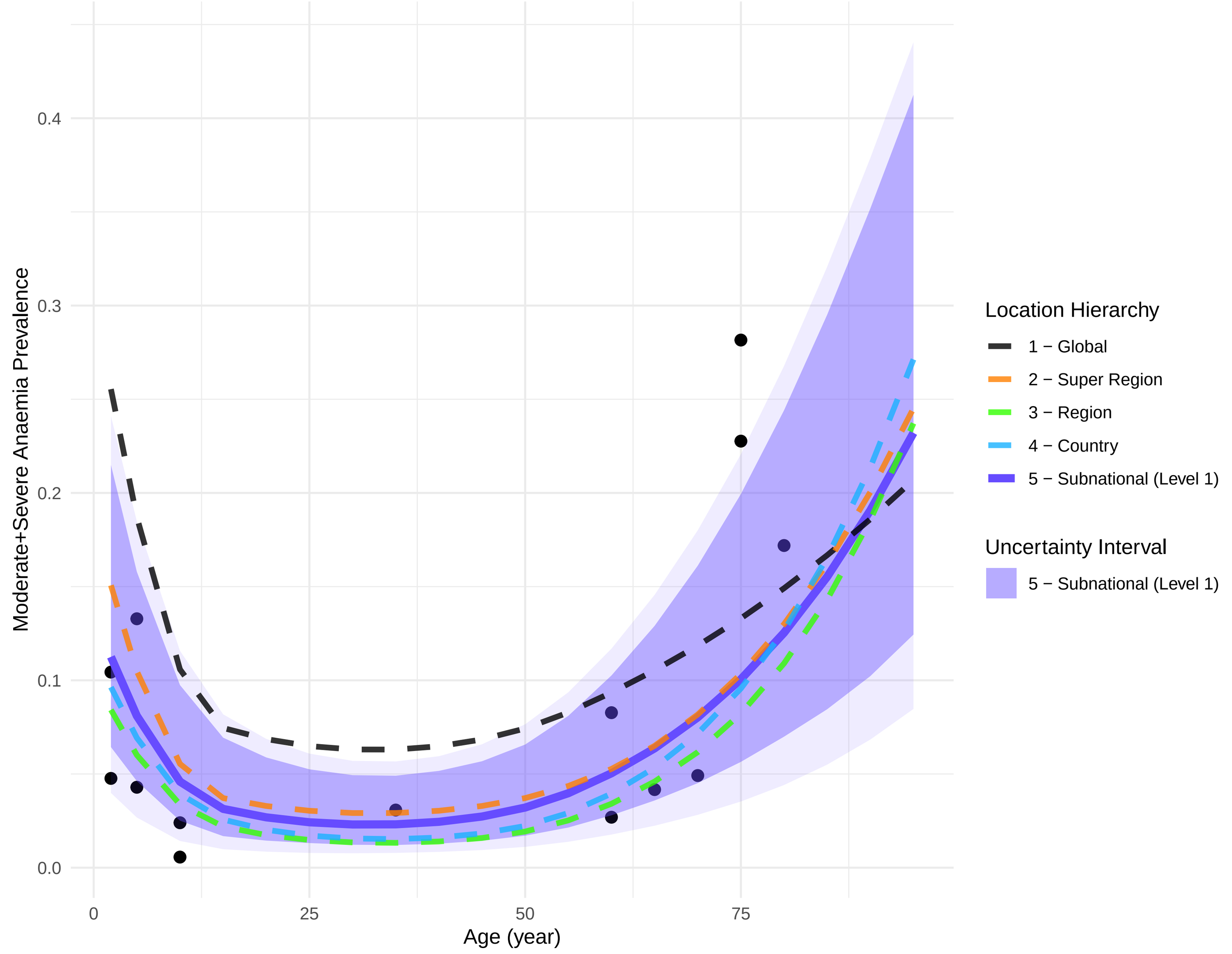

MR−BRT Age/Sex Fits − Males 2+ Years Old − Moderate+Severe Anaemia Prevalence − Jalisco
WHO−2001 Adjustment Method

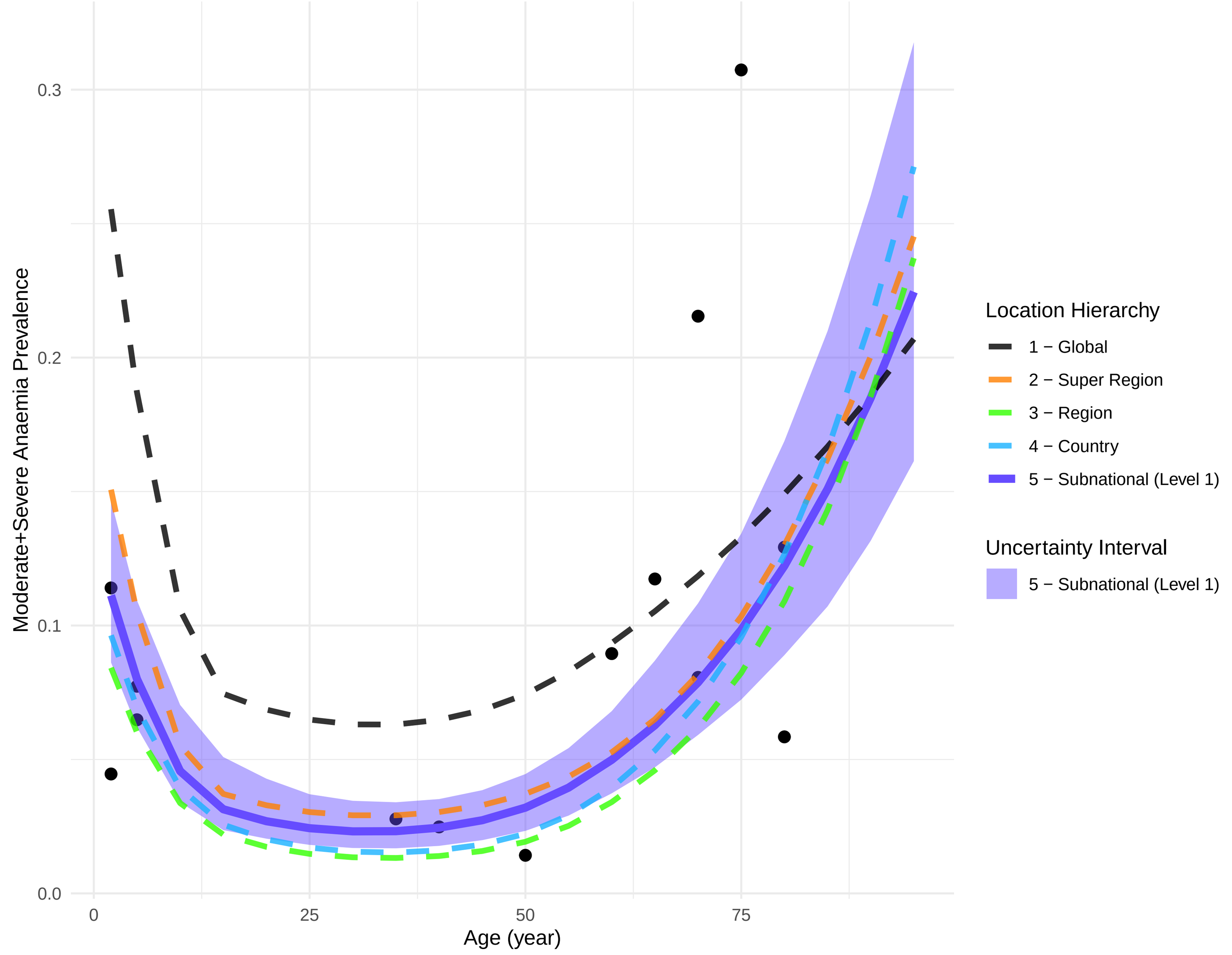

MR−BRT Age/Sex Fits − Males 2+ Years Old − Moderate+Severe Anaemia Prevalence − México
WHO−2001 Adjustment Method

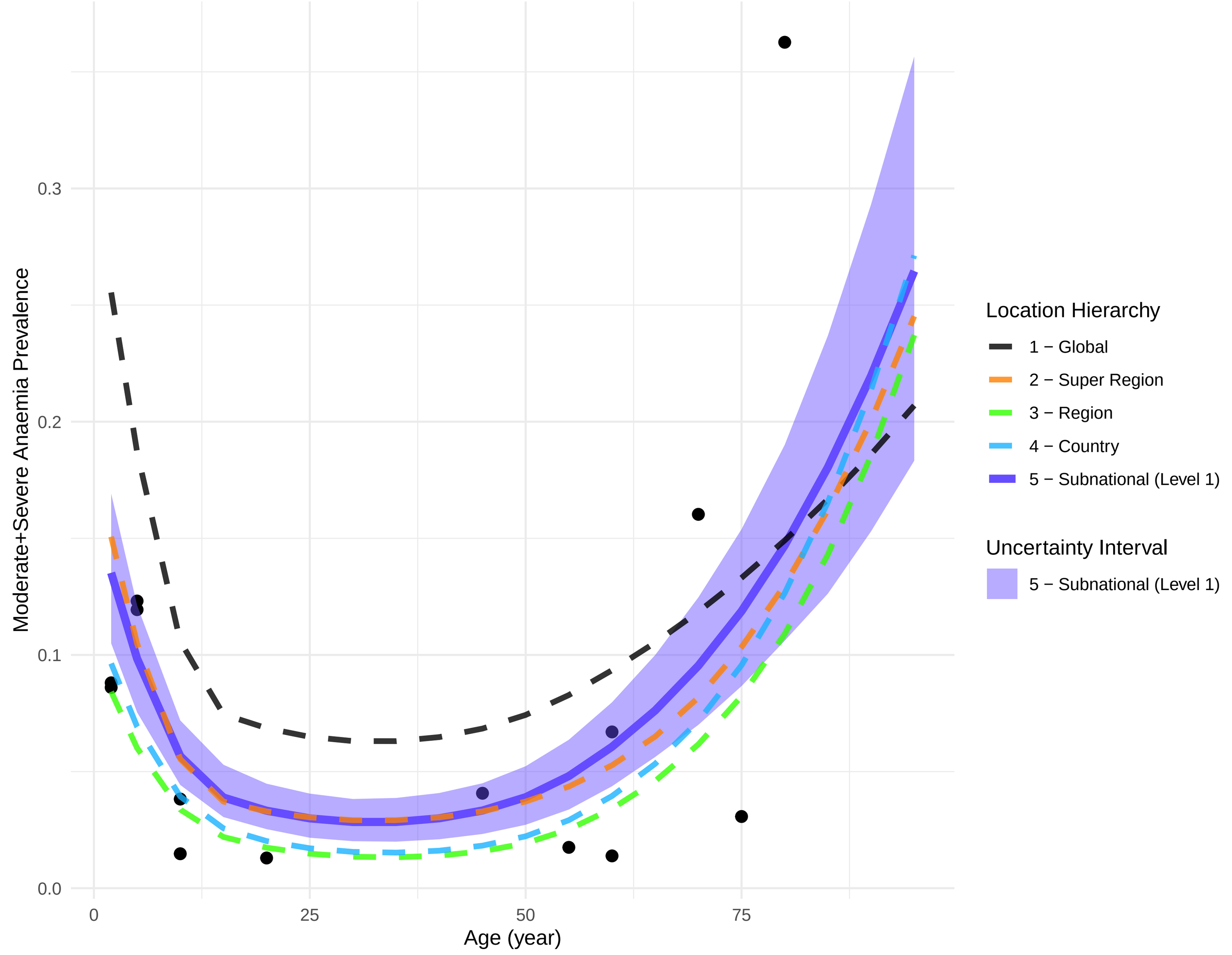

MR−BRT Age/Sex Fits − Males 2+ Years Old − Moderate+Severe Anaemia Prevalence − Mexico City
WHO−2001 Adjustment Method

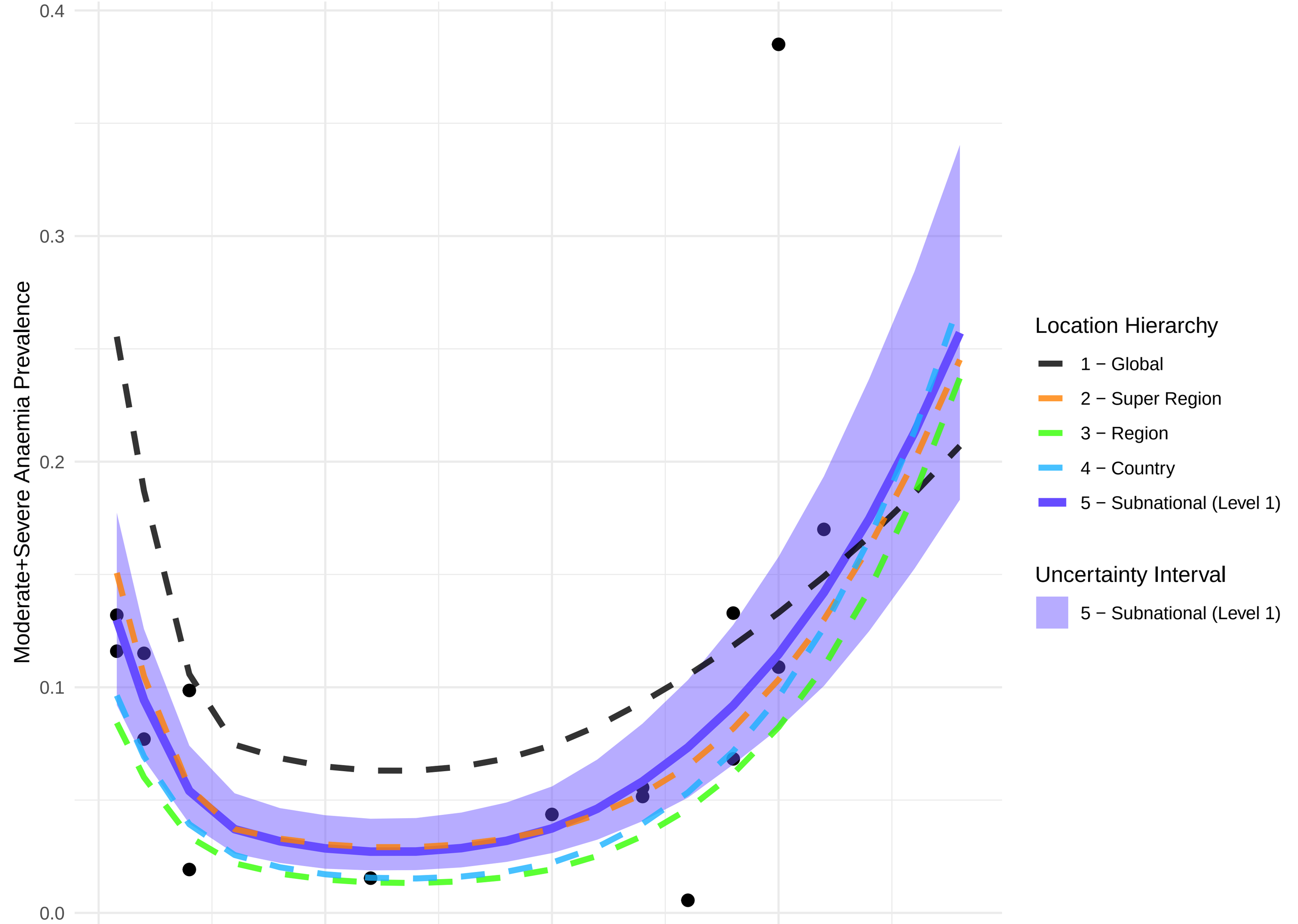

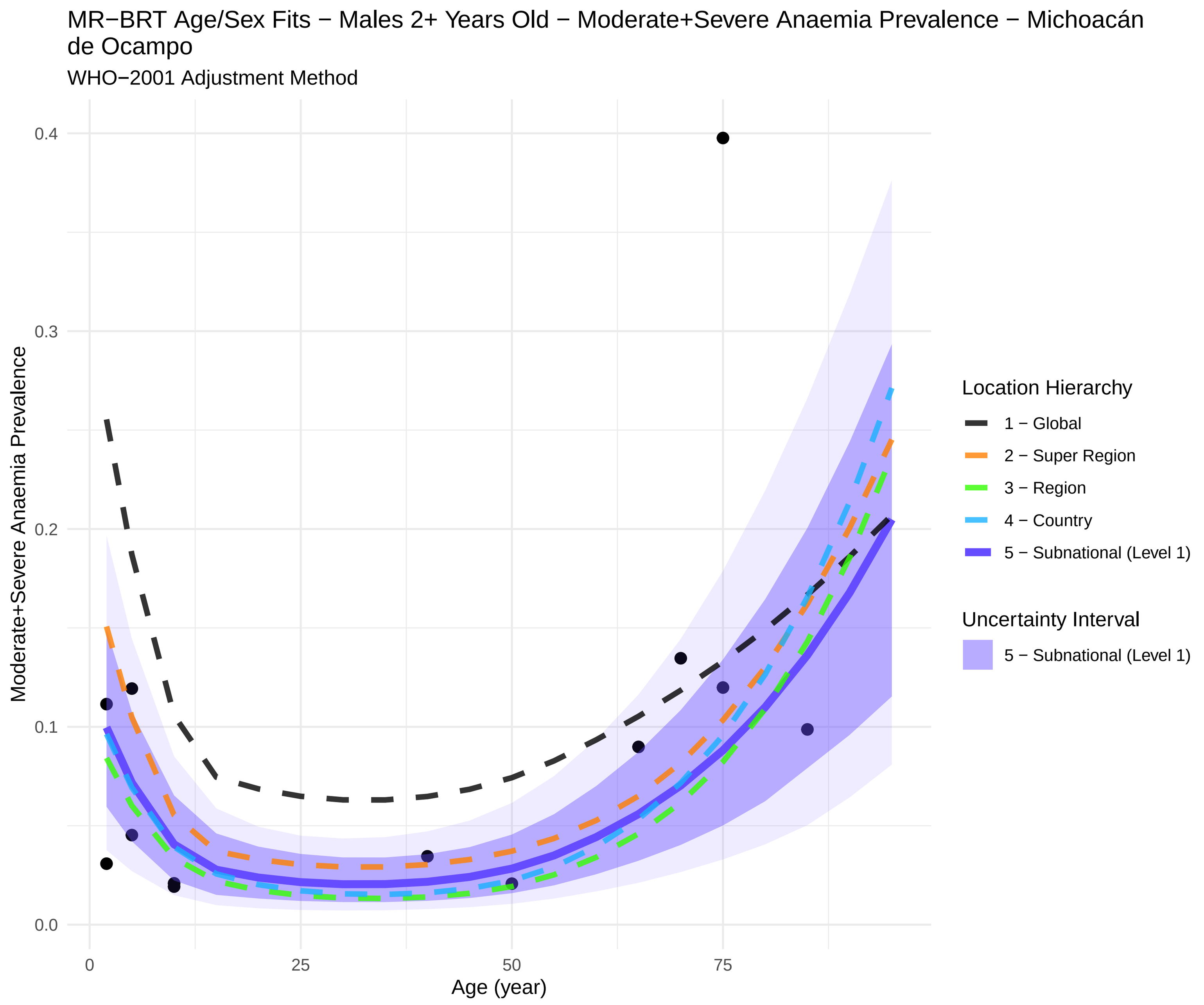
MR−BRT Age/Sex Fits − Males 2+ Years Old − Moderate+Severe Anaemia Prevalence − Michoacán de Ocampo
WHO−2001 Adjustment Method
Moderate+Severe Anaemia Prevalence
Age (year)
Location Hierarchy
1 − Global
2 − Super Region
3 − Region
4 − Country
5 − Subnational (Level 1)
Uncertainty Interval
5 − Subnational (Level 1)

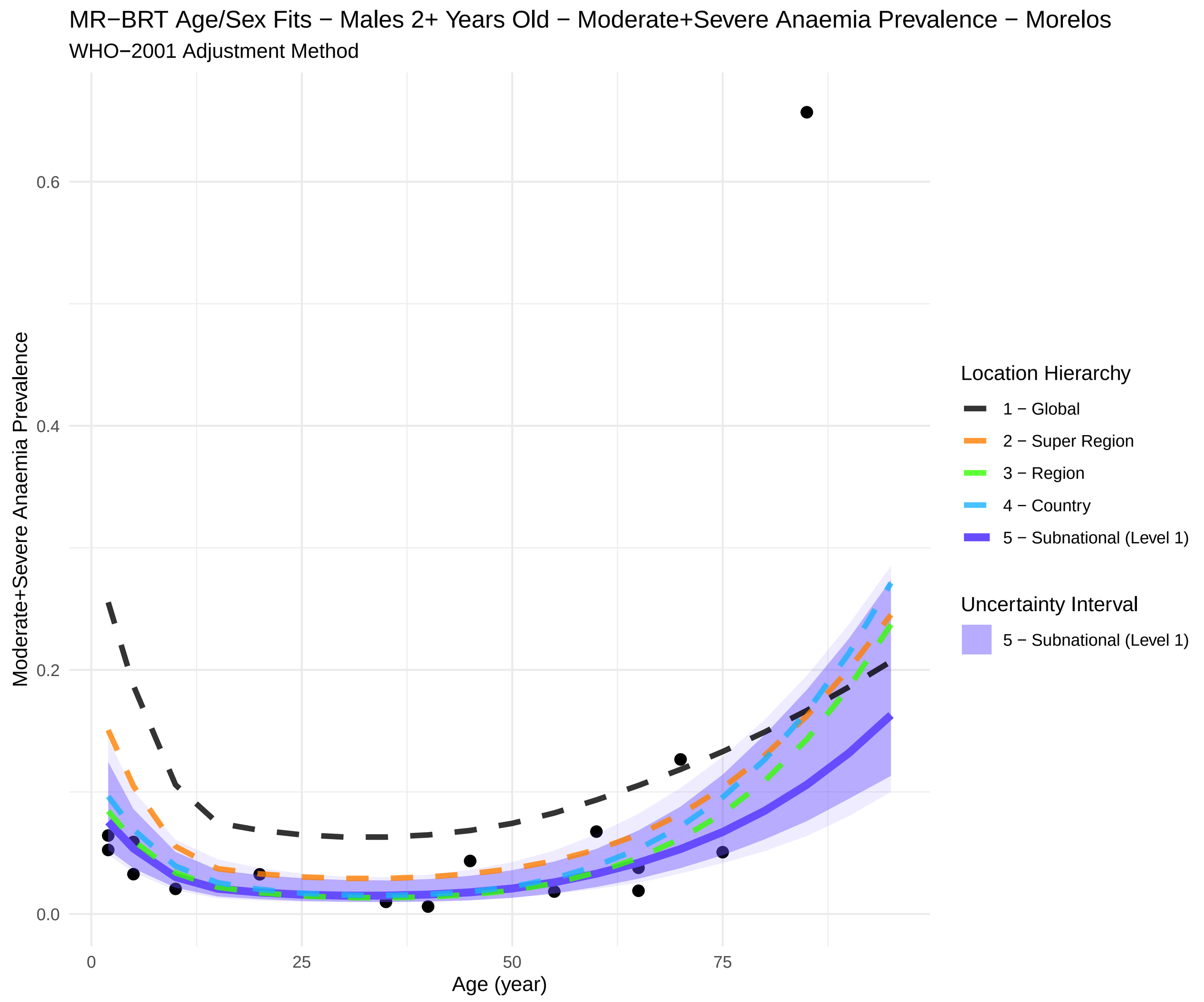
MR−BRT Age/Sex Fits − Males 2+ Years Old − Moderate+Severe Anaemia Prevalence − Morelos
WHO−2001 Adjustment Method
Moderate+Severe Anaemia Prevalence
Age (year)
Location Hierarchy
1 − Global
2 − Super Region
3 − Region
4 − Country
5 − Subnational (Level 1)
Uncertainty Interval
5 − Subnational (Level 1)

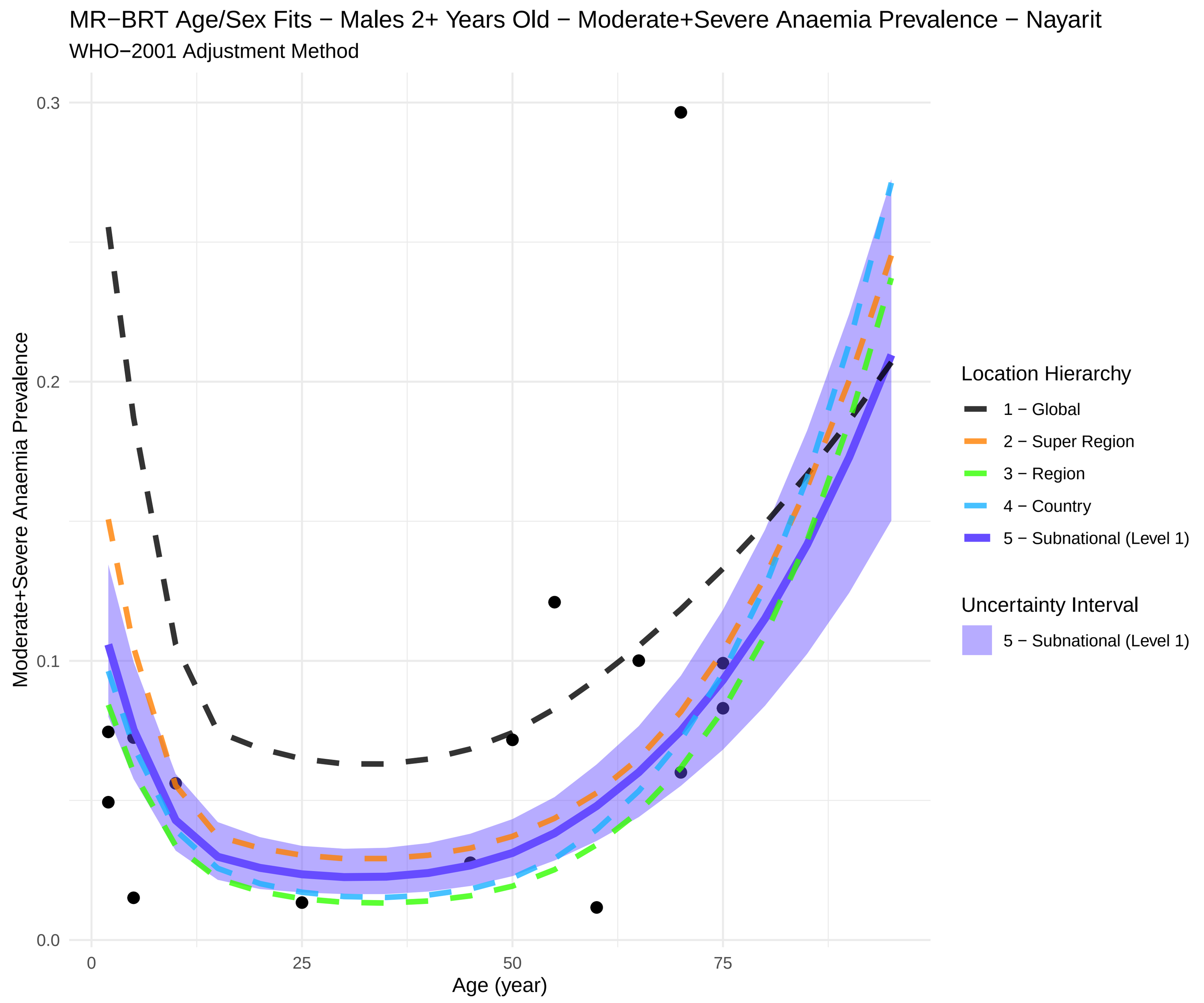
MR−BRT Age/Sex Fits − Males 2+ Years Old − Moderate+Severe Anaemia Prevalence − Nayarit
WHO−2001 Adjustment Method
Moderate+Severe Anaemia Prevalence
Age (year)
Location Hierarchy
1 − Global
2 − Super Region
3 − Region
4 − Country
5 − Subnational (Level 1)
Uncertainty Interval
5 − Subnational (Level 1)

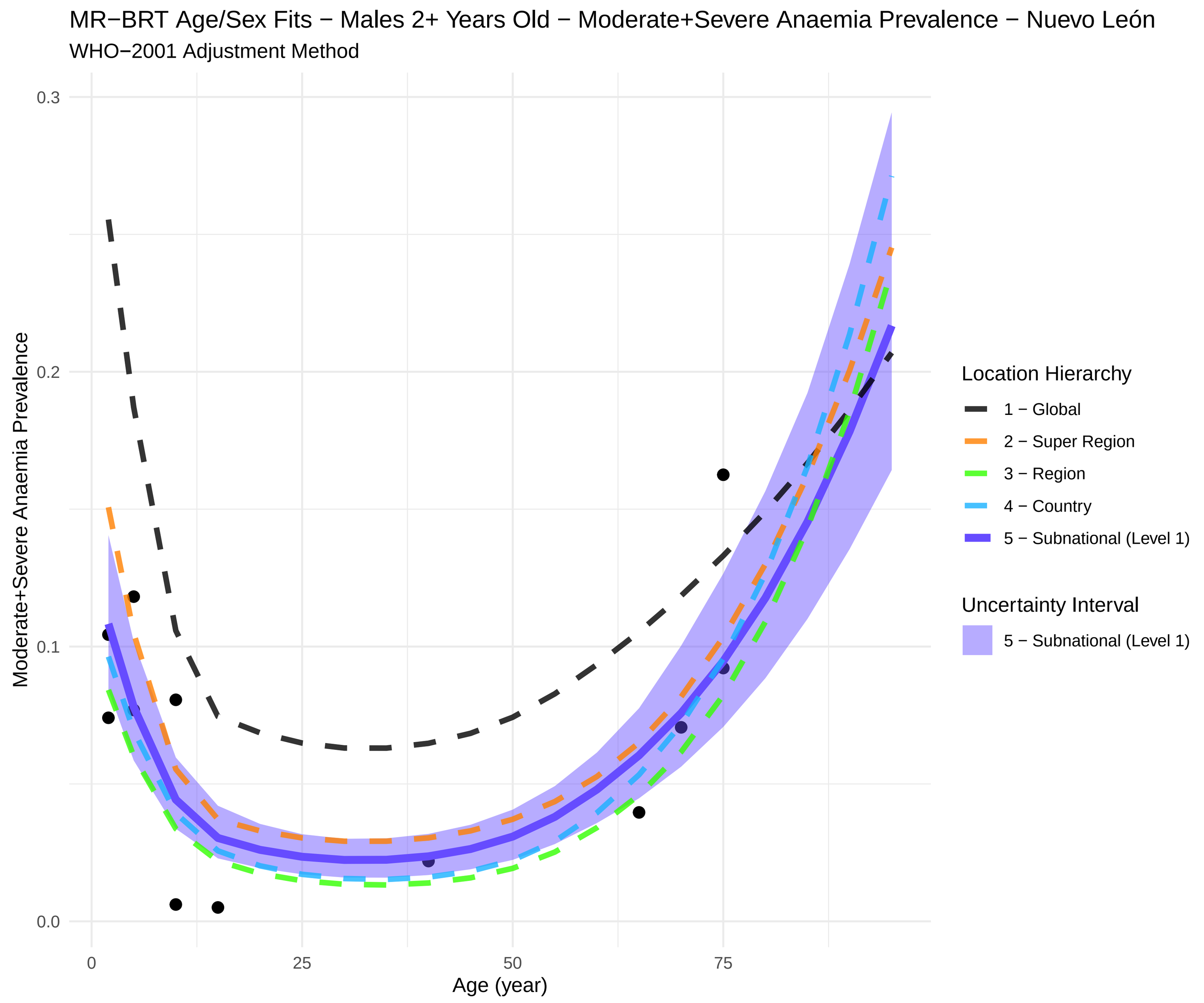
MR−BRT Age/Sex Fits − Males 2+ Years Old − Moderate+Severe Anaemia Prevalence − Nuevo León
WHO−2001 Adjustment Method
Moderate+Severe Anaemia Prevalence
Age (year)
Location Hierarchy
1 − Global
2 − Super Region
3 − Region
4 − Country
5 − Subnational (Level 1)
Uncertainty Interval
5 − Subnational (Level 1)

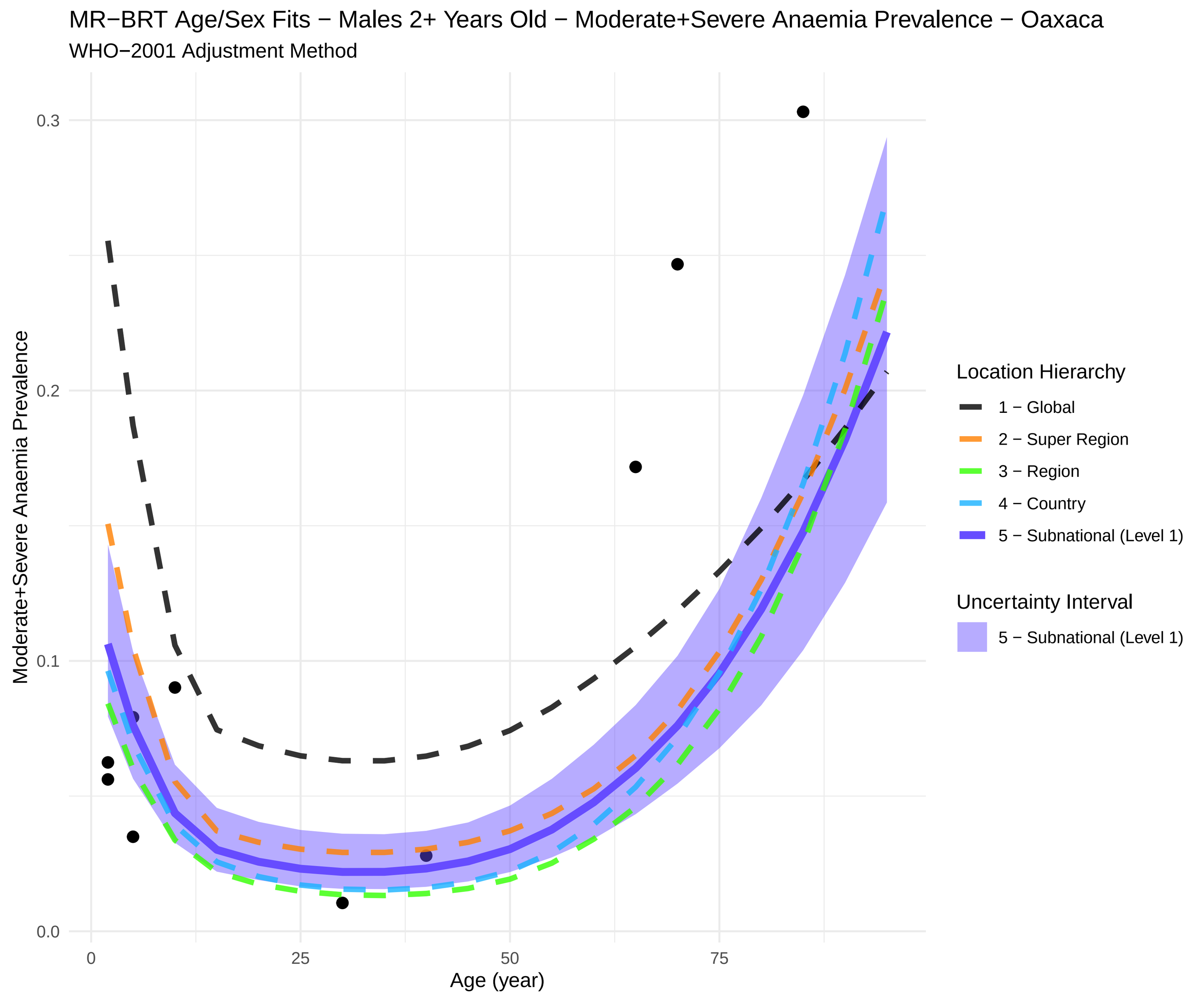
MR−BRT Age/Sex Fits − Males 2+ Years Old − Moderate+Severe Anaemia Prevalence − Oaxaca
WHO−2001 Adjustment Method
Moderate+Severe Anaemia Prevalence
Age (year)
Location Hierarchy
1 − Global
2 − Super Region
3 − Region
4 − Country
5 − Subnational (Level 1)
Uncertainty Interval
5 − Subnational (Level 1)

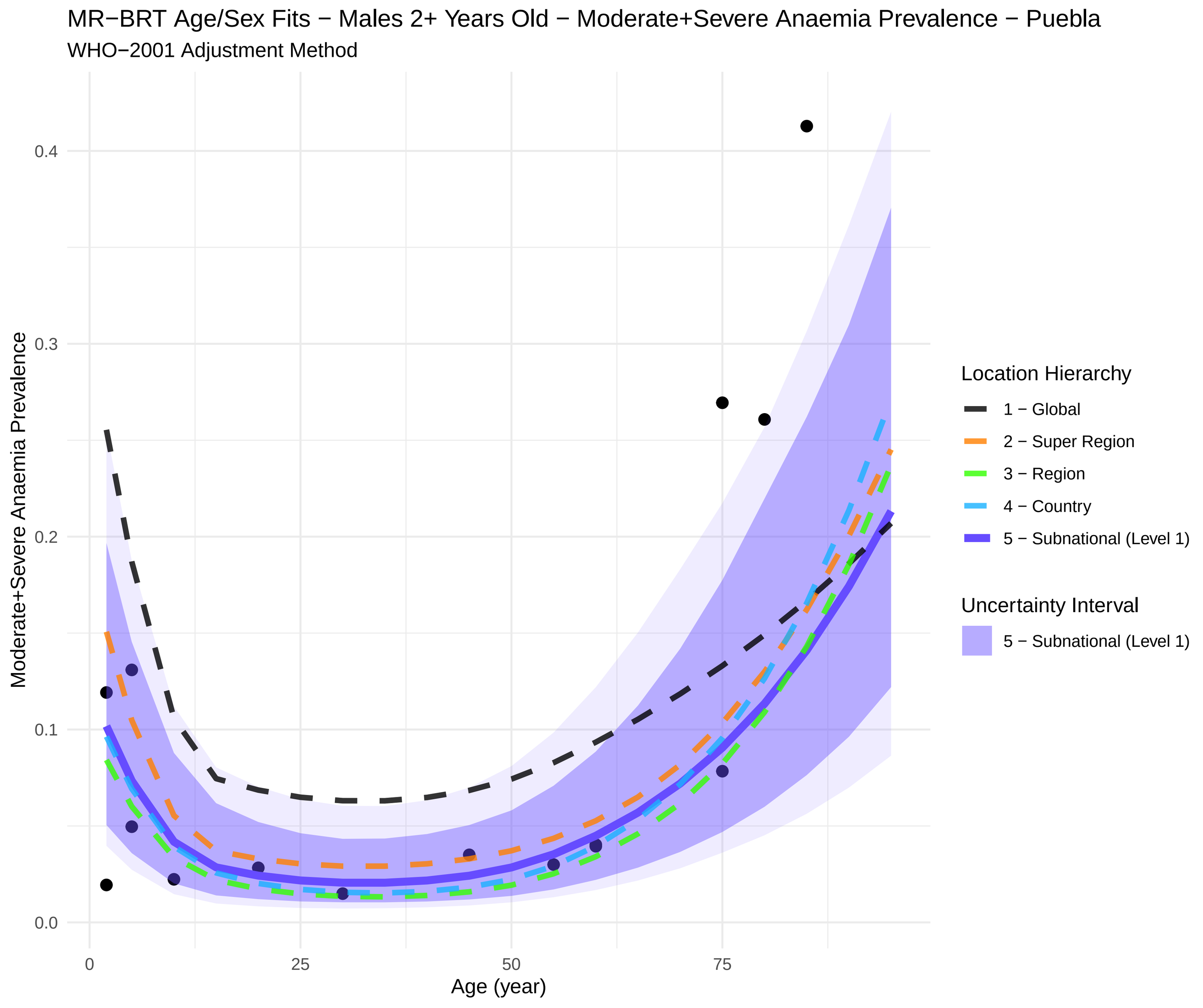
MR−BRT Age/Sex Fits − Males 2+ Years Old − Moderate+Severe Anaemia Prevalence − Puebla
WHO−2001 Adjustment Method
Moderate+Severe Anaemia Prevalence
Age (year)
Location Hierarchy
1 − Global
2 − Super Region
3 − Region
4 − Country
5 − Subnational (Level 1)
Uncertainty Interval
5 − Subnational (Level 1)

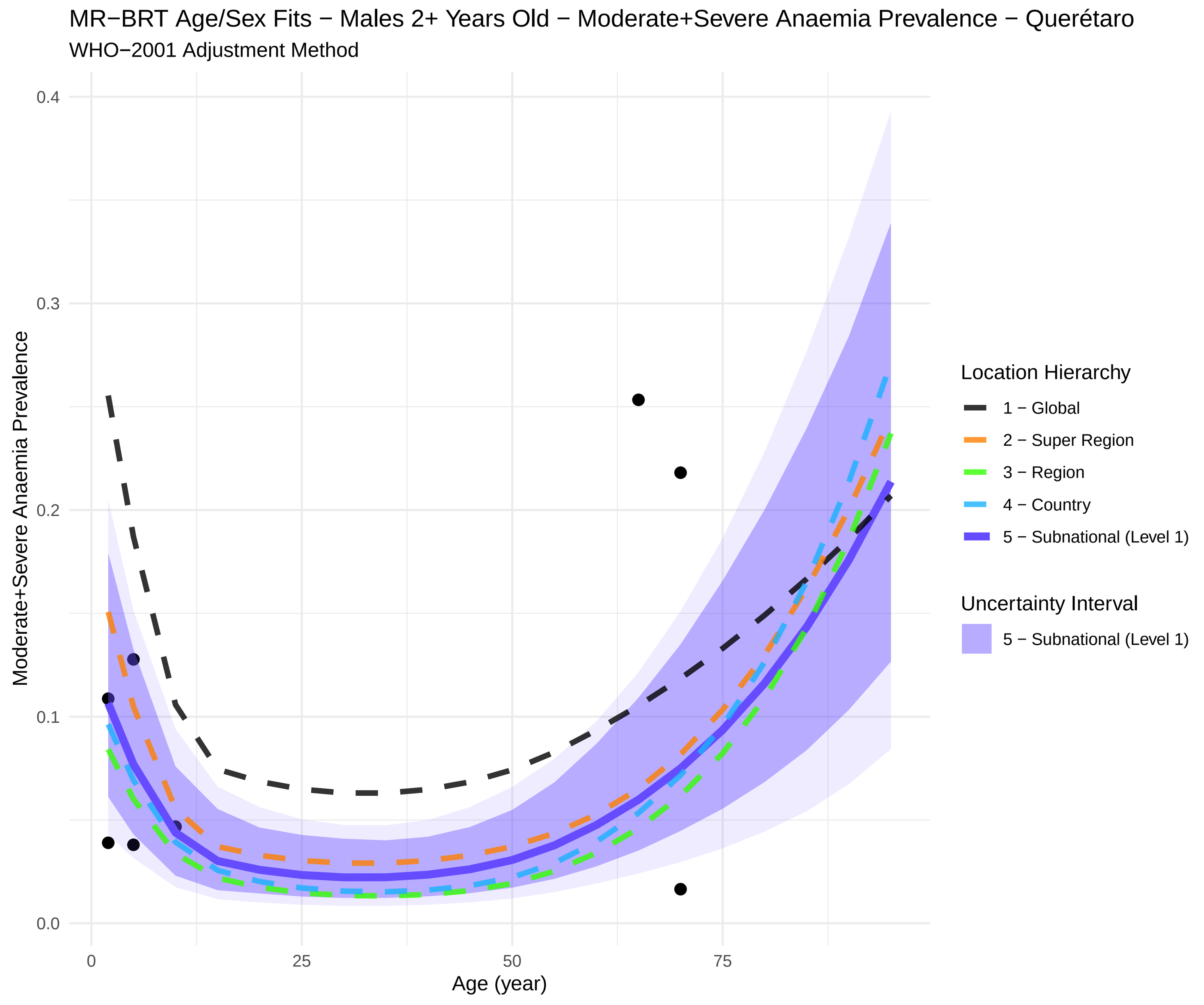
MR−BRT Age/Sex Fits − Males 2+ Years Old − Moderate+Severe Anaemia Prevalence − Querétaro
WHO−2001 Adjustment Method
Moderate+Severe Anaemia Prevalence
Age (year)
Location Hierarchy
1 − Global
2 − Super Region
3 − Region
4 − Country
5 − Subnational (Level 1)
Uncertainty Interval
5 − Subnational (Level 1)

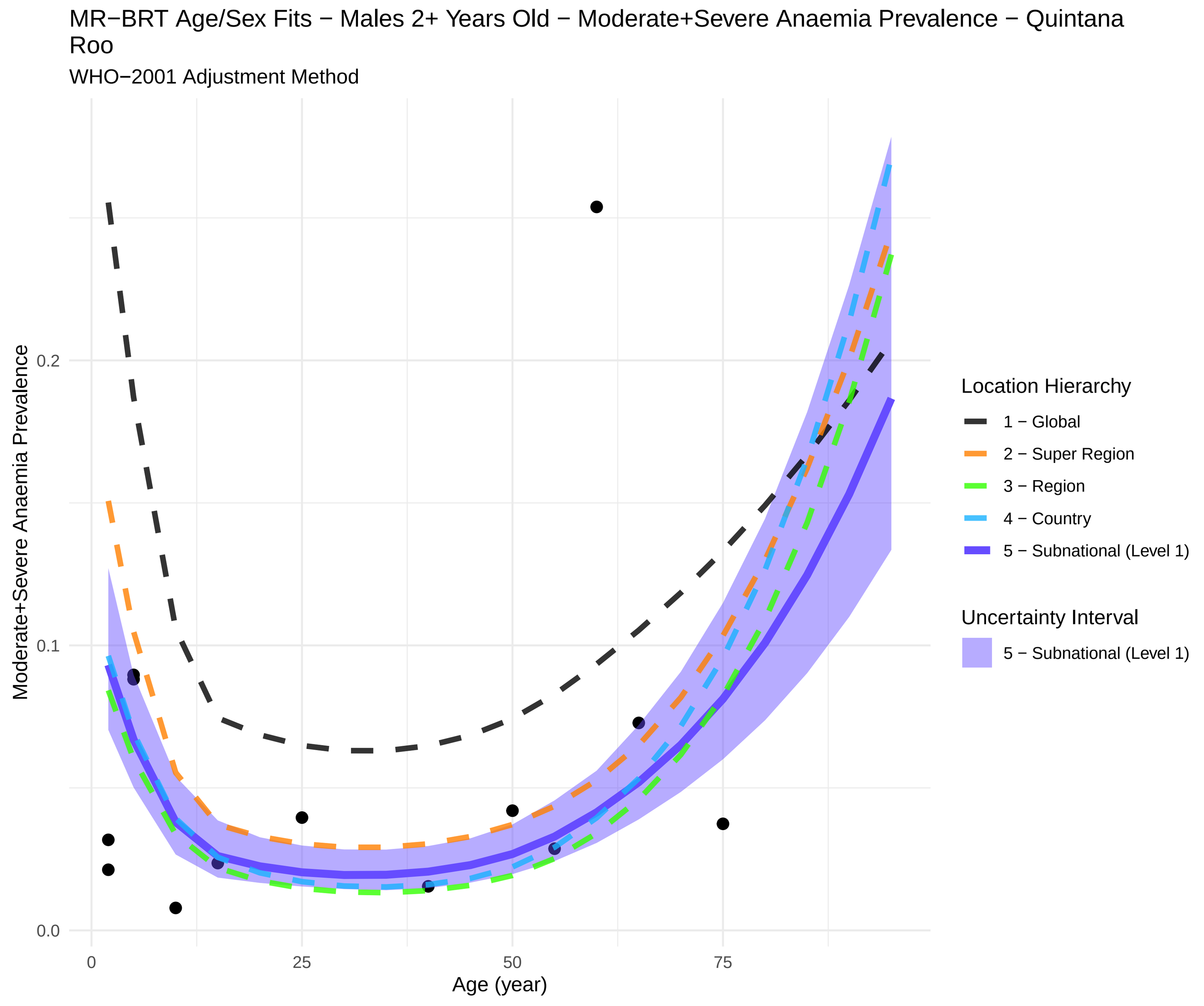
MR−BRT Age/Sex Fits − Males 2+ Years Old − Moderate+Severe Anaemia Prevalence − Quintana Roo
WHO−2001 Adjustment Method
Moderate+Severe Anaemia Prevalence
Age (year)
Location Hierarchy
1 − Global
2 − Super Region
3 − Region
4 − Country
5 − Subnational (Level 1)
Uncertainty Interval
5 − Subnational (Level 1)

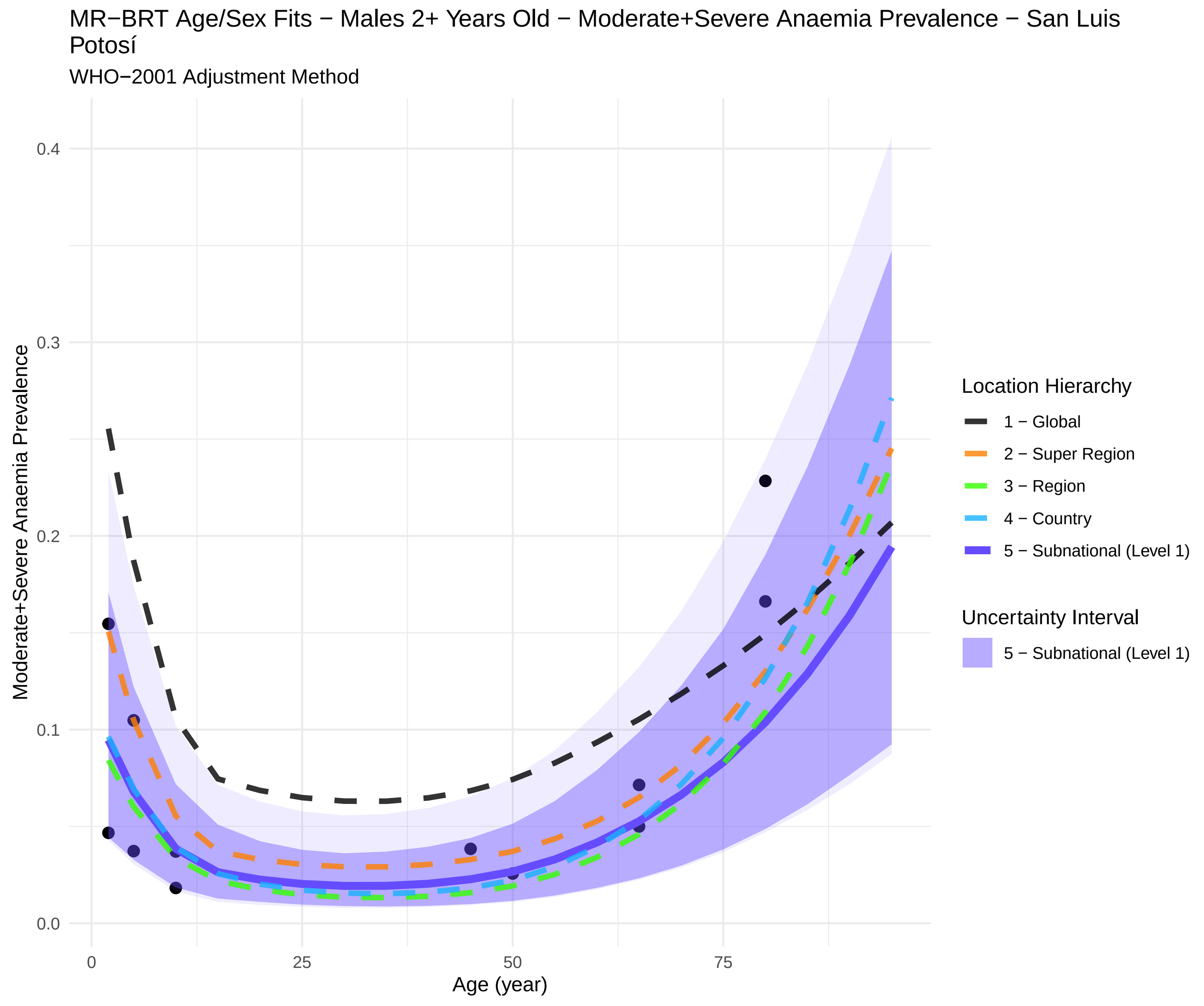
MR−BRT Age/Sex Fits − Males 2+ Years Old − Moderate+Severe Anaemia Prevalence − San Luis Potosí
WHO−2001 Adjustment Method
Moderate+Severe Anaemia Prevalence
Age (year)
Location Hierarchy
1 − Global
2 − Super Region
3 − Region
4 − Country
5 − Subnational (Level 1)
Uncertainty Interval
5 − Subnational (Level 1)

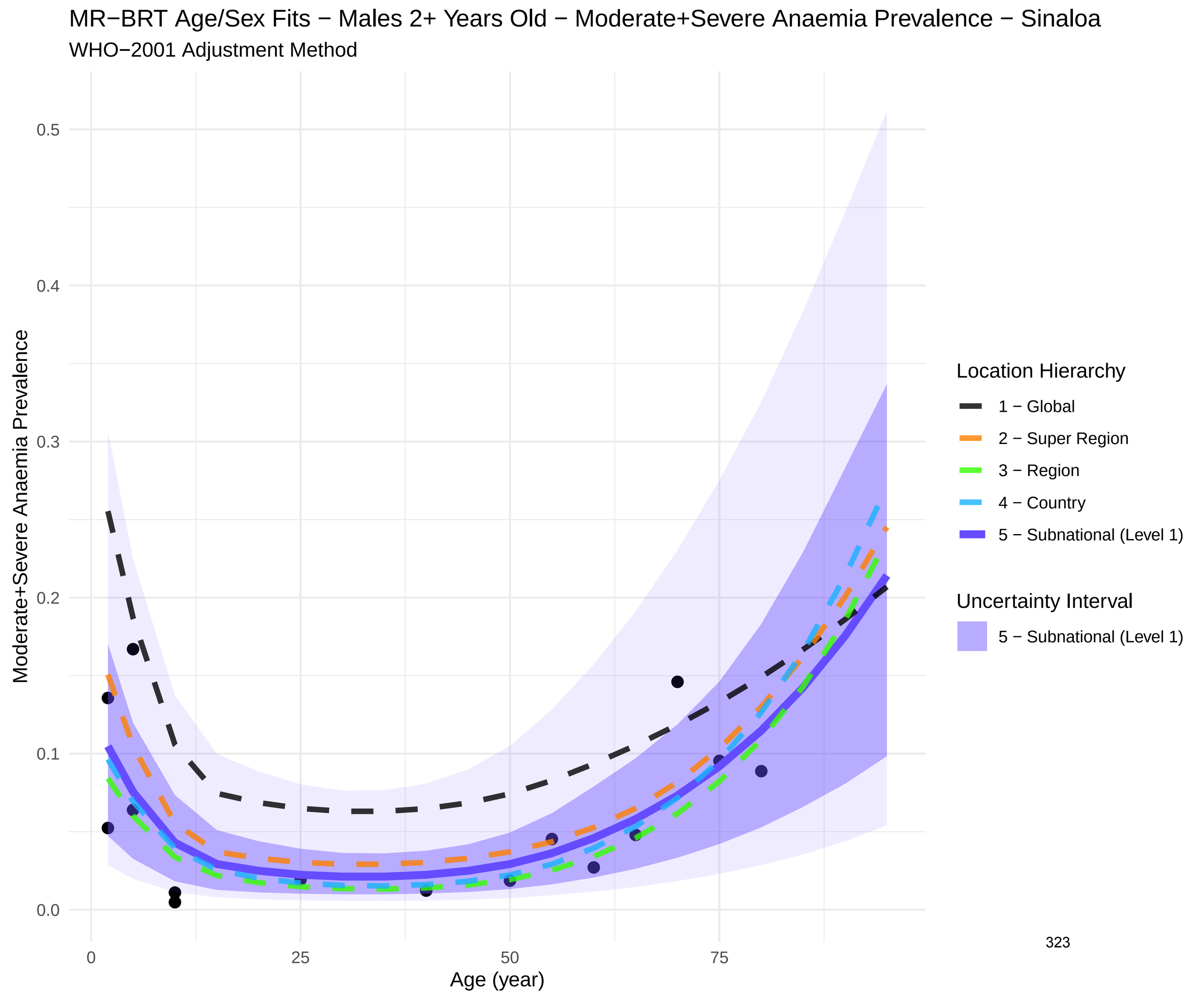
MR−BRT Age/Sex Fits − Males 2+ Years Old − Moderate+Severe Anaemia Prevalence − Sinaloa
WHO−2001 Adjustment Method
Moderate+Severe Anaemia Prevalence
Age (year)
Location Hierarchy
1 − Global
2 − Super Region
3 − Region
4 − Country
5 − Subnational (Level 1)
Uncertainty Interval
5 − Subnational (Level 1)

MR−BRT Age/Sex Fits − Males 2+ Years Old − Moderate+Severe Anaemia Prevalence − Sonora
WHO−2001 Adjustment Method

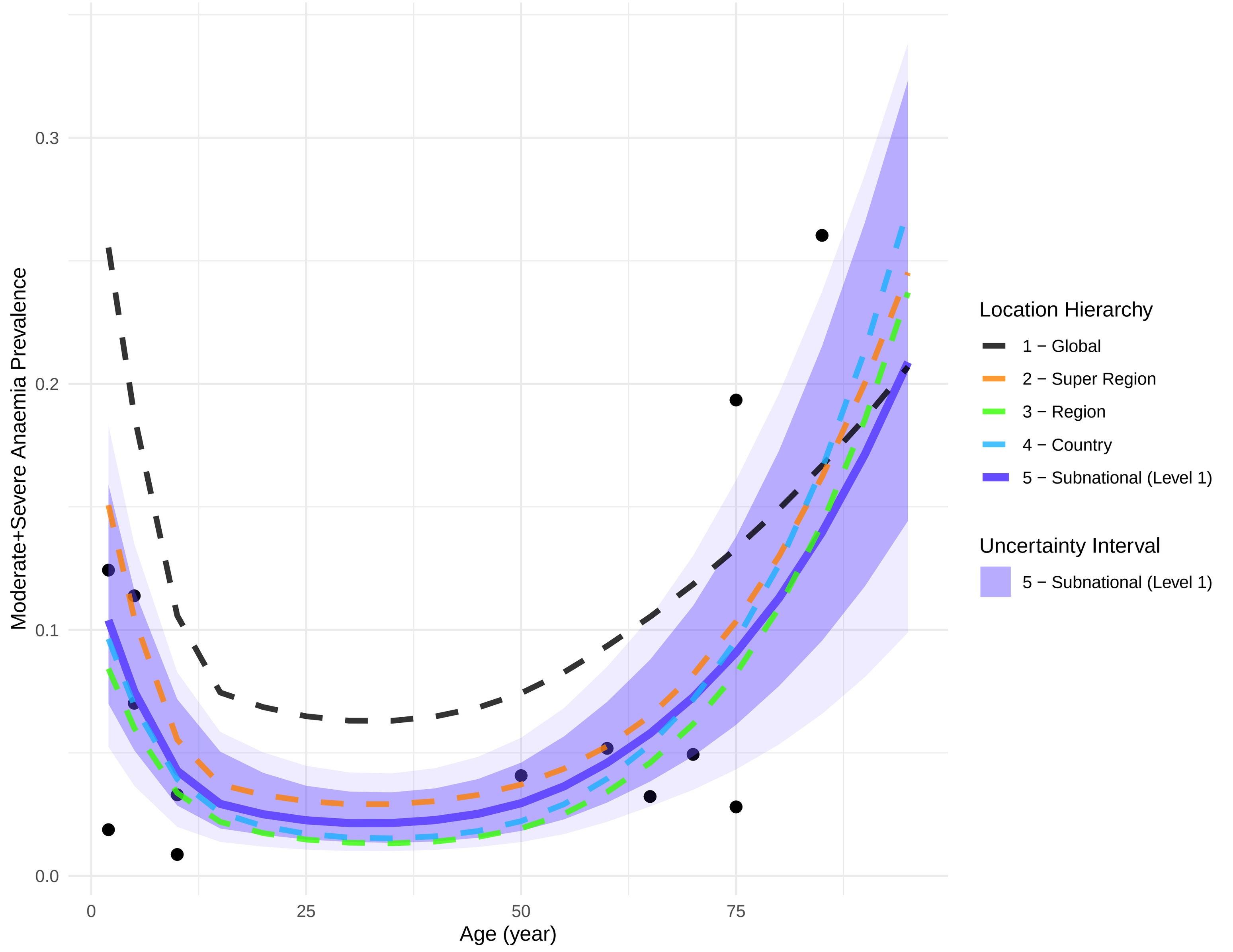

MR−BRT Age/Sex Fits − Males 2+ Years Old − Moderate+Severe Anaemia Prevalence − Tabasco
WHO−2001 Adjustment Method

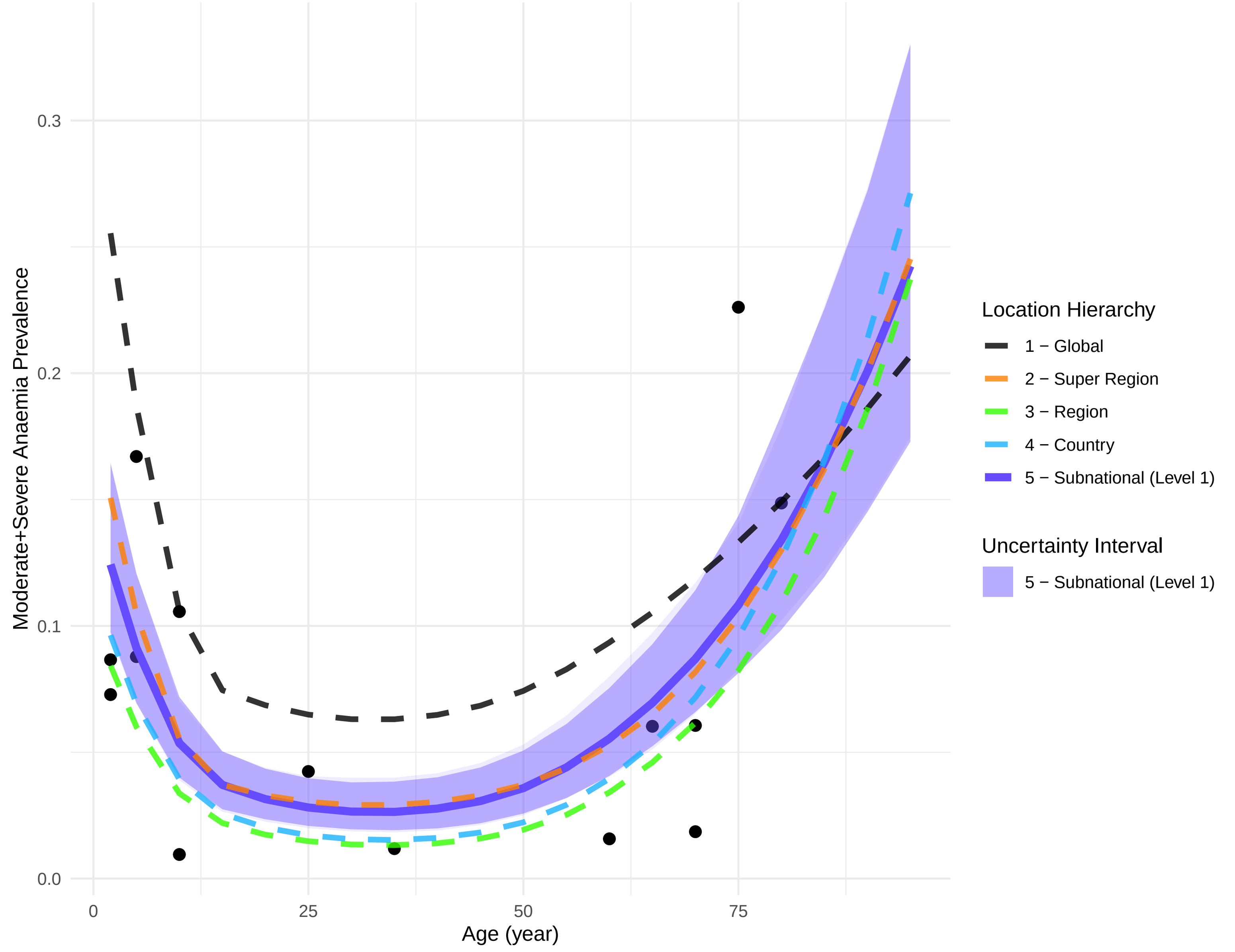

MR−BRT Age/Sex Fits − Males 2+ Years Old − Moderate+Severe Anaemia Prevalence − Tamaulipas
WHO−2001 Adjustment Method

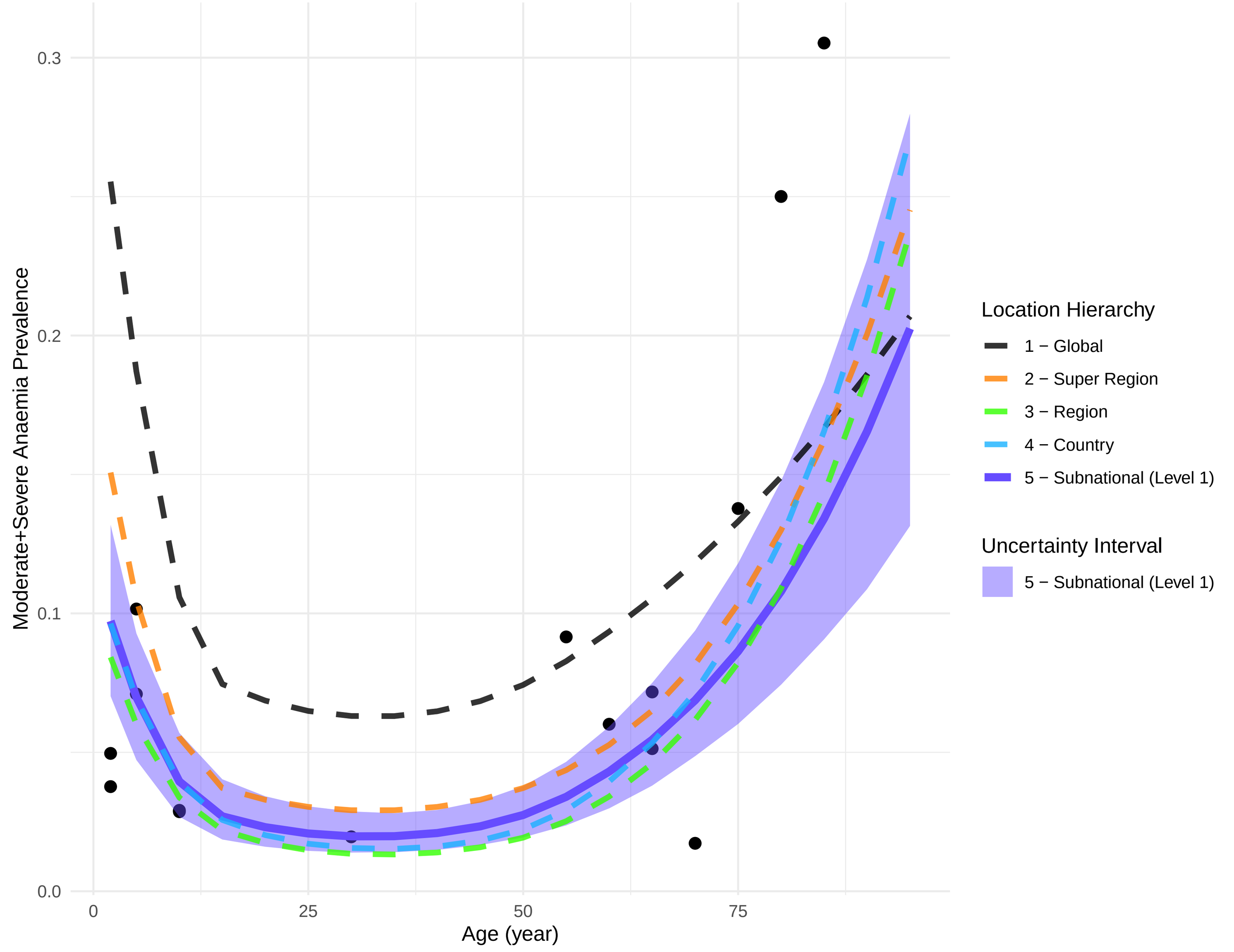

MR−BRT Age/Sex Fits − Males 2+ Years Old − Moderate+Severe Anaemia Prevalence − Tlaxcala
WHO−2001 Adjustment Method

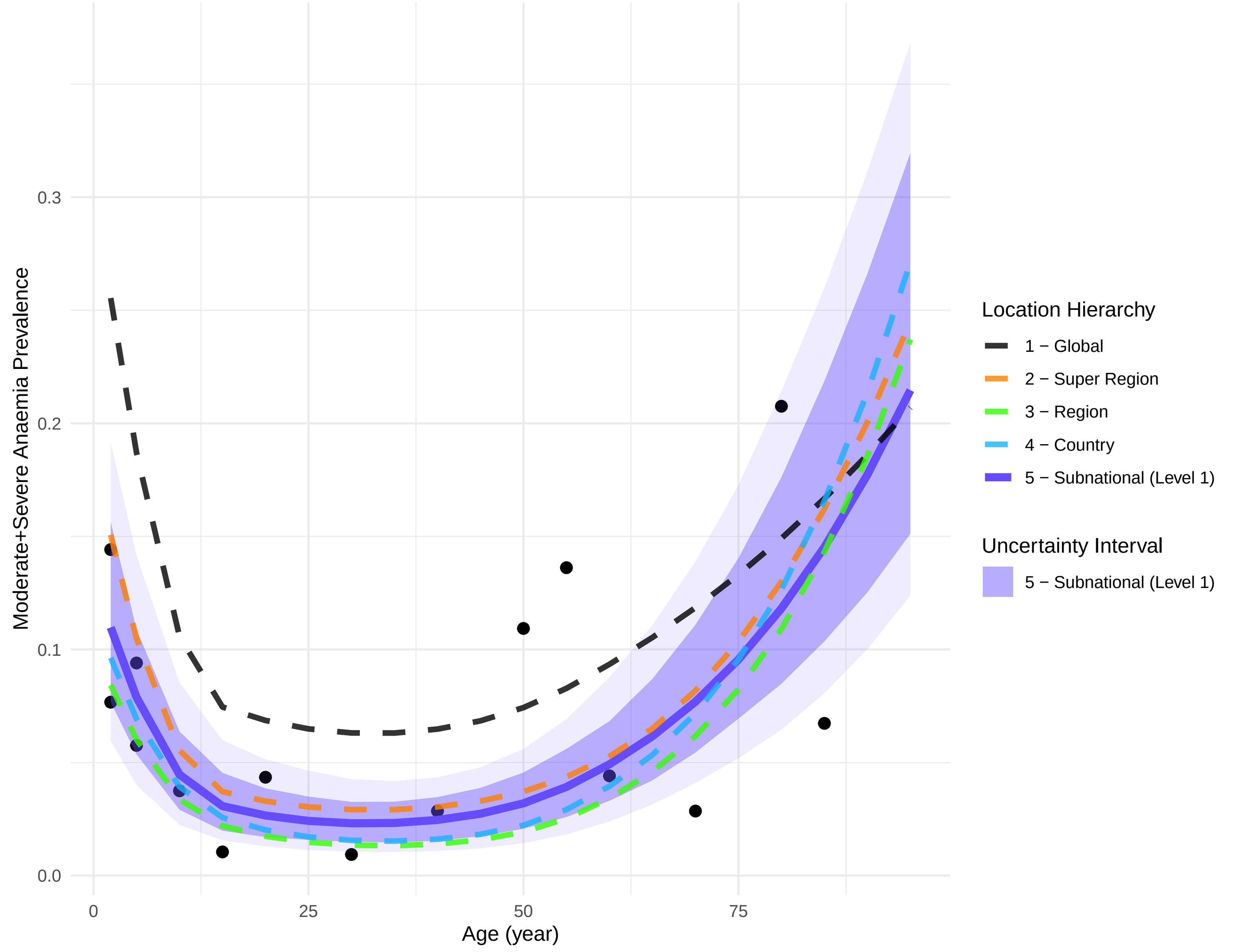

MR−BRT Age/Sex Fits − Males 2+ Years Old − Moderate+Severe Anaemia Prevalence − Veracruz de Ignacio de la Llave
WHO−2001 Adjustment Method

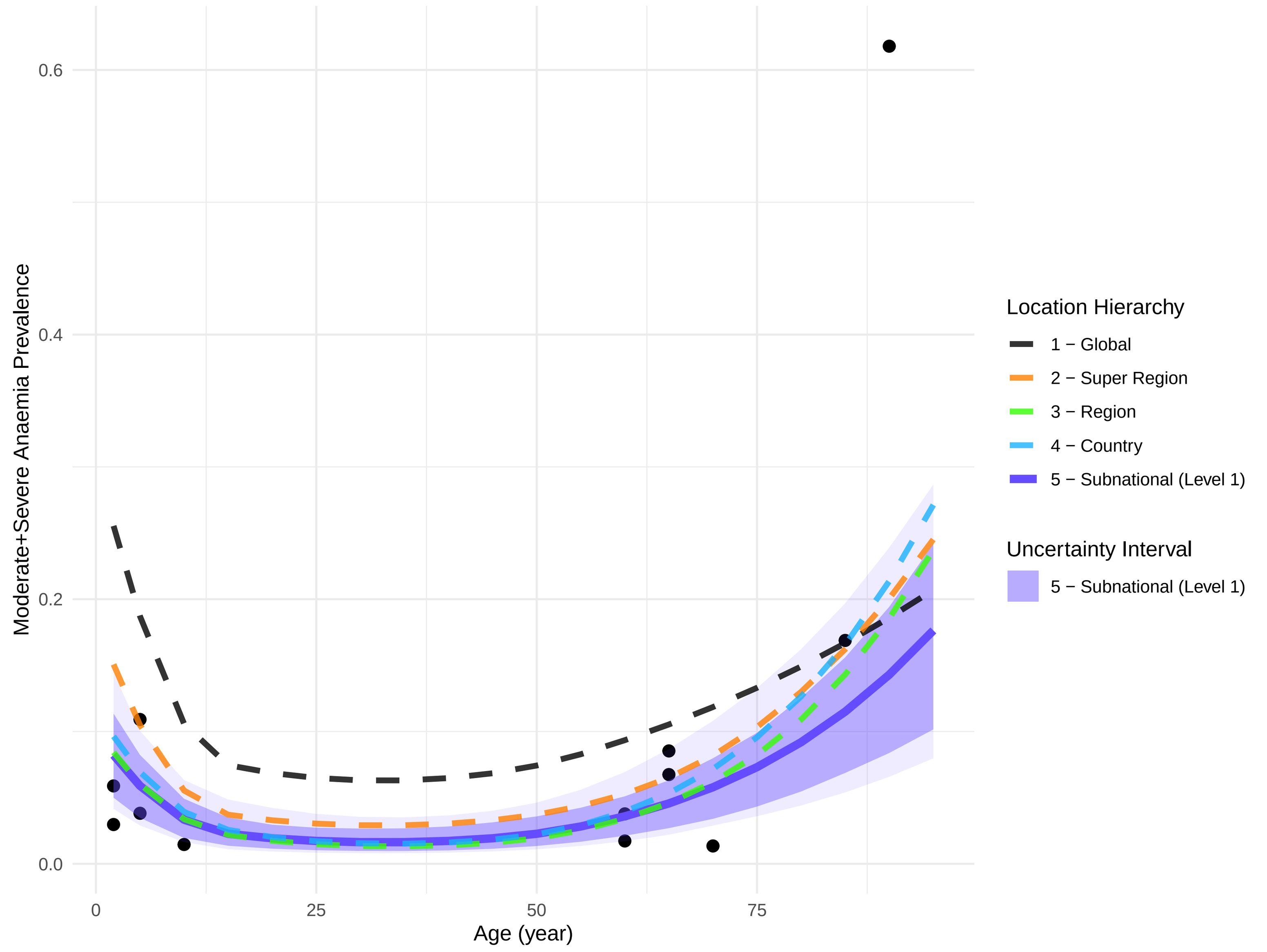

MR−BRT Age/Sex Fits − Males 2+ Years Old − Moderate+Severe Anaemia Prevalence − Yucatán
WHO−2001 Adjustment Method

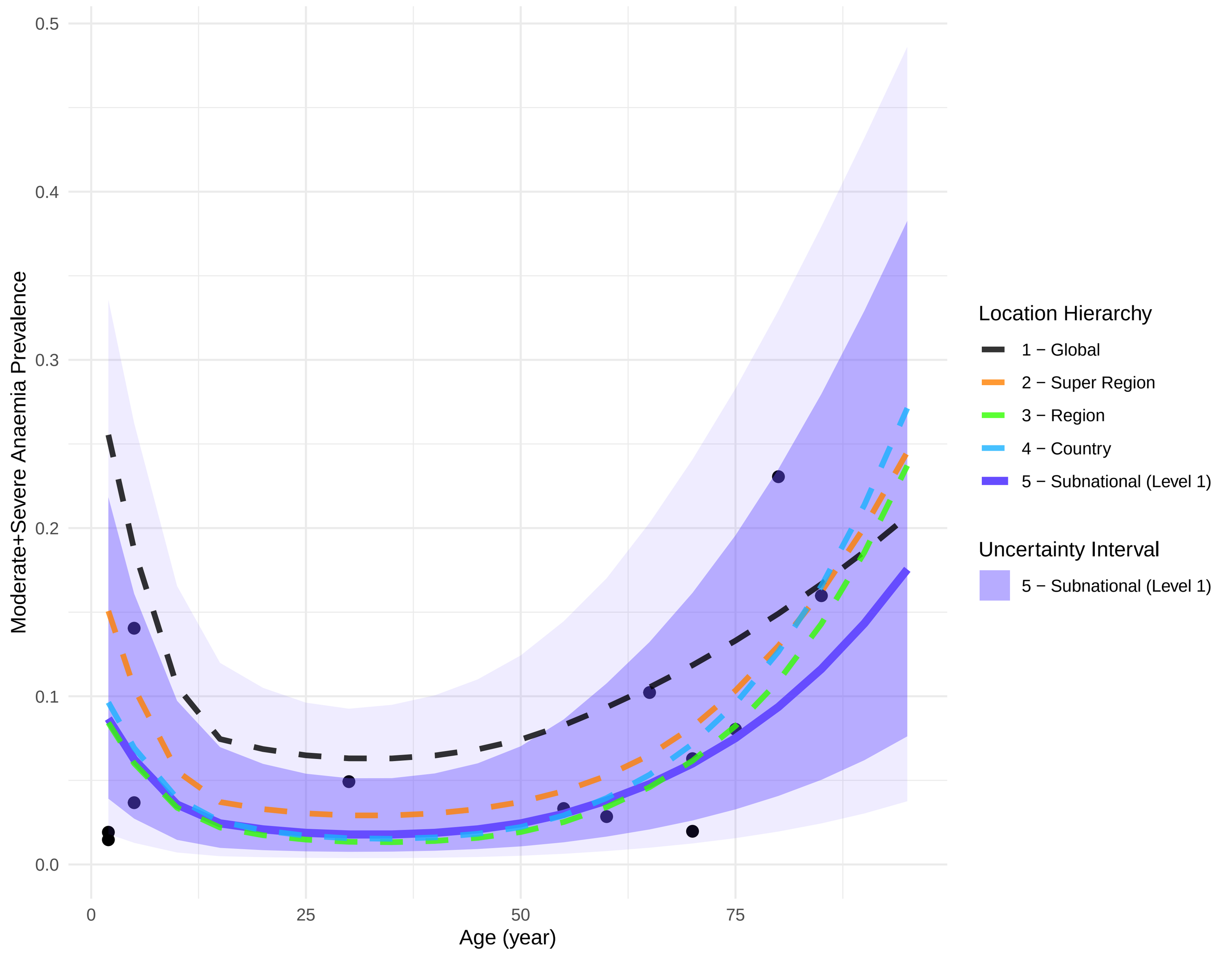

MR−BRT Age/Sex Fits − Males 2+ Years Old − Moderate+Severe Anaemia Prevalence − Zacatecas
WHO−2001 Adjustment Method

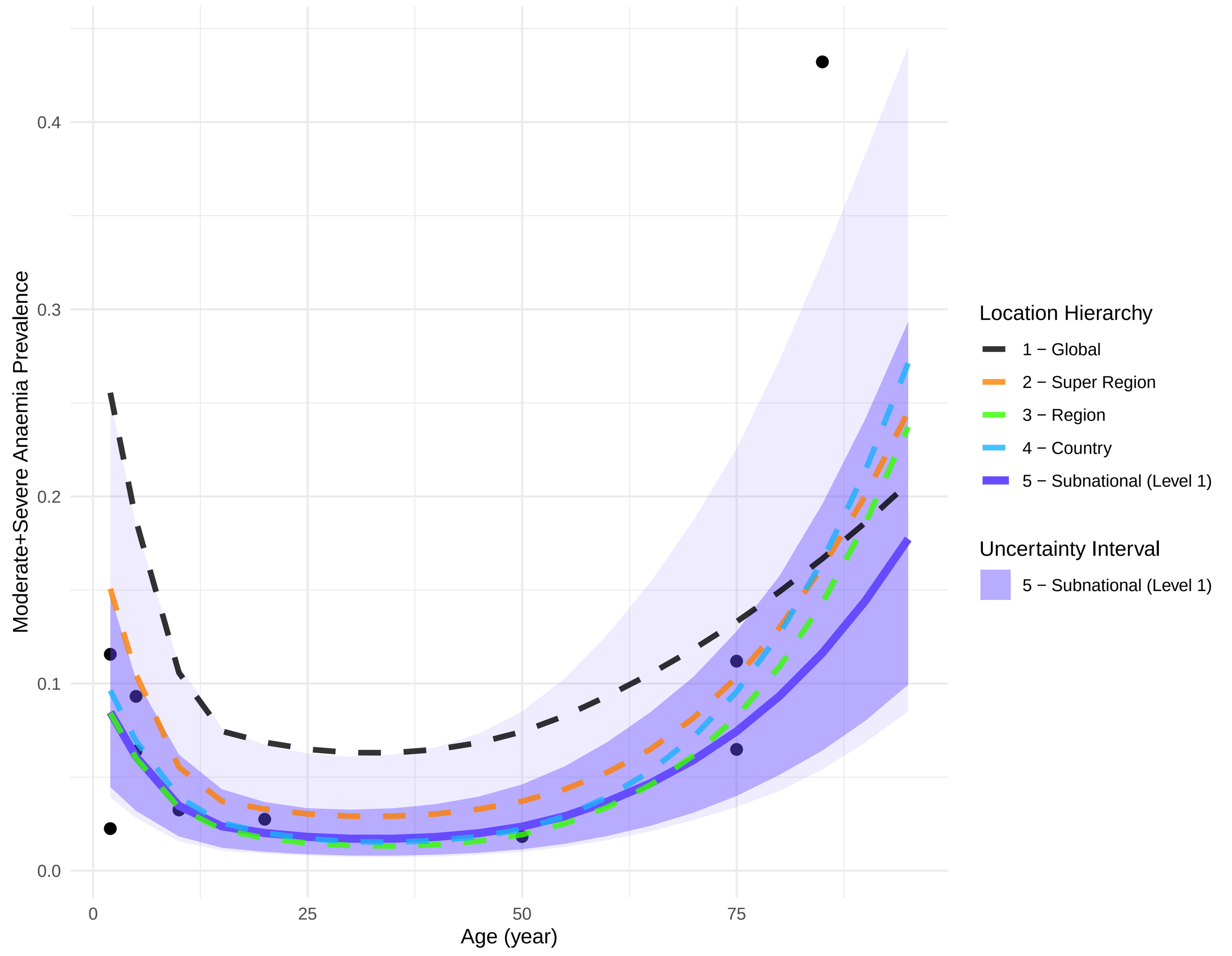

MR−BRT Age/Sex Fits − Males 2+ Years Old − Moderate+Severe Anaemia Prevalence − Tropical Latin America
WHO−2001 Adjustment Method

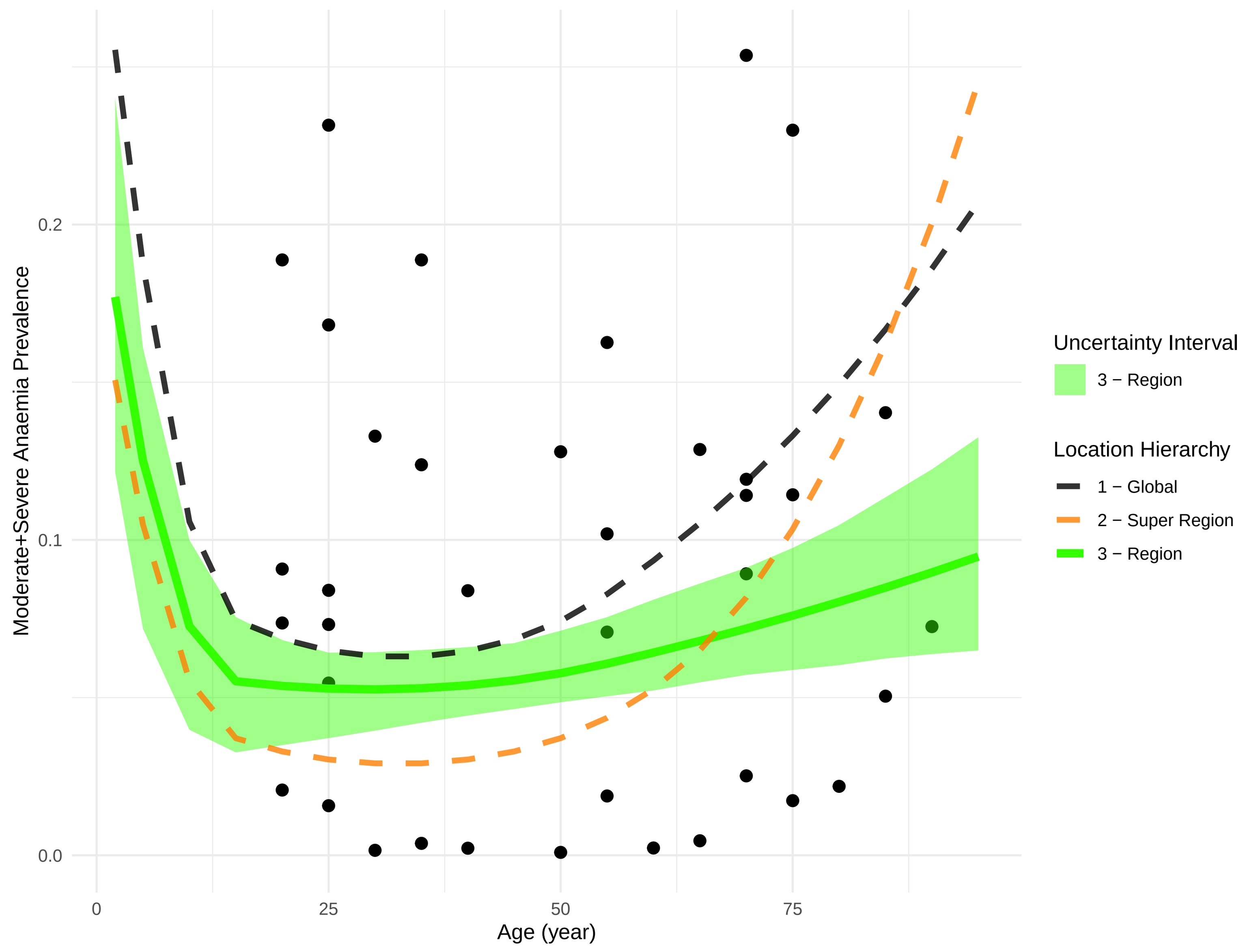

MR−BRT Age/Sex Fits − Males 2+ Years Old − Moderate+Severe Anaemia Prevalence − Brazil
WHO−2001 Adjustment Method

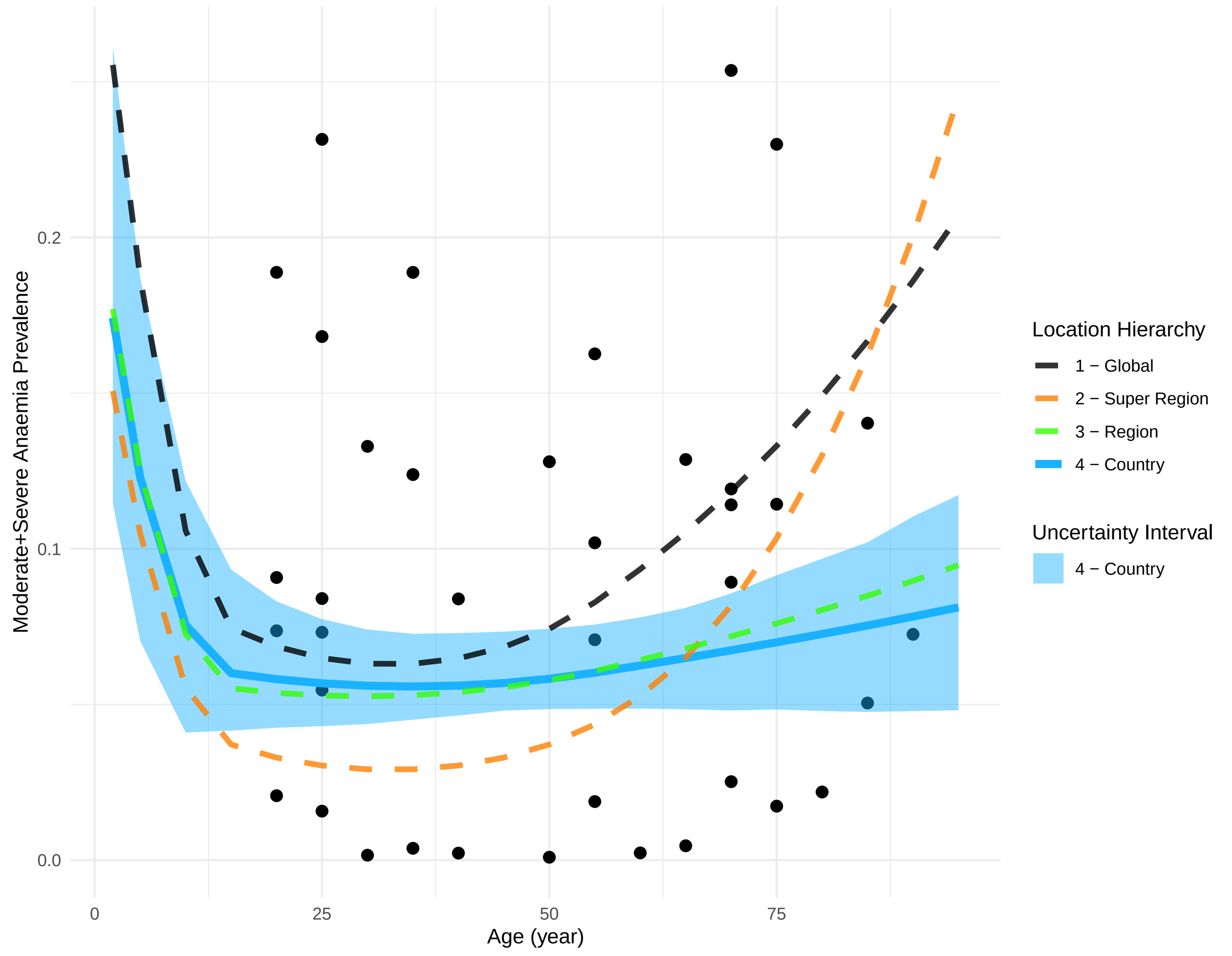

MR−BRT Age/Sex Fits − Males 2+ Years Old − Moderate+Severe Anaemia Prevalence − Acre
WHO−2001 Adjustment Method

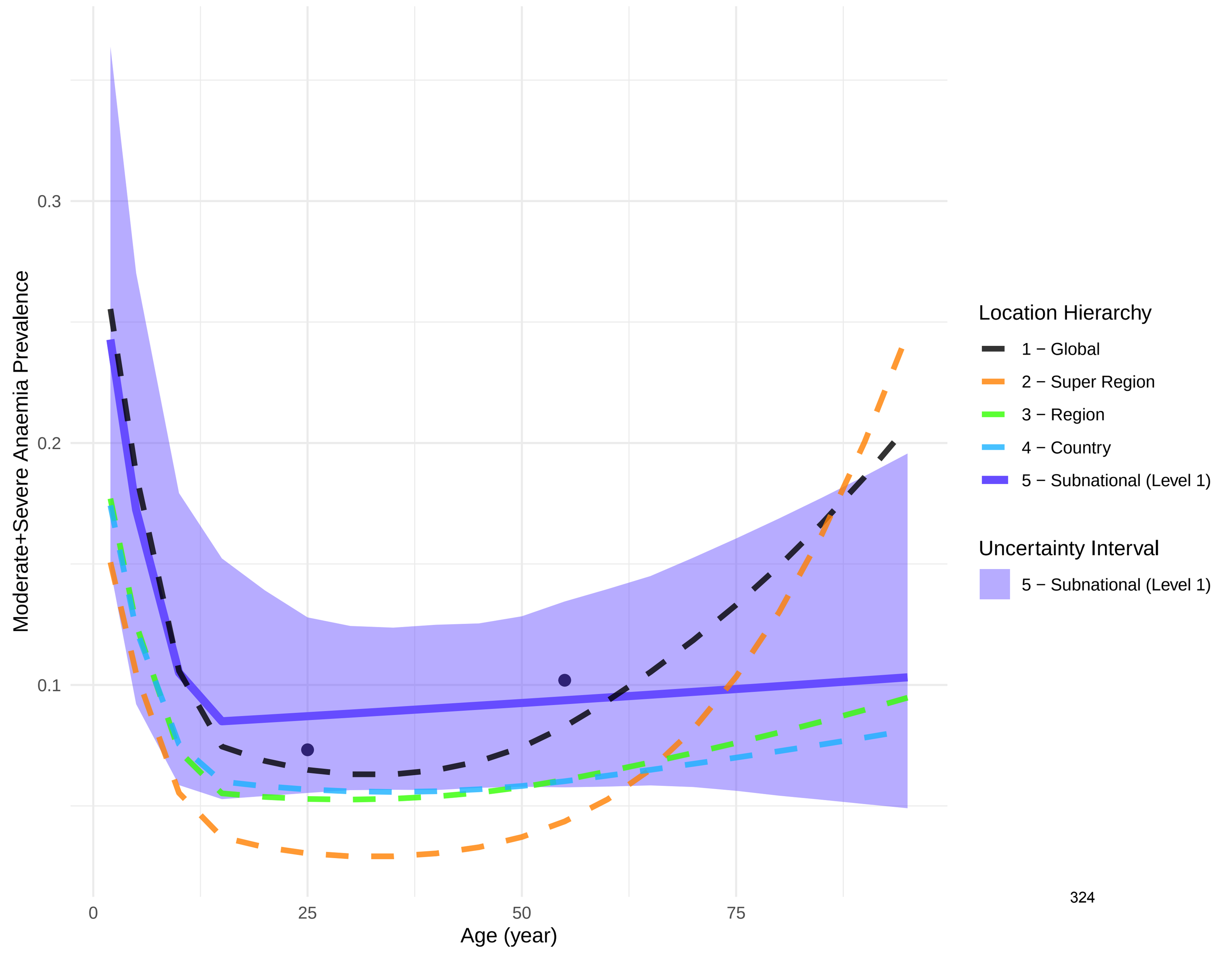

MR−BRT Age/Sex Fits − Males 2+ Years Old − Moderate+Severe Anaemia Prevalence − Alagoas
WHO−2001 Adjustment Method

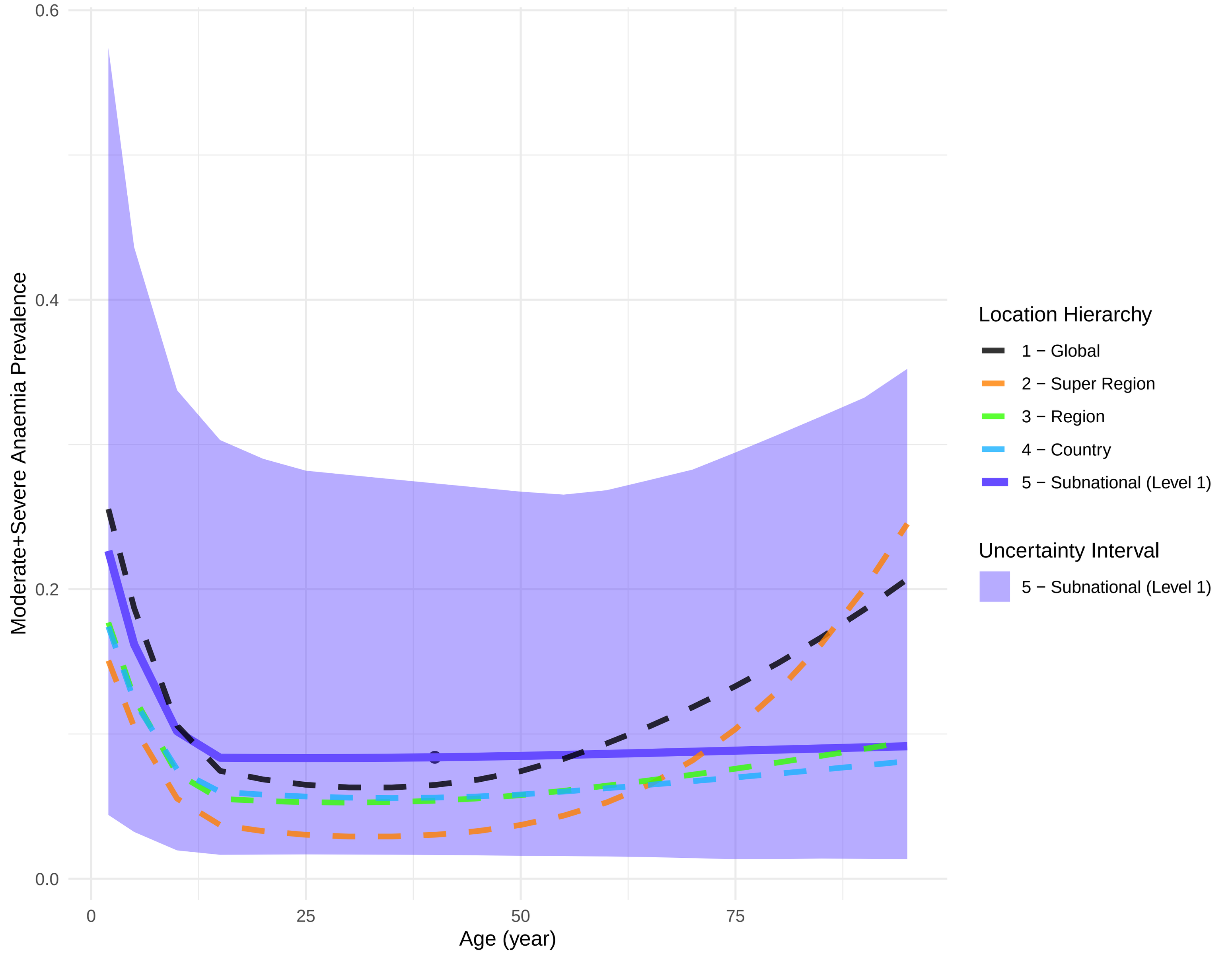

MR−BRT Age/Sex Fits − Males 2+ Years Old − Moderate+Severe Anaemia Prevalence − Amapá
WHO−2001 Adjustment Method

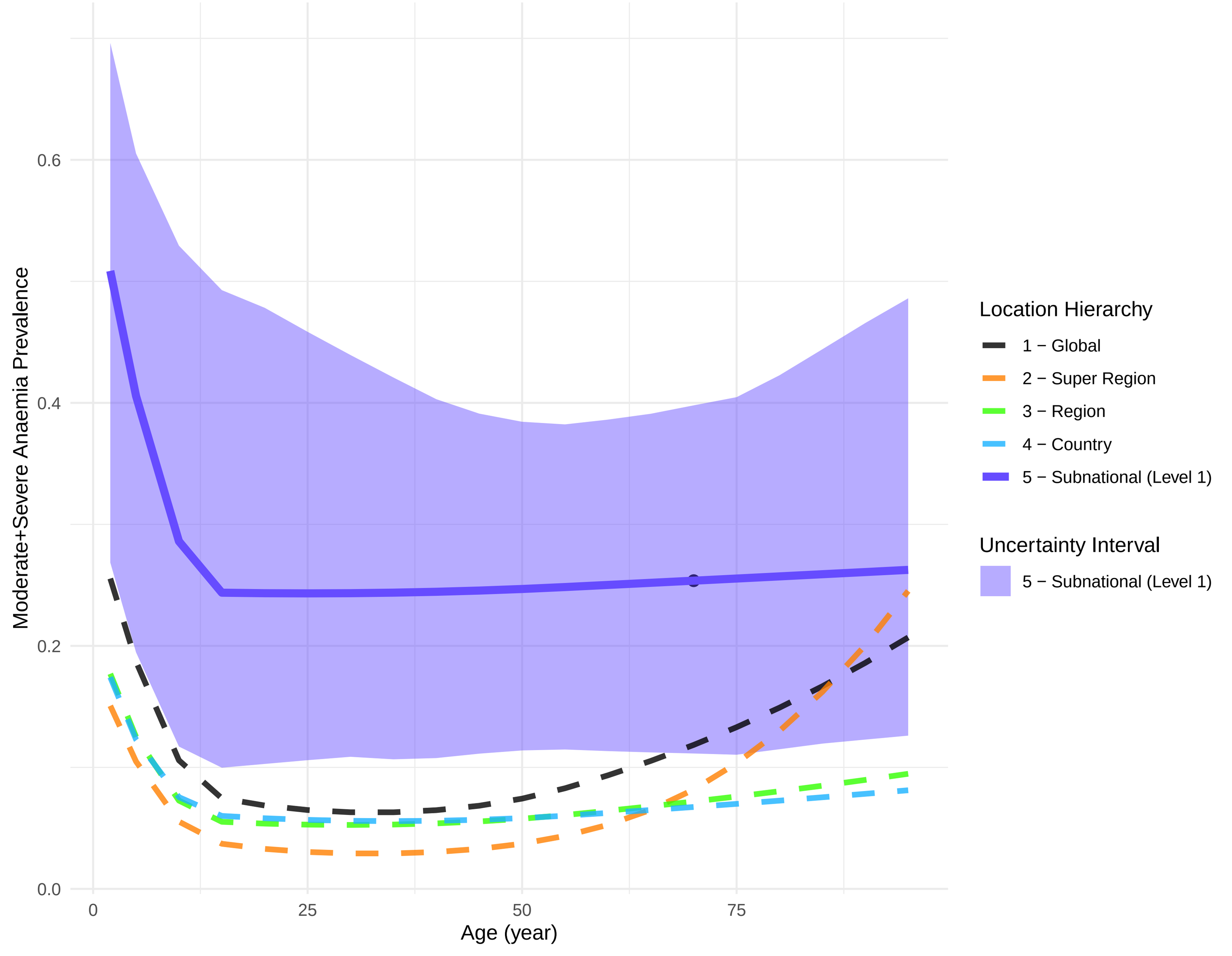

MR−BRT Age/Sex Fits − Males 2+ Years Old − Moderate+Severe Anaemia Prevalence − Amazonas
WHO−2001 Adjustment Method

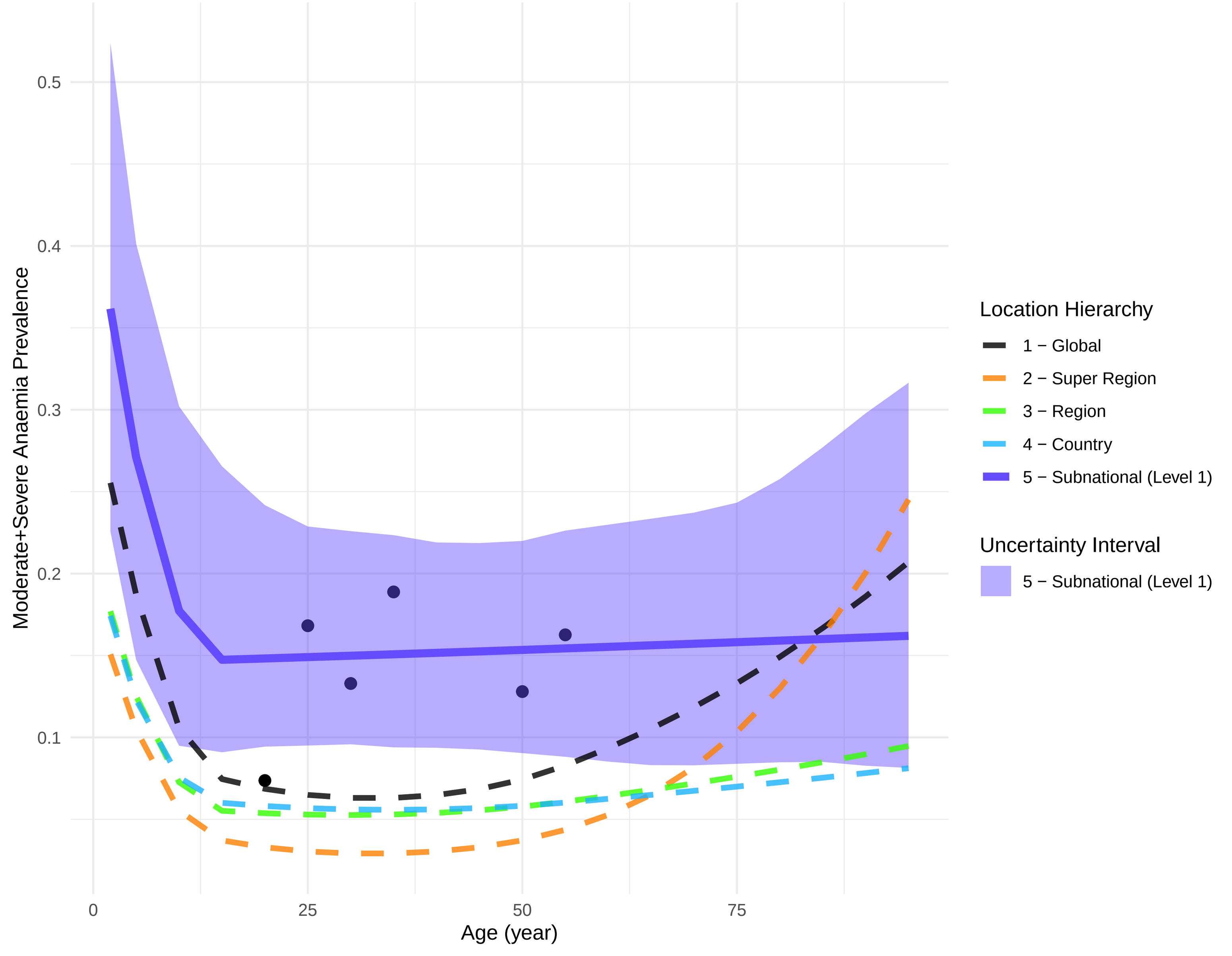

MR−BRT Age/Sex Fits − Males 2+ Years Old − Moderate+Severe Anaemia Prevalence − Minas Gerais
WHO−2001 Adjustment Method

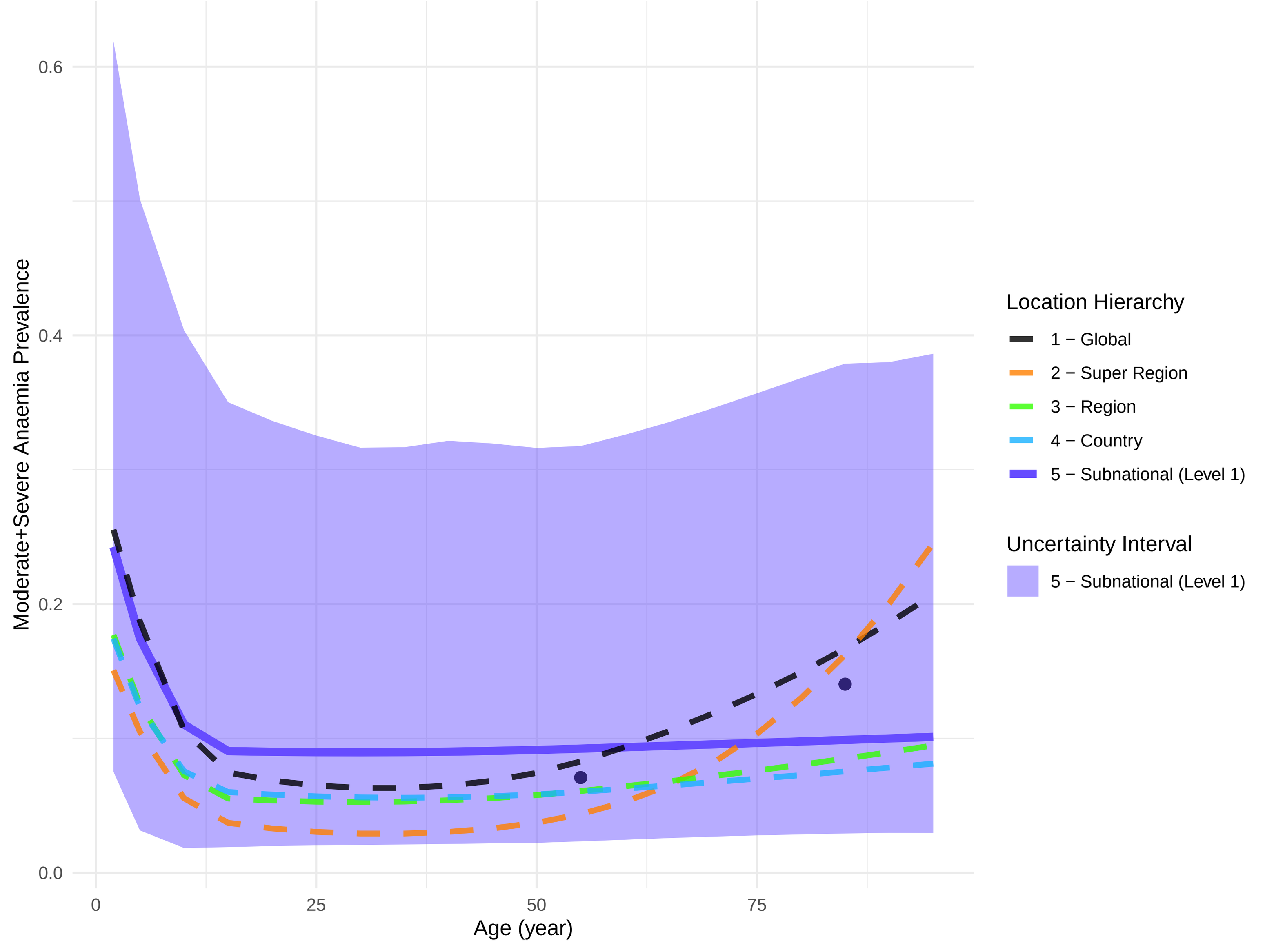

MR−BRT Age/Sex Fits − Males 2+ Years Old − Moderate+Severe Anaemia Prevalence − Paraiba
WHO−2001 Adjustment Method

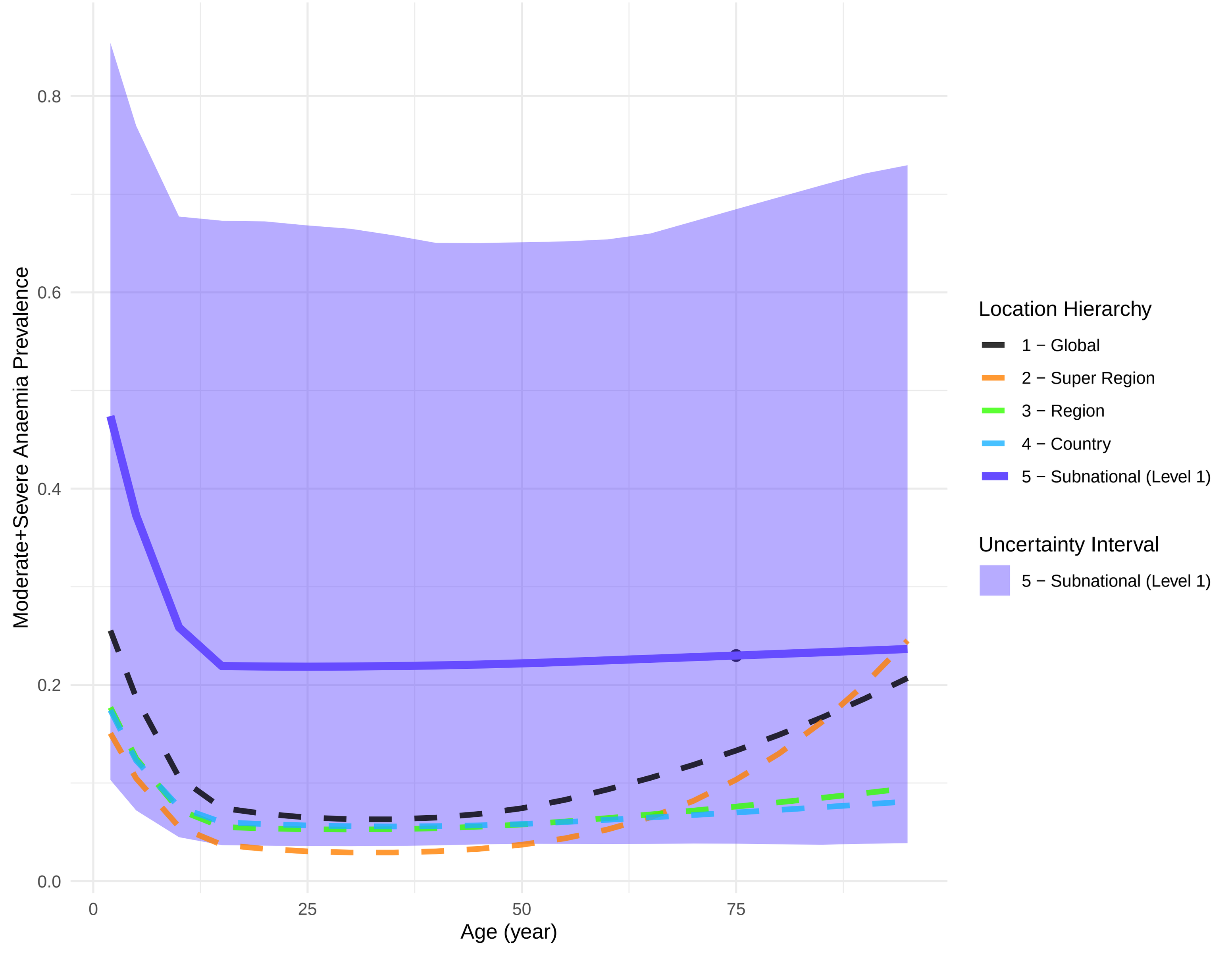

MR−BRT Age/Sex Fits − Males 2+ Years Old − Moderate+Severe Anaemia Prevalence − Paraná
WHO−2001 Adjustment Method

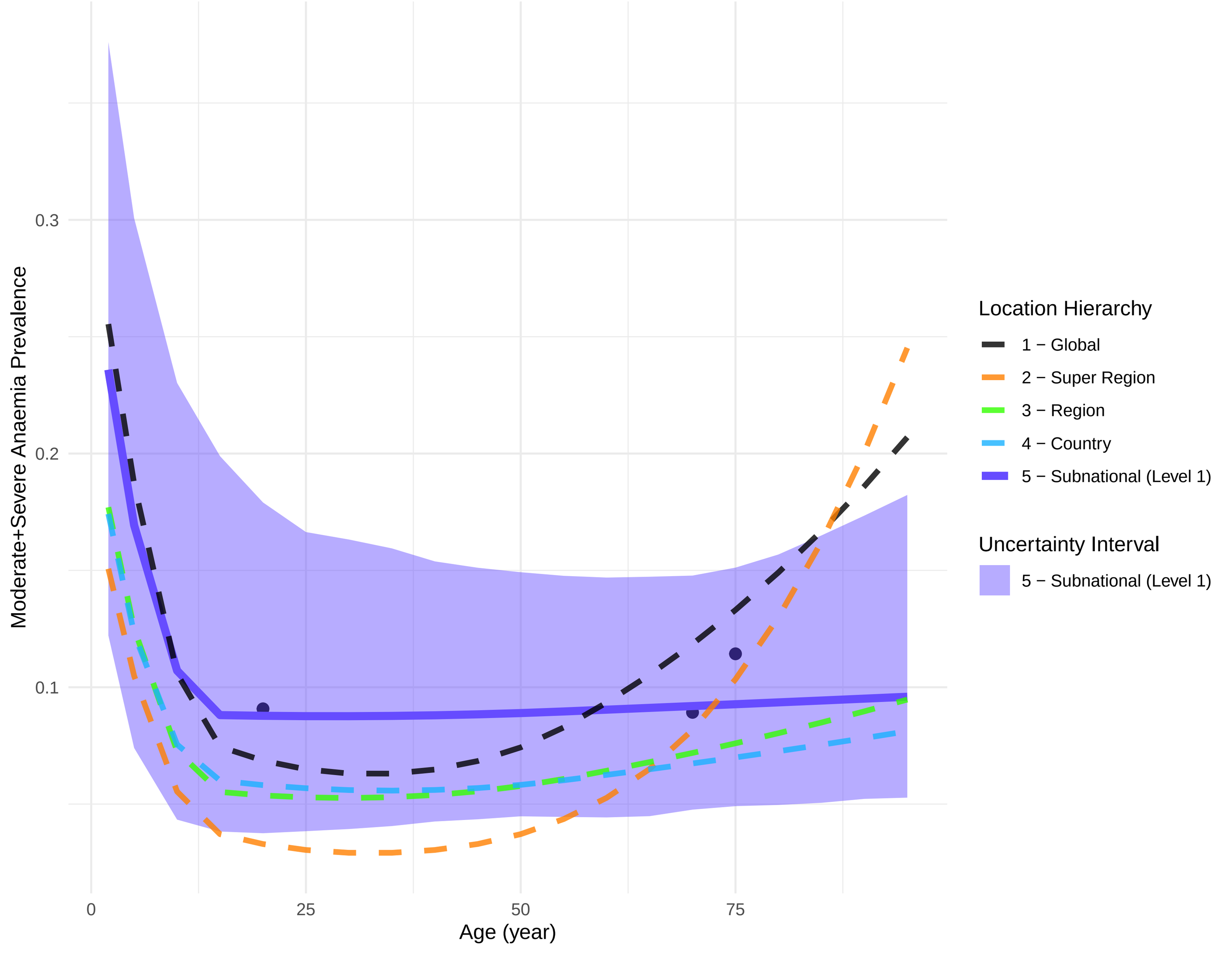

MR−BRT Age/Sex Fits − Males 2+ Years Old − Moderate+Severe Anaemia Prevalence − Piaui
WHO−2001 Adjustment Method

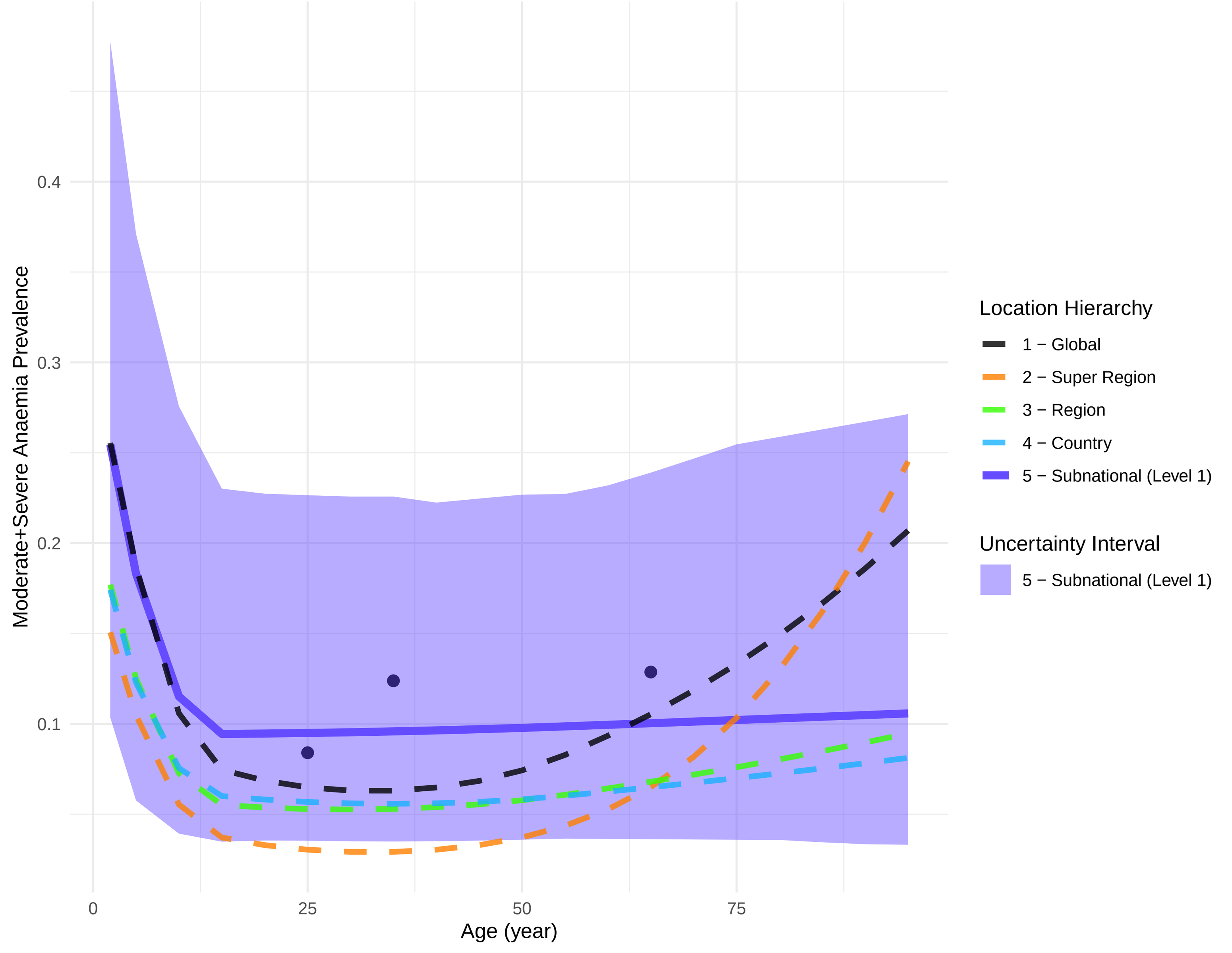

MR−BRT Age/Sex Fits − Males 2+ Years Old − Moderate+Severe Anaemia Prevalence − Rio de Janeiro
WHO−2001 Adjustment Method

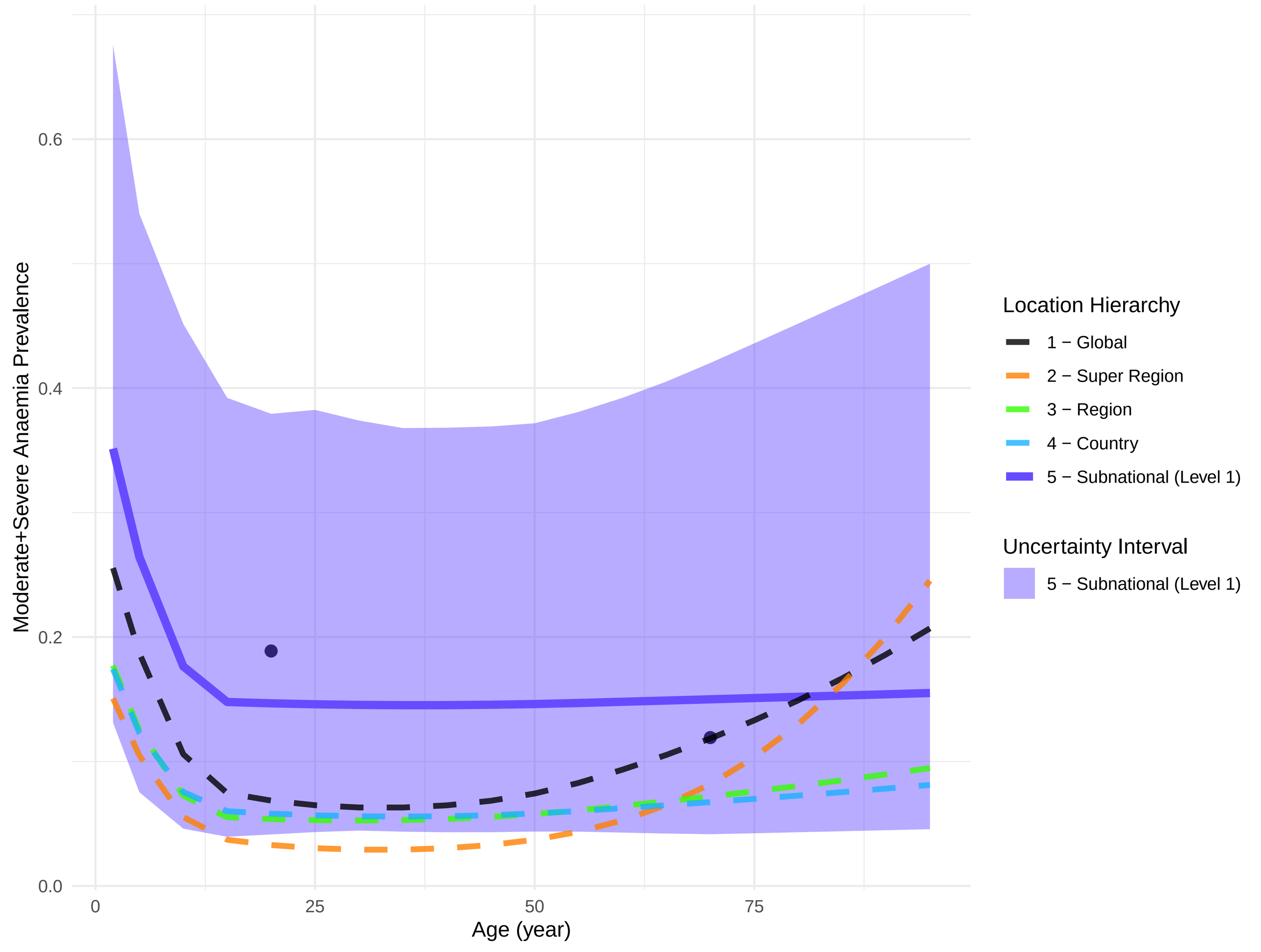

MR−BRT Age/Sex Fits − Males 2+ Years Old − Moderate+Severe Anaemia Prevalence − Rondônia
WHO−2001 Adjustment Method

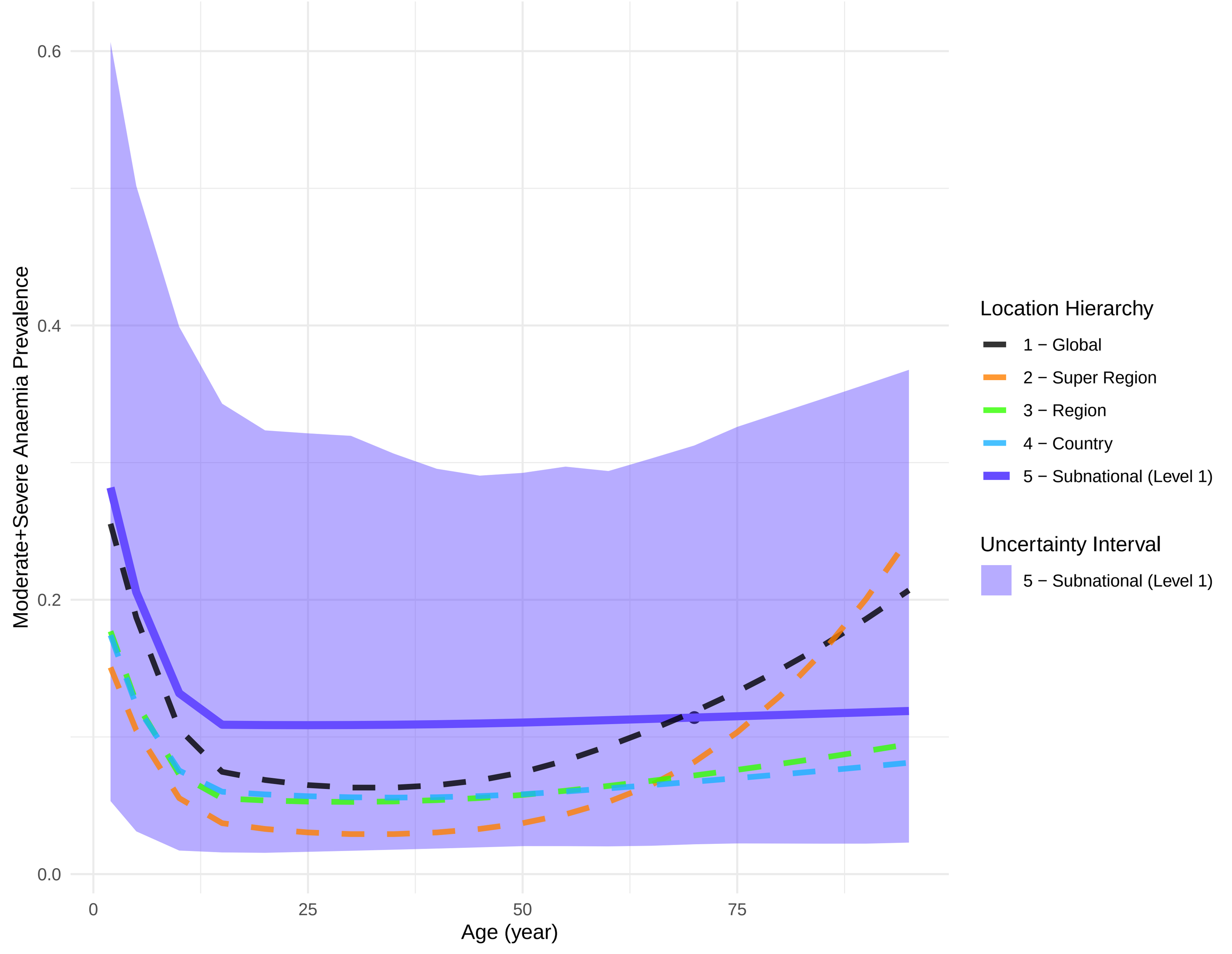

MR−BRT Age/Sex Fits − Males 2+ Years Old − Moderate+Severe Anaemia Prevalence − Roraima
WHO−2001 Adjustment Method

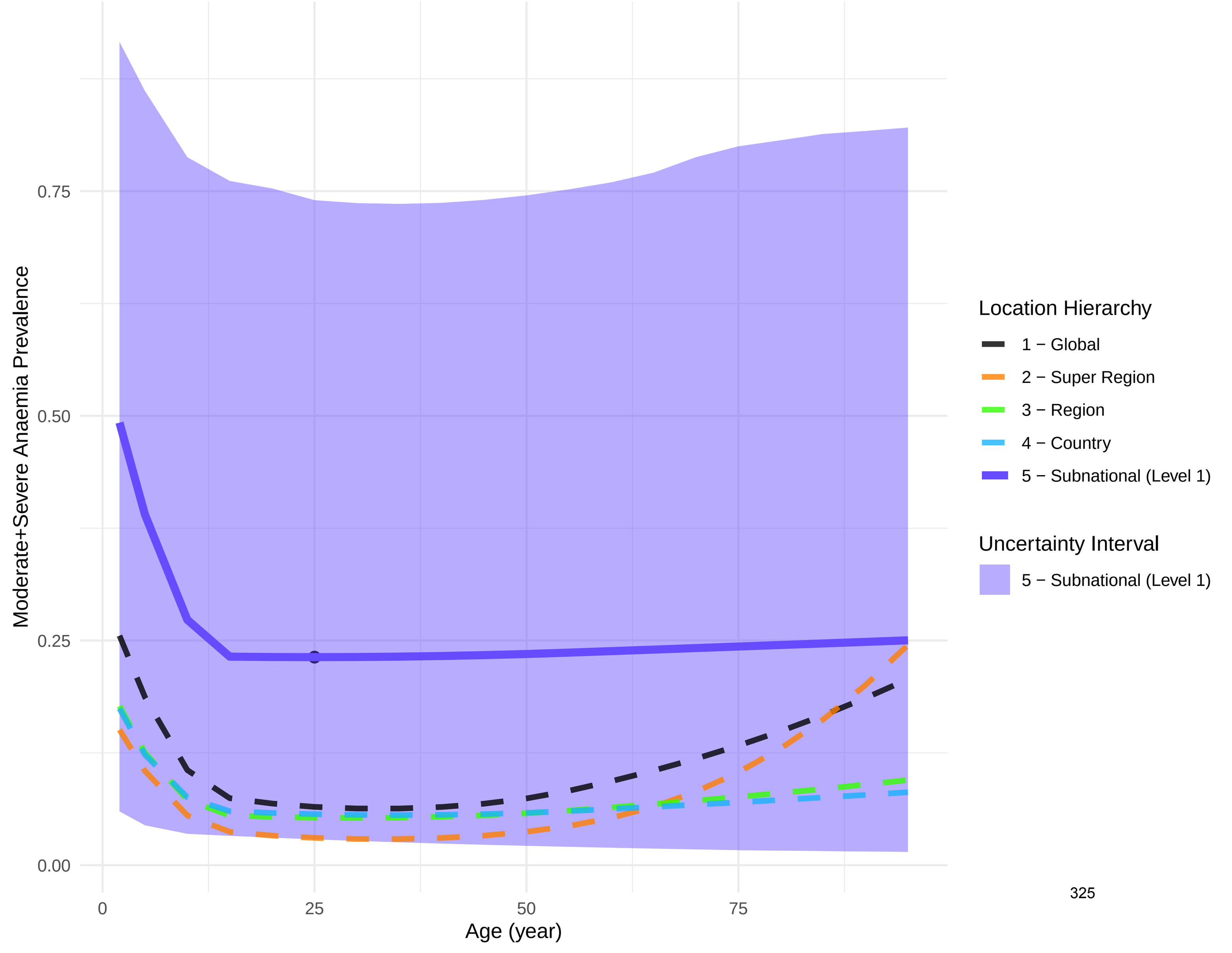

MR−BRT Age/Sex Fits − Males 2+ Years Old − Moderate+Severe Anaemia Prevalence − São Paulo
WHO−2001 Adjustment Method

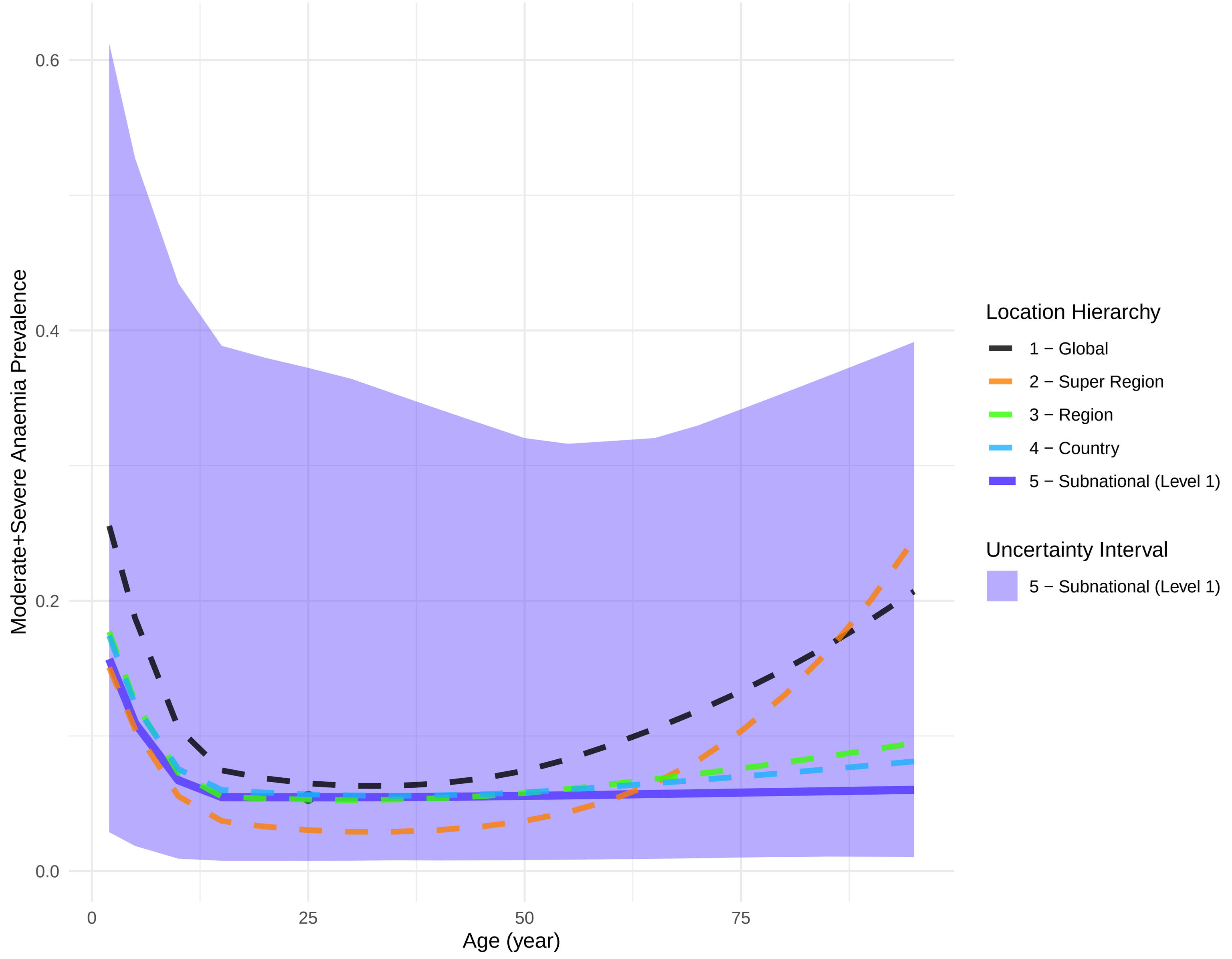

MR−BRT Age/Sex Fits − Males 2+ Years Old − Moderate+Severe Anaemia Prevalence − North Africa and Middle East
WHO−2001 Adjustment Method

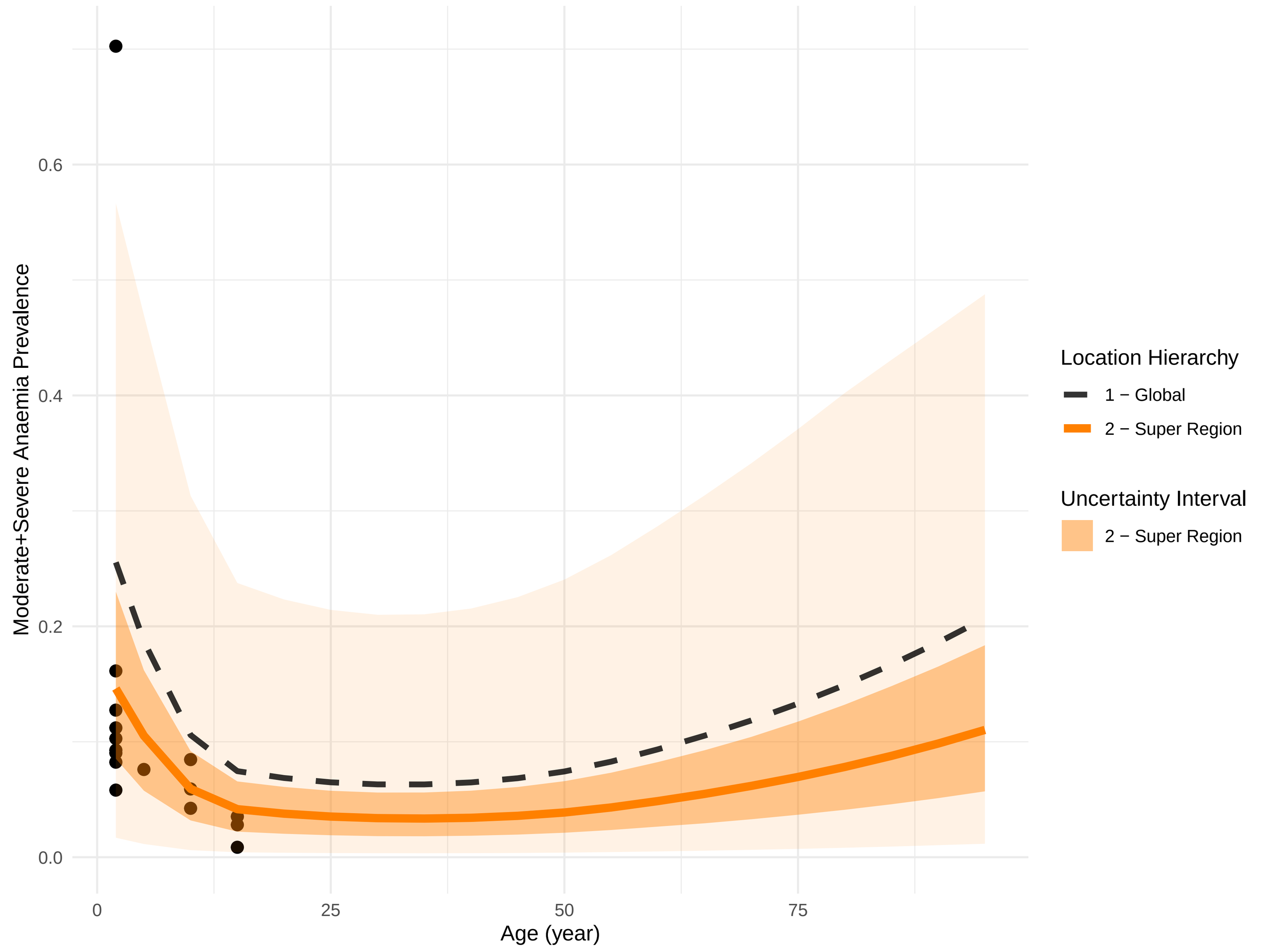

MR−BRT Age/Sex Fits − Males 2+ Years Old − Moderate+Severe Anaemia Prevalence − North Africa and Middle East
WHO−2001 Adjustment Method

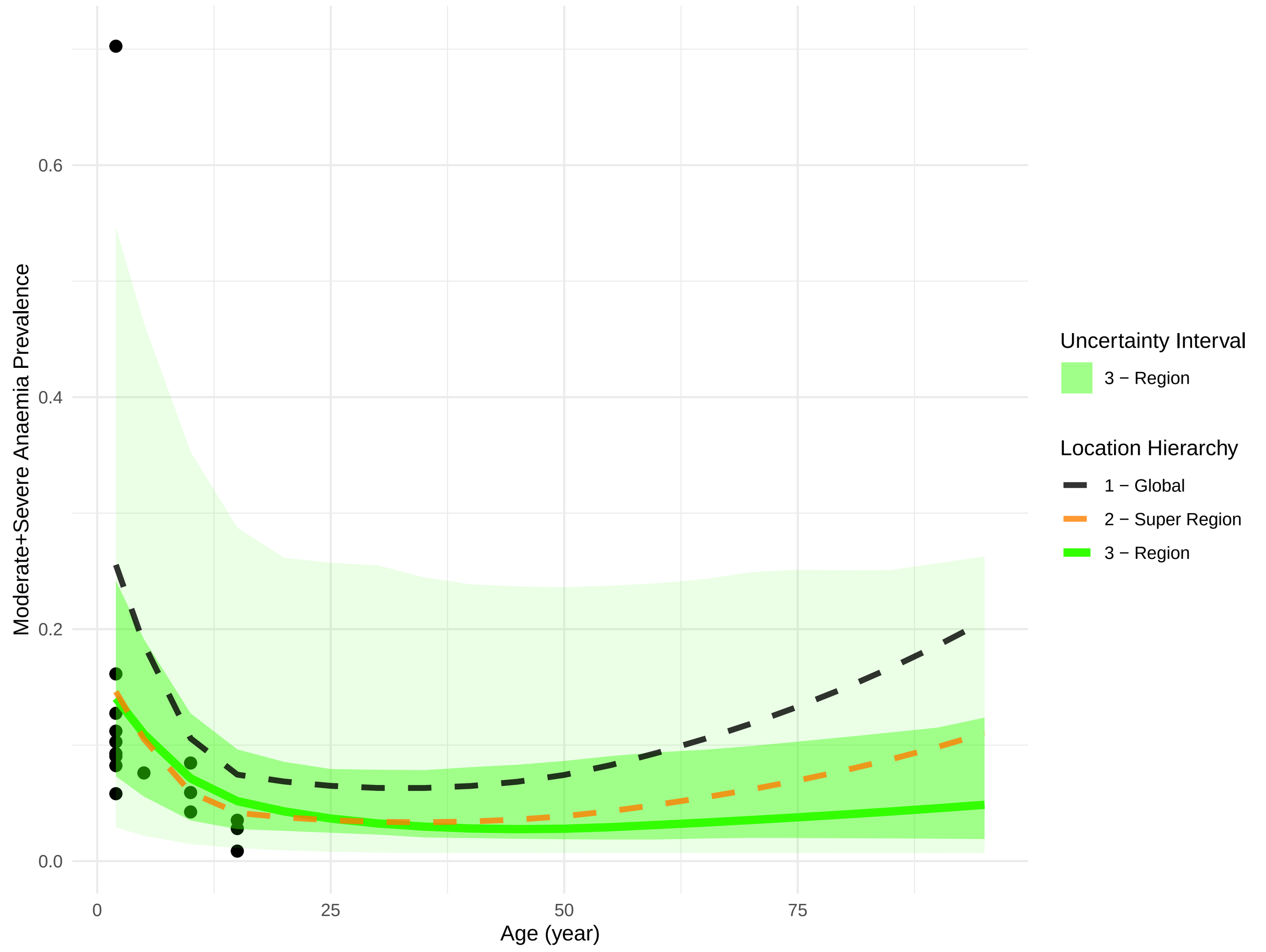

MR−BRT Age/Sex Fits − Males 2+ Years Old − Moderate+Severe Anaemia Prevalence − Egypt
WHO−2001 Adjustment Method

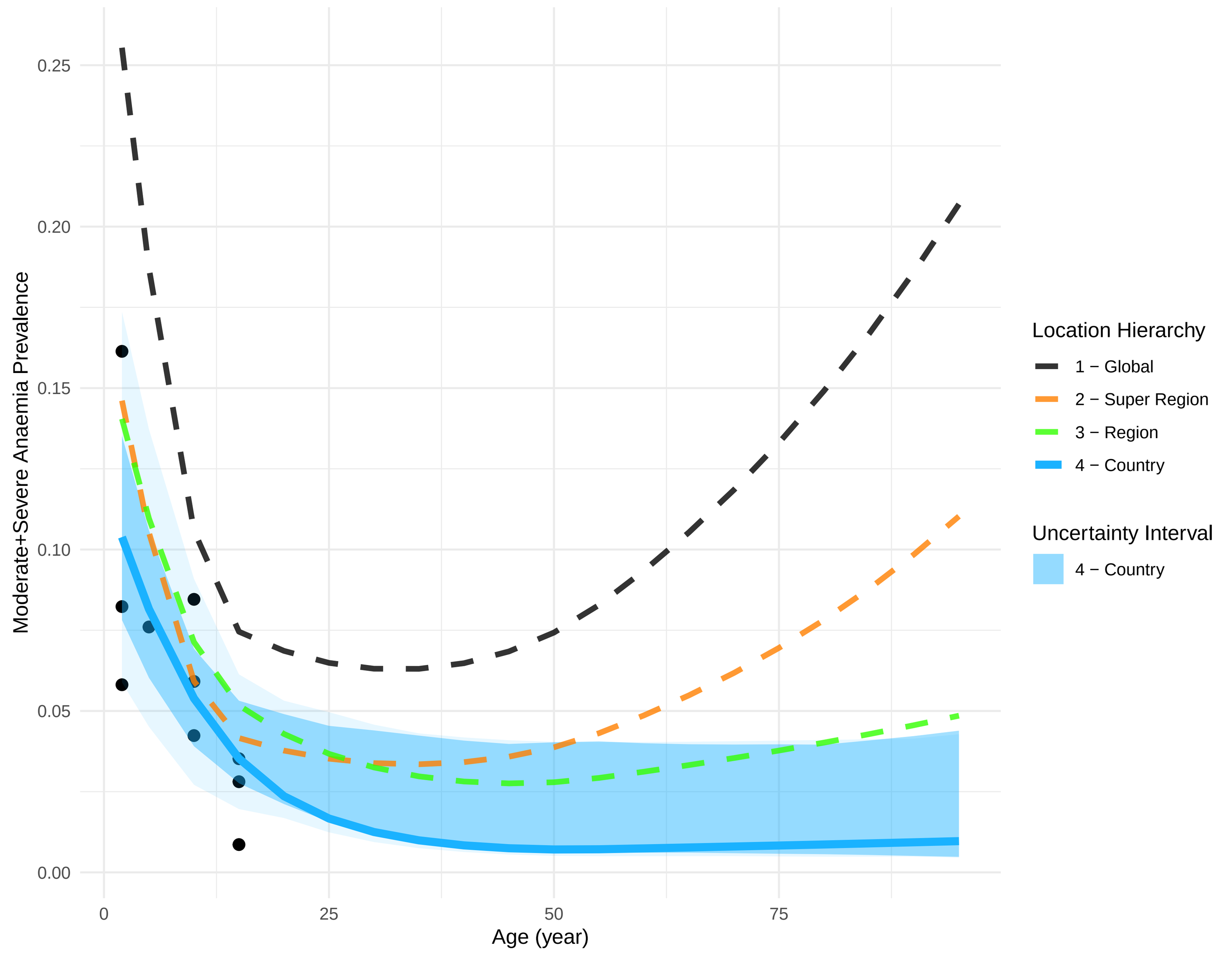

MR−BRT Age/Sex Fits − Males 2+ Years Old − Moderate+Severe Anaemia Prevalence − Jordan
WHO−2001 Adjustment Method

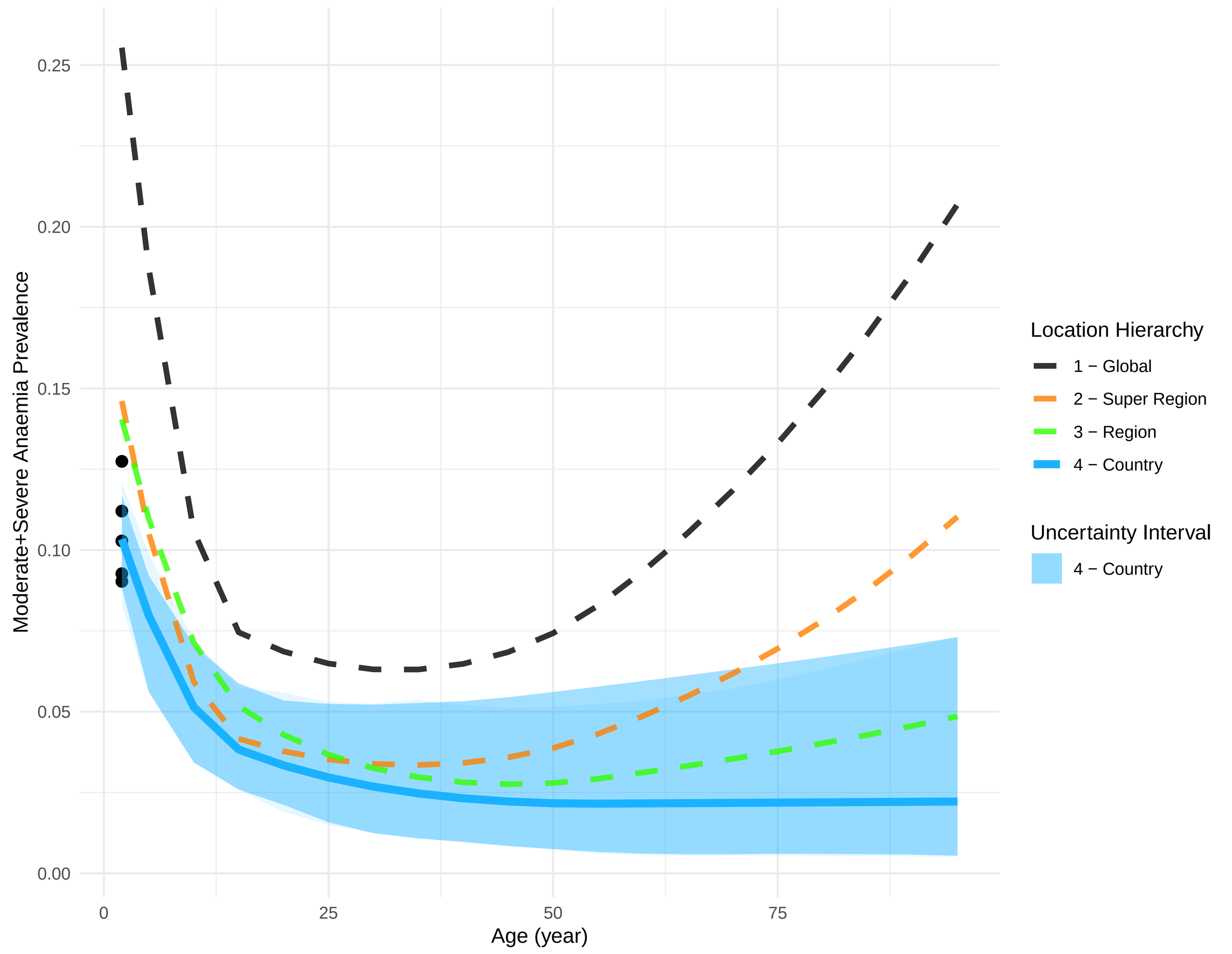

MR−BRT Age/Sex Fits − Males 2+ Years Old − Moderate+Severe Anaemia Prevalence − Yemen
WHO−2001 Adjustment Method

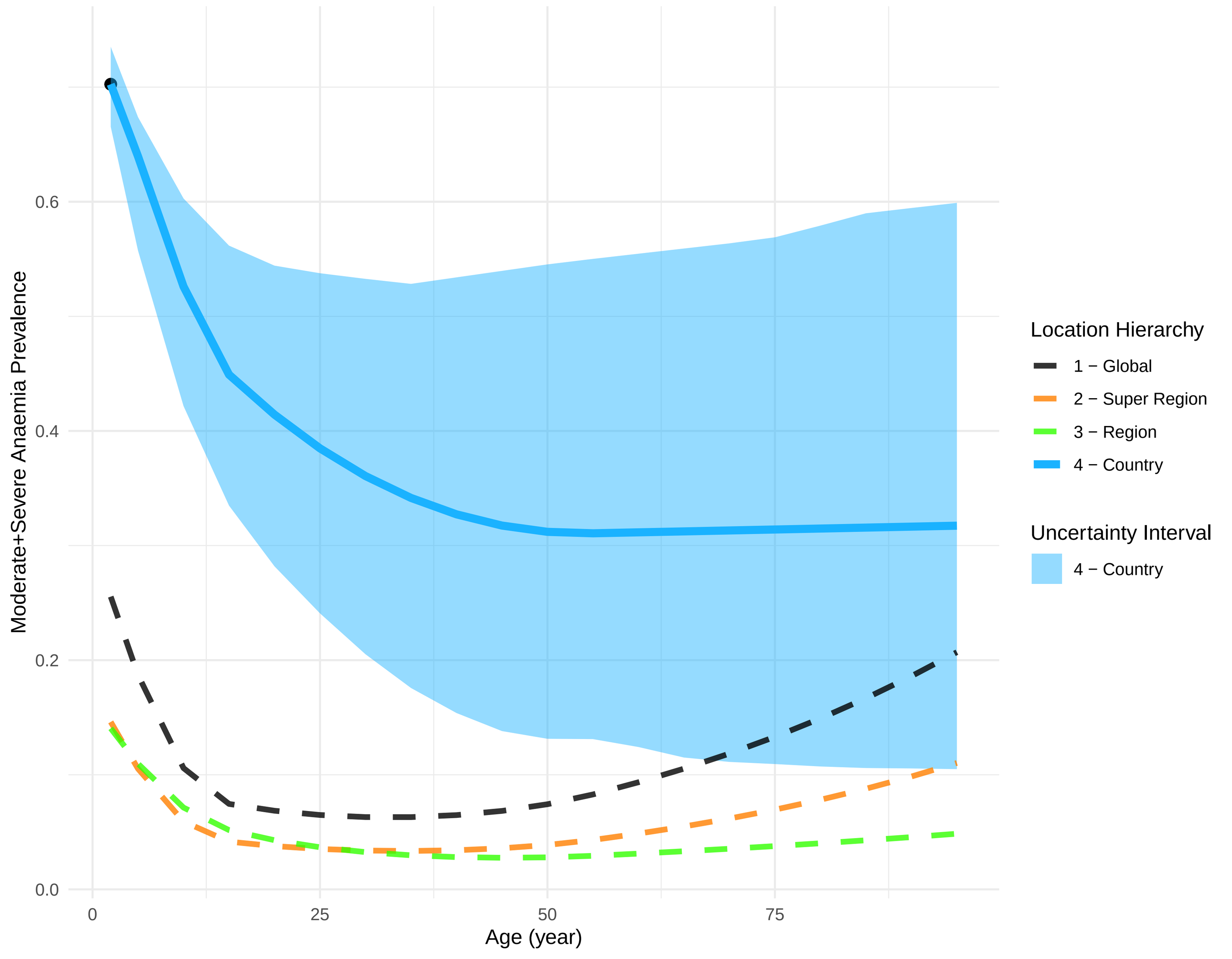

MR−BRT Age/Sex Fits − Males 2+ Years Old − Moderate+Severe Anaemia Prevalence − South Asia
WHO−2001 Adjustment Method

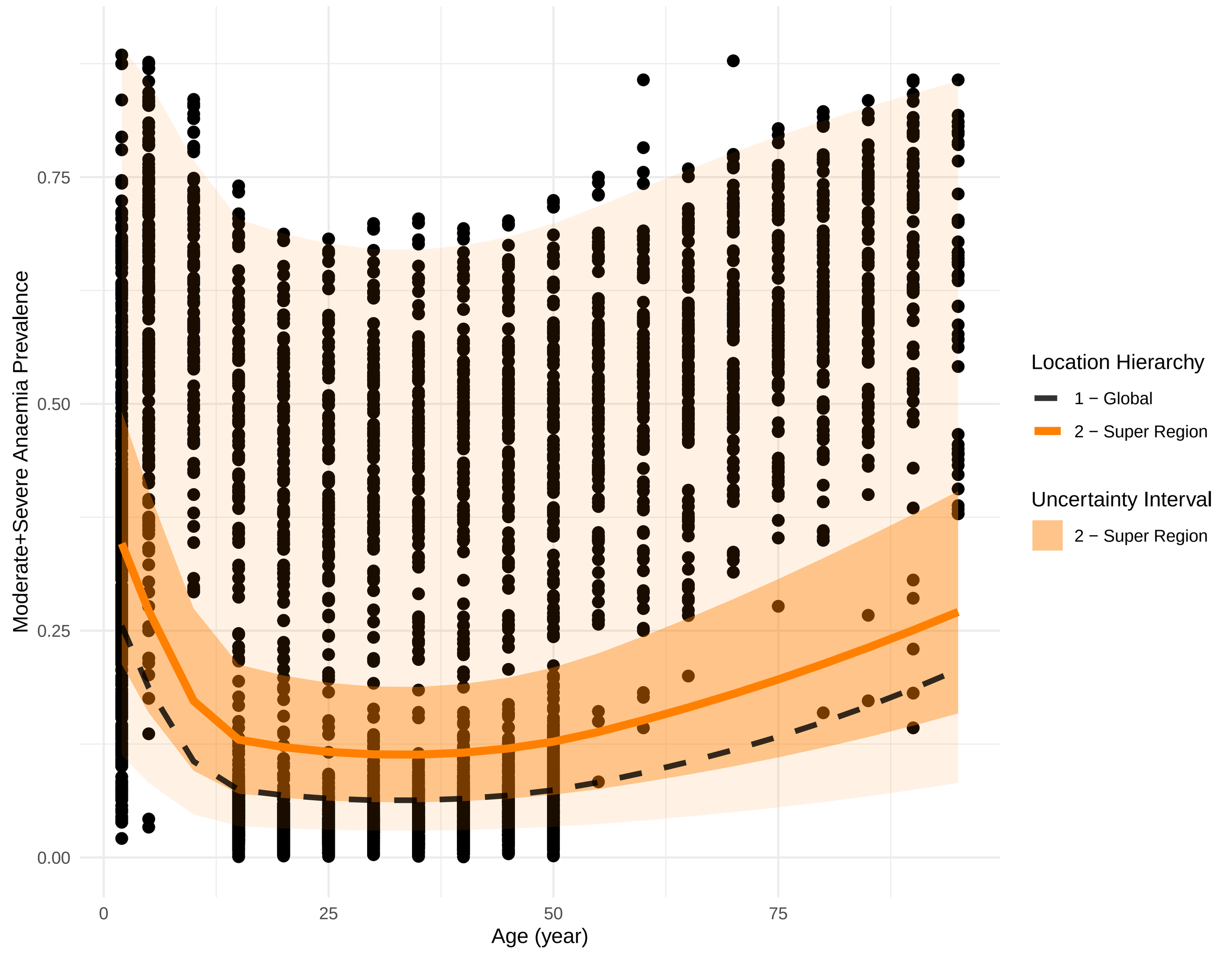

MR−BRT Age/Sex Fits − Males 2+ Years Old − Moderate+Severe Anaemia Prevalence − South Asia
WHO−2001 Adjustment Method

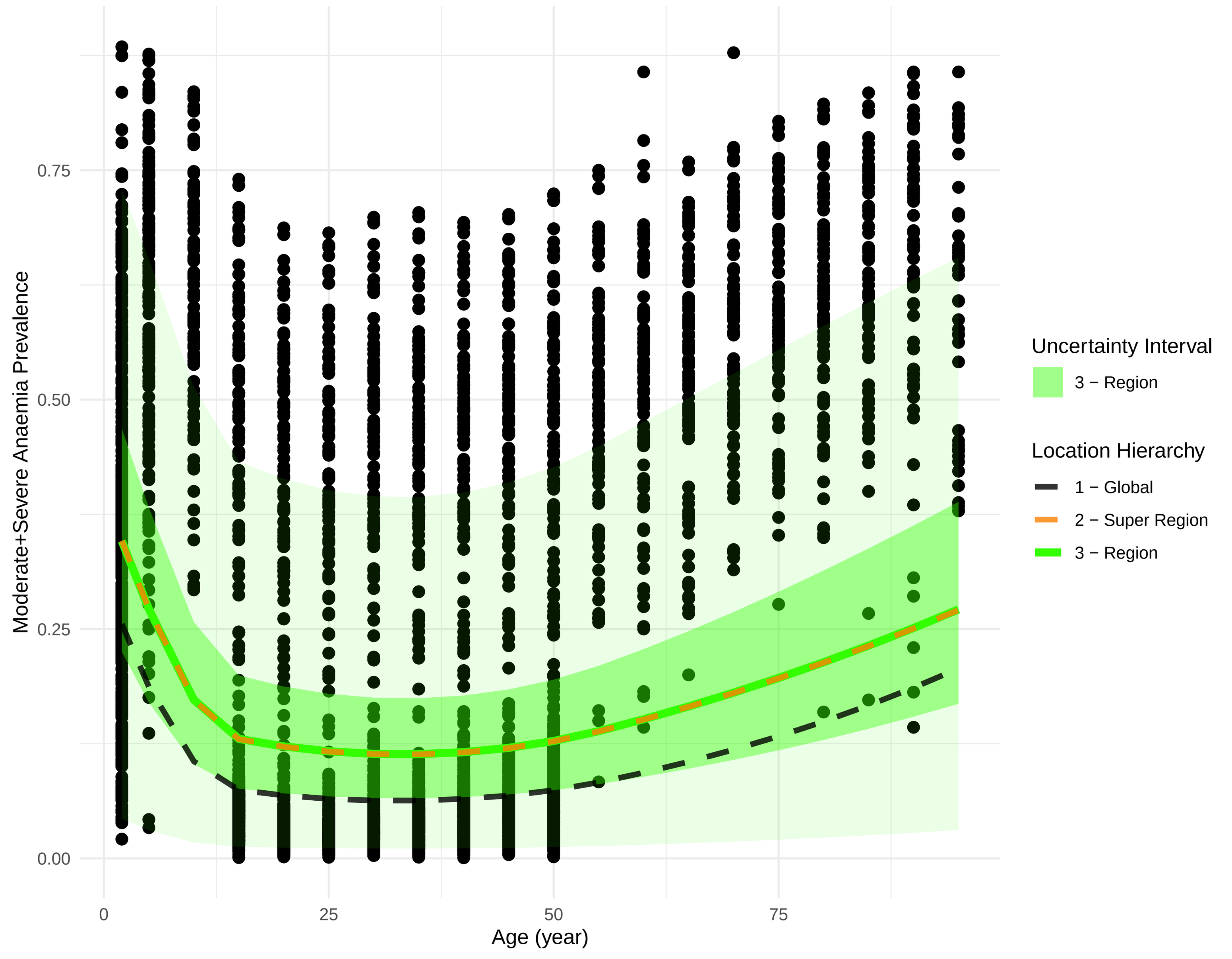

MR−BRT Age/Sex Fits − Males 2+ Years Old − Moderate+Severe Anaemia Prevalence − Bangladesh
WHO−2001 Adjustment Method

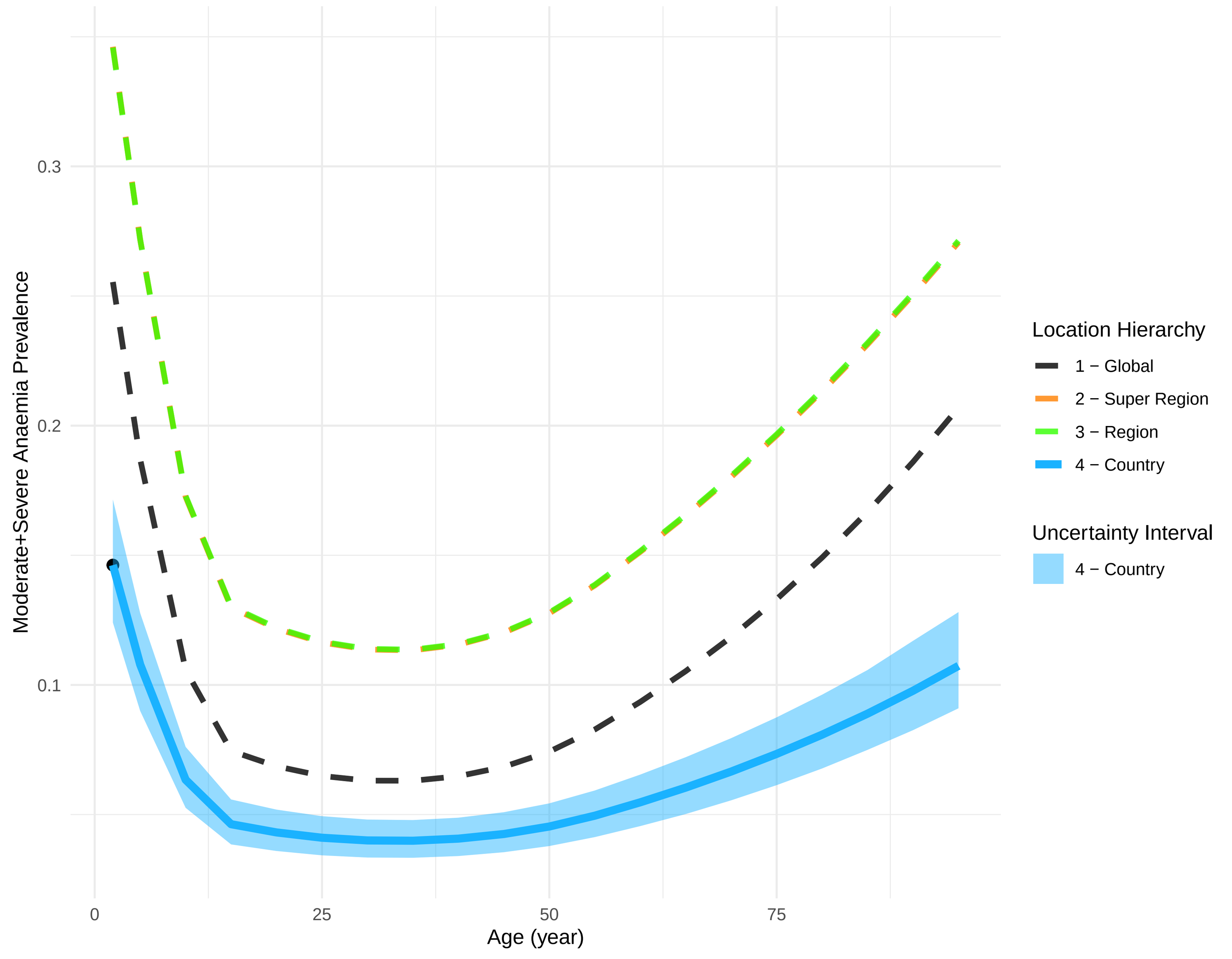

MR−BRT Age/Sex Fits − Males 2+ Years Old − Moderate+Severe Anaemia Prevalence − India
WHO−2001 Adjustment Method

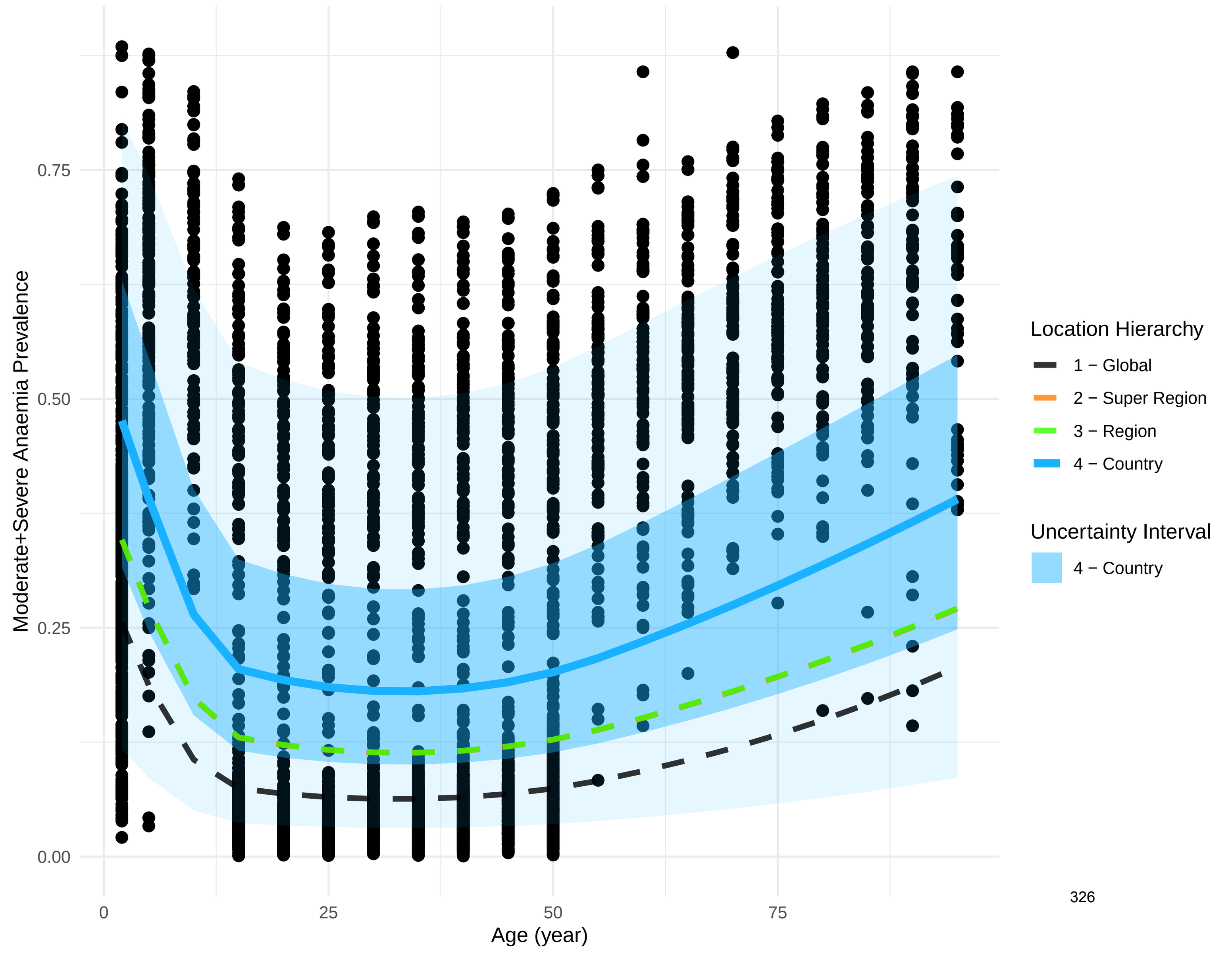

MR−BRT Age/Sex Fits − Males 2+ Years Old − Moderate+Severe Anaemia Prevalence − Andhra Pradesh
WHO−2001 Adjustment Method

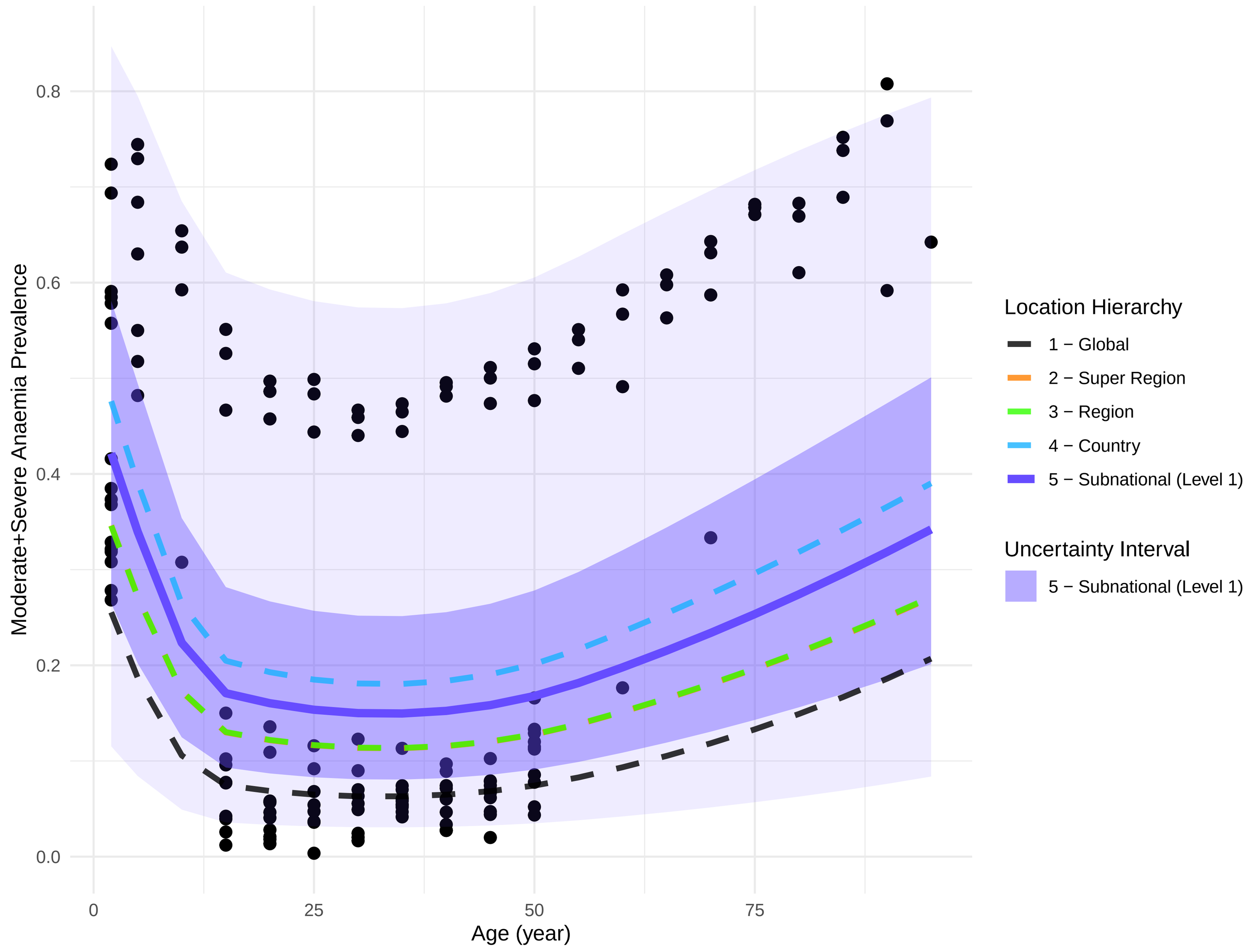

MR−BRT Age/Sex Fits − Males 2+ Years Old − Moderate+Severe Anaemia Prevalence − Andhra Pradesh, Rural
WHO−2001 Adjustment Method

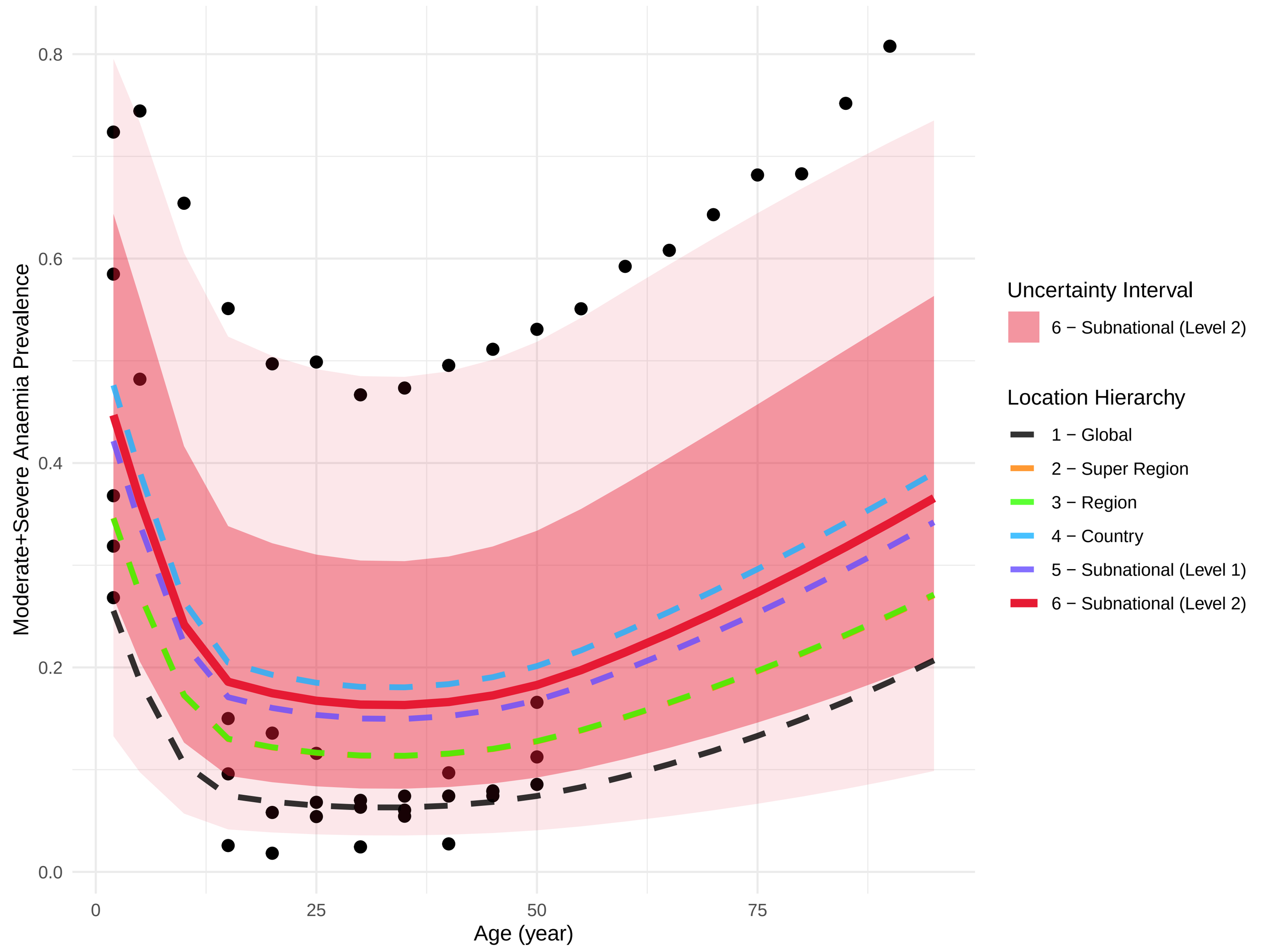

MR−BRT Age/Sex Fits − Males 2+ Years Old − Moderate+Severe Anaemia Prevalence − Andhra Pradesh, Urban
WHO−2001 Adjustment Method

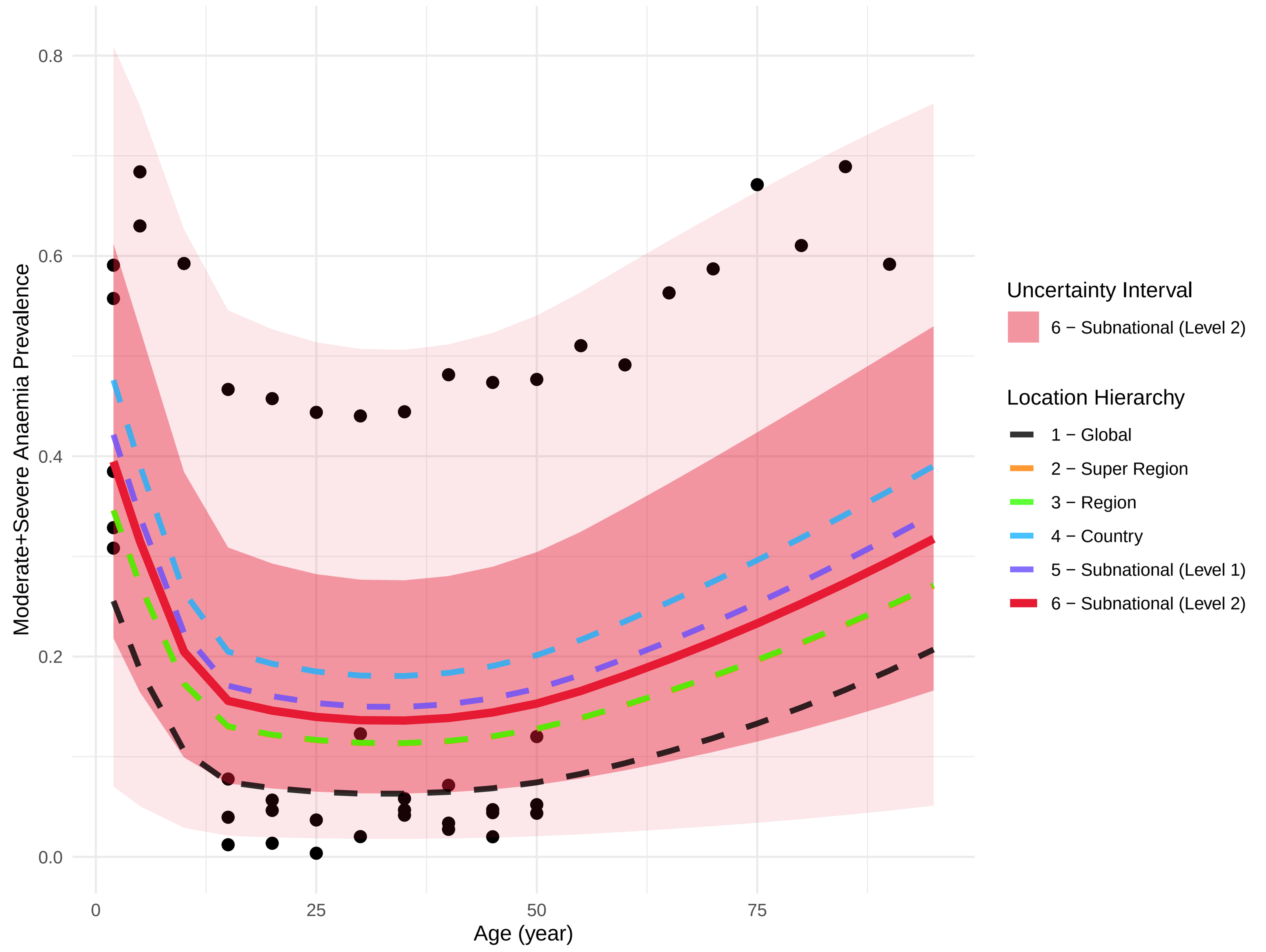

MR−BRT Age/Sex Fits − Males 2+ Years Old − Moderate+Severe Anaemia Prevalence − Arunachal Pradesh
WHO−2001 Adjustment Method

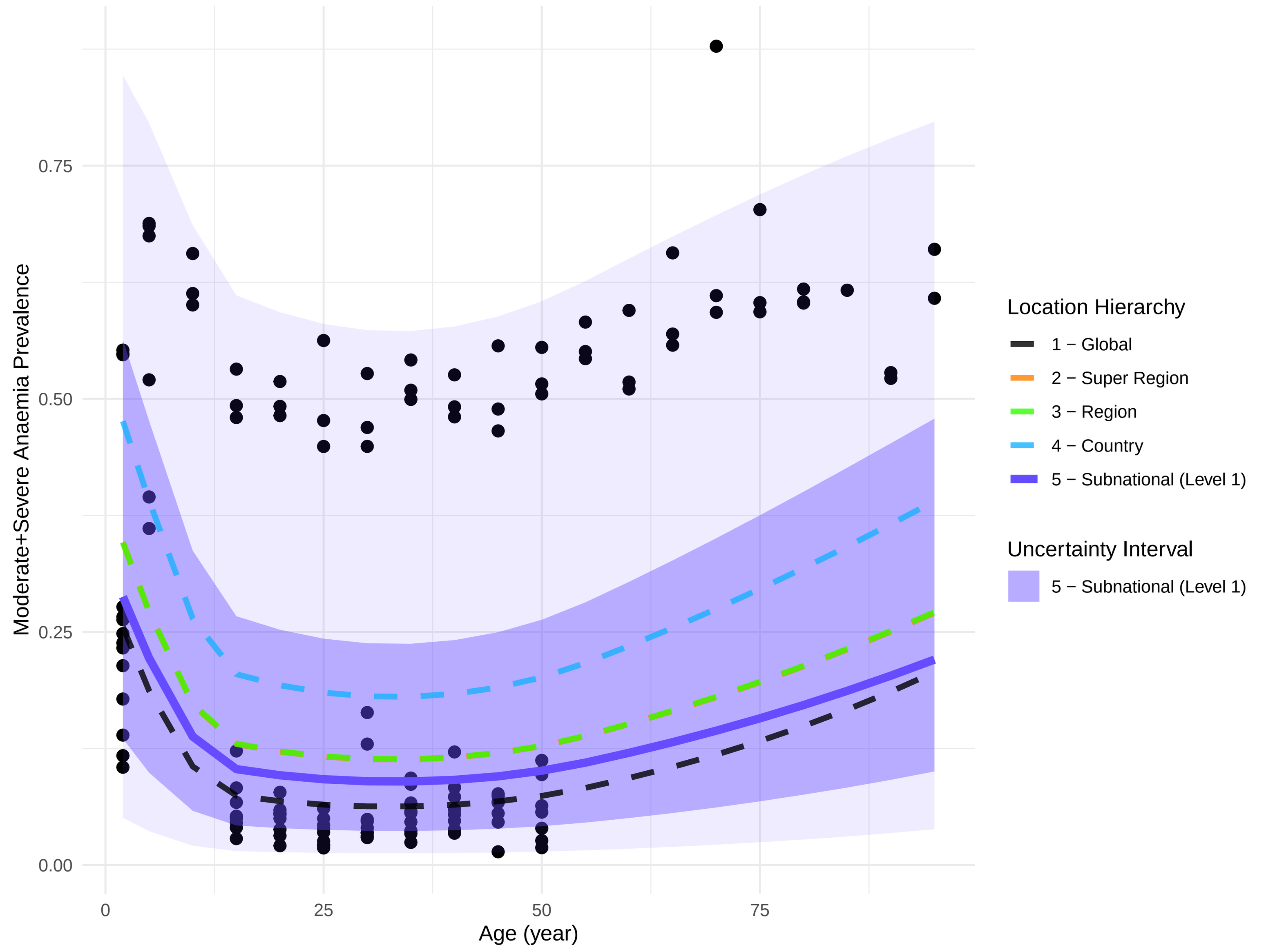

MR−BRT Age/Sex Fits − Males 2+ Years Old − Moderate+Severe Anaemia Prevalence − Arunachal Pradesh, Rural
WHO−2001 Adjustment Method

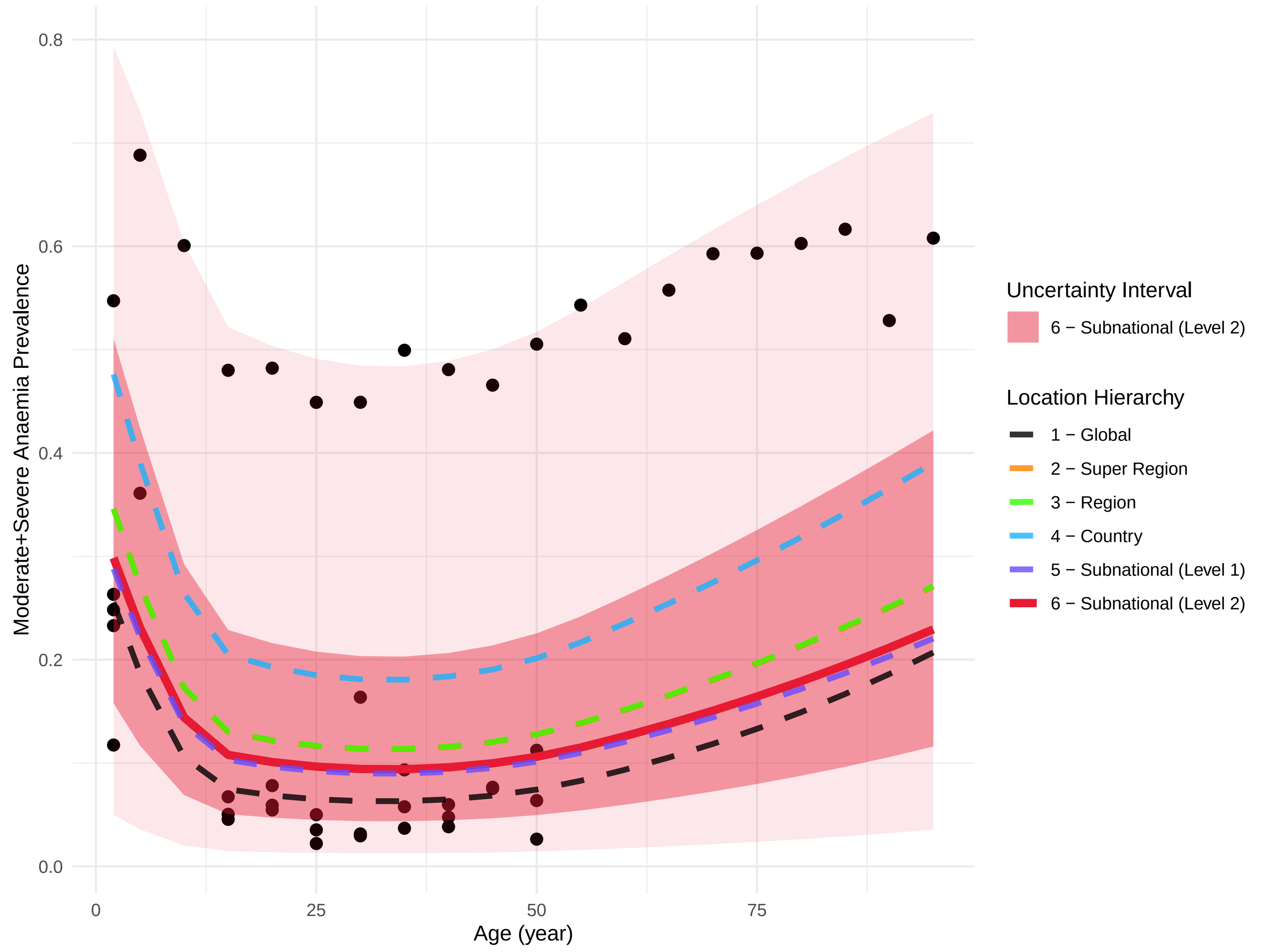

MR−BRT Age/Sex Fits − Males 2+ Years Old − Moderate+Severe Anaemia Prevalence − Arunachal Pradesh, Urban
WHO−2001 Adjustment Method

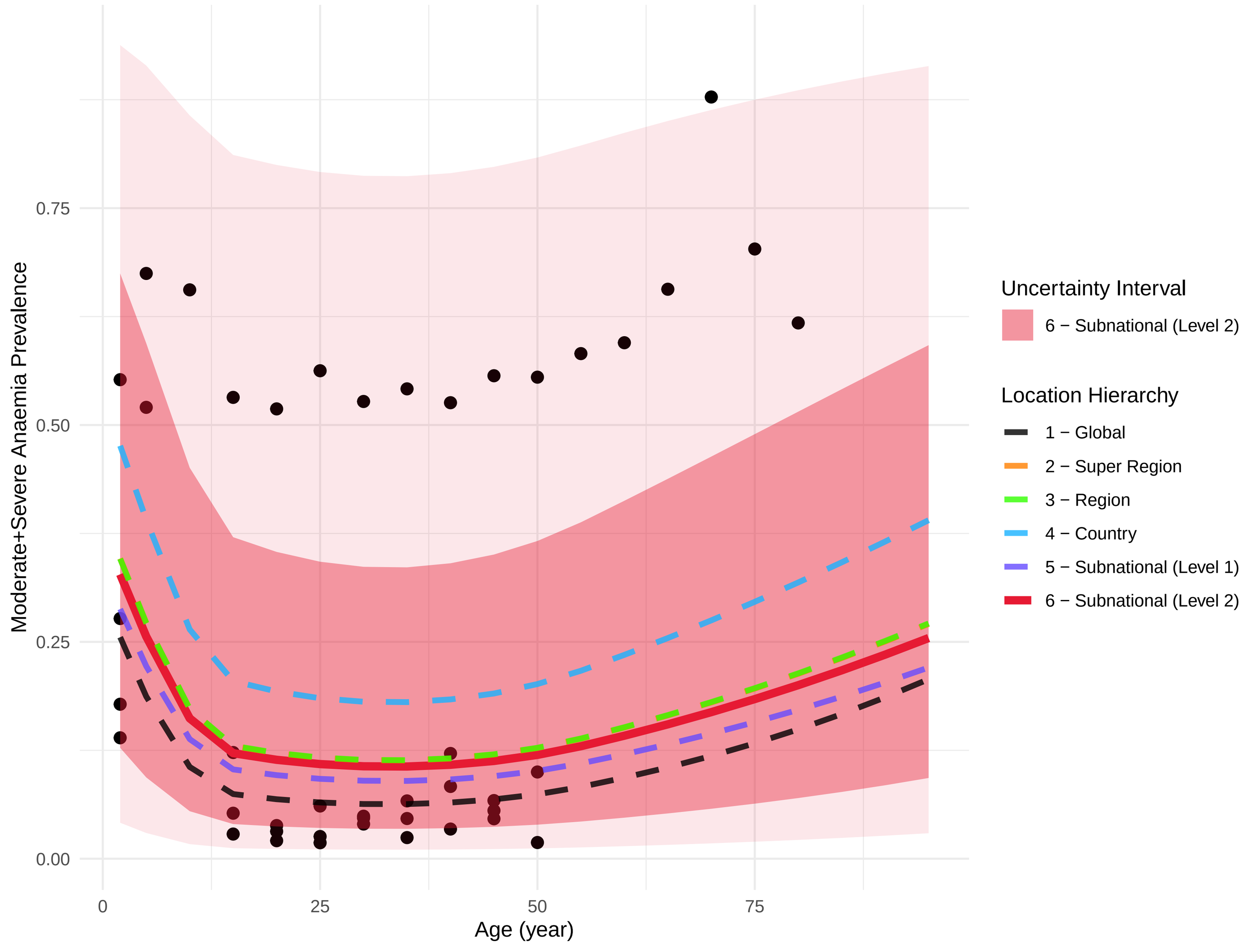

MR−BRT Age/Sex Fits − Males 2+ Years Old − Moderate+Severe Anaemia Prevalence − Assam
WHO−2001 Adjustment Method

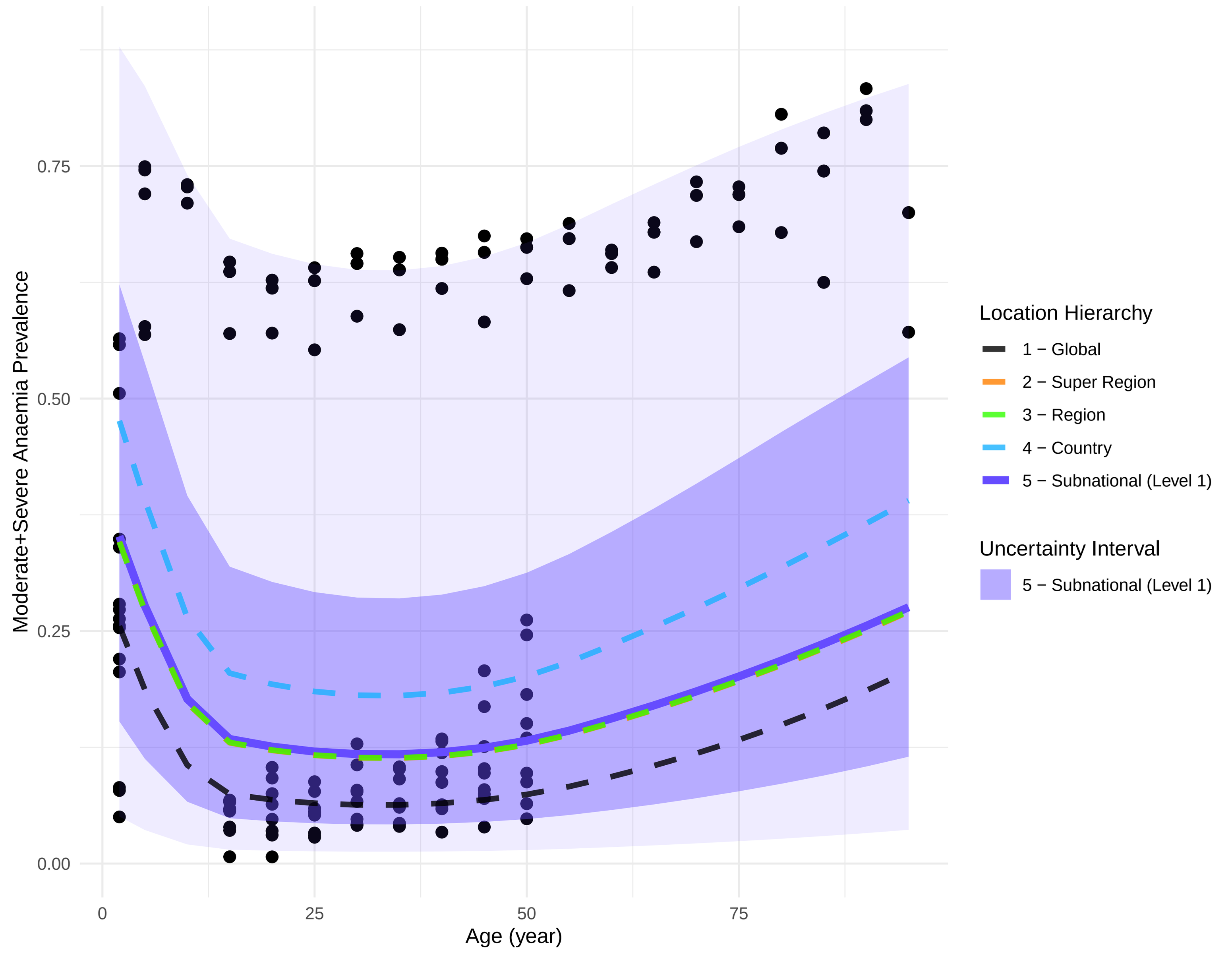

MR−BRT Age/Sex Fits − Males 2+ Years Old − Moderate+Severe Anaemia Prevalence − Assam, Rural
WHO−2001 Adjustment Method

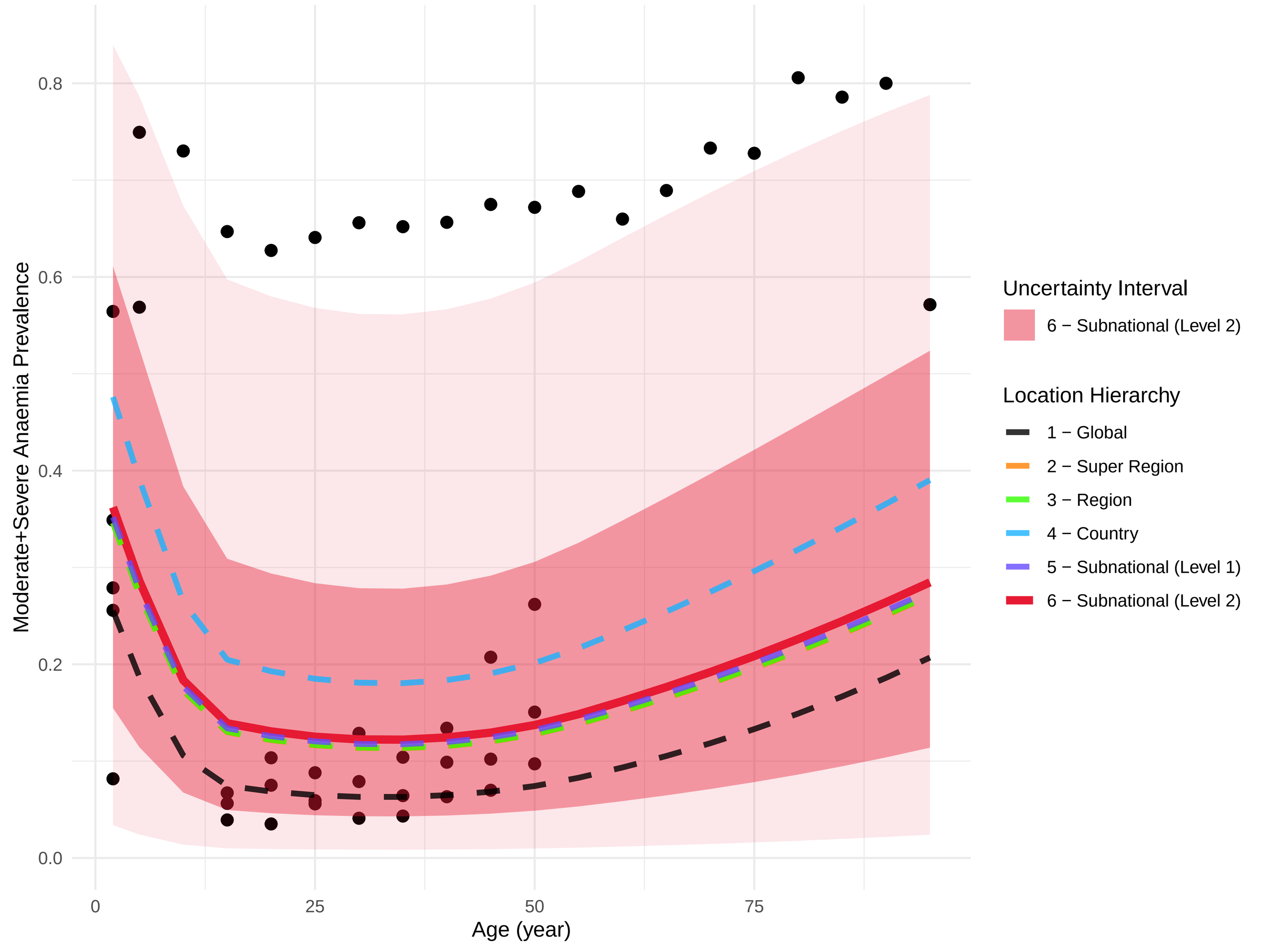

MR−BRT Age/Sex Fits − Males 2+ Years Old − Moderate+Severe Anaemia Prevalence − Assam, Urban
WHO−2001 Adjustment Method

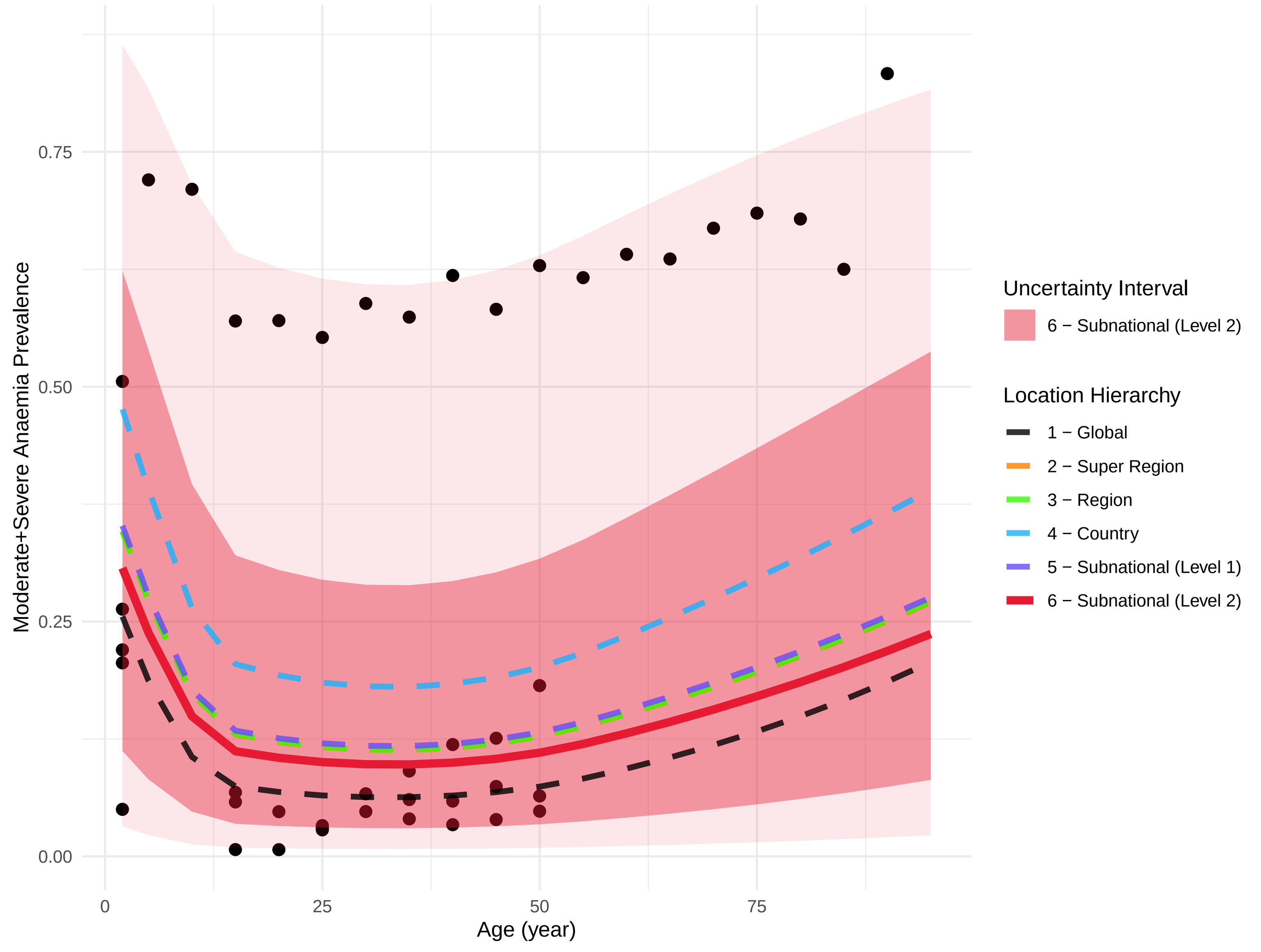

MR−BRT Age/Sex Fits − Males 2+ Years Old − Moderate+Severe Anaemia Prevalence − Bihar
WHO−2001 Adjustment Method

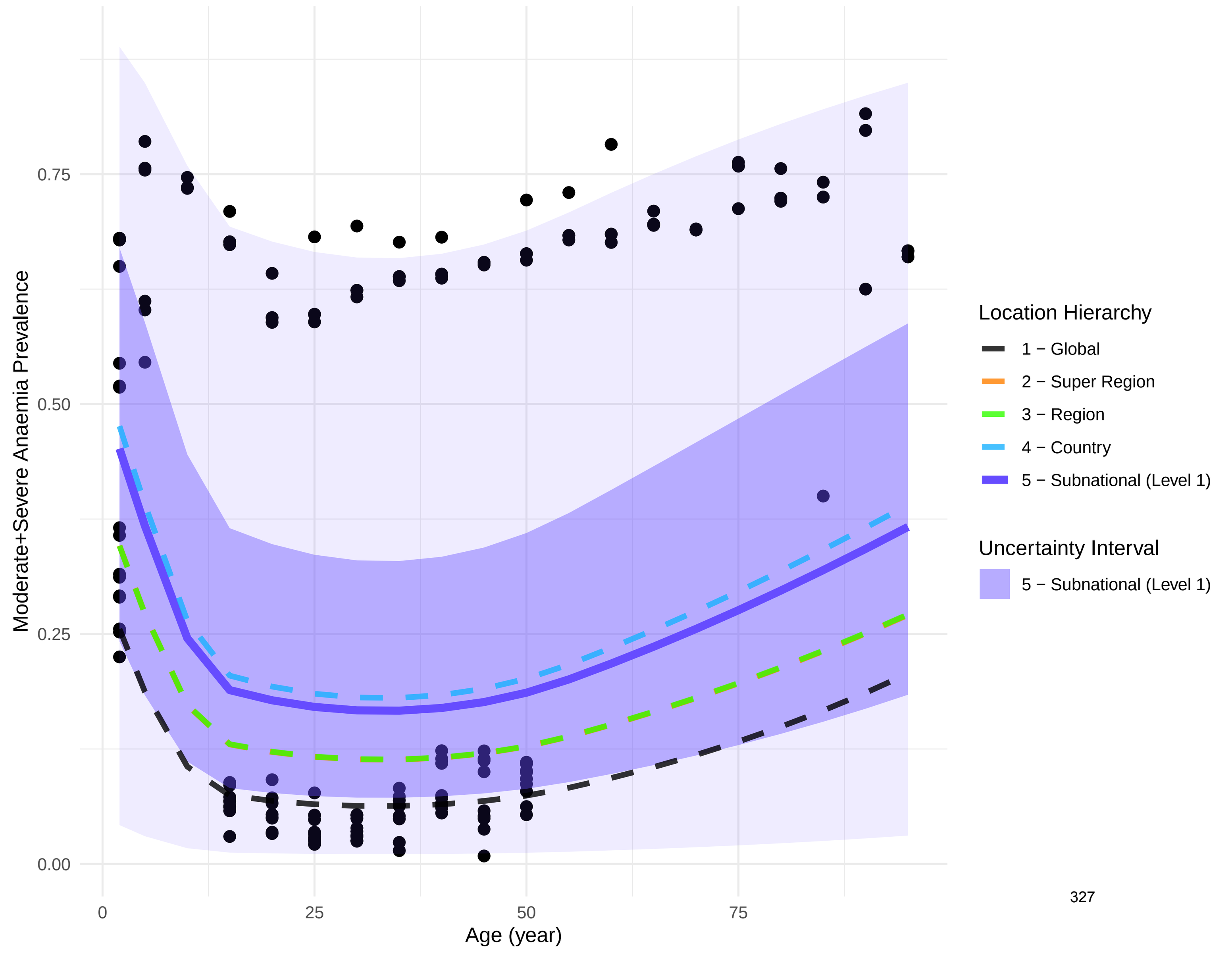

MR−BRT Age/Sex Fits − Males 2+ Years Old − Moderate+Severe Anaemia Prevalence − Bihar, Rural

WHO−2001 Adjustment Method

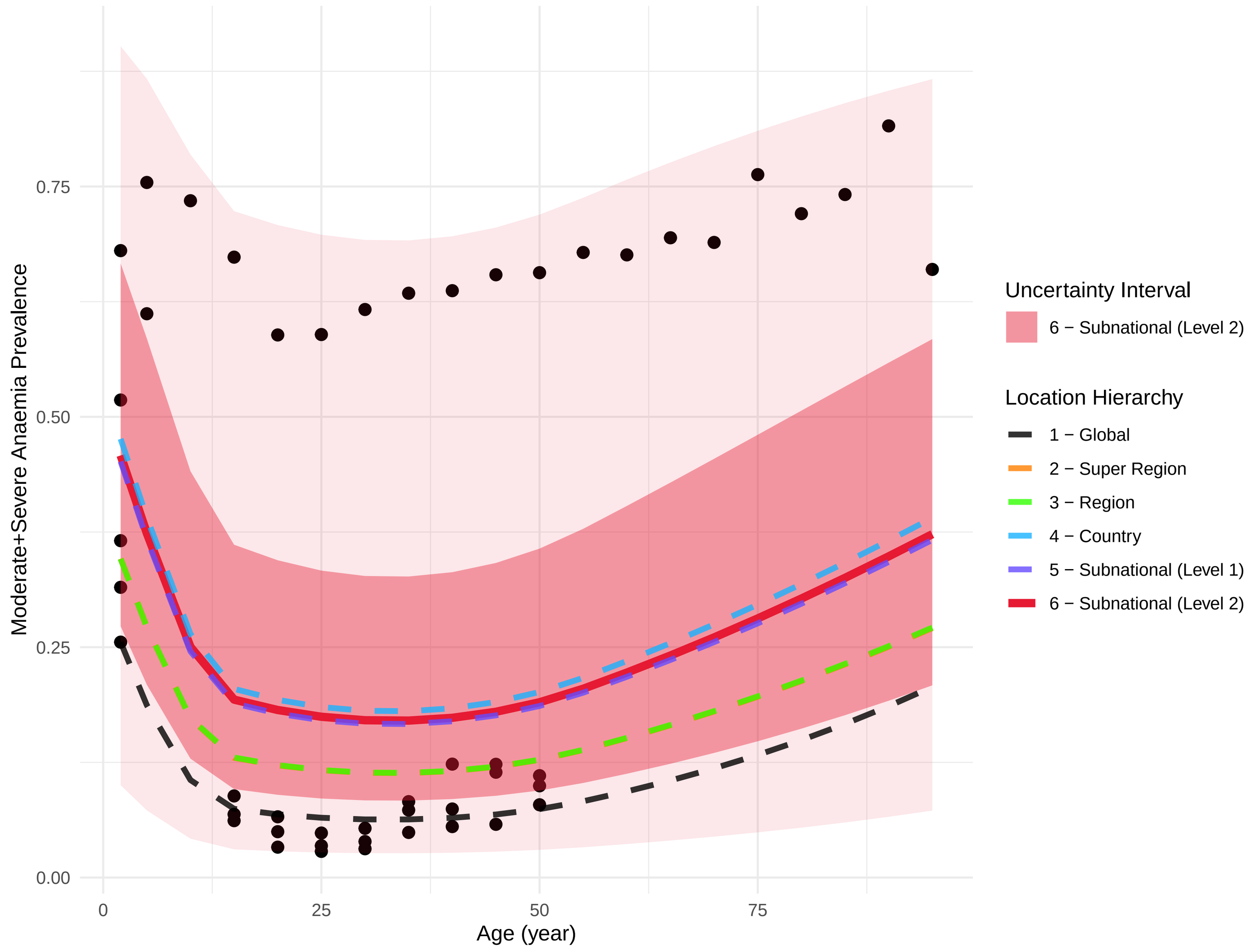

MR−BRT Age/Sex Fits − Males 2+ Years Old − Moderate+Severe Anaemia Prevalence − Bihar, Urban

WHO−2001 Adjustment Method

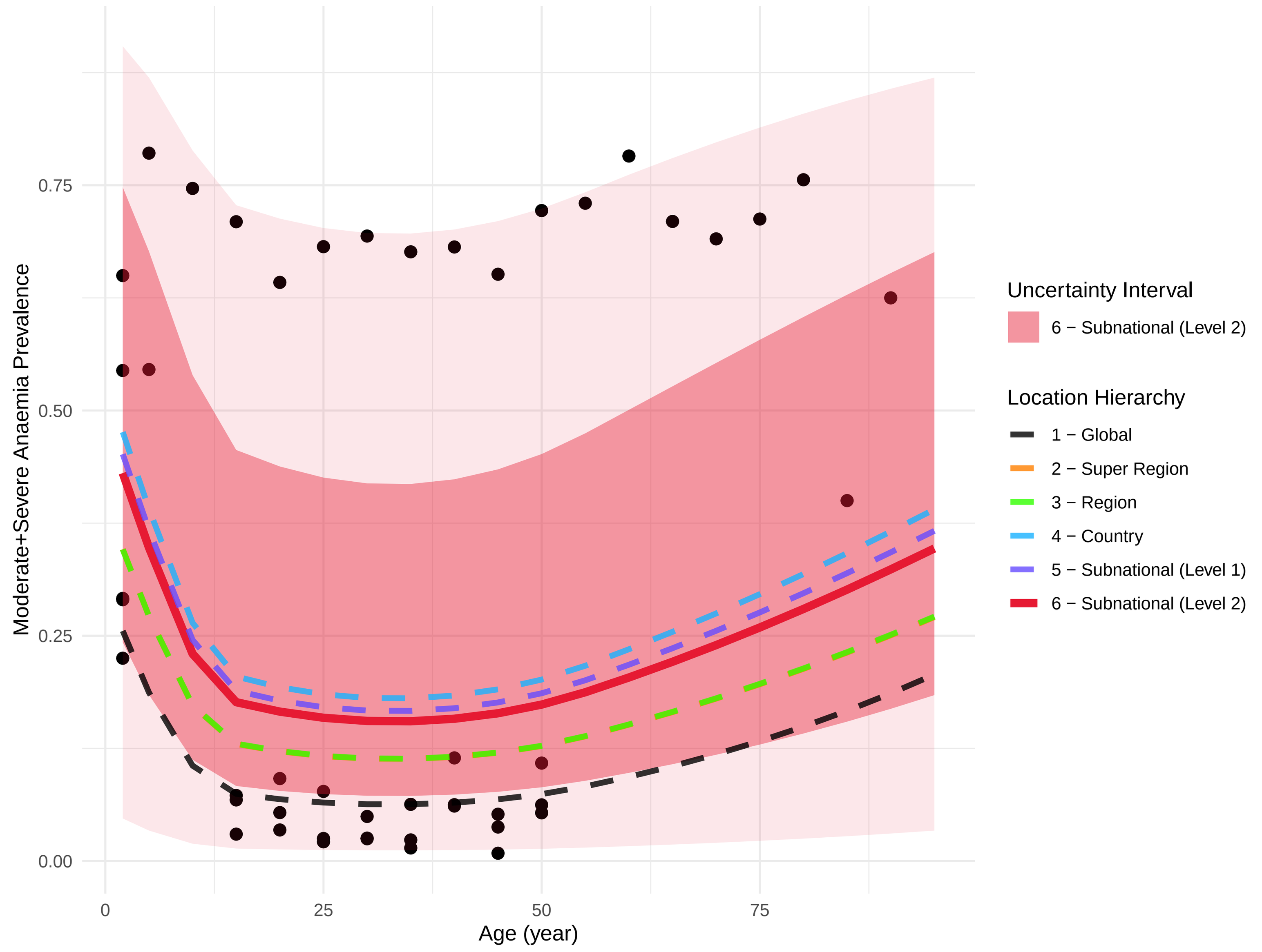

MR−BRT Age/Sex Fits − Males 2+ Years Old − Moderate+Severe Anaemia Prevalence − Chhattisgarh

WHO−2001 Adjustment Method

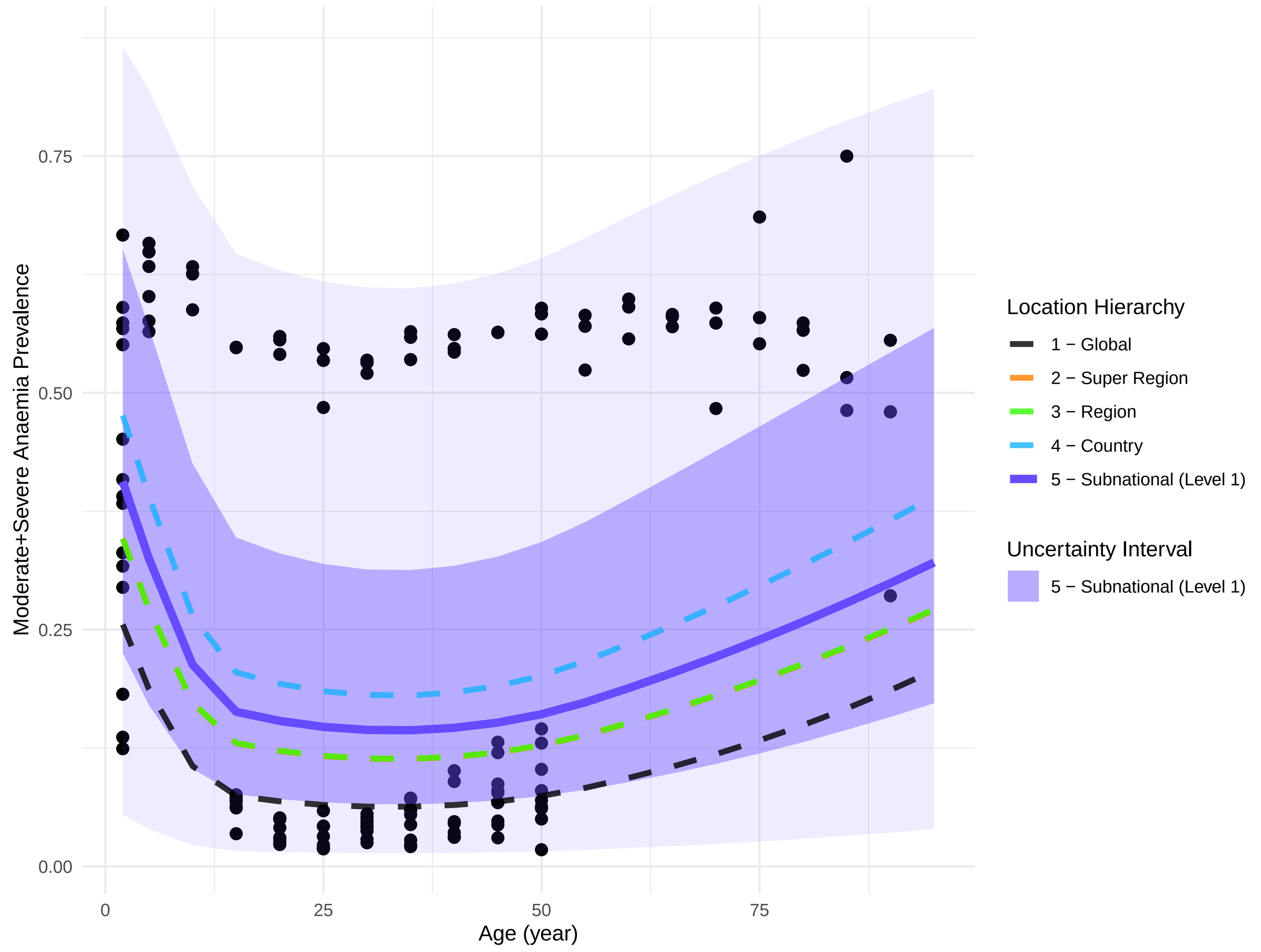

MR−BRT Age/Sex Fits − Males 2+ Years Old − Moderate+Severe Anaemia Prevalence − Chhattisgarh, Rural

WHO−2001 Adjustment Method

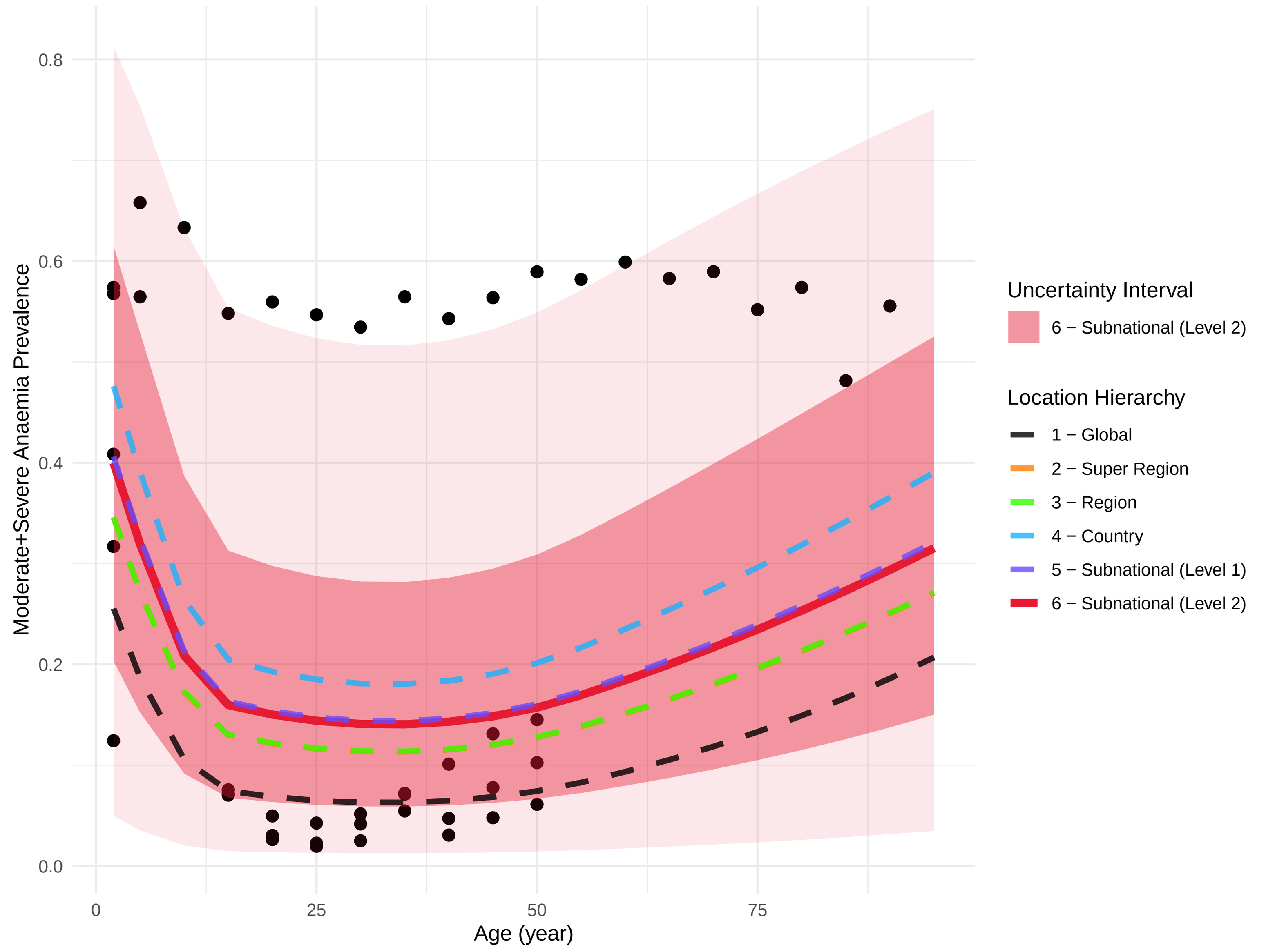

MR−BRT Age/Sex Fits − Males 2+ Years Old − Moderate+Severe Anaemia Prevalence − Chhattisgarh, Urban

WHO−2001 Adjustment Method

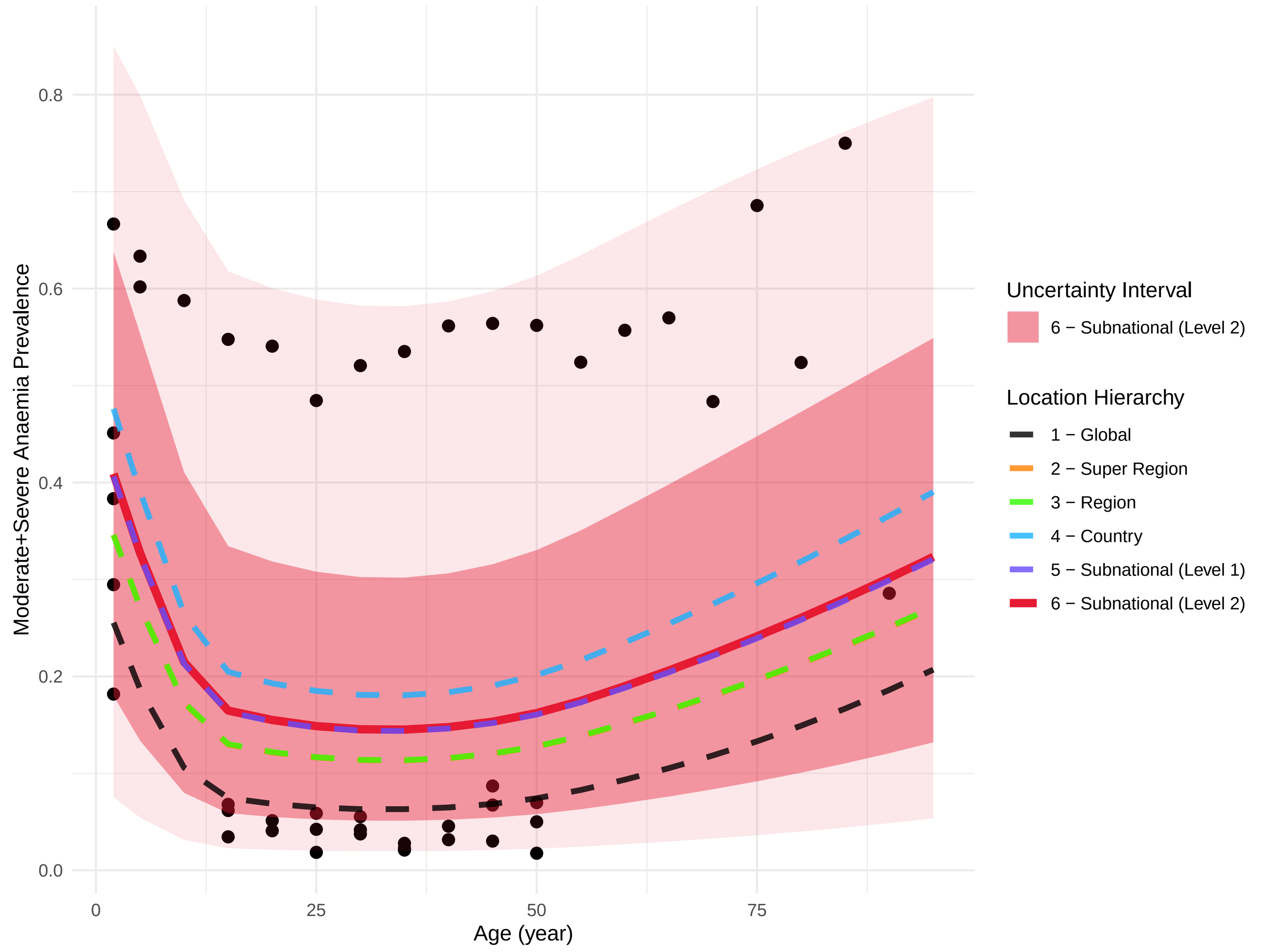

MR−BRT Age/Sex Fits − Males 2+ Years Old − Moderate+Severe Anaemia Prevalence − Delhi

WHO−2001 Adjustment Method

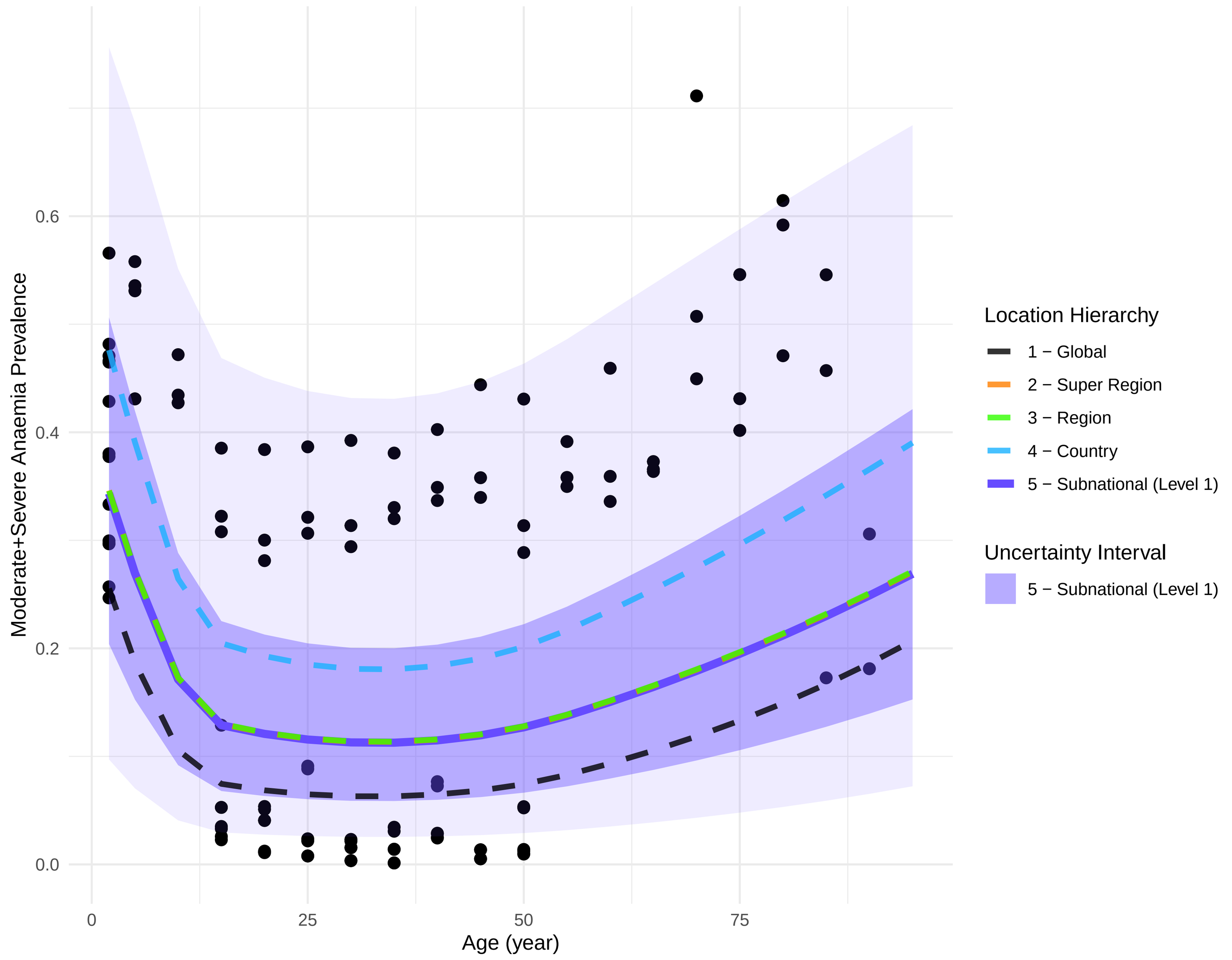

MR−BRT Age/Sex Fits − Males 2+ Years Old − Moderate+Severe Anaemia Prevalence − Delhi, Rural

WHO−2001 Adjustment Method

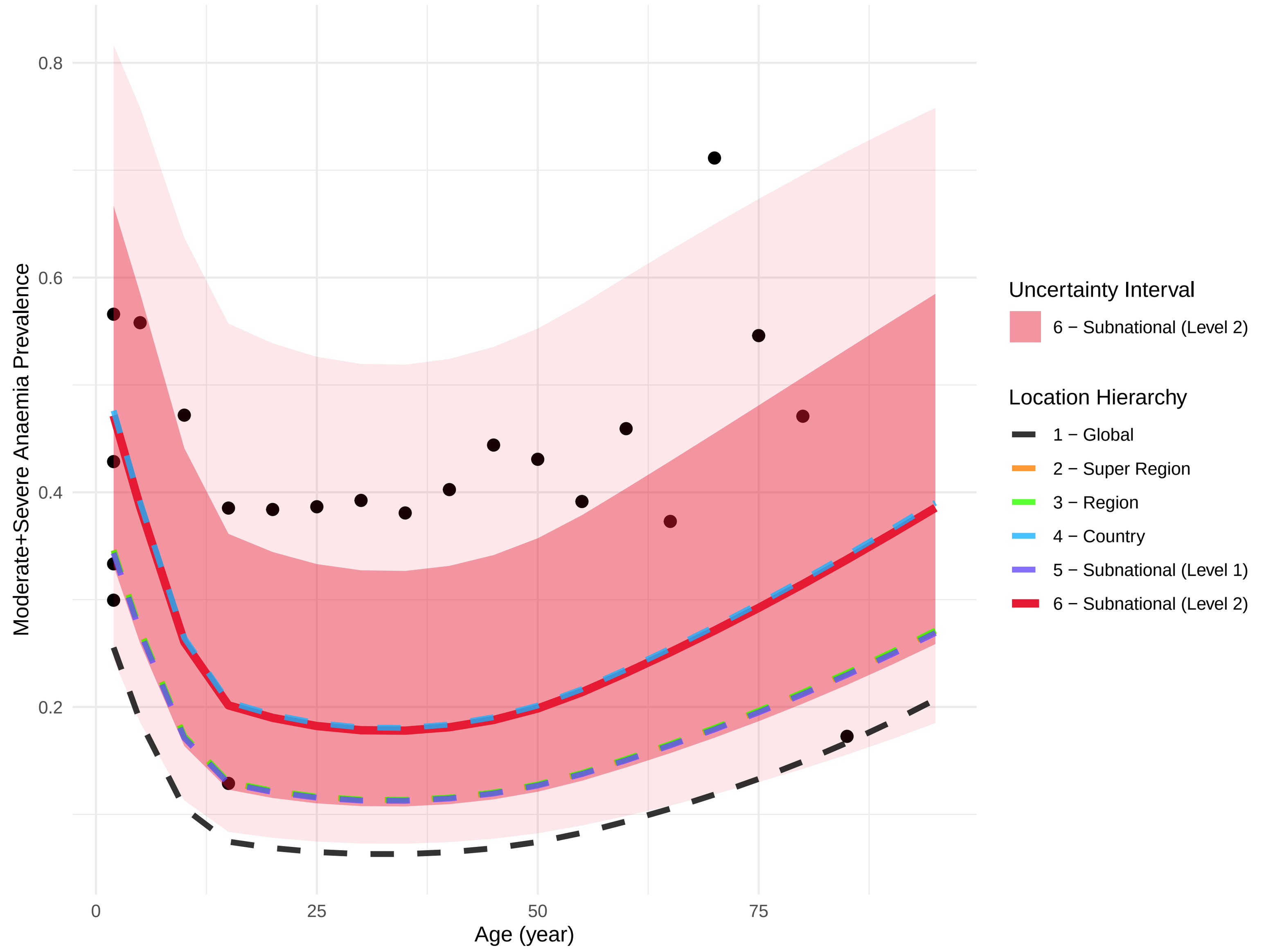

MR−BRT Age/Sex Fits − Males 2+ Years Old − Moderate+Severe Anaemia Prevalence − Delhi, Urban

WHO−2001 Adjustment Method

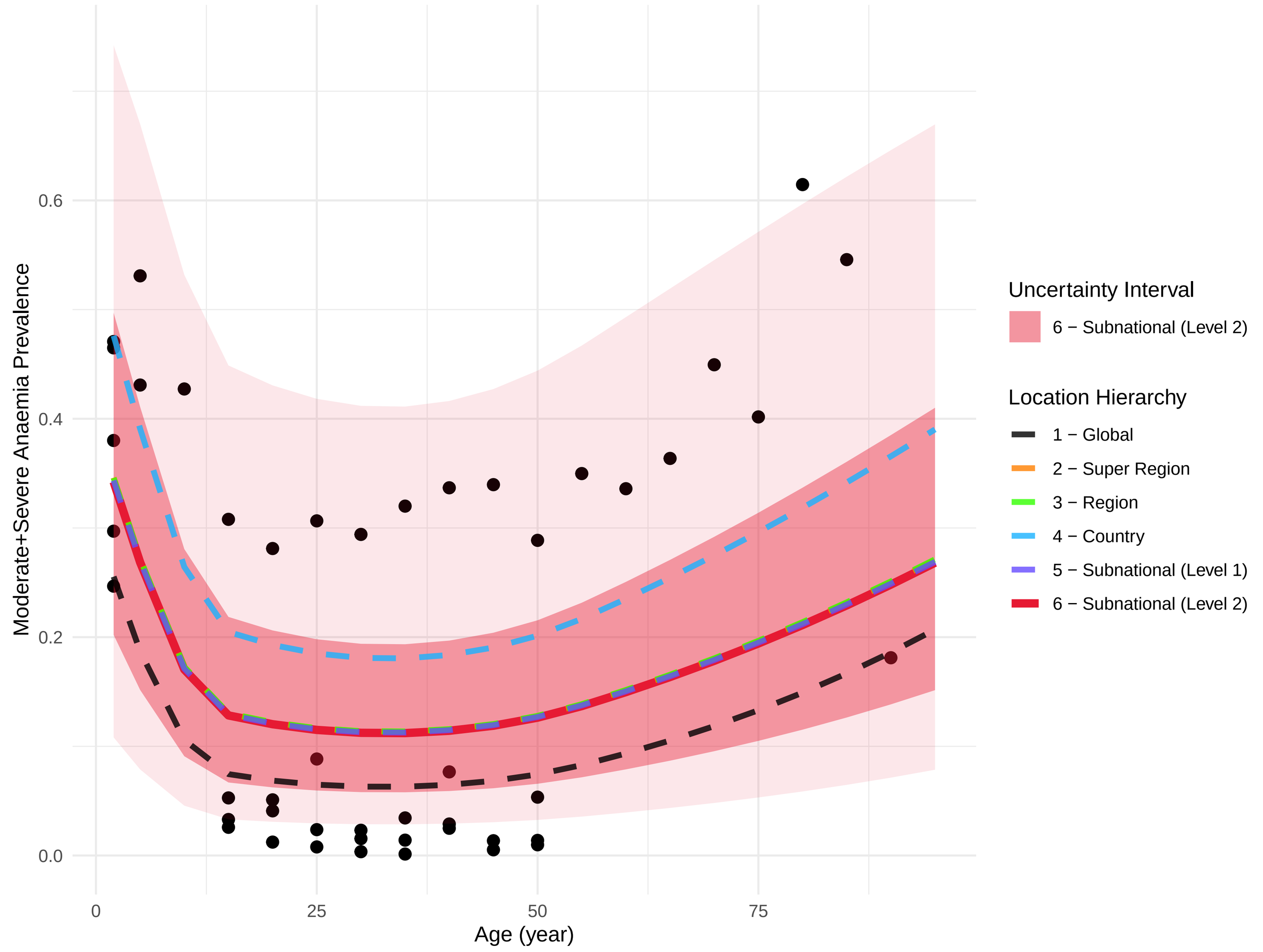

MR−BRT Age/Sex Fits − Males 2+ Years Old − Moderate+Severe Anaemia Prevalence − Goa

WHO−2001 Adjustment Method

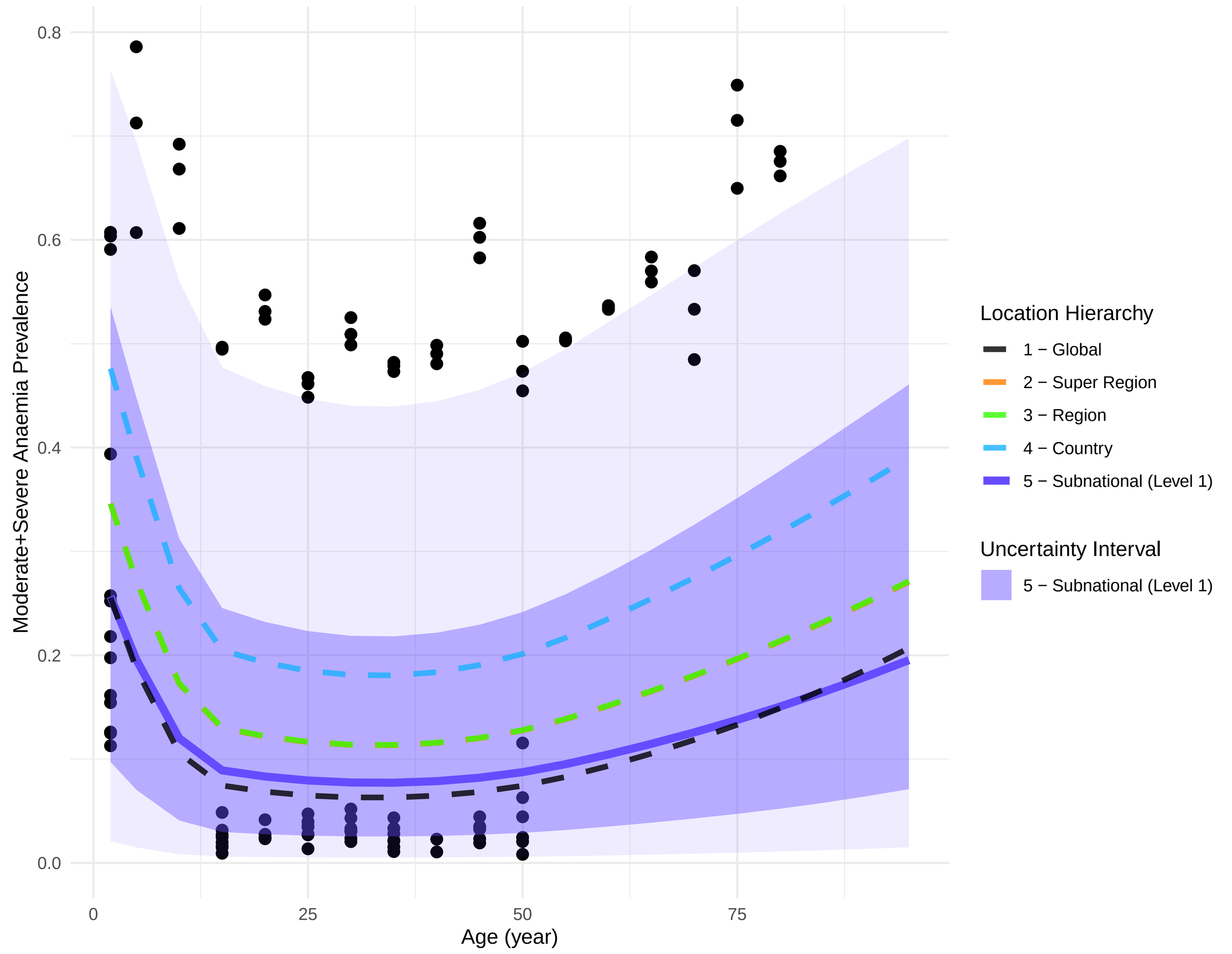

MR−BRT Age/Sex Fits − Males 2+ Years Old − Moderate+Severe Anaemia Prevalence − Goa, Rural

WHO−2001 Adjustment Method

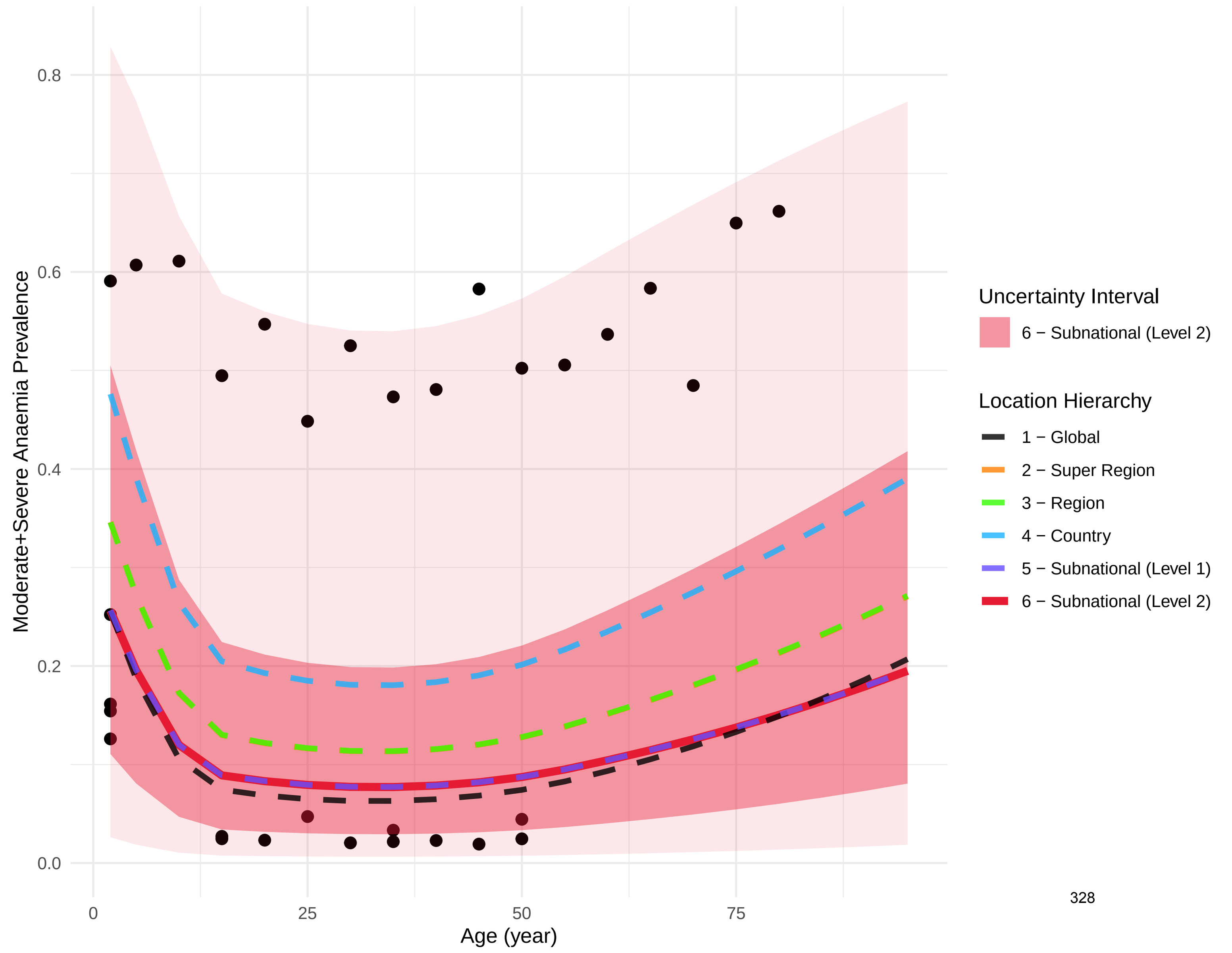

MR−BRT Age/Sex Fits − Males 2+ Years Old − Moderate+Severe Anaemia Prevalence − Goa, Urban
WHO−2001 Adjustment Method

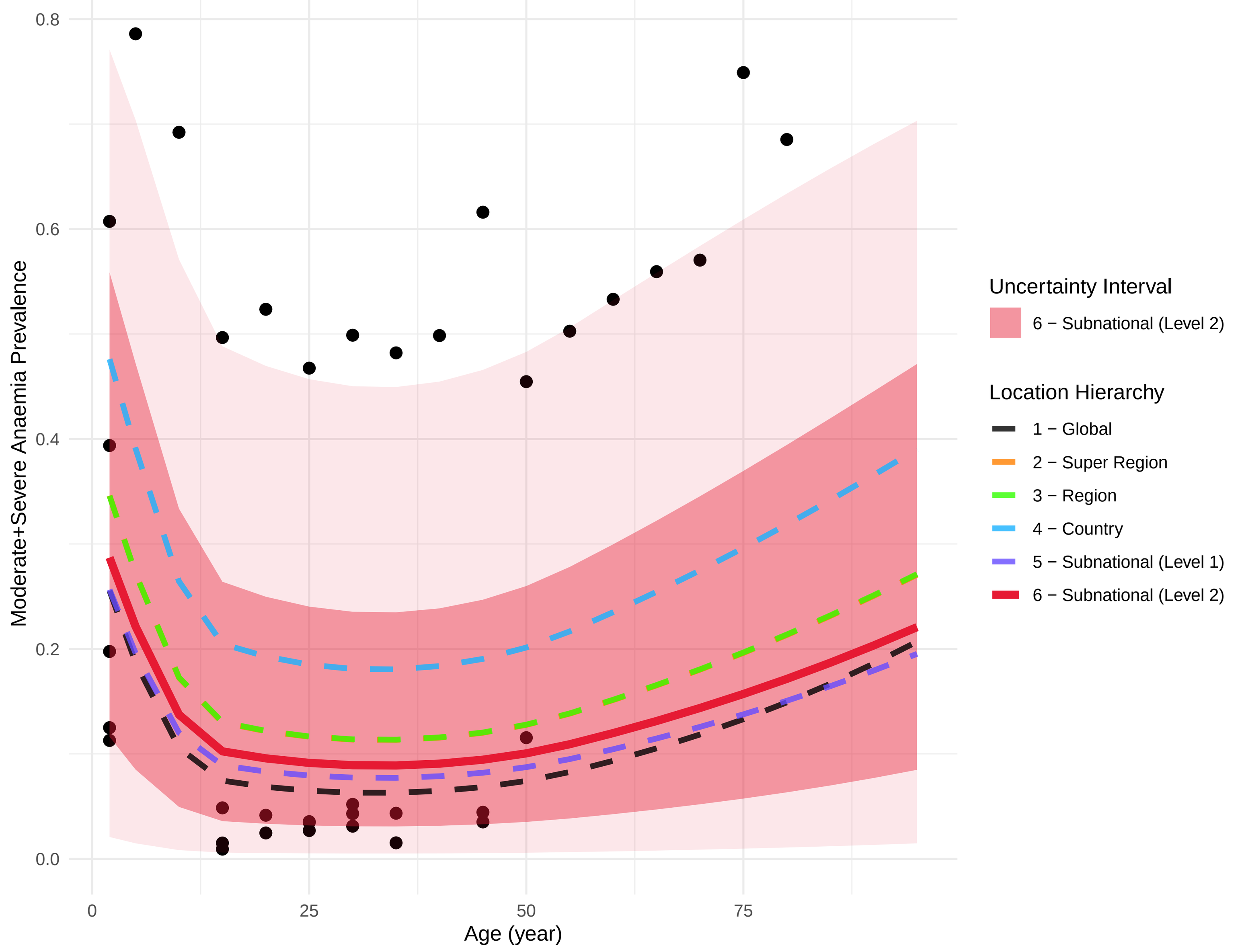

MR−BRT Age/Sex Fits − Males 2+ Years Old − Moderate+Severe Anaemia Prevalence − Gujarat
WHO−2001 Adjustment Method

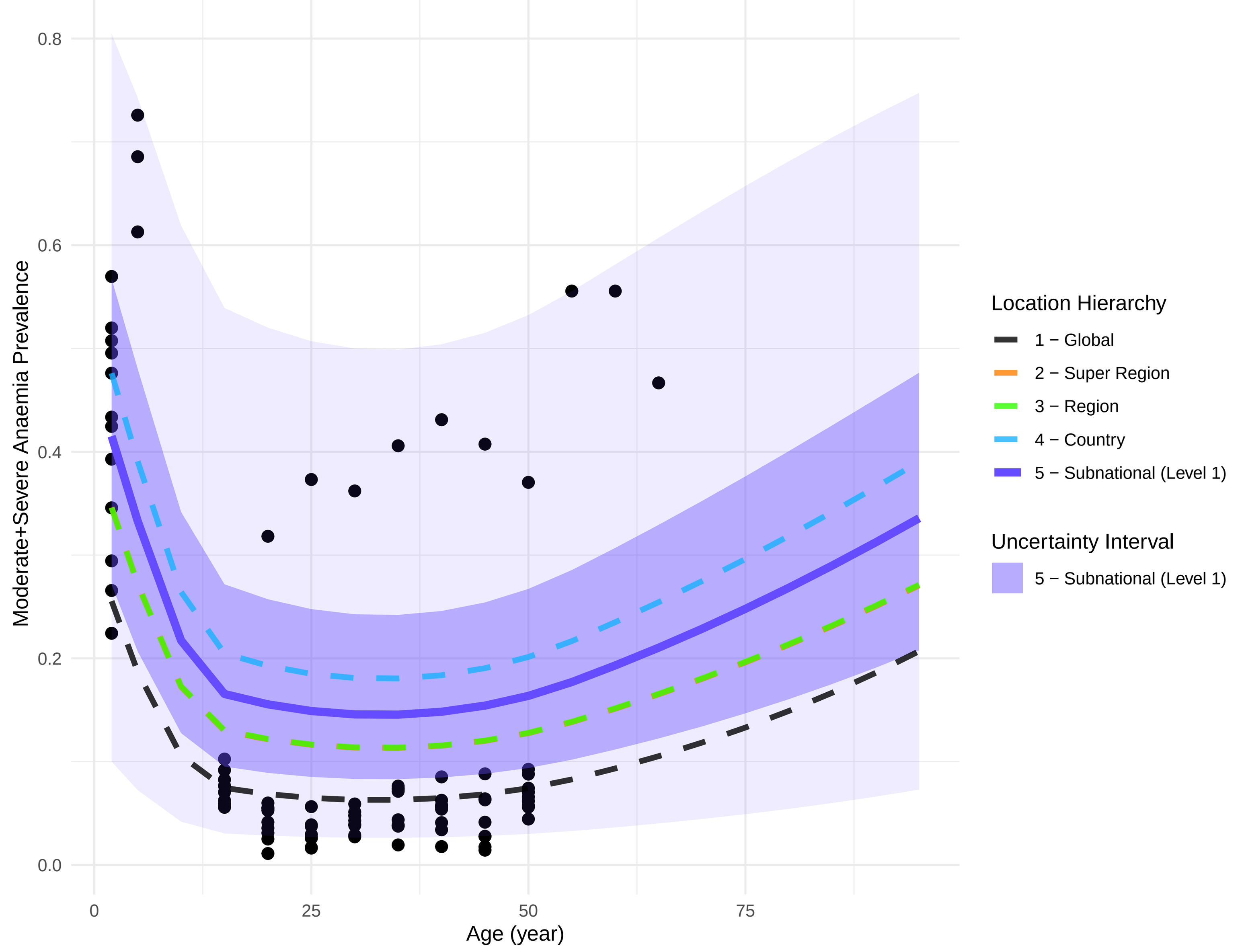

MR−BRT Age/Sex Fits − Males 2+ Years Old − Moderate+Severe Anaemia Prevalence − Gujarat, Rural
WHO−2001 Adjustment Method

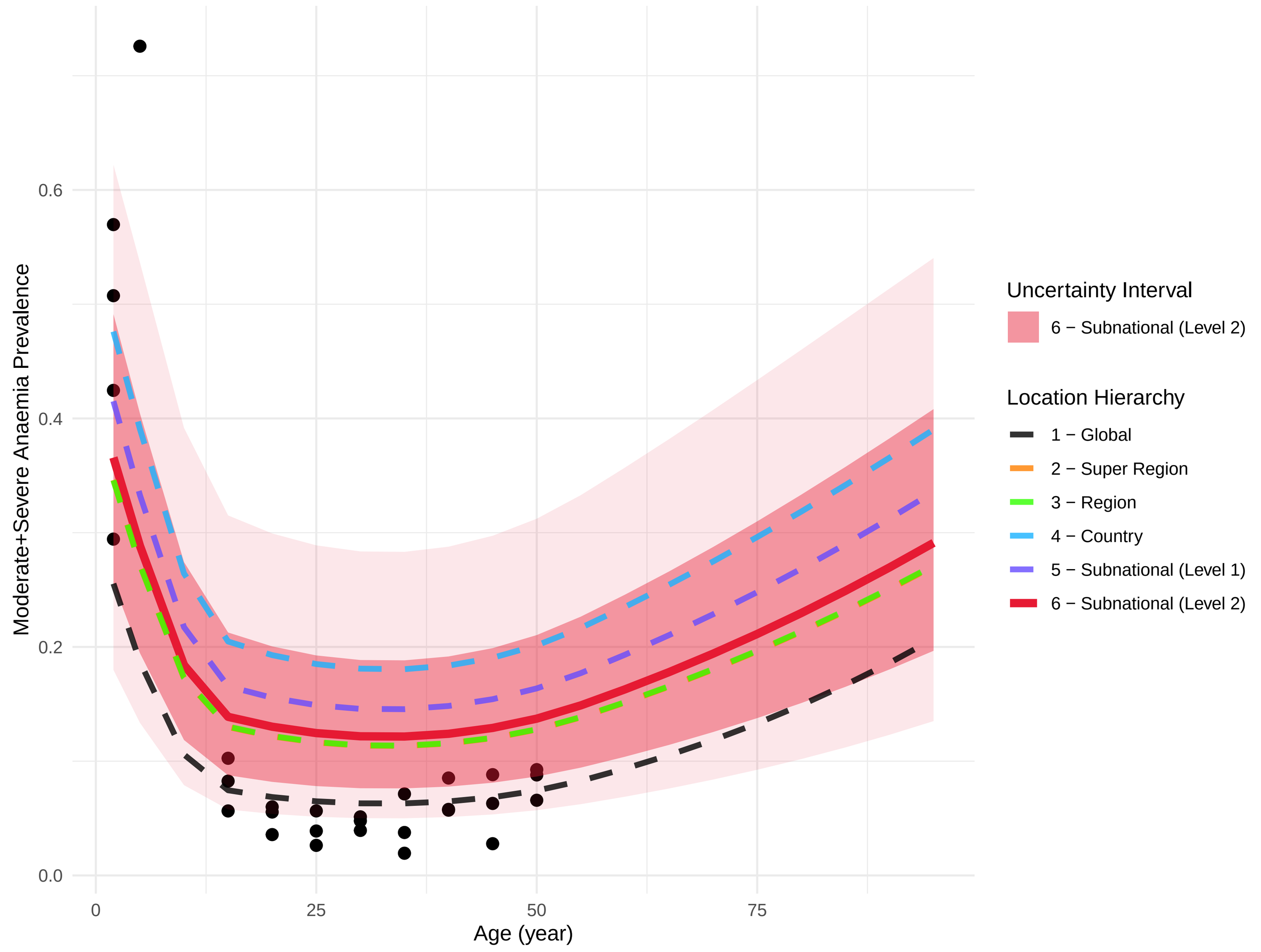

MR−BRT Age/Sex Fits − Males 2+ Years Old − Moderate+Severe Anaemia Prevalence − Gujarat, Urban
WHO−2001 Adjustment Method

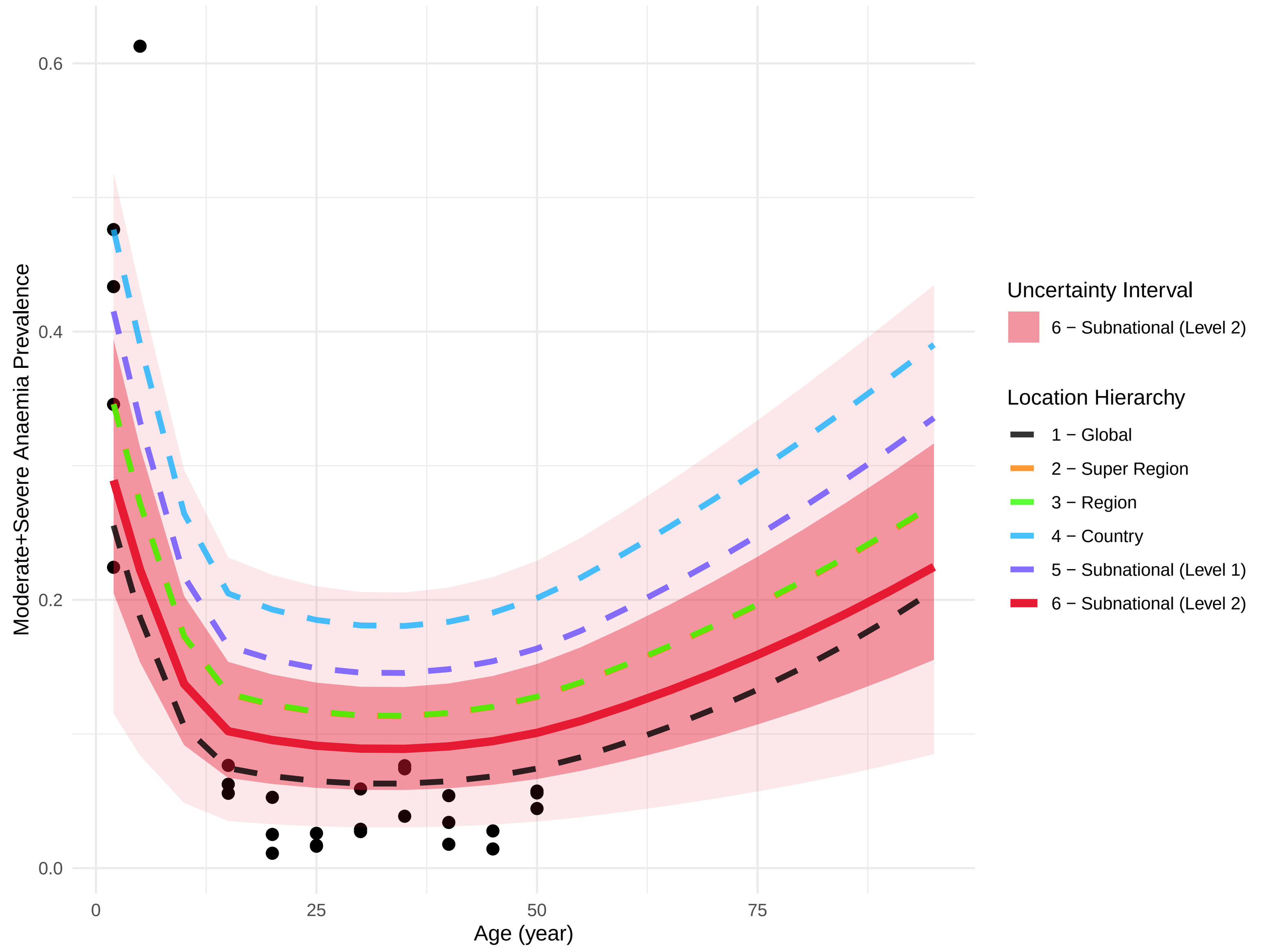

MR−BRT Age/Sex Fits − Males 2+ Years Old − Moderate+Severe Anaemia Prevalence − Haryana
WHO−2001 Adjustment Method

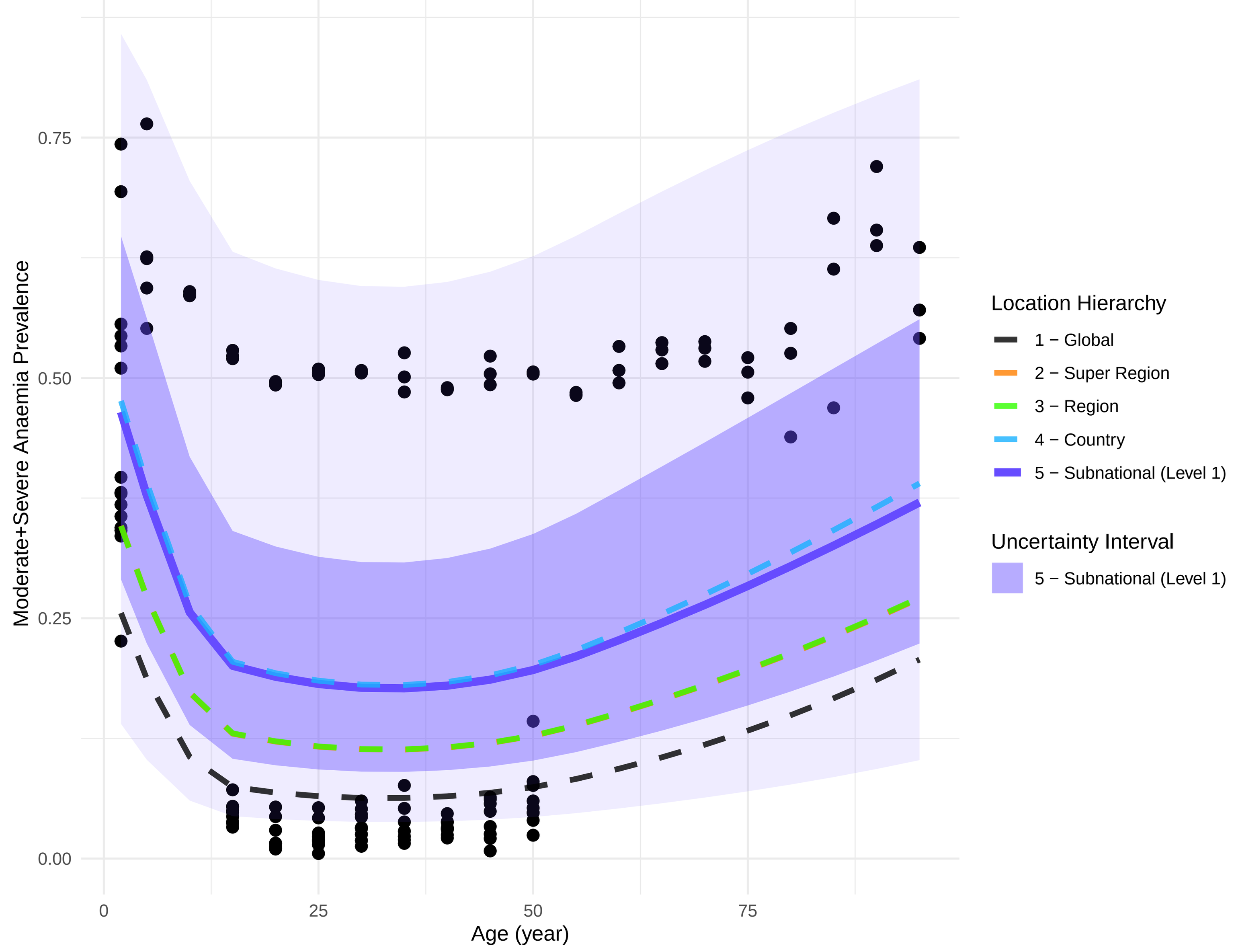

MR−BRT Age/Sex Fits − Males 2+ Years Old − Moderate+Severe Anaemia Prevalence − Haryana, Rural
WHO−2001 Adjustment Method

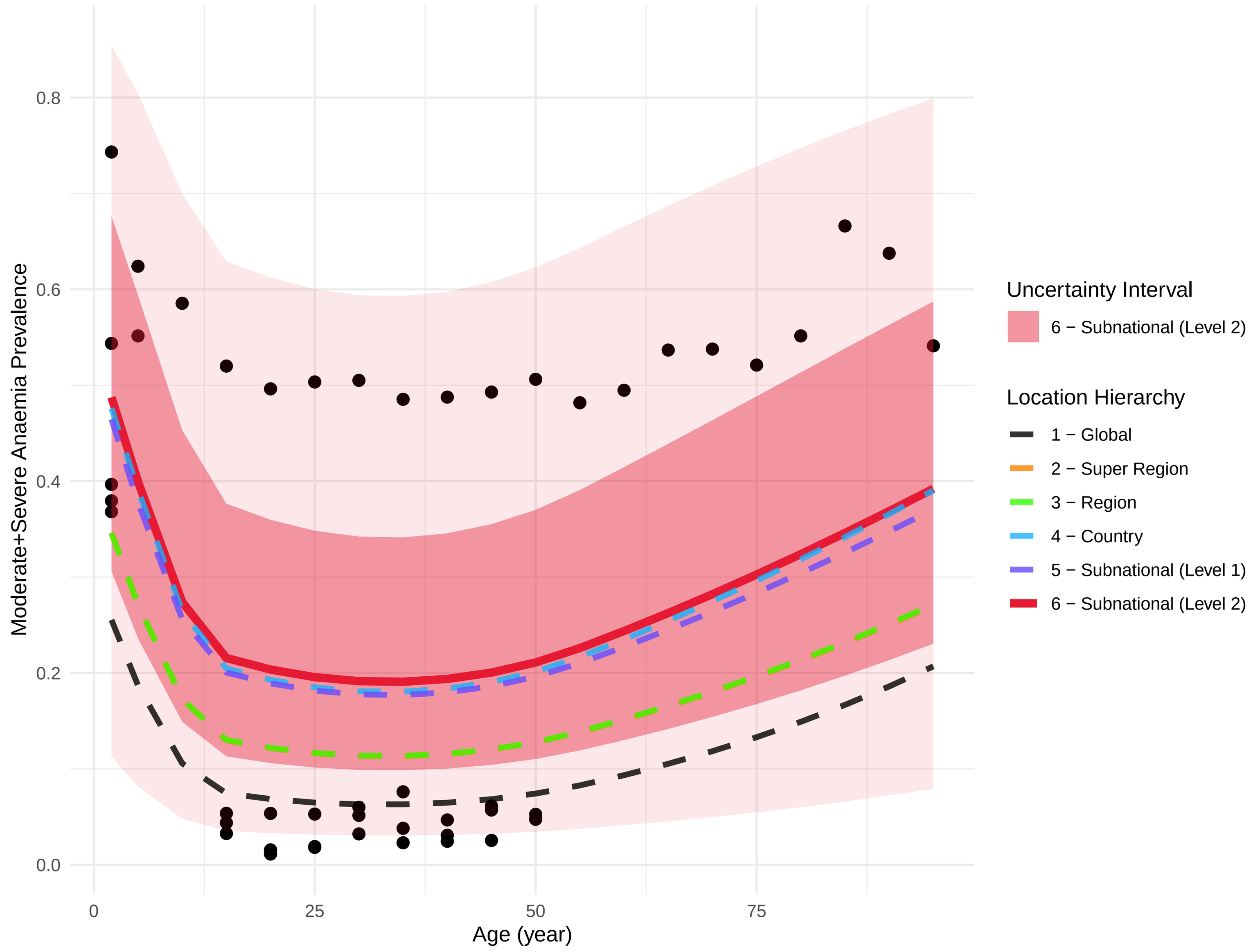

MR−BRT Age/Sex Fits − Males 2+ Years Old − Moderate+Severe Anaemia Prevalence − Haryana, Urban
WHO−2001 Adjustment Method

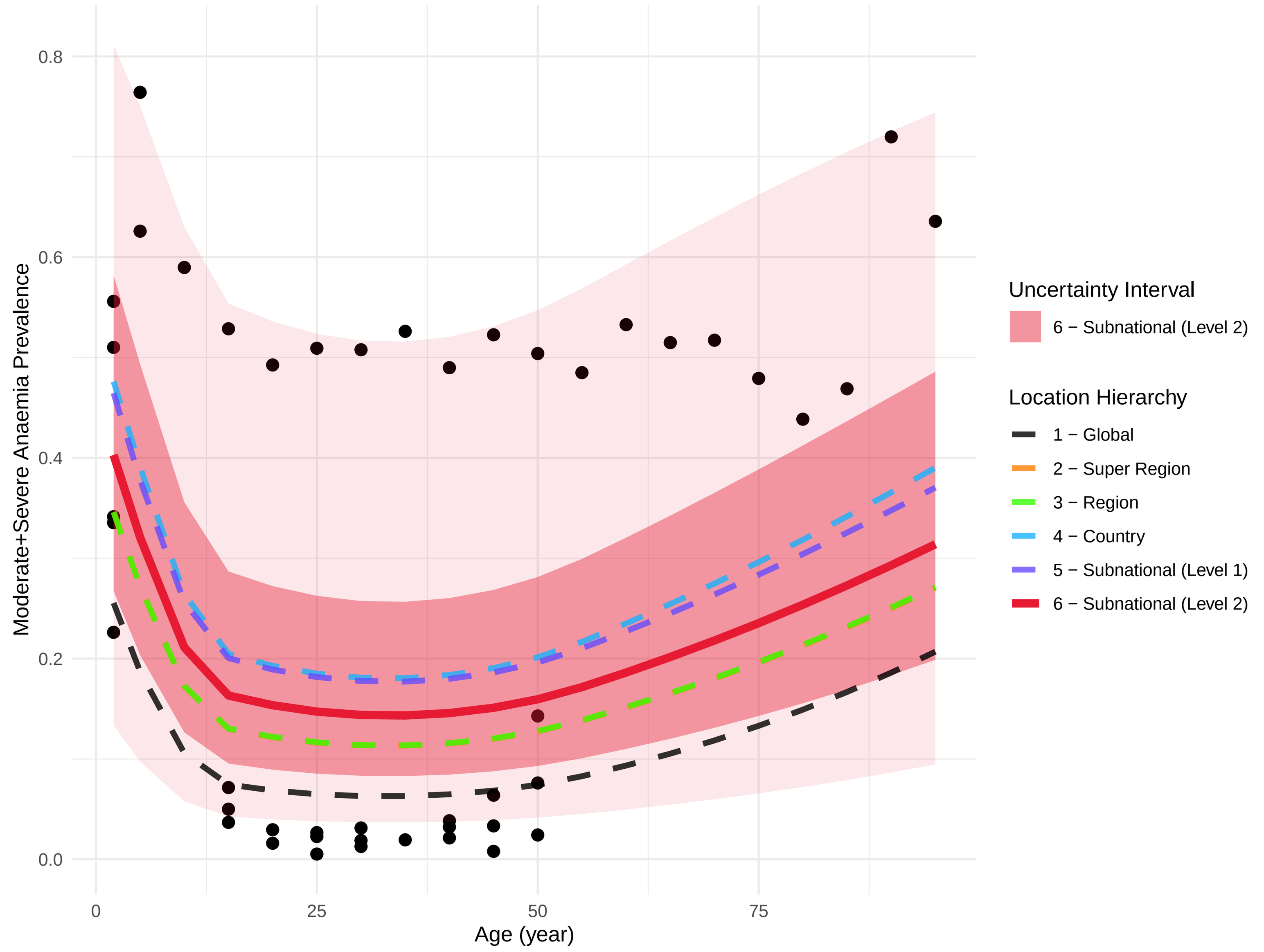

MR−BRT Age/Sex Fits − Males 2+ Years Old − Moderate+Severe Anaemia Prevalence − Himachal Pradesh
WHO−2001 Adjustment Method

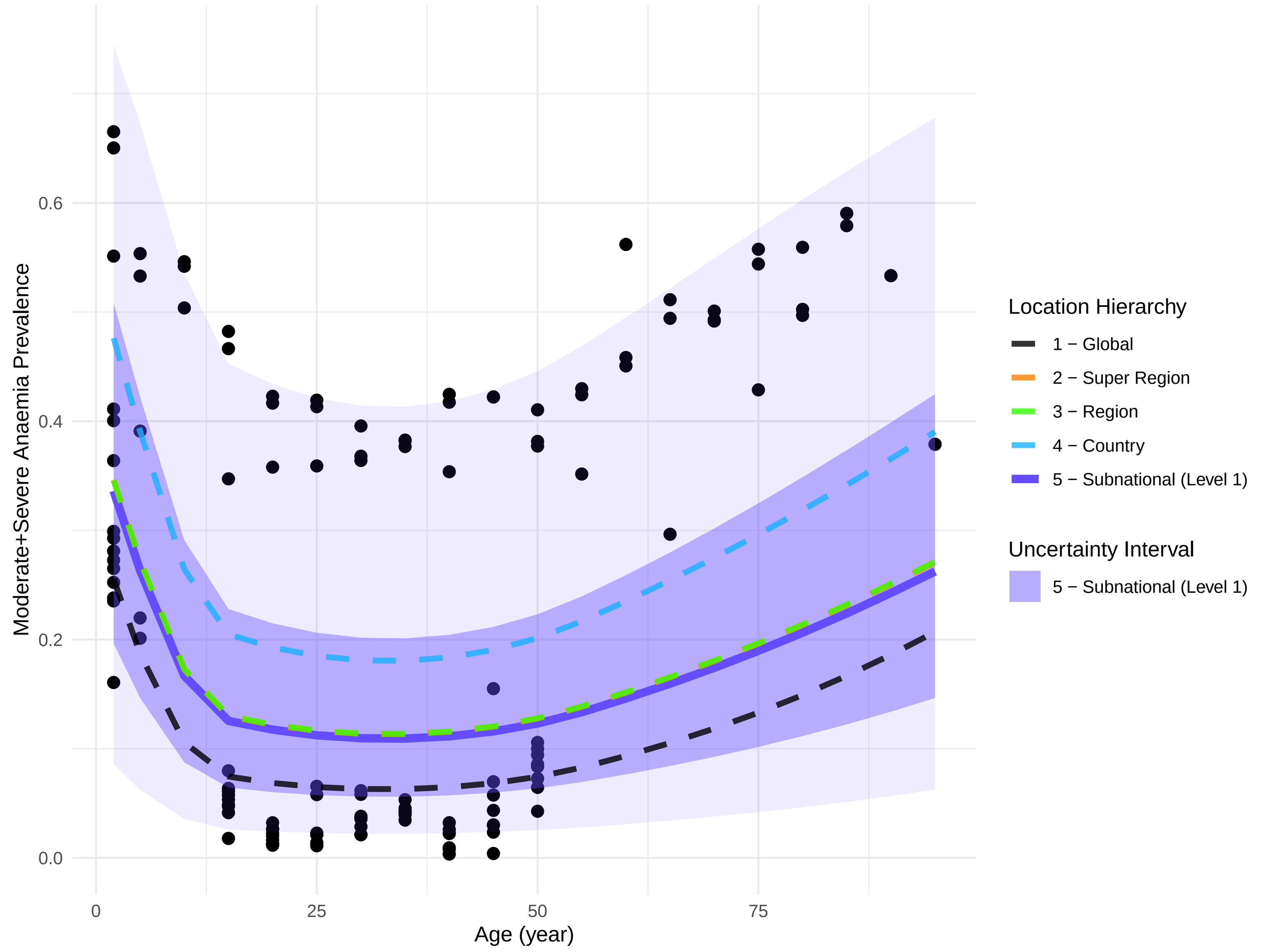

MR−BRT Age/Sex Fits − Males 2+ Years Old − Moderate+Severe Anaemia Prevalence − Himachal Pradesh, Rural
WHO−2001 Adjustment Method

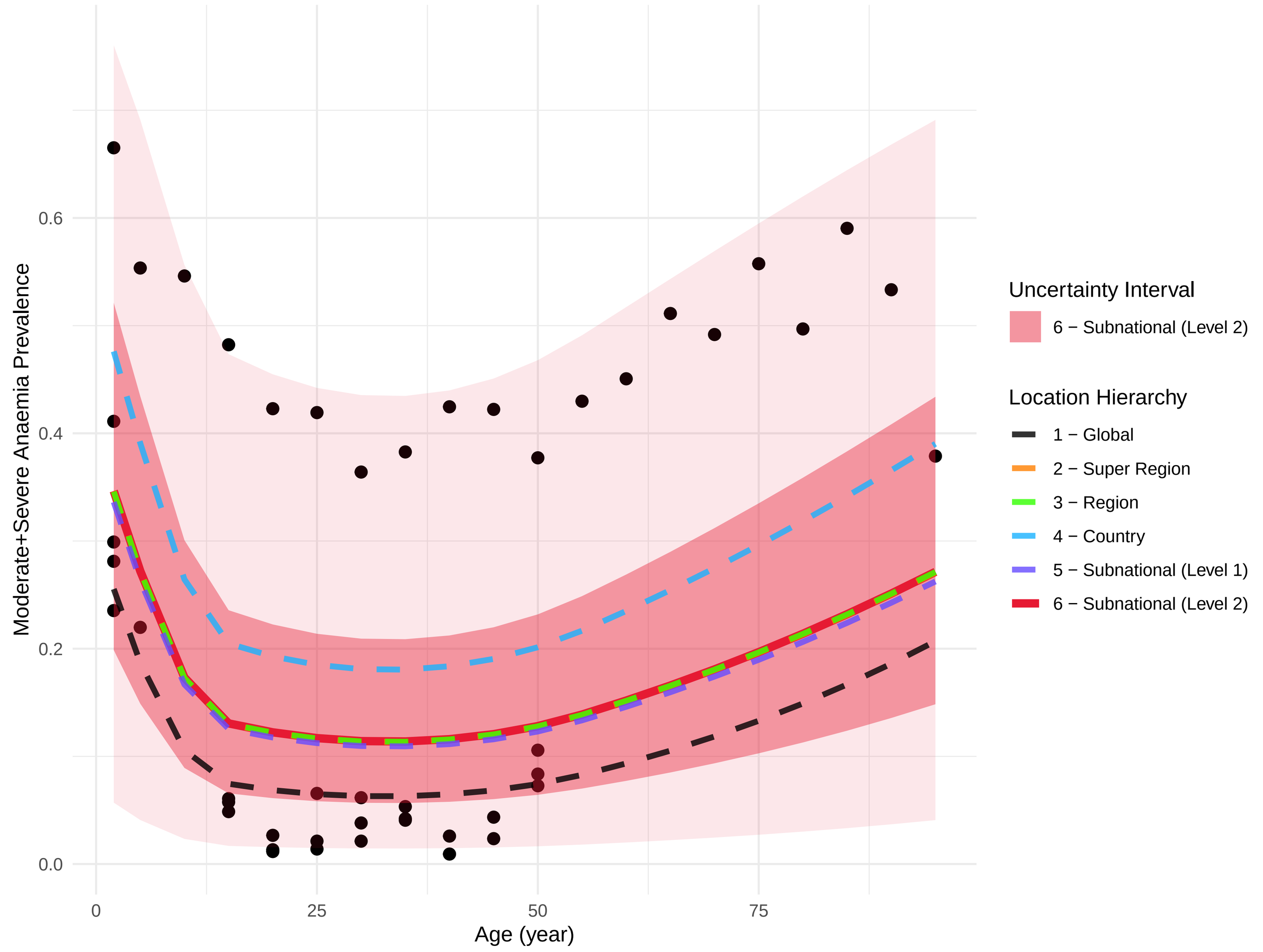

MR−BRT Age/Sex Fits − Males 2+ Years Old − Moderate+Severe Anaemia Prevalence − Himachal Pradesh, Urban
WHO−2001 Adjustment Method

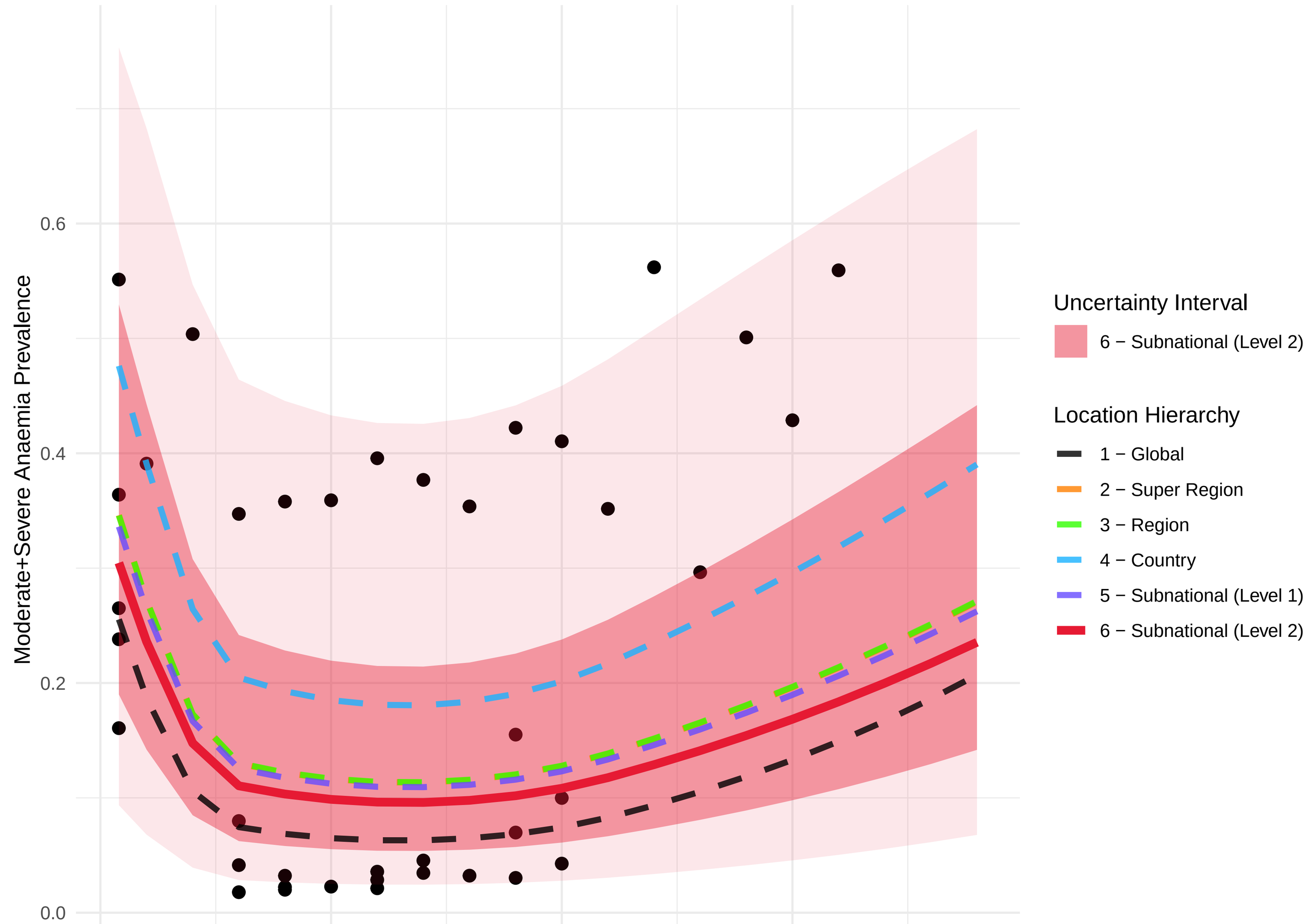

MR−BRT Age/Sex Fits − Males 2+ Years Old − Moderate+Severe Anaemia Prevalence − Jammu & Kashmir and Ladakh
WHO−2001 Adjustment Method

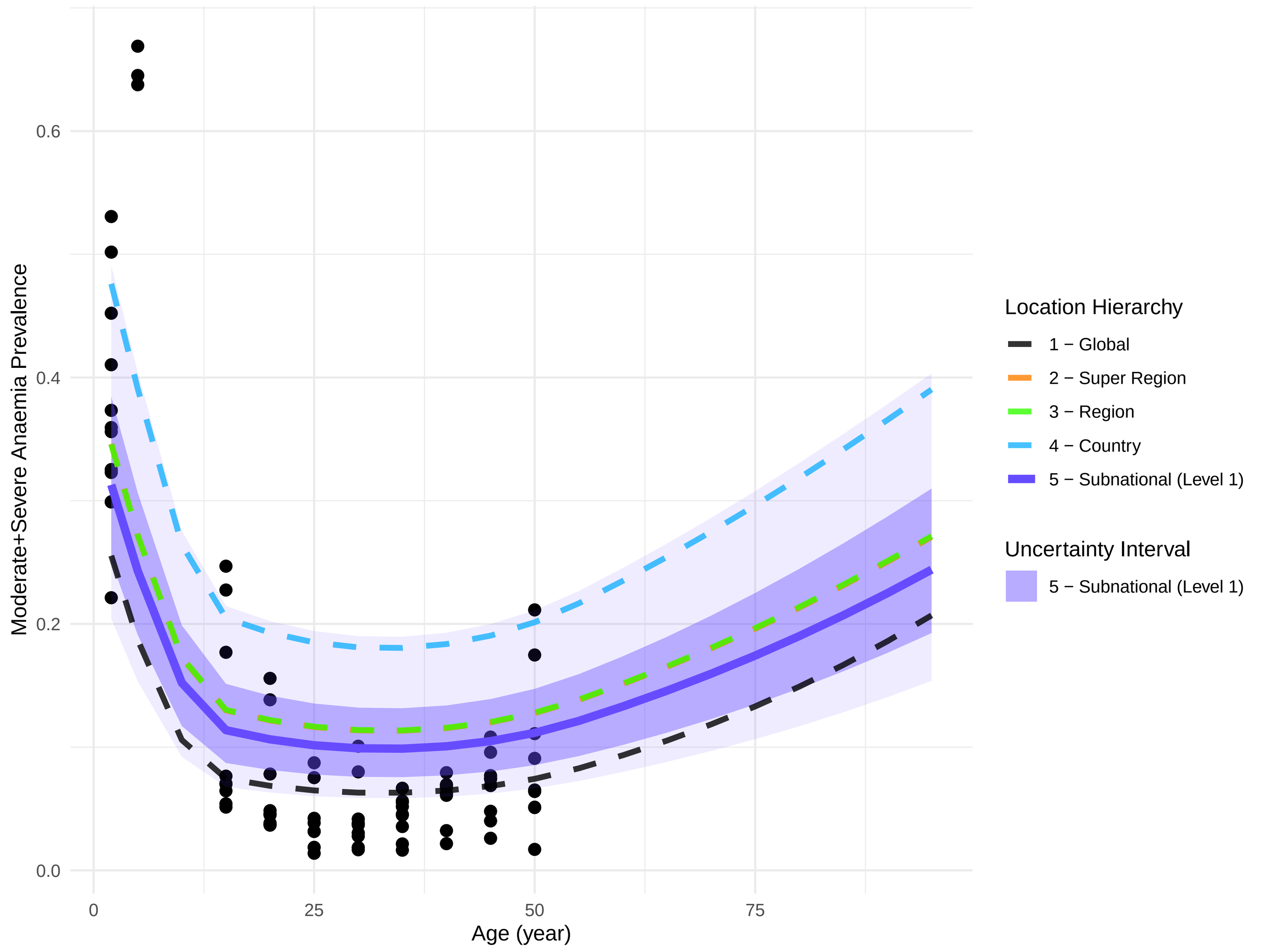

MR−BRT Age/Sex Fits − Males 2+ Years Old − Moderate+Severe Anaemia Prevalence − Jammu & Kashmir and Ladakh, Rural
WHO−2001 Adjustment Method

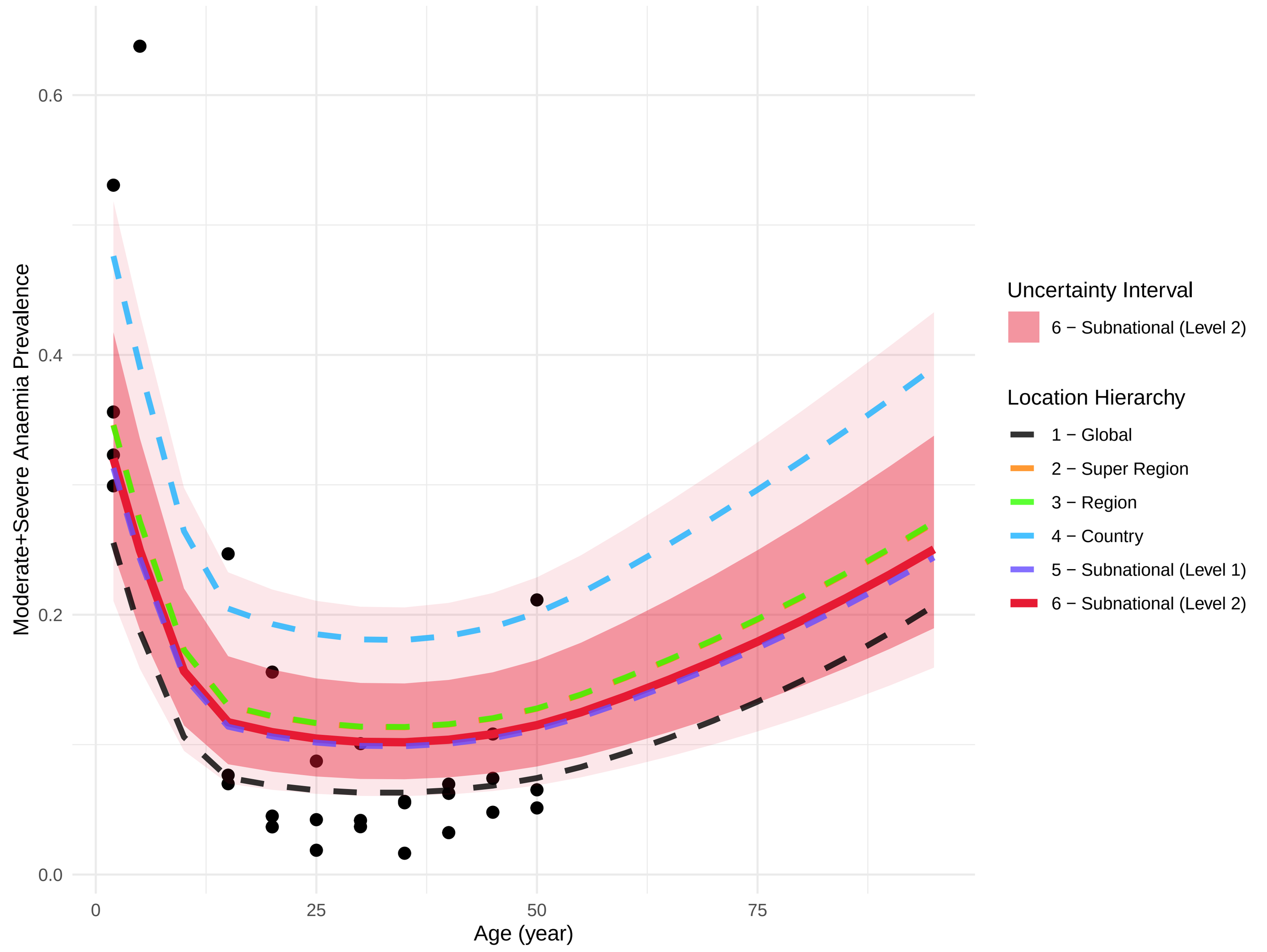

MR−BRT Age/Sex Fits − Males 2+ Years Old − Moderate+Severe Anaemia Prevalence − Jammu & Kashmir and Ladakh, Urban
WHO−2001 Adjustment Method

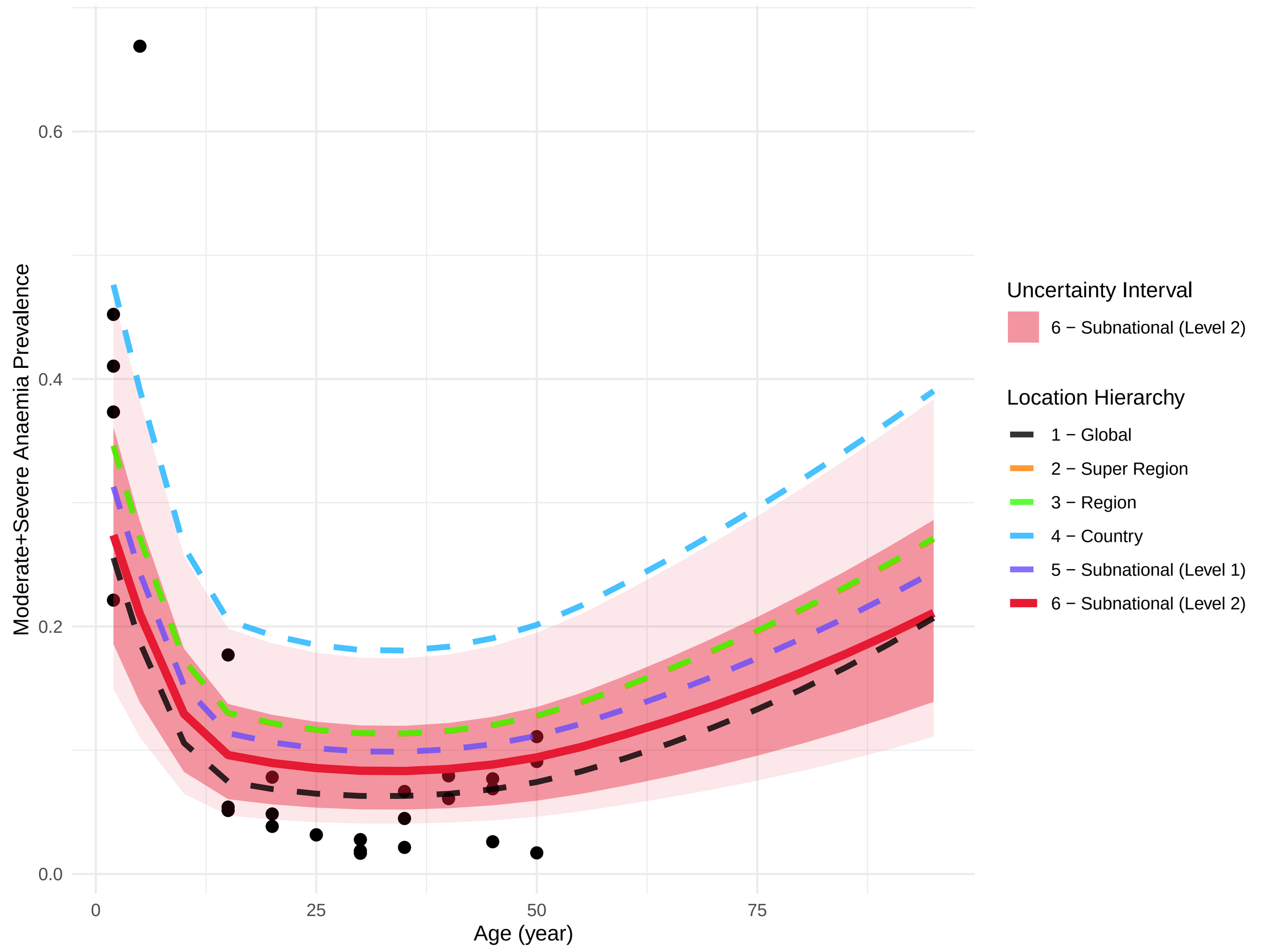

MR−BRT Age/Sex Fits − Males 2+ Years Old − Moderate+Severe Anaemia Prevalence − Jharkhand
WHO−2001 Adjustment Method

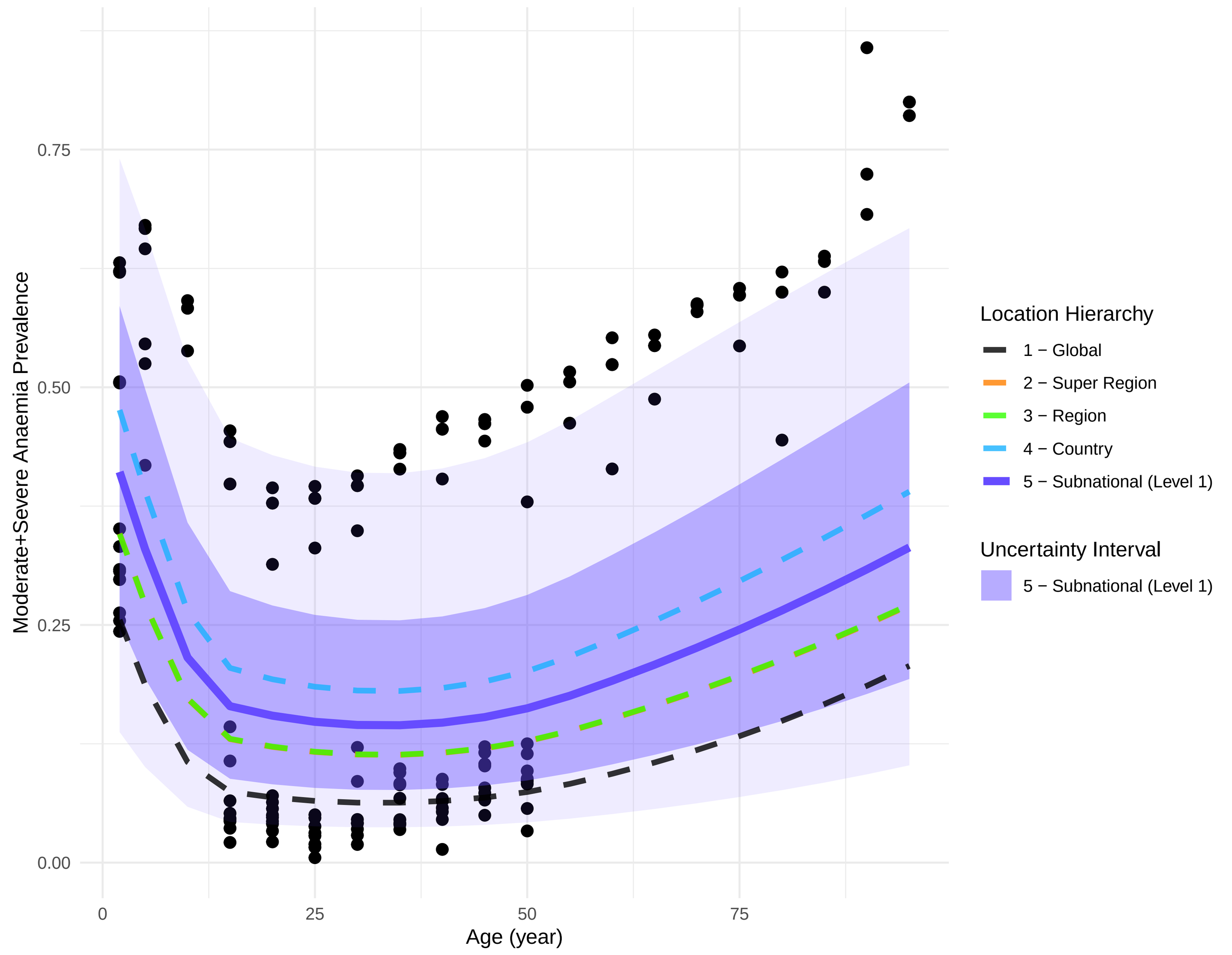

MR−BRT Age/Sex Fits − Males 2+ Years Old − Moderate+Severe Anaemia Prevalence − Jharkhand, Rural
WHO−2001 Adjustment Method

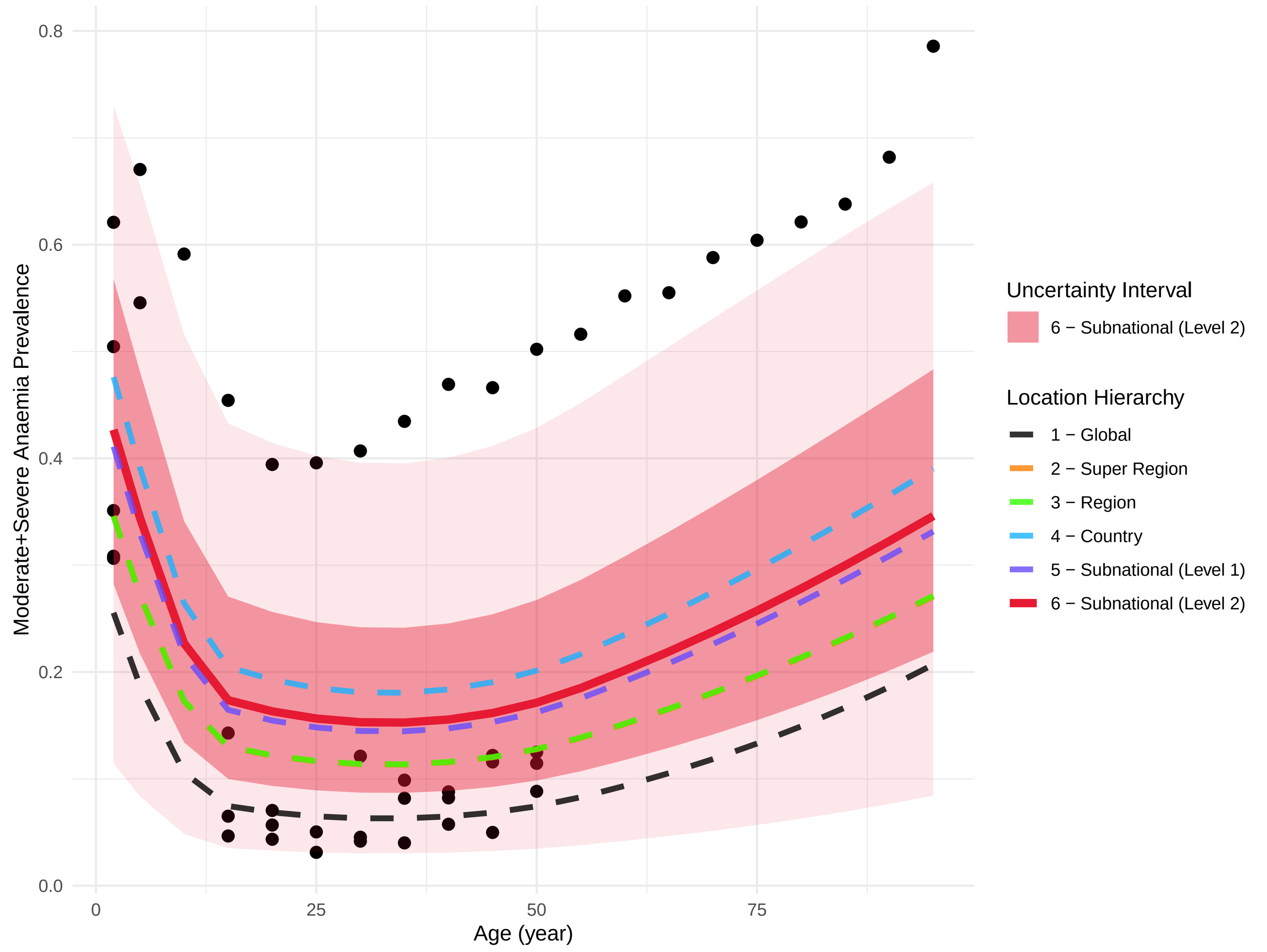

MR−BRT Age/Sex Fits − Males 2+ Years Old − Moderate+Severe Anaemia Prevalence − Jharkhand, Urban
WHO−2001 Adjustment Method

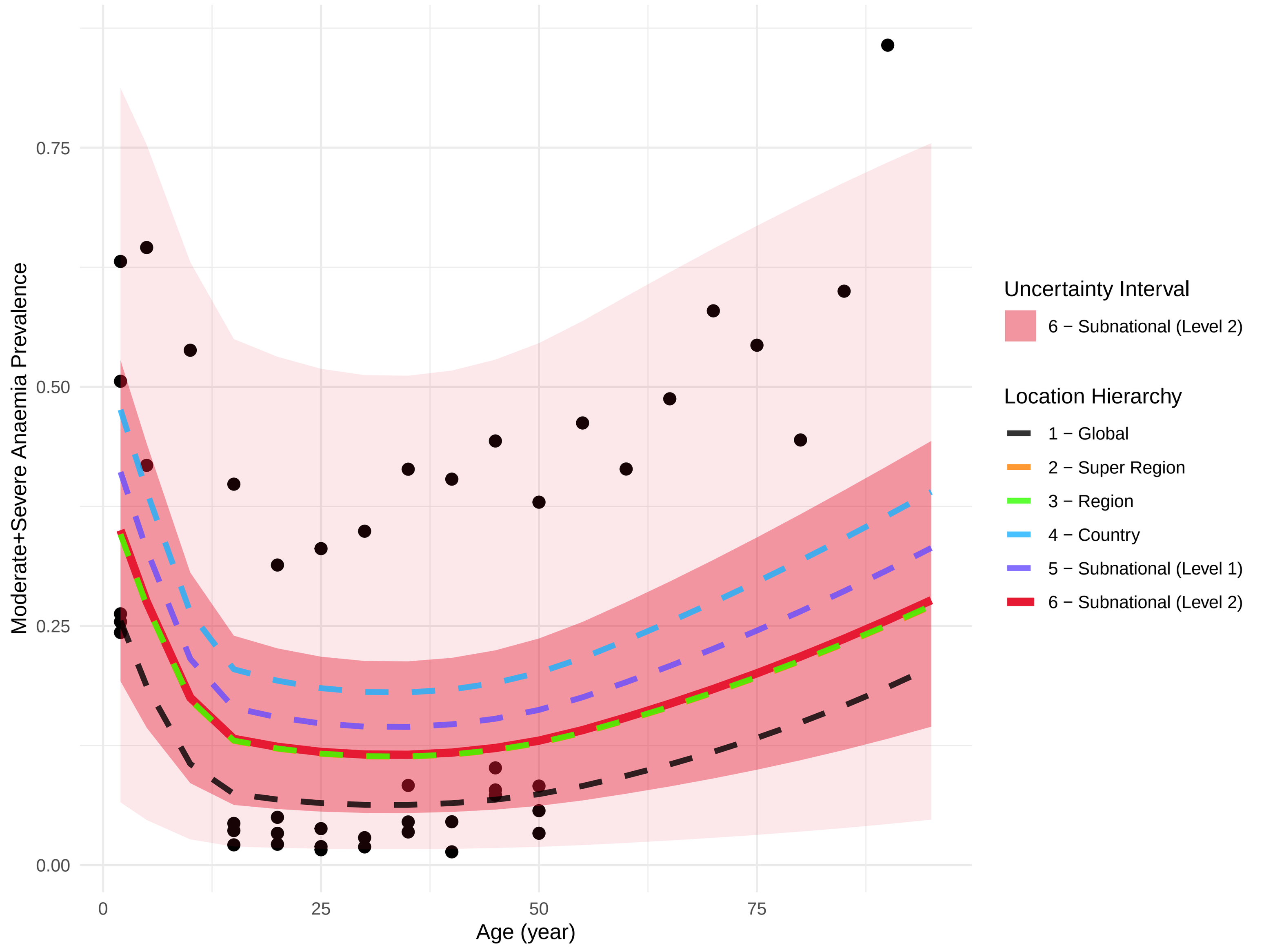

MR−BRT Age/Sex Fits − Males 2+ Years Old − Moderate+Severe Anaemia Prevalence − Karnataka
WHO−2001 Adjustment Method

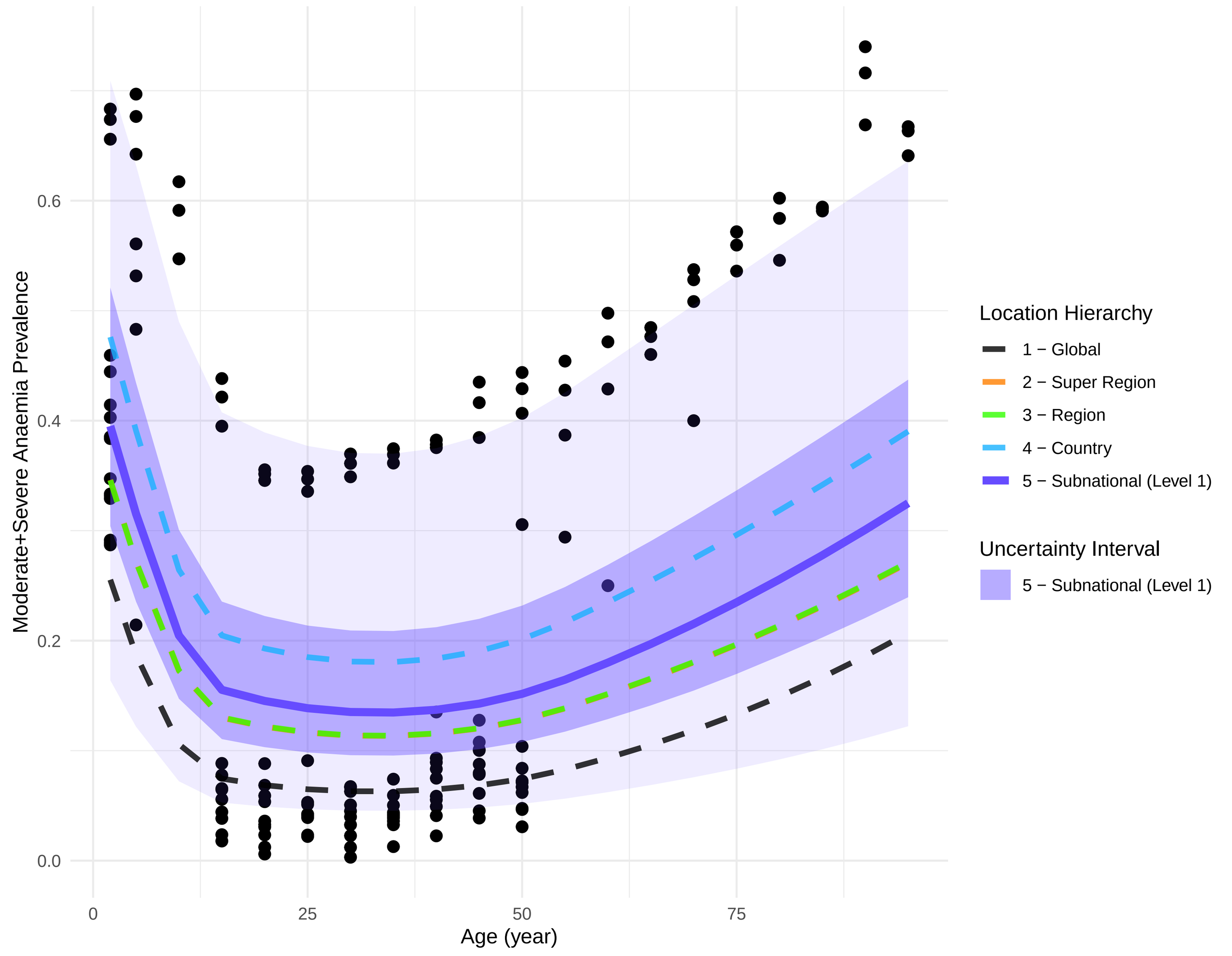

MR−BRT Age/Sex Fits − Males 2+ Years Old − Moderate+Severe Anaemia Prevalence − Karnataka, Rural
WHO−2001 Adjustment Method

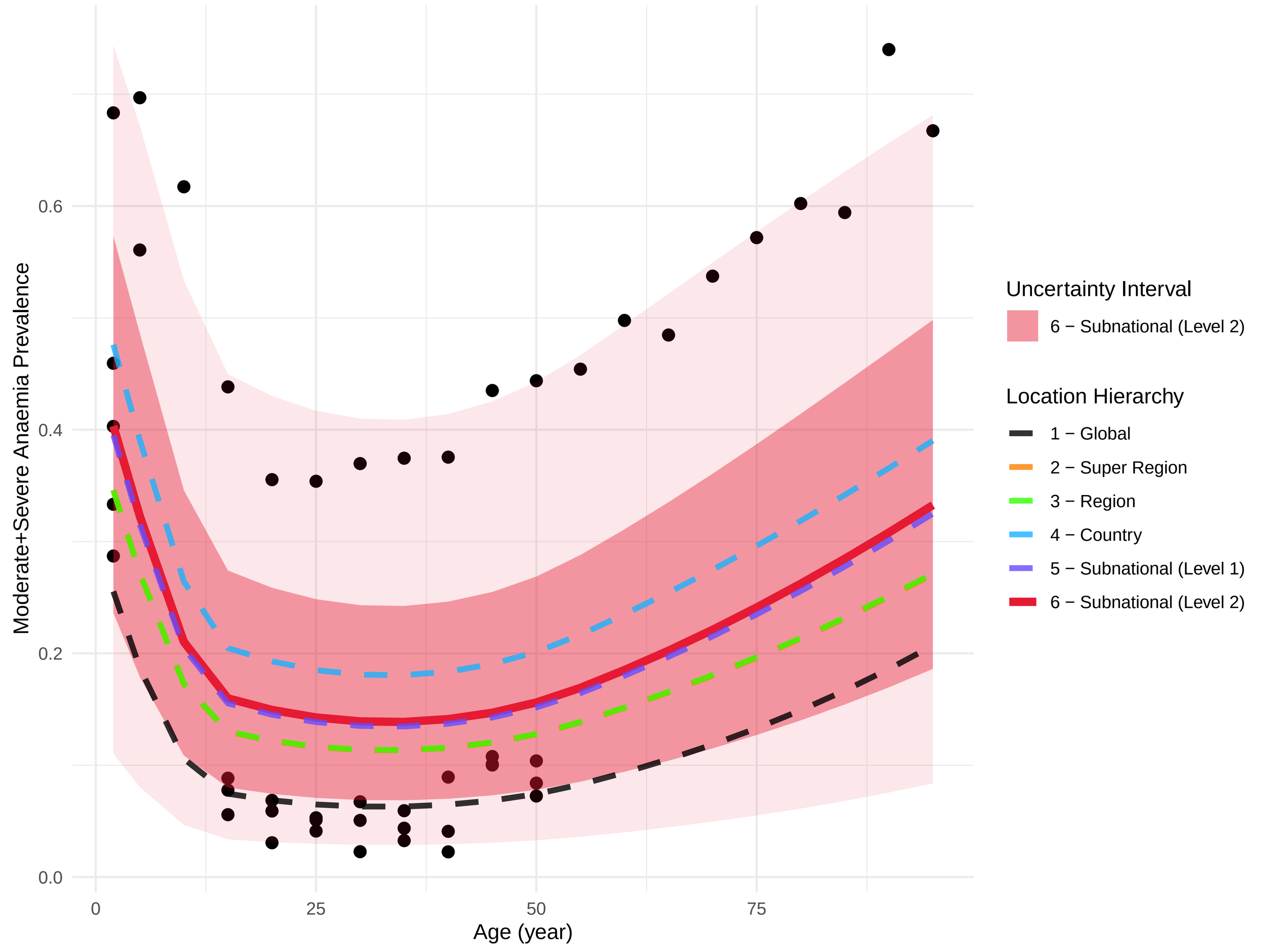

MR−BRT Age/Sex Fits − Males 2+ Years Old − Moderate+Severe Anaemia Prevalence − Karnataka, Urban
WHO−2001 Adjustment Method

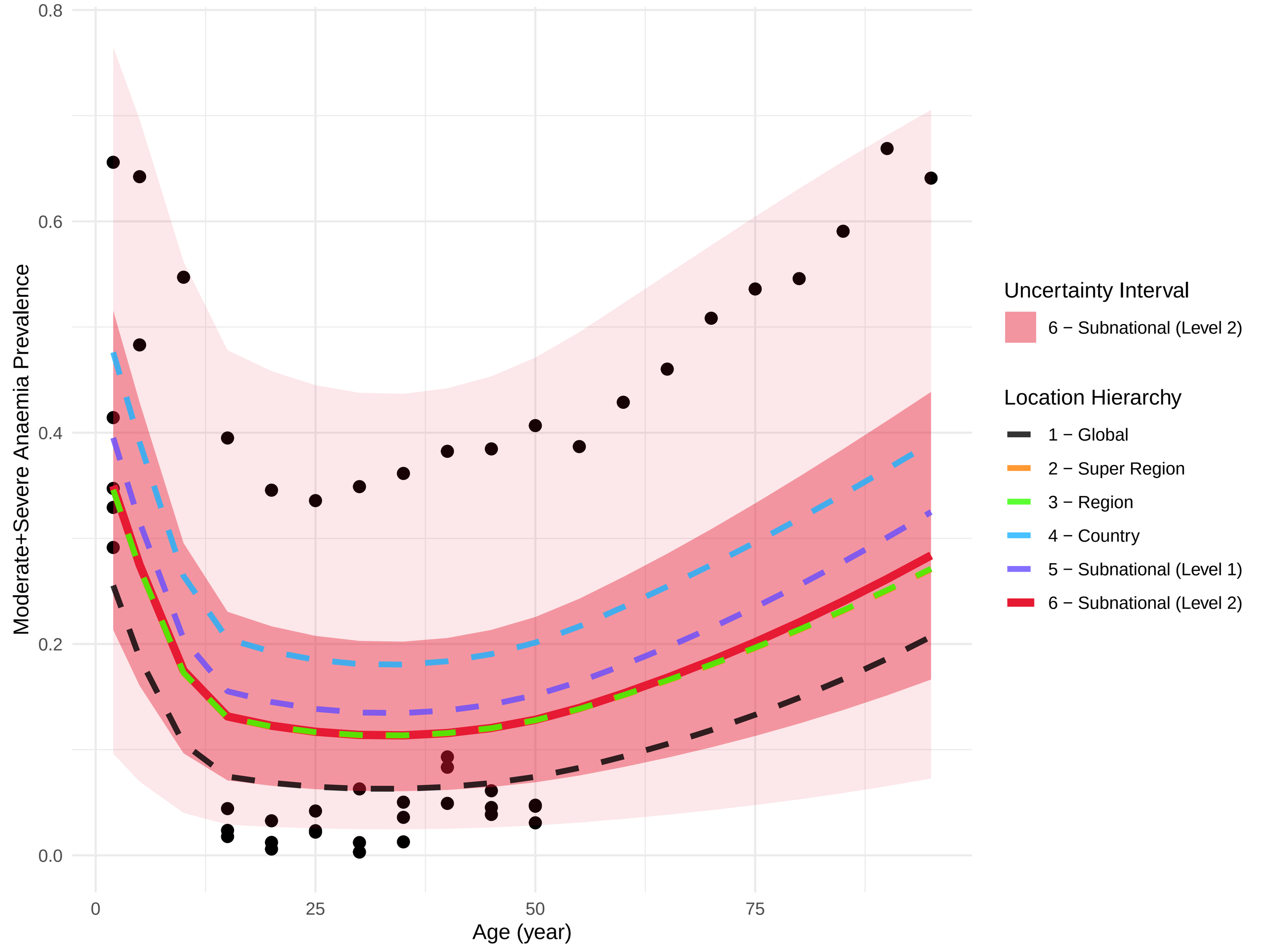

MR−BRT Age/Sex Fits − Males 2+ Years Old − Moderate+Severe Anaemia Prevalence − Kerala
WHO−2001 Adjustment Method

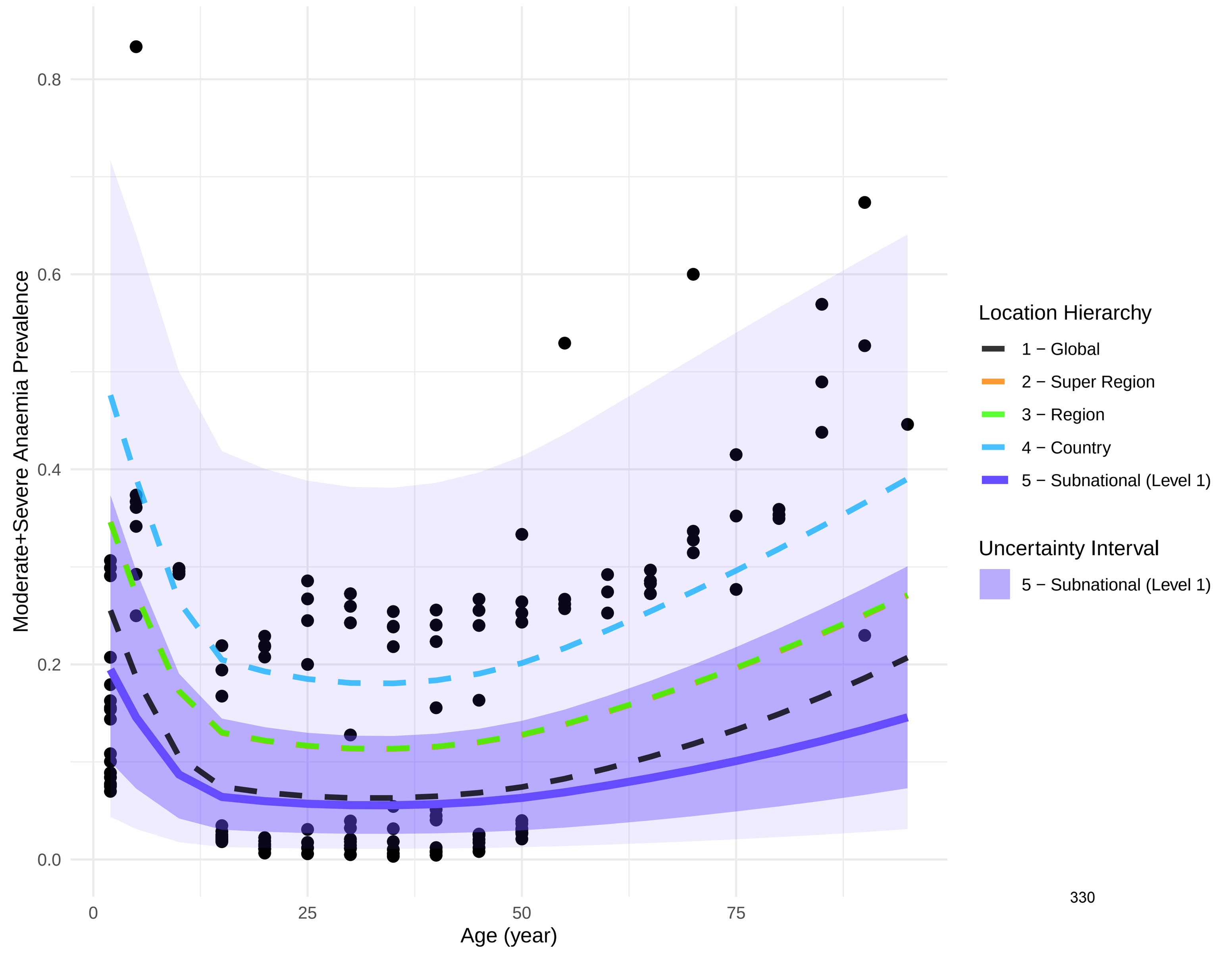

MR−BRT Age/Sex Fits − Males 2+ Years Old − Moderate+Severe Anaemia Prevalence − Kerala, Rural
WHO−2001 Adjustment Method

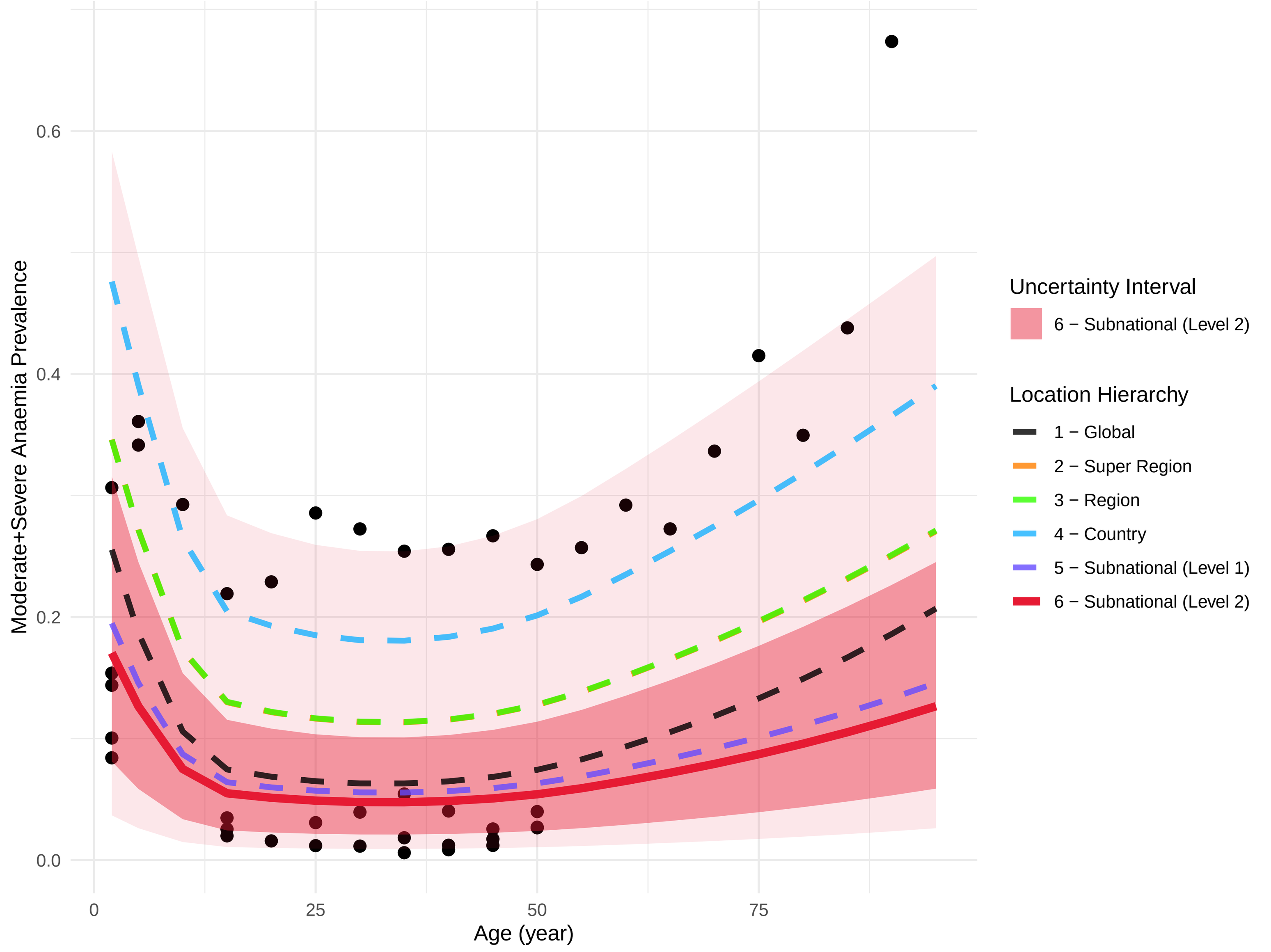

MR−BRT Age/Sex Fits − Males 2+ Years Old − Moderate+Severe Anaemia Prevalence − Kerala, Urban
WHO−2001 Adjustment Method

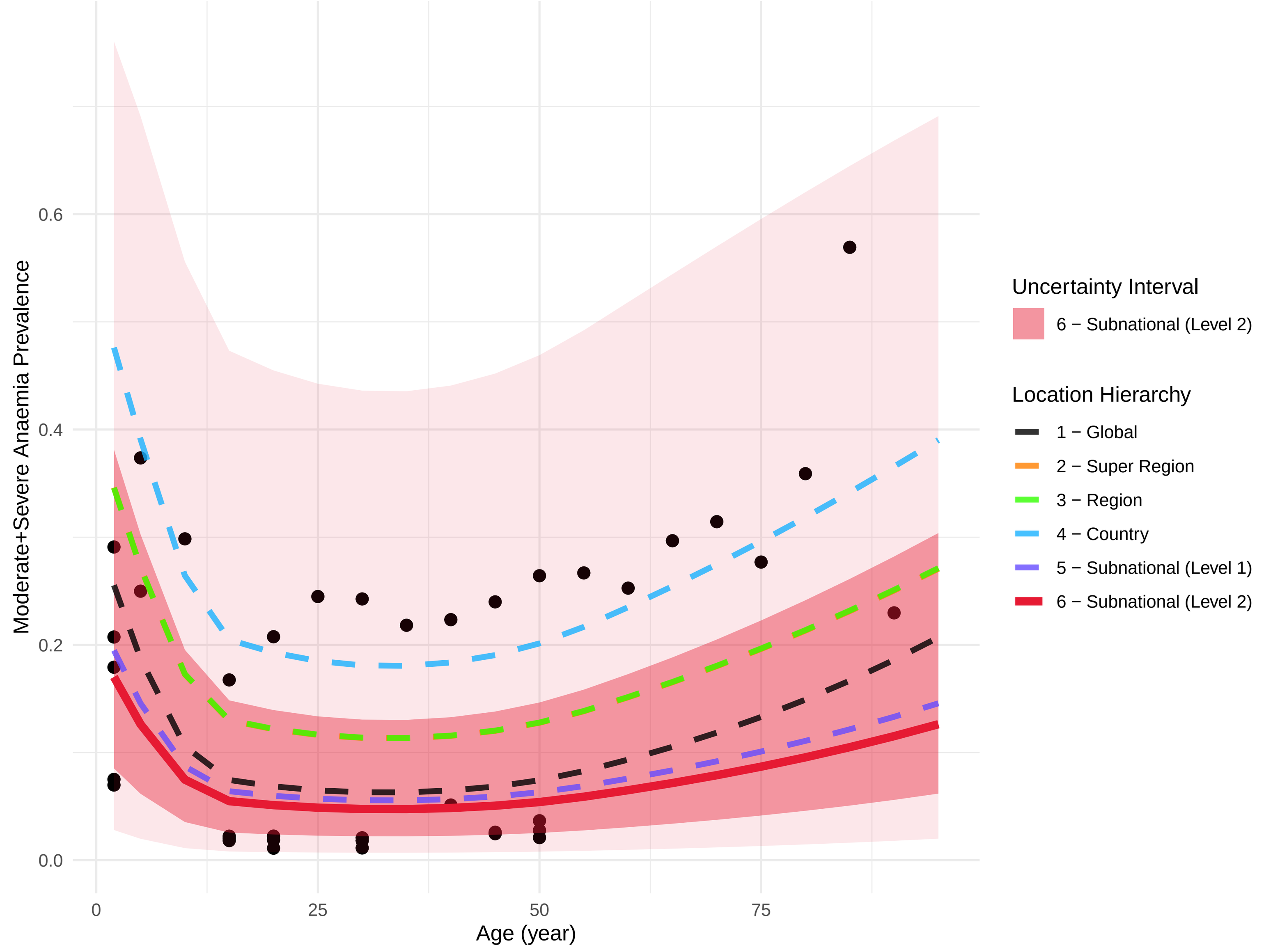

MR−BRT Age/Sex Fits − Males 2+ Years Old − Moderate+Severe Anaemia Prevalence − Madhya Pradesh
WHO−2001 Adjustment Method

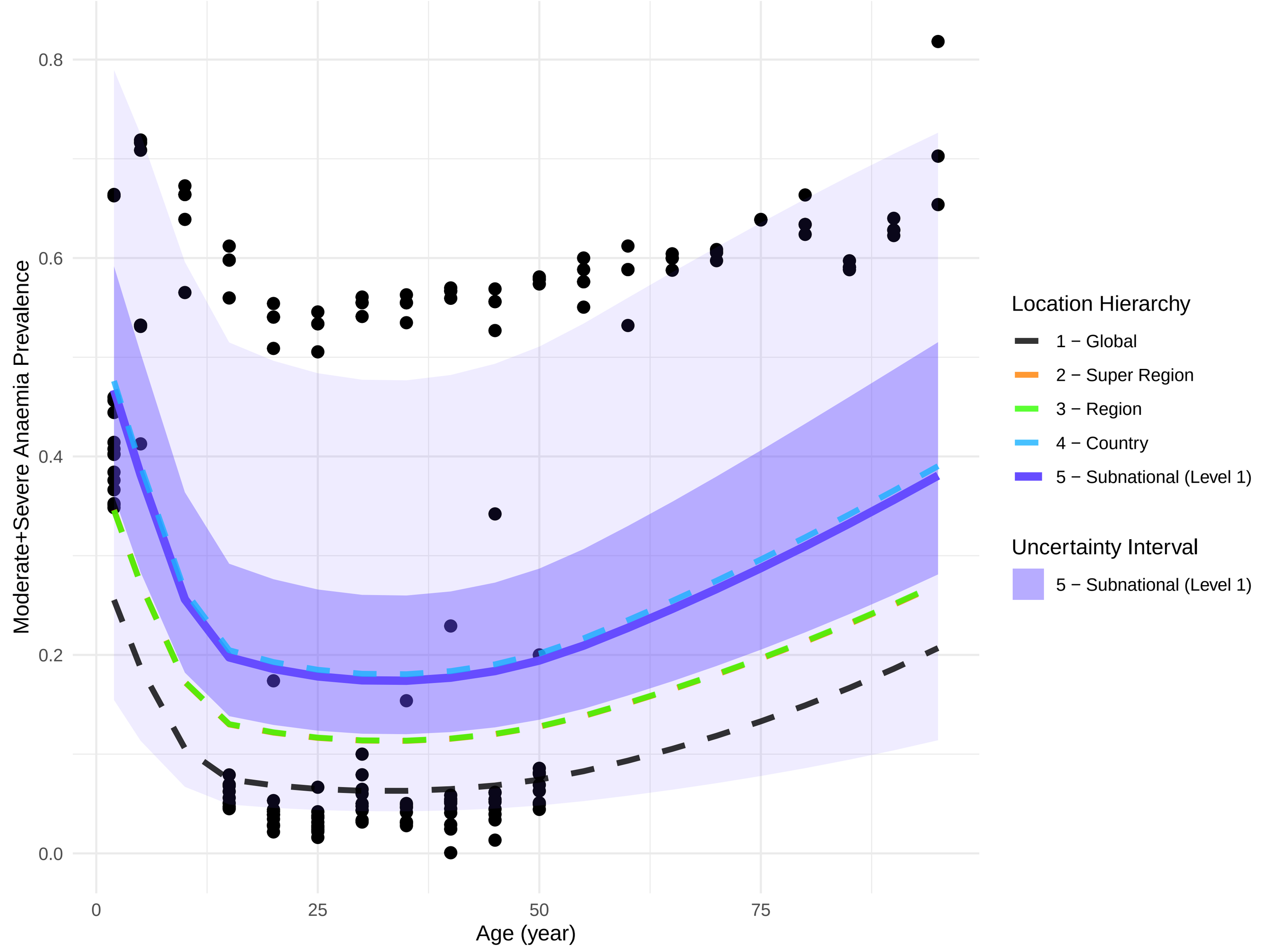

MR−BRT Age/Sex Fits − Males 2+ Years Old − Moderate+Severe Anaemia Prevalence − Madhya Pradesh, Rural
WHO−2001 Adjustment Method

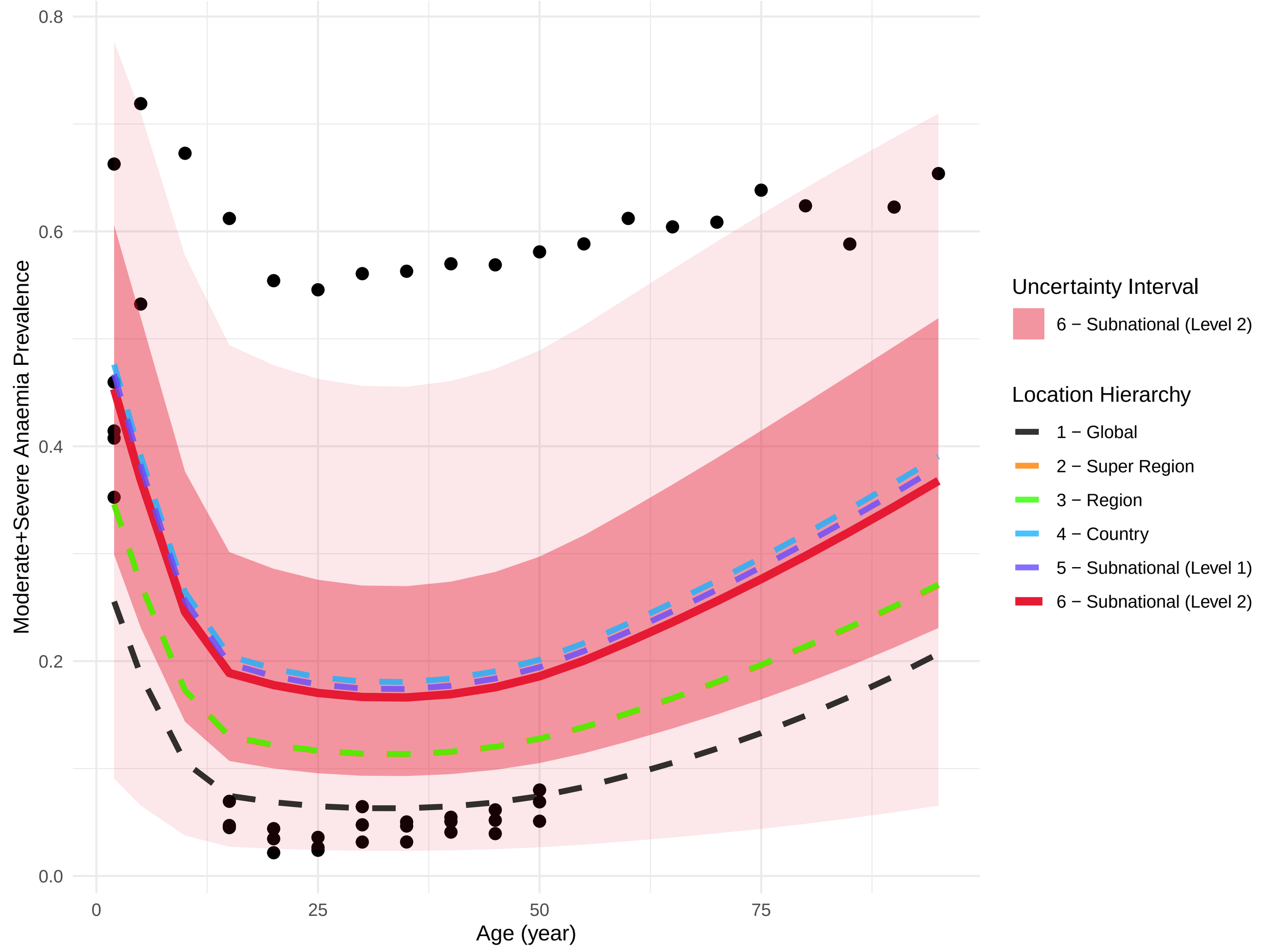

MR−BRT Age/Sex Fits − Males 2+ Years Old − Moderate+Severe Anaemia Prevalence − Madhya Pradesh, Urban
WHO−2001 Adjustment Method

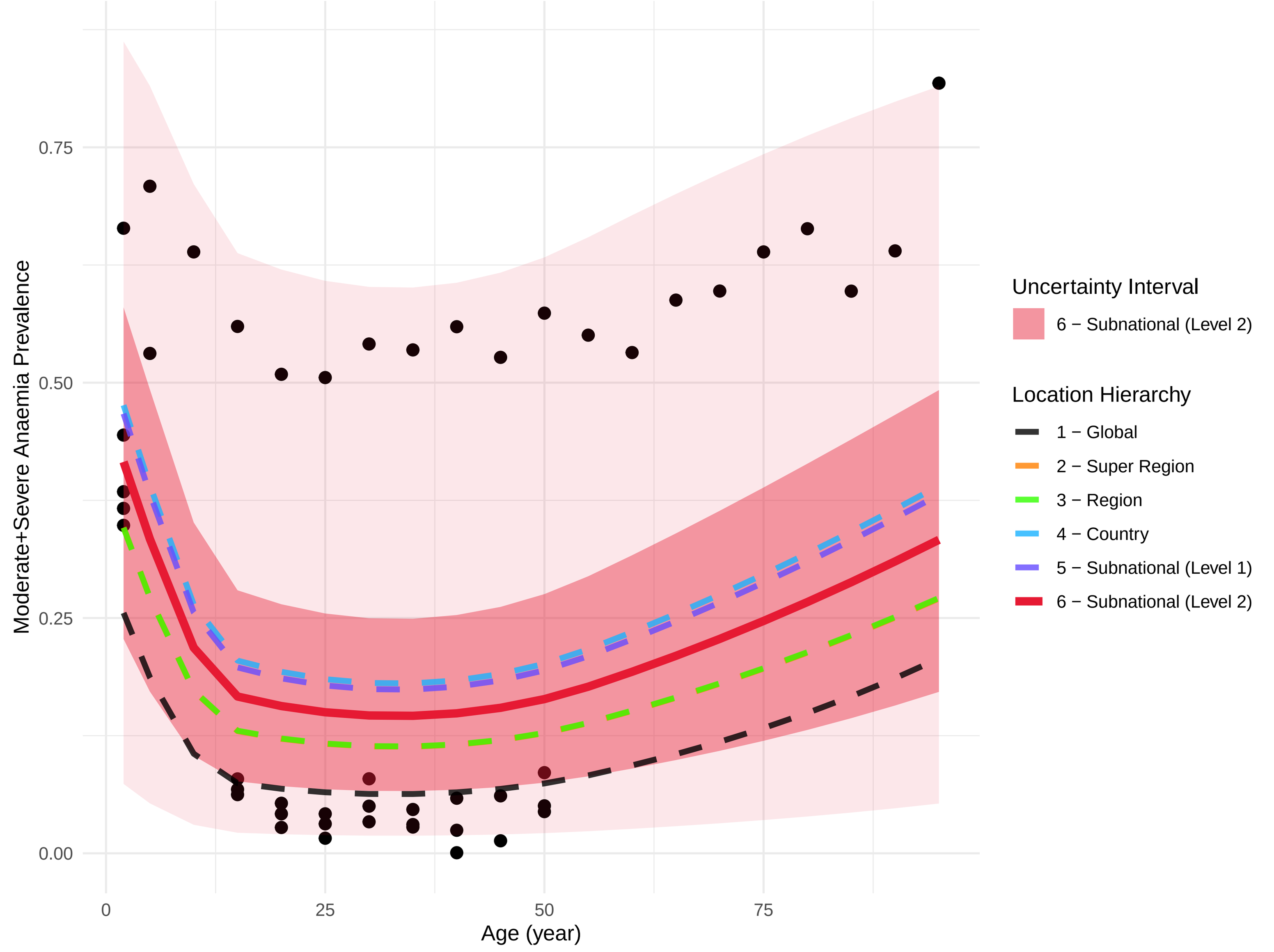

MR−BRT Age/Sex Fits − Males 2+ Years Old − Moderate+Severe Anaemia Prevalence − Maharashtra
WHO−2001 Adjustment Method

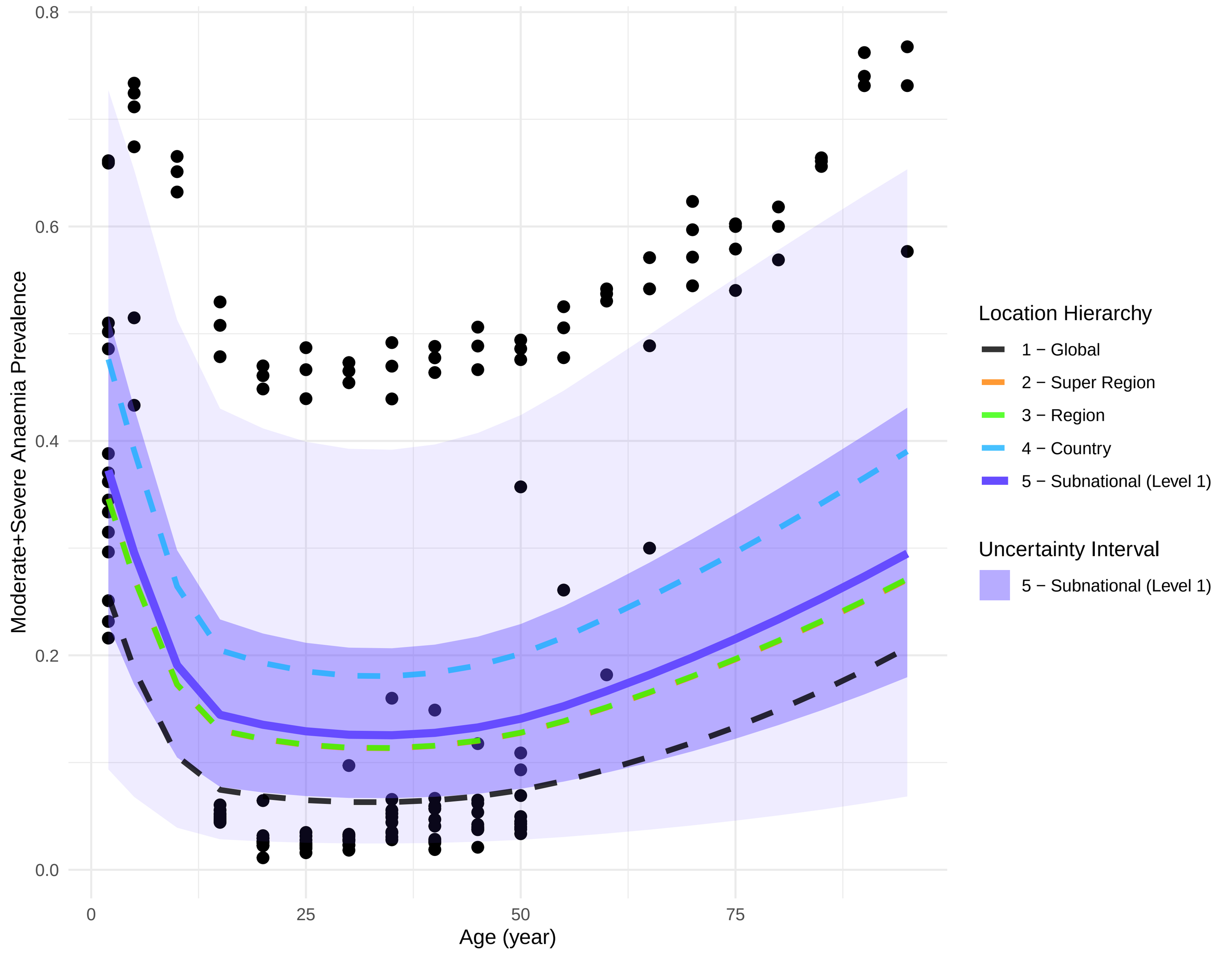

MR−BRT Age/Sex Fits − Males 2+ Years Old − Moderate+Severe Anaemia Prevalence − Maharashtra, Rural
WHO−2001 Adjustment Method

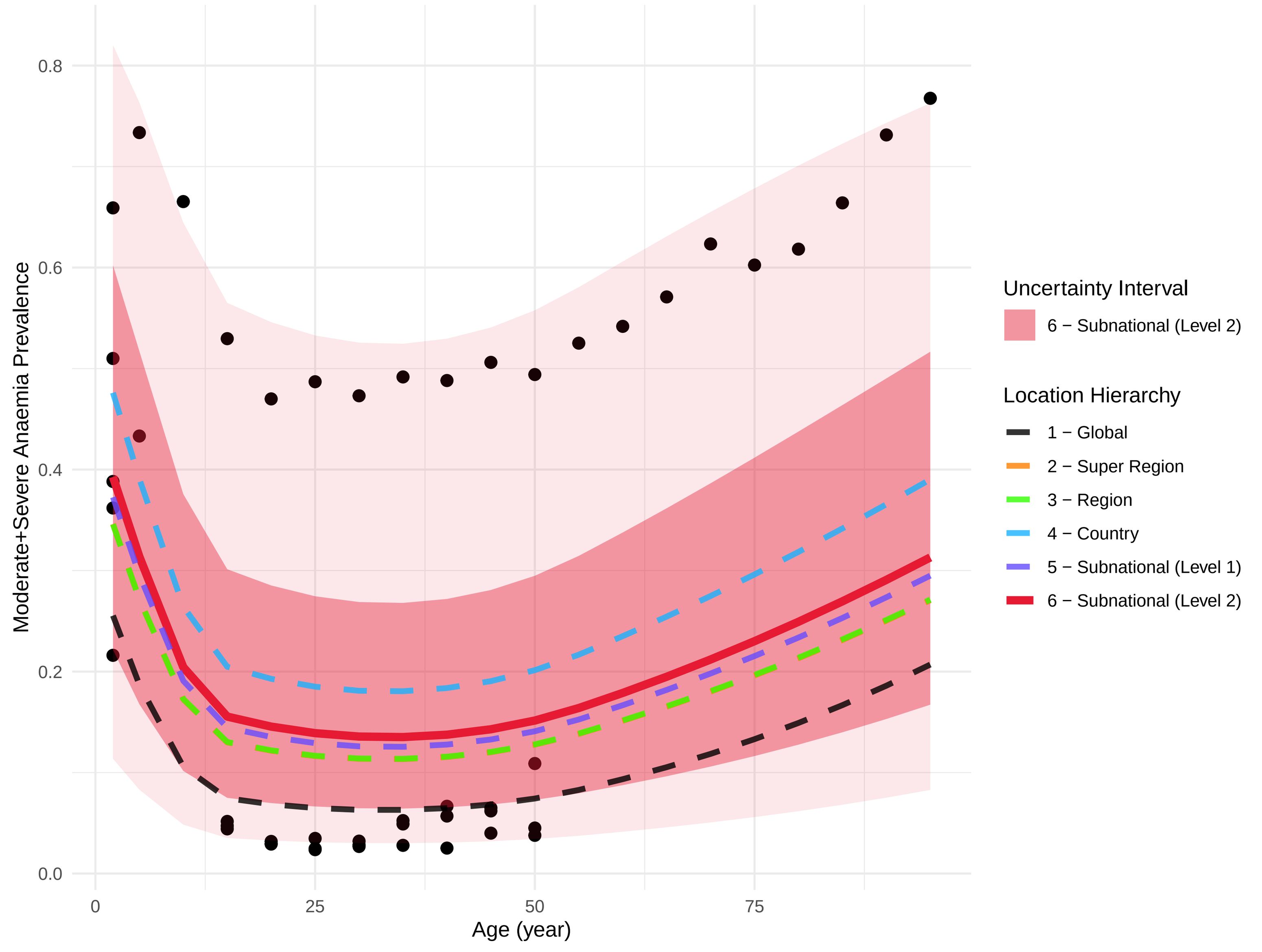

MR−BRT Age/Sex Fits − Males 2+ Years Old − Moderate+Severe Anaemia Prevalence − Maharashtra, Urban
WHO−2001 Adjustment Method

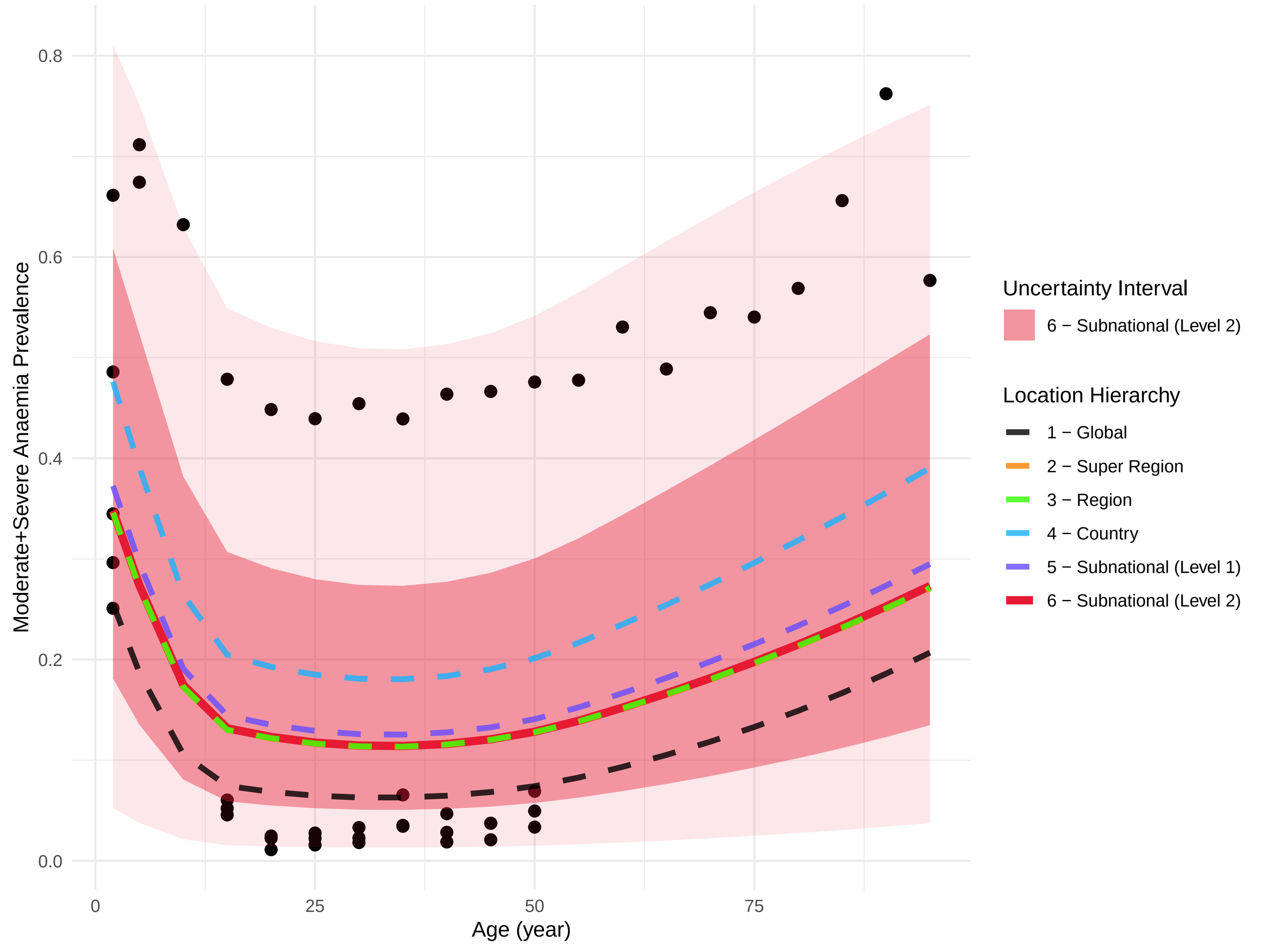

MR−BRT Age/Sex Fits − Males 2+ Years Old − Moderate+Severe Anaemia Prevalence − Manipur
WHO−2001 Adjustment Method

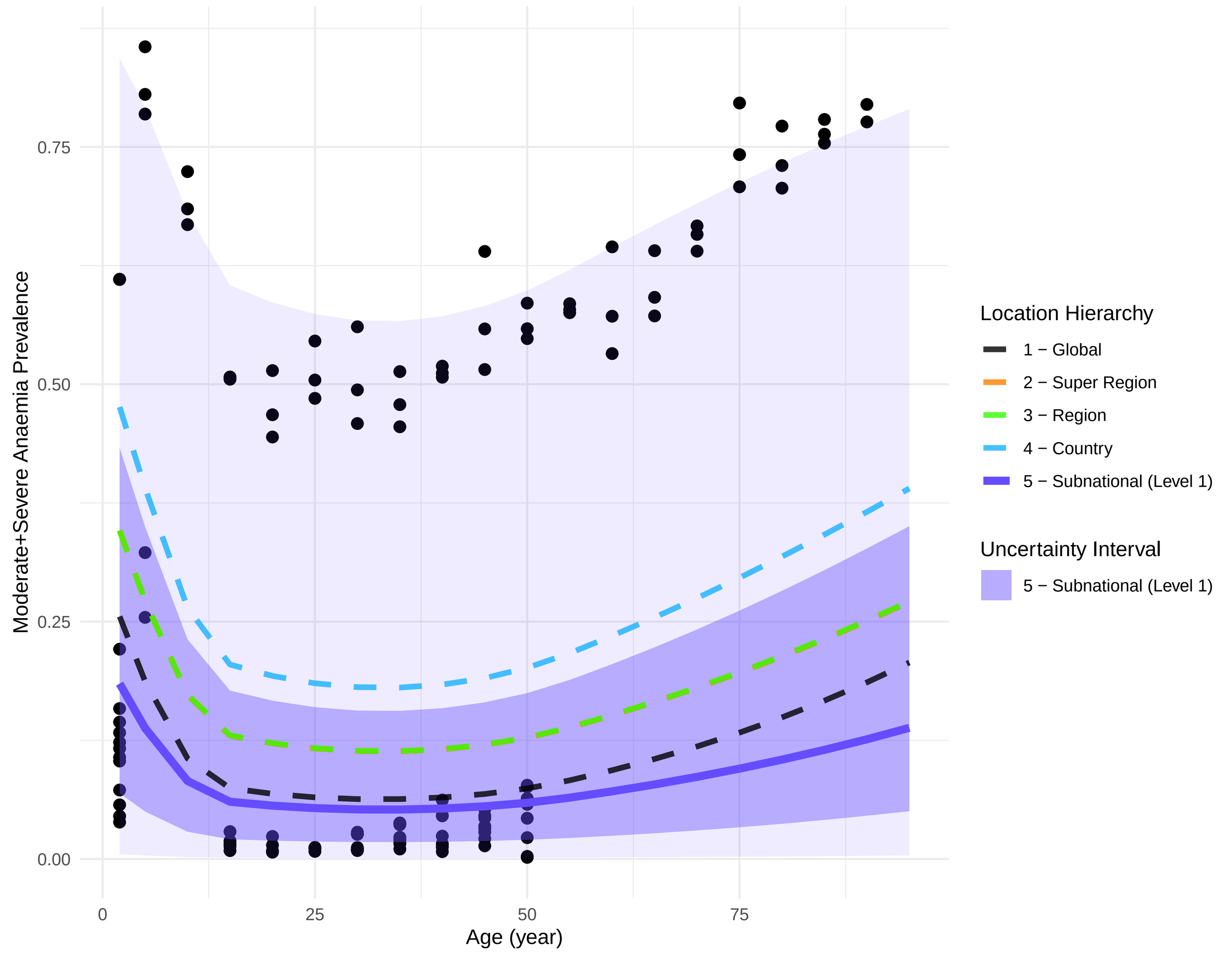

MR−BRT Age/Sex Fits − Males 2+ Years Old − Moderate+Severe Anaemia Prevalence − Manipur, Rural
WHO−2001 Adjustment Method

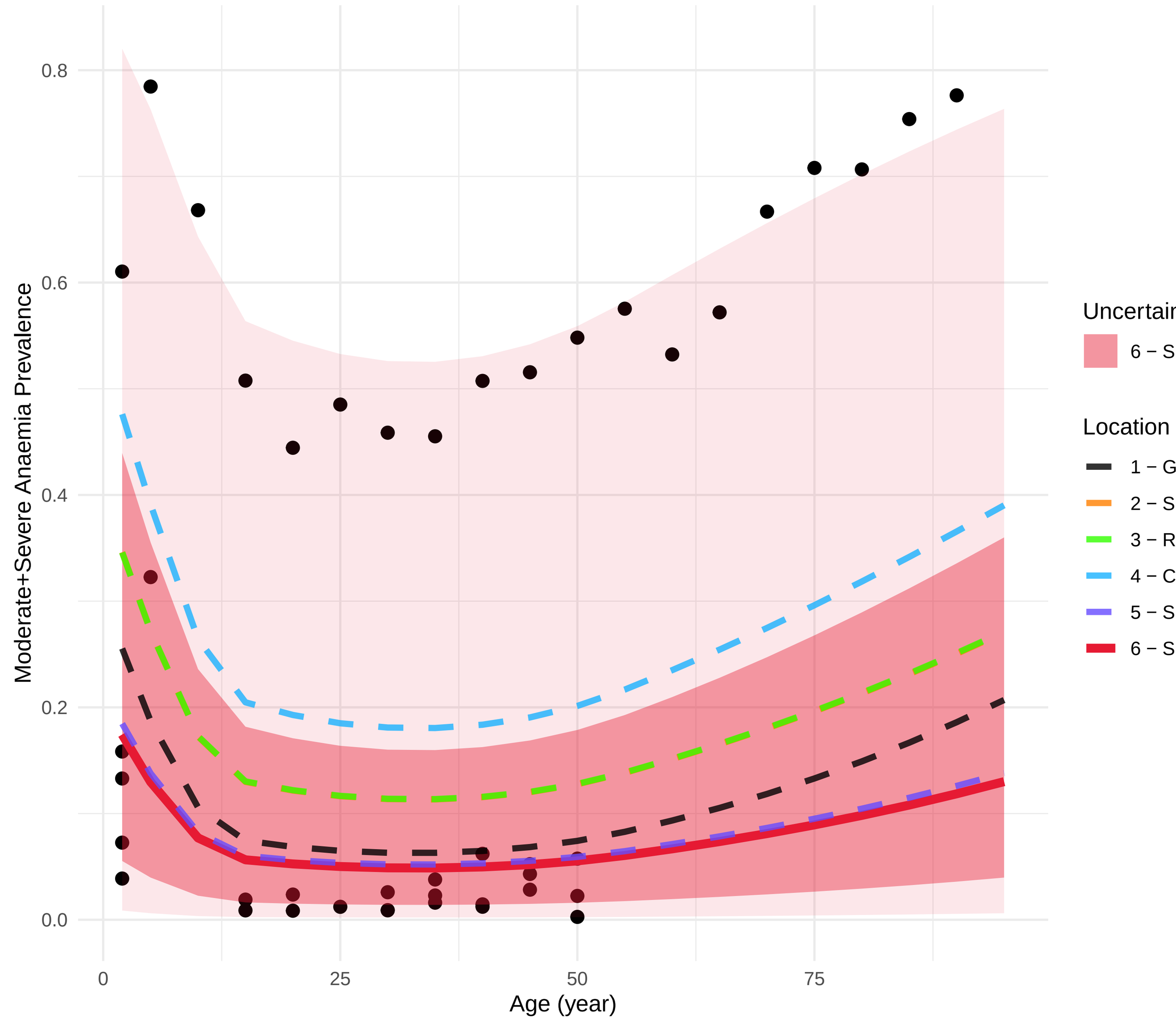

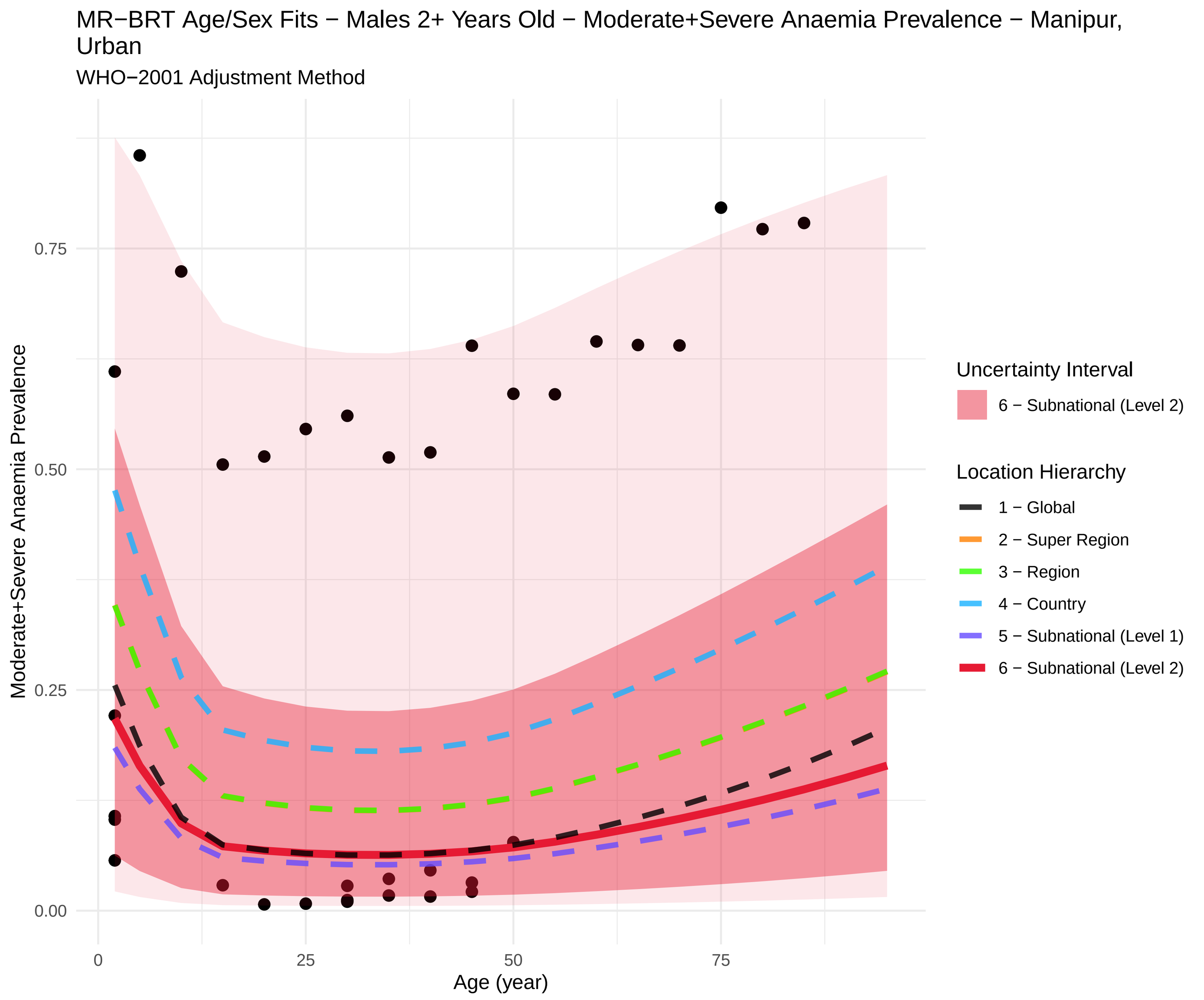
MR−BRT Age/Sex Fits − Males 2+ Years Old − Moderate+Severe Anaemia Prevalence − Manipur, Urban
WHO−2001 Adjustment Method
Moderate+Severe Anaemia Prevalence
Age (year)
Uncertainty Interval
6 − Subnational (Level 2)
Location Hierarchy
1 − Global
2 − Super Region
3 − Region
4 − Country
5 − Subnational (Level 1)
6 − Subnational (Level 2)

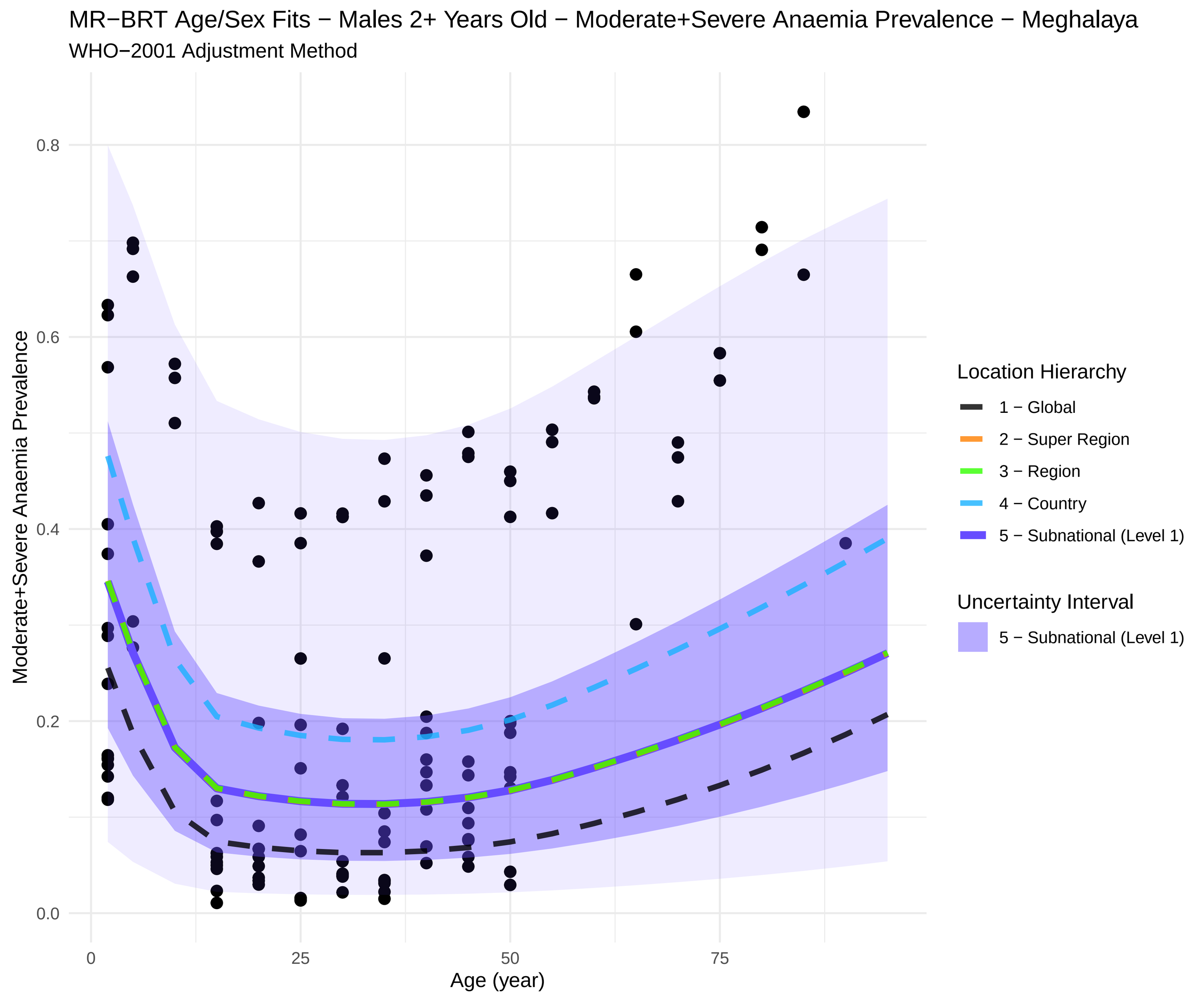
MR−BRT Age/Sex Fits − Males 2+ Years Old − Moderate+Severe Anaemia Prevalence − Meghalaya
WHO−2001 Adjustment Method
Moderate+Severe Anaemia Prevalence
Age (year)
Location Hierarchy
1 − Global
2 − Super Region
3 − Region
4 − Country
5 − Subnational (Level 1)
Uncertainty Interval
5 − Subnational (Level 1)

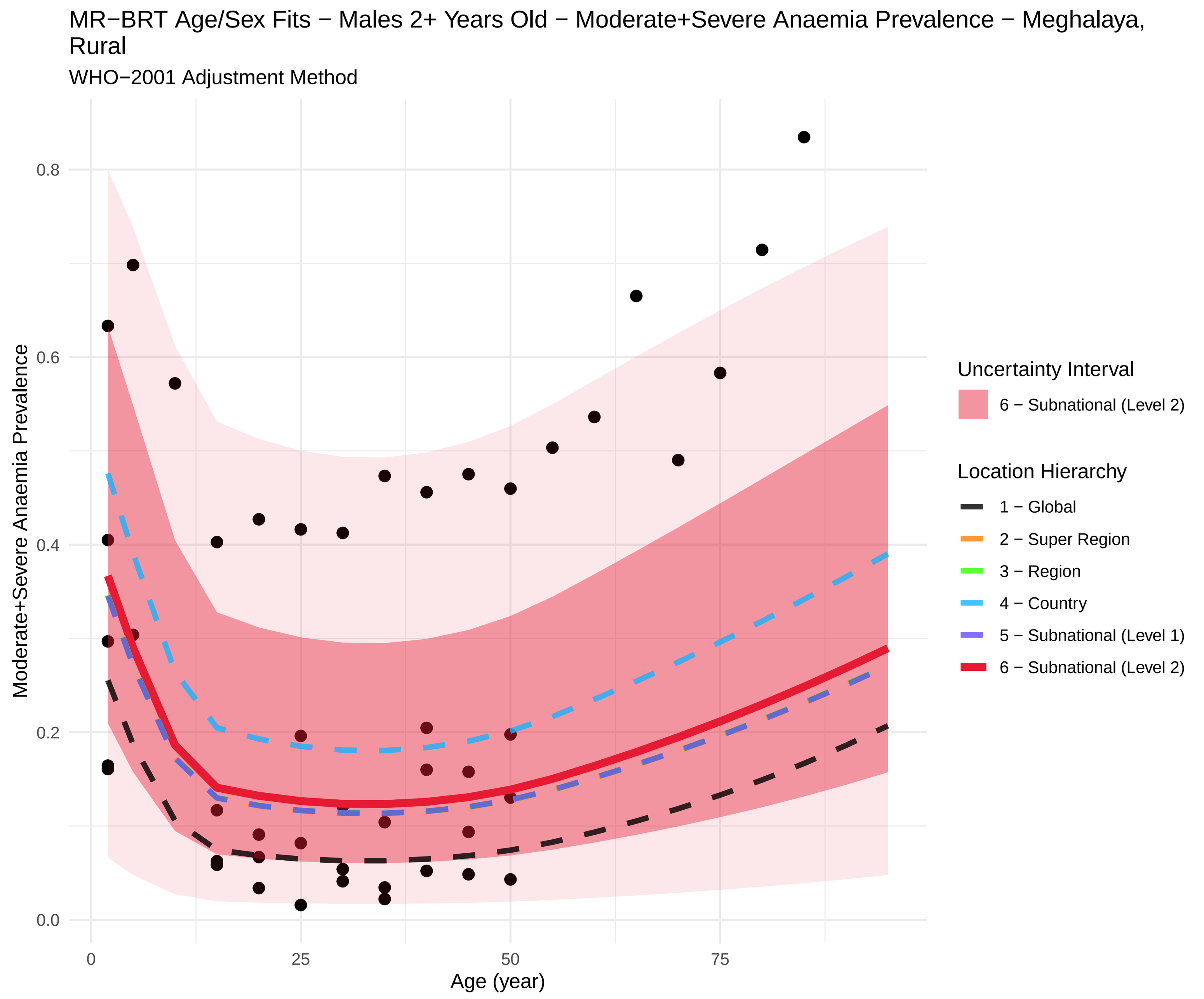
MR−BRT Age/Sex Fits − Males 2+ Years Old − Moderate+Severe Anaemia Prevalence − Meghalaya, Rural
WHO−2001 Adjustment Method
Moderate+Severe Anaemia Prevalence
Age (year)
Uncertainty Interval
6 − Subnational (Level 2)
Location Hierarchy
1 − Global
2 − Super Region
3 − Region
4 − Country
5 − Subnational (Level 1)
6 − Subnational (Level 2)

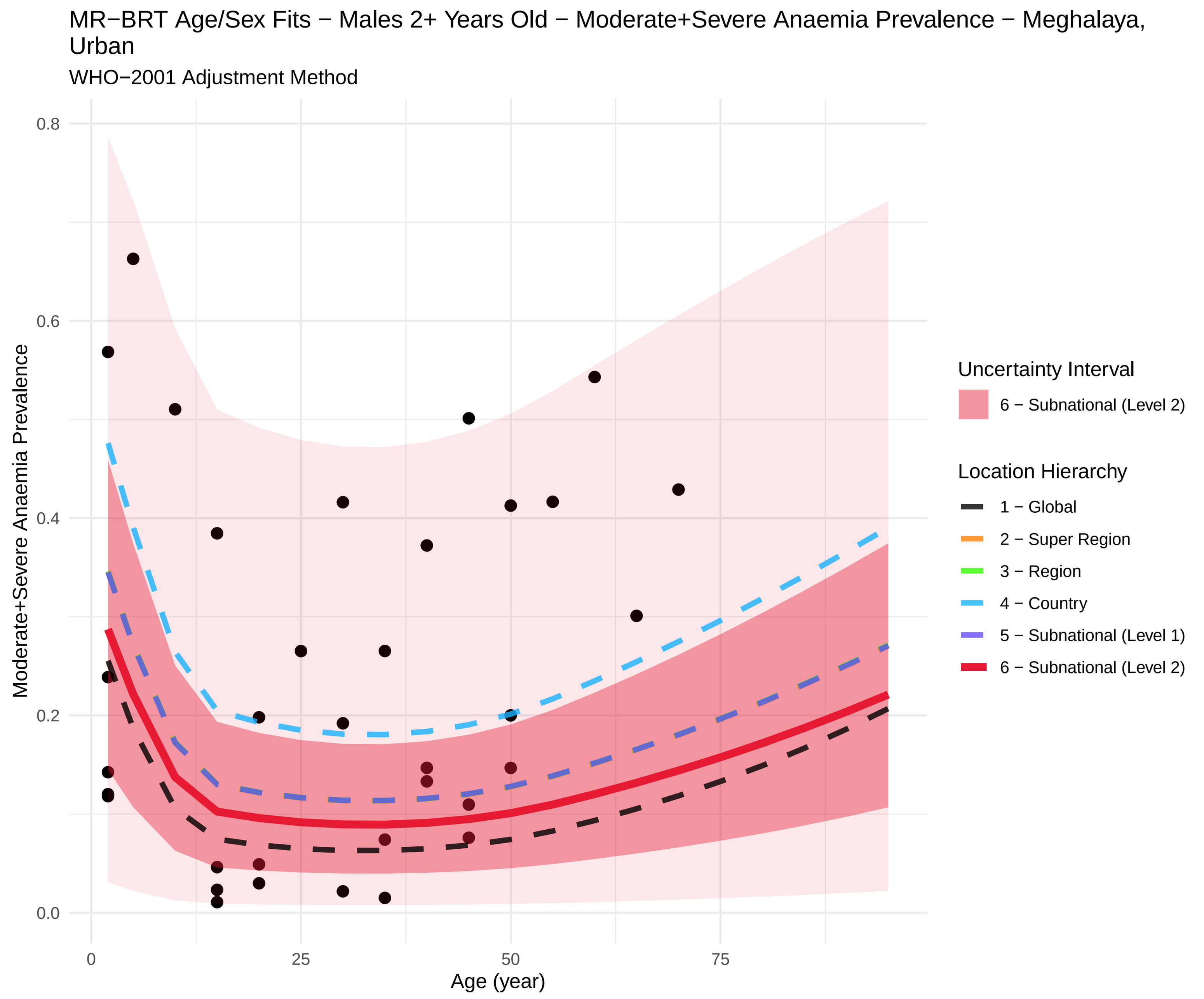
MR−BRT Age/Sex Fits − Males 2+ Years Old − Moderate+Severe Anaemia Prevalence − Meghalaya, Urban
WHO−2001 Adjustment Method
Moderate+Severe Anaemia Prevalence
Age (year)
Uncertainty Interval
6 − Subnational (Level 2)
Location Hierarchy
1 − Global
2 − Super Region
3 − Region
4 − Country
5 − Subnational (Level 1)
6 − Subnational (Level 2)

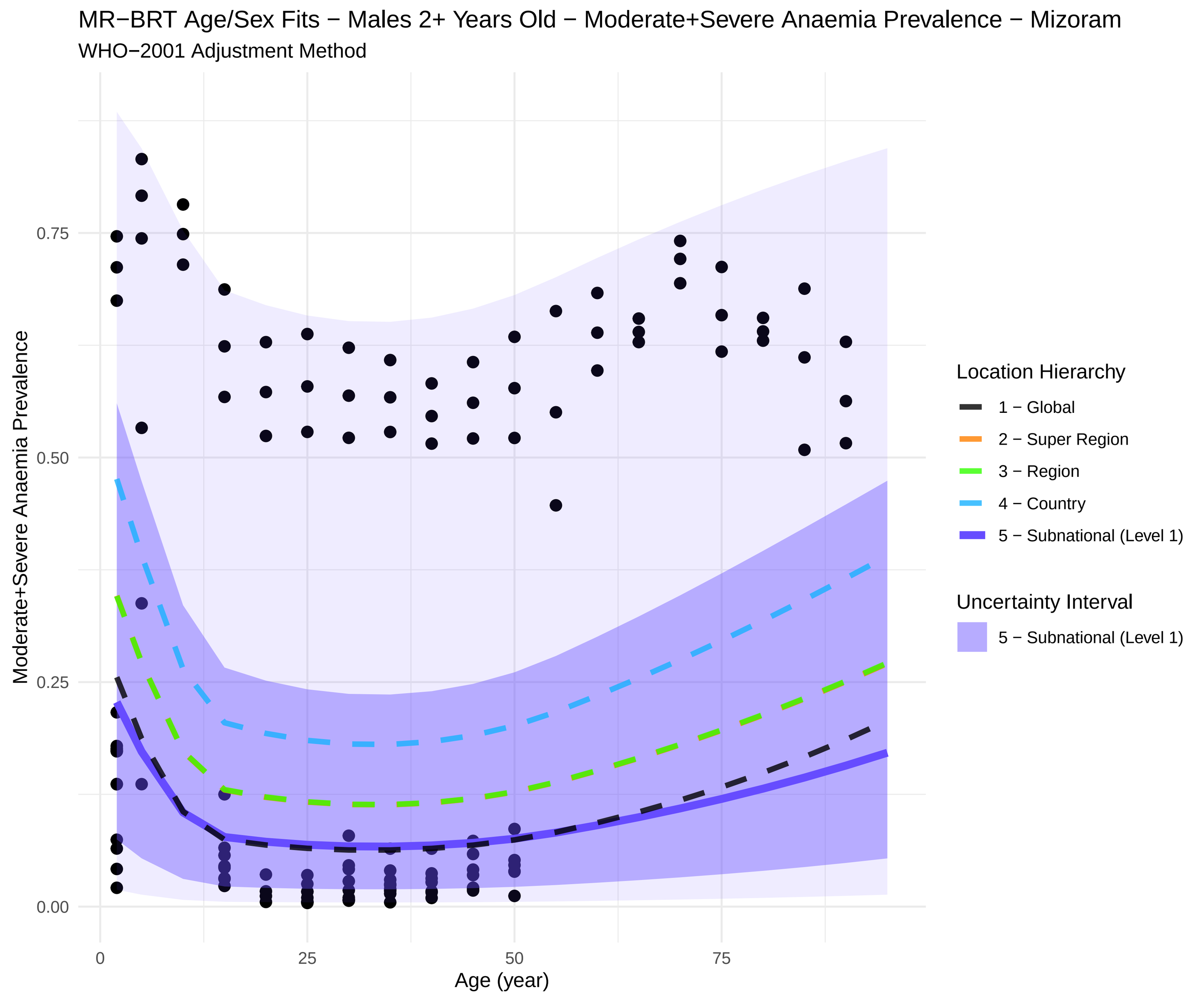
MR−BRT Age/Sex Fits − Males 2+ Years Old − Moderate+Severe Anaemia Prevalence − Mizoram
WHO−2001 Adjustment Method
Moderate+Severe Anaemia Prevalence
Age (year)
Location Hierarchy
1 − Global
2 − Super Region
3 − Region
4 − Country
5 − Subnational (Level 1)
Uncertainty Interval
5 − Subnational (Level 1)

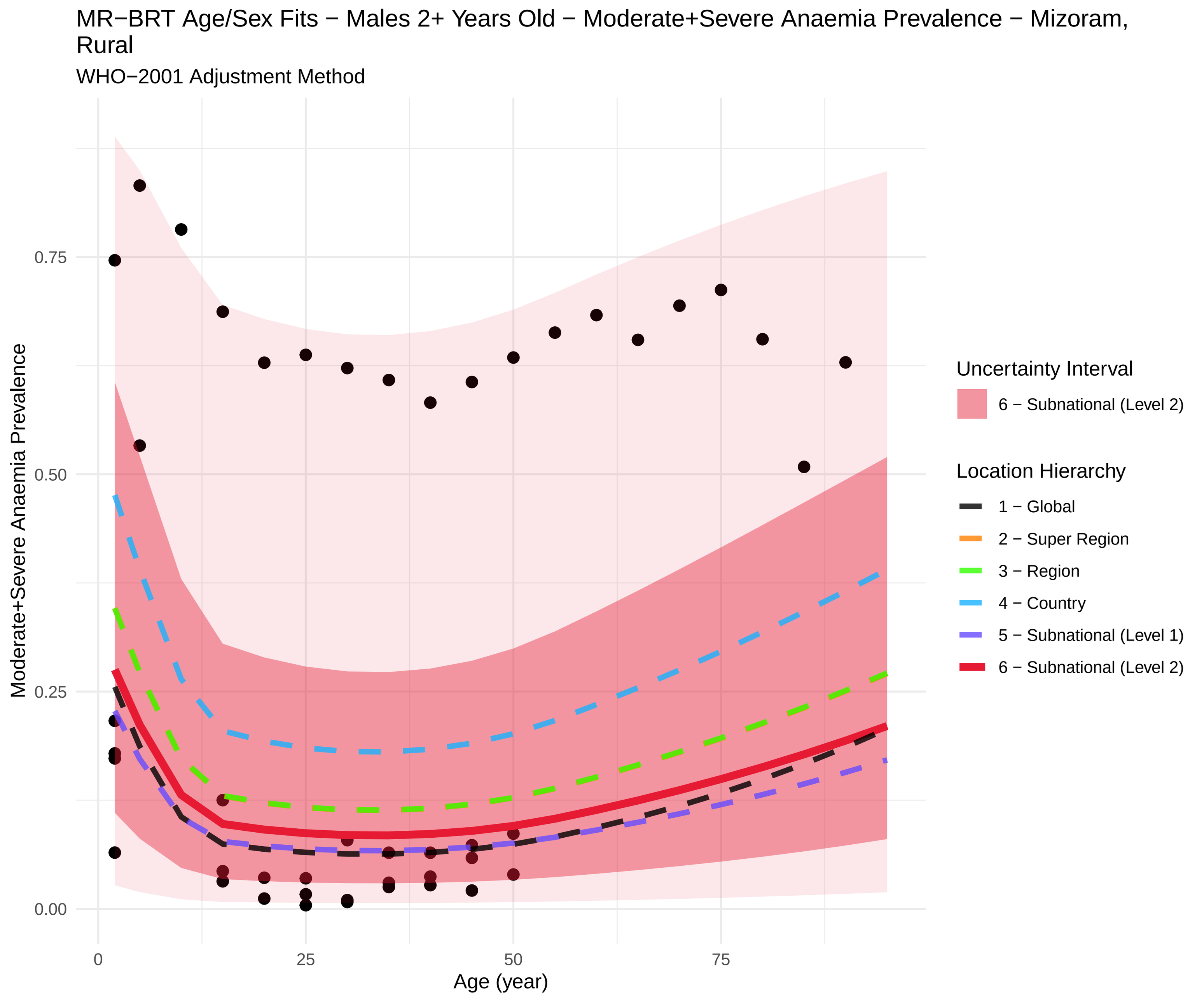
MR−BRT Age/Sex Fits − Males 2+ Years Old − Moderate+Severe Anaemia Prevalence − Mizoram, Rural
WHO−2001 Adjustment Method
Moderate+Severe Anaemia Prevalence
Age (year)
Uncertainty Interval
6 − Subnational (Level 2)
Location Hierarchy
1 − Global
2 − Super Region
3 − Region
4 − Country
5 − Subnational (Level 1)
6 − Subnational (Level 2)

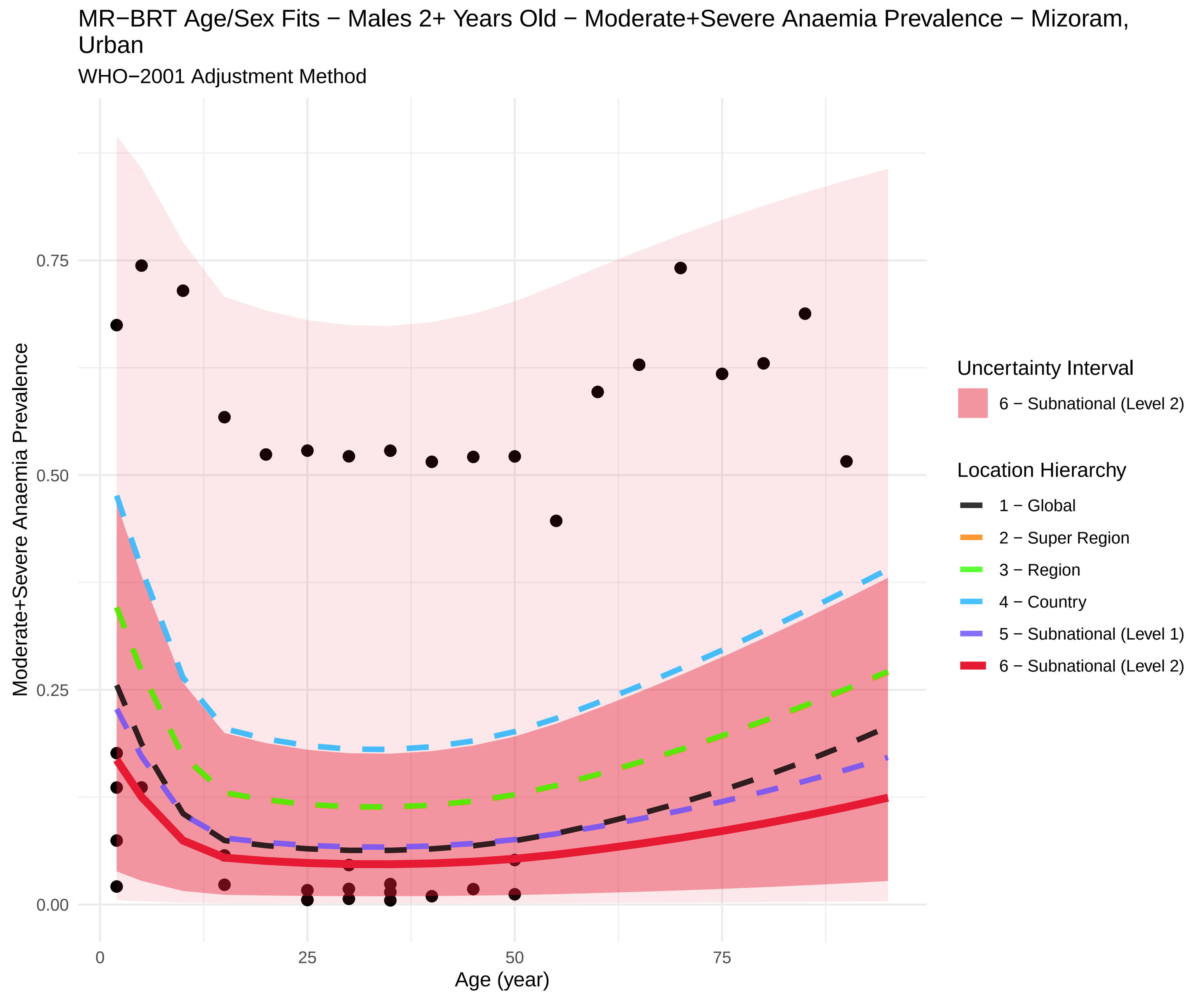
MR−BRT Age/Sex Fits − Males 2+ Years Old − Moderate+Severe Anaemia Prevalence − Mizoram, Urban
WHO−2001 Adjustment Method
Moderate+Severe Anaemia Prevalence
Age (year)
Uncertainty Interval
6 − Subnational (Level 2)
Location Hierarchy
1 − Global
2 − Super Region
3 − Region
4 − Country
5 − Subnational (Level 1)
6 − Subnational (Level 2)

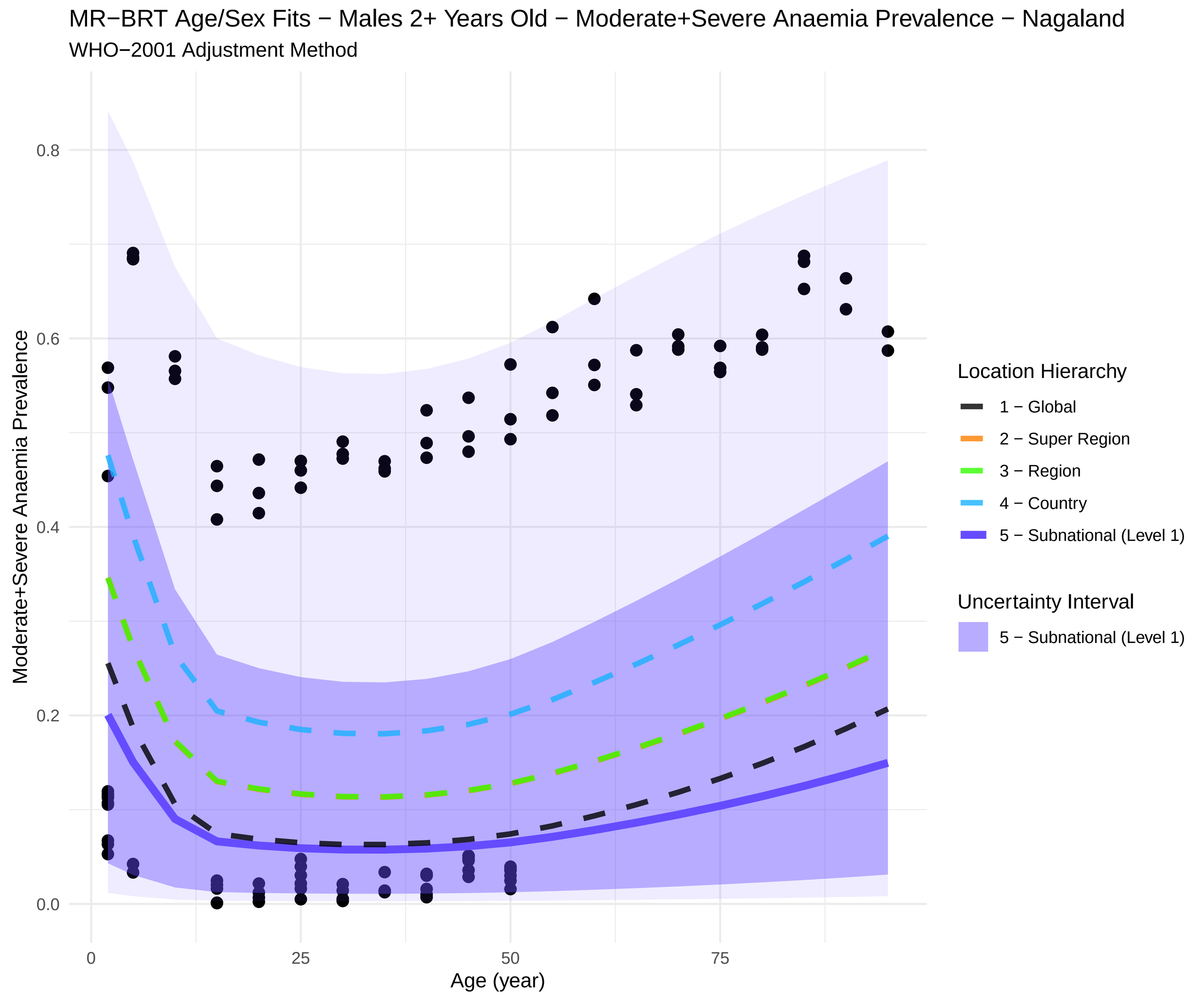
MR−BRT Age/Sex Fits − Males 2+ Years Old − Moderate+Severe Anaemia Prevalence − Nagaland
WHO−2001 Adjustment Method
Moderate+Severe Anaemia Prevalence
Age (year)
Location Hierarchy
1 − Global
2 − Super Region
3 − Region
4 − Country
5 − Subnational (Level 1)
Uncertainty Interval
5 − Subnational (Level 1)

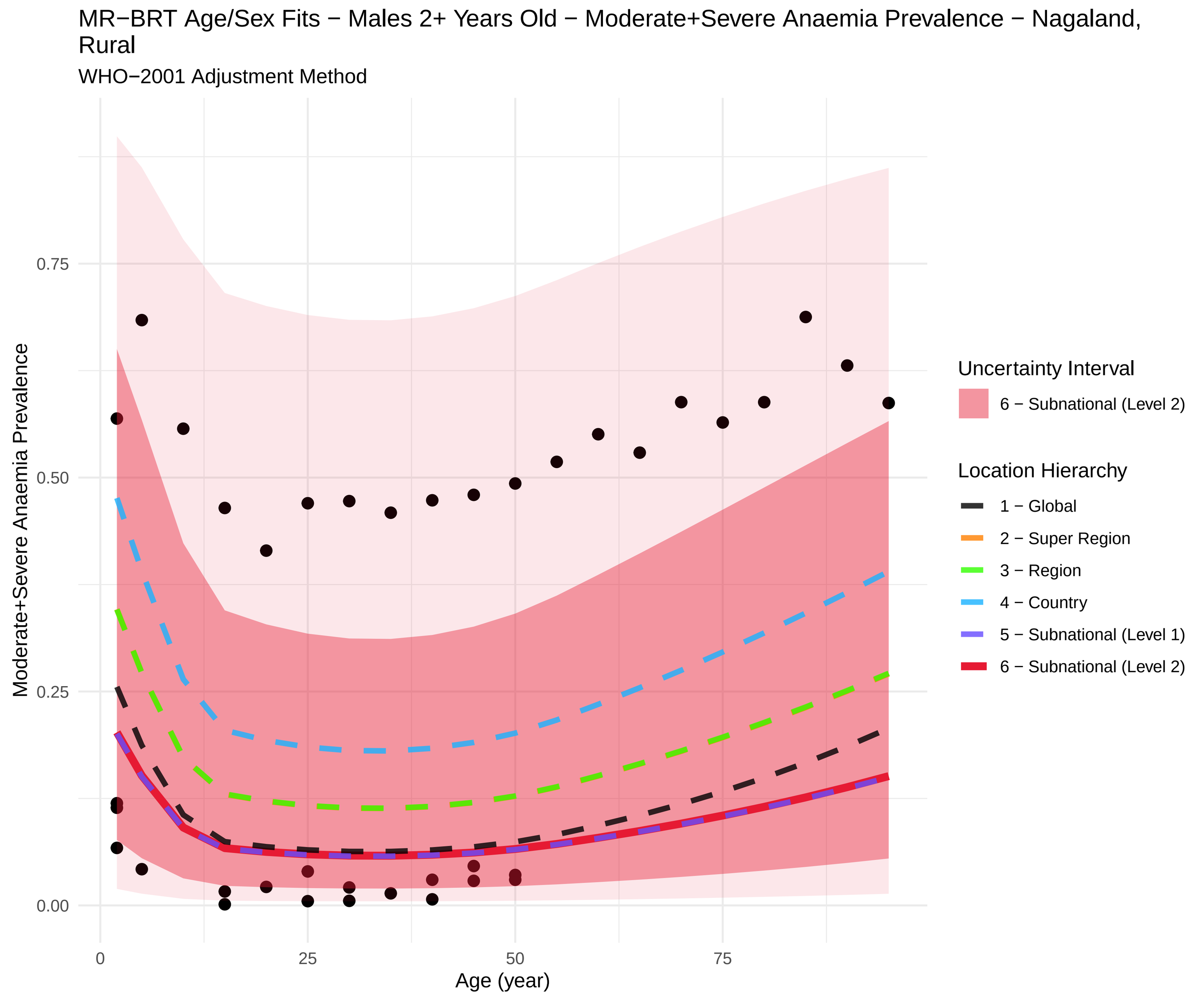
MR−BRT Age/Sex Fits − Males 2+ Years Old − Moderate+Severe Anaemia Prevalence − Nagaland, Rural
WHO−2001 Adjustment Method
Moderate+Severe Anaemia Prevalence
Age (year)
Uncertainty Interval
6 − Subnational (Level 2)
Location Hierarchy
1 − Global
2 − Super Region
3 − Region
4 − Country
5 − Subnational (Level 1)
6 − Subnational (Level 2)

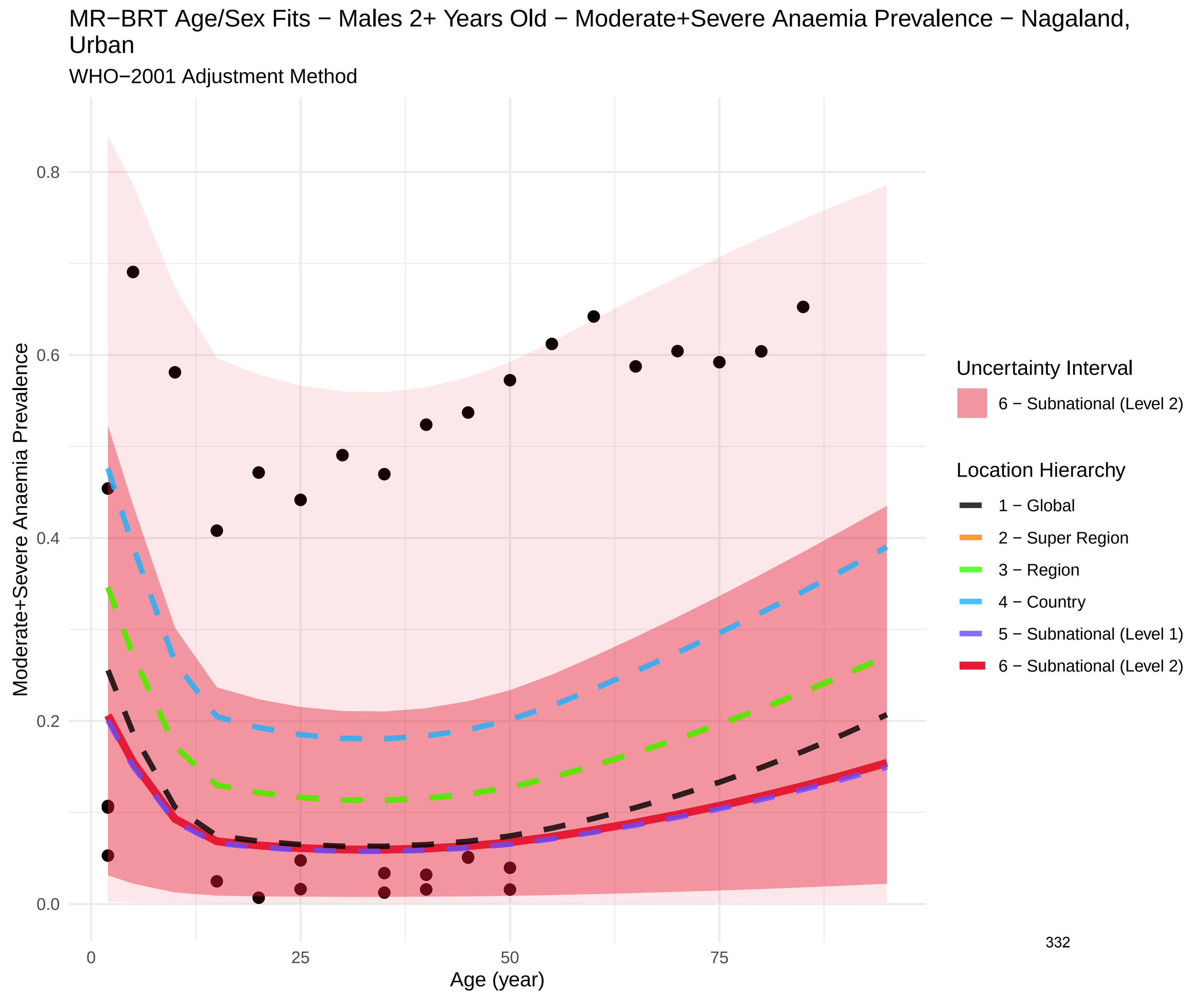
MR−BRT Age/Sex Fits − Males 2+ Years Old − Moderate+Severe Anaemia Prevalence − Nagaland, Urban
WHO−2001 Adjustment Method
Moderate+Severe Anaemia Prevalence
Age (year)
Uncertainty Interval
6 − Subnational (Level 2)
Location Hierarchy
1 − Global
2 − Super Region
3 − Region
4 − Country
5 − Subnational (Level 1)
6 − Subnational (Level 2)

MR−BRT Age/Sex Fits − Males 2+ Years Old − Moderate+Severe Anaemia Prevalence − Odisha
WHO−2001 Adjustment Method

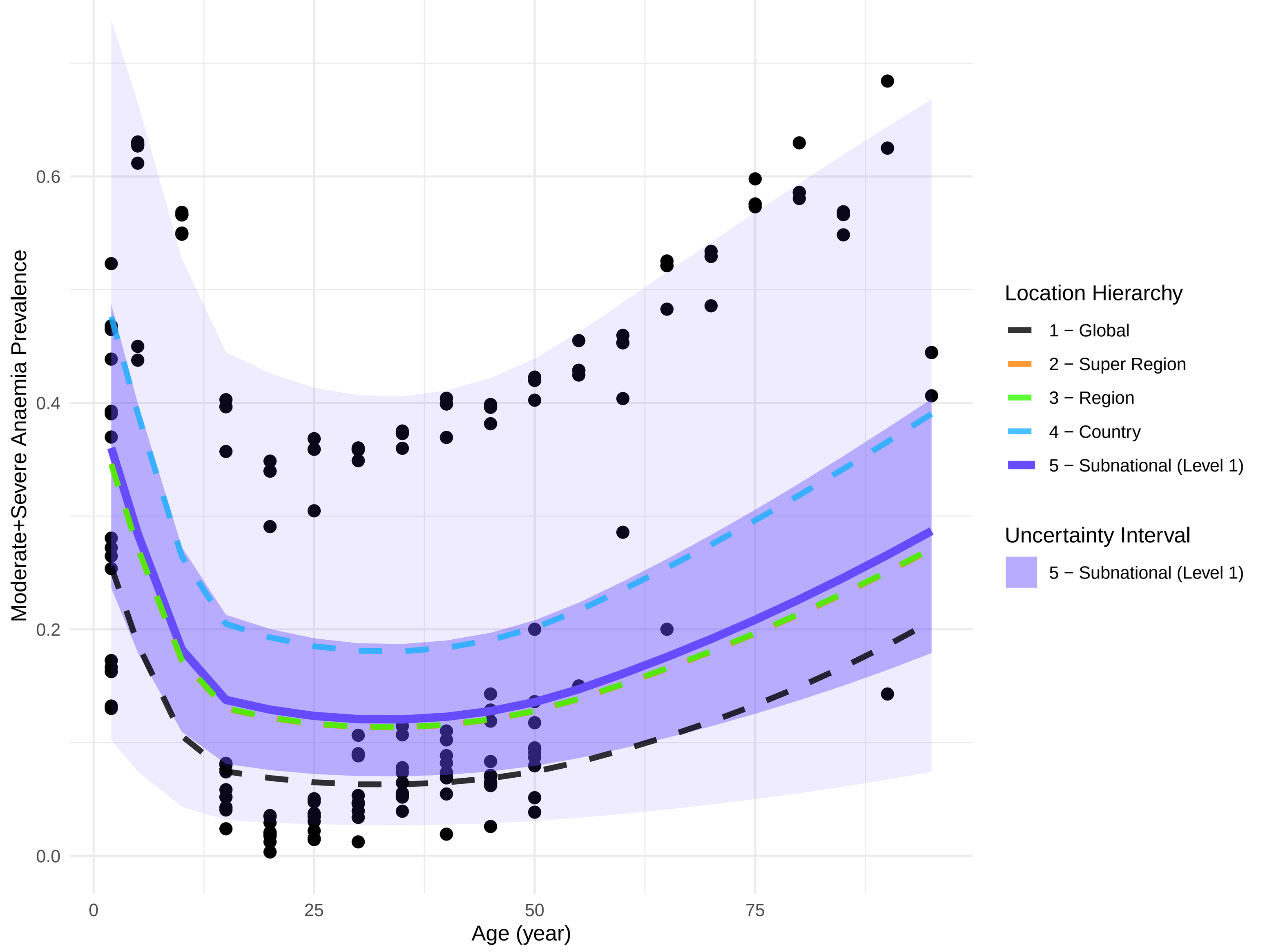

MR−BRT Age/Sex Fits − Males 2+ Years Old − Moderate+Severe Anaemia Prevalence − Odisha, Rural
WHO−2001 Adjustment Method

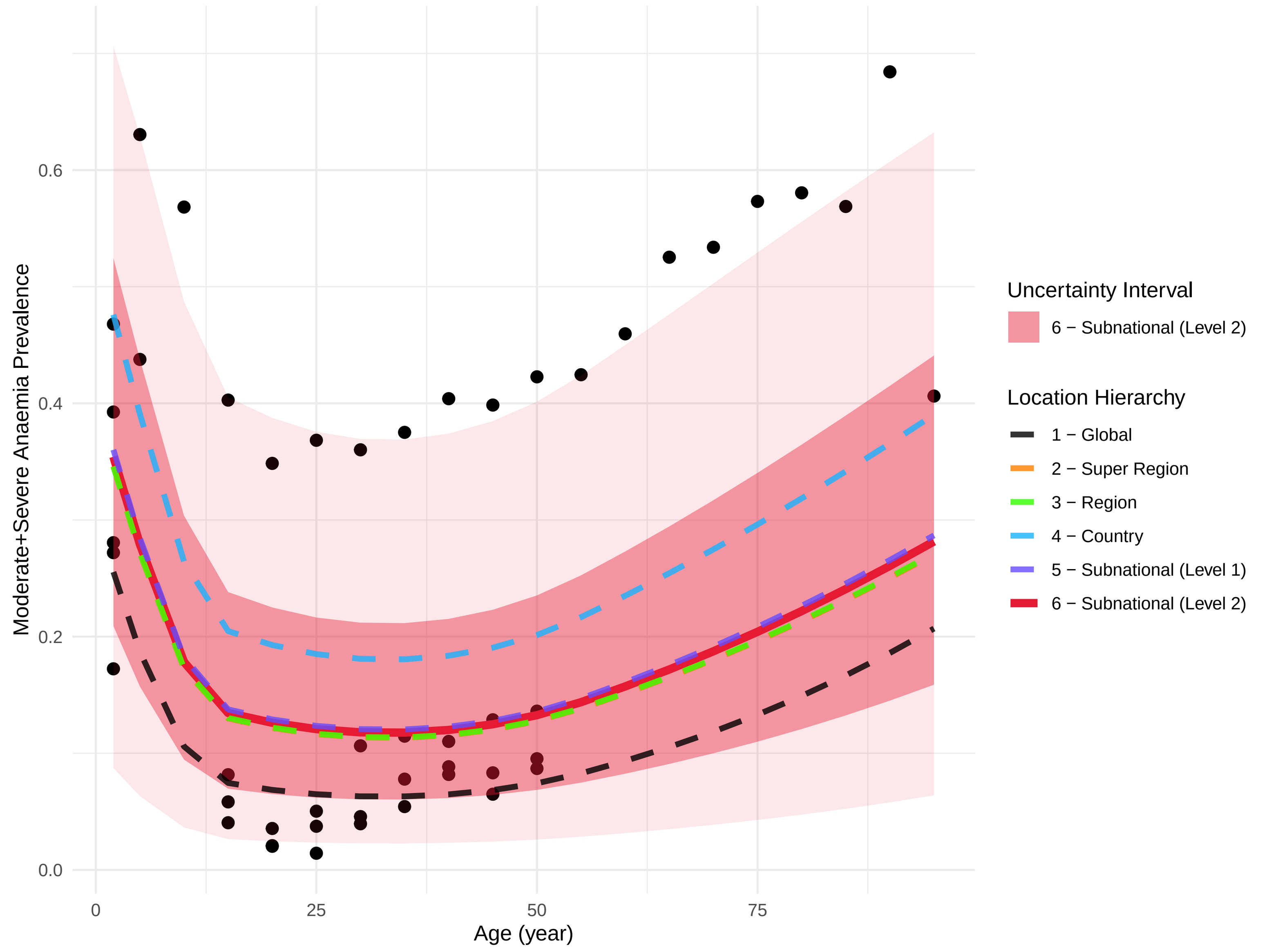

MR−BRT Age/Sex Fits − Males 2+ Years Old − Moderate+Severe Anaemia Prevalence − Odisha, Urban
WHO−2001 Adjustment Method

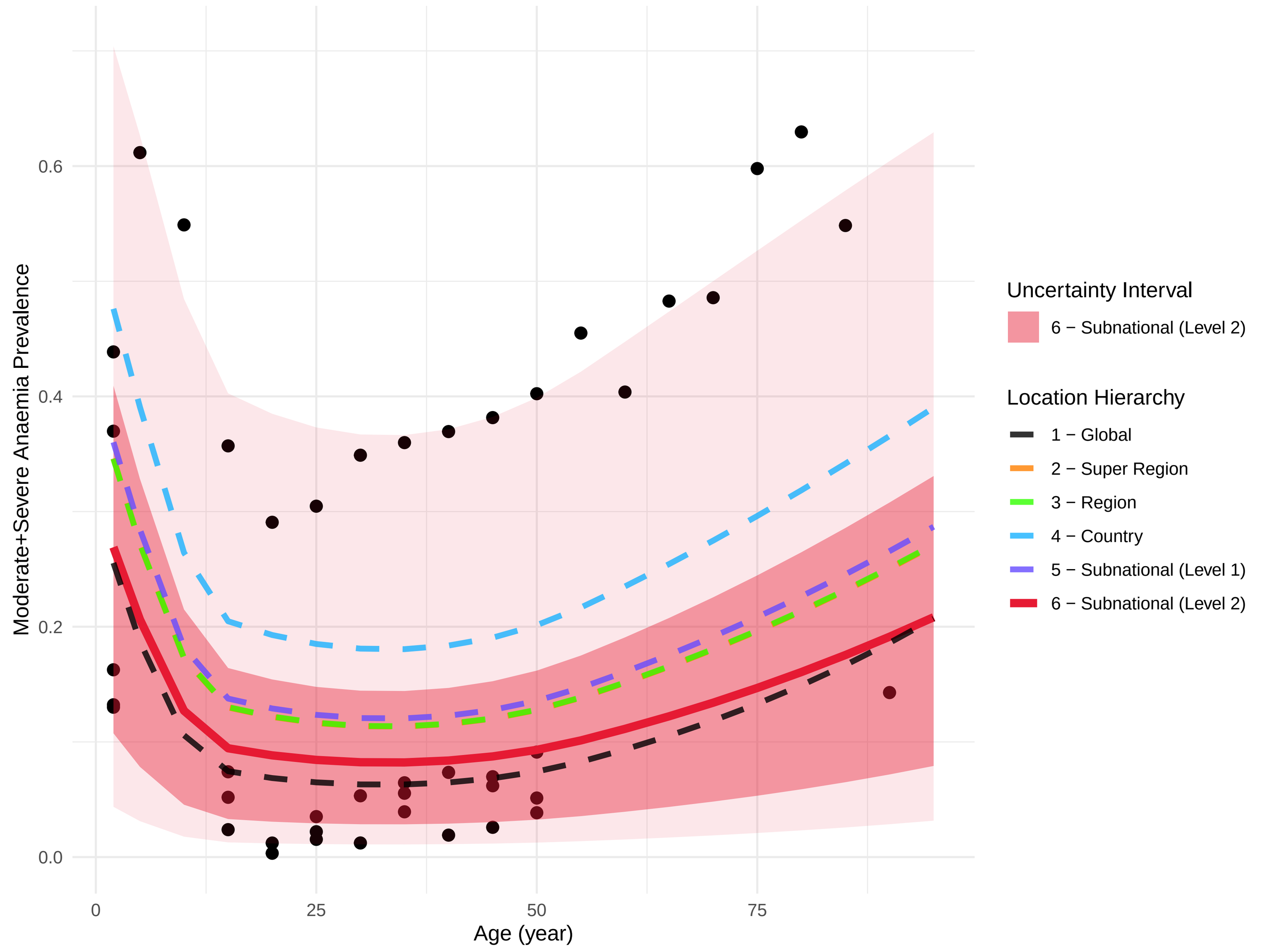

MR−BRT Age/Sex Fits − Males 2+ Years Old − Moderate+Severe Anaemia Prevalence − Other Union Territories
WHO−2001 Adjustment Method

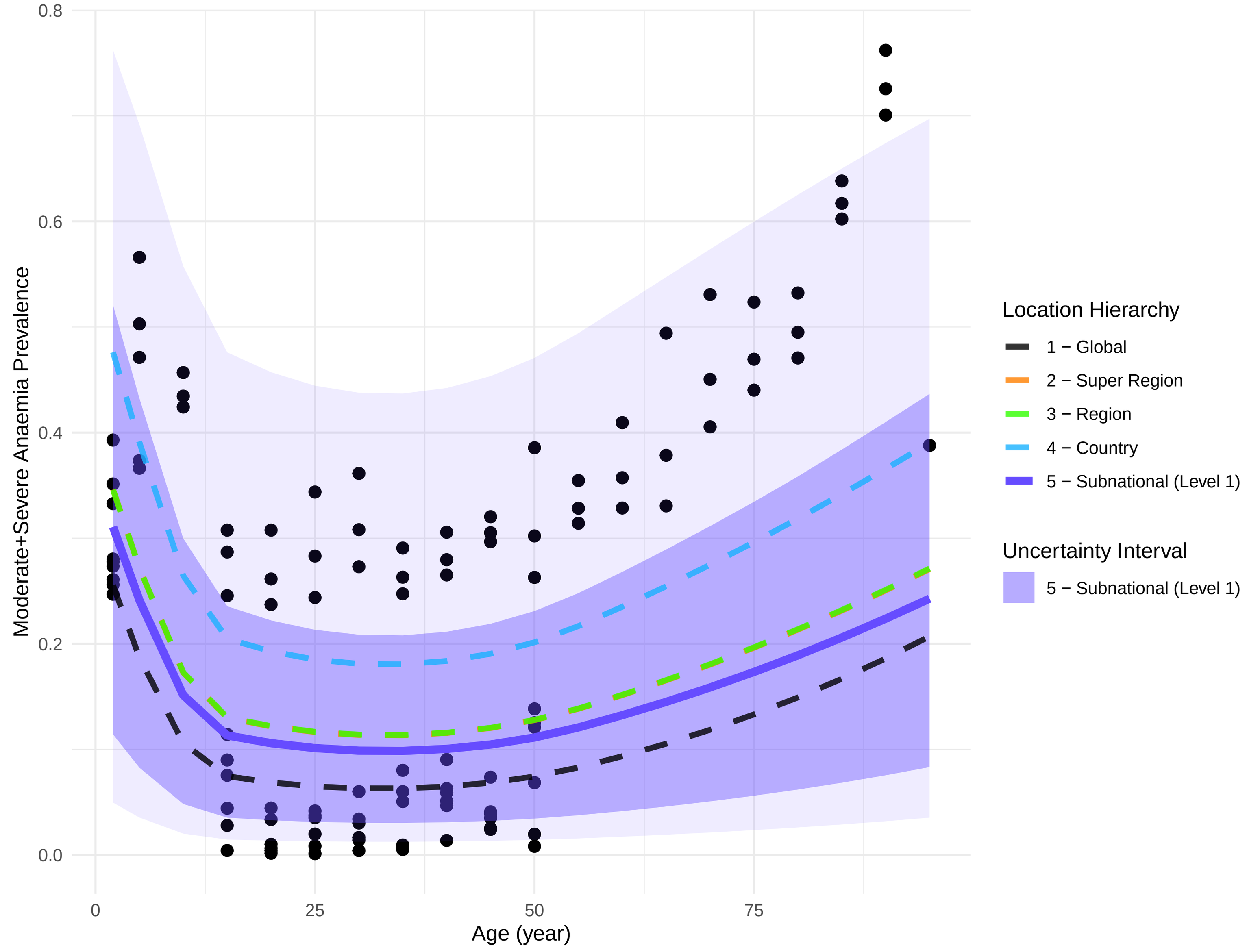

MR−BRT Age/Sex Fits − Males 2+ Years Old − Moderate+Severe Anaemia Prevalence − Other Union Territories, Rural
WHO−2001 Adjustment Method

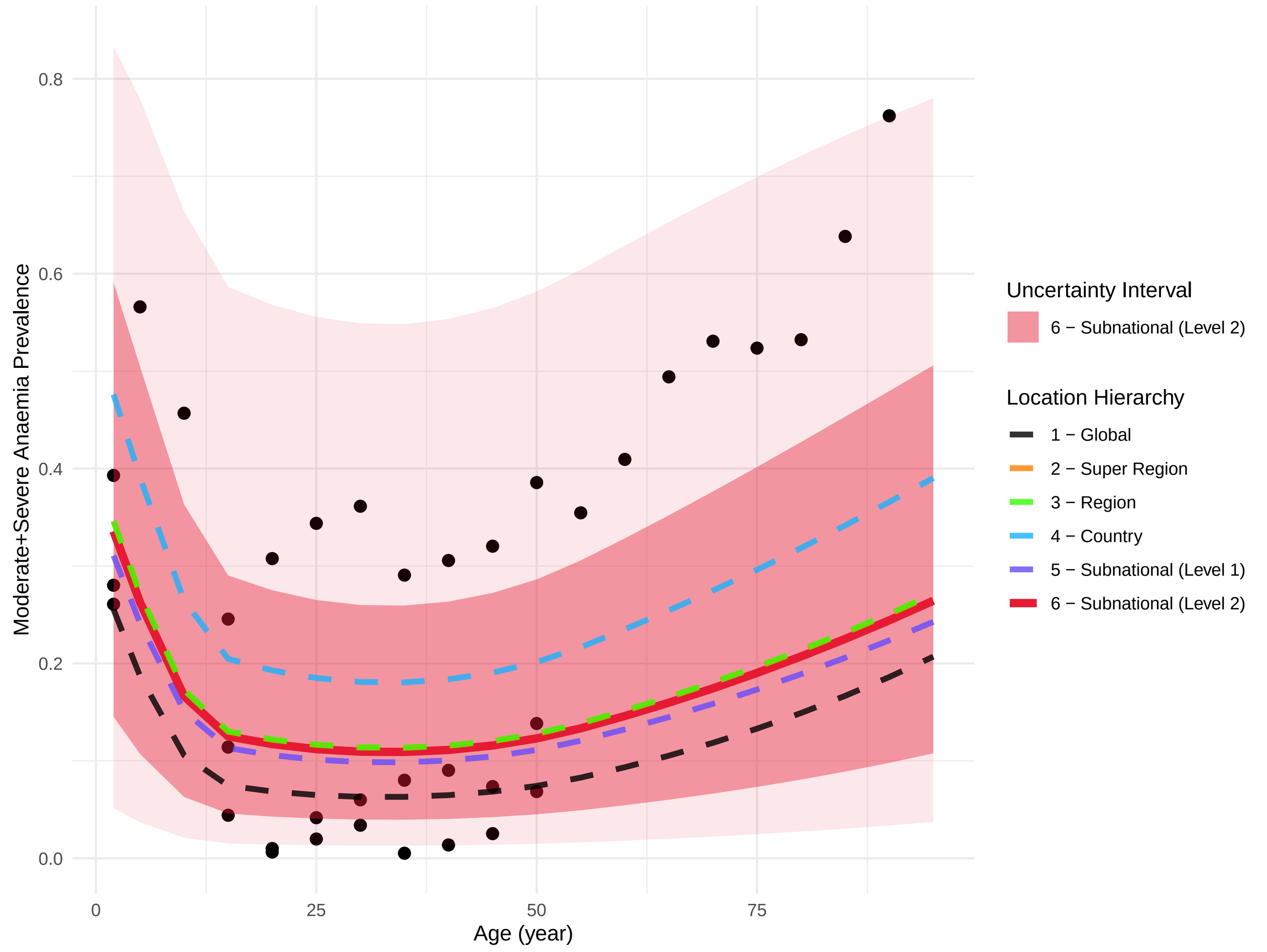

MR−BRT Age/Sex Fits − Males 2+ Years Old − Moderate+Severe Anaemia Prevalence − Other Union Territories, Urban
WHO−2001 Adjustment Method

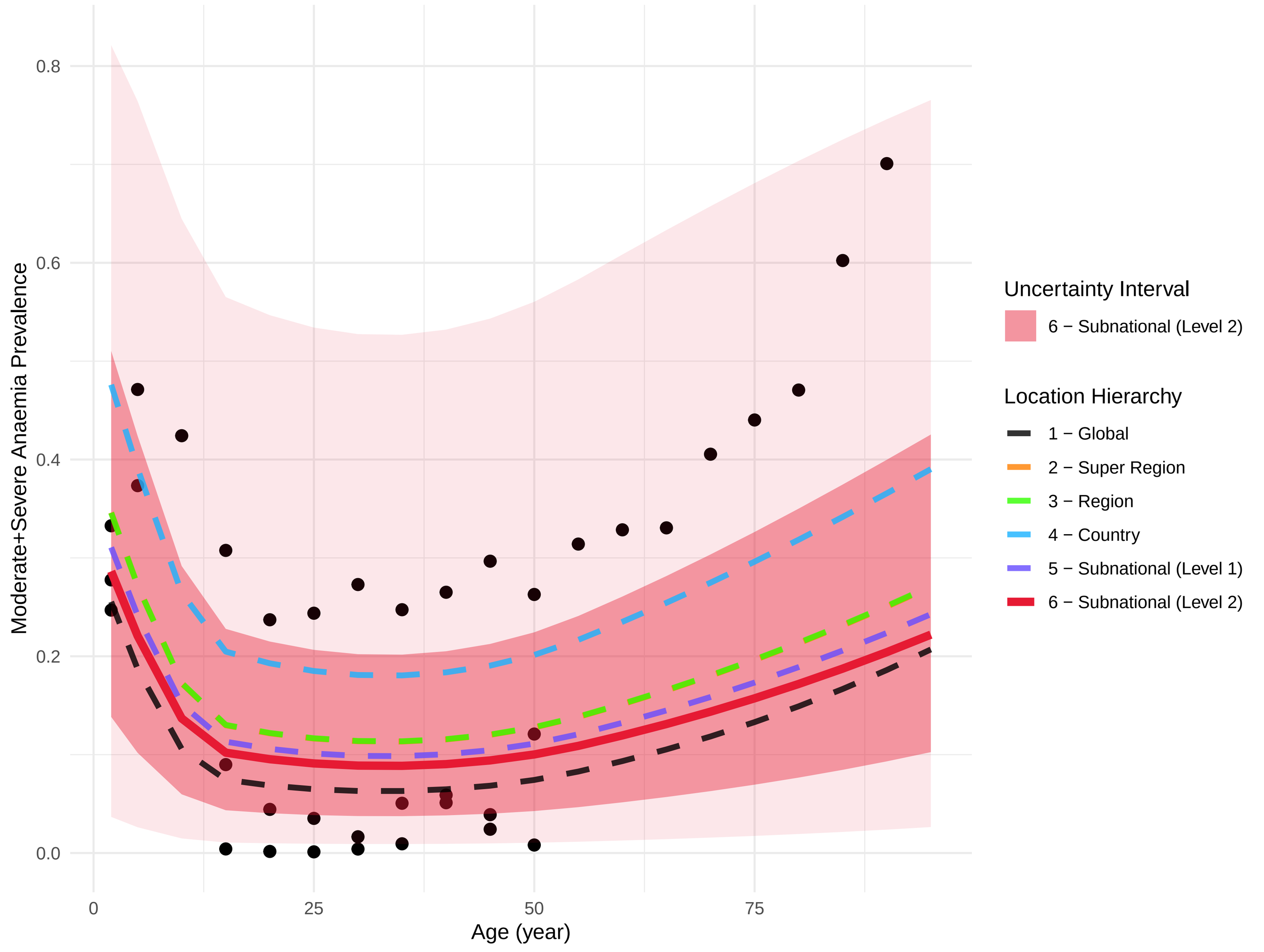

MR−BRT Age/Sex Fits − Males 2+ Years Old − Moderate+Severe Anaemia Prevalence − Punjab
WHO−2001 Adjustment Method

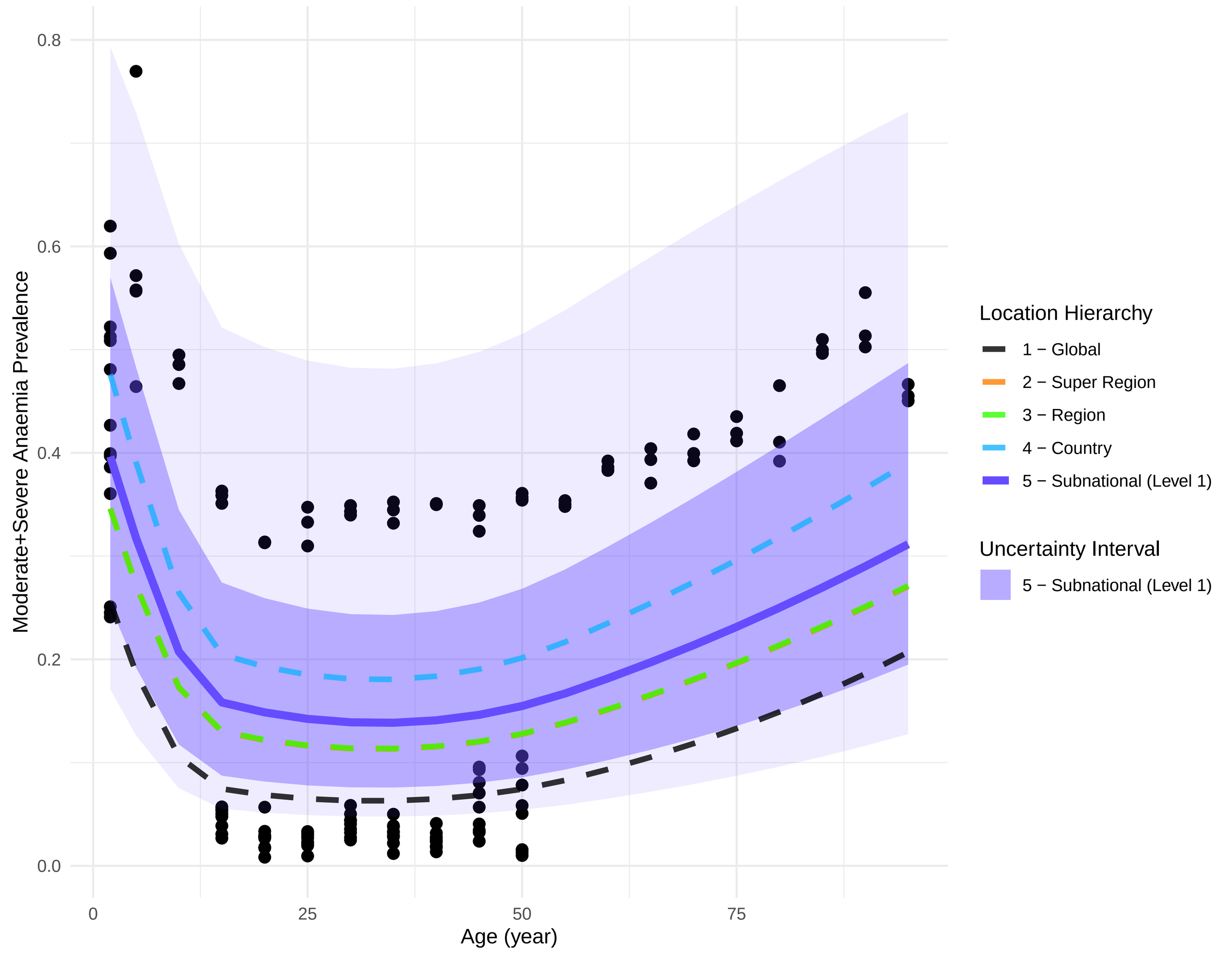

MR−BRT Age/Sex Fits − Males 2+ Years Old − Moderate+Severe Anaemia Prevalence − Punjab, Rural
WHO−2001 Adjustment Method

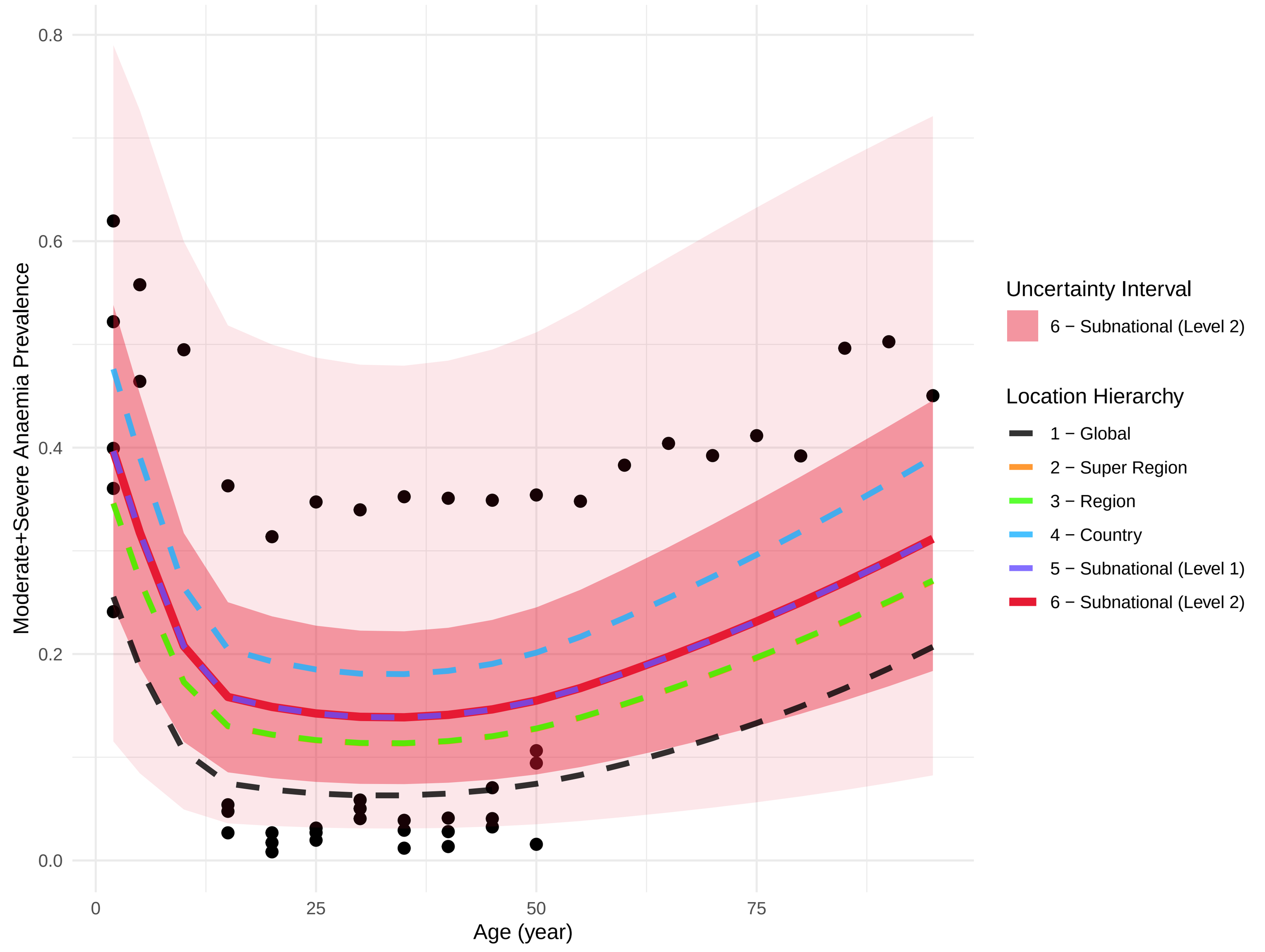

MR−BRT Age/Sex Fits − Males 2+ Years Old − Moderate+Severe Anaemia Prevalence − Punjab, Urban
WHO−2001 Adjustment Method

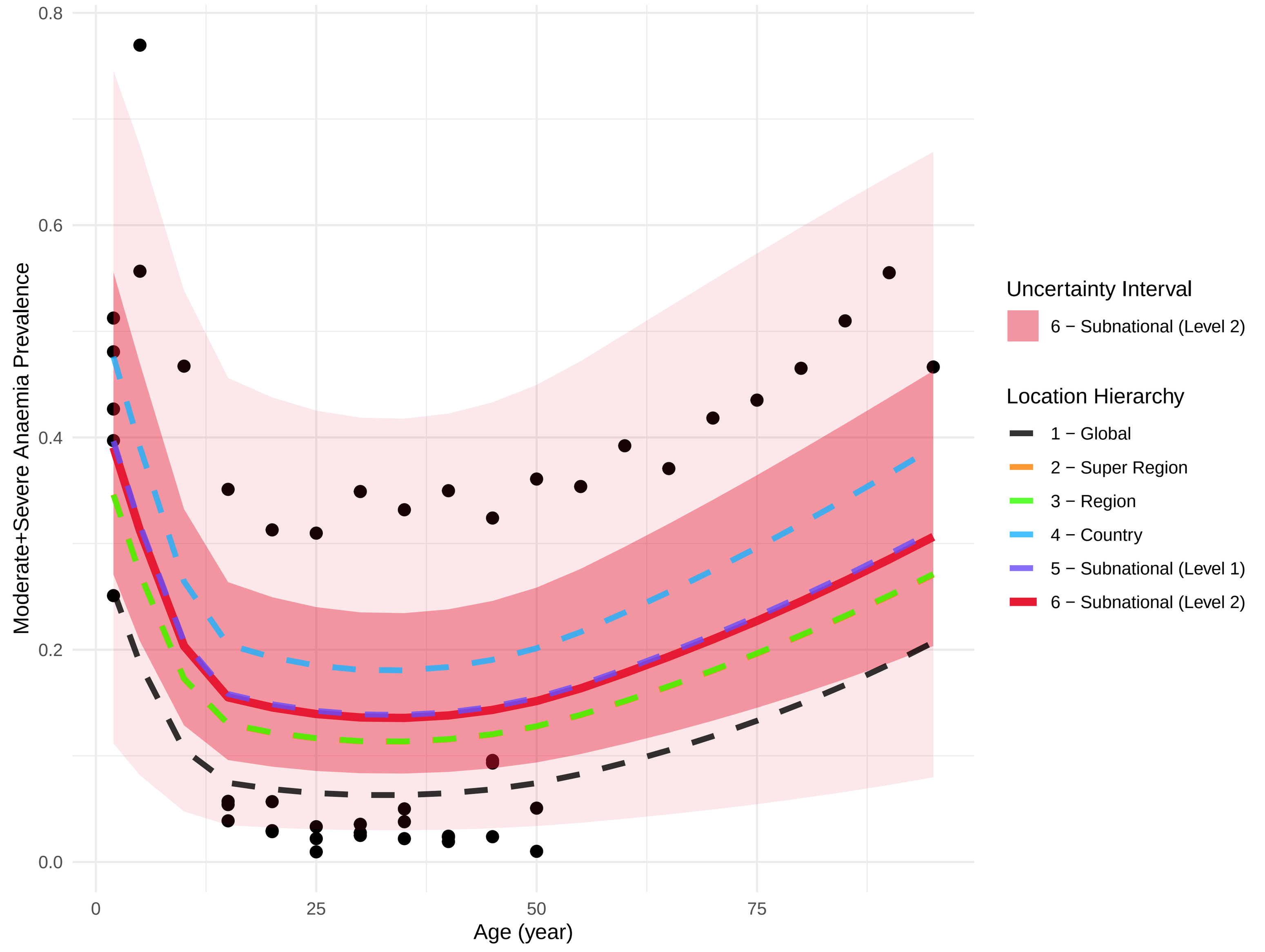

MR−BRT Age/Sex Fits − Males 2+ Years Old − Moderate+Severe Anaemia Prevalence − Rajasthan
WHO−2001 Adjustment Method

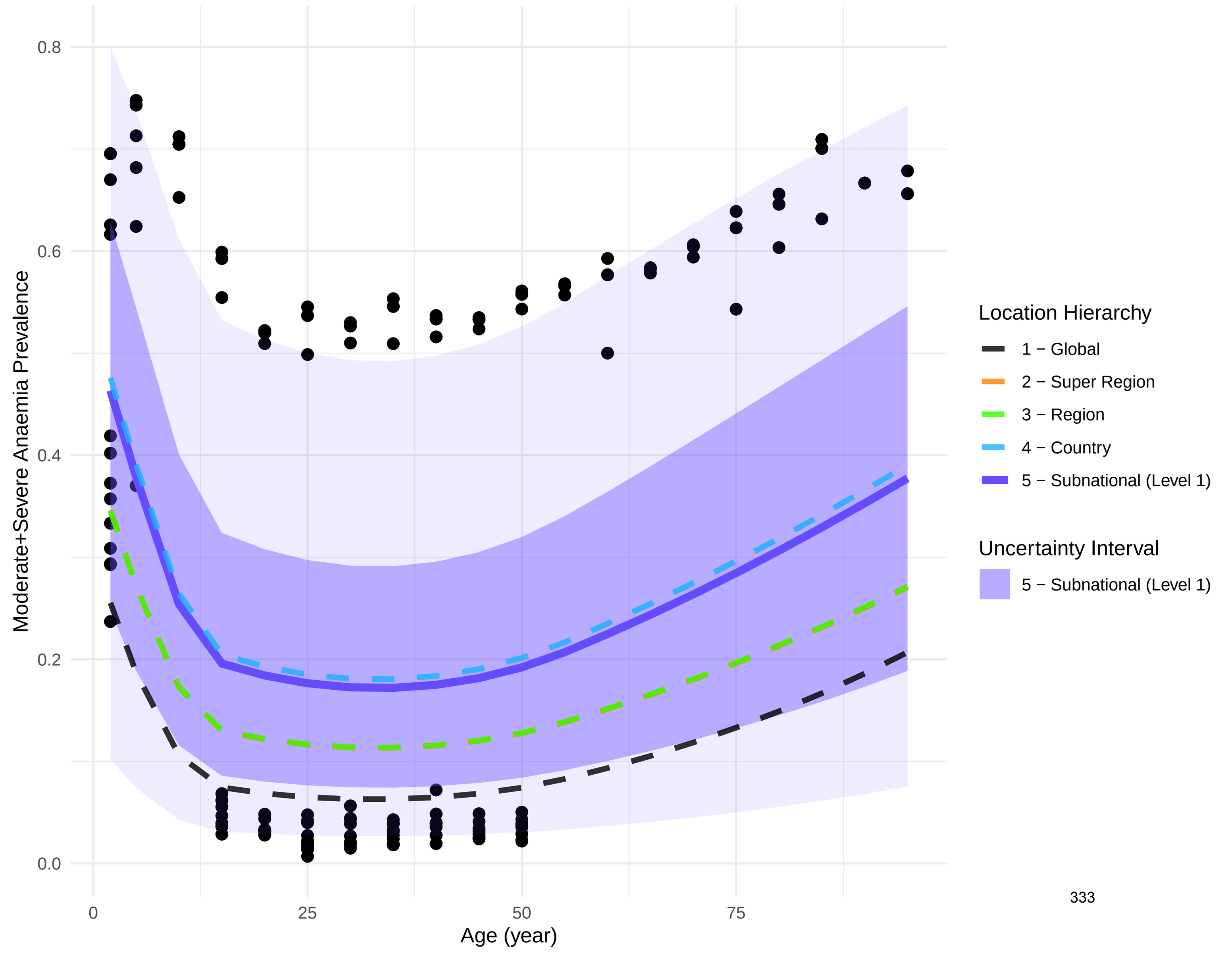

MR−BRT Age/Sex Fits − Males 2+ Years Old − Moderate+Severe Anaemia Prevalence − Rajasthan, Rural
WHO−2001 Adjustment Method

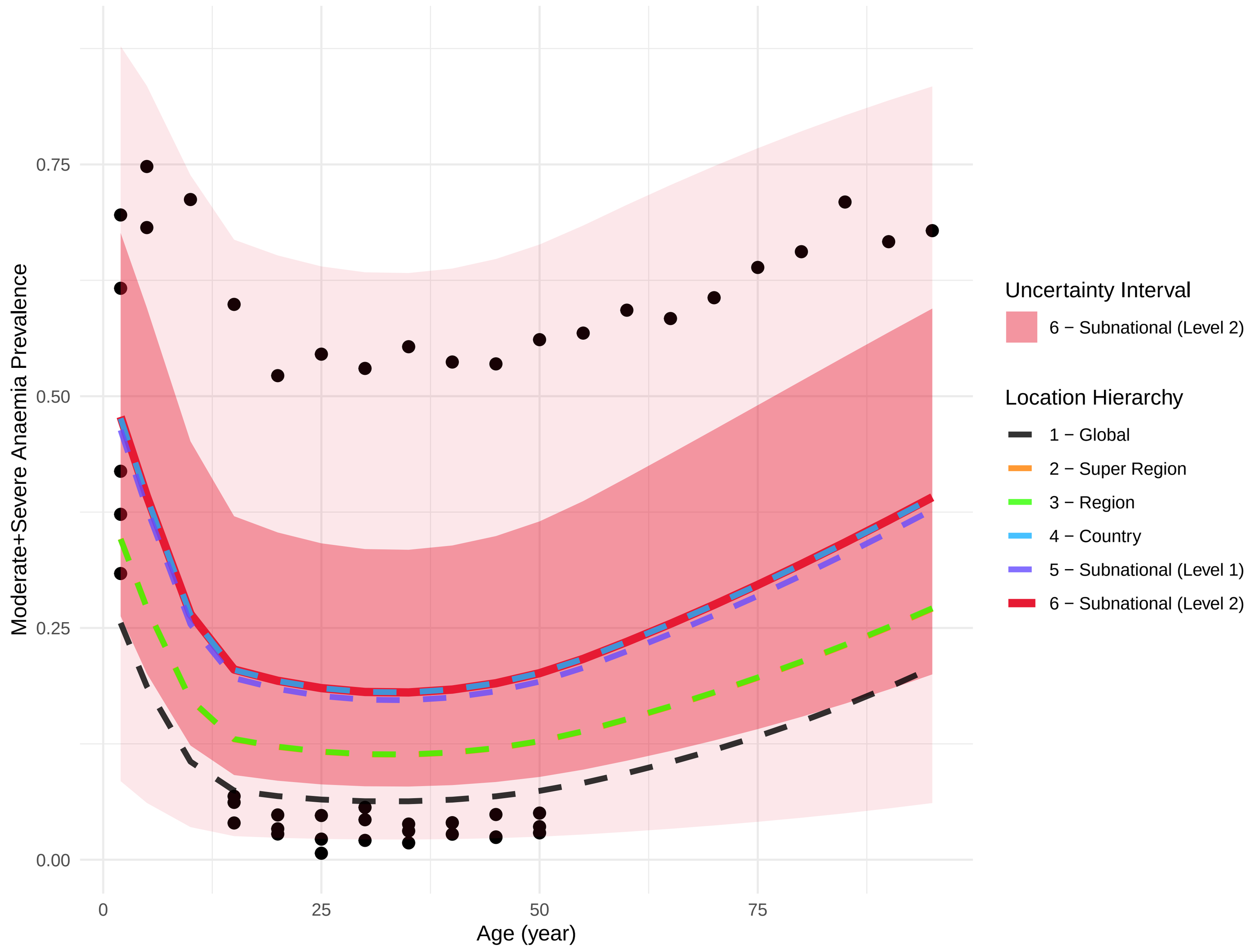

MR−BRT Age/Sex Fits − Males 2+ Years Old − Moderate+Severe Anaemia Prevalence − Rajasthan, Urban
WHO−2001 Adjustment Method

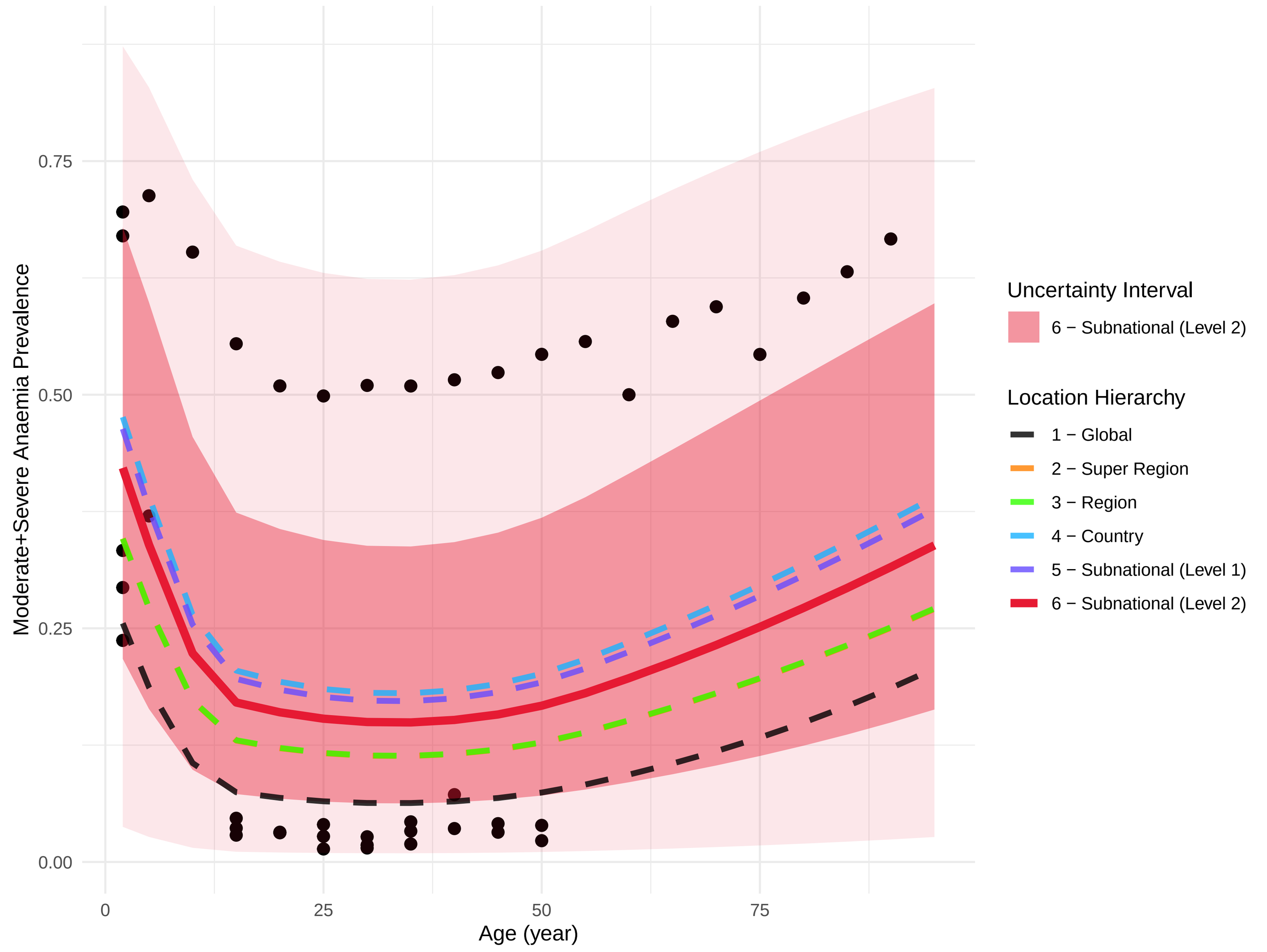

MR−BRT Age/Sex Fits − Males 2+ Years Old − Moderate+Severe Anaemia Prevalence − Sikkim
WHO−2001 Adjustment Method

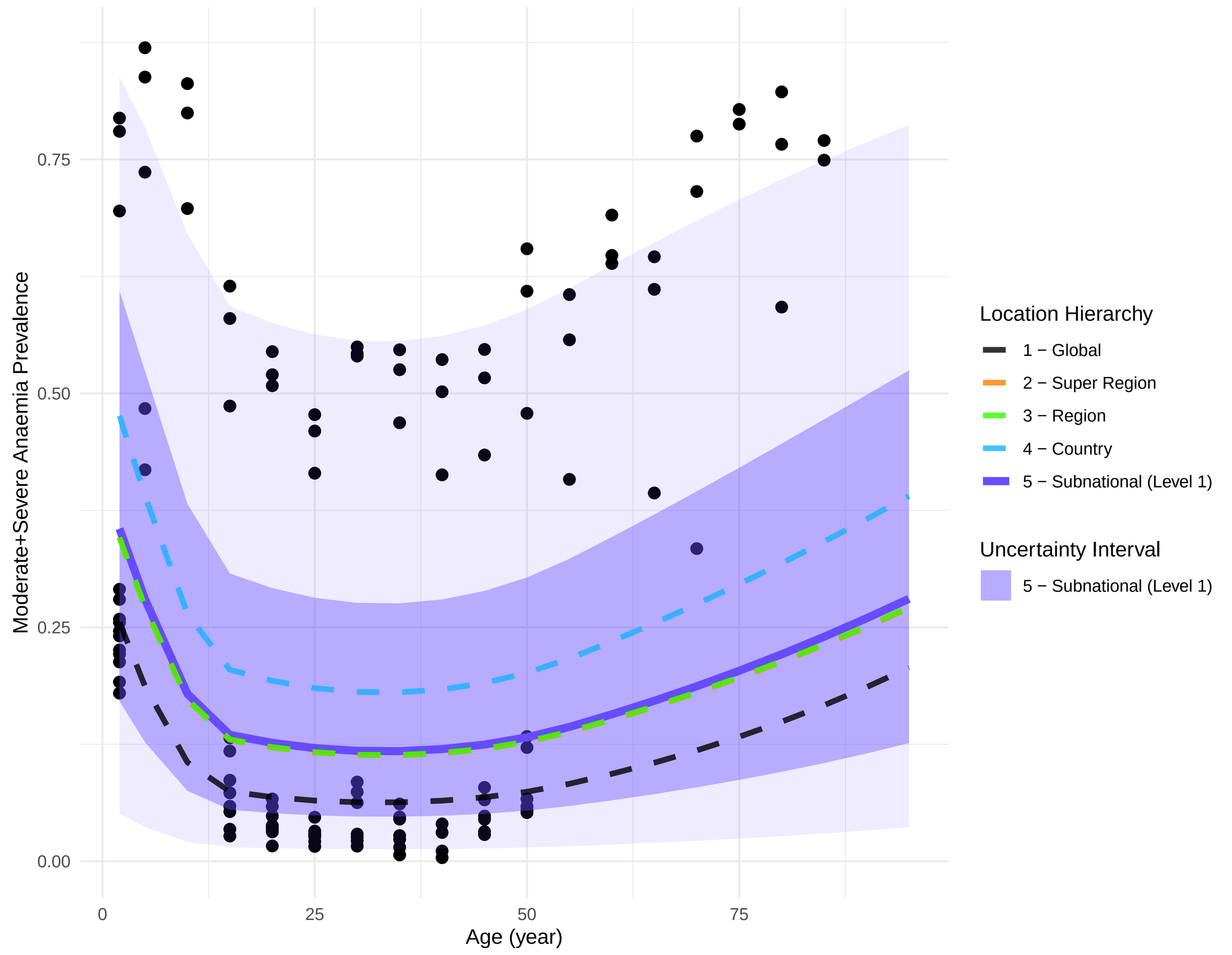

MR−BRT Age/Sex Fits − Males 2+ Years Old − Moderate+Severe Anaemia Prevalence − Sikkim, Rural
WHO−2001 Adjustment Method

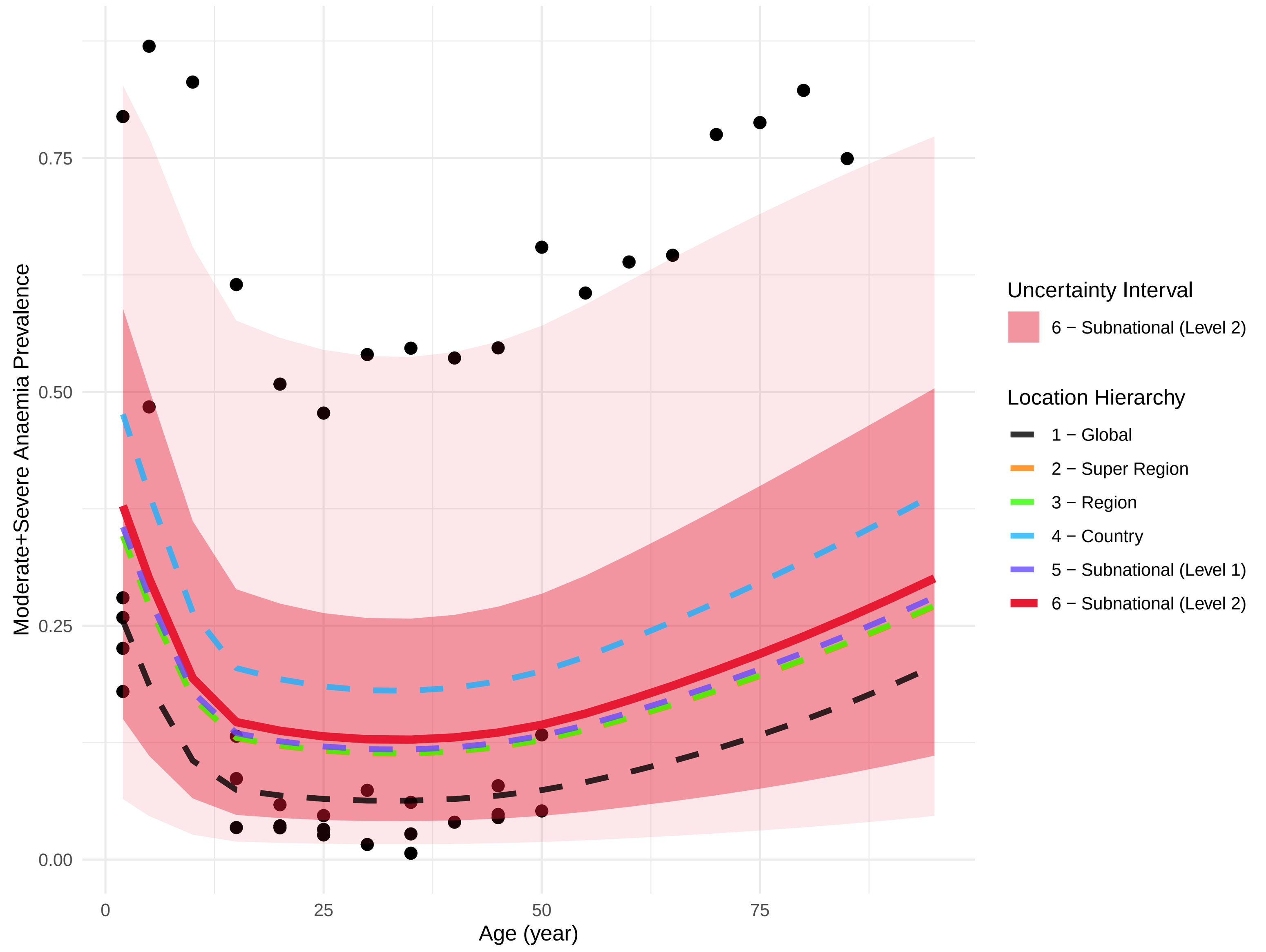

MR−BRT Age/Sex Fits − Males 2+ Years Old − Moderate+Severe Anaemia Prevalence − Sikkim, Urban
WHO−2001 Adjustment Method

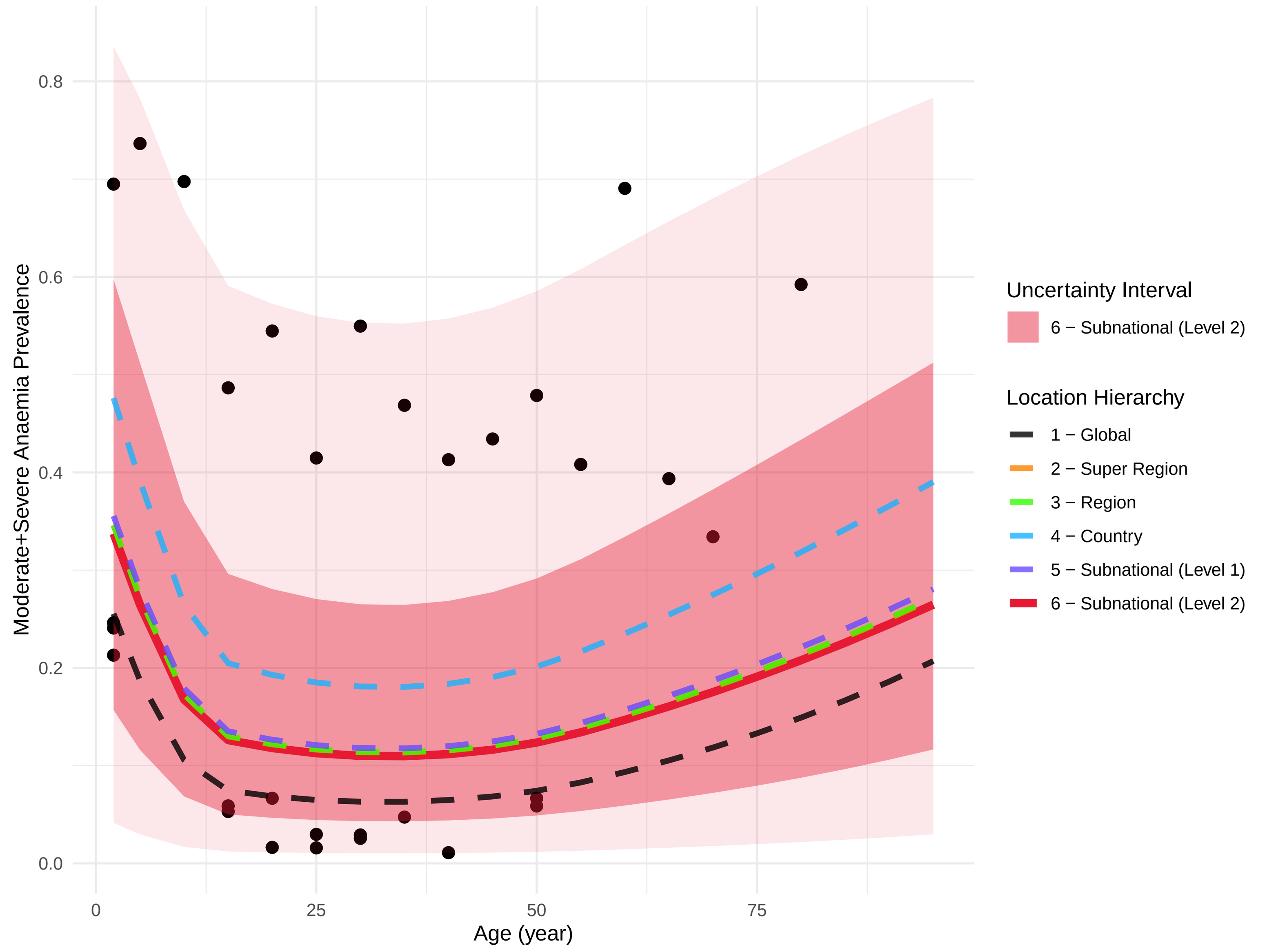

MR−BRT Age/Sex Fits − Males 2+ Years Old − Moderate+Severe Anaemia Prevalence − Tamil Nadu
WHO−2001 Adjustment Method

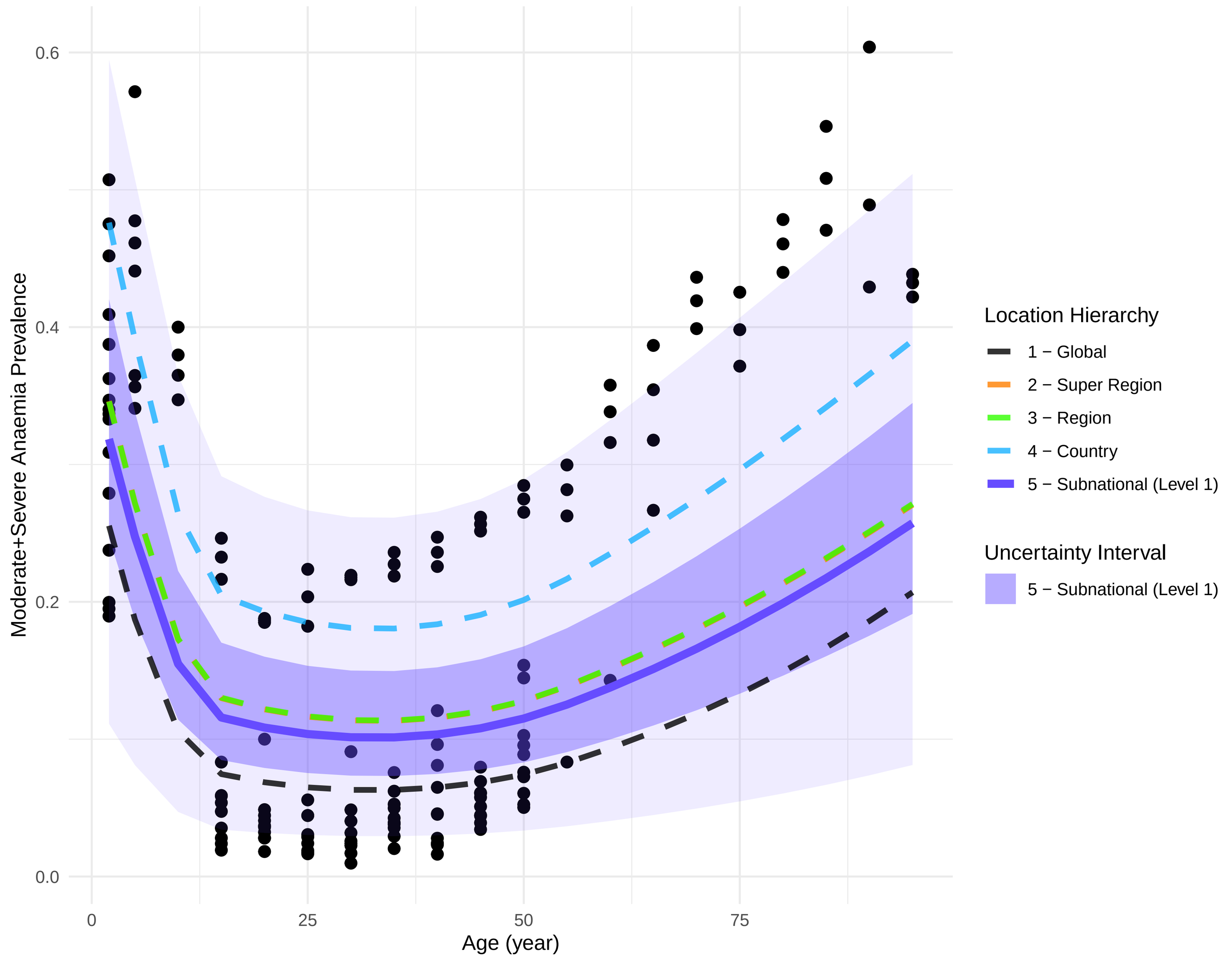

MR−BRT Age/Sex Fits − Males 2+ Years Old − Moderate+Severe Anaemia Prevalence − Tamil Nadu, Rural
WHO−2001 Adjustment Method

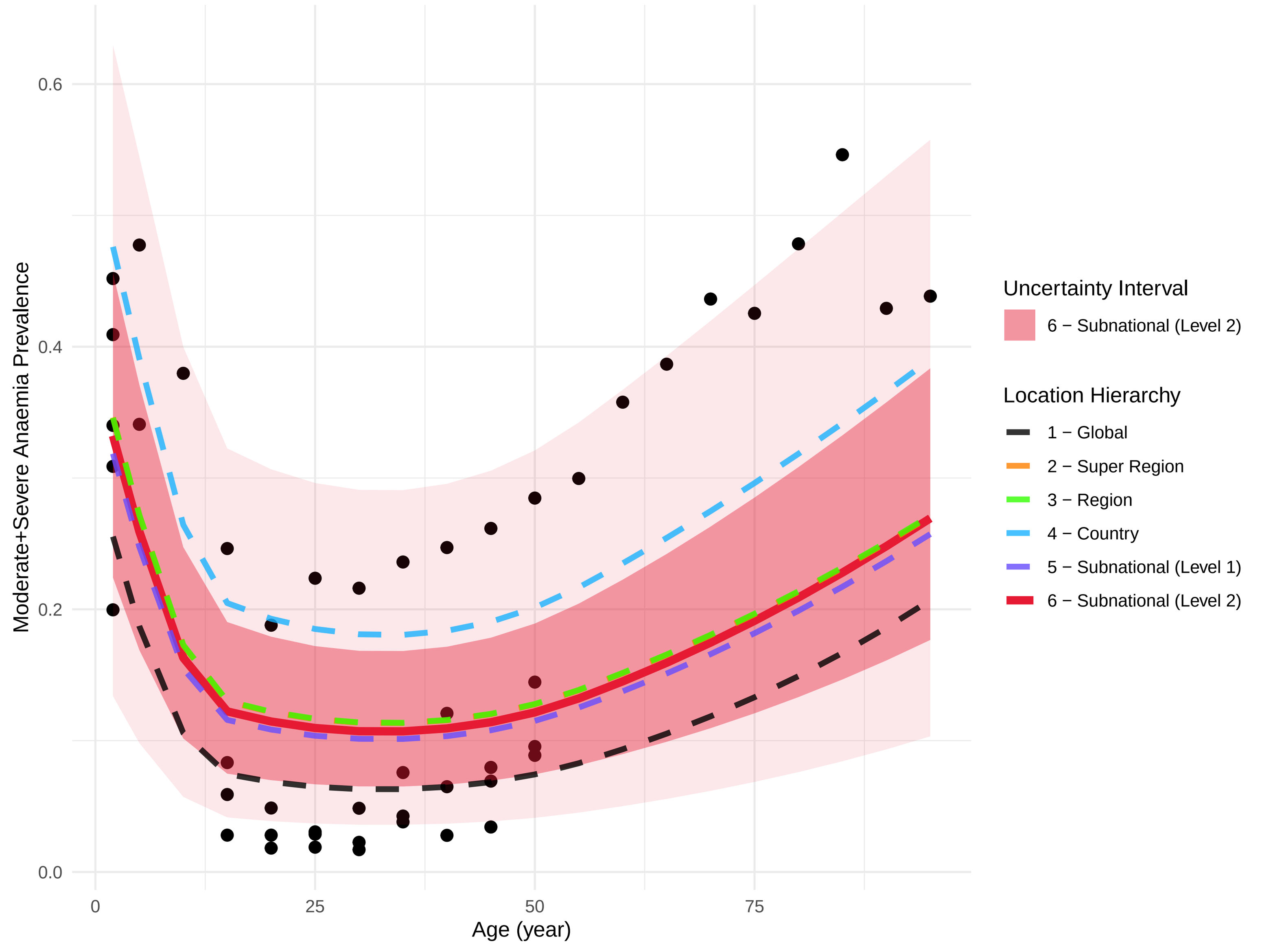

MR−BRT Age/Sex Fits − Males 2+ Years Old − Moderate+Severe Anaemia Prevalence − Tamil Nadu, Urban
WHO−2001 Adjustment Method

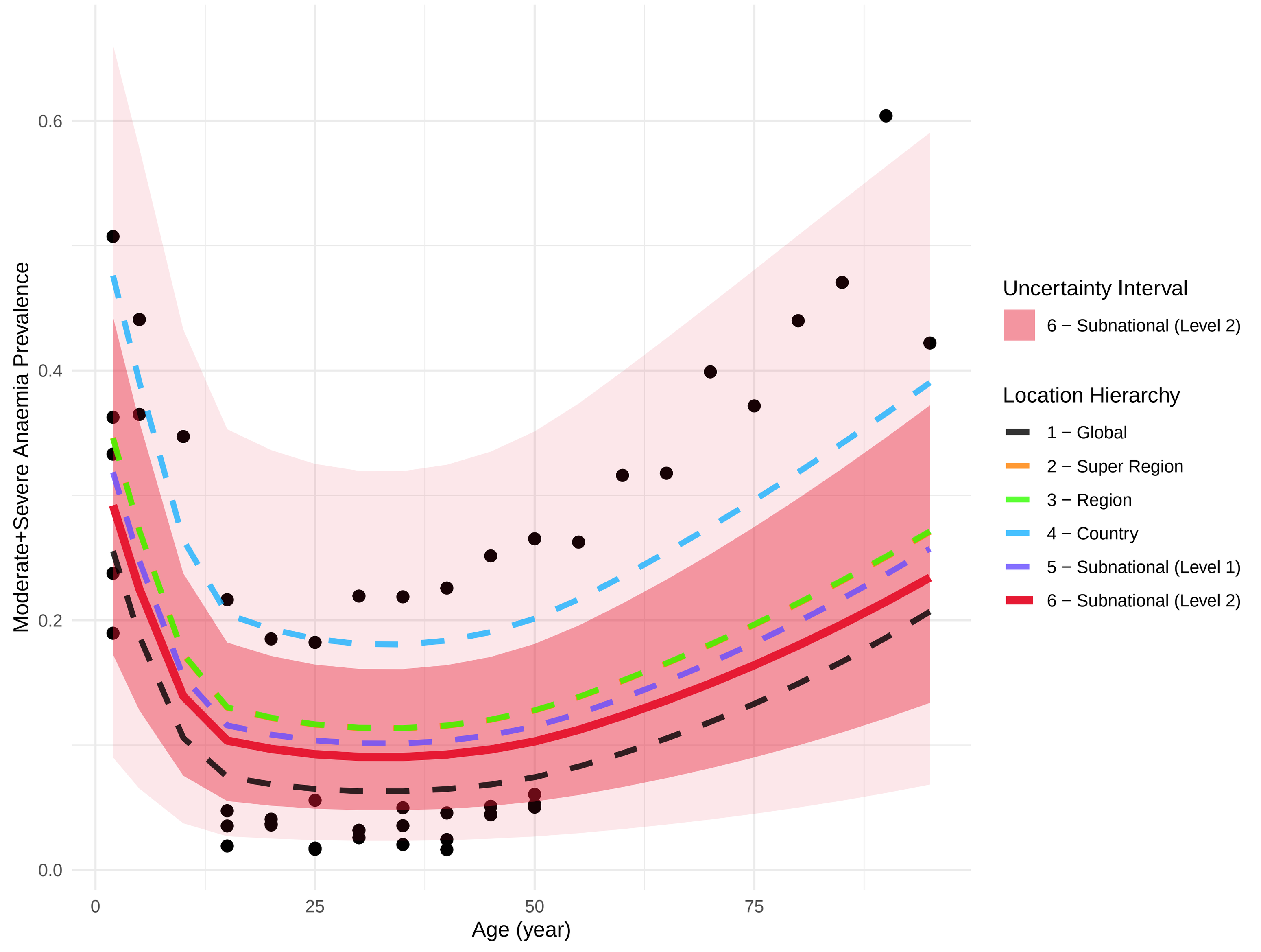

MR−BRT Age/Sex Fits − Males 2+ Years Old − Moderate+Severe Anaemia Prevalence − Telangana
WHO−2001 Adjustment Method

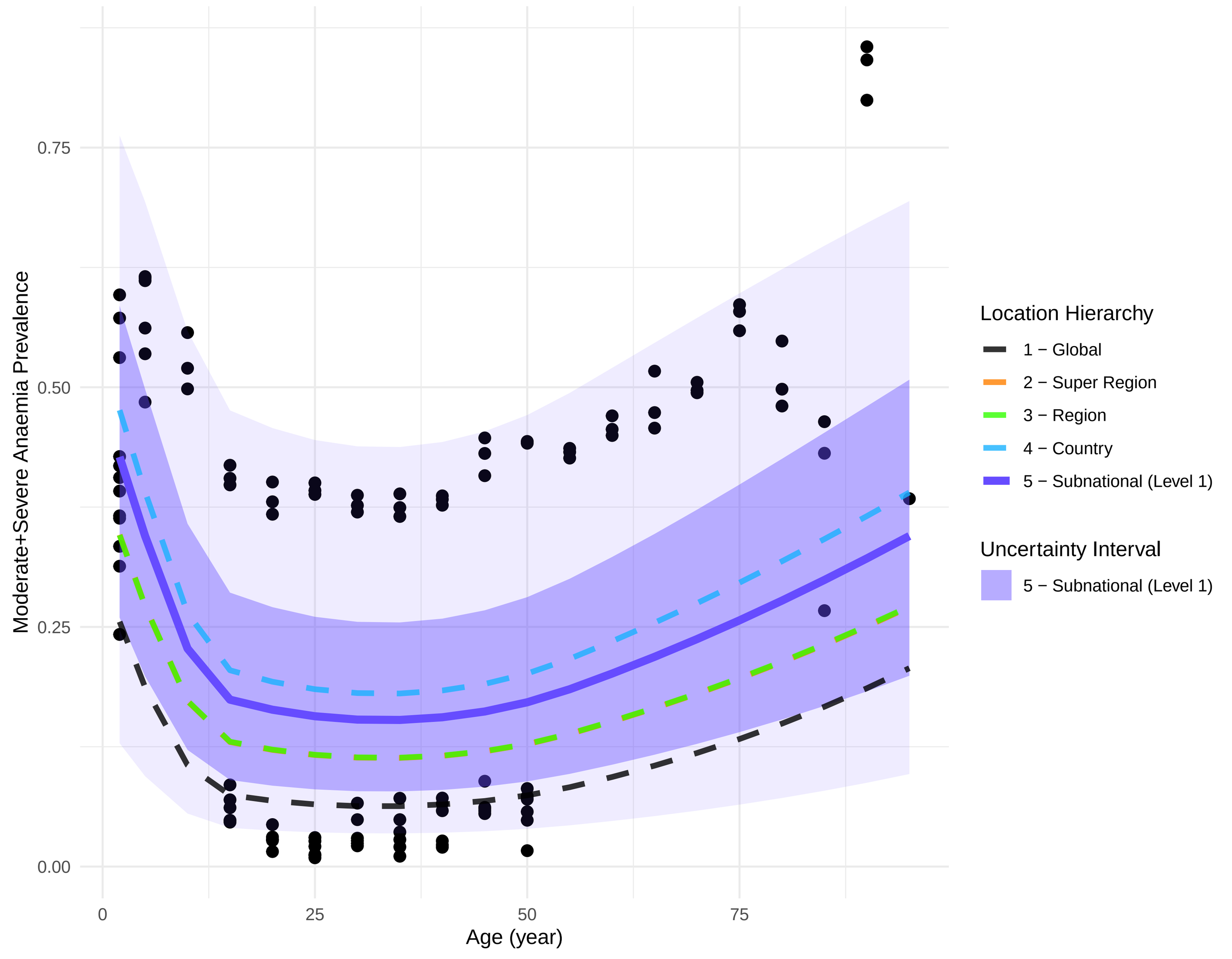

MR−BRT Age/Sex Fits − Males 2+ Years Old − Moderate+Severe Anaemia Prevalence − Telangana, Rural
WHO−2001 Adjustment Method

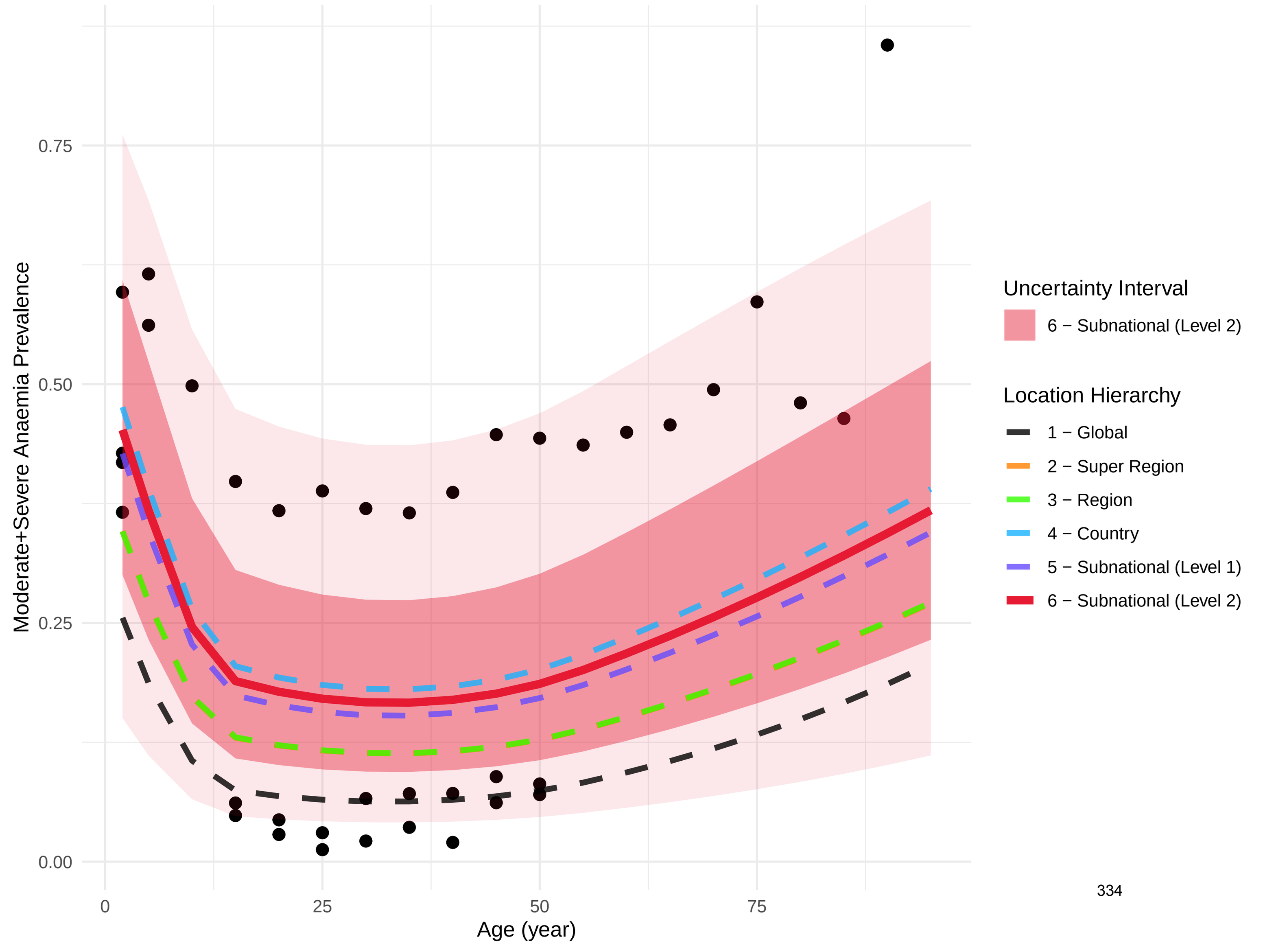

MR−BRT Age/Sex Fits − Males 2+ Years Old − Moderate+Severe Anaemia Prevalence − Telangana, Urban
WHO−2001 Adjustment Method

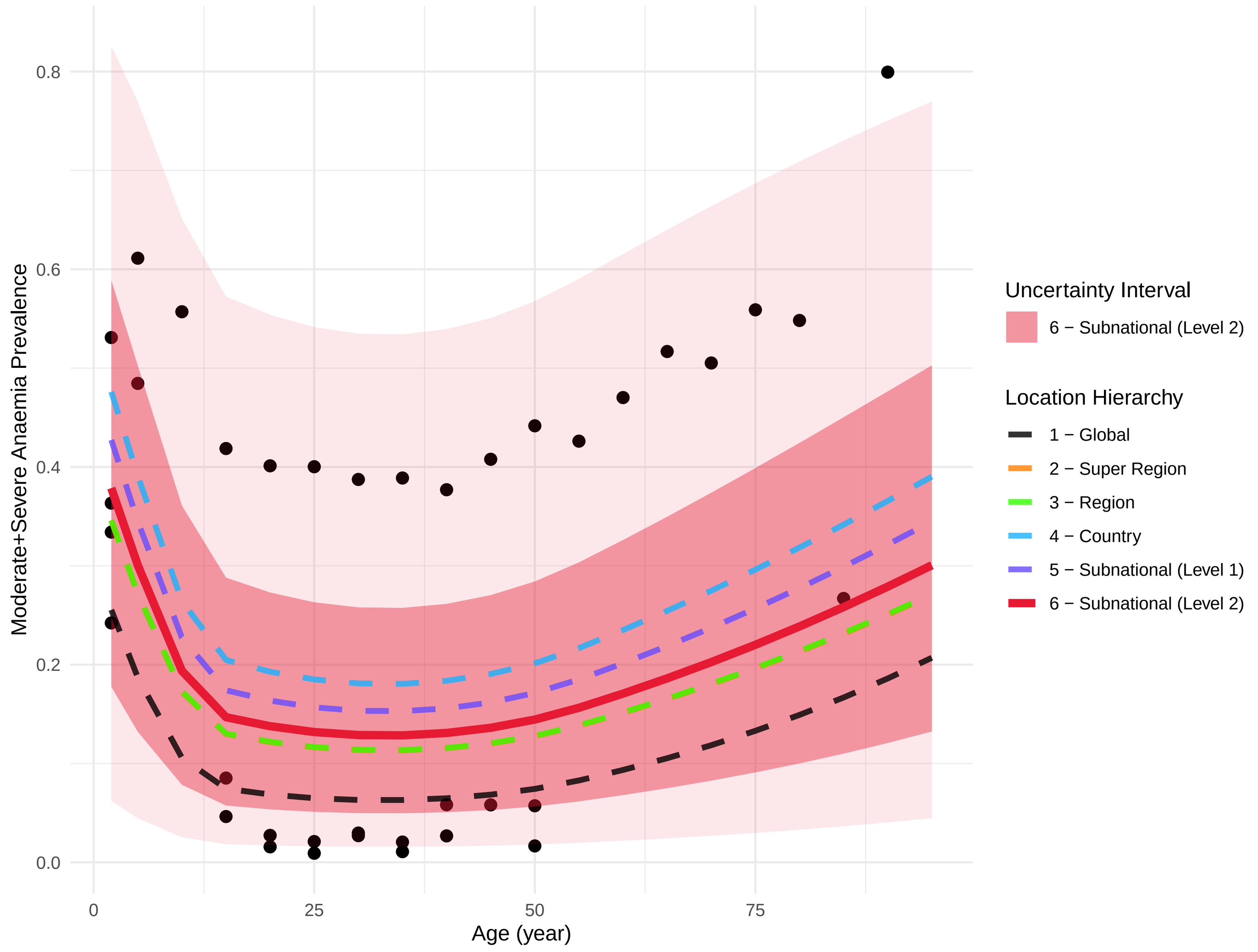

MR−BRT Age/Sex Fits − Males 2+ Years Old − Moderate+Severe Anaemia Prevalence − Tripura
WHO−2001 Adjustment Method

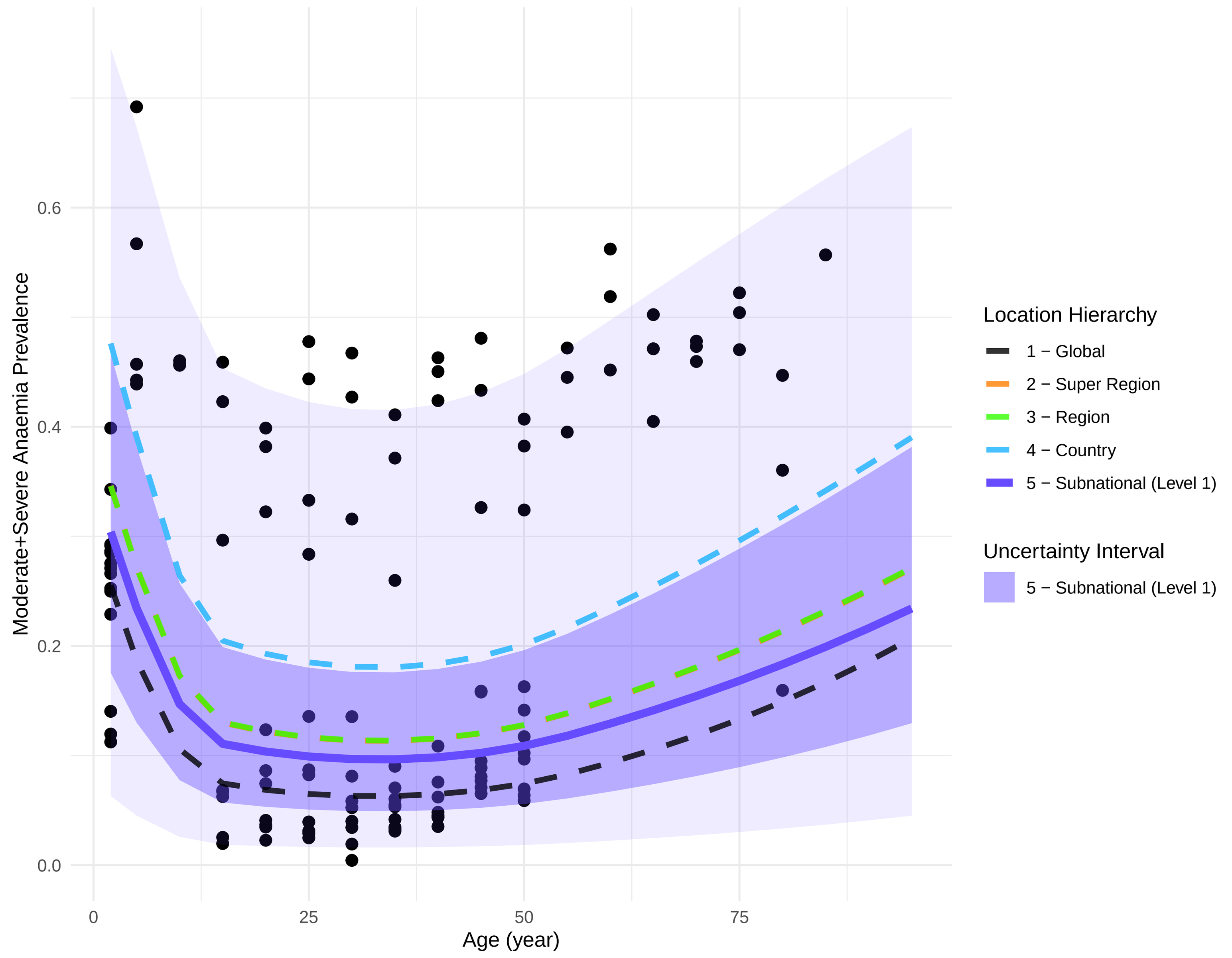

MR−BRT Age/Sex Fits − Males 2+ Years Old − Moderate+Severe Anaemia Prevalence − Tripura, Rural
WHO−2001 Adjustment Method

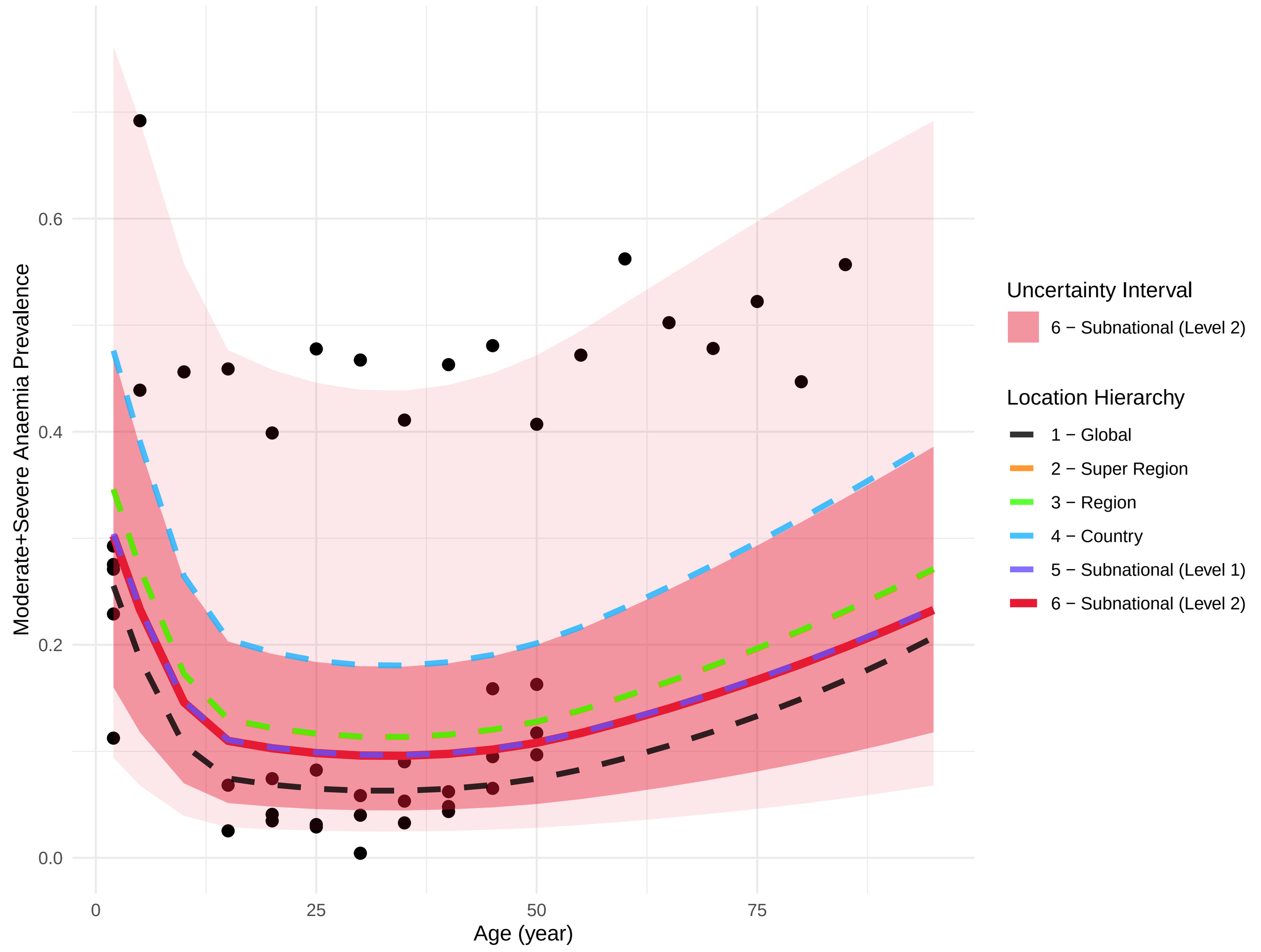

MR−BRT Age/Sex Fits − Males 2+ Years Old − Moderate+Severe Anaemia Prevalence − Tripura, Urban
WHO−2001 Adjustment Method

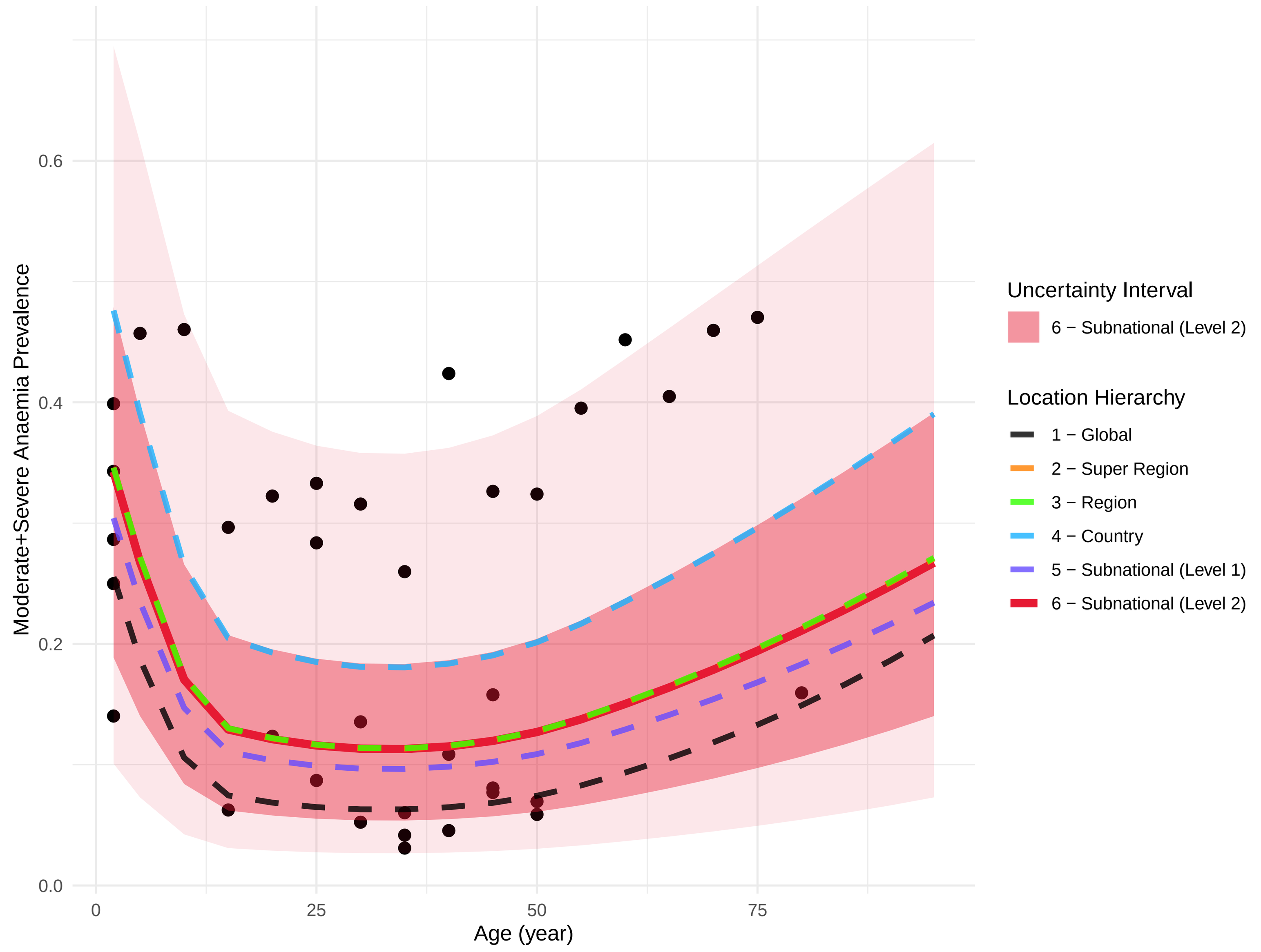

MR−BRT Age/Sex Fits − Males 2+ Years Old − Moderate+Severe Anaemia Prevalence − Uttar Pradesh
WHO−2001 Adjustment Method

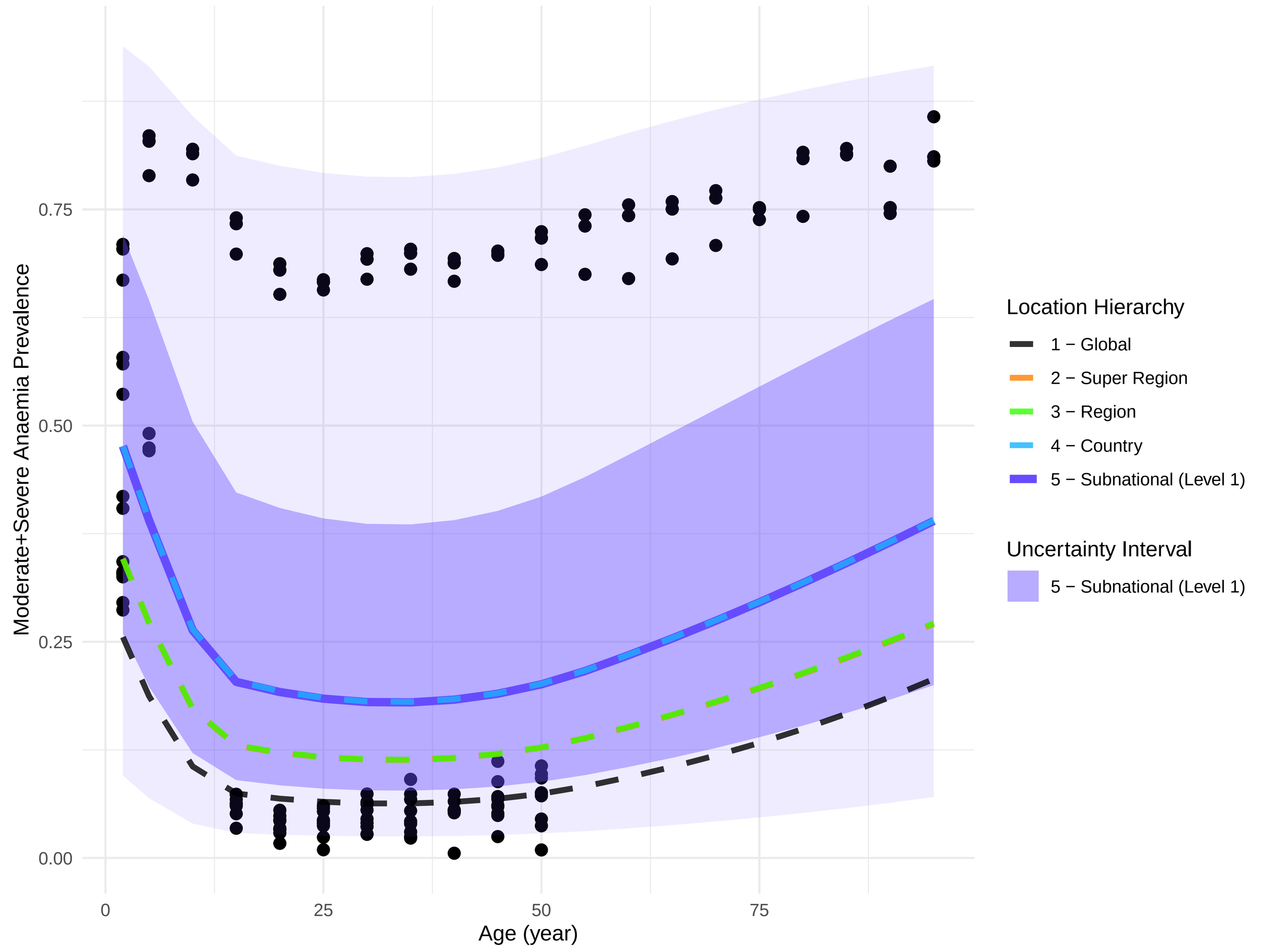

MR−BRT Age/Sex Fits − Males 2+ Years Old − Moderate+Severe Anaemia Prevalence − Uttar Pradesh, Rural
WHO−2001 Adjustment Method

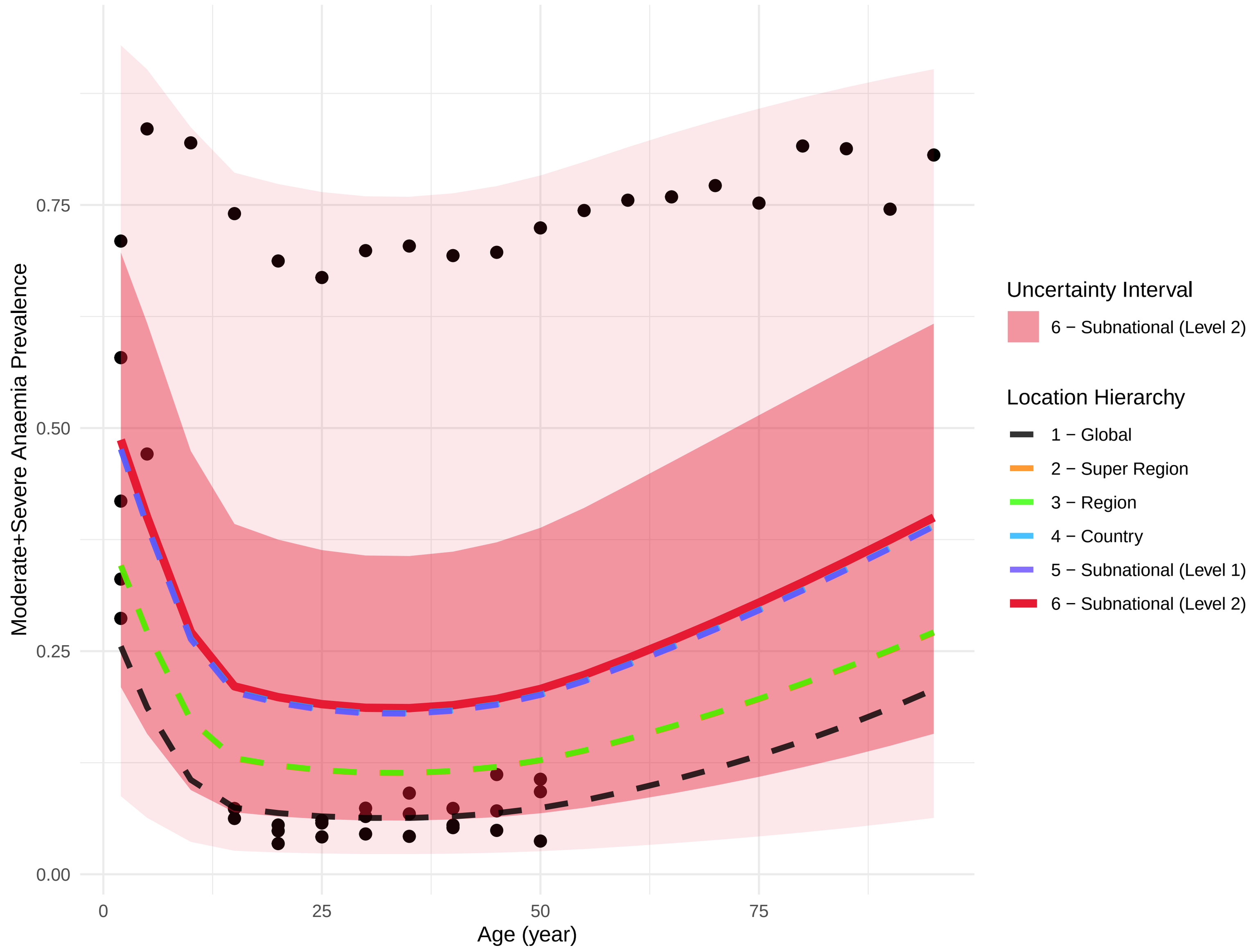

MR−BRT Age/Sex Fits − Males 2+ Years Old − Moderate+Severe Anaemia Prevalence − Uttar Pradesh, Urban
WHO−2001 Adjustment Method

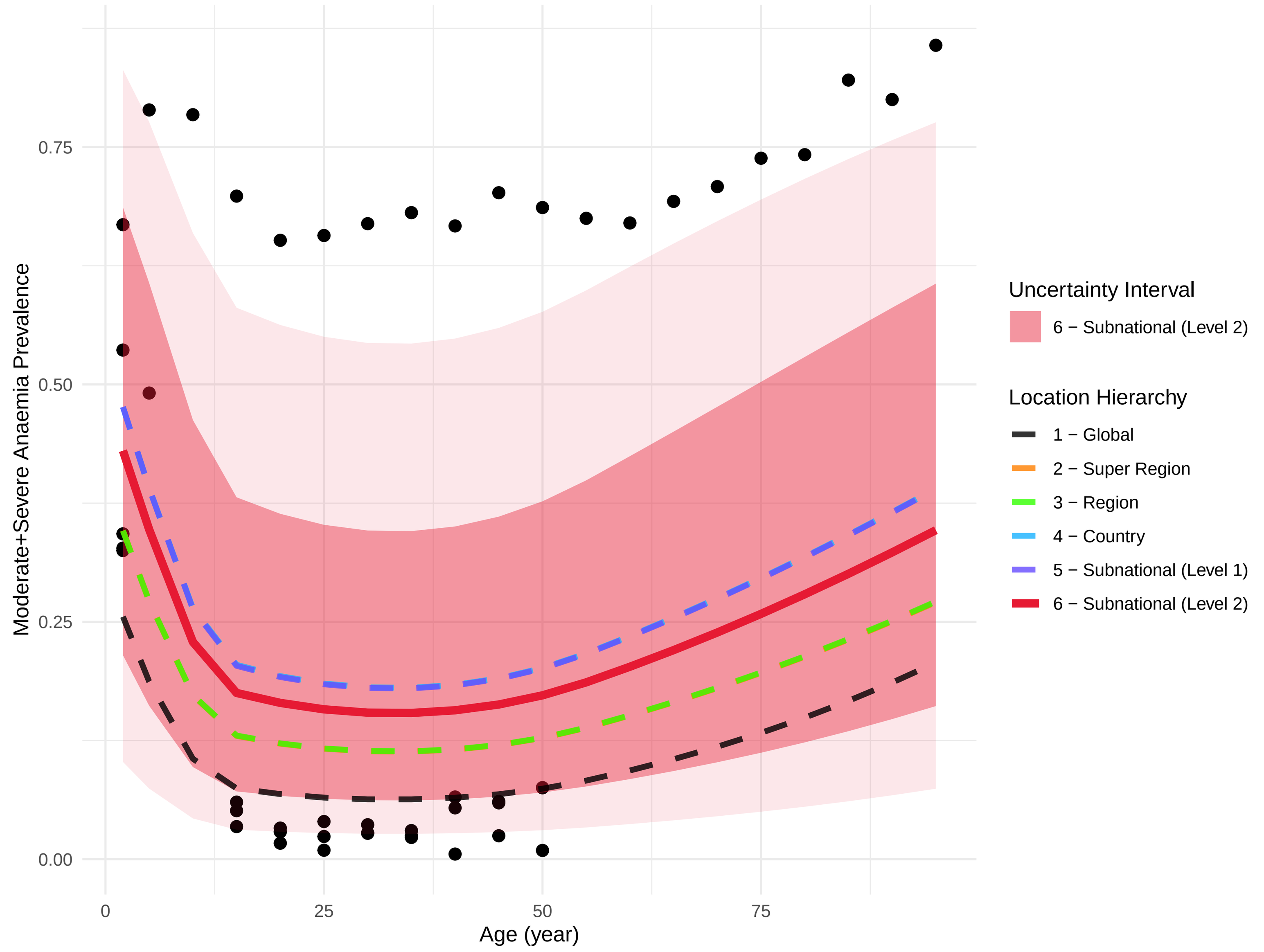

MR−BRT Age/Sex Fits − Males 2+ Years Old − Moderate+Severe Anaemia Prevalence − Uttarakhand
WHO−2001 Adjustment Method

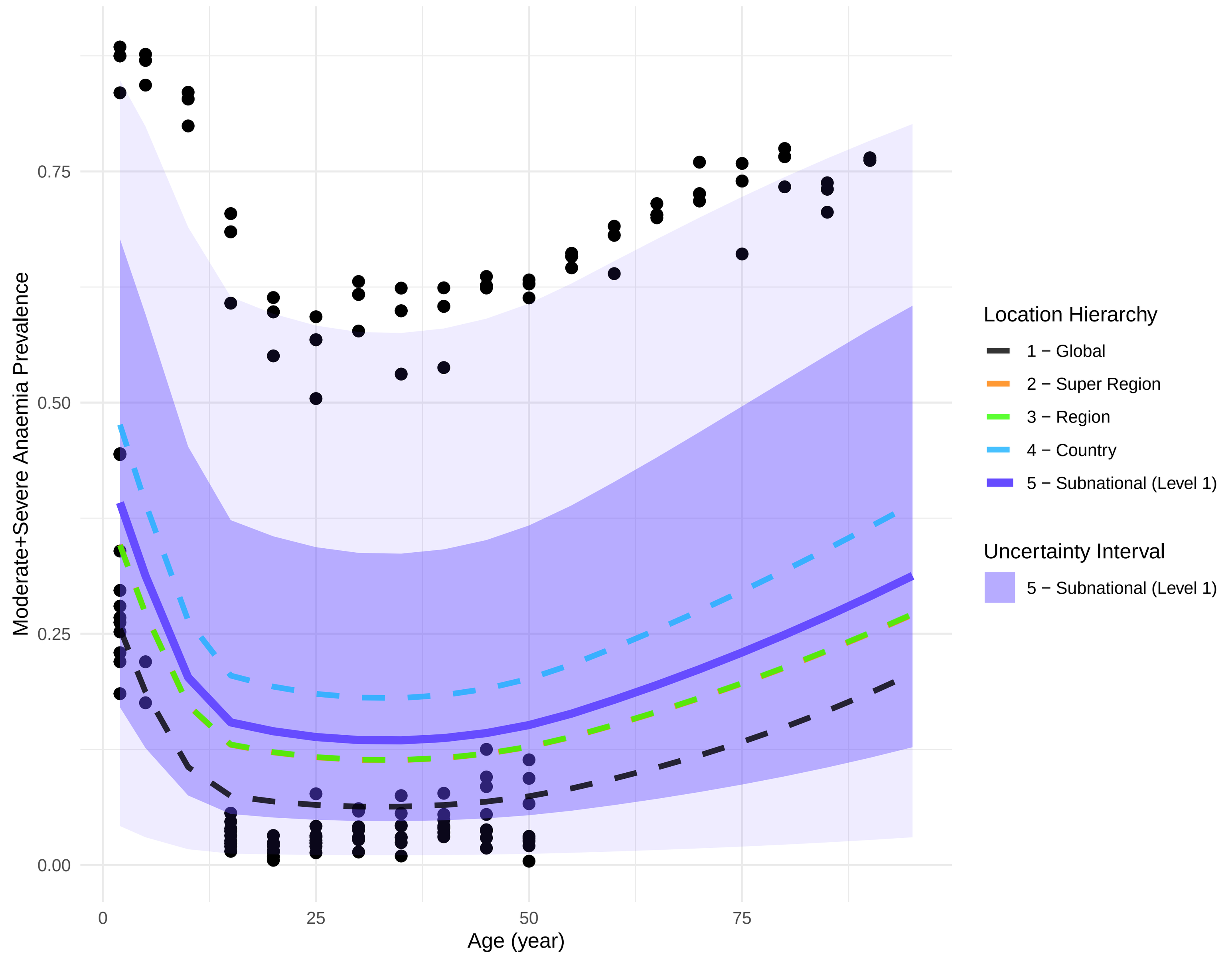

MR−BRT Age/Sex Fits − Males 2+ Years Old − Moderate+Severe Anaemia Prevalence − Uttarakhand, Rural
WHO−2001 Adjustment Method

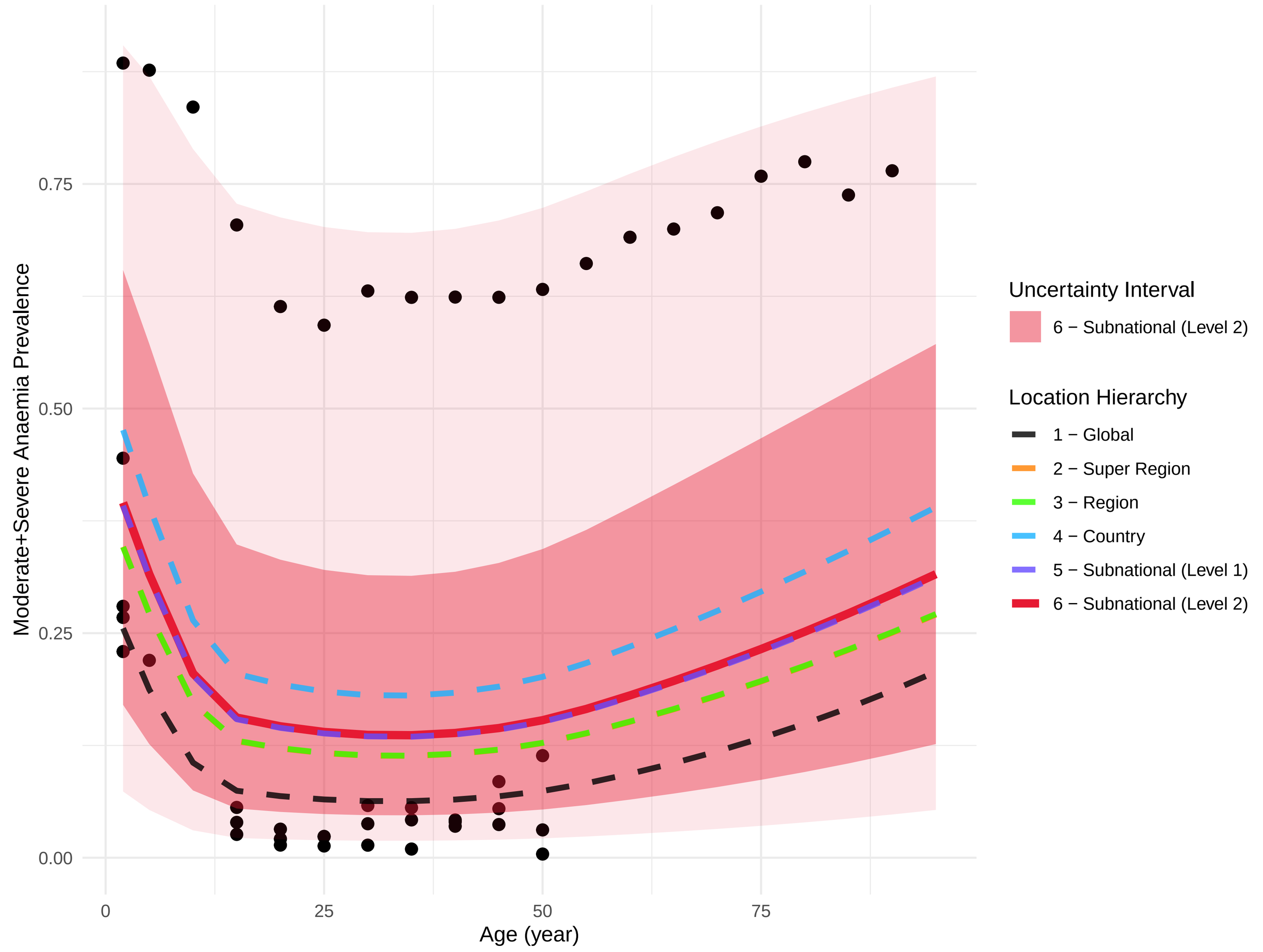

MR−BRT Age/Sex Fits − Males 2+ Years Old − Moderate+Severe Anaemia Prevalence − Uttarakhand, Urban
WHO−2001 Adjustment Method

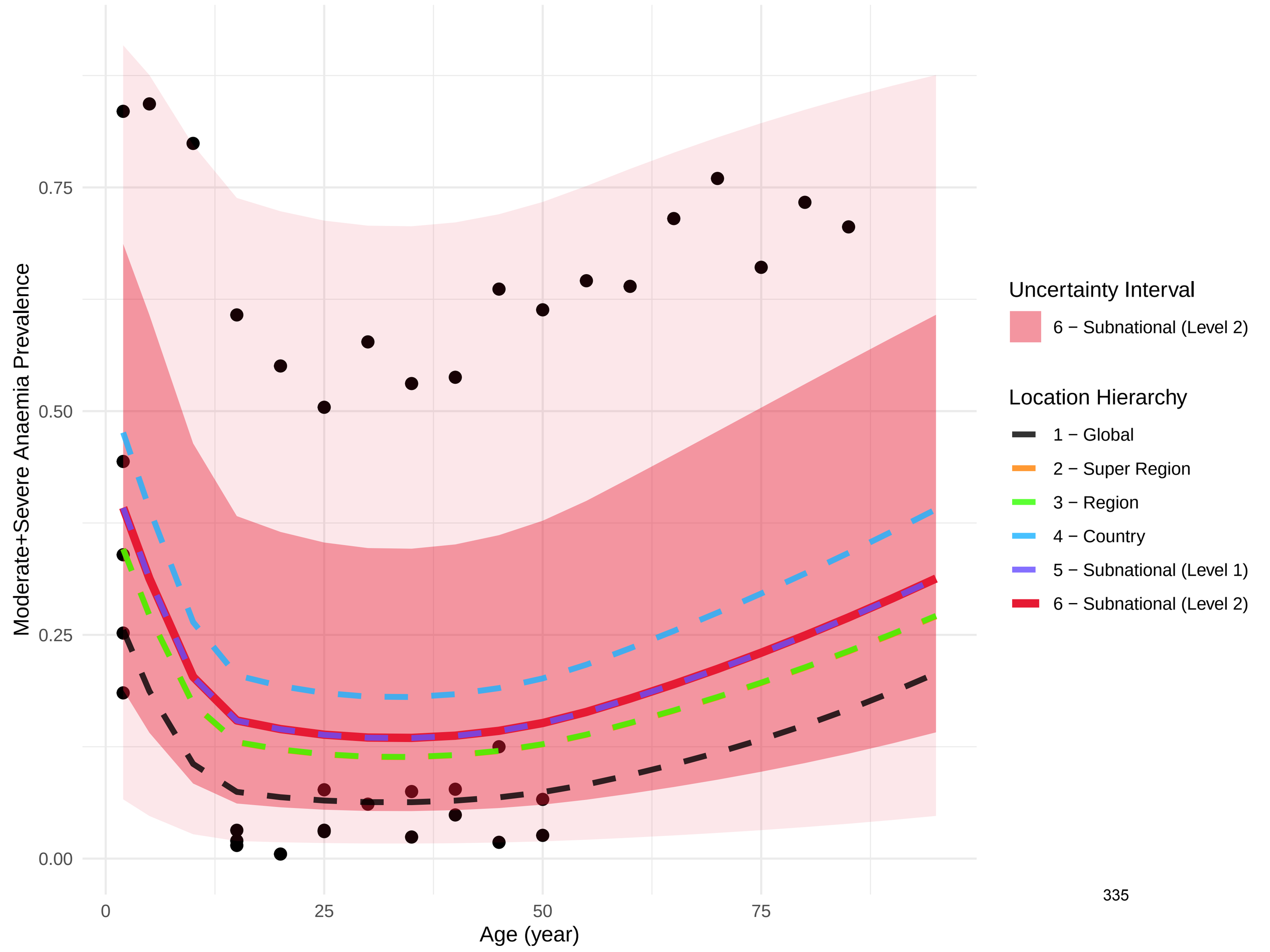

MR−BRT Age/Sex Fits − Males 2+ Years Old − Moderate+Severe Anaemia Prevalence − West Bengal
WHO−2001 Adjustment Method

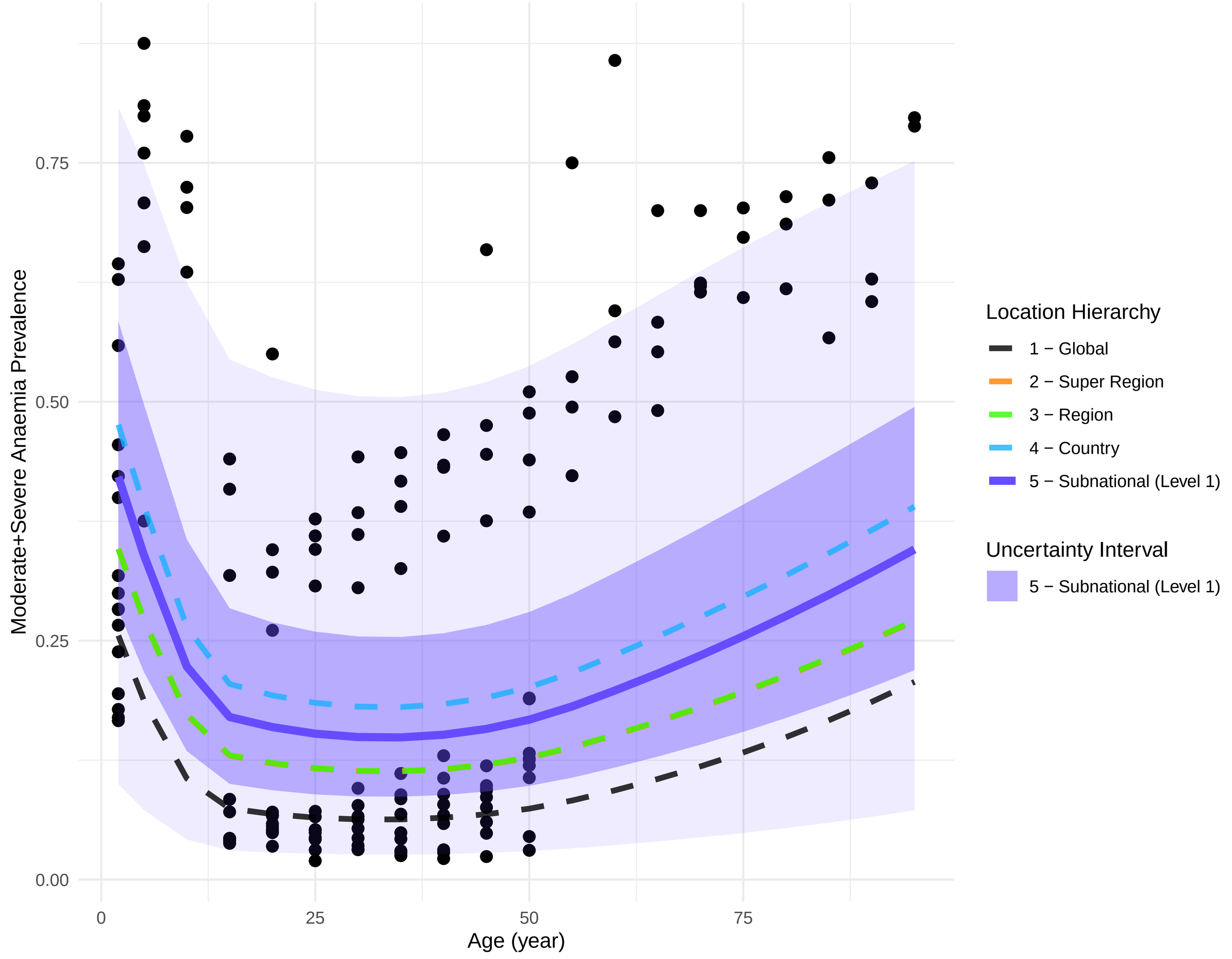

MR−BRT Age/Sex Fits − Males 2+ Years Old − Moderate+Severe Anaemia Prevalence − West Bengal, Rural
WHO−2001 Adjustment Method

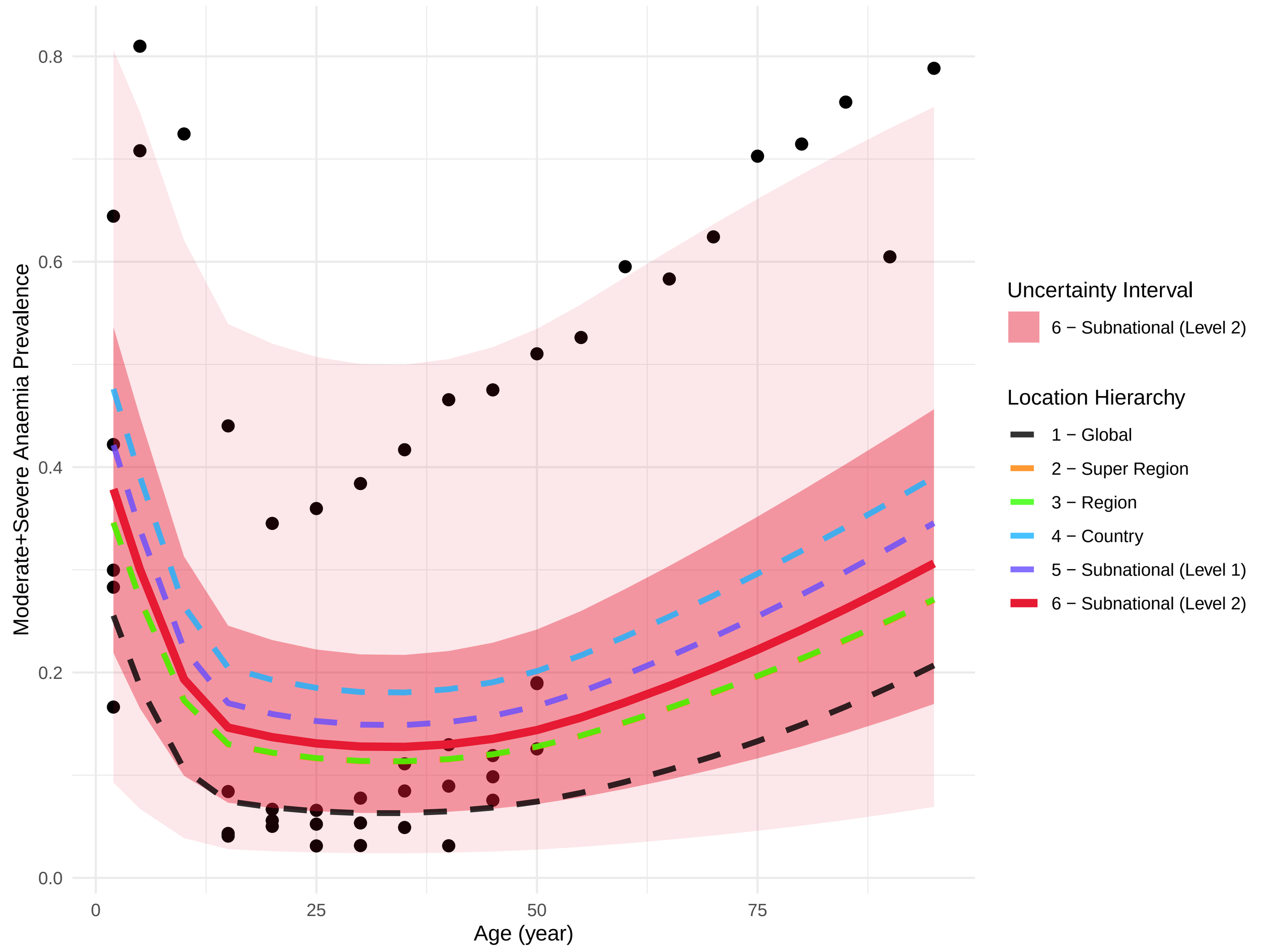

MR−BRT Age/Sex Fits − Males 2+ Years Old − Moderate+Severe Anaemia Prevalence − West Bengal, Urban
WHO−2001 Adjustment Method

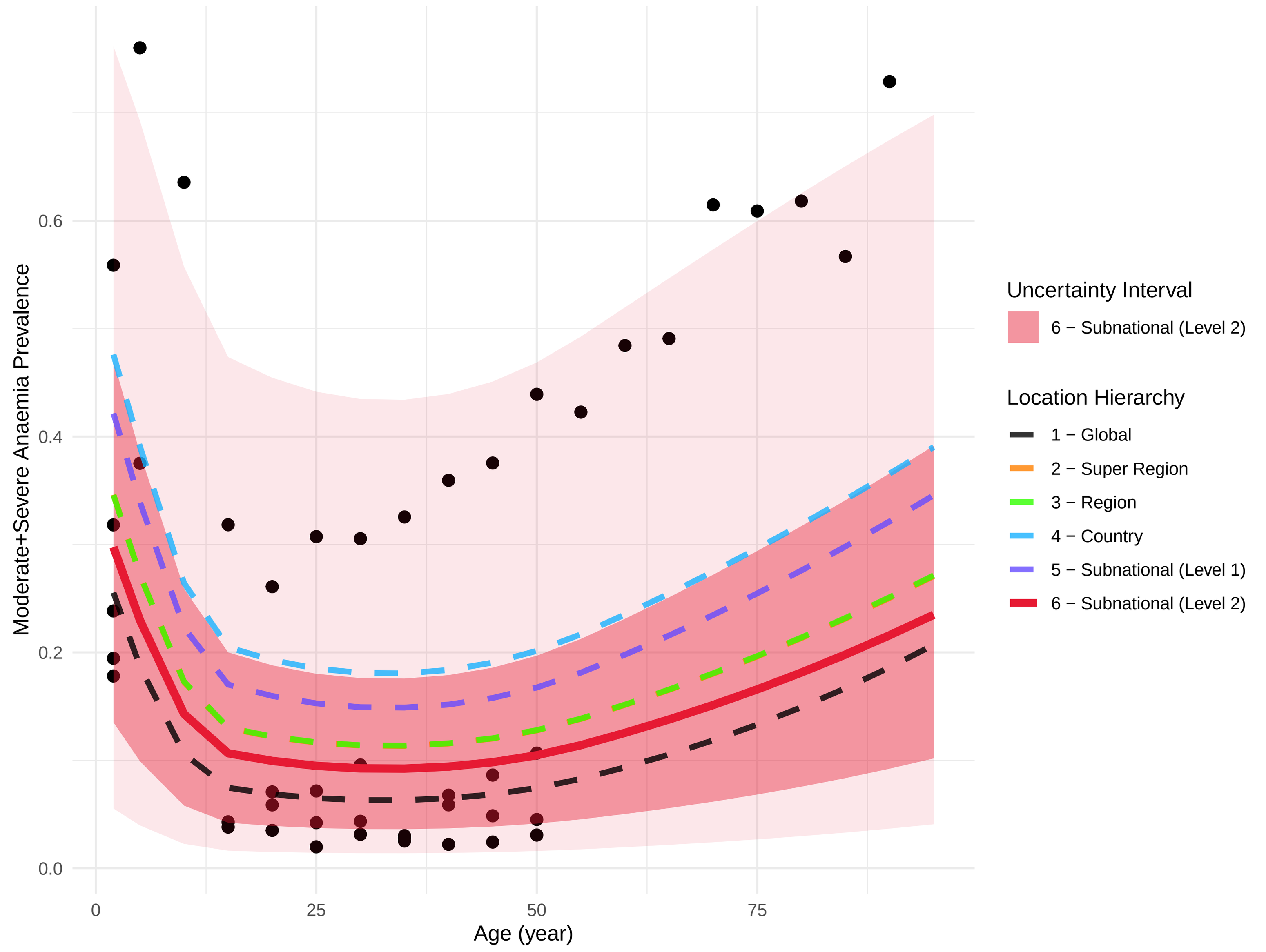

MR−BRT Age/Sex Fits − Males 2+ Years Old − Moderate+Severe Anaemia Prevalence − Nepal
WHO−2001 Adjustment Method

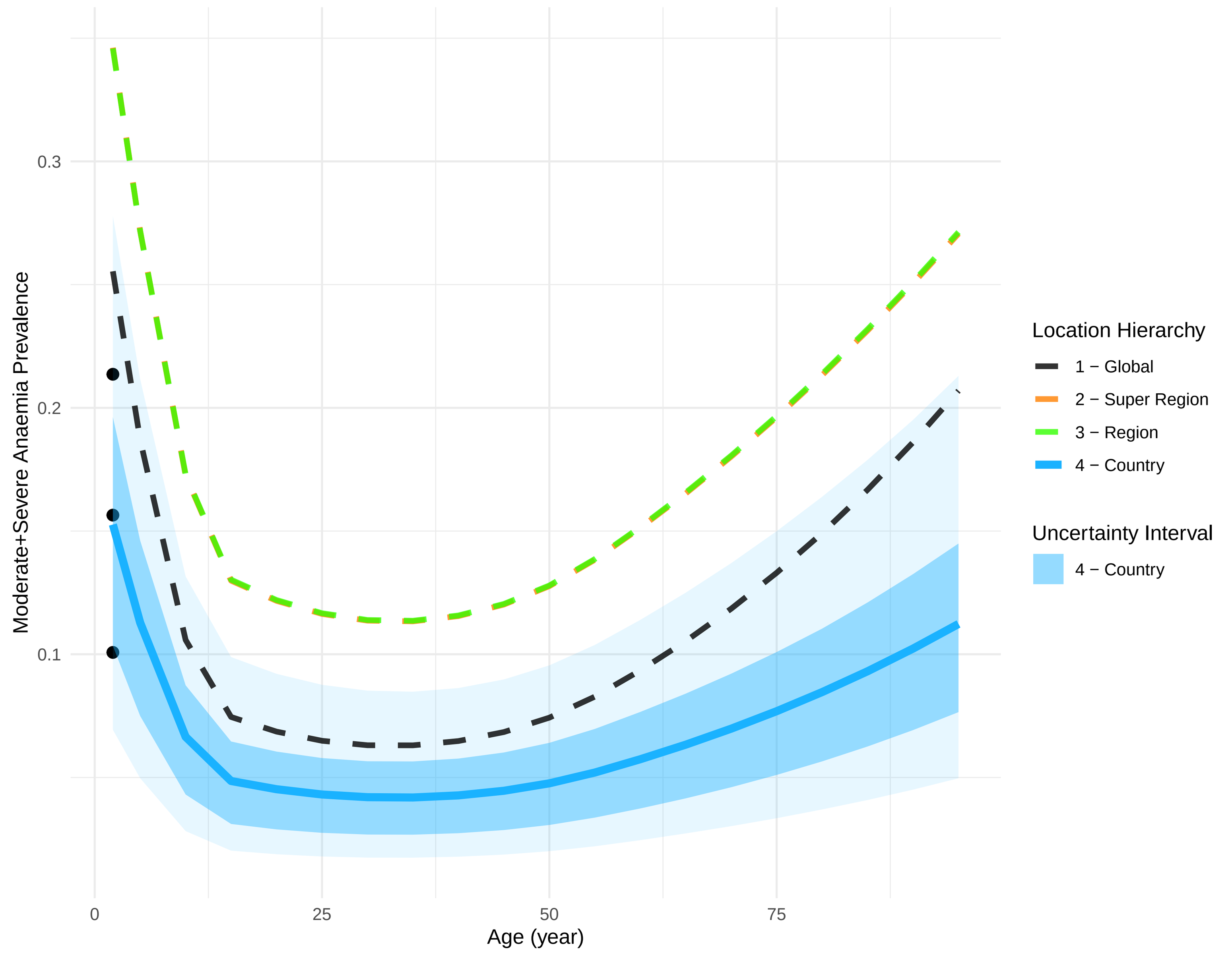

MR−BRT Age/Sex Fits − Males 2+ Years Old − Moderate+Severe Anaemia Prevalence − Southeast Asia, East Asia, and Oceania
WHO−2001 Adjustment Method

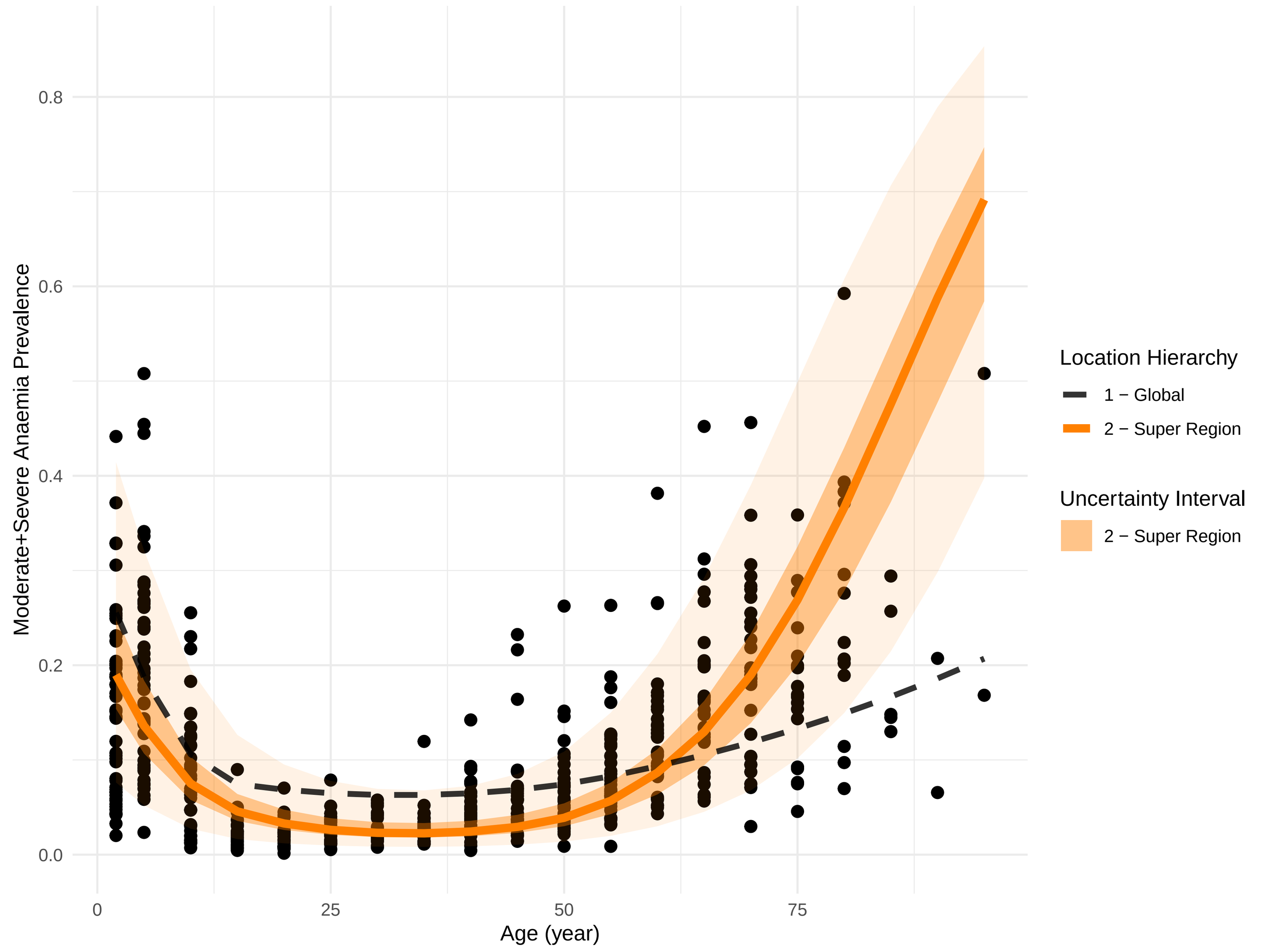

MR−BRT Age/Sex Fits − Males 2+ Years Old − Moderate+Severe Anaemia Prevalence − Southeast Asia
WHO−2001 Adjustment Method

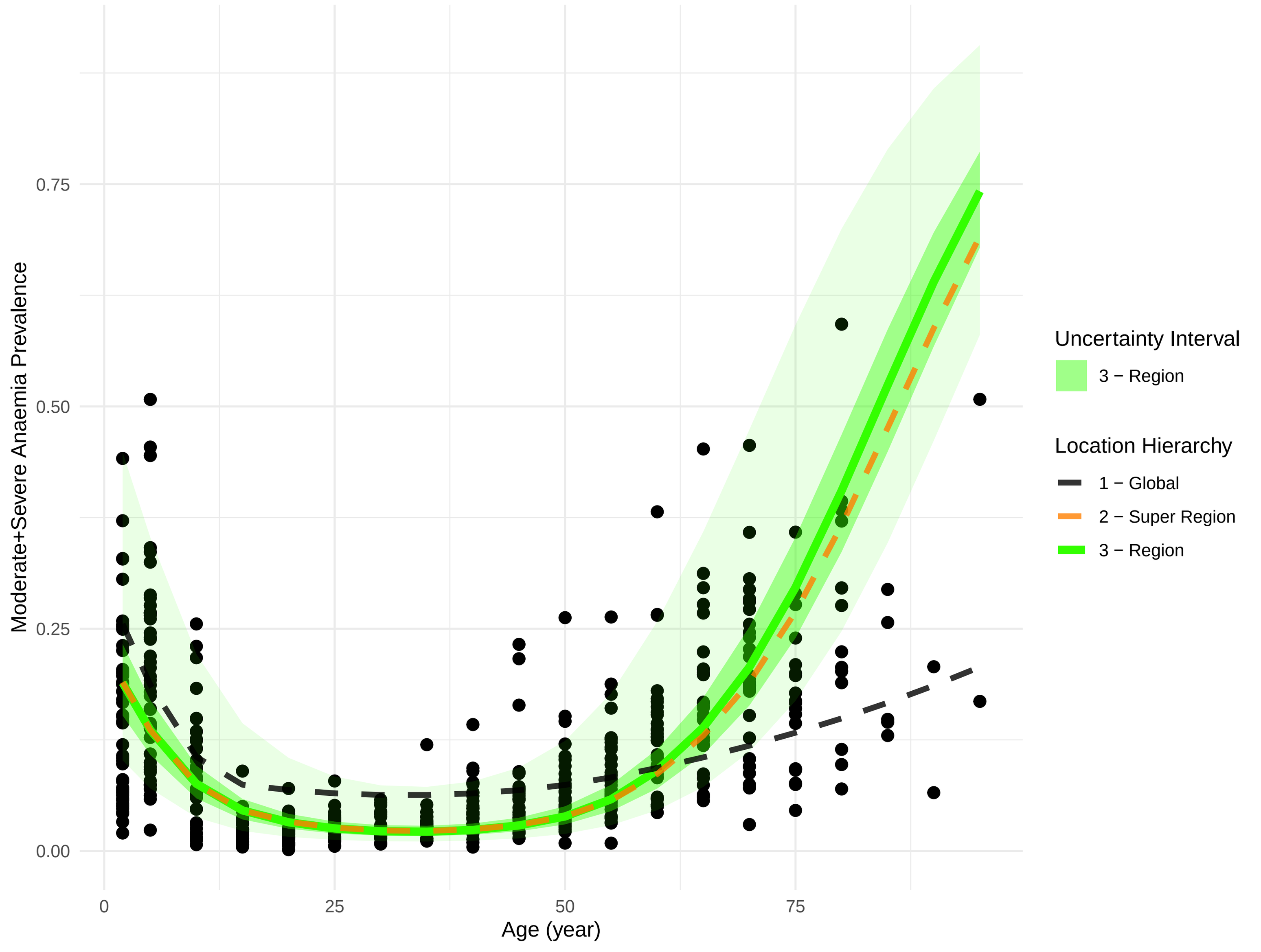

MR−BRT Age/Sex Fits − Males 2+ Years Old − Moderate+Severe Anaemia Prevalence − Cambodia
WHO−2001 Adjustment Method

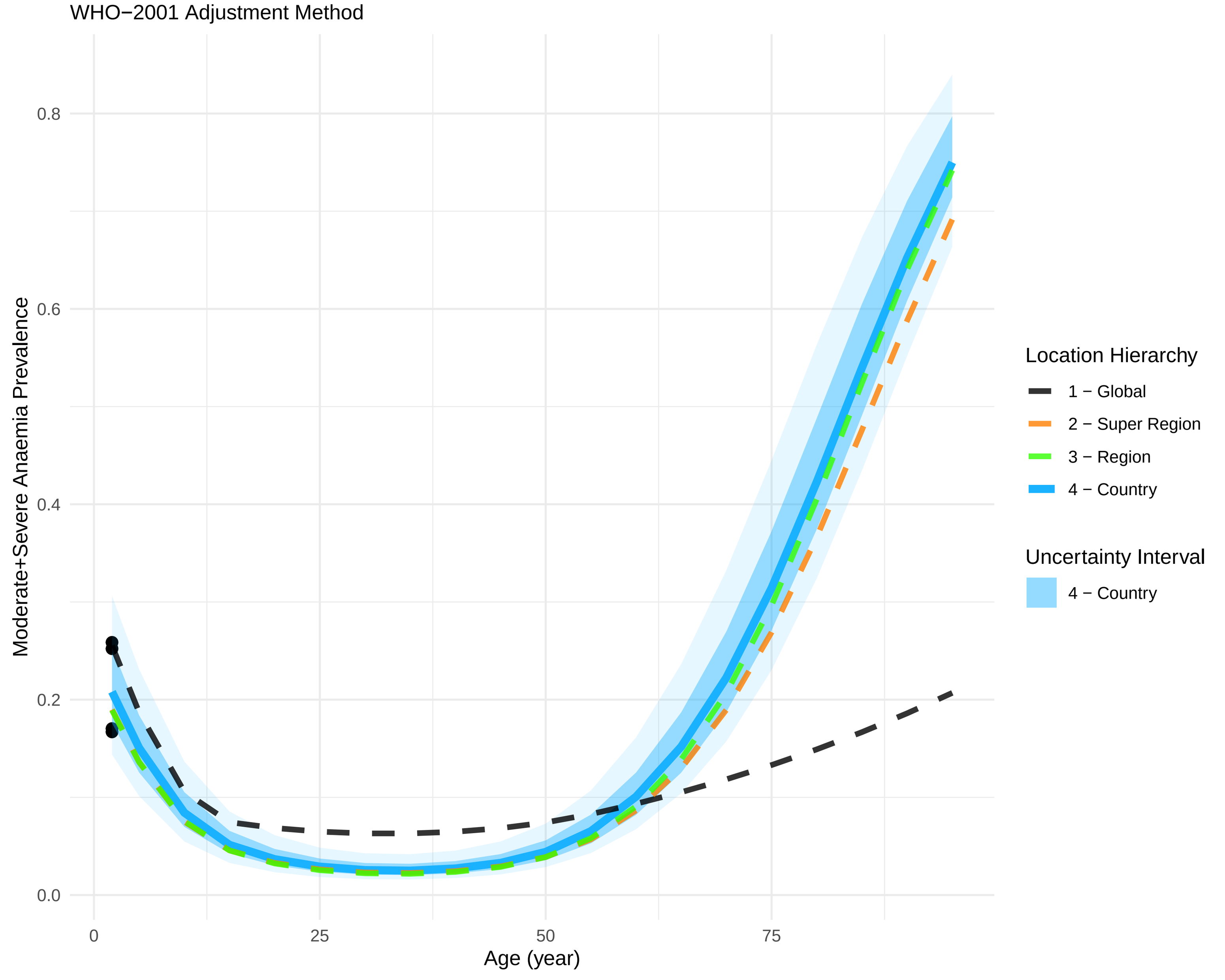

MR−BRT Age/Sex Fits − Males 2+ Years Old − Moderate+Severe Anaemia Prevalence − Indonesia
WHO−2001 Adjustment Method

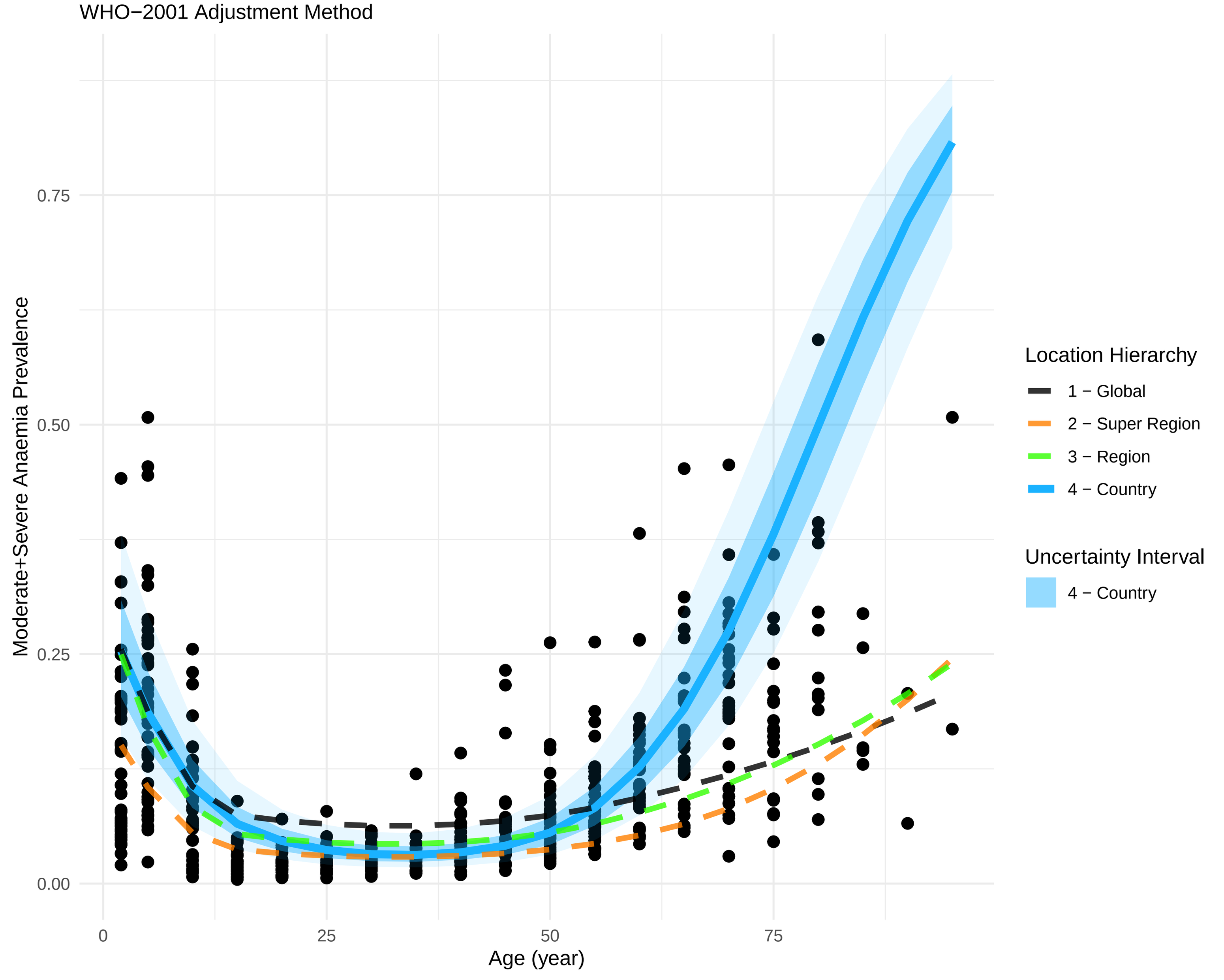

MR−BRT Age/Sex Fits − Males 2+ Years Old − Moderate+Severe Anaemia Prevalence − Bali
WHO−2001 Adjustment Method

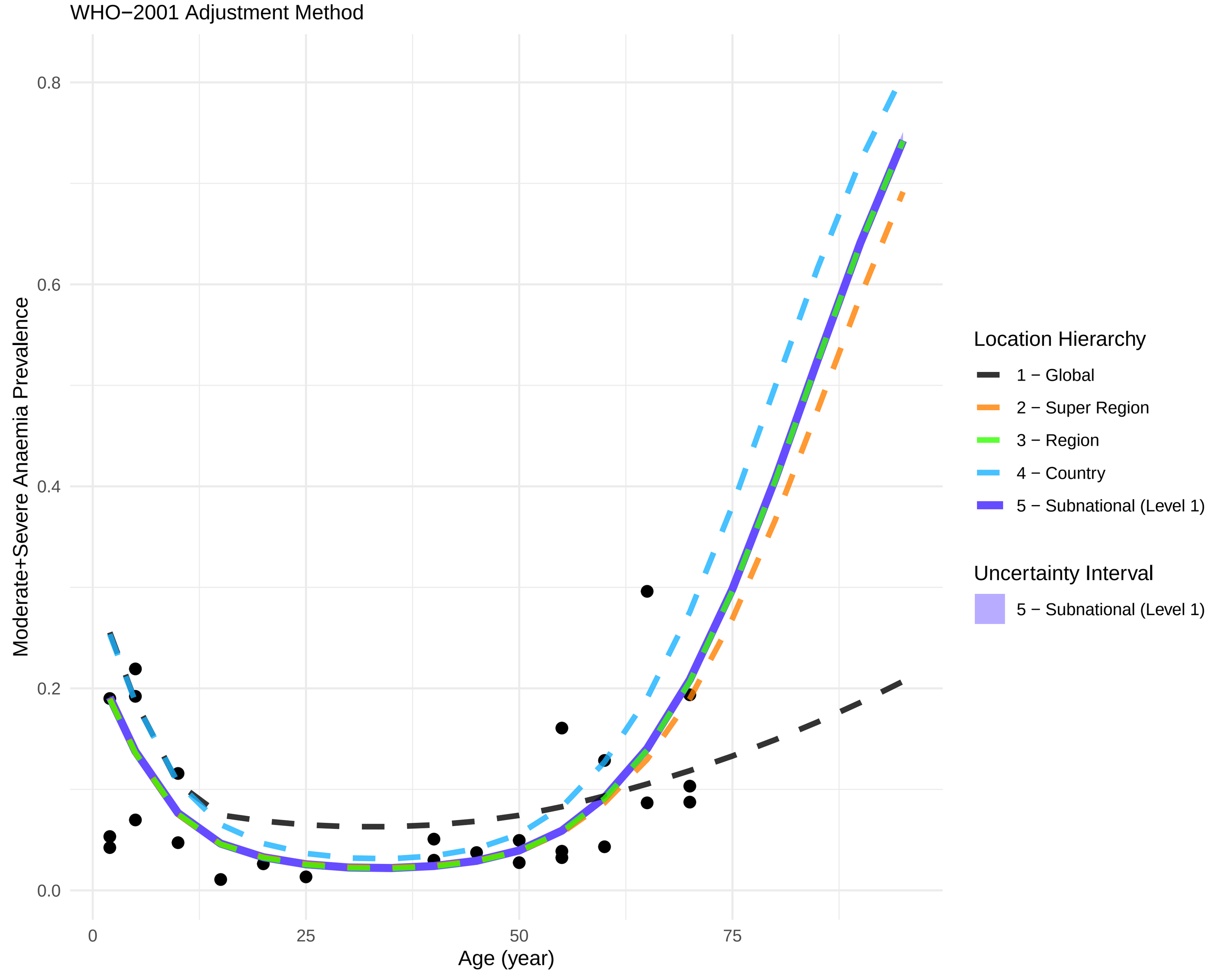

MR−BRT Age/Sex Fits − Males 2+ Years Old − Moderate+Severe Anaemia Prevalence − Bangka−Belitung Islands
WHO−2001 Adjustment Method

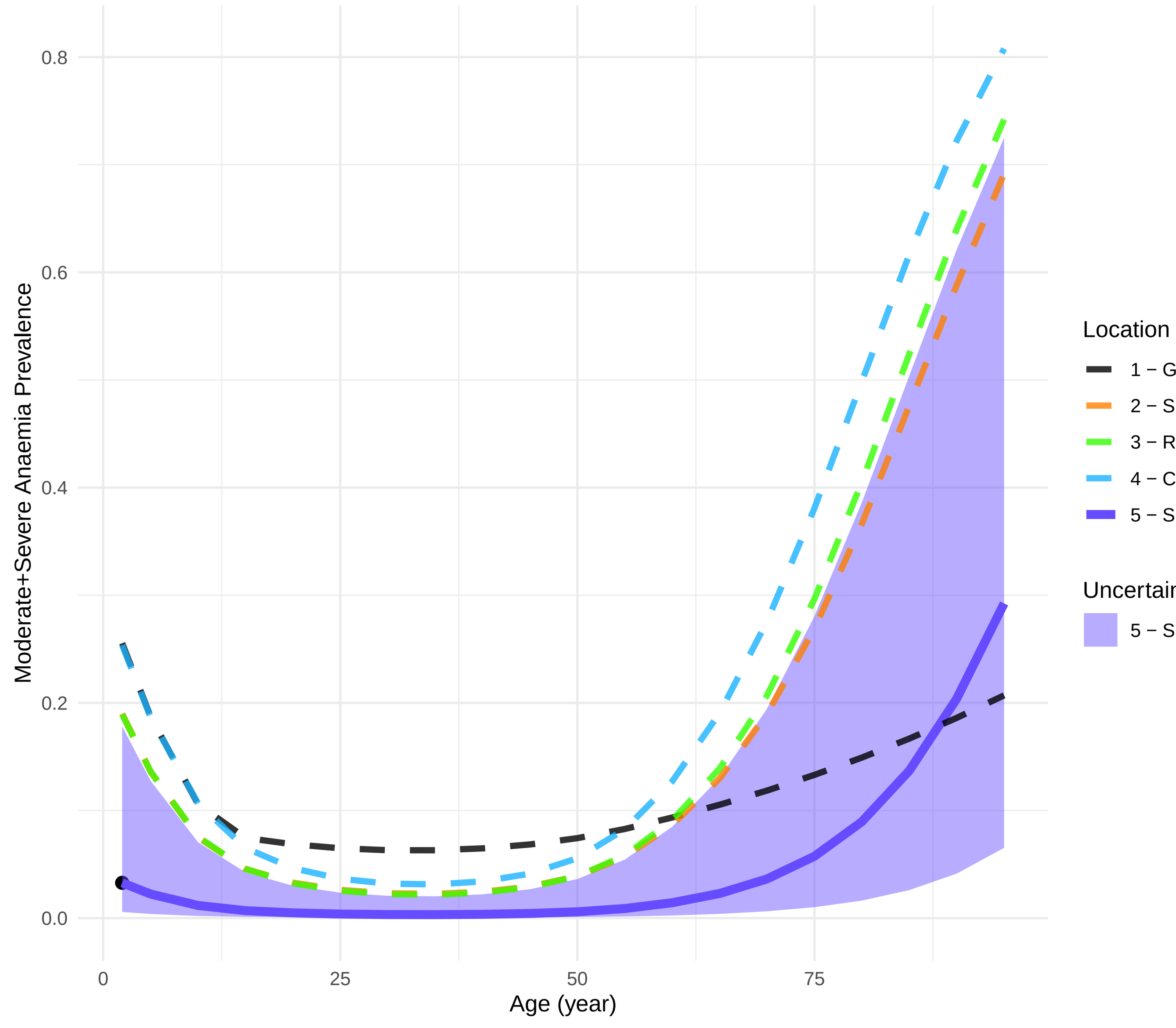

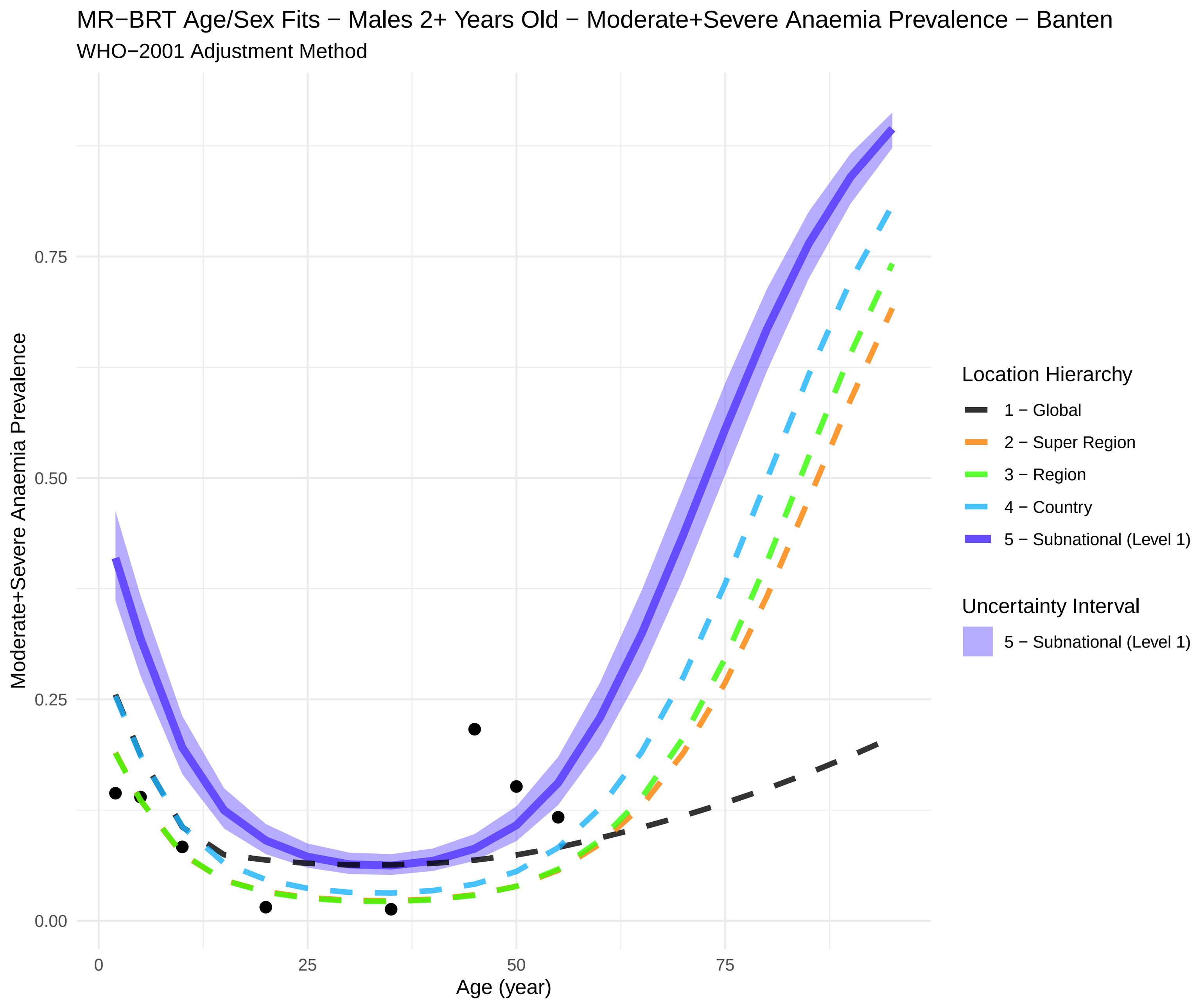
MR−BRT Age/Sex Fits − Males 2+ Years Old − Moderate+Severe Anaemia Prevalence − Banten
WHO−2001 Adjustment Method
Moderate+Severe Anaemia Prevalence
Age (year)
Location Hierarchy
1 − Global
2 − Super Region
3 − Region
4 − Country
5 − Subnational (Level 1)
Uncertainty Interval
5 − Subnational (Level 1)

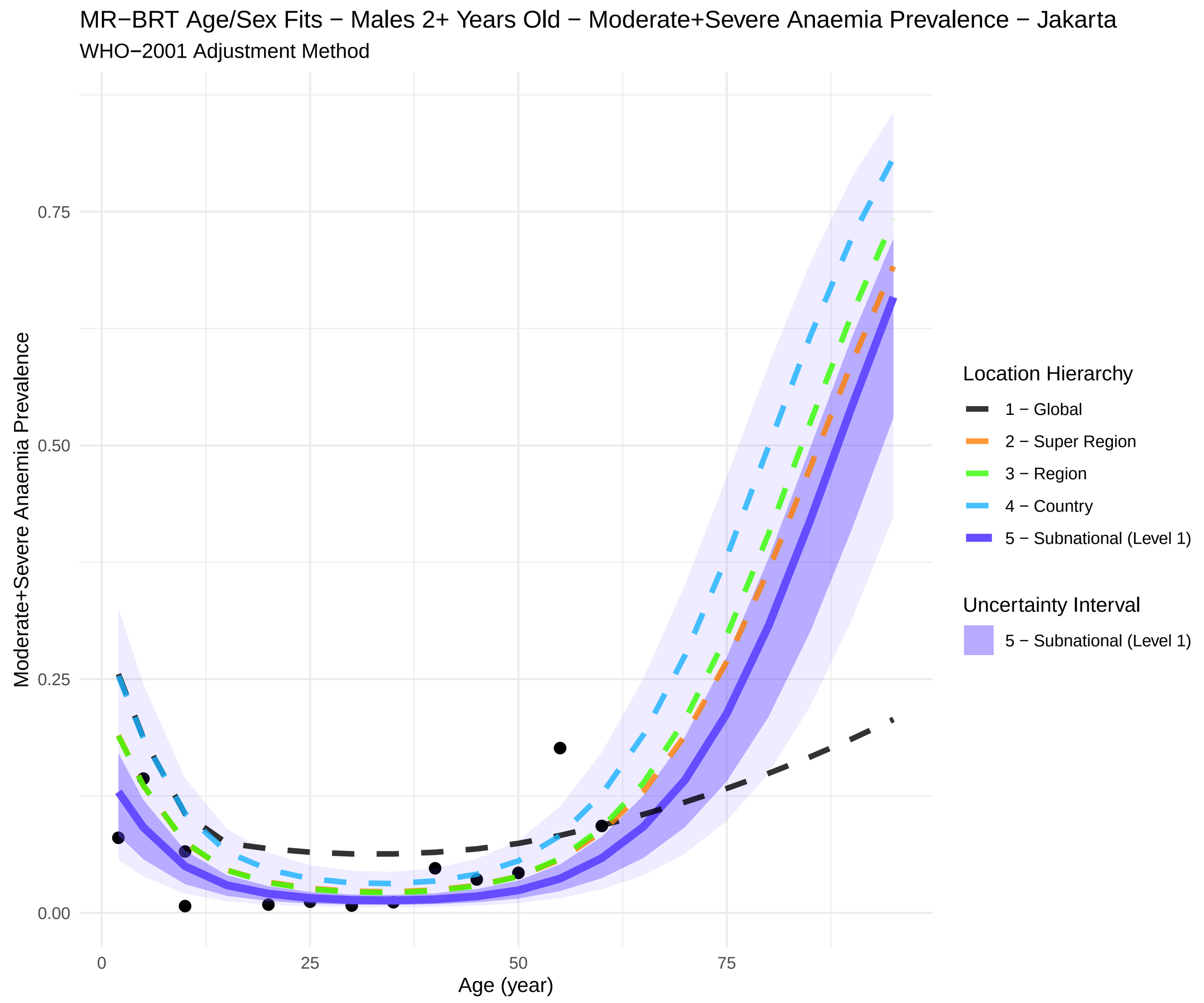
MR−BRT Age/Sex Fits − Males 2+ Years Old − Moderate+Severe Anaemia Prevalence − Jakarta
WHO−2001 Adjustment Method
Moderate+Severe Anaemia Prevalence
Age (year)
Location Hierarchy
1 − Global
2 − Super Region
3 − Region
4 − Country
5 − Subnational (Level 1)
Uncertainty Interval
5 − Subnational (Level 1)

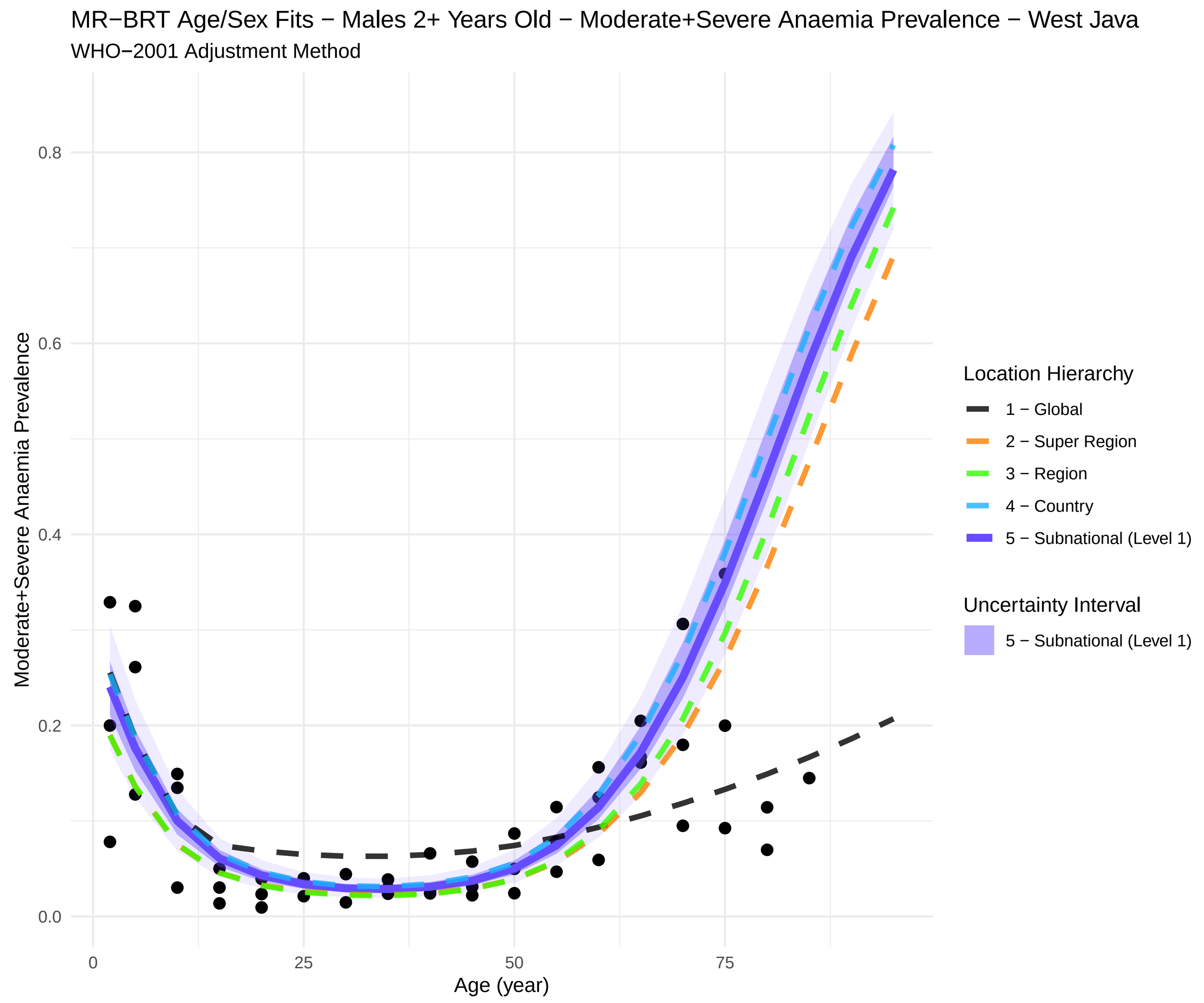
MR−BRT Age/Sex Fits − Males 2+ Years Old − Moderate+Severe Anaemia Prevalence − West Java
WHO−2001 Adjustment Method
Moderate+Severe Anaemia Prevalence
Age (year)
Location Hierarchy
1 − Global
2 − Super Region
3 − Region
4 − Country
5 − Subnational (Level 1)
Uncertainty Interval
5 − Subnational (Level 1)

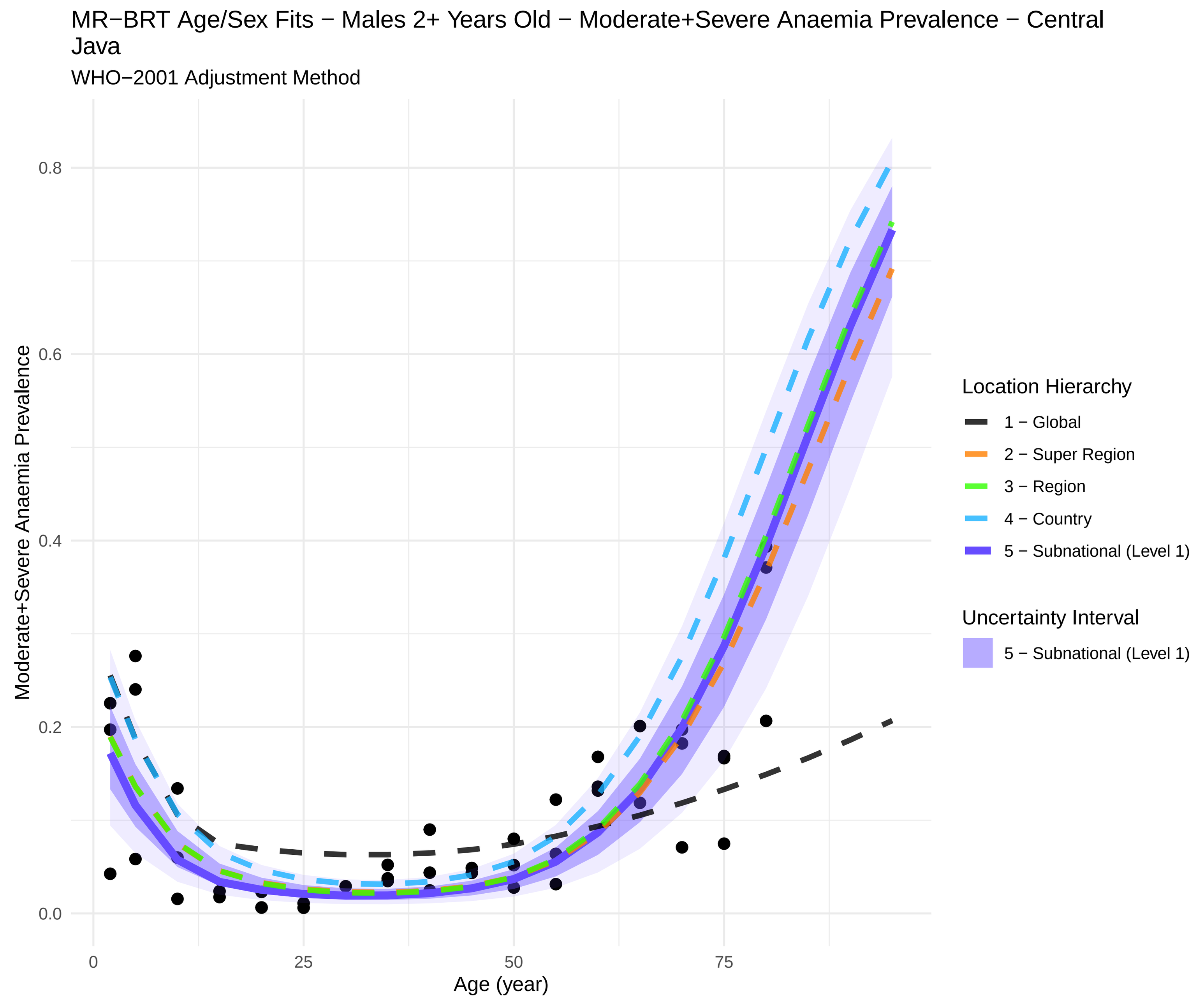
MR−BRT Age/Sex Fits − Males 2+ Years Old − Moderate+Severe Anaemia Prevalence − Central Java
WHO−2001 Adjustment Method
Moderate+Severe Anaemia Prevalence
Age (year)
Location Hierarchy
1 − Global
2 − Super Region
3 − Region
4 − Country
5 − Subnational (Level 1)
Uncertainty Interval
5 − Subnational (Level 1)

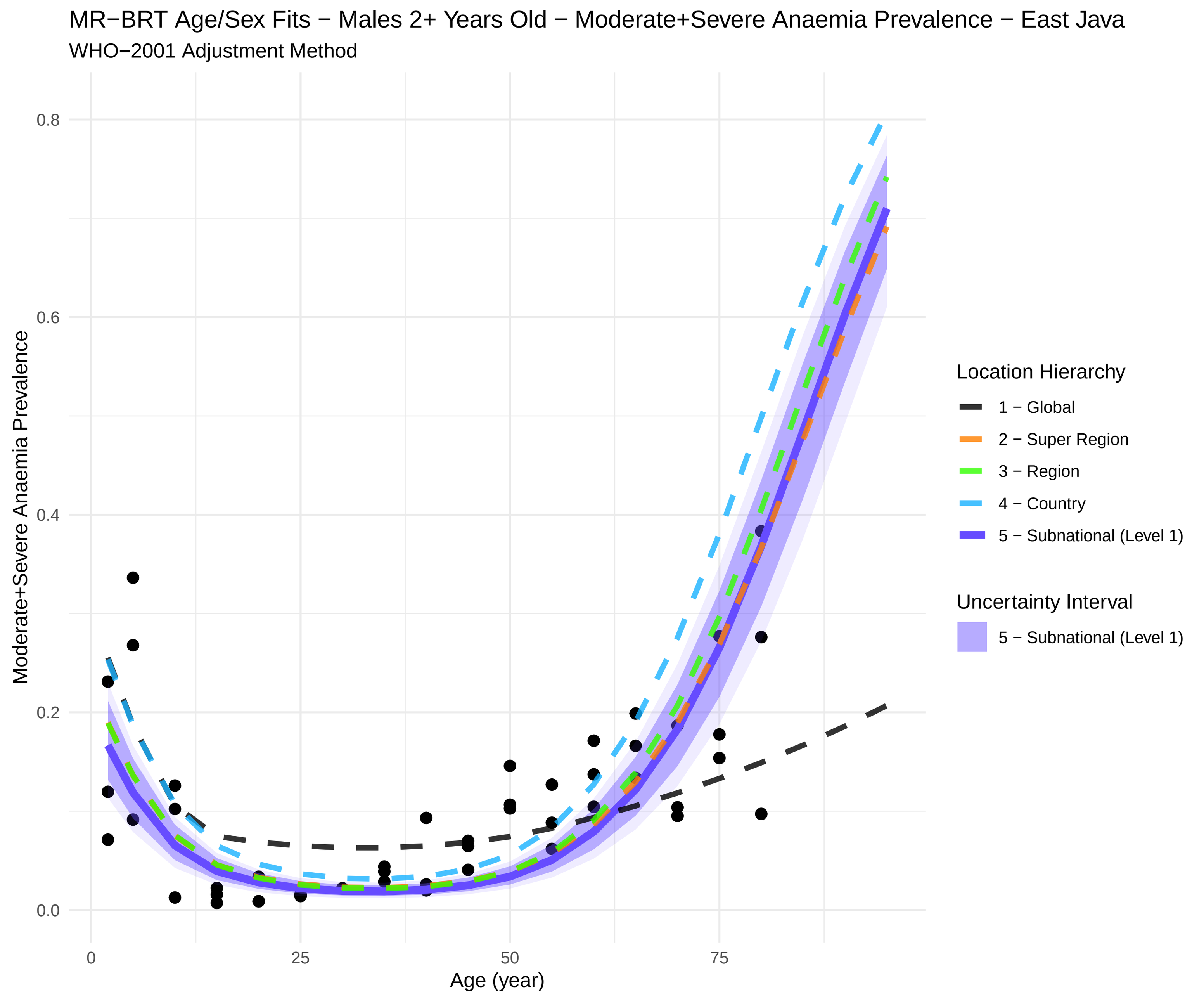
MR−BRT Age/Sex Fits − Males 2+ Years Old − Moderate+Severe Anaemia Prevalence − East Java
WHO−2001 Adjustment Method
Moderate+Severe Anaemia Prevalence
Age (year)
Location Hierarchy
1 − Global
2 − Super Region
3 − Region
4 − Country
5 − Subnational (Level 1)
Uncertainty Interval
5 − Subnational (Level 1)

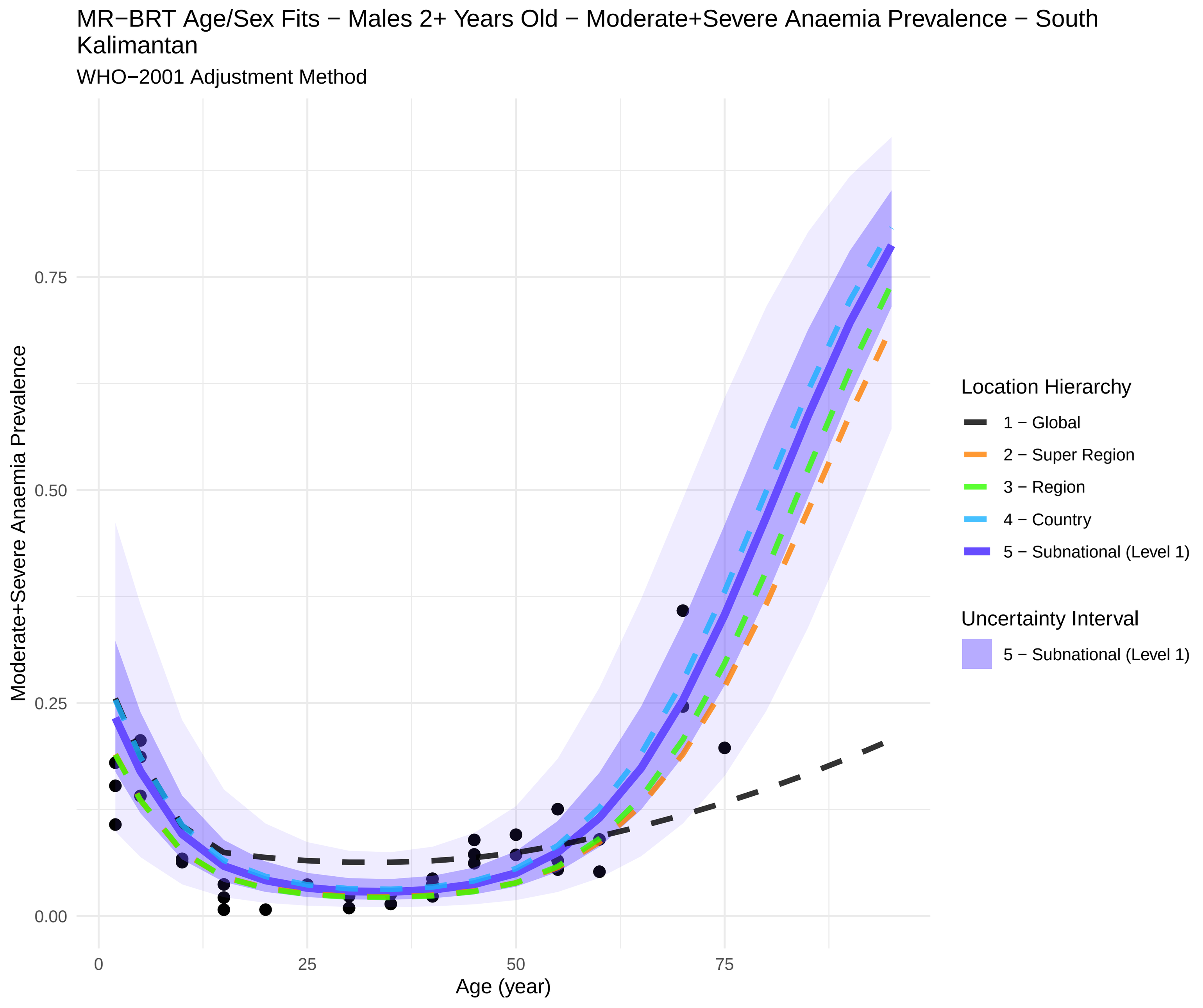
MR−BRT Age/Sex Fits − Males 2+ Years Old − Moderate+Severe Anaemia Prevalence − South Kalimantan
WHO−2001 Adjustment Method
Moderate+Severe Anaemia Prevalence
Age (year)
Location Hierarchy
1 − Global
2 − Super Region
3 − Region
4 − Country
5 − Subnational (Level 1)
Uncertainty Interval
5 − Subnational (Level 1)

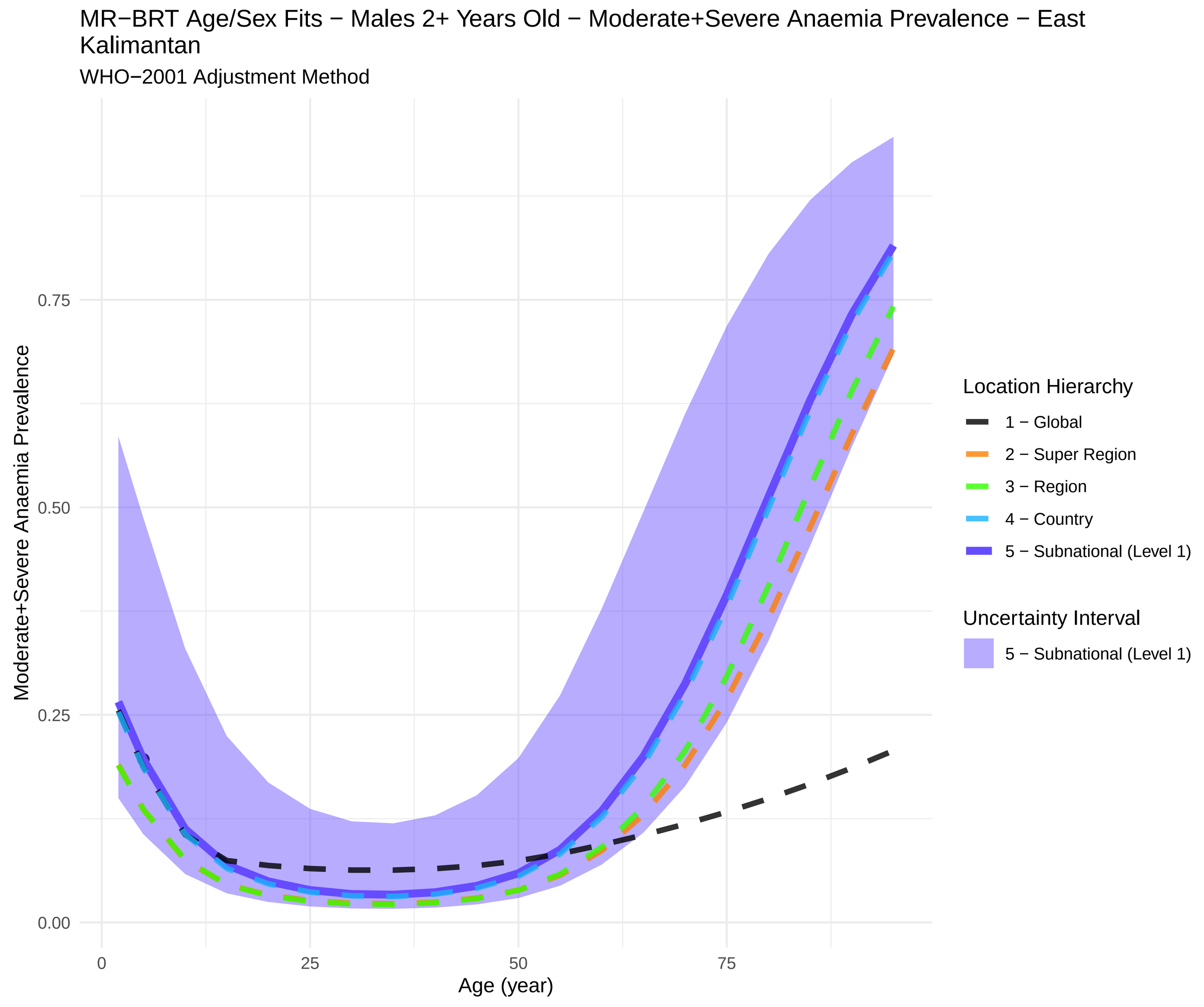
MR−BRT Age/Sex Fits − Males 2+ Years Old − Moderate+Severe Anaemia Prevalence − East Kalimantan
WHO−2001 Adjustment Method
Moderate+Severe Anaemia Prevalence
Age (year)
Location Hierarchy
1 − Global
2 − Super Region
3 − Region
4 − Country
5 − Subnational (Level 1)
Uncertainty Interval
5 − Subnational (Level 1)

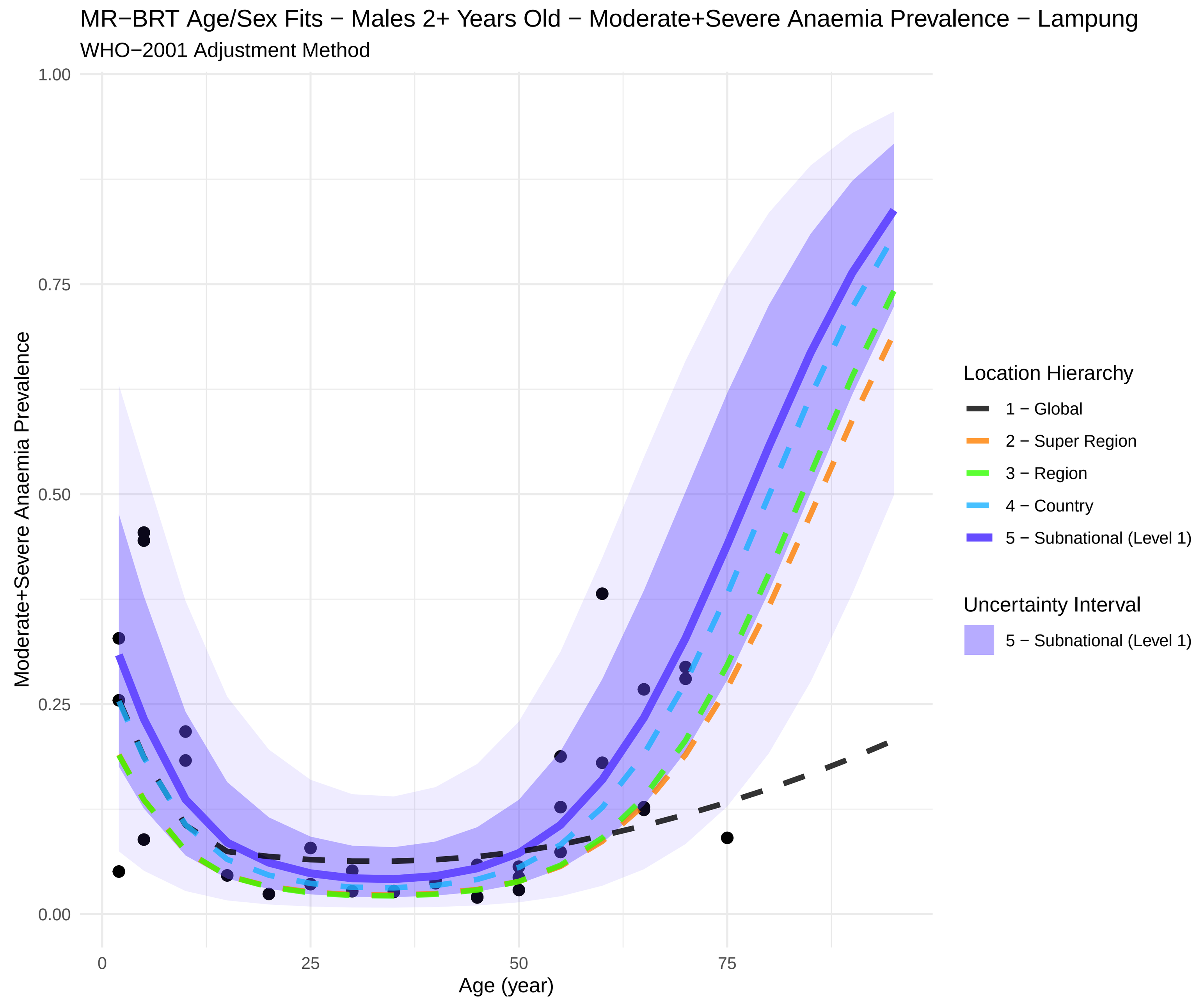
MR−BRT Age/Sex Fits − Males 2+ Years Old − Moderate+Severe Anaemia Prevalence − Lampung
WHO−2001 Adjustment Method
Moderate+Severe Anaemia Prevalence
Age (year)
Location Hierarchy
1 − Global
2 − Super Region
3 − Region
4 − Country
5 − Subnational (Level 1)
Uncertainty Interval
5 − Subnational (Level 1)

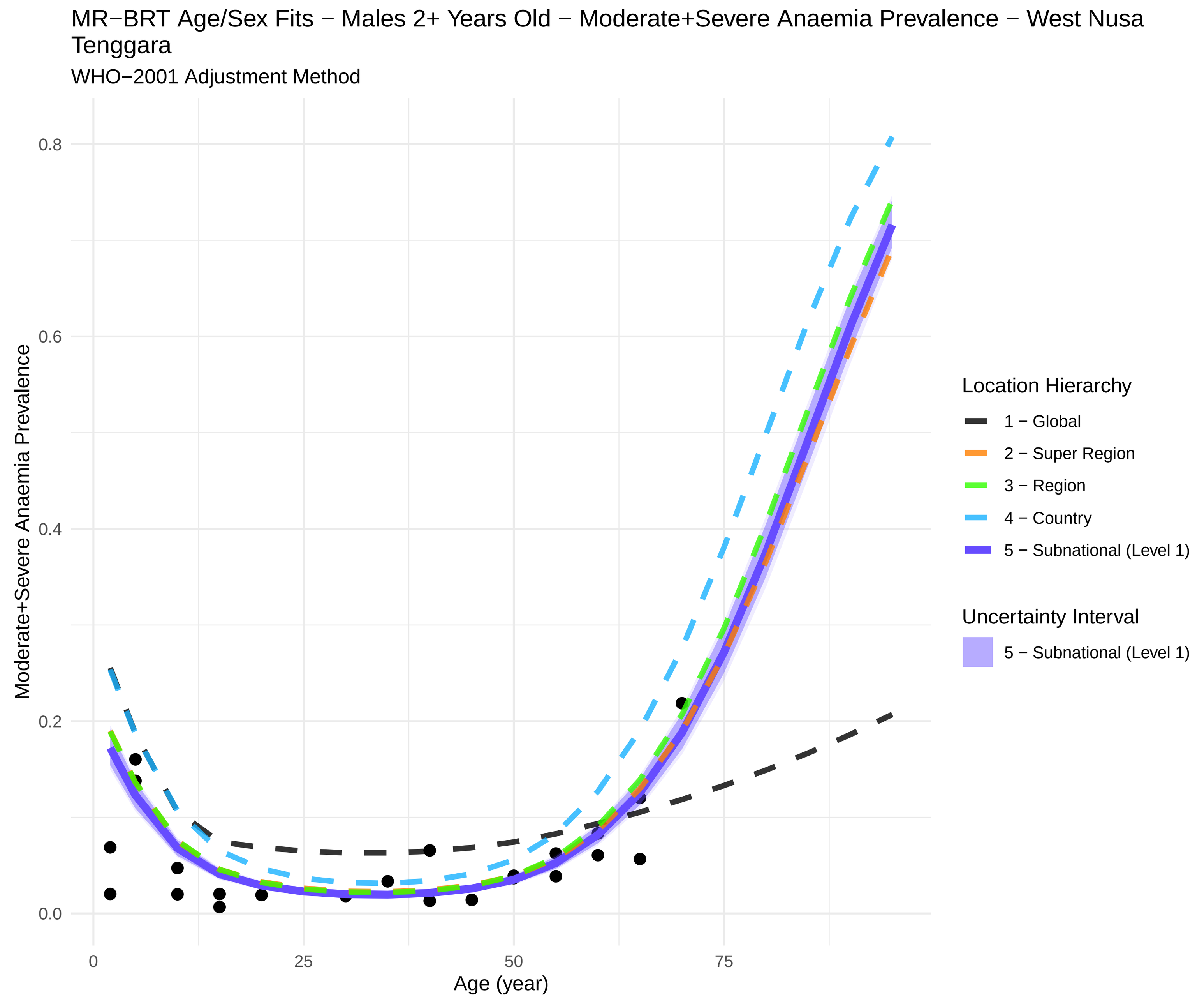
MR−BRT Age/Sex Fits − Males 2+ Years Old − Moderate+Severe Anaemia Prevalence − West Nusa Tenggara
WHO−2001 Adjustment Method
Moderate+Severe Anaemia Prevalence
Age (year)
Location Hierarchy
1 − Global
2 − Super Region
3 − Region
4 − Country
5 − Subnational (Level 1)
Uncertainty Interval
5 − Subnational (Level 1)

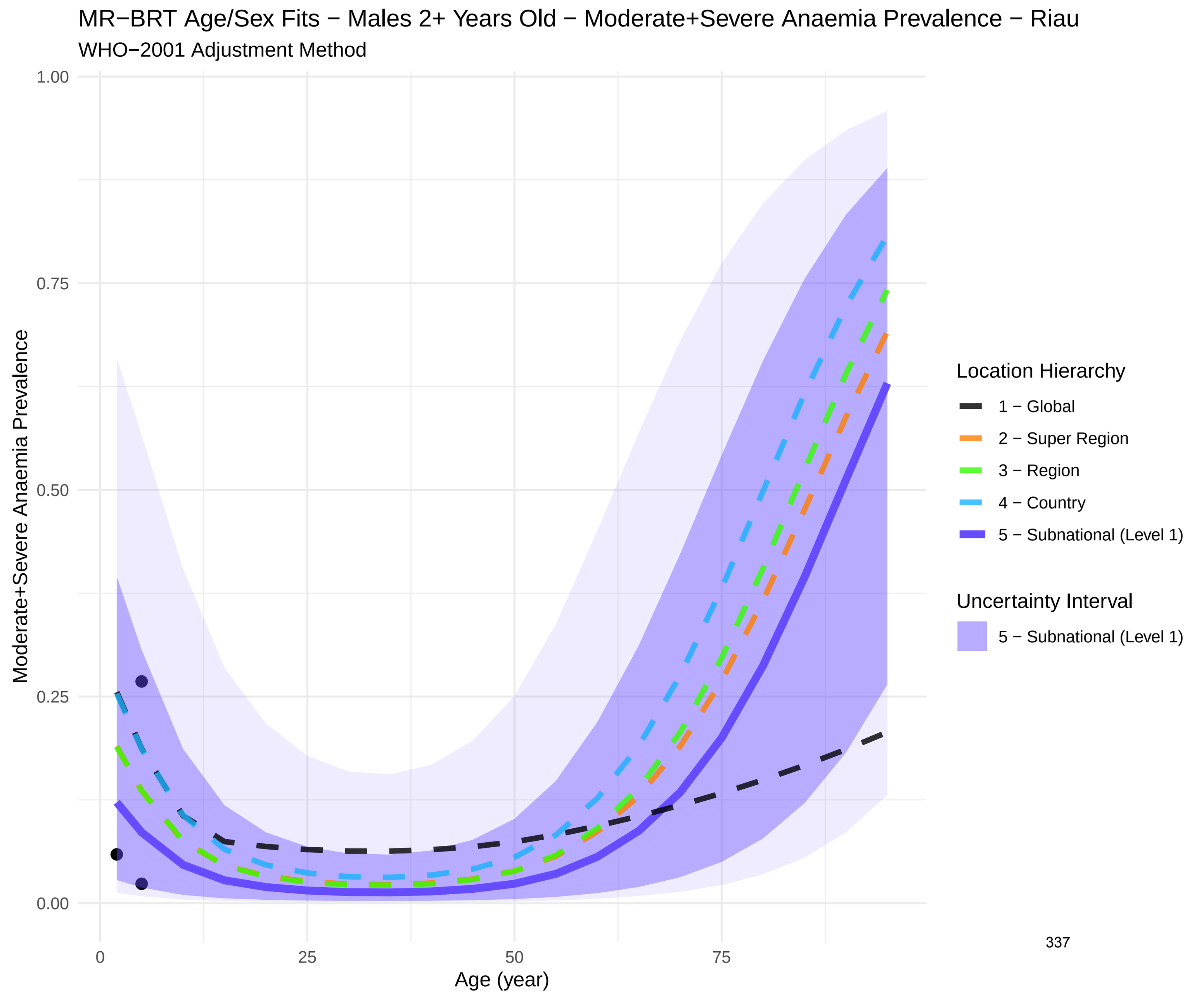
MR−BRT Age/Sex Fits − Males 2+ Years Old − Moderate+Severe Anaemia Prevalence − Riau
WHO−2001 Adjustment Method
Moderate+Severe Anaemia Prevalence
Age (year)
Location Hierarchy
1 − Global
2 − Super Region
3 − Region
4 − Country
5 − Subnational (Level 1)
Uncertainty Interval
5 − Subnational (Level 1)

MR−BRT Age/Sex Fits − Males 2+ Years Old − Moderate+Severe Anaemia Prevalence − West Sulawesi
WHO−2001 Adjustment Method

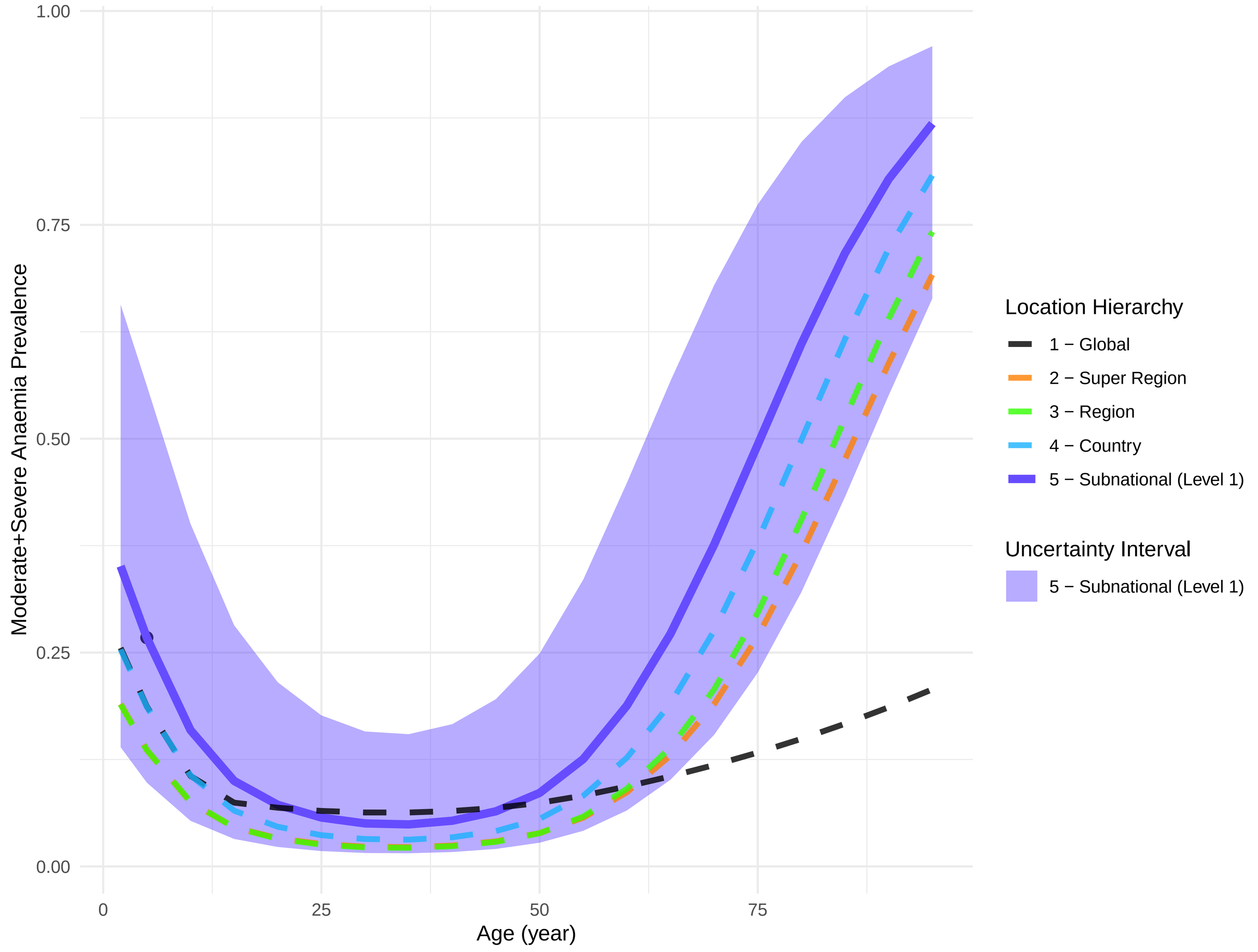

MR−BRT Age/Sex Fits − Males 2+ Years Old − Moderate+Severe Anaemia Prevalence − South Sulawesi
WHO−2001 Adjustment Method

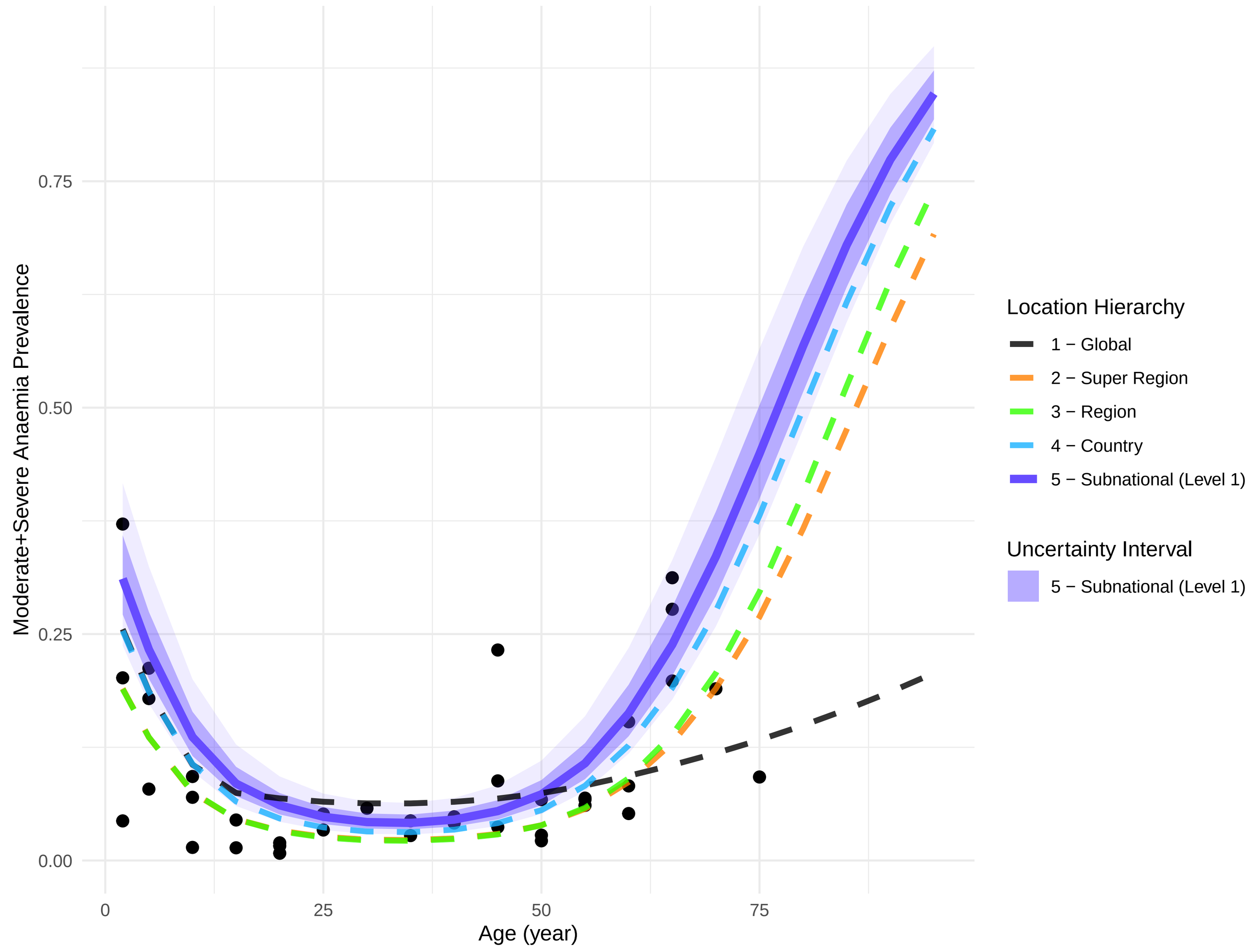

MR−BRT Age/Sex Fits − Males 2+ Years Old − Moderate+Severe Anaemia Prevalence − West Sumatra
WHO−2001 Adjustment Method

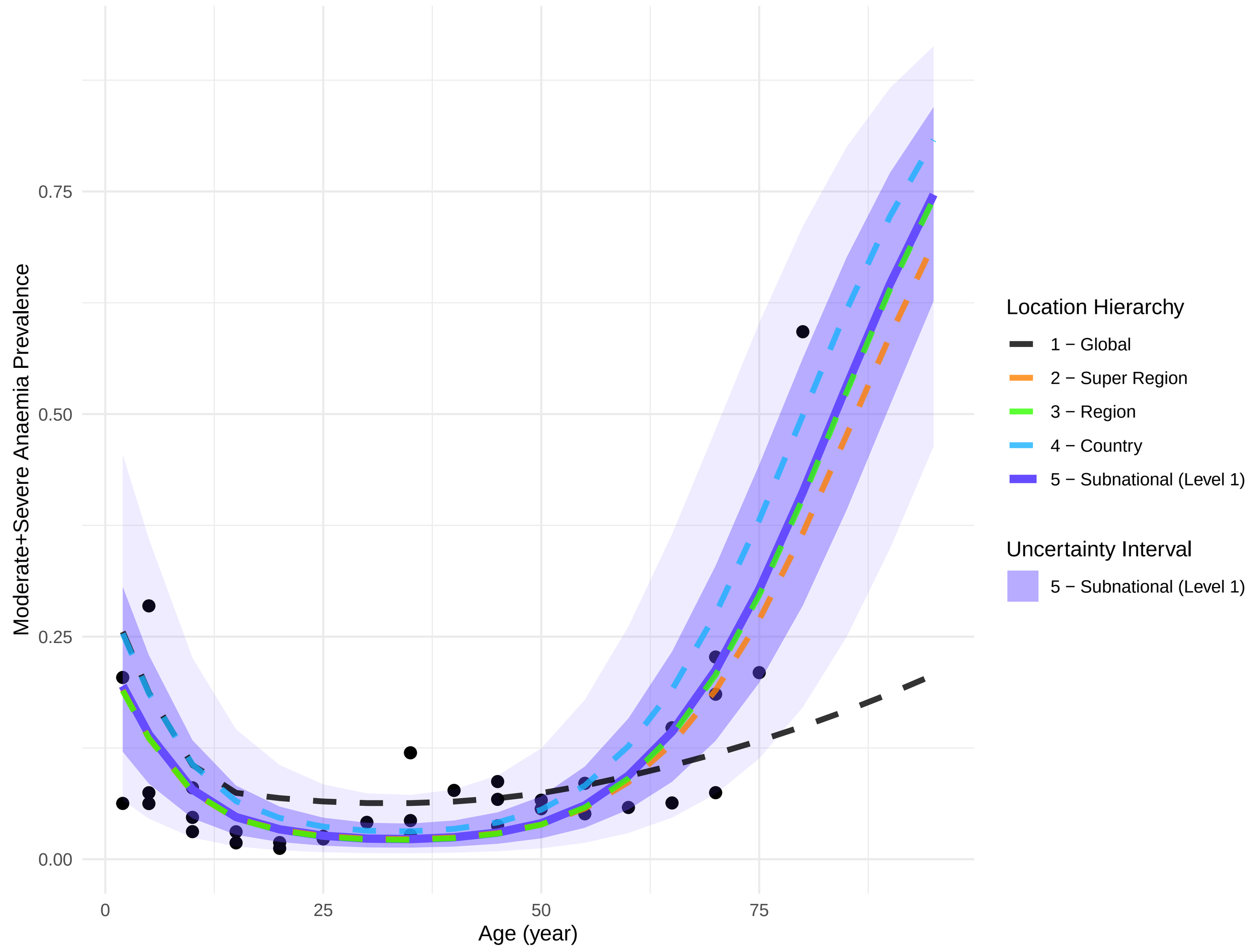

MR−BRT Age/Sex Fits − Males 2+ Years Old − Moderate+Severe Anaemia Prevalence − South Sumatra
WHO−2001 Adjustment Method

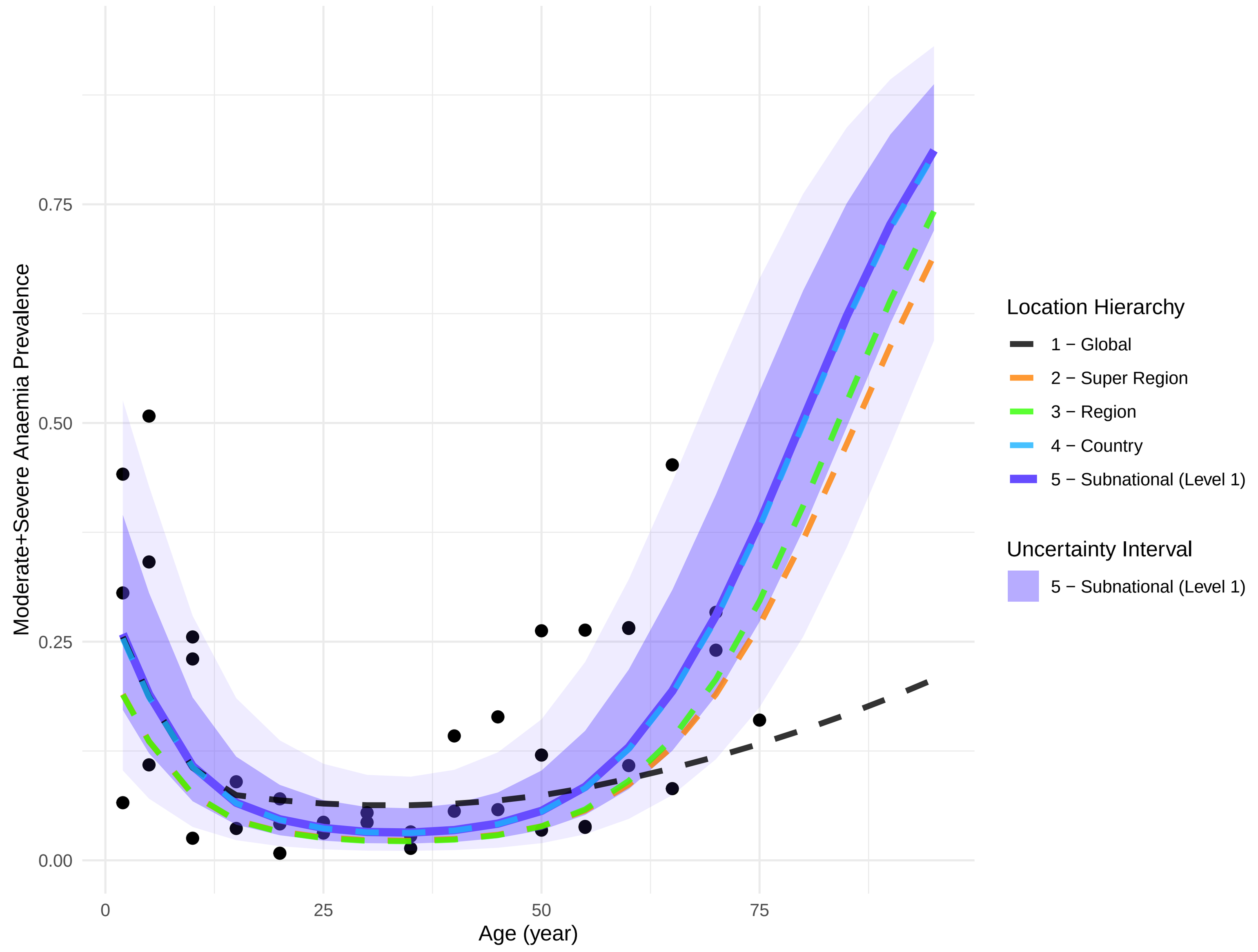

MR−BRT Age/Sex Fits − Males 2+ Years Old − Moderate+Severe Anaemia Prevalence − North Sumatra
WHO−2001 Adjustment Method

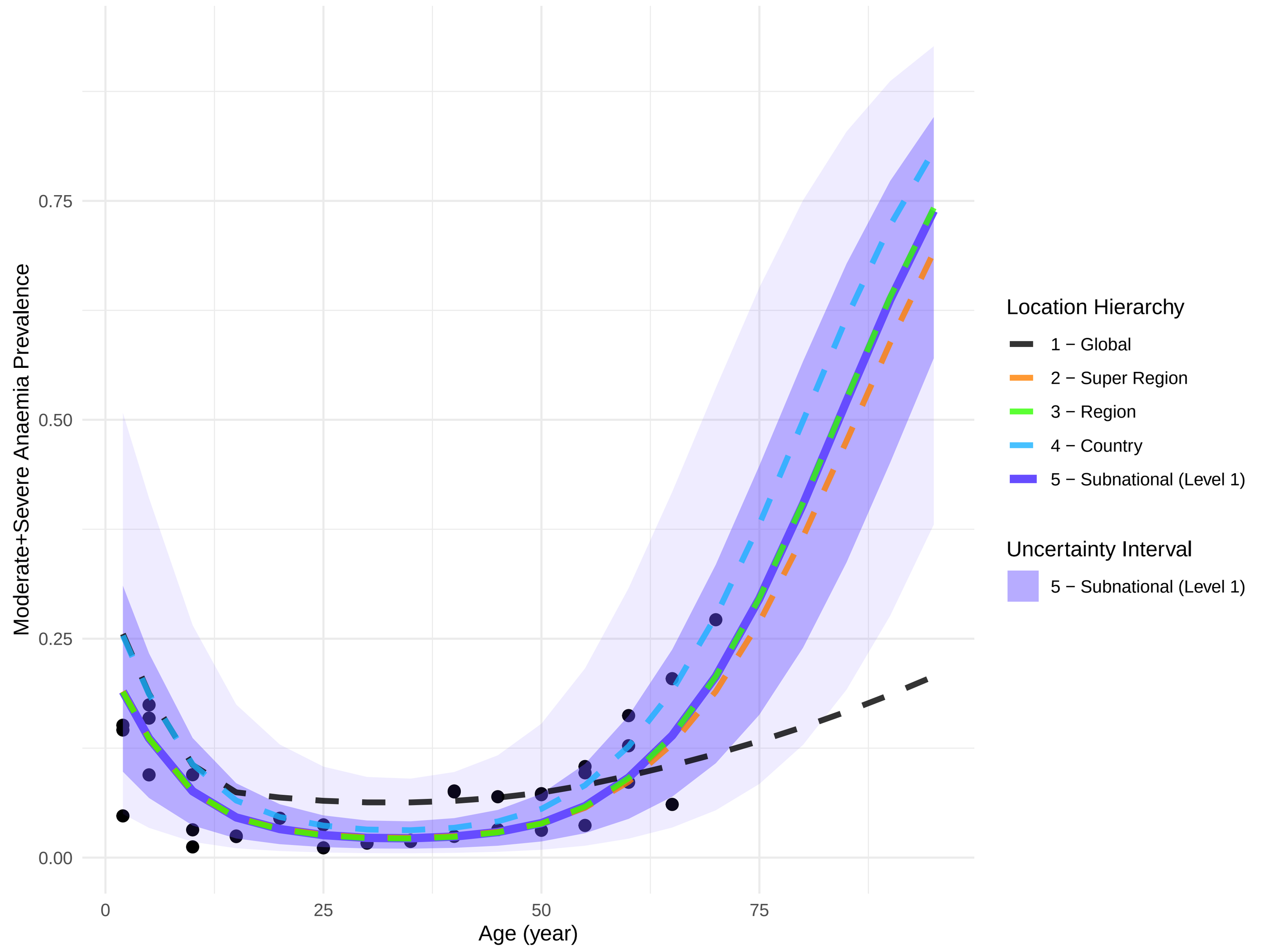

MR−BRT Age/Sex Fits − Males 2+ Years Old − Moderate+Severe Anaemia Prevalence − Yogyakarta
WHO−2001 Adjustment Method

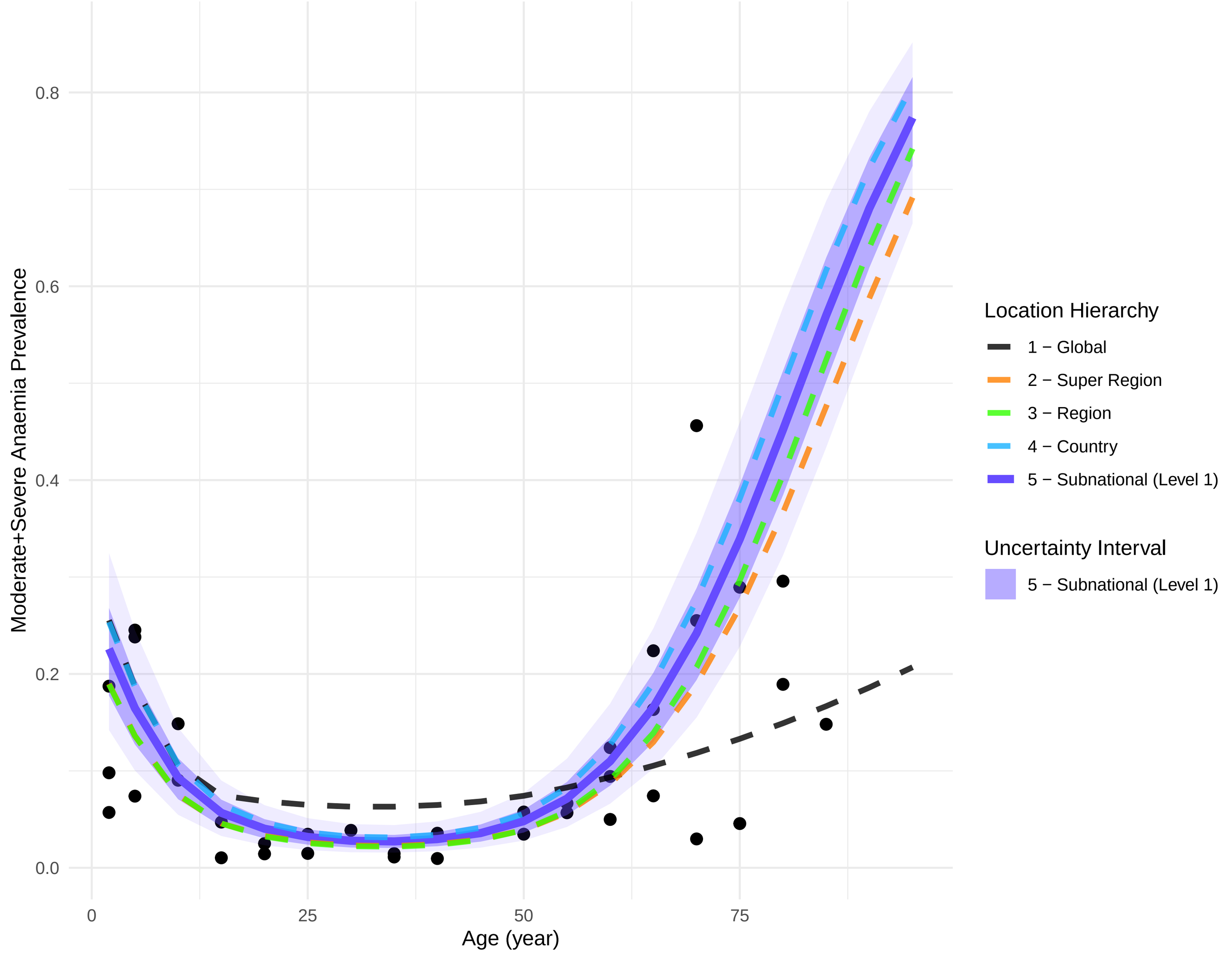

MR−BRT Age/Sex Fits − Males 2+ Years Old − Moderate+Severe Anaemia Prevalence − Myanmar
WHO−2001 Adjustment Method

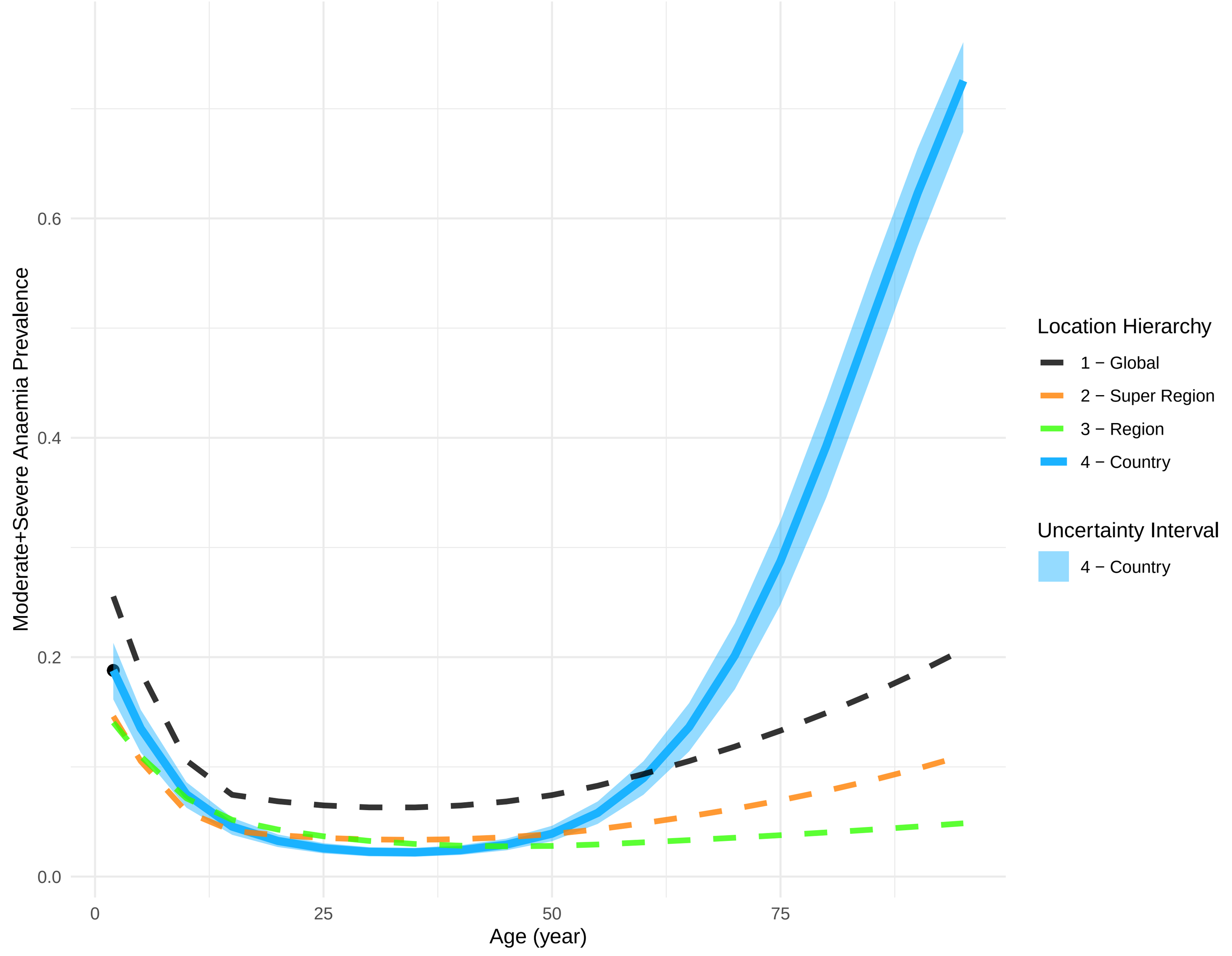

MR−BRT Age/Sex Fits − Males 2+ Years Old − Moderate+Severe Anaemia Prevalence − Timor−Leste
WHO−2001 Adjustment Method

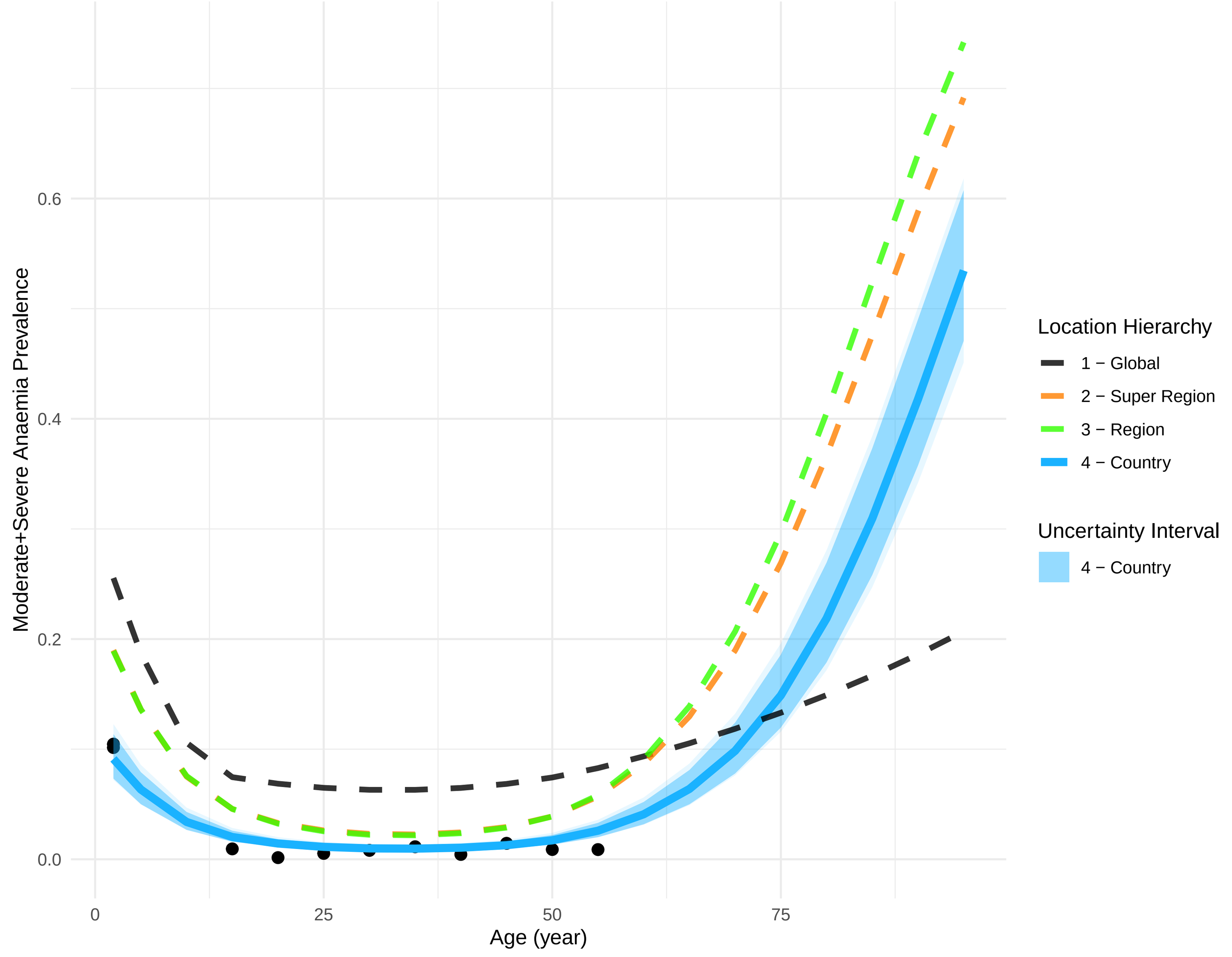

MR−BRT Age/Sex Fits − Males 2+ Years Old − Moderate+Severe Anaemia Prevalence − Sub−Saharan Africa
WHO−2001 Adjustment Method

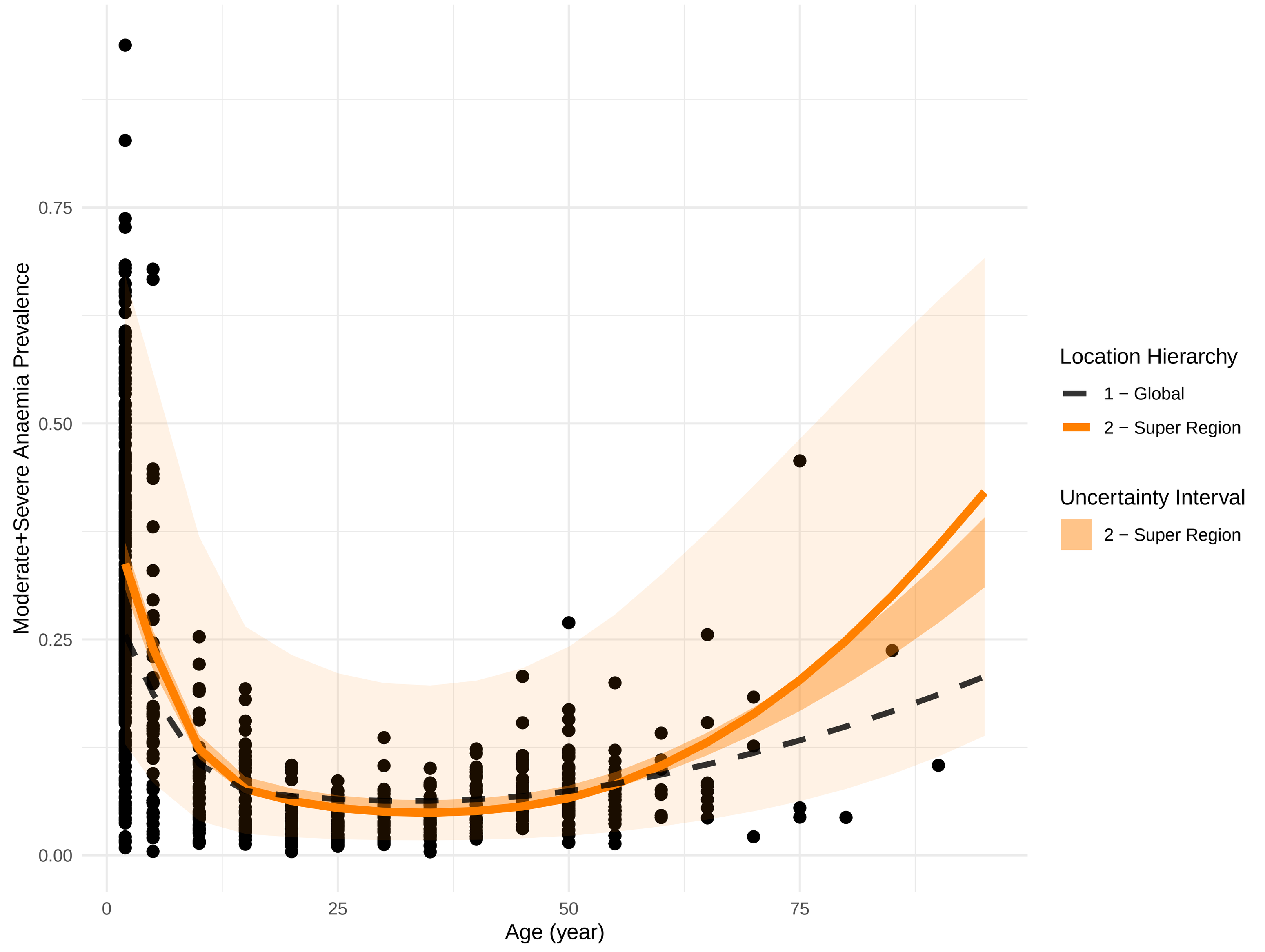

MR−BRT Age/Sex Fits − Males 2+ Years Old − Moderate+Severe Anaemia Prevalence − Central Sub−Saharan Africa
WHO−2001 Adjustment Method

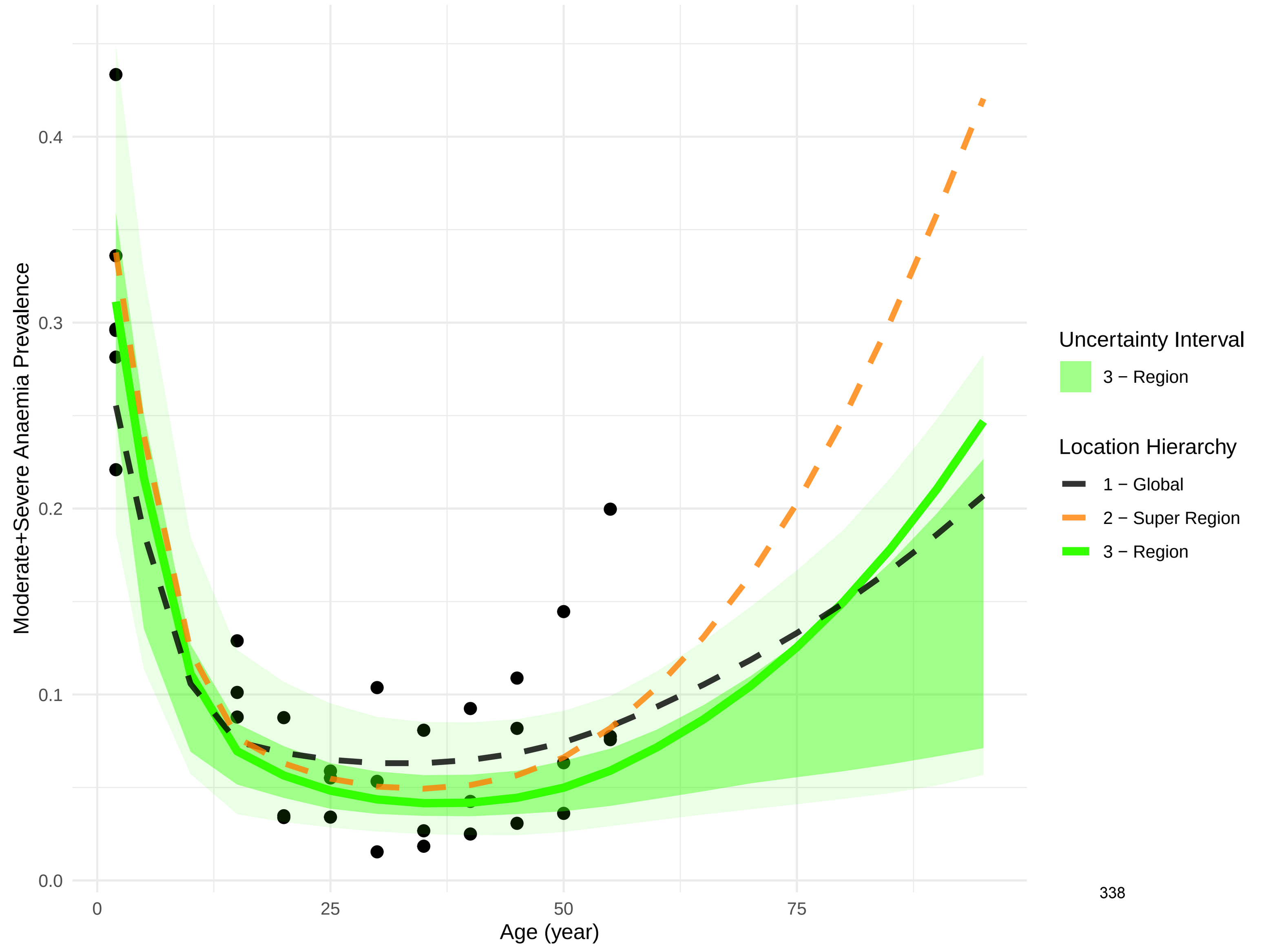

MR−BRT Age/Sex Fits − Males 2+ Years Old − Moderate+Severe Anaemia Prevalence − Angola
WHO−2001 Adjustment Method

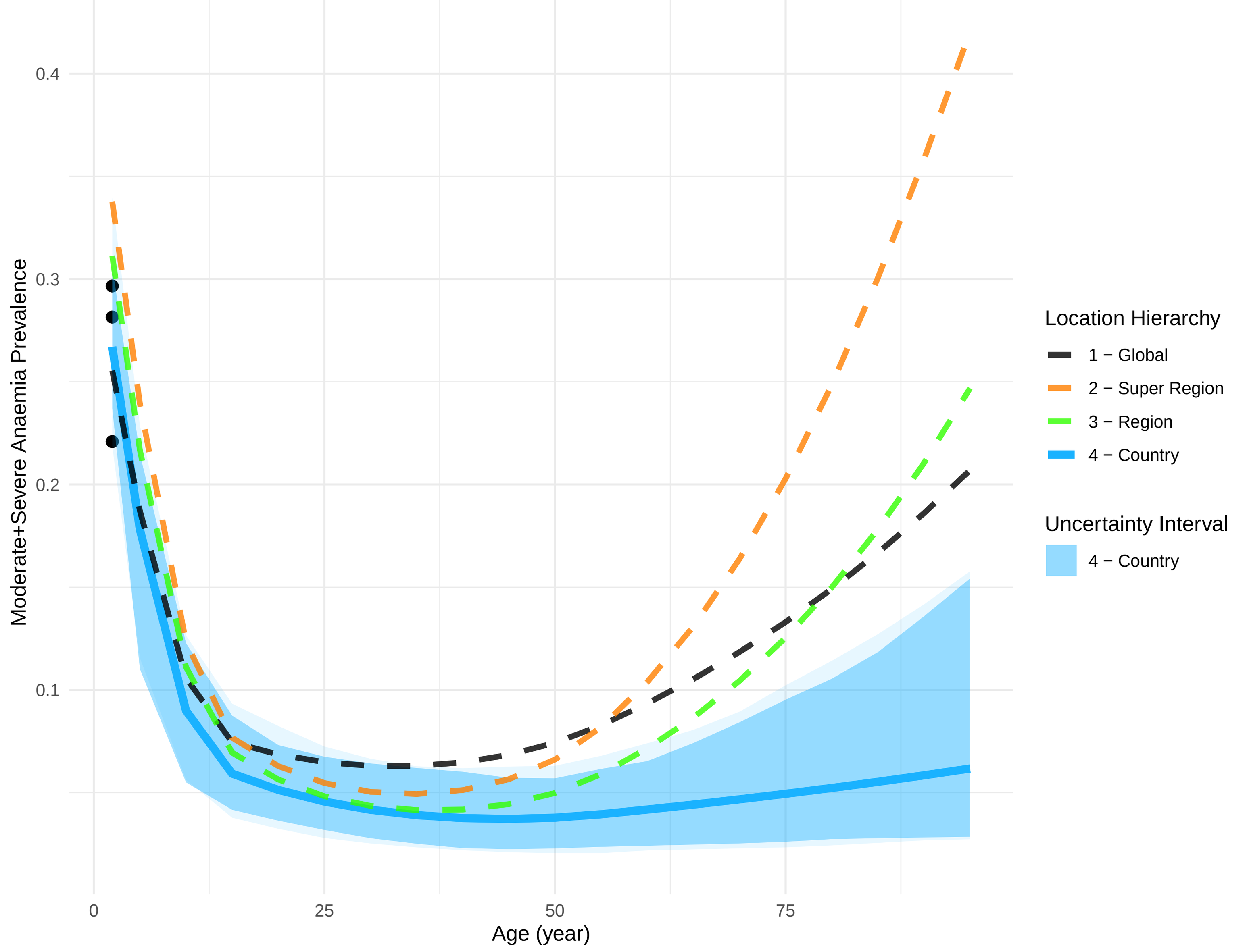

MR−BRT Age/Sex Fits − Males 2+ Years Old − Moderate+Severe Anaemia Prevalence − Democratic Republic of the Congo
WHO−2001 Adjustment Method

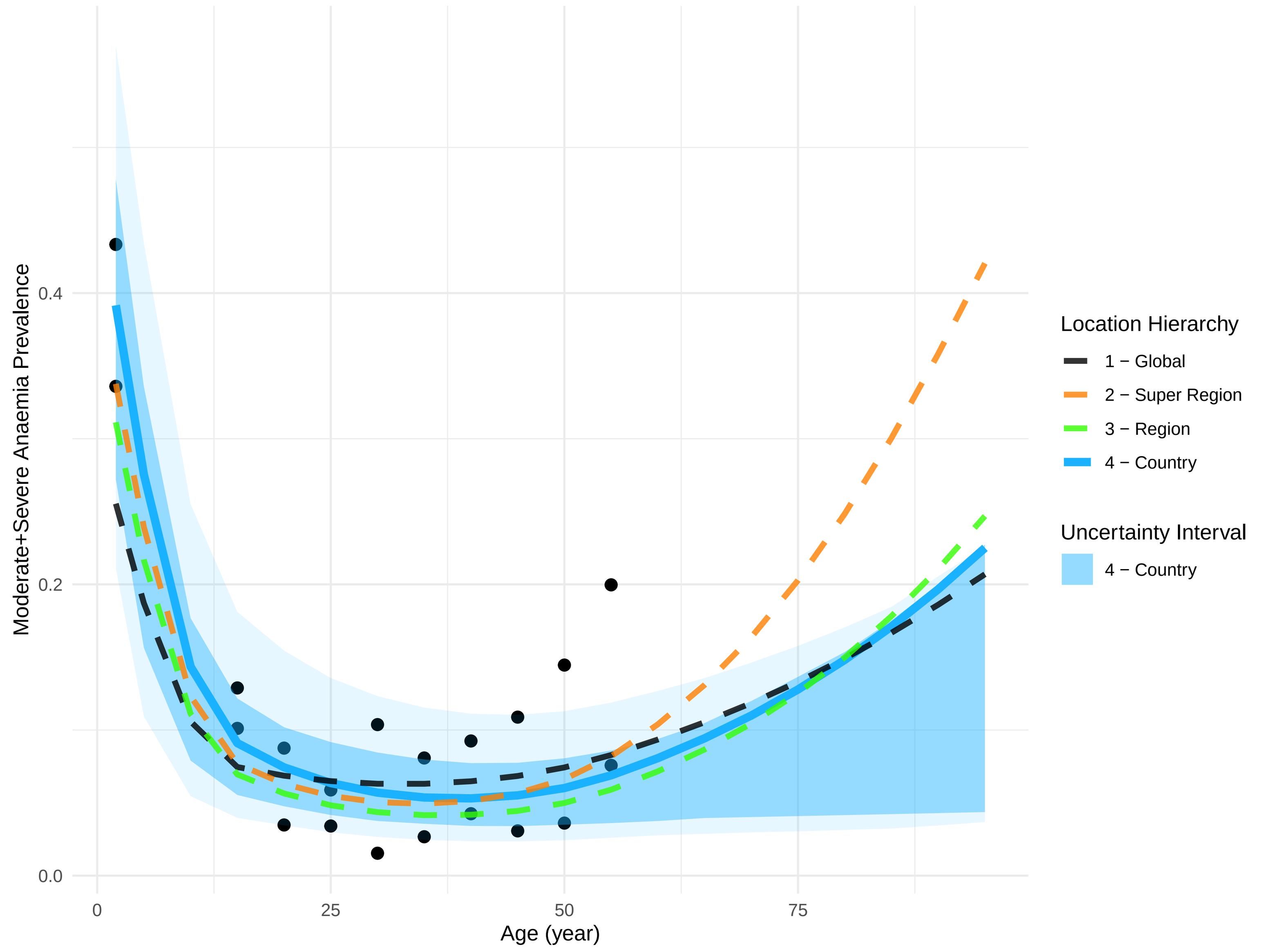

MR−BRT Age/Sex Fits − Males 2+ Years Old − Moderate+Severe Anaemia Prevalence − Gabon
WHO−2001 Adjustment Method

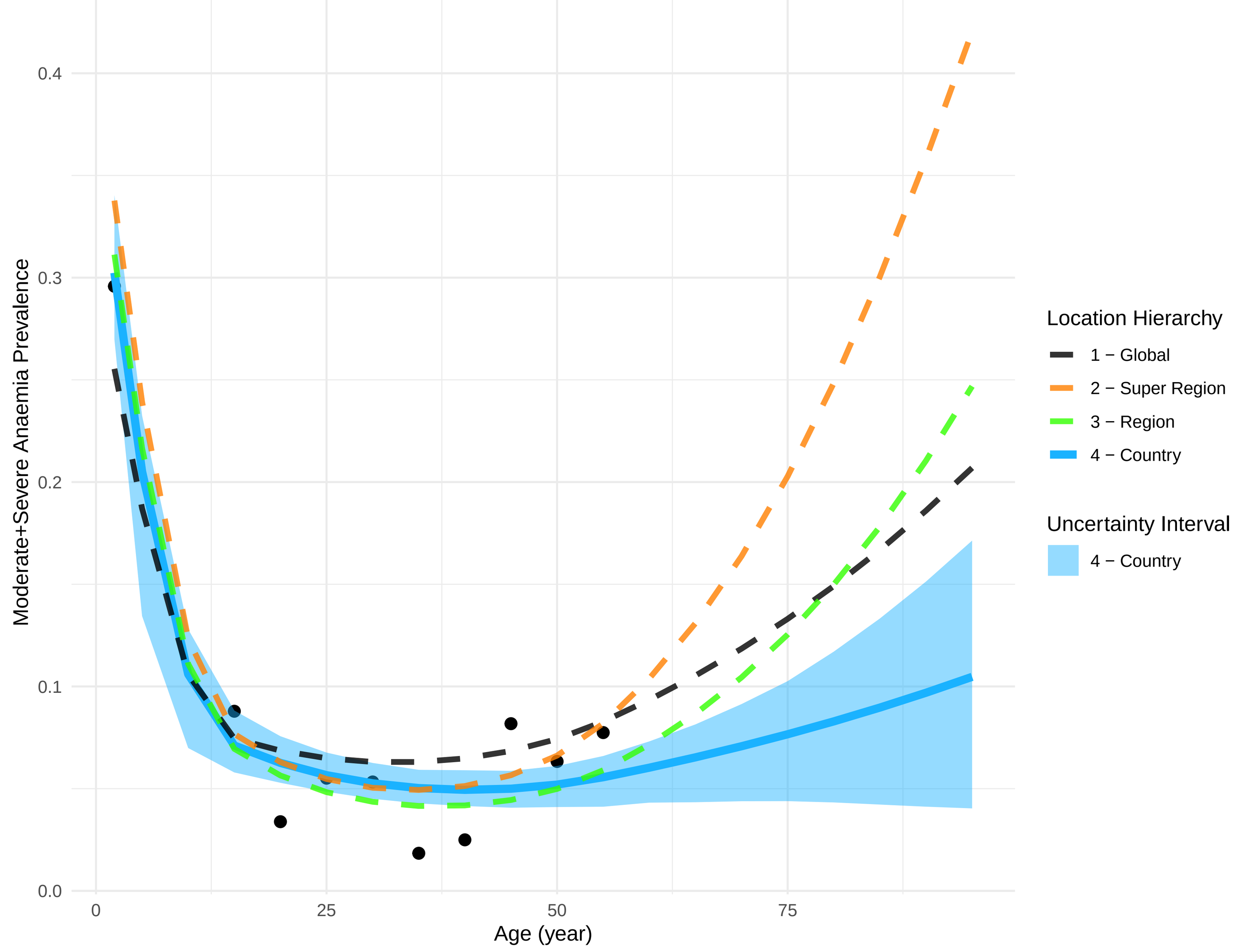

MR−BRT Age/Sex Fits − Males 2+ Years Old − Moderate+Severe Anaemia Prevalence − Eastern Sub−Saharan Africa
WHO−2001 Adjustment Method

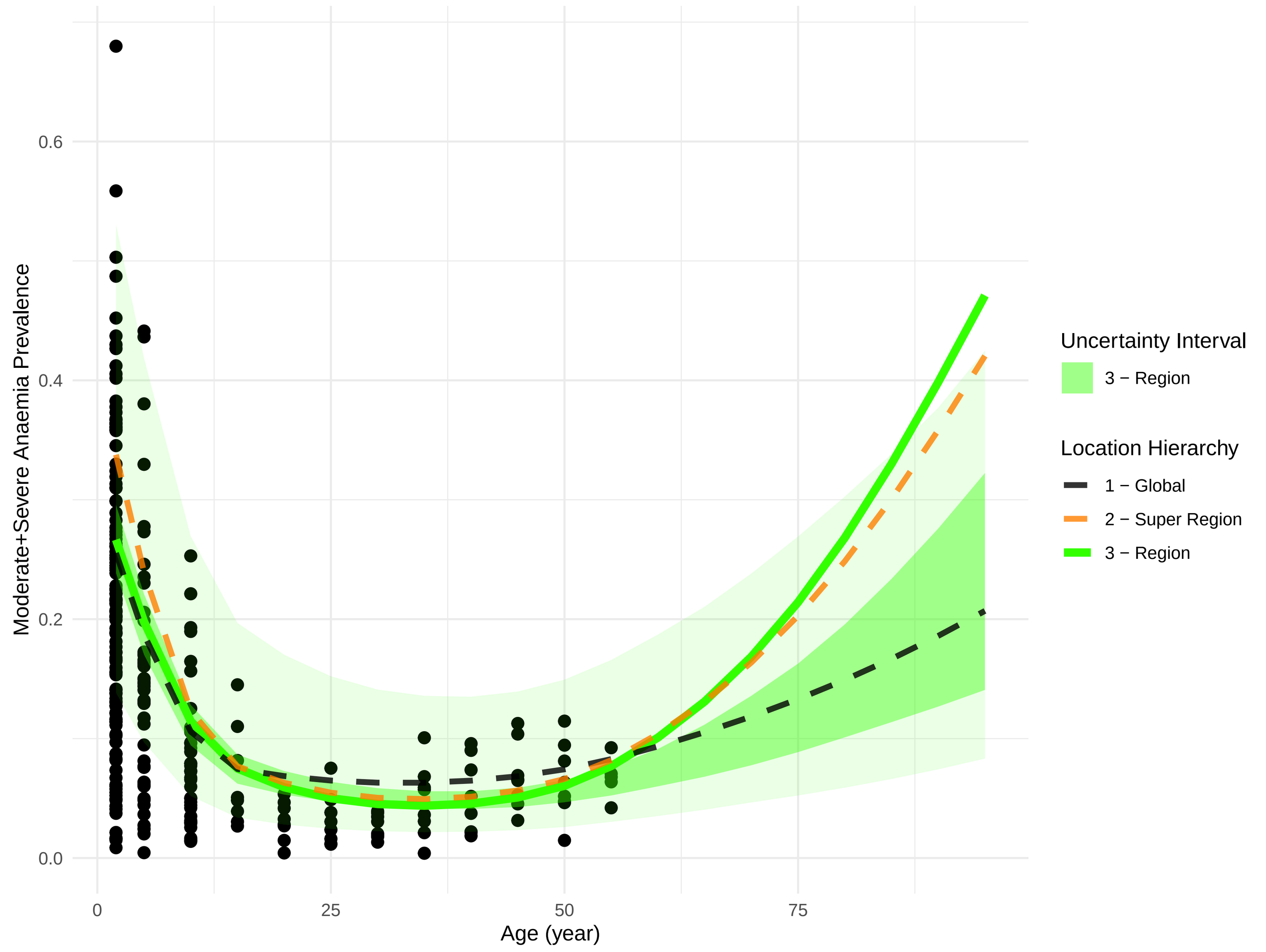

MR−BRT Age/Sex Fits − Males 2+ Years Old − Moderate+Severe Anaemia Prevalence − Burundi
WHO−2001 Adjustment Method

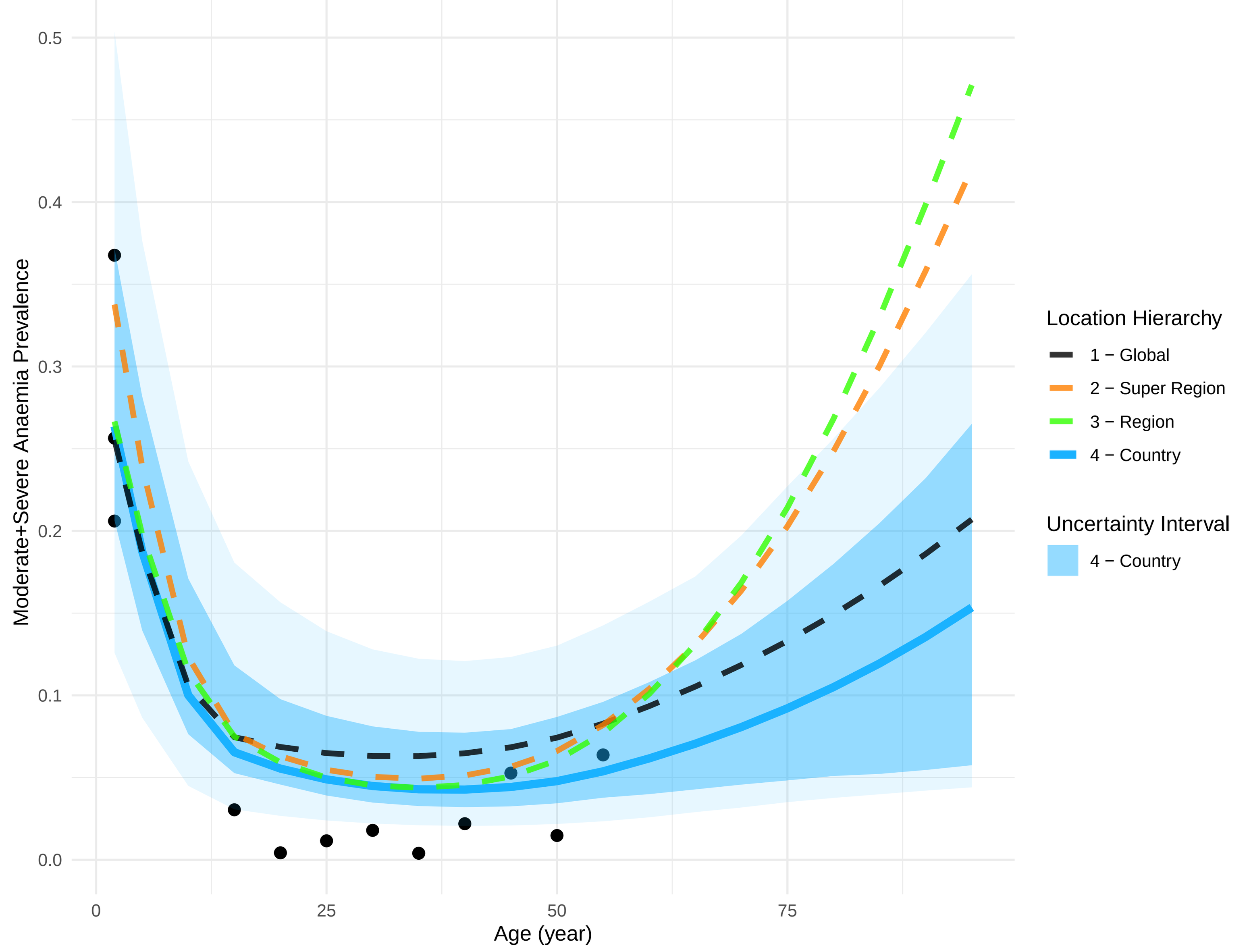

MR−BRT Age/Sex Fits − Males 2+ Years Old − Moderate+Severe Anaemia Prevalence − Ethiopia
WHO−2001 Adjustment Method

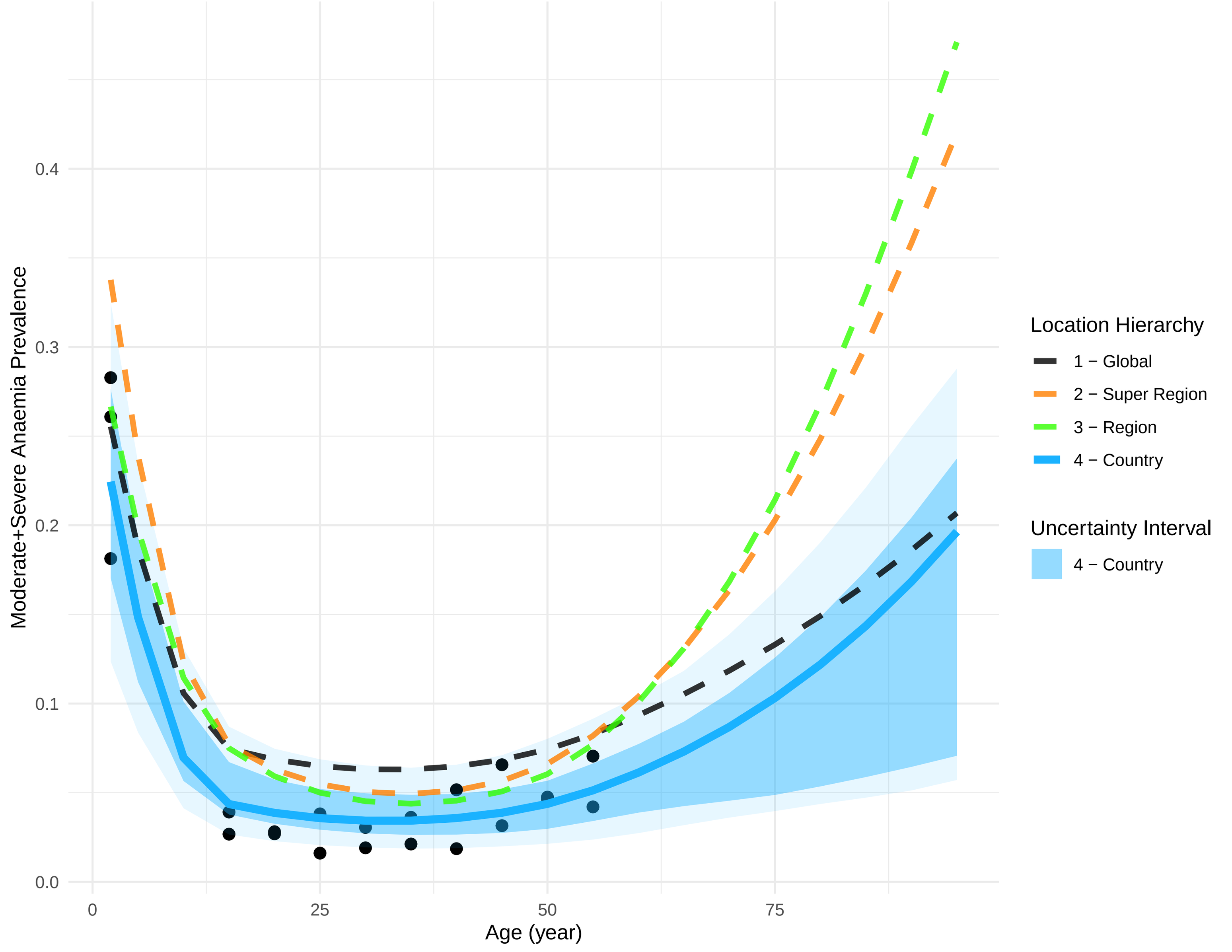

MR−BRT Age/Sex Fits − Males 2+ Years Old − Moderate+Severe Anaemia Prevalence − Kenya
WHO−2001 Adjustment Method

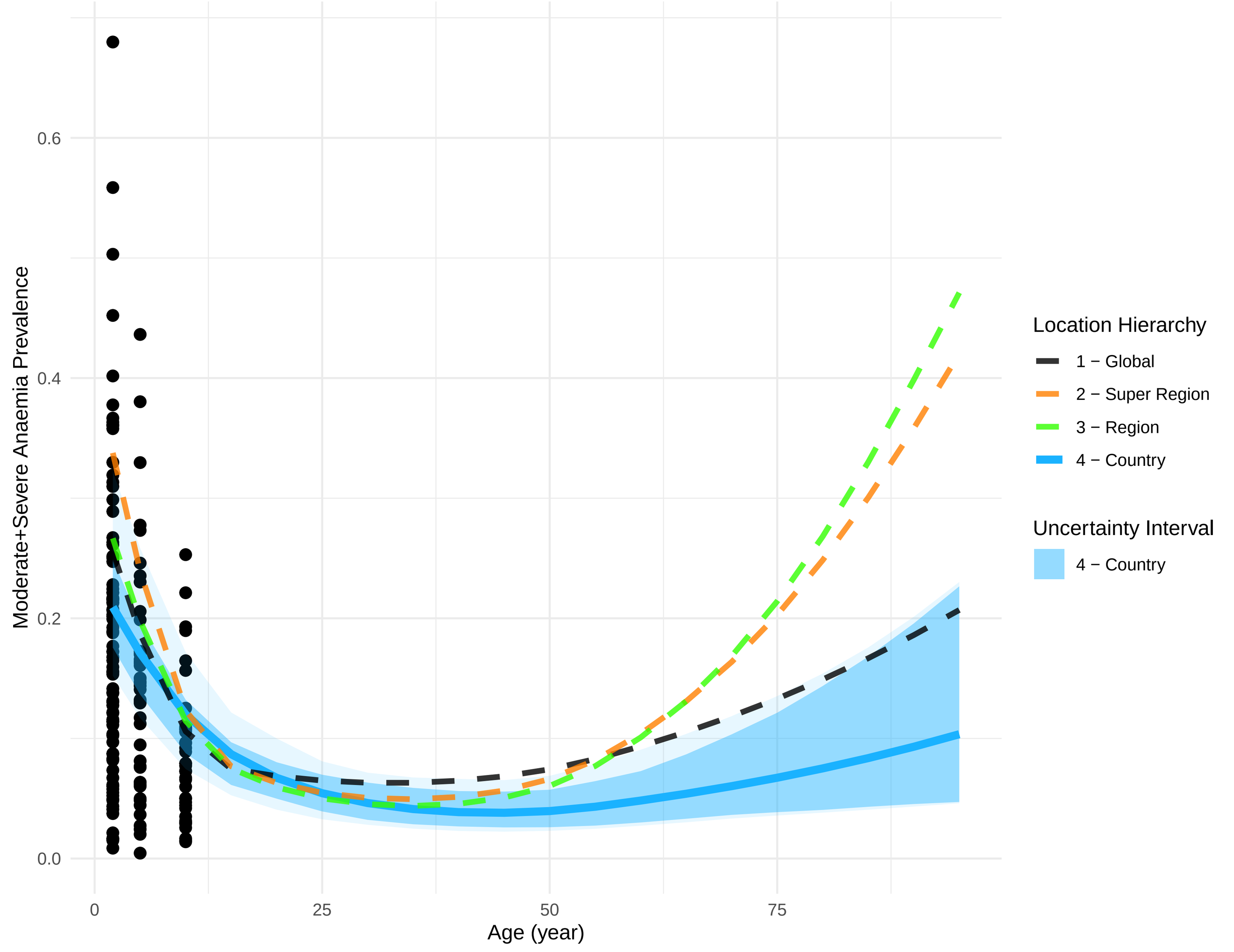

MR−BRT Age/Sex Fits − Males 2+ Years Old − Moderate+Severe Anaemia Prevalence − Baringo
WHO−2001 Adjustment Method

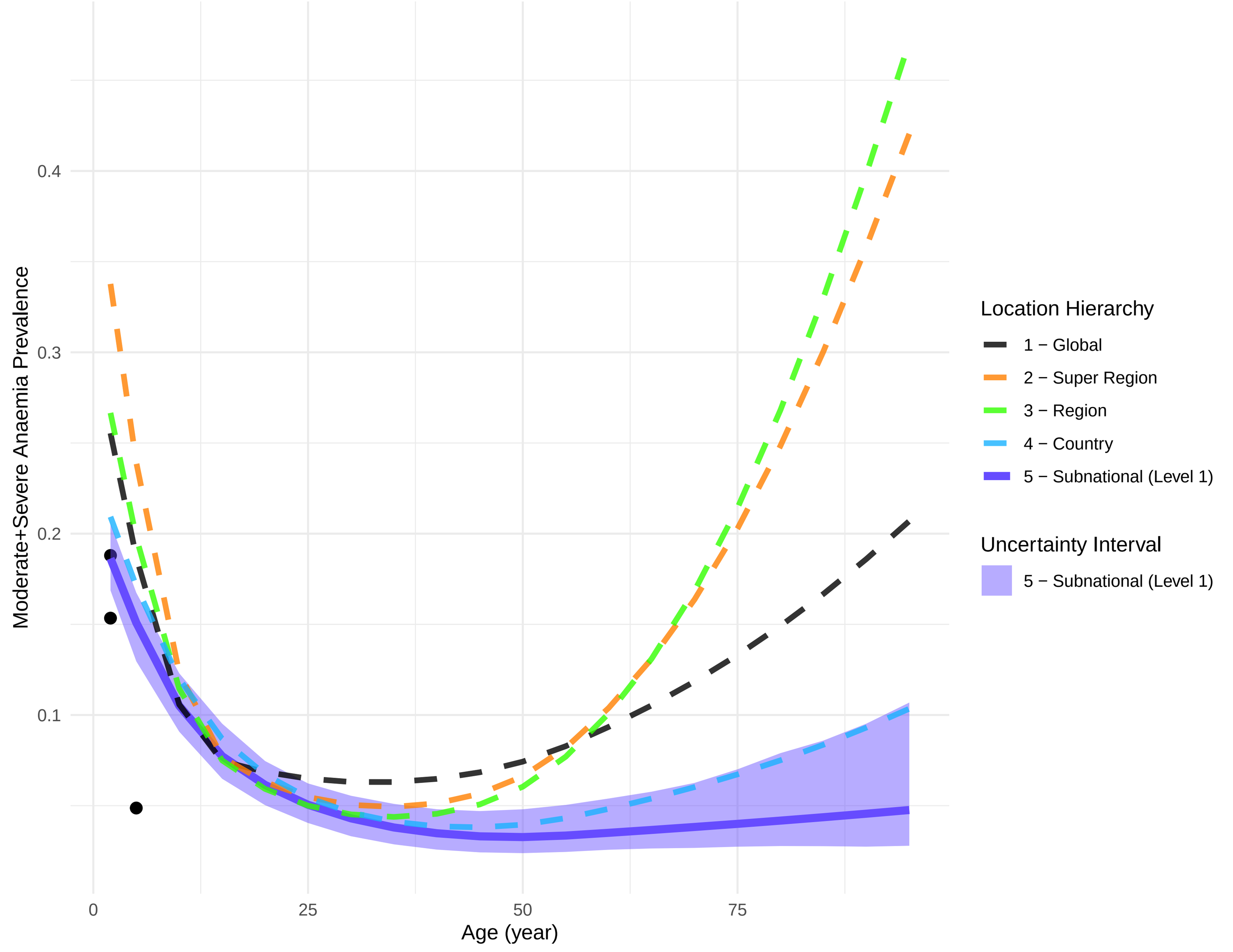

MR−BRT Age/Sex Fits − Males 2+ Years Old − Moderate+Severe Anaemia Prevalence − Bomet
WHO−2001 Adjustment Method

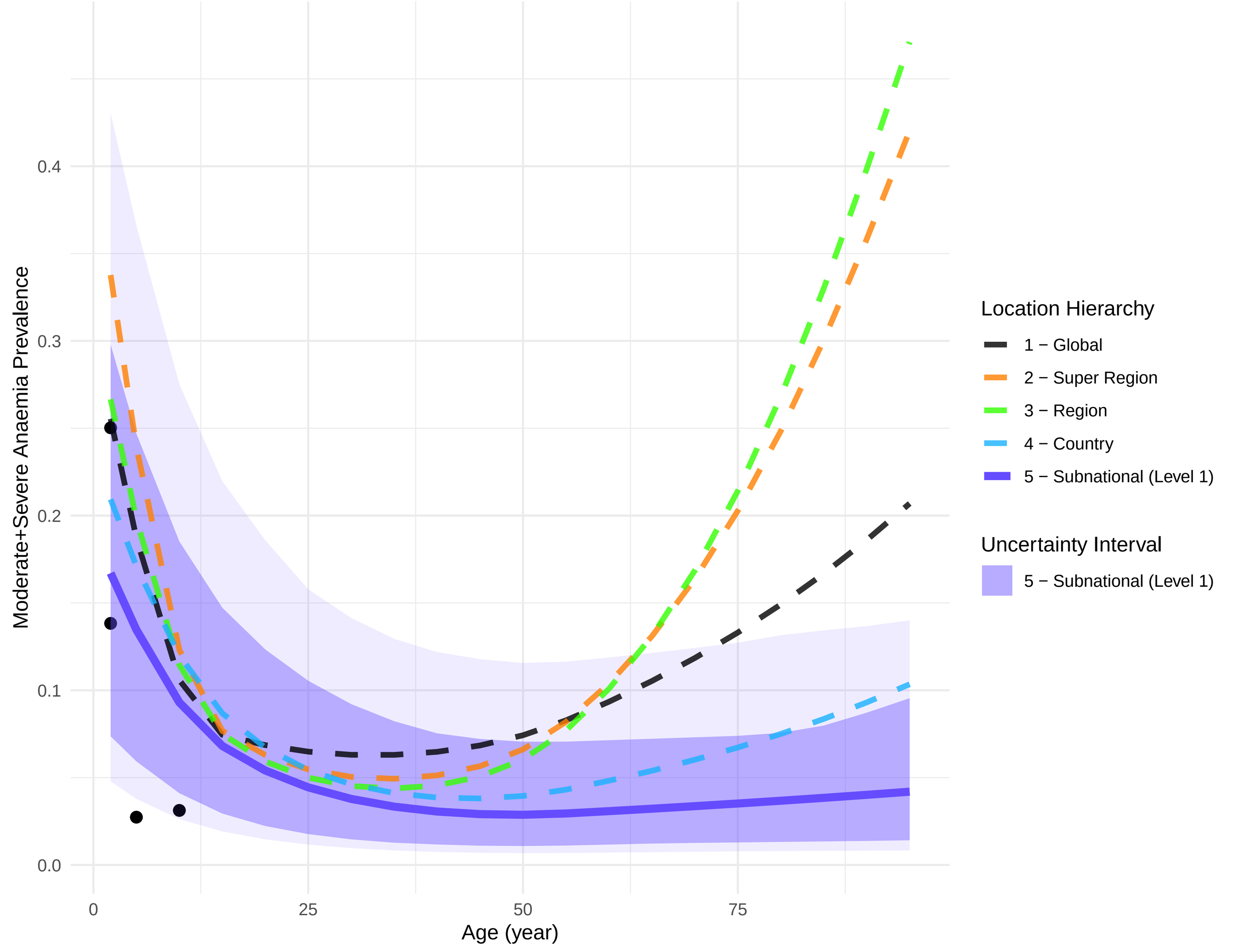

MR−BRT Age/Sex Fits − Males 2+ Years Old − Moderate+Severe Anaemia Prevalence − Bungoma
WHO−2001 Adjustment Method

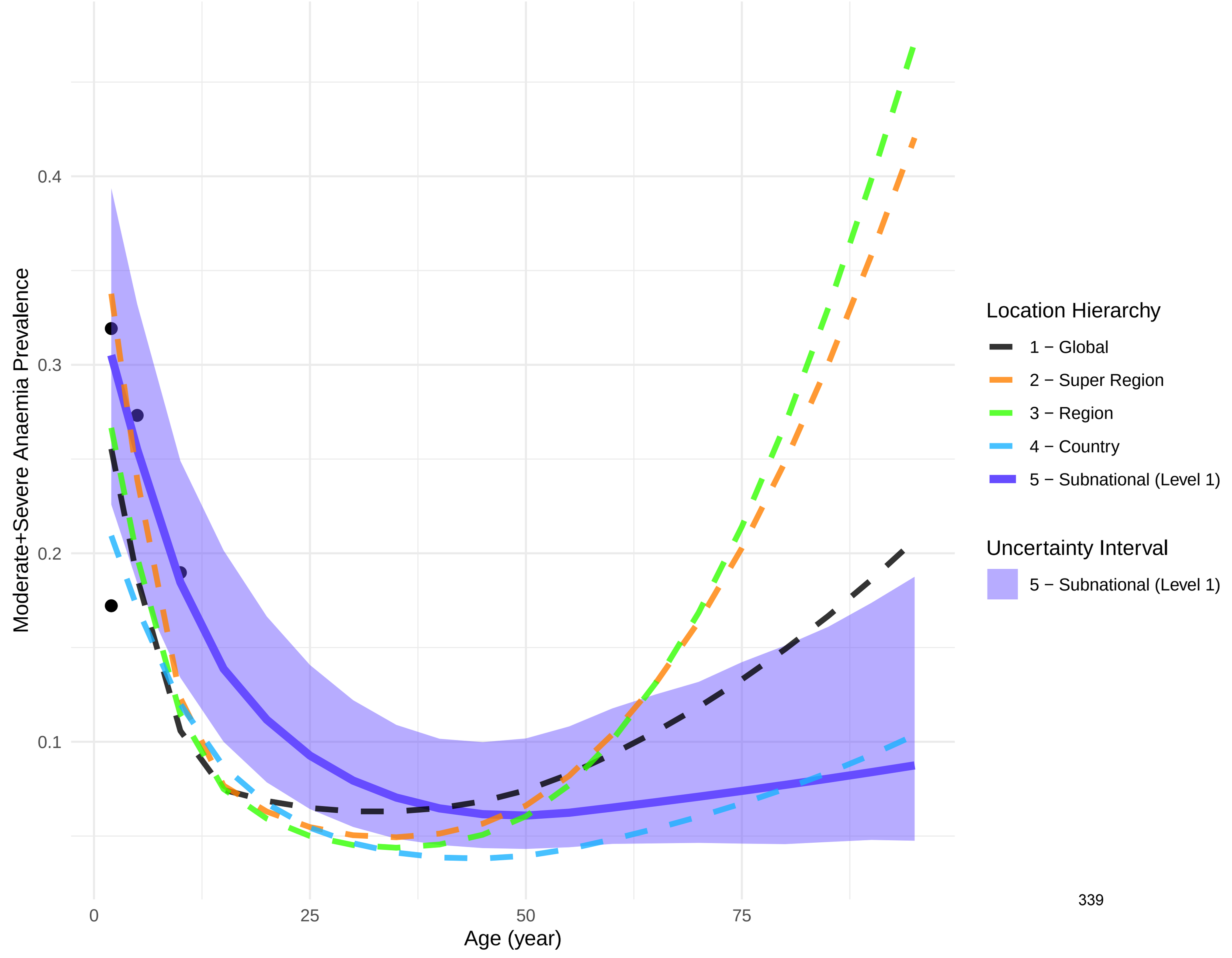

MR−BRT Age/Sex Fits − Males 2+ Years Old − Moderate+Severe Anaemia Prevalence − Busia
WHO−2001 Adjustment Method

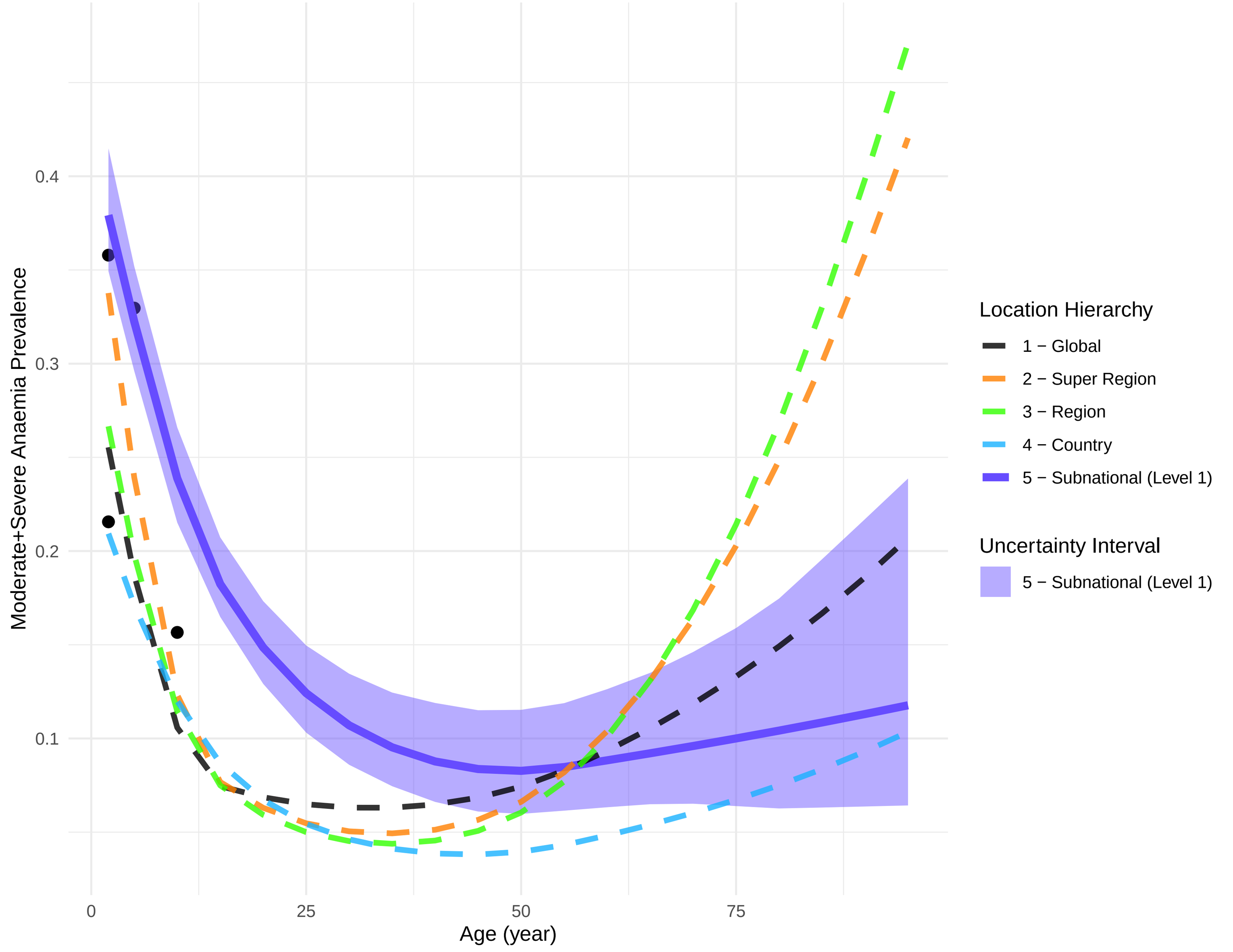

MR−BRT Age/Sex Fits − Males 2+ Years Old − Moderate+Severe Anaemia Prevalence − Elgeyo−Marakwet
WHO−2001 Adjustment Method

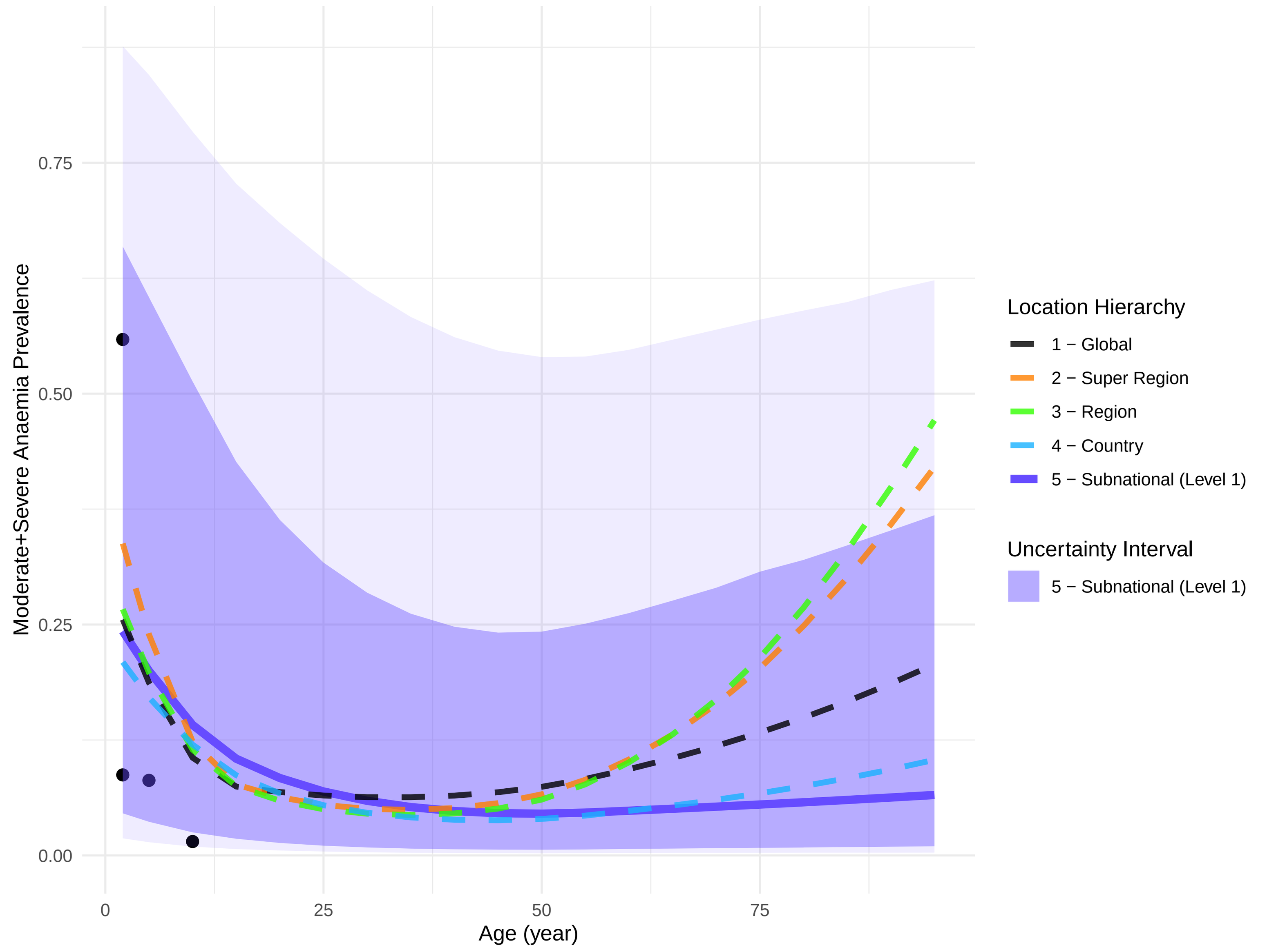

MR−BRT Age/Sex Fits − Males 2+ Years Old − Moderate+Severe Anaemia Prevalence − Embu
WHO−2001 Adjustment Method

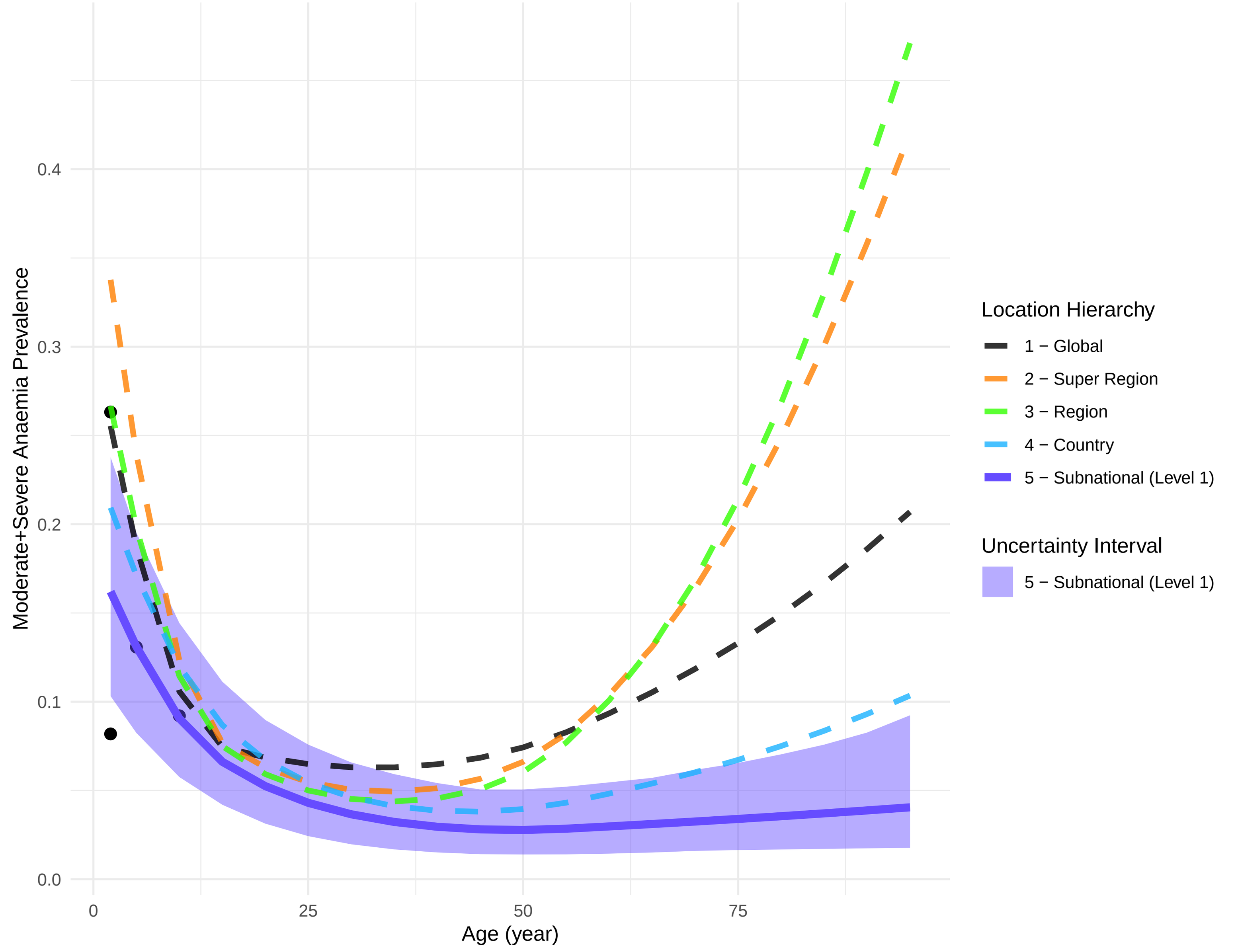

MR−BRT Age/Sex Fits − Males 2+ Years Old − Moderate+Severe Anaemia Prevalence − Garissa
WHO−2001 Adjustment Method

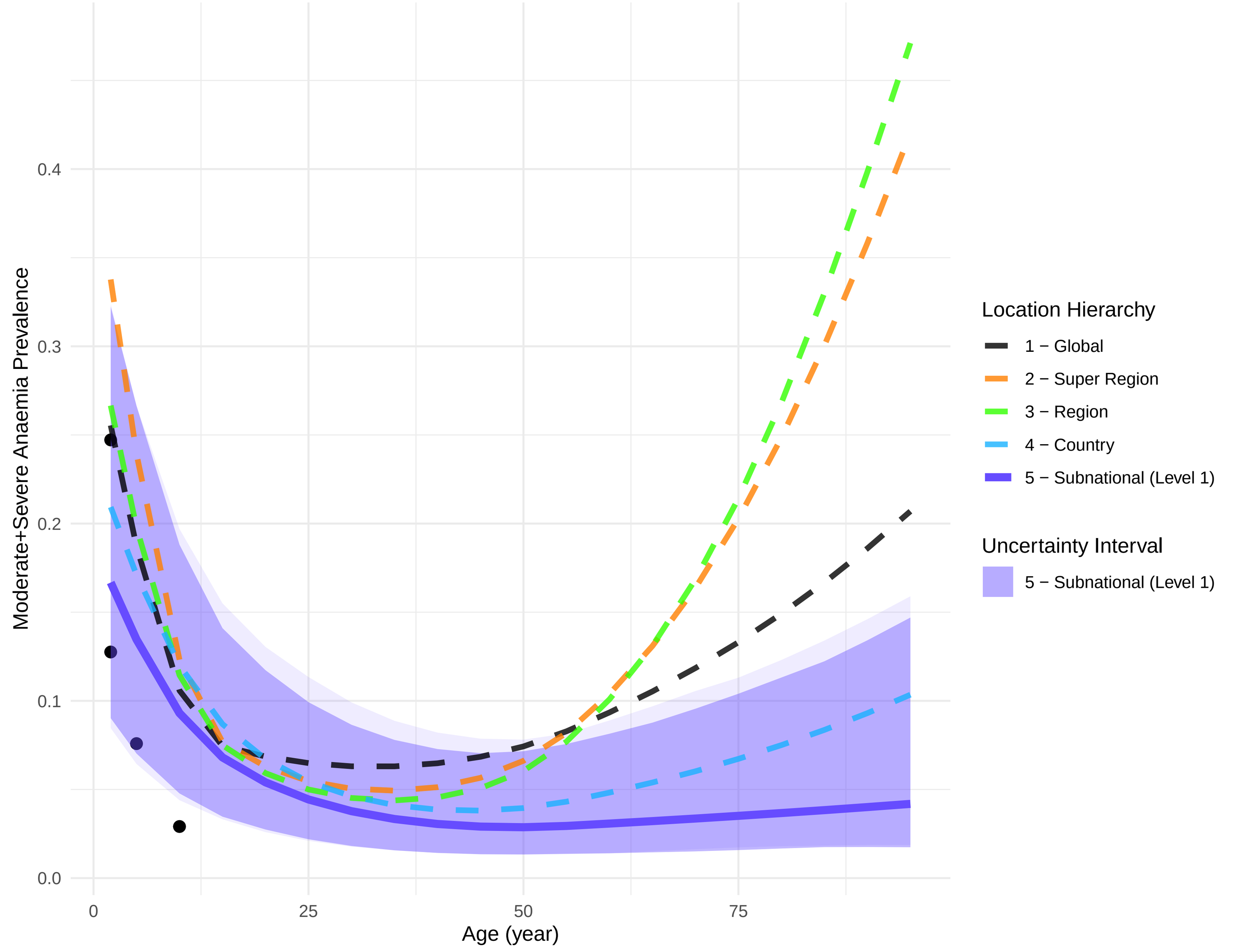

MR−BRT Age/Sex Fits − Males 2+ Years Old − Moderate+Severe Anaemia Prevalence − Homa Bay
WHO−2001 Adjustment Method

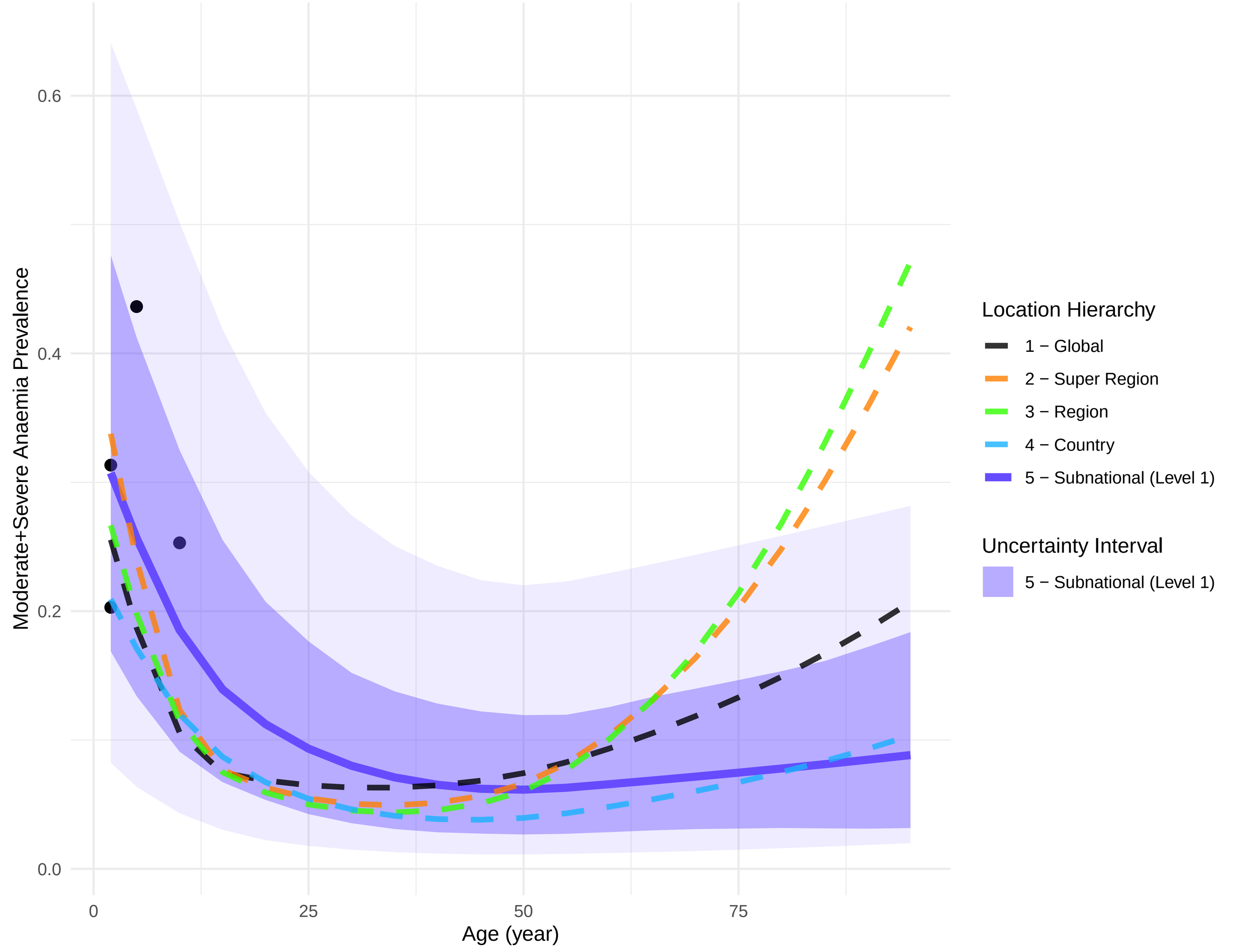

MR−BRT Age/Sex Fits − Males 2+ Years Old − Moderate+Severe Anaemia Prevalence − Isiolo
WHO−2001 Adjustment Method

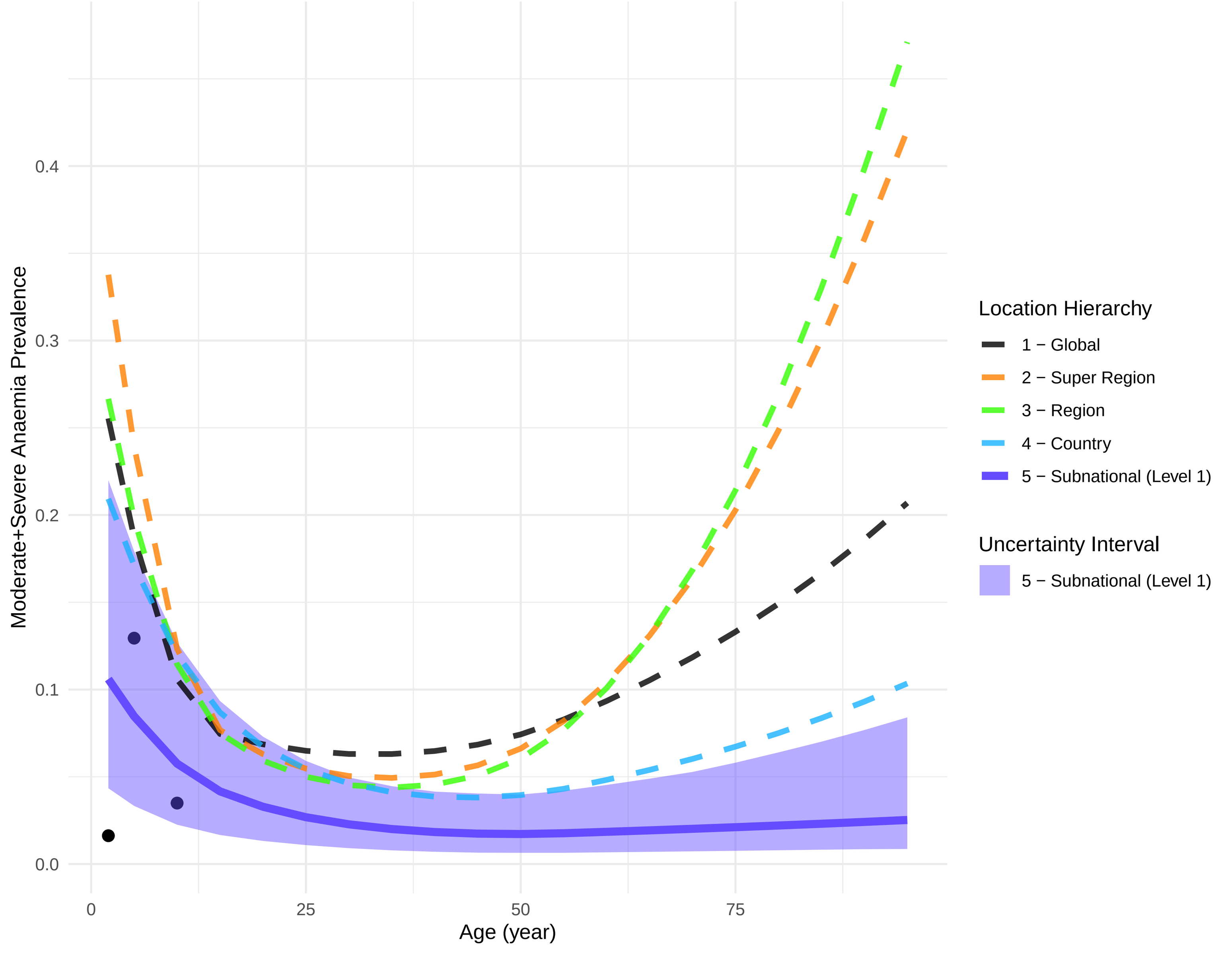

MR−BRT Age/Sex Fits − Males 2+ Years Old − Moderate+Severe Anaemia Prevalence − Kajiado
WHO−2001 Adjustment Method

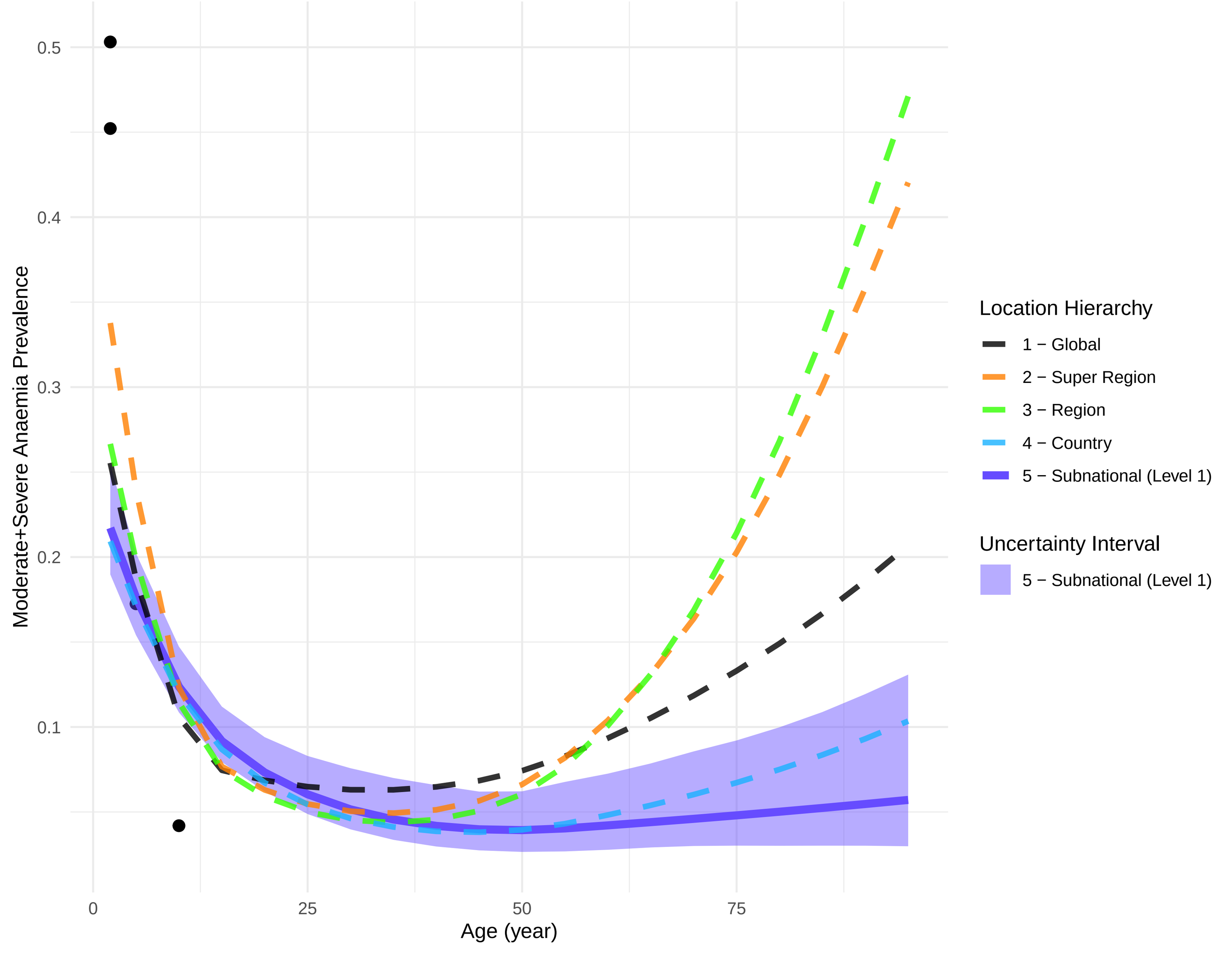

MR−BRT Age/Sex Fits − Males 2+ Years Old − Moderate+Severe Anaemia Prevalence − Kakamega
WHO−2001 Adjustment Method

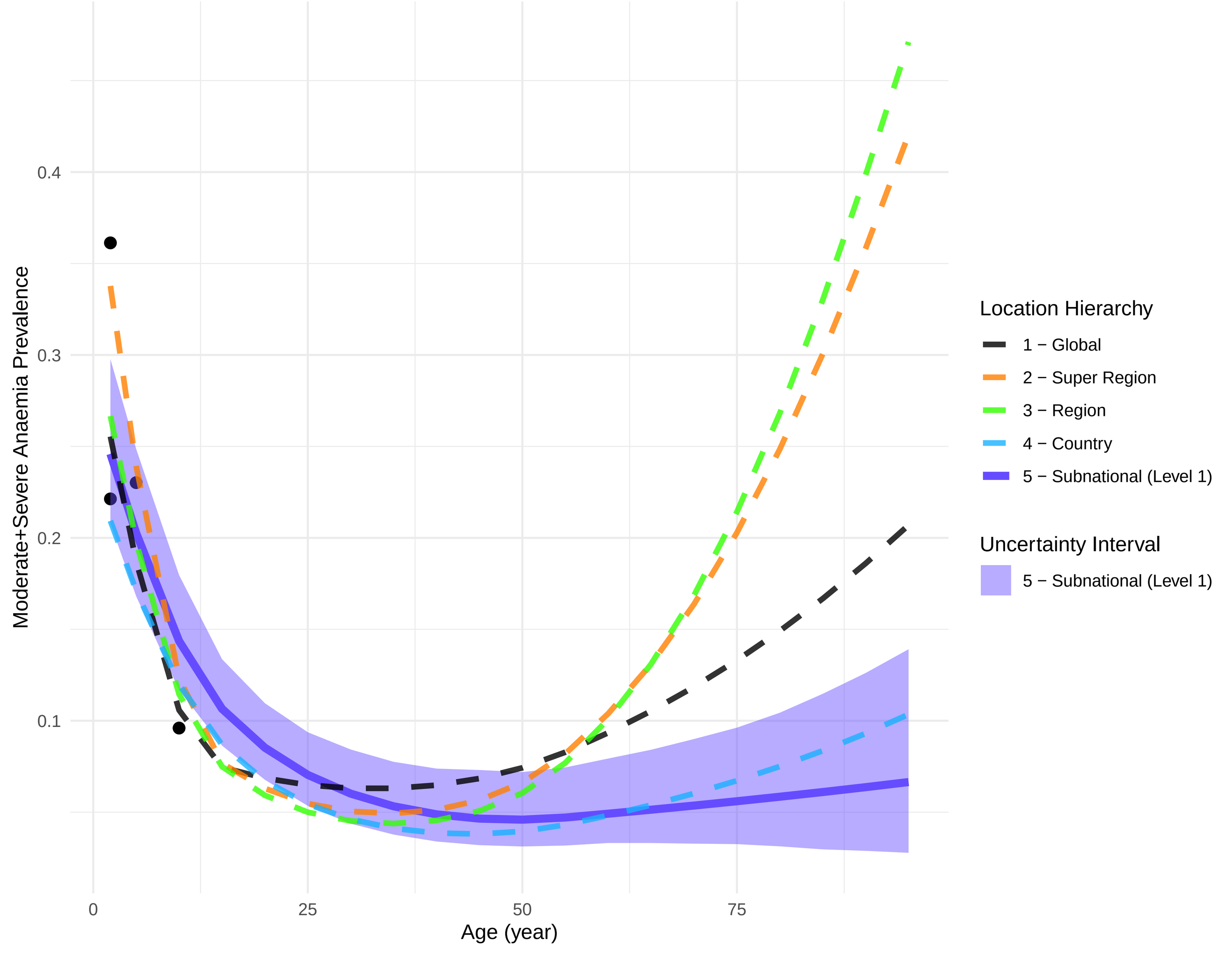

MR−BRT Age/Sex Fits − Males 2+ Years Old − Moderate+Severe Anaemia Prevalence − Kericho
WHO−2001 Adjustment Method

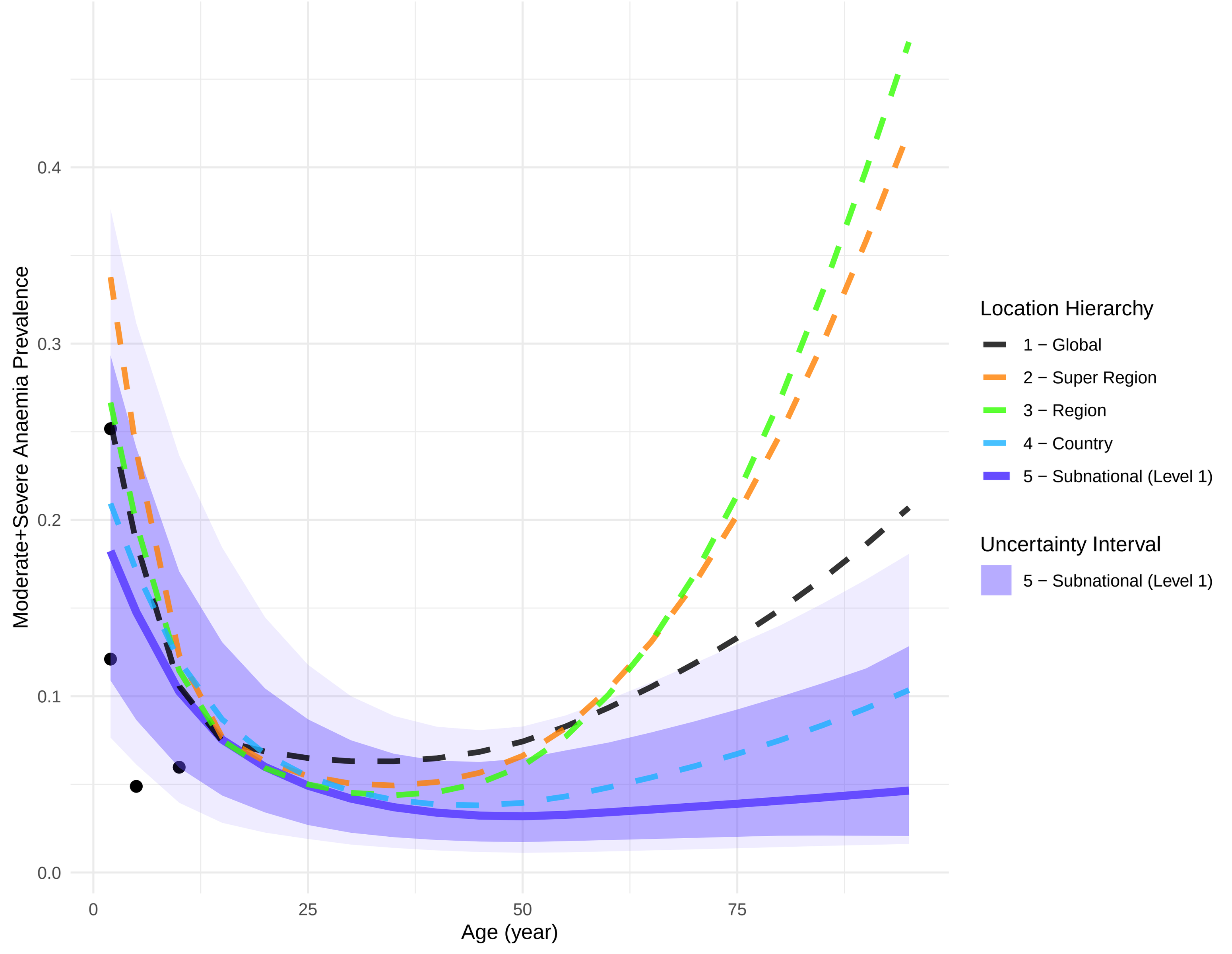

MR−BRT Age/Sex Fits − Males 2+ Years Old − Moderate+Severe Anaemia Prevalence − Kiambu
WHO−2001 Adjustment Method

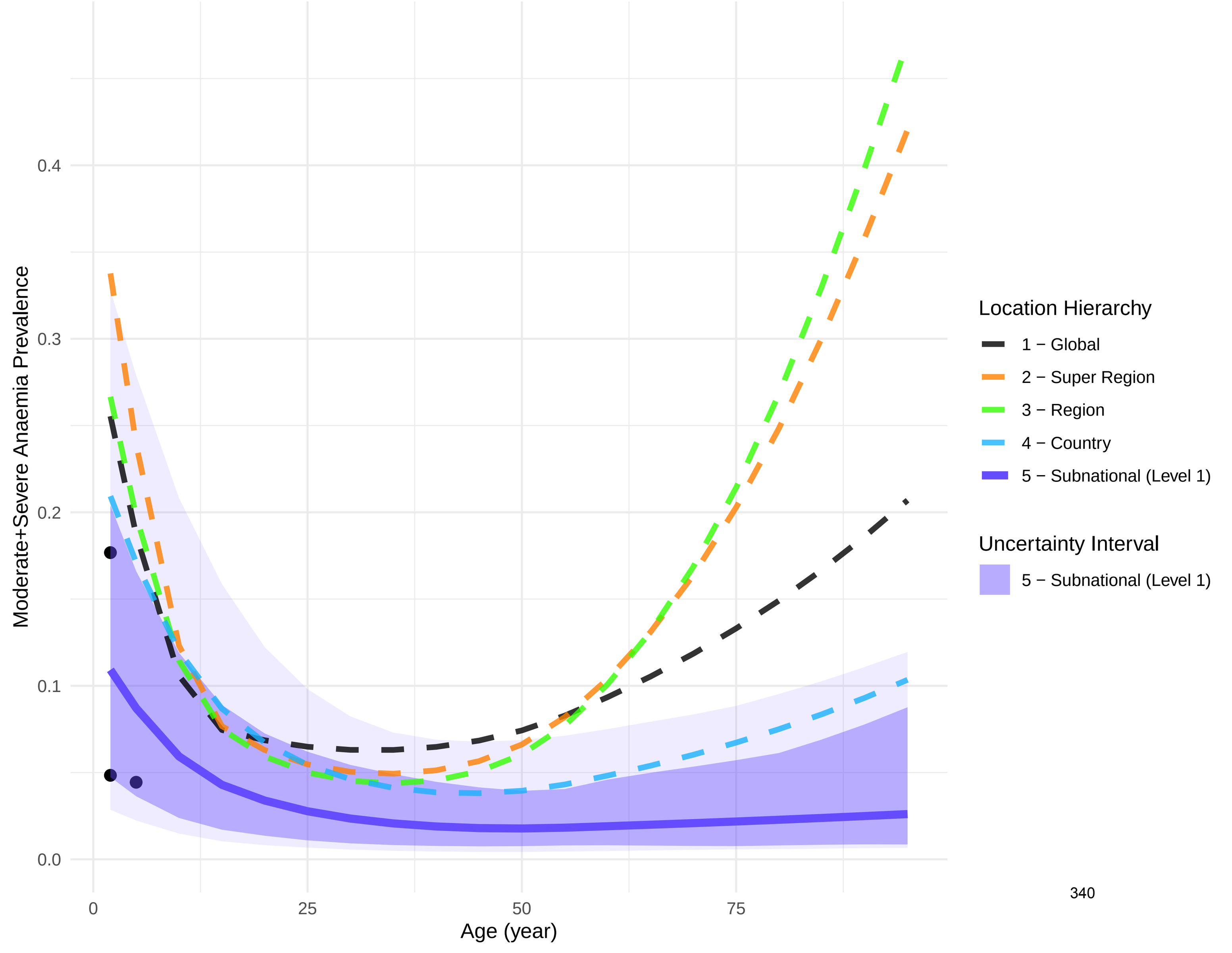

MR−BRT Age/Sex Fits − Males 2+ Years Old − Moderate+Severe Anaemia Prevalence − Kilifi
WHO−2001 Adjustment Method

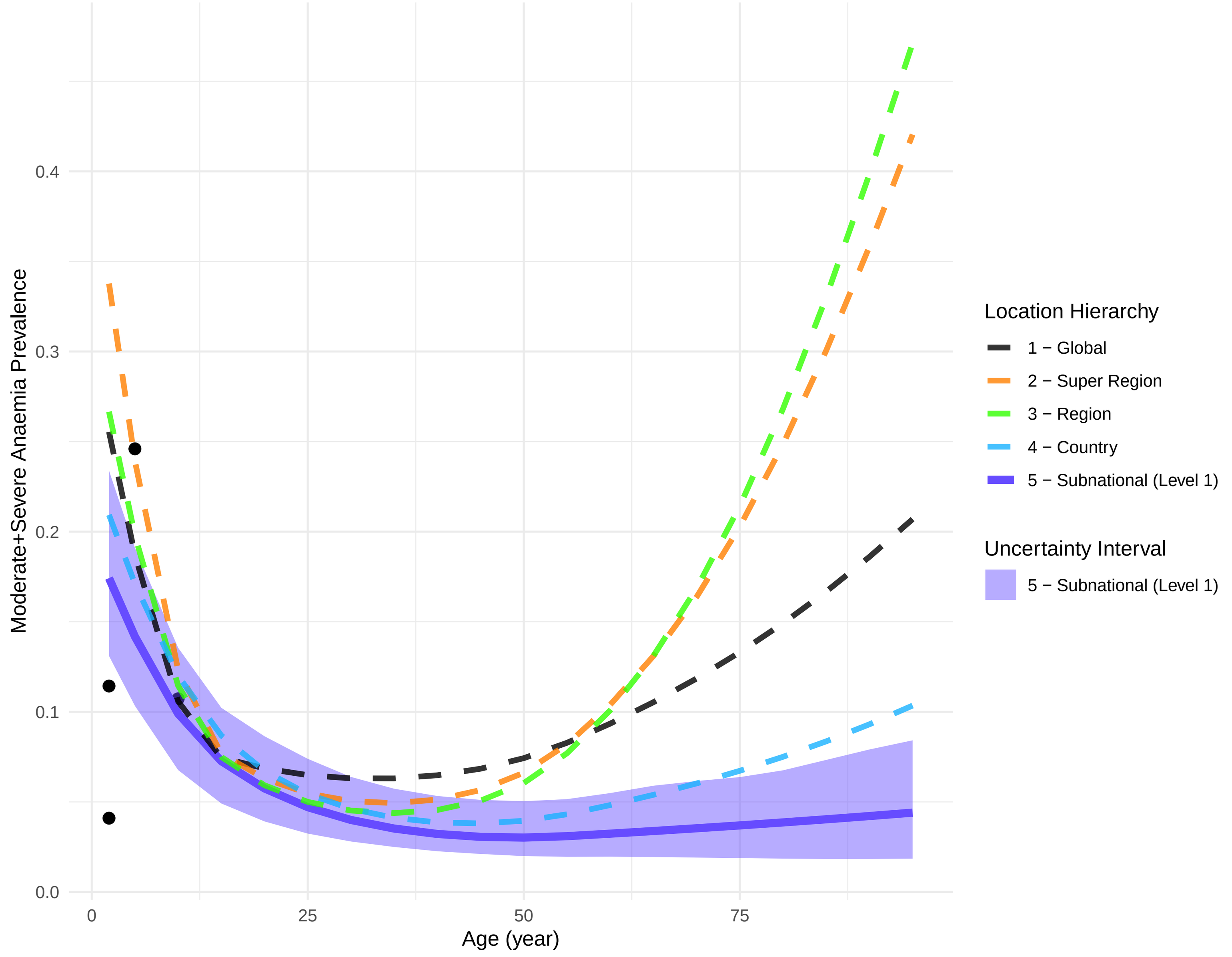

MR−BRT Age/Sex Fits − Males 2+ Years Old − Moderate+Severe Anaemia Prevalence − Kirinyaga
WHO−2001 Adjustment Method

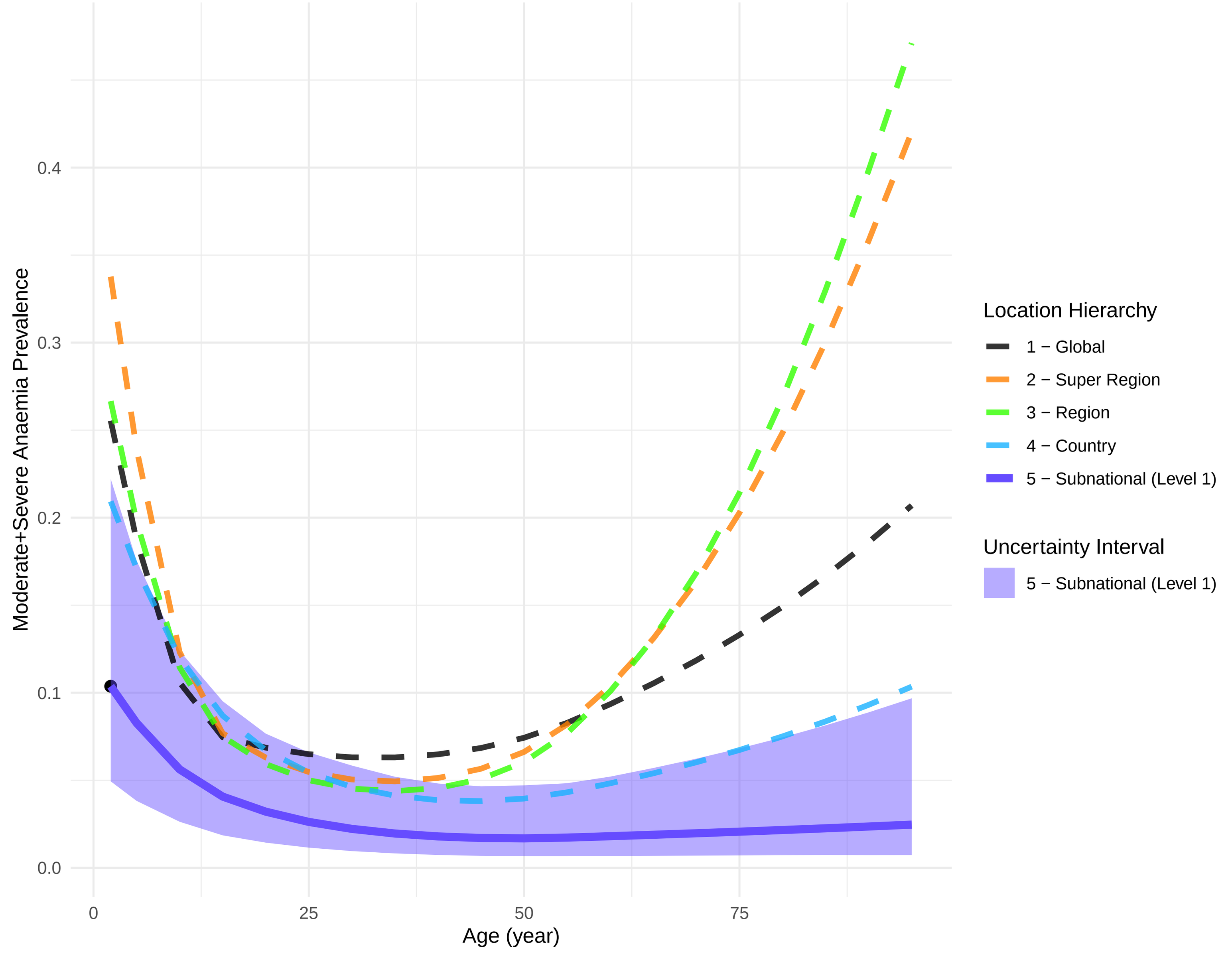

MR−BRT Age/Sex Fits − Males 2+ Years Old − Moderate+Severe Anaemia Prevalence − Kisii
WHO−2001 Adjustment Method

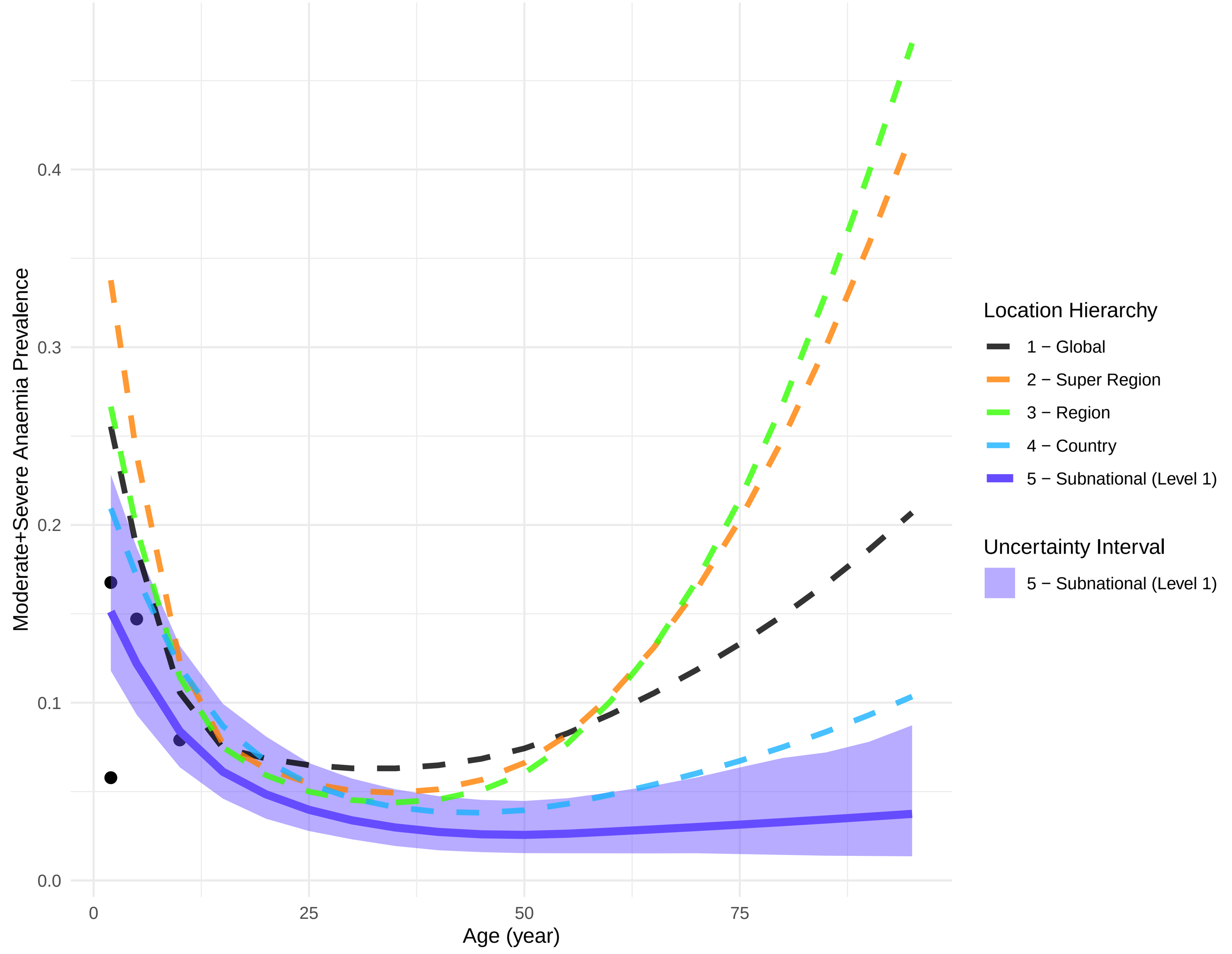

MR−BRT Age/Sex Fits − Males 2+ Years Old − Moderate+Severe Anaemia Prevalence − Kisumu
WHO−2001 Adjustment Method

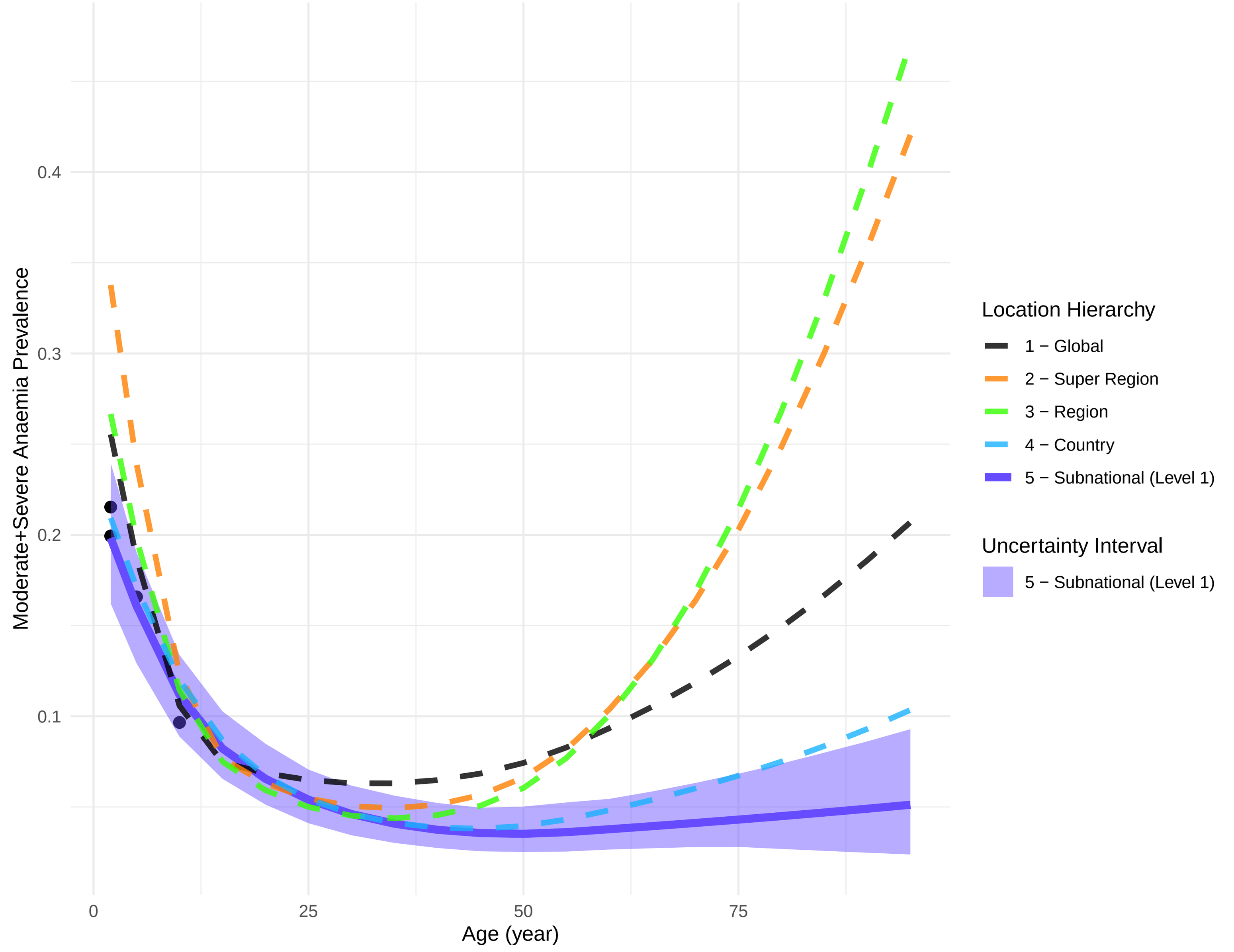

MR−BRT Age/Sex Fits − Males 2+ Years Old − Moderate+Severe Anaemia Prevalence − Kitui
WHO−2001 Adjustment Method

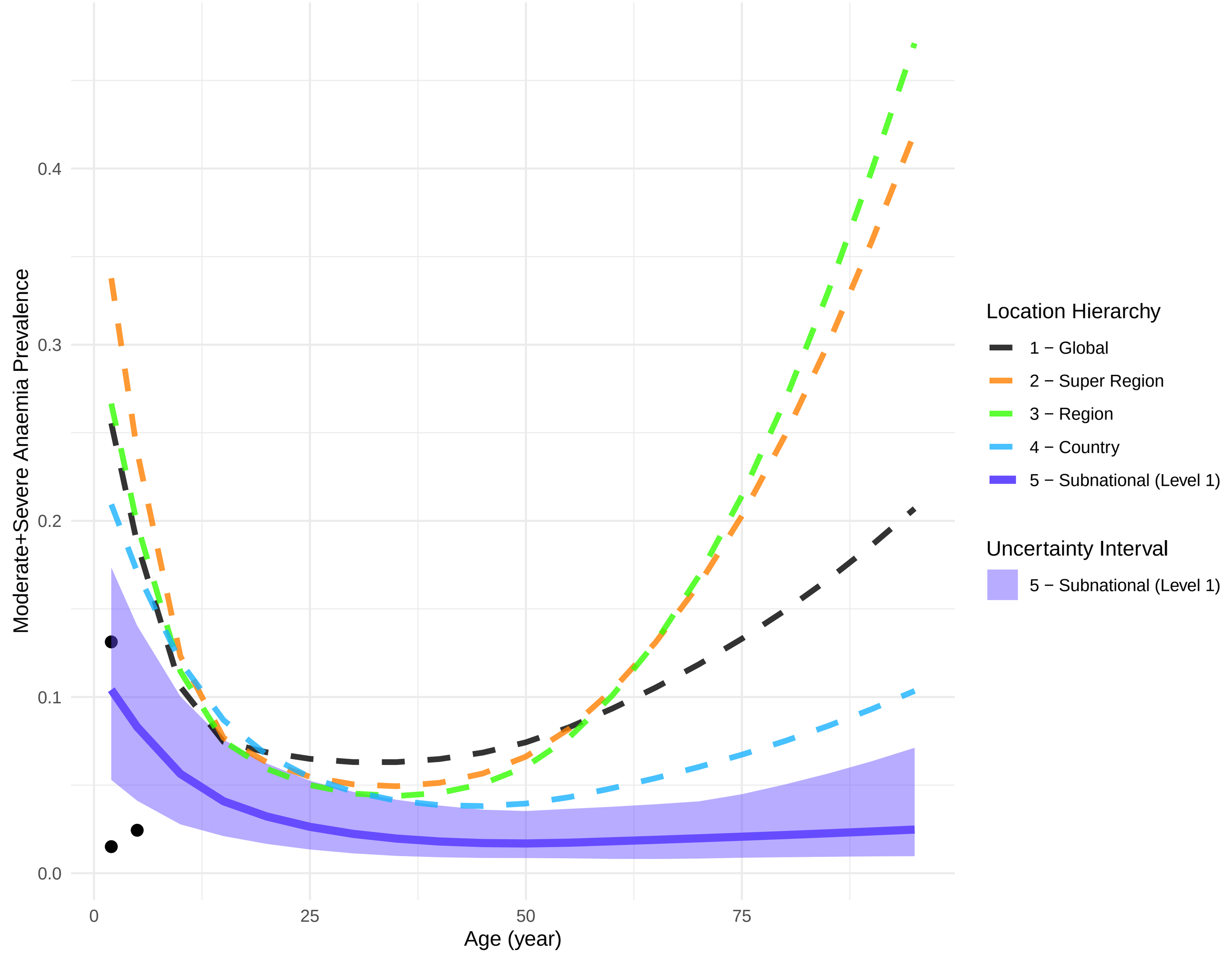

MR−BRT Age/Sex Fits − Males 2+ Years Old − Moderate+Severe Anaemia Prevalence − Kwale
WHO−2001 Adjustment Method

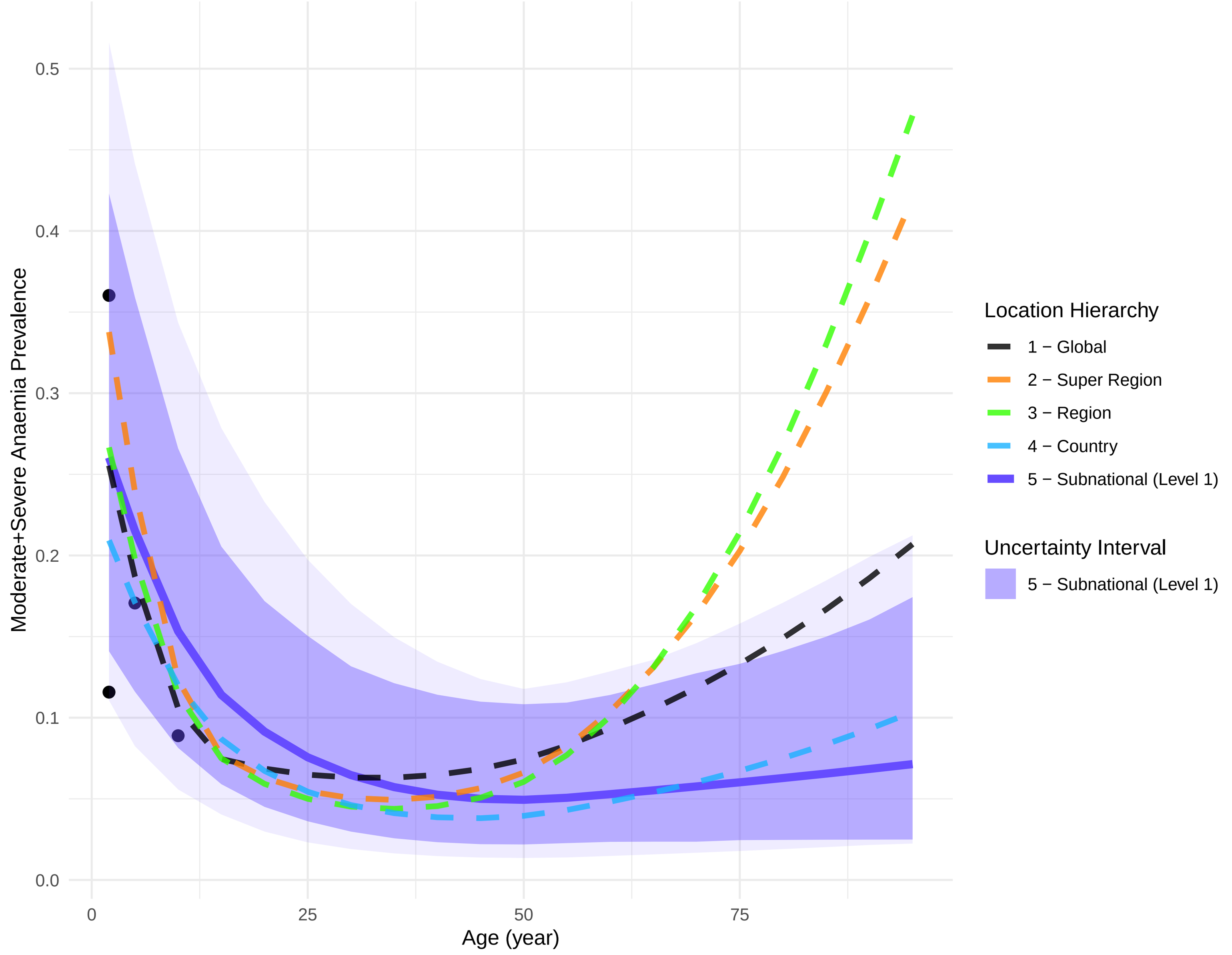

MR−BRT Age/Sex Fits − Males 2+ Years Old − Moderate+Severe Anaemia Prevalence − Laikipia
WHO−2001 Adjustment Method

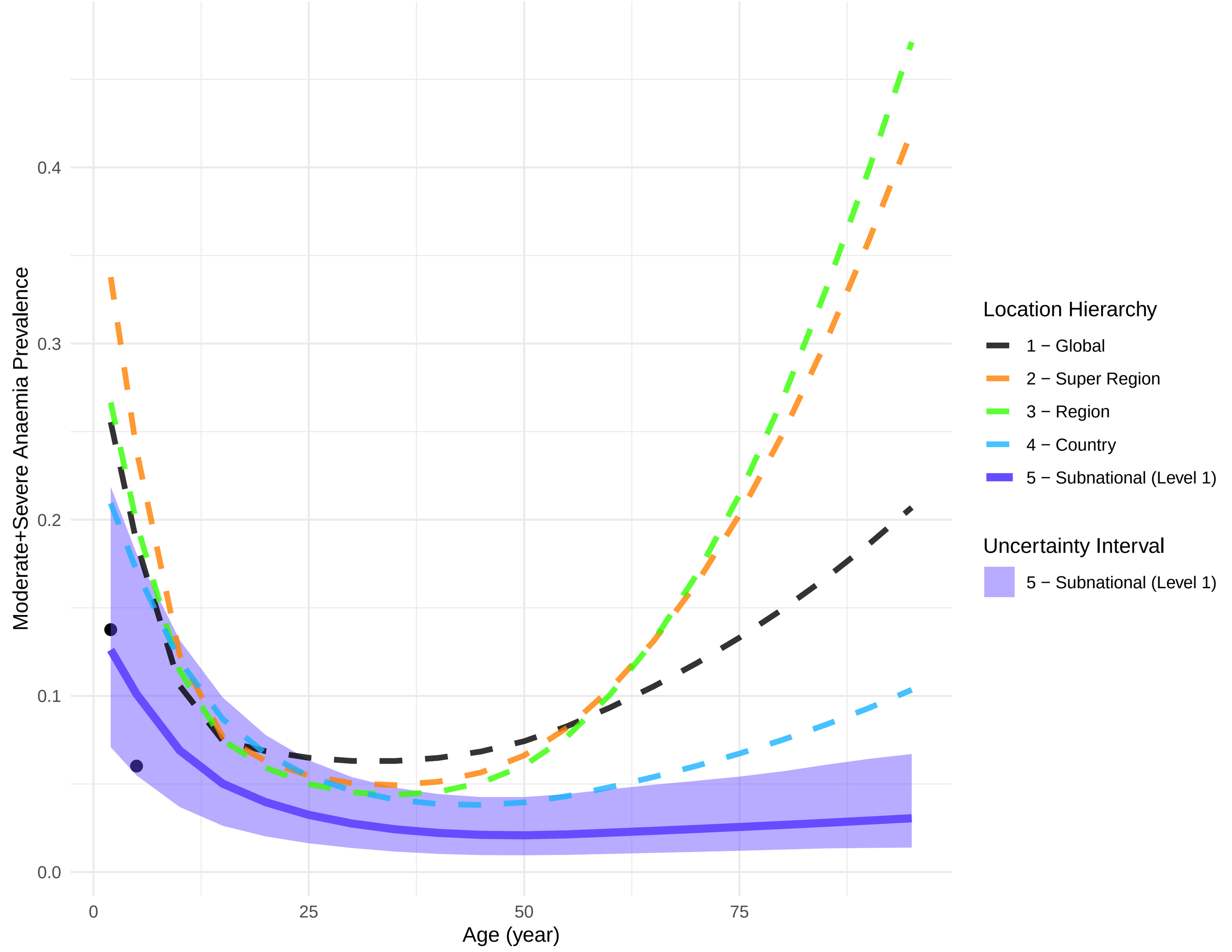

MR−BRT Age/Sex Fits − Males 2+ Years Old − Moderate+Severe Anaemia Prevalence − Lamu
WHO−2001 Adjustment Method

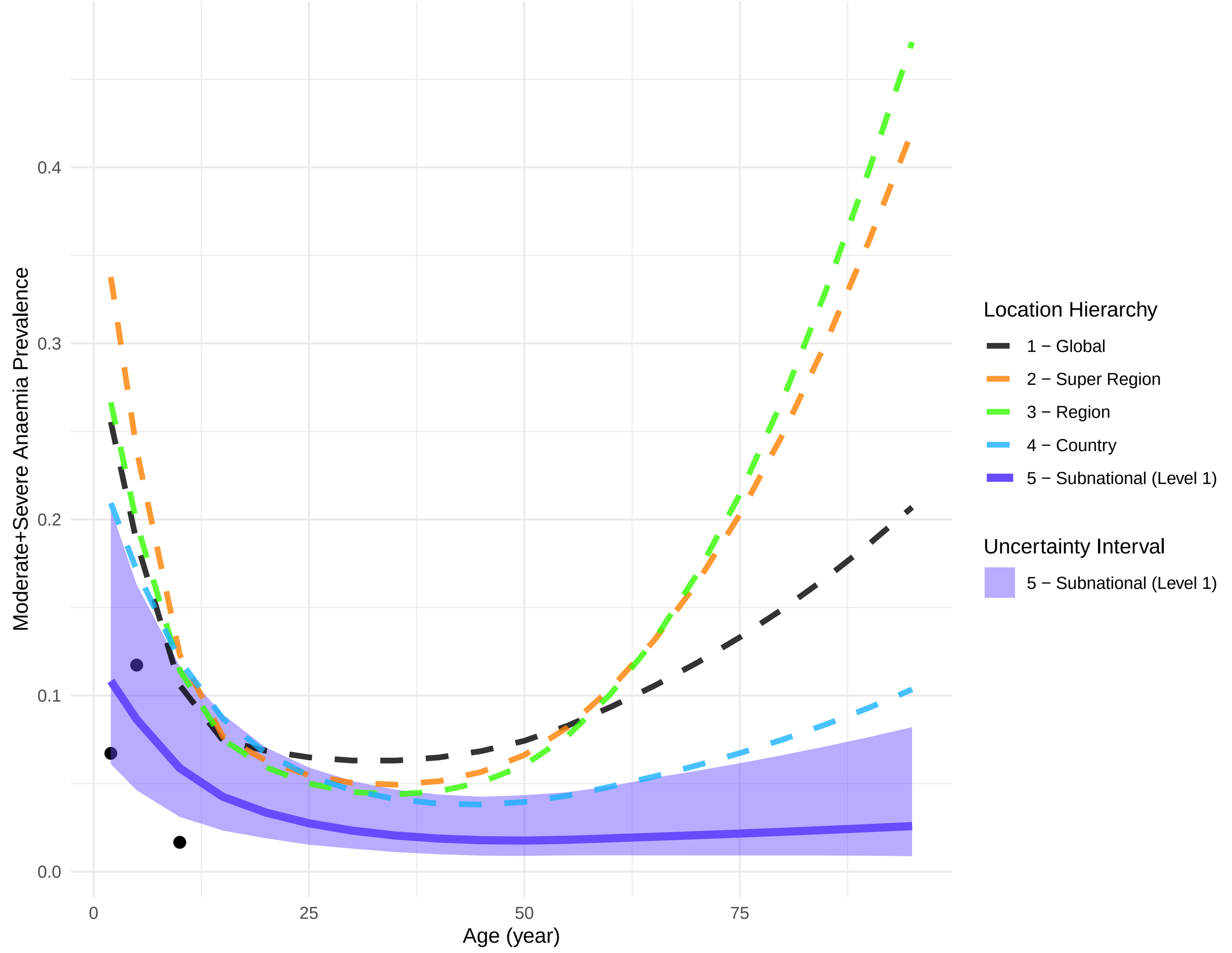

MR−BRT Age/Sex Fits − Males 2+ Years Old − Moderate+Severe Anaemia Prevalence − Machakos
WHO−2001 Adjustment Method

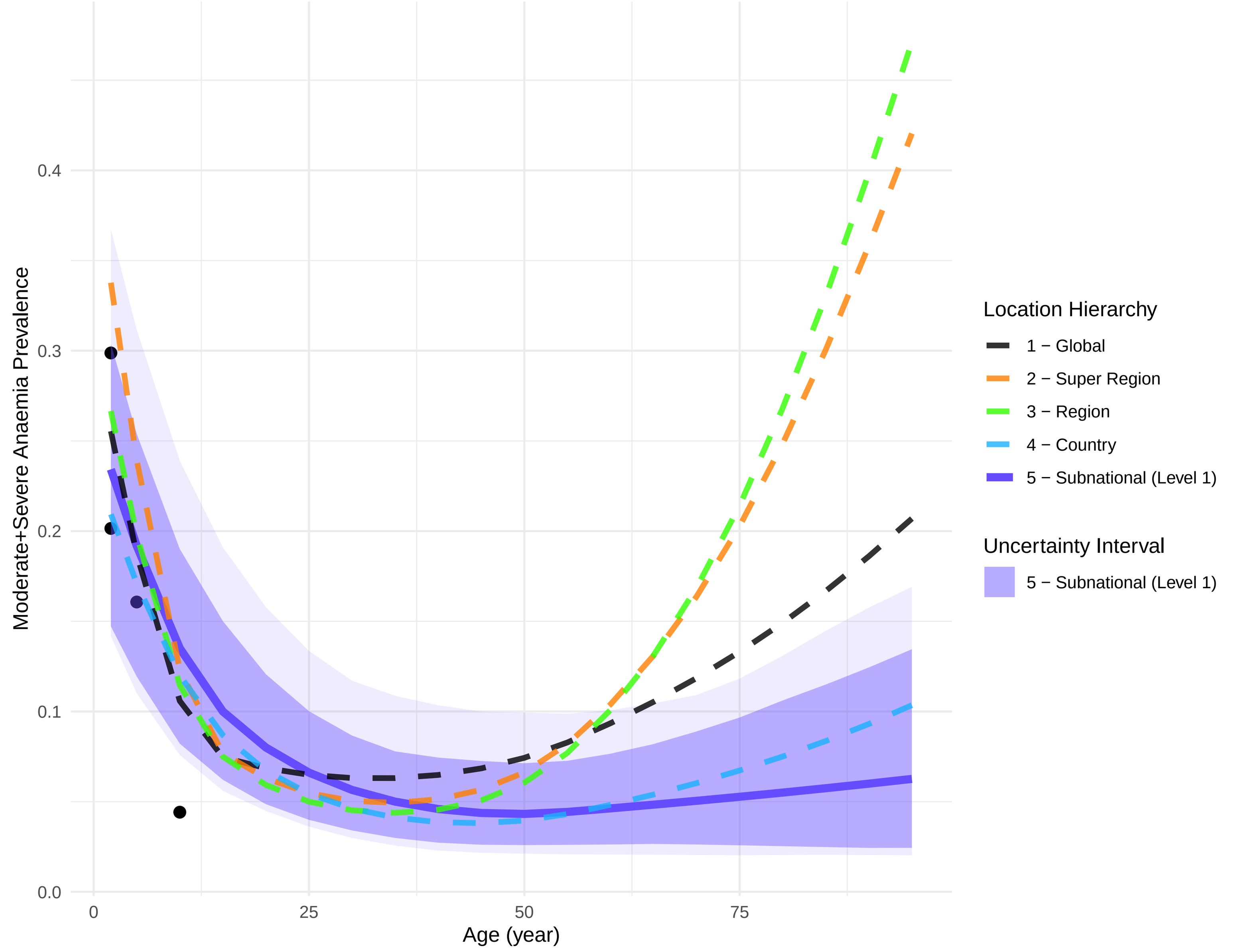

MR−BRT Age/Sex Fits − Males 2+ Years Old − Moderate+Severe Anaemia Prevalence − Makueni
WHO−2001 Adjustment Method

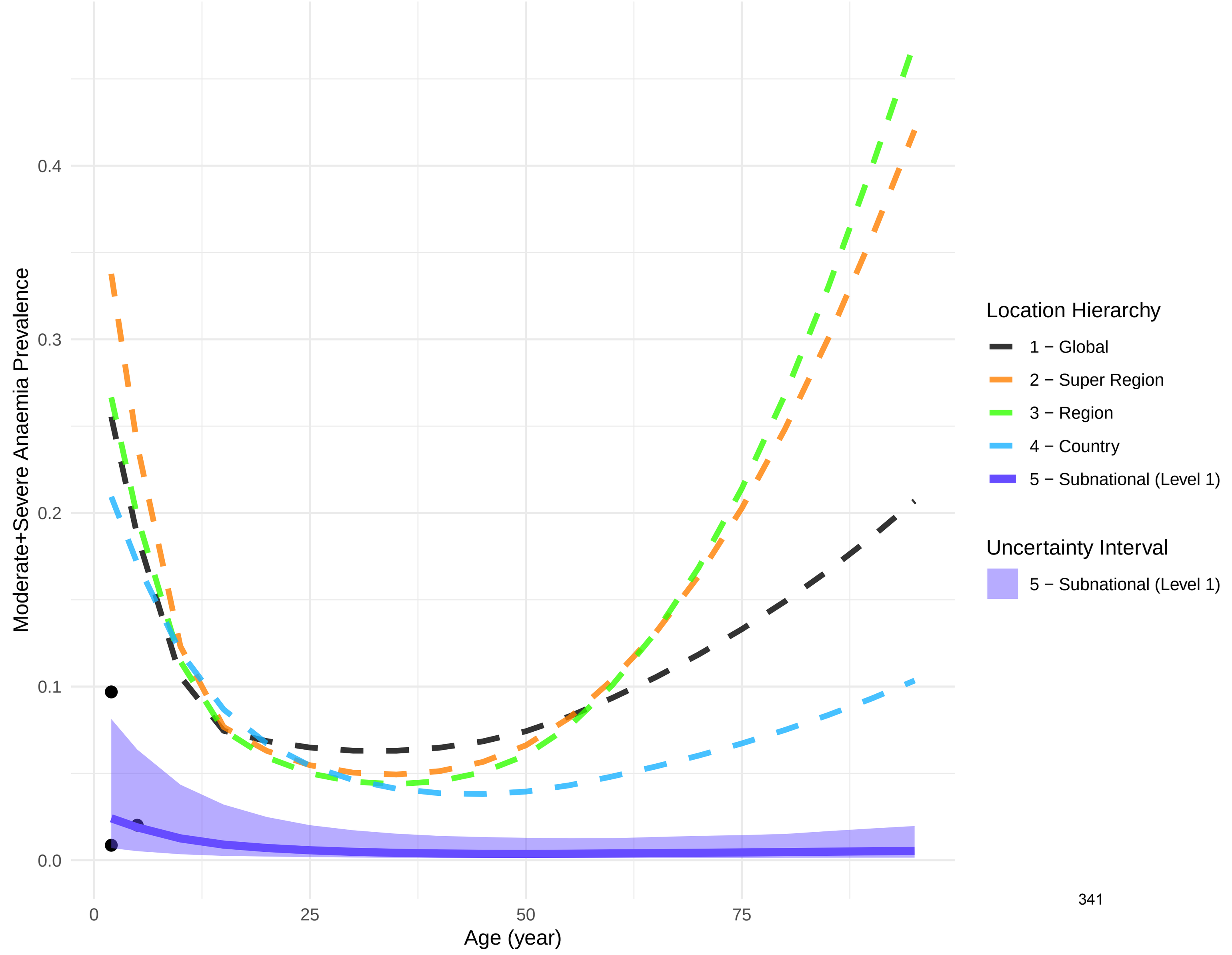

MR−BRT Age/Sex Fits − Males 2+ Years Old − Moderate+Severe Anaemia Prevalence − Mandera
WHO−2001 Adjustment Method

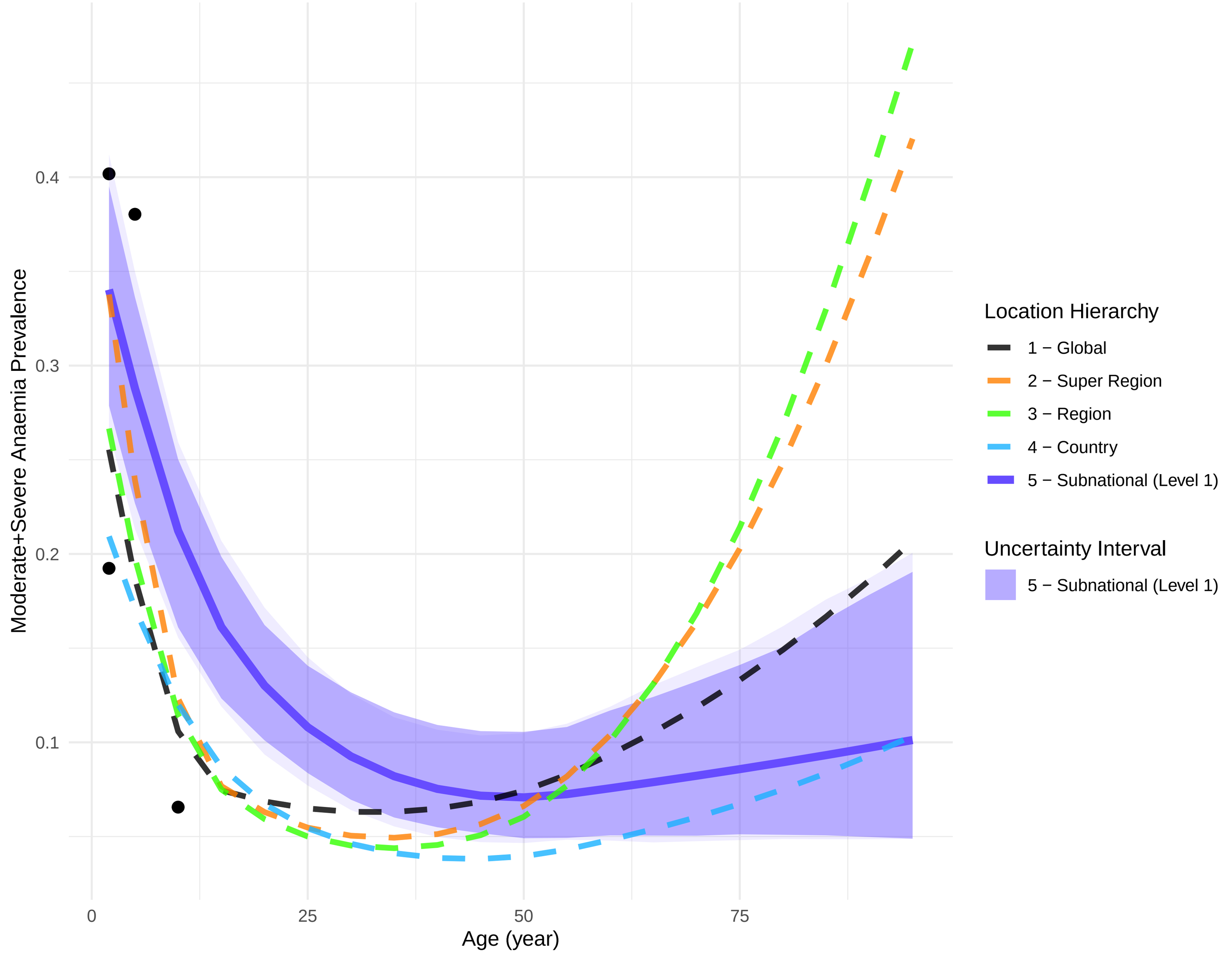

MR−BRT Age/Sex Fits − Males 2+ Years Old − Moderate+Severe Anaemia Prevalence − Marsabit
WHO−2001 Adjustment Method

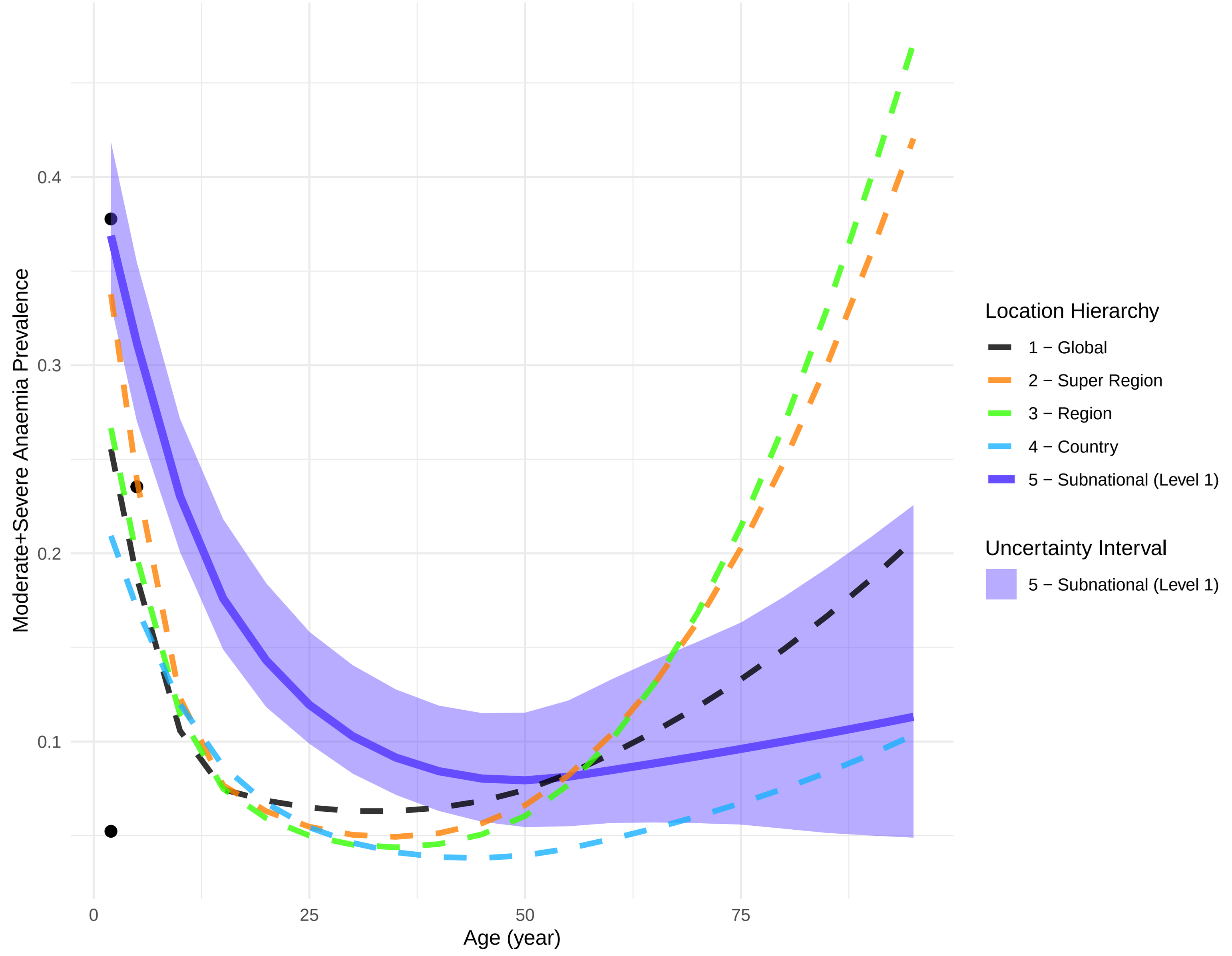

MR−BRT Age/Sex Fits − Males 2+ Years Old − Moderate+Severe Anaemia Prevalence − Meru
WHO−2001 Adjustment Method

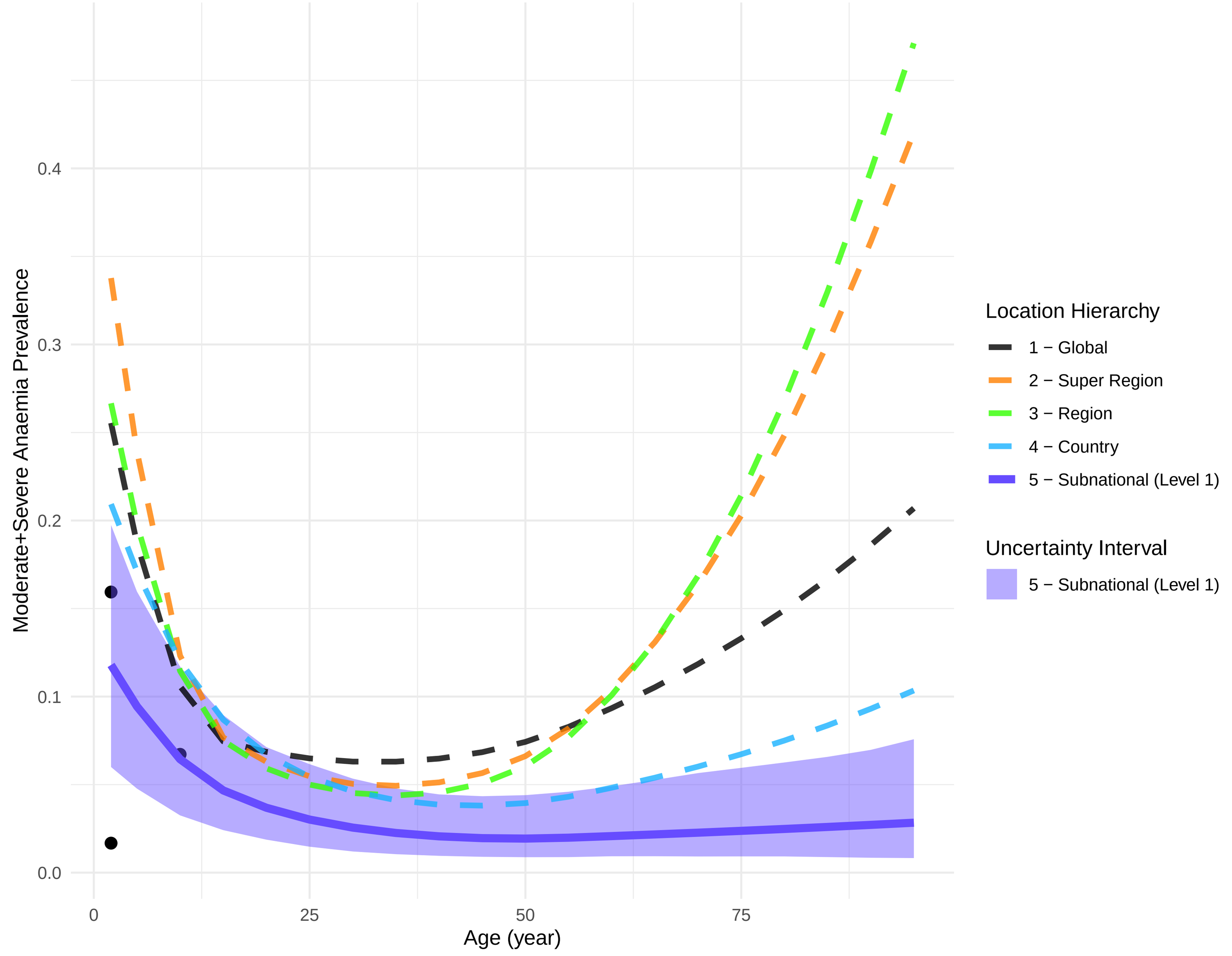

MR−BRT Age/Sex Fits − Males 2+ Years Old − Moderate+Severe Anaemia Prevalence − Migori
WHO−2001 Adjustment Method

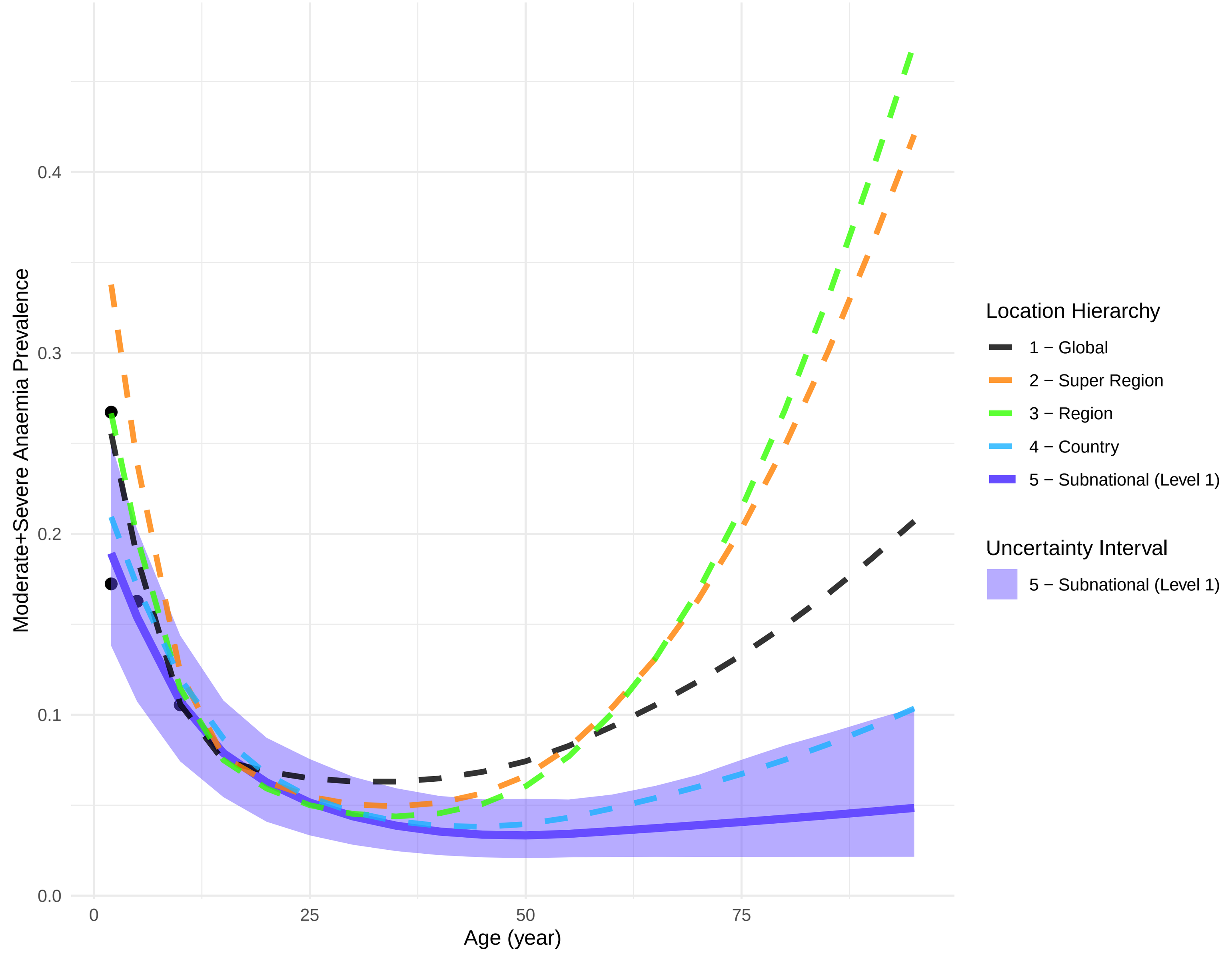

MR−BRT Age/Sex Fits − Males 2+ Years Old − Moderate+Severe Anaemia Prevalence − Mombasa
WHO−2001 Adjustment Method

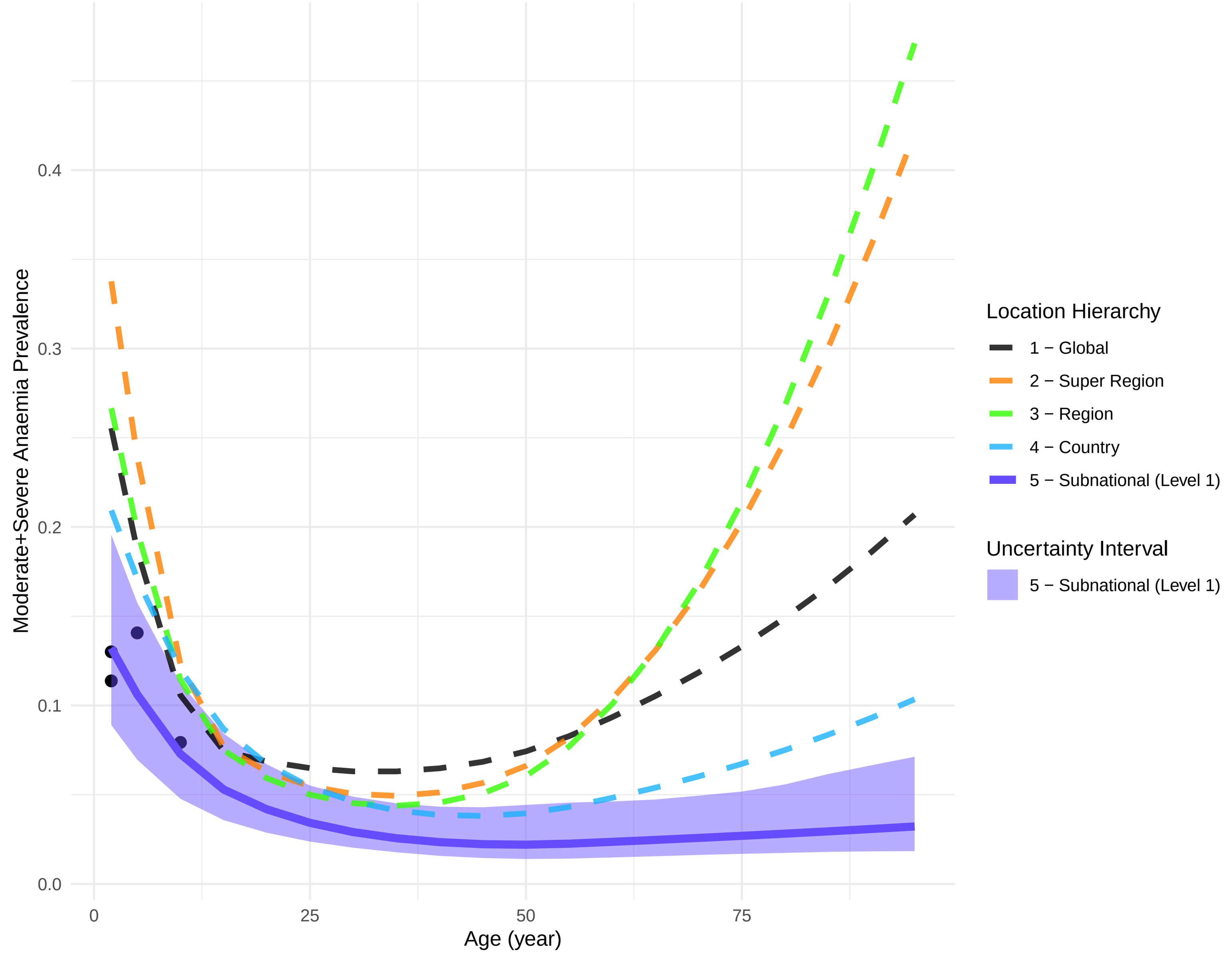

MR−BRT Age/Sex Fits − Males 2+ Years Old − Moderate+Severe Anaemia Prevalence − Murang'a
WHO−2001 Adjustment Method

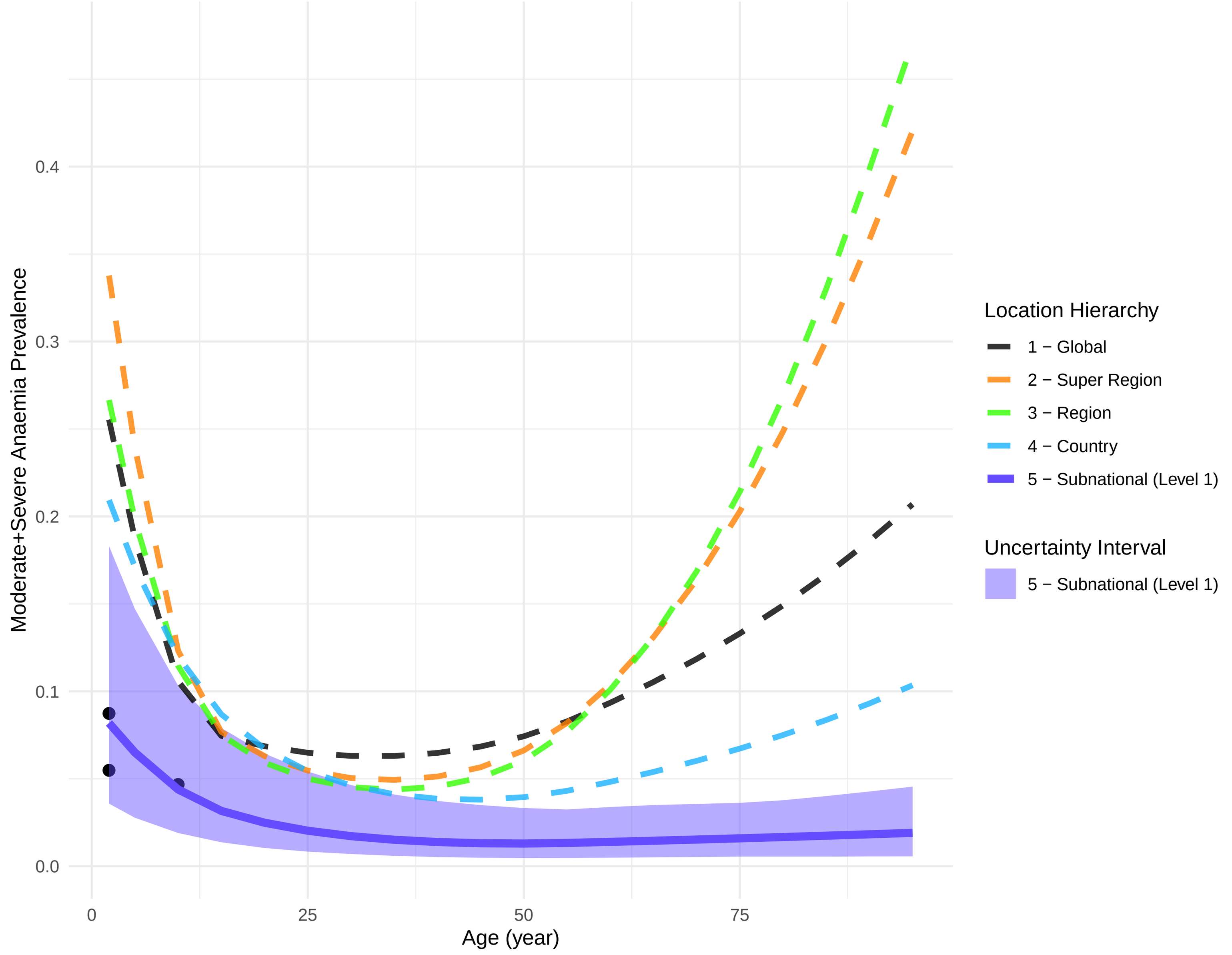

MR−BRT Age/Sex Fits − Males 2+ Years Old − Moderate+Severe Anaemia Prevalence − Nairobi
WHO−2001 Adjustment Method

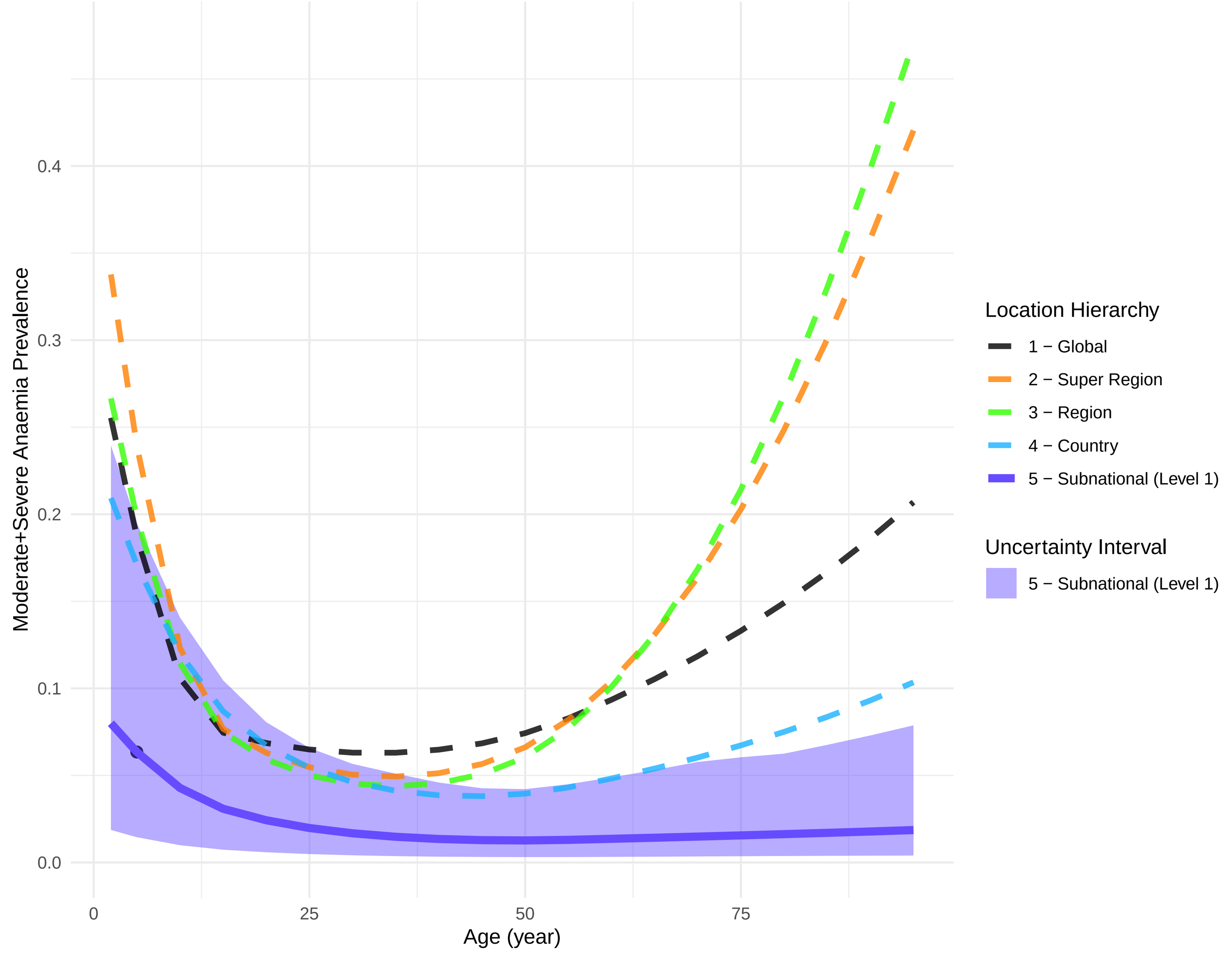

MR−BRT Age/Sex Fits − Males 2+ Years Old − Moderate+Severe Anaemia Prevalence − Nakuru
WHO−2001 Adjustment Method

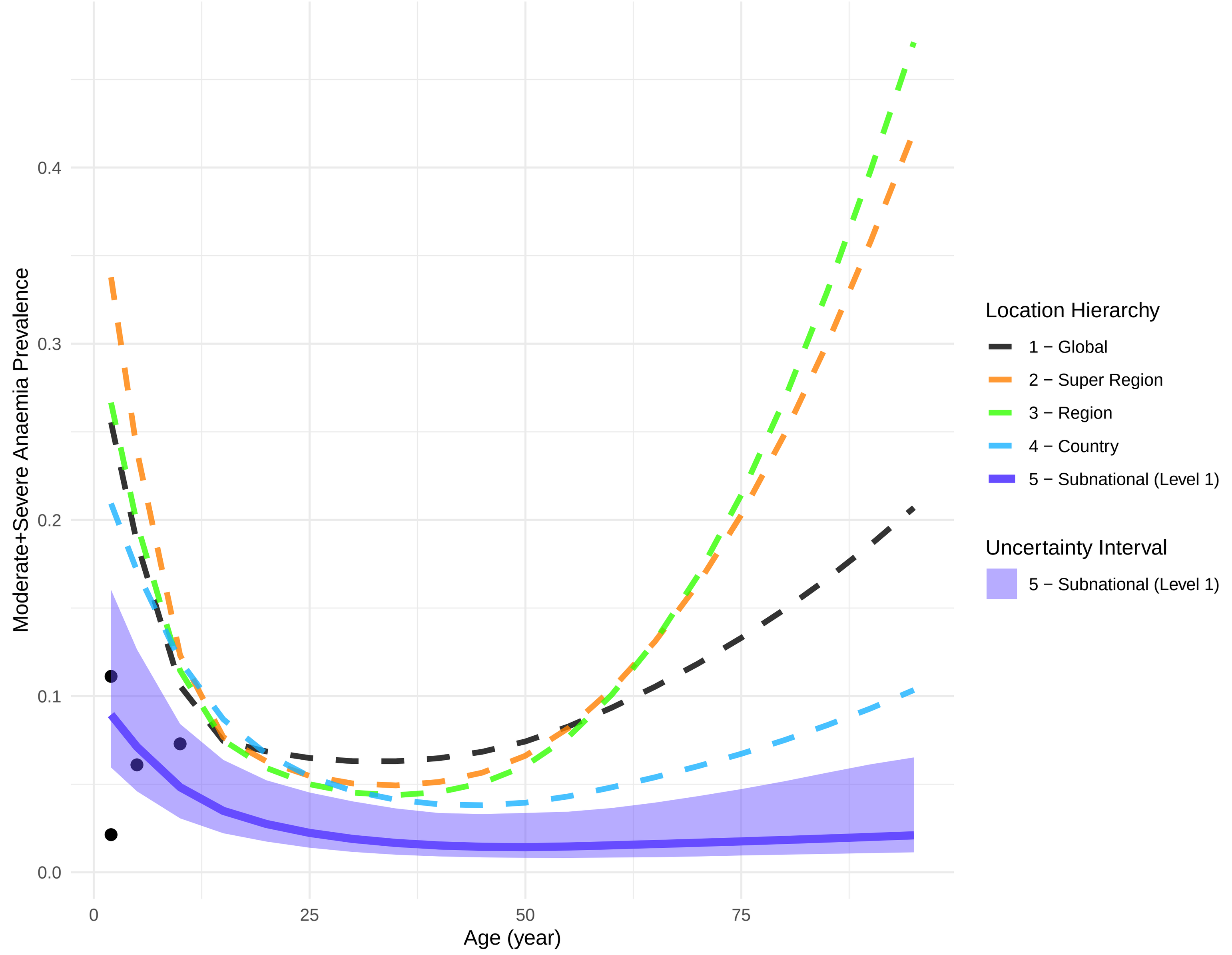

MR−BRT Age/Sex Fits − Males 2+ Years Old − Moderate+Severe Anaemia Prevalence − Nandi
WHO−2001 Adjustment Method

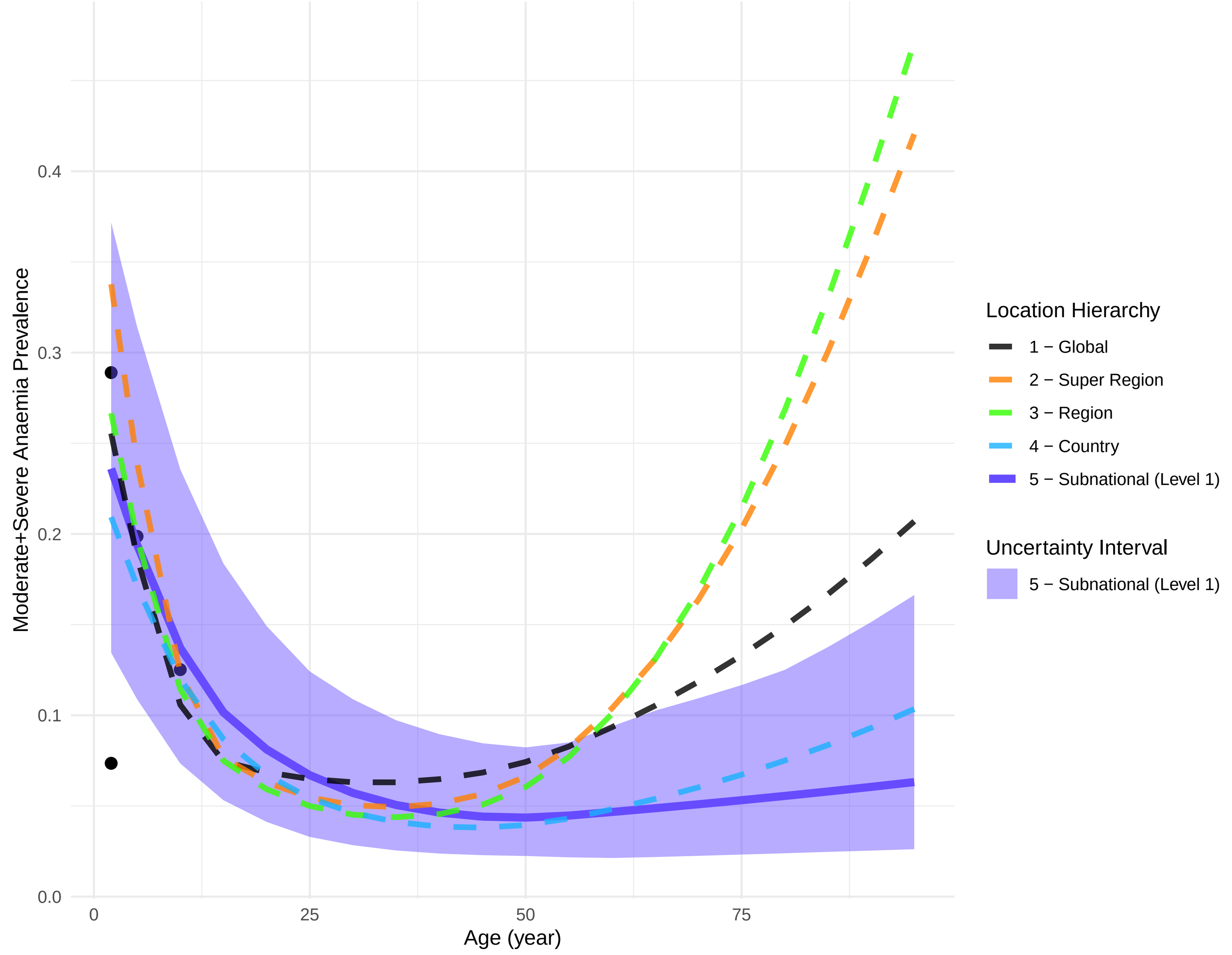

MR−BRT Age/Sex Fits − Males 2+ Years Old − Moderate+Severe Anaemia Prevalence − Narok
WHO−2001 Adjustment Method

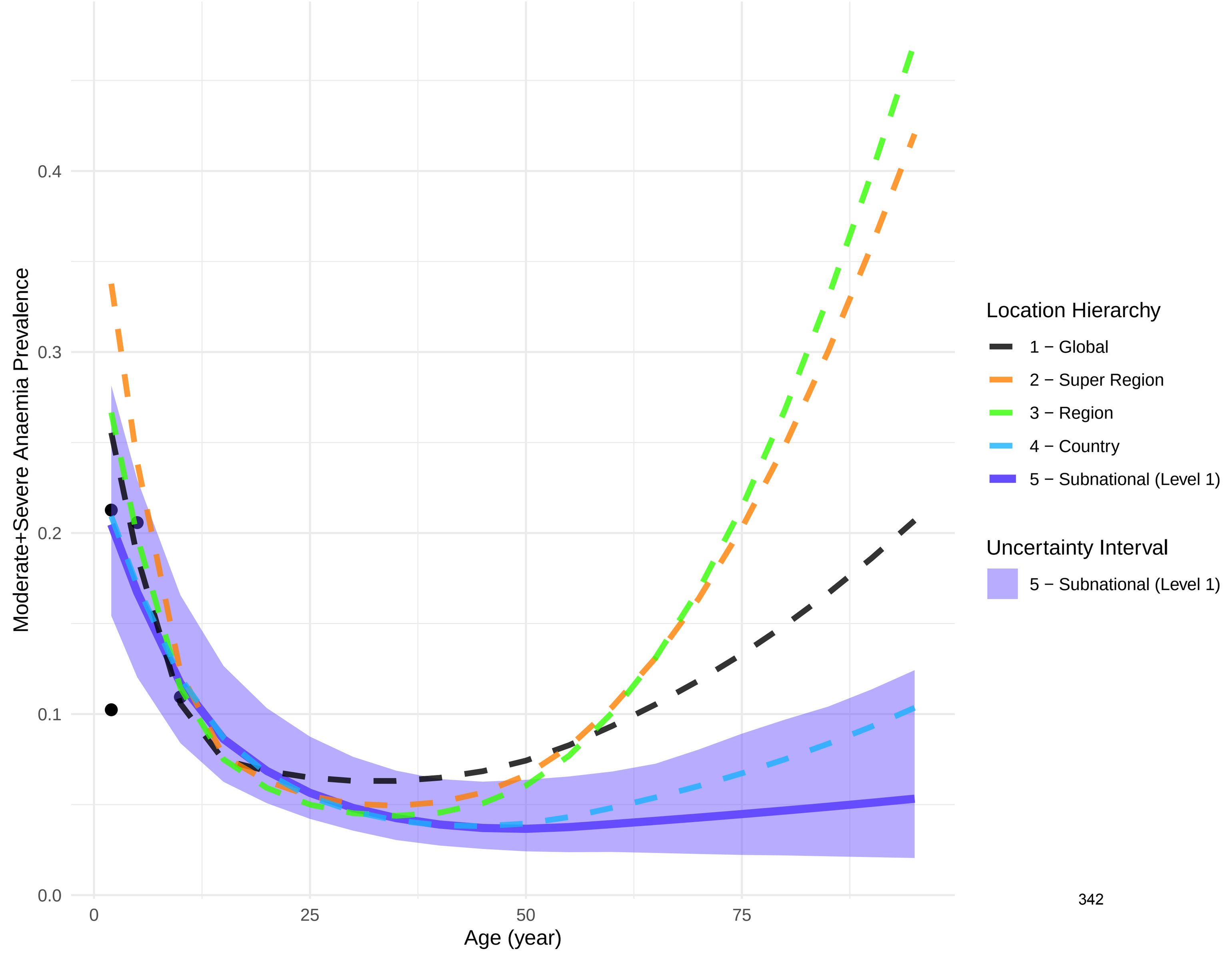

MR−BRT Age/Sex Fits − Males 2+ Years Old − Moderate+Severe Anaemia Prevalence − Nyamira
WHO−2001 Adjustment Method

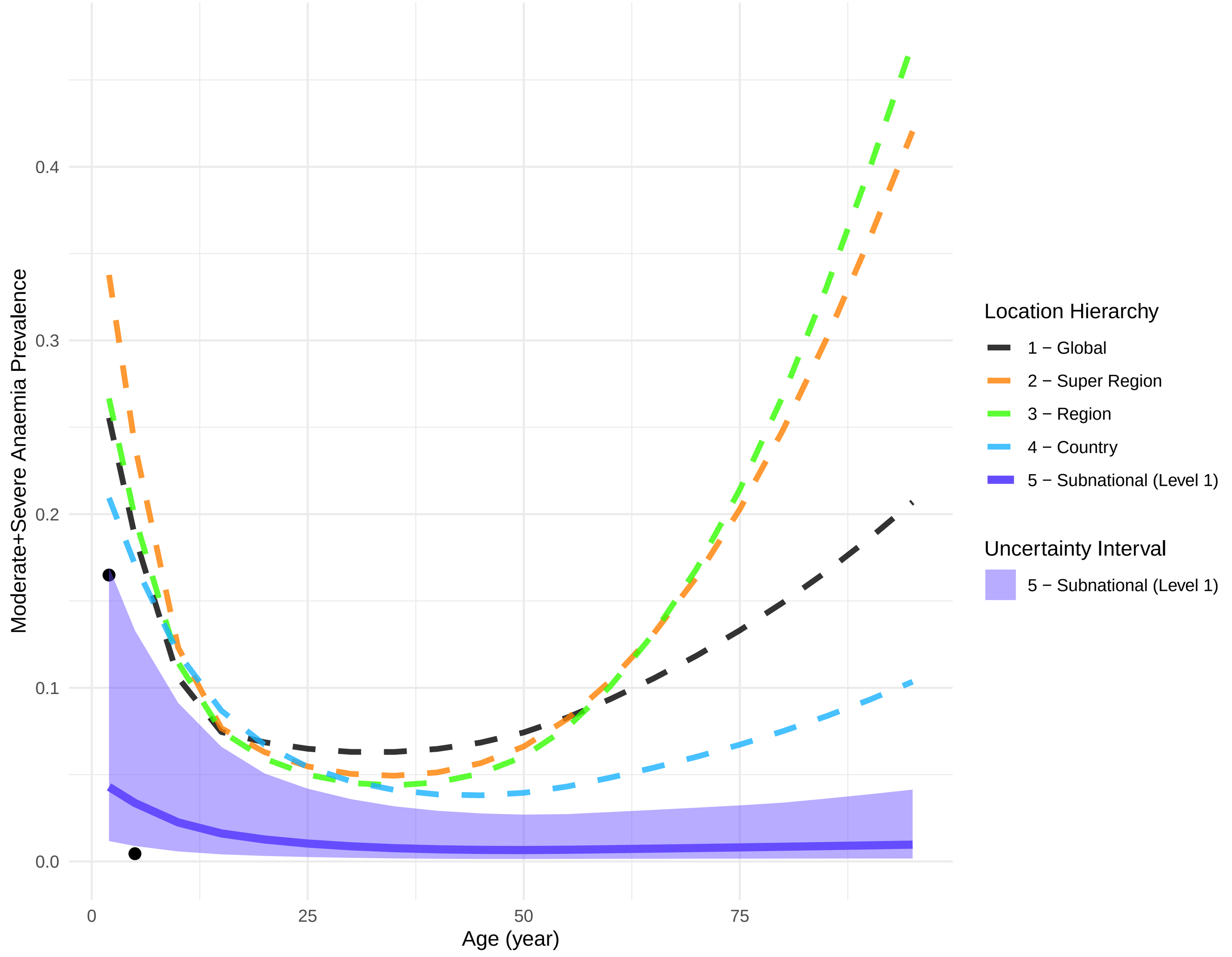

MR−BRT Age/Sex Fits − Males 2+ Years Old − Moderate+Severe Anaemia Prevalence − Nyandarua
WHO−2001 Adjustment Method

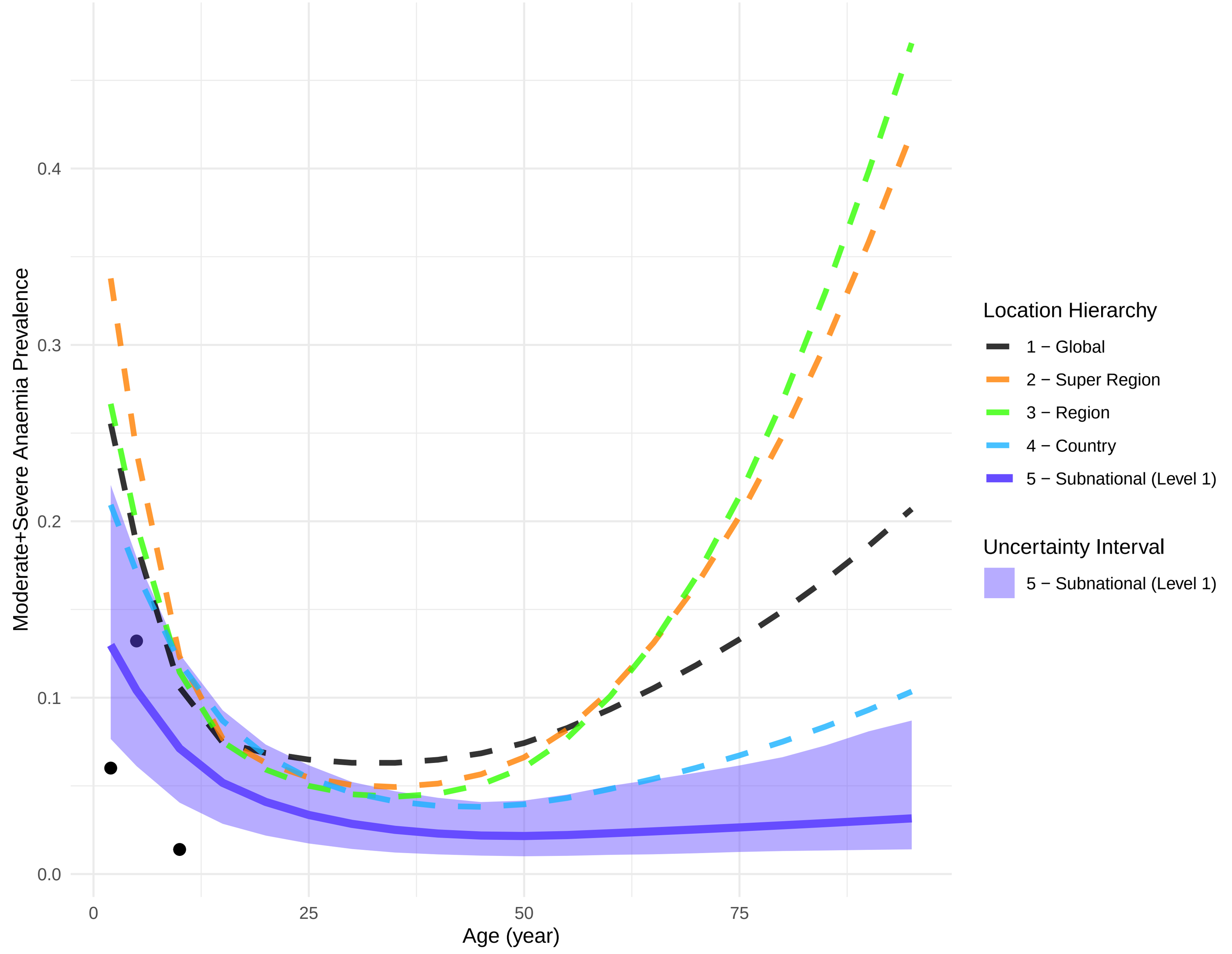

MR−BRT Age/Sex Fits − Males 2+ Years Old − Moderate+Severe Anaemia Prevalence − Nyeri
WHO−2001 Adjustment Method

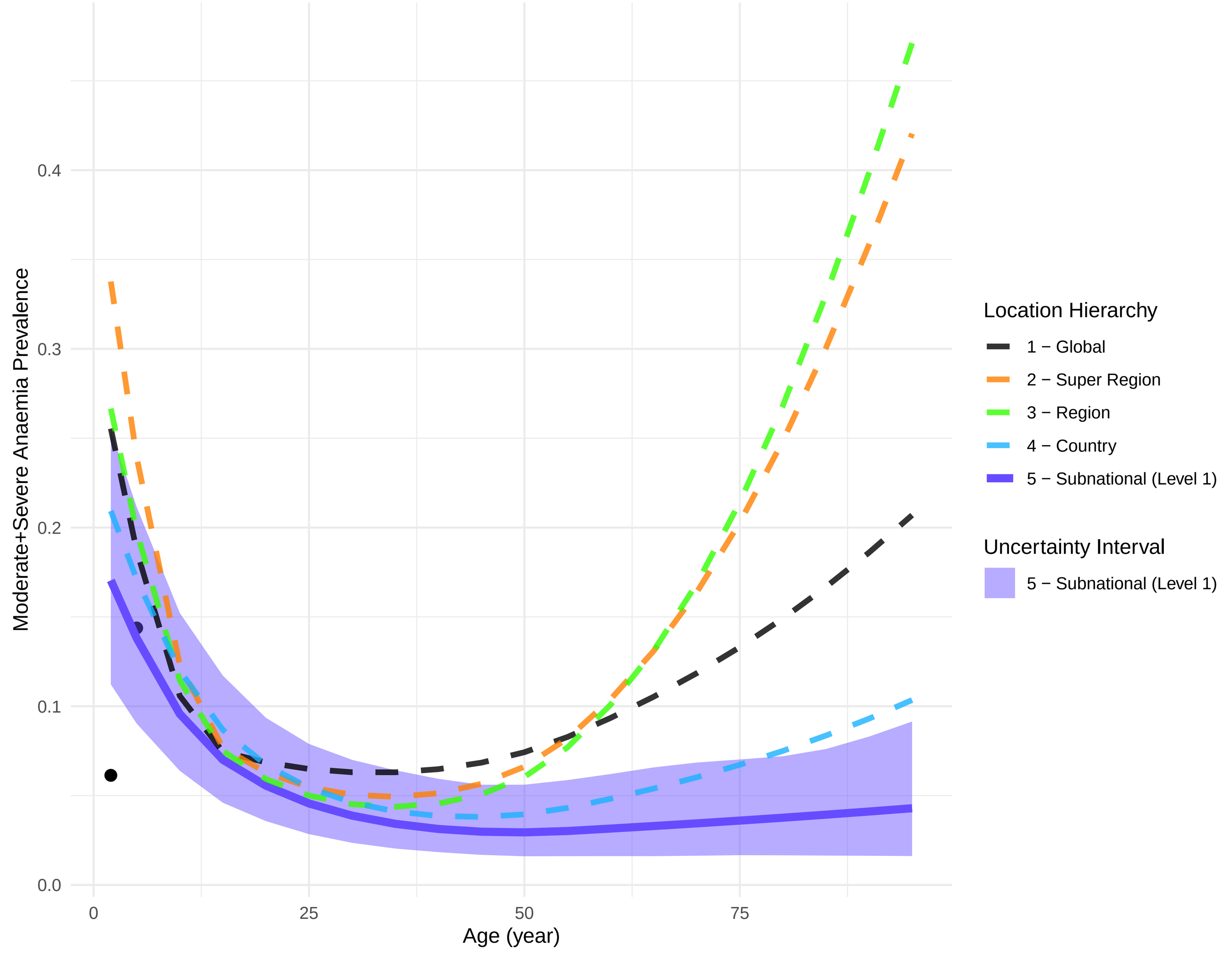

MR−BRT Age/Sex Fits − Males 2+ Years Old − Moderate+Severe Anaemia Prevalence − Samburu
WHO−2001 Adjustment Method

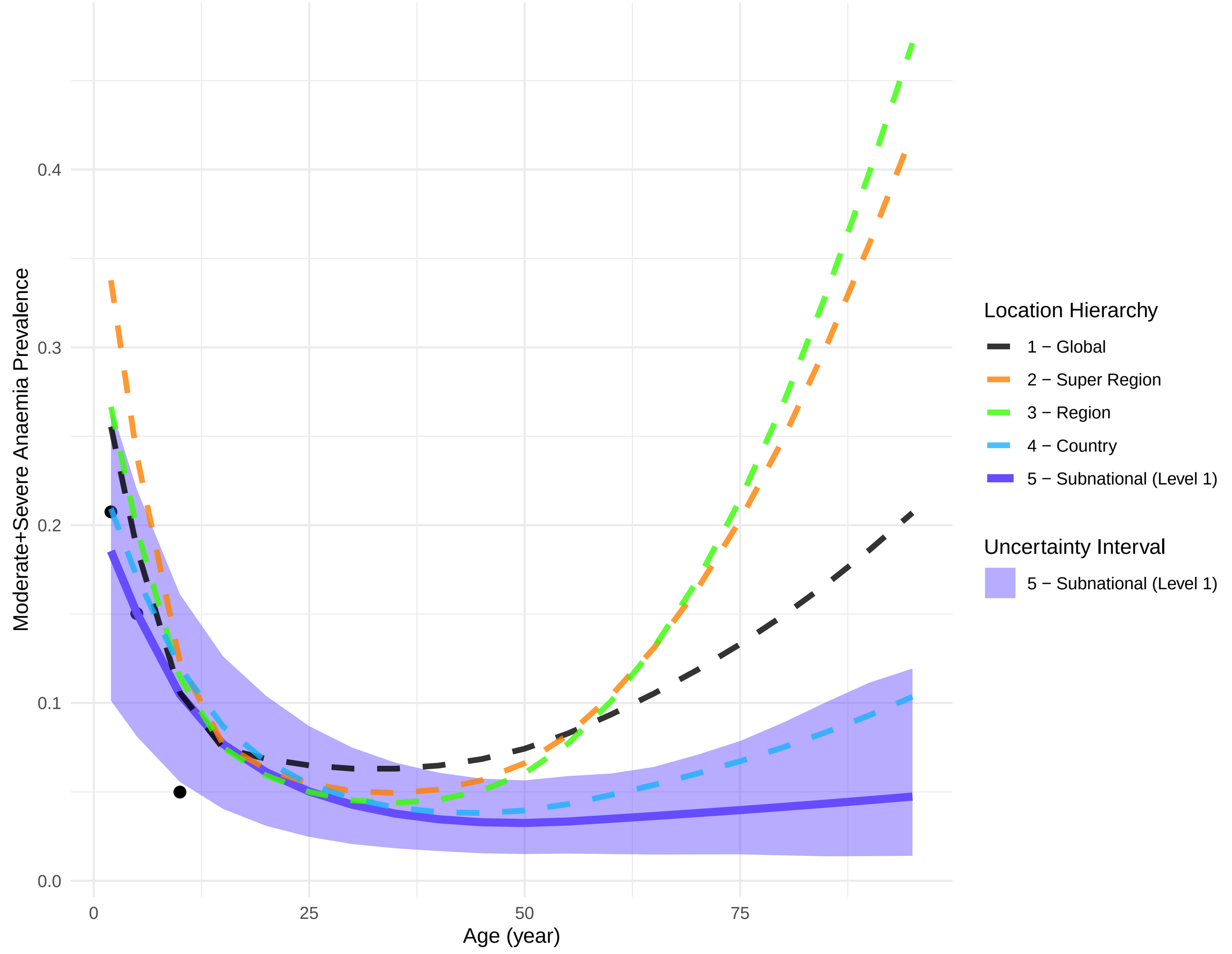

MR−BRT Age/Sex Fits − Males 2+ Years Old − Moderate+Severe Anaemia Prevalence − Siaya
WHO−2001 Adjustment Method

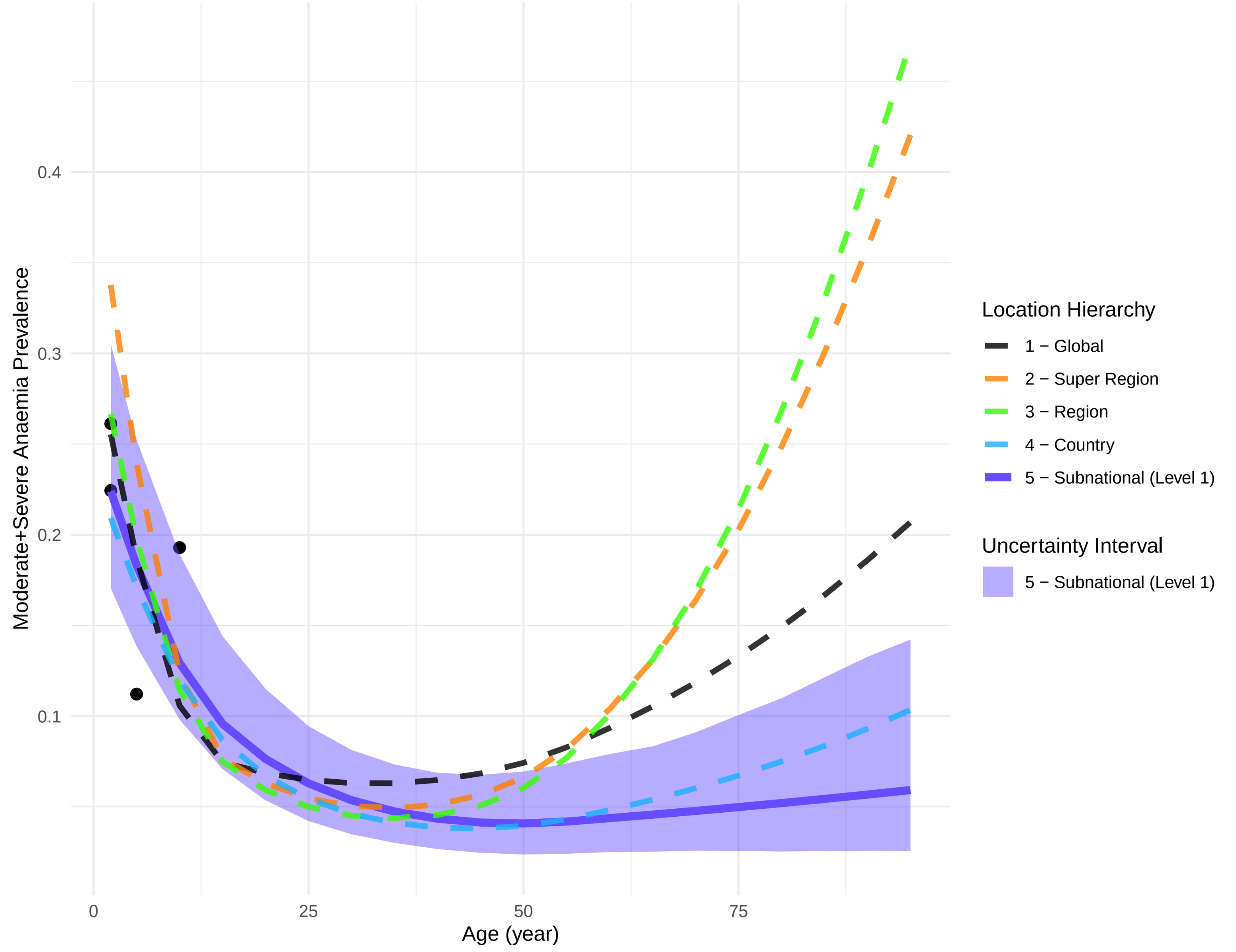

MR−BRT Age/Sex Fits − Males 2+ Years Old − Moderate+Severe Anaemia Prevalence − Taita Taveta
WHO−2001 Adjustment Method

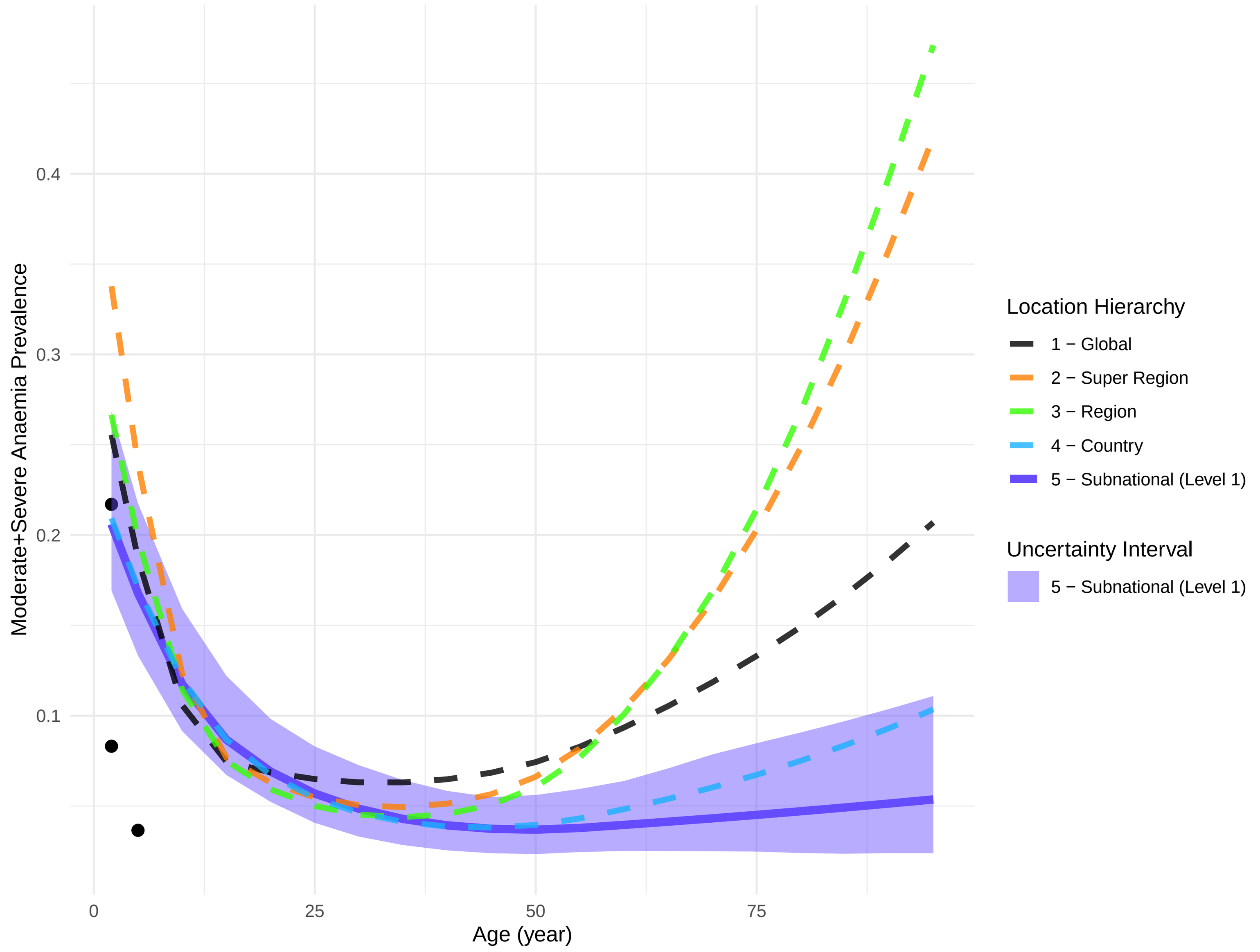

MR−BRT Age/Sex Fits − Males 2+ Years Old − Moderate+Severe Anaemia Prevalence − Tana River
WHO−2001 Adjustment Method

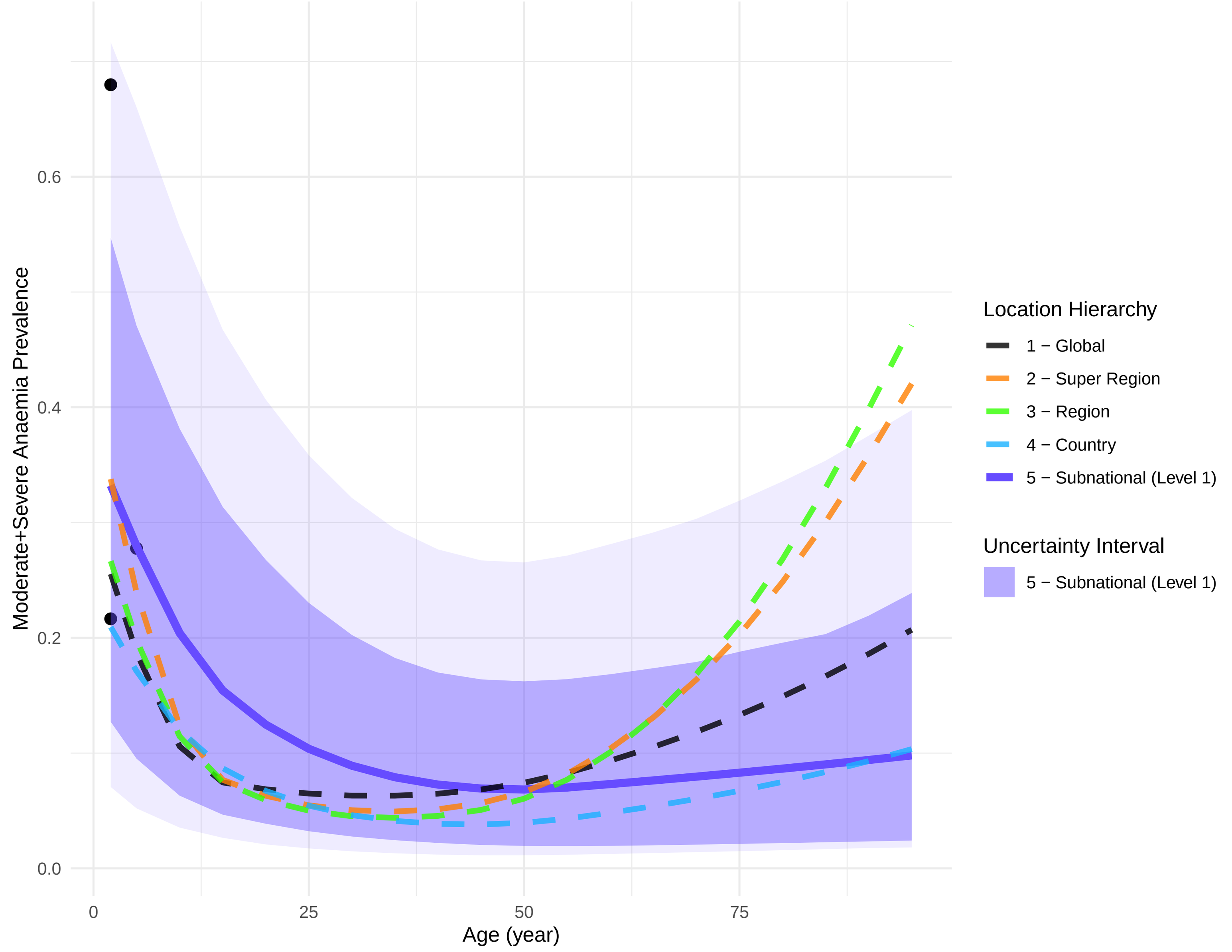

MR−BRT Age/Sex Fits − Males 2+ Years Old − Moderate+Severe Anaemia Prevalence − Tharaka Nithi
WHO−2001 Adjustment Method

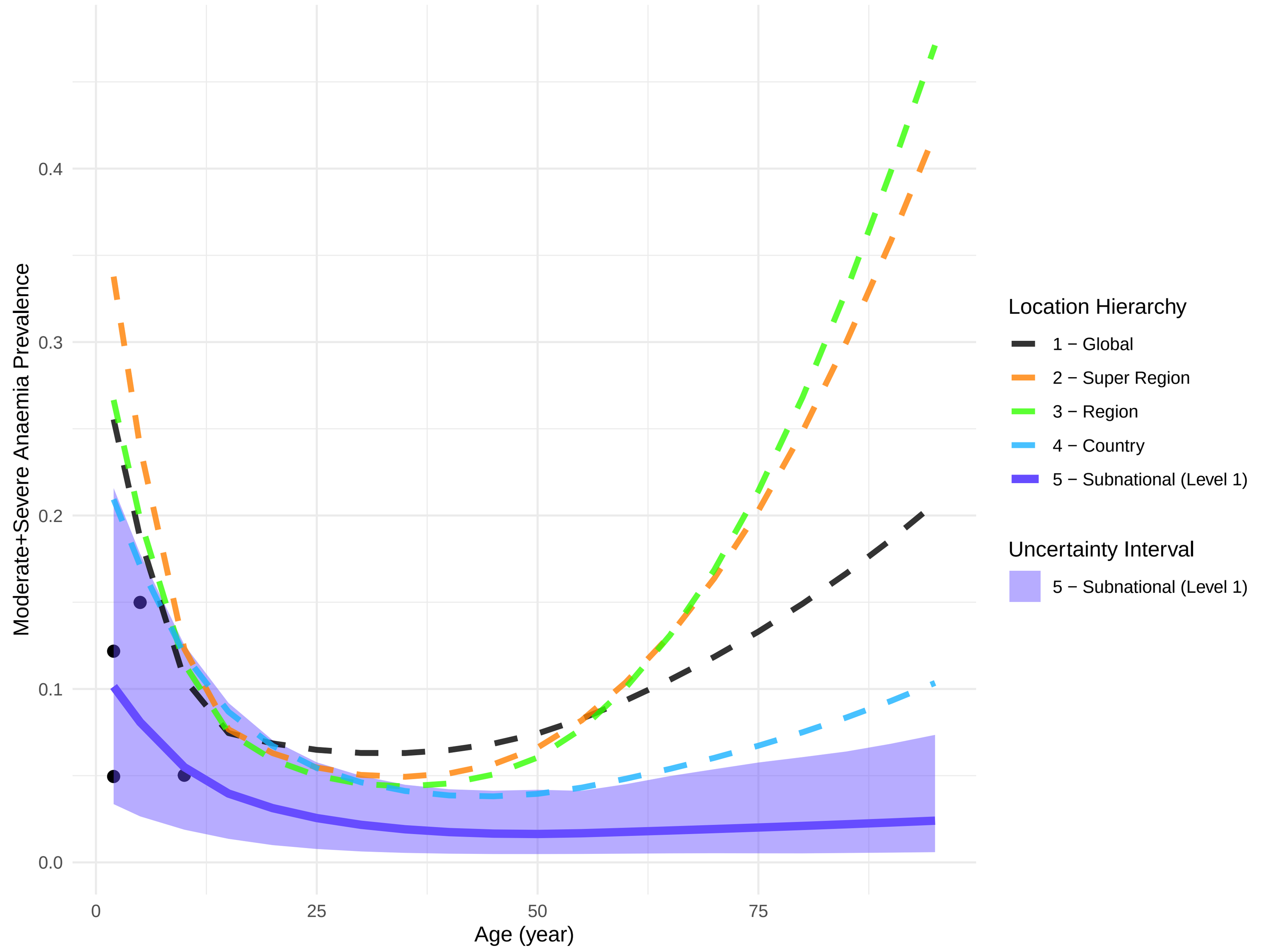

MR−BRT Age/Sex Fits − Males 2+ Years Old − Moderate+Severe Anaemia Prevalence − Trans Nzoia
WHO−2001 Adjustment Method

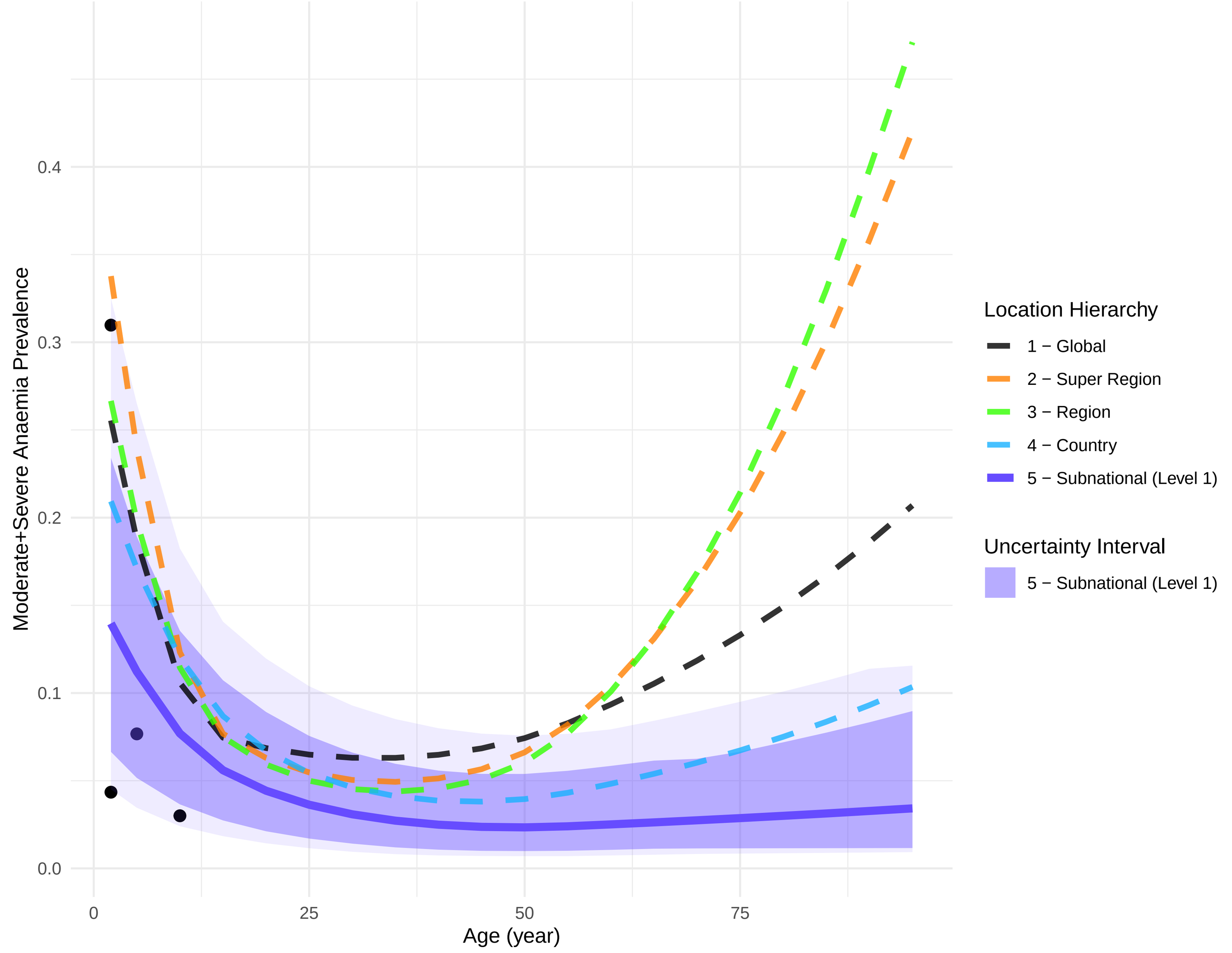

MR−BRT Age/Sex Fits − Males 2+ Years Old − Moderate+Severe Anaemia Prevalence − Turkana
WHO−2001 Adjustment Method

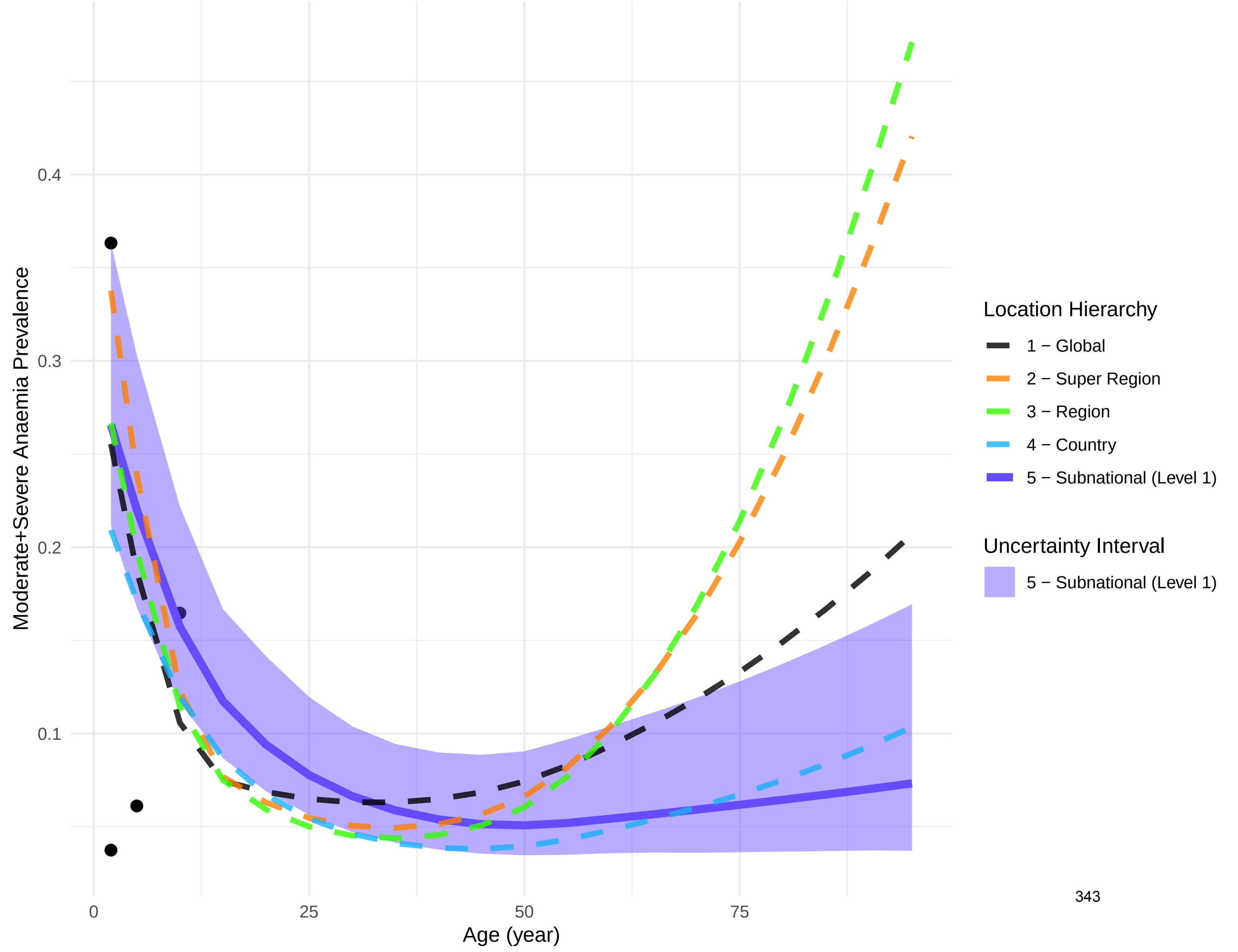

MR−BRT Age/Sex Fits − Males 2+ Years Old − Moderate+Severe Anaemia Prevalence − Uasin Gishu
WHO−2001 Adjustment Method

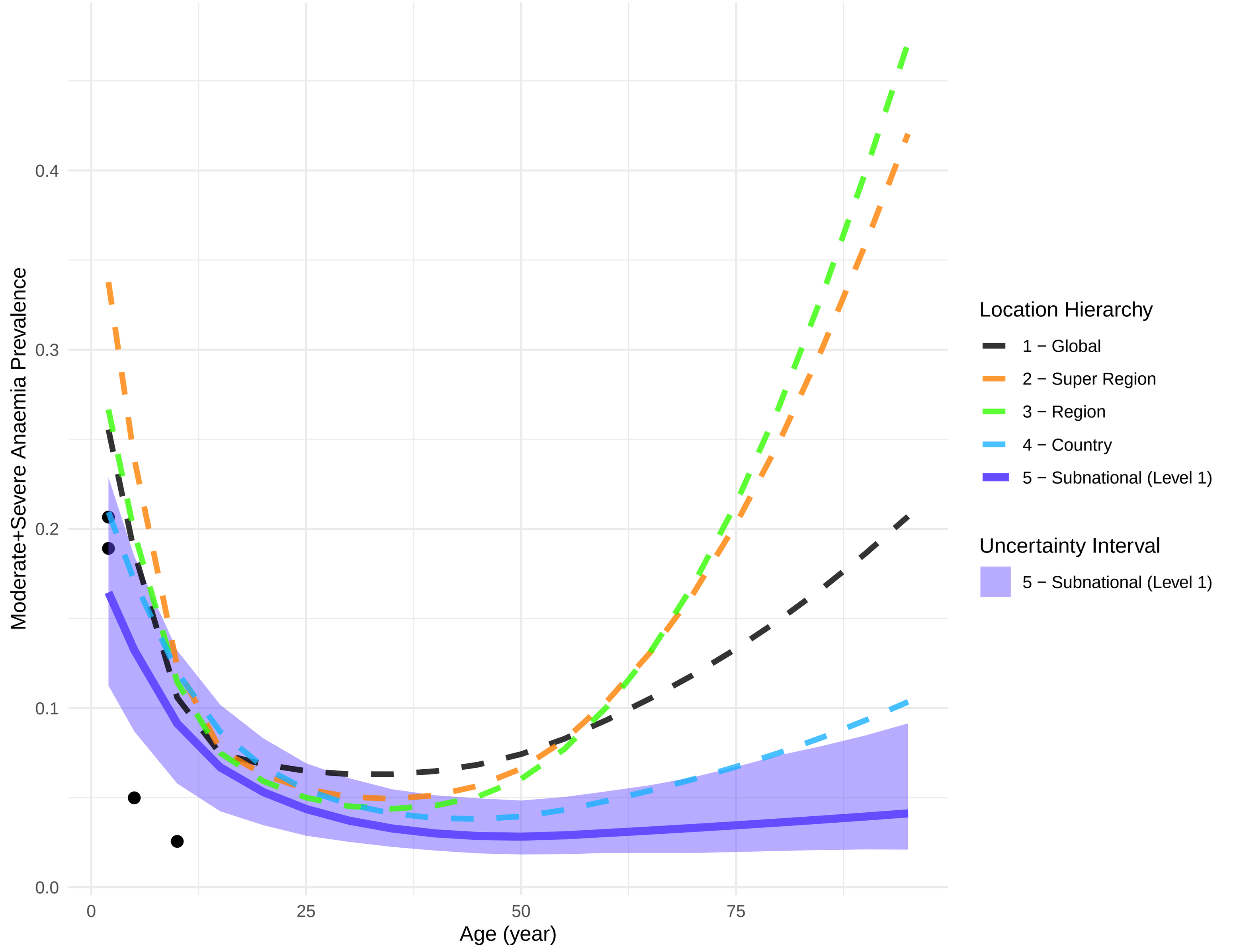

MR−BRT Age/Sex Fits − Males 2+ Years Old − Moderate+Severe Anaemia Prevalence − Vihiga
WHO−2001 Adjustment Method

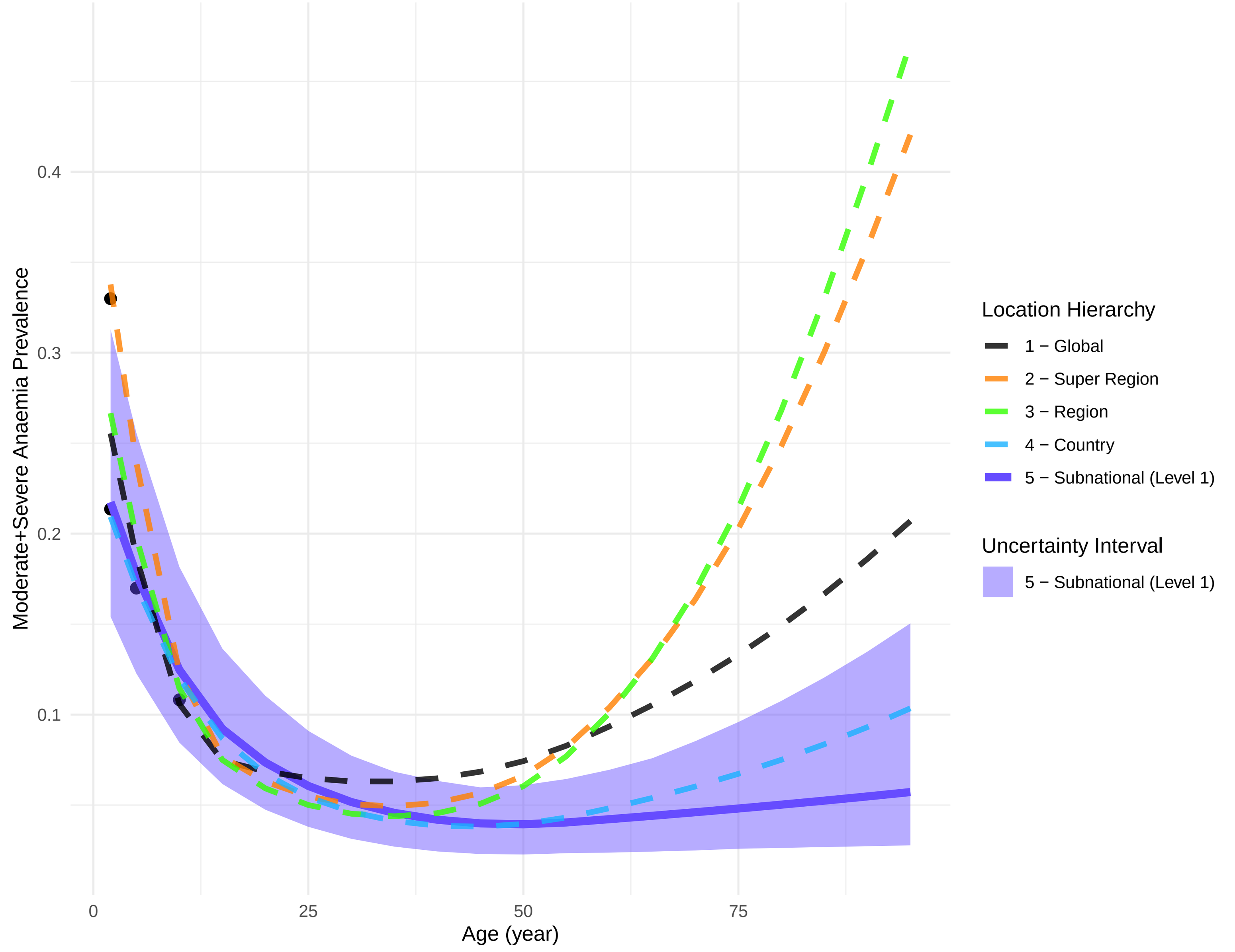

MR−BRT Age/Sex Fits − Males 2+ Years Old − Moderate+Severe Anaemia Prevalence − Wajir
WHO−2001 Adjustment Method

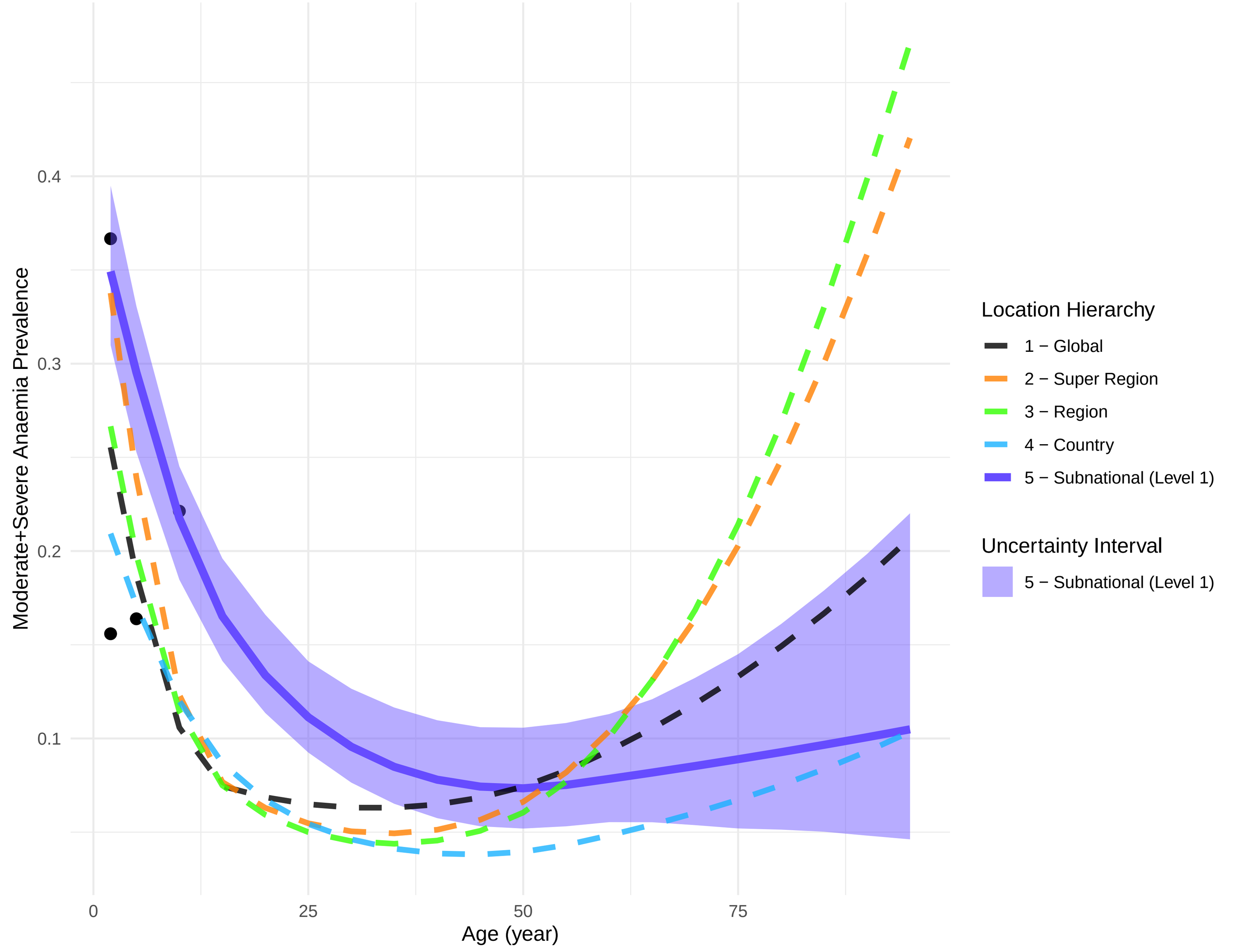

MR−BRT Age/Sex Fits − Males 2+ Years Old − Moderate+Severe Anaemia Prevalence − West Pokot
WHO−2001 Adjustment Method

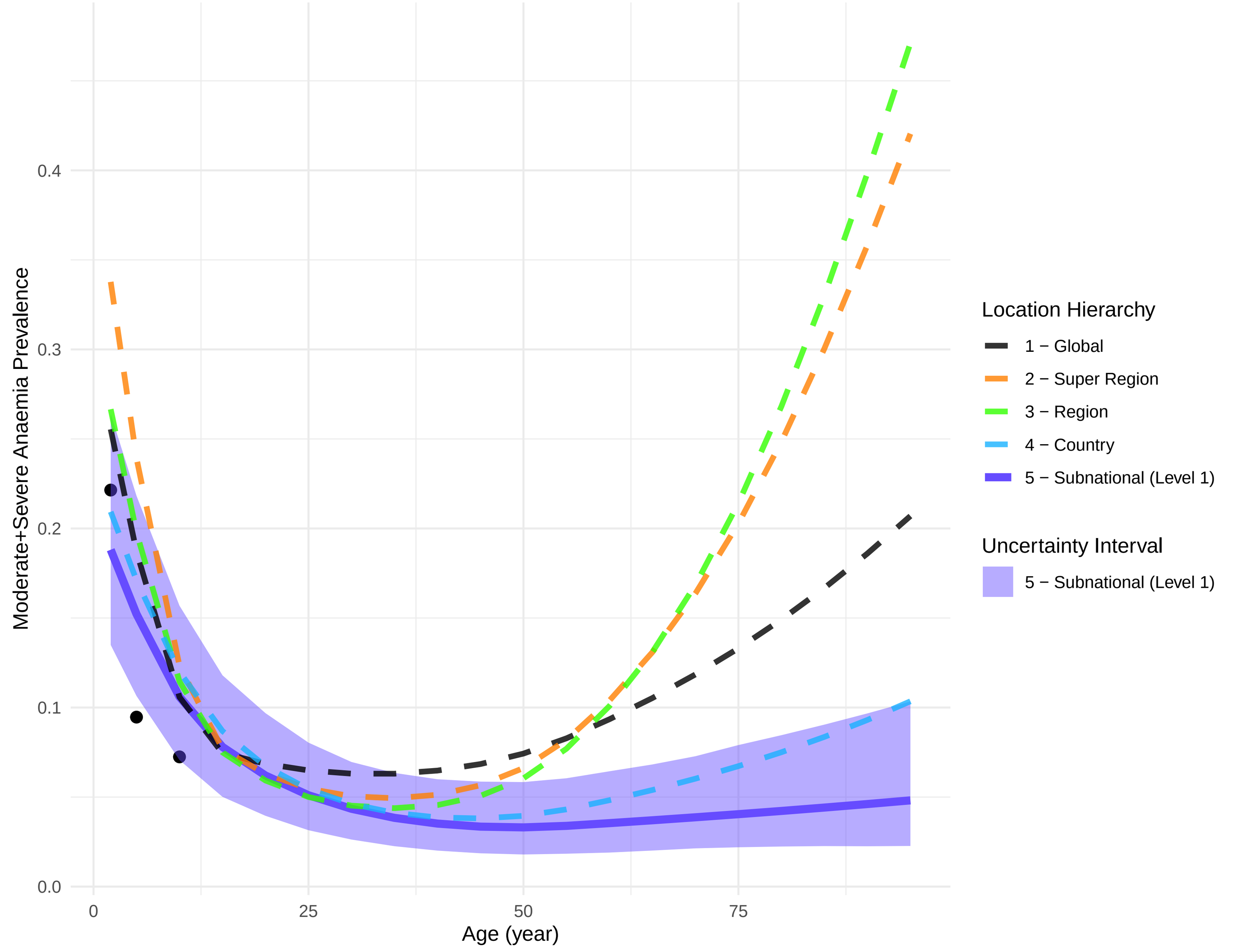

MR−BRT Age/Sex Fits − Males 2+ Years Old − Moderate+Severe Anaemia Prevalence − Madagascar
WHO−2001 Adjustment Method

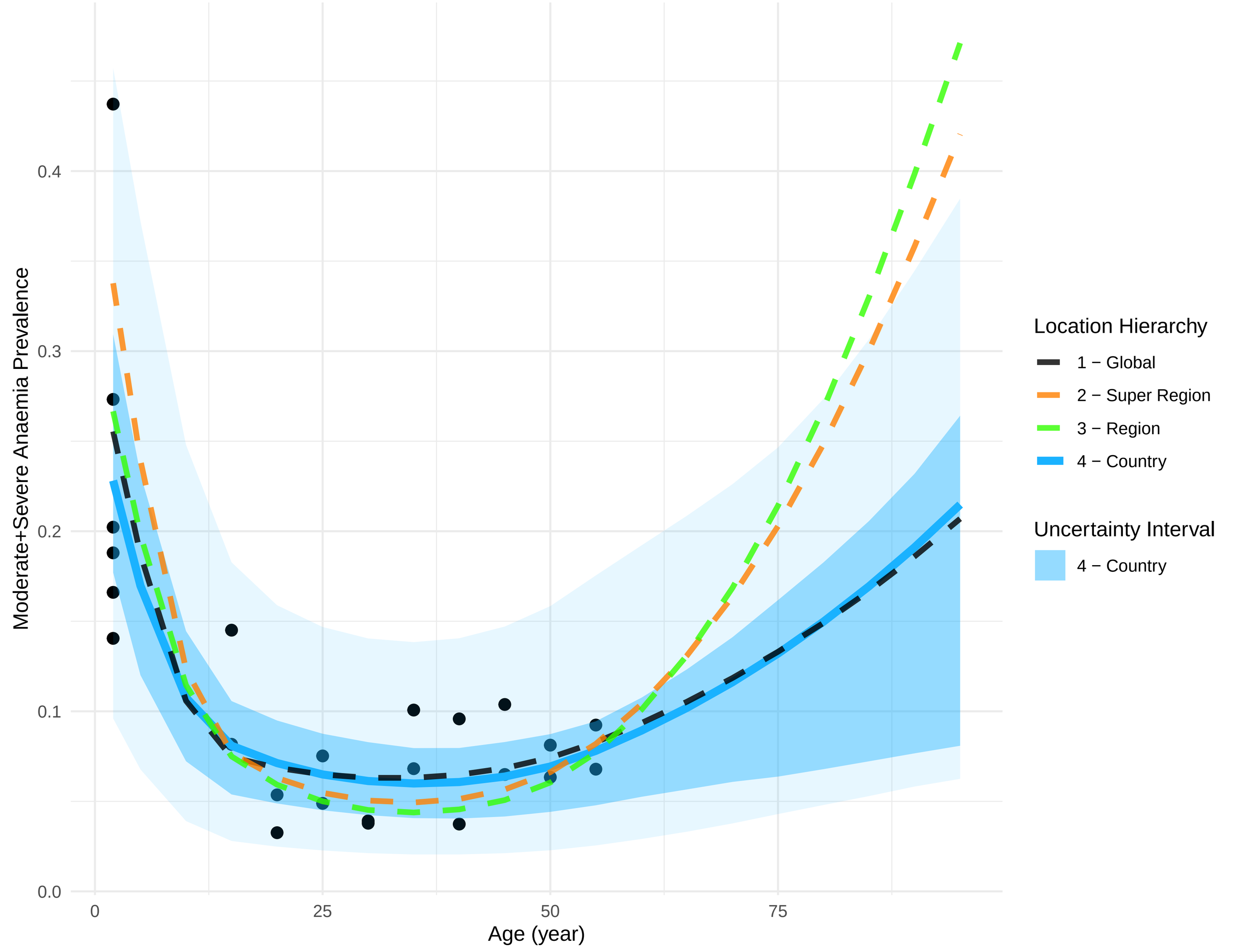

MR−BRT Age/Sex Fits − Males 2+ Years Old − Moderate+Severe Anaemia Prevalence − Malawi
WHO−2001 Adjustment Method

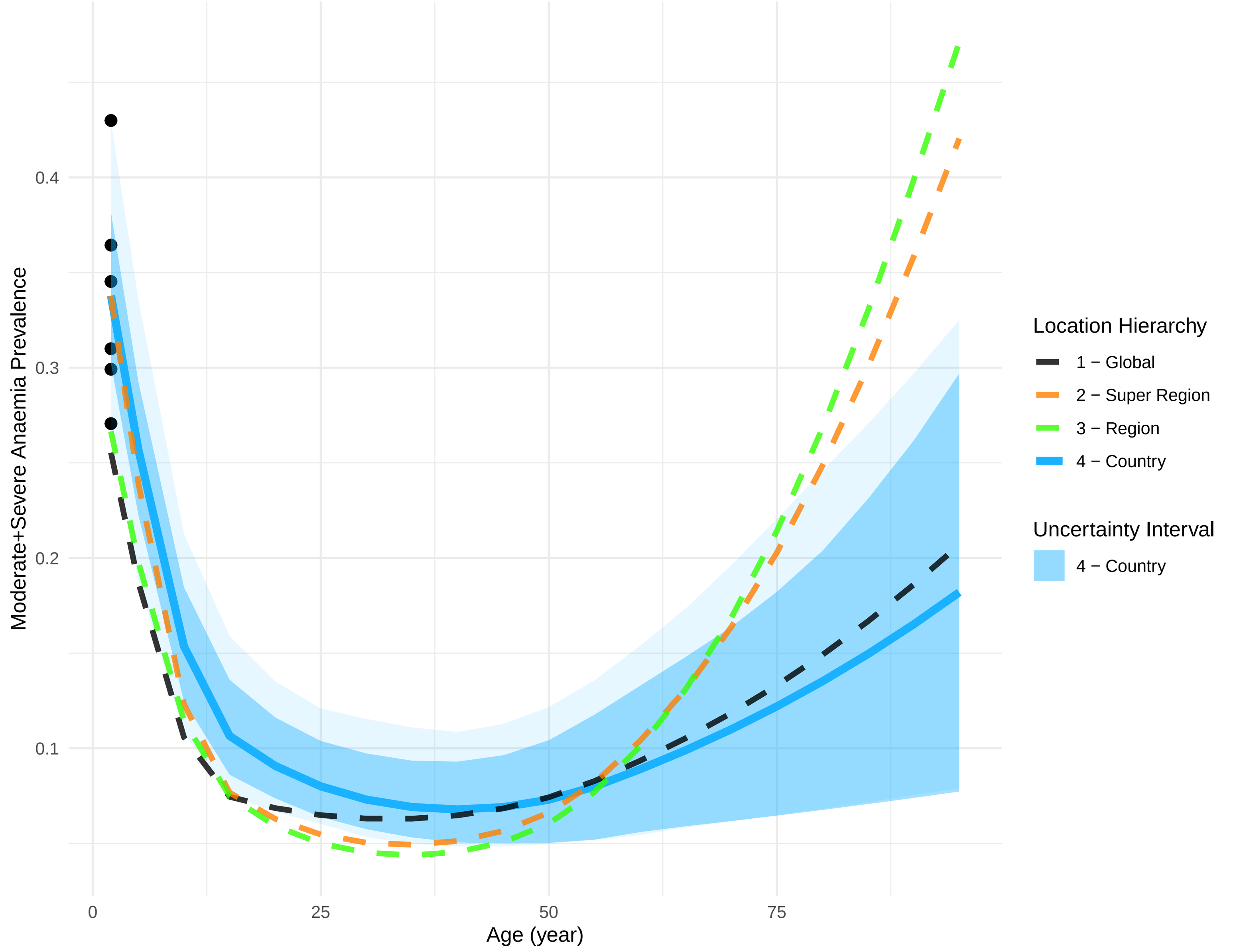

MR−BRT Age/Sex Fits − Males 2+ Years Old − Moderate+Severe Anaemia Prevalence − Mozambique
WHO−2001 Adjustment Method

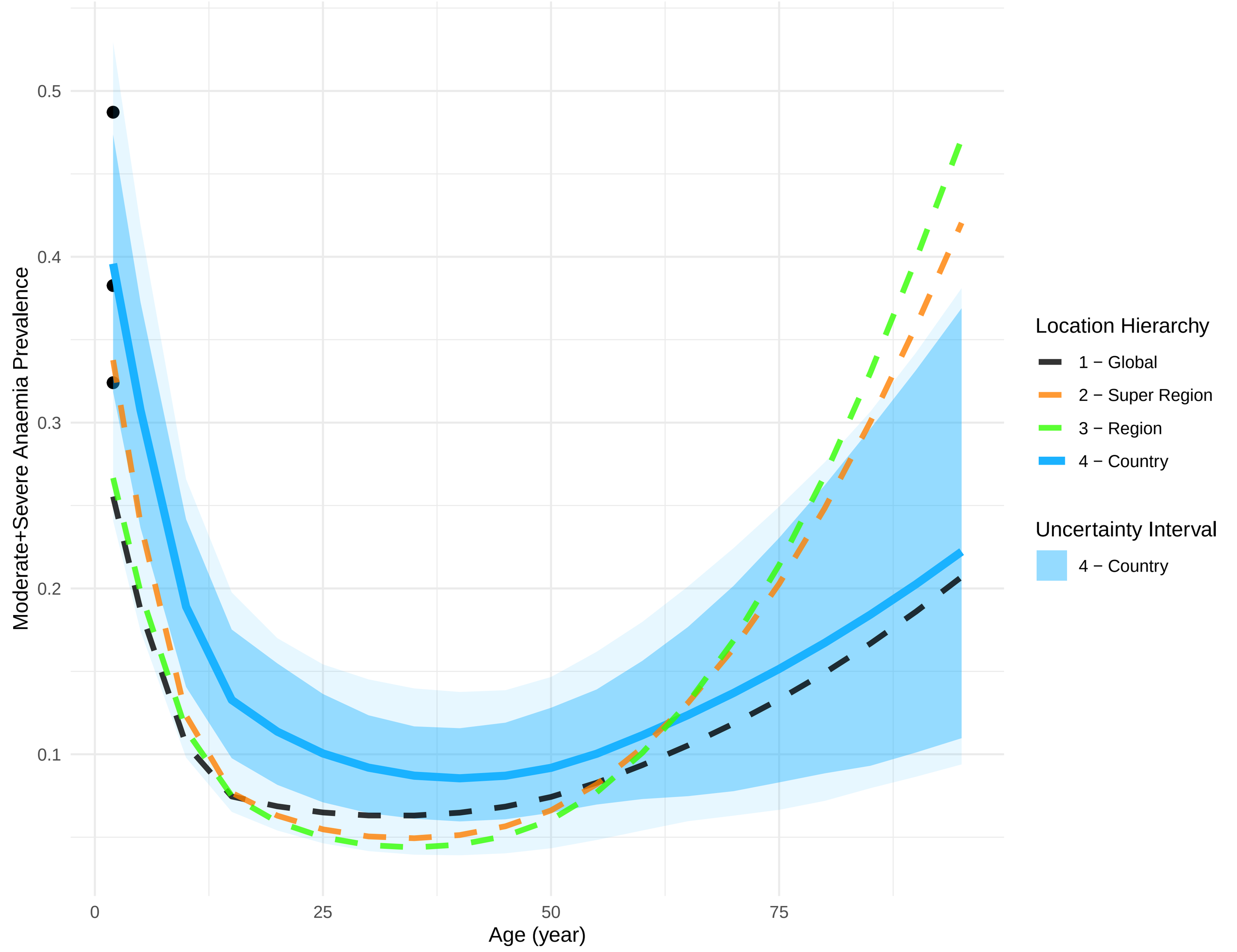

MR−BRT Age/Sex Fits − Males 2+ Years Old − Moderate+Severe Anaemia Prevalence − Rwanda
WHO−2001 Adjustment Method

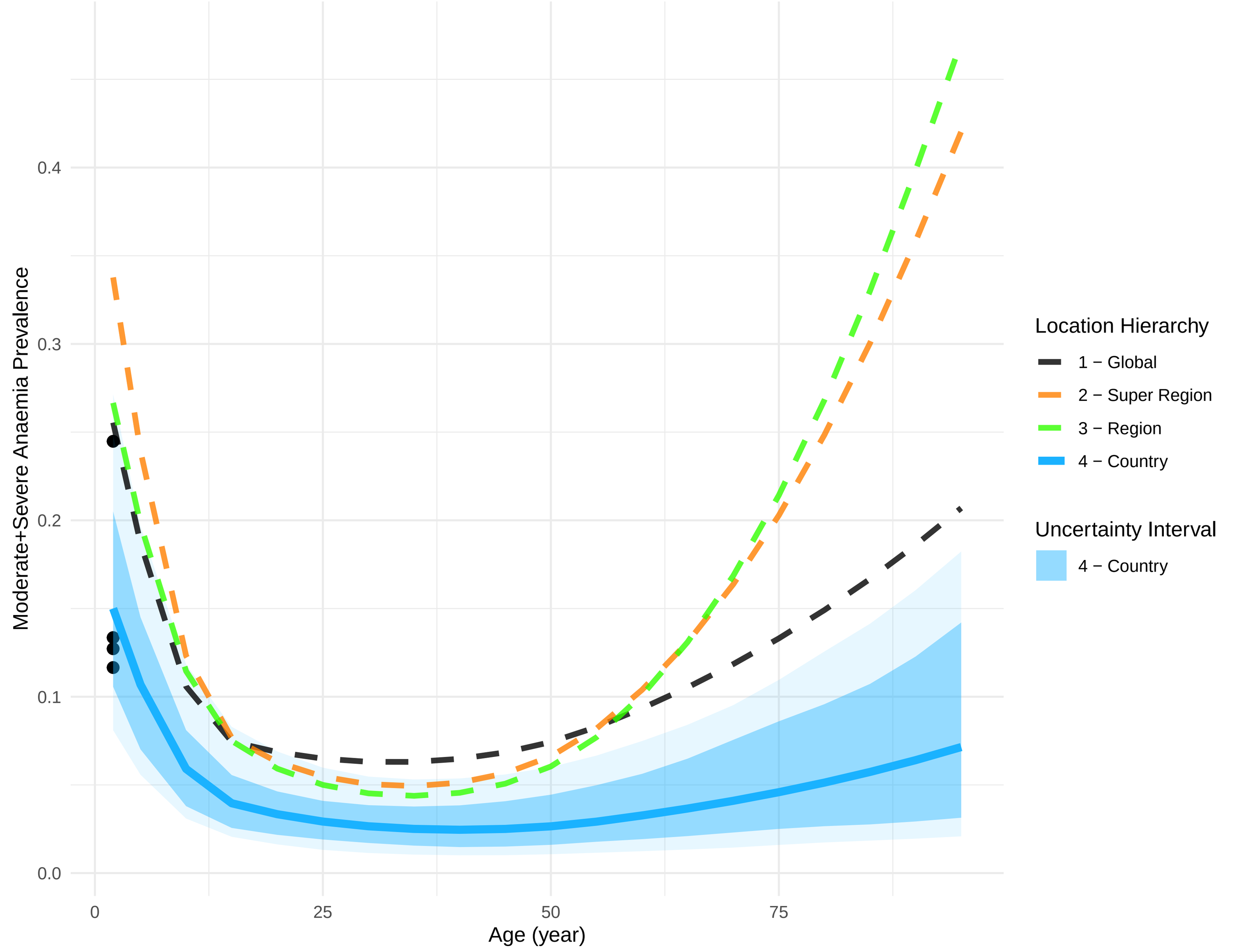

MR−BRT Age/Sex Fits − Males 2+ Years Old − Moderate+Severe Anaemia Prevalence − Uganda
WHO−2001 Adjustment Method

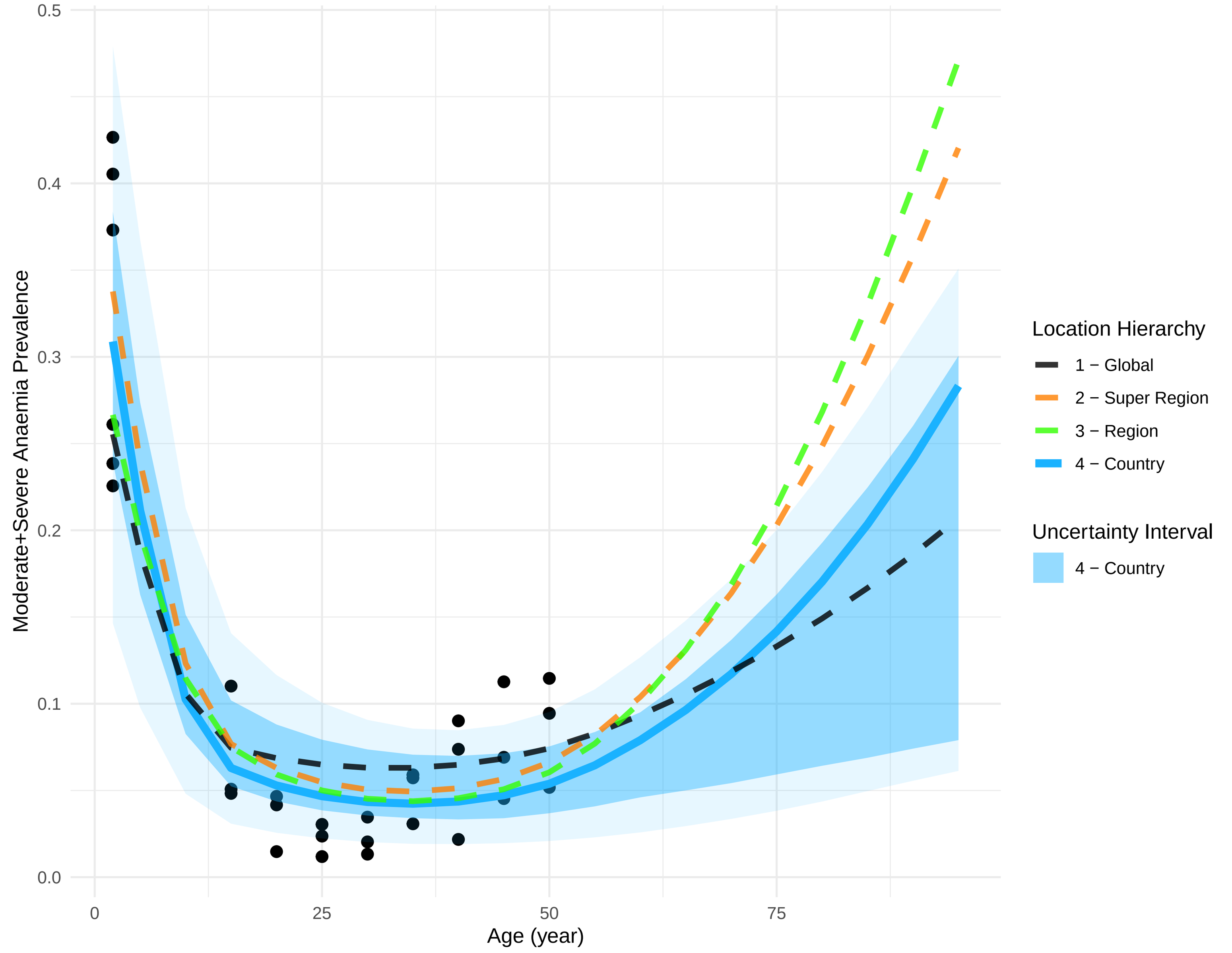

MR−BRT Age/Sex Fits − Males 2+ Years Old − Moderate+Severe Anaemia Prevalence − United Republic of Tanzania
WHO−2001 Adjustment Method

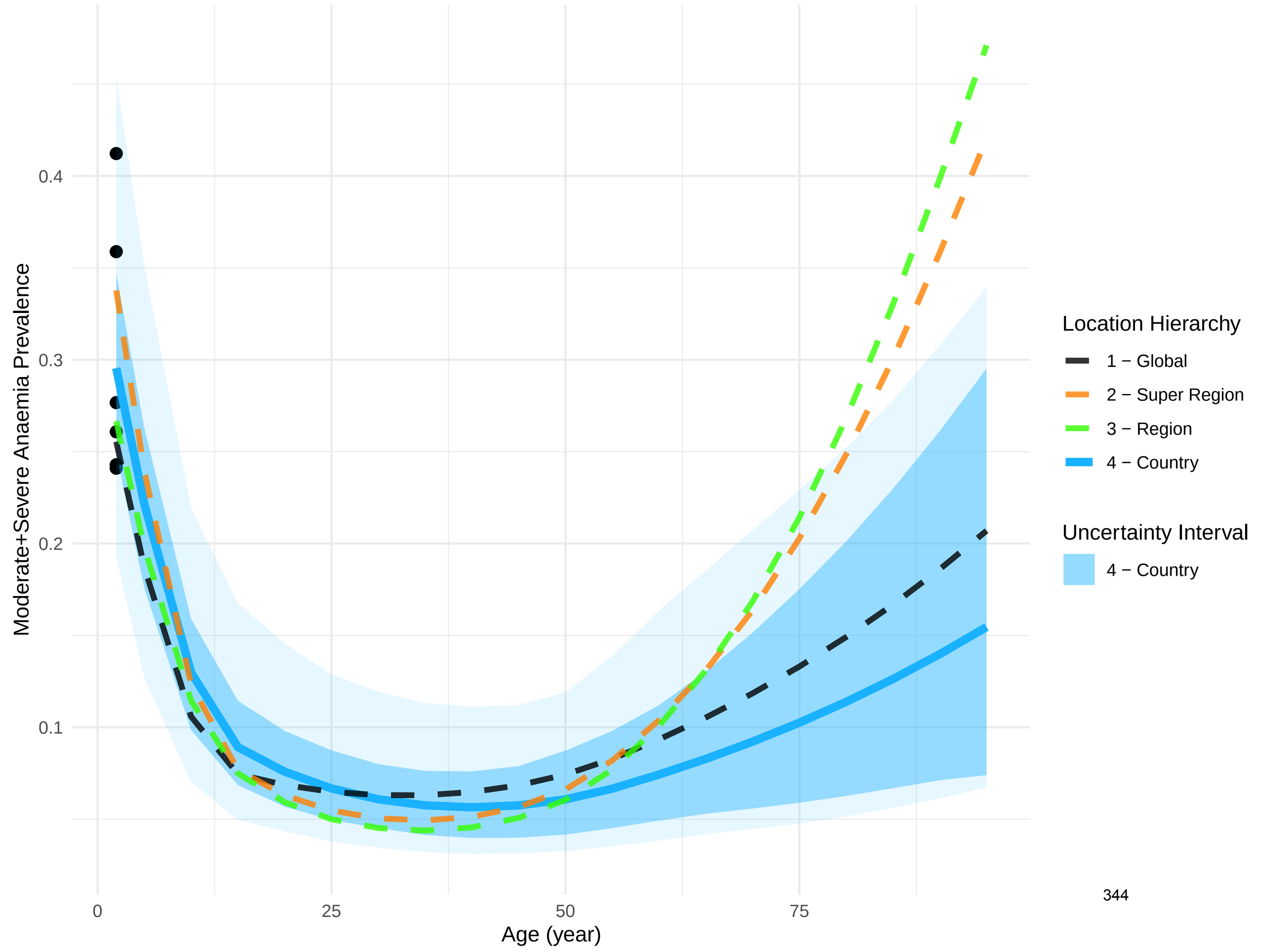

MR−BRT Age/Sex Fits − Males 2+ Years Old − Moderate+Severe Anaemia Prevalence − Zambia
WHO−2001 Adjustment Method

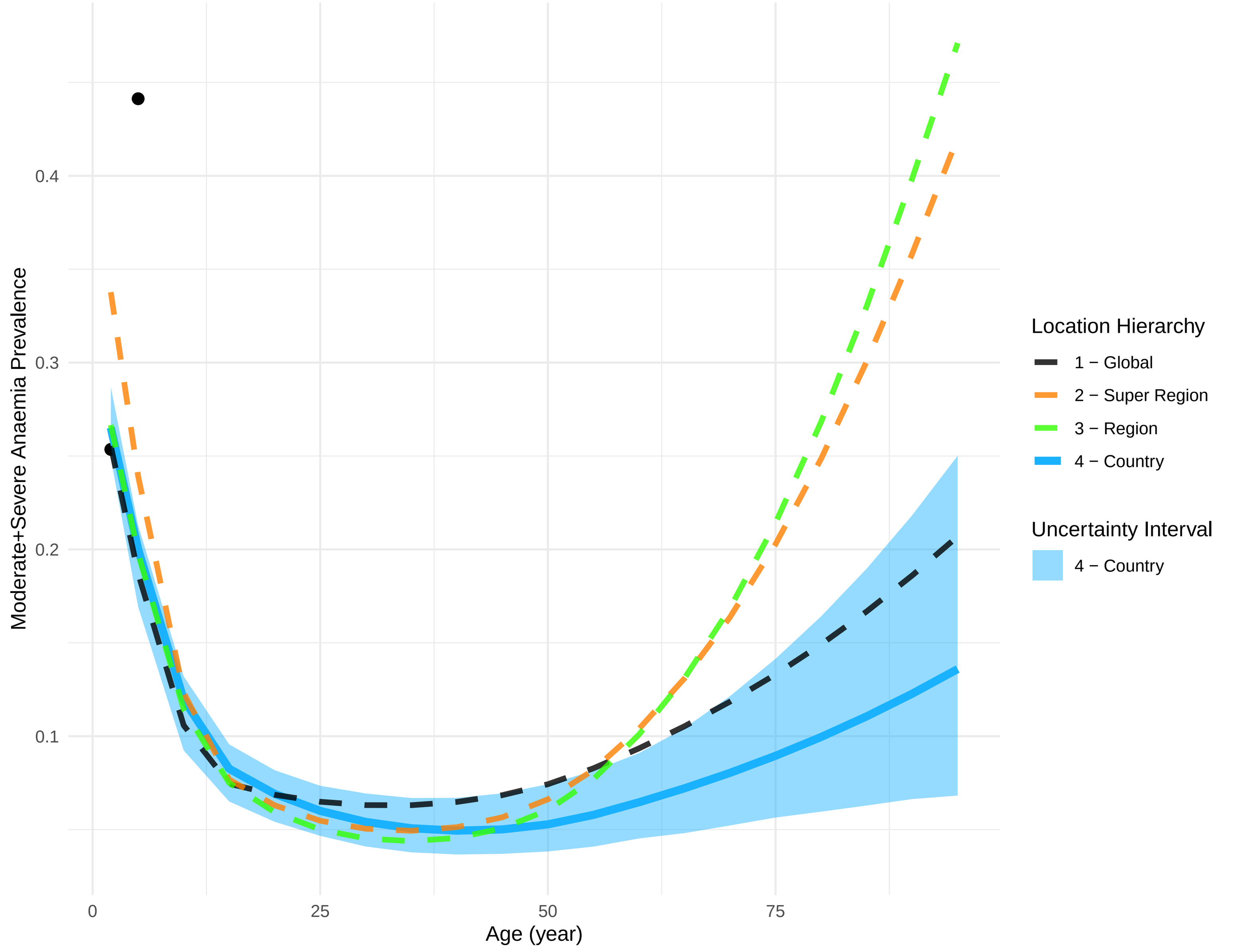

MR−BRT Age/Sex Fits − Males 2+ Years Old − Moderate+Severe Anaemia Prevalence − Southern Sub−Saharan Africa
WHO−2001 Adjustment Method

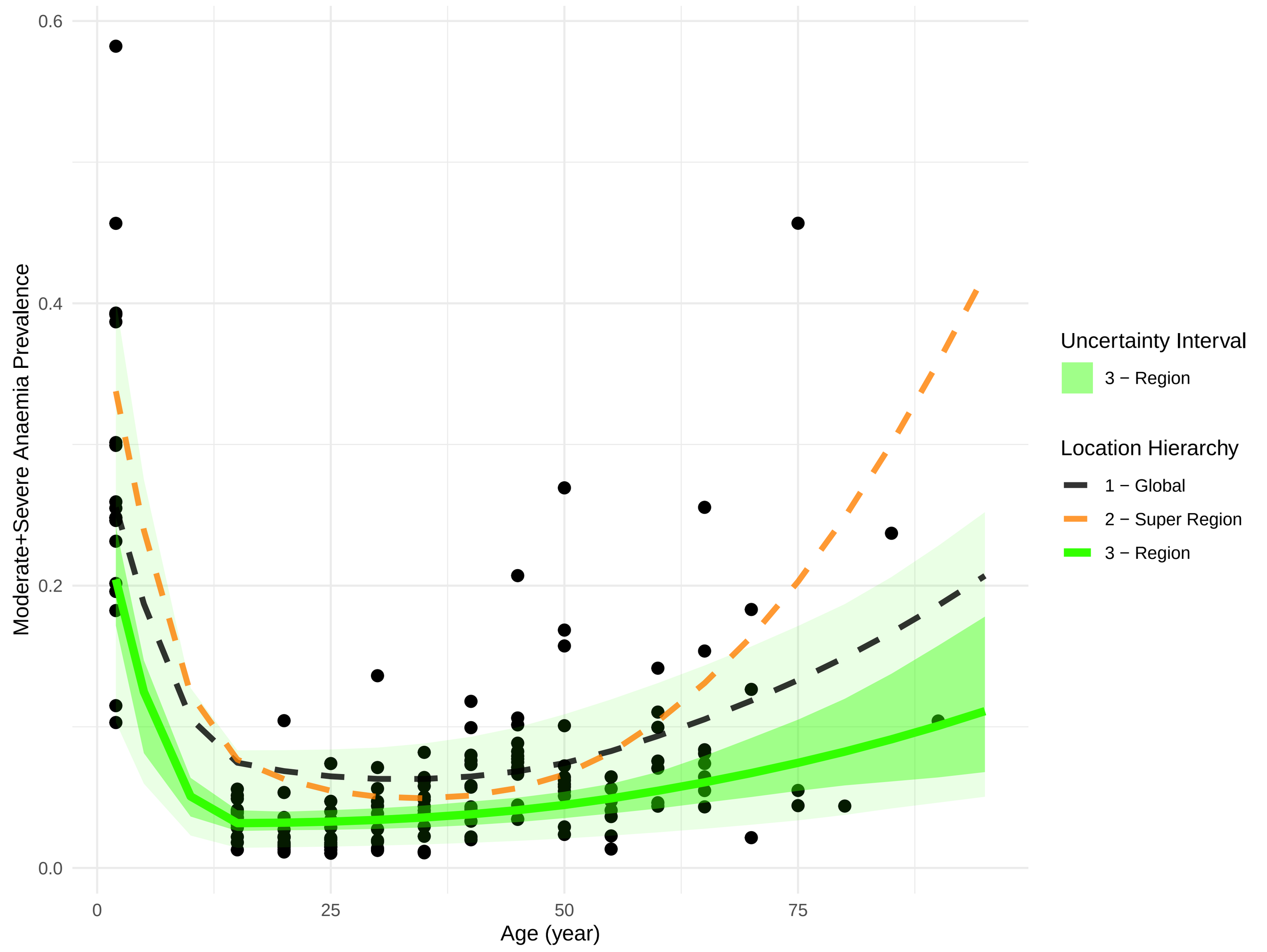

MR−BRT Age/Sex Fits − Males 2+ Years Old − Moderate+Severe Anaemia Prevalence − Eswatini
WHO−2001 Adjustment Method

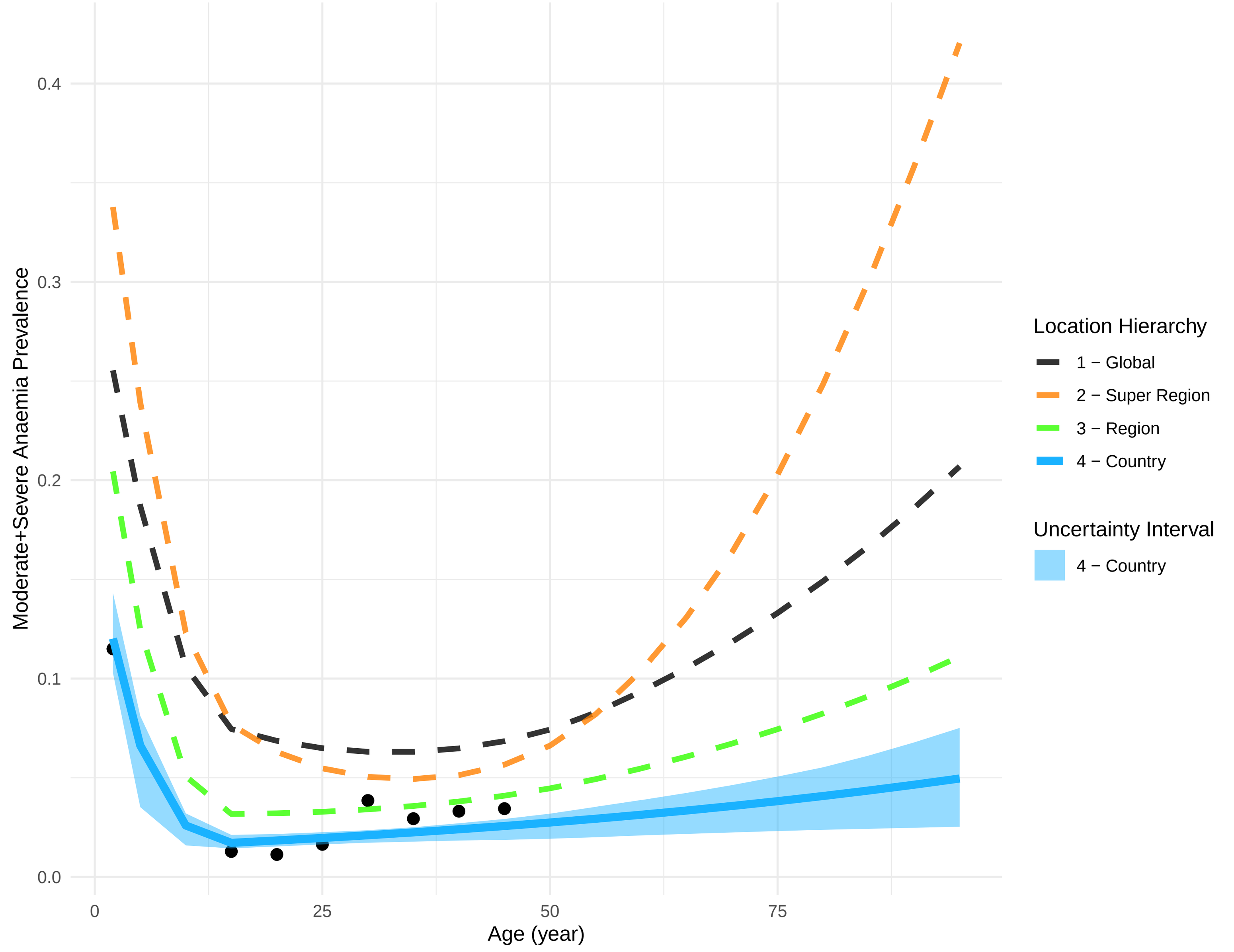

MR−BRT Age/Sex Fits − Males 2+ Years Old − Moderate+Severe Anaemia Prevalence − Lesotho
WHO−2001 Adjustment Method

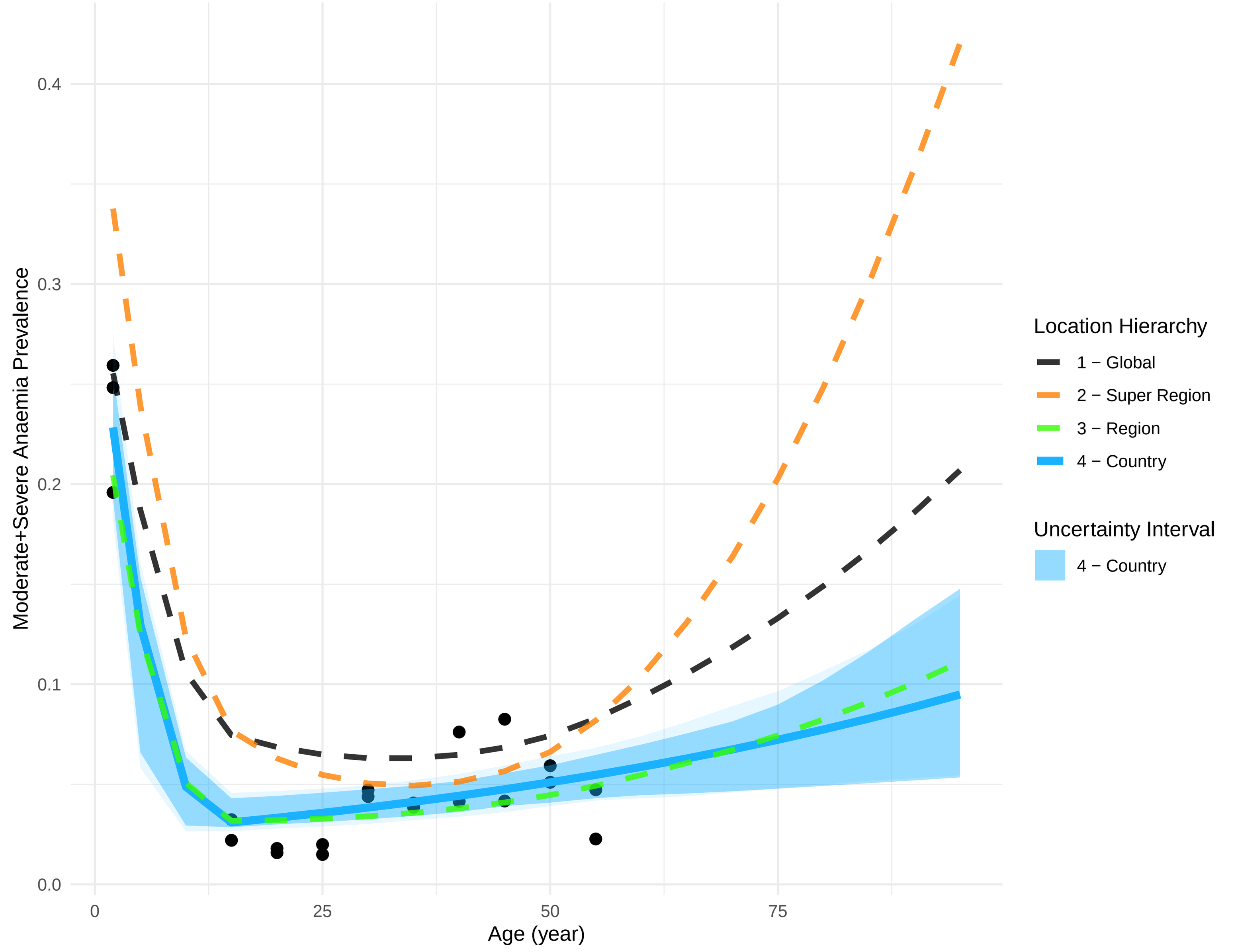

MR−BRT Age/Sex Fits − Males 2+ Years Old − Moderate+Severe Anaemia Prevalence − Namibia
WHO−2001 Adjustment Method

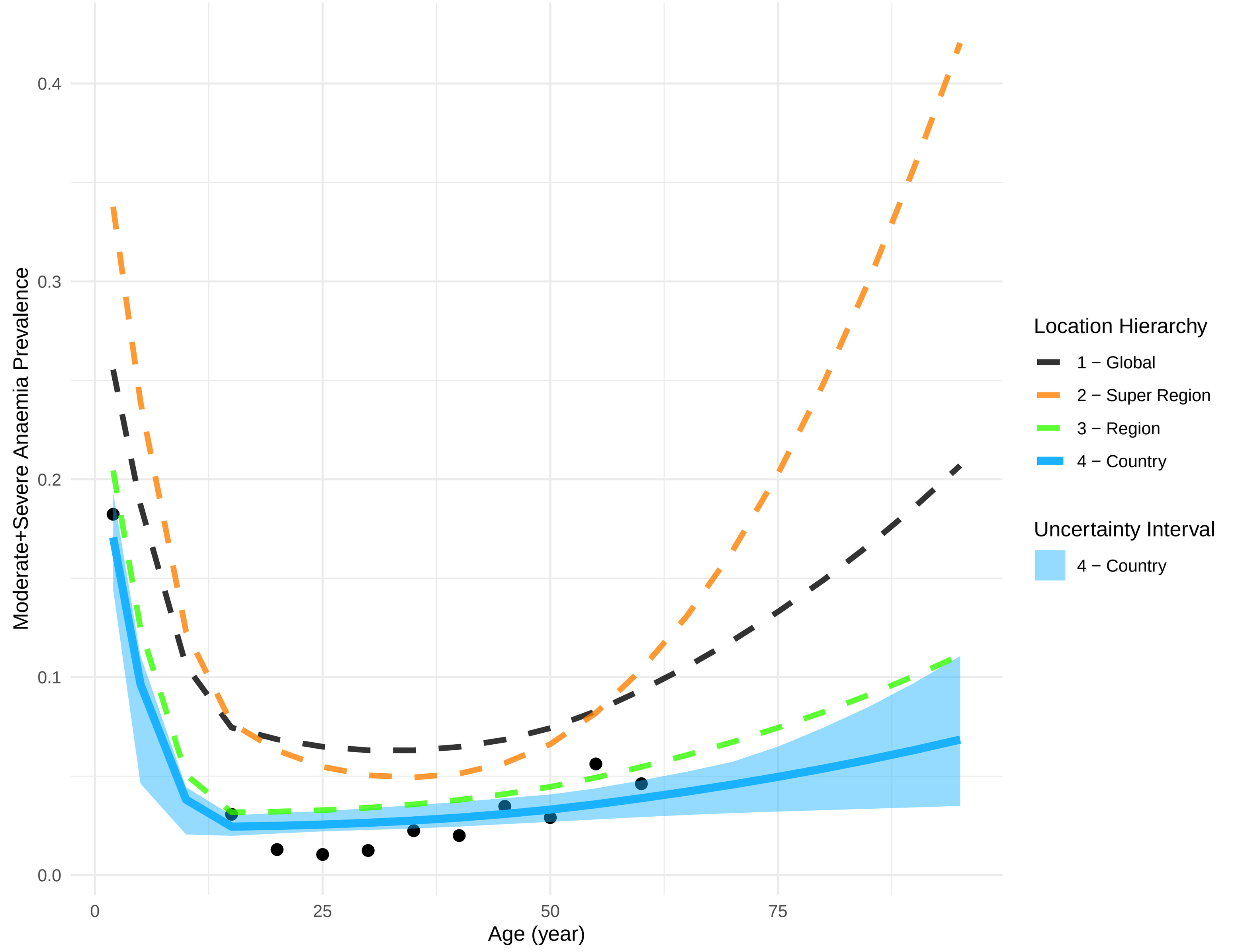

MR−BRT Age/Sex Fits − Males 2+ Years Old − Moderate+Severe Anaemia Prevalence − South Africa
WHO−2001 Adjustment Method

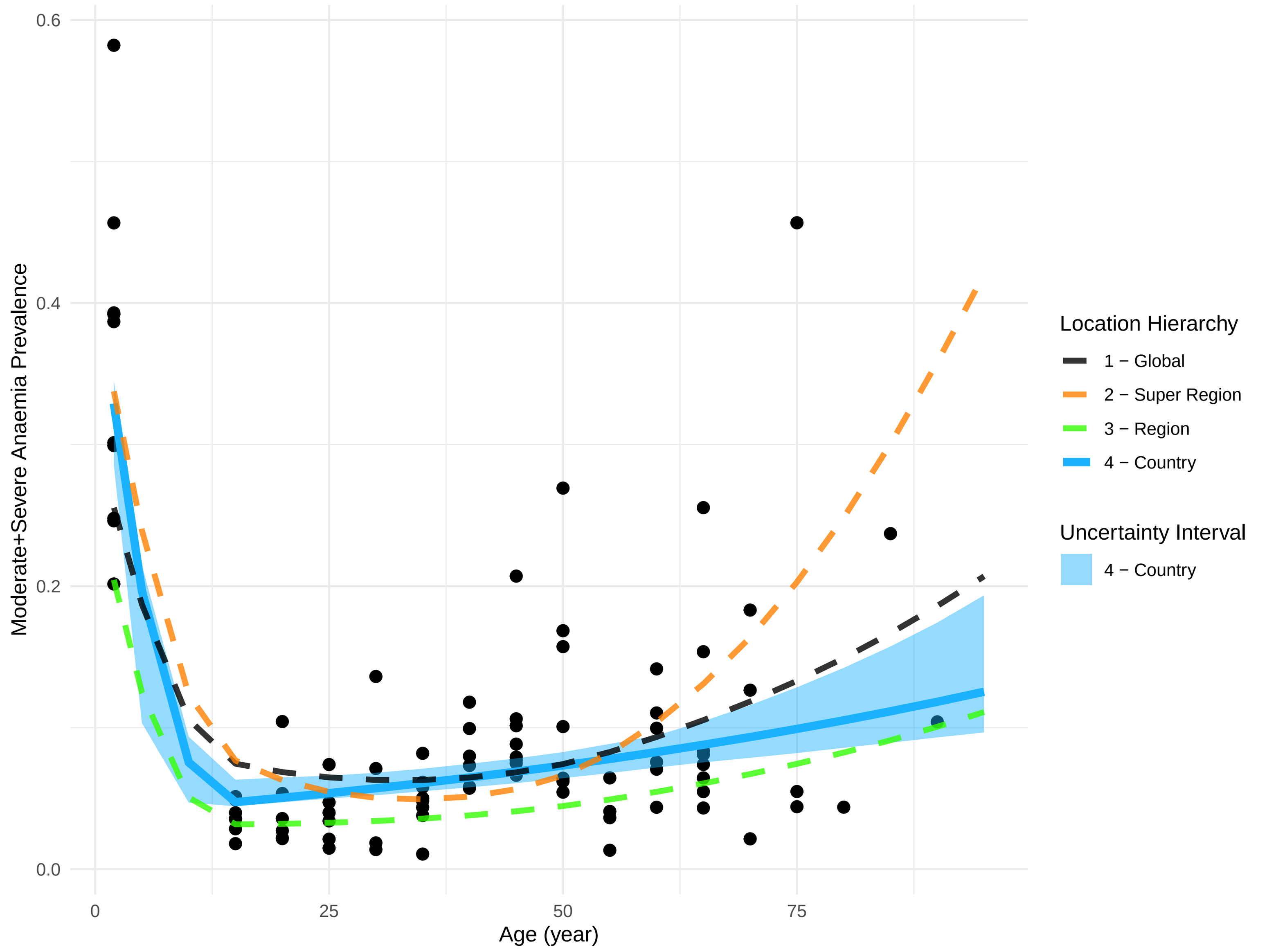

MR−BRT Age/Sex Fits − Males 2+ Years Old − Moderate+Severe Anaemia Prevalence − Eastern Cape
WHO−2001 Adjustment Method

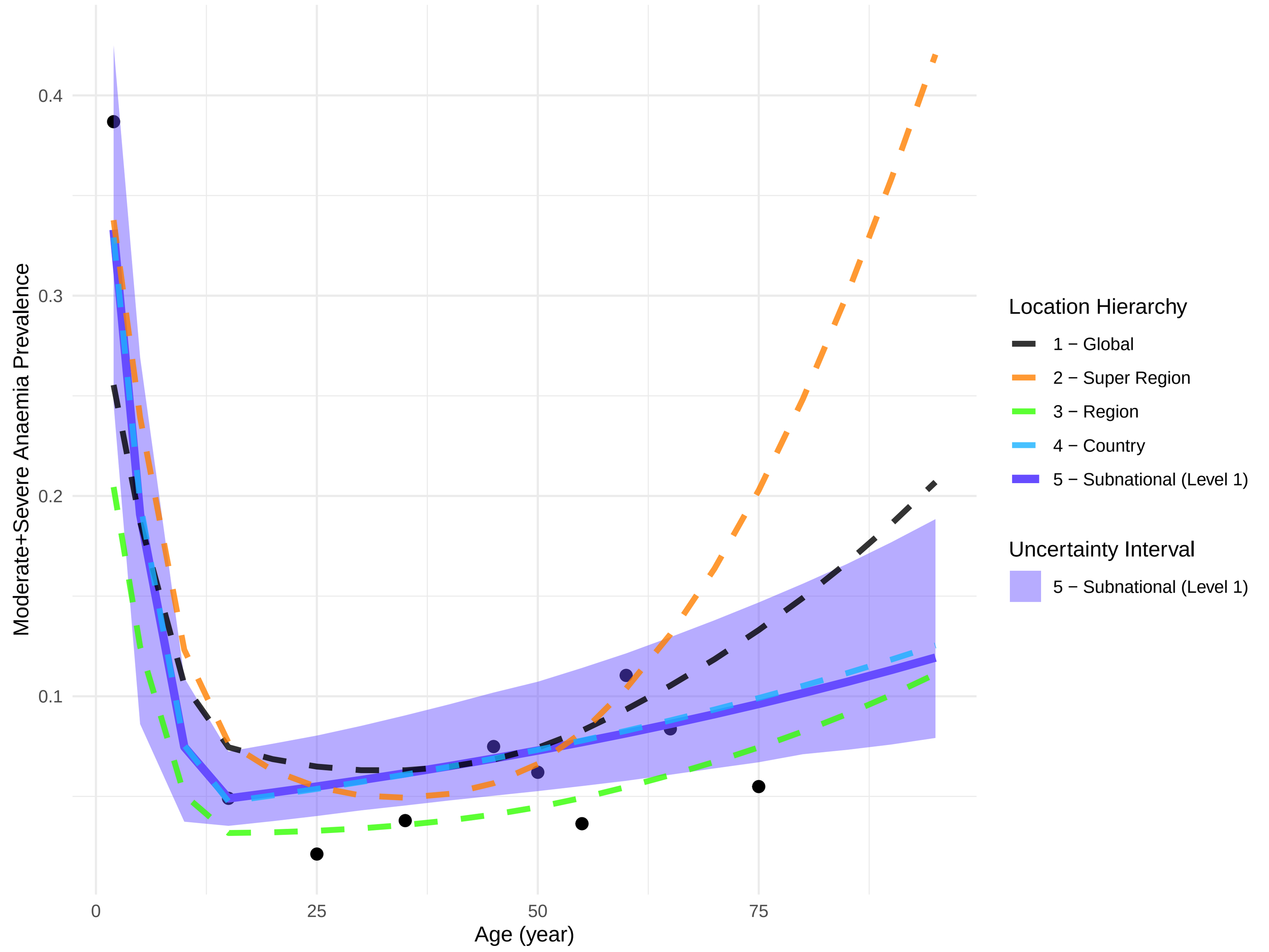

MR−BRT Age/Sex Fits − Males 2+ Years Old − Moderate+Severe Anaemia Prevalence − Free State
WHO−2001 Adjustment Method

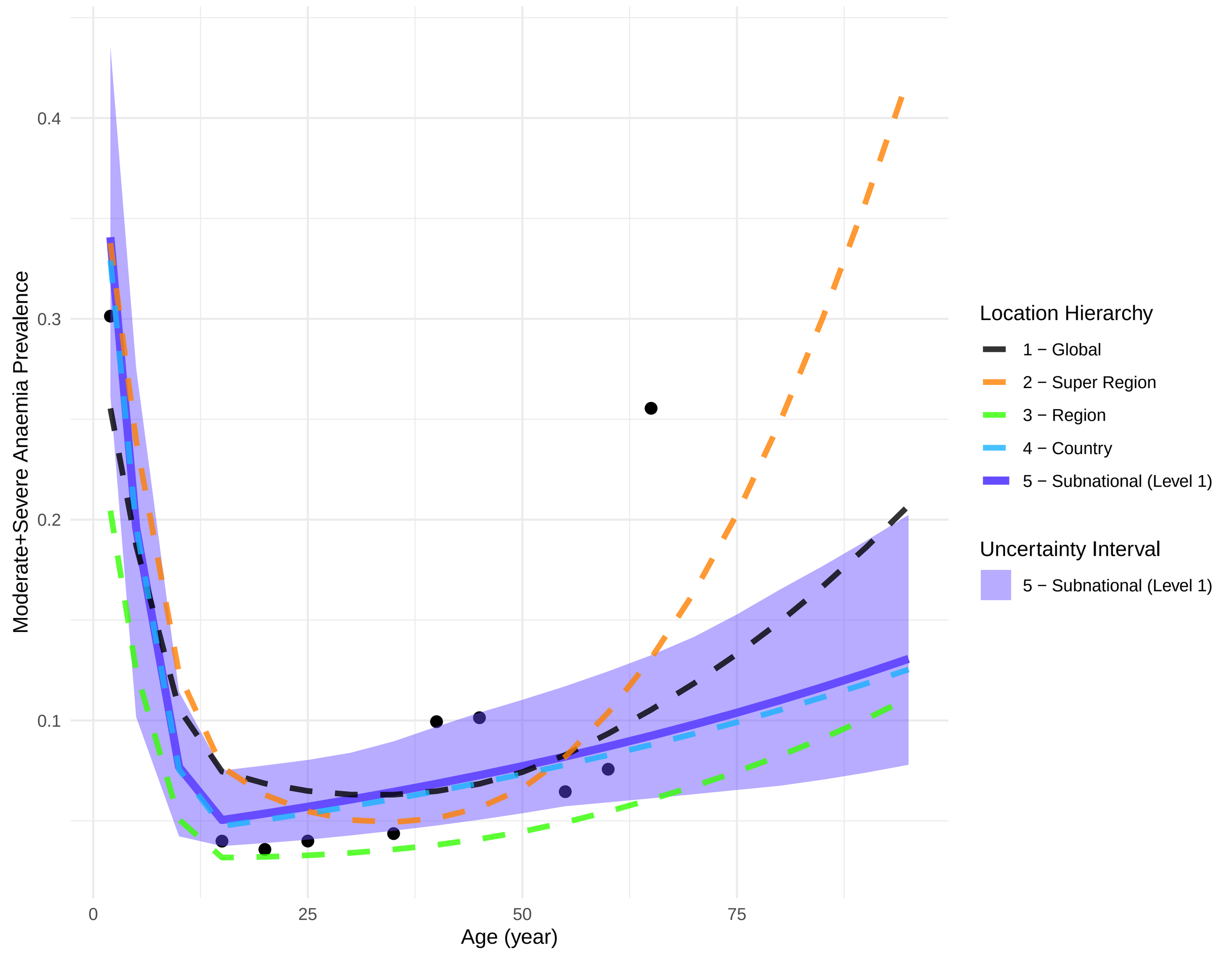

MR−BRT Age/Sex Fits − Males 2+ Years Old − Moderate+Severe Anaemia Prevalence − Gauteng
WHO−2001 Adjustment Method

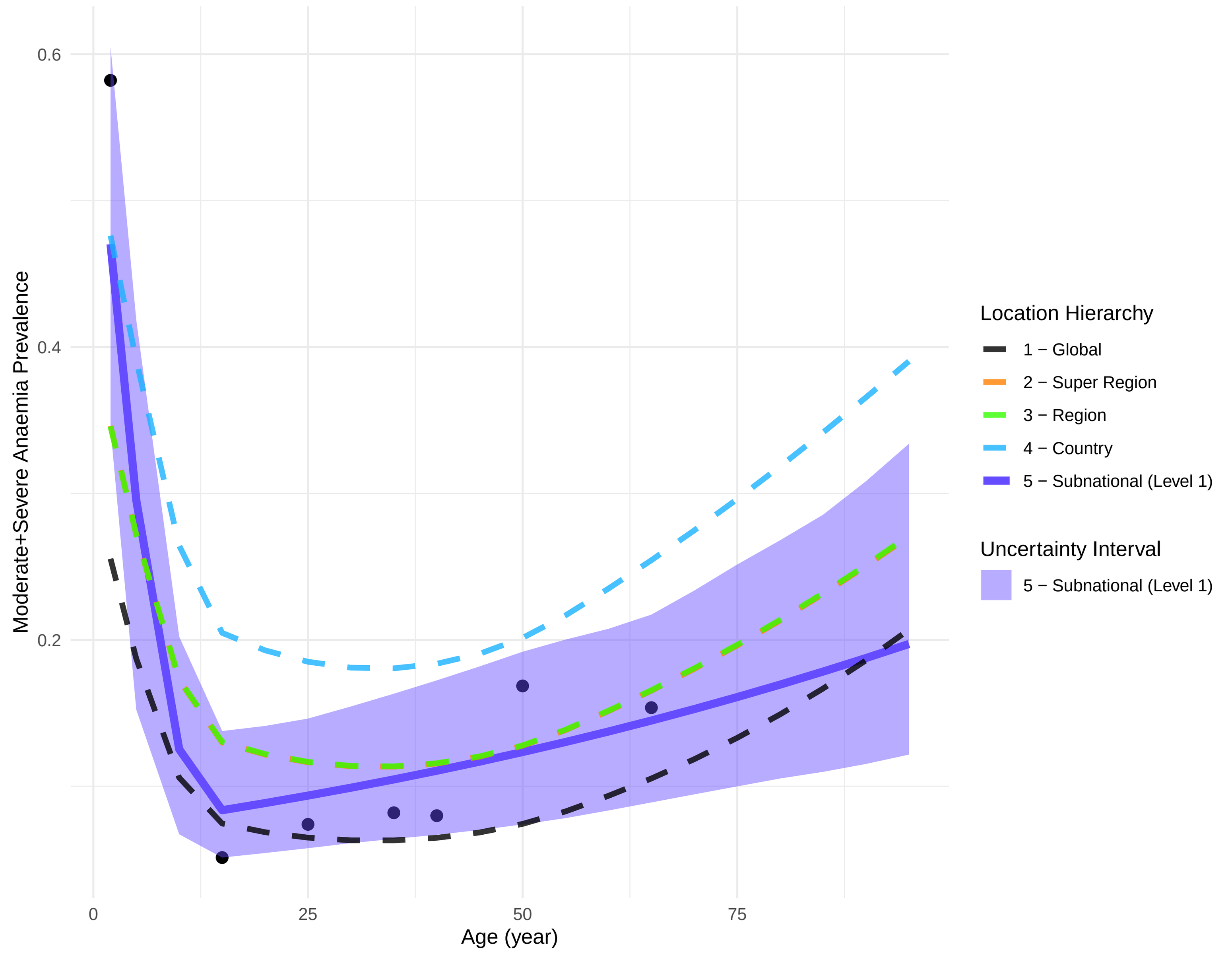

MR−BRT Age/Sex Fits − Males 2+ Years Old − Moderate+Severe Anaemia Prevalence − KwaZulu−Natal
WHO−2001 Adjustment Method

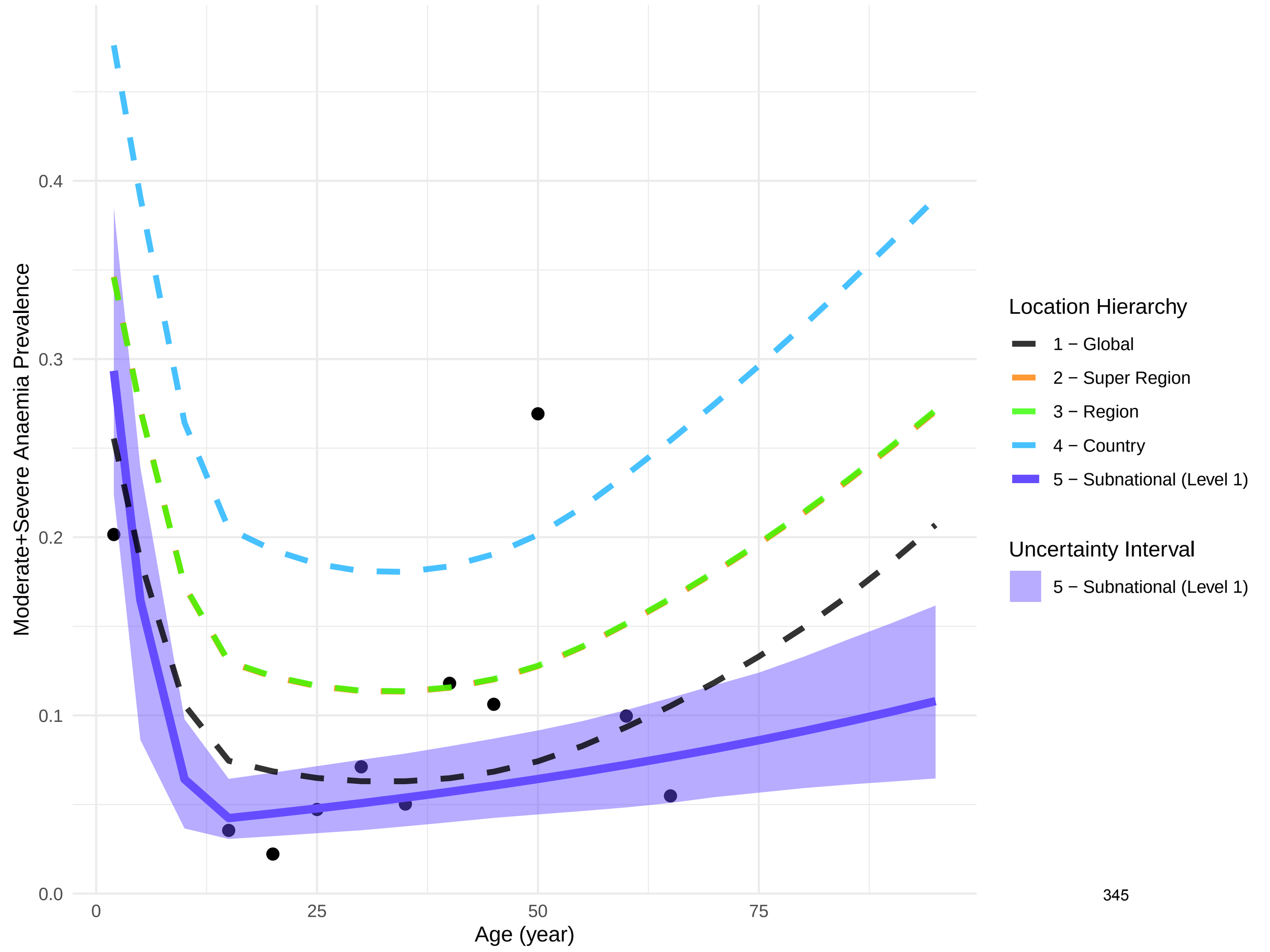

MR−BRT Age/Sex Fits − Males 2+ Years Old − Moderate+Severe Anaemia Prevalence − Limpopo
WHO−2001 Adjustment Method

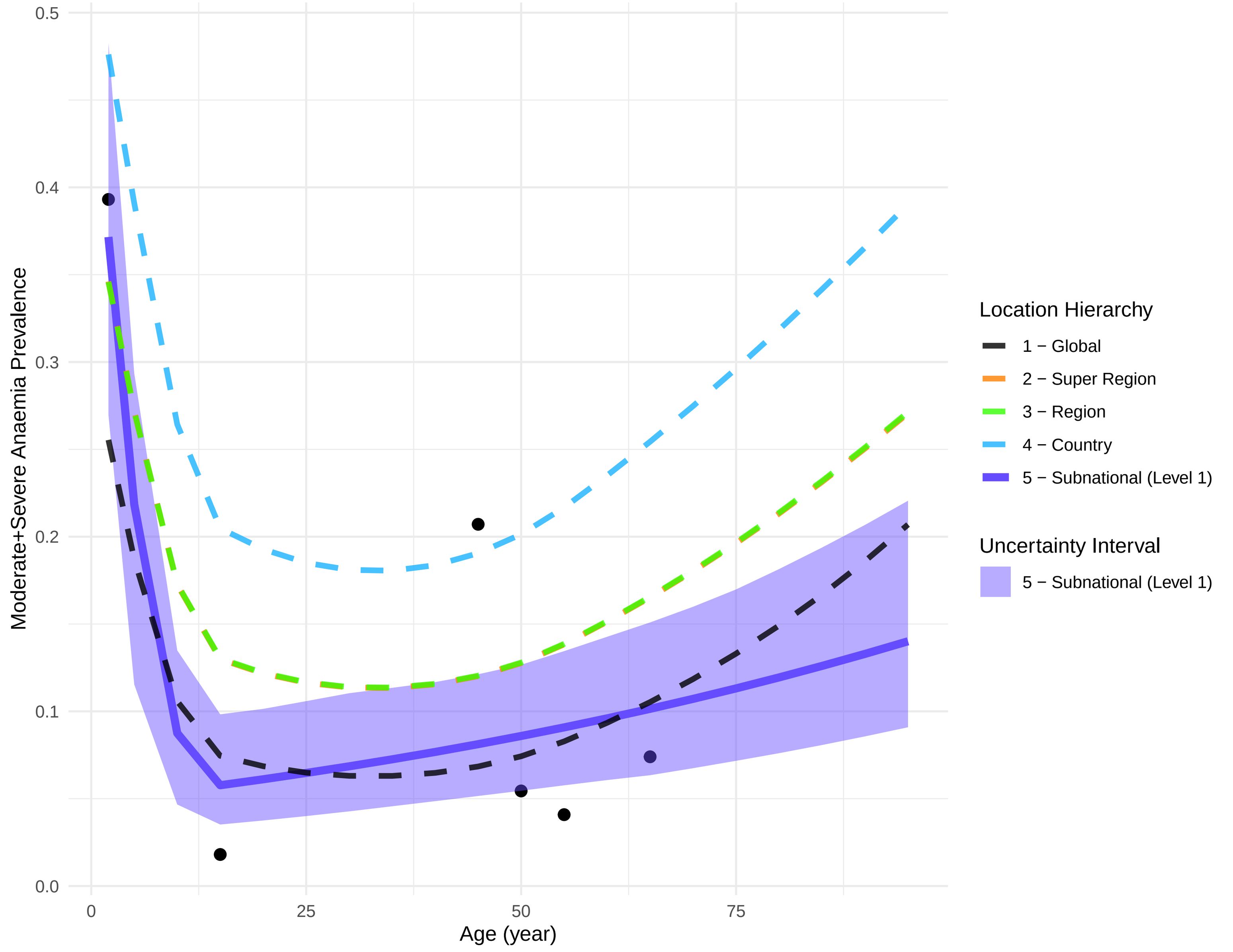

MR−BRT Age/Sex Fits − Males 2+ Years Old − Moderate+Severe Anaemia Prevalence − Mpumalanga
WHO−2001 Adjustment Method

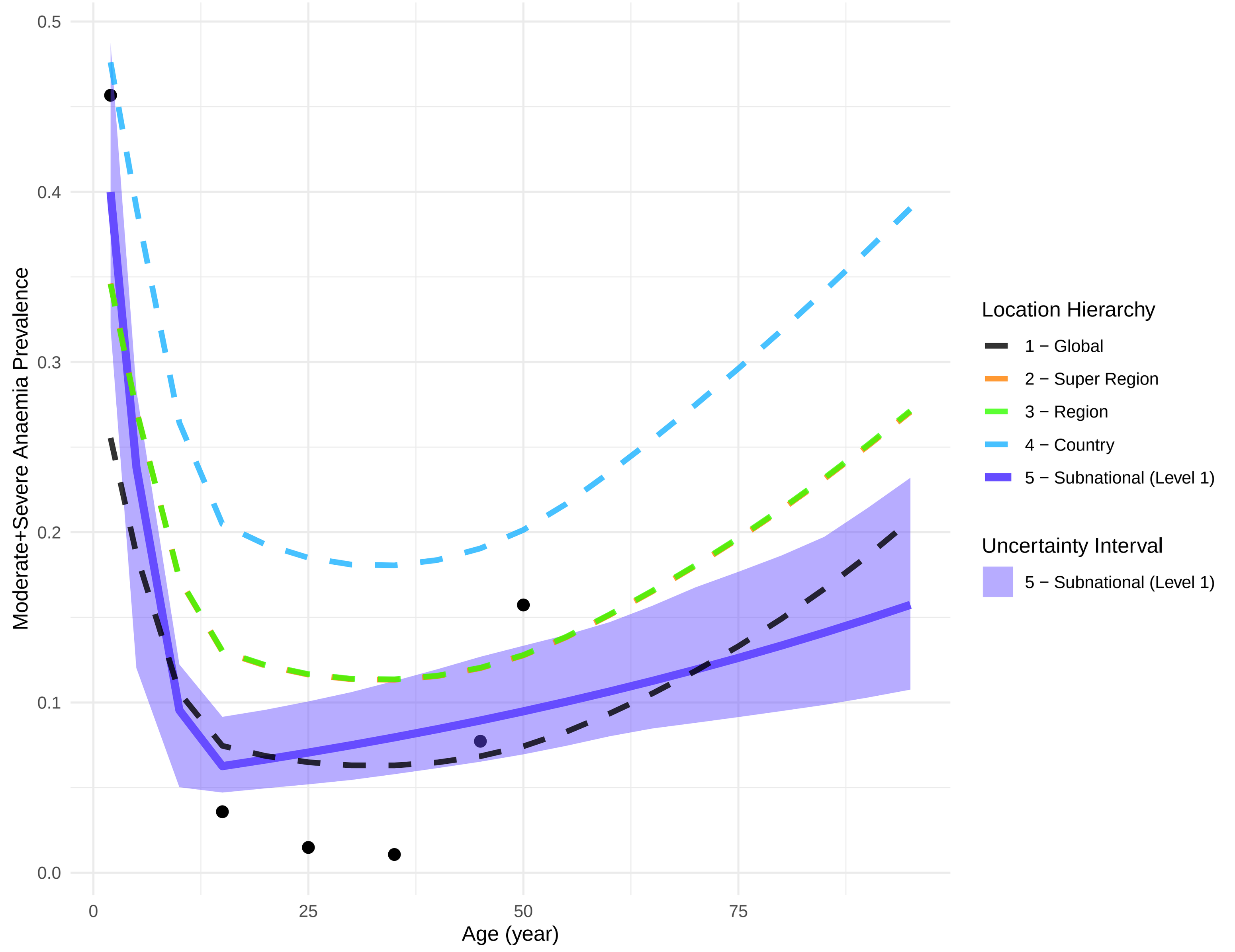

MR−BRT Age/Sex Fits − Males 2+ Years Old − Moderate+Severe Anaemia Prevalence − North−West
WHO−2001 Adjustment Method

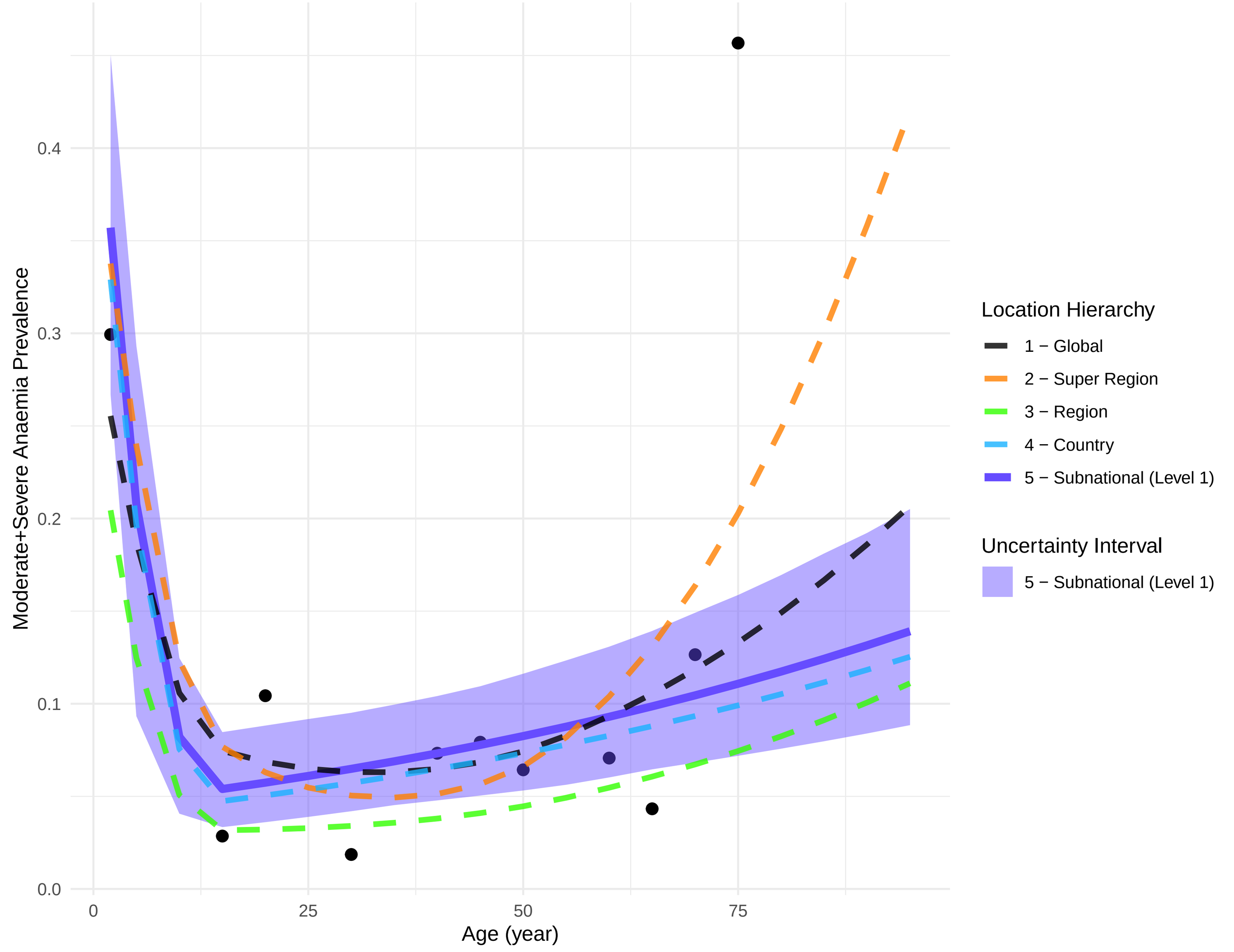

MR−BRT Age/Sex Fits − Males 2+ Years Old − Moderate+Severe Anaemia Prevalence − Northern Cape
WHO−2001 Adjustment Method

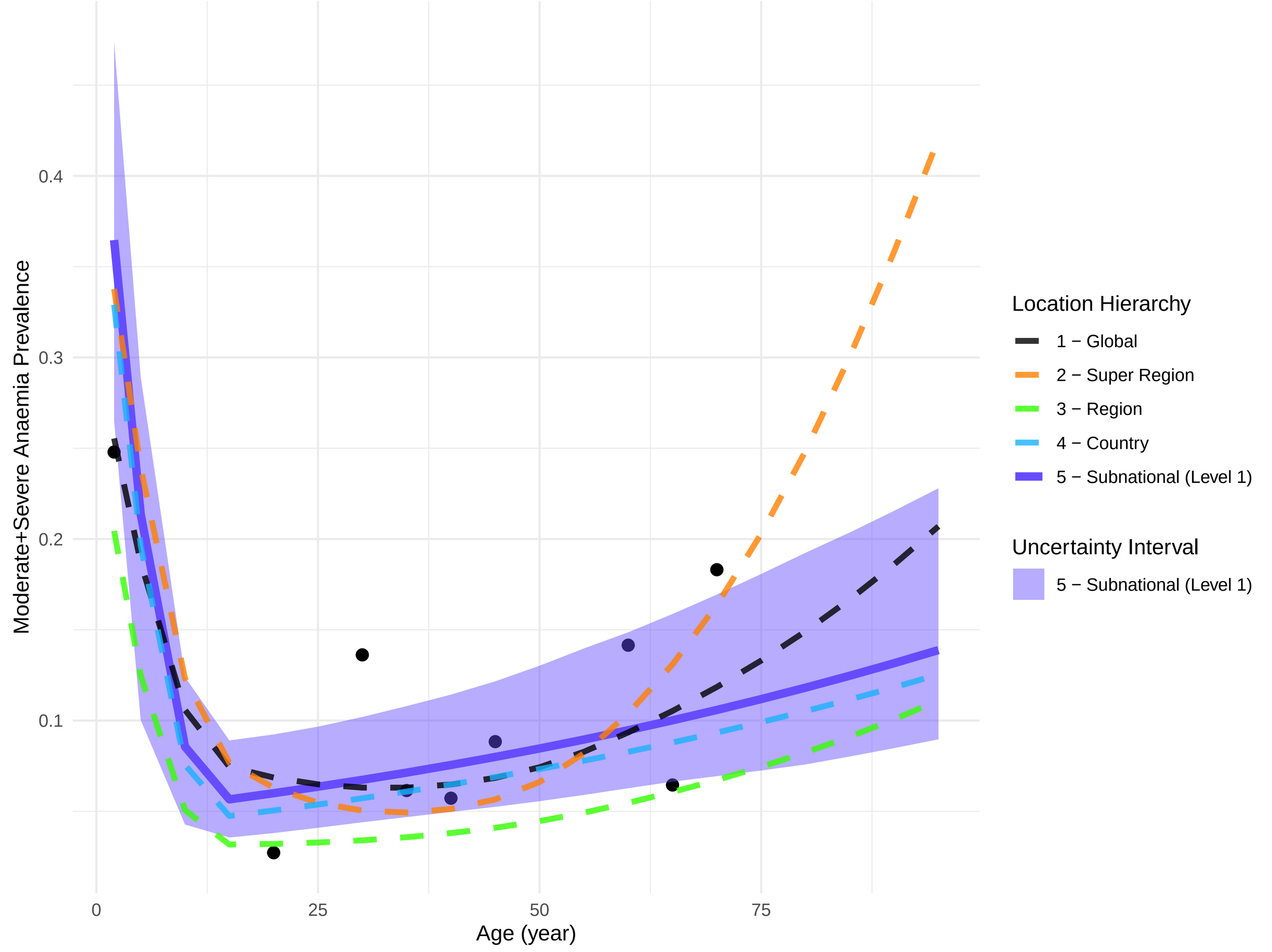

MR−BRT Age/Sex Fits − Males 2+ Years Old − Moderate+Severe Anaemia Prevalence − Western Cape
WHO−2001 Adjustment Method

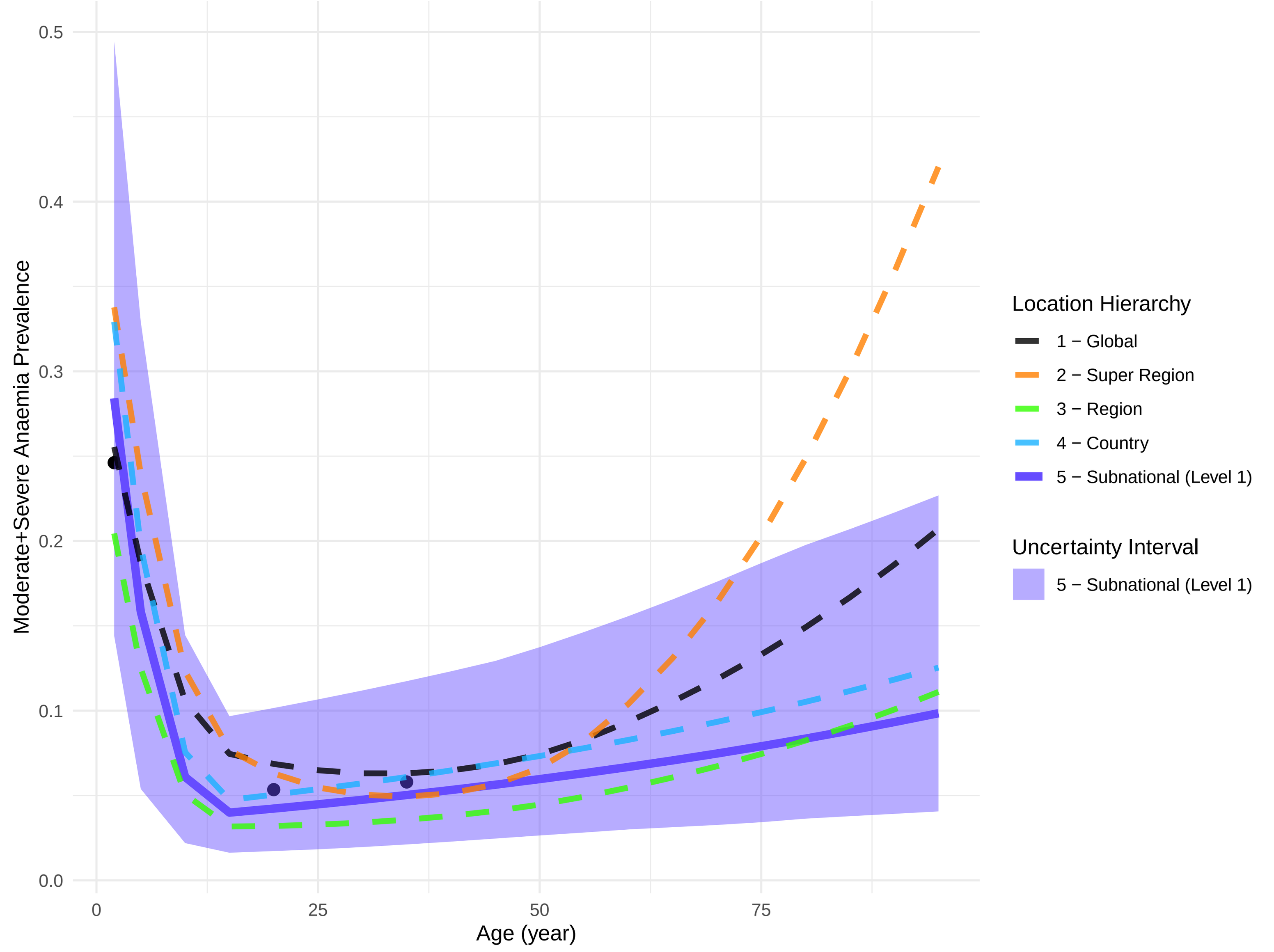

MR−BRT Age/Sex Fits − Males 2+ Years Old − Moderate+Severe Anaemia Prevalence − Zimbabwe
WHO−2001 Adjustment Method

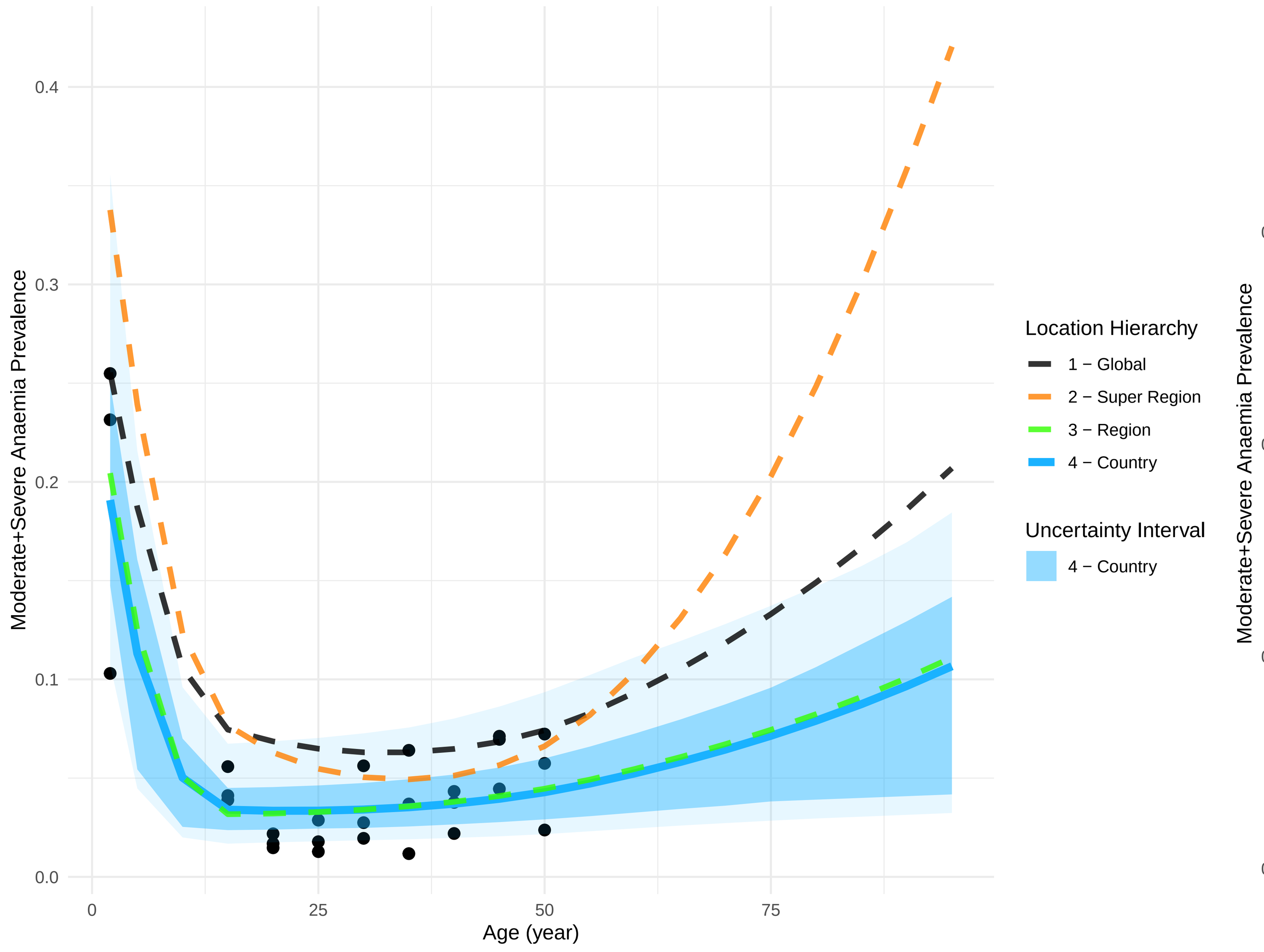

MR−BRT Age/Sex Fits − Males 2+ Years Old − Moderate+Severe Anaemia Prevalence − Western Sub−Saharan Africa
WHO−2001 Adjustment Method

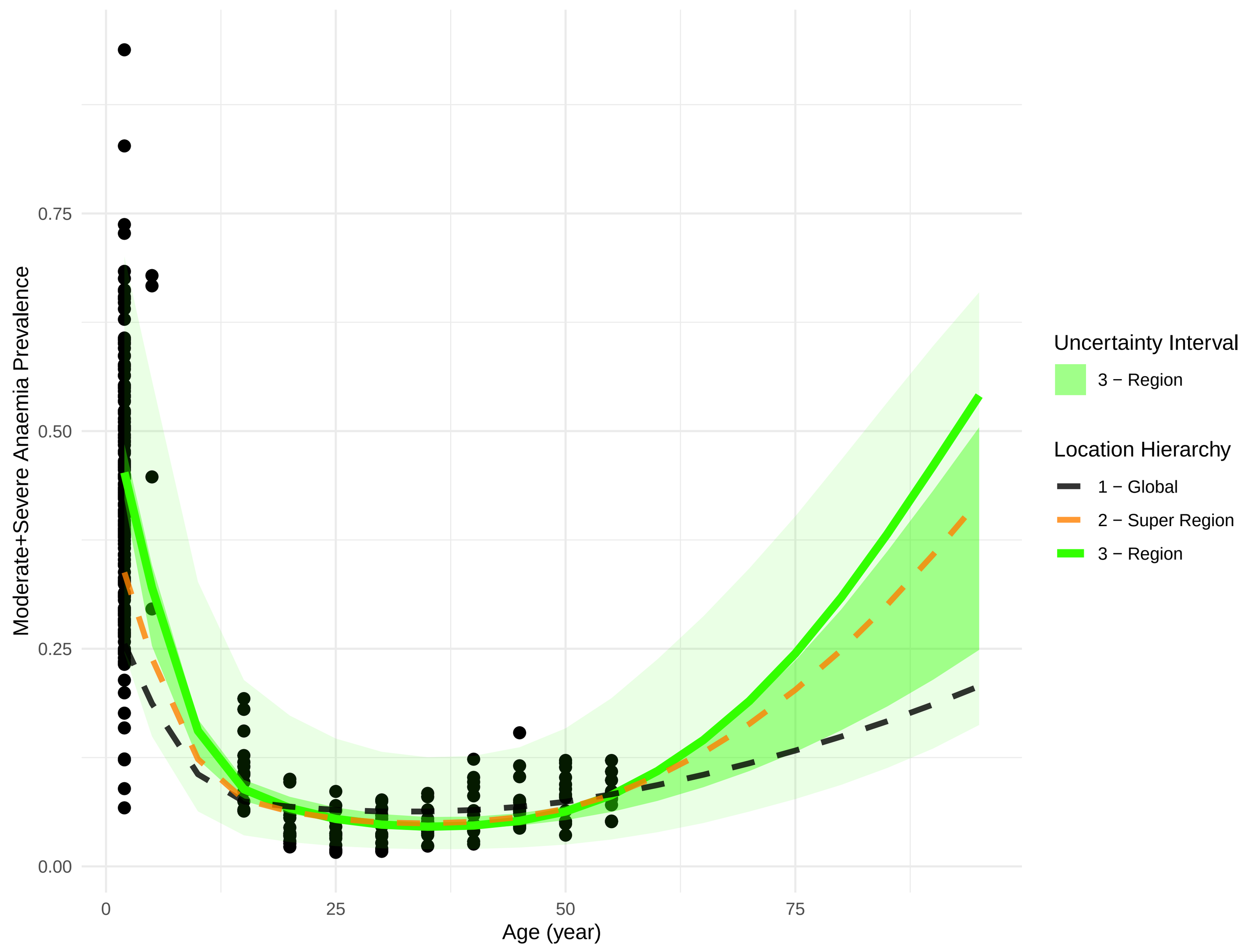

MR−BRT Age/Sex Fits − Males 2+ Years Old − Moderate+Severe Anaemia Prevalence − Benin
WHO−2001 Adjustment Method

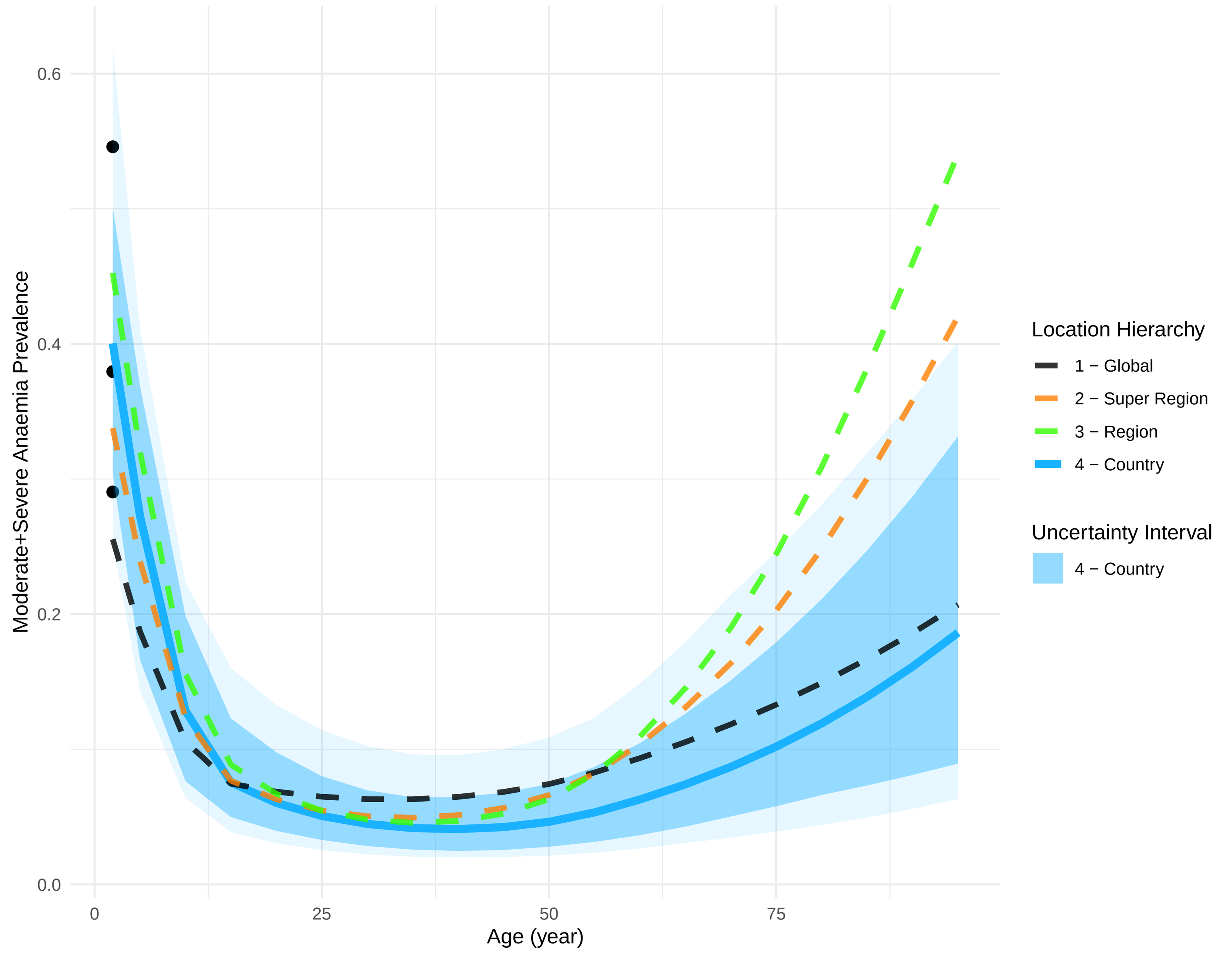

MR−BRT Age/Sex Fits − Males 2+ Years Old − Moderate+Severe Anaemia Prevalence − Burkina Faso
WHO−2001 Adjustment Method

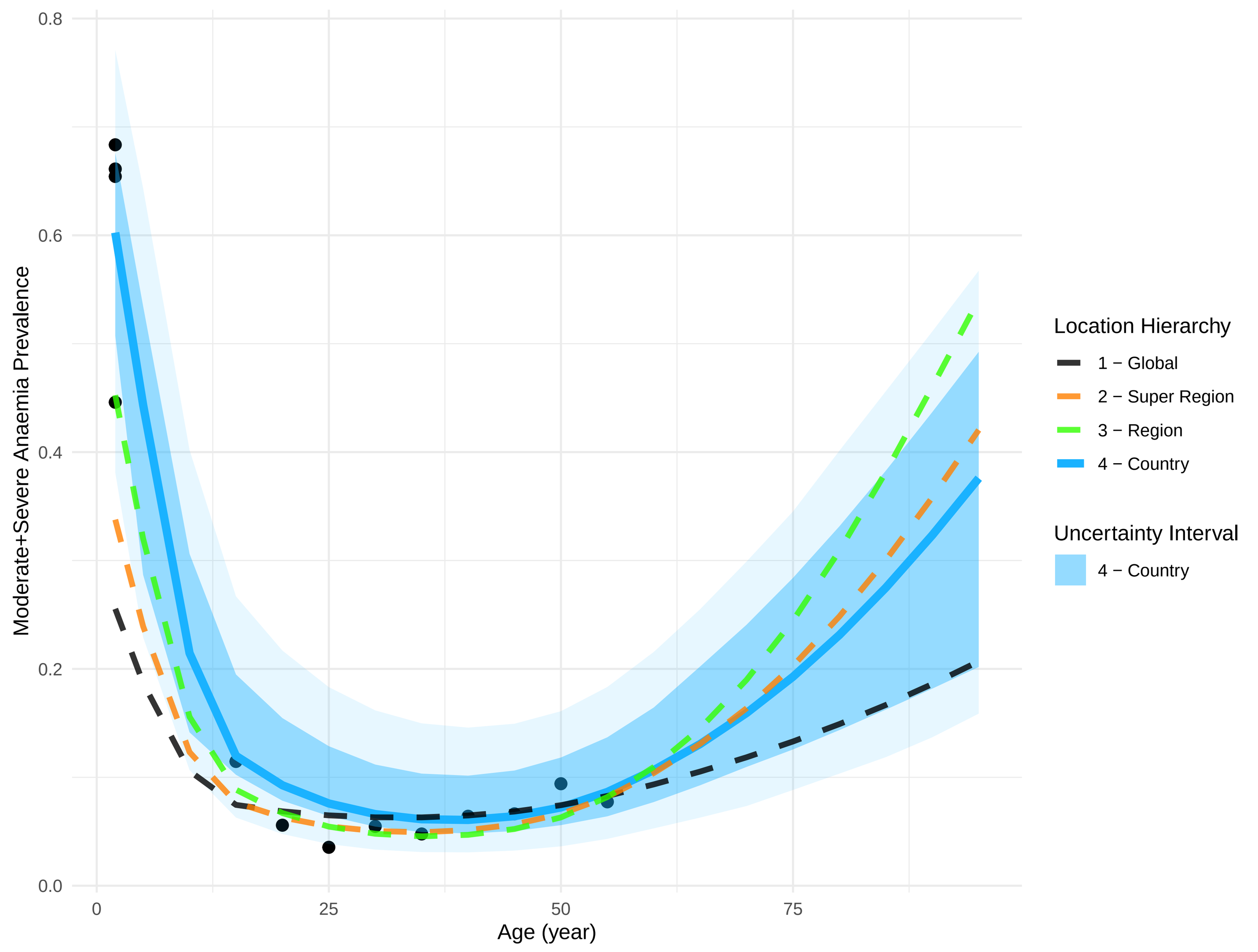

MR−BRT Age/Sex Fits − Males 2+ Years Old − Moderate+Severe Anaemia Prevalence − Cameroon
WHO−2001 Adjustment Method

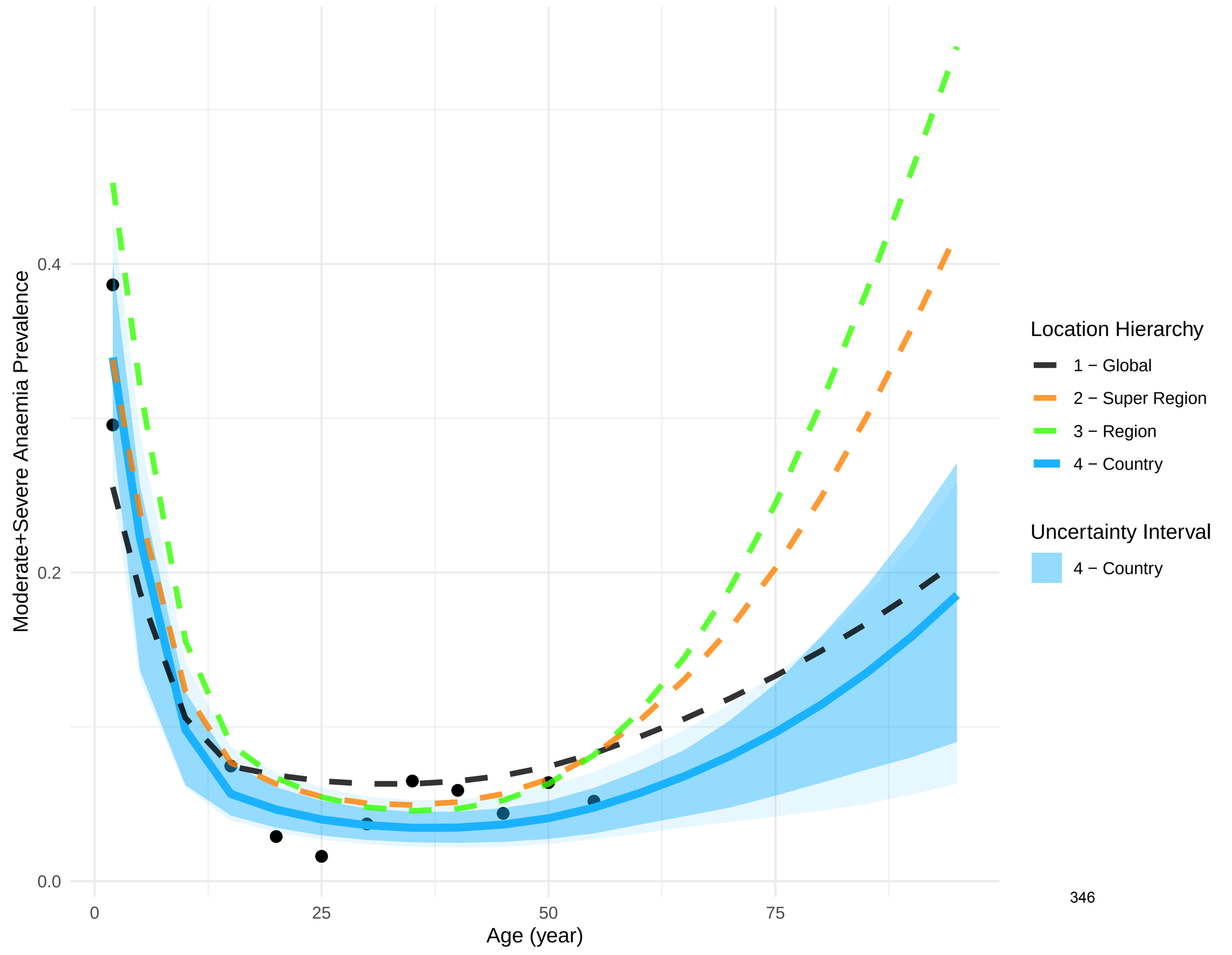

MR−BRT Age/Sex Fits − Males 2+ Years Old − Moderate+Severe Anaemia Prevalence − Côte d'Ivoire
WHO−2001 Adjustment Method

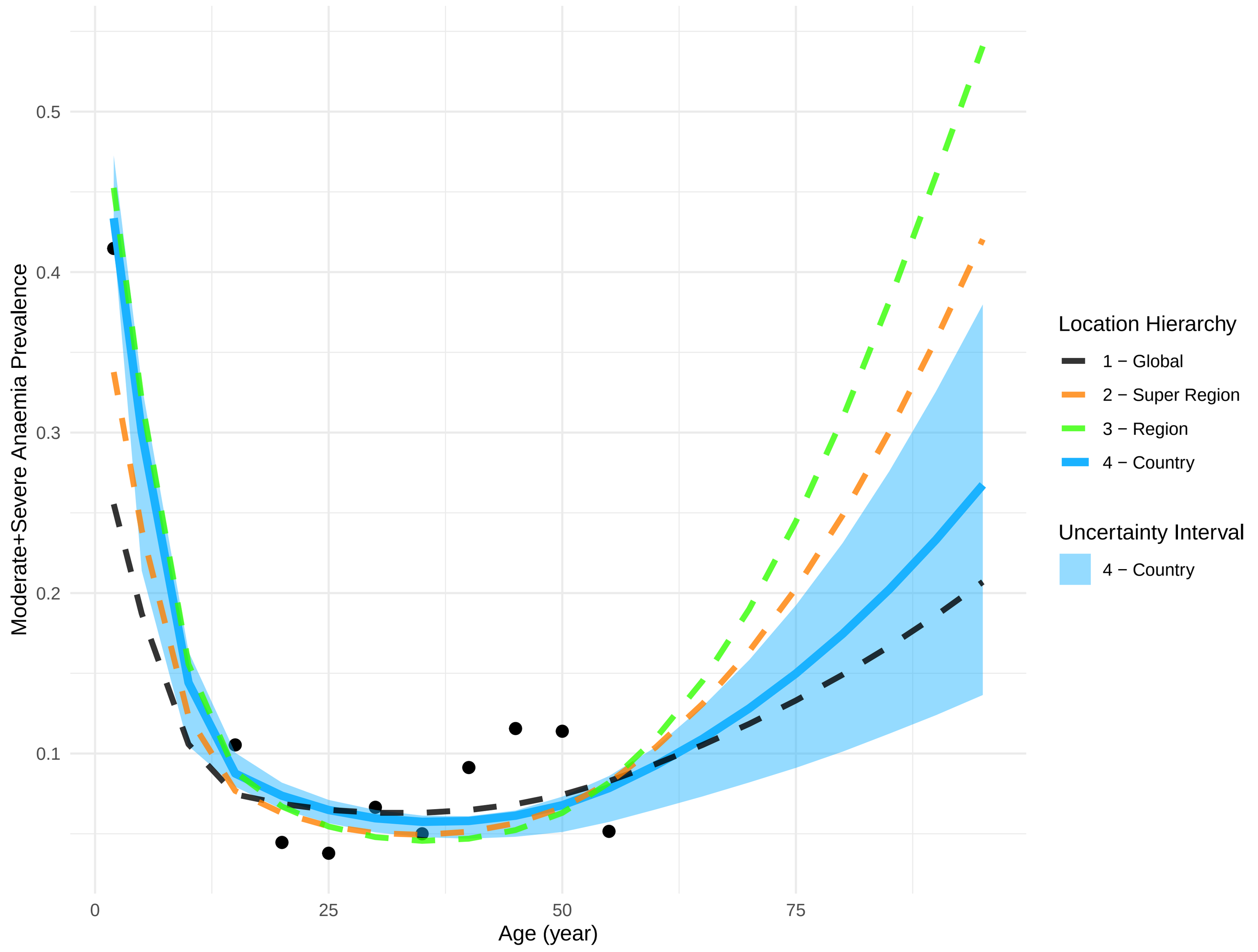

MR−BRT Age/Sex Fits − Males 2+ Years Old − Moderate+Severe Anaemia Prevalence − Gambia
WHO−2001 Adjustment Method

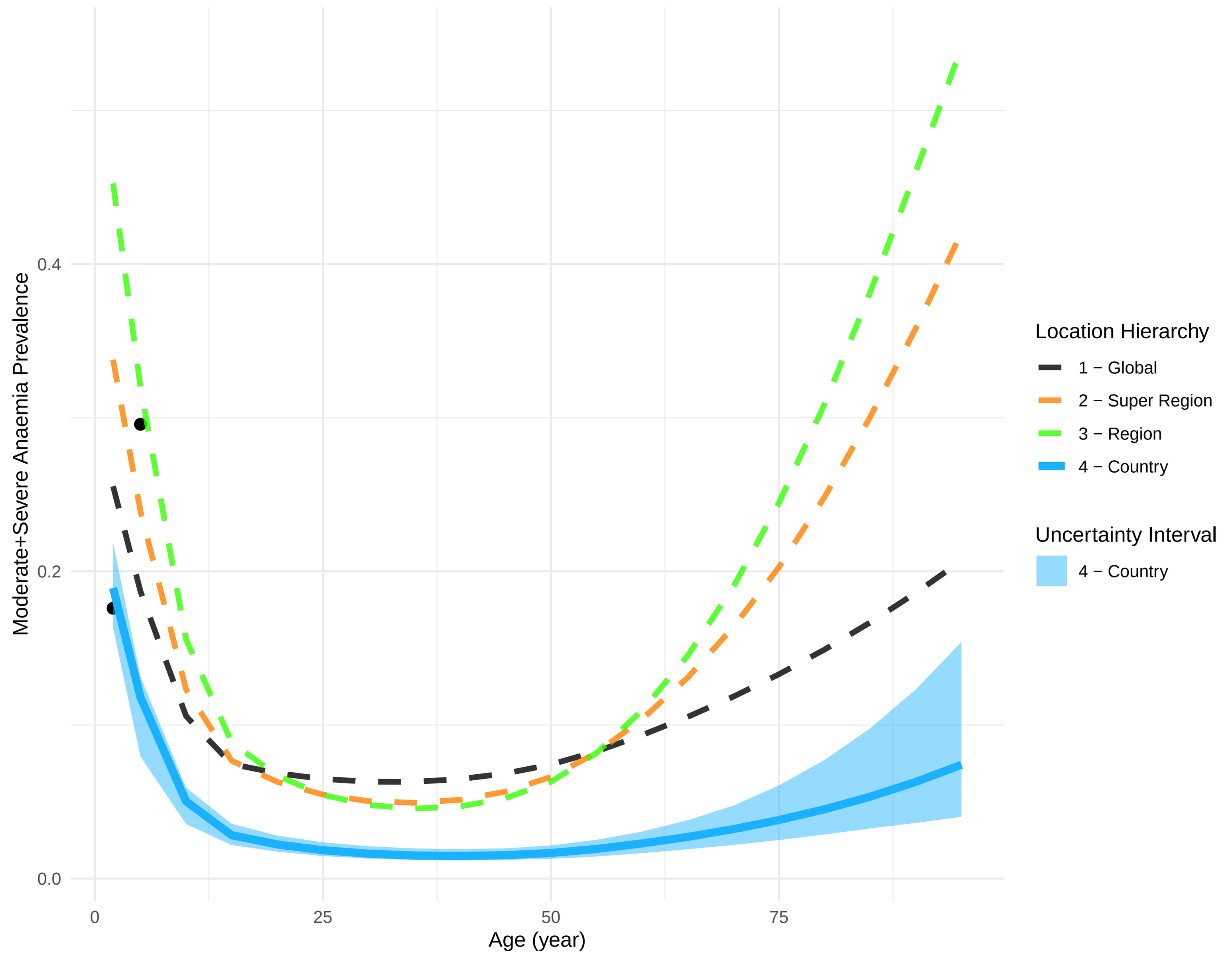

MR−BRT Age/Sex Fits − Males 2+ Years Old − Moderate+Severe Anaemia Prevalence − Ghana
WHO−2001 Adjustment Method

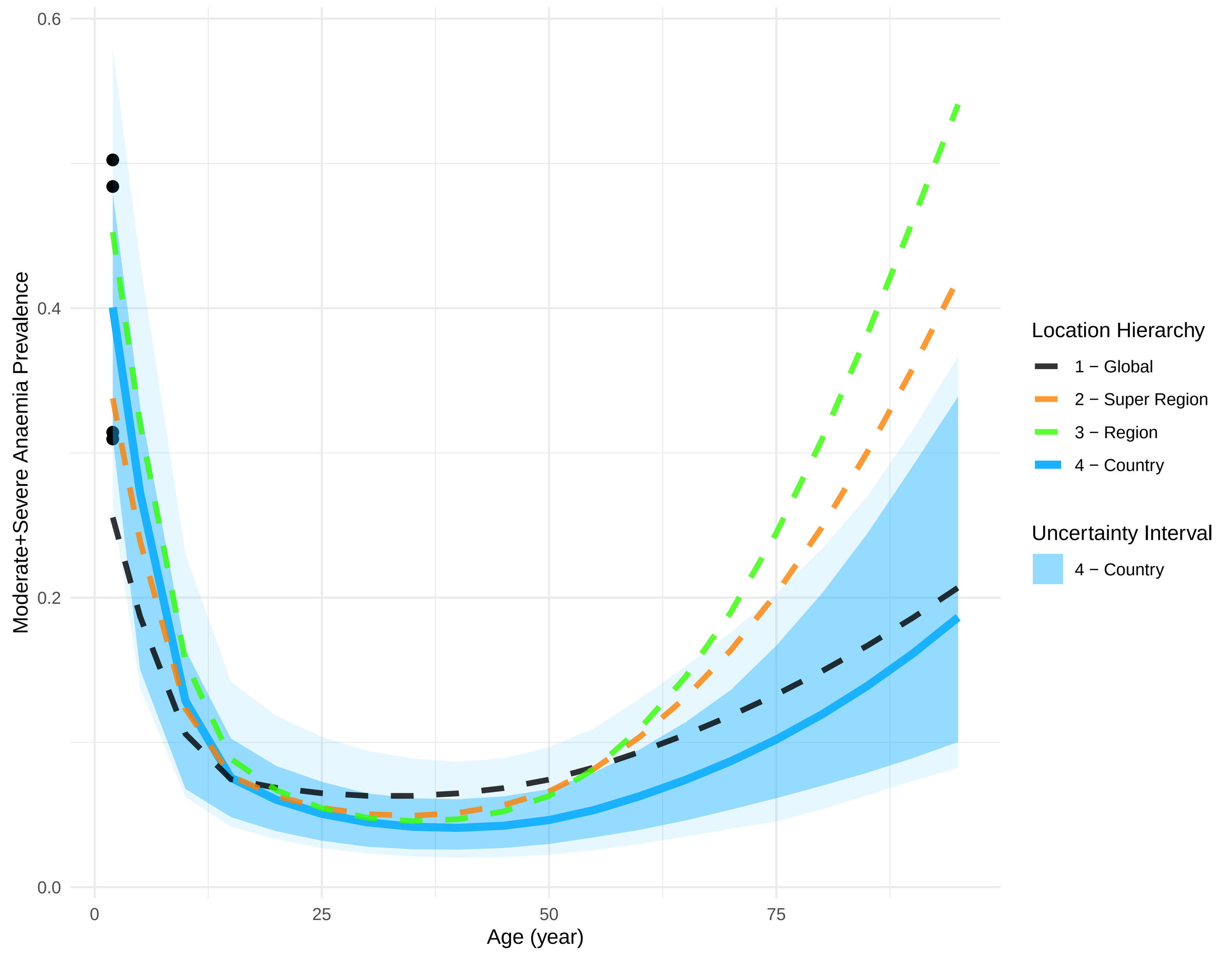

MR−BRT Age/Sex Fits − Males 2+ Years Old − Moderate+Severe Anaemia Prevalence − Guinea
WHO−2001 Adjustment Method

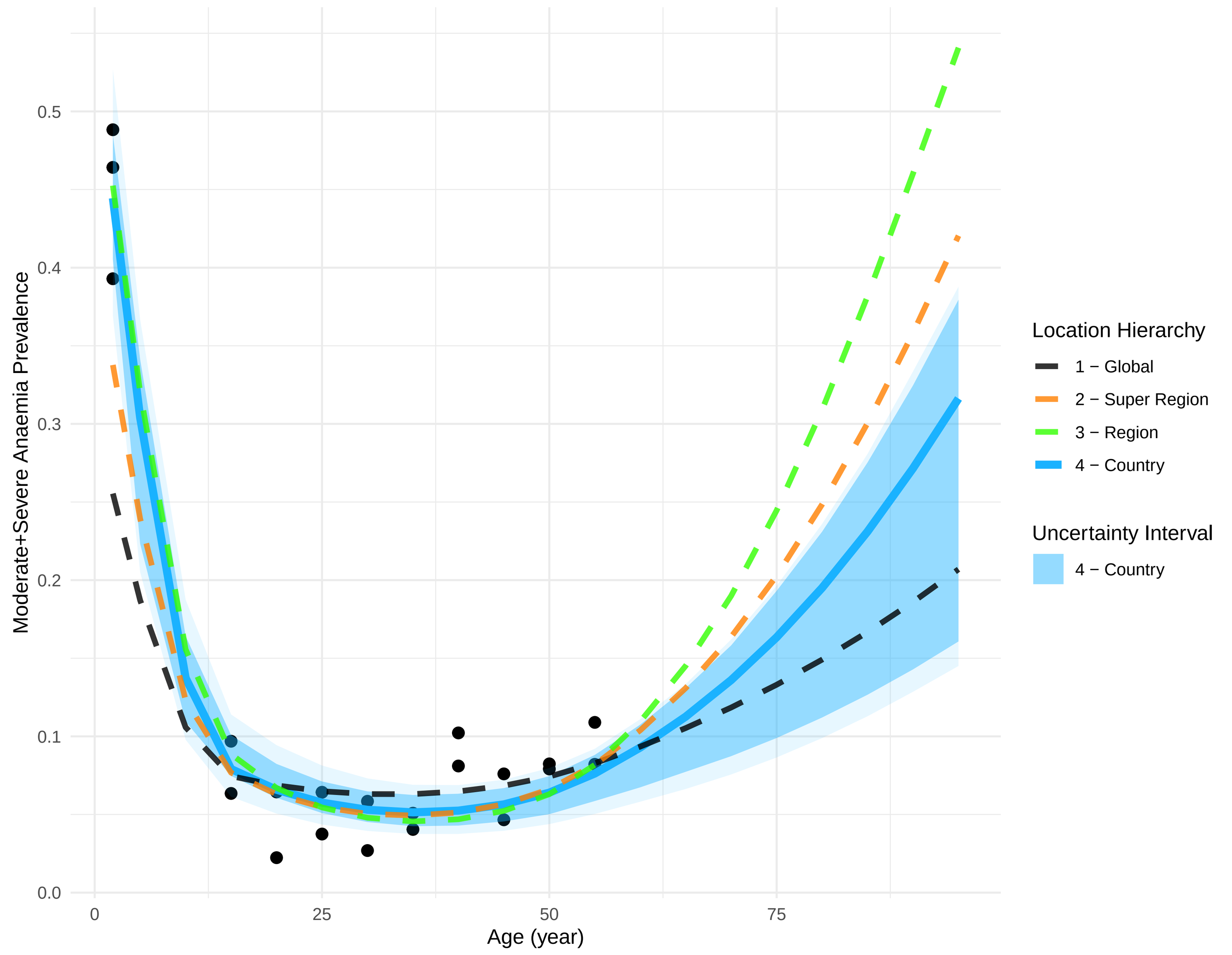

MR−BRT Age/Sex Fits − Males 2+ Years Old − Moderate+Severe Anaemia Prevalence − Liberia
WHO−2001 Adjustment Method

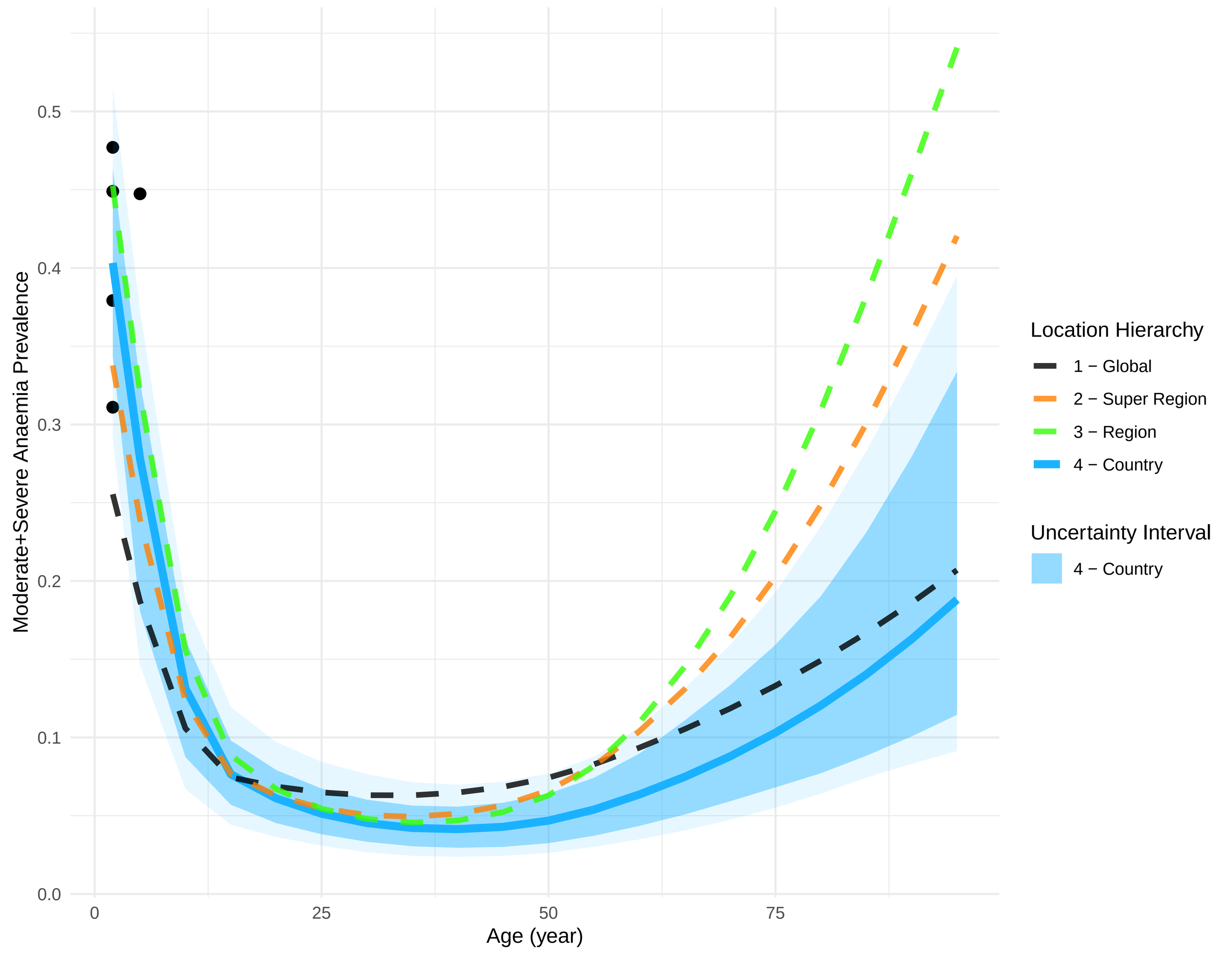

MR−BRT Age/Sex Fits − Males 2+ Years Old − Moderate+Severe Anaemia Prevalence − Mali
WHO−2001 Adjustment Method

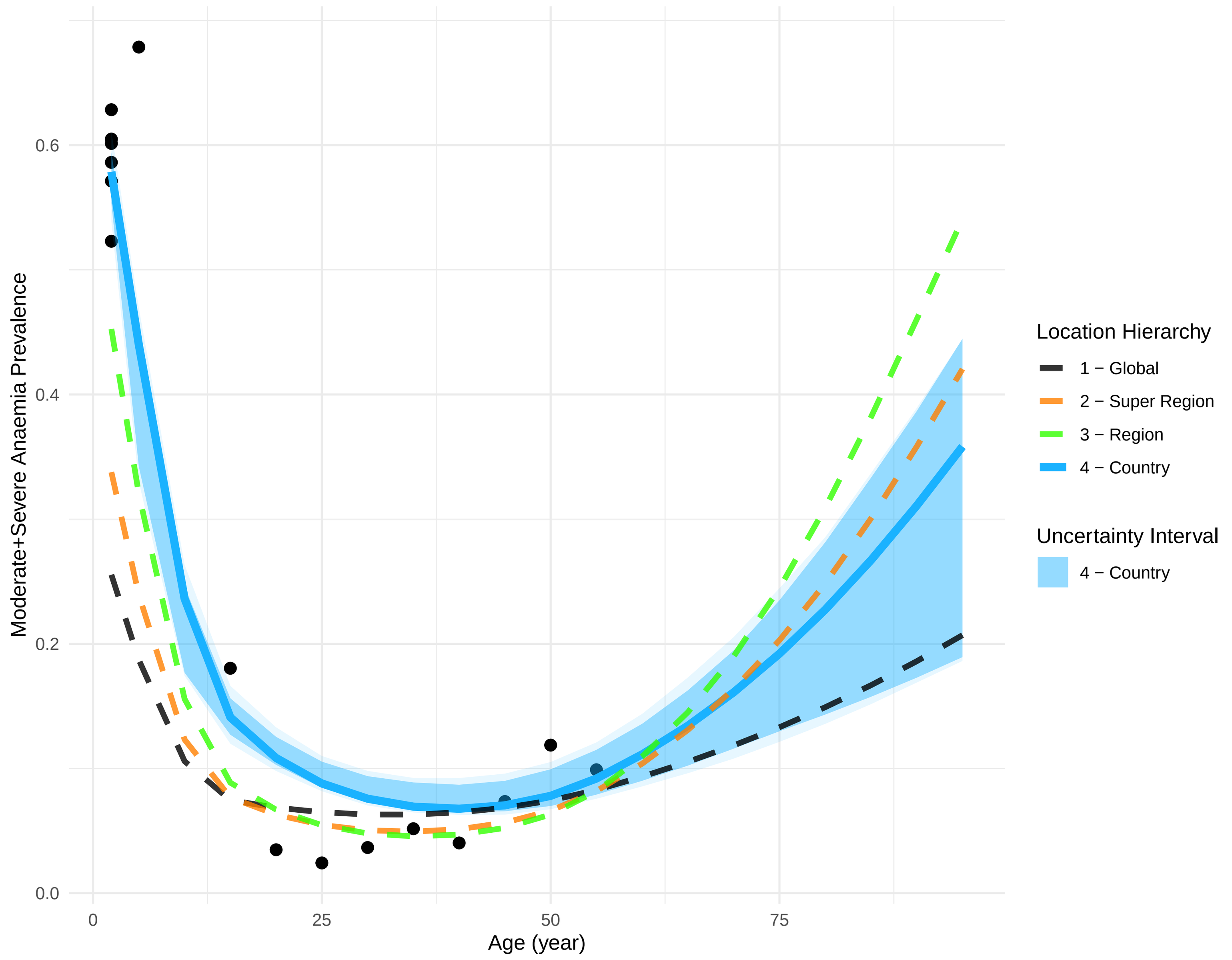

MR−BRT Age/Sex Fits − Males 2+ Years Old − Moderate+Severe Anaemia Prevalence − Niger
WHO−2001 Adjustment Method

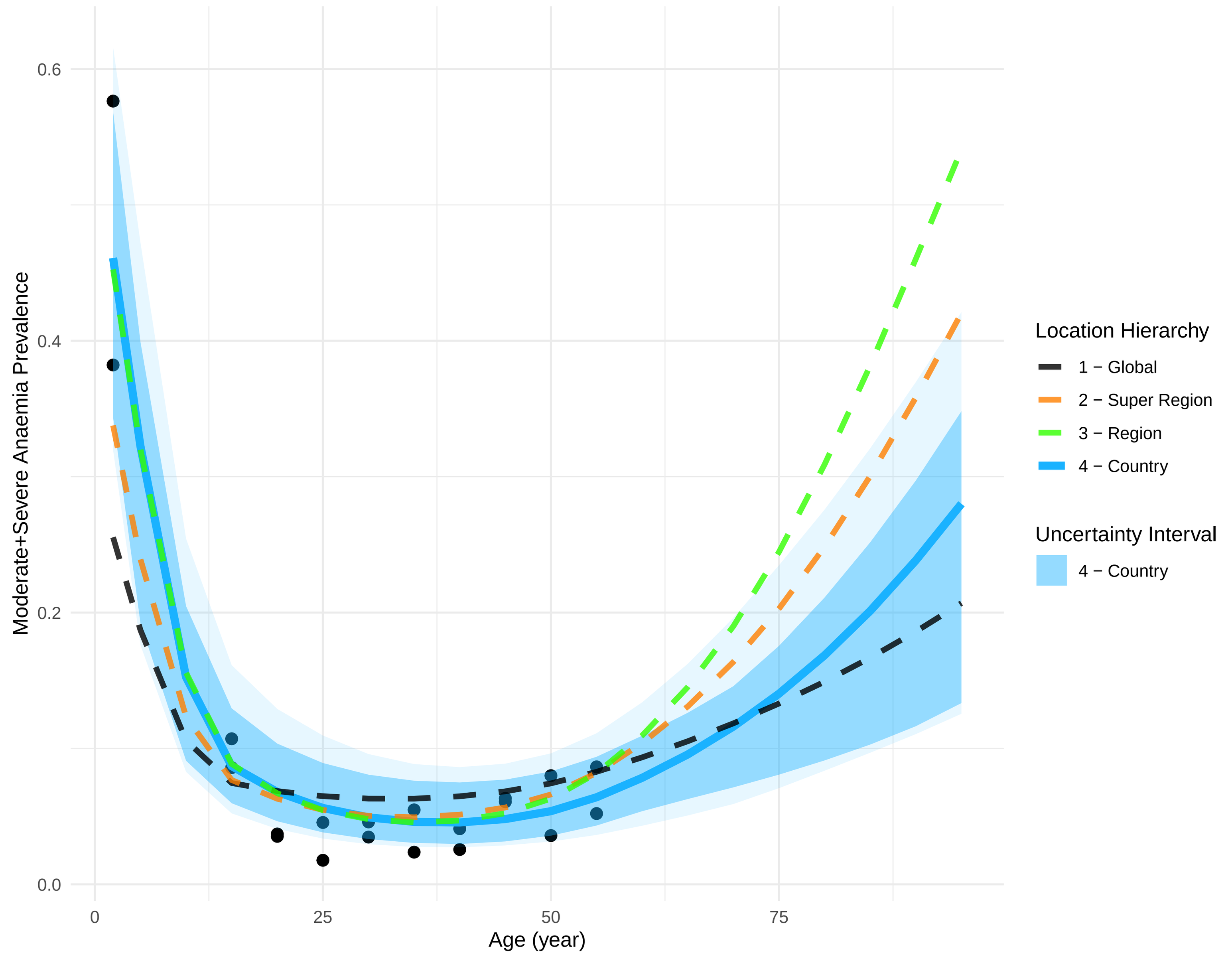

MR−BRT Age/Sex Fits − Males 2+ Years Old − Moderate+Severe Anaemia Prevalence − Nigeria
WHO−2001 Adjustment Method

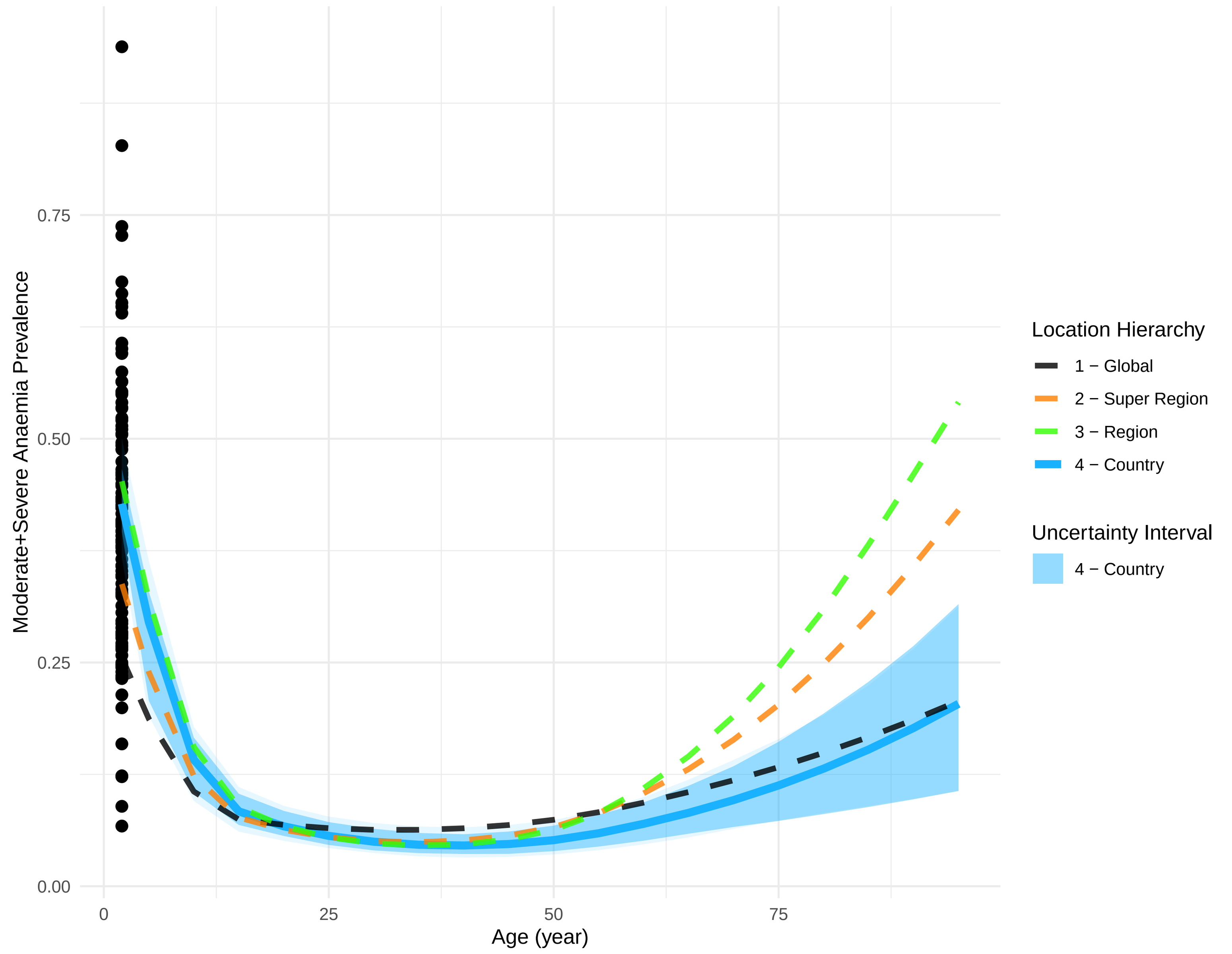

MR−BRT Age/Sex Fits − Males 2+ Years Old − Moderate+Severe Anaemia Prevalence − Abia
WHO−2001 Adjustment Method

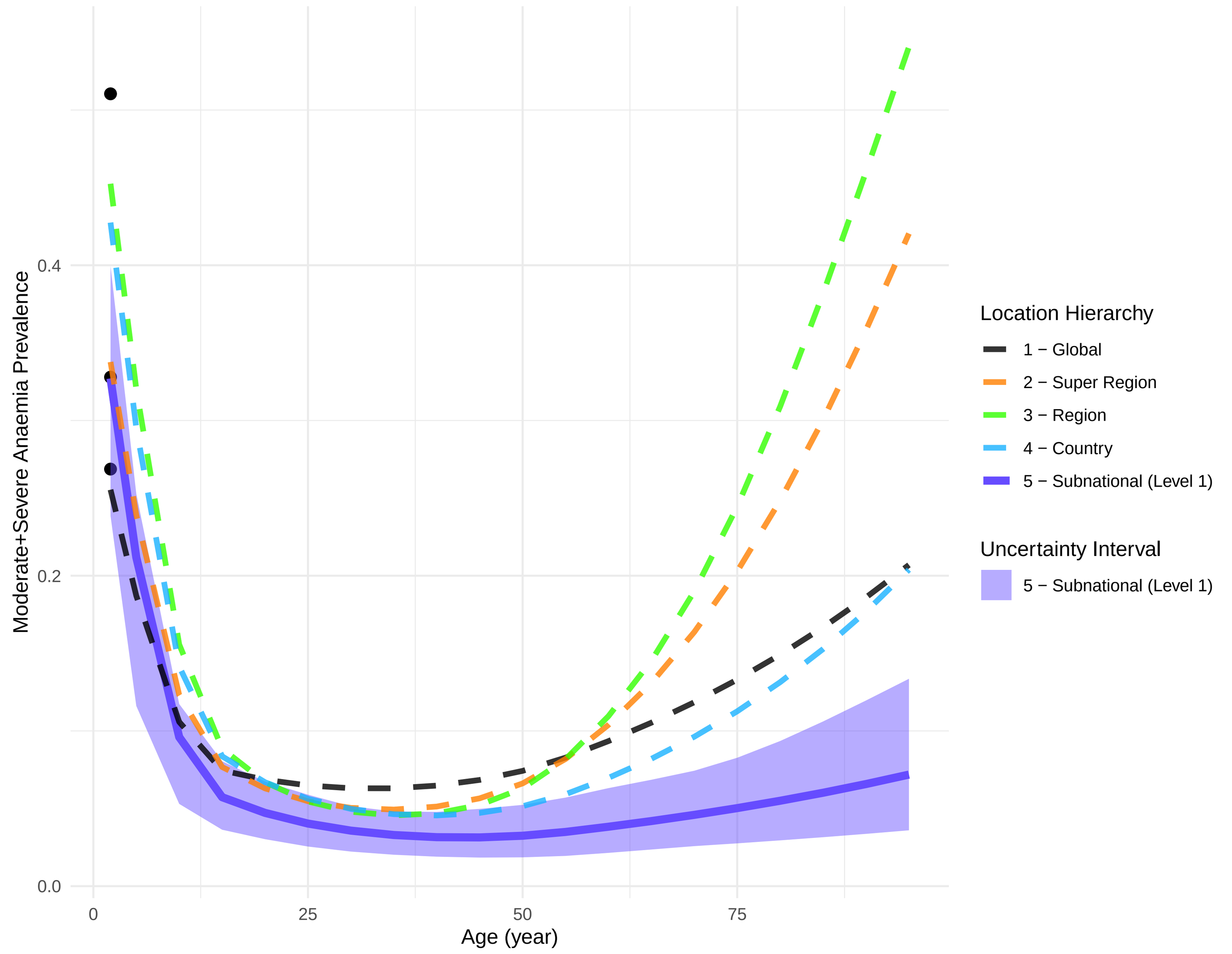

MR−BRT Age/Sex Fits − Males 2+ Years Old − Moderate+Severe Anaemia Prevalence − Adamawa
WHO−2001 Adjustment Method

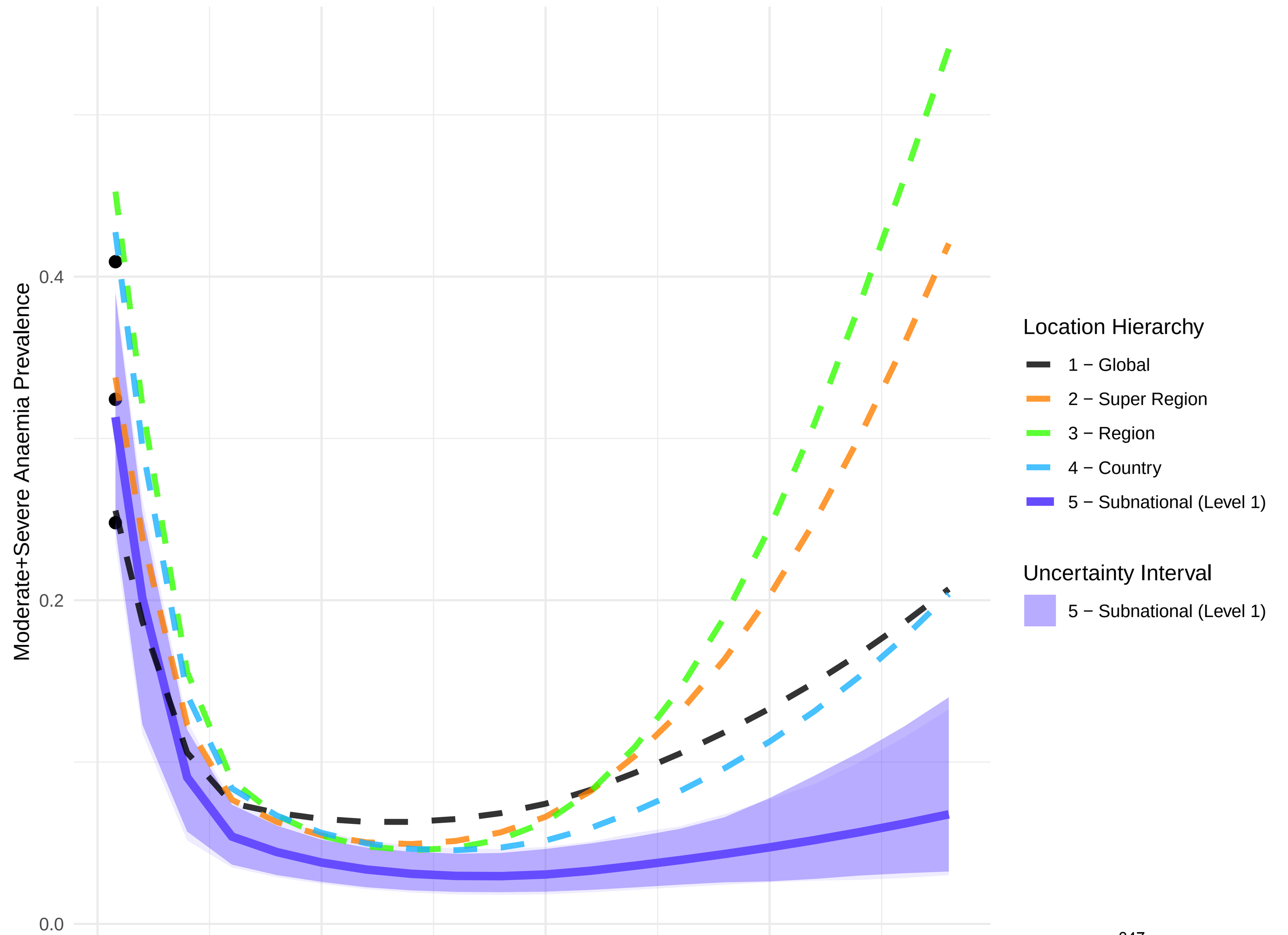

MR−BRT Age/Sex Fits − Males 2+ Years Old − Moderate+Severe Anaemia Prevalence − Akwa Ibom
WHO−2001 Adjustment Method

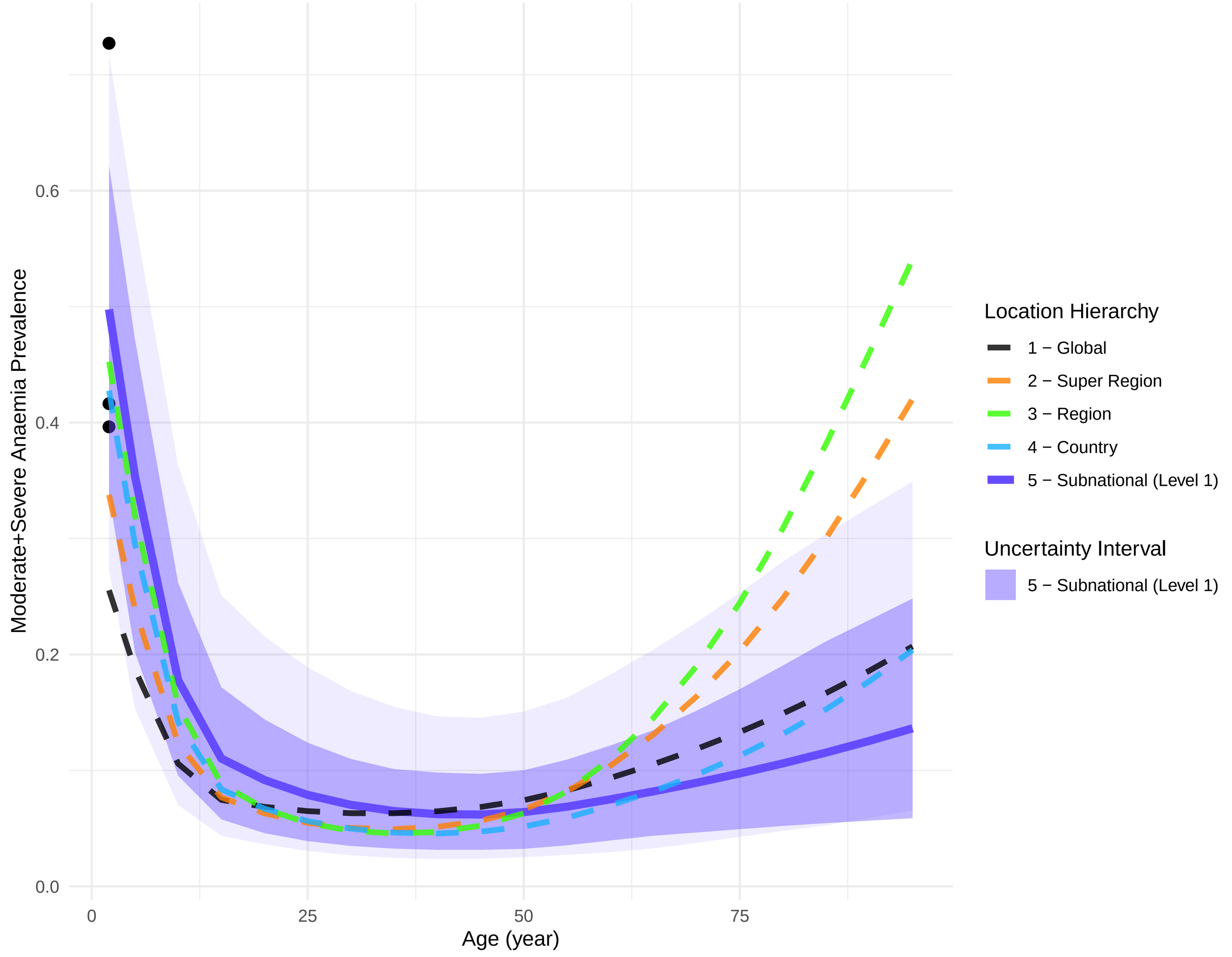

MR−BRT Age/Sex Fits − Males 2+ Years Old − Moderate+Severe Anaemia Prevalence − Anambra
WHO−2001 Adjustment Method

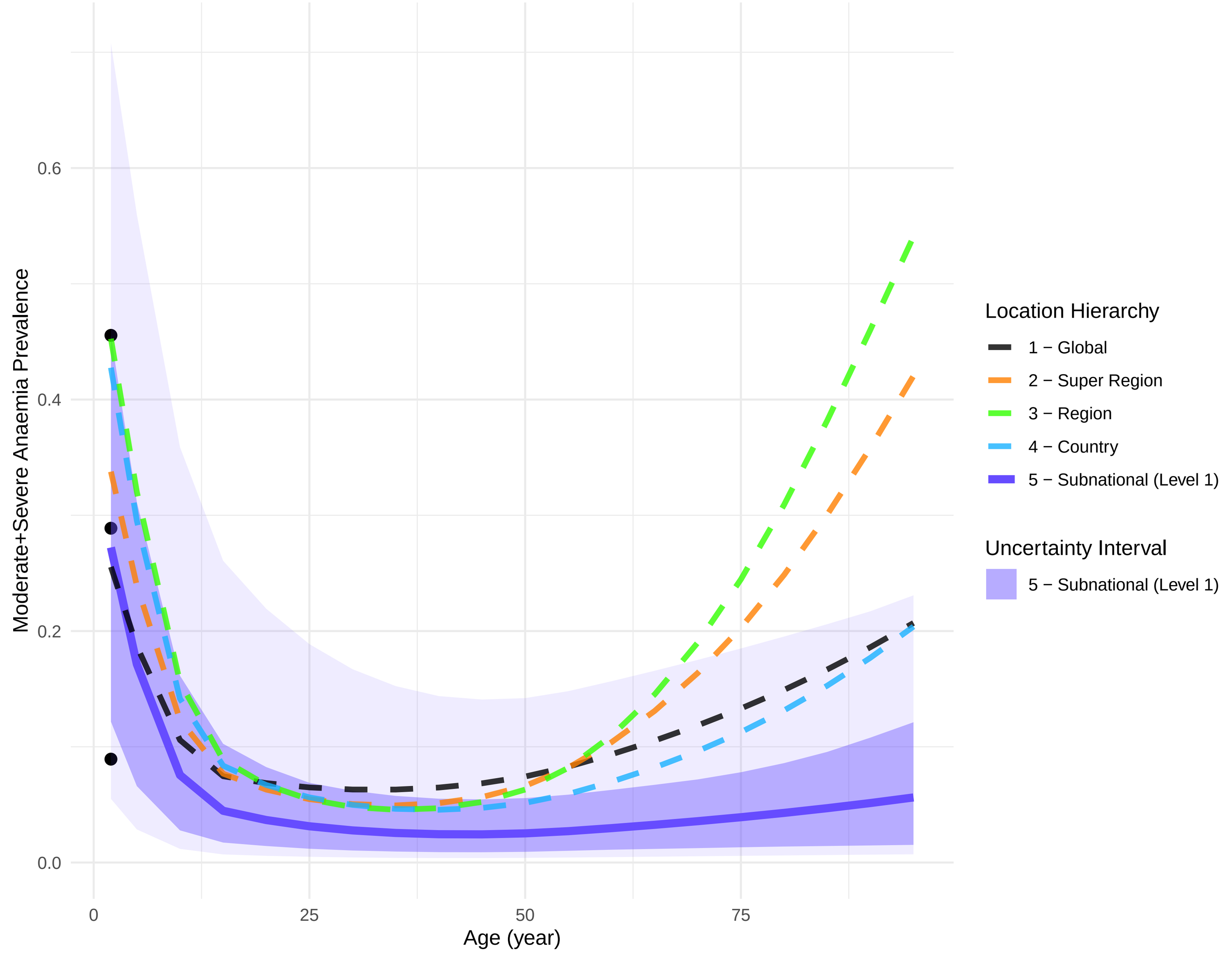

MR−BRT Age/Sex Fits − Males 2+ Years Old − Moderate+Severe Anaemia Prevalence − Bauchi
WHO−2001 Adjustment Method

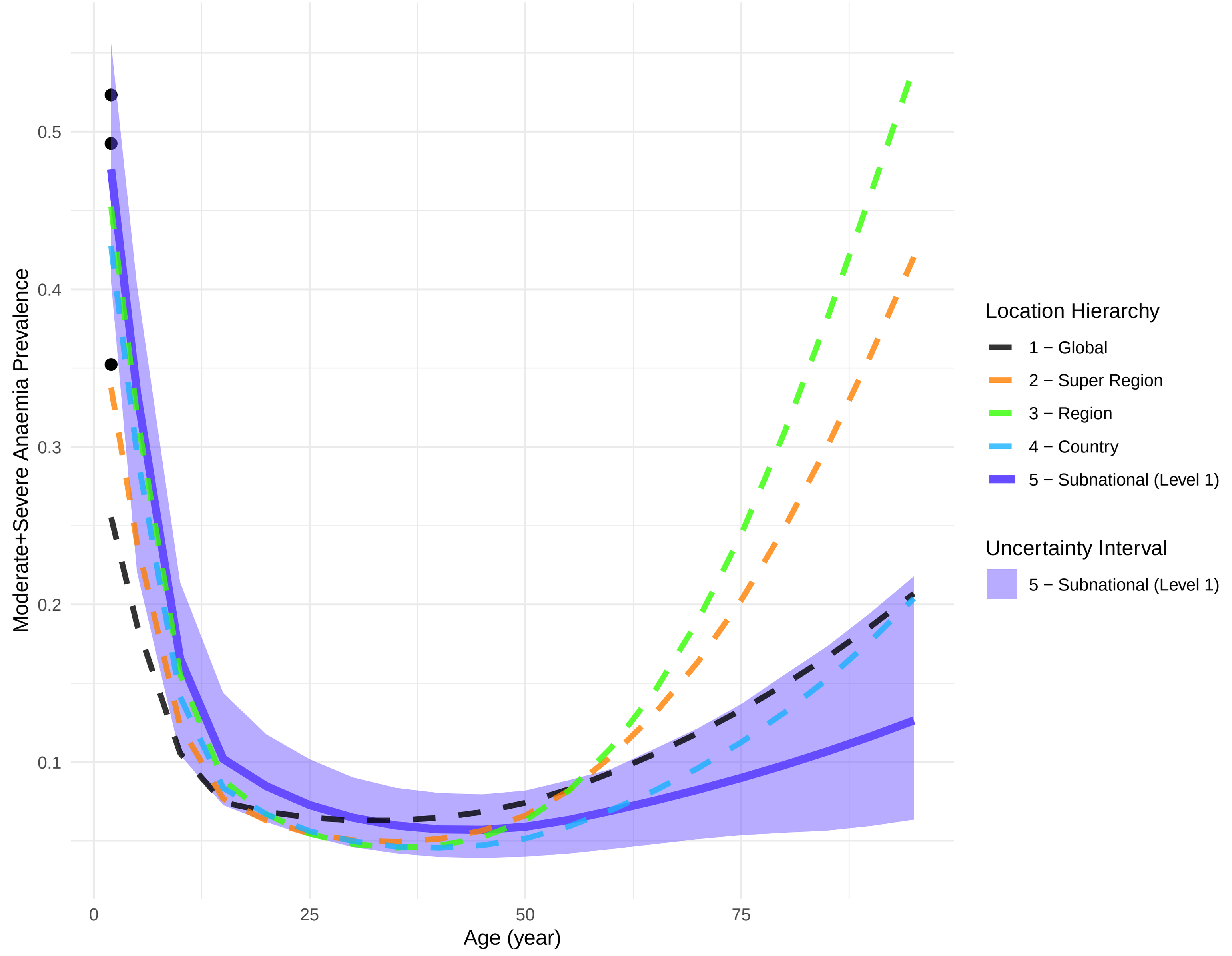

MR−BRT Age/Sex Fits − Males 2+ Years Old − Moderate+Severe Anaemia Prevalence − Bayelsa
WHO−2001 Adjustment Method

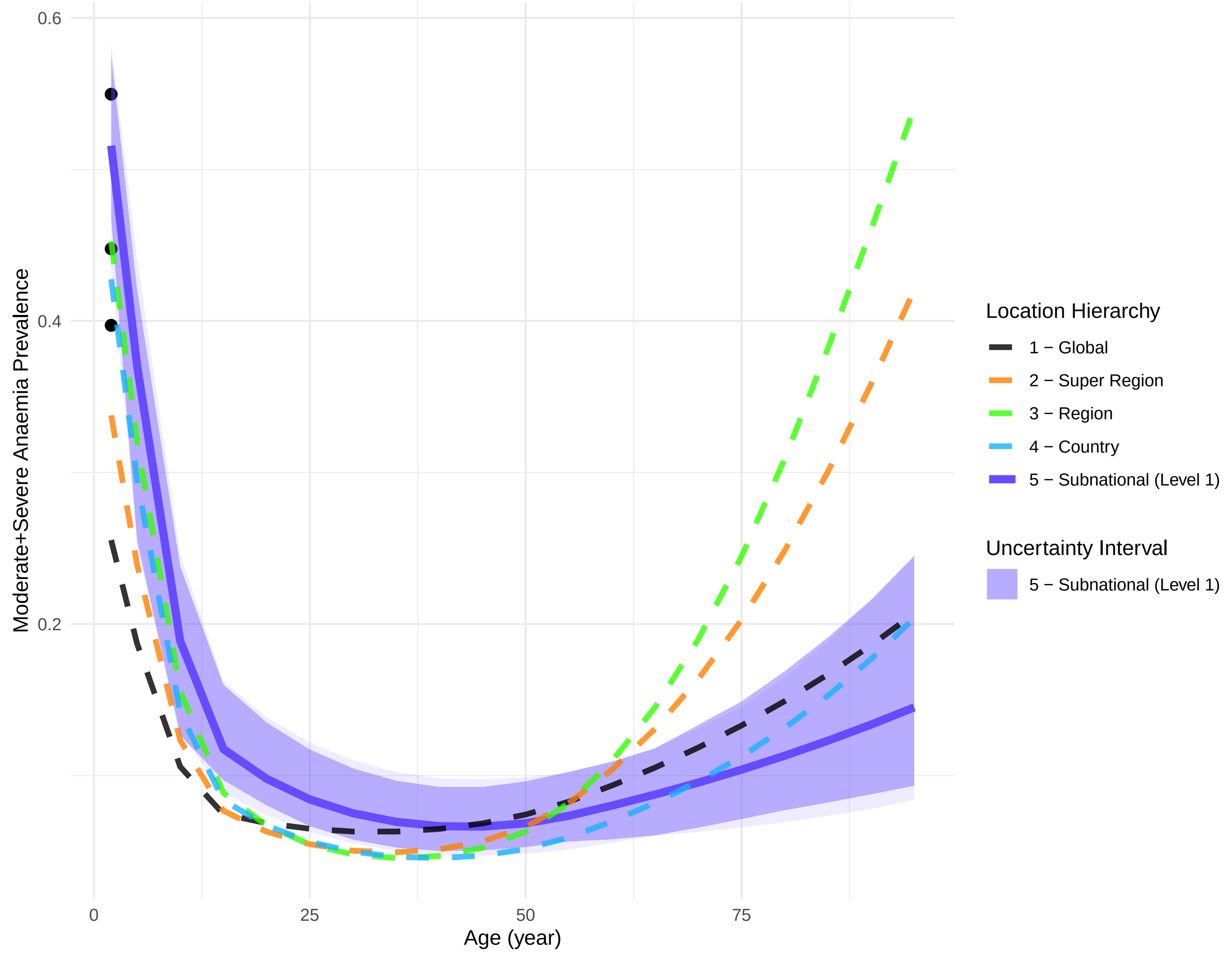

MR−BRT Age/Sex Fits − Males 2+ Years Old − Moderate+Severe Anaemia Prevalence − Benue
WHO−2001 Adjustment Method

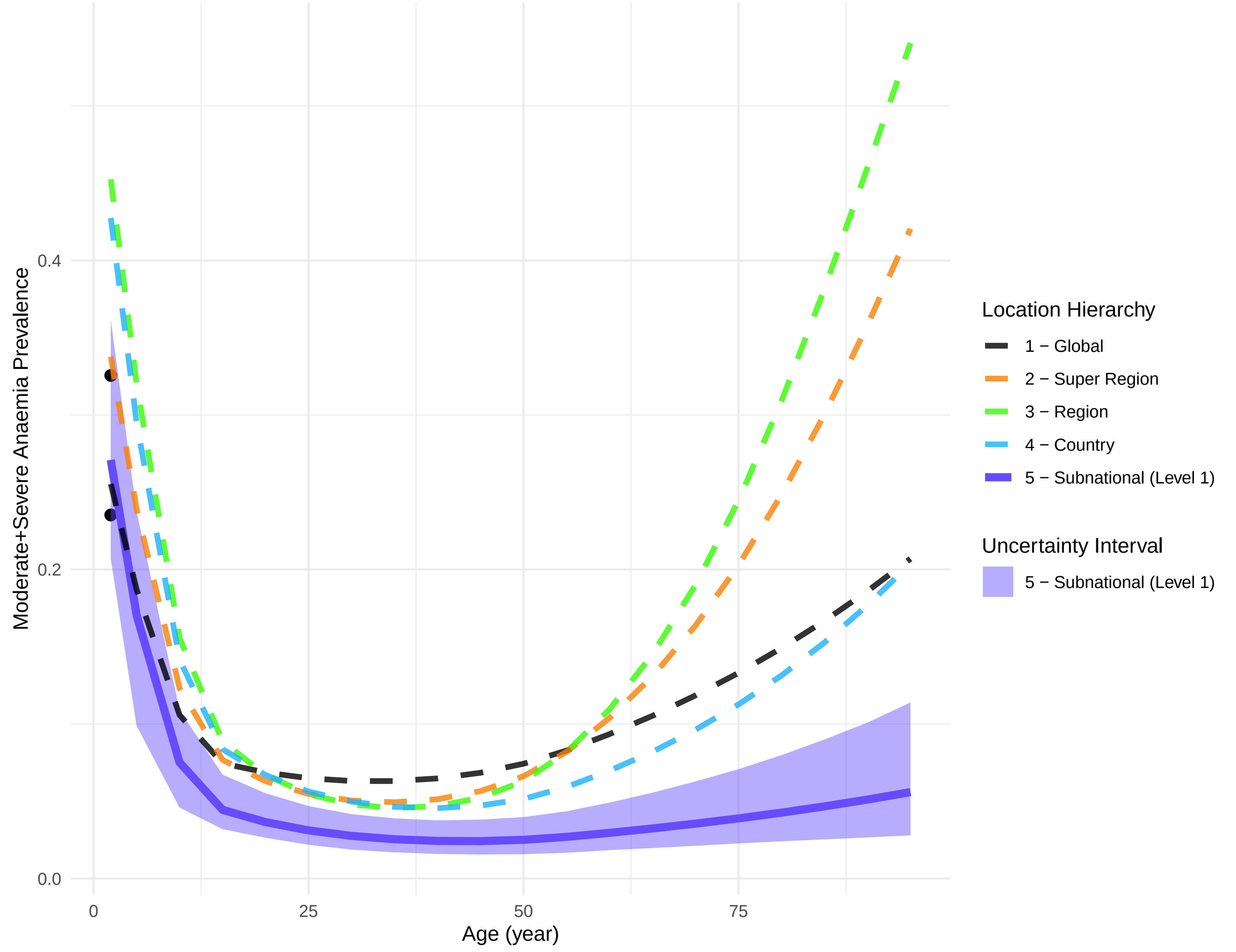

MR−BRT Age/Sex Fits − Males 2+ Years Old − Moderate+Severe Anaemia Prevalence − Borno
WHO−2001 Adjustment Method

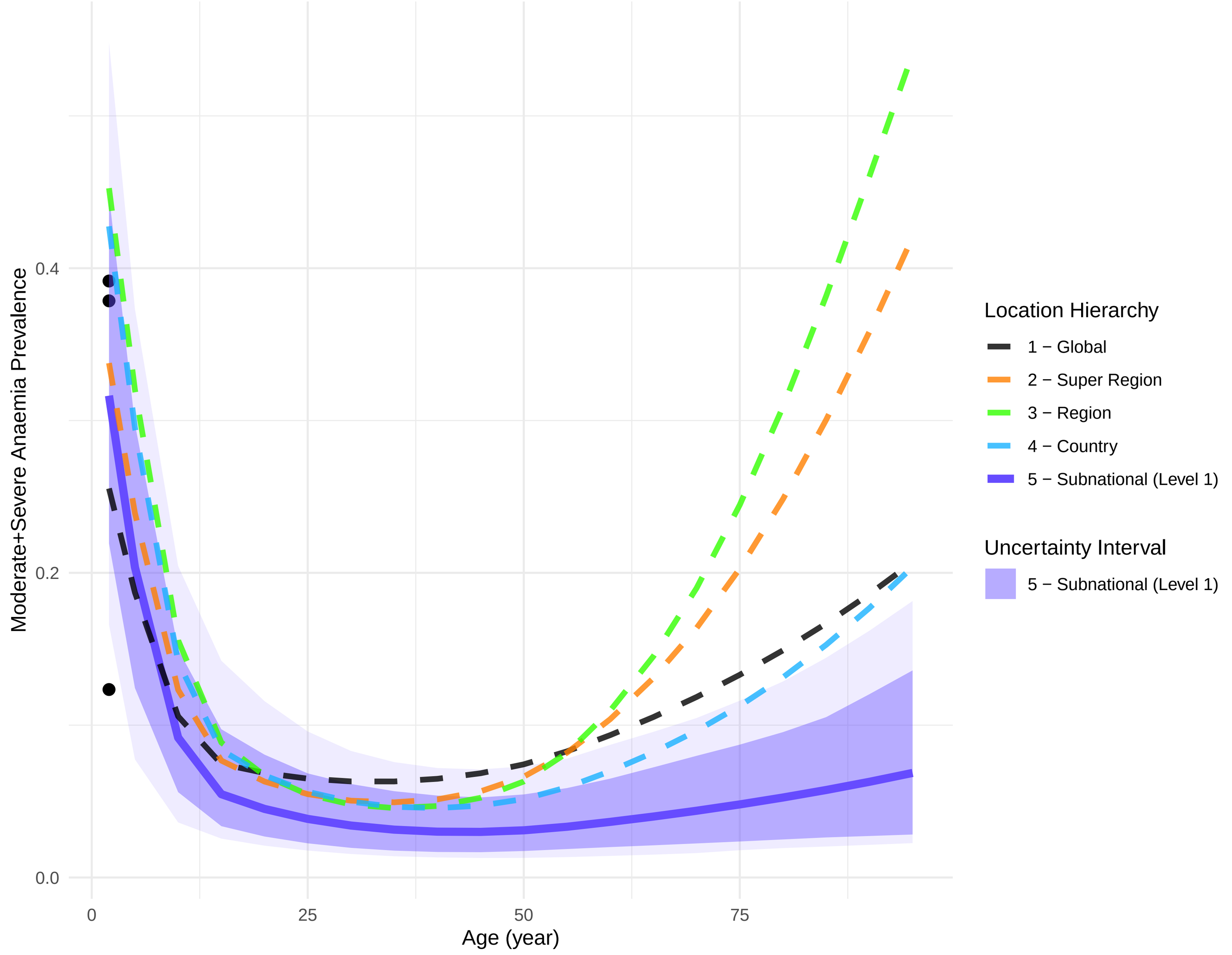

MR−BRT Age/Sex Fits − Males 2+ Years Old − Moderate+Severe Anaemia Prevalence − Cross River
WHO−2001 Adjustment Method

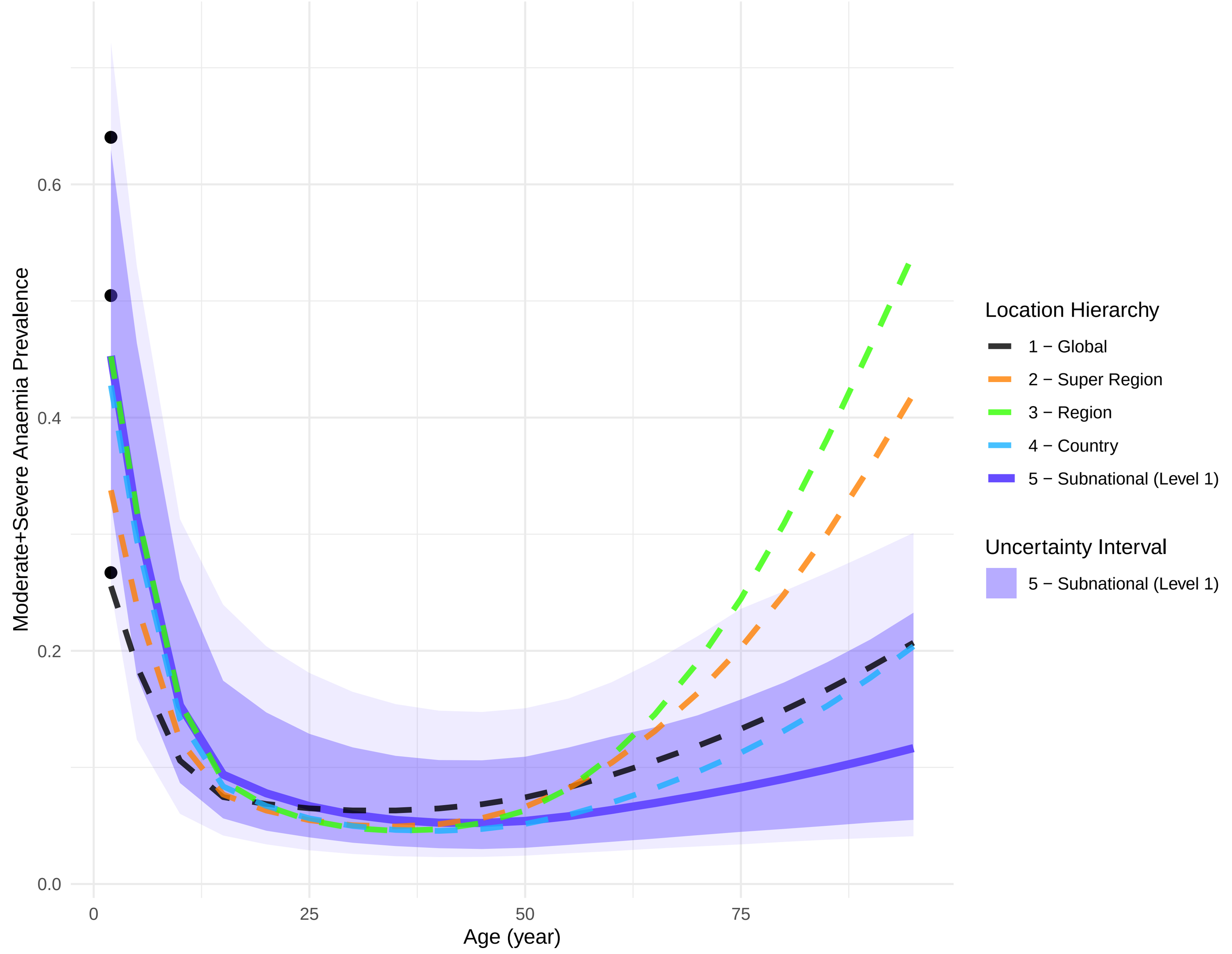

MR−BRT Age/Sex Fits − Males 2+ Years Old − Moderate+Severe Anaemia Prevalence − Delta
WHO−2001 Adjustment Method

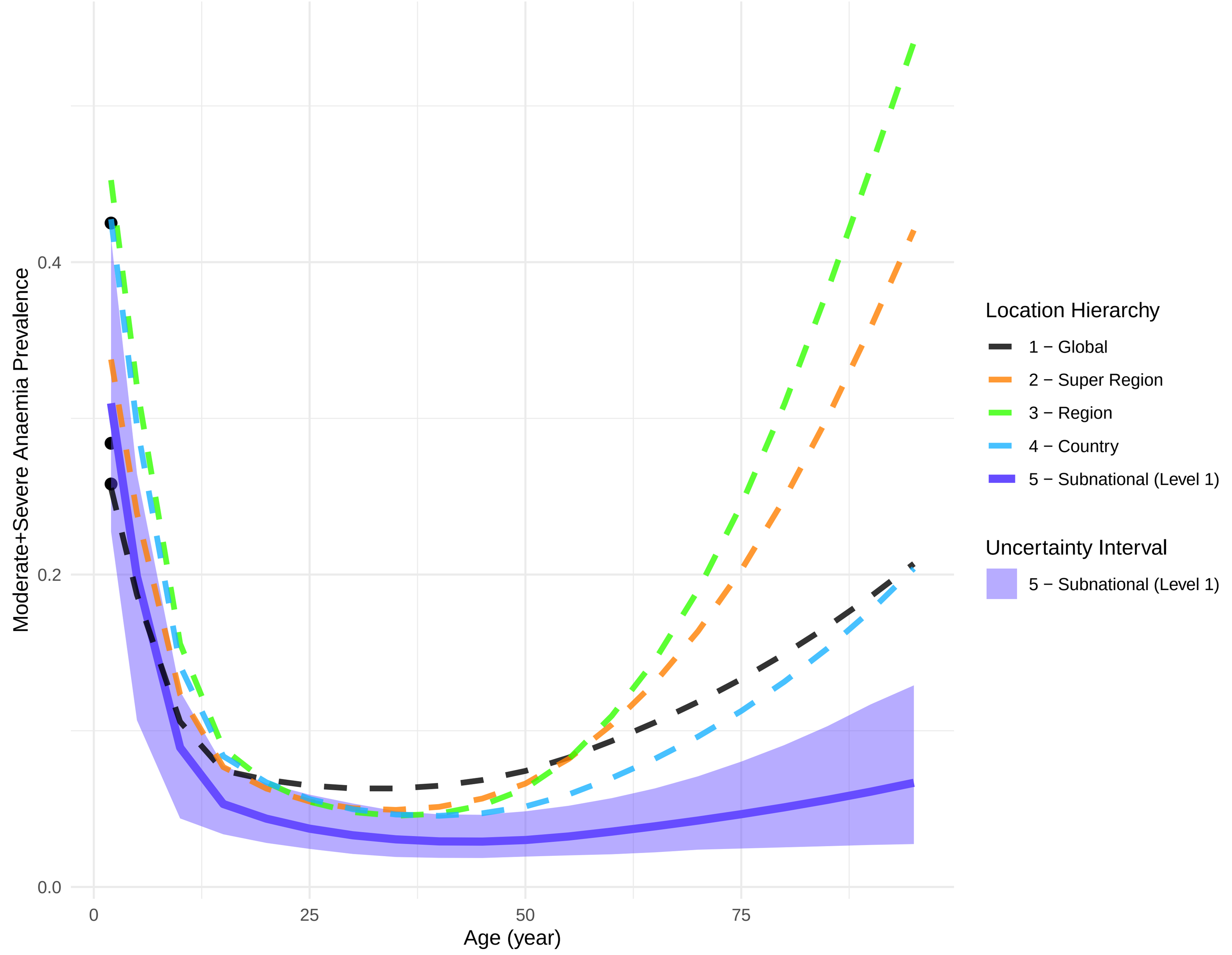

MR−BRT Age/Sex Fits − Males 2+ Years Old − Moderate+Severe Anaemia Prevalence − Ebonyi
WHO−2001 Adjustment Method

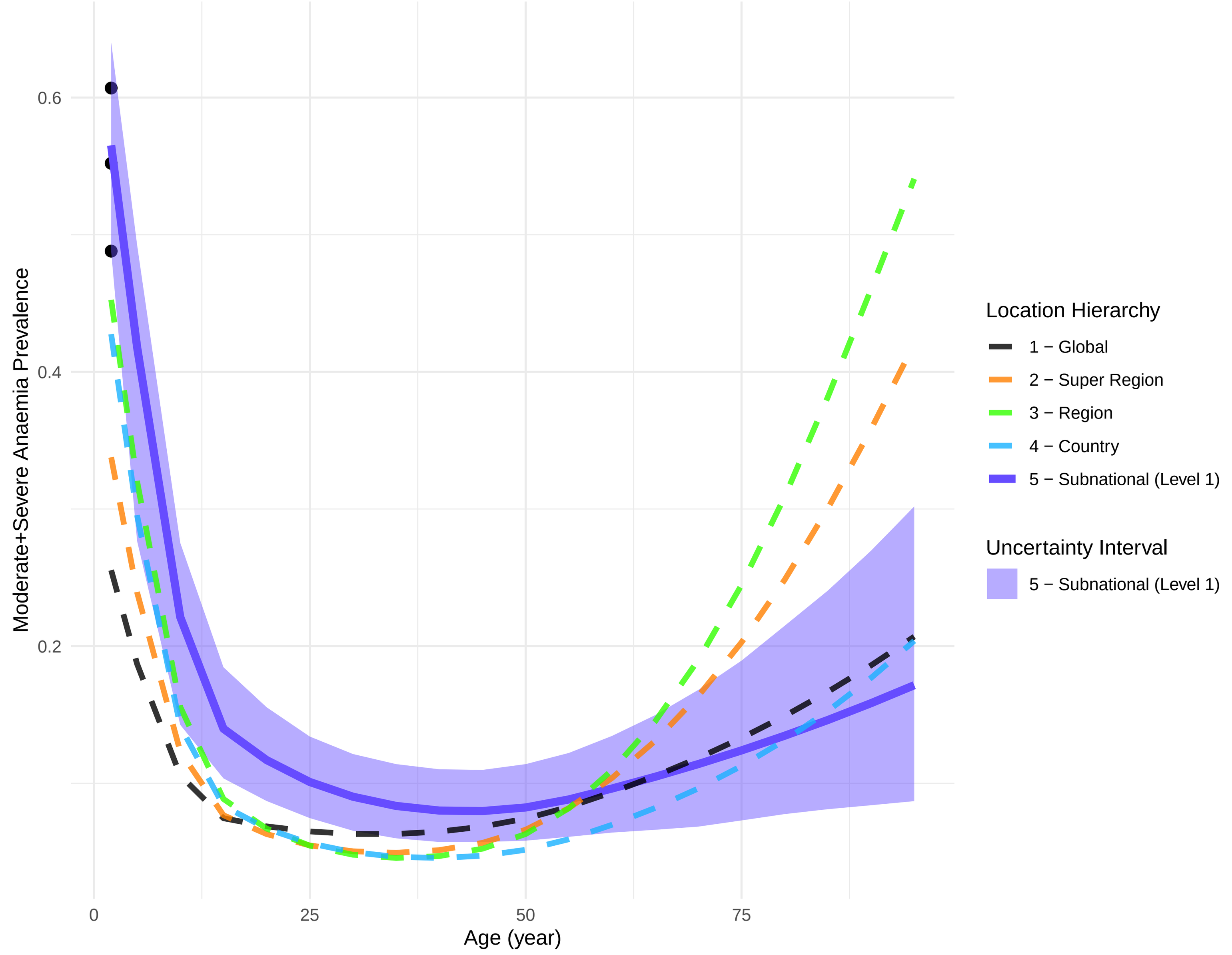

MR−BRT Age/Sex Fits − Males 2+ Years Old − Moderate+Severe Anaemia Prevalence − Edo
WHO−2001 Adjustment Method

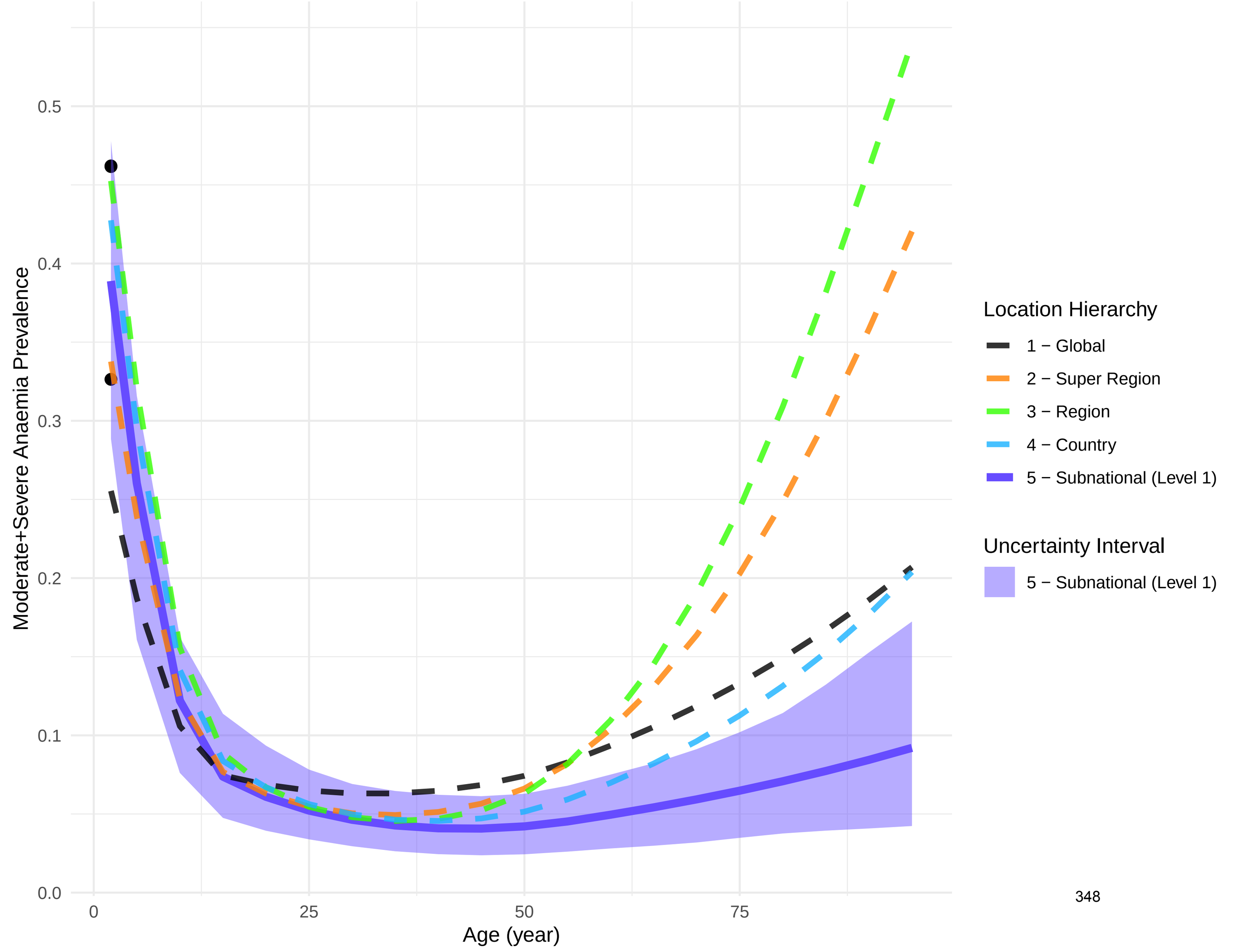

MR−BRT Age/Sex Fits − Males 2+ Years Old − Moderate+Severe Anaemia Prevalence − Ekiti
WHO−2001 Adjustment Method

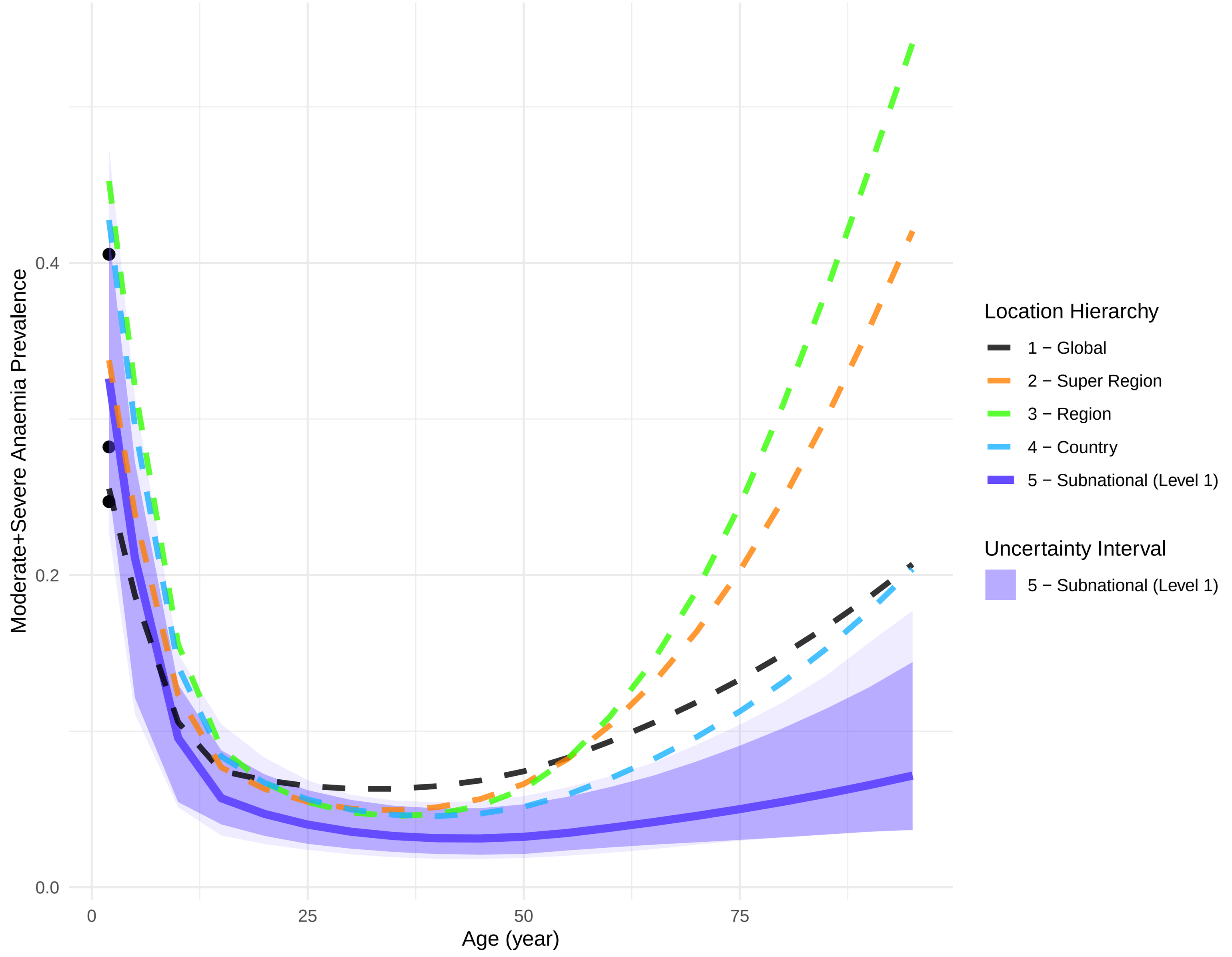

MR−BRT Age/Sex Fits − Males 2+ Years Old − Moderate+Severe Anaemia Prevalence − Enugu
WHO−2001 Adjustment Method

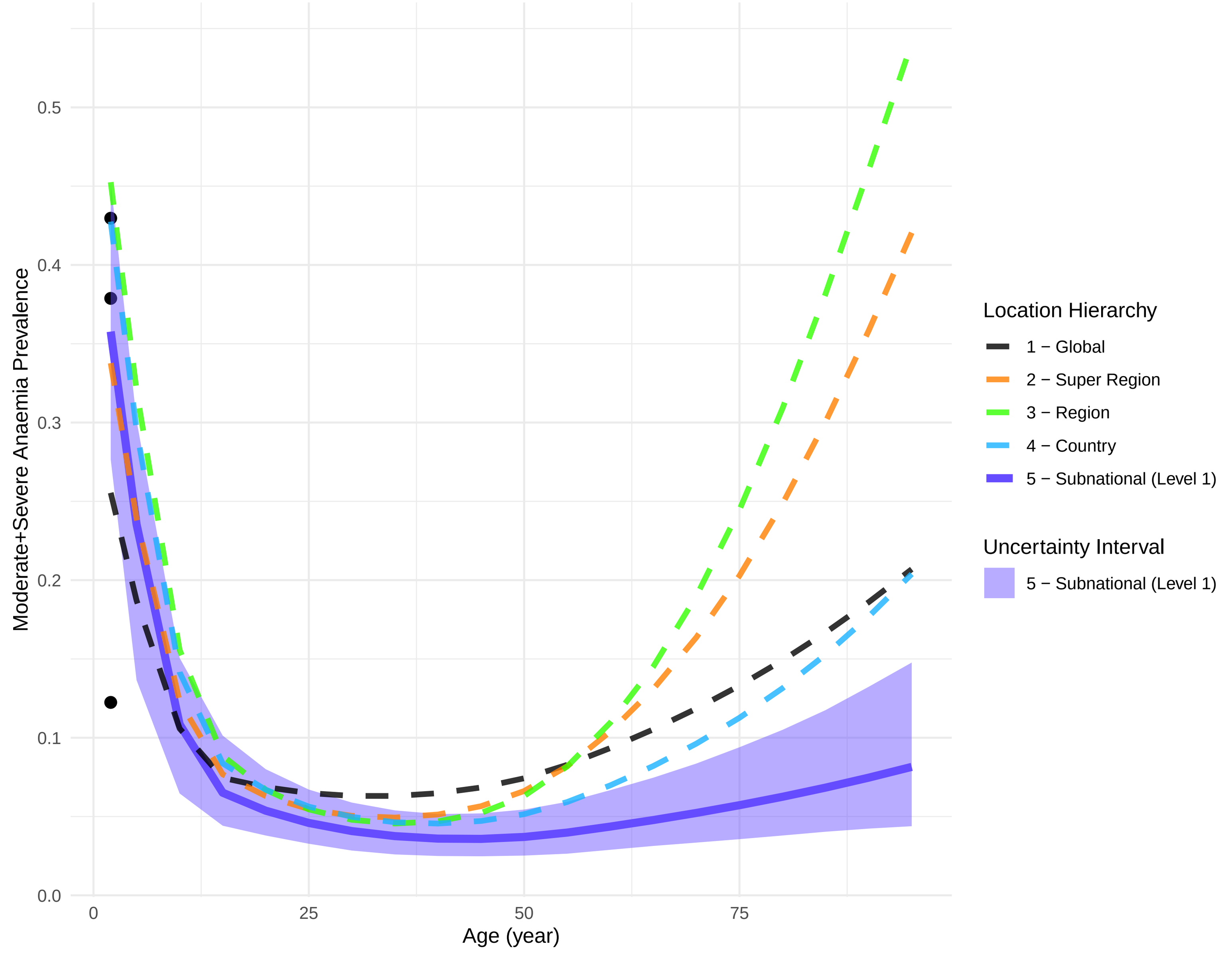

MR−BRT Age/Sex Fits − Males 2+ Years Old − Moderate+Severe Anaemia Prevalence − FCT (Abuja)
WHO−2001 Adjustment Method

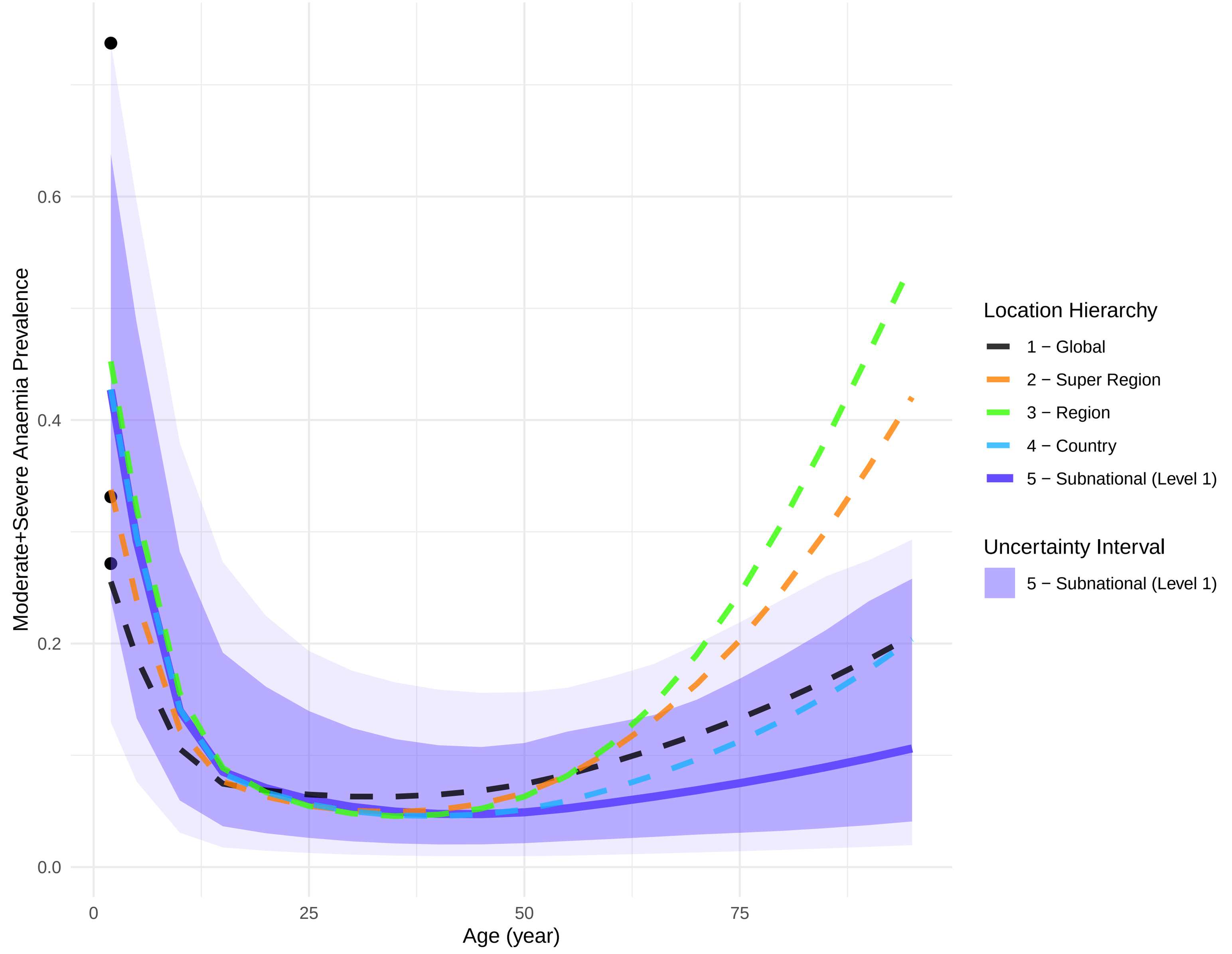

MR−BRT Age/Sex Fits − Males 2+ Years Old − Moderate+Severe Anaemia Prevalence − Gombe
WHO−2001 Adjustment Method

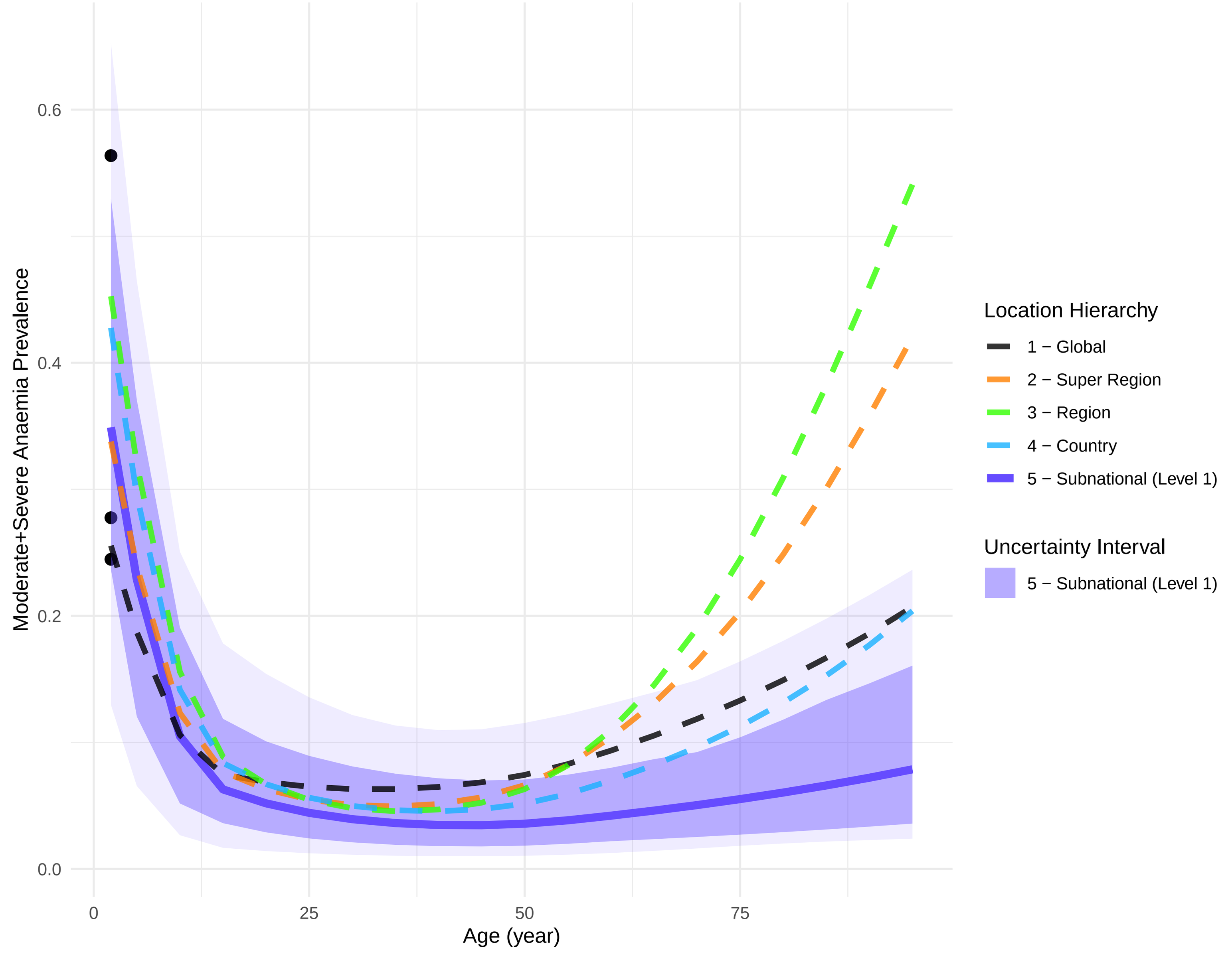

MR−BRT Age/Sex Fits − Males 2+ Years Old − Moderate+Severe Anaemia Prevalence − Imo
WHO−2001 Adjustment Method

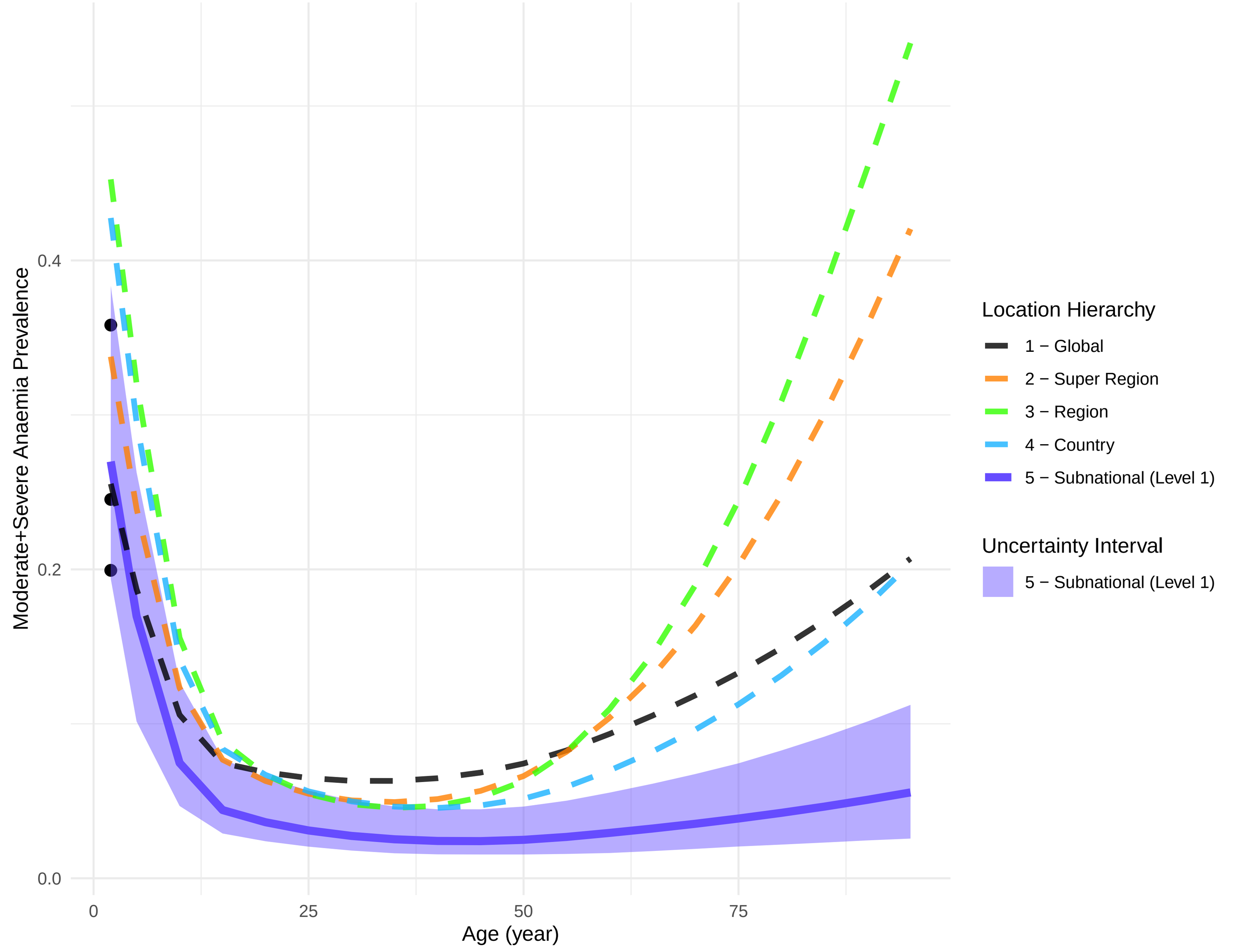

MR−BRT Age/Sex Fits − Males 2+ Years Old − Moderate+Severe Anaemia Prevalence − Jigawa
WHO−2001 Adjustment Method

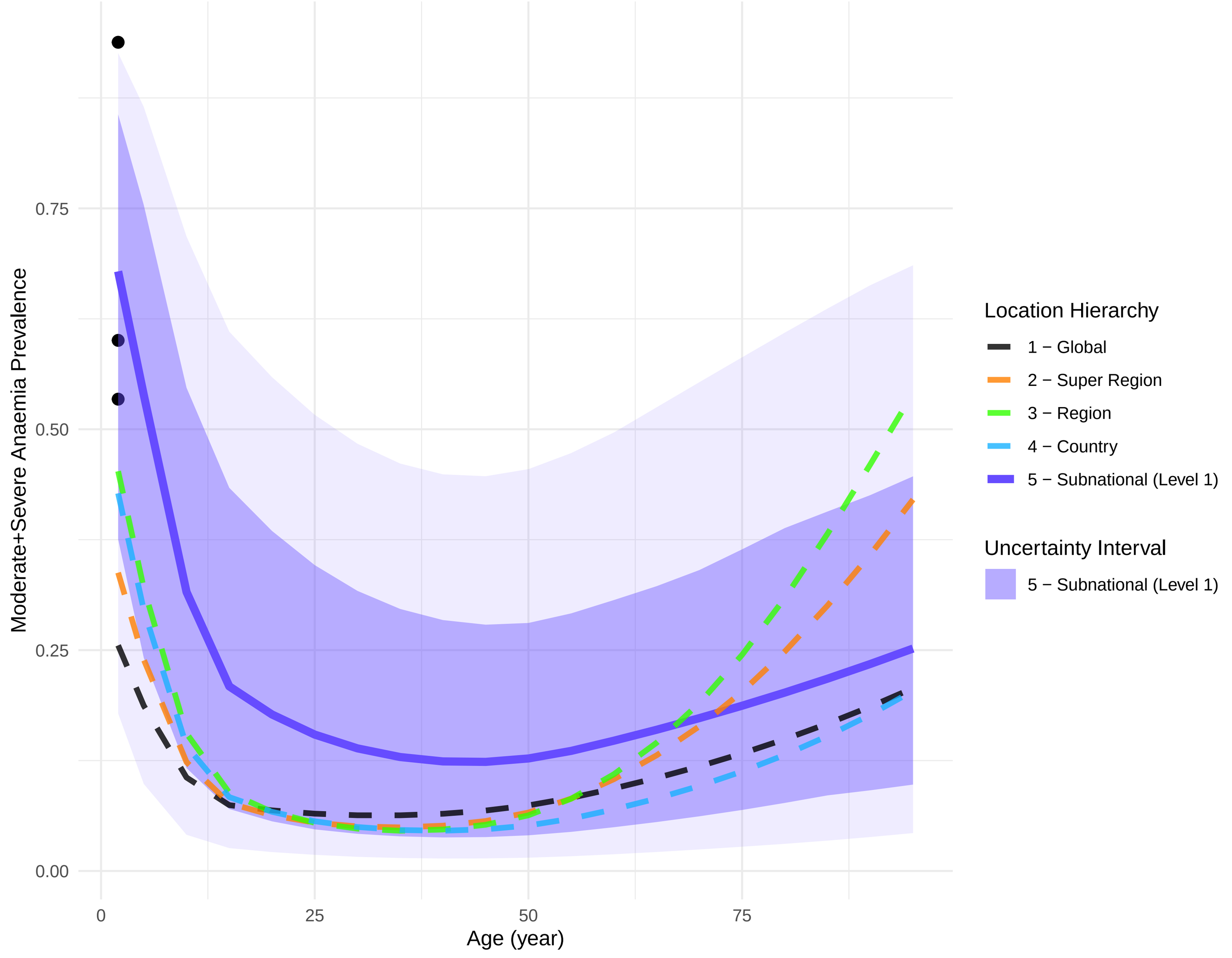

MR−BRT Age/Sex Fits − Males 2+ Years Old − Moderate+Severe Anaemia Prevalence − Kaduna
WHO−2001 Adjustment Method

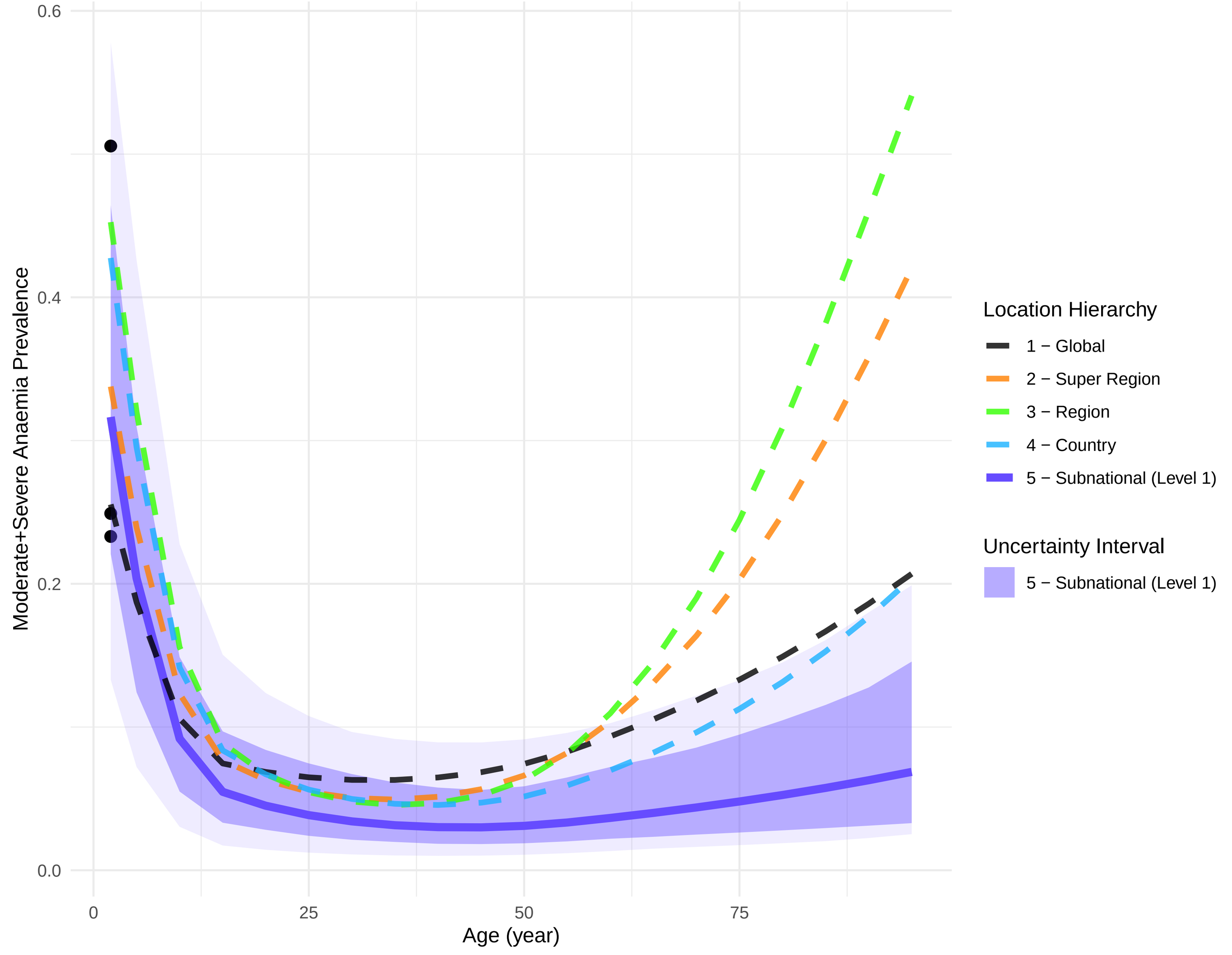

MR−BRT Age/Sex Fits − Males 2+ Years Old − Moderate+Severe Anaemia Prevalence − Kano
WHO−2001 Adjustment Method

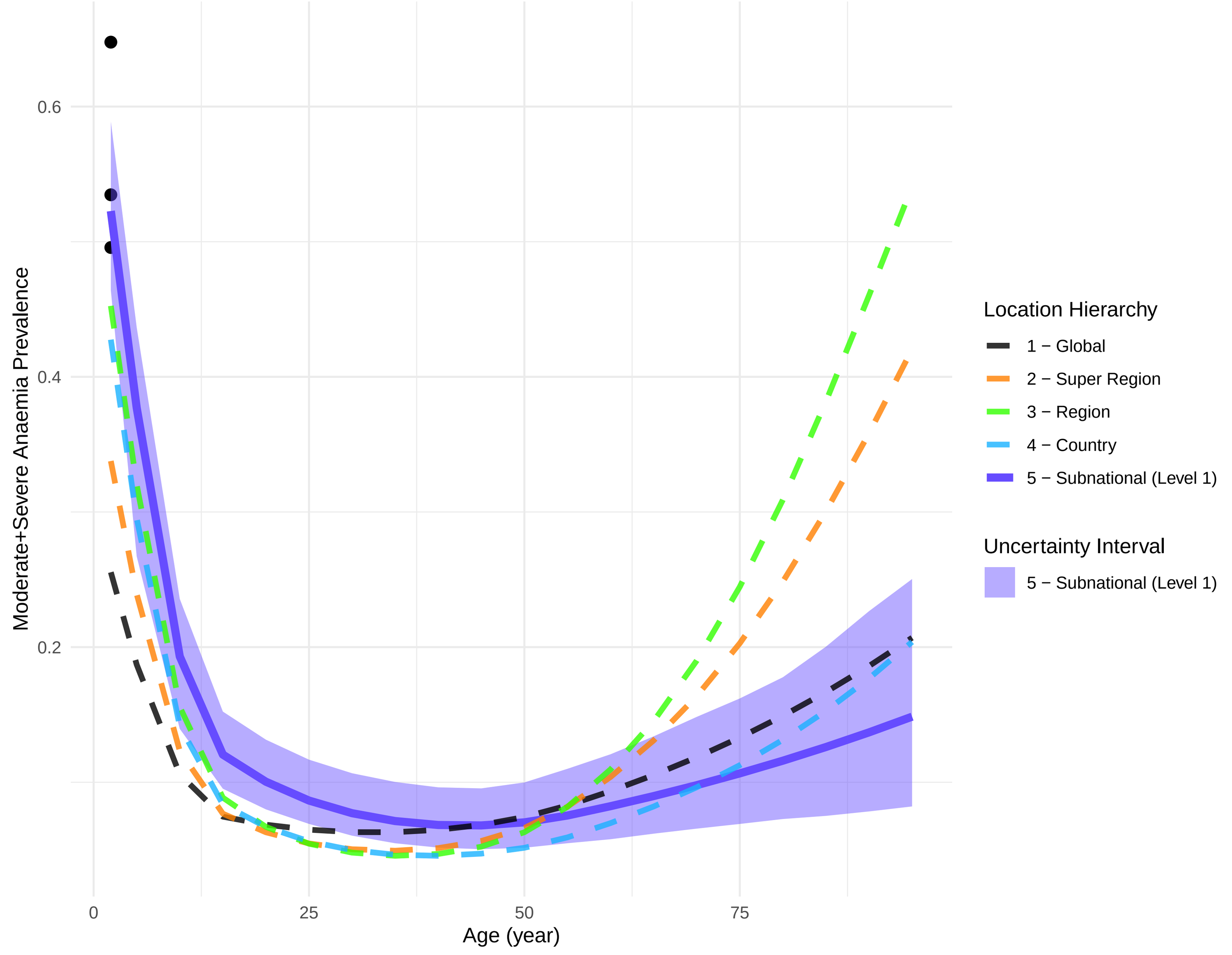

MR−BRT Age/Sex Fits − Males 2+ Years Old − Moderate+Severe Anaemia Prevalence − Katsina
WHO−2001 Adjustment Method

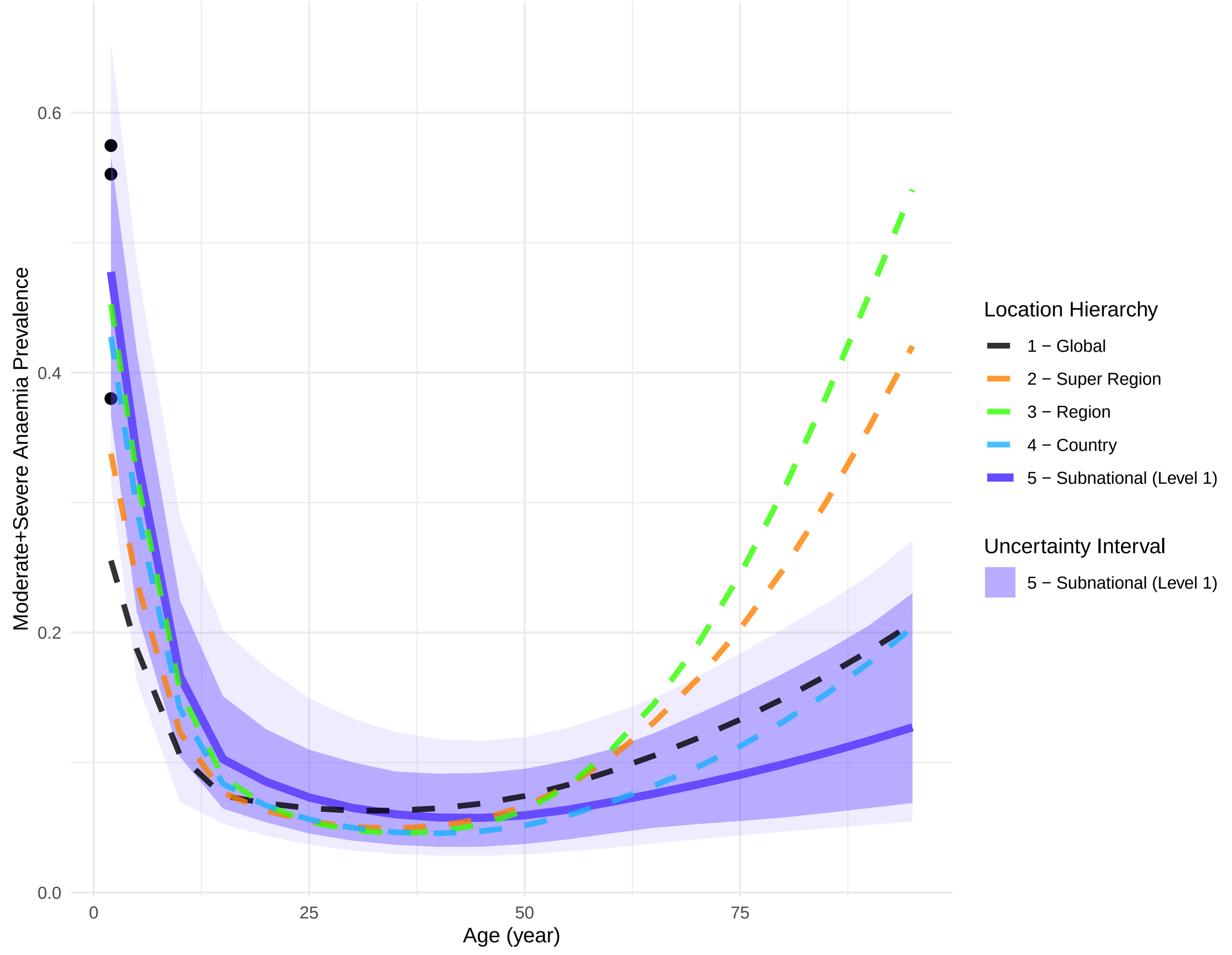

MR−BRT Age/Sex Fits − Males 2+ Years Old − Moderate+Severe Anaemia Prevalence − Kebbi
WHO−2001 Adjustment Method

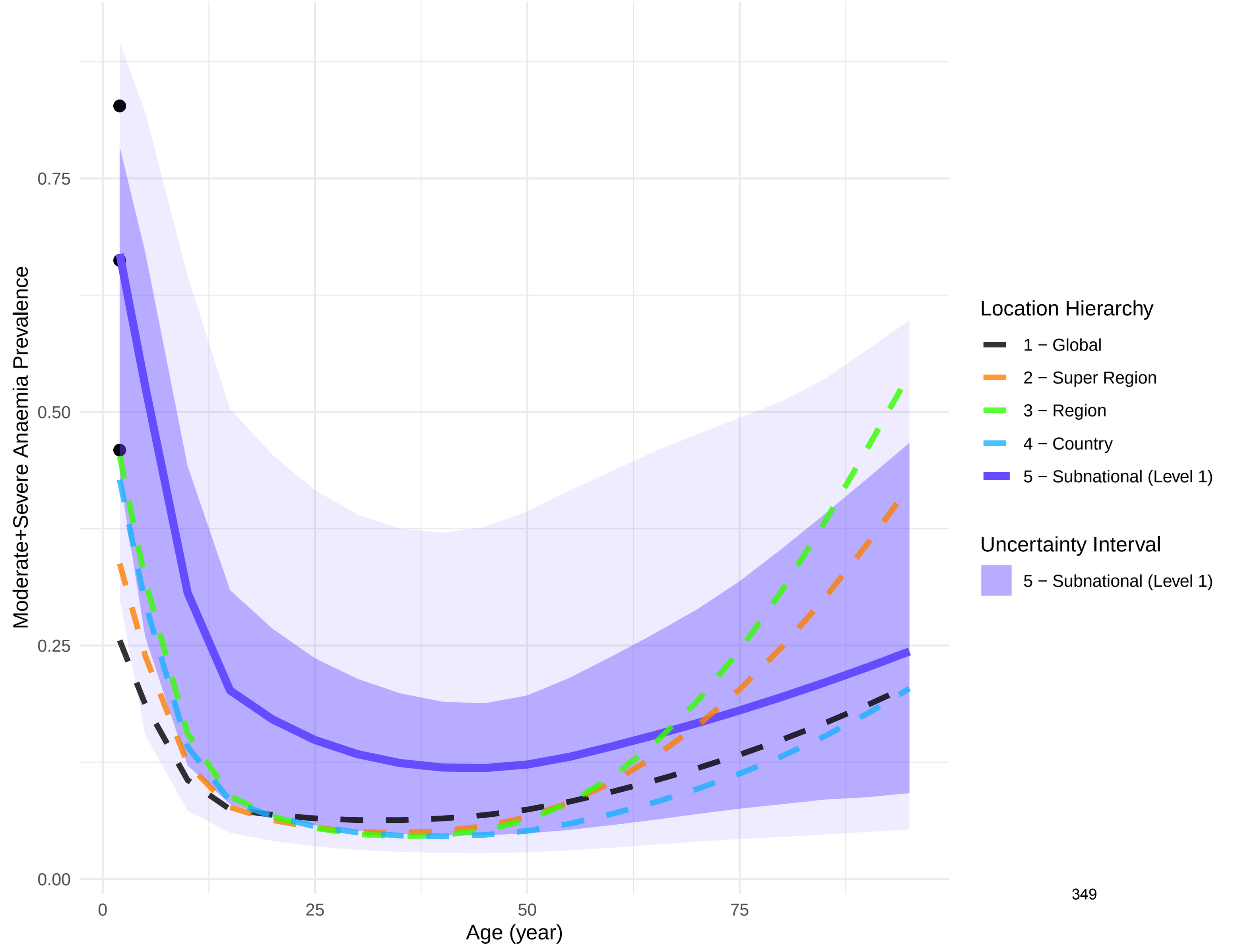

MR−BRT Age/Sex Fits − Males 2+ Years Old − Moderate+Severe Anaemia Prevalence − Kogi
WHO−2001 Adjustment Method

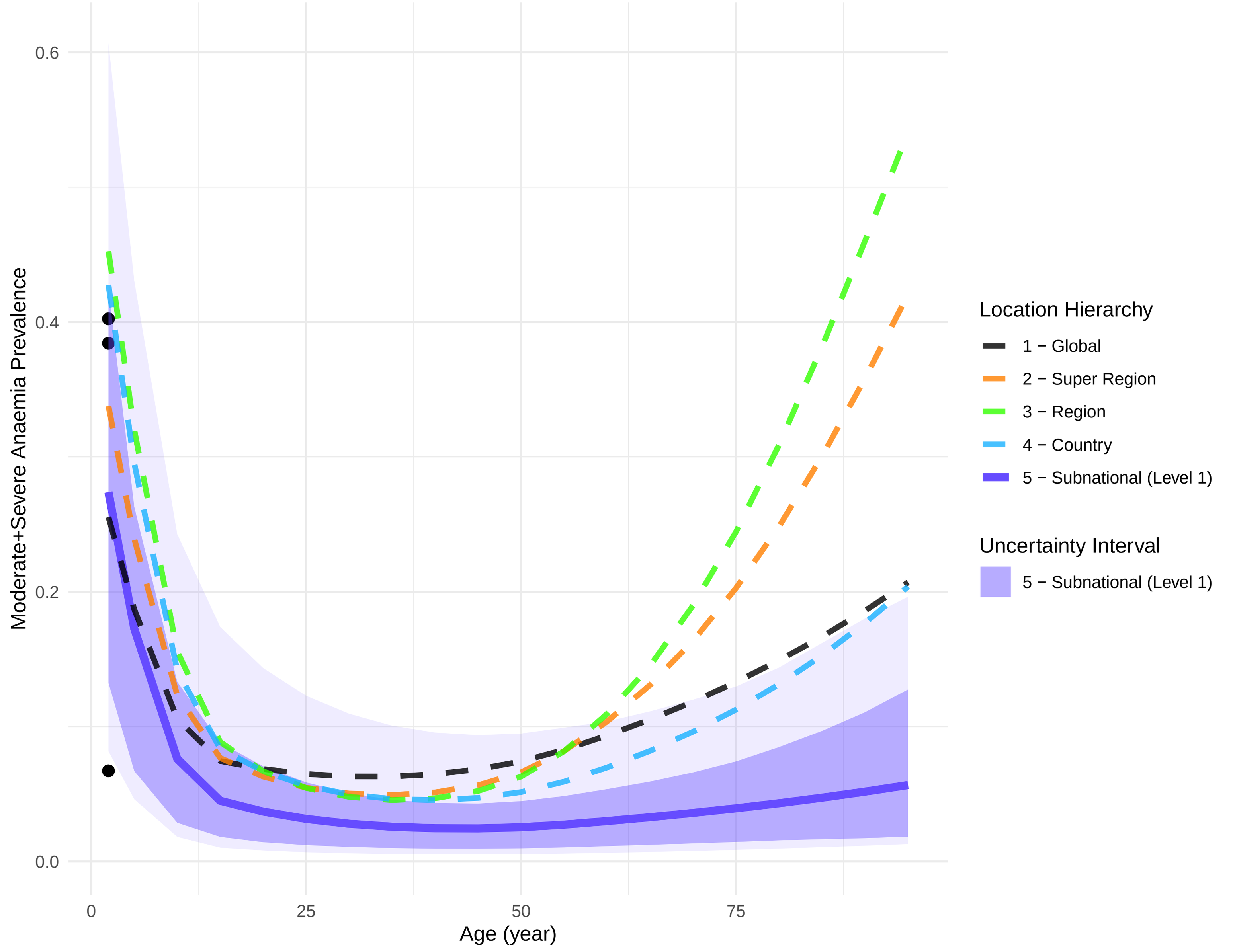

MR−BRT Age/Sex Fits − Males 2+ Years Old − Moderate+Severe Anaemia Prevalence − Kwara
WHO−2001 Adjustment Method

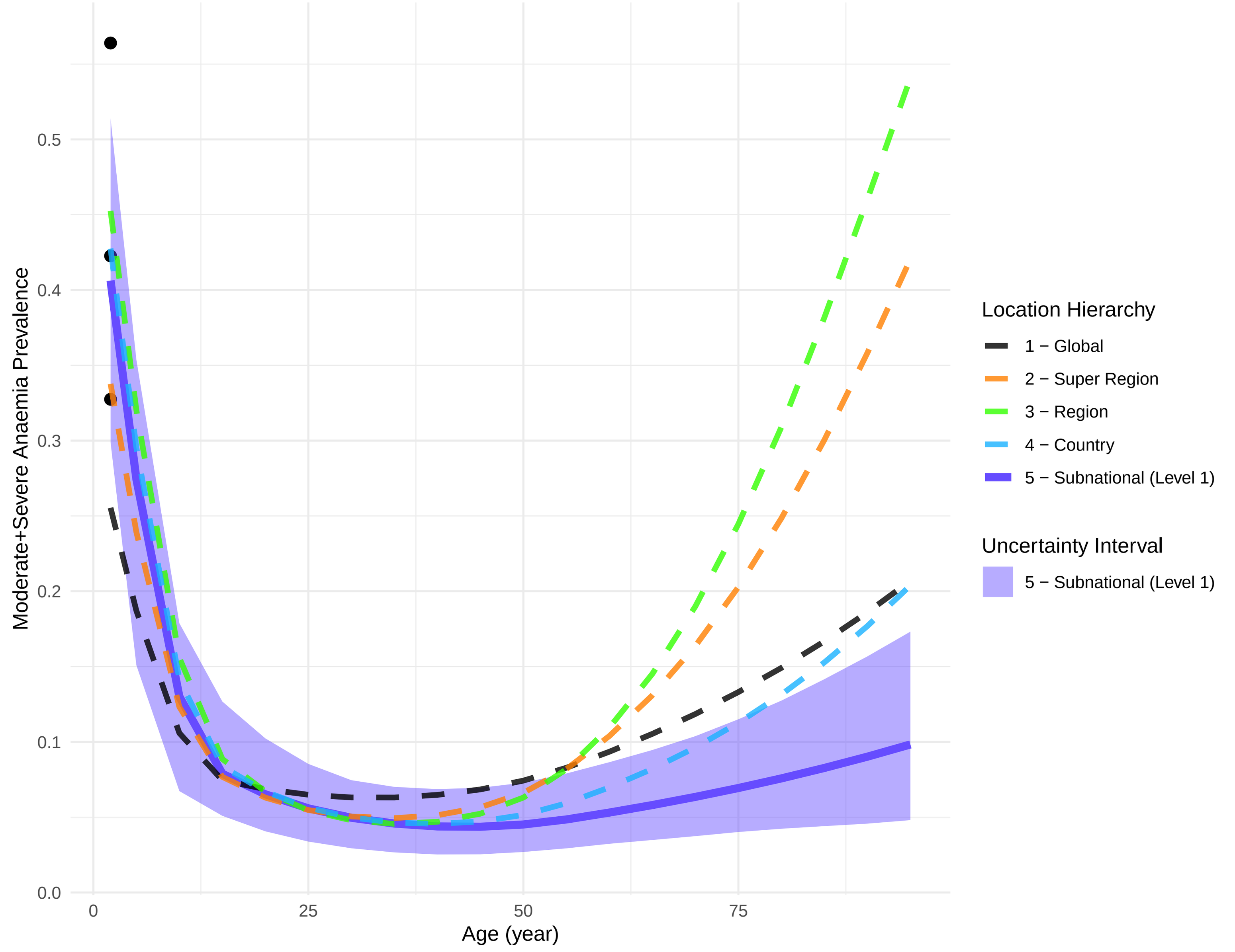

MR−BRT Age/Sex Fits − Males 2+ Years Old − Moderate+Severe Anaemia Prevalence − Lagos
WHO−2001 Adjustment Method

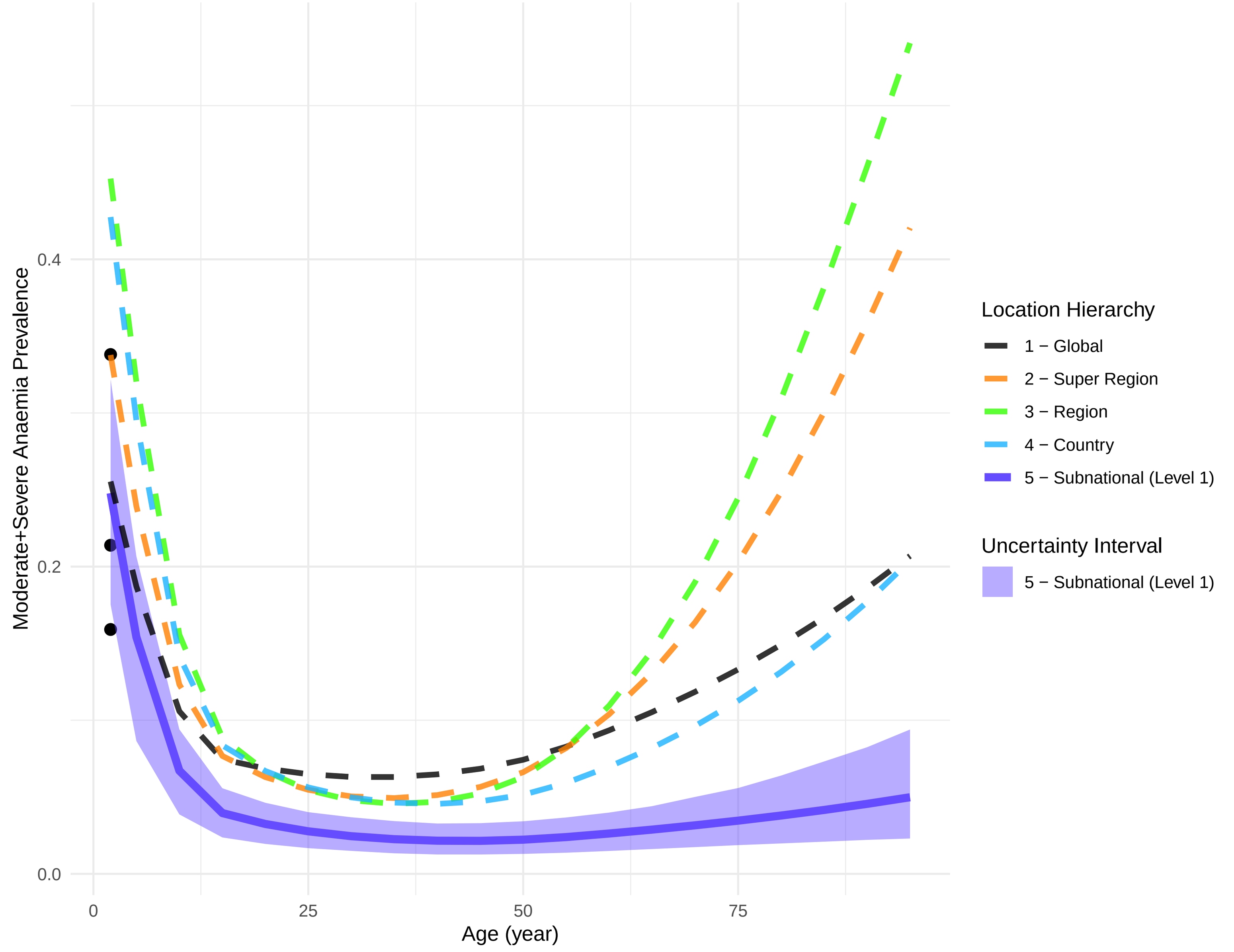

MR−BRT Age/Sex Fits − Males 2+ Years Old − Moderate+Severe Anaemia Prevalence − Nasarawa
WHO−2001 Adjustment Method

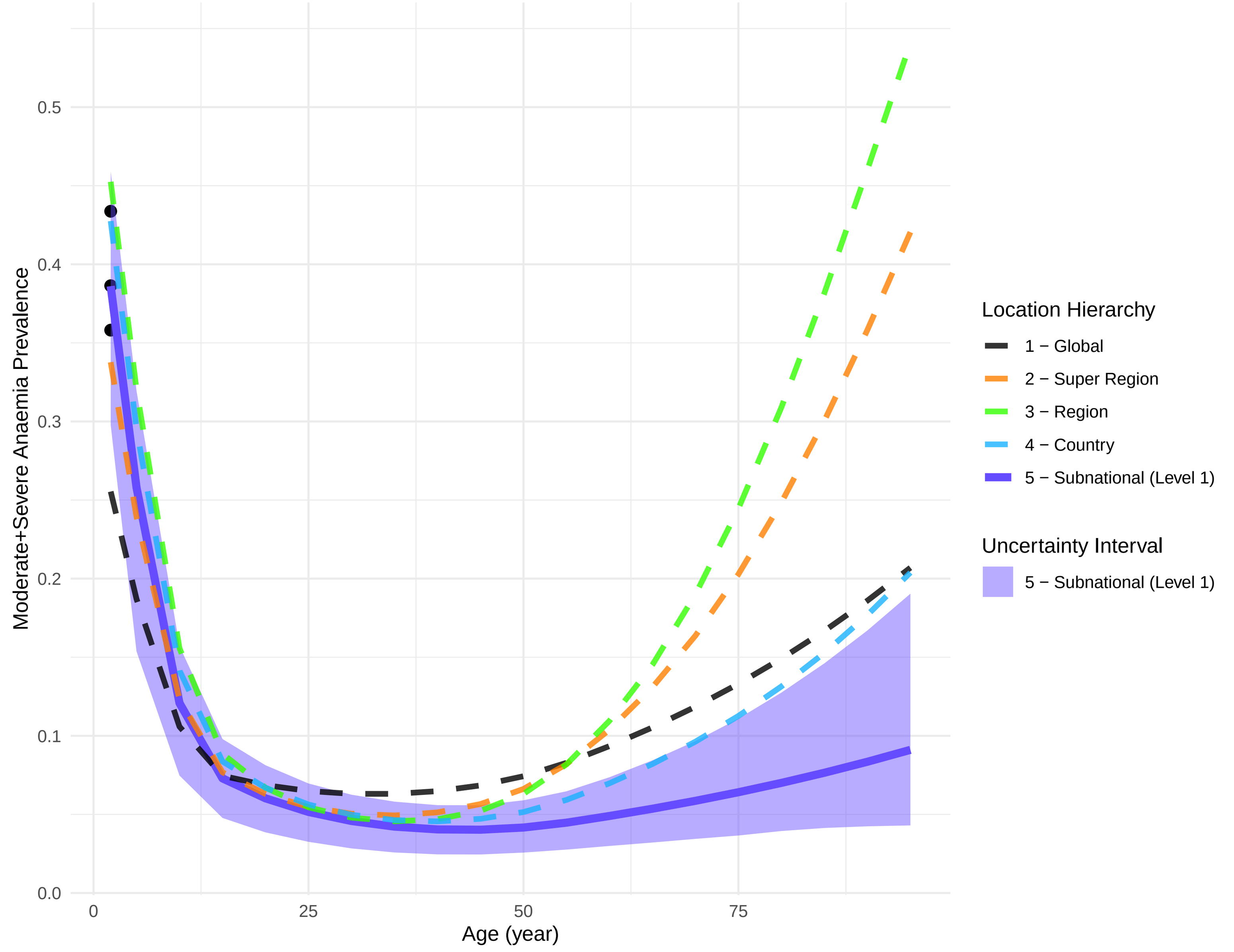

MR−BRT Age/Sex Fits − Males 2+ Years Old − Moderate+Severe Anaemia Prevalence − Niger
WHO−2001 Adjustment Method

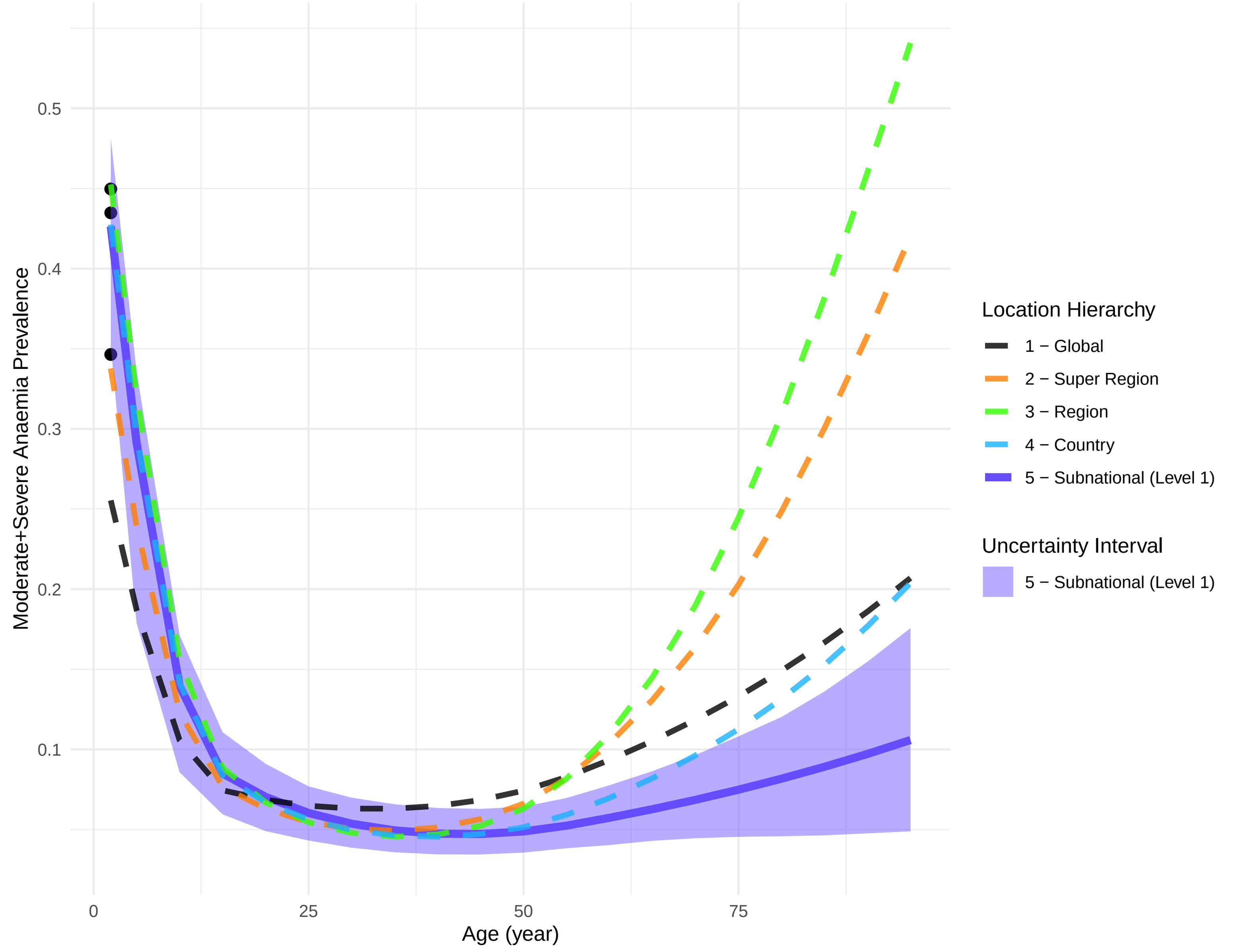

MR−BRT Age/Sex Fits − Males 2+ Years Old − Moderate+Severe Anaemia Prevalence − Ogun
WHO−2001 Adjustment Method

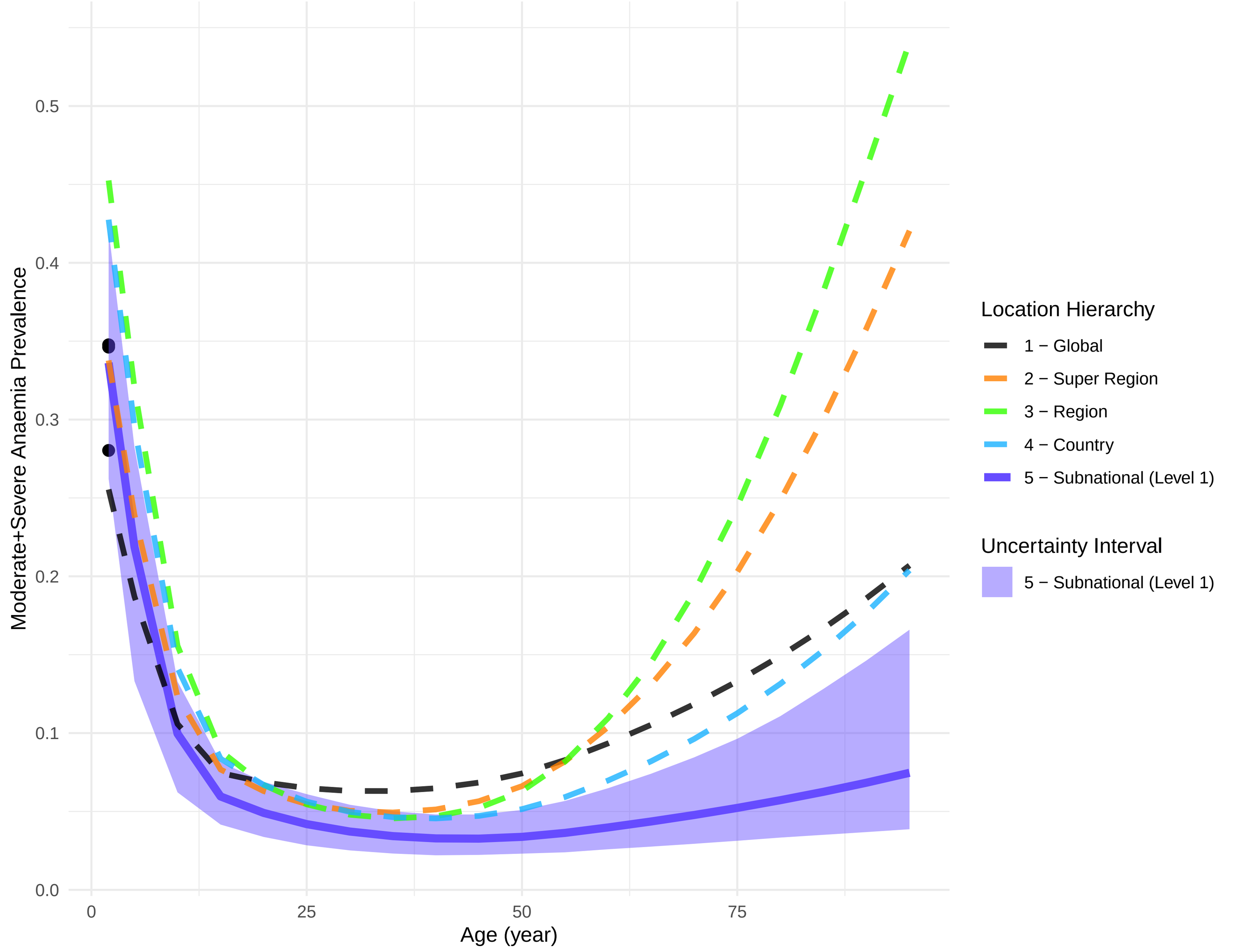

MR−BRT Age/Sex Fits − Males 2+ Years Old − Moderate+Severe Anaemia Prevalence − Ondo
WHO−2001 Adjustment Method

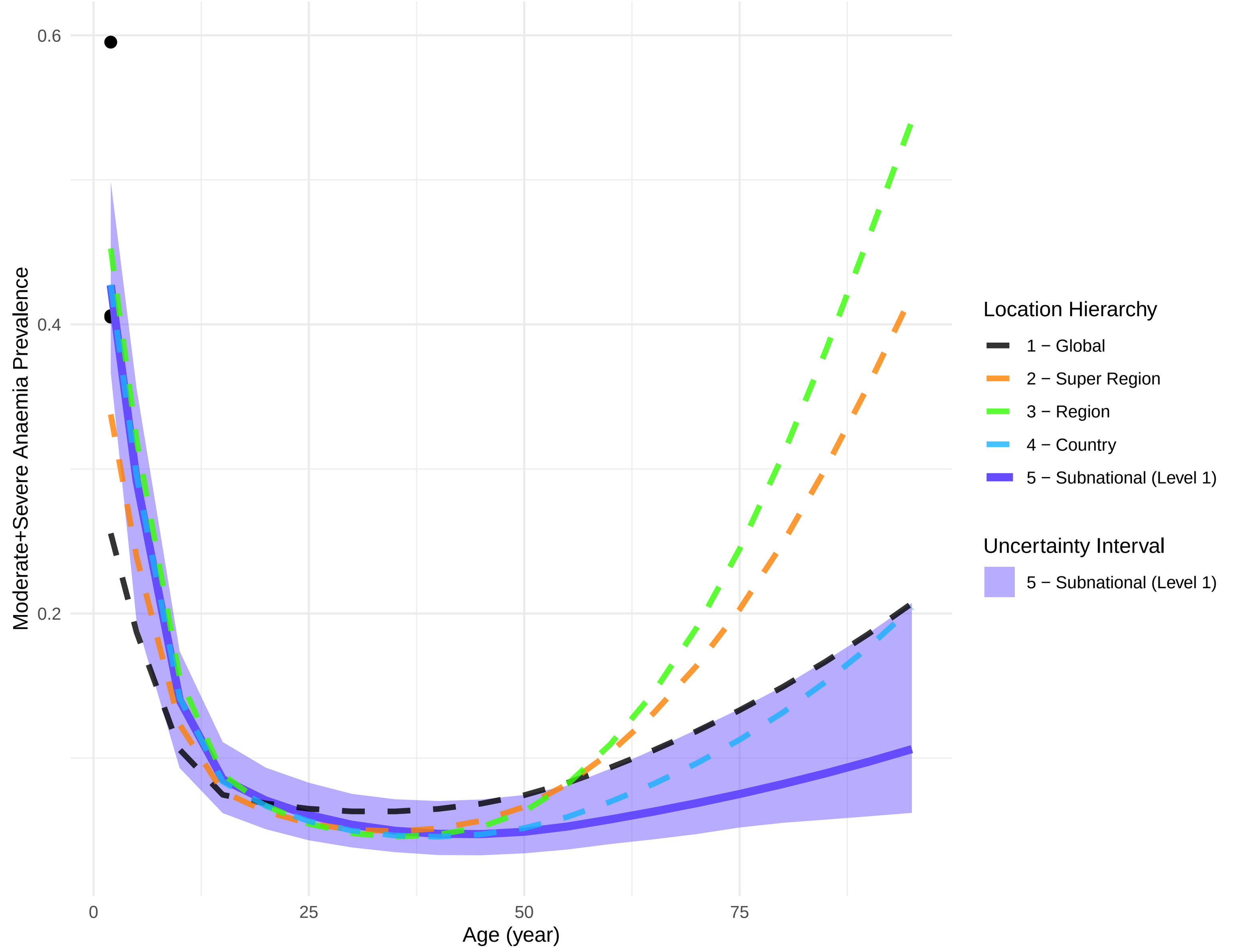

MR−BRT Age/Sex Fits − Males 2+ Years Old − Moderate+Severe Anaemia Prevalence − Osun
WHO−2001 Adjustment Method

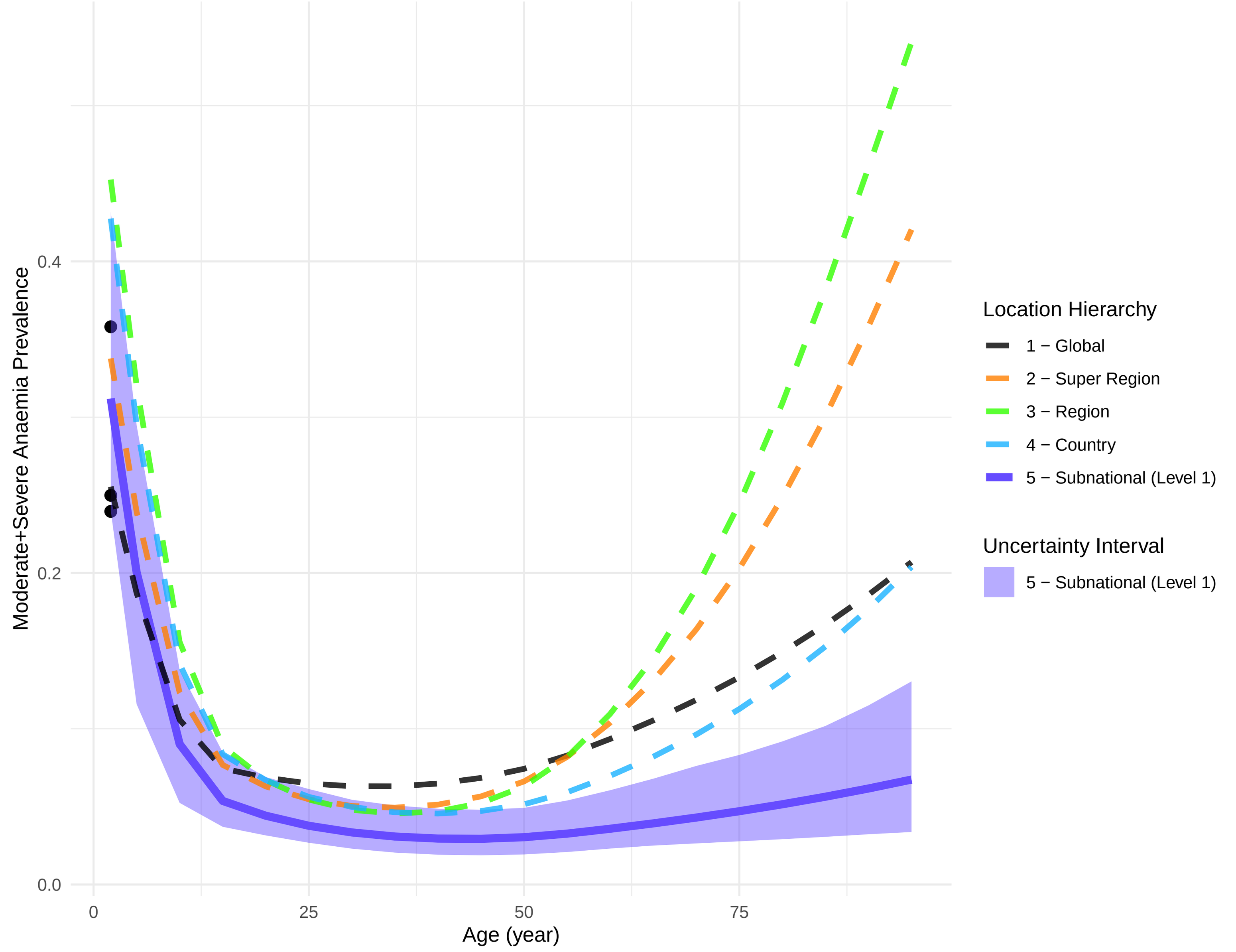

MR−BRT Age/Sex Fits − Males 2+ Years Old − Moderate+Severe Anaemia Prevalence − Oyo
WHO−2001 Adjustment Method

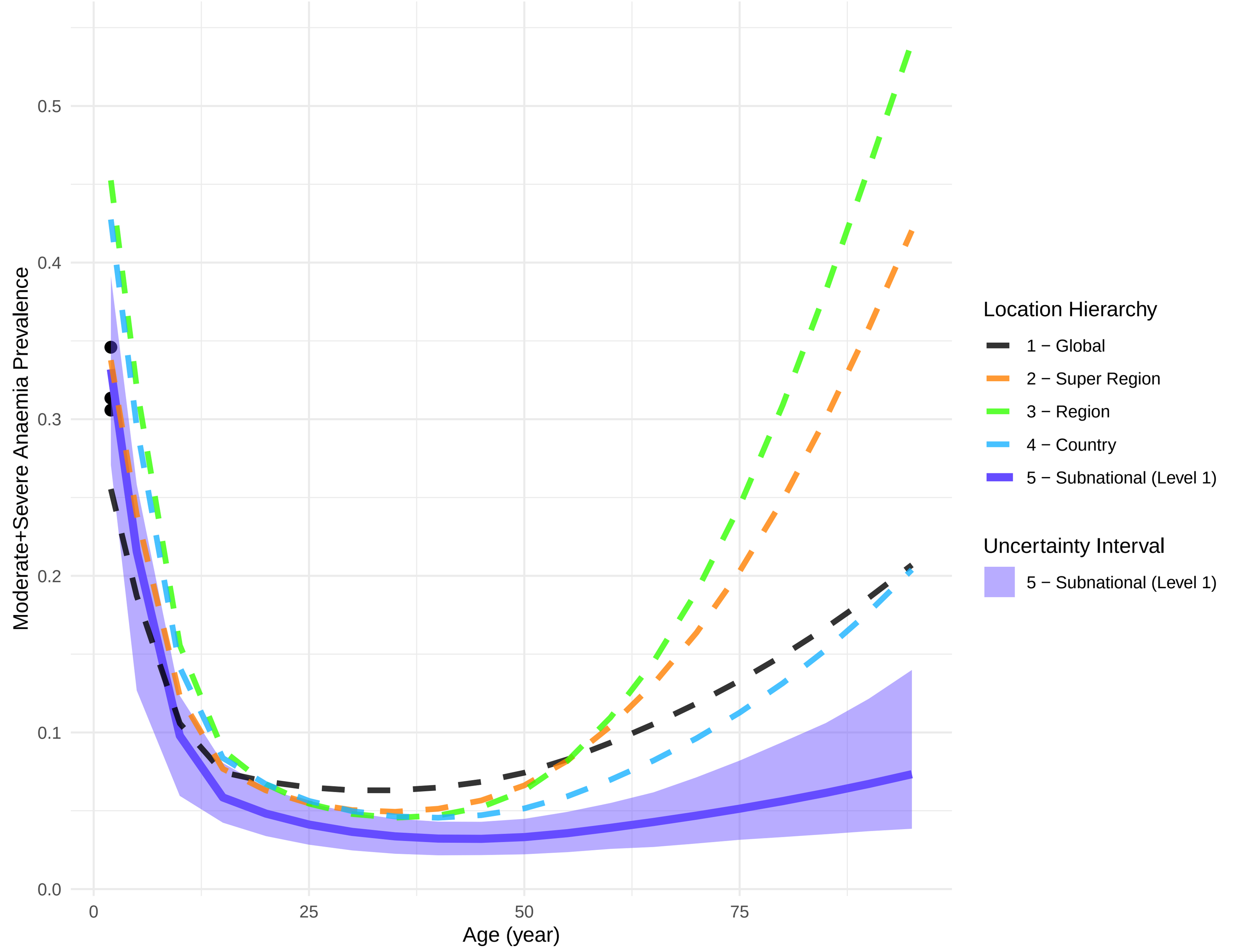

MR−BRT Age/Sex Fits − Males 2+ Years Old − Moderate+Severe Anaemia Prevalence − Plateau
WHO−2001 Adjustment Method

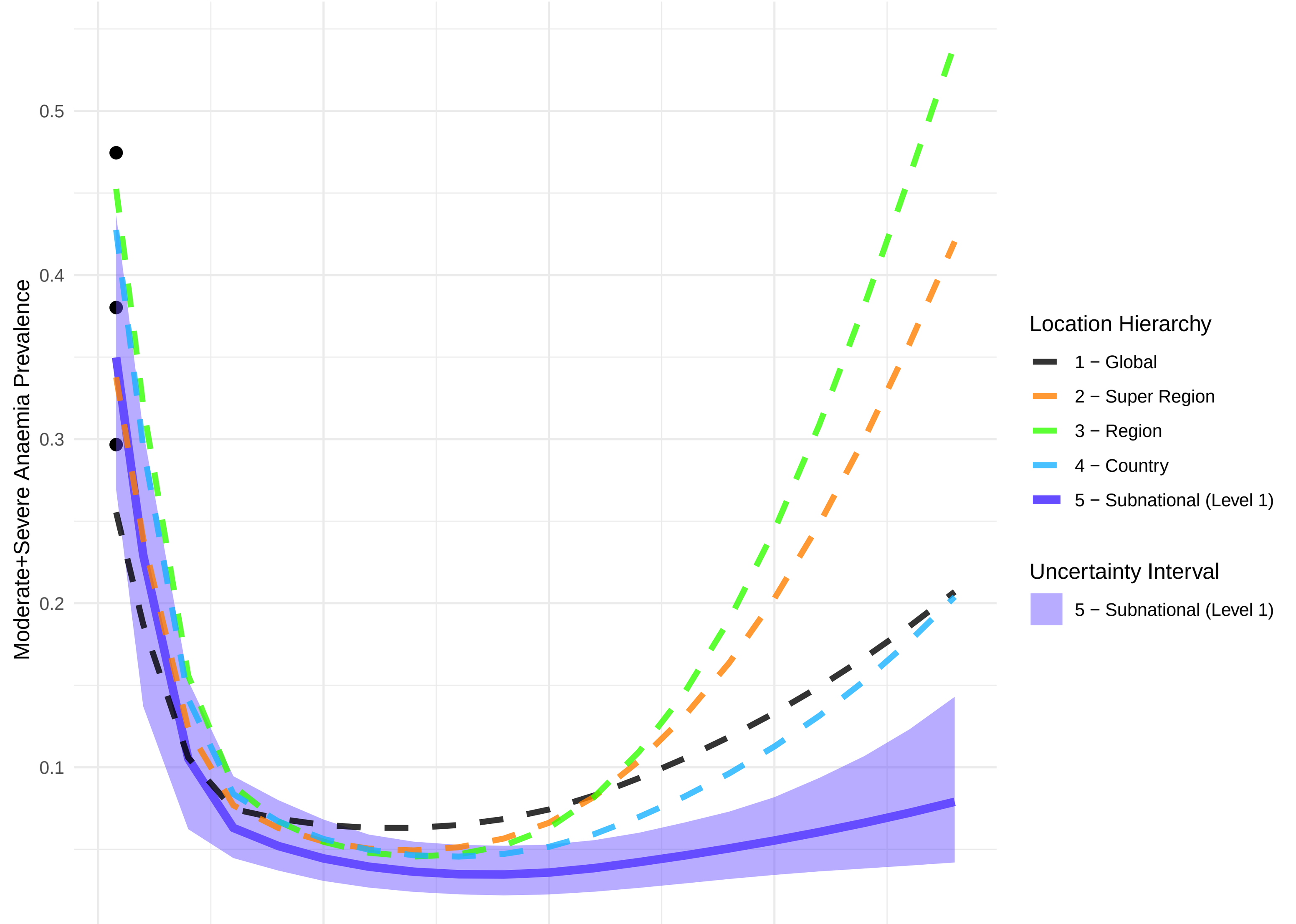

MR−BRT Age/Sex Fits − Males 2+ Years Old − Moderate+Severe Anaemia Prevalence − Rivers
WHO−2001 Adjustment Method

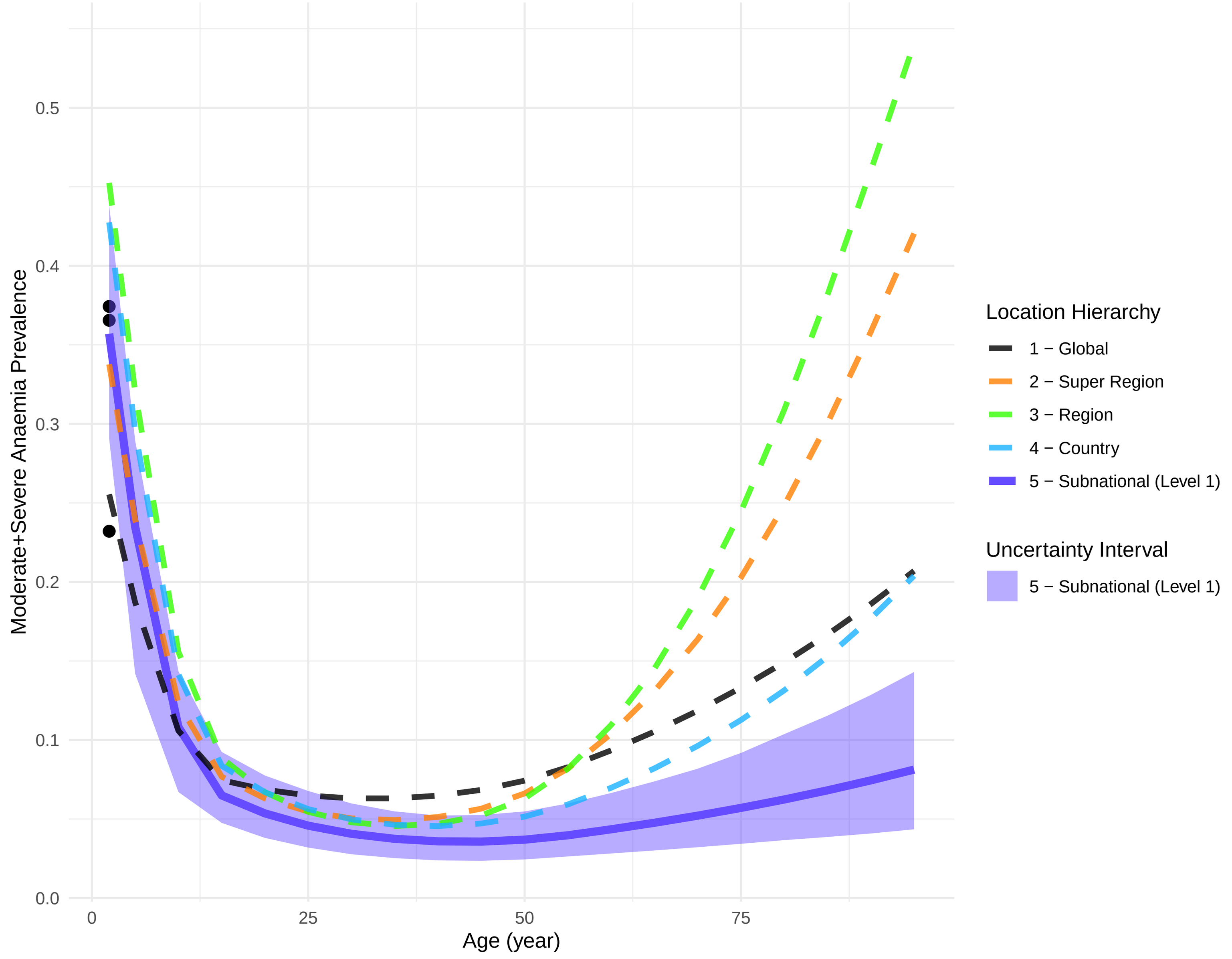

MR−BRT Age/Sex Fits − Males 2+ Years Old − Moderate+Severe Anaemia Prevalence − Sokoto
WHO−2001 Adjustment Method

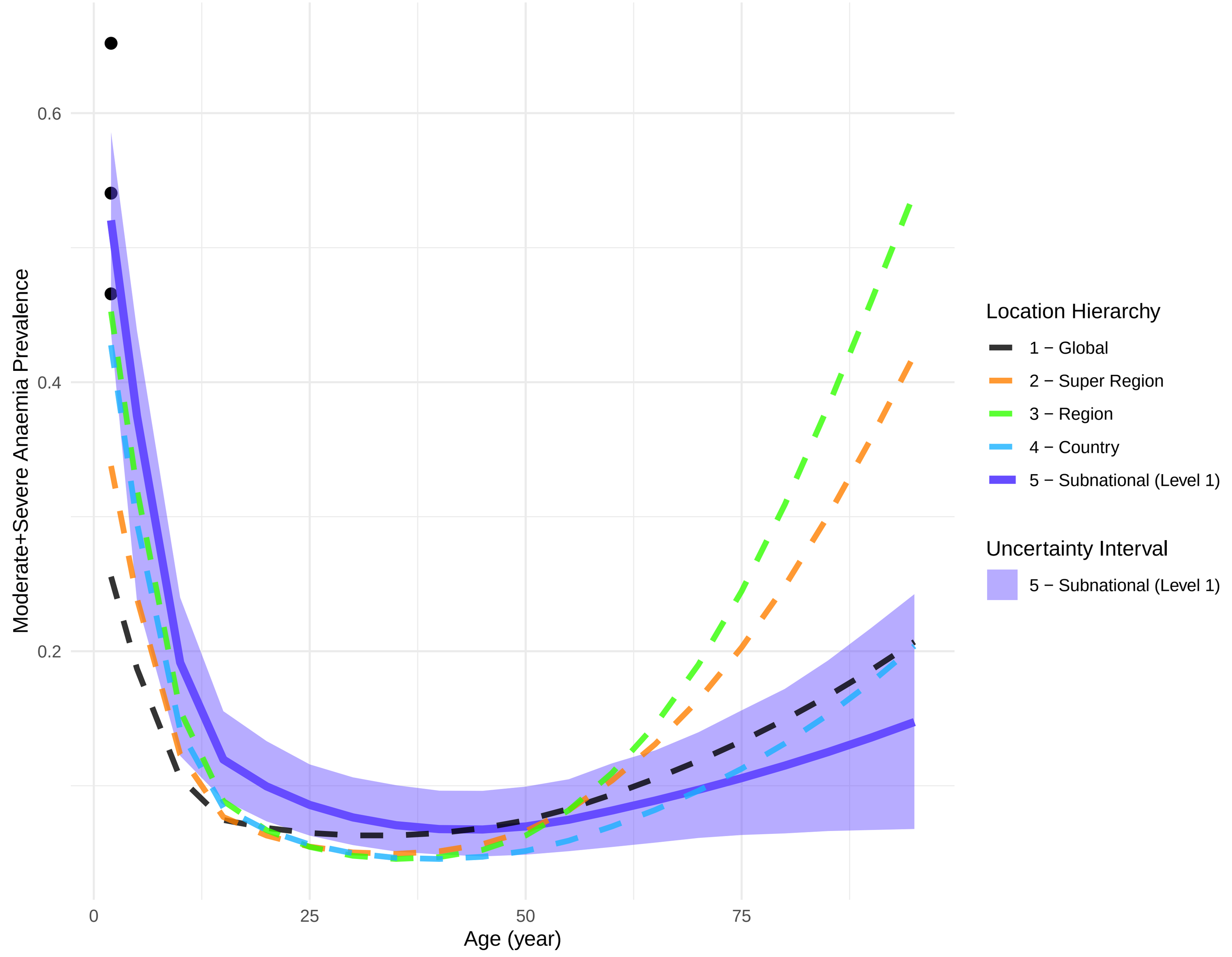

MR−BRT Age/Sex Fits − Males 2+ Years Old − Moderate+Severe Anaemia Prevalence − Taraba
WHO−2001 Adjustment Method

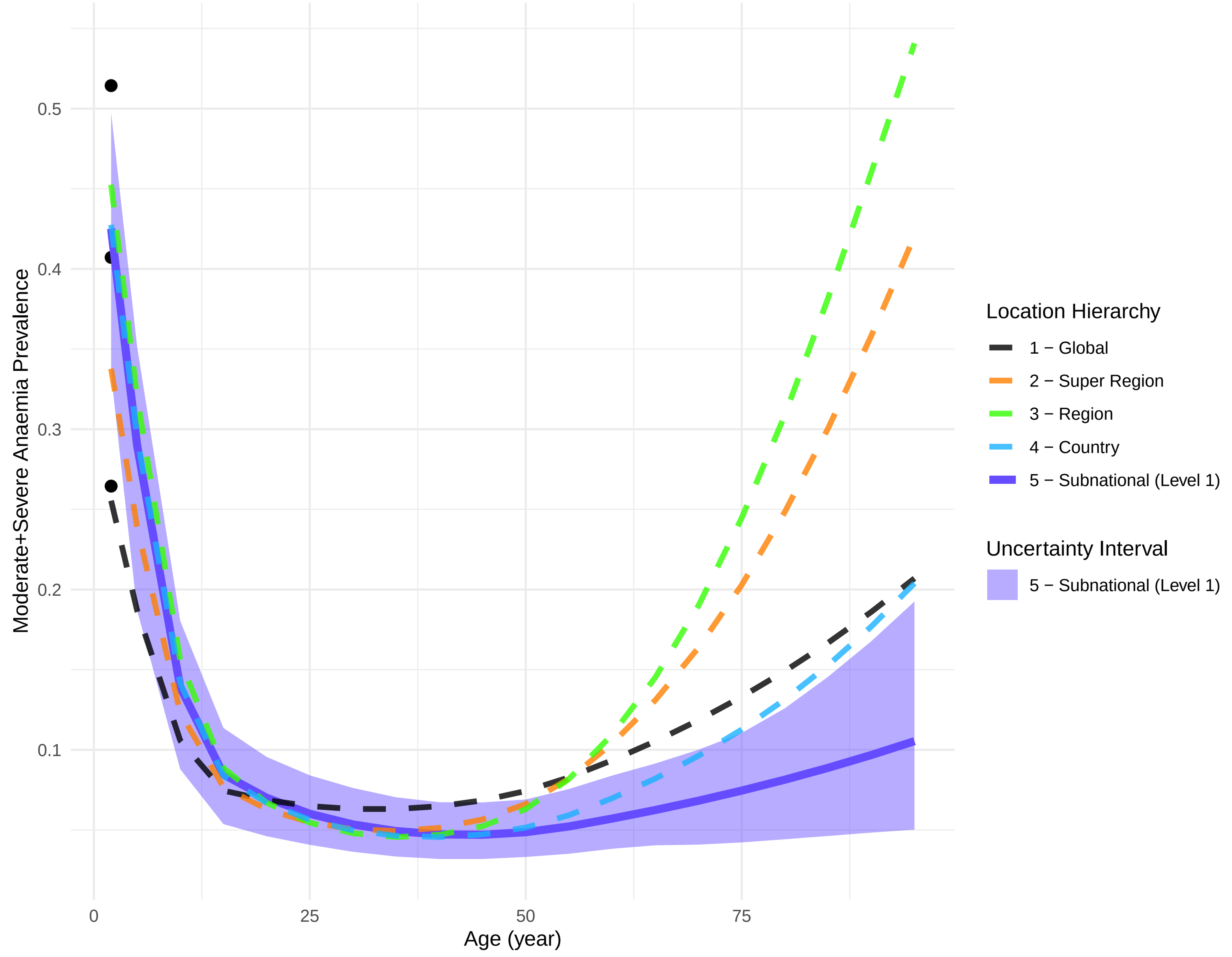

MR−BRT Age/Sex Fits − Males 2+ Years Old − Moderate+Severe Anaemia Prevalence − Yobe
WHO−2001 Adjustment Method

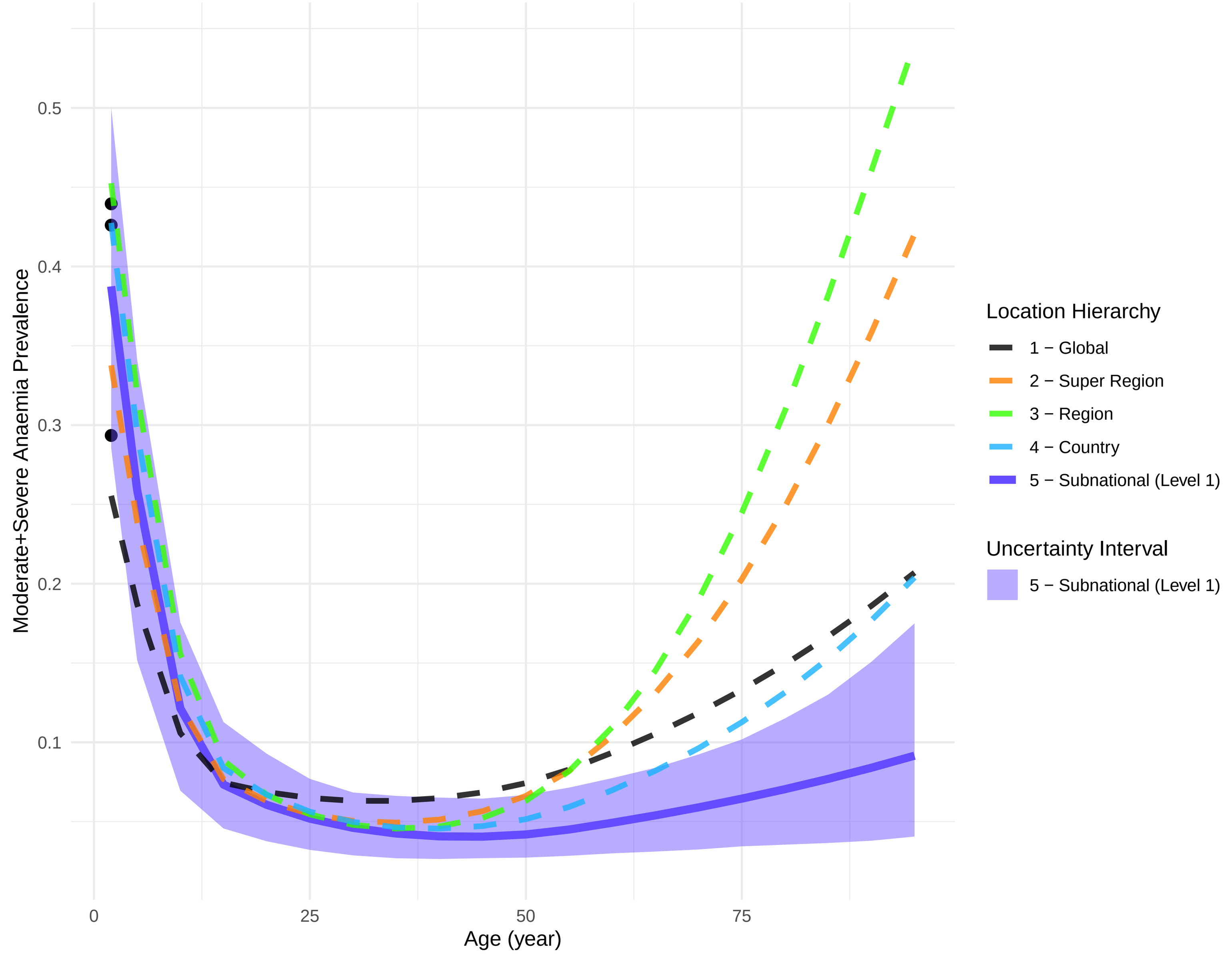

MR−BRT Age/Sex Fits − Males 2+ Years Old − Moderate+Severe Anaemia Prevalence − Zamfara
WHO−2001 Adjustment Method

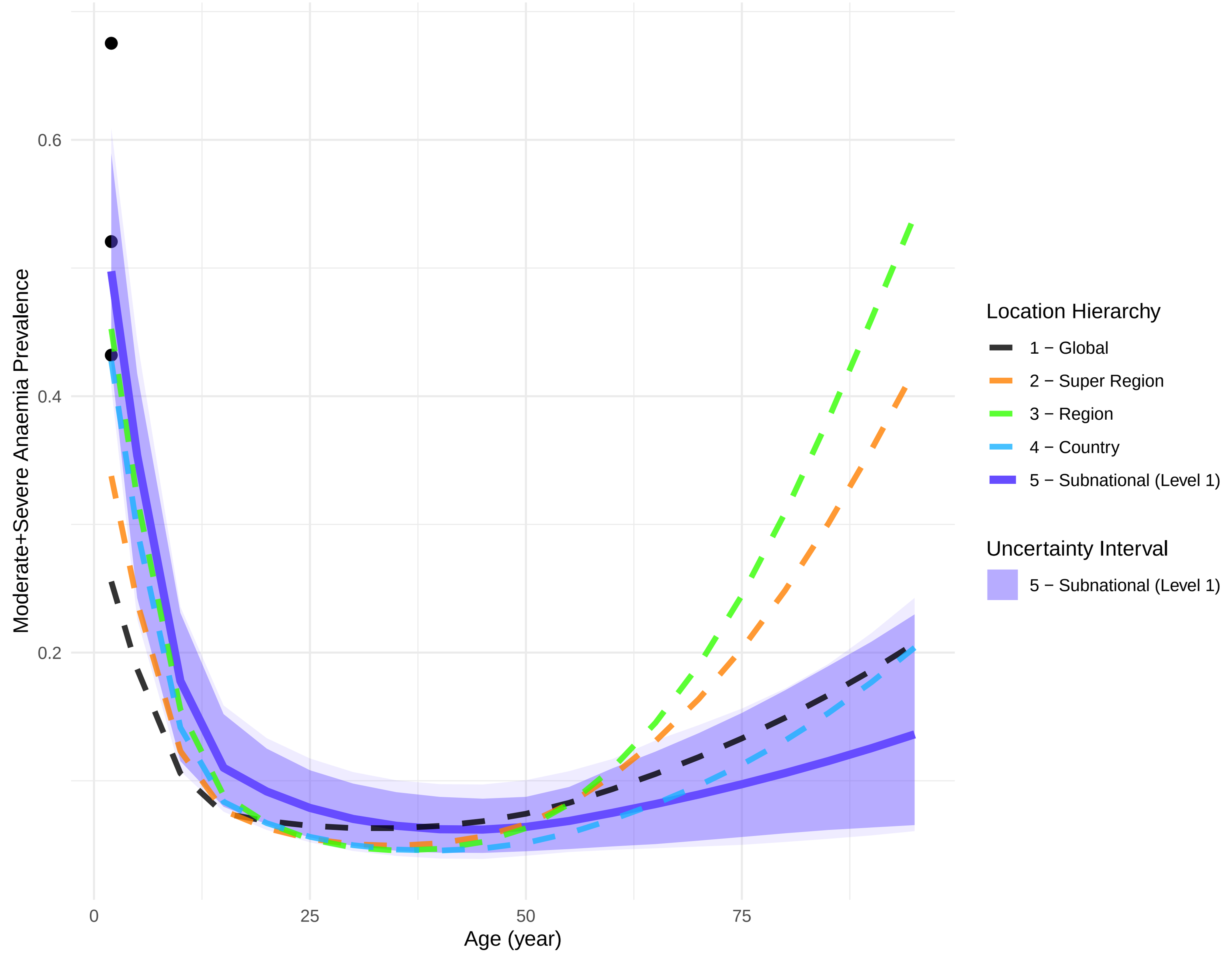

MR−BRT Age/Sex Fits − Males 2+ Years Old − Moderate+Severe Anaemia Prevalence − Senegal
WHO−2001 Adjustment Method

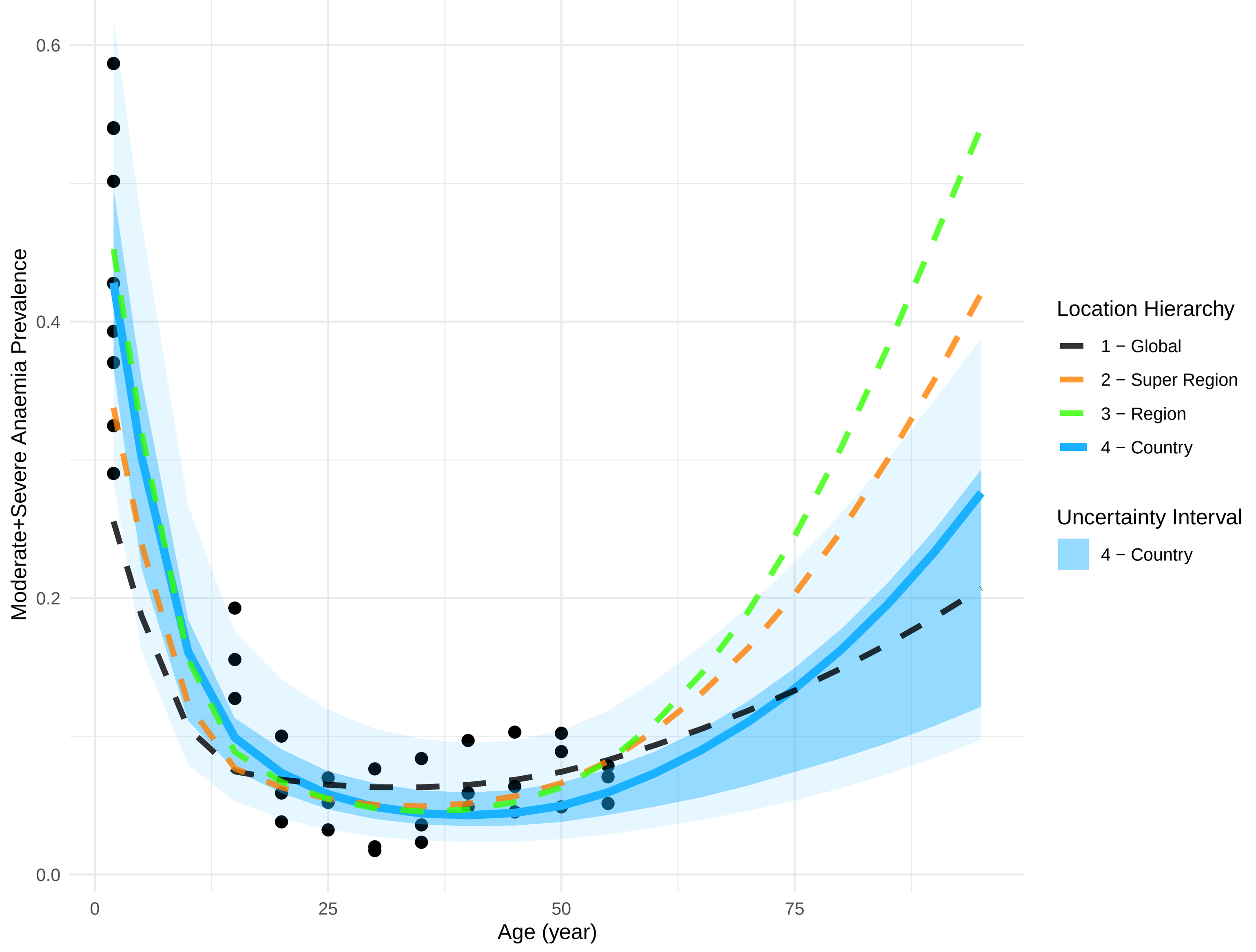

MR−BRT Age/Sex Fits − Males 2+ Years Old − Moderate+Severe Anaemia Prevalence − Sierra Leone
WHO−2001 Adjustment Method

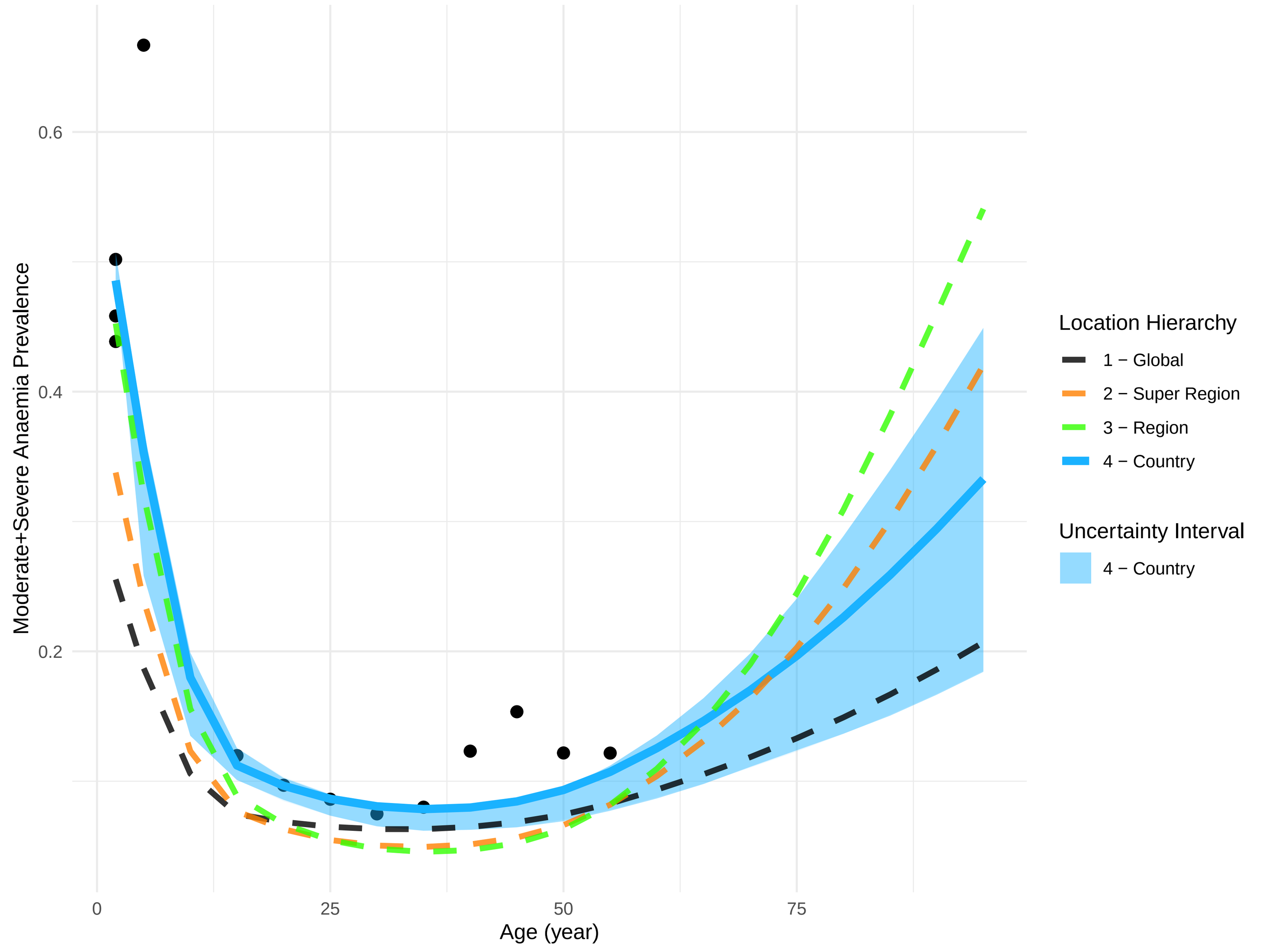

MR−BRT Age/Sex Fits − Males 2+ Years Old − Moderate+Severe Anaemia Prevalence − Togo
WHO−2001 Adjustment Method

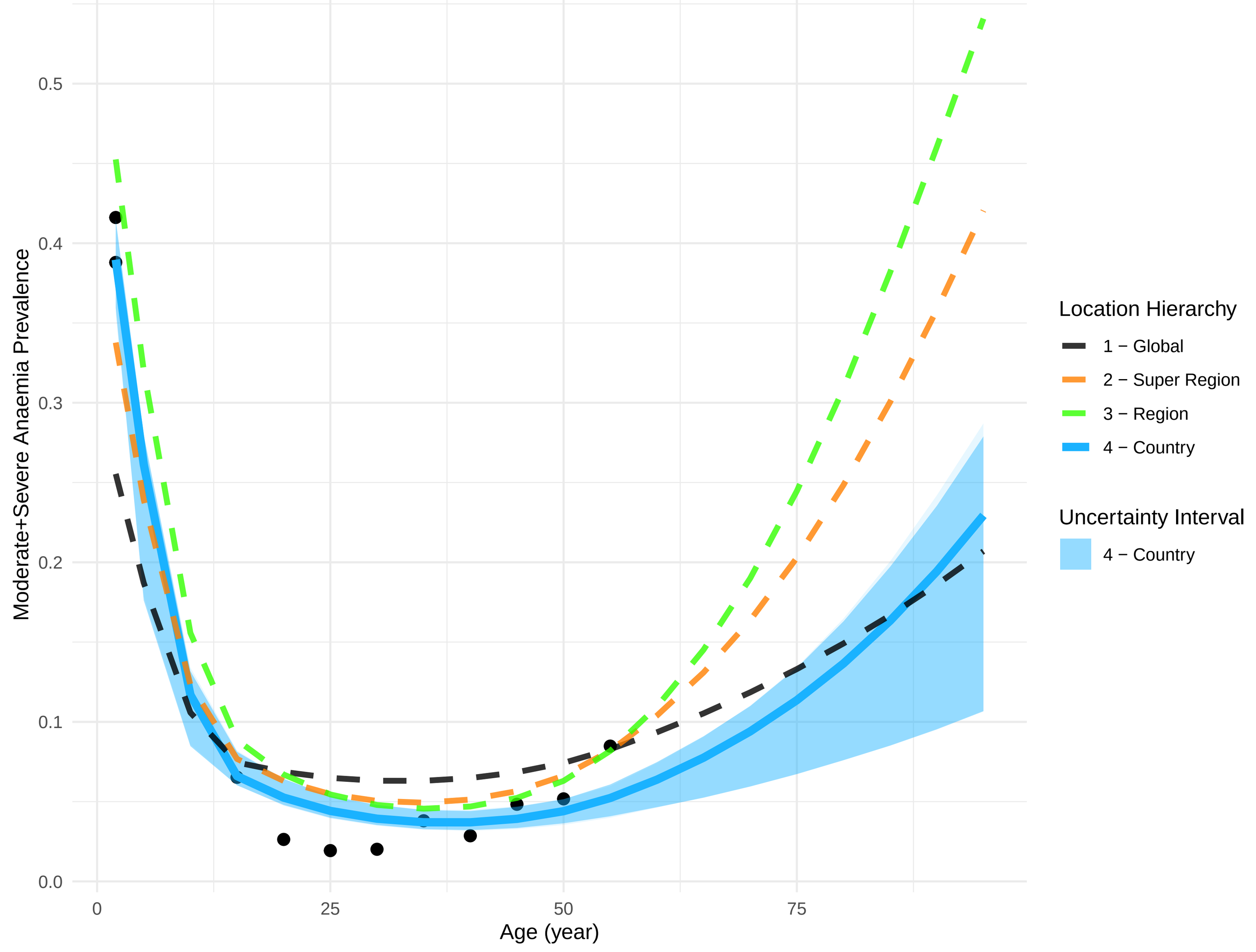

MR−BRT Age/Sex Fits − Non−pregnant Females 2+ Years Old − Moderate+Severe Anaemia Prevalence − Global
WHO−2001 Adjustment Method

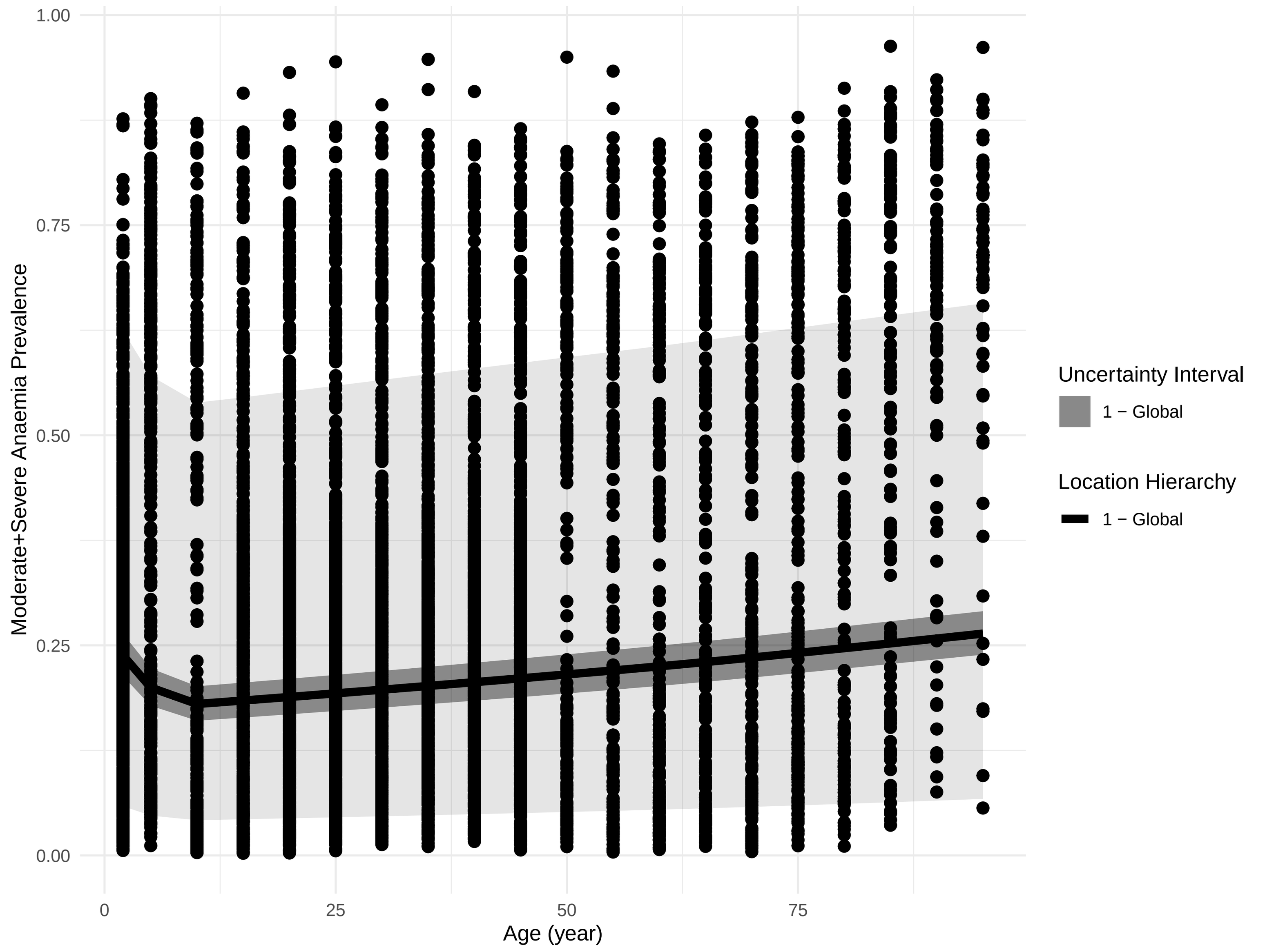

MR−BRT Age/Sex Fits − Non−pregnant Females 2+ Years Old − Moderate+Severe Anaemia Prevalence − Central Europe, Eastern Europe, and Central Asia
WHO−2001 Adjustment Method

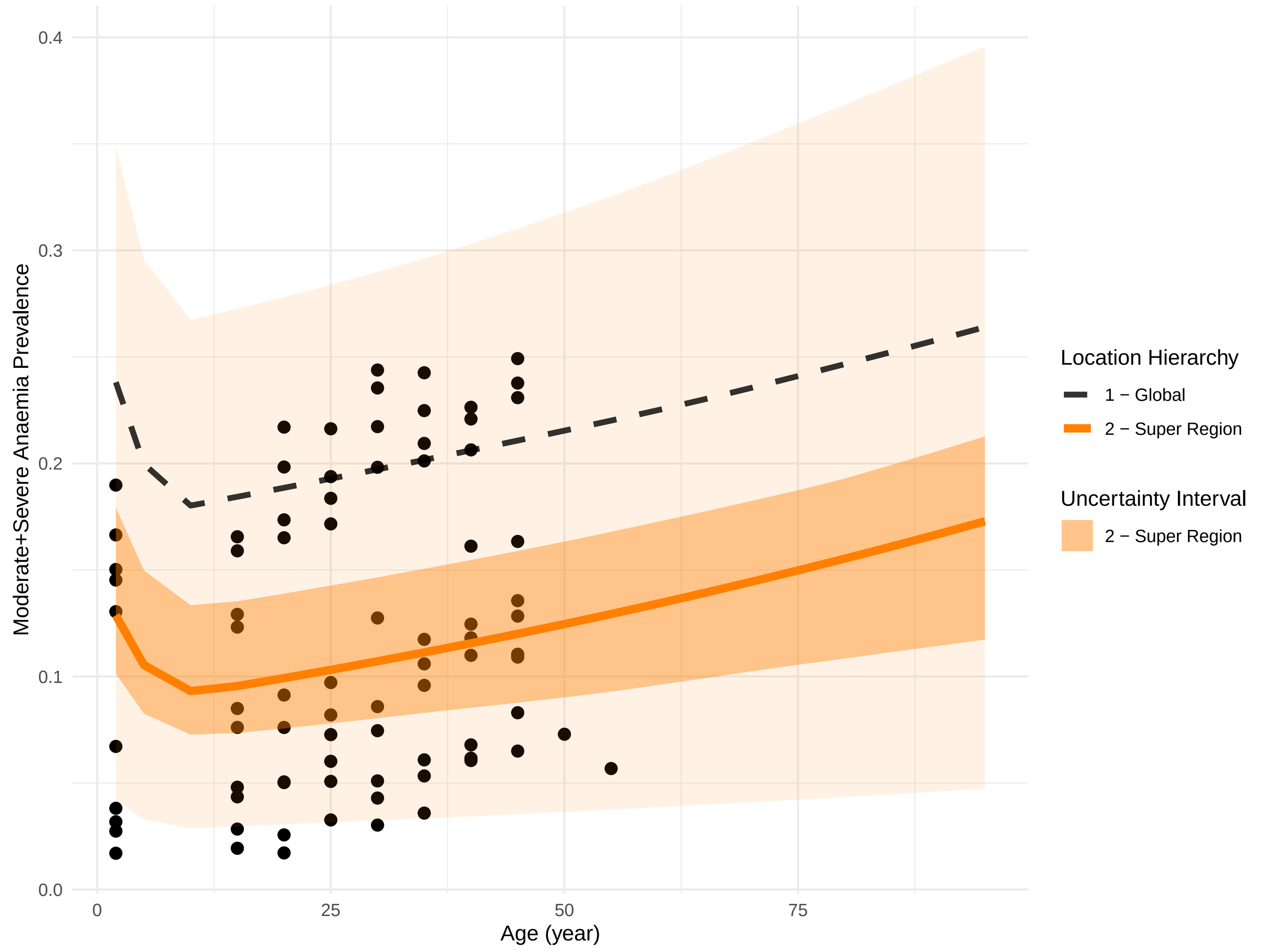

MR−BRT Age/Sex Fits − Non−pregnant Females 2+ Years Old − Moderate+Severe Anaemia Prevalence − Central Asia
WHO−2001 Adjustment Method

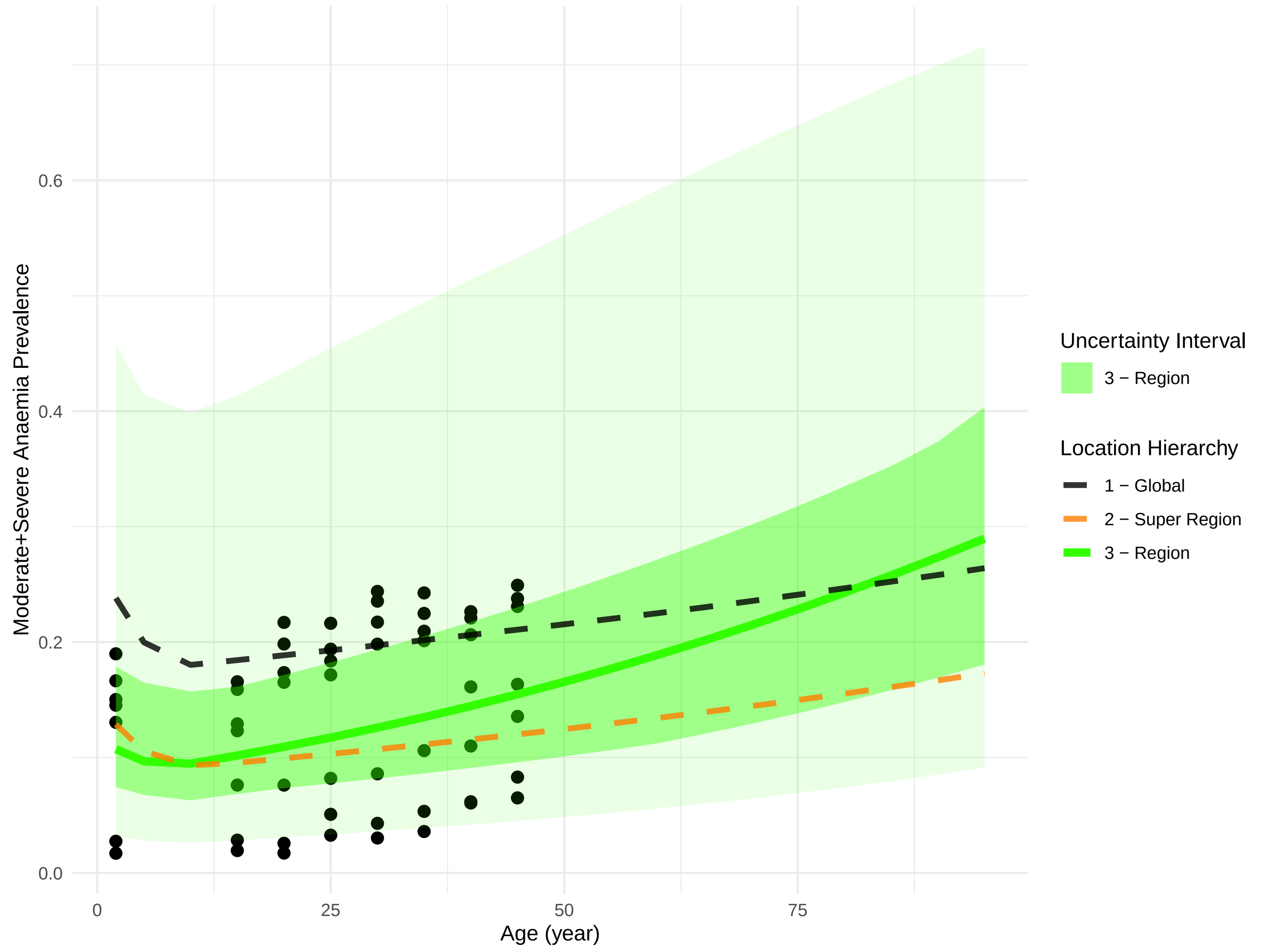

MR−BRT Age/Sex Fits − Non−pregnant Females 2+ Years Old − Moderate+Severe Anaemia Prevalence − Armenia
WHO−2001 Adjustment Method

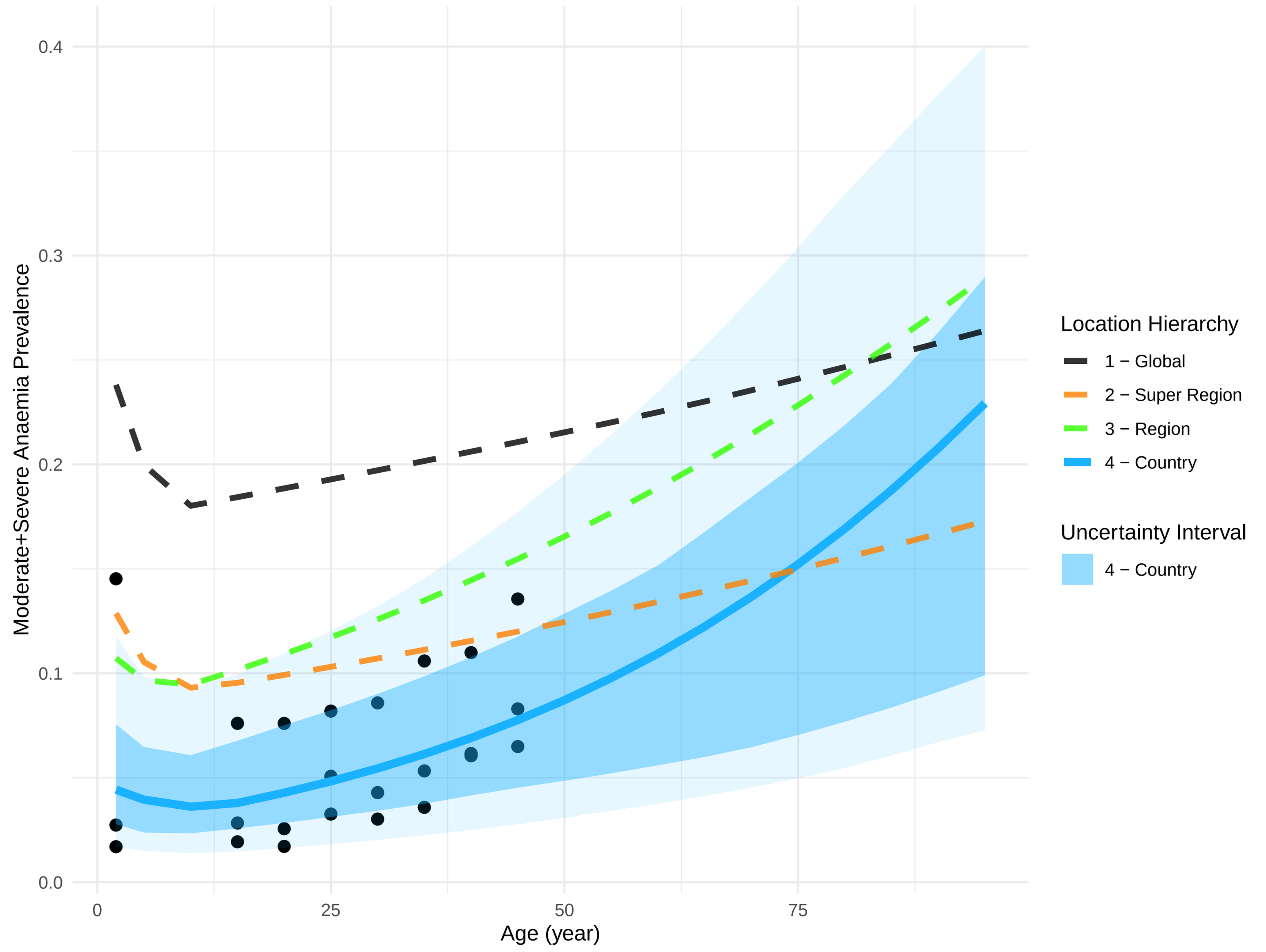

MR−BRT Age/Sex Fits − Non−pregnant Females 2+ Years Old − Moderate+Severe Anaemia Prevalence − Azerbaijan
WHO−2001 Adjustment Method

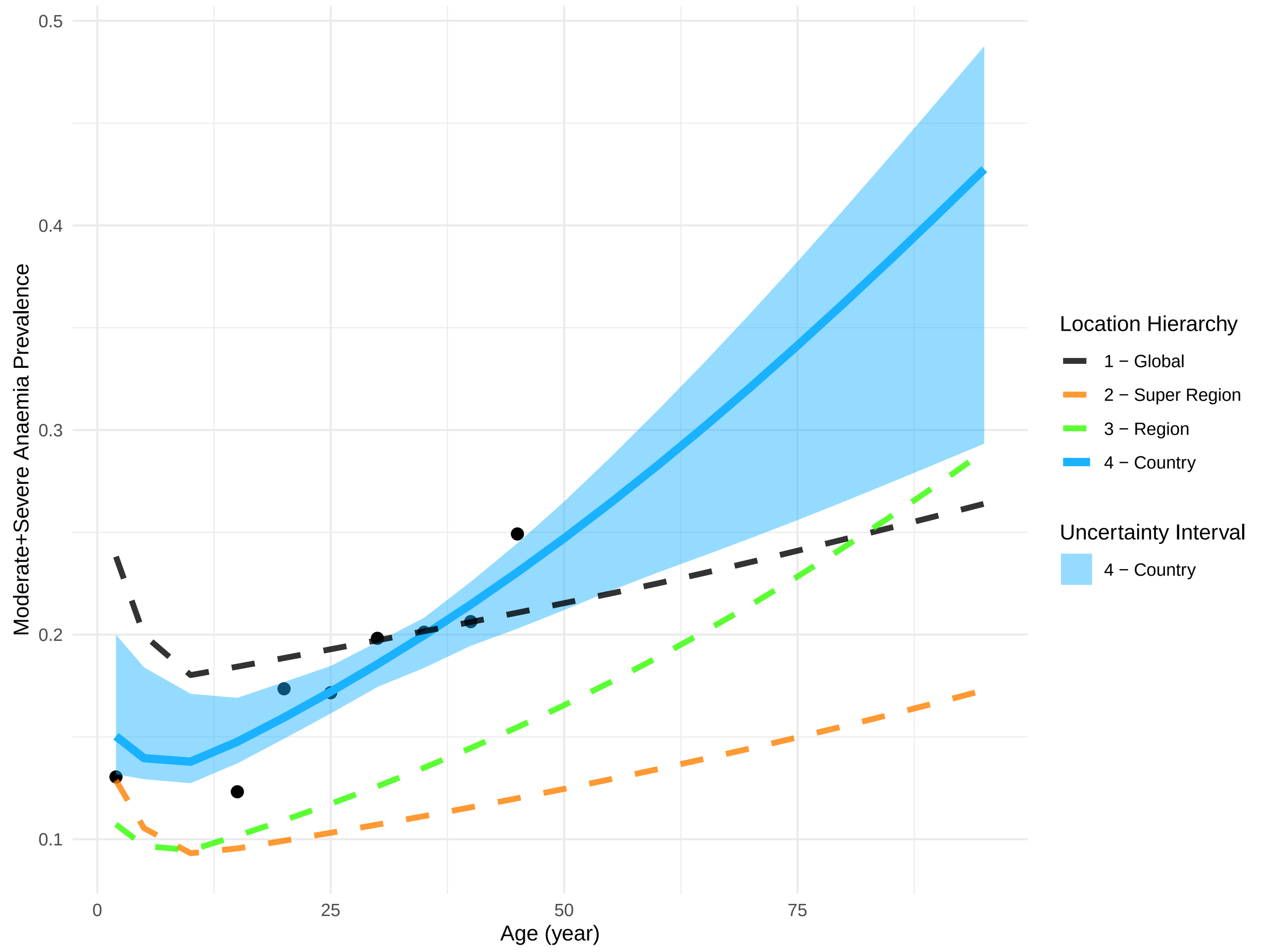

MR−BRT Age/Sex Fits − Non−pregnant Females 2+ Years Old − Moderate+Severe Anaemia Prevalence − Kyrgyzstan
WHO−2001 Adjustment Method

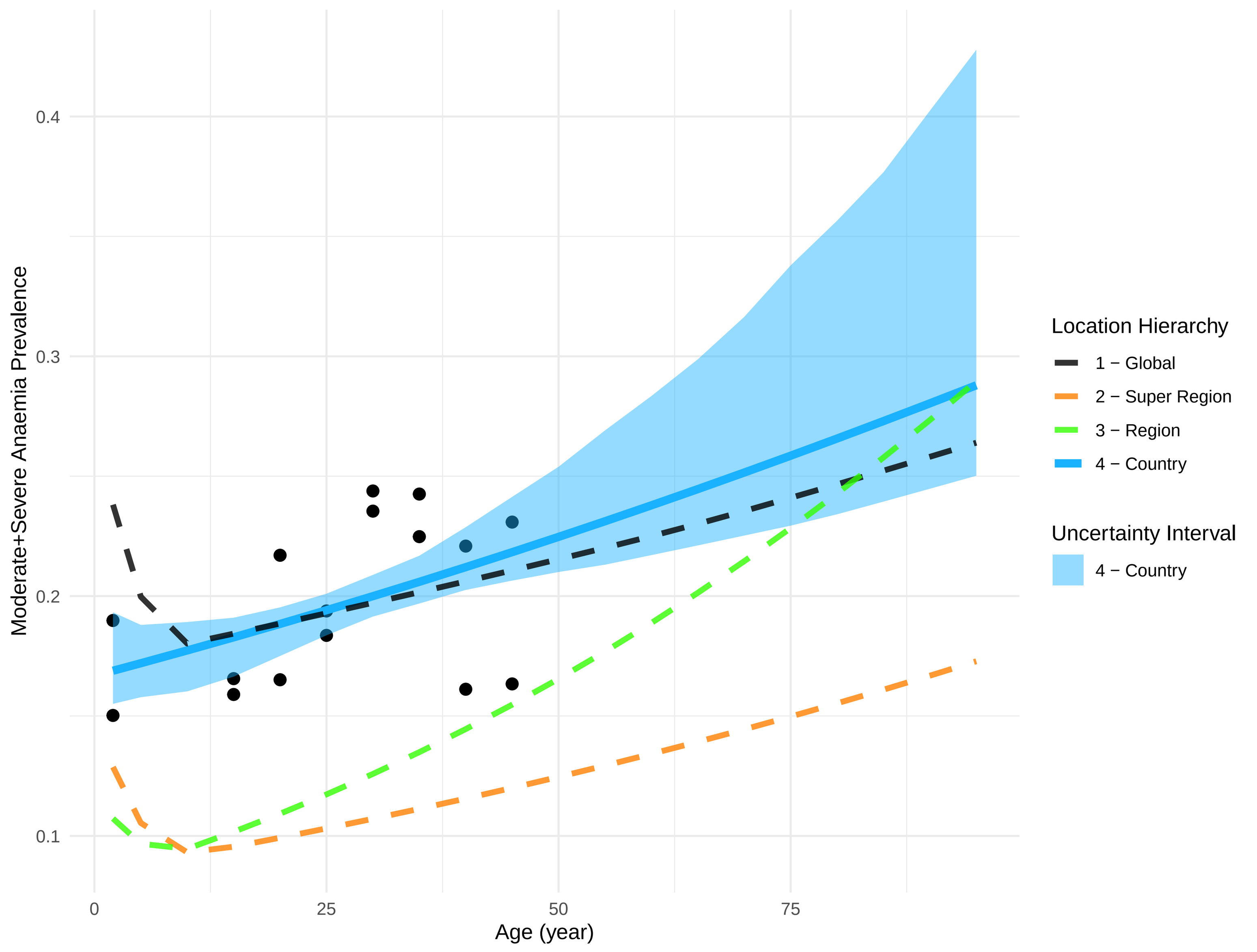

MR−BRT Age/Sex Fits − Non−pregnant Females 2+ Years Old − Moderate+Severe Anaemia Prevalence − Tajikistan
WHO−2001 Adjustment Method

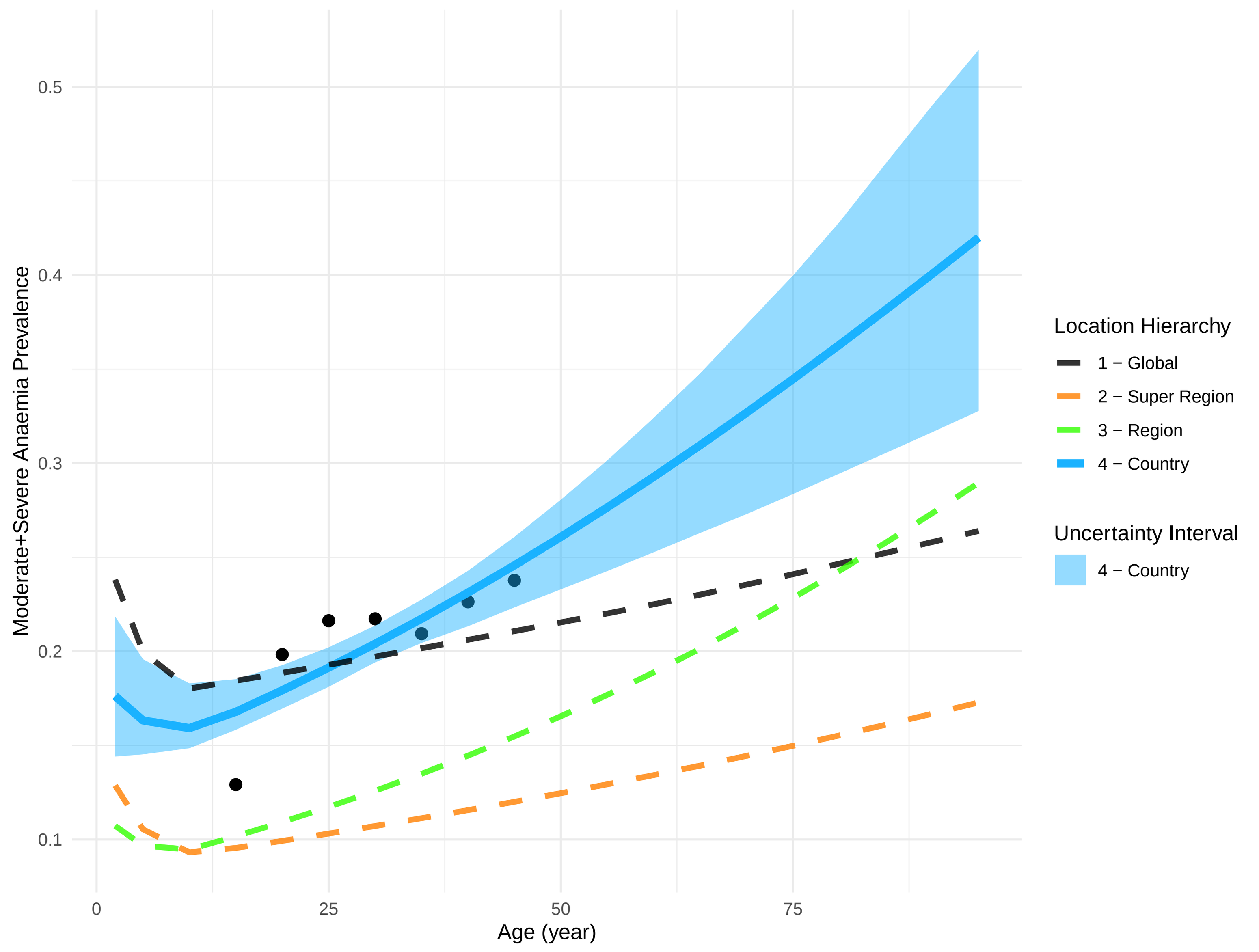

MR−BRT Age/Sex Fits − Non−pregnant Females 2+ Years Old − Moderate+Severe Anaemia Prevalence − Uzbekistan
WHO−2001 Adjustment Method

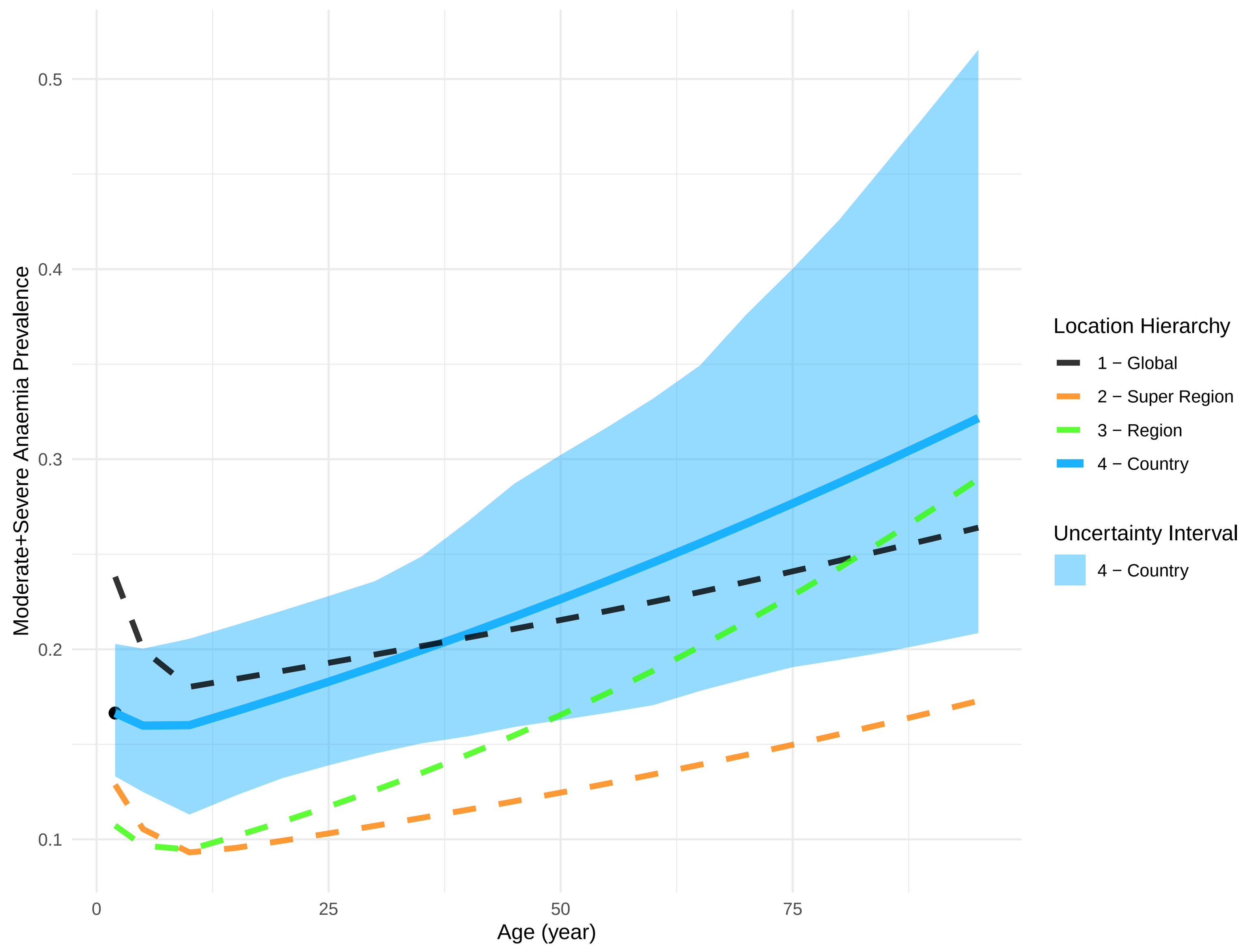

MR−BRT Age/Sex Fits − Non−pregnant Females 2+ Years Old − Moderate+Severe Anaemia Prevalence − Central Europe
WHO−2001 Adjustment Method

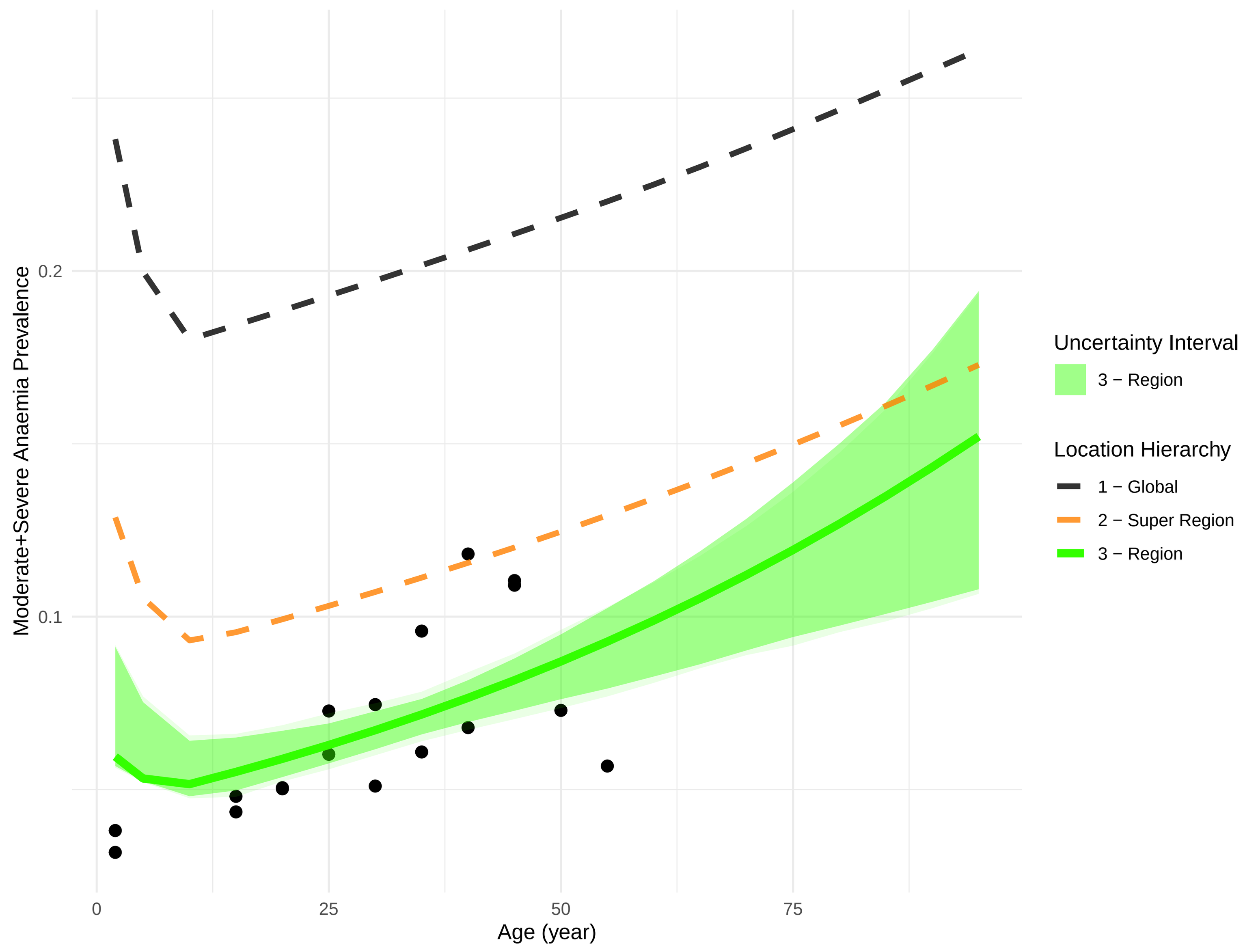

MR−BRT Age/Sex Fits − Non−pregnant Females 2+ Years Old − Moderate+Severe Anaemia Prevalence − Albania
WHO−2001 Adjustment Method

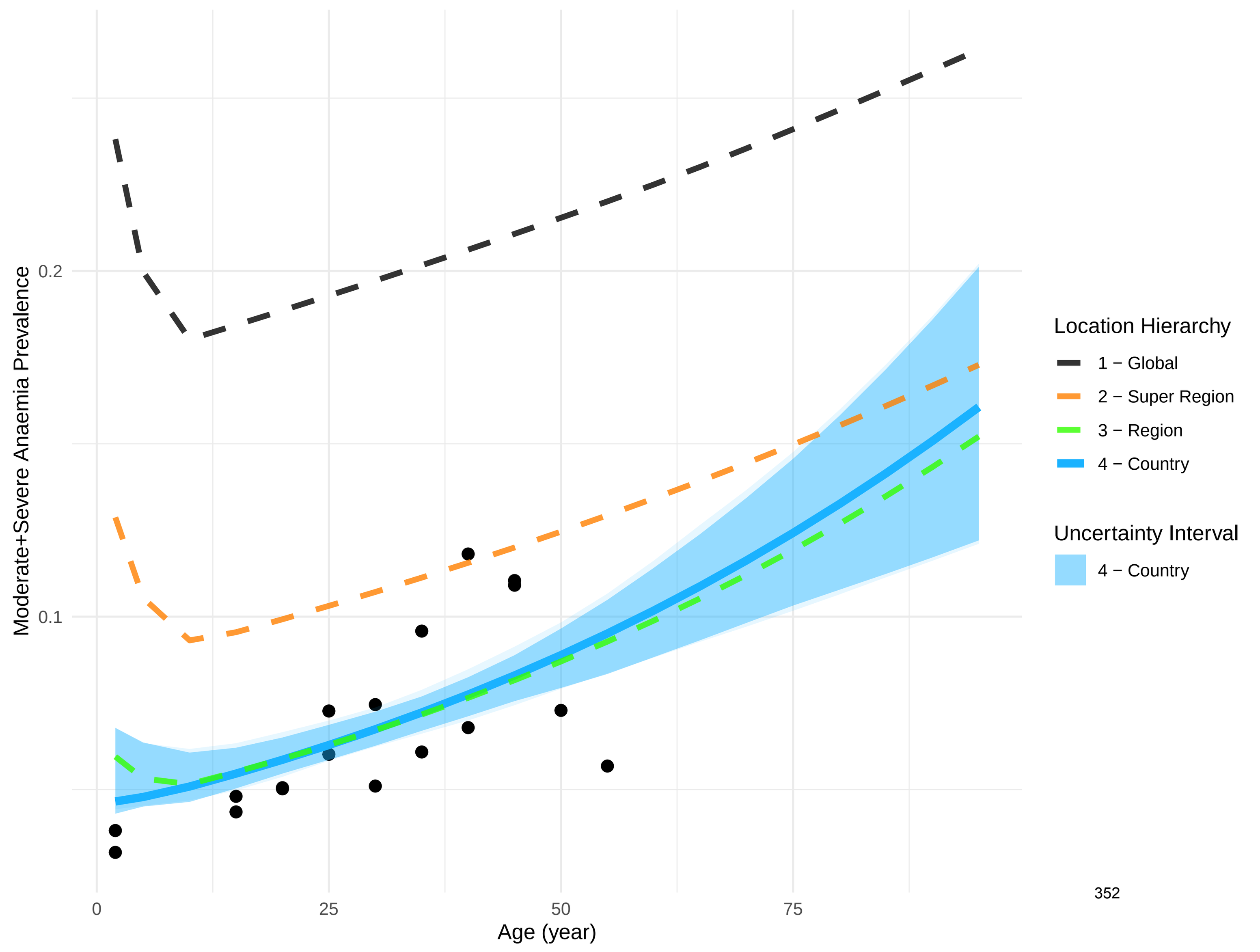

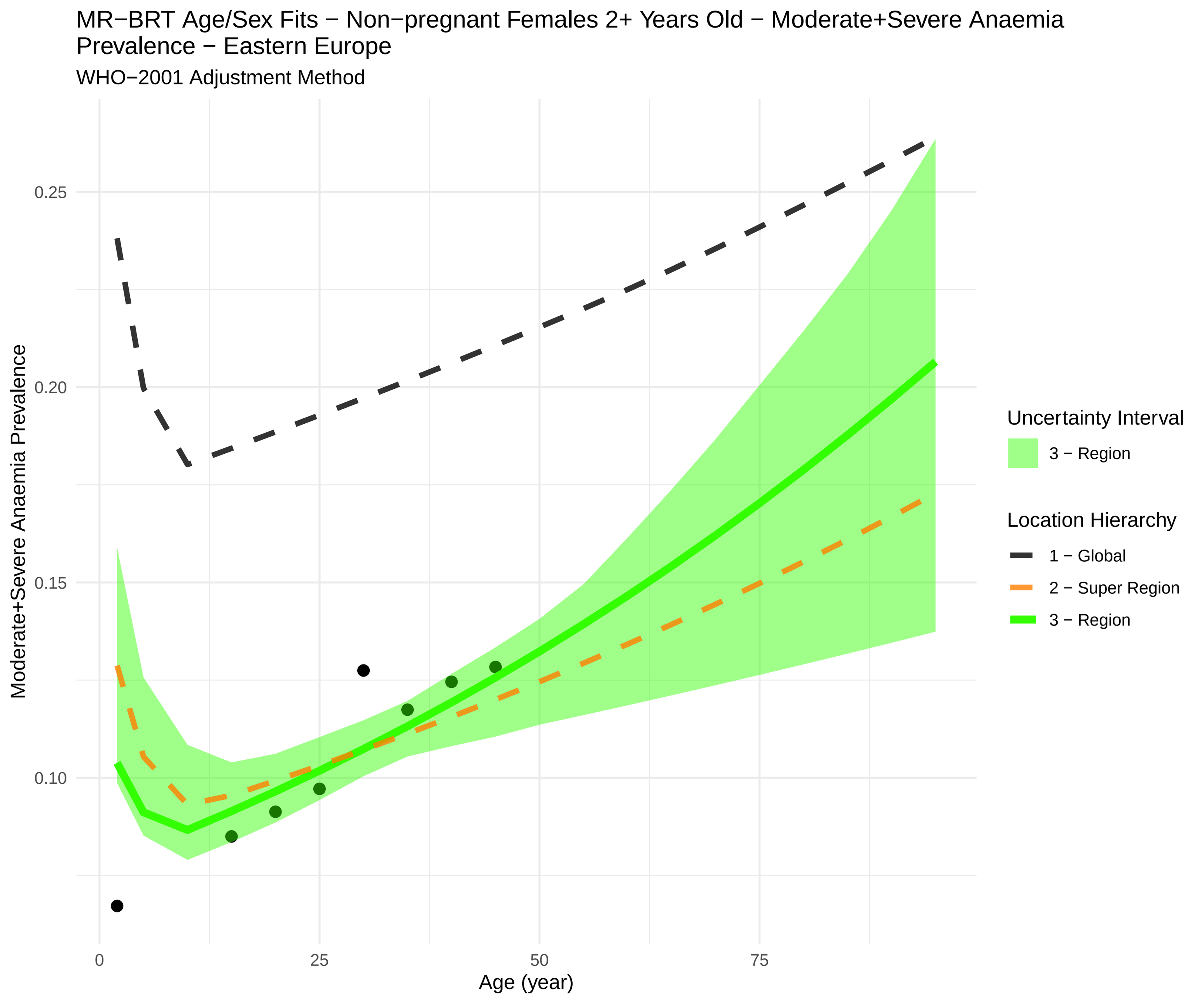
MR−BRT Age/Sex Fits − Non−pregnant Females 2+ Years Old − Moderate+Severe Anaemia Prevalence − Eastern Europe
WHO−2001 Adjustment Method
Moderate+Severe Anaemia Prevalence
Age (year)
Uncertainty Interval
3 − Region
Location Hierarchy
1 − Global
2 − Super Region
3 − Region

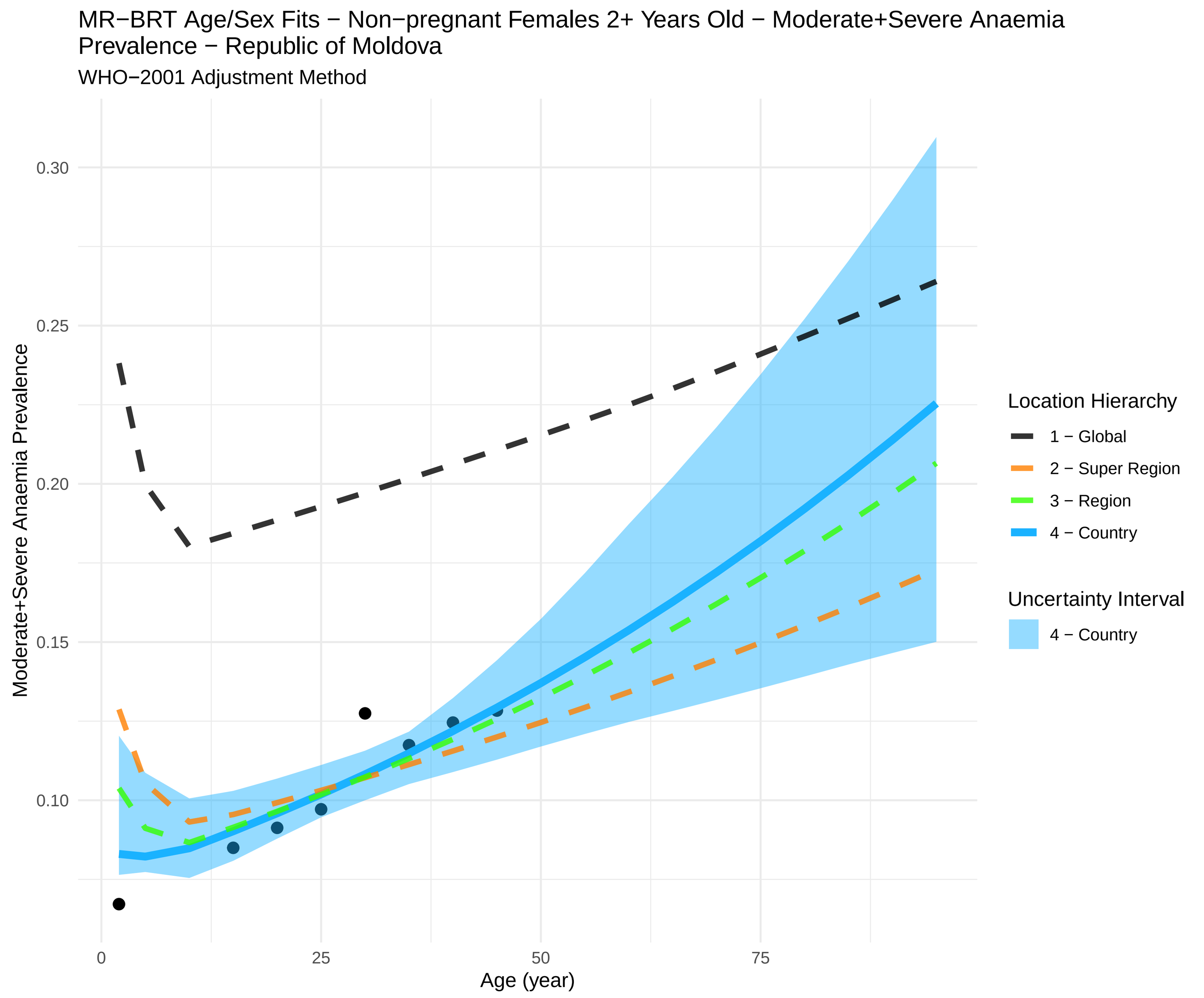
MR−BRT Age/Sex Fits − Non−pregnant Females 2+ Years Old − Moderate+Severe Anaemia Prevalence − Republic of Moldova
WHO−2001 Adjustment Method
Moderate+Severe Anaemia Prevalence
Age (year)
Location Hierarchy
1 − Global
2 − Super Region
3 − Region
4 − Country
Uncertainty Interval
4 − Country

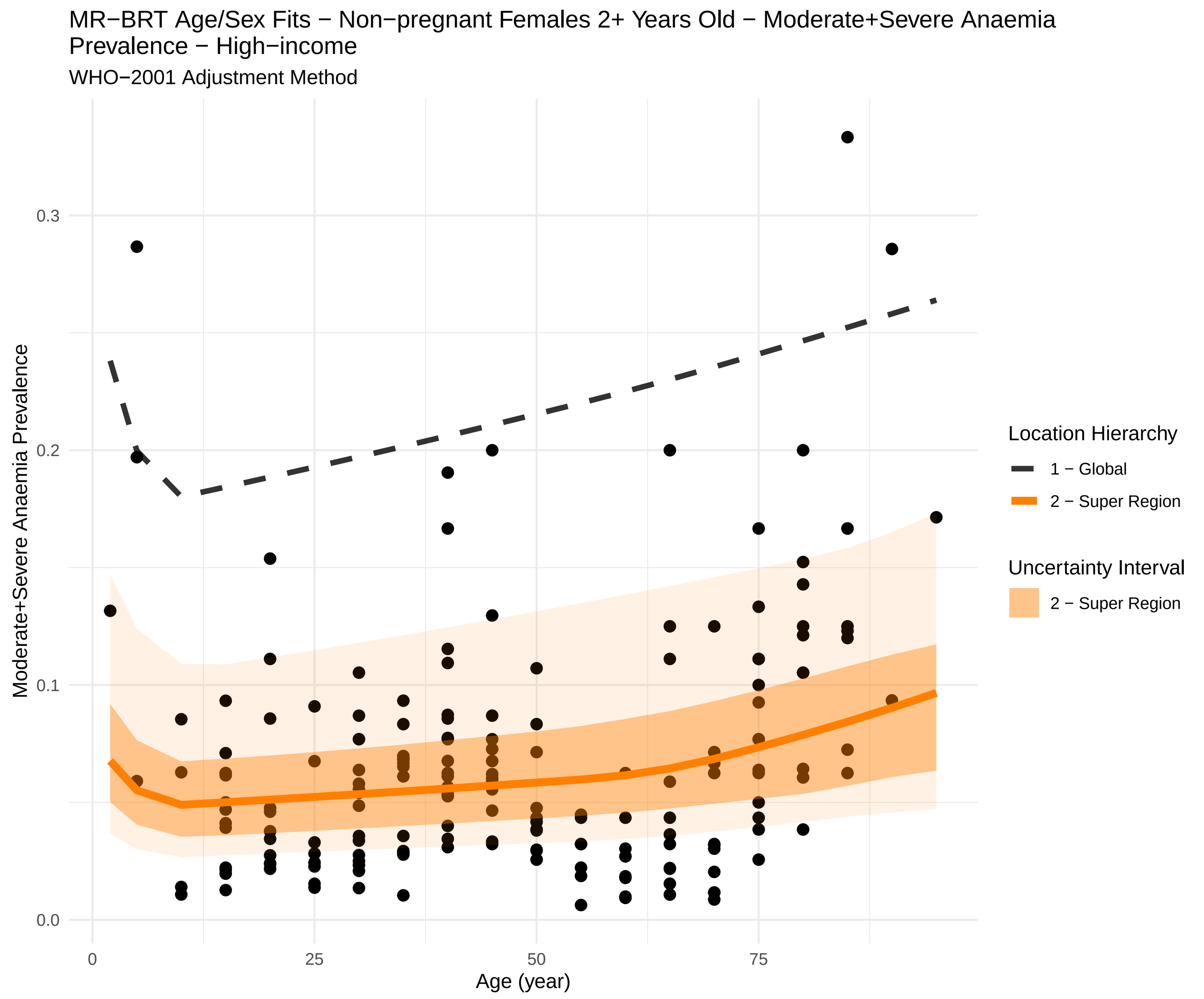
MR−BRT Age/Sex Fits − Non−pregnant Females 2+ Years Old − Moderate+Severe Anaemia Prevalence − High−income
WHO−2001 Adjustment Method
Moderate+Severe Anaemia Prevalence
Age (year)
Location Hierarchy
1 − Global
2 − Super Region
Uncertainty Interval
2 − Super Region

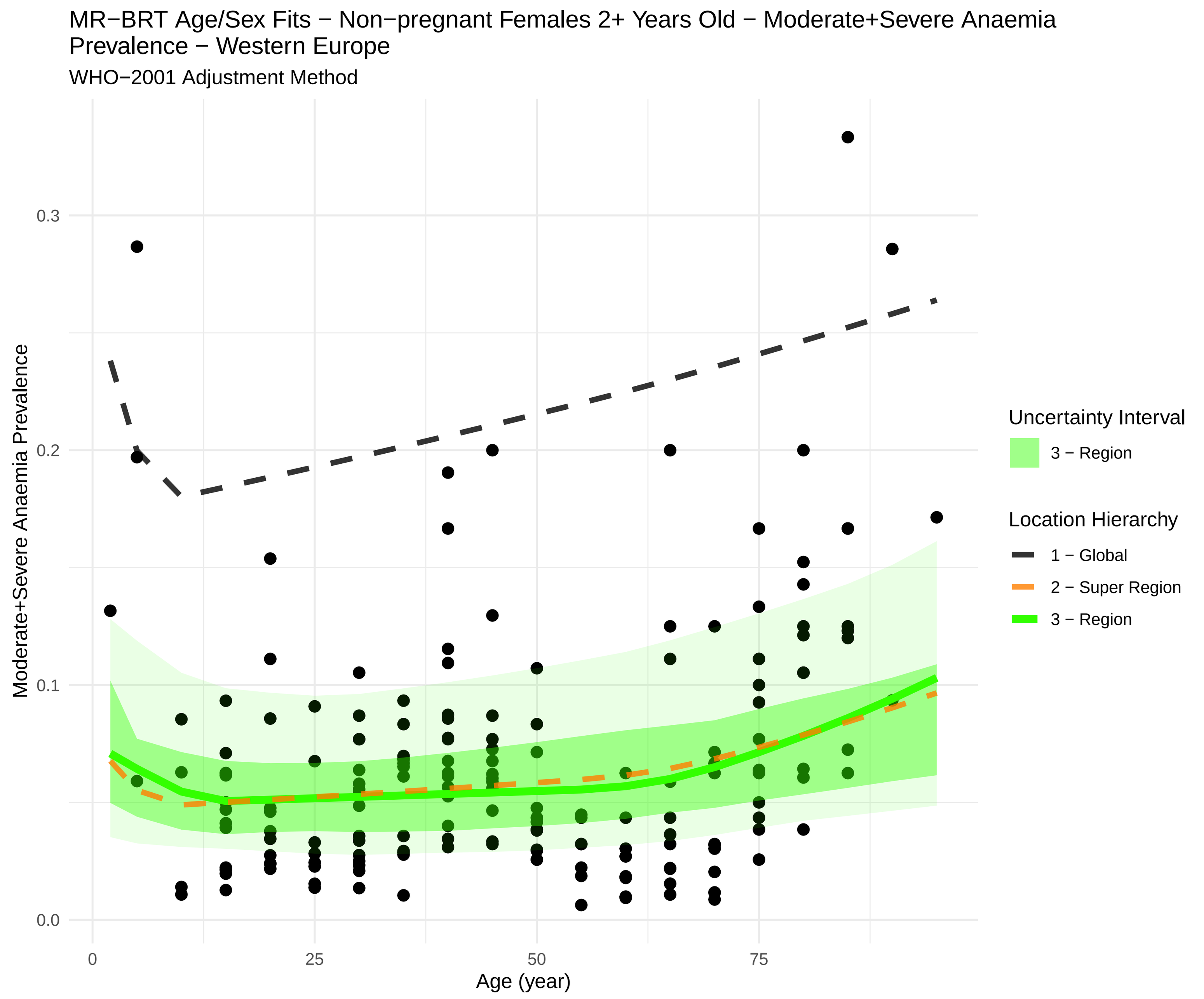
MR−BRT Age/Sex Fits − Non−pregnant Females 2+ Years Old − Moderate+Severe Anaemia Prevalence − Western Europe
WHO−2001 Adjustment Method
Moderate+Severe Anaemia Prevalence
Age (year)
Uncertainty Interval
3 − Region
Location Hierarchy
1 − Global
2 − Super Region
3 − Region

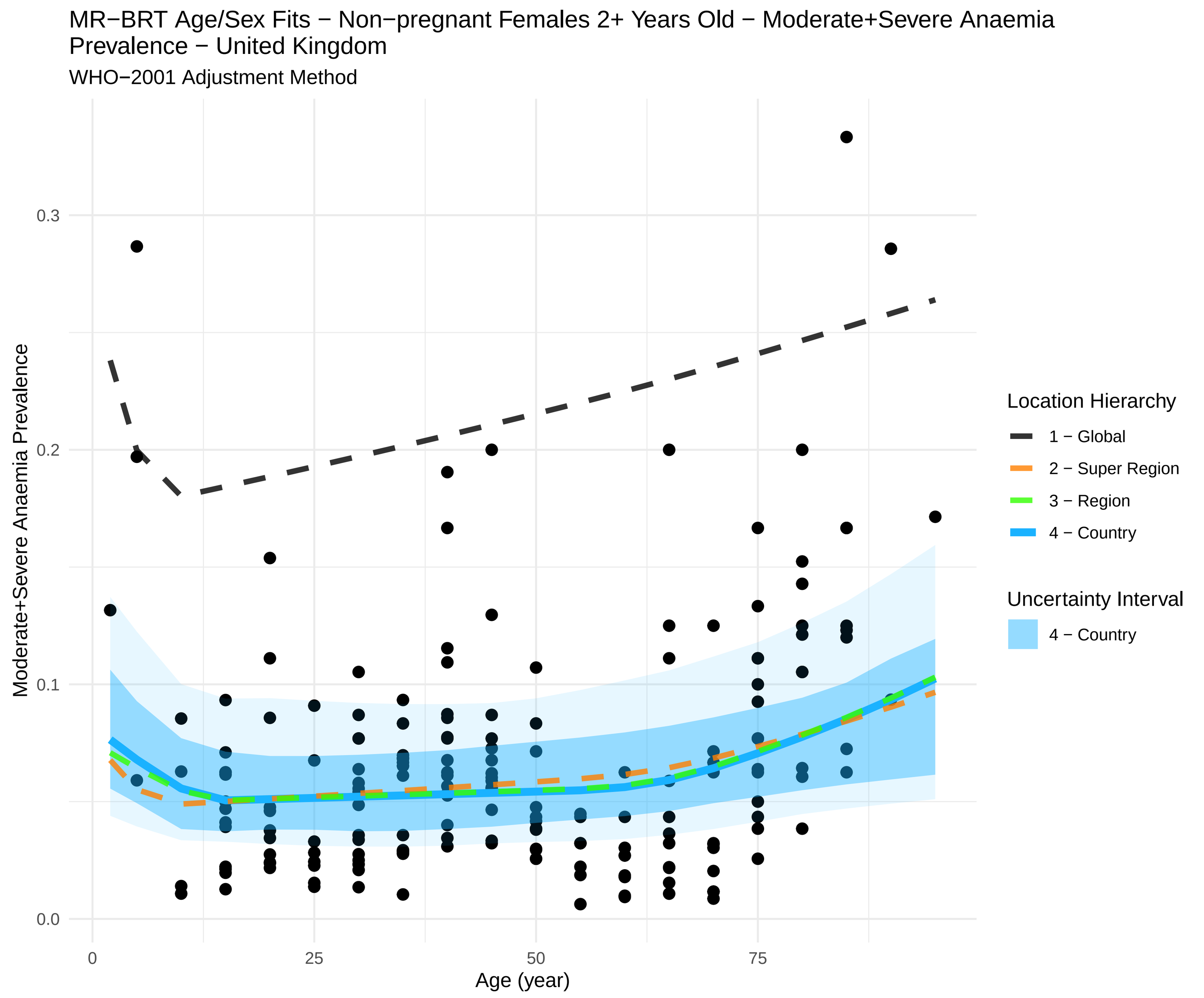
MR−BRT Age/Sex Fits − Non−pregnant Females 2+ Years Old − Moderate+Severe Anaemia Prevalence − United Kingdom
WHO−2001 Adjustment Method
Moderate+Severe Anaemia Prevalence
Age (year)
Location Hierarchy
1 − Global
2 − Super Region
3 − Region
4 − Country
Uncertainty Interval
4 − Country

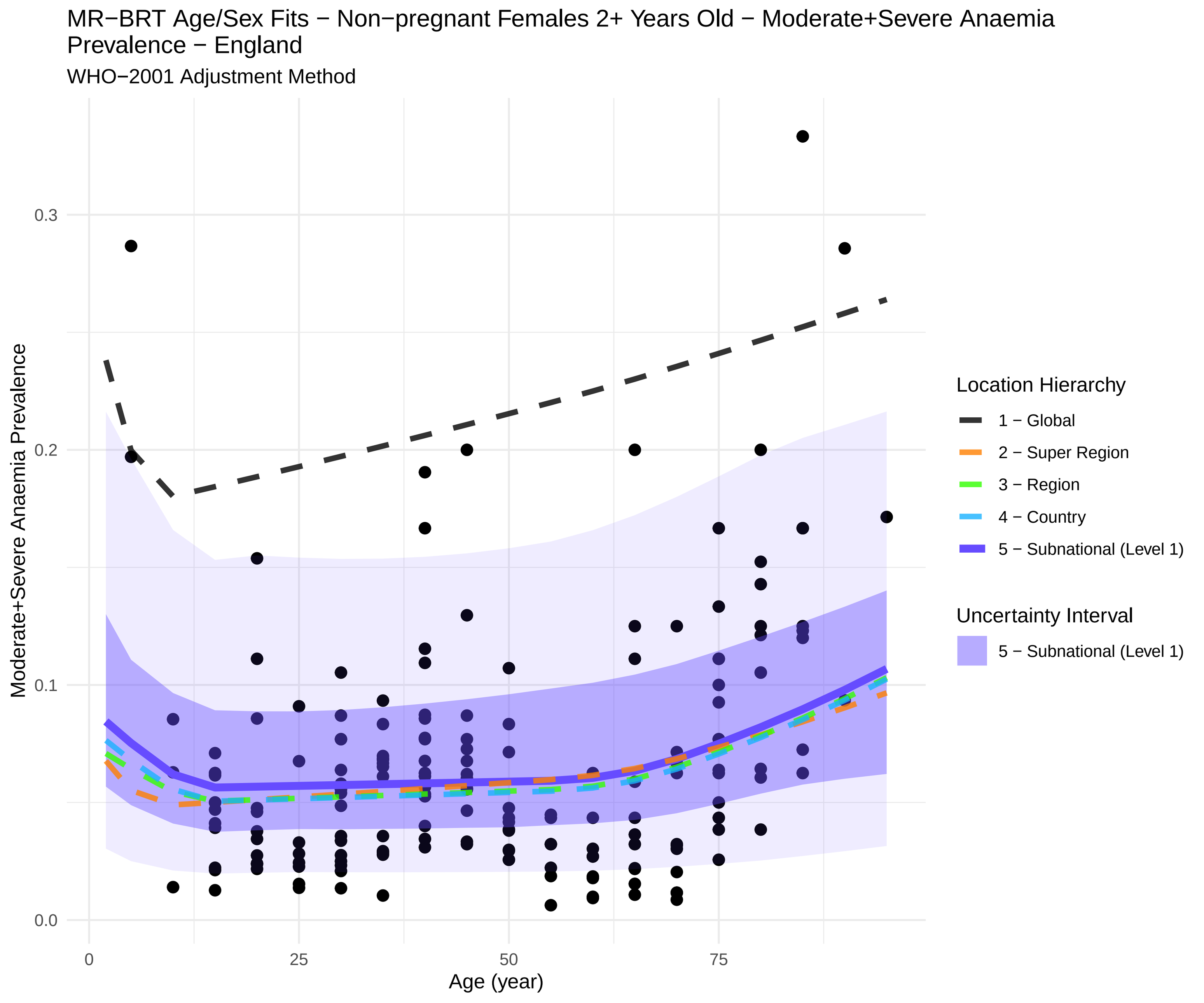
MR−BRT Age/Sex Fits − Non−pregnant Females 2+ Years Old − Moderate+Severe Anaemia Prevalence − England
WHO−2001 Adjustment Method
Moderate+Severe Anaemia Prevalence
Age (year)
Location Hierarchy
1 − Global
2 − Super Region
3 − Region
4 − Country
5 − Subnational (Level 1)
Uncertainty Interval
5 − Subnational (Level 1)

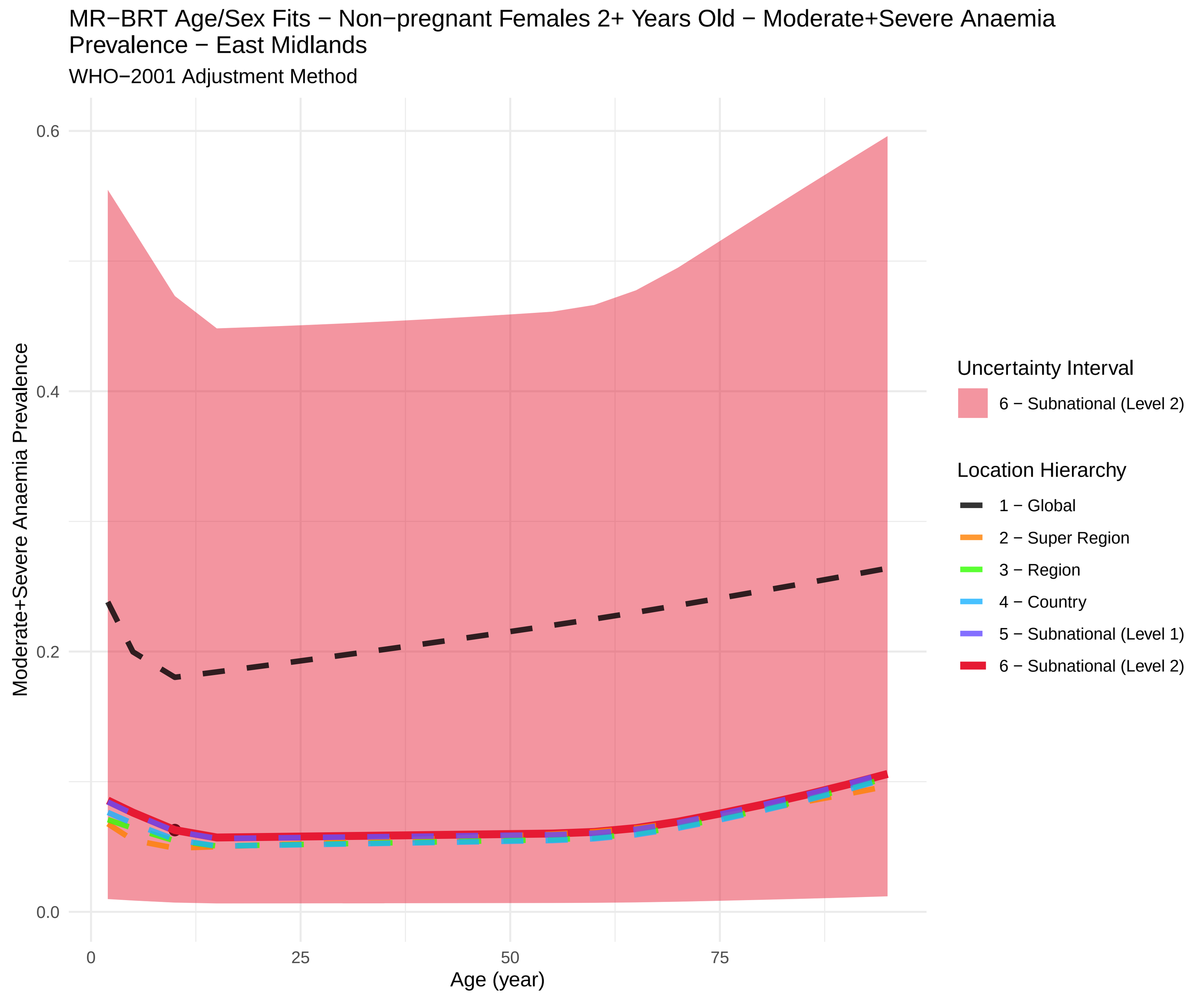
MR−BRT Age/Sex Fits − Non−pregnant Females 2+ Years Old − Moderate+Severe Anaemia Prevalence − East Midlands
WHO−2001 Adjustment Method
Moderate+Severe Anaemia Prevalence
Age (year)
Uncertainty Interval
6 − Subnational (Level 2)
Location Hierarchy
1 − Global
2 − Super Region
3 − Region
4 − Country
5 − Subnational (Level 1)
6 − Subnational (Level 2)

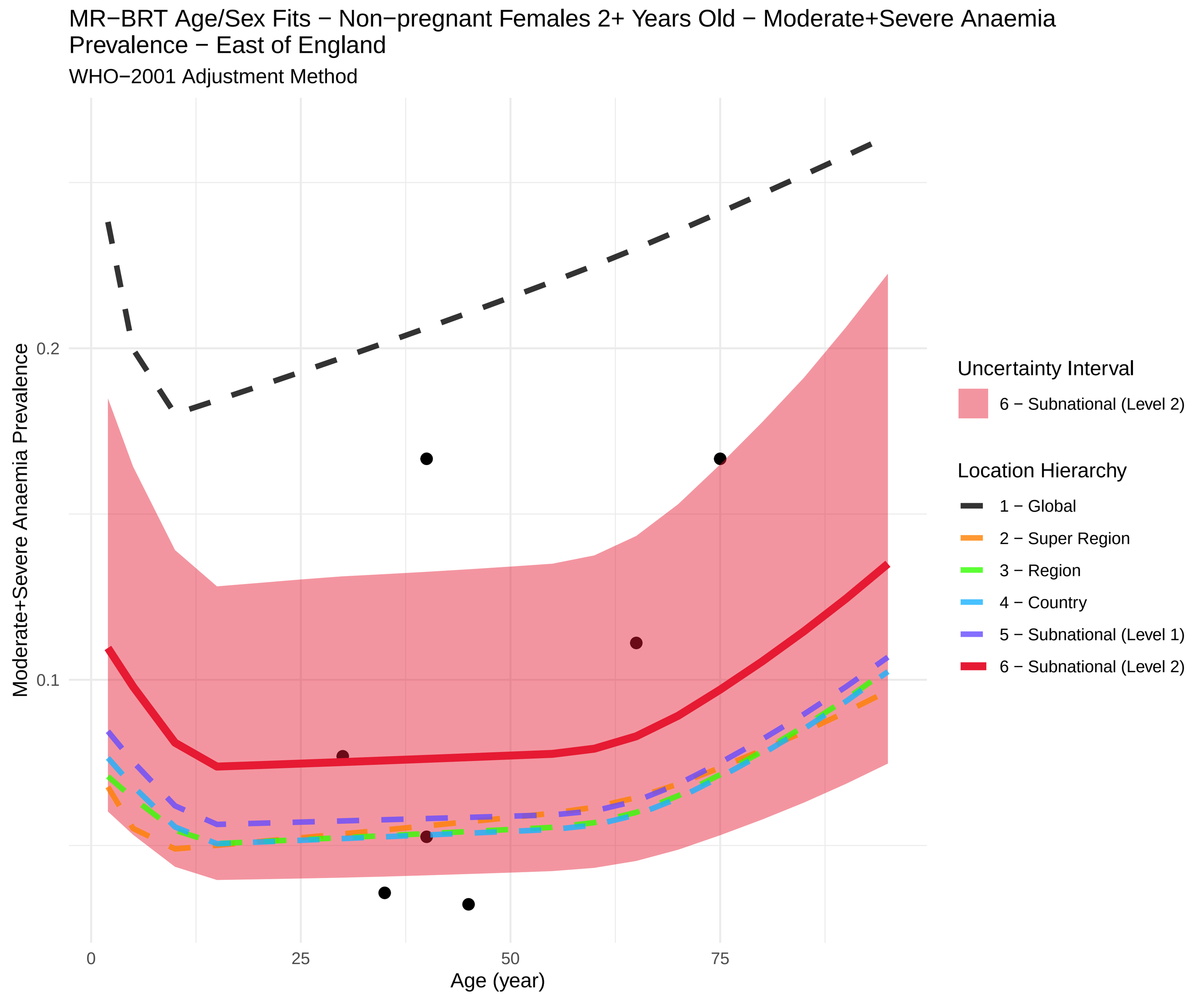
MR−BRT Age/Sex Fits − Non−pregnant Females 2+ Years Old − Moderate+Severe Anaemia Prevalence − East of England
WHO−2001 Adjustment Method
Moderate+Severe Anaemia Prevalence
Age (year)
Uncertainty Interval
6 − Subnational (Level 2)
Location Hierarchy
1 − Global
2 − Super Region
3 − Region
4 − Country
5 − Subnational (Level 1)
6 − Subnational (Level 2)

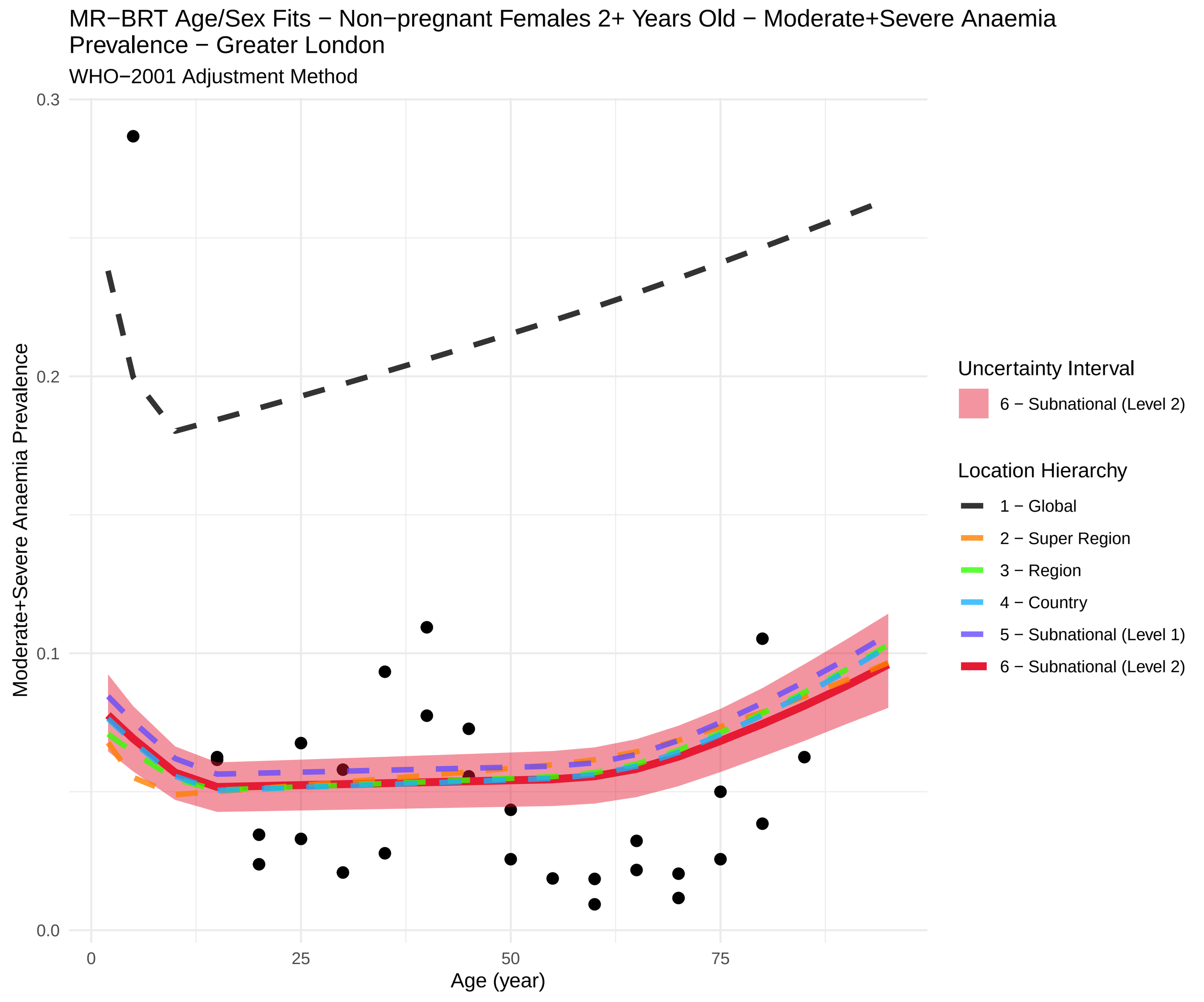
MR−BRT Age/Sex Fits − Non−pregnant Females 2+ Years Old − Moderate+Severe Anaemia Prevalence − Greater London
WHO−2001 Adjustment Method
Moderate+Severe Anaemia Prevalence
Age (year)
Uncertainty Interval
6 − Subnational (Level 2)
Location Hierarchy
1 − Global
2 − Super Region
3 − Region
4 − Country
5 − Subnational (Level 1)
6 − Subnational (Level 2)

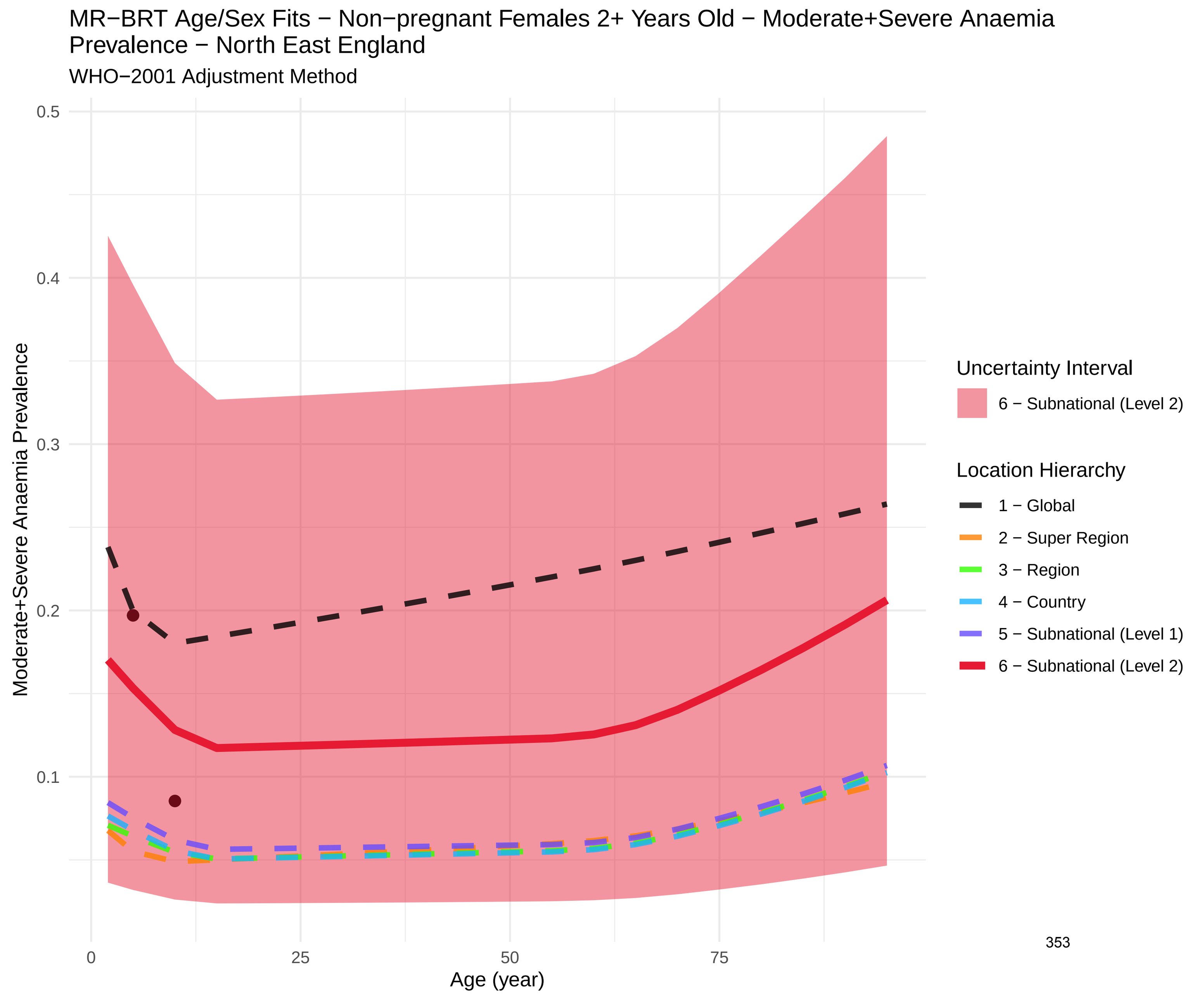
MR−BRT Age/Sex Fits − Non−pregnant Females 2+ Years Old − Moderate+Severe Anaemia Prevalence − North East England
WHO−2001 Adjustment Method
Moderate+Severe Anaemia Prevalence
Age (year)
Uncertainty Interval
6 − Subnational (Level 2)
Location Hierarchy
1 − Global
2 − Super Region
3 − Region
4 − Country
5 − Subnational (Level 1)
6 − Subnational (Level 2)

MR−BRT Age/Sex Fits − Non−pregnant Females 2+ Years Old − Moderate+Severe Anaemia Prevalence − North West England
WHO−2001 Adjustment Method

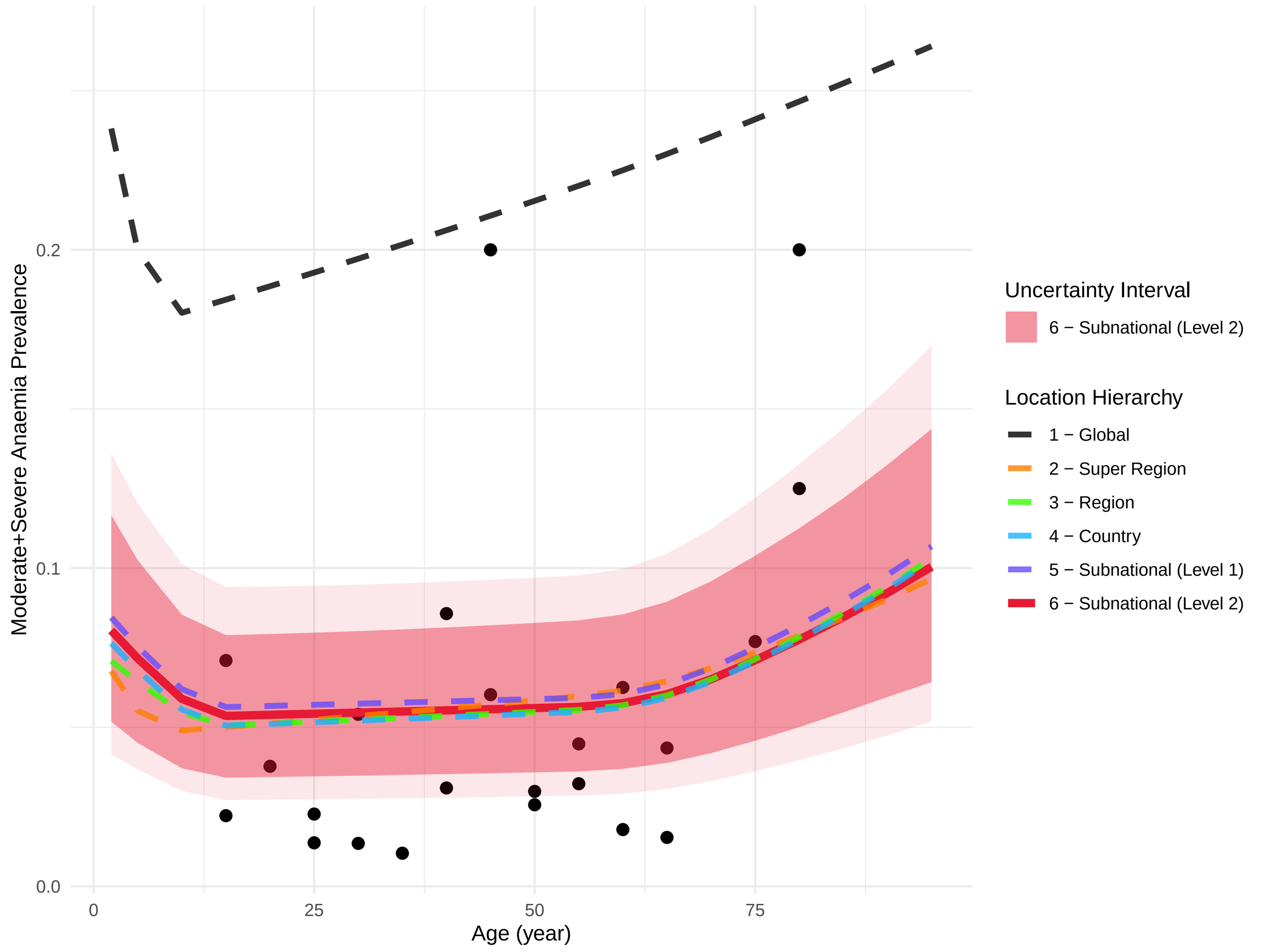

MR−BRT Age/Sex Fits − Non−pregnant Females 2+ Years Old − Moderate+Severe Anaemia Prevalence − South East England
WHO−2001 Adjustment Method

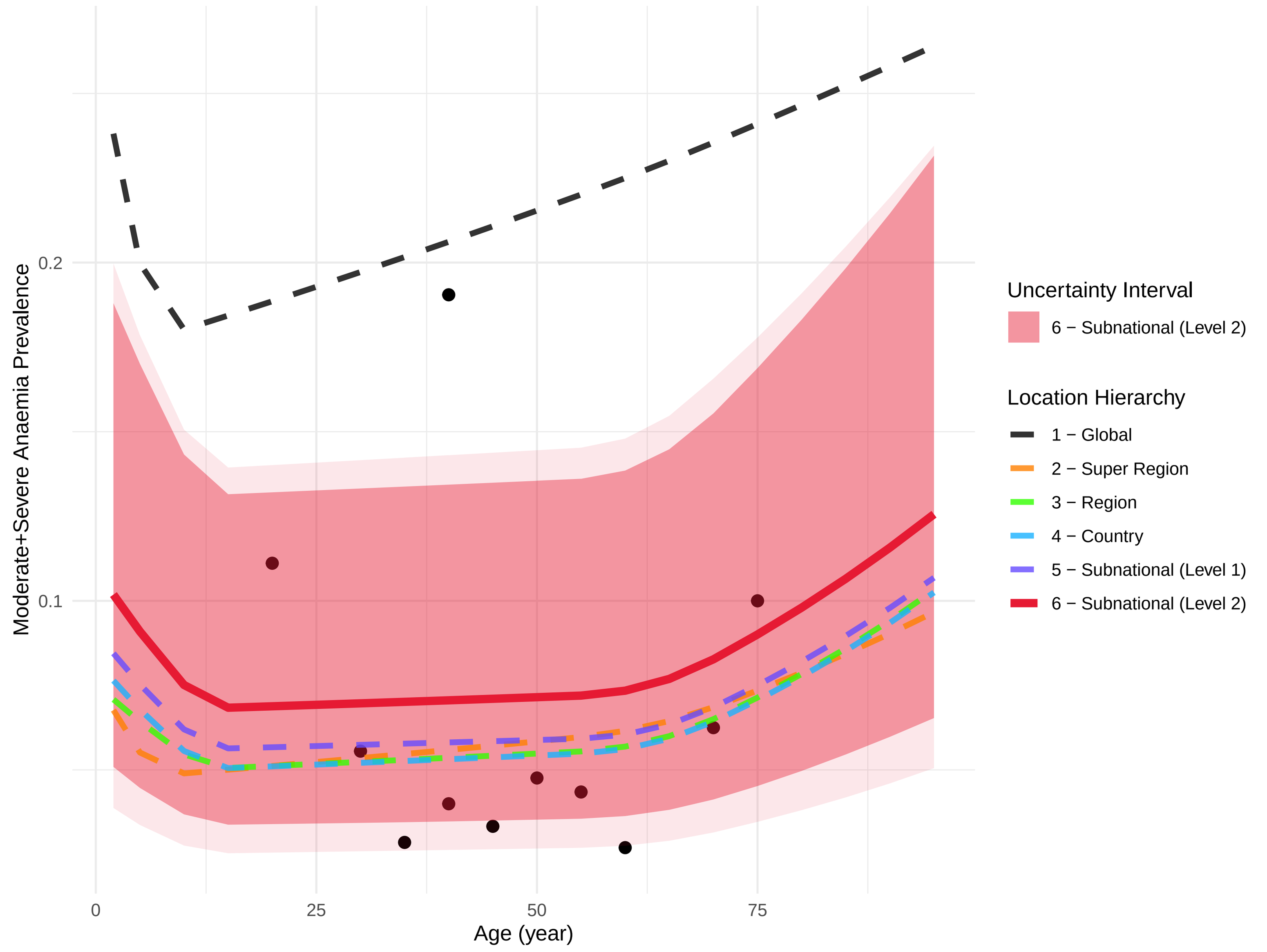

MR−BRT Age/Sex Fits − Non−pregnant Females 2+ Years Old − Moderate+Severe Anaemia Prevalence − South West England
WHO−2001 Adjustment Method

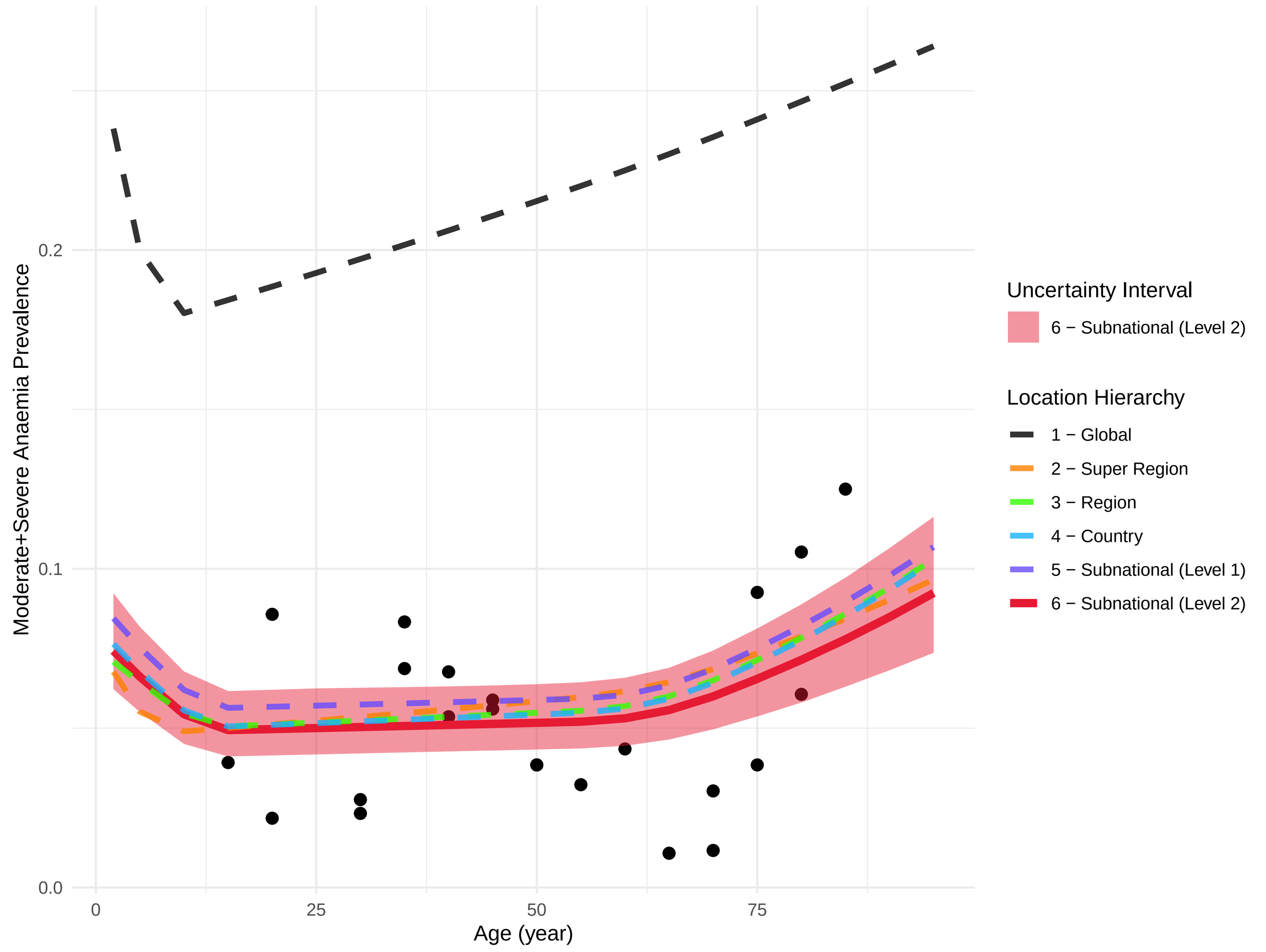

MR−BRT Age/Sex Fits − Non−pregnant Females 2+ Years Old − Moderate+Severe Anaemia Prevalence − West Midlands
WHO−2001 Adjustment Method

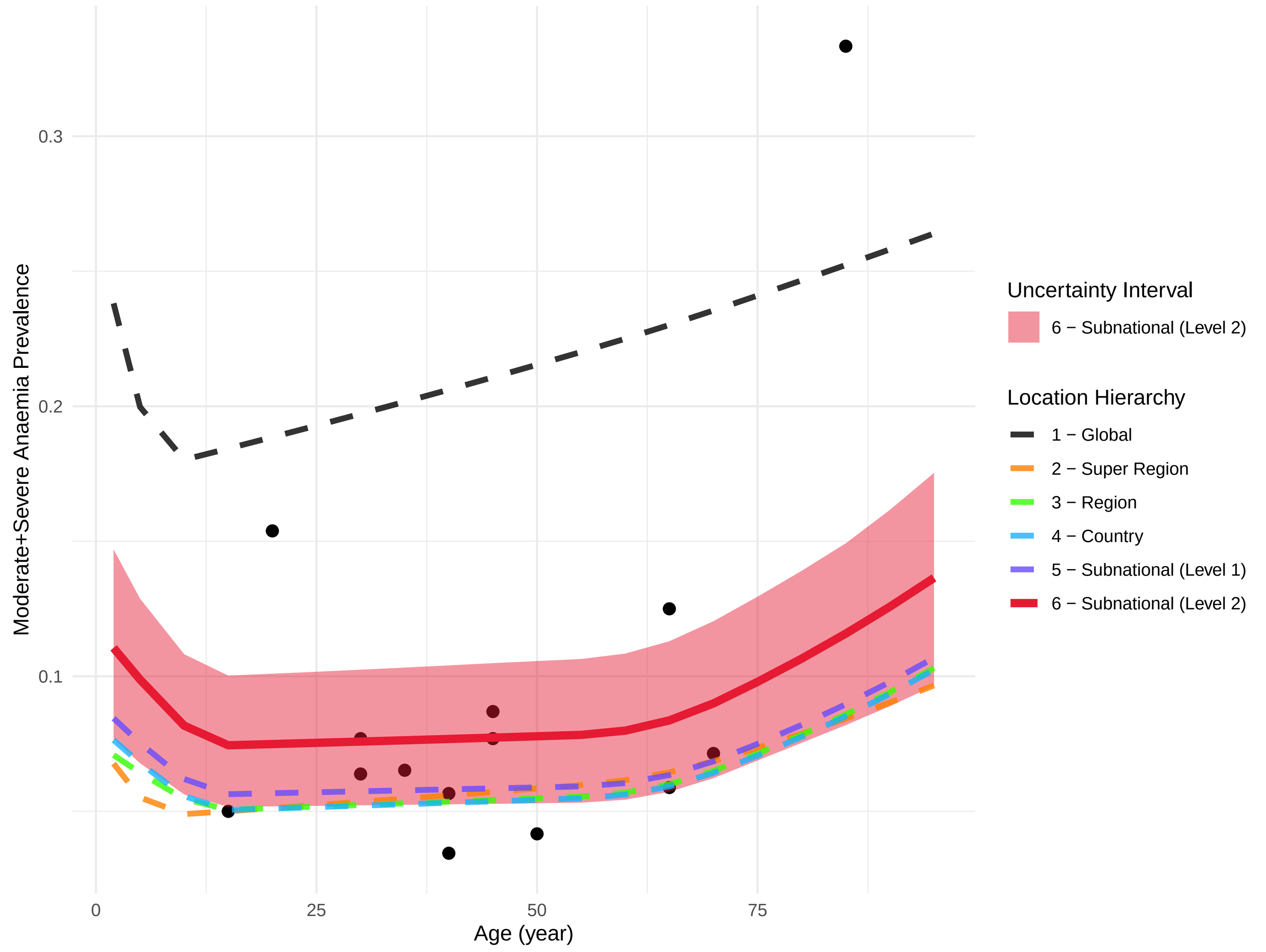

MR−BRT Age/Sex Fits − Non−pregnant Females 2+ Years Old − Moderate+Severe Anaemia Prevalence − Yorkshire and the Humber
WHO−2001 Adjustment Method

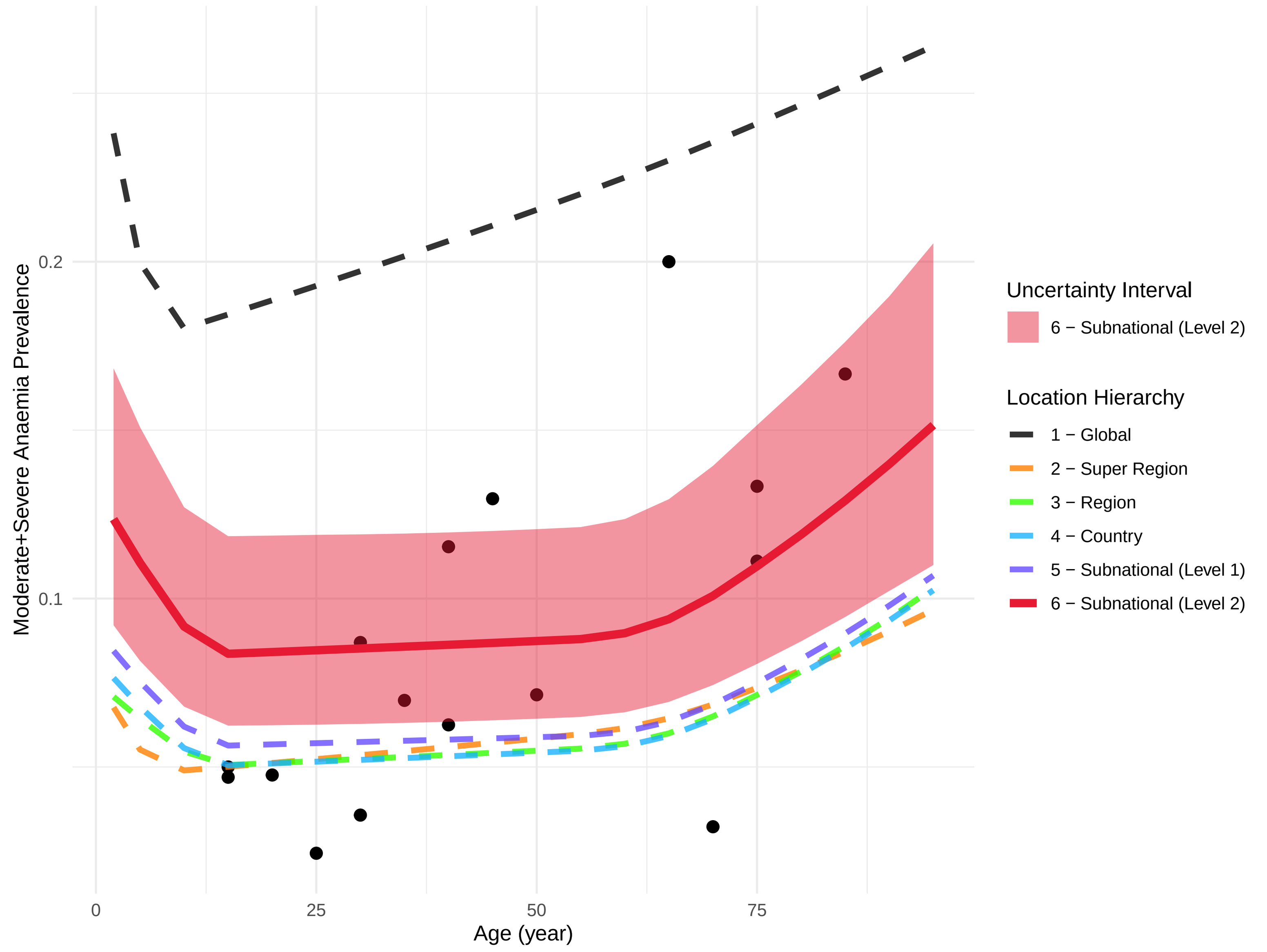

MR−BRT Age/Sex Fits − Non−pregnant Females 2+ Years Old − Moderate+Severe Anaemia Prevalence − Scotland
WHO−2001 Adjustment Method

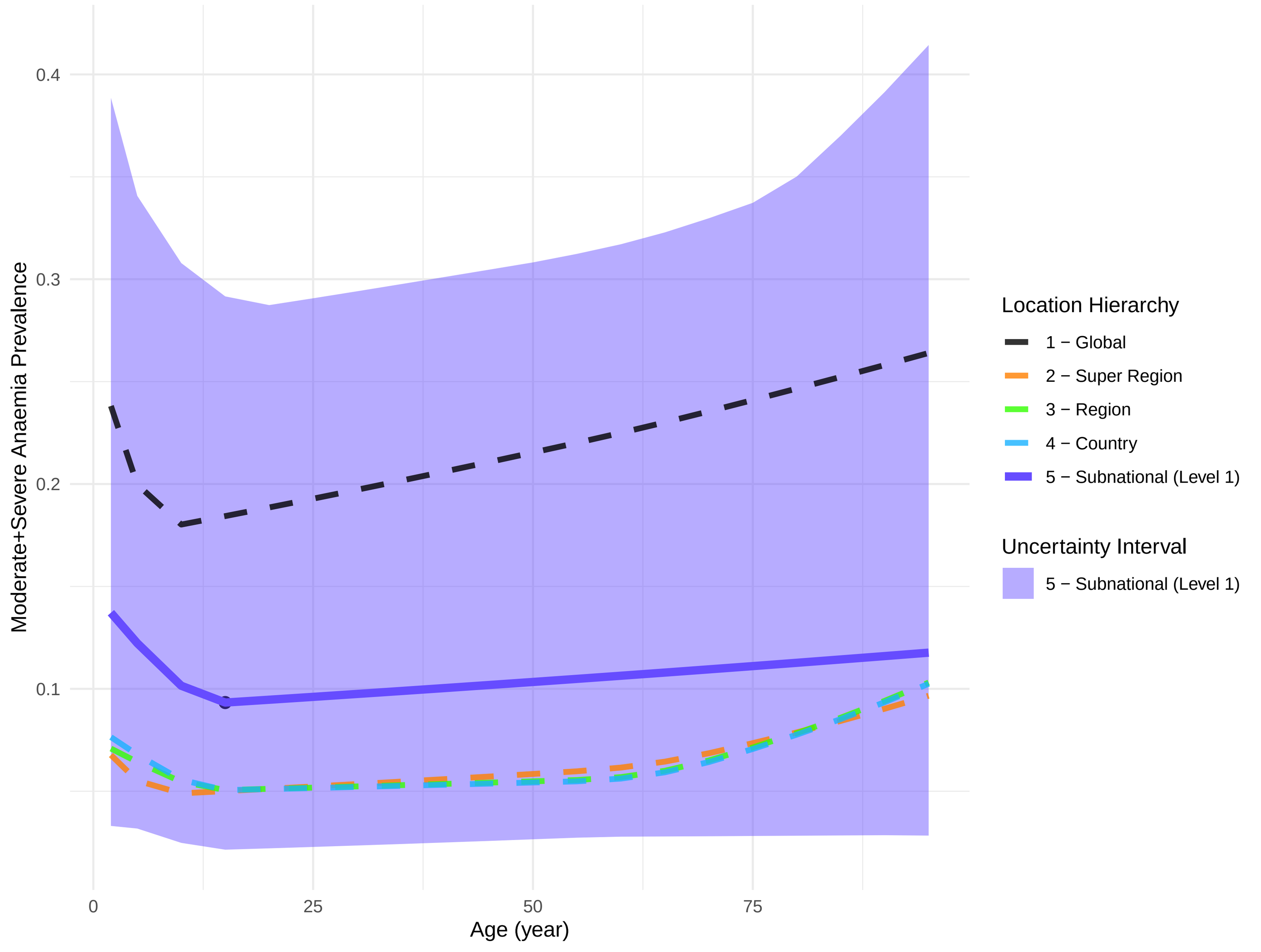

MR−BRT Age/Sex Fits − Non−pregnant Females 2+ Years Old − Moderate+Severe Anaemia Prevalence − Latin America and Caribbean
WHO−2001 Adjustment Method

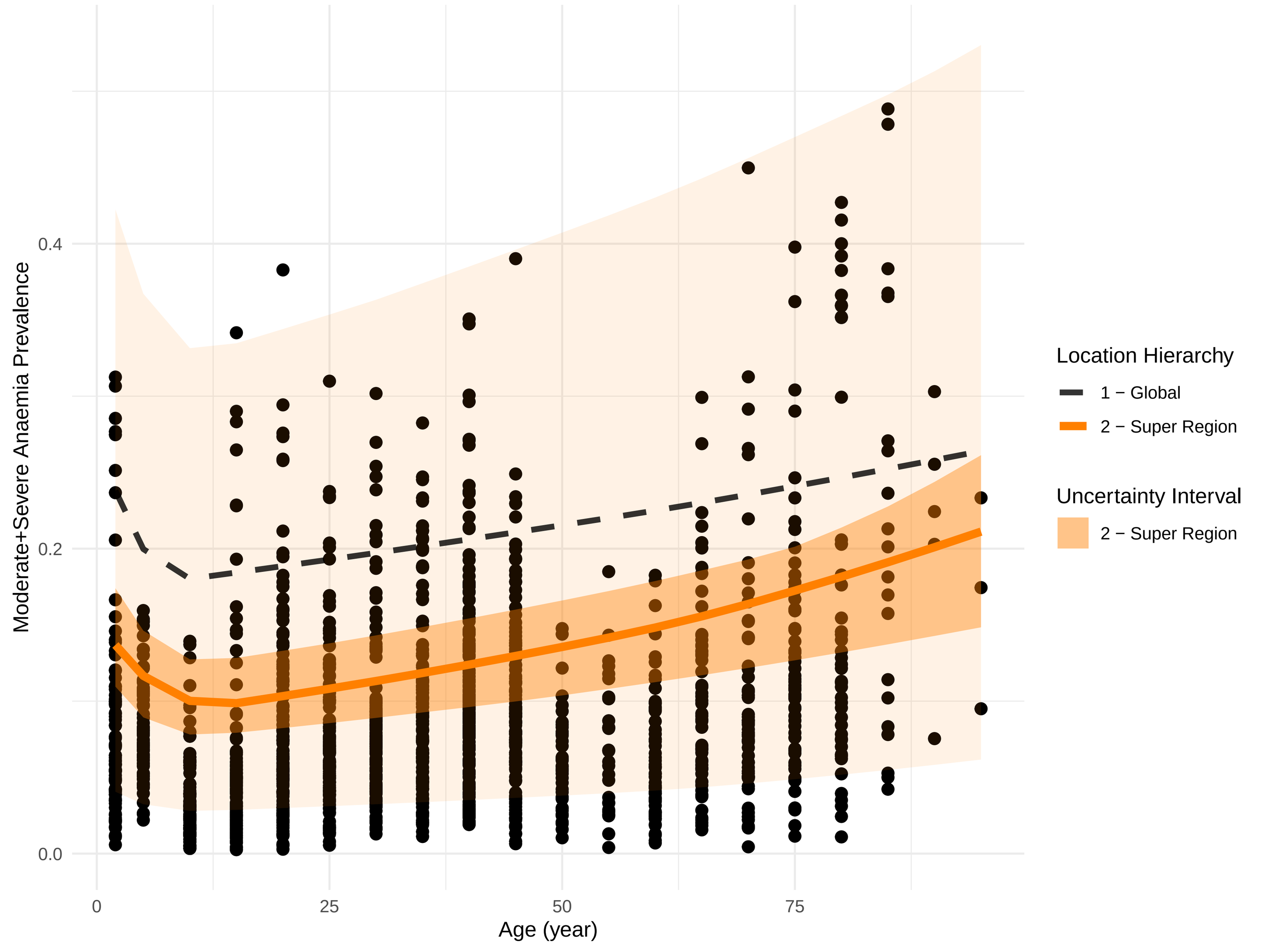

MR−BRT Age/Sex Fits − Non−pregnant Females 2+ Years Old − Moderate+Severe Anaemia Prevalence − Andean Latin America
WHO−2001 Adjustment Method

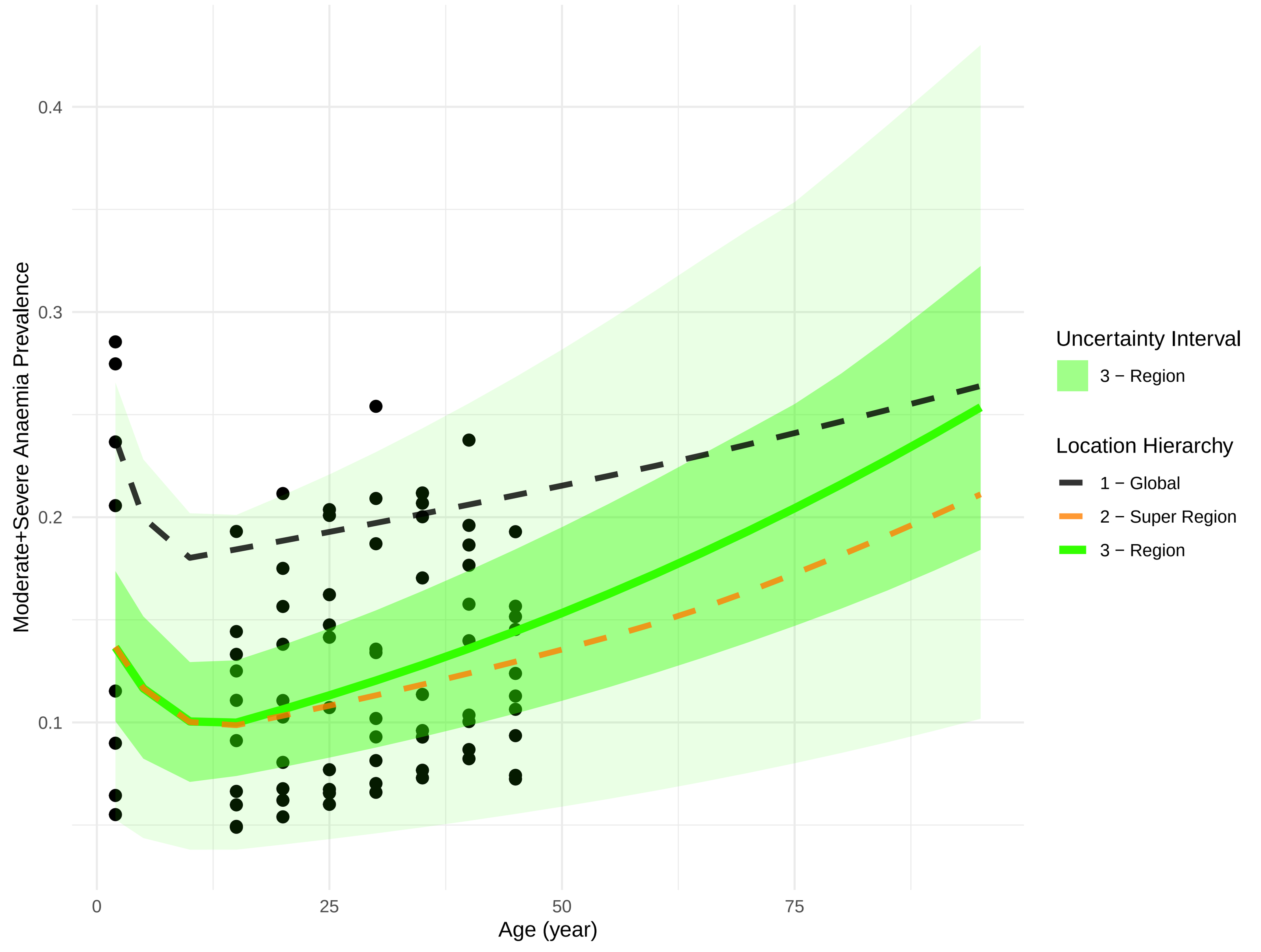

MR−BRT Age/Sex Fits − Non−pregnant Females 2+ Years Old − Moderate+Severe Anaemia Prevalence − Bolivia (Plurinational State of)
WHO−2001 Adjustment Method

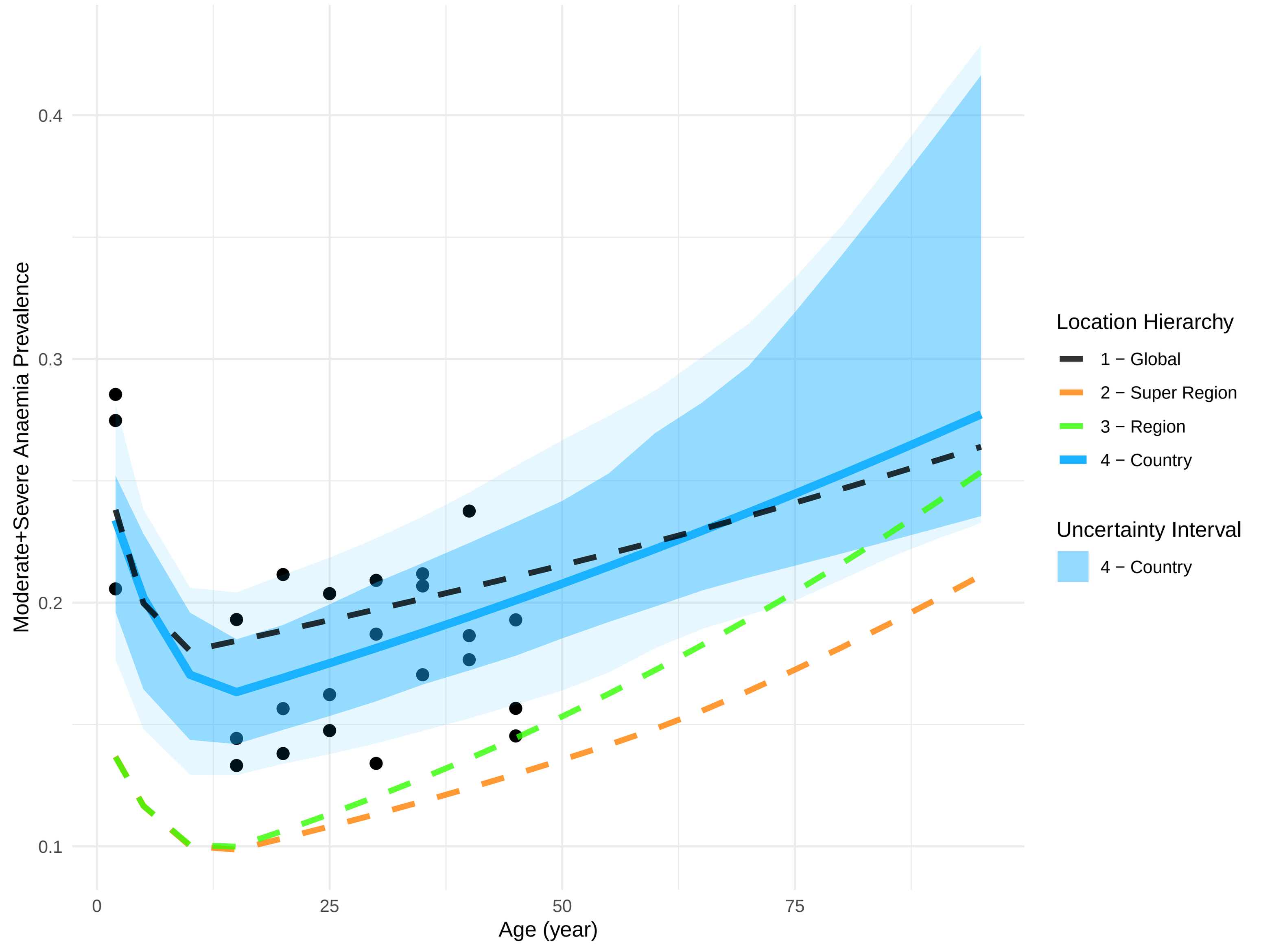

MR−BRT Age/Sex Fits − Non−pregnant Females 2+ Years Old − Moderate+Severe Anaemia Prevalence − Peru
WHO−2001 Adjustment Method

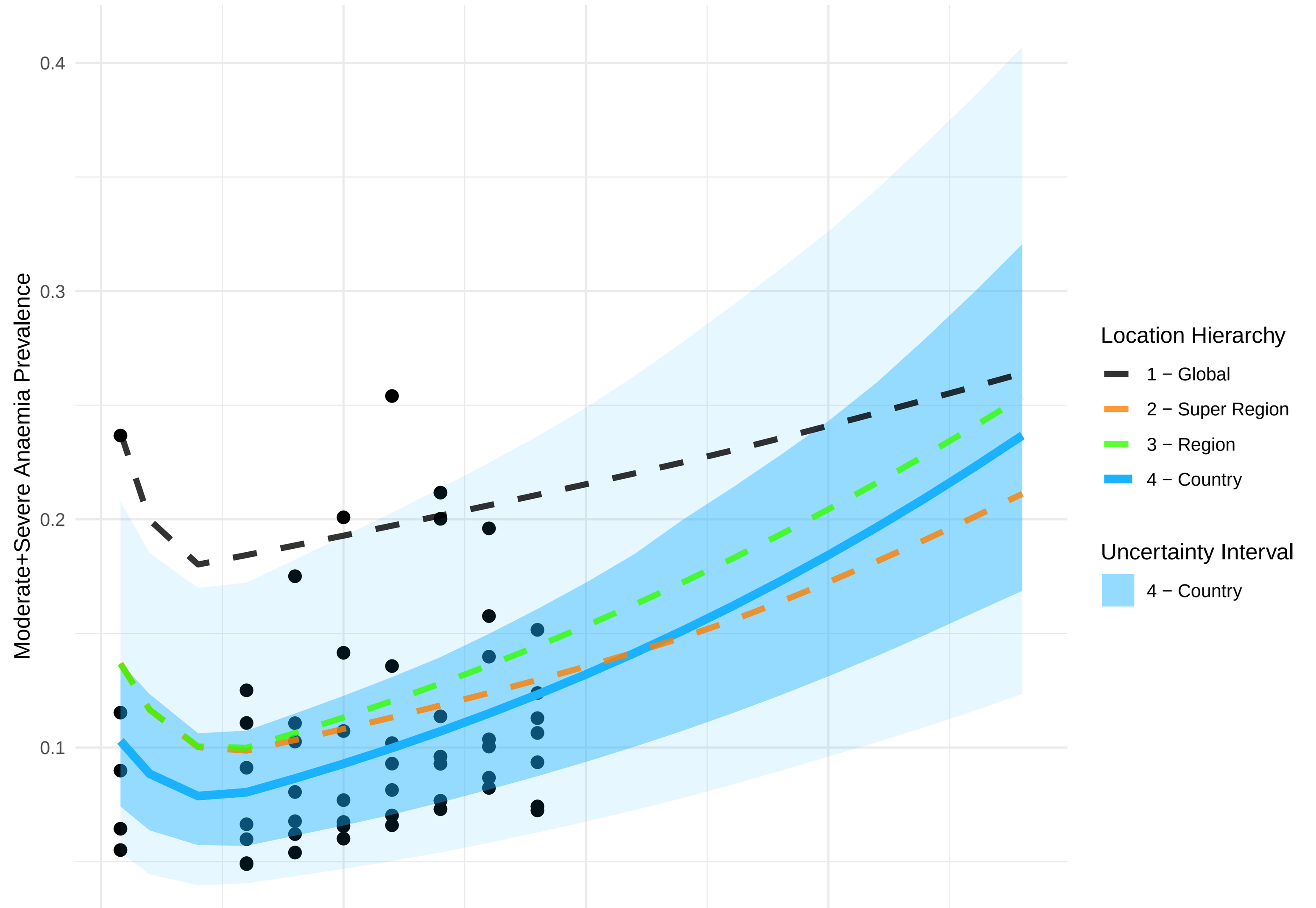

MR−BRT Age/Sex Fits − Non−pregnant Females 2+ Years Old − Moderate+Severe Anaemia Prevalence − Caribbean
WHO−2001 Adjustment Method

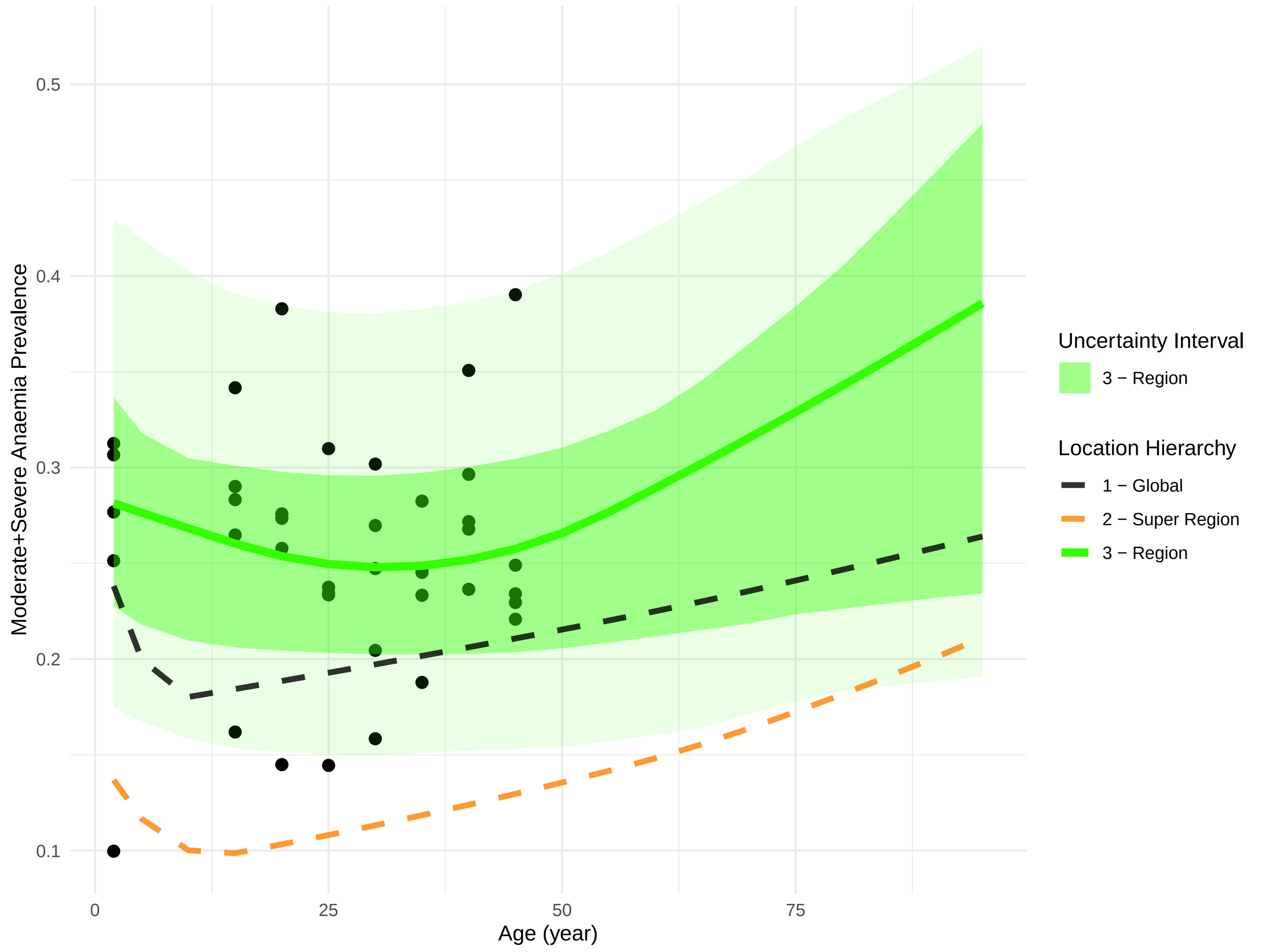

MR−BRT Age/Sex Fits − Non−pregnant Females 2+ Years Old − Moderate+Severe Anaemia Prevalence − Guyana
WHO−2001 Adjustment Method

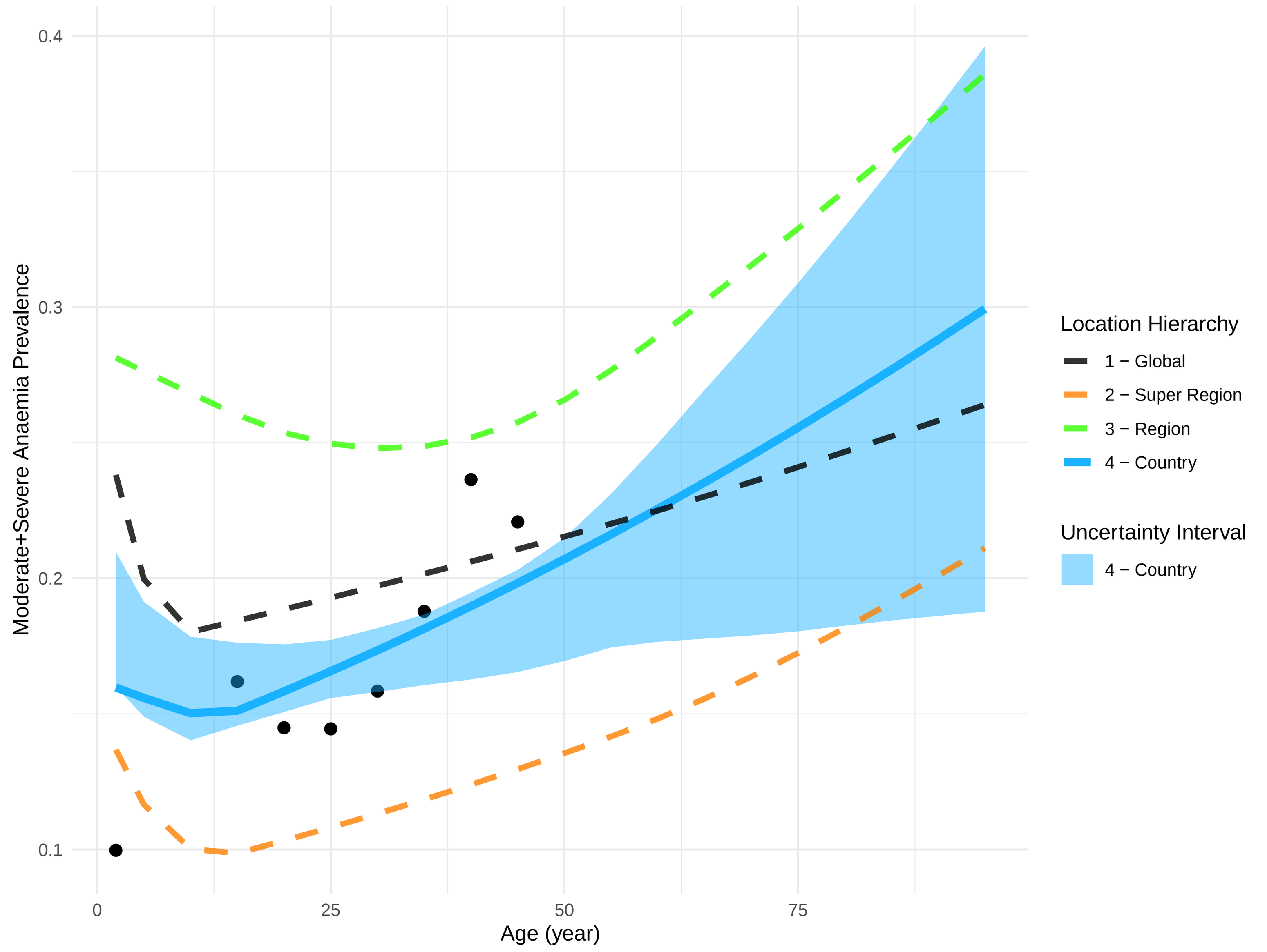

MR−BRT Age/Sex Fits − Non−pregnant Females 2+ Years Old − Moderate+Severe Anaemia Prevalence − Haiti
WHO−2001 Adjustment Method

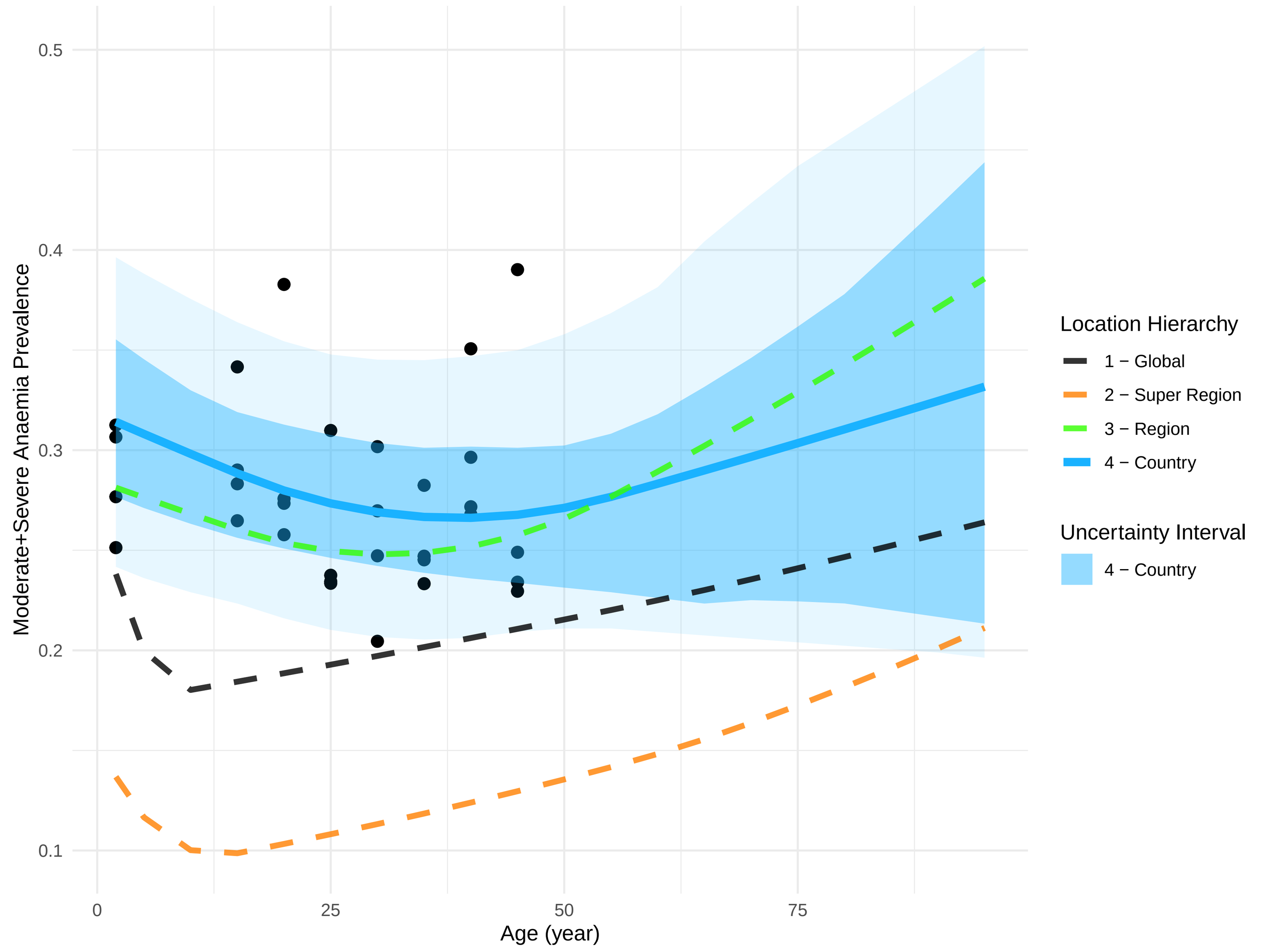

MR−BRT Age/Sex Fits − Non−pregnant Females 2+ Years Old − Moderate+Severe Anaemia Prevalence − Central Latin America
WHO−2001 Adjustment Method

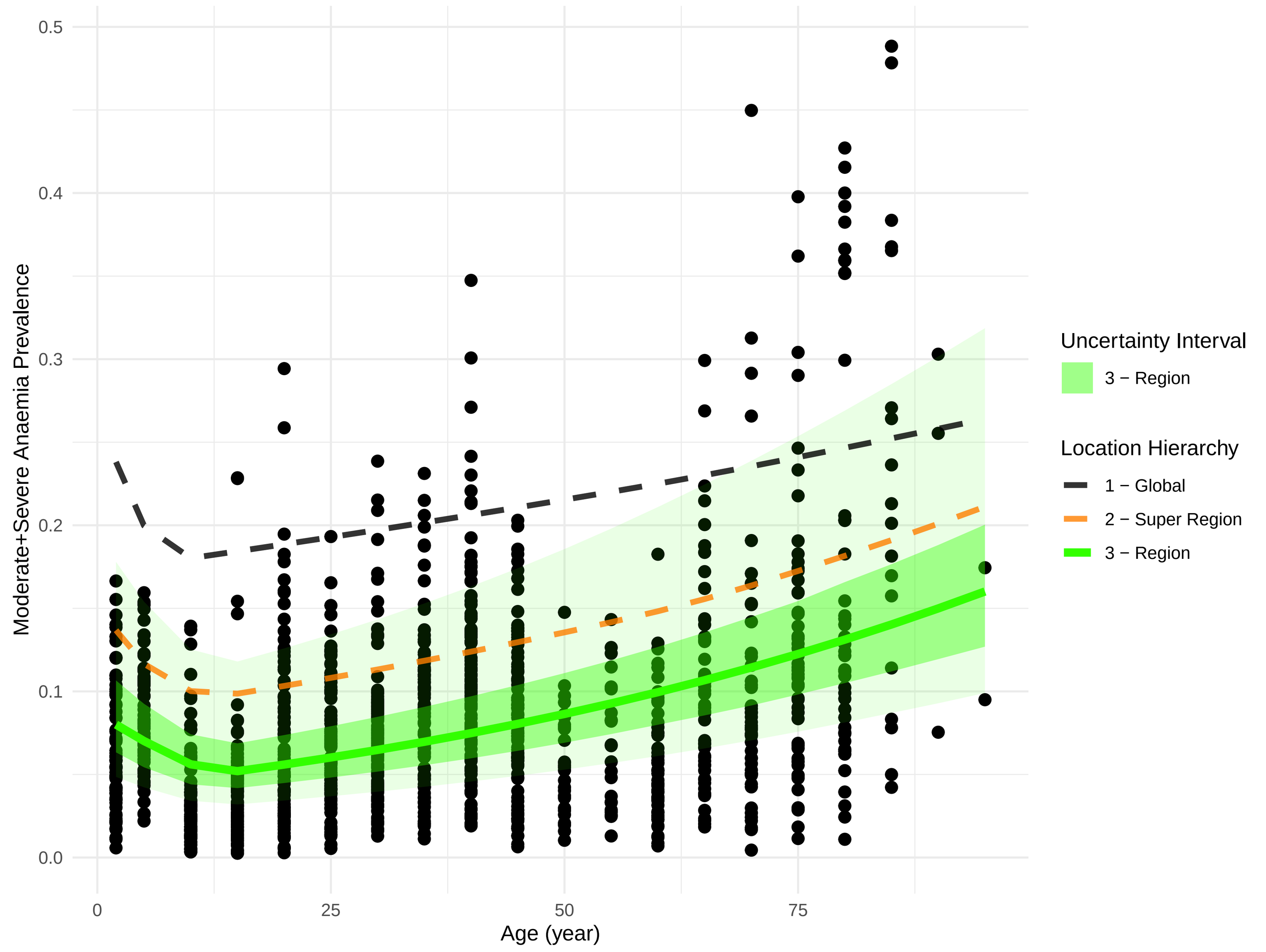

MR−BRT Age/Sex Fits − Non−pregnant Females 2+ Years Old − Moderate+Severe Anaemia Prevalence − El Salvador
WHO−2001 Adjustment Method

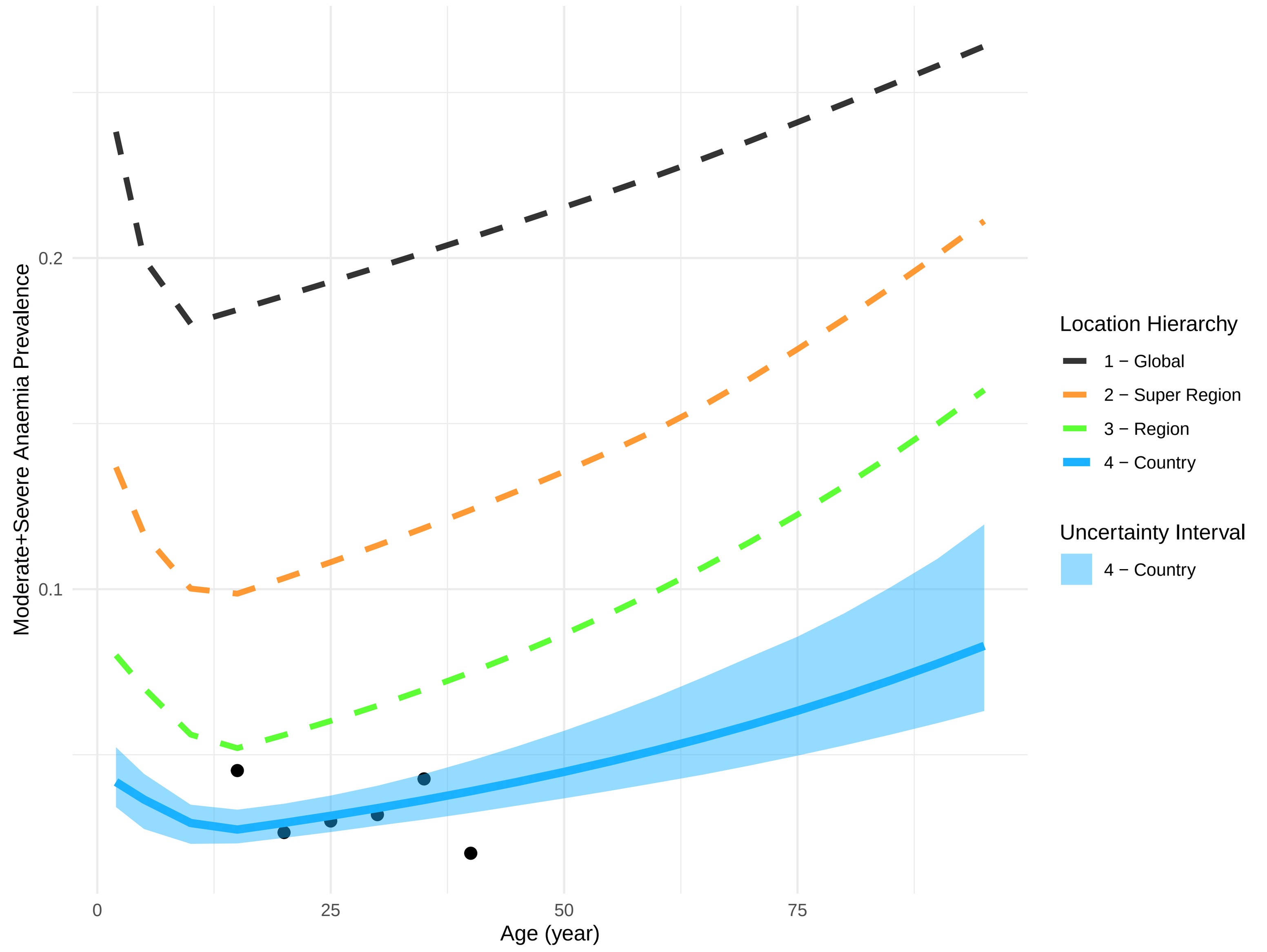

MR−BRT Age/Sex Fits − Non−pregnant Females 2+ Years Old − Moderate+Severe Anaemia Prevalence − Guatemala
WHO−2001 Adjustment Method

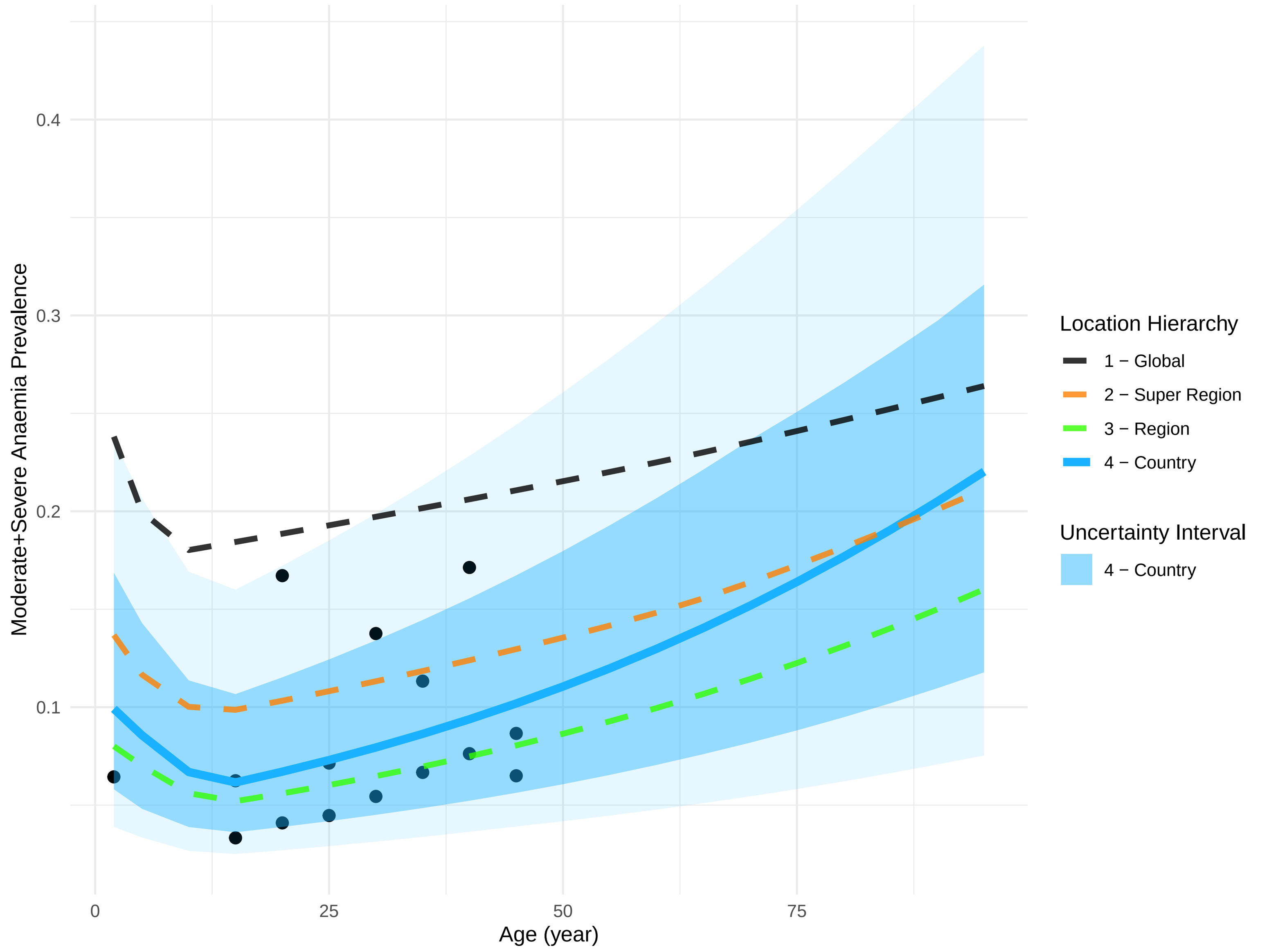

MR−BRT Age/Sex Fits − Non−pregnant Females 2+ Years Old − Moderate+Severe Anaemia Prevalence − Honduras
WHO−2001 Adjustment Method

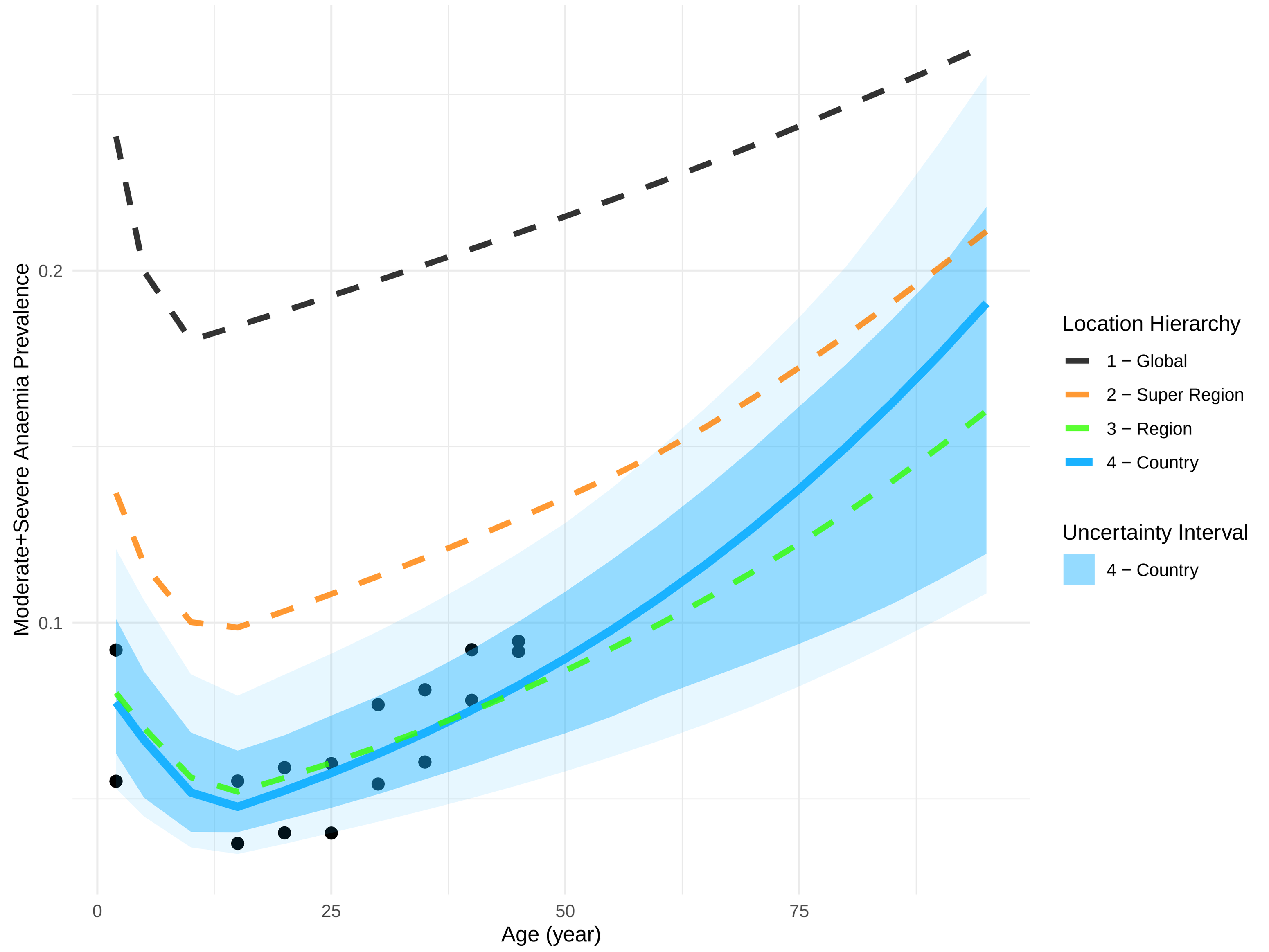

MR−BRT Age/Sex Fits − Non−pregnant Females 2+ Years Old − Moderate+Severe Anaemia Prevalence − Mexico
WHO−2001 Adjustment Method

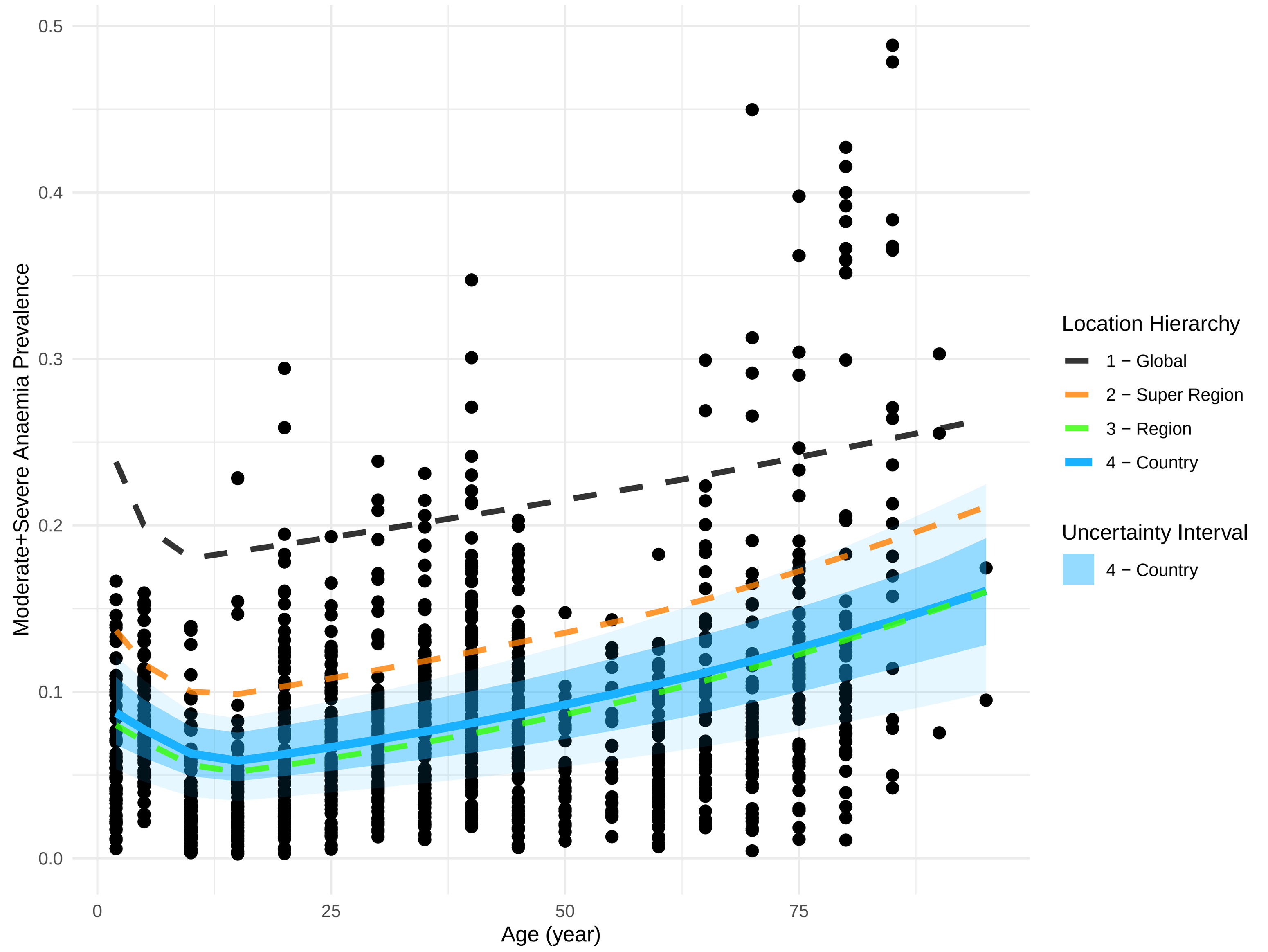

MR−BRT Age/Sex Fits − Non−pregnant Females 2+ Years Old − Moderate+Severe Anaemia Prevalence − Aguascalientes
WHO−2001 Adjustment Method

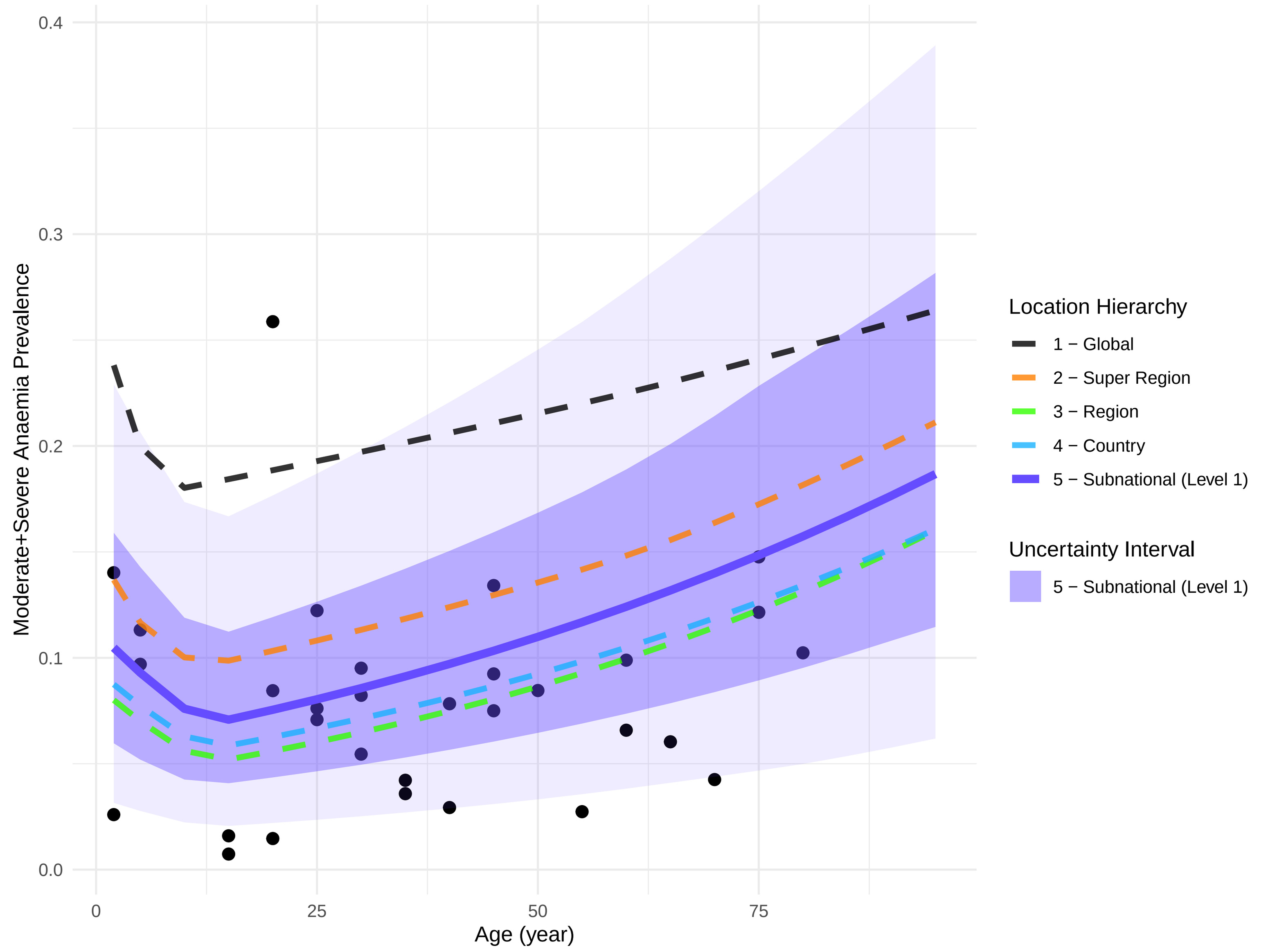

MR−BRT Age/Sex Fits − Non−pregnant Females 2+ Years Old − Moderate+Severe Anaemia Prevalence − Baja California
WHO−2001 Adjustment Method

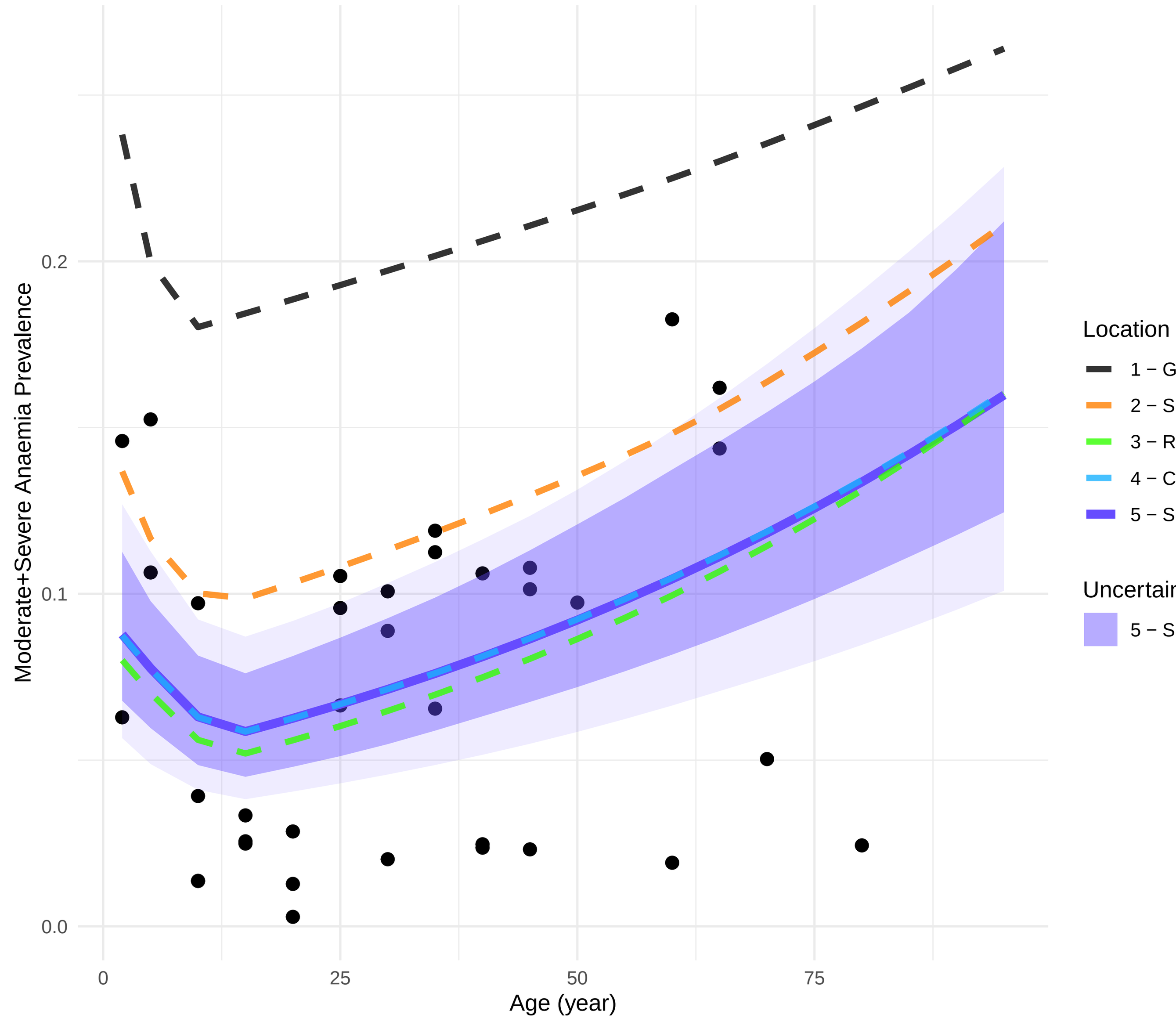

MR−BRT Age/Sex Fits − Non−pregnant Females 2+ Years Old − Moderate+Severe Anaemia Prevalence − Baja California Sur
WHO−2001 Adjustment Method

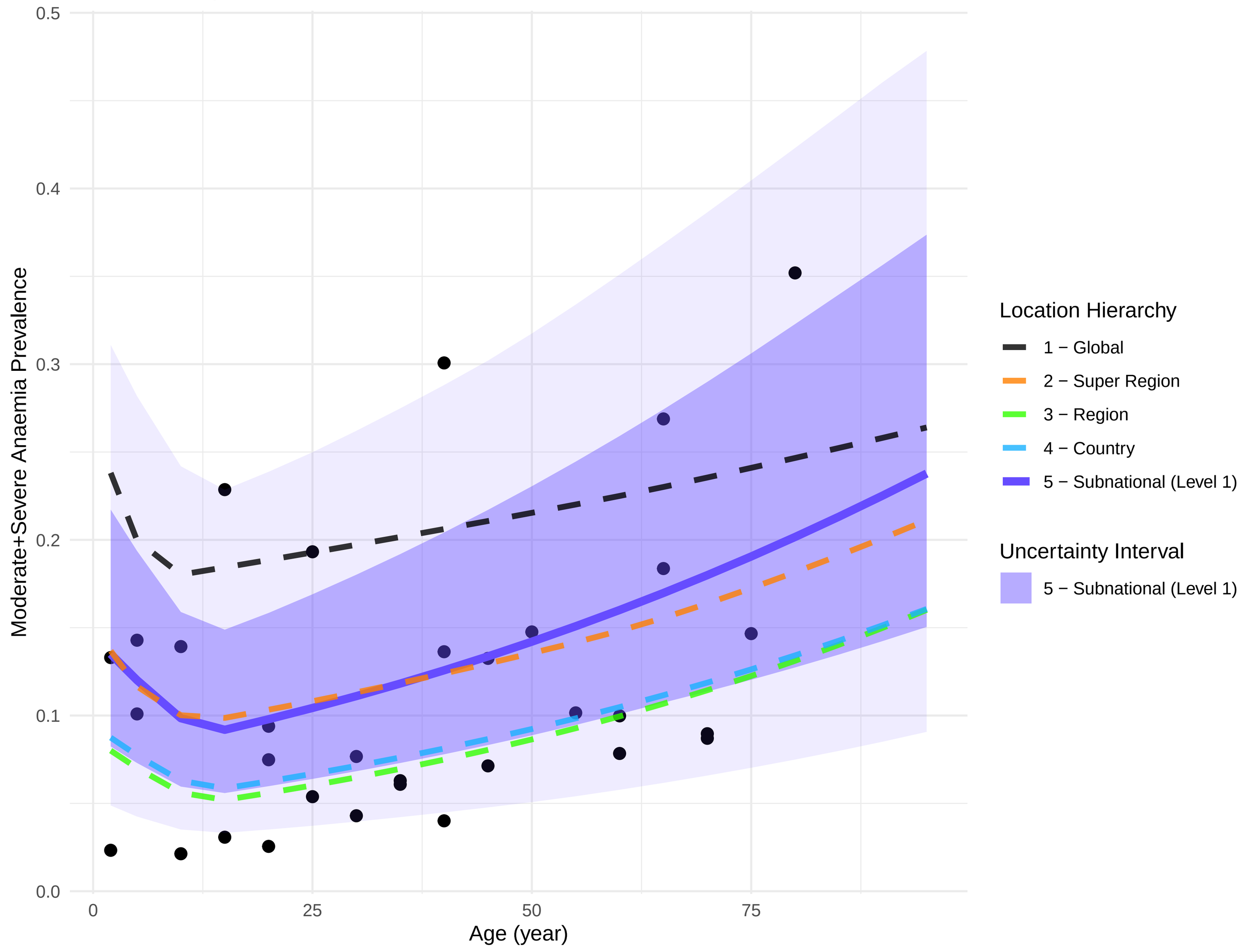

MR−BRT Age/Sex Fits − Non−pregnant Females 2+ Years Old − Moderate+Severe Anaemia Prevalence − Campeche
WHO−2001 Adjustment Method

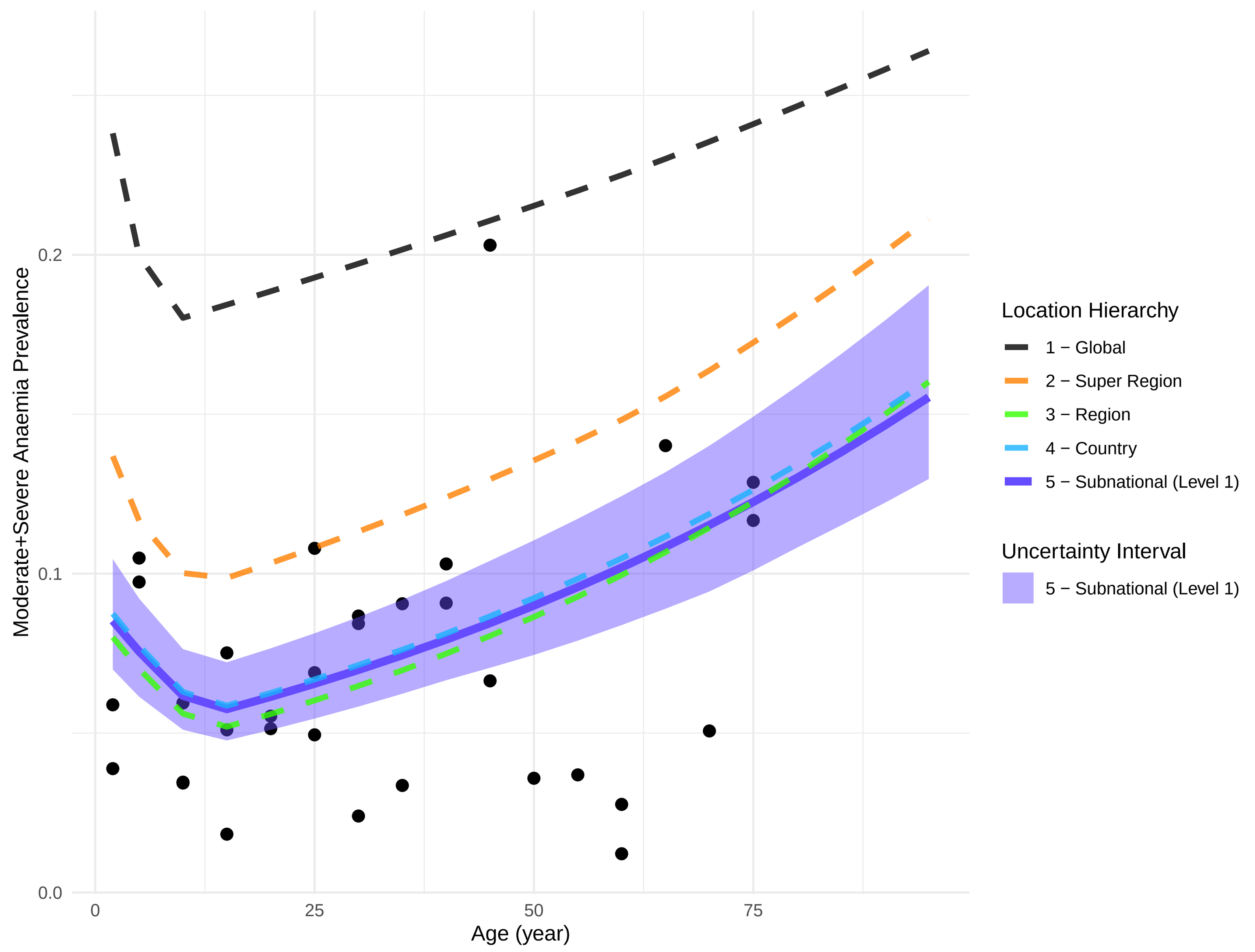

MR−BRT Age/Sex Fits − Non−pregnant Females 2+ Years Old − Moderate+Severe Anaemia Prevalence − Chiapas
WHO−2001 Adjustment Method

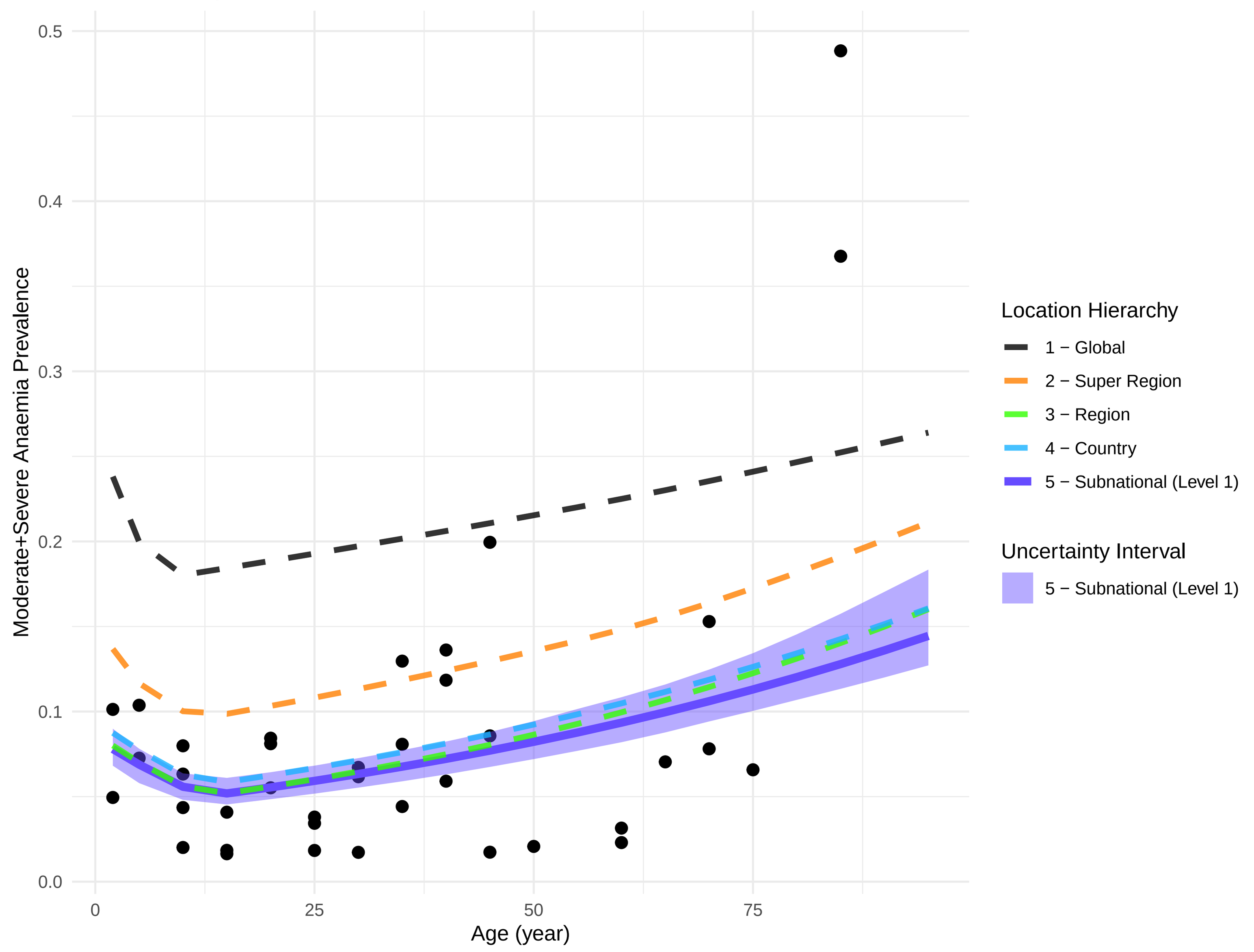

MR−BRT Age/Sex Fits − Non−pregnant Females 2+ Years Old − Moderate+Severe Anaemia Prevalence − Chihuahua
WHO−2001 Adjustment Method

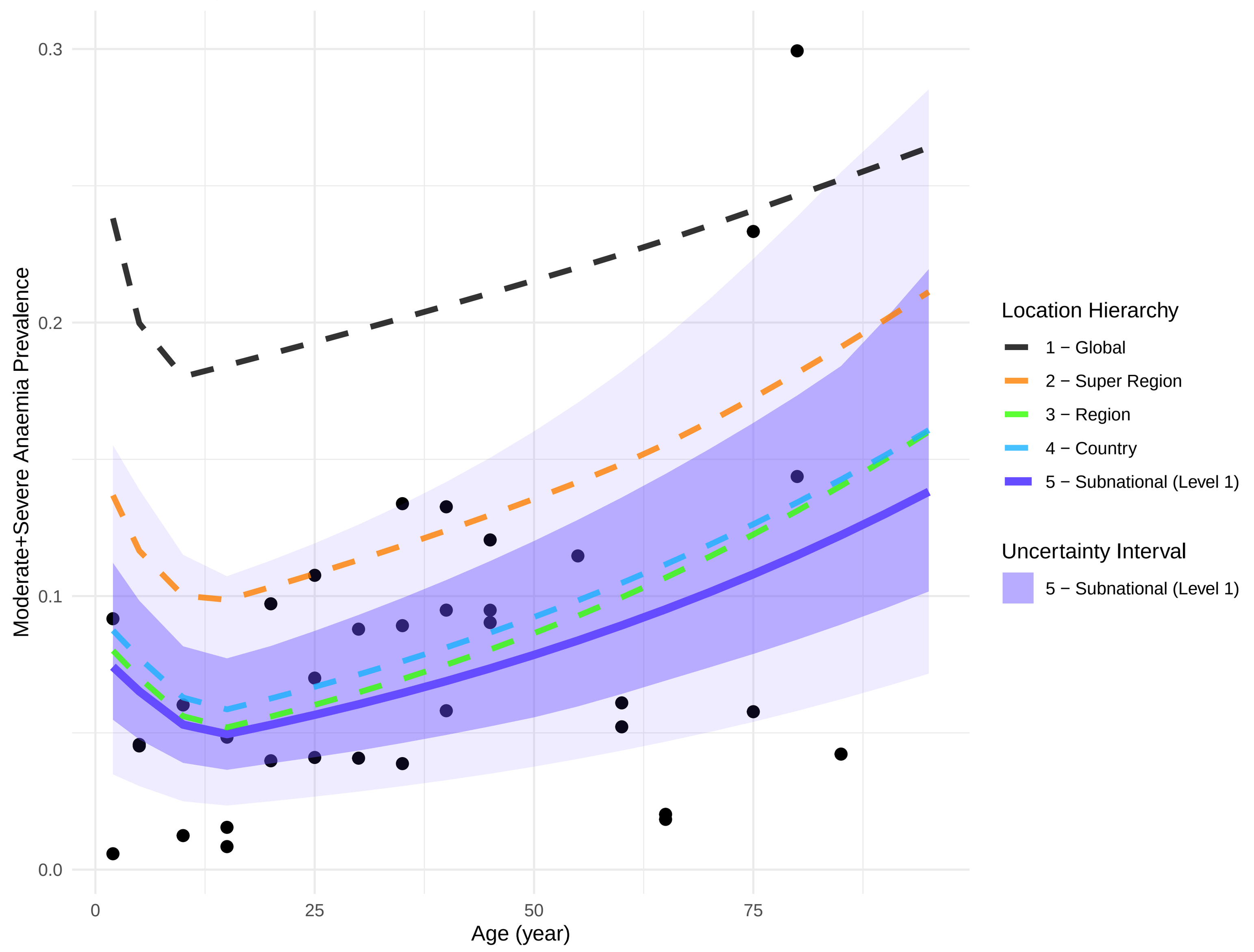

MR−BRT Age/Sex Fits − Non−pregnant Females 2+ Years Old − Moderate+Severe Anaemia Prevalence − Coahuila
WHO−2001 Adjustment Method

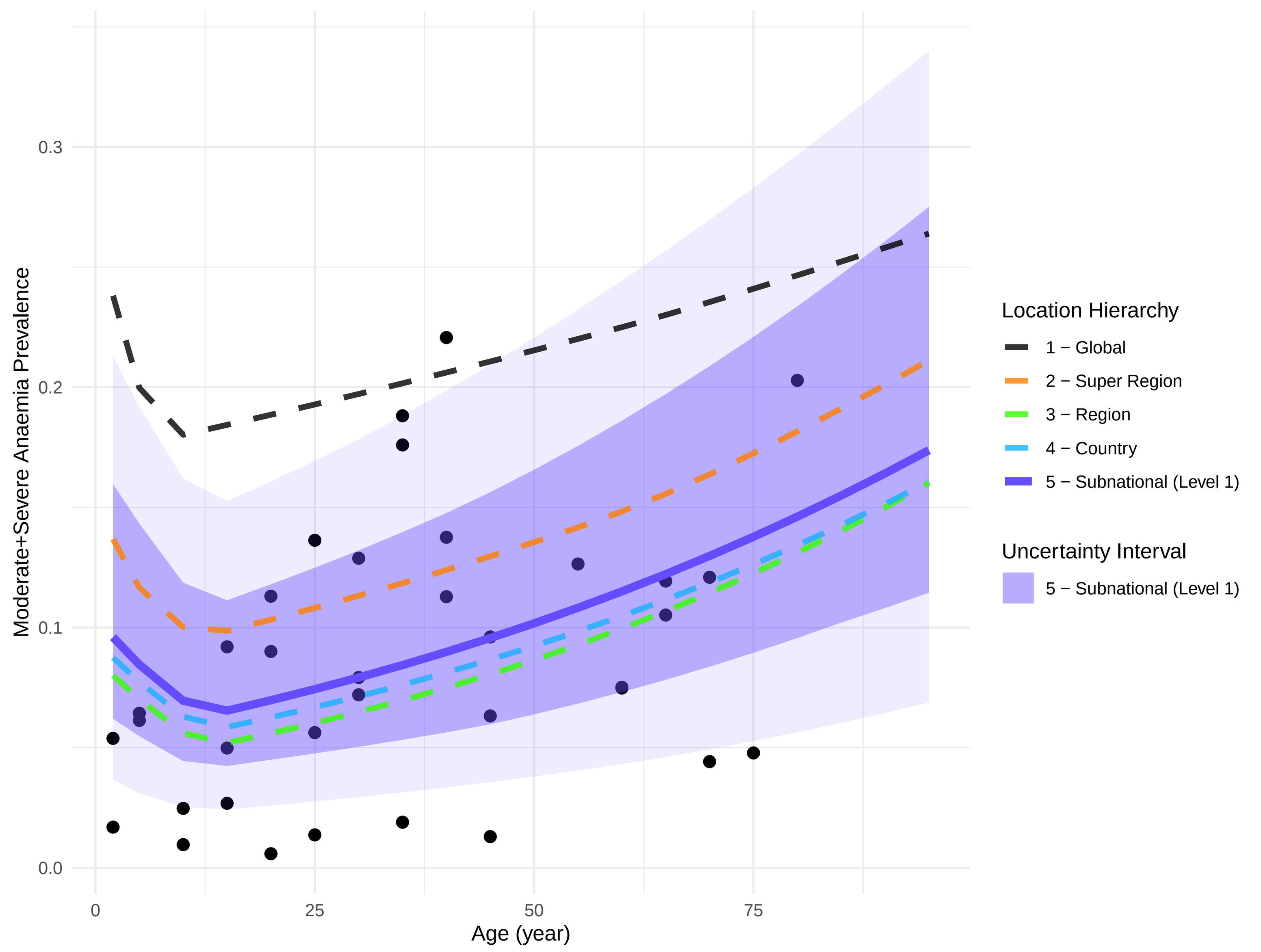

MR−BRT Age/Sex Fits − Non−pregnant Females 2+ Years Old − Moderate+Severe Anaemia Prevalence − Colima
WHO−2001 Adjustment Method

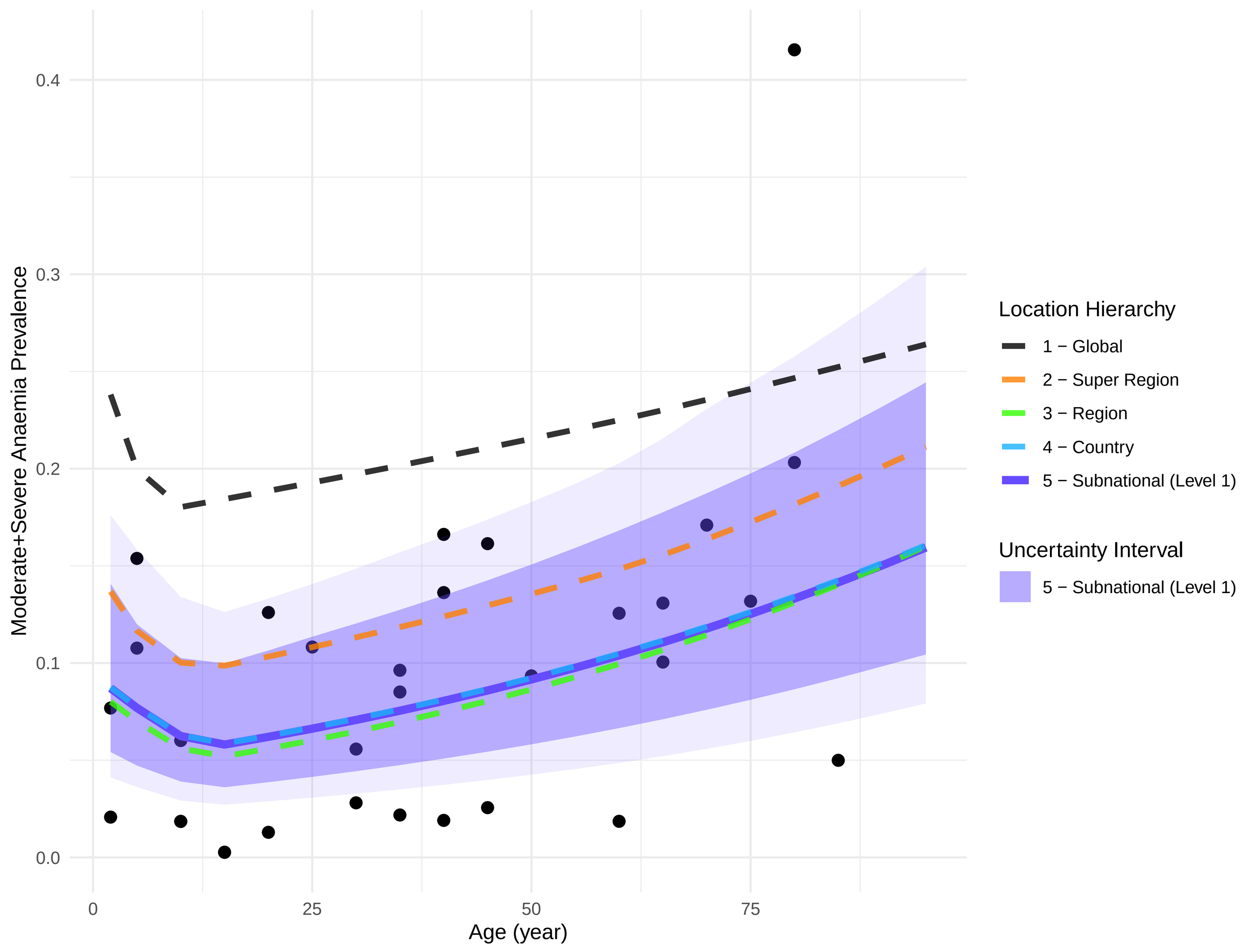

MR−BRT Age/Sex Fits − Non−pregnant Females 2+ Years Old − Moderate+Severe Anaemia Prevalence − Durango
WHO−2001 Adjustment Method

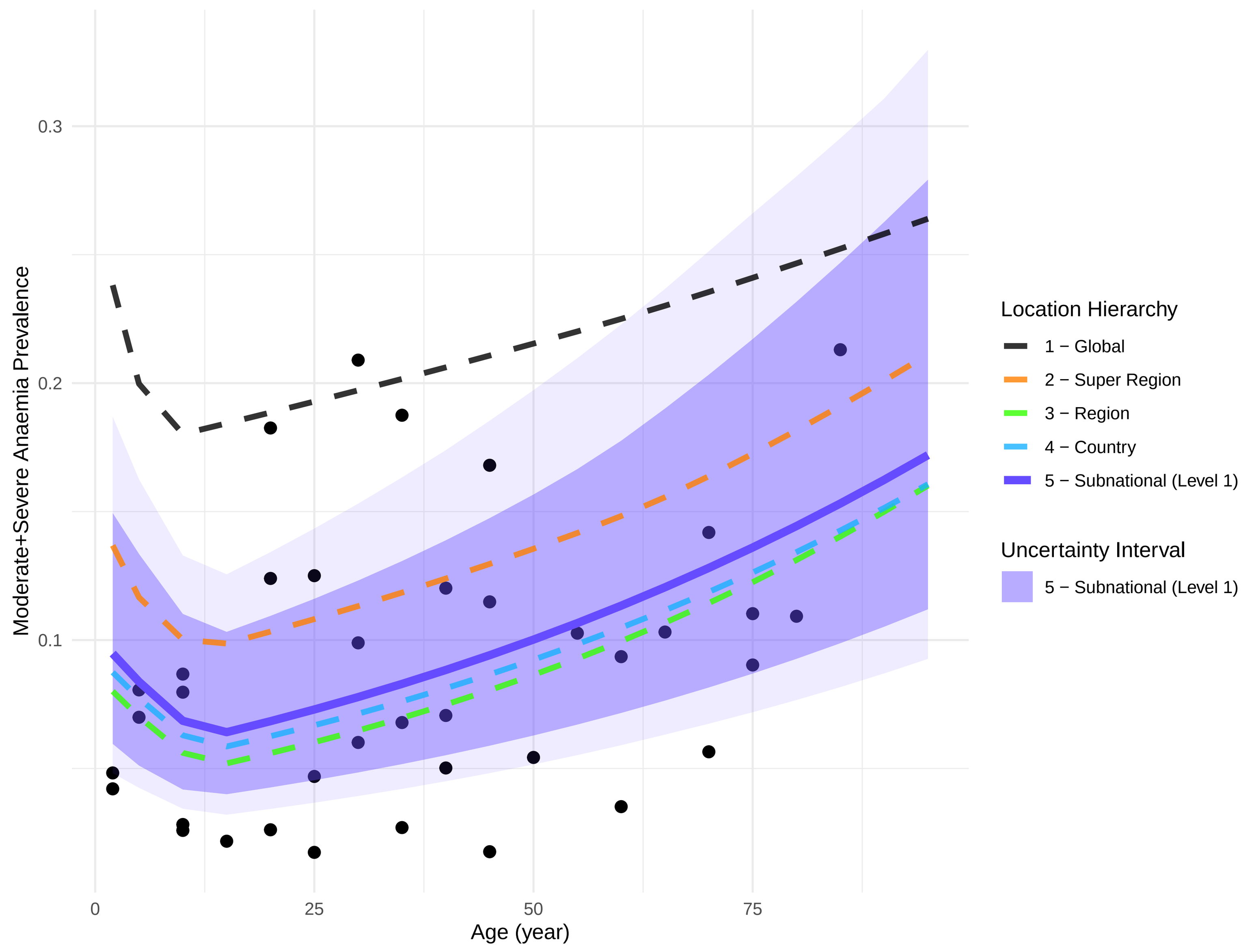

MR−BRT Age/Sex Fits − Non−pregnant Females 2+ Years Old − Moderate+Severe Anaemia Prevalence − Guanajuato
WHO−2001 Adjustment Method

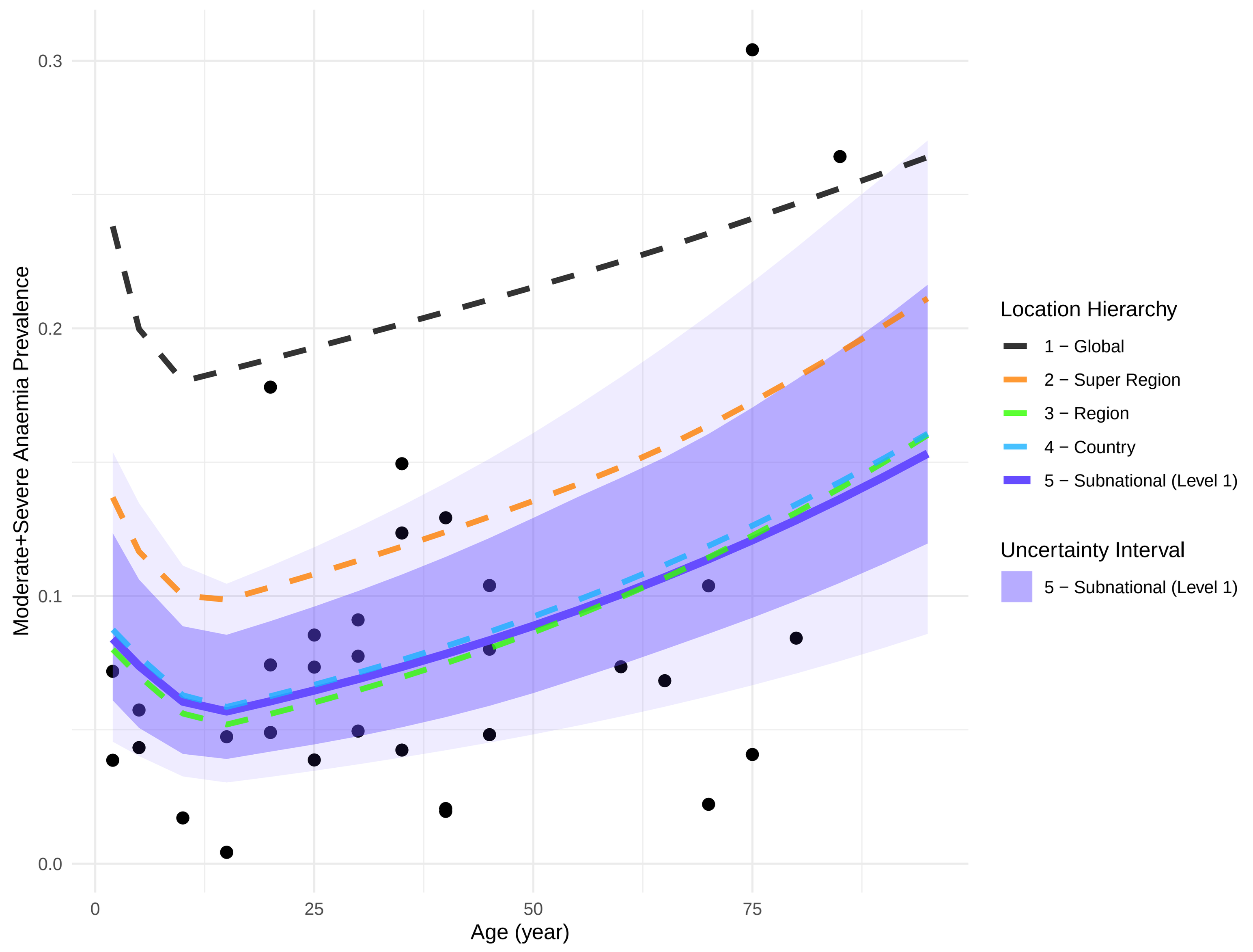

MR−BRT Age/Sex Fits − Non−pregnant Females 2+ Years Old − Moderate+Severe Anaemia Prevalence − Guerrero
WHO−2001 Adjustment Method

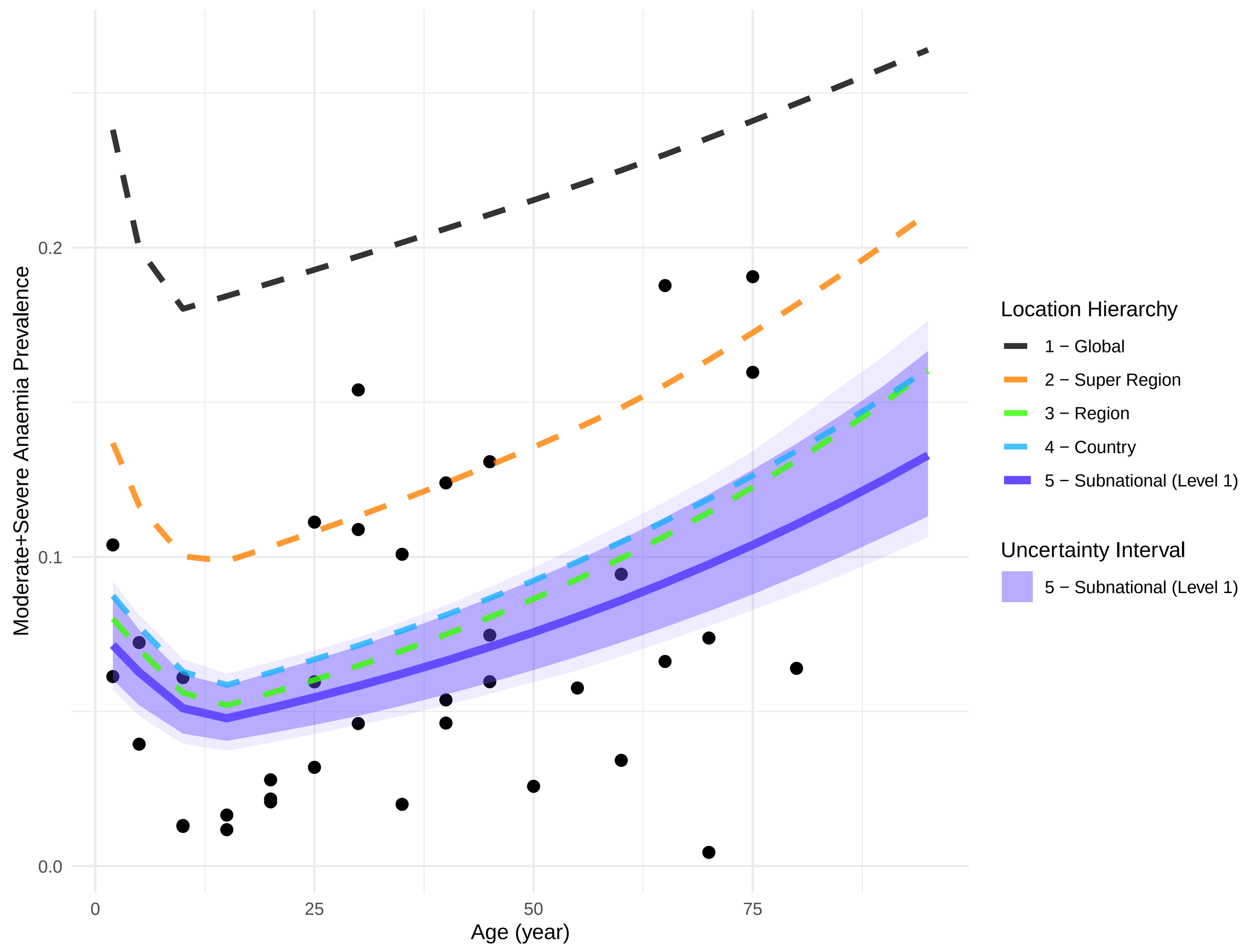

MR−BRT Age/Sex Fits − Non−pregnant Females 2+ Years Old − Moderate+Severe Anaemia Prevalence − Hidalgo
WHO−2001 Adjustment Method

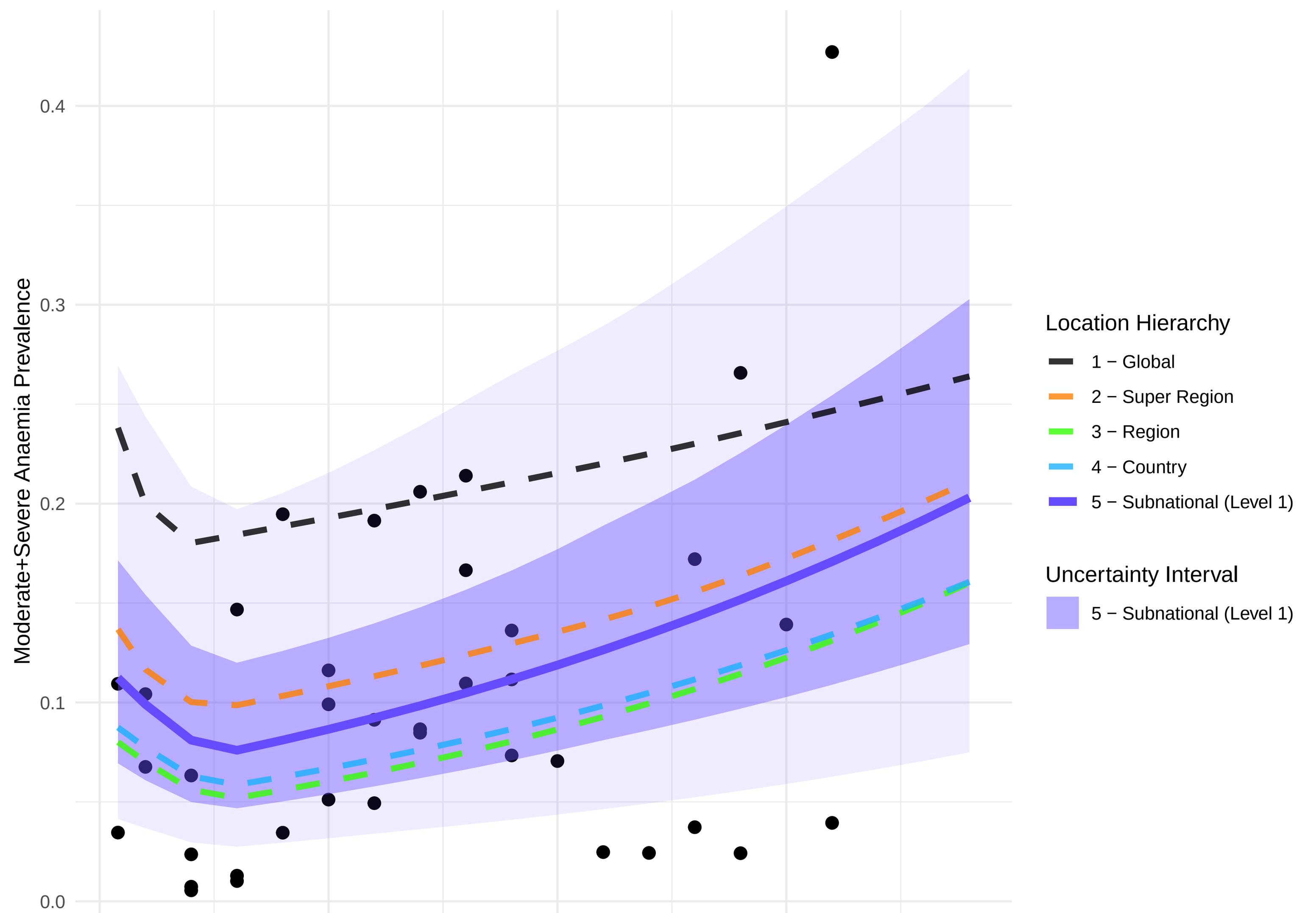

MR−BRT Age/Sex Fits − Non−pregnant Females 2+ Years Old − Moderate+Severe Anaemia Prevalence − Jalisco
WHO−2001 Adjustment Method

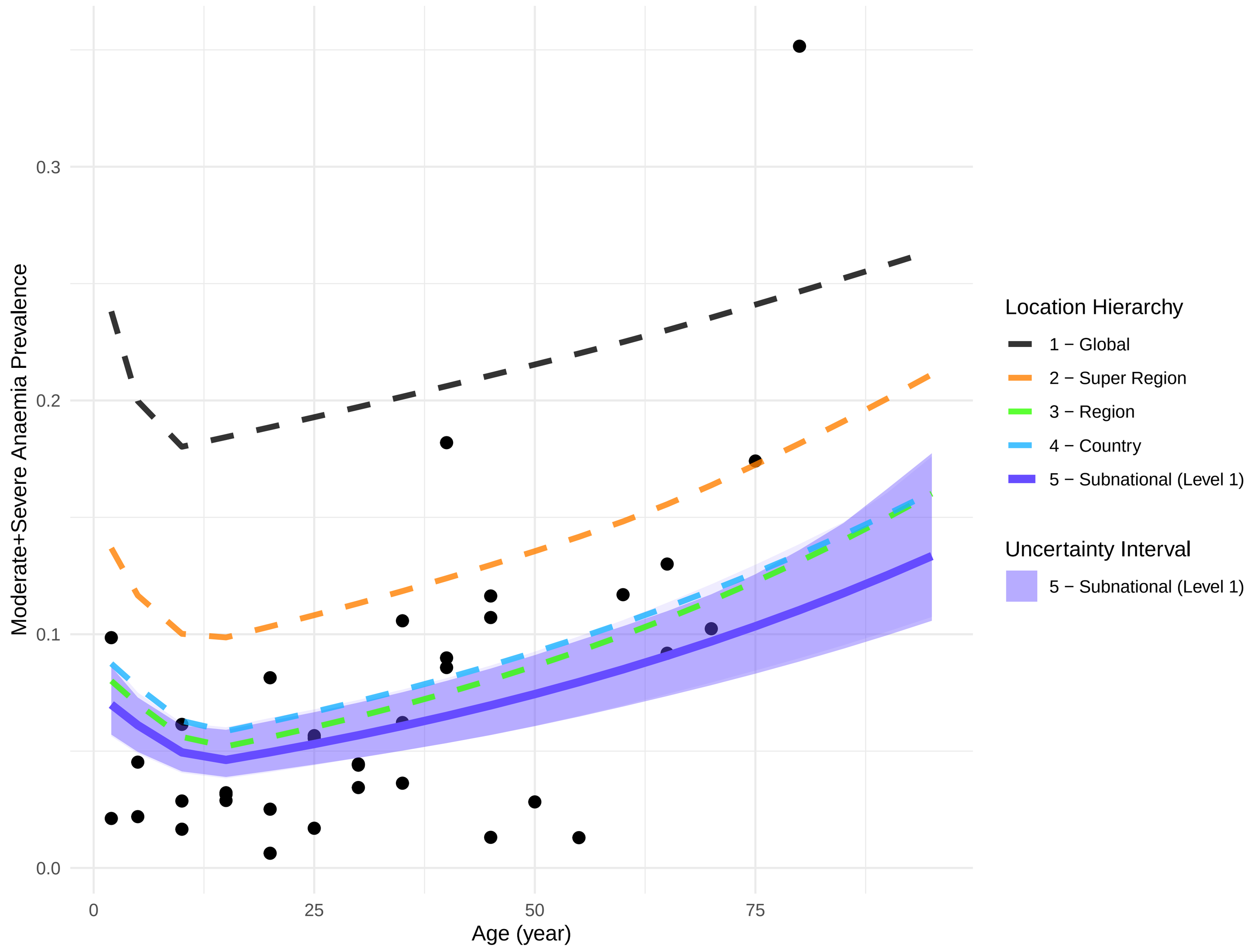

MR−BRT Age/Sex Fits − Non−pregnant Females 2+ Years Old − Moderate+Severe Anaemia Prevalence − México
WHO−2001 Adjustment Method

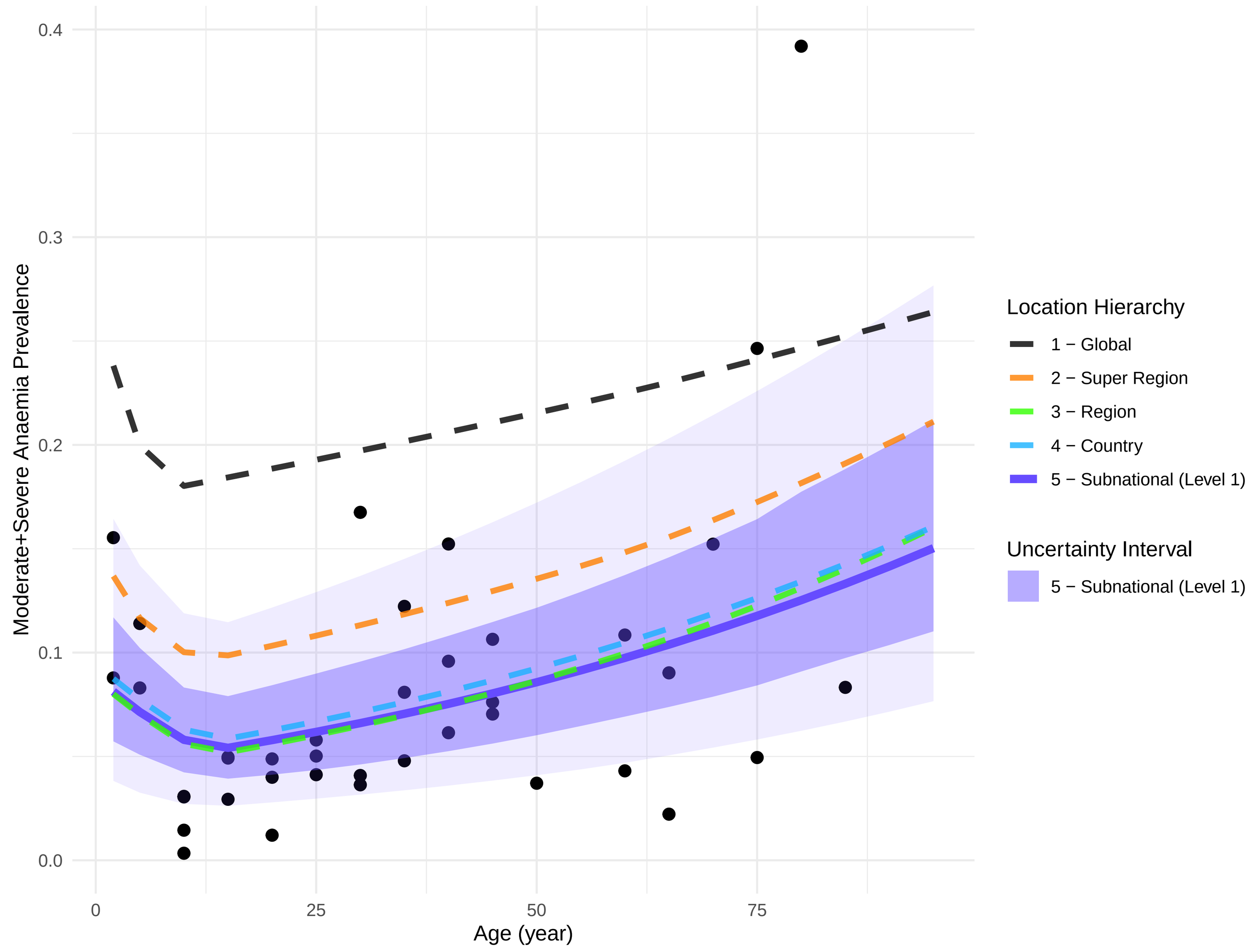

MR−BRT Age/Sex Fits − Non−pregnant Females 2+ Years Old − Moderate+Severe Anaemia Prevalence − Mexico City
WHO−2001 Adjustment Method

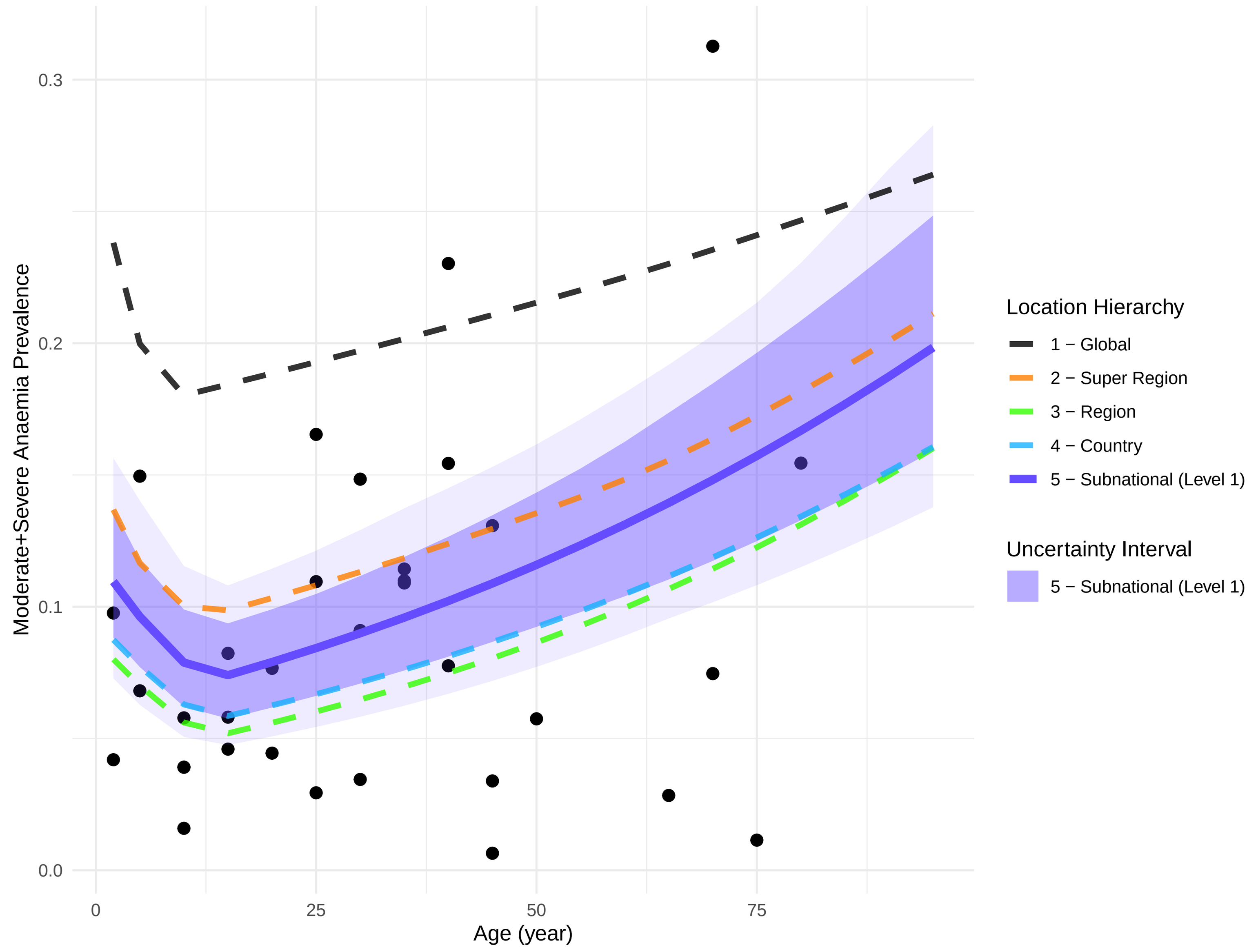

MR−BRT Age/Sex Fits − Non−pregnant Females 2+ Years Old − Moderate+Severe Anaemia Prevalence − Michoacán de Ocampo
WHO−2001 Adjustment Method

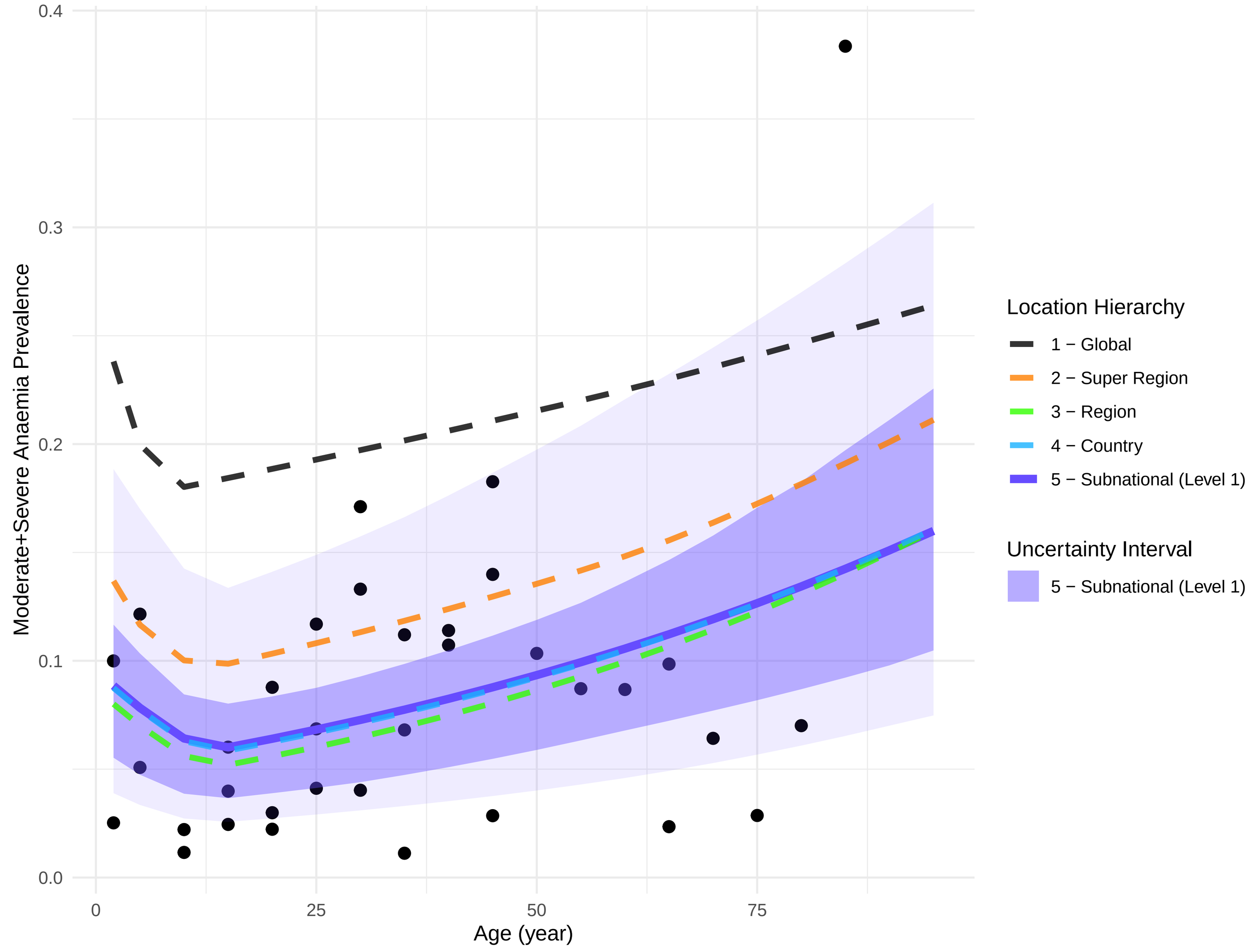

MR−BRT Age/Sex Fits − Non−pregnant Females 2+ Years Old − Moderate+Severe Anaemia Prevalence − Morelos
WHO−2001 Adjustment Method

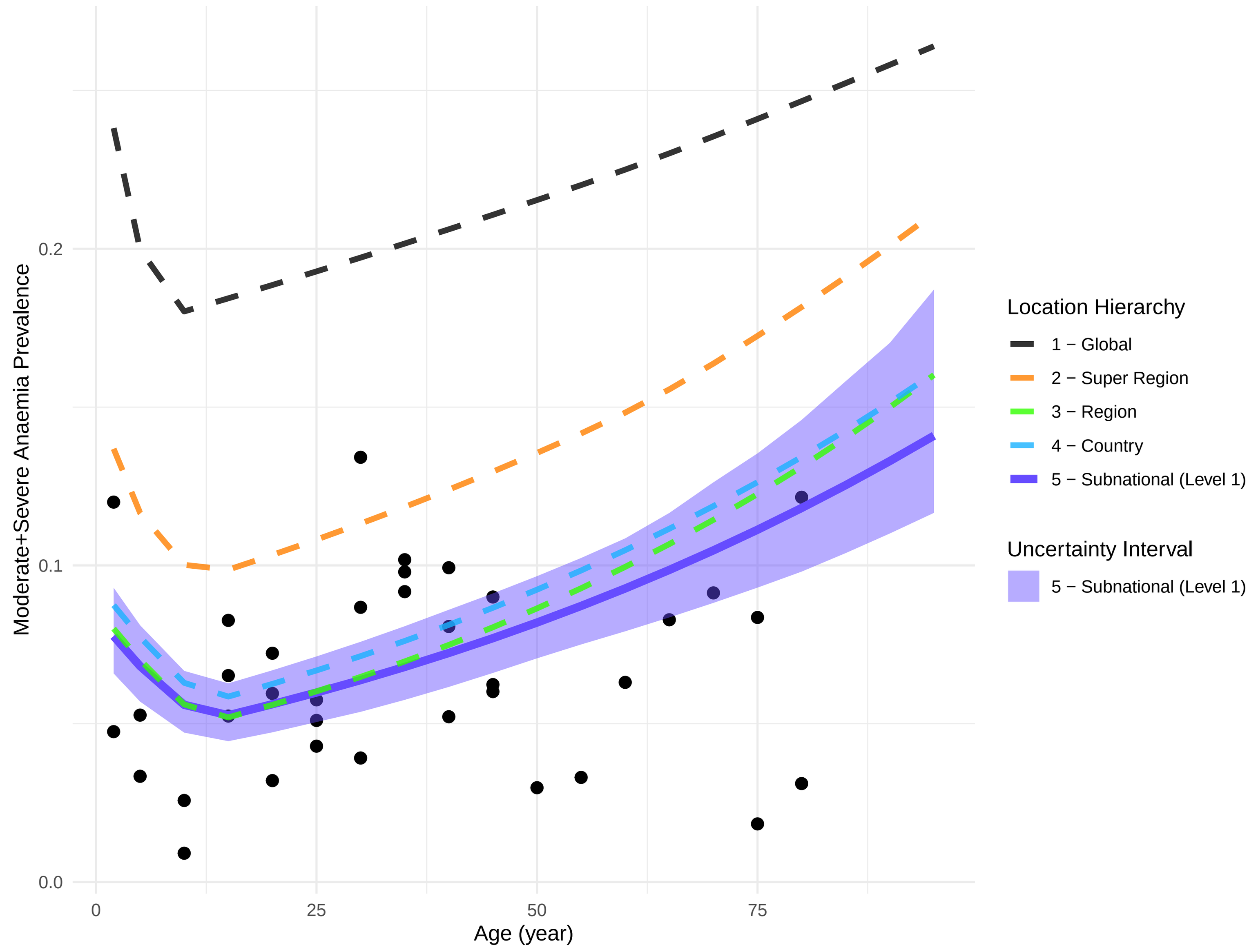

MR−BRT Age/Sex Fits − Non−pregnant Females 2+ Years Old − Moderate+Severe Anaemia Prevalence − Nayarit
WHO−2001 Adjustment Method

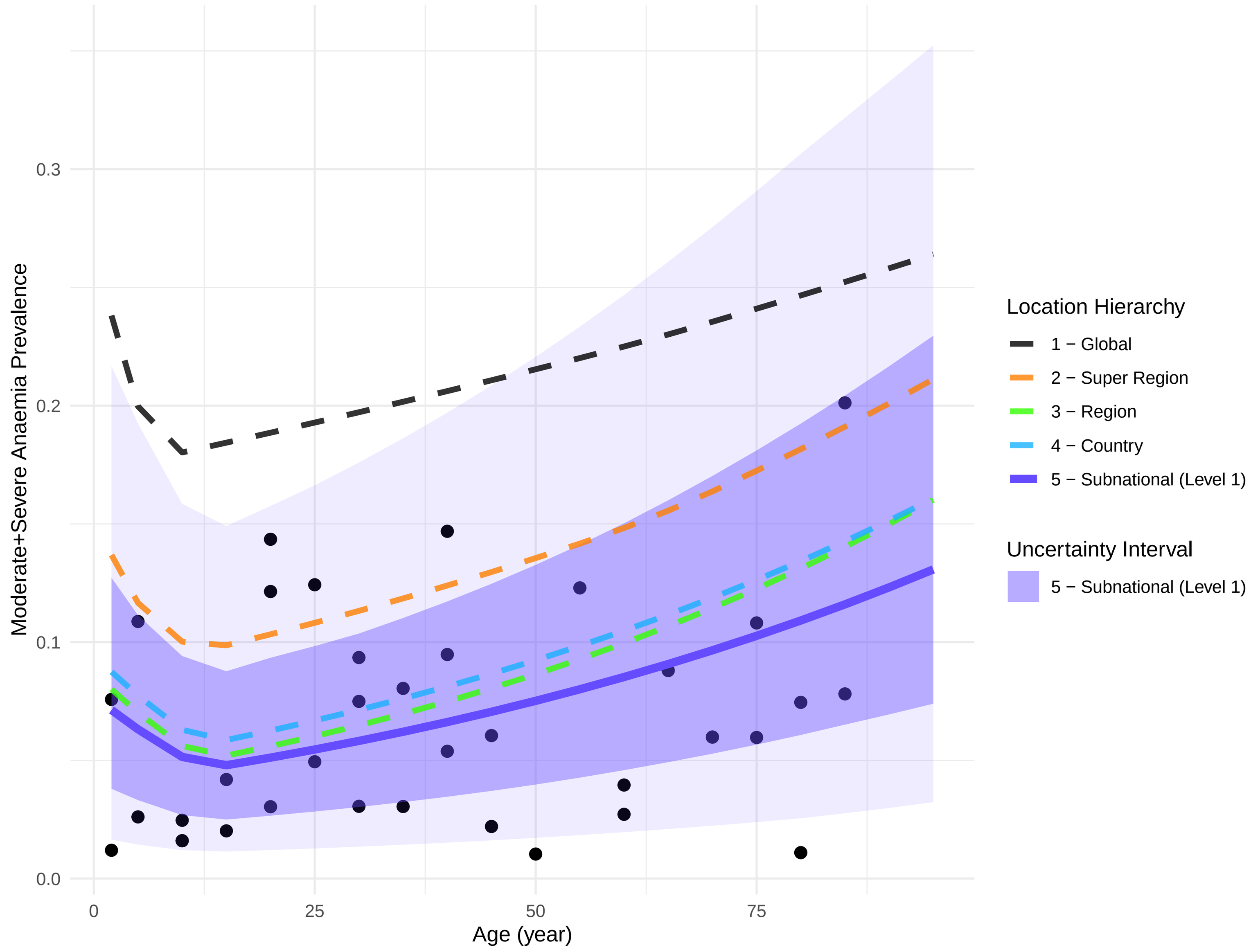

MR−BRT Age/Sex Fits − Non−pregnant Females 2+ Years Old − Moderate+Severe Anaemia Prevalence − Nuevo León
WHO−2001 Adjustment Method

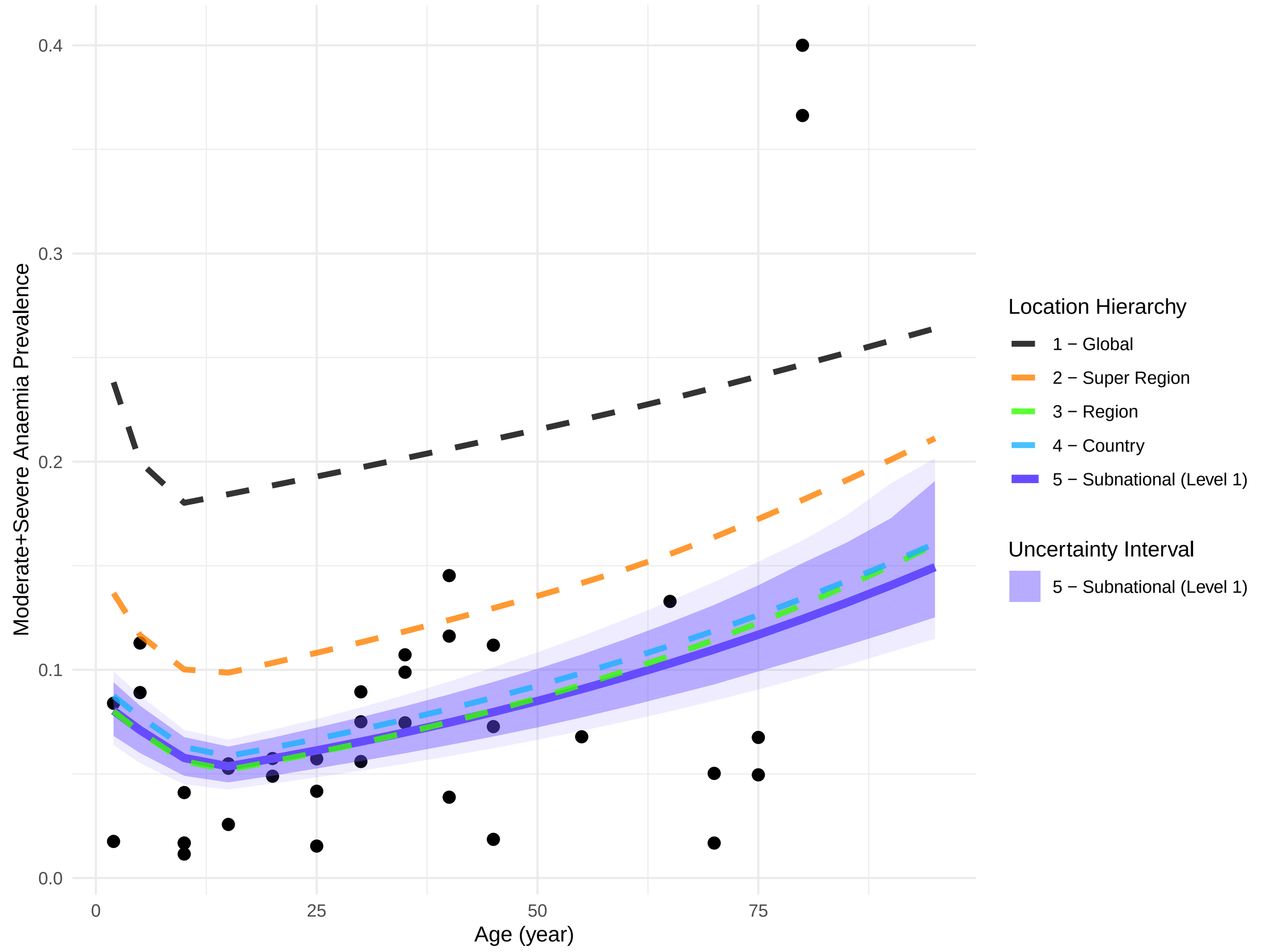

MR−BRT Age/Sex Fits − Non−pregnant Females 2+ Years Old − Moderate+Severe Anaemia Prevalence − Oaxaca
WHO−2001 Adjustment Method

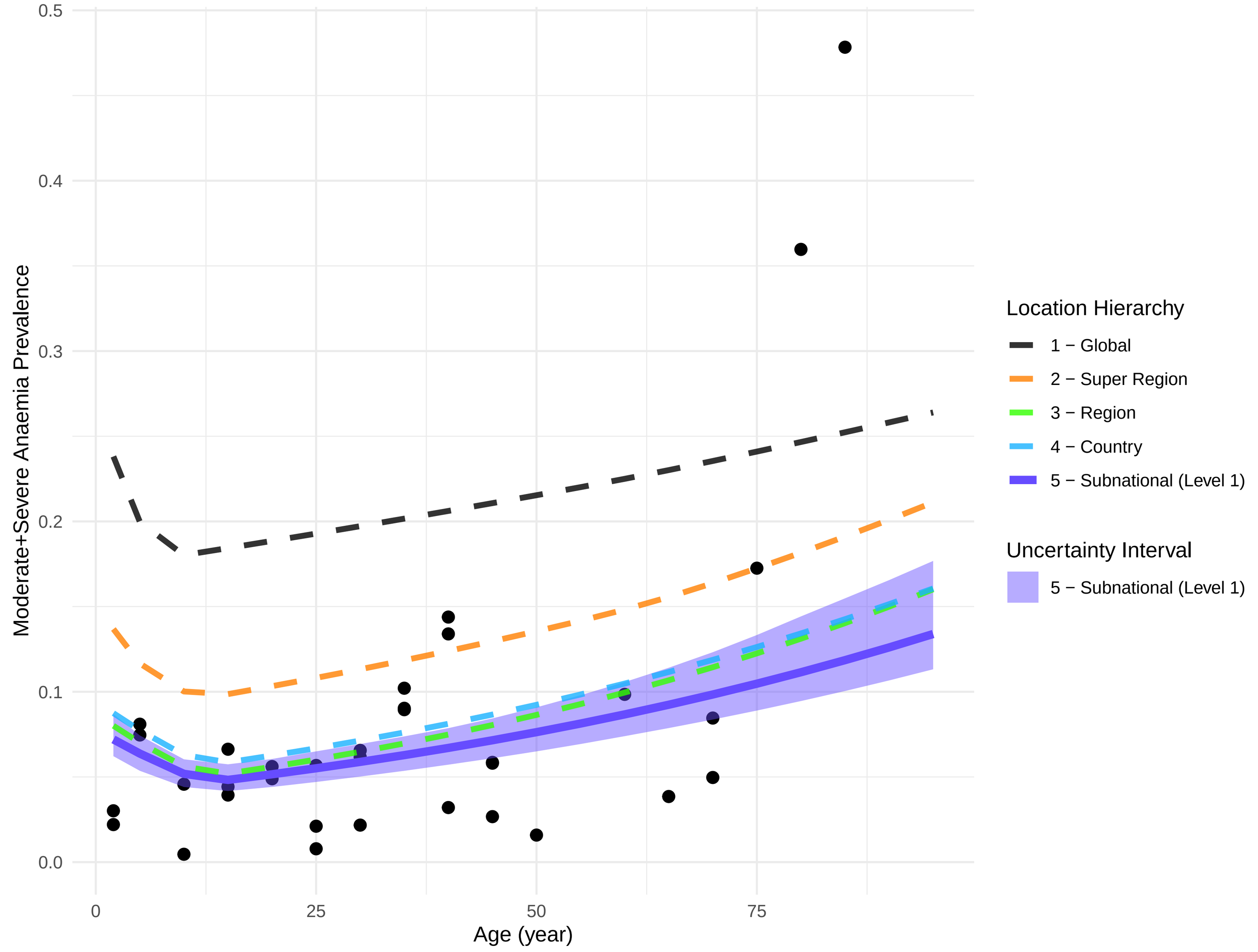

MR−BRT Age/Sex Fits − Non−pregnant Females 2+ Years Old − Moderate+Severe Anaemia Prevalence − Puebla
WHO−2001 Adjustment Method

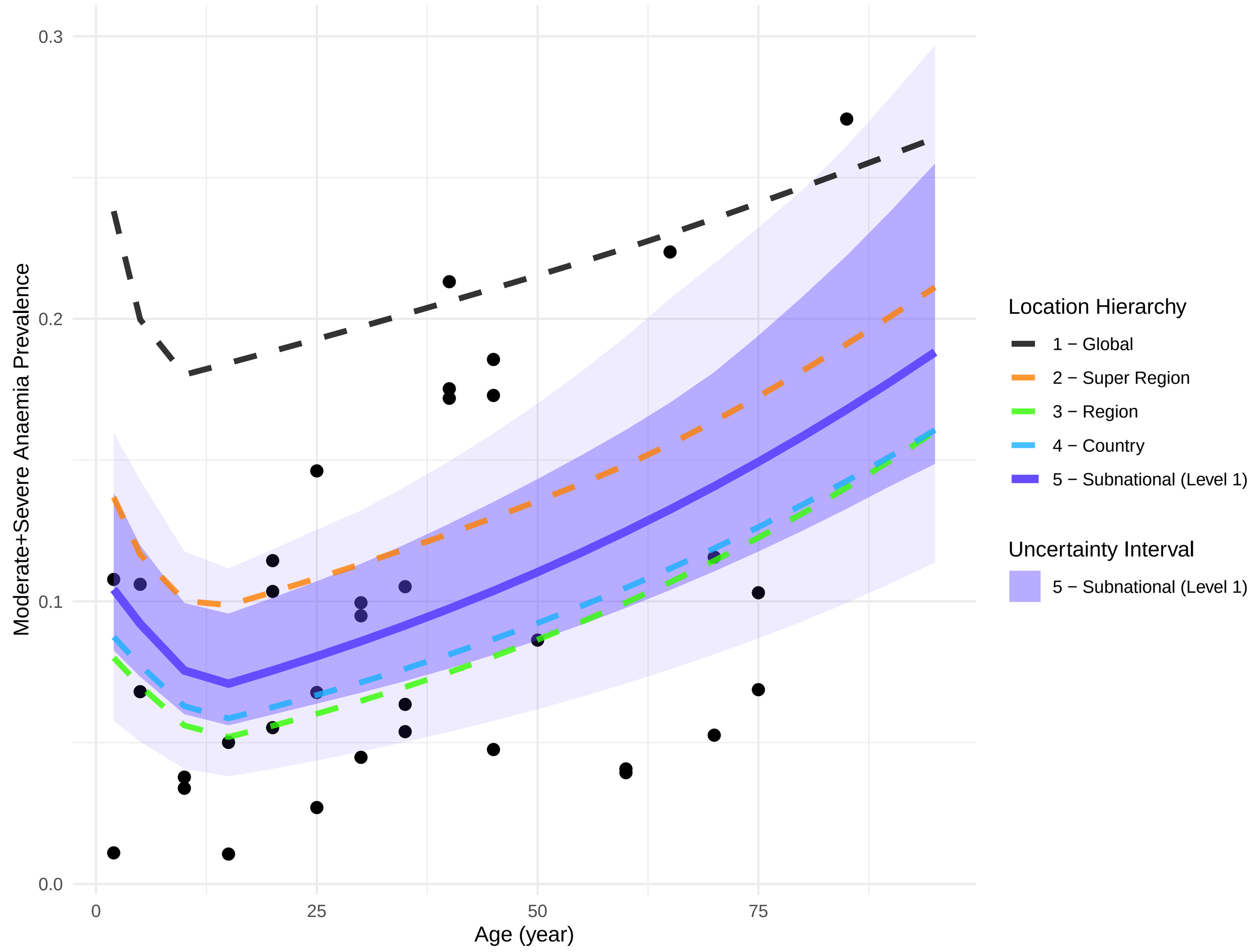

MR−BRT Age/Sex Fits − Non−pregnant Females 2+ Years Old − Moderate+Severe Anaemia Prevalence − Querétaro
WHO−2001 Adjustment Method

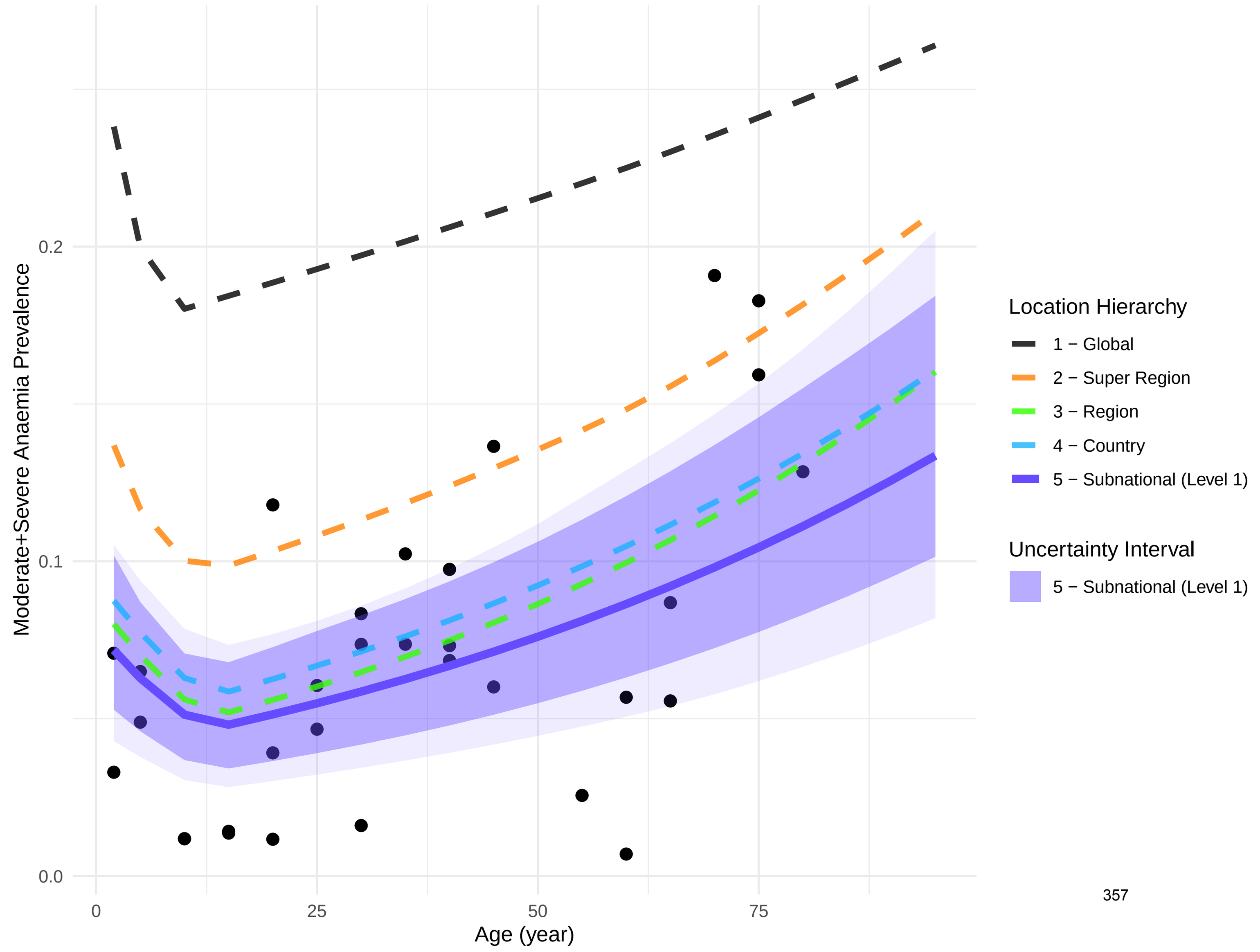

MR−BRT Age/Sex Fits − Non−pregnant Females 2+ Years Old − Moderate+Severe Anaemia Prevalence − Quintana Roo
WHO−2001 Adjustment Method

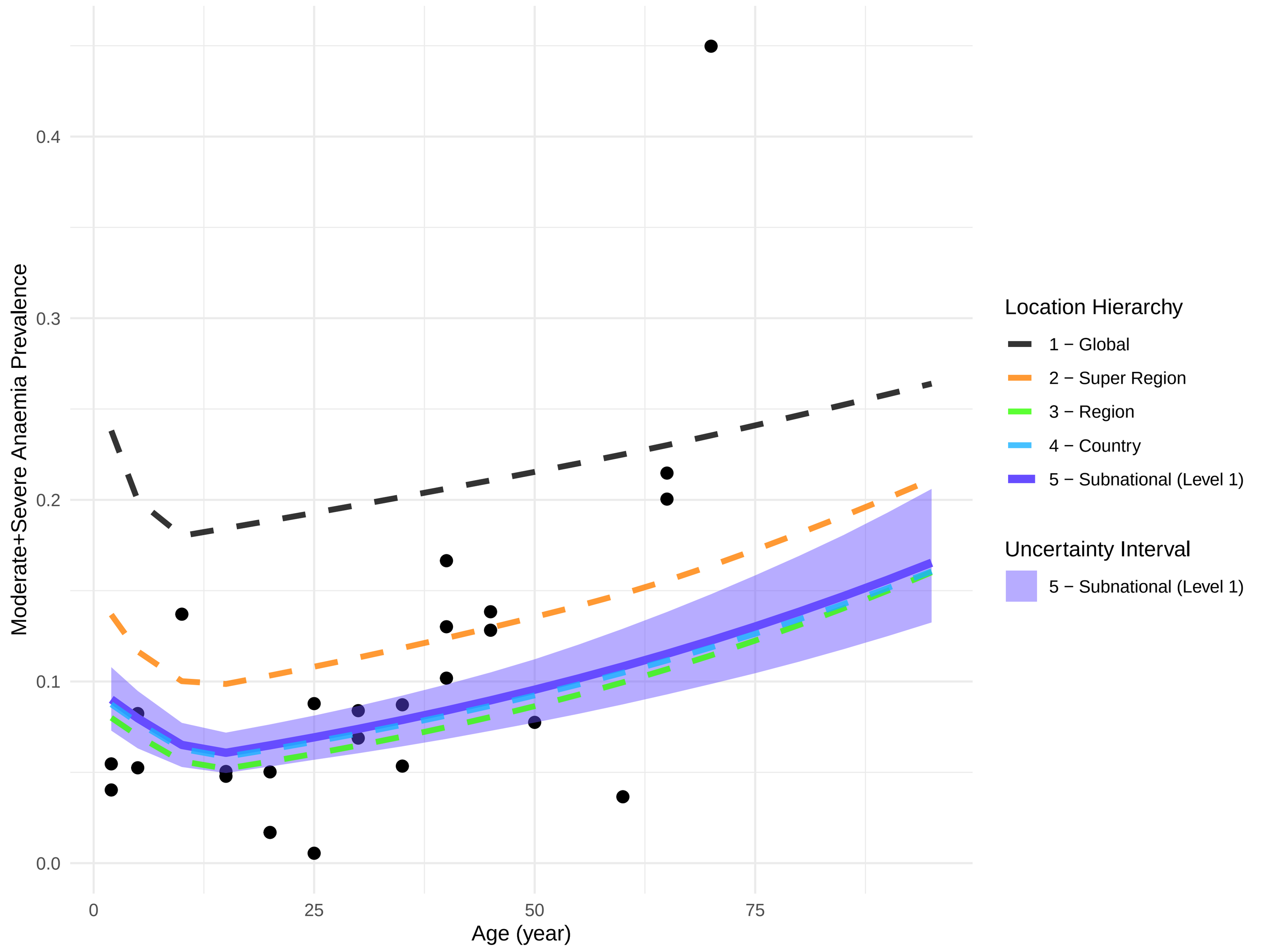

MR−BRT Age/Sex Fits − Non−pregnant Females 2+ Years Old − Moderate+Severe Anaemia Prevalence − San Luis Potosí
WHO−2001 Adjustment Method

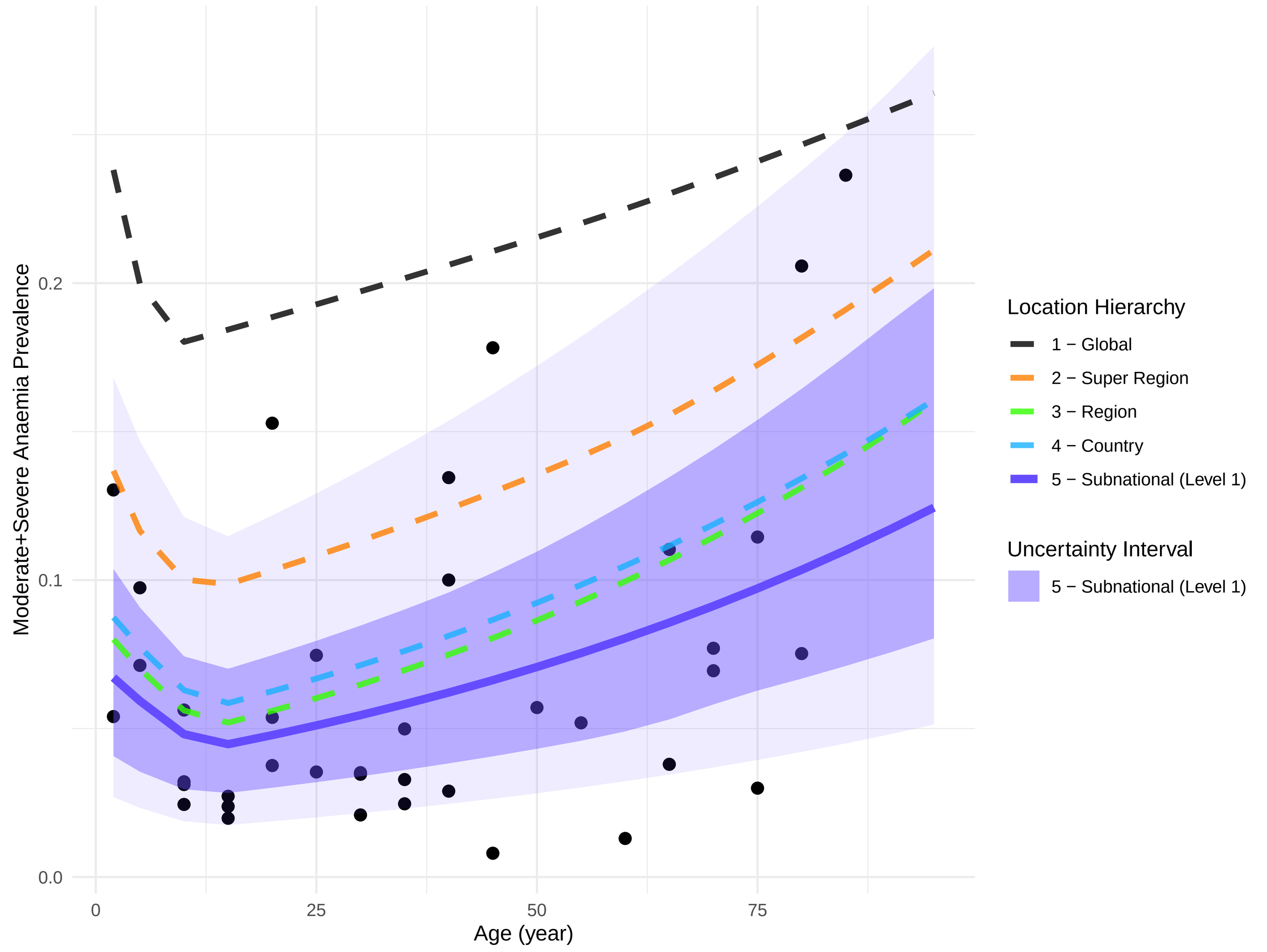

MR−BRT Age/Sex Fits − Non−pregnant Females 2+ Years Old − Moderate+Severe Anaemia Prevalence − Sinaloa
WHO−2001 Adjustment Method

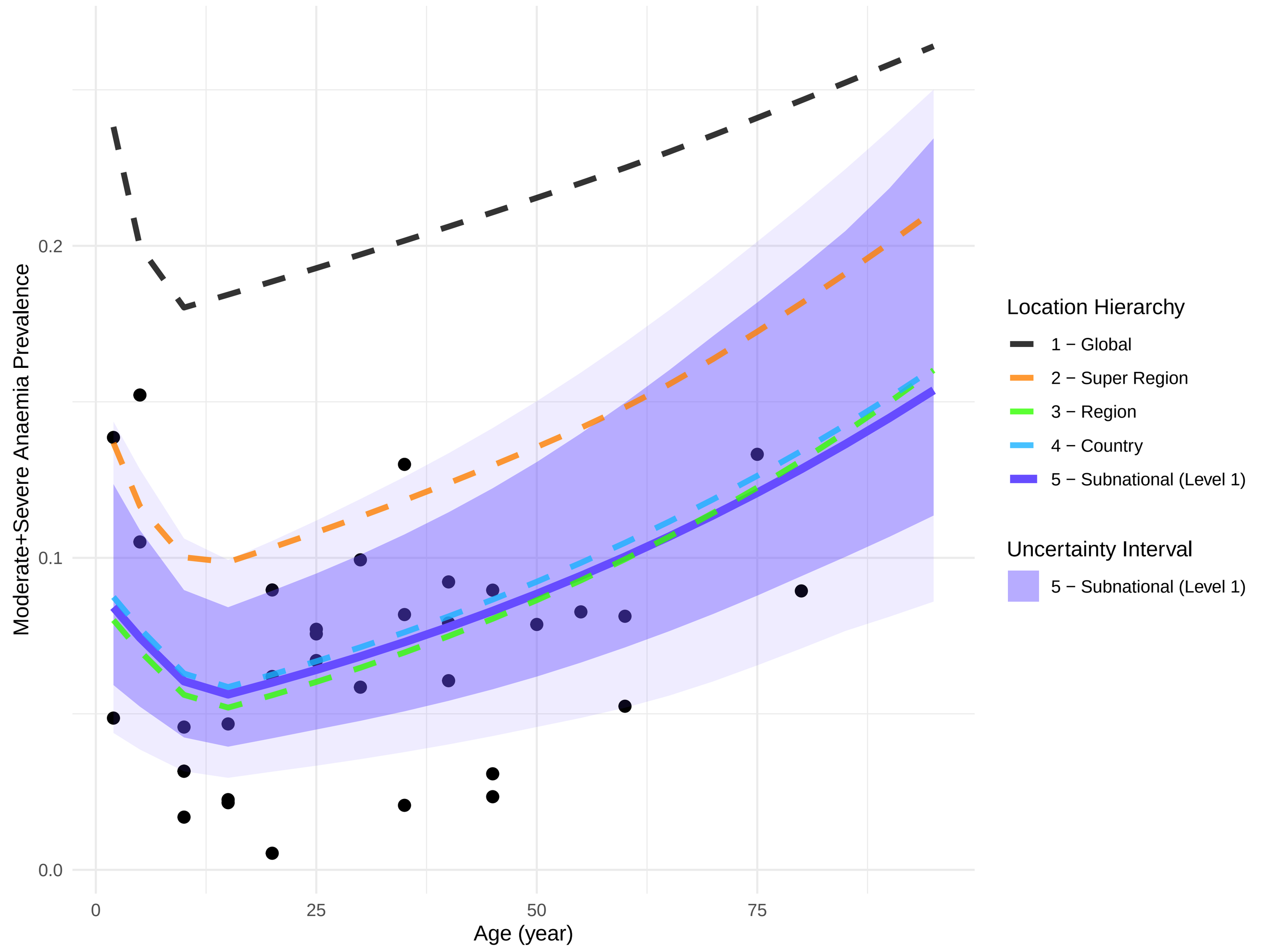

MR−BRT Age/Sex Fits − Non−pregnant Females 2+ Years Old − Moderate+Severe Anaemia Prevalence − Sonora
WHO−2001 Adjustment Method

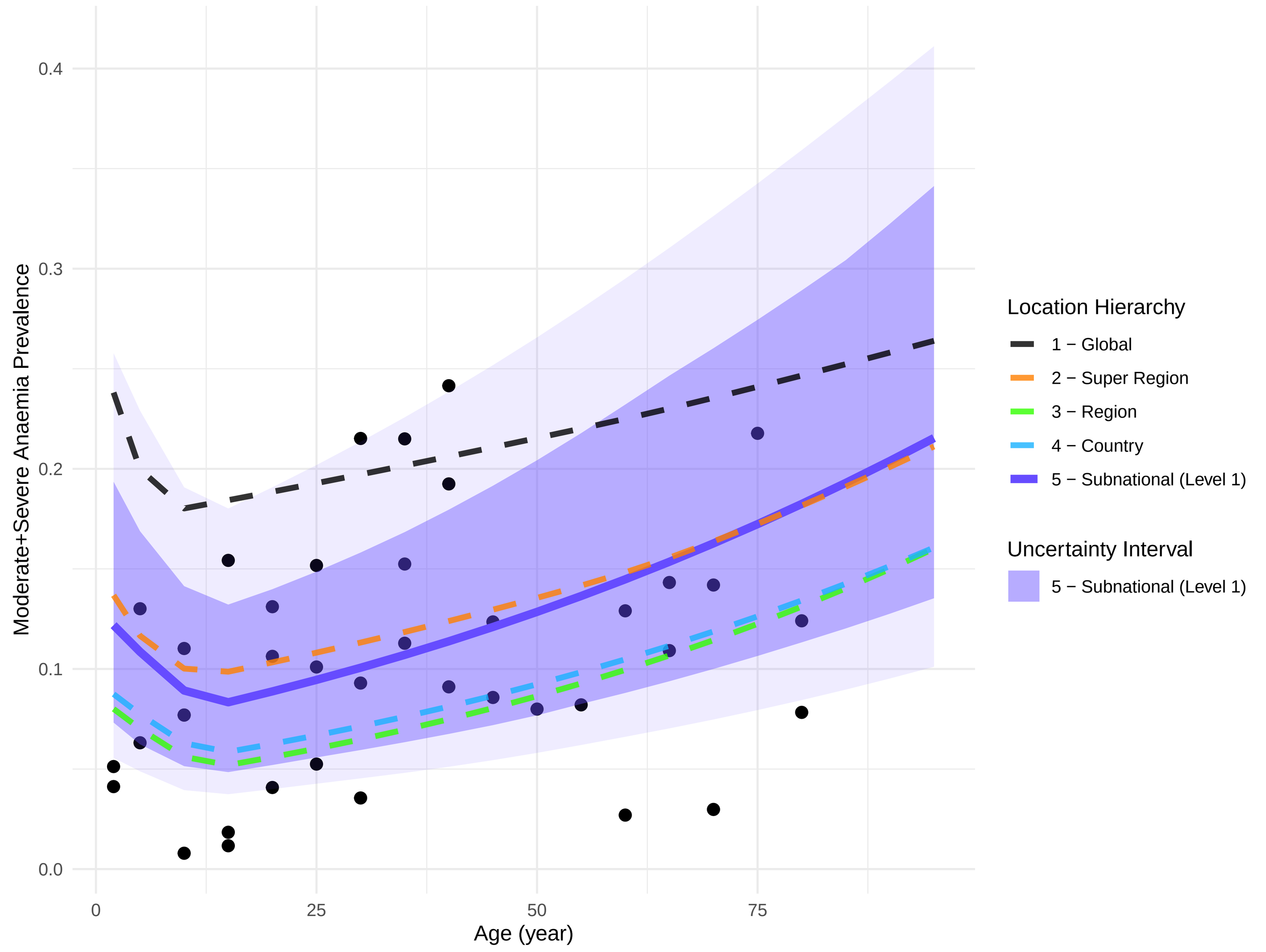

MR−BRT Age/Sex Fits − Non−pregnant Females 2+ Years Old − Moderate+Severe Anaemia Prevalence − Tabasco
WHO−2001 Adjustment Method

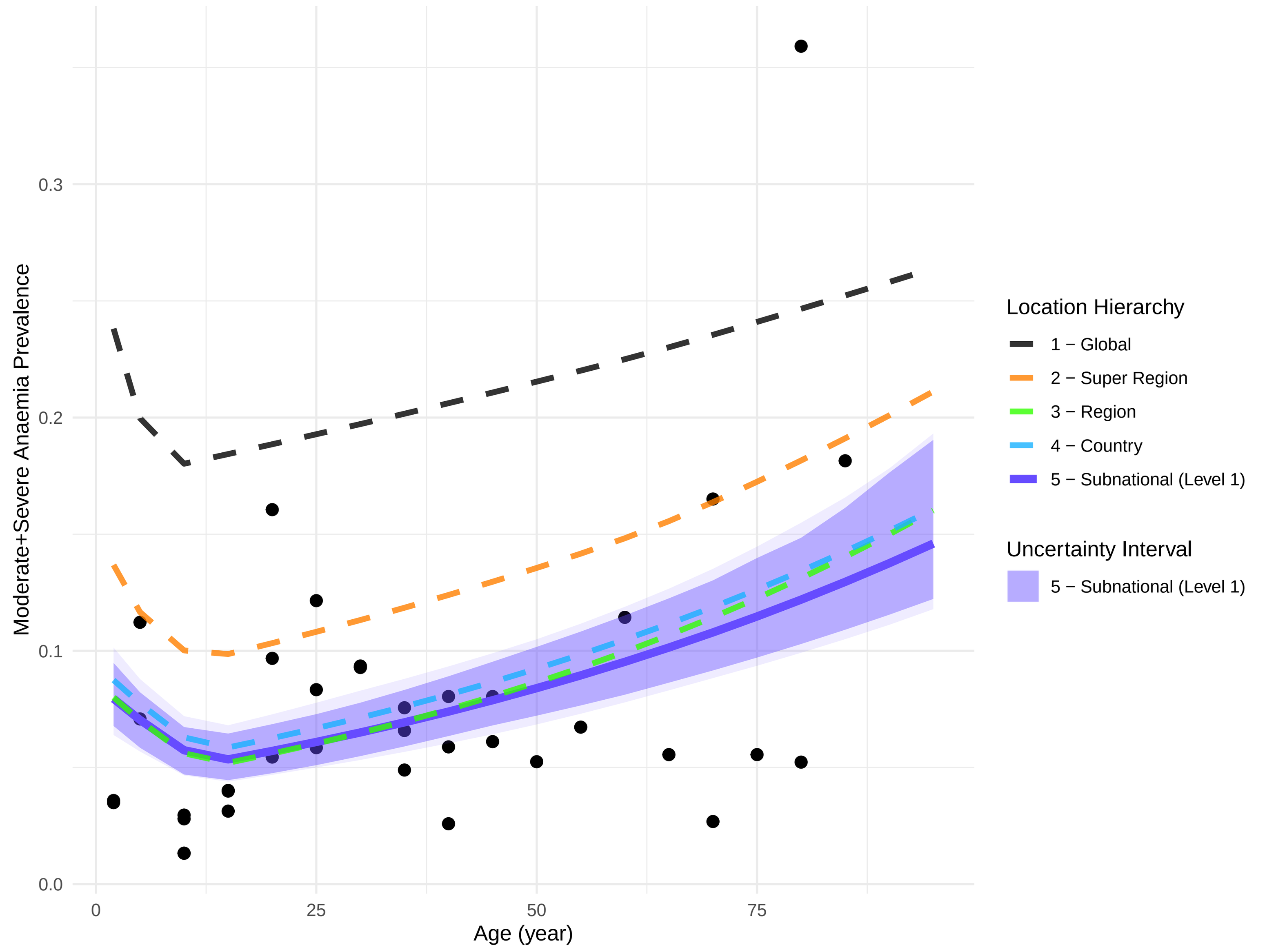

MR−BRT Age/Sex Fits − Non−pregnant Females 2+ Years Old − Moderate+Severe Anaemia Prevalence − Tamaulipas
WHO−2001 Adjustment Method

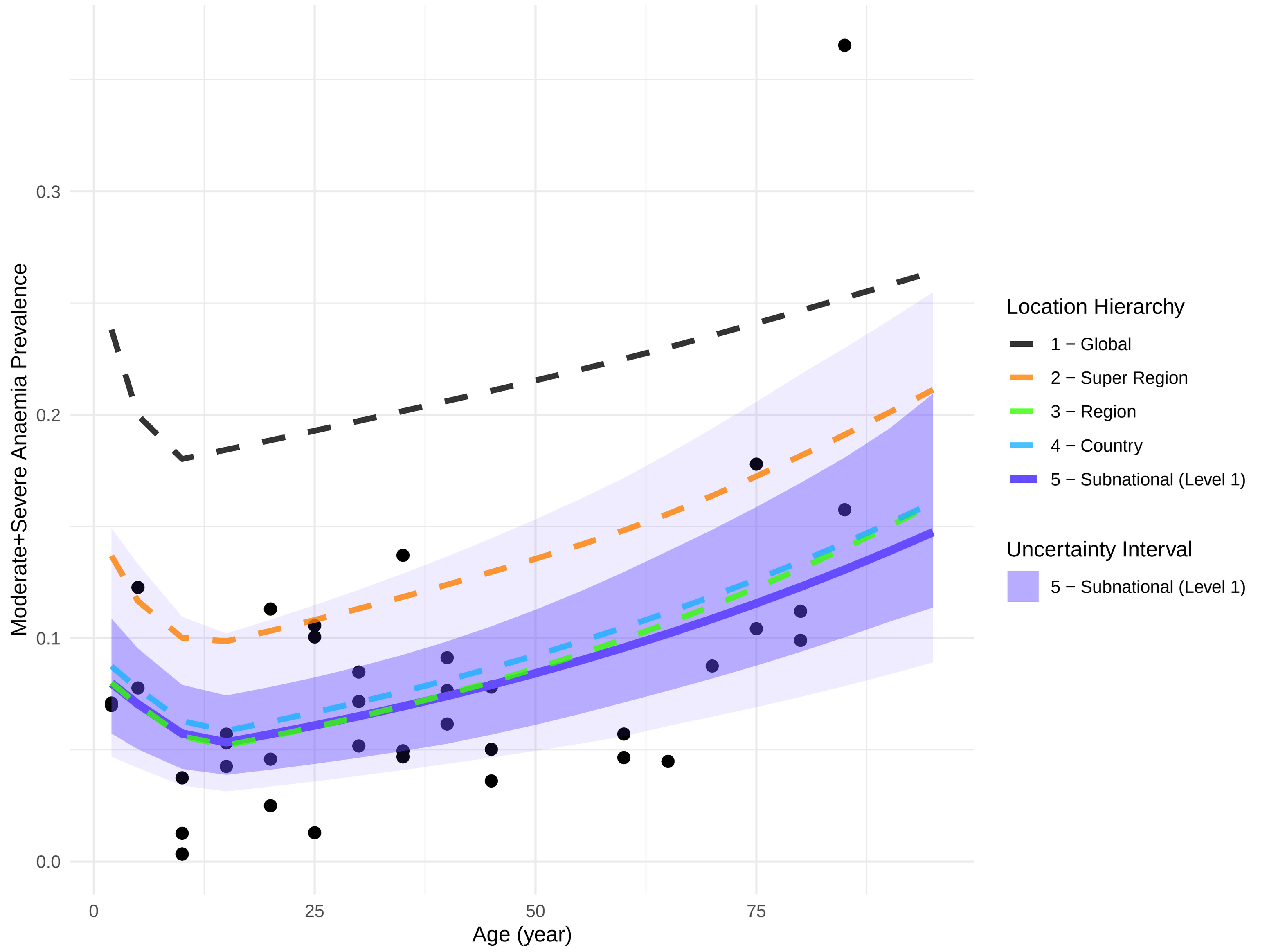

MR−BRT Age/Sex Fits − Non−pregnant Females 2+ Years Old − Moderate+Severe Anaemia Prevalence − Tlaxcala
WHO−2001 Adjustment Method

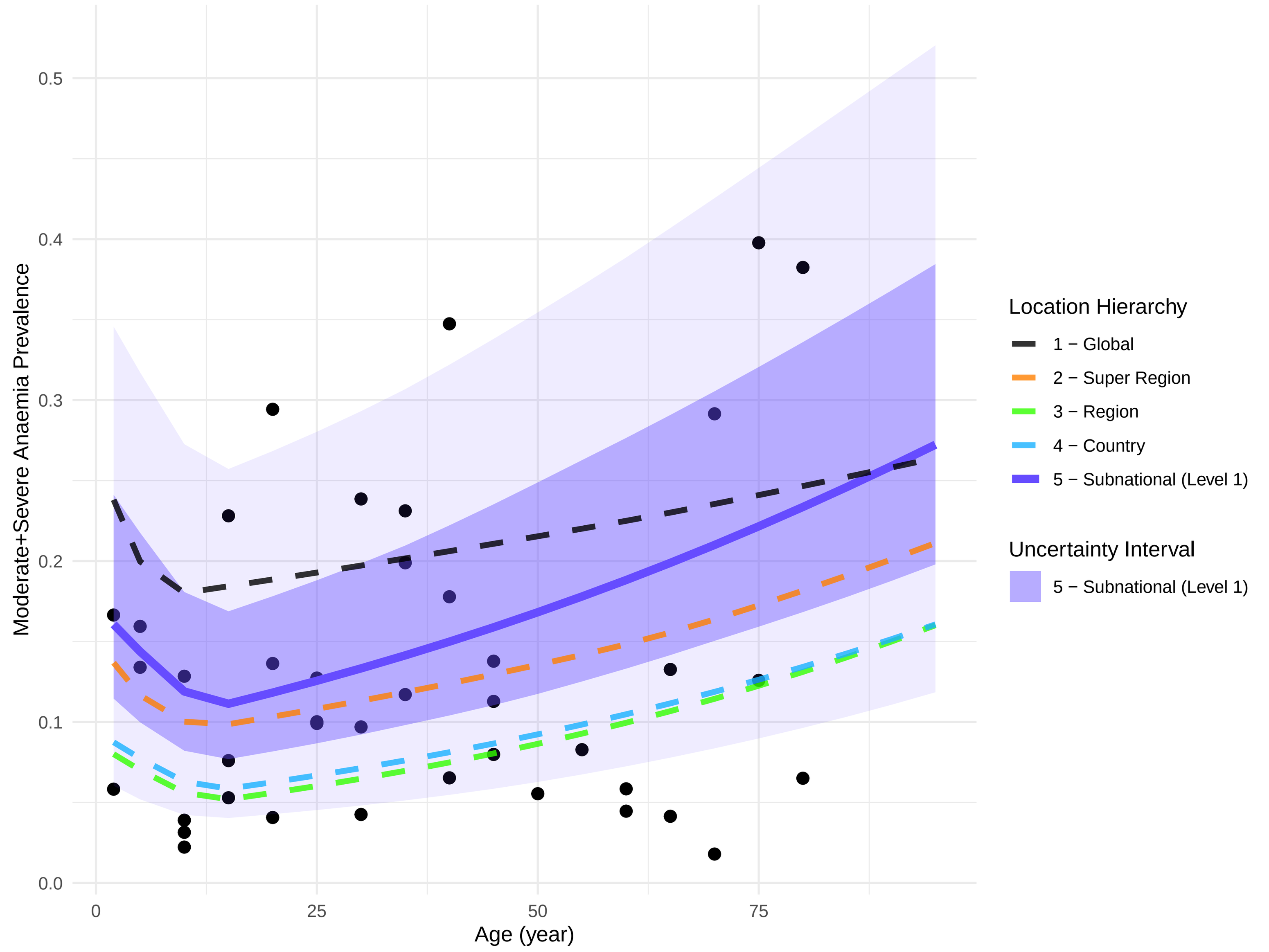

MR−BRT Age/Sex Fits − Non−pregnant Females 2+ Years Old − Moderate+Severe Anaemia Prevalence − Veracruz de Ignacio de la Llave
WHO−2001 Adjustment Method

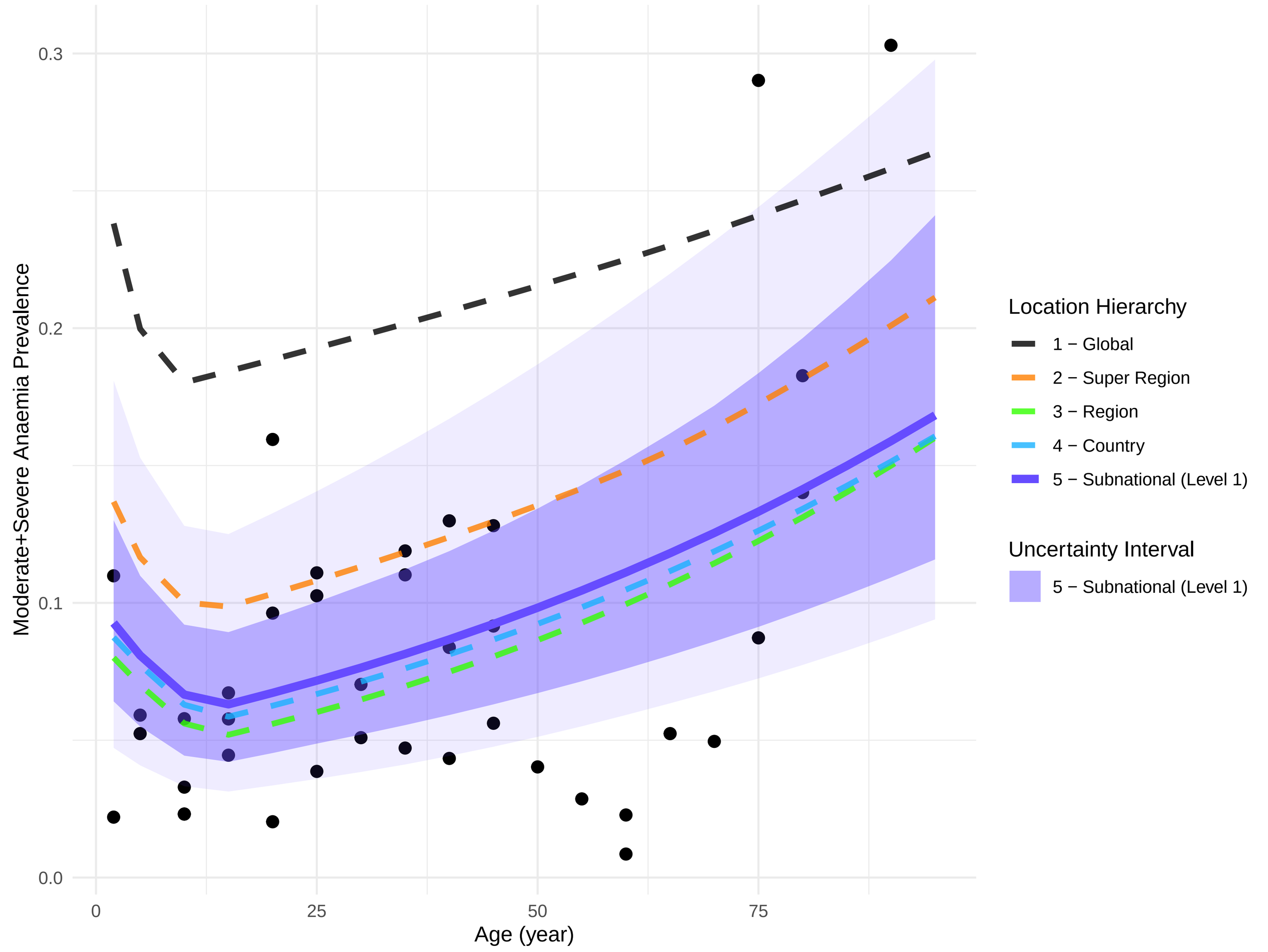

MR−BRT Age/Sex Fits − Non−pregnant Females 2+ Years Old − Moderate+Severe Anaemia Prevalence − Yucatán
WHO−2001 Adjustment Method

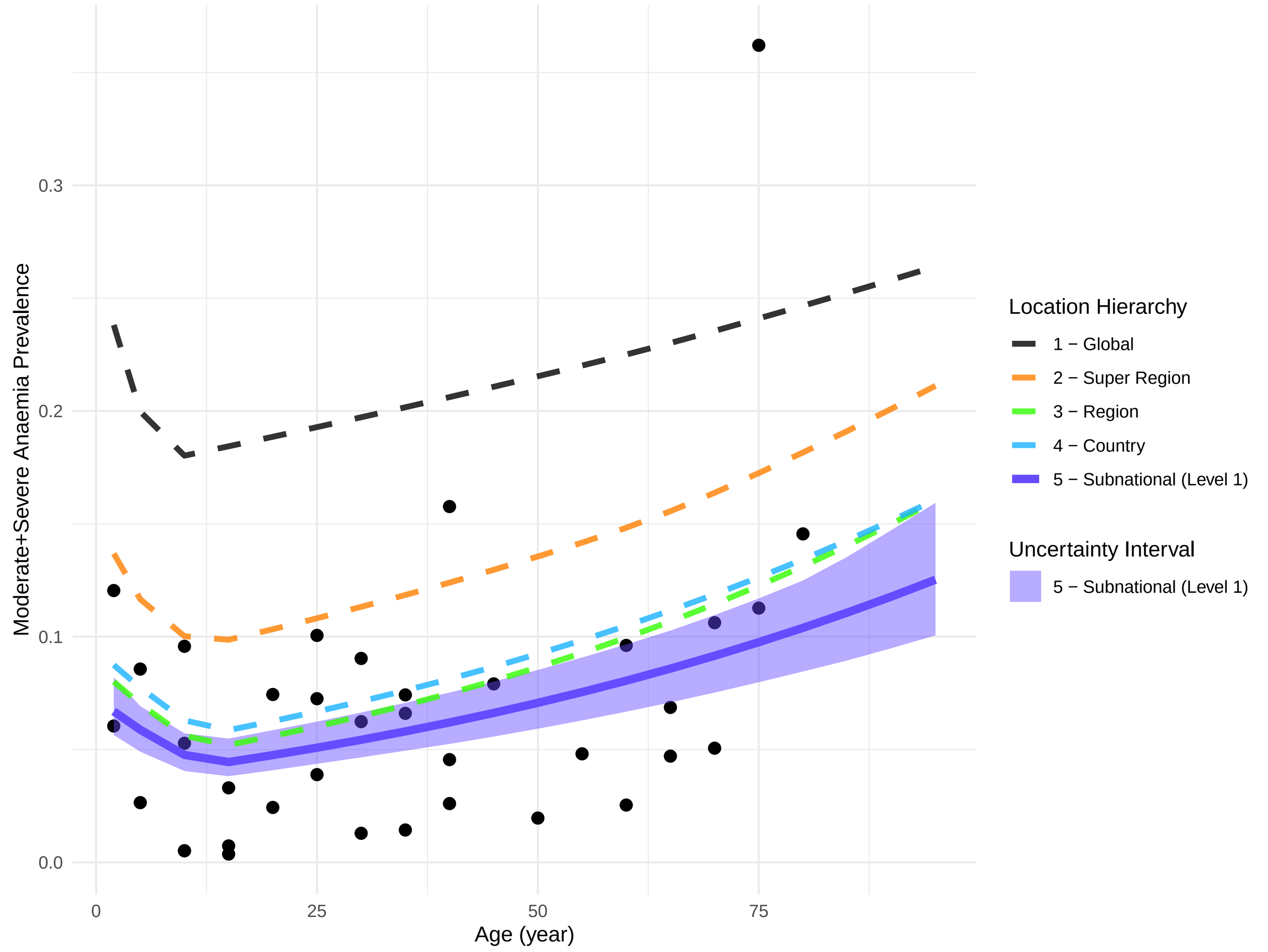

MR−BRT Age/Sex Fits − Non−pregnant Females 2+ Years Old − Moderate+Severe Anaemia Prevalence − Zacatecas
WHO−2001 Adjustment Method

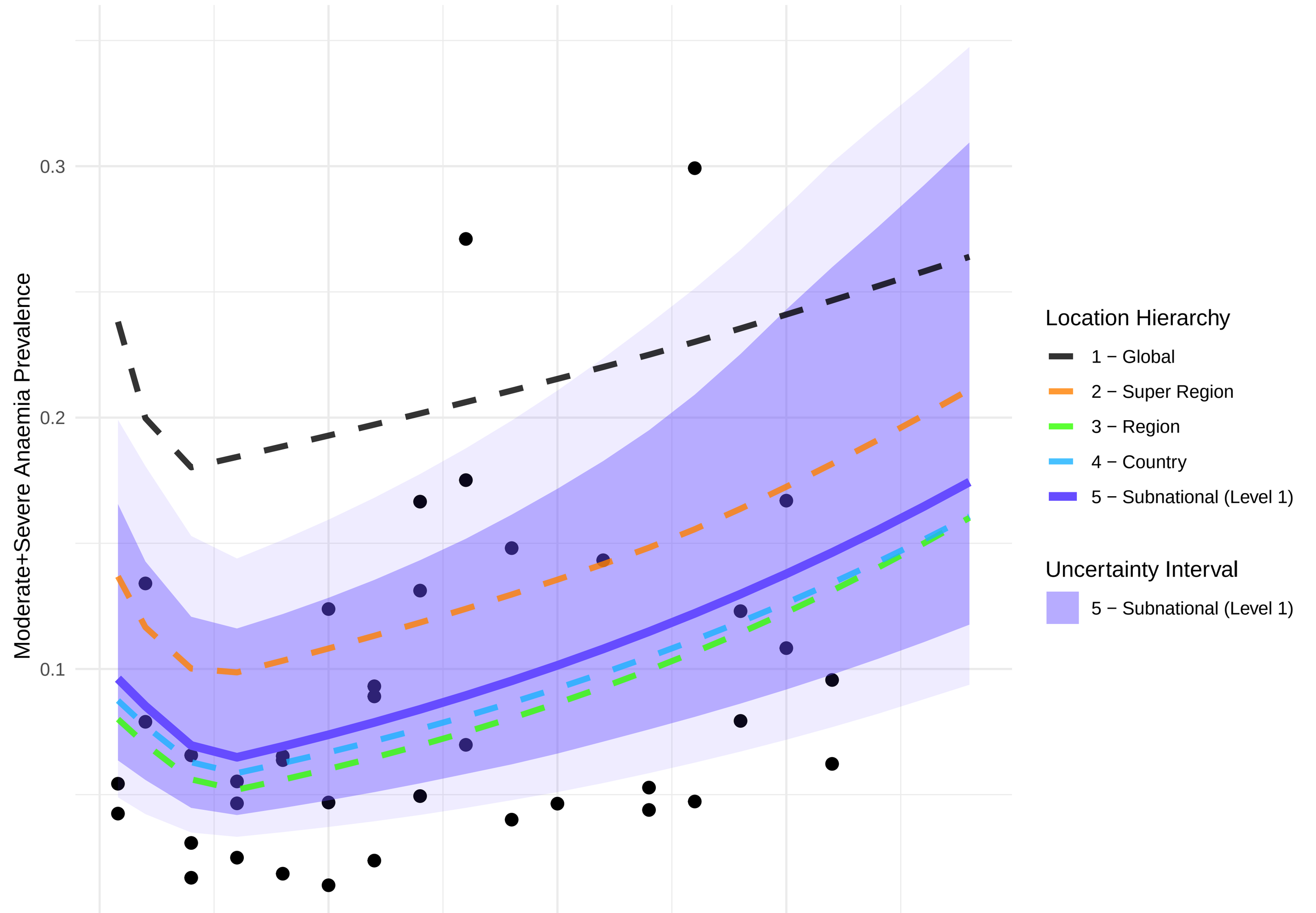

MR−BRT Age/Sex Fits − Non−pregnant Females 2+ Years Old − Moderate+Severe Anaemia Prevalence − Tropical Latin America
WHO−2001 Adjustment Method

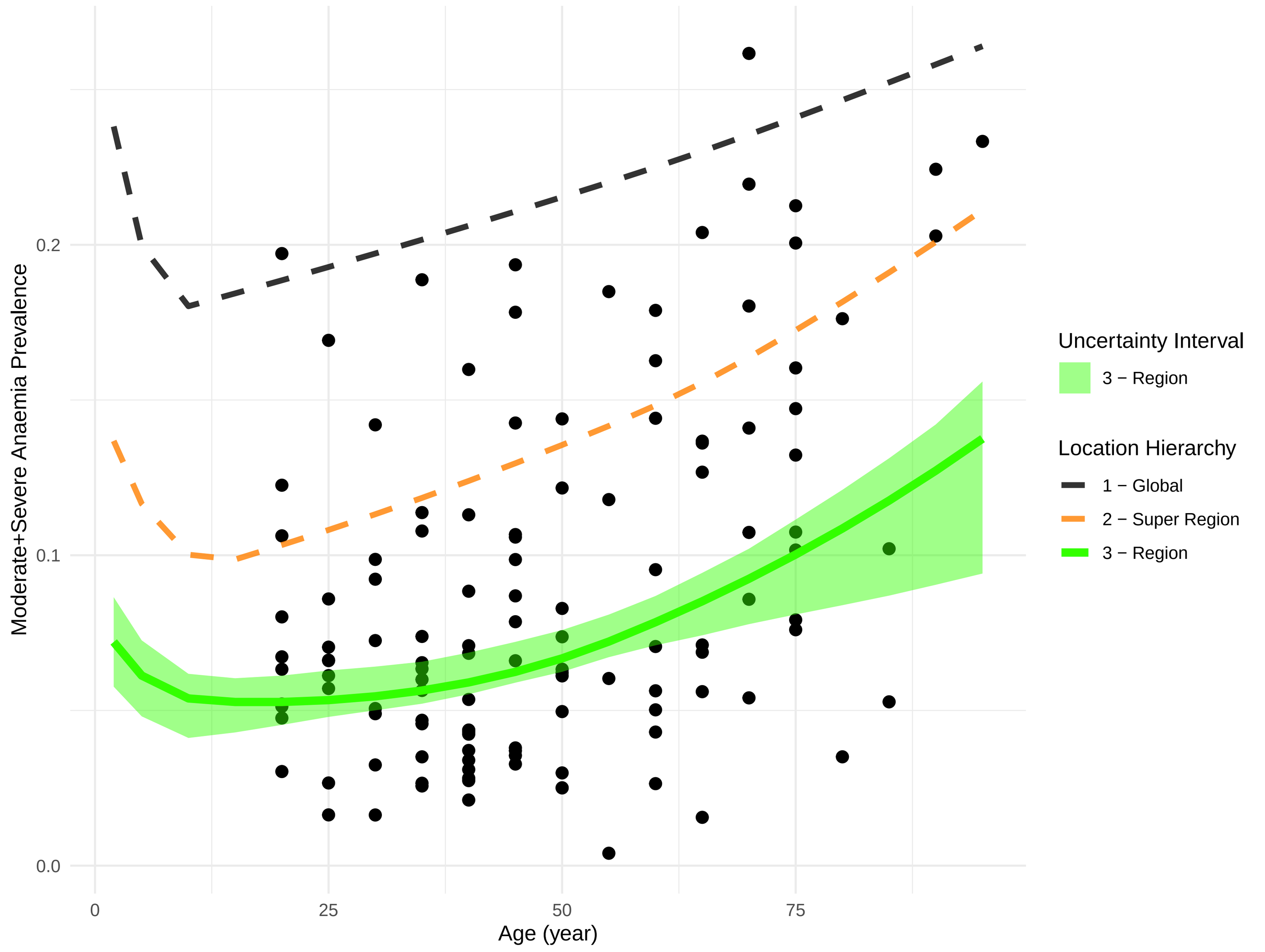

MR−BRT Age/Sex Fits − Non−pregnant Females 2+ Years Old − Moderate+Severe Anaemia Prevalence − Brazil
WHO−2001 Adjustment Method

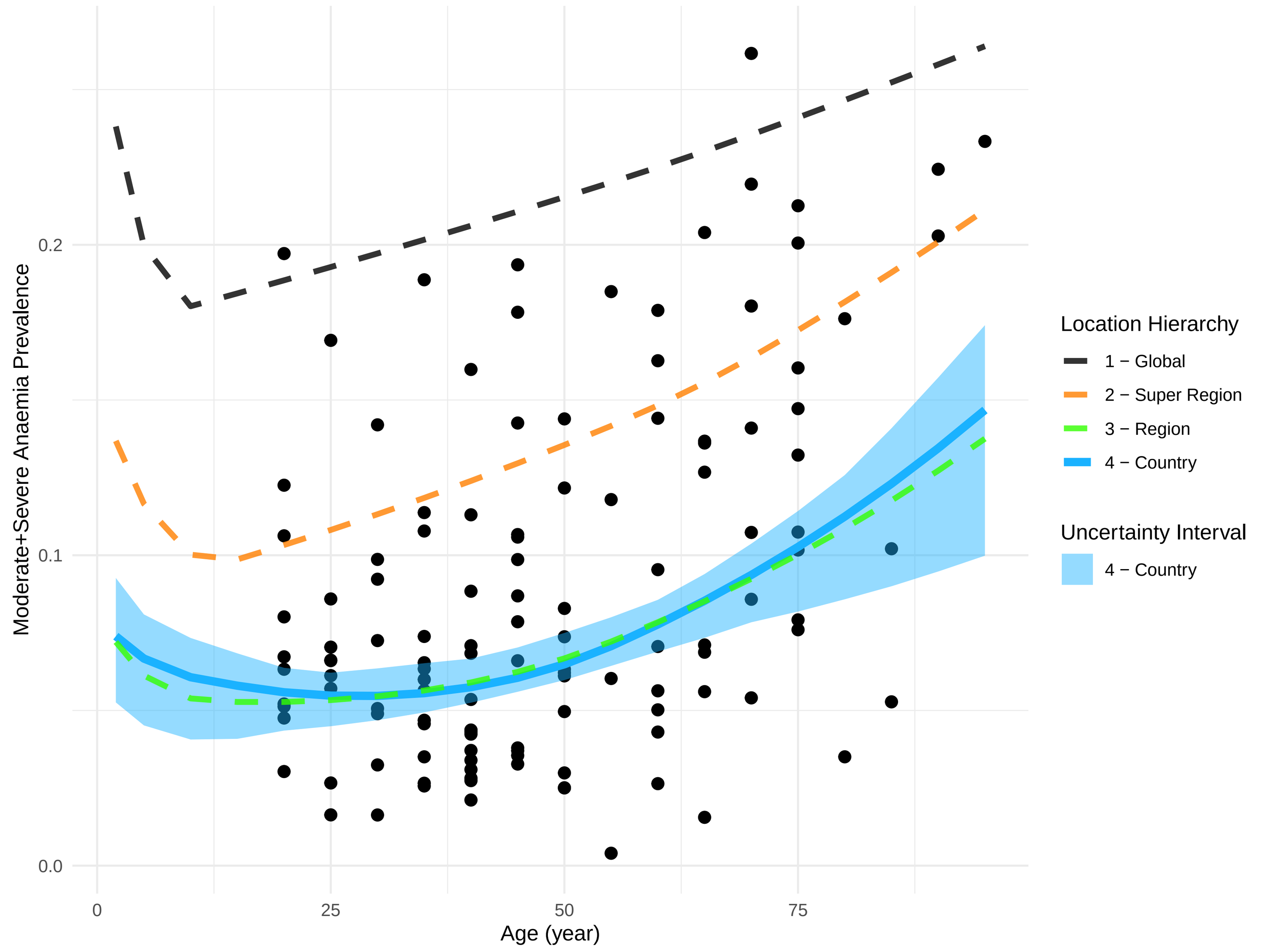

MR−BRT Age/Sex Fits − Non−pregnant Females 2+ Years Old − Moderate+Severe Anaemia Prevalence − Acre
WHO−2001 Adjustment Method

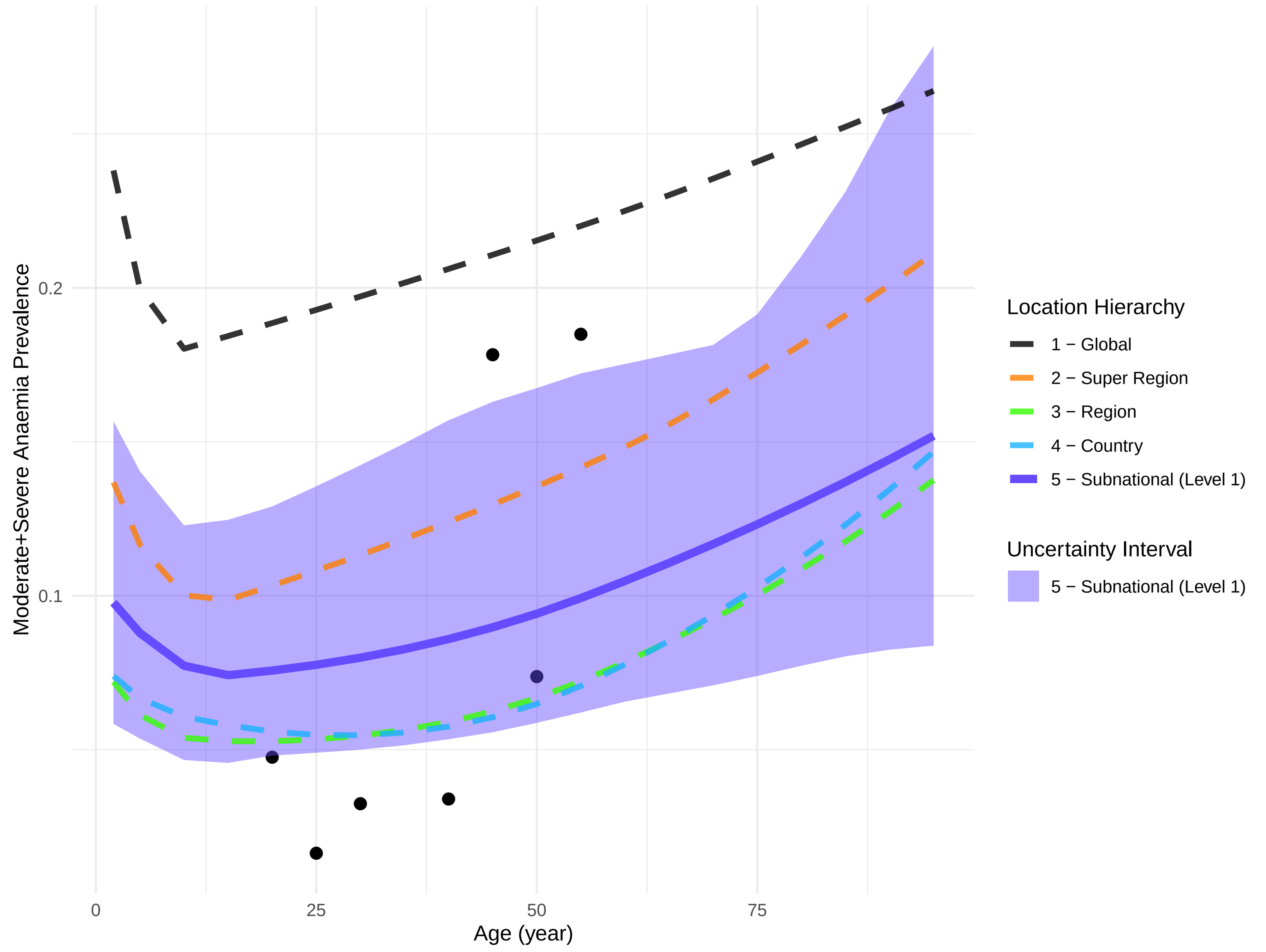

MR−BRT Age/Sex Fits − Non−pregnant Females 2+ Years Old − Moderate+Severe Anaemia Prevalence − Alagoas
WHO−2001 Adjustment Method

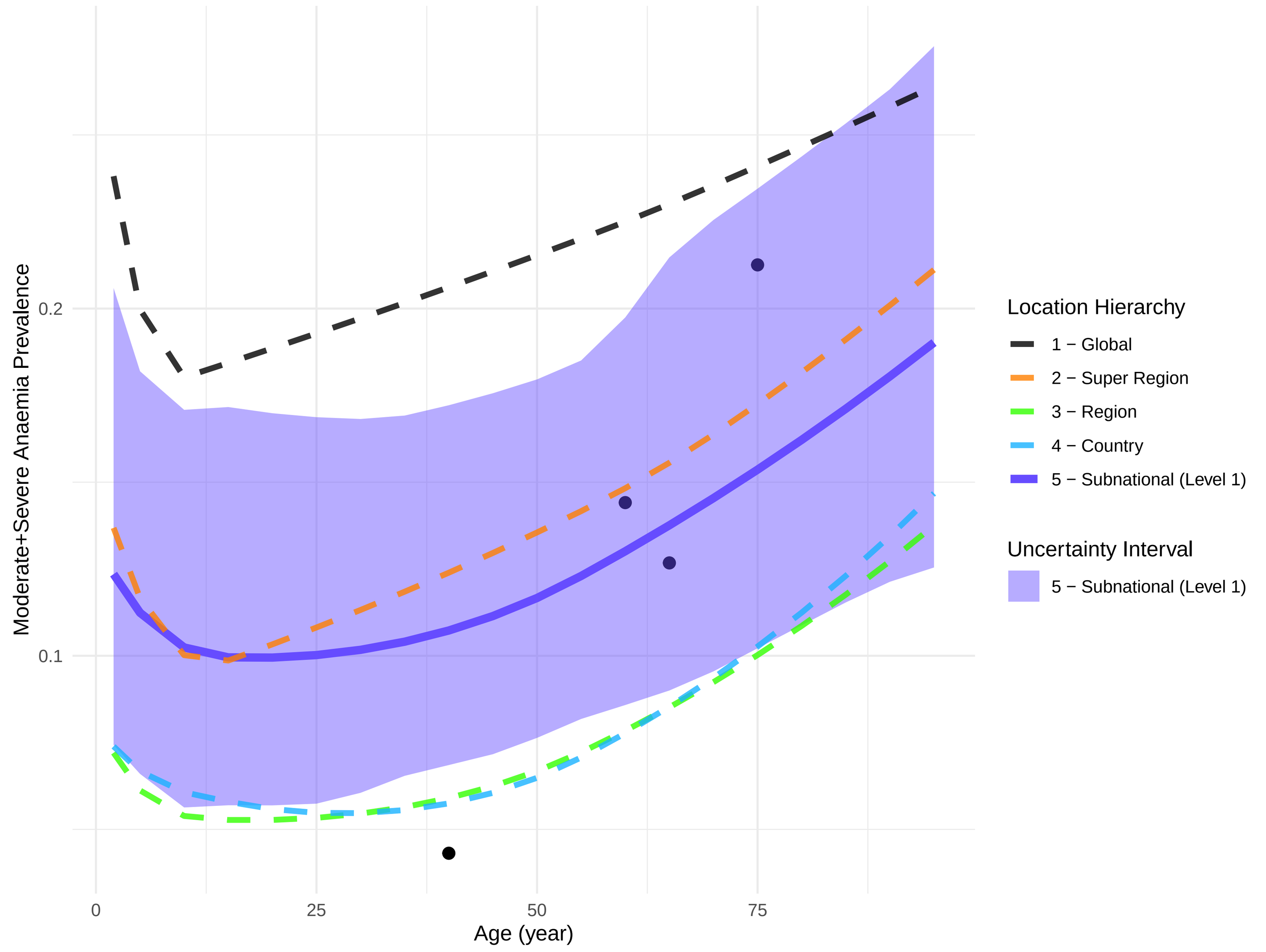

MR−BRT Age/Sex Fits − Non−pregnant Females 2+ Years Old − Moderate+Severe Anaemia Prevalence − Amapá
WHO−2001 Adjustment Method

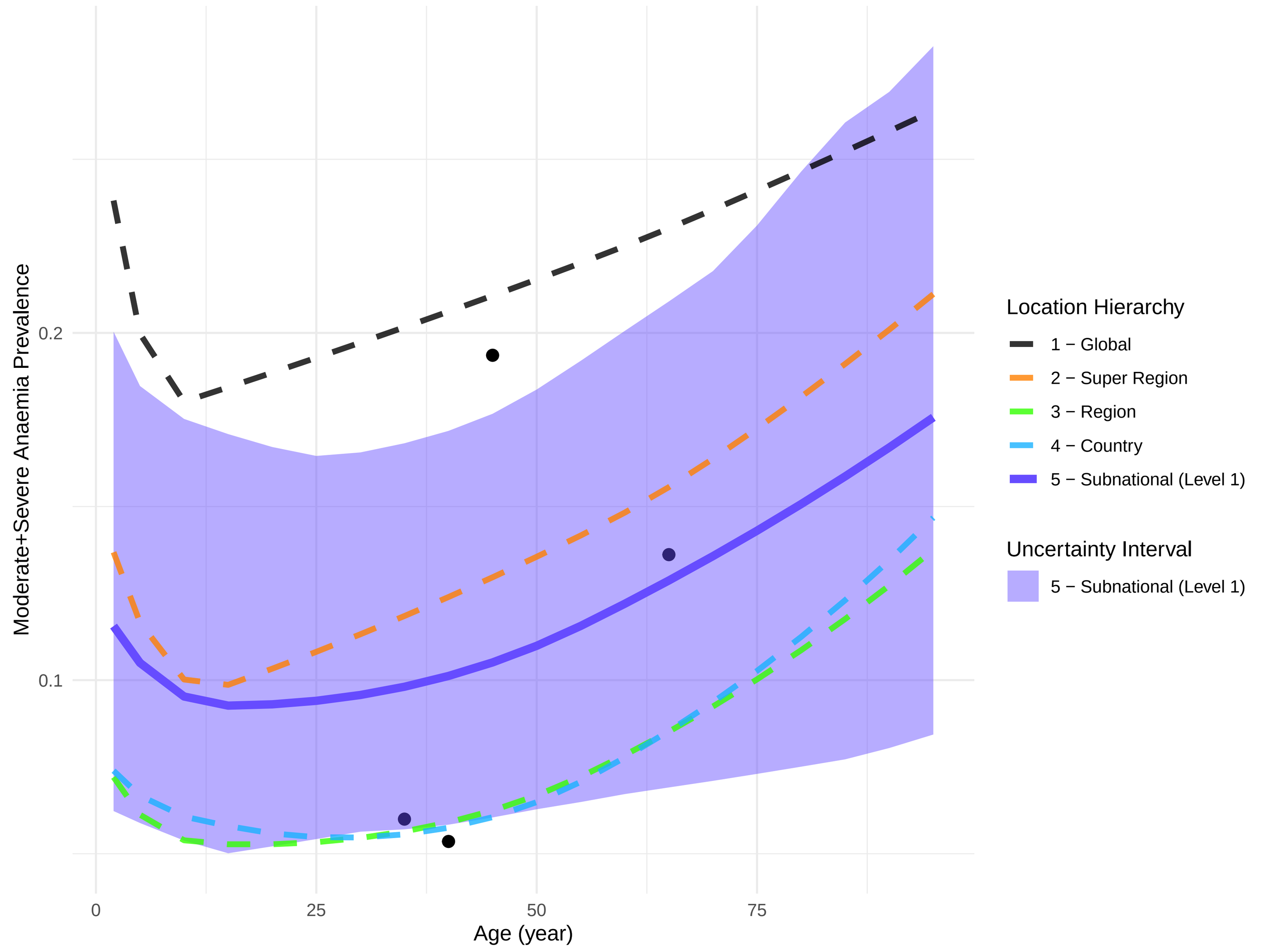

MR−BRT Age/Sex Fits − Non−pregnant Females 2+ Years Old − Moderate+Severe Anaemia Prevalence − Amazonas
WHO−2001 Adjustment Method

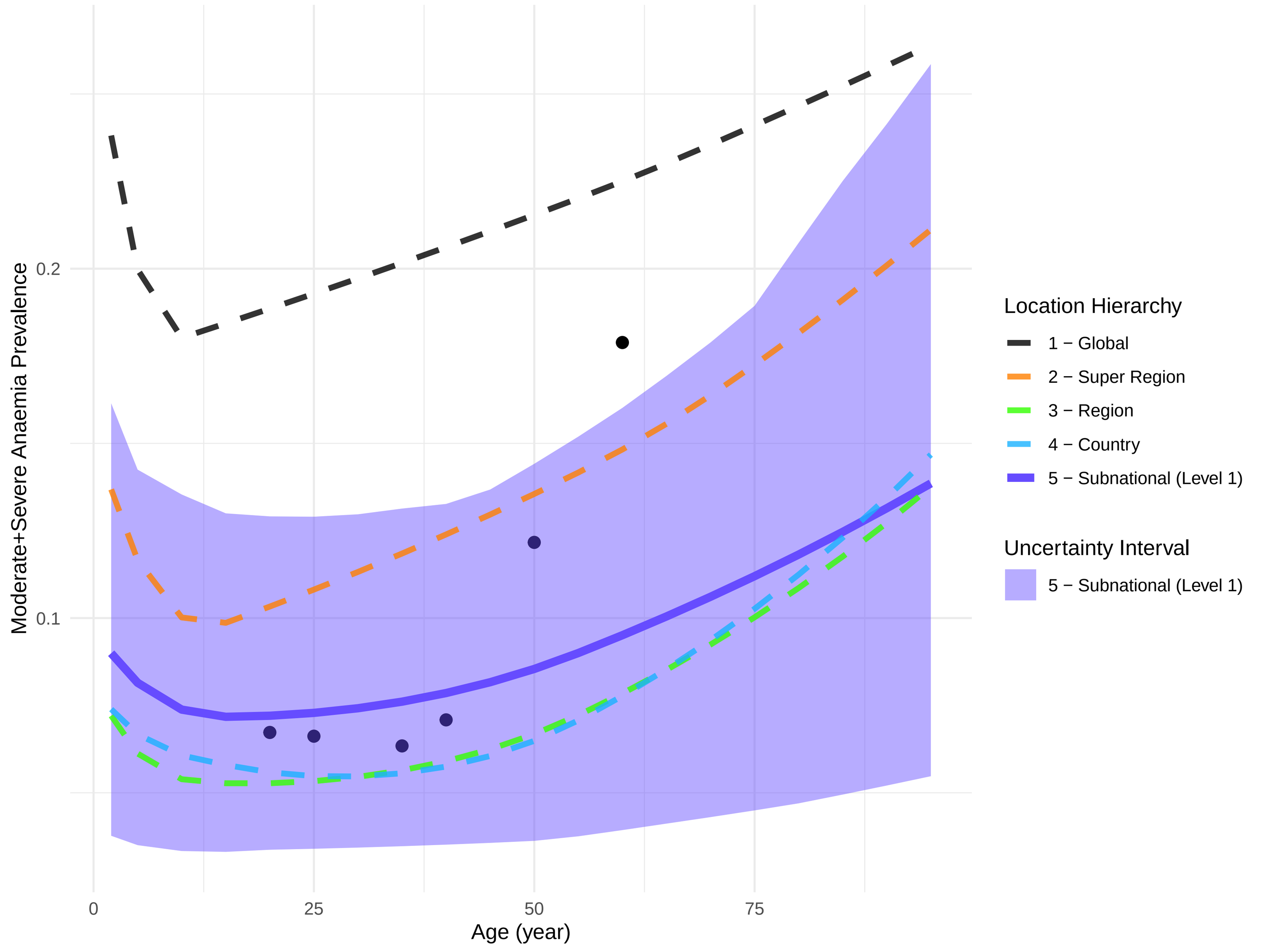

MR−BRT Age/Sex Fits − Non−pregnant Females 2+ Years Old − Moderate+Severe Anaemia Prevalence − Bahia
WHO−2001 Adjustment Method

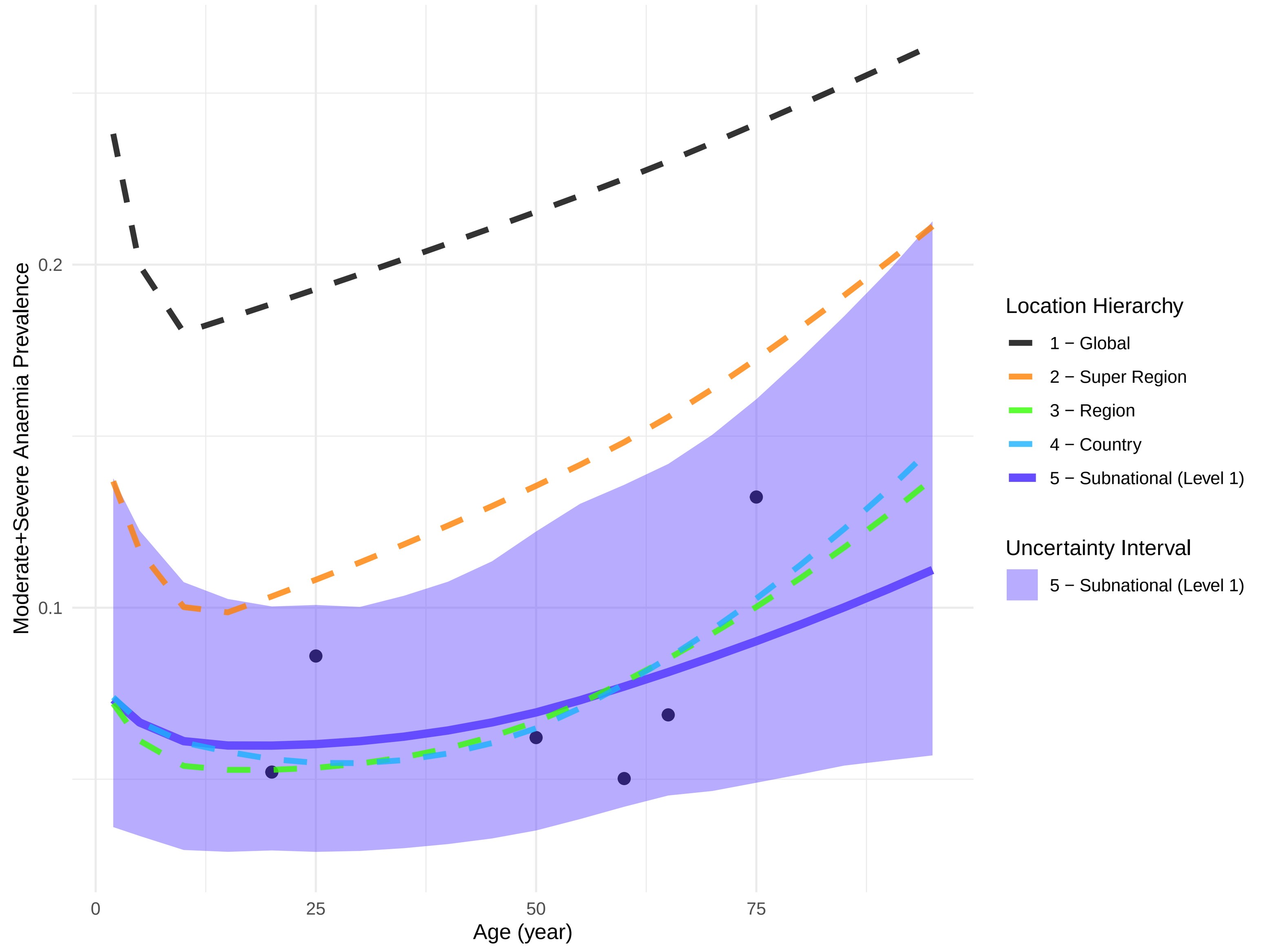

MR−BRT Age/Sex Fits − Non−pregnant Females 2+ Years Old − Moderate+Severe Anaemia Prevalence − Ceará
WHO−2001 Adjustment Method

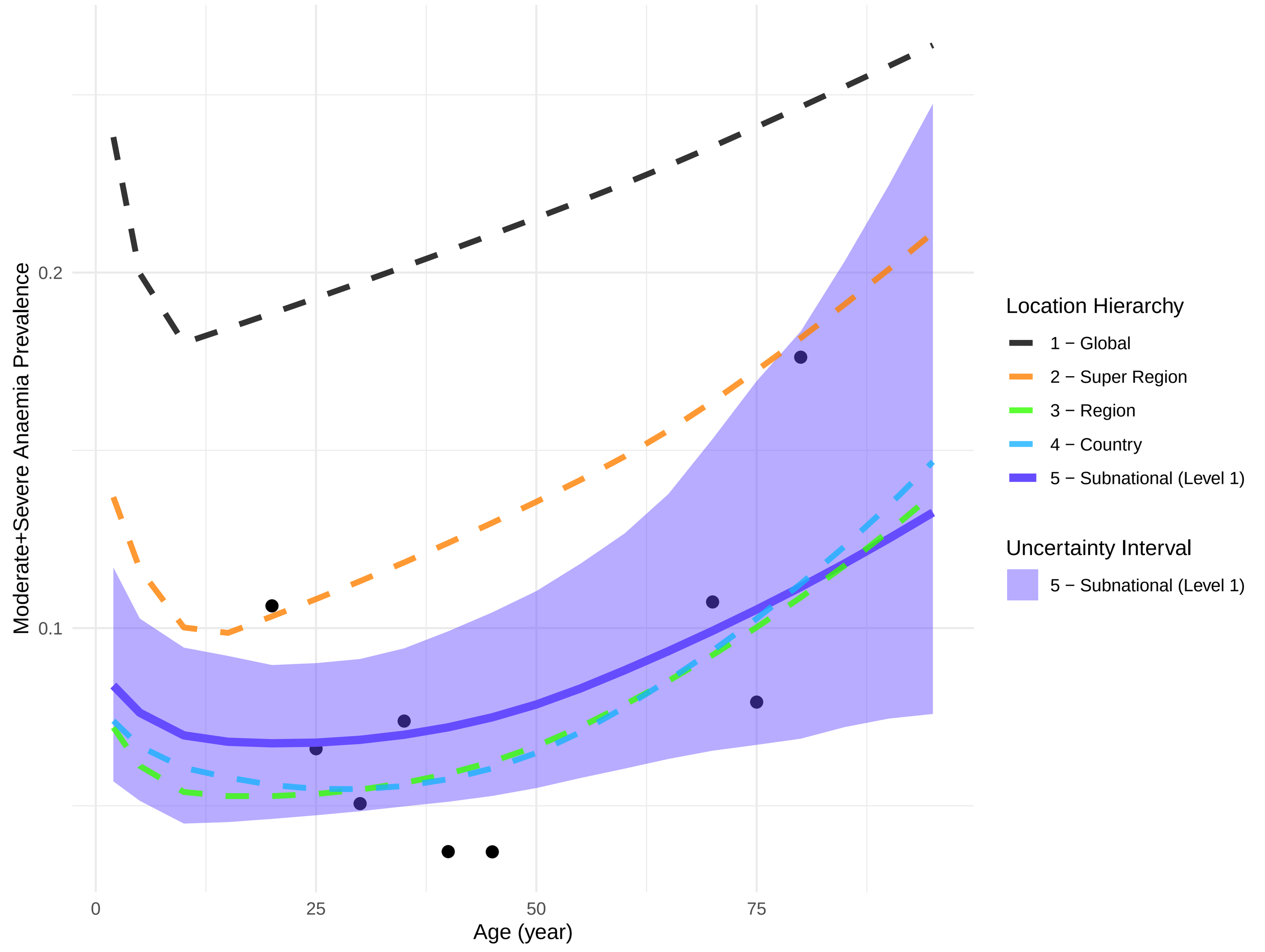

MR−BRT Age/Sex Fits − Non−pregnant Females 2+ Years Old − Moderate+Severe Anaemia Prevalence − Distrito Federal
WHO−2001 Adjustment Method

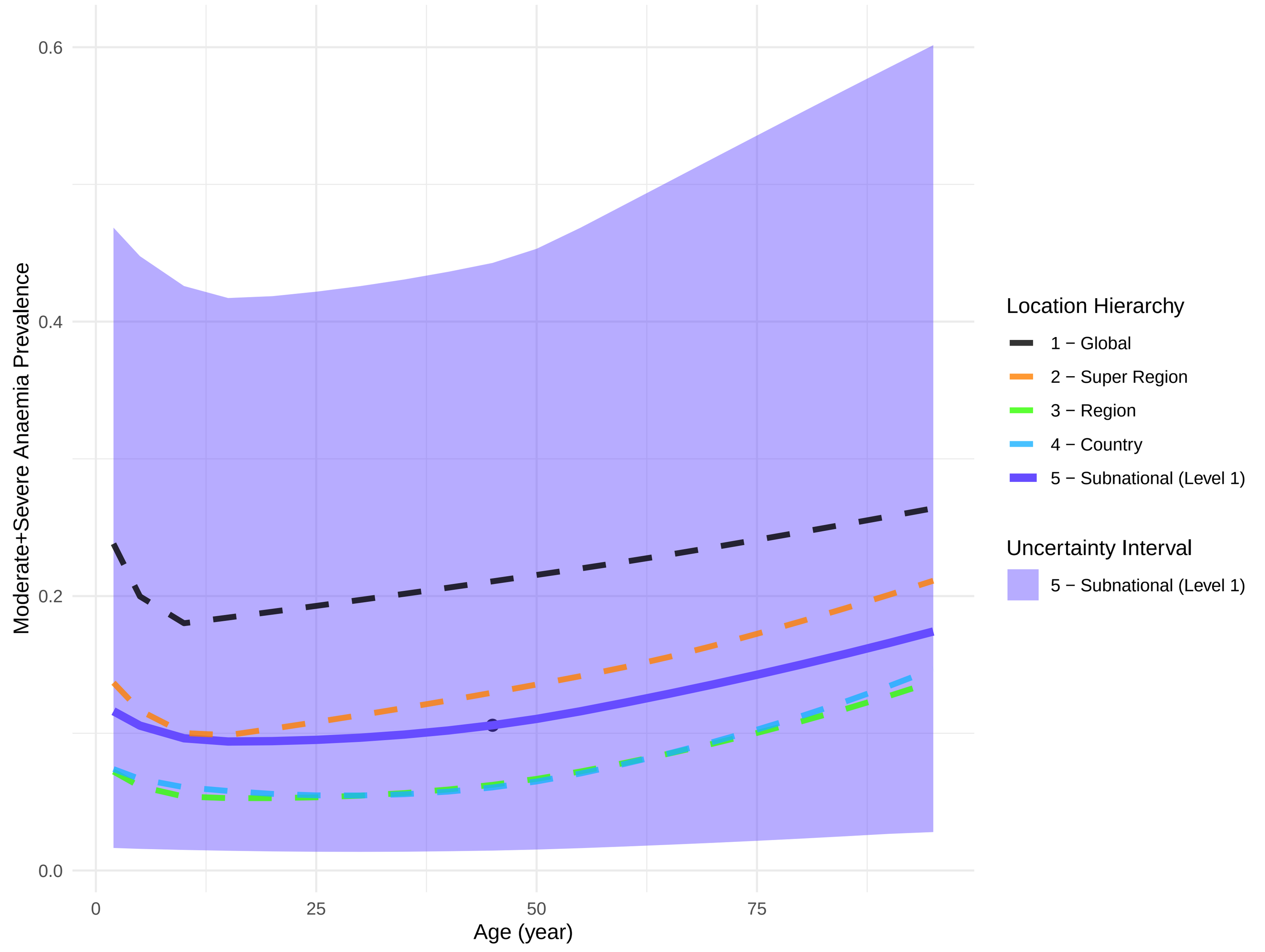

MR−BRT Age/Sex Fits − Non−pregnant Females 2+ Years Old − Moderate+Severe Anaemia Prevalence − Espírito Santo
WHO−2001 Adjustment Method

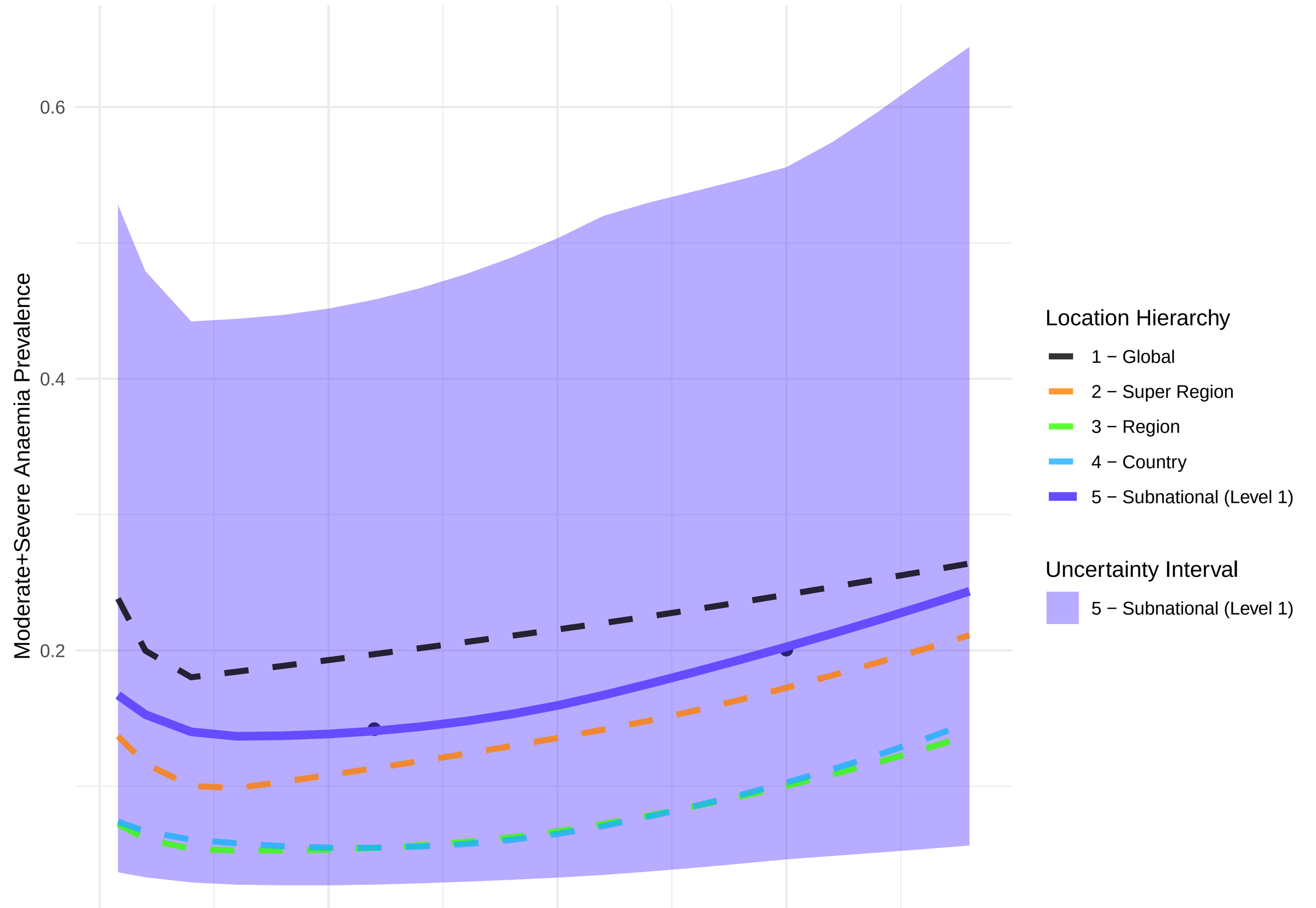

MR−BRT Age/Sex Fits − Non−pregnant Females 2+ Years Old − Moderate+Severe Anaemia Prevalence − Goiás
WHO−2001 Adjustment Method

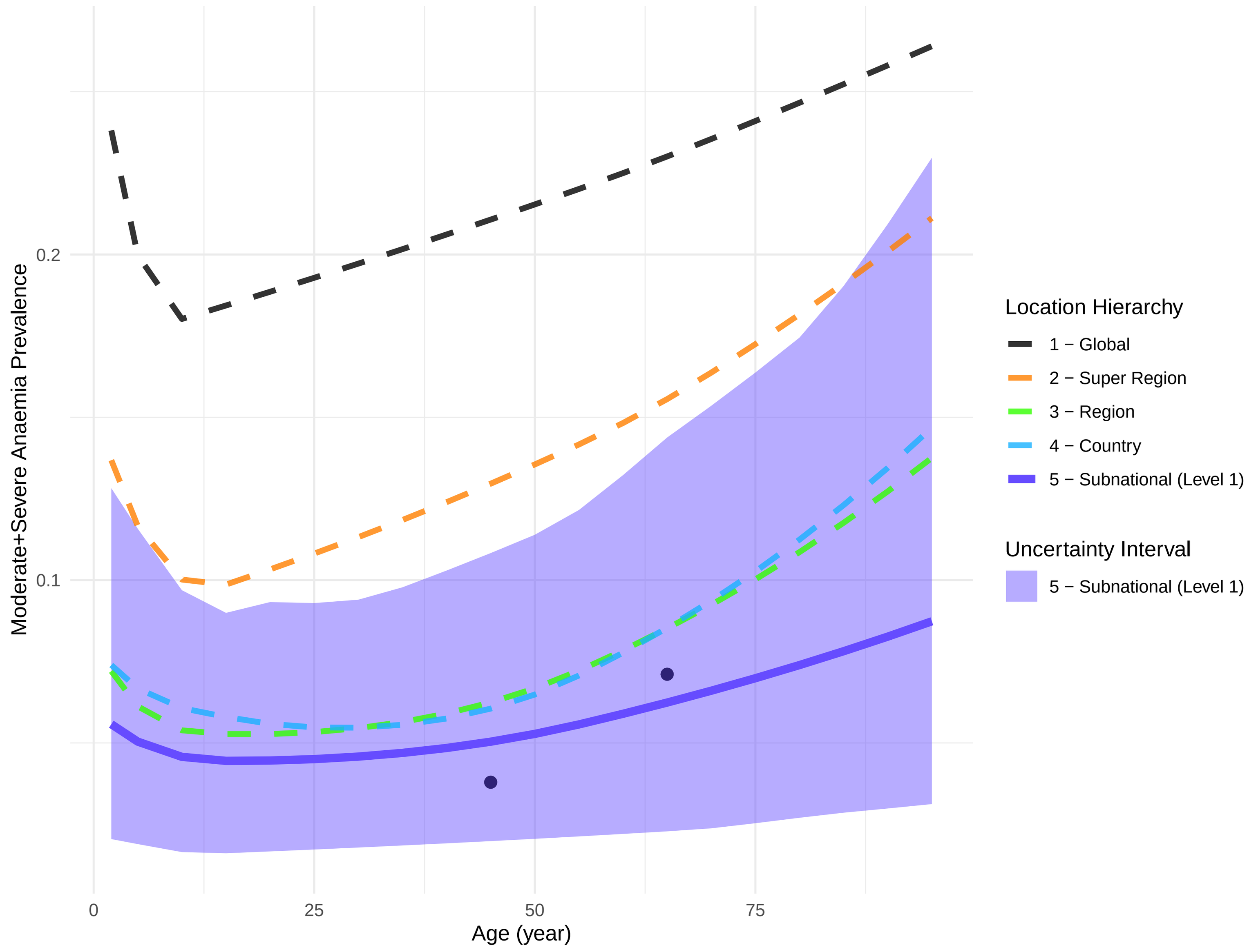

MR−BRT Age/Sex Fits − Non−pregnant Females 2+ Years Old − Moderate+Severe Anaemia Prevalence − Maranhão
WHO−2001 Adjustment Method

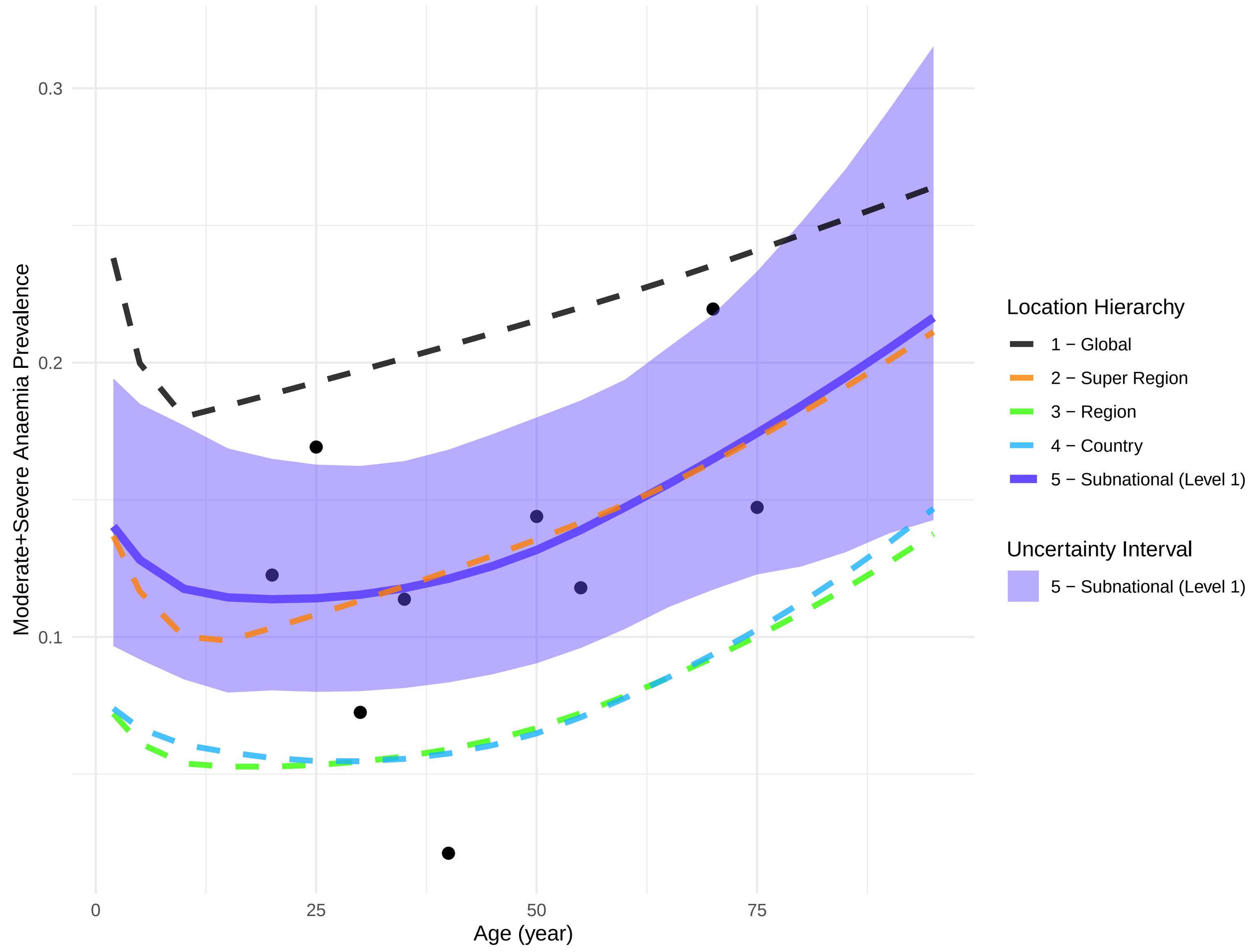

MR−BRT Age/Sex Fits − Non−pregnant Females 2+ Years Old − Moderate+Severe Anaemia Prevalence − Mato Grosso
WHO−2001 Adjustment Method

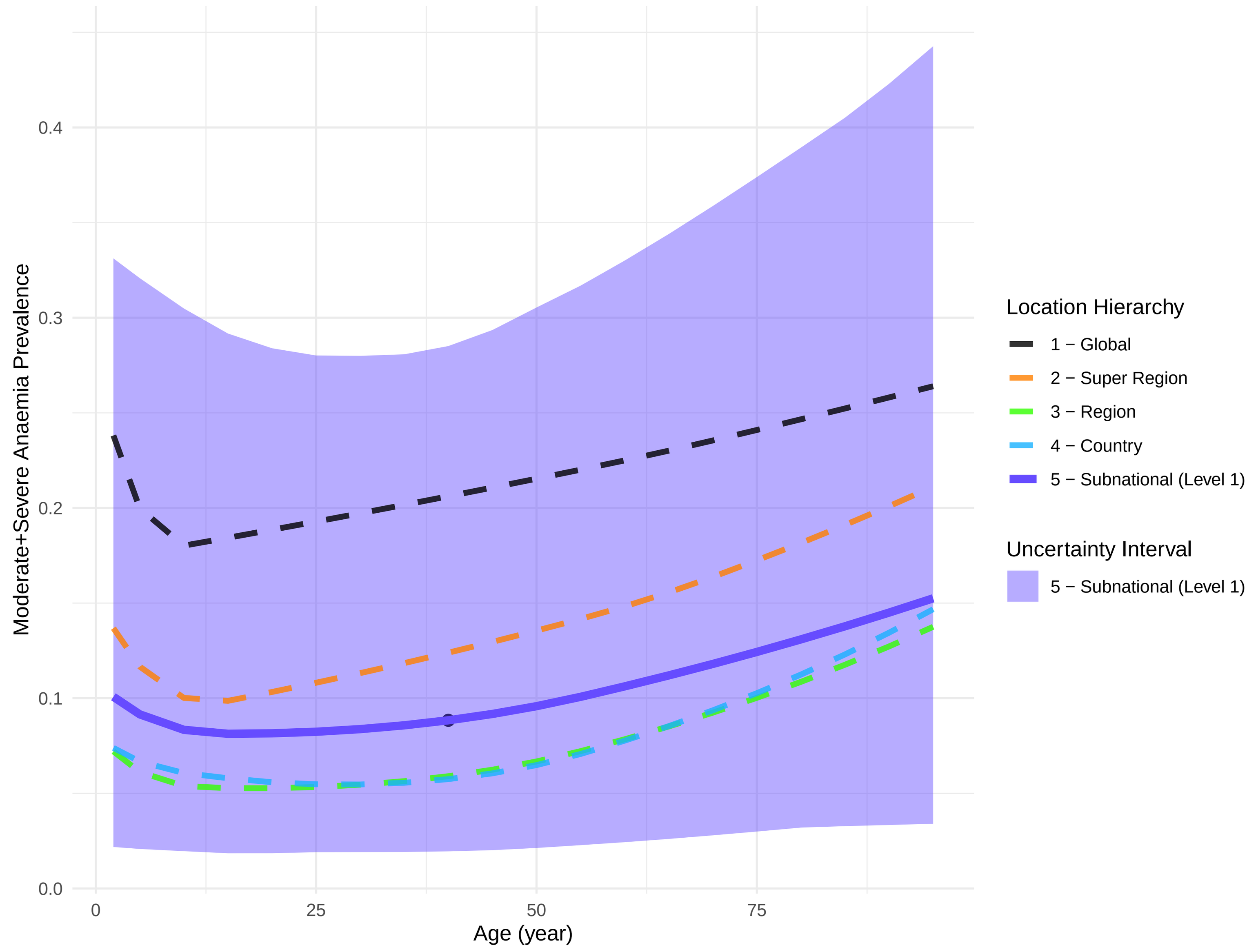

MR−BRT Age/Sex Fits − Non−pregnant Females 2+ Years Old − Moderate+Severe Anaemia Prevalence − Minas Gerais
WHO−2001 Adjustment Method

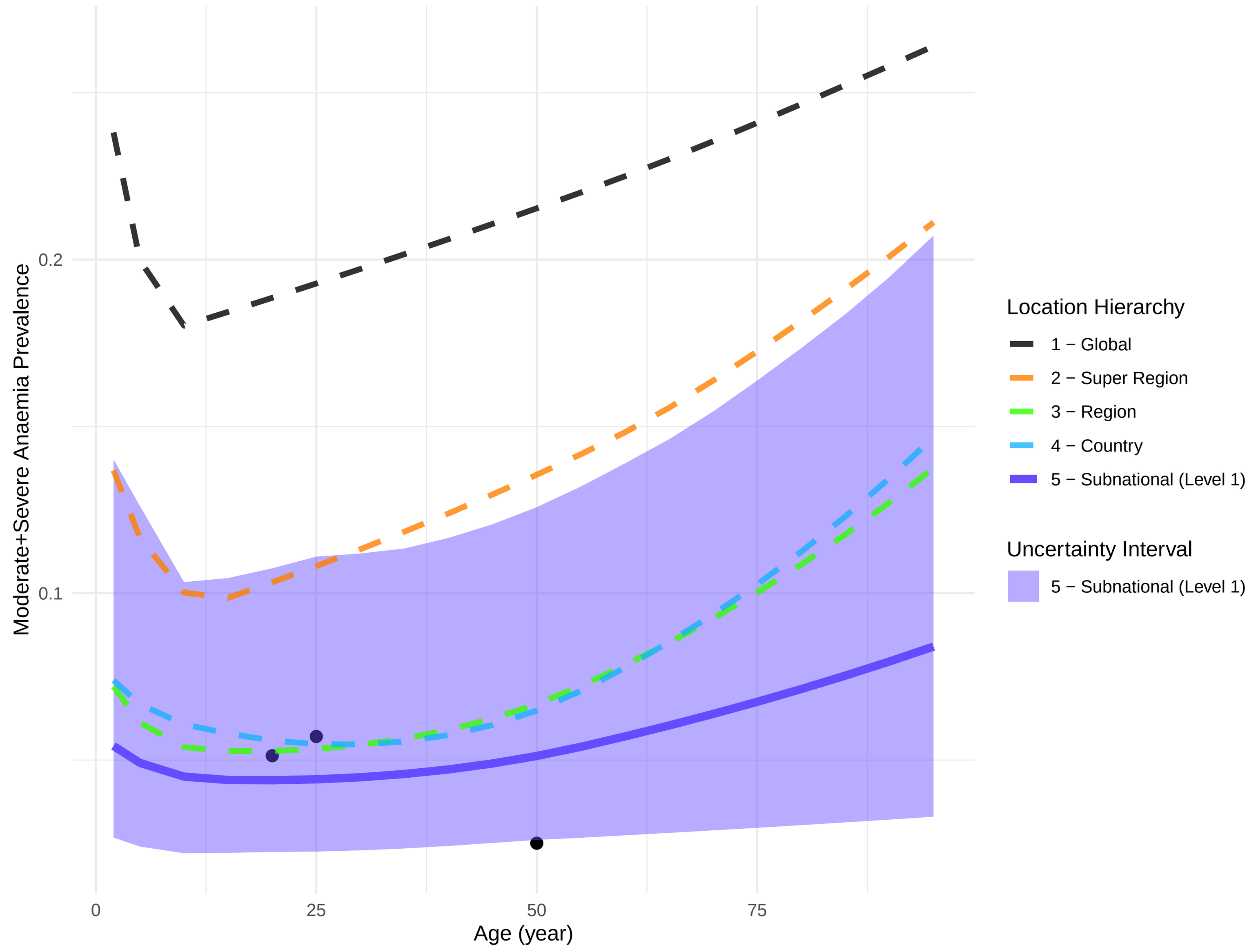

MR−BRT Age/Sex Fits − Non−pregnant Females 2+ Years Old − Moderate+Severe Anaemia Prevalence − Pará
WHO−2001 Adjustment Method

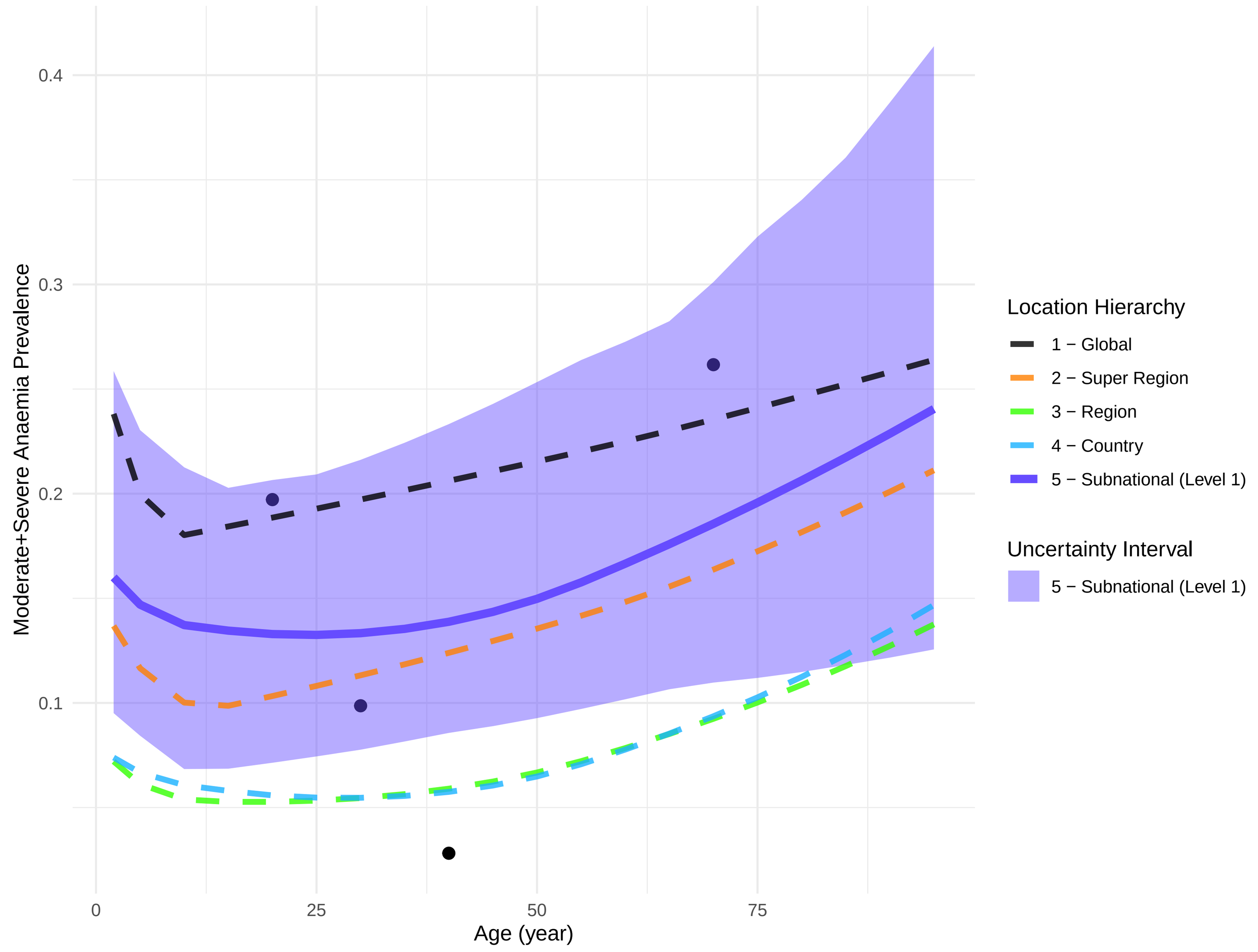

MR−BRT Age/Sex Fits − Non−pregnant Females 2+ Years Old − Moderate+Severe Anaemia Prevalence − Paraíba
WHO−2001 Adjustment Method

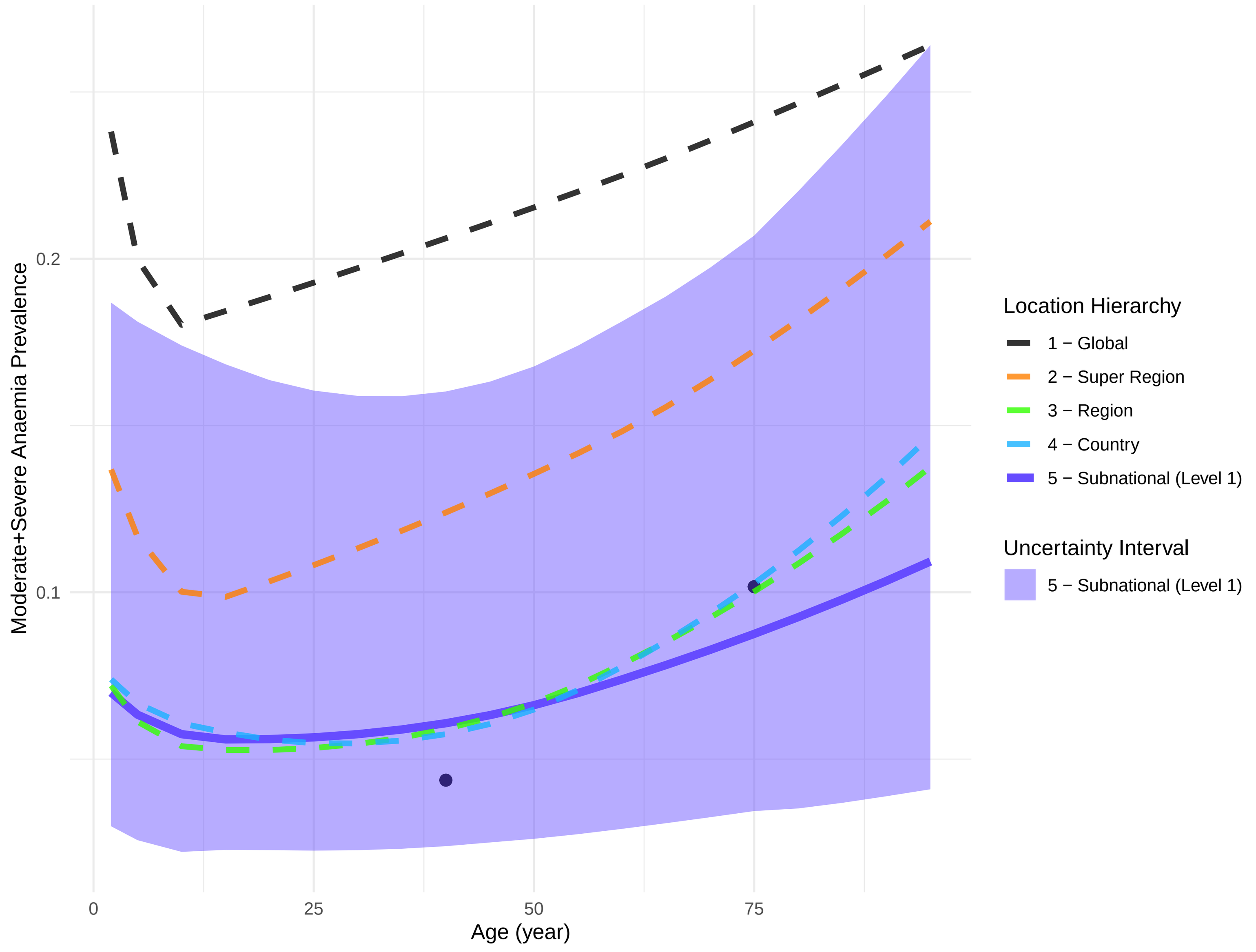

MR−BRT Age/Sex Fits − Non−pregnant Females 2+ Years Old − Moderate+Severe Anaemia Prevalence − Paraná
WHO−2001 Adjustment Method

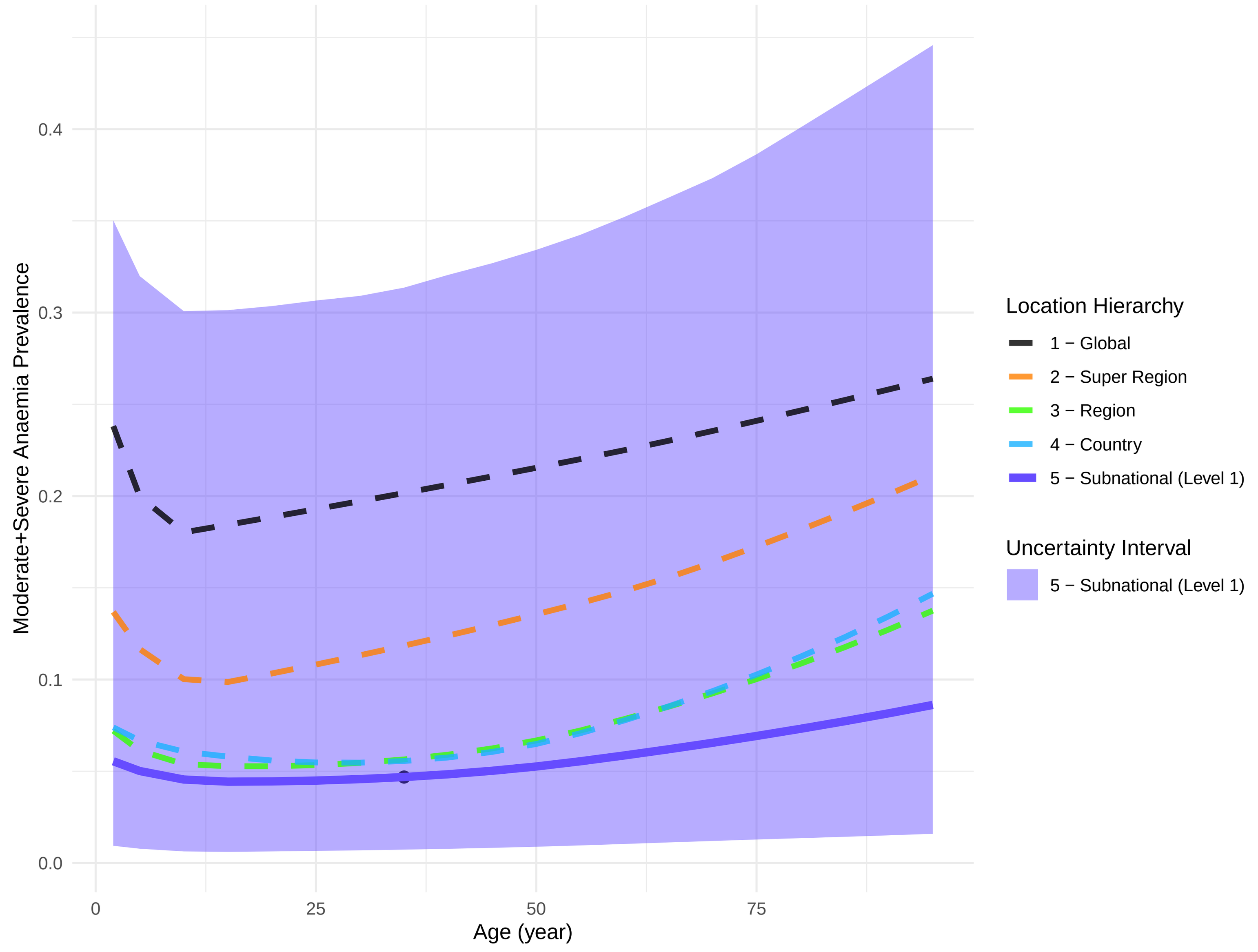

MR−BRT Age/Sex Fits − Non−pregnant Females 2+ Years Old − Moderate+Severe Anaemia Prevalence − Pernambuco
WHO−2001 Adjustment Method

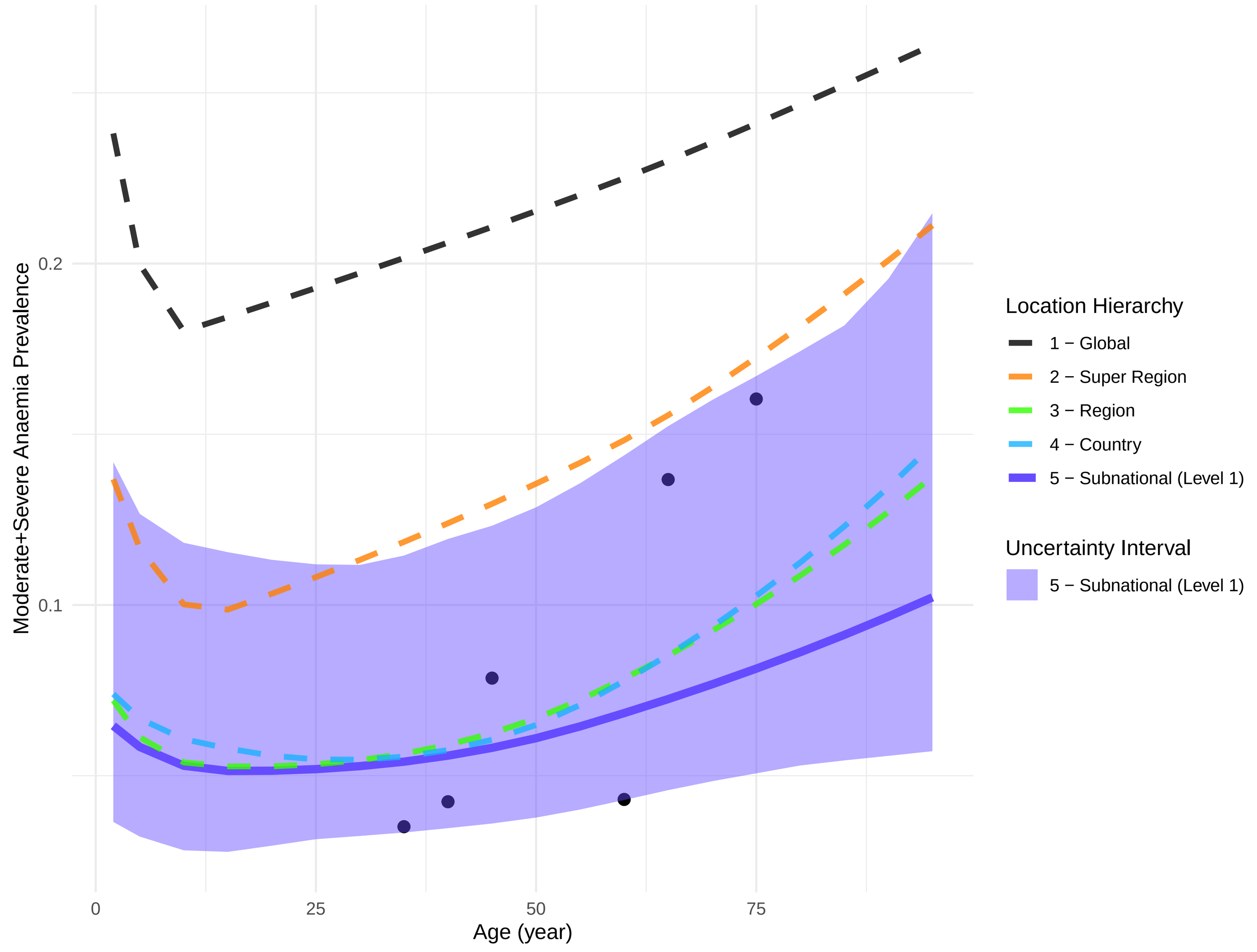

MR−BRT Age/Sex Fits − Non−pregnant Females 2+ Years Old − Moderate+Severe Anaemia Prevalence − Piauí
WHO−2001 Adjustment Method

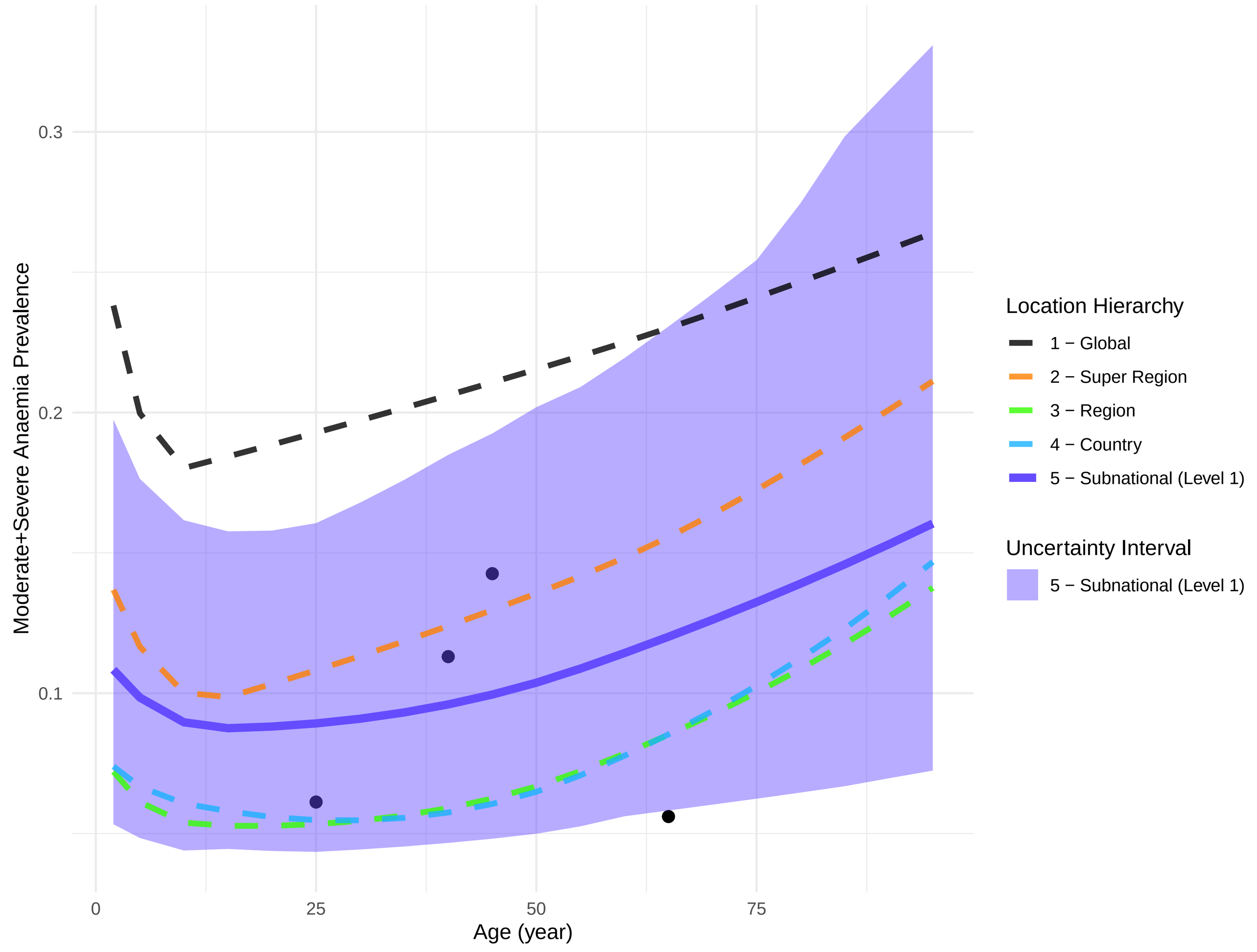

MR−BRT Age/Sex Fits − Non−pregnant Females 2+ Years Old − Moderate+Severe Anaemia Prevalence − Rio de Janeiro
WHO−2001 Adjustment Method

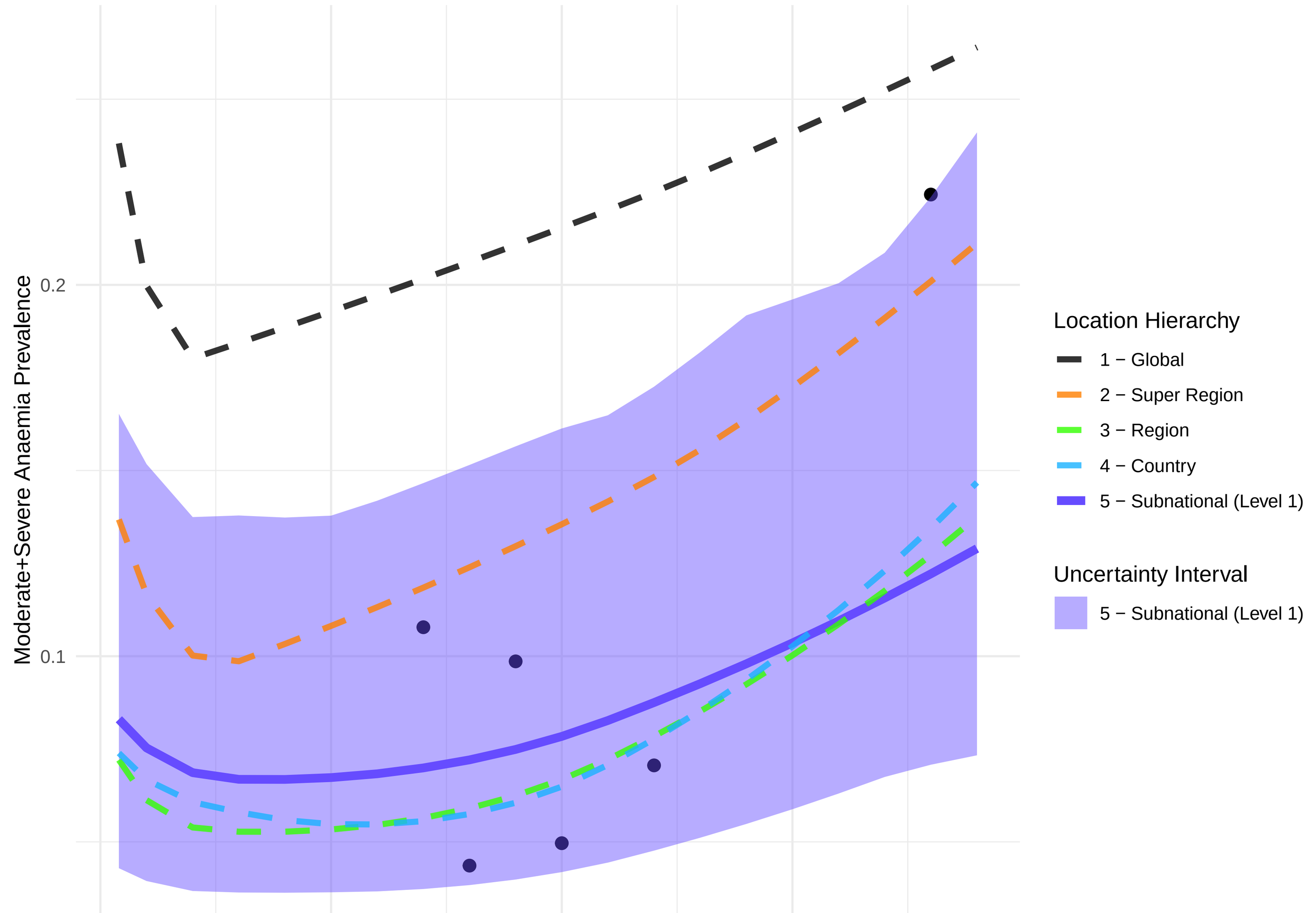

MR−BRT Age/Sex Fits − Non−pregnant Females 2+ Years Old − Moderate+Severe Anaemia Prevalence − Rio Grande do Norte
WHO−2001 Adjustment Method

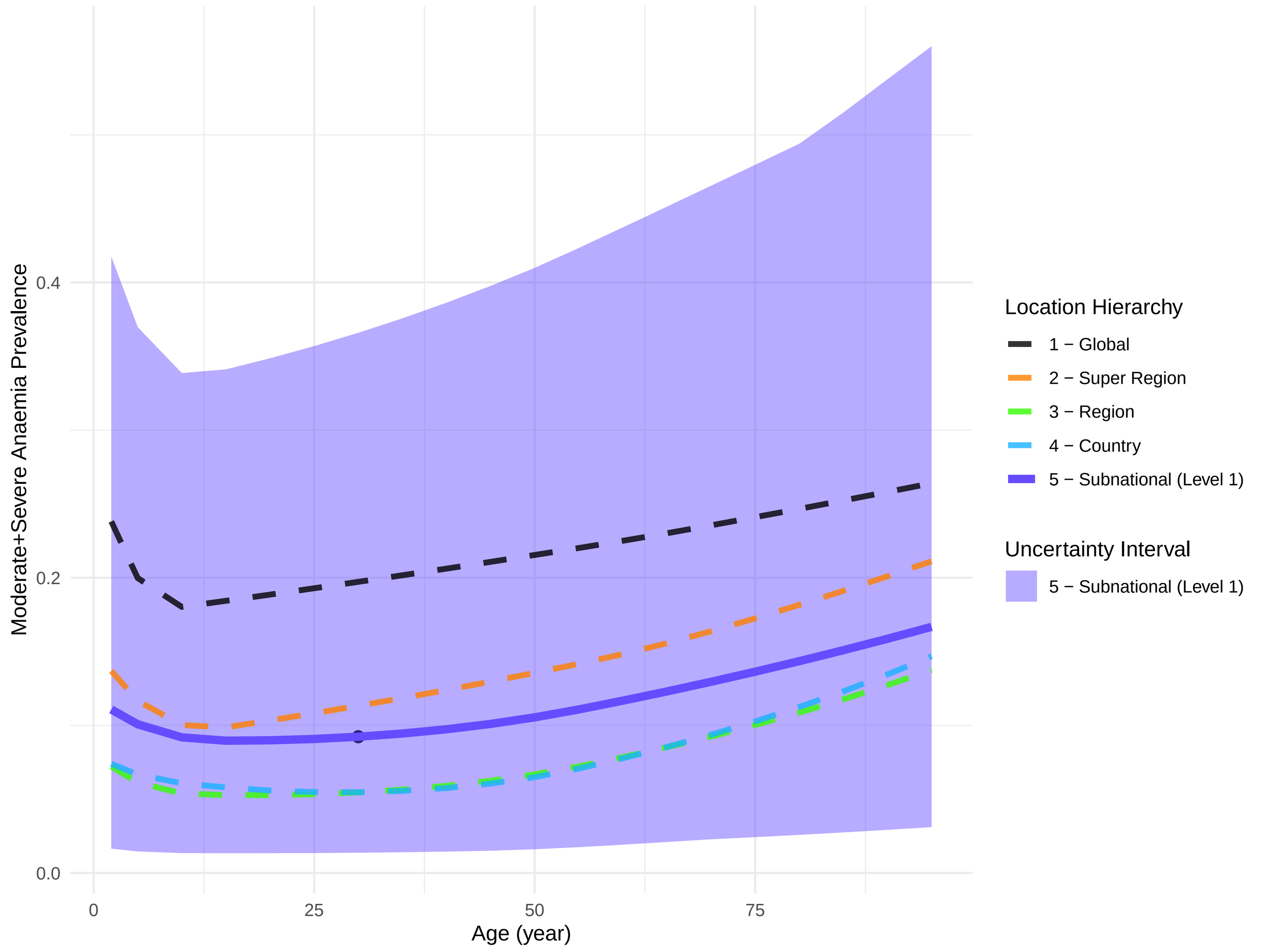

MR−BRT Age/Sex Fits − Non−pregnant Females 2+ Years Old − Moderate+Severe Anaemia Prevalence − Rio Grande do Sul
WHO−2001 Adjustment Method

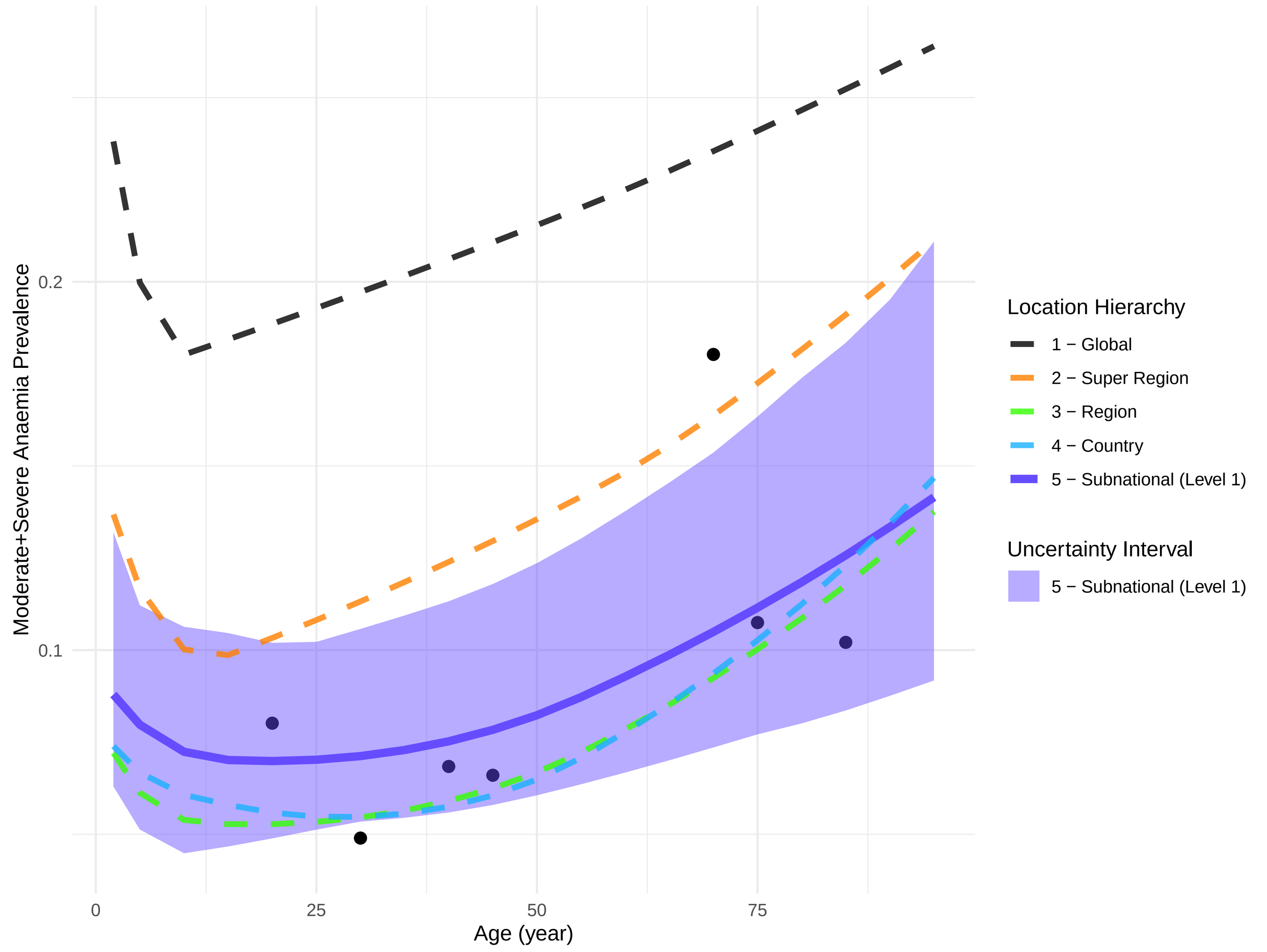

MR−BRT Age/Sex Fits − Non−pregnant Females 2+ Years Old − Moderate+Severe Anaemia Prevalence − Rondônia
WHO−2001 Adjustment Method

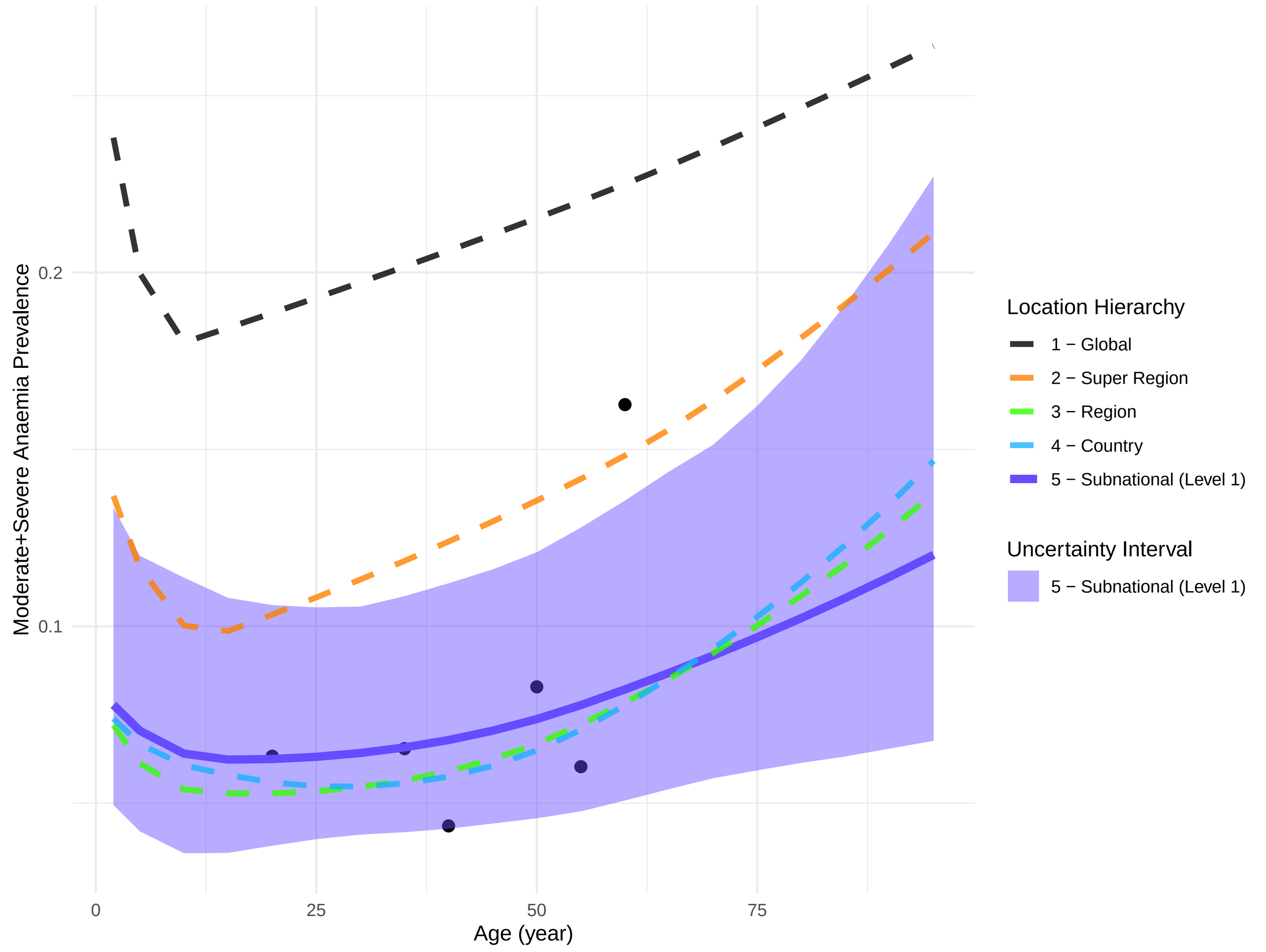

MR−BRT Age/Sex Fits − Non−pregnant Females 2+ Years Old − Moderate+Severe Anaemia Prevalence − Roraima
WHO−2001 Adjustment Method

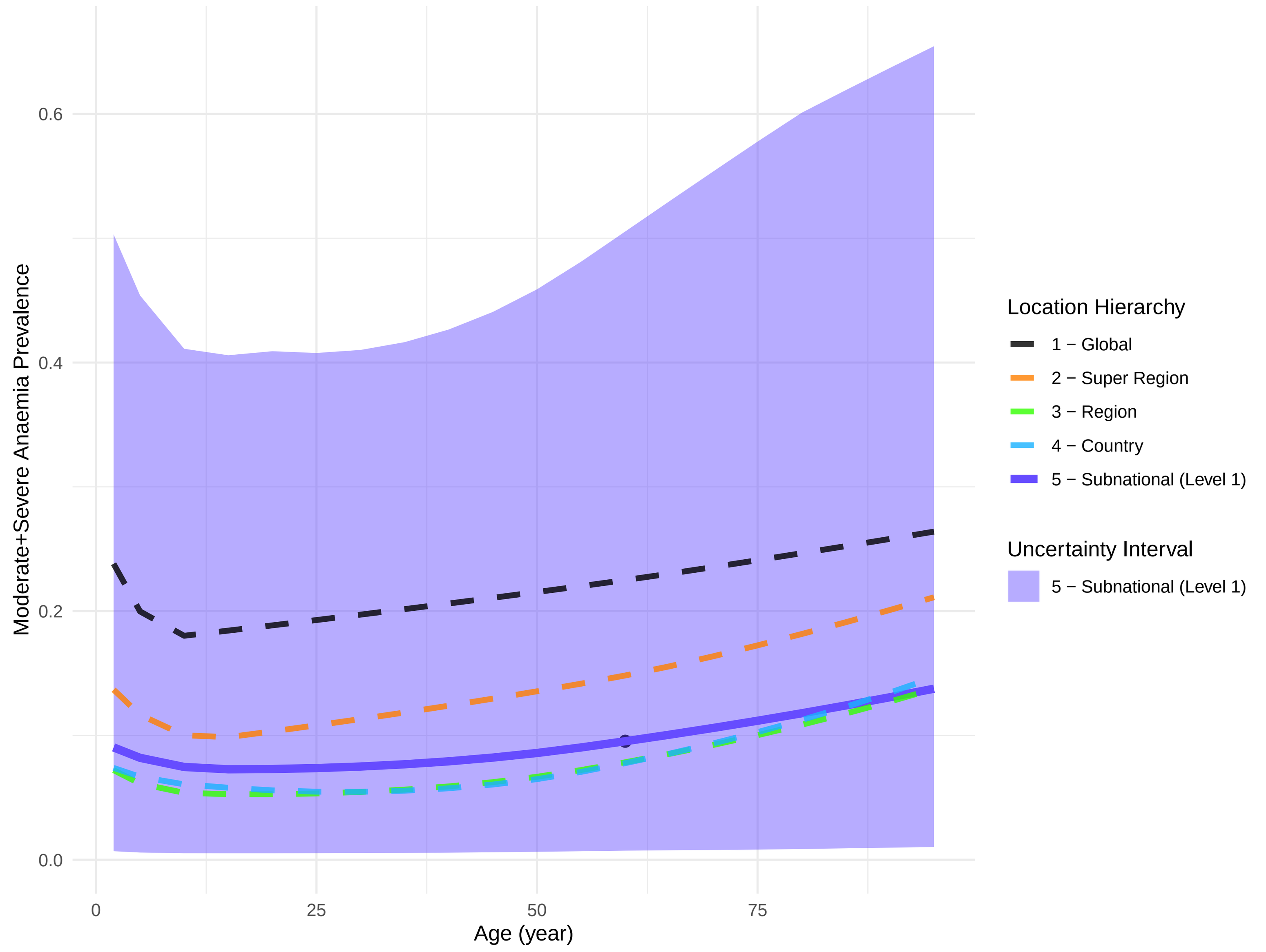

MR−BRT Age/Sex Fits − Non−pregnant Females 2+ Years Old − Moderate+Severe Anaemia Prevalence − Santa Catarina
WHO−2001 Adjustment Method

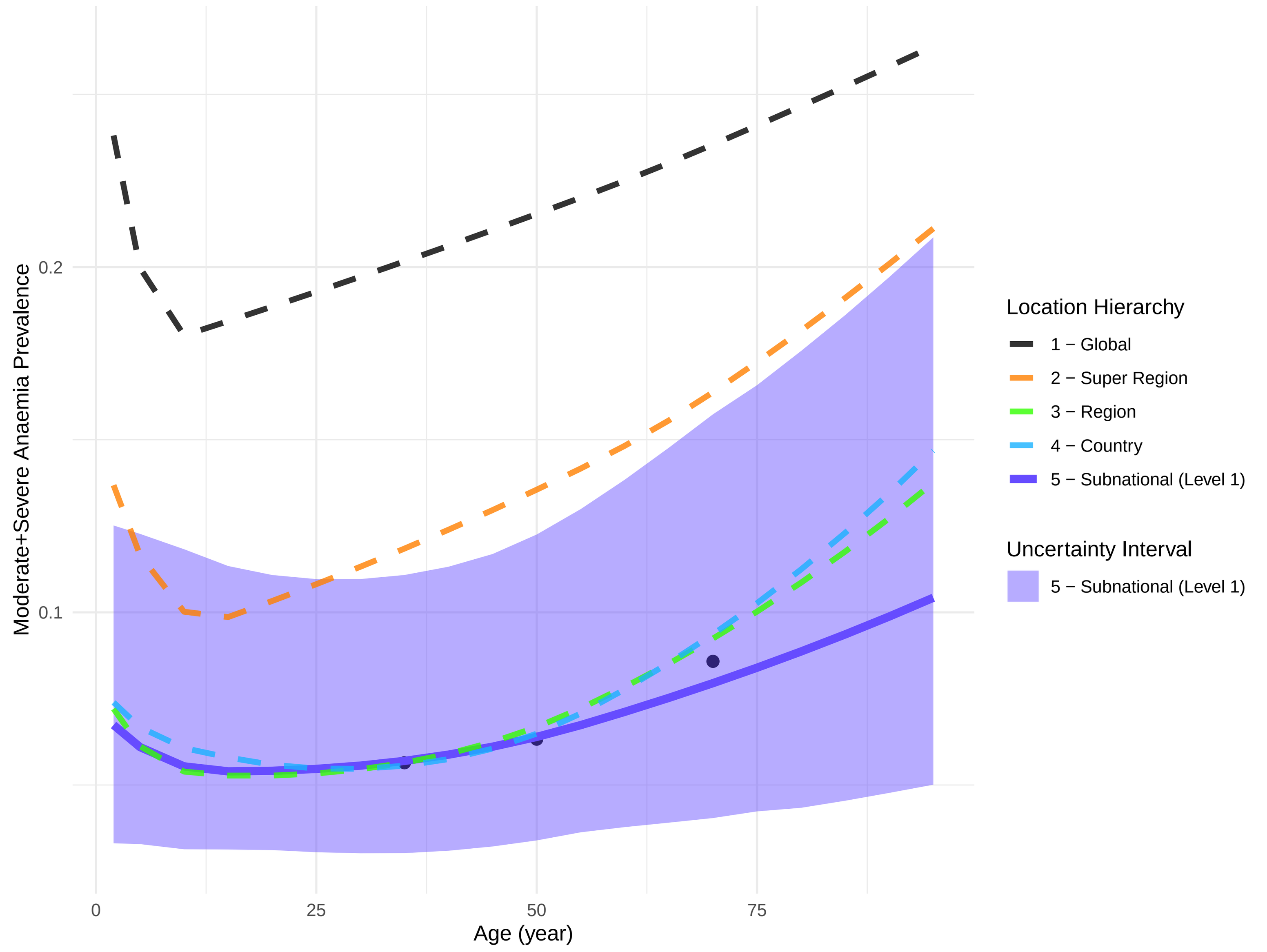

MR−BRT Age/Sex Fits − Non−pregnant Females 2+ Years Old − Moderate+Severe Anaemia Prevalence − São Paulo
WHO−2001 Adjustment Method

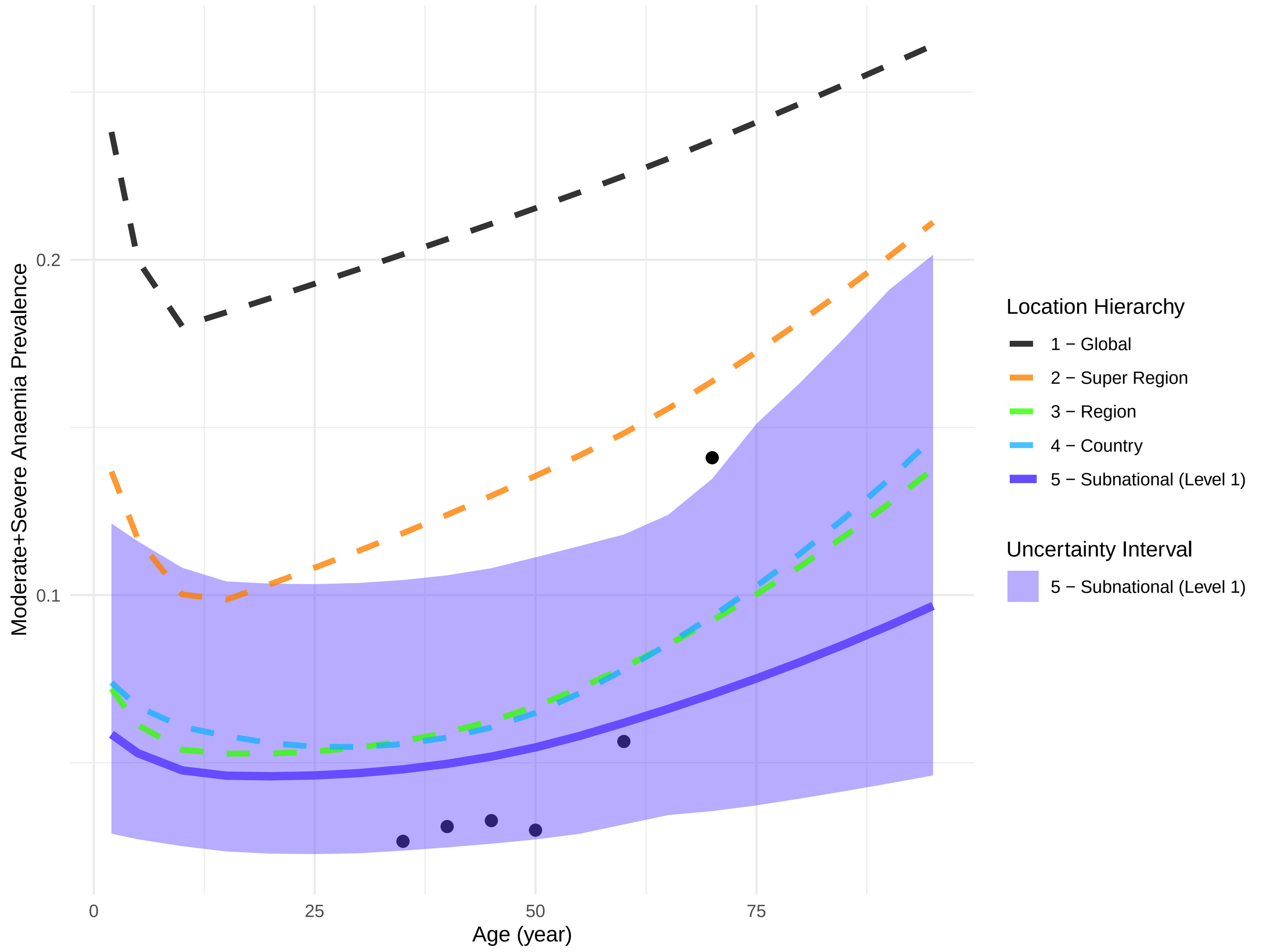

MR−BRT Age/Sex Fits − Non−pregnant Females 2+ Years Old − Moderate+Severe Anaemia Prevalence − Sergipe
WHO−2001 Adjustment Method

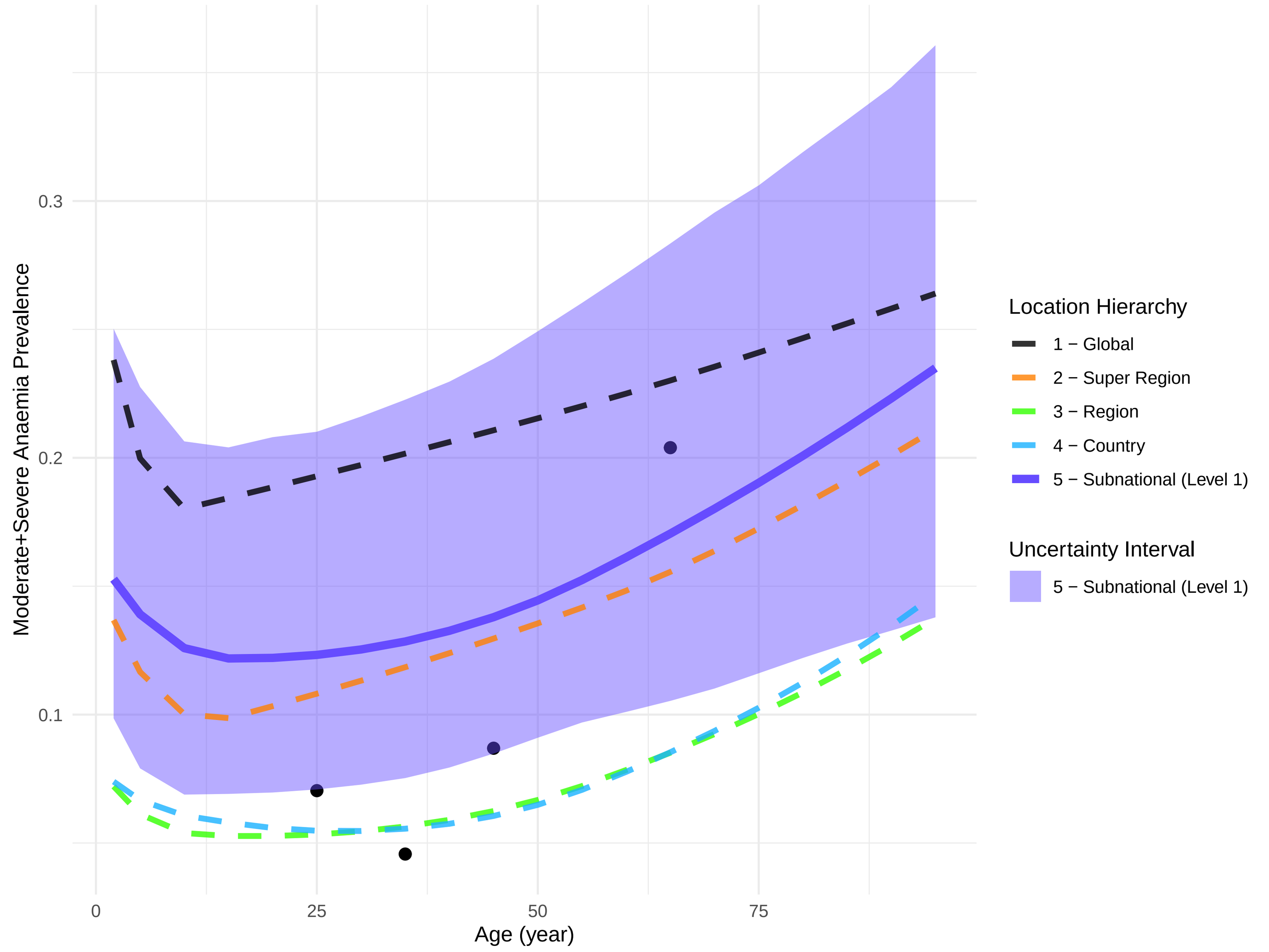

MR−BRT Age/Sex Fits − Non−pregnant Females 2+ Years Old − Moderate+Severe Anaemia Prevalence − Tocantins
WHO−2001 Adjustment Method

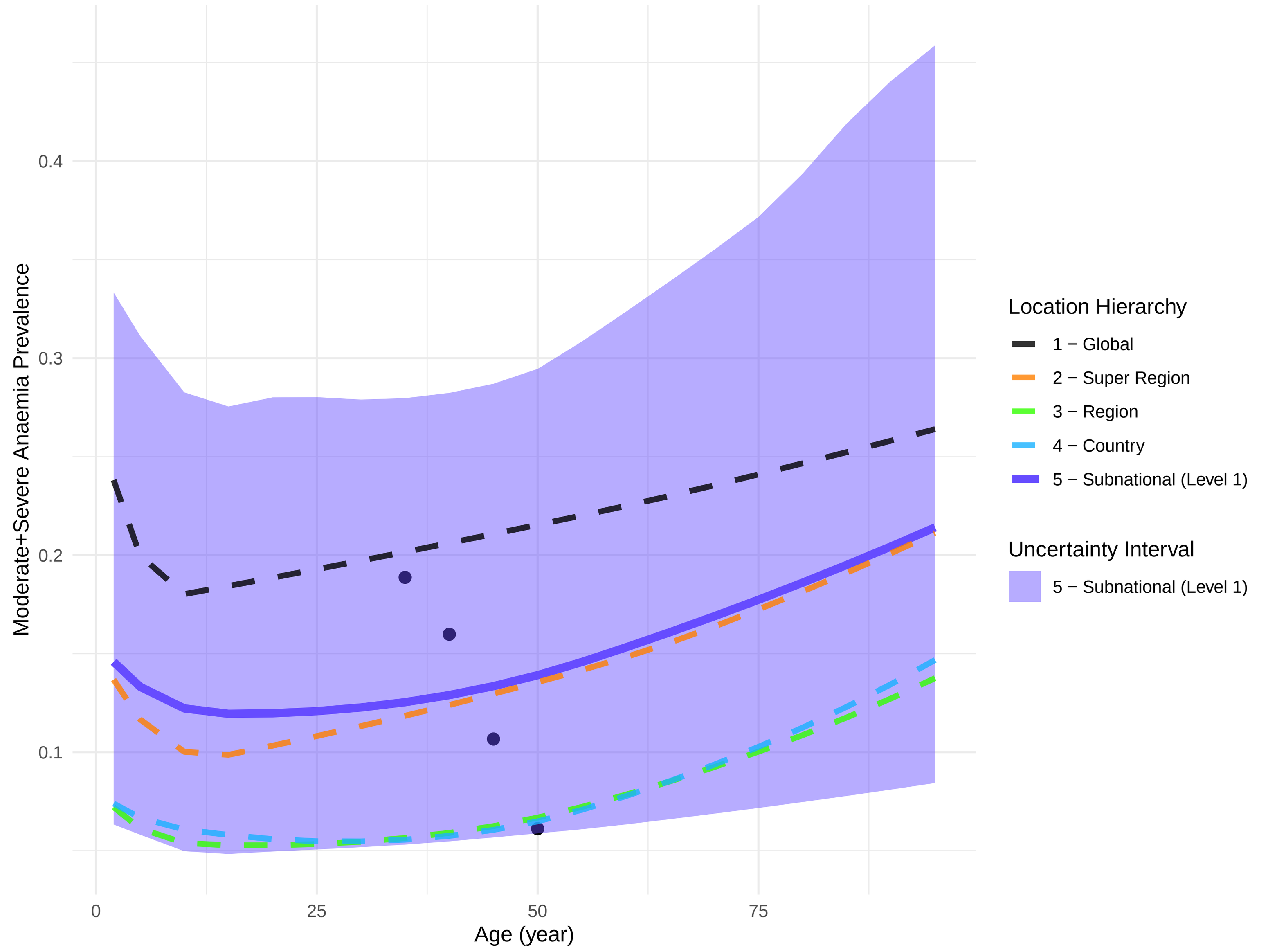

MR−BRT Age/Sex Fits − Non−pregnant Females 2+ Years Old − Moderate+Severe Anaemia Prevalence − North Africa and Middle East
WHO−2001 Adjustment Method

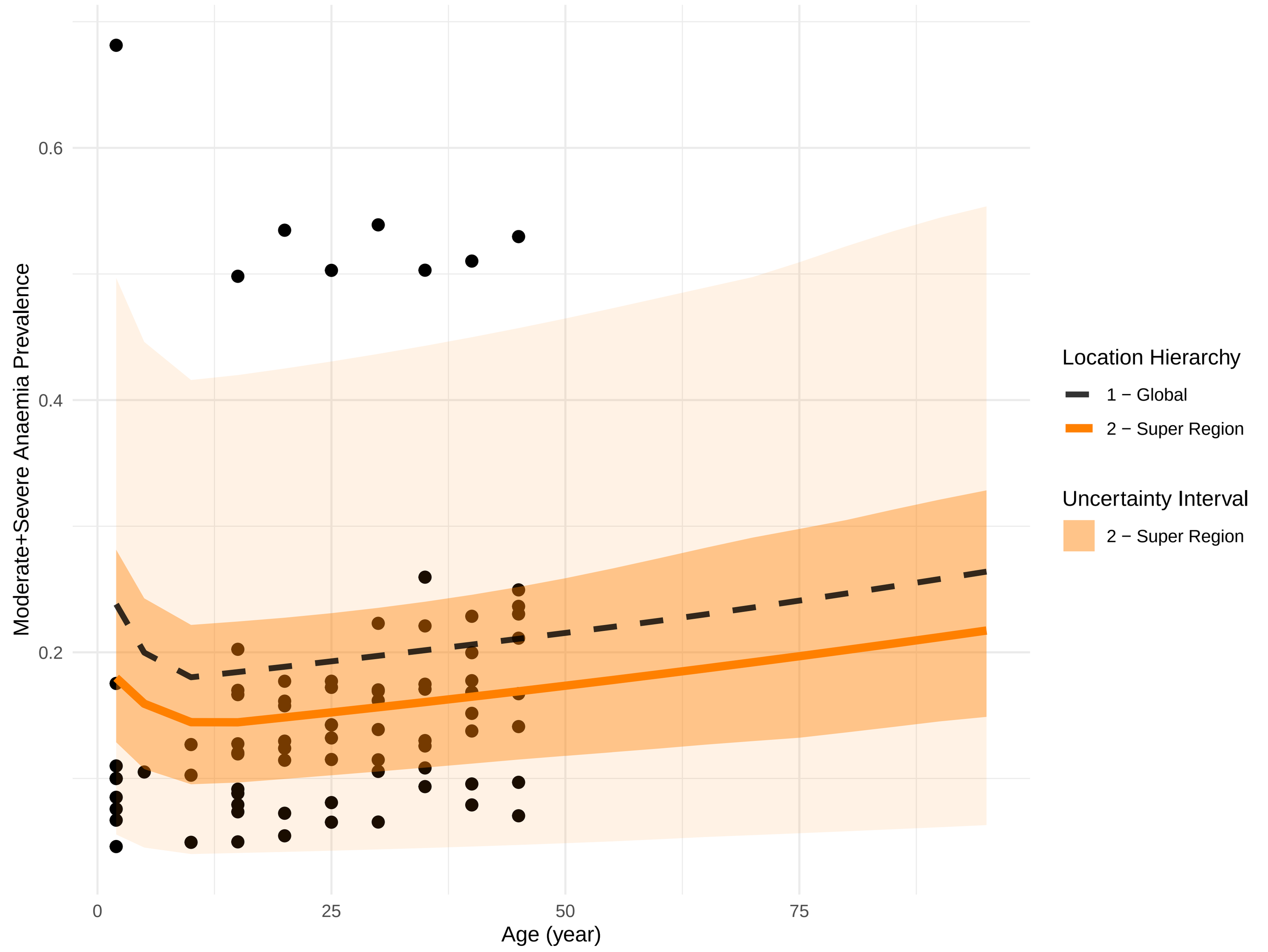

MR−BRT Age/Sex Fits − Non−pregnant Females 2+ Years Old − Moderate+Severe Anaemia Prevalence − North Africa and Middle East
WHO−2001 Adjustment Method

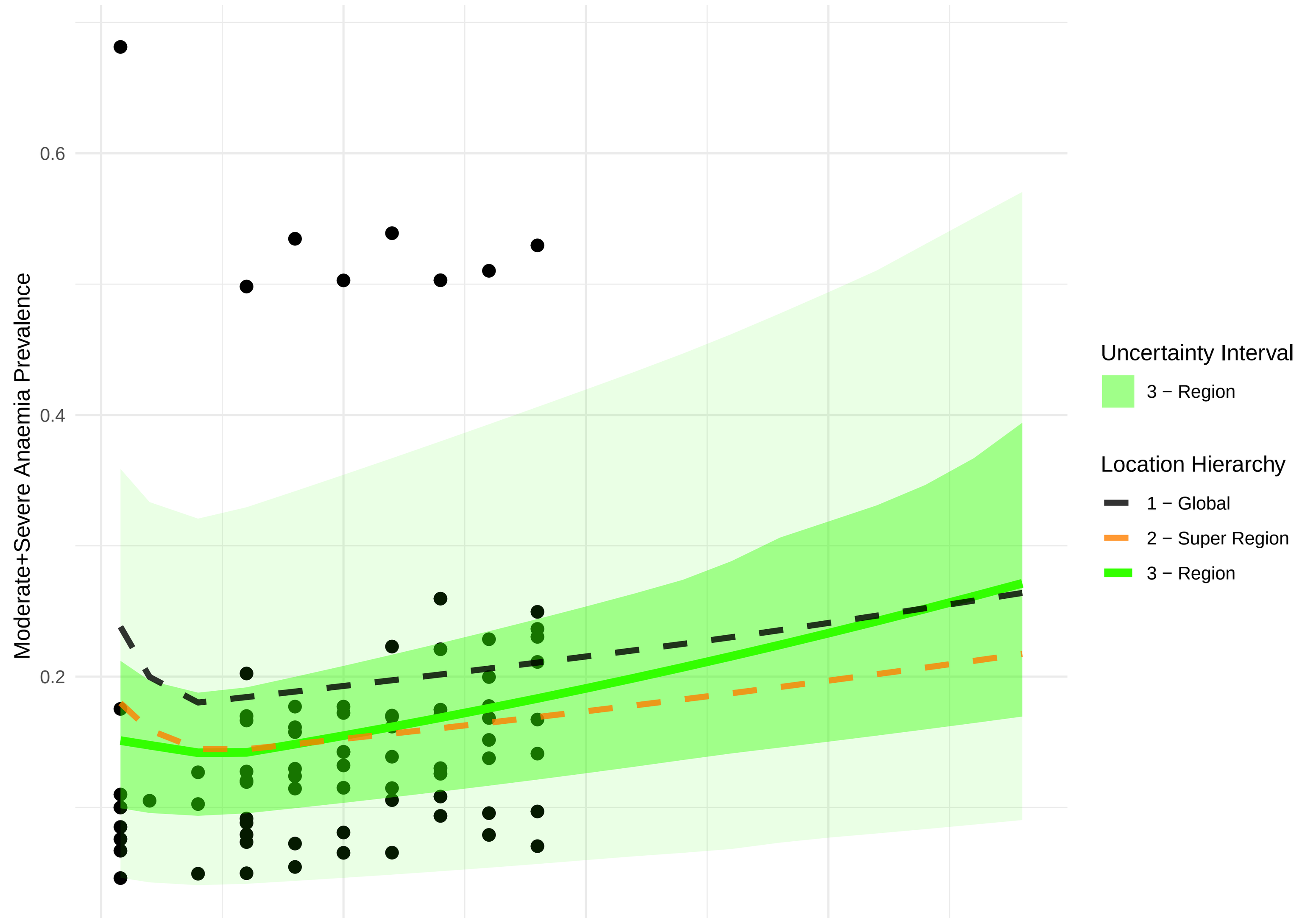

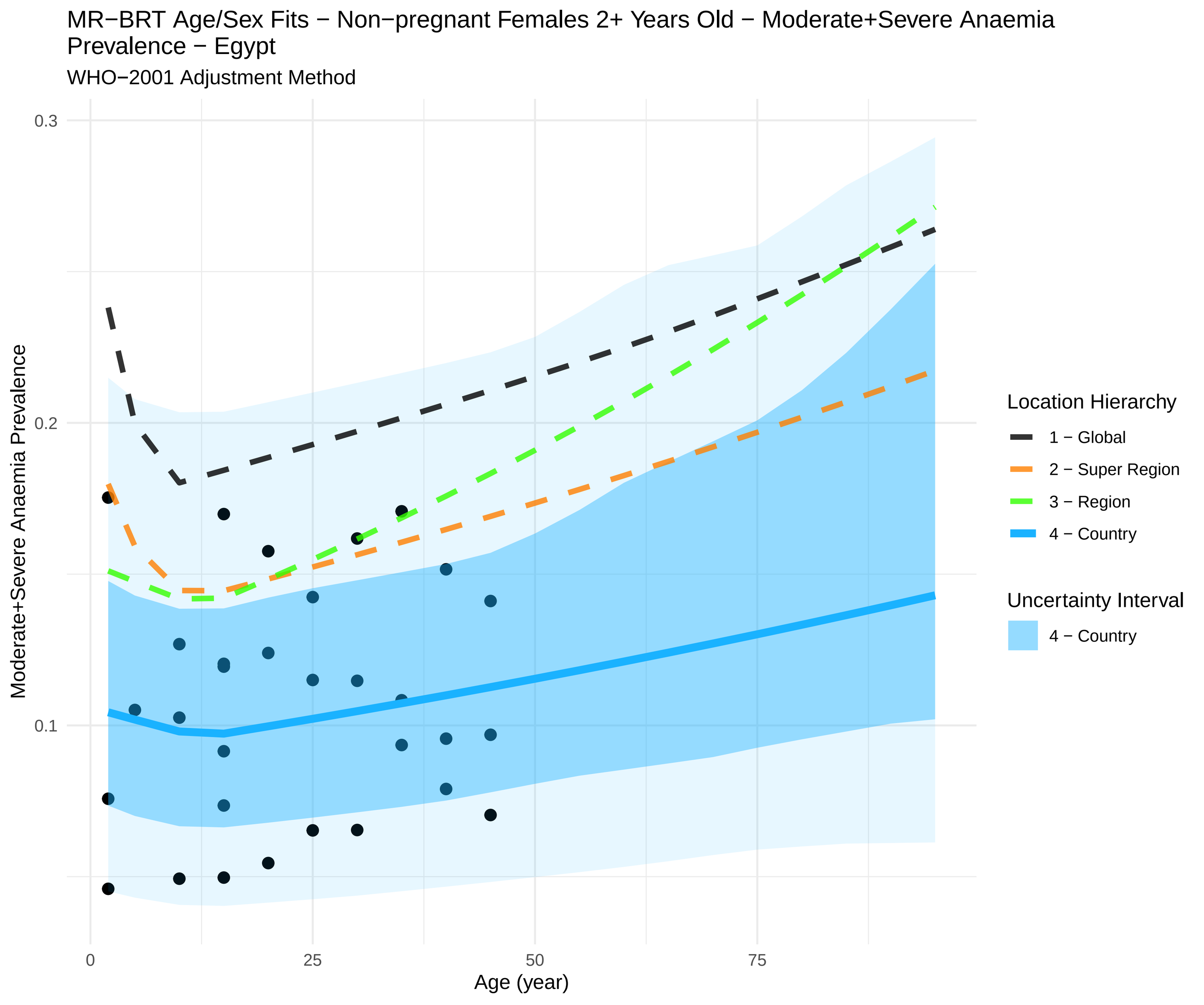
MR−BRT Age/Sex Fits − Non−pregnant Females 2+ Years Old − Moderate+Severe Anaemia Prevalence − Egypt
WHO−2001 Adjustment Method
Moderate+Severe Anaemia Prevalence
Age (year)
Location Hierarchy
1 − Global
2 − Super Region
3 − Region
4 − Country
Uncertainty Interval
4 − Country

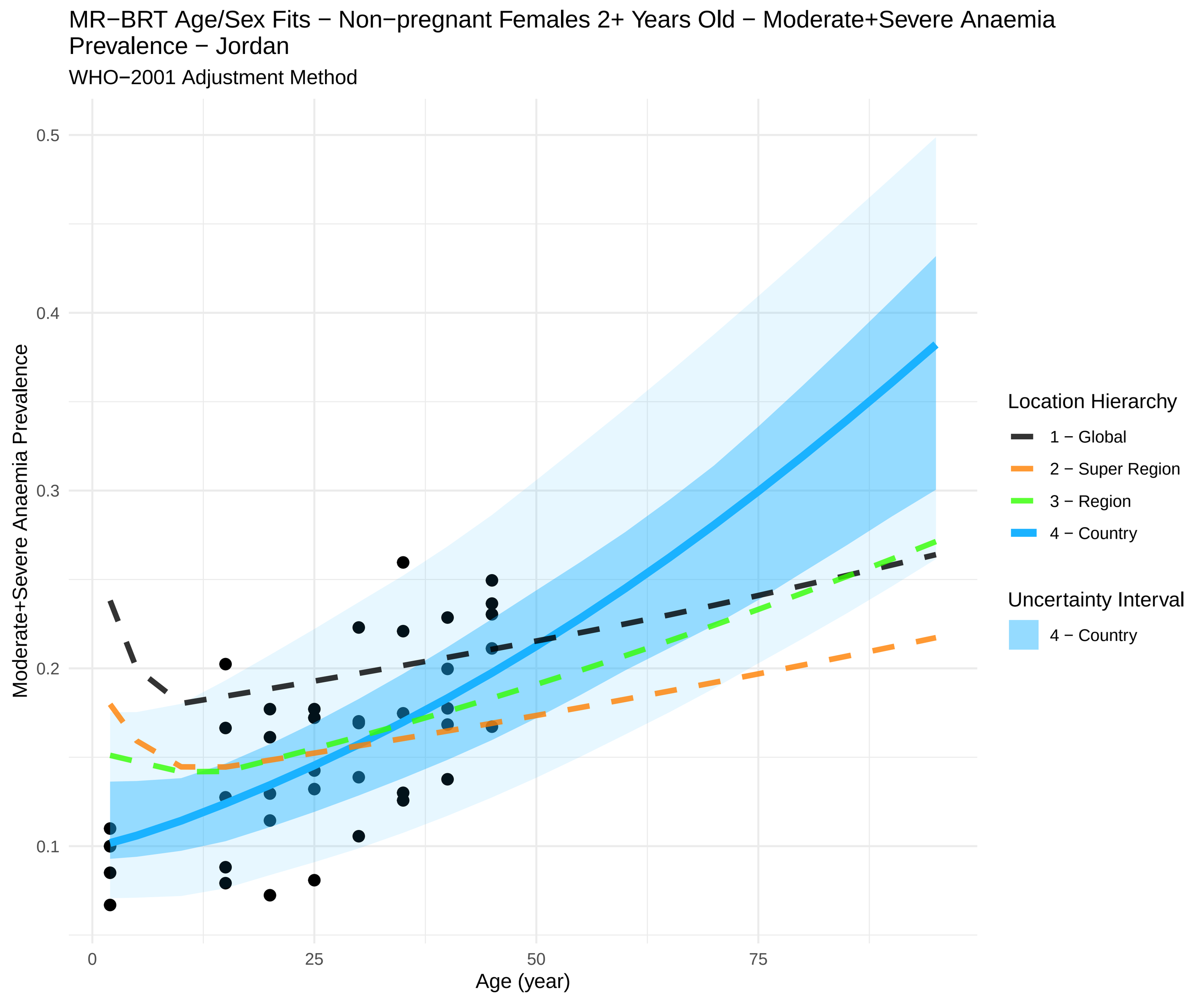
MR−BRT Age/Sex Fits − Non−pregnant Females 2+ Years Old − Moderate+Severe Anaemia Prevalence − Jordan
WHO−2001 Adjustment Method
Moderate+Severe Anaemia Prevalence
Age (year)
Location Hierarchy
1 − Global
2 − Super Region
3 − Region
4 − Country
Uncertainty Interval
4 − Country

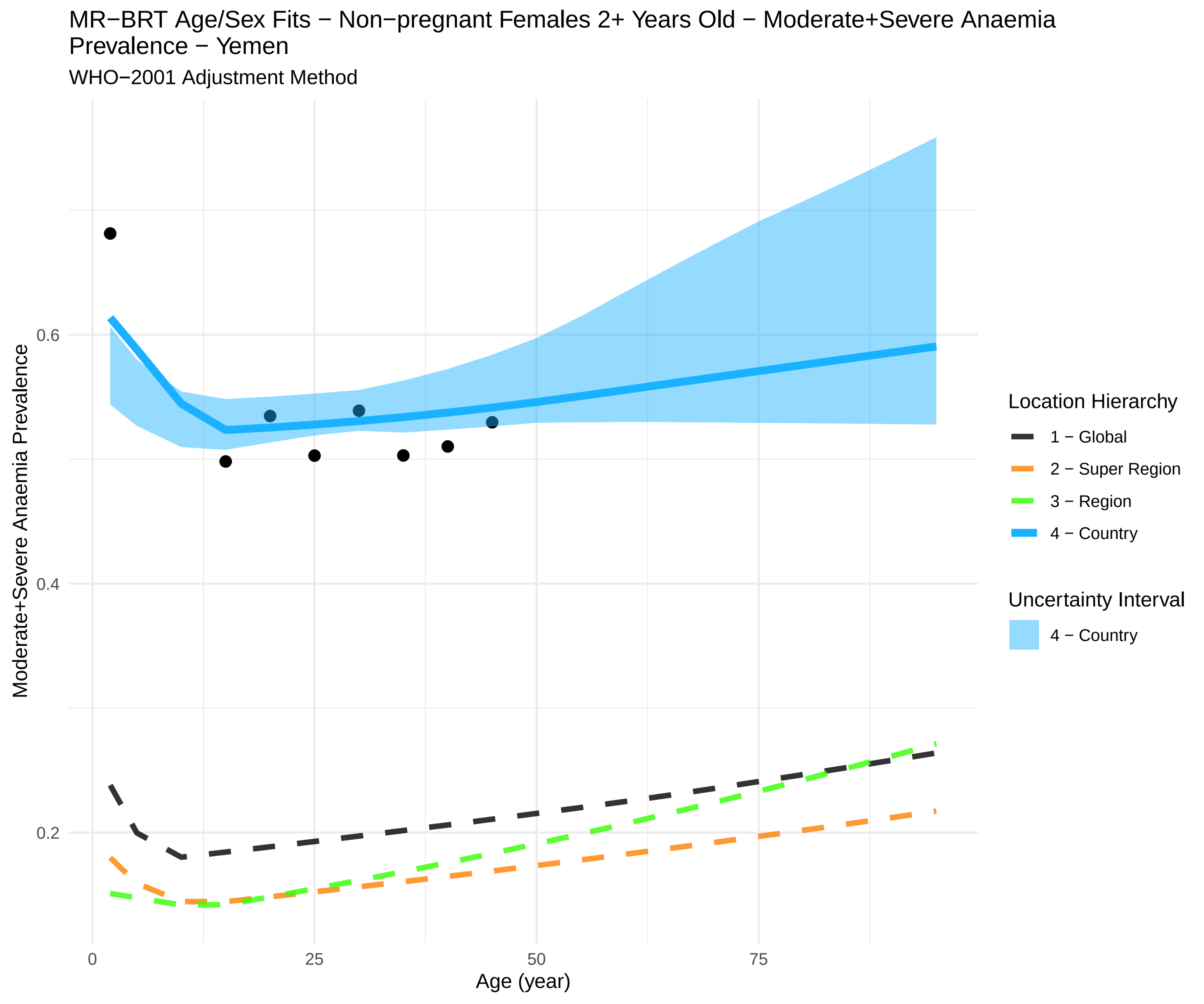
MR−BRT Age/Sex Fits − Non−pregnant Females 2+ Years Old − Moderate+Severe Anaemia Prevalence − Yemen
WHO−2001 Adjustment Method
Moderate+Severe Anaemia Prevalence
Age (year)
Location Hierarchy
1 − Global
2 − Super Region
3 − Region
4 − Country
Uncertainty Interval
4 − Country

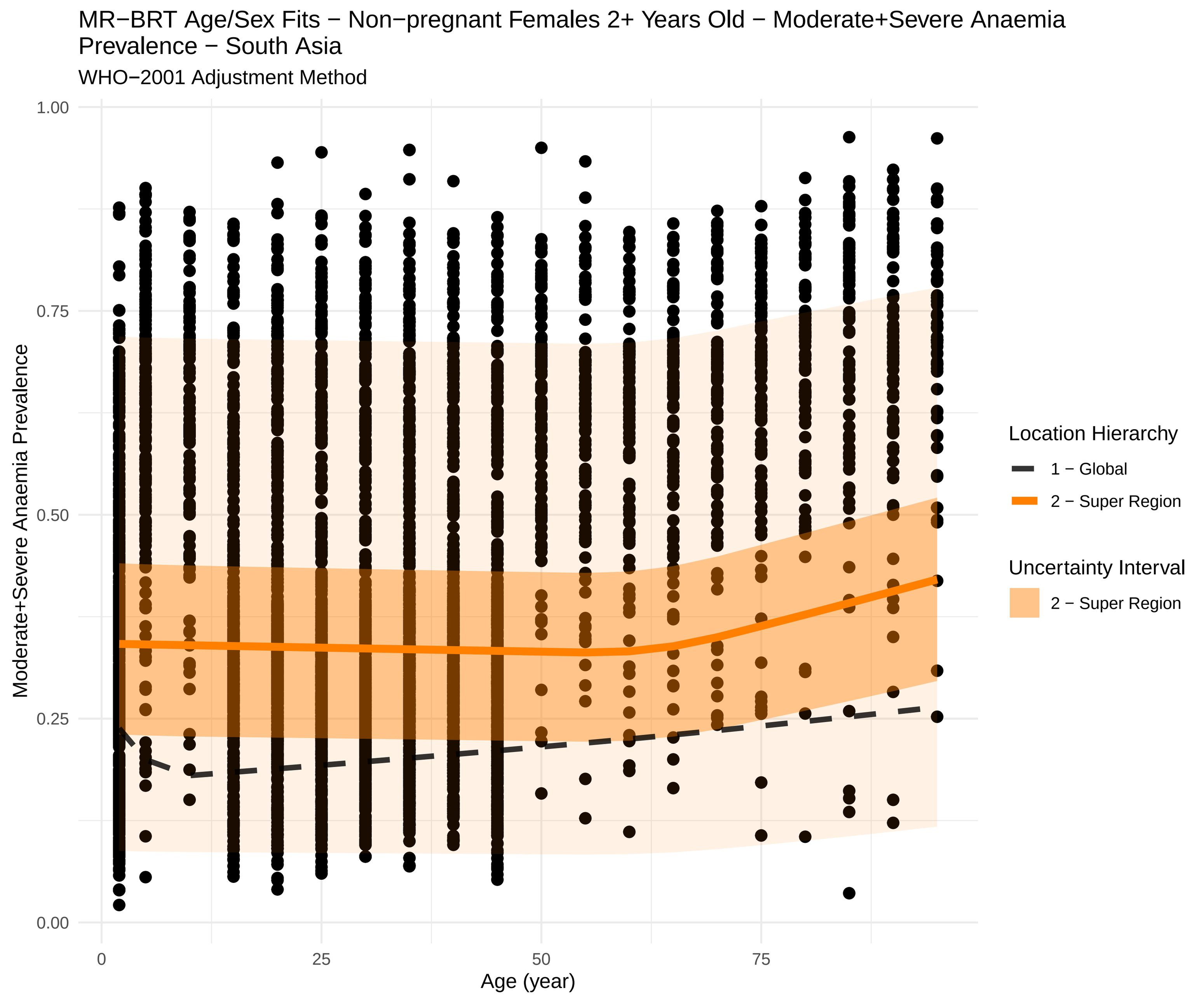
MR−BRT Age/Sex Fits − Non−pregnant Females 2+ Years Old − Moderate+Severe Anaemia Prevalence − South Asia
WHO−2001 Adjustment Method
Moderate+Severe Anaemia Prevalence
Age (year)
Location Hierarchy
1 − Global
2 − Super Region
Uncertainty Interval
2 − Super Region

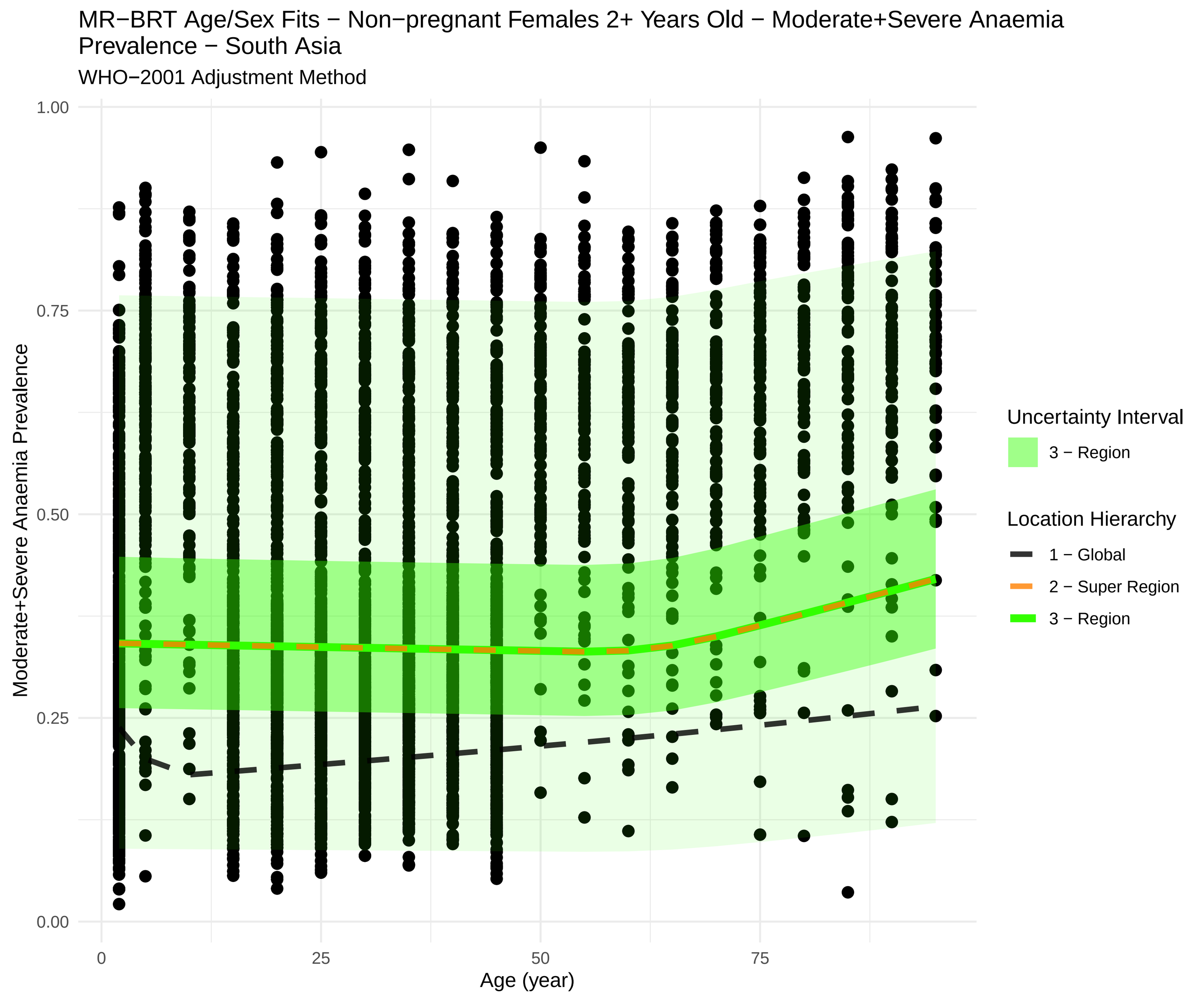
MR−BRT Age/Sex Fits − Non−pregnant Females 2+ Years Old − Moderate+Severe Anaemia Prevalence − South Asia
WHO−2001 Adjustment Method
Moderate+Severe Anaemia Prevalence
Age (year)
Uncertainty Interval
3 − Region
Location Hierarchy
1 − Global
2 − Super Region
3 − Region

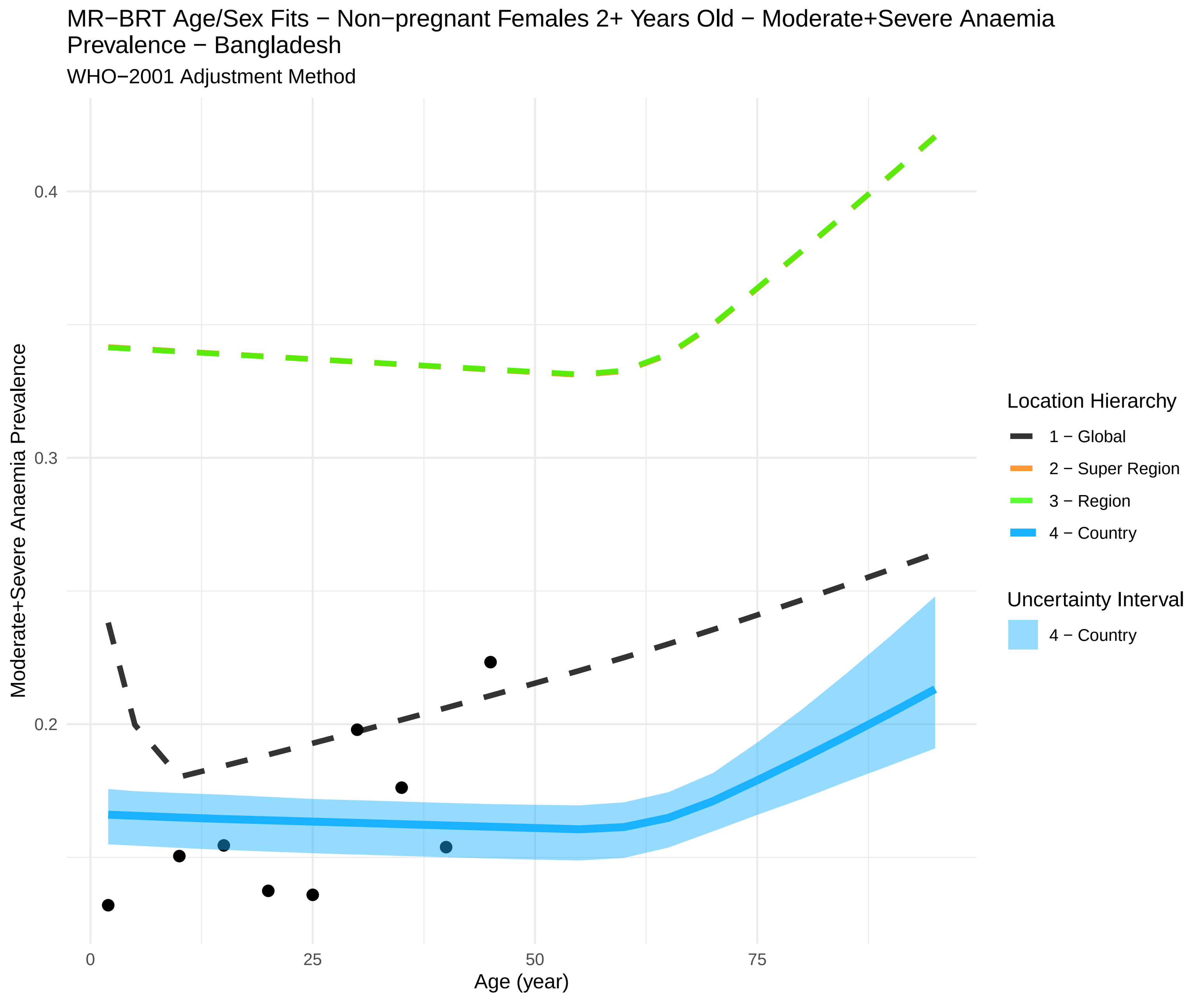
MR−BRT Age/Sex Fits − Non−pregnant Females 2+ Years Old − Moderate+Severe Anaemia Prevalence − Bangladesh
WHO−2001 Adjustment Method
Moderate+Severe Anaemia Prevalence
Age (year)
Location Hierarchy
1 − Global
2 − Super Region
3 − Region
4 − Country
Uncertainty Interval
4 − Country

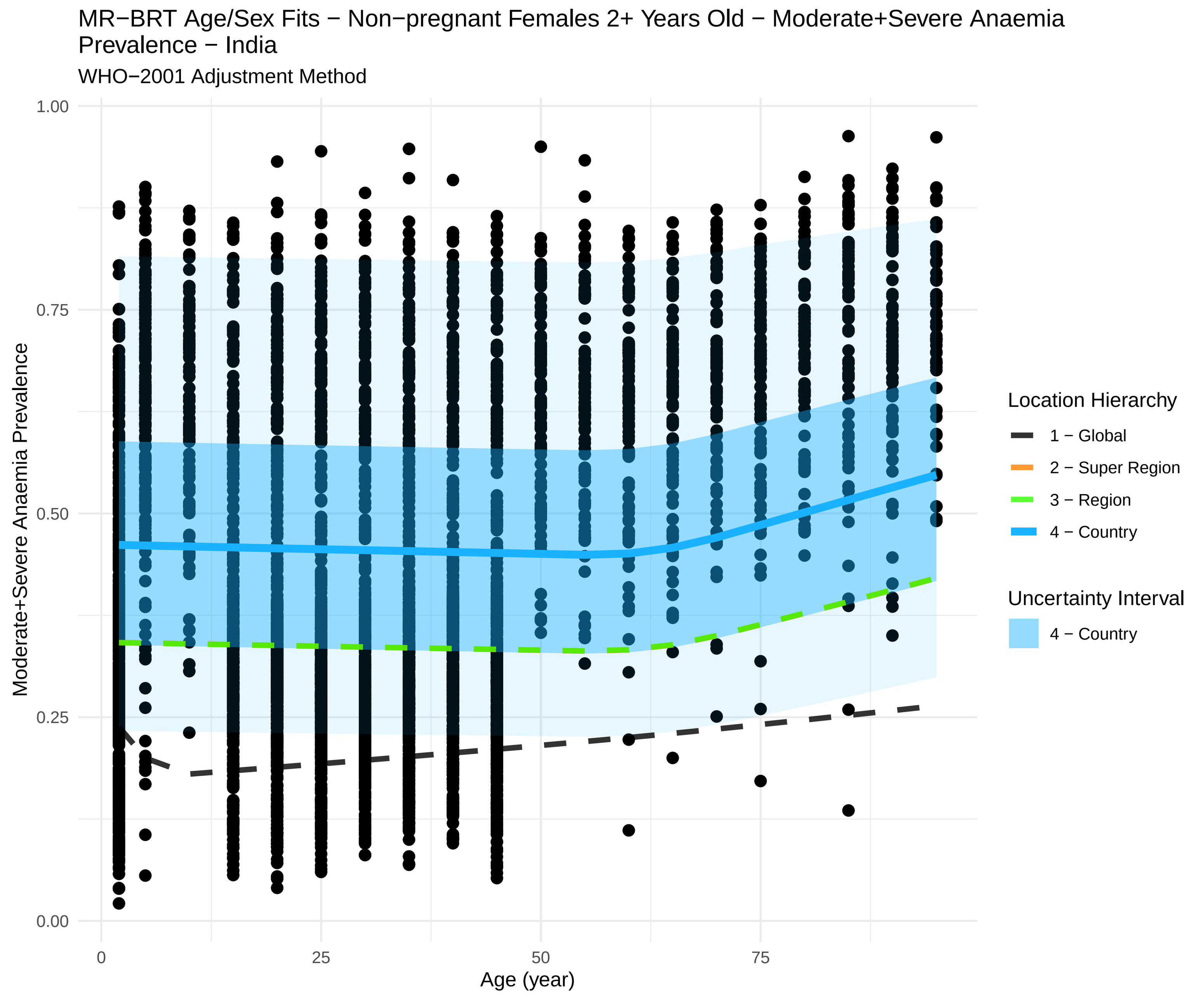
MR−BRT Age/Sex Fits − Non−pregnant Females 2+ Years Old − Moderate+Severe Anaemia Prevalence − India
WHO−2001 Adjustment Method
Moderate+Severe Anaemia Prevalence
Age (year)
Location Hierarchy
1 − Global
2 − Super Region
3 − Region
4 − Country
Uncertainty Interval
4 − Country

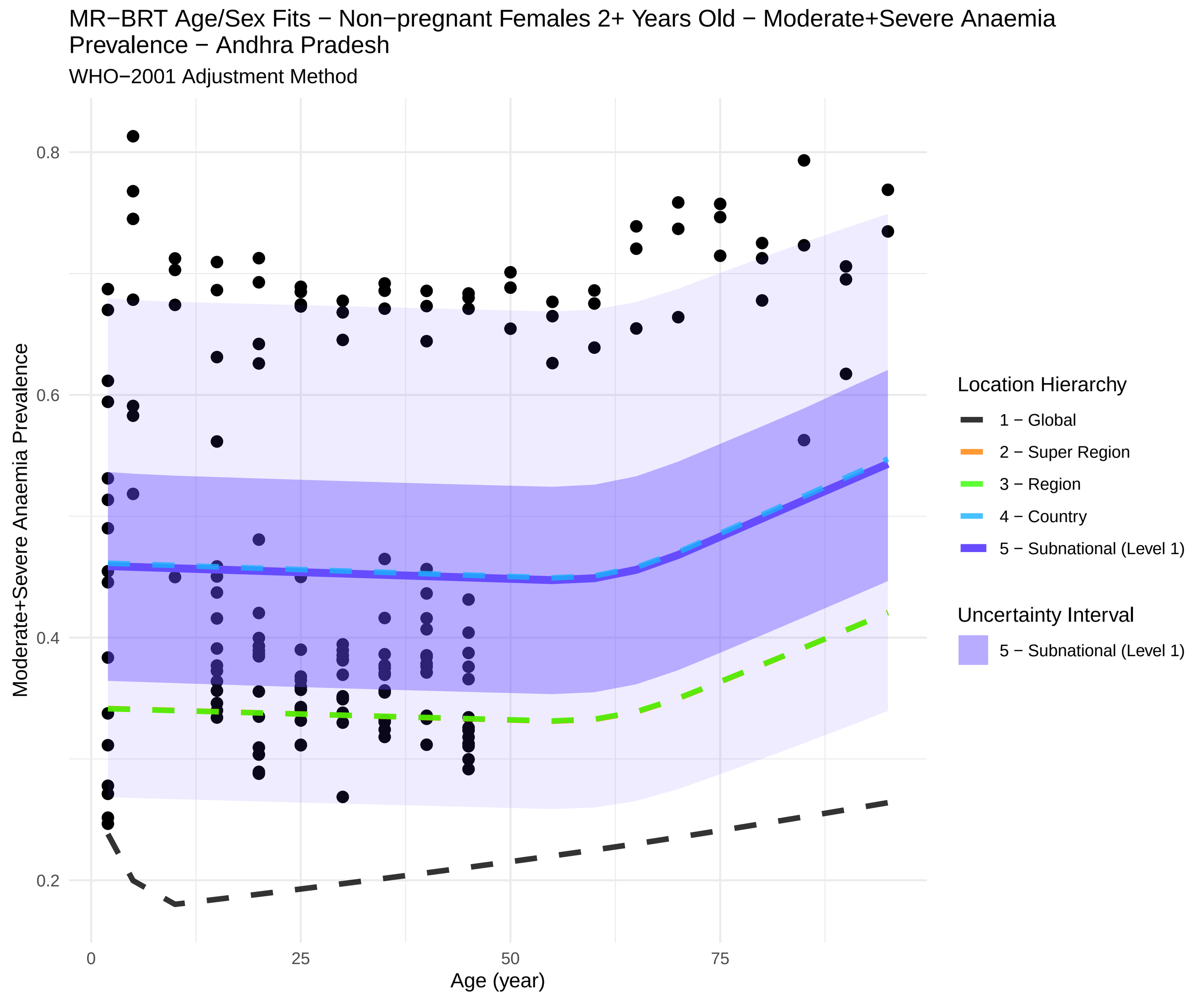
MR−BRT Age/Sex Fits − Non−pregnant Females 2+ Years Old − Moderate+Severe Anaemia Prevalence − Andhra Pradesh
WHO−2001 Adjustment Method
Moderate+Severe Anaemia Prevalence
Age (year)
Location Hierarchy
1 − Global
2 − Super Region
3 − Region
4 − Country
5 − Subnational (Level 1)
Uncertainty Interval
5 − Subnational (Level 1)

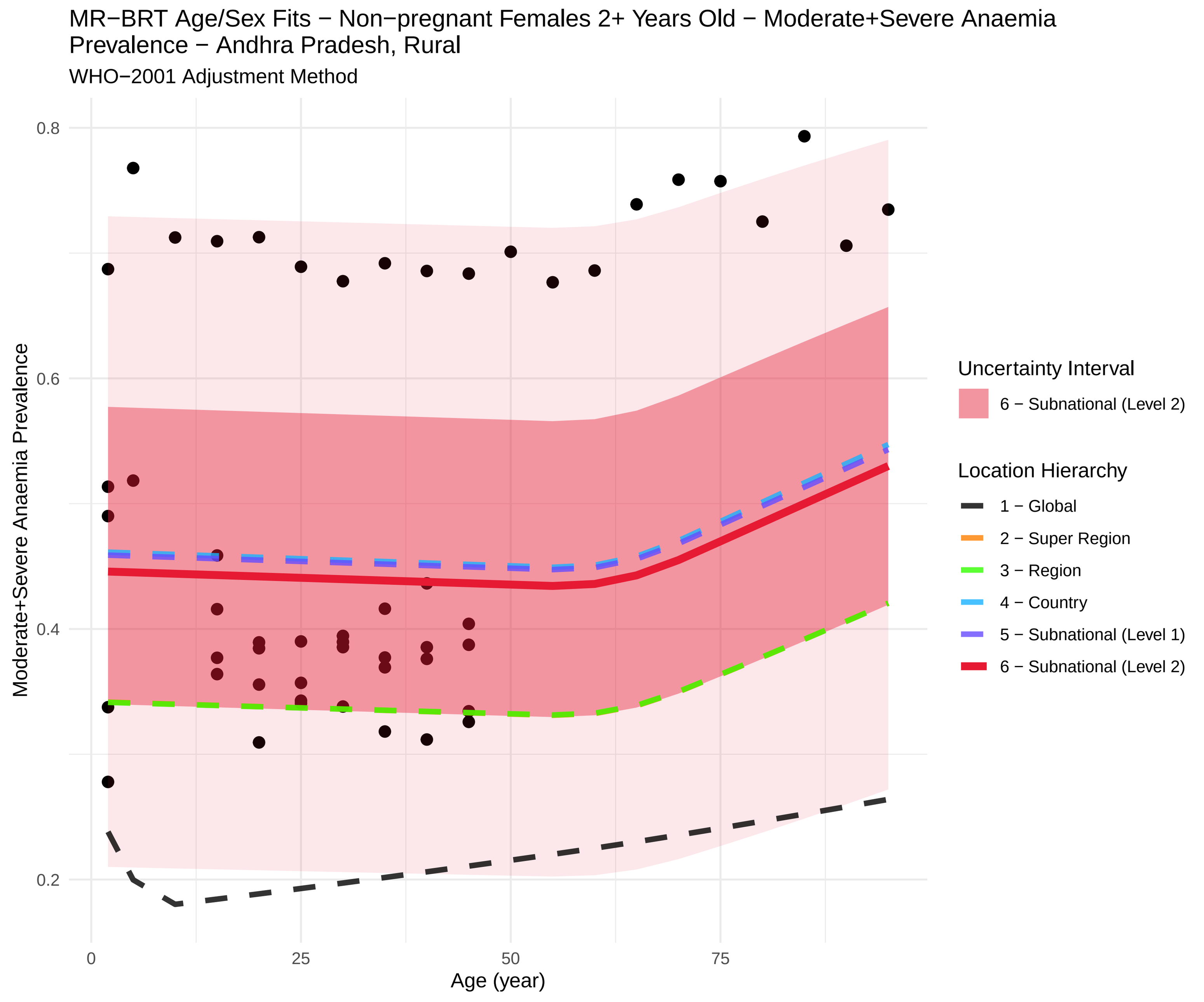
MR−BRT Age/Sex Fits − Non−pregnant Females 2+ Years Old − Moderate+Severe Anaemia Prevalence − Andhra Pradesh, Rural
WHO−2001 Adjustment Method
Moderate+Severe Anaemia Prevalence
Age (year)
Uncertainty Interval
6 − Subnational (Level 2)
Location Hierarchy
1 − Global
2 − Super Region
3 − Region
4 − Country
5 − Subnational (Level 1)
6 − Subnational (Level 2)

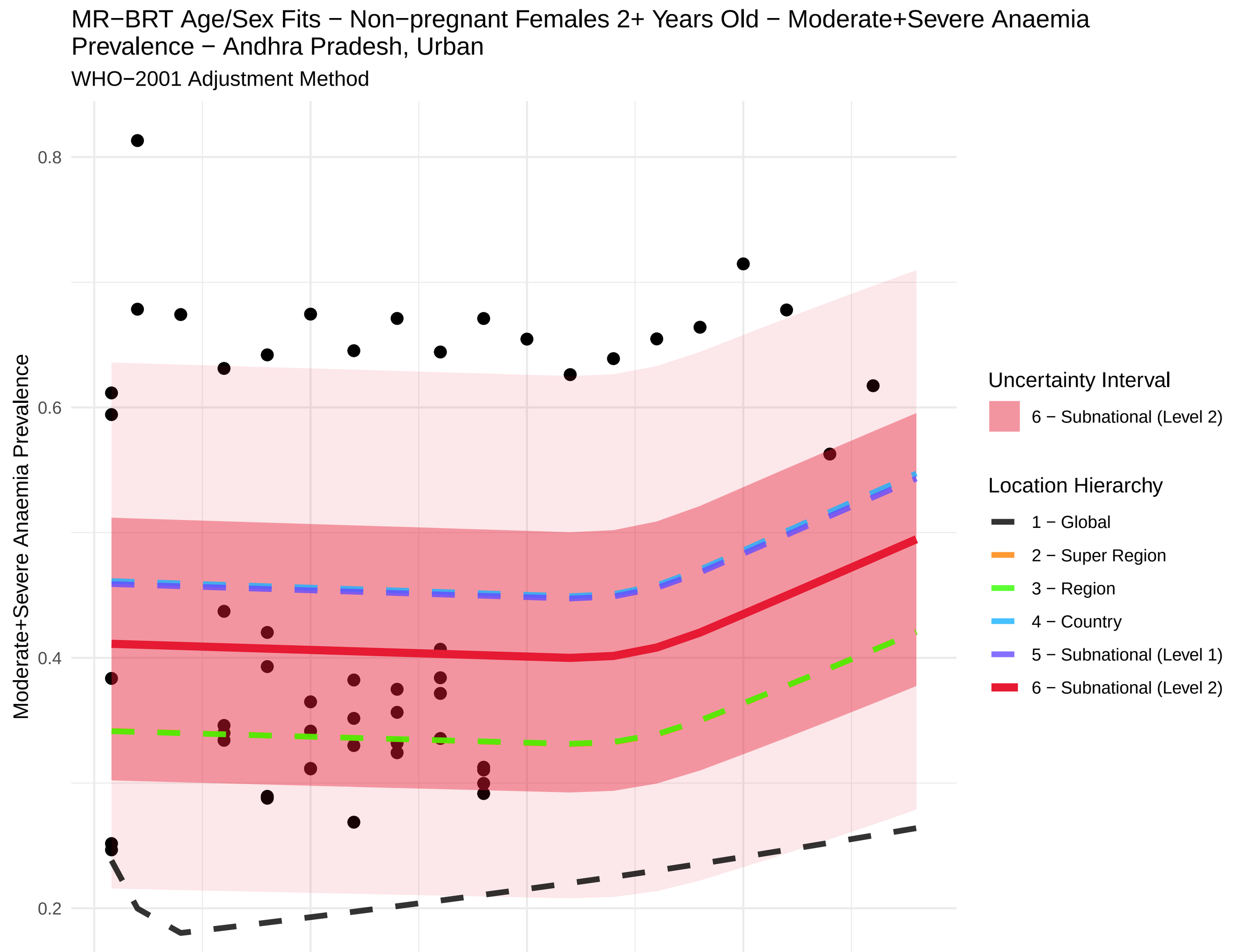
MR−BRT Age/Sex Fits − Non−pregnant Females 2+ Years Old − Moderate+Severe Anaemia Prevalence − Andhra Pradesh, Urban
WHO−2001 Adjustment Method
Moderate+Severe Anaemia Prevalence
Age (year)
Uncertainty Interval
6 − Subnational (Level 2)
Location Hierarchy
1 − Global
2 − Super Region
3 − Region
4 − Country
5 − Subnational (Level 1)
6 − Subnational (Level 2)

MR−BRT Age/Sex Fits − Non−pregnant Females 2+ Years Old − Moderate+Severe Anaemia Prevalence − Arunachal Pradesh
WHO−2001 Adjustment Method

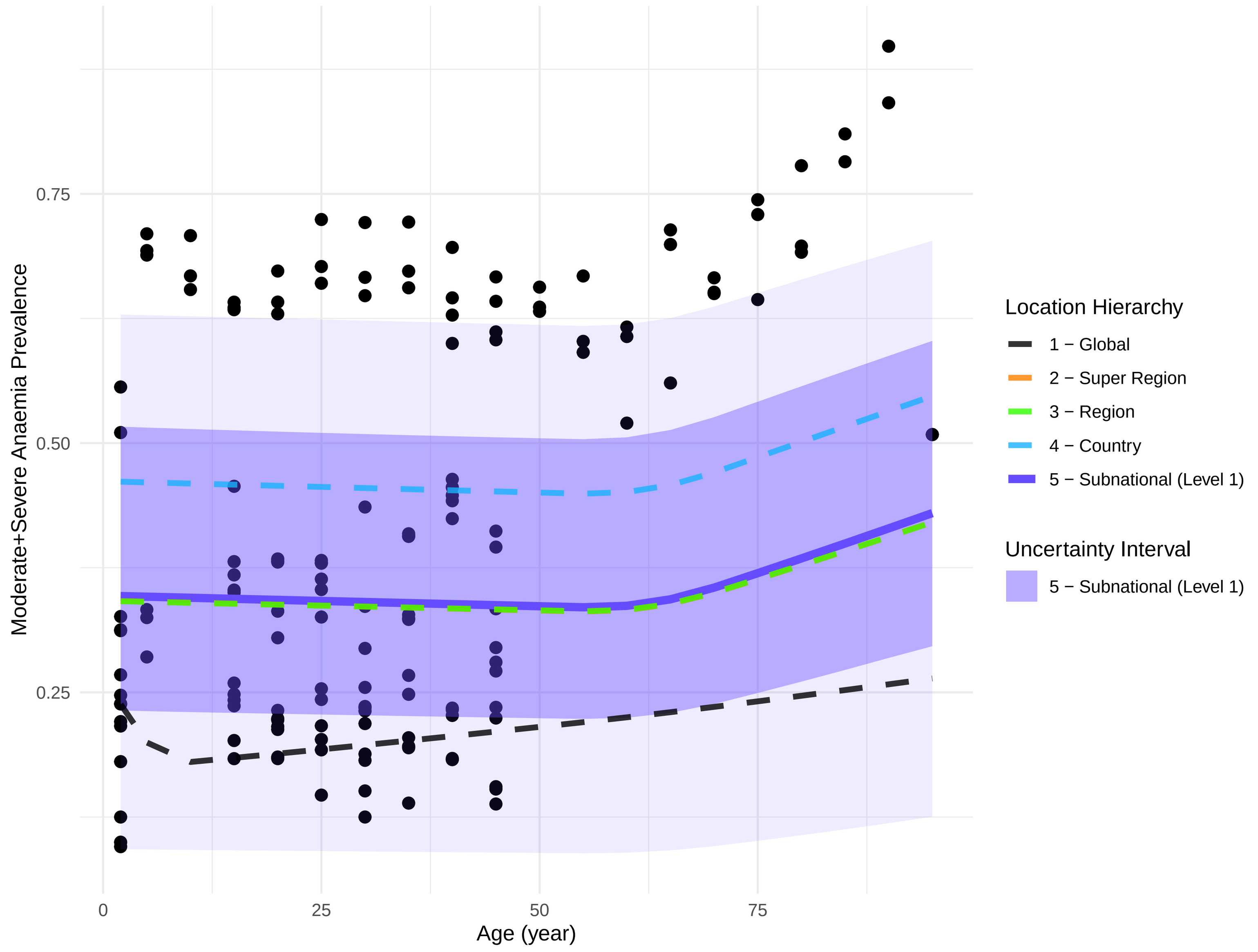

MR−BRT Age/Sex Fits − Non−pregnant Females 2+ Years Old − Moderate+Severe Anaemia Prevalence − Arunachal Pradesh, Rural
WHO−2001 Adjustment Method

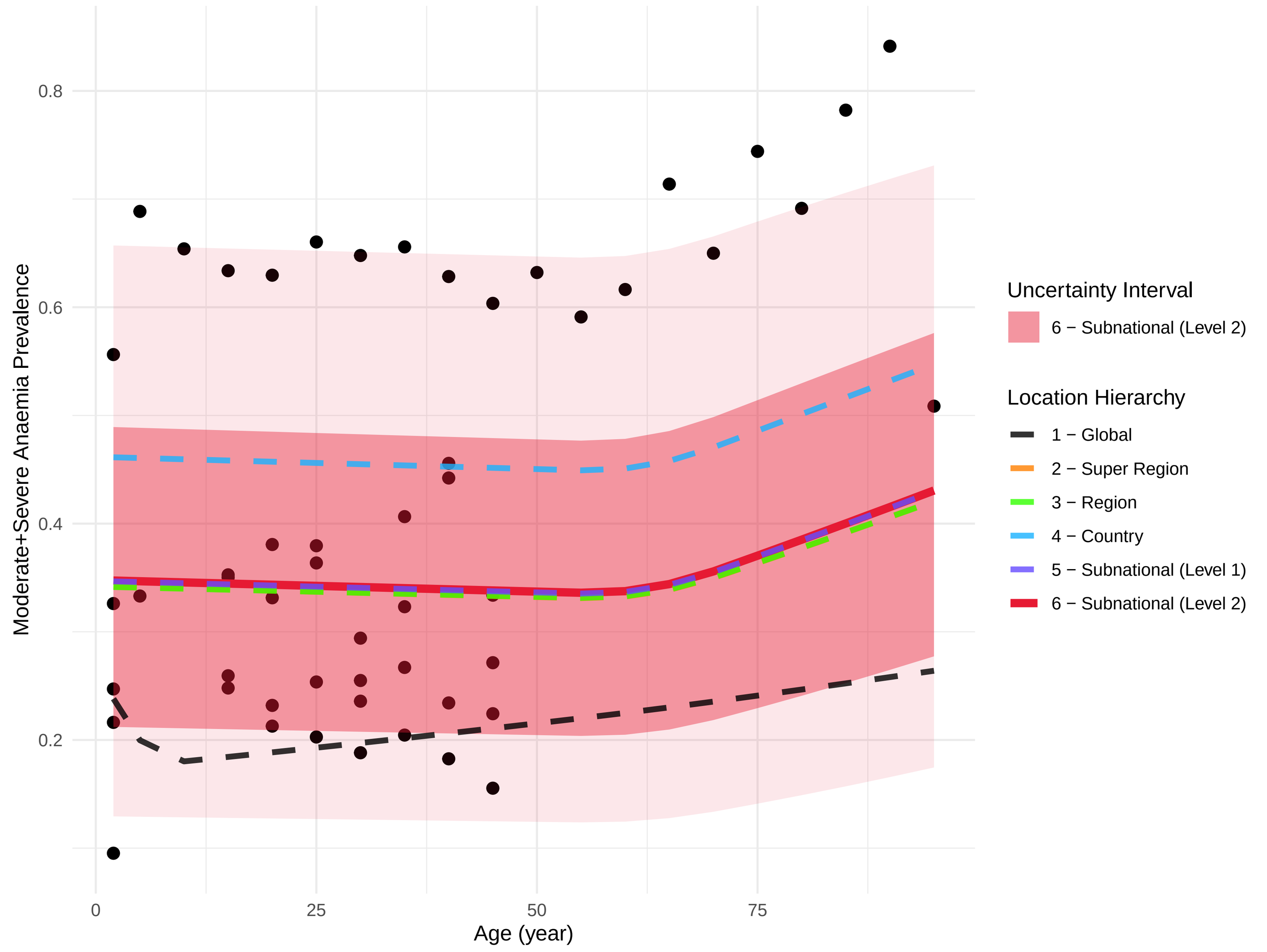

MR−BRT Age/Sex Fits − Non−pregnant Females 2+ Years Old − Moderate+Severe Anaemia Prevalence − Arunachal Pradesh, Urban
WHO−2001 Adjustment Method

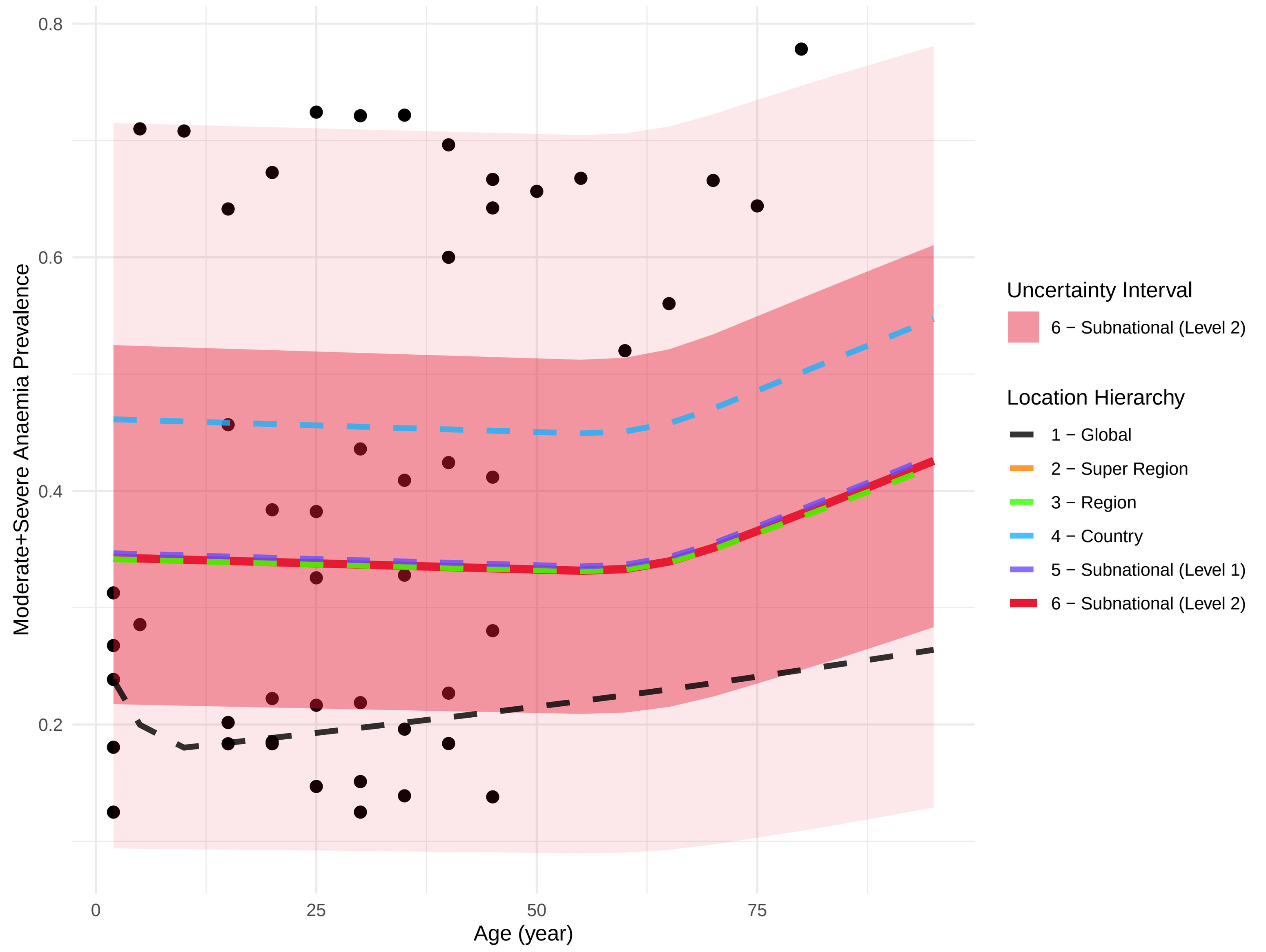

MR−BRT Age/Sex Fits − Non−pregnant Females 2+ Years Old − Moderate+Severe Anaemia Prevalence − Assam
WHO−2001 Adjustment Method

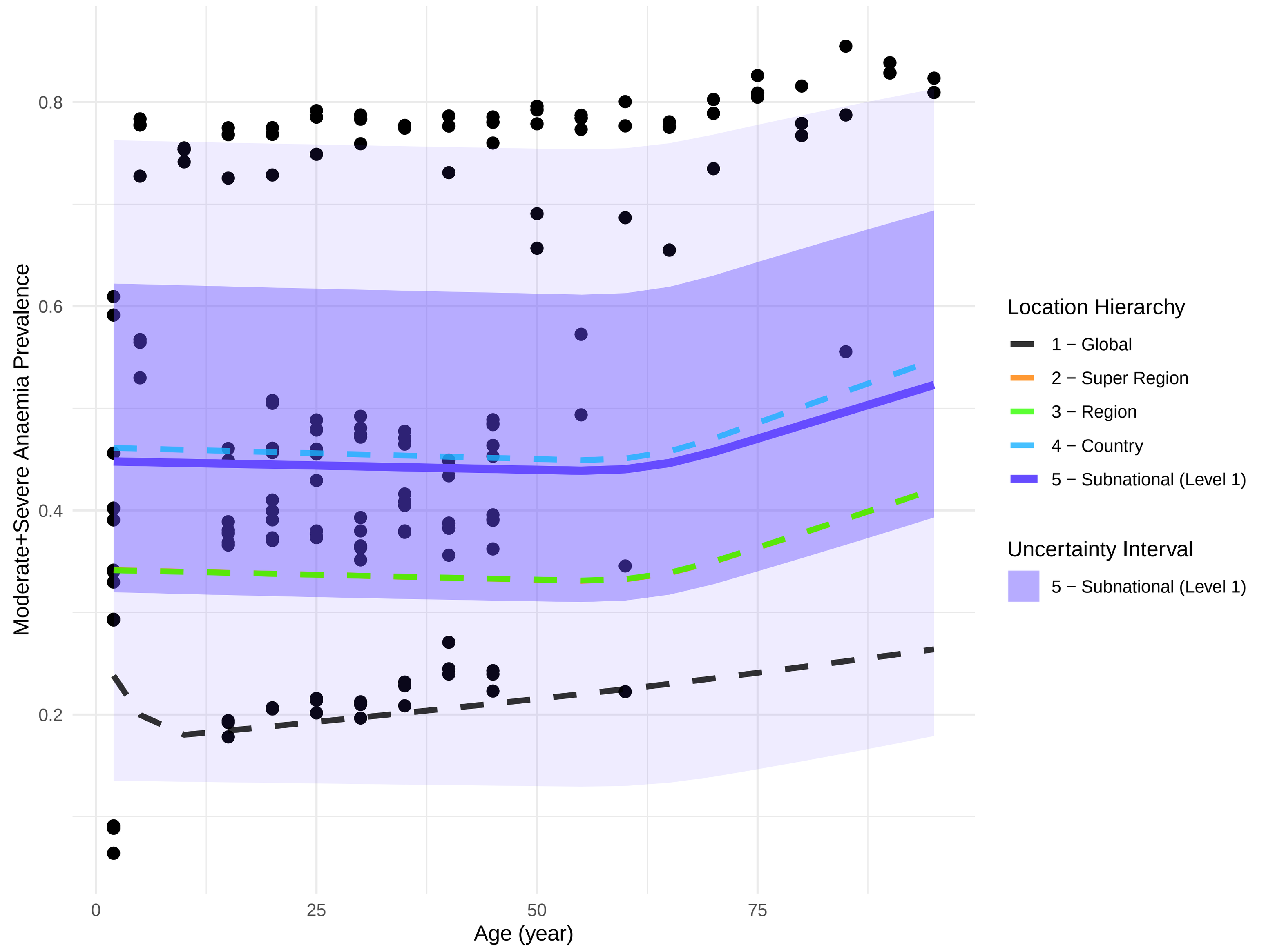

MR−BRT Age/Sex Fits − Non−pregnant Females 2+ Years Old − Moderate+Severe Anaemia Prevalence − Assam, Rural
WHO−2001 Adjustment Method

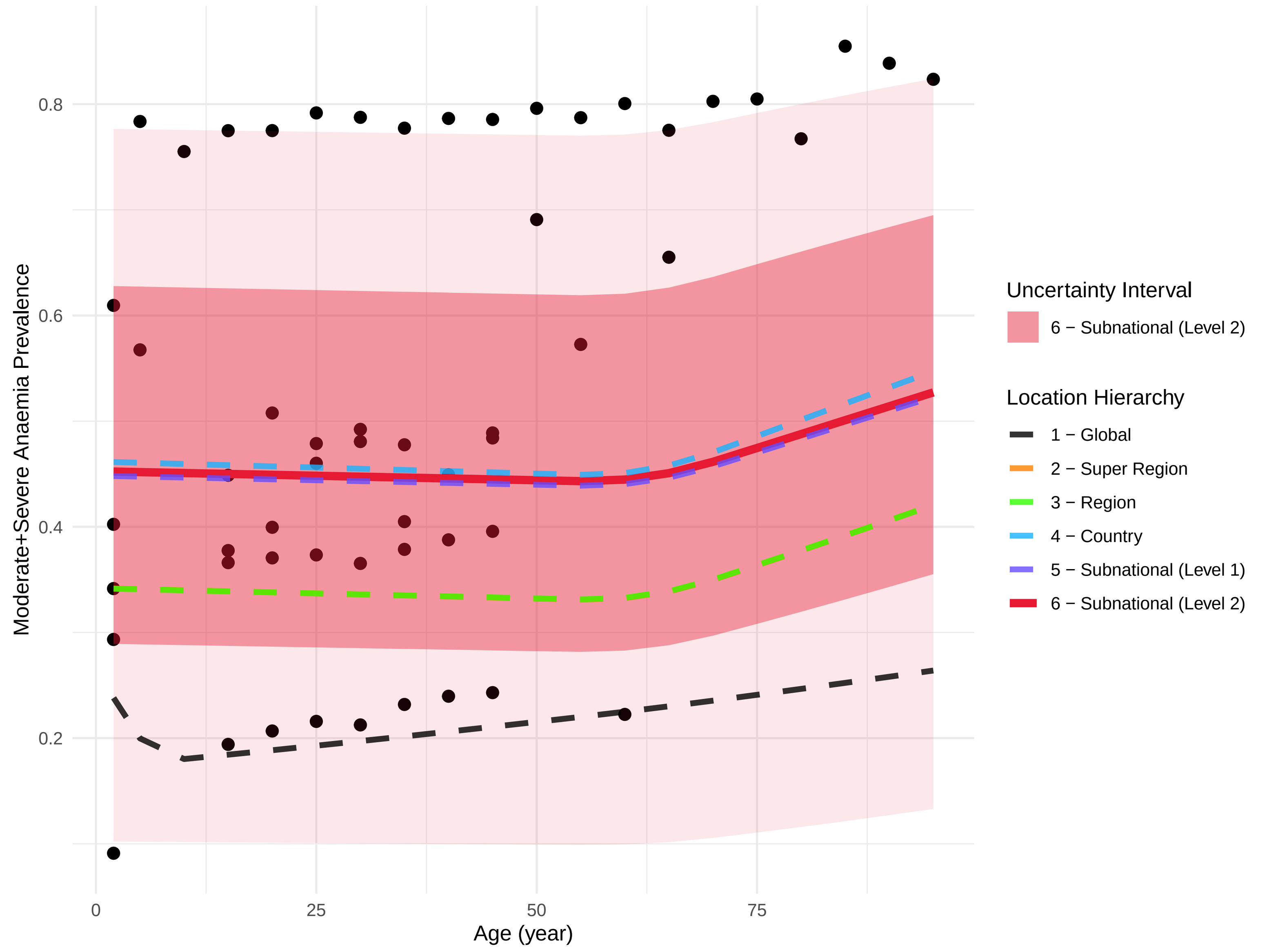

MR−BRT Age/Sex Fits − Non−pregnant Females 2+ Years Old − Moderate+Severe Anaemia Prevalence − Assam, Urban
WHO−2001 Adjustment Method

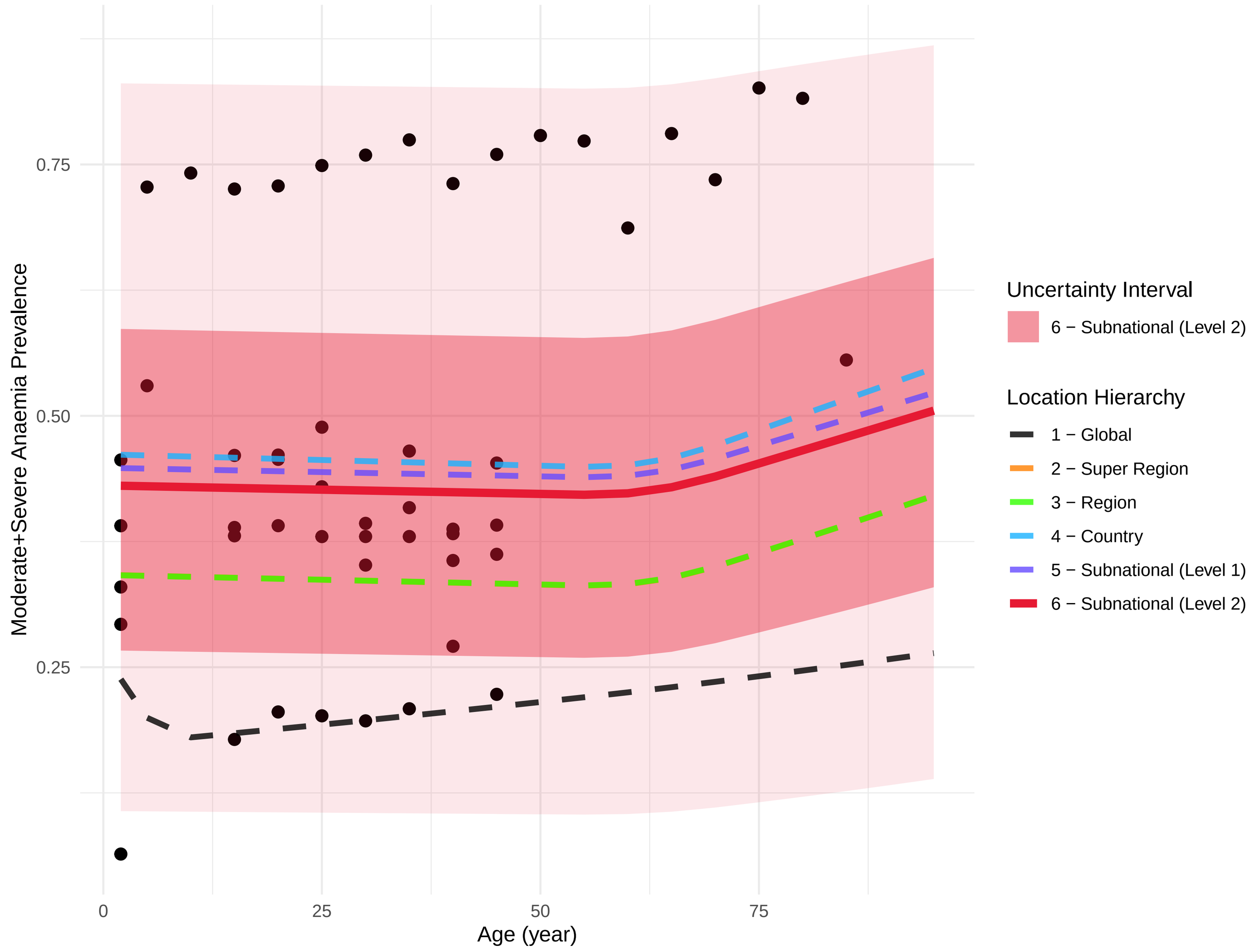

MR−BRT Age/Sex Fits − Non−pregnant Females 2+ Years Old − Moderate+Severe Anaemia Prevalence − Bihar
WHO−2001 Adjustment Method

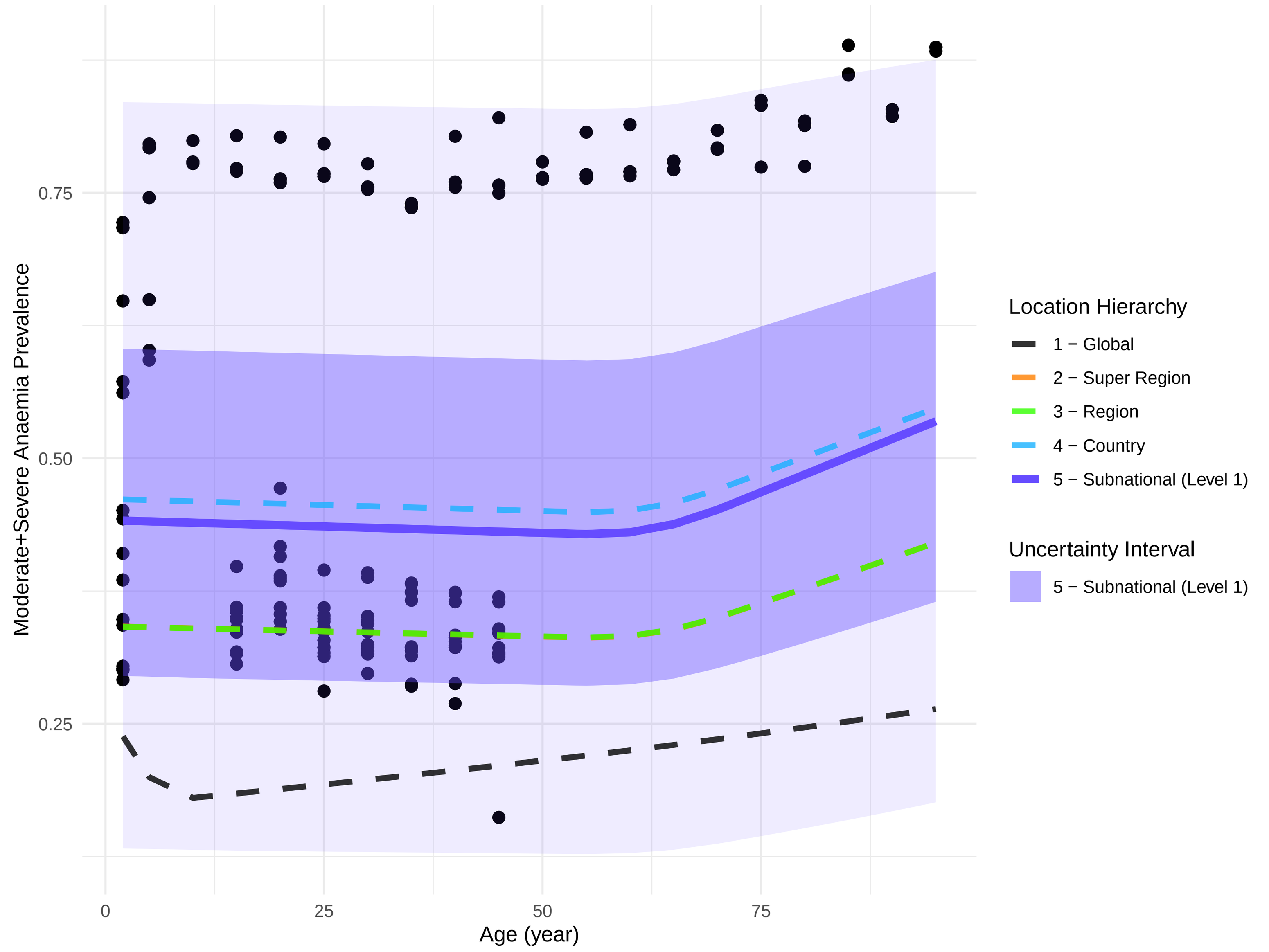

MR−BRT Age/Sex Fits − Non−pregnant Females 2+ Years Old − Moderate+Severe Anaemia Prevalence − Bihar, Rural
WHO−2001 Adjustment Method

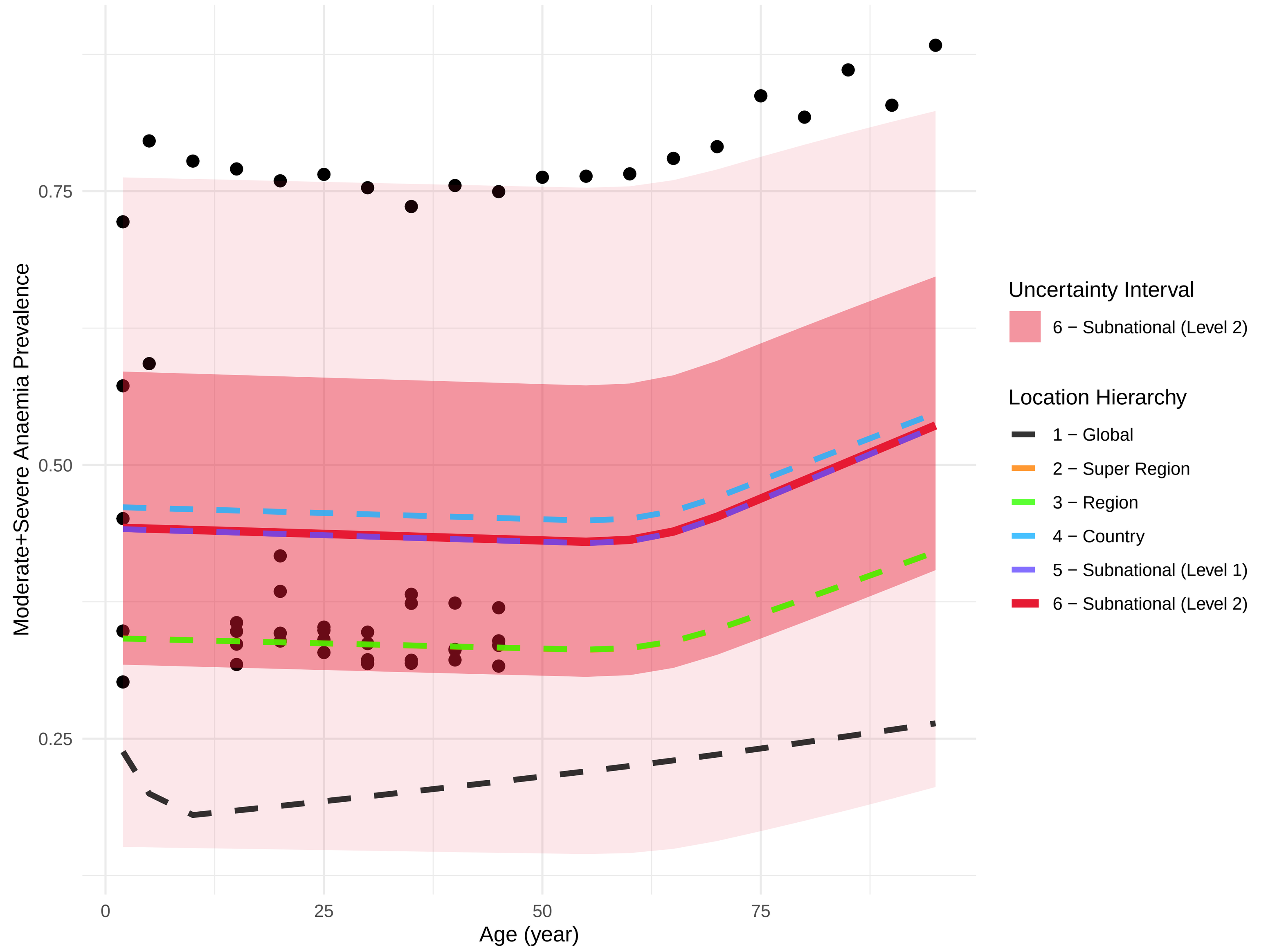

MR−BRT Age/Sex Fits − Non−pregnant Females 2+ Years Old − Moderate+Severe Anaemia Prevalence − Bihar, Urban
WHO−2001 Adjustment Method

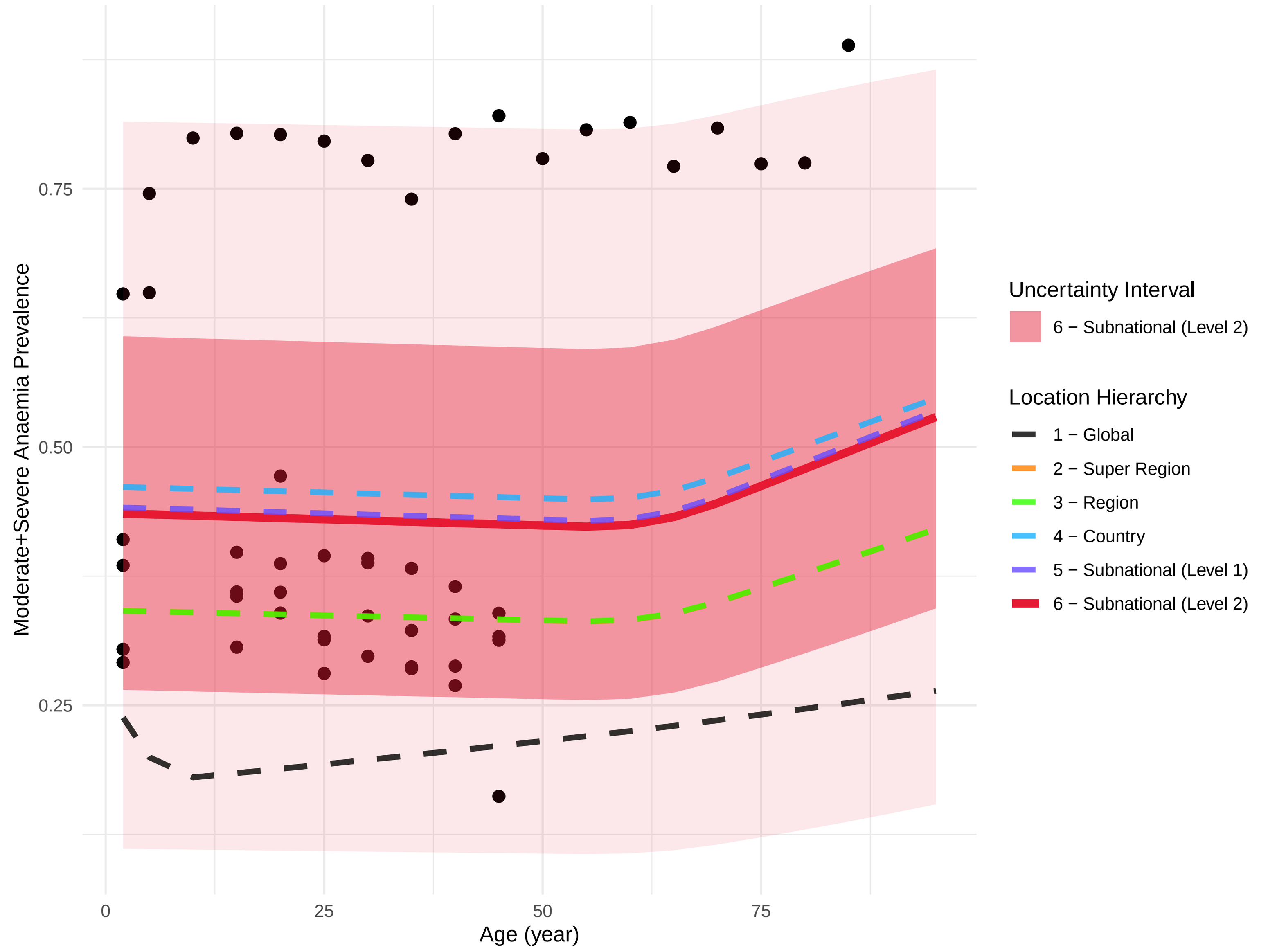

MR−BRT Age/Sex Fits − Non−pregnant Females 2+ Years Old − Moderate+Severe Anaemia Prevalence − Chhattisgarh
WHO−2001 Adjustment Method

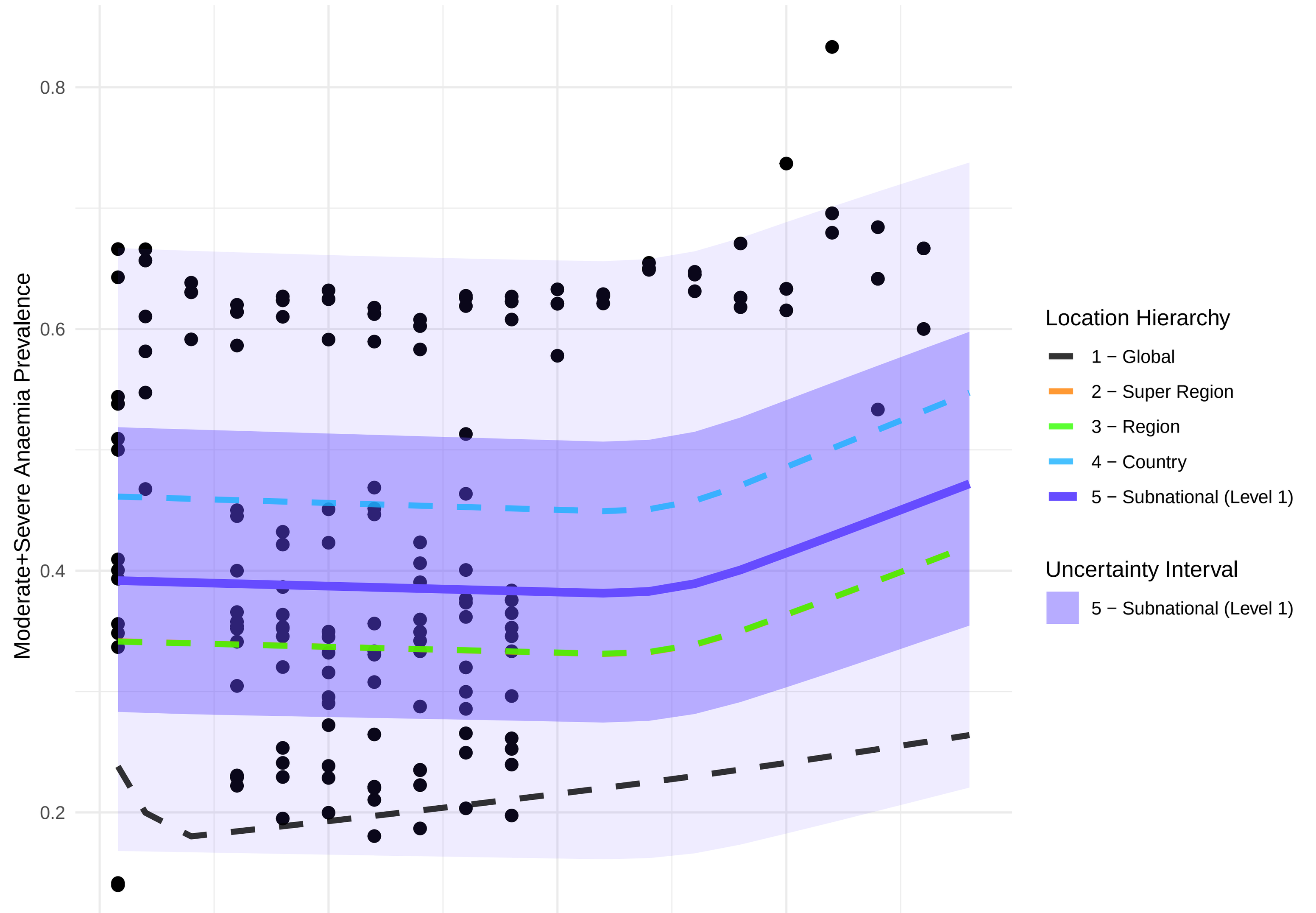

MR−BRT Age/Sex Fits − Non−pregnant Females 2+ Years Old − Moderate+Severe Anaemia Prevalence − Chhattisgarh, Rural
WHO−2001 Adjustment Method

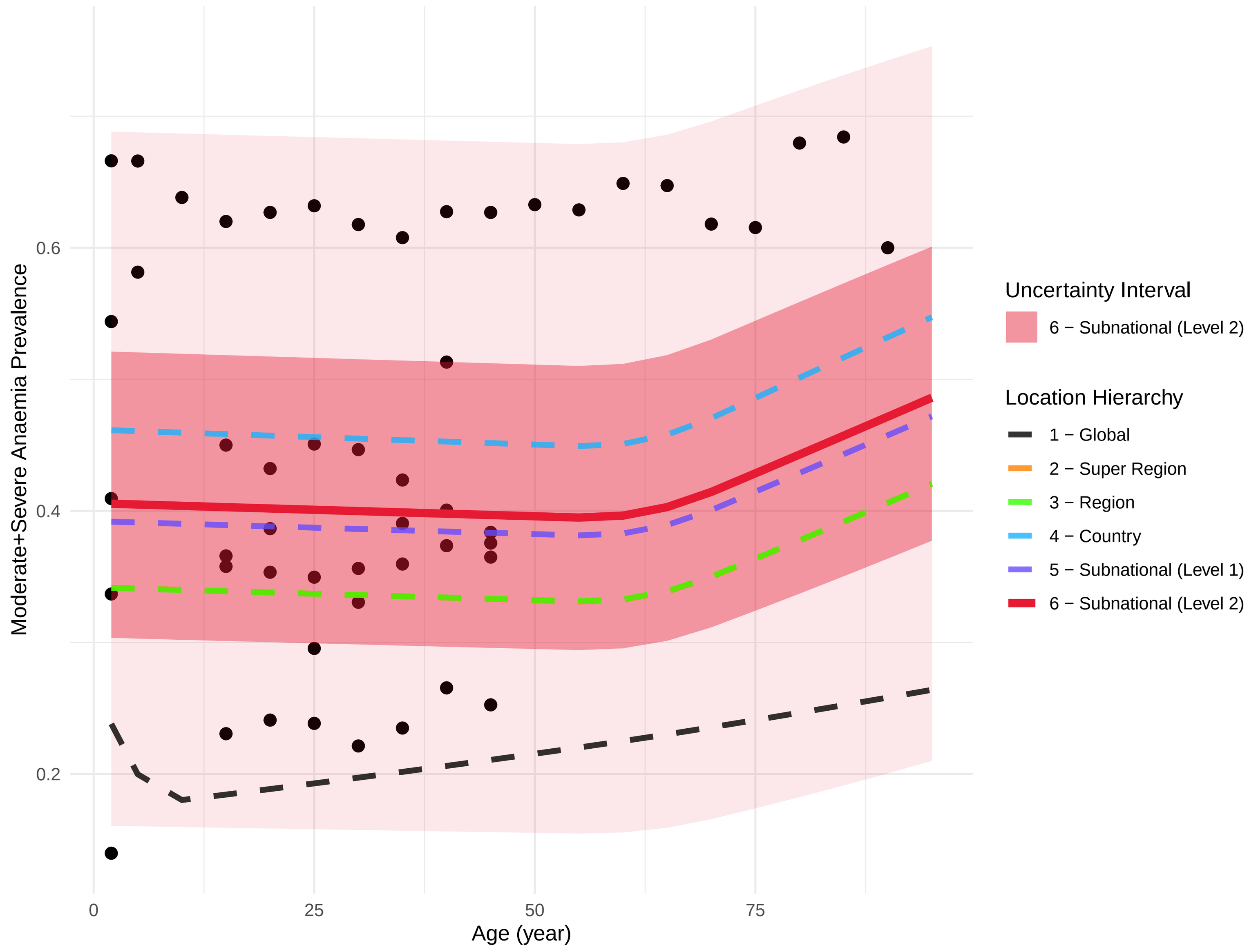

MR−BRT Age/Sex Fits − Non−pregnant Females 2+ Years Old − Moderate+Severe Anaemia Prevalence − Chhattisgarh, Urban
WHO−2001 Adjustment Method

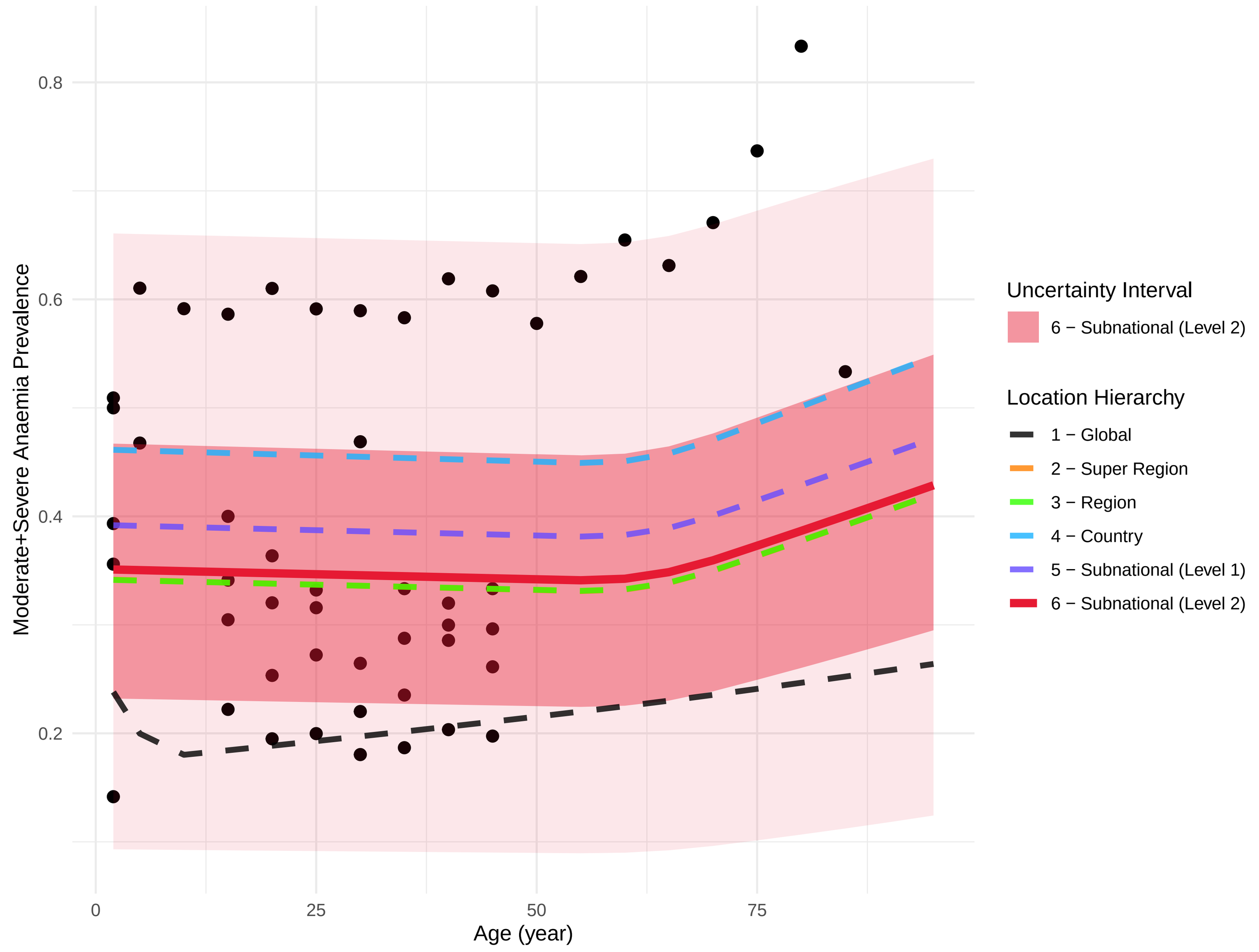

MR−BRT Age/Sex Fits − Non−pregnant Females 2+ Years Old − Moderate+Severe Anaemia Prevalence − Delhi
WHO−2001 Adjustment Method

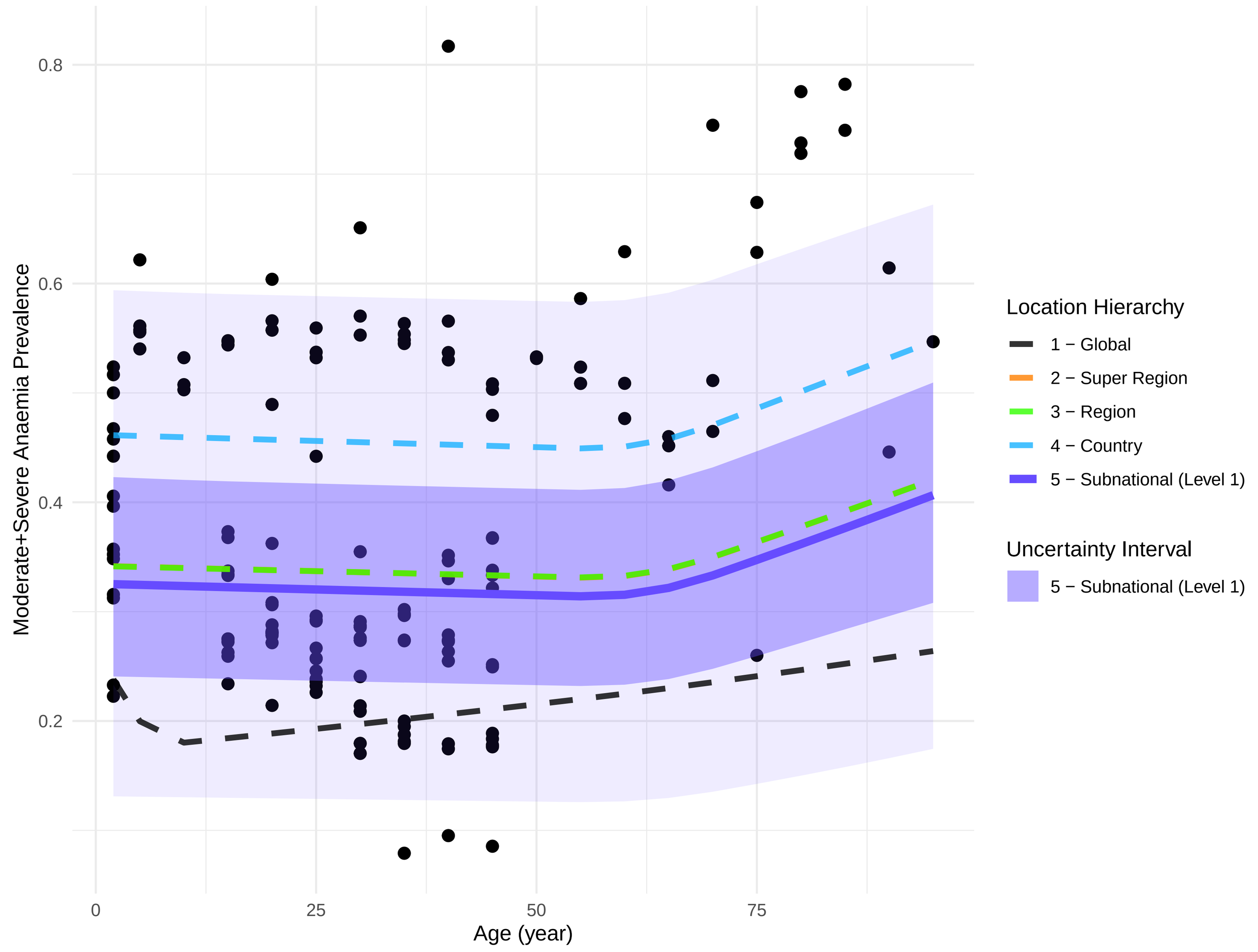

MR−BRT Age/Sex Fits − Non−pregnant Females 2+ Years Old − Moderate+Severe Anaemia Prevalence − Delhi, Rural
WHO−2001 Adjustment Method

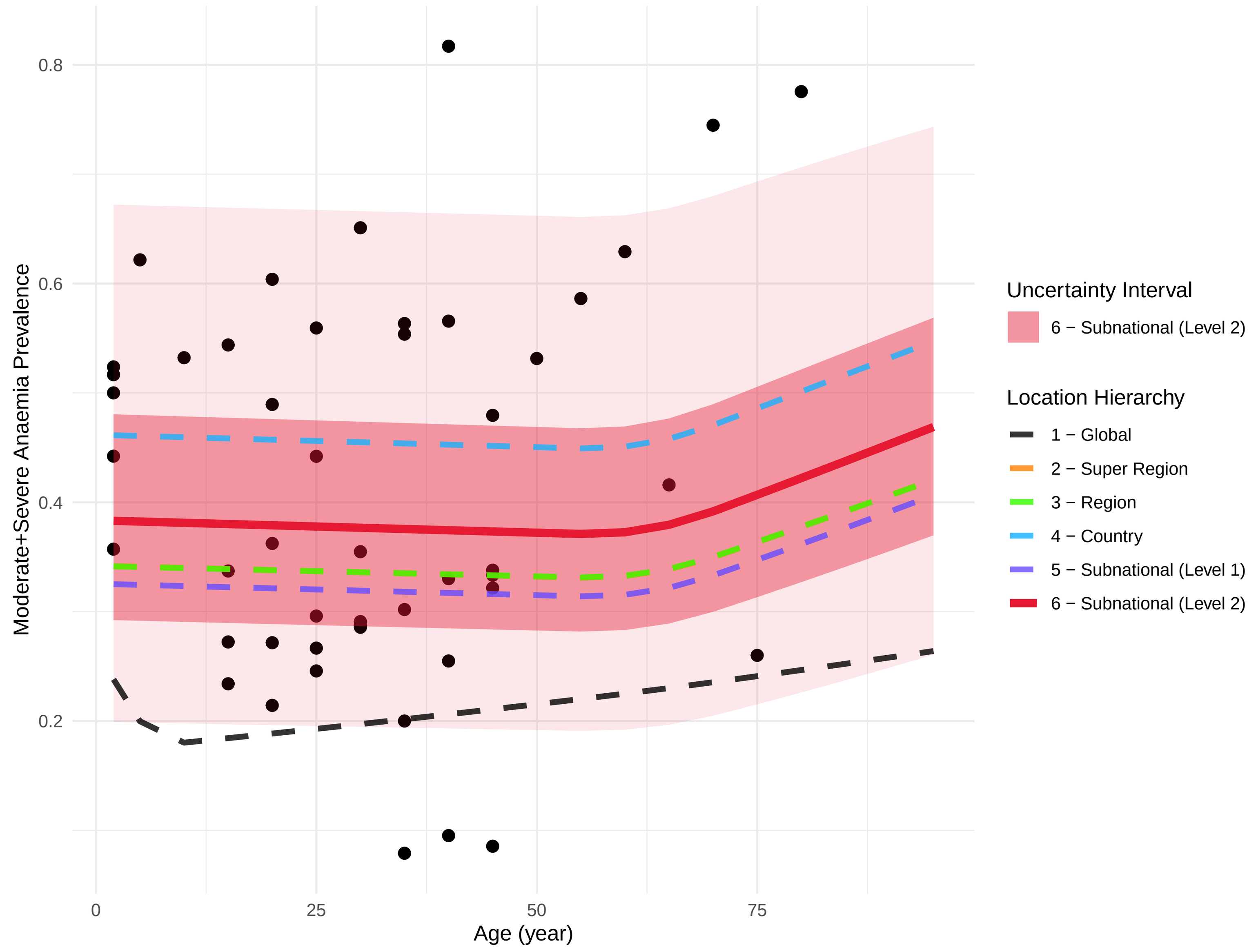

MR−BRT Age/Sex Fits − Non−pregnant Females 2+ Years Old − Moderate+Severe Anaemia Prevalence − Delhi, Urban
WHO−2001 Adjustment Method

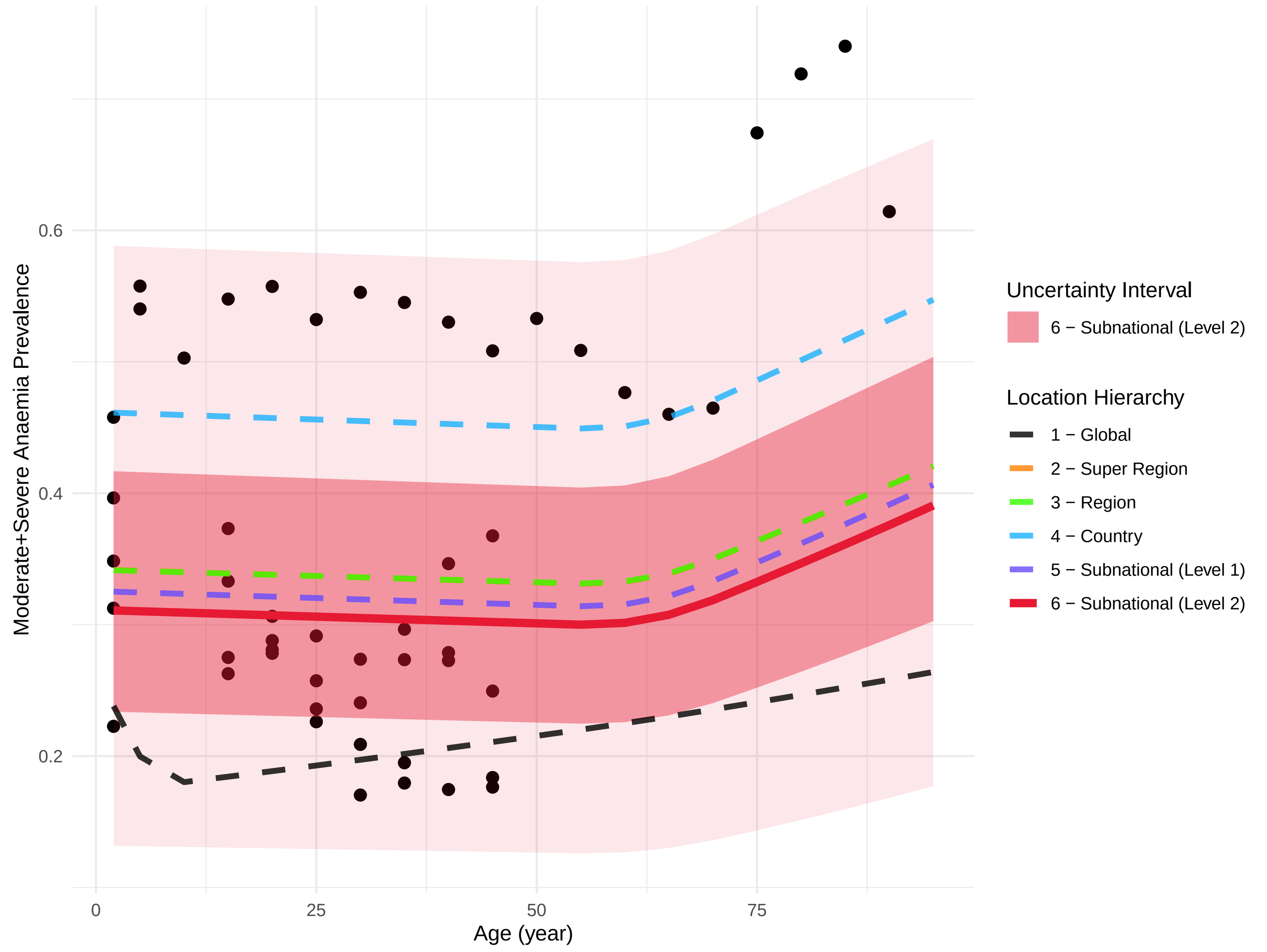

MR−BRT Age/Sex Fits − Non−pregnant Females 2+ Years Old − Moderate+Severe Anaemia Prevalence − Goa
WHO−2001 Adjustment Method

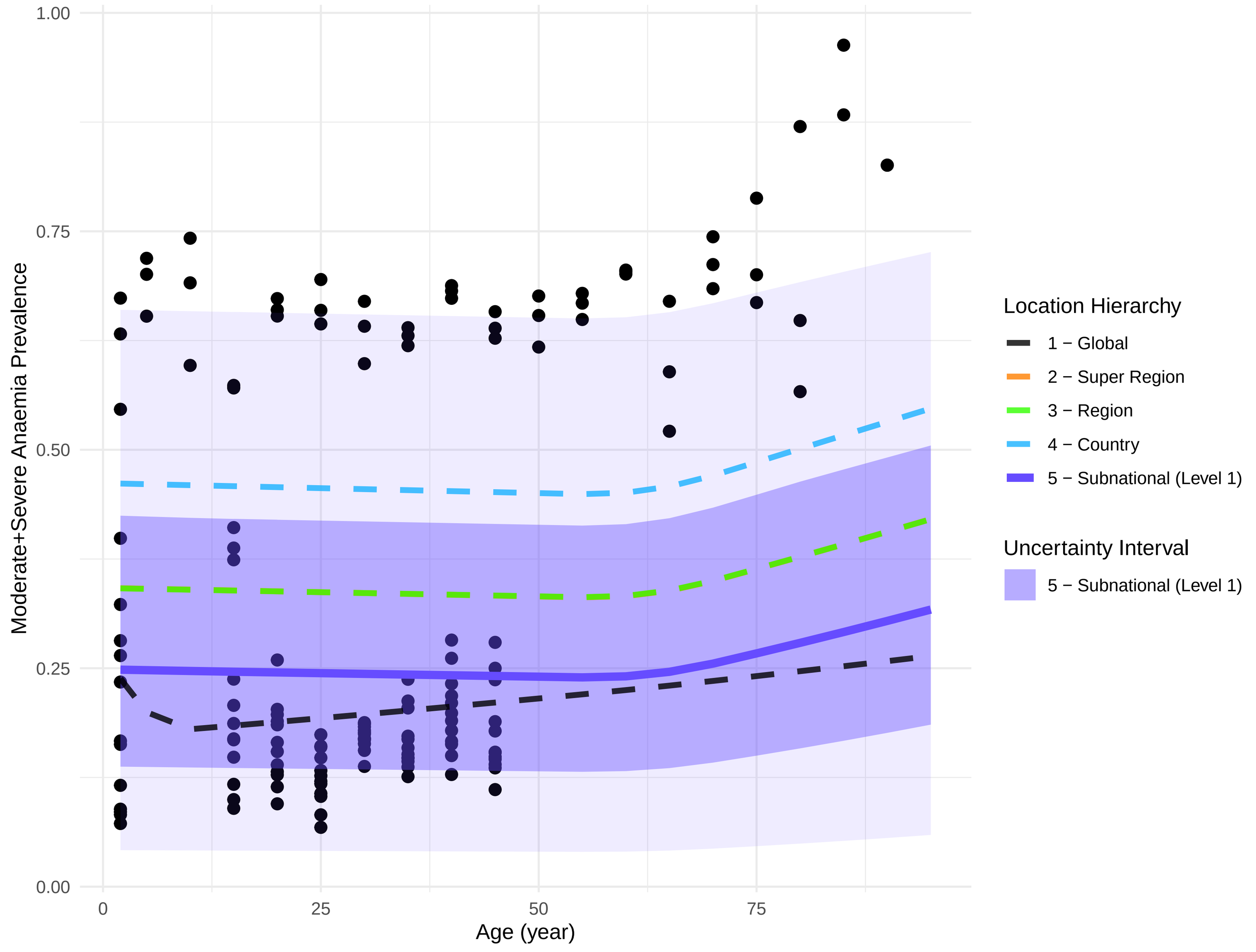

MR−BRT Age/Sex Fits − Non−pregnant Females 2+ Years Old − Moderate+Severe Anaemia Prevalence − Goa, Rural
WHO−2001 Adjustment Method

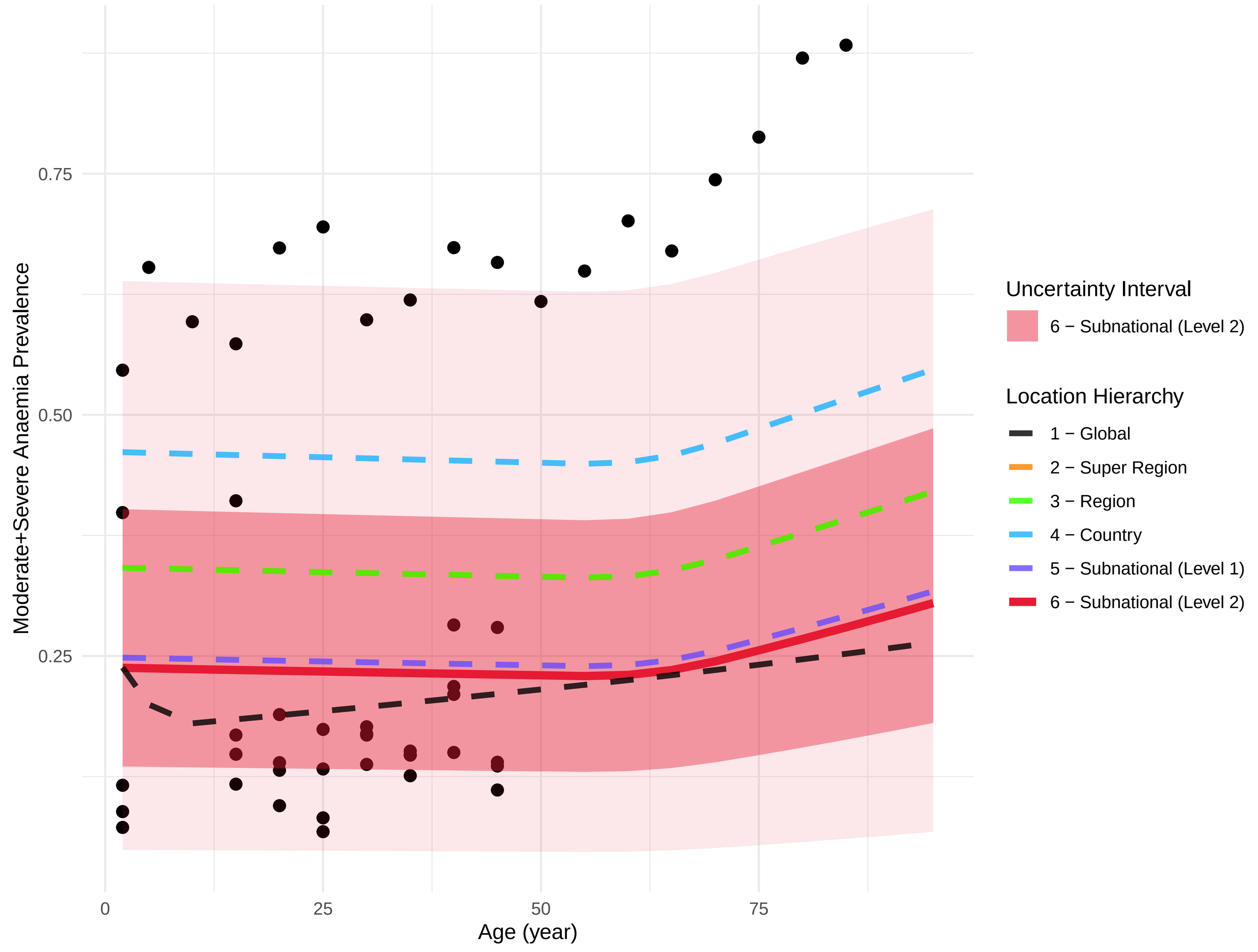

MR−BRT Age/Sex Fits − Non−pregnant Females 2+ Years Old − Moderate+Severe Anaemia Prevalence − Goa, Urban
WHO−2001 Adjustment Method

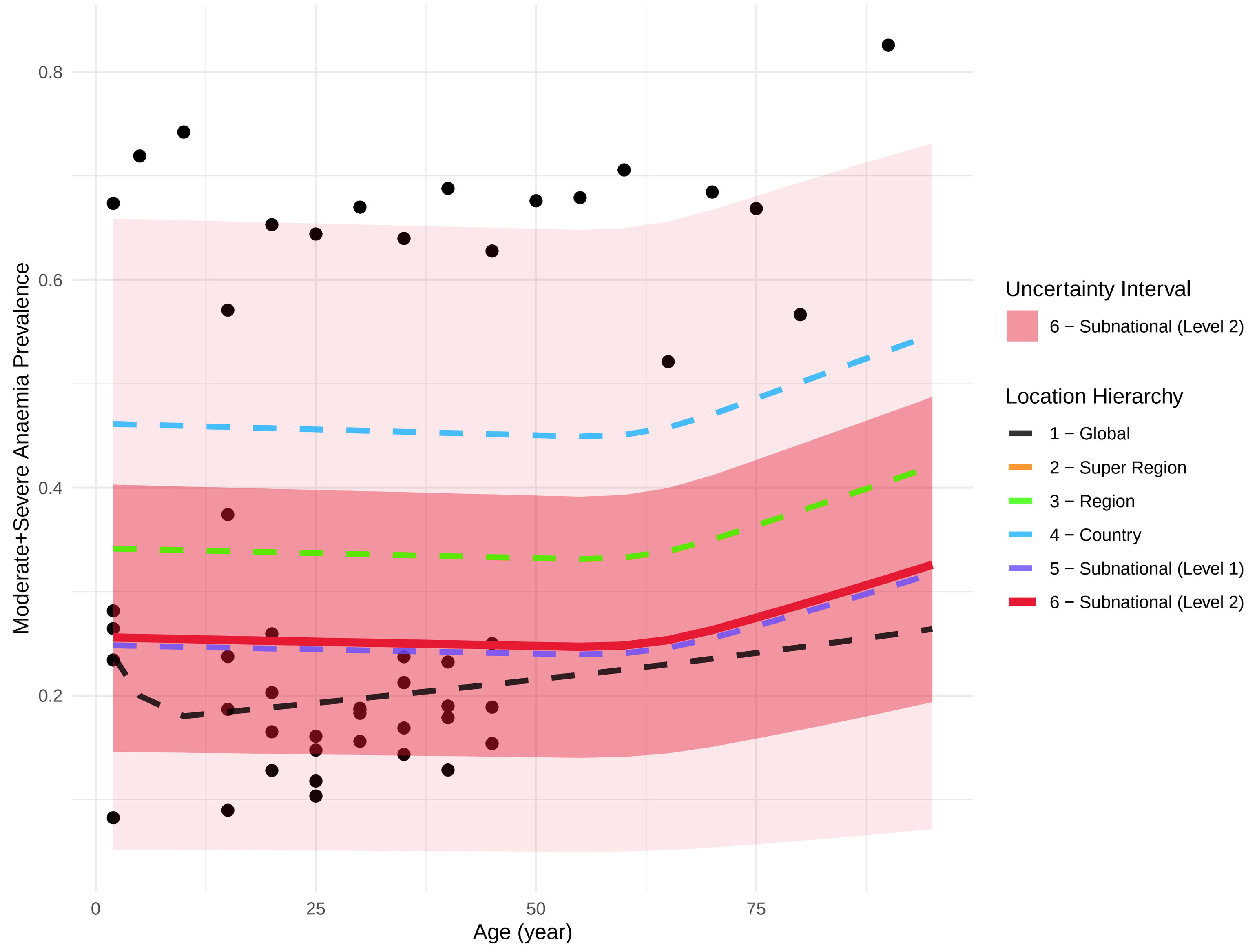

MR−BRT Age/Sex Fits − Non−pregnant Females 2+ Years Old − Moderate+Severe Anaemia Prevalence − Gujarat
WHO−2001 Adjustment Method

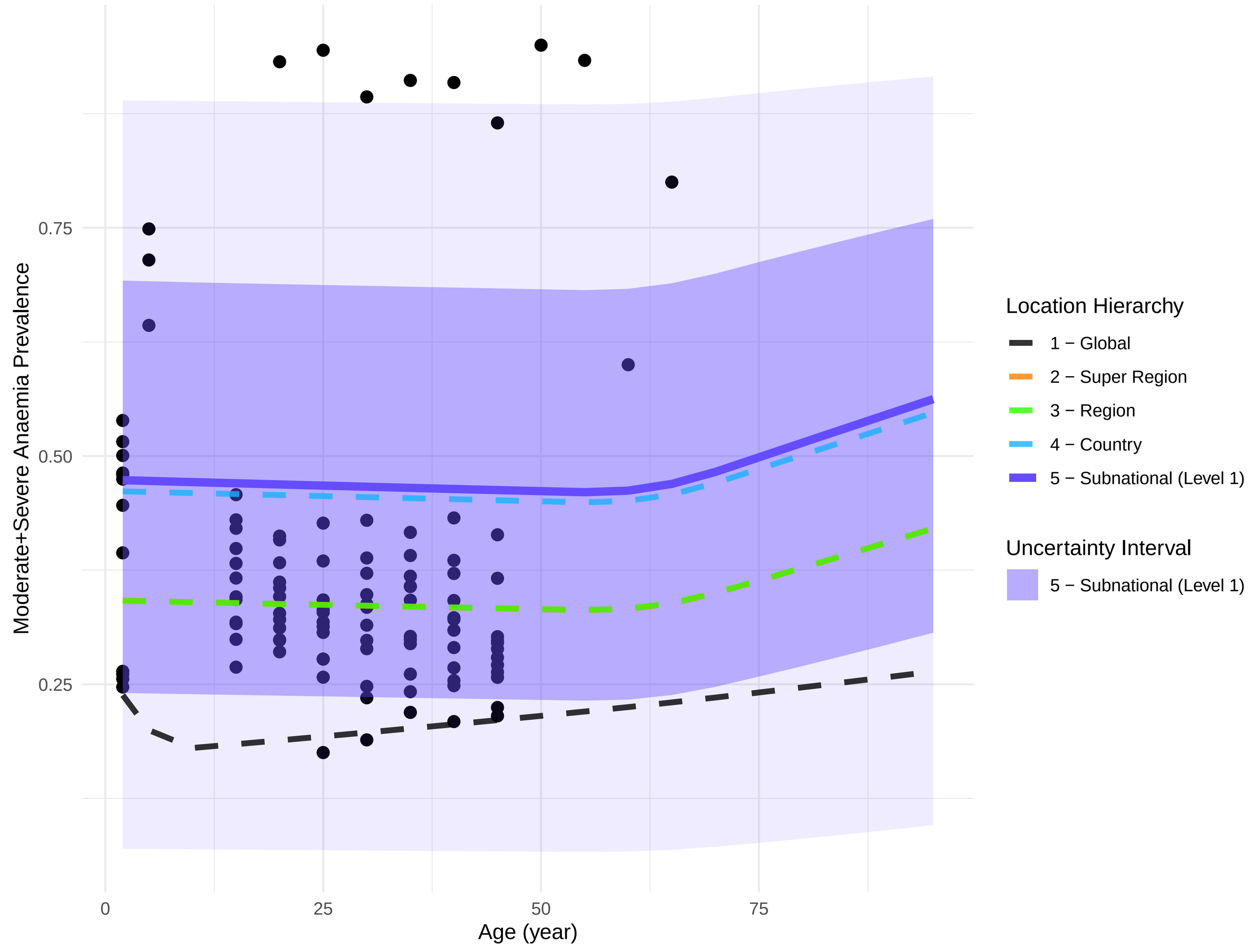

MR−BRT Age/Sex Fits − Non−pregnant Females 2+ Years Old − Moderate+Severe Anaemia Prevalence − Gujarat, Rural
WHO−2001 Adjustment Method

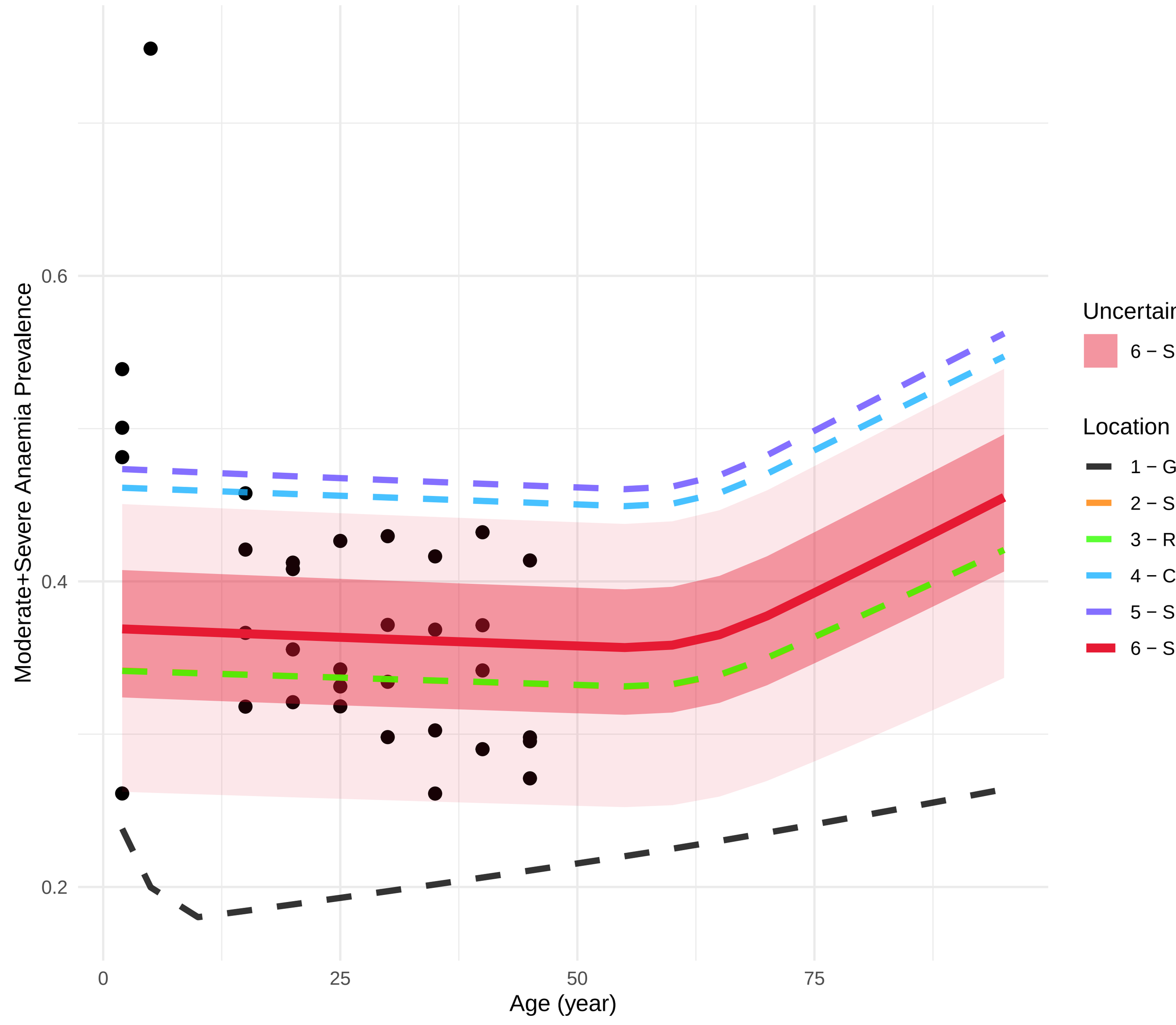

MR−BRT Age/Sex Fits − Non−pregnant Females 2+ Years Old − Moderate+Severe Anaemia Prevalence − Gujarat, Urban
WHO−2001 Adjustment Method

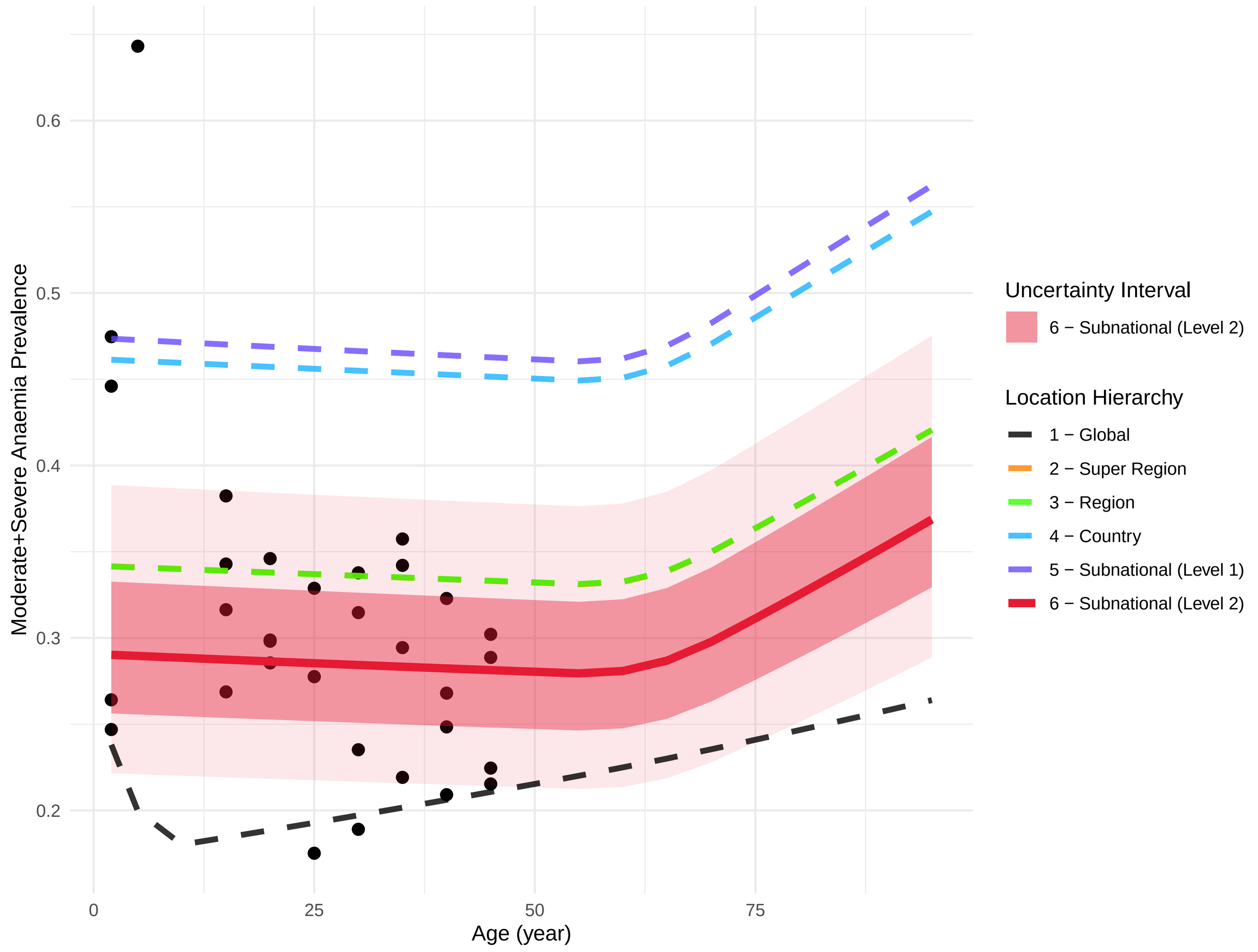

MR−BRT Age/Sex Fits − Non−pregnant Females 2+ Years Old − Moderate+Severe Anaemia Prevalence − Haryana
WHO−2001 Adjustment Method

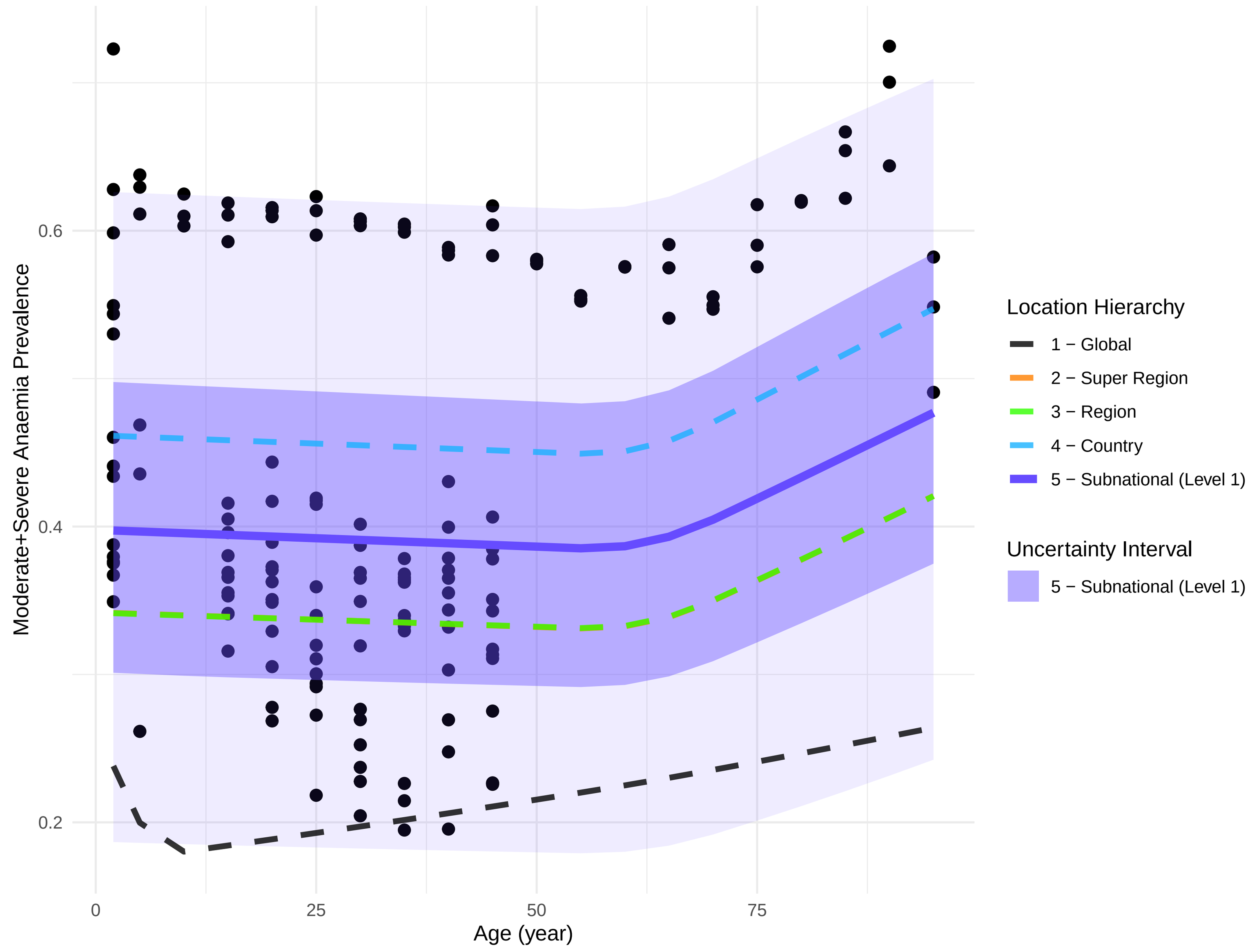

MR−BRT Age/Sex Fits − Non−pregnant Females 2+ Years Old − Moderate+Severe Anaemia Prevalence − Haryana, Rural
WHO−2001 Adjustment Method

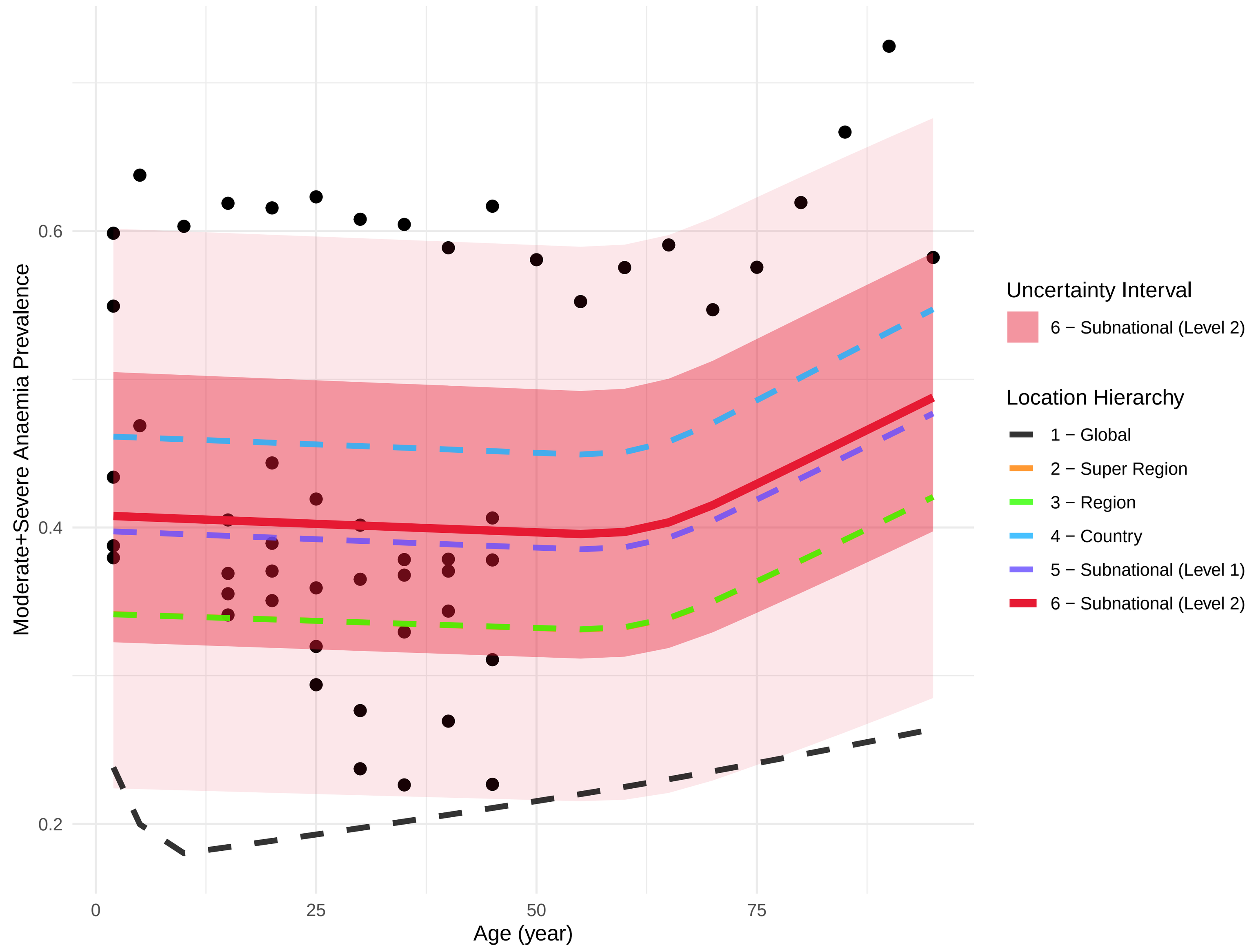

MR−BRT Age/Sex Fits − Non−pregnant Females 2+ Years Old − Moderate+Severe Anaemia Prevalence − Haryana, Urban
WHO−2001 Adjustment Method

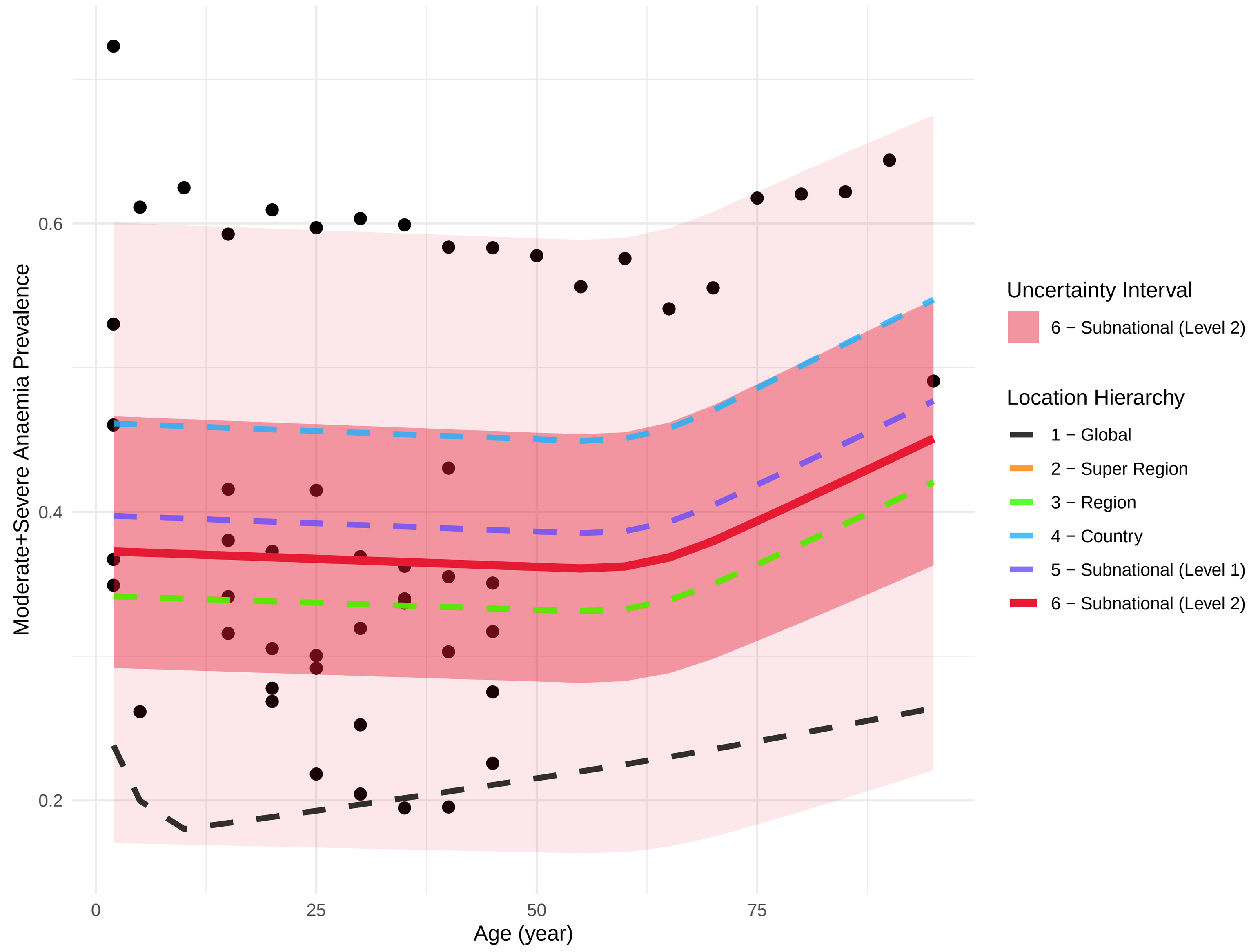

MR−BRT Age/Sex Fits − Non−pregnant Females 2+ Years Old − Moderate+Severe Anaemia Prevalence − Himachal Pradesh
WHO−2001 Adjustment Method

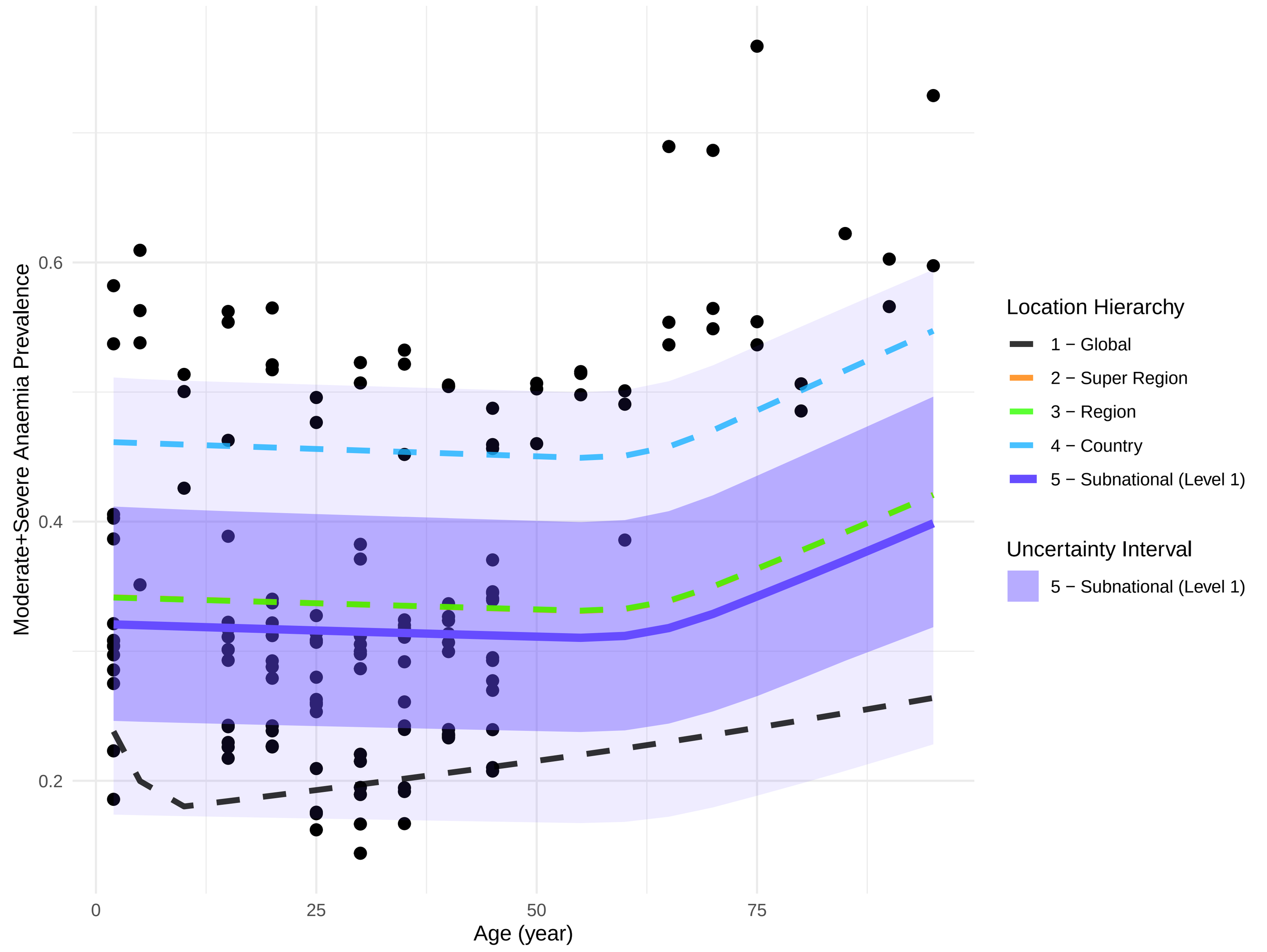

MR−BRT Age/Sex Fits − Non−pregnant Females 2+ Years Old − Moderate+Severe Anaemia Prevalence − Himachal Pradesh, Rural
WHO−2001 Adjustment Method

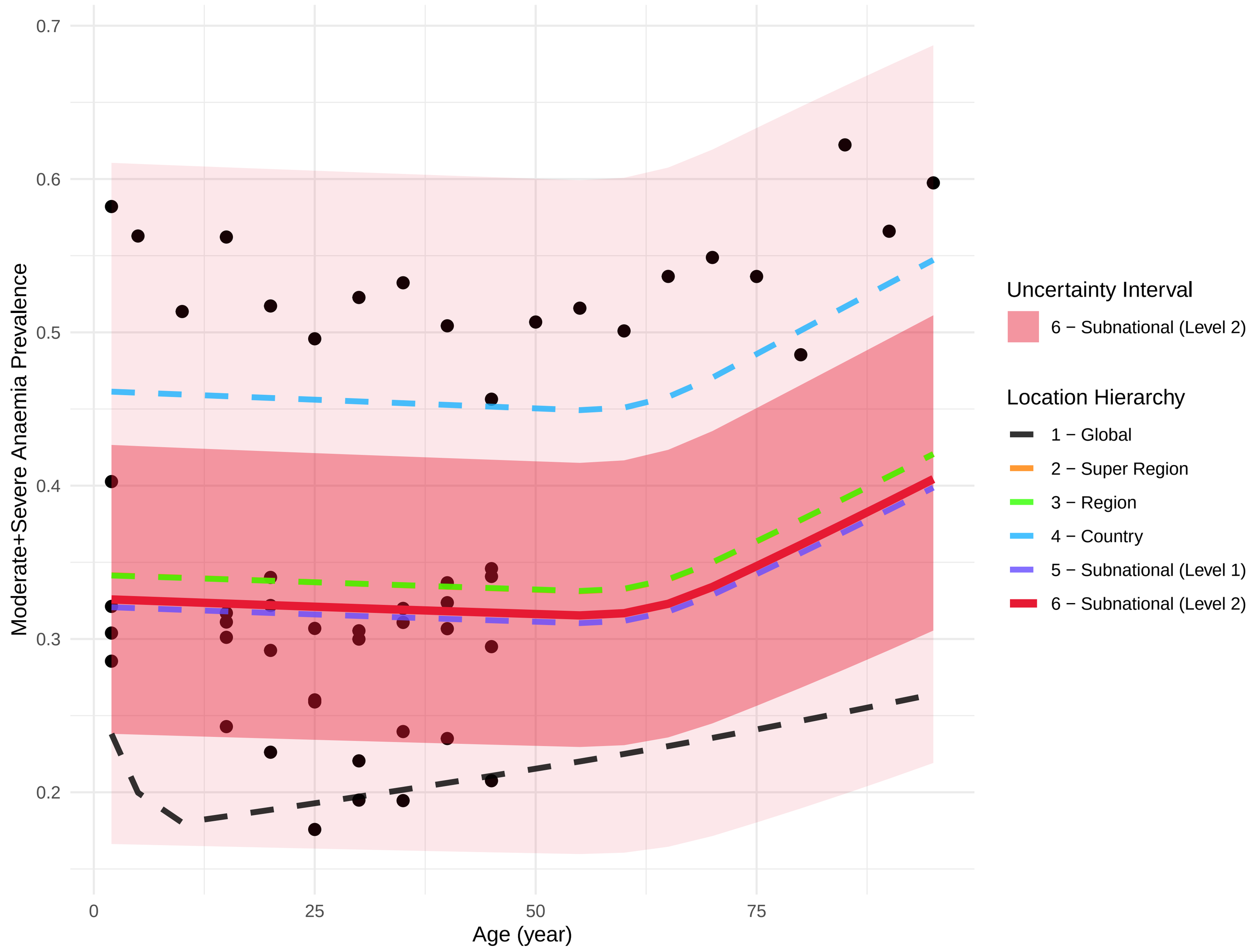

MR−BRT Age/Sex Fits − Non−pregnant Females 2+ Years Old − Moderate+Severe Anaemia Prevalence − Himachal Pradesh, Urban
WHO−2001 Adjustment Method

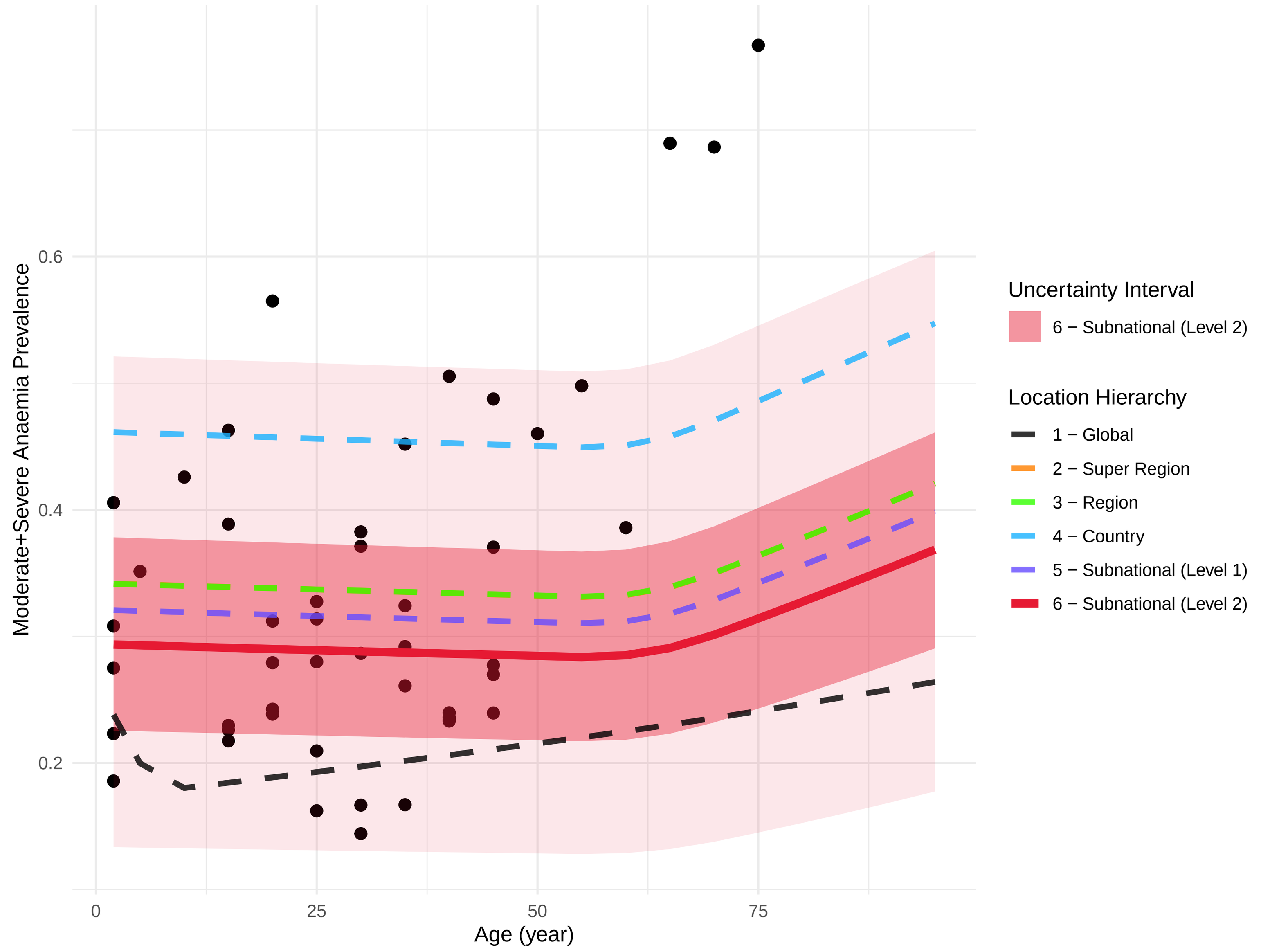

MR−BRT Age/Sex Fits − Non−pregnant Females 2+ Years Old − Moderate+Severe Anaemia Prevalence − Jammu & Kashmir and Ladakh
WHO−2001 Adjustment Method

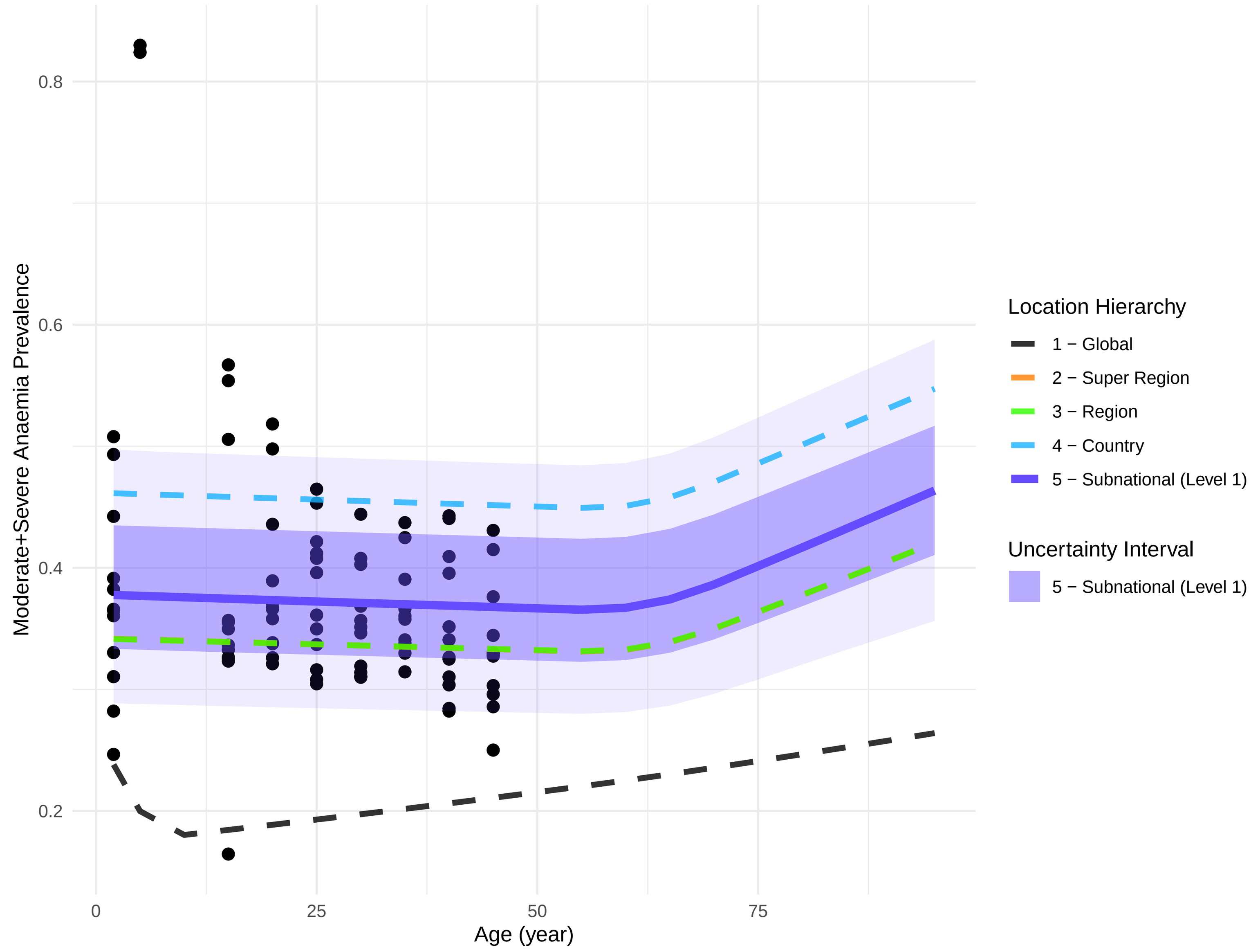

MR−BRT Age/Sex Fits − Non−pregnant Females 2+ Years Old − Moderate+Severe Anaemia Prevalence − Jammu & Kashmir and Ladakh, Rural
WHO−2001 Adjustment Method

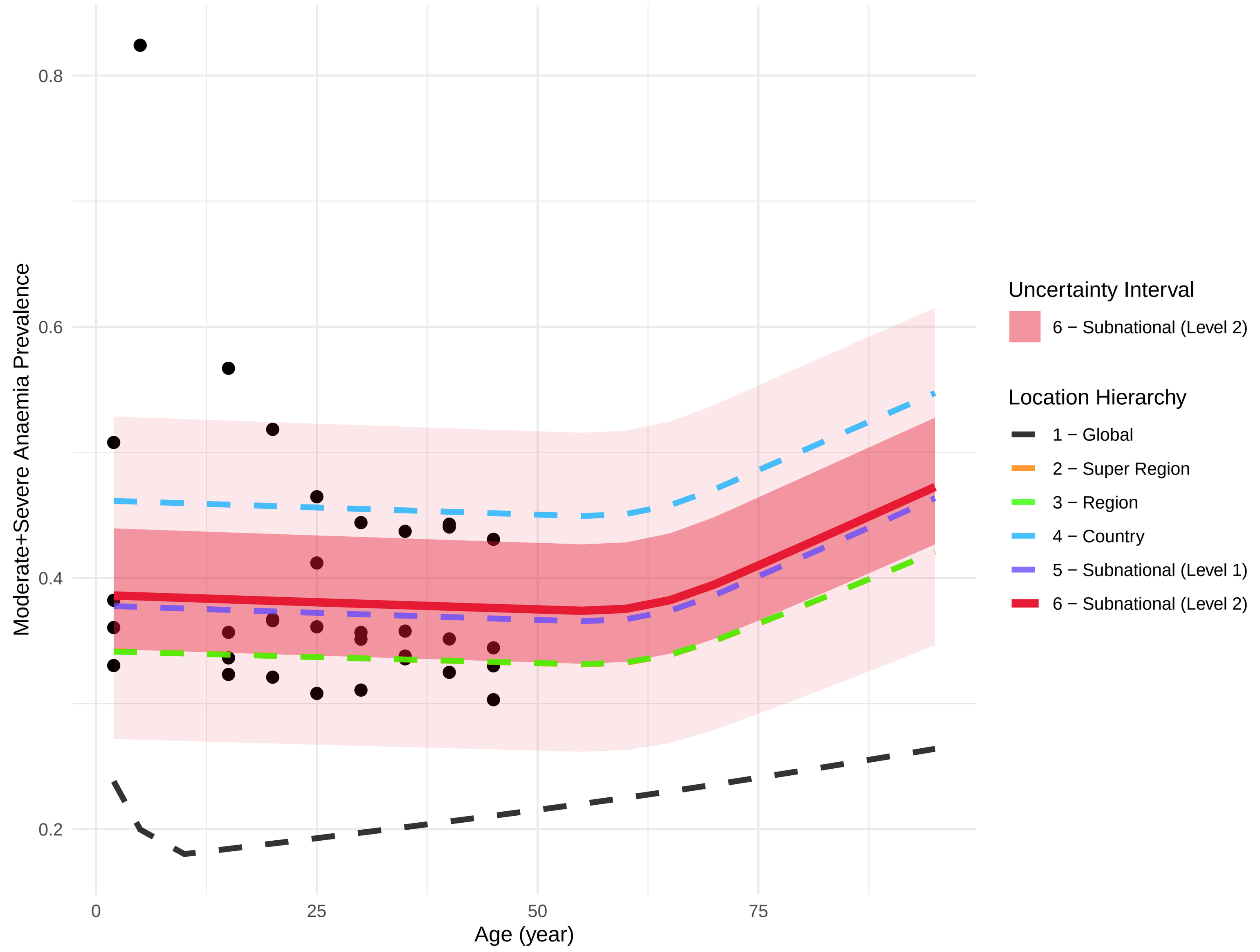

MR−BRT Age/Sex Fits − Non−pregnant Females 2+ Years Old − Moderate+Severe Anaemia Prevalence − Jammu & Kashmir and Ladakh, Urban
WHO−2001 Adjustment Method

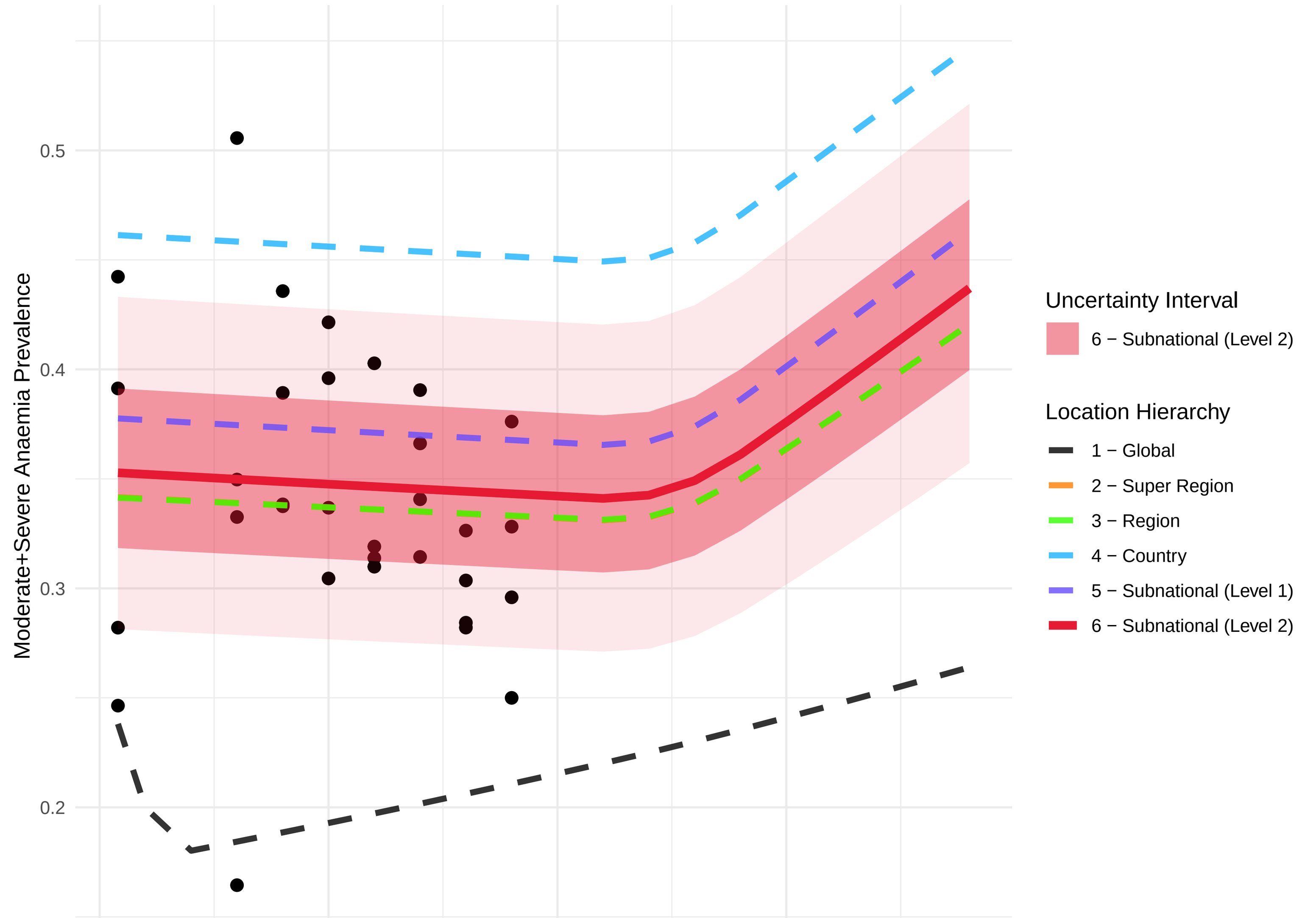

MR−BRT Age/Sex Fits − Non−pregnant Females 2+ Years Old − Moderate+Severe Anaemia Prevalence − Jharkhand
WHO−2001 Adjustment Method

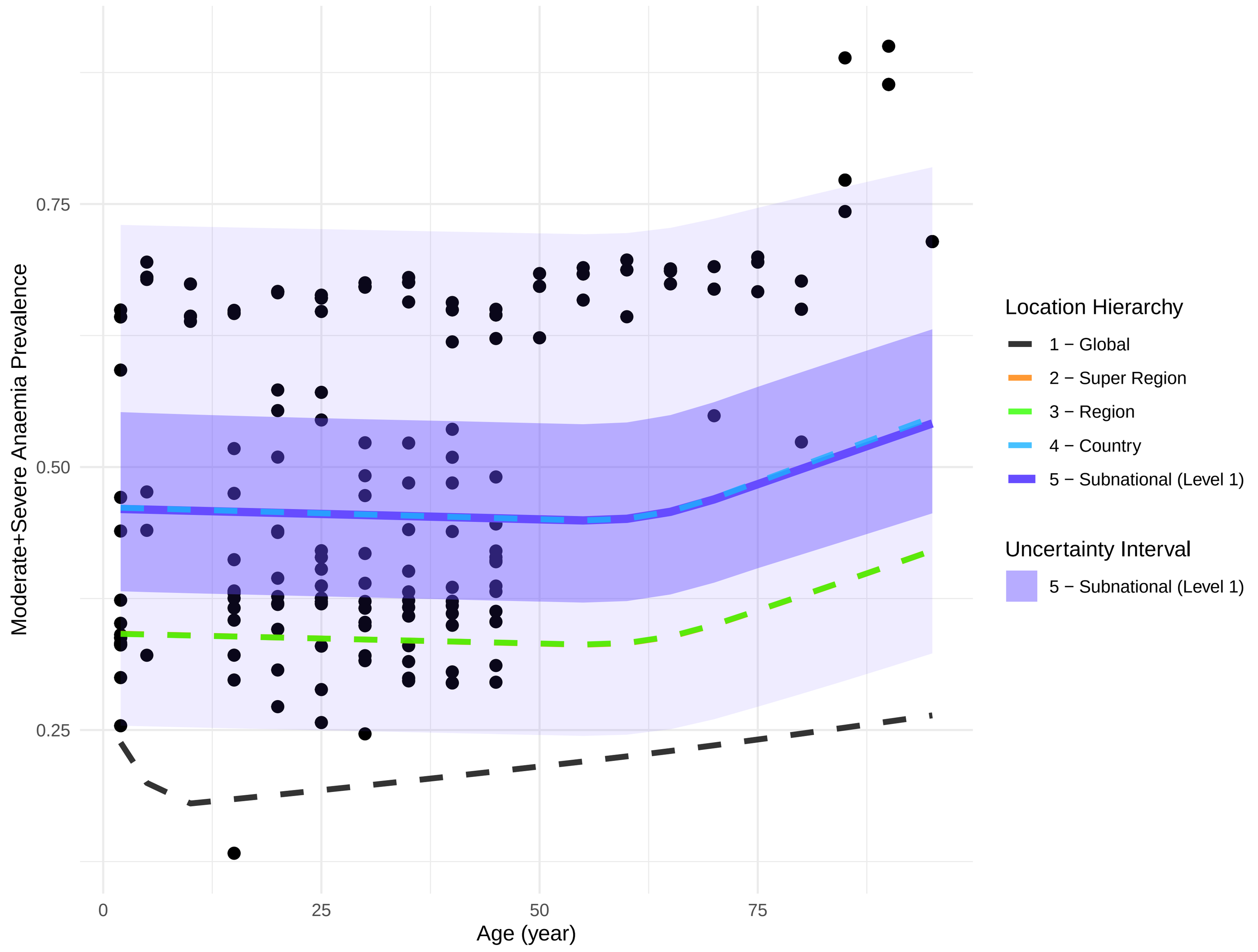

MR−BRT Age/Sex Fits − Non−pregnant Females 2+ Years Old − Moderate+Severe Anaemia Prevalence − Jharkhand, Rural
WHO−2001 Adjustment Method

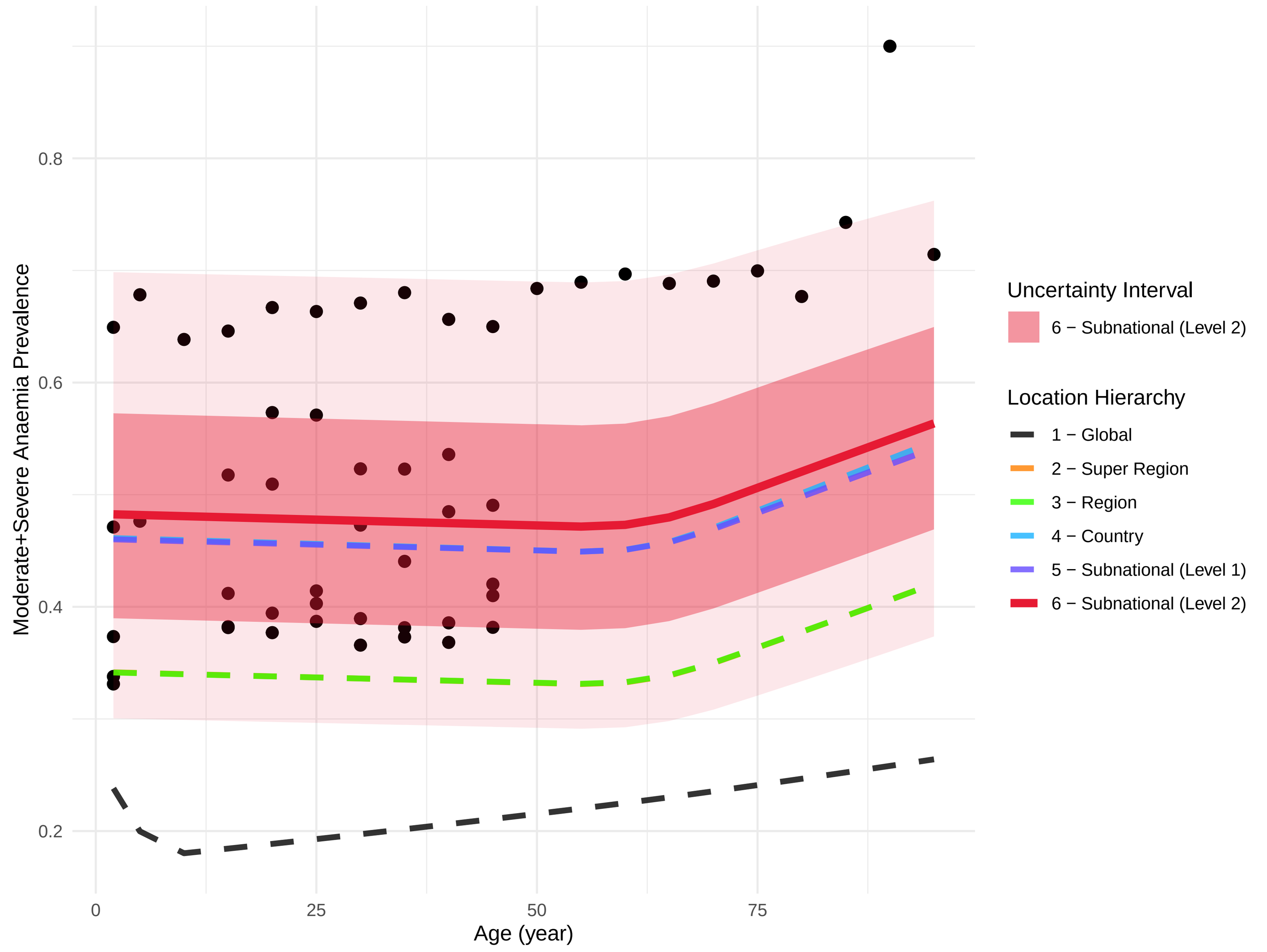

MR−BRT Age/Sex Fits − Non−pregnant Females 2+ Years Old − Moderate+Severe Anaemia Prevalence − Jharkhand, Urban
WHO−2001 Adjustment Method

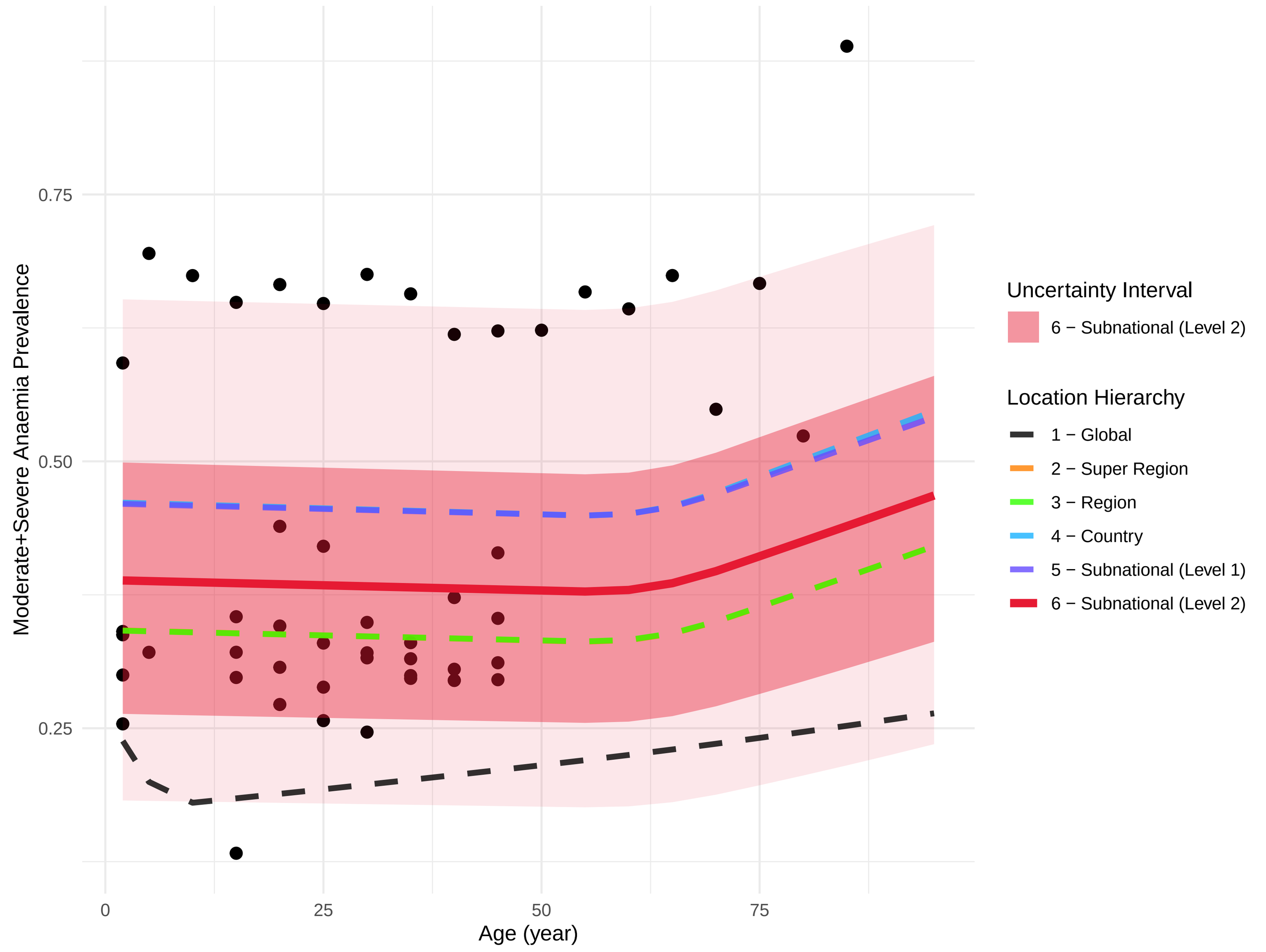

MR−BRT Age/Sex Fits − Non−pregnant Females 2+ Years Old − Moderate+Severe Anaemia Prevalence − Karnataka
WHO−2001 Adjustment Method

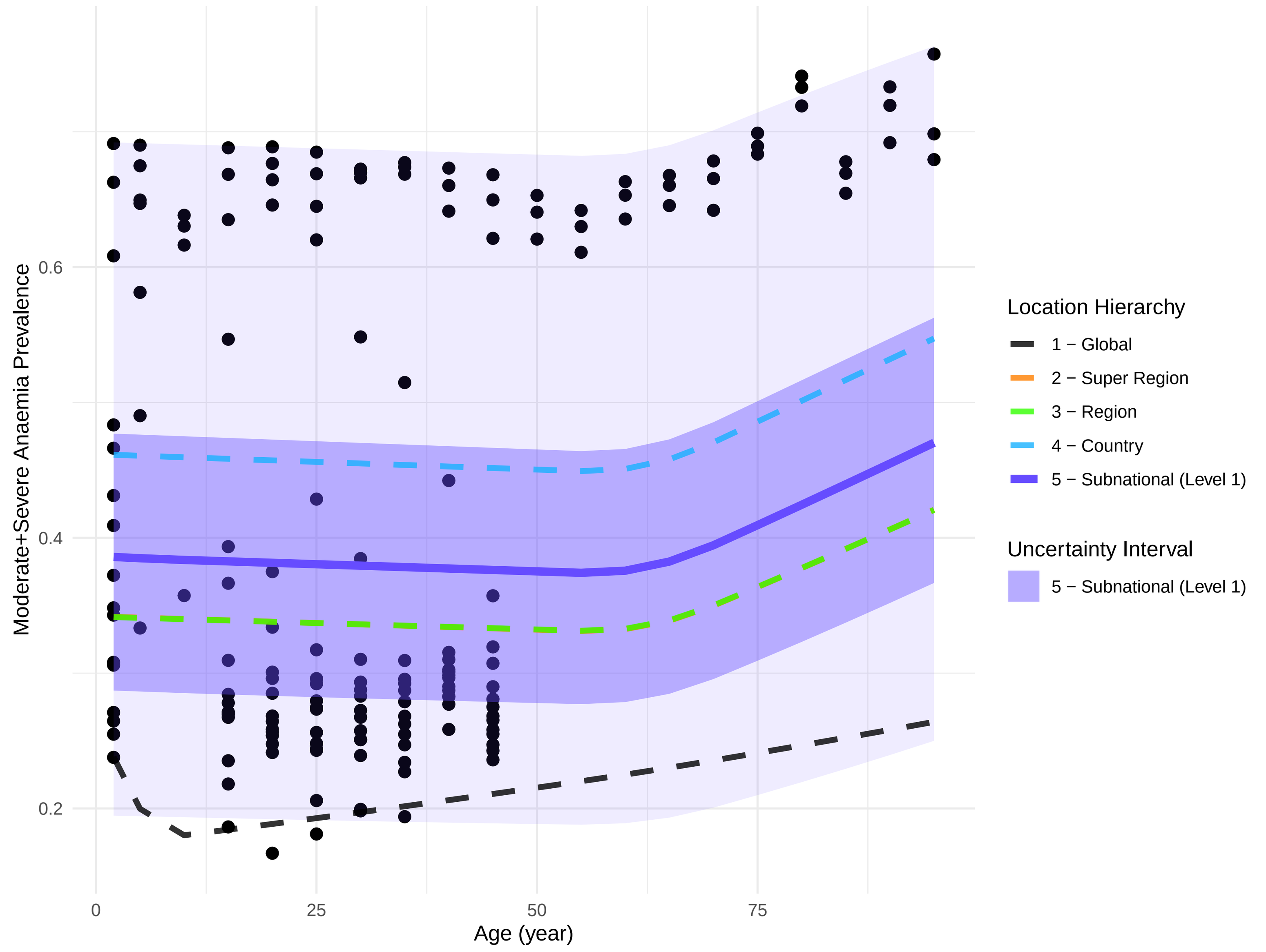

MR−BRT Age/Sex Fits − Non−pregnant Females 2+ Years Old − Moderate+Severe Anaemia Prevalence − Karnataka, Rural
WHO−2001 Adjustment Method

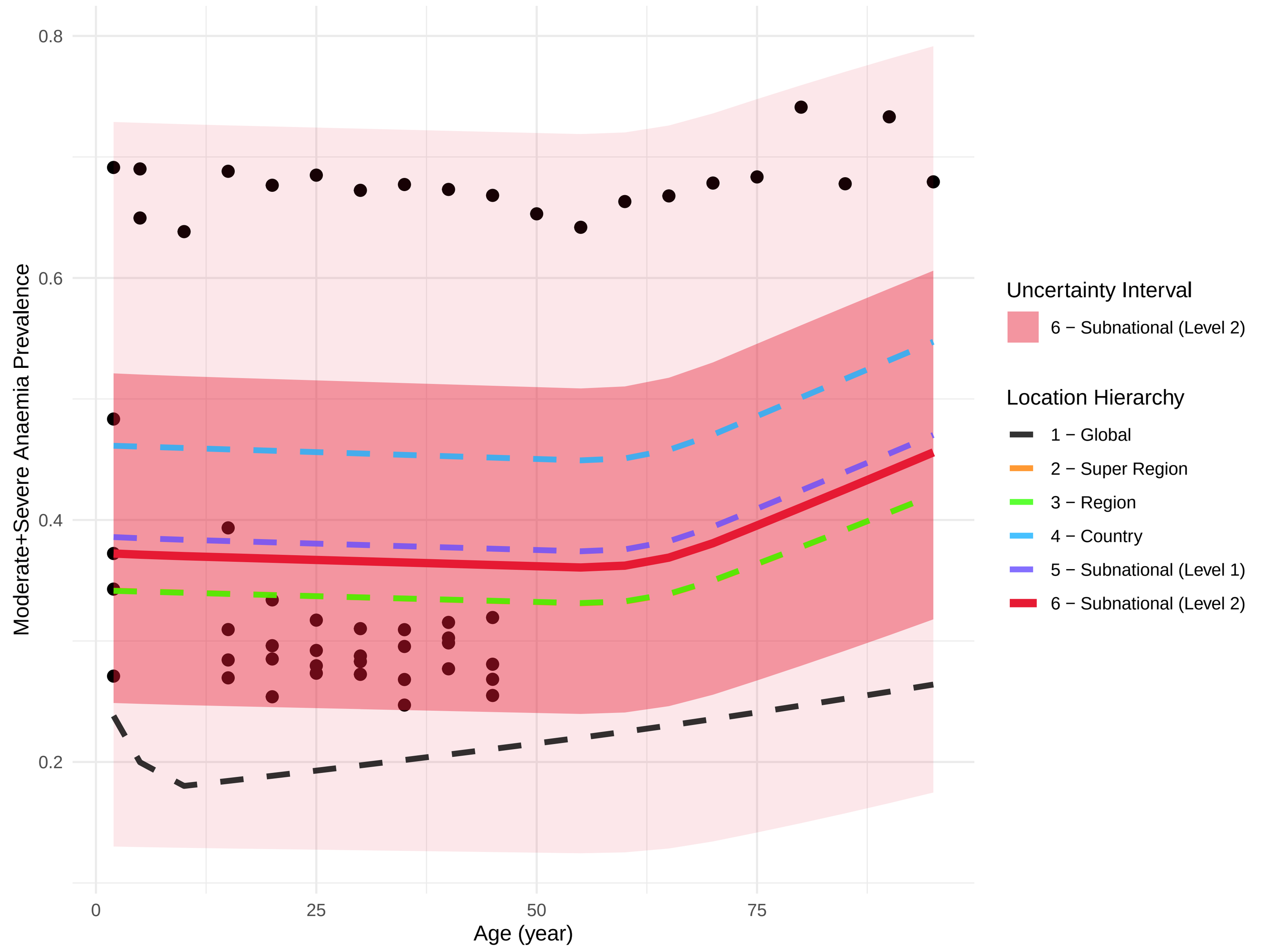

MR−BRT Age/Sex Fits − Non−pregnant Females 2+ Years Old − Moderate+Severe Anaemia Prevalence − Karnataka, Urban
WHO−2001 Adjustment Method

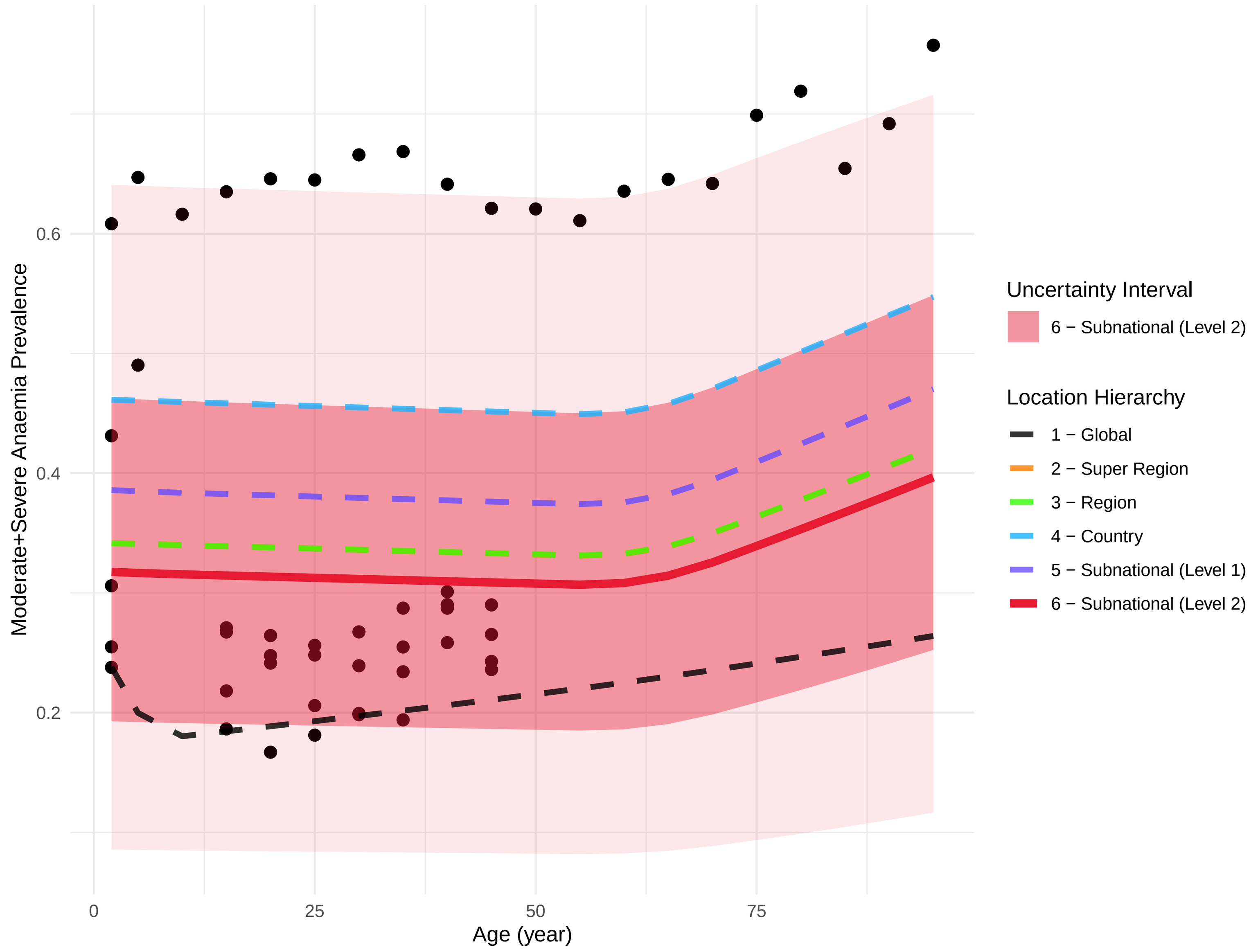

MR−BRT Age/Sex Fits − Non−pregnant Females 2+ Years Old − Moderate+Severe Anaemia Prevalence − Kerala
WHO−2001 Adjustment Method

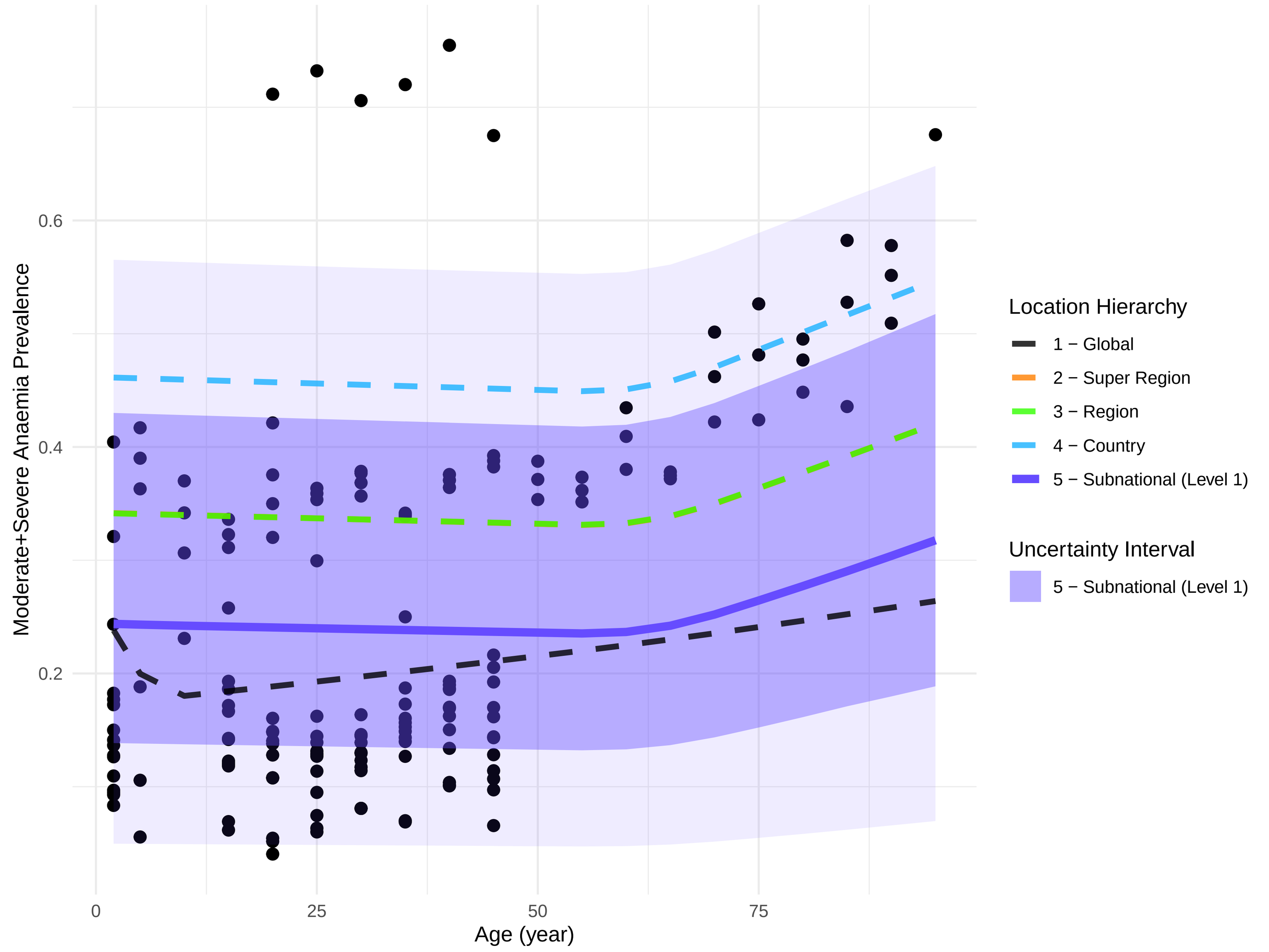

MR−BRT Age/Sex Fits − Non−pregnant Females 2+ Years Old − Moderate+Severe Anaemia Prevalence − Kerala, Rural
WHO−2001 Adjustment Method

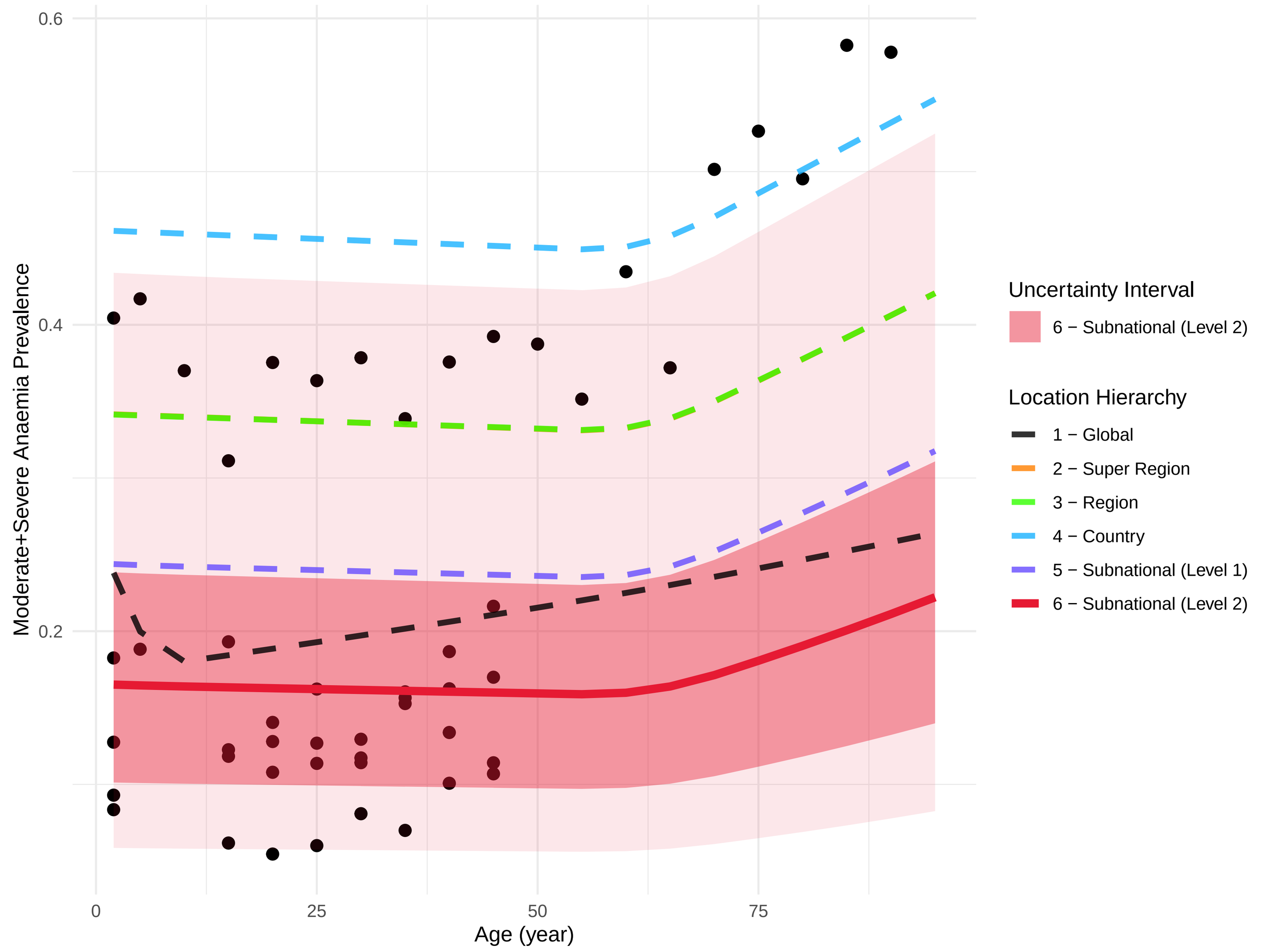

MR−BRT Age/Sex Fits − Non−pregnant Females 2+ Years Old − Moderate+Severe Anaemia Prevalence − Kerala, Urban
WHO−2001 Adjustment Method

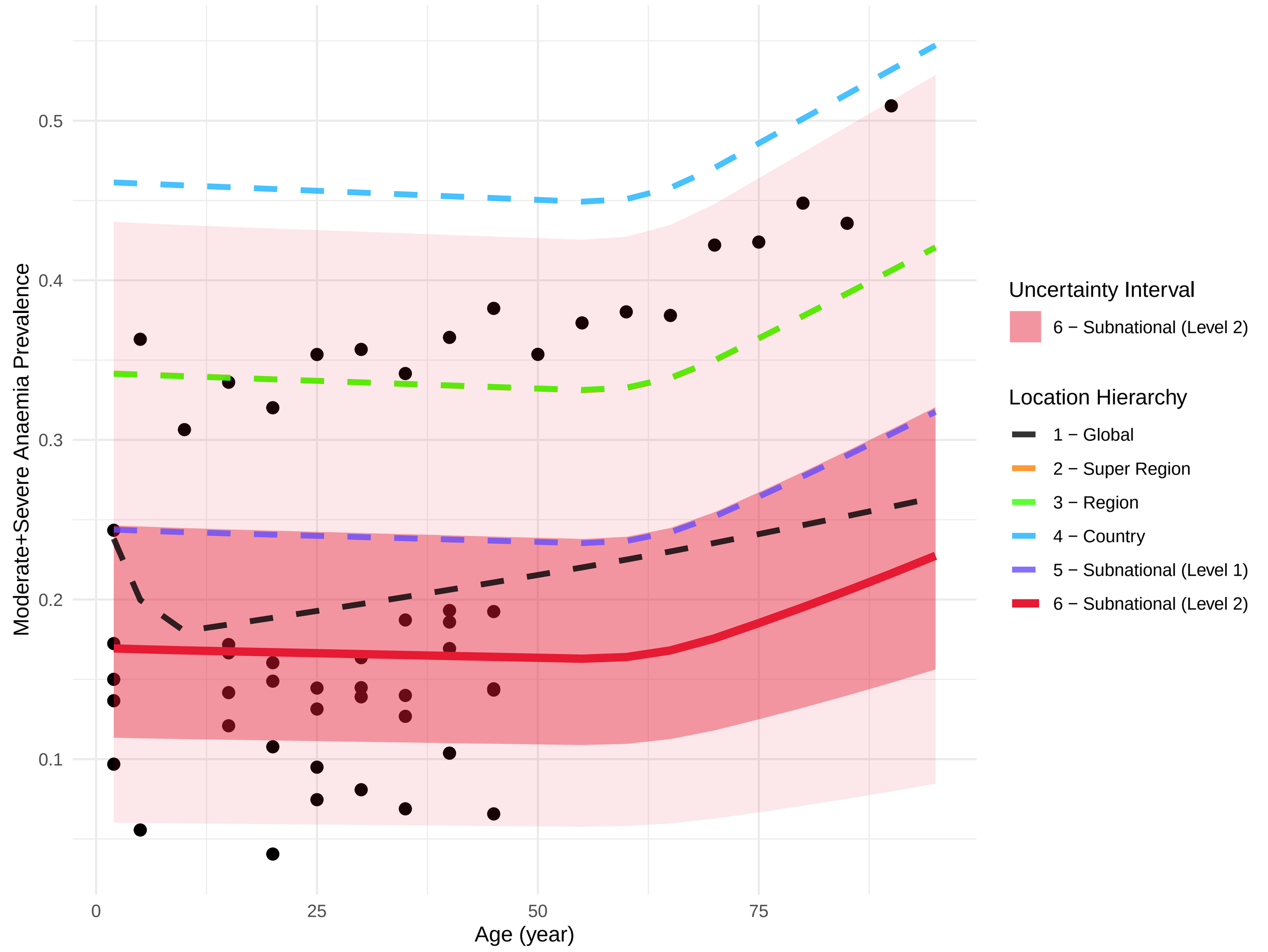

MR−BRT Age/Sex Fits − Non−pregnant Females 2+ Years Old − Moderate+Severe Anaemia Prevalence − Madhya Pradesh
WHO−2001 Adjustment Method

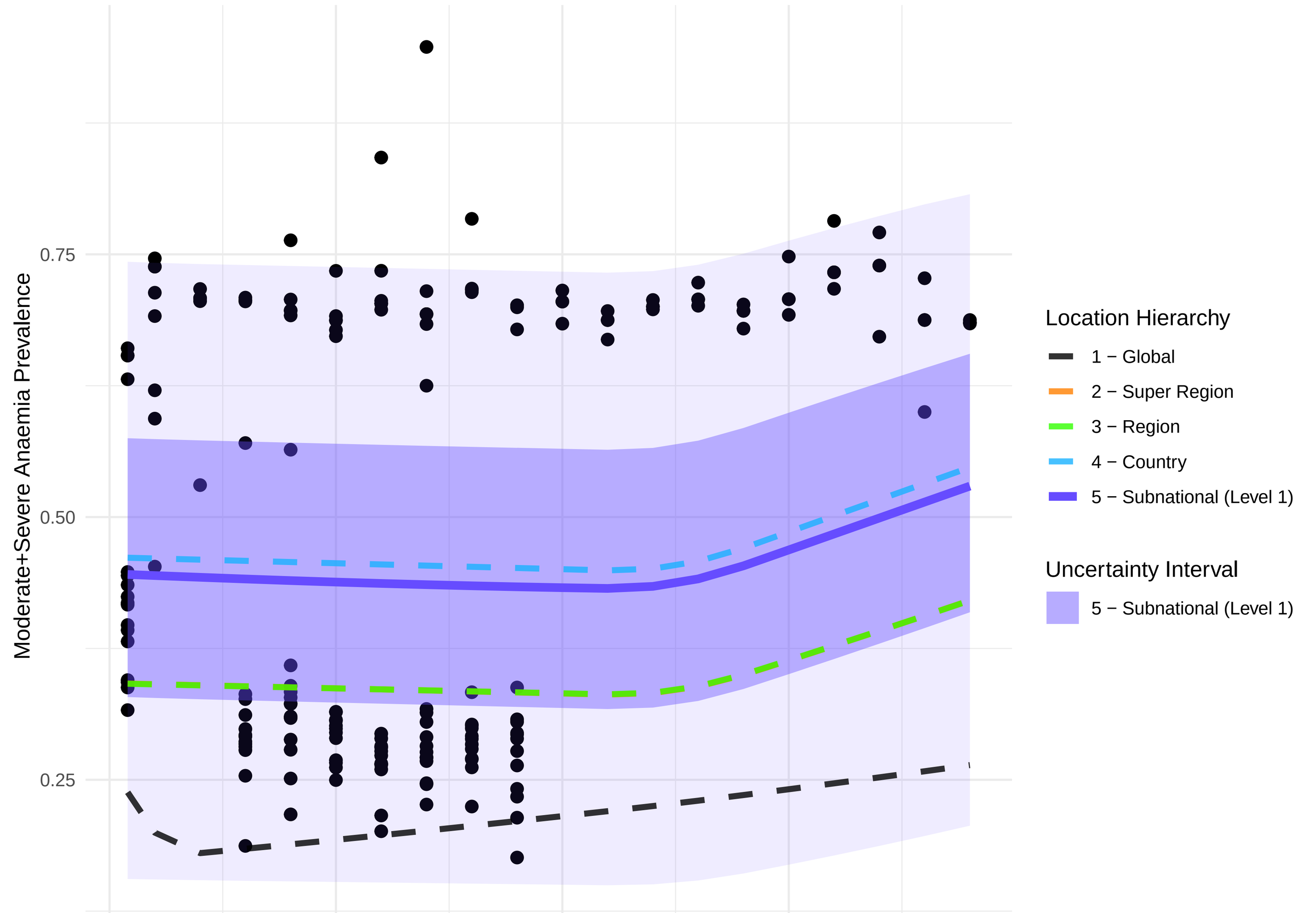

MR−BRT Age/Sex Fits − Non−pregnant Females 2+ Years Old − Moderate+Severe Anaemia Prevalence − Madhya Pradesh, Rural
WHO−2001 Adjustment Method

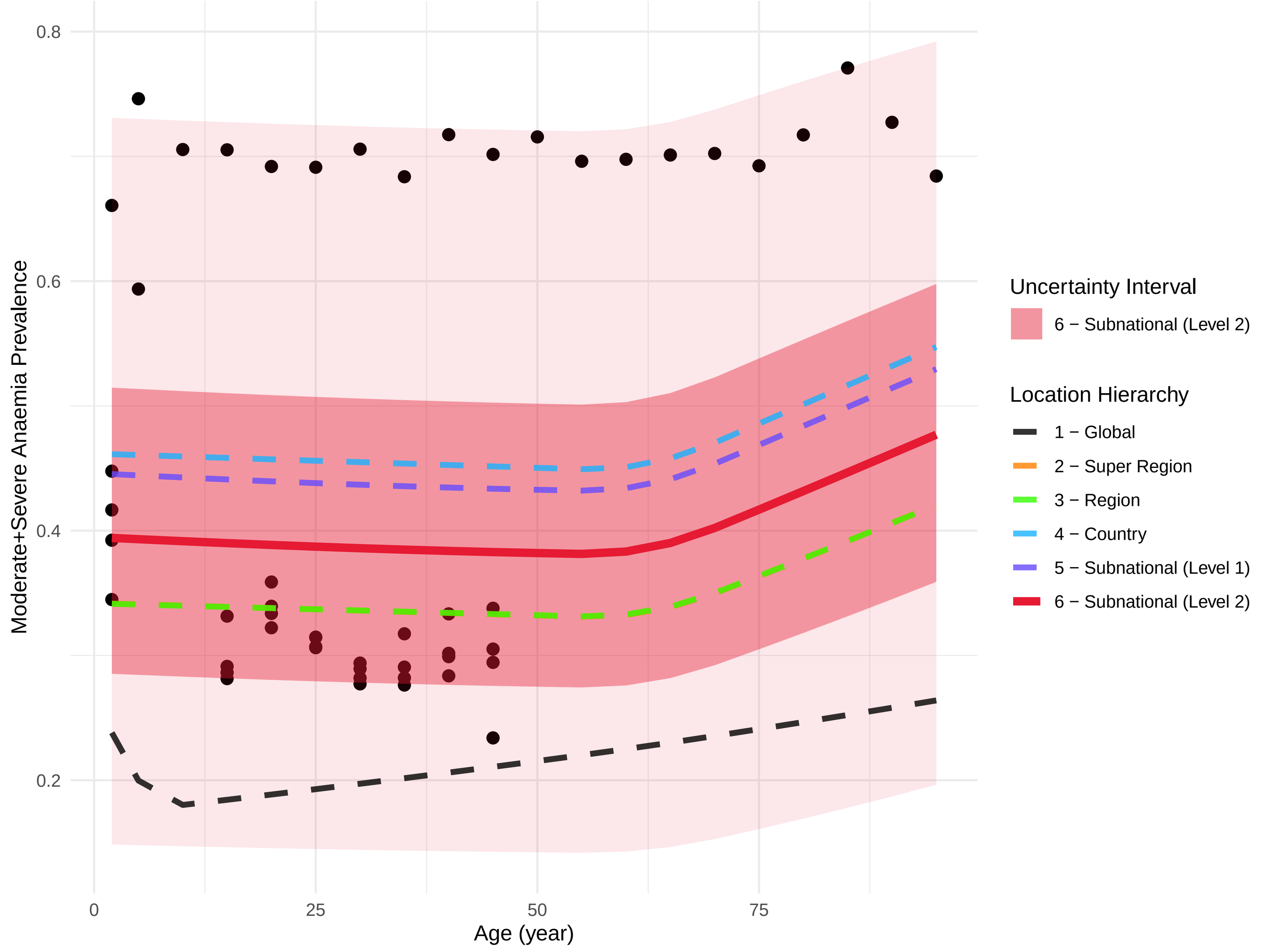

MR−BRT Age/Sex Fits − Non−pregnant Females 2+ Years Old − Moderate+Severe Anaemia Prevalence − Madhya Pradesh, Urban
WHO−2001 Adjustment Method

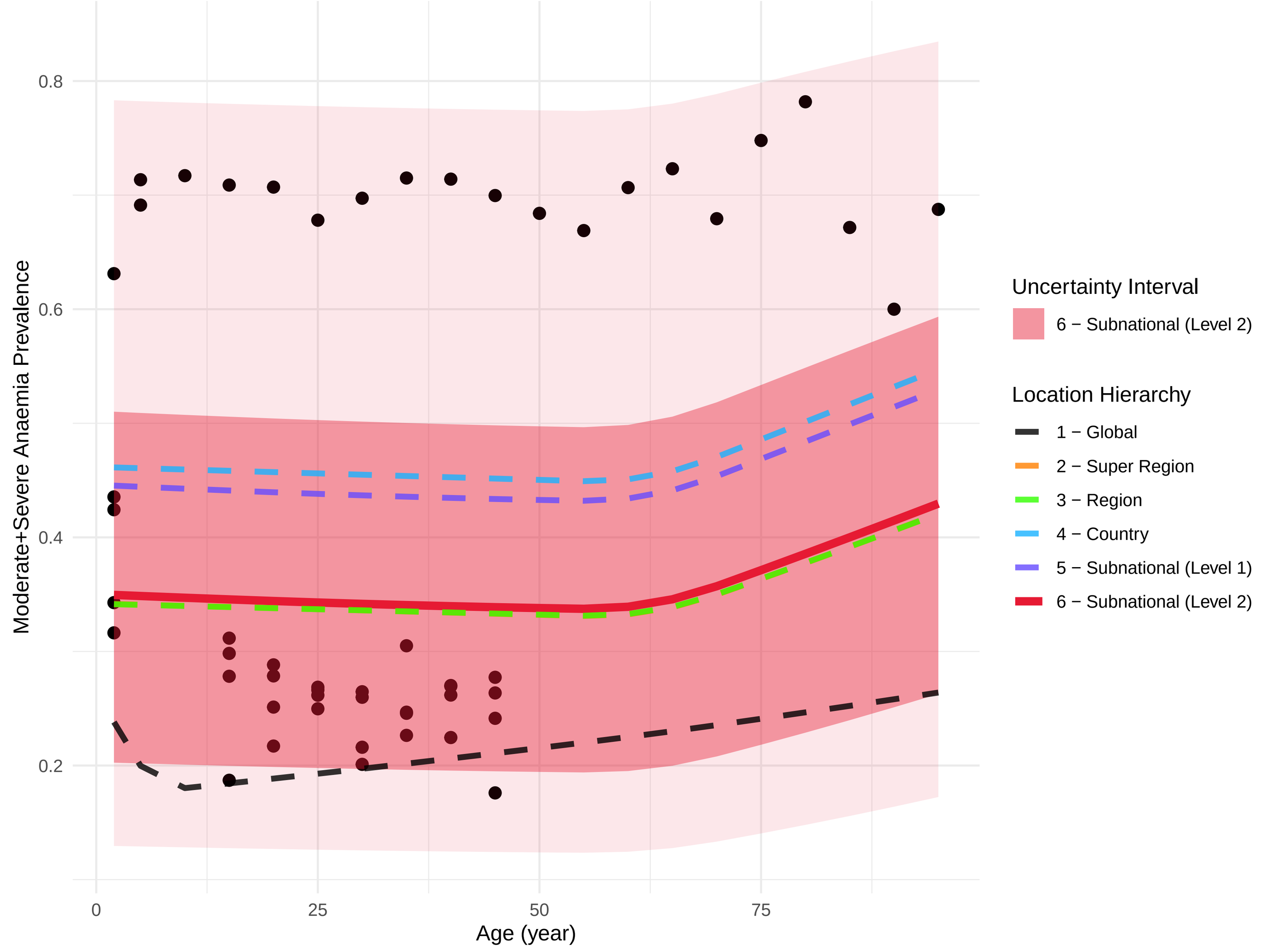

MR−BRT Age/Sex Fits − Non−pregnant Females 2+ Years Old − Moderate+Severe Anaemia Prevalence − Maharashtra
WHO−2001 Adjustment Method

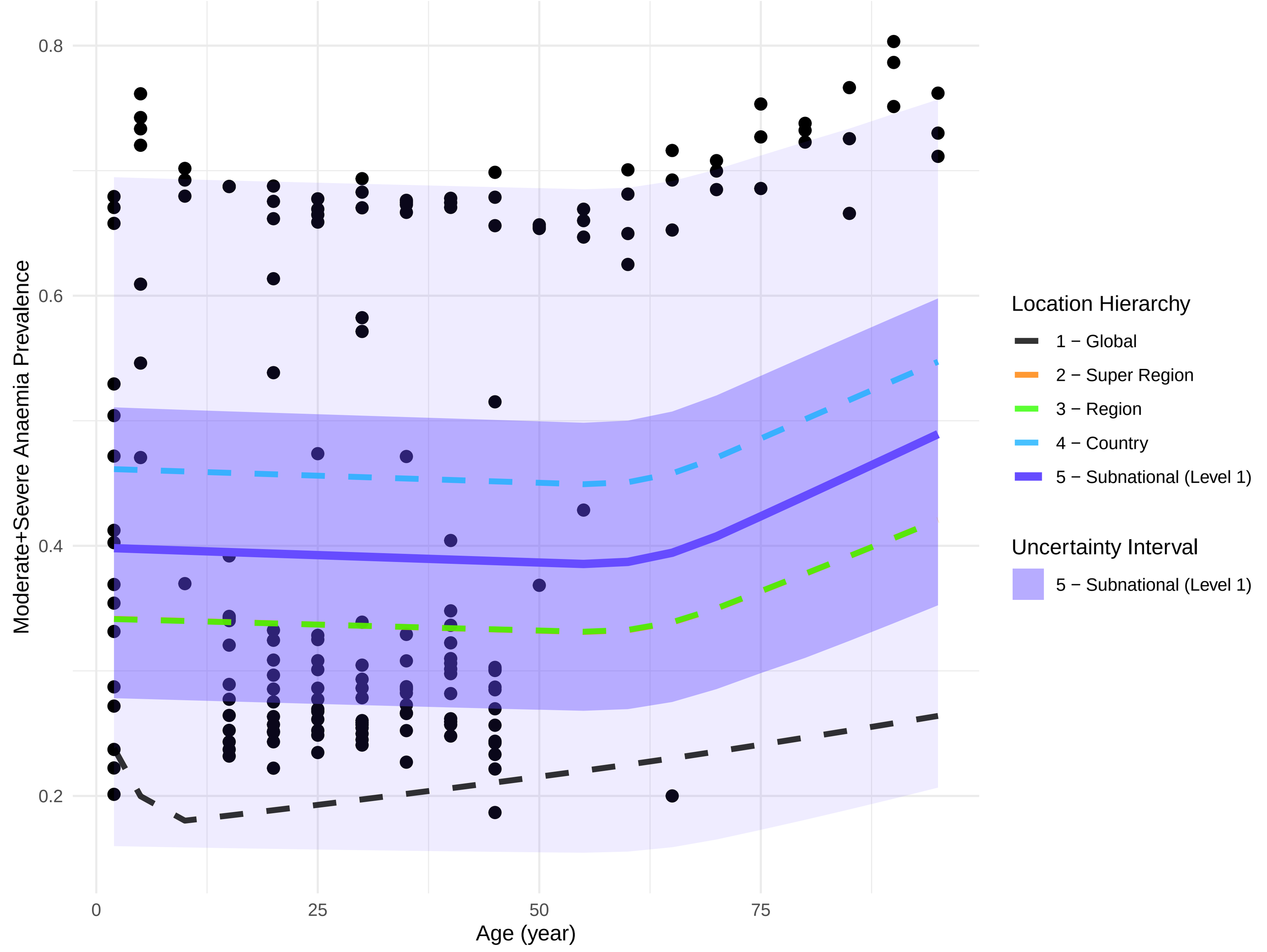

MR−BRT Age/Sex Fits − Non−pregnant Females 2+ Years Old − Moderate+Severe Anaemia Prevalence − Maharashtra, Rural
WHO−2001 Adjustment Method

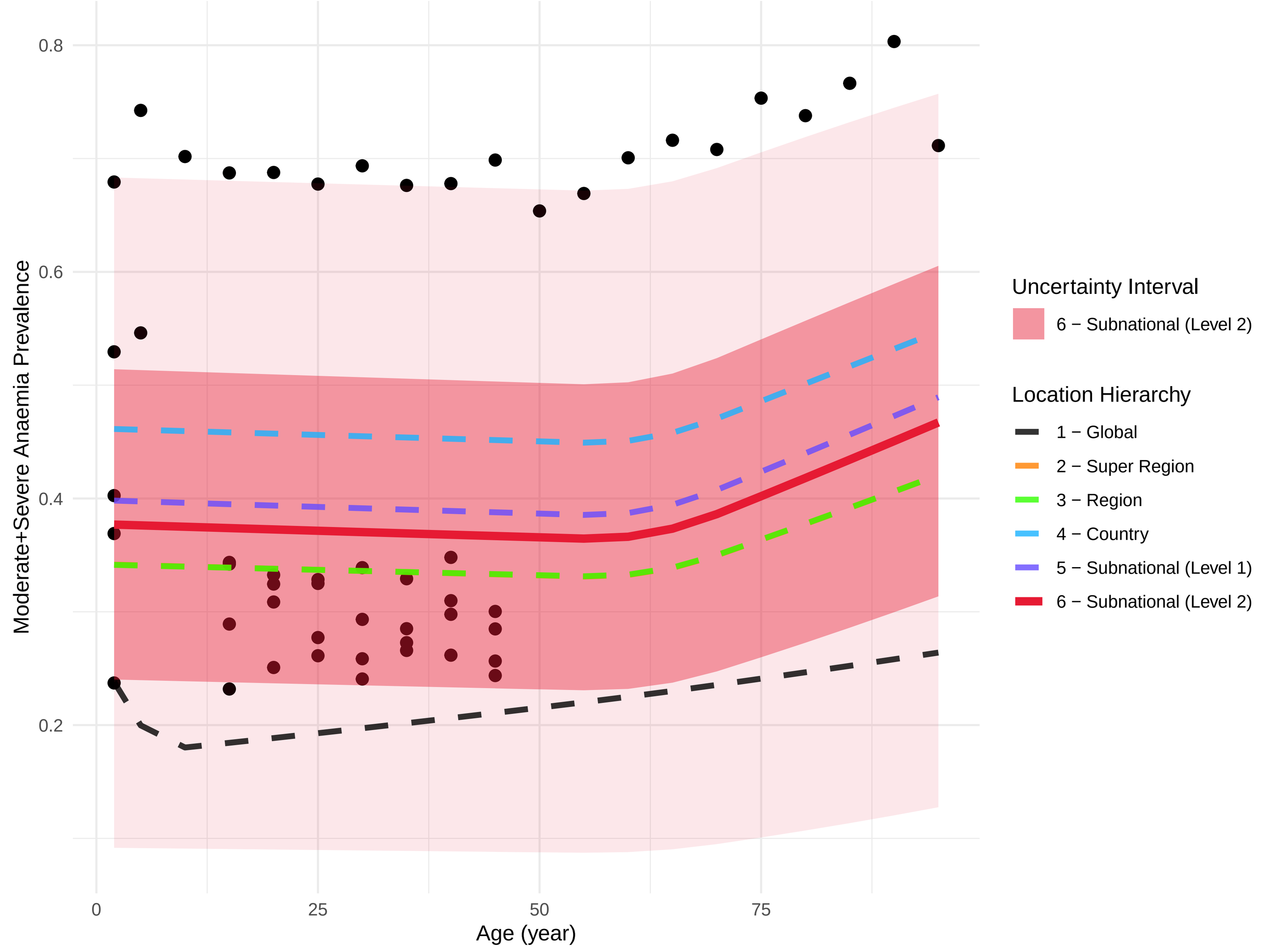

MR−BRT Age/Sex Fits − Non−pregnant Females 2+ Years Old − Moderate+Severe Anaemia Prevalence − Maharashtra, Urban
WHO−2001 Adjustment Method

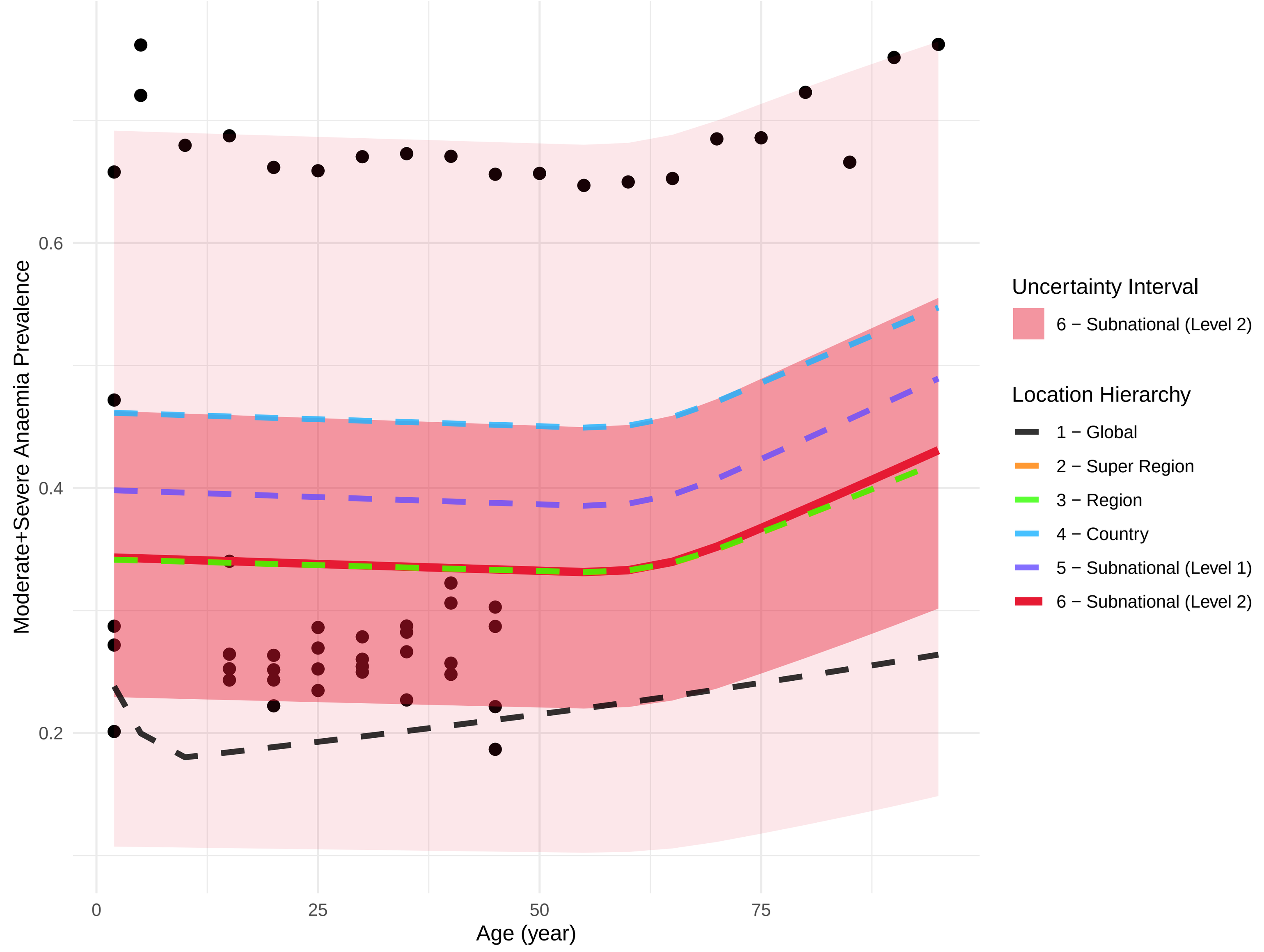

MR−BRT Age/Sex Fits − Non−pregnant Females 2+ Years Old − Moderate+Severe Anaemia Prevalence − Manipur
WHO−2001 Adjustment Method

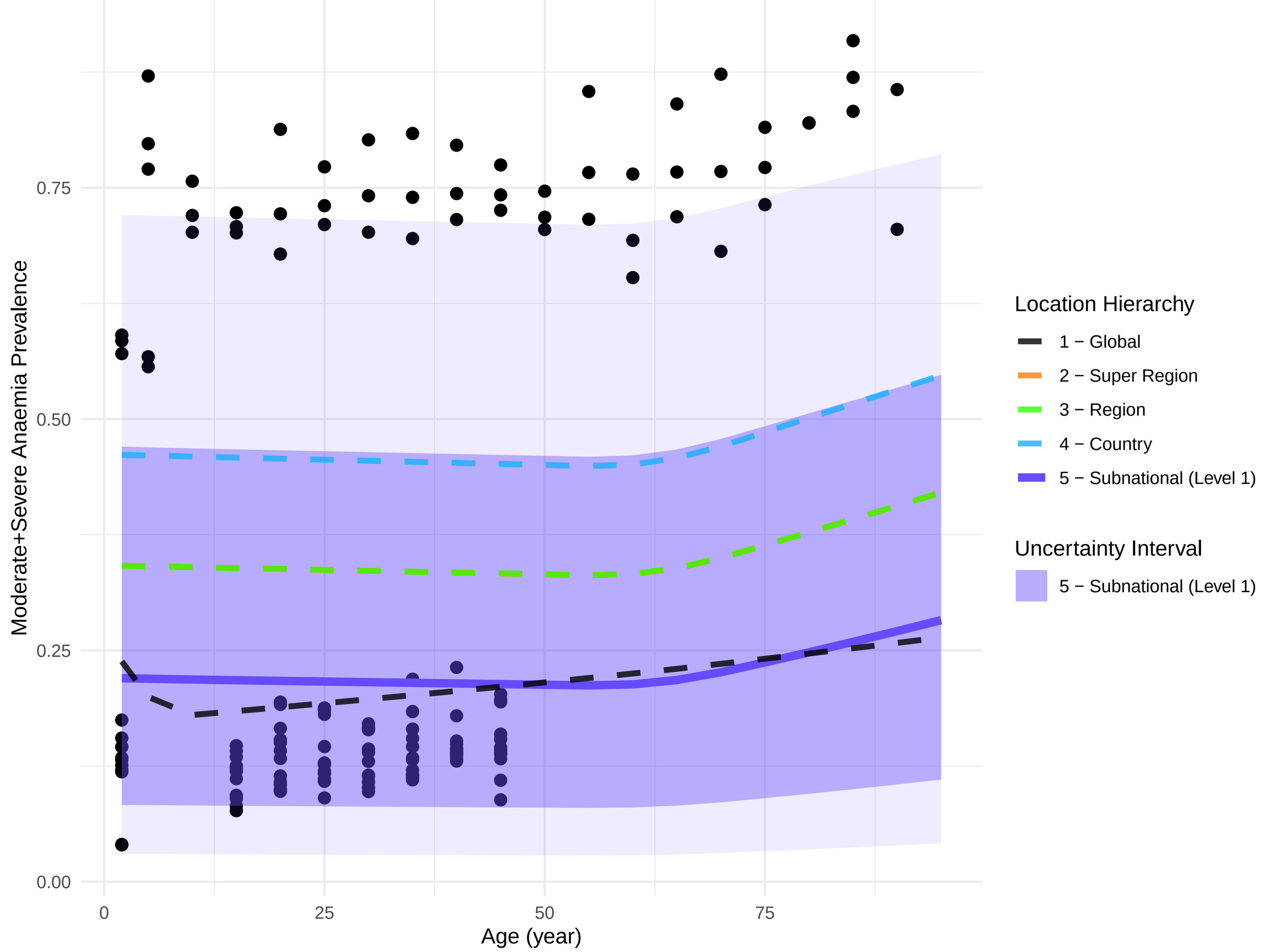

MR−BRT Age/Sex Fits − Non−pregnant Females 2+ Years Old − Moderate+Severe Anaemia Prevalence − Manipur, Rural
WHO−2001 Adjustment Method

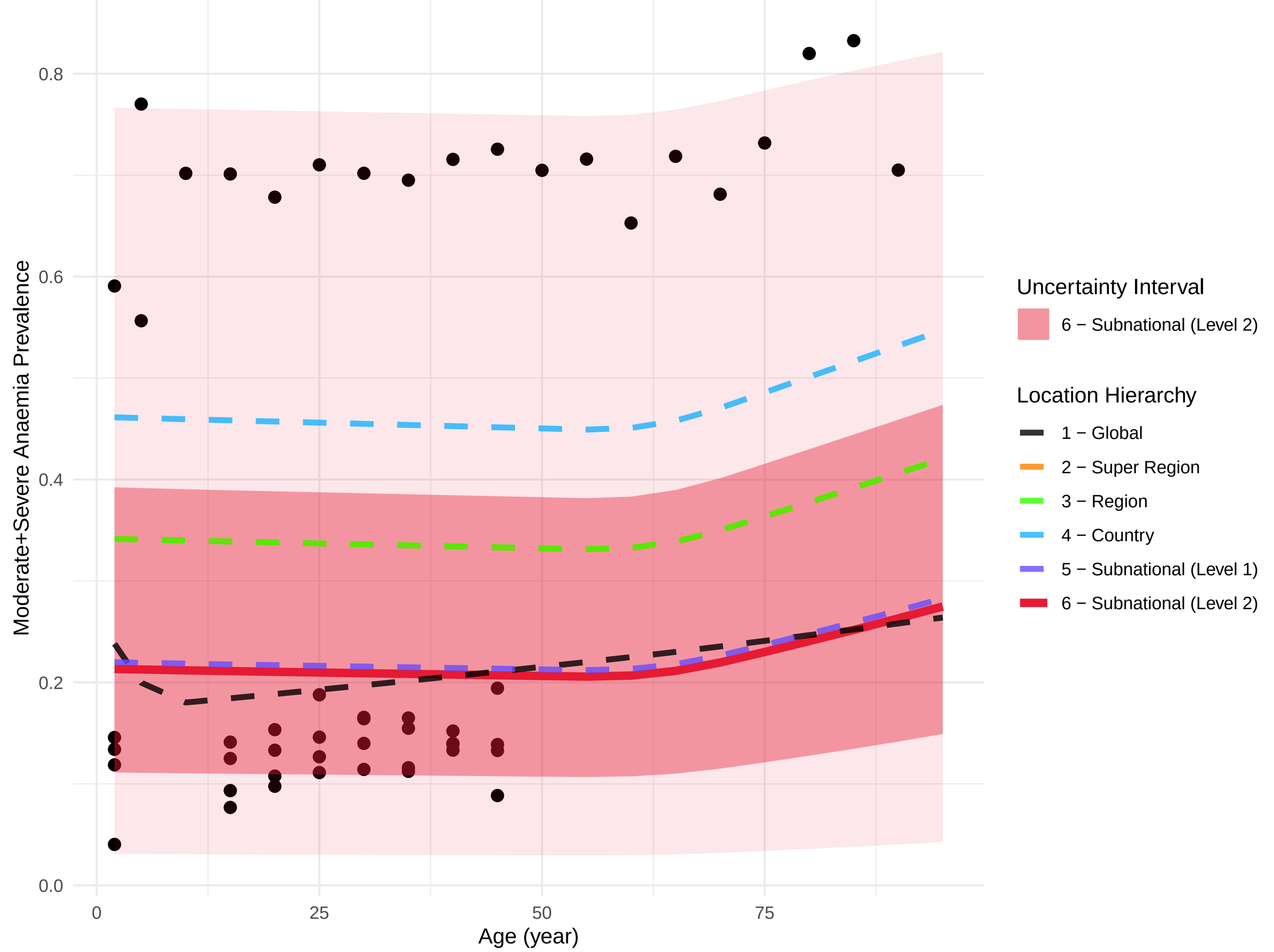

MR−BRT Age/Sex Fits − Non−pregnant Females 2+ Years Old − Moderate+Severe Anaemia Prevalence − Manipur, Urban
WHO−2001 Adjustment Method

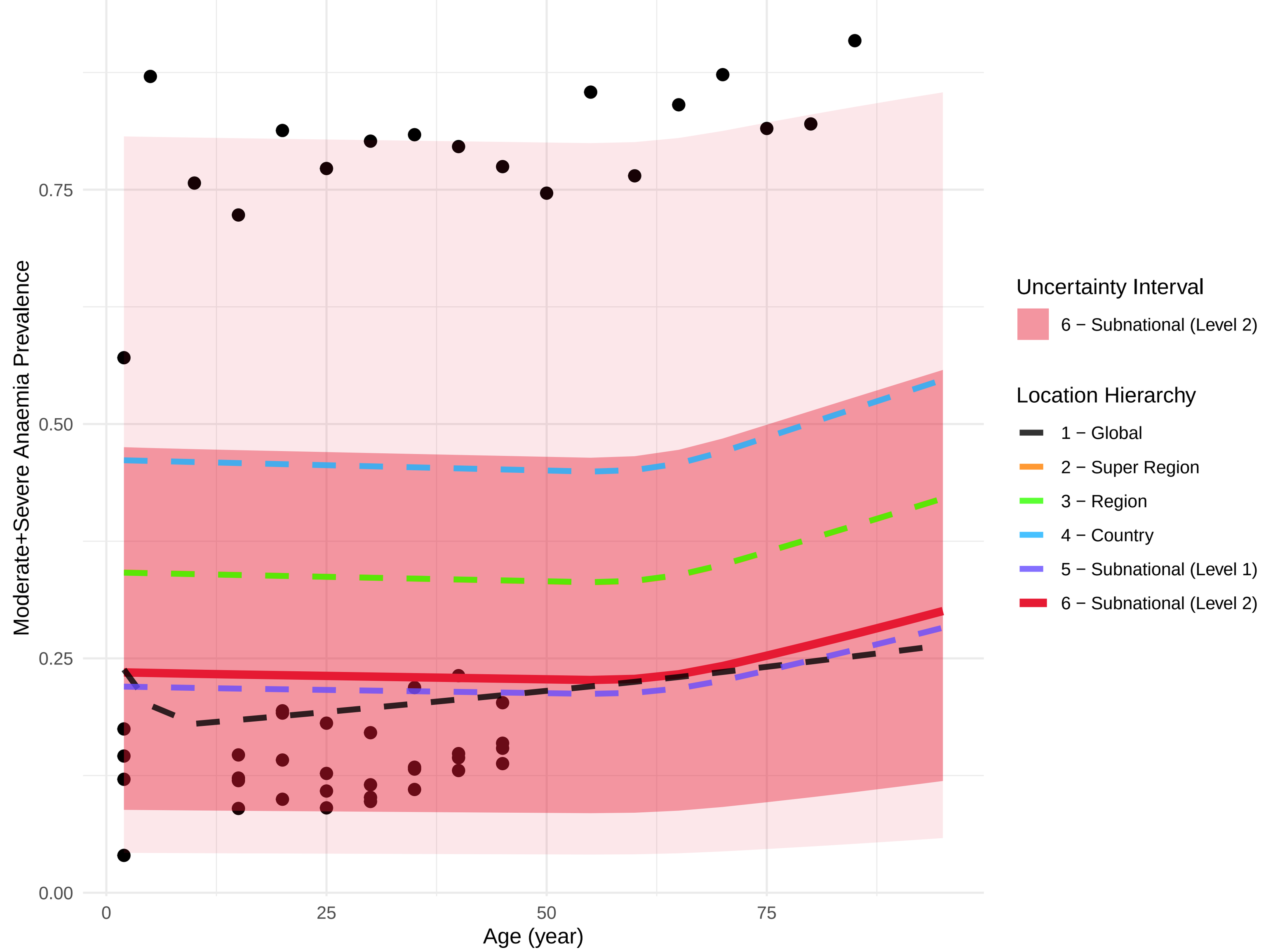

MR−BRT Age/Sex Fits − Non−pregnant Females 2+ Years Old − Moderate+Severe Anaemia Prevalence − Meghalaya
WHO−2001 Adjustment Method

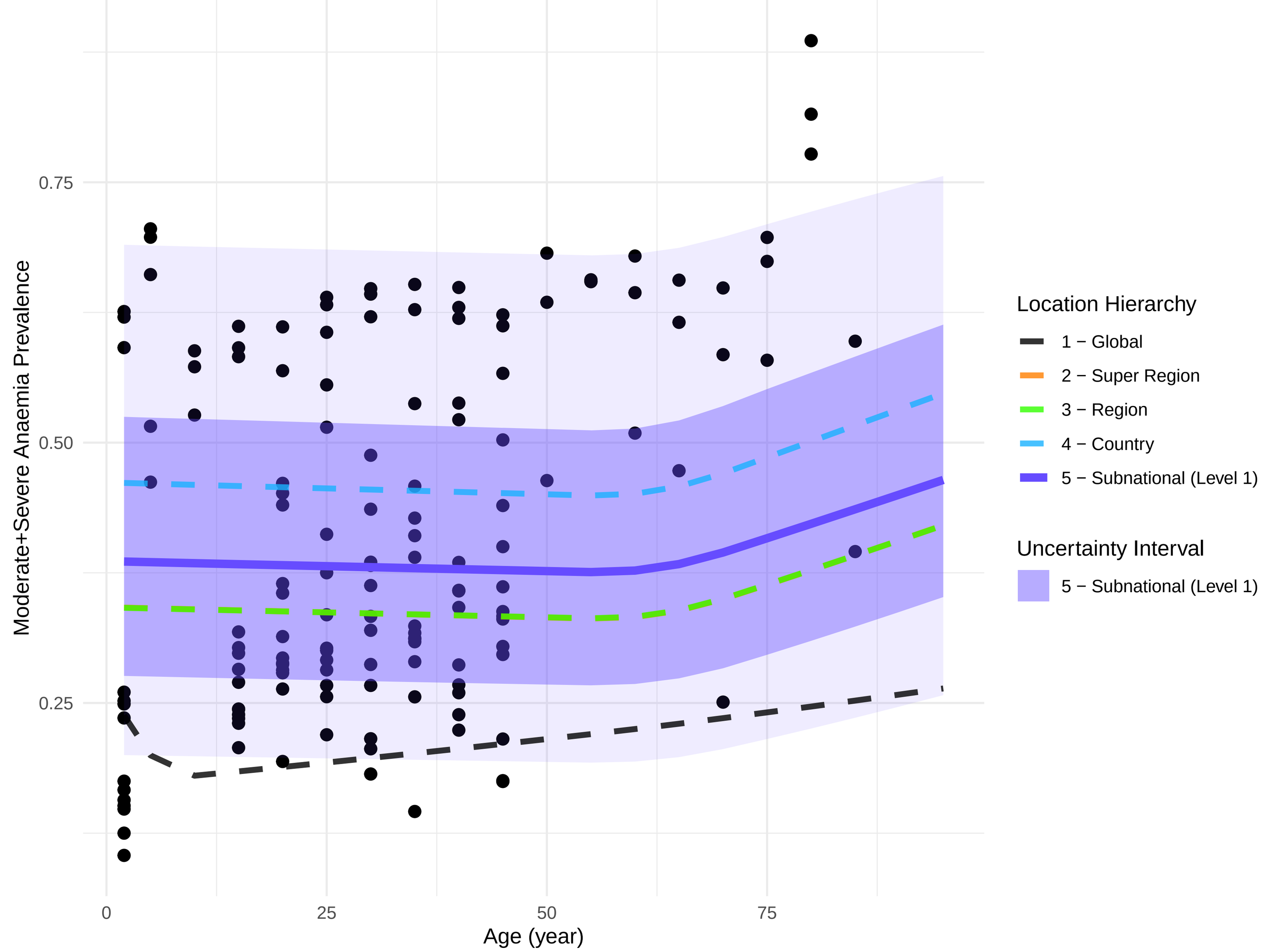

MR−BRT Age/Sex Fits − Non−pregnant Females 2+ Years Old − Moderate+Severe Anaemia Prevalence − Meghalaya, Rural
WHO−2001 Adjustment Method

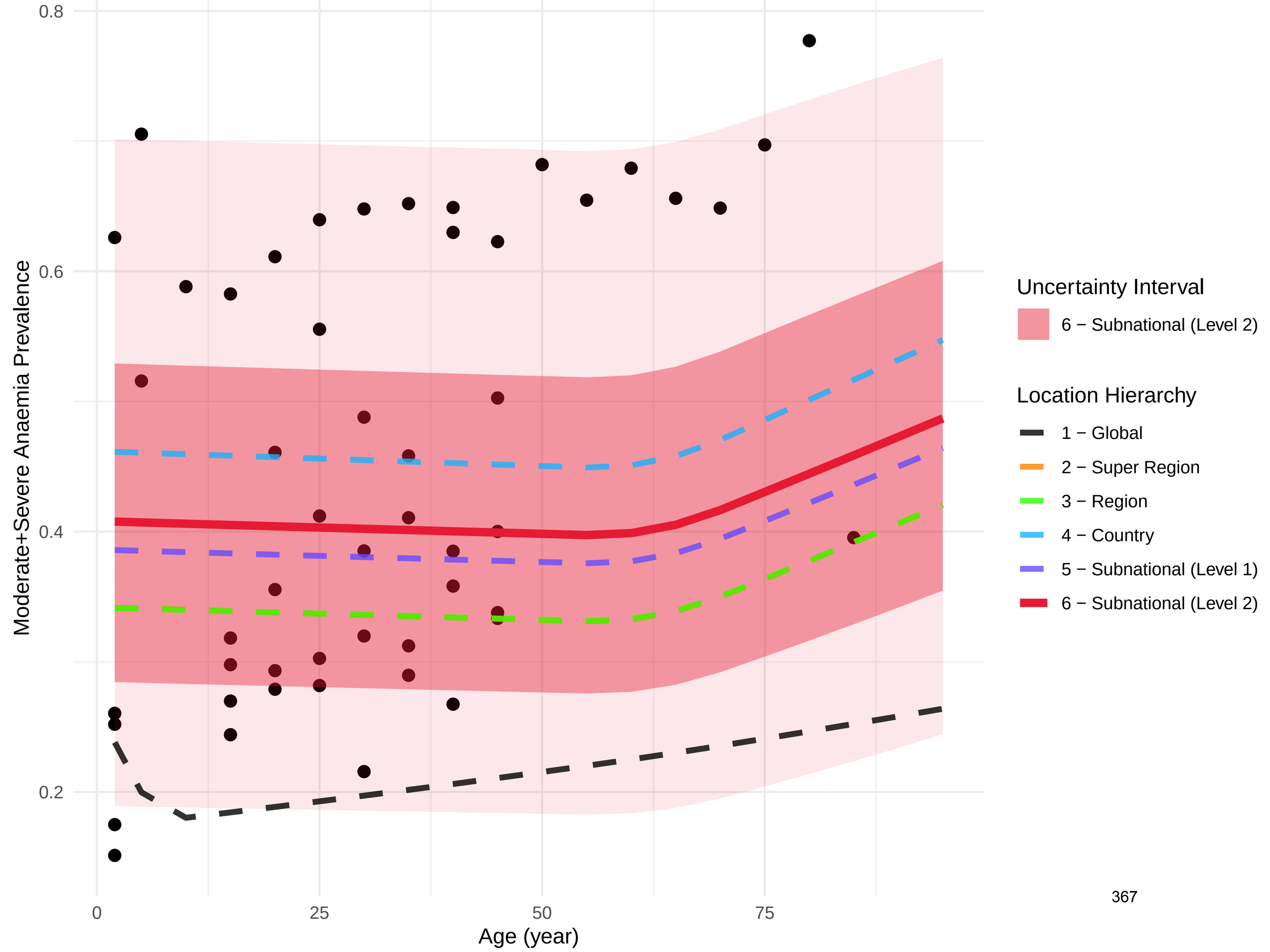

MR−BRT Age/Sex Fits − Non−pregnant Females 2+ Years Old − Moderate+Severe Anaemia Prevalence − Meghalaya, Urban
WHO−2001 Adjustment Method

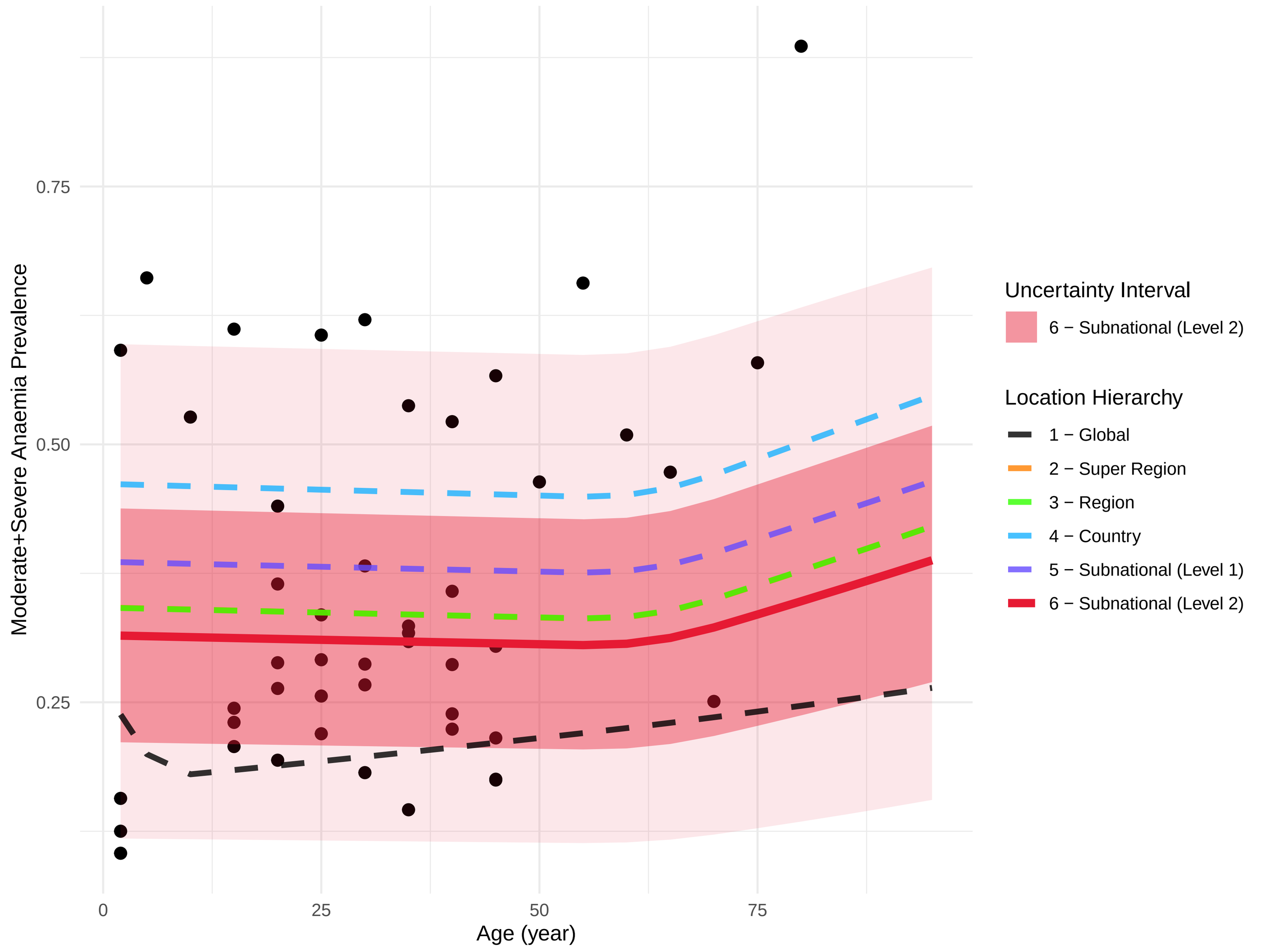

MR−BRT Age/Sex Fits − Non−pregnant Females 2+ Years Old − Moderate+Severe Anaemia Prevalence − Mizoram
WHO−2001 Adjustment Method

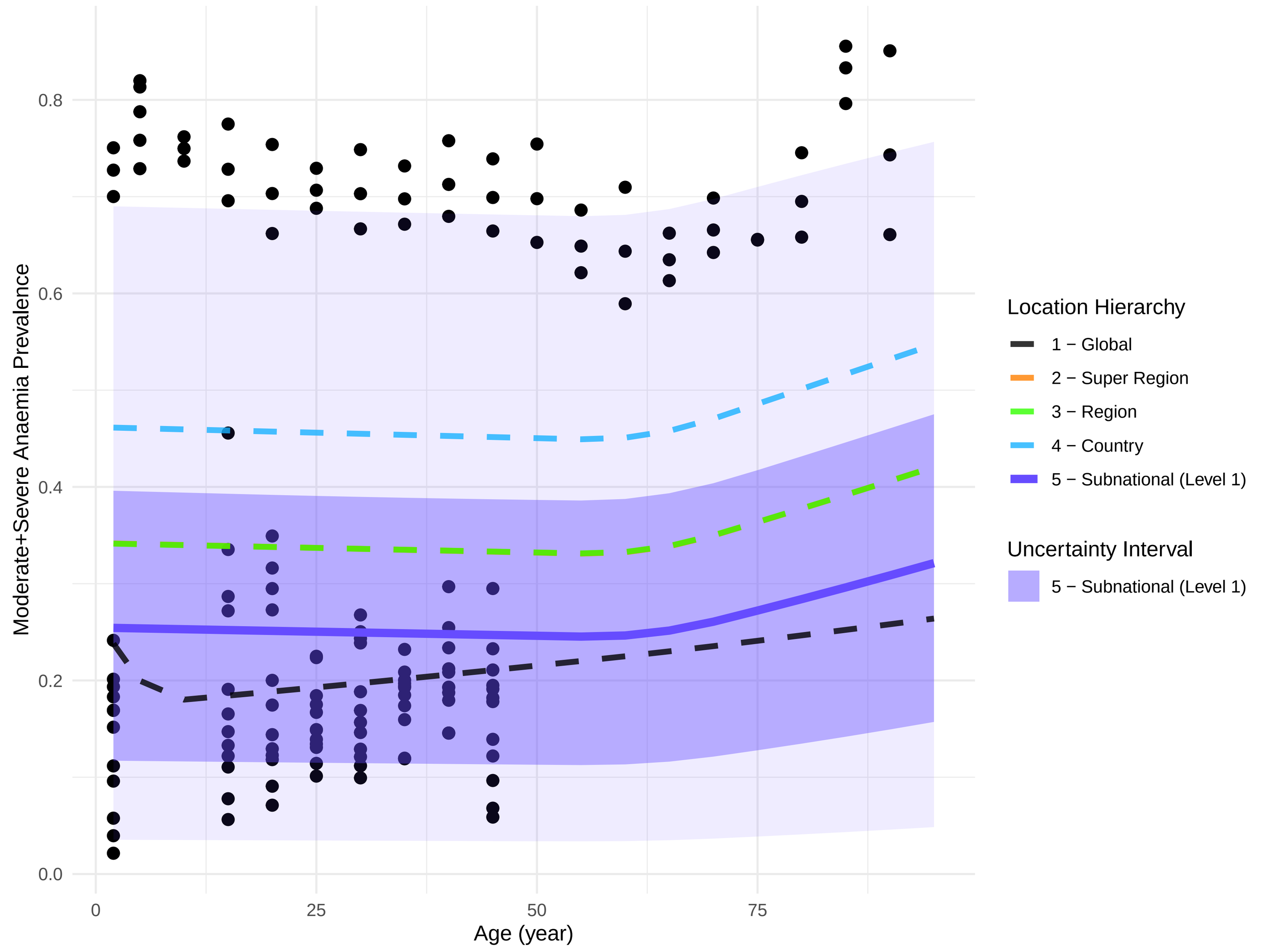

MR−BRT Age/Sex Fits − Non−pregnant Females 2+ Years Old − Moderate+Severe Anaemia Prevalence − Mizoram, Rural
WHO−2001 Adjustment Method

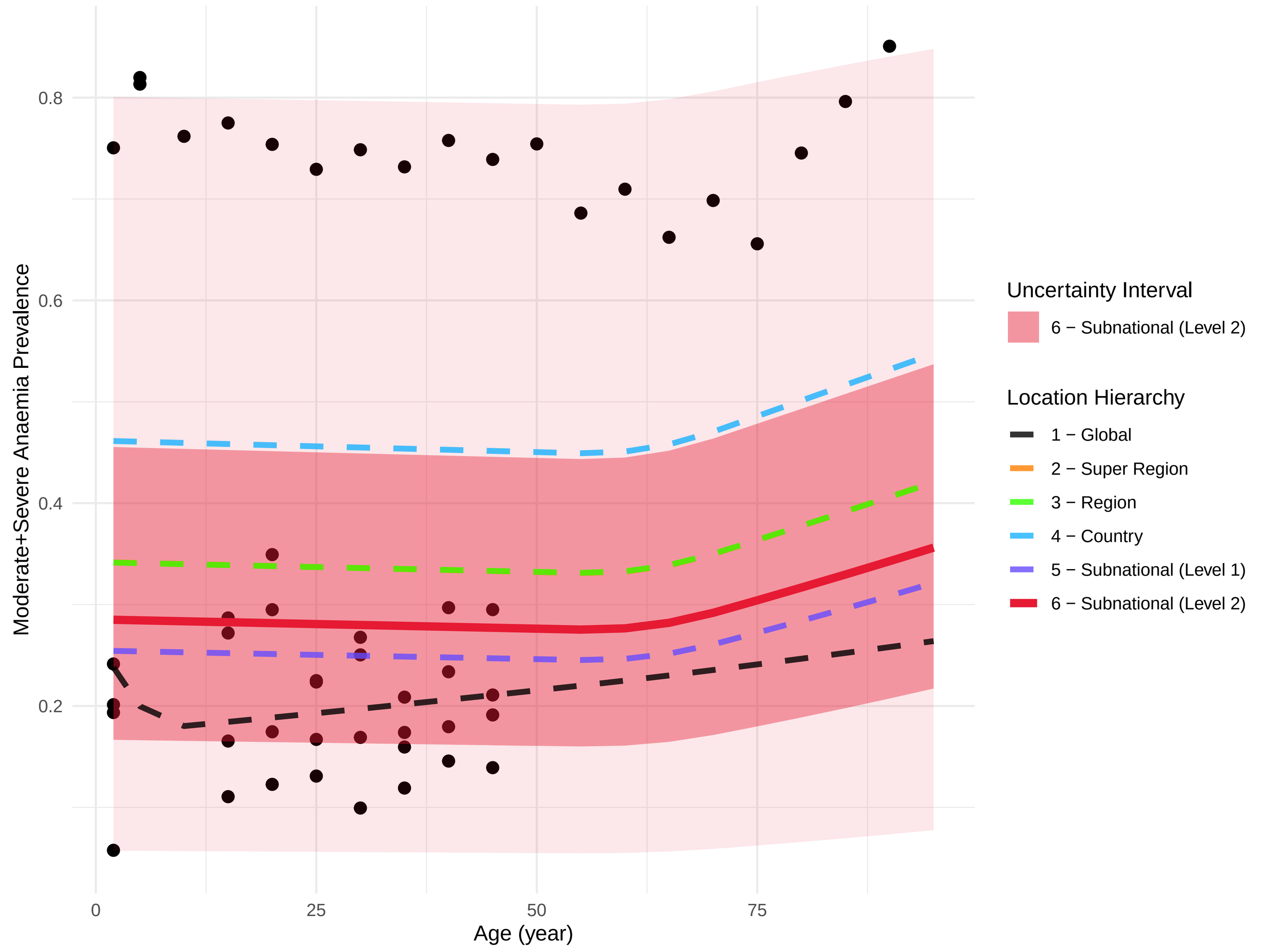

MR−BRT Age/Sex Fits − Non−pregnant Females 2+ Years Old − Moderate+Severe Anaemia Prevalence − Mizoram, Urban
WHO−2001 Adjustment Method

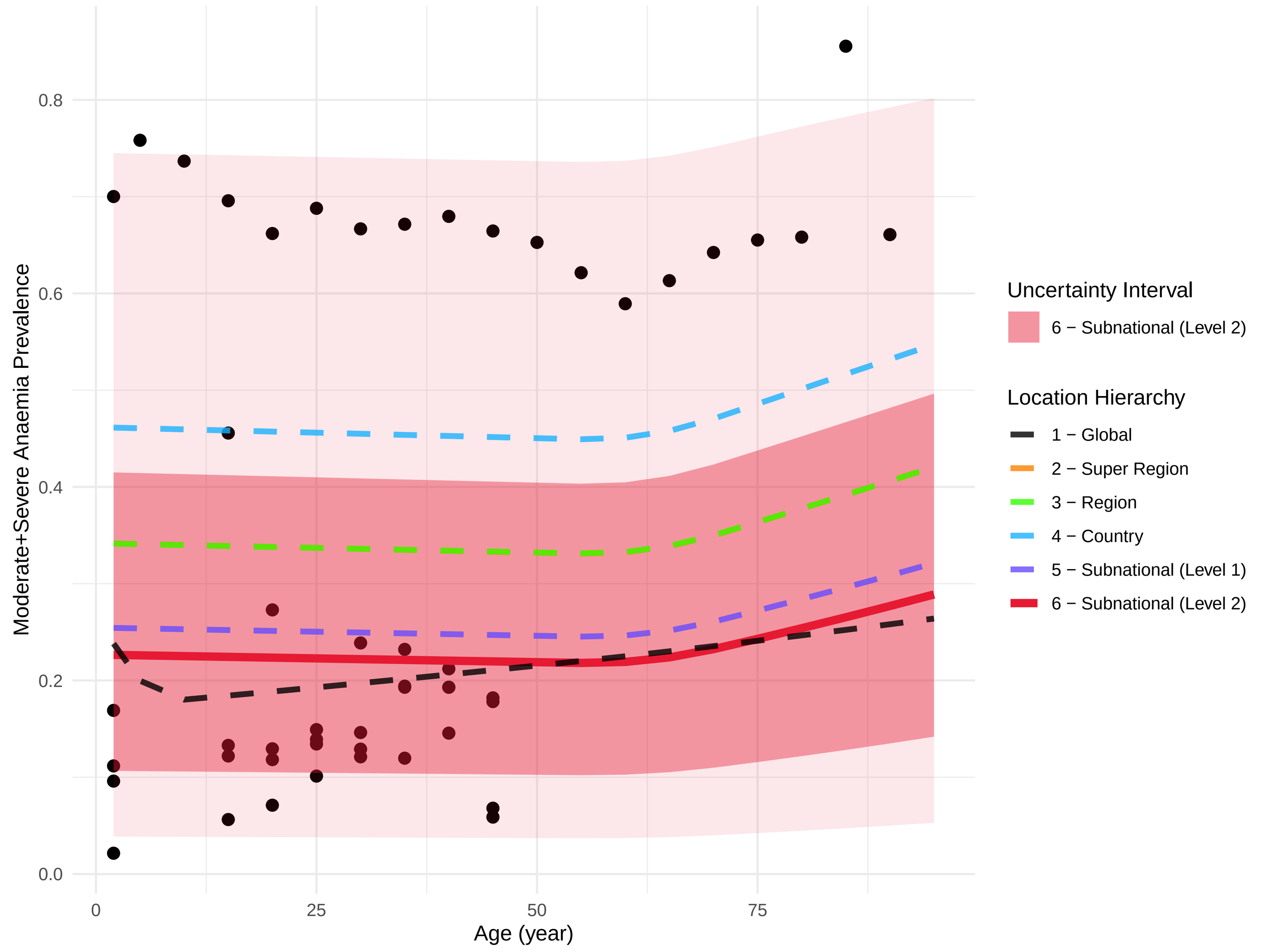

MR−BRT Age/Sex Fits − Non−pregnant Females 2+ Years Old − Moderate+Severe Anaemia Prevalence − Nagaland
WHO−2001 Adjustment Method

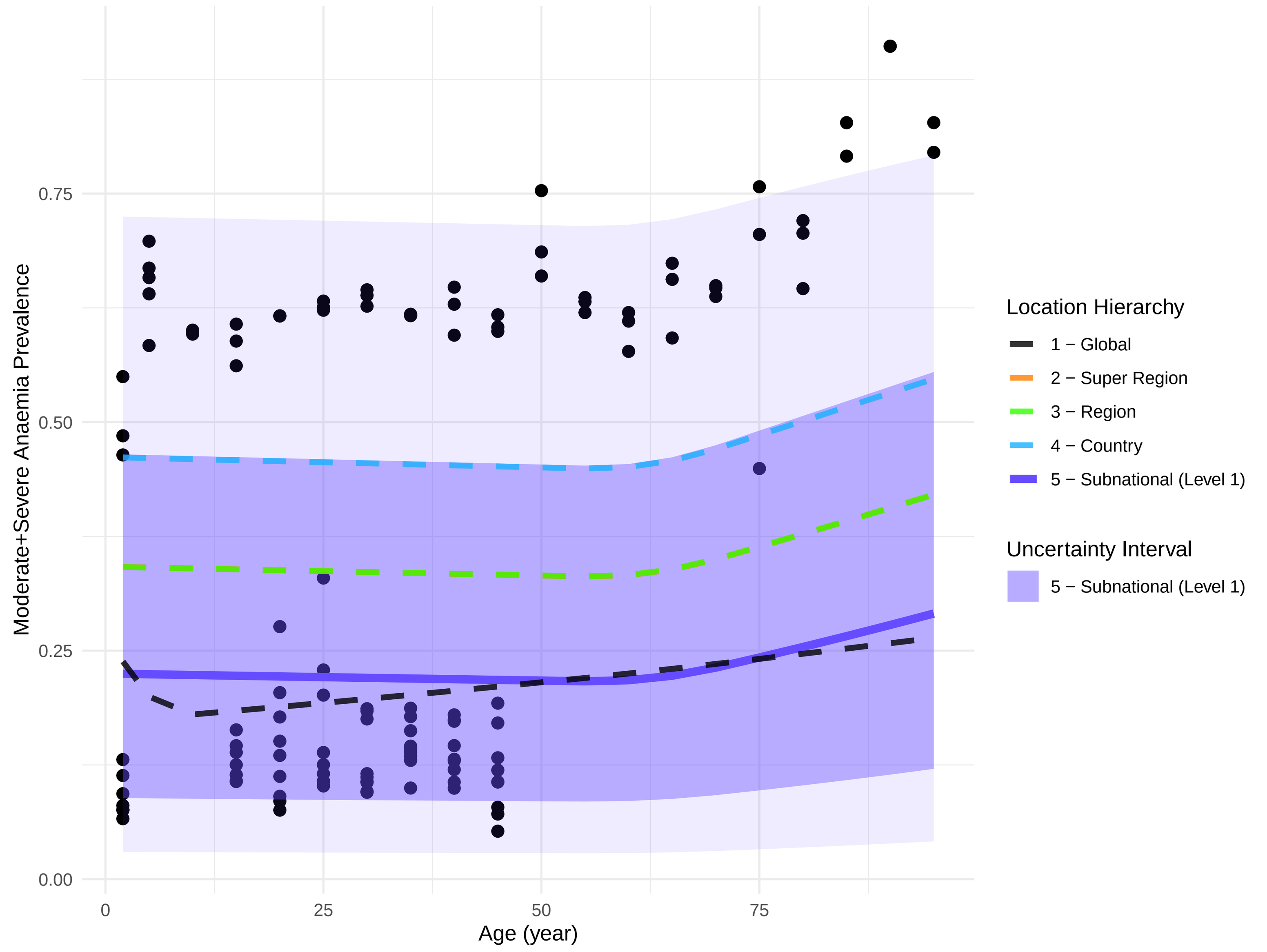

MR−BRT Age/Sex Fits − Non−pregnant Females 2+ Years Old − Moderate+Severe Anaemia Prevalence − Nagaland, Rural
WHO−2001 Adjustment Method

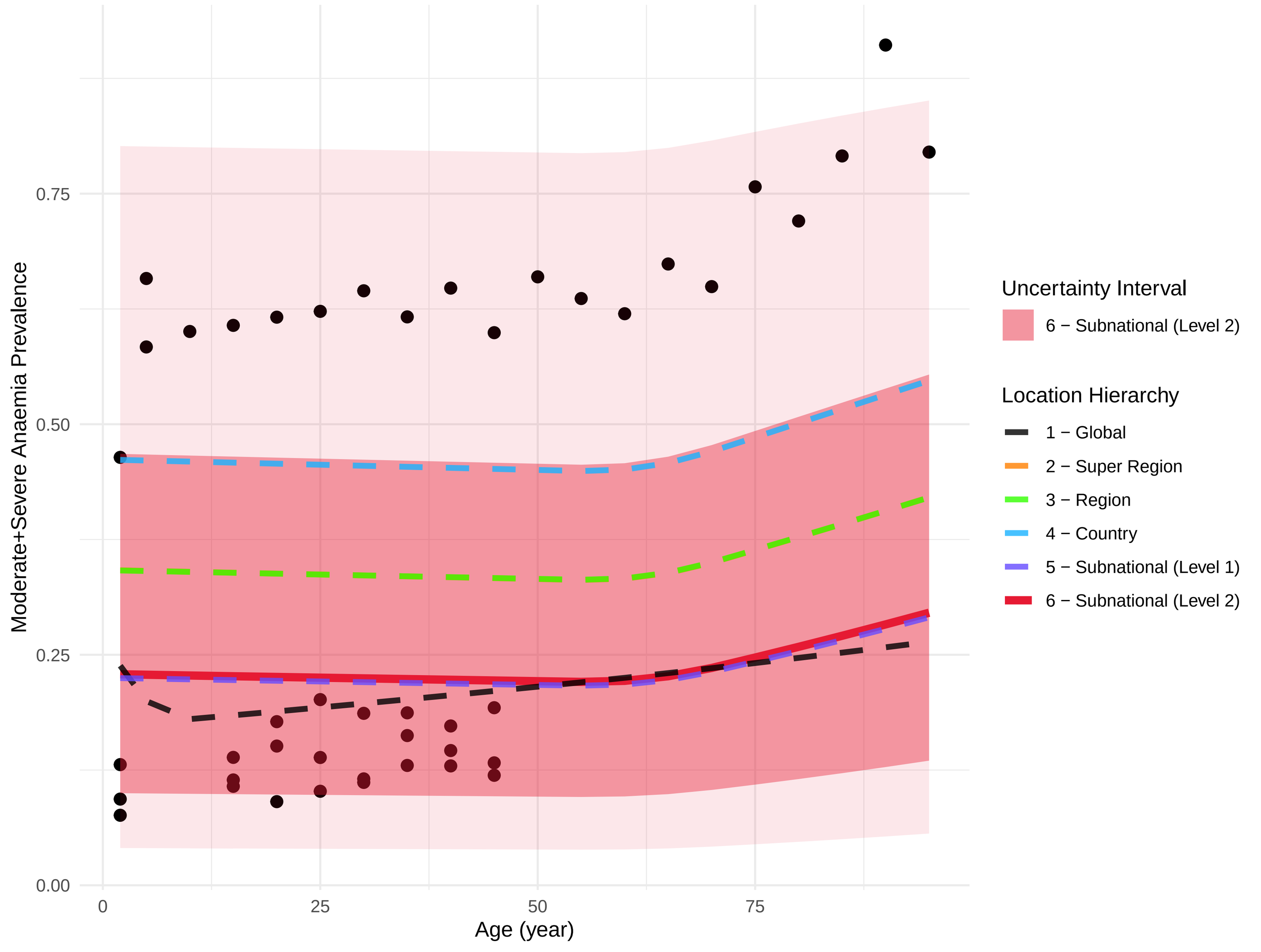

MR−BRT Age/Sex Fits − Non−pregnant Females 2+ Years Old − Moderate+Severe Anaemia Prevalence − Nagaland, Urban
WHO−2001 Adjustment Method

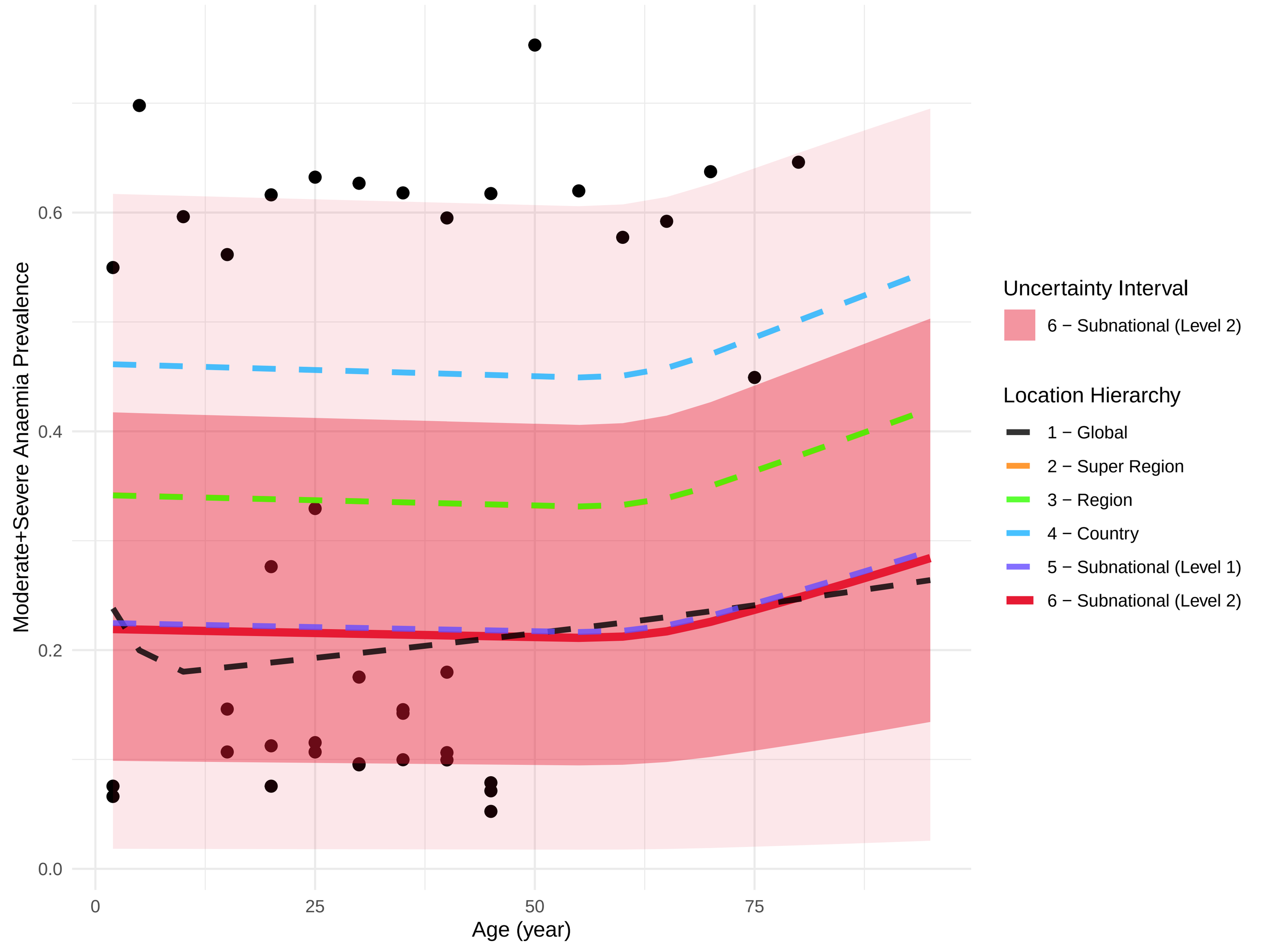

MR−BRT Age/Sex Fits − Non−pregnant Females 2+ Years Old − Moderate+Severe Anaemia Prevalence − Odisha
WHO−2001 Adjustment Method

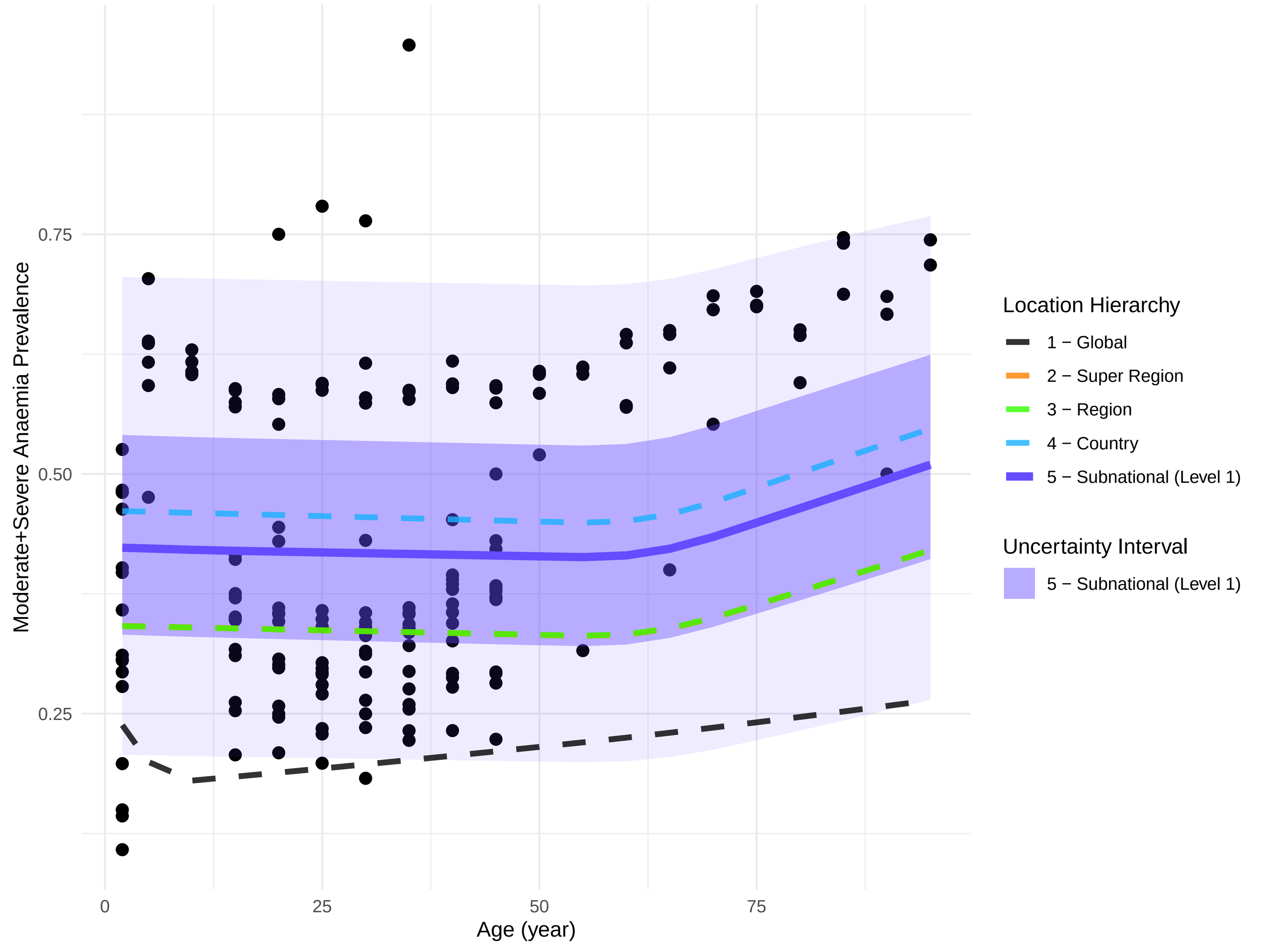

MR−BRT Age/Sex Fits − Non−pregnant Females 2+ Years Old − Moderate+Severe Anaemia Prevalence − Odisha, Rural
WHO−2001 Adjustment Method

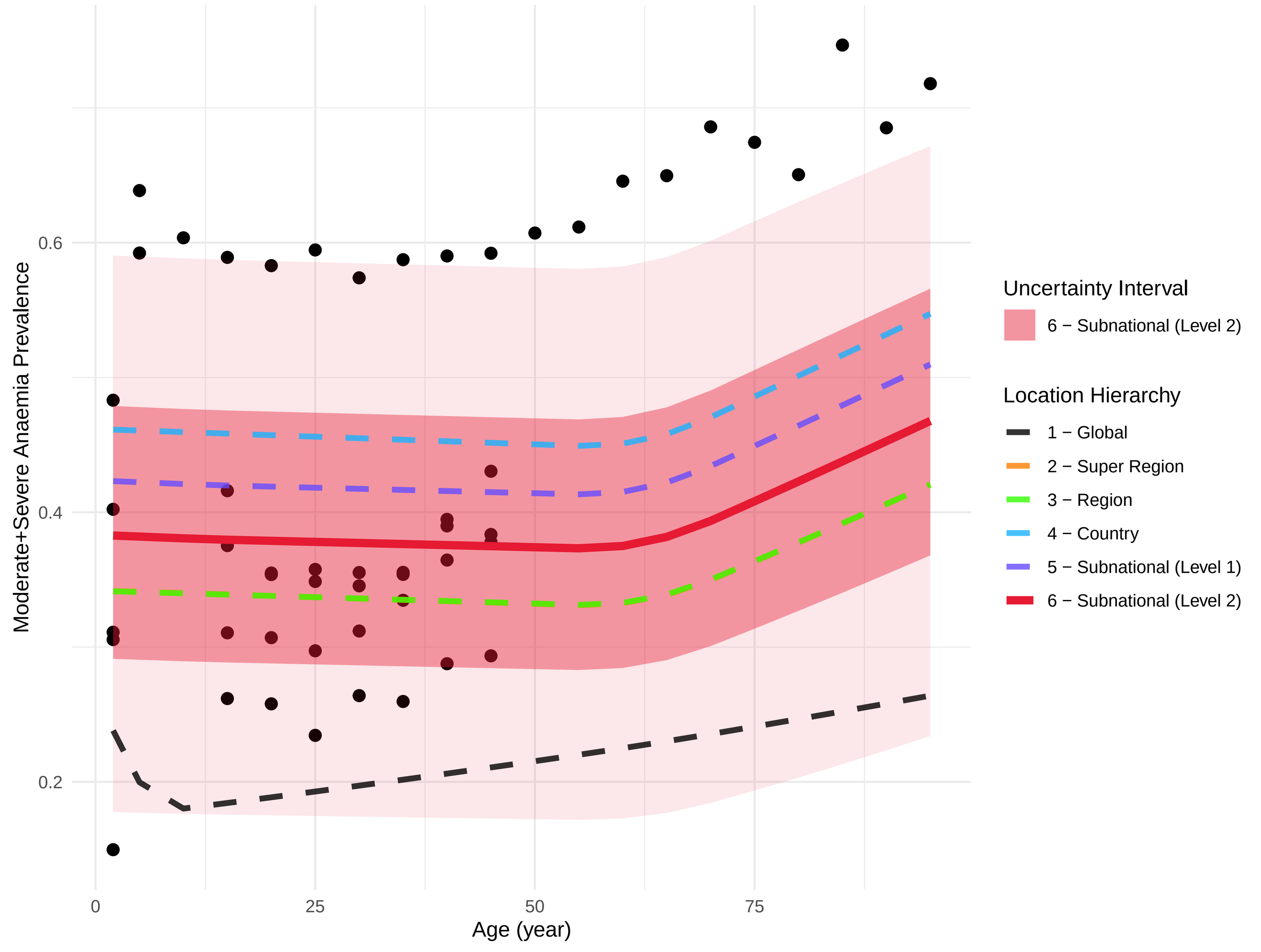

MR−BRT Age/Sex Fits − Non−pregnant Females 2+ Years Old − Moderate+Severe Anaemia Prevalence − Odisha, Urban
WHO−2001 Adjustment Method

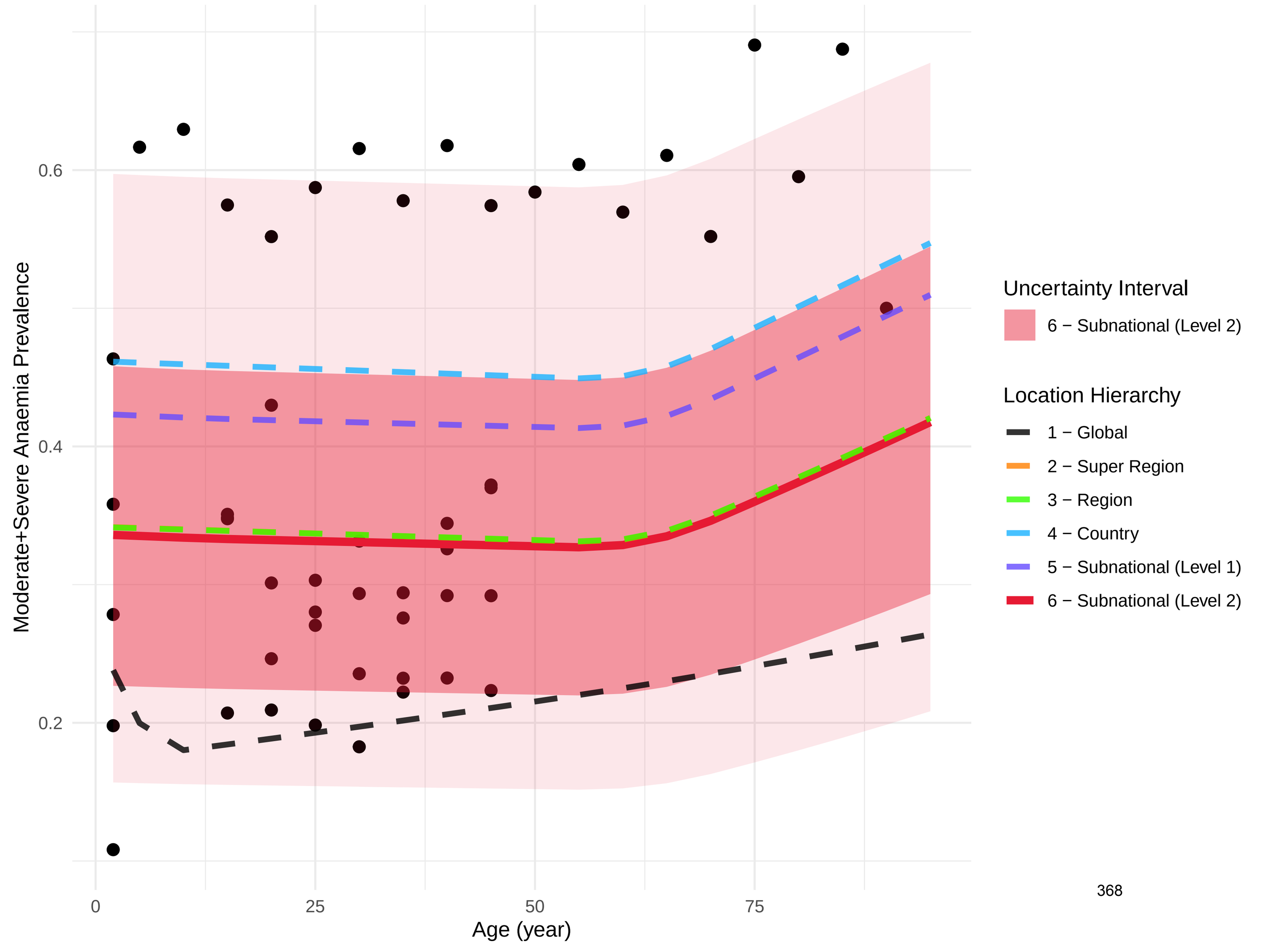

MR−BRT Age/Sex Fits − Non−pregnant Females 2+ Years Old − Moderate+Severe Anaemia Prevalence − Other Union Territories
WHO−2001 Adjustment Method

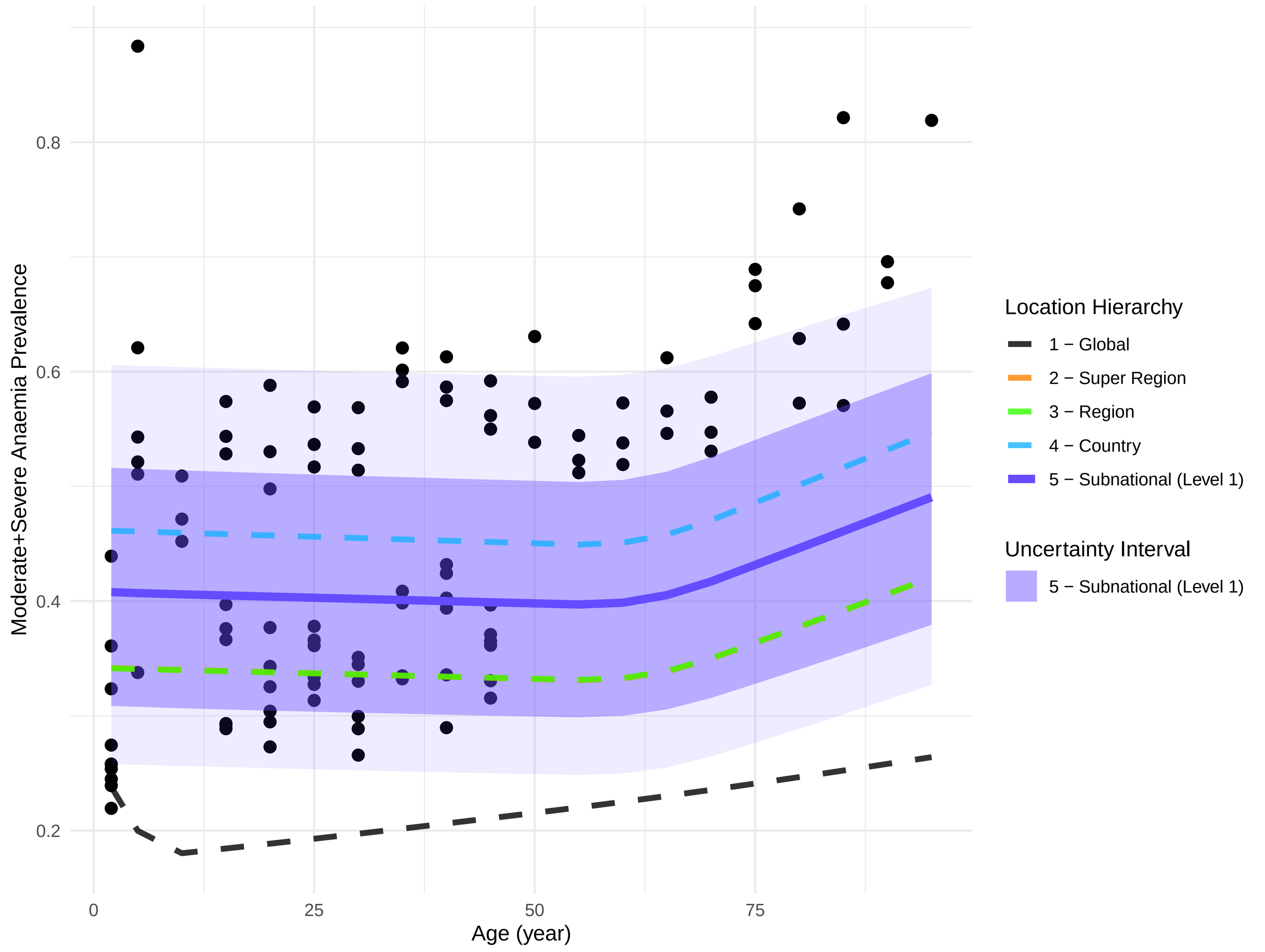

MR−BRT Age/Sex Fits − Non−pregnant Females 2+ Years Old − Moderate+Severe Anaemia Prevalence − Other Union Territories, Rural
WHO−2001 Adjustment Method

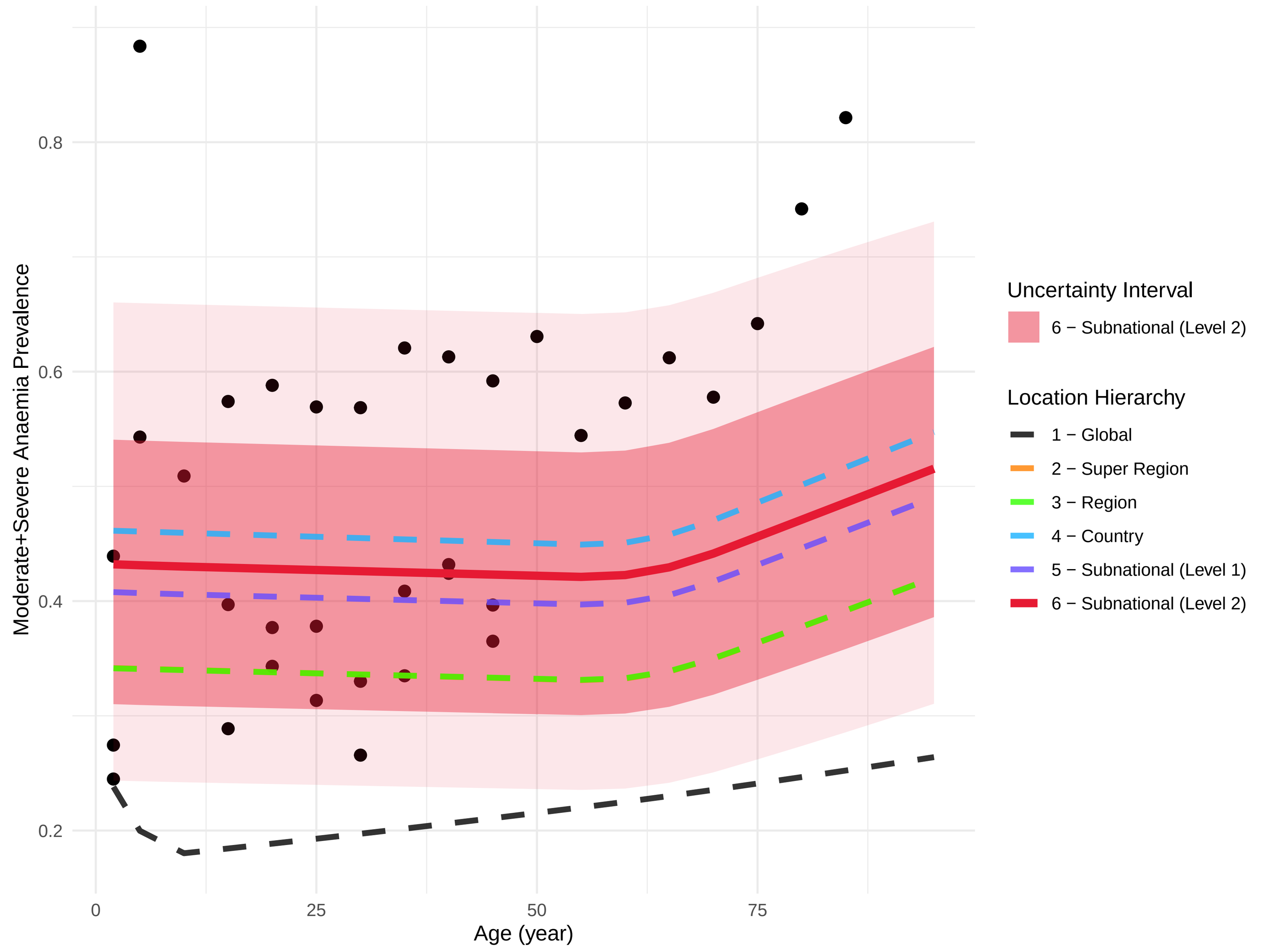

MR−BRT Age/Sex Fits − Non−pregnant Females 2+ Years Old − Moderate+Severe Anaemia Prevalence − Other Union Territories, Urban
WHO−2001 Adjustment Method

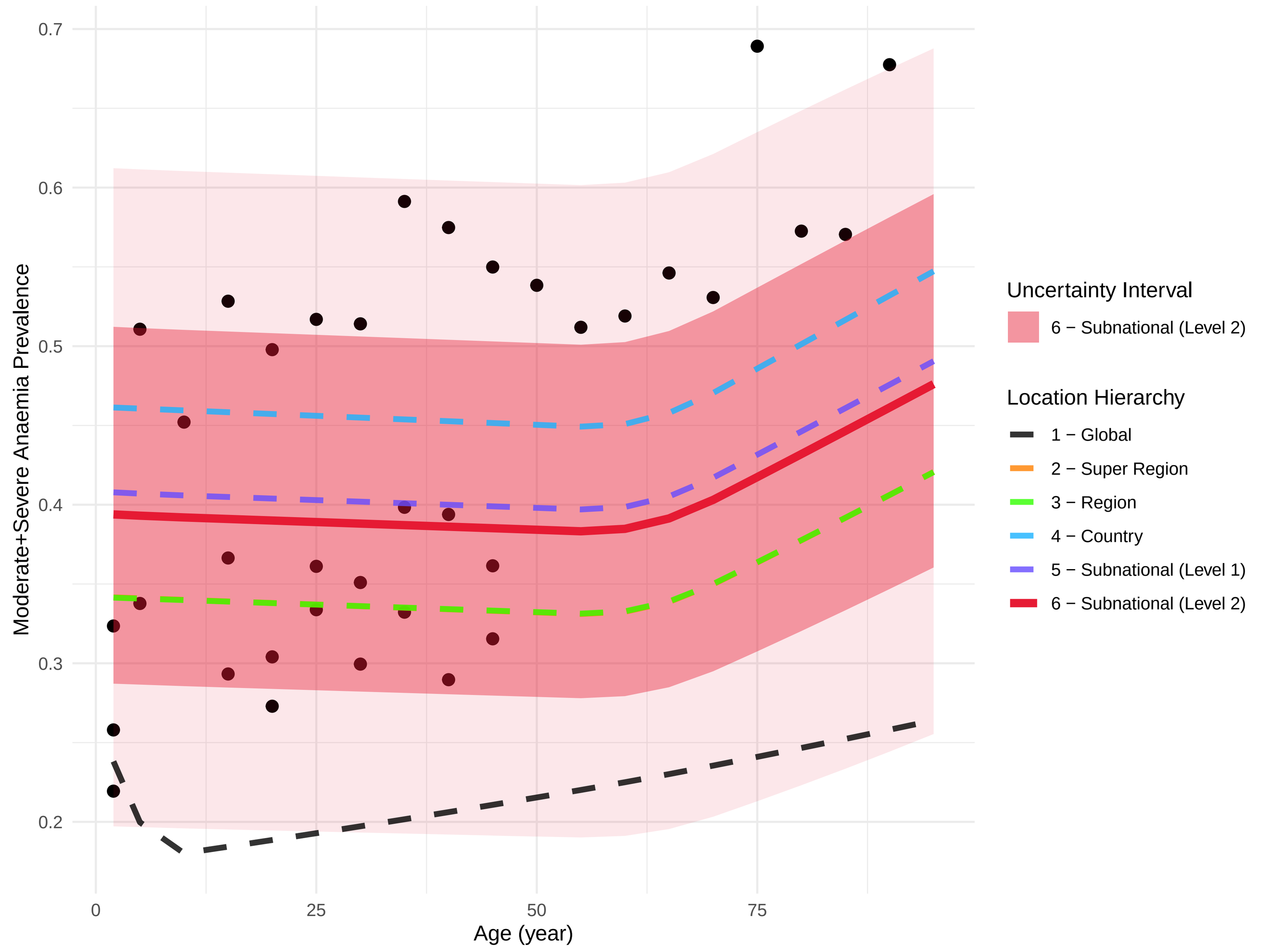

MR−BRT Age/Sex Fits − Non−pregnant Females 2+ Years Old − Moderate+Severe Anaemia Prevalence − Punjab
WHO−2001 Adjustment Method

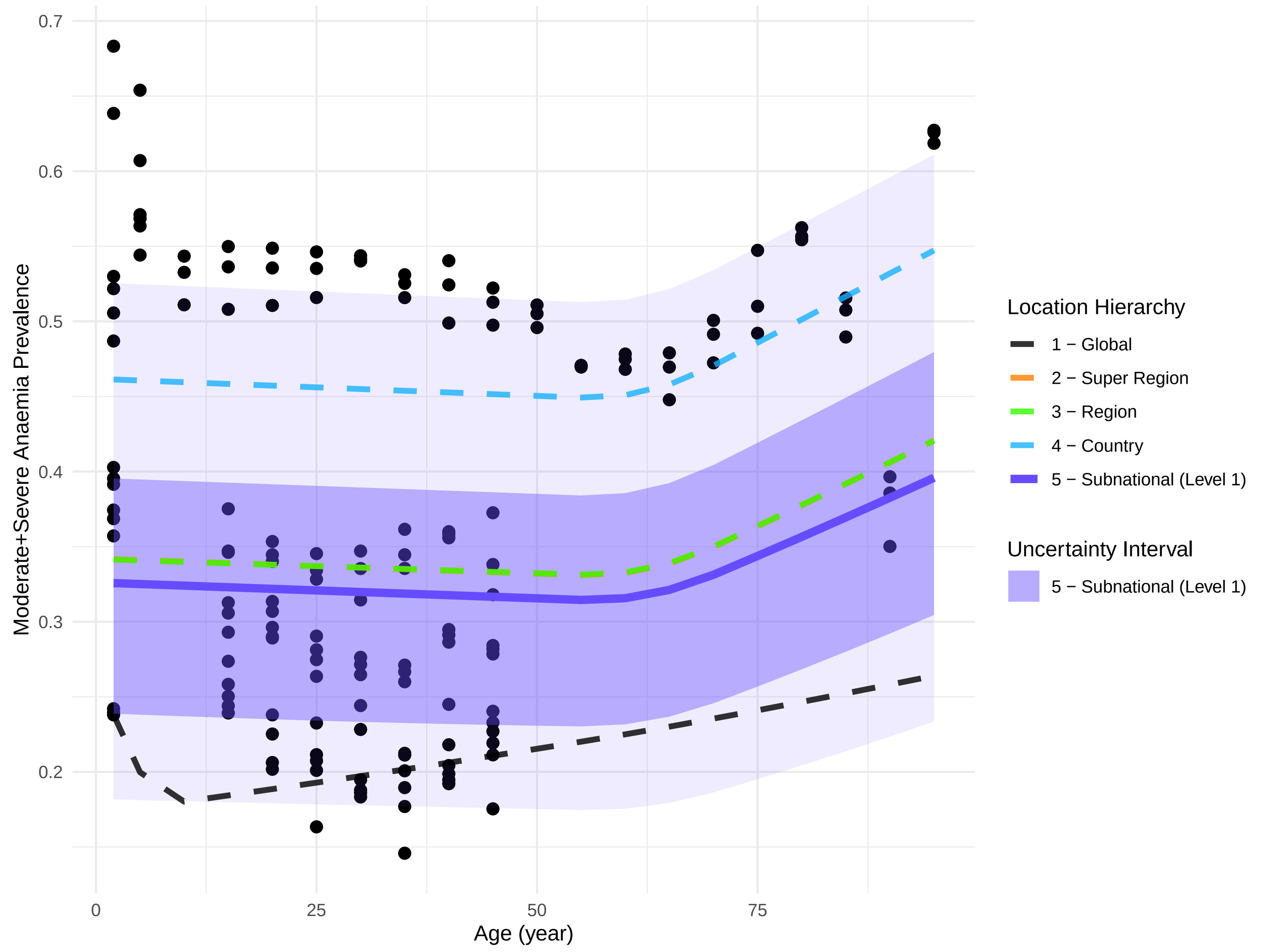

MR−BRT Age/Sex Fits − Non−pregnant Females 2+ Years Old − Moderate+Severe Anaemia Prevalence − Punjab, Rural
WHO−2001 Adjustment Method

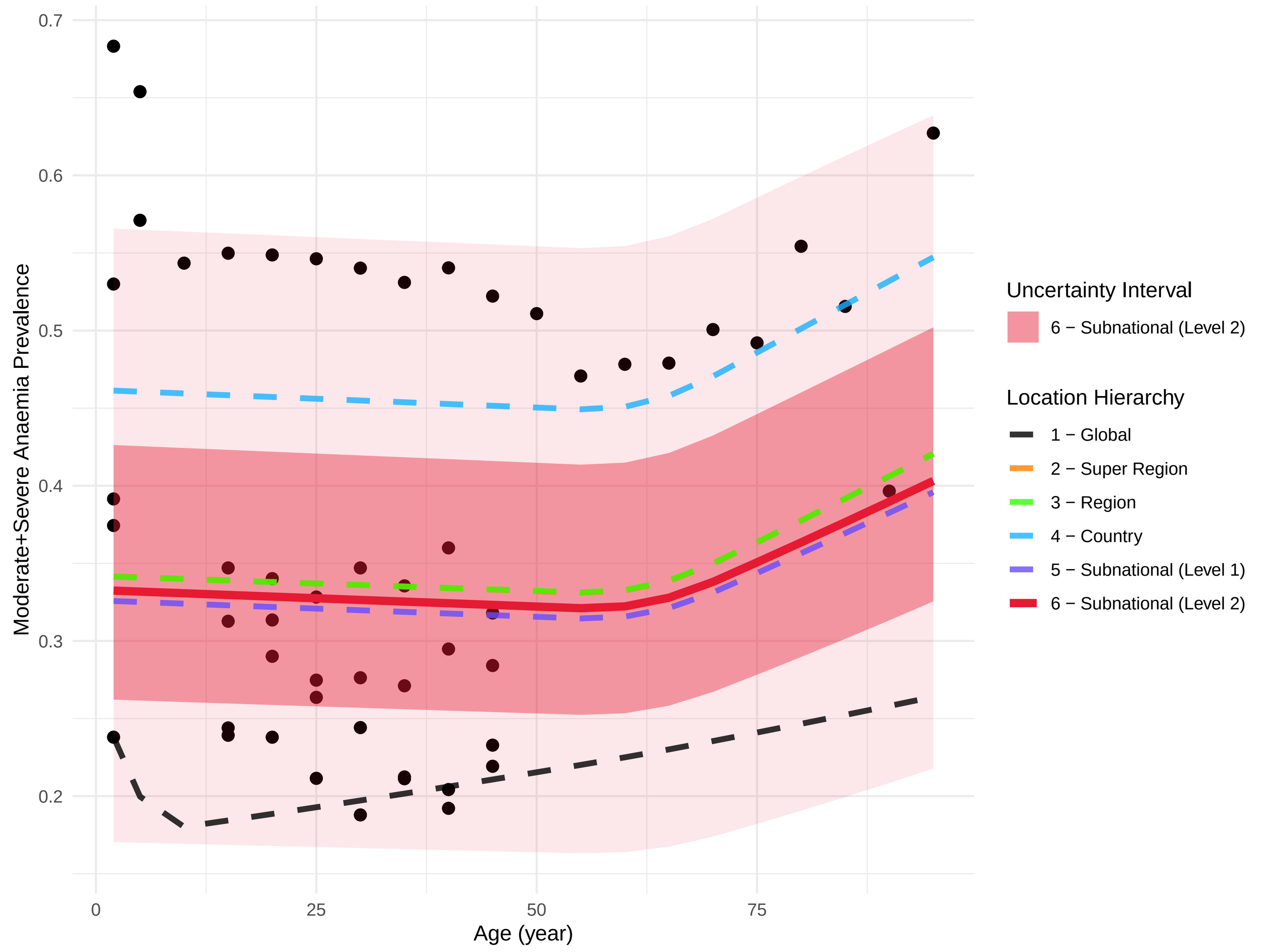

MR−BRT Age/Sex Fits − Non−pregnant Females 2+ Years Old − Moderate+Severe Anaemia Prevalence − Punjab, Urban
WHO−2001 Adjustment Method

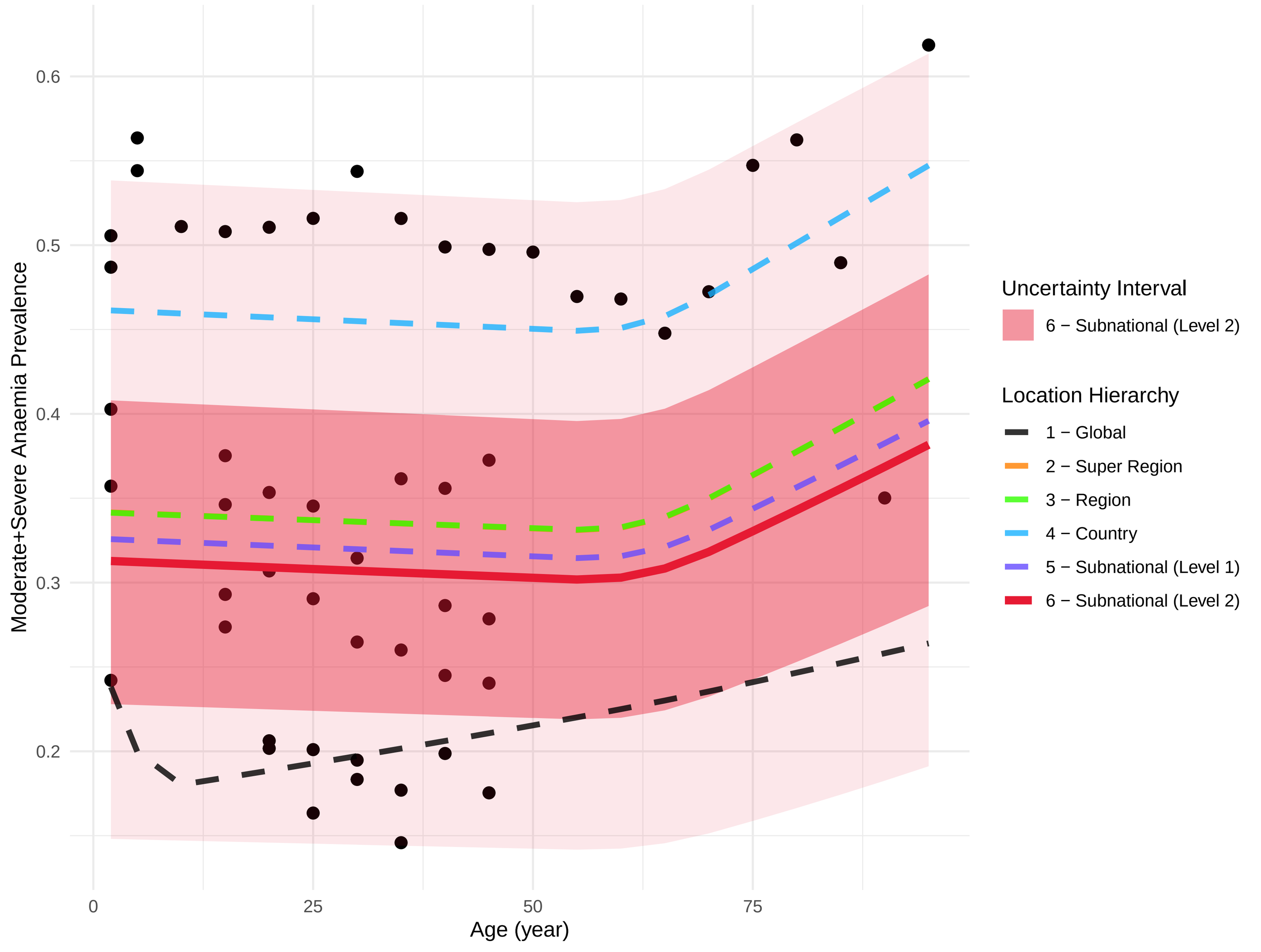

MR−BRT Age/Sex Fits − Non−pregnant Females 2+ Years Old − Moderate+Severe Anaemia Prevalence − Rajasthan
WHO−2001 Adjustment Method

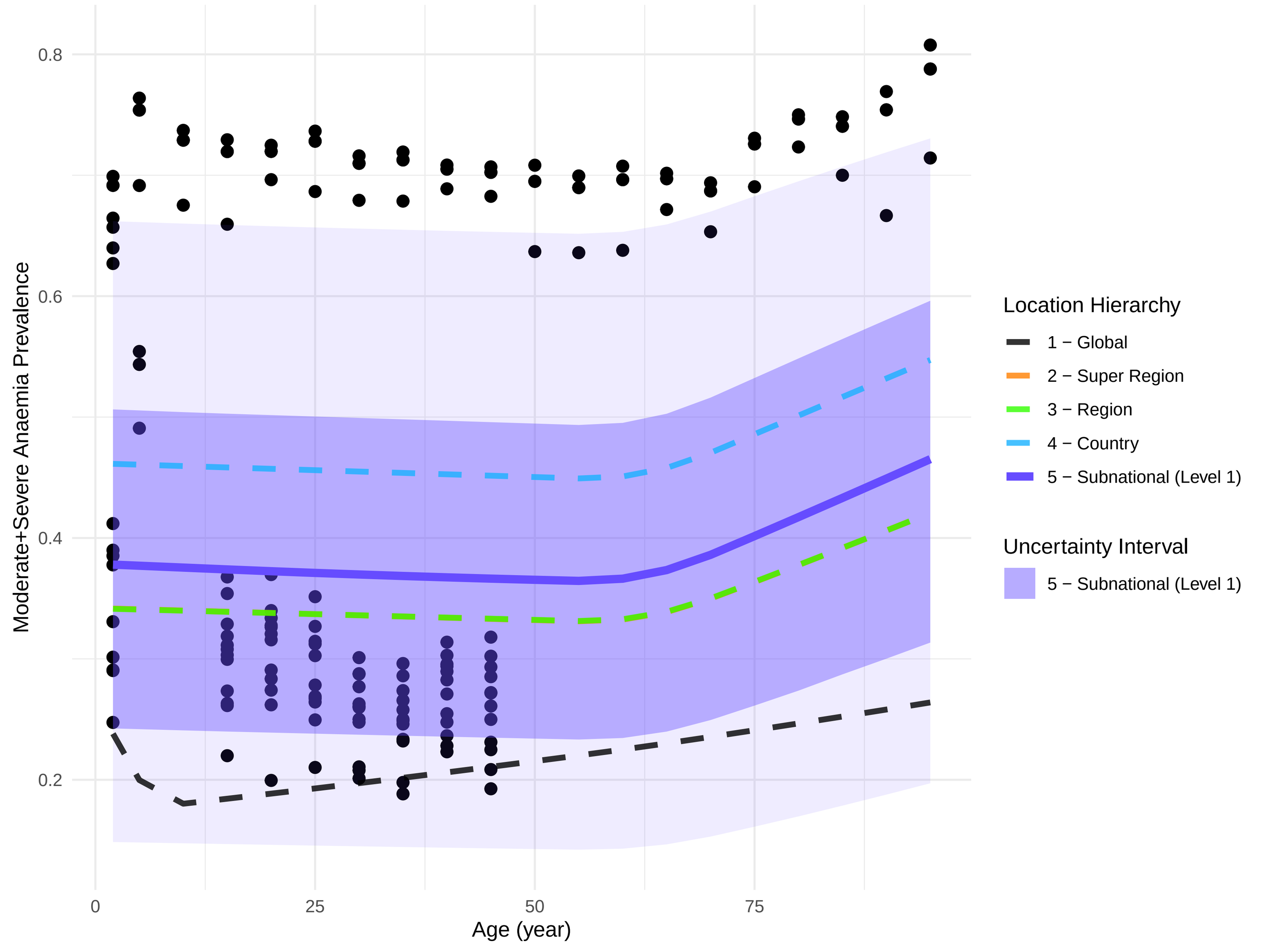

MR−BRT Age/Sex Fits − Non−pregnant Females 2+ Years Old − Moderate+Severe Anaemia Prevalence − Rajasthan, Rural
WHO−2001 Adjustment Method

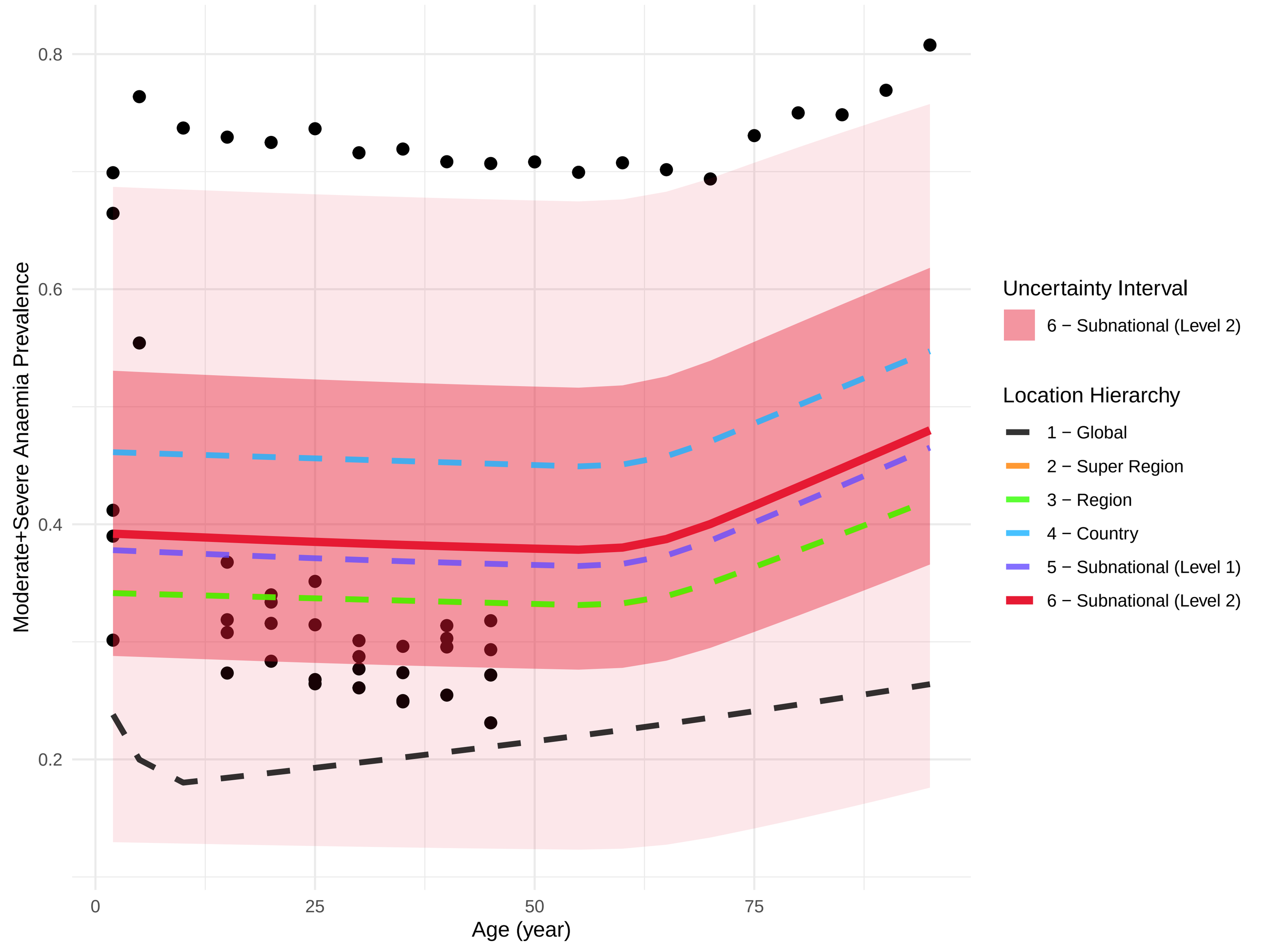

MR−BRT Age/Sex Fits − Non−pregnant Females 2+ Years Old − Moderate+Severe Anaemia Prevalence − Rajasthan, Urban
WHO−2001 Adjustment Method

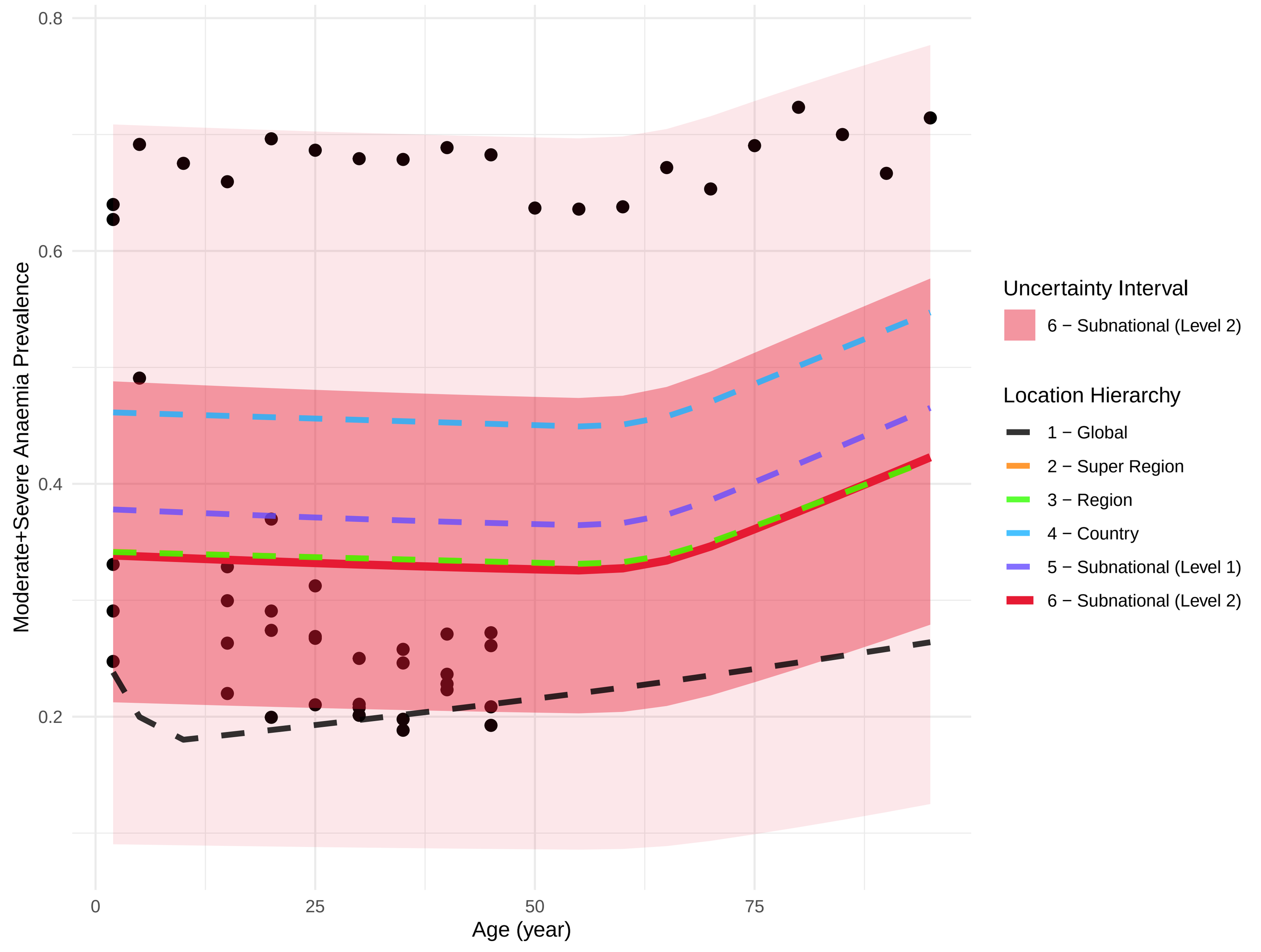

MR−BRT Age/Sex Fits − Non−pregnant Females 2+ Years Old − Moderate+Severe Anaemia Prevalence − Sikkim
WHO−2001 Adjustment Method

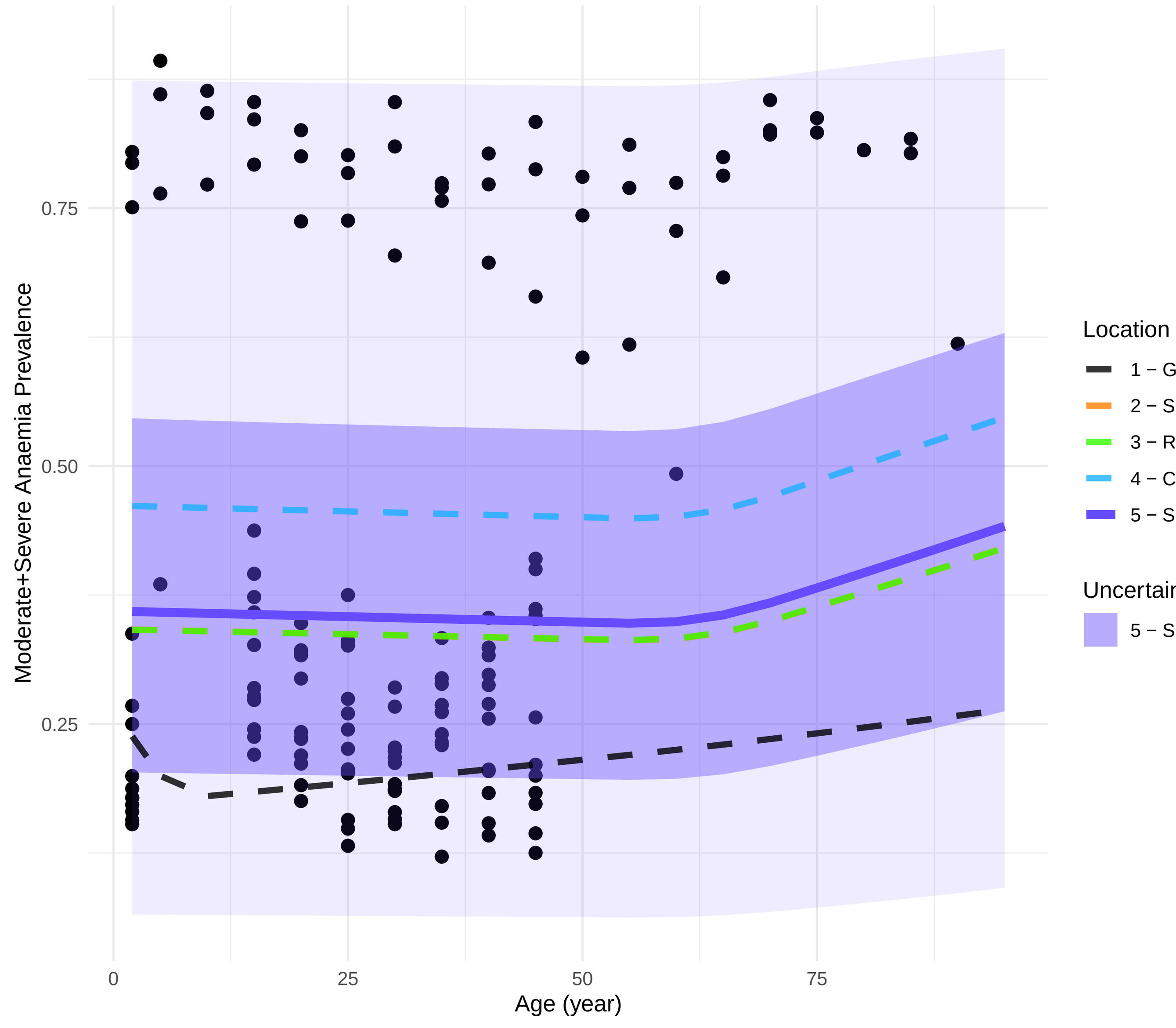

MR−BRT Age/Sex Fits − Non−pregnant Females 2+ Years Old − Moderate+Severe Anaemia Prevalence − Sikkim, Rural
WHO−2001 Adjustment Method

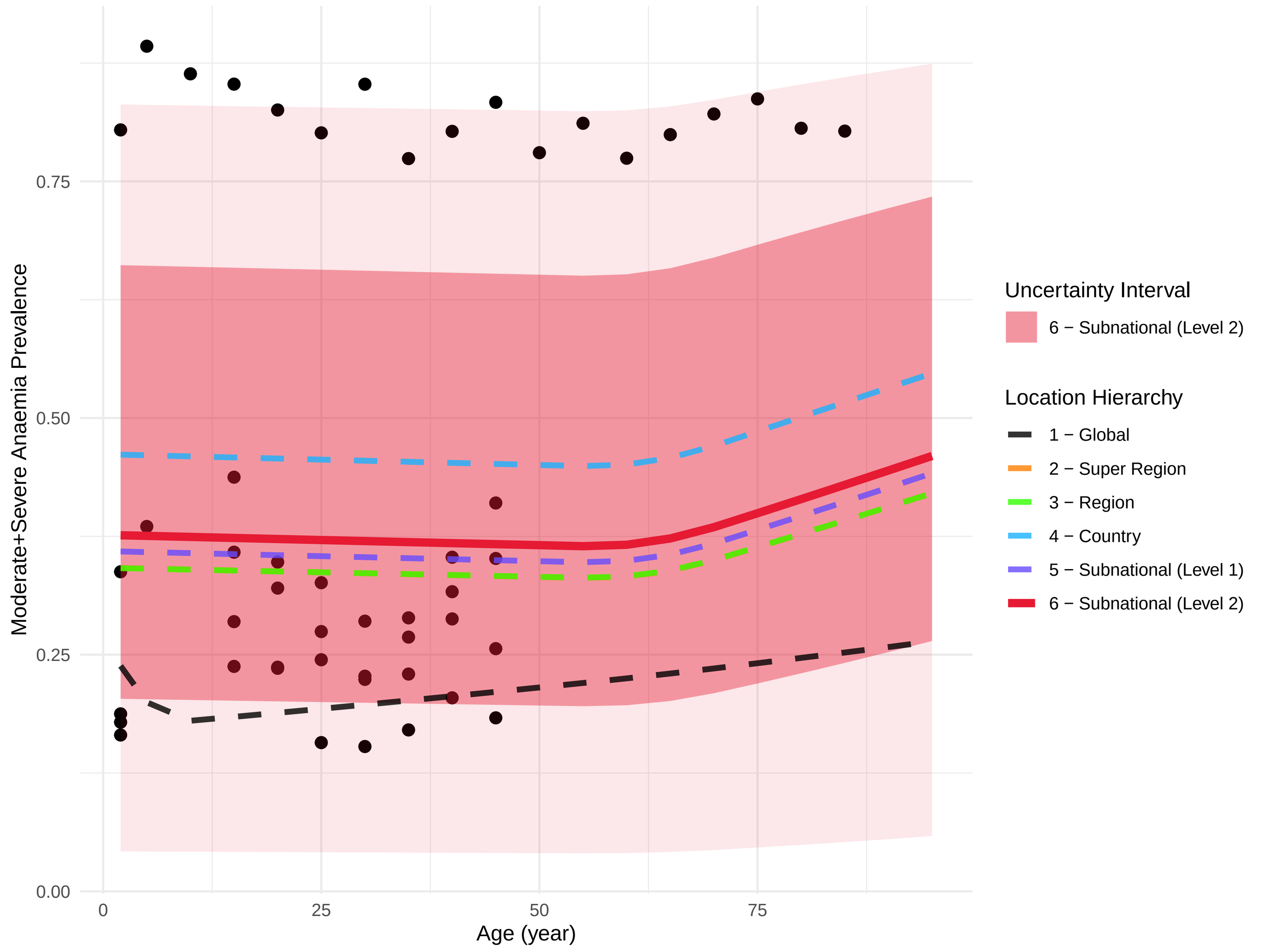

MR−BRT Age/Sex Fits − Non−pregnant Females 2+ Years Old − Moderate+Severe Anaemia Prevalence − Sikkim, Urban
WHO−2001 Adjustment Method

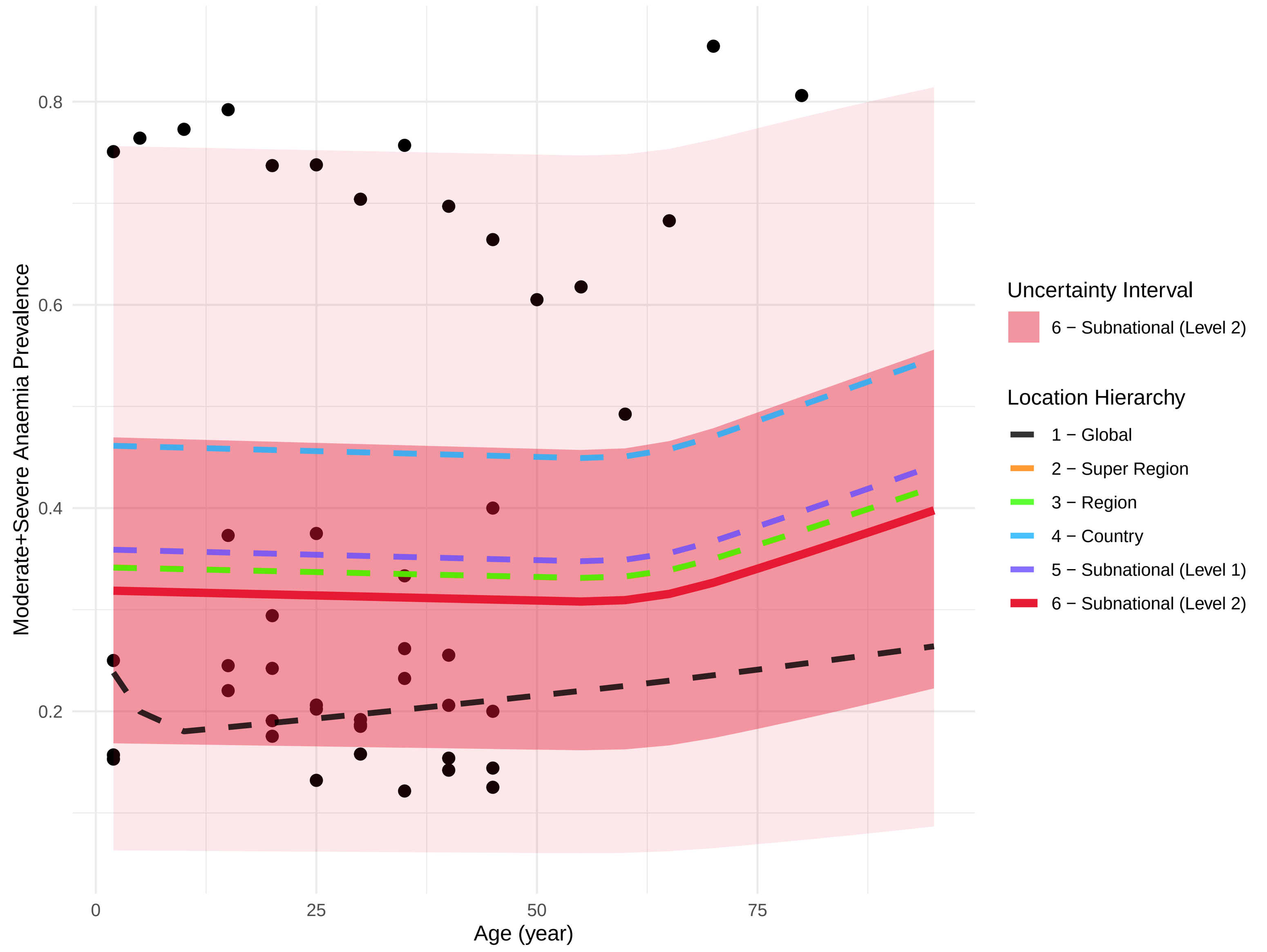

MR−BRT Age/Sex Fits − Non−pregnant Females 2+ Years Old − Moderate+Severe Anaemia Prevalence − Tamil Nadu
WHO−2001 Adjustment Method

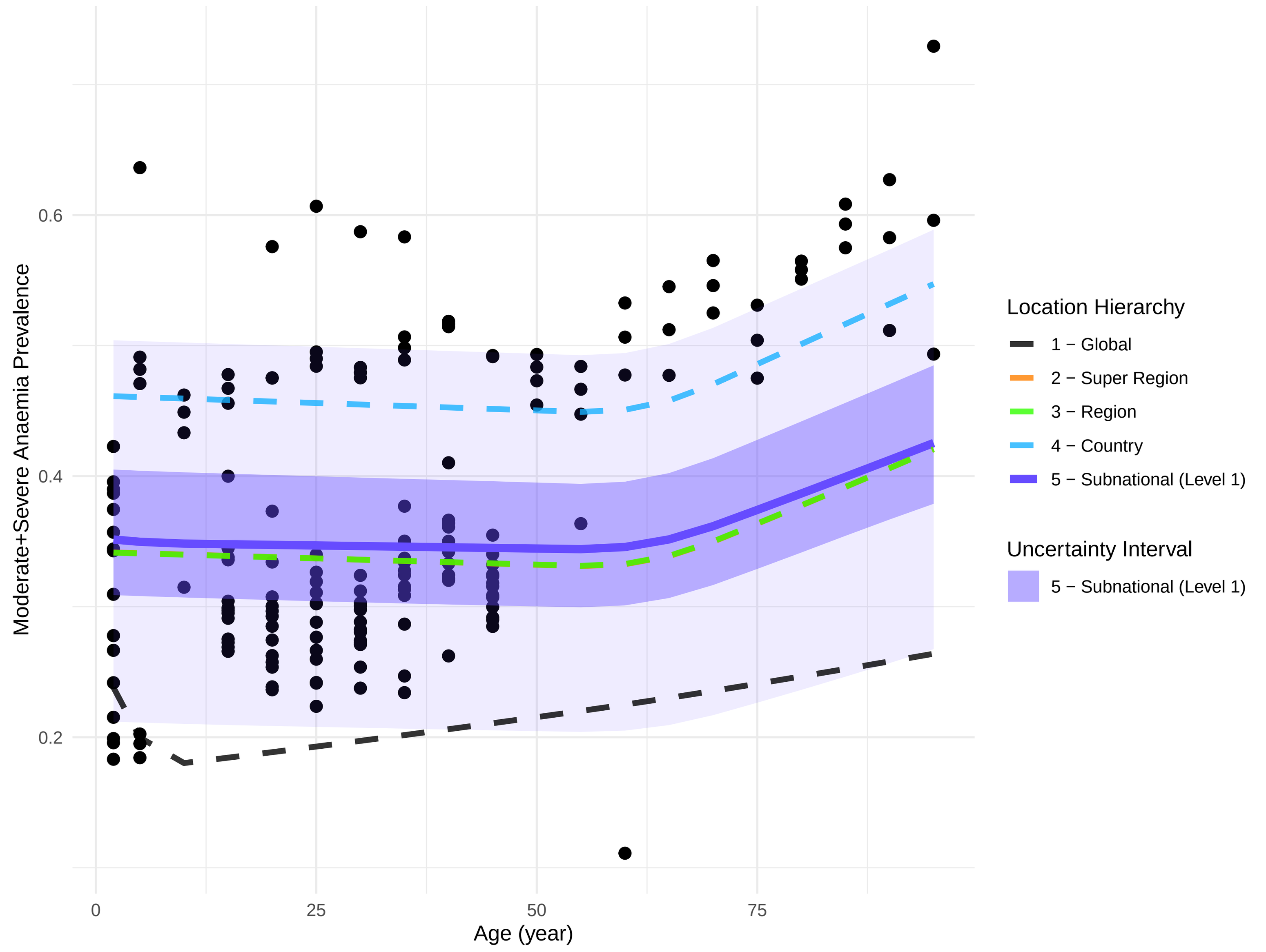

MR−BRT Age/Sex Fits − Non−pregnant Females 2+ Years Old − Moderate+Severe Anaemia Prevalence − Tamil Nadu, Rural
WHO−2001 Adjustment Method

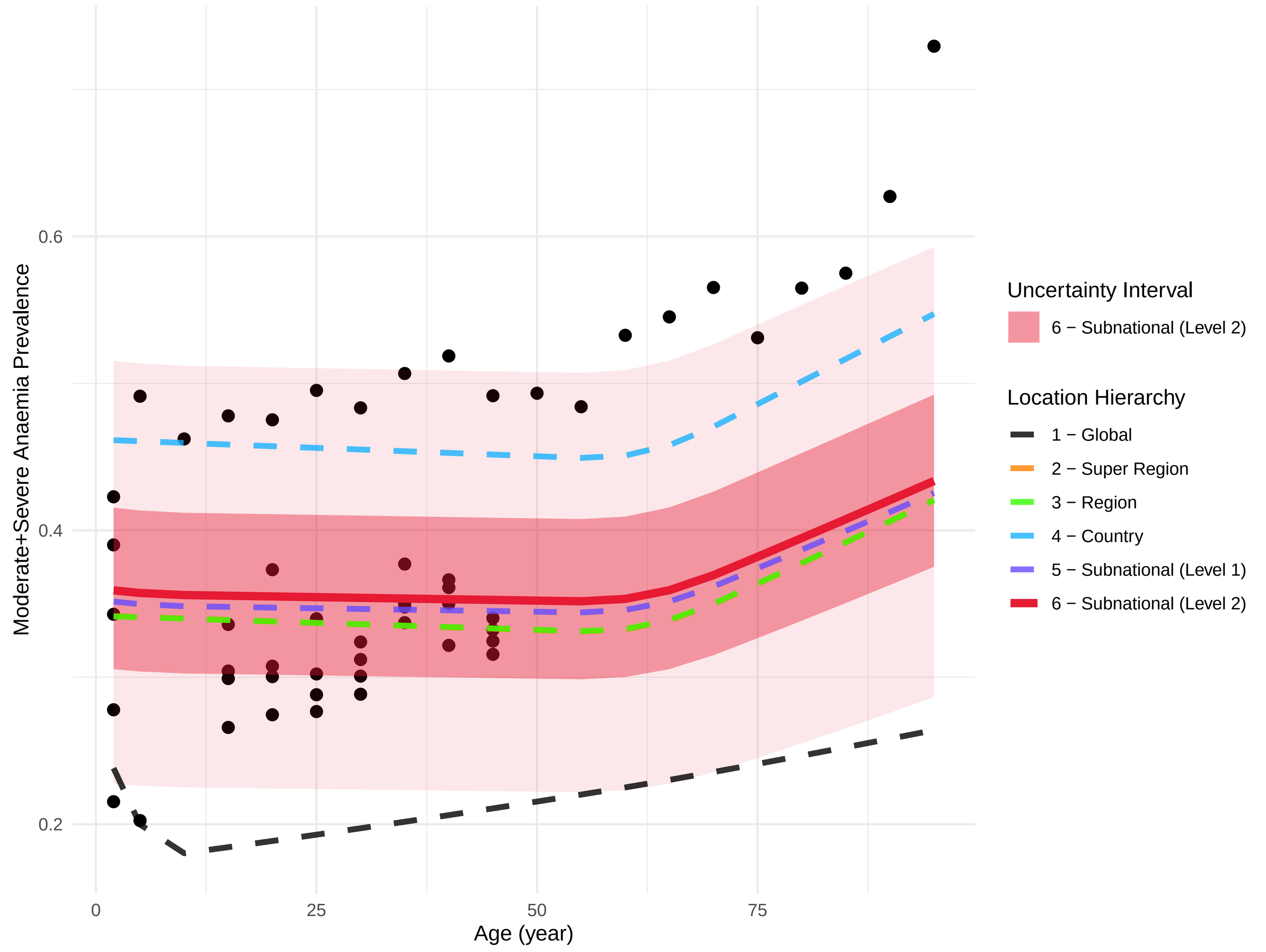

MR−BRT Age/Sex Fits − Non−pregnant Females 2+ Years Old − Moderate+Severe Anaemia Prevalence − Tamil Nadu, Urban
WHO−2001 Adjustment Method

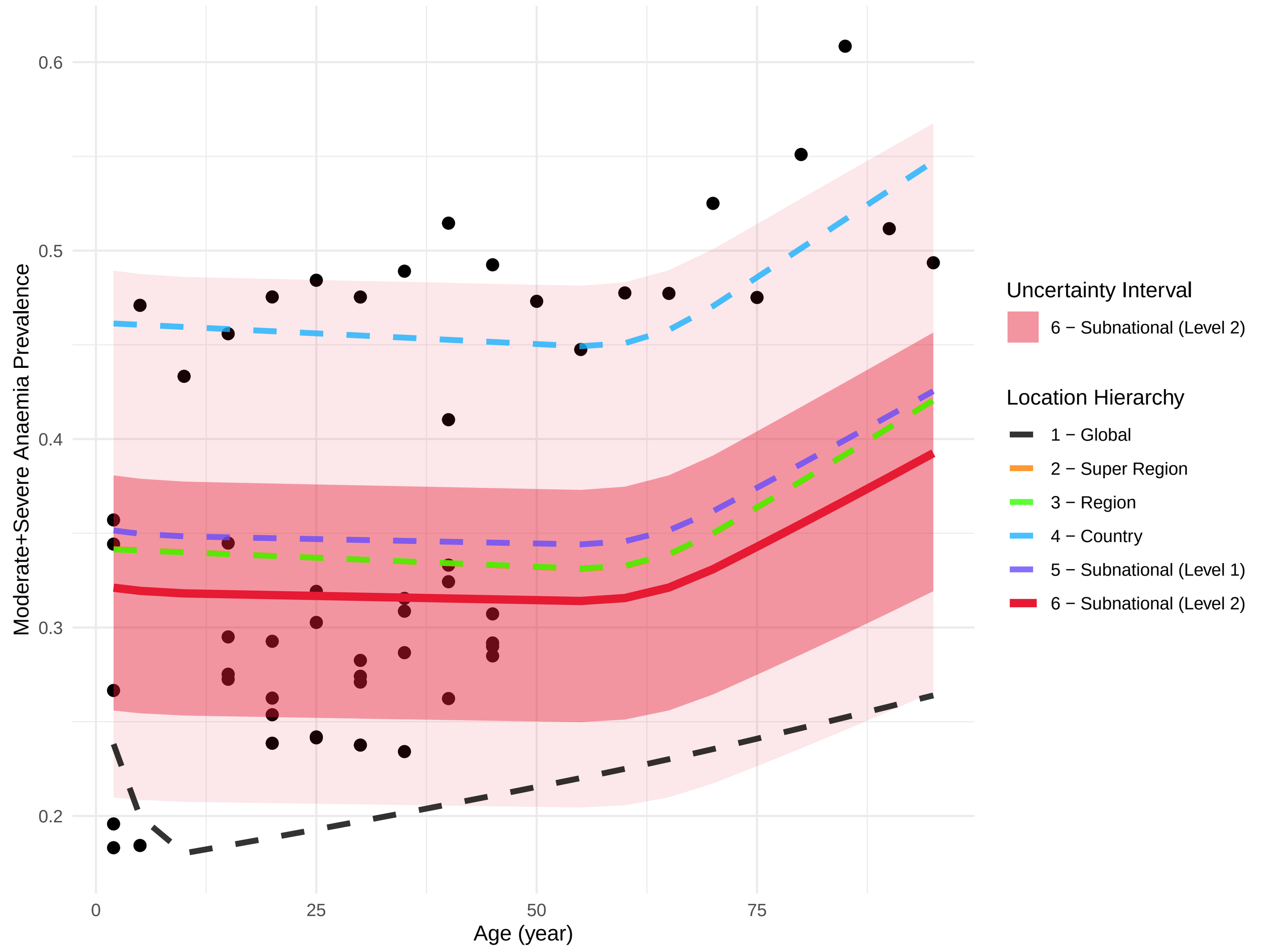

MR−BRT Age/Sex Fits − Non−pregnant Females 2+ Years Old − Moderate+Severe Anaemia Prevalence − Telangana
WHO−2001 Adjustment Method

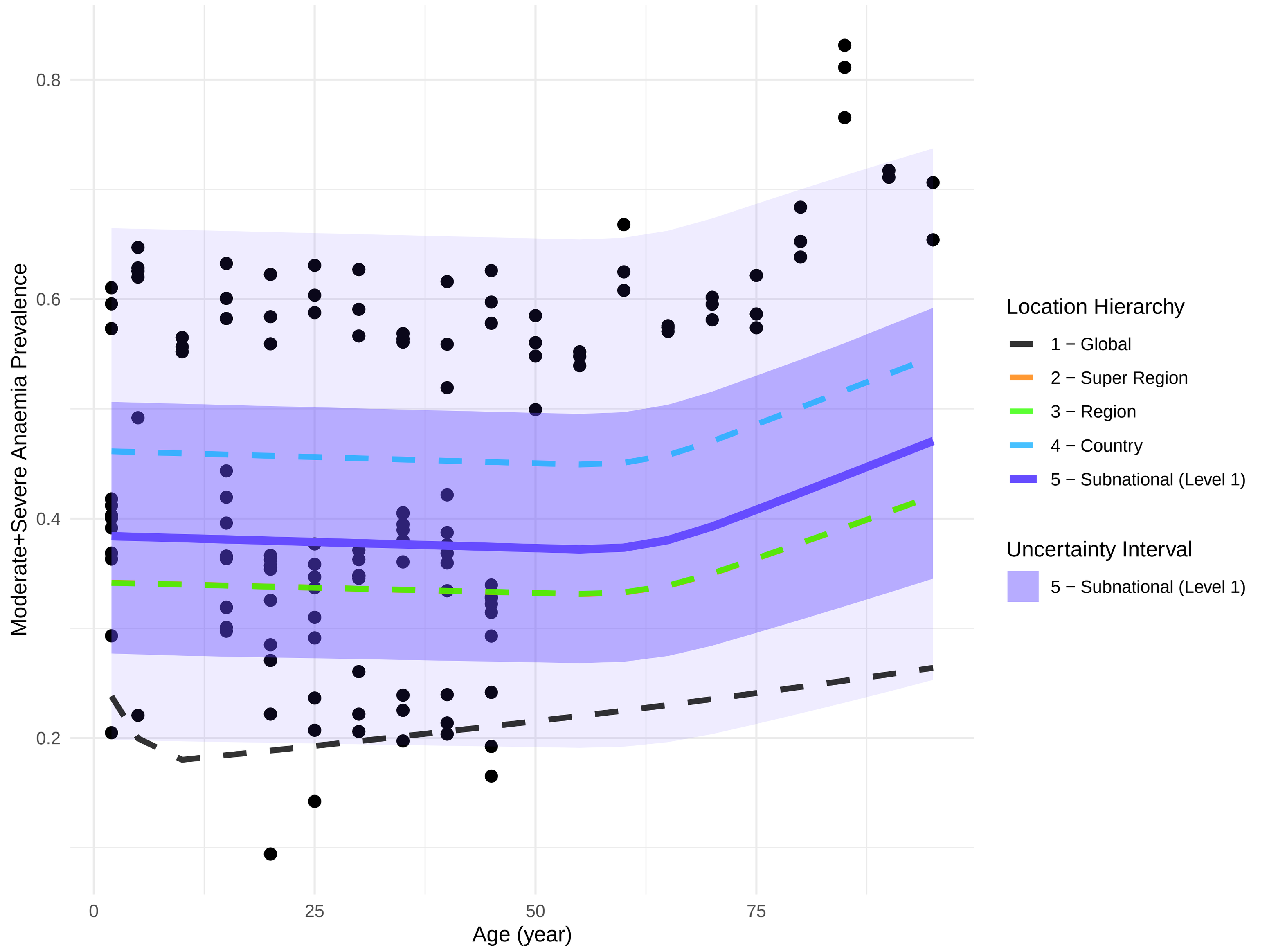

MR−BRT Age/Sex Fits − Non−pregnant Females 2+ Years Old − Moderate+Severe Anaemia Prevalence − Telangana, Rural
WHO−2001 Adjustment Method

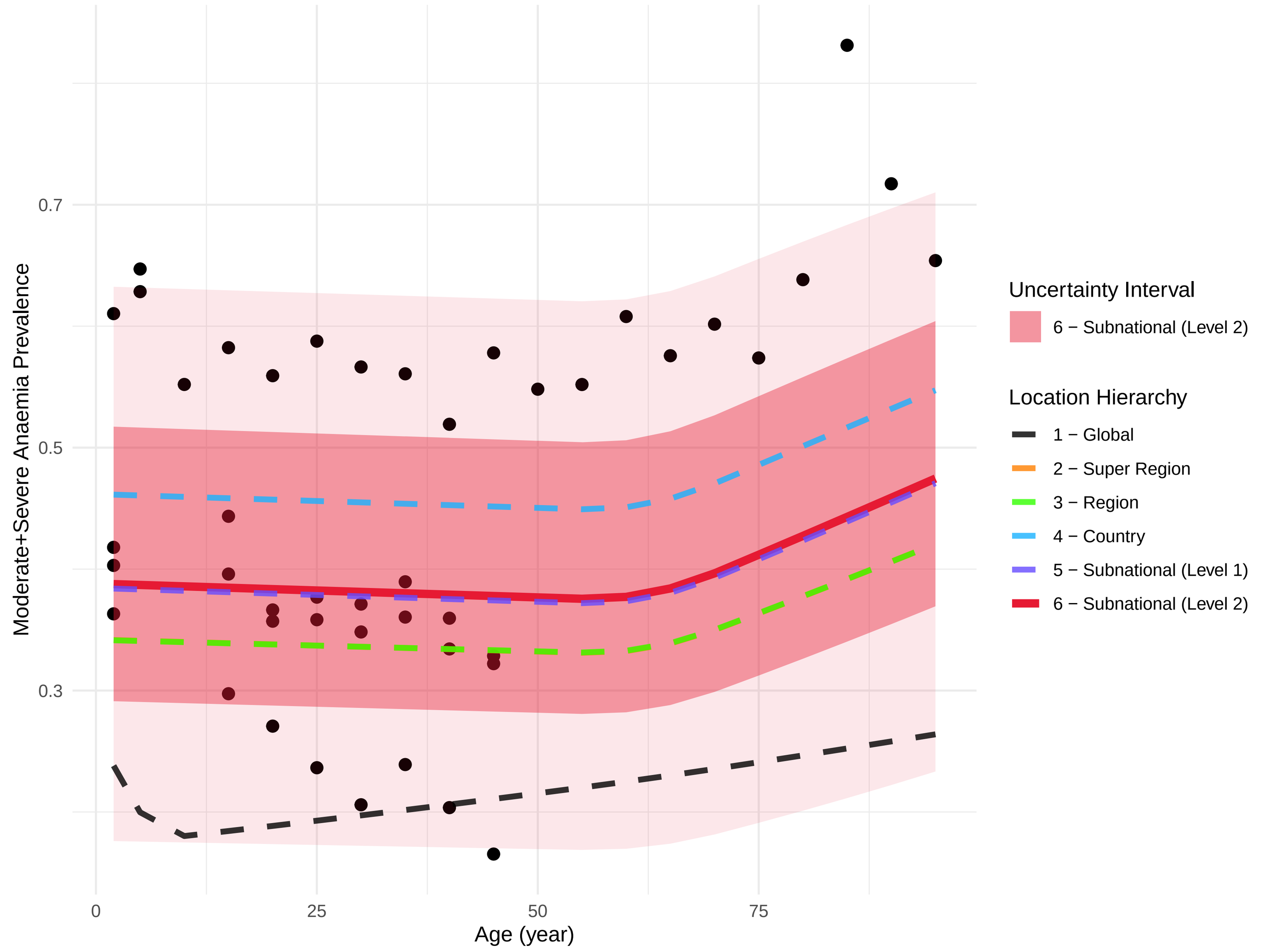

MR−BRT Age/Sex Fits − Non−pregnant Females 2+ Years Old − Moderate+Severe Anaemia Prevalence − Telangana, Urban
WHO−2001 Adjustment Method

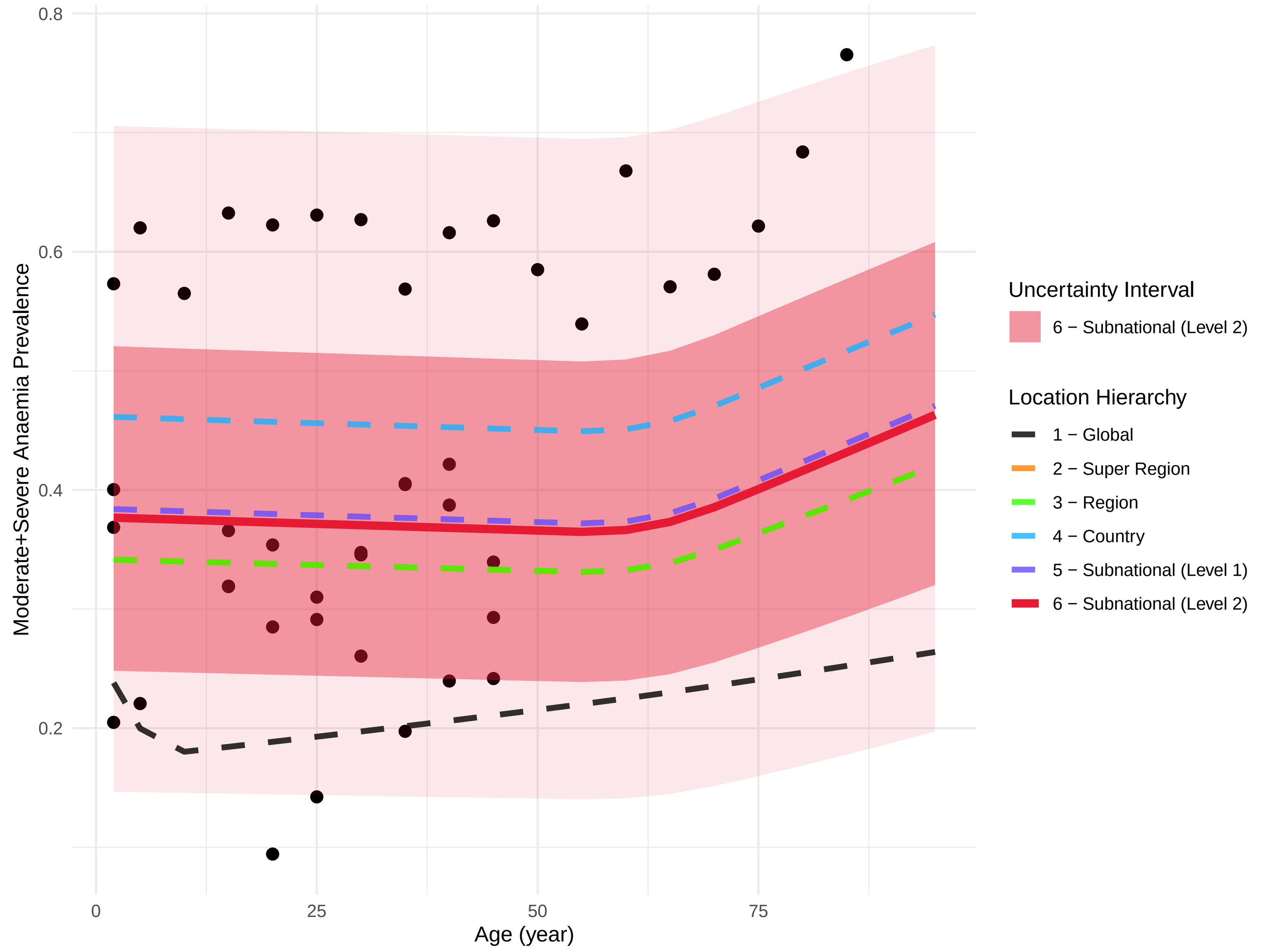

MR−BRT Age/Sex Fits − Non−pregnant Females 2+ Years Old − Moderate+Severe Anaemia Prevalence − Tripura
WHO−2001 Adjustment Method

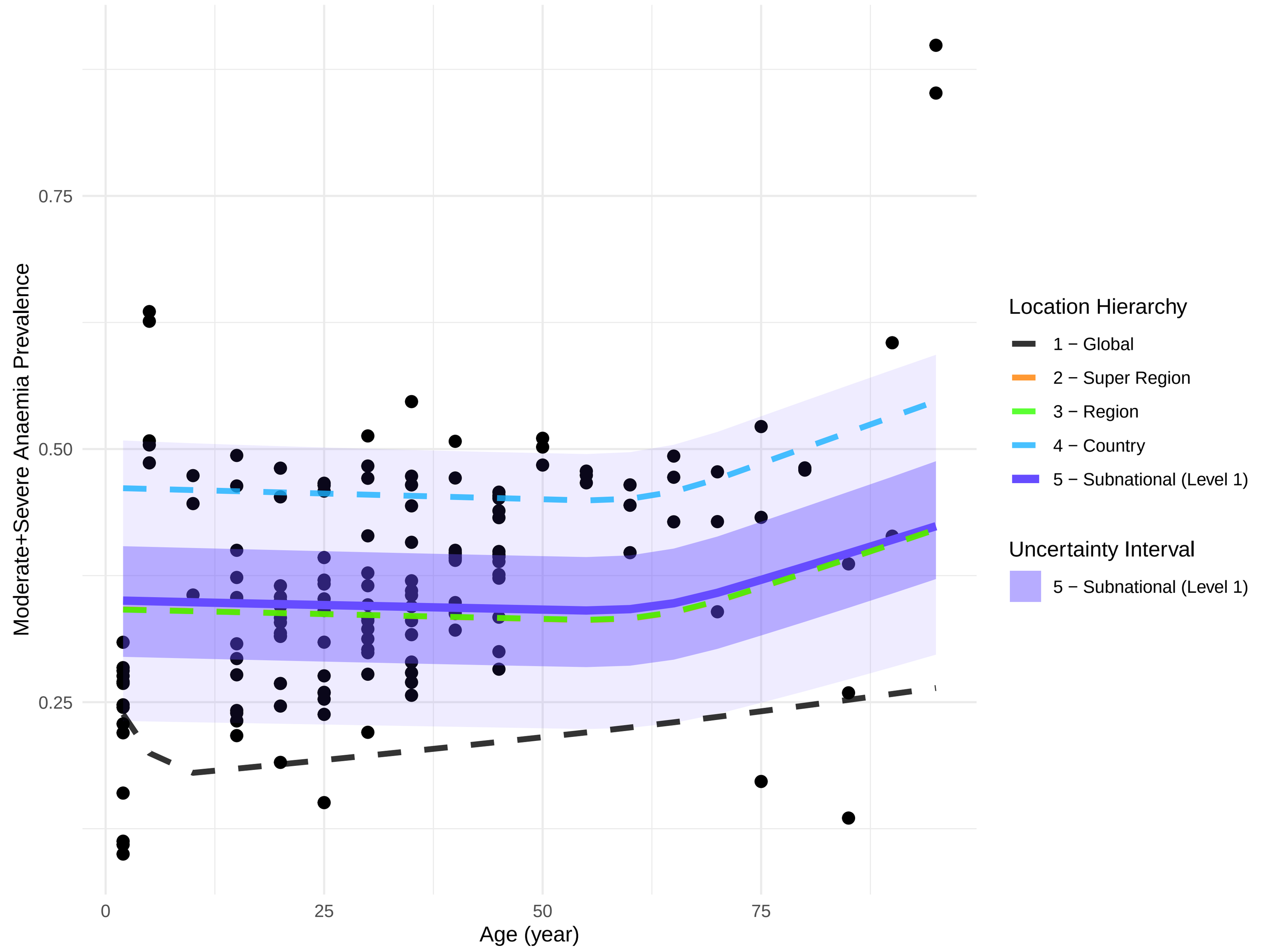

MR−BRT Age/Sex Fits − Non−pregnant Females 2+ Years Old − Moderate+Severe Anaemia Prevalence − Tripura, Rural
WHO−2001 Adjustment Method

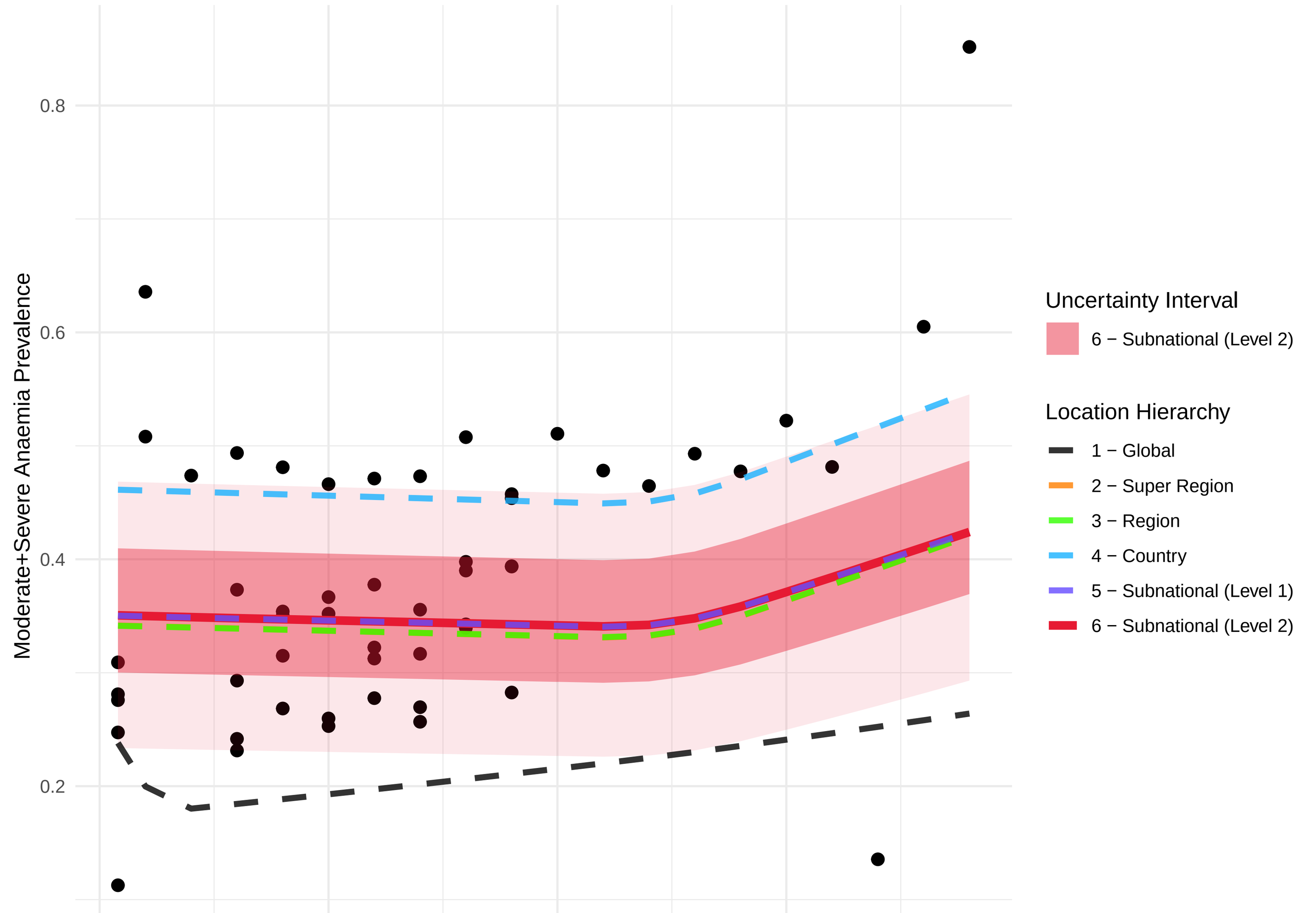

MR−BRT Age/Sex Fits − Non−pregnant Females 2+ Years Old − Moderate+Severe Anaemia Prevalence − Tripura, Urban
WHO−2001 Adjustment Method

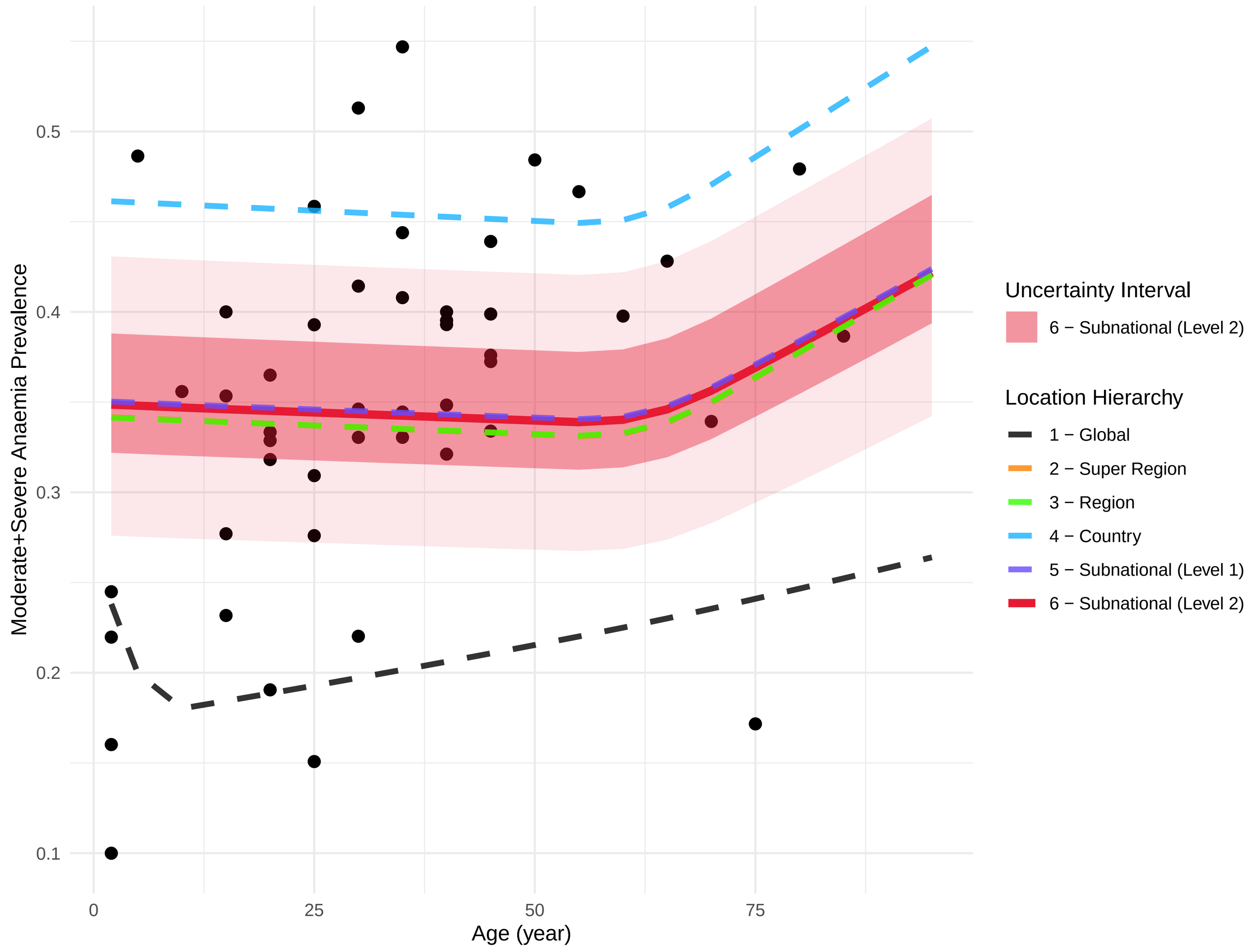

MR−BRT Age/Sex Fits − Non−pregnant Females 2+ Years Old − Moderate+Severe Anaemia Prevalence − Uttar Pradesh
WHO−2001 Adjustment Method

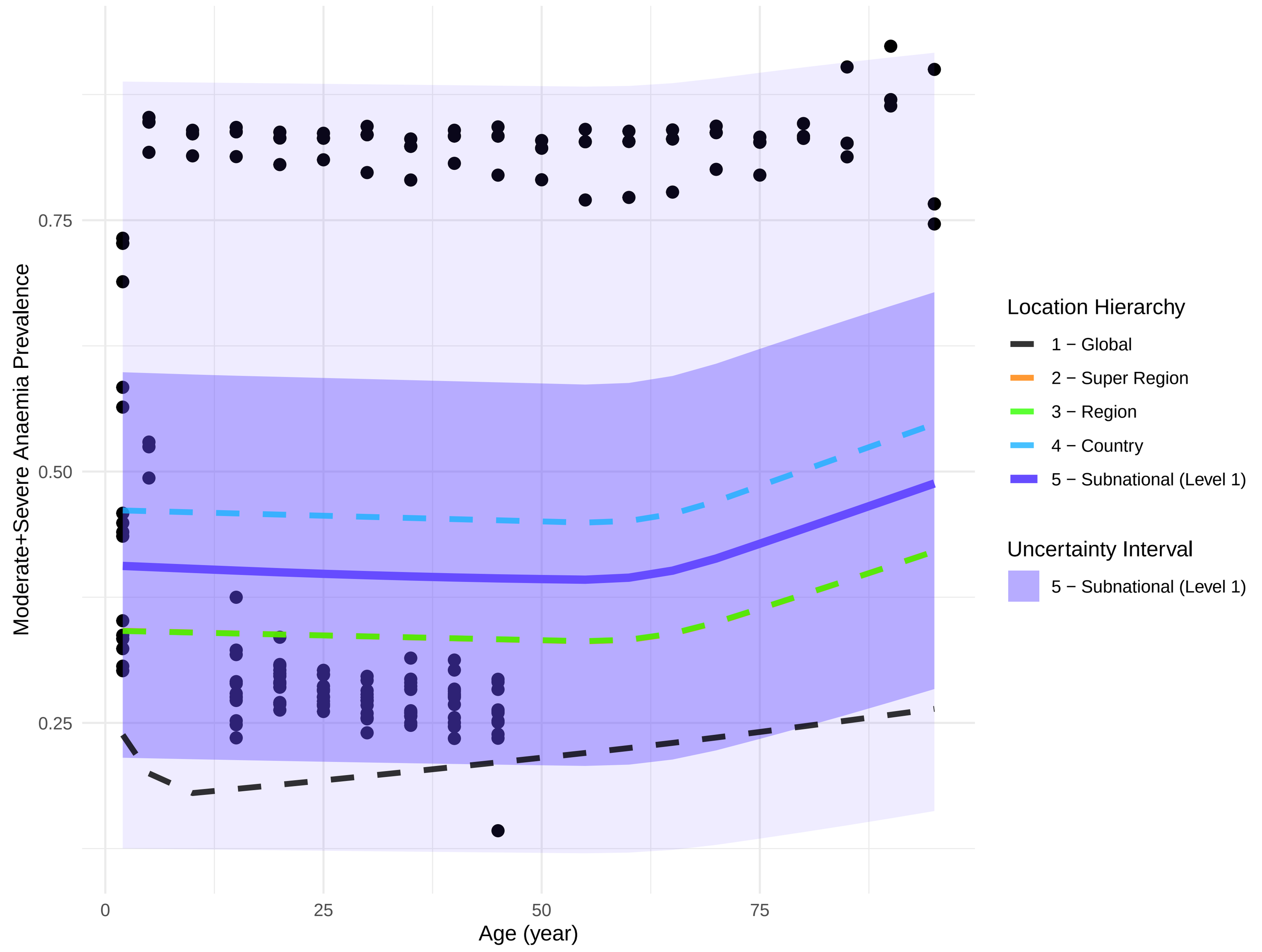

MR−BRT Age/Sex Fits − Non−pregnant Females 2+ Years Old − Moderate+Severe Anaemia Prevalence − Uttar Pradesh, Rural
WHO−2001 Adjustment Method

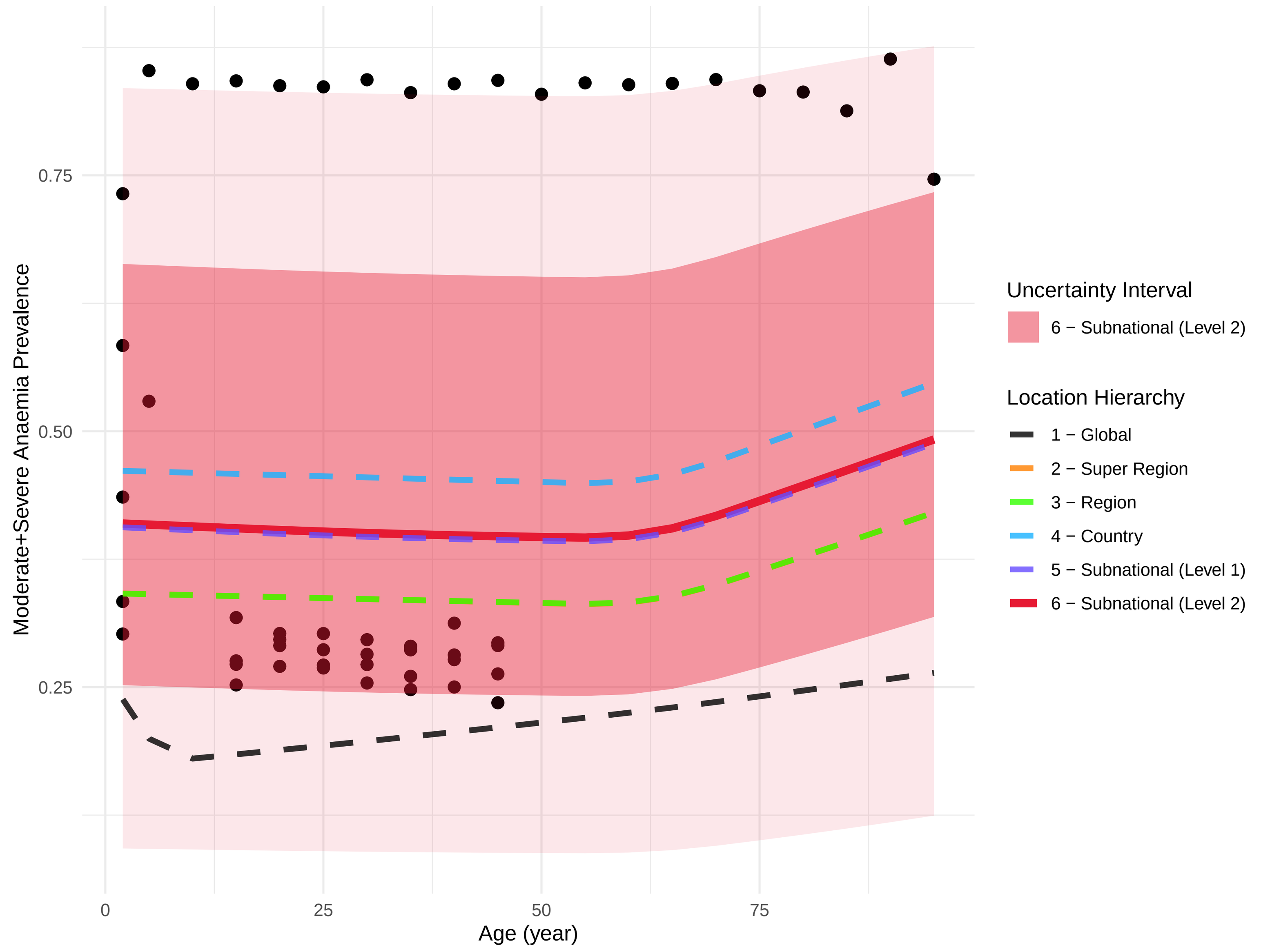

MR−BRT Age/Sex Fits − Non−pregnant Females 2+ Years Old − Moderate+Severe Anaemia Prevalence − Uttar Pradesh, Urban
WHO−2001 Adjustment Method

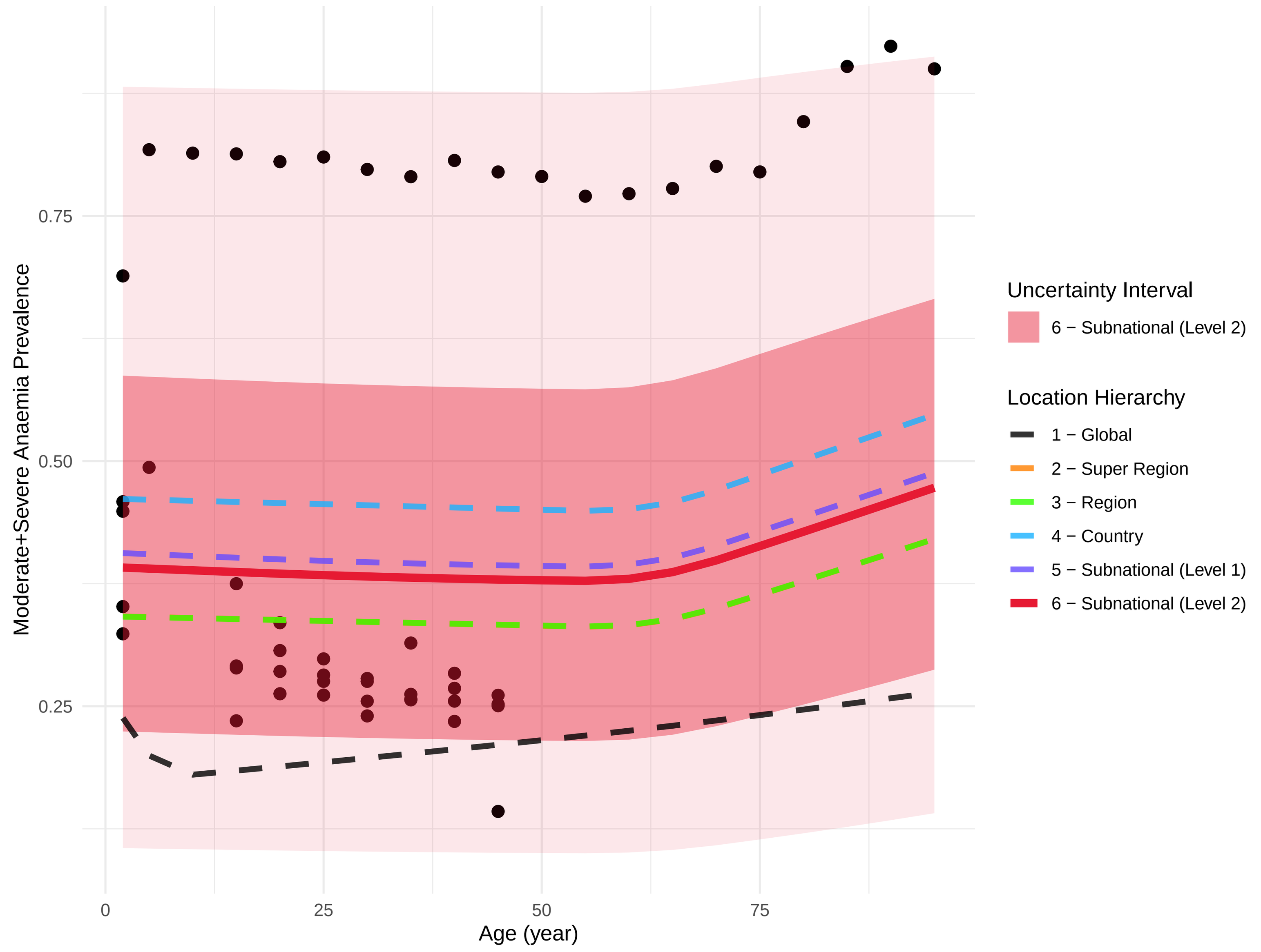

MR−BRT Age/Sex Fits − Non−pregnant Females 2+ Years Old − Moderate+Severe Anaemia Prevalence − Uttarakhand
WHO−2001 Adjustment Method

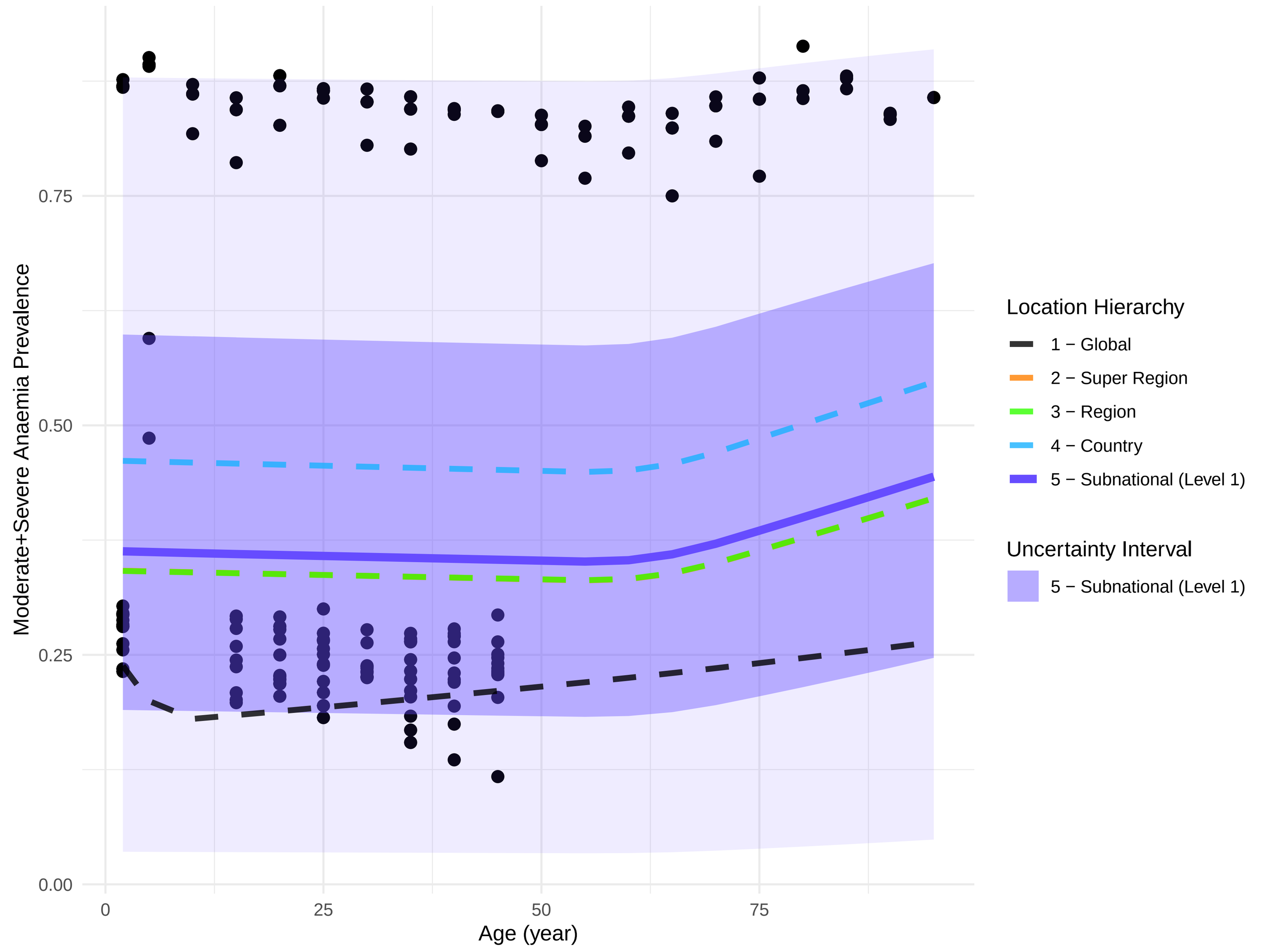

MR−BRT Age/Sex Fits − Non−pregnant Females 2+ Years Old − Moderate+Severe Anaemia Prevalence − Uttarakhand, Rural
WHO−2001 Adjustment Method

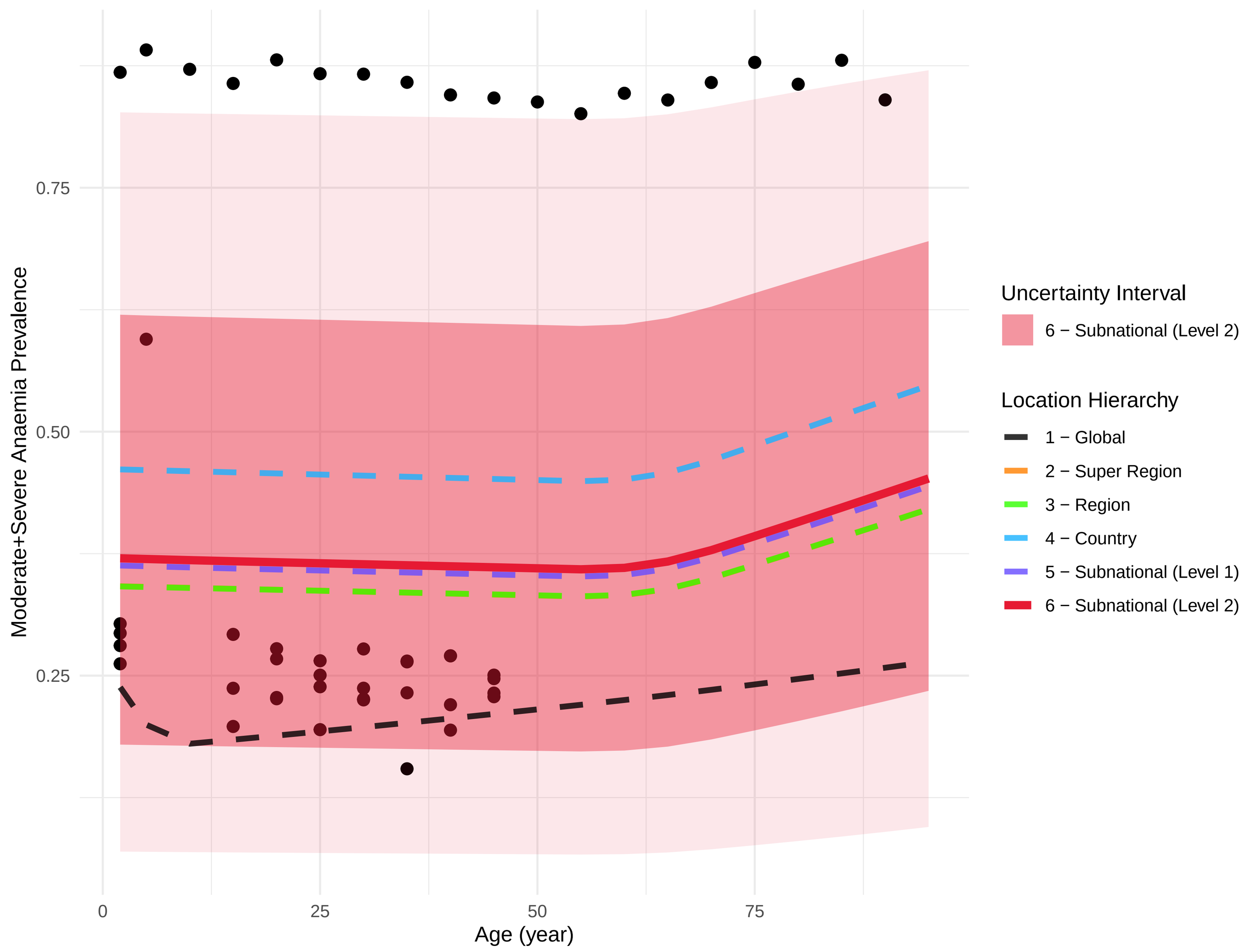

MR−BRT Age/Sex Fits − Non−pregnant Females 2+ Years Old − Moderate+Severe Anaemia Prevalence − Uttarakhand, Urban
WHO−2001 Adjustment Method

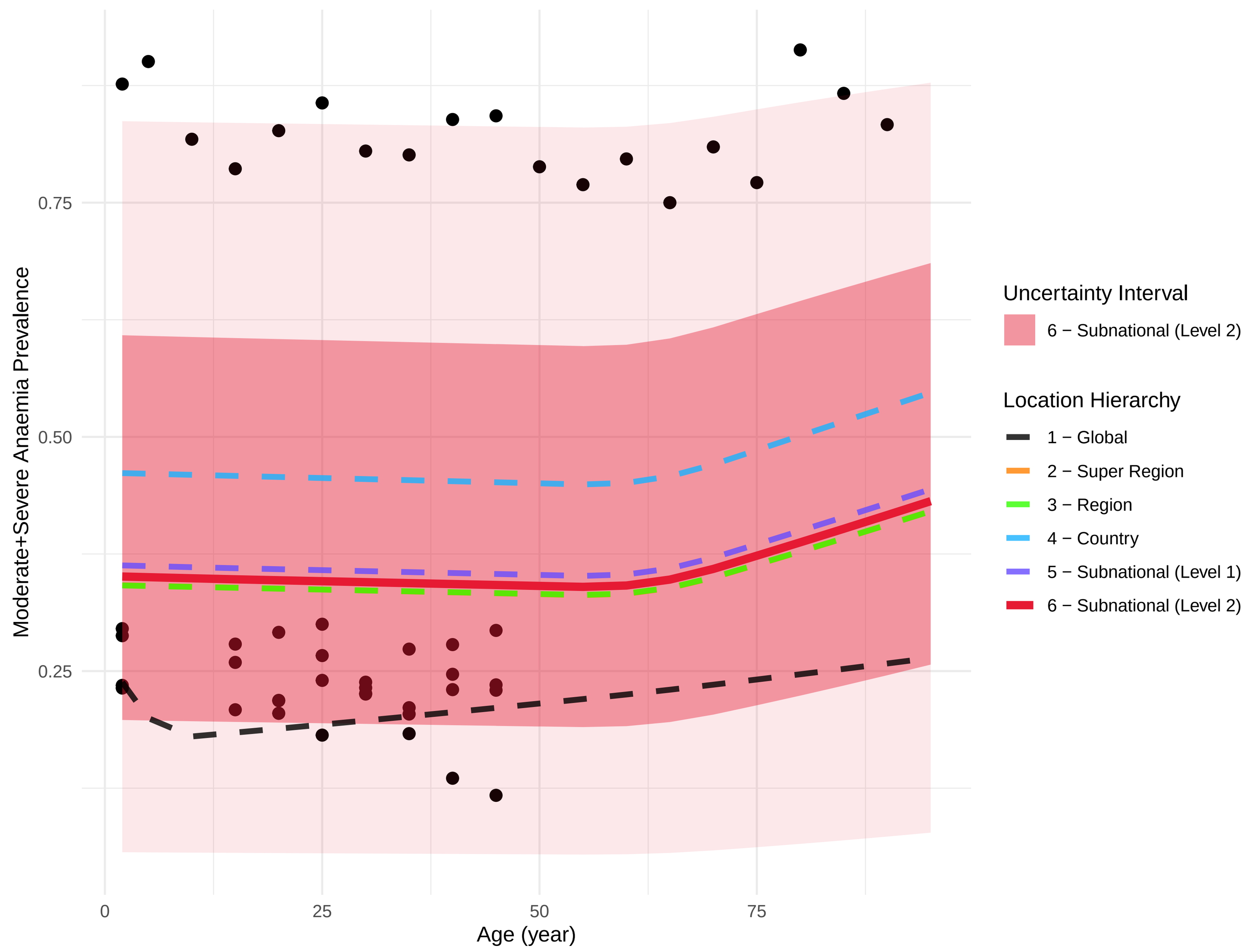

MR−BRT Age/Sex Fits − Non−pregnant Females 2+ Years Old − Moderate+Severe Anaemia Prevalence − West Bengal
WHO−2001 Adjustment Method

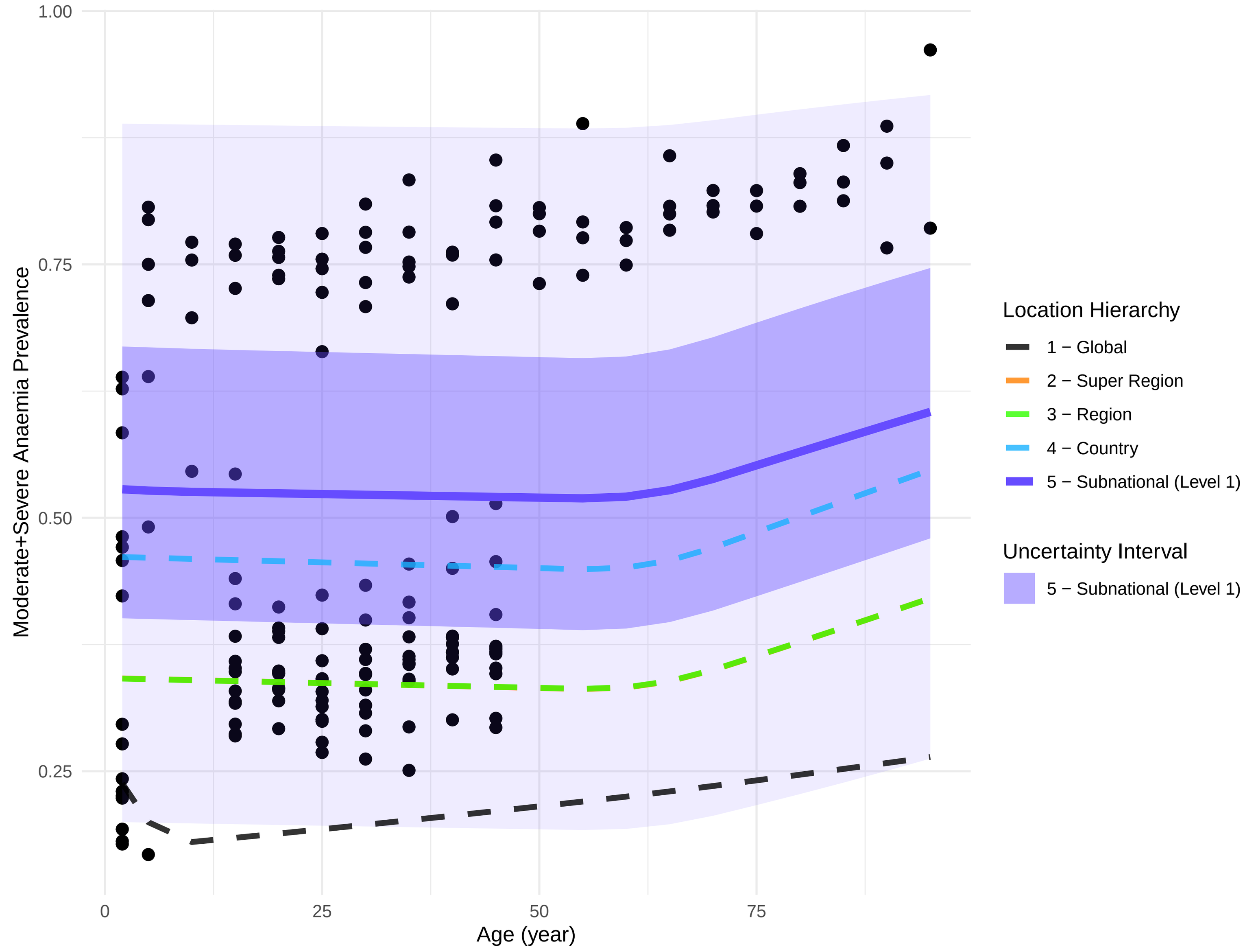

MR−BRT Age/Sex Fits − Non−pregnant Females 2+ Years Old − Moderate+Severe Anaemia Prevalence − West Bengal, Rural
WHO−2001 Adjustment Method

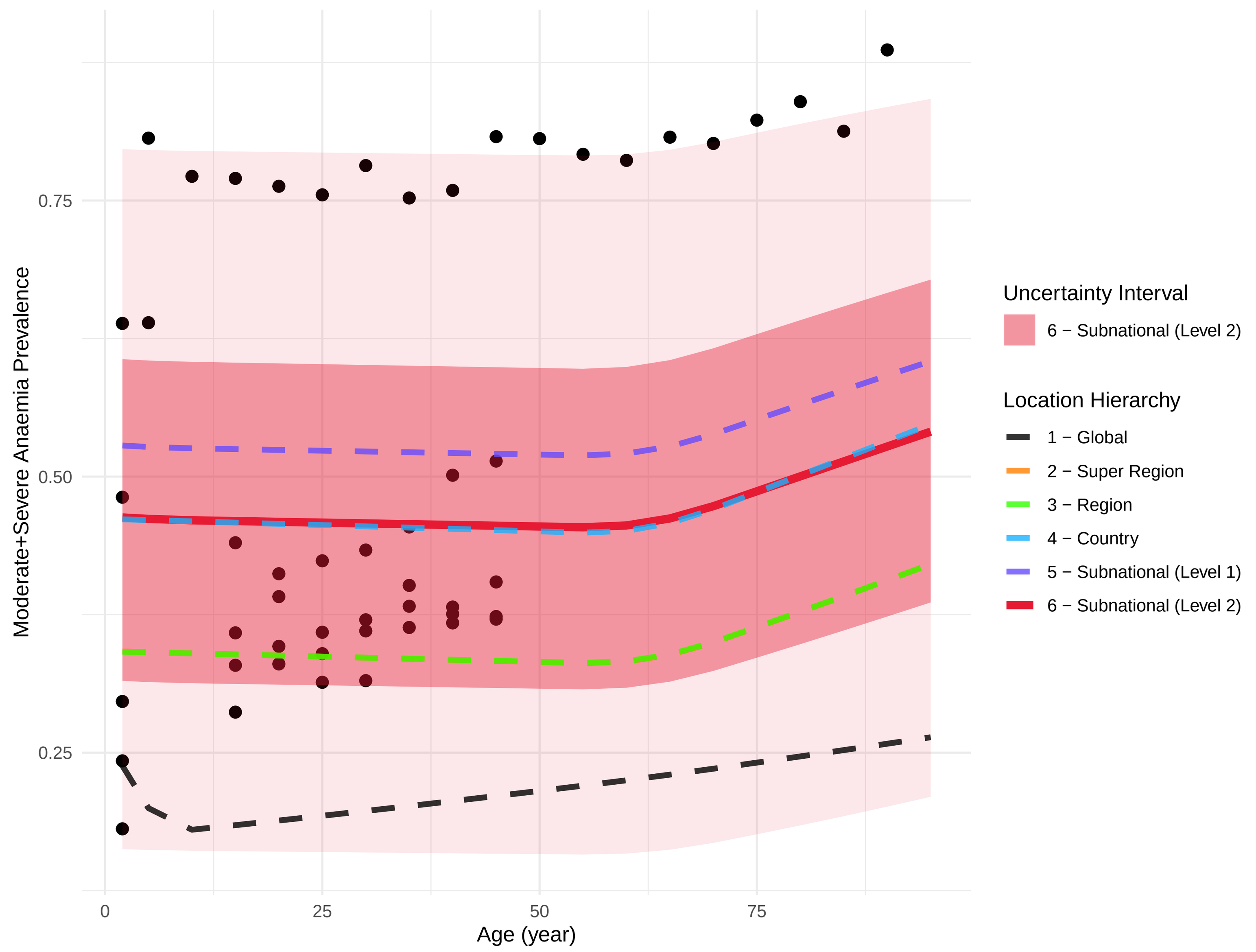

MR−BRT Age/Sex Fits − Non−pregnant Females 2+ Years Old − Moderate+Severe Anaemia Prevalence − West Bengal, Urban
WHO−2001 Adjustment Method

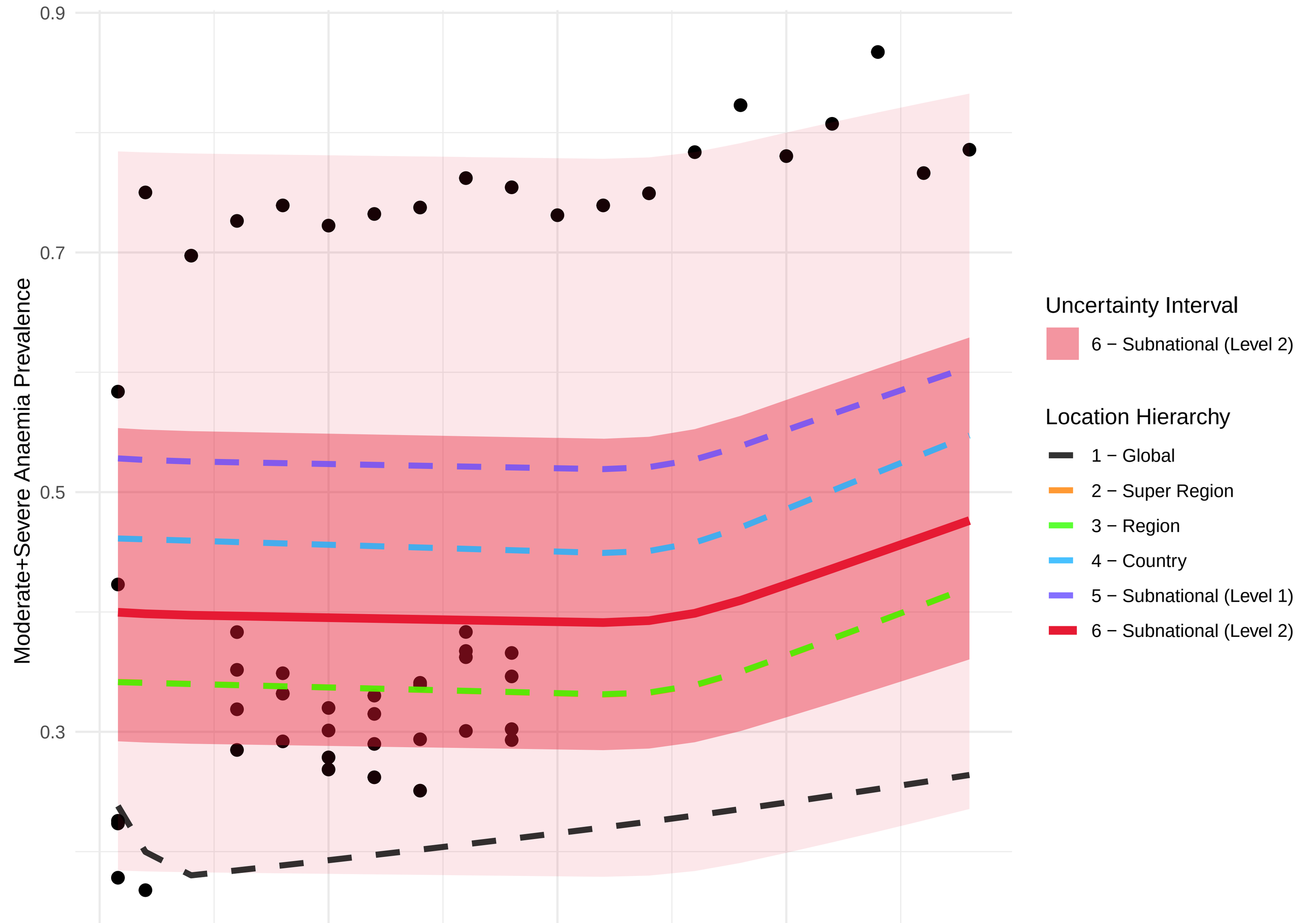

MR−BRT Age/Sex Fits − Non−pregnant Females 2+ Years Old − Moderate+Severe Anaemia Prevalence − Nepal
WHO−2001 Adjustment Method

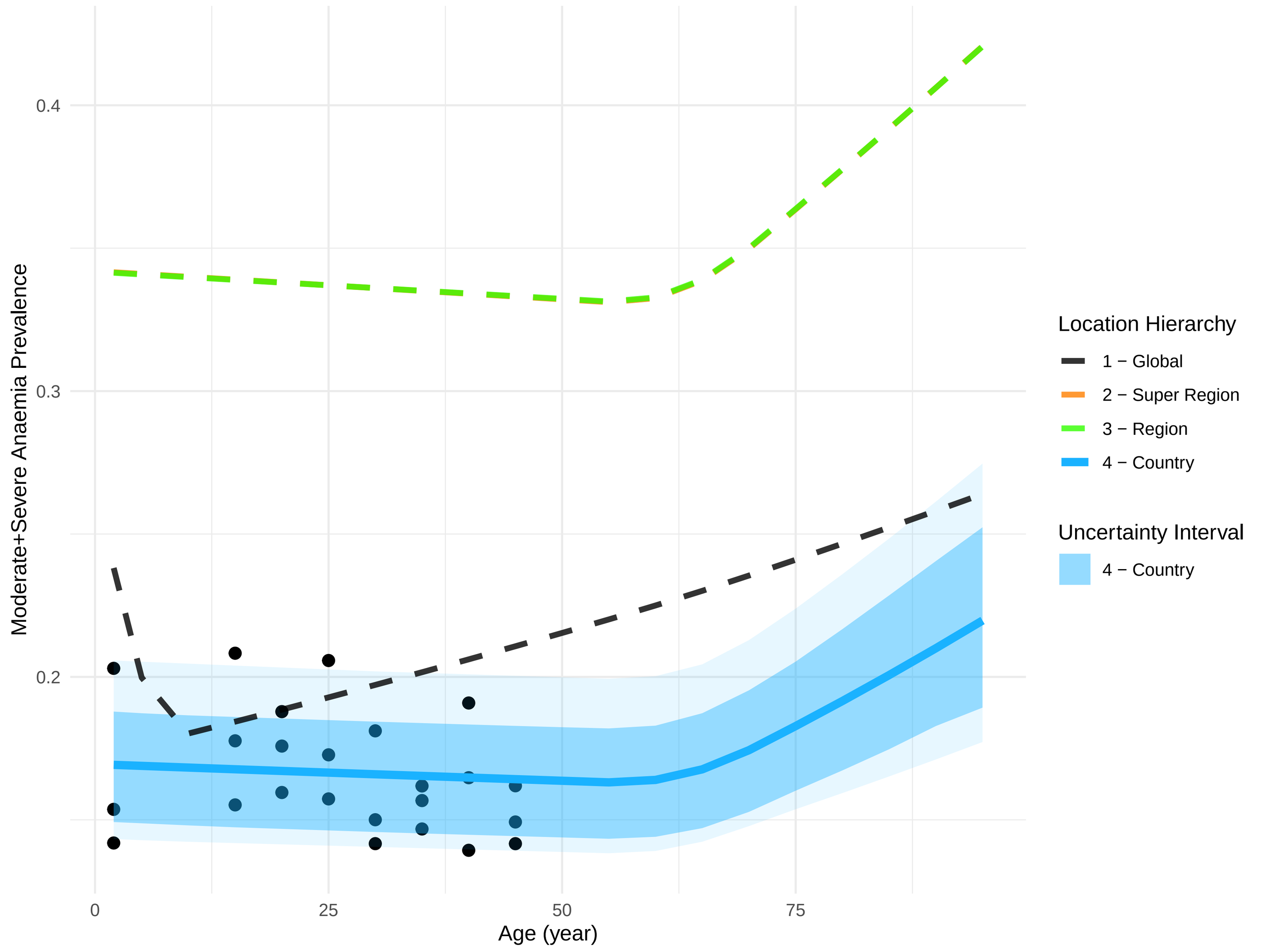

MR−BRT Age/Sex Fits − Non−pregnant Females 2+ Years Old − Moderate+Severe Anaemia Prevalence − Pakistan
WHO−2001 Adjustment Method

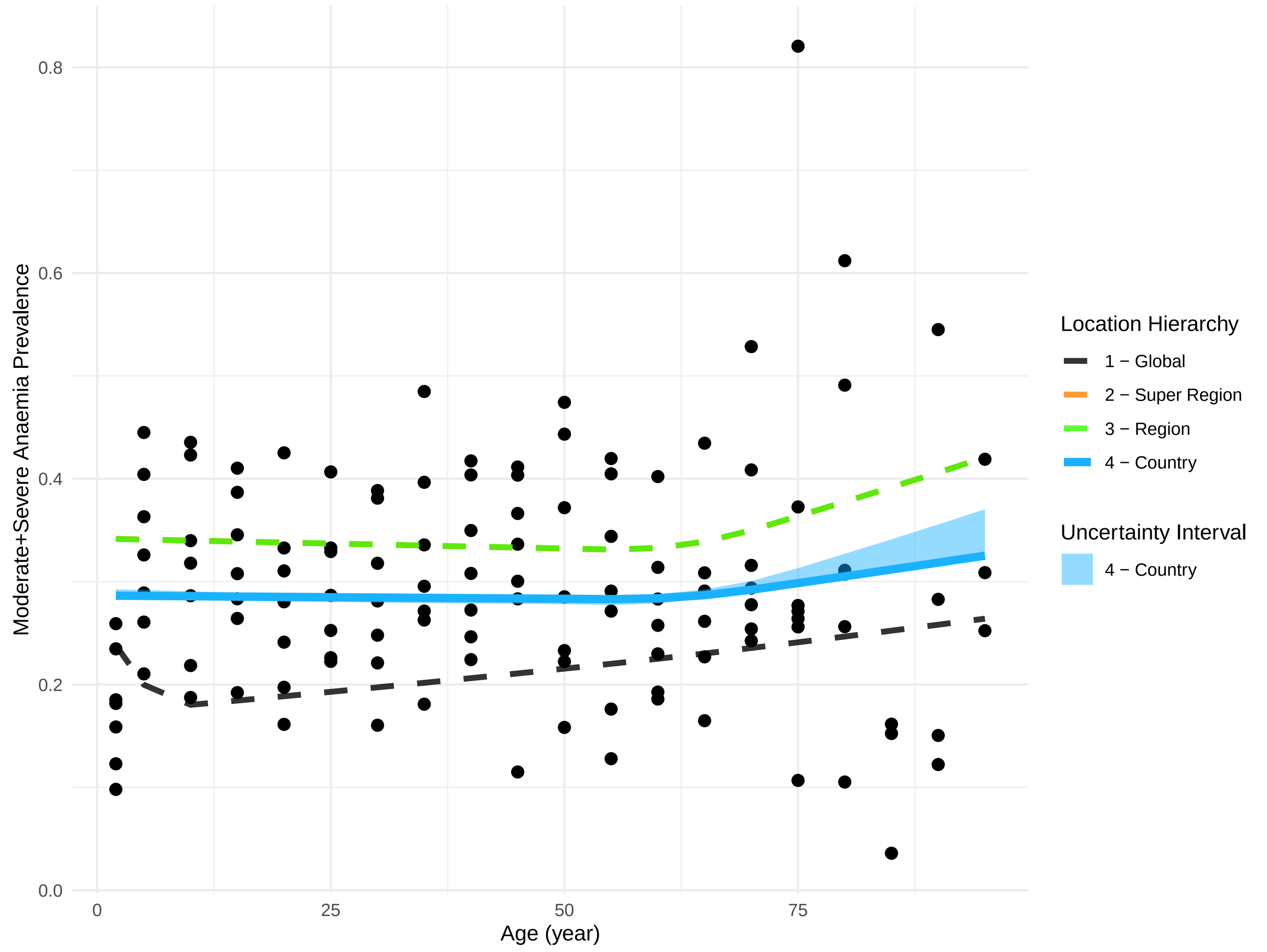

MR−BRT Age/Sex Fits − Non−pregnant Females 2+ Years Old − Moderate+Severe Anaemia Prevalence − Azad Jammu & Kashmir
WHO−2001 Adjustment Method

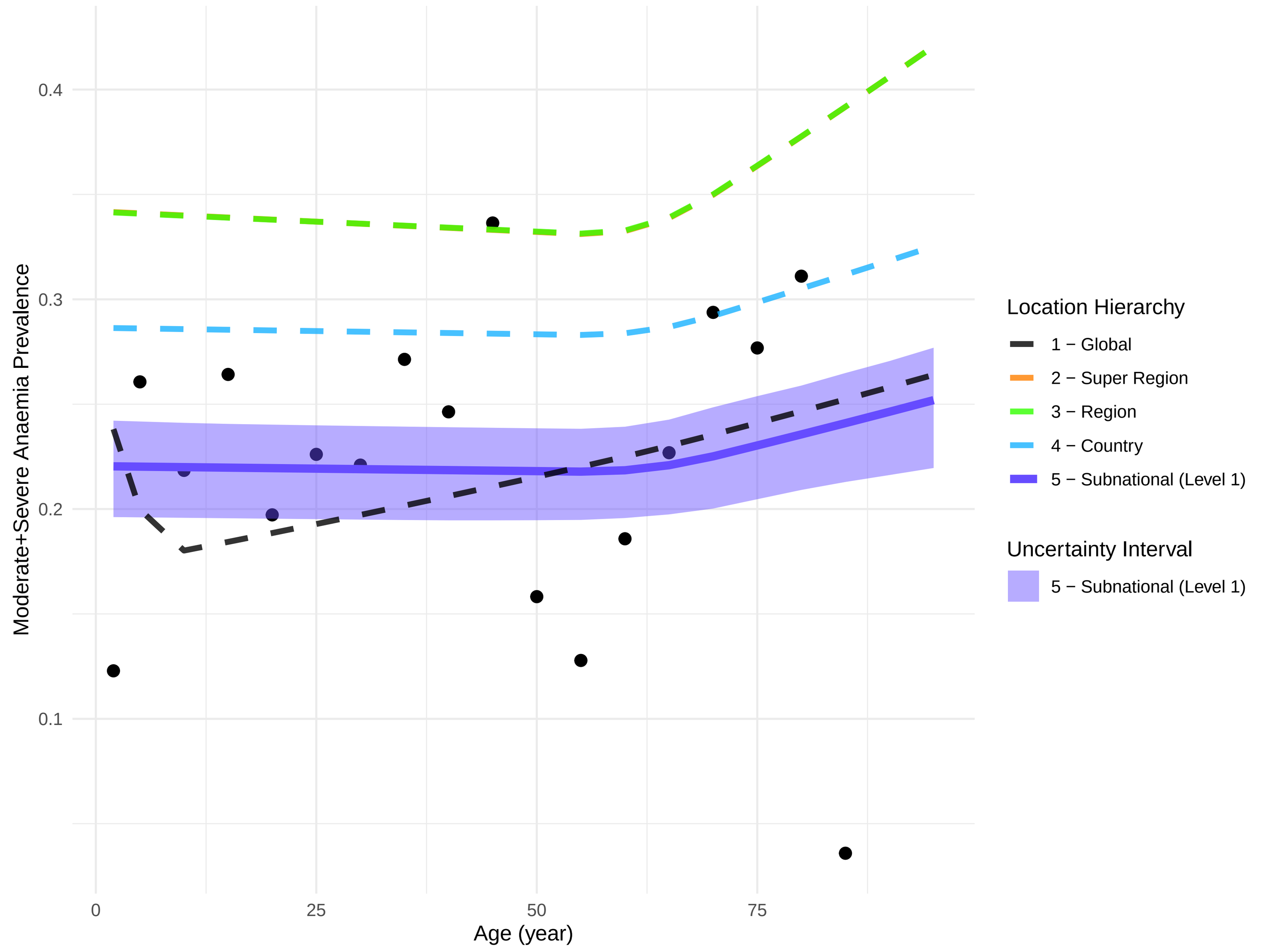

MR−BRT Age/Sex Fits − Non−pregnant Females 2+ Years Old − Moderate+Severe Anaemia Prevalence − Balochistan
WHO−2001 Adjustment Method

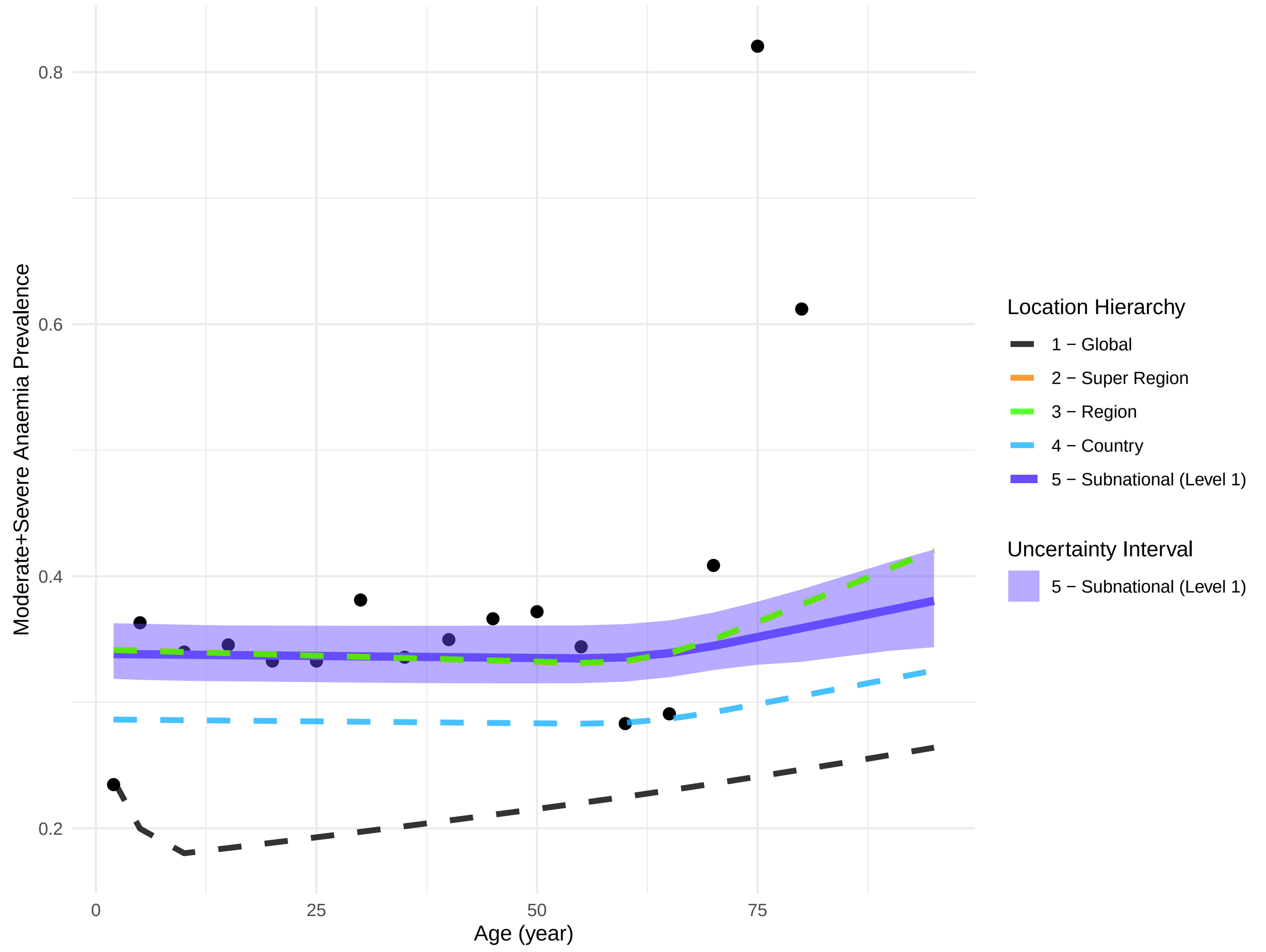

MR−BRT Age/Sex Fits − Non−pregnant Females 2+ Years Old − Moderate+Severe Anaemia Prevalence − Gilgit−Baltistan
WHO−2001 Adjustment Method

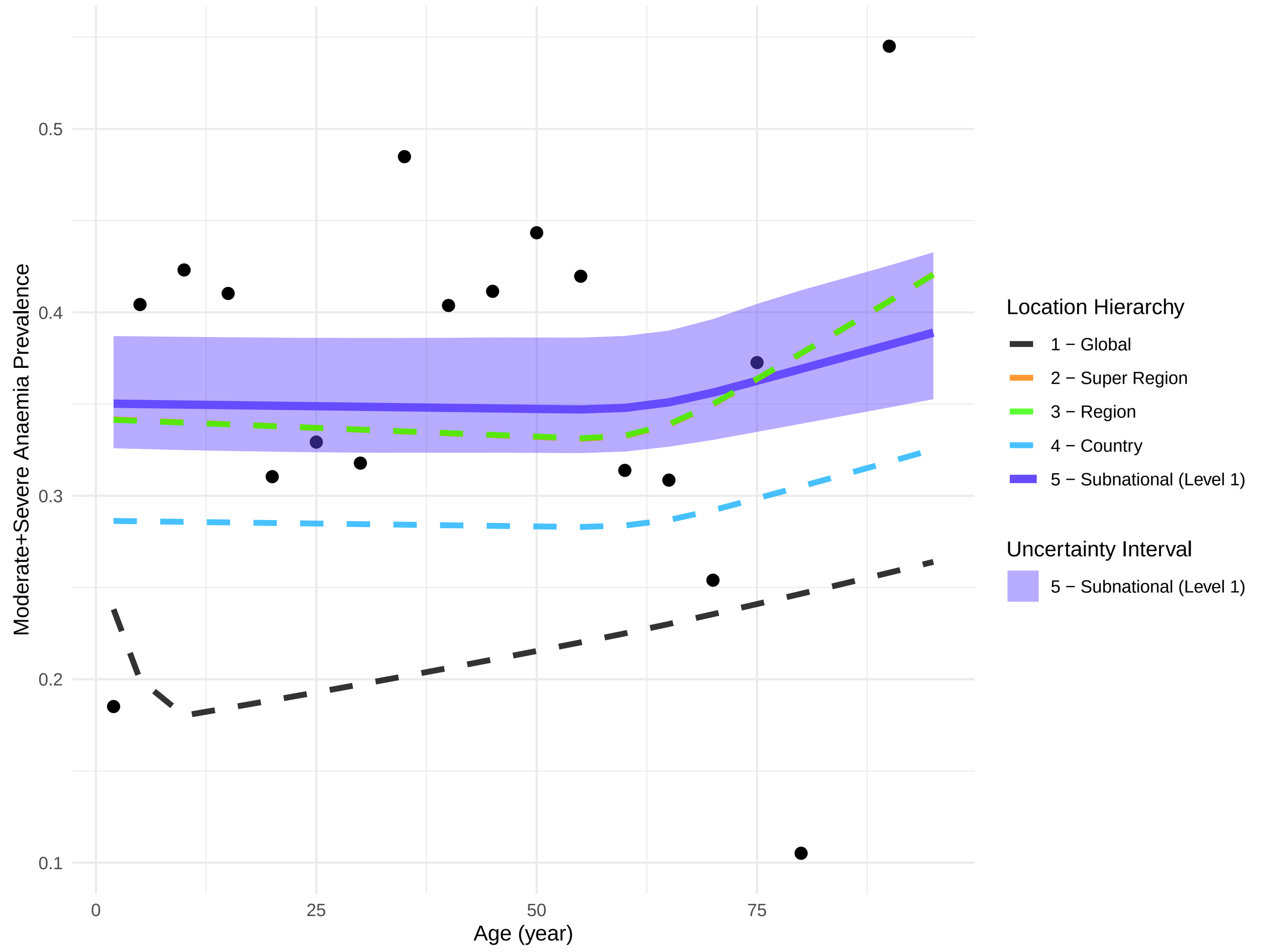

MR−BRT Age/Sex Fits − Non−pregnant Females 2+ Years Old − Moderate+Severe Anaemia Prevalence − Khyber Pakhtunkhwa
WHO−2001 Adjustment Method

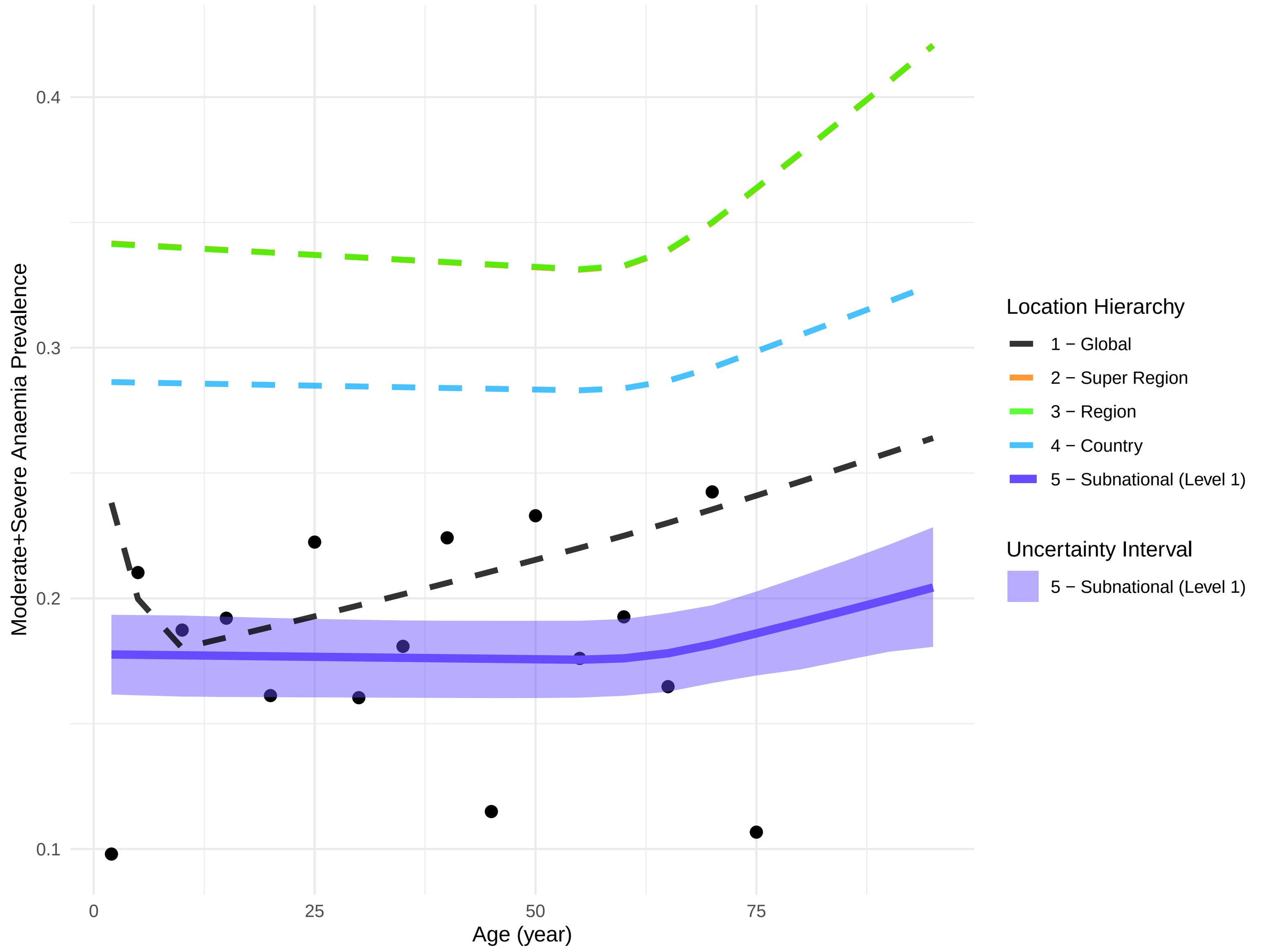

MR−BRT Age/Sex Fits − Non−pregnant Females 2+ Years Old − Moderate+Severe Anaemia Prevalence − Punjab
WHO−2001 Adjustment Method

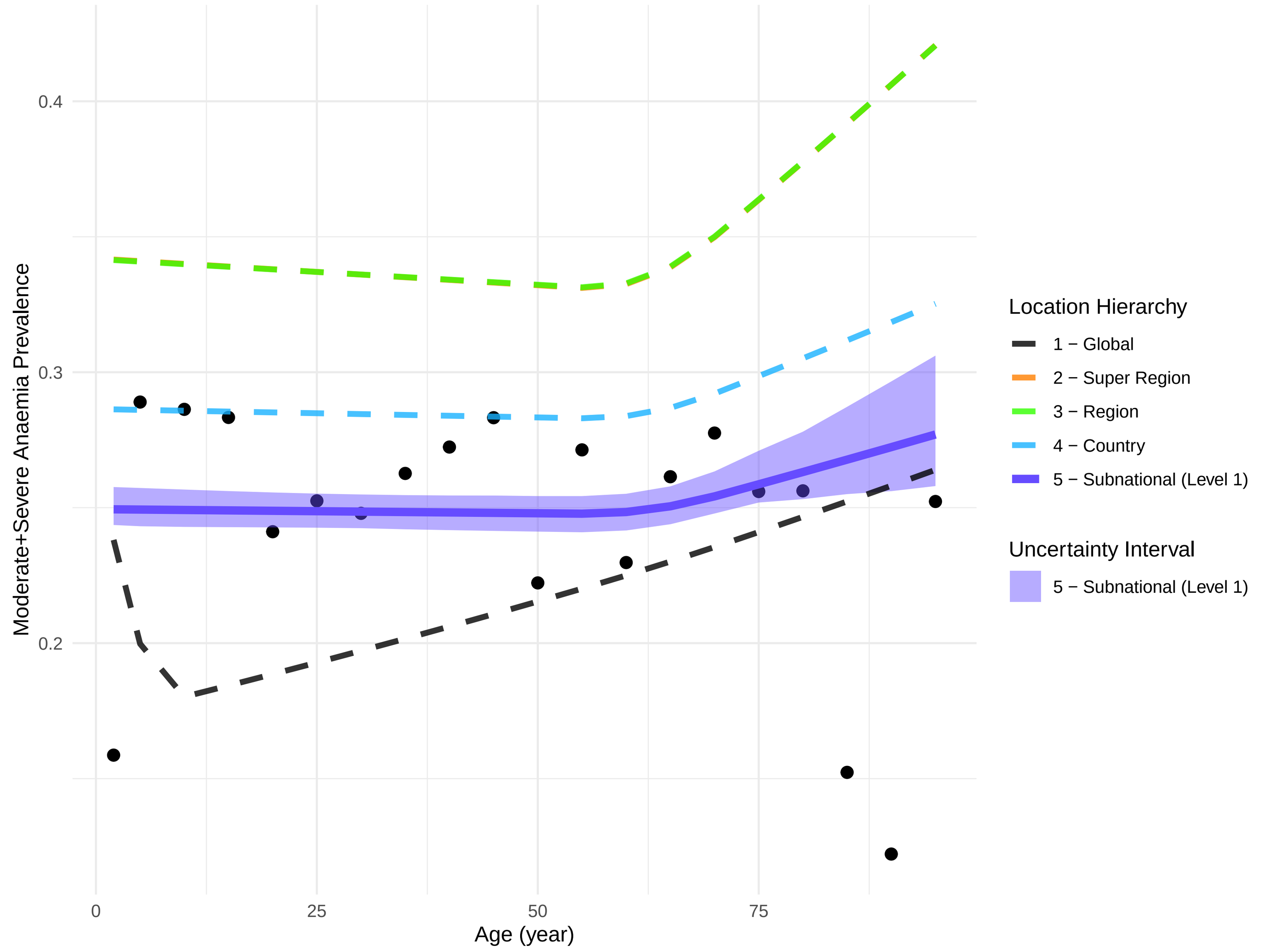

MR−BRT Age/Sex Fits − Non−pregnant Females 2+ Years Old − Moderate+Severe Anaemia Prevalence − Sindh
WHO−2001 Adjustment Method

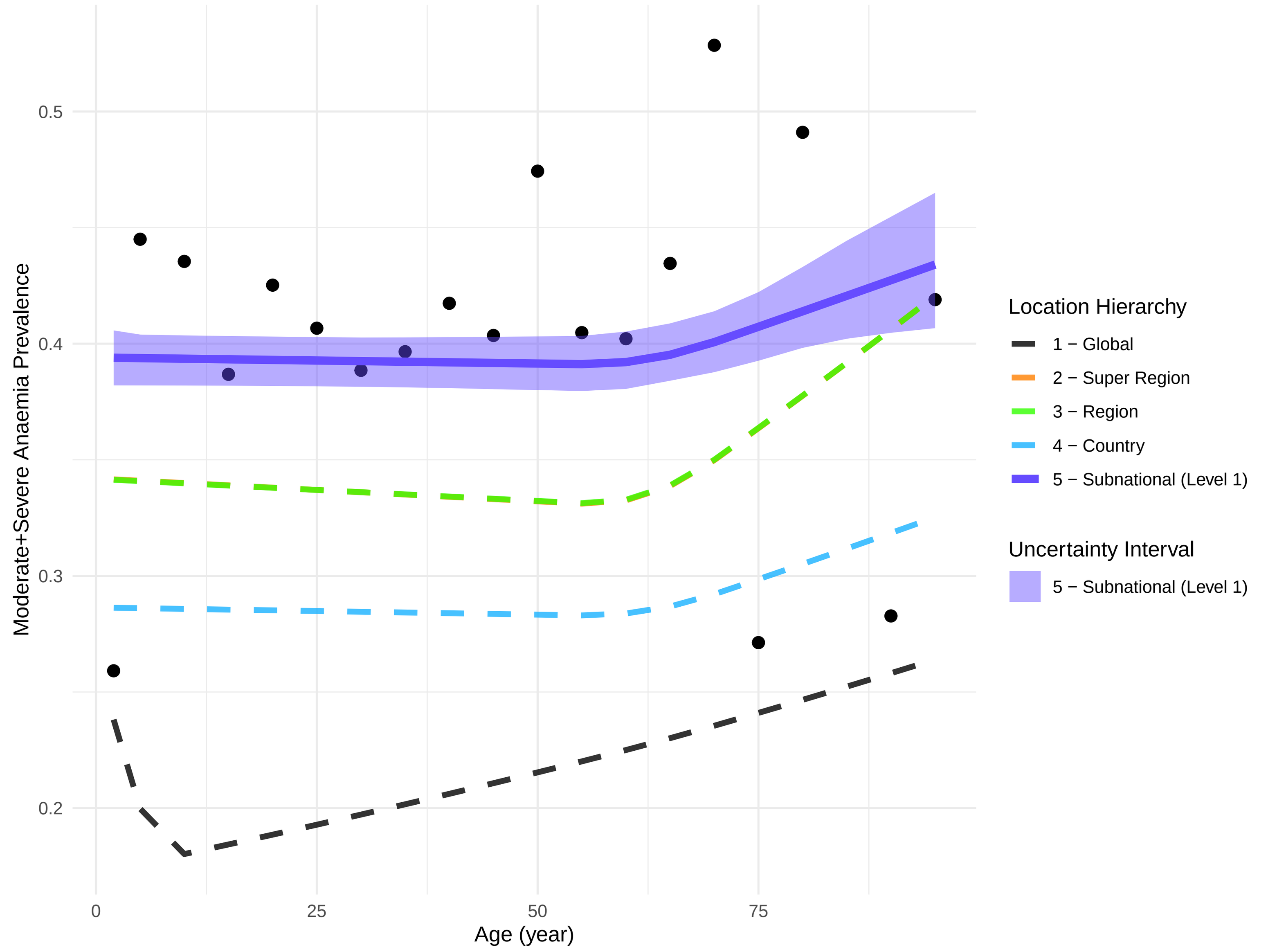

MR−BRT Age/Sex Fits − Non−pregnant Females 2+ Years Old − Moderate+Severe Anaemia Prevalence − Southeast Asia, East Asia, and Oceania
WHO−2001 Adjustment Method

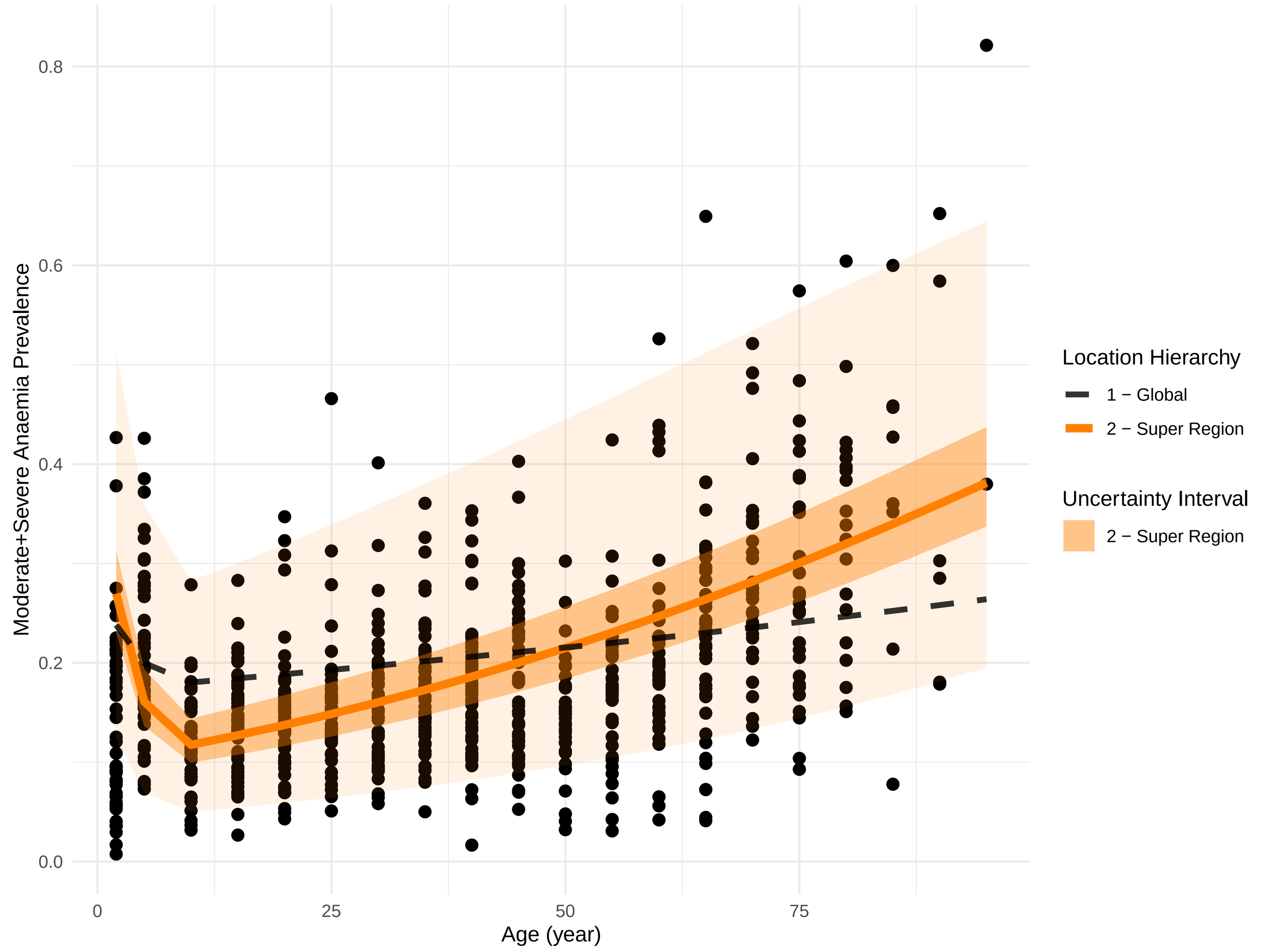

MR−BRT Age/Sex Fits − Non−pregnant Females 2+ Years Old − Moderate+Severe Anaemia Prevalence − Oceania
WHO−2001 Adjustment Method

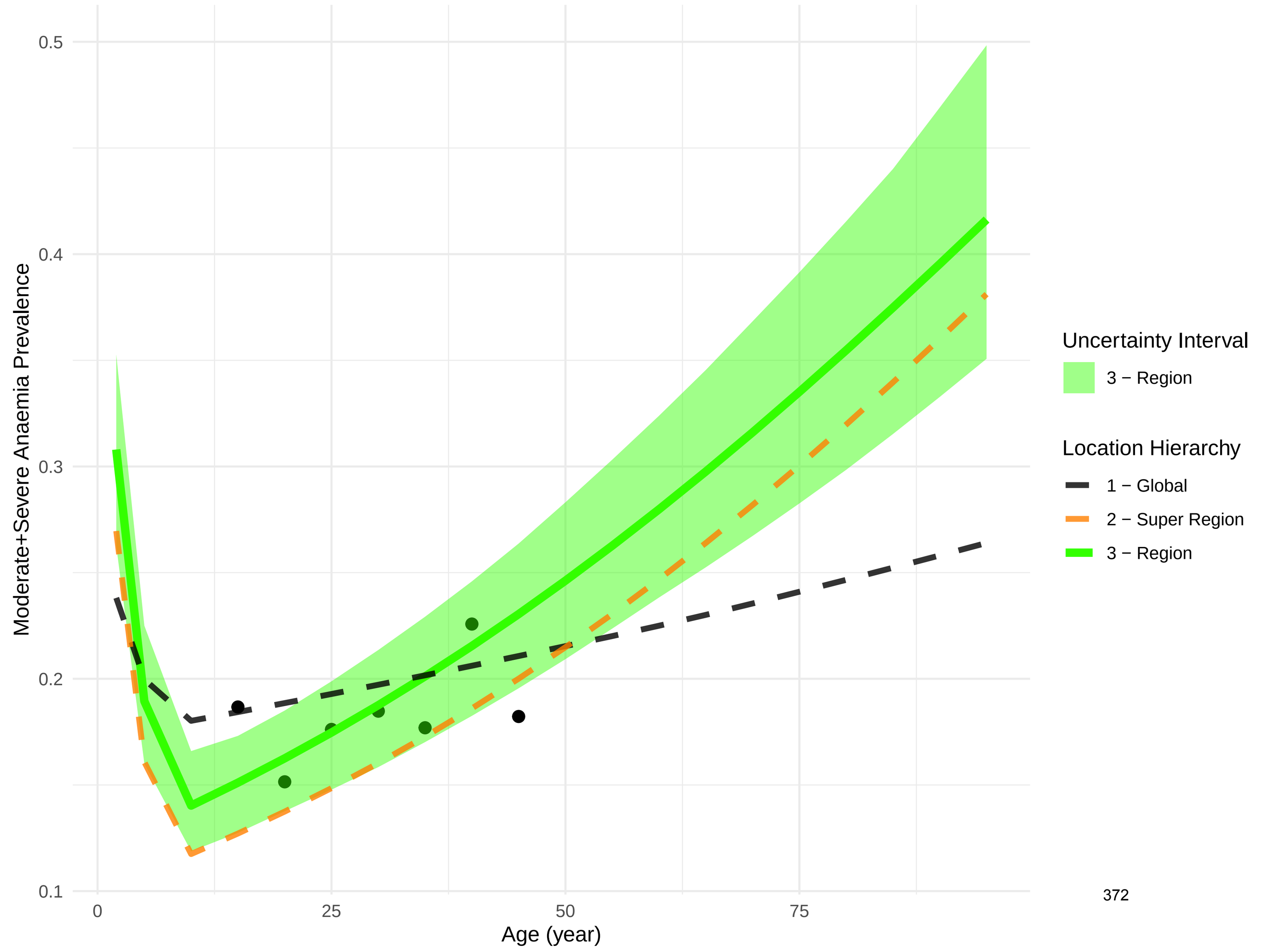

MR−BRT Age/Sex Fits − Non−pregnant Females 2+ Years Old − Moderate+Severe Anaemia Prevalence − Papua New Guinea
WHO−2001 Adjustment Method

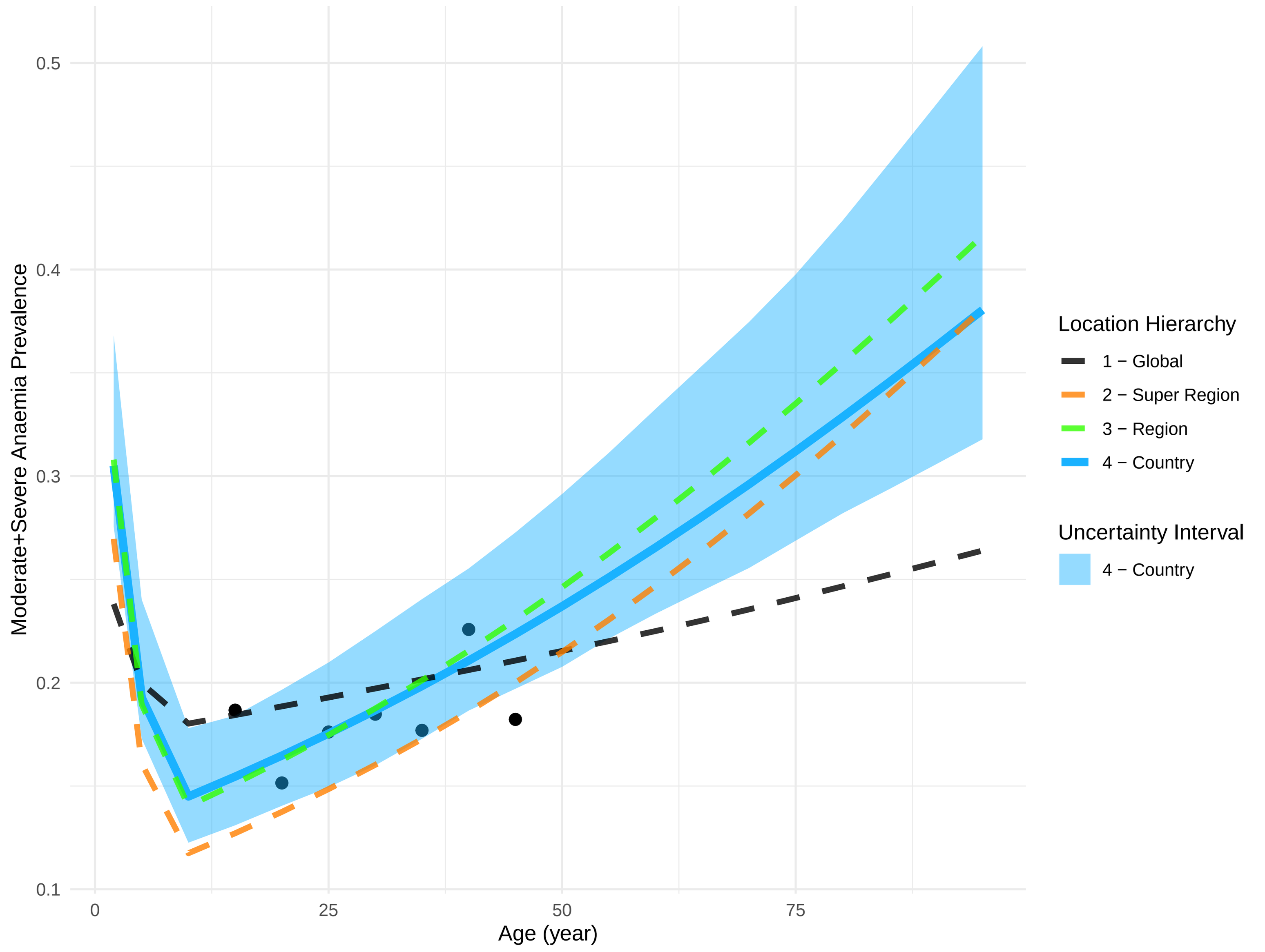

MR−BRT Age/Sex Fits − Non−pregnant Females 2+ Years Old − Moderate+Severe Anaemia Prevalence − Southeast Asia
WHO−2001 Adjustment Method

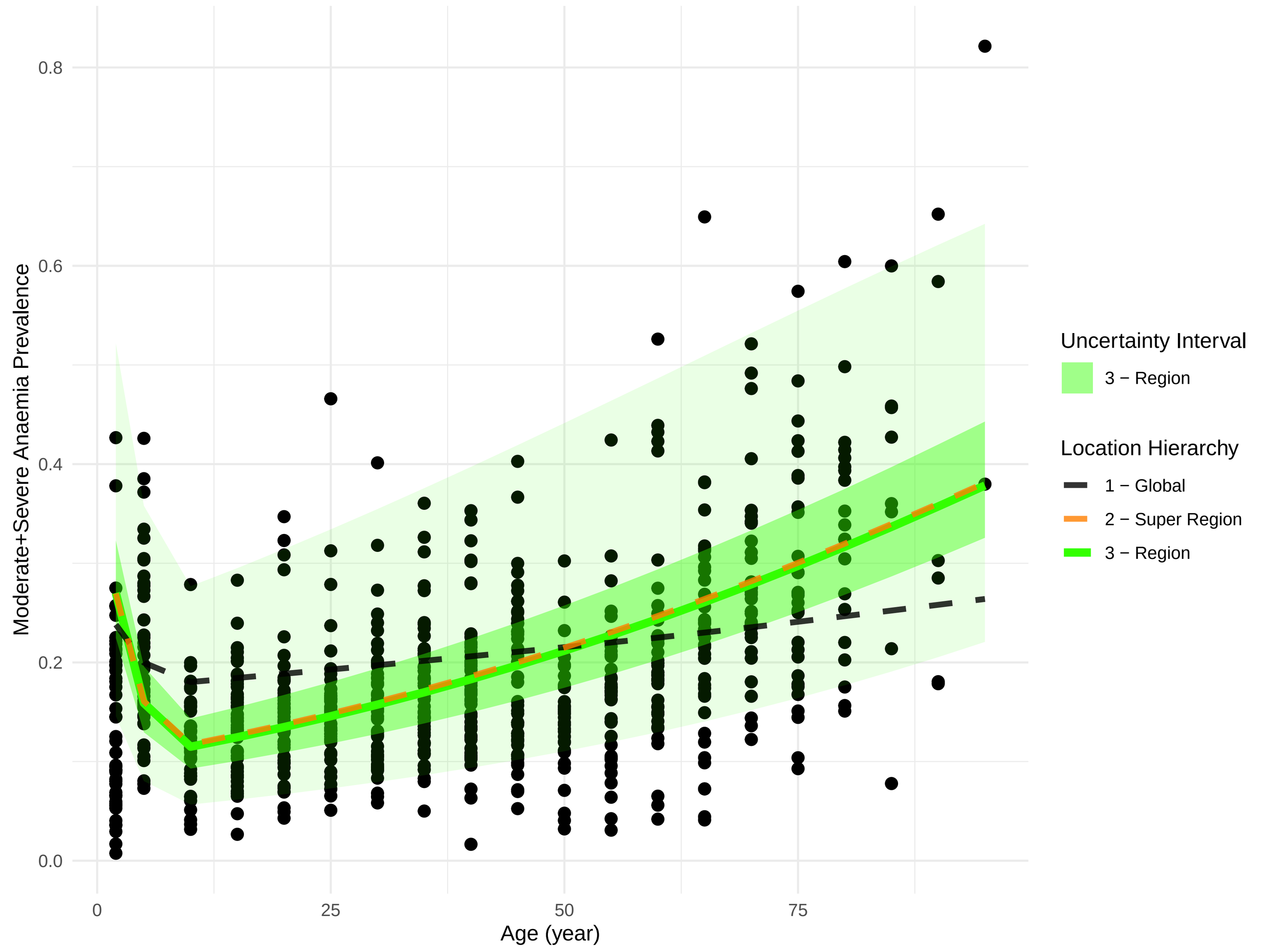

MR−BRT Age/Sex Fits − Non−pregnant Females 2+ Years Old − Moderate+Severe Anaemia Prevalence − Cambodia
WHO−2001 Adjustment Method

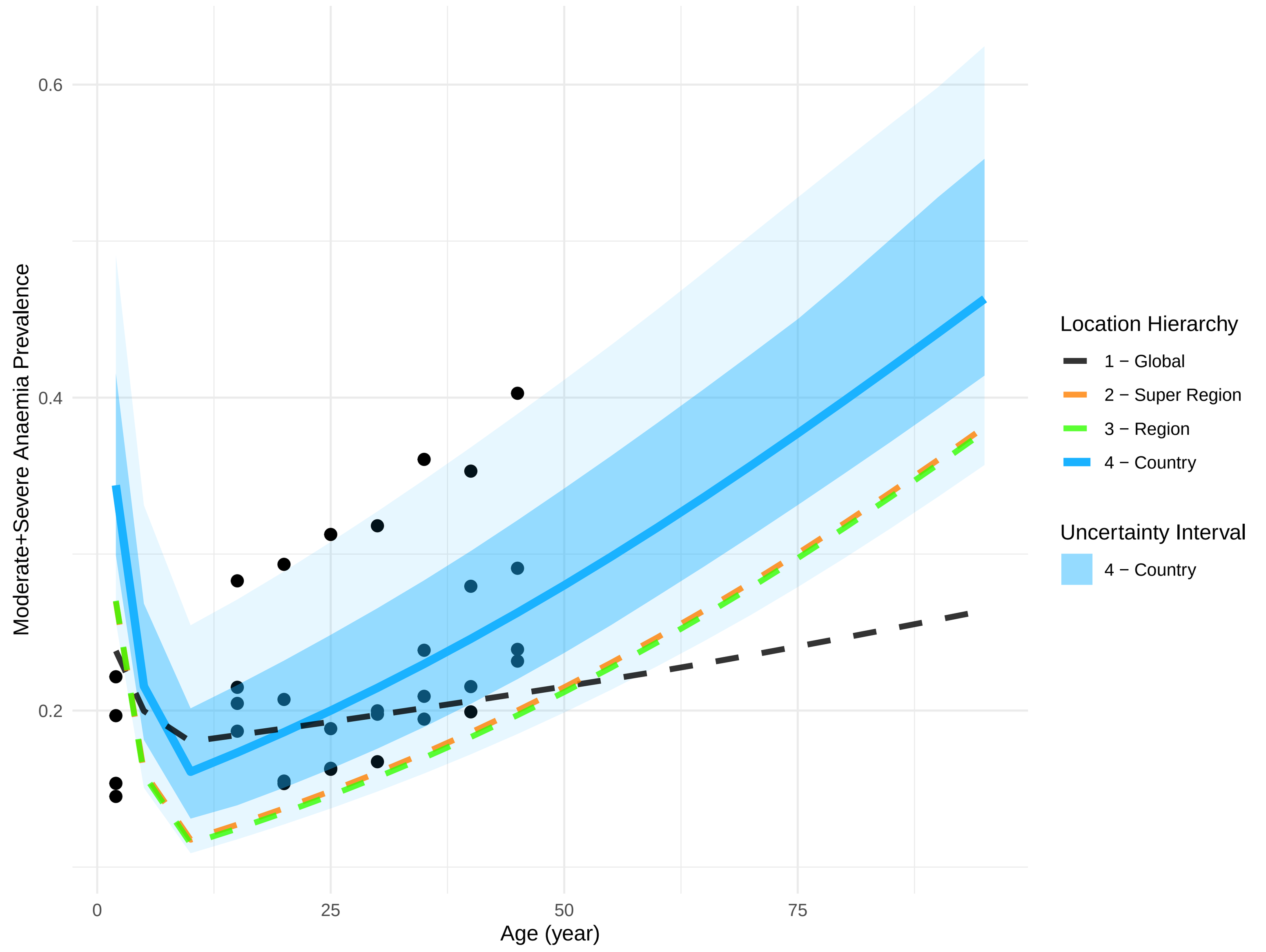

MR−BRT Age/Sex Fits − Non−pregnant Females 2+ Years Old − Moderate+Severe Anaemia Prevalence − Indonesia
WHO−2001 Adjustment Method

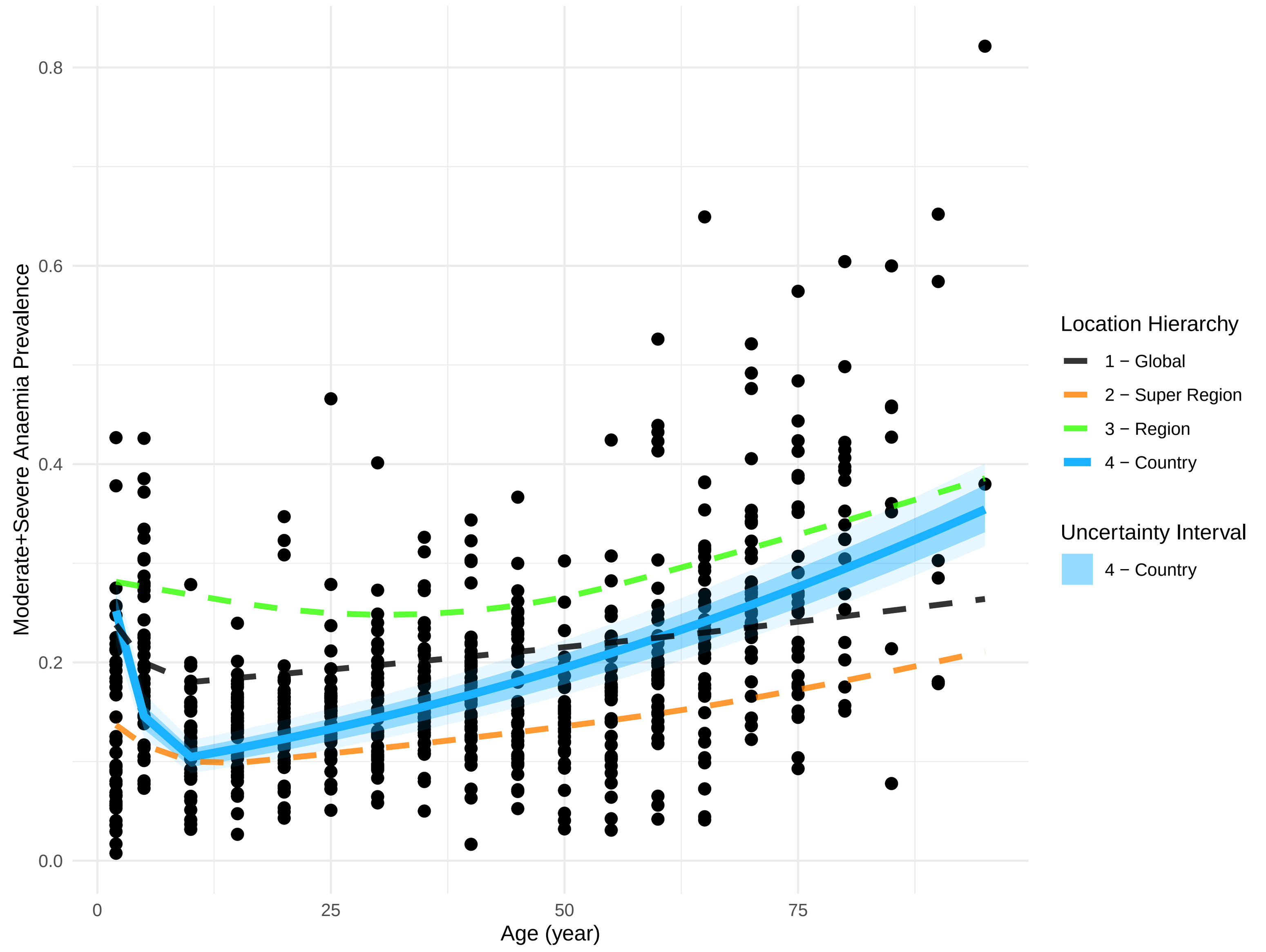

MR−BRT Age/Sex Fits − Non−pregnant Females 2+ Years Old − Moderate+Severe Anaemia Prevalence − Bali
WHO−2001 Adjustment Method

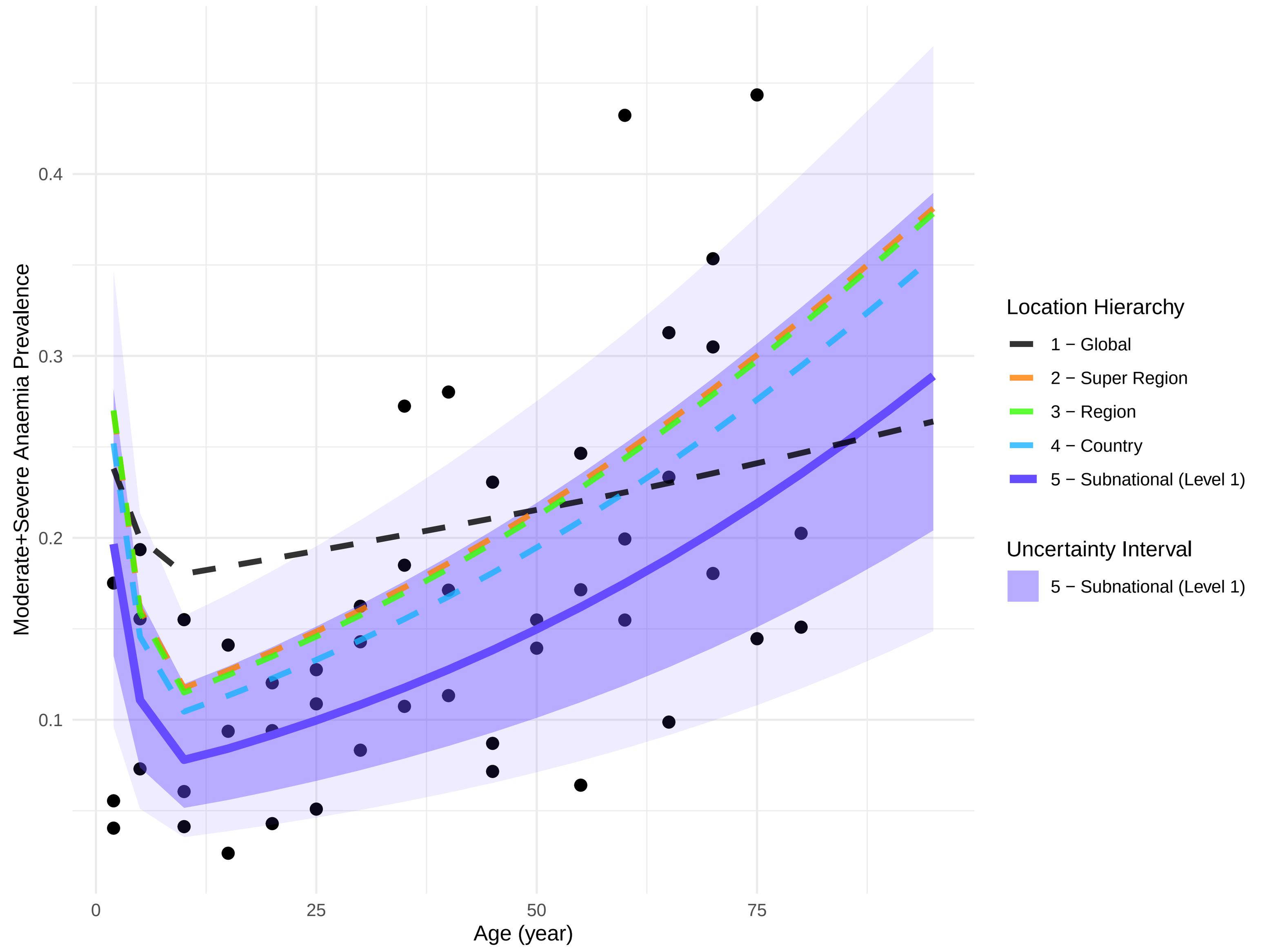

MR−BRT Age/Sex Fits − Non−pregnant Females 2+ Years Old − Moderate+Severe Anaemia Prevalence − Bangka−Belitung Islands
WHO−2001 Adjustment Method

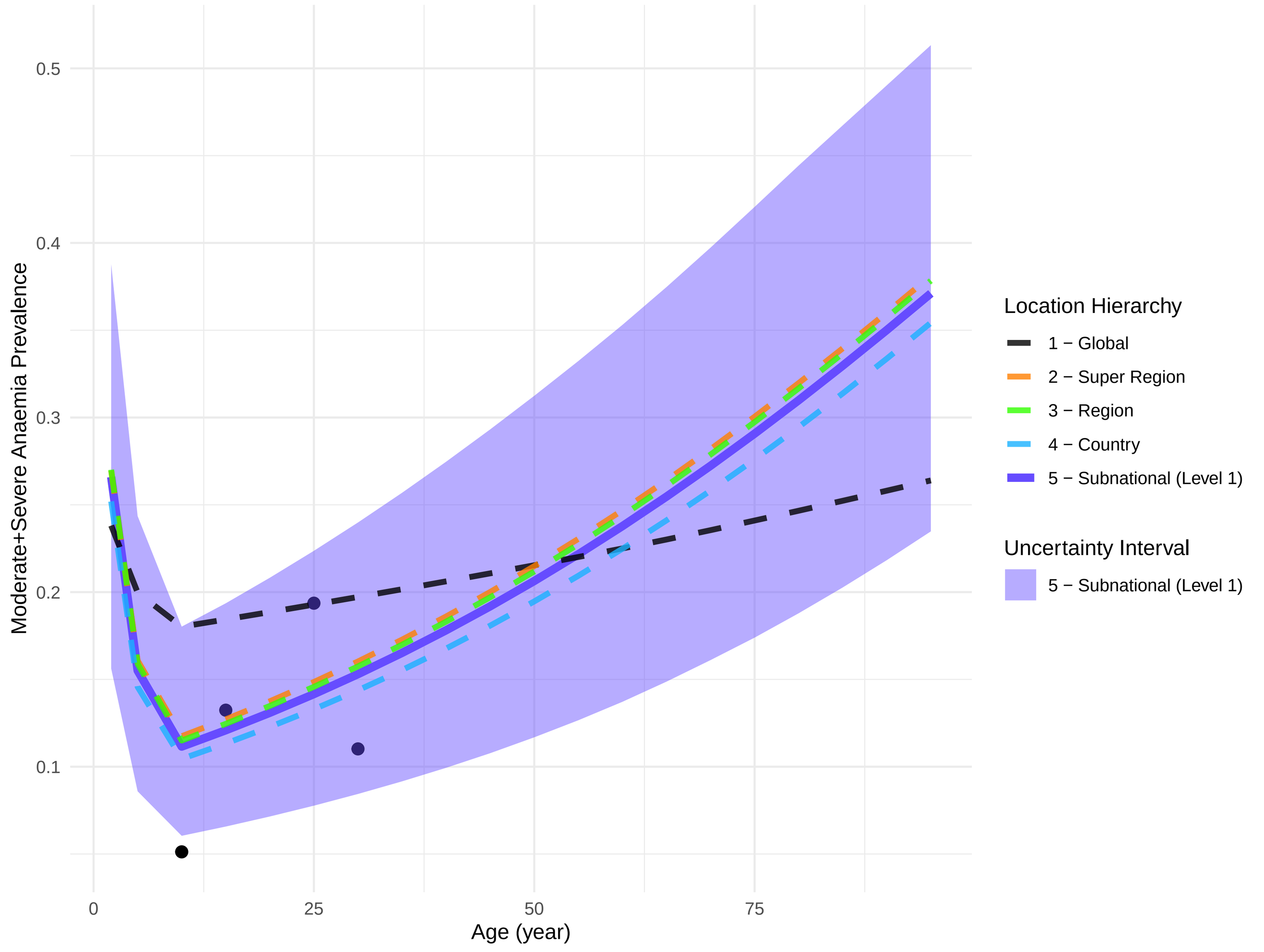

MR−BRT Age/Sex Fits − Non−pregnant Females 2+ Years Old − Moderate+Severe Anaemia Prevalence − Banten
WHO−2001 Adjustment Method

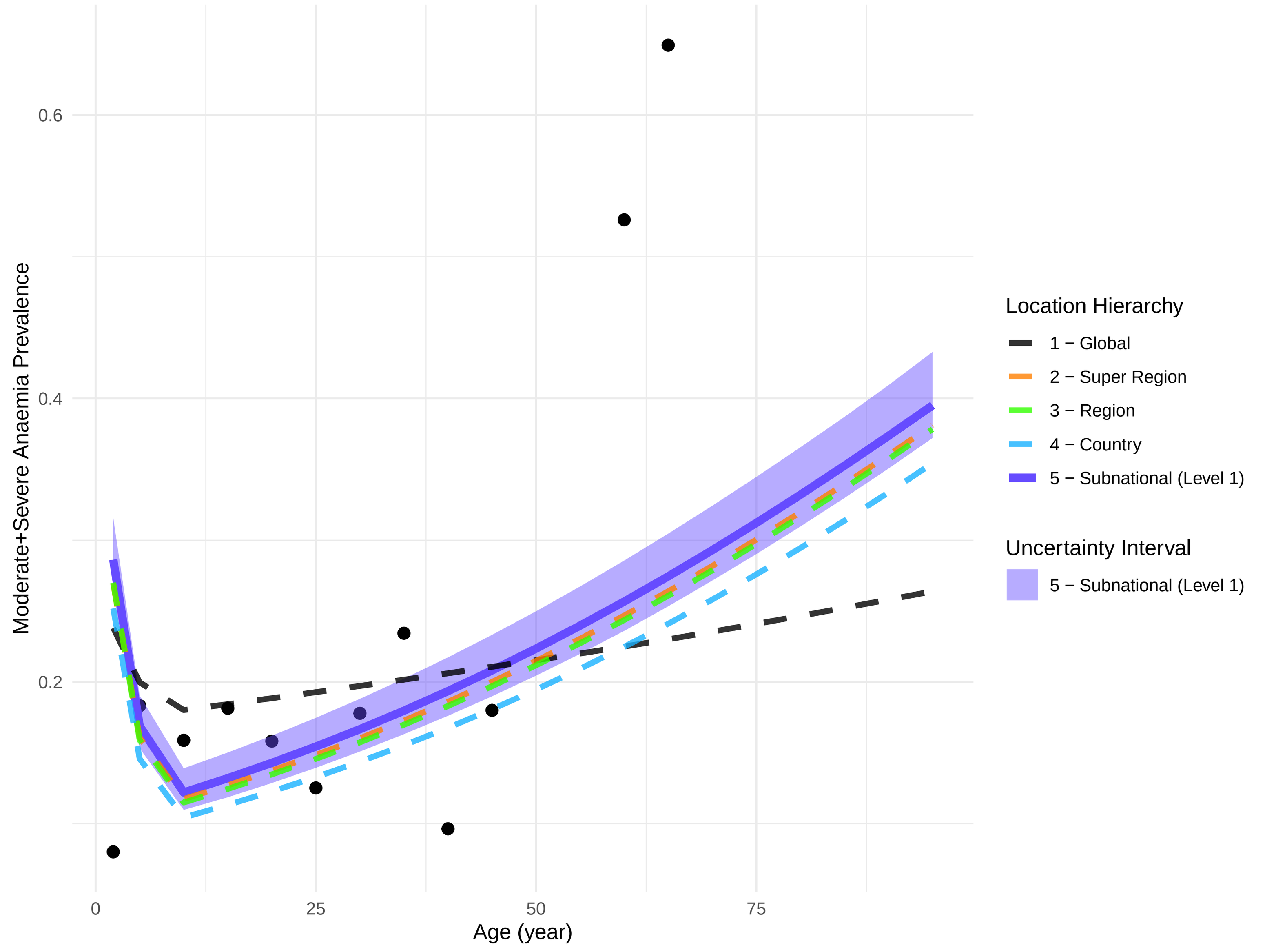

MR−BRT Age/Sex Fits − Non−pregnant Females 2+ Years Old − Moderate+Severe Anaemia Prevalence − Jakarta
WHO−2001 Adjustment Method

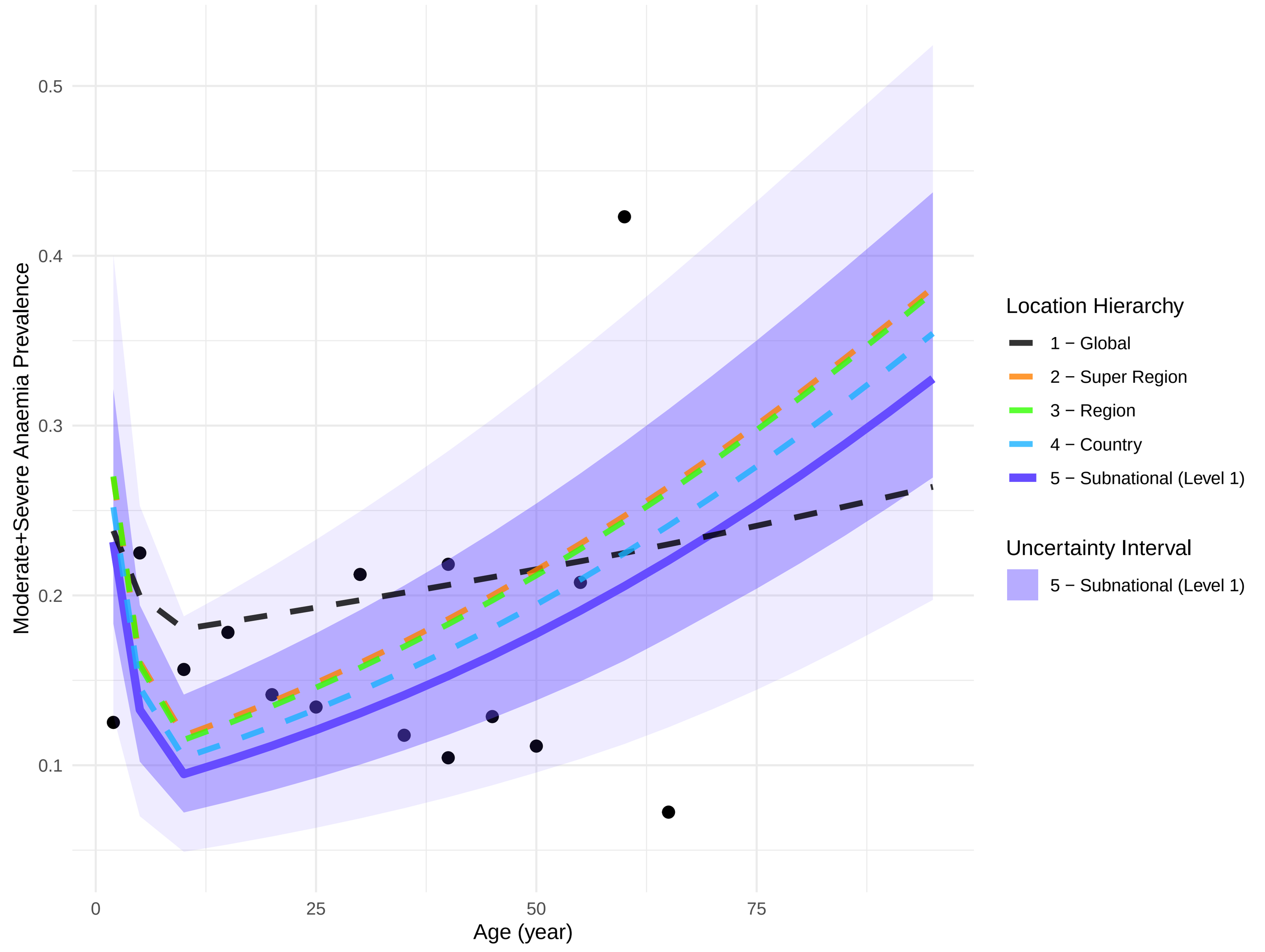

MR−BRT Age/Sex Fits − Non−pregnant Females 2+ Years Old − Moderate+Severe Anaemia Prevalence − West Java
WHO−2001 Adjustment Method

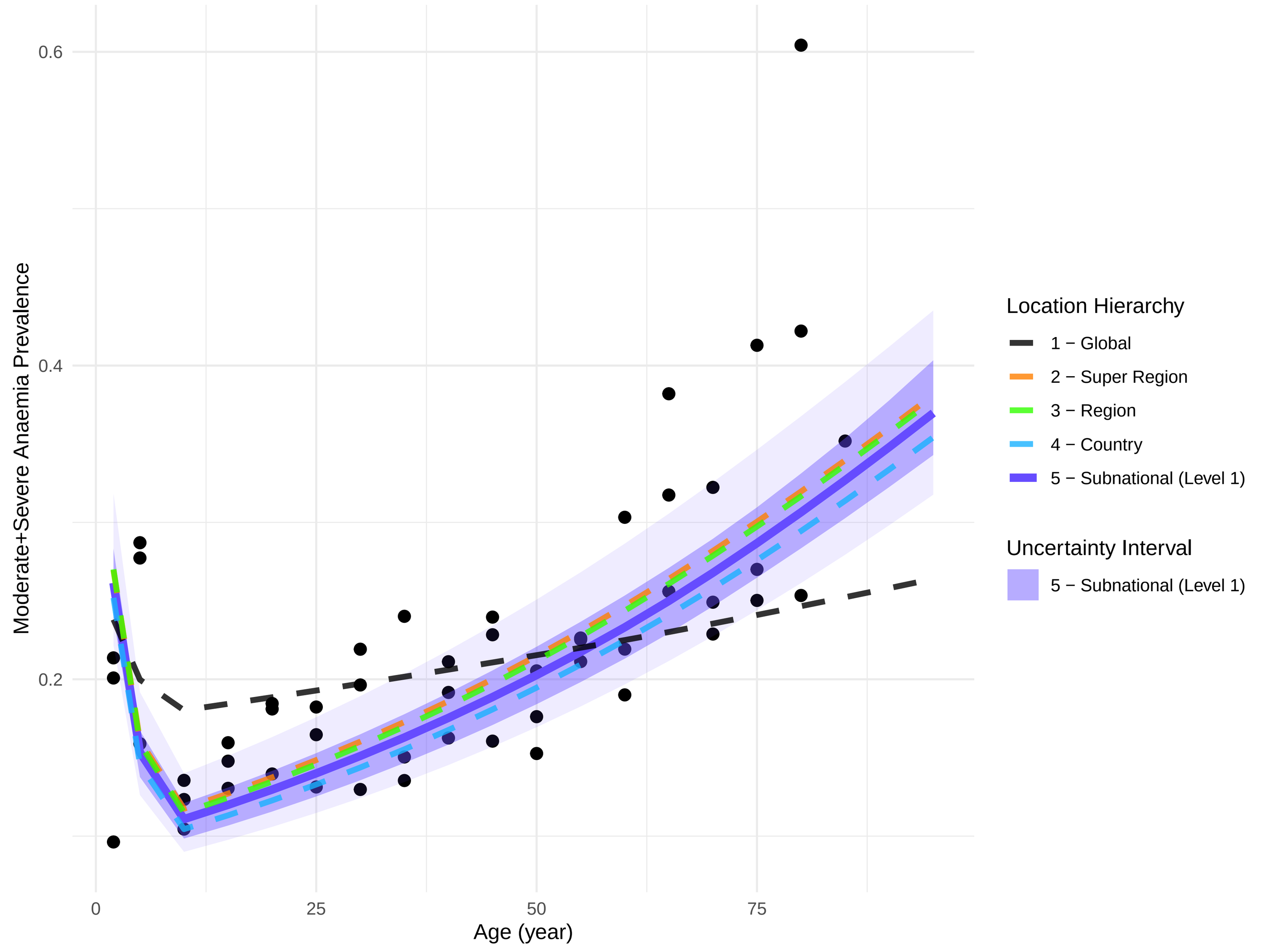

MR−BRT Age/Sex Fits − Non−pregnant Females 2+ Years Old − Moderate+Severe Anaemia Prevalence − Central Java
WHO−2001 Adjustment Method

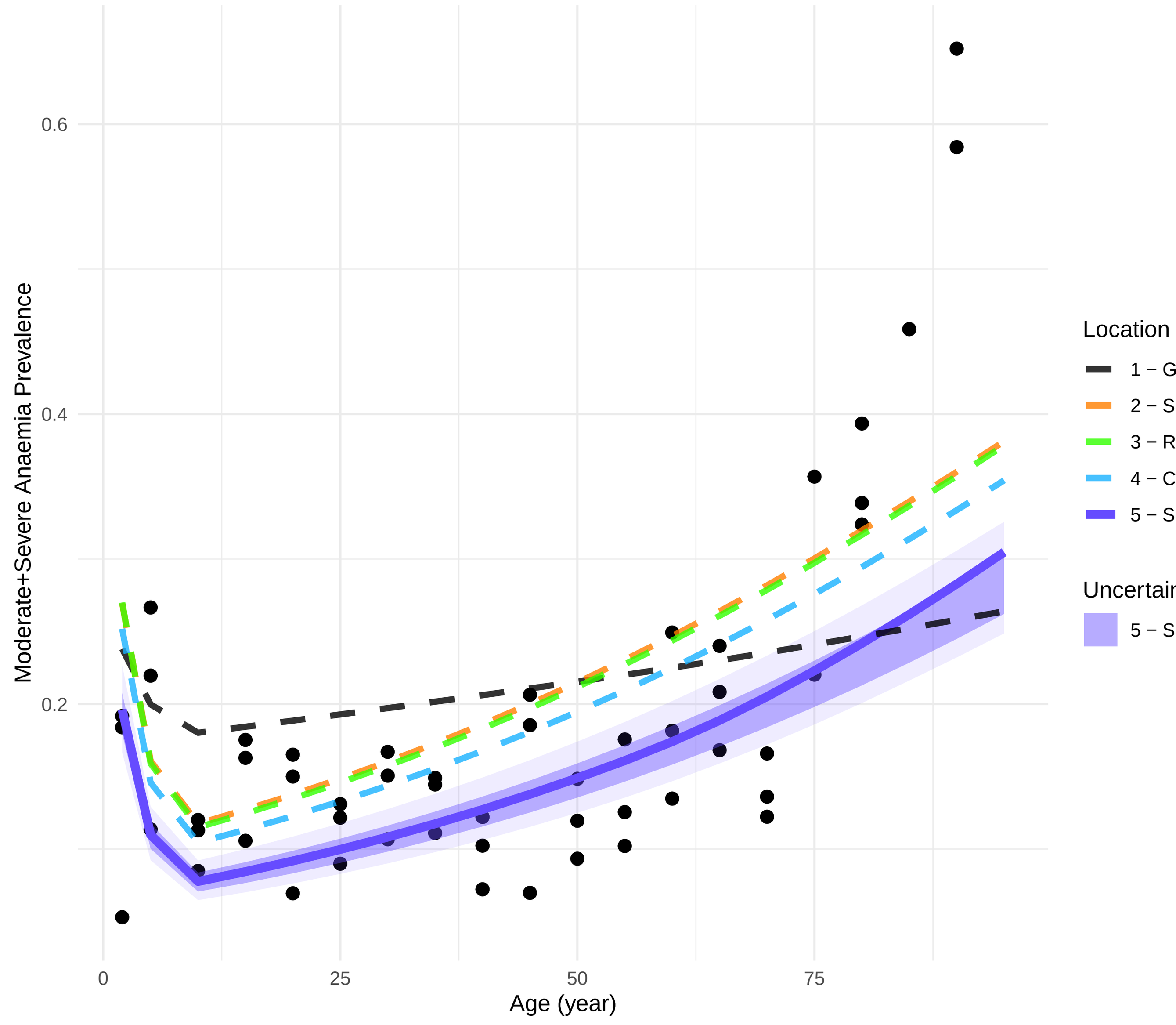

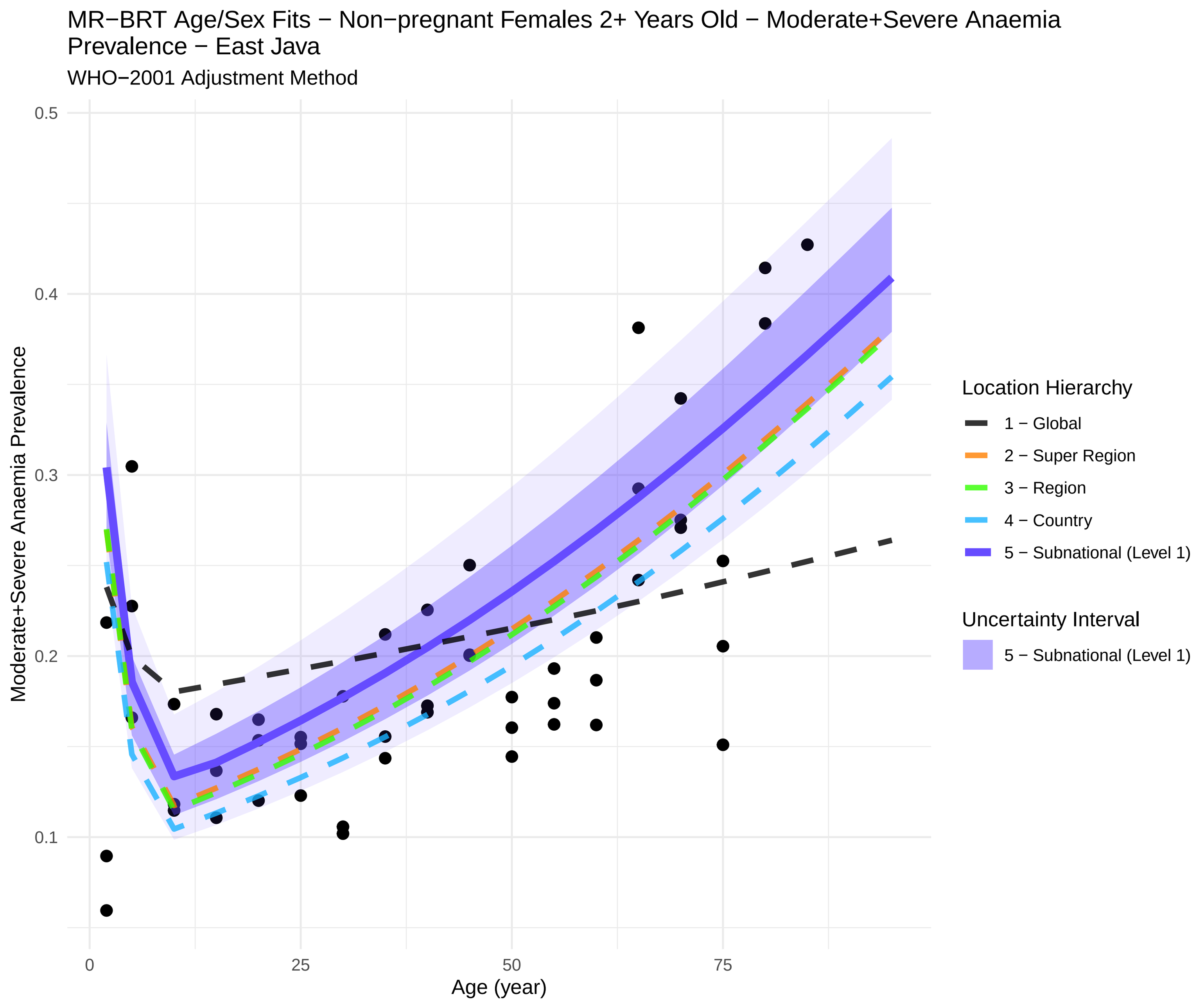
MR−BRT Age/Sex Fits − Non−pregnant Females 2+ Years Old − Moderate+Severe Anaemia Prevalence − East Java
WHO−2001 Adjustment Method
Moderate+Severe Anaemia Prevalence
Age (year)
Location Hierarchy
1 − Global
2 − Super Region
3 − Region
4 − Country
5 − Subnational (Level 1)
Uncertainty Interval
5 − Subnational (Level 1)

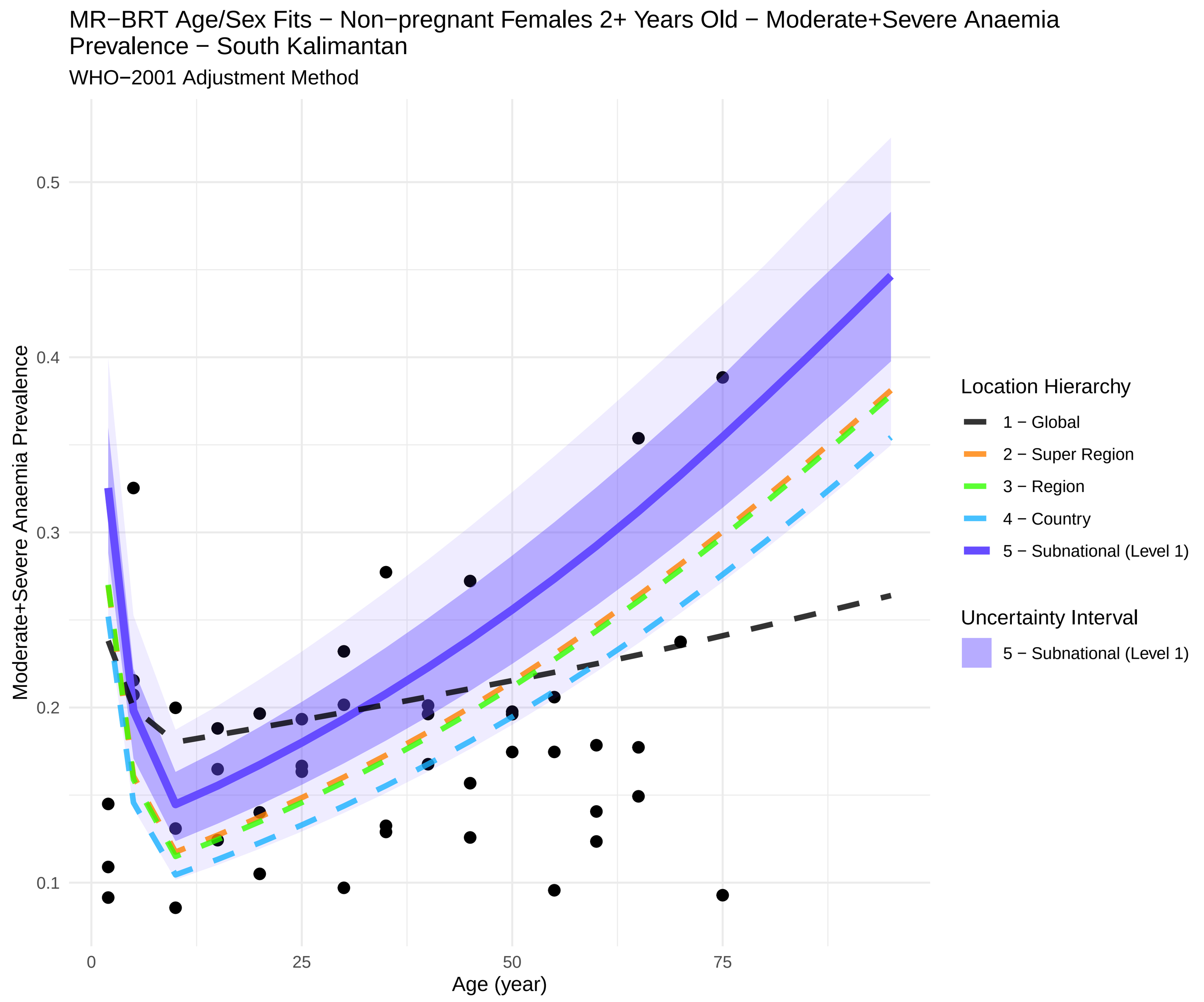
MR−BRT Age/Sex Fits − Non−pregnant Females 2+ Years Old − Moderate+Severe Anaemia Prevalence − South Kalimantan
WHO−2001 Adjustment Method
Moderate+Severe Anaemia Prevalence
Age (year)
Location Hierarchy
1 − Global
2 − Super Region
3 − Region
4 − Country
5 − Subnational (Level 1)
Uncertainty Interval
5 − Subnational (Level 1)

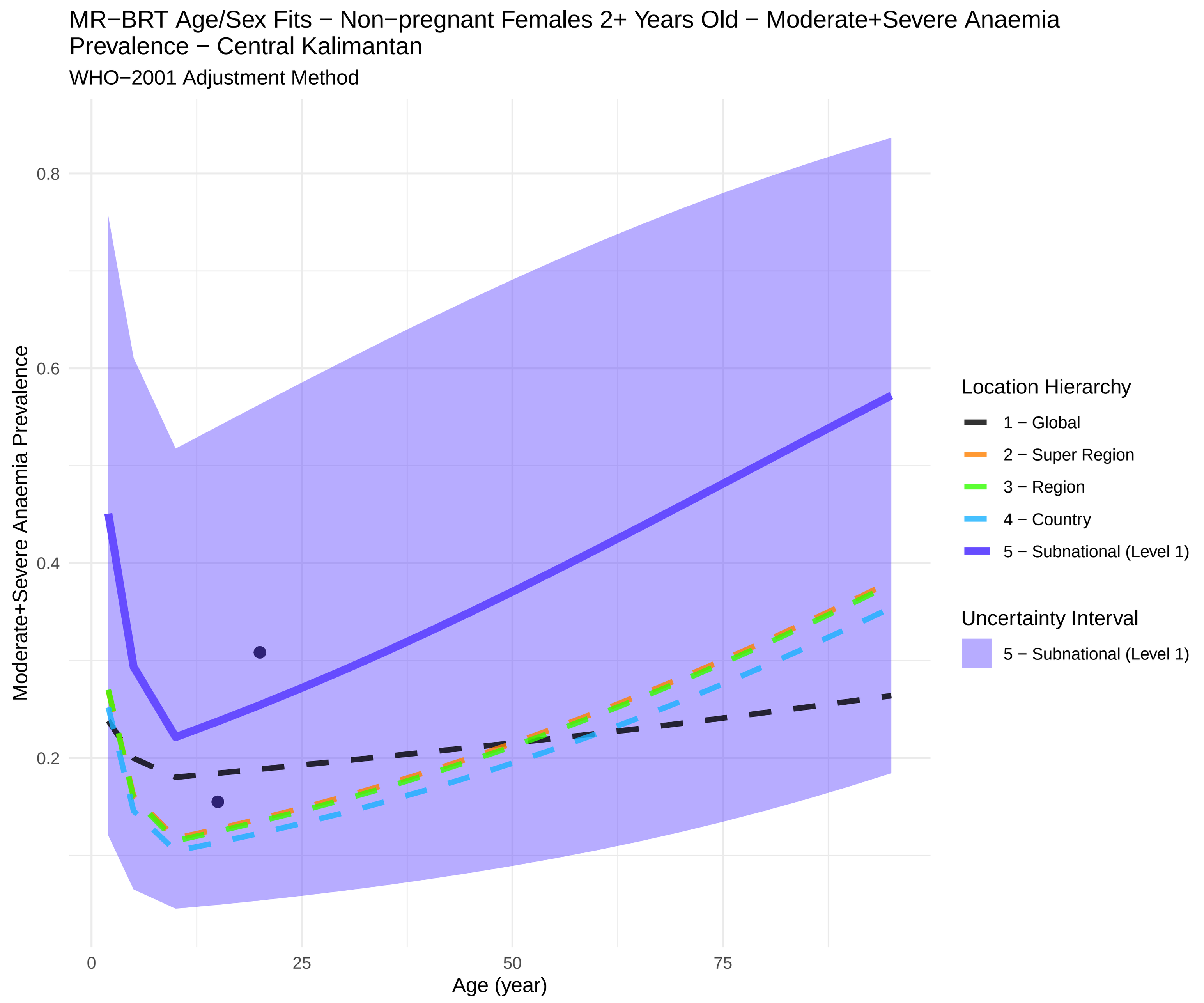
MR−BRT Age/Sex Fits − Non−pregnant Females 2+ Years Old − Moderate+Severe Anaemia Prevalence − Central Kalimantan
WHO−2001 Adjustment Method
Moderate+Severe Anaemia Prevalence
Age (year)
Location Hierarchy
1 − Global
2 − Super Region
3 − Region
4 − Country
5 − Subnational (Level 1)
Uncertainty Interval
5 − Subnational (Level 1)

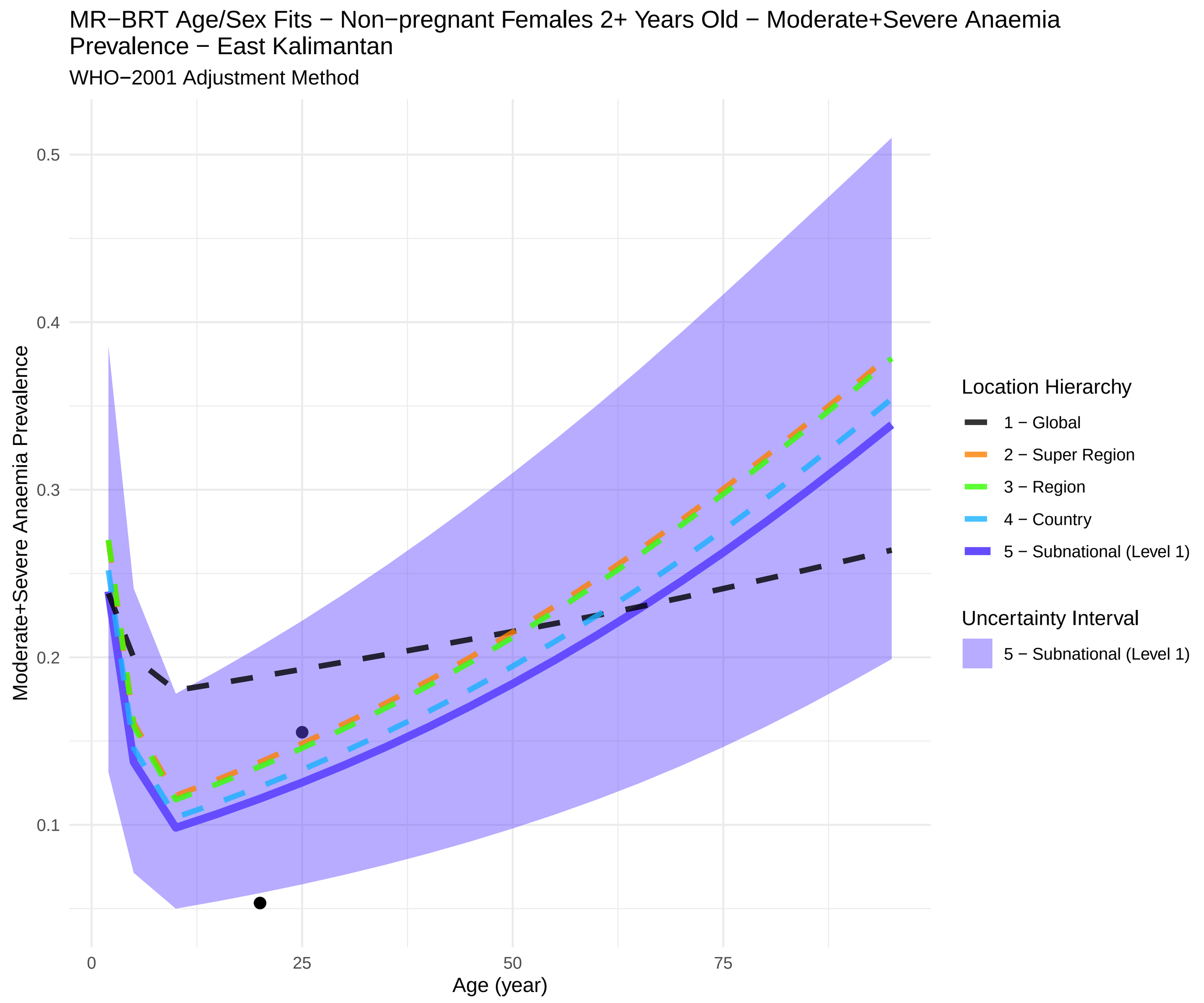
MR−BRT Age/Sex Fits − Non−pregnant Females 2+ Years Old − Moderate+Severe Anaemia Prevalence − East Kalimantan
WHO−2001 Adjustment Method
Moderate+Severe Anaemia Prevalence
Age (year)
Location Hierarchy
1 − Global
2 − Super Region
3 − Region
4 − Country
5 − Subnational (Level 1)
Uncertainty Interval
5 − Subnational (Level 1)

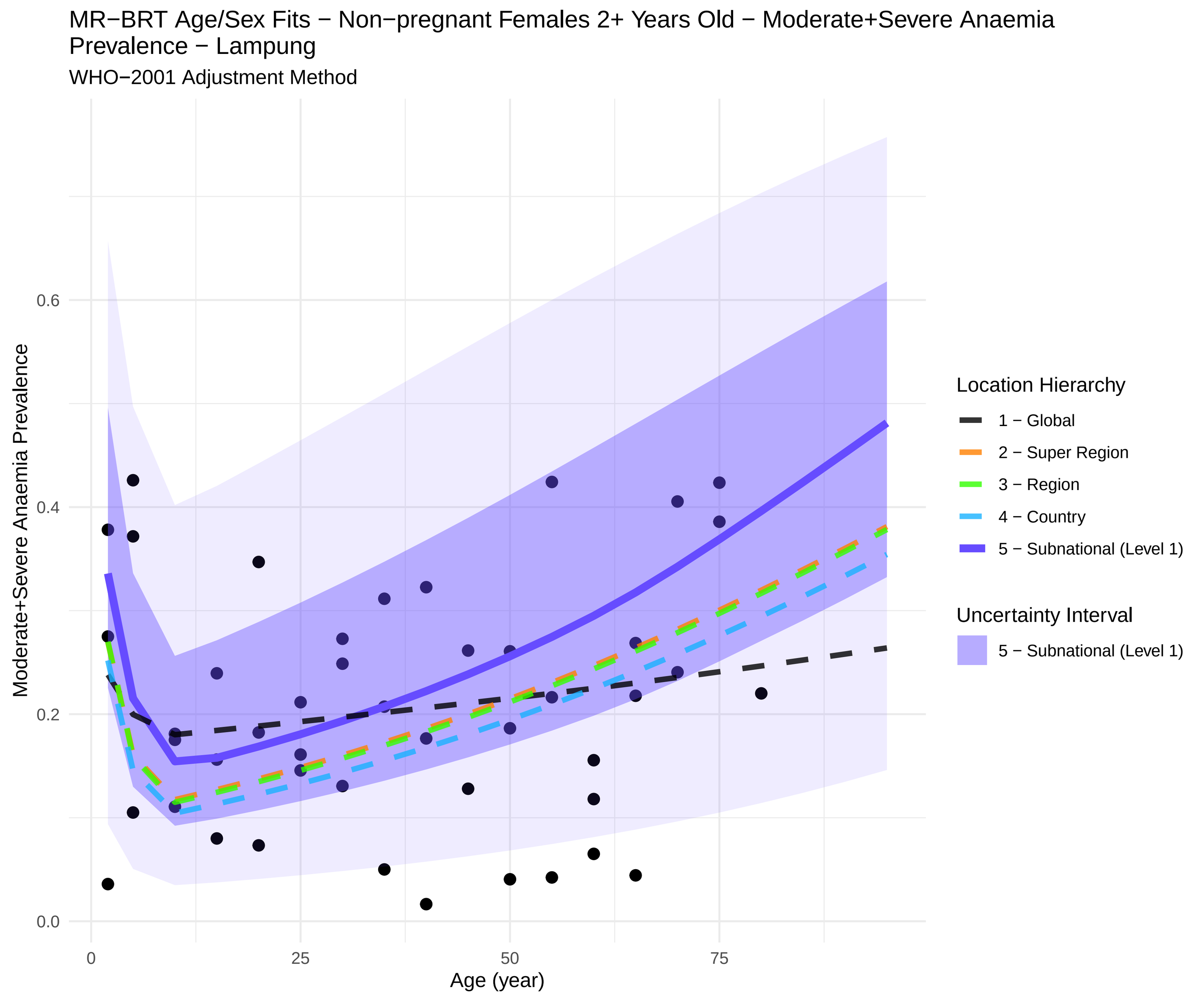
MR−BRT Age/Sex Fits − Non−pregnant Females 2+ Years Old − Moderate+Severe Anaemia Prevalence − Lampung
WHO−2001 Adjustment Method
Moderate+Severe Anaemia Prevalence
Age (year)
Location Hierarchy
1 − Global
2 − Super Region
3 − Region
4 − Country
5 − Subnational (Level 1)
Uncertainty Interval
5 − Subnational (Level 1)

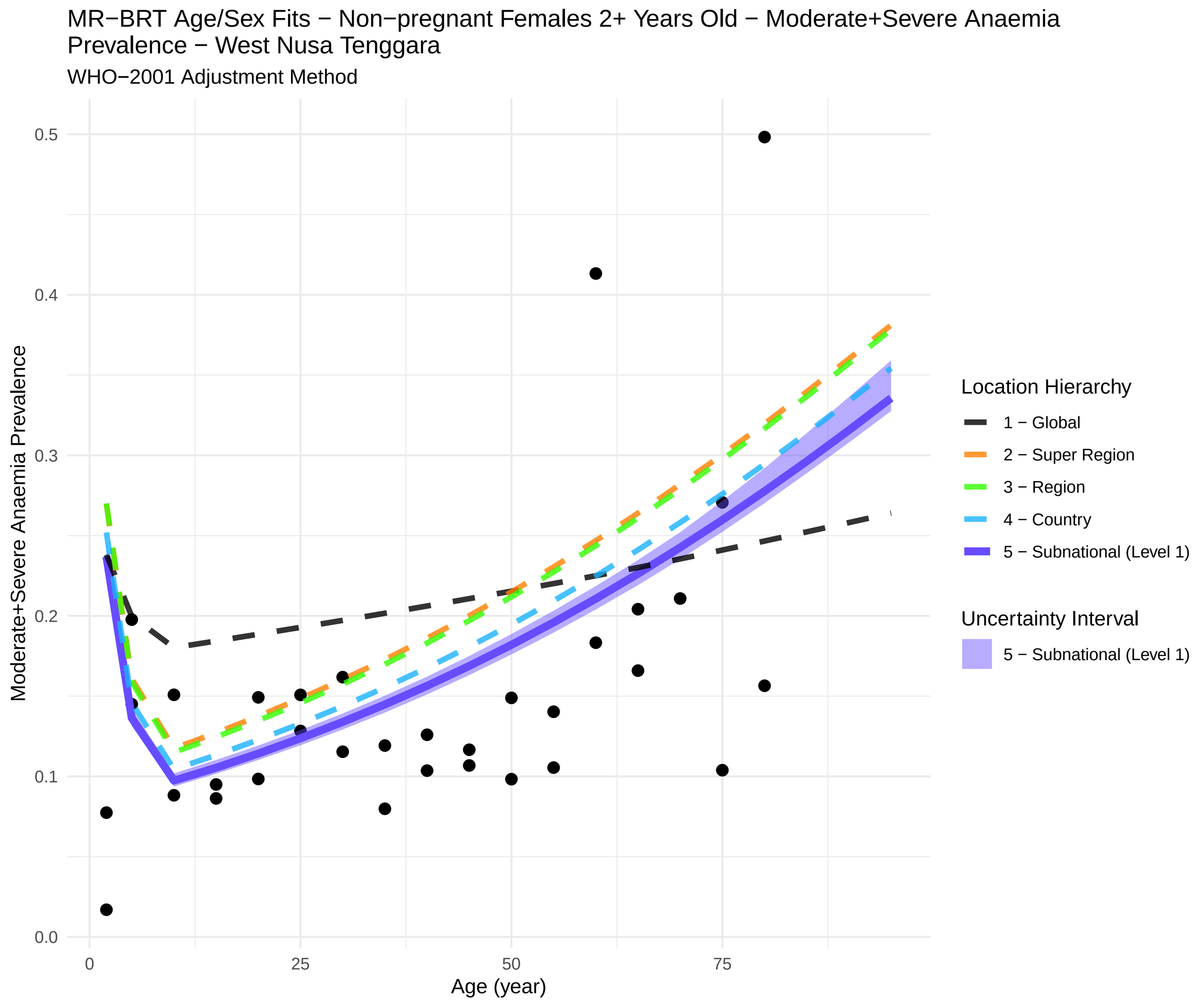
MR−BRT Age/Sex Fits − Non−pregnant Females 2+ Years Old − Moderate+Severe Anaemia Prevalence − West Nusa Tenggara
WHO−2001 Adjustment Method
Moderate+Severe Anaemia Prevalence
Age (year)
Location Hierarchy
1 − Global
2 − Super Region
3 − Region
4 − Country
5 − Subnational (Level 1)
Uncertainty Interval
5 − Subnational (Level 1)

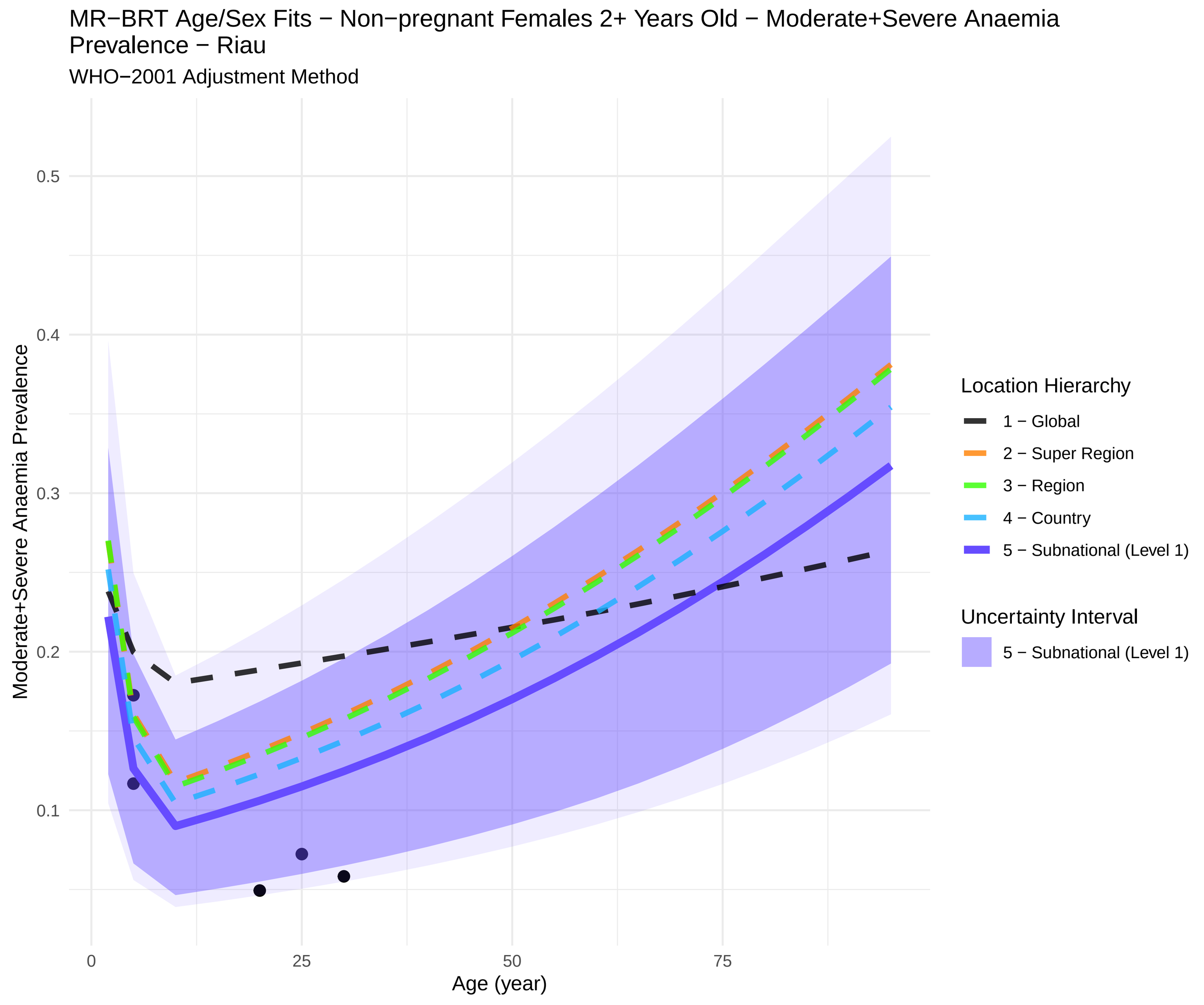
MR−BRT Age/Sex Fits − Non−pregnant Females 2+ Years Old − Moderate+Severe Anaemia Prevalence − Riau
WHO−2001 Adjustment Method
Moderate+Severe Anaemia Prevalence
Age (year)
Location Hierarchy
1 − Global
2 − Super Region
3 − Region
4 − Country
5 − Subnational (Level 1)
Uncertainty Interval
5 − Subnational (Level 1)

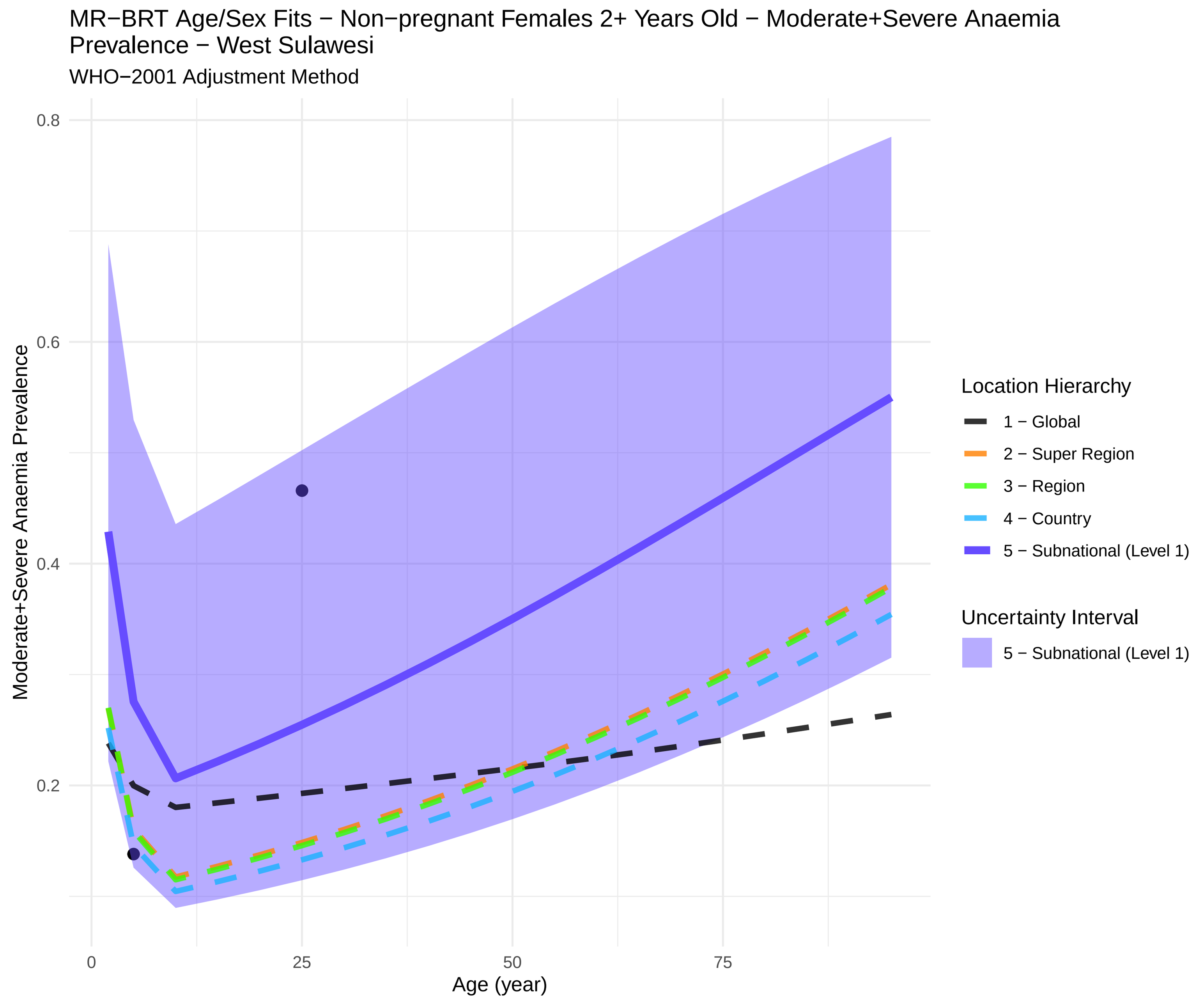
MR−BRT Age/Sex Fits − Non−pregnant Females 2+ Years Old − Moderate+Severe Anaemia Prevalence − West Sulawesi
WHO−2001 Adjustment Method
Moderate+Severe Anaemia Prevalence
Age (year)
Location Hierarchy
1 − Global
2 − Super Region
3 − Region
4 − Country
5 − Subnational (Level 1)
Uncertainty Interval
5 − Subnational (Level 1)

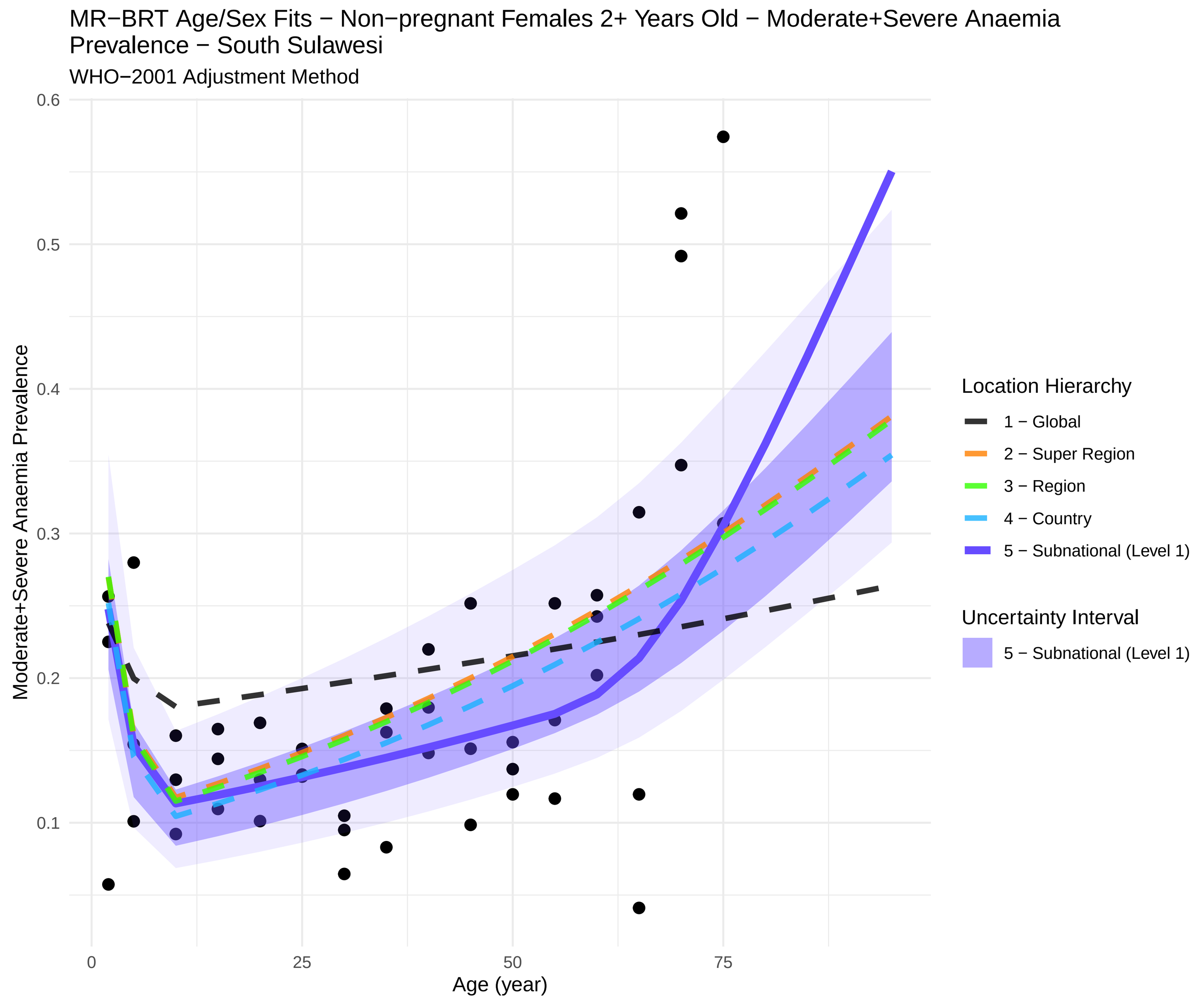
MR−BRT Age/Sex Fits − Non−pregnant Females 2+ Years Old − Moderate+Severe Anaemia Prevalence − South Sulawesi
WHO−2001 Adjustment Method
Moderate+Severe Anaemia Prevalence
Age (year)
Location Hierarchy
1 − Global
2 − Super Region
3 − Region
4 − Country
5 − Subnational (Level 1)
Uncertainty Interval
5 − Subnational (Level 1)

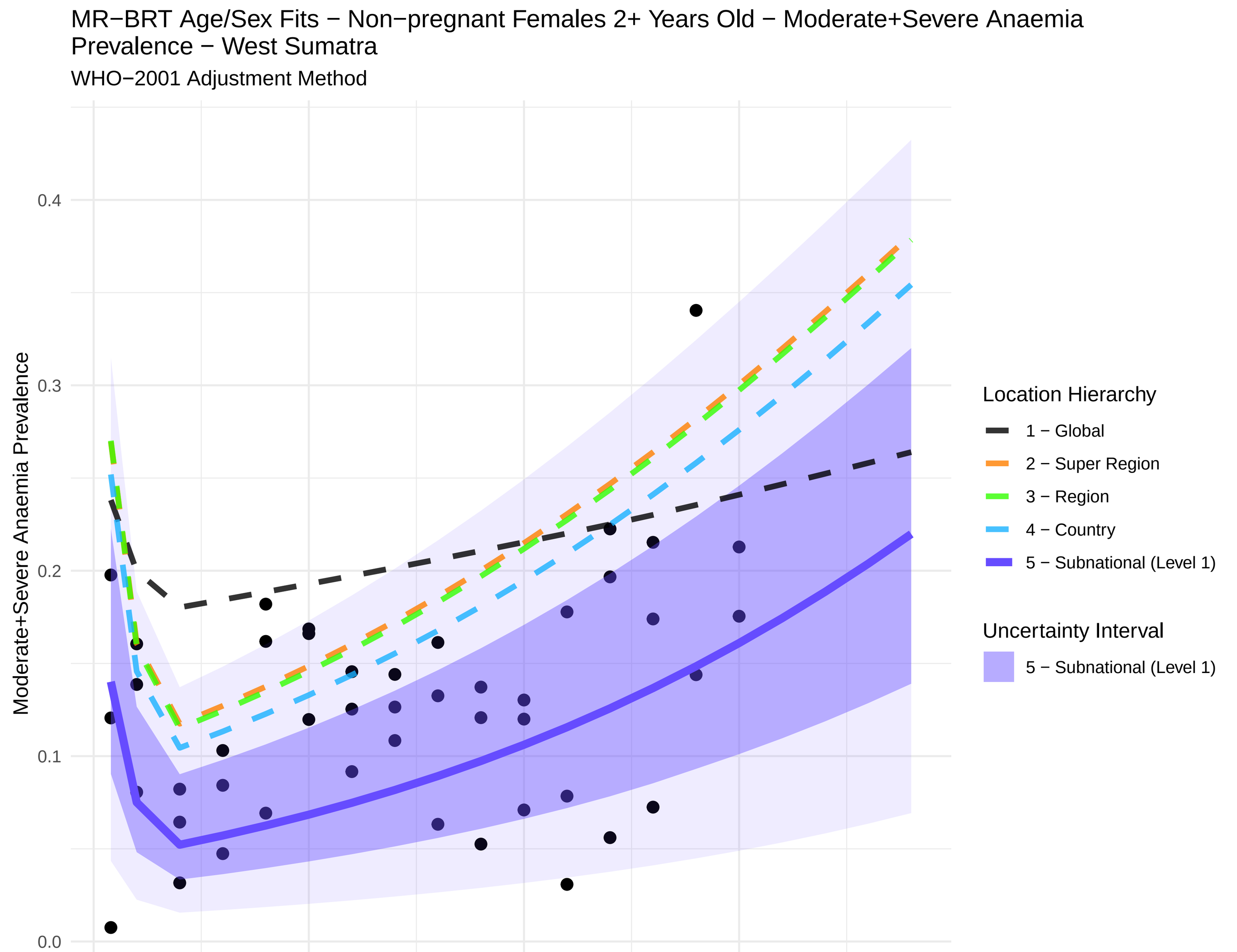
MR−BRT Age/Sex Fits − Non−pregnant Females 2+ Years Old − Moderate+Severe Anaemia Prevalence − West Sumatra
WHO−2001 Adjustment Method
Moderate+Severe Anaemia Prevalence
Age (year)
Location Hierarchy
1 − Global
2 − Super Region
3 − Region
4 − Country
5 − Subnational (Level 1)
Uncertainty Interval
5 − Subnational (Level 1)

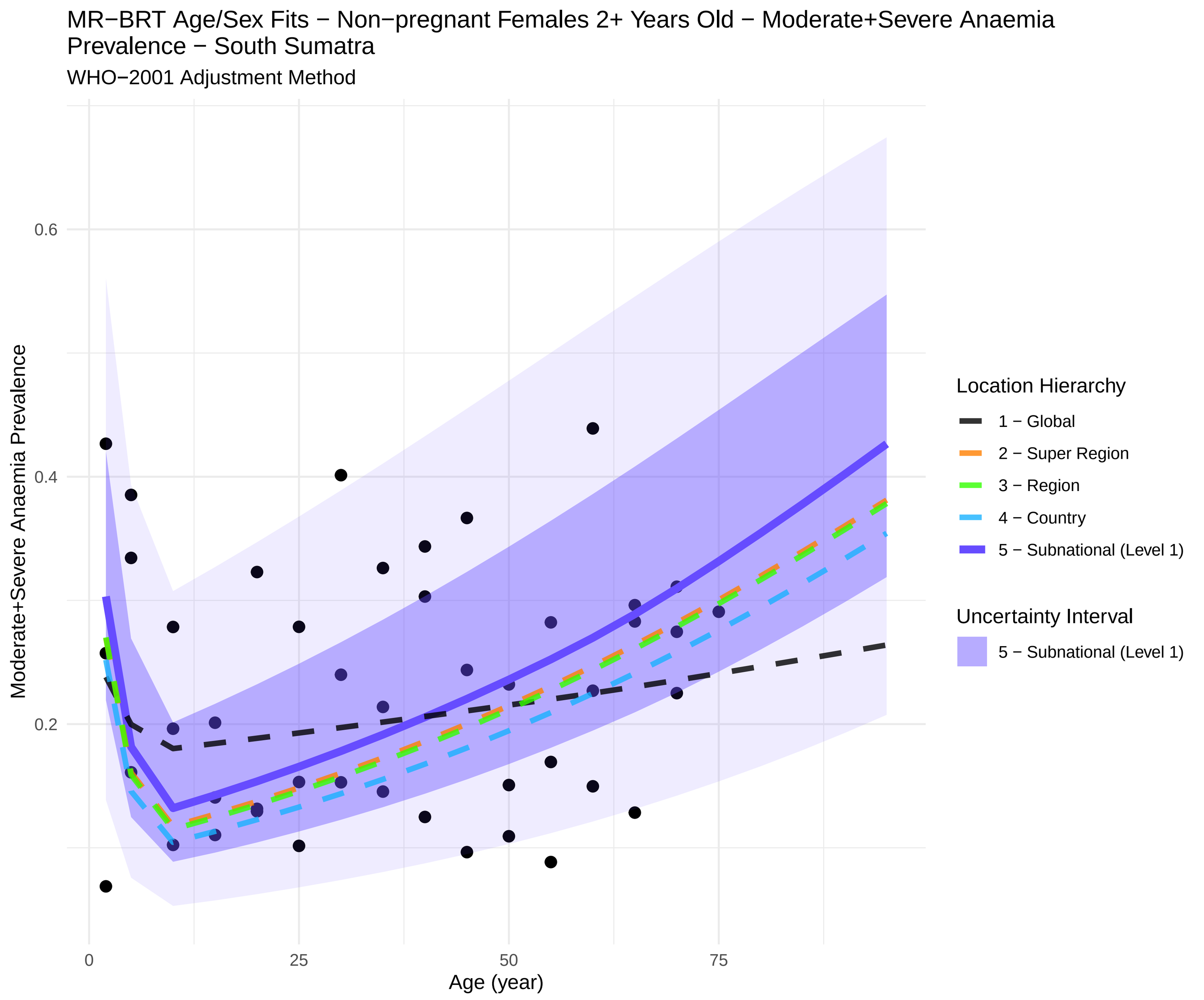
MR−BRT Age/Sex Fits − Non−pregnant Females 2+ Years Old − Moderate+Severe Anaemia Prevalence − South Sumatra
WHO−2001 Adjustment Method
Moderate+Severe Anaemia Prevalence
Age (year)
Location Hierarchy
1 − Global
2 − Super Region
3 − Region
4 − Country
5 − Subnational (Level 1)
Uncertainty Interval
5 − Subnational (Level 1)

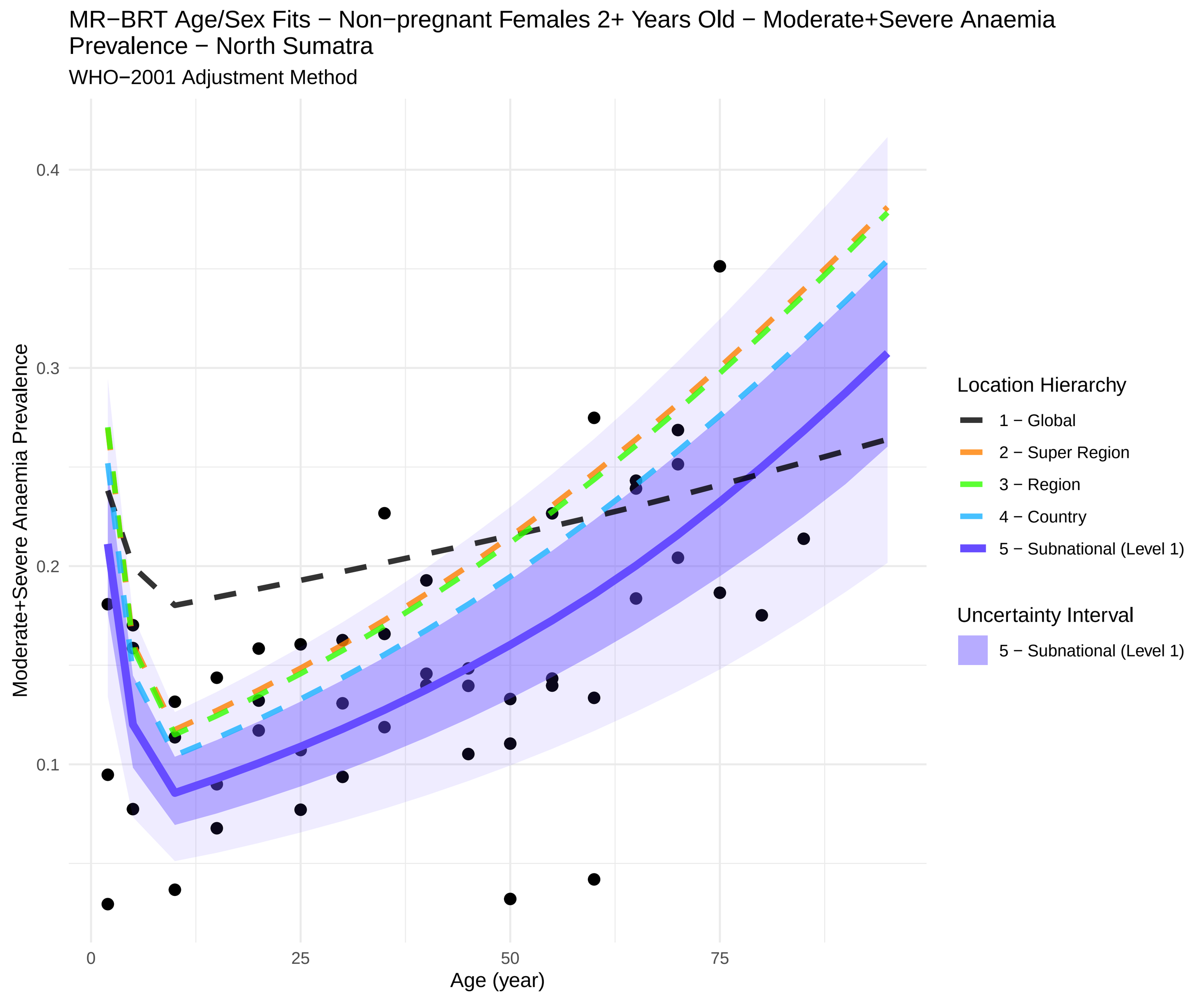
MR−BRT Age/Sex Fits − Non−pregnant Females 2+ Years Old − Moderate+Severe Anaemia Prevalence − North Sumatra
WHO−2001 Adjustment Method
Moderate+Severe Anaemia Prevalence
Age (year)
Location Hierarchy
1 − Global
2 − Super Region
3 − Region
4 − Country
5 − Subnational (Level 1)
Uncertainty Interval
5 − Subnational (Level 1)

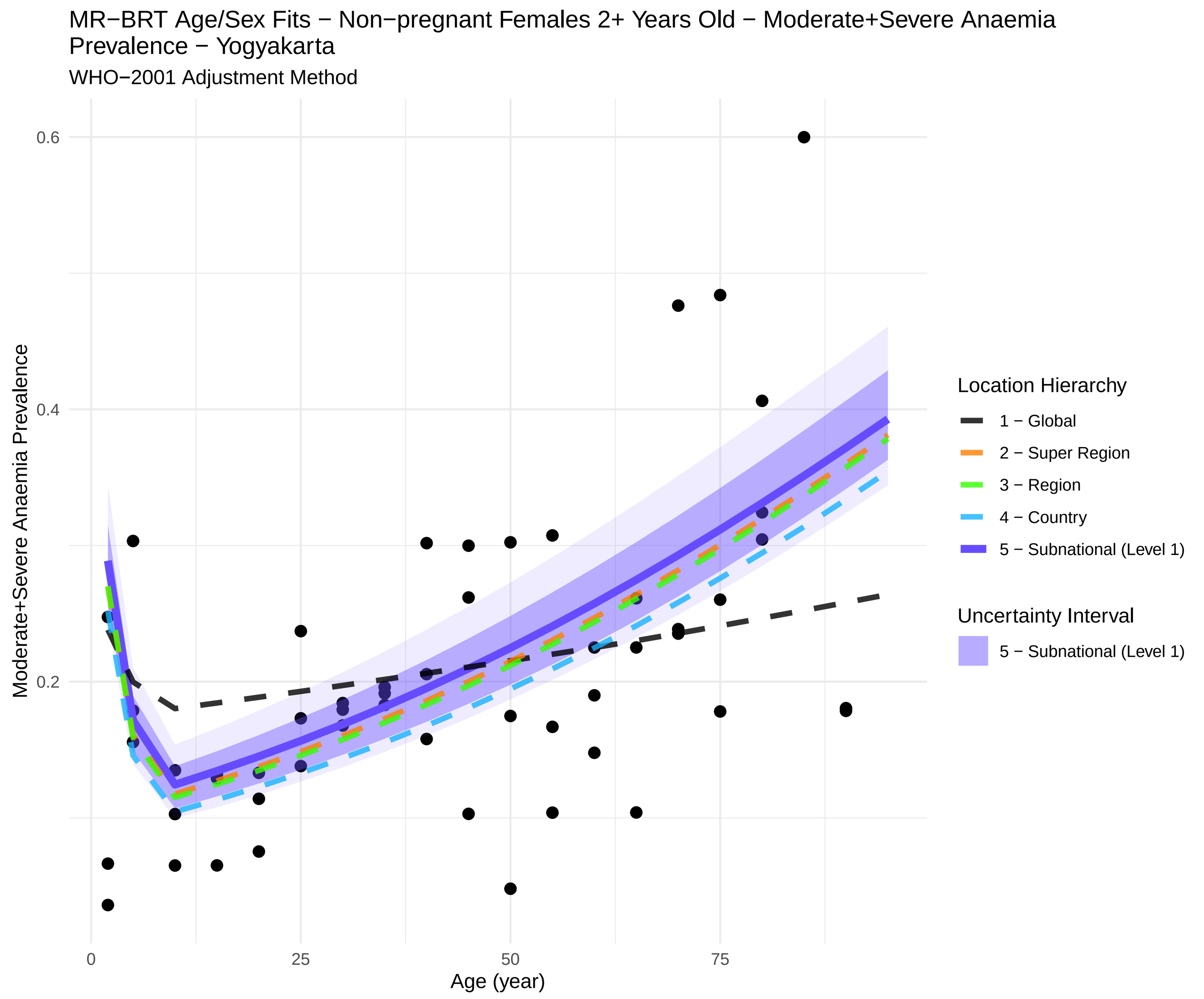
MR−BRT Age/Sex Fits − Non−pregnant Females 2+ Years Old − Moderate+Severe Anaemia Prevalence − Yogyakarta
WHO−2001 Adjustment Method
Moderate+Severe Anaemia Prevalence
Age (year)
Location Hierarchy
1 − Global
2 − Super Region
3 − Region
4 − Country
5 − Subnational (Level 1)
Uncertainty Interval
5 − Subnational (Level 1)

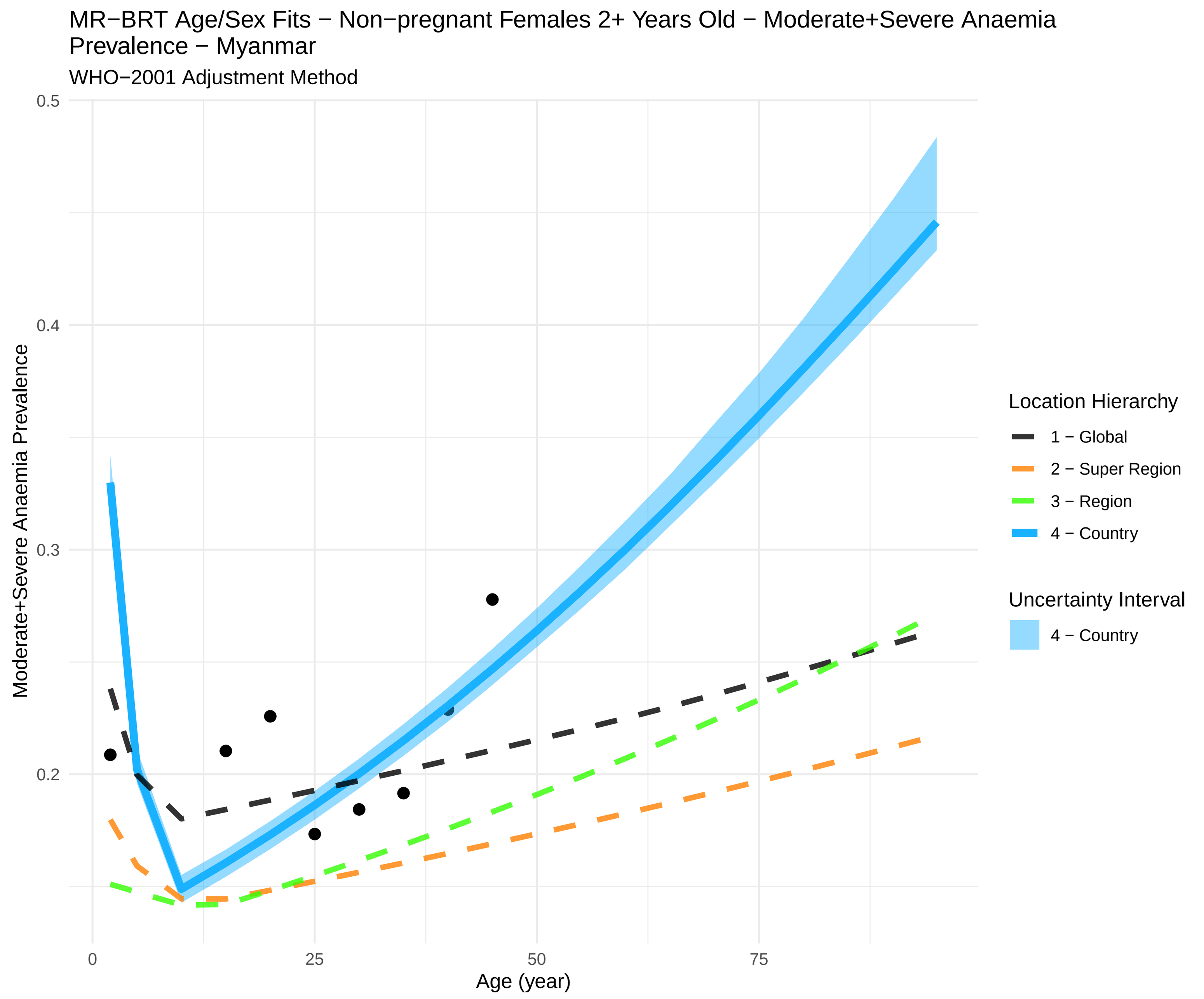
MR−BRT Age/Sex Fits − Non−pregnant Females 2+ Years Old − Moderate+Severe Anaemia Prevalence − Myanmar
WHO−2001 Adjustment Method
Moderate+Severe Anaemia Prevalence
Age (year)
Location Hierarchy
1 − Global
2 − Super Region
3 − Region
4 − Country
Uncertainty Interval
4 − Country

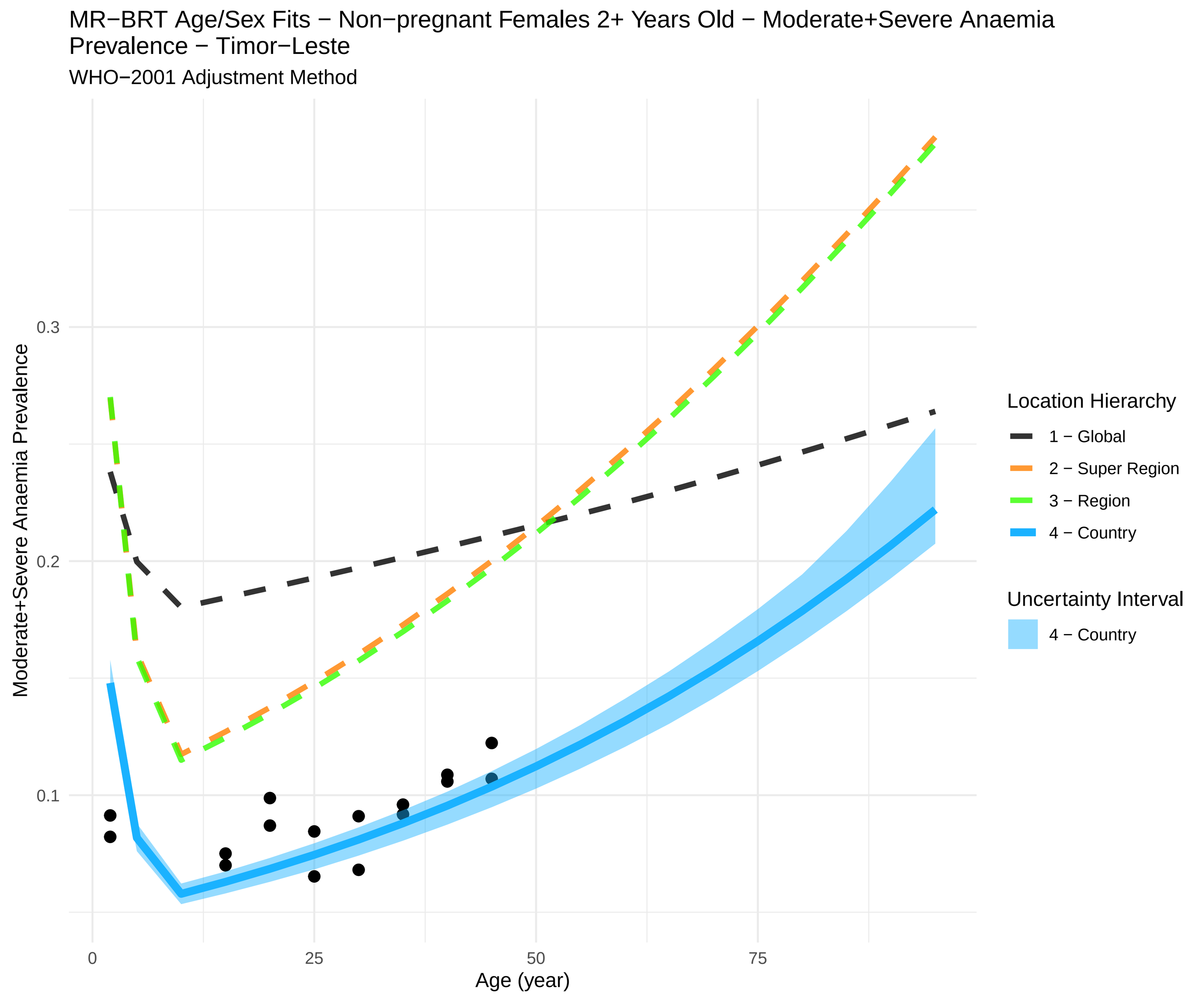
MR−BRT Age/Sex Fits − Non−pregnant Females 2+ Years Old − Moderate+Severe Anaemia Prevalence − Timor−Leste
WHO−2001 Adjustment Method
Moderate+Severe Anaemia Prevalence
Age (year)
Location Hierarchy
1 − Global
2 − Super Region
3 − Region
4 − Country
Uncertainty Interval
4 − Country

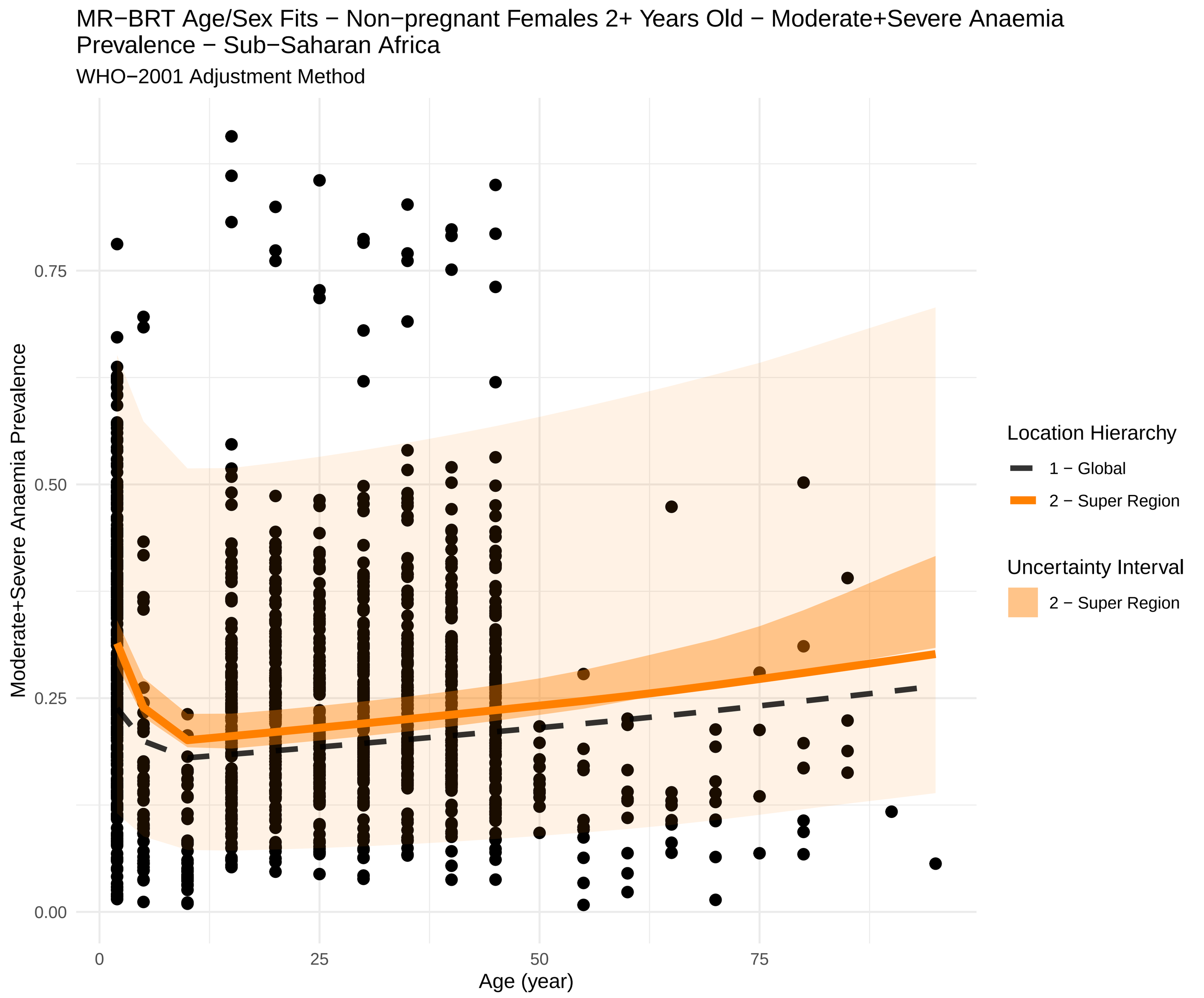
MR−BRT Age/Sex Fits − Non−pregnant Females 2+ Years Old − Moderate+Severe Anaemia Prevalence − Sub−Saharan Africa
WHO−2001 Adjustment Method
Moderate+Severe Anaemia Prevalence
Age (year)
Location Hierarchy
1 − Global
2 − Super Region
Uncertainty Interval
2 − Super Region

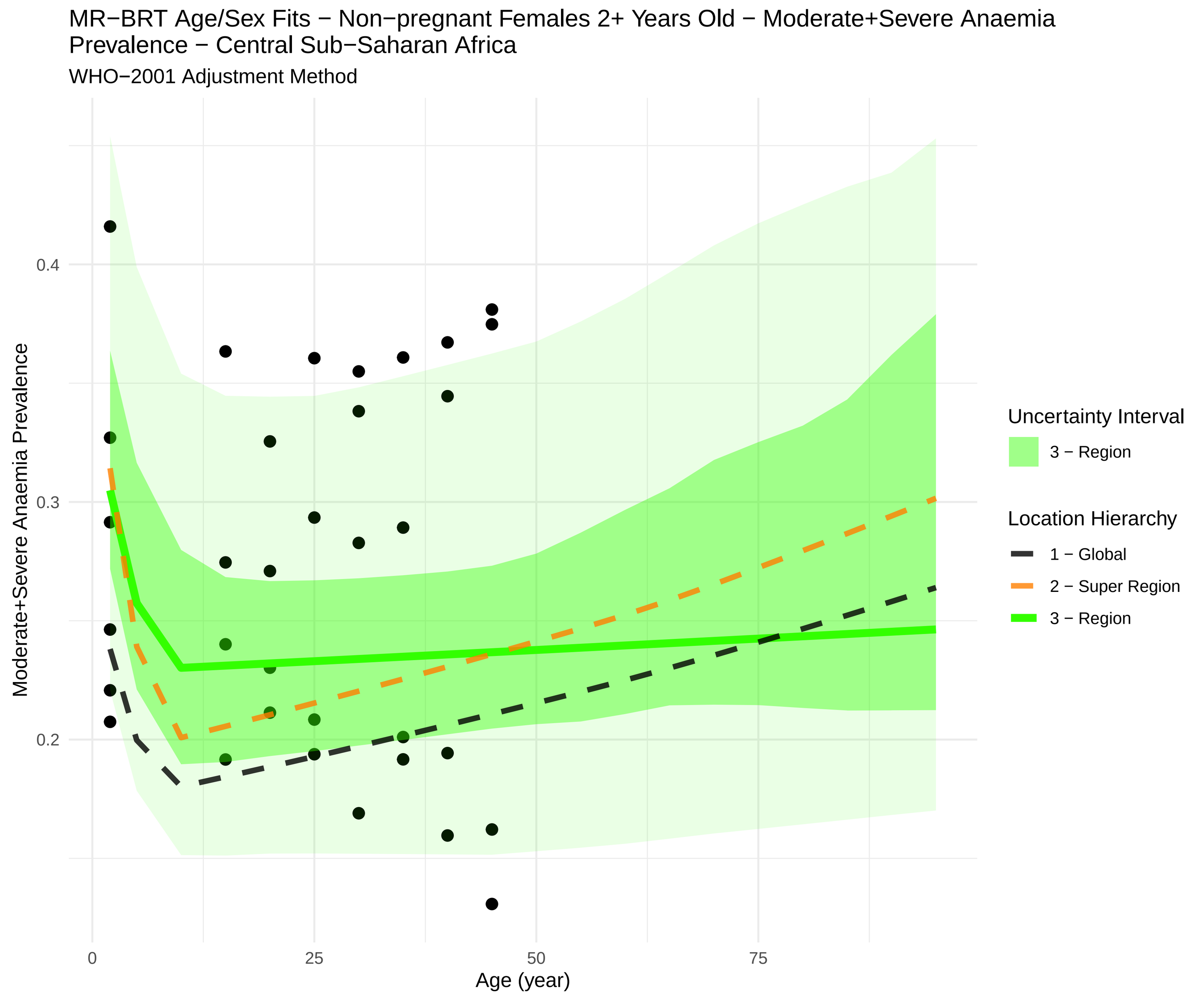
MR−BRT Age/Sex Fits − Non−pregnant Females 2+ Years Old − Moderate+Severe Anaemia Prevalence − Central Sub−Saharan Africa
WHO−2001 Adjustment Method
Moderate+Severe Anaemia Prevalence
Age (year)
Uncertainty Interval
3 − Region
Location Hierarchy
1 − Global
2 − Super Region
3 − Region

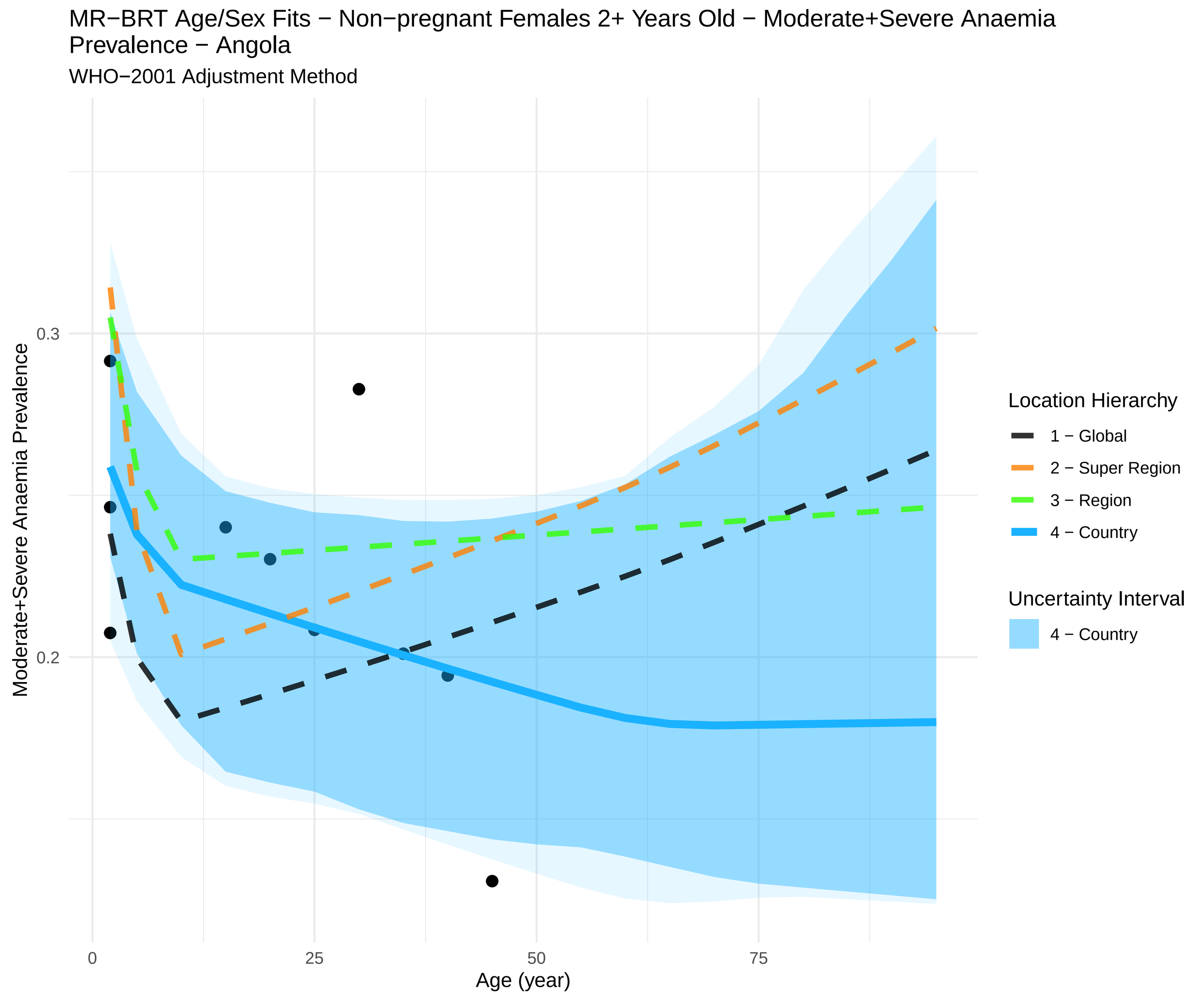
MR−BRT Age/Sex Fits − Non−pregnant Females 2+ Years Old − Moderate+Severe Anaemia Prevalence − Angola
WHO−2001 Adjustment Method
Moderate+Severe Anaemia Prevalence
Age (year)
Location Hierarchy
1 − Global
2 − Super Region
3 − Region
4 − Country
Uncertainty Interval
4 − Country

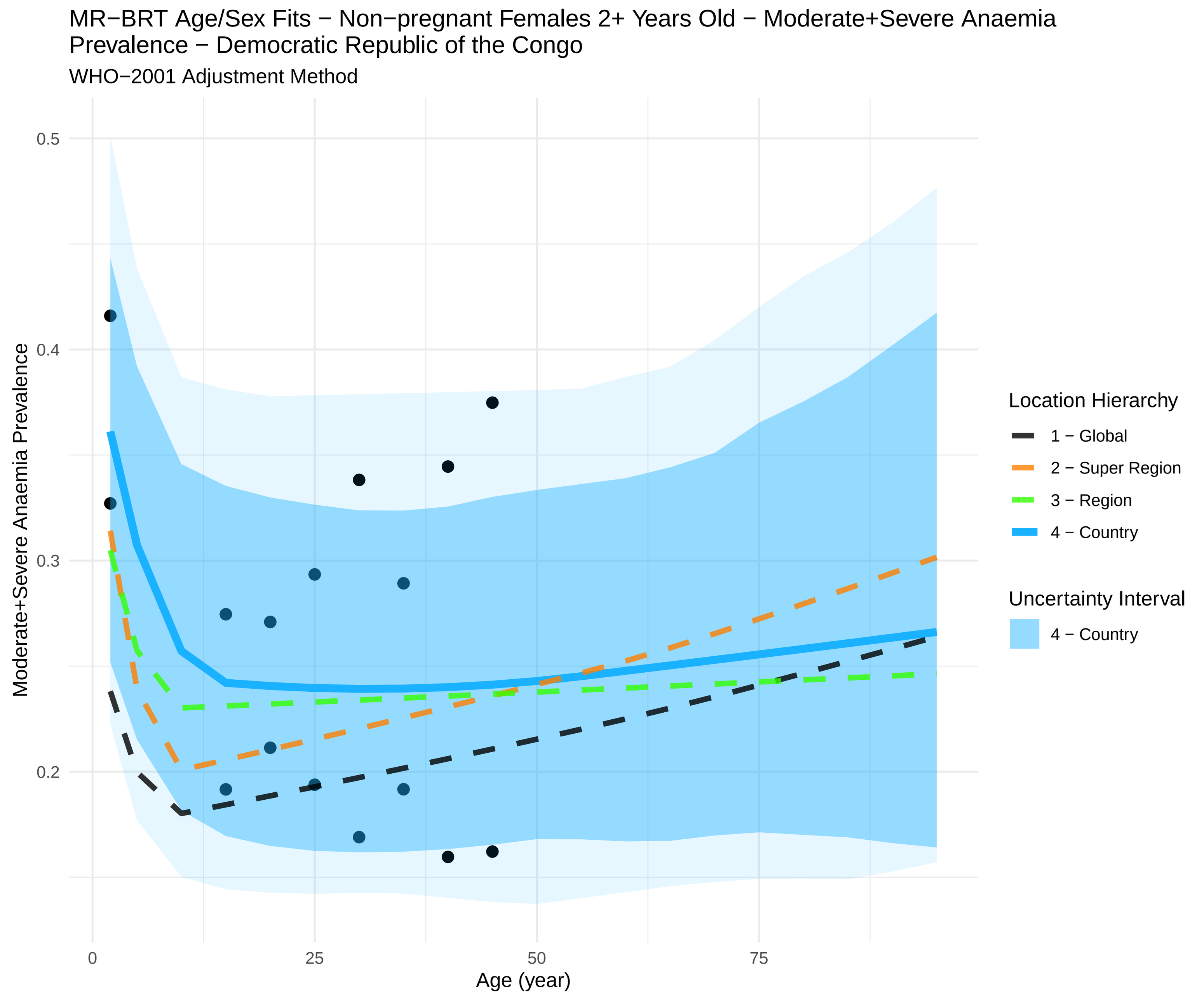
MR−BRT Age/Sex Fits − Non−pregnant Females 2+ Years Old − Moderate+Severe Anaemia Prevalence − Democratic Republic of the Congo
WHO−2001 Adjustment Method
Moderate+Severe Anaemia Prevalence
Age (year)
Location Hierarchy
1 − Global
2 − Super Region
3 − Region
4 − Country
Uncertainty Interval
4 − Country

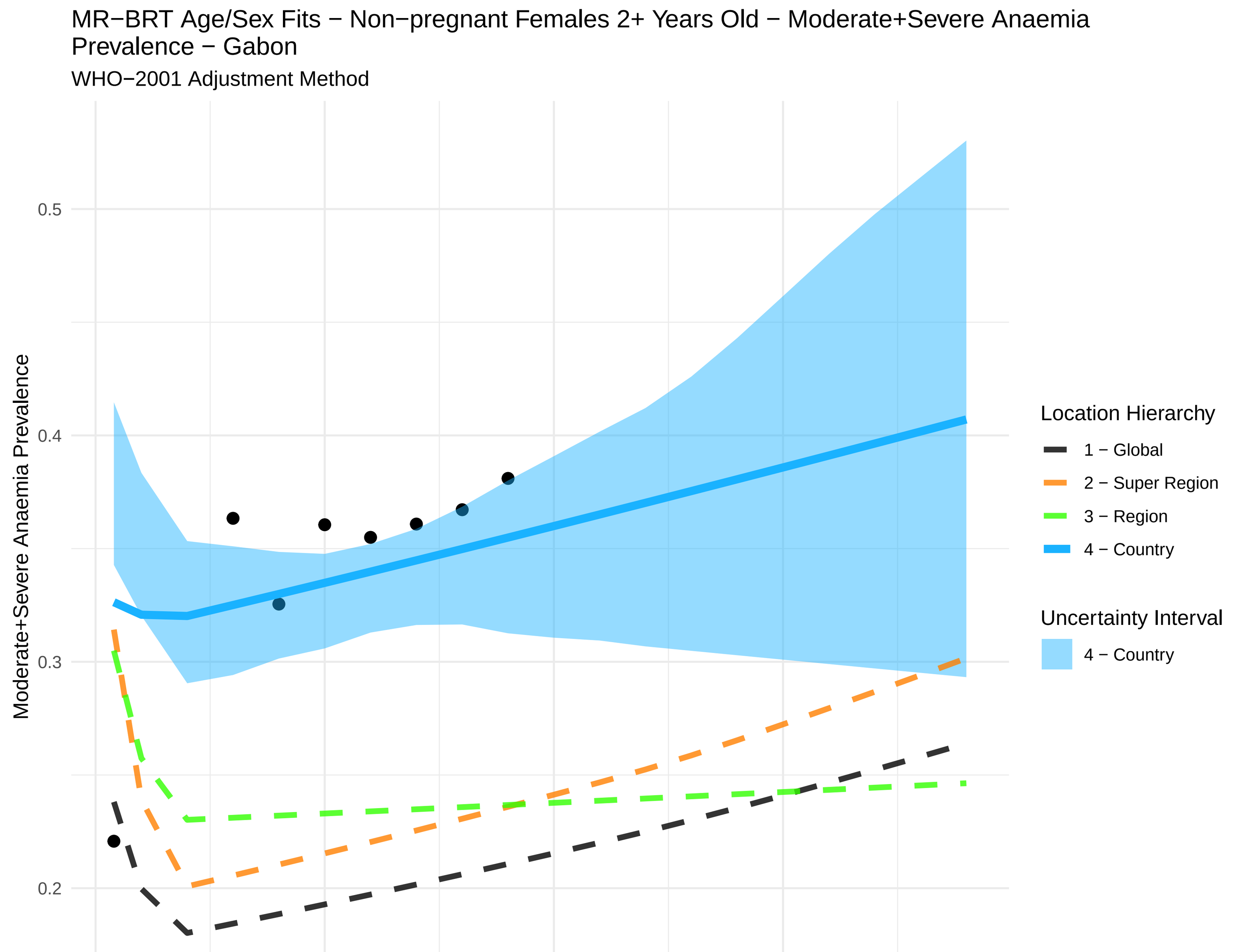
MR−BRT Age/Sex Fits − Non−pregnant Females 2+ Years Old − Moderate+Severe Anaemia Prevalence − Gabon
WHO−2001 Adjustment Method
Moderate+Severe Anaemia Prevalence
Age (year)
Location Hierarchy
1 − Global
2 − Super Region
3 − Region
4 − Country
Uncertainty Interval
4 − Country

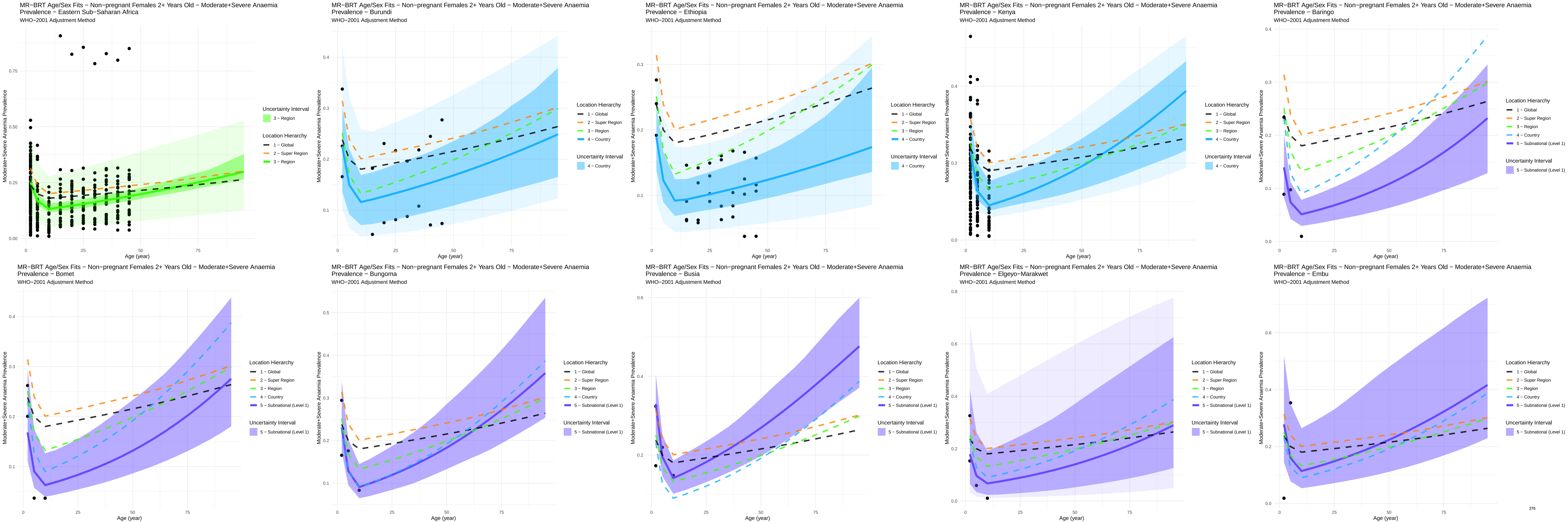
MR−BRT Age/Sex Fits − Non−pregnant Females 2+ Years Old − Moderate+Severe Anaemia Prevalence − Eastern Sub−Saharan Africa
WHO−2001 Adjustment Method
Moderate+Severe Anaemia Prevalence
Age (year)
Uncertainty Interval
3 − Region
Location Hierarchy
1 − Global
2 − Super Region
3 − Region
MR−BRT Age/Sex Fits − Non−pregnant Females 2+ Years Old − Moderate+Severe Anaemia Prevalence − Burundi
WHO−2001 Adjustment Method
Moderate+Severe Anaemia Prevalence
Age (year)
Location Hierarchy
1 − Global
2 − Super Region
3 − Region
4 − Country
Uncertainty Interval
4 − Country
MR−BRT Age/Sex Fits − Non−pregnant Females 2+ Years Old − Moderate+Severe Anaemia Prevalence − Ethiopia
WHO−2001 Adjustment Method
Moderate+Severe Anaemia Prevalence
Age (year)
Location Hierarchy
1 − Global
2 − Super Region
3 − Region
4 − Country
Uncertainty Interval
4 − Country
MR−BRT Age/Sex Fits − Non−pregnant Females 2+ Years Old − Moderate+Severe Anaemia Prevalence − Kenya
WHO−2001 Adjustment Method
Moderate+Severe Anaemia Prevalence
Age (year)
Location Hierarchy
1 − Global
2 − Super Region
3 − Region
4 − Country
Uncertainty Interval
4 − Country
MR−BRT Age/Sex Fits − Non−pregnant Females 2+ Years Old − Moderate+Severe Anaemia Prevalence − Baringo
WHO−2001 Adjustment Method
Moderate+Severe Anaemia Prevalence
Age (year)
Location Hierarchy
1 − Global
2 − Super Region
3 − Region
4 − Country
5 − Subnational (Level 1)
Uncertainty Interval
5 − Subnational (Level 1)
MR−BRT Age/Sex Fits − Non−pregnant Females 2+ Years Old − Moderate+Severe Anaemia Prevalence − Bomet
WHO−2001 Adjustment Method
Moderate+Severe Anaemia Prevalence
Age (year)
Location Hierarchy
1 − Global
2 − Super Region
3 − Region
4 − Country
5 − Subnational (Level 1)
Uncertainty Interval
5 − Subnational (Level 1)
MR−BRT Age/Sex Fits − Non−pregnant Females 2+ Years Old − Moderate+Severe Anaemia Prevalence − Bungoma
WHO−2001 Adjustment Method
Moderate+Severe Anaemia Prevalence
Age (year)
Location Hierarchy
1 − Global
2 − Super Region
3 − Region
4 − Country
5 − Subnational (Level 1)
Uncertainty Interval
5 − Subnational (Level 1)
MR−BRT Age/Sex Fits − Non−pregnant Females 2+ Years Old − Moderate+Severe Anaemia Prevalence − Busia
WHO−2001 Adjustment Method
Moderate+Severe Anaemia Prevalence
Age (year)
Location Hierarchy
1 − Global
2 − Super Region
3 − Region
4 − Country
5 − Subnational (Level 1)
Uncertainty Interval
5 − Subnational (Level 1)
MR−BRT Age/Sex Fits − Non−pregnant Females 2+ Years Old − Moderate+Severe Anaemia Prevalence − Elgeyo−Marakwet
WHO−2001 Adjustment Method
Moderate+Severe Anaemia Prevalence
Age (year)
Location Hierarchy
1 − Global
2 − Super Region
3 − Region
4 − Country
5 − Subnational (Level 1)
Uncertainty Interval
5 − Subnational (Level 1)
MR−BRT Age/Sex Fits − Non−pregnant Females 2+ Years Old − Moderate+Severe Anaemia Prevalence − Embu
WHO−2001 Adjustment Method
Moderate+Severe Anaemia Prevalence
Age (year)
Location Hierarchy
1 − Global
2 − Super Region
3 − Region
4 − Country
5 − Subnational (Level 1)
Uncertainty Interval
5 − Subnational (Level 1)

MR−BRT Age/Sex Fits − Non−pregnant Females 2+ Years Old − Moderate+Severe Anaemia Prevalence − Garissa
WHO−2001 Adjustment Method

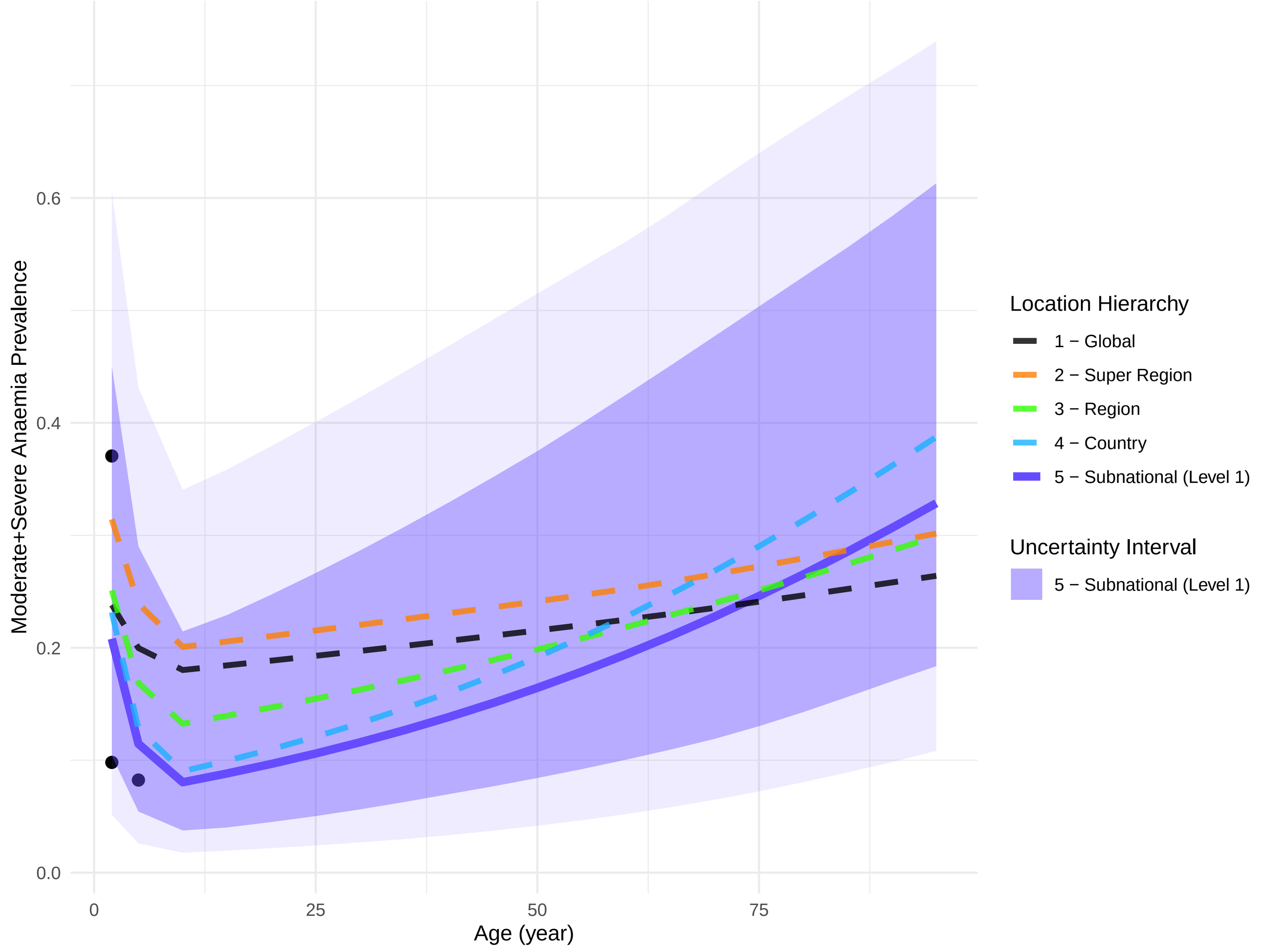

MR−BRT Age/Sex Fits − Non−pregnant Females 2+ Years Old − Moderate+Severe Anaemia Prevalence − Homa Bay
WHO−2001 Adjustment Method

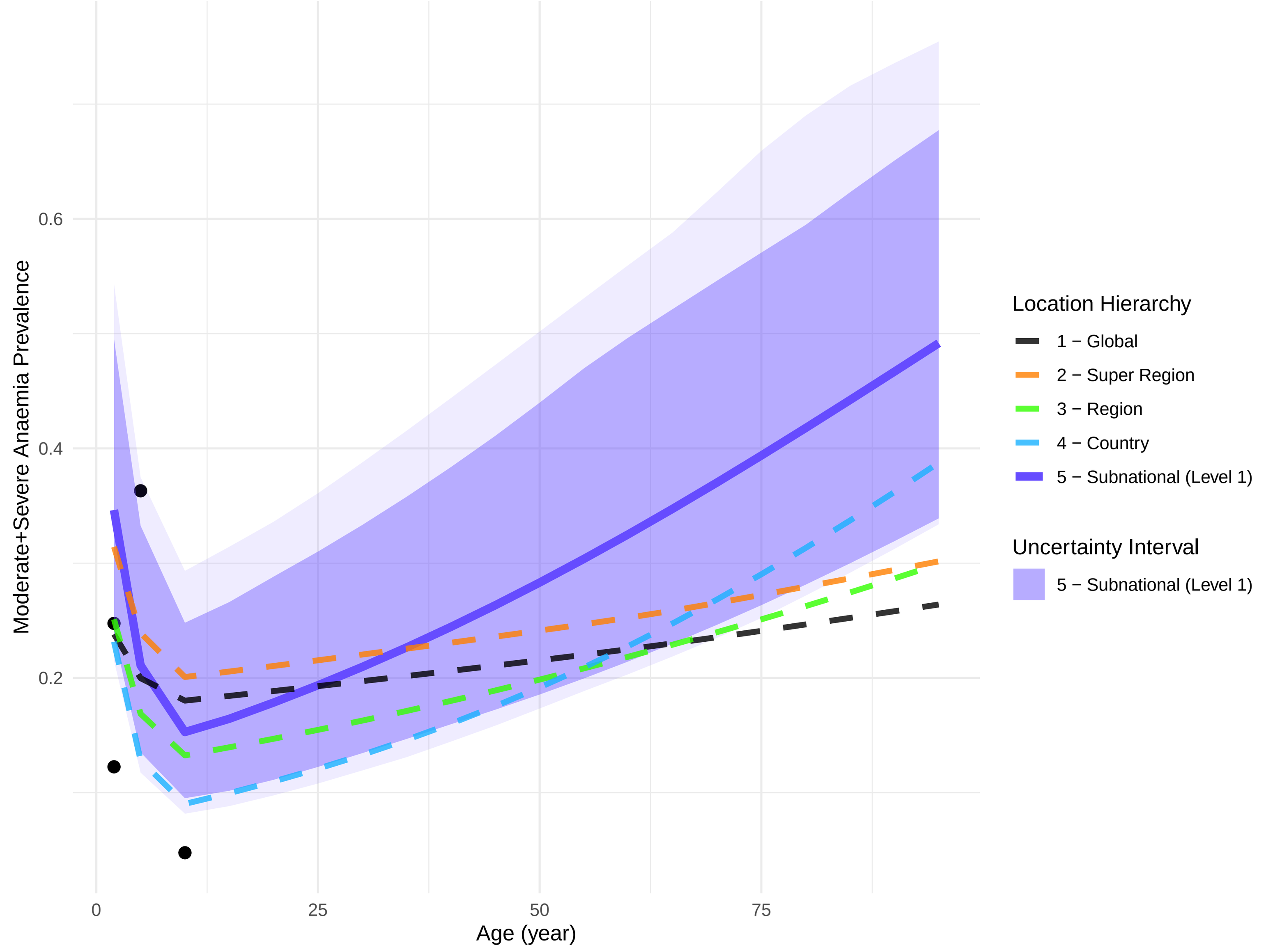

MR−BRT Age/Sex Fits − Non−pregnant Females 2+ Years Old − Moderate+Severe Anaemia Prevalence − Isiolo
WHO−2001 Adjustment Method

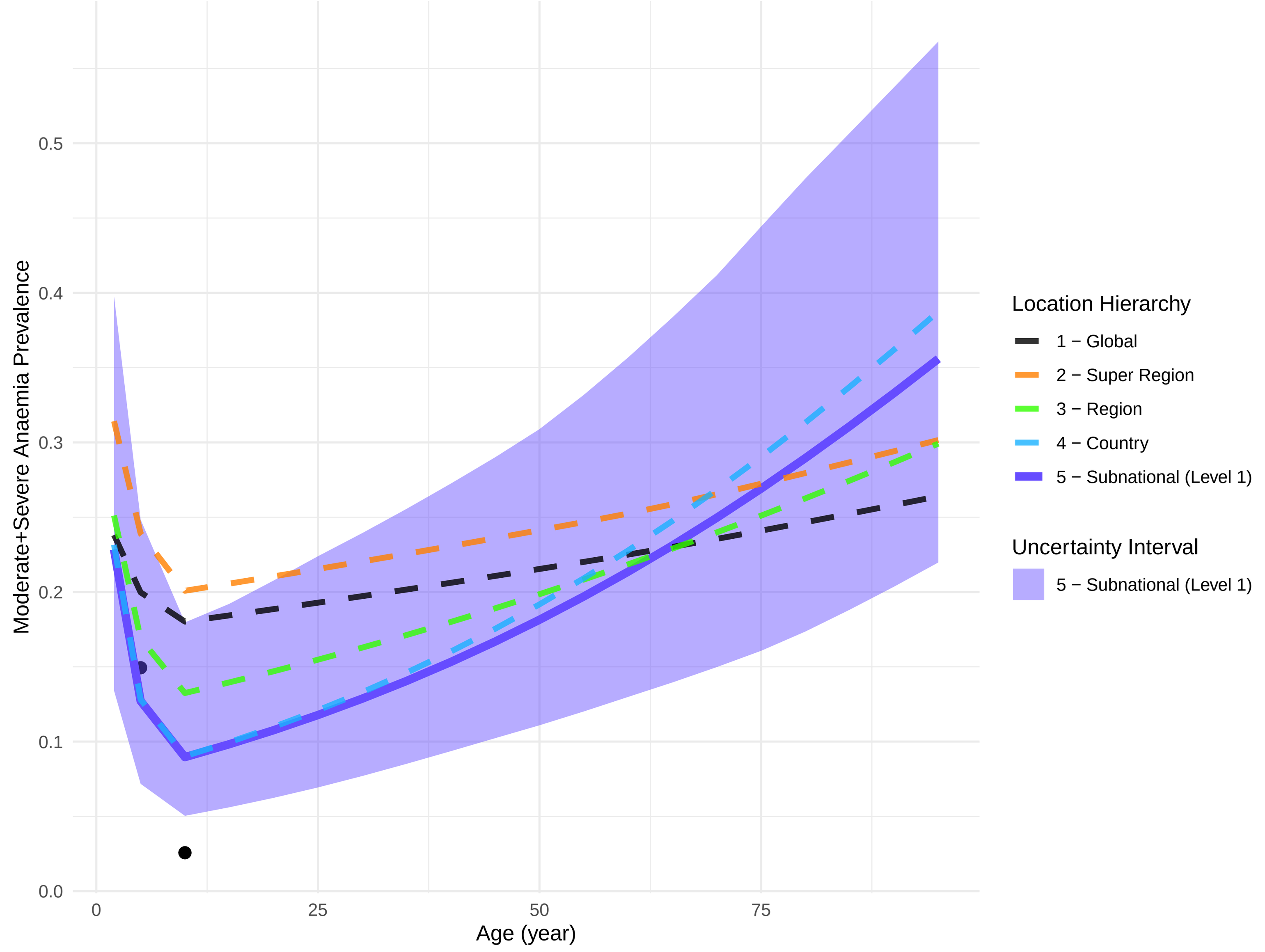

MR−BRT Age/Sex Fits − Non−pregnant Females 2+ Years Old − Moderate+Severe Anaemia Prevalence − Kajiado
WHO−2001 Adjustment Method

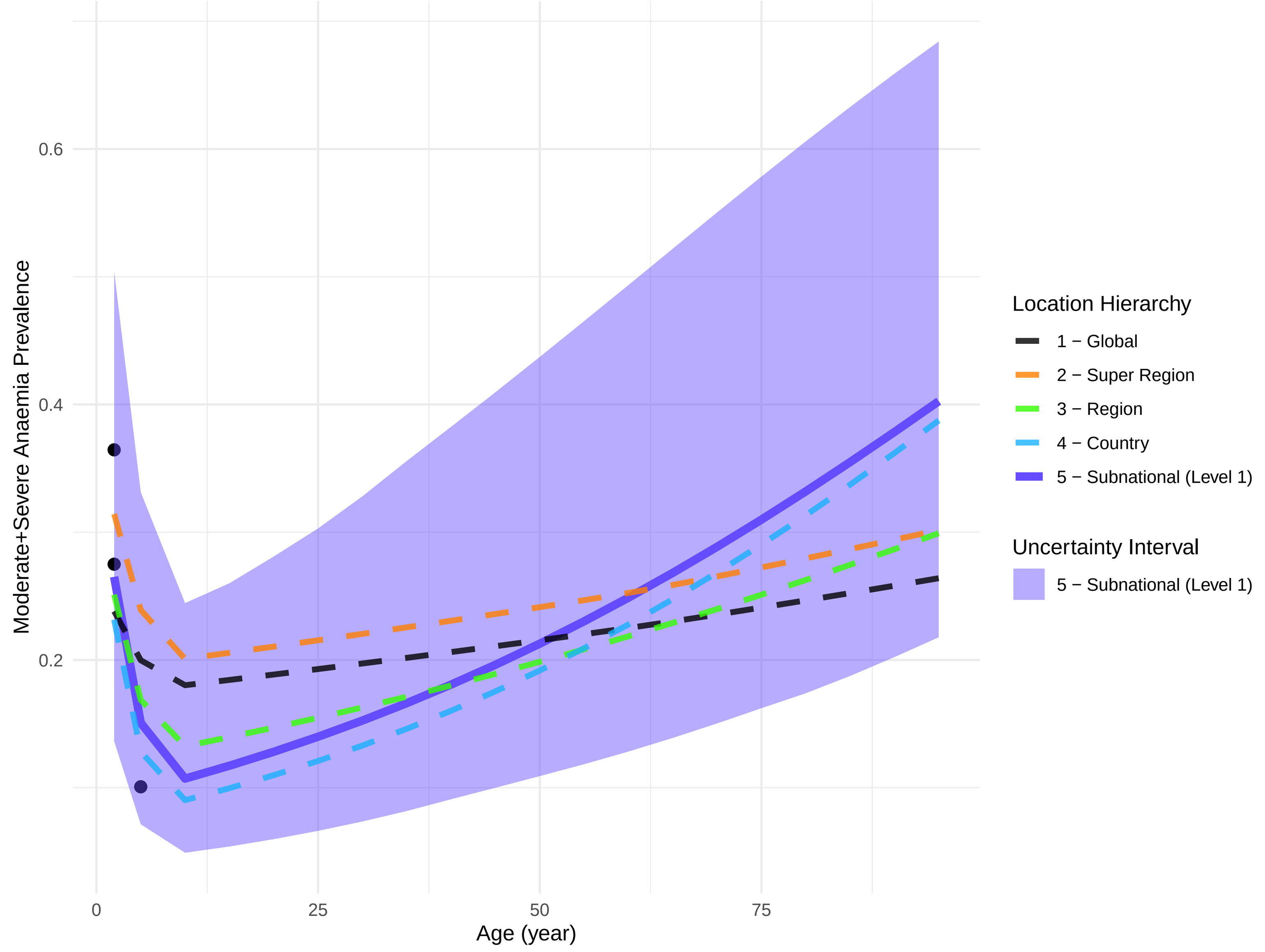

MR−BRT Age/Sex Fits − Non−pregnant Females 2+ Years Old − Moderate+Severe Anaemia Prevalence − Kakamega
WHO−2001 Adjustment Method

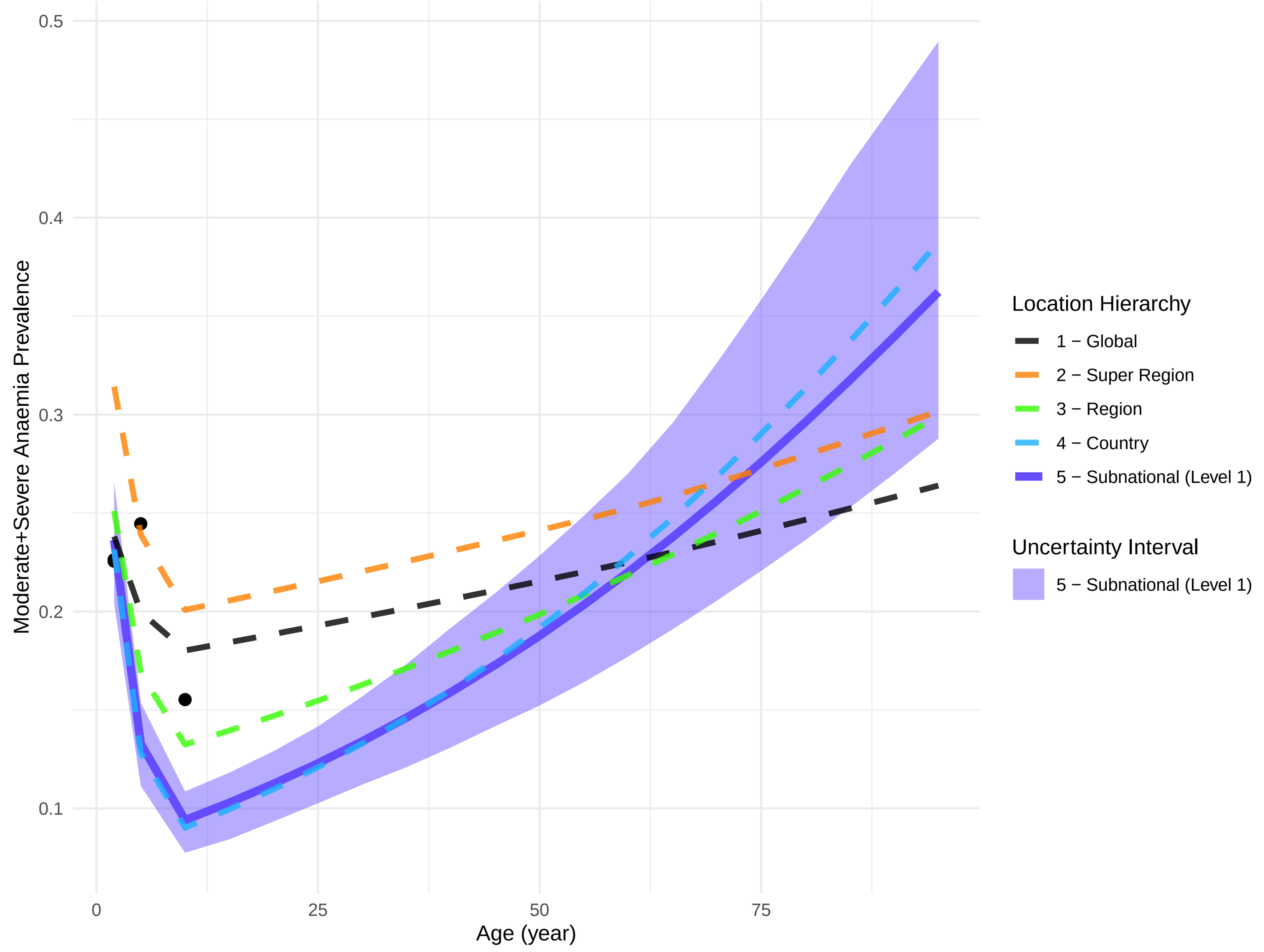

MR−BRT Age/Sex Fits − Non−pregnant Females 2+ Years Old − Moderate+Severe Anaemia Prevalence − Kericho
WHO−2001 Adjustment Method

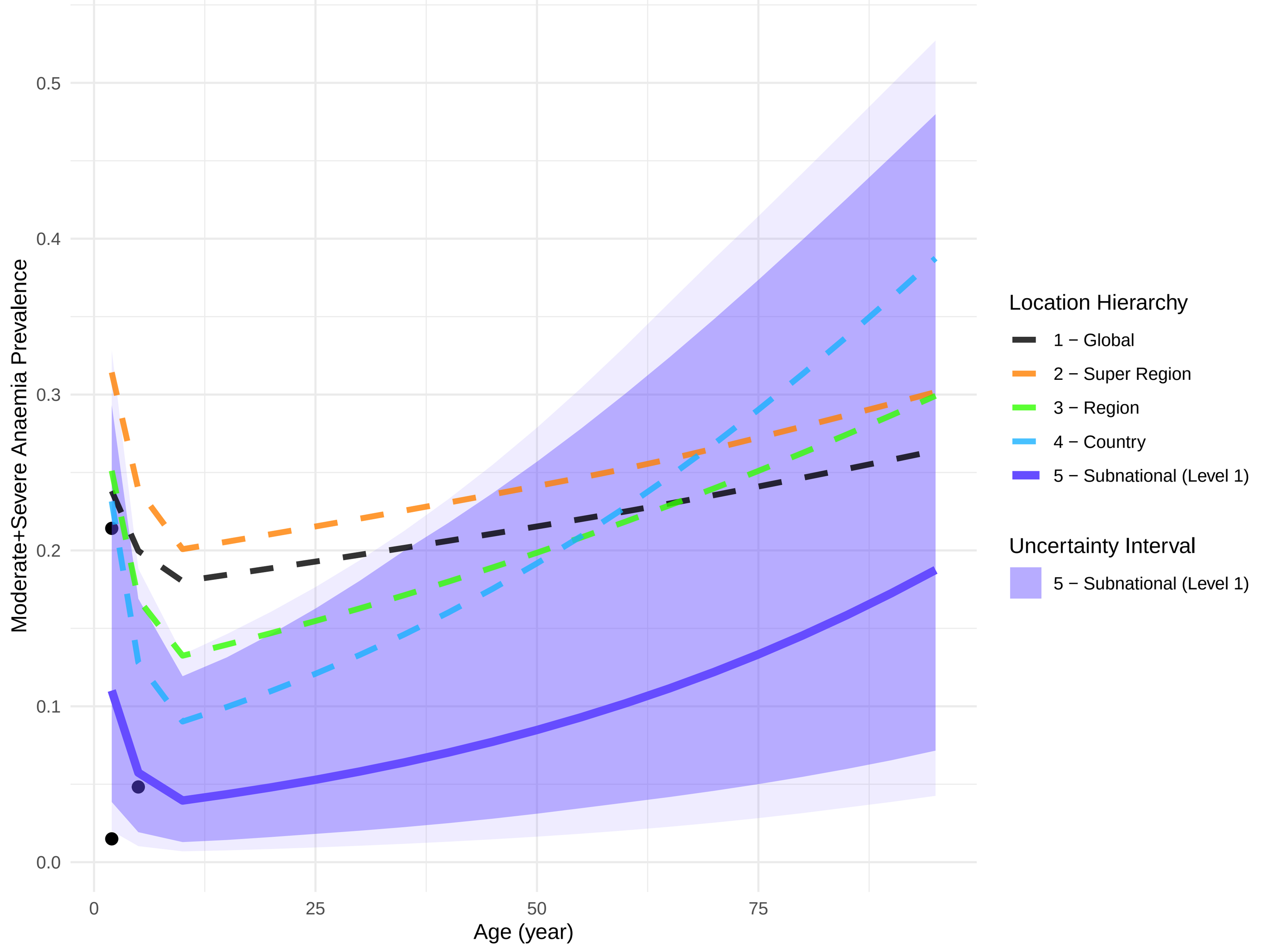

MR−BRT Age/Sex Fits − Non−pregnant Females 2+ Years Old − Moderate+Severe Anaemia Prevalence − Kiambu
WHO−2001 Adjustment Method

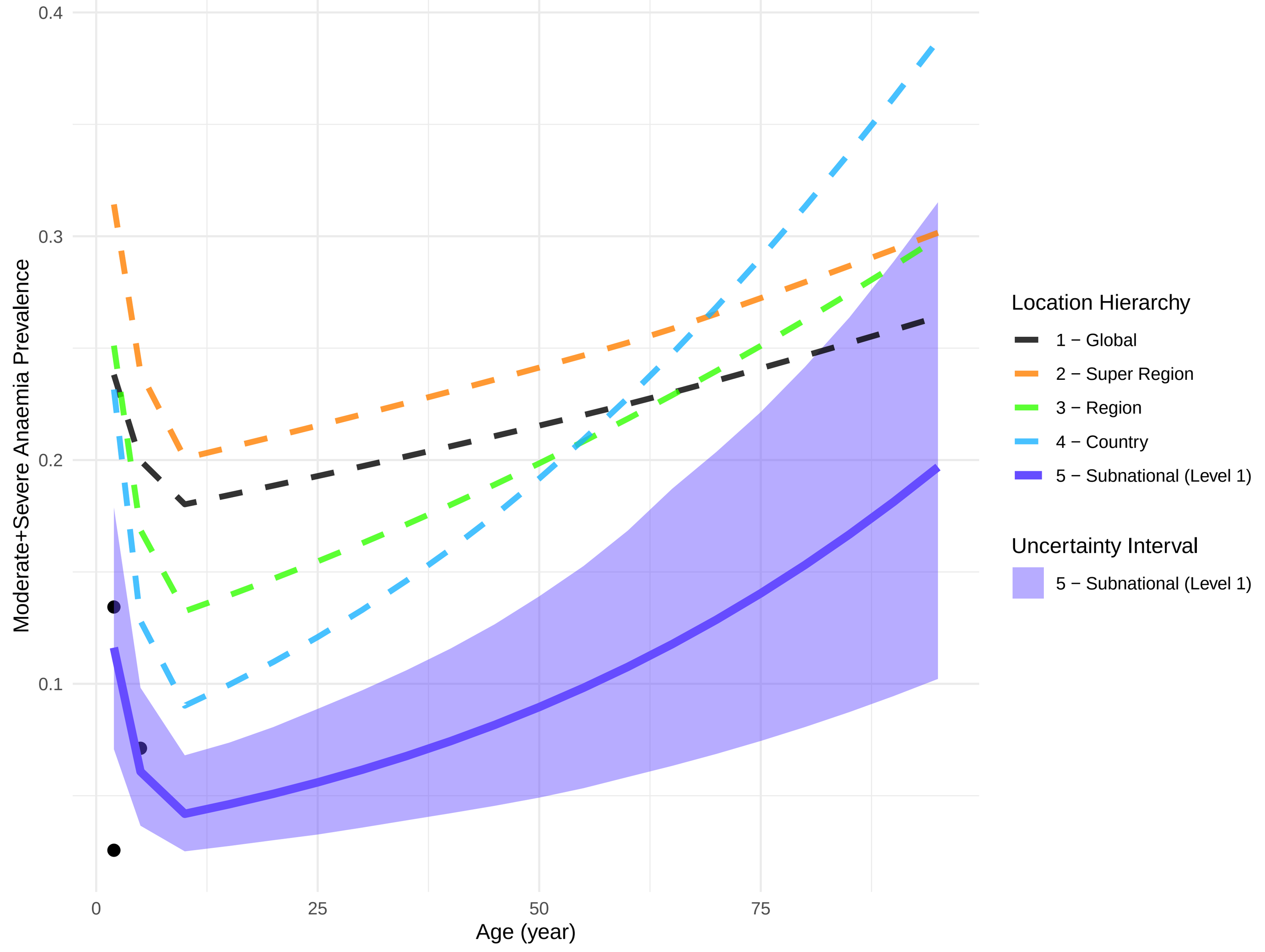

MR−BRT Age/Sex Fits − Non−pregnant Females 2+ Years Old − Moderate+Severe Anaemia Prevalence − Kilifi
WHO−2001 Adjustment Method

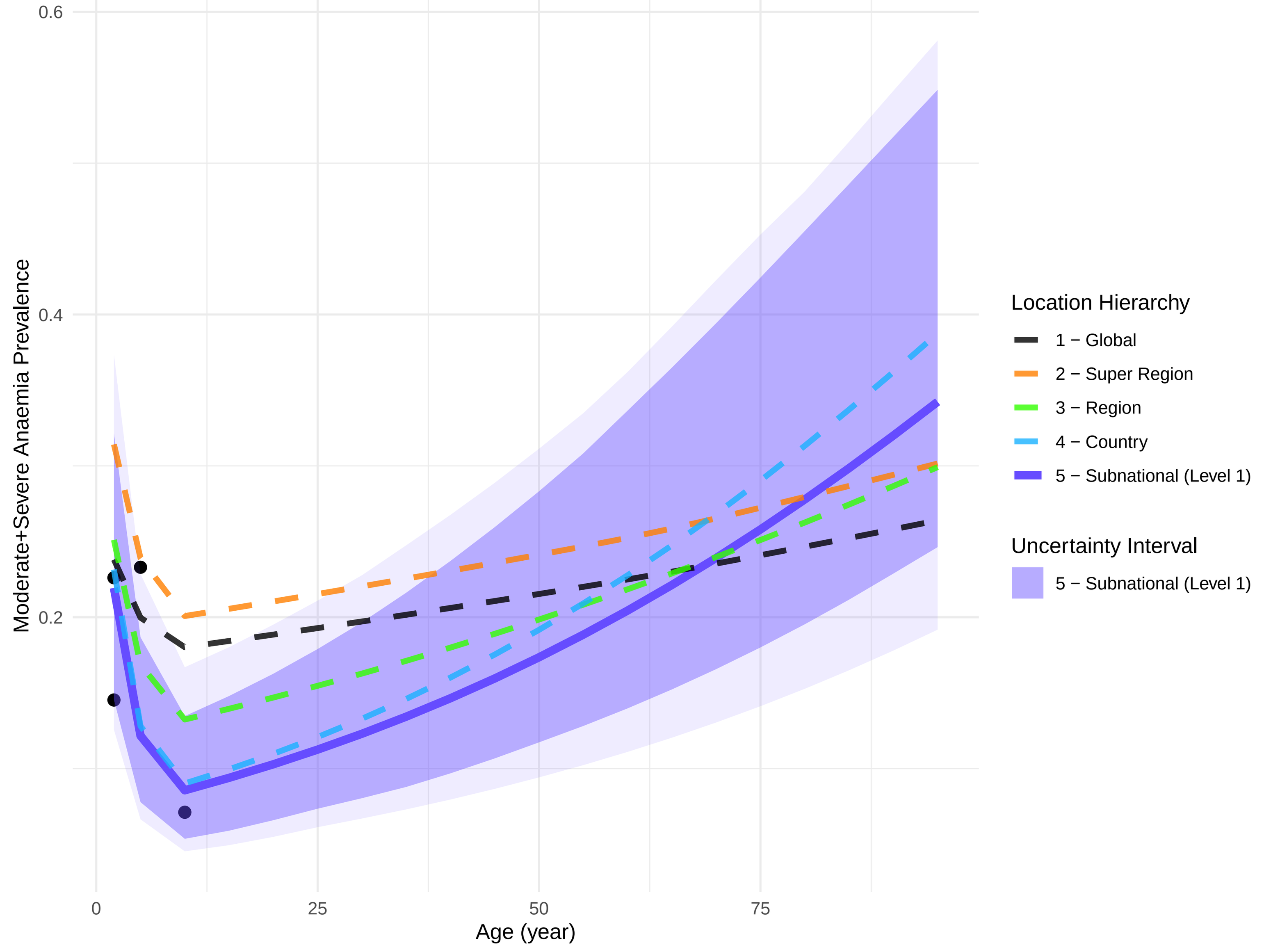

MR−BRT Age/Sex Fits − Non−pregnant Females 2+ Years Old − Moderate+Severe Anaemia Prevalence − Kisii
WHO−2001 Adjustment Method

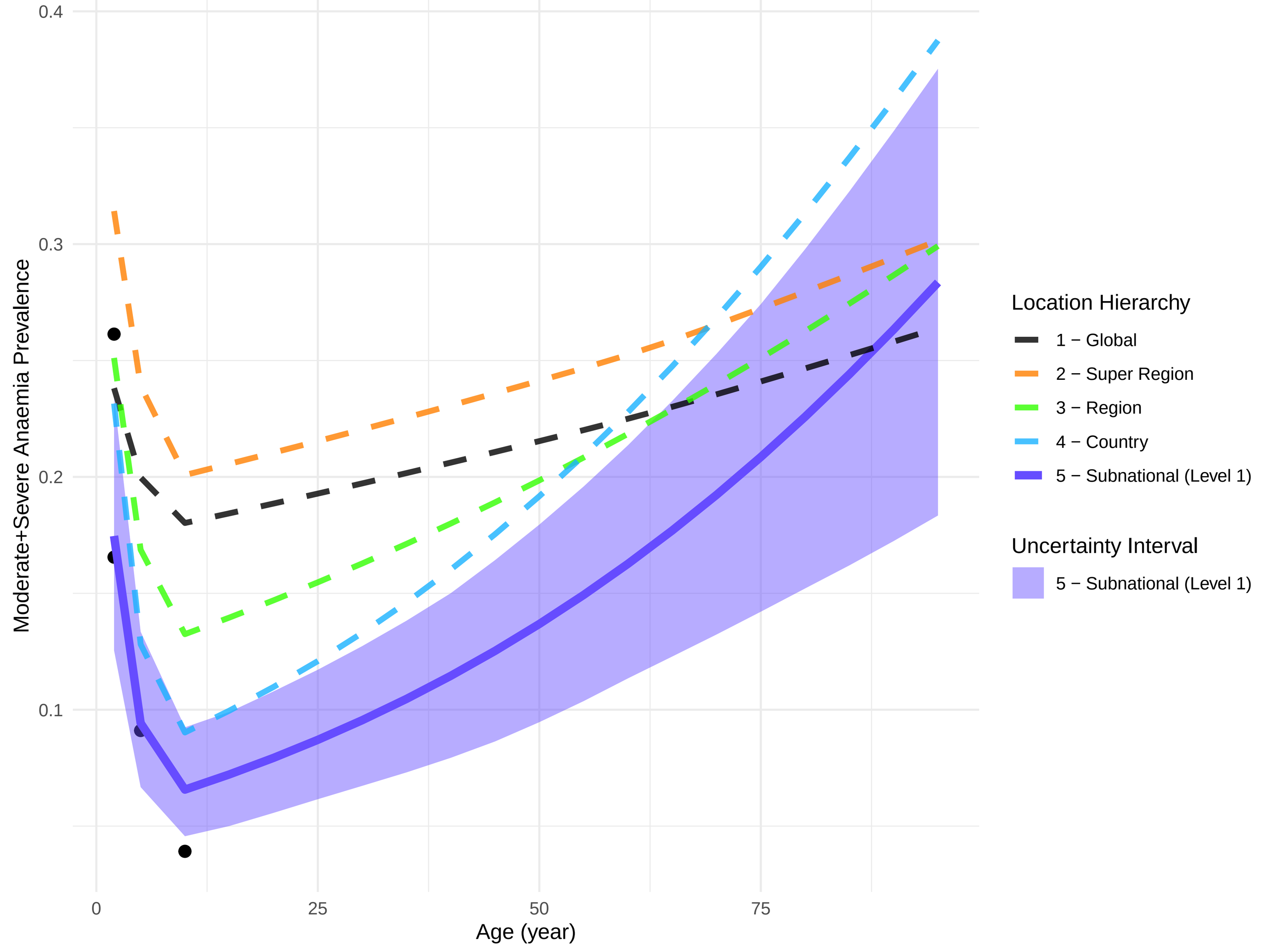

MR−BRT Age/Sex Fits − Non−pregnant Females 2+ Years Old − Moderate+Severe Anaemia Prevalence − Kisumu
WHO−2001 Adjustment Method

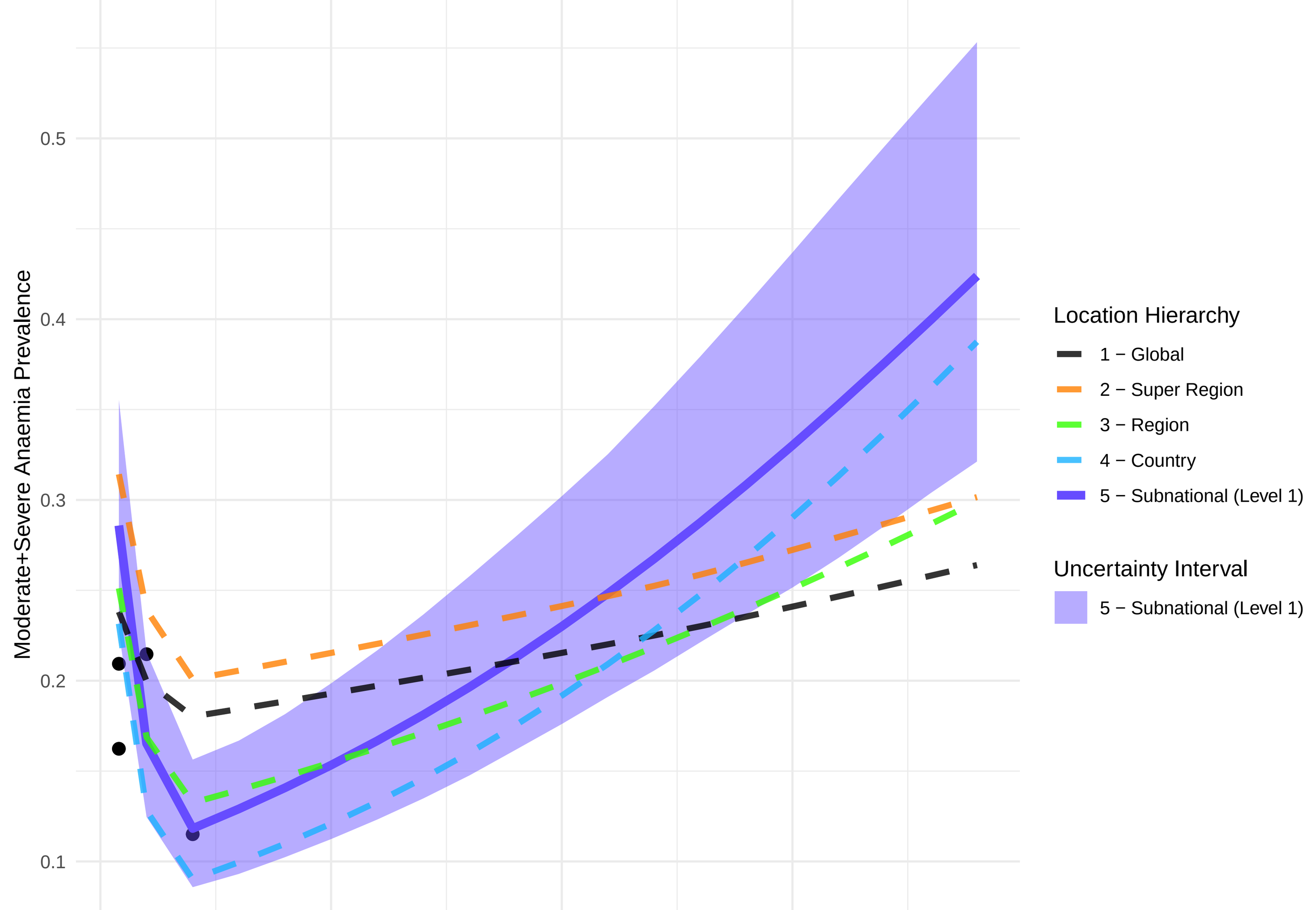

MR−BRT Age/Sex Fits − Non−pregnant Females 2+ Years Old − Moderate+Severe Anaemia Prevalence − Kitui
WHO−2001 Adjustment Method

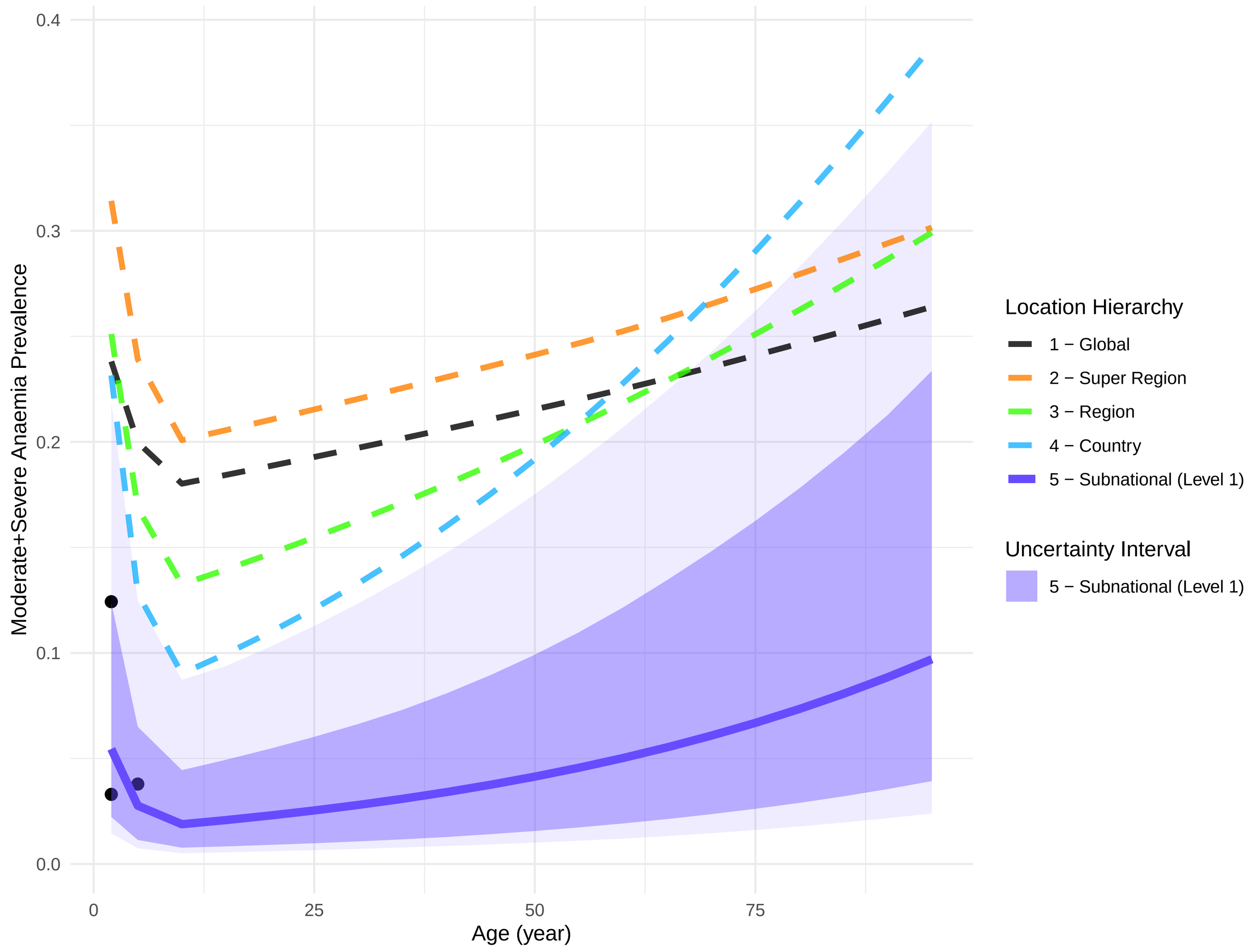

MR−BRT Age/Sex Fits − Non−pregnant Females 2+ Years Old − Moderate+Severe Anaemia Prevalence − Kwale
WHO−2001 Adjustment Method

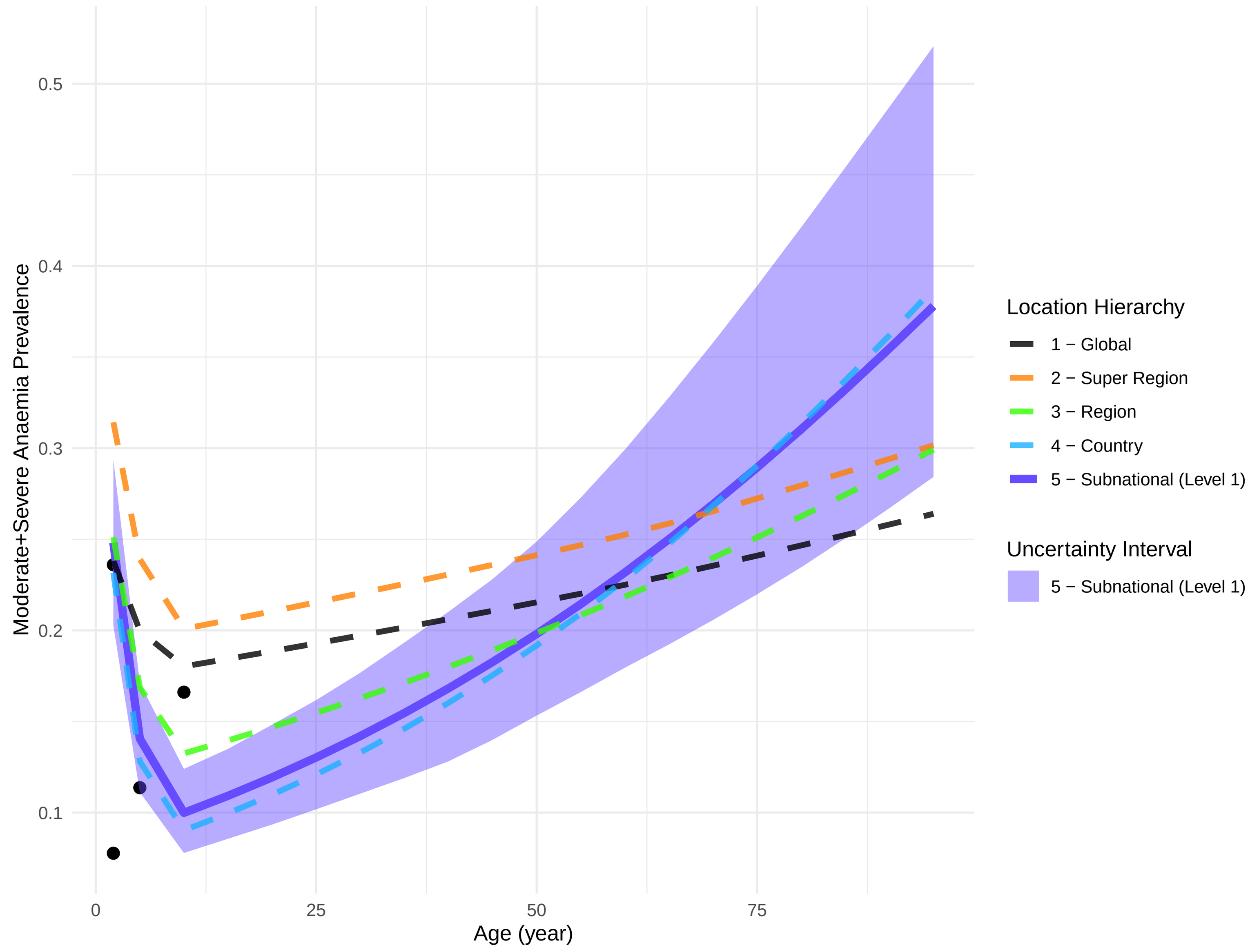

MR−BRT Age/Sex Fits − Non−pregnant Females 2+ Years Old − Moderate+Severe Anaemia Prevalence − Laikipia
WHO−2001 Adjustment Method

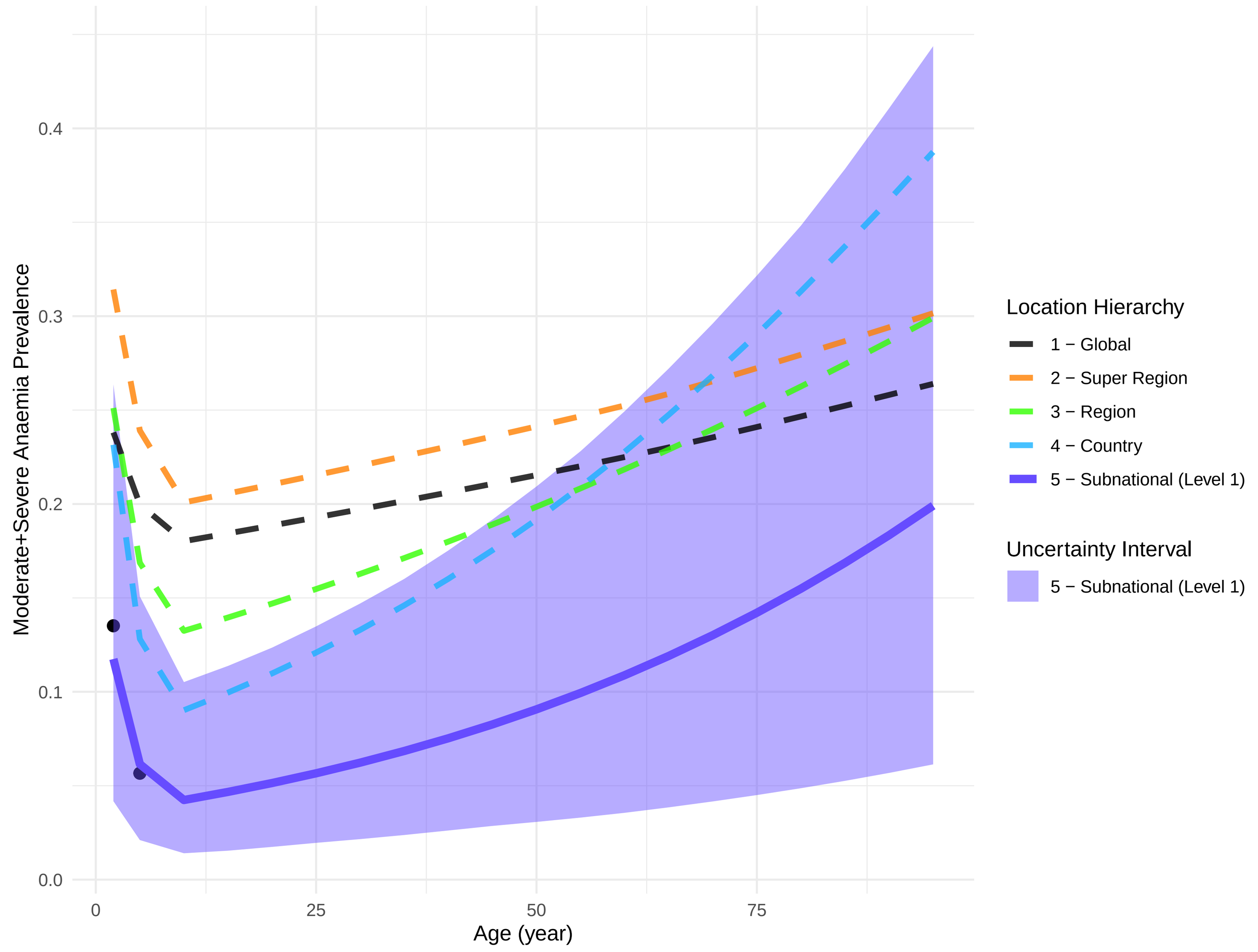

MR−BRT Age/Sex Fits − Non−pregnant Females 2+ Years Old − Moderate+Severe Anaemia Prevalence − Lamu
WHO−2001 Adjustment Method

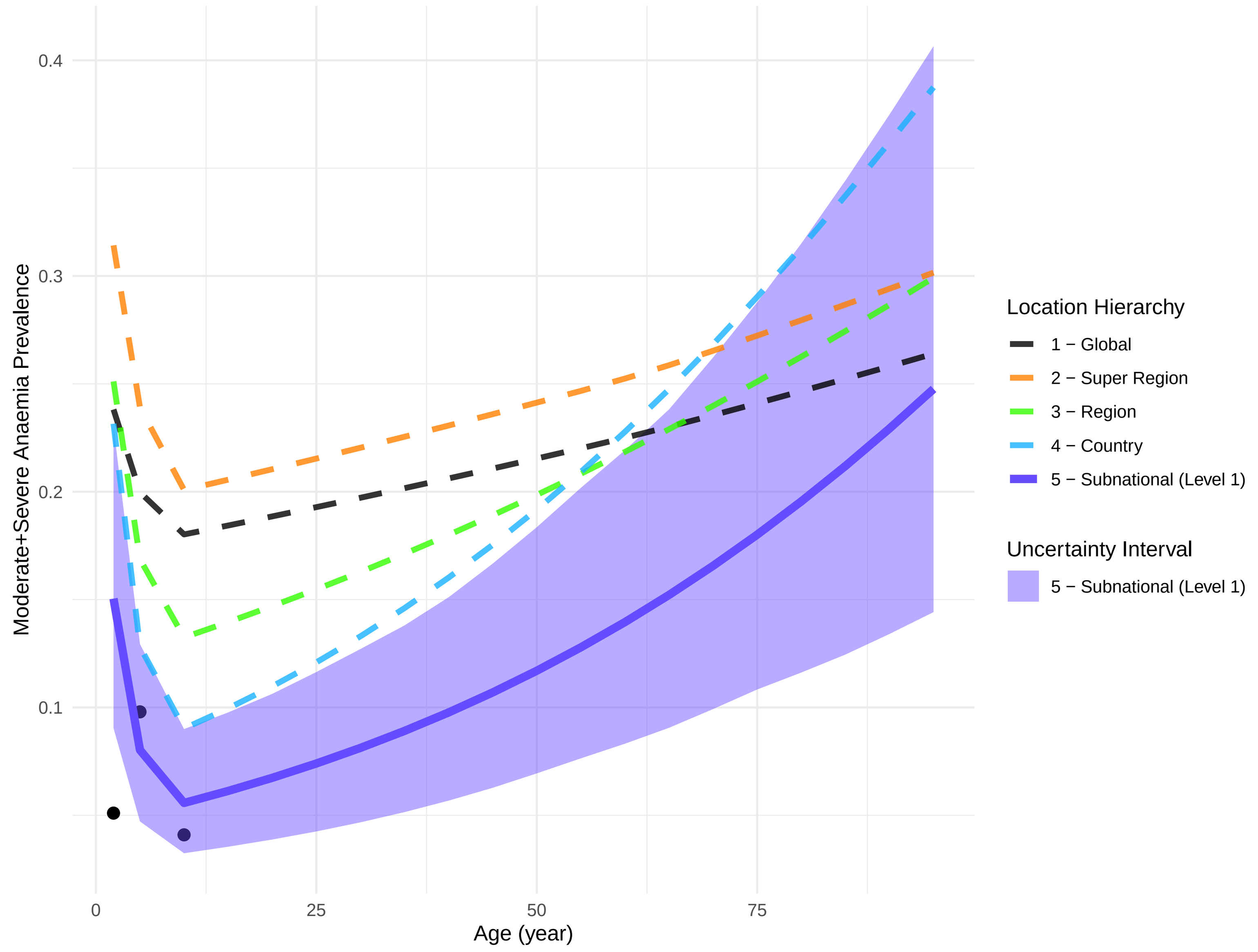

MR−BRT Age/Sex Fits − Non−pregnant Females 2+ Years Old − Moderate+Severe Anaemia Prevalence − Machakos
WHO−2001 Adjustment Method

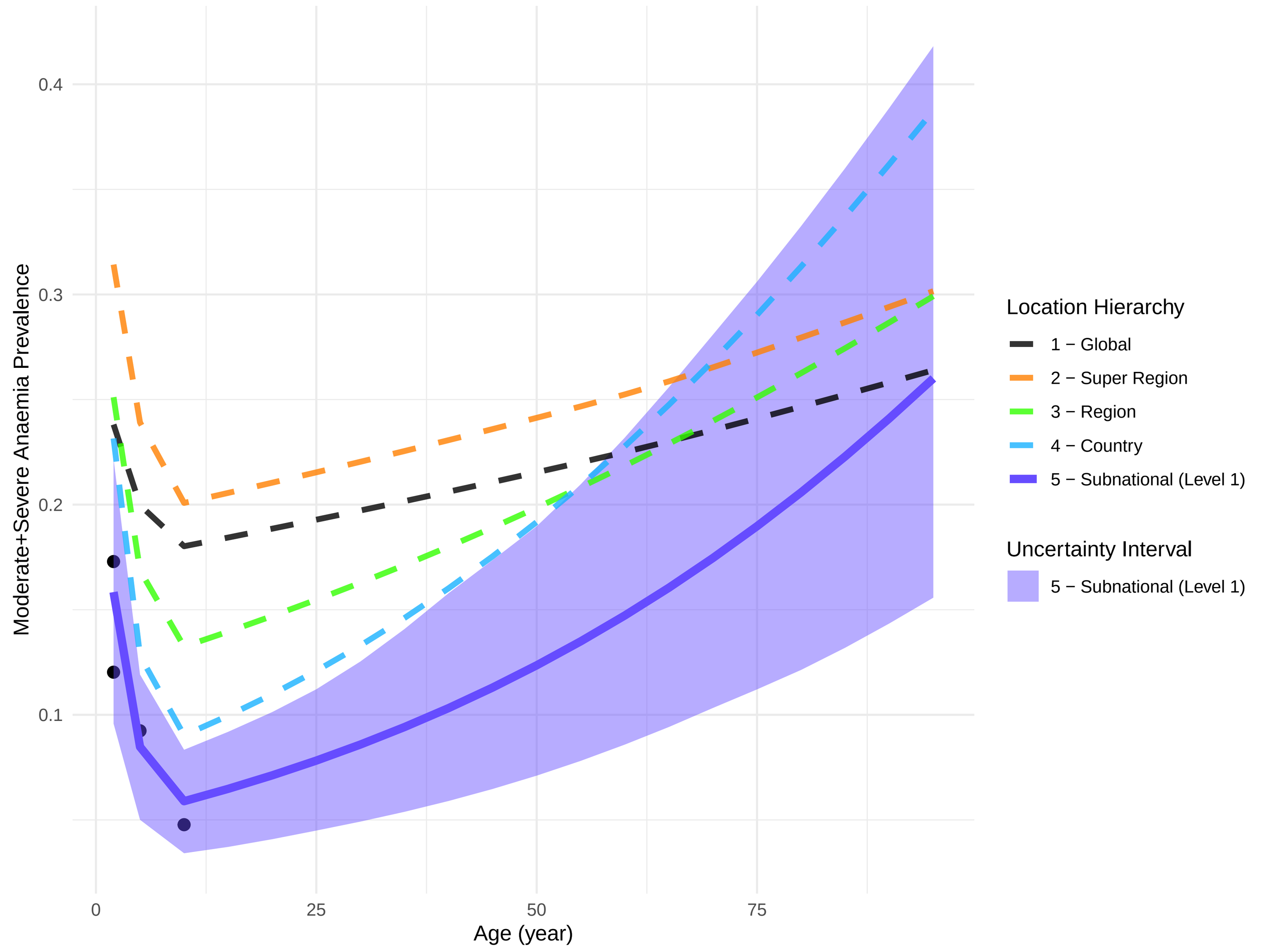

MR−BRT Age/Sex Fits − Non−pregnant Females 2+ Years Old − Moderate+Severe Anaemia Prevalence − Makueni
WHO−2001 Adjustment Method

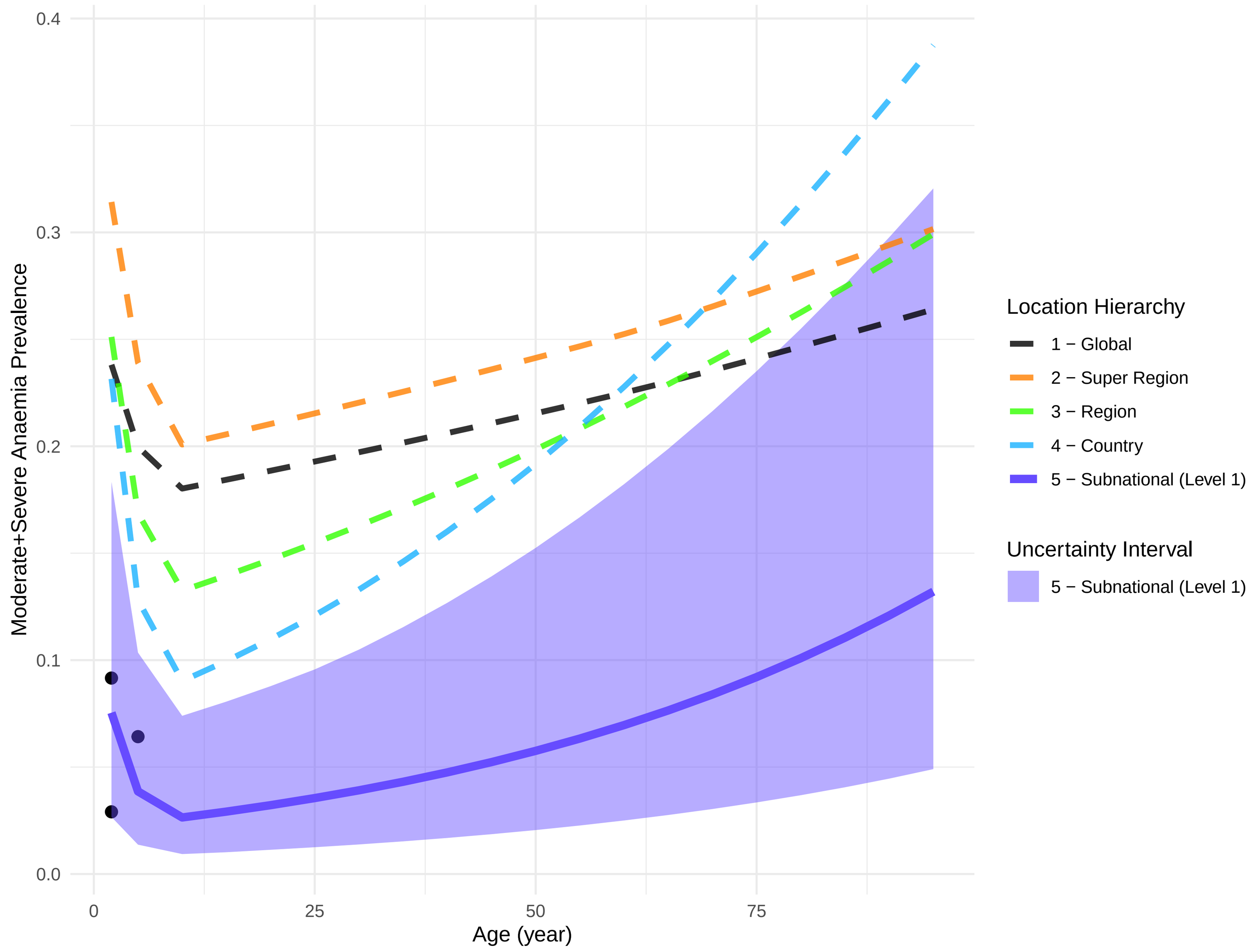

MR−BRT Age/Sex Fits − Non−pregnant Females 2+ Years Old − Moderate+Severe Anaemia Prevalence − Mandera
WHO−2001 Adjustment Method

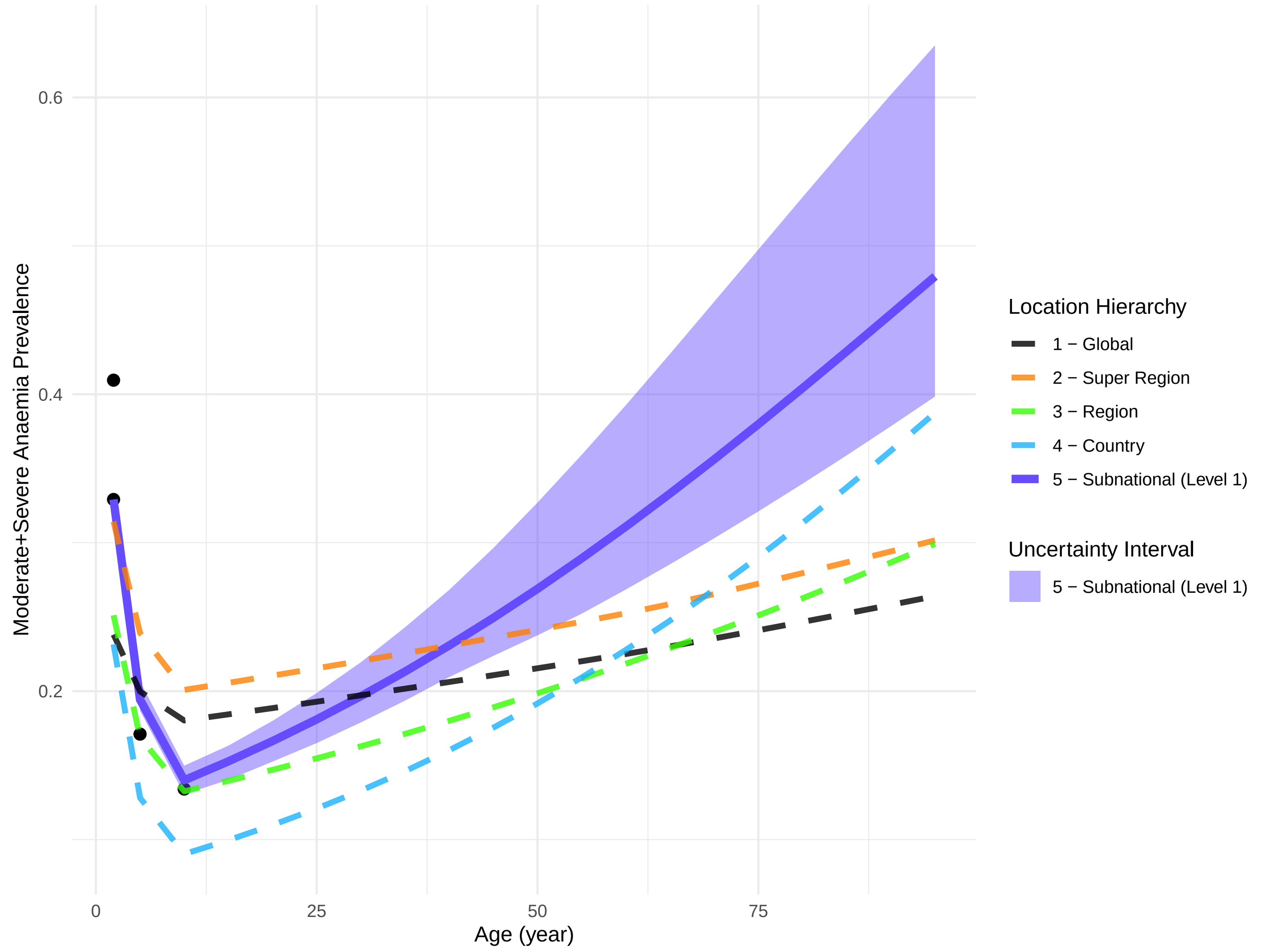

MR−BRT Age/Sex Fits − Non−pregnant Females 2+ Years Old − Moderate+Severe Anaemia Prevalence − Marsabit
WHO−2001 Adjustment Method

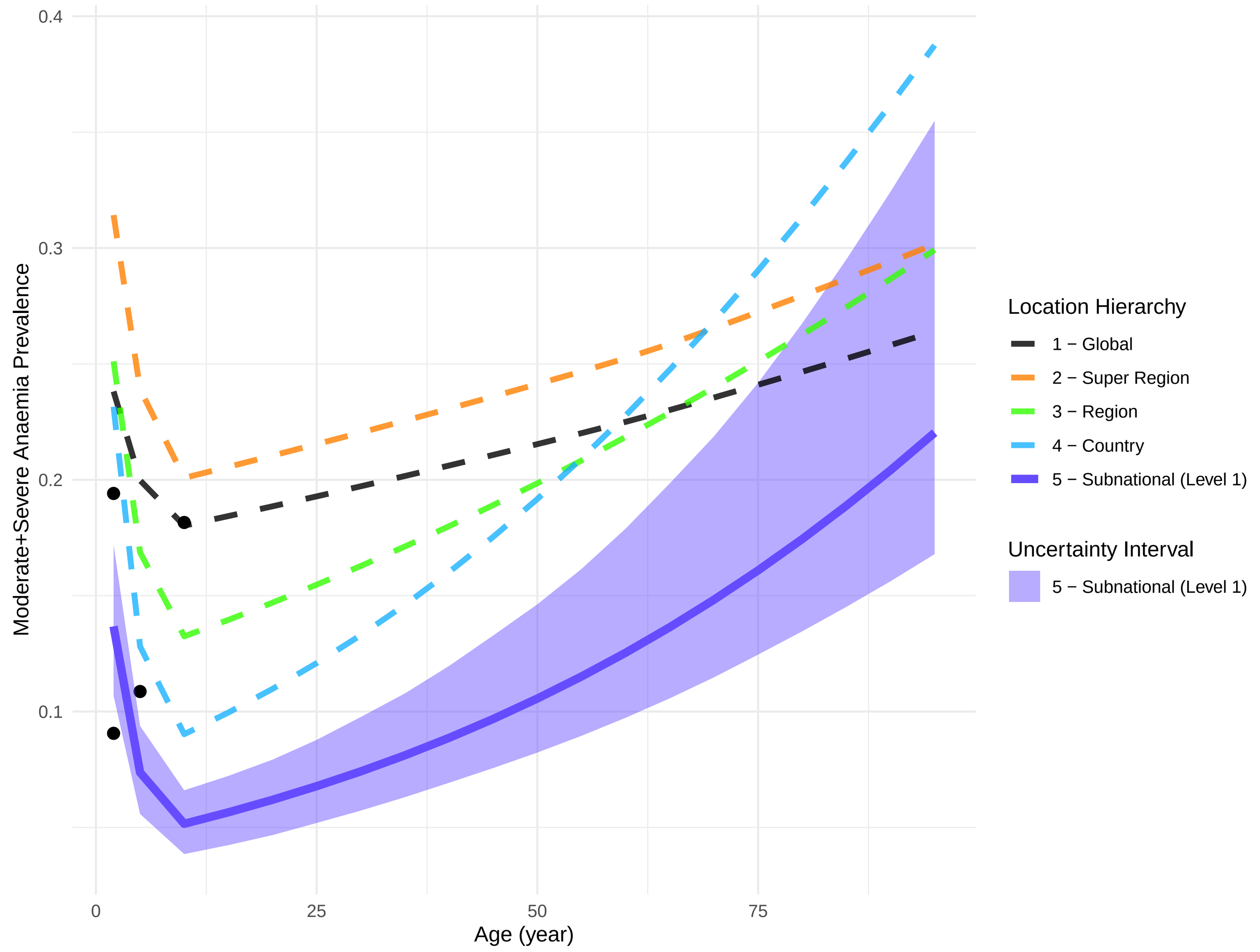

MR−BRT Age/Sex Fits − Non−pregnant Females 2+ Years Old − Moderate+Severe Anaemia Prevalence − Meru
WHO−2001 Adjustment Method

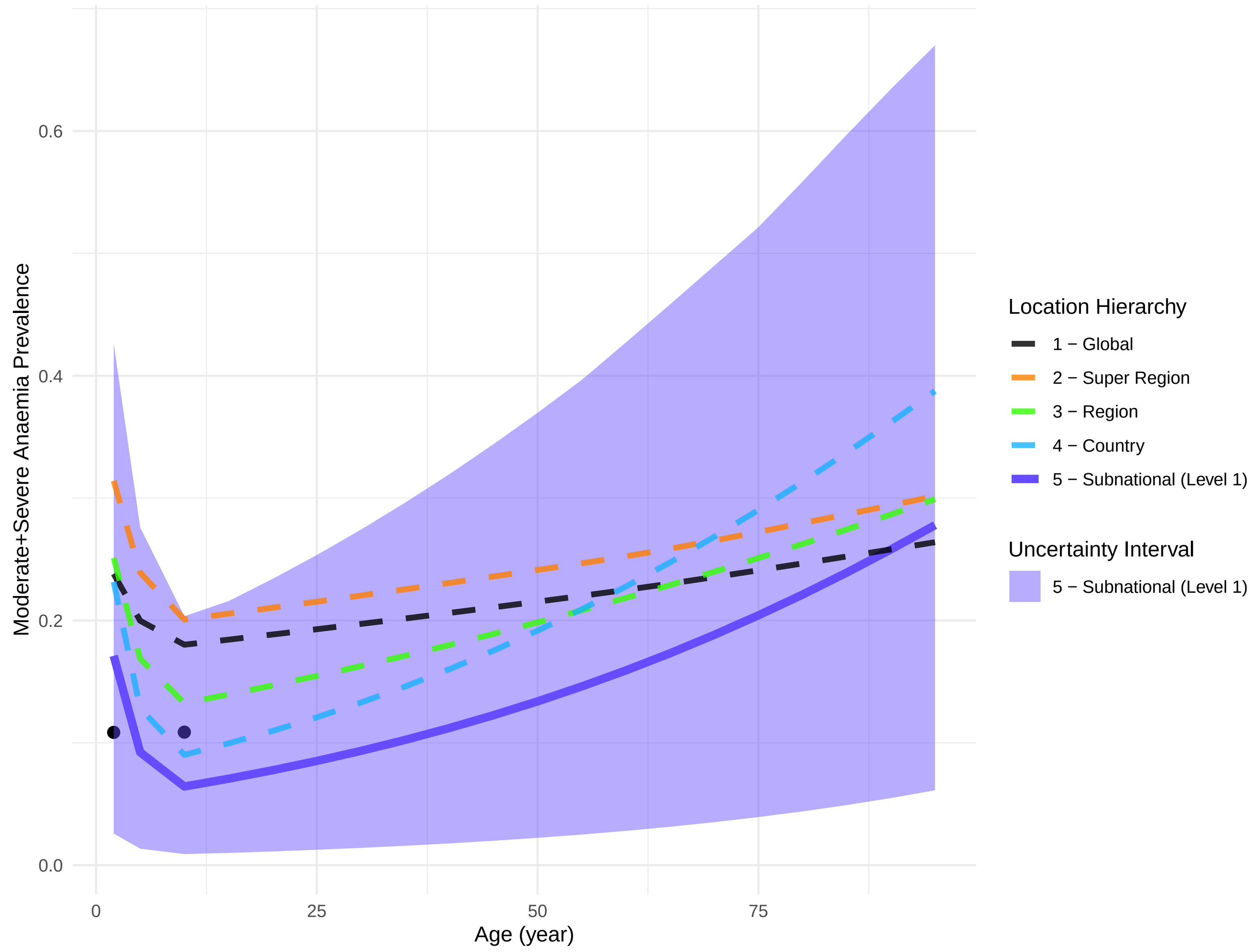

MR−BRT Age/Sex Fits − Non−pregnant Females 2+ Years Old − Moderate+Severe Anaemia Prevalence − Migori
WHO−2001 Adjustment Method

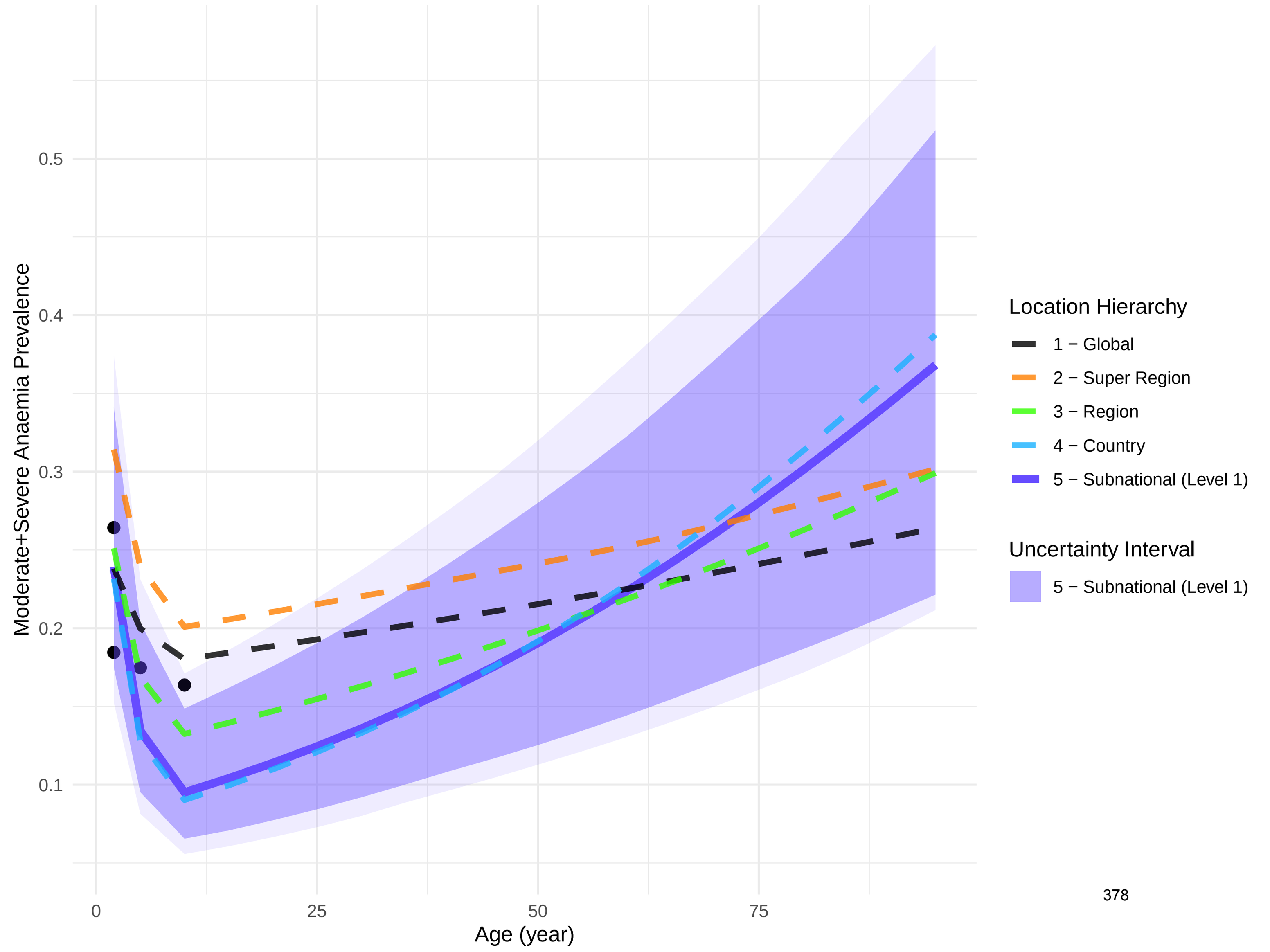

MR−BRT Age/Sex Fits − Non−pregnant Females 2+ Years Old − Moderate+Severe Anaemia Prevalence − Mombasa
WHO−2001 Adjustment Method

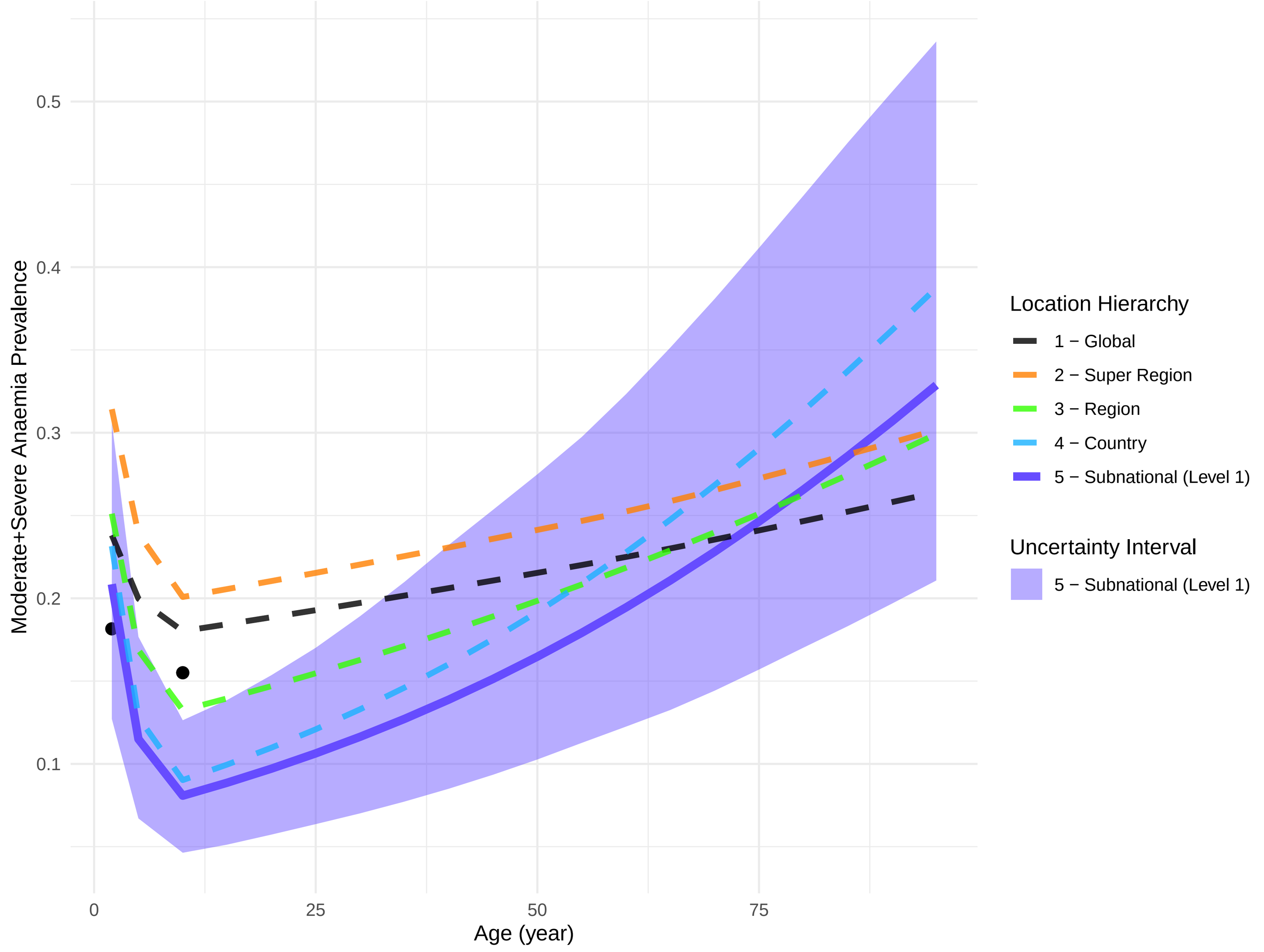

MR−BRT Age/Sex Fits − Non−pregnant Females 2+ Years Old − Moderate+Severe Anaemia Prevalence − Murang'a
WHO−2001 Adjustment Method

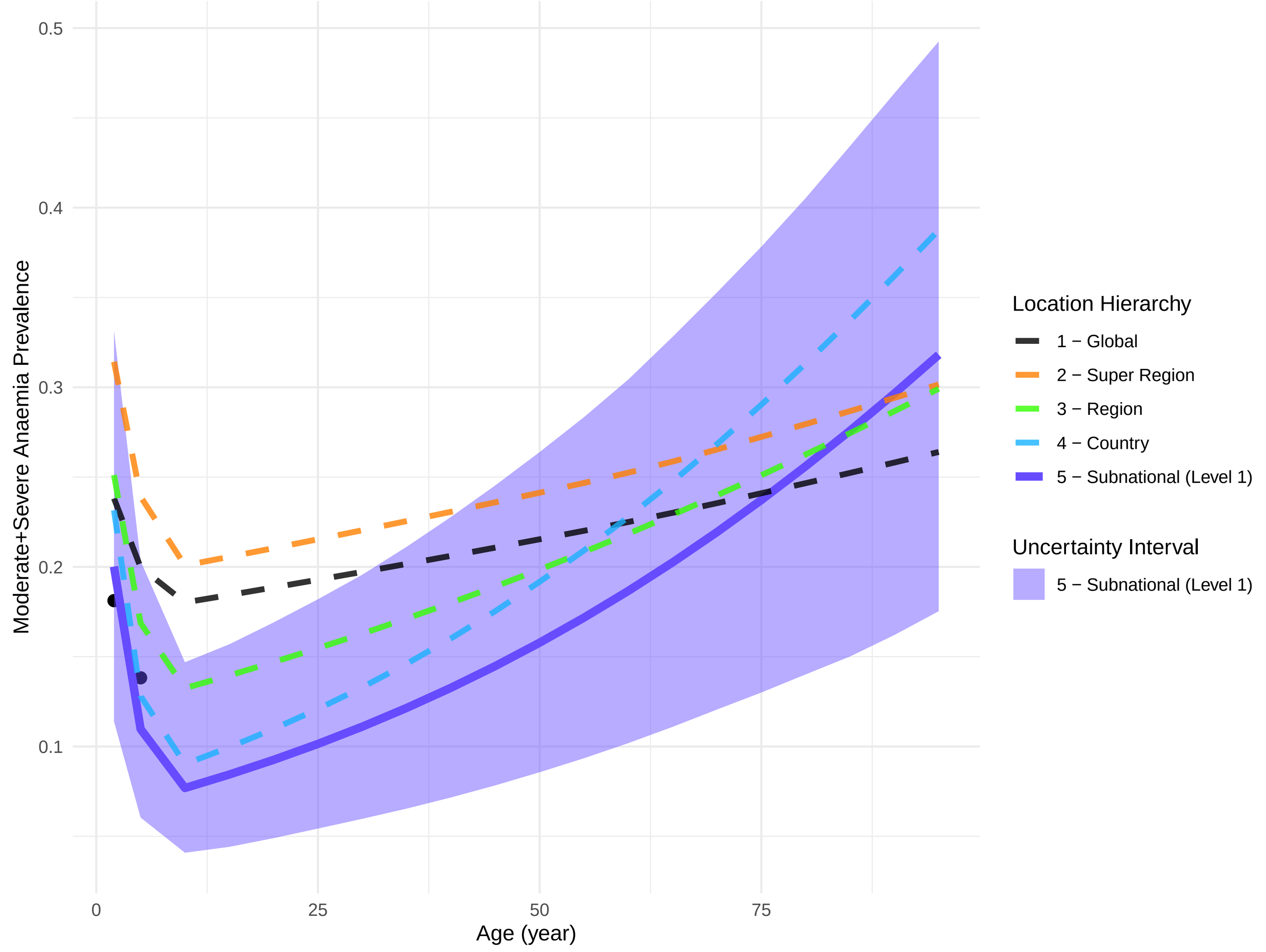

MR−BRT Age/Sex Fits − Non−pregnant Females 2+ Years Old − Moderate+Severe Anaemia Prevalence − Nairobi
WHO−2001 Adjustment Method

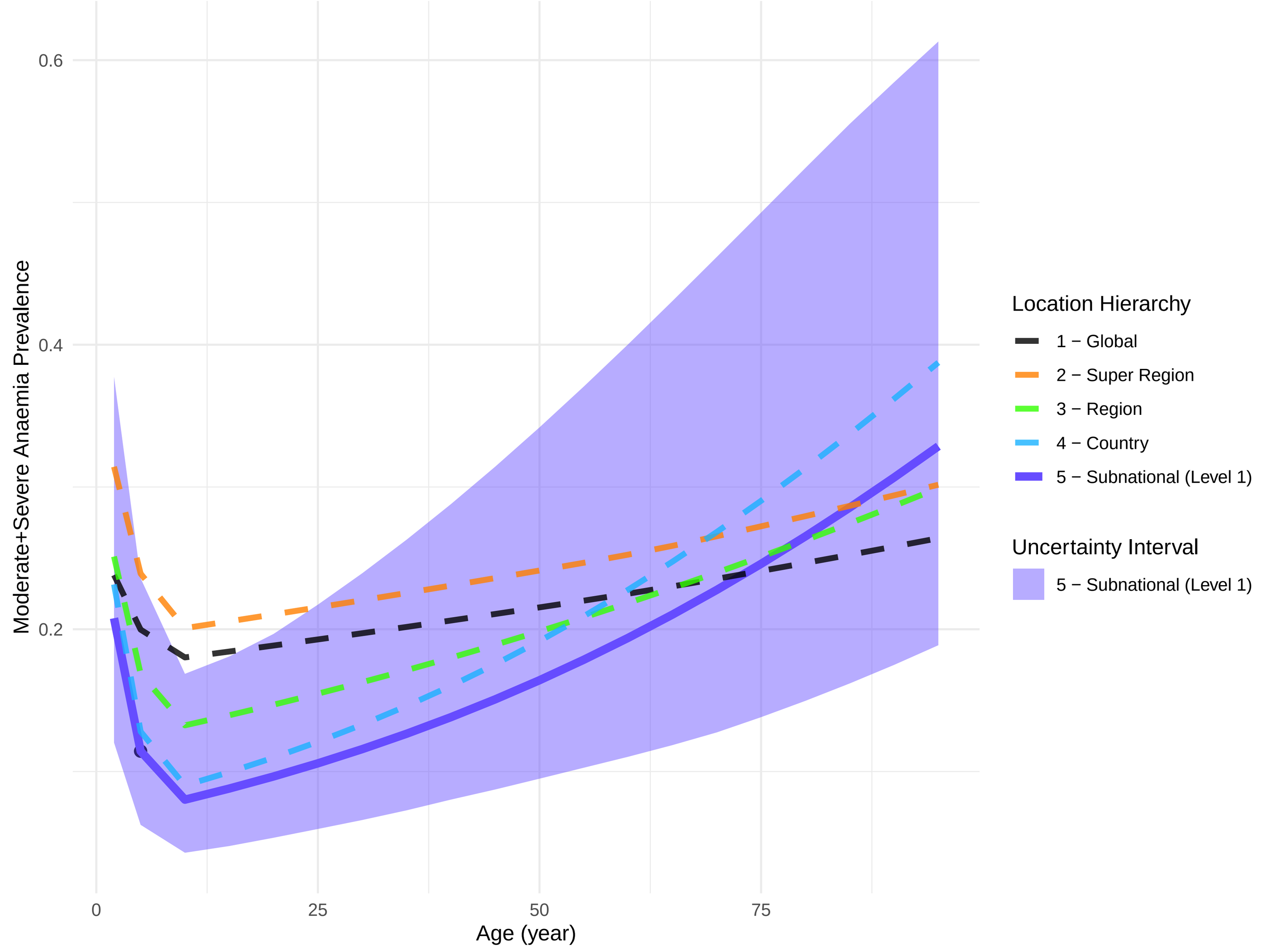

MR−BRT Age/Sex Fits − Non−pregnant Females 2+ Years Old − Moderate+Severe Anaemia Prevalence − Nakuru
WHO−2001 Adjustment Method

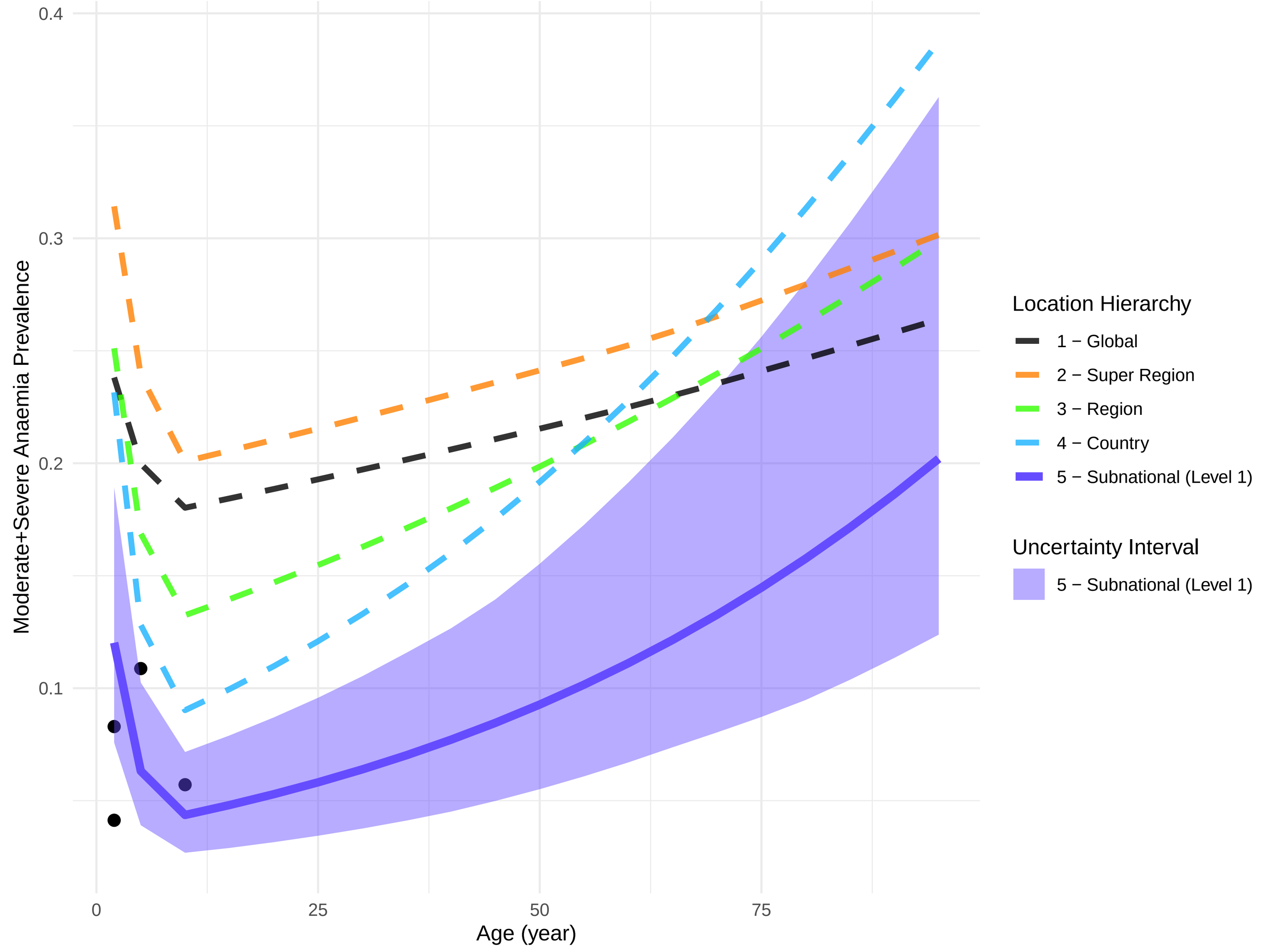

MR−BRT Age/Sex Fits − Non−pregnant Females 2+ Years Old − Moderate+Severe Anaemia Prevalence − Nandi
WHO−2001 Adjustment Method

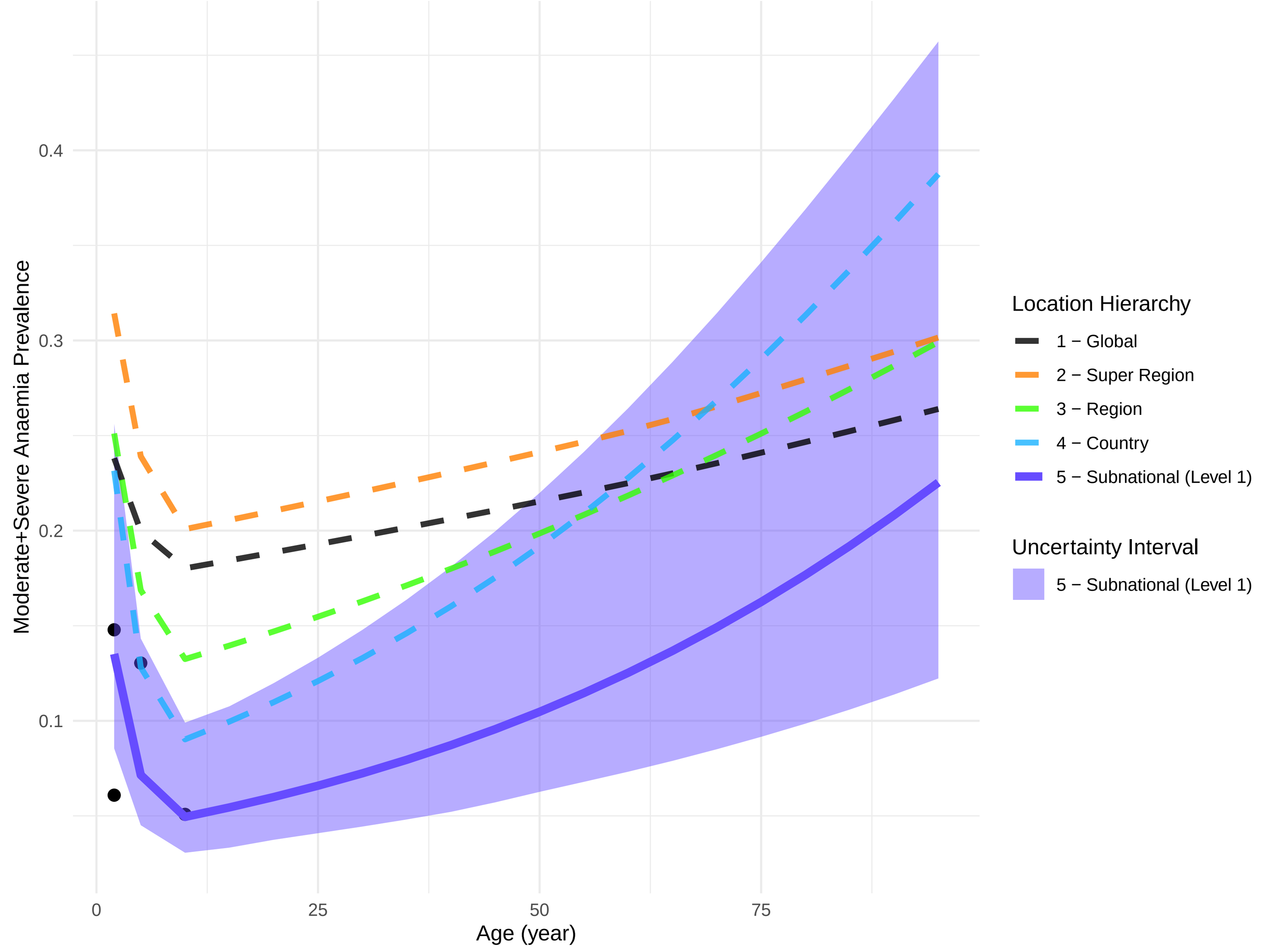

MR−BRT Age/Sex Fits − Non−pregnant Females 2+ Years Old − Moderate+Severe Anaemia Prevalence − Narok
WHO−2001 Adjustment Method

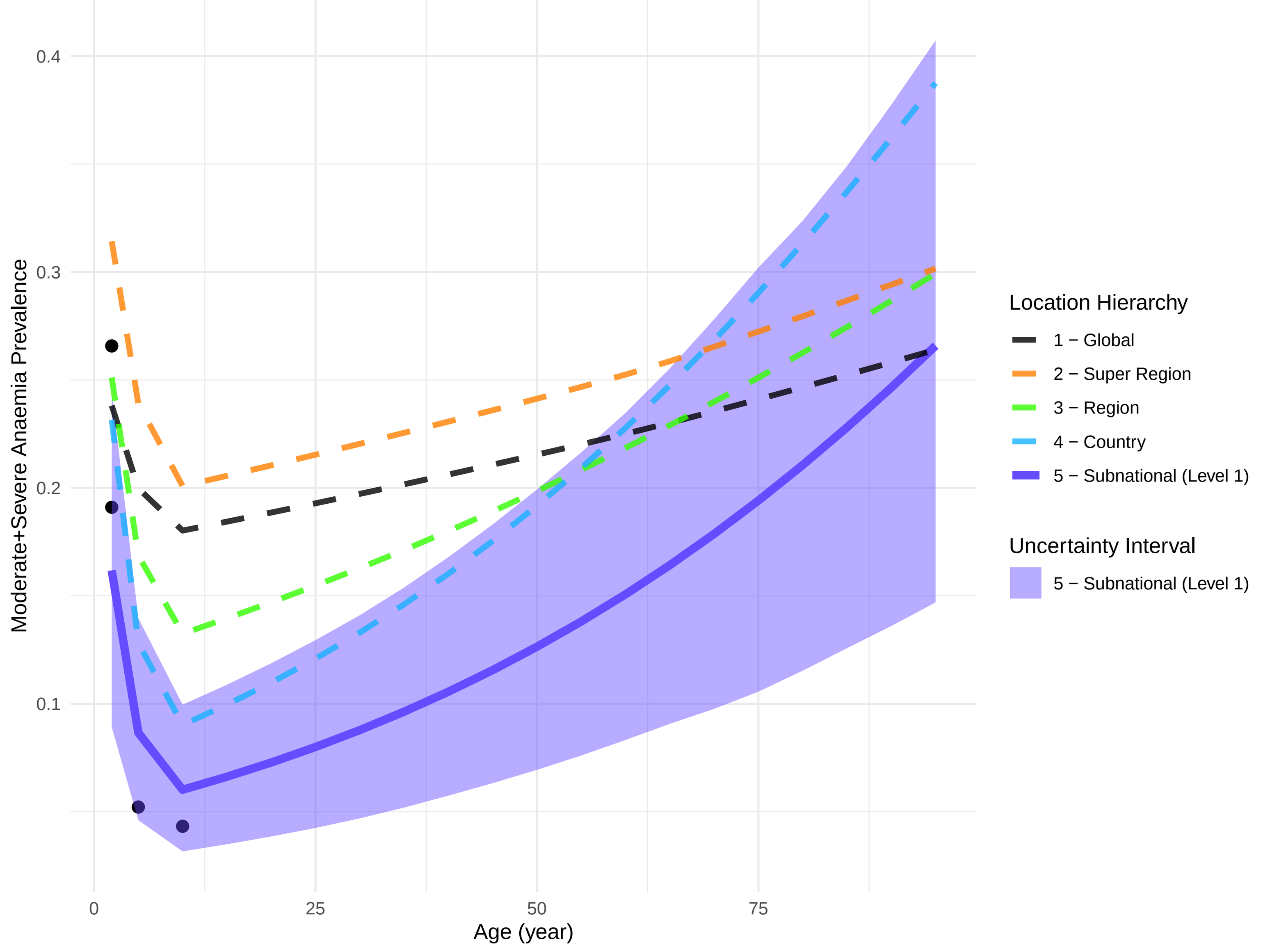

MR−BRT Age/Sex Fits − Non−pregnant Females 2+ Years Old − Moderate+Severe Anaemia Prevalence − Nyamira
WHO−2001 Adjustment Method

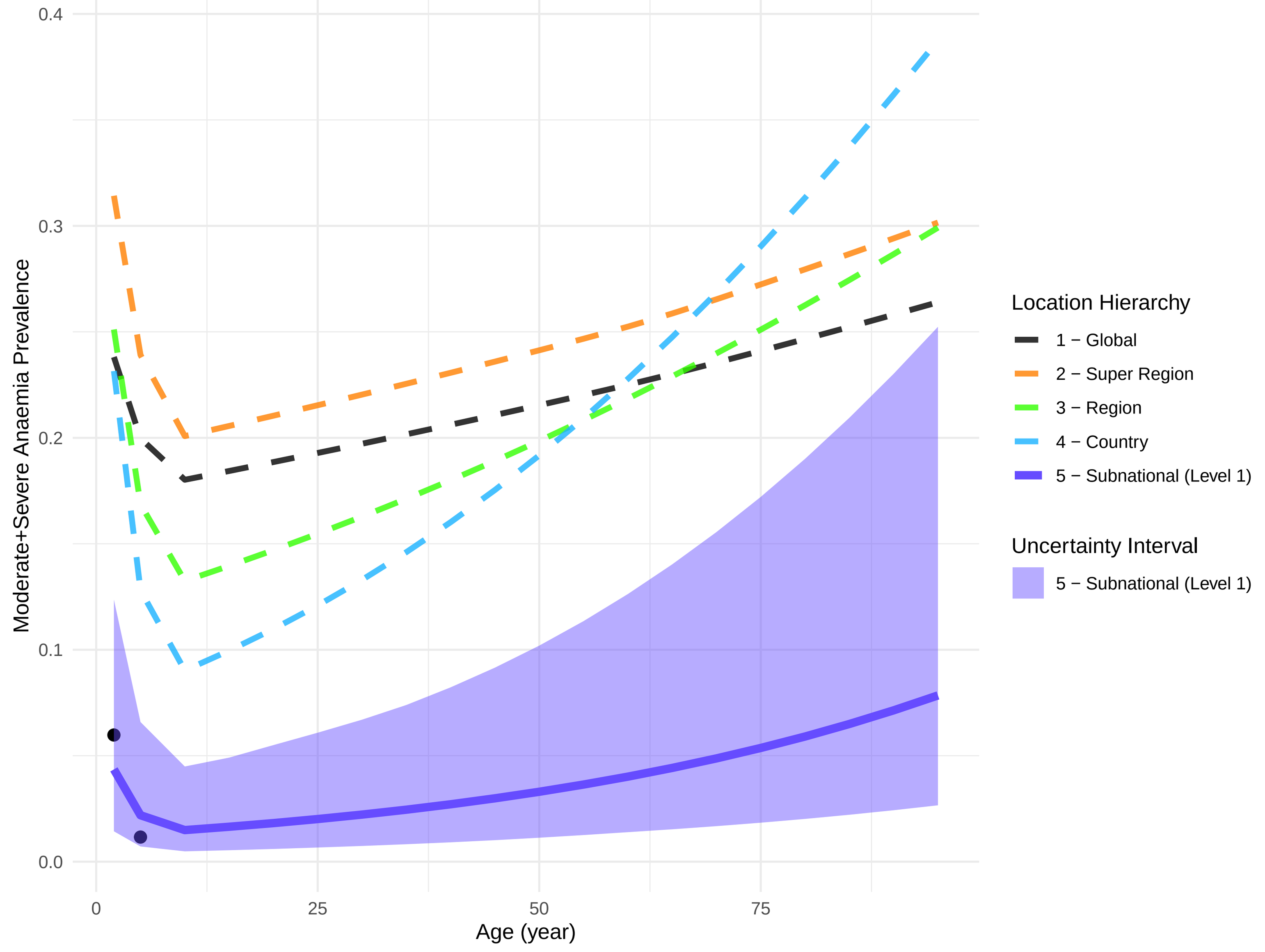

MR−BRT Age/Sex Fits − Non−pregnant Females 2+ Years Old − Moderate+Severe Anaemia Prevalence − Nyandarua
WHO−2001 Adjustment Method

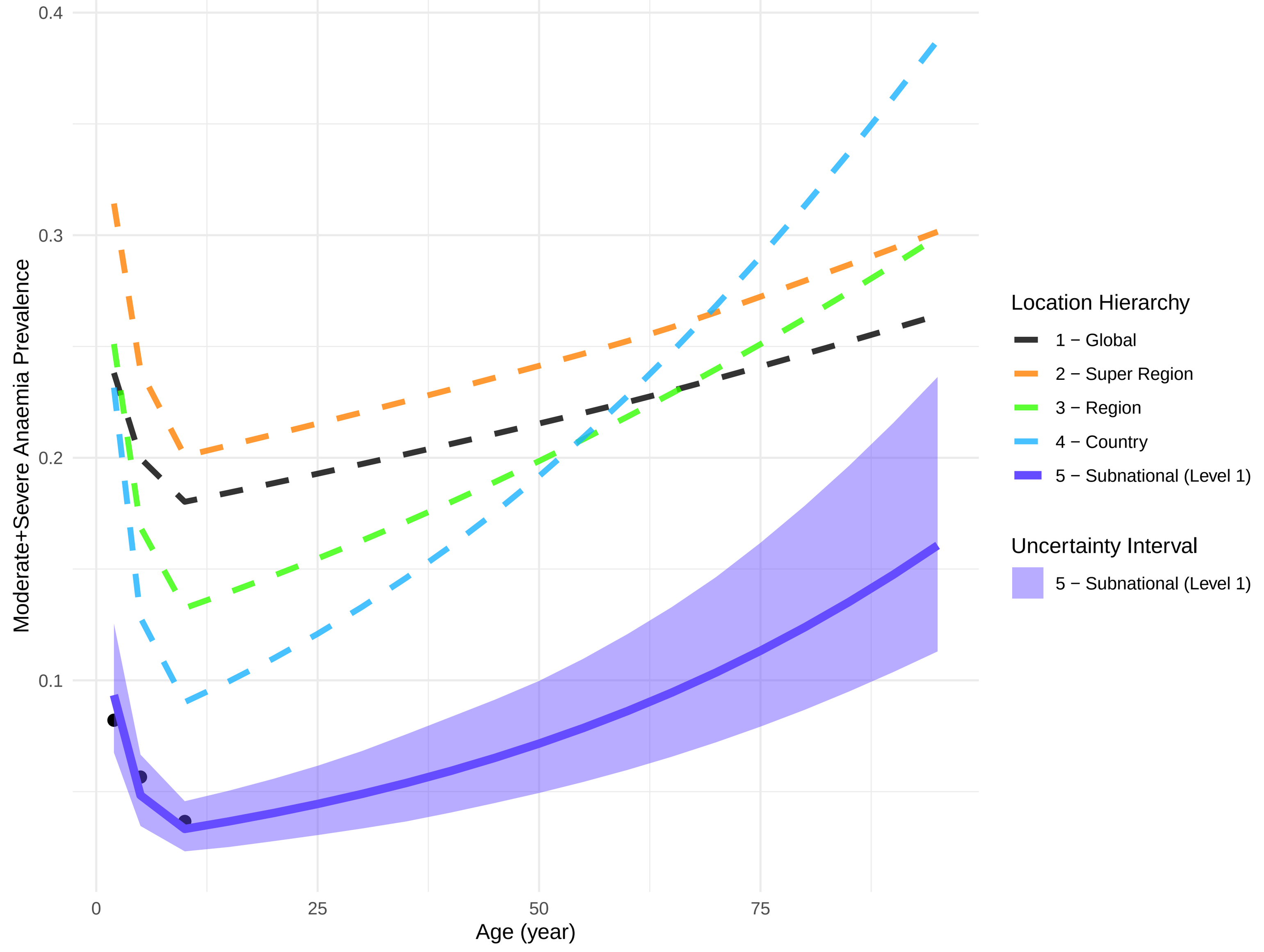

MR−BRT Age/Sex Fits − Non−pregnant Females 2+ Years Old − Moderate+Severe Anaemia Prevalence − Nyeri
WHO−2001 Adjustment Method

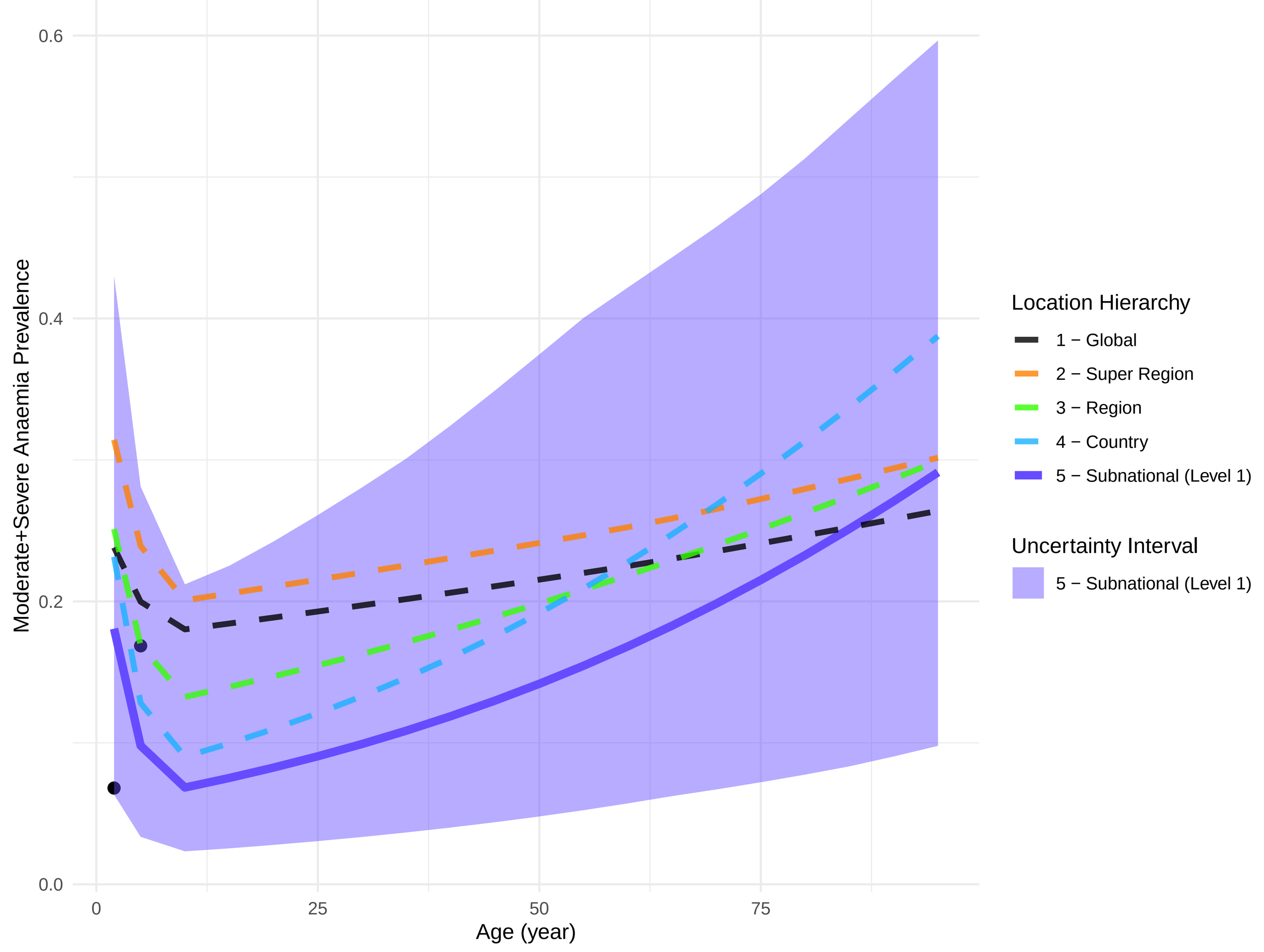

MR−BRT Age/Sex Fits − Non−pregnant Females 2+ Years Old − Moderate+Severe Anaemia Prevalence − Samburu
WHO−2001 Adjustment Method

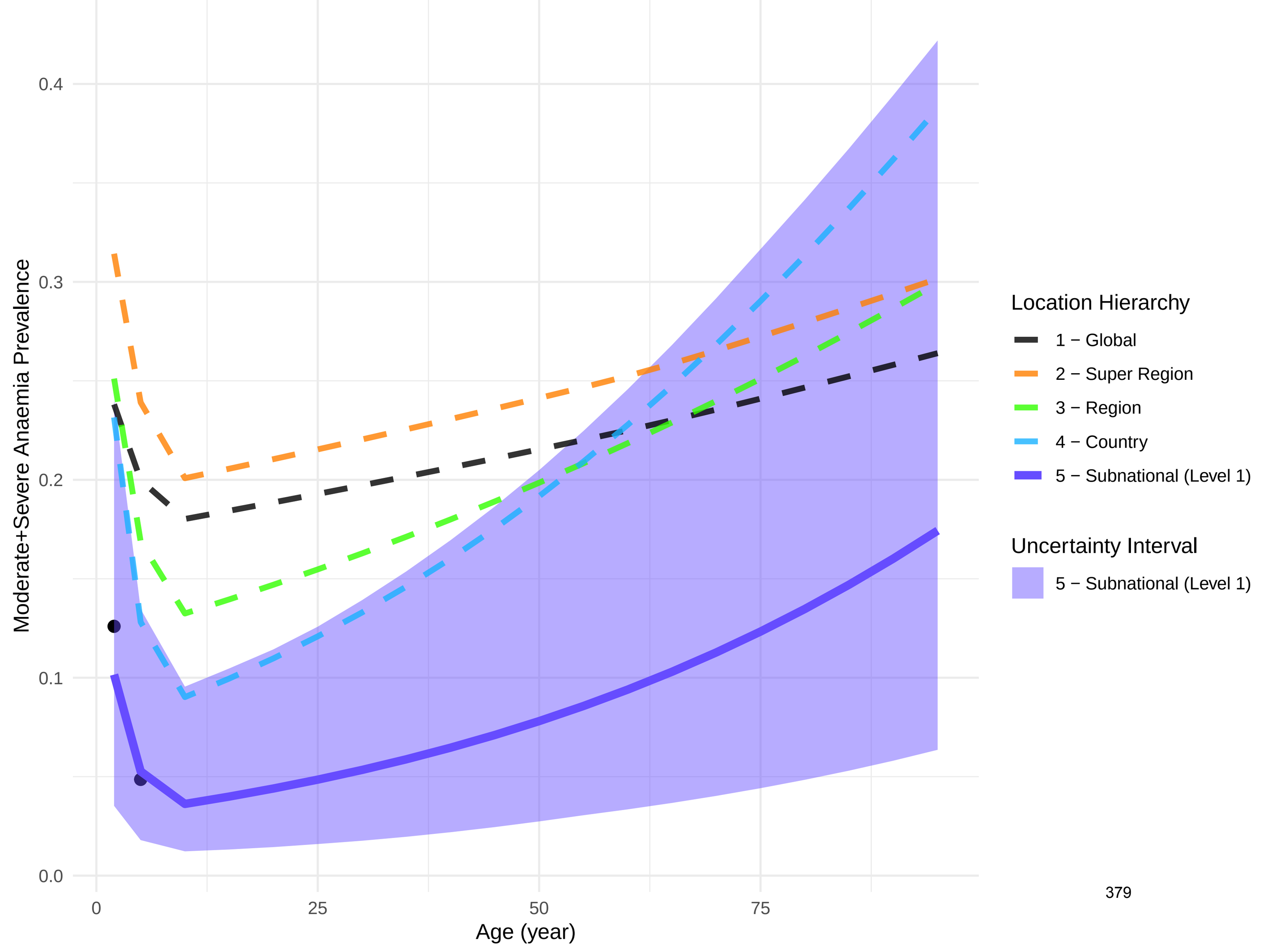

MR−BRT Age/Sex Fits − Non−pregnant Females 2+ Years Old − Moderate+Severe Anaemia Prevalence − Siaya
WHO−2001 Adjustment Method

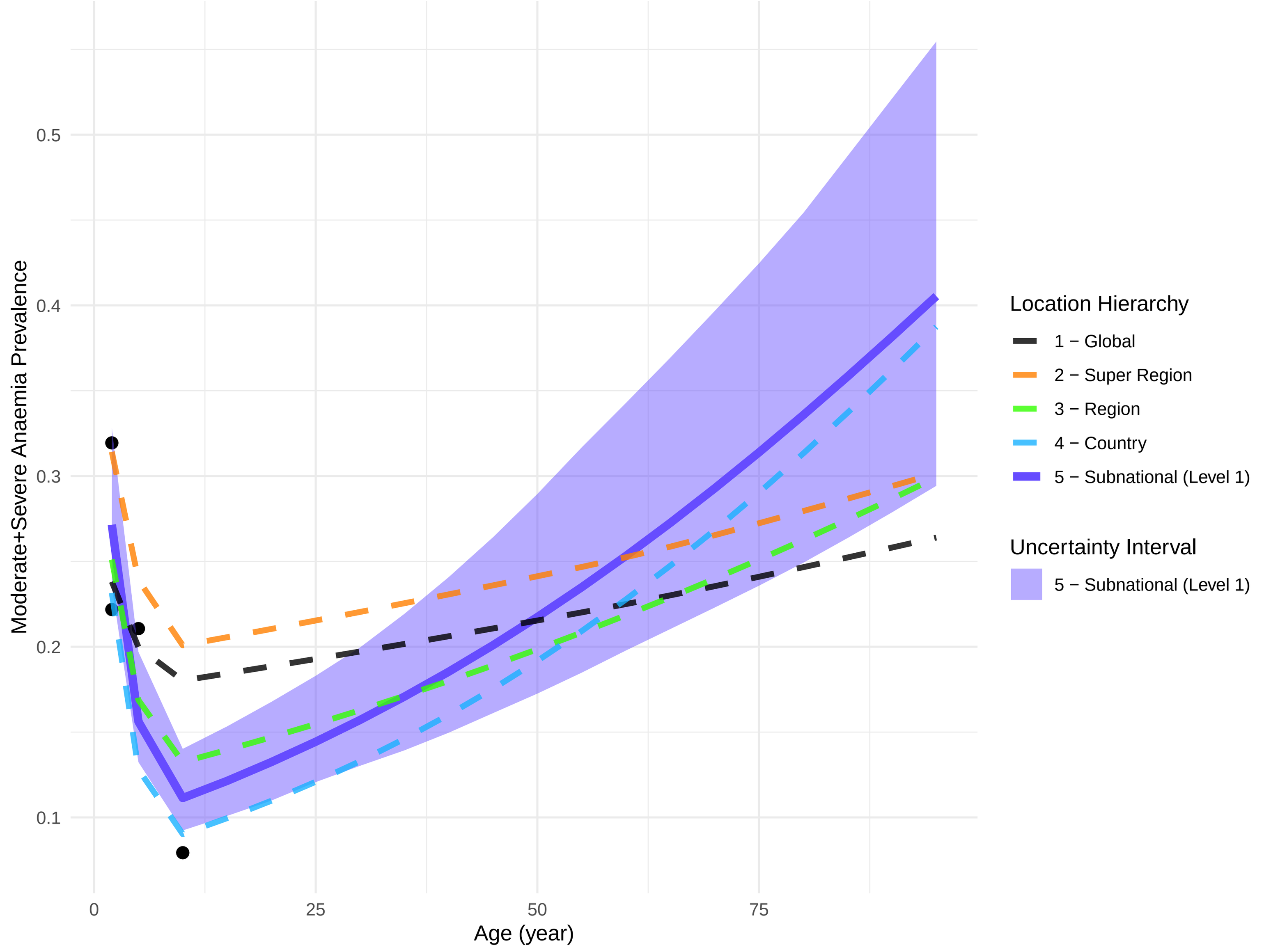

MR−BRT Age/Sex Fits − Non−pregnant Females 2+ Years Old − Moderate+Severe Anaemia Prevalence − Taita Taveta
WHO−2001 Adjustment Method

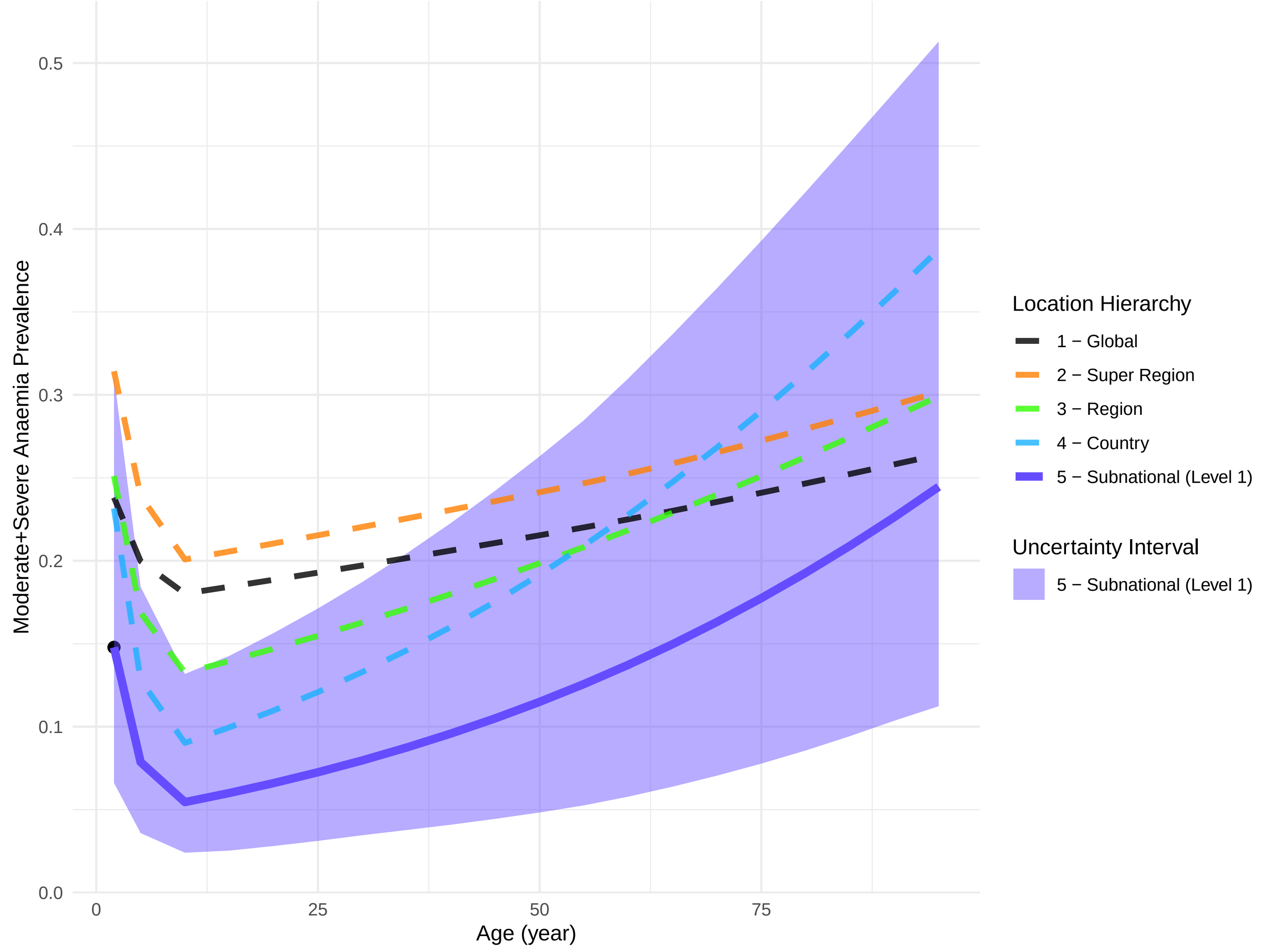

MR−BRT Age/Sex Fits − Non−pregnant Females 2+ Years Old − Moderate+Severe Anaemia Prevalence − Tana River
WHO−2001 Adjustment Method

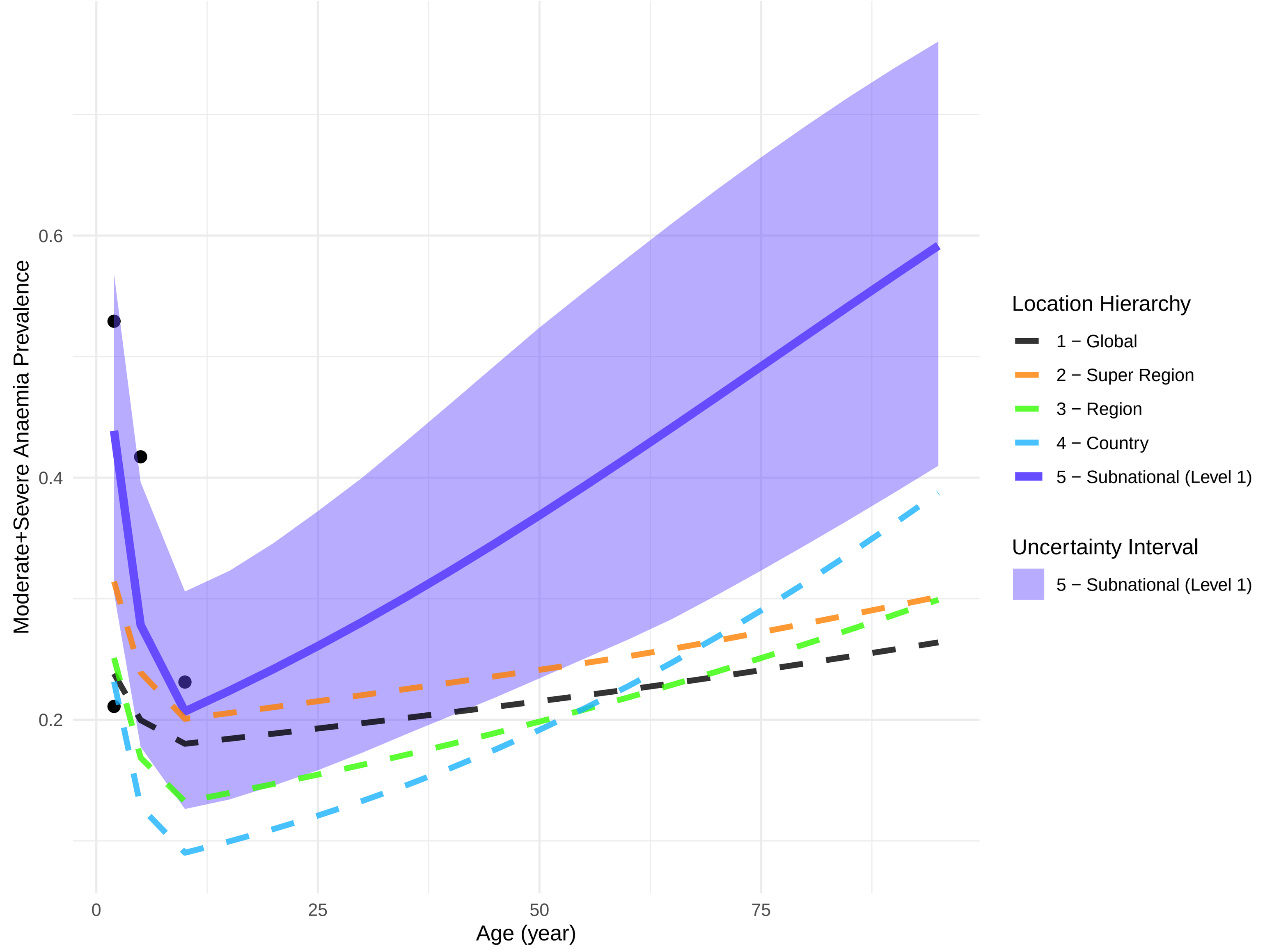

MR−BRT Age/Sex Fits − Non−pregnant Females 2+ Years Old − Moderate+Severe Anaemia Prevalence − Tharaka Nithi
WHO−2001 Adjustment Method

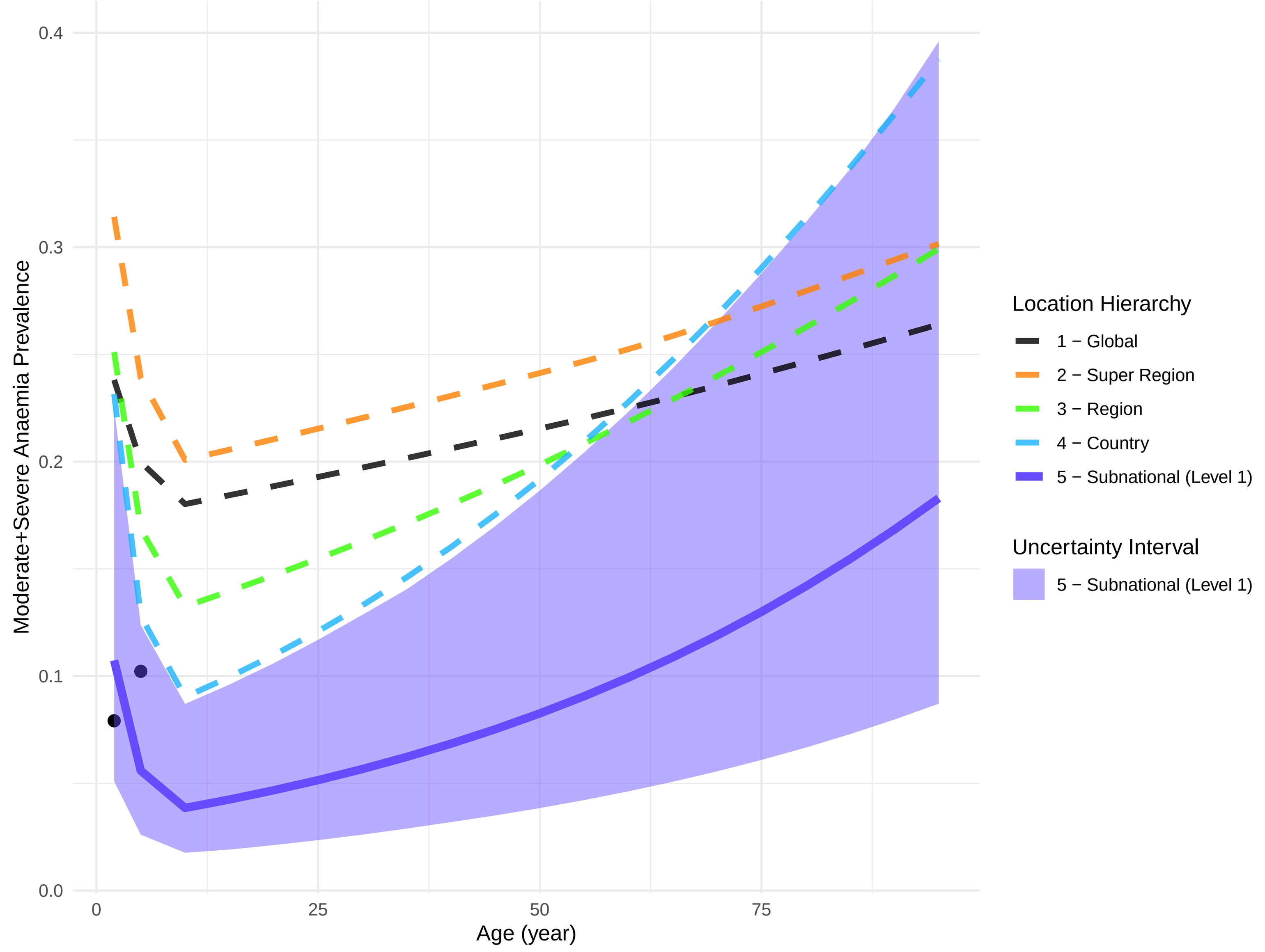

MR−BRT Age/Sex Fits − Non−pregnant Females 2+ Years Old − Moderate+Severe Anaemia Prevalence − Trans Nzoia
WHO−2001 Adjustment Method

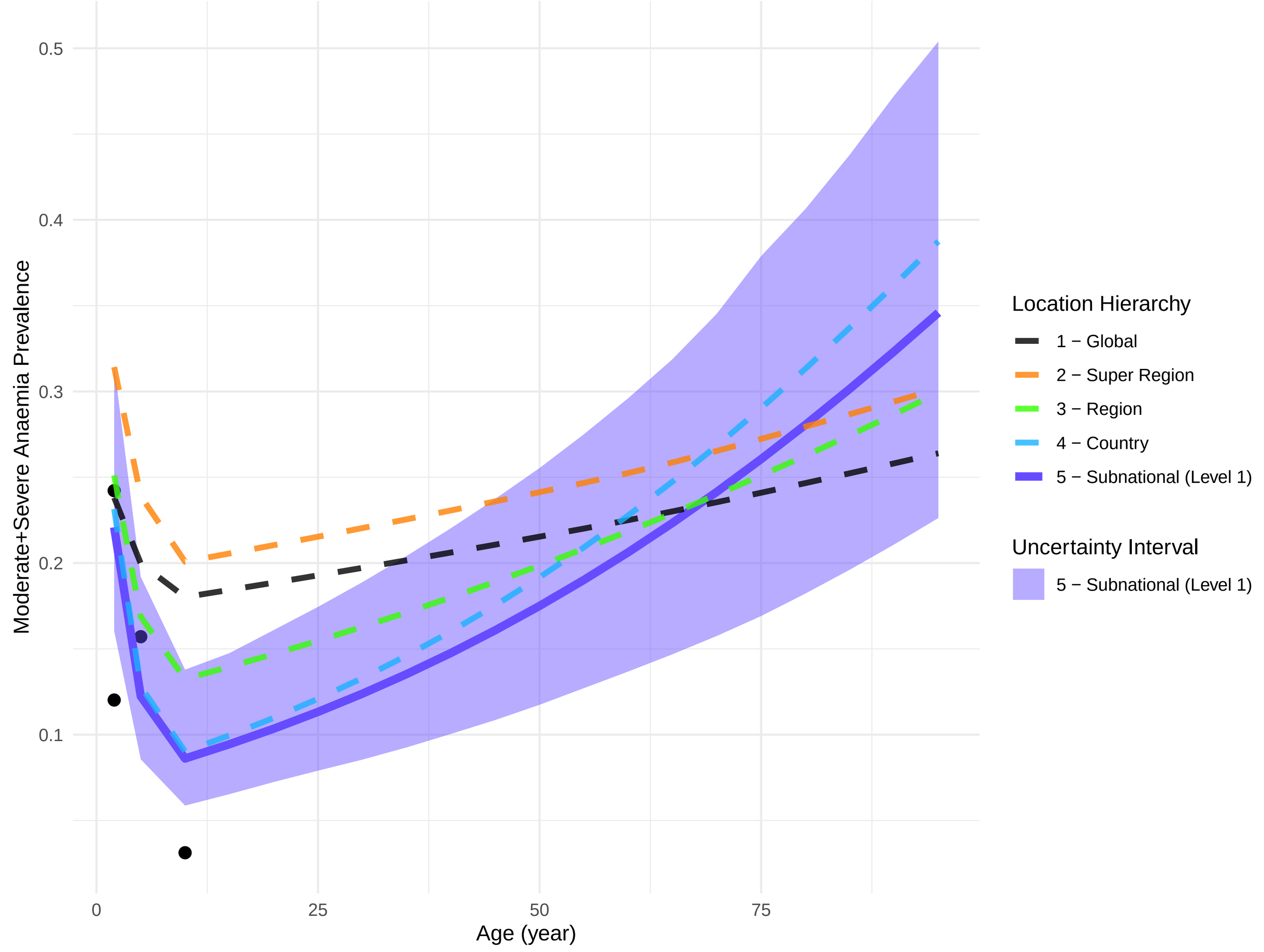

MR−BRT Age/Sex Fits − Non−pregnant Females 2+ Years Old − Moderate+Severe Anaemia Prevalence − Turkana
WHO−2001 Adjustment Method

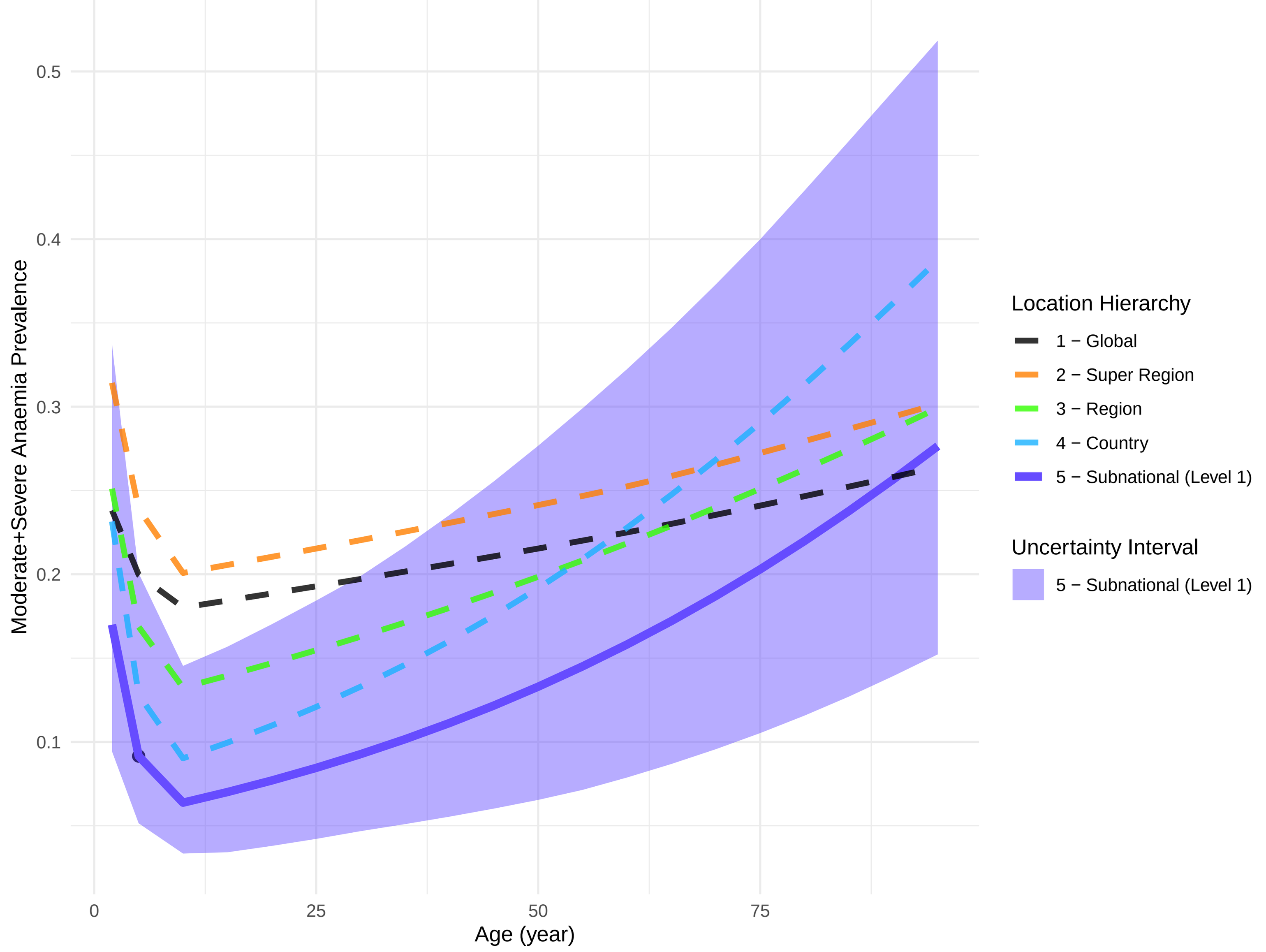

MR−BRT Age/Sex Fits − Non−pregnant Females 2+ Years Old − Moderate+Severe Anaemia Prevalence − Uasin Gishu
WHO−2001 Adjustment Method

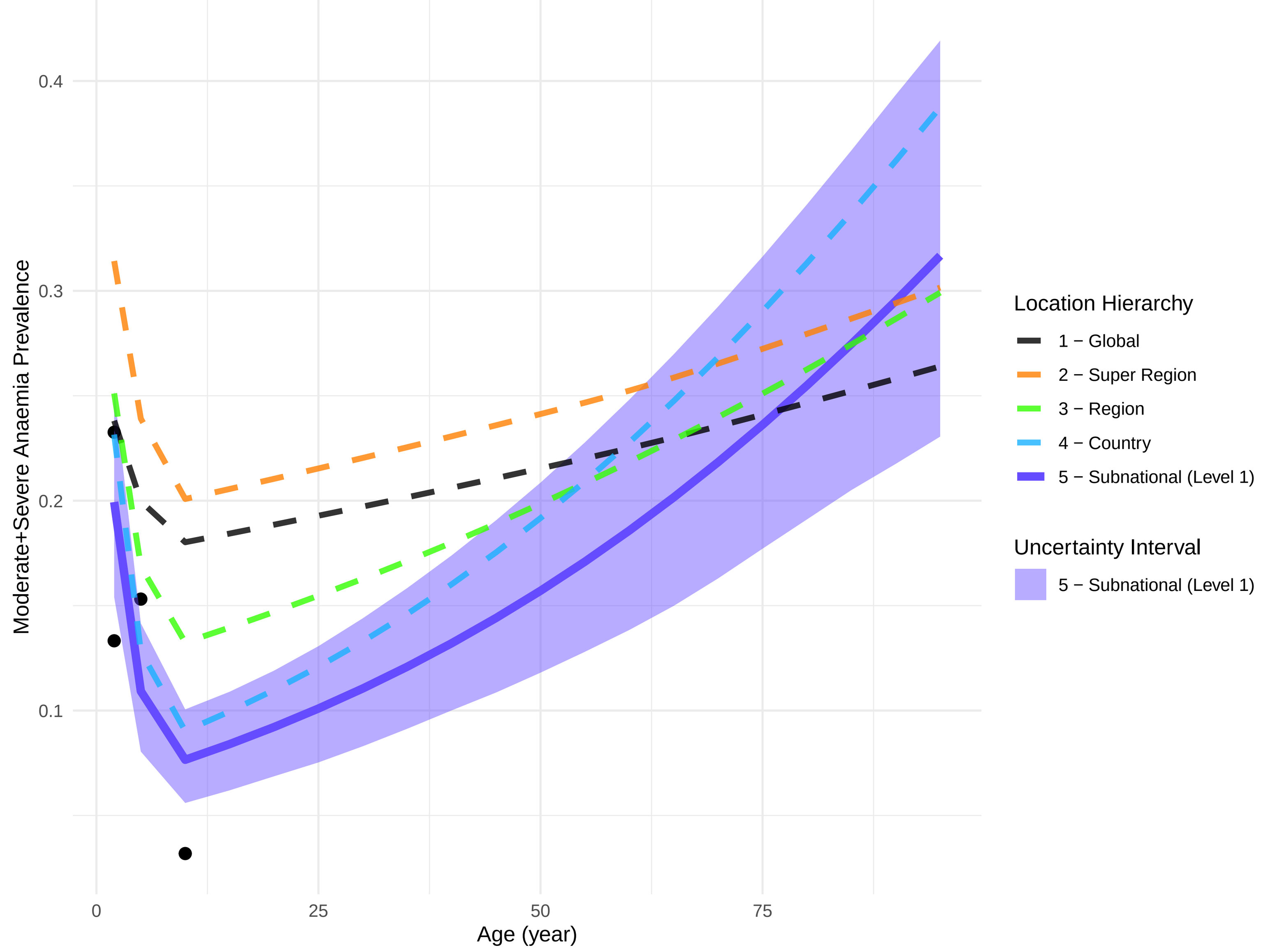

MR−BRT Age/Sex Fits − Non−pregnant Females 2+ Years Old − Moderate+Severe Anaemia Prevalence − Vihiga
WHO−2001 Adjustment Method

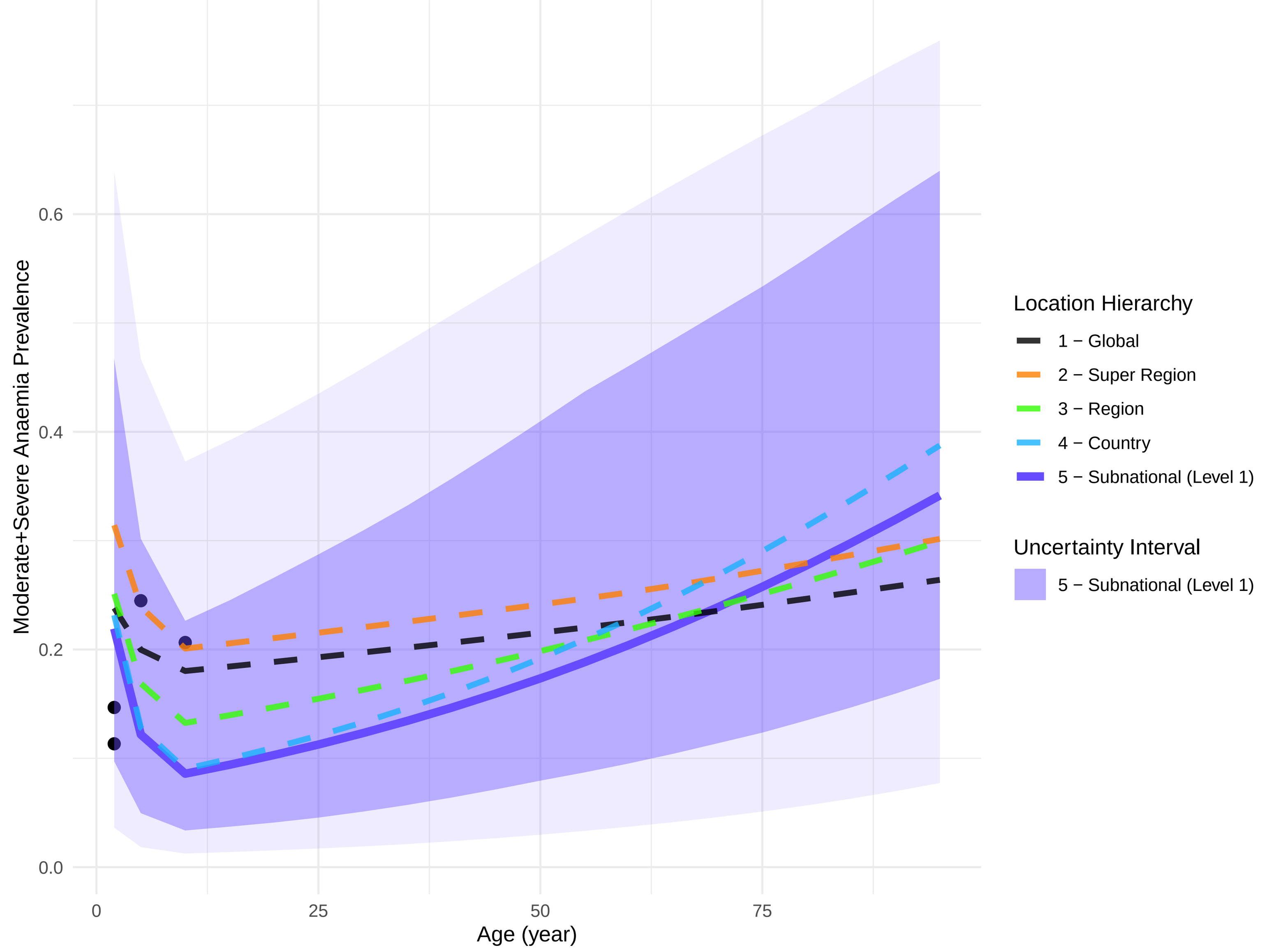

MR−BRT Age/Sex Fits − Non−pregnant Females 2+ Years Old − Moderate+Severe Anaemia Prevalence − Wajir
WHO−2001 Adjustment Method

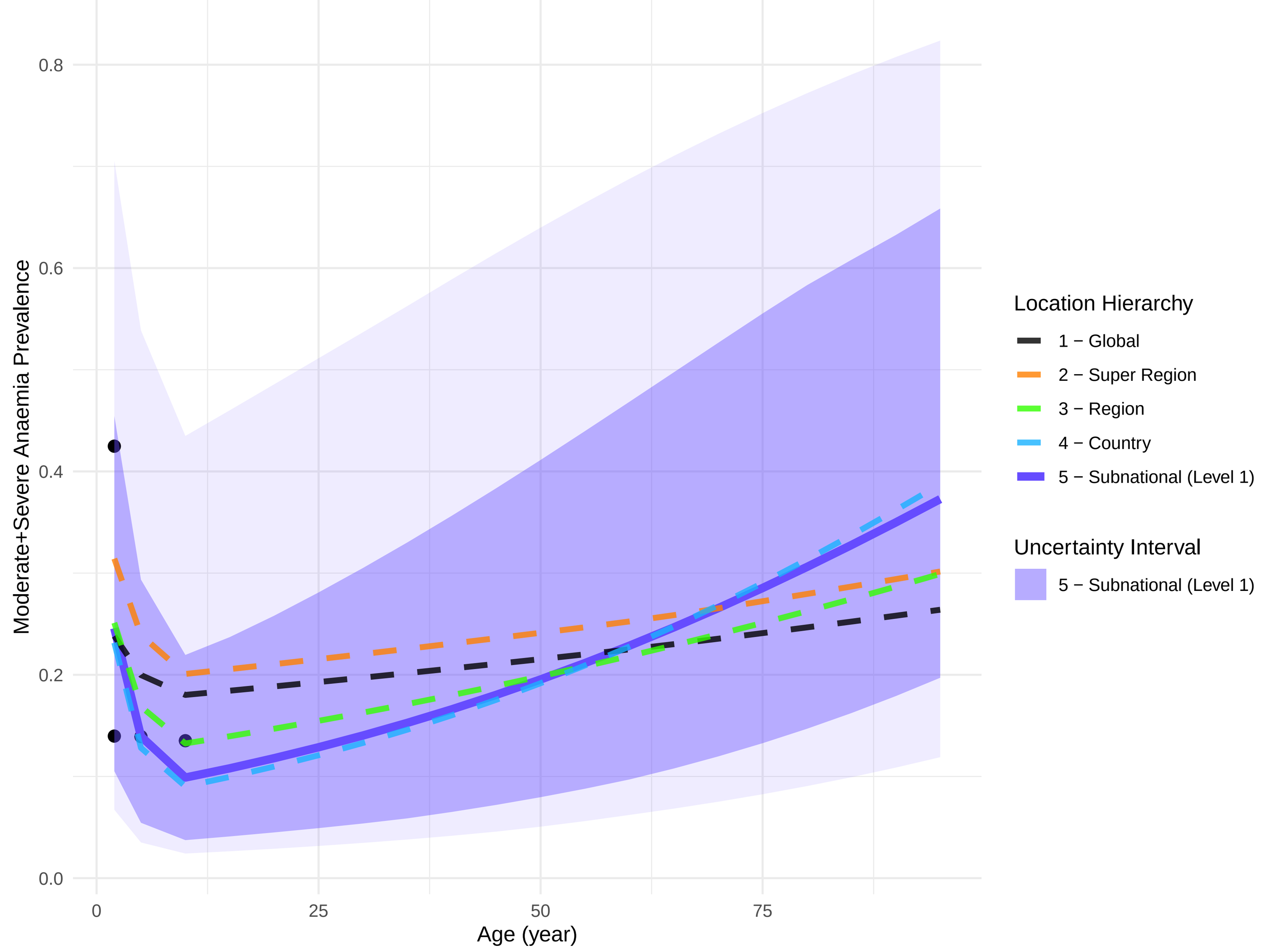

MR−BRT Age/Sex Fits − Non−pregnant Females 2+ Years Old − Moderate+Severe Anaemia Prevalence − West Pokot
WHO−2001 Adjustment Method

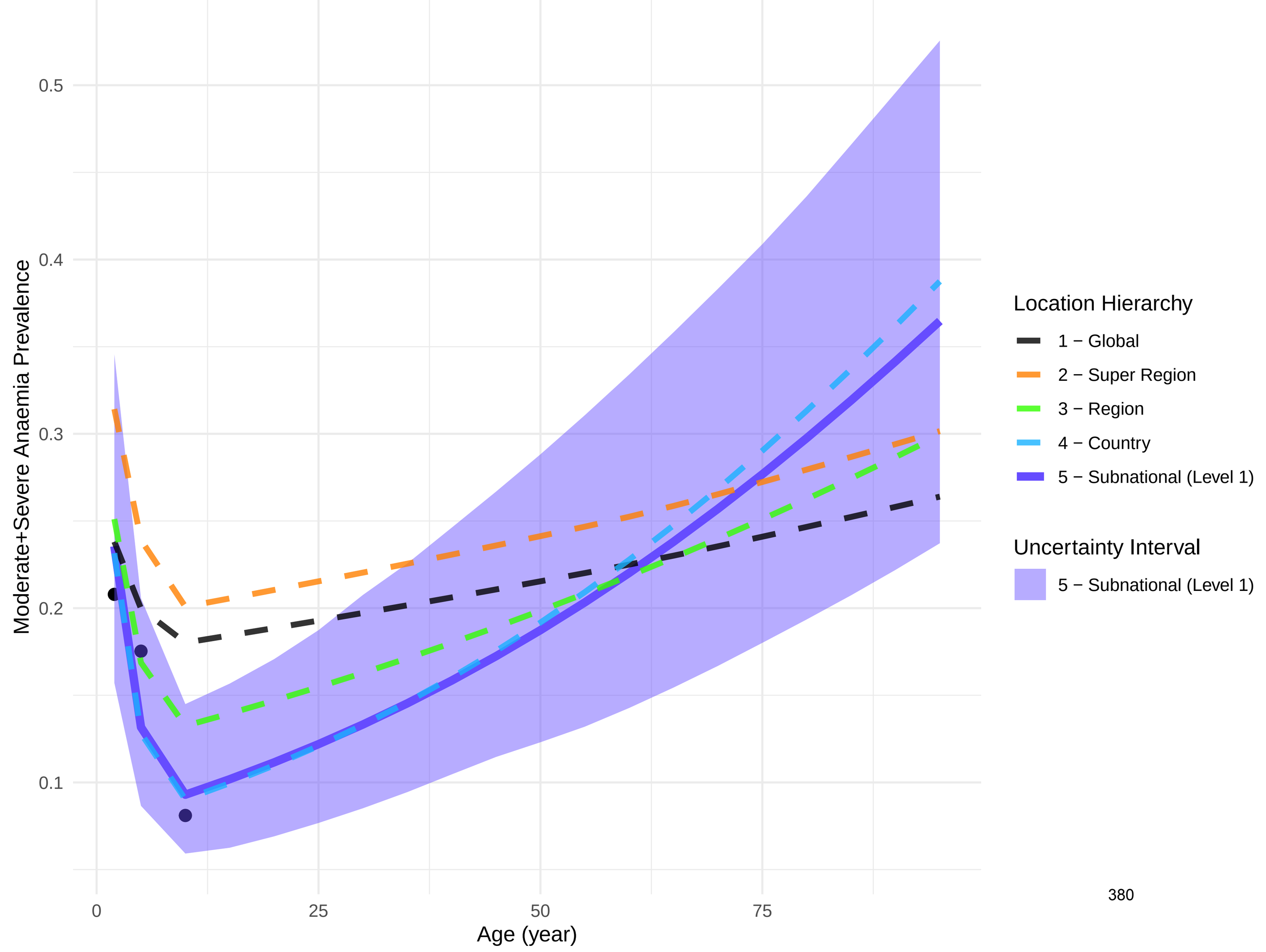

MR−BRT Age/Sex Fits − Non−pregnant Females 2+ Years Old − Moderate+Severe Anaemia Prevalence − Madagascar
WHO−2001 Adjustment Method

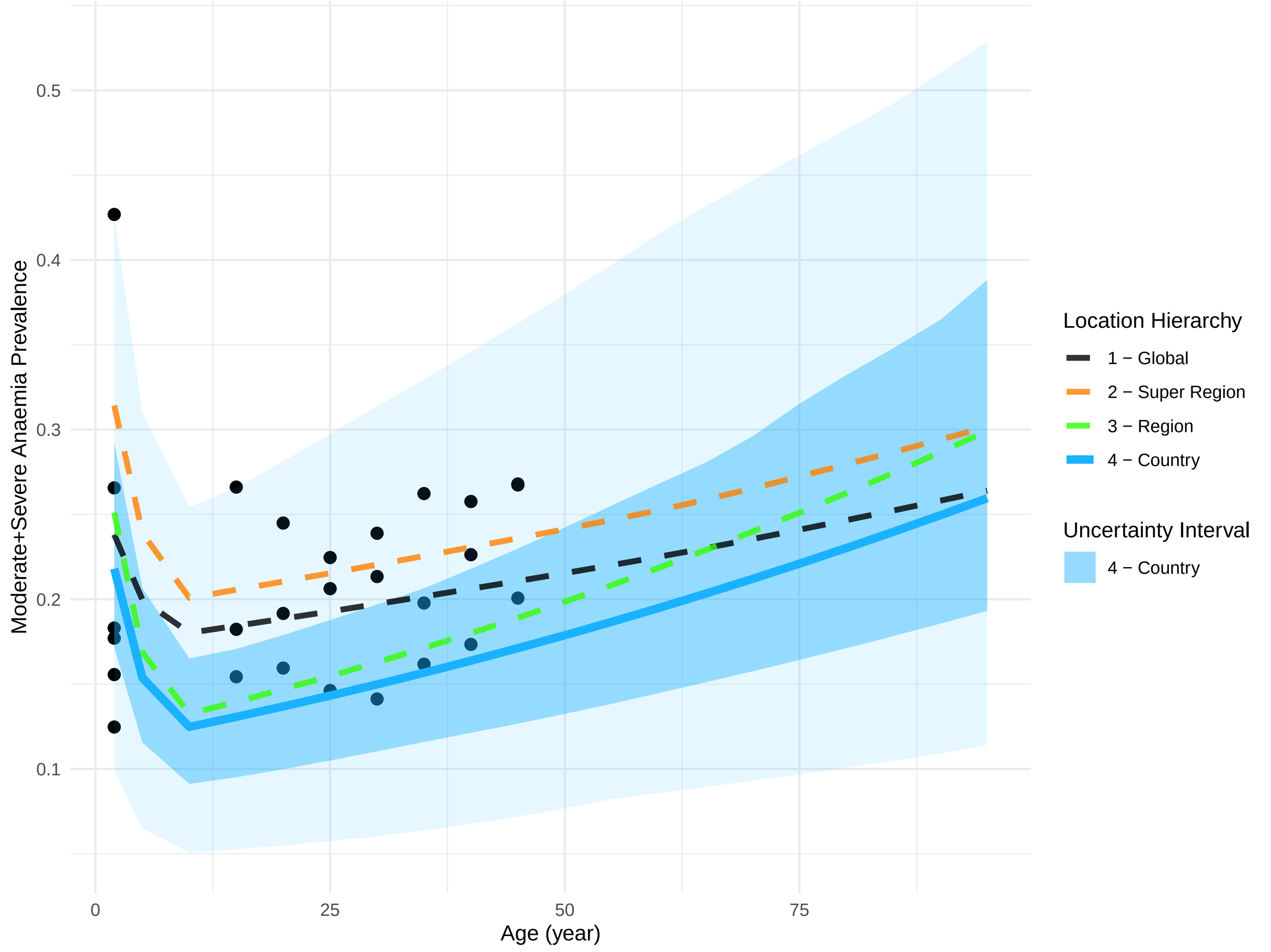

MR−BRT Age/Sex Fits − Non−pregnant Females 2+ Years Old − Moderate+Severe Anaemia Prevalence − Malawi
WHO−2001 Adjustment Method

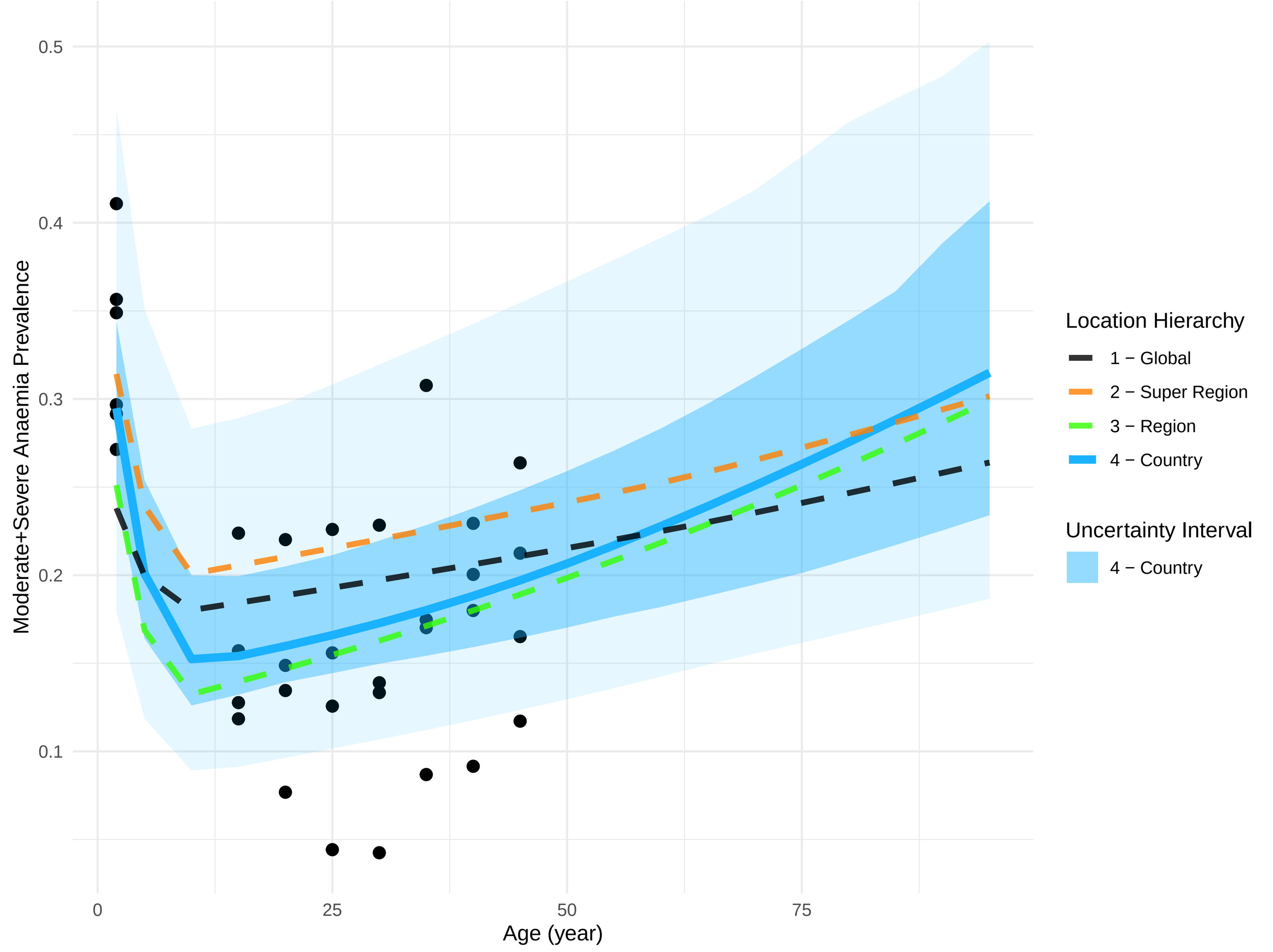

MR−BRT Age/Sex Fits − Non−pregnant Females 2+ Years Old − Moderate+Severe Anaemia Prevalence − Mozambique
WHO−2001 Adjustment Method

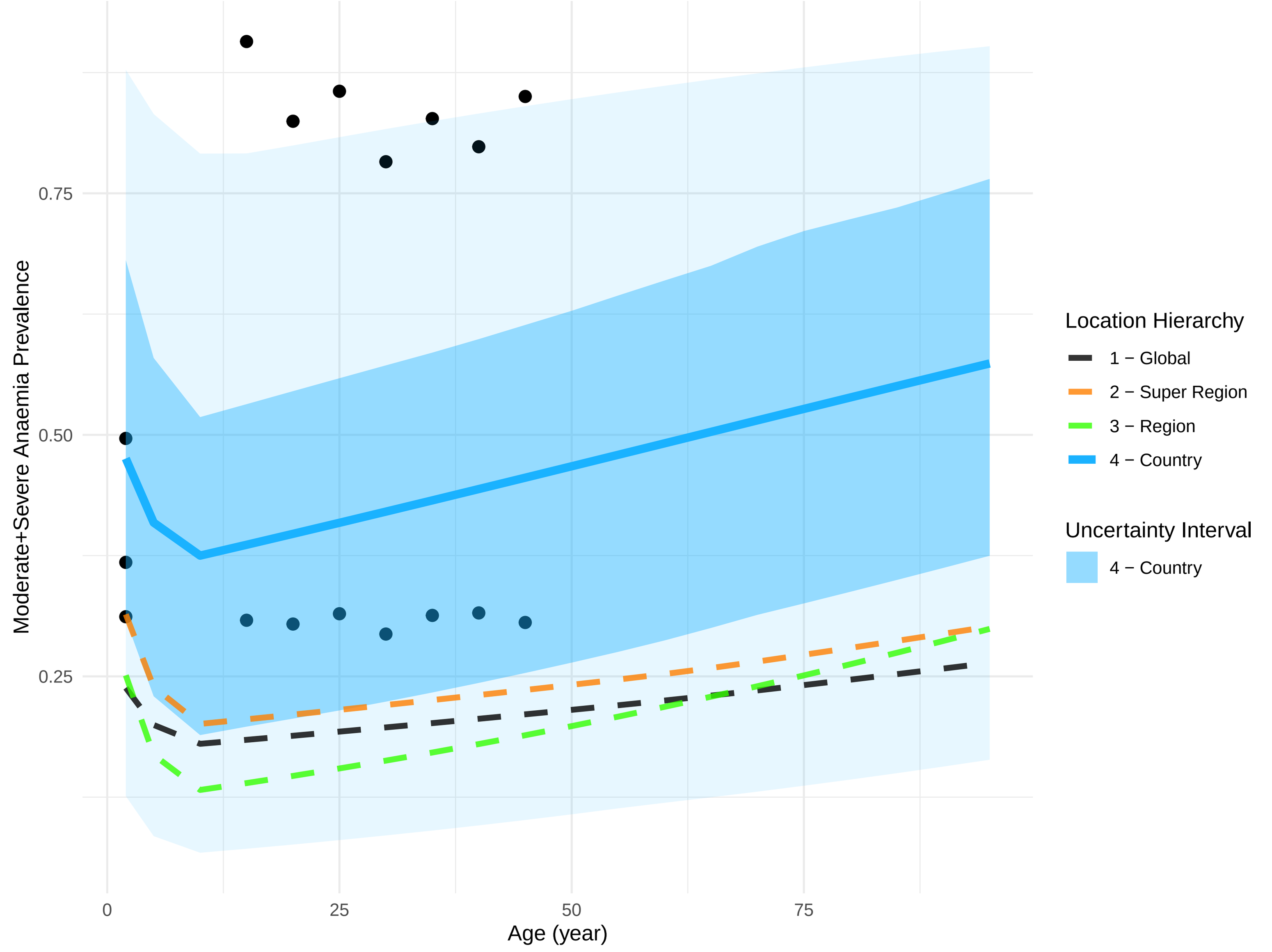

MR−BRT Age/Sex Fits − Non−pregnant Females 2+ Years Old − Moderate+Severe Anaemia Prevalence − Rwanda
WHO−2001 Adjustment Method

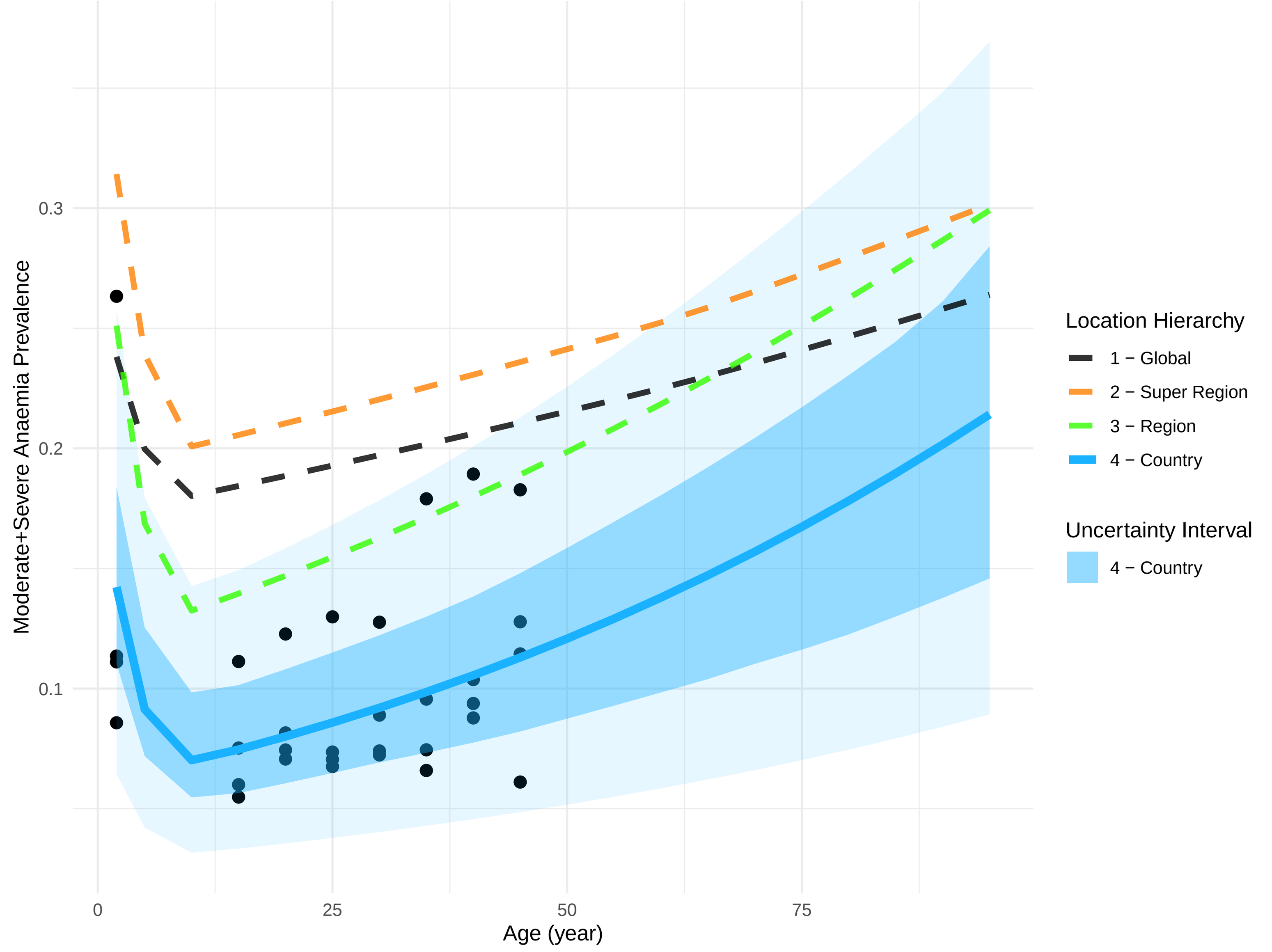

MR−BRT Age/Sex Fits − Non−pregnant Females 2+ Years Old − Moderate+Severe Anaemia Prevalence − Uganda
WHO−2001 Adjustment Method

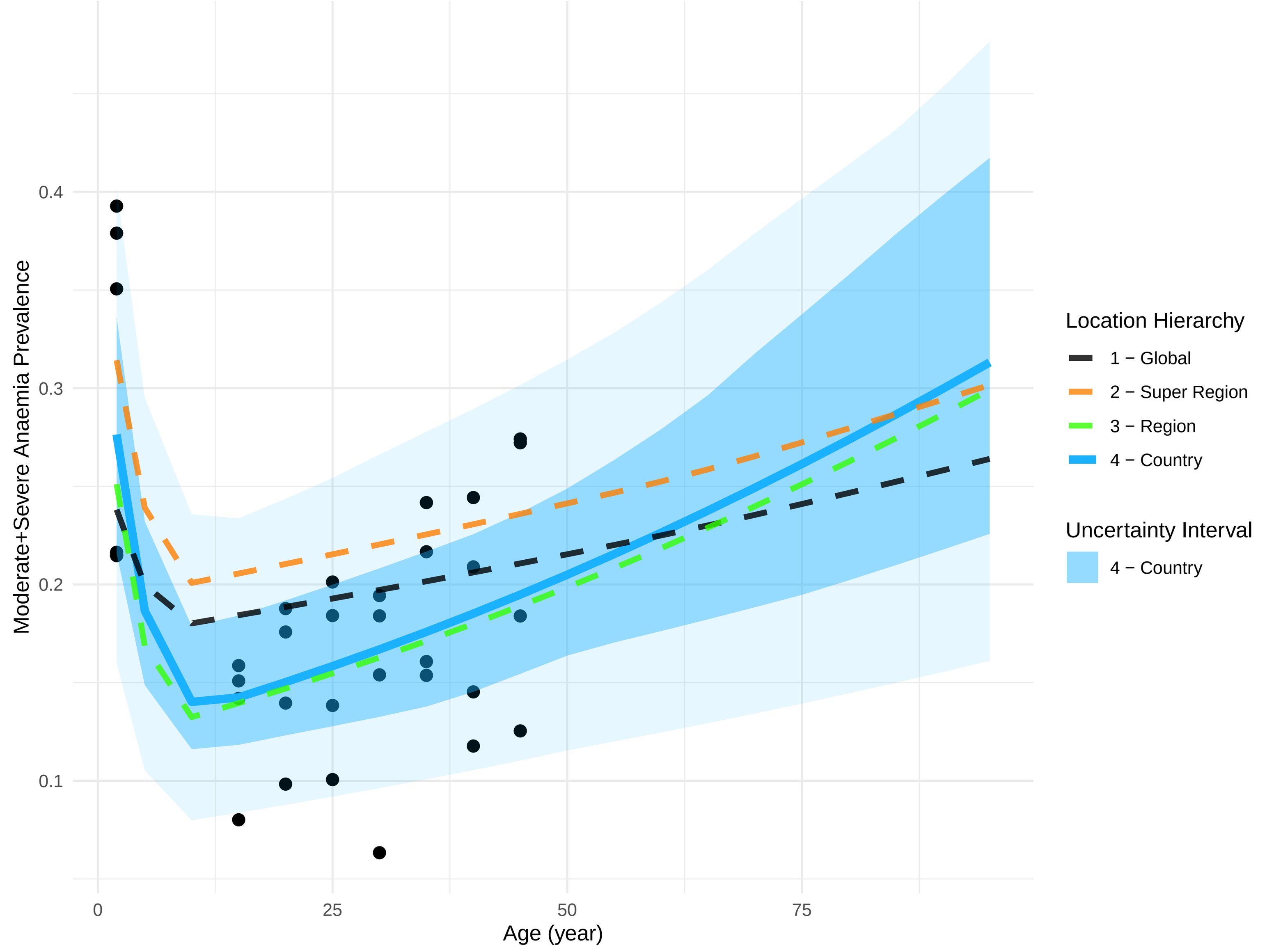

MR−BRT Age/Sex Fits − Non−pregnant Females 2+ Years Old − Moderate+Severe Anaemia Prevalence − United Republic of Tanzania
WHO−2001 Adjustment Method

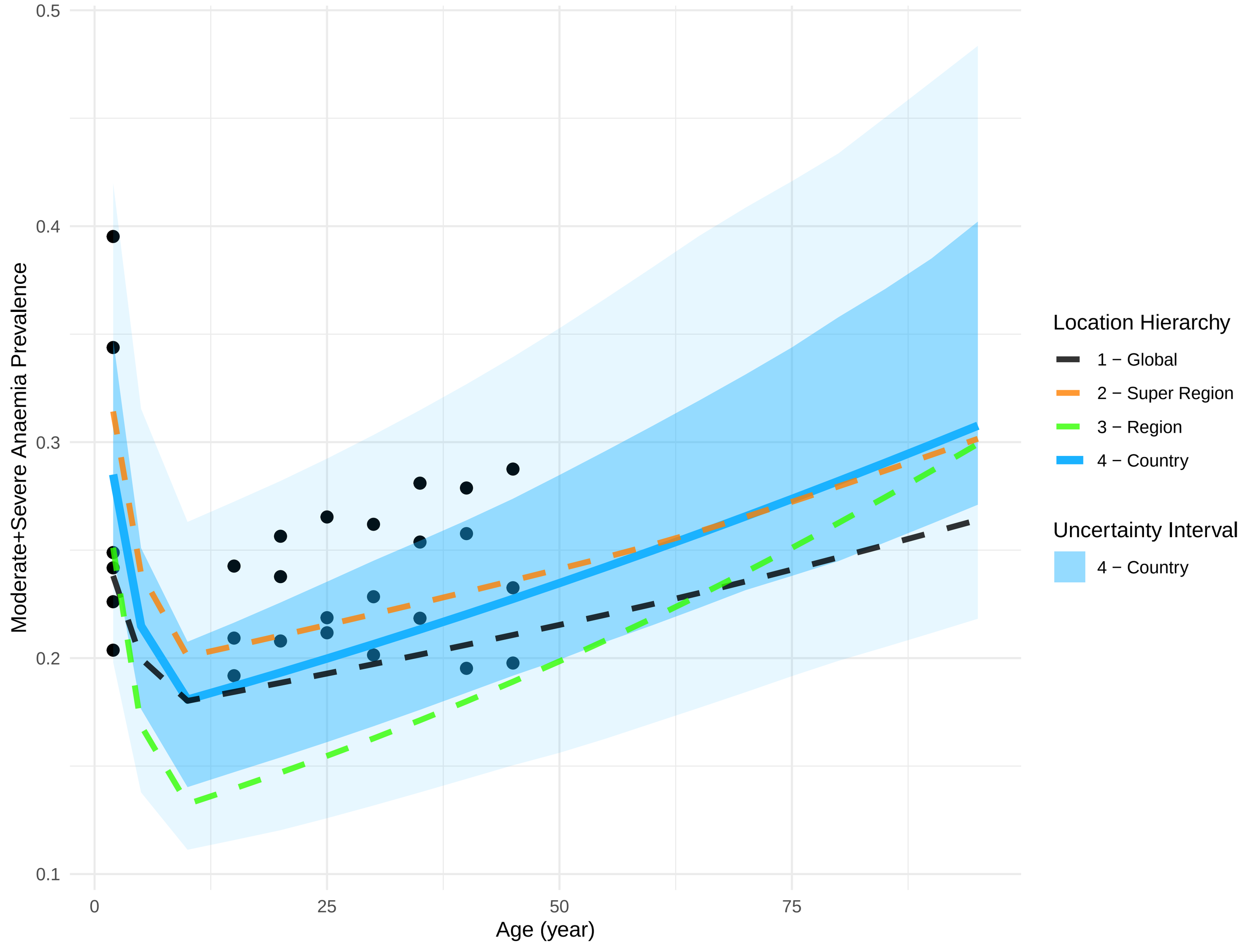

MR−BRT Age/Sex Fits − Non−pregnant Females 2+ Years Old − Moderate+Severe Anaemia Prevalence − Zambia
WHO−2001 Adjustment Method

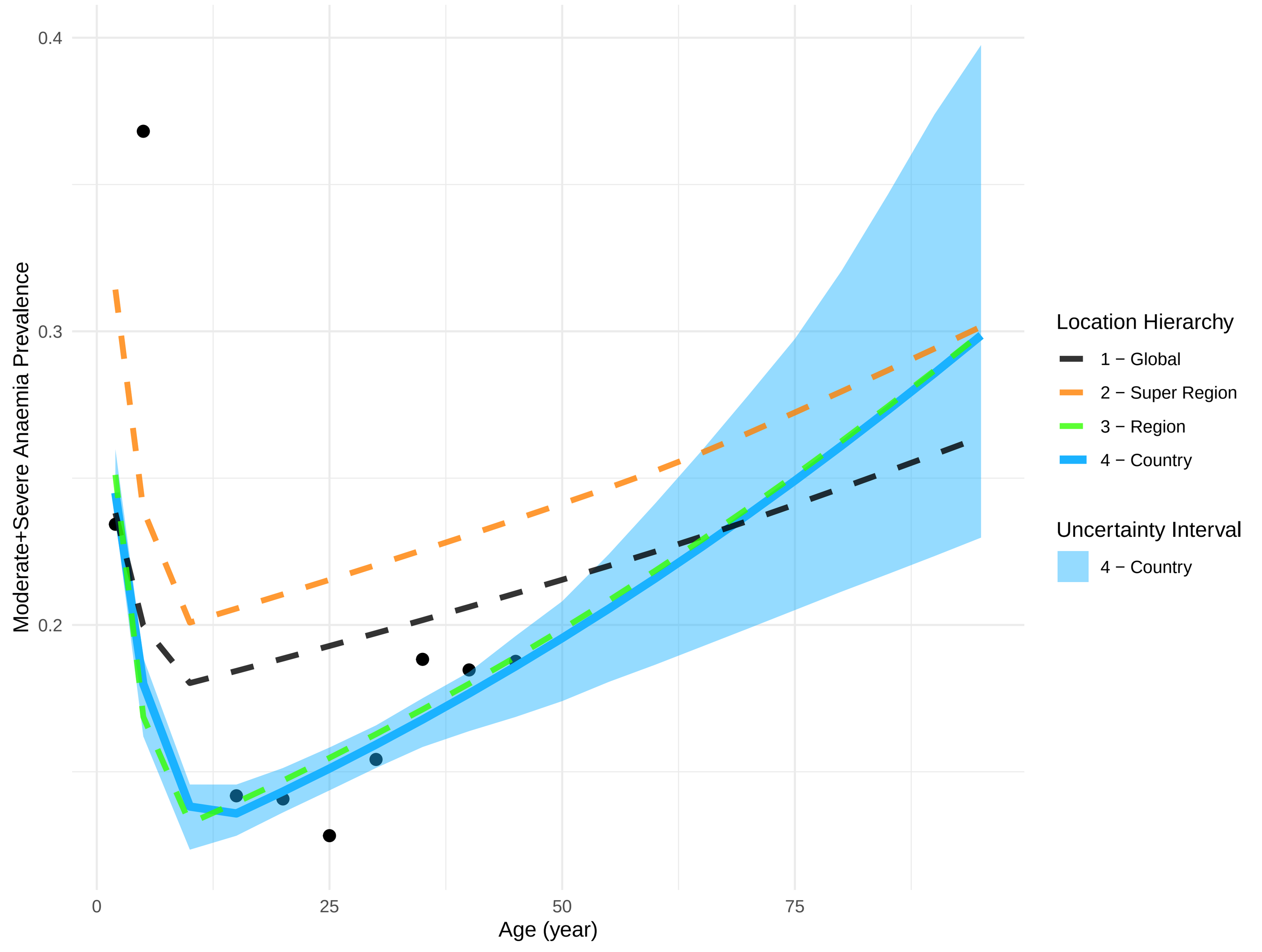

MR−BRT Age/Sex Fits − Non−pregnant Females 2+ Years Old − Moderate+Severe Anaemia Prevalence − Southern Sub−Saharan Africa
WHO−2001 Adjustment Method

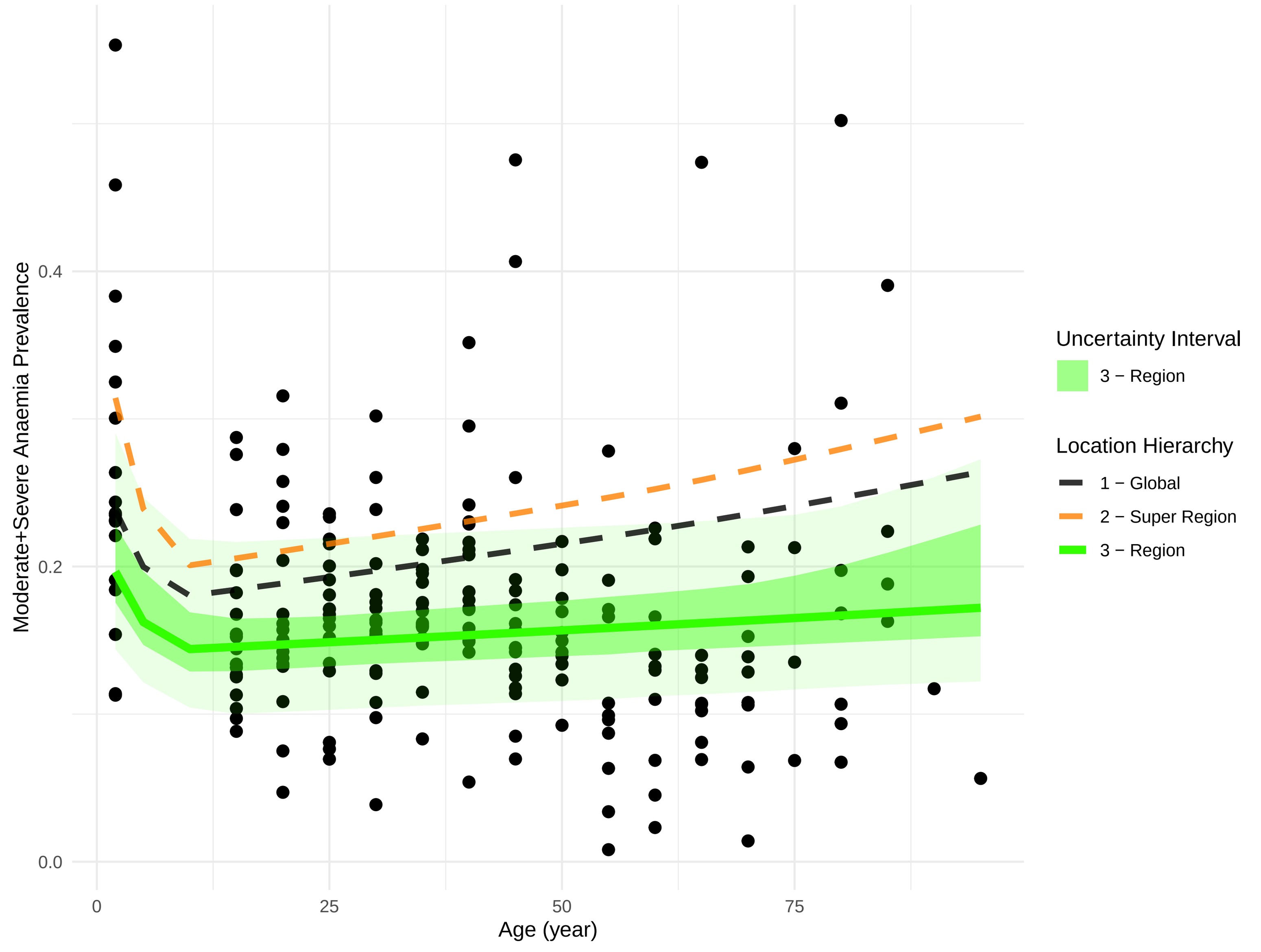

MR−BRT Age/Sex Fits − Non−pregnant Females 2+ Years Old − Moderate+Severe Anaemia Prevalence − Eswatini
WHO−2001 Adjustment Method

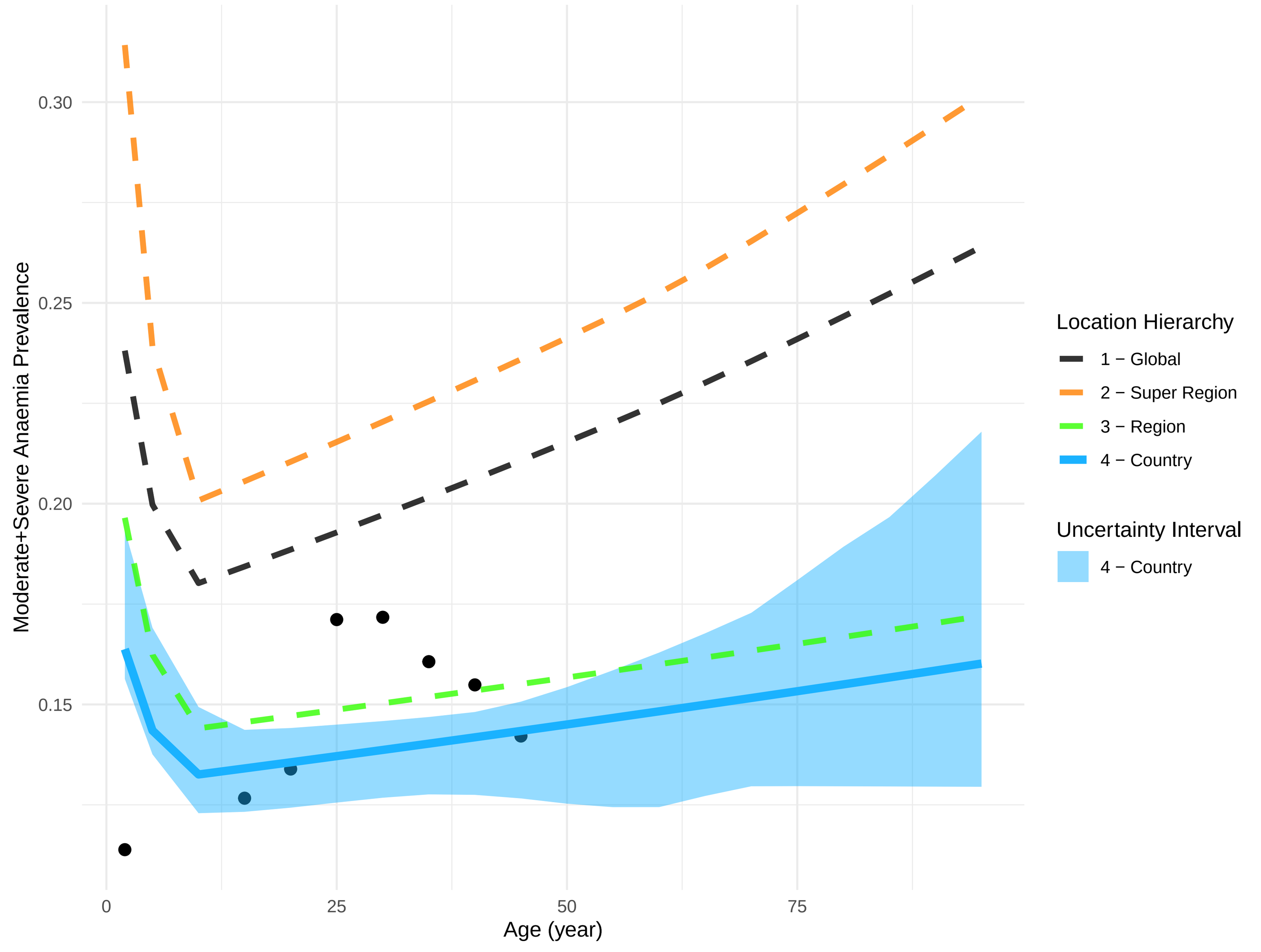

MR−BRT Age/Sex Fits − Non−pregnant Females 2+ Years Old − Moderate+Severe Anaemia Prevalence − Lesotho
WHO−2001 Adjustment Method

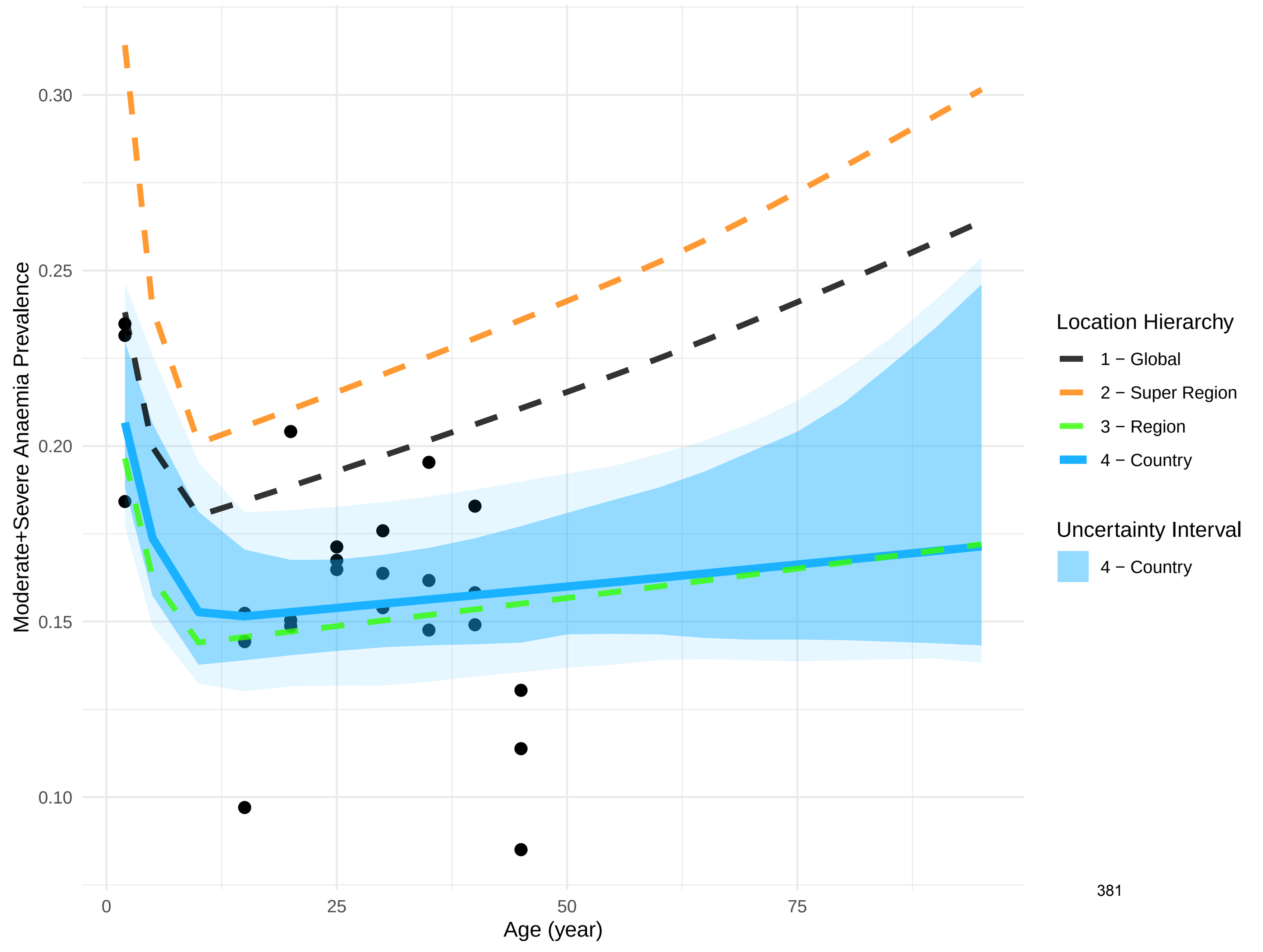

MR−BRT Age/Sex Fits − Non−pregnant Females 2+ Years Old − Moderate+Severe Anaemia Prevalence − Namibia
WHO−2001 Adjustment Method

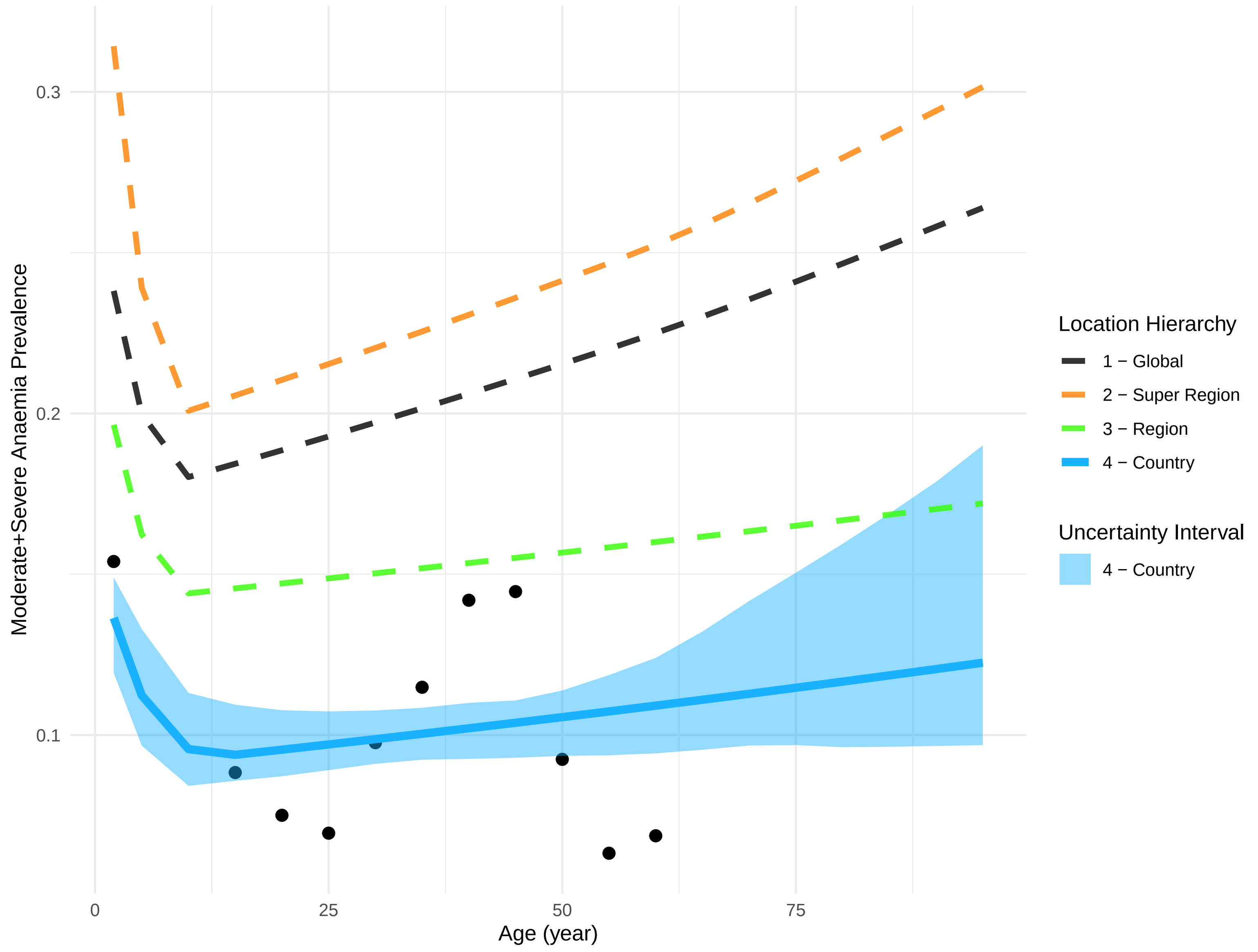

MR−BRT Age/Sex Fits − Non−pregnant Females 2+ Years Old − Moderate+Severe Anaemia Prevalence − South Africa
WHO−2001 Adjustment Method

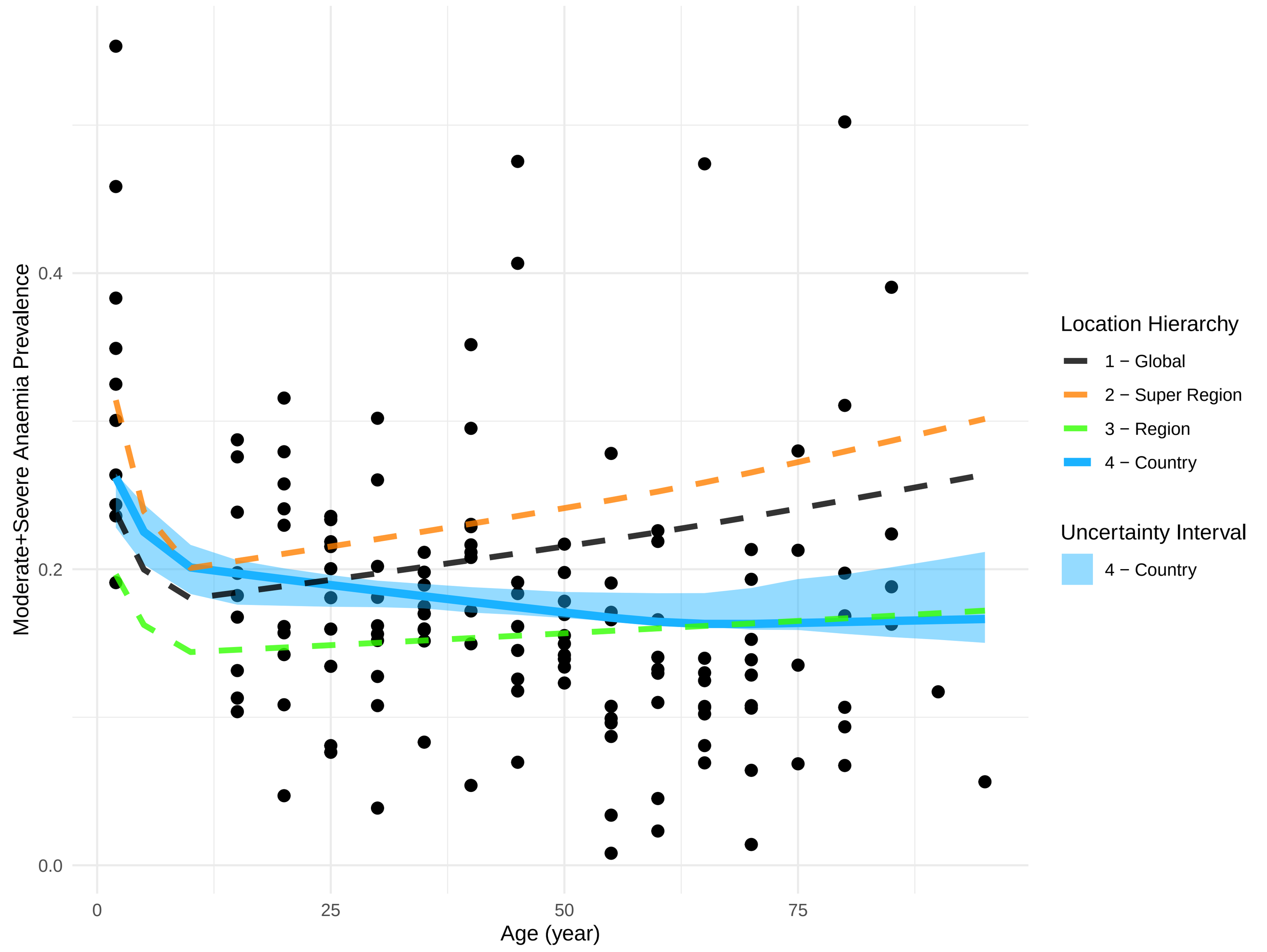

MR−BRT Age/Sex Fits − Non−pregnant Females 2+ Years Old − Moderate+Severe Anaemia Prevalence − Eastern Cape
WHO−2001 Adjustment Method

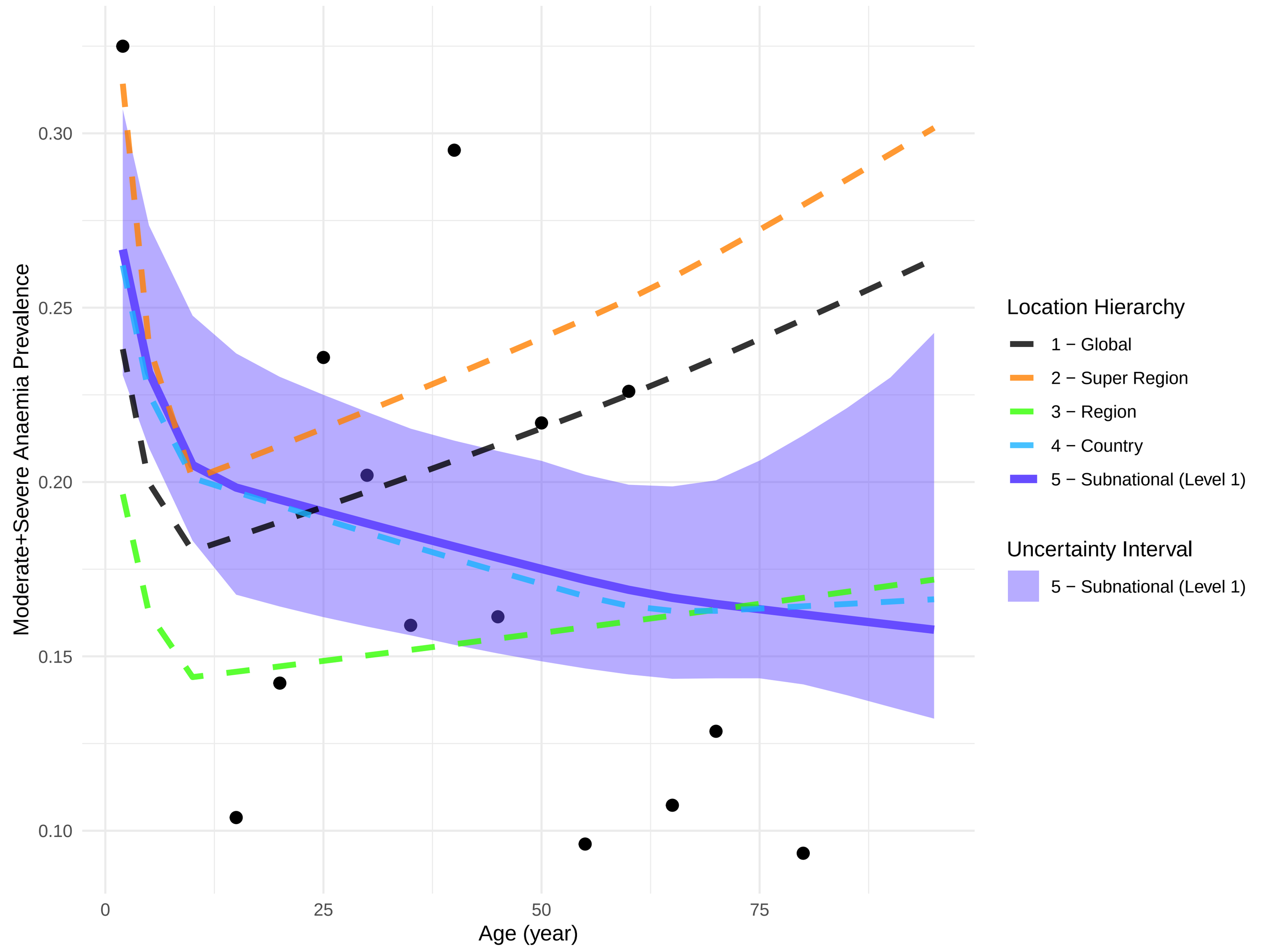

MR−BRT Age/Sex Fits − Non−pregnant Females 2+ Years Old − Moderate+Severe Anaemia Prevalence − Free State
WHO−2001 Adjustment Method

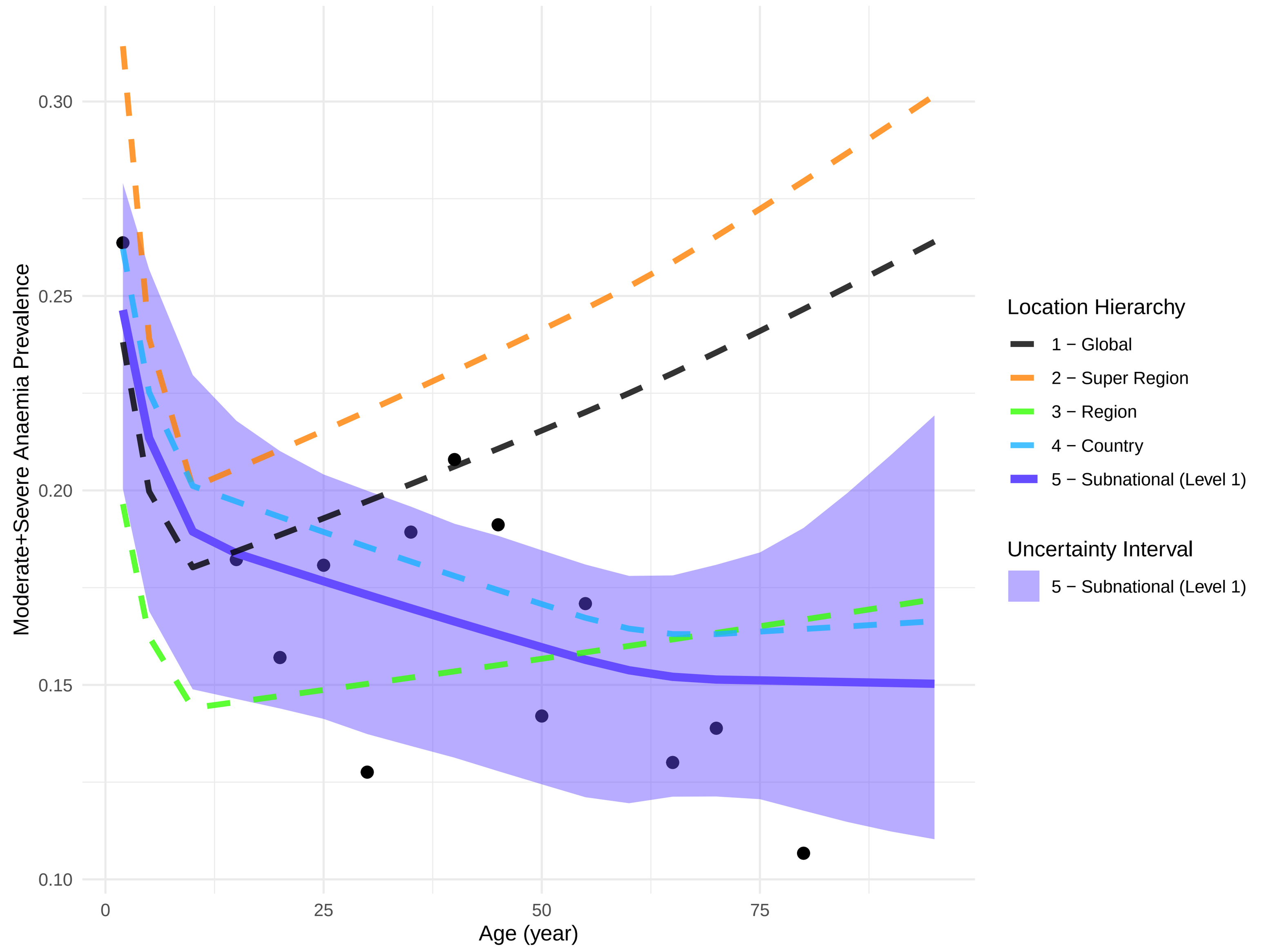

MR−BRT Age/Sex Fits − Non−pregnant Females 2+ Years Old − Moderate+Severe Anaemia Prevalence − Gauteng
WHO−2001 Adjustment Method

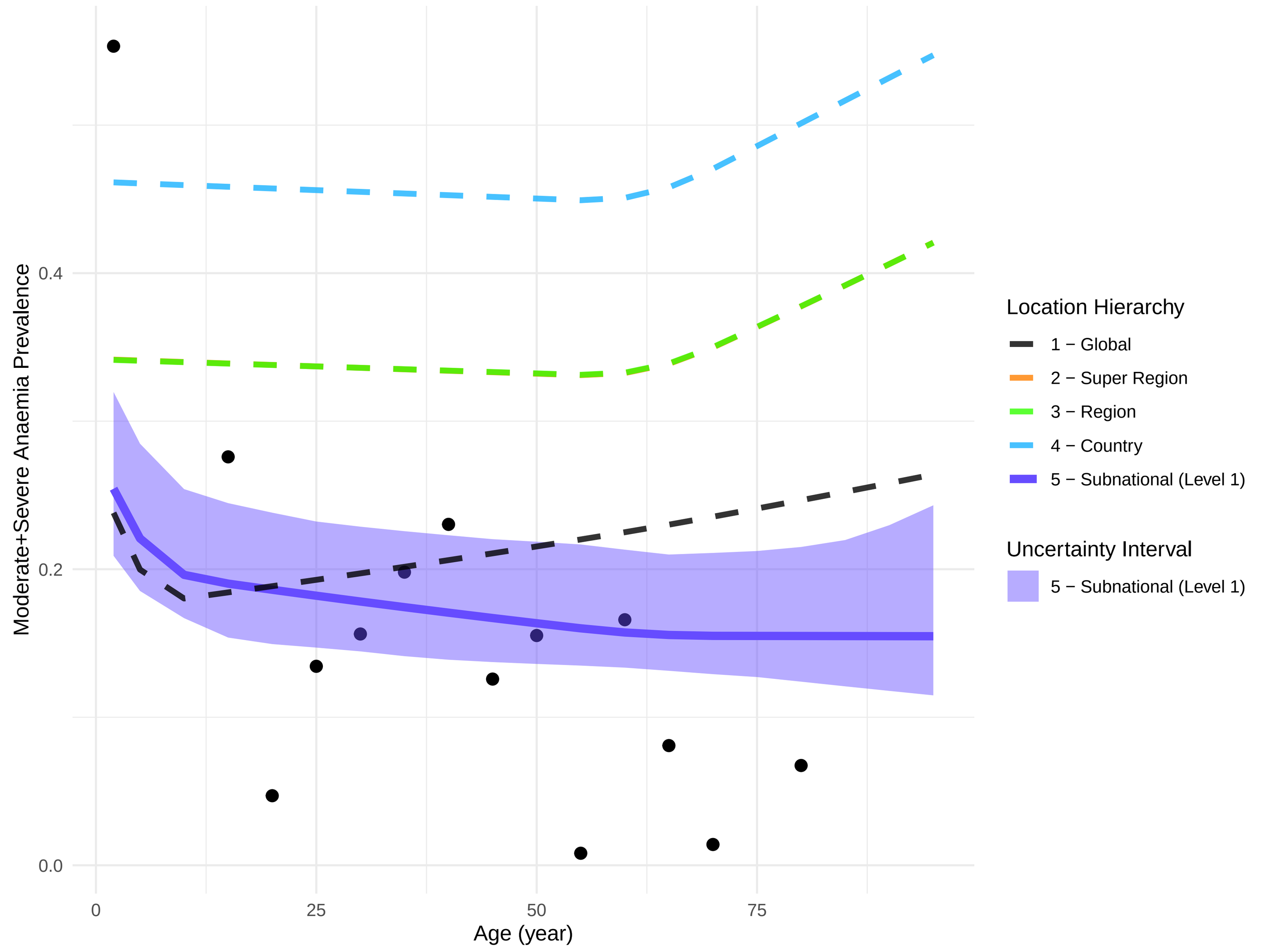

MR−BRT Age/Sex Fits − Non−pregnant Females 2+ Years Old − Moderate+Severe Anaemia Prevalence − KwaZulu−Natal
WHO−2001 Adjustment Method

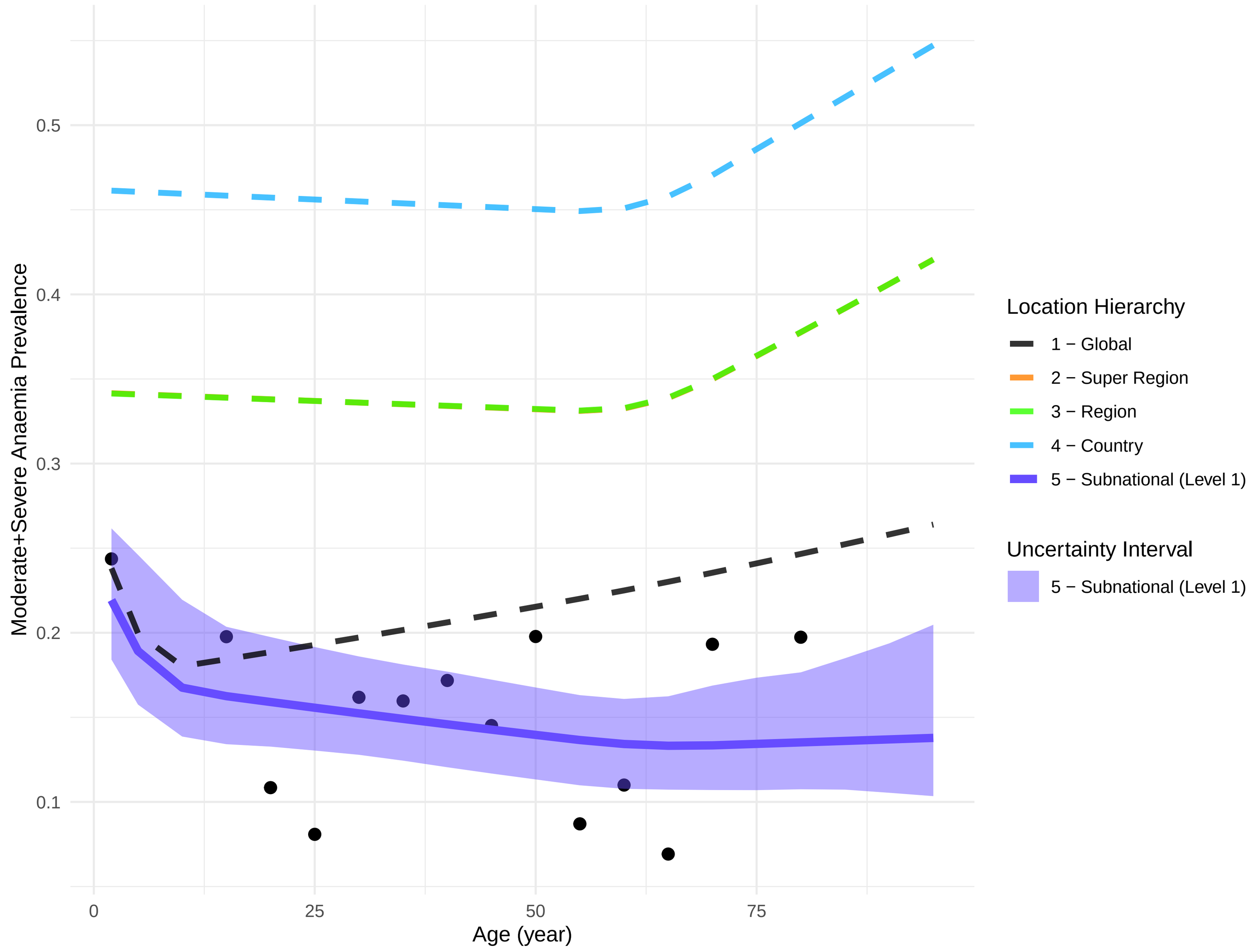

MR−BRT Age/Sex Fits − Non−pregnant Females 2+ Years Old − Moderate+Severe Anaemia Prevalence − Limpopo
WHO−2001 Adjustment Method

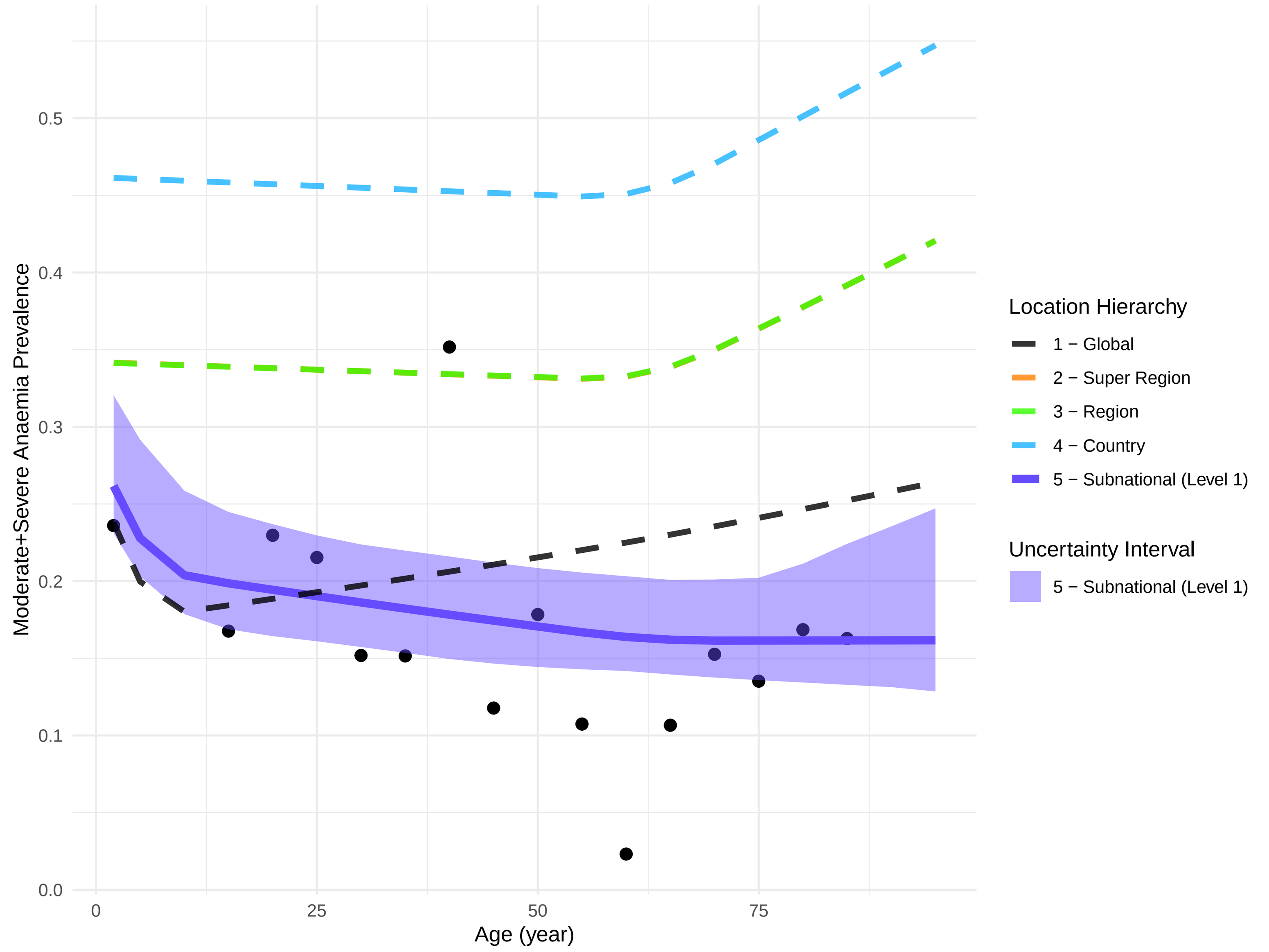

MR−BRT Age/Sex Fits − Non−pregnant Females 2+ Years Old − Moderate+Severe Anaemia Prevalence − Mpumalanga
WHO−2001 Adjustment Method

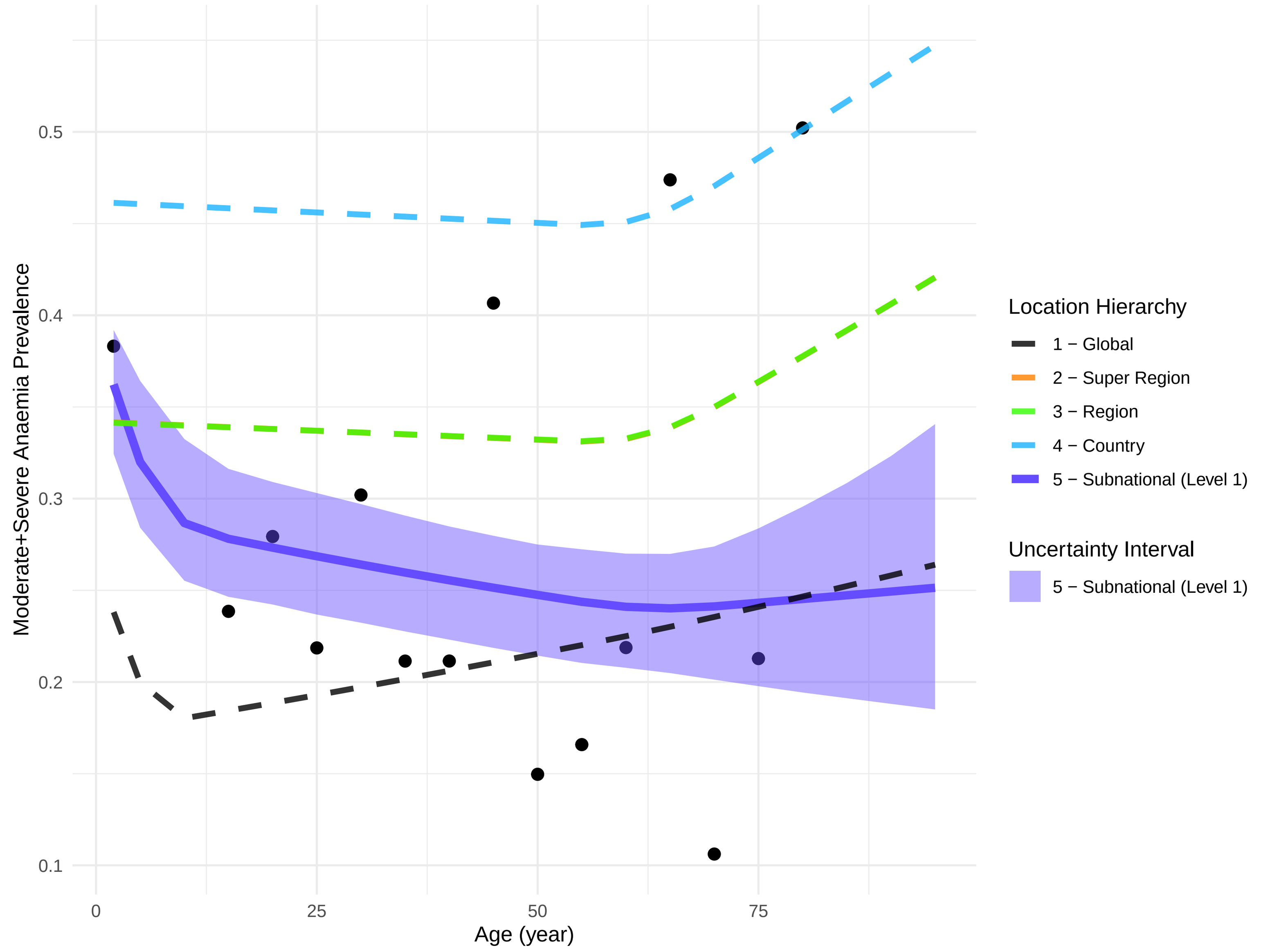

MR−BRT Age/Sex Fits − Non−pregnant Females 2+ Years Old − Moderate+Severe Anaemia Prevalence − North−West
WHO−2001 Adjustment Method

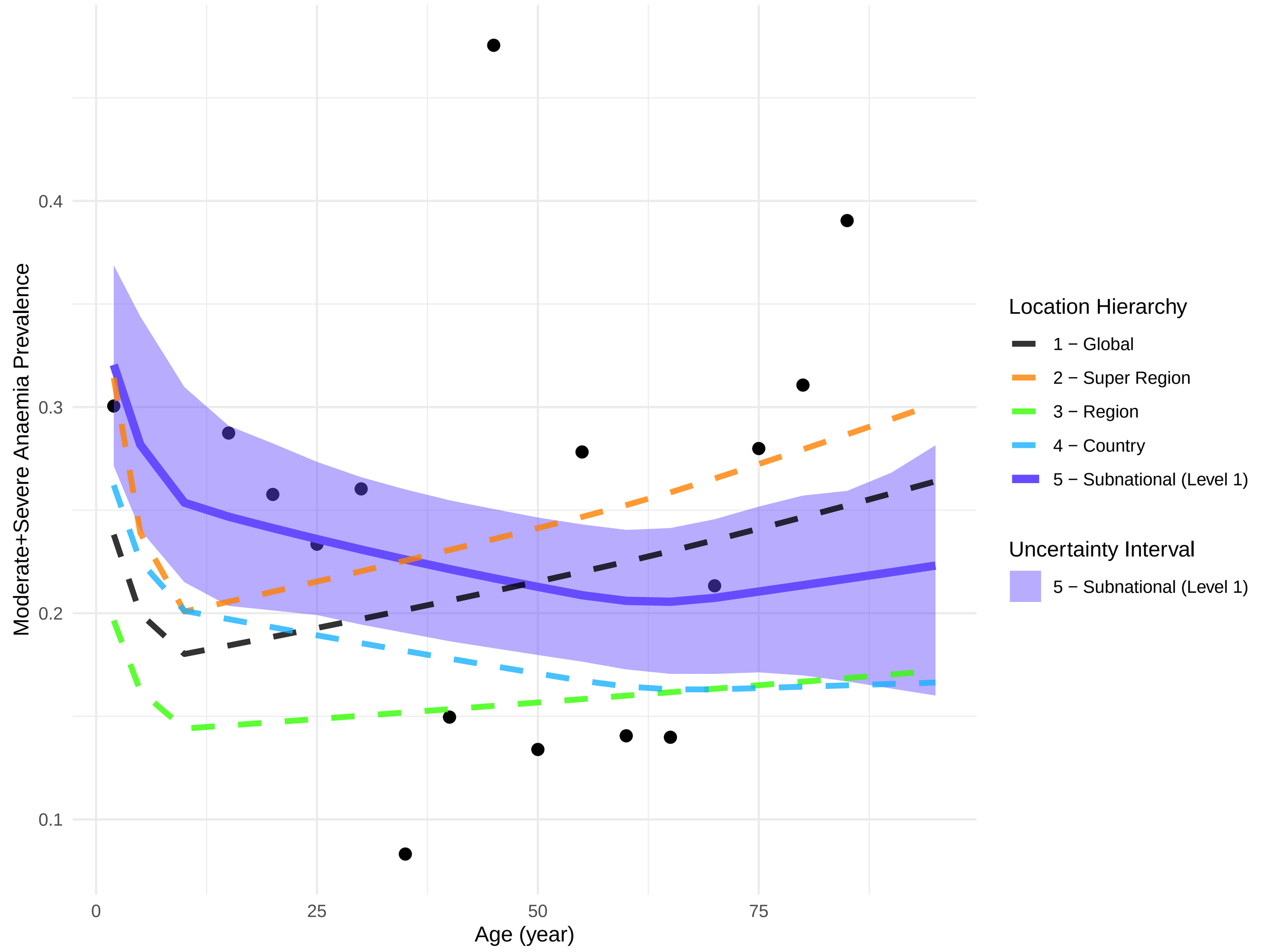

MR−BRT Age/Sex Fits − Non−pregnant Females 2+ Years Old − Moderate+Severe Anaemia Prevalence − Northern Cape
WHO−2001 Adjustment Method

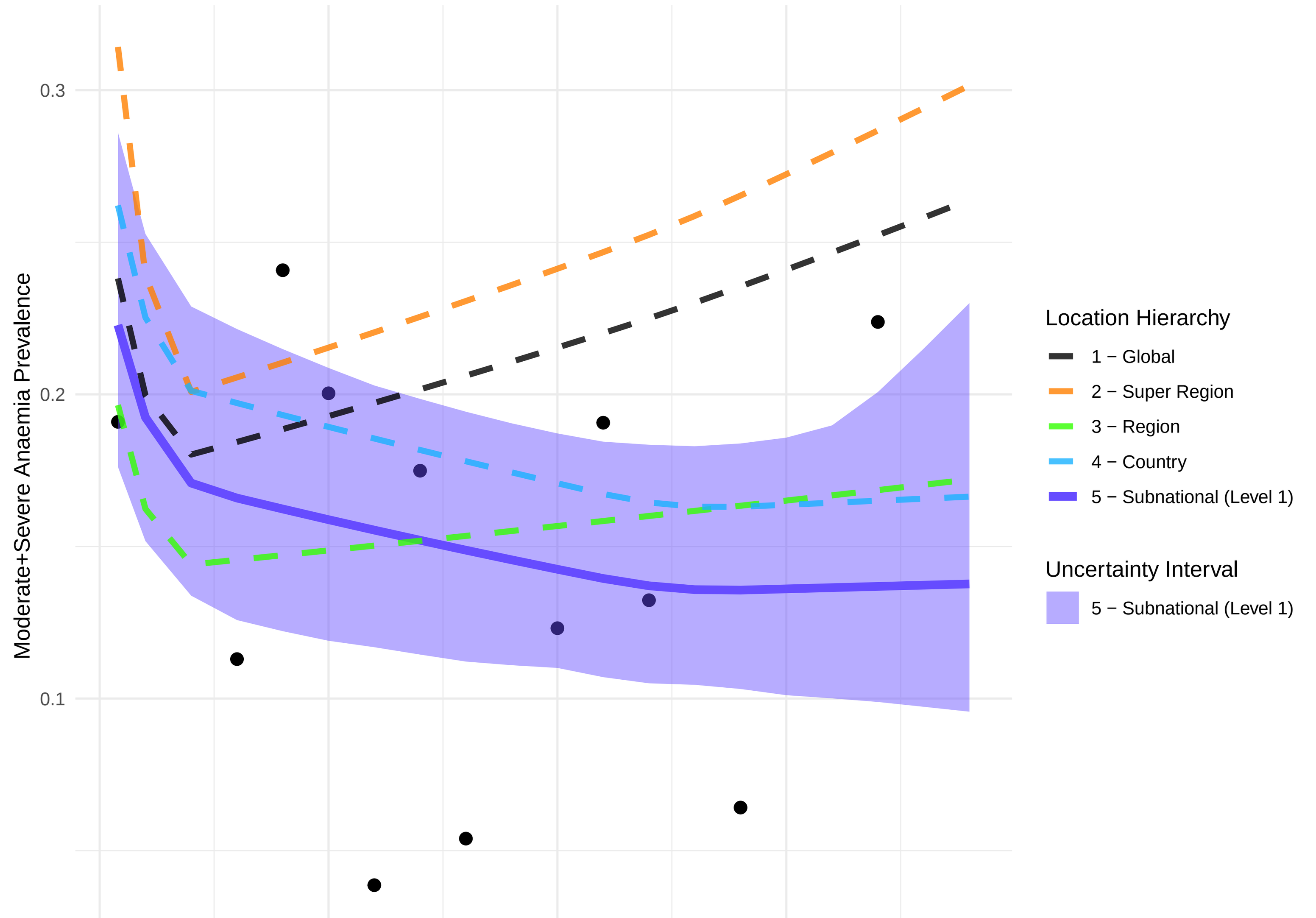

MR−BRT Age/Sex Fits − Non−pregnant Females 2+ Years Old − Moderate+Severe Anaemia Prevalence − Western Cape
WHO−2001 Adjustment Method

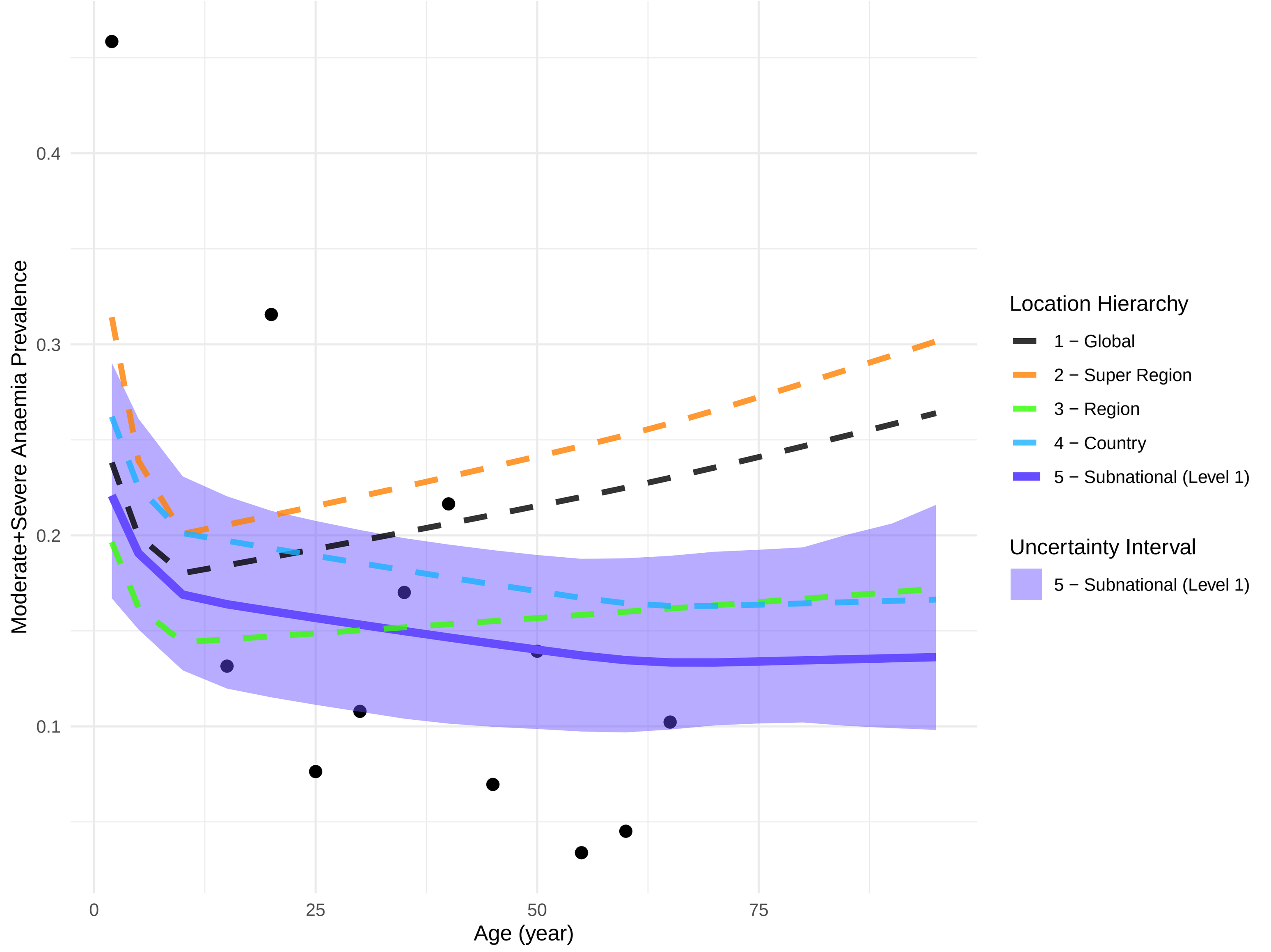

MR−BRT Age/Sex Fits − Non−pregnant Females 2+ Years Old − Moderate+Severe Anaemia Prevalence − Zimbabwe
WHO−2001 Adjustment Method

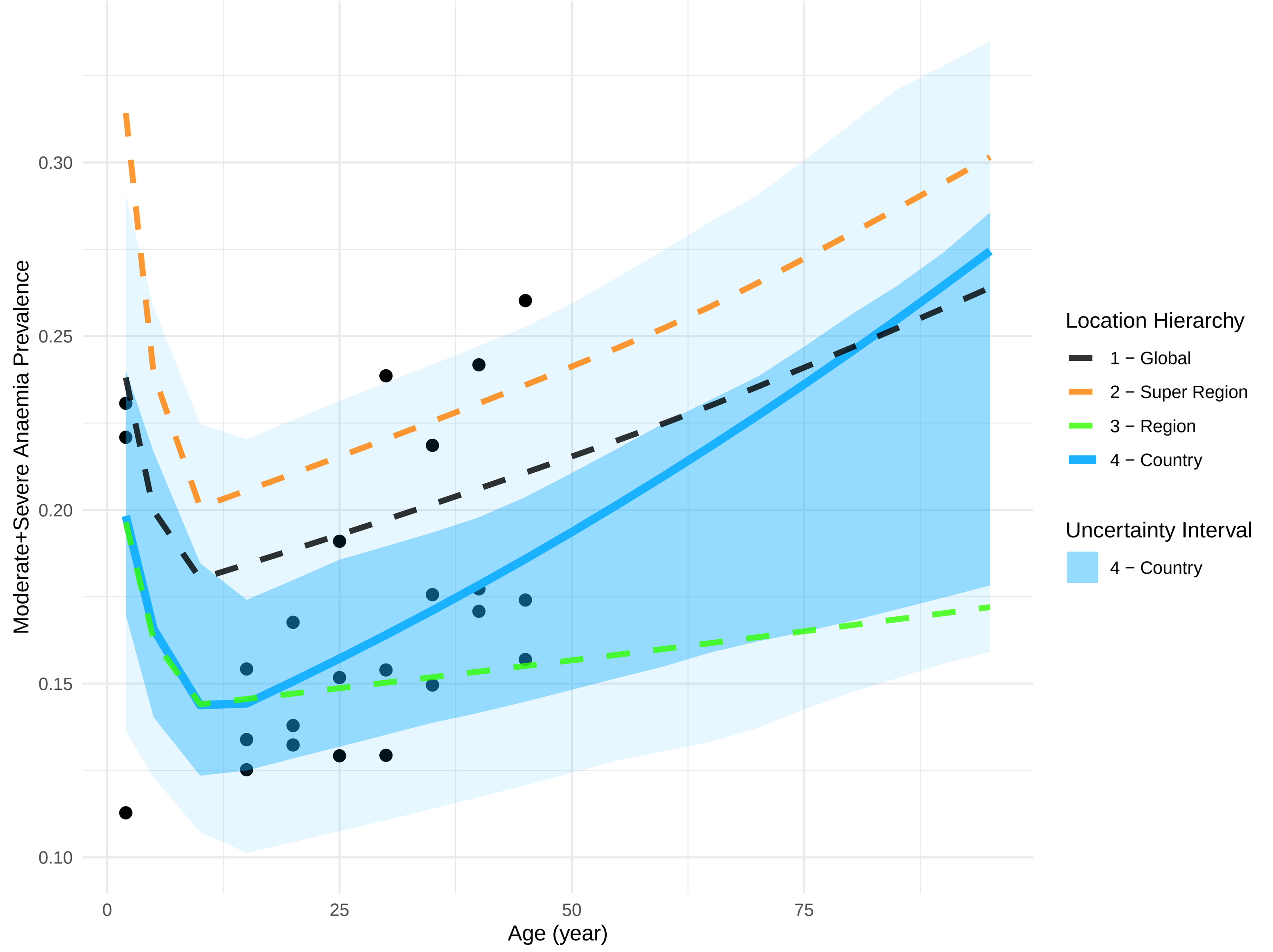

MR−BRT Age/Sex Fits − Non−pregnant Females 2+ Years Old − Moderate+Severe Anaemia Prevalence − Western Sub−Saharan Africa
WHO−2001 Adjustment Method

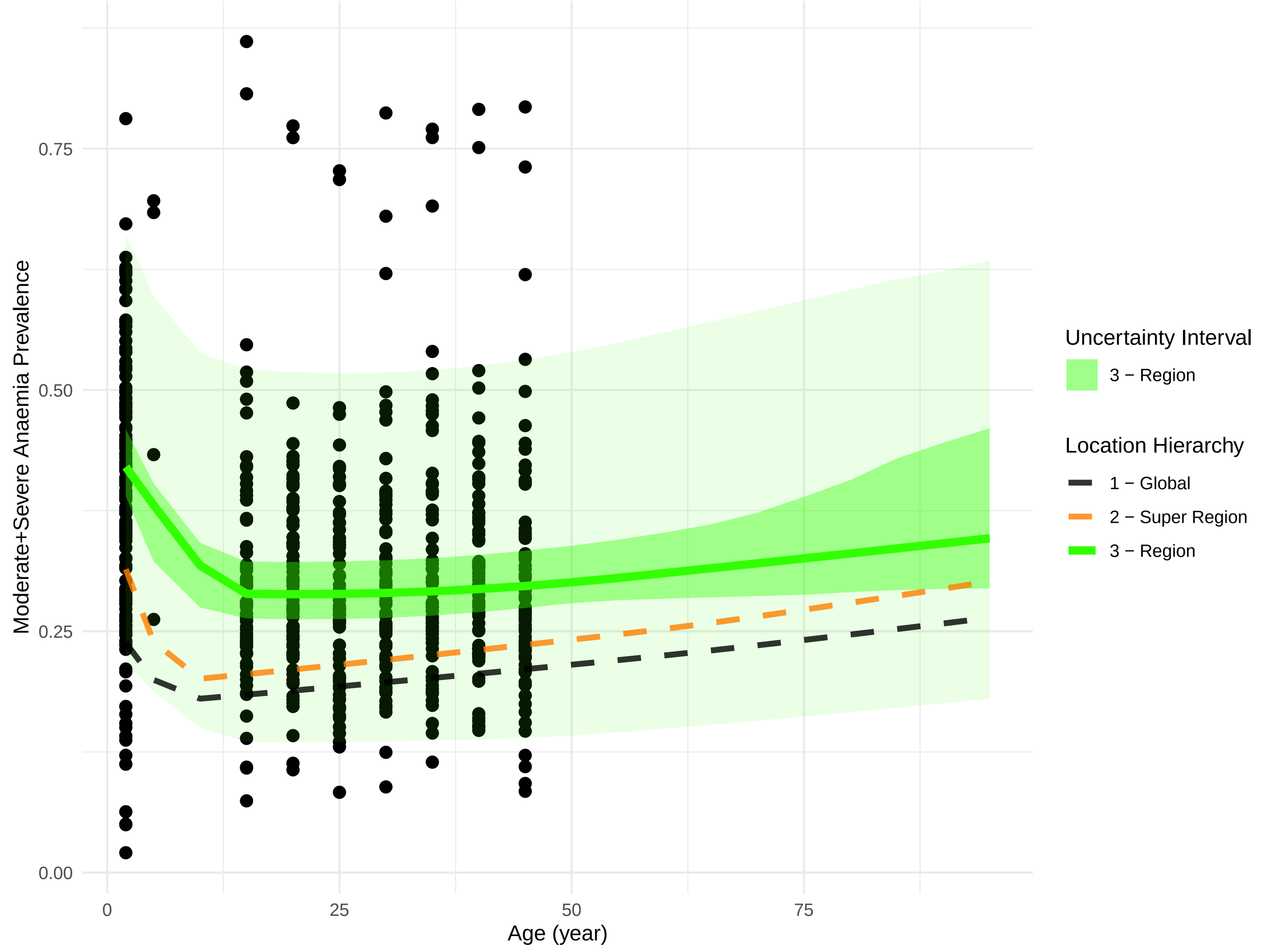

MR−BRT Age/Sex Fits − Non−pregnant Females 2+ Years Old − Moderate+Severe Anaemia Prevalence − Benin
WHO−2001 Adjustment Method

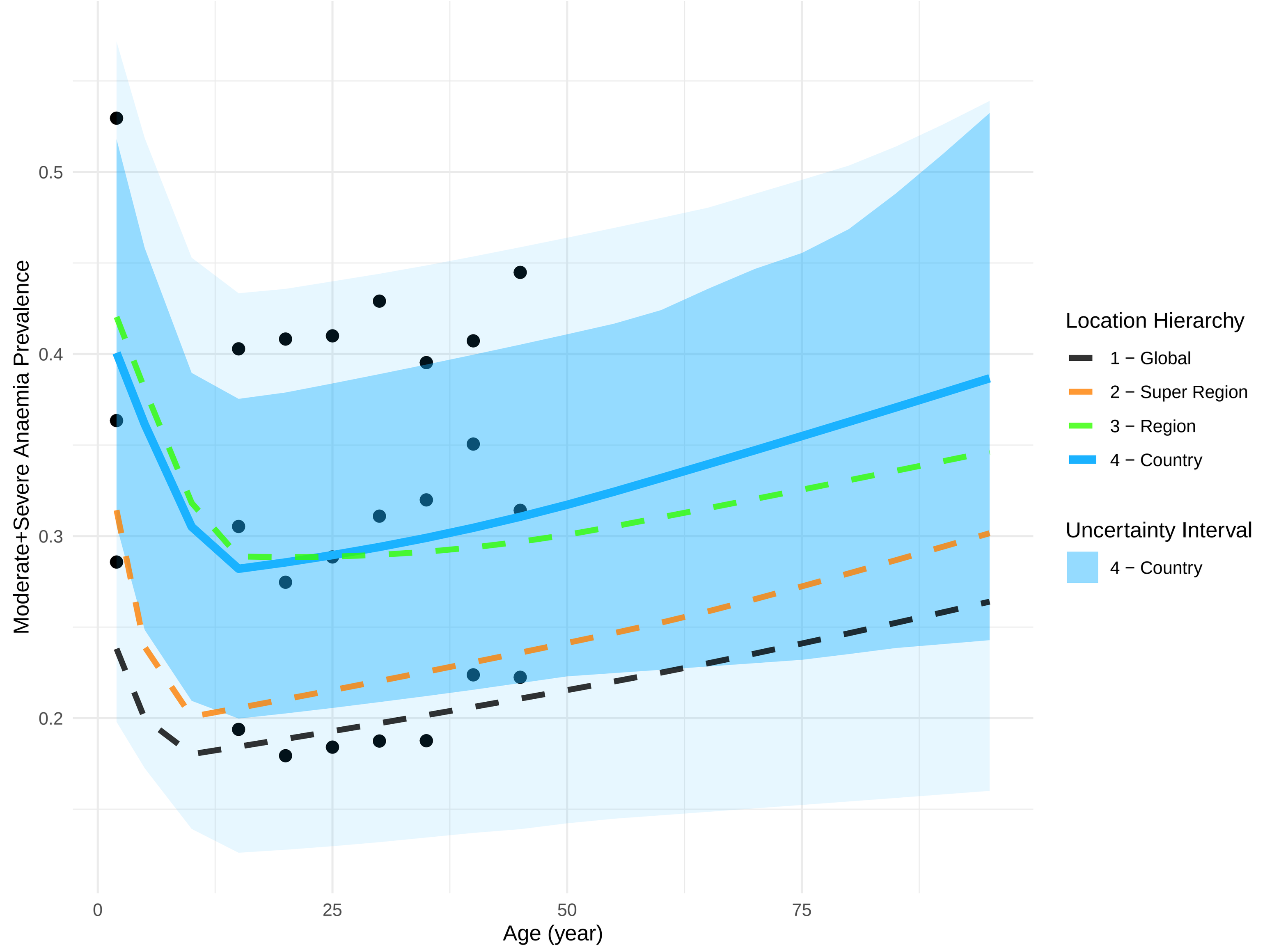

MR−BRT Age/Sex Fits − Non−pregnant Females 2+ Years Old − Moderate+Severe Anaemia Prevalence − Burkina Faso
WHO−2001 Adjustment Method

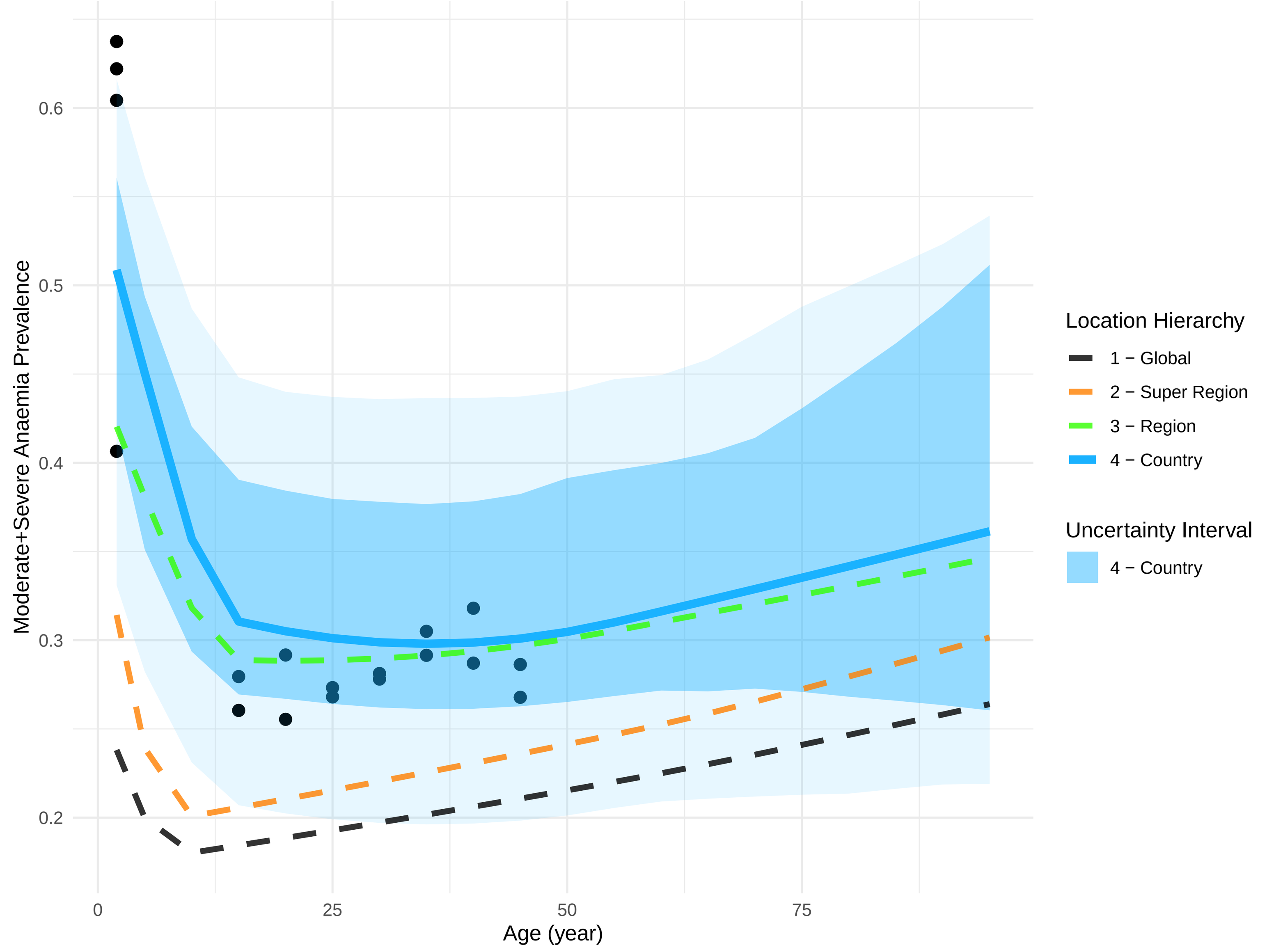

MR−BRT Age/Sex Fits − Non−pregnant Females 2+ Years Old − Moderate+Severe Anaemia Prevalence − Cameroon
WHO−2001 Adjustment Method

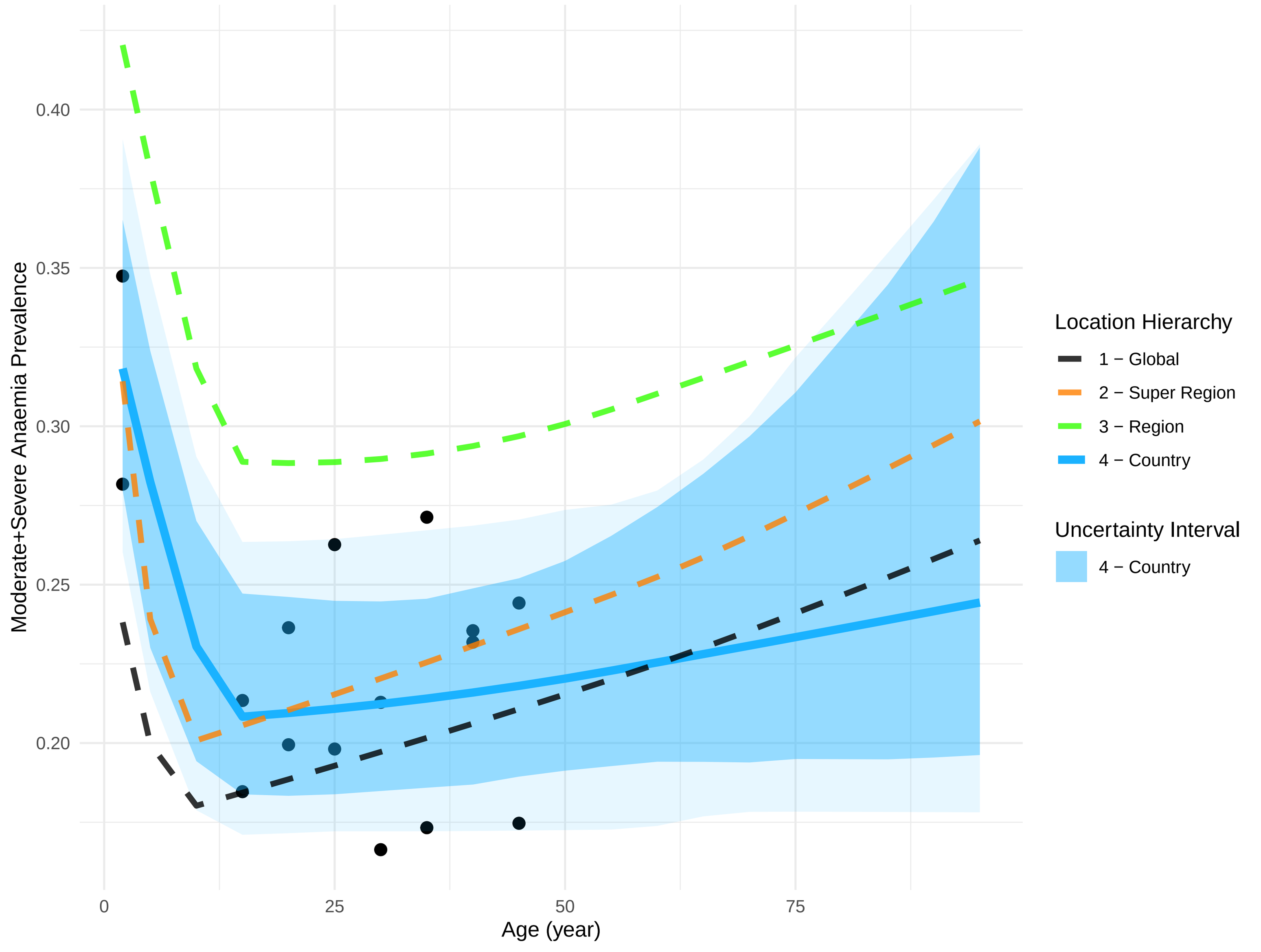

MR−BRT Age/Sex Fits − Non−pregnant Females 2+ Years Old − Moderate+Severe Anaemia Prevalence − Côte d'Ivoire
WHO−2001 Adjustment Method

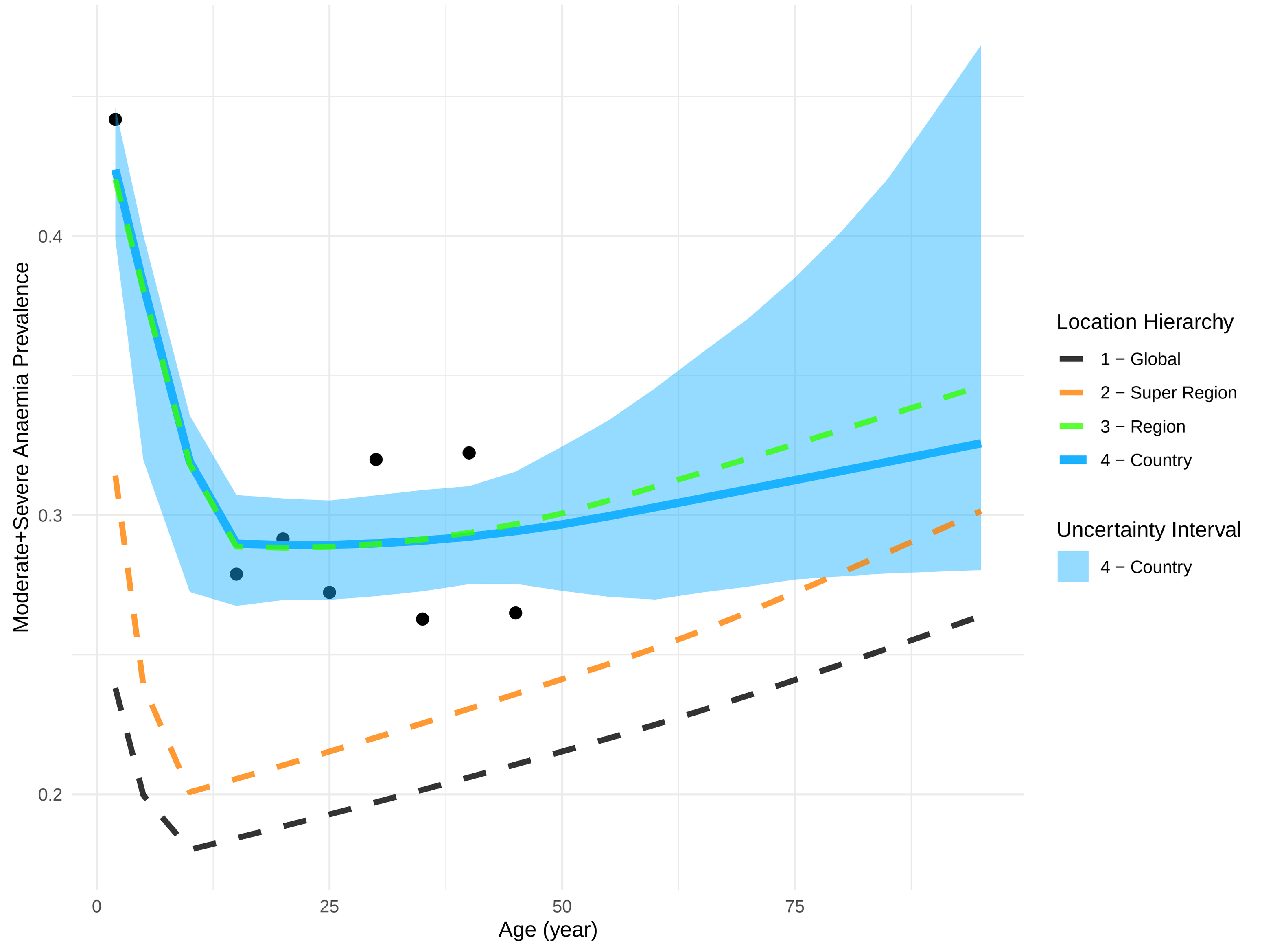

MR−BRT Age/Sex Fits − Non−pregnant Females 2+ Years Old − Moderate+Severe Anaemia Prevalence − Gambia
WHO−2001 Adjustment Method

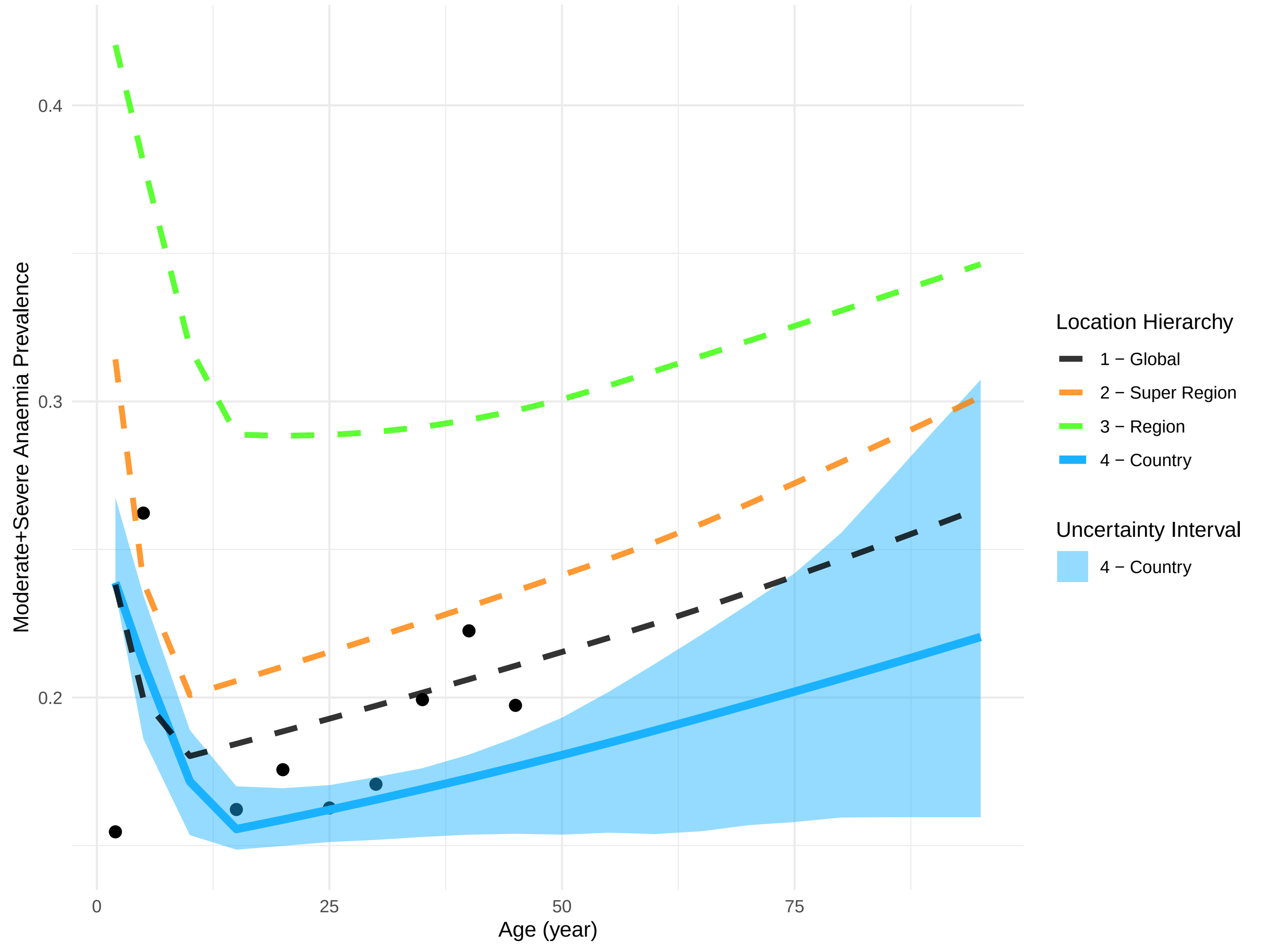

MR−BRT Age/Sex Fits − Non−pregnant Females 2+ Years Old − Moderate+Severe Anaemia Prevalence − Ghana
WHO−2001 Adjustment Method

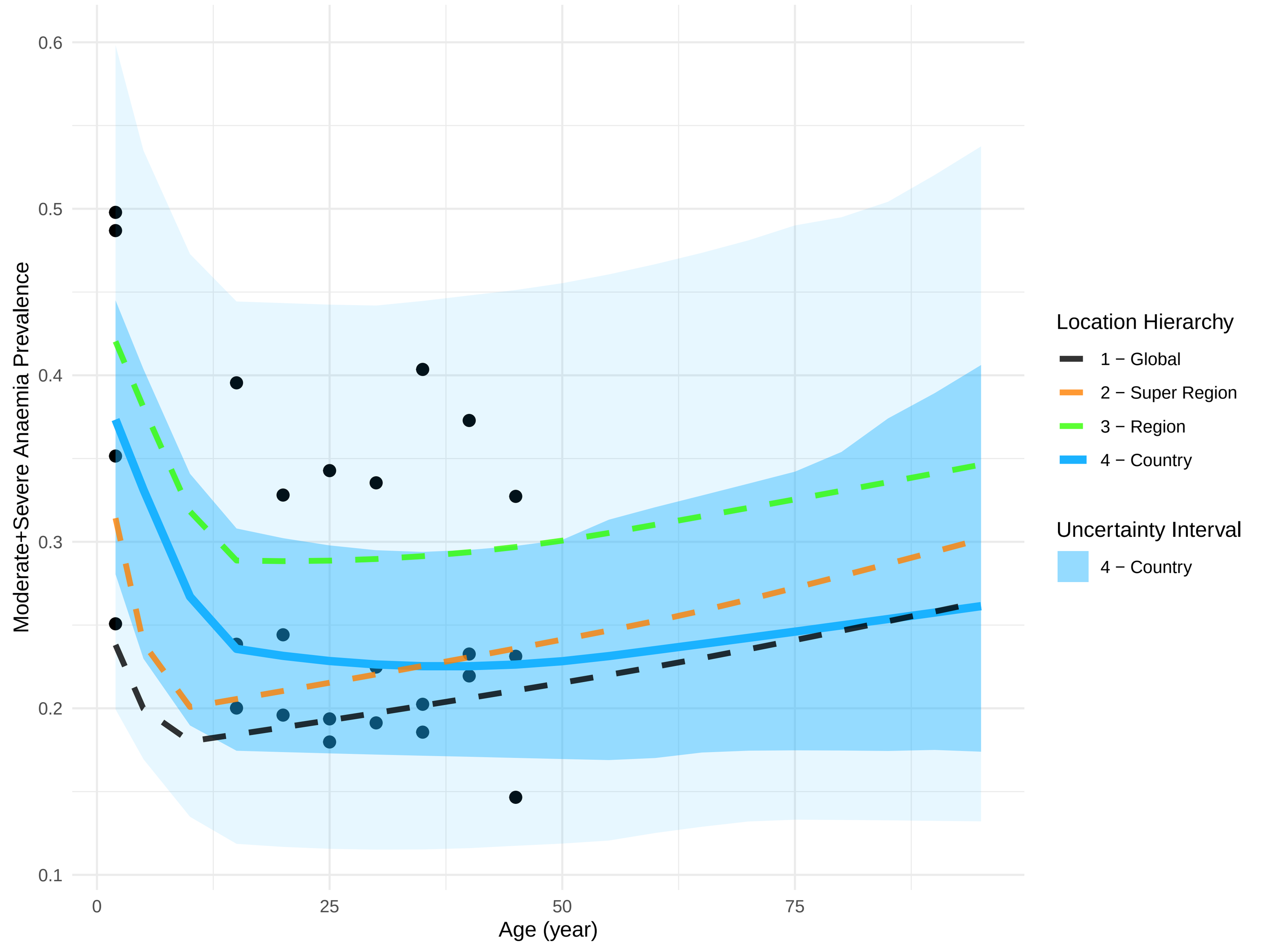

MR−BRT Age/Sex Fits − Non−pregnant Females 2+ Years Old − Moderate+Severe Anaemia Prevalence − Guinea
WHO−2001 Adjustment Method

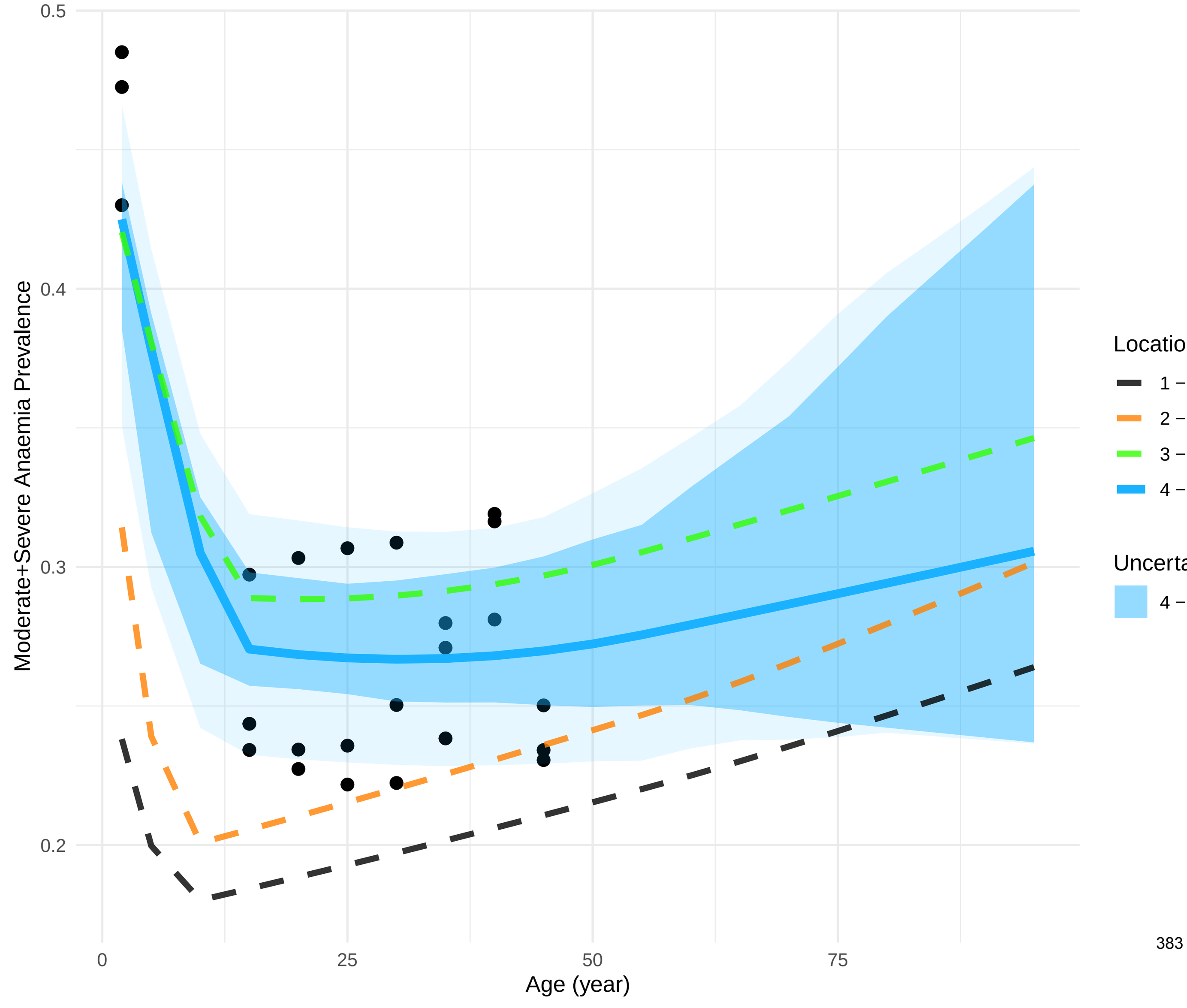

MR−BRT Age/Sex Fits − Non−pregnant Females 2+ Years Old − Moderate+Severe Anaemia Prevalence − Liberia
WHO−2001 Adjustment Method

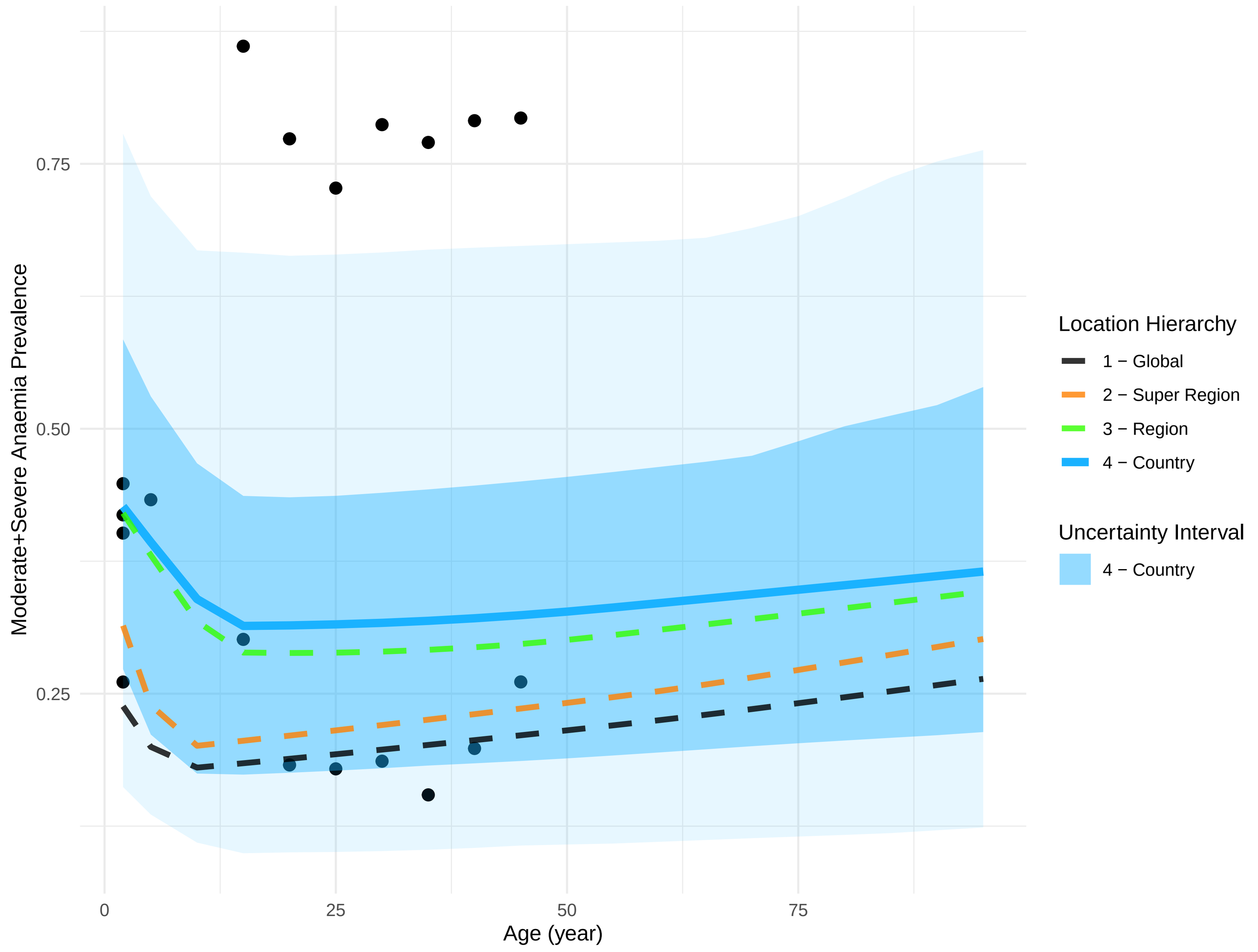

MR−BRT Age/Sex Fits − Non−pregnant Females 2+ Years Old − Moderate+Severe Anaemia Prevalence − Mali
WHO−2001 Adjustment Method

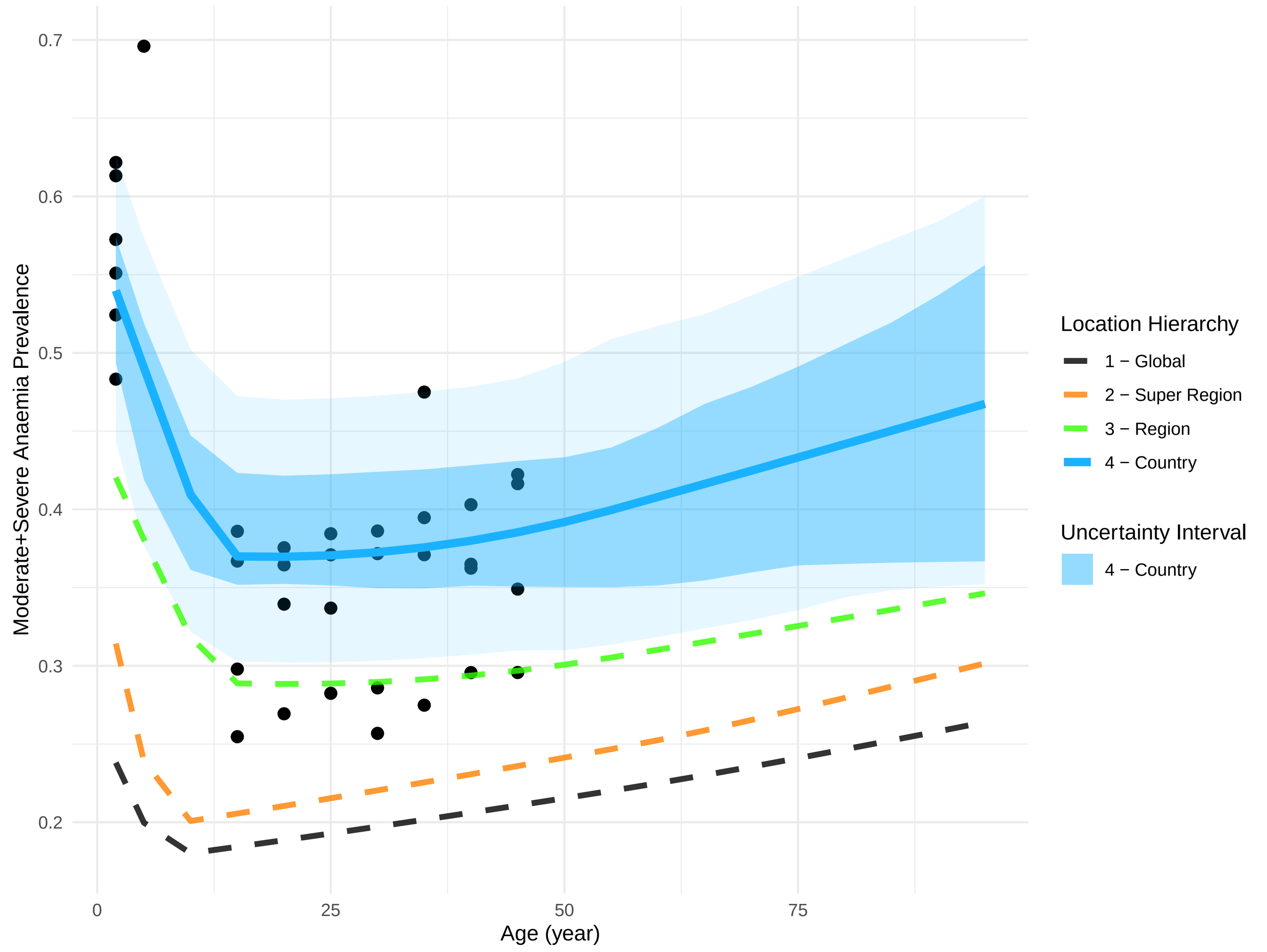

MR−BRT Age/Sex Fits − Non−pregnant Females 2+ Years Old − Moderate+Severe Anaemia Prevalence − Niger
WHO−2001 Adjustment Method

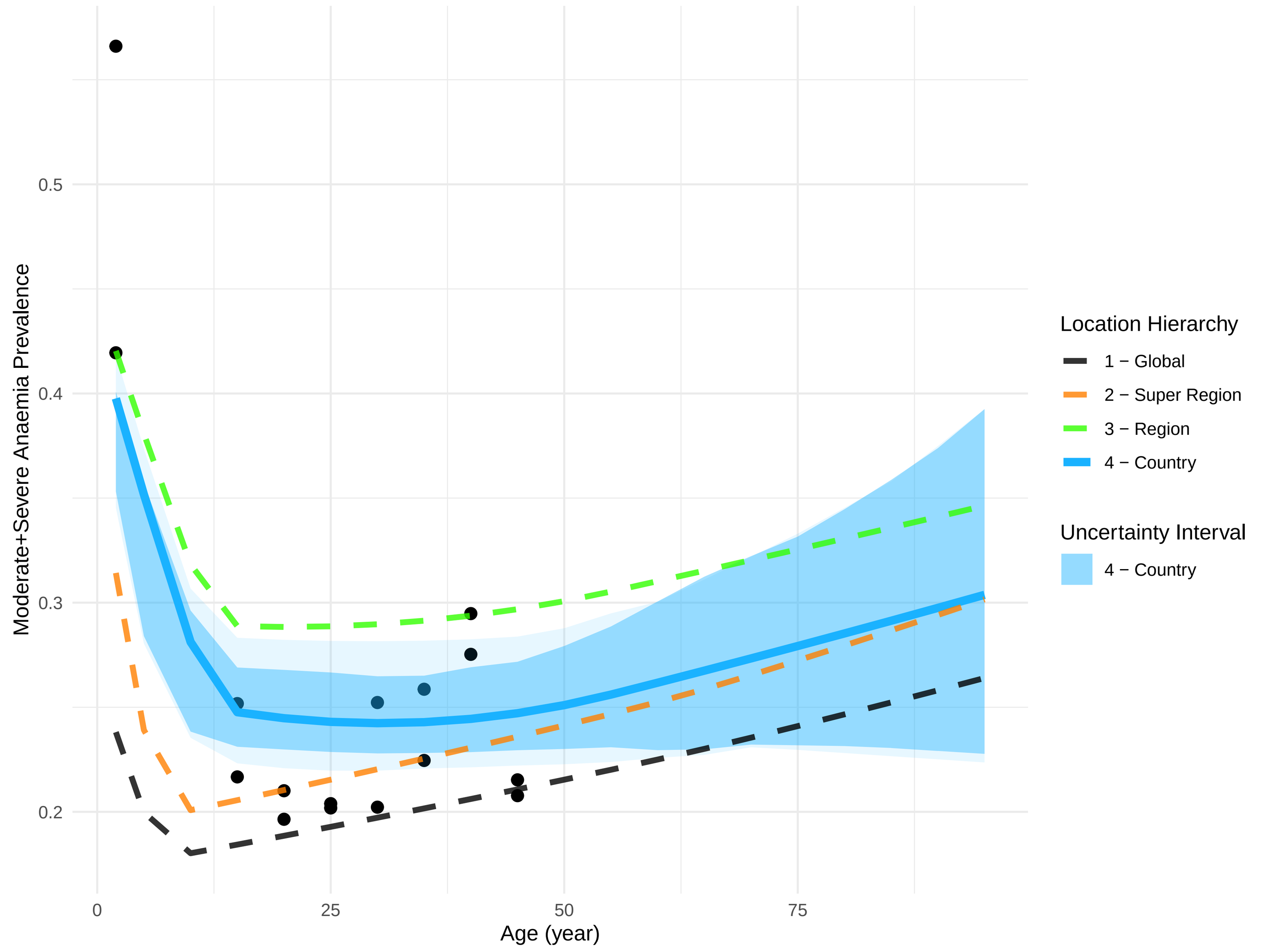

MR−BRT Age/Sex Fits − Non−pregnant Females 2+ Years Old − Moderate+Severe Anaemia Prevalence − Nigeria
WHO−2001 Adjustment Method

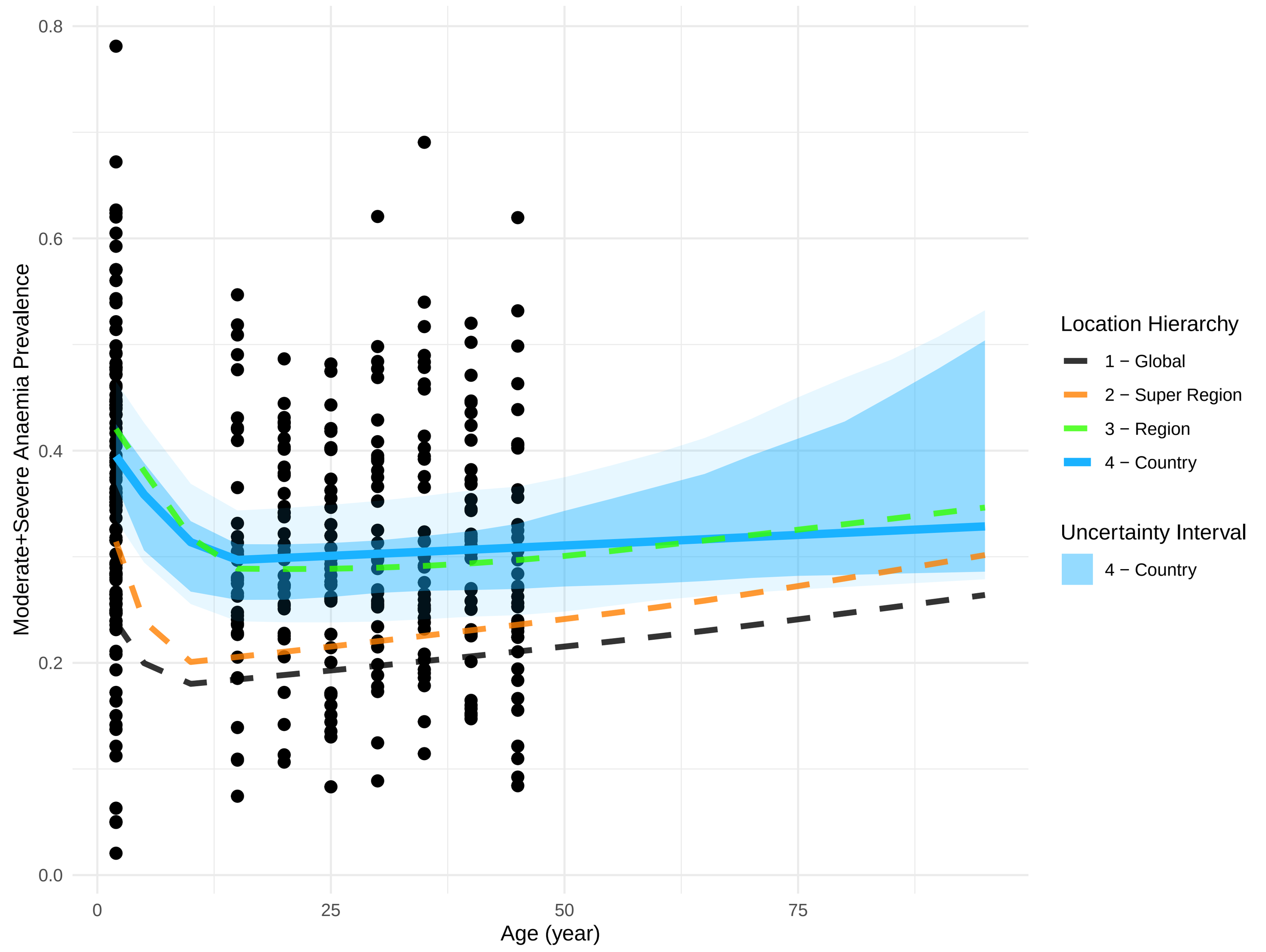

MR−BRT Age/Sex Fits − Non−pregnant Females 2+ Years Old − Moderate+Severe Anaemia Prevalence − Abia
WHO−2001 Adjustment Method

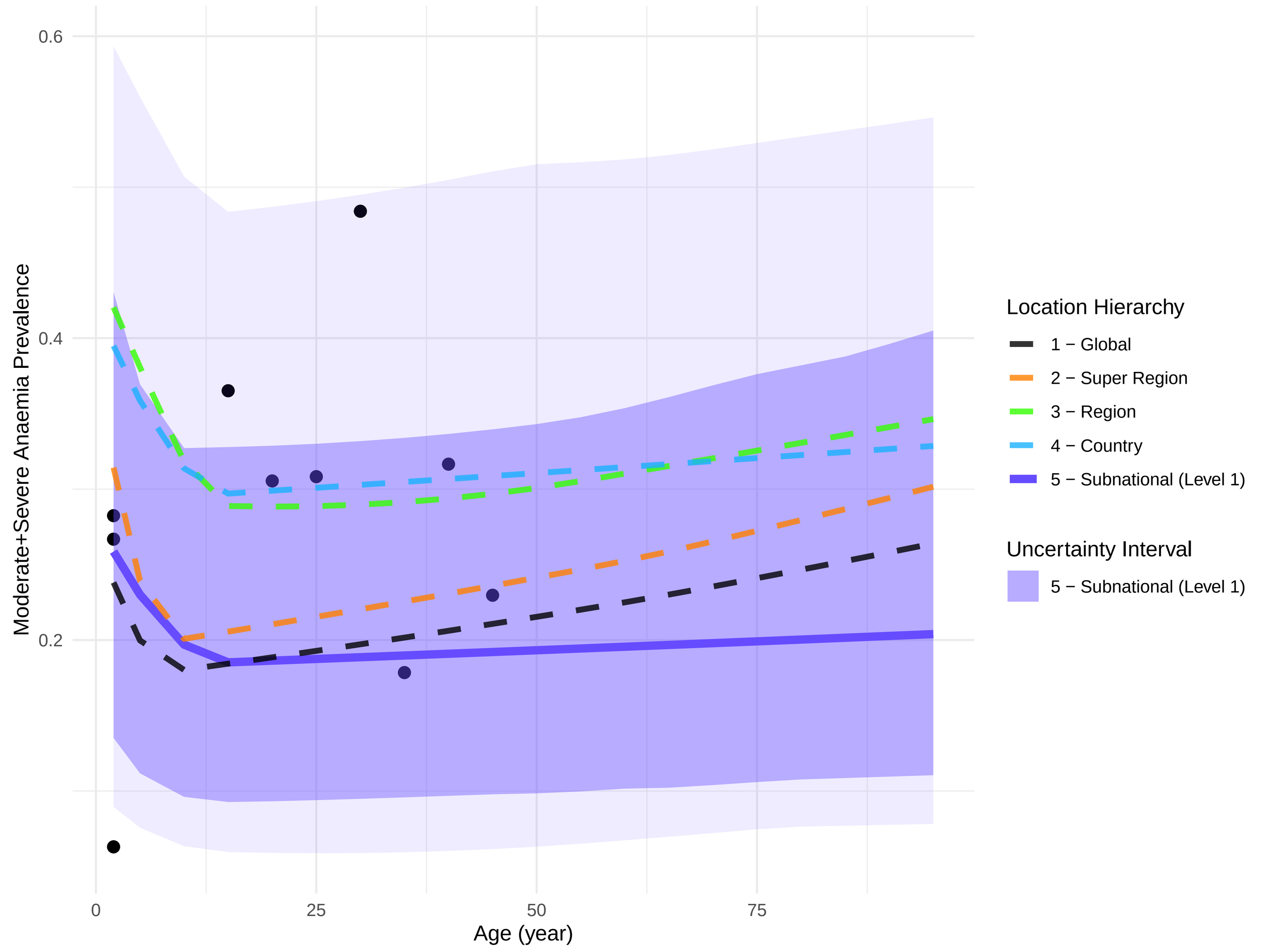

MR−BRT Age/Sex Fits − Non−pregnant Females 2+ Years Old − Moderate+Severe Anaemia Prevalence − Adamawa
WHO−2001 Adjustment Method

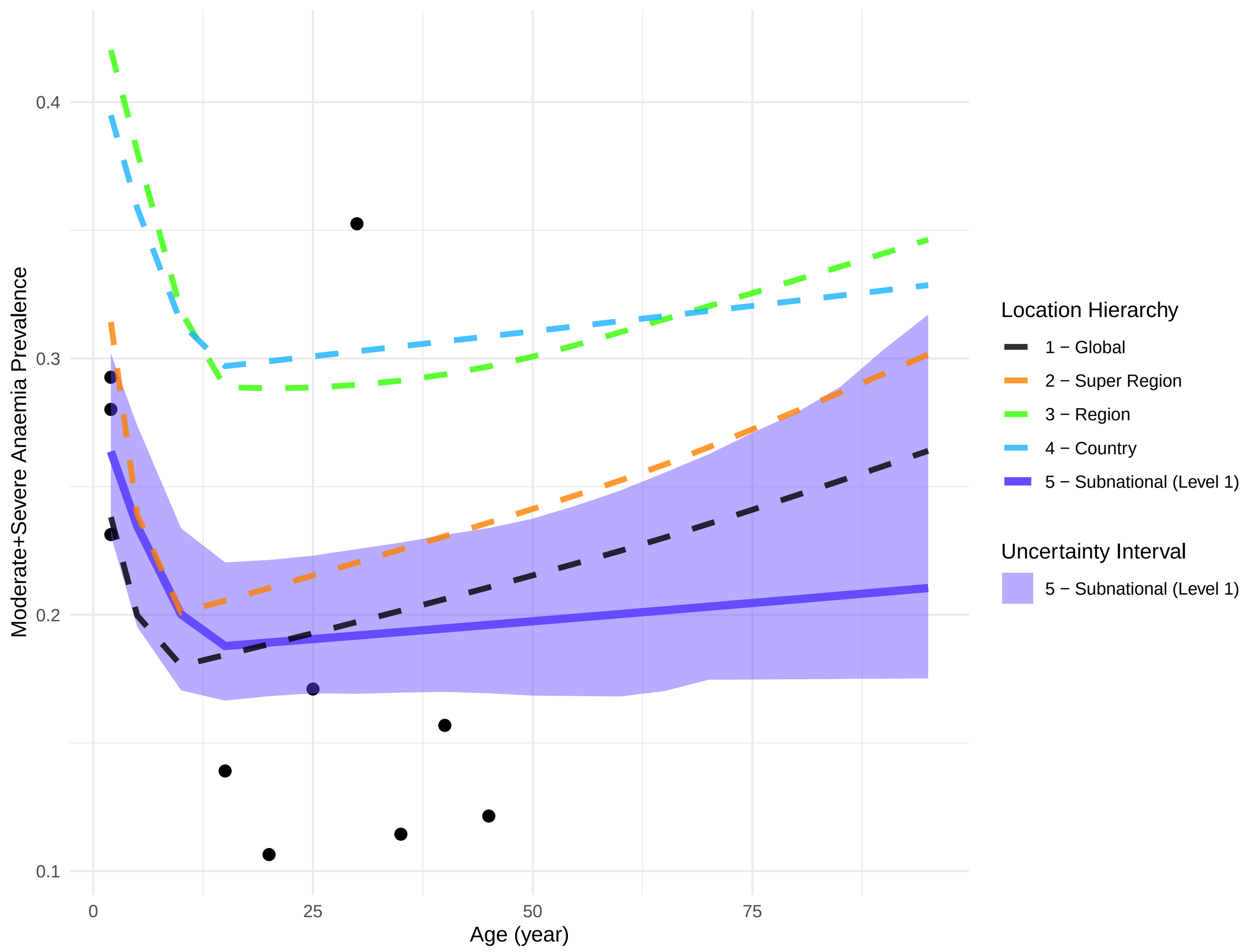

MR−BRT Age/Sex Fits − Non−pregnant Females 2+ Years Old − Moderate+Severe Anaemia Prevalence − Akwa Ibom
WHO−2001 Adjustment Method

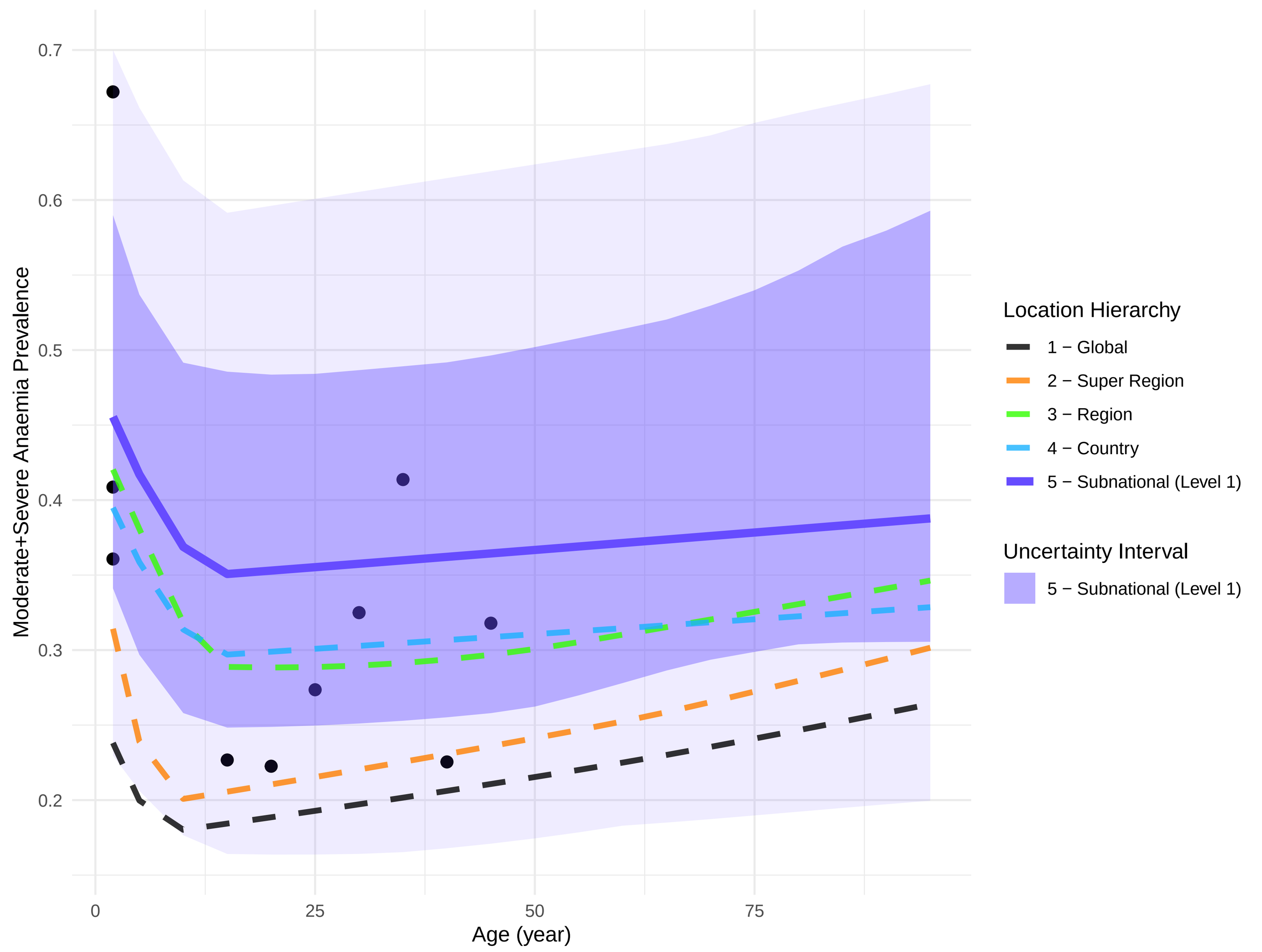

MR−BRT Age/Sex Fits − Non−pregnant Females 2+ Years Old − Moderate+Severe Anaemia Prevalence − Anambra
WHO−2001 Adjustment Method

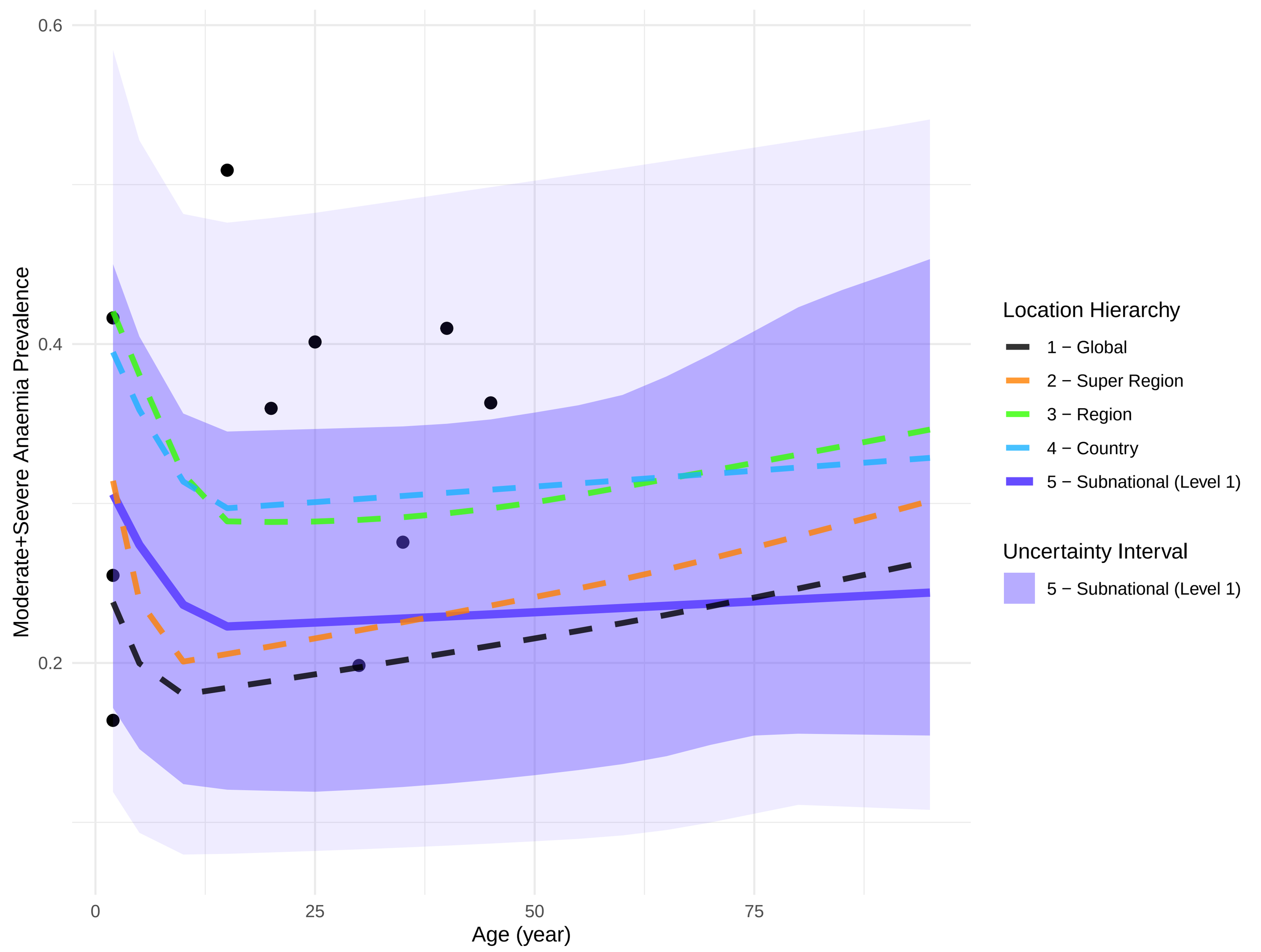

MR−BRT Age/Sex Fits − Non−pregnant Females 2+ Years Old − Moderate+Severe Anaemia Prevalence − Bauchi
WHO−2001 Adjustment Method

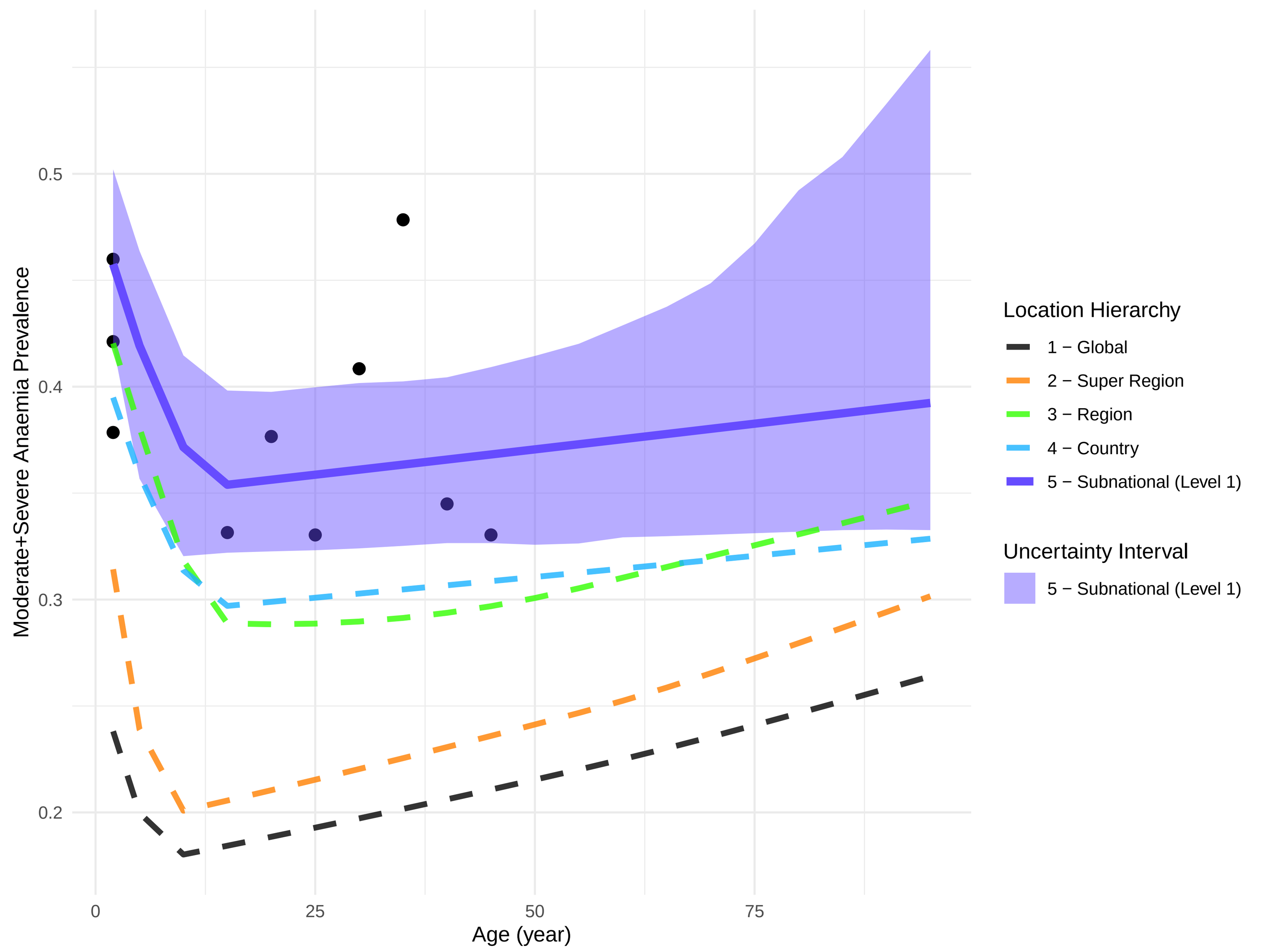

MR−BRT Age/Sex Fits − Non−pregnant Females 2+ Years Old − Moderate+Severe Anaemia Prevalence − Bayelsa
WHO−2001 Adjustment Method

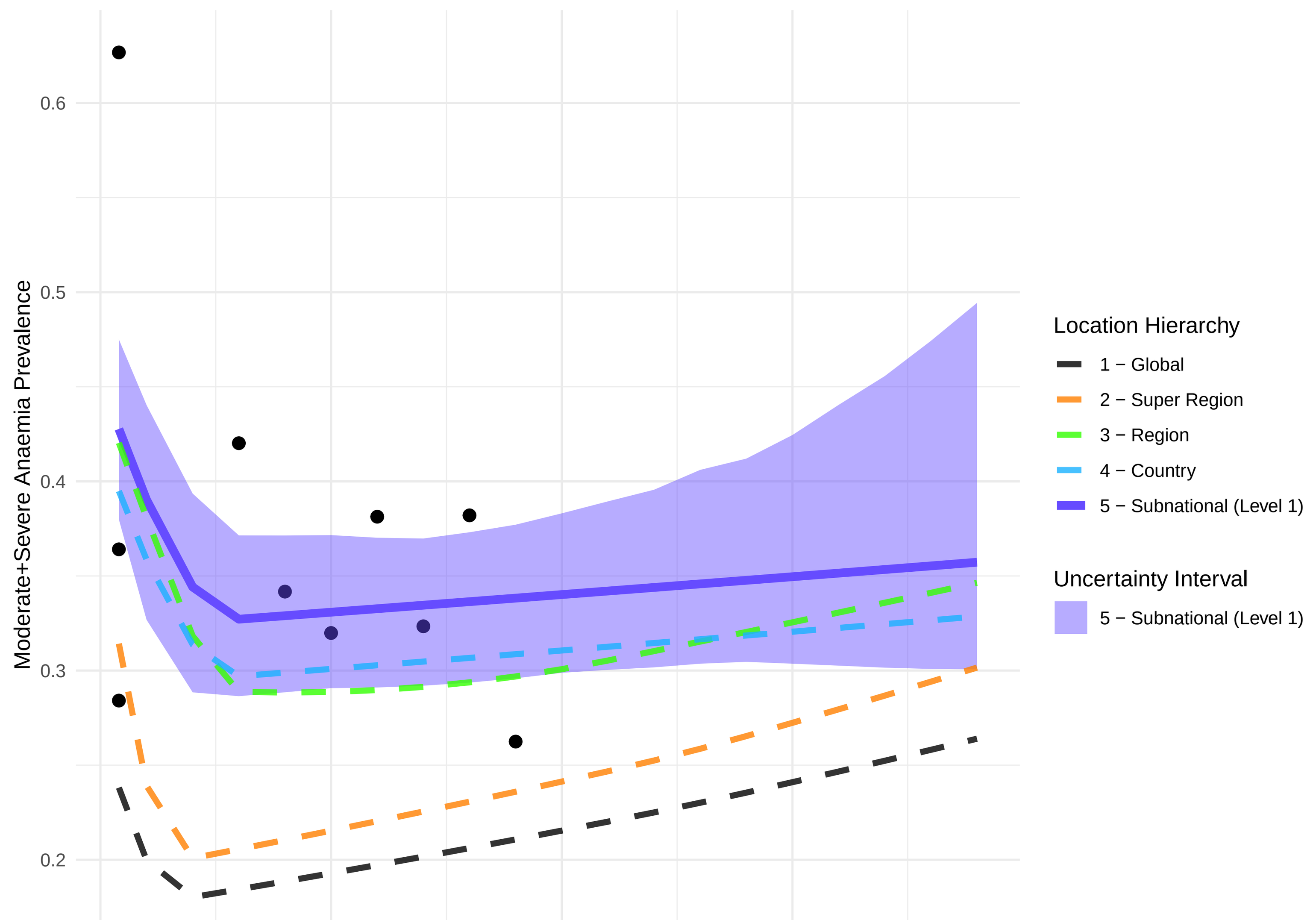

MR−BRT Age/Sex Fits − Non−pregnant Females 2+ Years Old − Moderate+Severe Anaemia Prevalence − Benue
WHO−2001 Adjustment Method

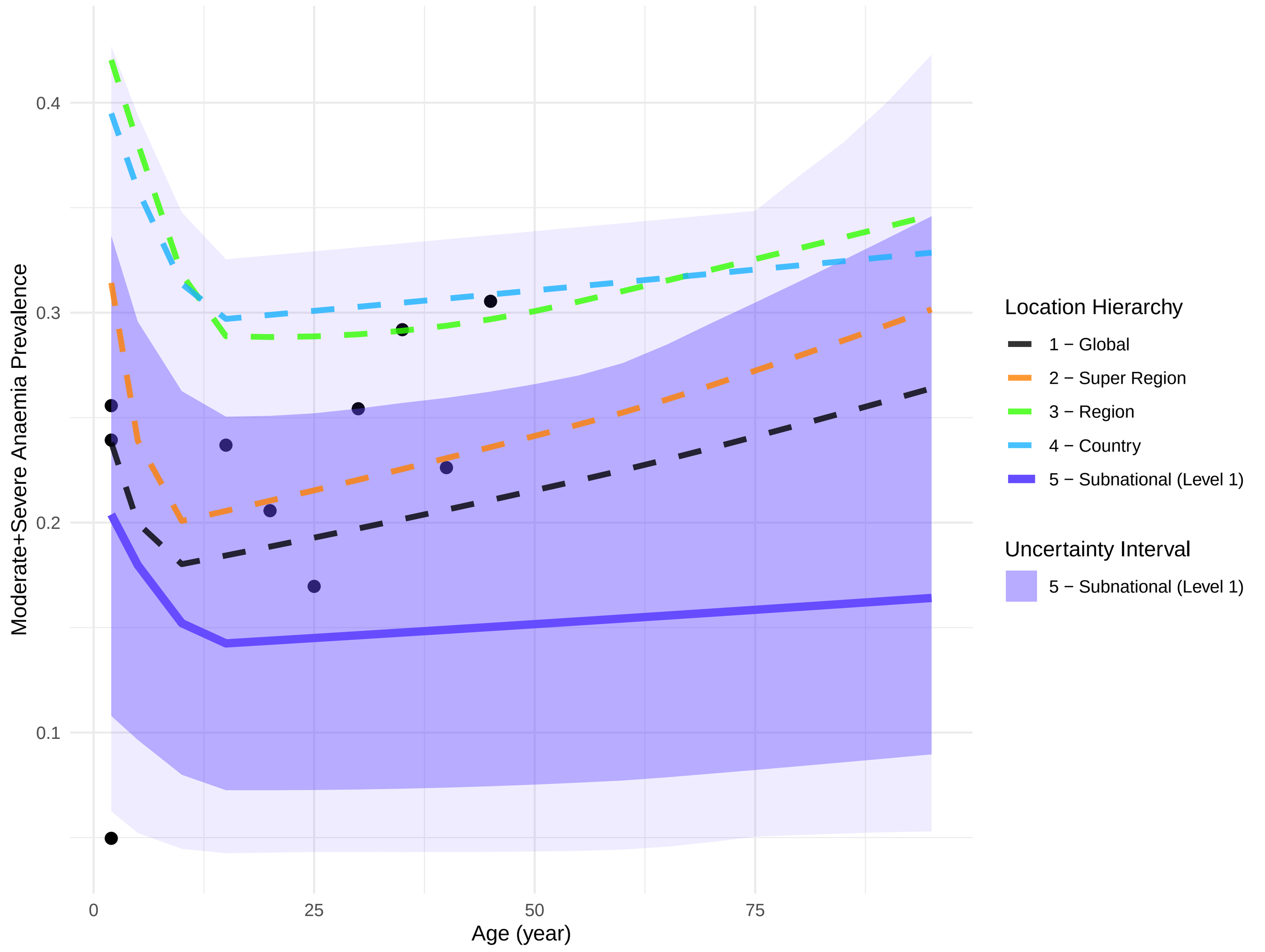

MR−BRT Age/Sex Fits − Non−pregnant Females 2+ Years Old − Moderate+Severe Anaemia Prevalence − Borno
WHO−2001 Adjustment Method

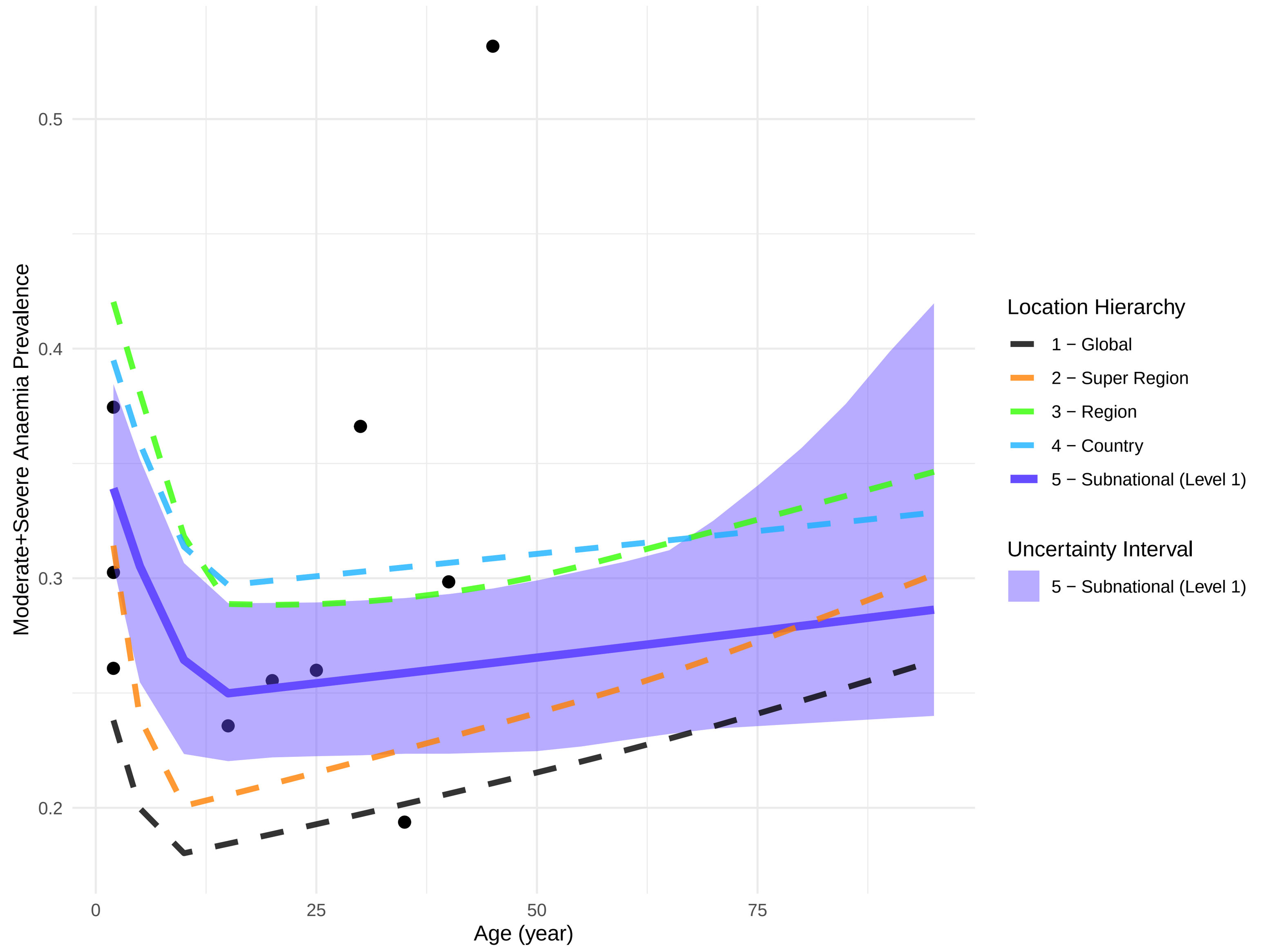

MR−BRT Age/Sex Fits − Non−pregnant Females 2+ Years Old − Moderate+Severe Anaemia Prevalence − Cross River
WHO−2001 Adjustment Method

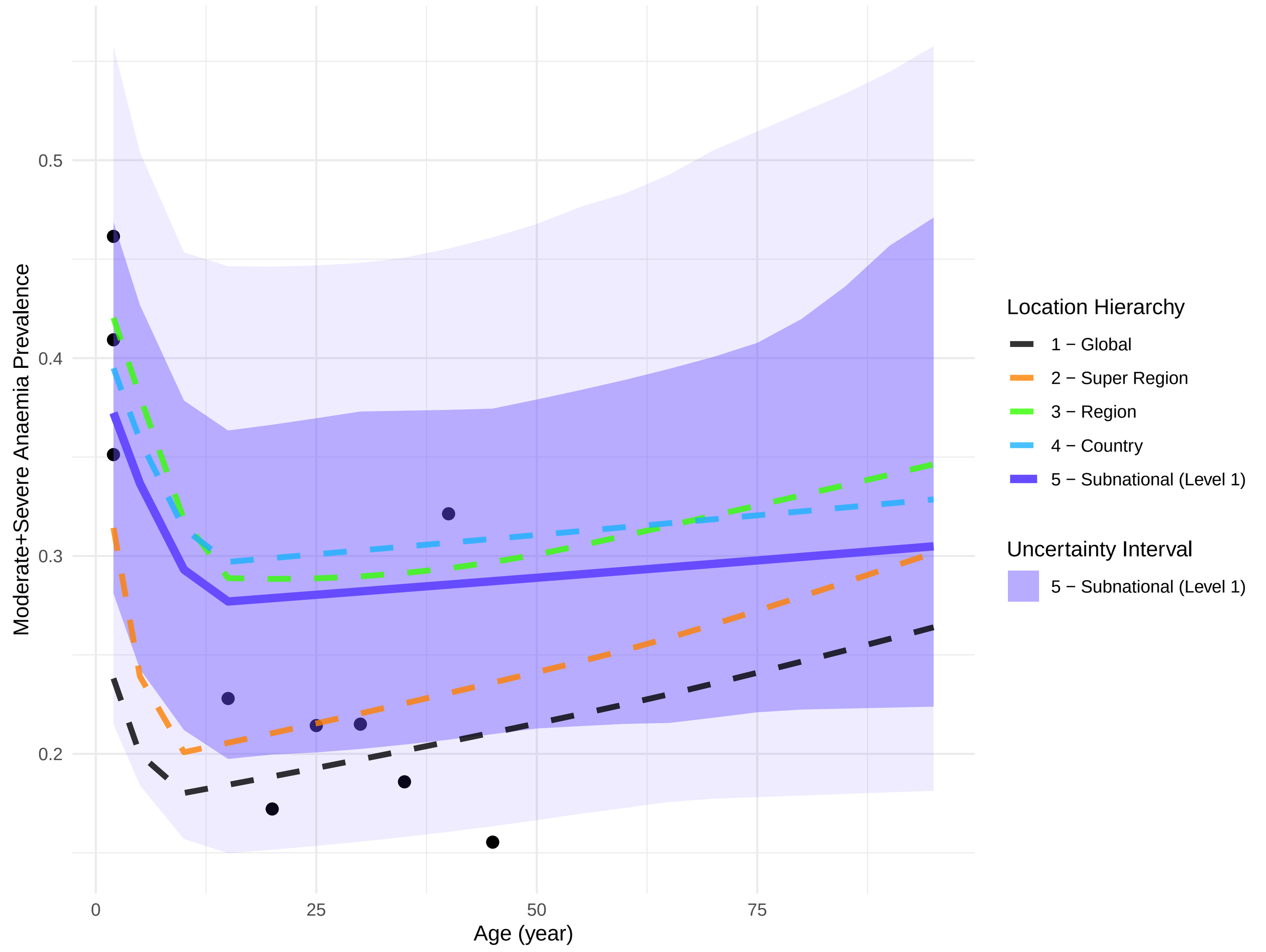

MR−BRT Age/Sex Fits − Non−pregnant Females 2+ Years Old − Moderate+Severe Anaemia Prevalence − Delta
WHO−2001 Adjustment Method

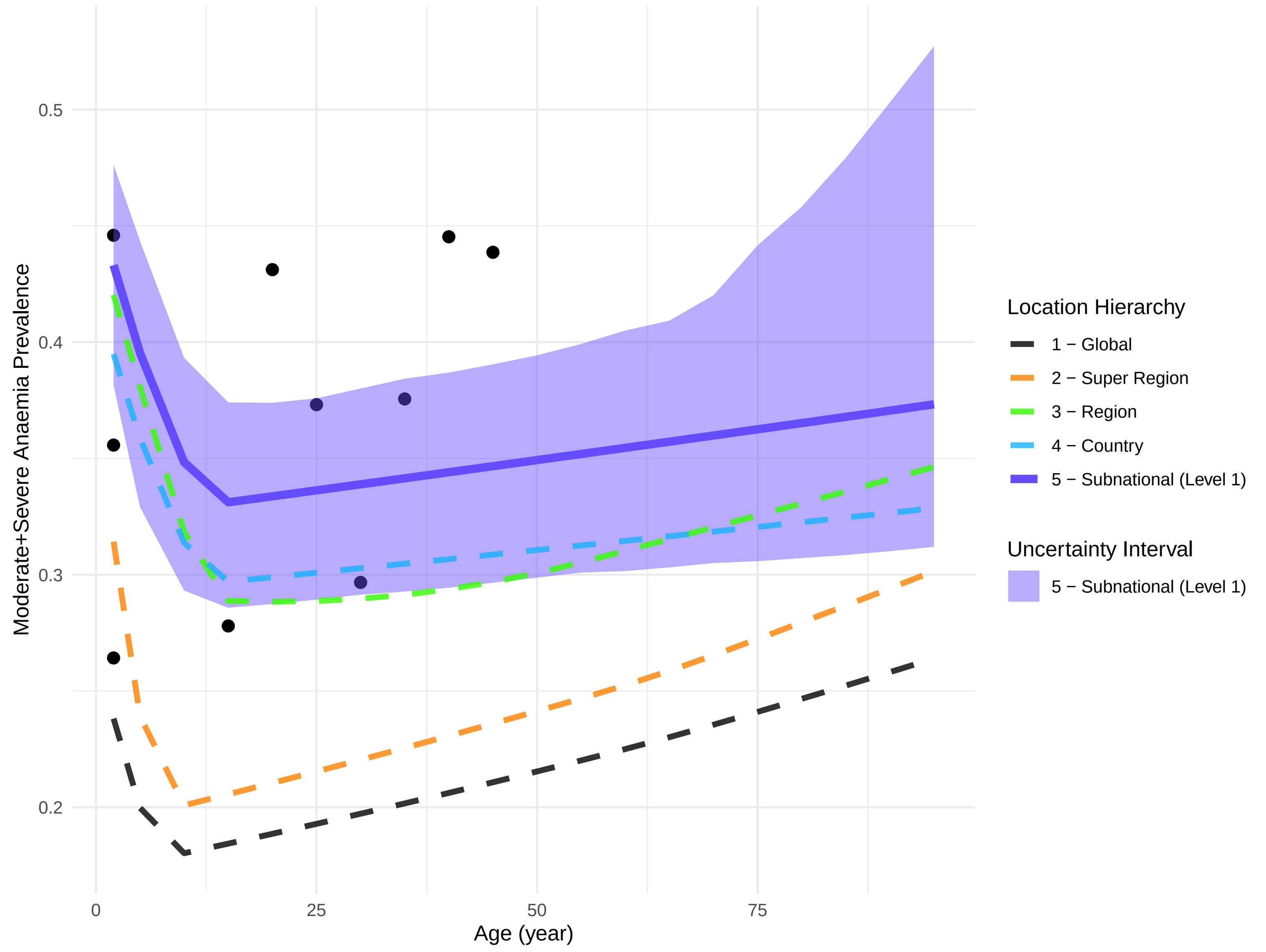

MR−BRT Age/Sex Fits − Non−pregnant Females 2+ Years Old − Moderate+Severe Anaemia Prevalence − Ebonyi
WHO−2001 Adjustment Method

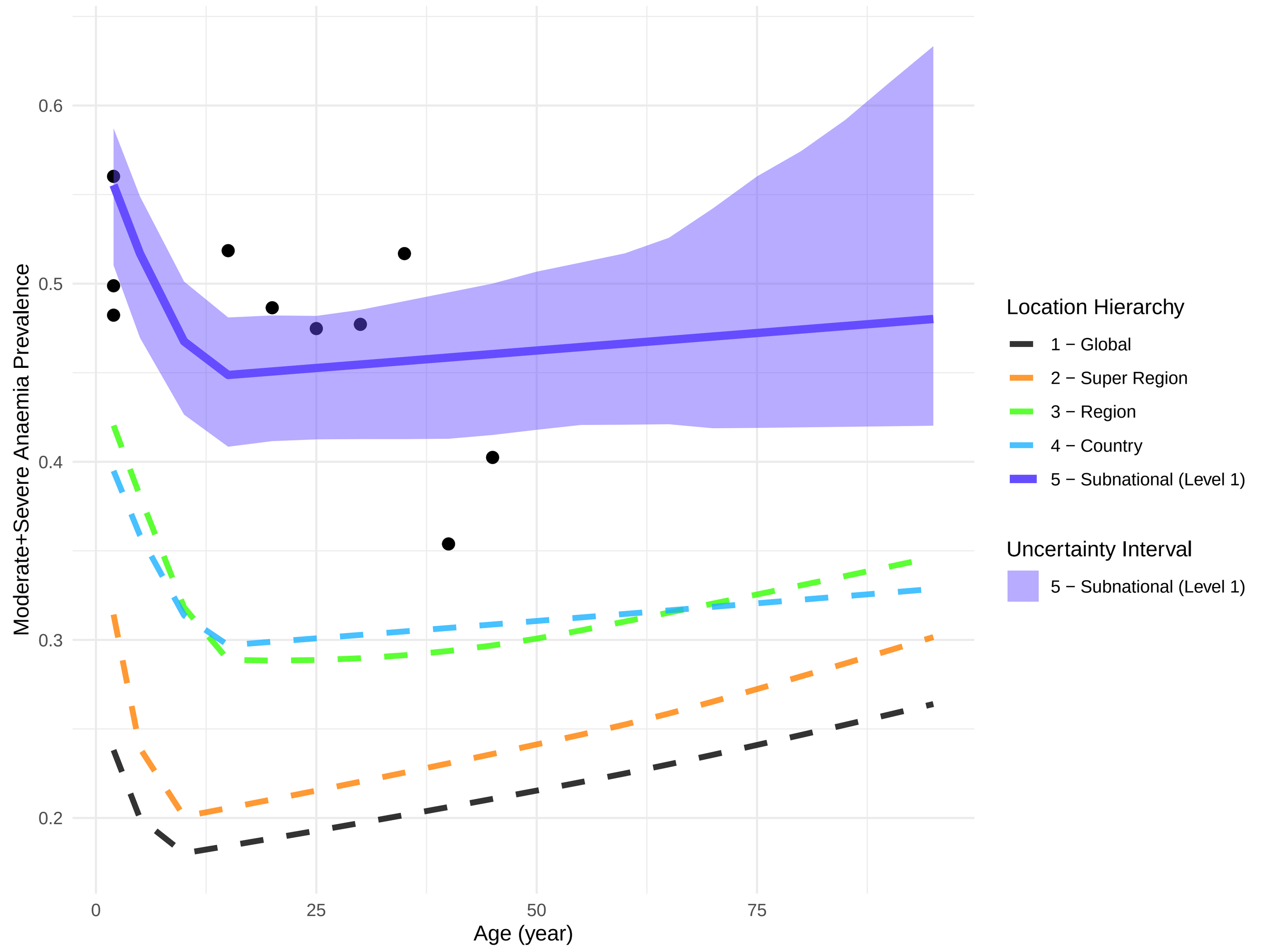

MR−BRT Age/Sex Fits − Non−pregnant Females 2+ Years Old − Moderate+Severe Anaemia Prevalence − Edo
WHO−2001 Adjustment Method

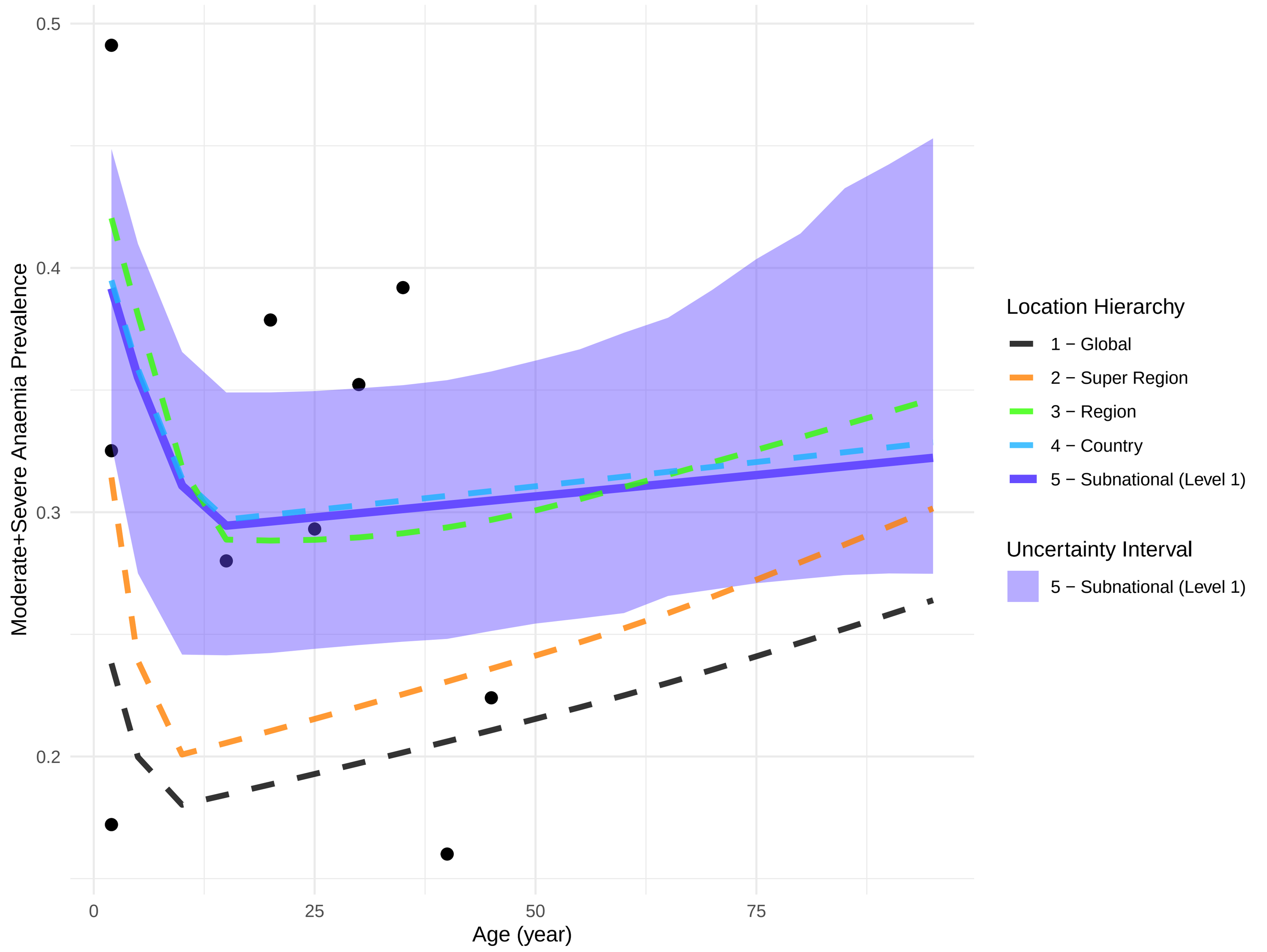

MR−BRT Age/Sex Fits − Non−pregnant Females 2+ Years Old − Moderate+Severe Anaemia Prevalence − Ekiti
WHO−2001 Adjustment Method

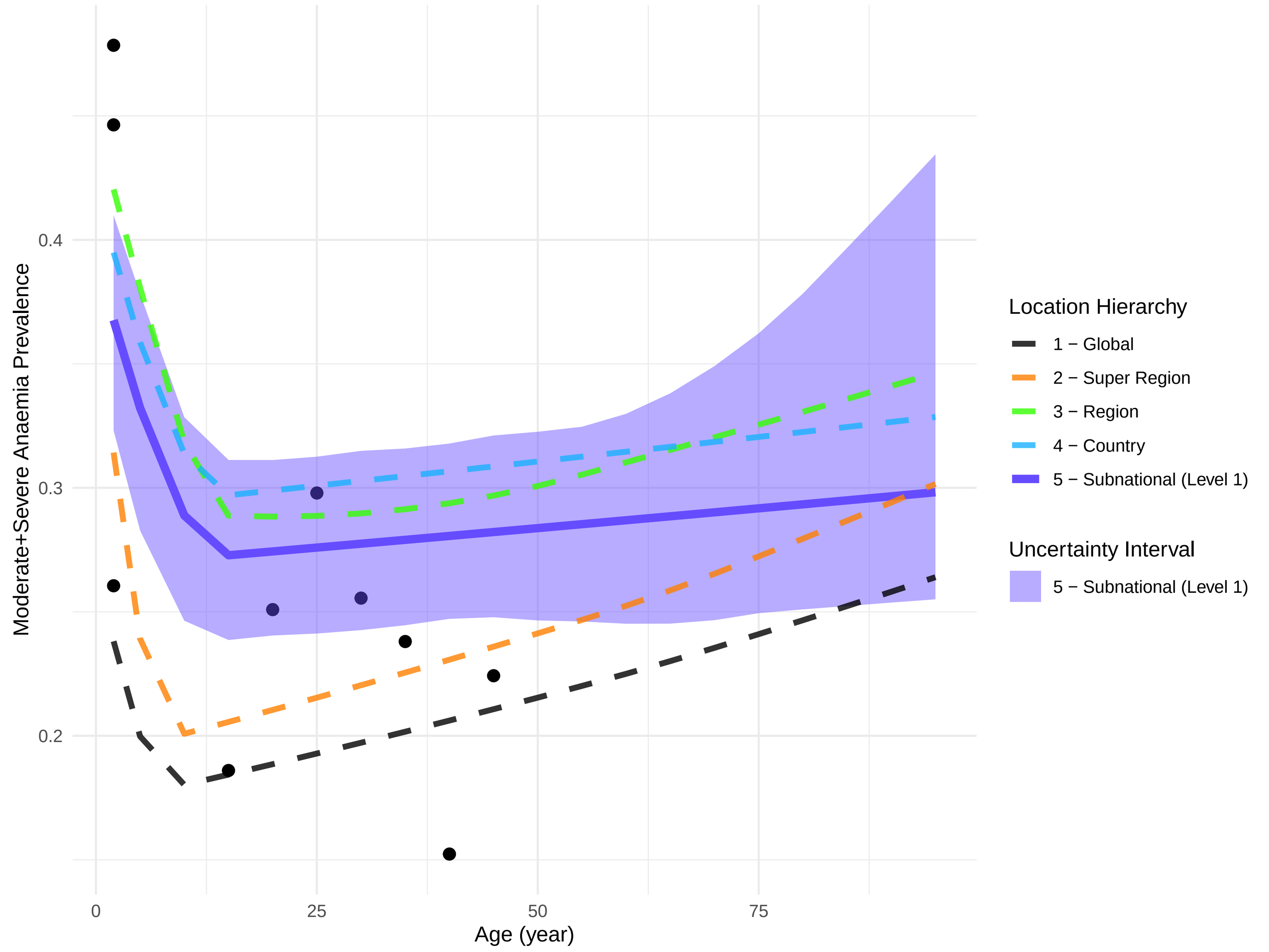

MR−BRT Age/Sex Fits − Non−pregnant Females 2+ Years Old − Moderate+Severe Anaemia Prevalence − Enugu
WHO−2001 Adjustment Method

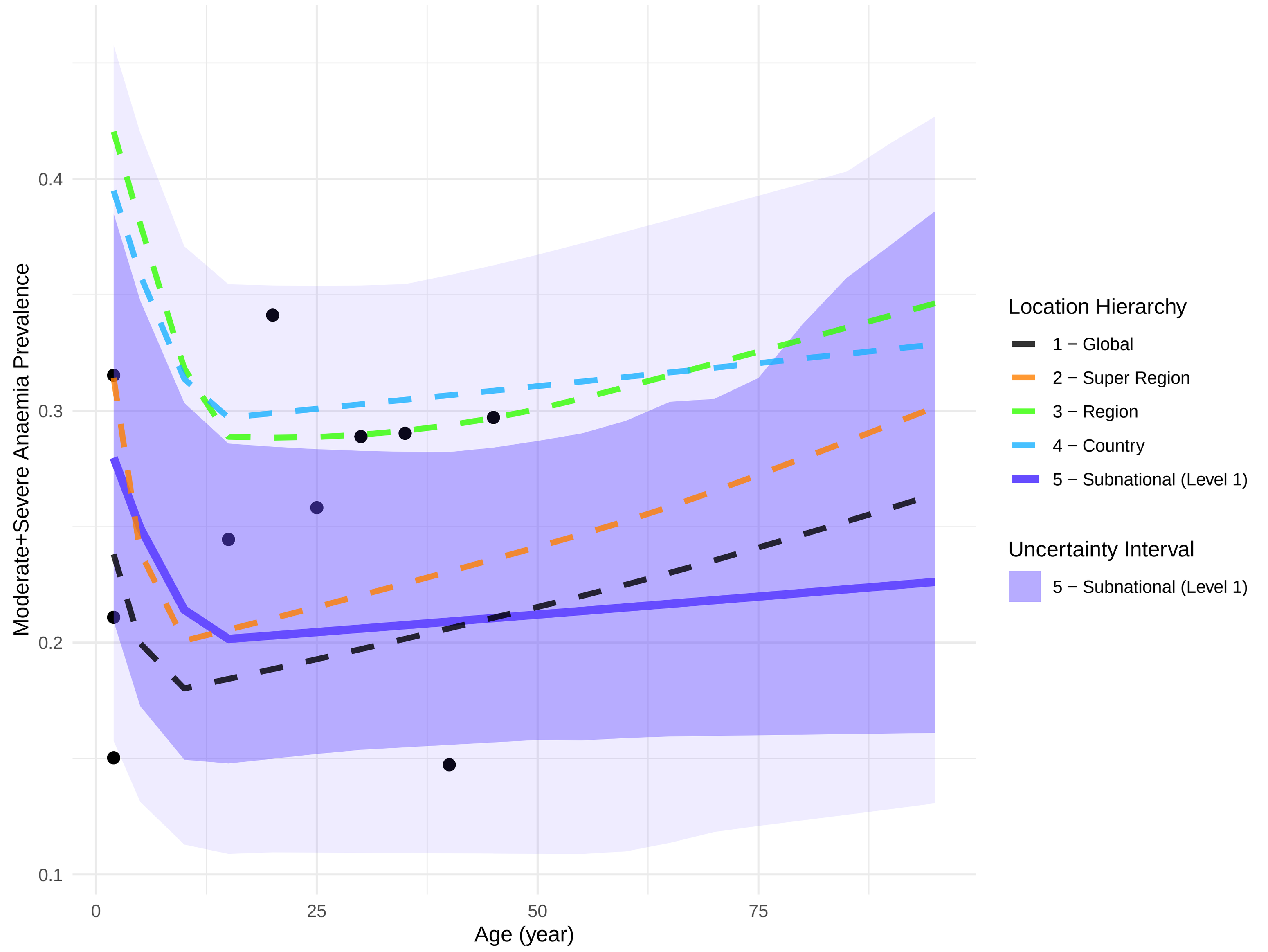

MR−BRT Age/Sex Fits − Non−pregnant Females 2+ Years Old − Moderate+Severe Anaemia Prevalence − FCT (Abuja)
WHO−2001 Adjustment Method

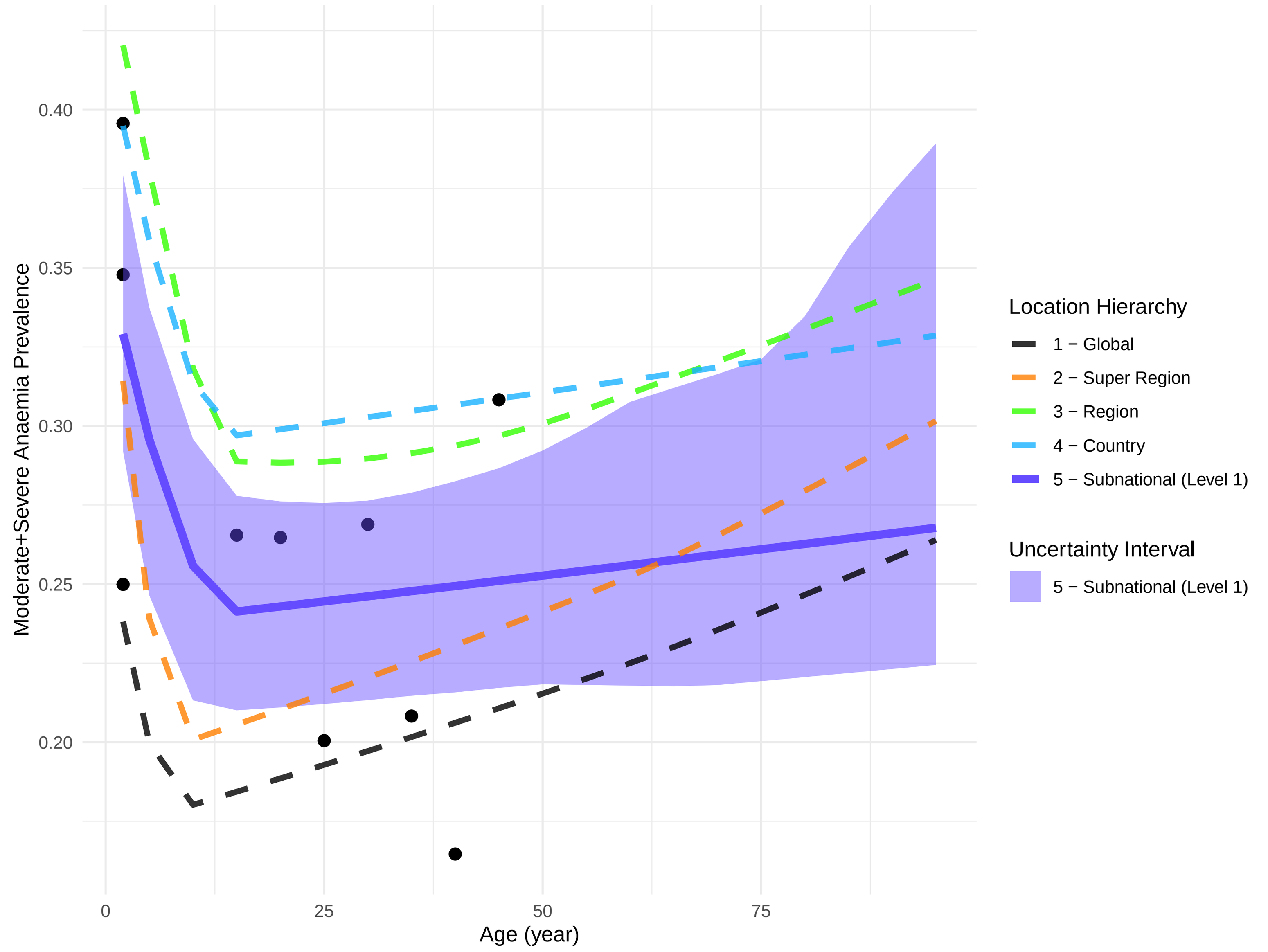

MR−BRT Age/Sex Fits − Non−pregnant Females 2+ Years Old − Moderate+Severe Anaemia Prevalence − Gombe
WHO−2001 Adjustment Method

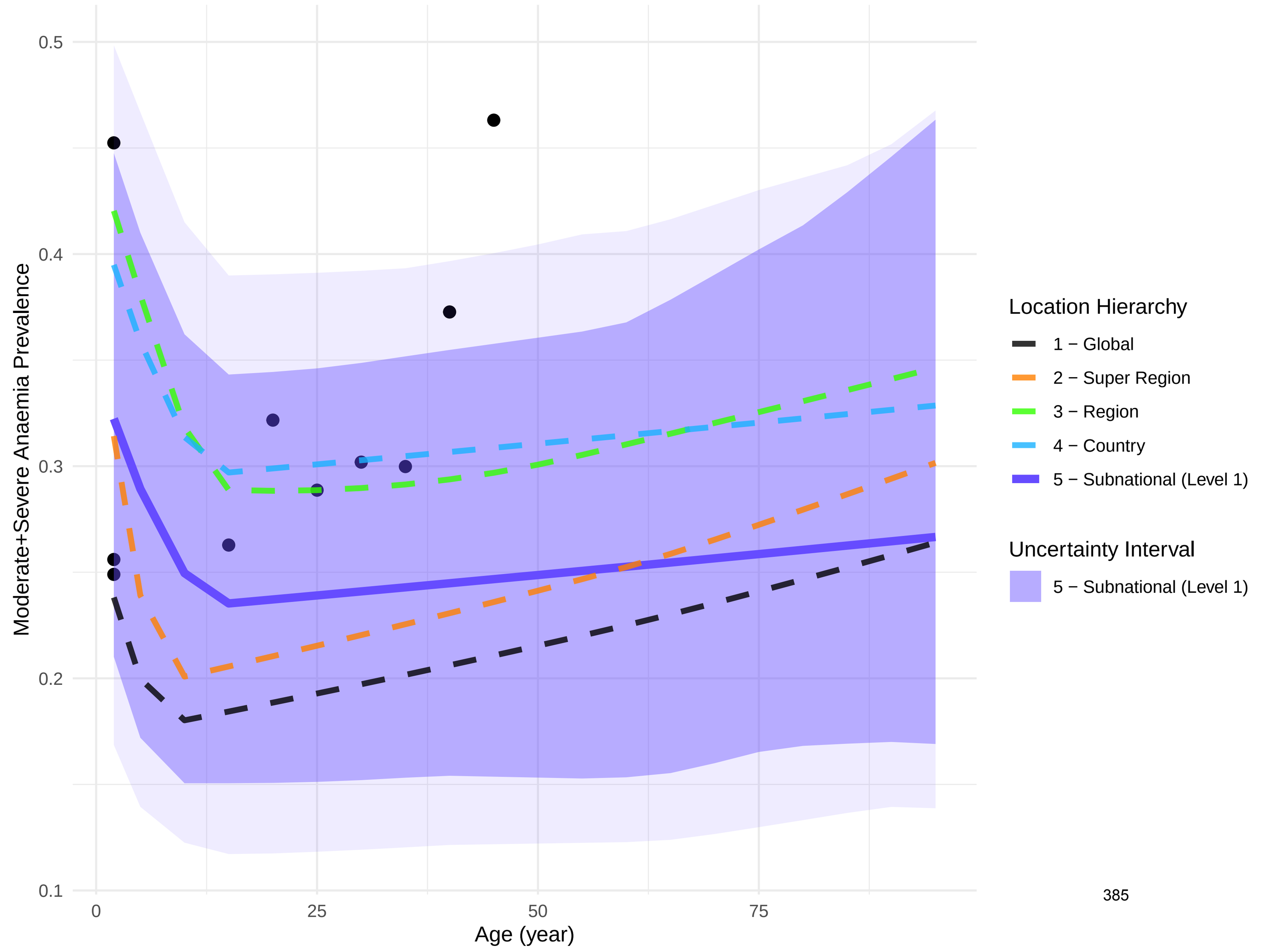

MR−BRT Age/Sex Fits − Non−pregnant Females 2+ Years Old − Moderate+Severe Anaemia Prevalence − Imo
WHO−2001 Adjustment Method

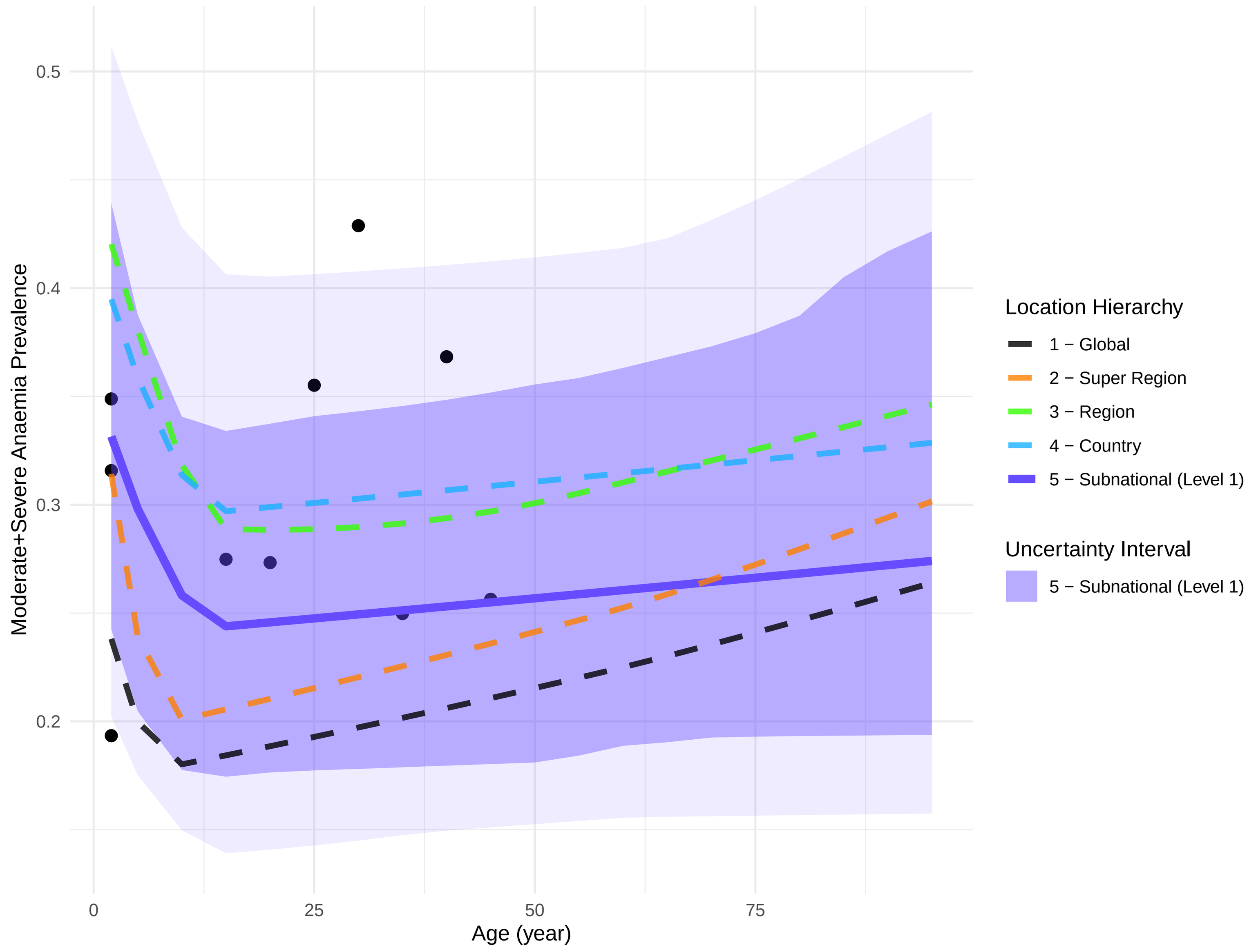

MR−BRT Age/Sex Fits − Non−pregnant Females 2+ Years Old − Moderate+Severe Anaemia Prevalence − Jigawa
WHO−2001 Adjustment Method

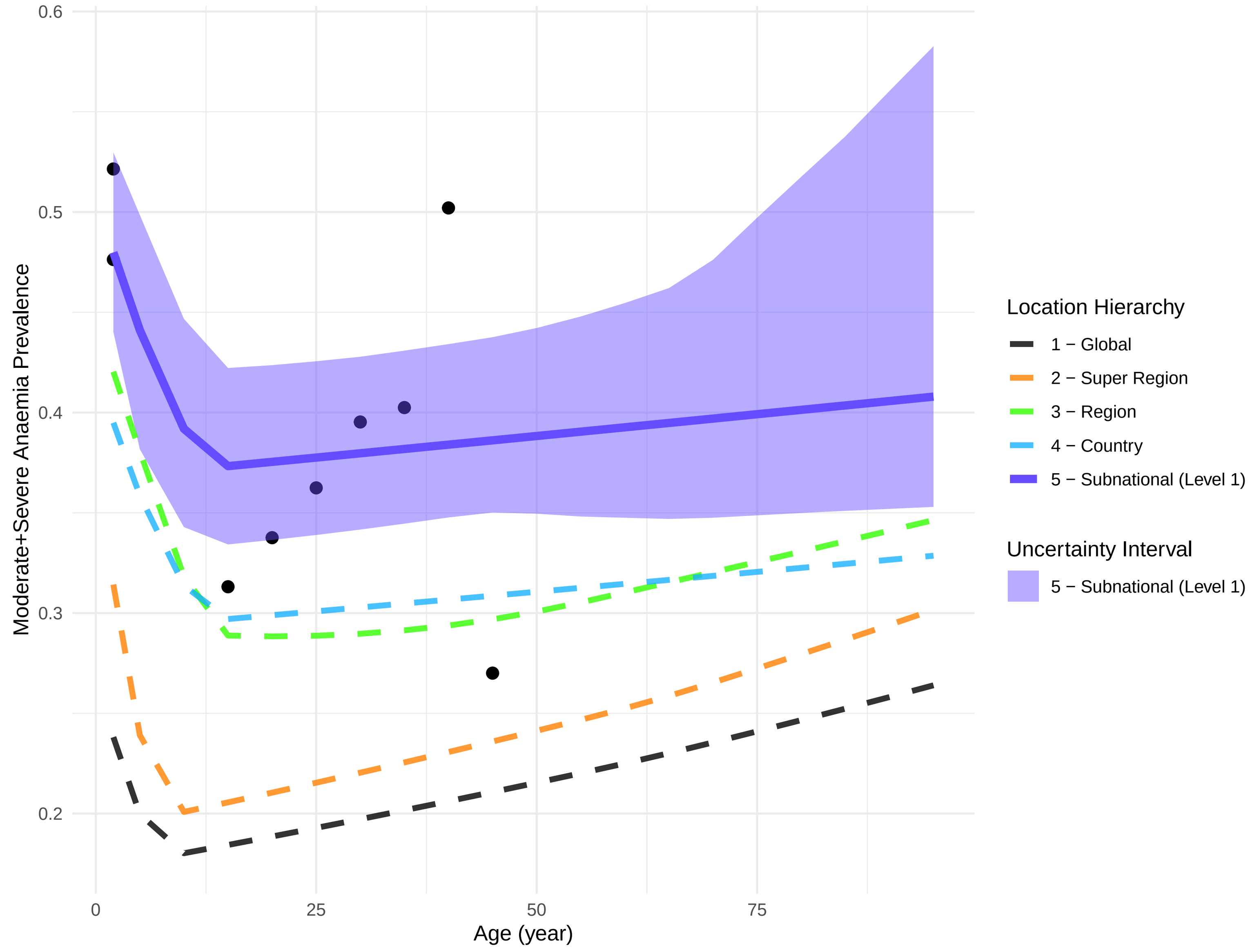

MR−BRT Age/Sex Fits − Non−pregnant Females 2+ Years Old − Moderate+Severe Anaemia Prevalence − Kaduna
WHO−2001 Adjustment Method

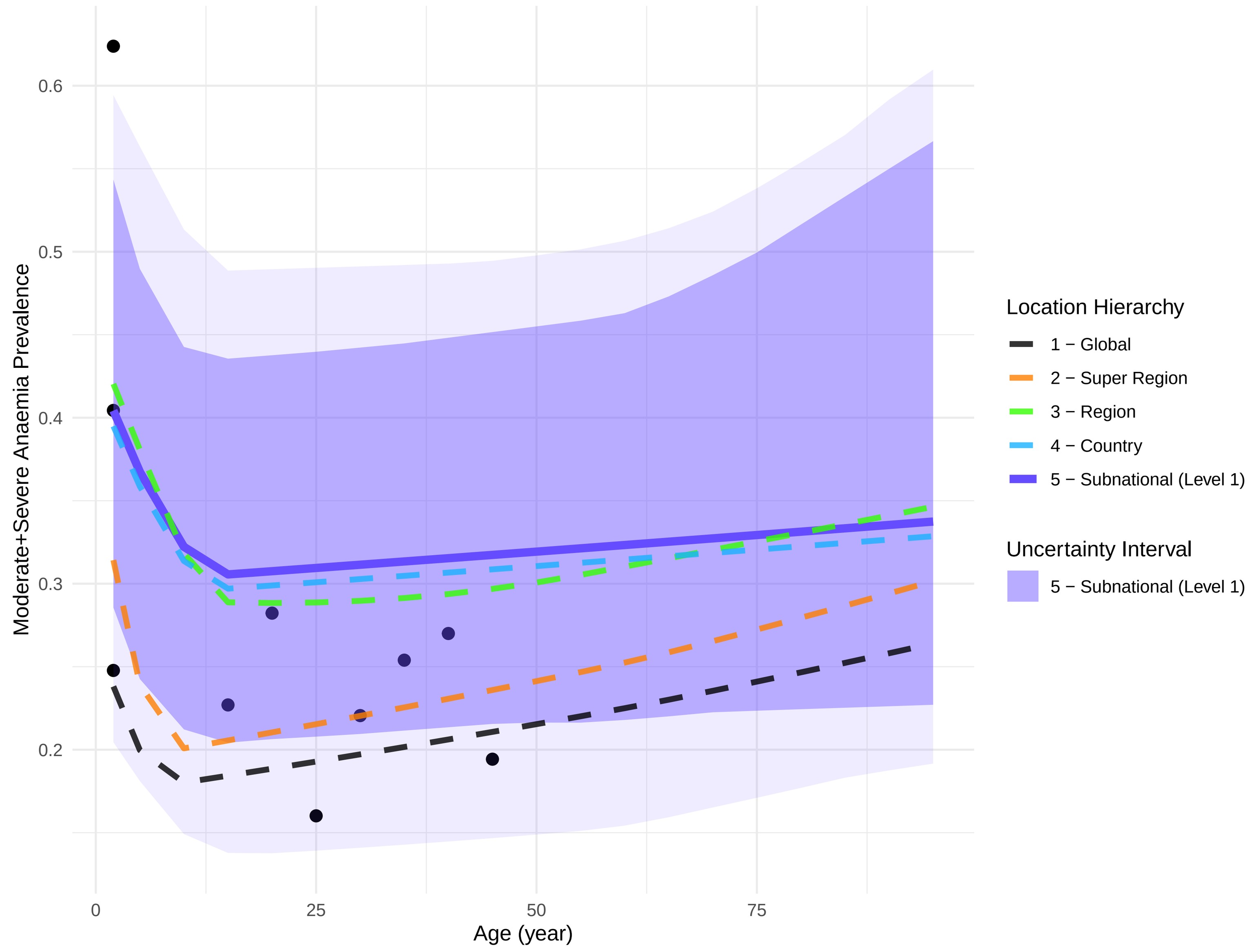

MR−BRT Age/Sex Fits − Non−pregnant Females 2+ Years Old − Moderate+Severe Anaemia Prevalence − Kano
WHO−2001 Adjustment Method

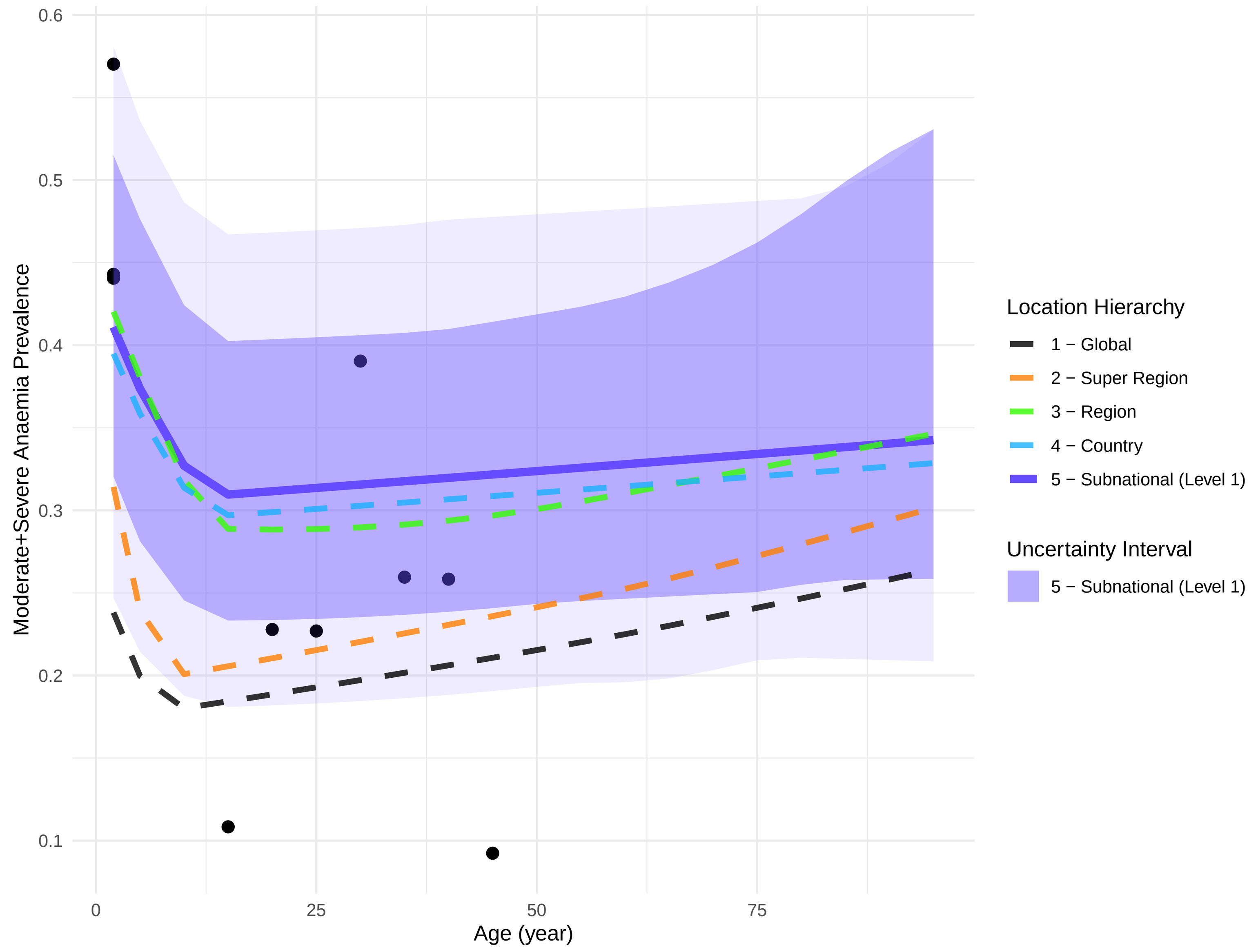

MR−BRT Age/Sex Fits − Non−pregnant Females 2+ Years Old − Moderate+Severe Anaemia Prevalence − Katsina
WHO−2001 Adjustment Method

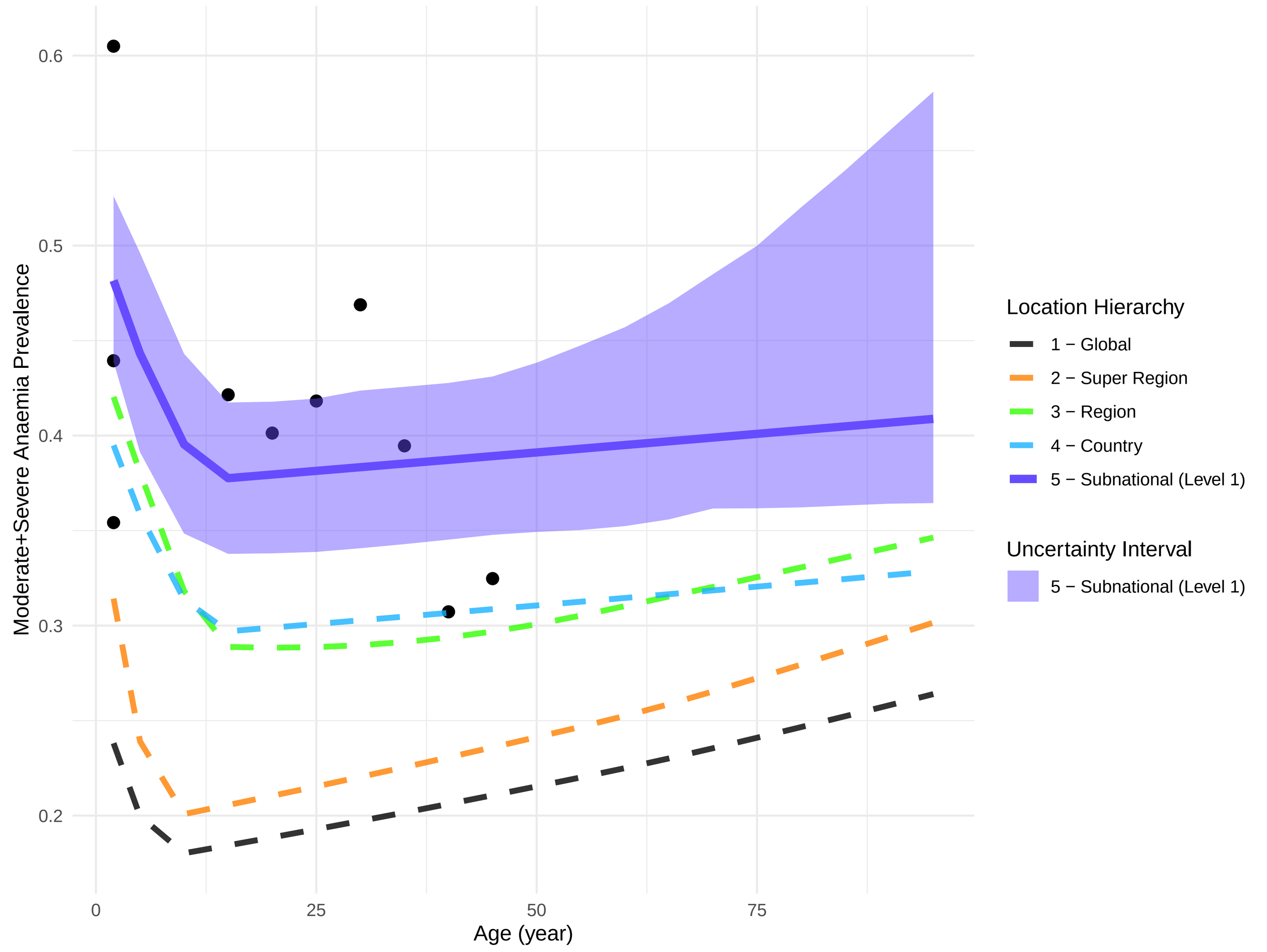

MR−BRT Age/Sex Fits − Non−pregnant Females 2+ Years Old − Moderate+Severe Anaemia Prevalence − Kebbi
WHO−2001 Adjustment Method

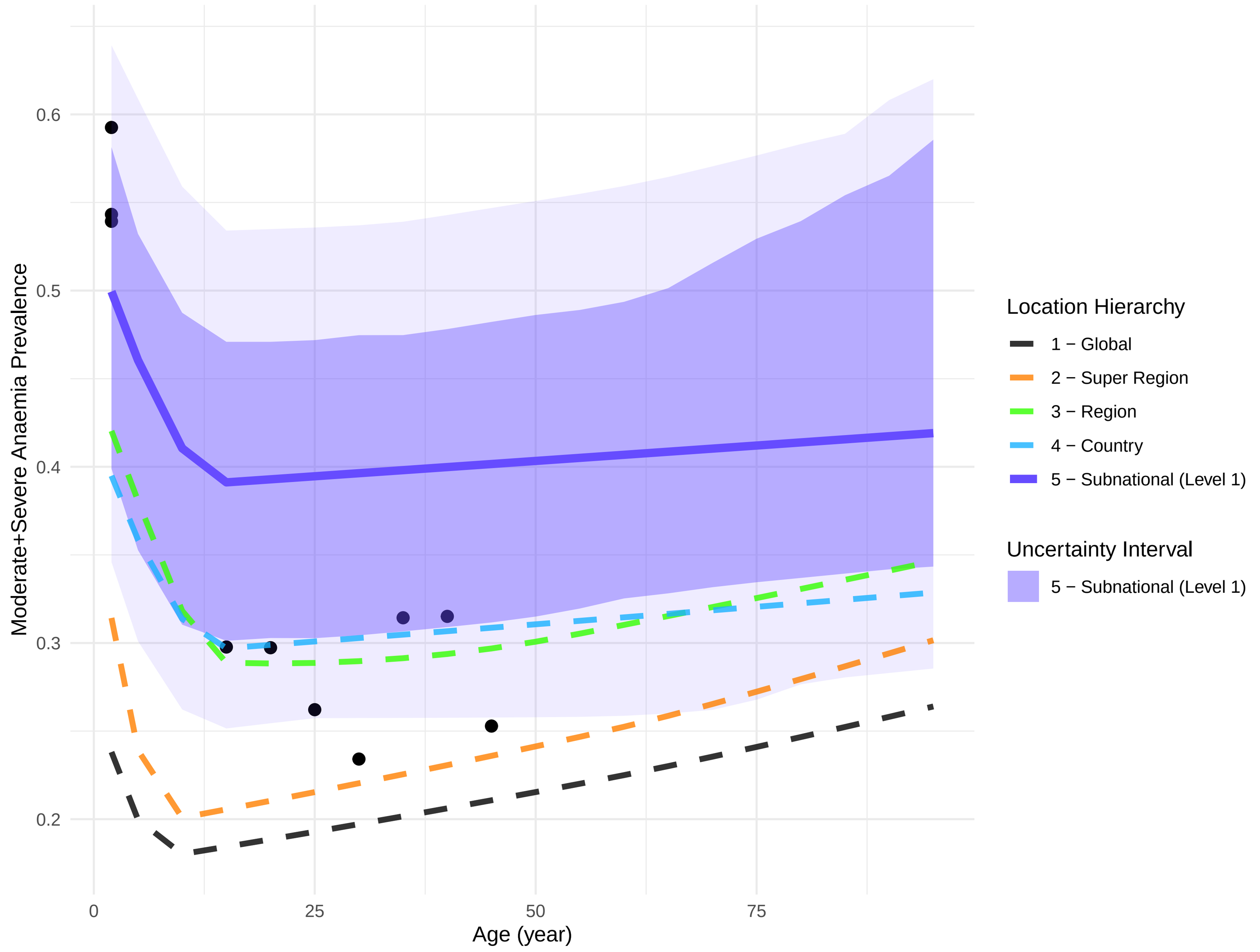

MR−BRT Age/Sex Fits − Non−pregnant Females 2+ Years Old − Moderate+Severe Anaemia Prevalence − Kogi
WHO−2001 Adjustment Method

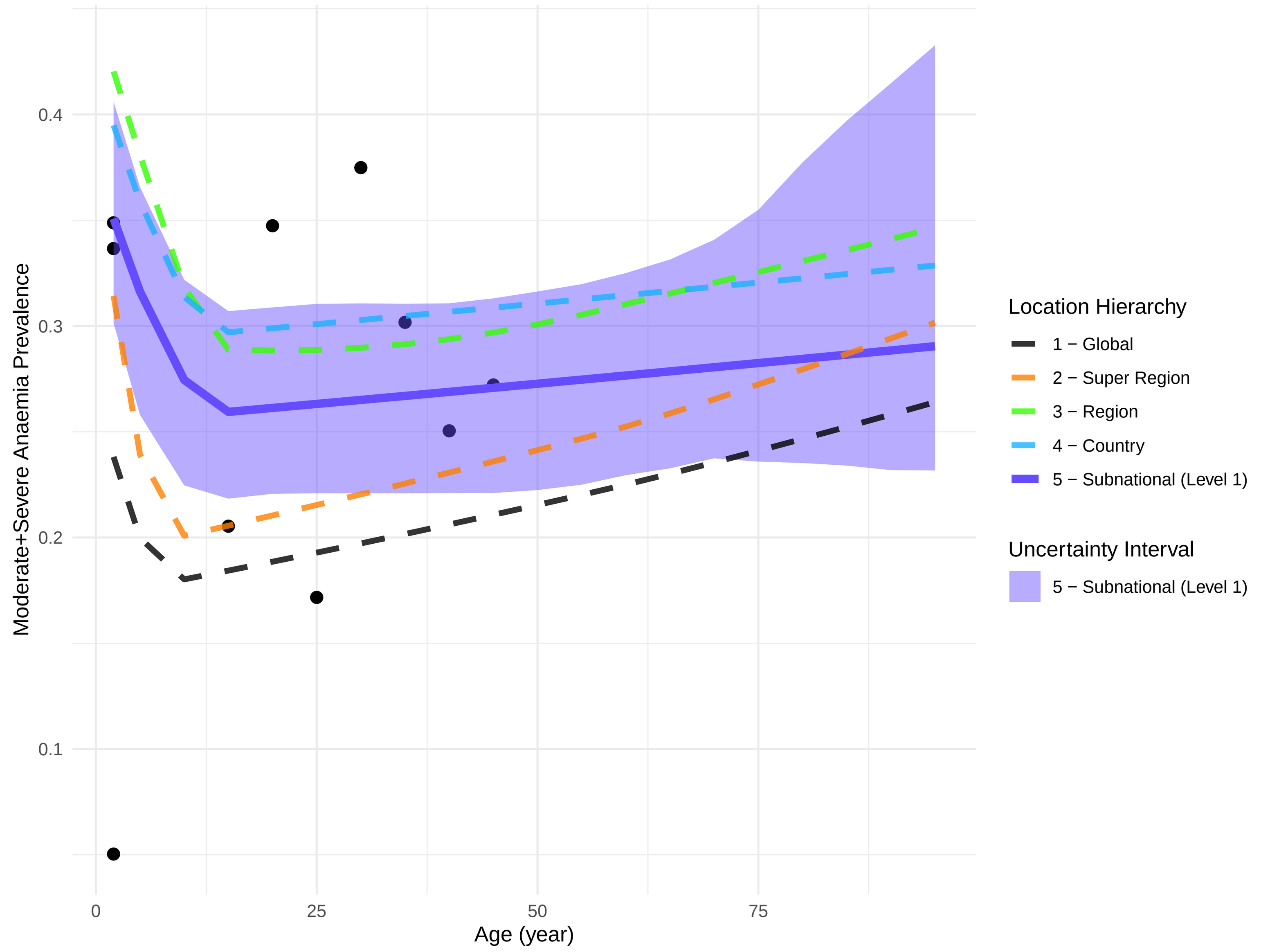

MR−BRT Age/Sex Fits − Non−pregnant Females 2+ Years Old − Moderate+Severe Anaemia Prevalence − Kwara
WHO−2001 Adjustment Method

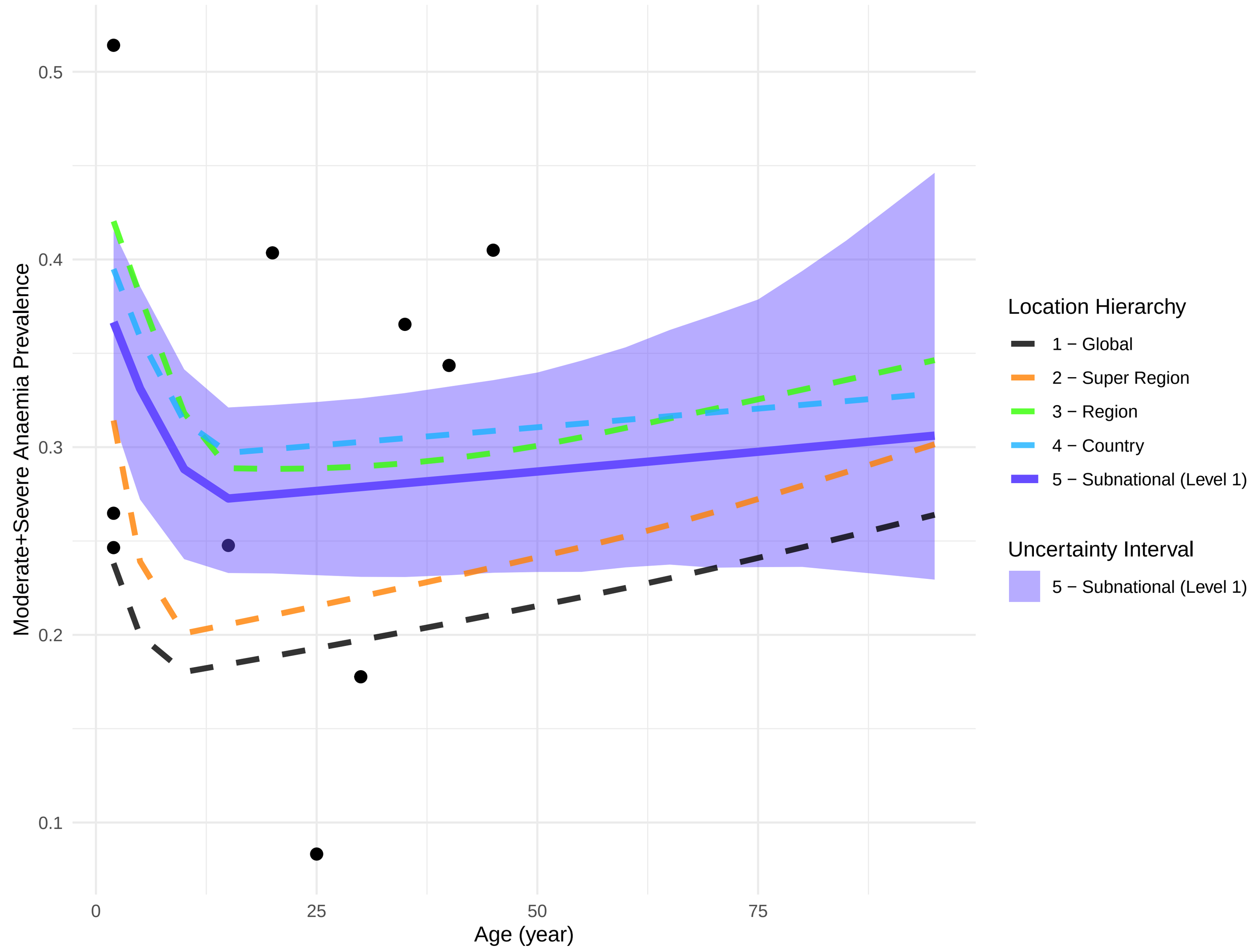

MR−BRT Age/Sex Fits − Non−pregnant Females 2+ Years Old − Moderate+Severe Anaemia Prevalence − Lagos
WHO−2001 Adjustment Method

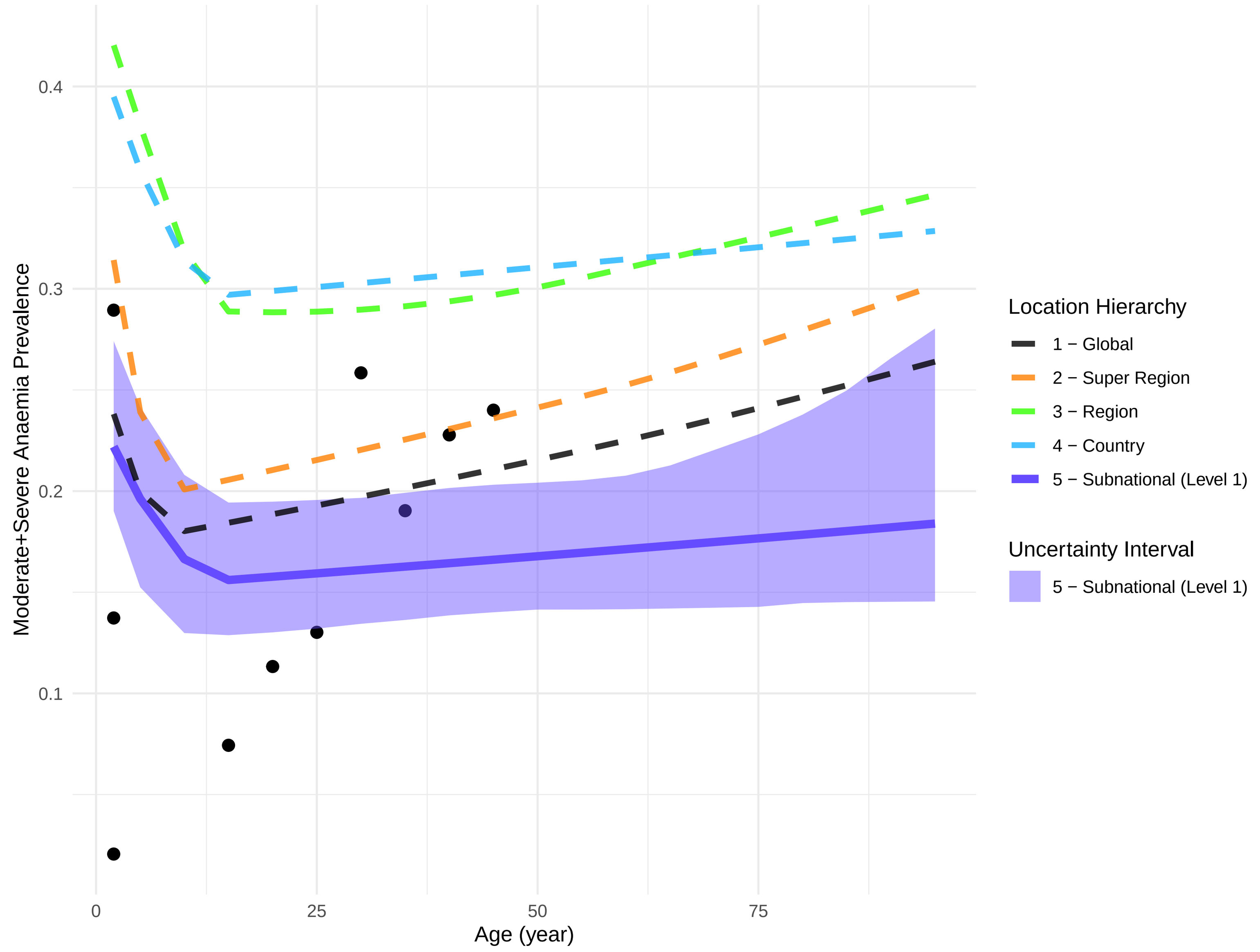

MR−BRT Age/Sex Fits − Non−pregnant Females 2+ Years Old − Moderate+Severe Anaemia Prevalence − Nasarawa
WHO−2001 Adjustment Method

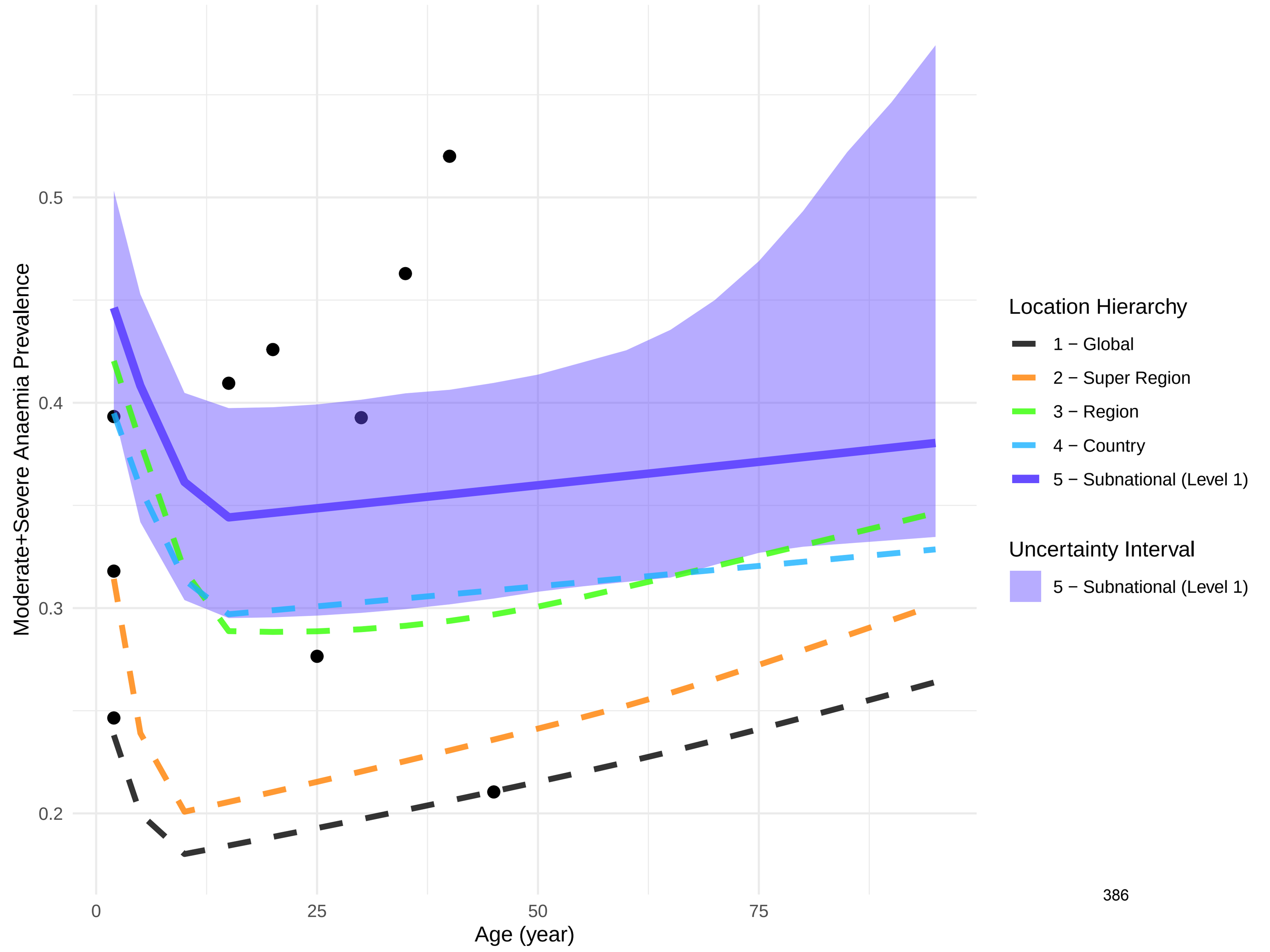

MR−BRT Age/Sex Fits − Non−pregnant Females 2+ Years Old − Moderate+Severe Anaemia Prevalence − Niger
WHO−2001 Adjustment Method

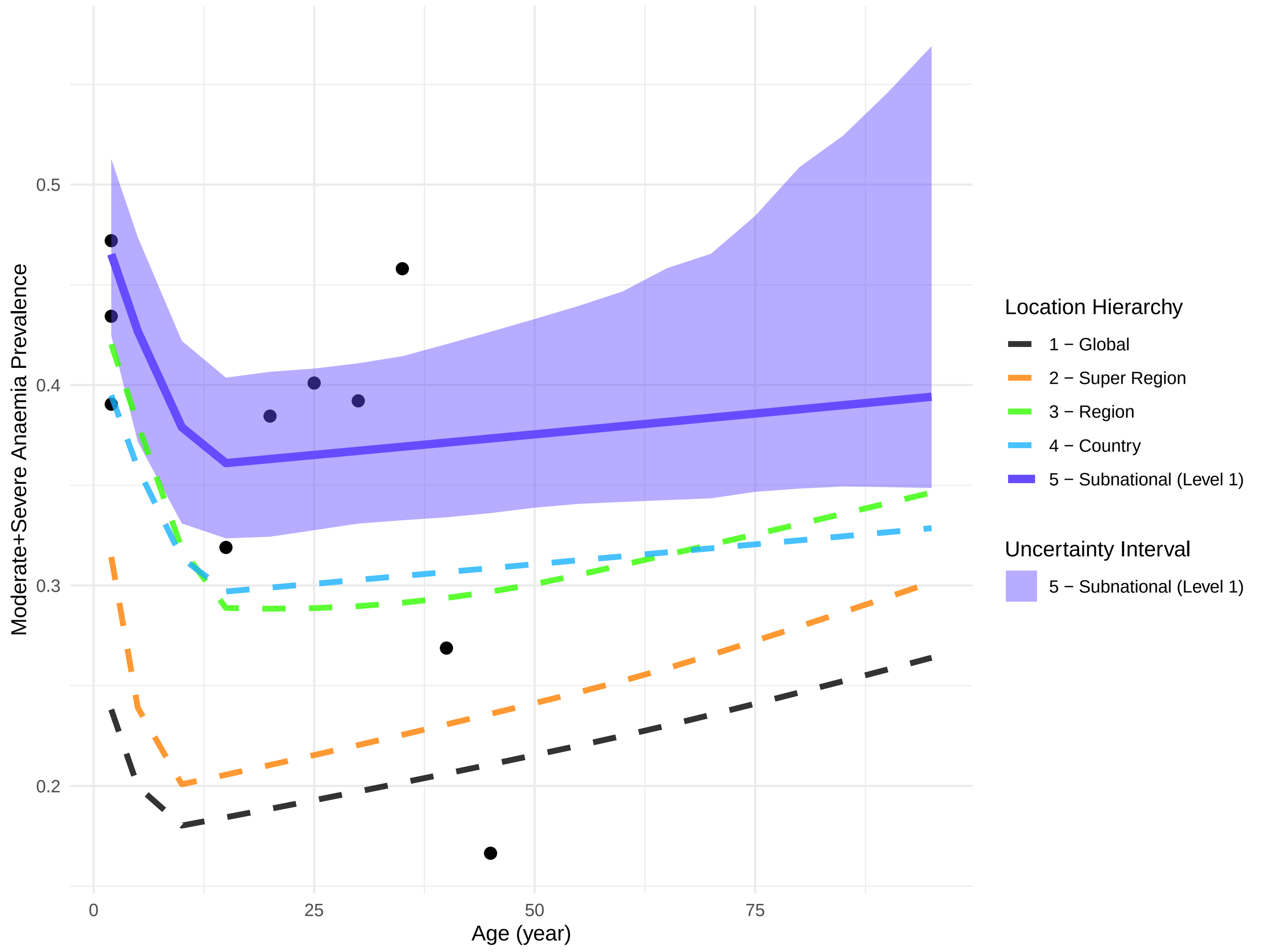

MR−BRT Age/Sex Fits − Non−pregnant Females 2+ Years Old − Moderate+Severe Anaemia Prevalence − Ogun
WHO−2001 Adjustment Method

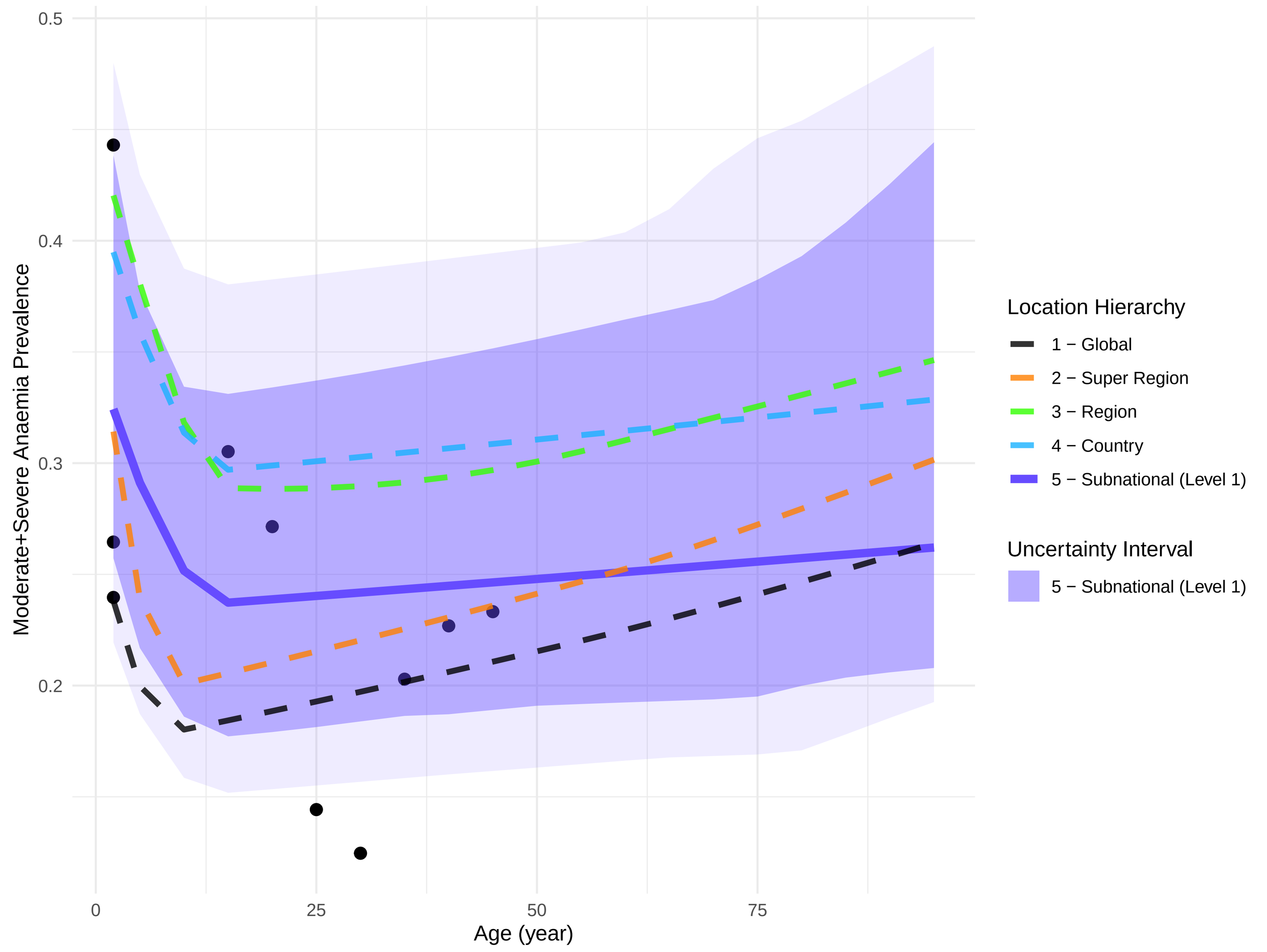

MR−BRT Age/Sex Fits − Non−pregnant Females 2+ Years Old − Moderate+Severe Anaemia Prevalence − Ondo
WHO−2001 Adjustment Method

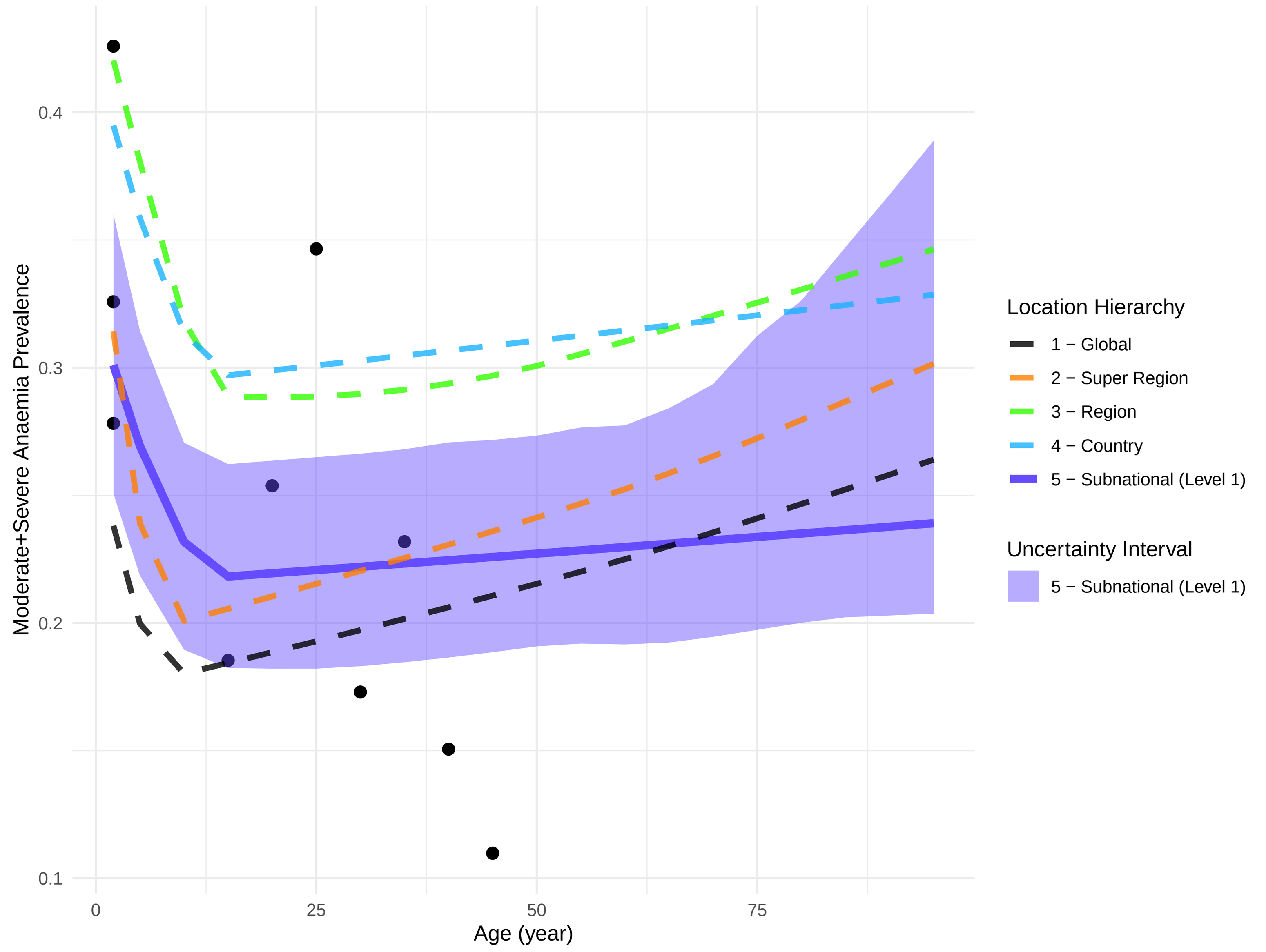

MR−BRT Age/Sex Fits − Non−pregnant Females 2+ Years Old − Moderate+Severe Anaemia Prevalence − Osun
WHO−2001 Adjustment Method

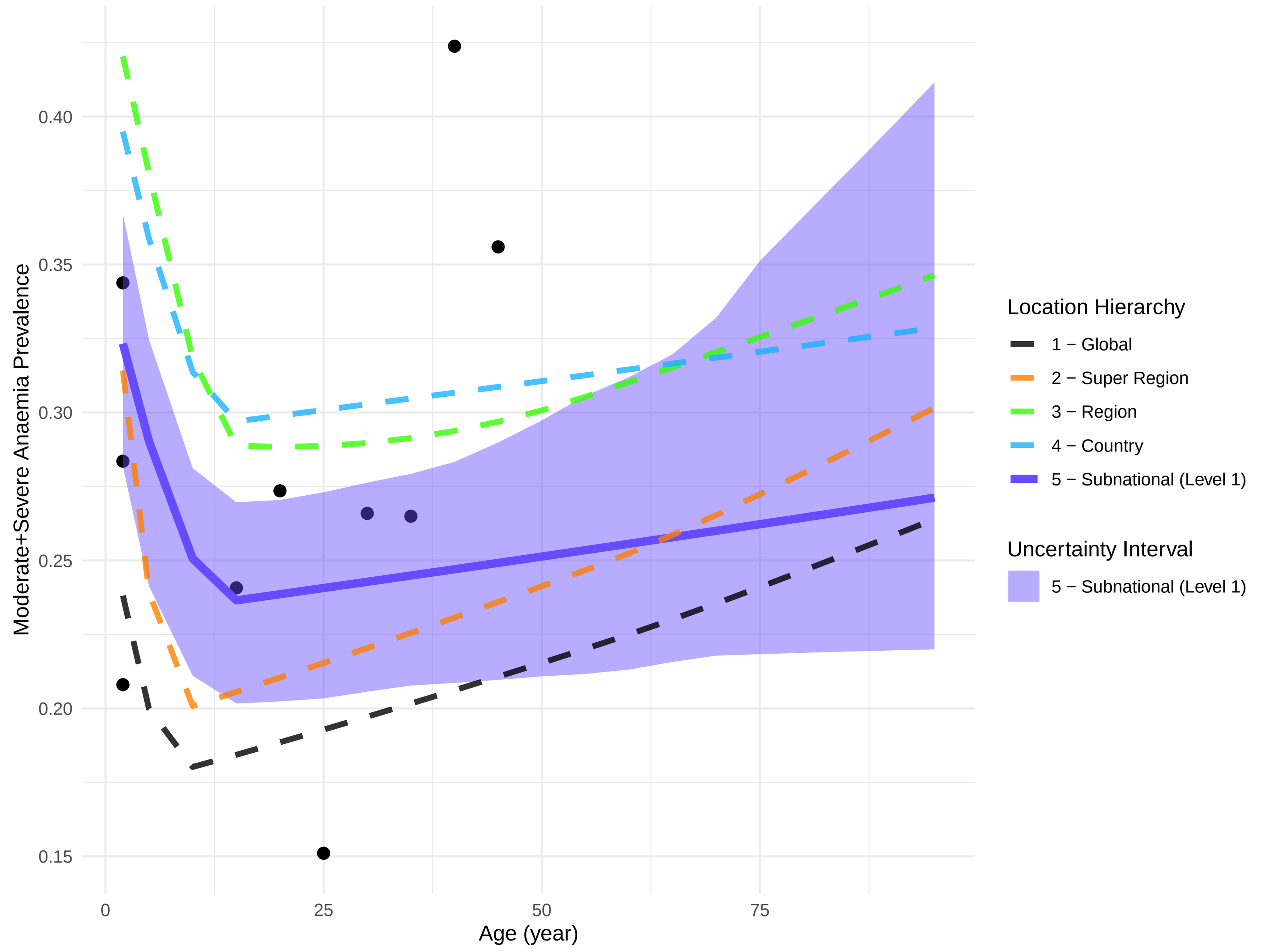

MR−BRT Age/Sex Fits − Non−pregnant Females 2+ Years Old − Moderate+Severe Anaemia Prevalence − Oyo
WHO−2001 Adjustment Method

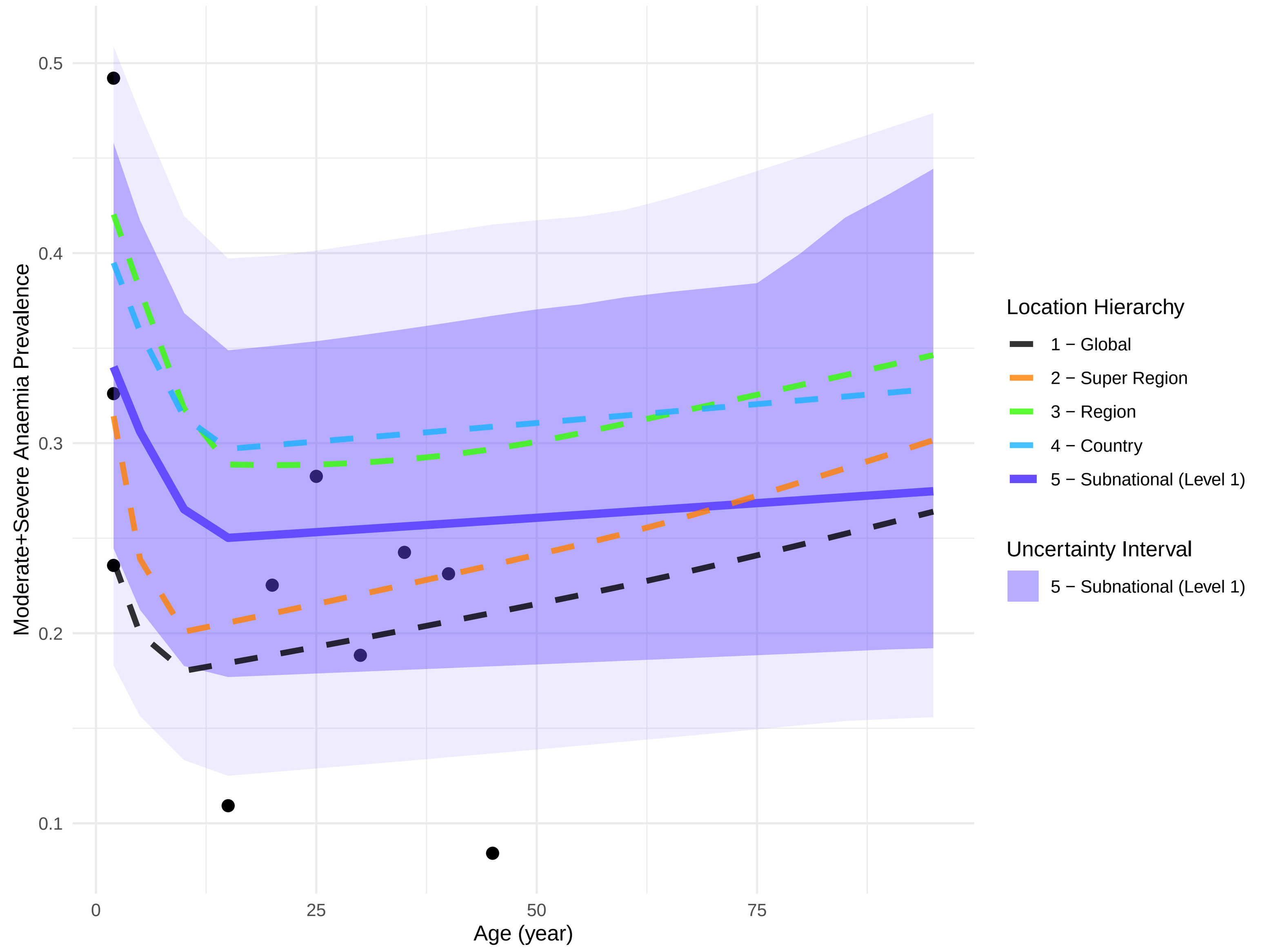

MR−BRT Age/Sex Fits − Non−pregnant Females 2+ Years Old − Moderate+Severe Anaemia Prevalence − Plateau
WHO−2001 Adjustment Method

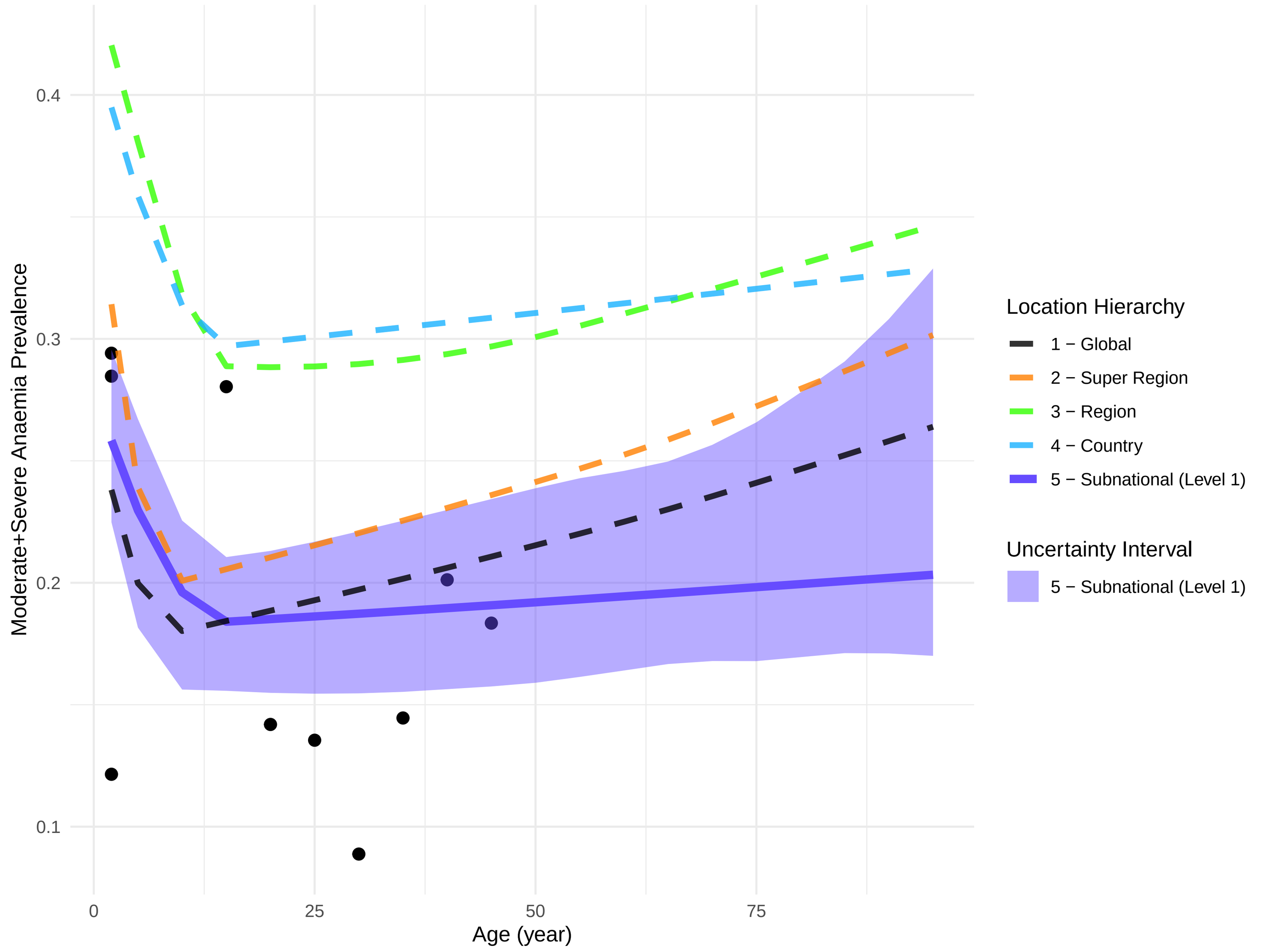

MR−BRT Age/Sex Fits − Non−pregnant Females 2+ Years Old − Moderate+Severe Anaemia Prevalence − Rivers
WHO−2001 Adjustment Method

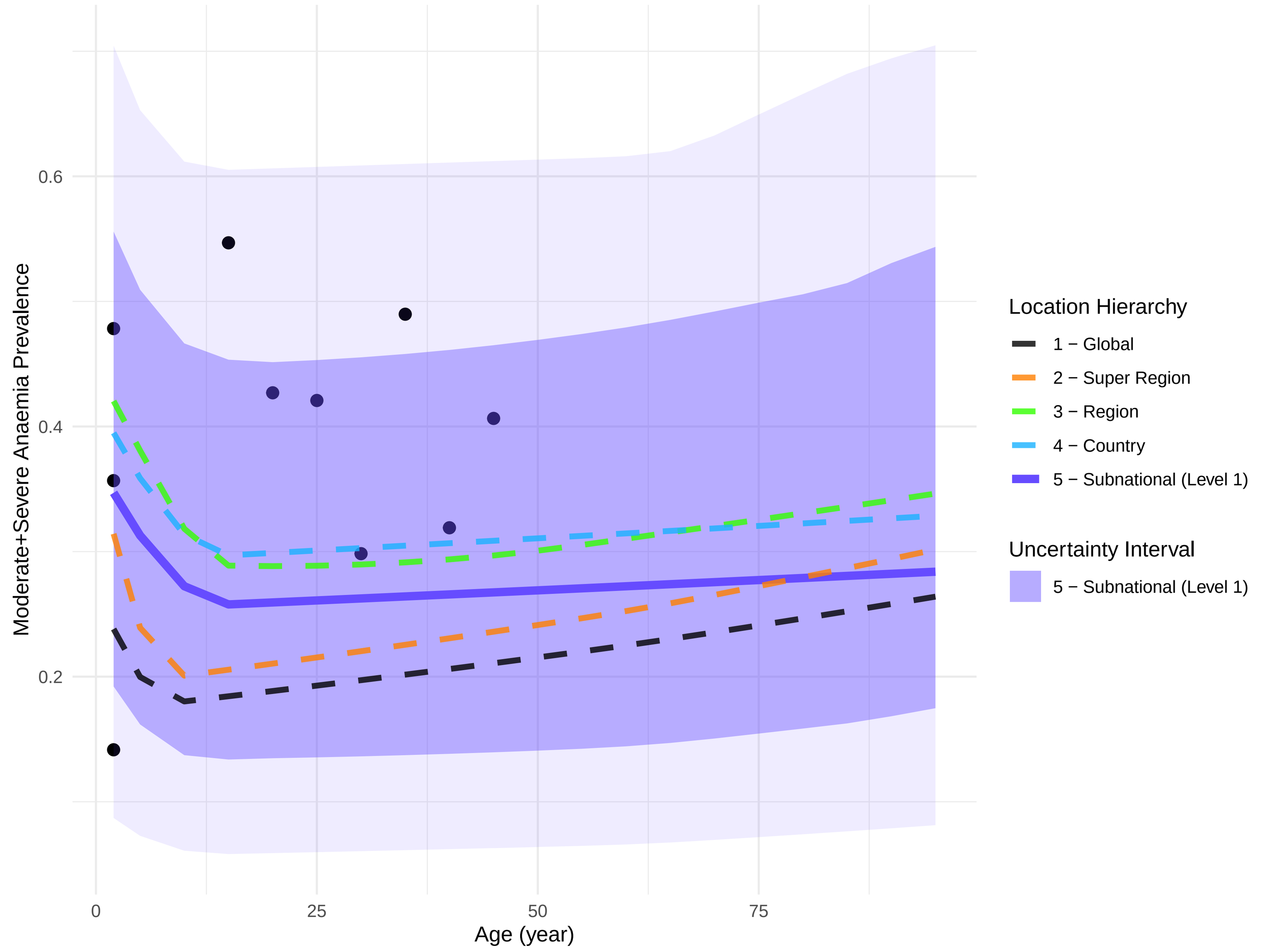

MR−BRT Age/Sex Fits − Non−pregnant Females 2+ Years Old − Moderate+Severe Anaemia Prevalence − Sokoto
WHO−2001 Adjustment Method

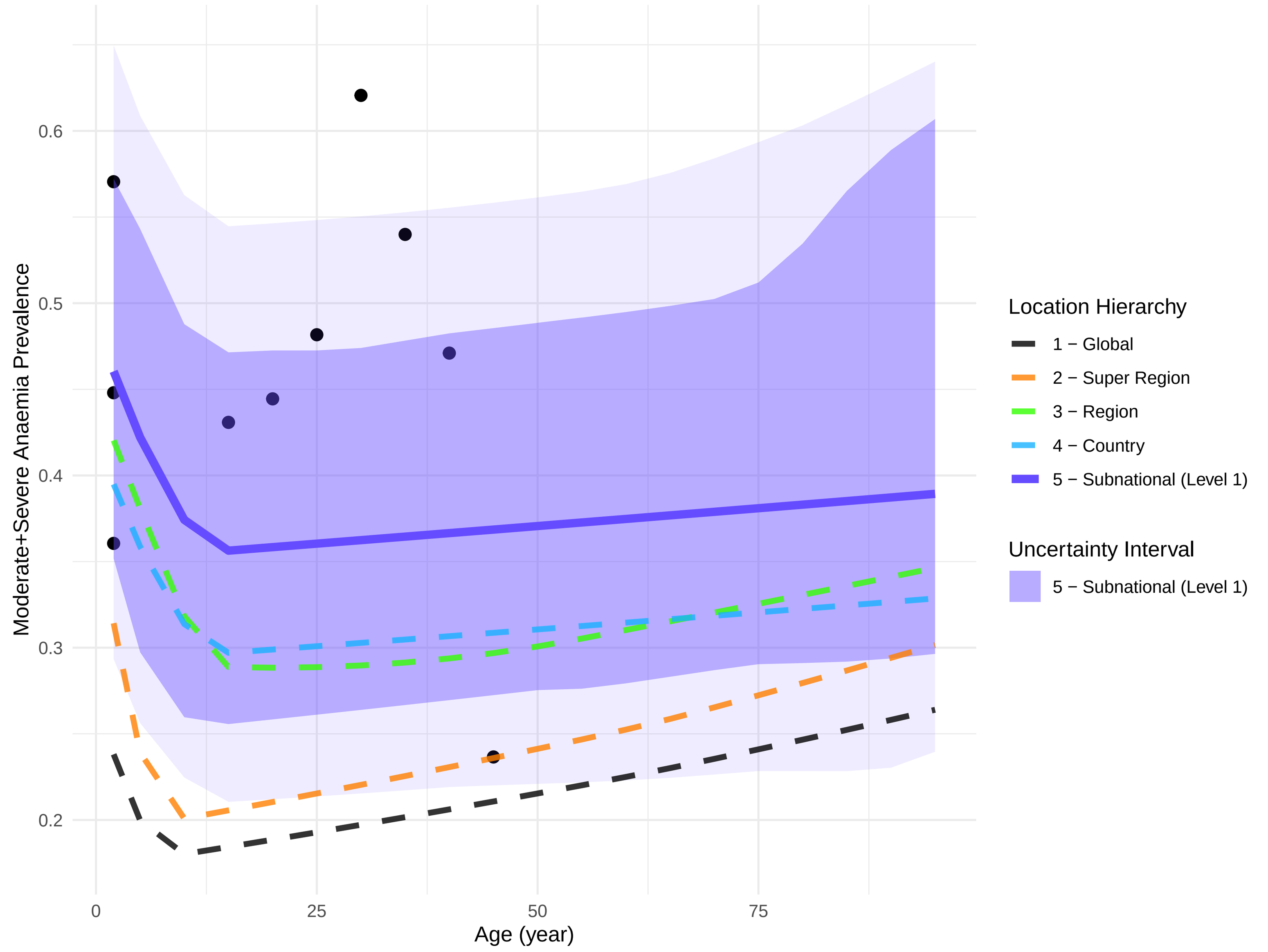

MR−BRT Age/Sex Fits − Non−pregnant Females 2+ Years Old − Moderate+Severe Anaemia Prevalence − Taraba
WHO−2001 Adjustment Method

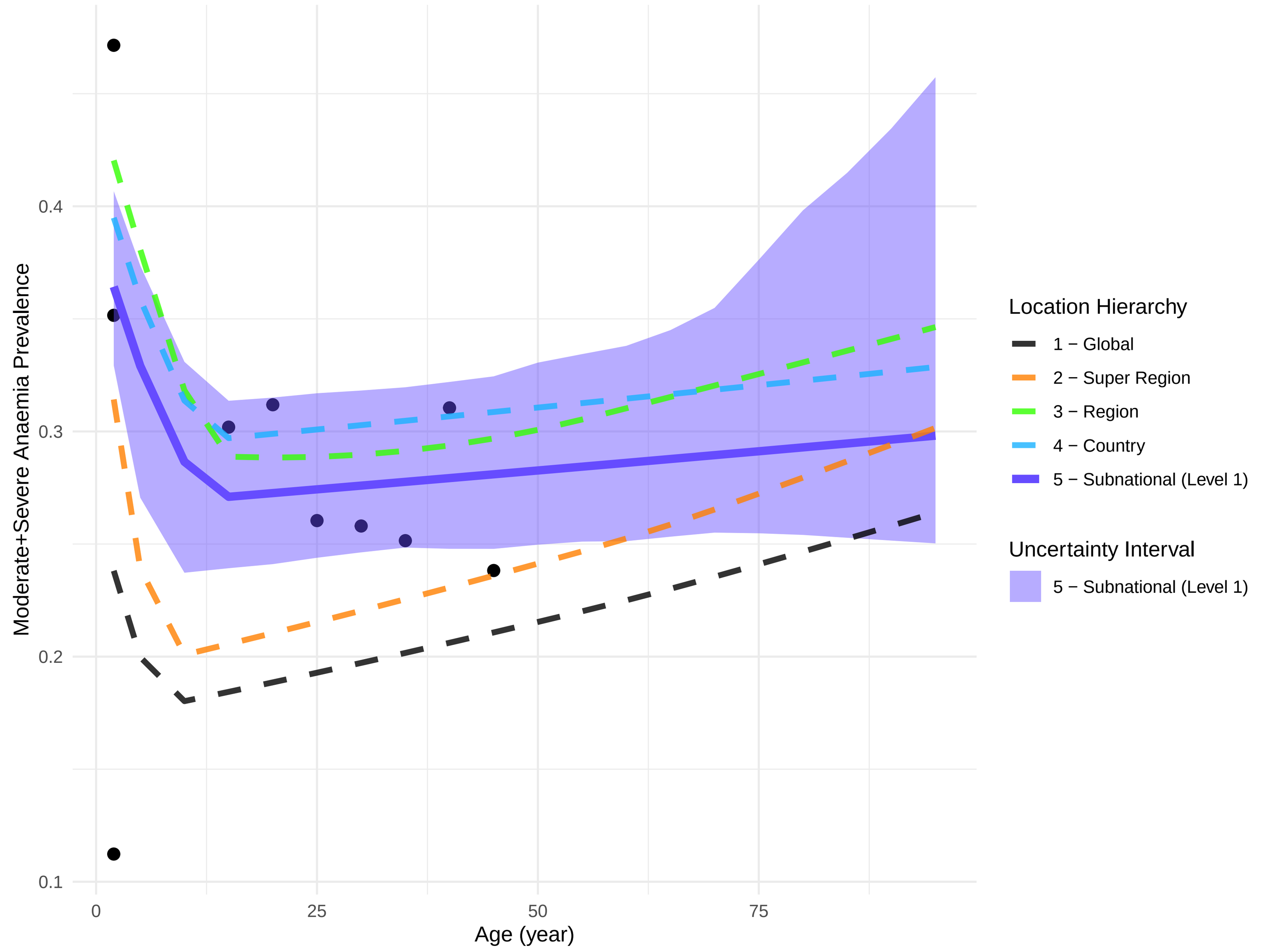

MR−BRT Age/Sex Fits − Non−pregnant Females 2+ Years Old − Moderate+Severe Anaemia Prevalence − Yobe
WHO−2001 Adjustment Method

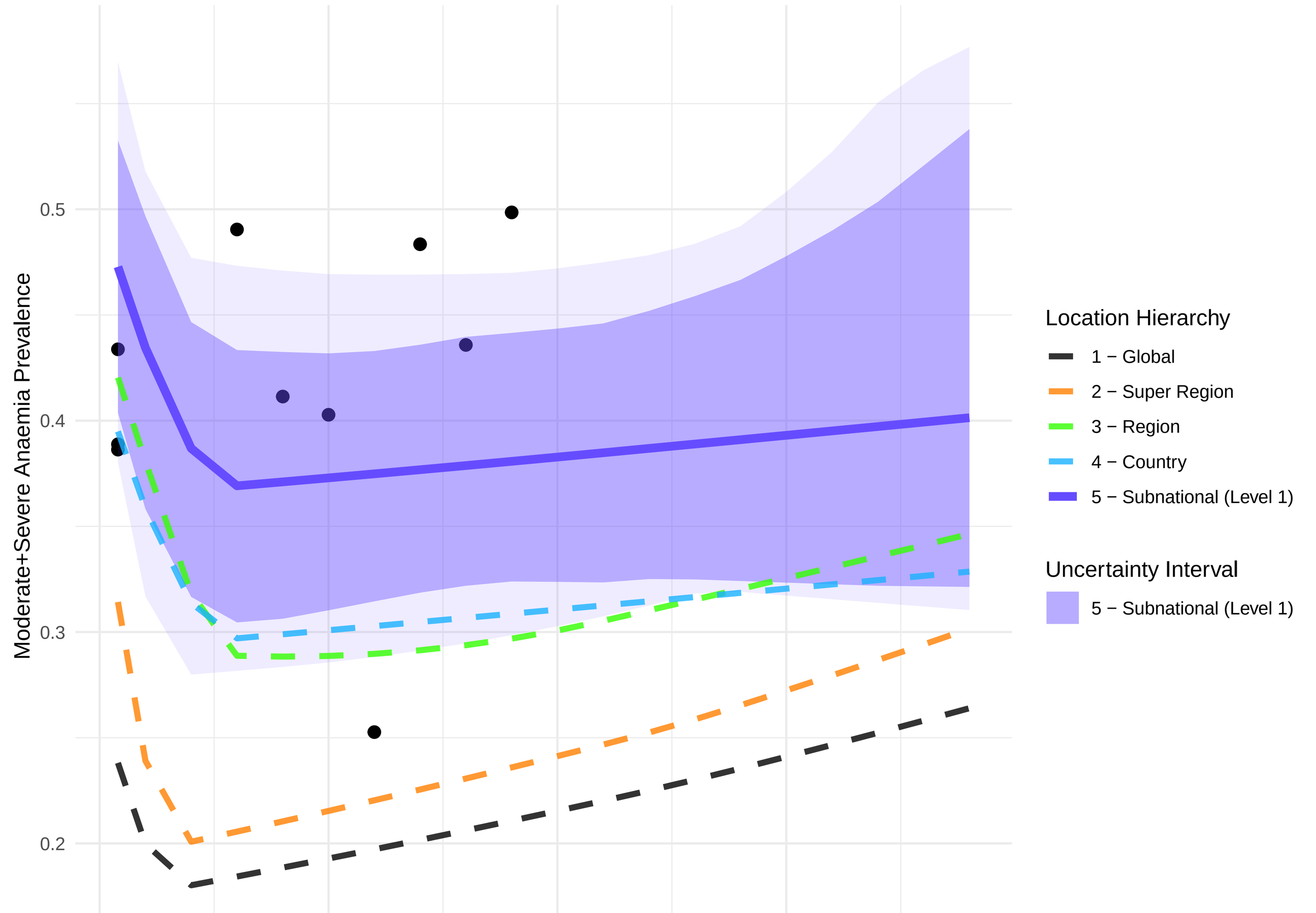

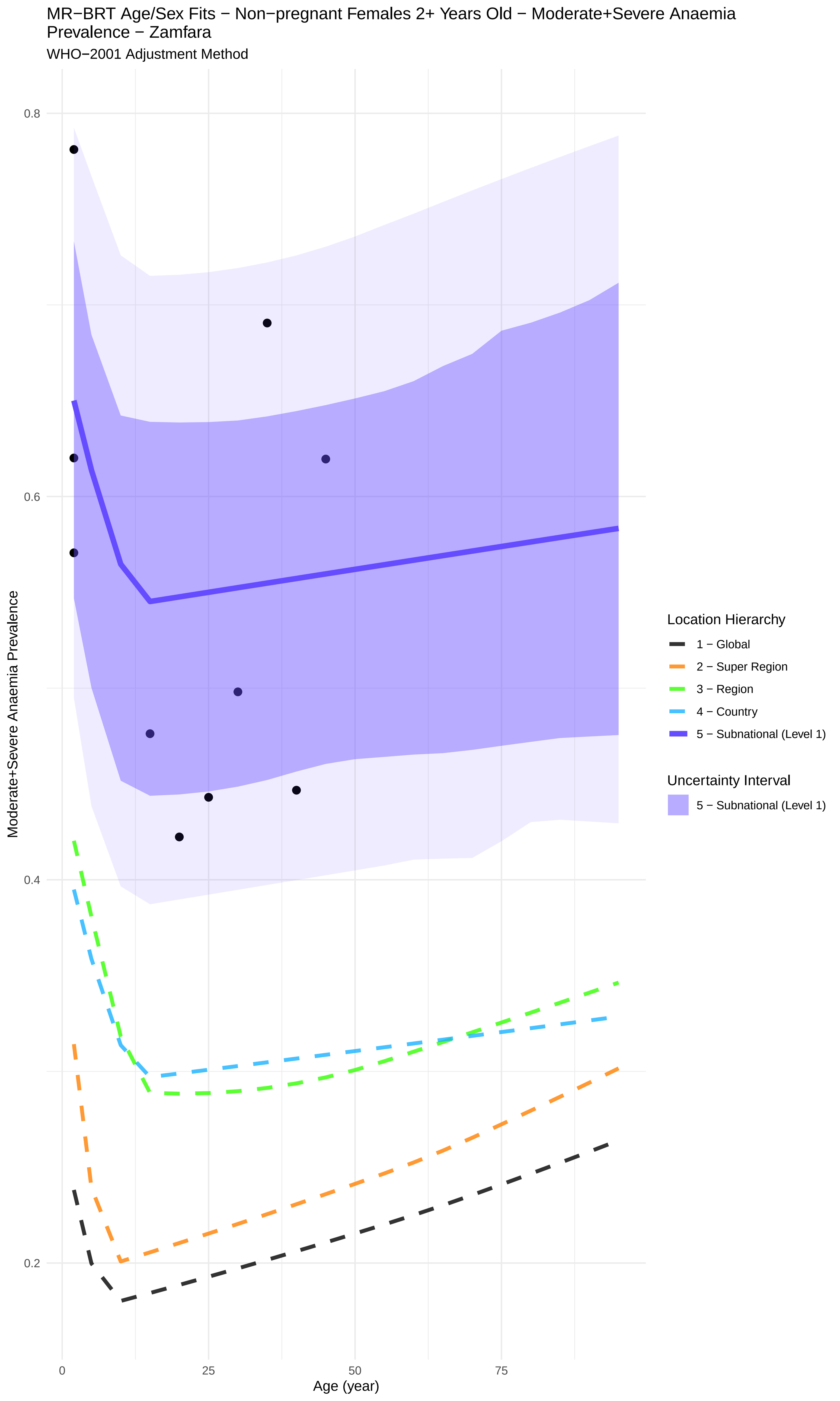
MR−BRT Age/Sex Fits − Non−pregnant Females 2+ Years Old − Moderate+Severe Anaemia Prevalence − Zamfara
WHO−2001 Adjustment Method
Moderate+Severe Anaemia Prevalence
Age (year)
Location Hierarchy
1 − Global
2 − Super Region
3 − Region
4 − Country
5 − Subnational (Level 1)
Uncertainty Interval
5 − Subnational (Level 1)

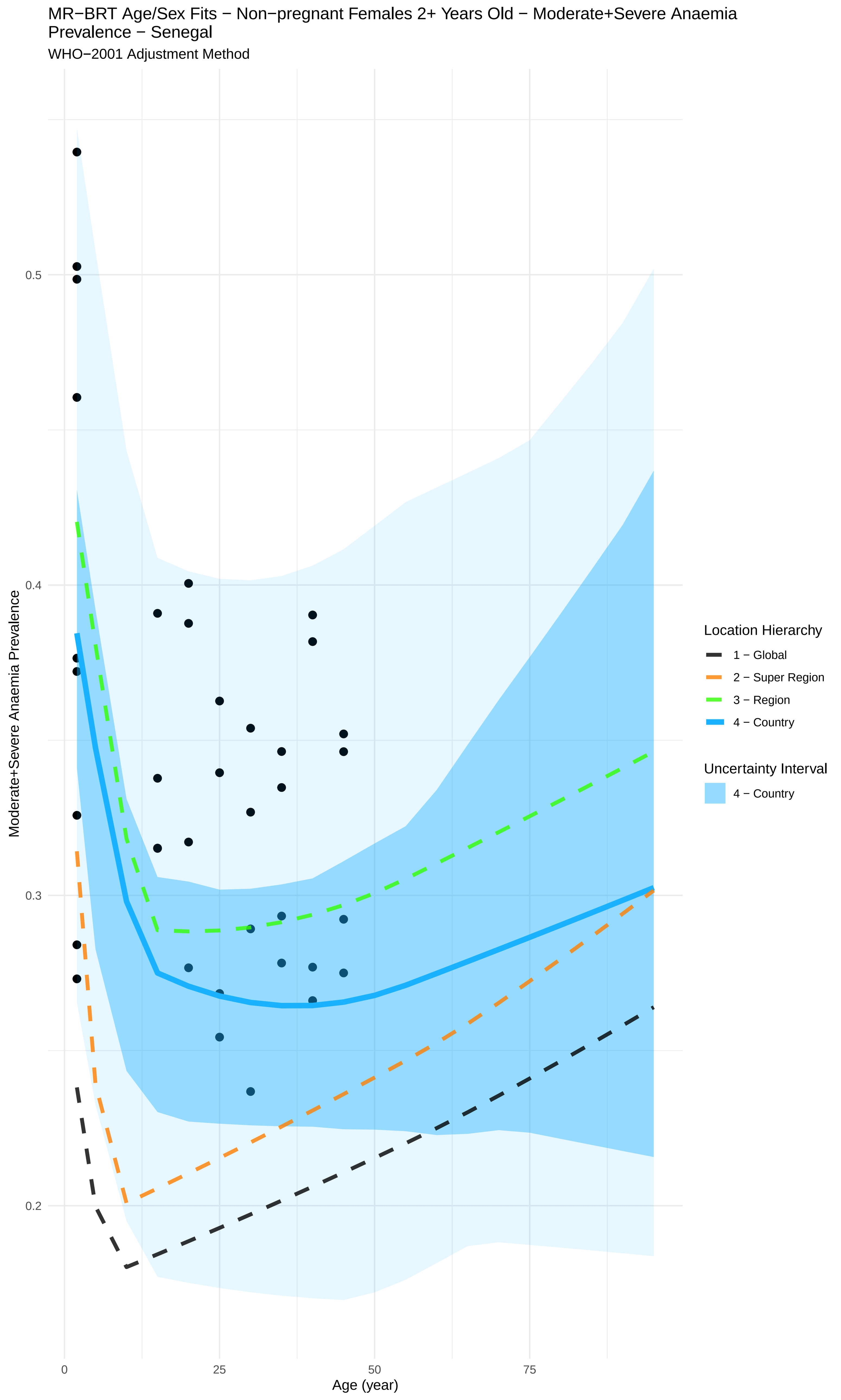
MR−BRT Age/Sex Fits − Non−pregnant Females 2+ Years Old − Moderate+Severe Anaemia Prevalence − Senegal
WHO−2001 Adjustment Method
Moderate+Severe Anaemia Prevalence
Age (year)
Location Hierarchy
1 − Global
2 − Super Region
3 − Region
4 − Country
Uncertainty Interval
4 − Country

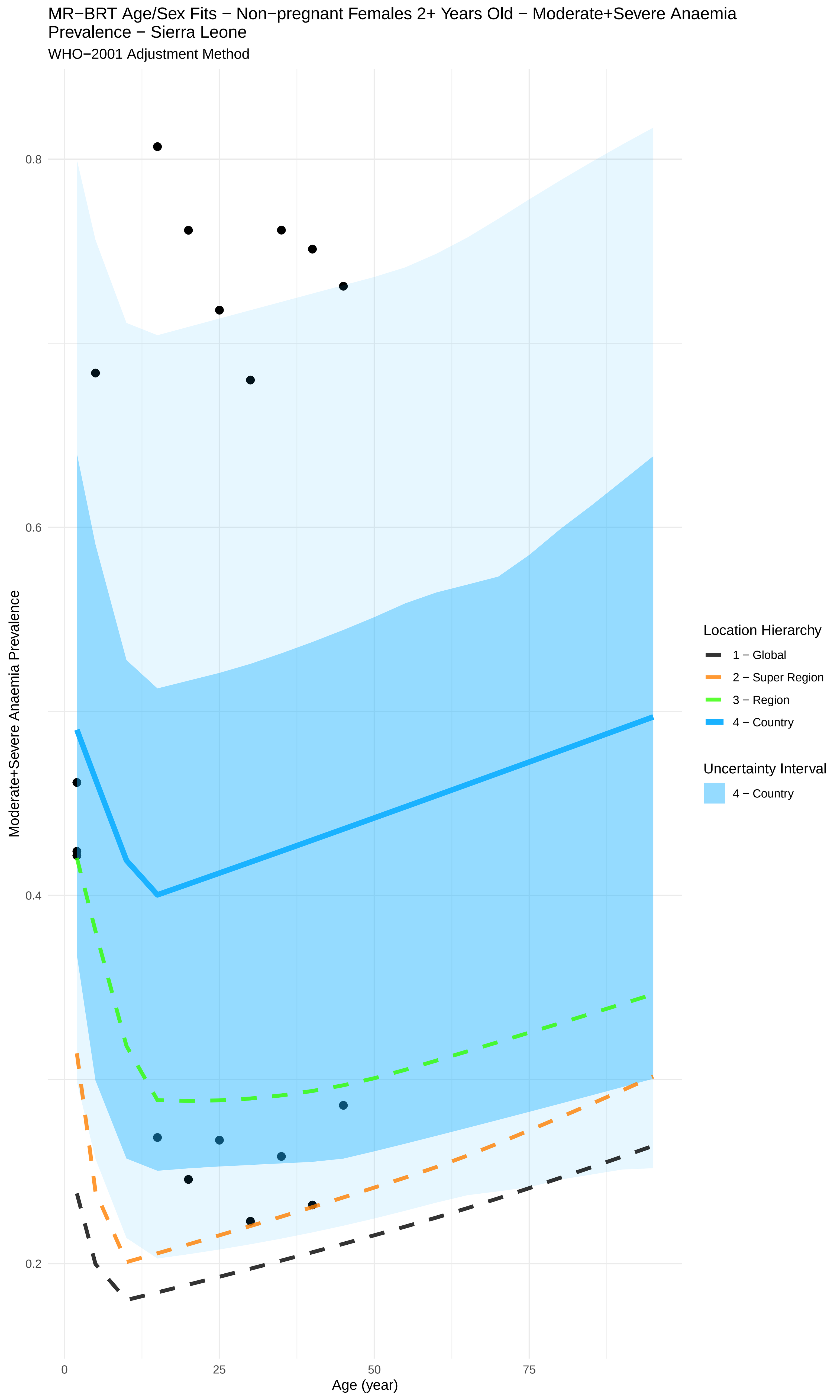
MR−BRT Age/Sex Fits − Non−pregnant Females 2+ Years Old − Moderate+Severe Anaemia Prevalence − Sierra Leone
WHO−2001 Adjustment Method
Moderate+Severe Anaemia Prevalence
Age (year)
Location Hierarchy
1 − Global
2 − Super Region
3 − Region
4 − Country
Uncertainty Interval
4 − Country

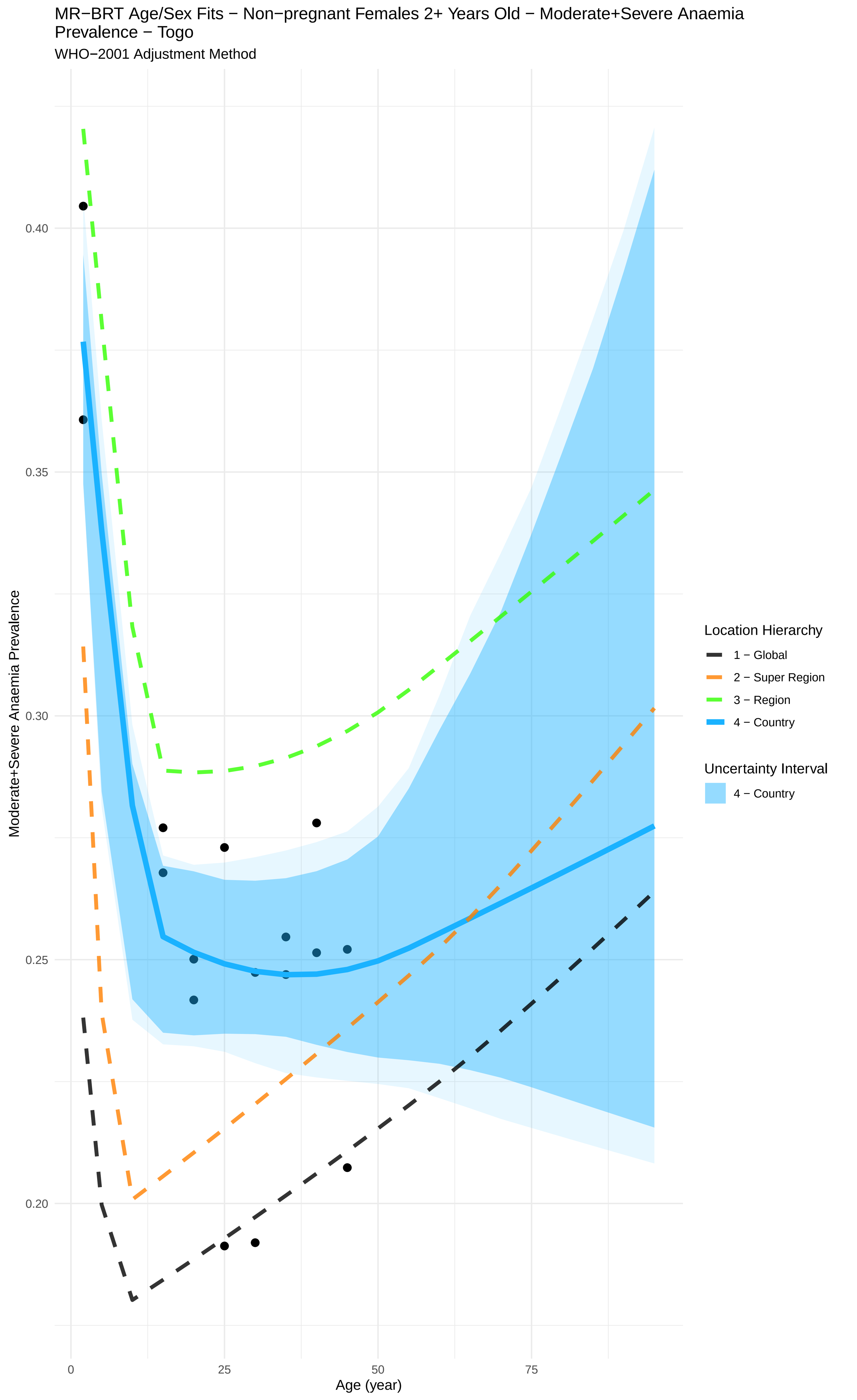
MR−BRT Age/Sex Fits − Non−pregnant Females 2+ Years Old − Moderate+Severe Anaemia Prevalence − Togo
WHO−2001 Adjustment Method
Moderate+Severe Anaemia Prevalence
Age (year)
Location Hierarchy
1 − Global
2 − Super Region
3 − Region
4 − Country
Uncertainty Interval
4 − Country

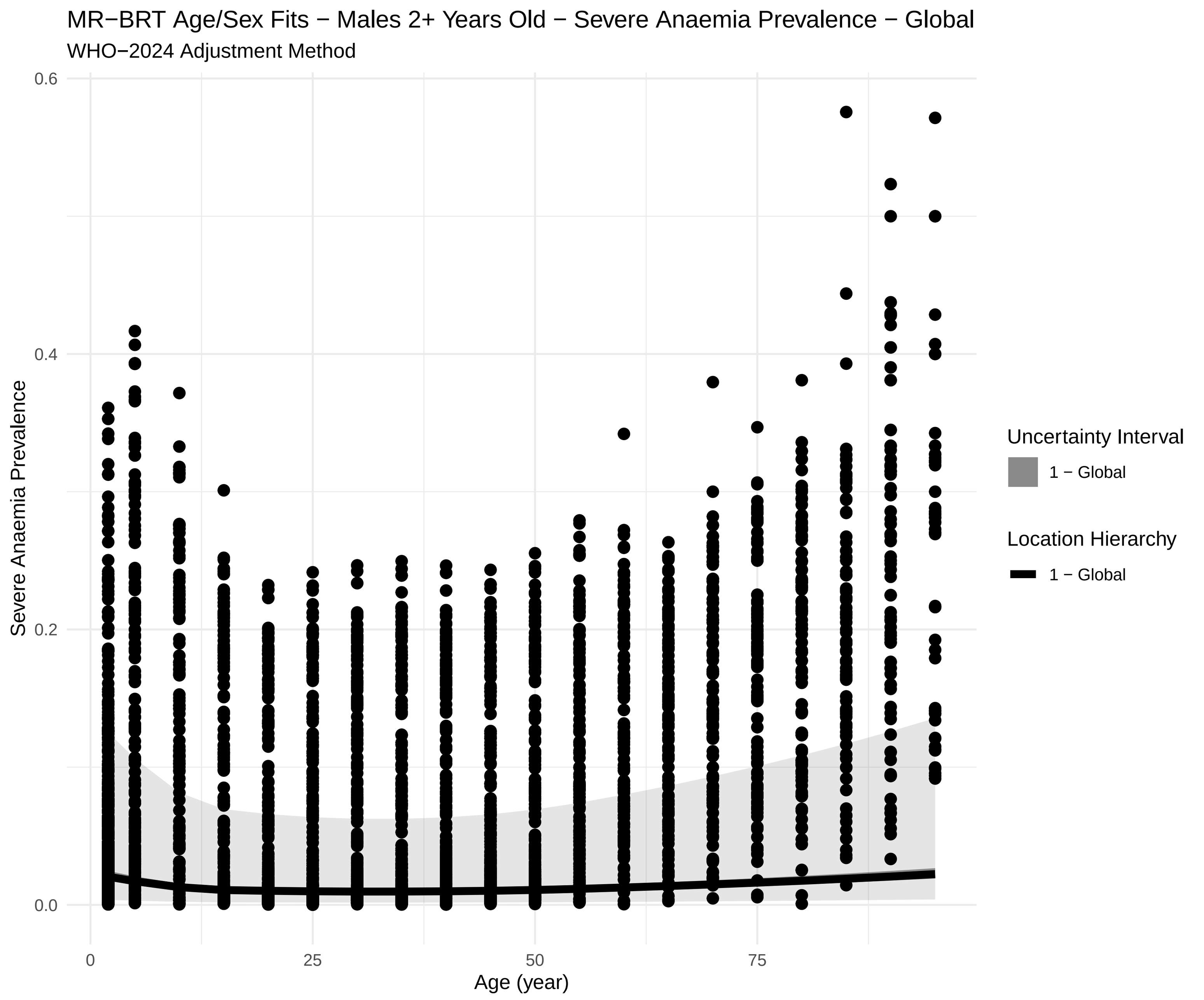
MR−BRT Age/Sex Fits − Males 2+ Years Old − Severe Anaemia Prevalence − Global
WHO−2024 Adjustment Method
Severe Anaemia Prevalence
Age (year)
Uncertainty Interval
1 − Global
Location Hierarchy
1 − Global

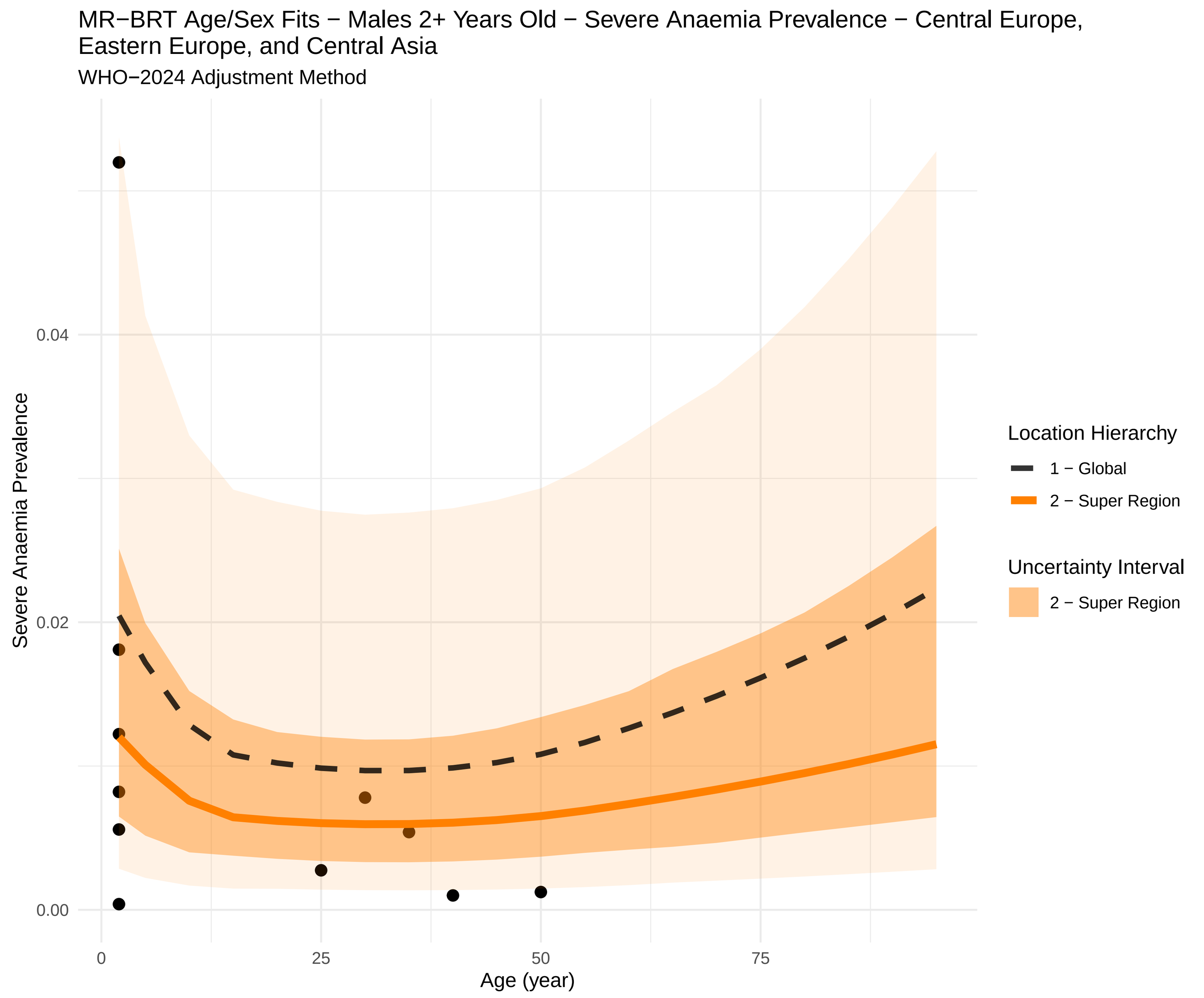
MR−BRT Age/Sex Fits − Males 2+ Years Old − Severe Anaemia Prevalence − Central Europe, Eastern Europe, and Central Asia
WHO−2024 Adjustment Method
Severe Anaemia Prevalence
Age (year)
Location Hierarchy
1 − Global
2 − Super Region
Uncertainty Interval
2 − Super Region

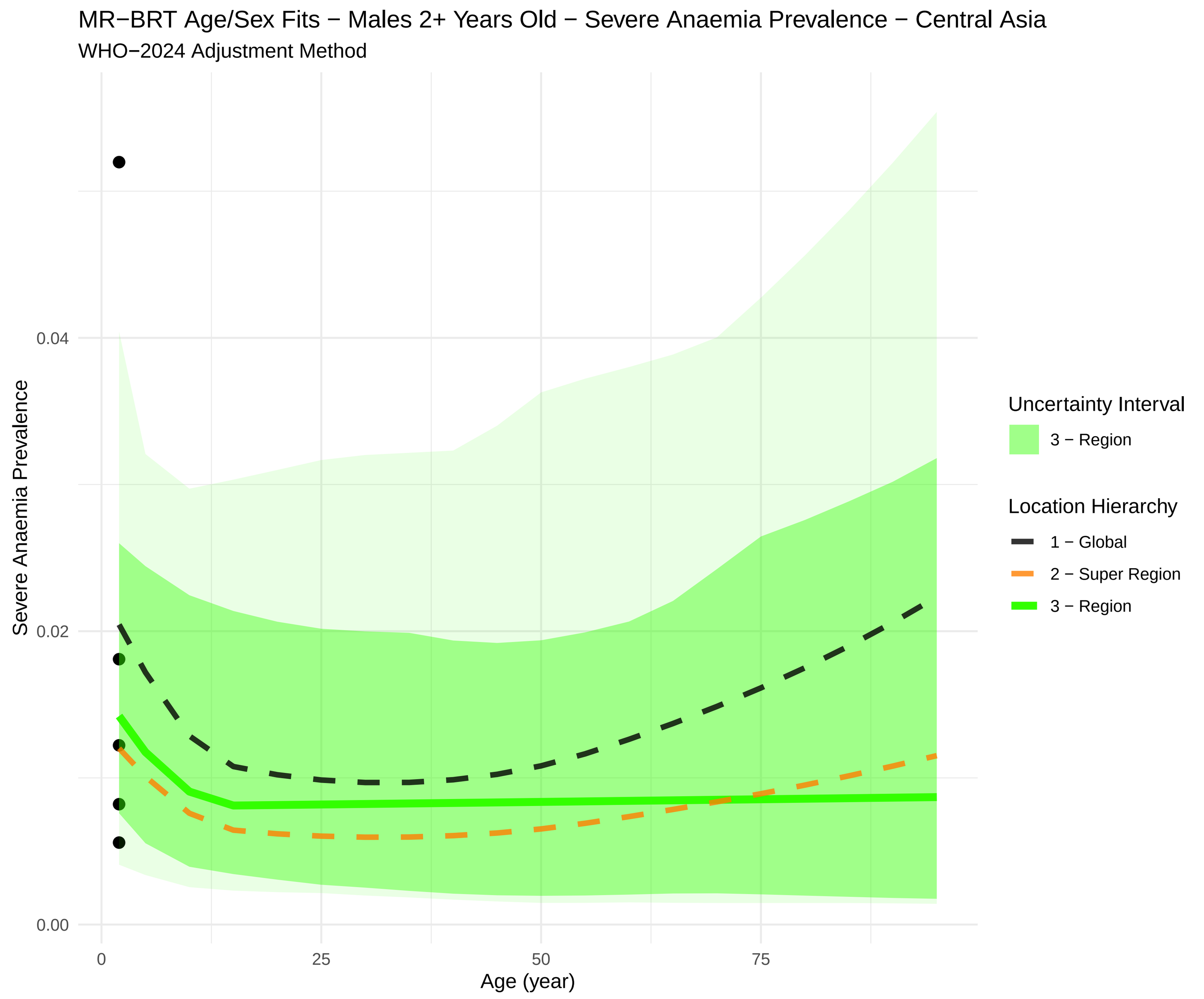
MR−BRT Age/Sex Fits − Males 2+ Years Old − Severe Anaemia Prevalence − Central Asia
WHO−2024 Adjustment Method
Severe Anaemia Prevalence
Age (year)
Uncertainty Interval
3 − Region
Location Hierarchy
1 − Global
2 − Super Region
3 − Region

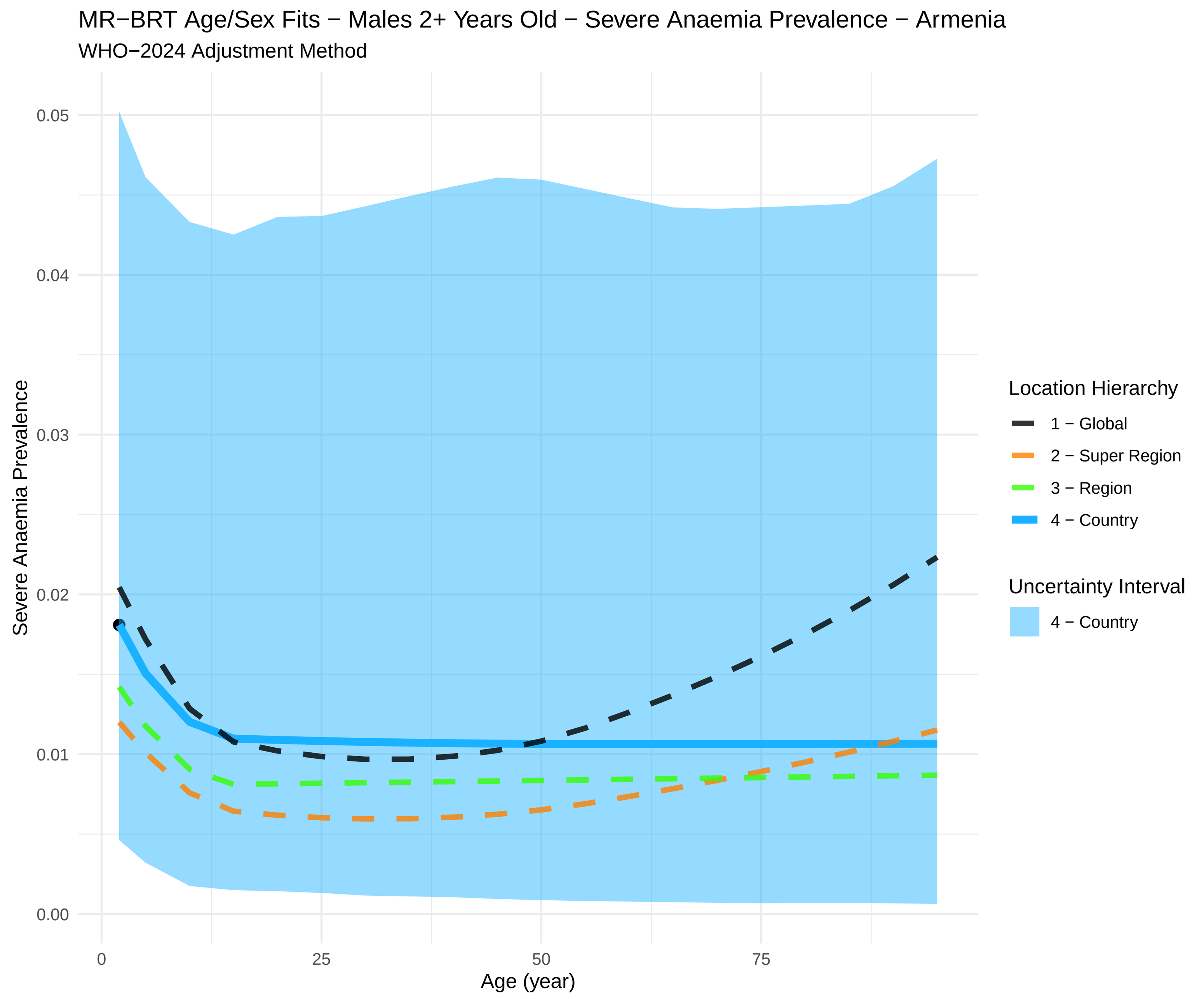
MR−BRT Age/Sex Fits − Males 2+ Years Old − Severe Anaemia Prevalence − Armenia
WHO−2024 Adjustment Method
Severe Anaemia Prevalence
Age (year)
Location Hierarchy
1 − Global
2 − Super Region
3 − Region
4 − Country
Uncertainty Interval
4 − Country

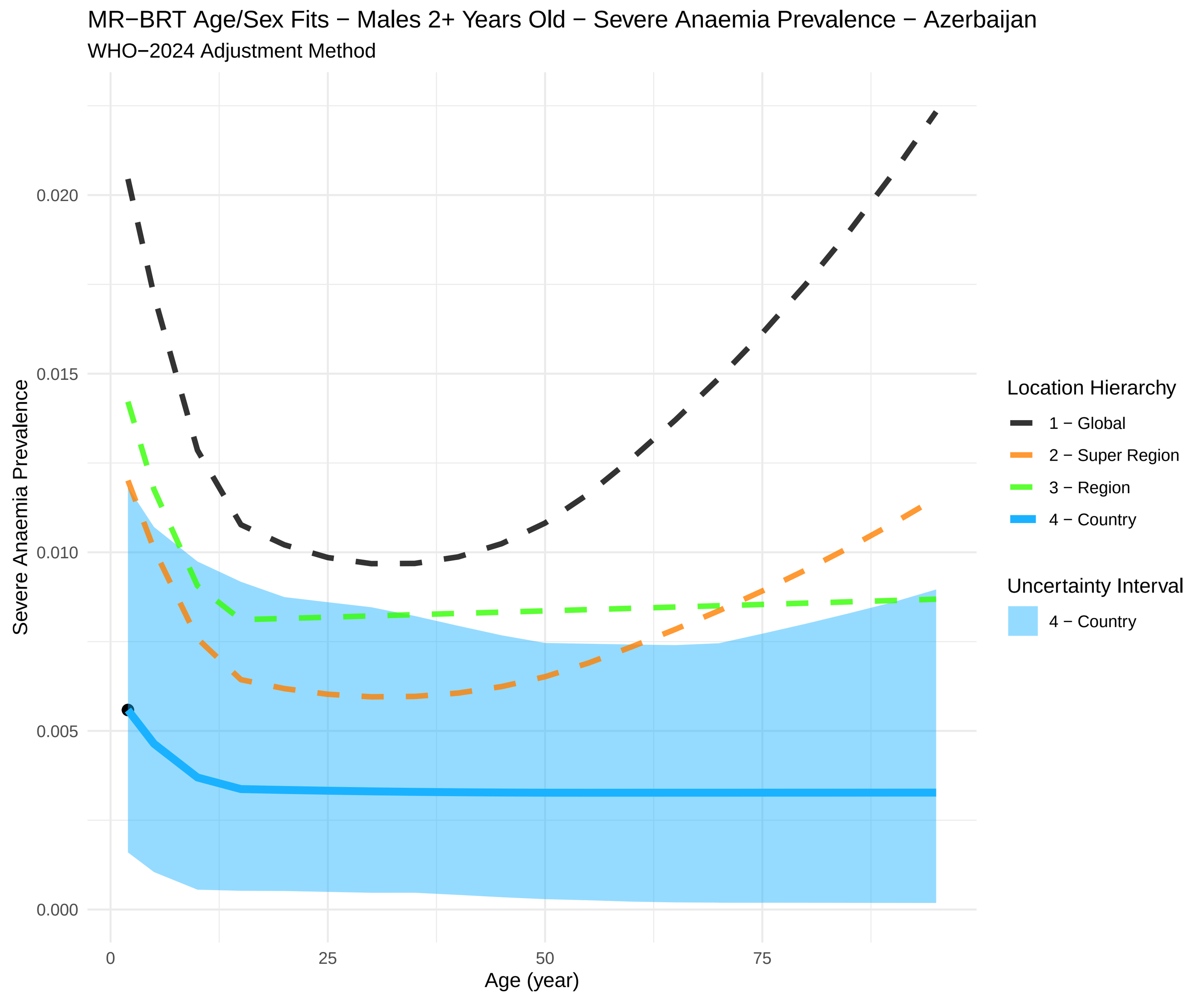
MR−BRT Age/Sex Fits − Males 2+ Years Old − Severe Anaemia Prevalence − Azerbaijan
WHO−2024 Adjustment Method
Severe Anaemia Prevalence
Age (year)
Location Hierarchy
1 − Global
2 − Super Region
3 − Region
4 − Country
Uncertainty Interval
4 − Country

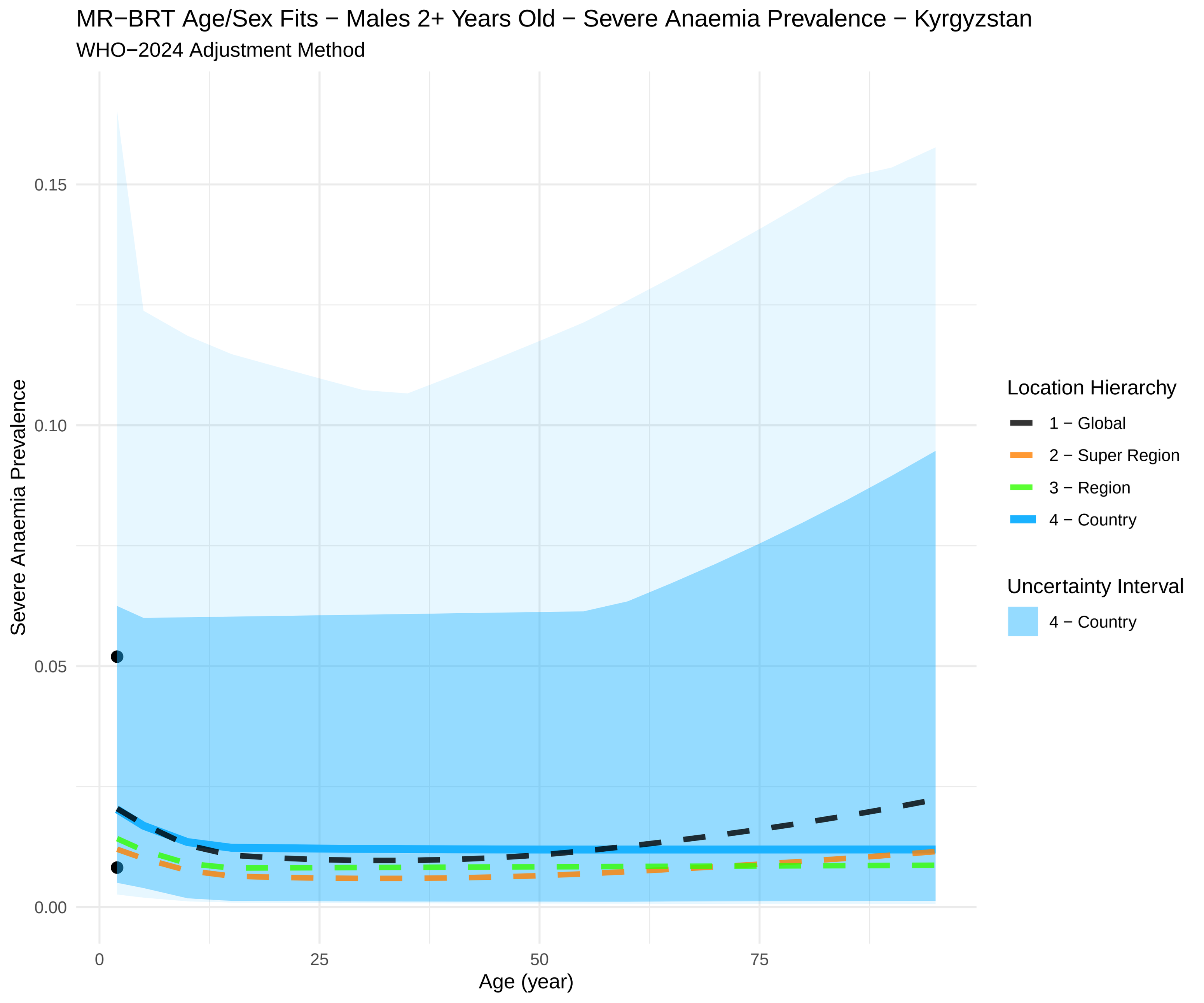
MR−BRT Age/Sex Fits − Males 2+ Years Old − Severe Anaemia Prevalence − Kyrgyzstan
WHO−2024 Adjustment Method
Severe Anaemia Prevalence
Age (year)
Location Hierarchy
1 − Global
2 − Super Region
3 − Region
4 − Country
Uncertainty Interval
4 − Country

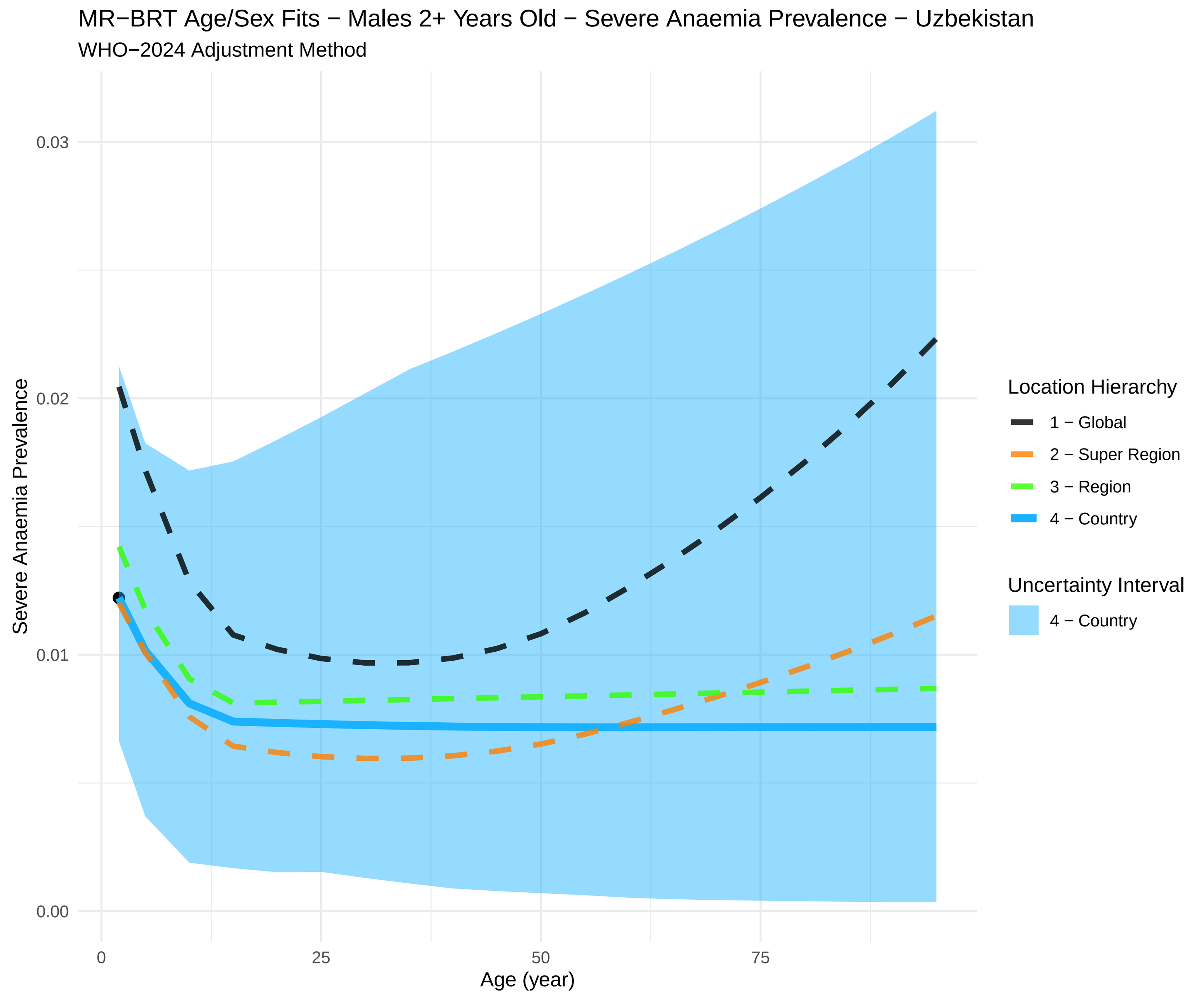
MR−BRT Age/Sex Fits − Males 2+ Years Old − Severe Anaemia Prevalence − Uzbekistan
WHO−2024 Adjustment Method
Severe Anaemia Prevalence
Age (year)
Location Hierarchy
1 − Global
2 − Super Region
3 − Region
4 − Country
Uncertainty Interval
4 − Country

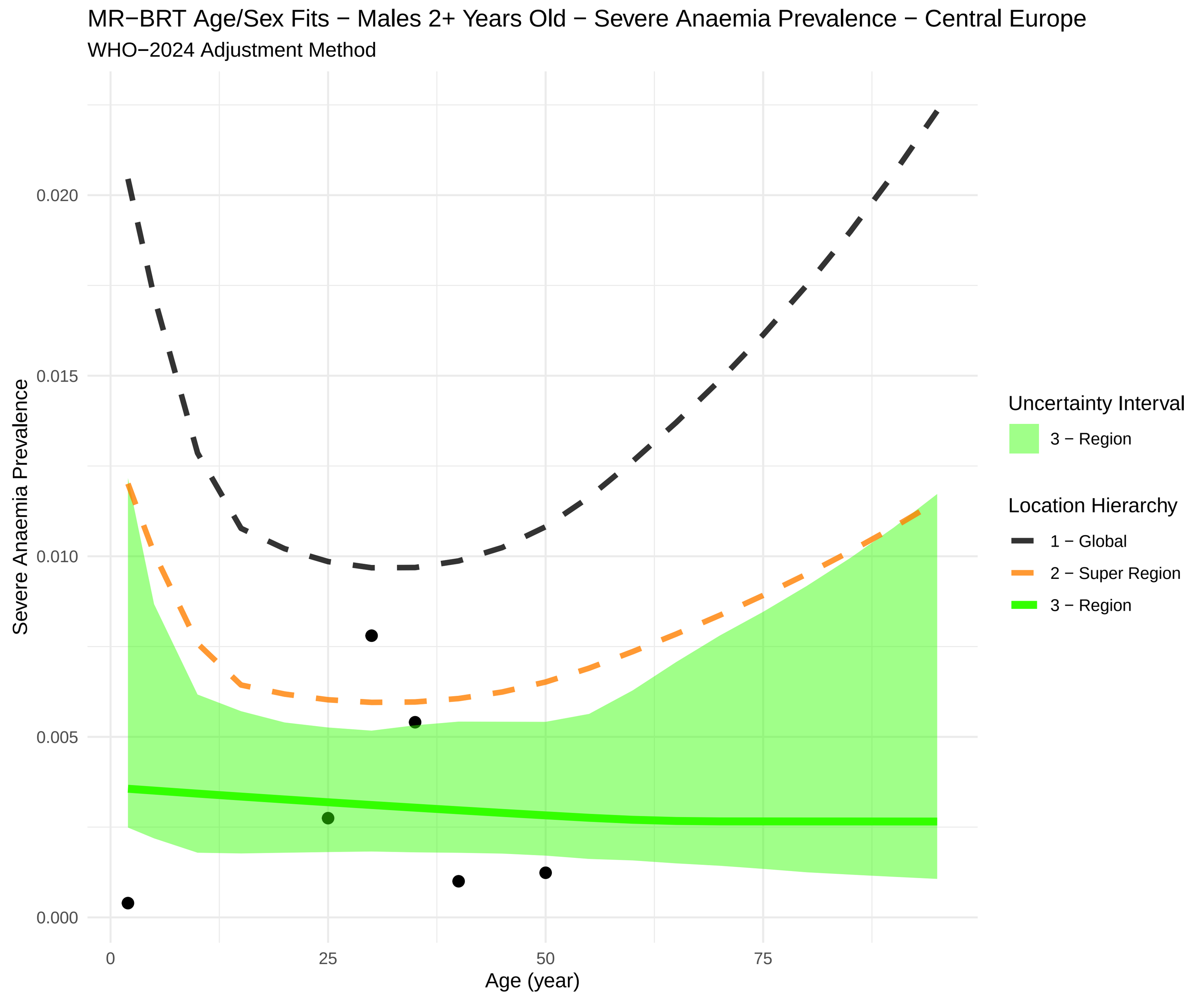
MR−BRT Age/Sex Fits − Males 2+ Years Old − Severe Anaemia Prevalence − Central Europe
WHO−2024 Adjustment Method
Severe Anaemia Prevalence
Age (year)
Uncertainty Interval
3 − Region
Location Hierarchy
1 − Global
2 − Super Region
3 − Region

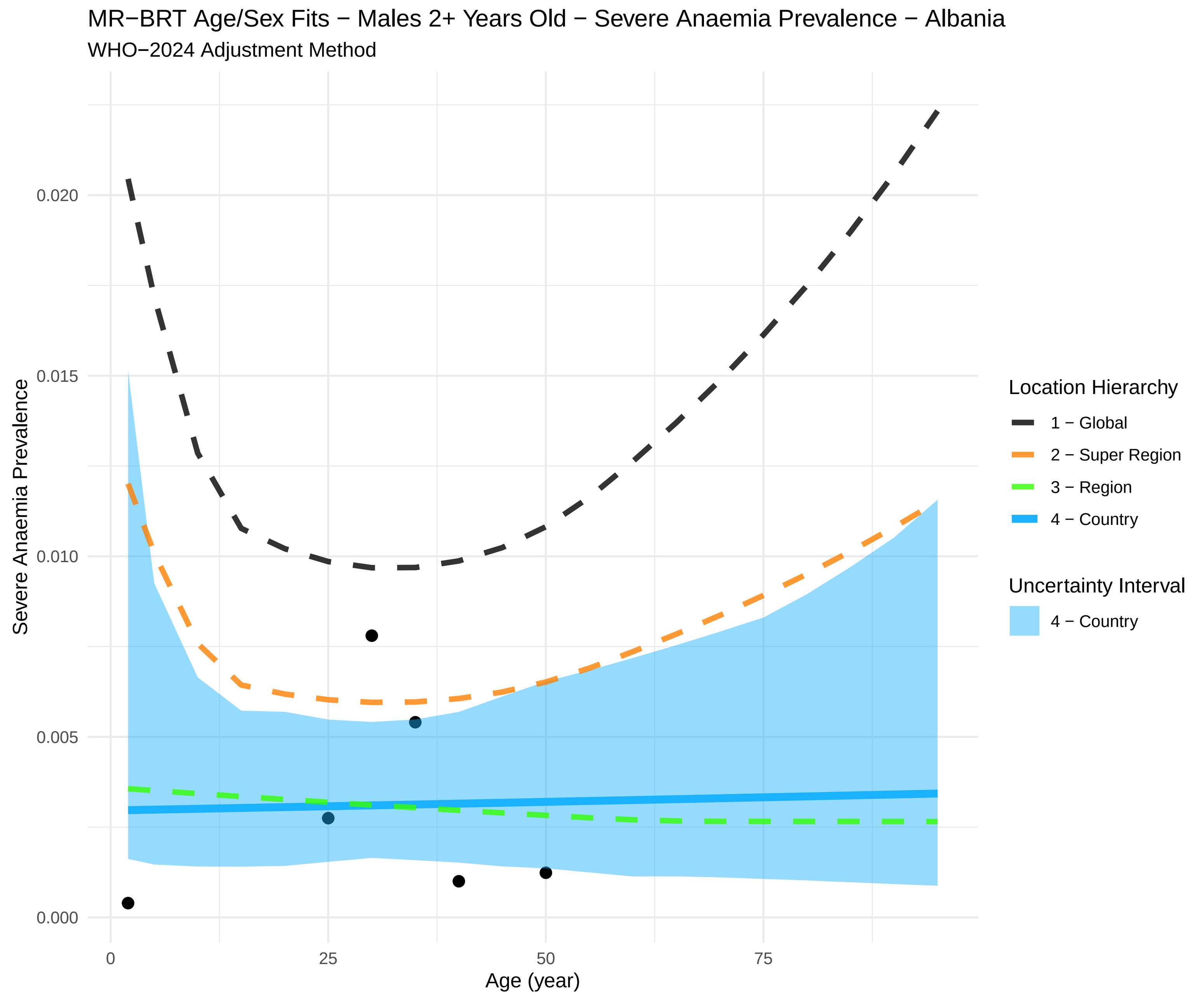
MR−BRT Age/Sex Fits − Males 2+ Years Old − Severe Anaemia Prevalence − Albania
WHO−2024 Adjustment Method
Severe Anaemia Prevalence
Age (year)
Location Hierarchy
1 − Global
2 − Super Region
3 − Region
4 − Country
Uncertainty Interval
4 − Country

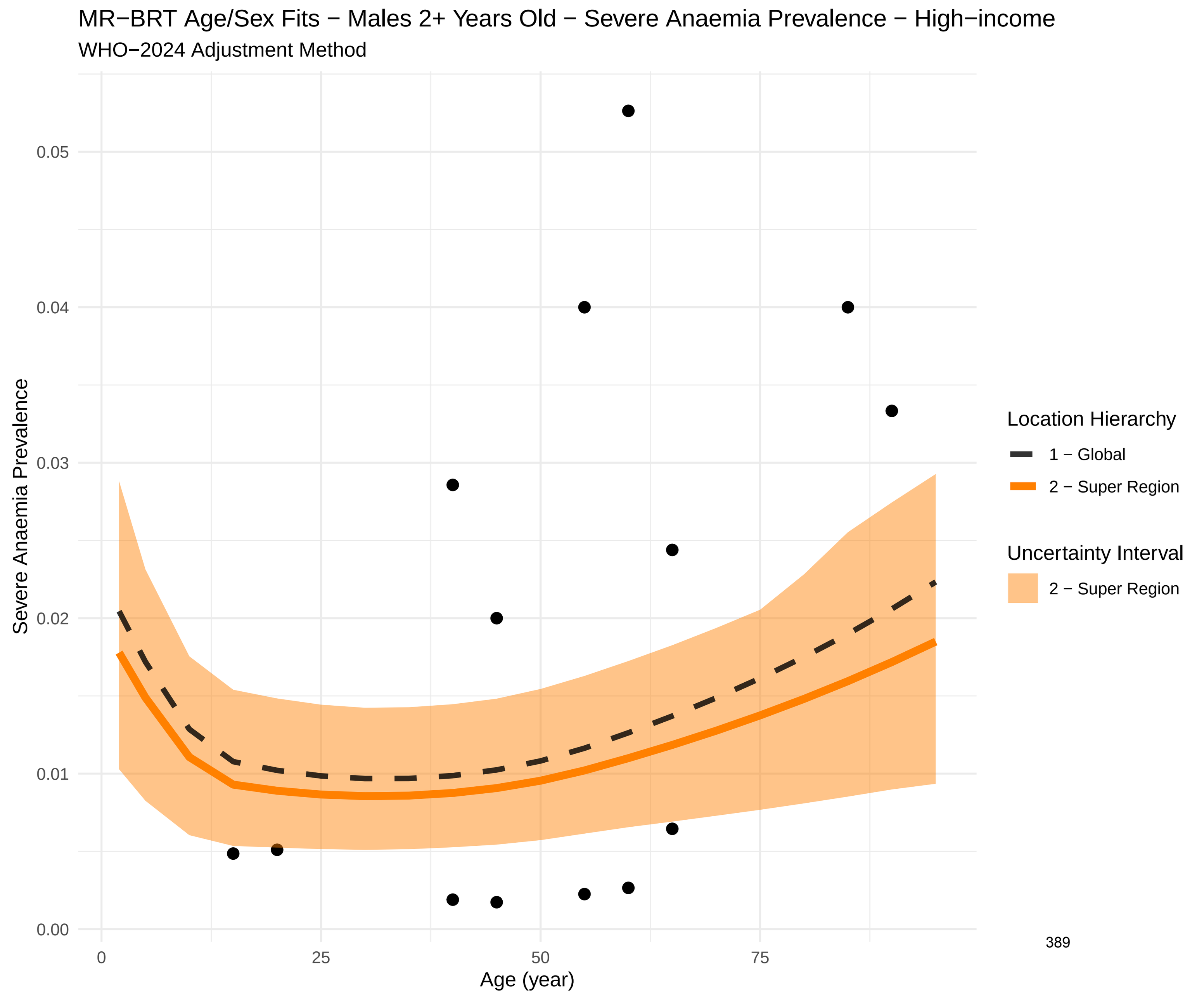
MR−BRT Age/Sex Fits − Males 2+ Years Old − Severe Anaemia Prevalence − High−income
WHO−2024 Adjustment Method
Severe Anaemia Prevalence
Age (year)
Location Hierarchy
1 − Global
2 − Super Region
Uncertainty Interval
2 − Super Region

MR−BRT Age/Sex Fits − Males 2+ Years Old − Severe Anaemia Prevalence − Western Europe
WHO−2024 Adjustment Method

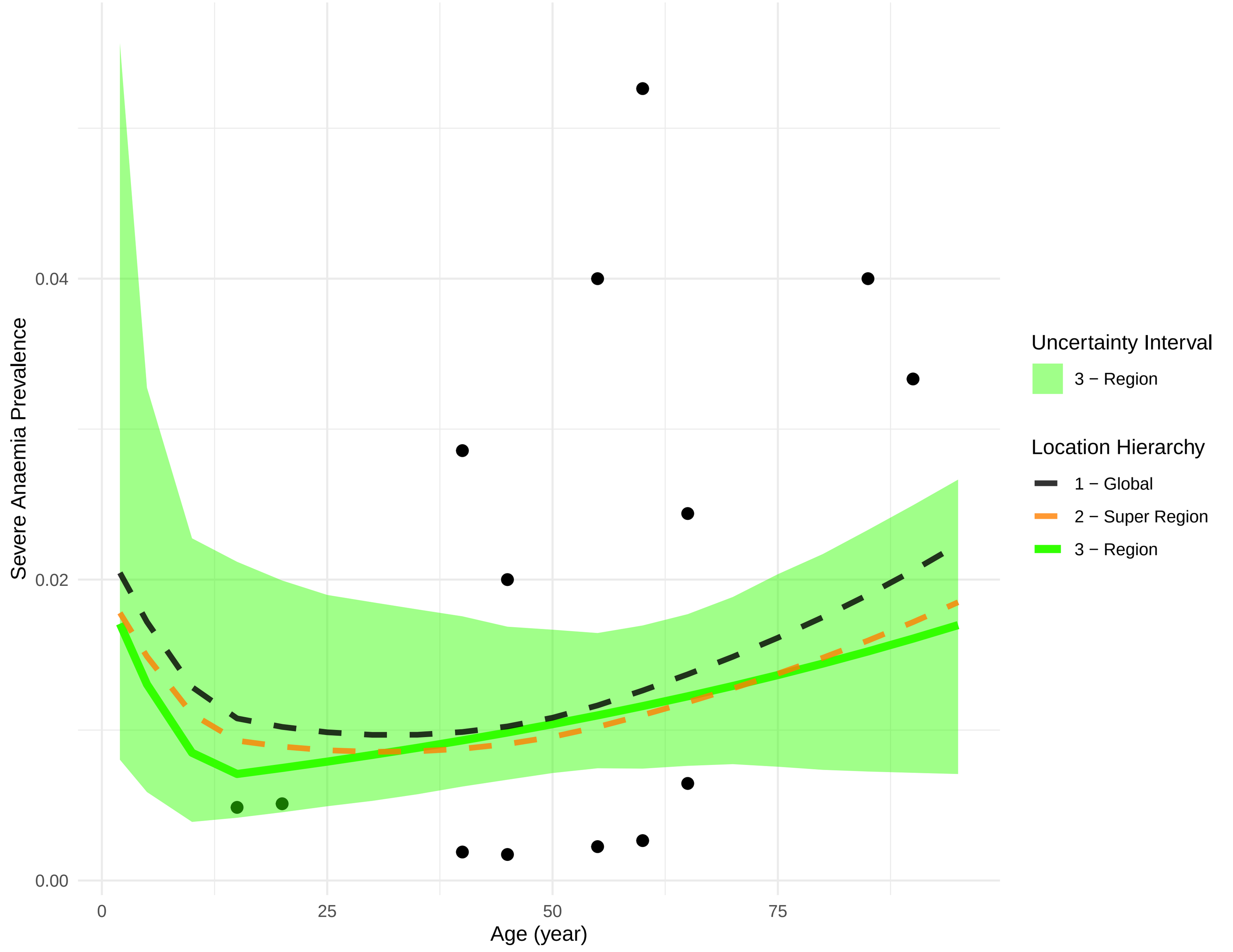

MR−BRT Age/Sex Fits − Males 2+ Years Old − Severe Anaemia Prevalence − United Kingdom
WHO−2024 Adjustment Method

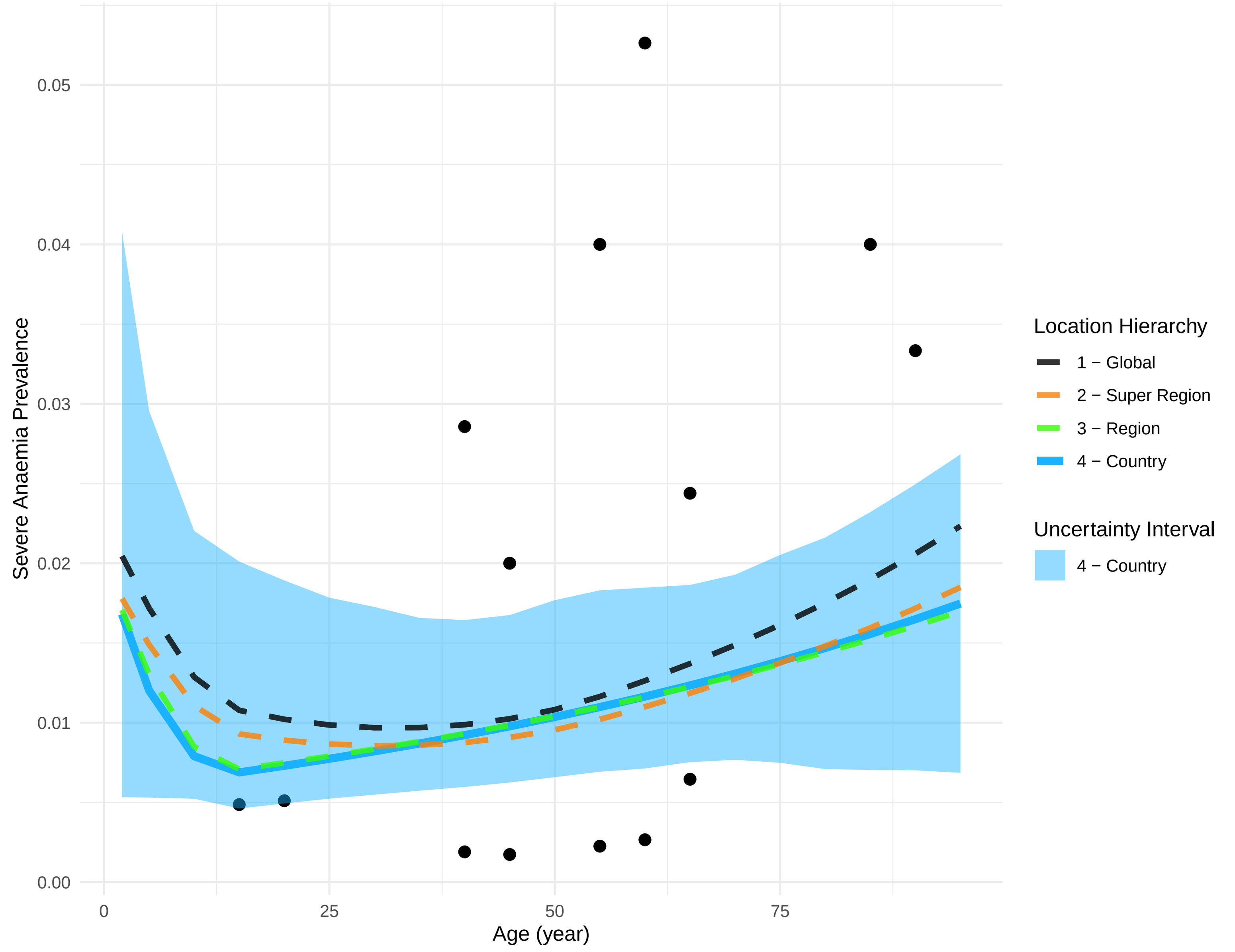

MR−BRT Age/Sex Fits − Males 2+ Years Old − Severe Anaemia Prevalence − England
WHO−2024 Adjustment Method

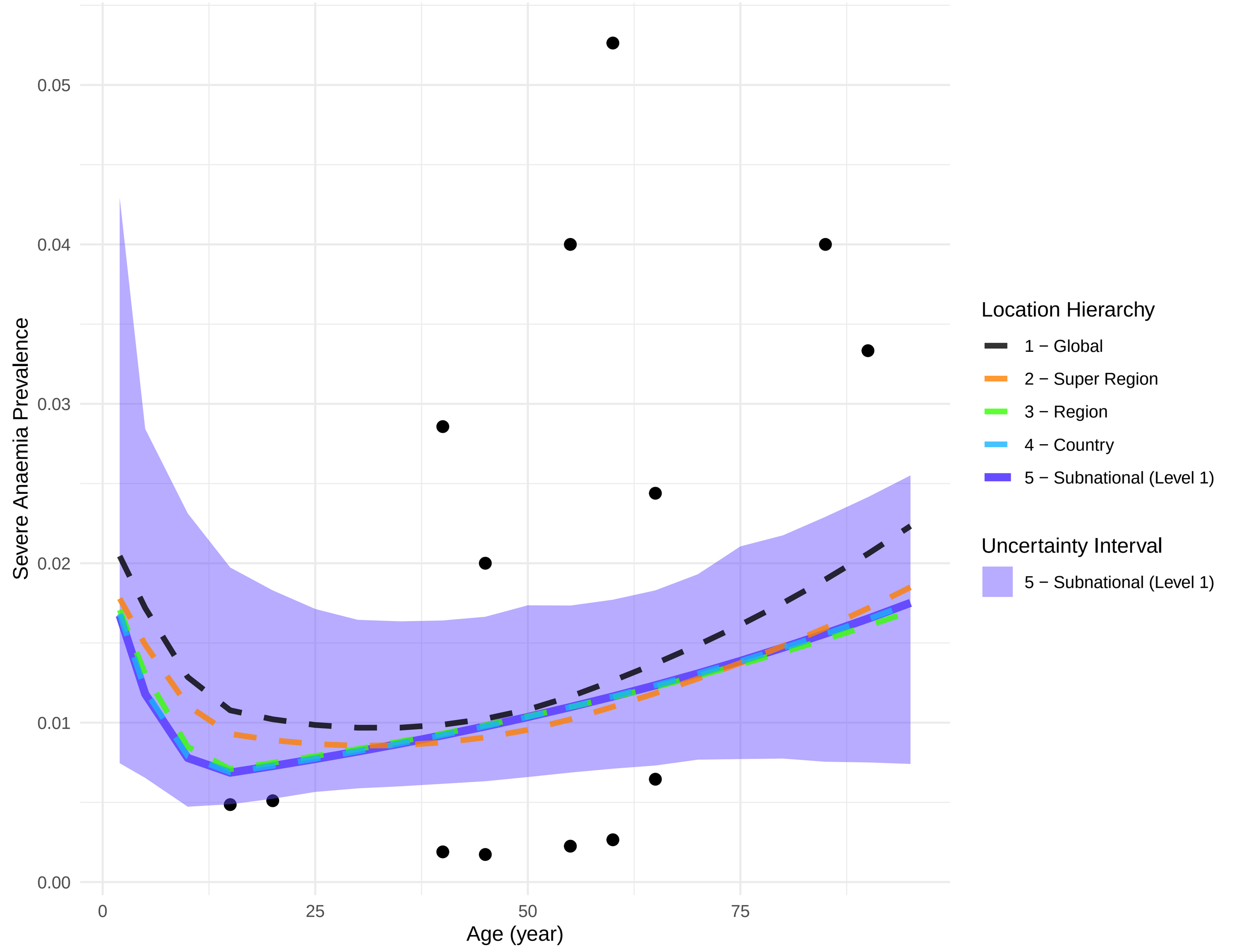

MR−BRT Age/Sex Fits − Males 2+ Years Old − Severe Anaemia Prevalence − East of England
WHO−2024 Adjustment Method

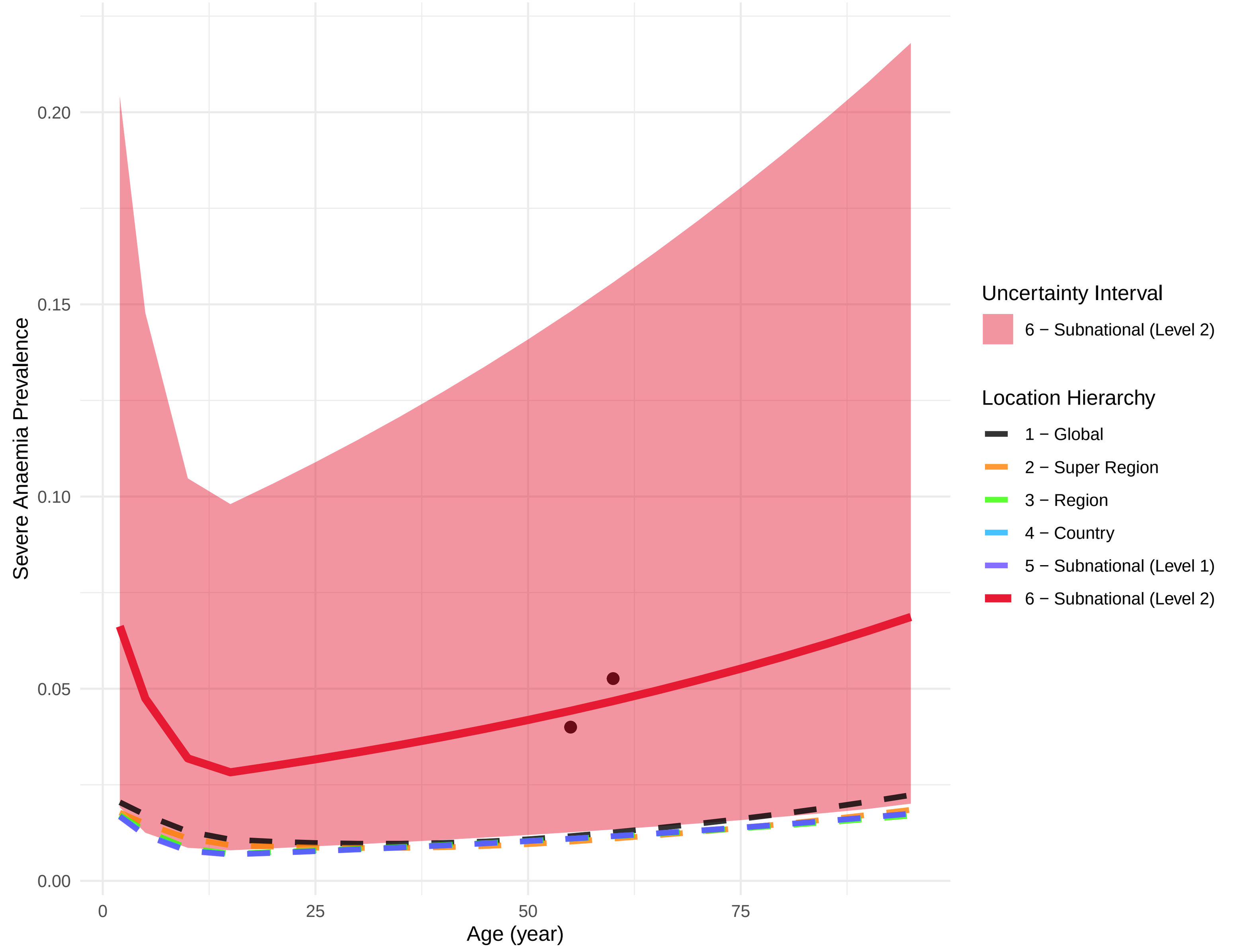

MR−BRT Age/Sex Fits − Males 2+ Years Old − Severe Anaemia Prevalence − South West England
WHO−2024 Adjustment Method

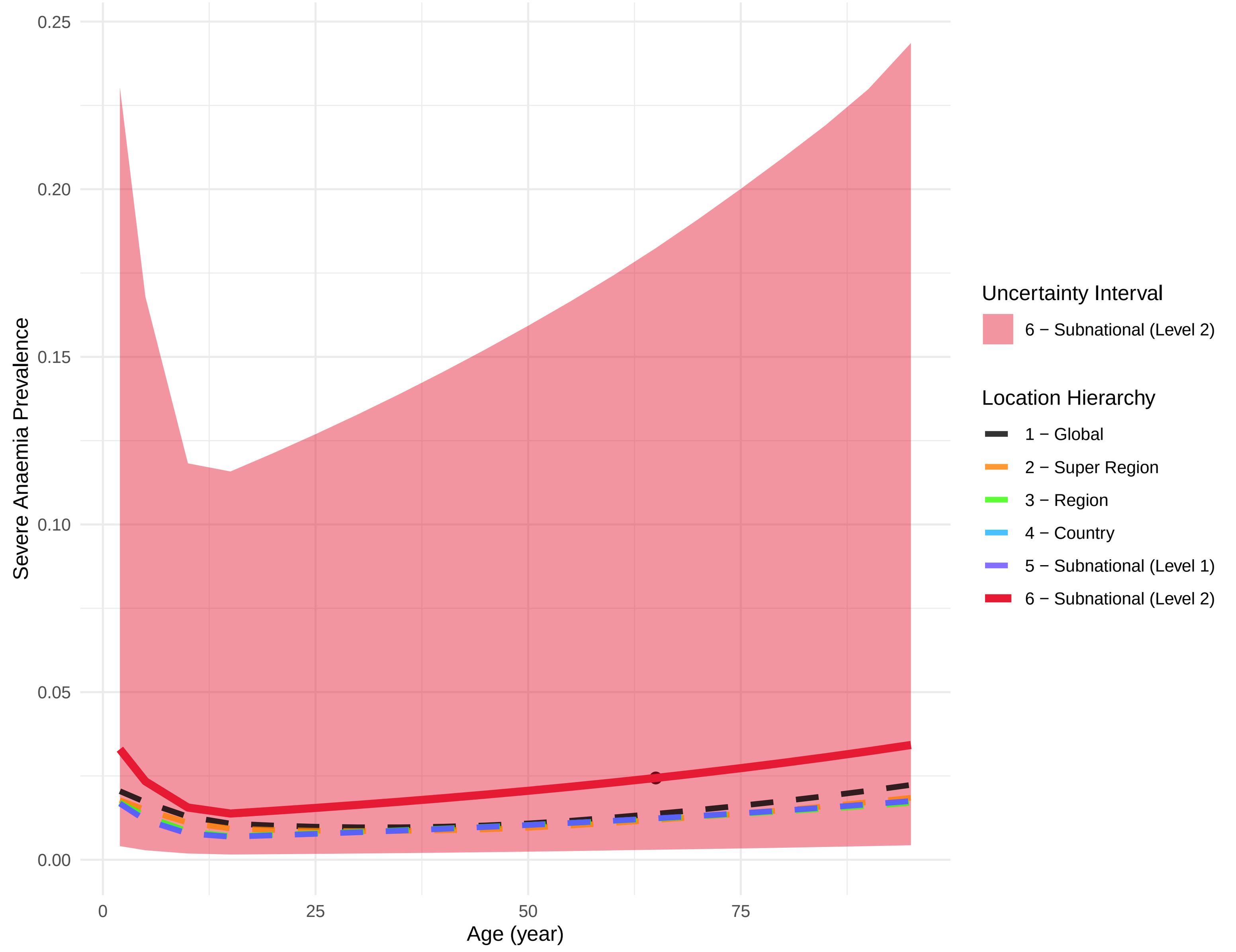

MR−BRT Age/Sex Fits − Males 2+ Years Old − Severe Anaemia Prevalence − West Midlands
WHO−2024 Adjustment Method

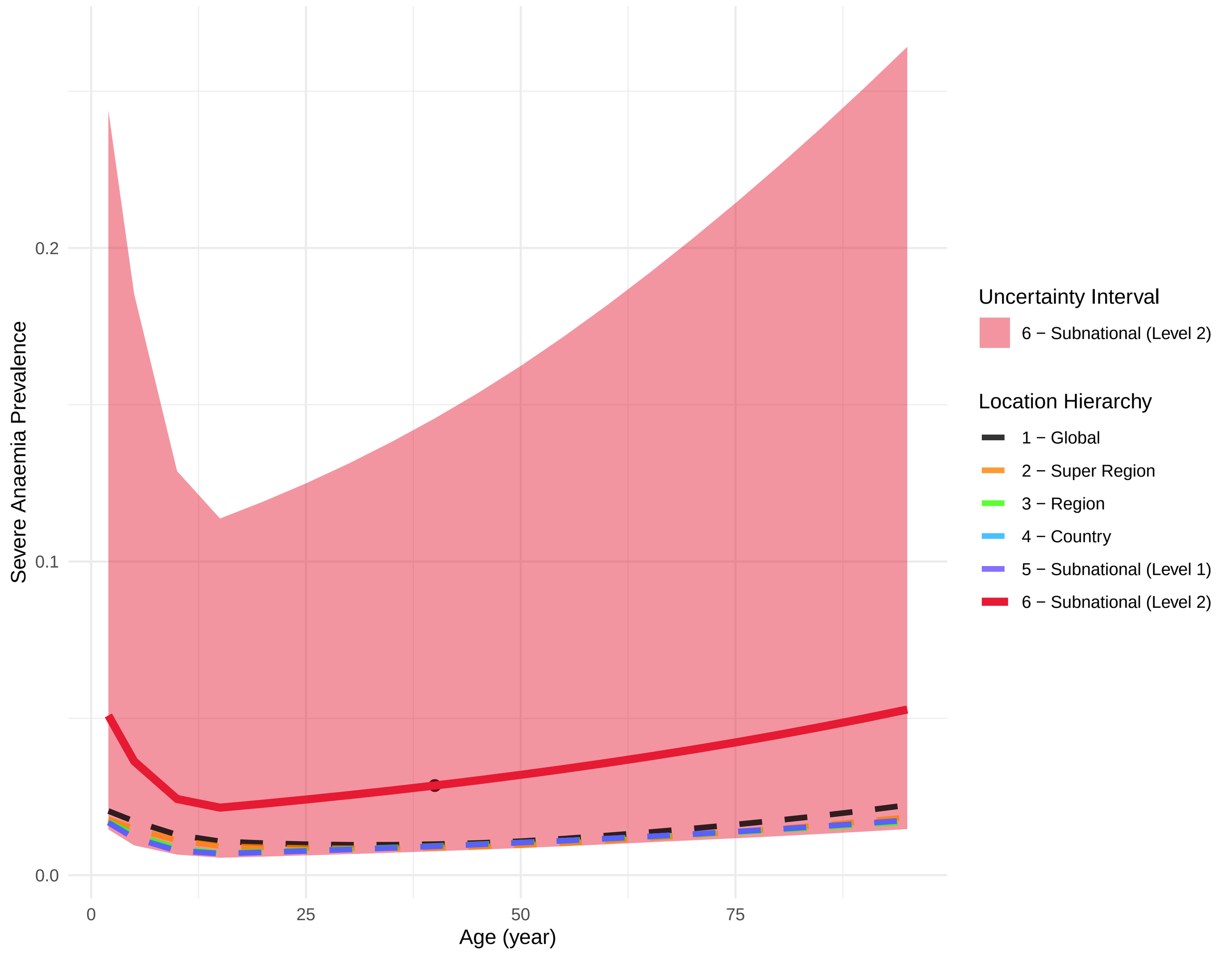

MR−BRT Age/Sex Fits − Males 2+ Years Old − Severe Anaemia Prevalence − Yorkshire and the Humber
WHO−2024 Adjustment Method

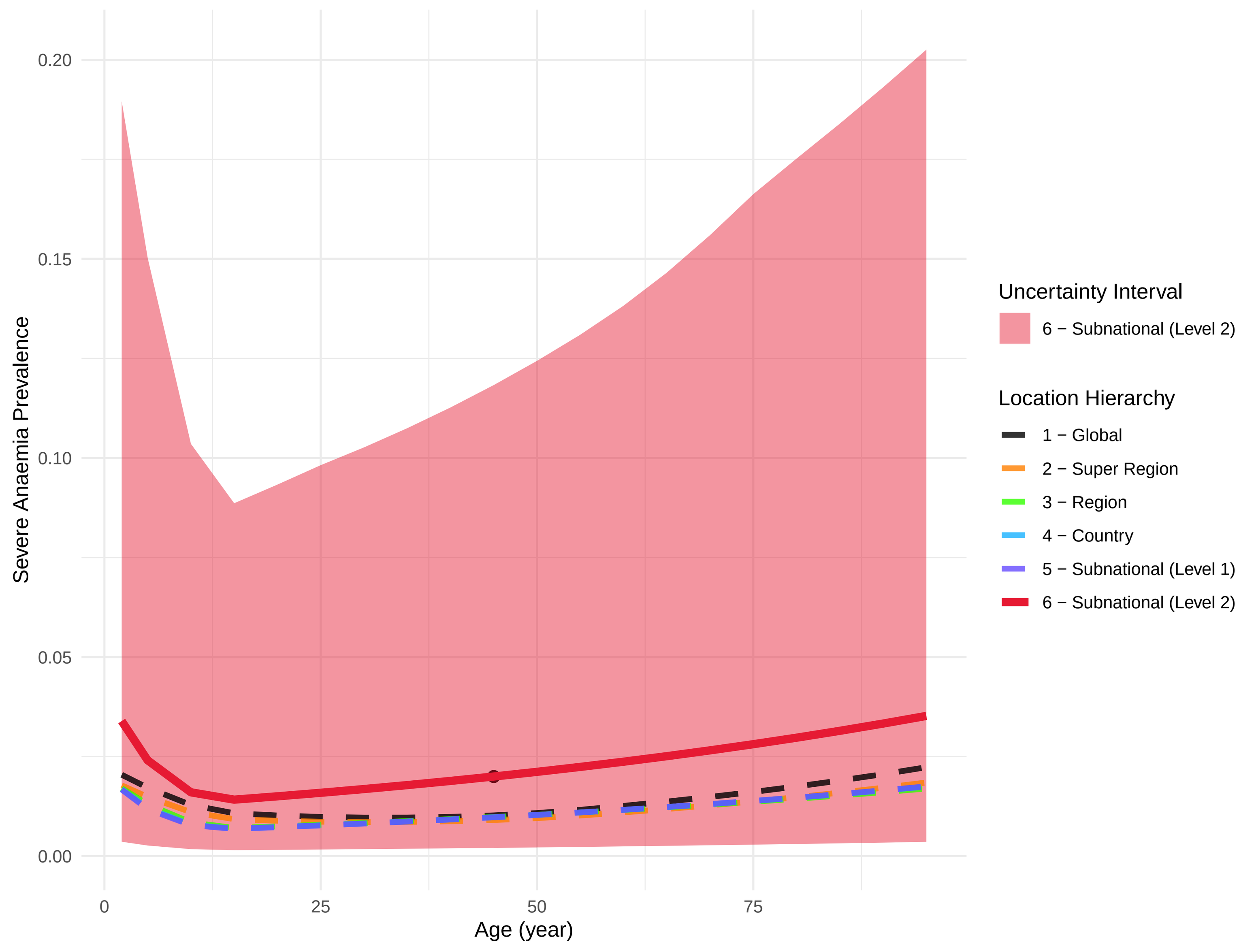

MR−BRT Age/Sex Fits − Males 2+ Years Old − Severe Anaemia Prevalence − Latin America and Caribbean
WHO−2024 Adjustment Method

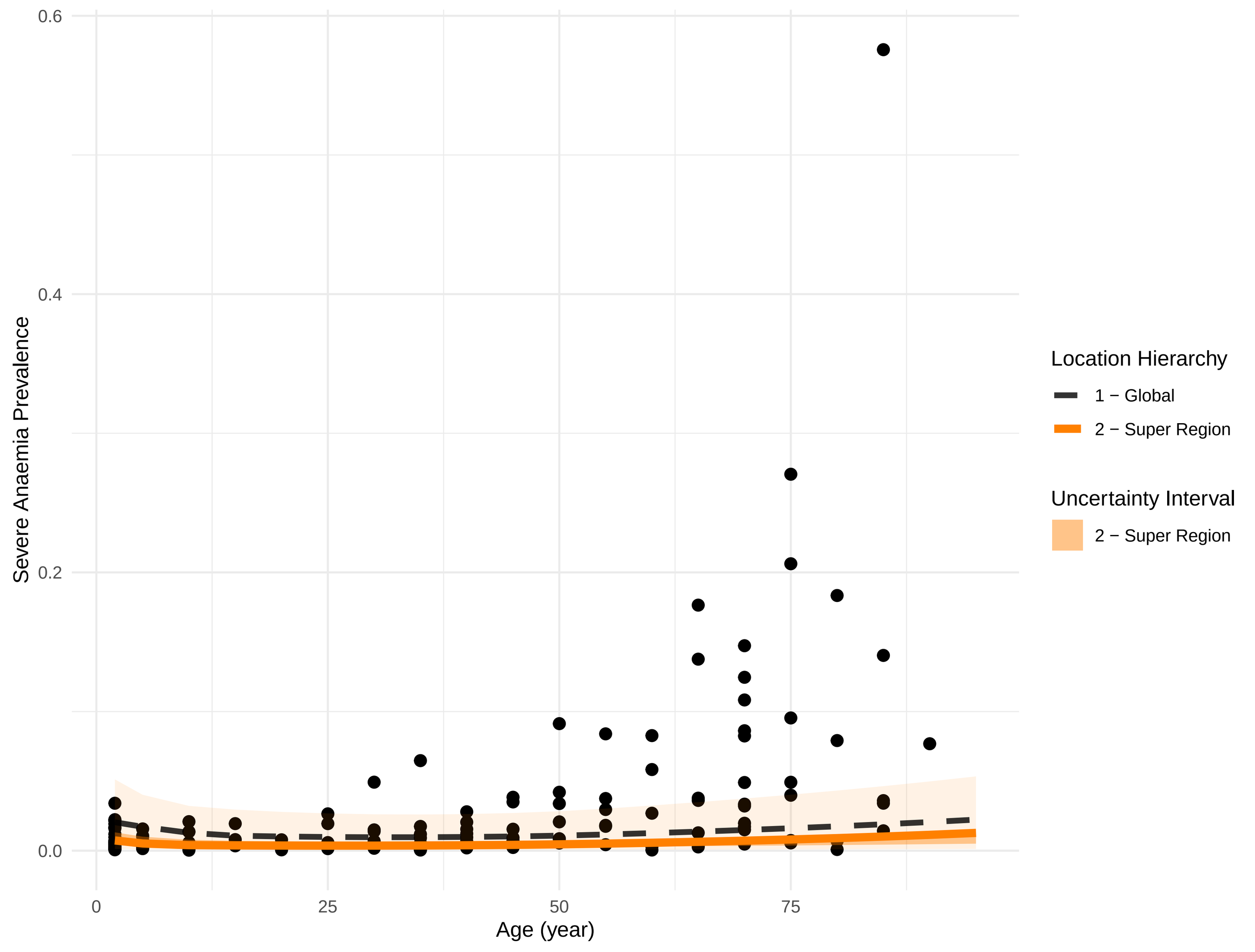

MR−BRT Age/Sex Fits − Males 2+ Years Old − Severe Anaemia Prevalence − Andean Latin America
WHO−2024 Adjustment Method

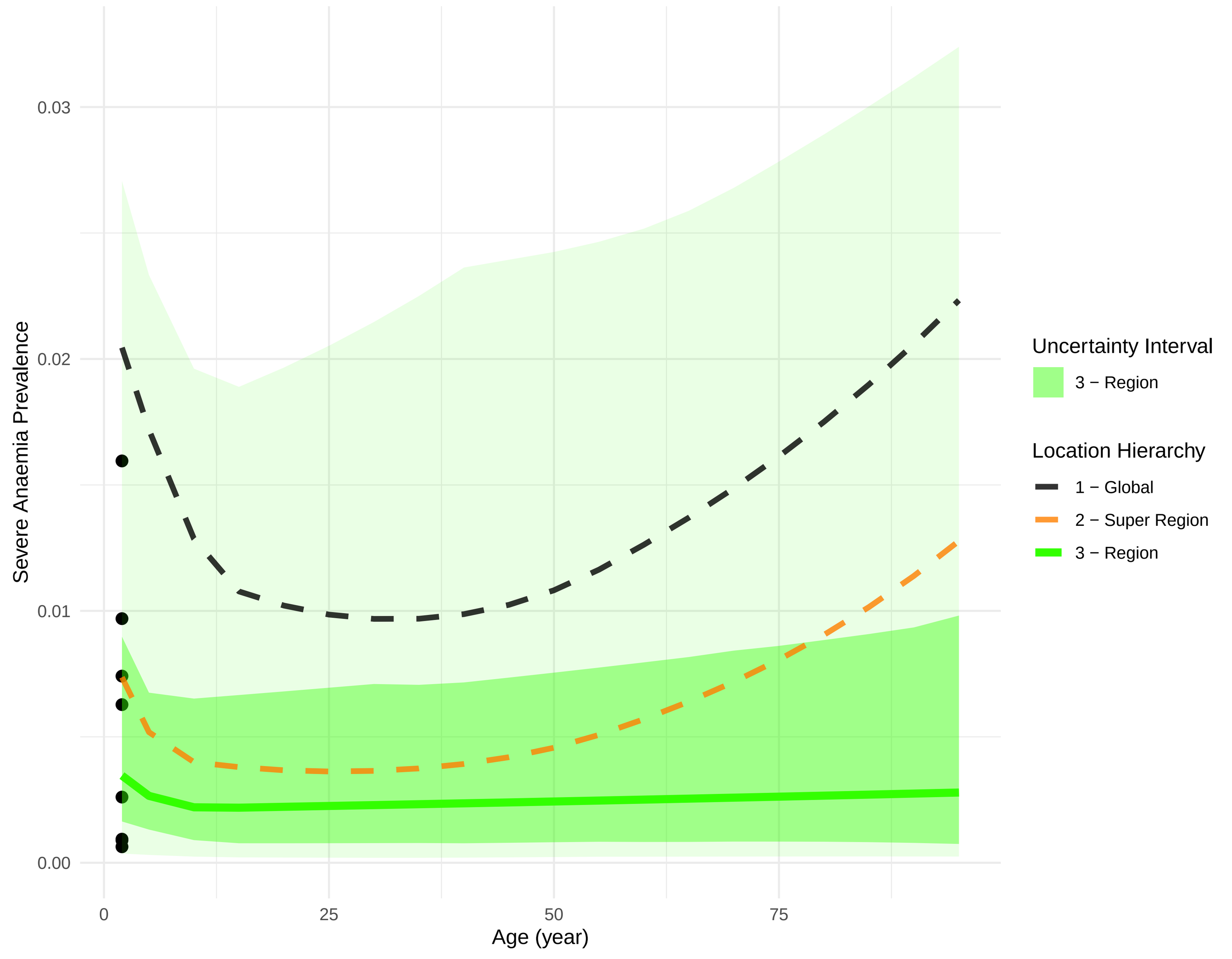

MR−BRT Age/Sex Fits − Males 2+ Years Old − Severe Anaemia Prevalence − Bolivia (Plurinational State of)
WHO−2024 Adjustment Method

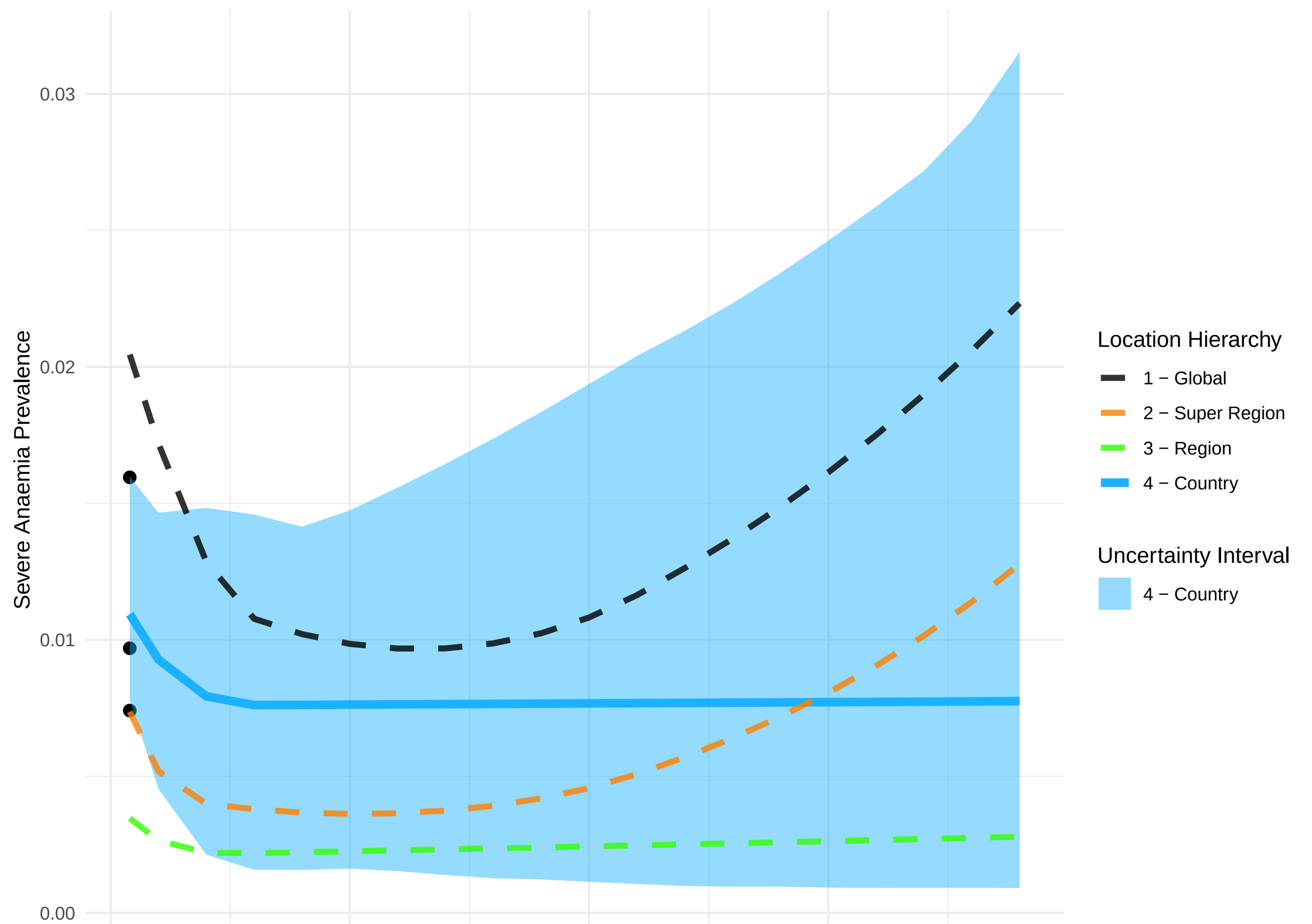

MR−BRT Age/Sex Fits − Males 2+ Years Old − Severe Anaemia Prevalence − Peru
WHO−2024 Adjustment Method

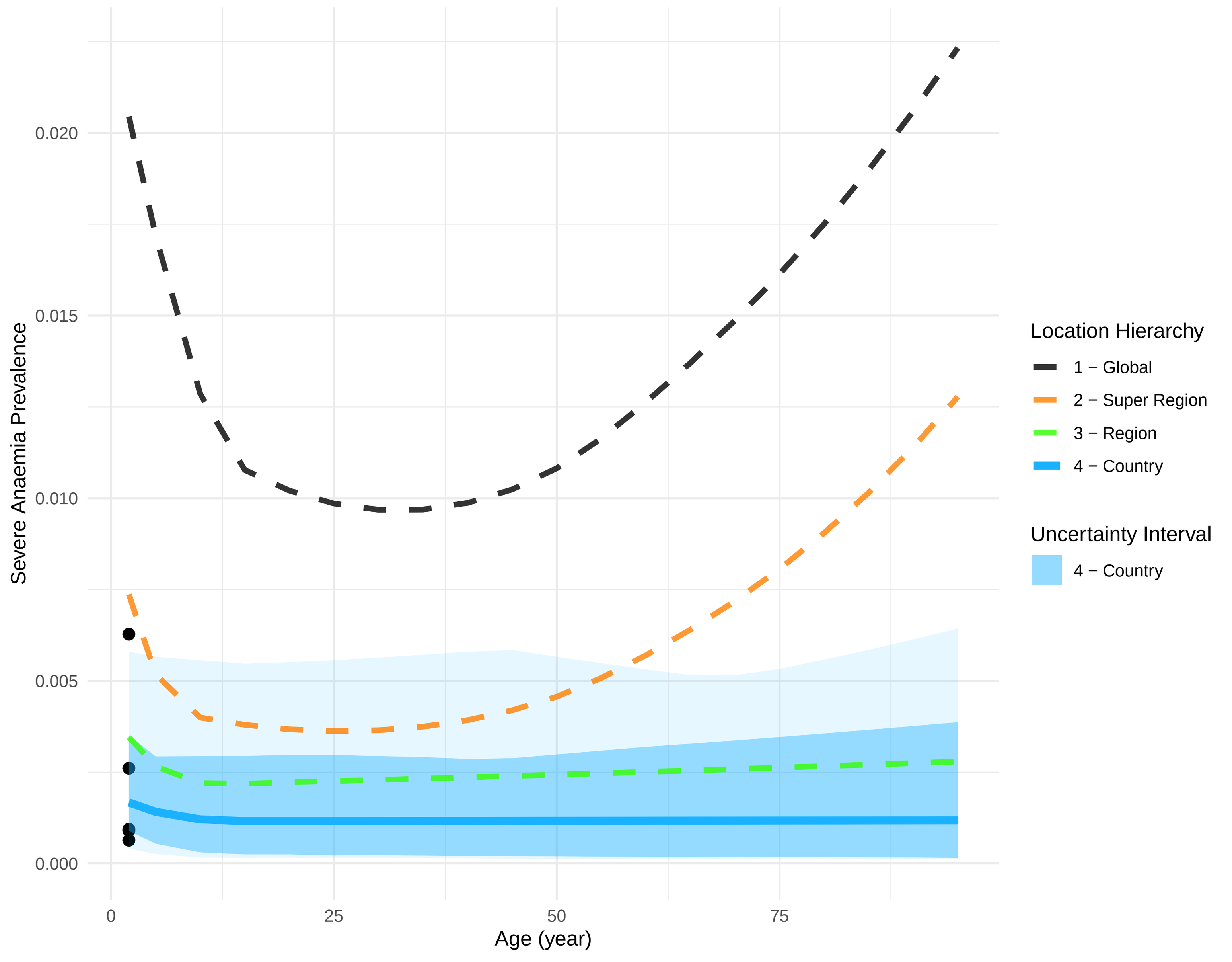

MR−BRT Age/Sex Fits − Males 2+ Years Old − Severe Anaemia Prevalence − Caribbean
WHO−2024 Adjustment Method

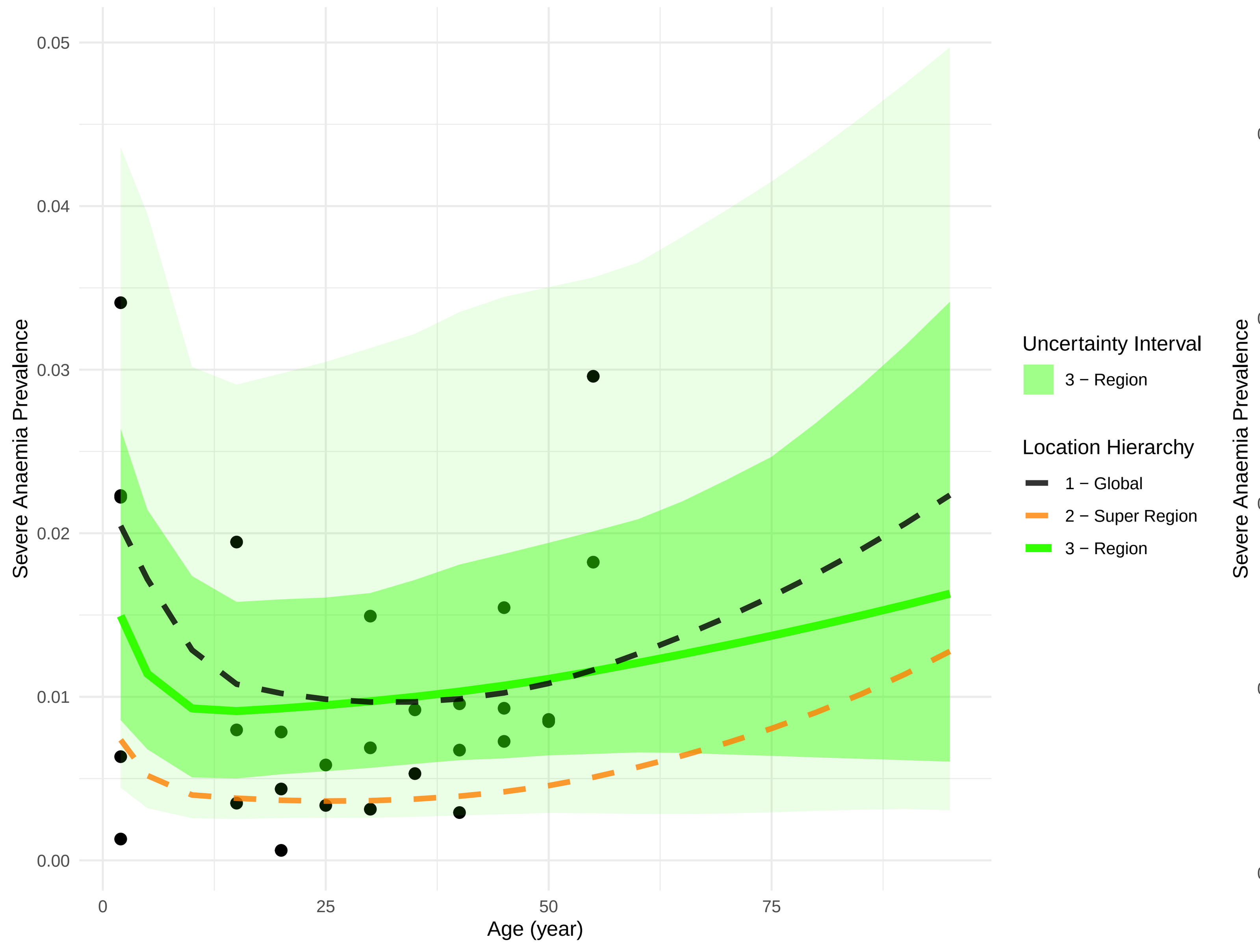

MR−BRT Age/Sex Fits − Males 2+ Years Old − Severe Anaemia Prevalence − Guyana
WHO−2024 Adjustment Method

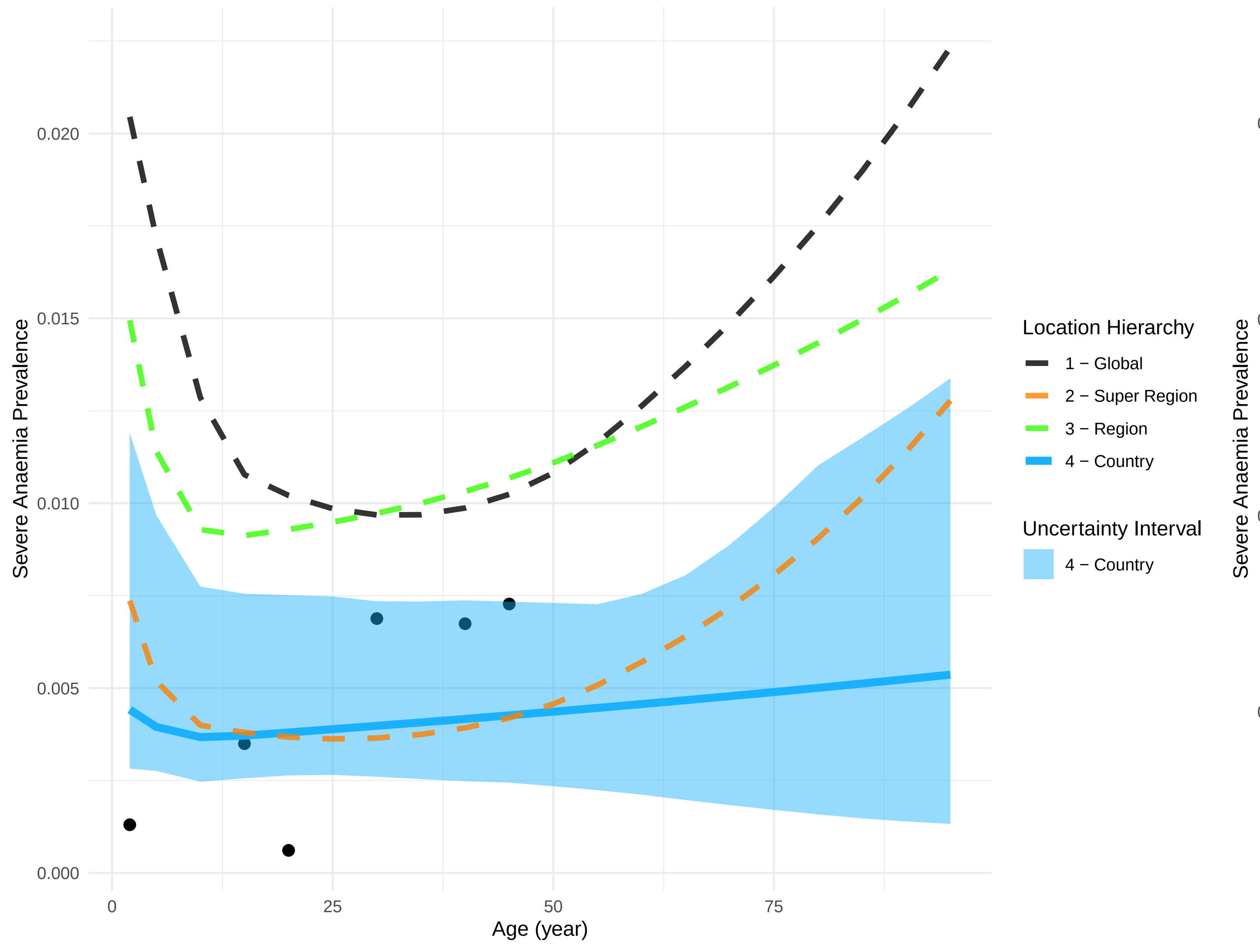

MR−BRT Age/Sex Fits − Males 2+ Years Old − Severe Anaemia Prevalence − Haiti
WHO−2024 Adjustment Method

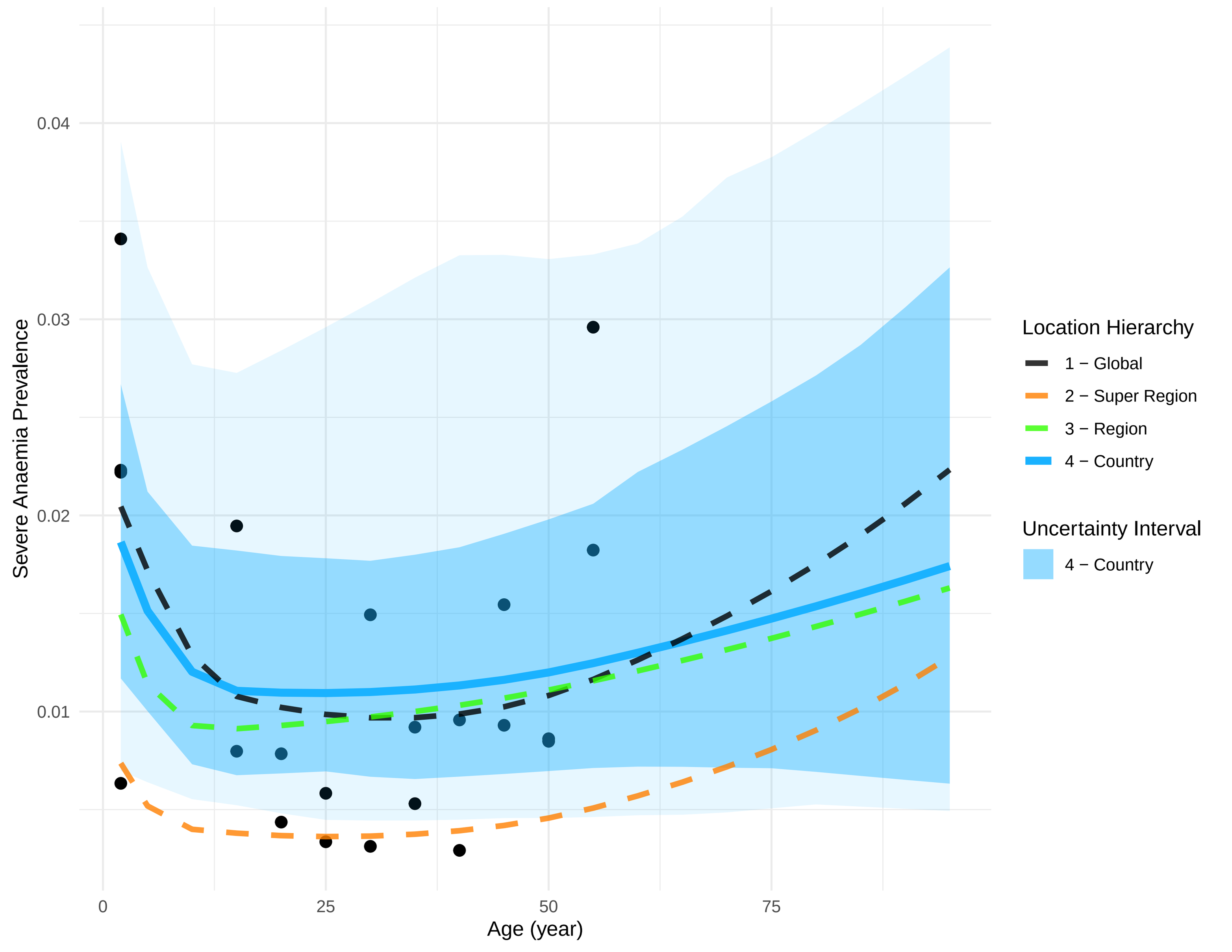

MR−BRT Age/Sex Fits − Males 2+ Years Old − Severe Anaemia Prevalence − Central Latin America
WHO−2024 Adjustment Method

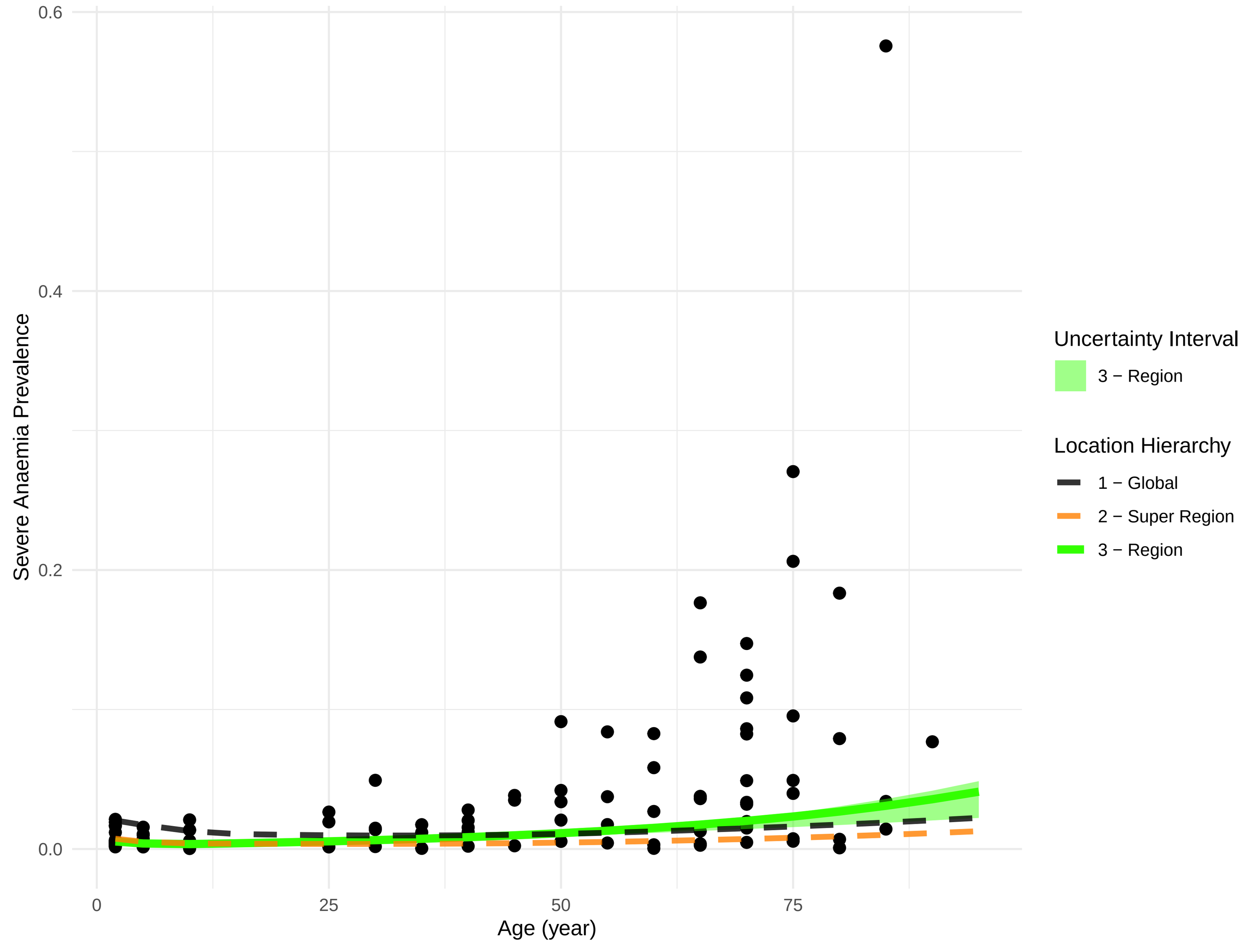

MR−BRT Age/Sex Fits − Males 2+ Years Old − Severe Anaemia Prevalence − Honduras
WHO−2024 Adjustment Method

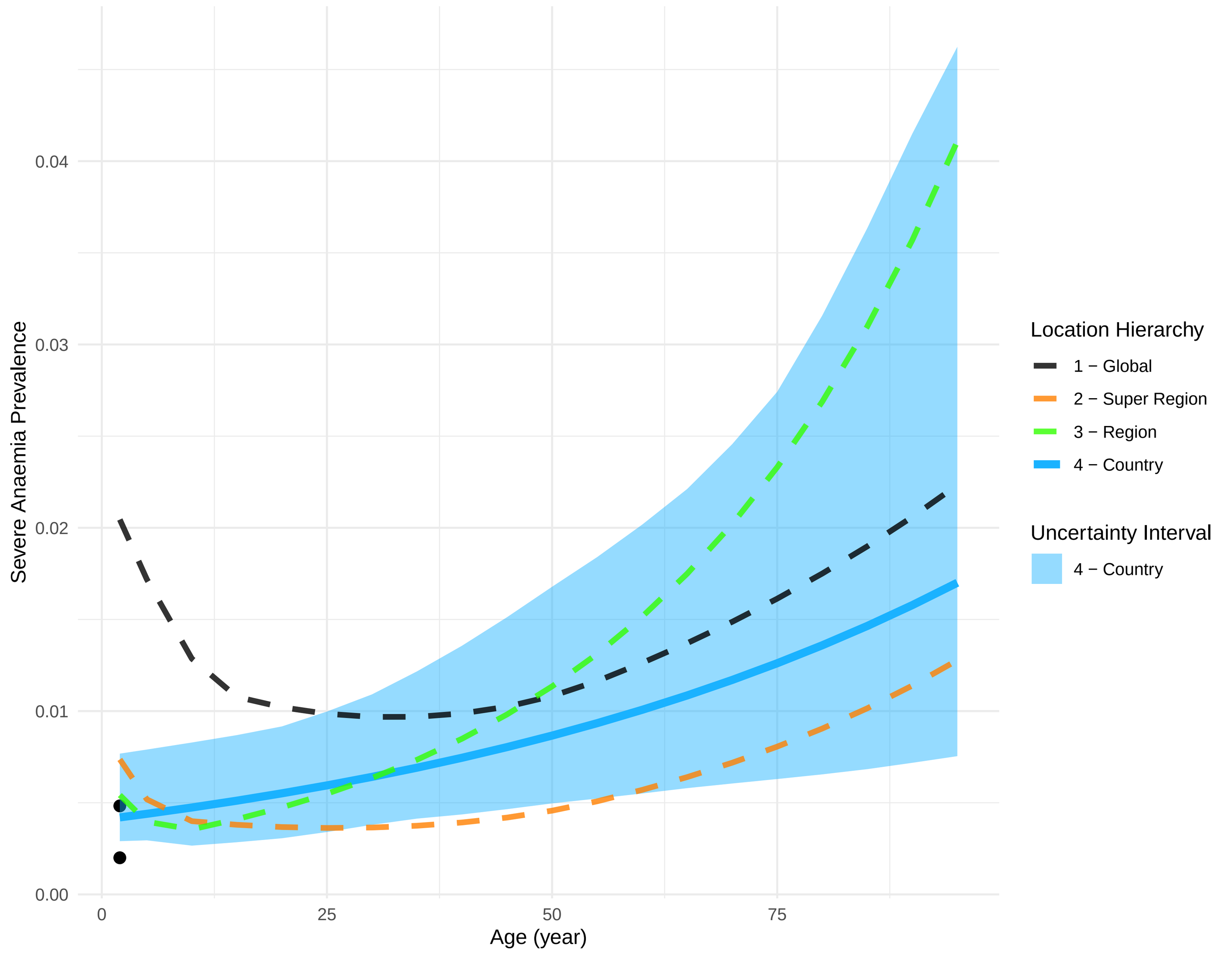

MR−BRT Age/Sex Fits − Males 2+ Years Old − Severe Anaemia Prevalence − Mexico
WHO−2024 Adjustment Method

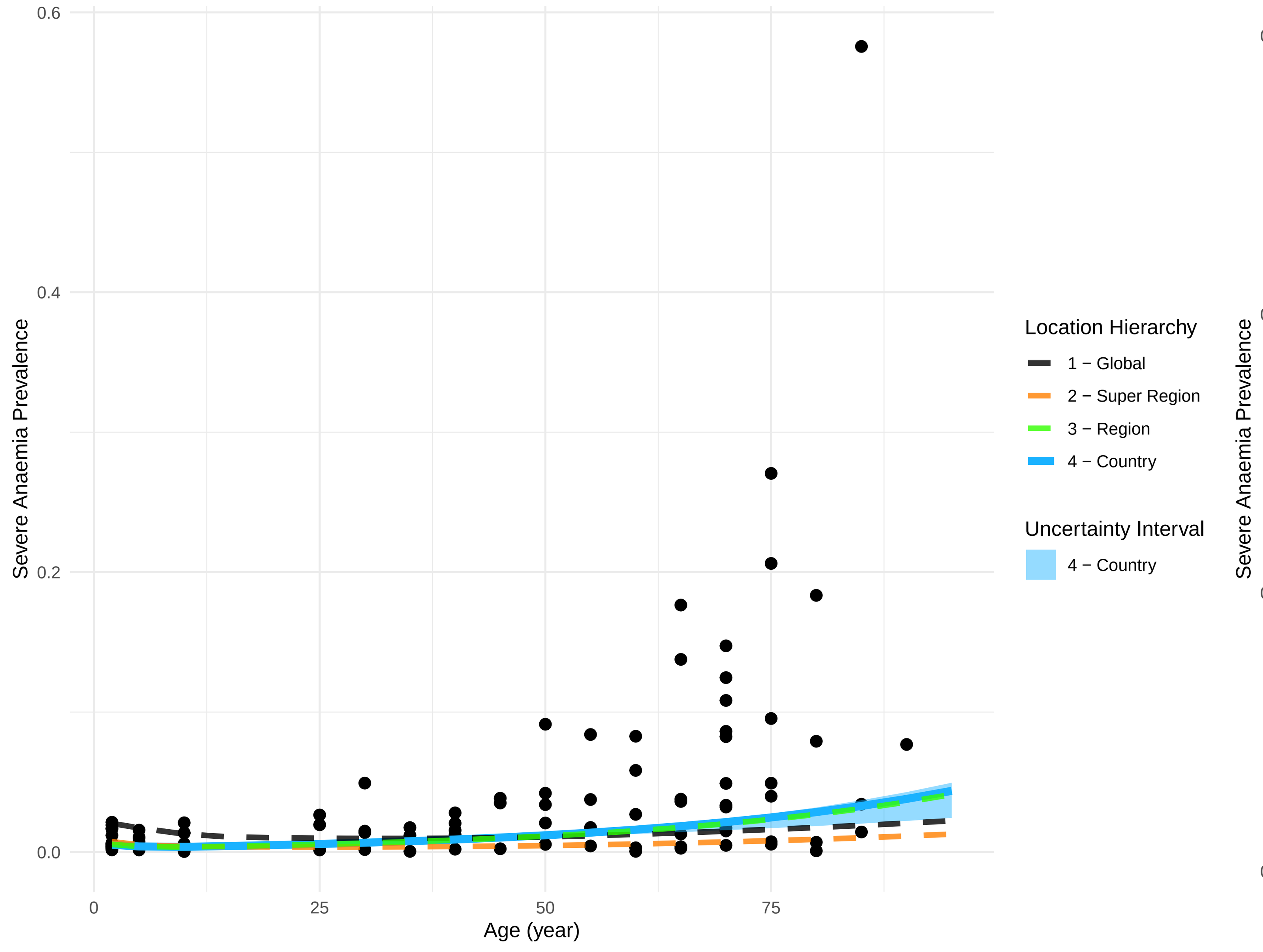

MR−BRT Age/Sex Fits − Males 2+ Years Old − Severe Anaemia Prevalence − Aguascalientes
WHO−2024 Adjustment Method

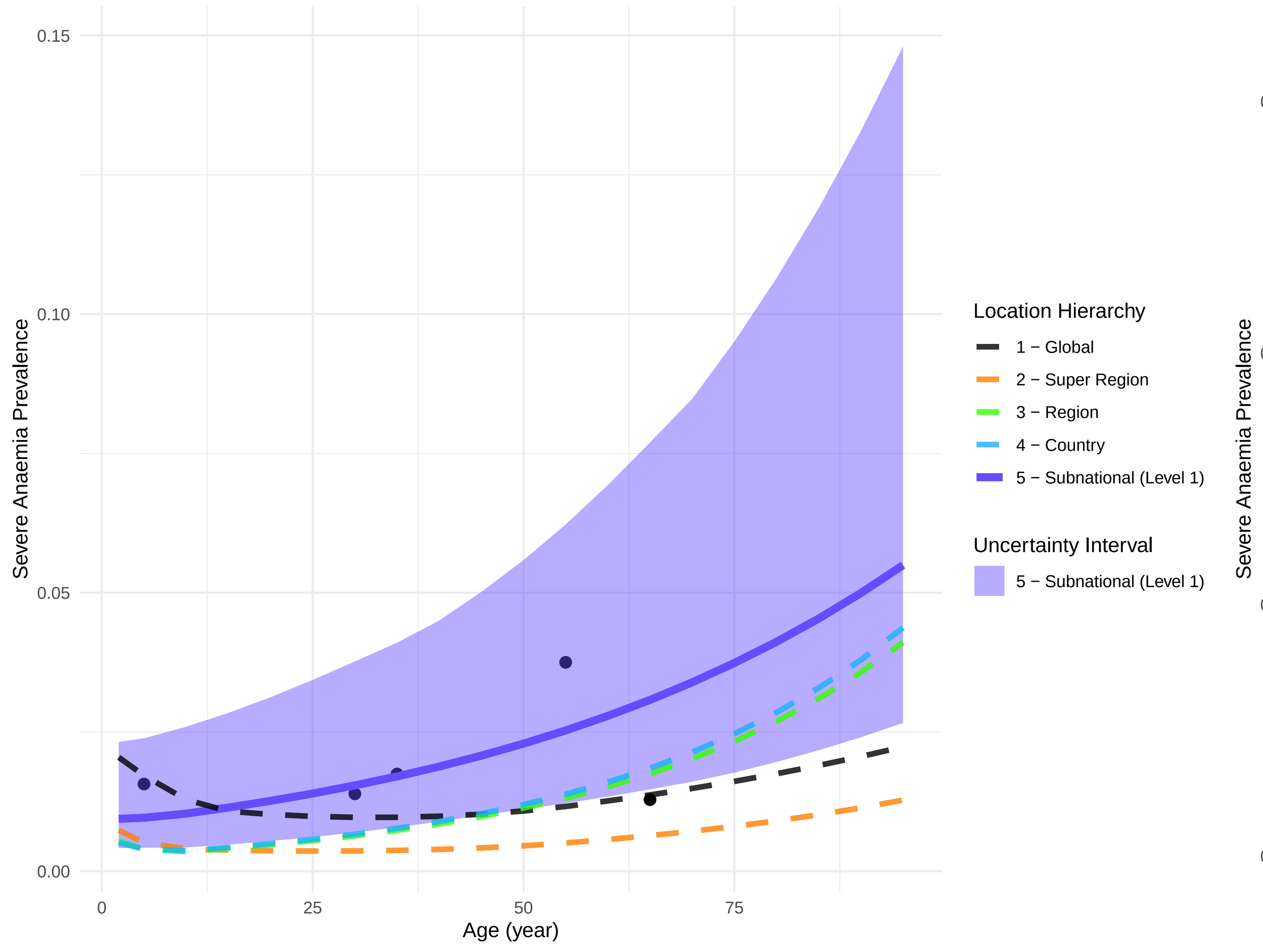

MR−BRT Age/Sex Fits − Males 2+ Years Old − Severe Anaemia Prevalence − Baja California
WHO−2024 Adjustment Method

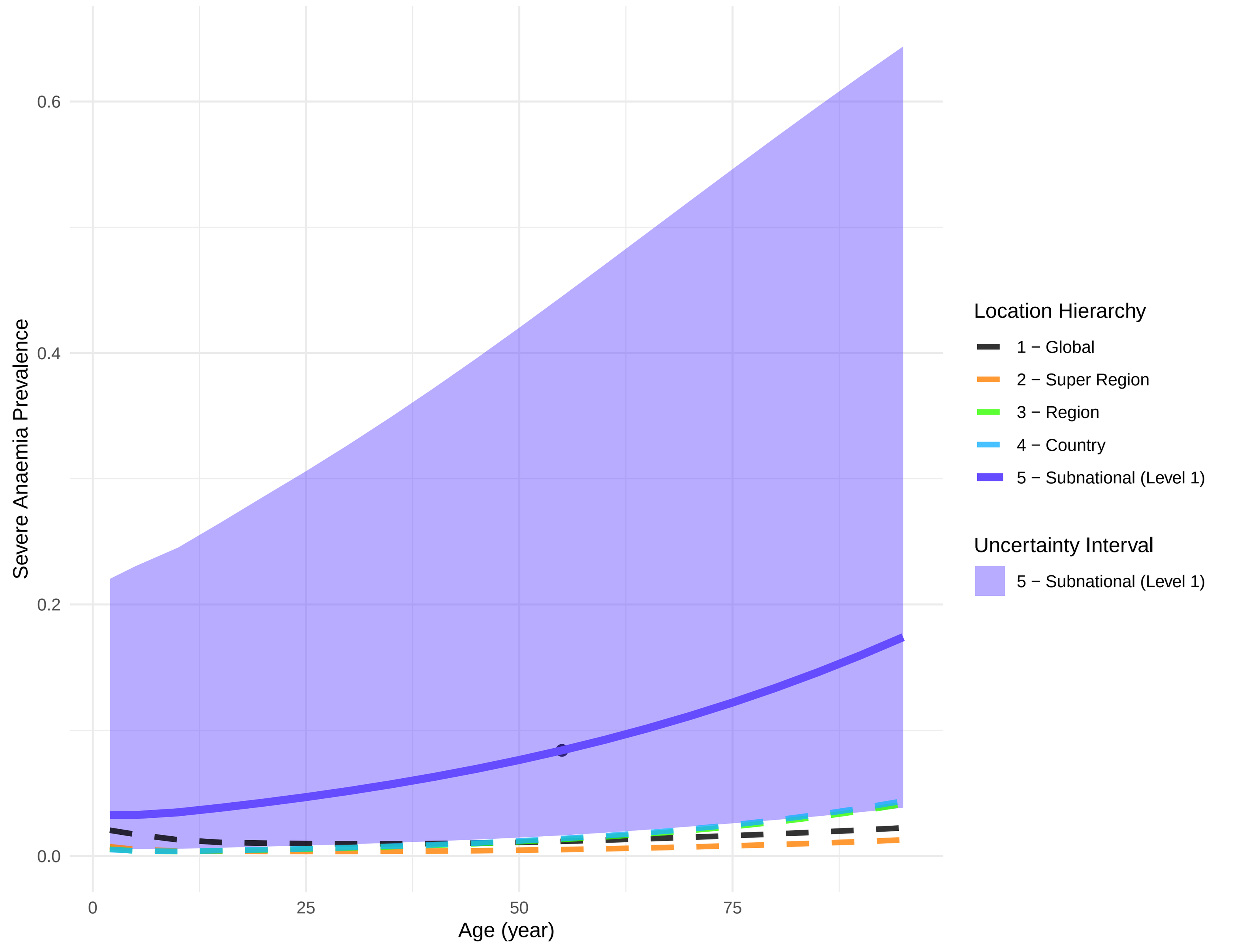

MR−BRT Age/Sex Fits − Males 2+ Years Old − Severe Anaemia Prevalence − Campeche
WHO−2024 Adjustment Method

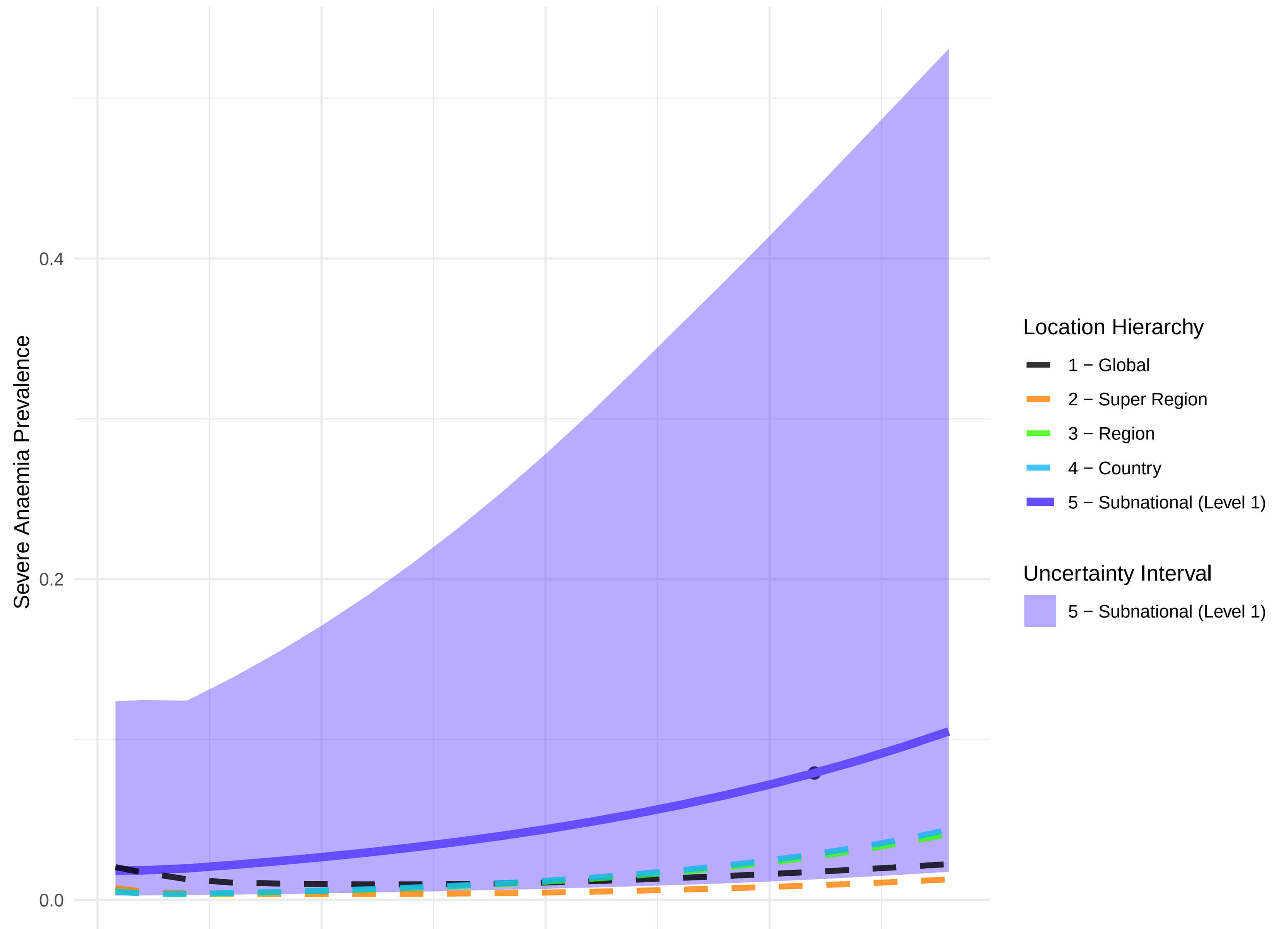

MR−BRT Age/Sex Fits − Males 2+ Years Old − Severe Anaemia Prevalence − Chiapas
WHO−2024 Adjustment Method

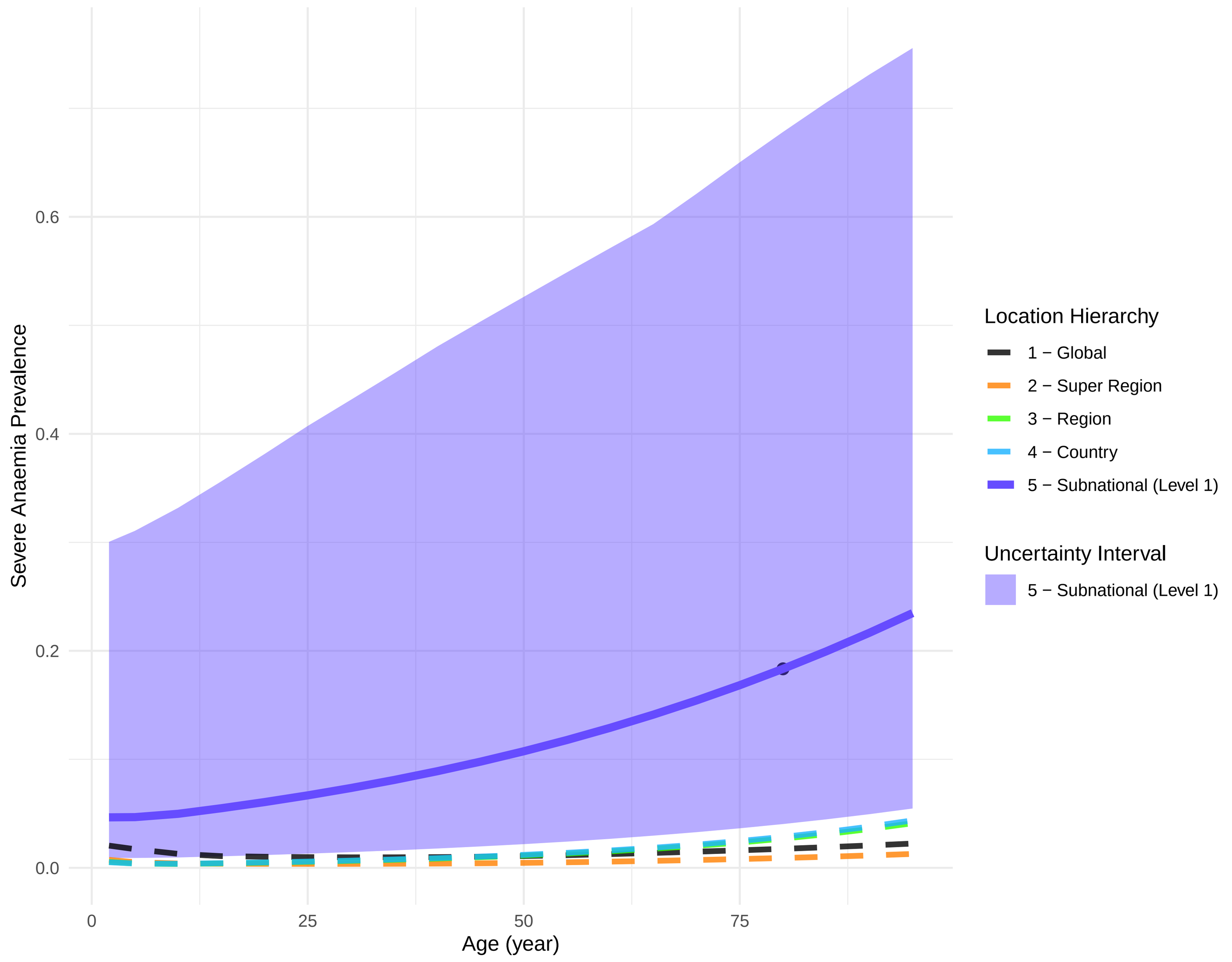

MR−BRT Age/Sex Fits − Males 2+ Years Old − Severe Anaemia Prevalence − Chihuahua
WHO−2024 Adjustment Method

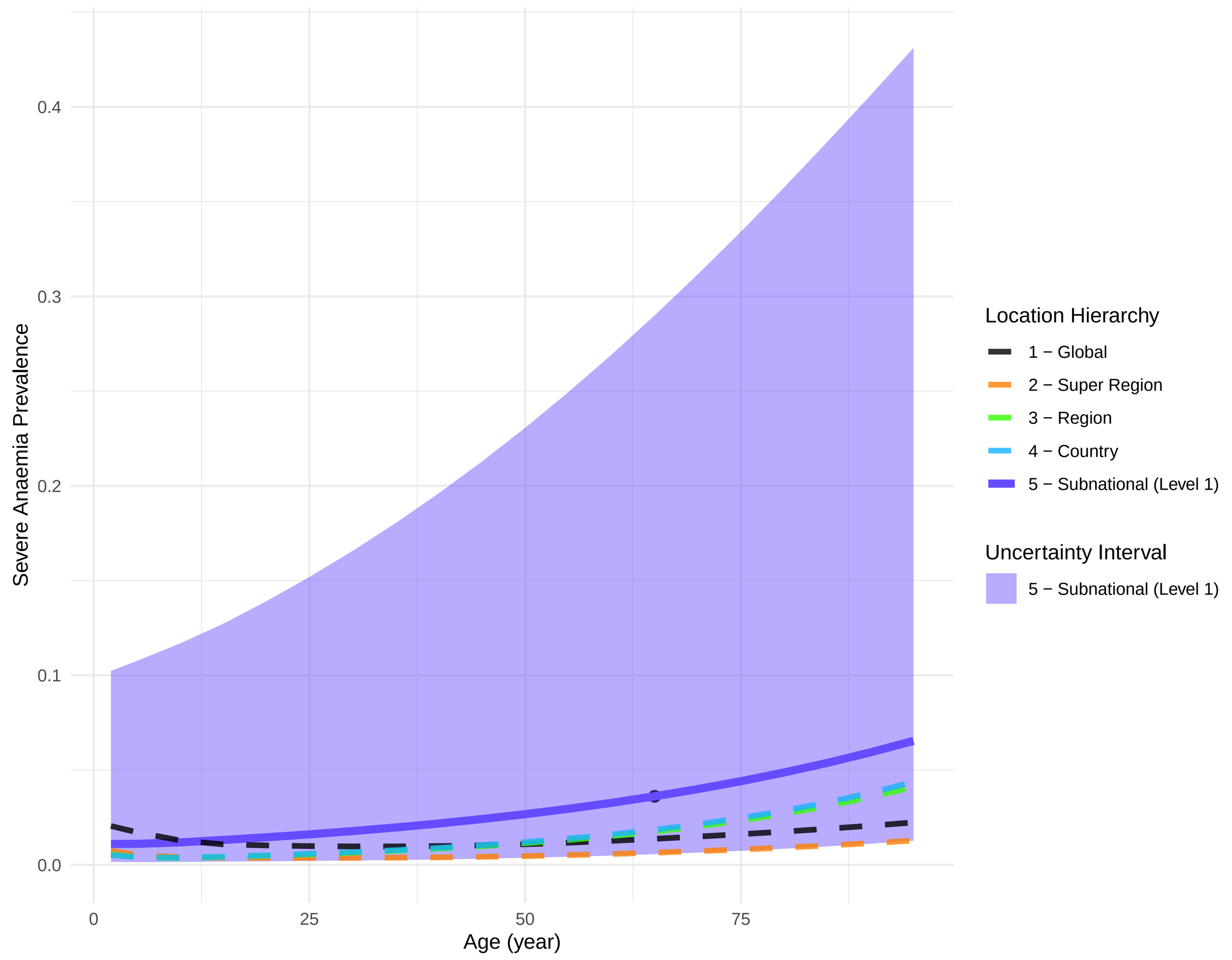

MR−BRT Age/Sex Fits − Males 2+ Years Old − Severe Anaemia Prevalence − Coahuila
WHO−2024 Adjustment Method

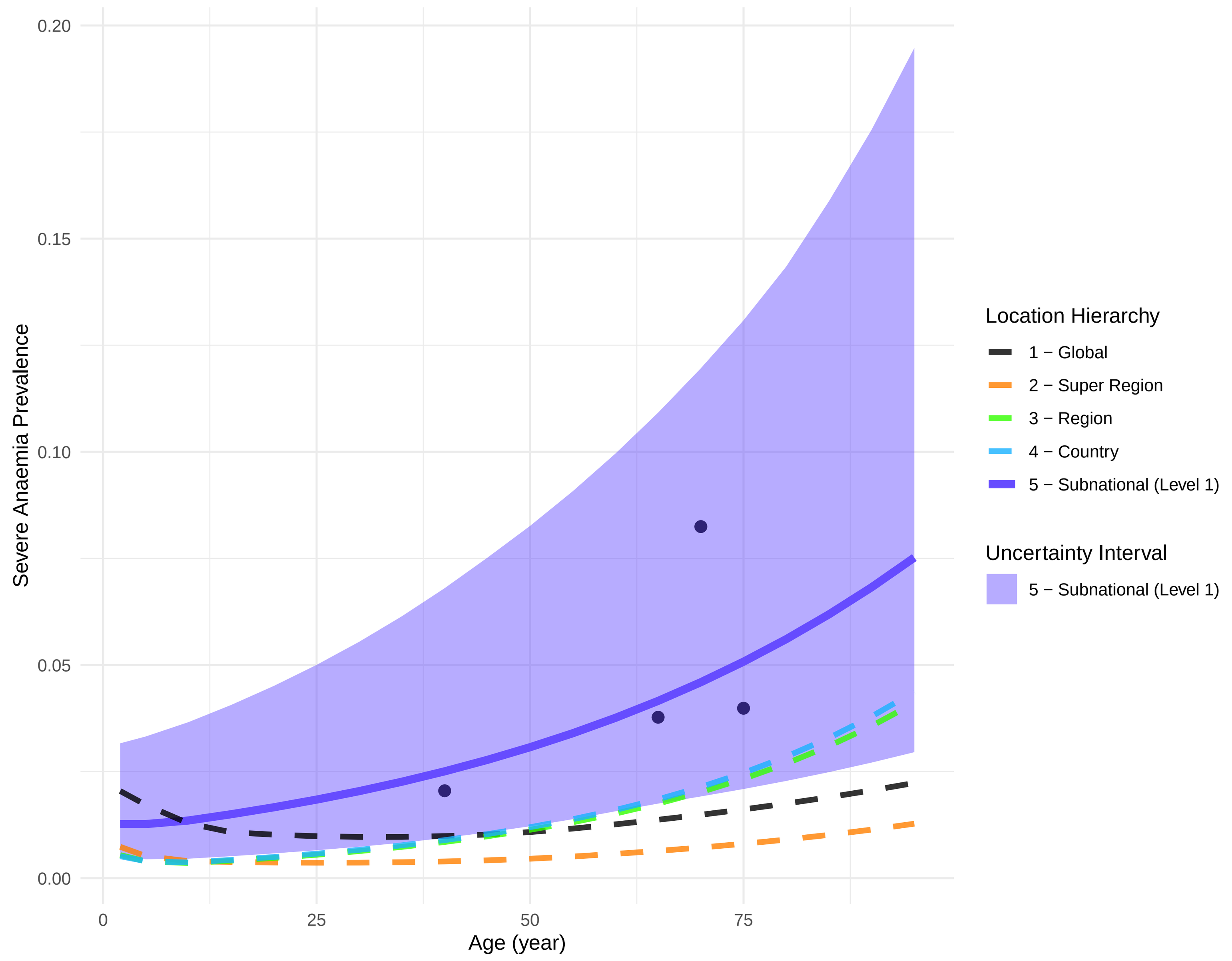

MR−BRT Age/Sex Fits − Males 2+ Years Old − Severe Anaemia Prevalence − Colima
WHO−2024 Adjustment Method

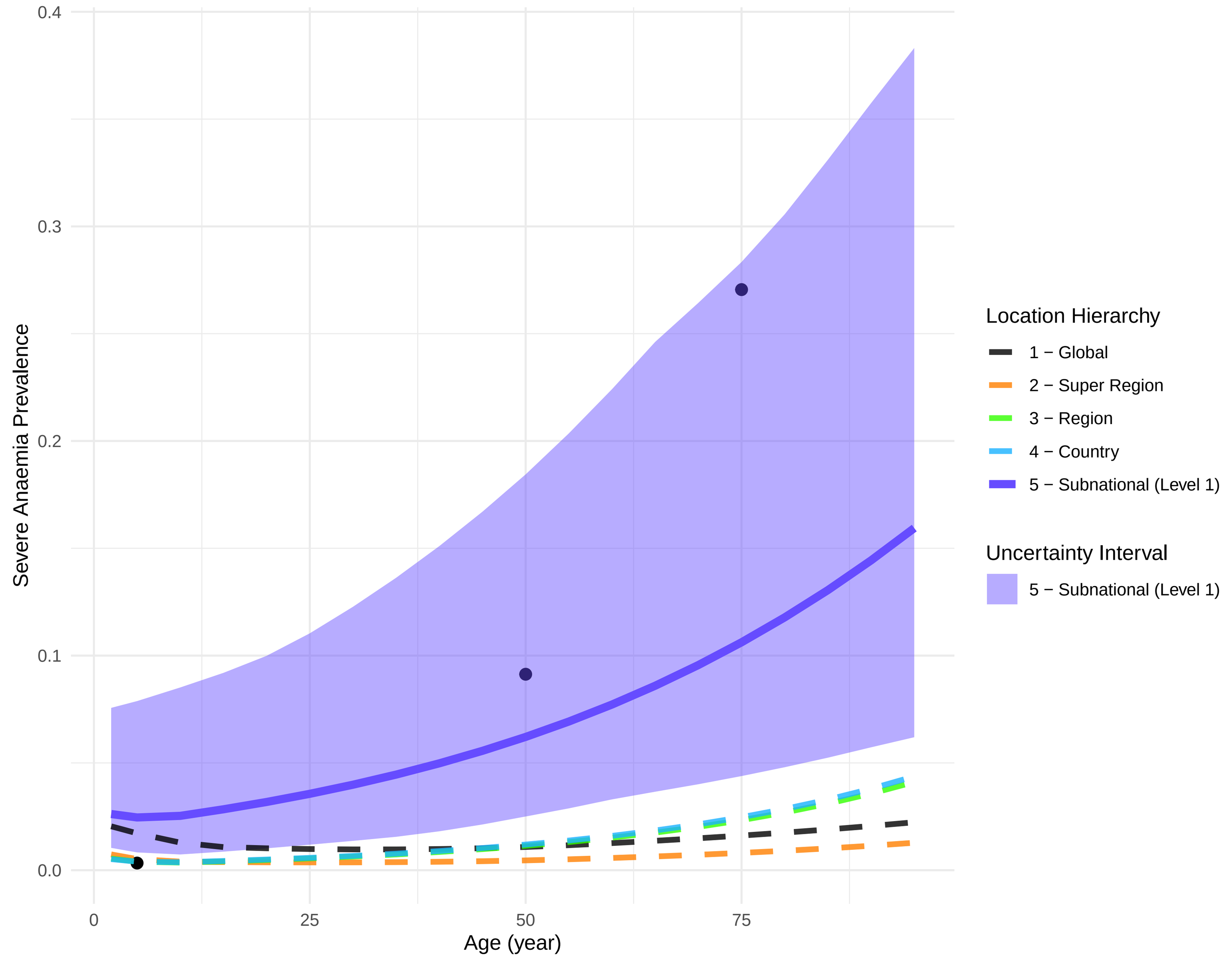

MR−BRT Age/Sex Fits − Males 2+ Years Old − Severe Anaemia Prevalence − Durango
WHO−2024 Adjustment Method

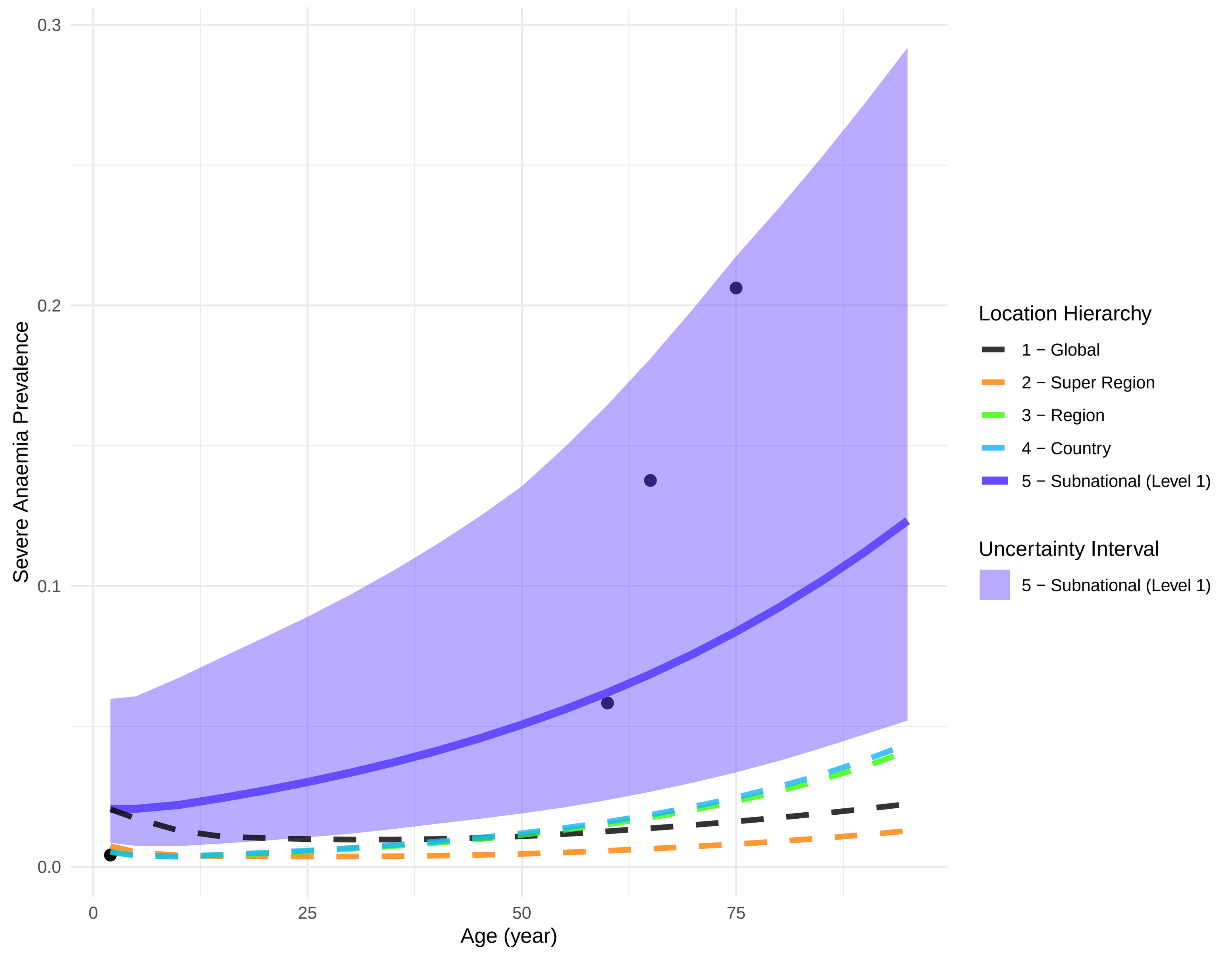

MR−BRT Age/Sex Fits − Males 2+ Years Old − Severe Anaemia Prevalence − Guanajuato
WHO−2024 Adjustment Method

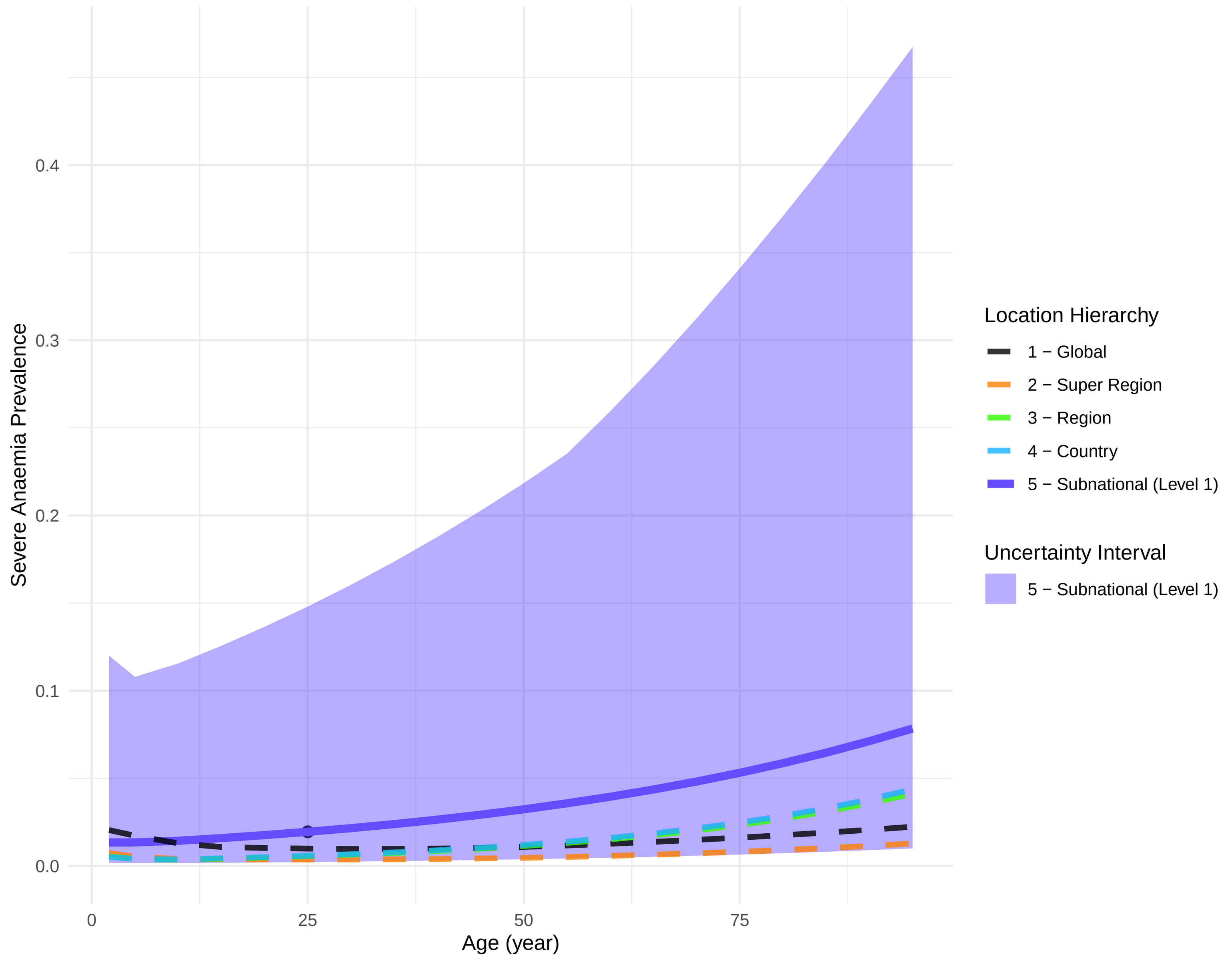

MR−BRT Age/Sex Fits − Males 2+ Years Old − Severe Anaemia Prevalence − Guerrero
WHO−2024 Adjustment Method

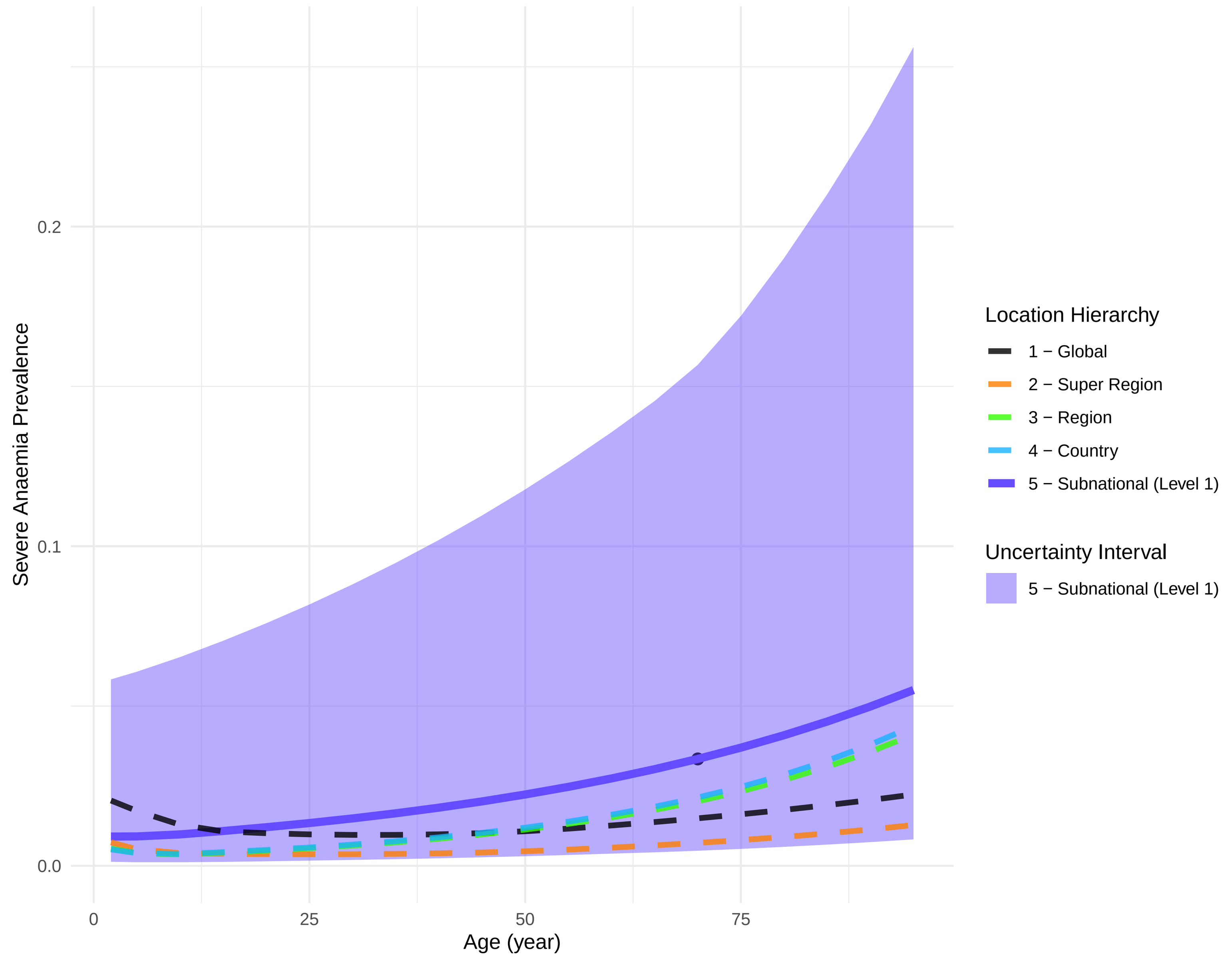

MR−BRT Age/Sex Fits − Males 2+ Years Old − Severe Anaemia Prevalence − Hidalgo
WHO−2024 Adjustment Method

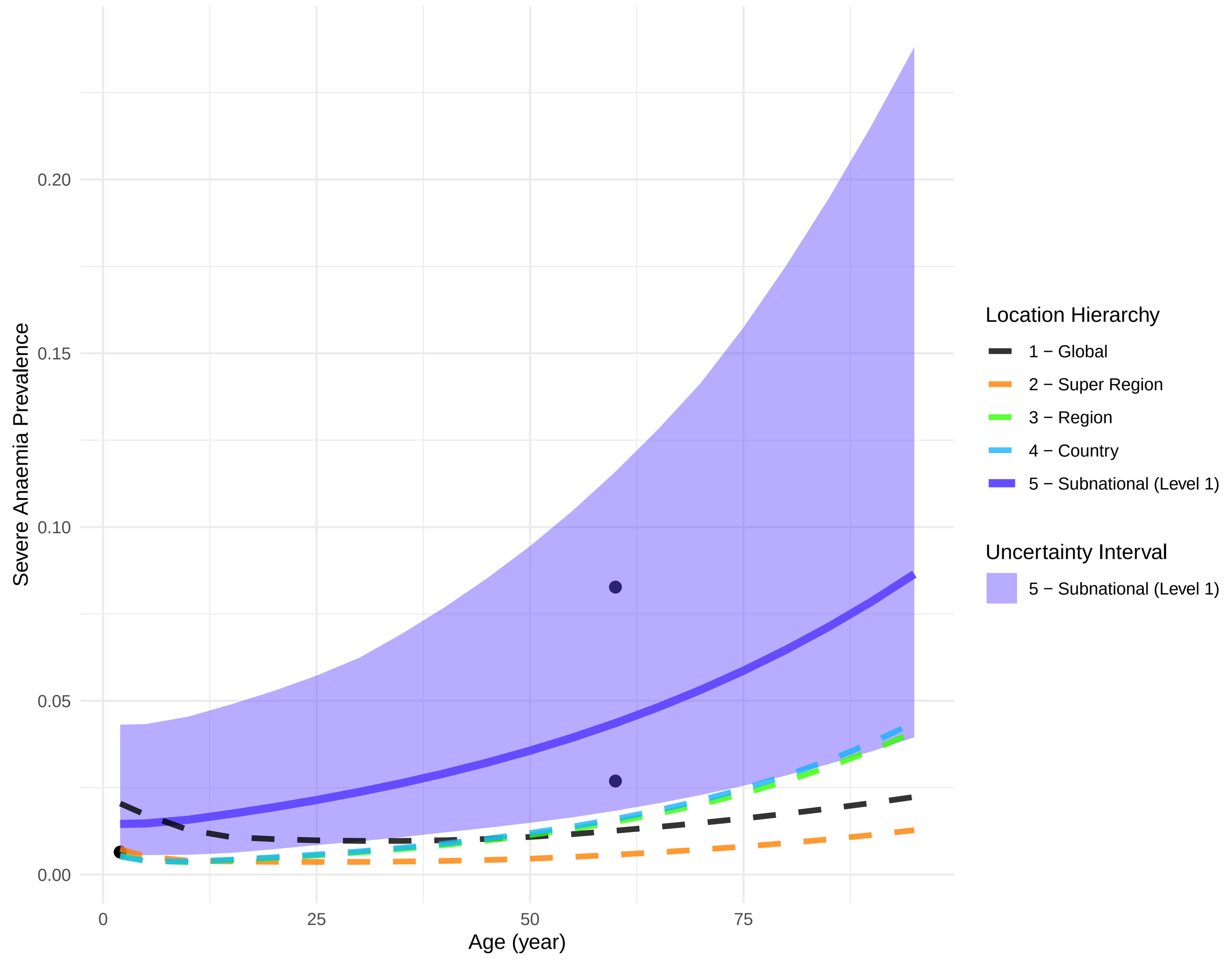

MR−BRT Age/Sex Fits − Males 2+ Years Old − Severe Anaemia Prevalence − Jalisco
WHO−2024 Adjustment Method

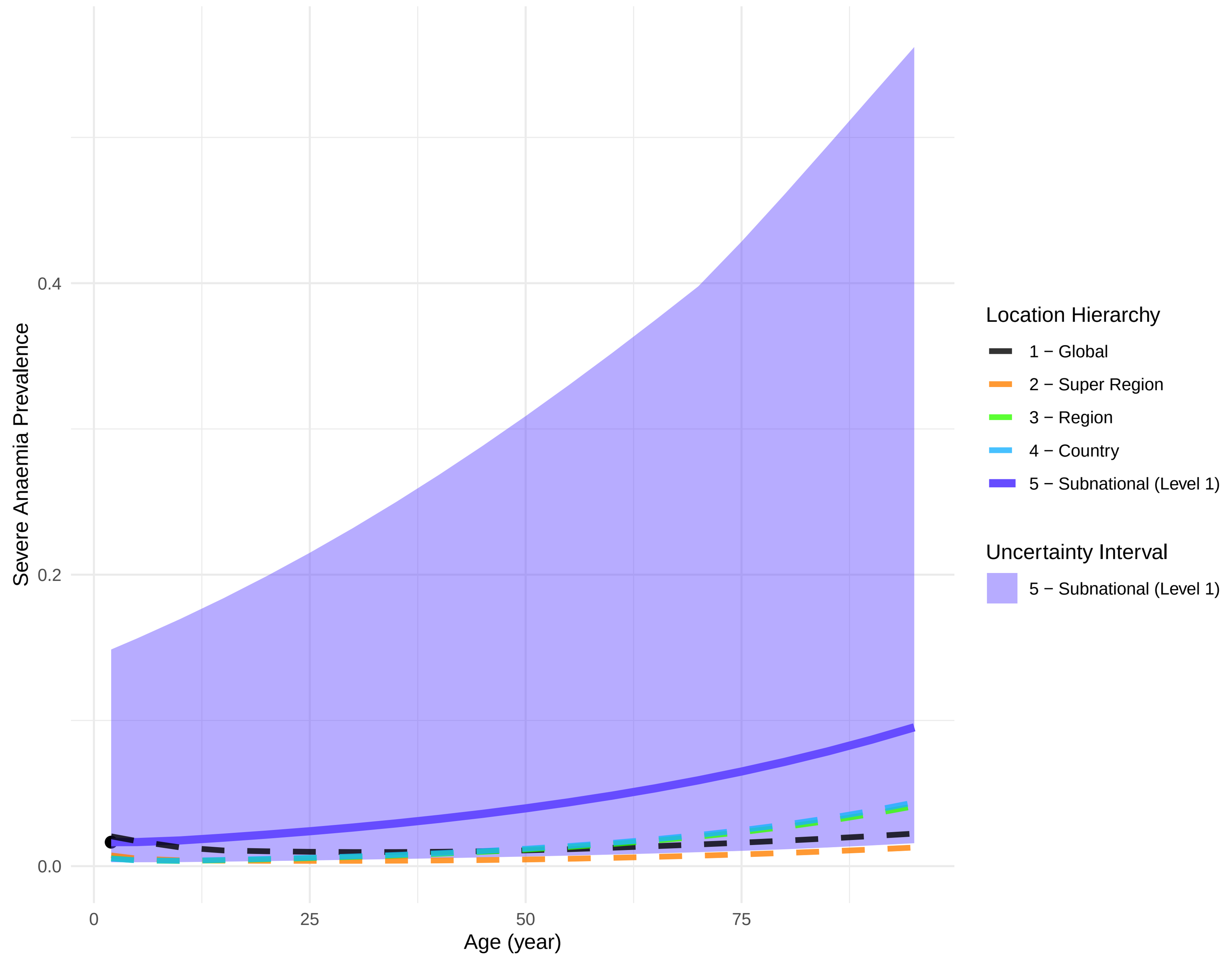

MR−BRT Age/Sex Fits − Males 2+ Years Old − Severe Anaemia Prevalence − México
WHO−2024 Adjustment Method

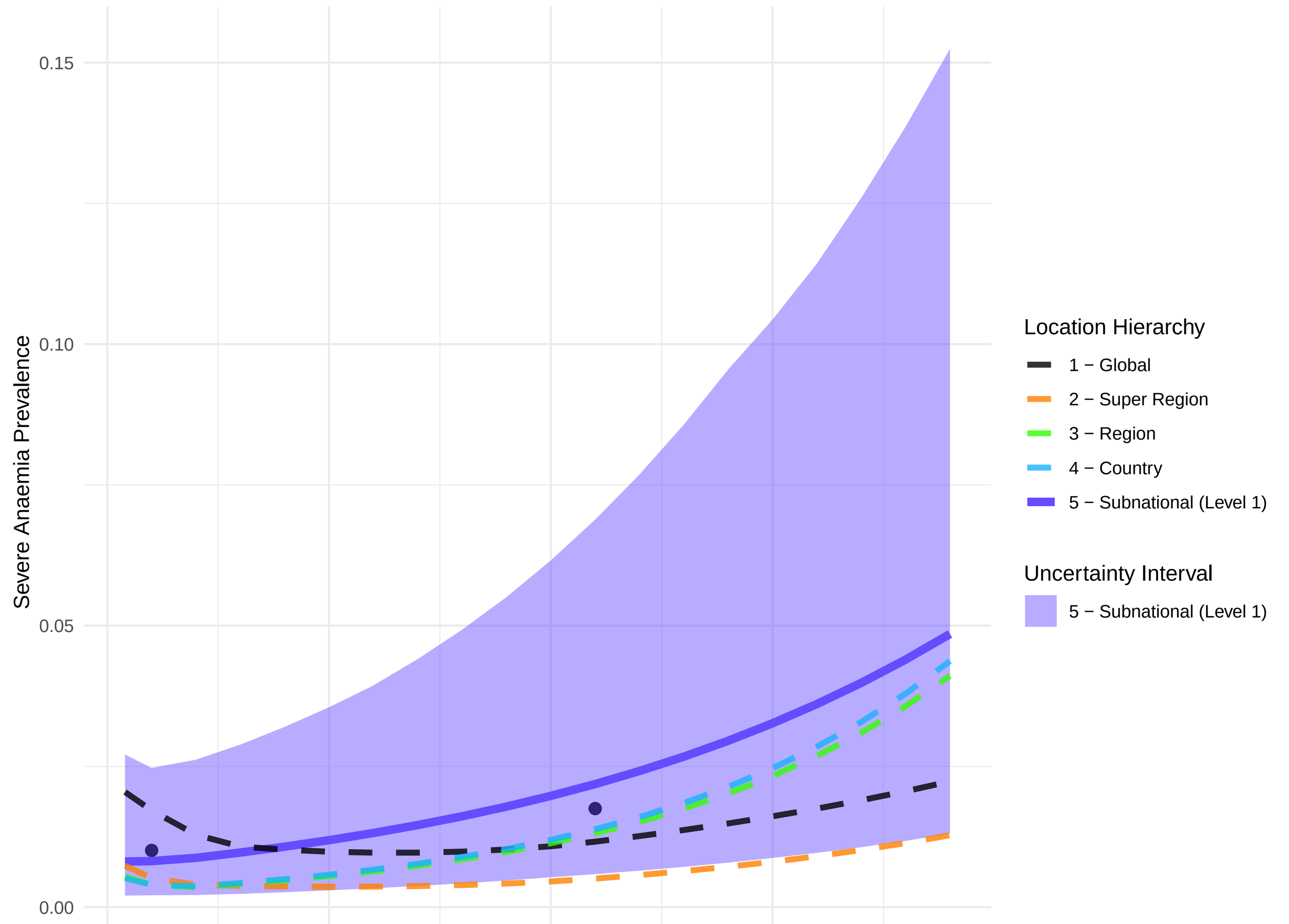

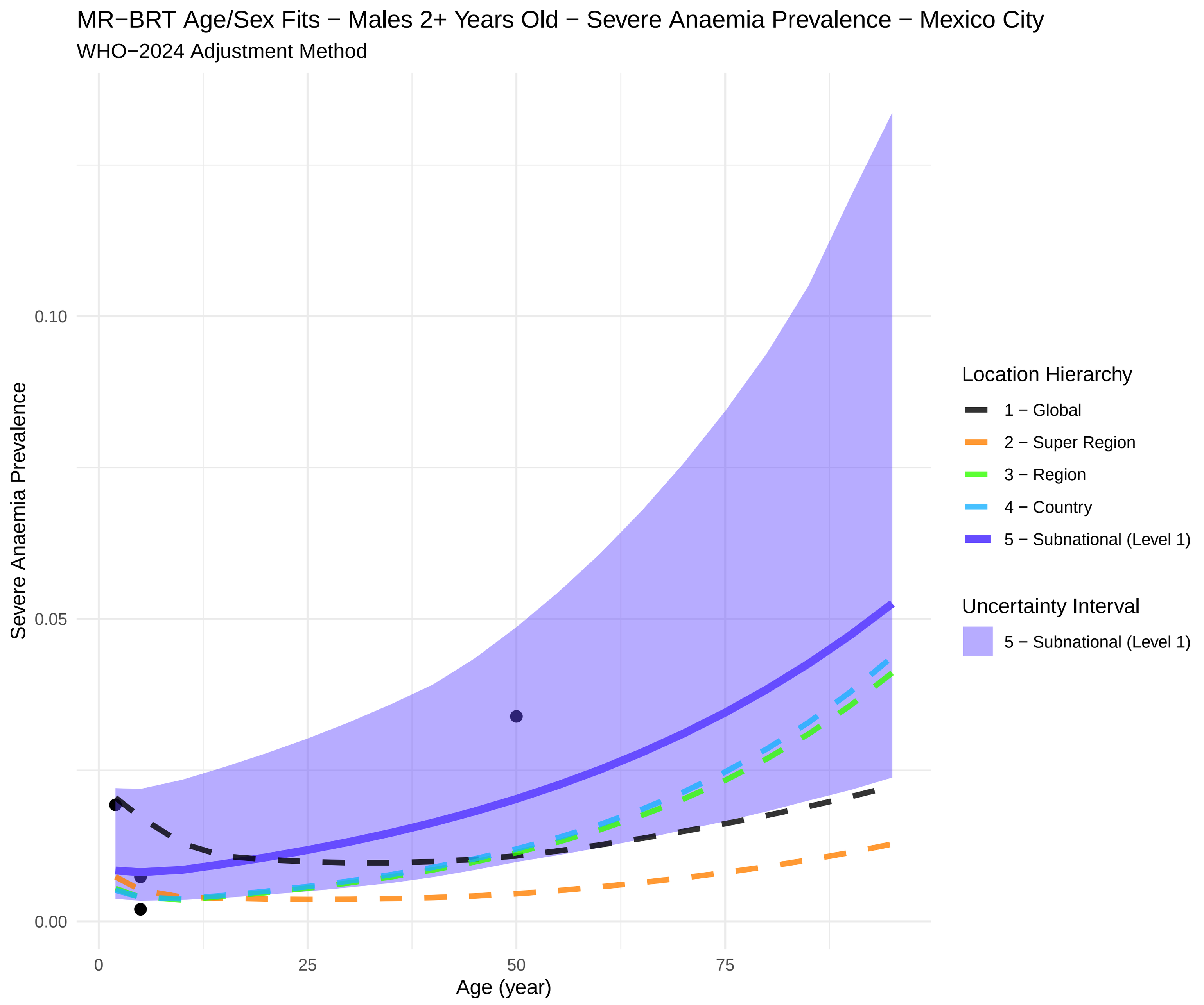
MR−BRT Age/Sex Fits − Males 2+ Years Old − Severe Anaemia Prevalence − Mexico City
WHO−2024 Adjustment Method
Severe Anaemia Prevalence
Age (year)
Location Hierarchy
1 − Global
2 − Super Region
3 − Region
4 − Country
5 − Subnational (Level 1)
Uncertainty Interval
5 − Subnational (Level 1)

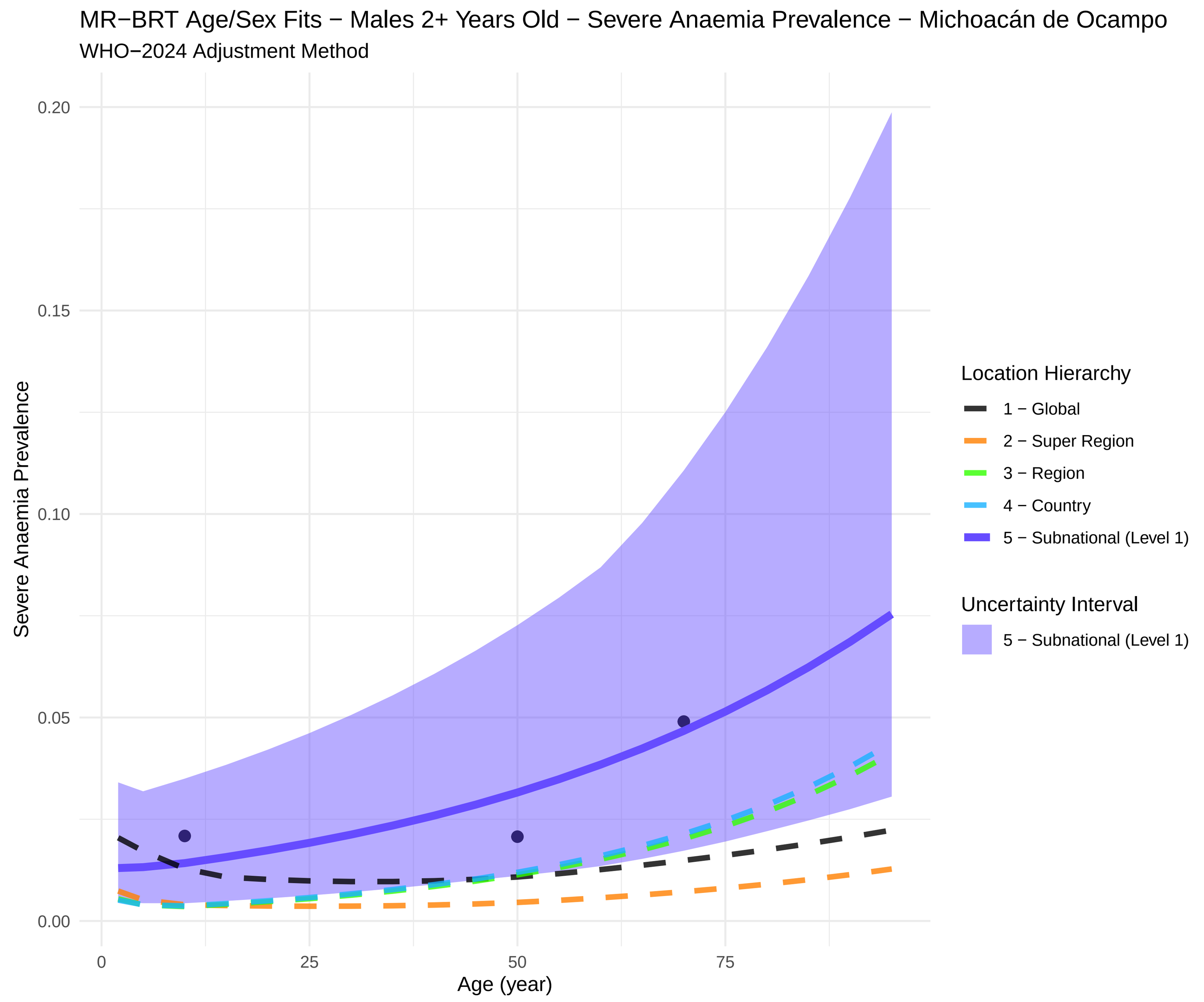
MR−BRT Age/Sex Fits − Males 2+ Years Old − Severe Anaemia Prevalence − Michoacán de Ocampo
WHO−2024 Adjustment Method
Severe Anaemia Prevalence
Age (year)
Location Hierarchy
1 − Global
2 − Super Region
3 − Region
4 − Country
5 − Subnational (Level 1)
Uncertainty Interval
5 − Subnational (Level 1)

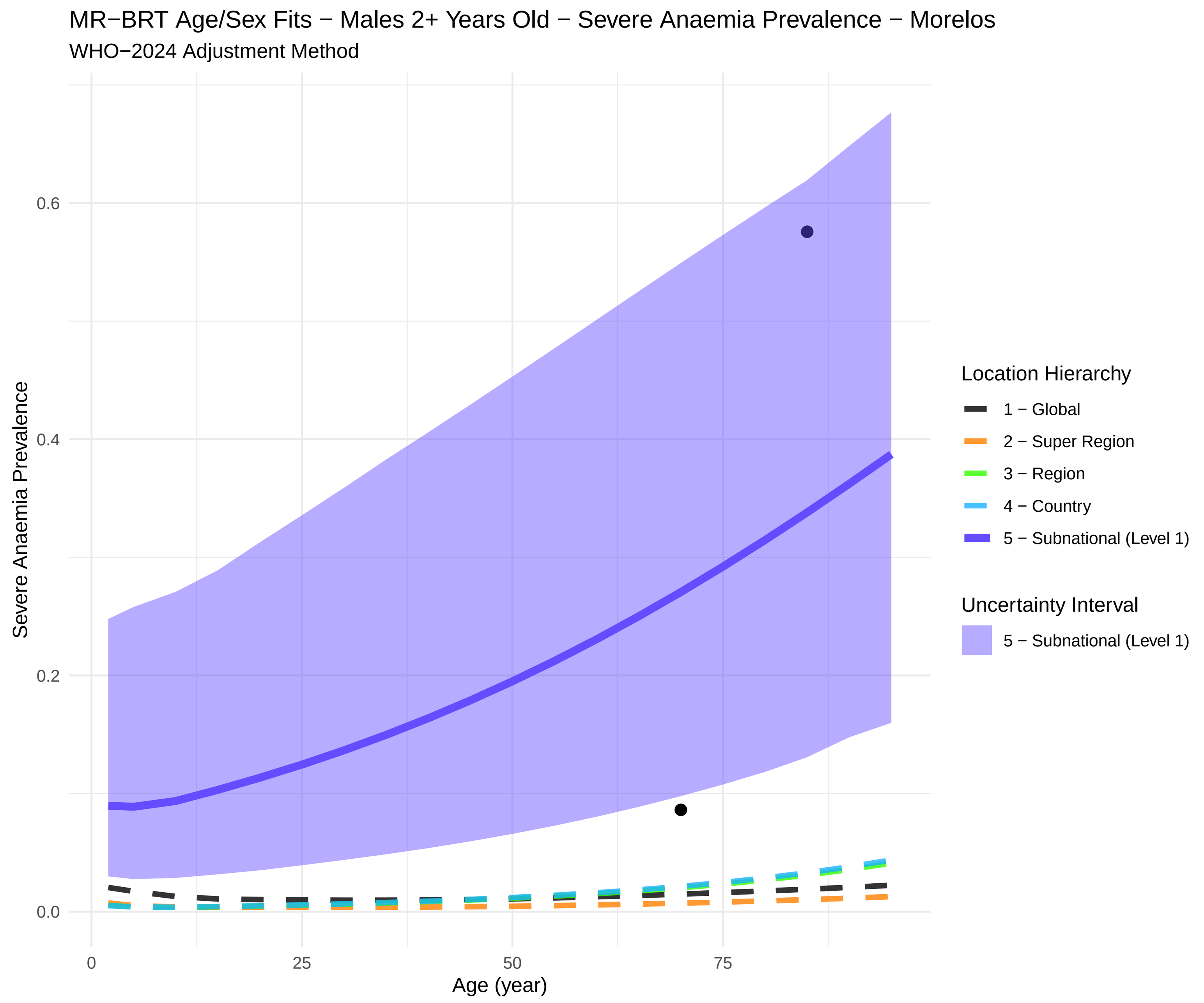
MR−BRT Age/Sex Fits − Males 2+ Years Old − Severe Anaemia Prevalence − Morelos
WHO−2024 Adjustment Method
Severe Anaemia Prevalence
Age (year)
Location Hierarchy
1 − Global
2 − Super Region
3 − Region
4 − Country
5 − Subnational (Level 1)
Uncertainty Interval
5 − Subnational (Level 1)

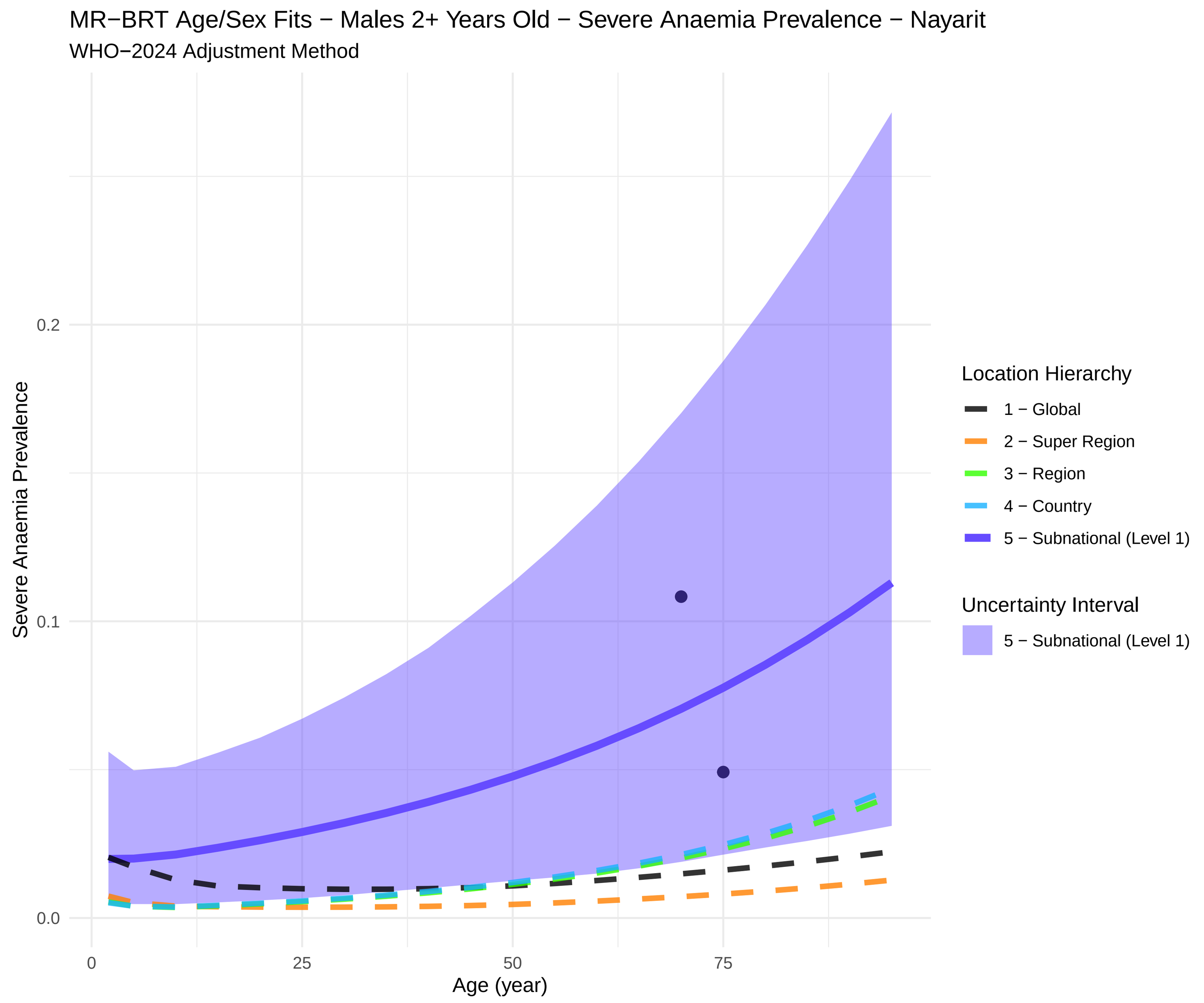
MR−BRT Age/Sex Fits − Males 2+ Years Old − Severe Anaemia Prevalence − Nayarit
WHO−2024 Adjustment Method
Severe Anaemia Prevalence
Age (year)
Location Hierarchy
1 − Global
2 − Super Region
3 − Region
4 − Country
5 − Subnational (Level 1)
Uncertainty Interval
5 − Subnational (Level 1)

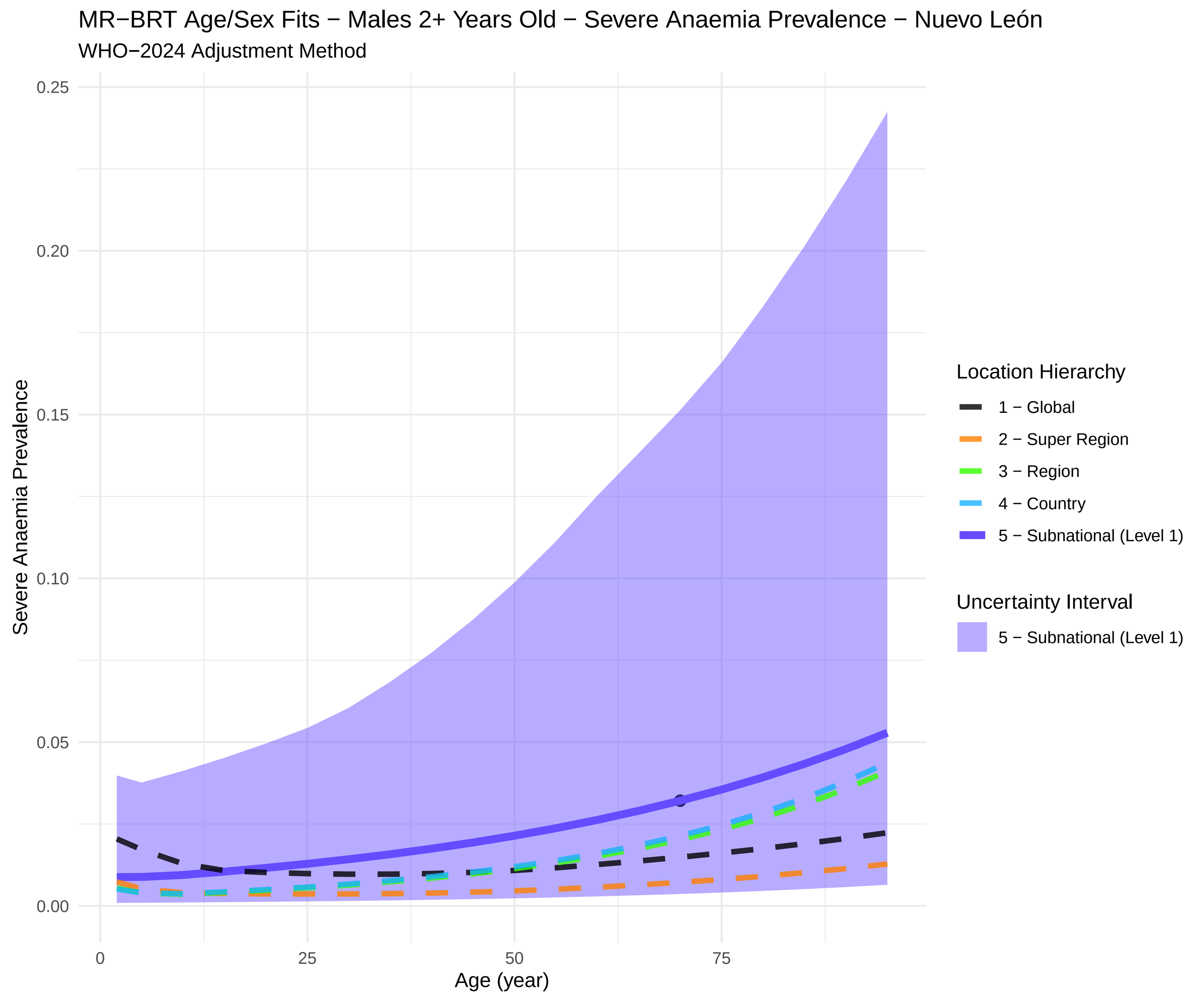
MR−BRT Age/Sex Fits − Males 2+ Years Old − Severe Anaemia Prevalence − Nuevo León
WHO−2024 Adjustment Method
Severe Anaemia Prevalence
Age (year)
Location Hierarchy
1 − Global
2 − Super Region
3 − Region
4 − Country
5 − Subnational (Level 1)
Uncertainty Interval
5 − Subnational (Level 1)

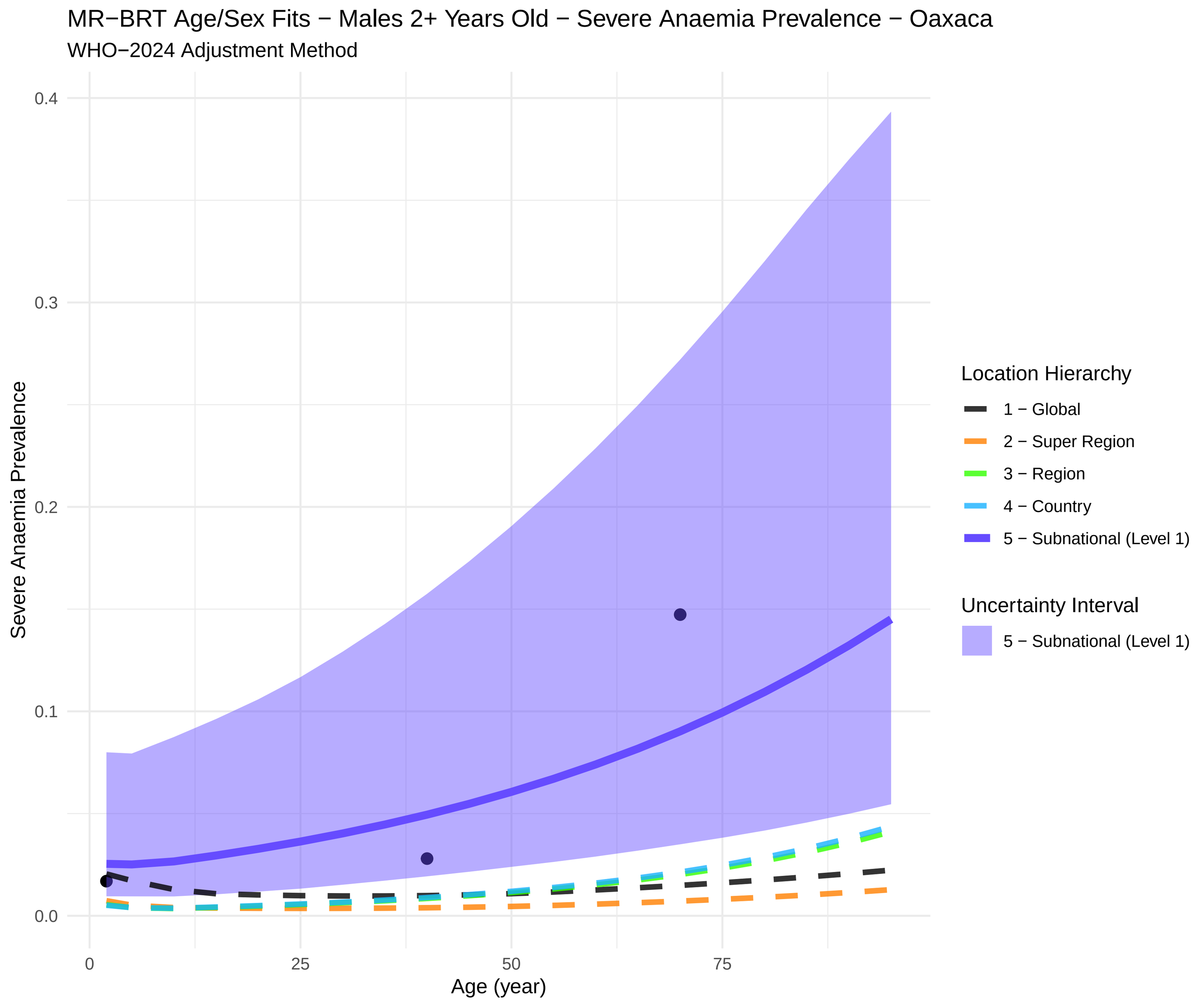
MR−BRT Age/Sex Fits − Males 2+ Years Old − Severe Anaemia Prevalence − Oaxaca
WHO−2024 Adjustment Method
Severe Anaemia Prevalence
Age (year)
Location Hierarchy
1 − Global
2 − Super Region
3 − Region
4 − Country
5 − Subnational (Level 1)
Uncertainty Interval
5 − Subnational (Level 1)

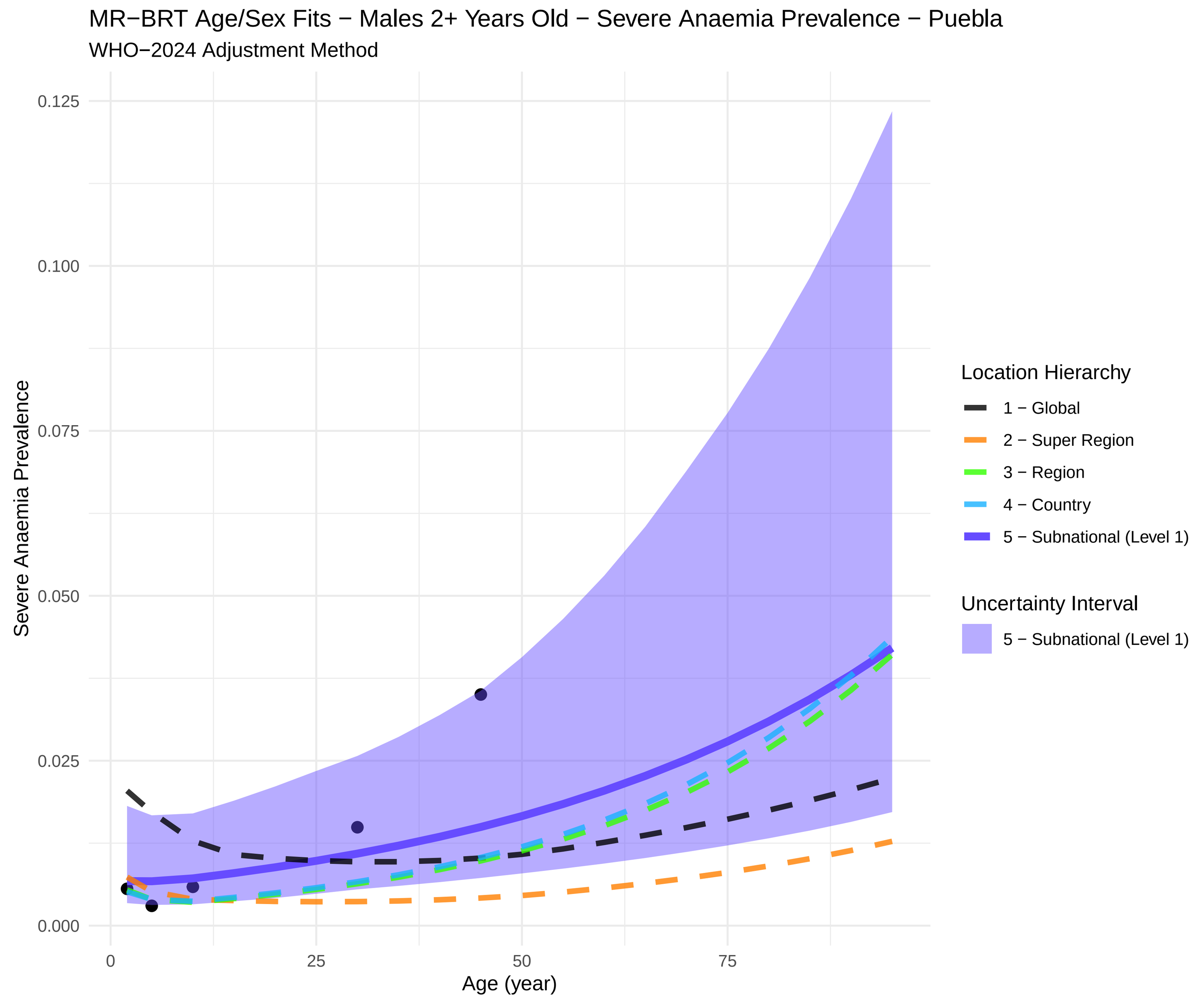
MR−BRT Age/Sex Fits − Males 2+ Years Old − Severe Anaemia Prevalence − Puebla
WHO−2024 Adjustment Method
Severe Anaemia Prevalence
Age (year)
Location Hierarchy
1 − Global
2 − Super Region
3 − Region
4 − Country
5 − Subnational (Level 1)
Uncertainty Interval
5 − Subnational (Level 1)

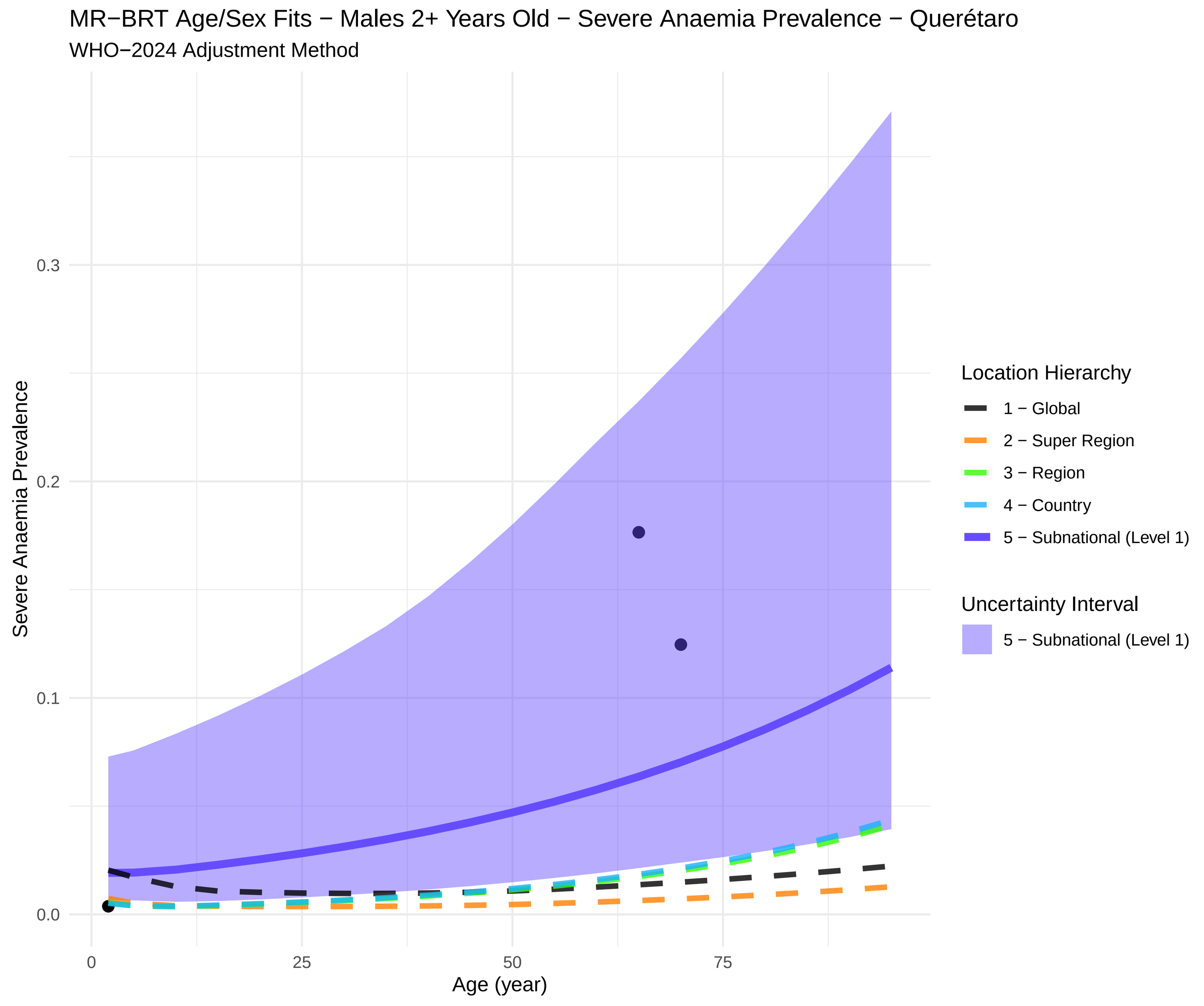
MR−BRT Age/Sex Fits − Males 2+ Years Old − Severe Anaemia Prevalence − Querétaro
WHO−2024 Adjustment Method
Severe Anaemia Prevalence
Age (year)
Location Hierarchy
1 − Global
2 − Super Region
3 − Region
4 − Country
5 − Subnational (Level 1)
Uncertainty Interval
5 − Subnational (Level 1)

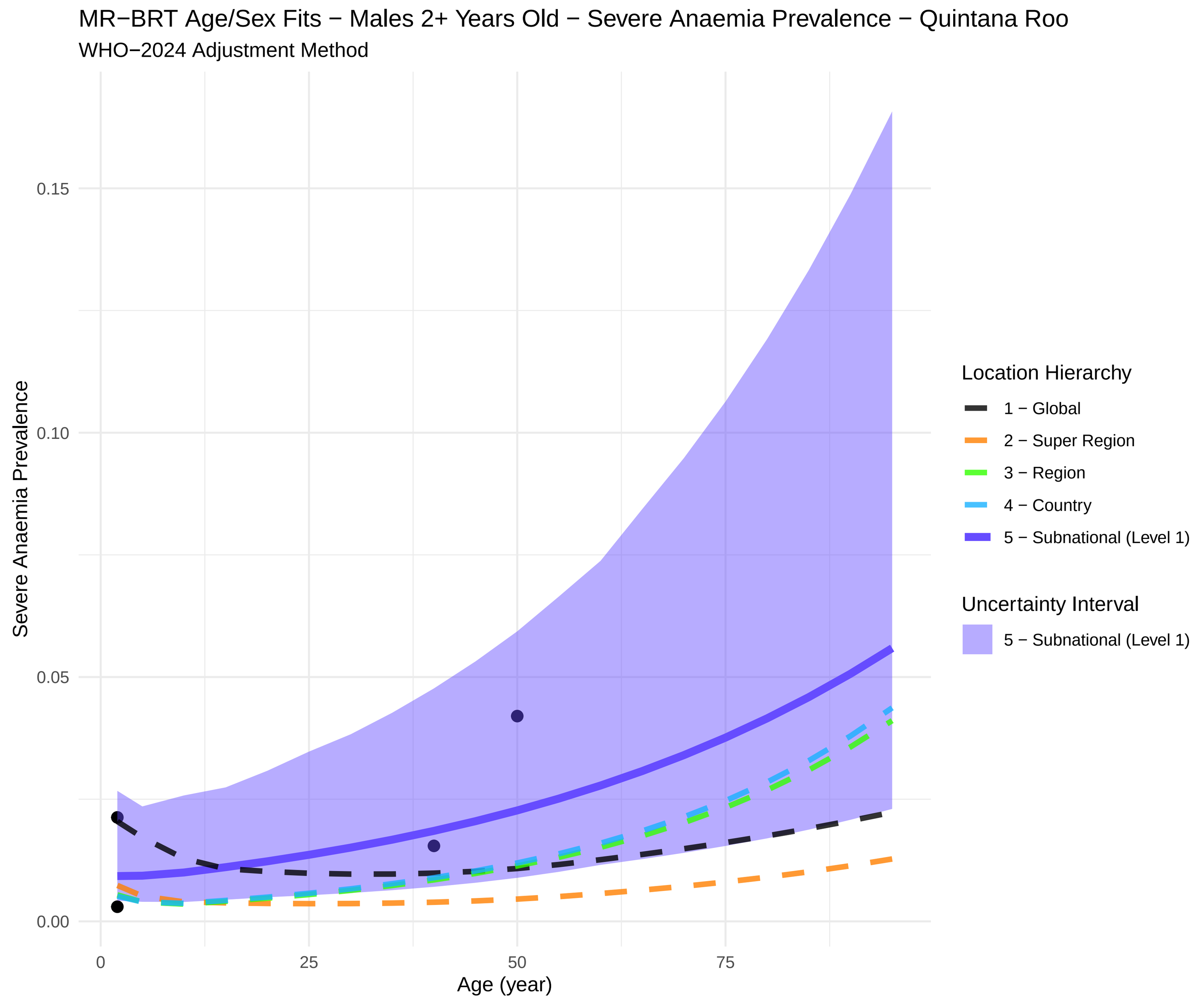
MR−BRT Age/Sex Fits − Males 2+ Years Old − Severe Anaemia Prevalence − Quintana Roo
WHO−2024 Adjustment Method
Severe Anaemia Prevalence
Age (year)
Location Hierarchy
1 − Global
2 − Super Region
3 − Region
4 − Country
5 − Subnational (Level 1)
Uncertainty Interval
5 − Subnational (Level 1)

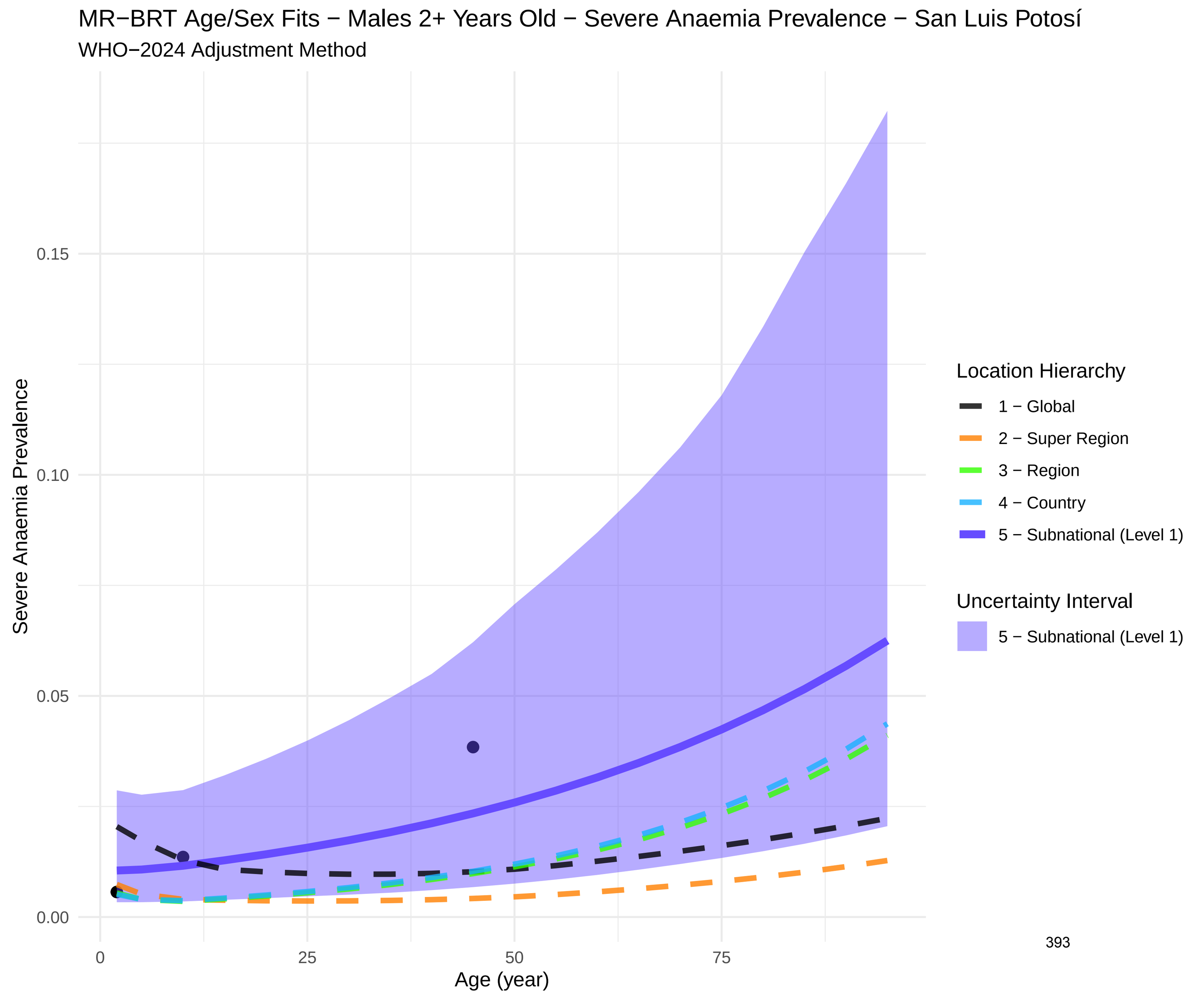
MR−BRT Age/Sex Fits − Males 2+ Years Old − Severe Anaemia Prevalence − San Luis Potosí
WHO−2024 Adjustment Method
Severe Anaemia Prevalence
Age (year)
Location Hierarchy
1 − Global
2 − Super Region
3 − Region
4 − Country
5 − Subnational (Level 1)
Uncertainty Interval
5 − Subnational (Level 1)

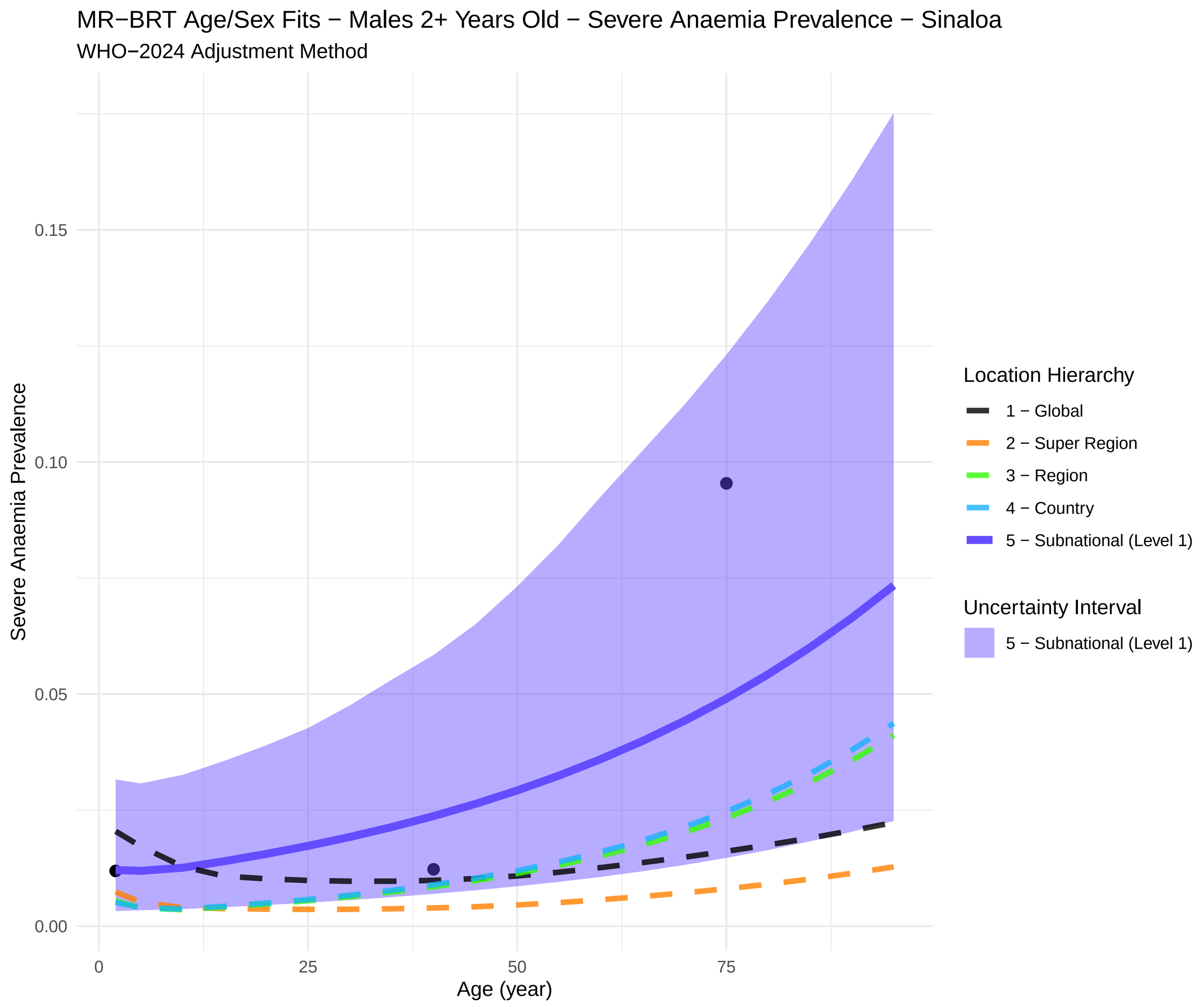
MR−BRT Age/Sex Fits − Males 2+ Years Old − Severe Anaemia Prevalence − Sinaloa
WHO−2024 Adjustment Method
Severe Anaemia Prevalence
Age (year)
Location Hierarchy
1 − Global
2 − Super Region
3 − Region
4 − Country
5 − Subnational (Level 1)
Uncertainty Interval
5 − Subnational (Level 1)

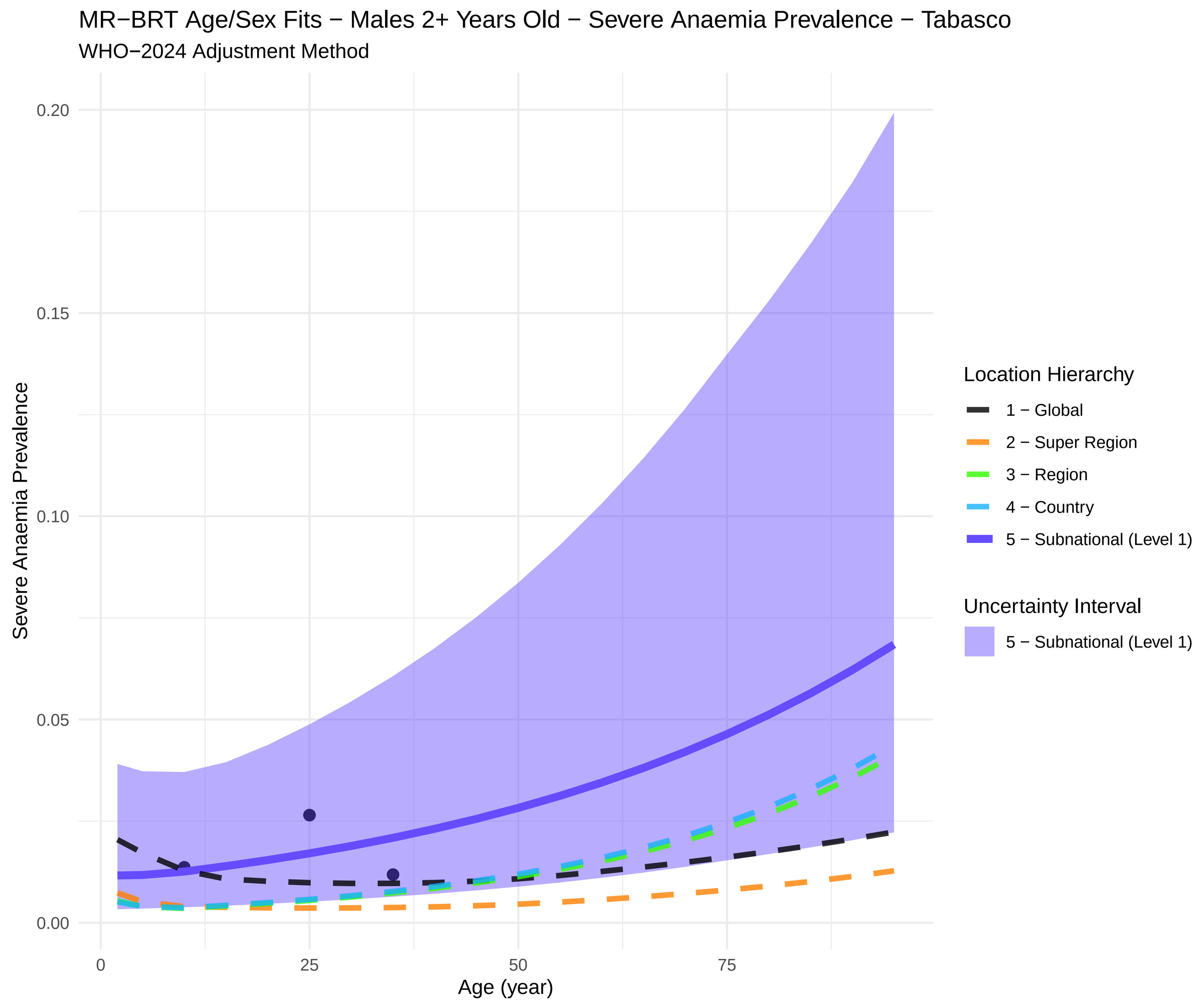
MR−BRT Age/Sex Fits − Males 2+ Years Old − Severe Anaemia Prevalence − Tabasco
WHO−2024 Adjustment Method
Severe Anaemia Prevalence
Age (year)
Location Hierarchy
1 − Global
2 − Super Region
3 − Region
4 − Country
5 − Subnational (Level 1)
Uncertainty Interval
5 − Subnational (Level 1)

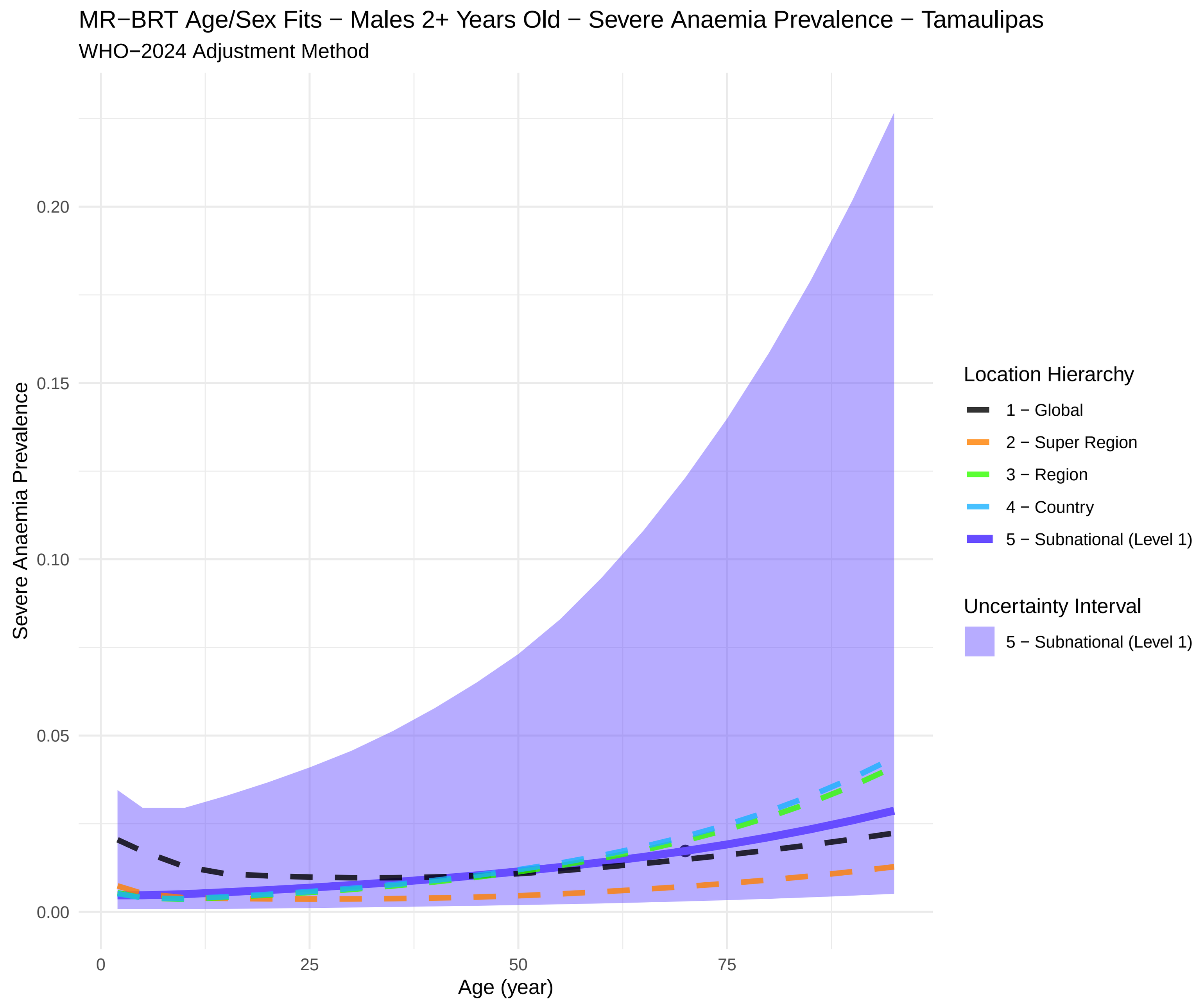
MR−BRT Age/Sex Fits − Males 2+ Years Old − Severe Anaemia Prevalence − Tamaulipas
WHO−2024 Adjustment Method
Severe Anaemia Prevalence
Age (year)
Location Hierarchy
1 − Global
2 − Super Region
3 − Region
4 − Country
5 − Subnational (Level 1)
Uncertainty Interval
5 − Subnational (Level 1)

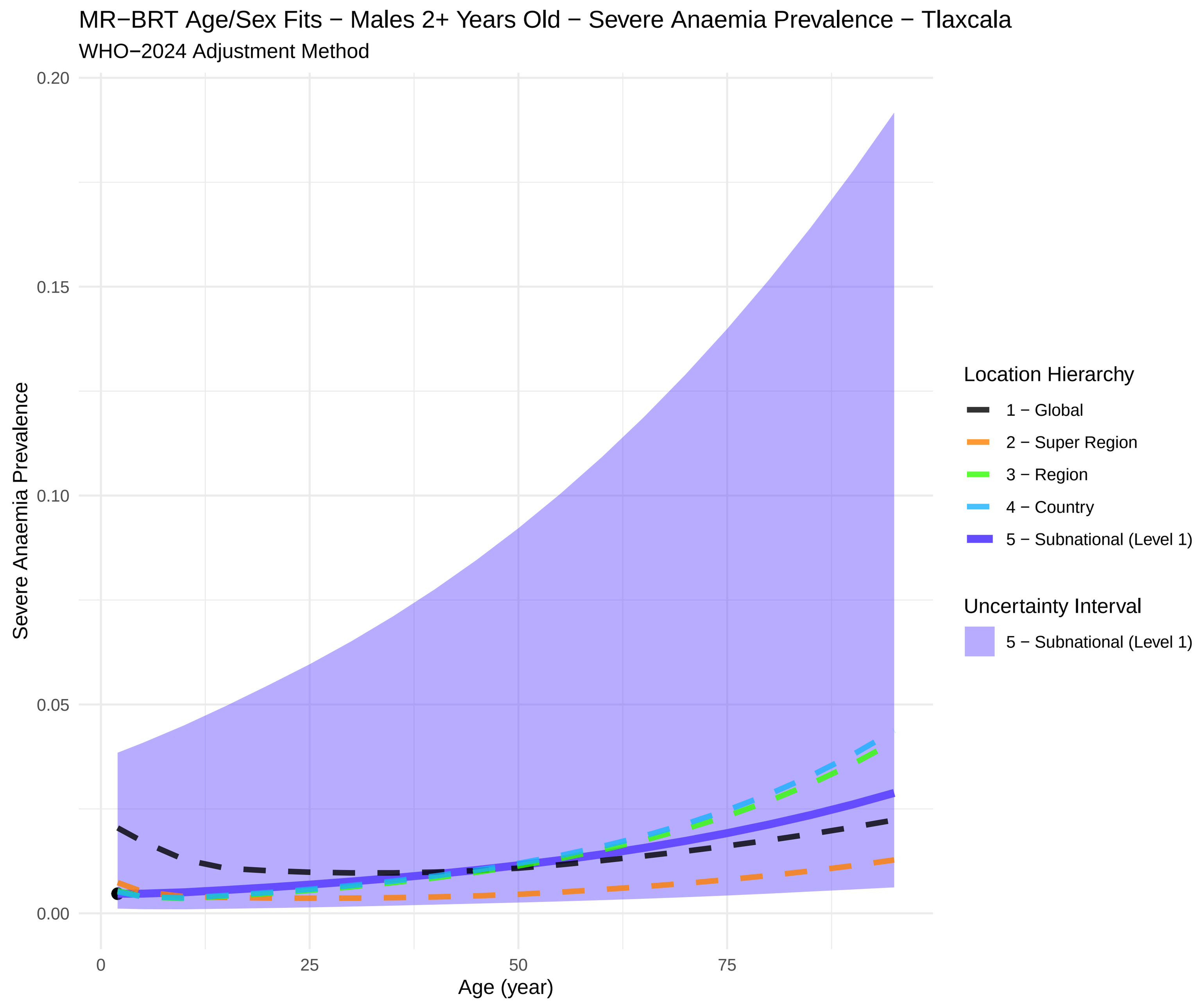
MR−BRT Age/Sex Fits − Males 2+ Years Old − Severe Anaemia Prevalence − Tlaxcala
WHO−2024 Adjustment Method
Severe Anaemia Prevalence
Age (year)
Location Hierarchy
1 − Global
2 − Super Region
3 − Region
4 − Country
5 − Subnational (Level 1)
Uncertainty Interval
5 − Subnational (Level 1)

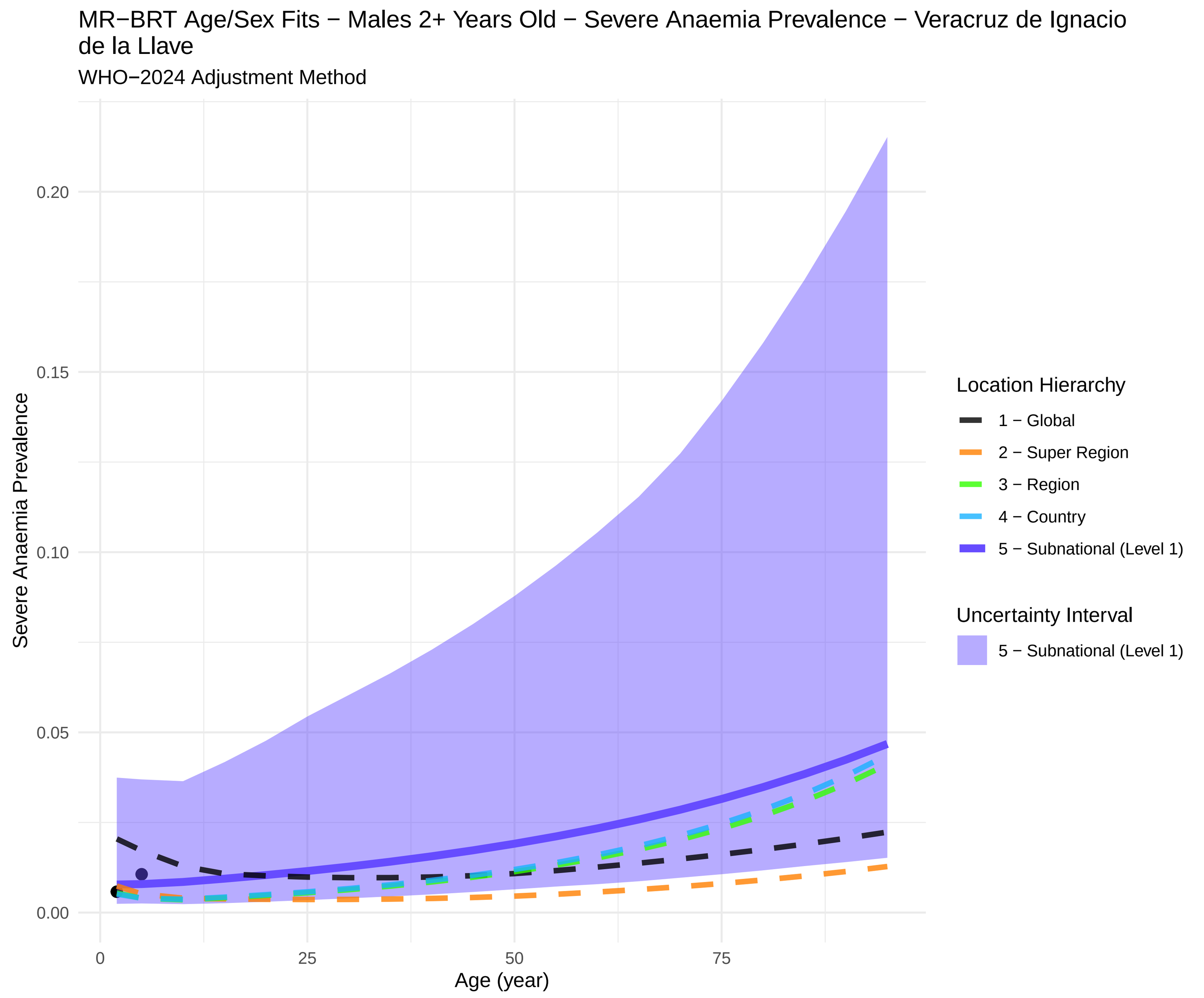
MR−BRT Age/Sex Fits − Males 2+ Years Old − Severe Anaemia Prevalence − Veracruz de Ignacio de la Llave
WHO−2024 Adjustment Method
Severe Anaemia Prevalence
Age (year)
Location Hierarchy
1 − Global
2 − Super Region
3 − Region
4 − Country
5 − Subnational (Level 1)
Uncertainty Interval
5 − Subnational (Level 1)

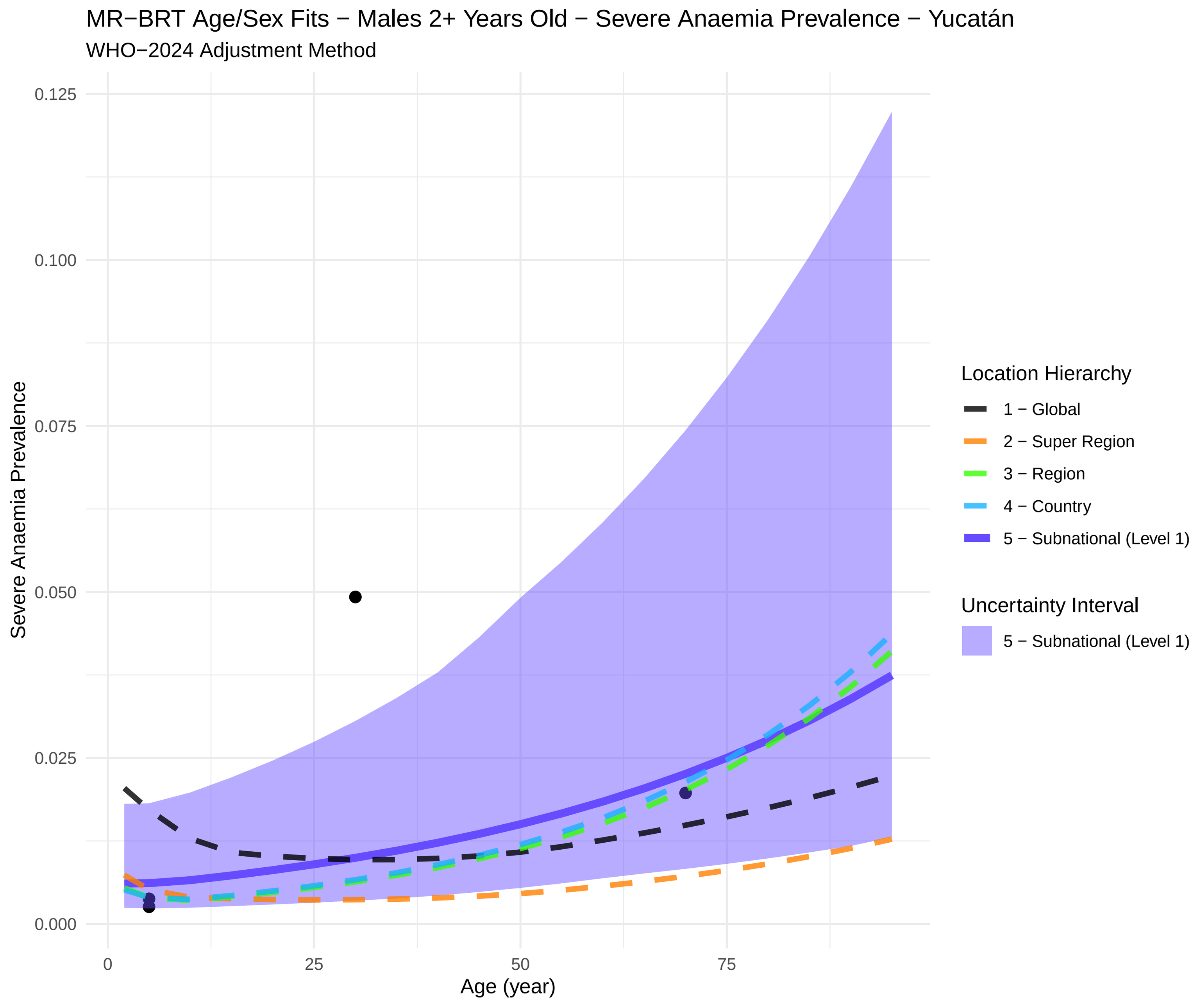
MR−BRT Age/Sex Fits − Males 2+ Years Old − Severe Anaemia Prevalence − Yucatán
WHO−2024 Adjustment Method
Severe Anaemia Prevalence
Age (year)
Location Hierarchy
1 − Global
2 − Super Region
3 − Region
4 − Country
5 − Subnational (Level 1)
Uncertainty Interval
5 − Subnational (Level 1)

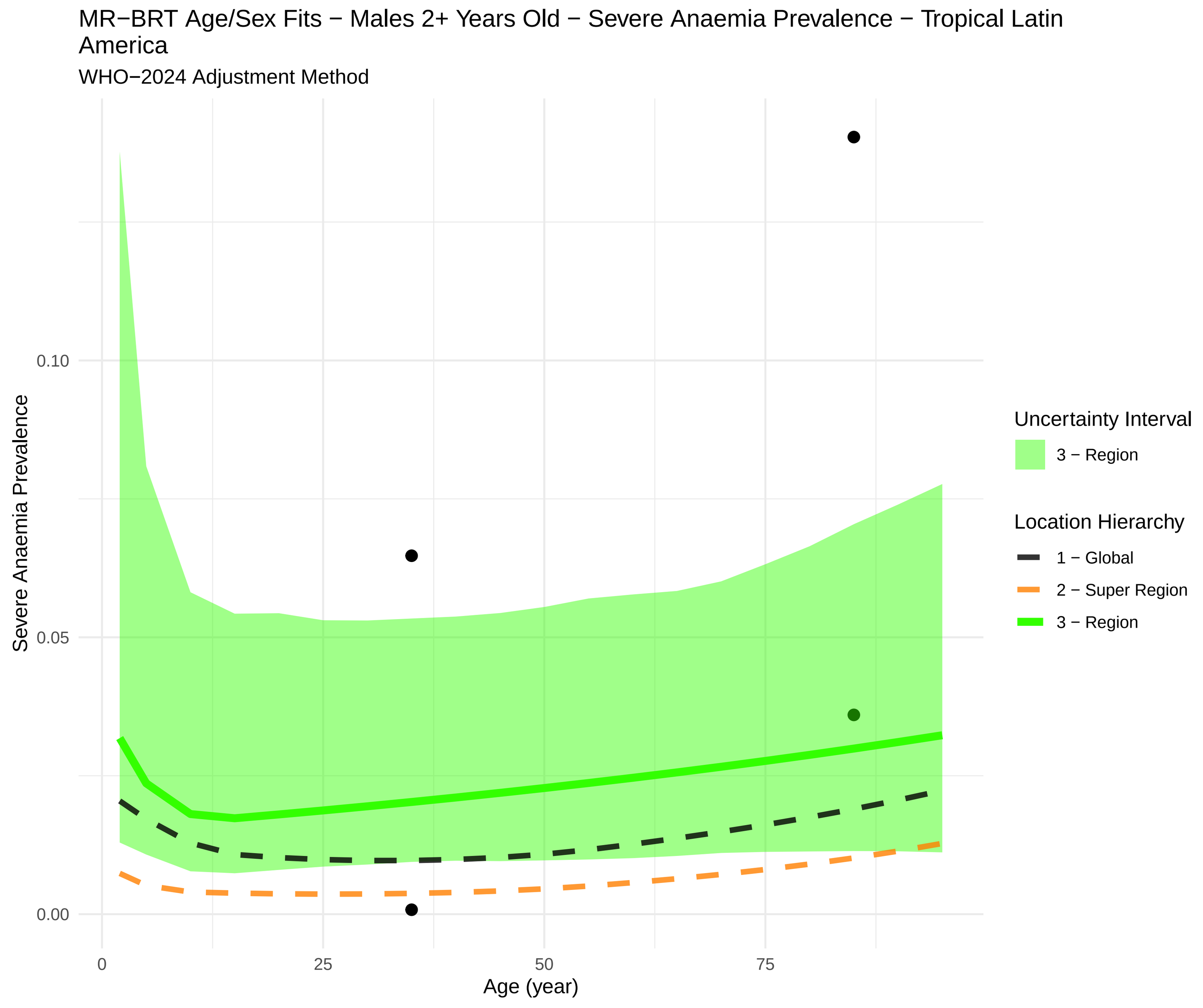
MR−BRT Age/Sex Fits − Males 2+ Years Old − Severe Anaemia Prevalence − Tropical Latin America
WHO−2024 Adjustment Method
Severe Anaemia Prevalence
Age (year)
Uncertainty Interval
3 − Region
Location Hierarchy
1 − Global
2 − Super Region
3 − Region

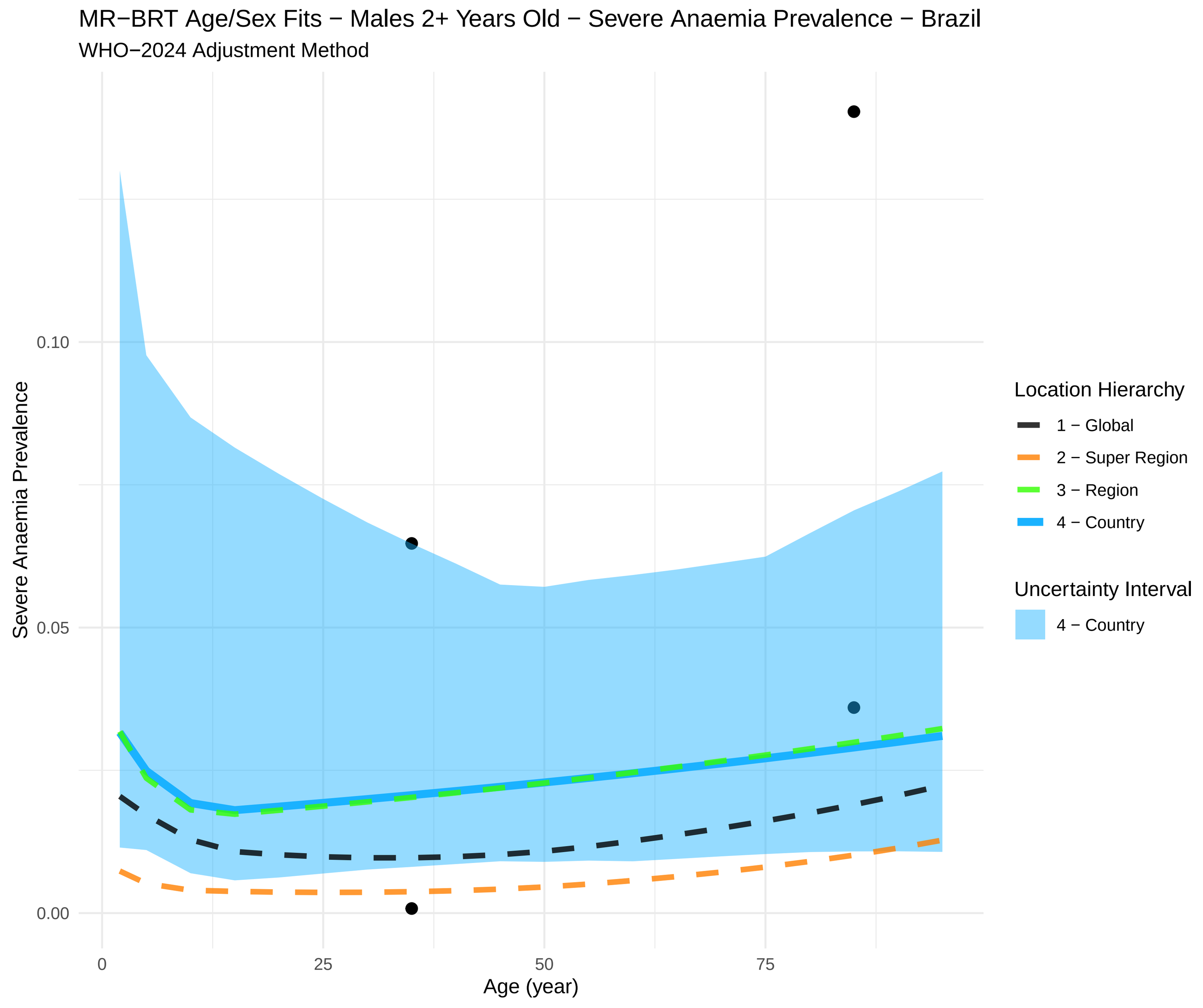
MR−BRT Age/Sex Fits − Males 2+ Years Old − Severe Anaemia Prevalence − Brazil
WHO−2024 Adjustment Method
Severe Anaemia Prevalence
Age (year)
Location Hierarchy
1 − Global
2 − Super Region
3 − Region
4 − Country
Uncertainty Interval
4 − Country

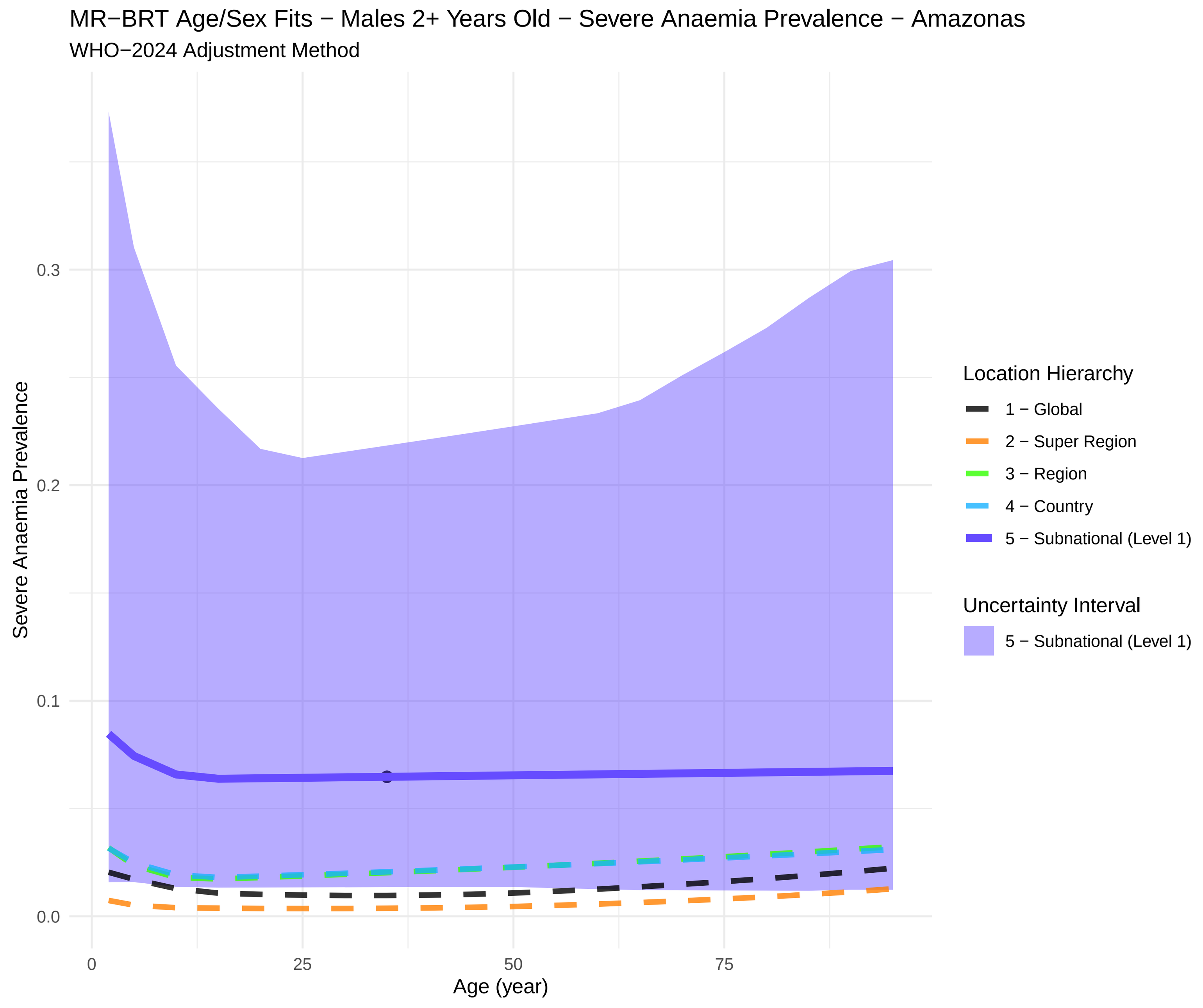
MR−BRT Age/Sex Fits − Males 2+ Years Old − Severe Anaemia Prevalence − Amazonas
WHO−2024 Adjustment Method
Severe Anaemia Prevalence
Age (year)
Location Hierarchy
1 − Global
2 − Super Region
3 − Region
4 − Country
5 − Subnational (Level 1)
Uncertainty Interval
5 − Subnational (Level 1)

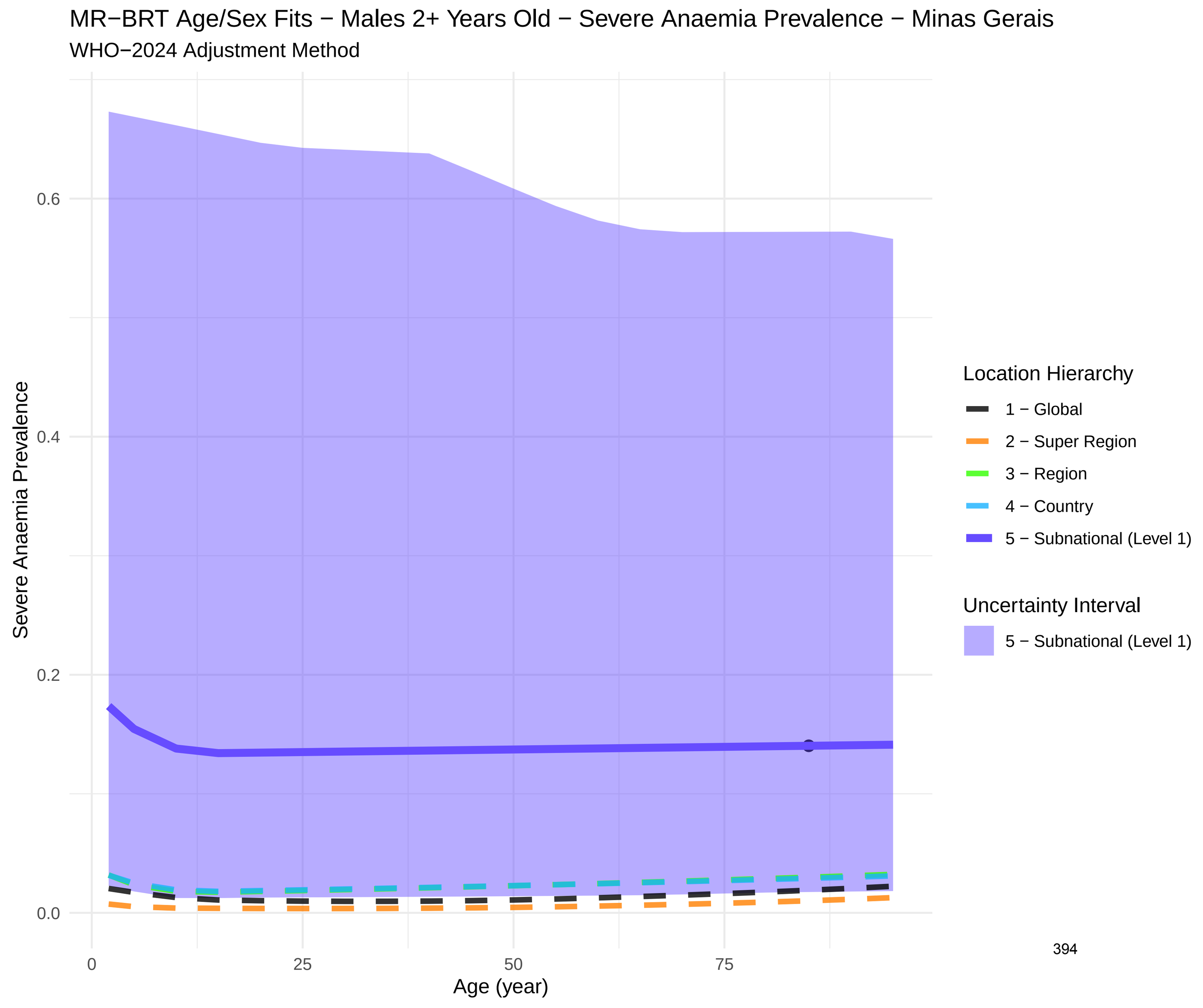
MR−BRT Age/Sex Fits − Males 2+ Years Old − Severe Anaemia Prevalence − Minas Gerais
WHO−2024 Adjustment Method
Severe Anaemia Prevalence
Age (year)
Location Hierarchy
1 − Global
2 − Super Region
3 − Region
4 − Country
5 − Subnational (Level 1)
Uncertainty Interval
5 − Subnational (Level 1)

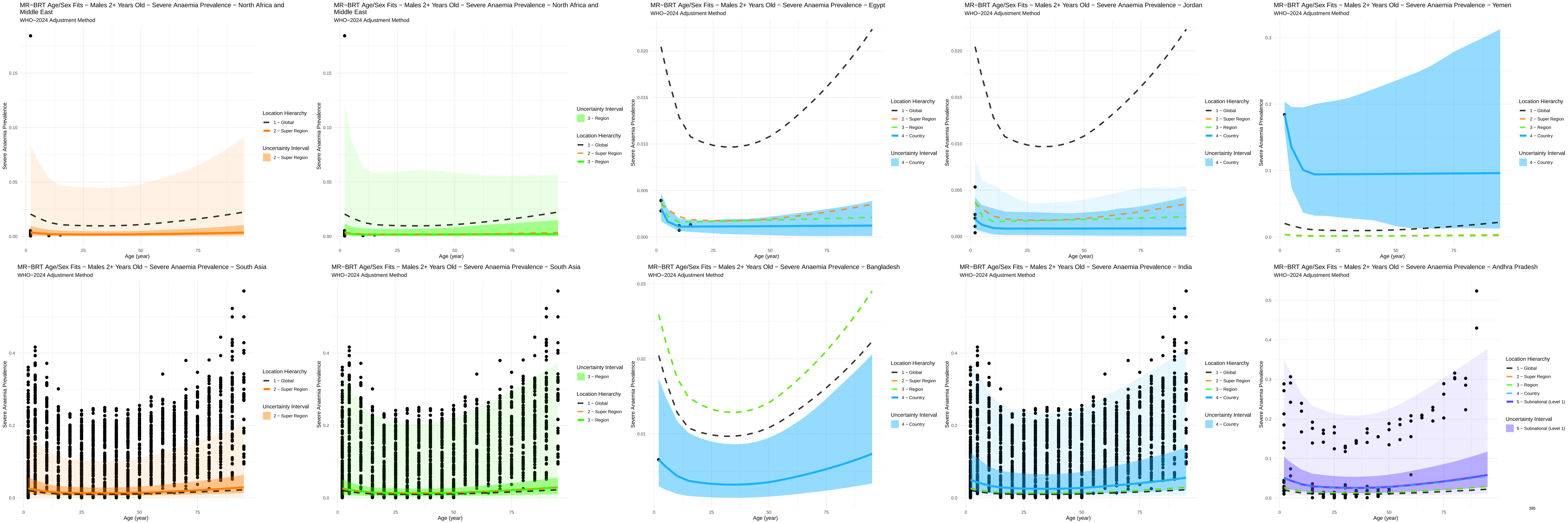
MR−BRT Age/Sex Fits − Males 2+ Years Old − Severe Anaemia Prevalence − North Africa and Middle East
WHO−2024 Adjustment Method
Severe Anaemia Prevalence
Age (year)
Location Hierarchy
1 − Global
2 − Super Region
Uncertainty Interval
2 − Super Region
MR−BRT Age/Sex Fits − Males 2+ Years Old − Severe Anaemia Prevalence − North Africa and Middle East
WHO−2024 Adjustment Method
Uncertainty Interval
3 − Region
Location Hierarchy
1 − Global
2 − Super Region
3 − Region
MR−BRT Age/Sex Fits − Males 2+ Years Old − Severe Anaemia Prevalence − Egypt
WHO−2024 Adjustment Method
Location Hierarchy
1 − Global
2 − Super Region
3 − Region
4 − Country
Uncertainty Interval
4 − Country
MR−BRT Age/Sex Fits − Males 2+ Years Old − Severe Anaemia Prevalence − Jordan
WHO−2024 Adjustment Method
MR−BRT Age/Sex Fits − Males 2+ Years Old − Severe Anaemia Prevalence − Yemen
WHO−2024 Adjustment Method
MR−BRT Age/Sex Fits − Males 2+ Years Old − Severe Anaemia Prevalence − South Asia
WHO−2024 Adjustment Method
MR−BRT Age/Sex Fits − Males 2+ Years Old − Severe Anaemia Prevalence − Bangladesh
WHO−2024 Adjustment Method
MR−BRT Age/Sex Fits − Males 2+ Years Old − Severe Anaemia Prevalence − India
WHO−2024 Adjustment Method
MR−BRT Age/Sex Fits − Males 2+ Years Old − Severe Anaemia Prevalence − Andhra Pradesh
WHO−2024 Adjustment Method
Location Hierarchy
1 − Global
2 − Super Region
3 − Region
4 − Country
5 − Subnational (Level 1)
Uncertainty Interval
5 − Subnational (Level 1)

MR−BRT Age/Sex Fits − Males 2+ Years Old − Severe Anaemia Prevalence − Andhra Pradesh, Rural
WHO−2024 Adjustment Method

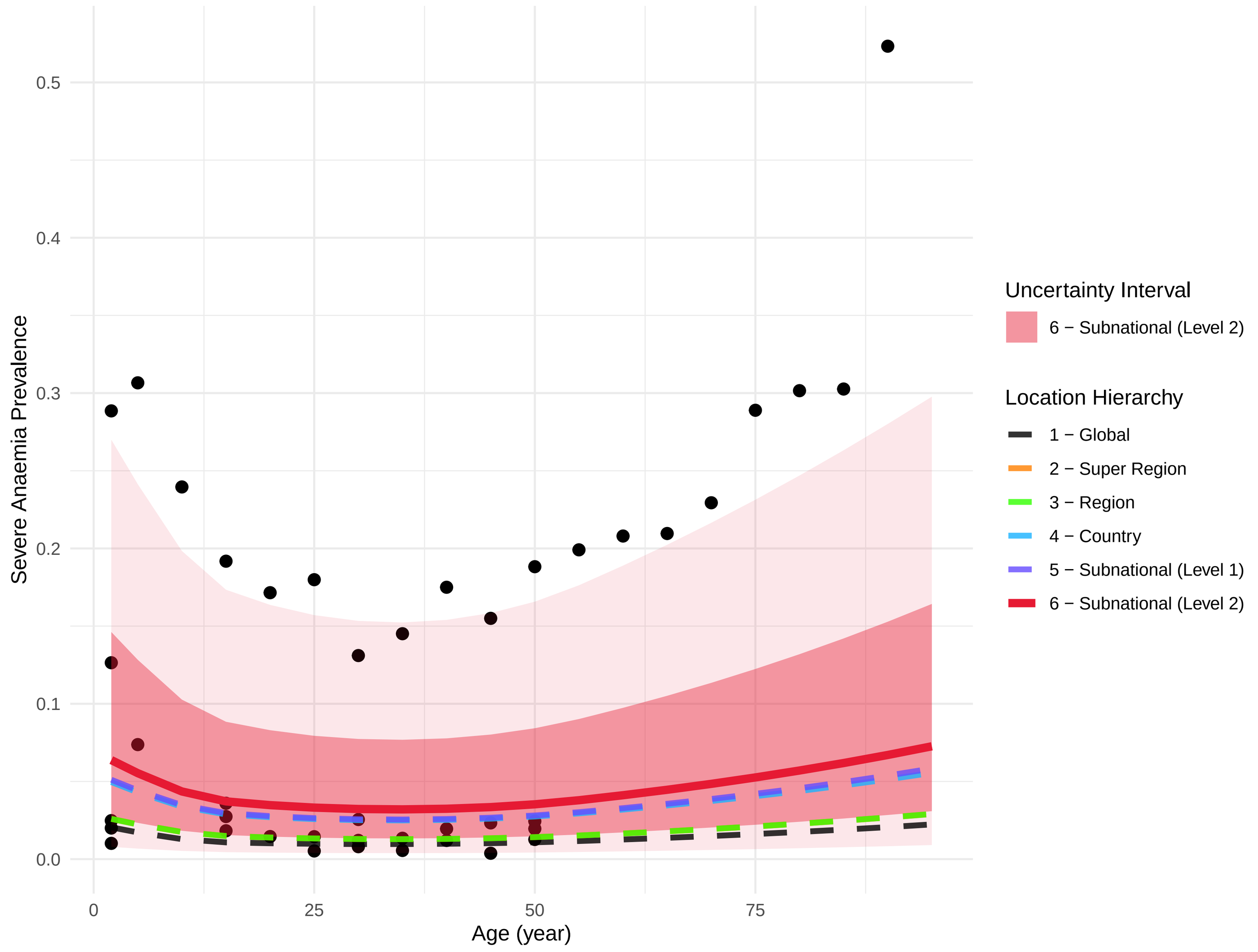

MR−BRT Age/Sex Fits − Males 2+ Years Old − Severe Anaemia Prevalence − Andhra Pradesh, Urban
WHO−2024 Adjustment Method

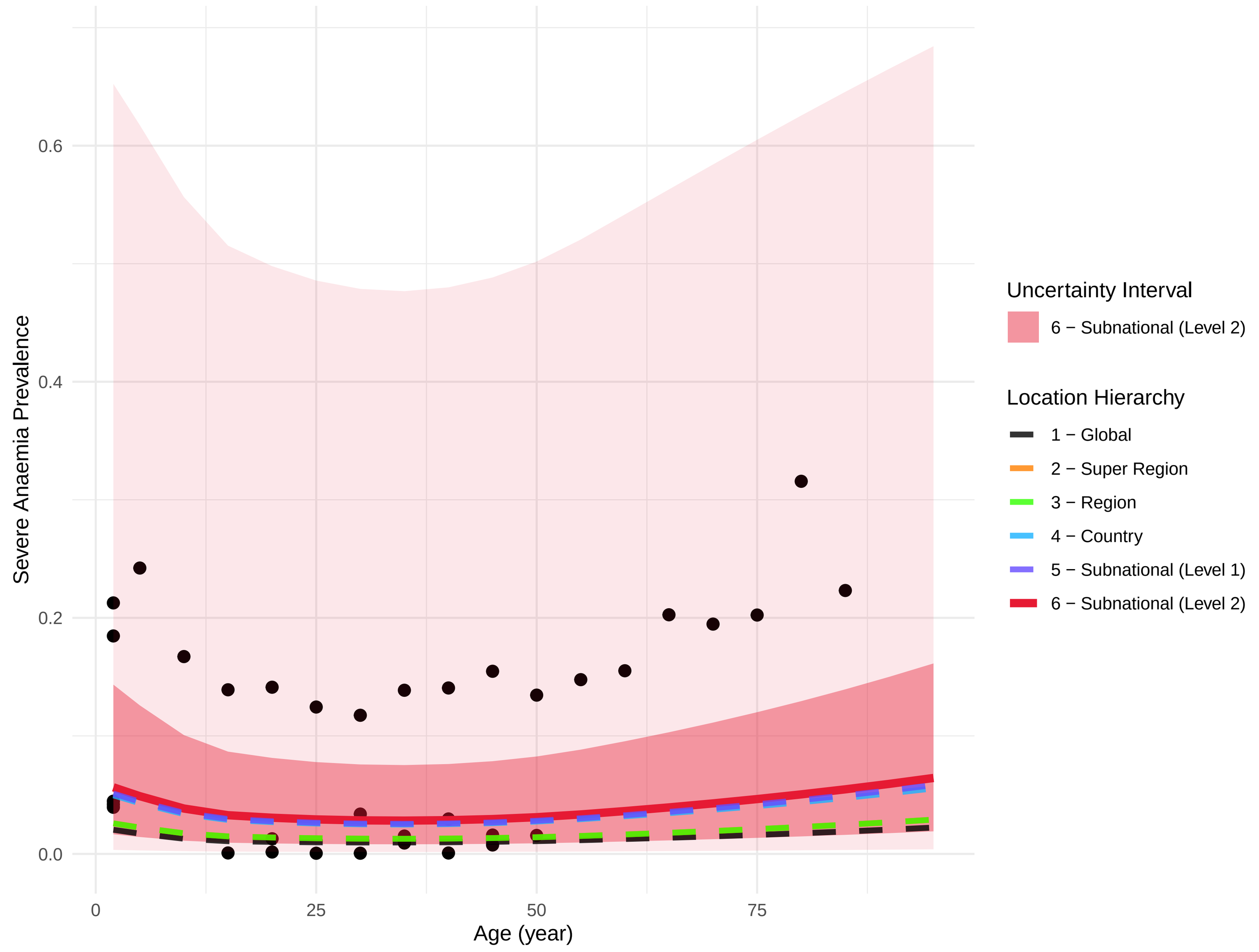

MR−BRT Age/Sex Fits − Males 2+ Years Old − Severe Anaemia Prevalence − Arunachal Pradesh
WHO−2024 Adjustment Method

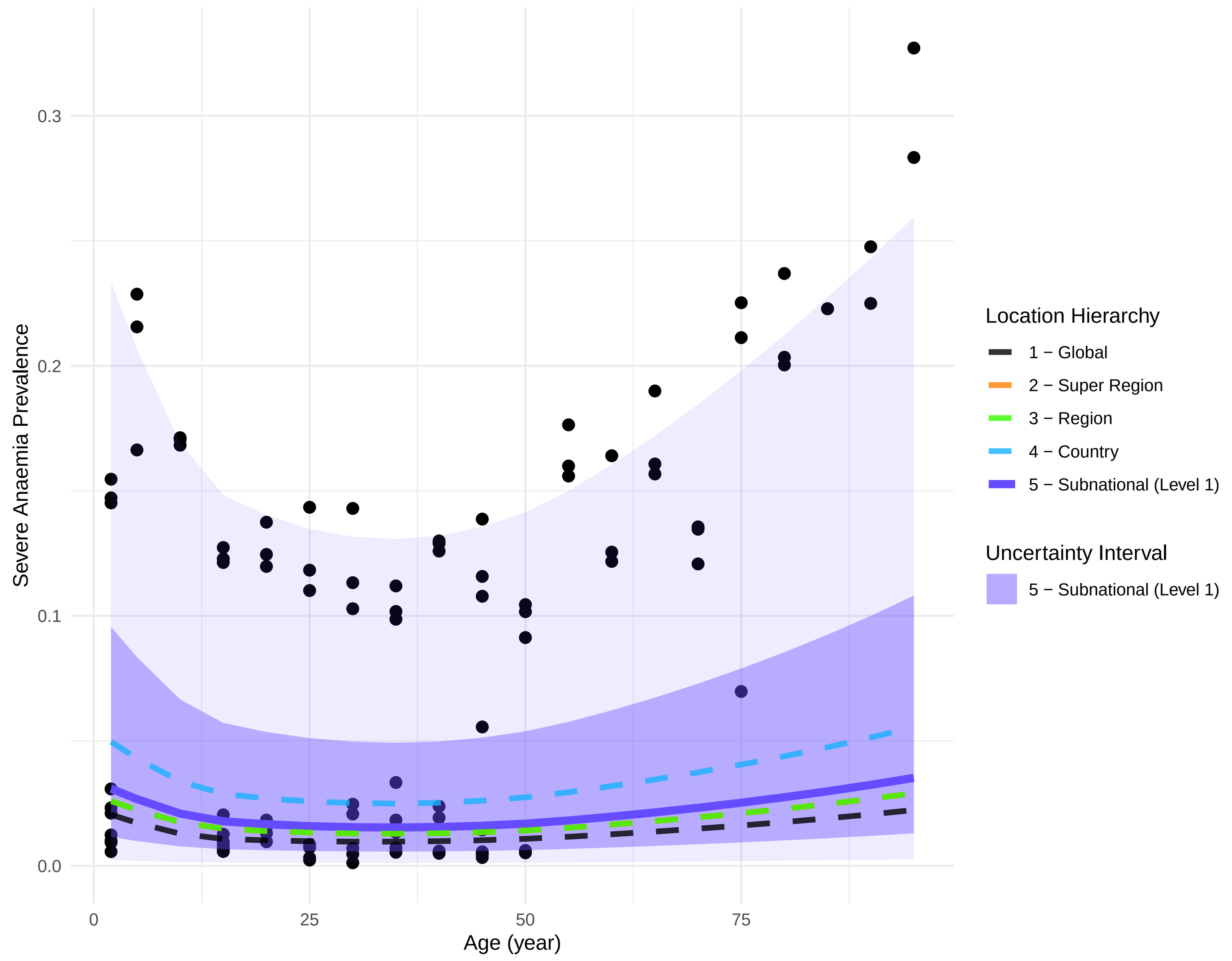

MR−BRT Age/Sex Fits − Males 2+ Years Old − Severe Anaemia Prevalence − Arunachal Pradesh, Rural
WHO−2024 Adjustment Method

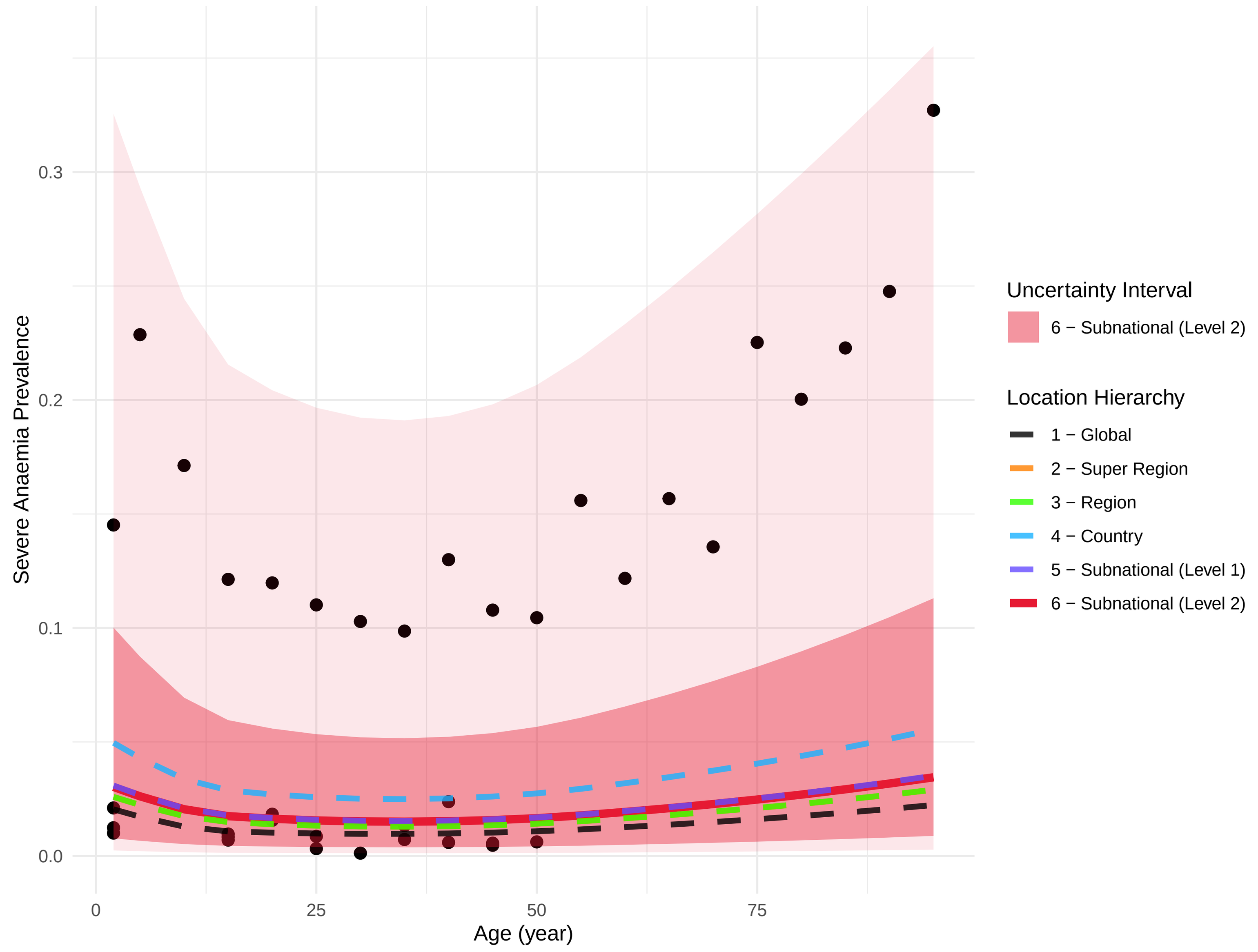

MR−BRT Age/Sex Fits − Males 2+ Years Old − Severe Anaemia Prevalence − Arunachal Pradesh, Urban
WHO−2024 Adjustment Method

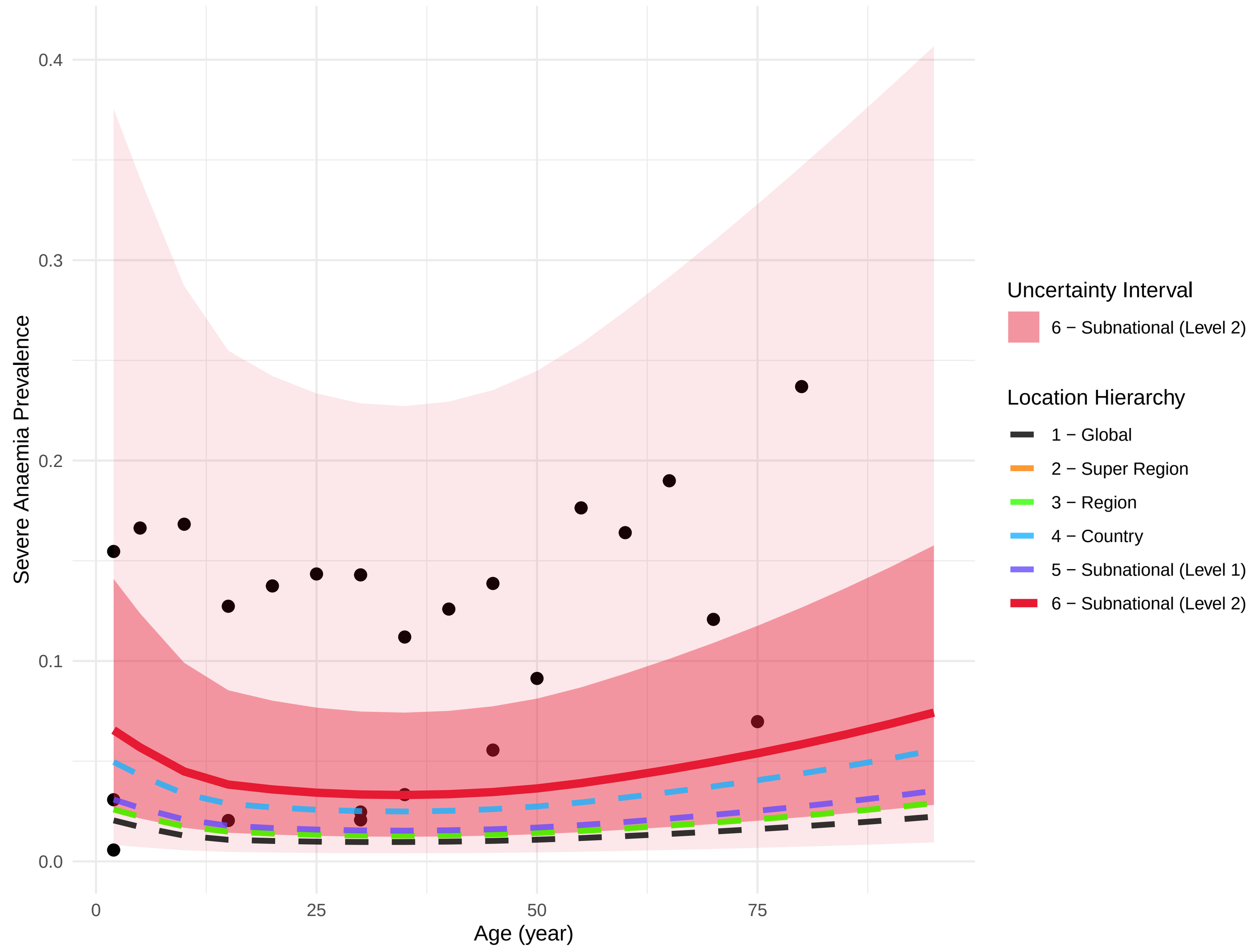

MR−BRT Age/Sex Fits − Males 2+ Years Old − Severe Anaemia Prevalence − Assam
WHO−2024 Adjustment Method

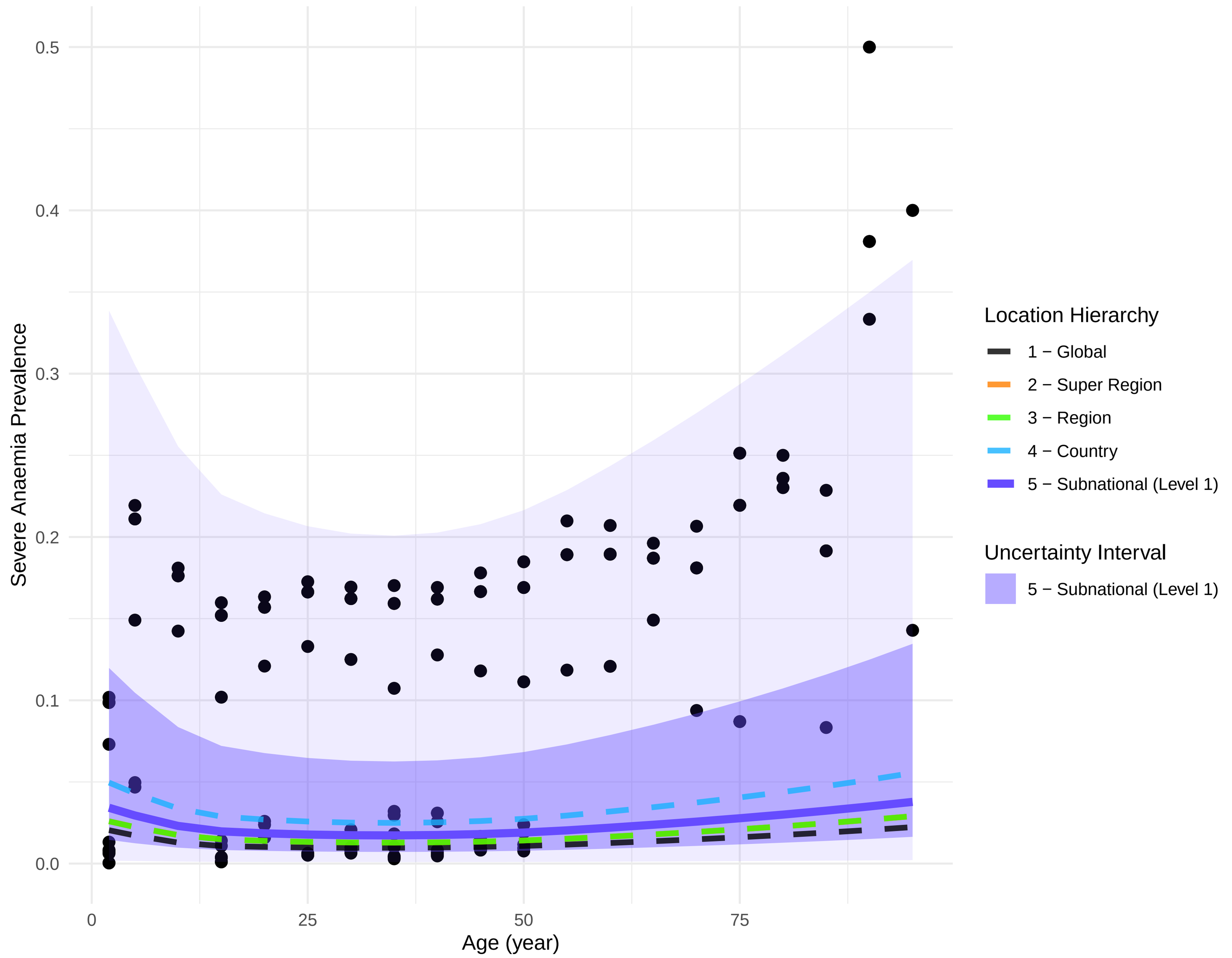

MR−BRT Age/Sex Fits − Males 2+ Years Old − Severe Anaemia Prevalence − Assam, Rural
WHO−2024 Adjustment Method

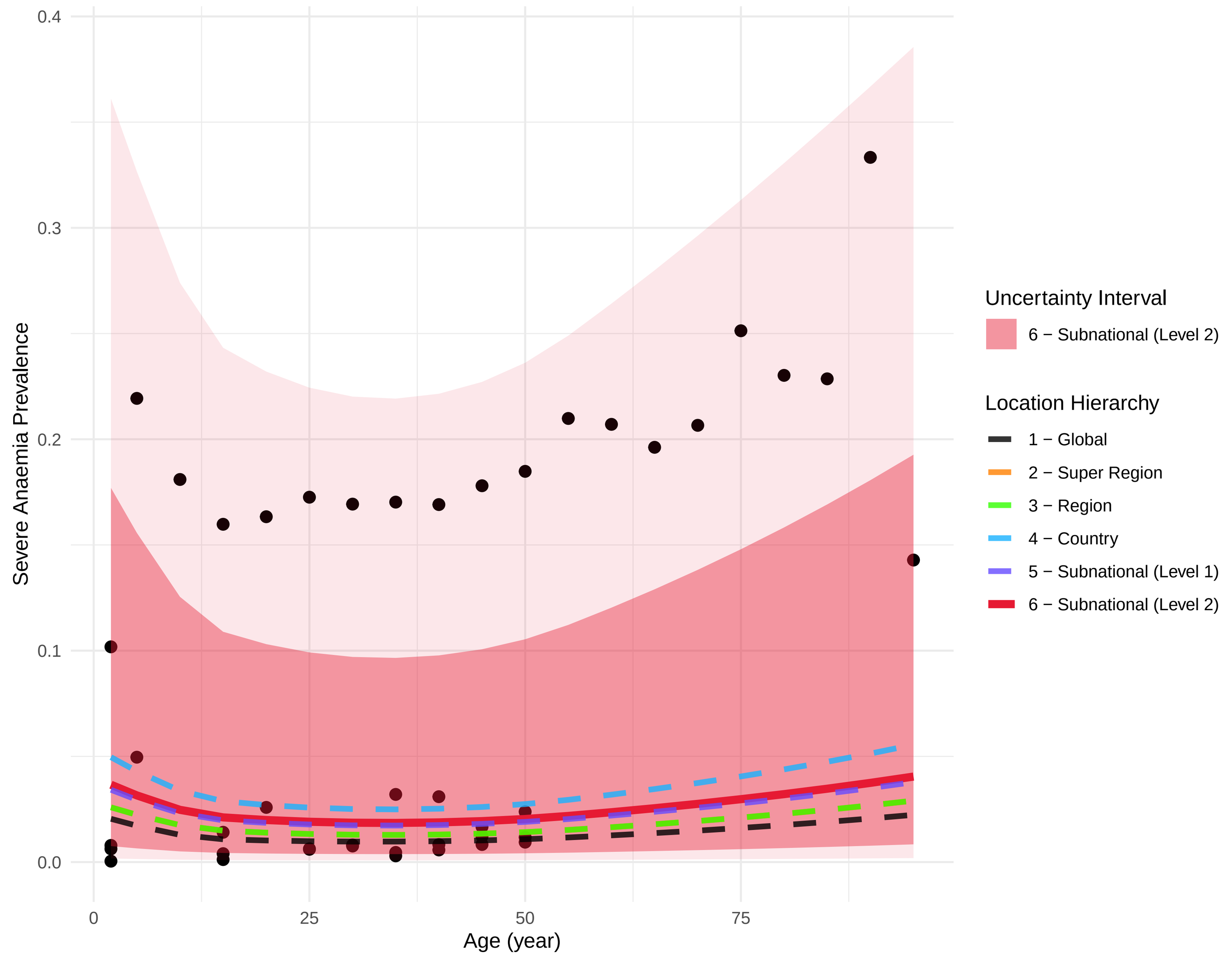

MR−BRT Age/Sex Fits − Males 2+ Years Old − Severe Anaemia Prevalence − Assam, Urban
WHO−2024 Adjustment Method

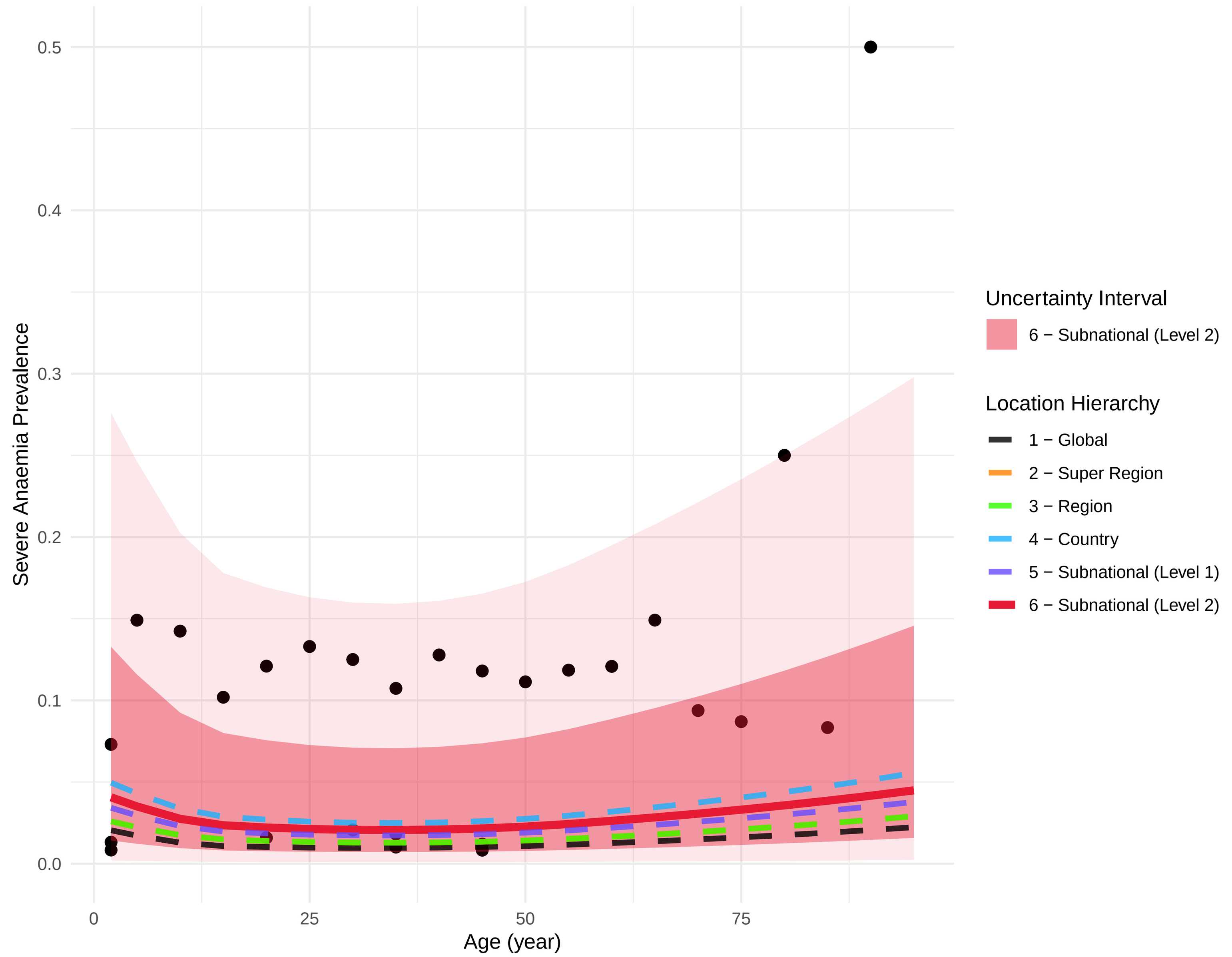

MR−BRT Age/Sex Fits − Males 2+ Years Old − Severe Anaemia Prevalence − Bihar
WHO−2024 Adjustment Method

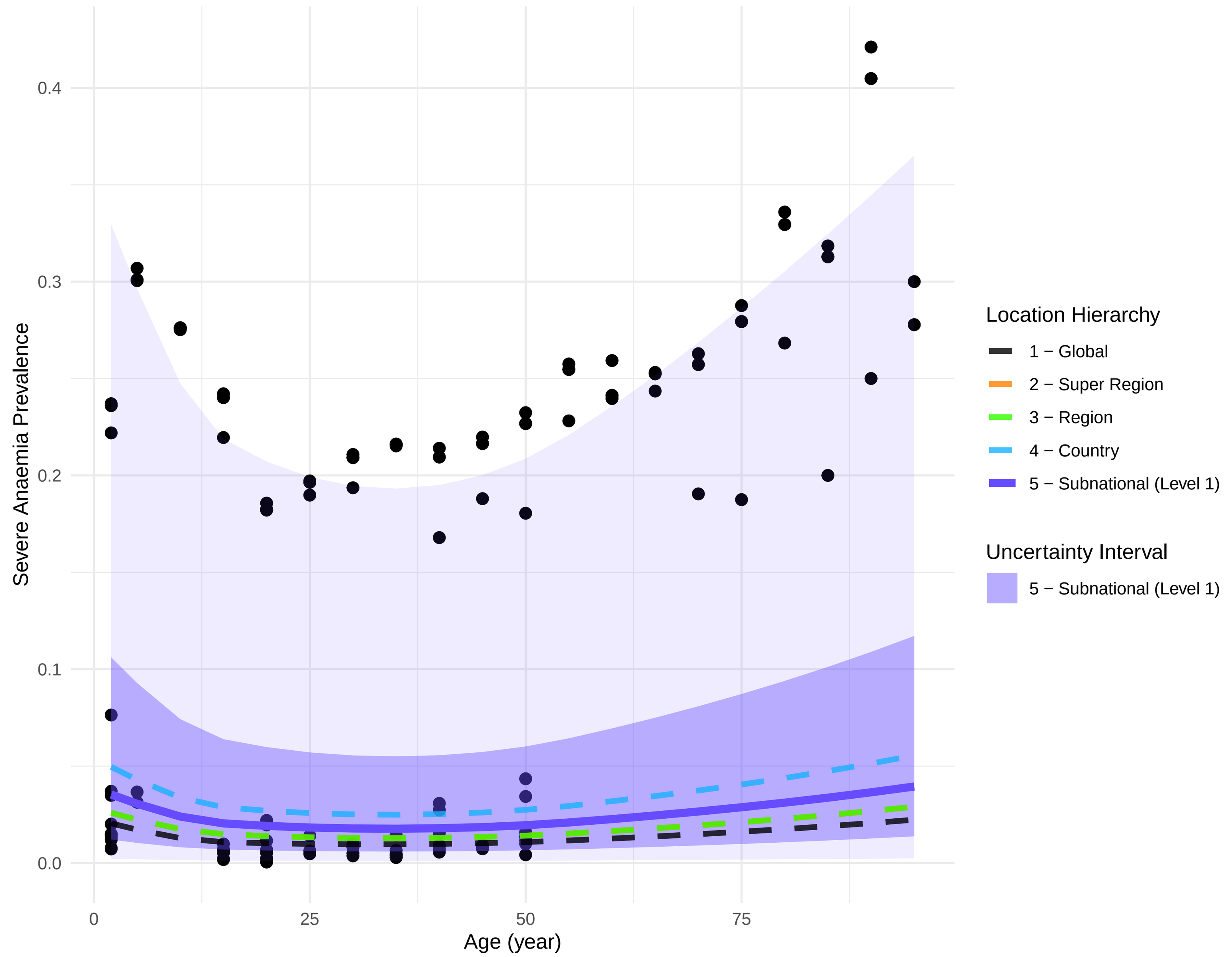

MR−BRT Age/Sex Fits − Males 2+ Years Old − Severe Anaemia Prevalence − Bihar, Rural
WHO−2024 Adjustment Method

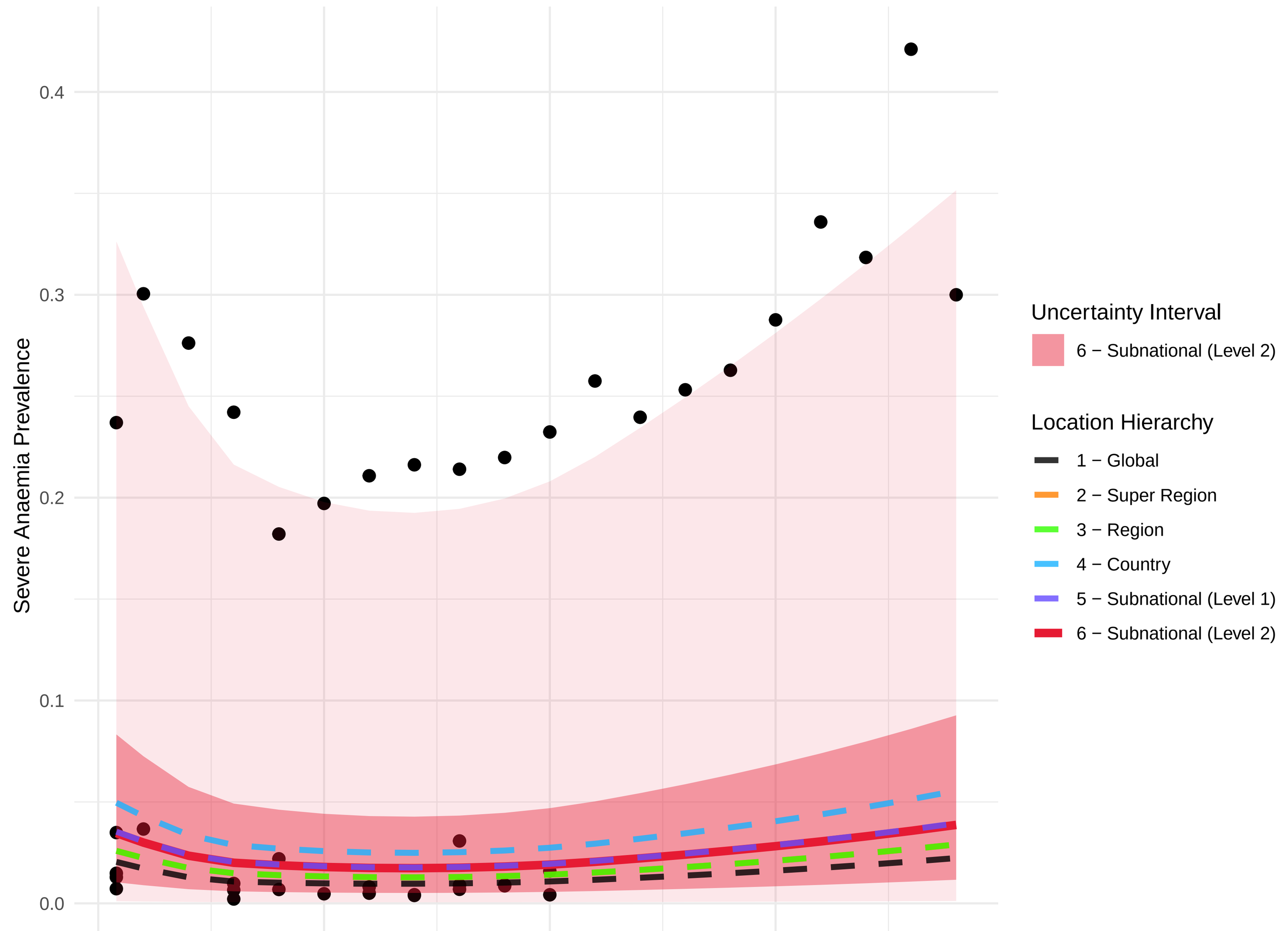

MR−BRT Age/Sex Fits − Males 2+ Years Old − Severe Anaemia Prevalence − Bihar, Urban
WHO−2024 Adjustment Method

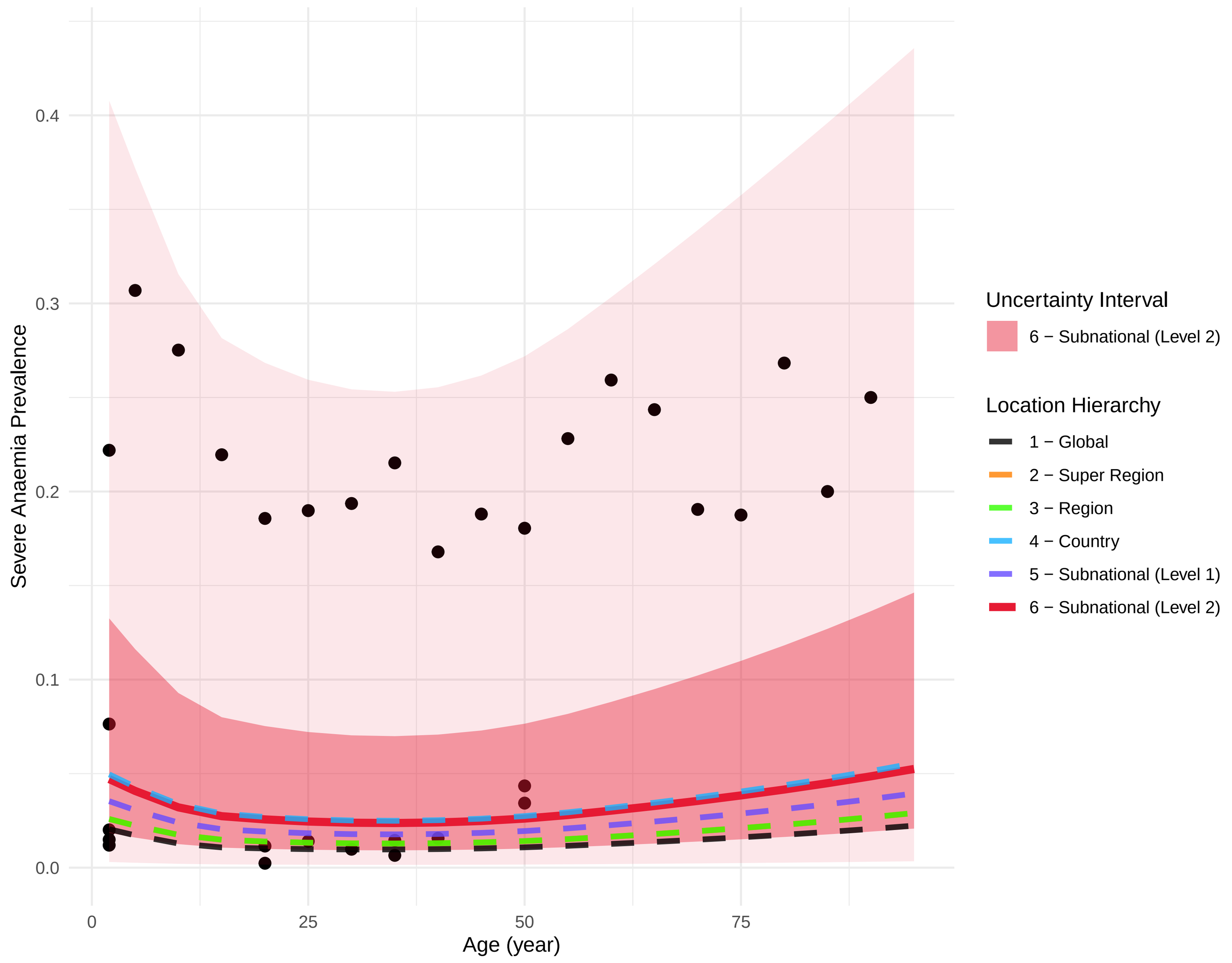

MR−BRT Age/Sex Fits − Males 2+ Years Old − Severe Anaemia Prevalence − Chhattisgarh
WHO−2024 Adjustment Method

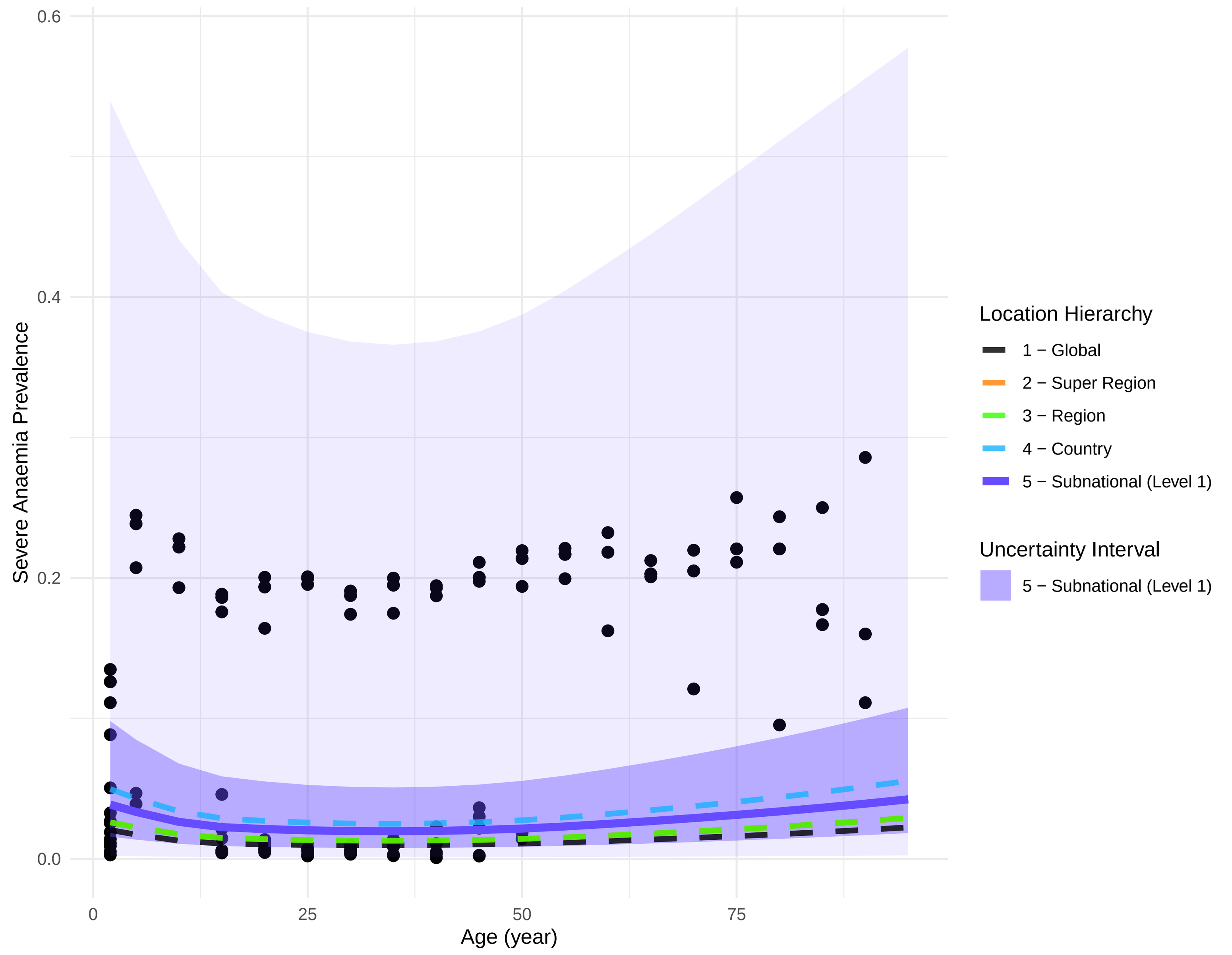

MR−BRT Age/Sex Fits − Males 2+ Years Old − Severe Anaemia Prevalence − Chhattisgarh, Rural
WHO−2024 Adjustment Method

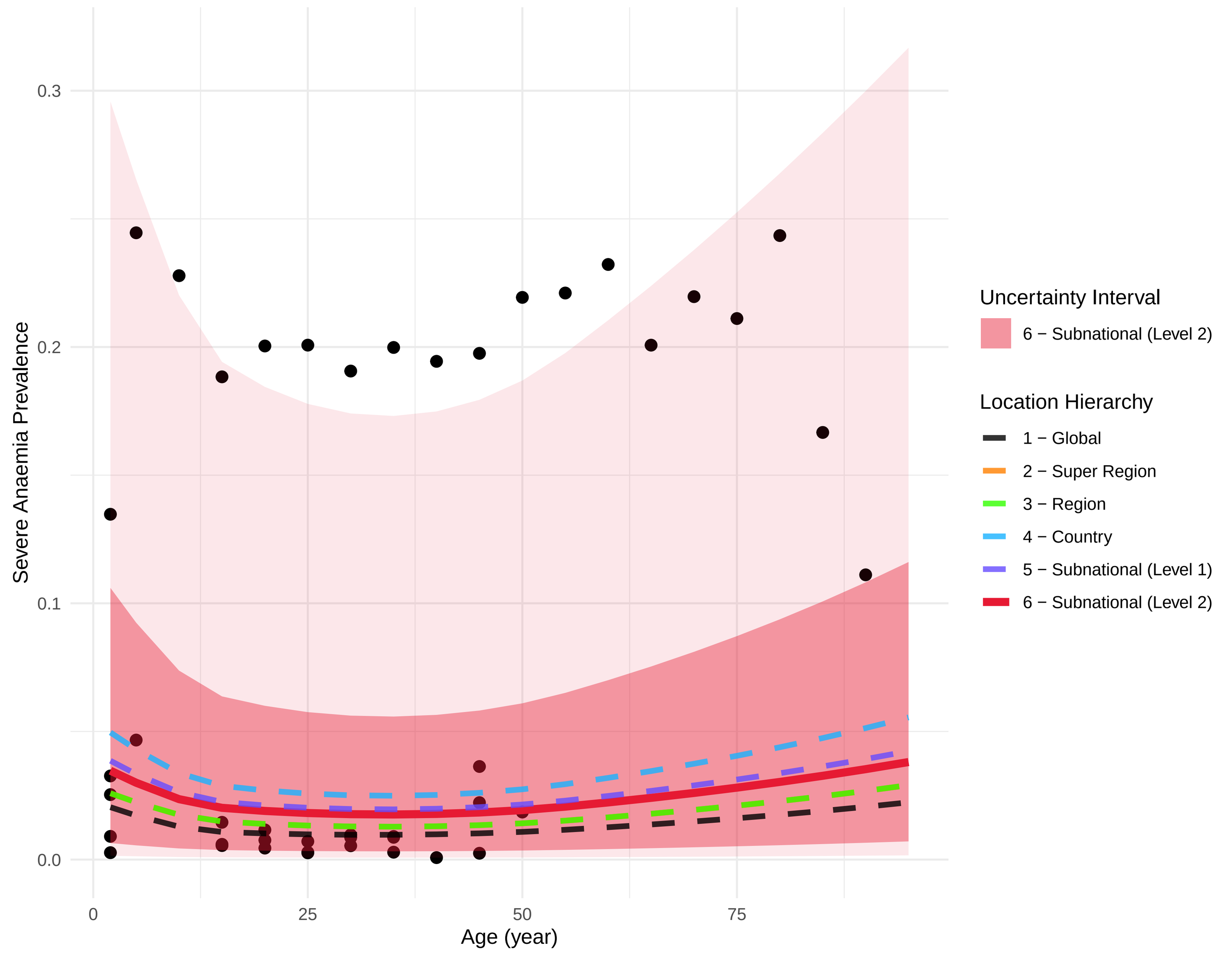

MR−BRT Age/Sex Fits − Males 2+ Years Old − Severe Anaemia Prevalence − Chhattisgarh, Urban
WHO−2024 Adjustment Method

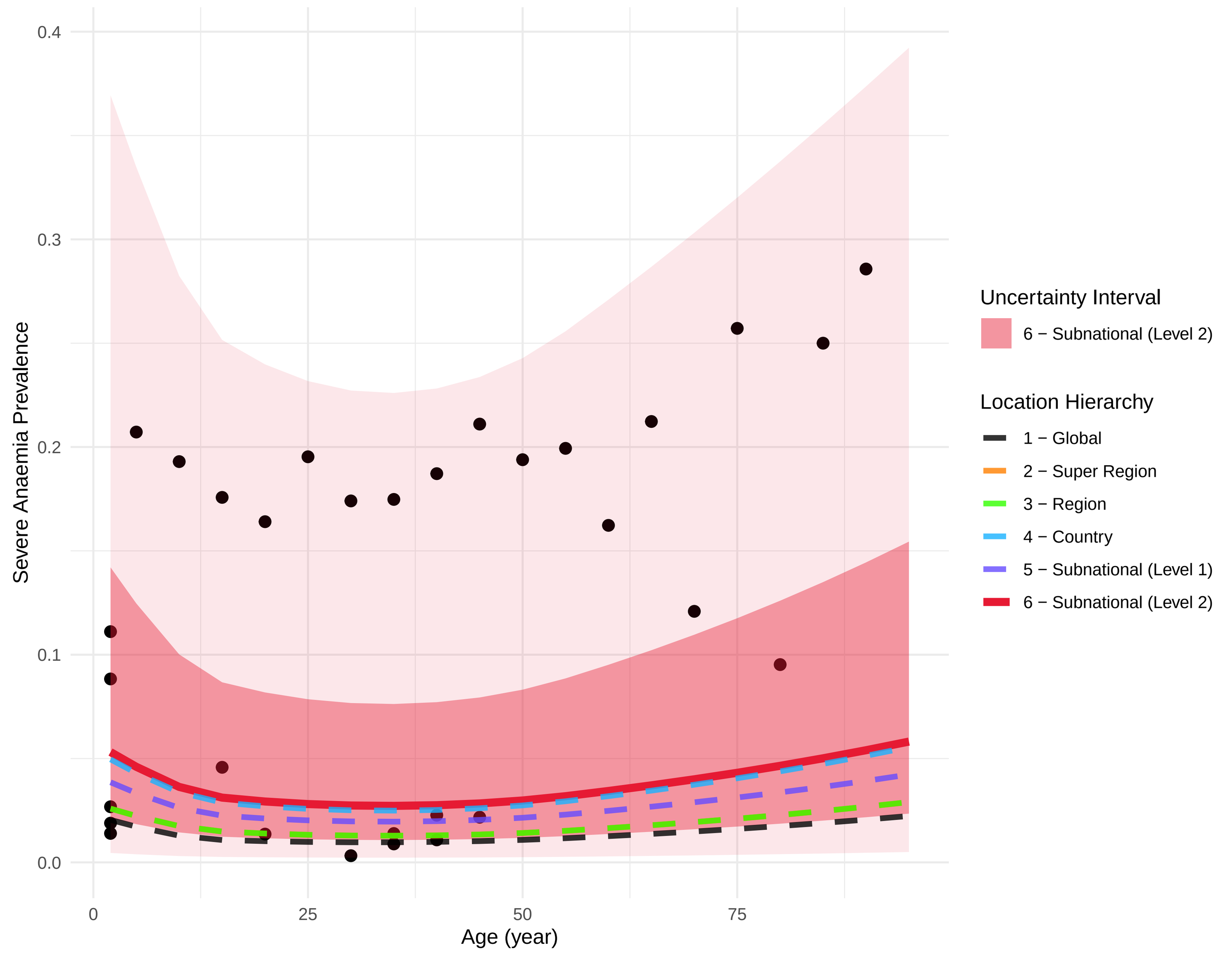

MR−BRT Age/Sex Fits − Males 2+ Years Old − Severe Anaemia Prevalence − Delhi
WHO−2024 Adjustment Method

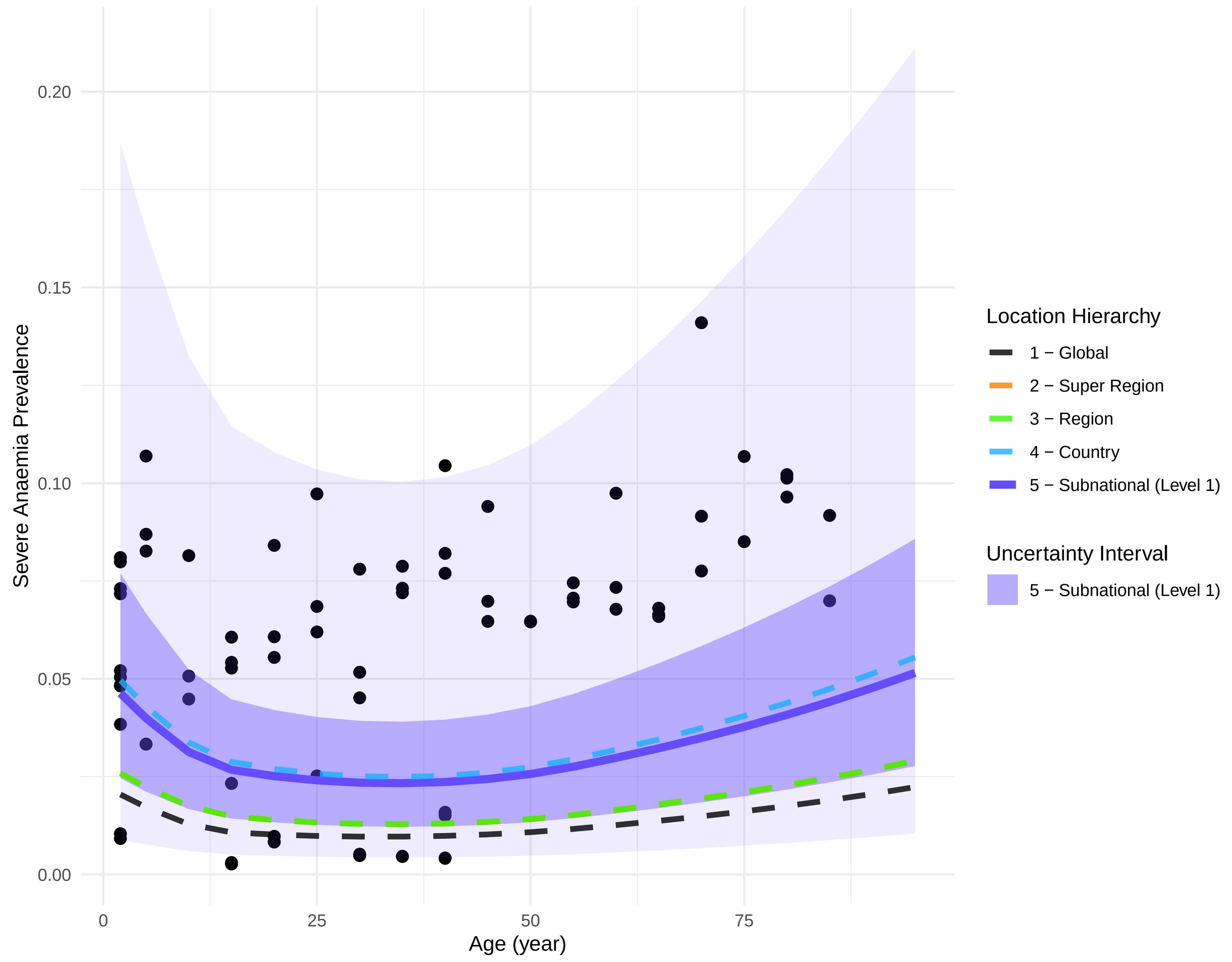

MR−BRT Age/Sex Fits − Males 2+ Years Old − Severe Anaemia Prevalence − Delhi, Rural
WHO−2024 Adjustment Method

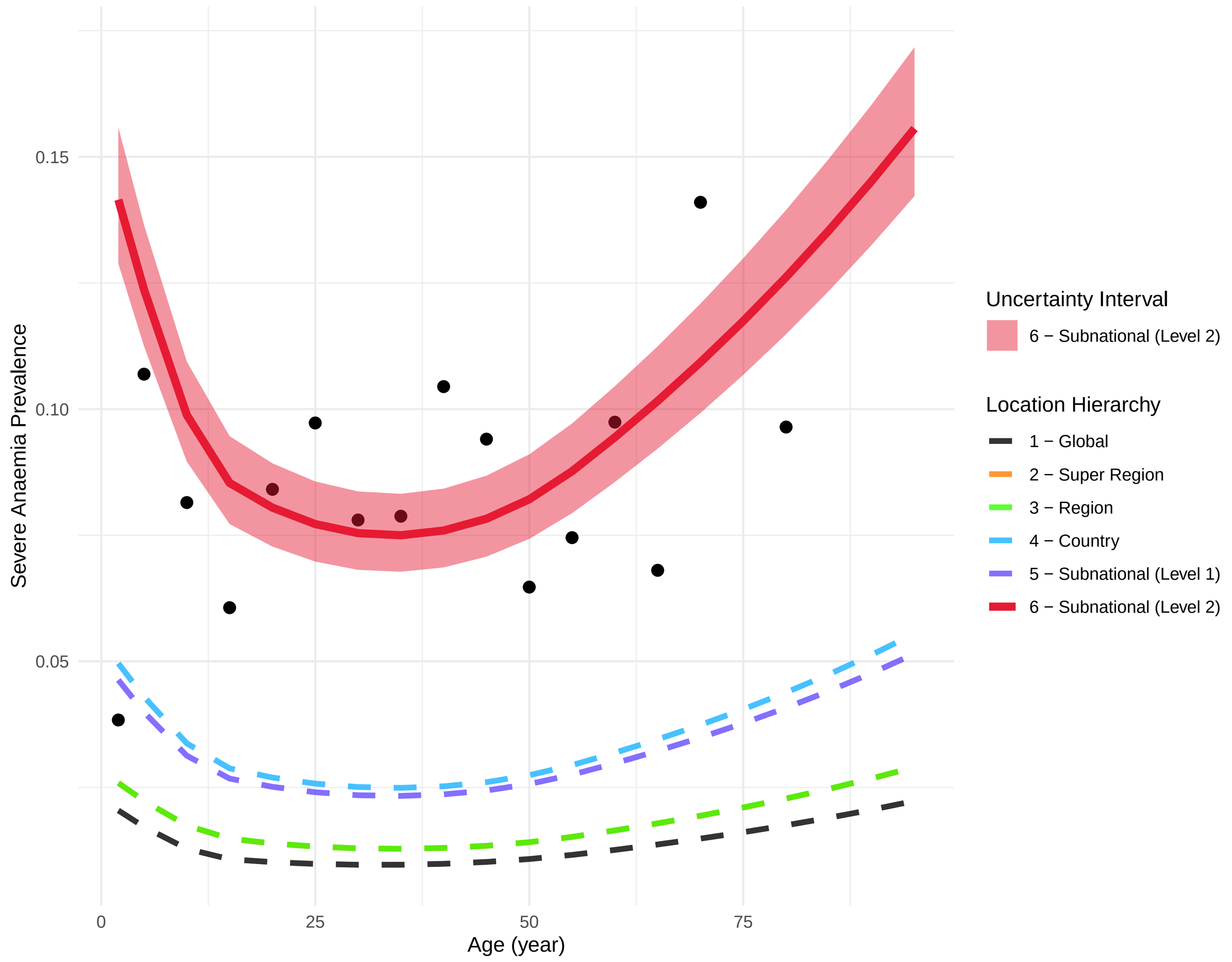

MR−BRT Age/Sex Fits − Males 2+ Years Old − Severe Anaemia Prevalence − Delhi, Urban
WHO−2024 Adjustment Method

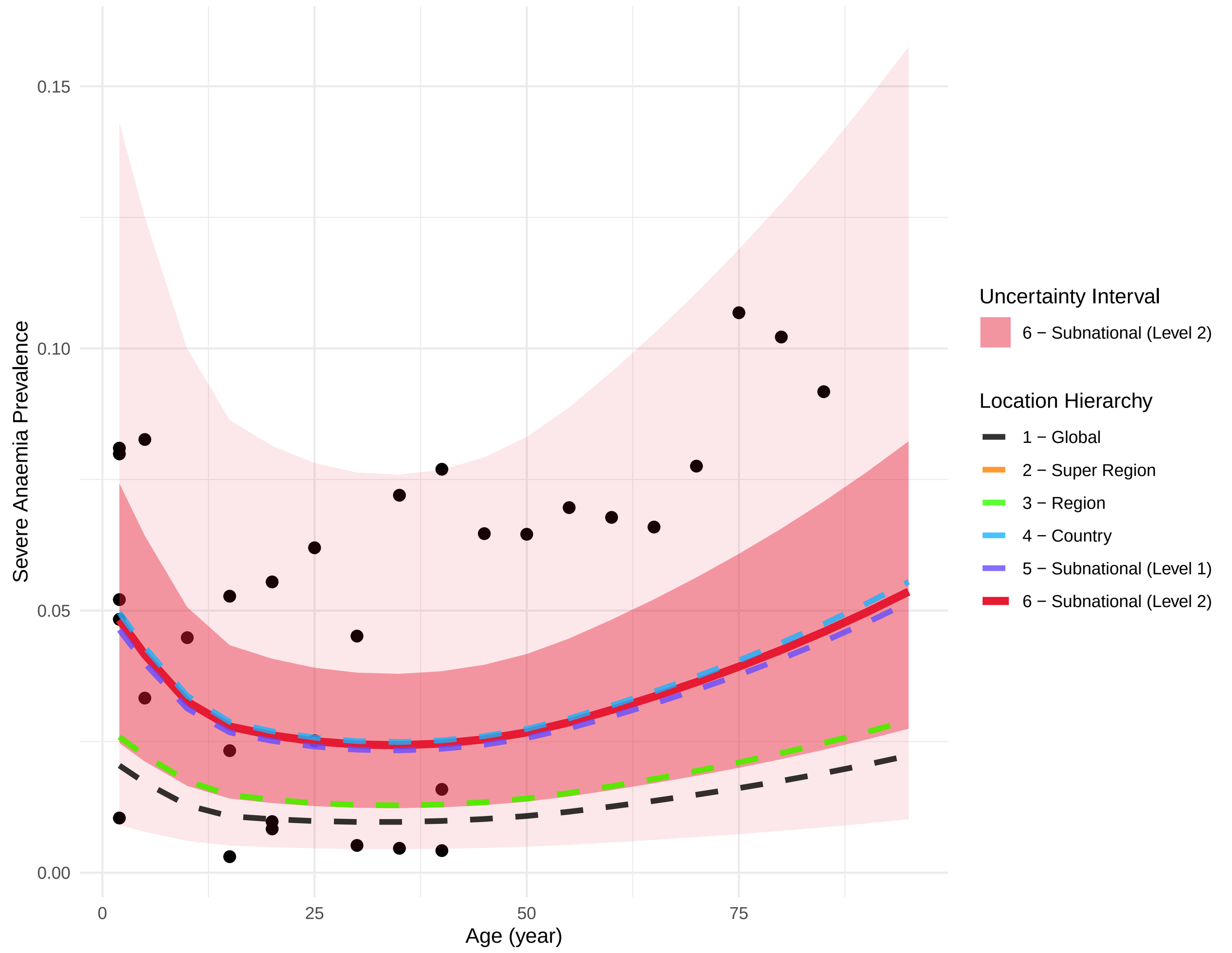

MR−BRT Age/Sex Fits − Males 2+ Years Old − Severe Anaemia Prevalence − Goa
WHO−2024 Adjustment Method

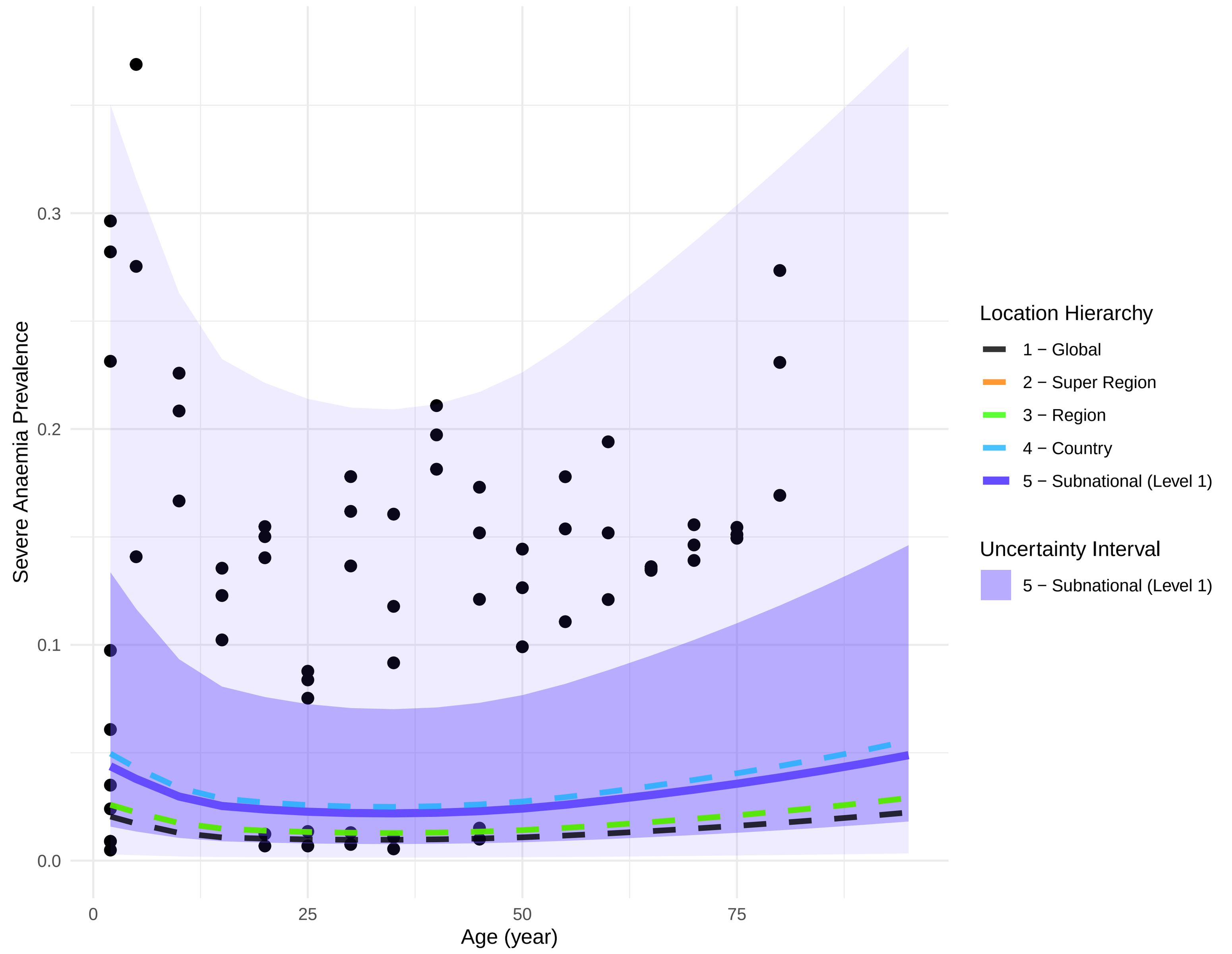

MR−BRT Age/Sex Fits − Males 2+ Years Old − Severe Anaemia Prevalence − Goa, Rural
WHO−2024 Adjustment Method

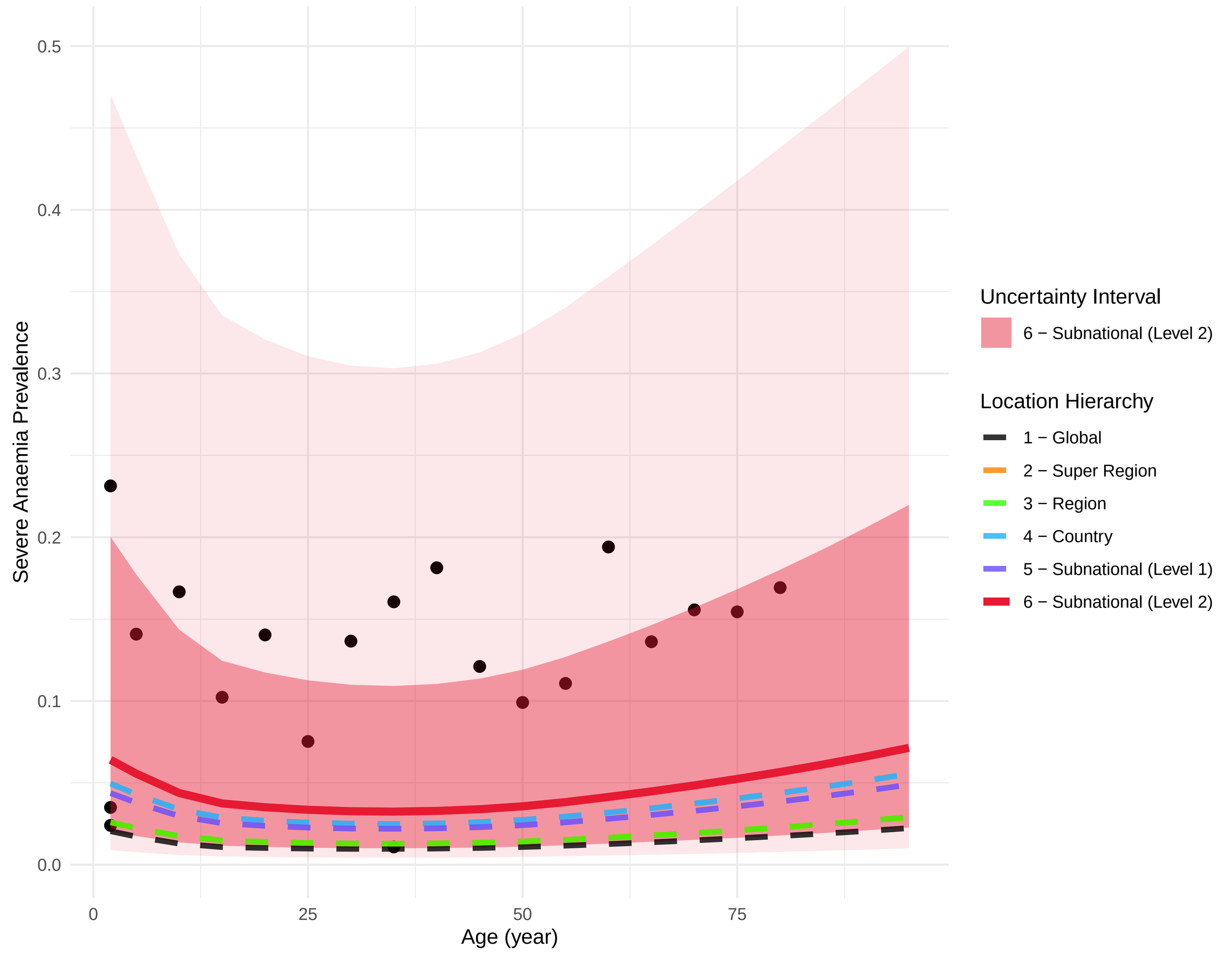

MR−BRT Age/Sex Fits − Males 2+ Years Old − Severe Anaemia Prevalence − Goa, Urban
WHO−2024 Adjustment Method

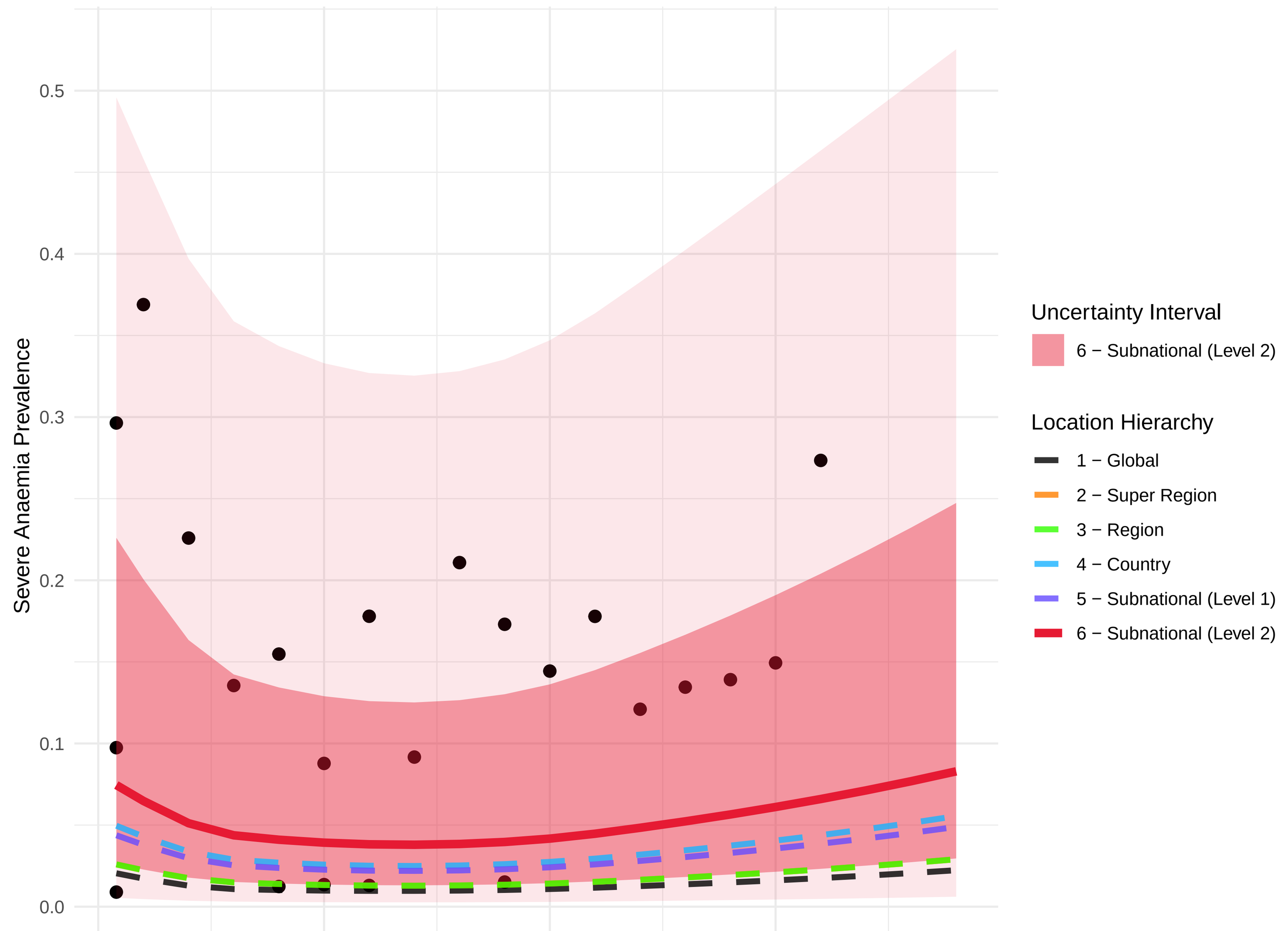

MR−BRT Age/Sex Fits − Males 2+ Years Old − Severe Anaemia Prevalence − Gujarat
WHO−2024 Adjustment Method

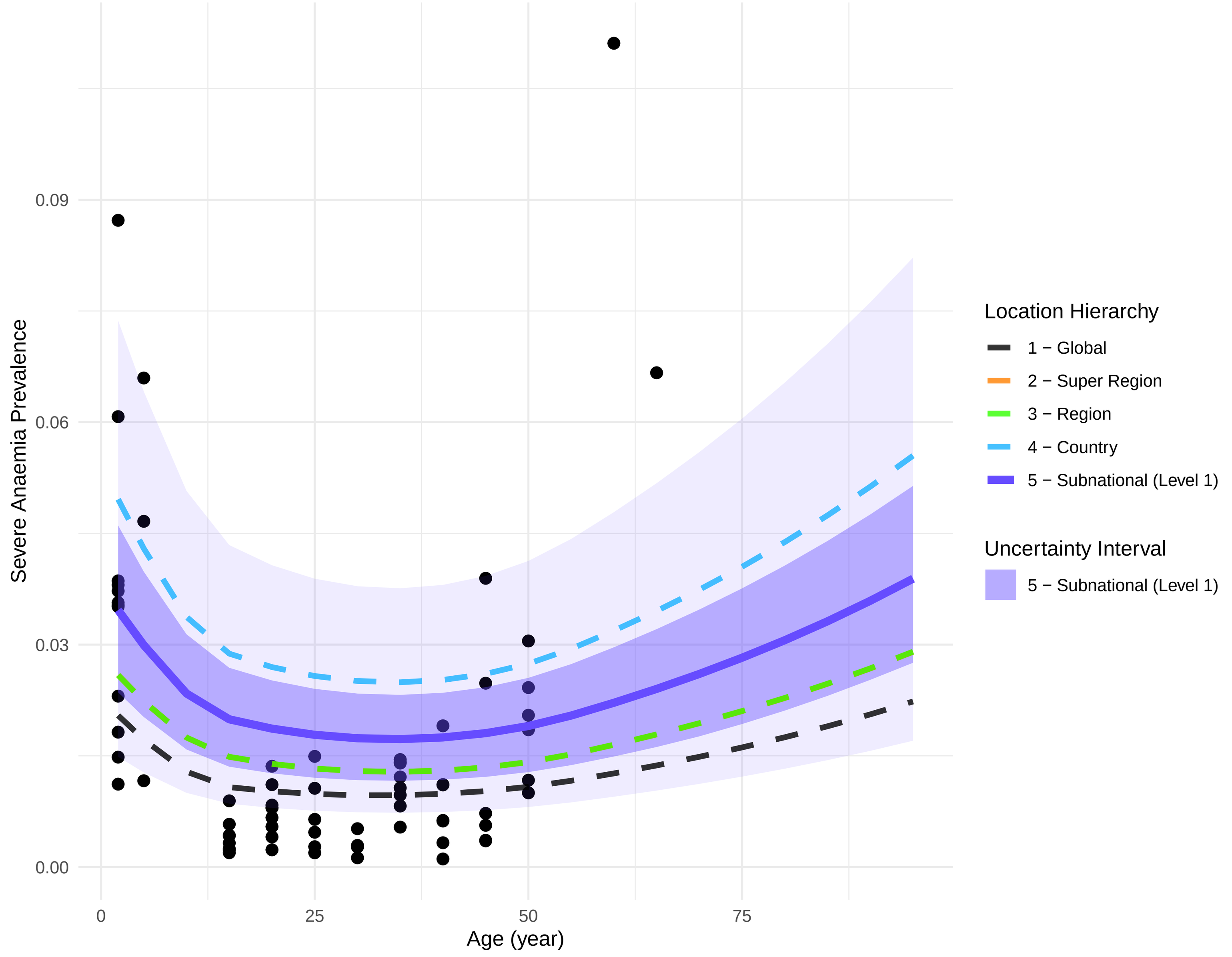

MR−BRT Age/Sex Fits − Males 2+ Years Old − Severe Anaemia Prevalence − Gujarat, Rural
WHO−2024 Adjustment Method

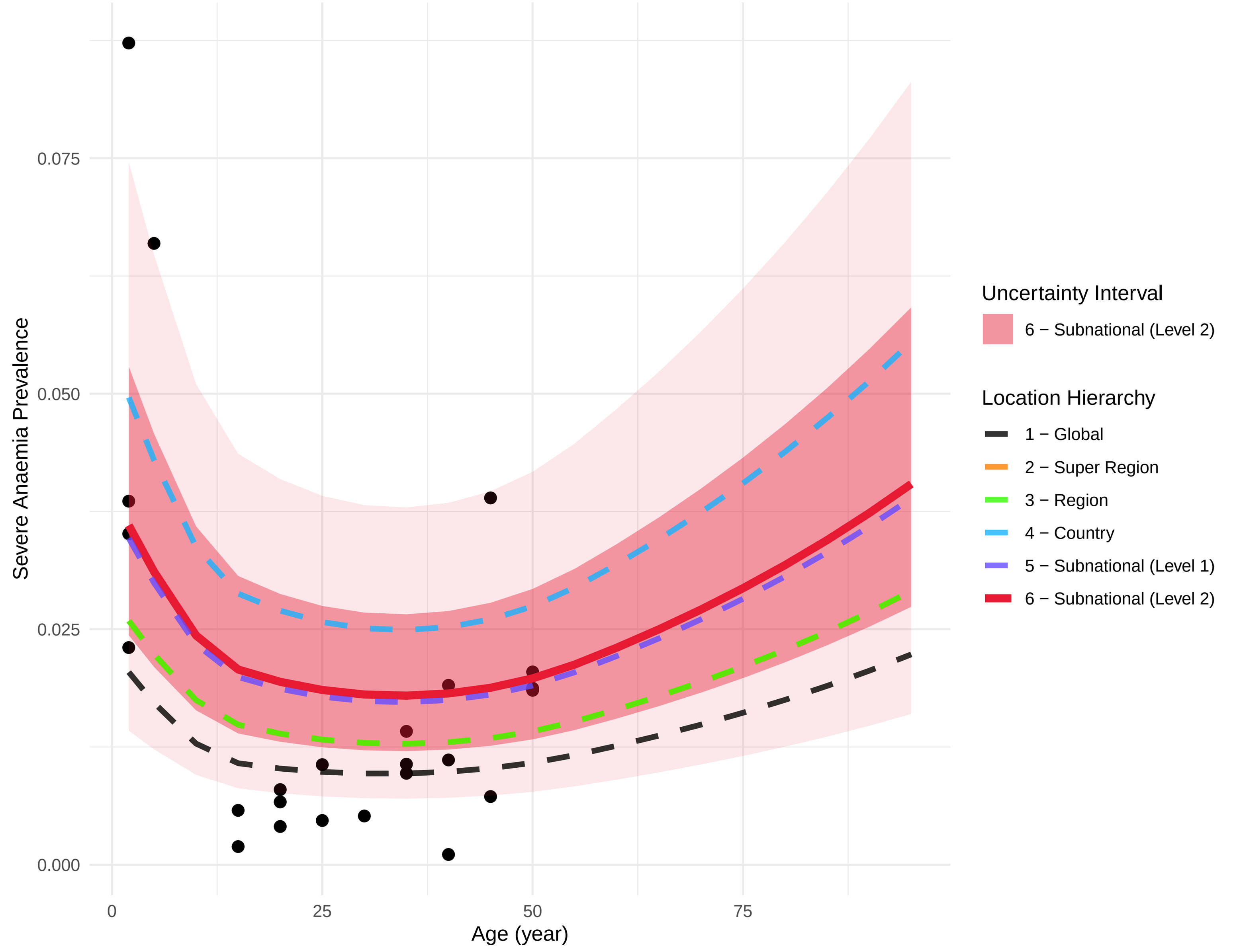

MR−BRT Age/Sex Fits − Males 2+ Years Old − Severe Anaemia Prevalence − Gujarat, Urban
WHO−2024 Adjustment Method

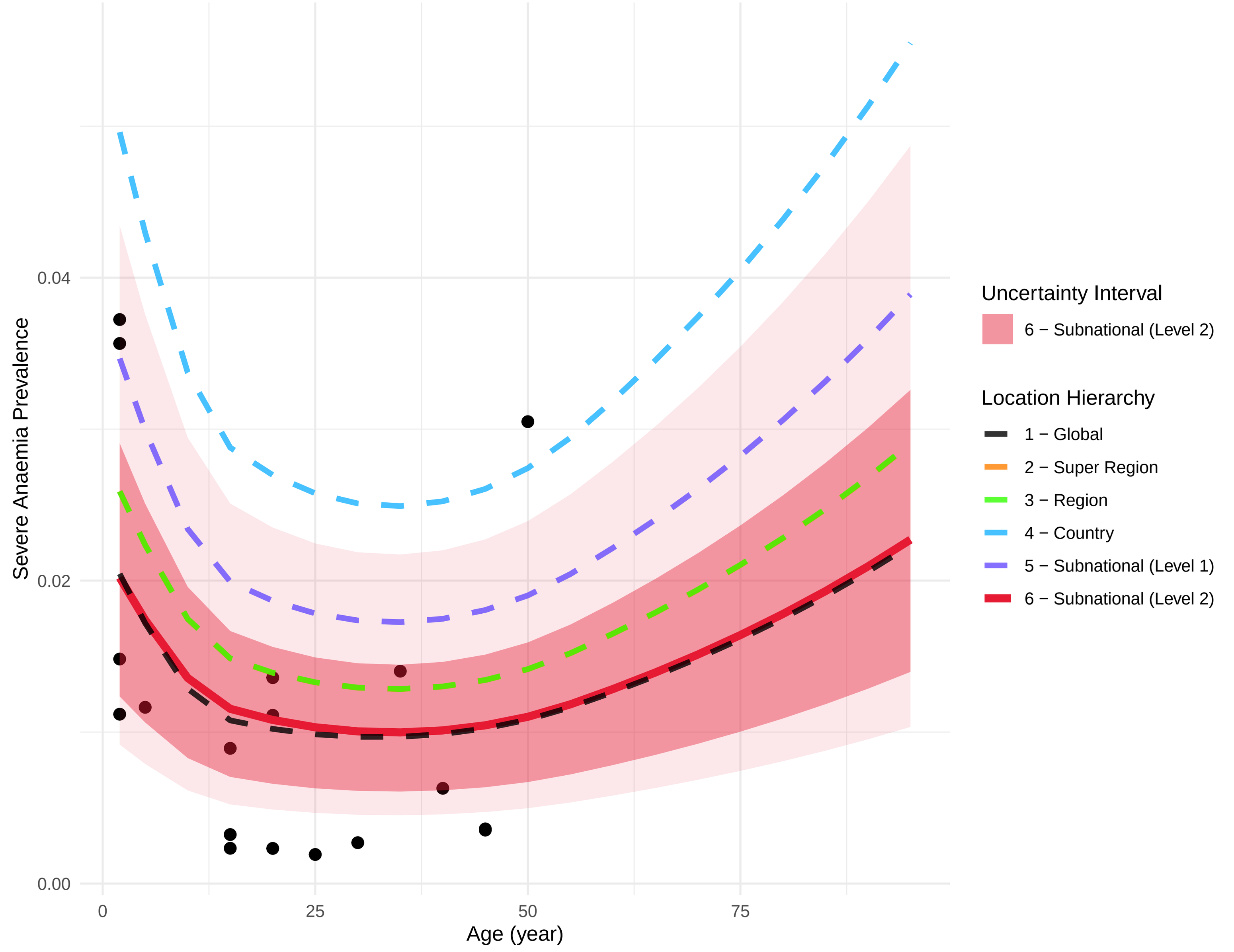

MR−BRT Age/Sex Fits − Males 2+ Years Old − Severe Anaemia Prevalence − Haryana
WHO−2024 Adjustment Method

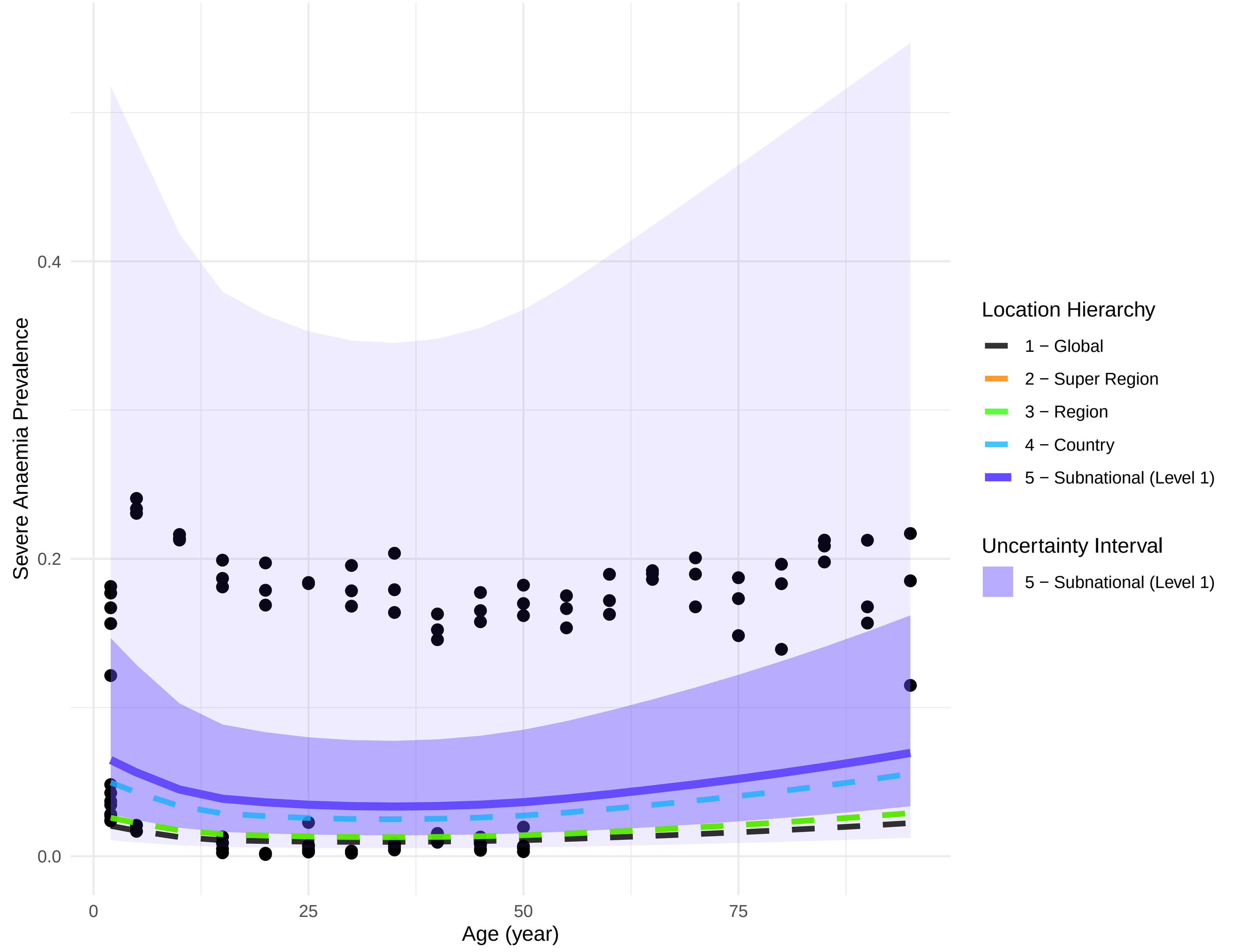

MR−BRT Age/Sex Fits − Males 2+ Years Old − Severe Anaemia Prevalence − Haryana, Rural
WHO−2024 Adjustment Method

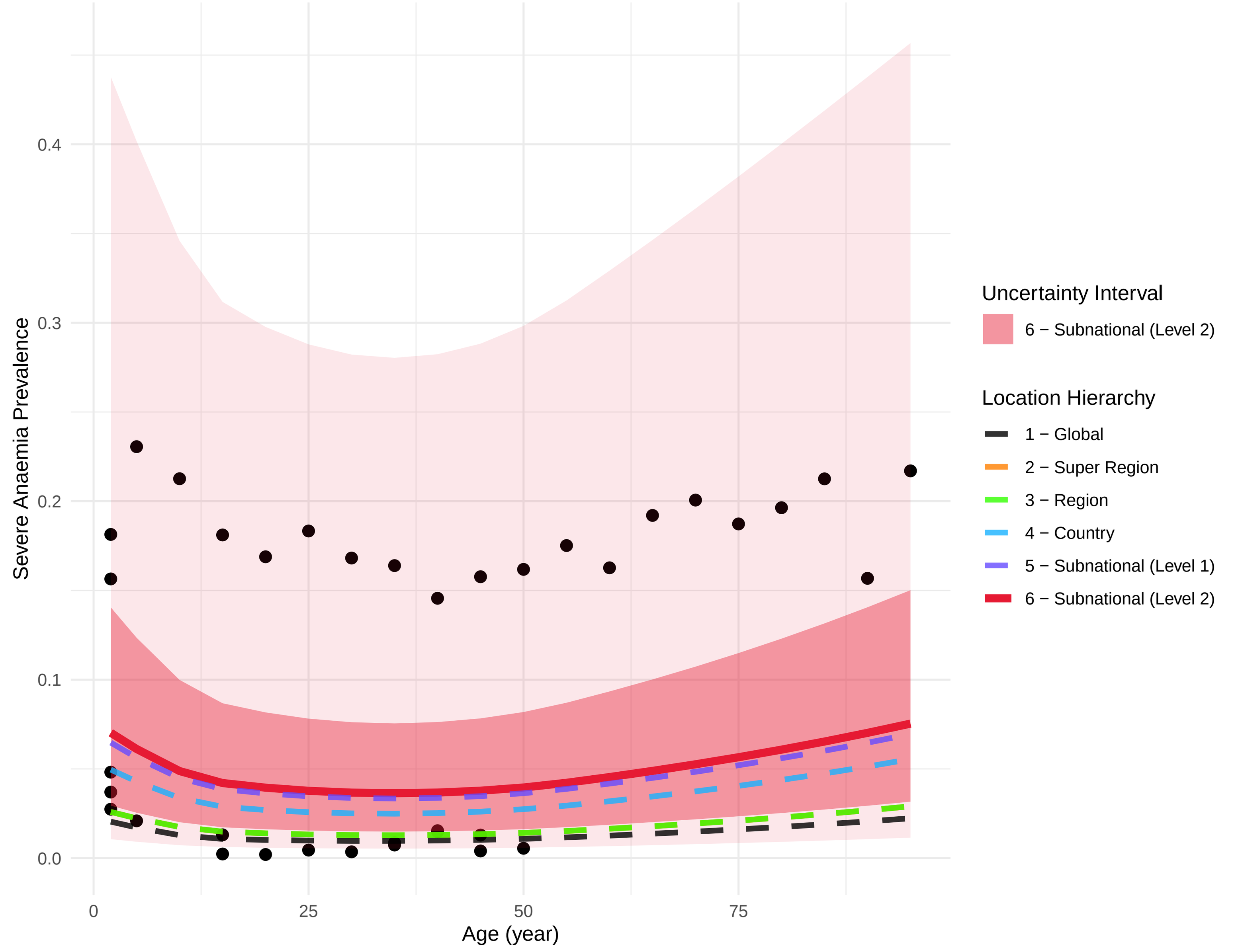

MR−BRT Age/Sex Fits − Males 2+ Years Old − Severe Anaemia Prevalence − Haryana, Urban
WHO−2024 Adjustment Method

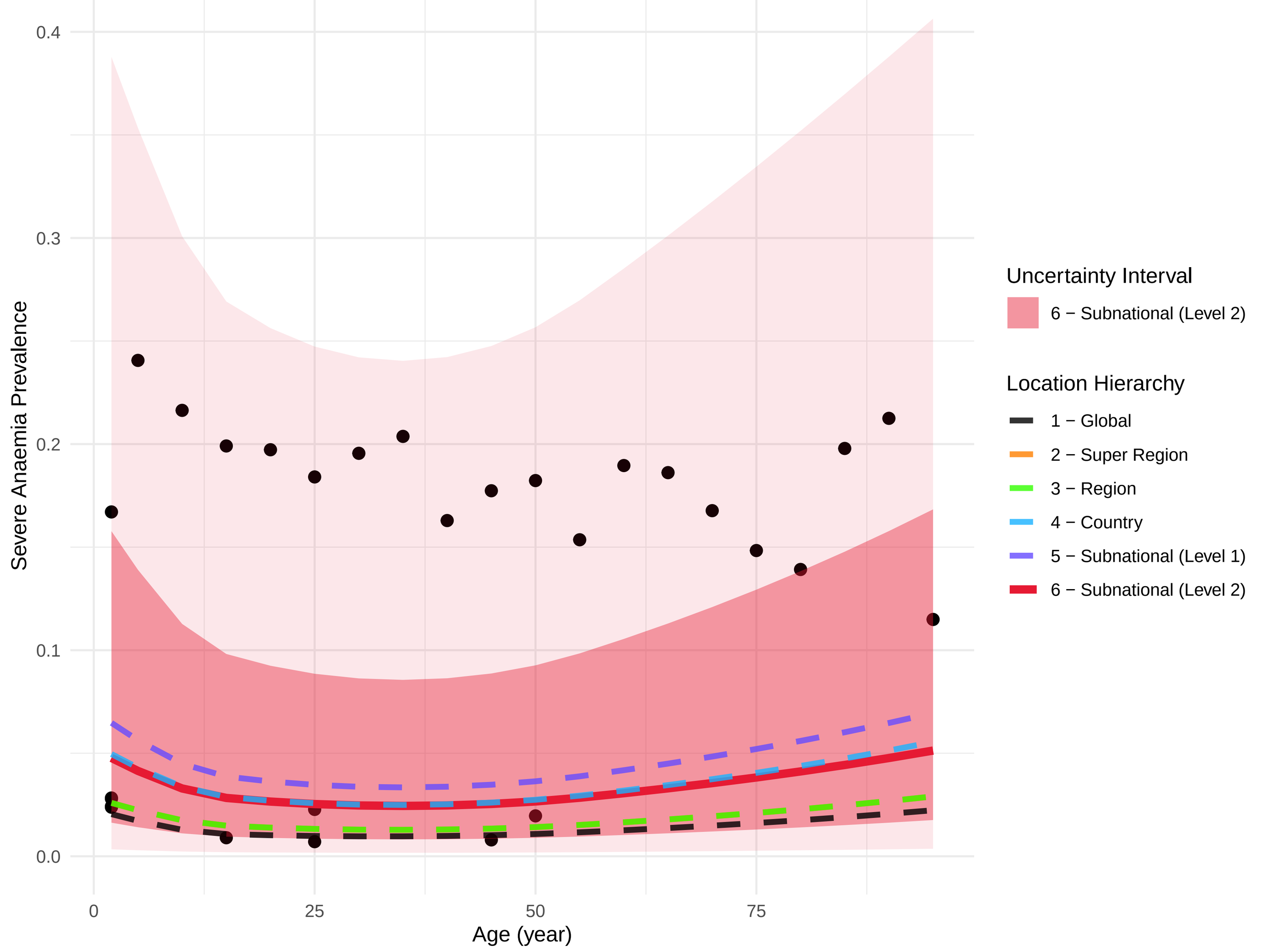

MR−BRT Age/Sex Fits − Males 2+ Years Old − Severe Anaemia Prevalence − Himachal Pradesh
WHO−2024 Adjustment Method

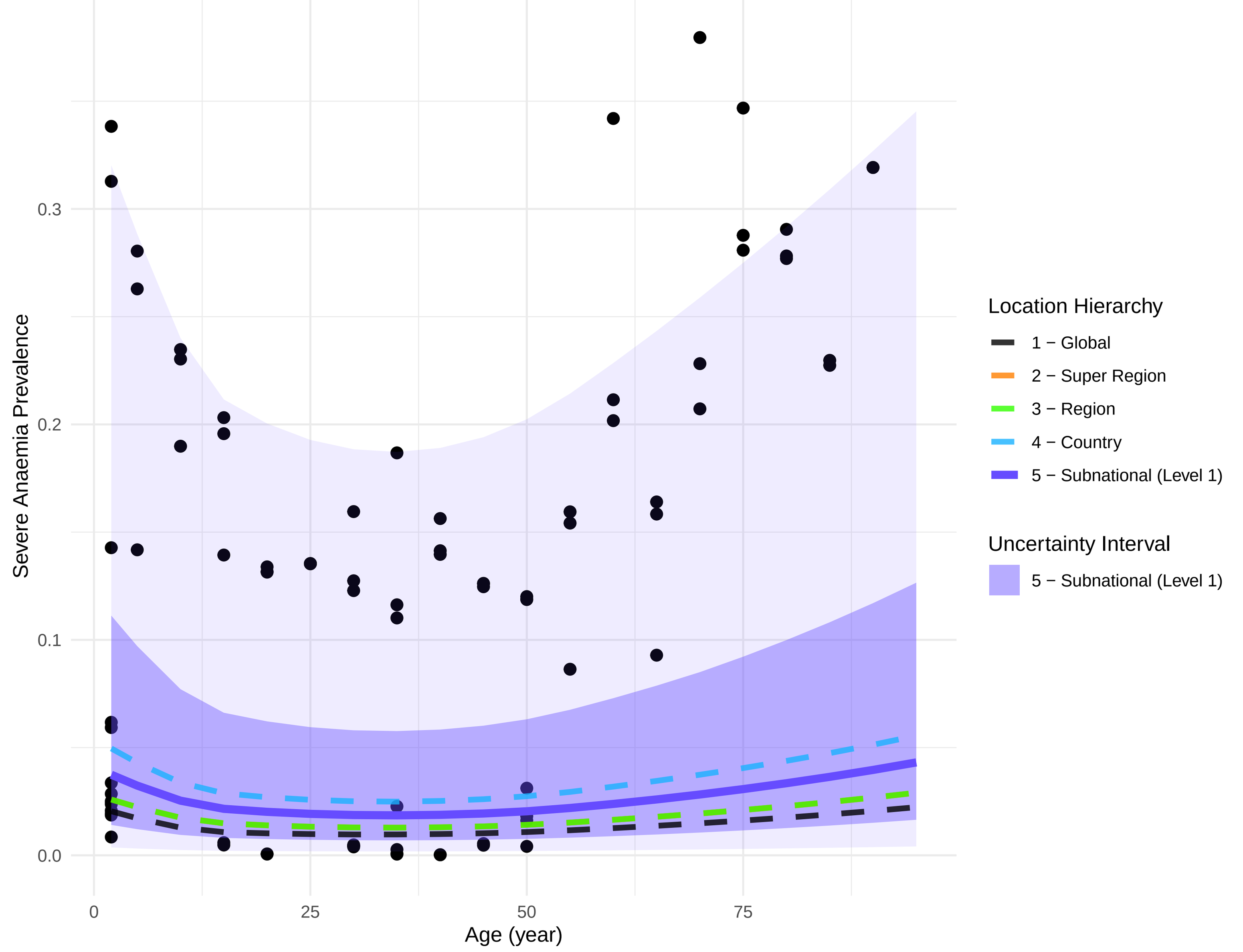

MR−BRT Age/Sex Fits − Males 2+ Years Old − Severe Anaemia Prevalence − Himachal Pradesh, Rural
WHO−2024 Adjustment Method

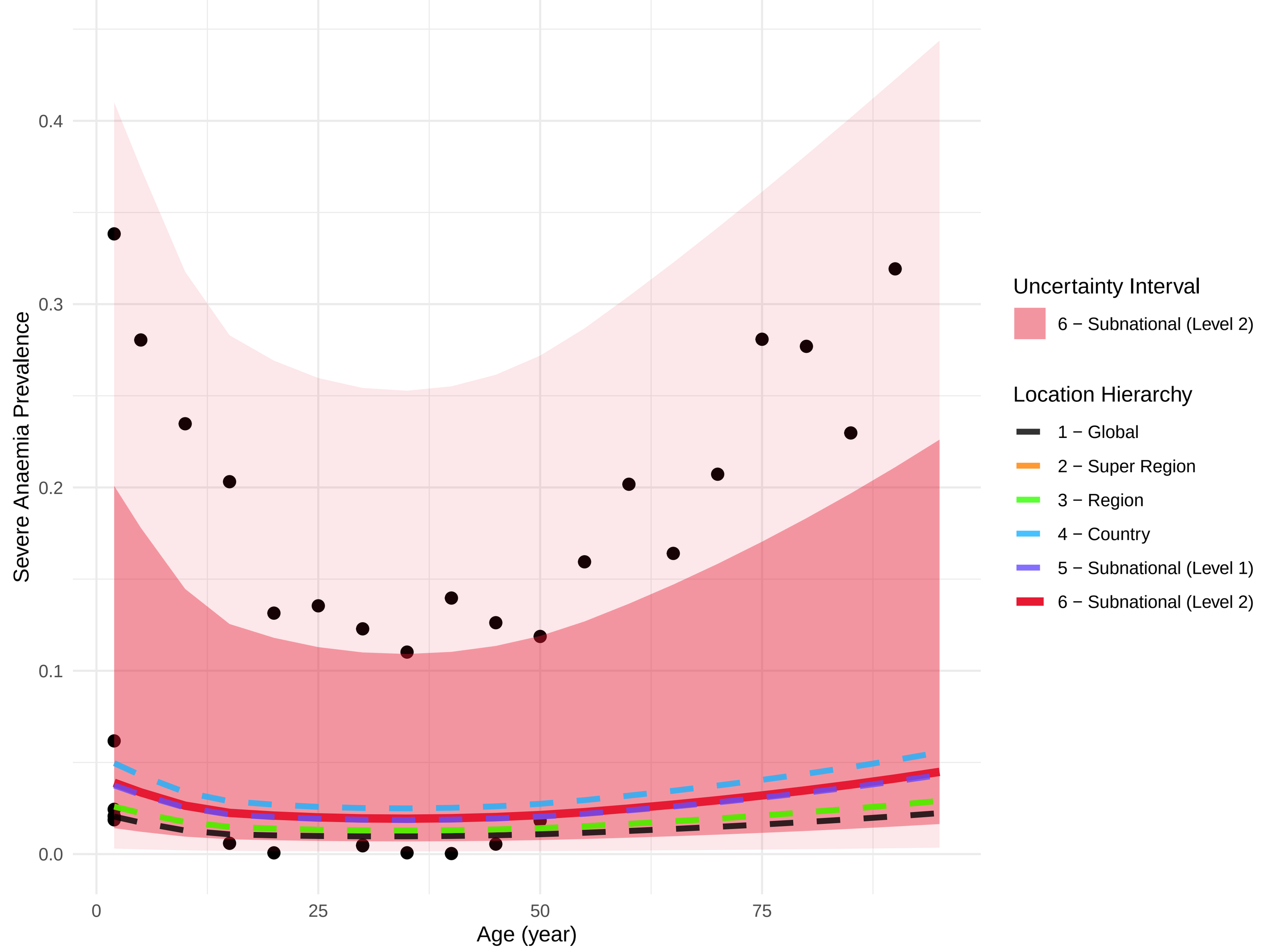

MR−BRT Age/Sex Fits − Males 2+ Years Old − Severe Anaemia Prevalence − Himachal Pradesh, Urban
WHO−2024 Adjustment Method

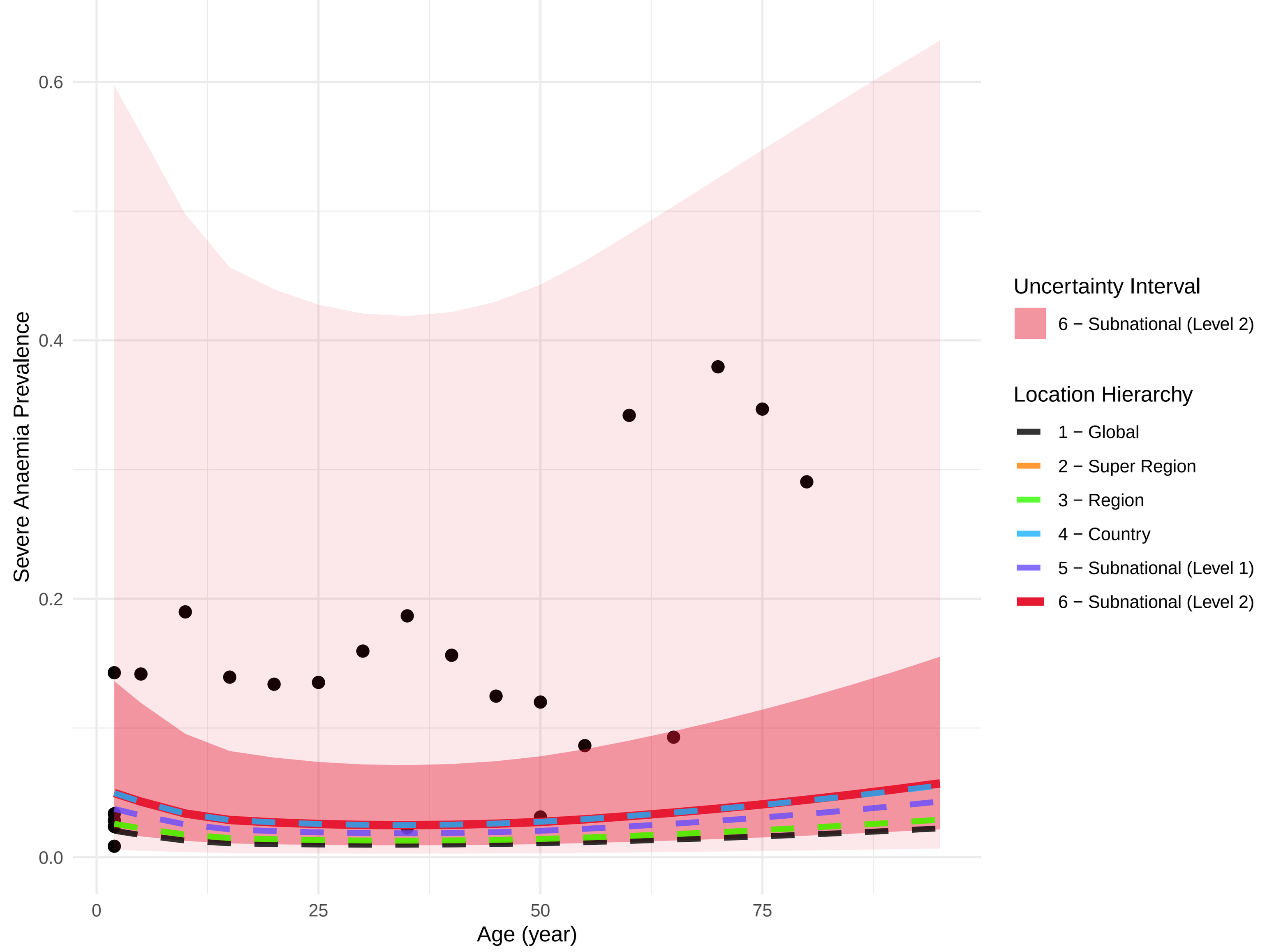

MR−BRT Age/Sex Fits − Males 2+ Years Old − Severe Anaemia Prevalence − Jammu & Kashmir and Ladakh
WHO−2024 Adjustment Method

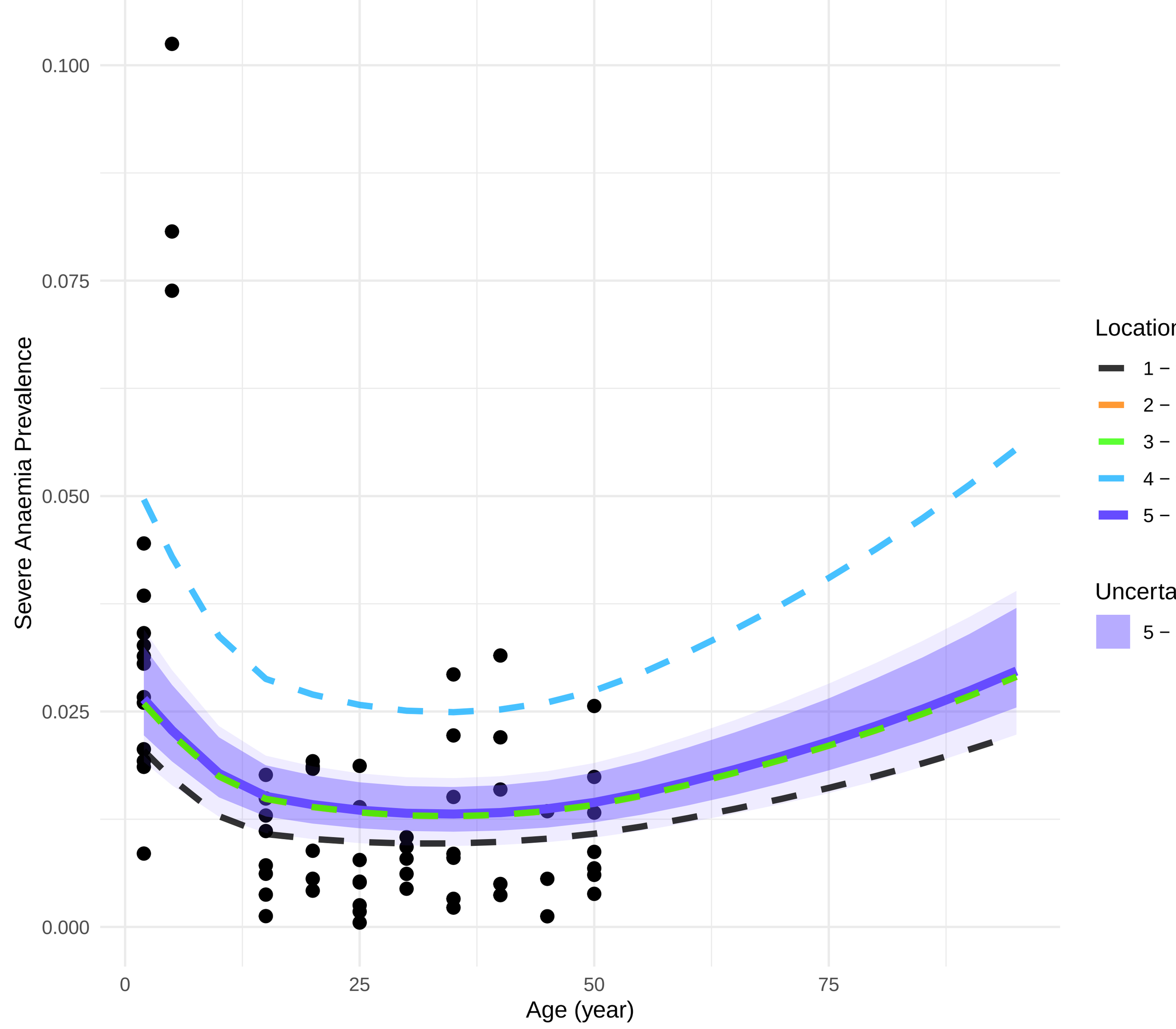

MR−BRT Age/Sex Fits − Males 2+ Years Old − Severe Anaemia Prevalence − Jammu & Kashmir and Ladakh, Rural
WHO−2024 Adjustment Method

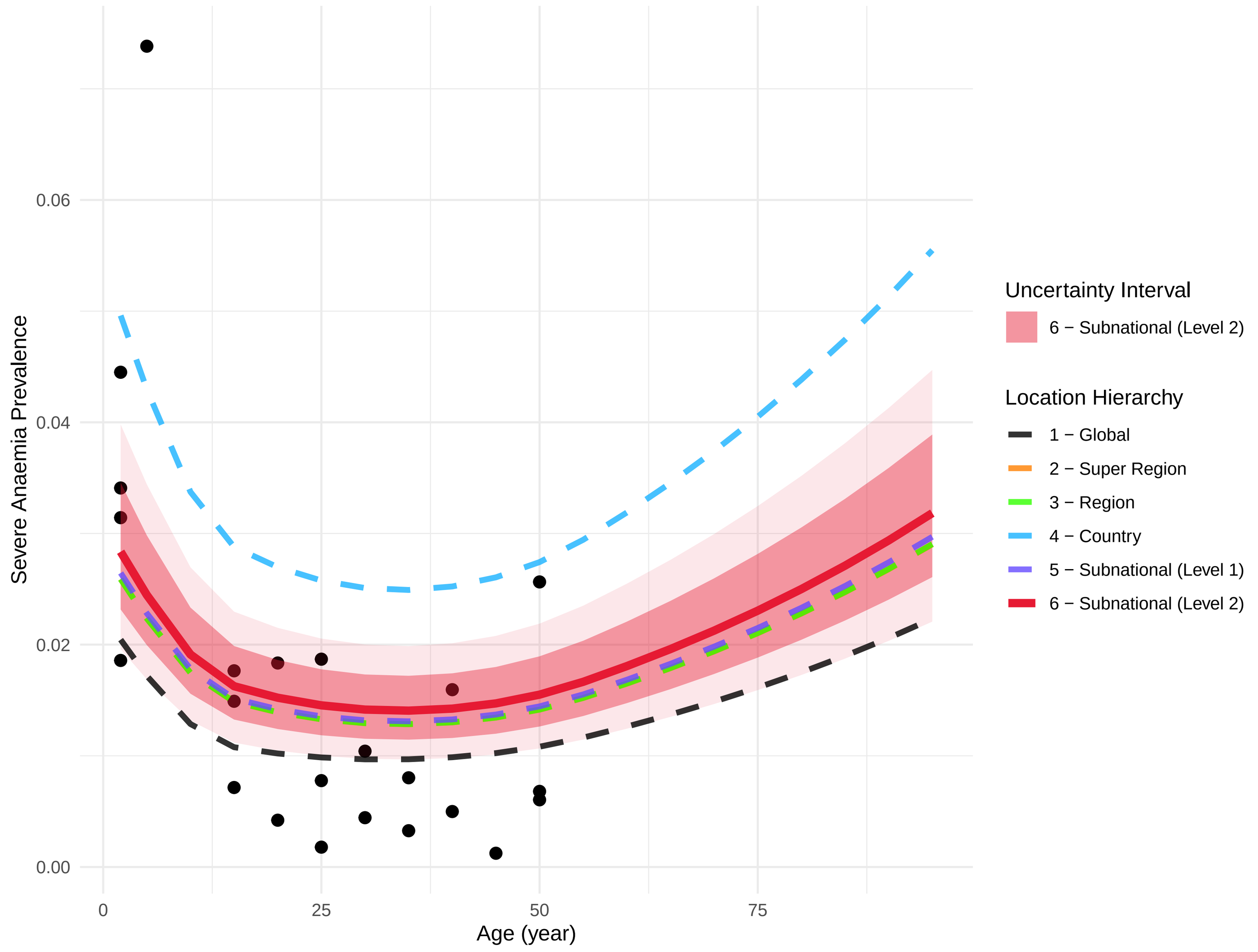

MR−BRT Age/Sex Fits − Males 2+ Years Old − Severe Anaemia Prevalence − Jammu & Kashmir and Ladakh, Urban
WHO−2024 Adjustment Method

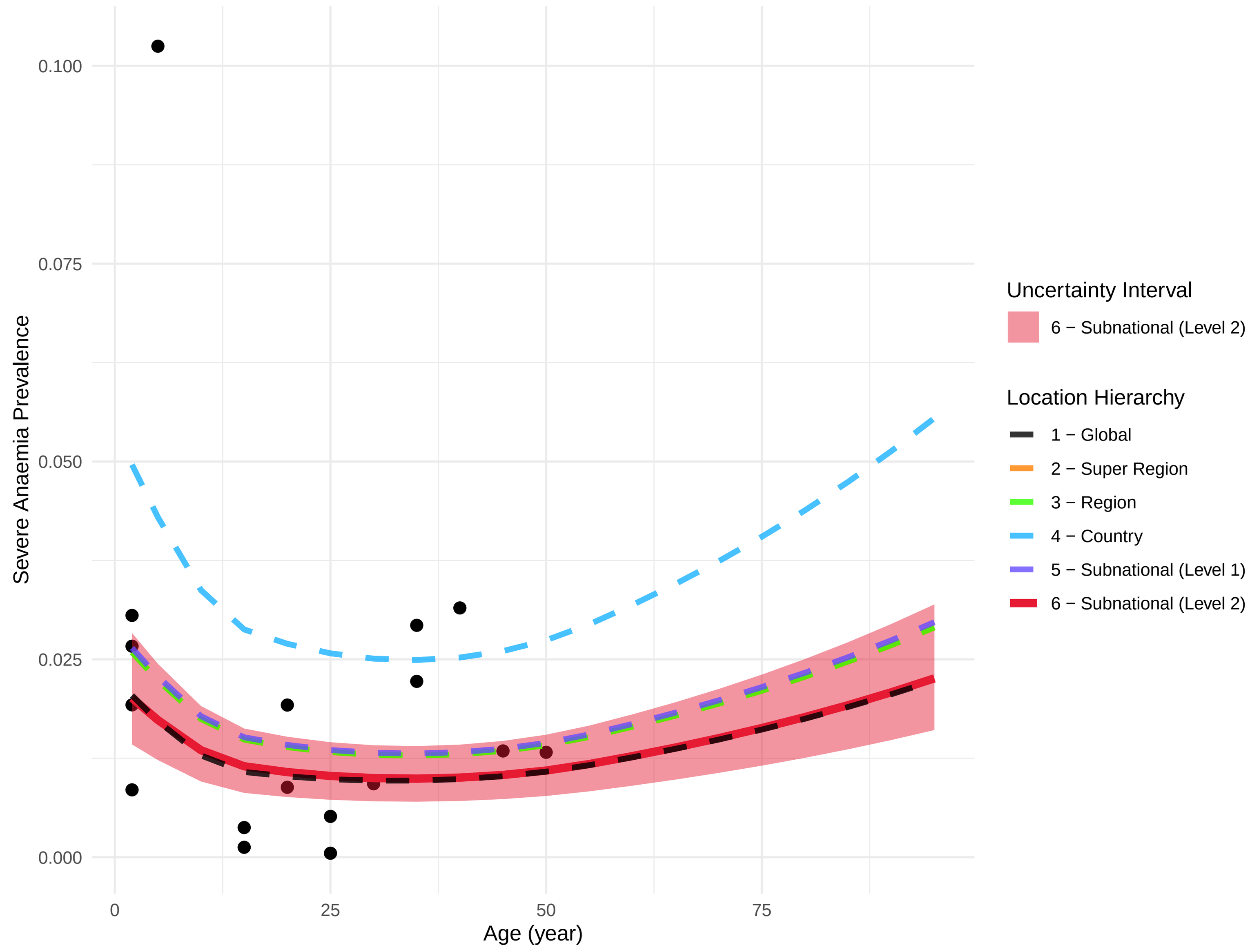

MR−BRT Age/Sex Fits − Males 2+ Years Old − Severe Anaemia Prevalence − Jharkhand
WHO−2024 Adjustment Method

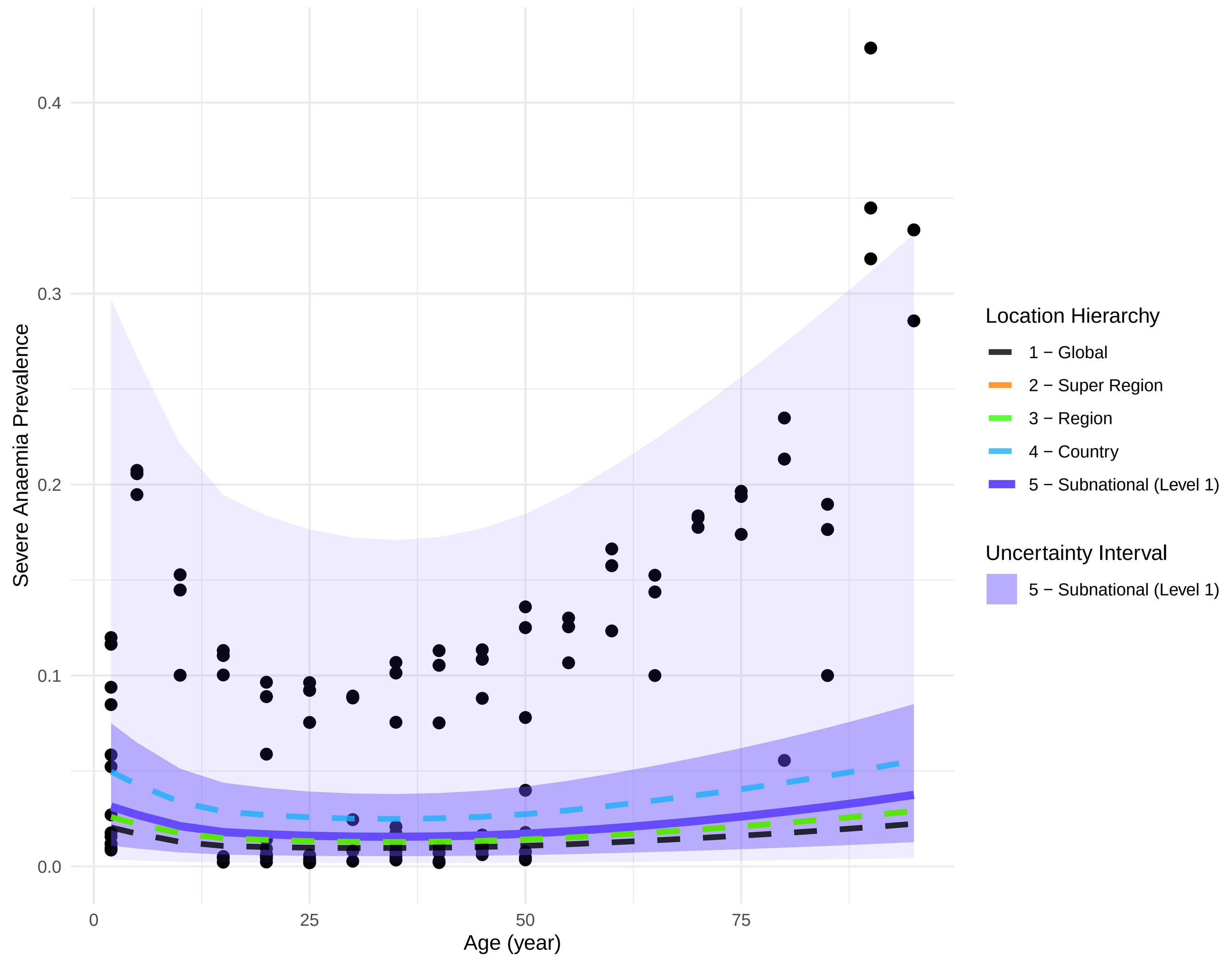

MR−BRT Age/Sex Fits − Males 2+ Years Old − Severe Anaemia Prevalence − Jharkhand, Rural
WHO−2024 Adjustment Method

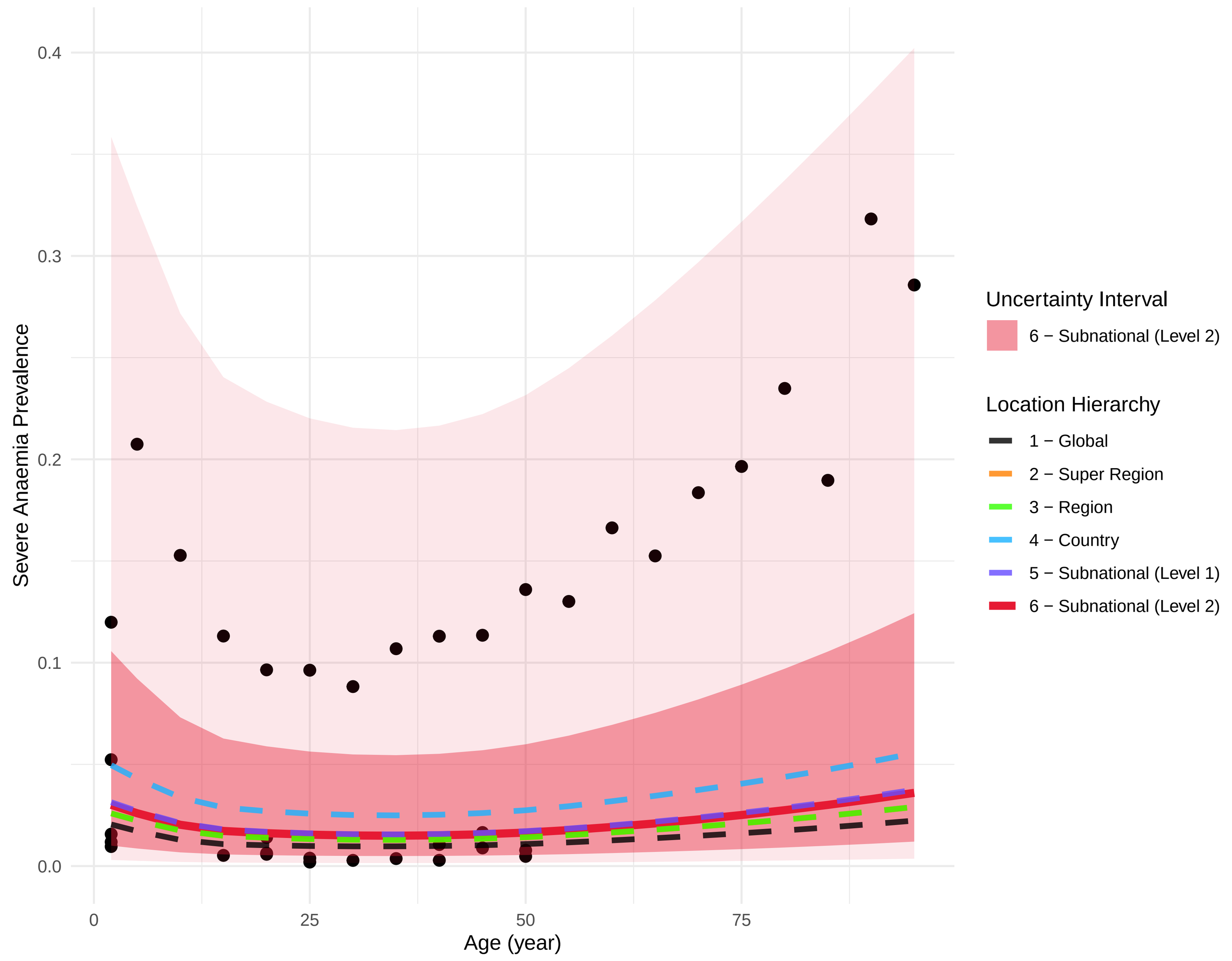

MR−BRT Age/Sex Fits − Males 2+ Years Old − Severe Anaemia Prevalence − Jharkhand, Urban
WHO−2024 Adjustment Method

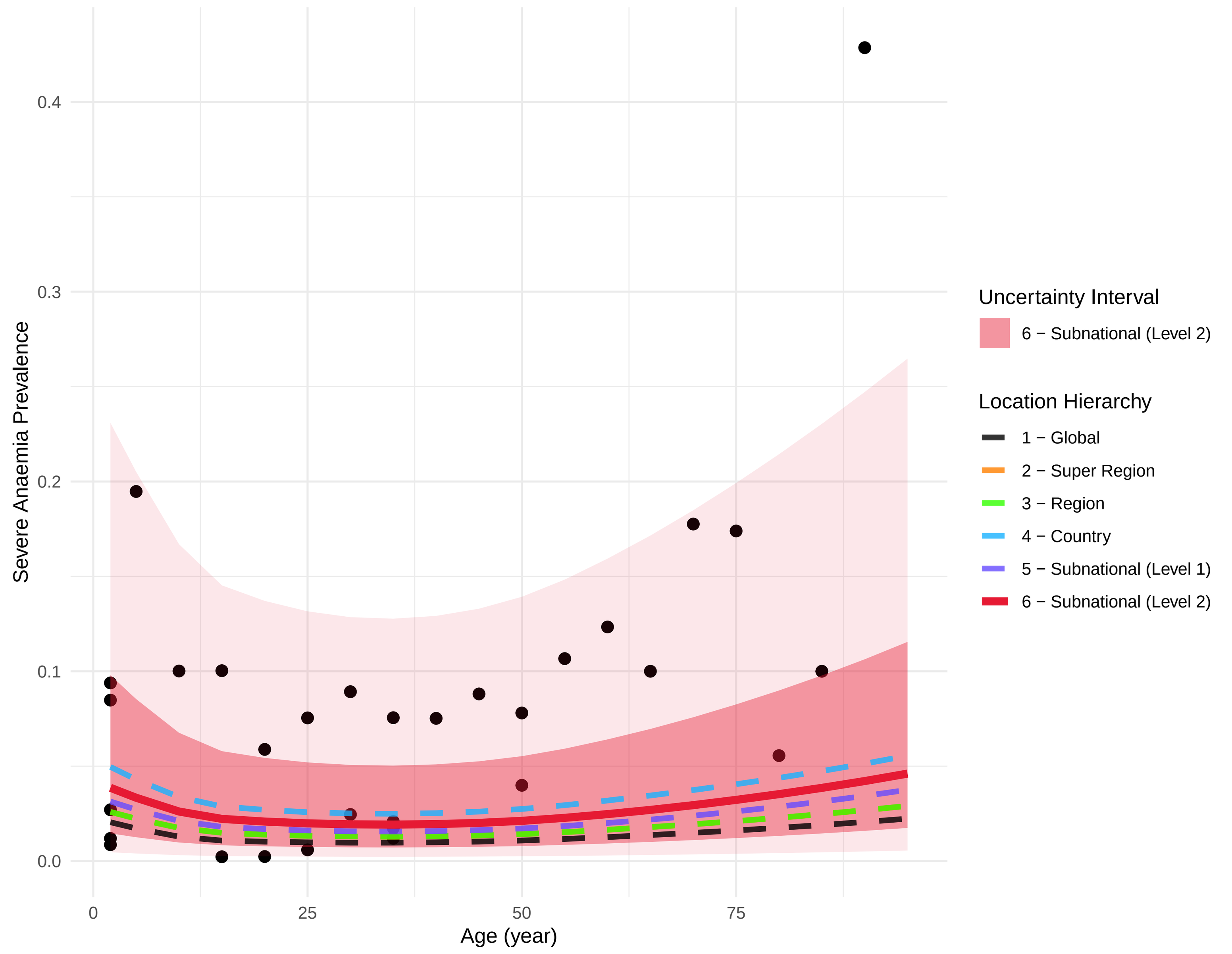

MR−BRT Age/Sex Fits − Males 2+ Years Old − Severe Anaemia Prevalence − Karnataka
WHO−2024 Adjustment Method

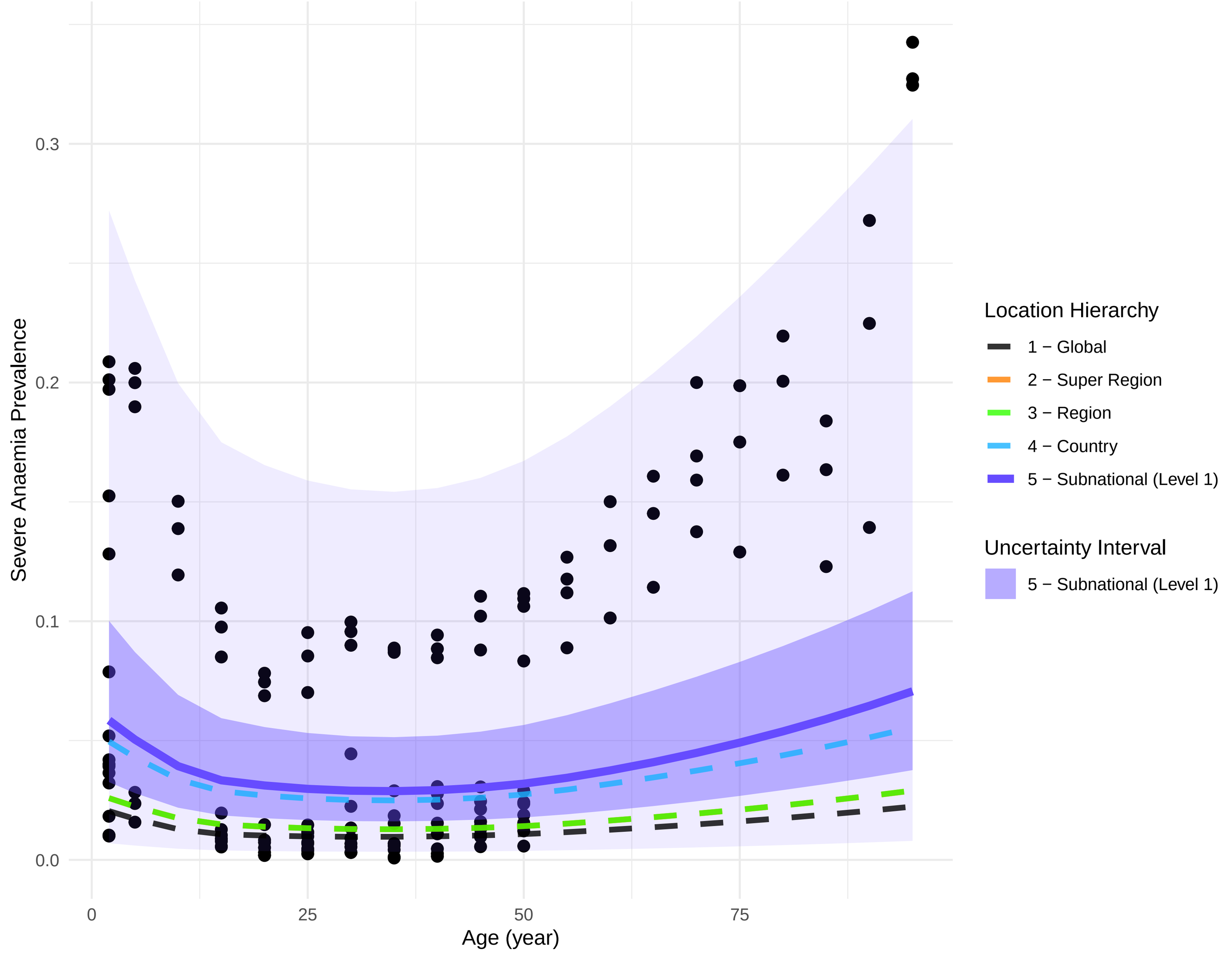

MR−BRT Age/Sex Fits − Males 2+ Years Old − Severe Anaemia Prevalence − Karnataka, Rural
WHO−2024 Adjustment Method

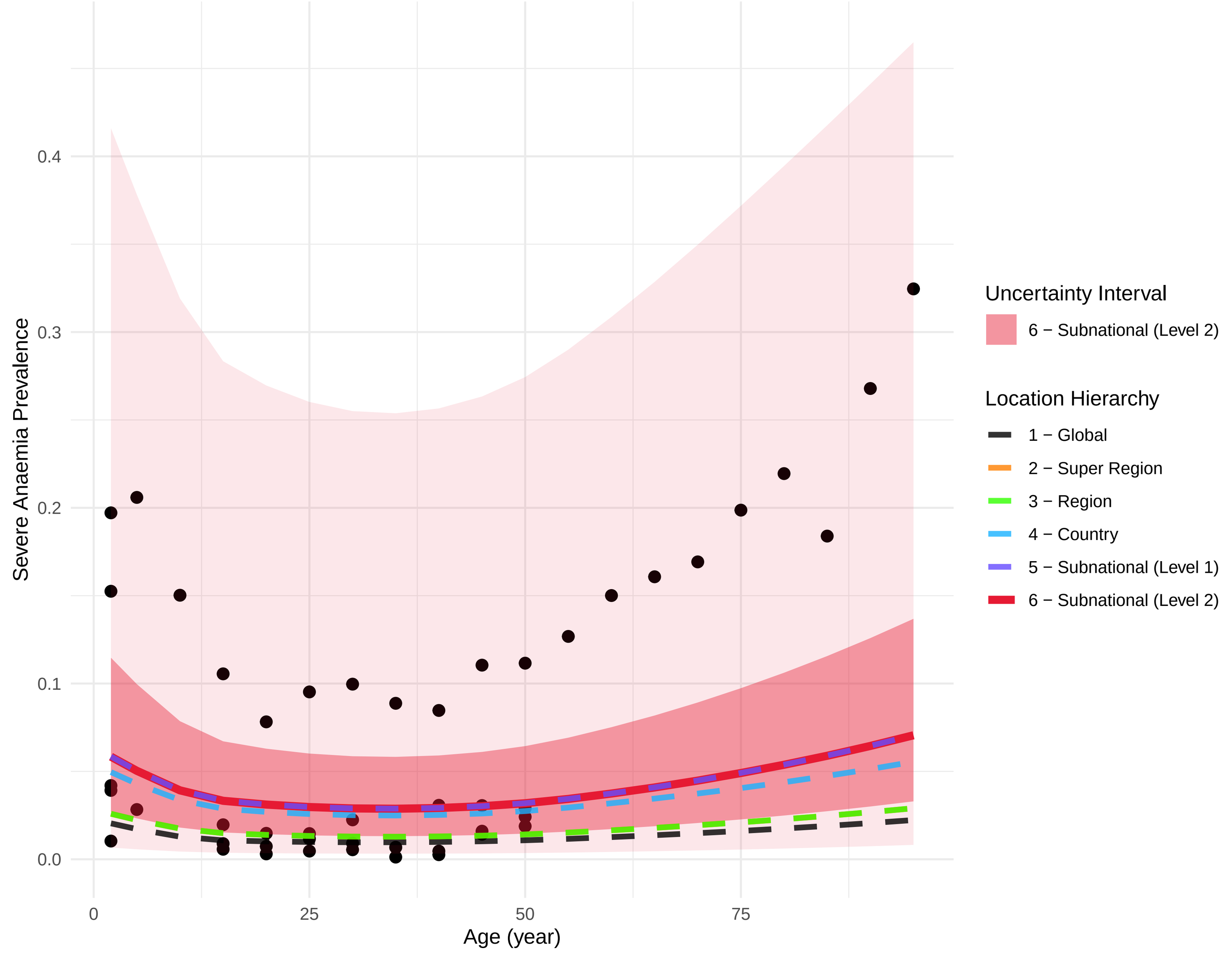

MR−BRT Age/Sex Fits − Males 2+ Years Old − Severe Anaemia Prevalence − Karnataka, Urban
WHO−2024 Adjustment Method

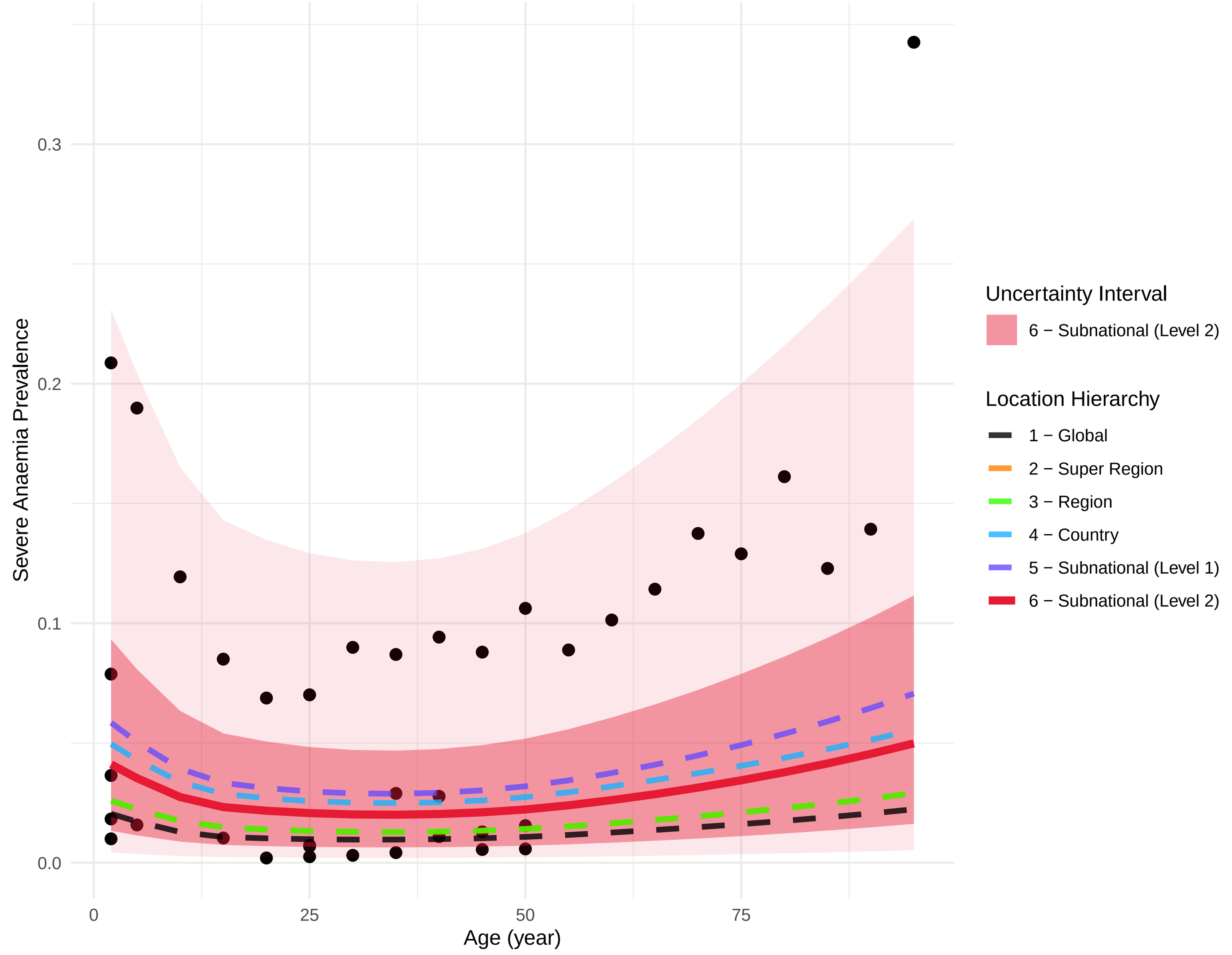

MR−BRT Age/Sex Fits − Males 2+ Years Old − Severe Anaemia Prevalence − Kerala
WHO−2024 Adjustment Method

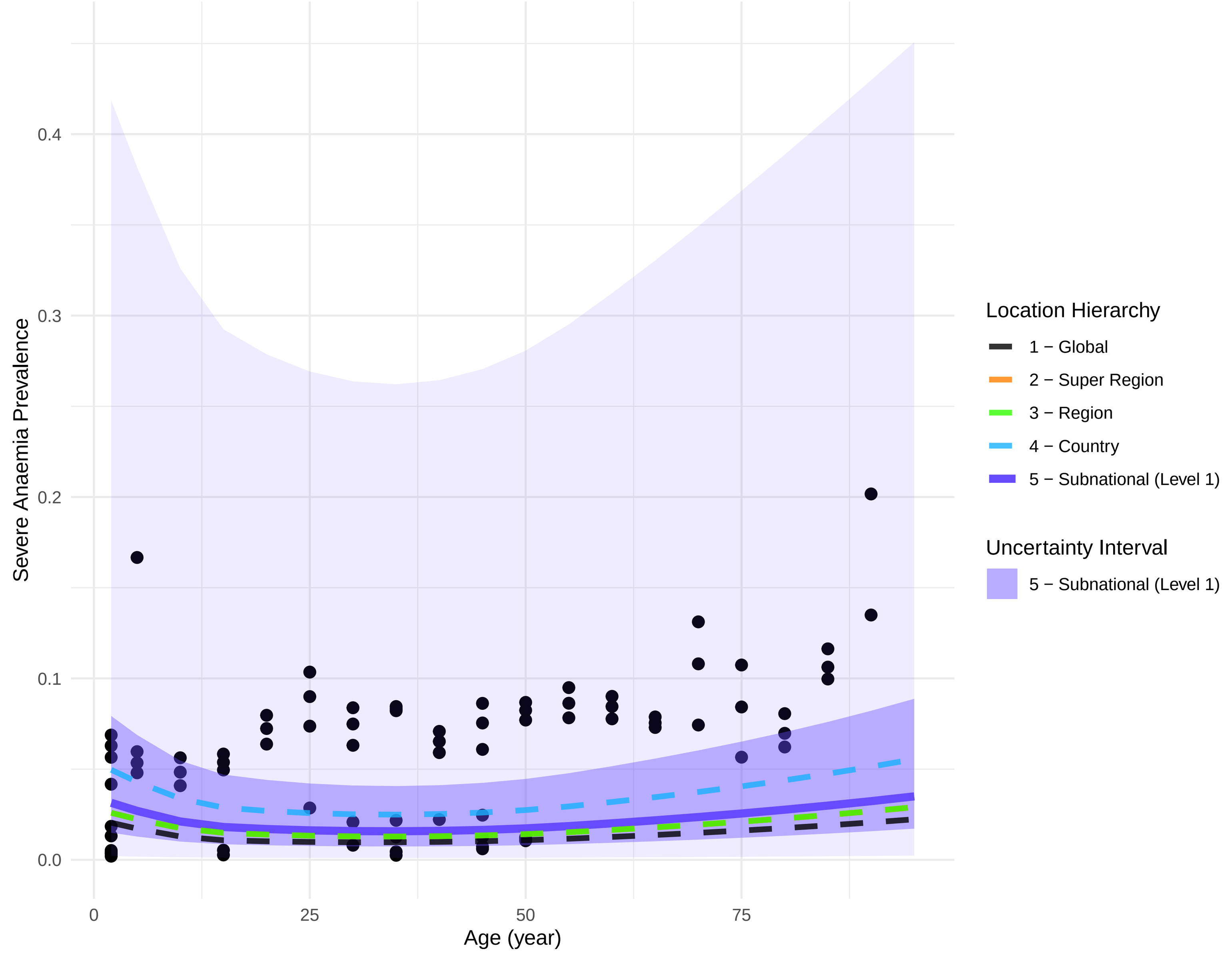

MR−BRT Age/Sex Fits − Males 2+ Years Old − Severe Anaemia Prevalence − Kerala, Rural
WHO−2024 Adjustment Method

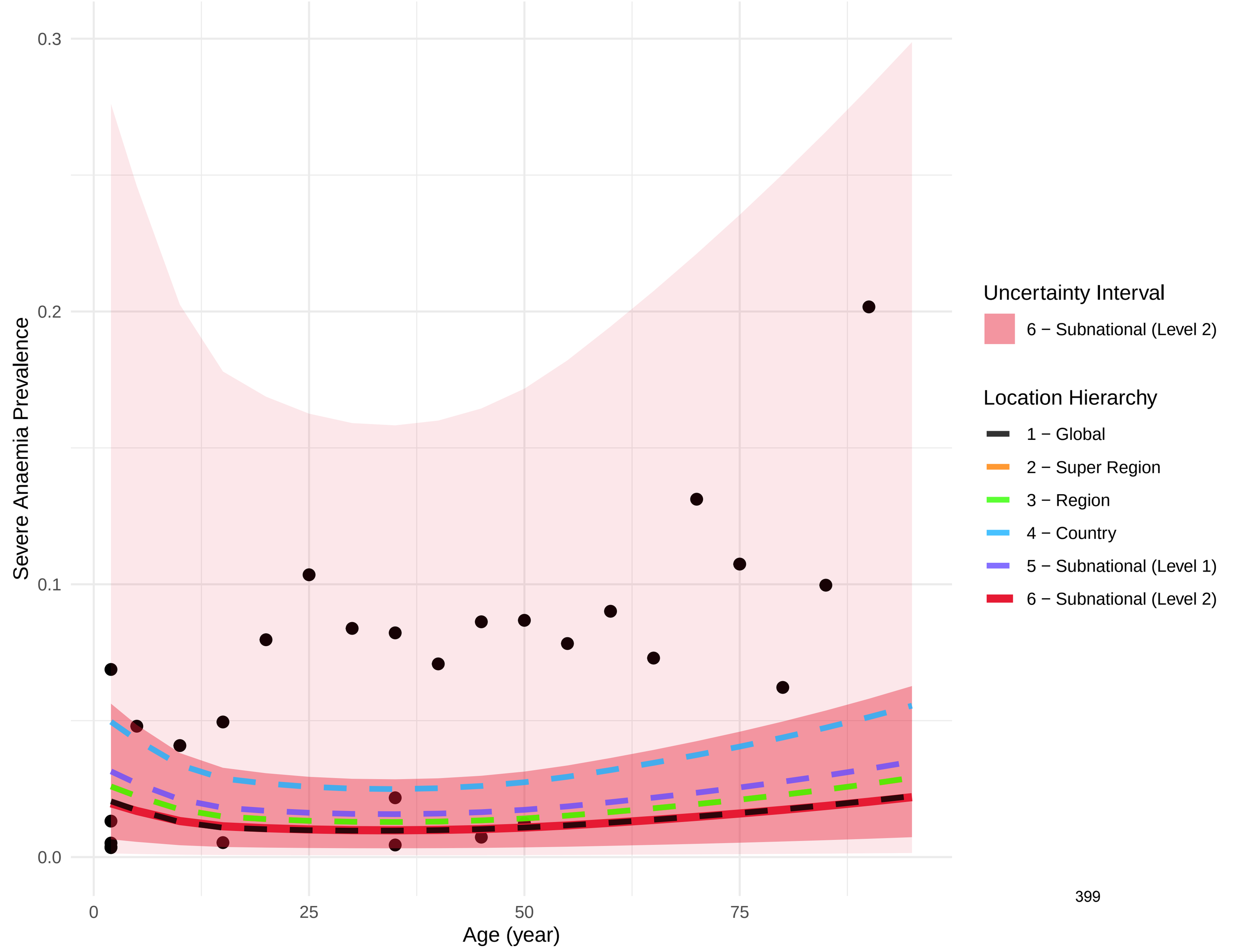

MR−BRT Age/Sex Fits − Males 2+ Years Old − Severe Anaemia Prevalence − Kerala, Urban
WHO−2024 Adjustment Method

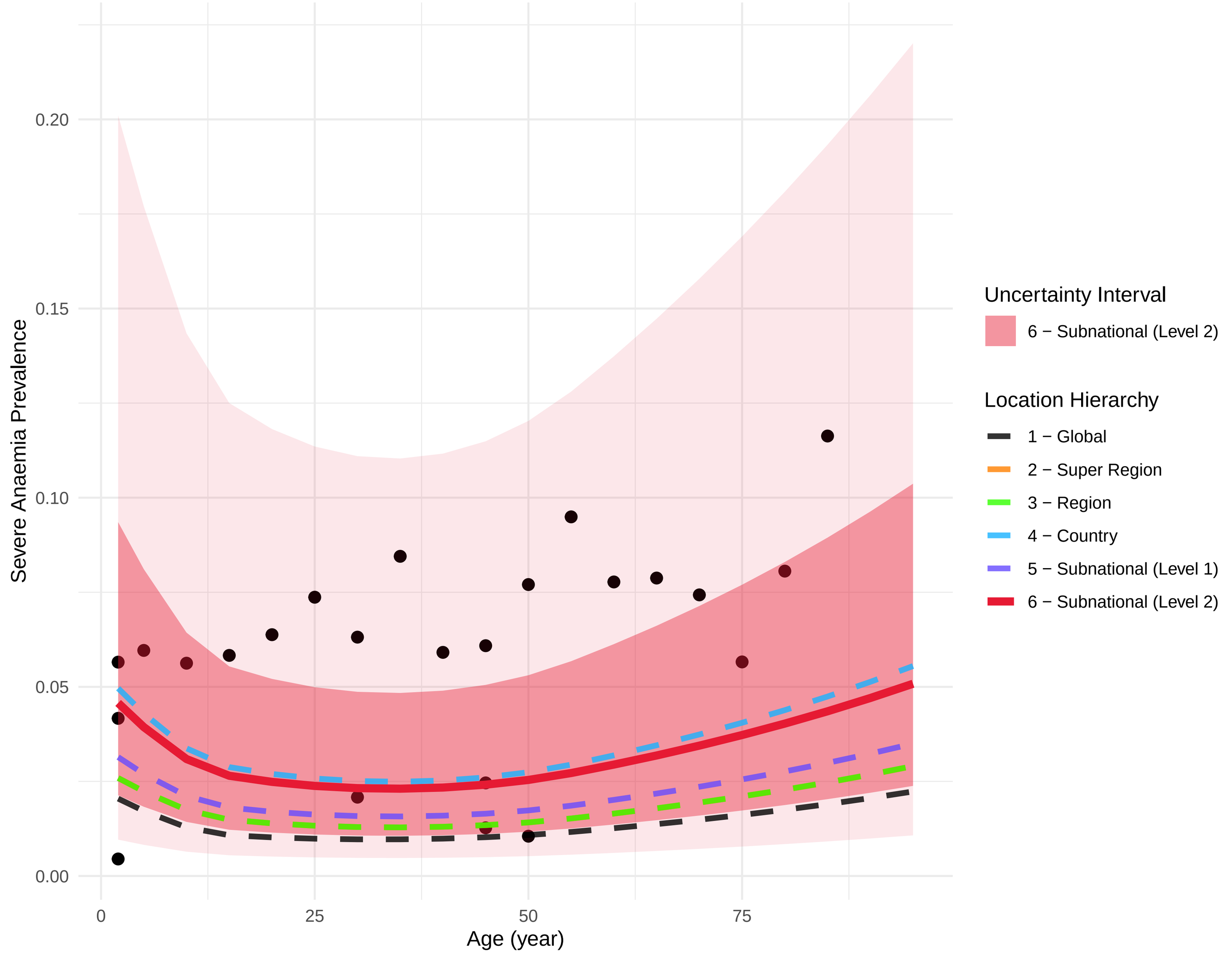

MR−BRT Age/Sex Fits − Males 2+ Years Old − Severe Anaemia Prevalence − Madhya Pradesh
WHO−2024 Adjustment Method

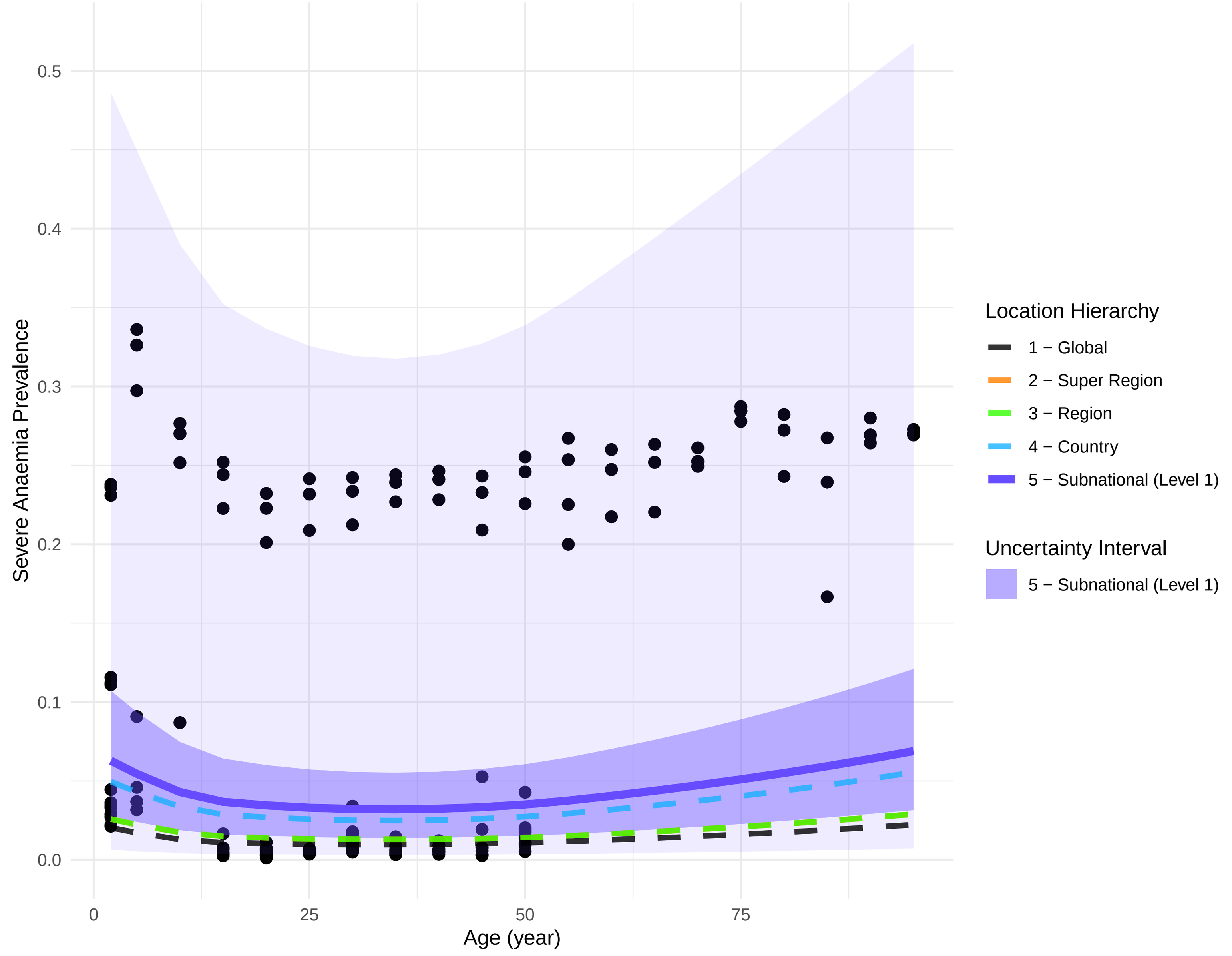

MR−BRT Age/Sex Fits − Males 2+ Years Old − Severe Anaemia Prevalence − Madhya Pradesh, Rural
WHO−2024 Adjustment Method

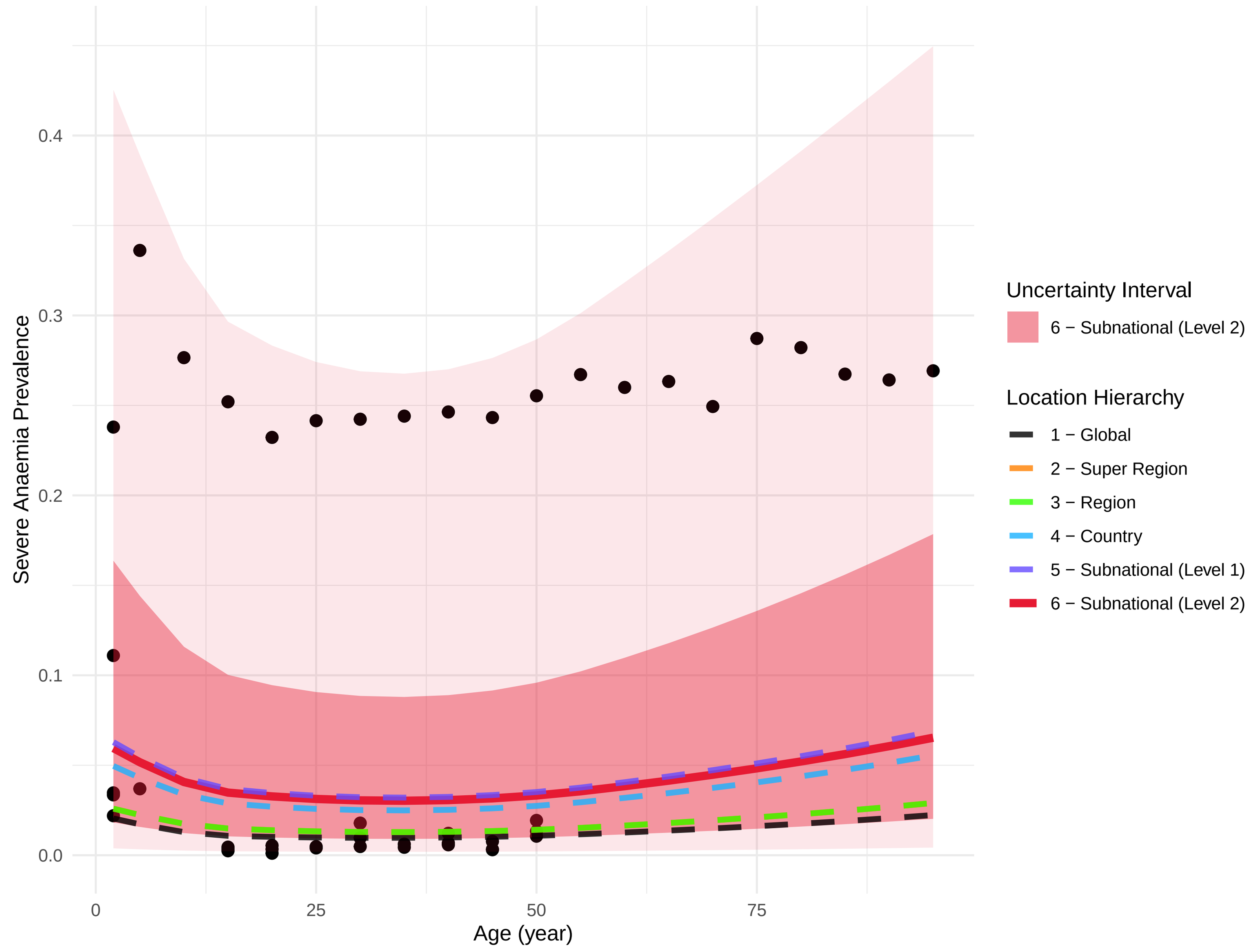

MR−BRT Age/Sex Fits − Males 2+ Years Old − Severe Anaemia Prevalence − Madhya Pradesh, Urban
WHO−2024 Adjustment Method

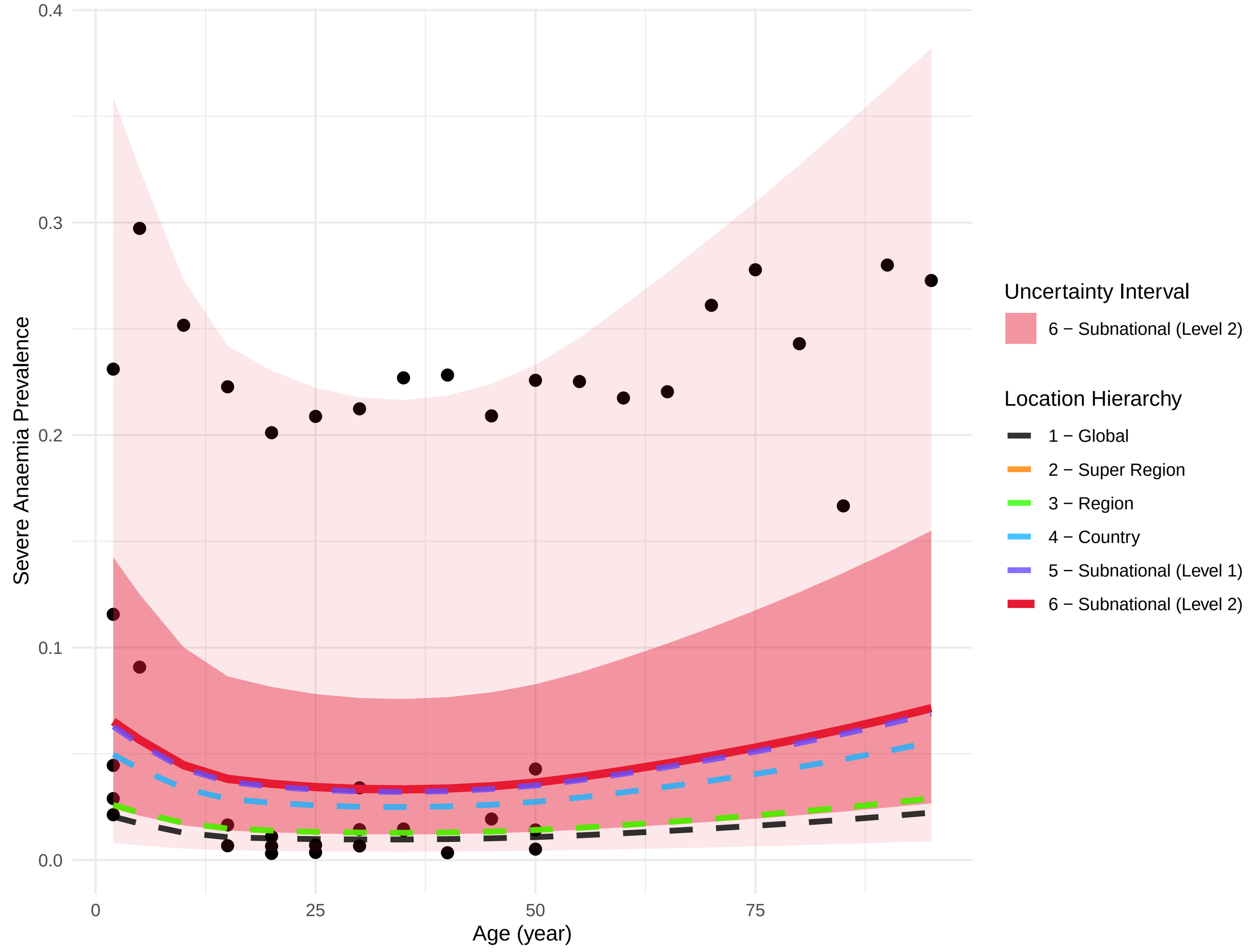

MR−BRT Age/Sex Fits − Males 2+ Years Old − Severe Anaemia Prevalence − Maharashtra
WHO−2024 Adjustment Method

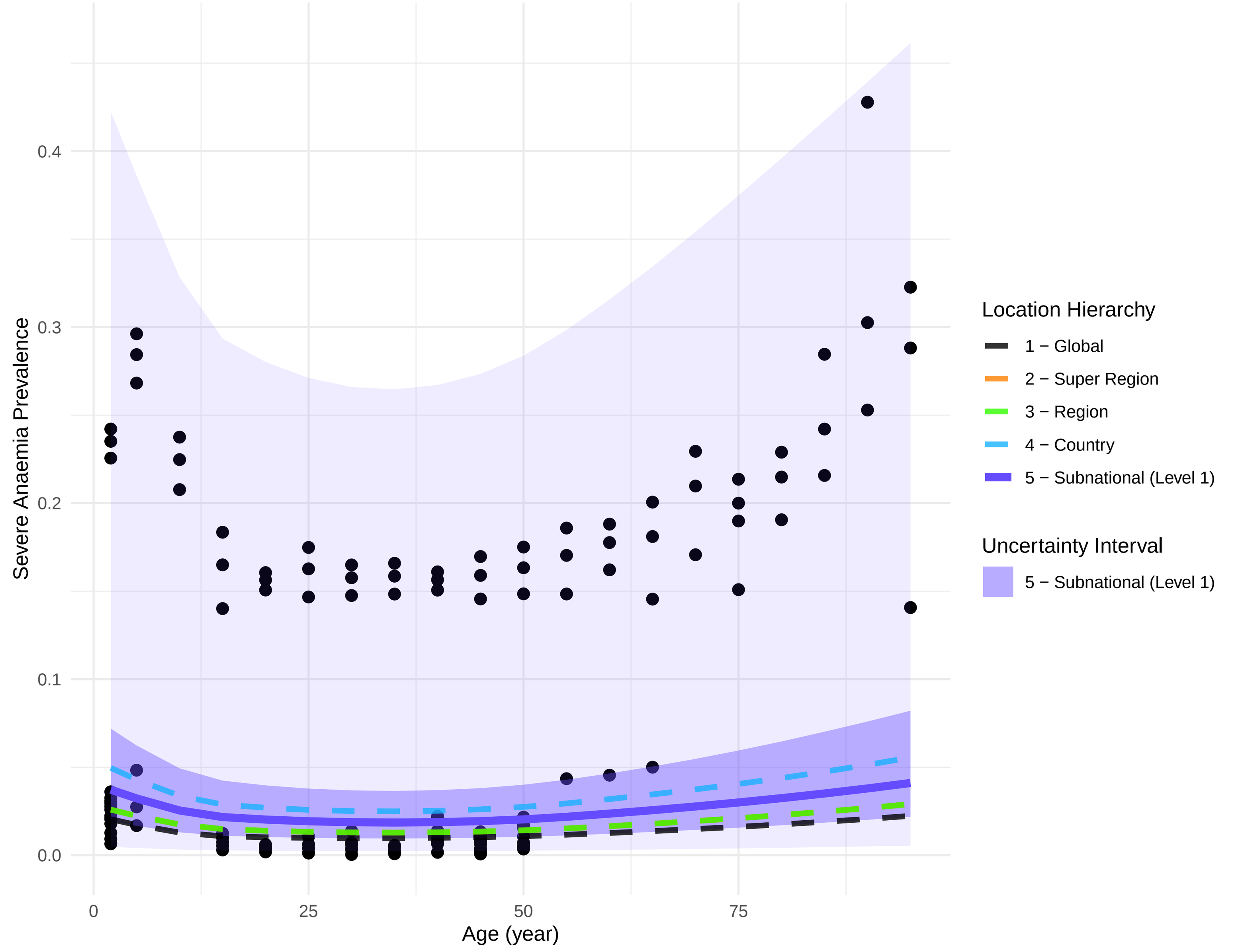

MR−BRT Age/Sex Fits − Males 2+ Years Old − Severe Anaemia Prevalence − Maharashtra, Rural
WHO−2024 Adjustment Method

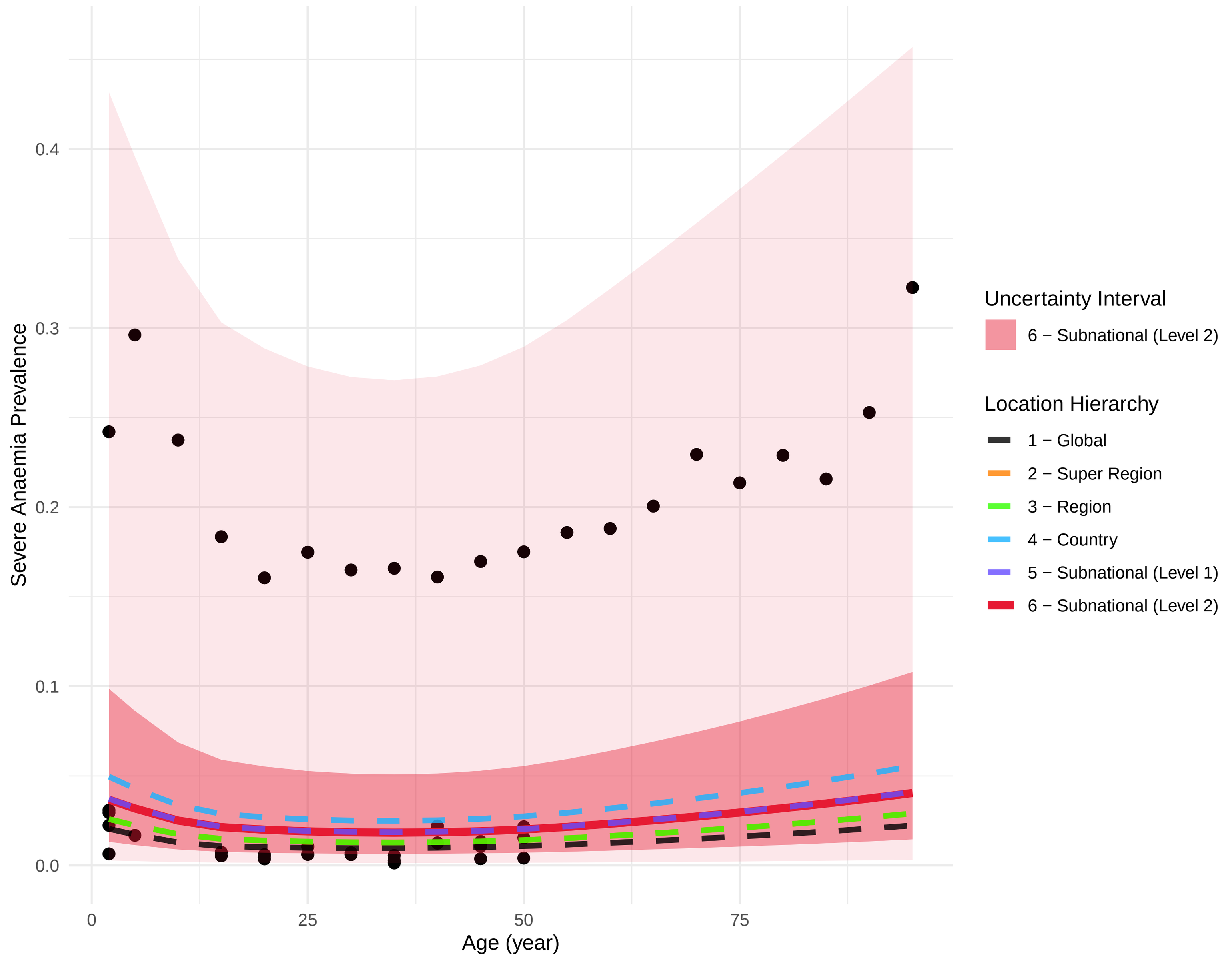

MR−BRT Age/Sex Fits − Males 2+ Years Old − Severe Anaemia Prevalence − Maharashtra, Urban
WHO−2024 Adjustment Method

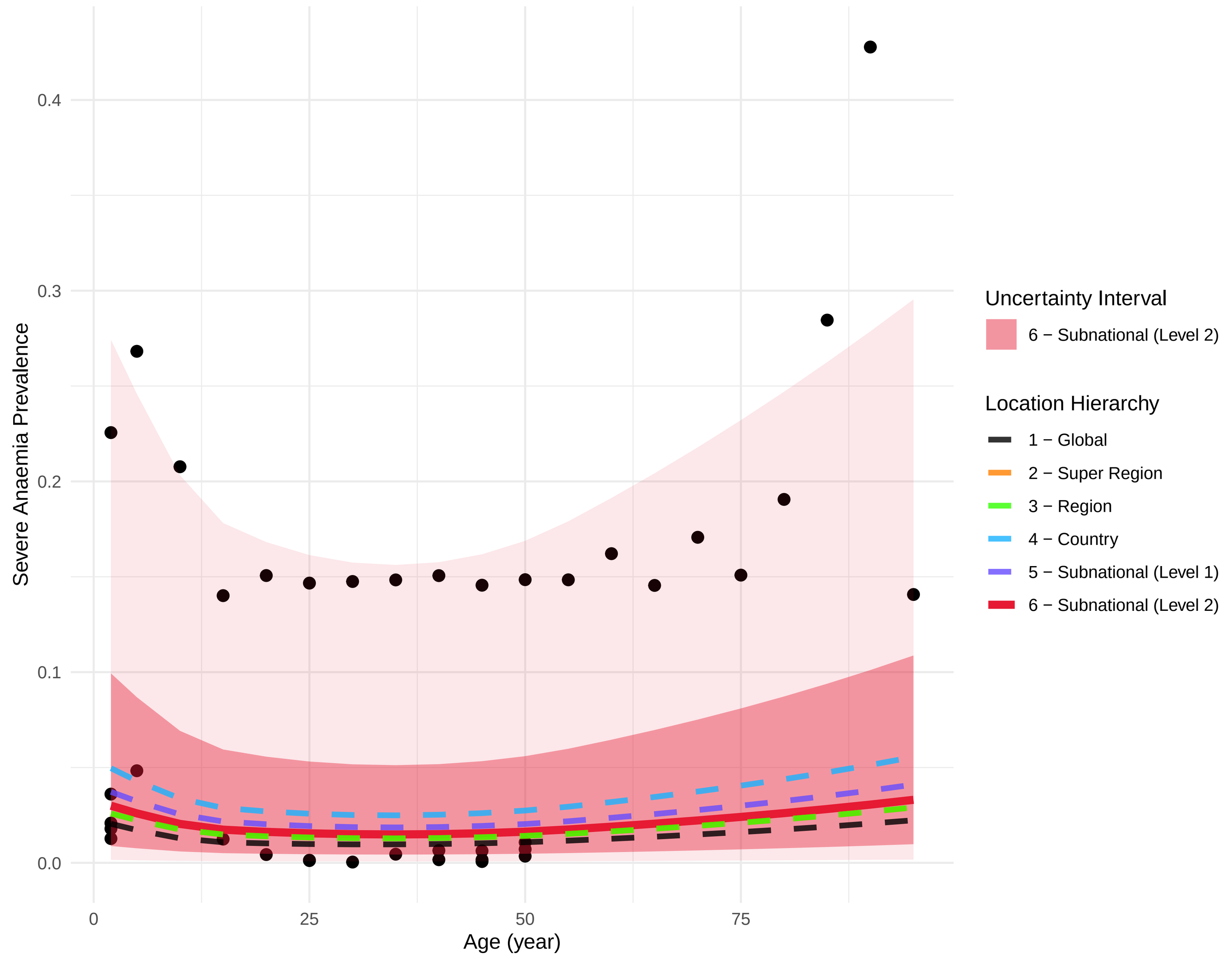

MR−BRT Age/Sex Fits − Males 2+ Years Old − Severe Anaemia Prevalence − Manipur
WHO−2024 Adjustment Method

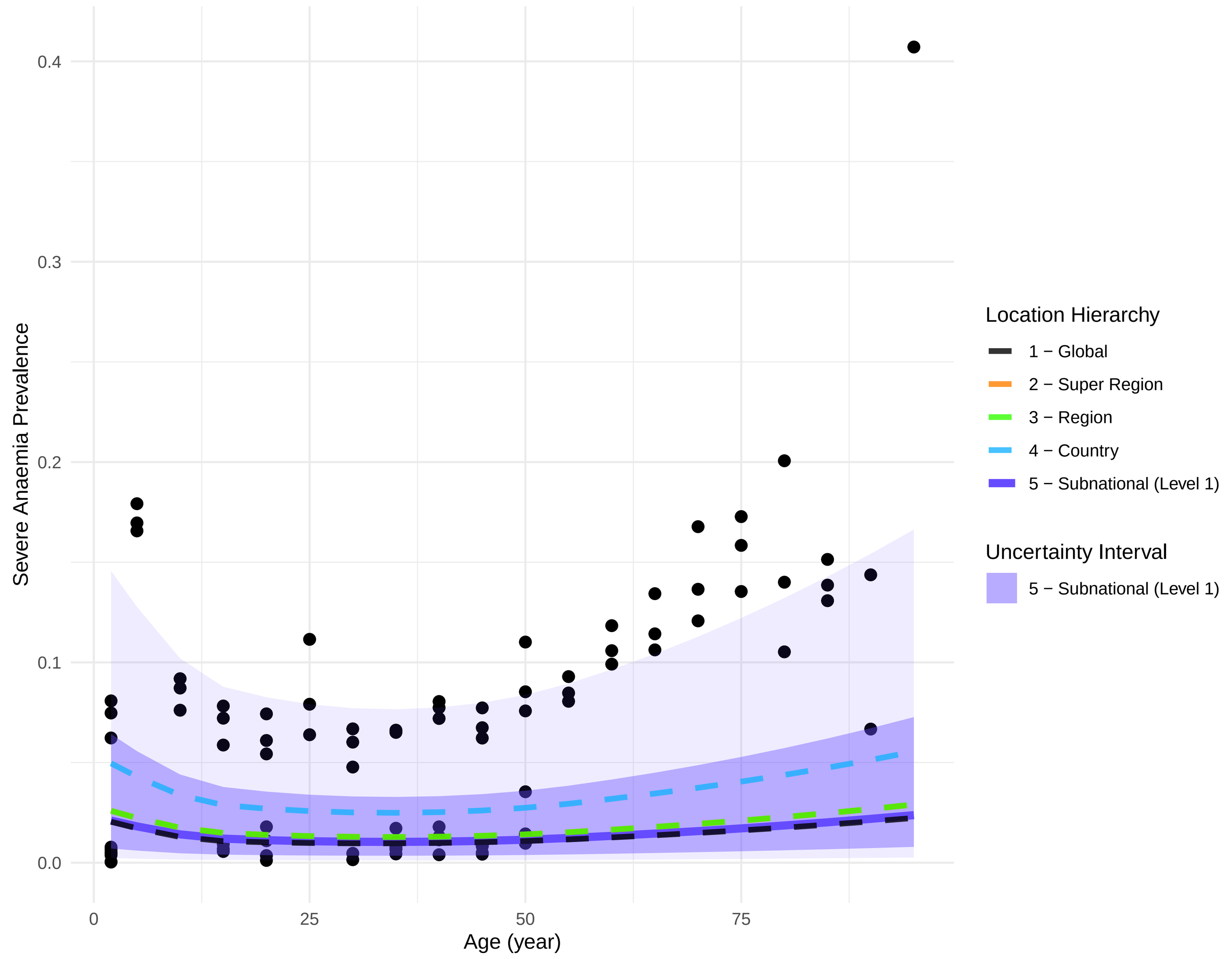

MR−BRT Age/Sex Fits − Males 2+ Years Old − Severe Anaemia Prevalence − Manipur, Rural
WHO−2024 Adjustment Method

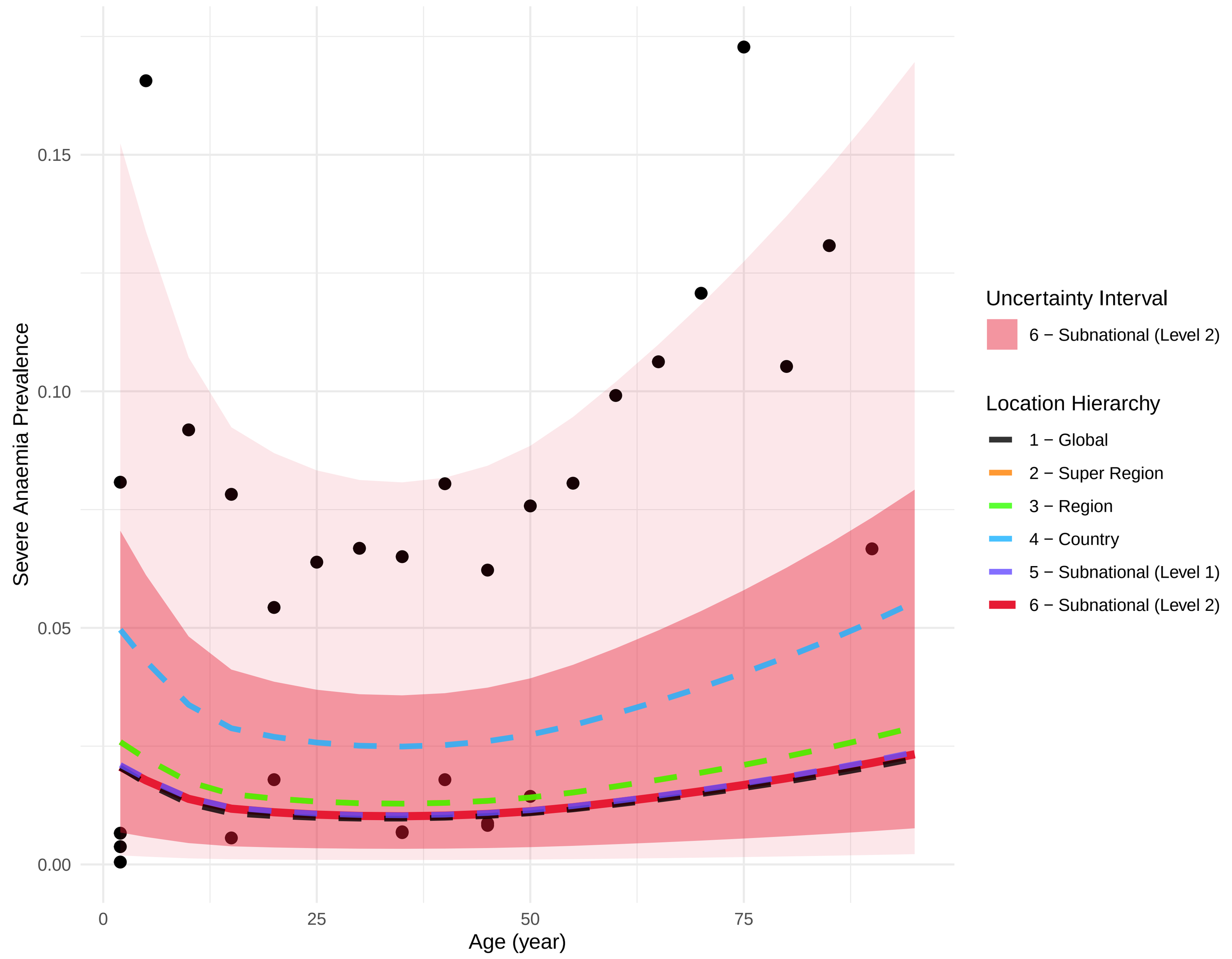

MR−BRT Age/Sex Fits − Males 2+ Years Old − Severe Anaemia Prevalence − Manipur, Urban
WHO−2024 Adjustment Method

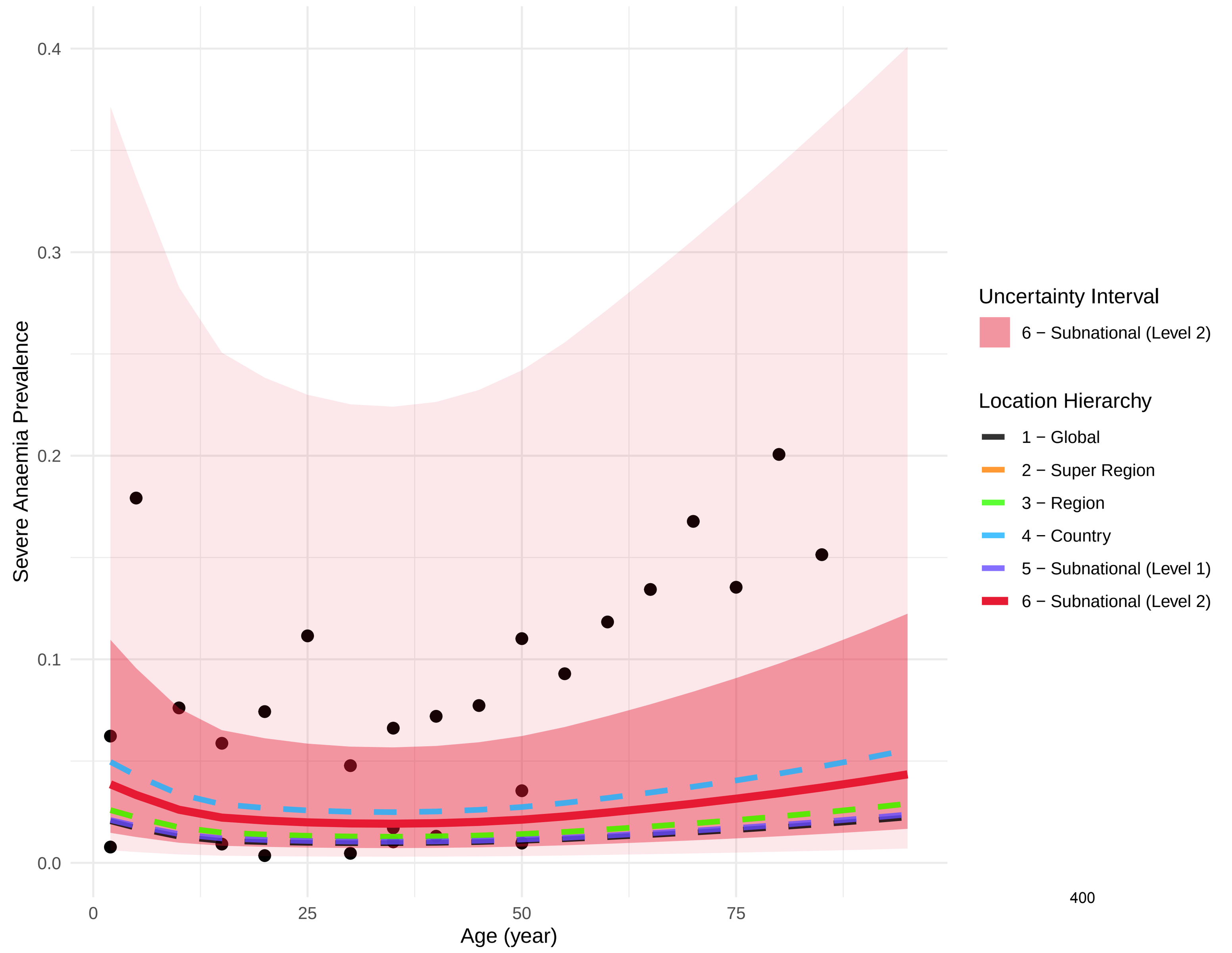

MR−BRT Age/Sex Fits − Males 2+ Years Old − Severe Anaemia Prevalence − Meghalaya
WHO−2024 Adjustment Method

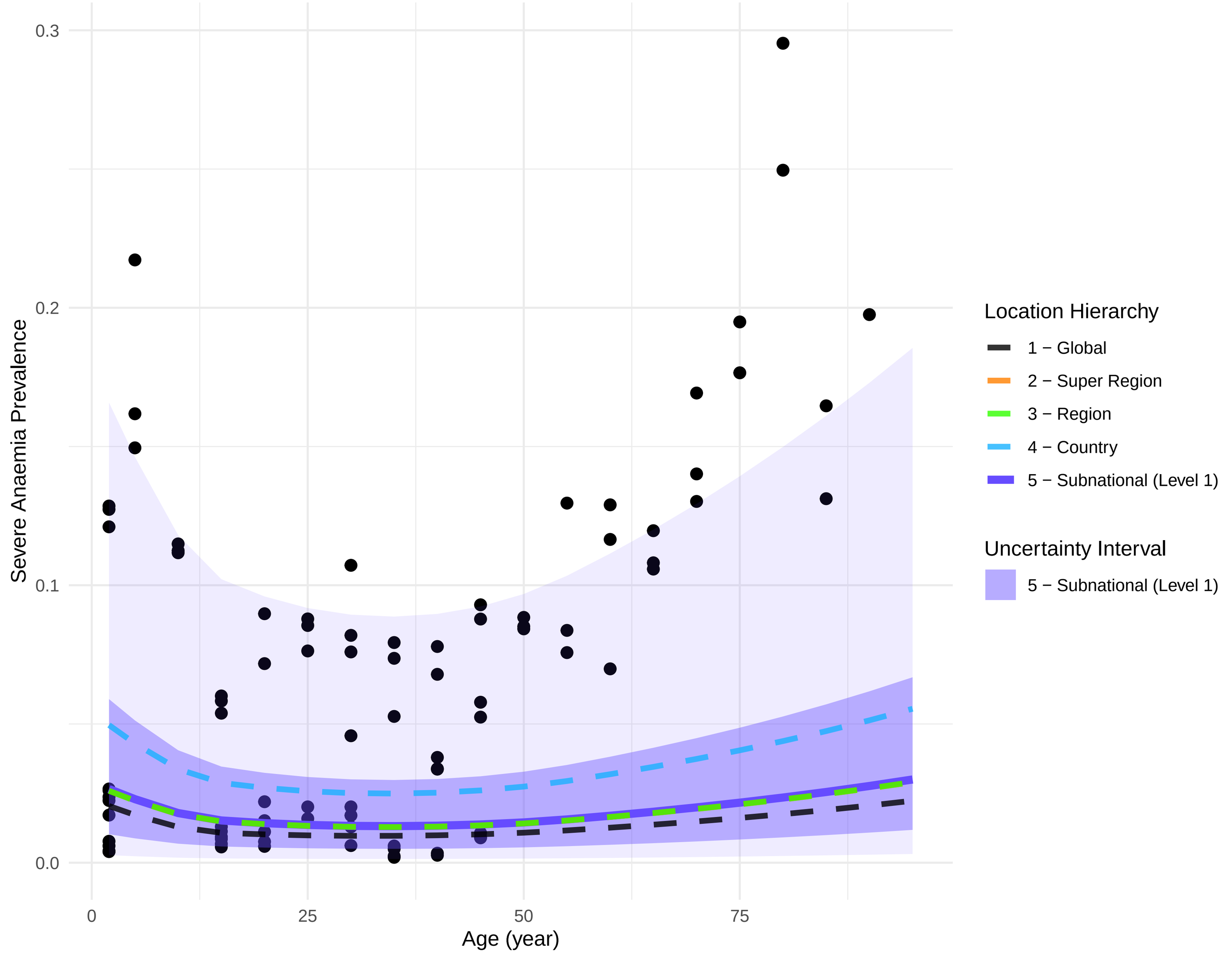

MR−BRT Age/Sex Fits − Males 2+ Years Old − Severe Anaemia Prevalence − Meghalaya, Rural
WHO−2024 Adjustment Method

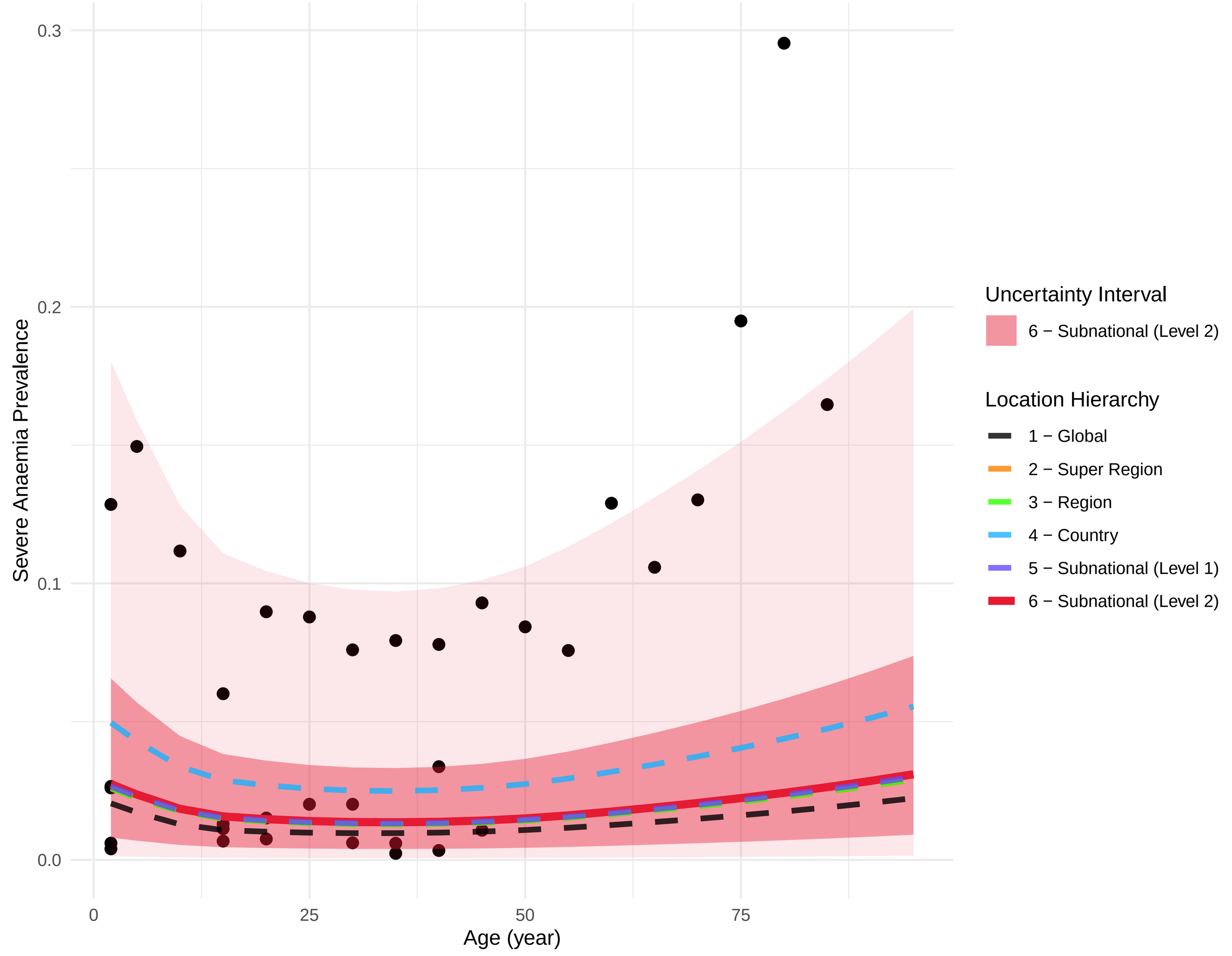

MR−BRT Age/Sex Fits − Males 2+ Years Old − Severe Anaemia Prevalence − Meghalaya, Urban
WHO−2024 Adjustment Method

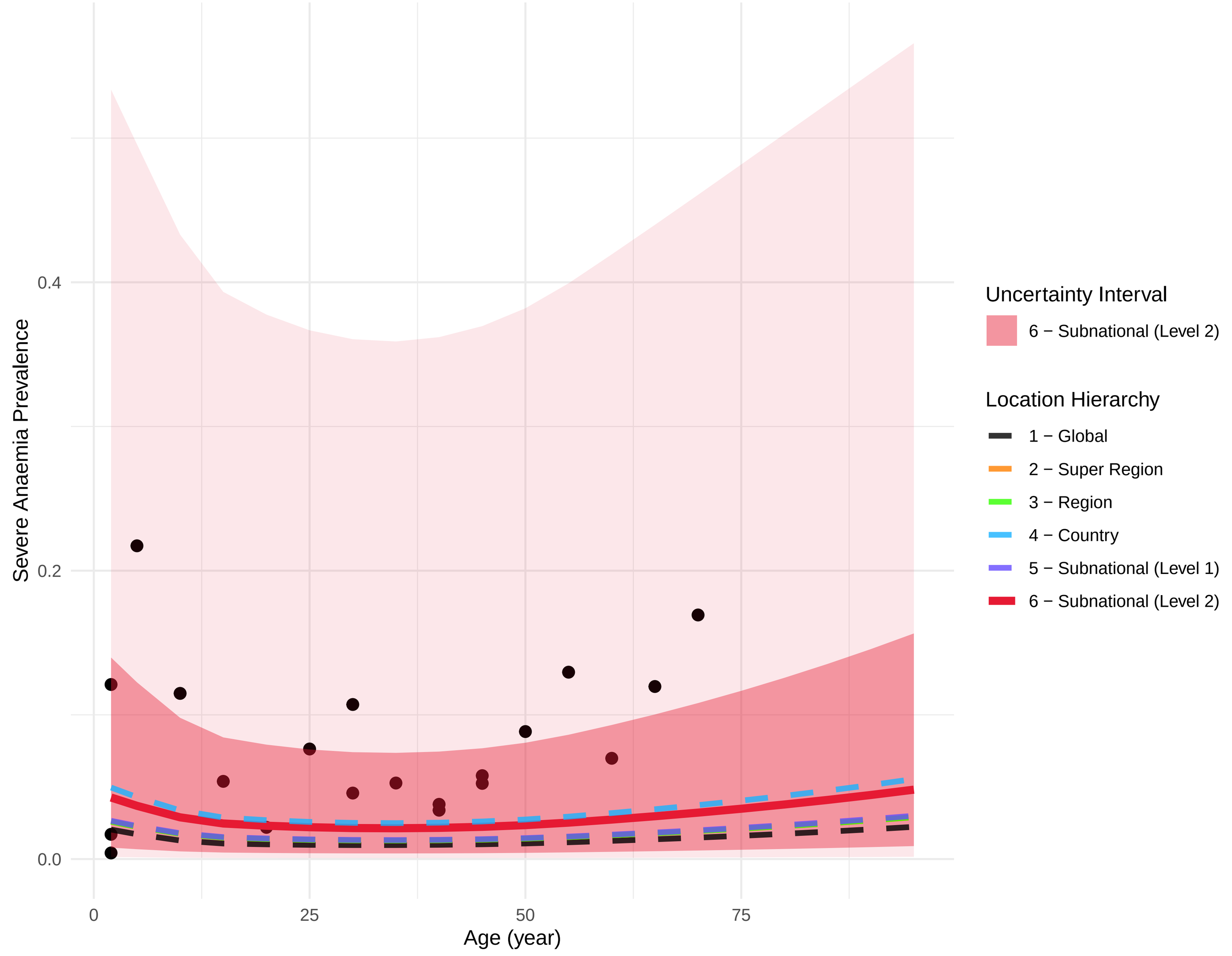

MR−BRT Age/Sex Fits − Males 2+ Years Old − Severe Anaemia Prevalence − Mizoram
WHO−2024 Adjustment Method

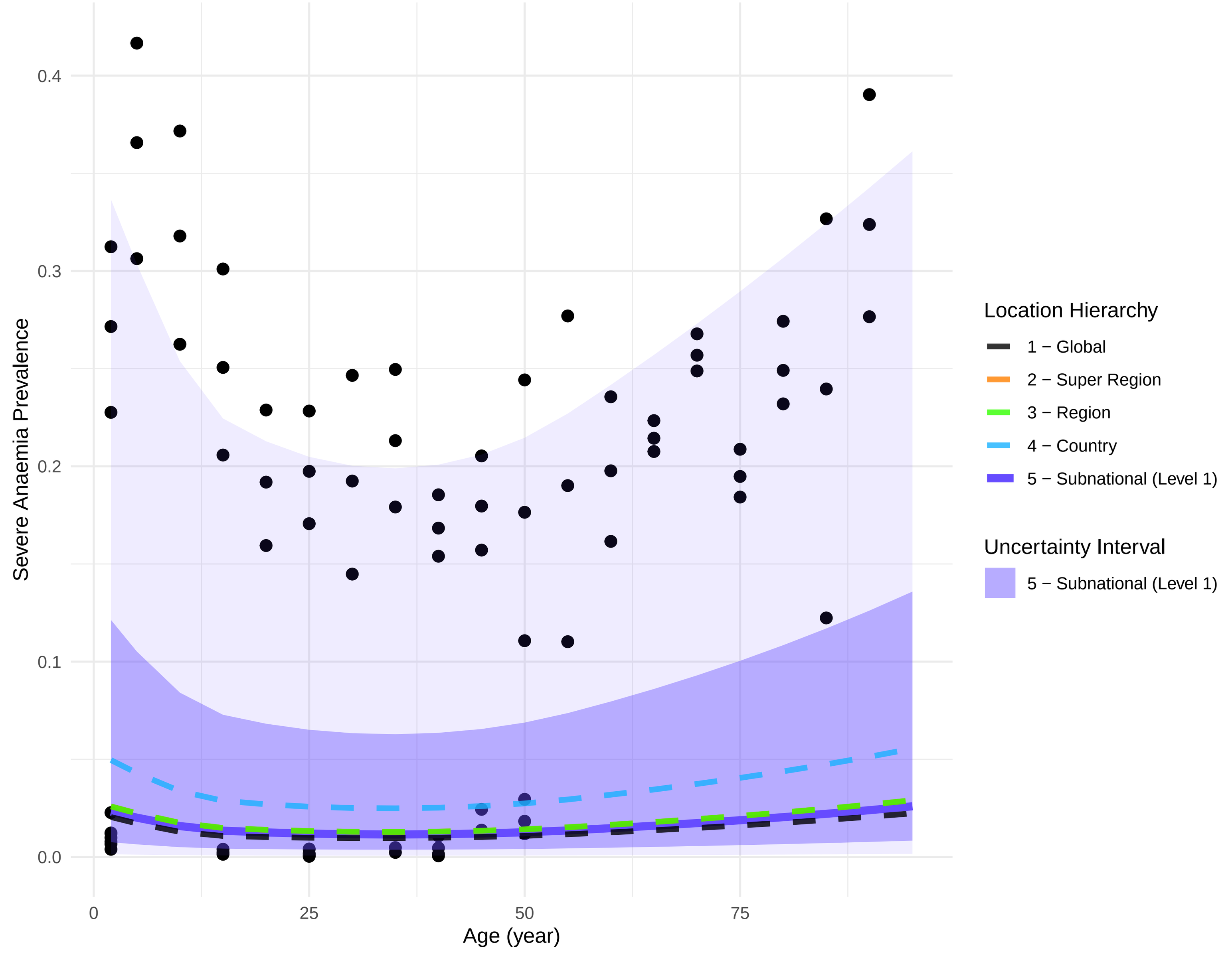

MR−BRT Age/Sex Fits − Males 2+ Years Old − Severe Anaemia Prevalence − Mizoram, Rural
WHO−2024 Adjustment Method

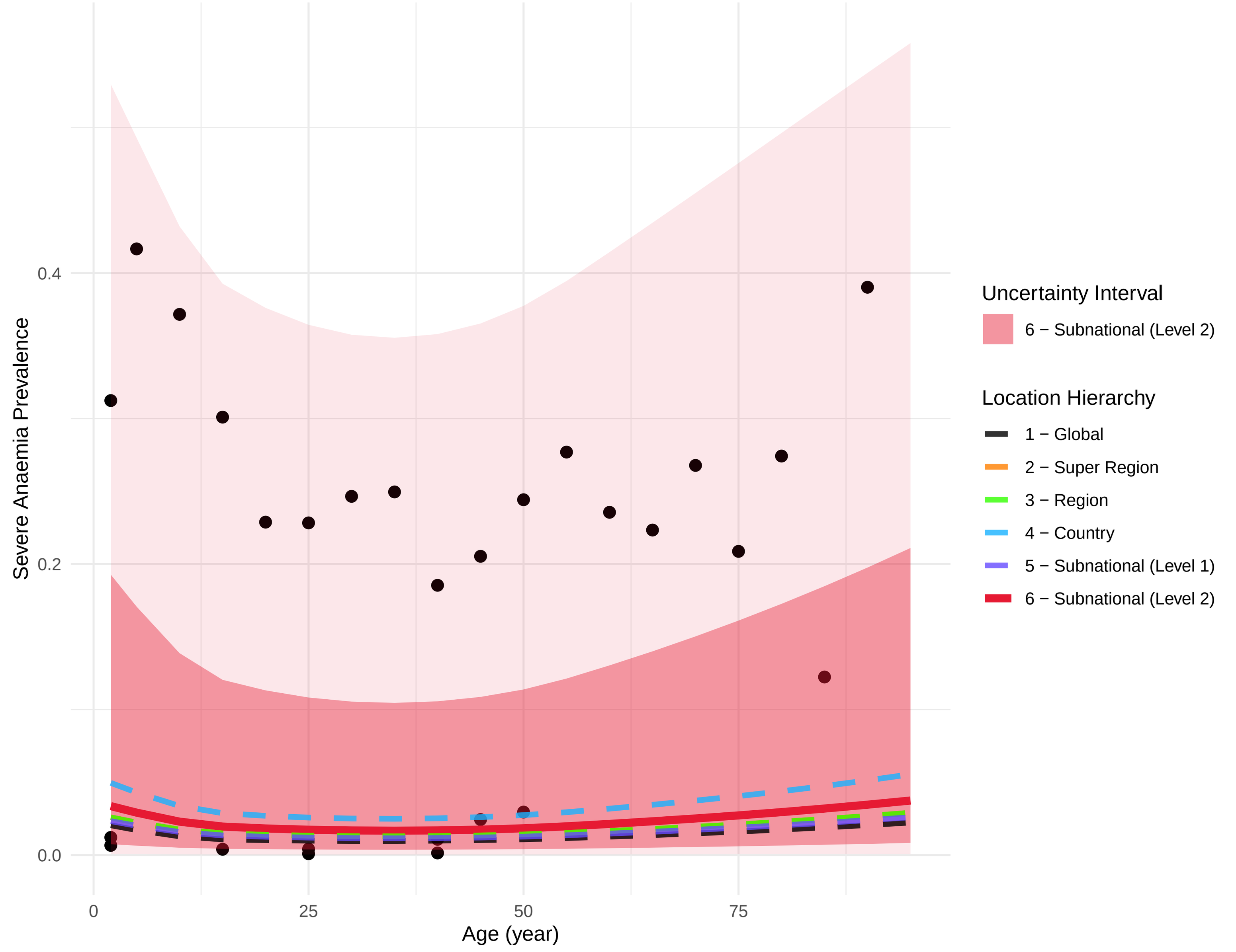

MR−BRT Age/Sex Fits − Males 2+ Years Old − Severe Anaemia Prevalence − Mizoram, Urban
WHO−2024 Adjustment Method

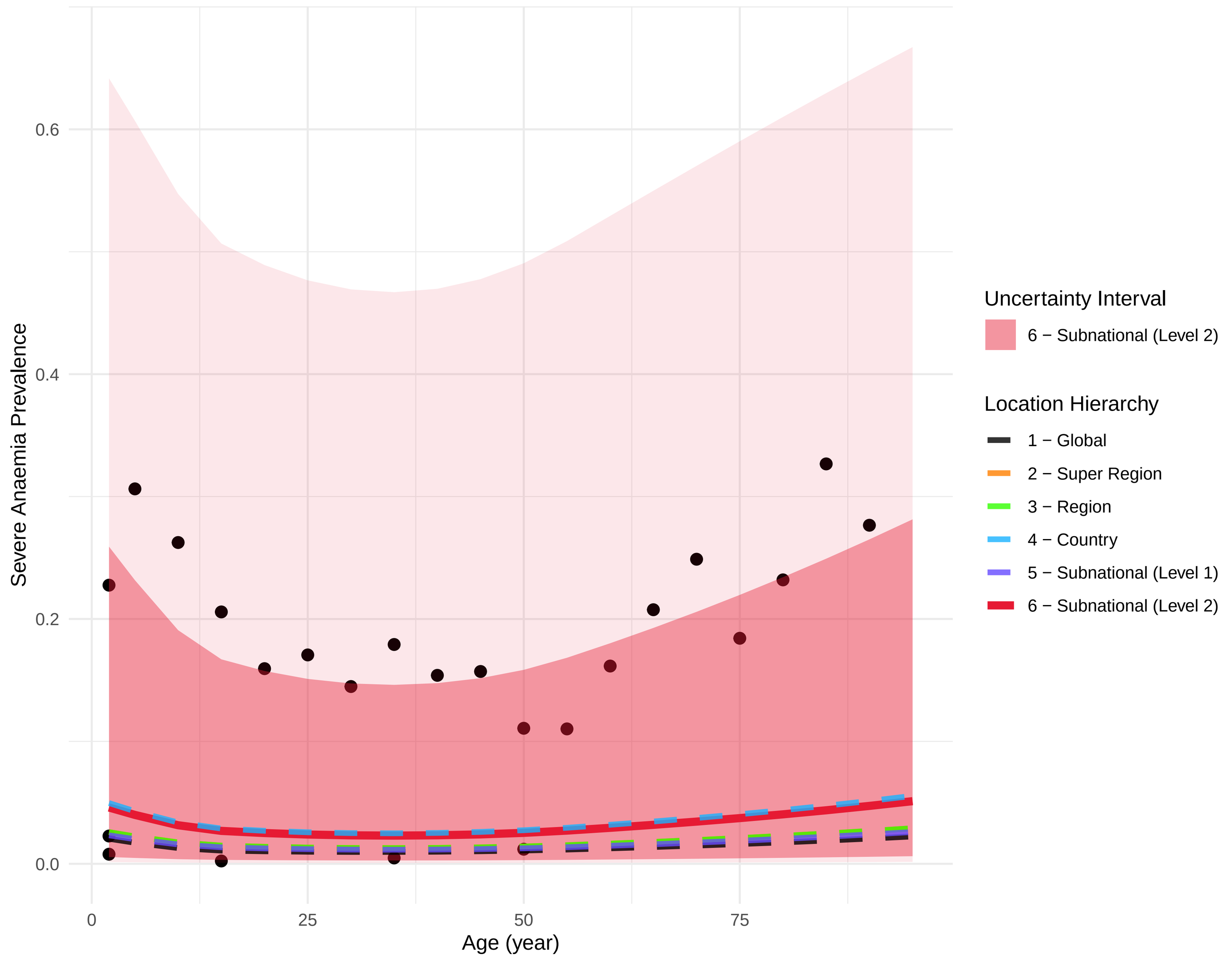

MR−BRT Age/Sex Fits − Males 2+ Years Old − Severe Anaemia Prevalence − Nagaland
WHO−2024 Adjustment Method

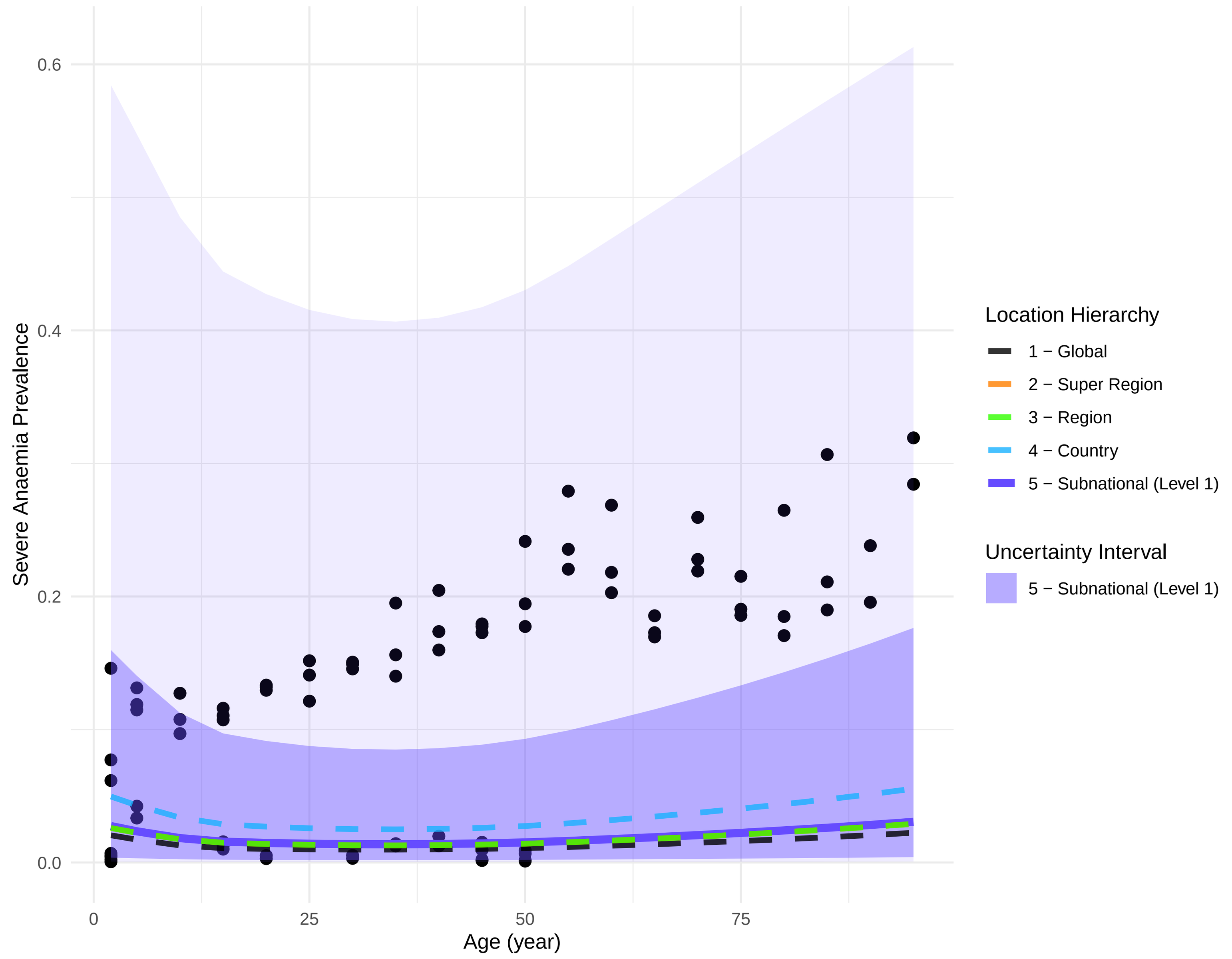

MR−BRT Age/Sex Fits − Males 2+ Years Old − Severe Anaemia Prevalence − Nagaland, Rural
WHO−2024 Adjustment Method

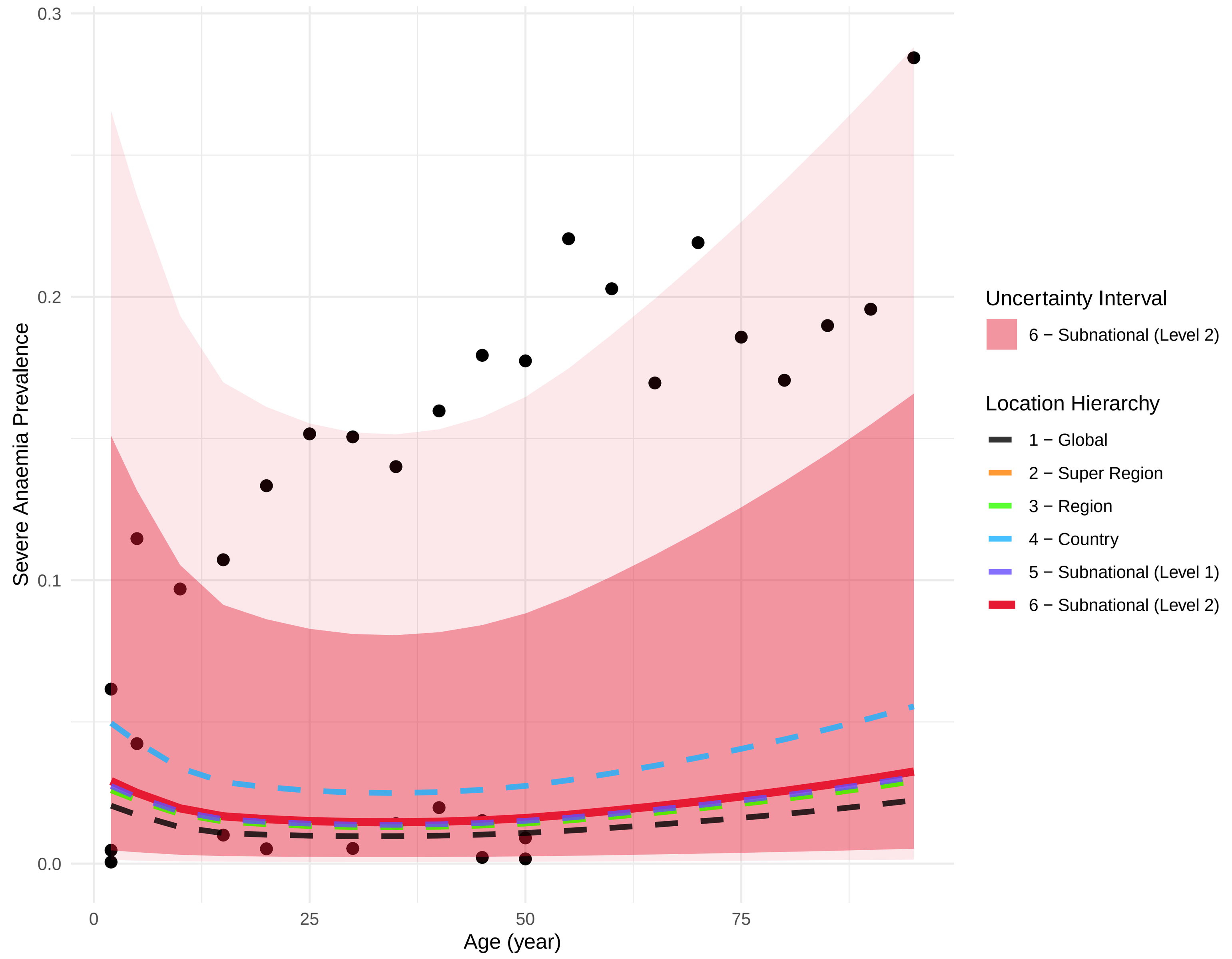

MR−BRT Age/Sex Fits − Males 2+ Years Old − Severe Anaemia Prevalence − Nagaland, Urban
WHO−2024 Adjustment Method

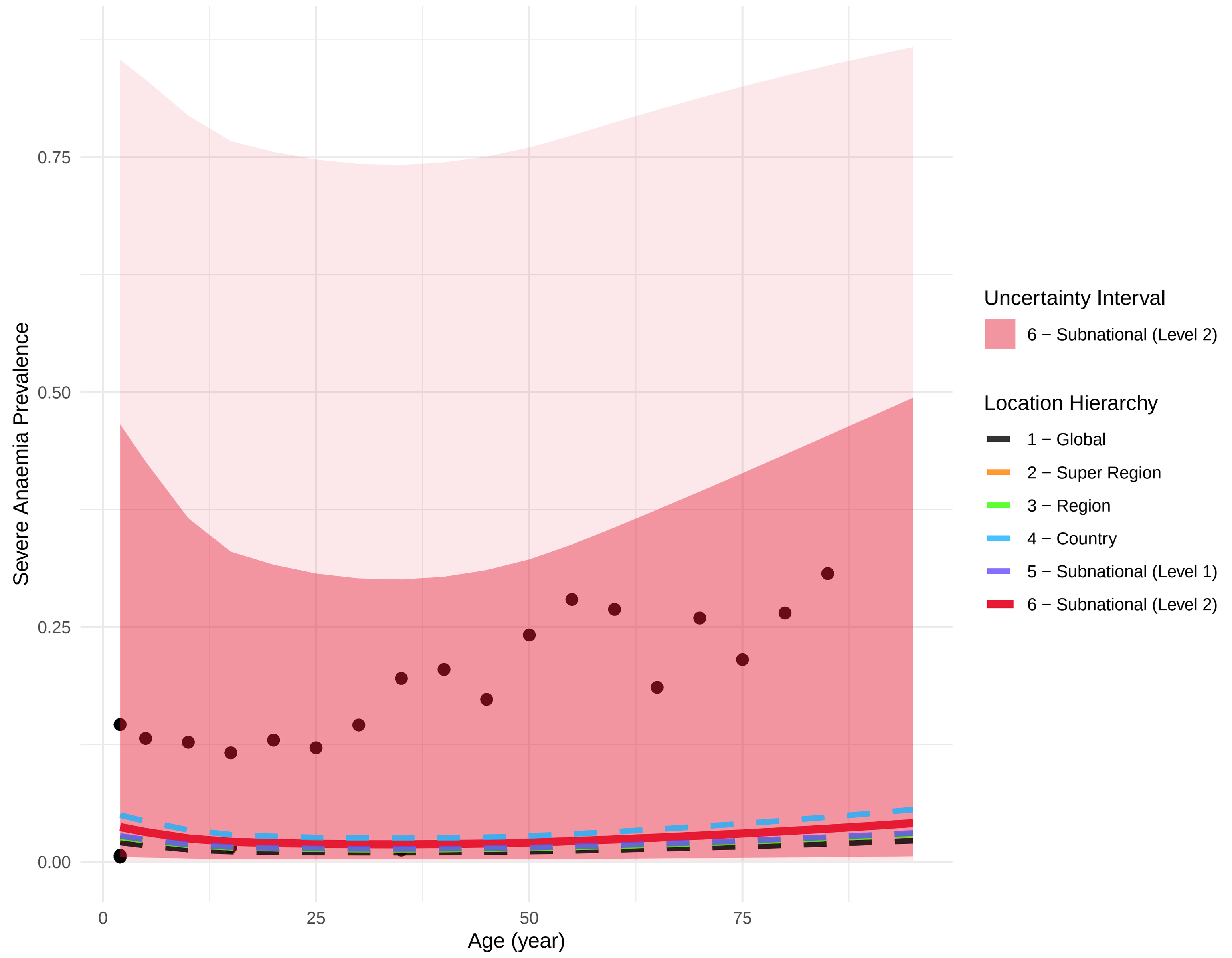

MR−BRT Age/Sex Fits − Males 2+ Years Old − Severe Anaemia Prevalence − Odisha
WHO−2024 Adjustment Method

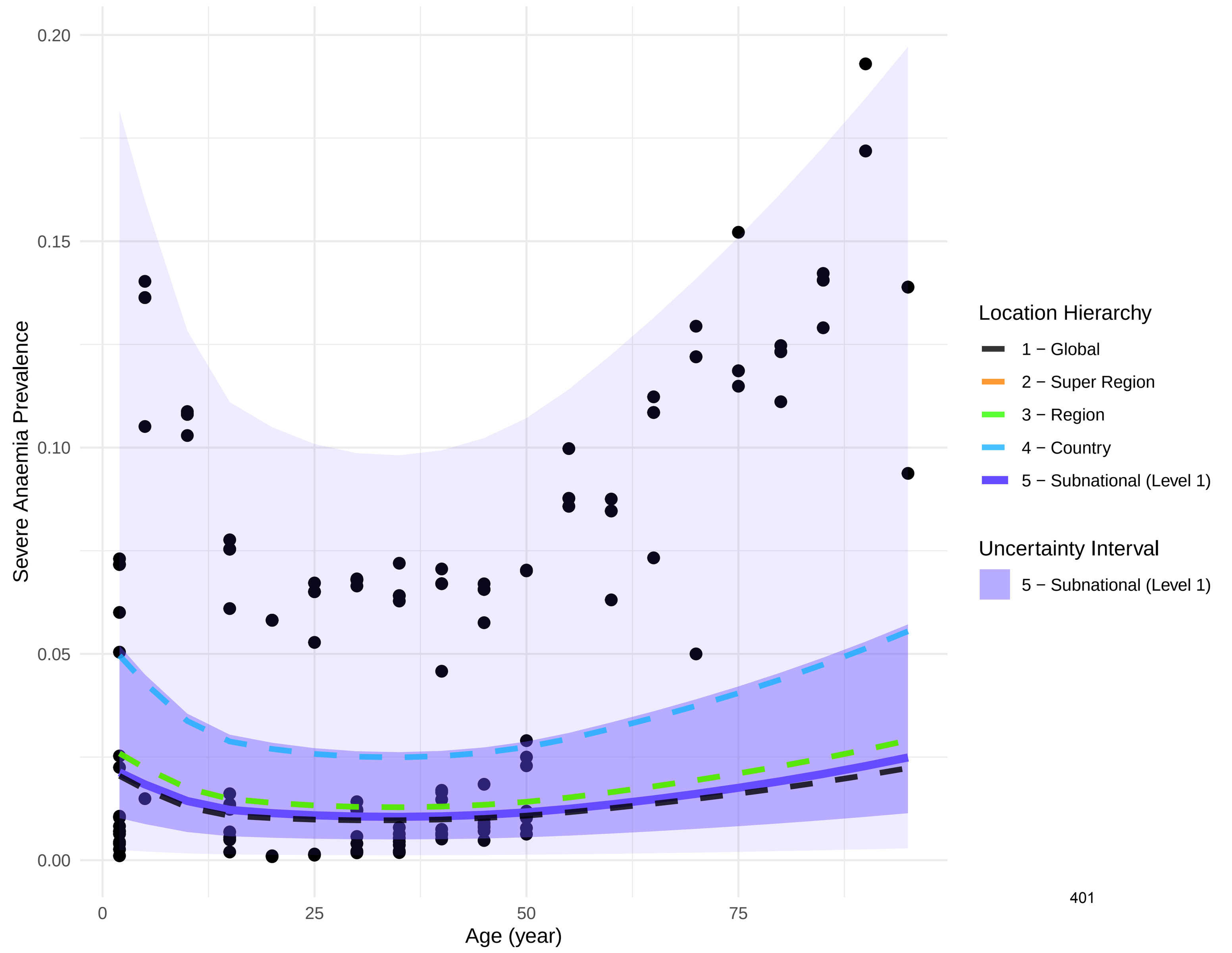

MR−BRT Age/Sex Fits − Males 2+ Years Old − Severe Anaemia Prevalence − Odisha, Rural
WHO−2024 Adjustment Method

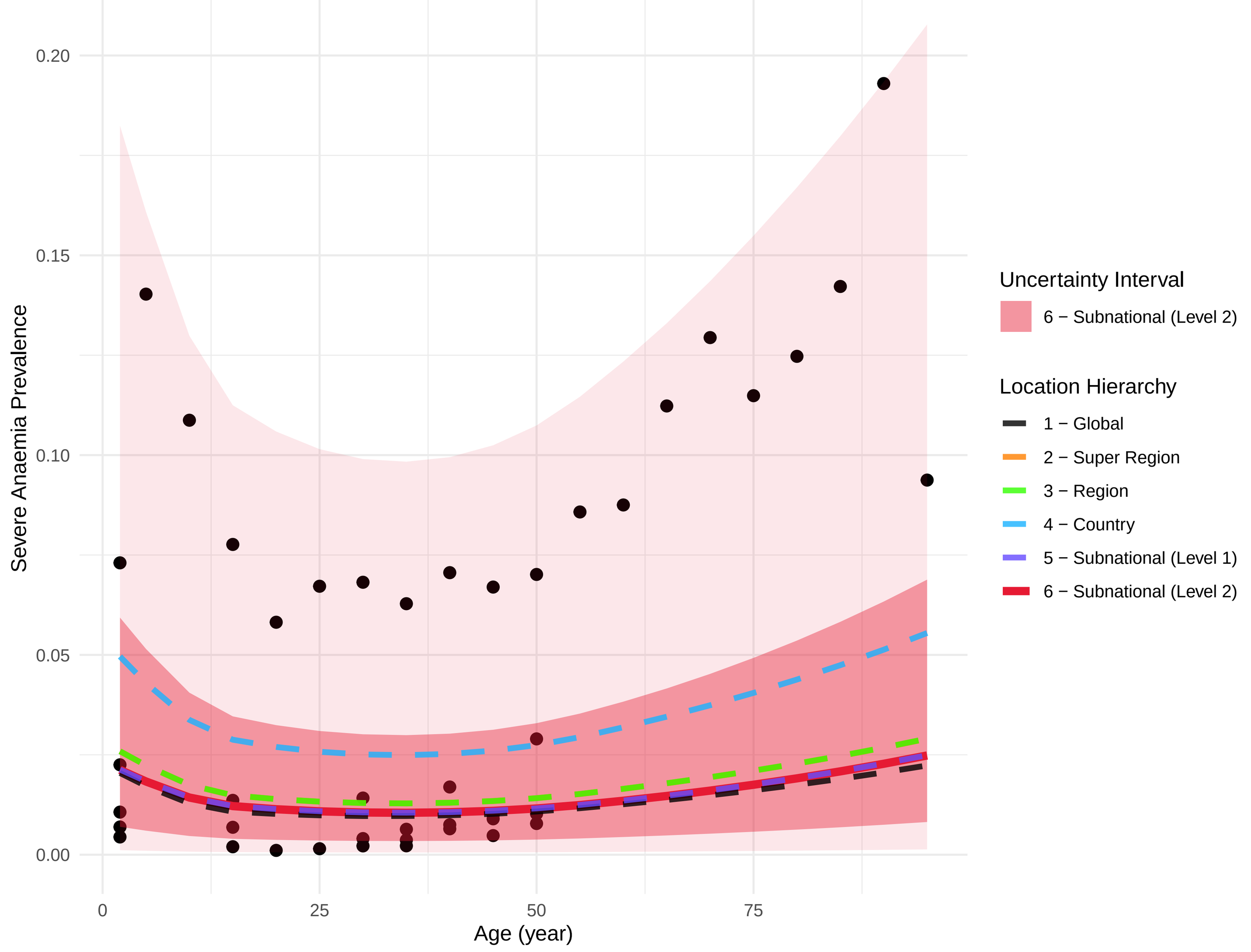

MR−BRT Age/Sex Fits − Males 2+ Years Old − Severe Anaemia Prevalence − Odisha, Urban
WHO−2024 Adjustment Method

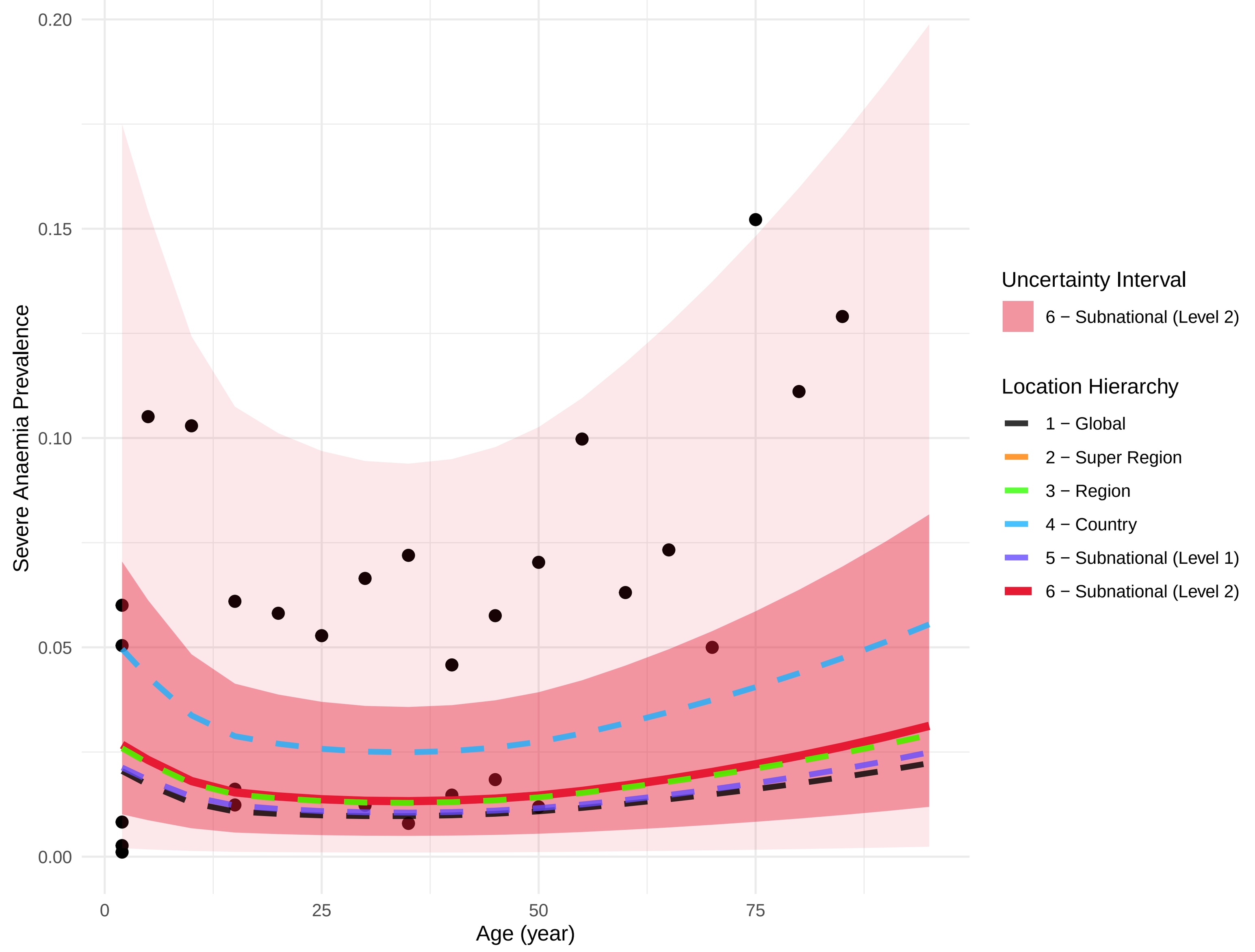

MR−BRT Age/Sex Fits − Males 2+ Years Old − Severe Anaemia Prevalence − Other Union Territories
WHO−2024 Adjustment Method

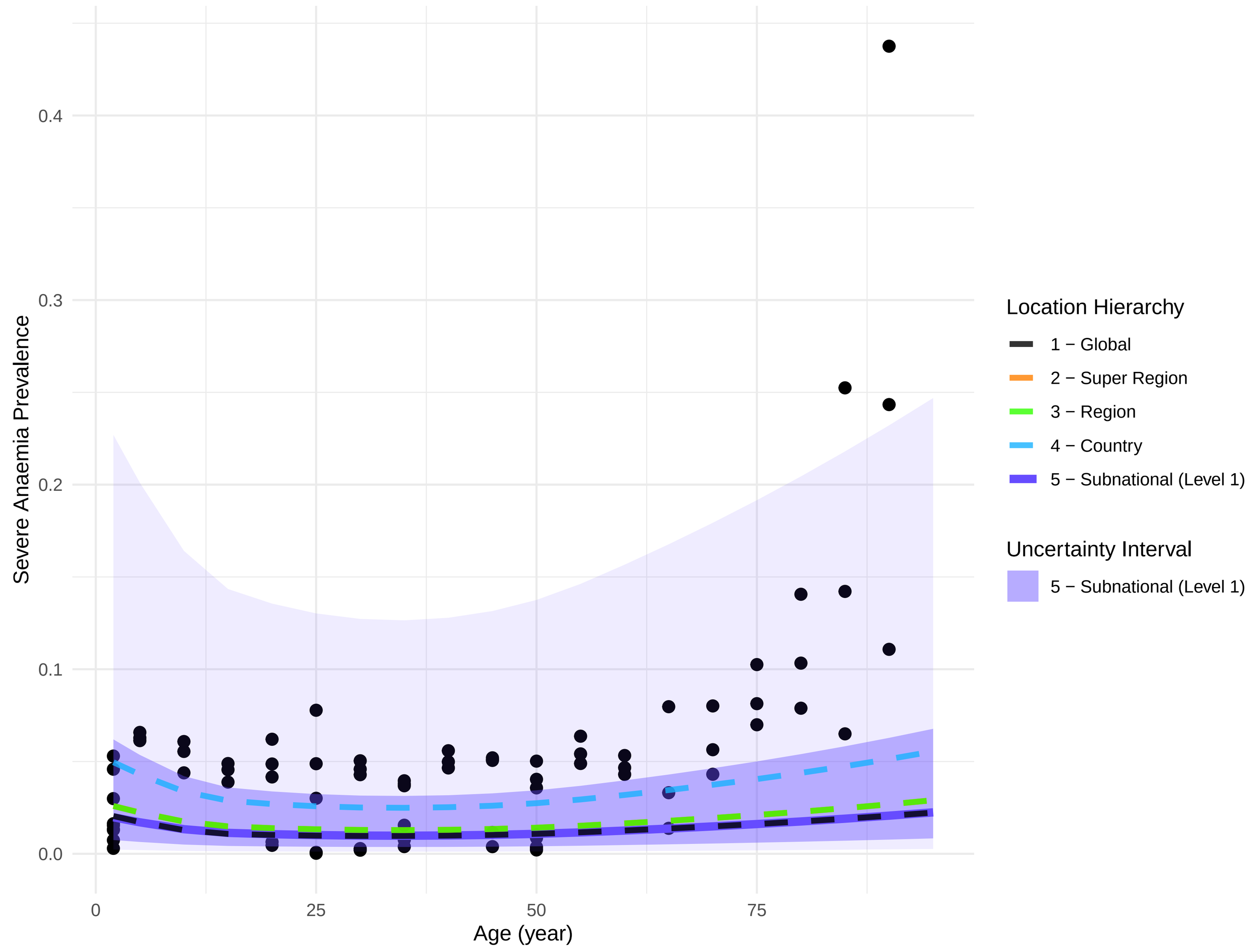

MR−BRT Age/Sex Fits − Males 2+ Years Old − Severe Anaemia Prevalence − Other Union Territories, Rural
WHO−2024 Adjustment Method

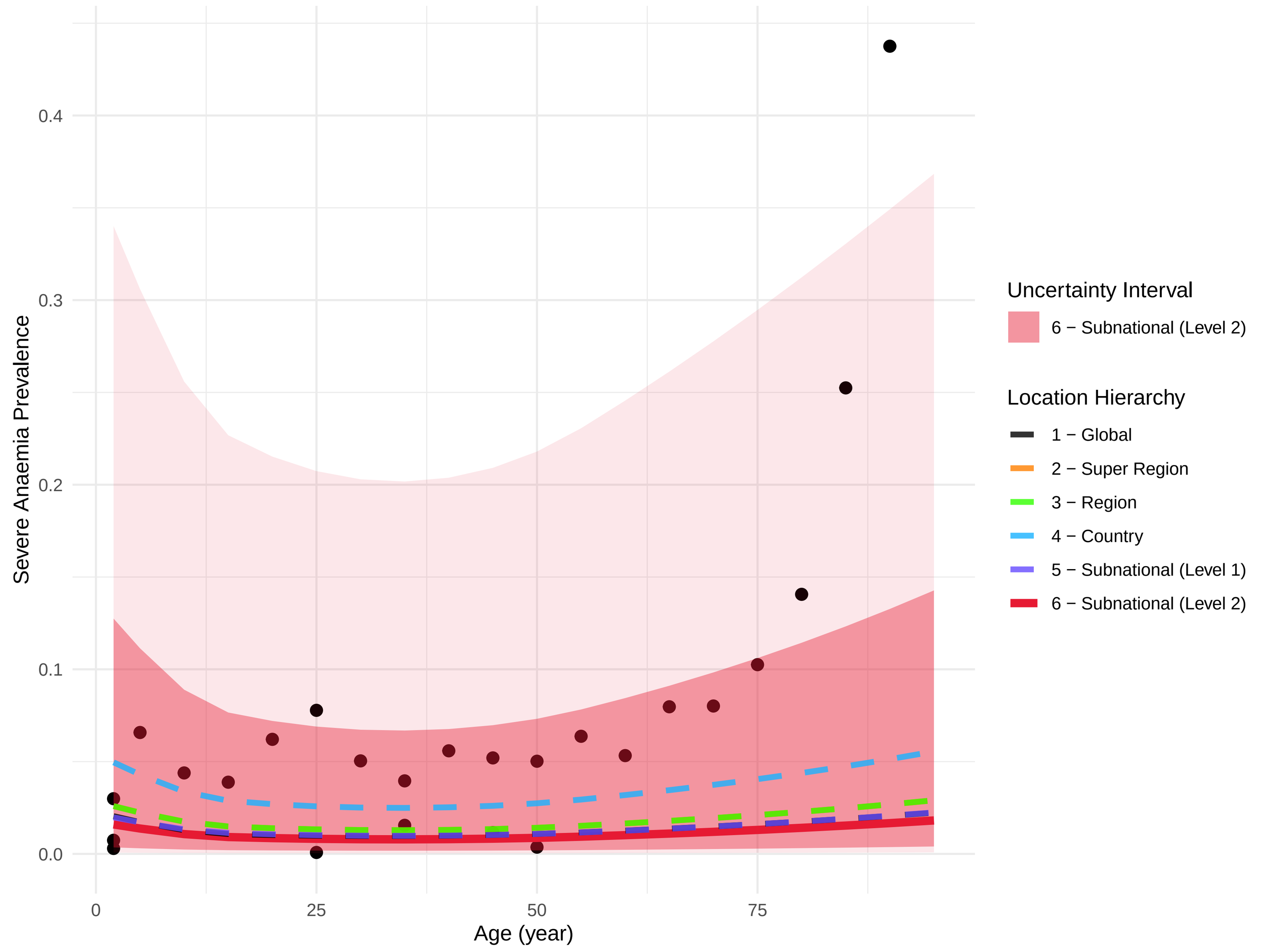

MR−BRT Age/Sex Fits − Males 2+ Years Old − Severe Anaemia Prevalence − Other Union Territories, Urban
WHO−2024 Adjustment Method

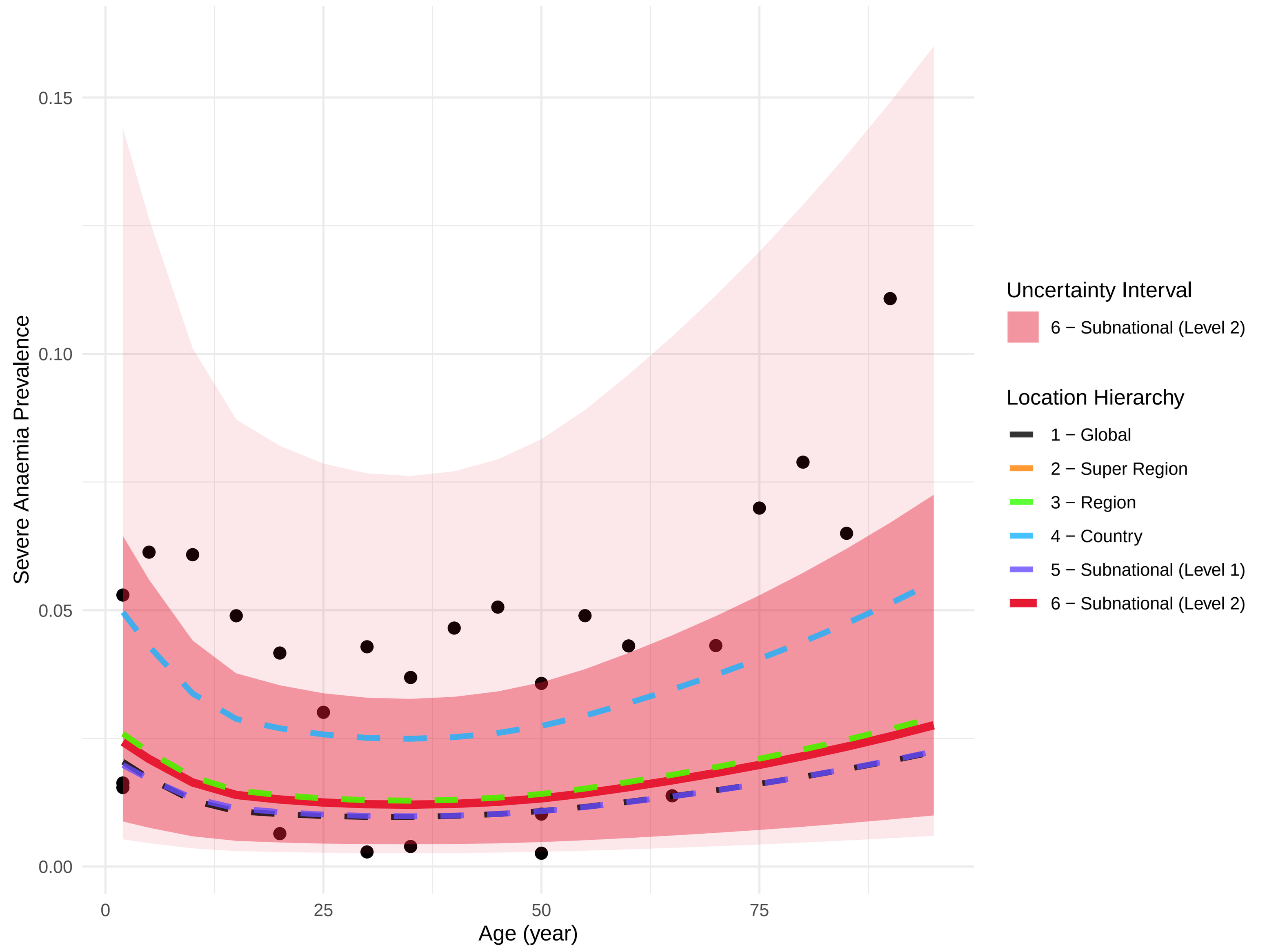

MR−BRT Age/Sex Fits − Males 2+ Years Old − Severe Anaemia Prevalence − Punjab
WHO−2024 Adjustment Method

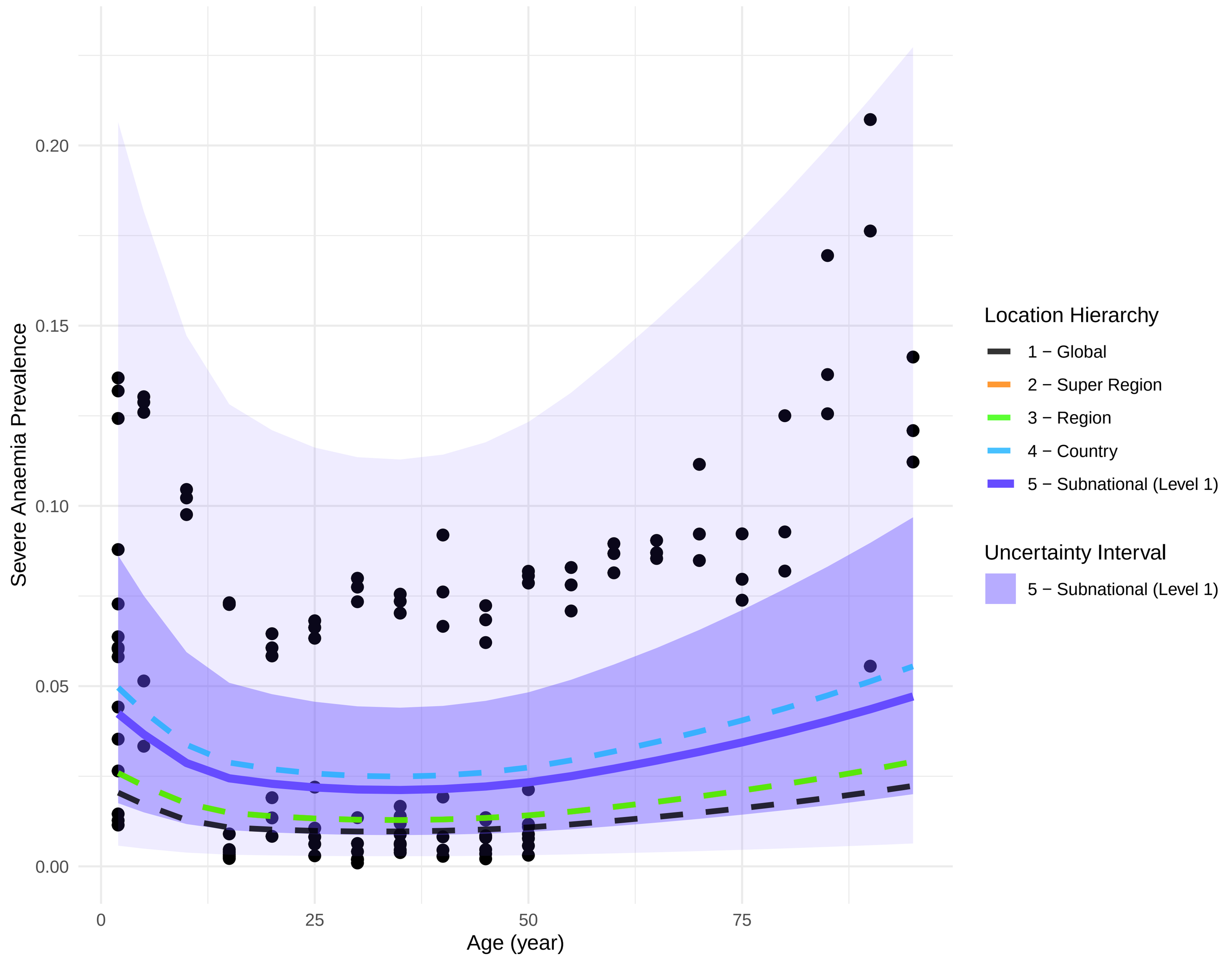

MR−BRT Age/Sex Fits − Males 2+ Years Old − Severe Anaemia Prevalence − Punjab, Rural
WHO−2024 Adjustment Method

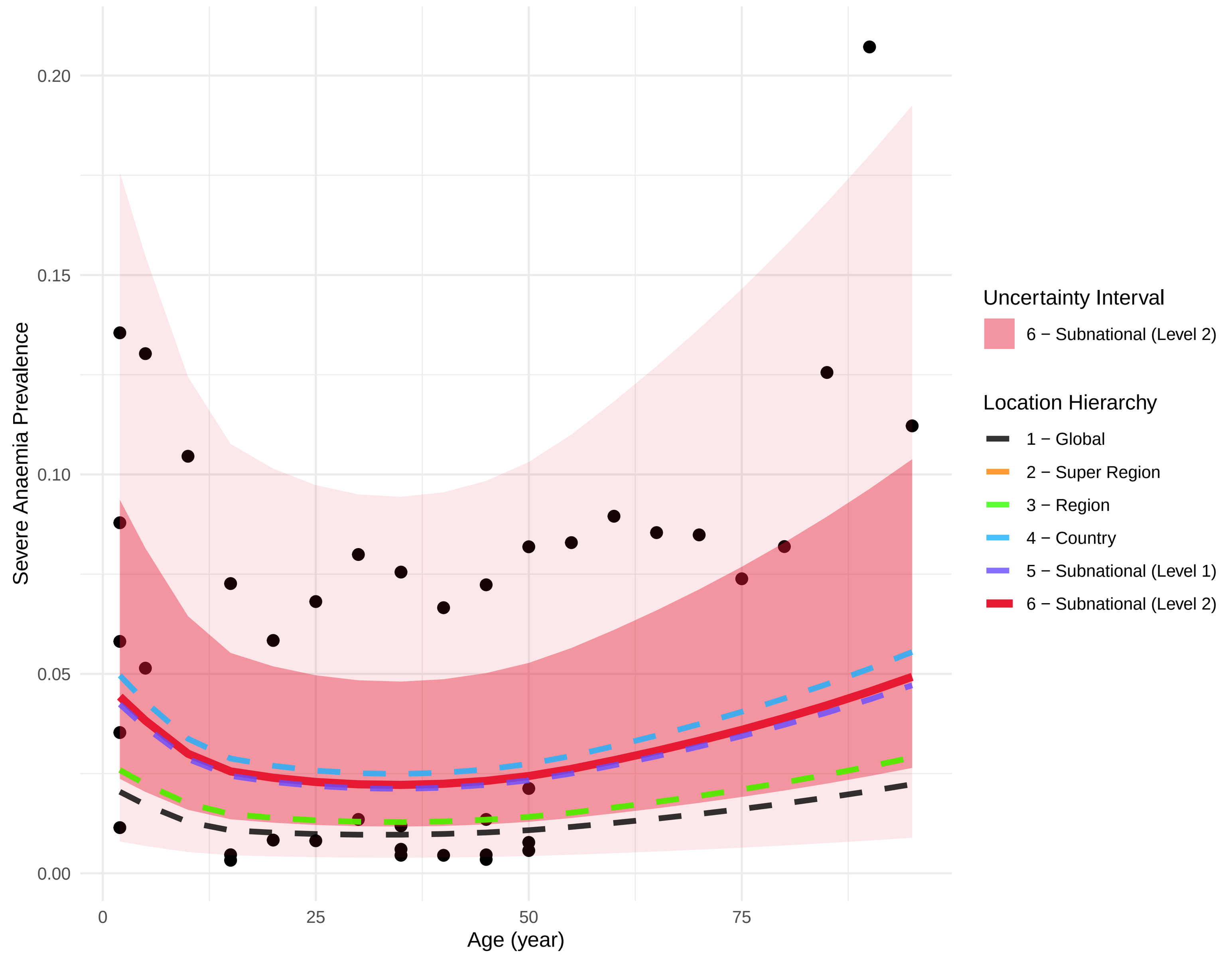

MR−BRT Age/Sex Fits − Males 2+ Years Old − Severe Anaemia Prevalence − Punjab, Urban
WHO−2024 Adjustment Method

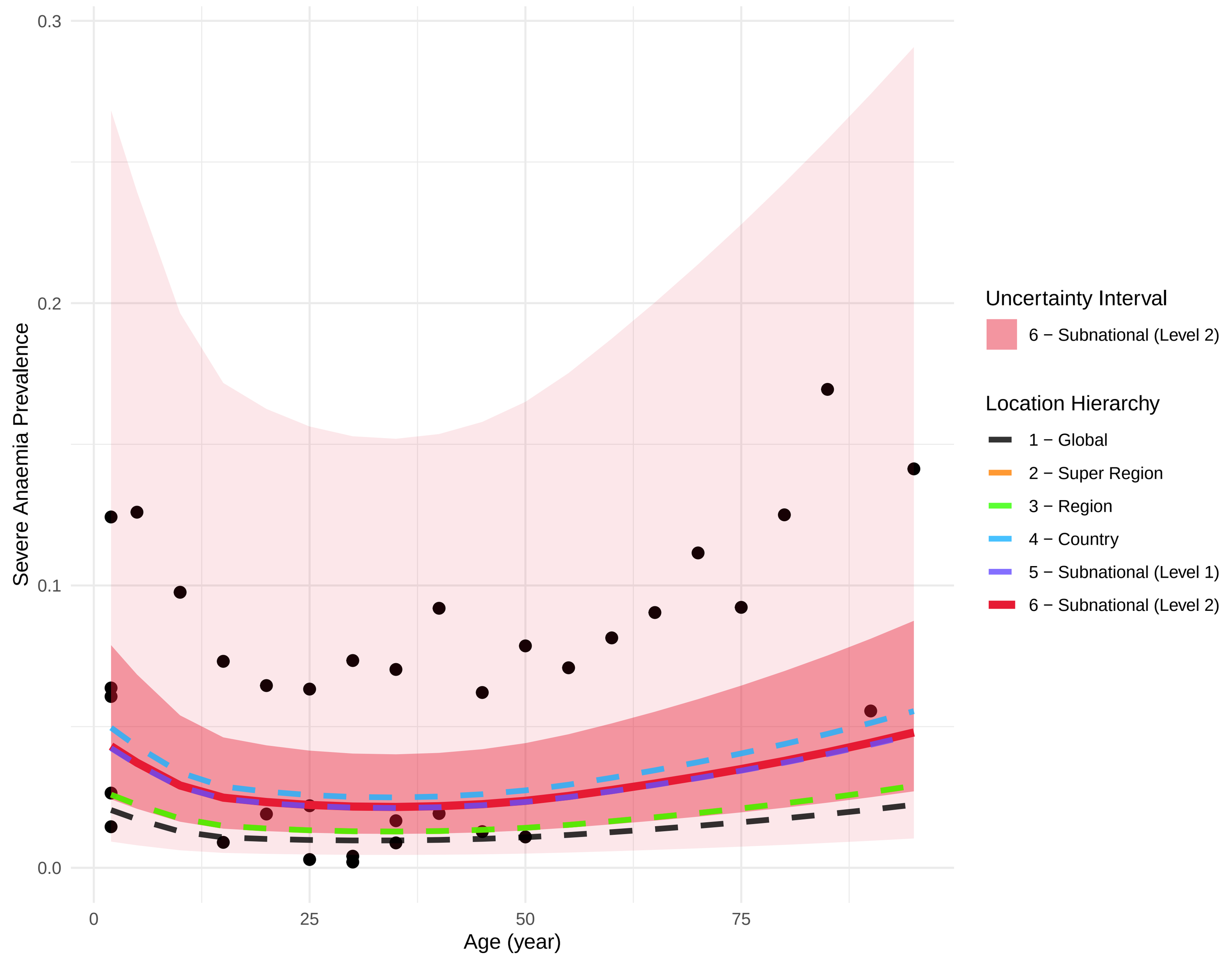

MR−BRT Age/Sex Fits − Males 2+ Years Old − Severe Anaemia Prevalence − Rajasthan
WHO−2024 Adjustment Method

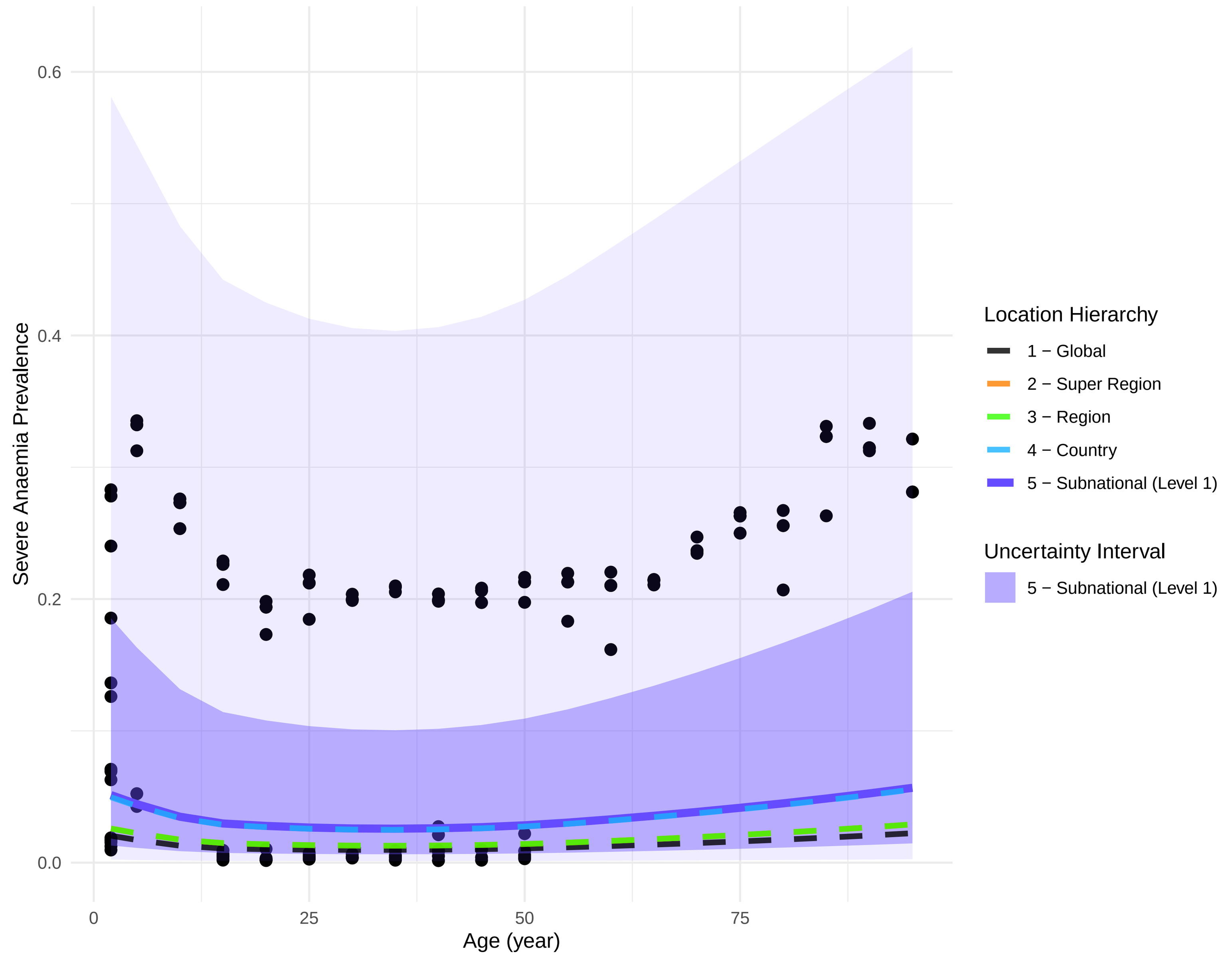

MR−BRT Age/Sex Fits − Males 2+ Years Old − Severe Anaemia Prevalence − Rajasthan, Rural
WHO−2024 Adjustment Method

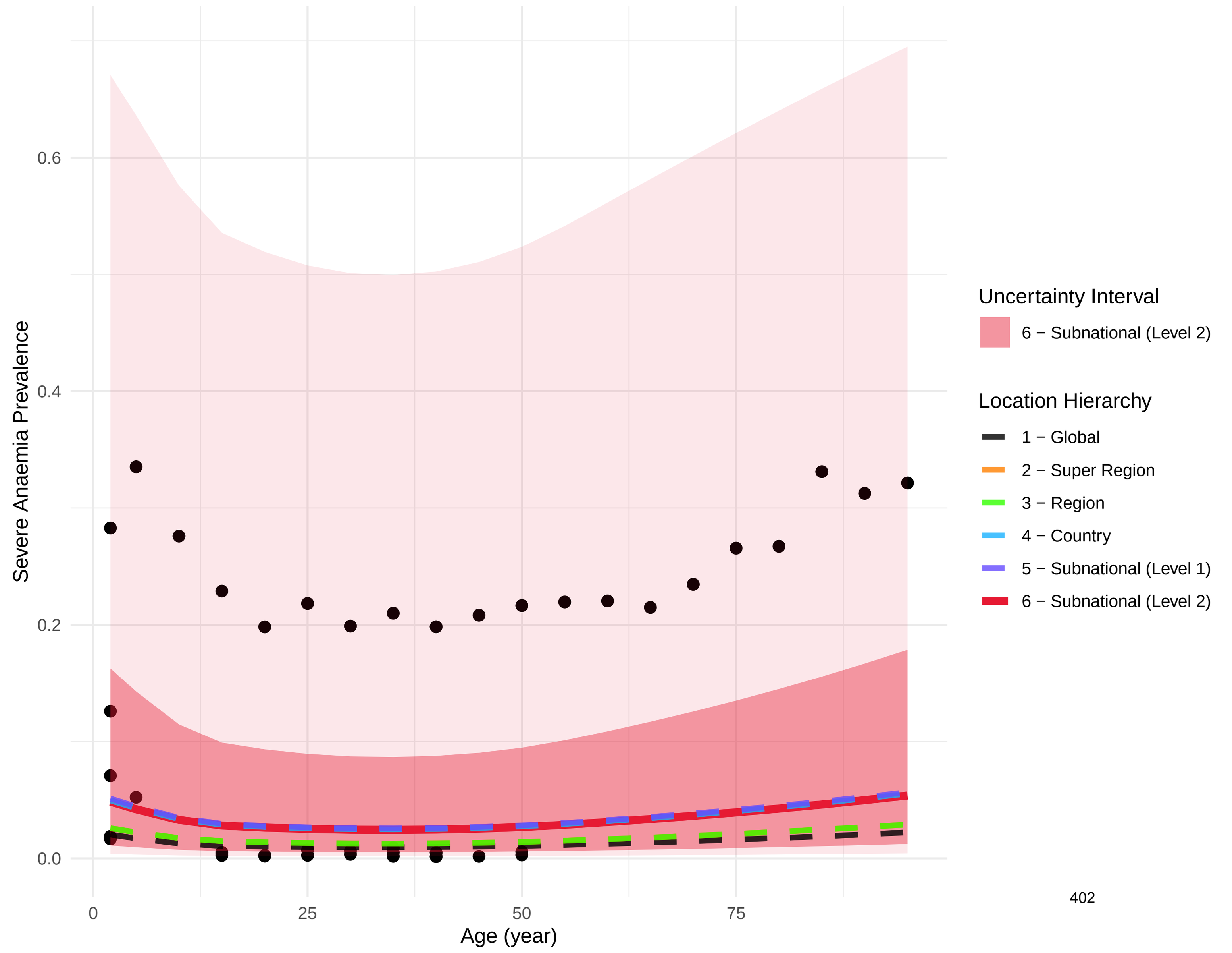

MR−BRT Age/Sex Fits − Males 2+ Years Old − Severe Anaemia Prevalence − Rajasthan, Urban
WHO−2024 Adjustment Method

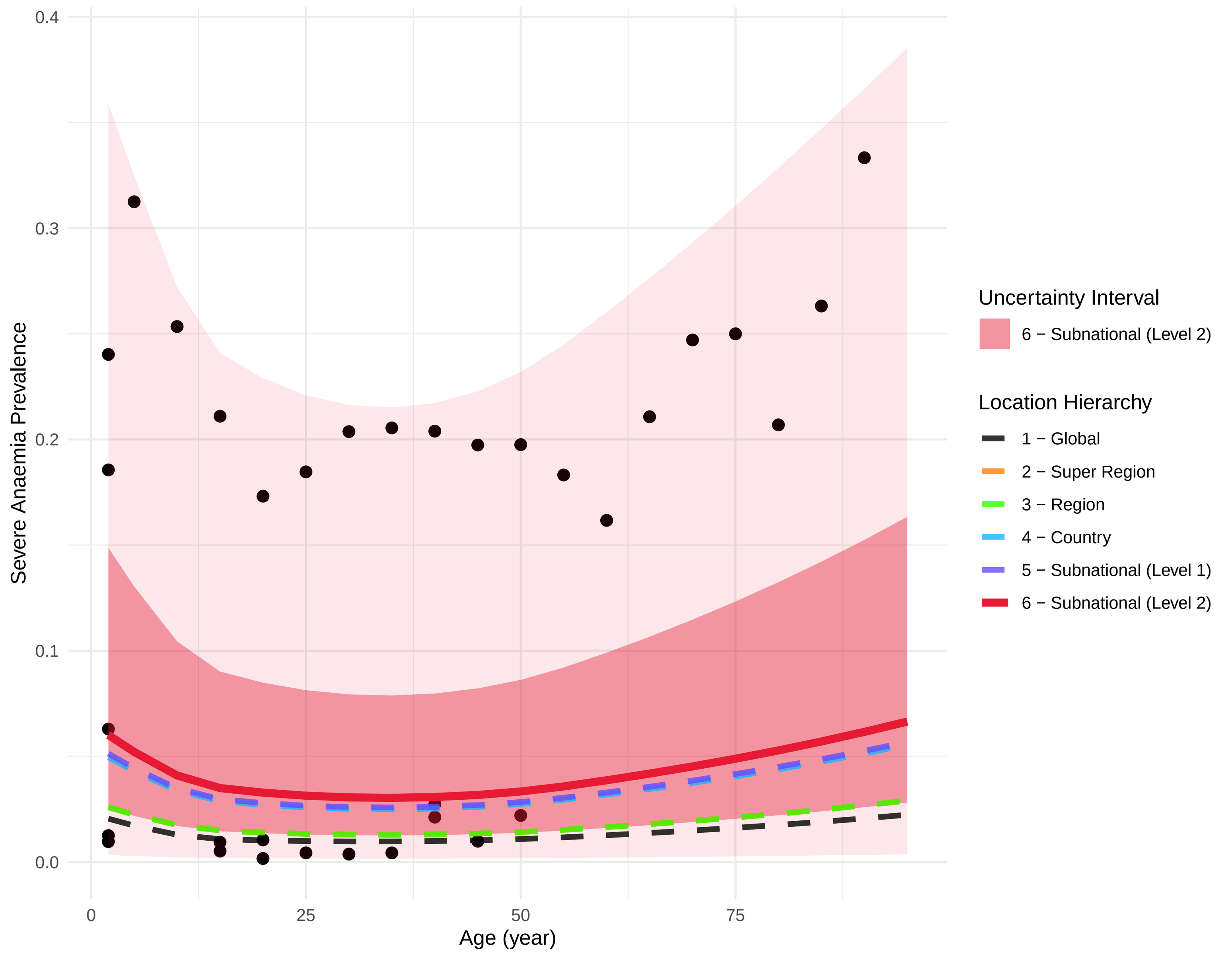

MR−BRT Age/Sex Fits − Males 2+ Years Old − Severe Anaemia Prevalence − Sikkim
WHO−2024 Adjustment Method

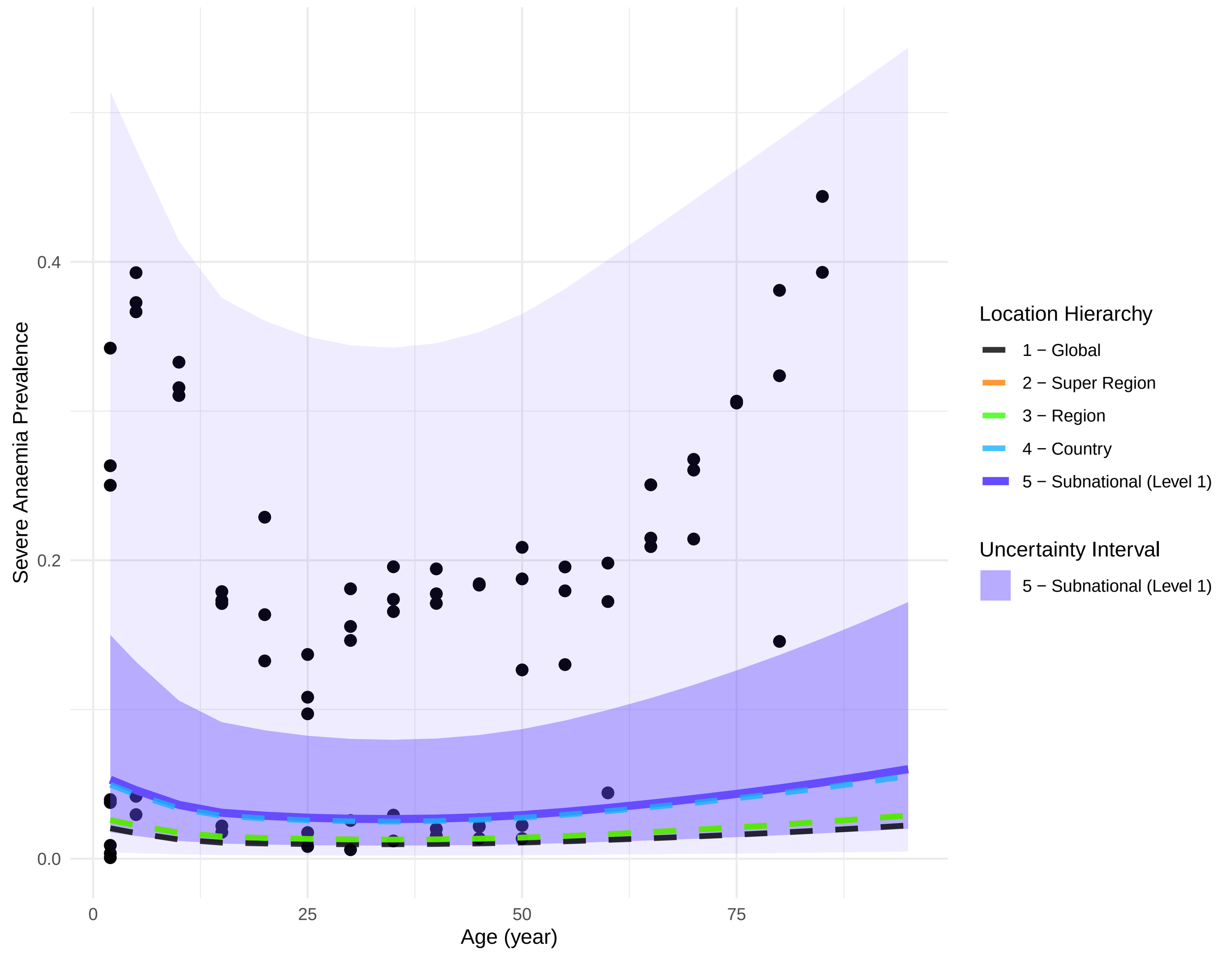

MR−BRT Age/Sex Fits − Males 2+ Years Old − Severe Anaemia Prevalence − Sikkim, Rural
WHO−2024 Adjustment Method

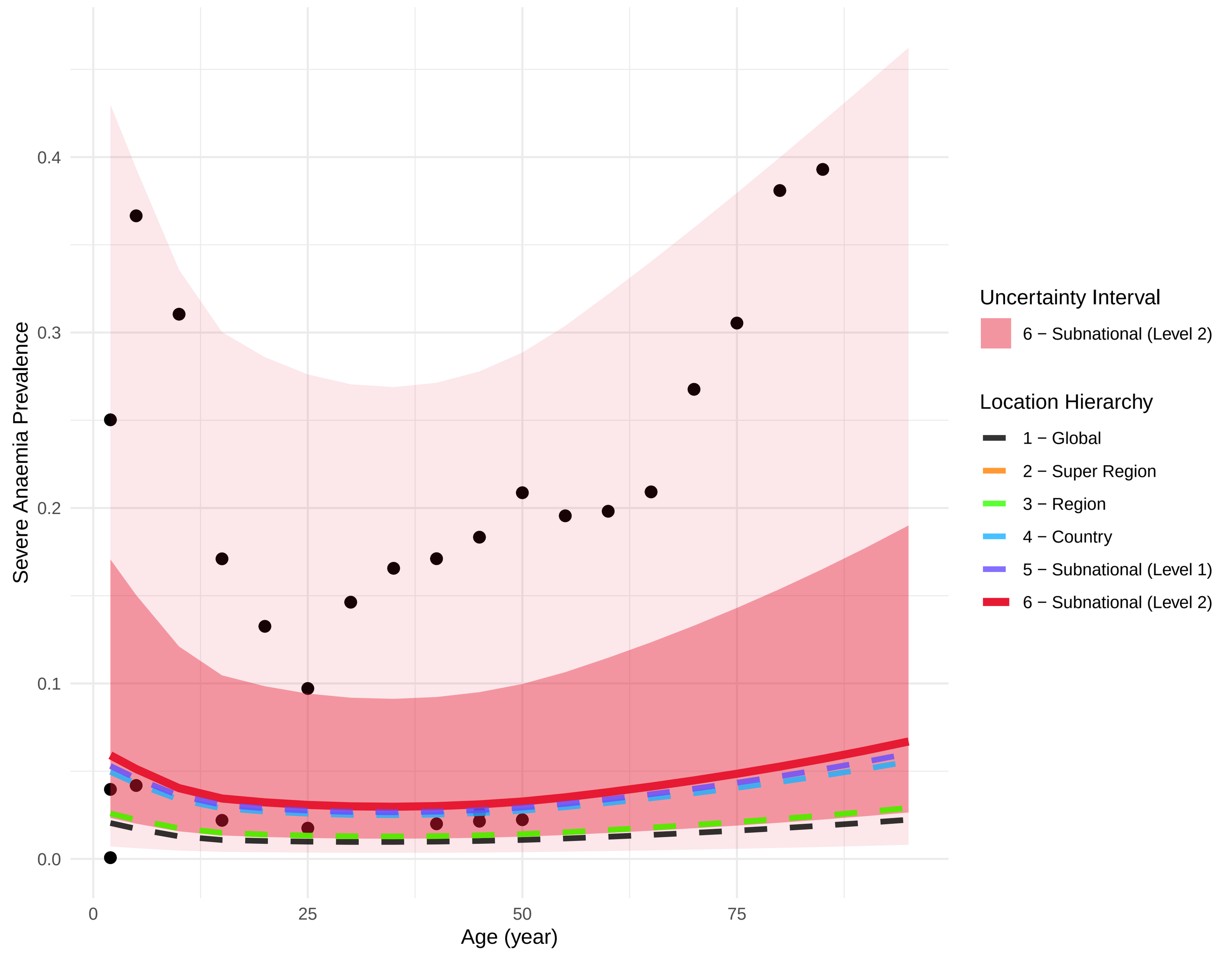

MR−BRT Age/Sex Fits − Males 2+ Years Old − Severe Anaemia Prevalence − Sikkim, Urban
WHO−2024 Adjustment Method

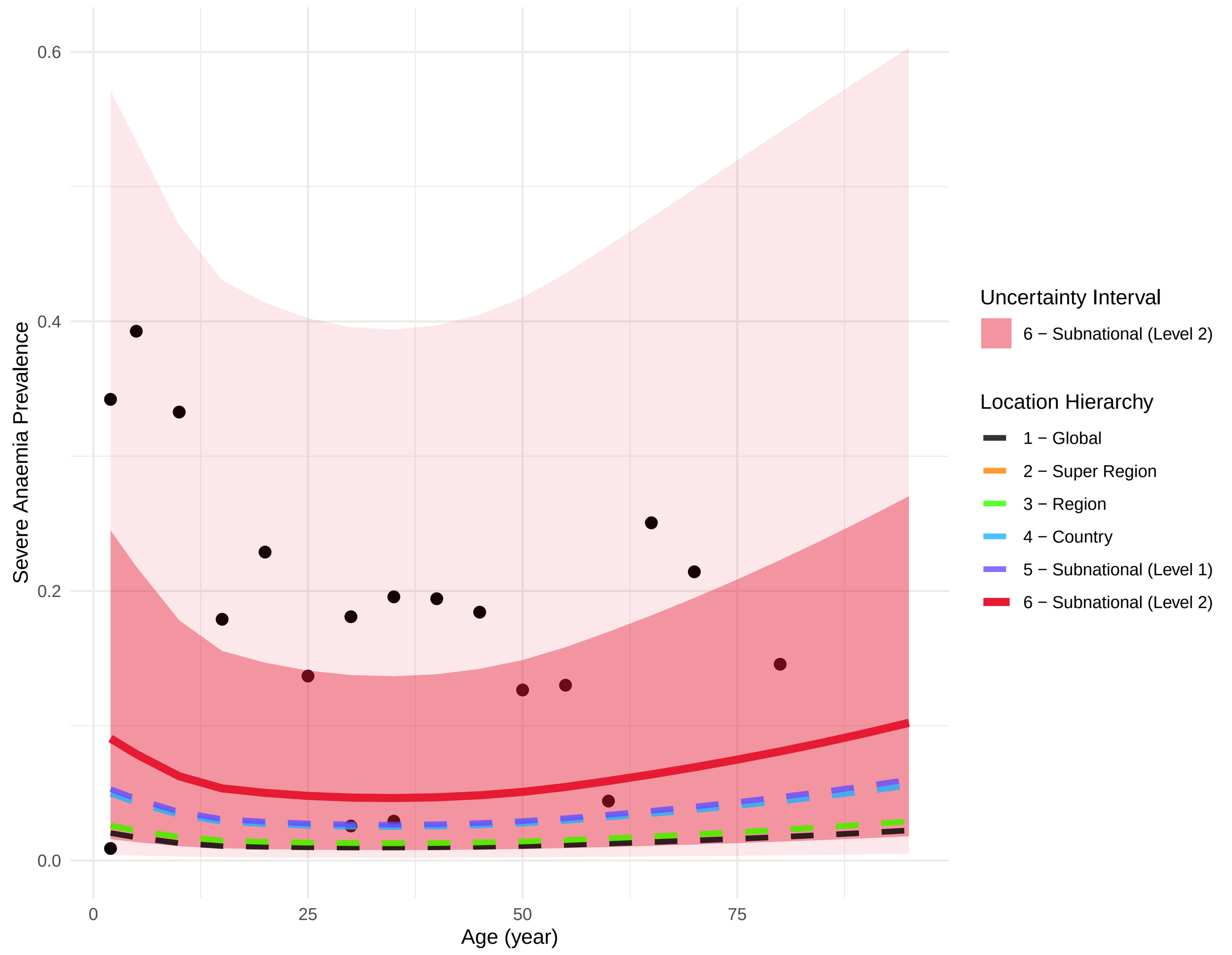

MR−BRT Age/Sex Fits − Males 2+ Years Old − Severe Anaemia Prevalence − Tamil Nadu
WHO−2024 Adjustment Method

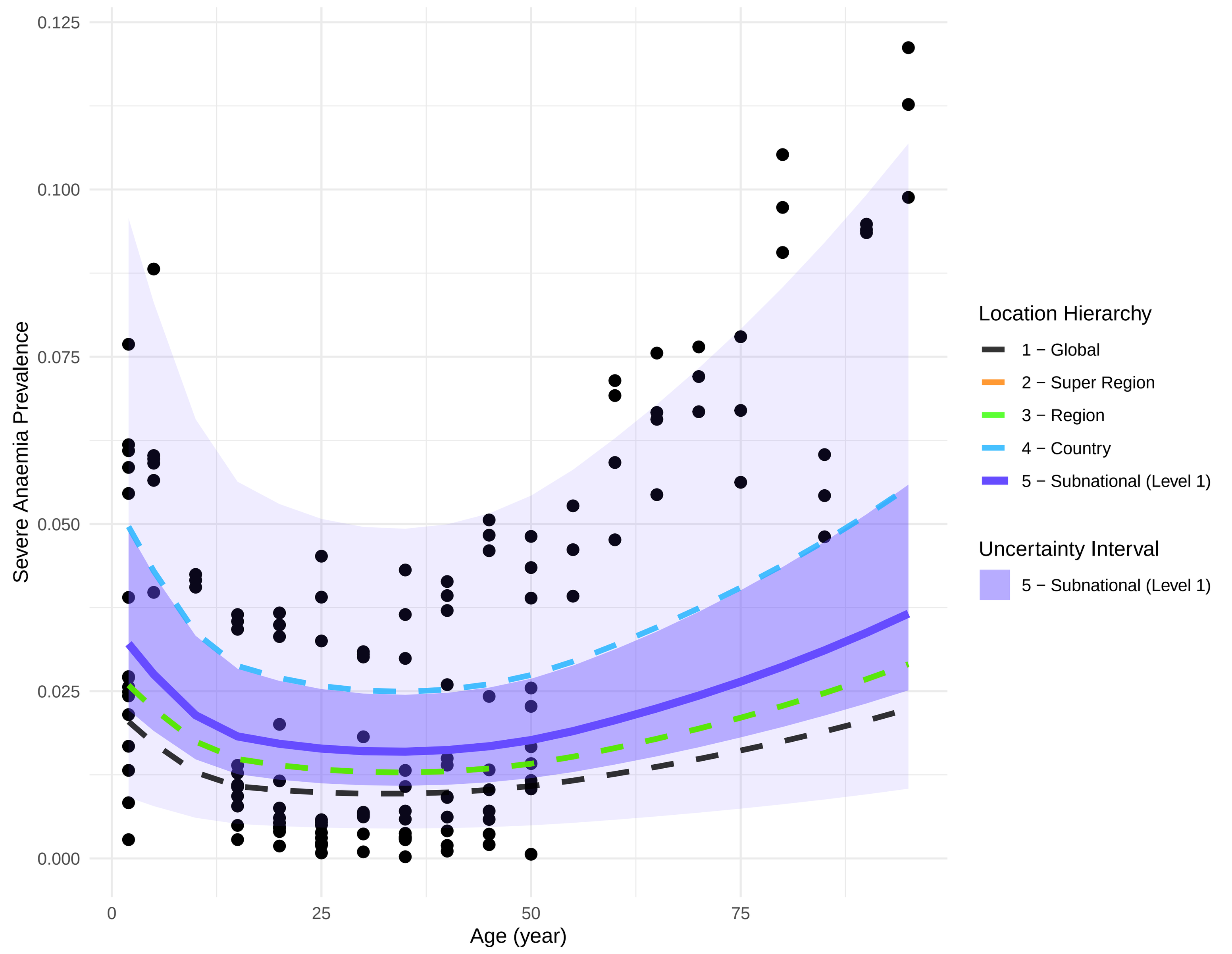

MR−BRT Age/Sex Fits − Males 2+ Years Old − Severe Anaemia Prevalence − Tamil Nadu, Rural
WHO−2024 Adjustment Method

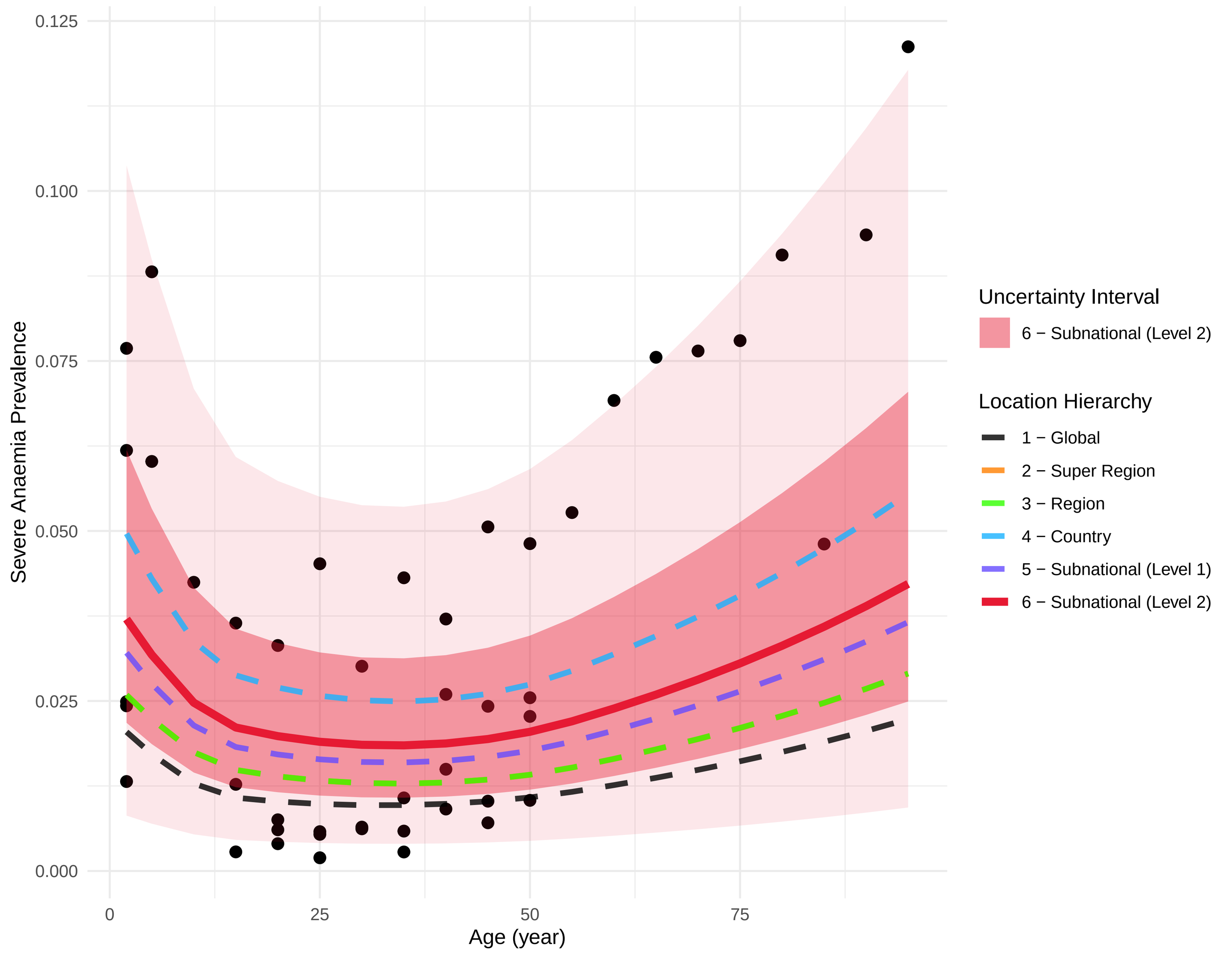

MR−BRT Age/Sex Fits − Males 2+ Years Old − Severe Anaemia Prevalence − Tamil Nadu, Urban
WHO−2024 Adjustment Method

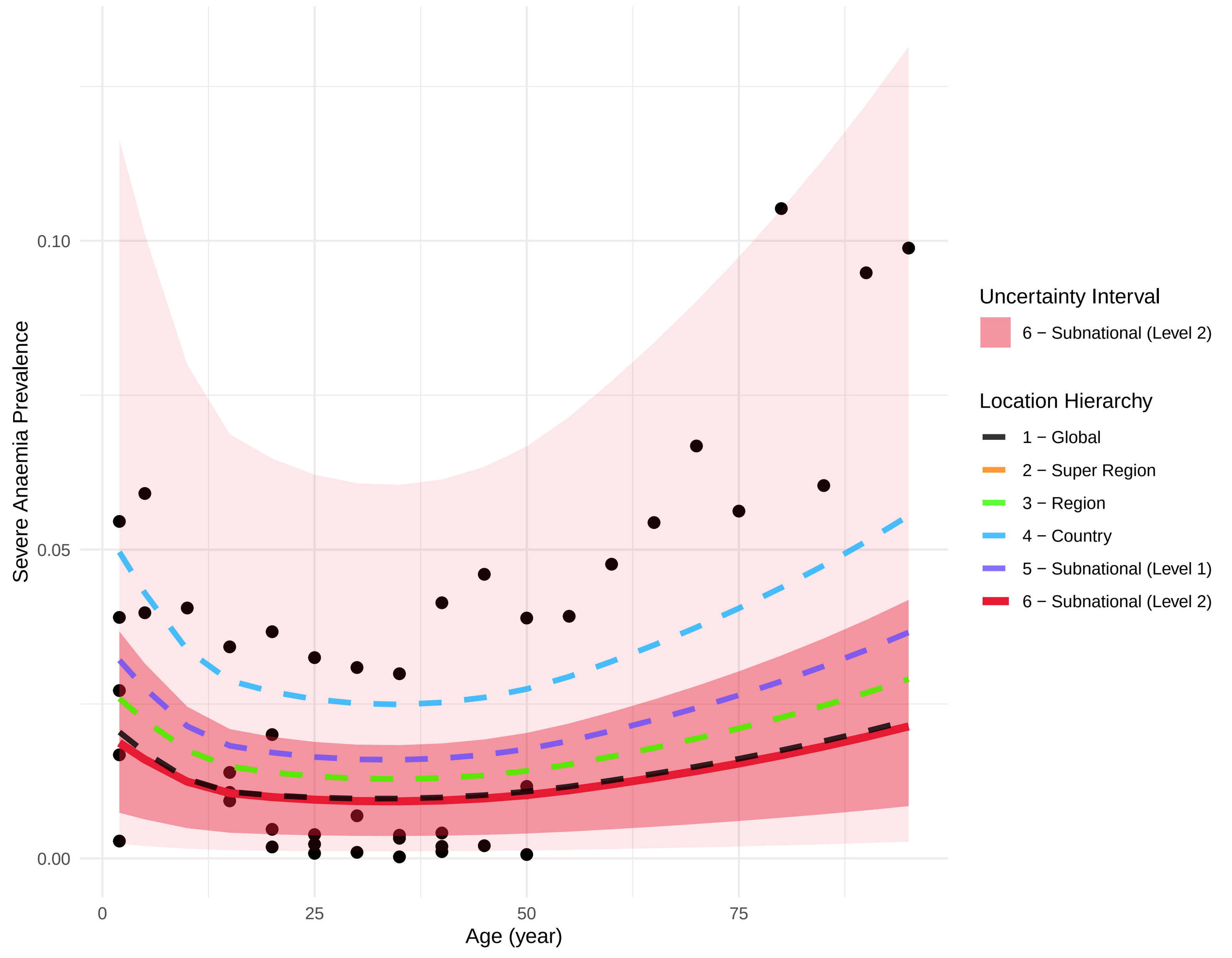

MR−BRT Age/Sex Fits − Males 2+ Years Old − Severe Anaemia Prevalence − Telangana
WHO−2024 Adjustment Method

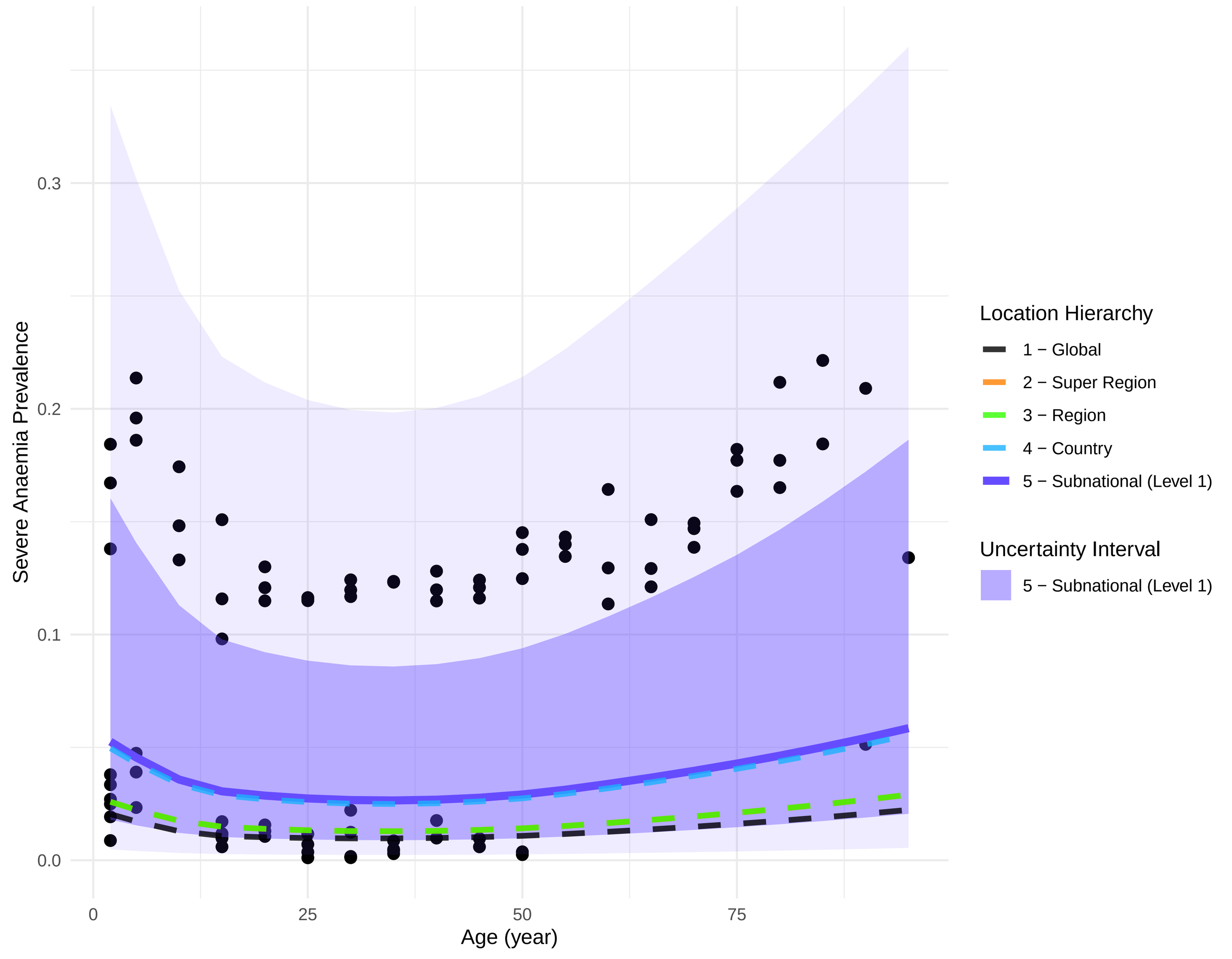

MR−BRT Age/Sex Fits − Males 2+ Years Old − Severe Anaemia Prevalence − Telangana, Rural
WHO−2024 Adjustment Method

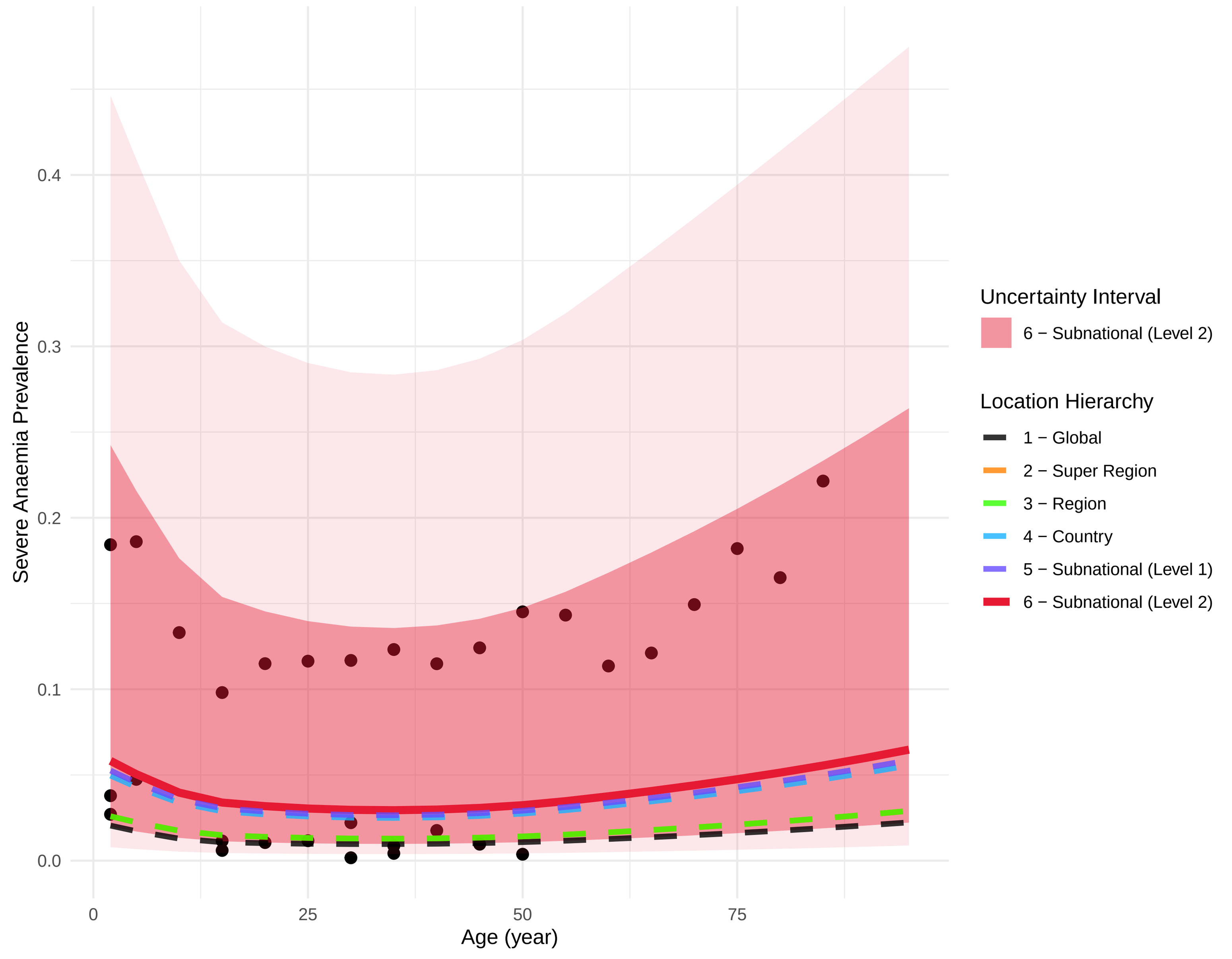

MR−BRT Age/Sex Fits − Males 2+ Years Old − Severe Anaemia Prevalence − Telangana, Urban
WHO−2024 Adjustment Method

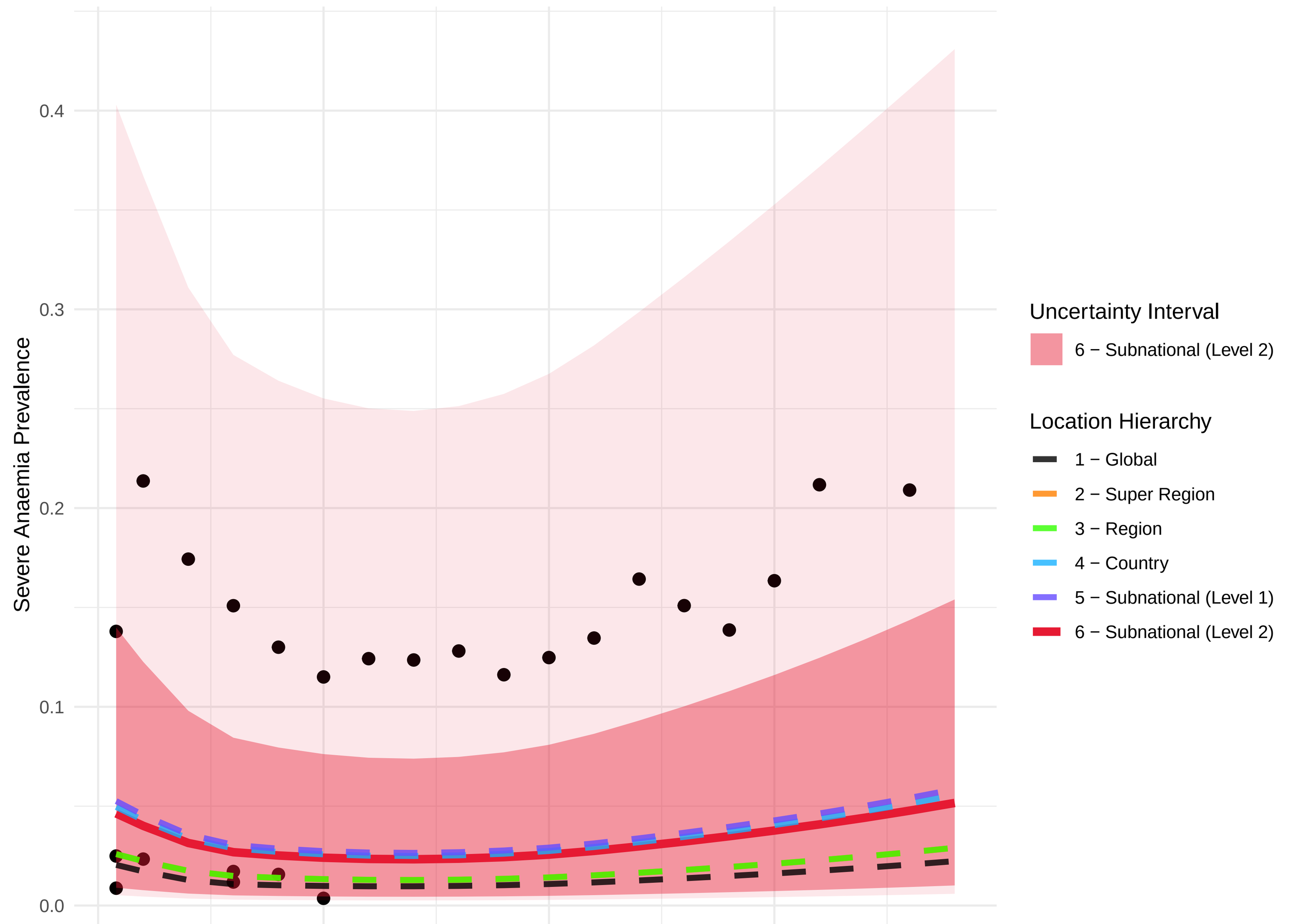

MR−BRT Age/Sex Fits − Males 2+ Years Old − Severe Anaemia Prevalence − Tripura
WHO−2024 Adjustment Method

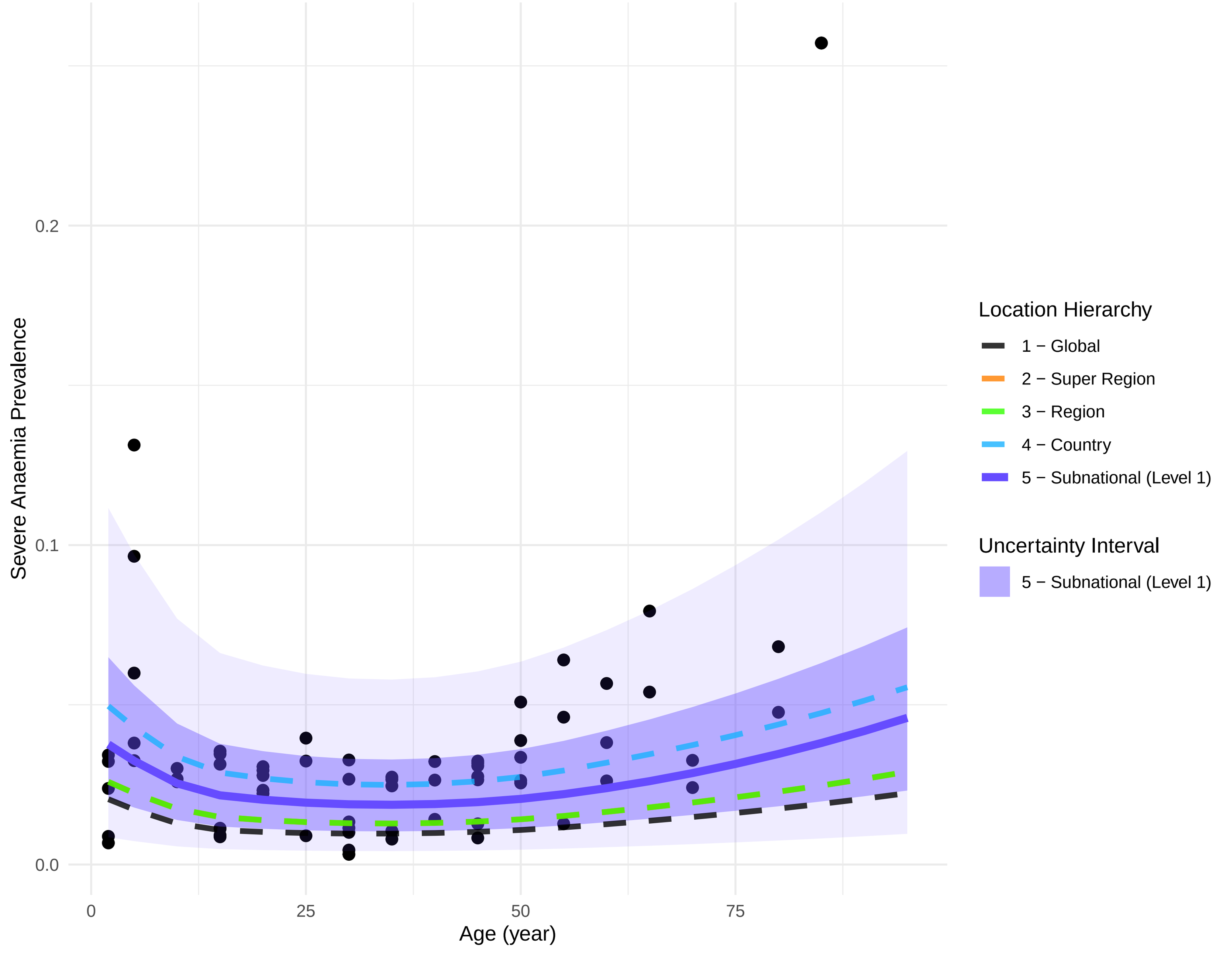

MR−BRT Age/Sex Fits − Males 2+ Years Old − Severe Anaemia Prevalence − Tripura, Rural
WHO−2024 Adjustment Method

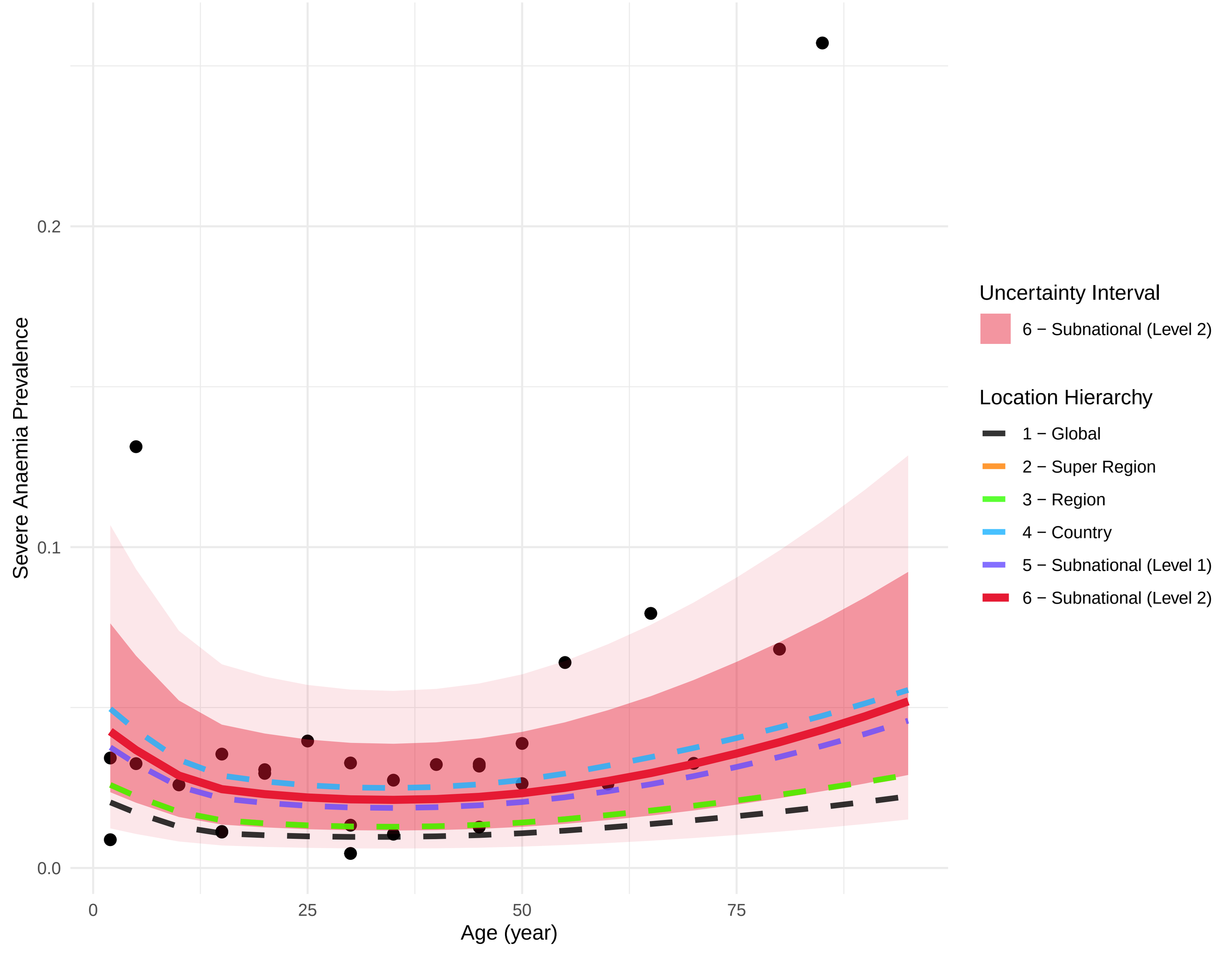

MR−BRT Age/Sex Fits − Males 2+ Years Old − Severe Anaemia Prevalence − Tripura, Urban
WHO−2024 Adjustment Method

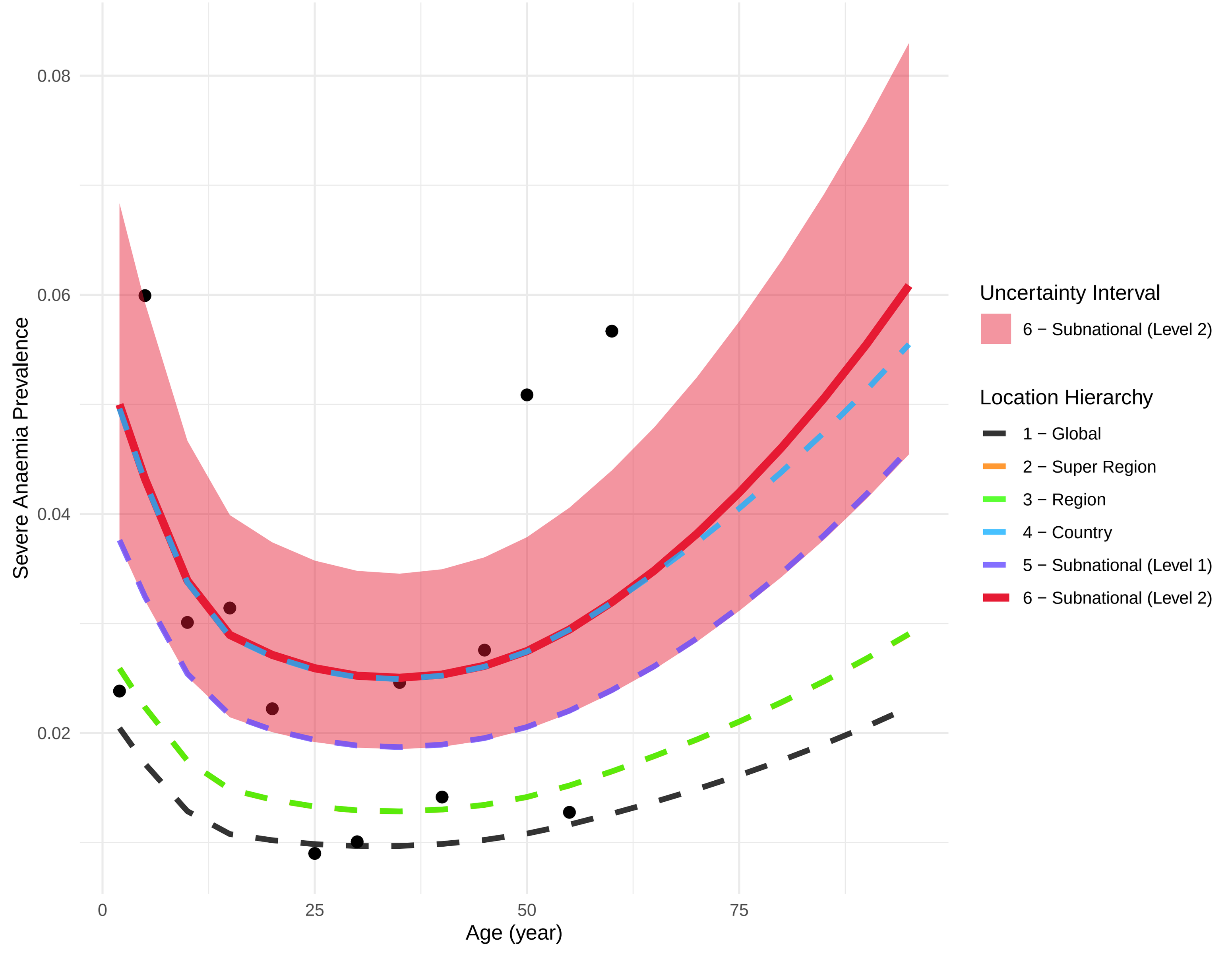

MR−BRT Age/Sex Fits − Males 2+ Years Old − Severe Anaemia Prevalence − Uttar Pradesh
WHO−2024 Adjustment Method

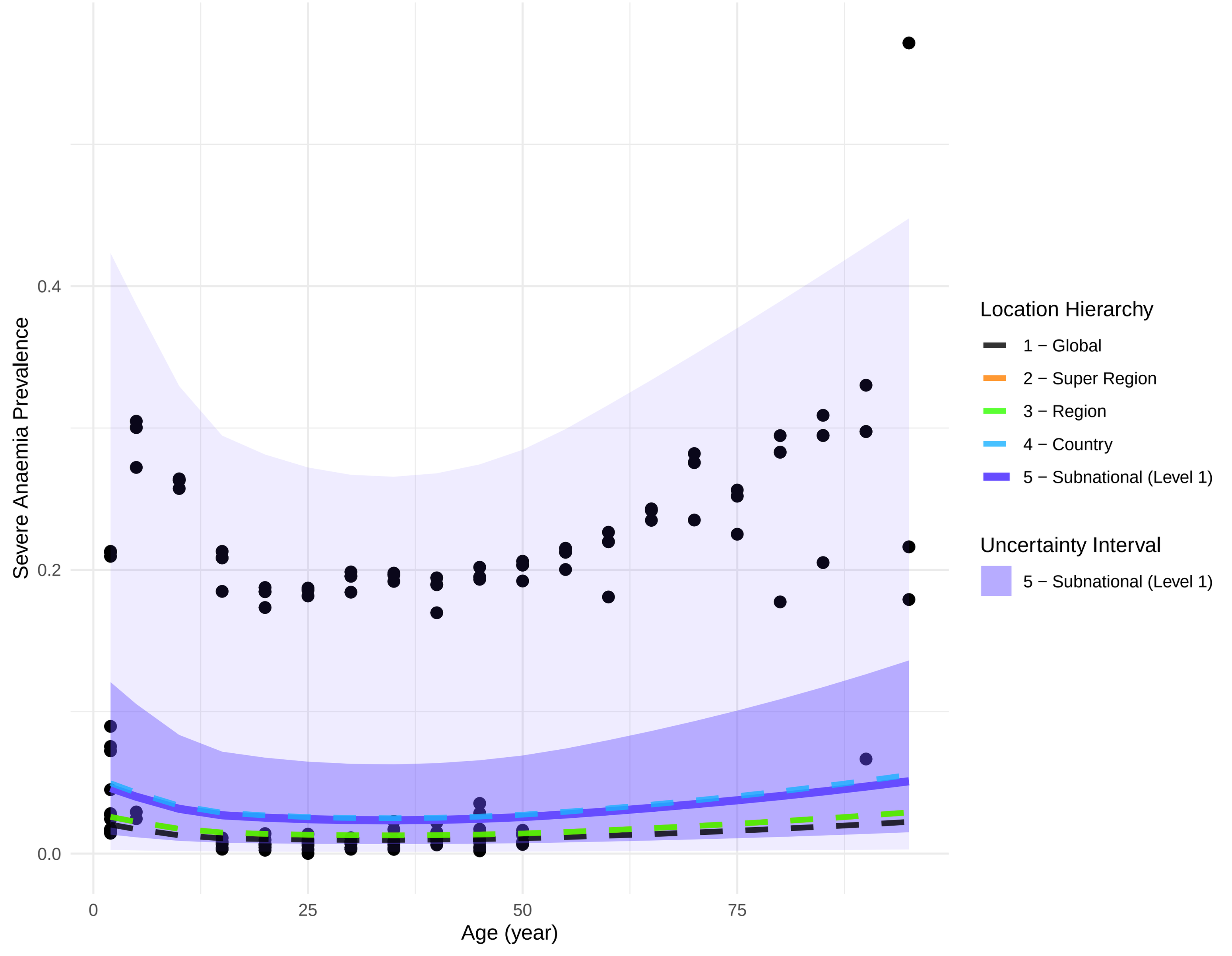

MR−BRT Age/Sex Fits − Males 2+ Years Old − Severe Anaemia Prevalence − Uttar Pradesh, Rural
WHO−2024 Adjustment Method

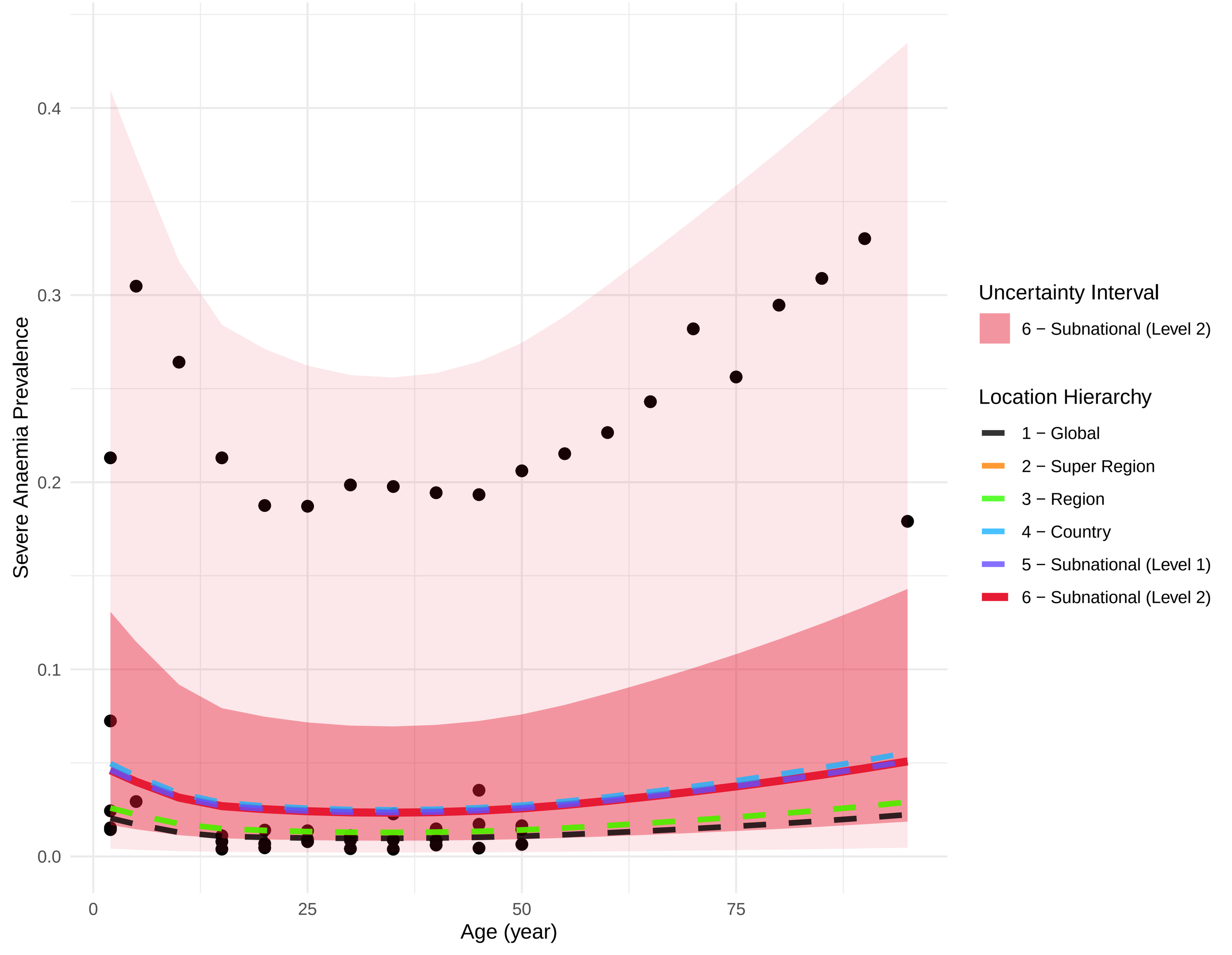

MR−BRT Age/Sex Fits − Males 2+ Years Old − Severe Anaemia Prevalence − Uttar Pradesh, Urban
WHO−2024 Adjustment Method

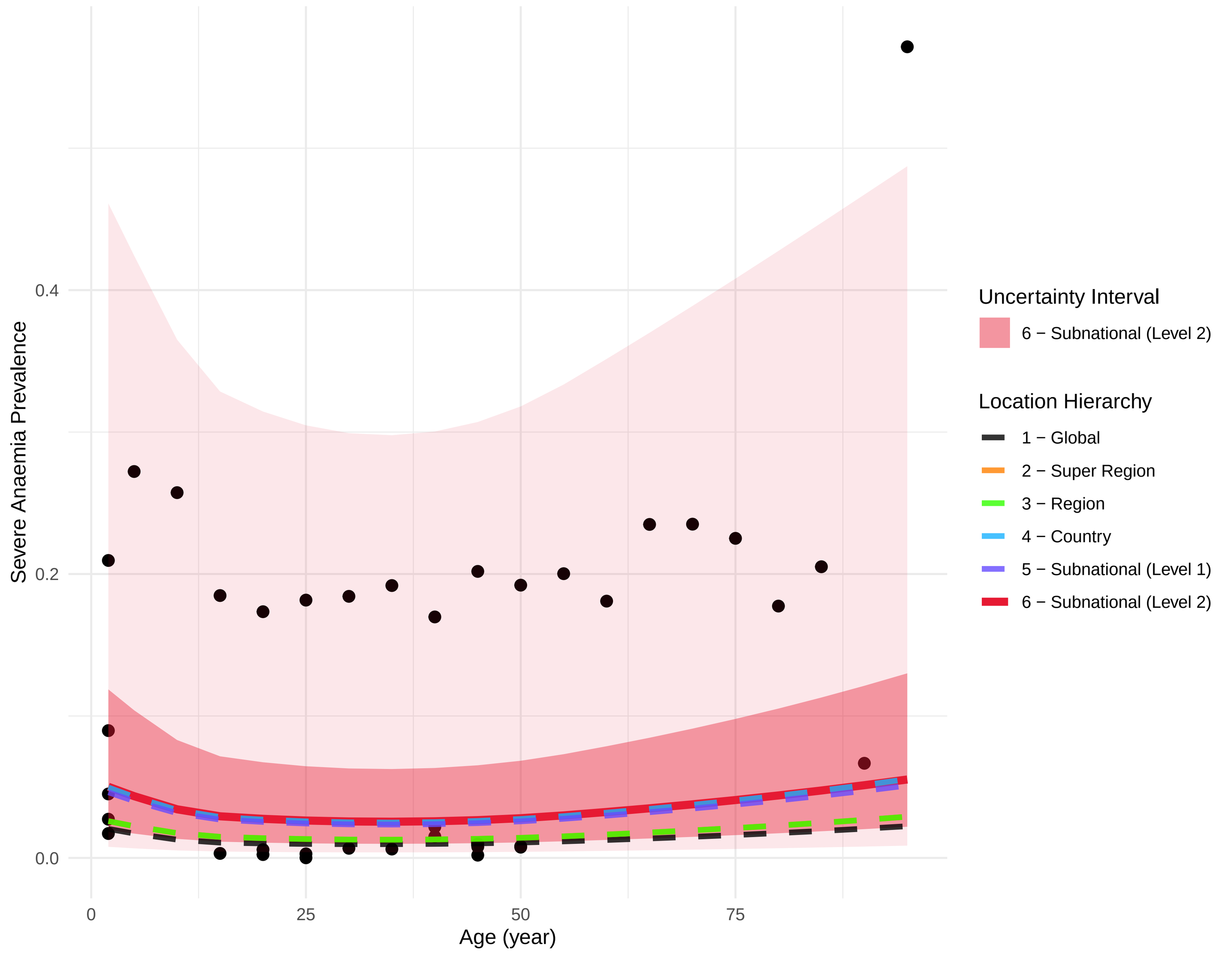

MR−BRT Age/Sex Fits − Males 2+ Years Old − Severe Anaemia Prevalence − Uttarakhand
WHO−2024 Adjustment Method

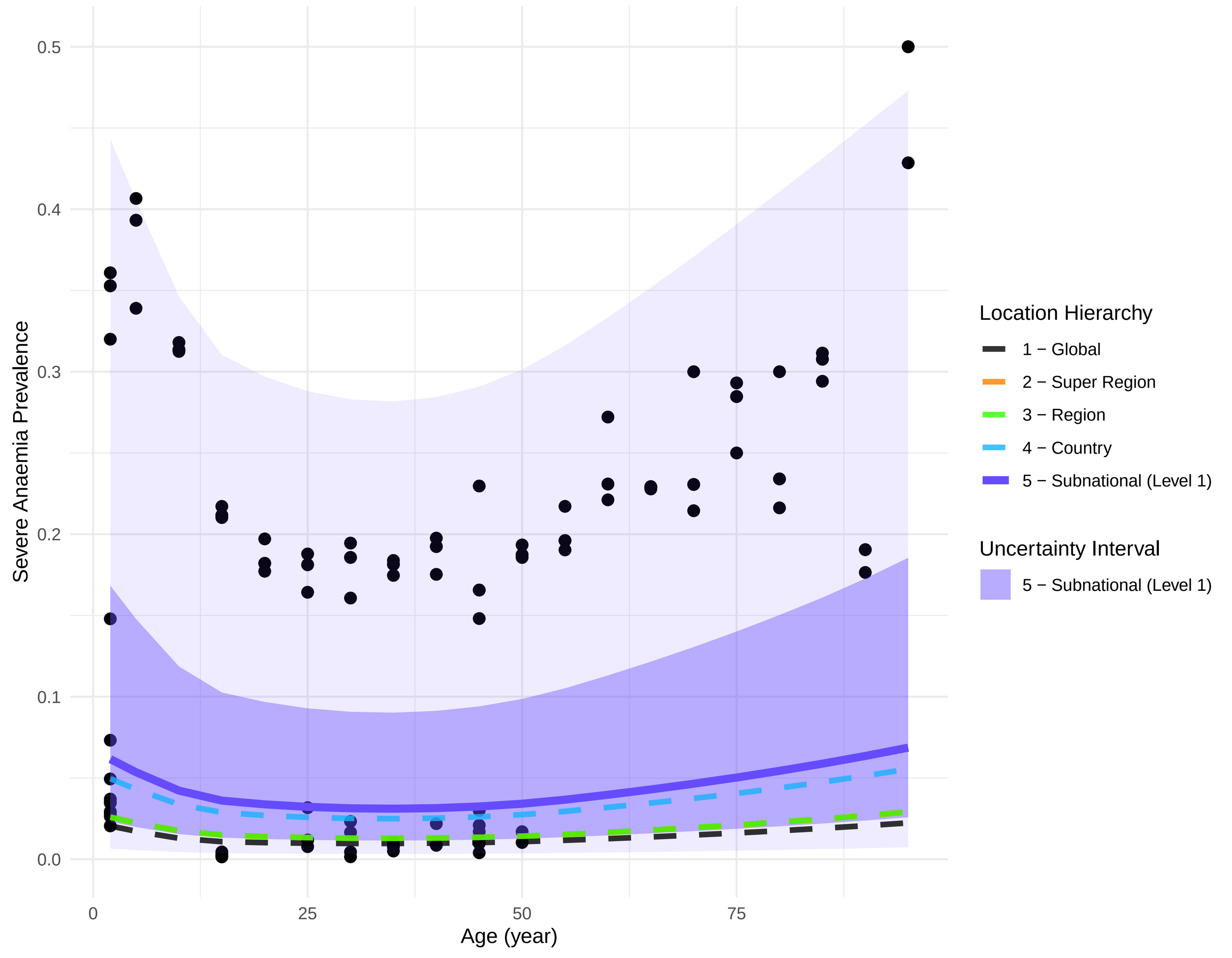

MR−BRT Age/Sex Fits − Males 2+ Years Old − Severe Anaemia Prevalence − Uttarakhand, Rural
WHO−2024 Adjustment Method

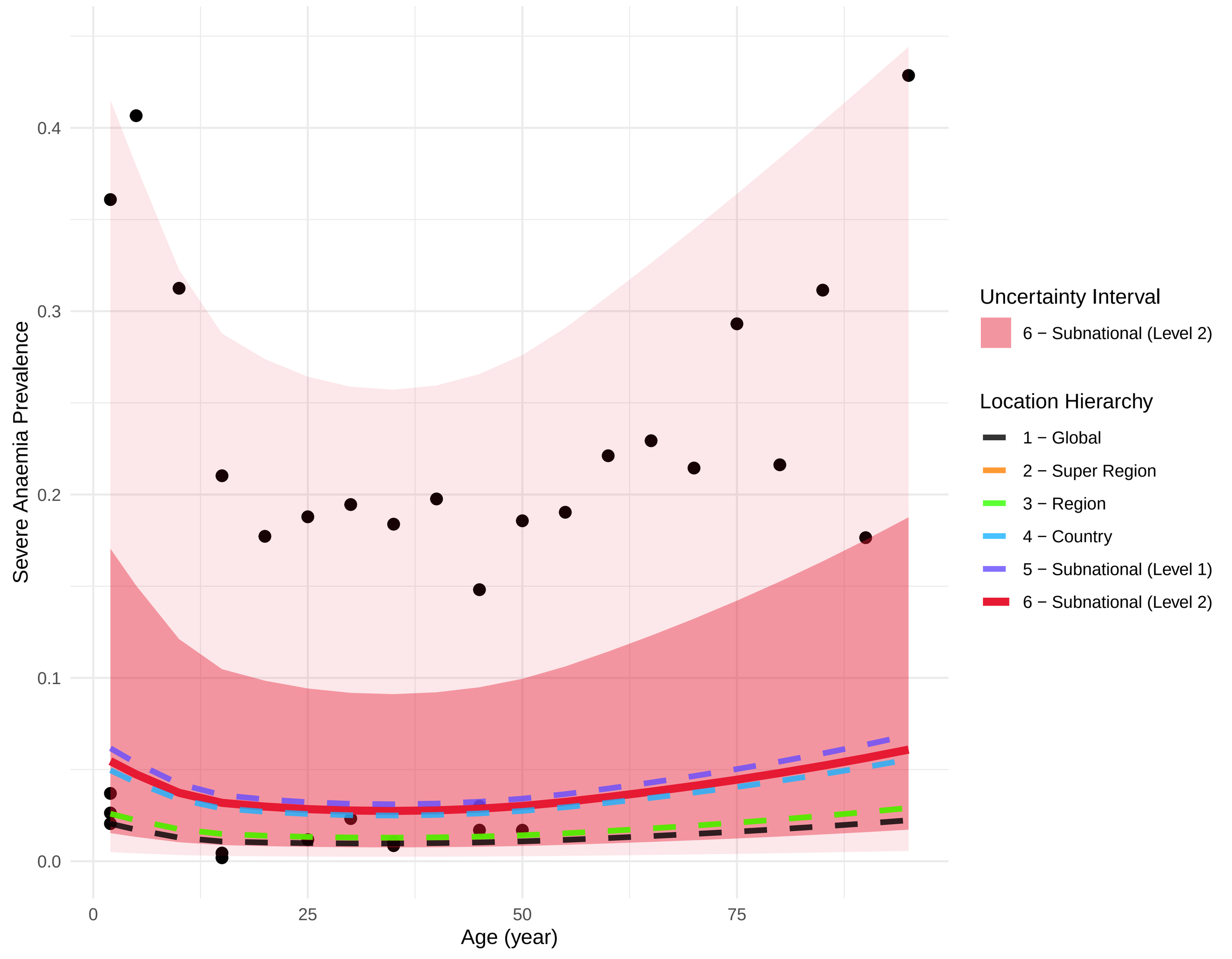

MR−BRT Age/Sex Fits − Males 2+ Years Old − Severe Anaemia Prevalence − Uttarakhand, Urban
WHO−2024 Adjustment Method

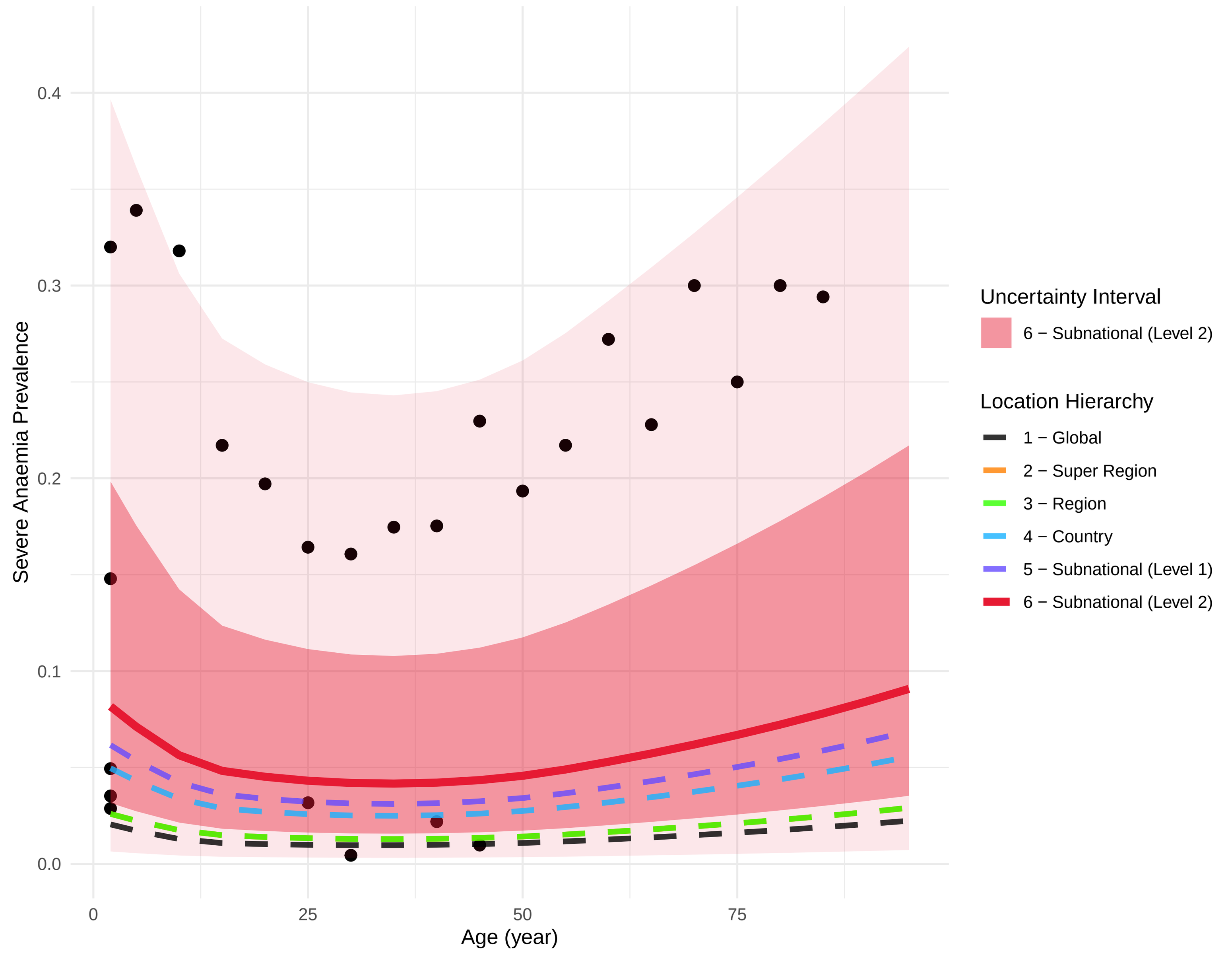

MR−BRT Age/Sex Fits − Males 2+ Years Old − Severe Anaemia Prevalence − West Bengal
WHO−2024 Adjustment Method

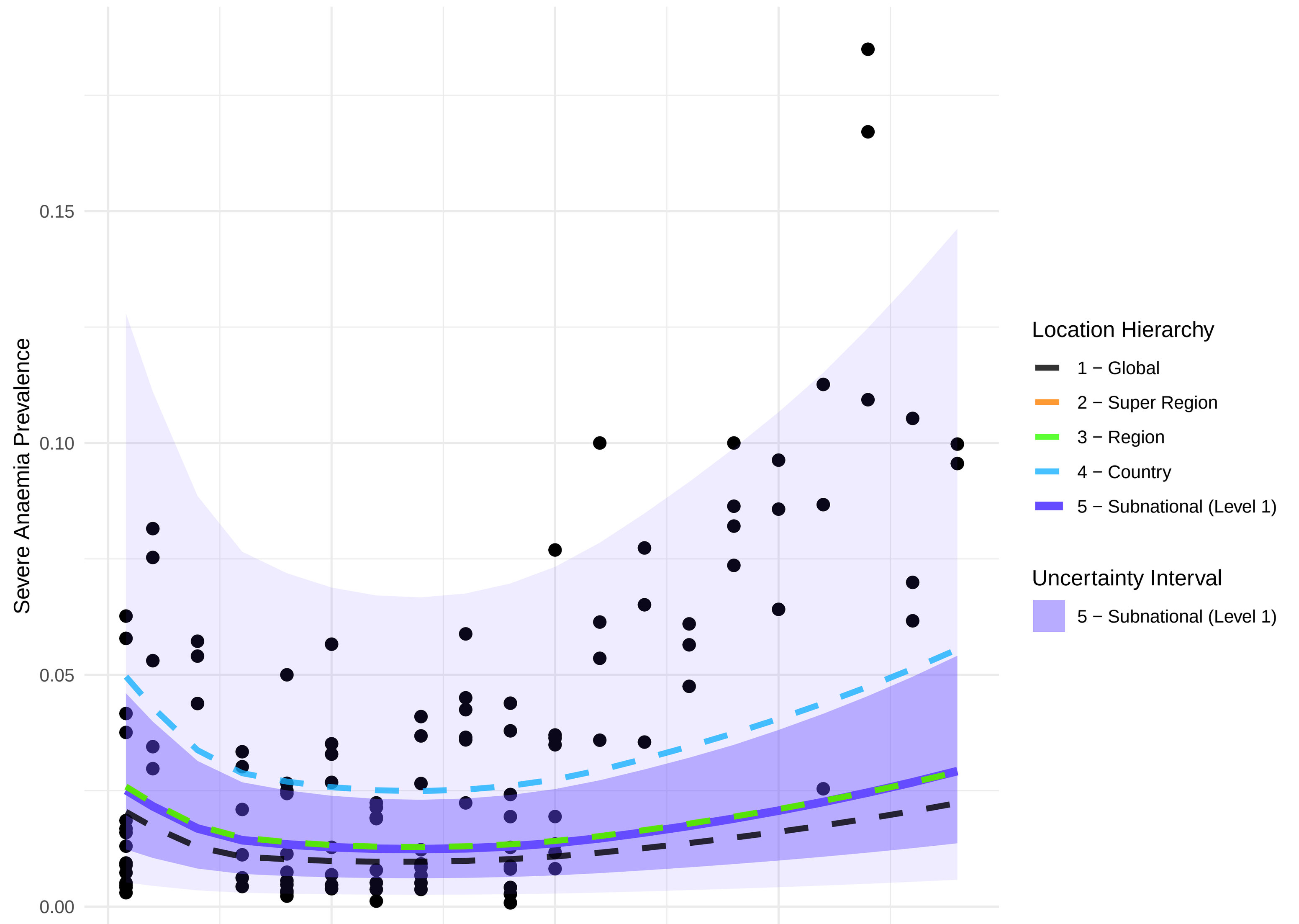

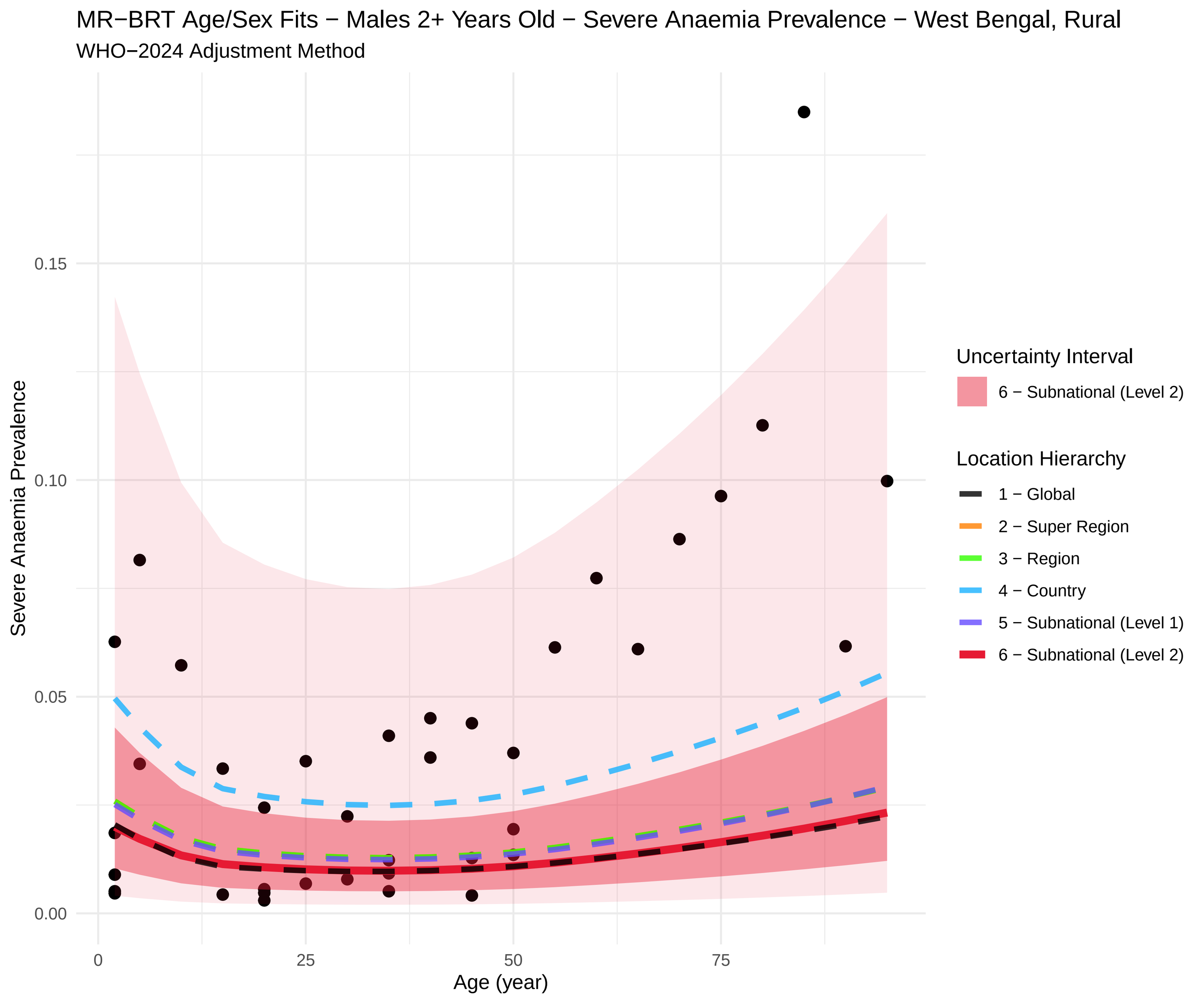
MR−BRT Age/Sex Fits − Males 2+ Years Old − Severe Anaemia Prevalence − West Bengal, Rural
WHO−2024 Adjustment Method
Severe Anaemia Prevalence
Age (year)
Uncertainty Interval
6 − Subnational (Level 2)
Location Hierarchy
1 − Global
2 − Super Region
3 − Region
4 − Country
5 − Subnational (Level 1)
6 − Subnational (Level 2)

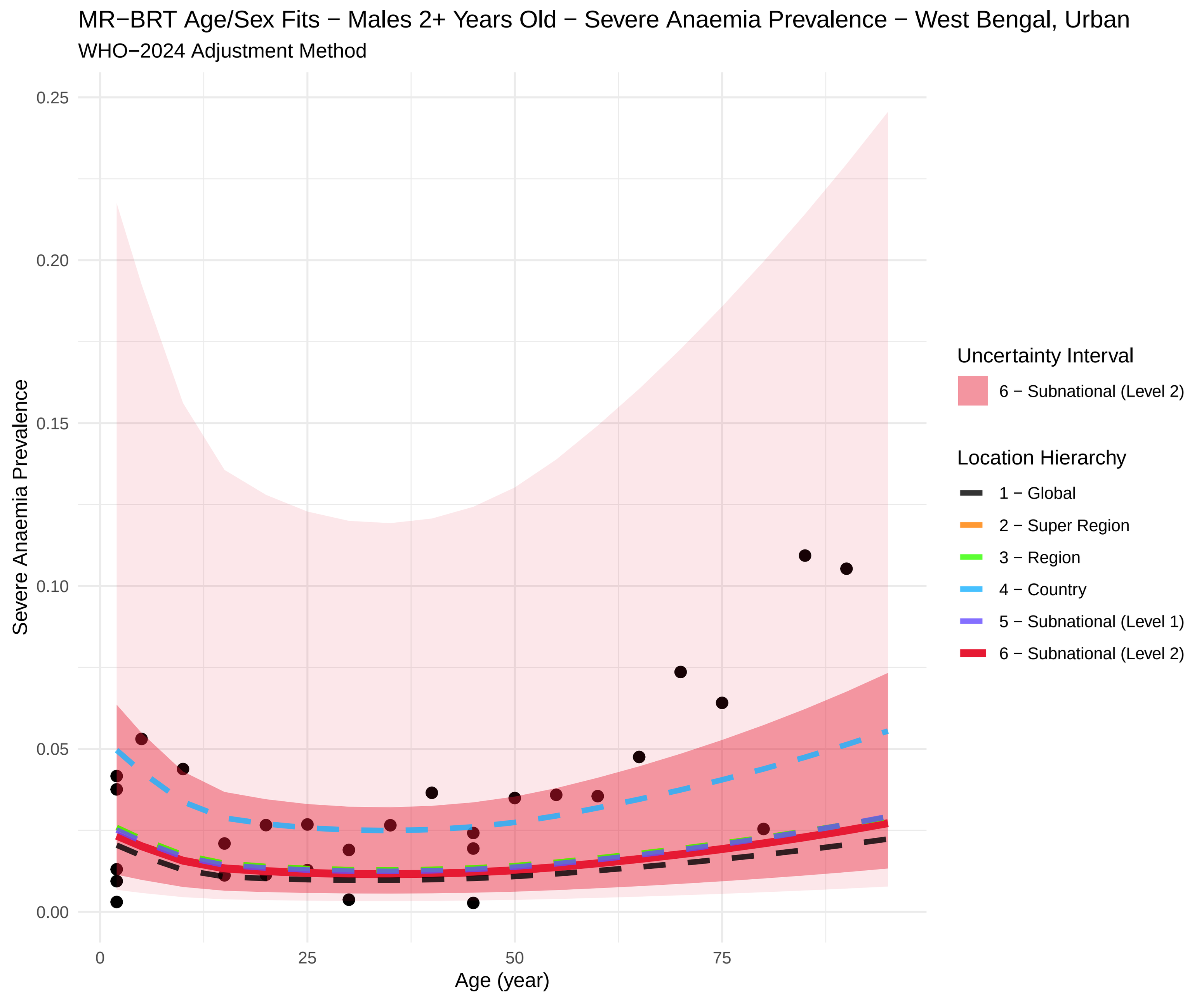
MR−BRT Age/Sex Fits − Males 2+ Years Old − Severe Anaemia Prevalence − West Bengal, Urban
WHO−2024 Adjustment Method
Severe Anaemia Prevalence
Age (year)
Uncertainty Interval
6 − Subnational (Level 2)
Location Hierarchy
1 − Global
2 − Super Region
3 − Region
4 − Country
5 − Subnational (Level 1)
6 − Subnational (Level 2)

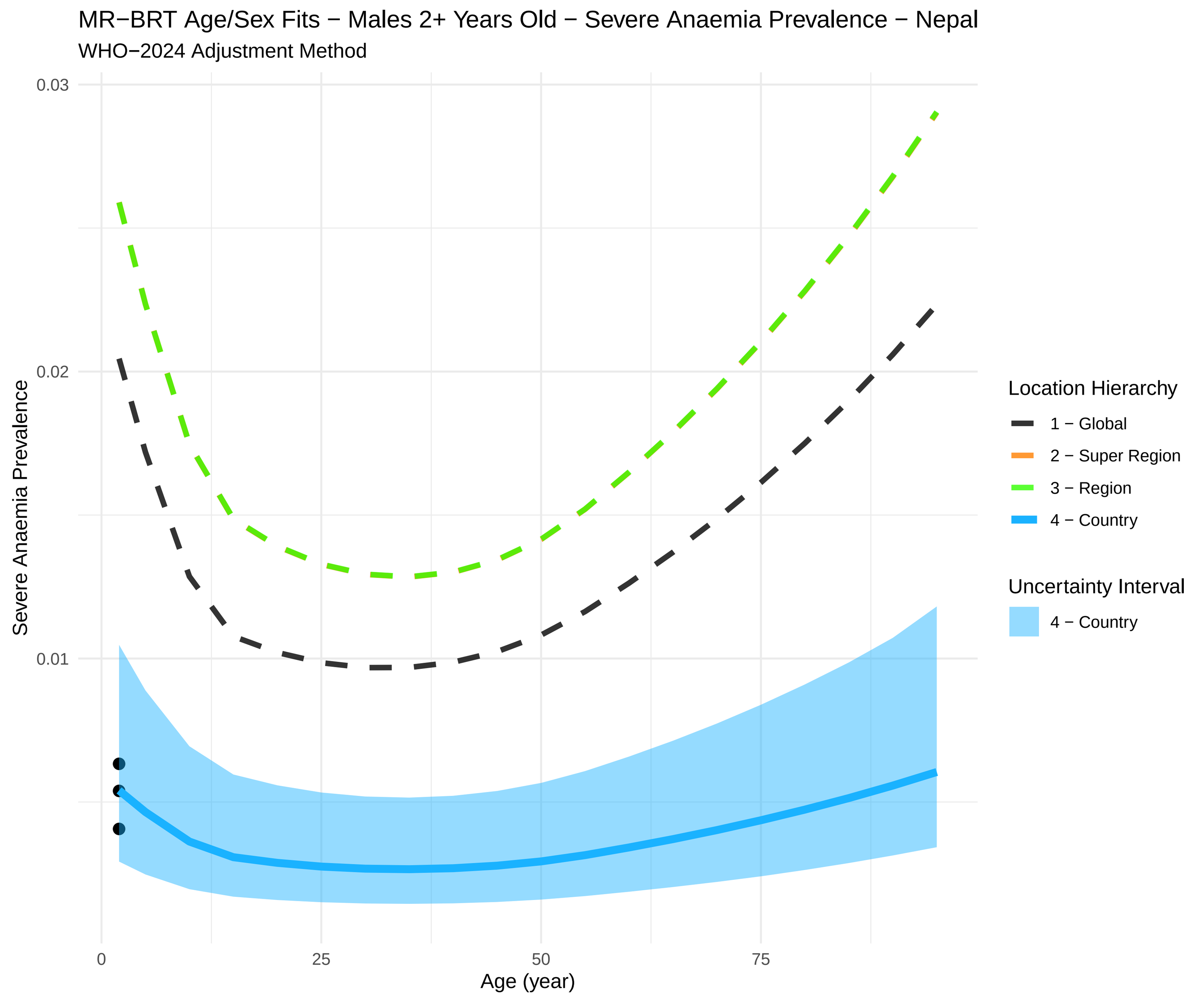
MR−BRT Age/Sex Fits − Males 2+ Years Old − Severe Anaemia Prevalence − Nepal
WHO−2024 Adjustment Method
Severe Anaemia Prevalence
Age (year)
Location Hierarchy
1 − Global
2 − Super Region
3 − Region
4 − Country
Uncertainty Interval
4 − Country

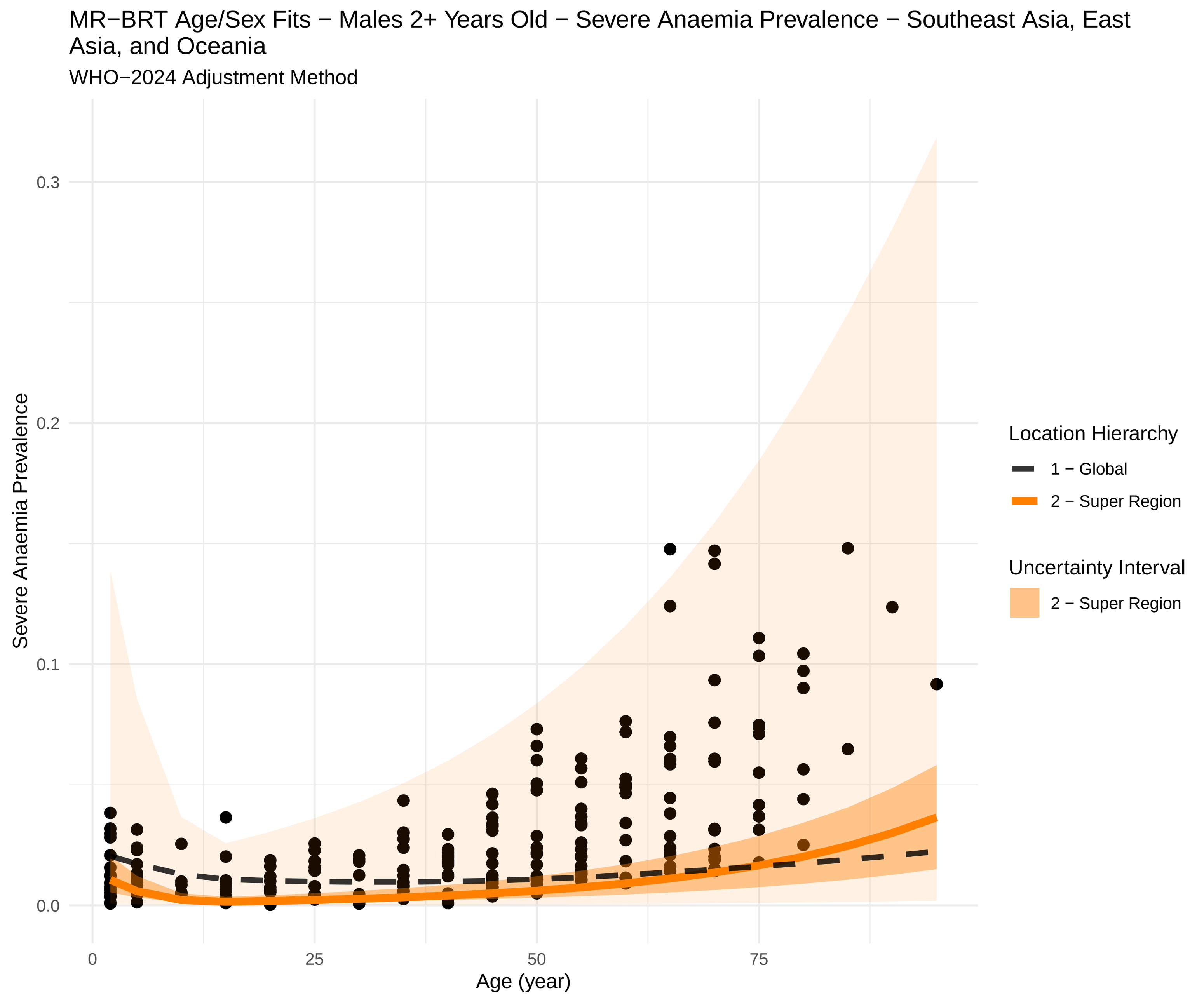
MR−BRT Age/Sex Fits − Males 2+ Years Old − Severe Anaemia Prevalence − Southeast Asia, East Asia, and Oceania
WHO−2024 Adjustment Method
Severe Anaemia Prevalence
Age (year)
Location Hierarchy
1 − Global
2 − Super Region
Uncertainty Interval
2 − Super Region

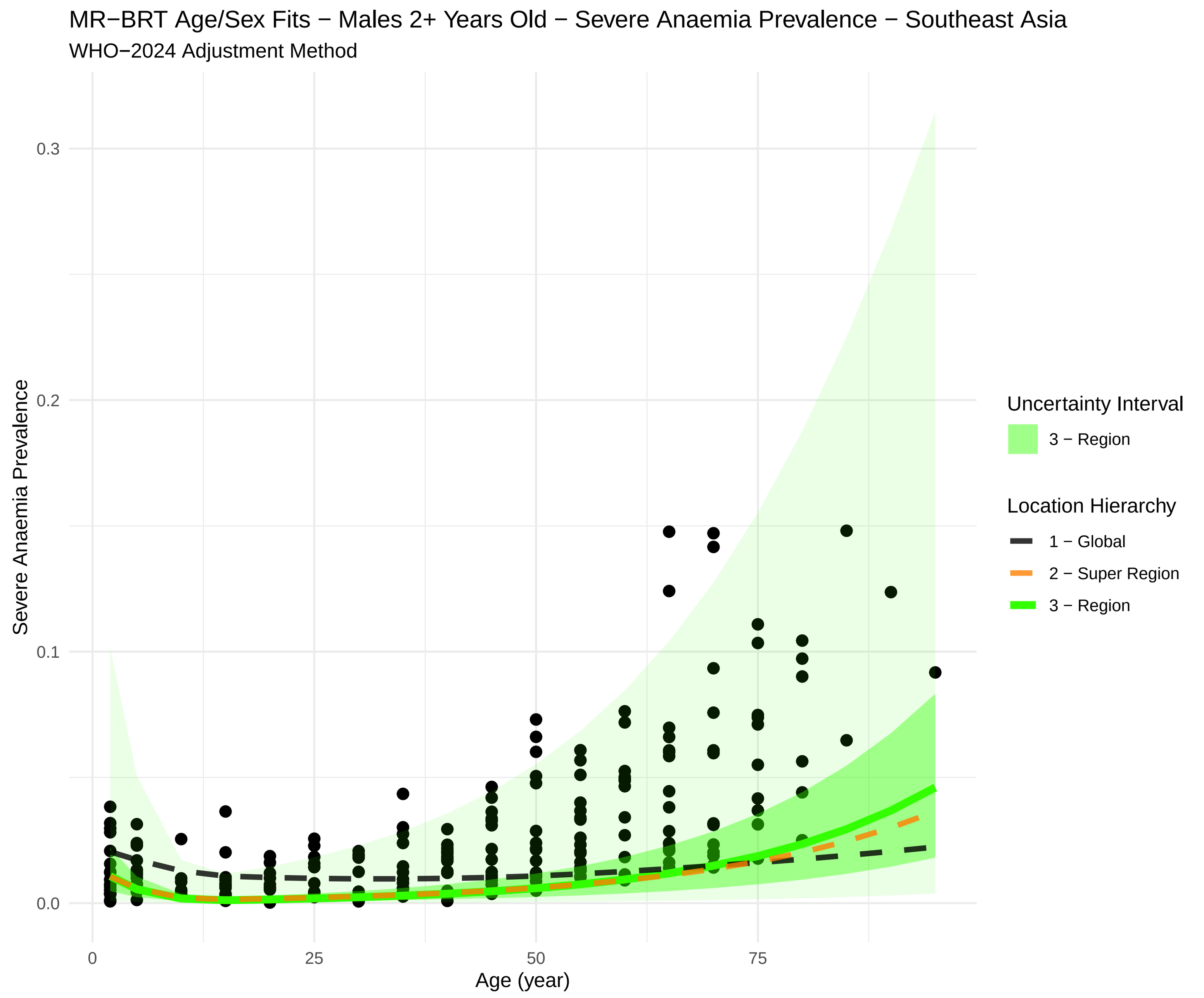
MR−BRT Age/Sex Fits − Males 2+ Years Old − Severe Anaemia Prevalence − Southeast Asia
WHO−2024 Adjustment Method
Severe Anaemia Prevalence
Age (year)
Uncertainty Interval
3 − Region
Location Hierarchy
1 − Global
2 − Super Region
3 − Region

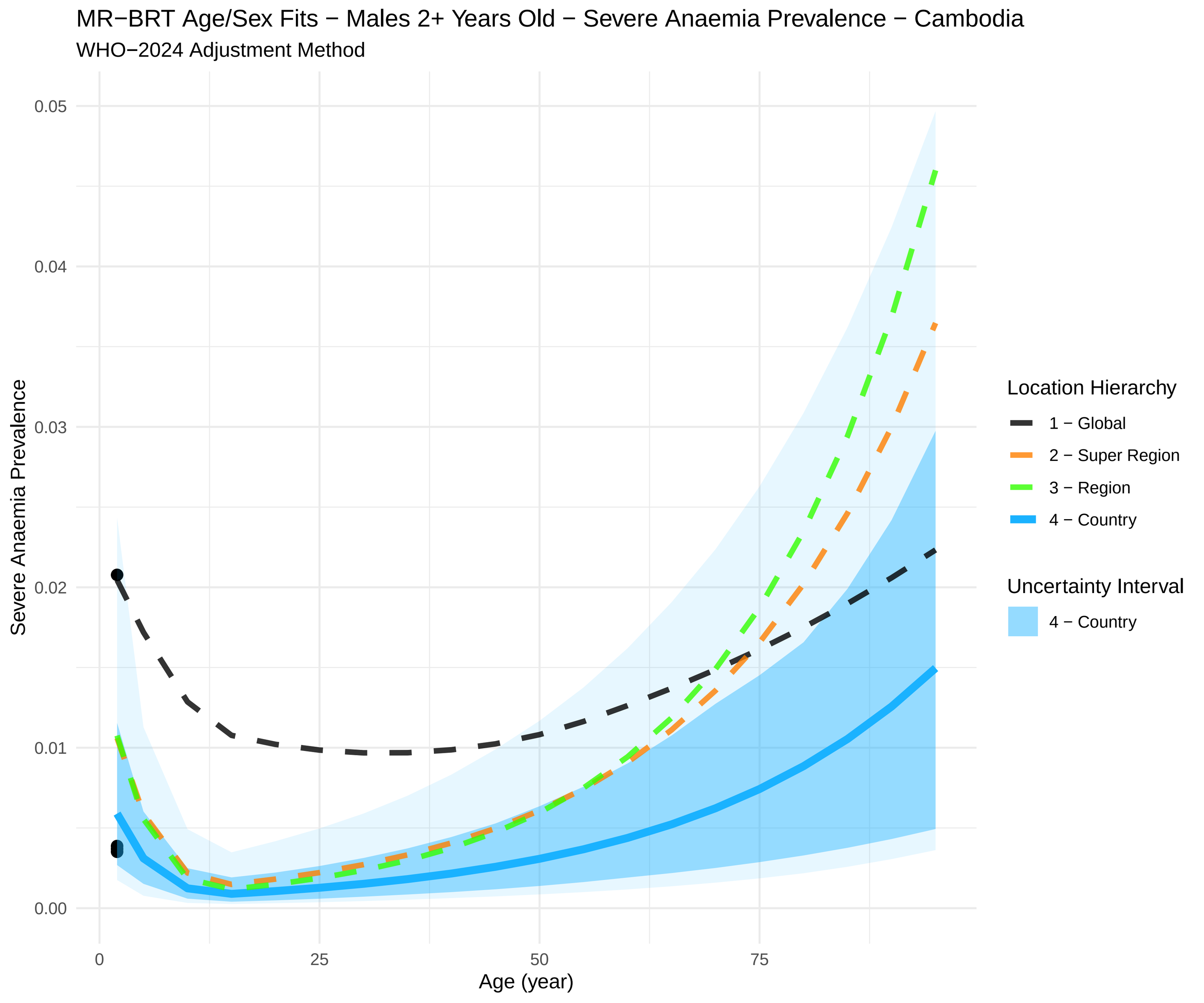
MR−BRT Age/Sex Fits − Males 2+ Years Old − Severe Anaemia Prevalence − Cambodia
WHO−2024 Adjustment Method
Severe Anaemia Prevalence
Age (year)
Location Hierarchy
1 − Global
2 − Super Region
3 − Region
4 − Country
Uncertainty Interval
4 − Country

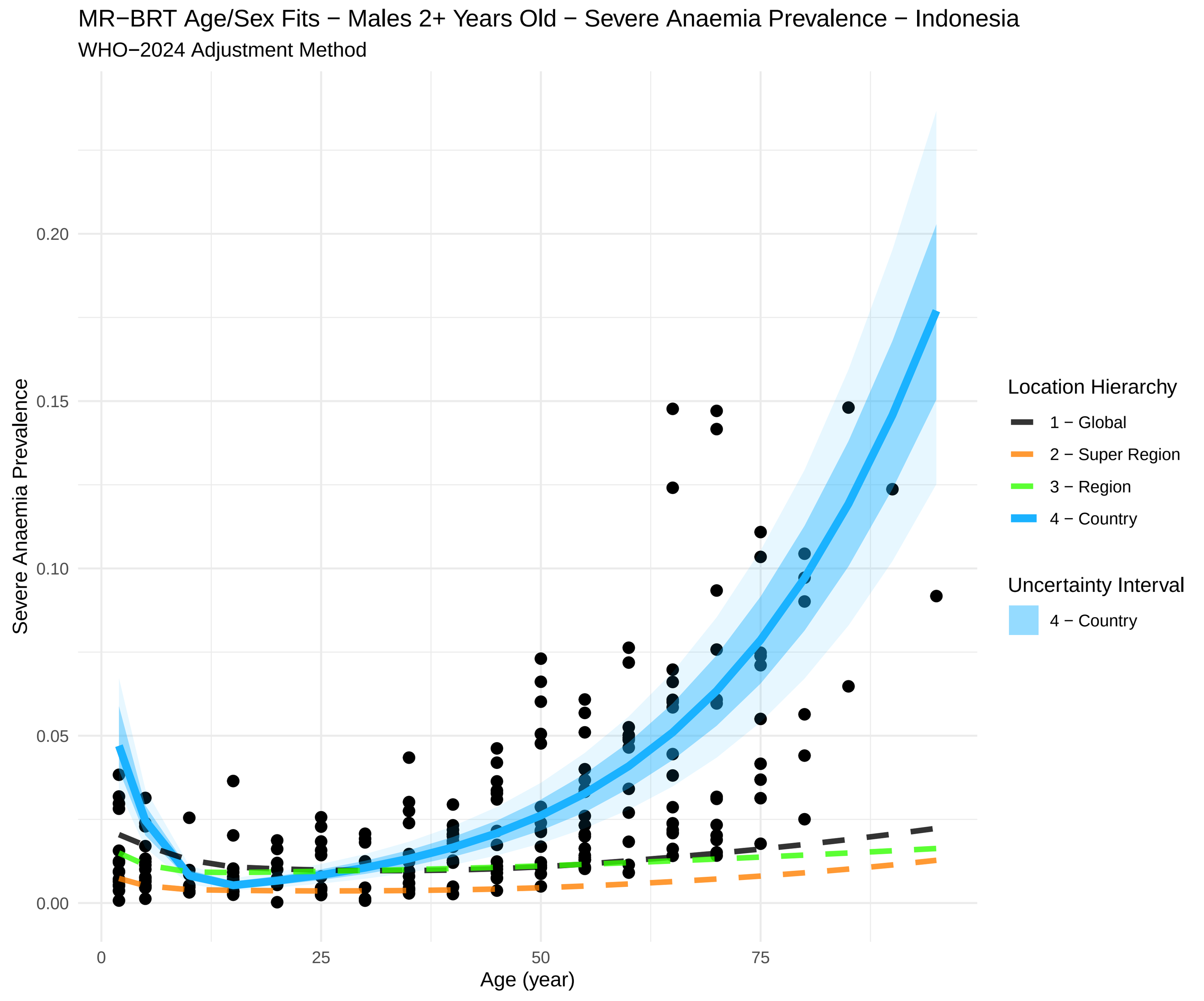
MR−BRT Age/Sex Fits − Males 2+ Years Old − Severe Anaemia Prevalence − Indonesia
WHO−2024 Adjustment Method
Severe Anaemia Prevalence
Age (year)
Location Hierarchy
1 − Global
2 − Super Region
3 − Region
4 − Country
Uncertainty Interval
4 − Country

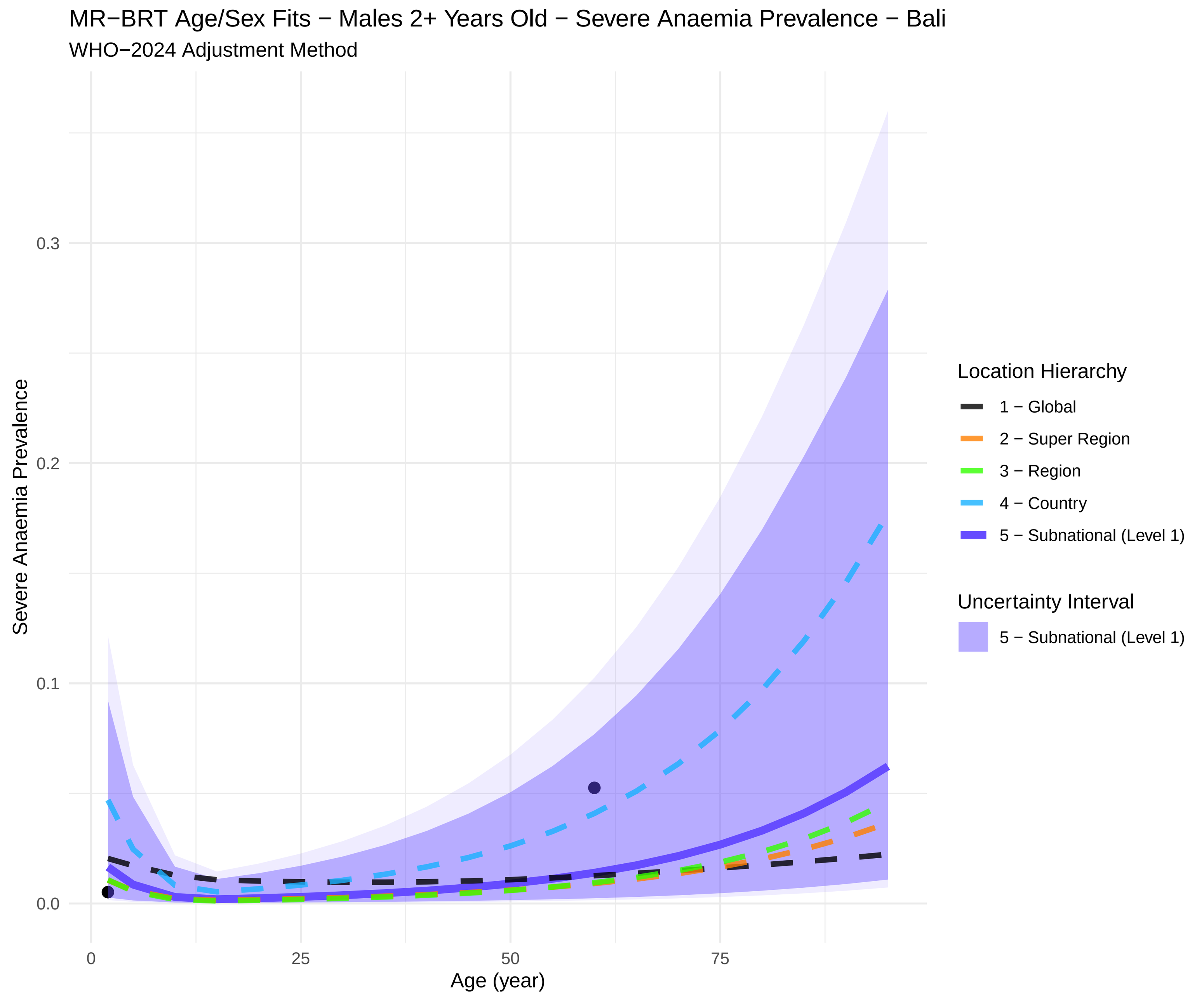
MR−BRT Age/Sex Fits − Males 2+ Years Old − Severe Anaemia Prevalence − Bali
WHO−2024 Adjustment Method
Severe Anaemia Prevalence
Age (year)
Location Hierarchy
1 − Global
2 − Super Region
3 − Region
4 − Country
5 − Subnational (Level 1)
Uncertainty Interval
5 − Subnational (Level 1)

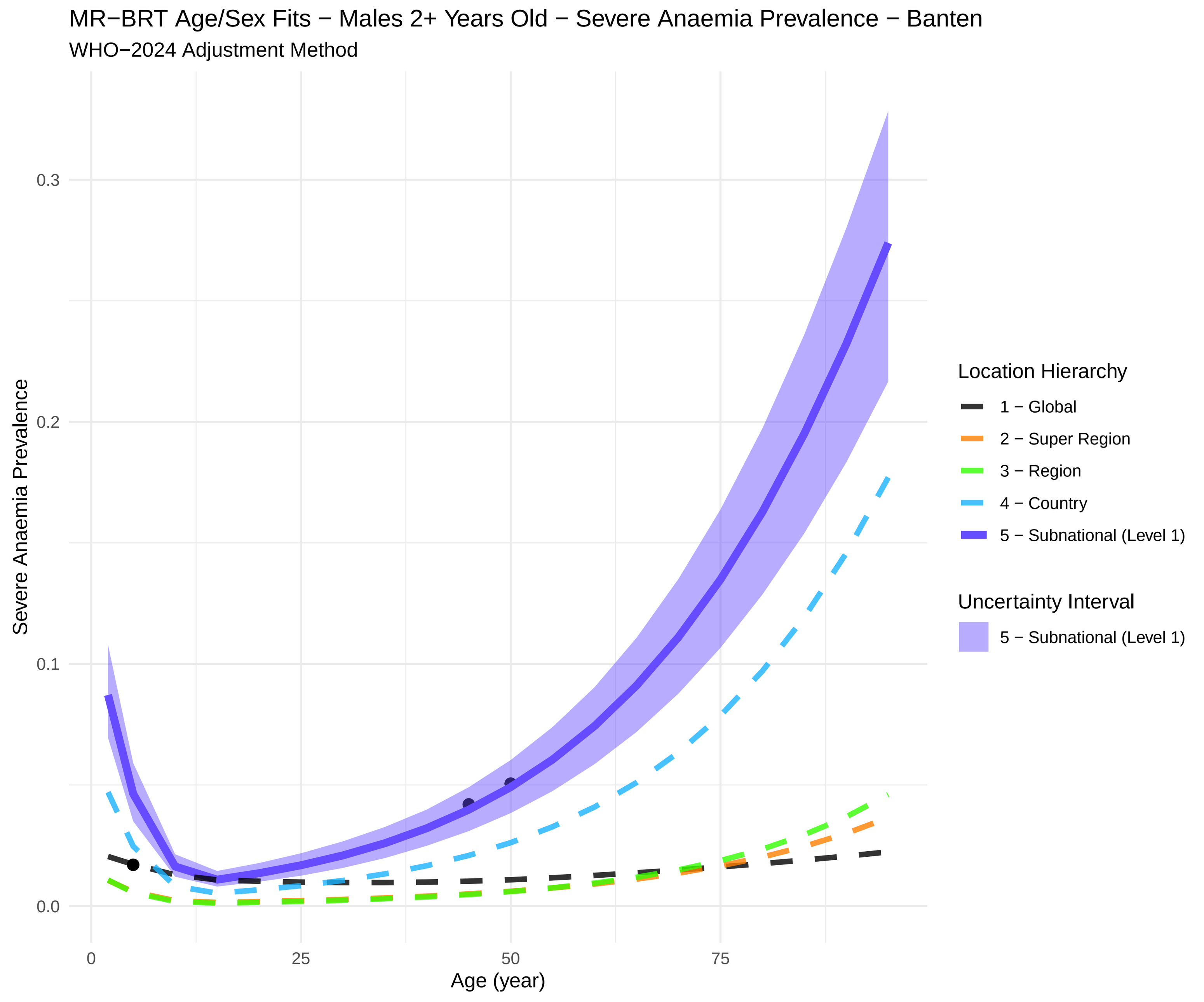
MR−BRT Age/Sex Fits − Males 2+ Years Old − Severe Anaemia Prevalence − Banten
WHO−2024 Adjustment Method
Severe Anaemia Prevalence
Age (year)
Location Hierarchy
1 − Global
2 − Super Region
3 − Region
4 − Country
5 − Subnational (Level 1)
Uncertainty Interval
5 − Subnational (Level 1)

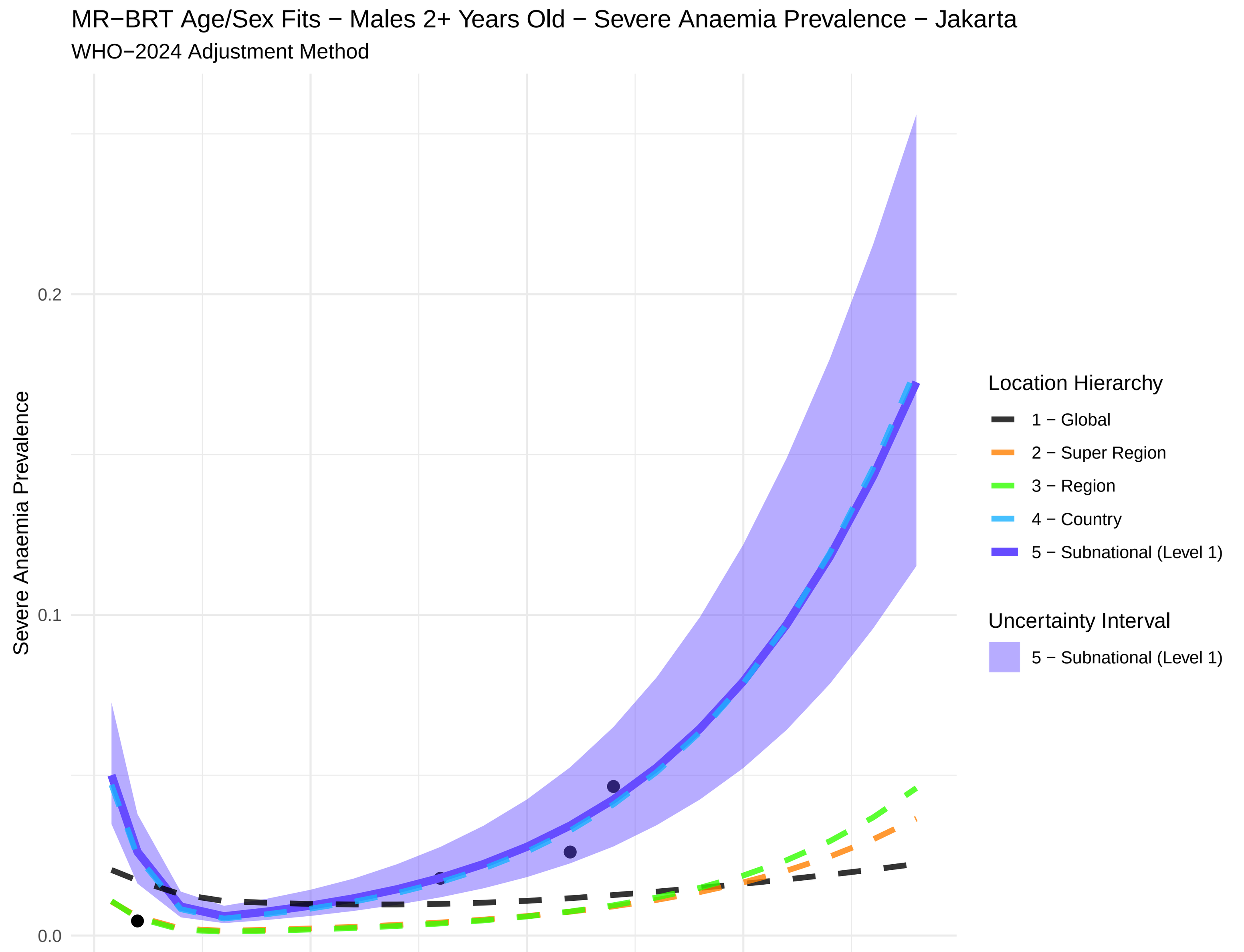
MR−BRT Age/Sex Fits − Males 2+ Years Old − Severe Anaemia Prevalence − Jakarta
WHO−2024 Adjustment Method
Severe Anaemia Prevalence
Age (year)
Location Hierarchy
1 − Global
2 − Super Region
3 − Region
4 − Country
5 − Subnational (Level 1)
Uncertainty Interval
5 − Subnational (Level 1)

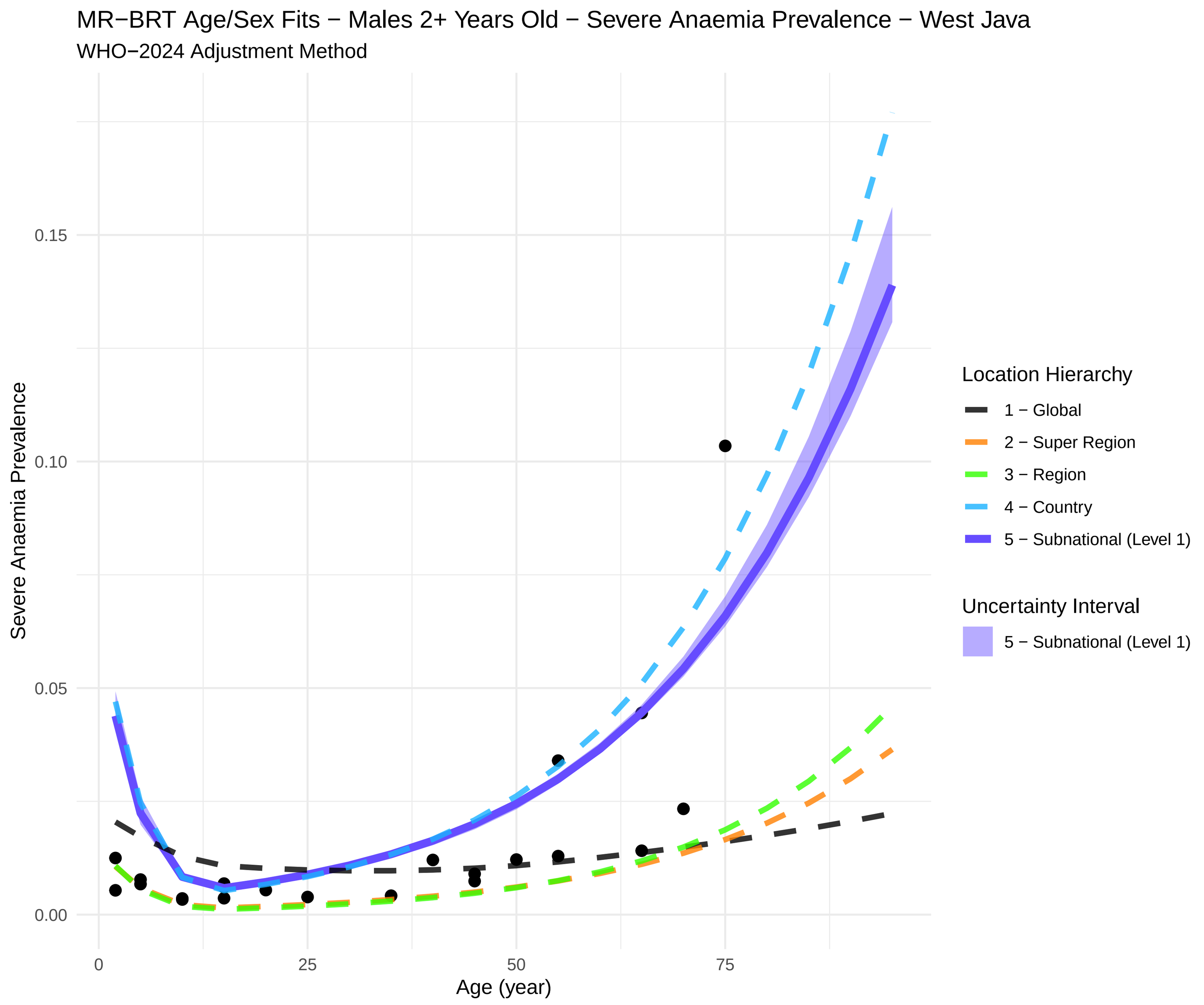
MR−BRT Age/Sex Fits − Males 2+ Years Old − Severe Anaemia Prevalence − West Java
WHO−2024 Adjustment Method
Severe Anaemia Prevalence
Age (year)
Location Hierarchy
1 − Global
2 − Super Region
3 − Region
4 − Country
5 − Subnational (Level 1)
Uncertainty Interval
5 − Subnational (Level 1)

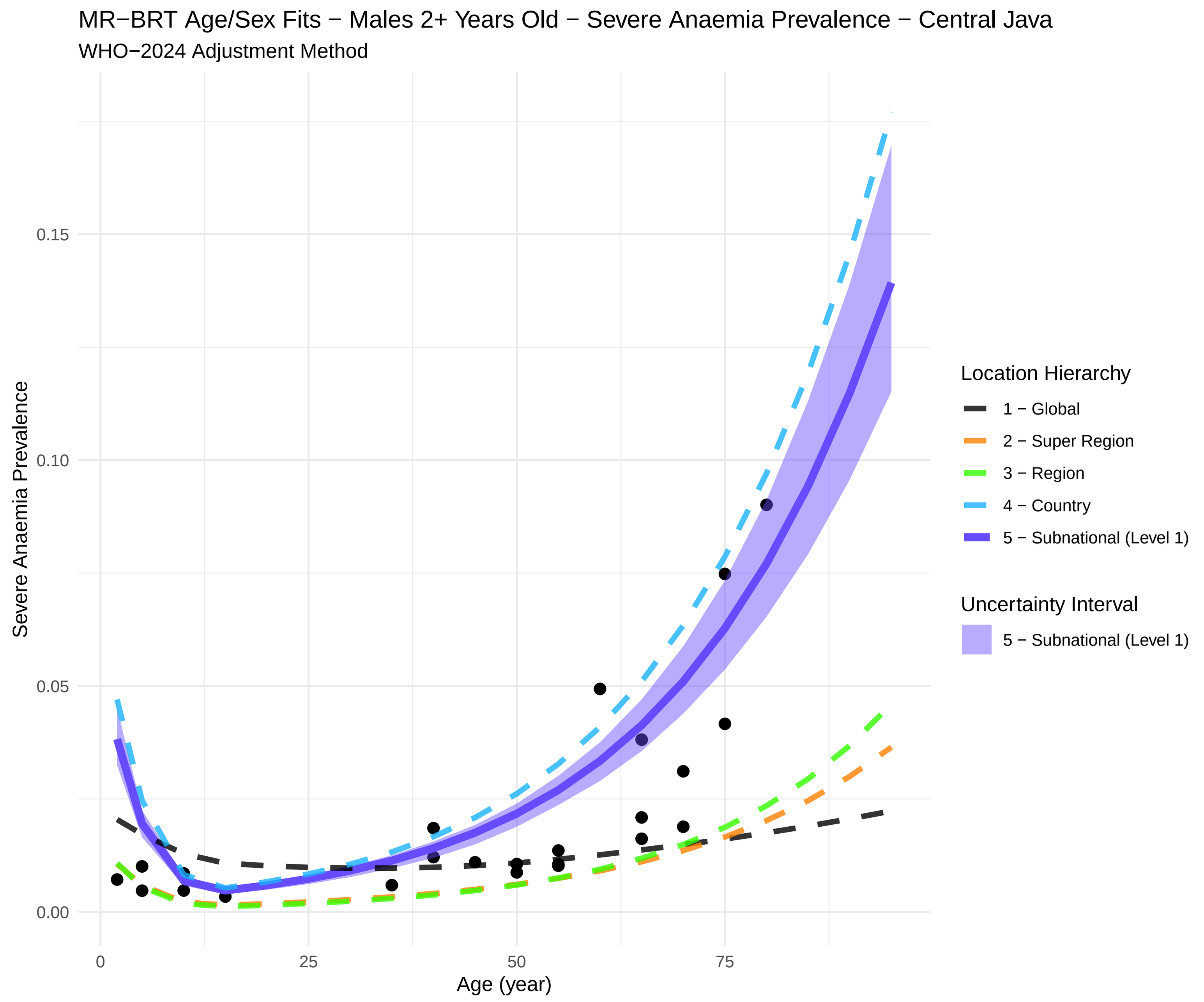
MR−BRT Age/Sex Fits − Males 2+ Years Old − Severe Anaemia Prevalence − Central Java
WHO−2024 Adjustment Method
Severe Anaemia Prevalence
Age (year)
Location Hierarchy
1 − Global
2 − Super Region
3 − Region
4 − Country
5 − Subnational (Level 1)
Uncertainty Interval
5 − Subnational (Level 1)

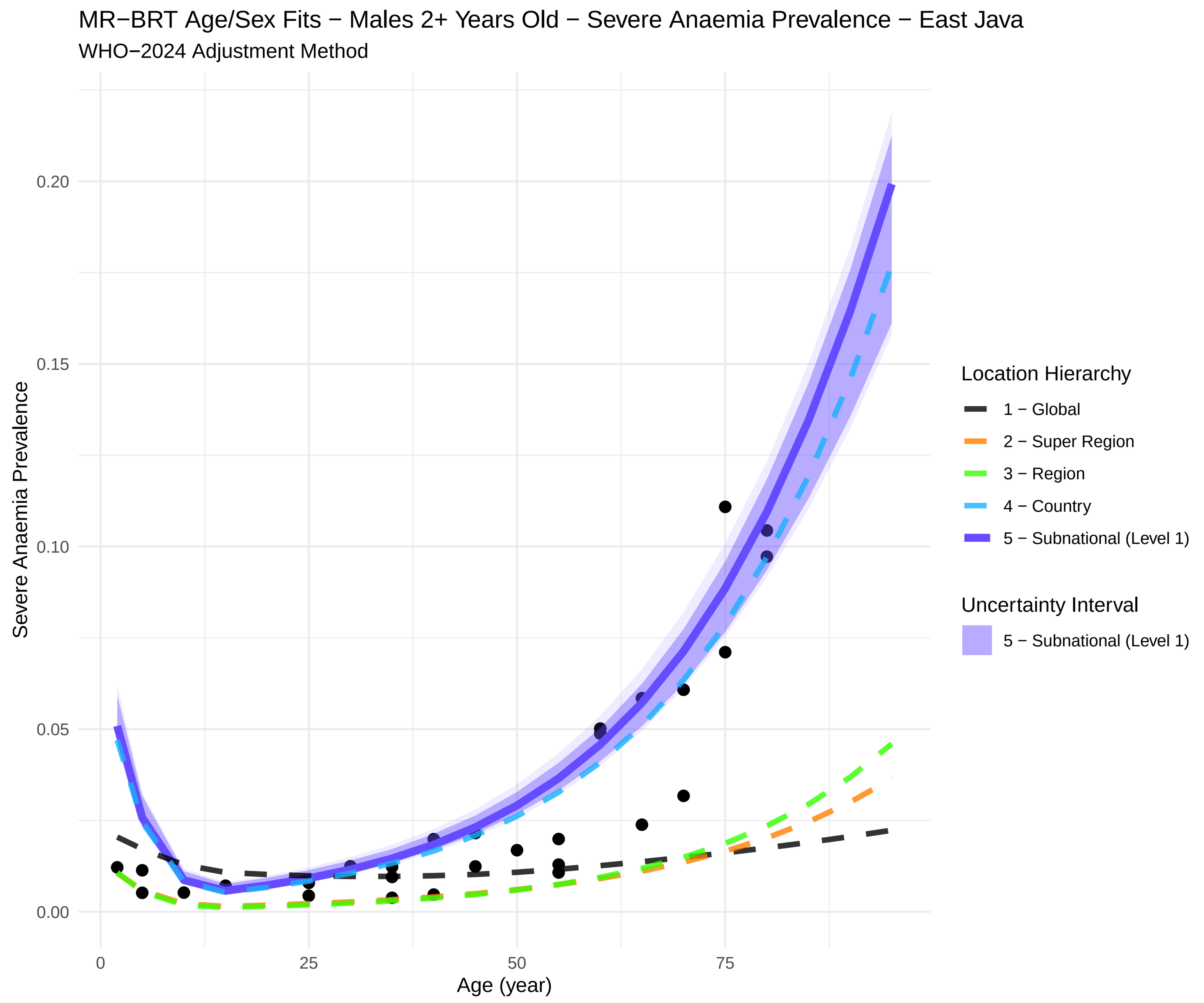
MR−BRT Age/Sex Fits − Males 2+ Years Old − Severe Anaemia Prevalence − East Java
WHO−2024 Adjustment Method
Severe Anaemia Prevalence
Age (year)
Location Hierarchy
1 − Global
2 − Super Region
3 − Region
4 − Country
5 − Subnational (Level 1)
Uncertainty Interval
5 − Subnational (Level 1)

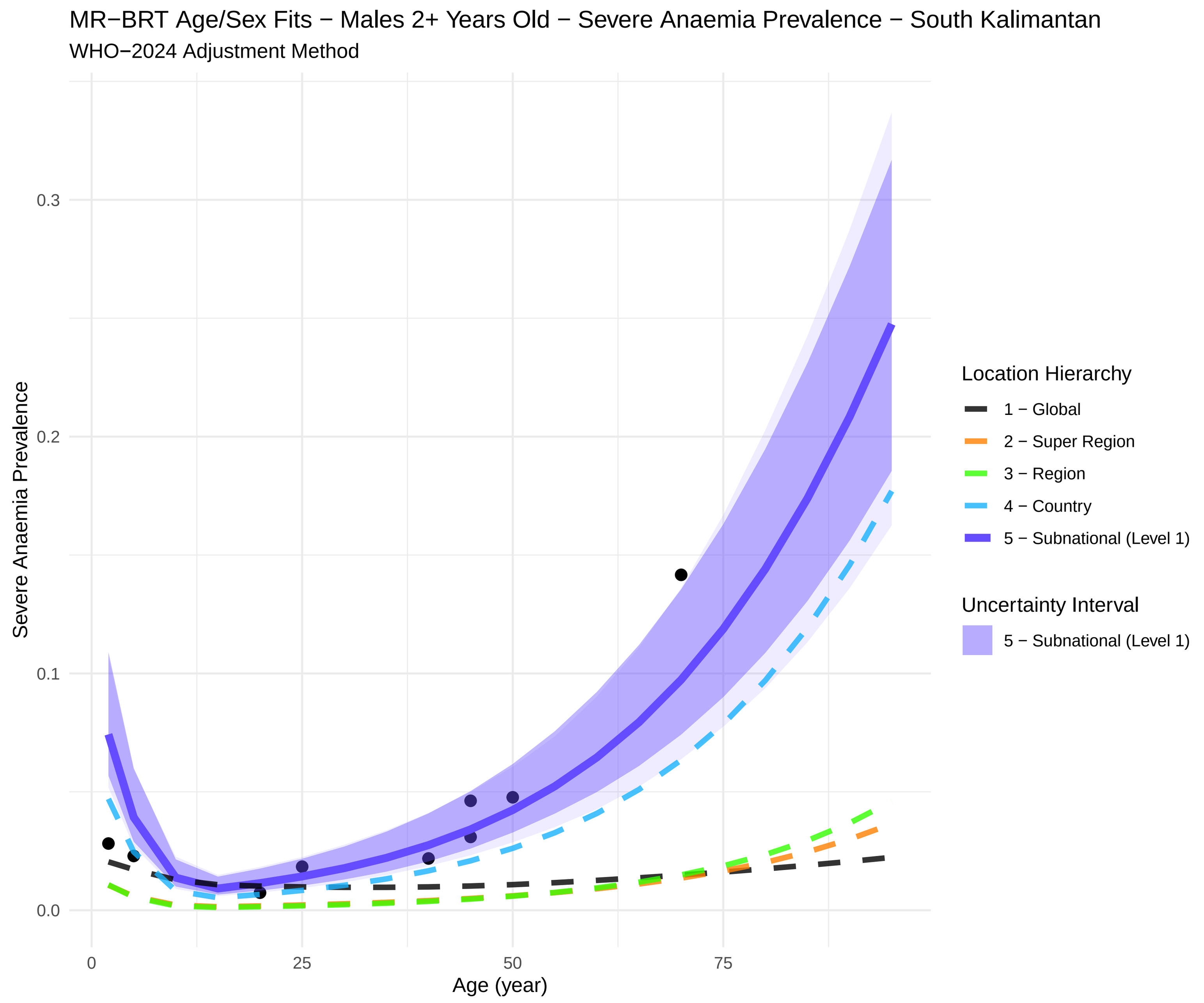
MR−BRT Age/Sex Fits − Males 2+ Years Old − Severe Anaemia Prevalence − South Kalimantan
WHO−2024 Adjustment Method
Severe Anaemia Prevalence
Age (year)
Location Hierarchy
1 − Global
2 − Super Region
3 − Region
4 − Country
5 − Subnational (Level 1)
Uncertainty Interval
5 − Subnational (Level 1)

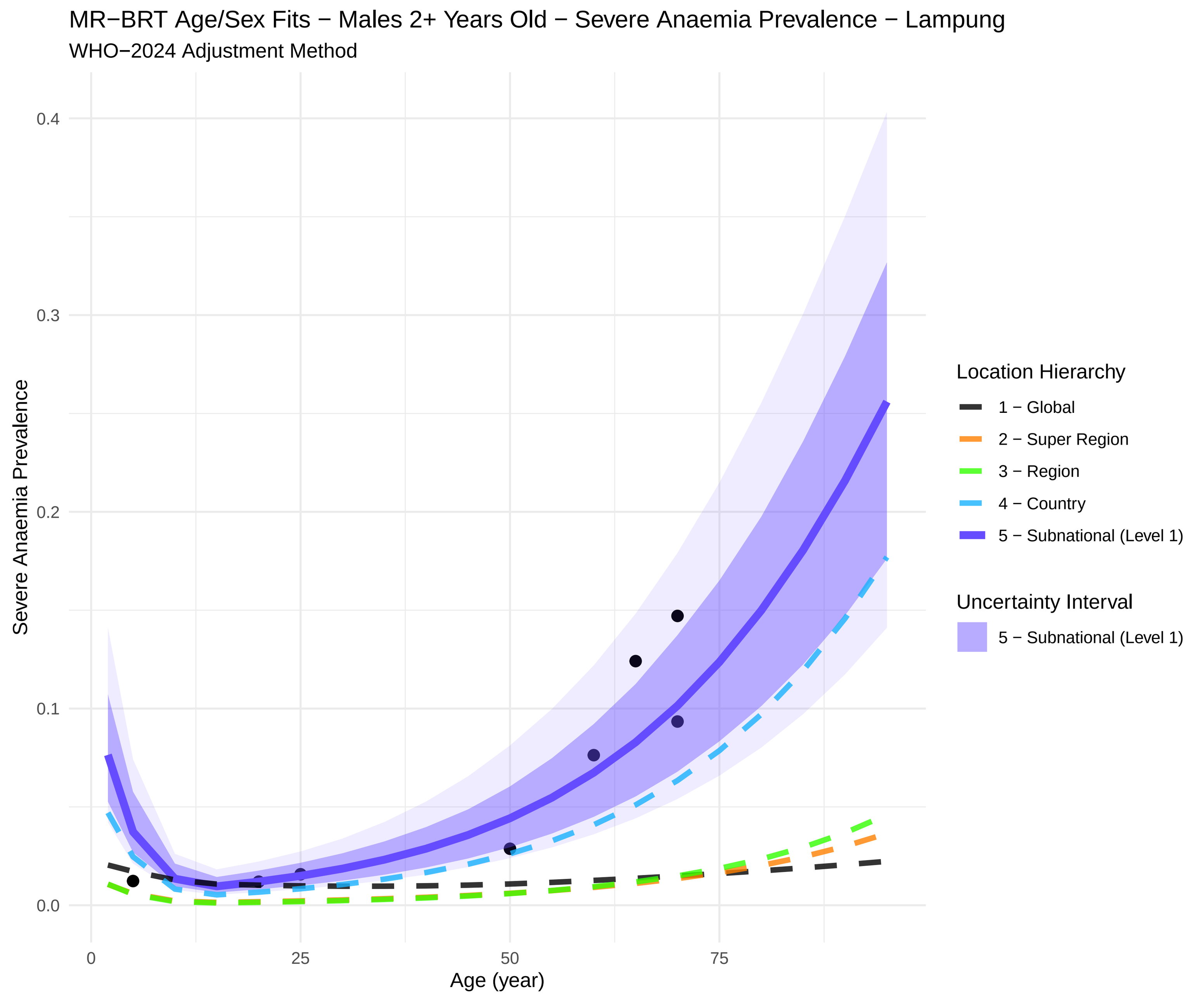
MR−BRT Age/Sex Fits − Males 2+ Years Old − Severe Anaemia Prevalence − Lampung
WHO−2024 Adjustment Method
Severe Anaemia Prevalence
Age (year)
Location Hierarchy
1 − Global
2 − Super Region
3 − Region
4 − Country
5 − Subnational (Level 1)
Uncertainty Interval
5 − Subnational (Level 1)

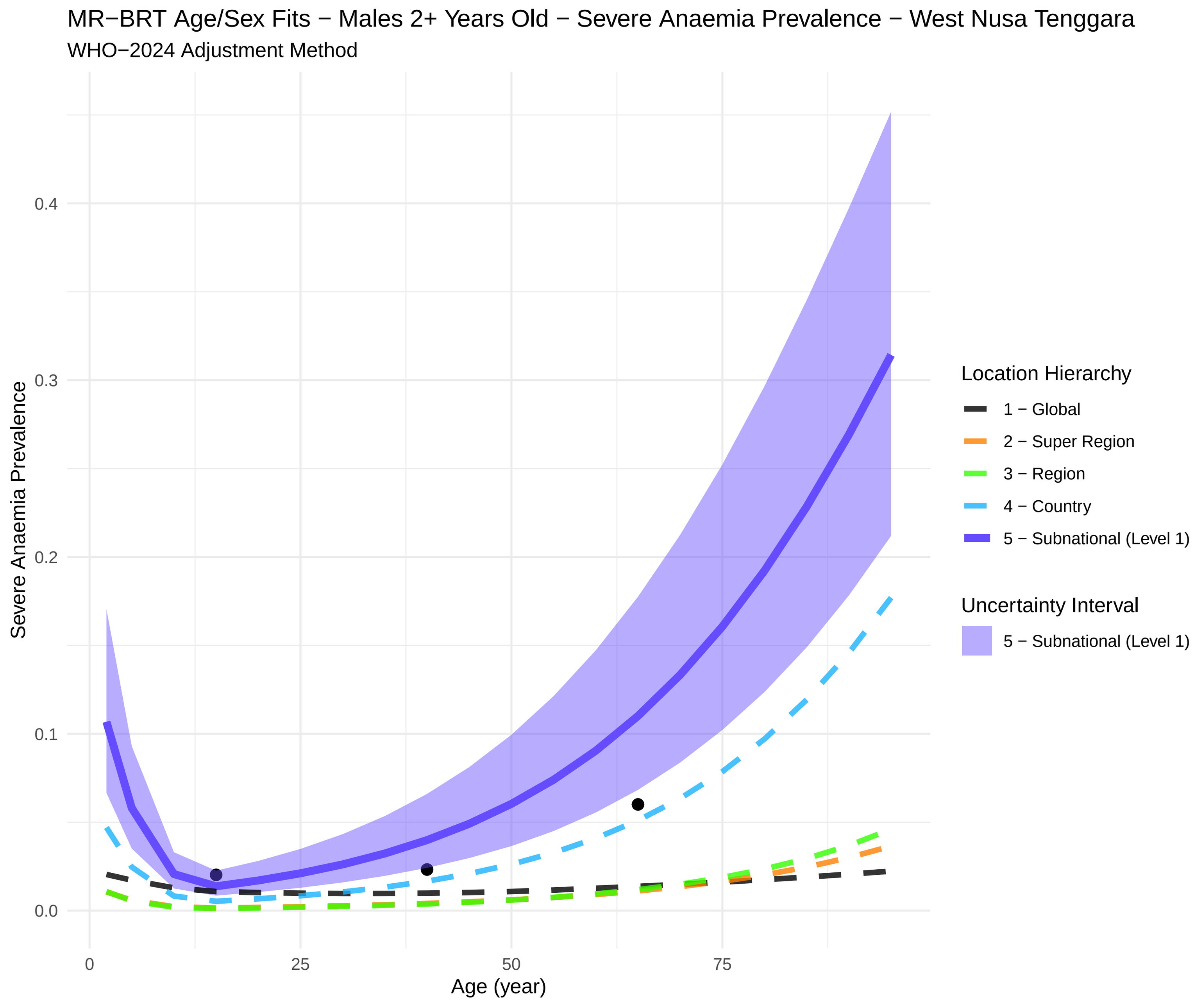
MR−BRT Age/Sex Fits − Males 2+ Years Old − Severe Anaemia Prevalence − West Nusa Tenggara
WHO−2024 Adjustment Method
Severe Anaemia Prevalence
Age (year)
Location Hierarchy
1 − Global
2 − Super Region
3 − Region
4 − Country
5 − Subnational (Level 1)
Uncertainty Interval
5 − Subnational (Level 1)

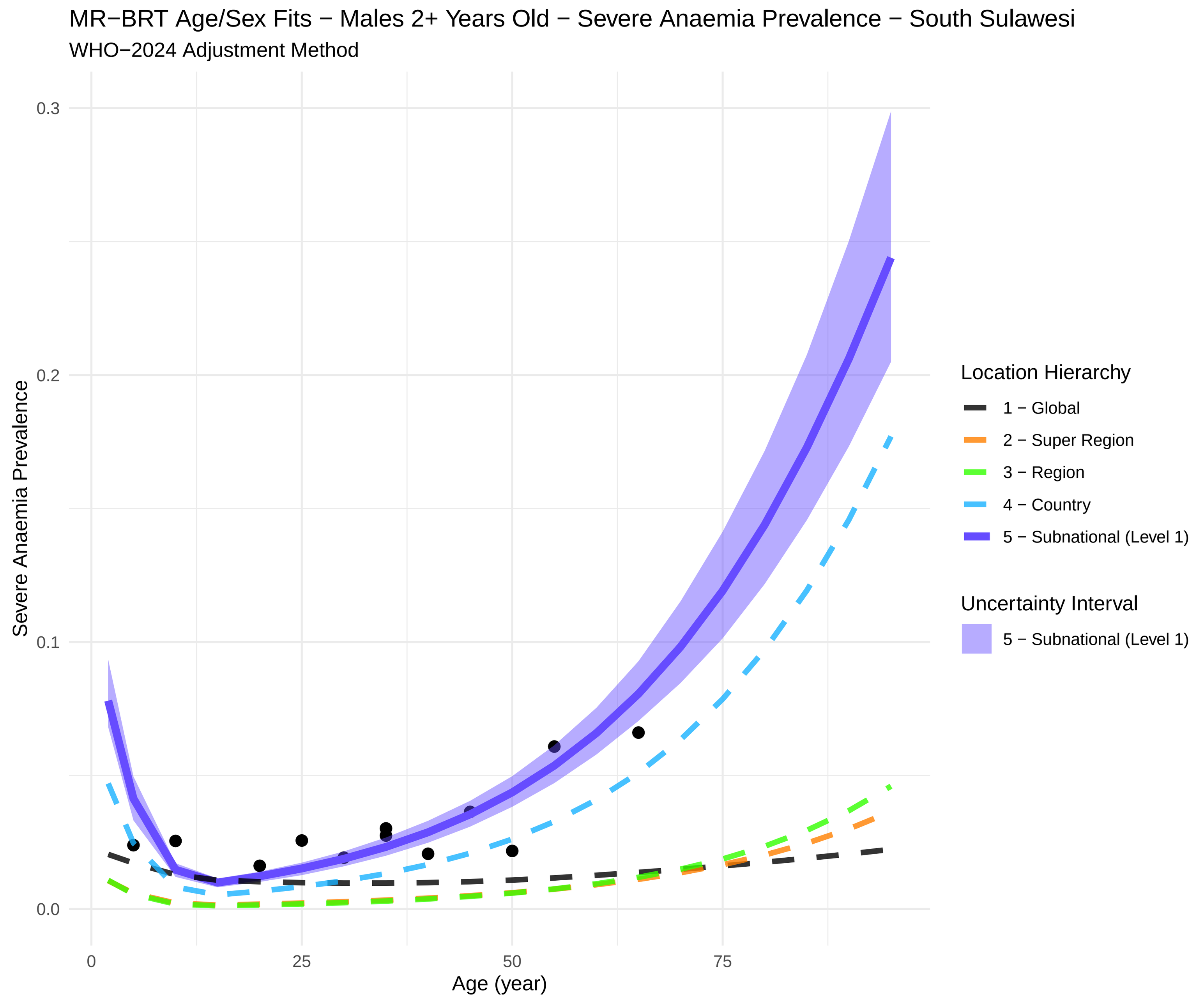
MR−BRT Age/Sex Fits − Males 2+ Years Old − Severe Anaemia Prevalence − South Sulawesi
WHO−2024 Adjustment Method
Severe Anaemia Prevalence
Age (year)
Location Hierarchy
1 − Global
2 − Super Region
3 − Region
4 − Country
5 − Subnational (Level 1)
Uncertainty Interval
5 − Subnational (Level 1)

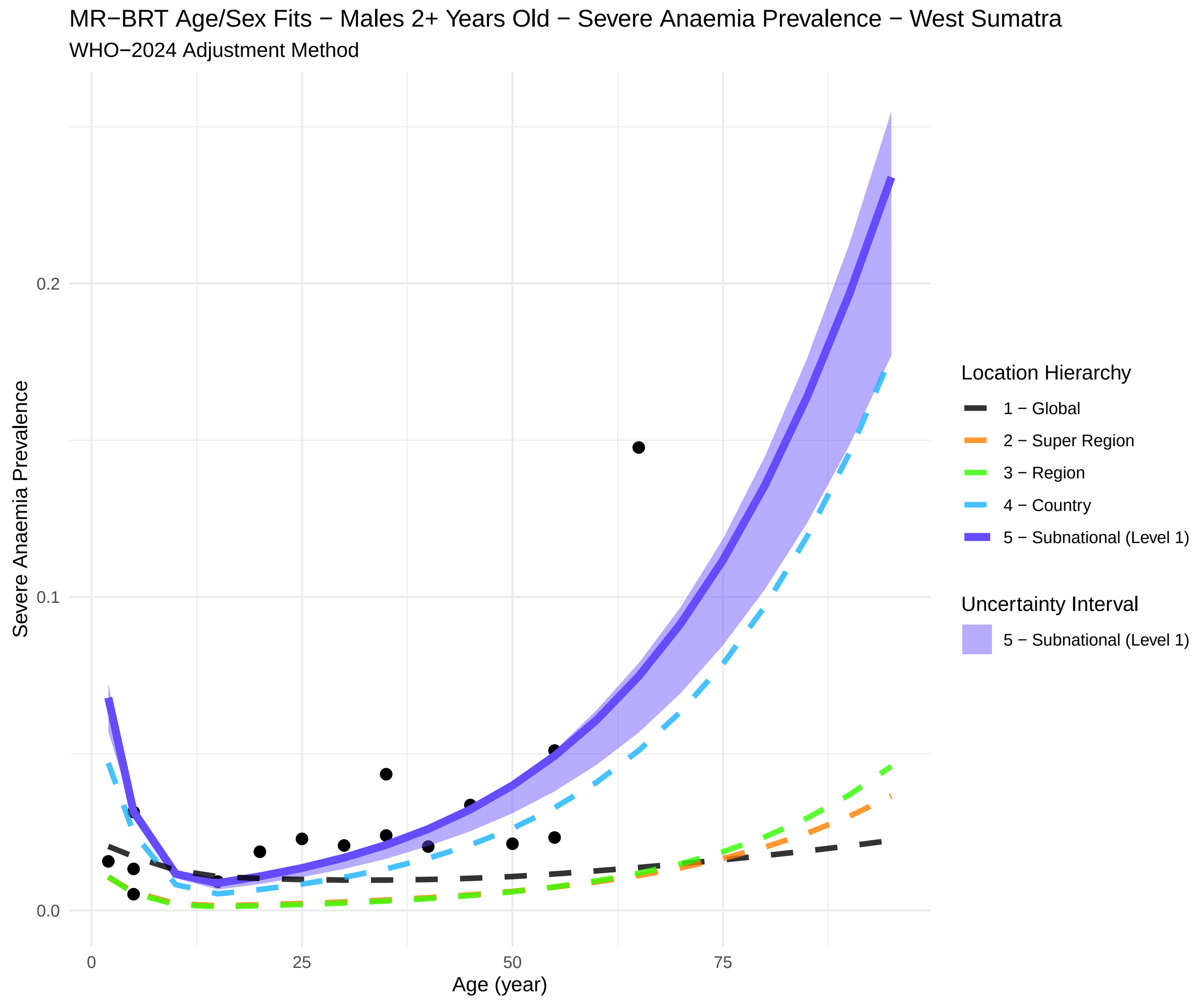
MR−BRT Age/Sex Fits − Males 2+ Years Old − Severe Anaemia Prevalence − West Sumatra
WHO−2024 Adjustment Method
Severe Anaemia Prevalence
Age (year)
Location Hierarchy
1 − Global
2 − Super Region
3 − Region
4 − Country
5 − Subnational (Level 1)
Uncertainty Interval
5 − Subnational (Level 1)

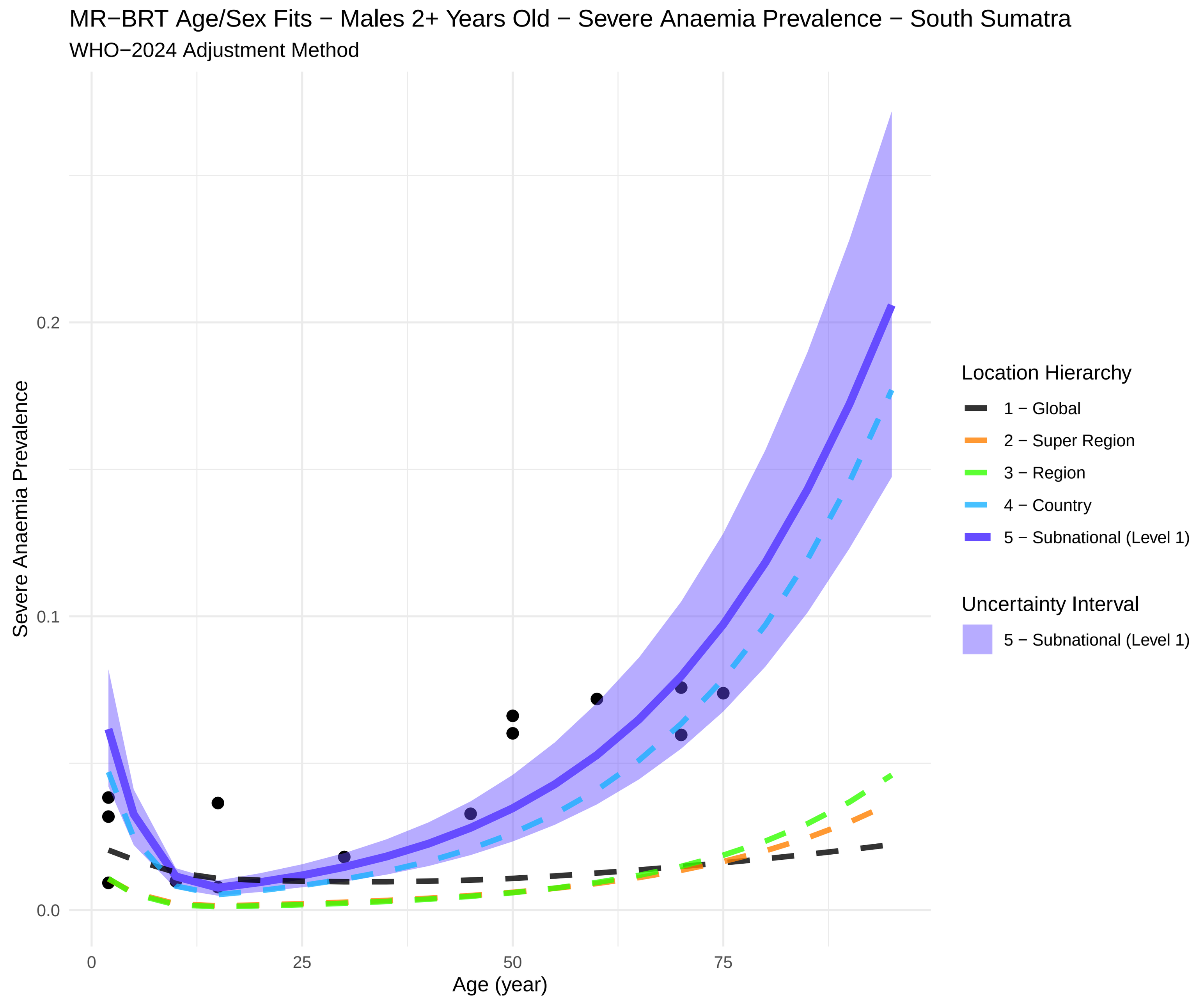
MR−BRT Age/Sex Fits − Males 2+ Years Old − Severe Anaemia Prevalence − South Sumatra
WHO−2024 Adjustment Method
Severe Anaemia Prevalence
Age (year)
Location Hierarchy
1 − Global
2 − Super Region
3 − Region
4 − Country
5 − Subnational (Level 1)
Uncertainty Interval
5 − Subnational (Level 1)

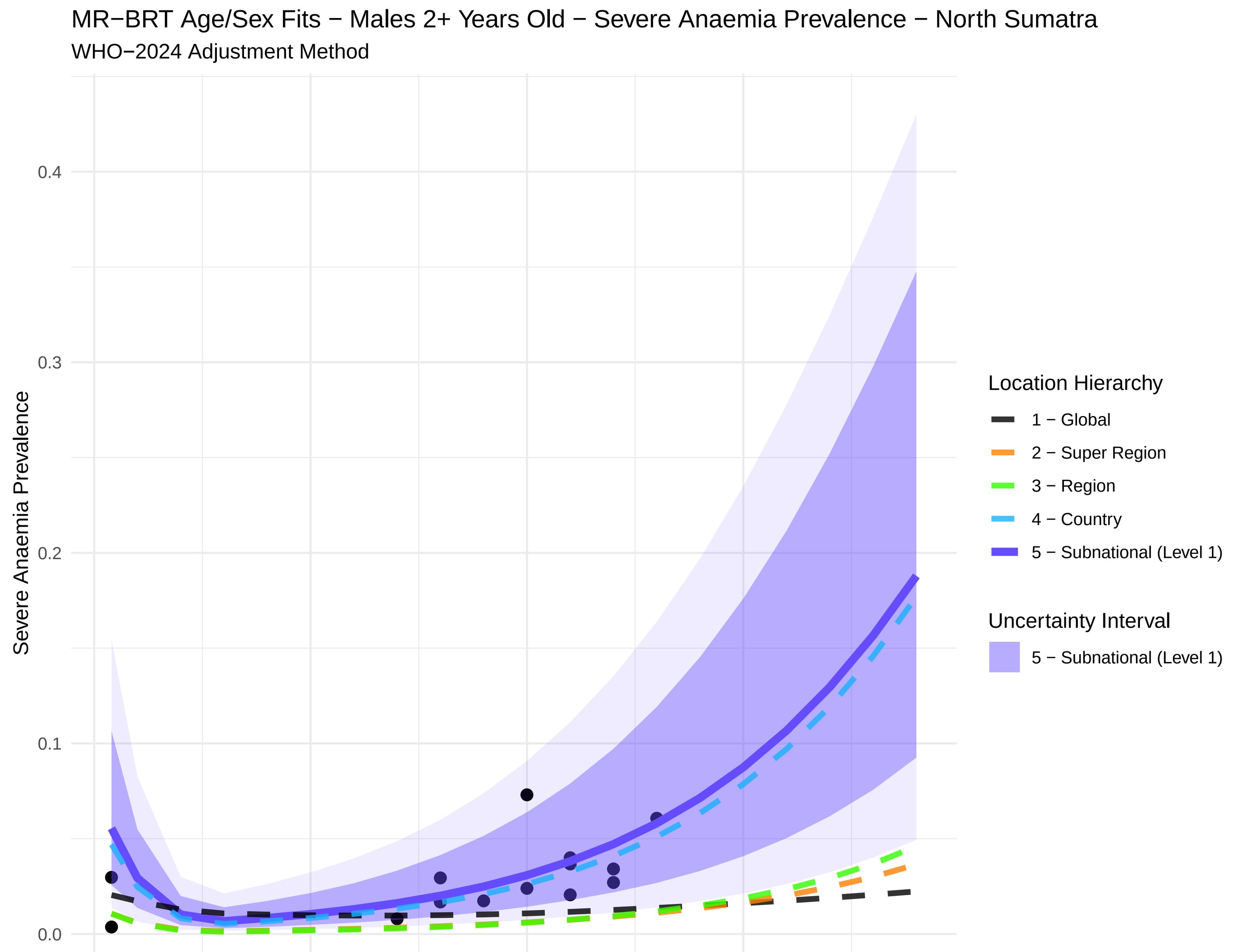
MR−BRT Age/Sex Fits − Males 2+ Years Old − Severe Anaemia Prevalence − North Sumatra
WHO−2024 Adjustment Method
Severe Anaemia Prevalence
Age (year)
Location Hierarchy
1 − Global
2 − Super Region
3 − Region
4 − Country
5 − Subnational (Level 1)
Uncertainty Interval
5 − Subnational (Level 1)

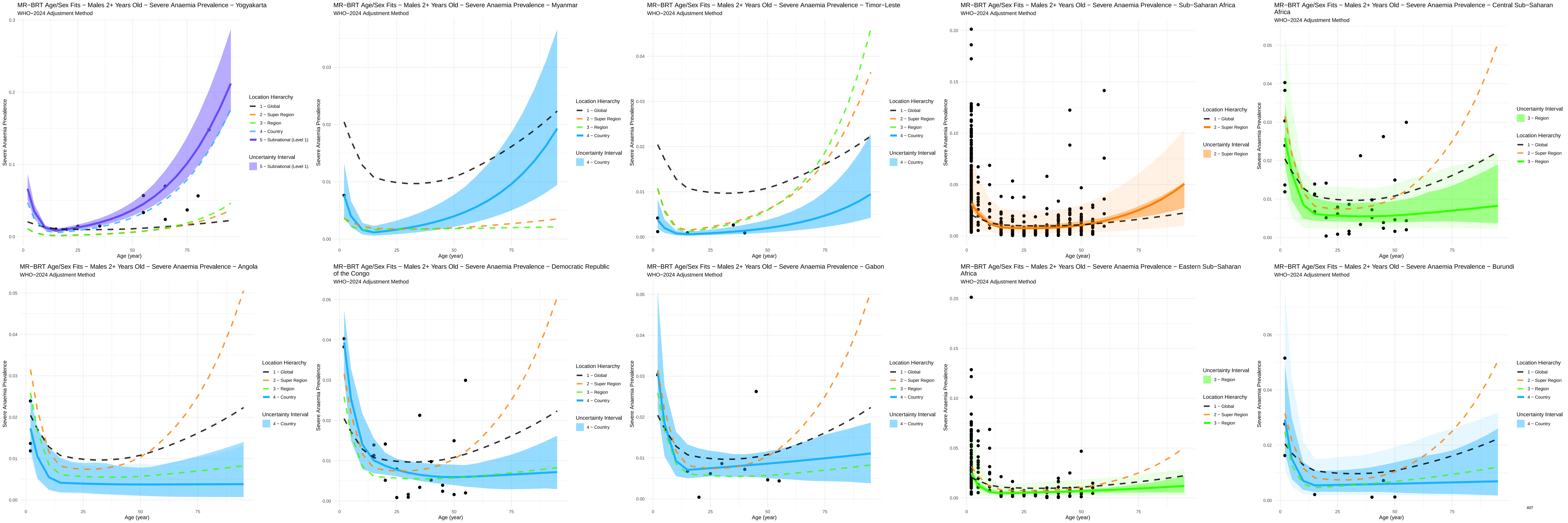
MR−BRT Age/Sex Fits − Males 2+ Years Old − Severe Anaemia Prevalence − Yogyakarta
WHO−2024 Adjustment Method
Location Hierarchy
1 − Global
2 − Super Region
3 − Region
4 − Country
5 − Subnational (Level 1)
Uncertainty Interval
5 − Subnational (Level 1)
MR−BRT Age/Sex Fits − Males 2+ Years Old − Severe Anaemia Prevalence − Myanmar
WHO−2024 Adjustment Method
Location Hierarchy
1 − Global
2 − Super Region
3 − Region
4 − Country
Uncertainty Interval
4 − Country
MR−BRT Age/Sex Fits − Males 2+ Years Old − Severe Anaemia Prevalence − Timor-Leste
WHO−2024 Adjustment Method
MR−BRT Age/Sex Fits − Males 2+ Years Old − Severe Anaemia Prevalence − Sub-Saharan Africa
WHO−2024 Adjustment Method
Location Hierarchy
1 − Global
2 − Super Region
Uncertainty Interval
2 − Super Region
MR−BRT Age/Sex Fits − Males 2+ Years Old − Severe Anaemia Prevalence − Central Sub-Saharan Africa
WHO−2024 Adjustment Method
Uncertainty Interval
3 − Region
Location Hierarchy
1 − Global
2 − Super Region
3 − Region
MR−BRT Age/Sex Fits − Males 2+ Years Old − Severe Anaemia Prevalence − Angola
WHO−2024 Adjustment Method
MR−BRT Age/Sex Fits − Males 2+ Years Old − Severe Anaemia Prevalence − Democratic Republic of the Congo
WHO−2024 Adjustment Method
MR−BRT Age/Sex Fits − Males 2+ Years Old − Severe Anaemia Prevalence − Gabon
WHO−2024 Adjustment Method
MR−BRT Age/Sex Fits − Males 2+ Years Old − Severe Anaemia Prevalence − Eastern Sub-Saharan Africa
WHO−2024 Adjustment Method
MR−BRT Age/Sex Fits − Males 2+ Years Old − Severe Anaemia Prevalence − Burundi
WHO−2024 Adjustment Method
Severe Anaemia Prevalence
Age (year)

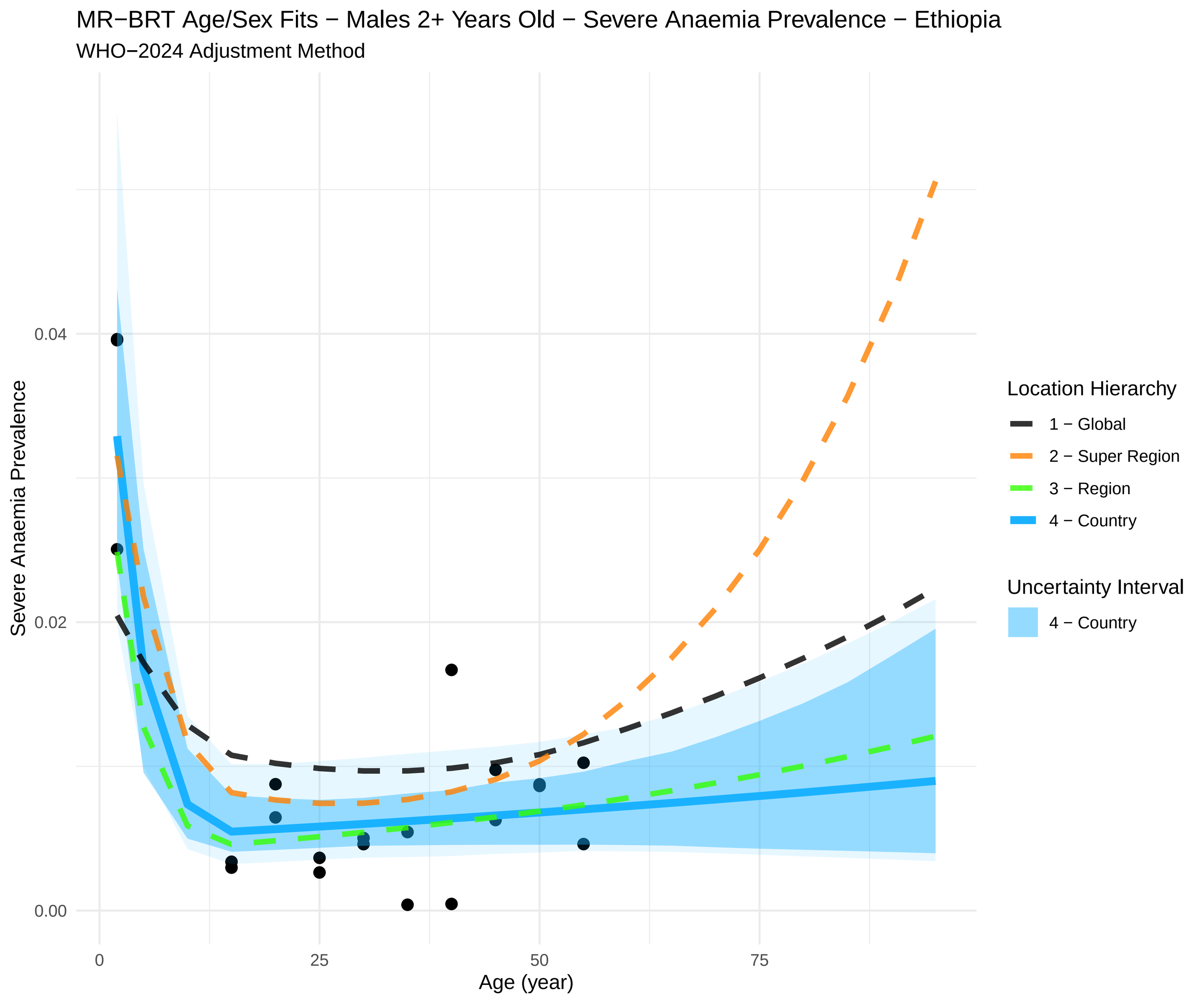
MR−BRT Age/Sex Fits − Males 2+ Years Old − Severe Anaemia Prevalence − Ethiopia
WHO−2024 Adjustment Method
Severe Anaemia Prevalence
Age (year)
Location Hierarchy
1 − Global
2 − Super Region
3 − Region
4 − Country
Uncertainty Interval
4 − Country

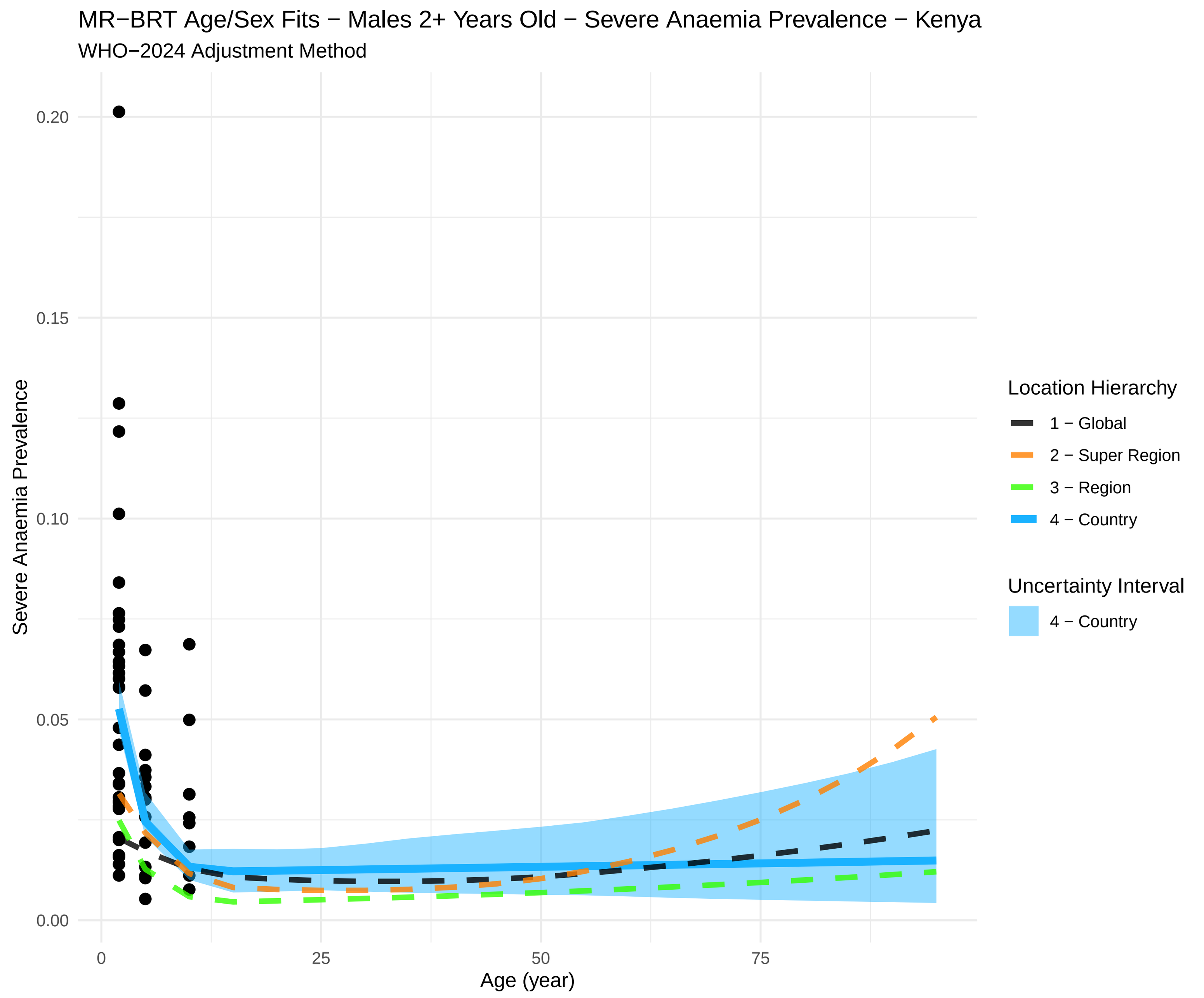
MR−BRT Age/Sex Fits − Males 2+ Years Old − Severe Anaemia Prevalence − Kenya
WHO−2024 Adjustment Method
Severe Anaemia Prevalence
Age (year)
Location Hierarchy
1 − Global
2 − Super Region
3 − Region
4 − Country
Uncertainty Interval
4 − Country

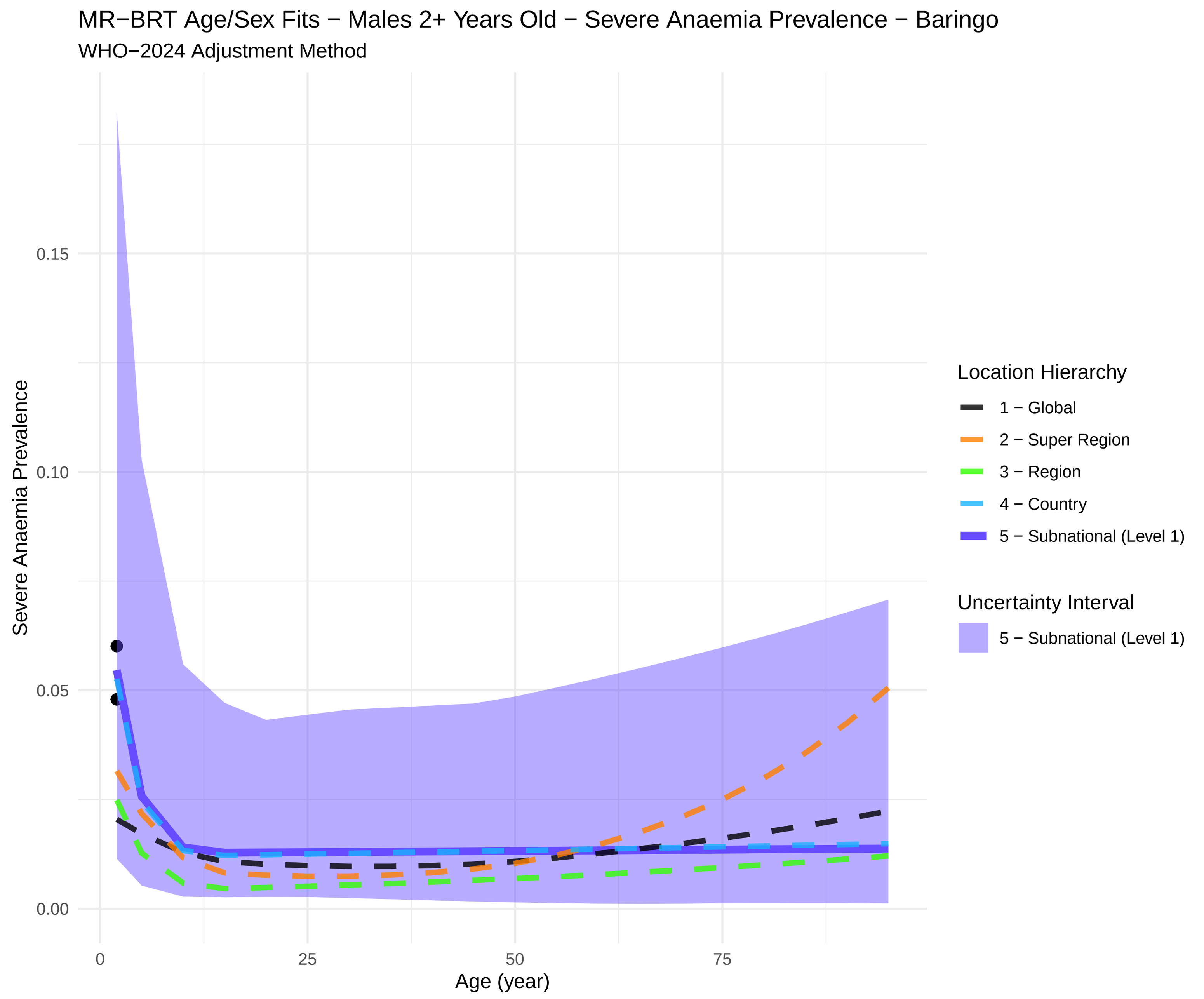
MR−BRT Age/Sex Fits − Males 2+ Years Old − Severe Anaemia Prevalence − Baringo
WHO−2024 Adjustment Method
Severe Anaemia Prevalence
Age (year)
Location Hierarchy
1 − Global
2 − Super Region
3 − Region
4 − Country
5 − Subnational (Level 1)
Uncertainty Interval
5 − Subnational (Level 1)

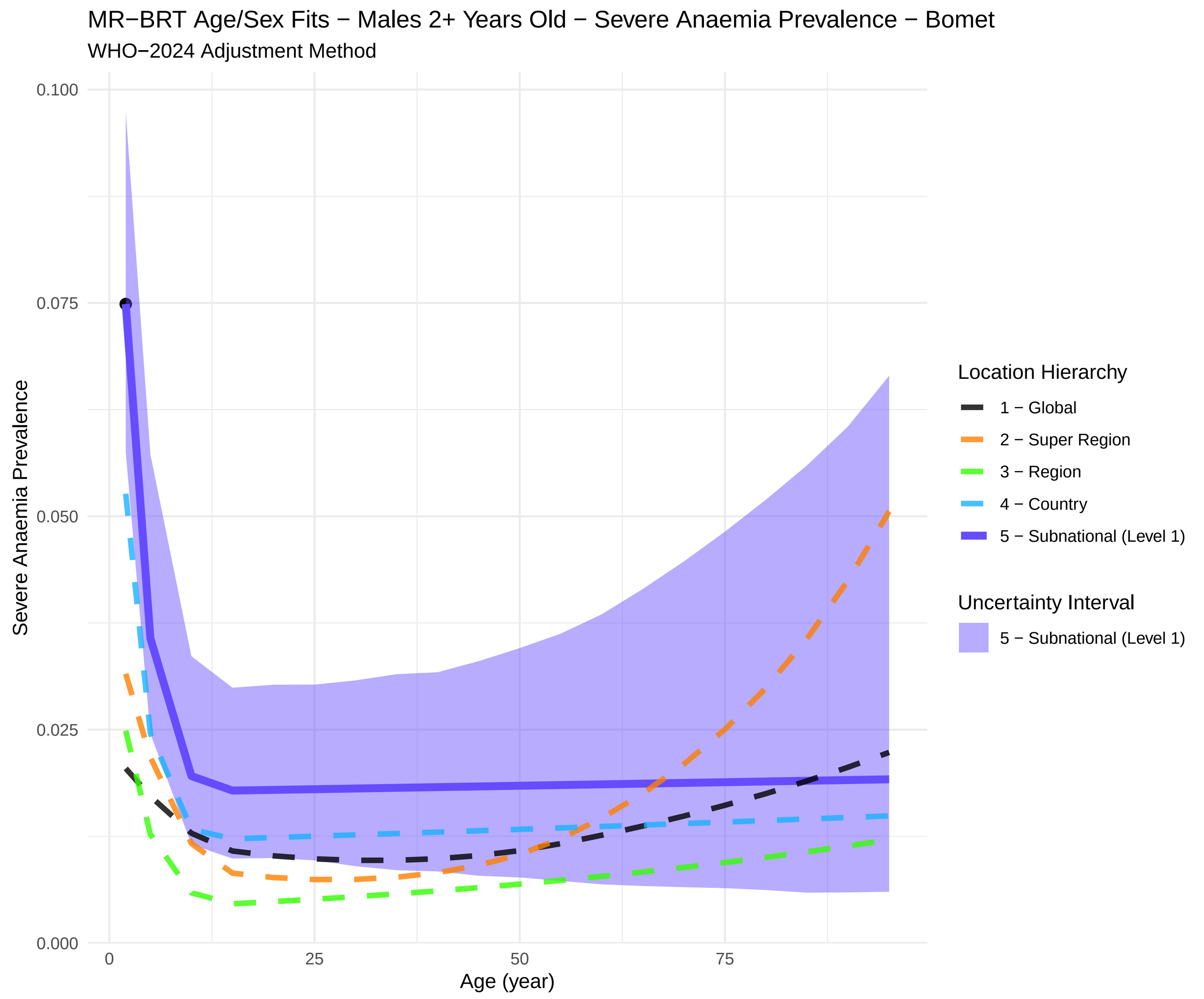
MR−BRT Age/Sex Fits − Males 2+ Years Old − Severe Anaemia Prevalence − Bomet
WHO−2024 Adjustment Method
Severe Anaemia Prevalence
Age (year)
Location Hierarchy
1 − Global
2 − Super Region
3 − Region
4 − Country
5 − Subnational (Level 1)
Uncertainty Interval
5 − Subnational (Level 1)

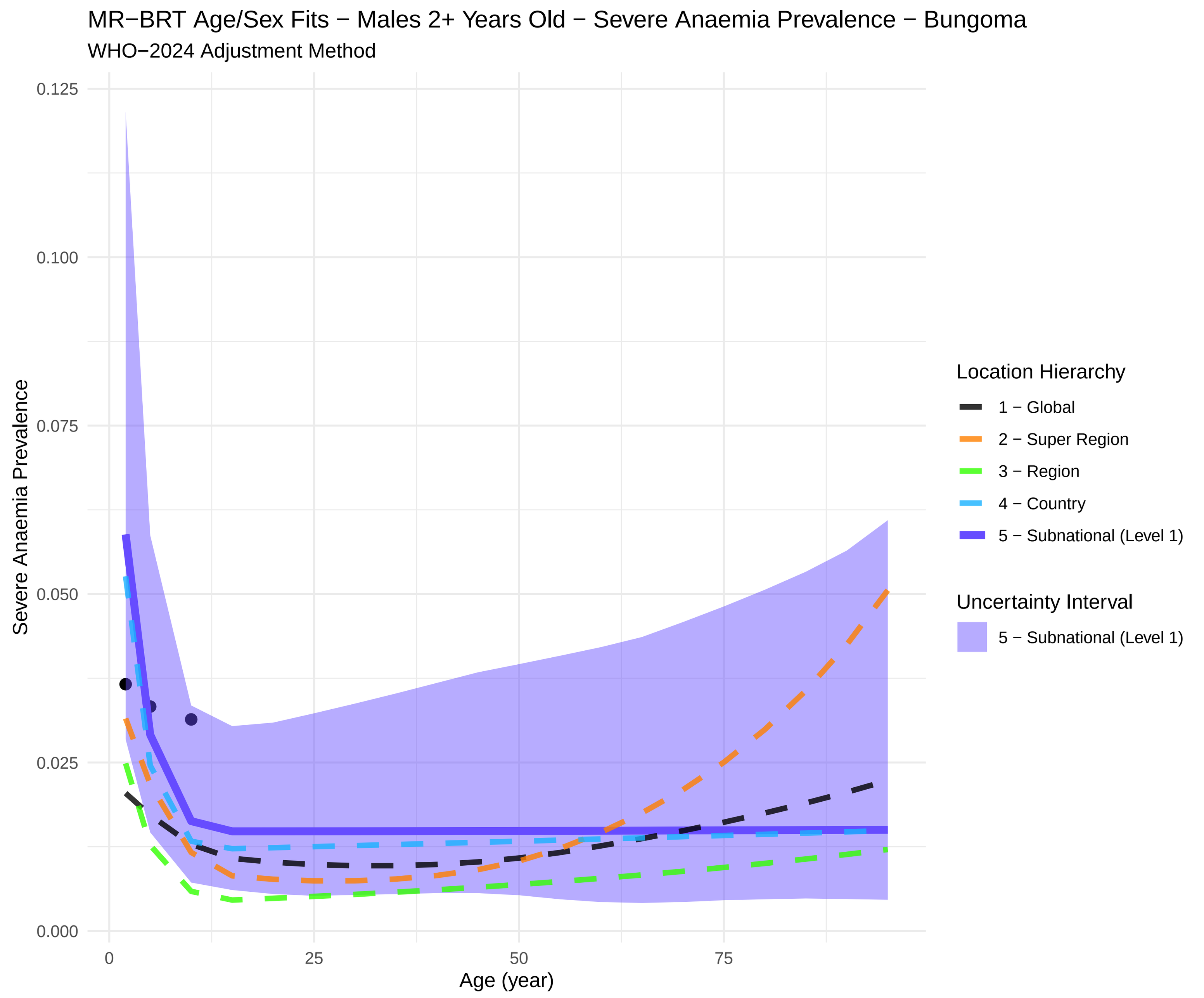
MR−BRT Age/Sex Fits − Males 2+ Years Old − Severe Anaemia Prevalence − Bungoma
WHO−2024 Adjustment Method
Severe Anaemia Prevalence
Age (year)
Location Hierarchy
1 − Global
2 − Super Region
3 − Region
4 − Country
5 − Subnational (Level 1)
Uncertainty Interval
5 − Subnational (Level 1)

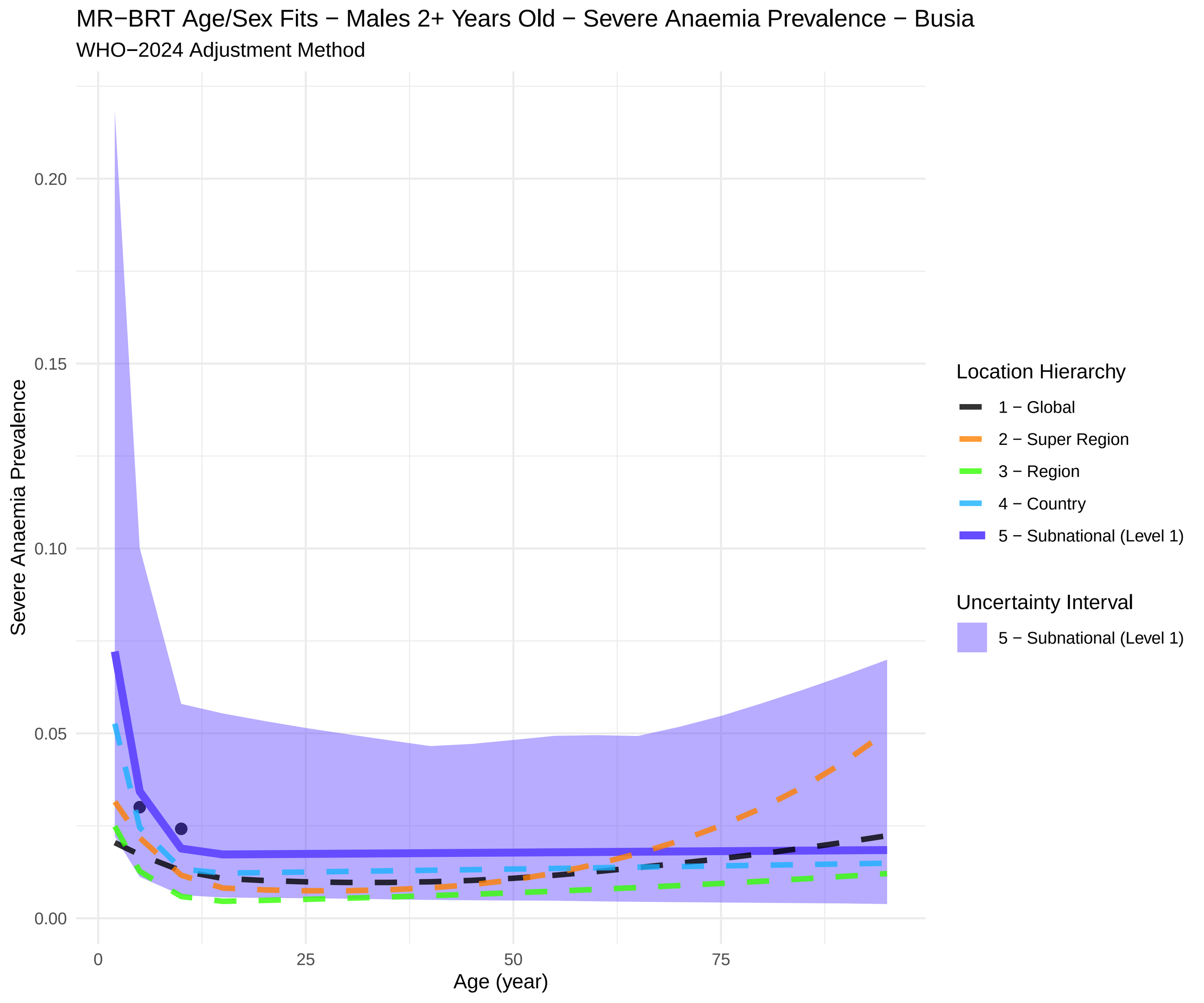
MR−BRT Age/Sex Fits − Males 2+ Years Old − Severe Anaemia Prevalence − Busia
WHO−2024 Adjustment Method
Severe Anaemia Prevalence
Age (year)
Location Hierarchy
1 − Global
2 − Super Region
3 − Region
4 − Country
5 − Subnational (Level 1)
Uncertainty Interval
5 − Subnational (Level 1)

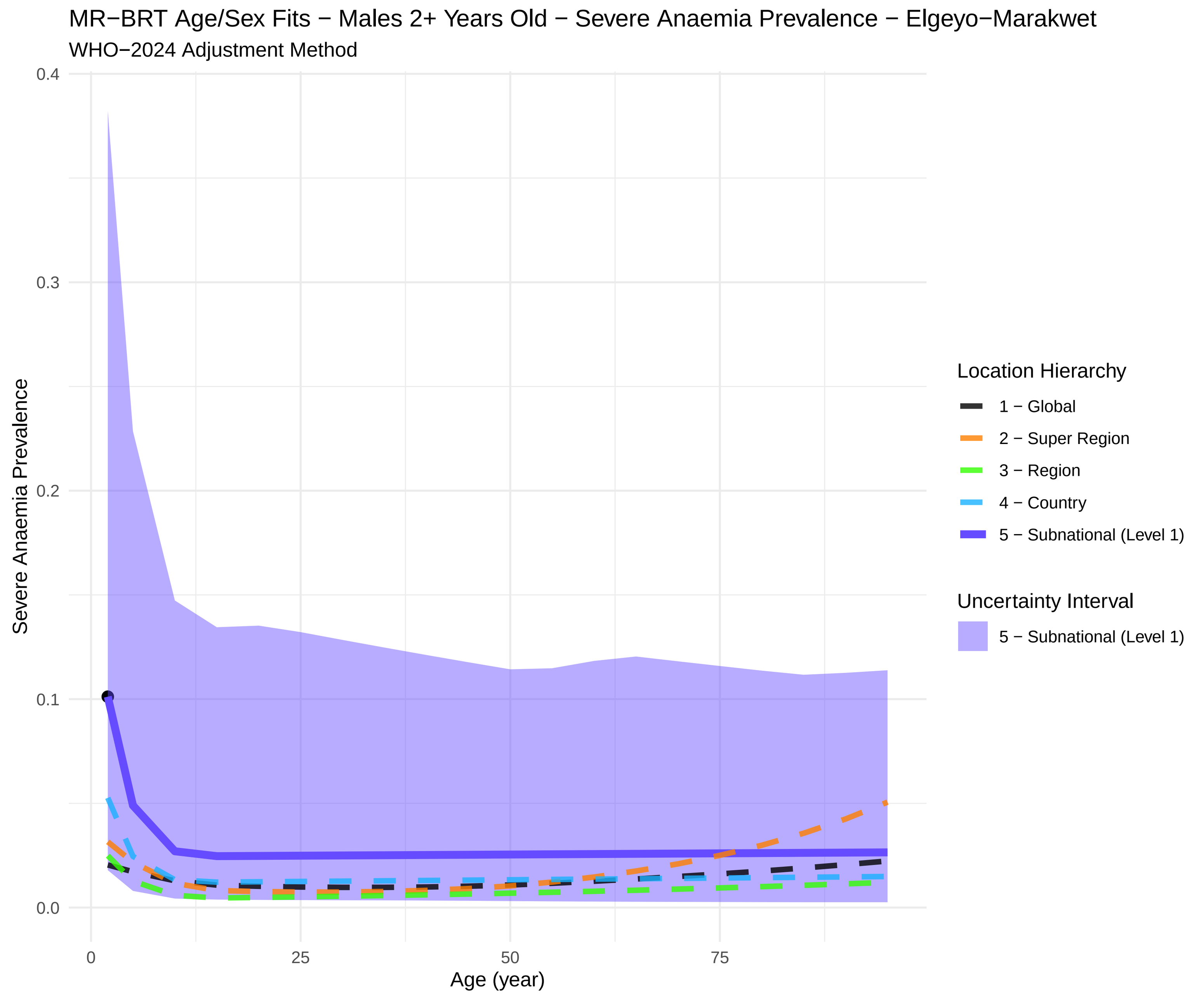
MR−BRT Age/Sex Fits − Males 2+ Years Old − Severe Anaemia Prevalence − Elgeyo−Marakwet
WHO−2024 Adjustment Method
Severe Anaemia Prevalence
Age (year)
Location Hierarchy
1 − Global
2 − Super Region
3 − Region
4 − Country
5 − Subnational (Level 1)
Uncertainty Interval
5 − Subnational (Level 1)

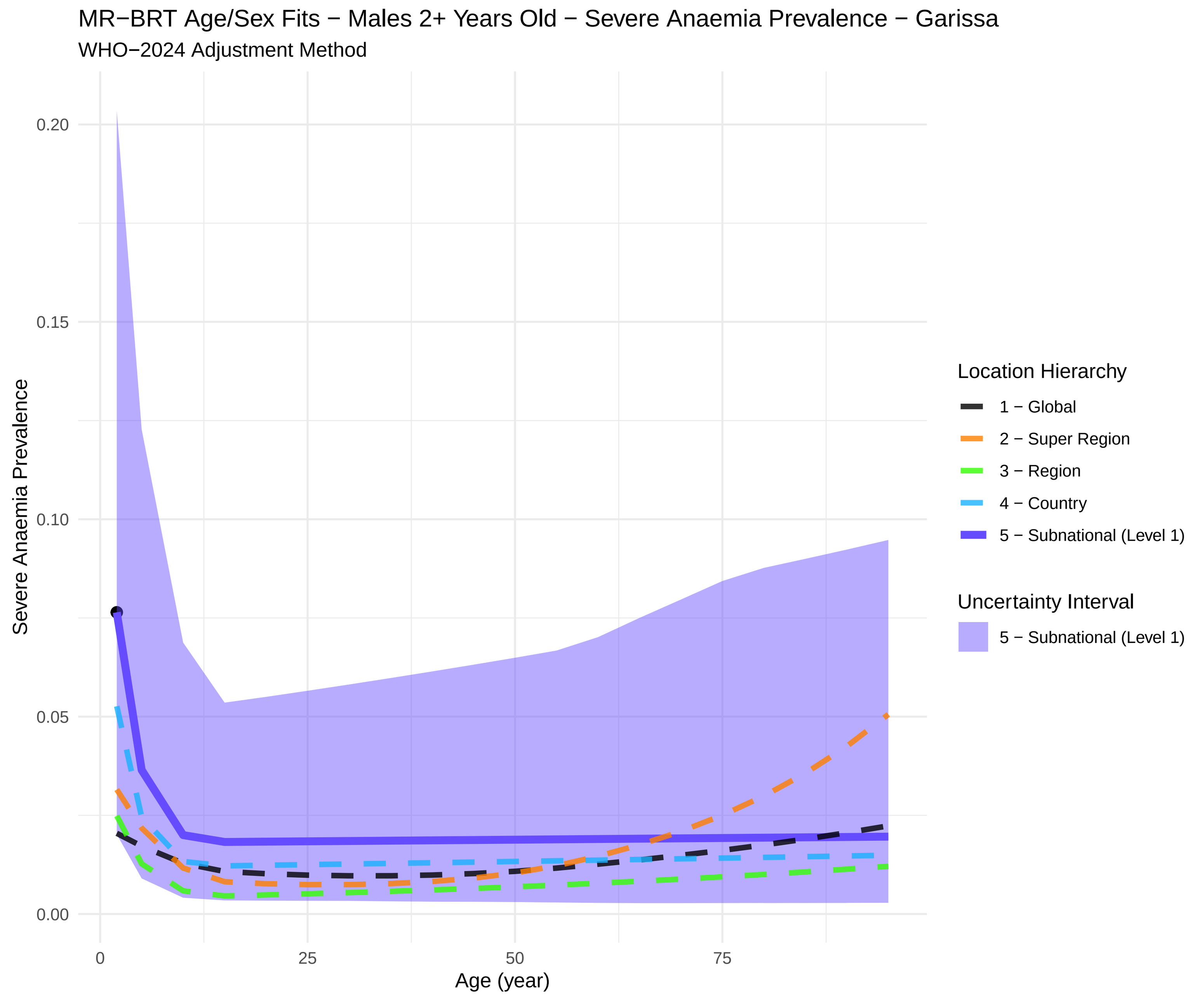
MR−BRT Age/Sex Fits − Males 2+ Years Old − Severe Anaemia Prevalence − Garissa
WHO−2024 Adjustment Method
Severe Anaemia Prevalence
Age (year)
Location Hierarchy
1 − Global
2 − Super Region
3 − Region
4 − Country
5 − Subnational (Level 1)
Uncertainty Interval
5 − Subnational (Level 1)

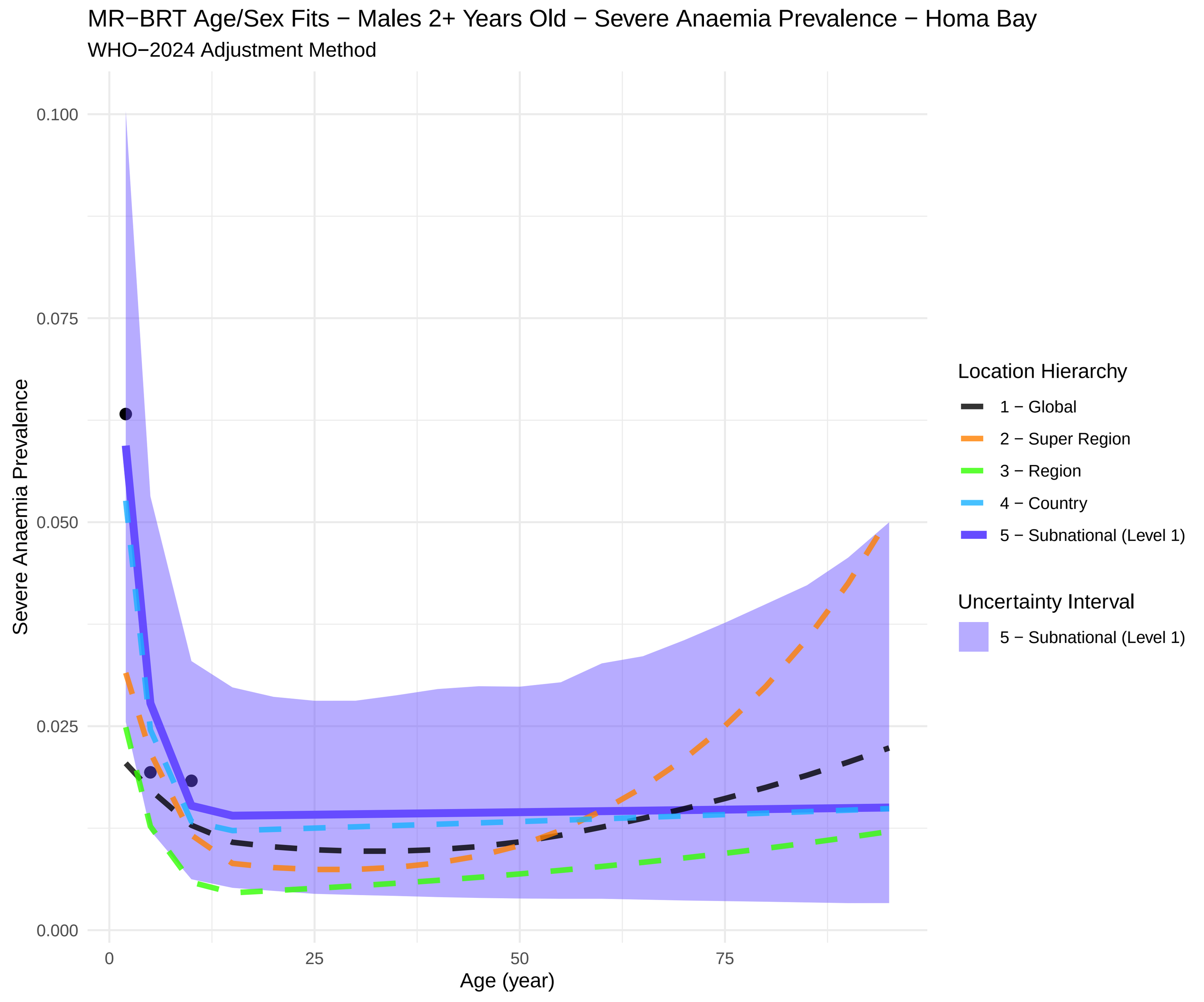
MR−BRT Age/Sex Fits − Males 2+ Years Old − Severe Anaemia Prevalence − Homa Bay
WHO−2024 Adjustment Method
Severe Anaemia Prevalence
Age (year)
Location Hierarchy
1 − Global
2 − Super Region
3 − Region
4 − Country
5 − Subnational (Level 1)
Uncertainty Interval
5 − Subnational (Level 1)

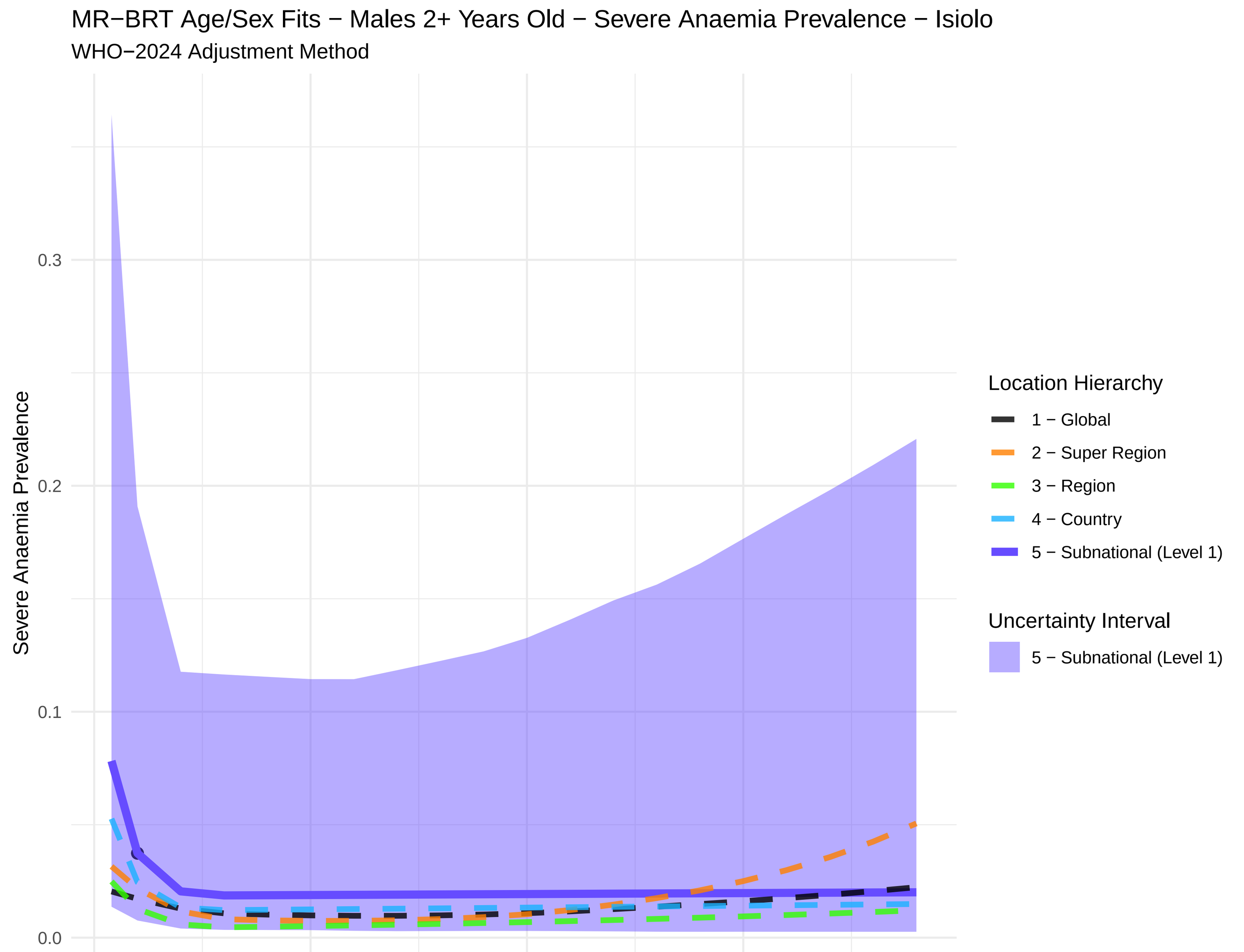
MR−BRT Age/Sex Fits − Males 2+ Years Old − Severe Anaemia Prevalence − Isiolo
WHO−2024 Adjustment Method
Severe Anaemia Prevalence
Age (year)
Location Hierarchy
1 − Global
2 − Super Region
3 − Region
4 − Country
5 − Subnational (Level 1)
Uncertainty Interval
5 − Subnational (Level 1)

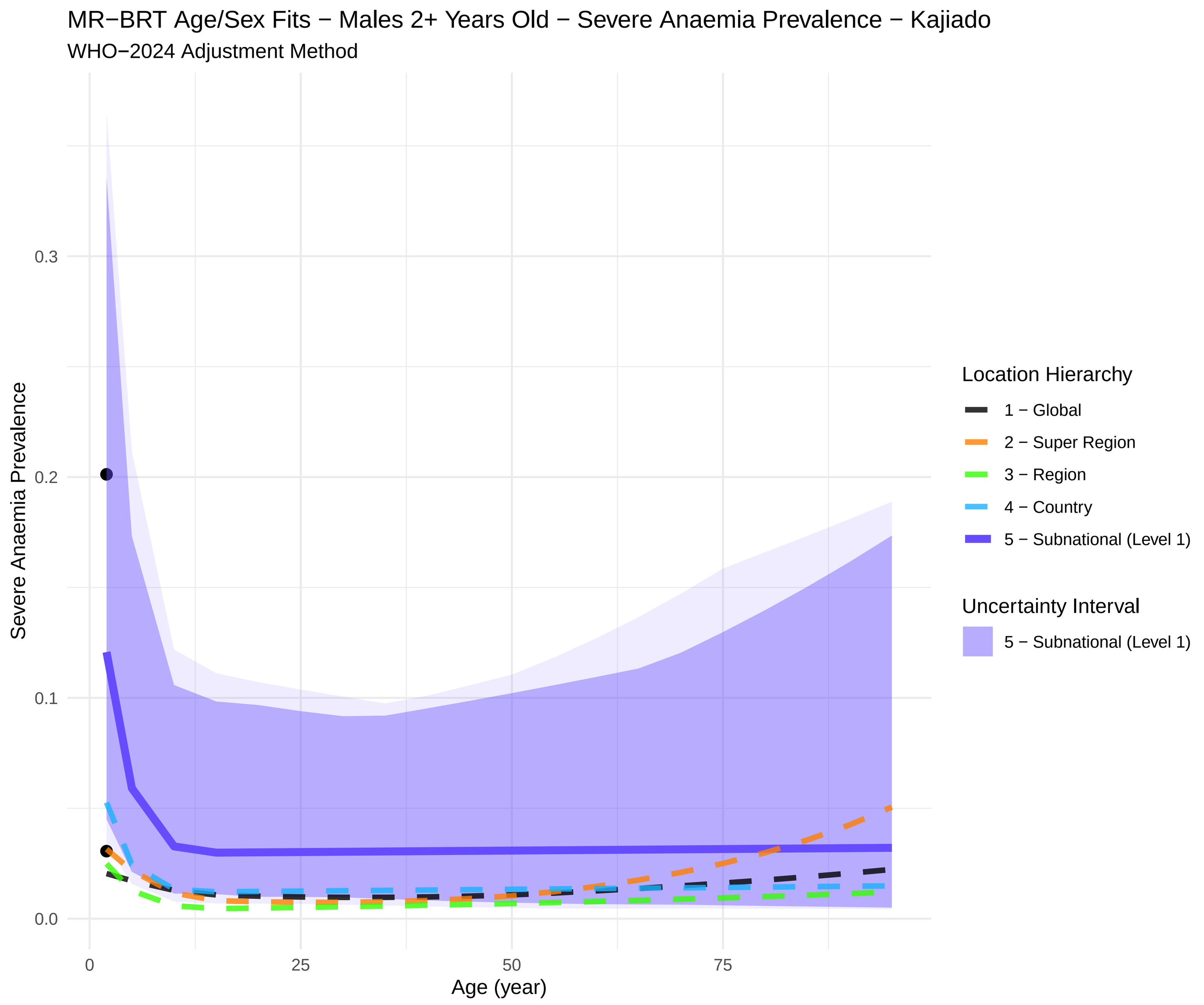
MR−BRT Age/Sex Fits − Males 2+ Years Old − Severe Anaemia Prevalence − Kajiado
WHO−2024 Adjustment Method
Severe Anaemia Prevalence
Age (year)
Location Hierarchy
1 − Global
2 − Super Region
3 − Region
4 − Country
5 − Subnational (Level 1)
Uncertainty Interval
5 − Subnational (Level 1)

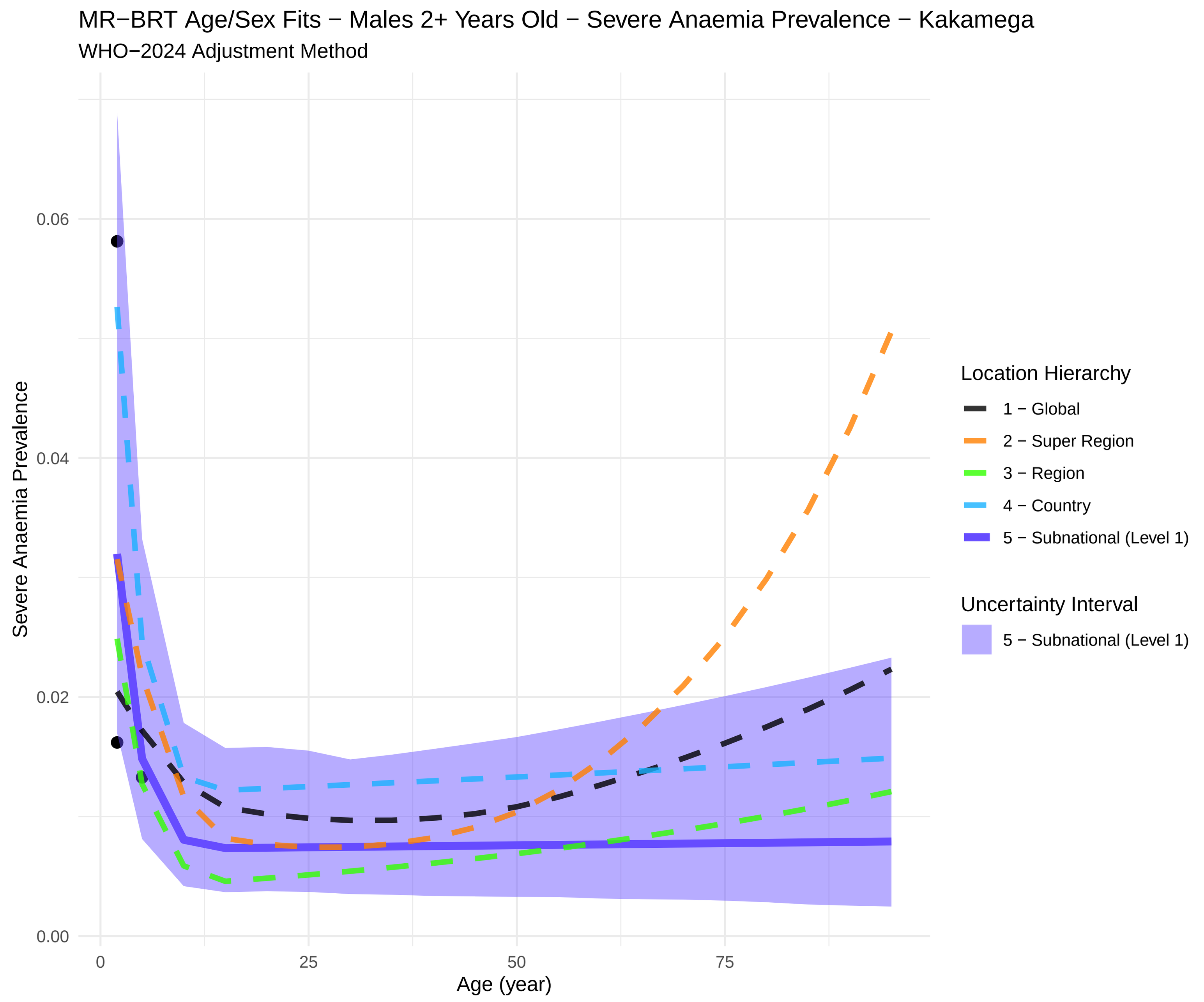
MR−BRT Age/Sex Fits − Males 2+ Years Old − Severe Anaemia Prevalence − Kakamega
WHO−2024 Adjustment Method
Severe Anaemia Prevalence
Age (year)
Location Hierarchy
1 − Global
2 − Super Region
3 − Region
4 − Country
5 − Subnational (Level 1)
Uncertainty Interval
5 − Subnational (Level 1)

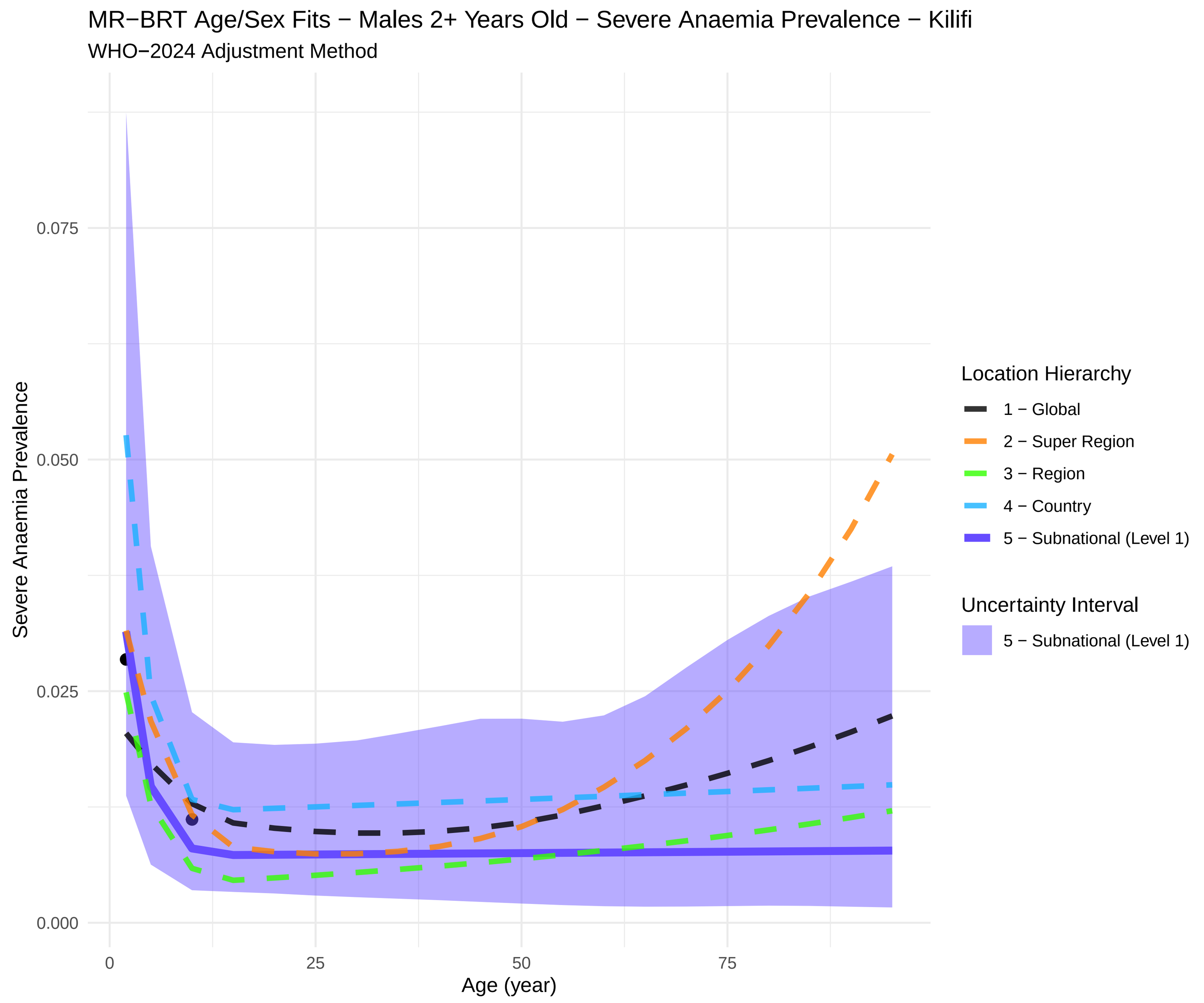
MR−BRT Age/Sex Fits − Males 2+ Years Old − Severe Anaemia Prevalence − Kilifi
WHO−2024 Adjustment Method
Severe Anaemia Prevalence
Age (year)
Location Hierarchy
1 − Global
2 − Super Region
3 − Region
4 − Country
5 − Subnational (Level 1)
Uncertainty Interval
5 − Subnational (Level 1)

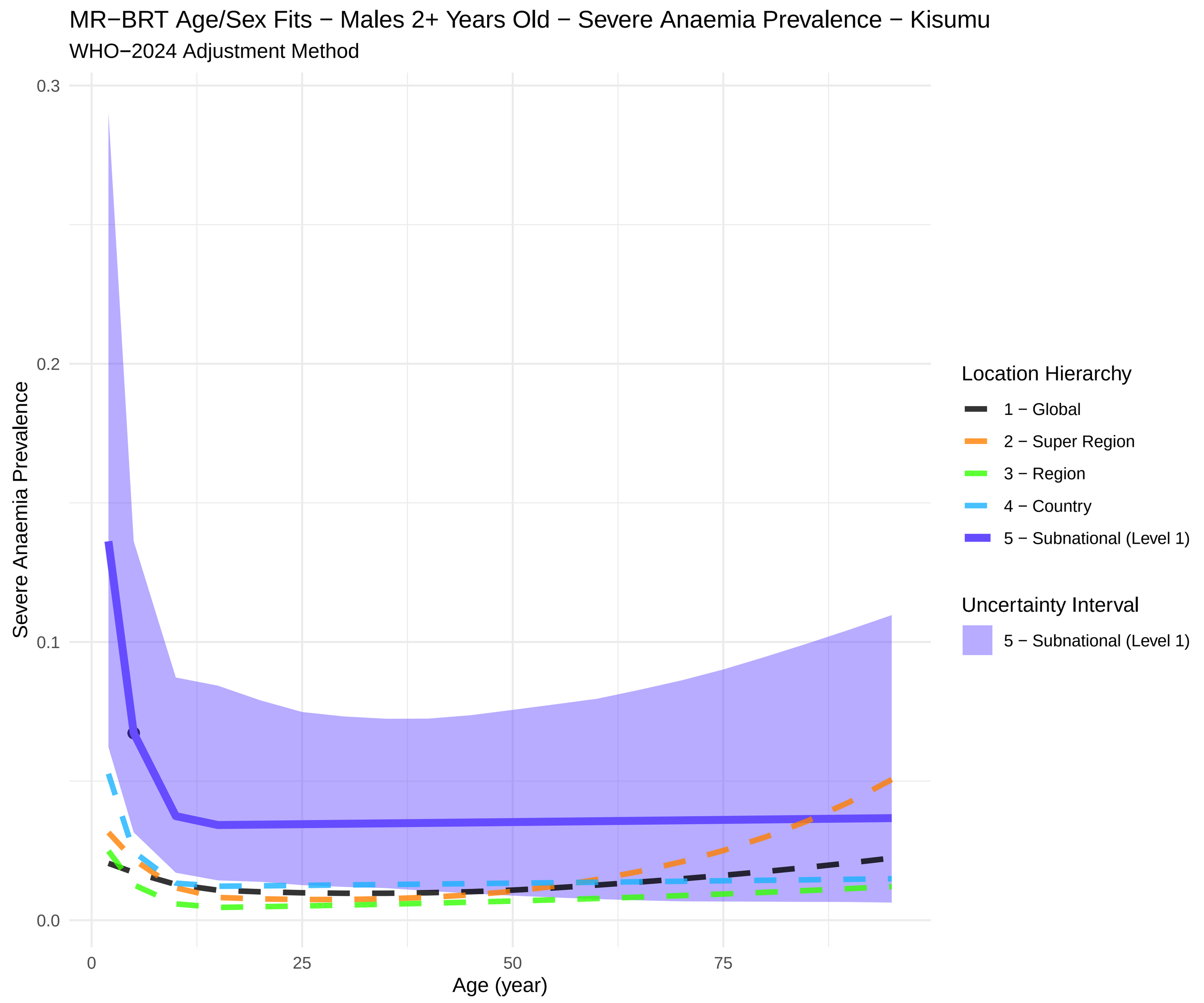
MR−BRT Age/Sex Fits − Males 2+ Years Old − Severe Anaemia Prevalence − Kisumu
WHO−2024 Adjustment Method
Severe Anaemia Prevalence
Age (year)
Location Hierarchy
1 − Global
2 − Super Region
3 − Region
4 − Country
5 − Subnational (Level 1)
Uncertainty Interval
5 − Subnational (Level 1)

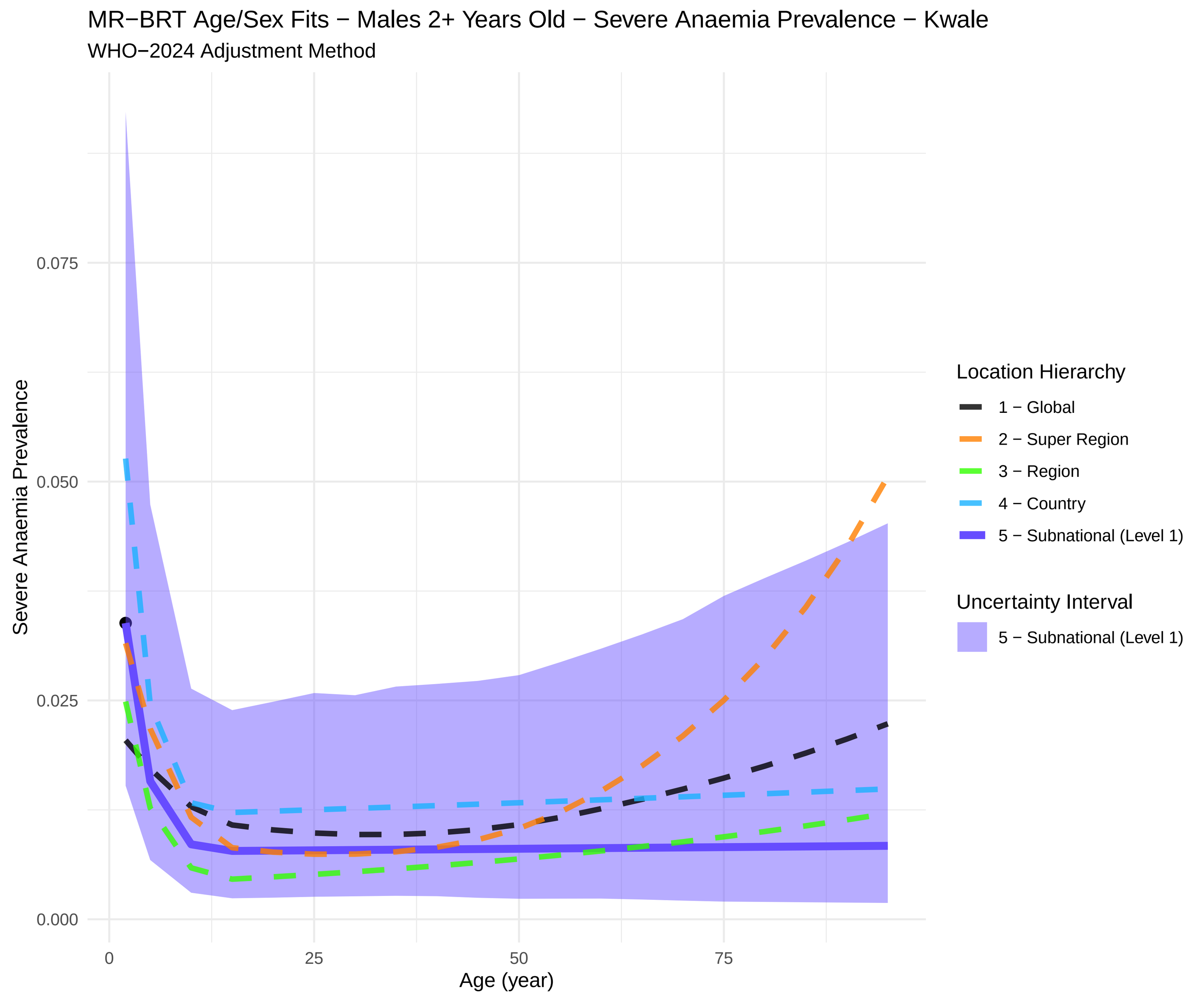
MR−BRT Age/Sex Fits − Males 2+ Years Old − Severe Anaemia Prevalence − Kwale
WHO−2024 Adjustment Method
Severe Anaemia Prevalence
Age (year)
Location Hierarchy
1 − Global
2 − Super Region
3 − Region
4 − Country
5 − Subnational (Level 1)
Uncertainty Interval
5 − Subnational (Level 1)

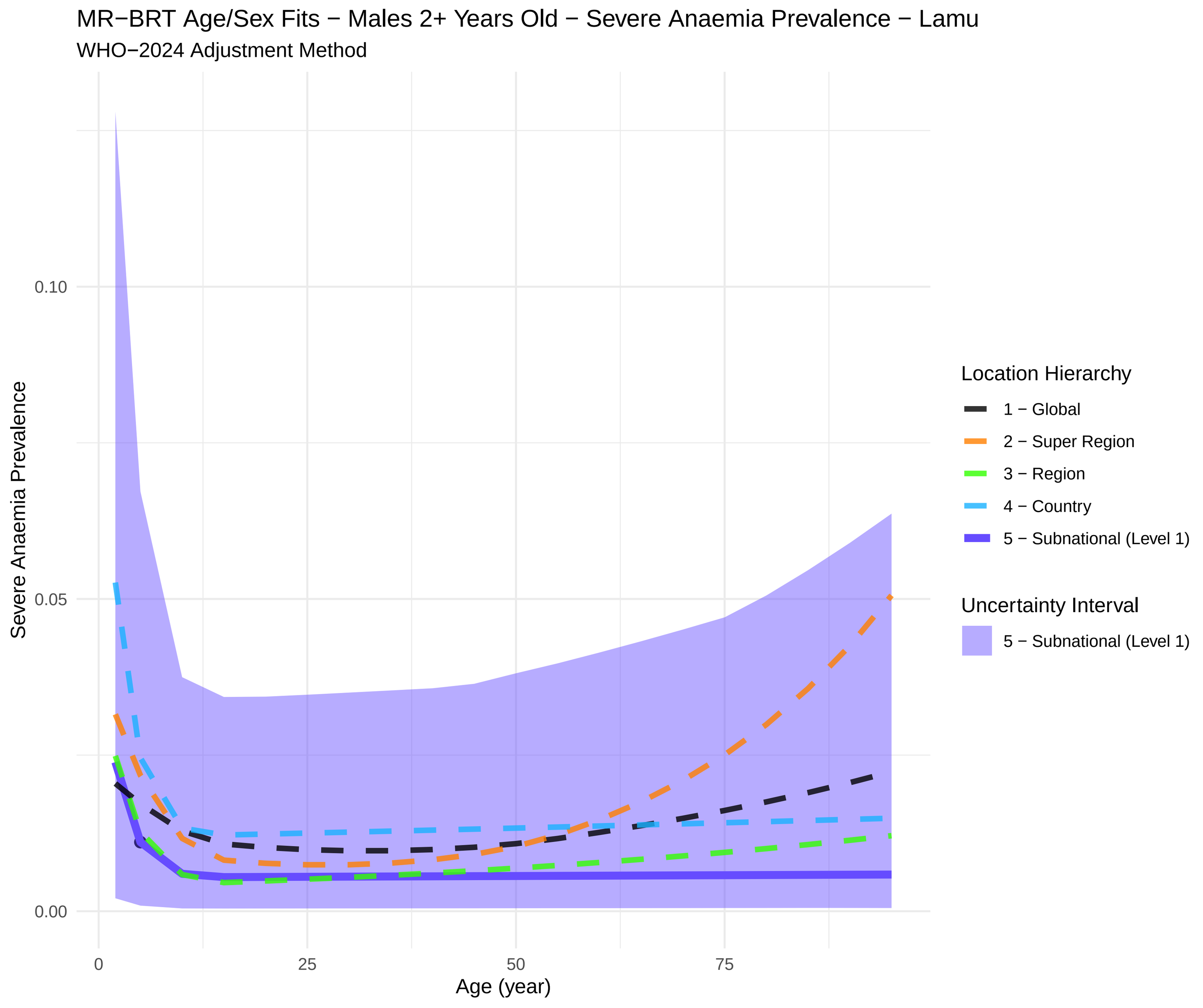
MR−BRT Age/Sex Fits − Males 2+ Years Old − Severe Anaemia Prevalence − Lamu
WHO−2024 Adjustment Method
Severe Anaemia Prevalence
Age (year)
Location Hierarchy
1 − Global
2 − Super Region
3 − Region
4 − Country
5 − Subnational (Level 1)
Uncertainty Interval
5 − Subnational (Level 1)

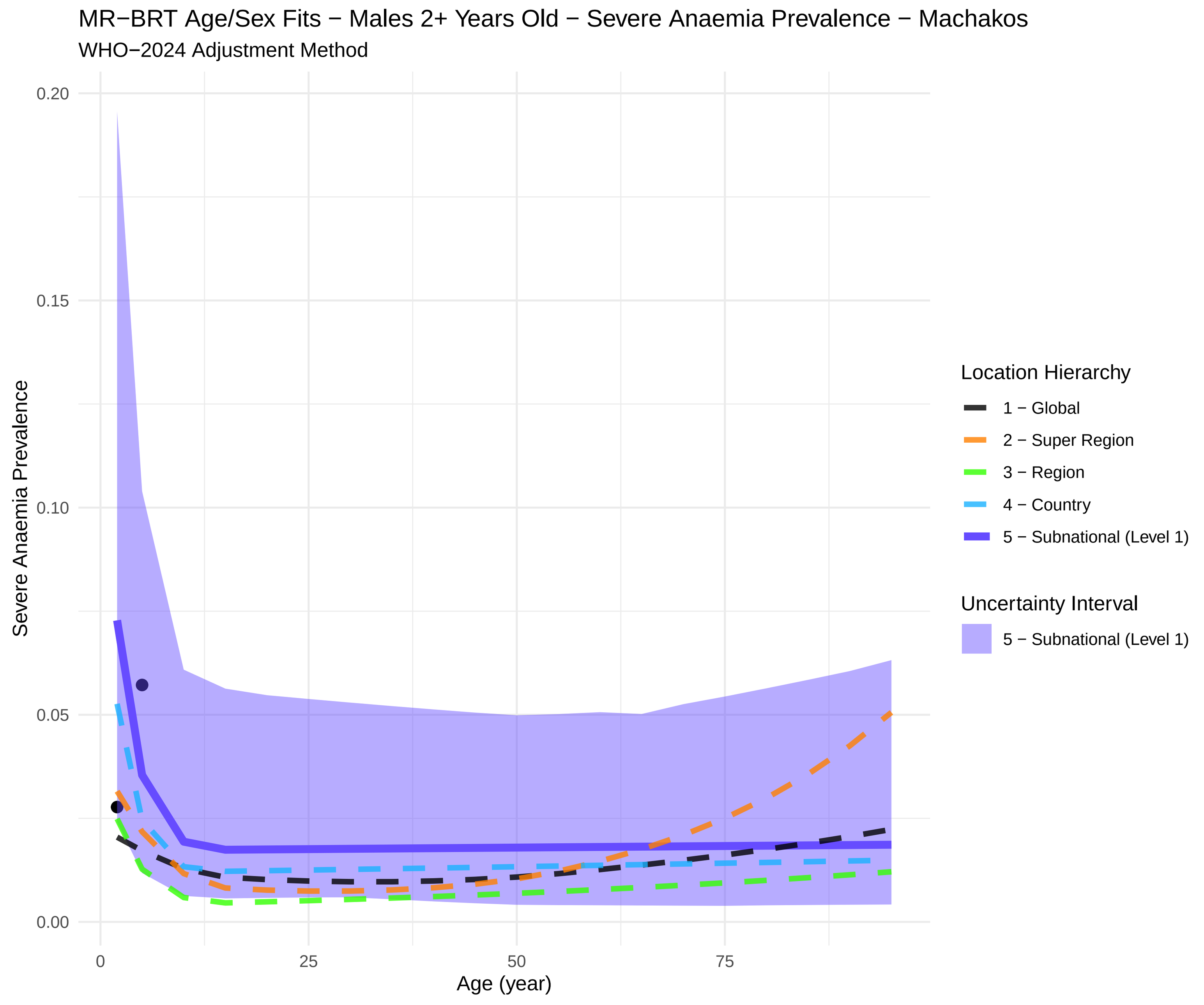
MR−BRT Age/Sex Fits − Males 2+ Years Old − Severe Anaemia Prevalence − Machakos
WHO−2024 Adjustment Method
Severe Anaemia Prevalence
Age (year)
Location Hierarchy
1 − Global
2 − Super Region
3 − Region
4 − Country
5 − Subnational (Level 1)
Uncertainty Interval
5 − Subnational (Level 1)

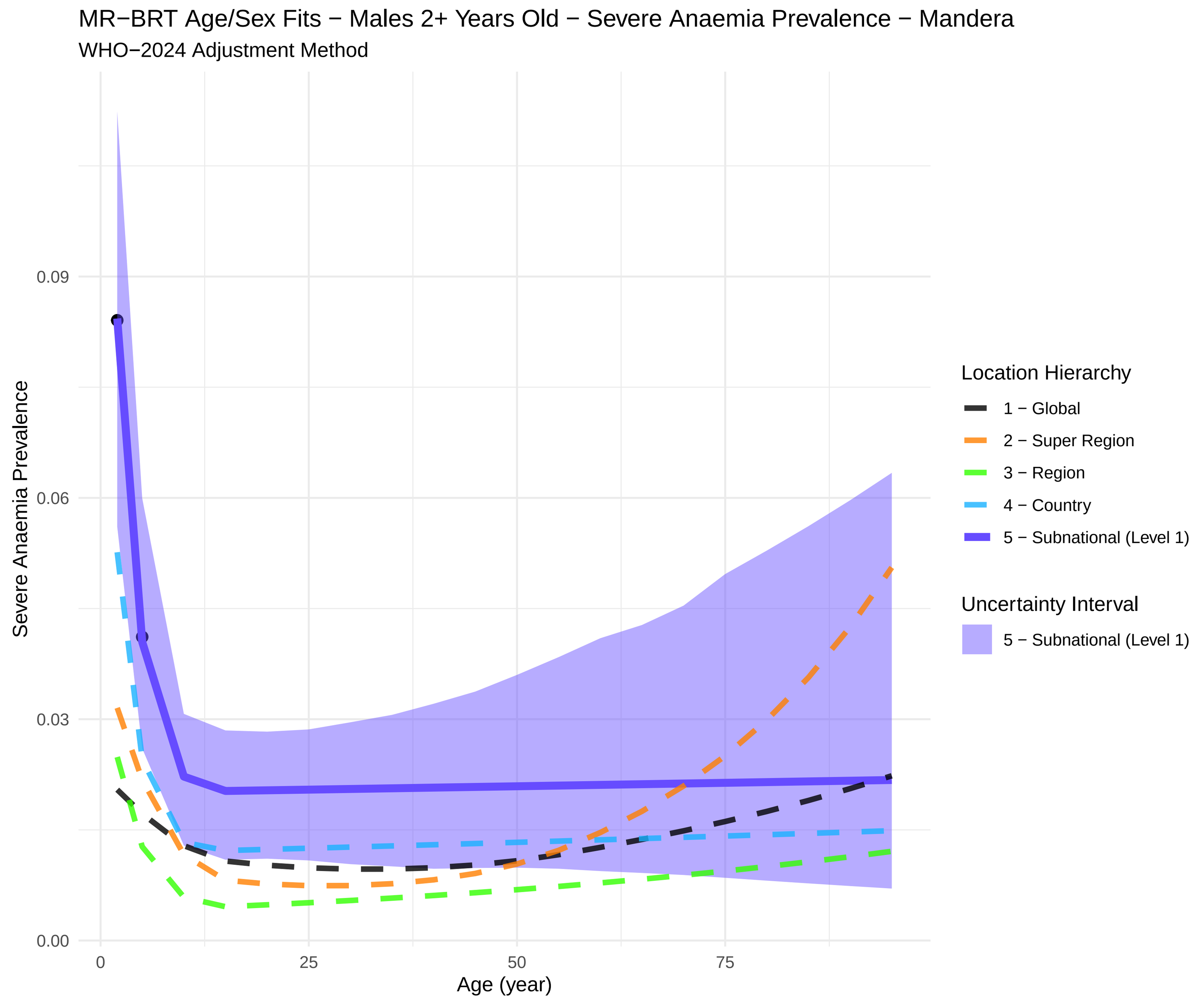
MR−BRT Age/Sex Fits − Males 2+ Years Old − Severe Anaemia Prevalence − Mandera
WHO−2024 Adjustment Method
Severe Anaemia Prevalence
Age (year)
Location Hierarchy
1 − Global
2 − Super Region
3 − Region
4 − Country
5 − Subnational (Level 1)
Uncertainty Interval
5 − Subnational (Level 1)

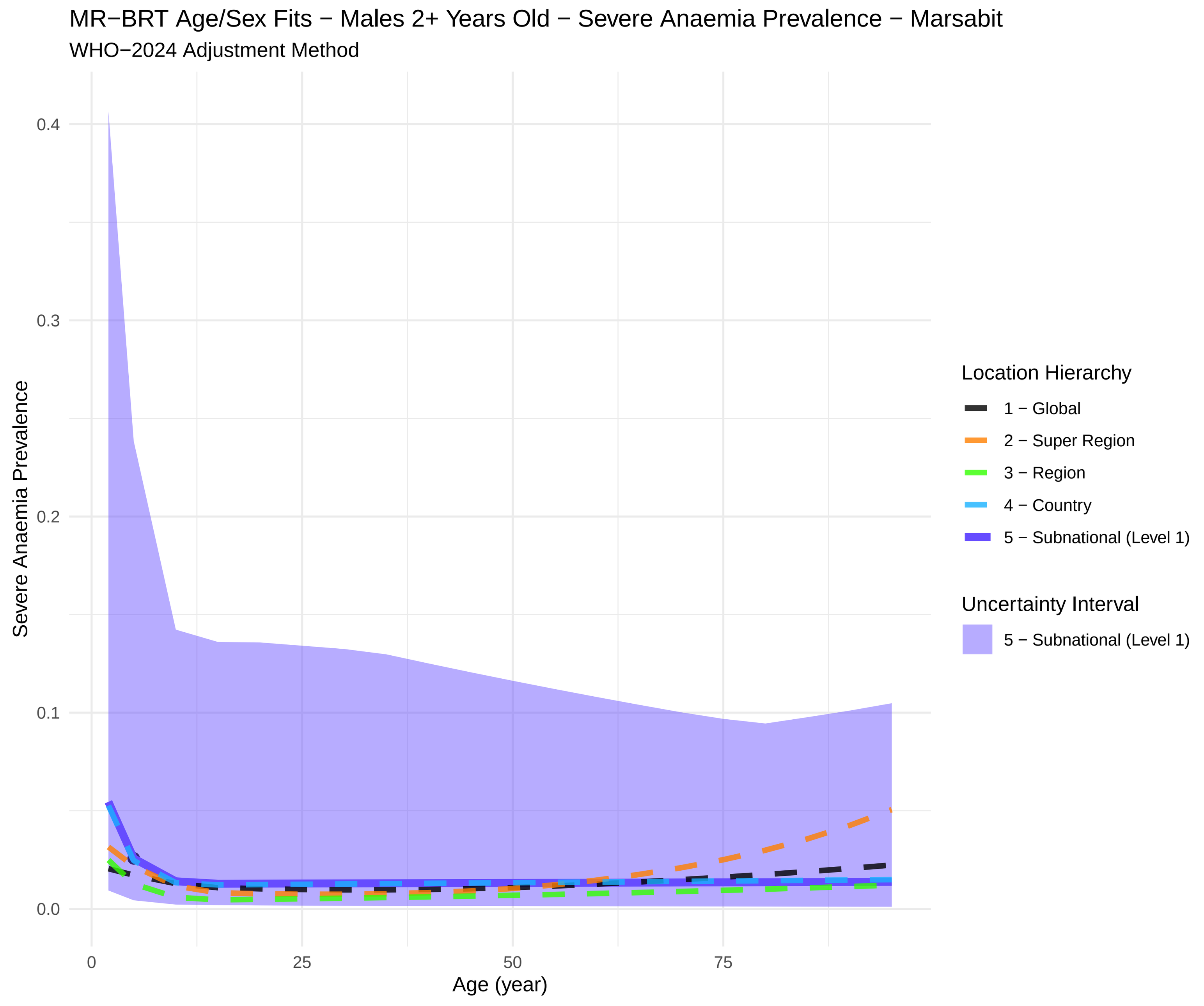
MR−BRT Age/Sex Fits − Males 2+ Years Old − Severe Anaemia Prevalence − Marsabit
WHO−2024 Adjustment Method
Severe Anaemia Prevalence
Age (year)
Location Hierarchy
1 − Global
2 − Super Region
3 − Region
4 − Country
5 − Subnational (Level 1)
Uncertainty Interval
5 − Subnational (Level 1)

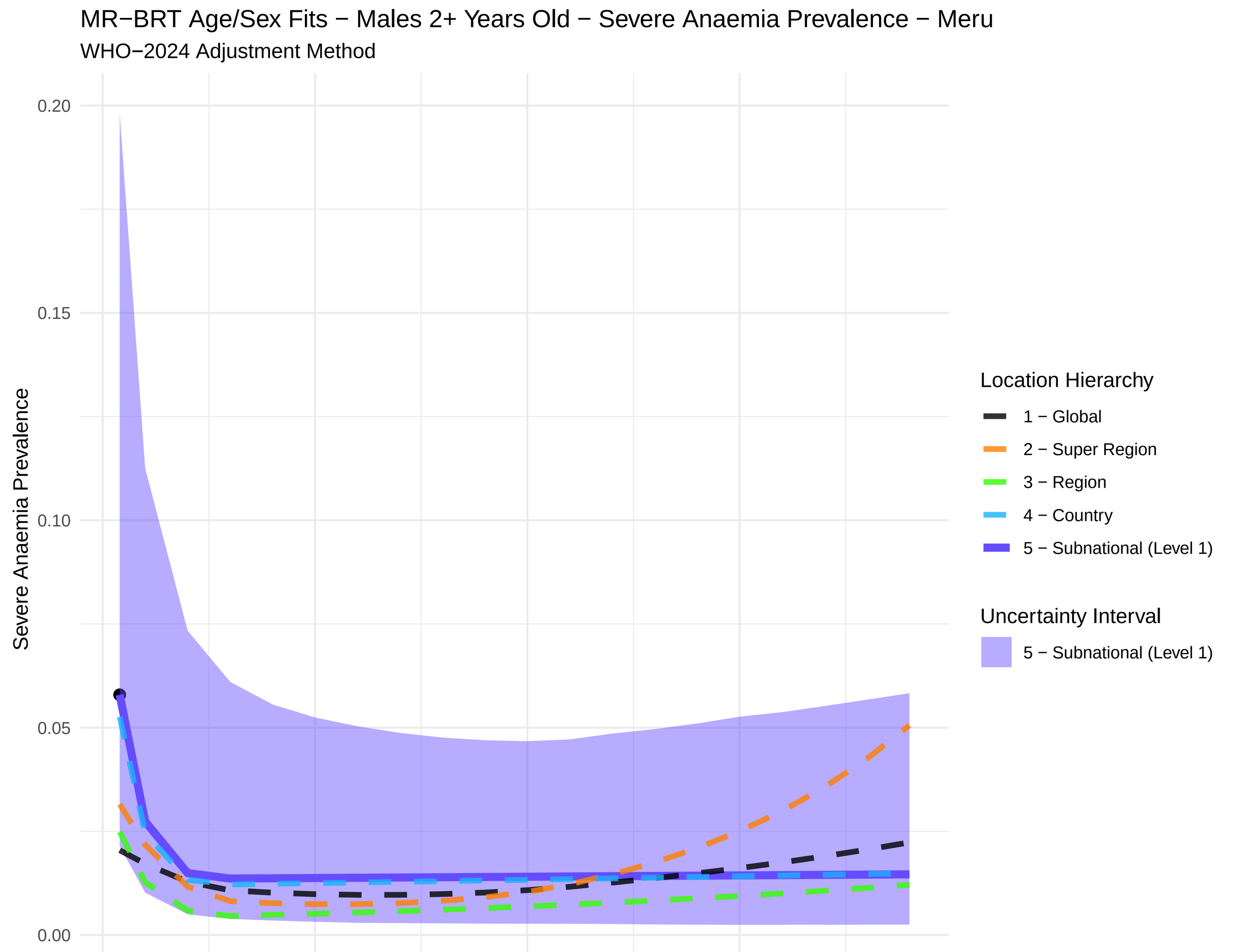
MR−BRT Age/Sex Fits − Males 2+ Years Old − Severe Anaemia Prevalence − Meru
WHO−2024 Adjustment Method
Severe Anaemia Prevalence
Age (year)
Location Hierarchy
1 − Global
2 − Super Region
3 − Region
4 − Country
5 − Subnational (Level 1)
Uncertainty Interval
5 − Subnational (Level 1)

MR−BRT Age/Sex Fits − Males 2+ Years Old − Severe Anaemia Prevalence − Migori
WHO−2024 Adjustment Method

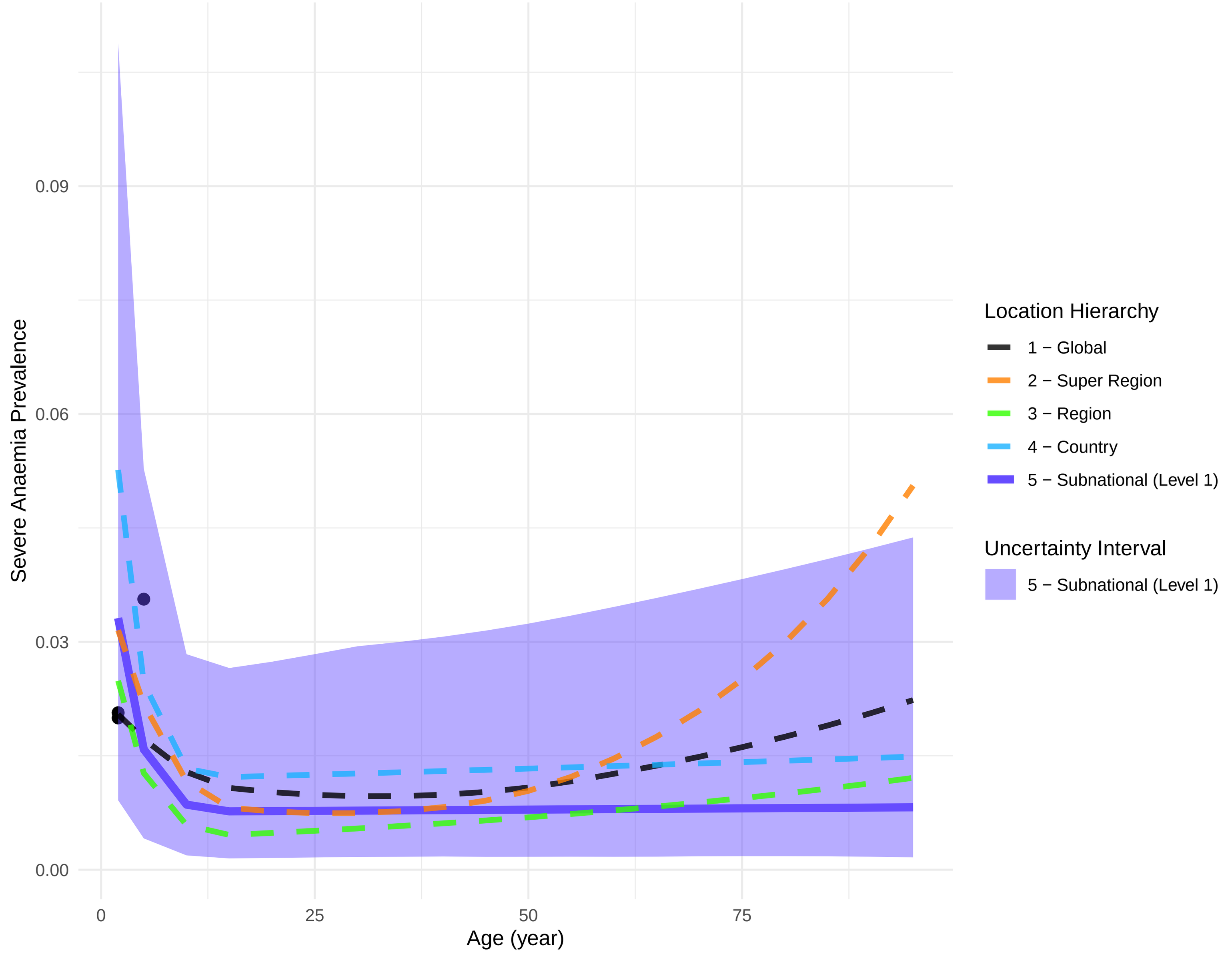

MR−BRT Age/Sex Fits − Males 2+ Years Old − Severe Anaemia Prevalence − Nakuru
WHO−2024 Adjustment Method

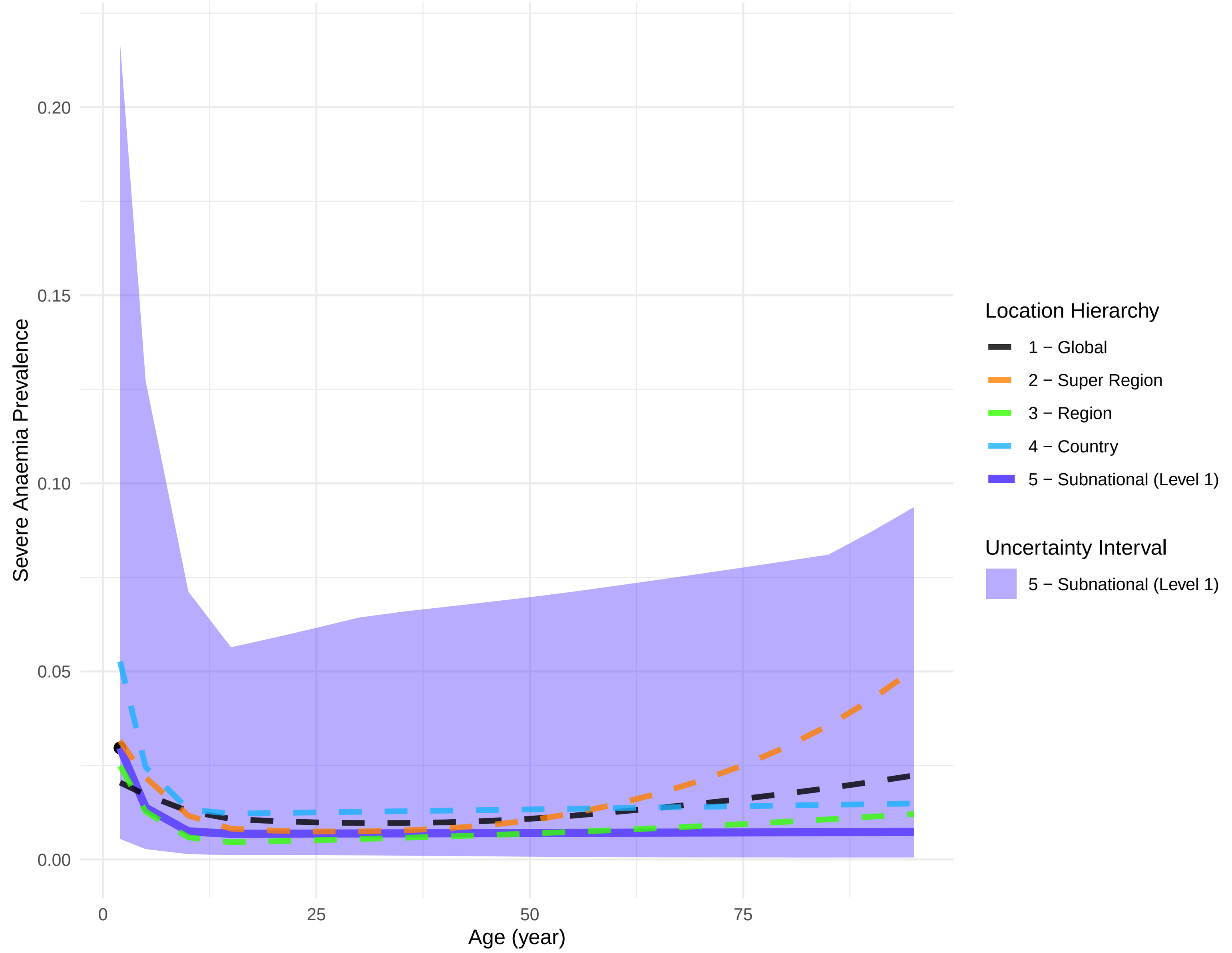

MR−BRT Age/Sex Fits − Males 2+ Years Old − Severe Anaemia Prevalence − Narok
WHO−2024 Adjustment Method

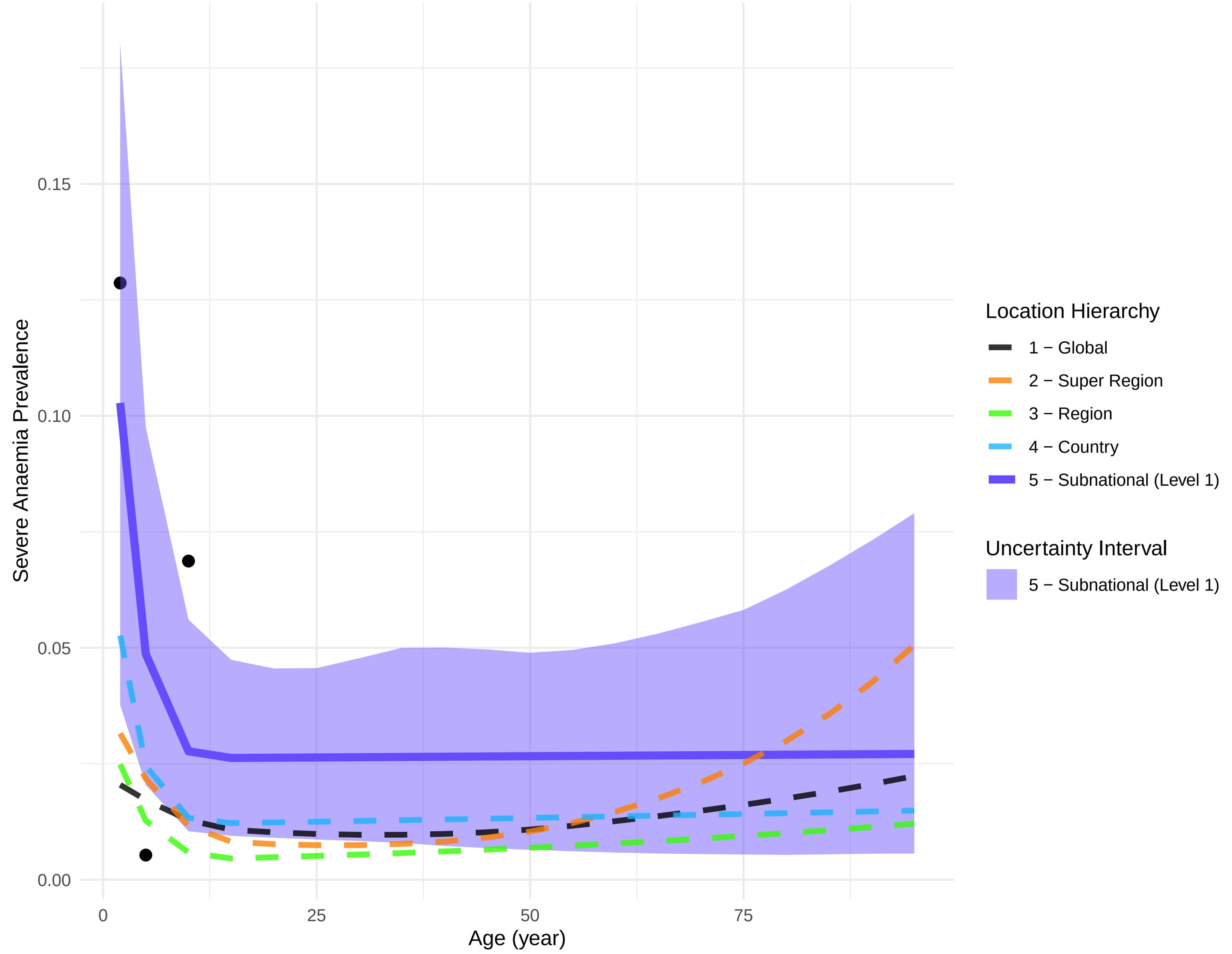

MR−BRT Age/Sex Fits − Males 2+ Years Old − Severe Anaemia Prevalence − Samburu
WHO−2024 Adjustment Method

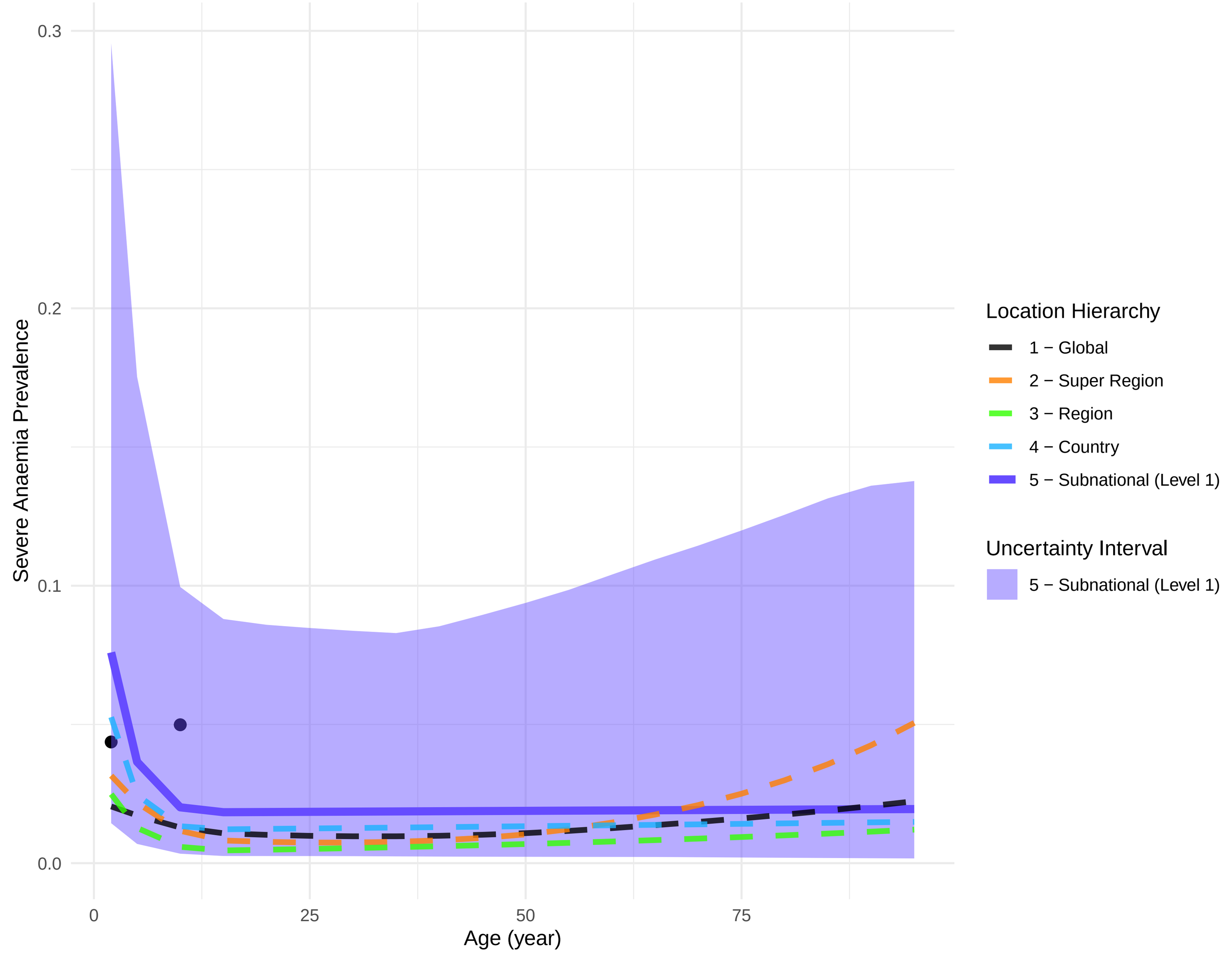

MR−BRT Age/Sex Fits − Males 2+ Years Old − Severe Anaemia Prevalence − Siaya
WHO−2024 Adjustment Method

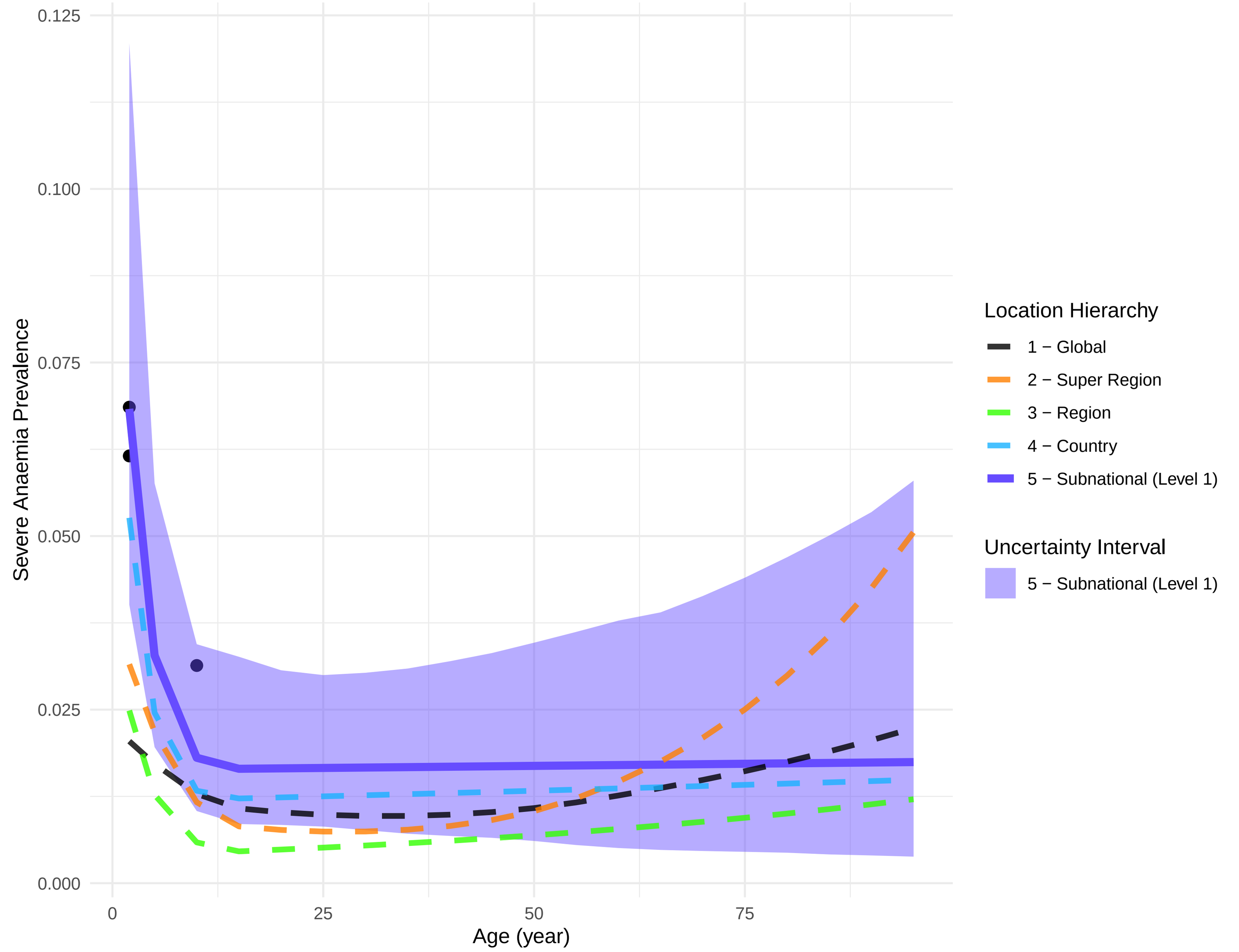

MR−BRT Age/Sex Fits − Males 2+ Years Old − Severe Anaemia Prevalence − Tana River
WHO−2024 Adjustment Method

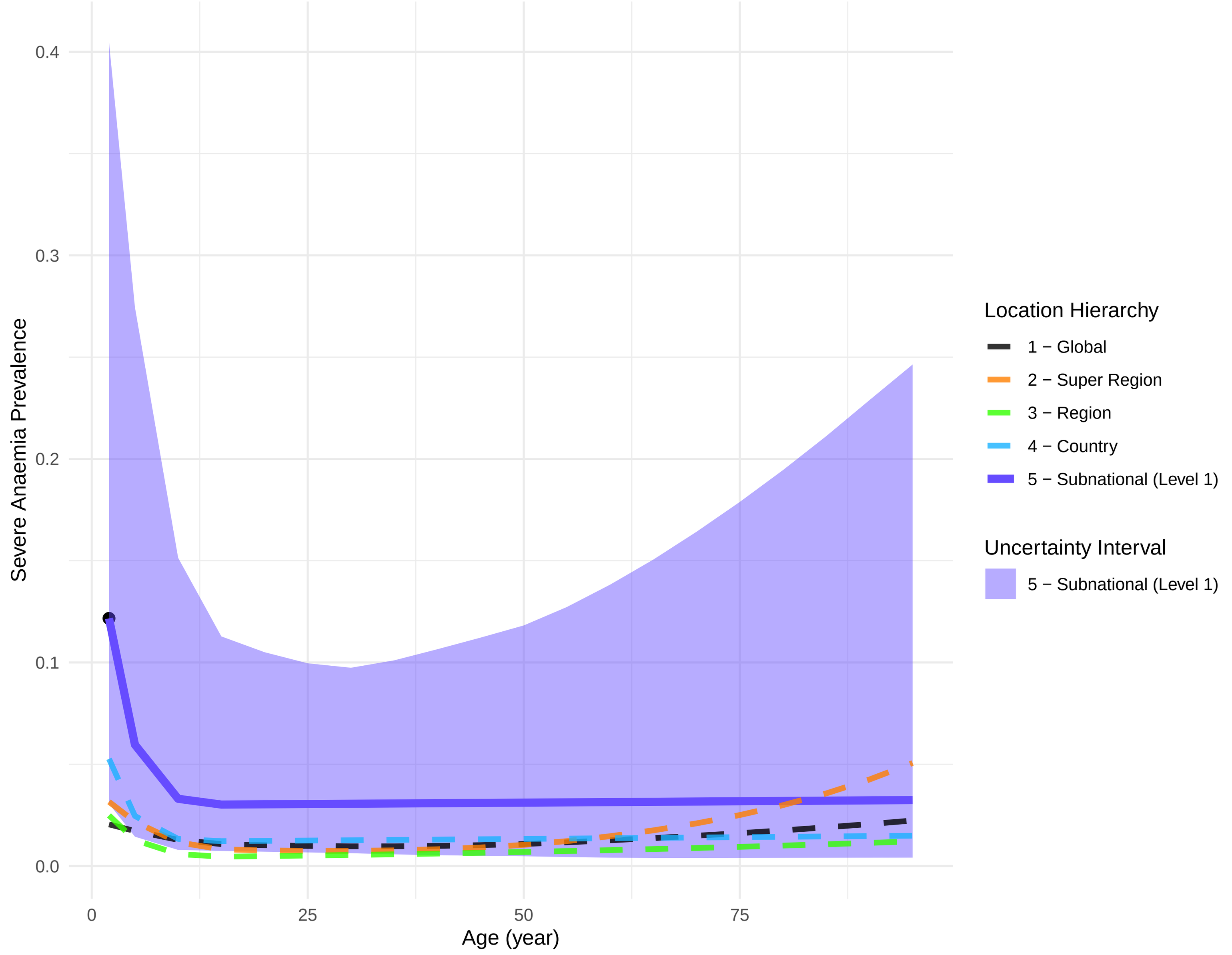

MR−BRT Age/Sex Fits − Males 2+ Years Old − Severe Anaemia Prevalence − Trans Nzoia
WHO−2024 Adjustment Method

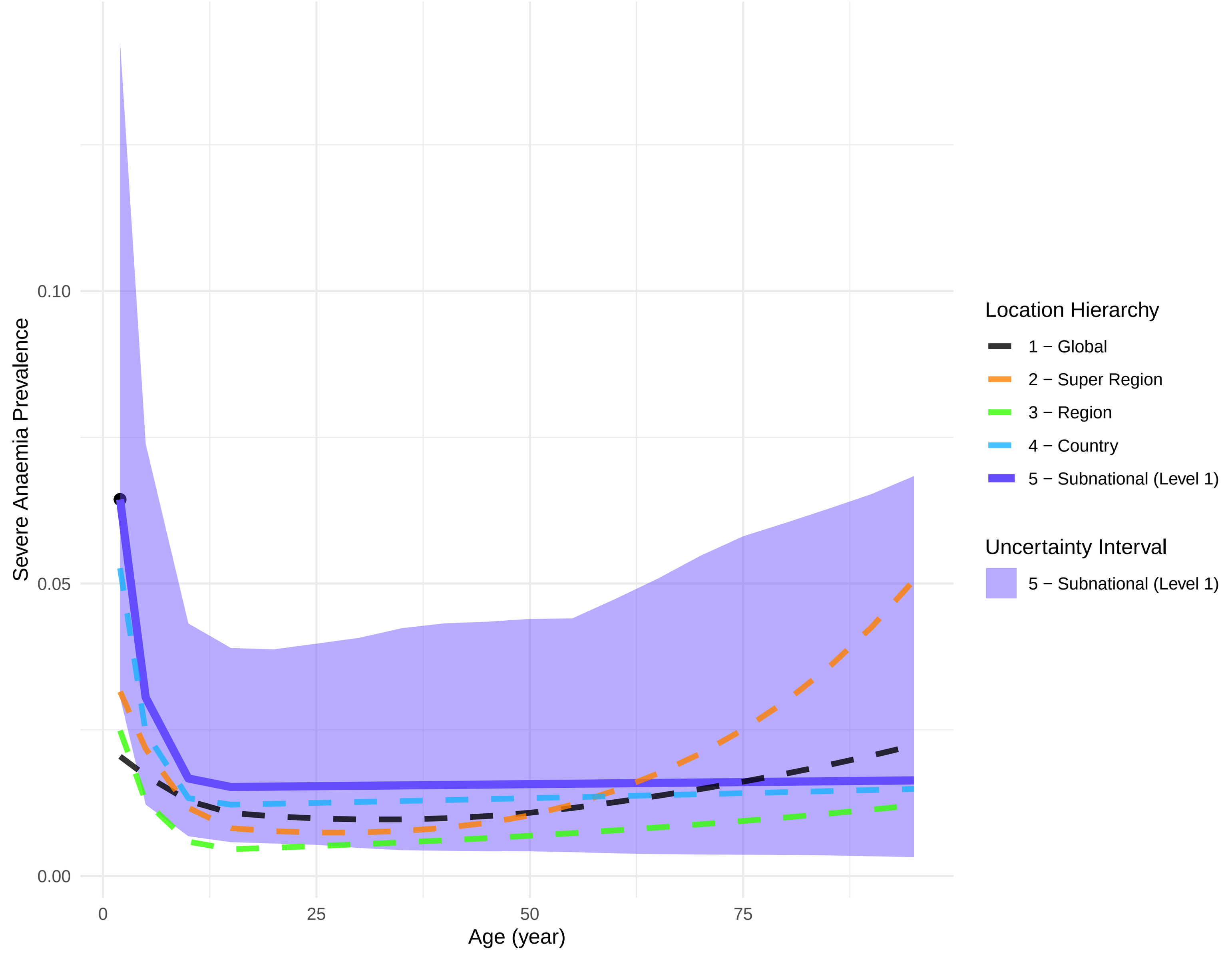

MR−BRT Age/Sex Fits − Males 2+ Years Old − Severe Anaemia Prevalence − Turkana
WHO−2024 Adjustment Method

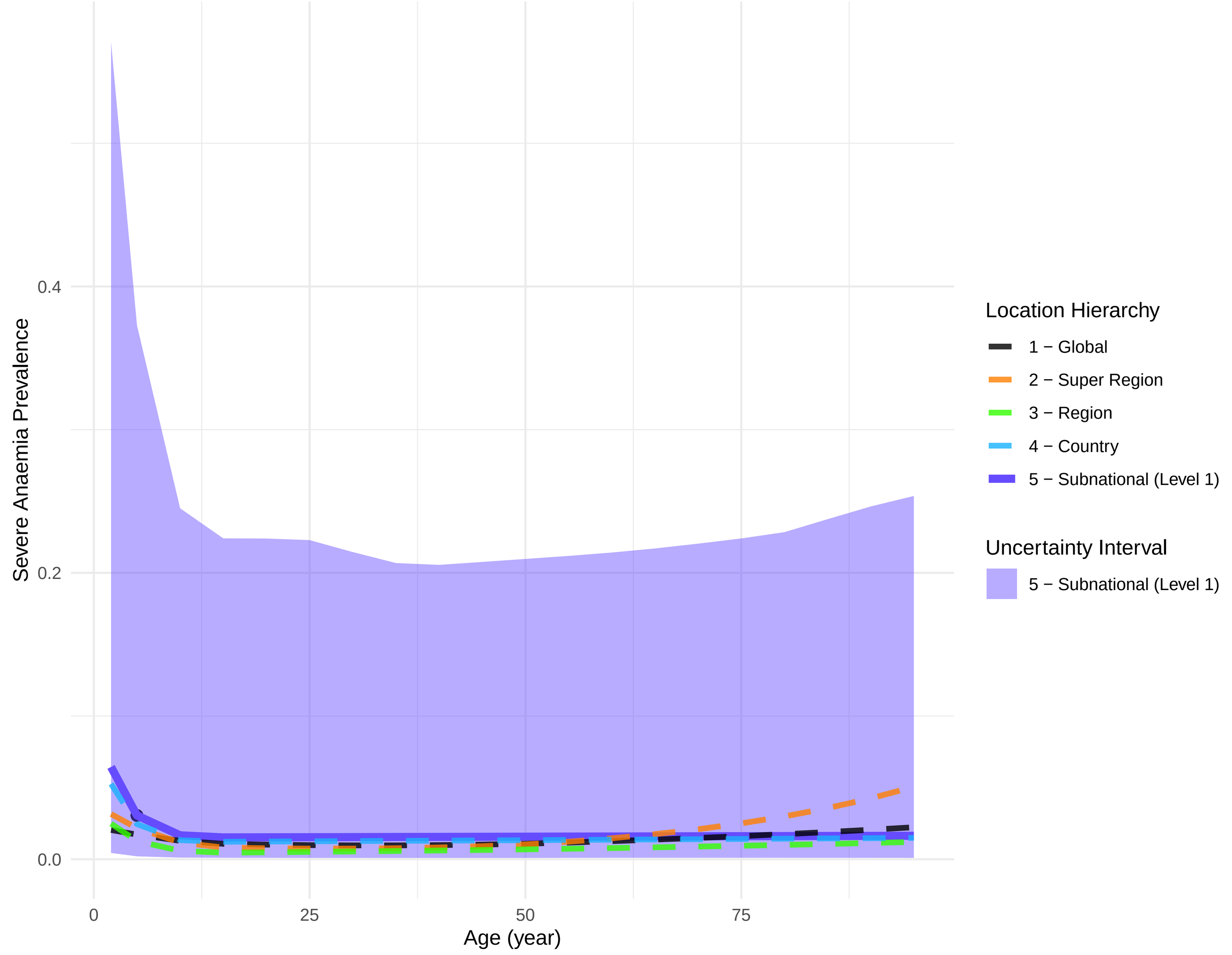

MR−BRT Age/Sex Fits − Males 2+ Years Old − Severe Anaemia Prevalence − Uasin Gishu
WHO−2024 Adjustment Method

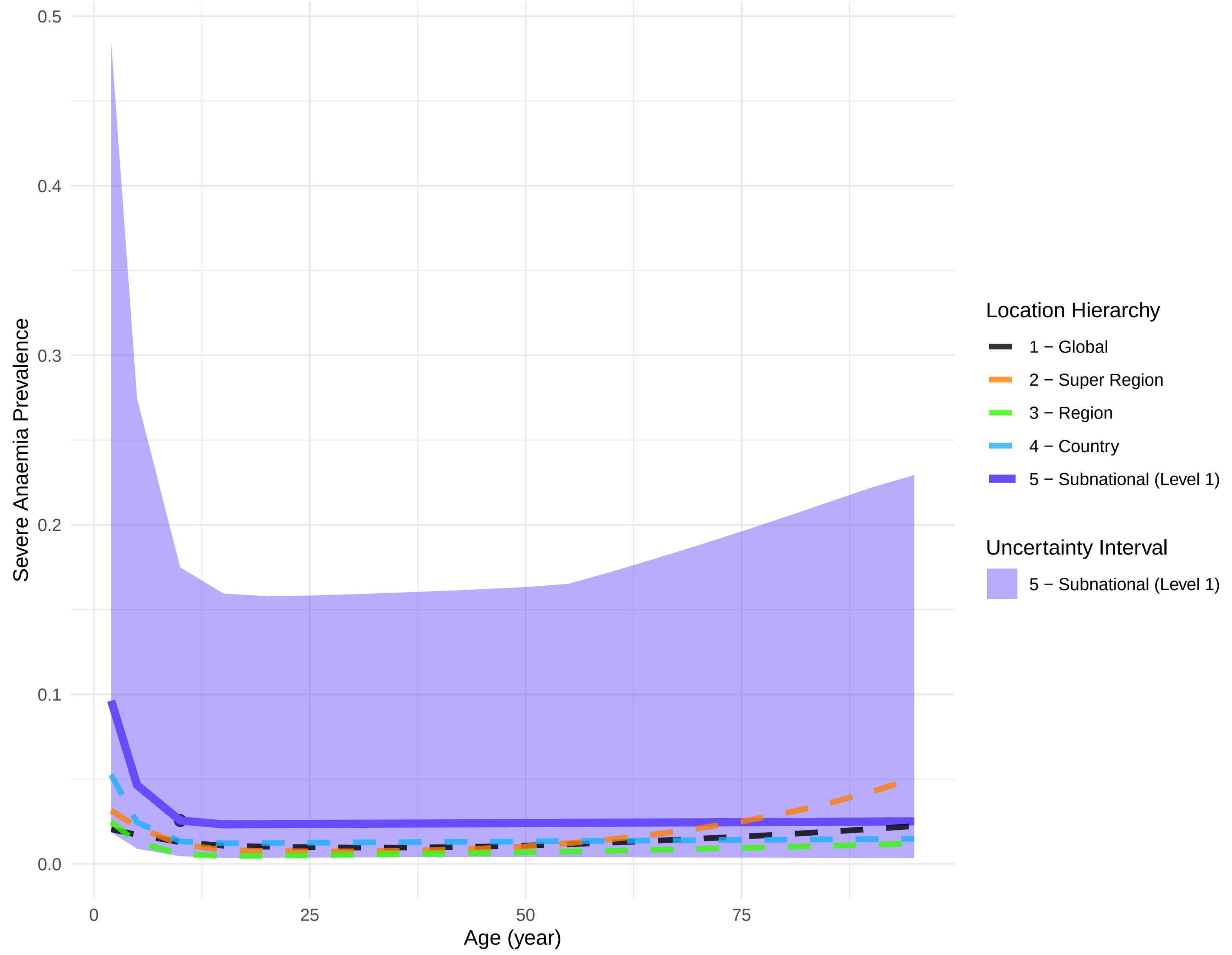

MR−BRT Age/Sex Fits − Males 2+ Years Old − Severe Anaemia Prevalence − Vihiga
WHO−2024 Adjustment Method

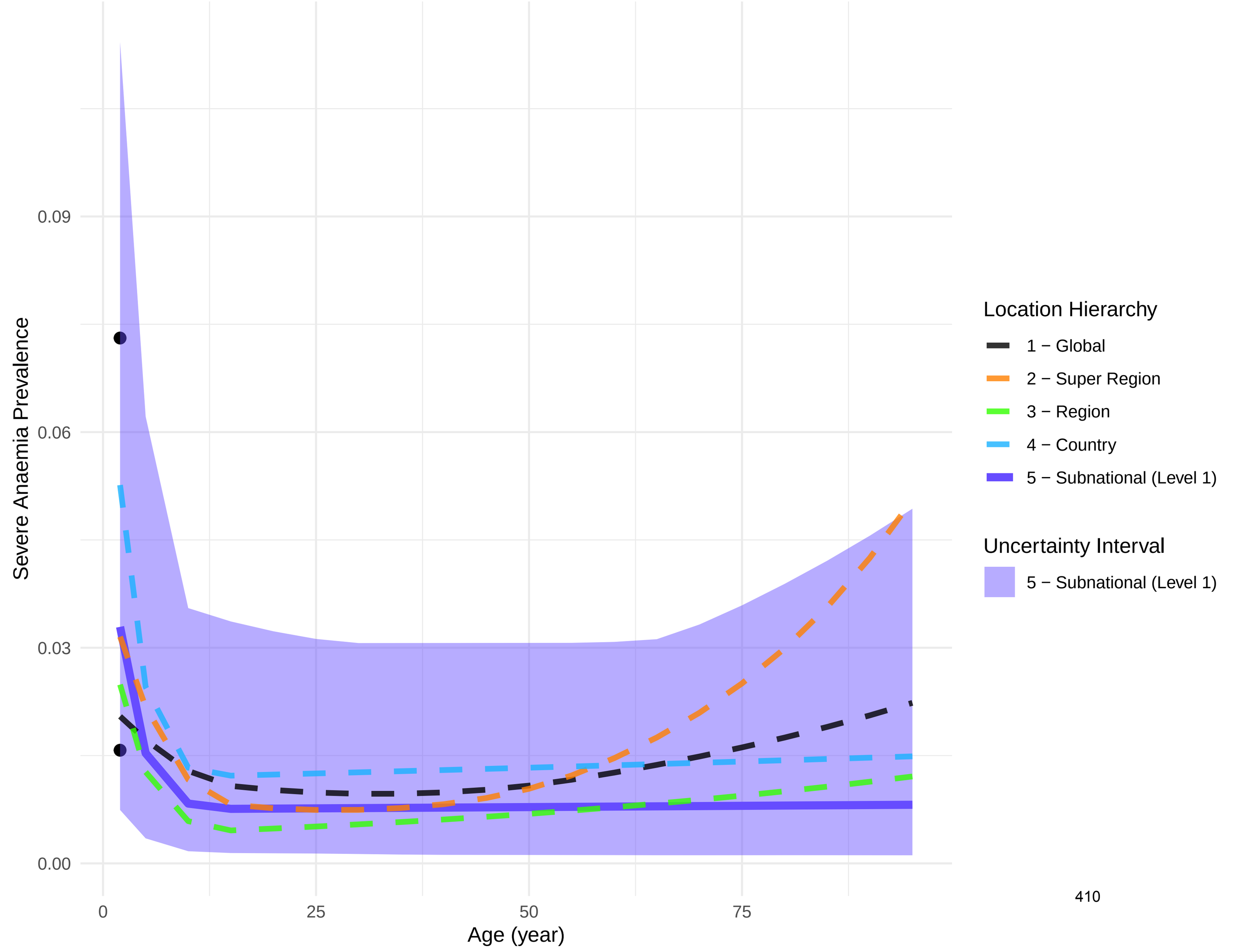

MR−BRT Age/Sex Fits − Males 2+ Years Old − Severe Anaemia Prevalence − Wajir
WHO−2024 Adjustment Method

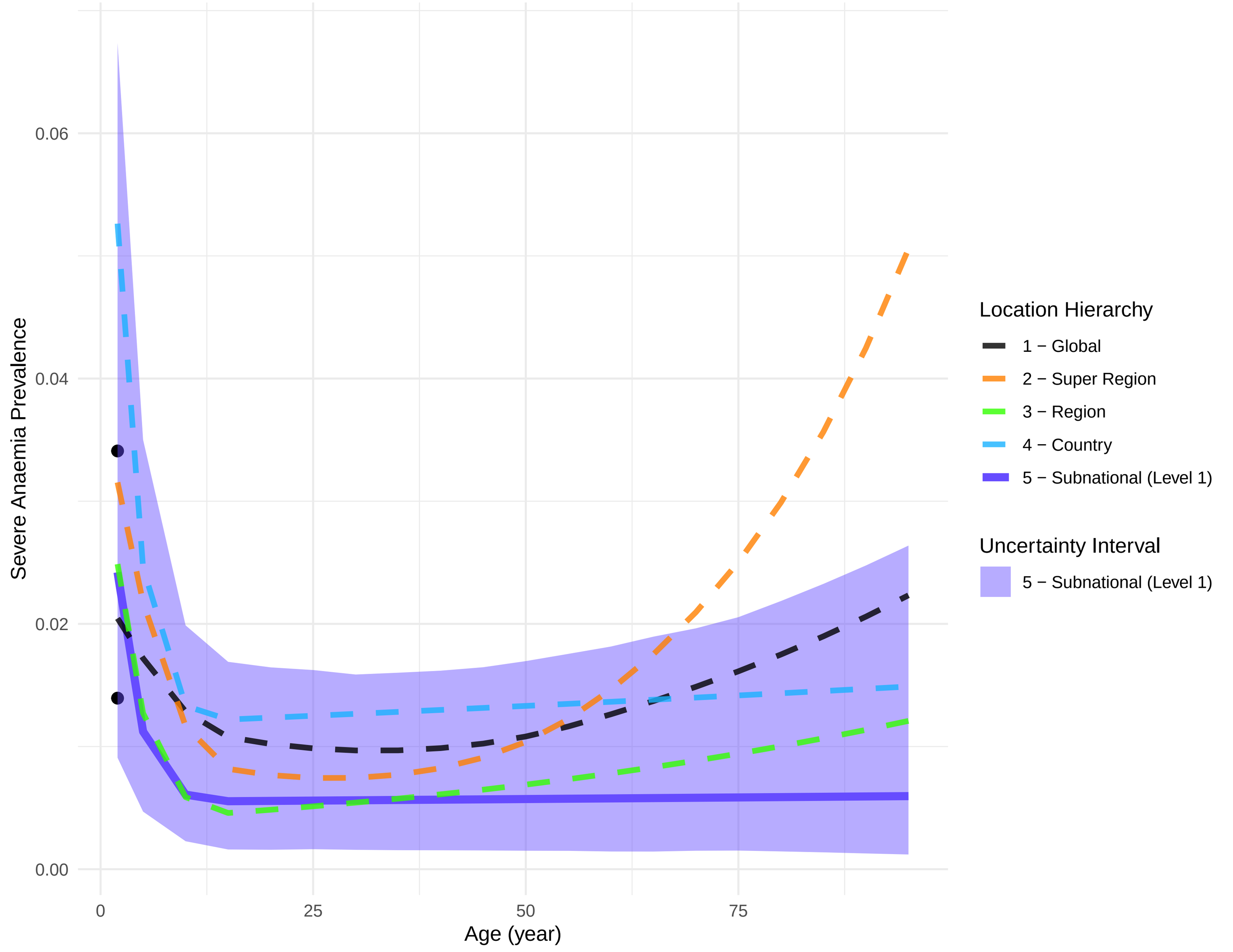

MR−BRT Age/Sex Fits − Males 2+ Years Old − Severe Anaemia Prevalence − West Pokot
WHO−2024 Adjustment Method

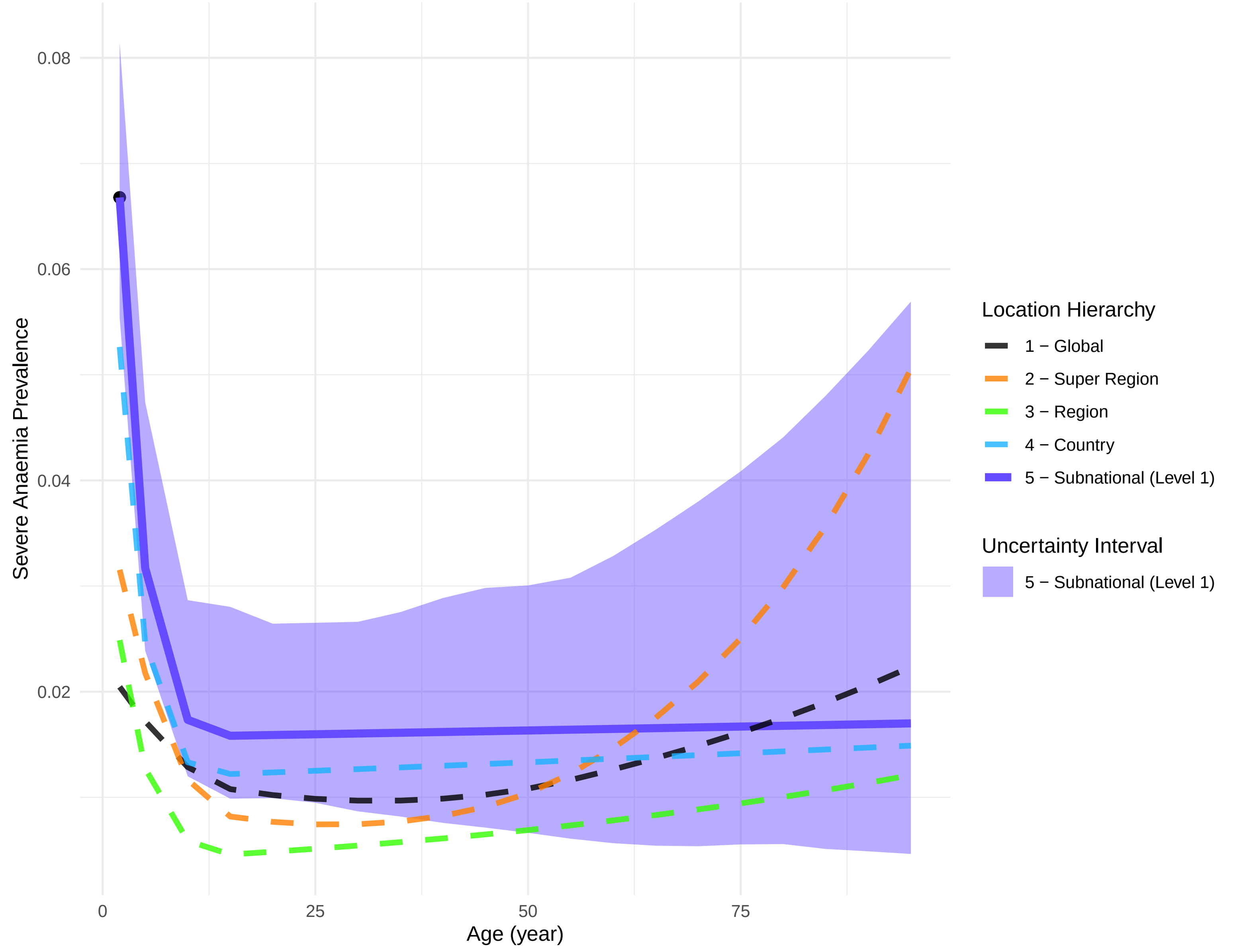

MR−BRT Age/Sex Fits − Males 2+ Years Old − Severe Anaemia Prevalence − Madagascar
WHO−2024 Adjustment Method

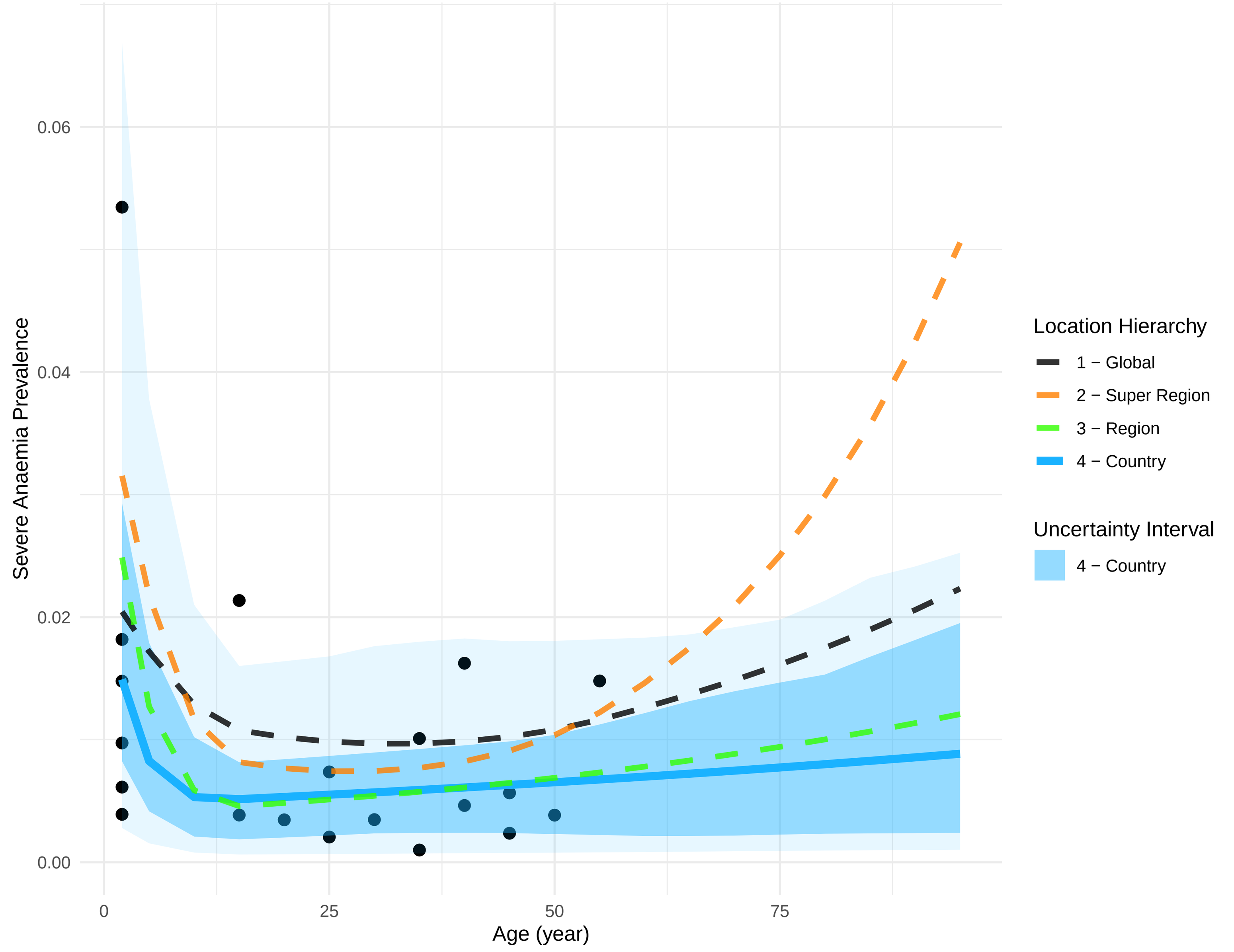

MR−BRT Age/Sex Fits − Males 2+ Years Old − Severe Anaemia Prevalence − Malawi
WHO−2024 Adjustment Method

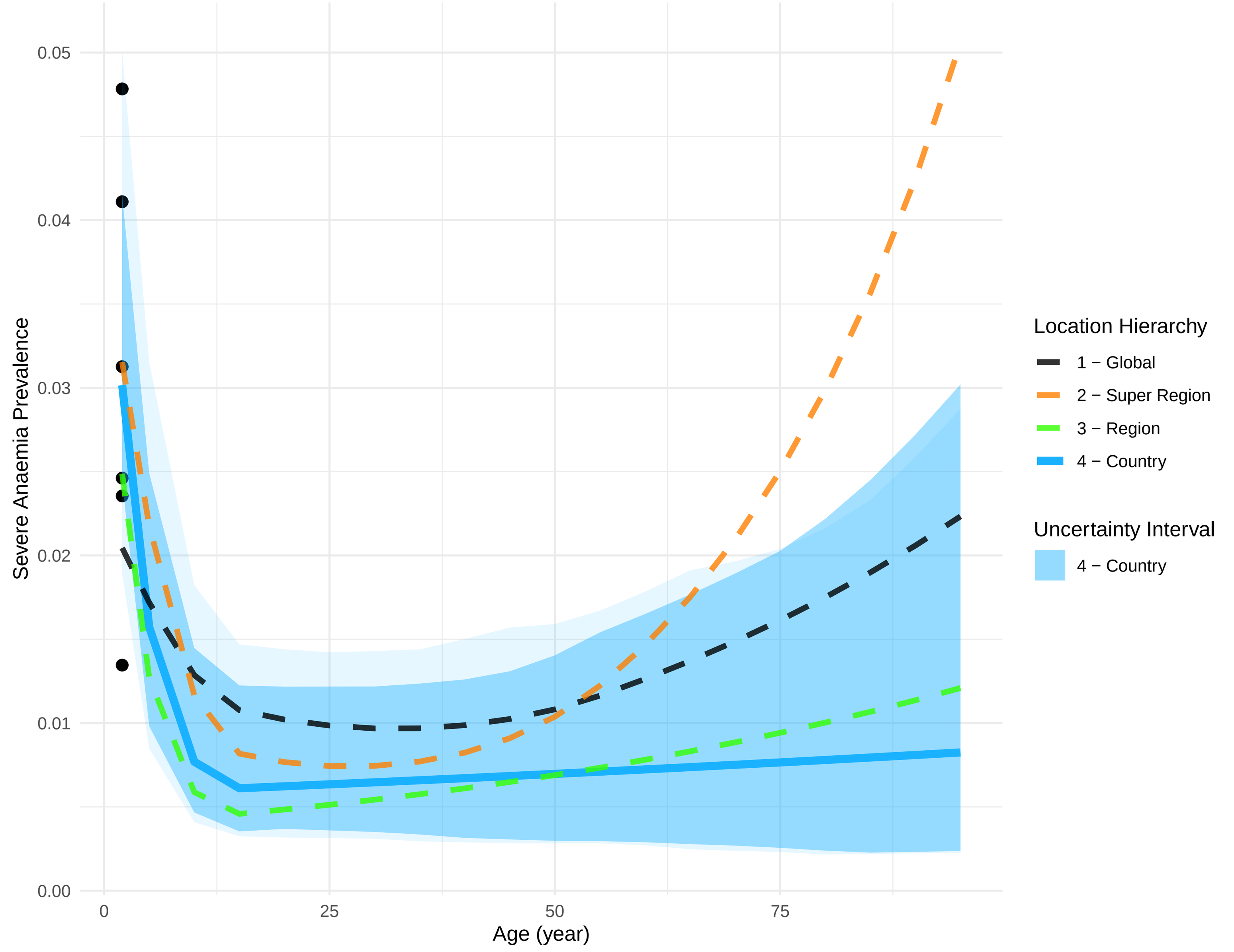

MR−BRT Age/Sex Fits − Males 2+ Years Old − Severe Anaemia Prevalence − Mozambique
WHO−2024 Adjustment Method

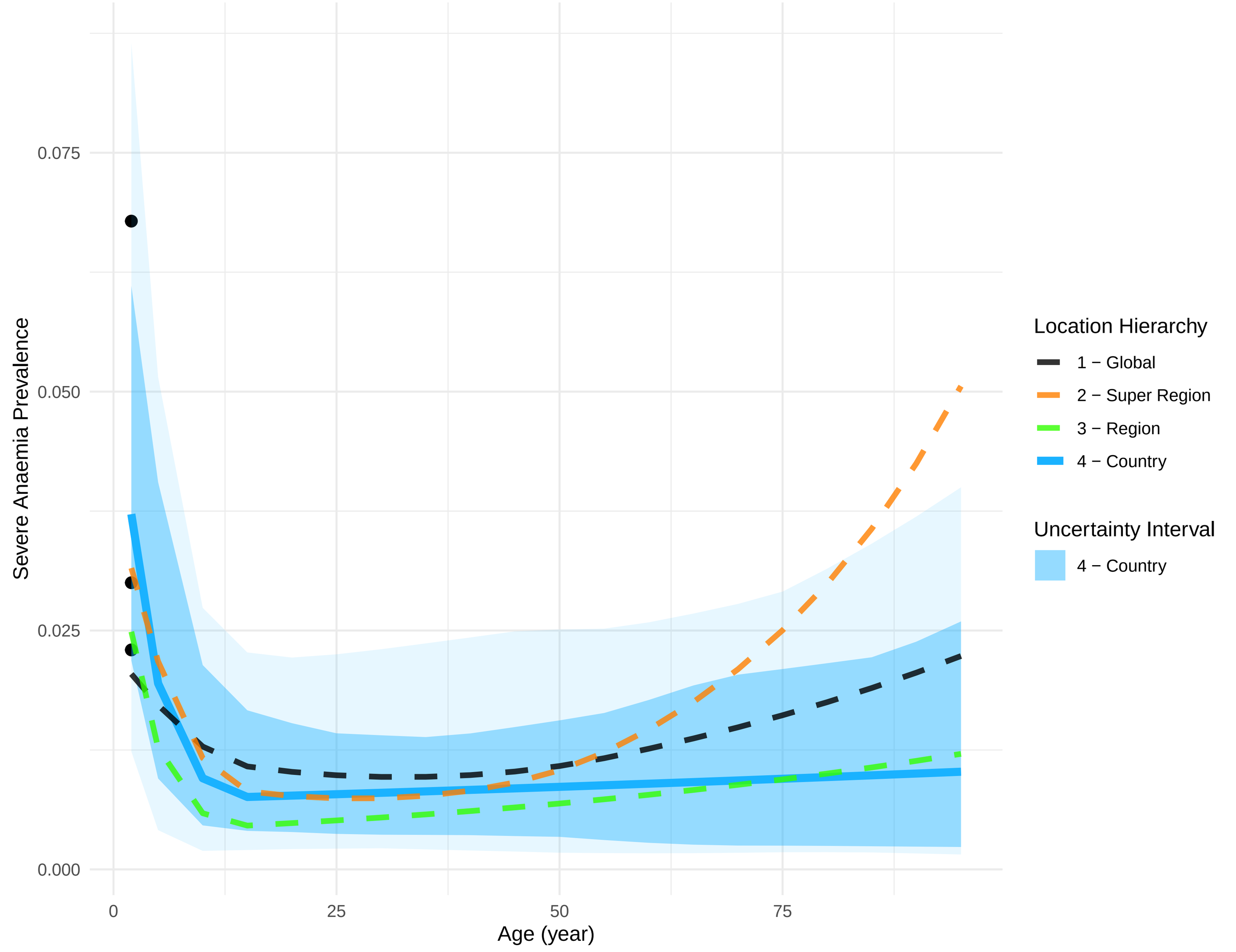

MR−BRT Age/Sex Fits − Males 2+ Years Old − Severe Anaemia Prevalence − Rwanda
WHO−2024 Adjustment Method

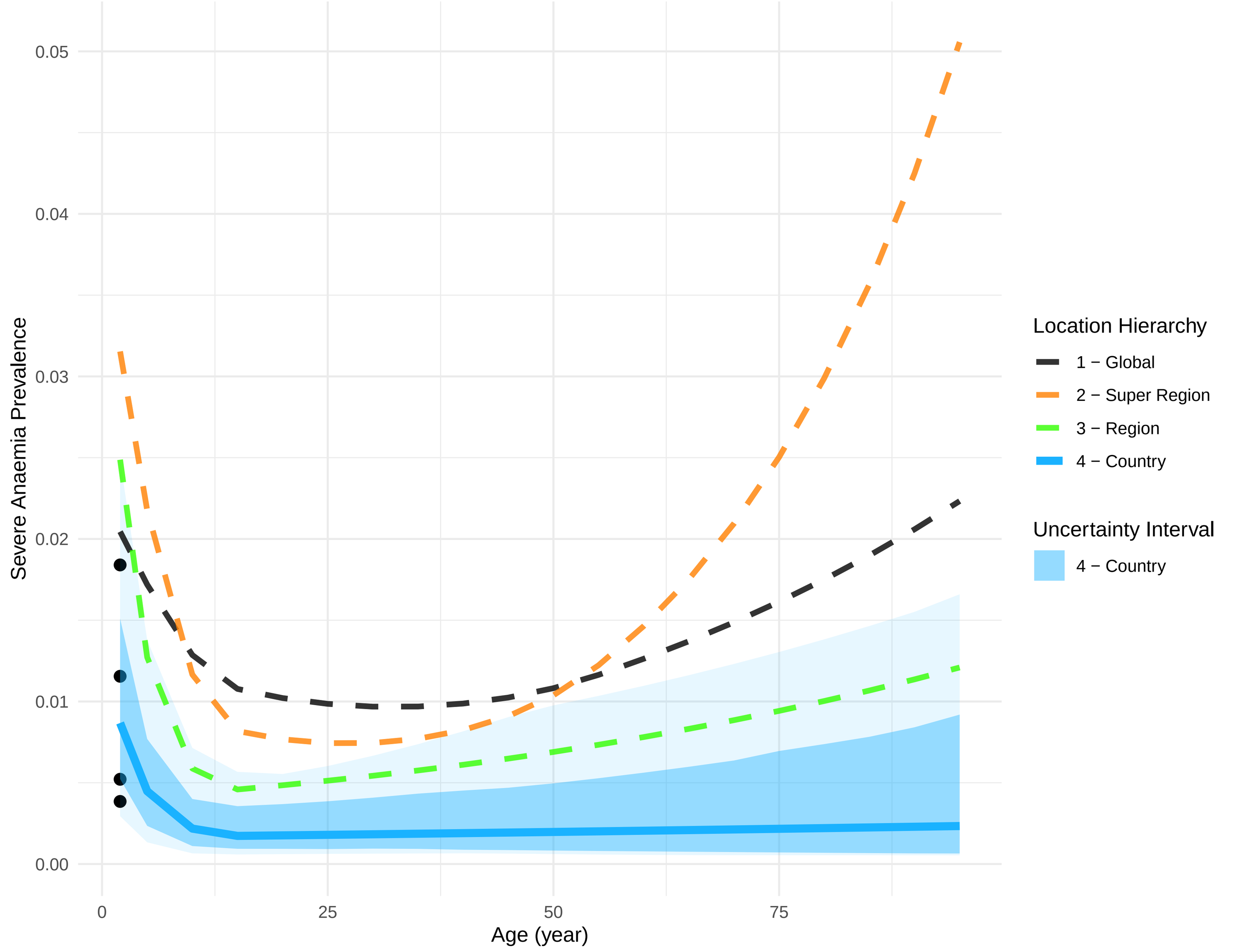

MR−BRT Age/Sex Fits − Males 2+ Years Old − Severe Anaemia Prevalence − Uganda
WHO−2024 Adjustment Method

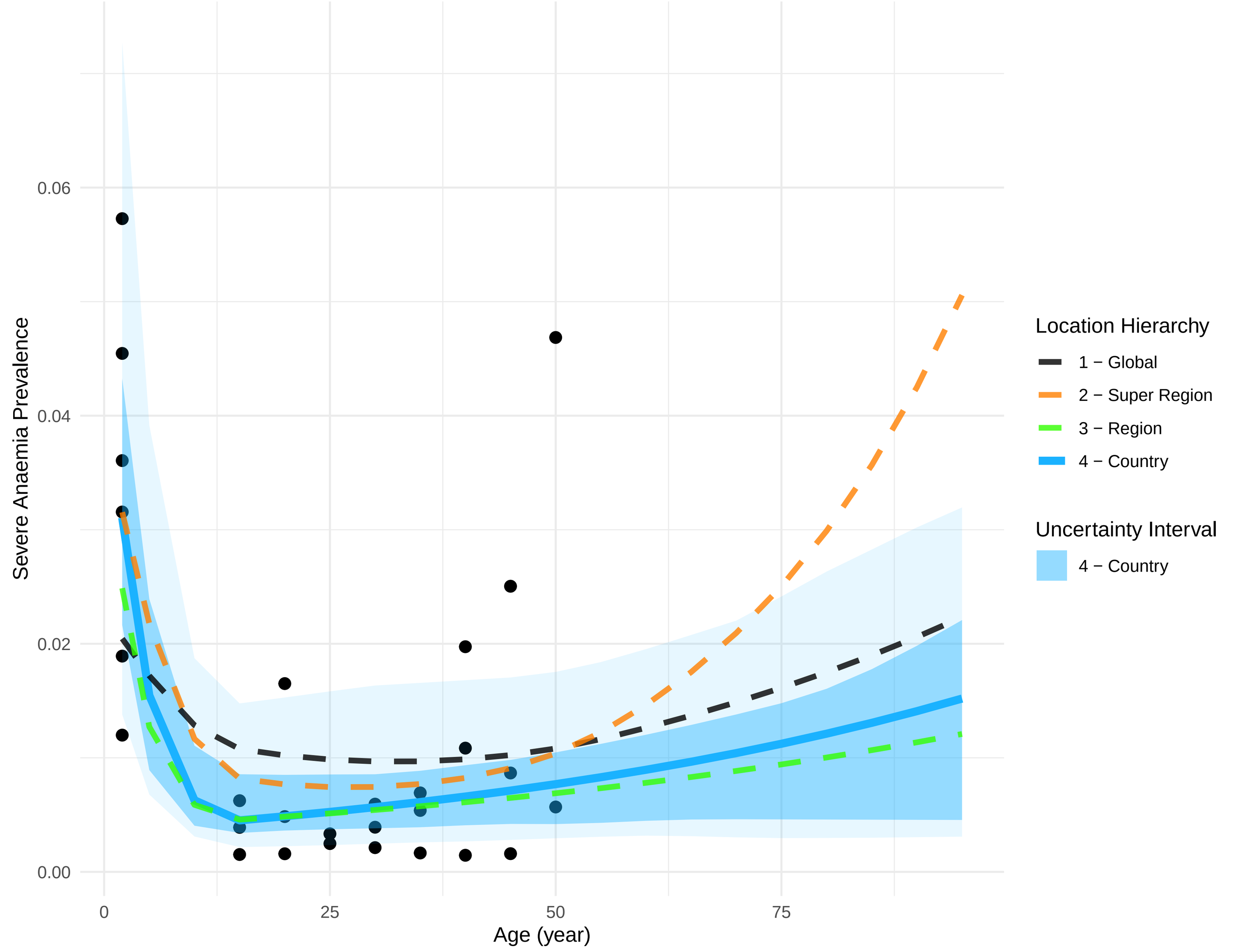

MR−BRT Age/Sex Fits − Males 2+ Years Old − Severe Anaemia Prevalence − United Republic of Tanzania
WHO−2024 Adjustment Method

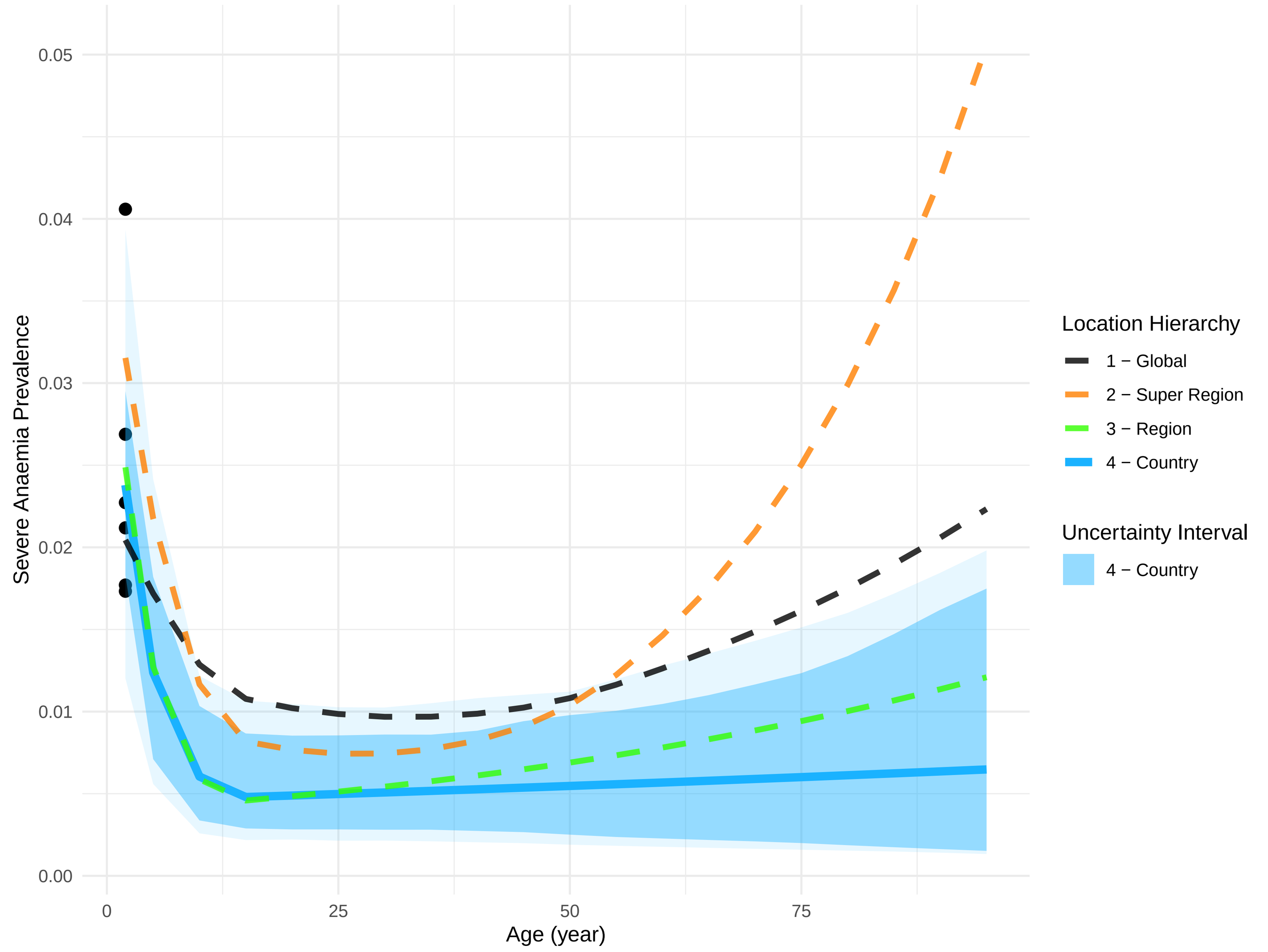

MR−BRT Age/Sex Fits − Males 2+ Years Old − Severe Anaemia Prevalence − Zambia
WHO−2024 Adjustment Method

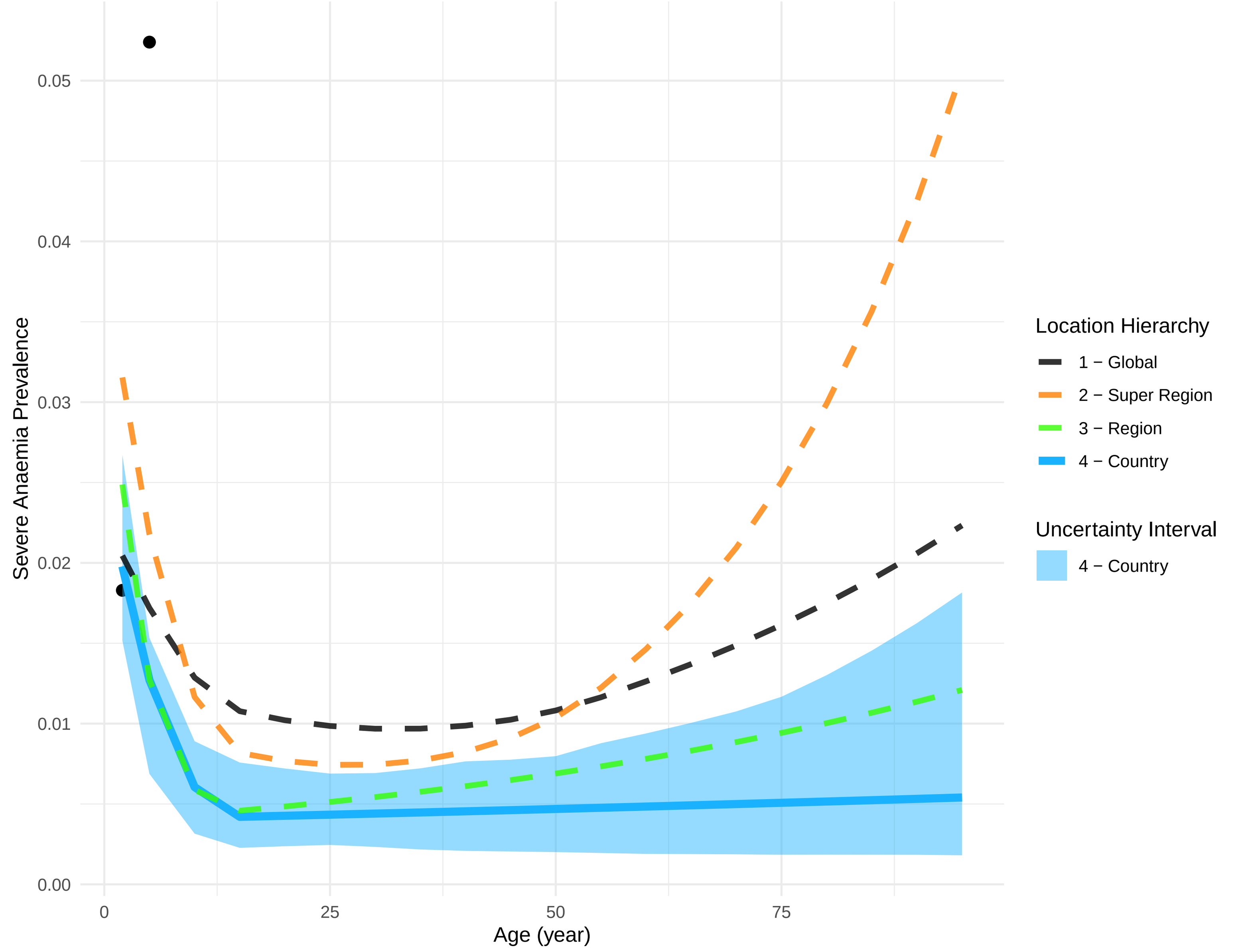

MR−BRT Age/Sex Fits − Males 2+ Years Old − Severe Anaemia Prevalence − Southern Sub−Saharan Africa
WHO−2024 Adjustment Method

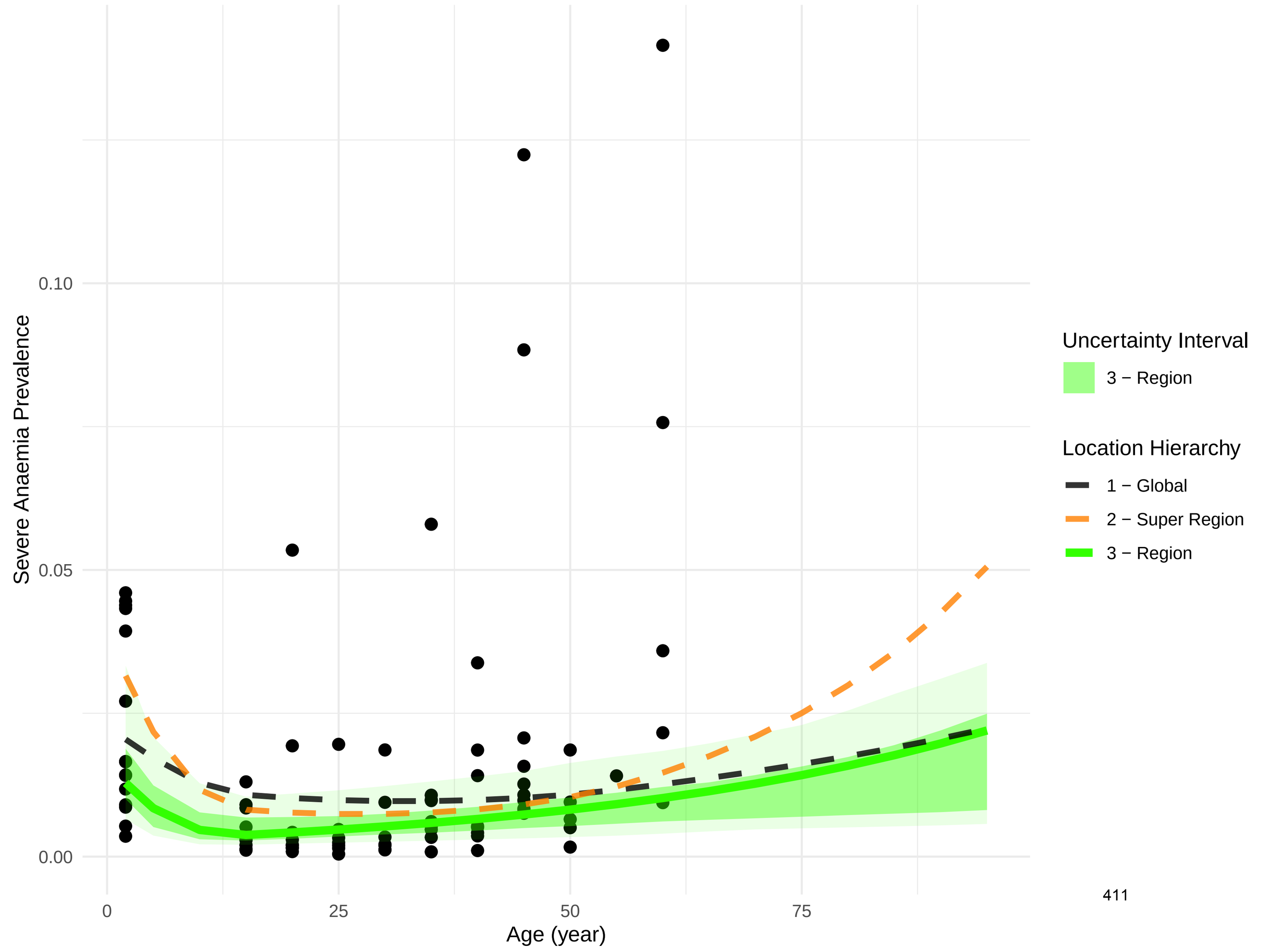

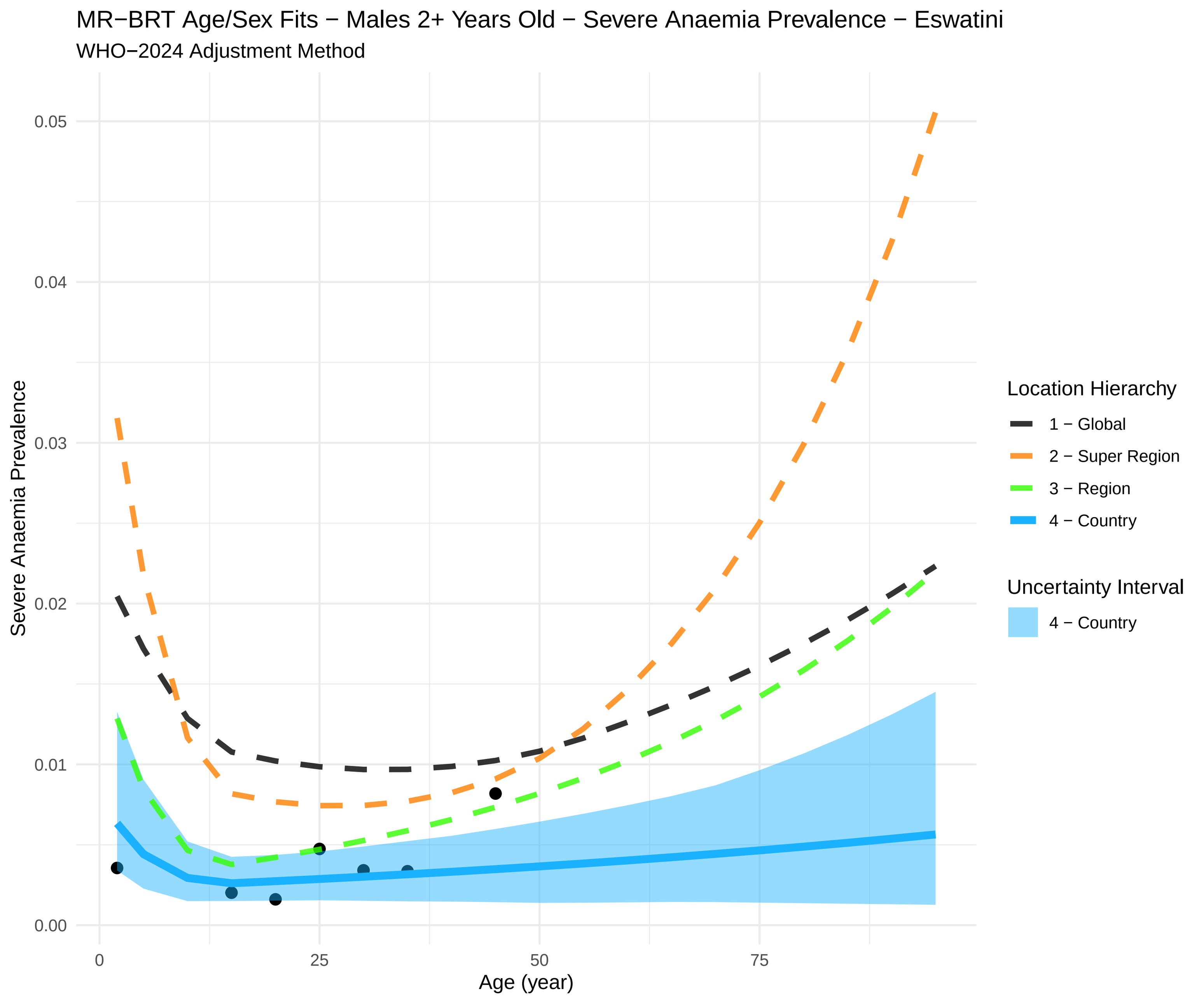
MR−BRT Age/Sex Fits − Males 2+ Years Old − Severe Anaemia Prevalence − Eswatini
WHO−2024 Adjustment Method
Severe Anaemia Prevalence
Age (year)
Location Hierarchy
1 − Global
2 − Super Region
3 − Region
4 − Country
Uncertainty Interval
4 − Country

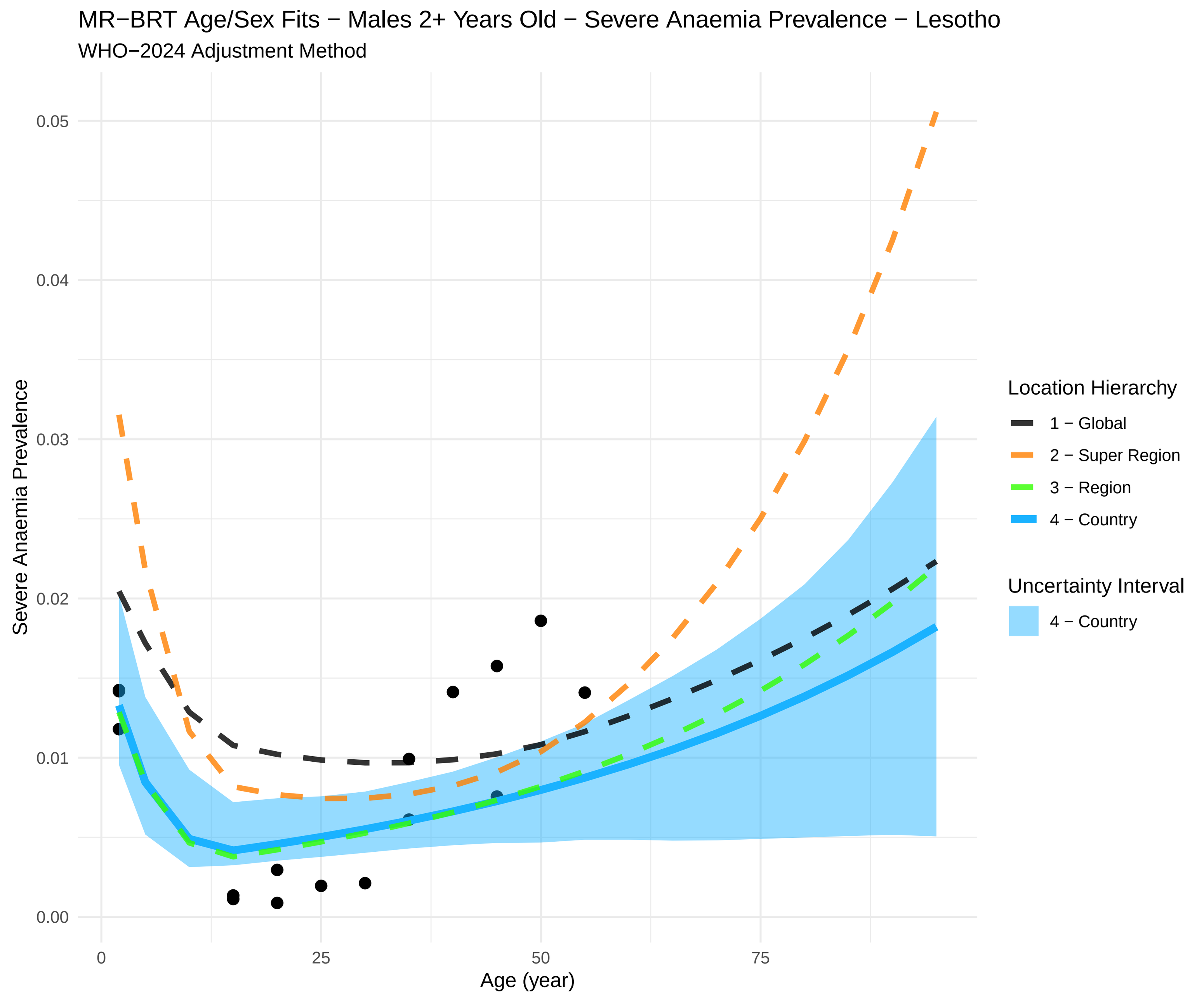
MR−BRT Age/Sex Fits − Males 2+ Years Old − Severe Anaemia Prevalence − Lesotho
WHO−2024 Adjustment Method
Severe Anaemia Prevalence
Age (year)
Location Hierarchy
1 − Global
2 − Super Region
3 − Region
4 − Country
Uncertainty Interval
4 − Country

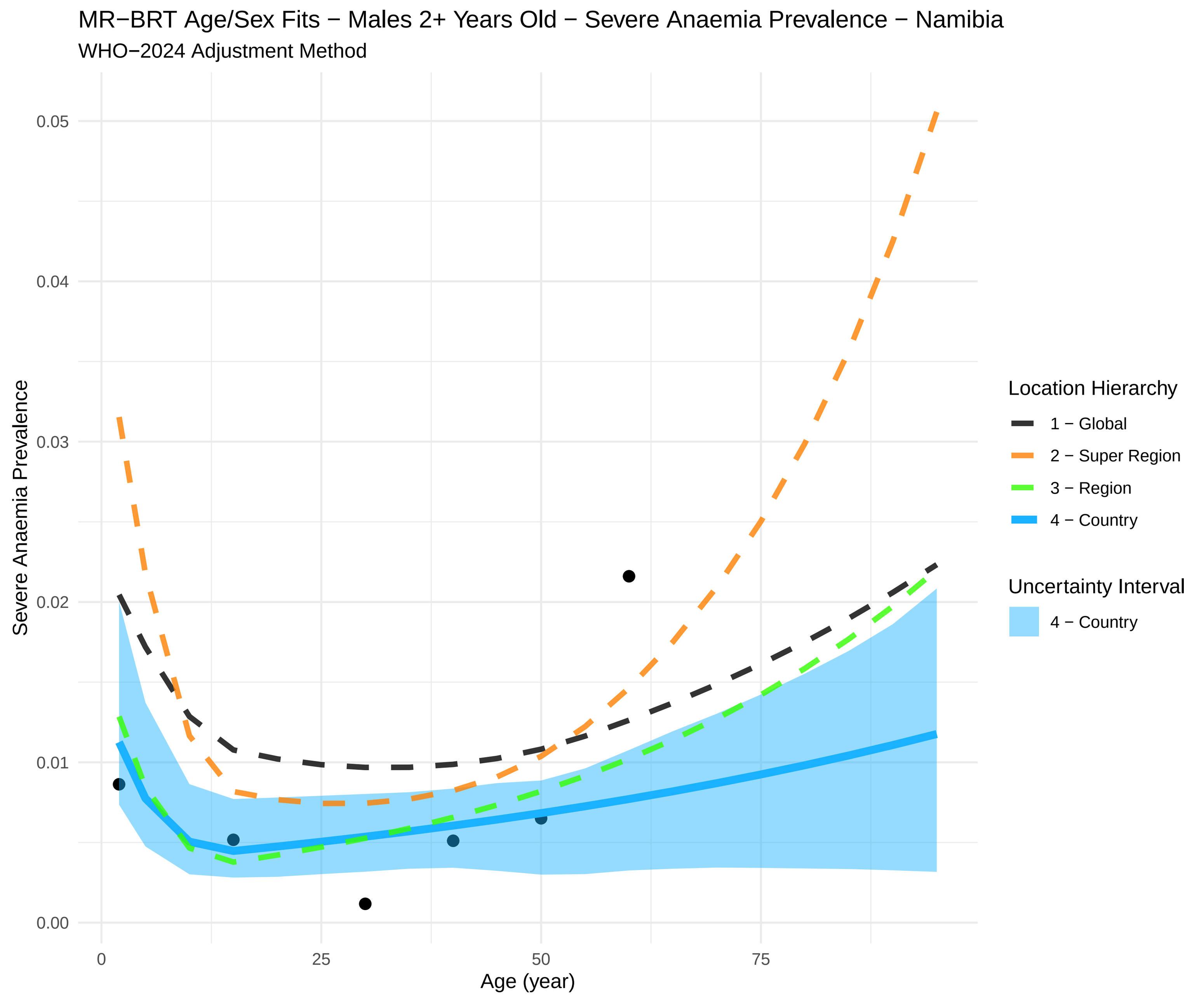
MR−BRT Age/Sex Fits − Males 2+ Years Old − Severe Anaemia Prevalence − Namibia
WHO−2024 Adjustment Method
Severe Anaemia Prevalence
Age (year)
Location Hierarchy
1 − Global
2 − Super Region
3 − Region
4 − Country
Uncertainty Interval
4 − Country

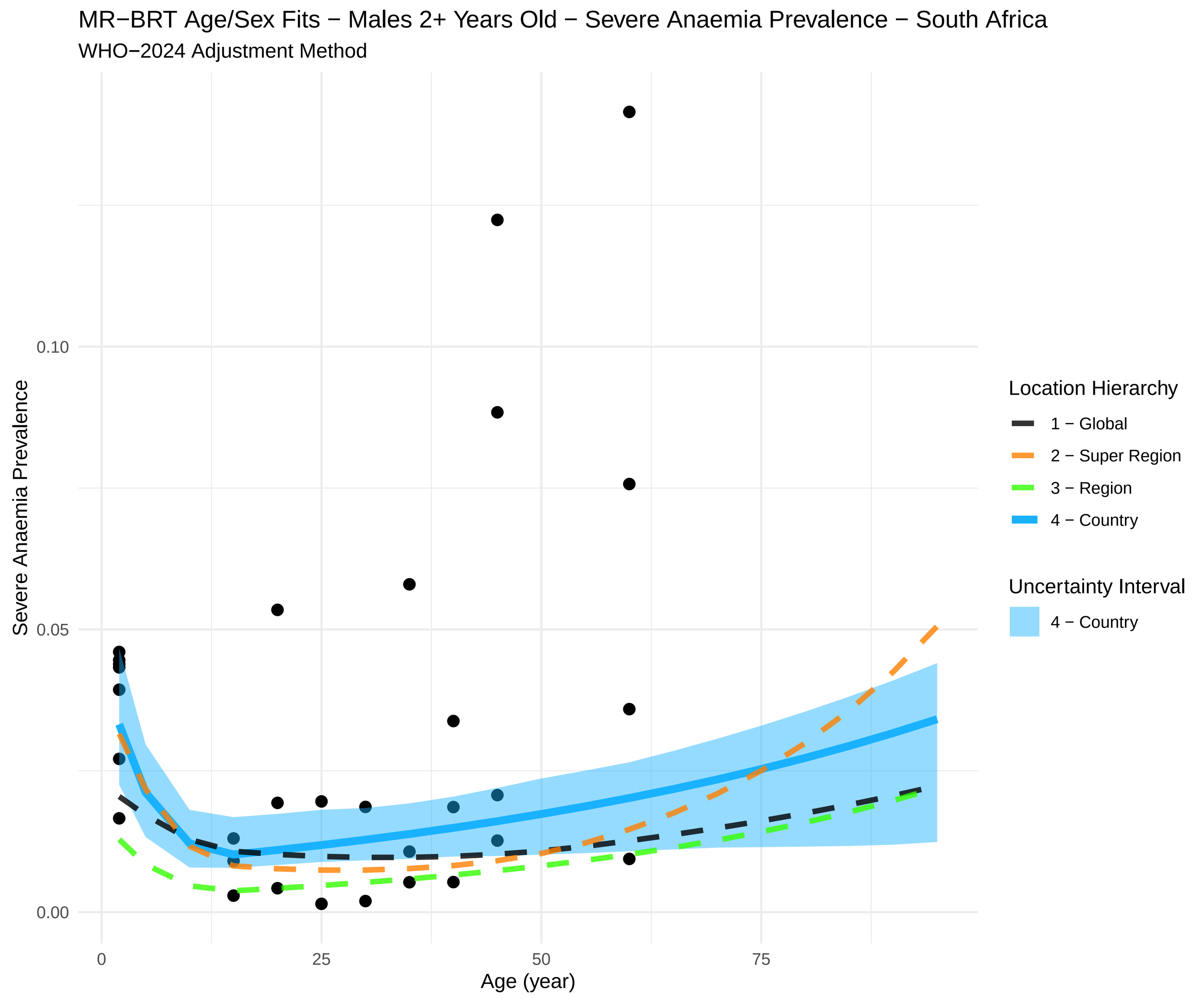
MR−BRT Age/Sex Fits − Males 2+ Years Old − Severe Anaemia Prevalence − South Africa
WHO−2024 Adjustment Method
Severe Anaemia Prevalence
Age (year)
Location Hierarchy
1 − Global
2 − Super Region
3 − Region
4 − Country
Uncertainty Interval
4 − Country

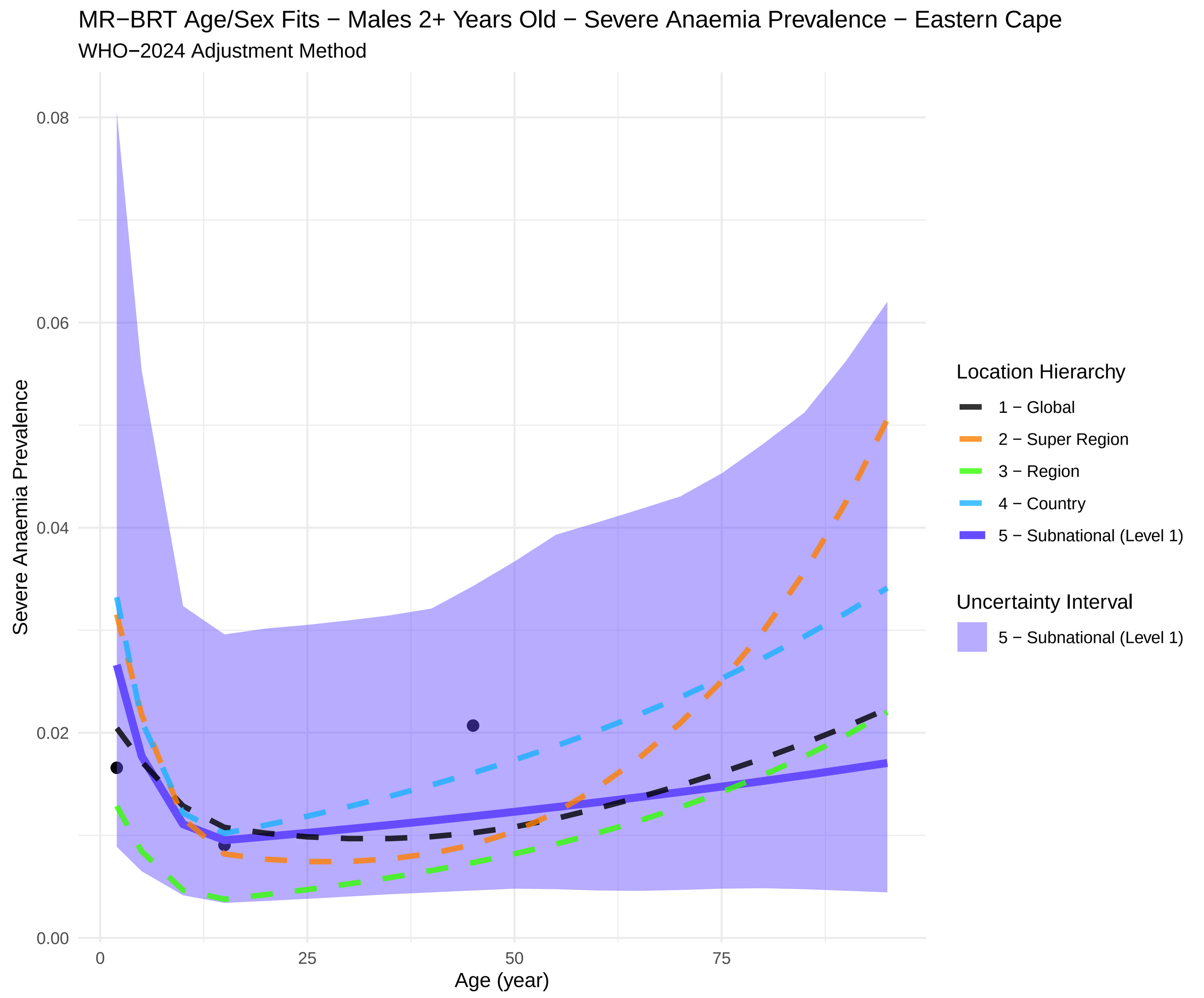
MR−BRT Age/Sex Fits − Males 2+ Years Old − Severe Anaemia Prevalence − Eastern Cape
WHO−2024 Adjustment Method
Severe Anaemia Prevalence
Age (year)
Location Hierarchy
1 − Global
2 − Super Region
3 − Region
4 − Country
5 − Subnational (Level 1)
Uncertainty Interval
5 − Subnational (Level 1)

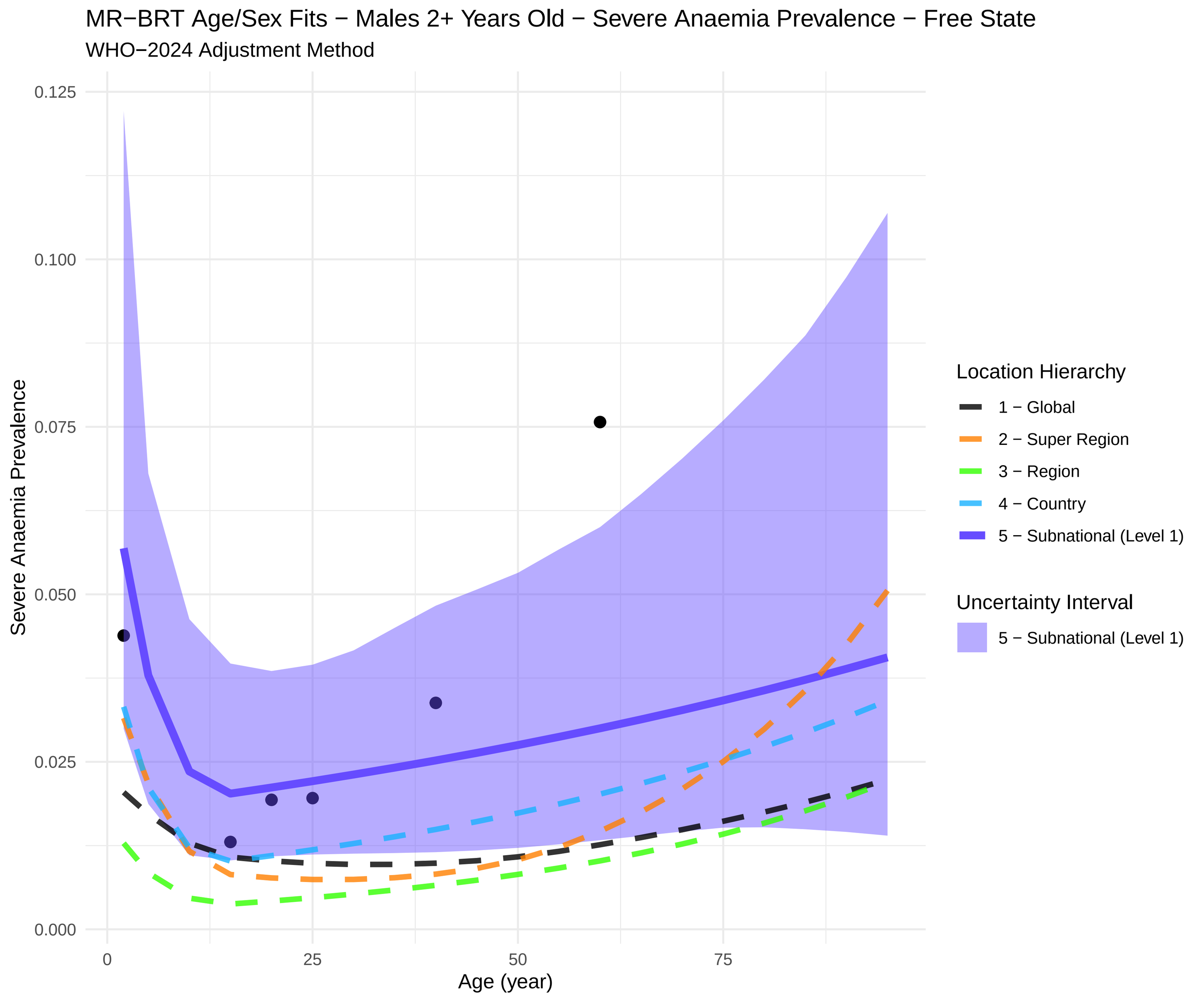
MR−BRT Age/Sex Fits − Males 2+ Years Old − Severe Anaemia Prevalence − Free State
WHO−2024 Adjustment Method
Severe Anaemia Prevalence
Age (year)
Location Hierarchy
1 − Global
2 − Super Region
3 − Region
4 − Country
5 − Subnational (Level 1)
Uncertainty Interval
5 − Subnational (Level 1)

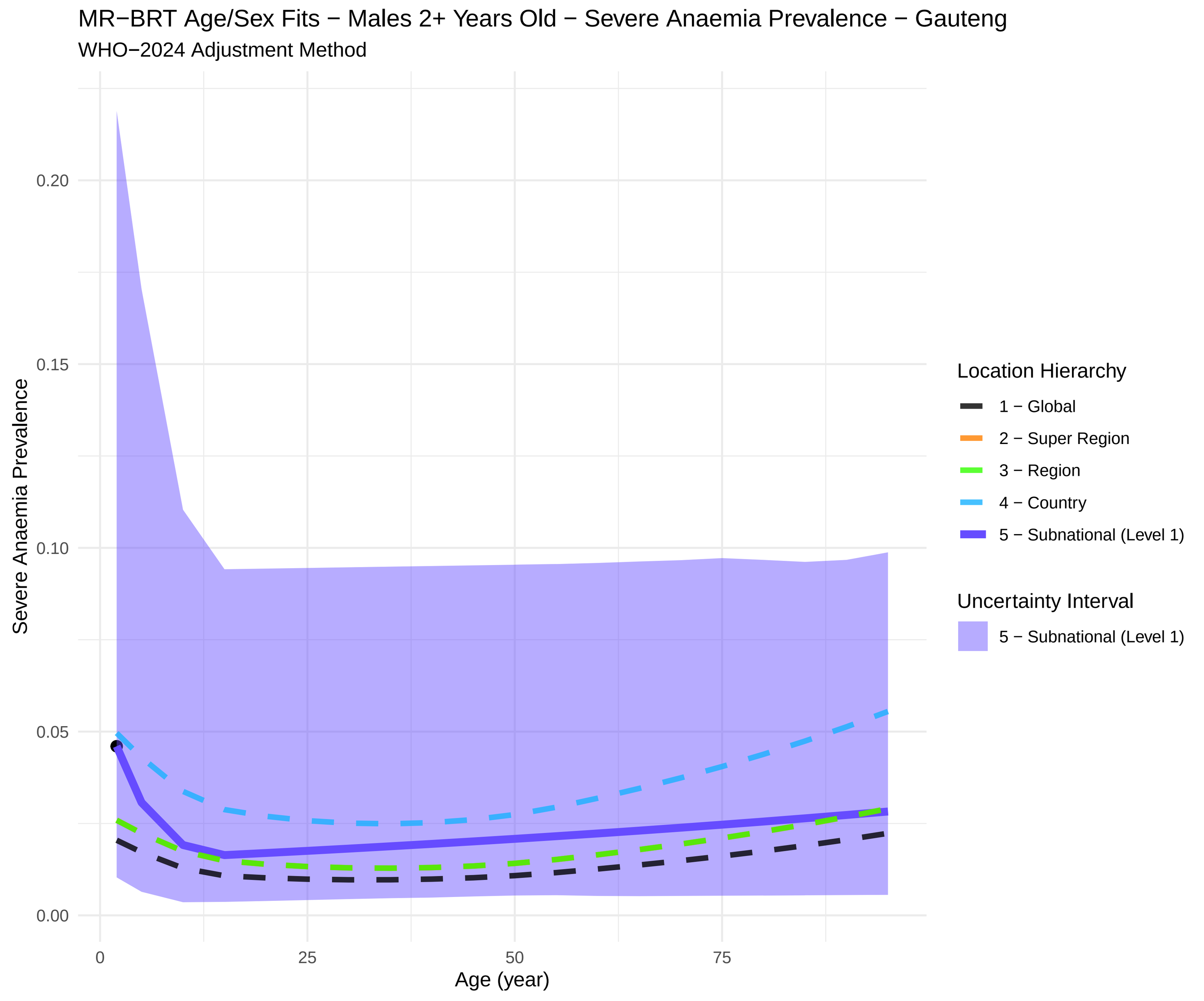
MR−BRT Age/Sex Fits − Males 2+ Years Old − Severe Anaemia Prevalence − Gauteng
WHO−2024 Adjustment Method
Severe Anaemia Prevalence
Age (year)
Location Hierarchy
1 − Global
2 − Super Region
3 − Region
4 − Country
5 − Subnational (Level 1)
Uncertainty Interval
5 − Subnational (Level 1)

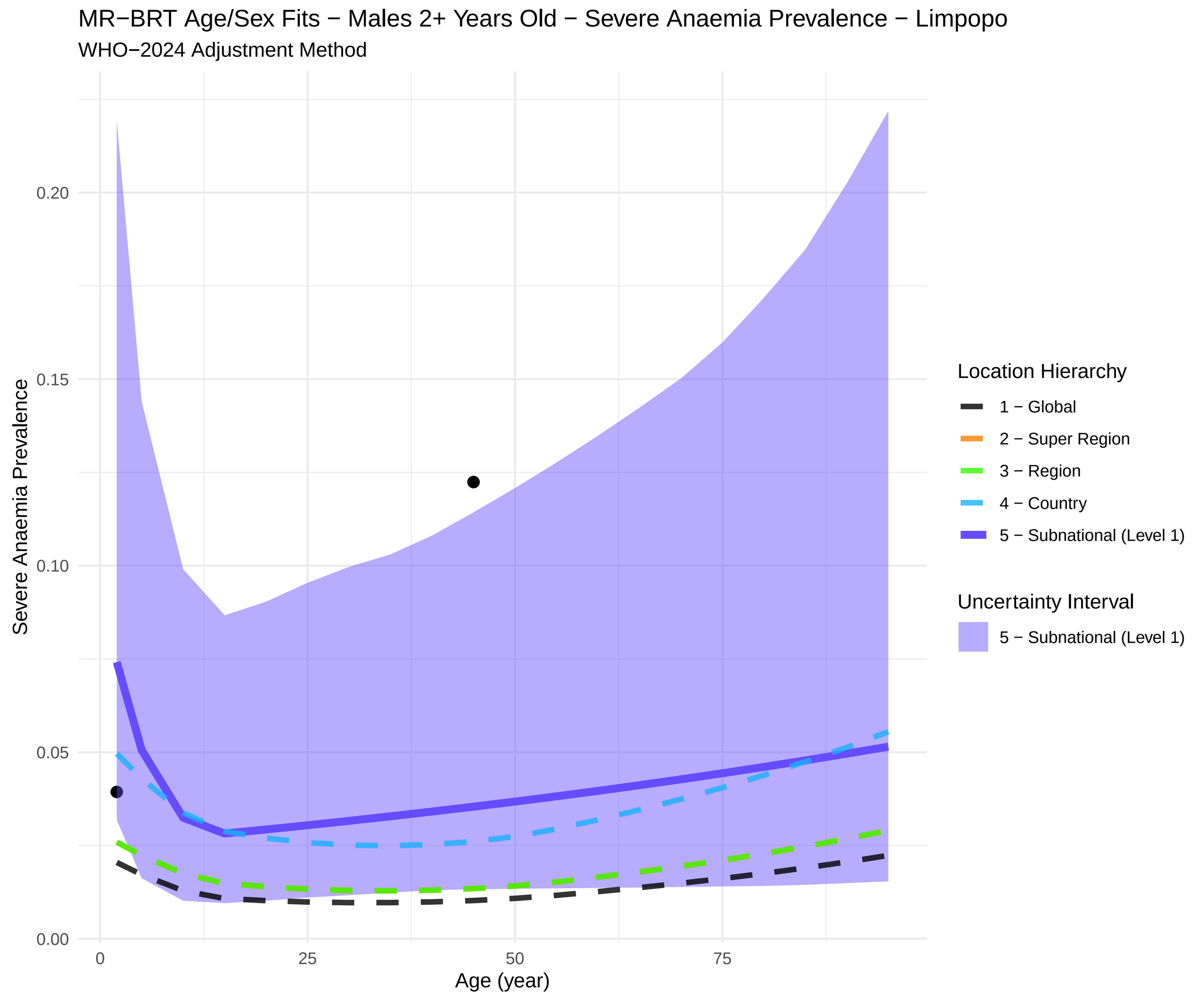
MR−BRT Age/Sex Fits − Males 2+ Years Old − Severe Anaemia Prevalence − Limpopo
WHO−2024 Adjustment Method
Severe Anaemia Prevalence
Age (year)
Location Hierarchy
1 − Global
2 − Super Region
3 − Region
4 − Country
5 − Subnational (Level 1)
Uncertainty Interval
5 − Subnational (Level 1)

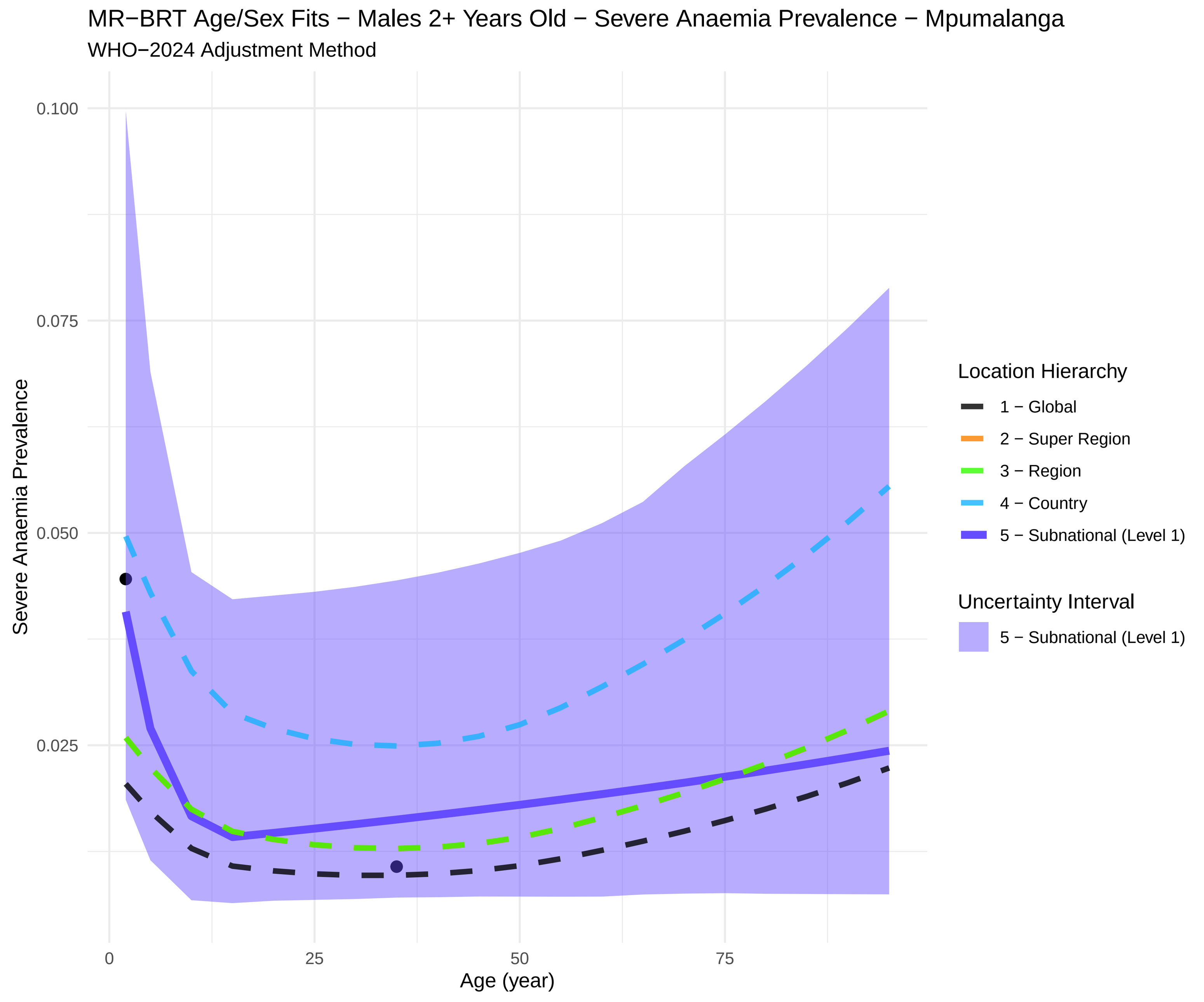
MR−BRT Age/Sex Fits − Males 2+ Years Old − Severe Anaemia Prevalence − Mpumalanga
WHO−2024 Adjustment Method
Severe Anaemia Prevalence
Age (year)
Location Hierarchy
1 − Global
2 − Super Region
3 − Region
4 − Country
5 − Subnational (Level 1)
Uncertainty Interval
5 − Subnational (Level 1)

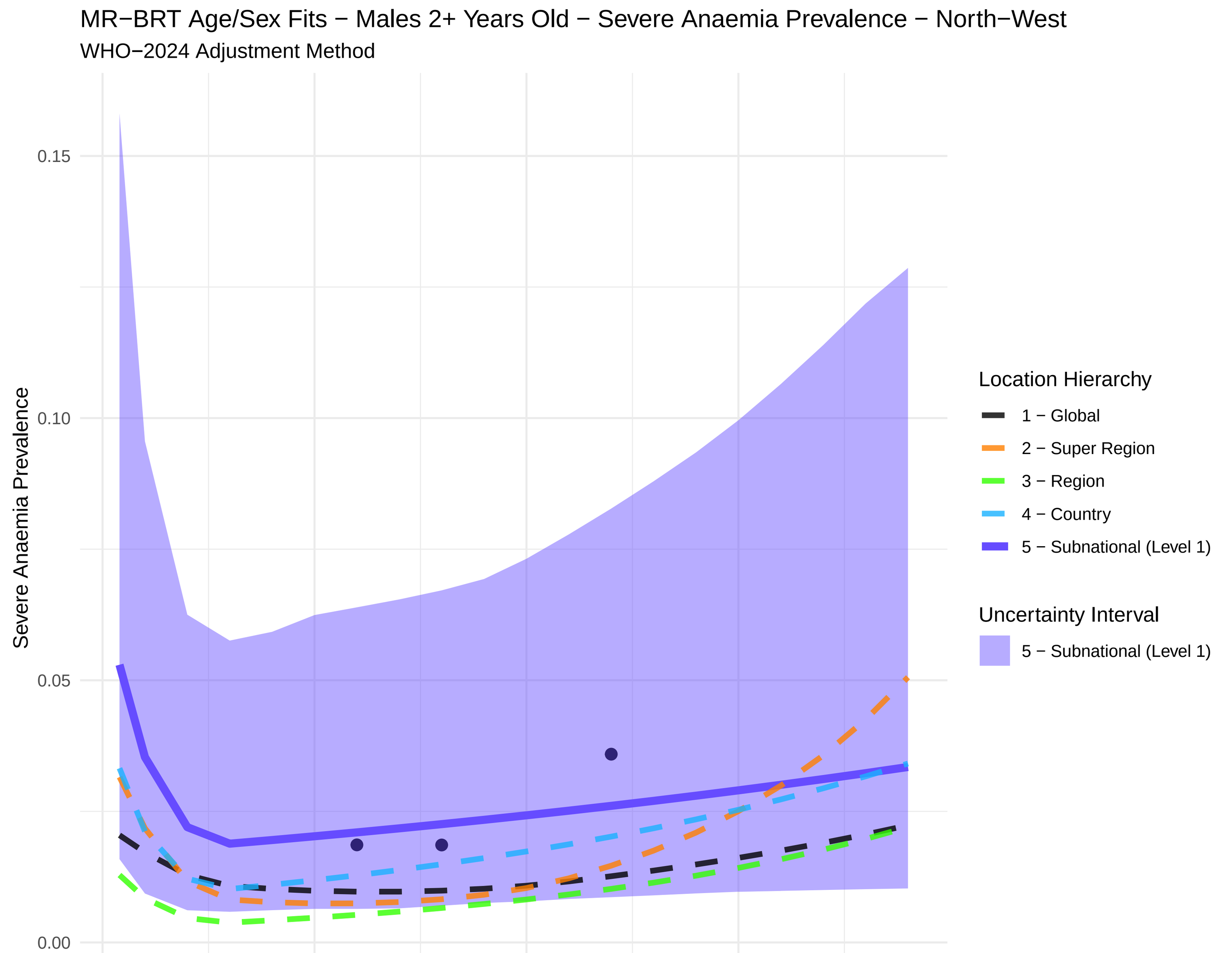
MR−BRT Age/Sex Fits − Males 2+ Years Old − Severe Anaemia Prevalence − North−West
WHO−2024 Adjustment Method
Severe Anaemia Prevalence
Age (year)
Location Hierarchy
1 − Global
2 − Super Region
3 − Region
4 − Country
5 − Subnational (Level 1)
Uncertainty Interval
5 − Subnational (Level 1)

MR−BRT Age/Sex Fits − Males 2+ Years Old − Severe Anaemia Prevalence − Northern Cape
WHO−2024 Adjustment Method

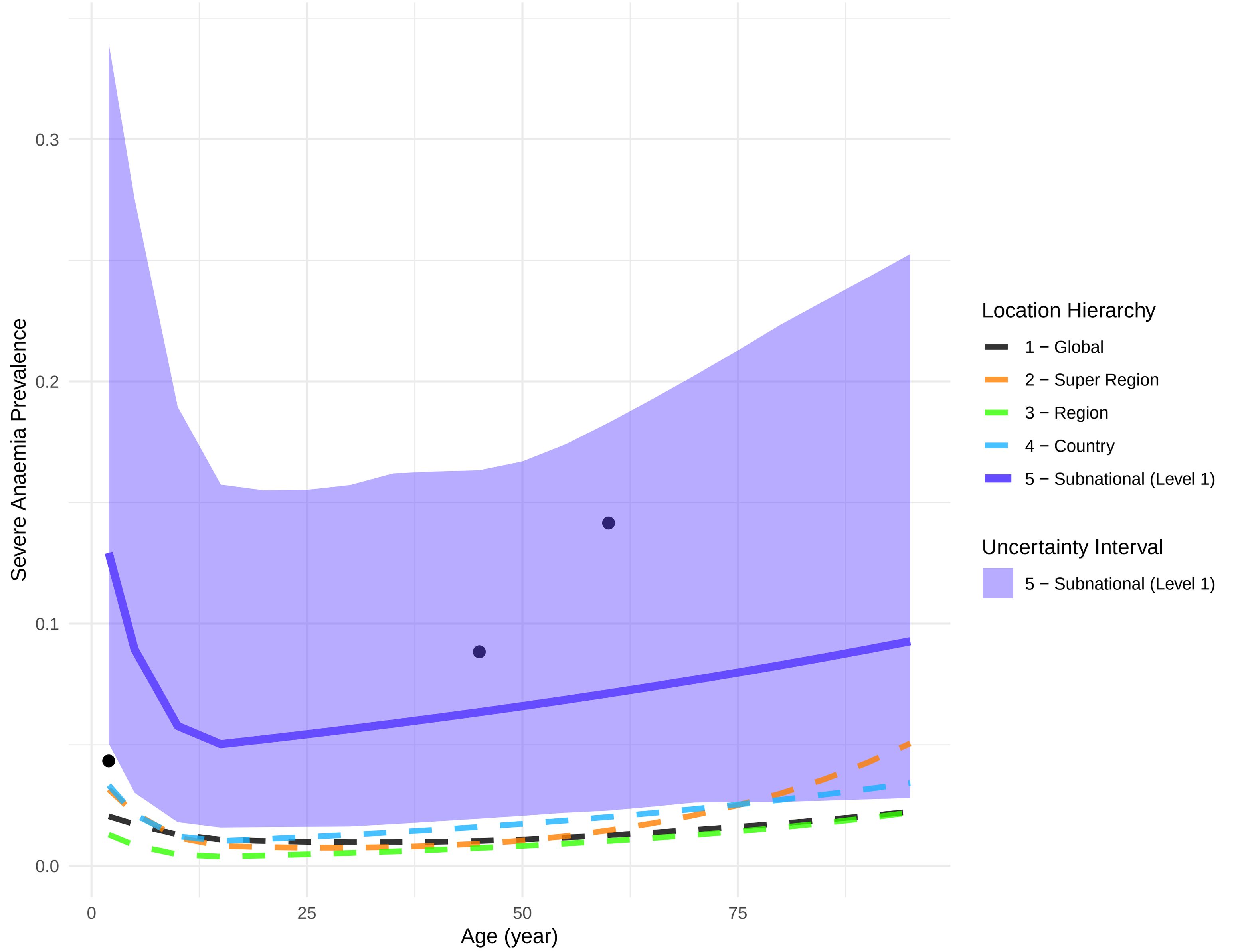

MR−BRT Age/Sex Fits − Males 2+ Years Old − Severe Anaemia Prevalence − Western Cape
WHO−2024 Adjustment Method

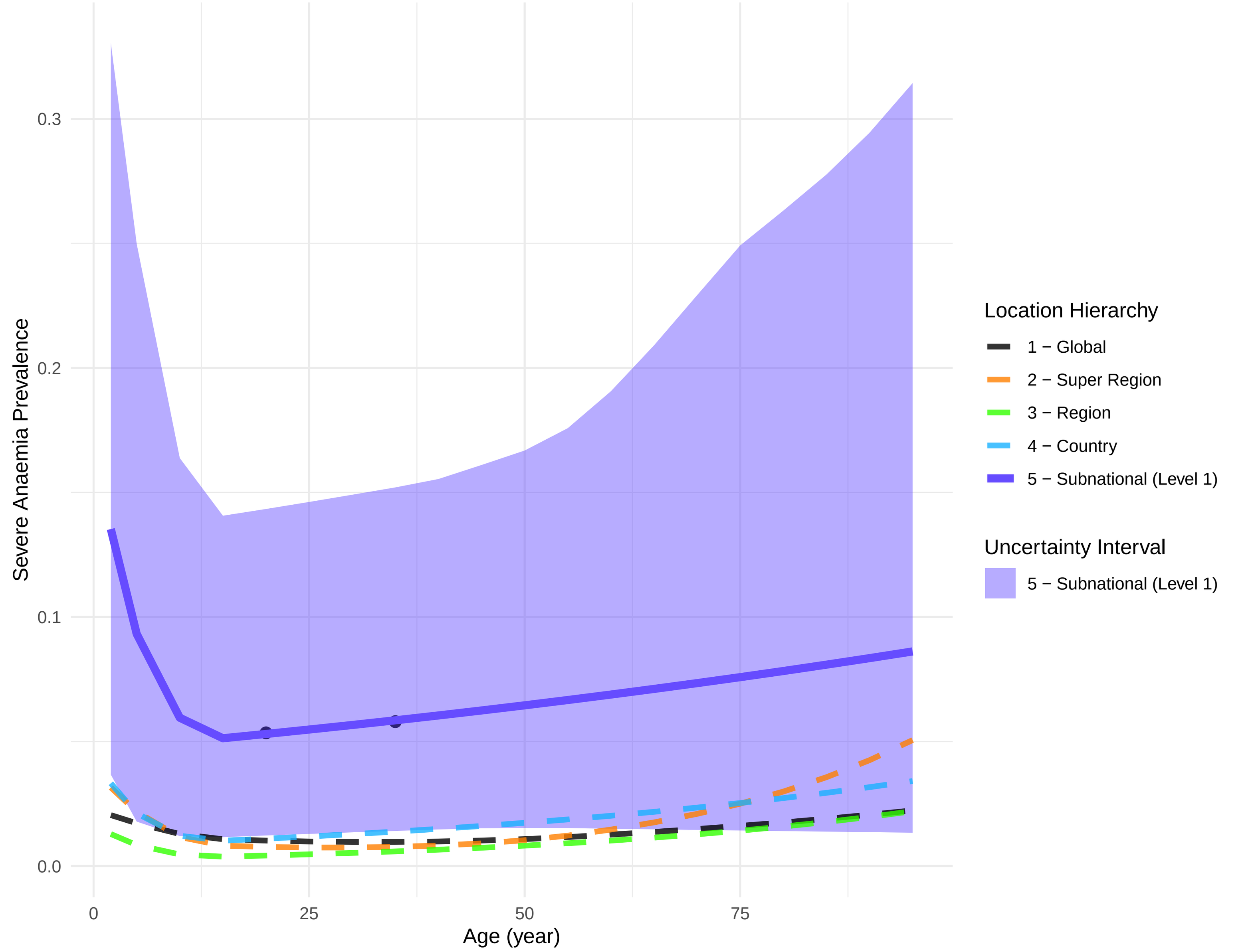

MR−BRT Age/Sex Fits − Males 2+ Years Old − Severe Anaemia Prevalence − Zimbabwe
WHO−2024 Adjustment Method

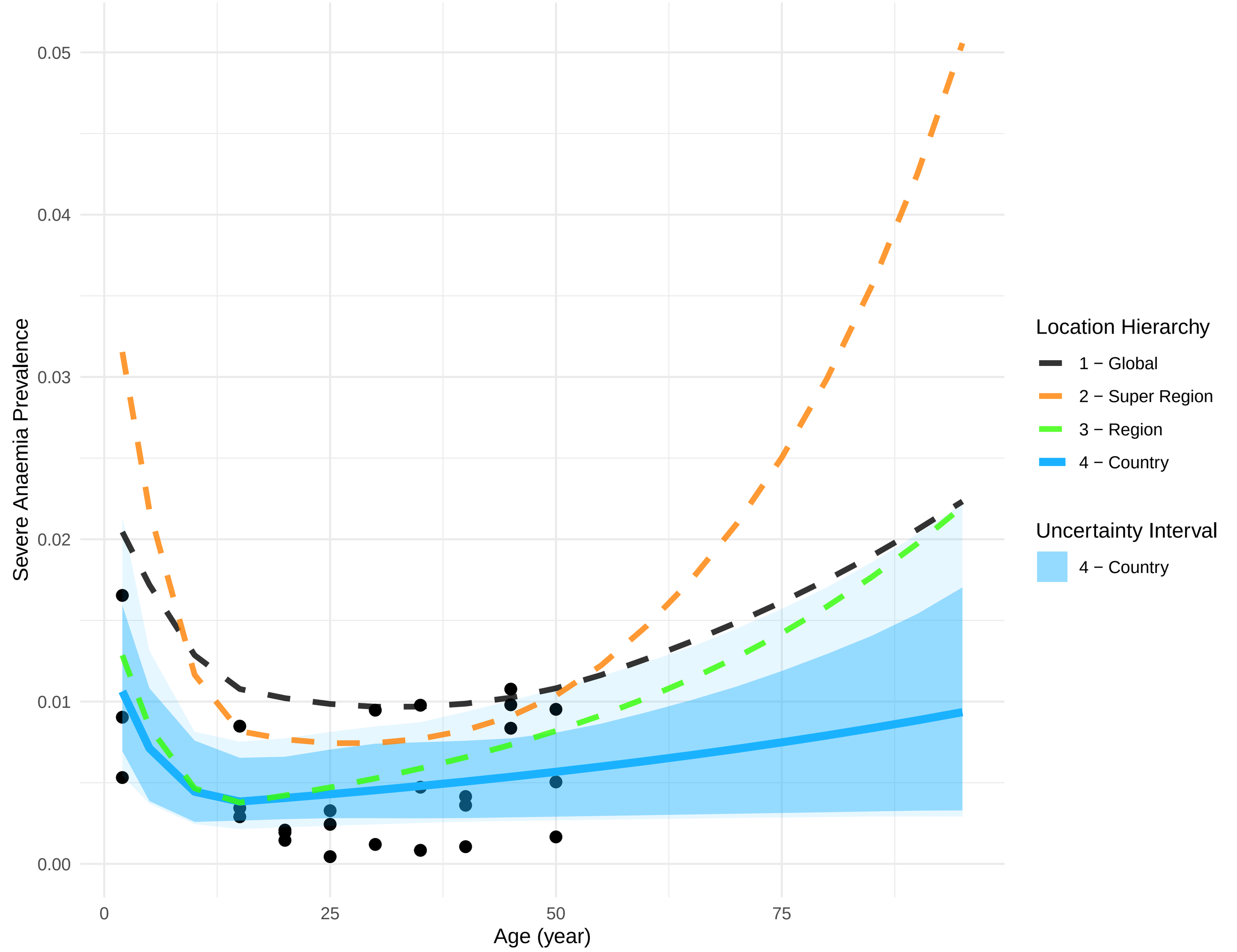

MR−BRT Age/Sex Fits − Males 2+ Years Old − Severe Anaemia Prevalence − Western Sub−Saharan Africa
WHO−2024 Adjustment Method

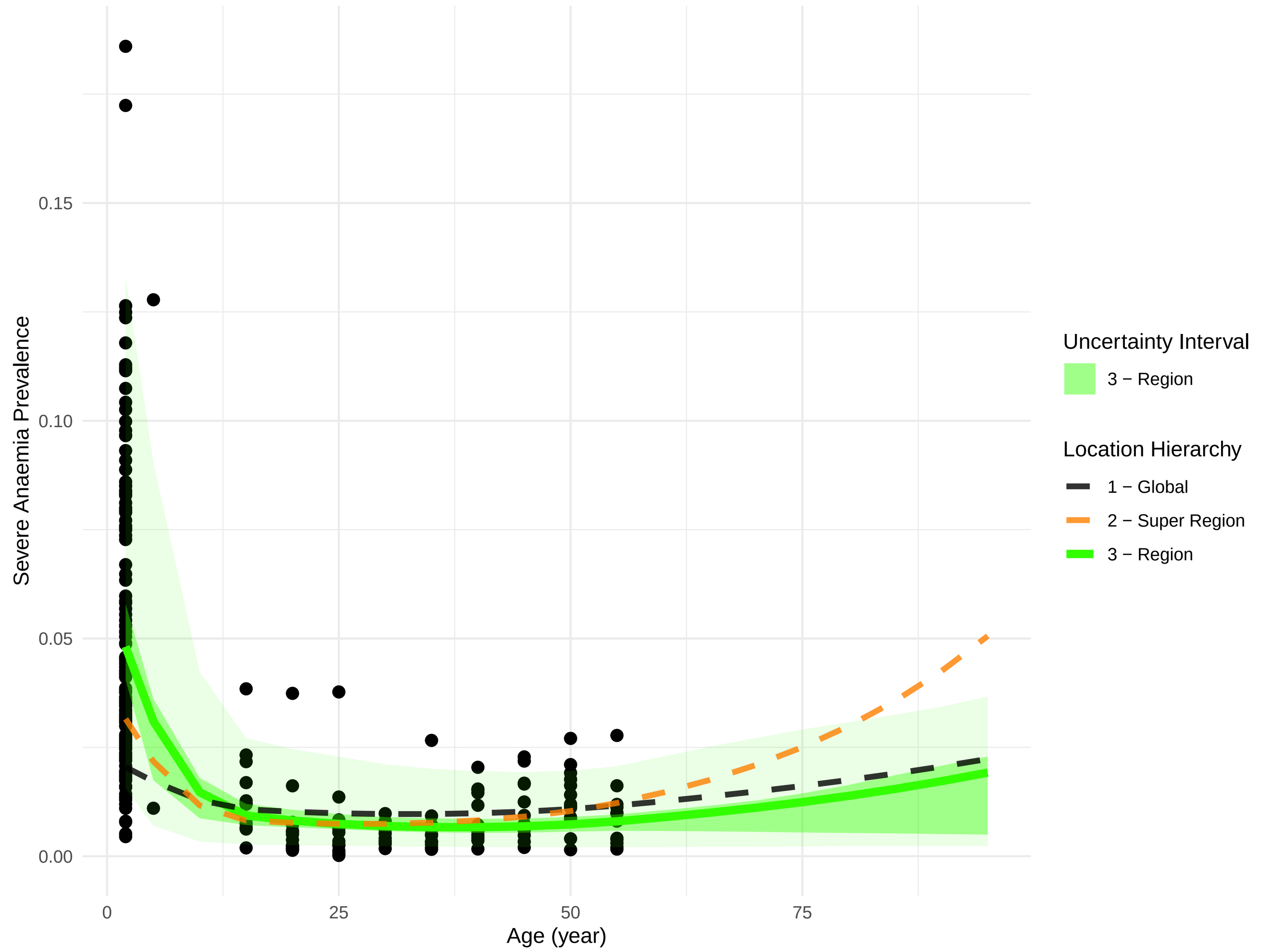

MR−BRT Age/Sex Fits − Males 2+ Years Old − Severe Anaemia Prevalence − Benin
WHO−2024 Adjustment Method

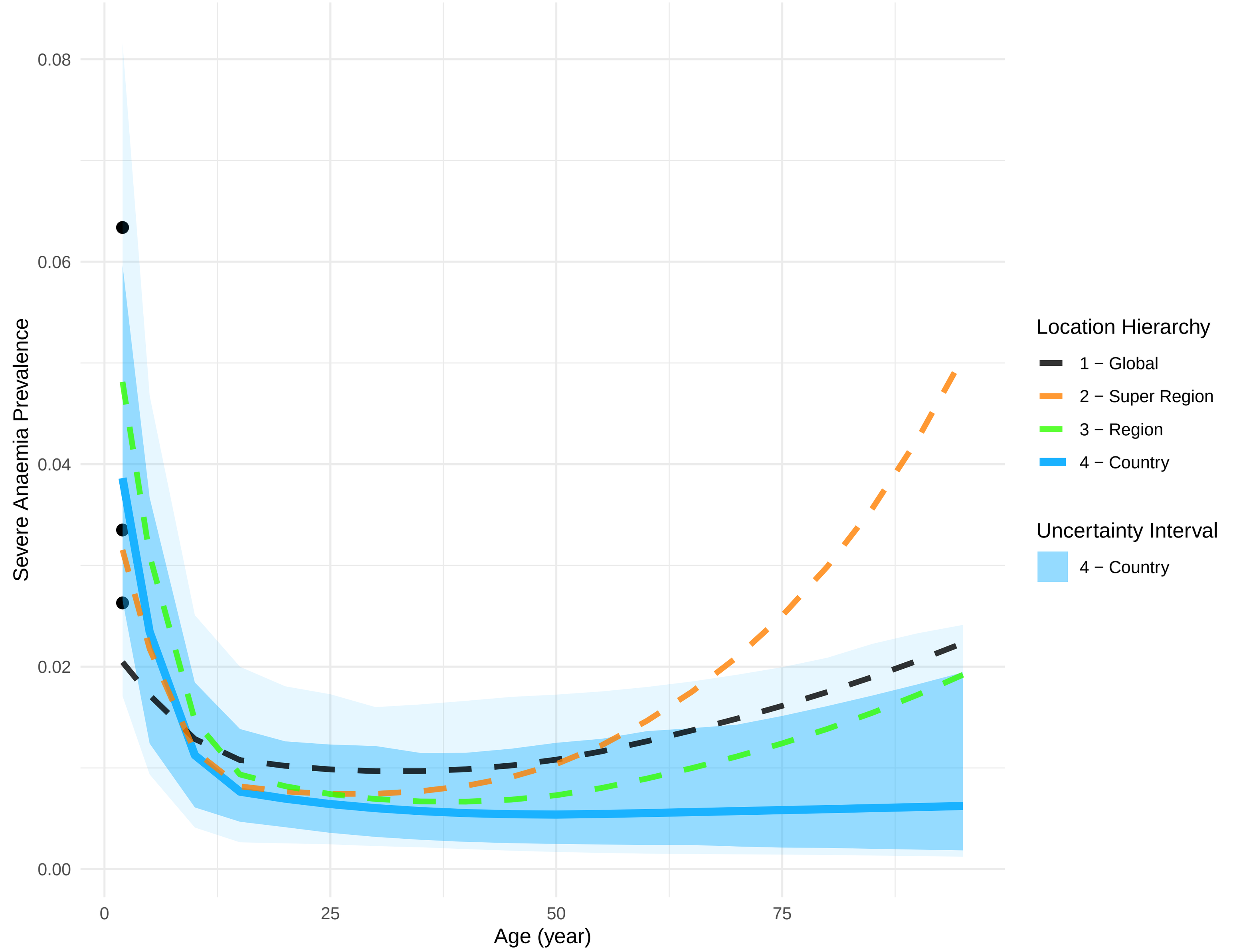

MR−BRT Age/Sex Fits − Males 2+ Years Old − Severe Anaemia Prevalence − Burkina Faso
WHO−2024 Adjustment Method

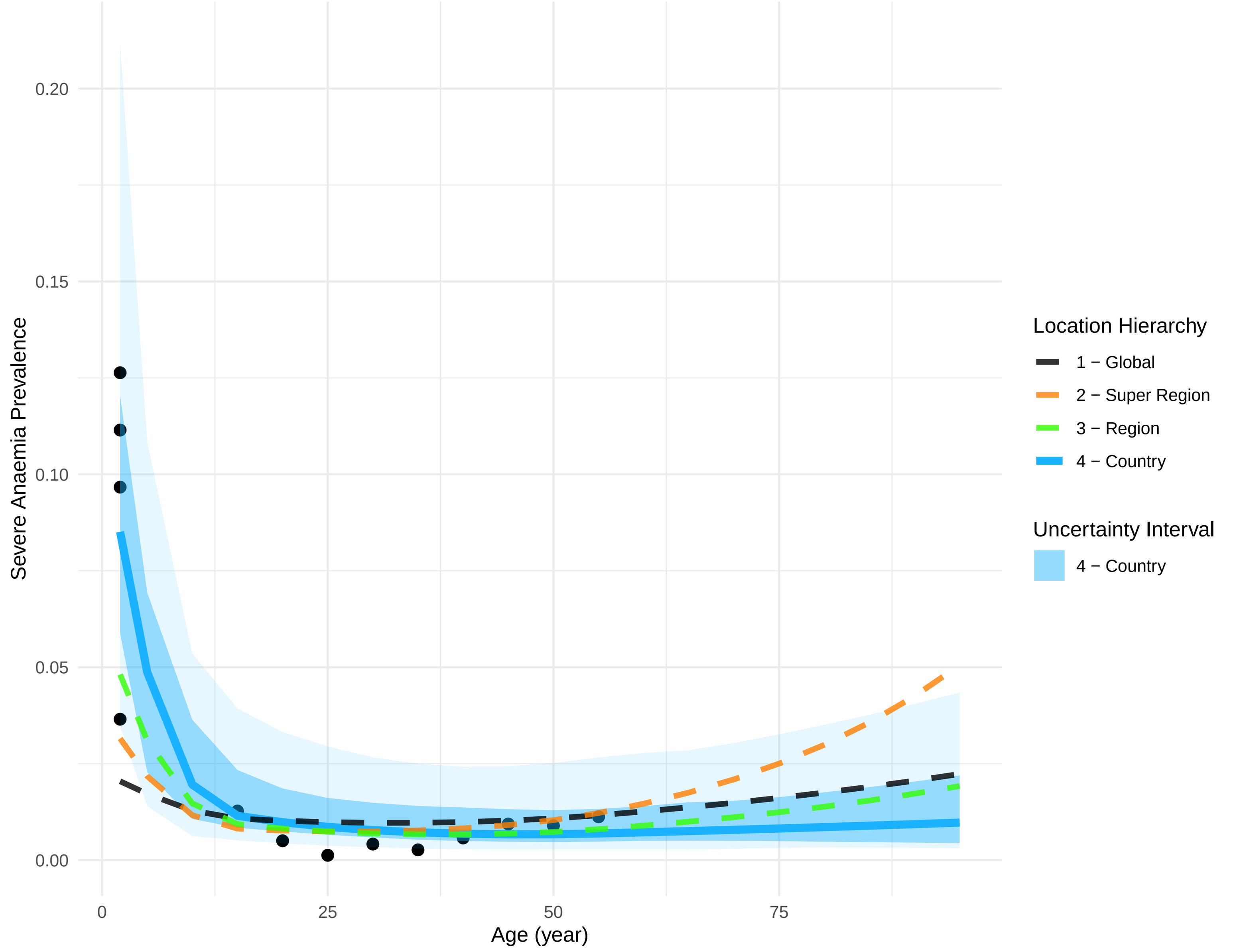

MR−BRT Age/Sex Fits − Males 2+ Years Old − Severe Anaemia Prevalence − Cameroon
WHO−2024 Adjustment Method

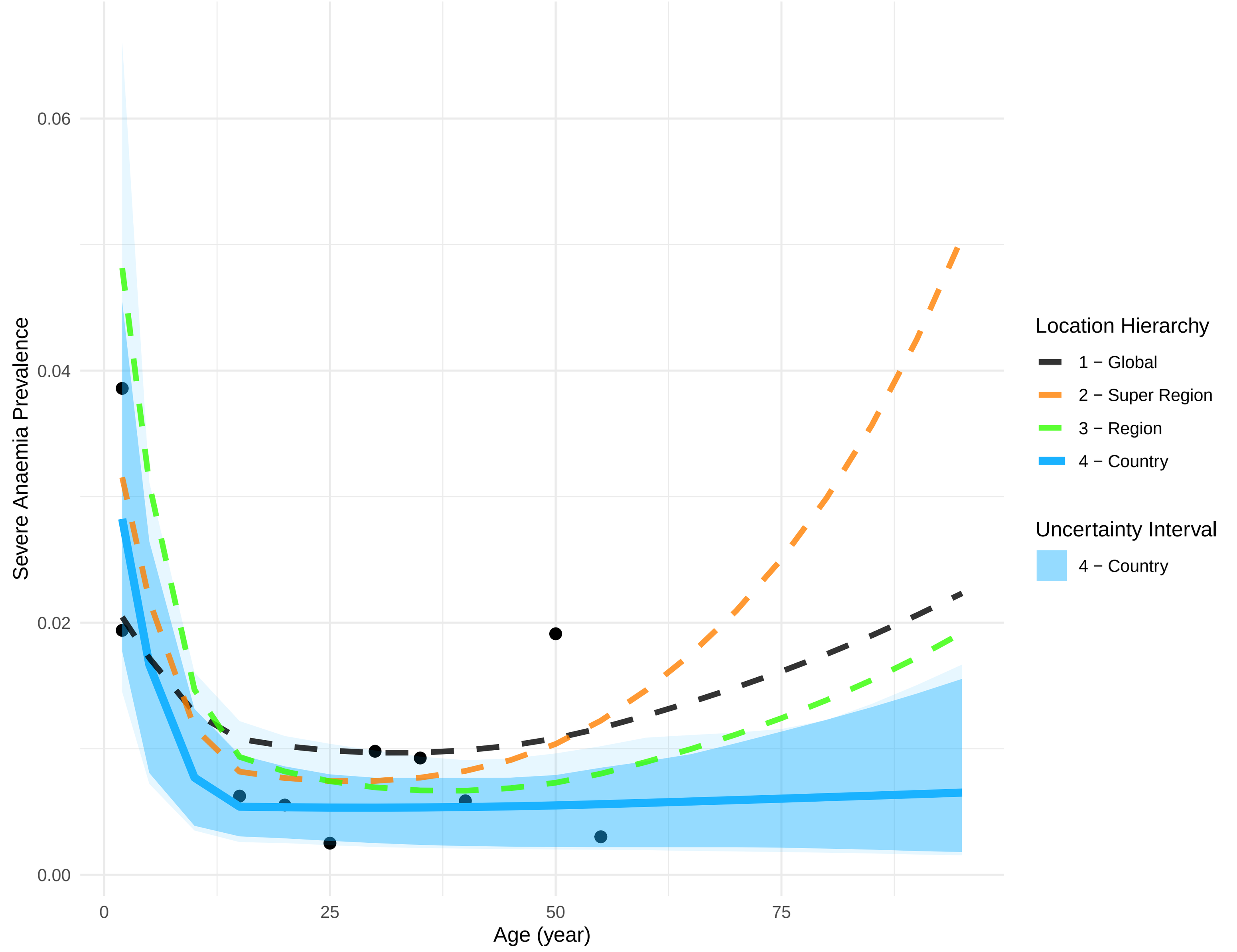

MR−BRT Age/Sex Fits − Males 2+ Years Old − Severe Anaemia Prevalence − Côte d'Ivoire
WHO−2024 Adjustment Method

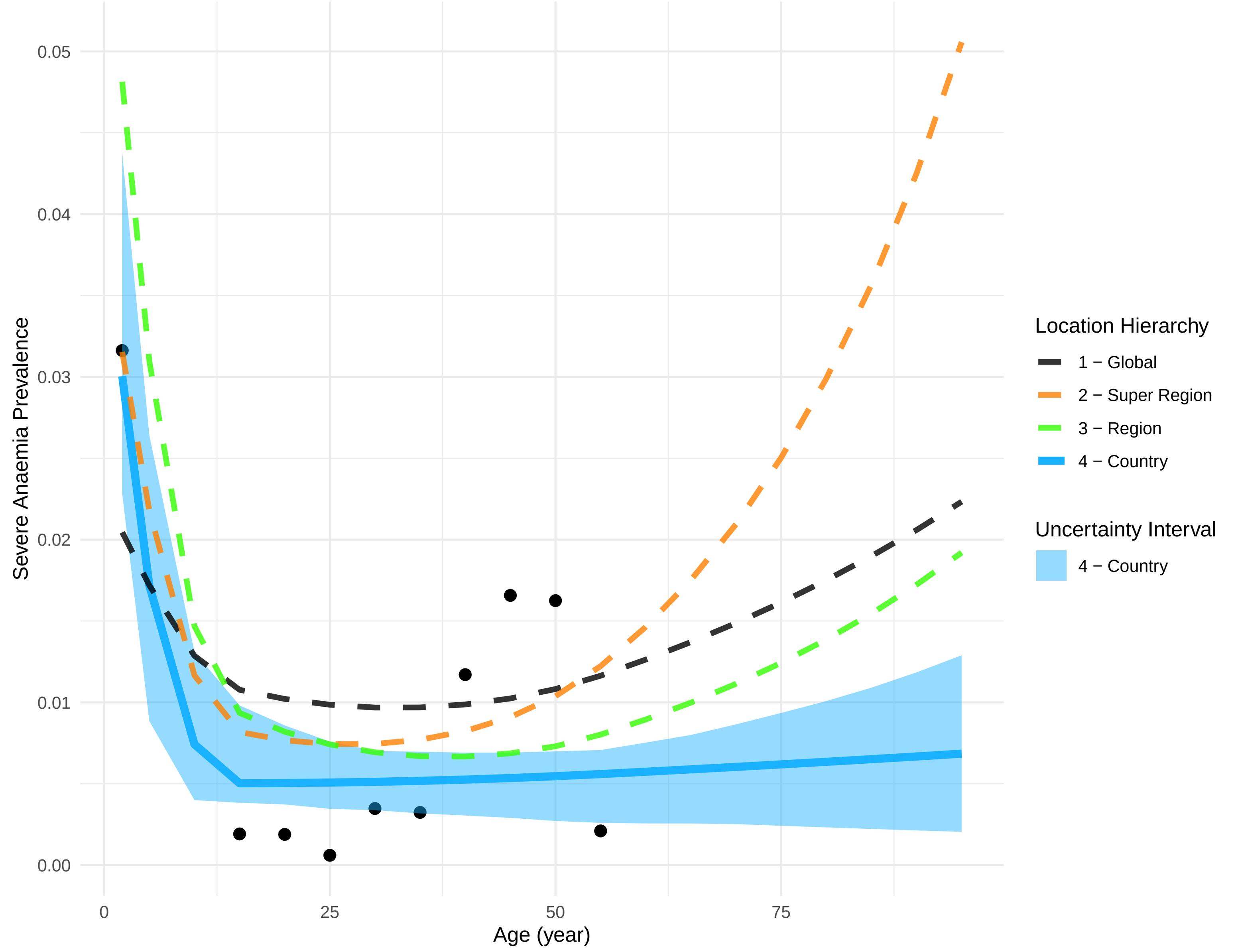

MR−BRT Age/Sex Fits − Males 2+ Years Old − Severe Anaemia Prevalence − Gambia
WHO−2024 Adjustment Method

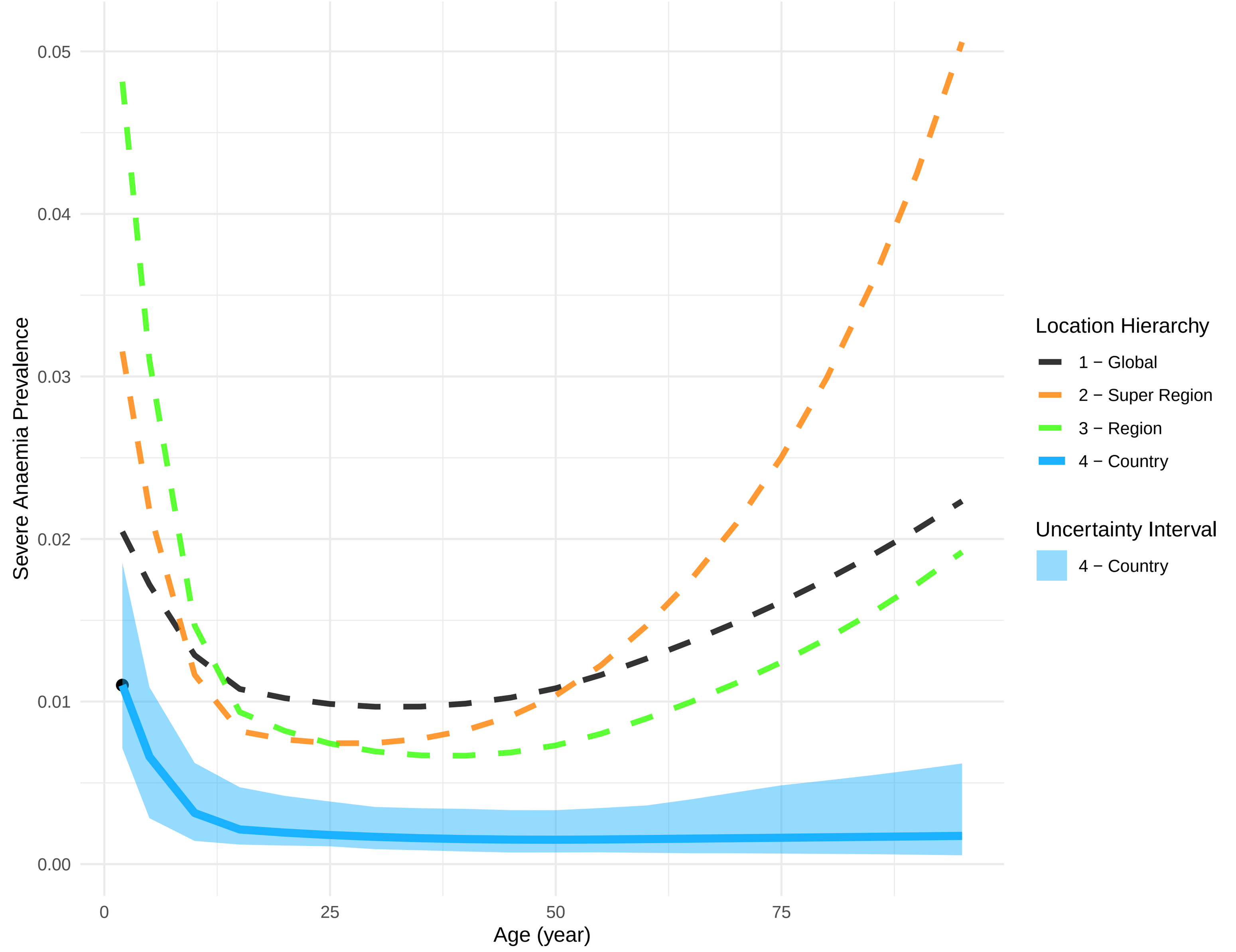

MR−BRT Age/Sex Fits − Males 2+ Years Old − Severe Anaemia Prevalence − Ghana
WHO−2024 Adjustment Method

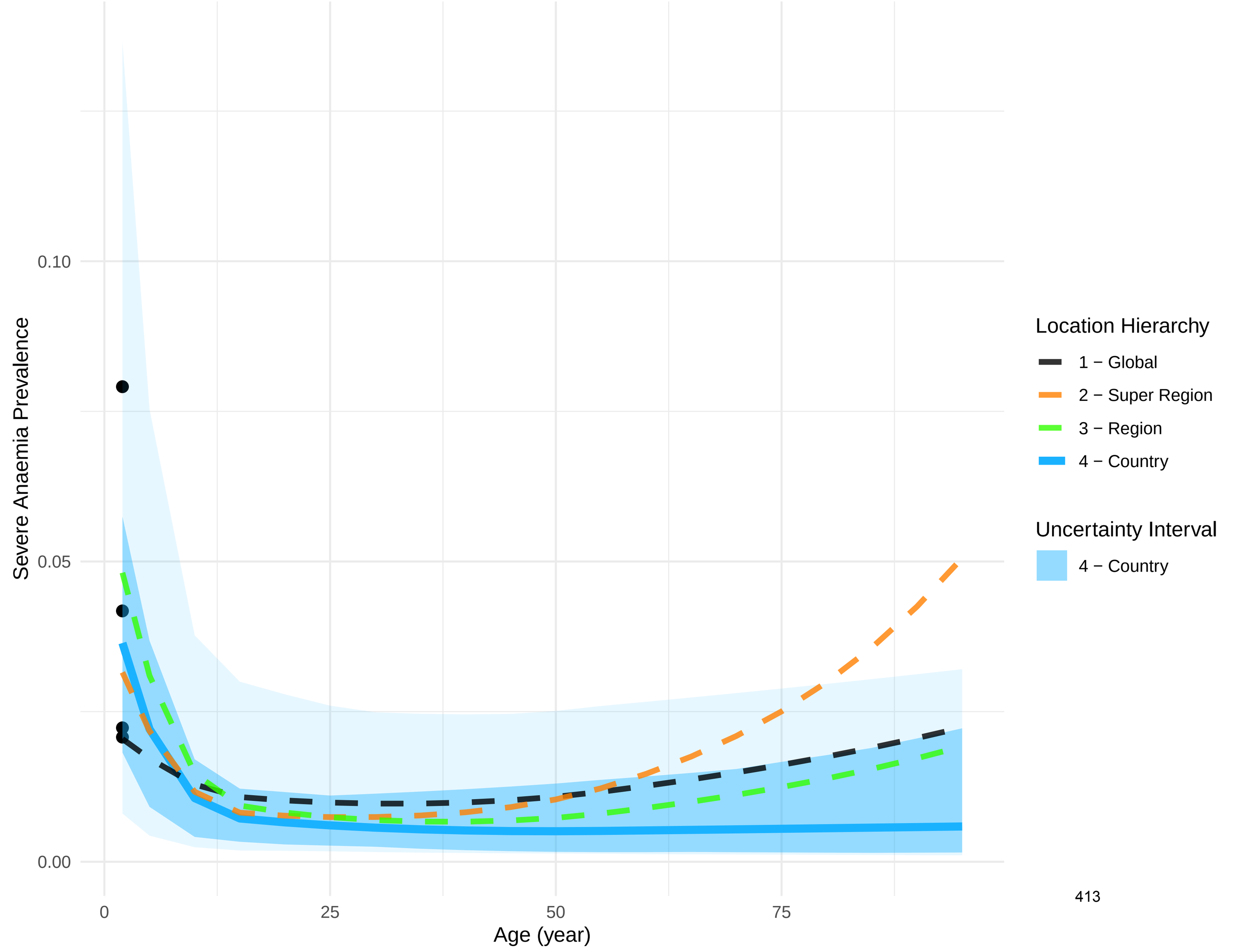

MR−BRT Age/Sex Fits − Males 2+ Years Old − Severe Anaemia Prevalence − Guinea
WHO−2024 Adjustment Method

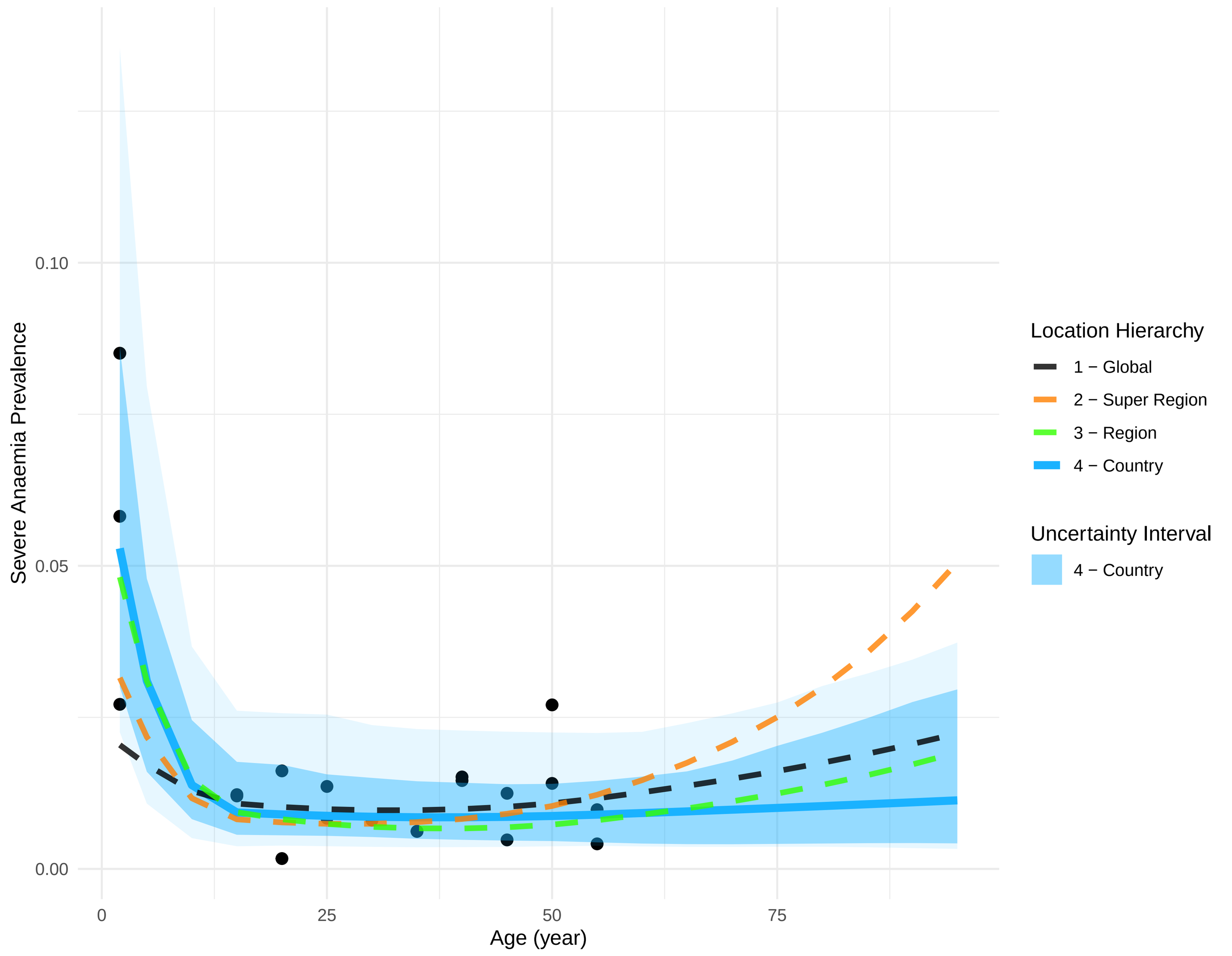

MR−BRT Age/Sex Fits − Males 2+ Years Old − Severe Anaemia Prevalence − Liberia
WHO−2024 Adjustment Method

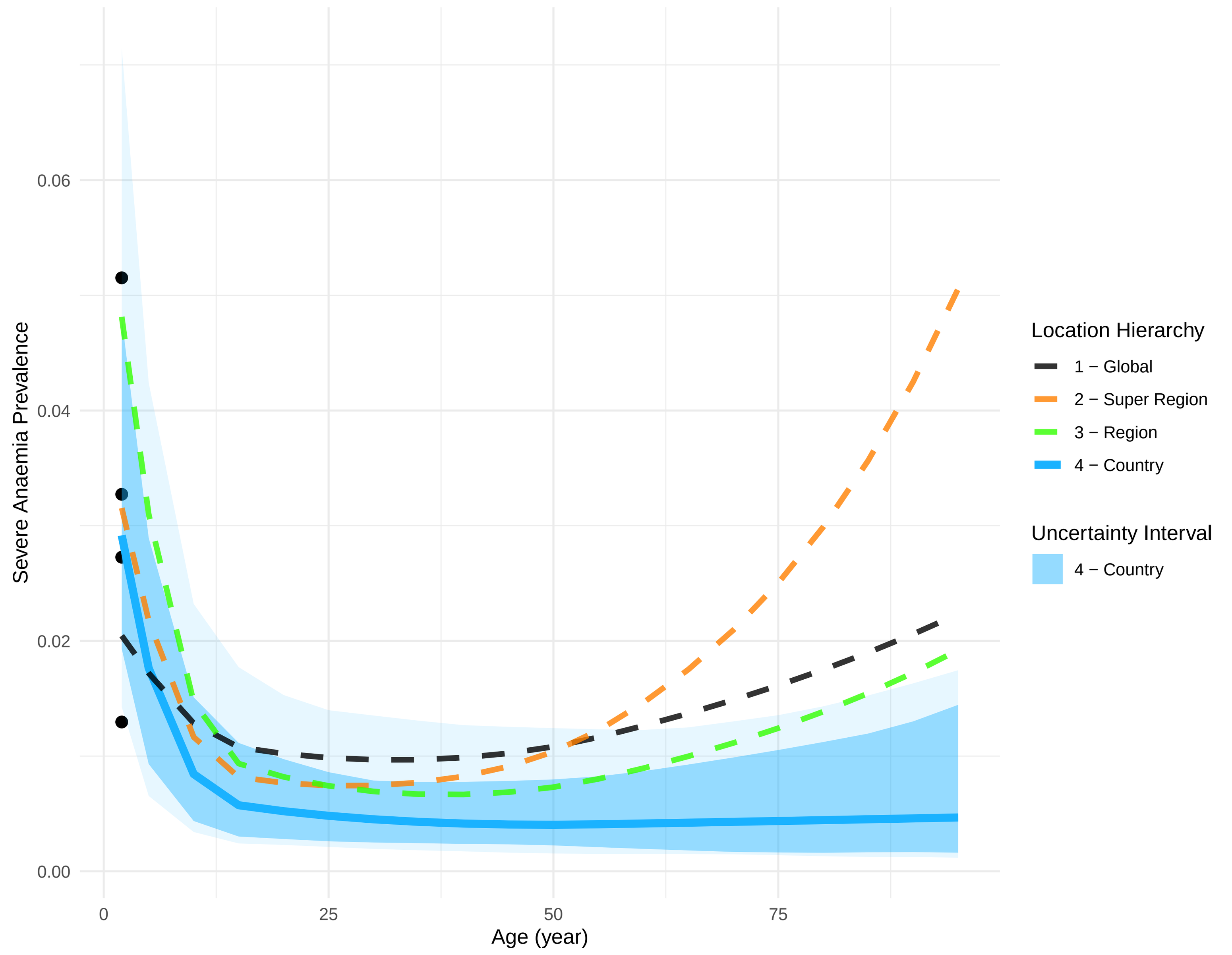

MR−BRT Age/Sex Fits − Males 2+ Years Old − Severe Anaemia Prevalence − Mali
WHO−2024 Adjustment Method

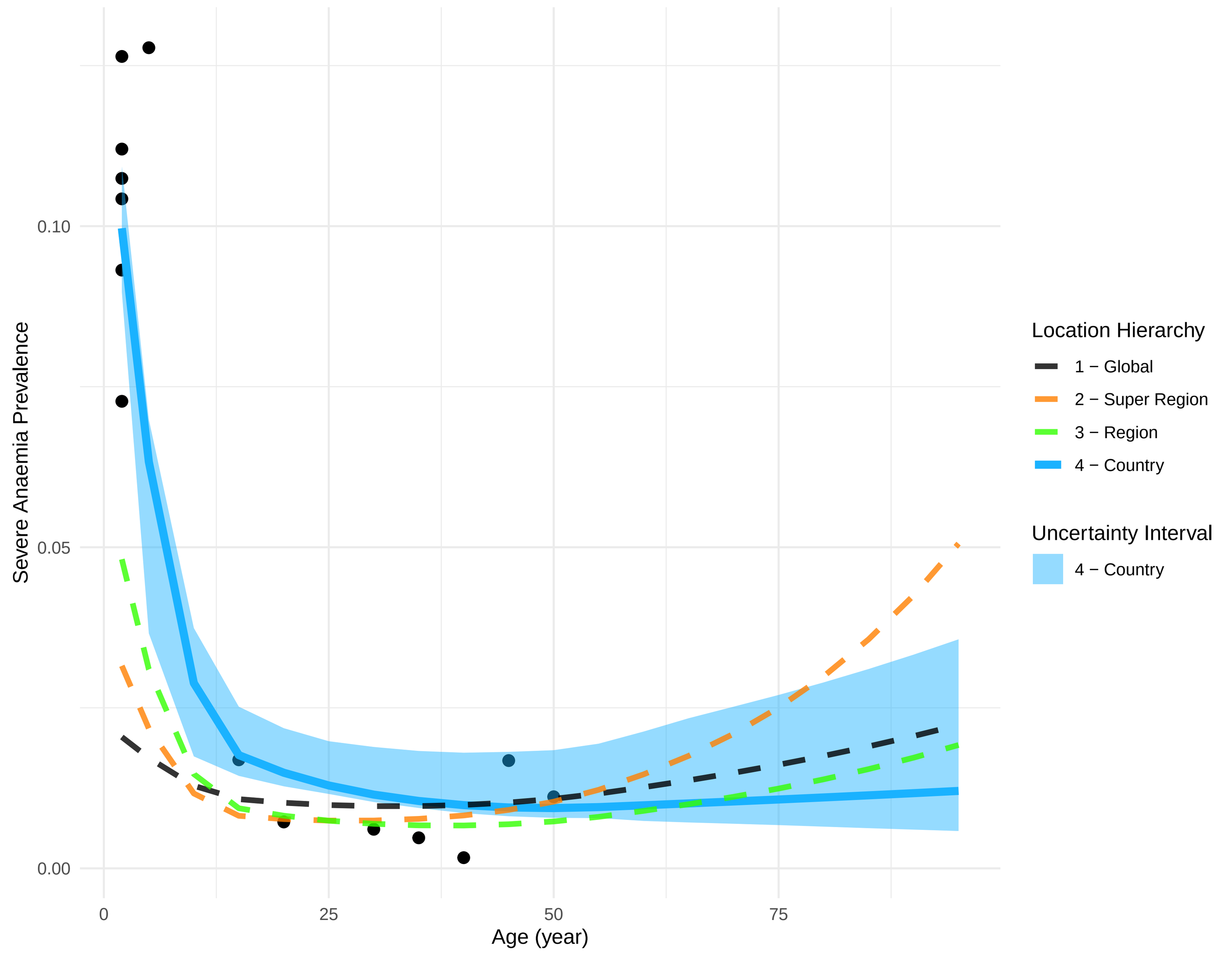

MR−BRT Age/Sex Fits − Males 2+ Years Old − Severe Anaemia Prevalence − Niger
WHO−2024 Adjustment Method

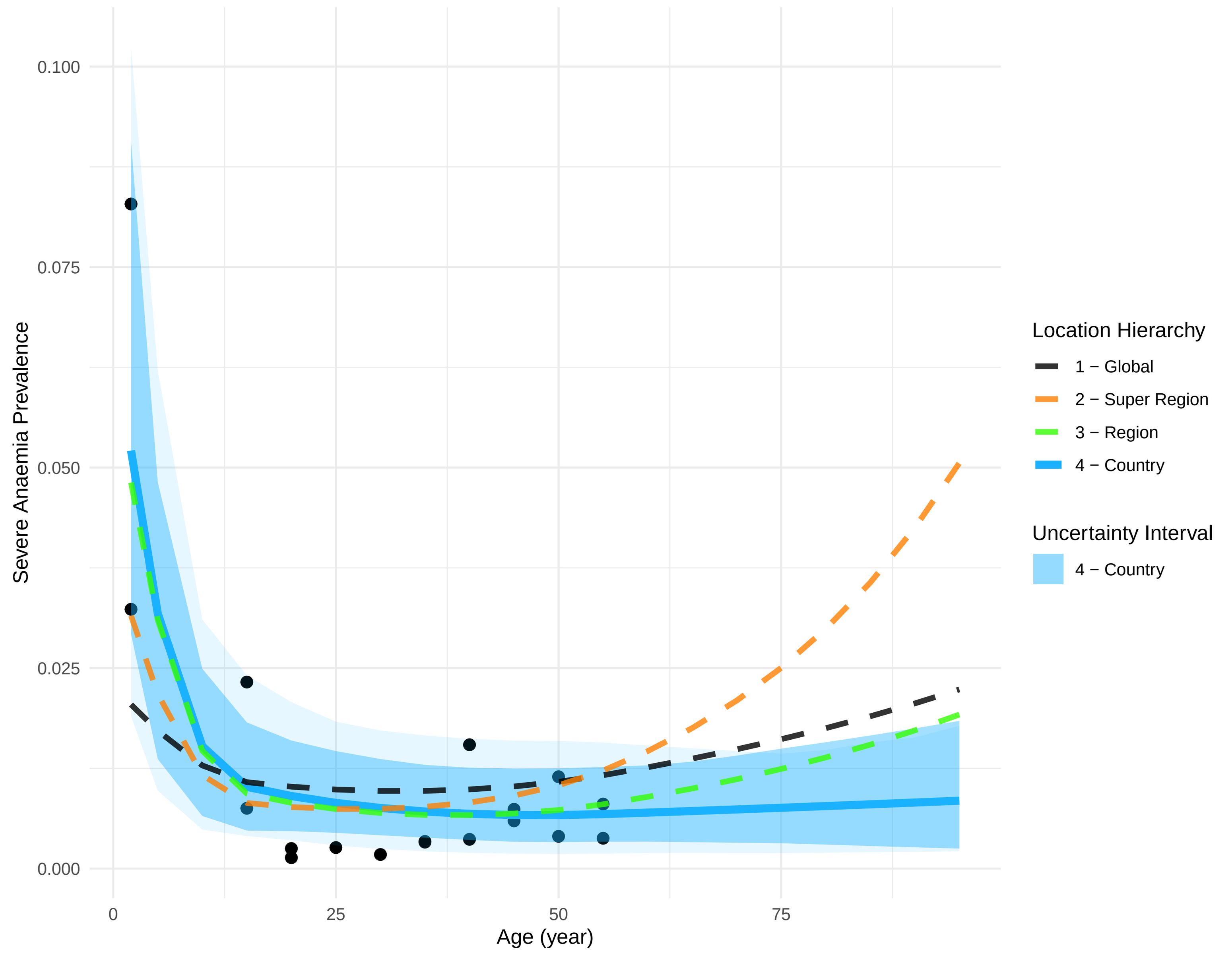

MR−BRT Age/Sex Fits − Males 2+ Years Old − Severe Anaemia Prevalence − Nigeria
WHO−2024 Adjustment Method

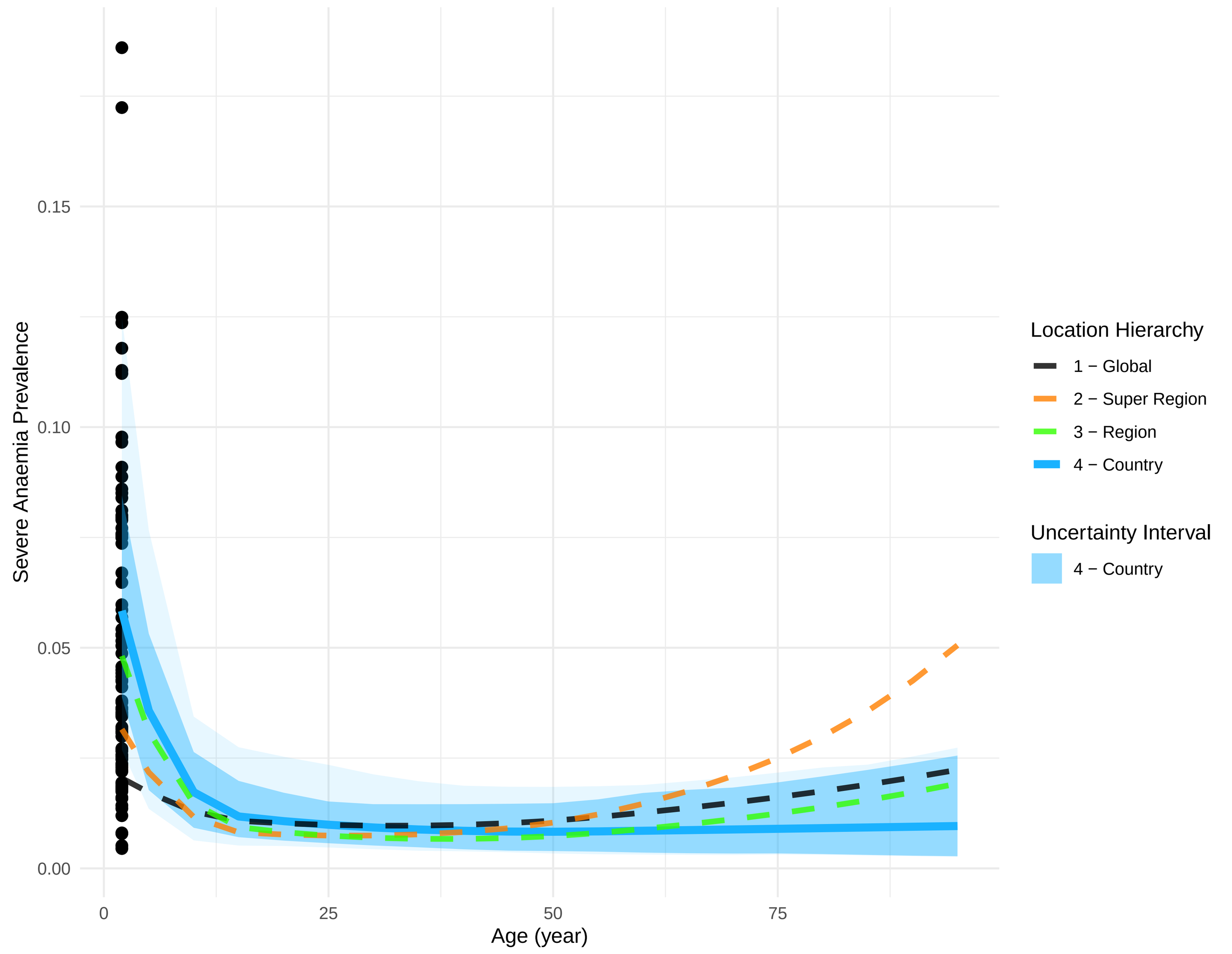

MR−BRT Age/Sex Fits − Males 2+ Years Old − Severe Anaemia Prevalence − Abia
WHO−2024 Adjustment Method

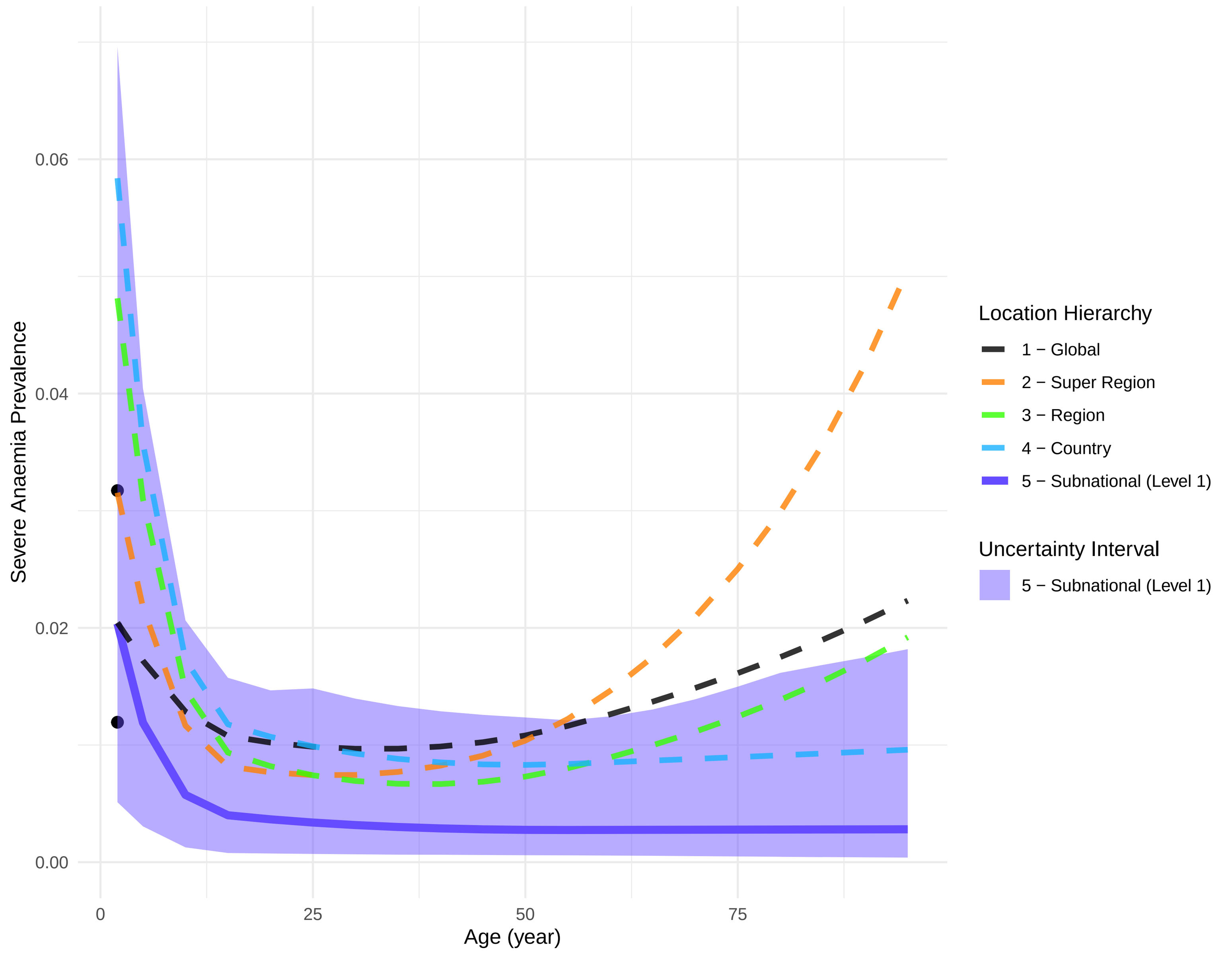

MR−BRT Age/Sex Fits − Males 2+ Years Old − Severe Anaemia Prevalence − Adamawa
WHO−2024 Adjustment Method

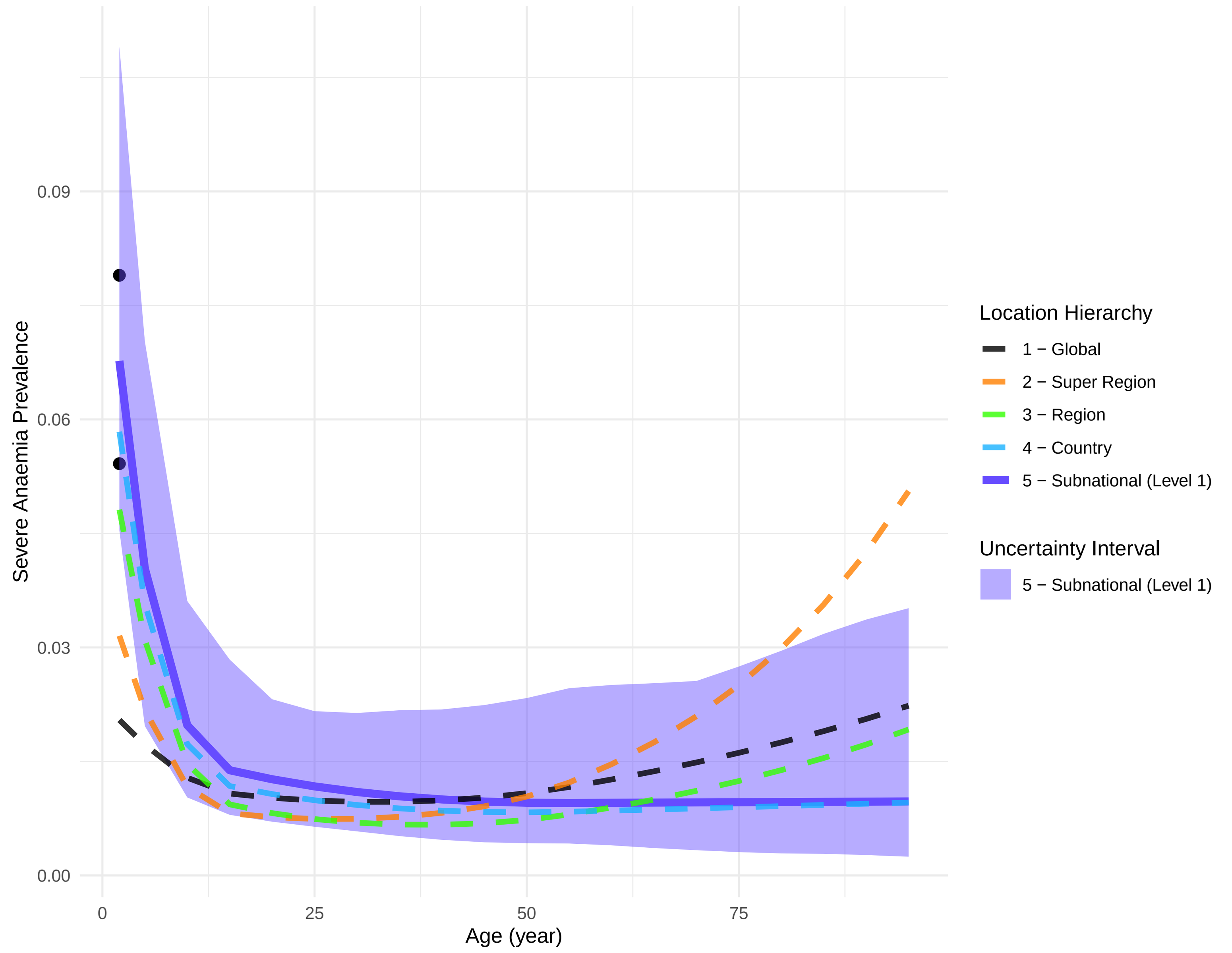

MR−BRT Age/Sex Fits − Males 2+ Years Old − Severe Anaemia Prevalence − Akwa Ibom
WHO−2024 Adjustment Method

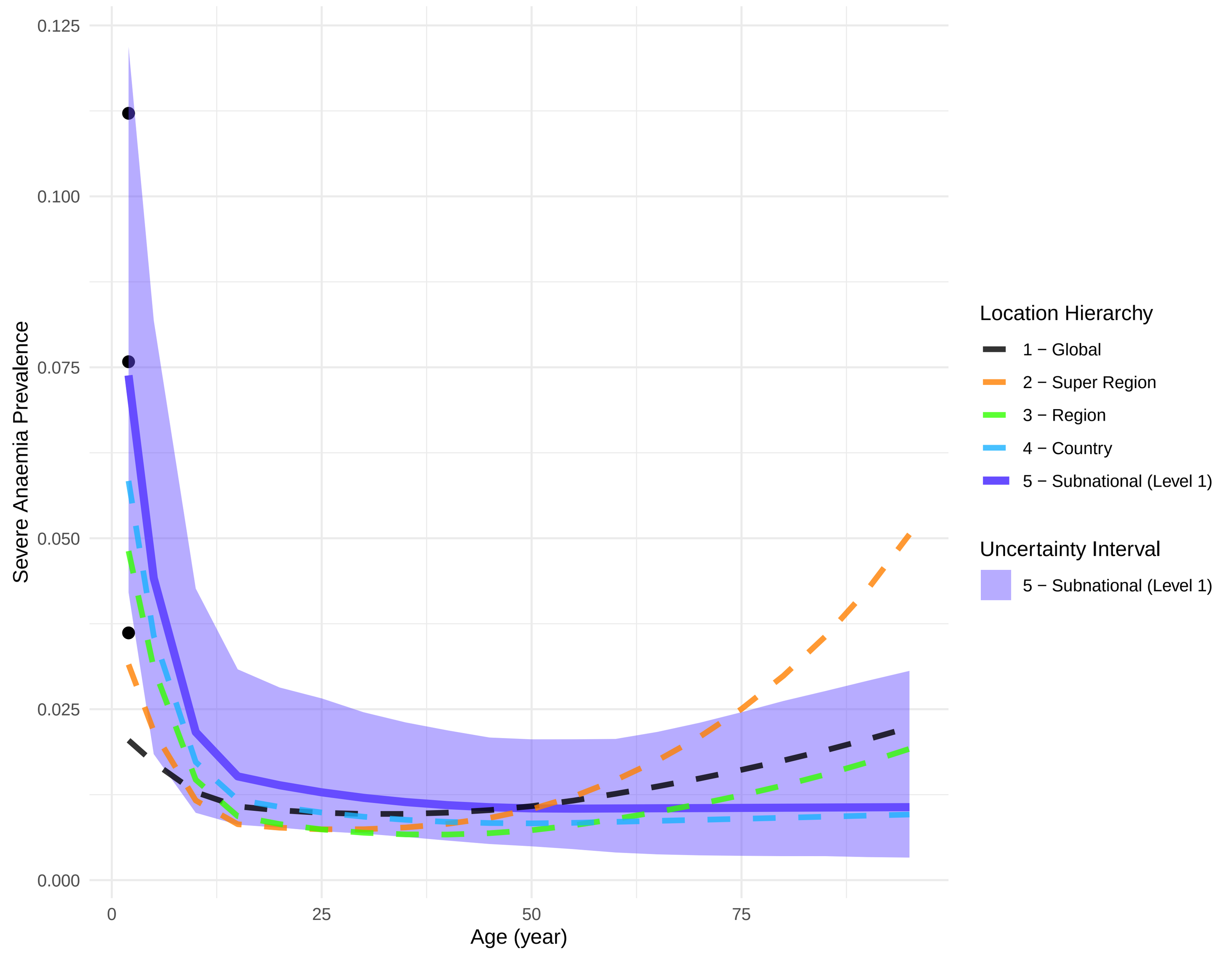

MR−BRT Age/Sex Fits − Males 2+ Years Old − Severe Anaemia Prevalence − Anambra
WHO−2024 Adjustment Method

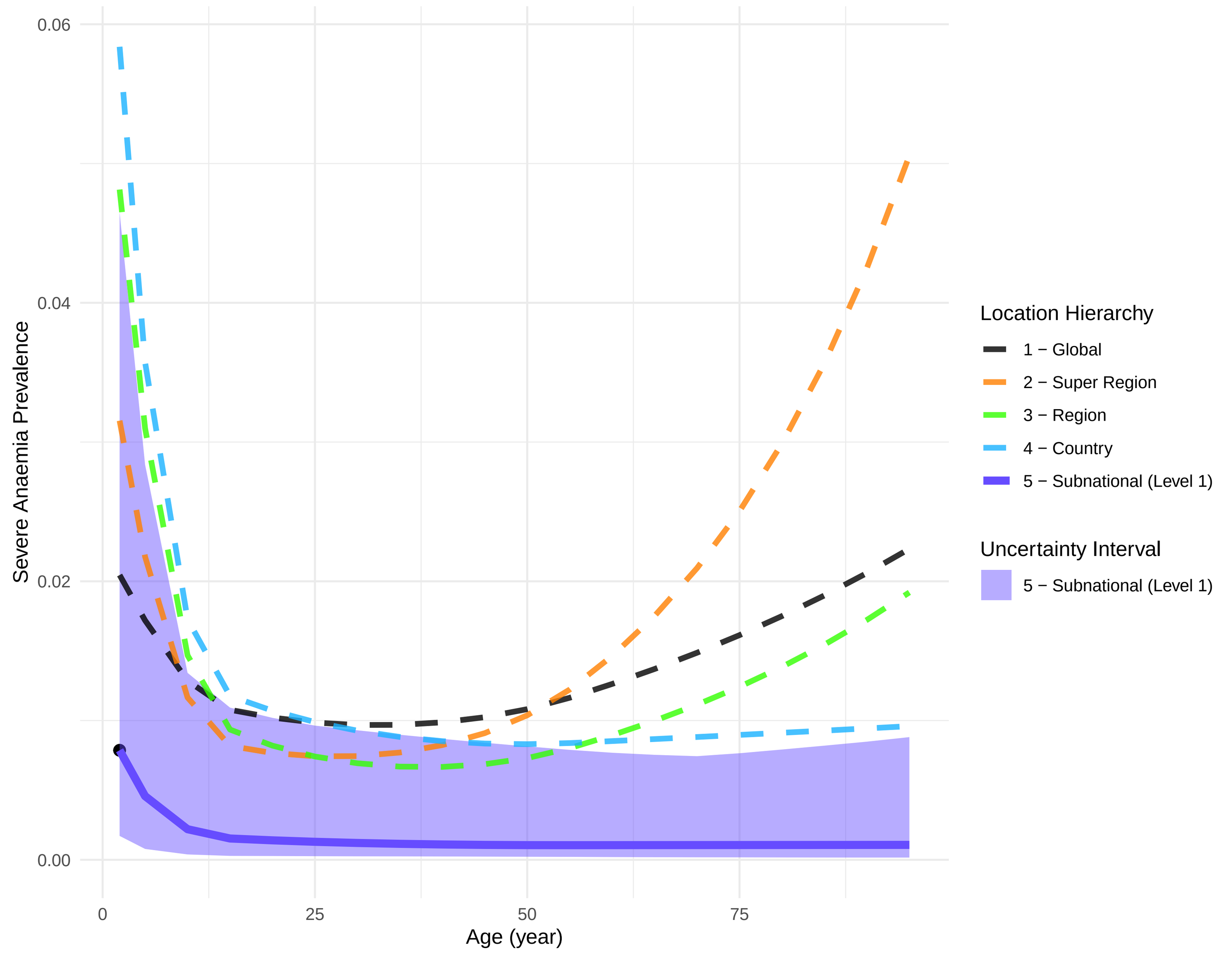

MR−BRT Age/Sex Fits − Males 2+ Years Old − Severe Anaemia Prevalence − Bauchi
WHO−2024 Adjustment Method

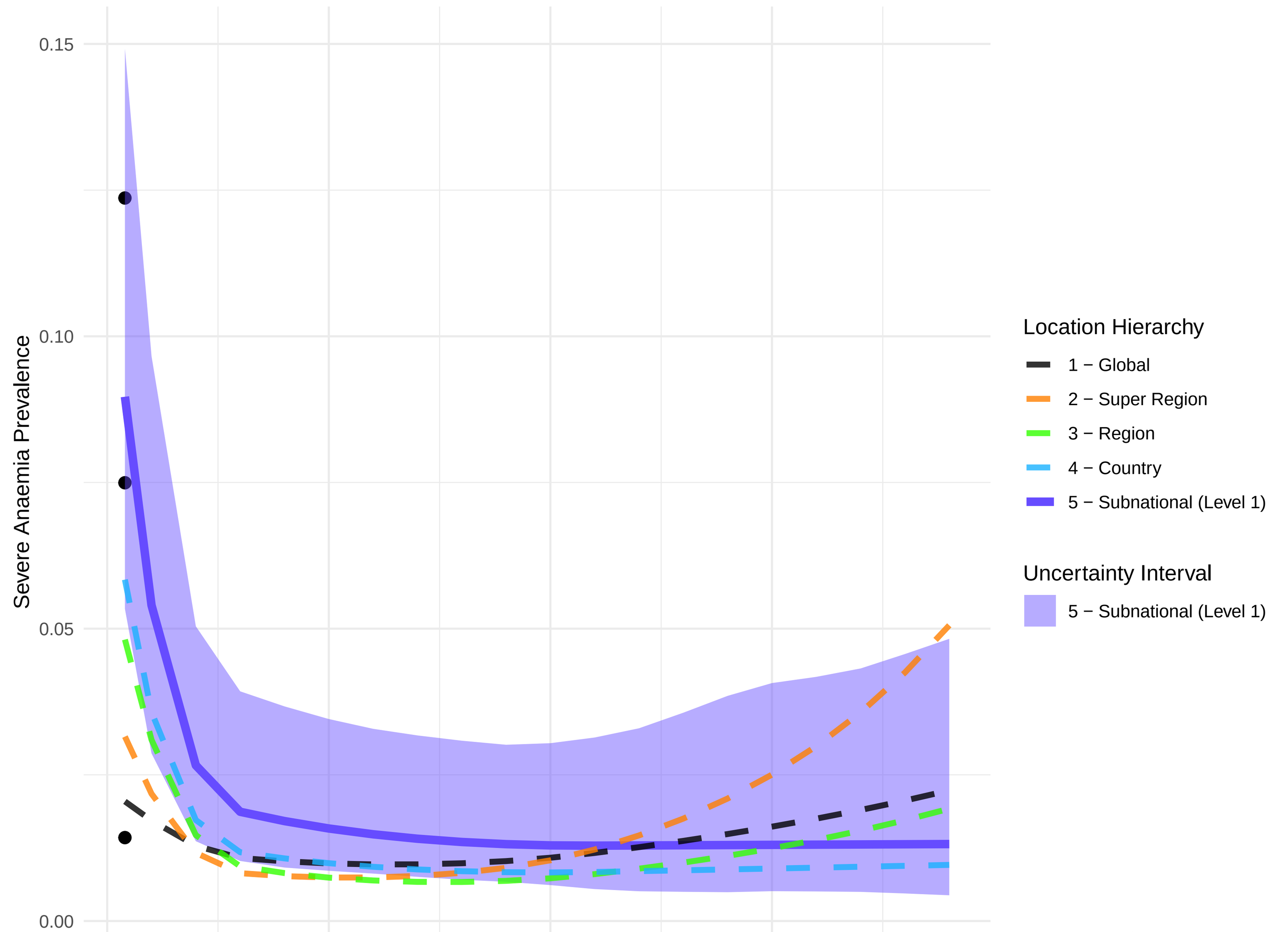

MR−BRT Age/Sex Fits − Males 2+ Years Old − Severe Anaemia Prevalence − Bayelsa

WHO−2024 Adjustment Method

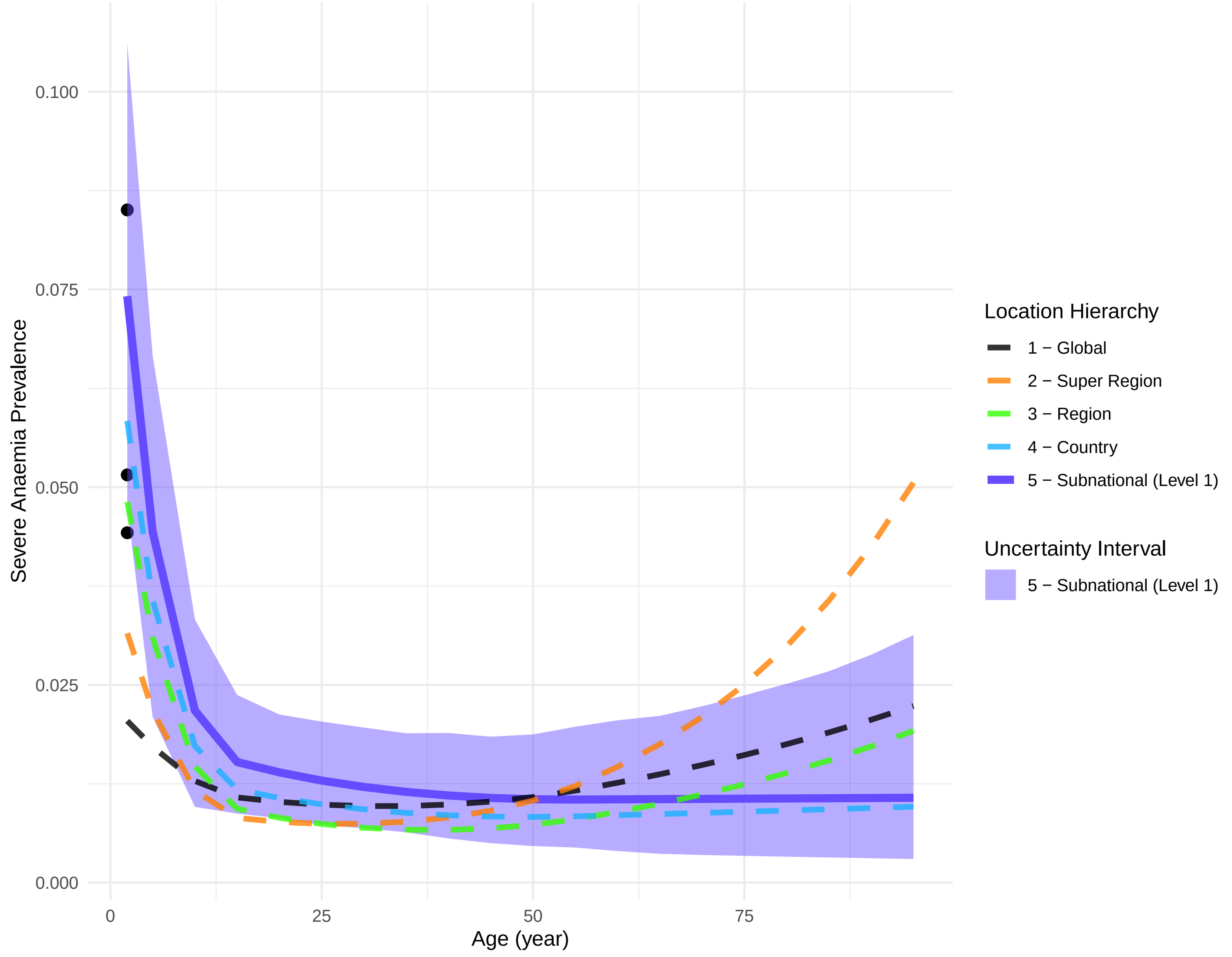

MR−BRT Age/Sex Fits − Males 2+ Years Old − Severe Anaemia Prevalence − Benue

WHO−2024 Adjustment Method

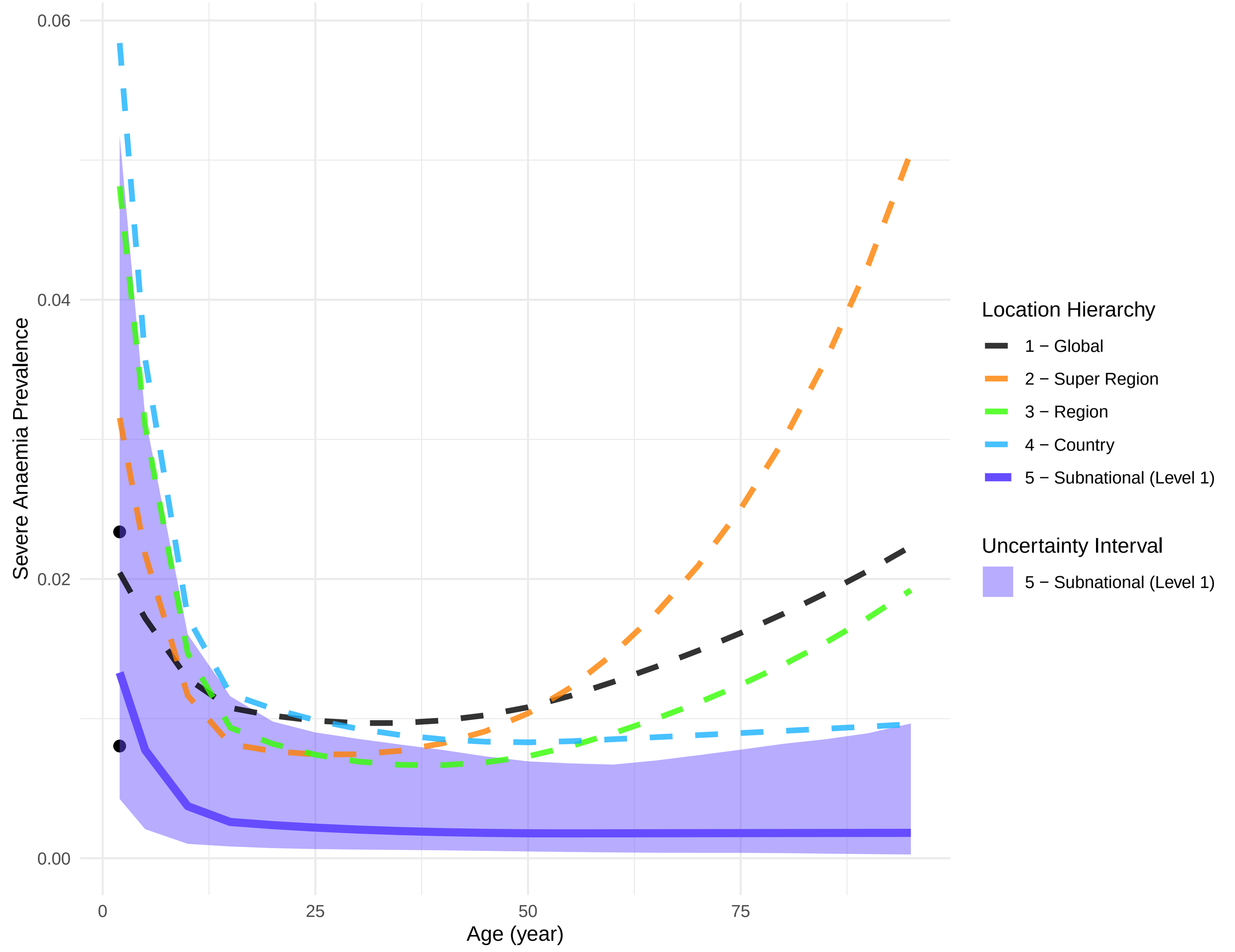

MR−BRT Age/Sex Fits − Males 2+ Years Old − Severe Anaemia Prevalence − Borno

WHO−2024 Adjustment Method

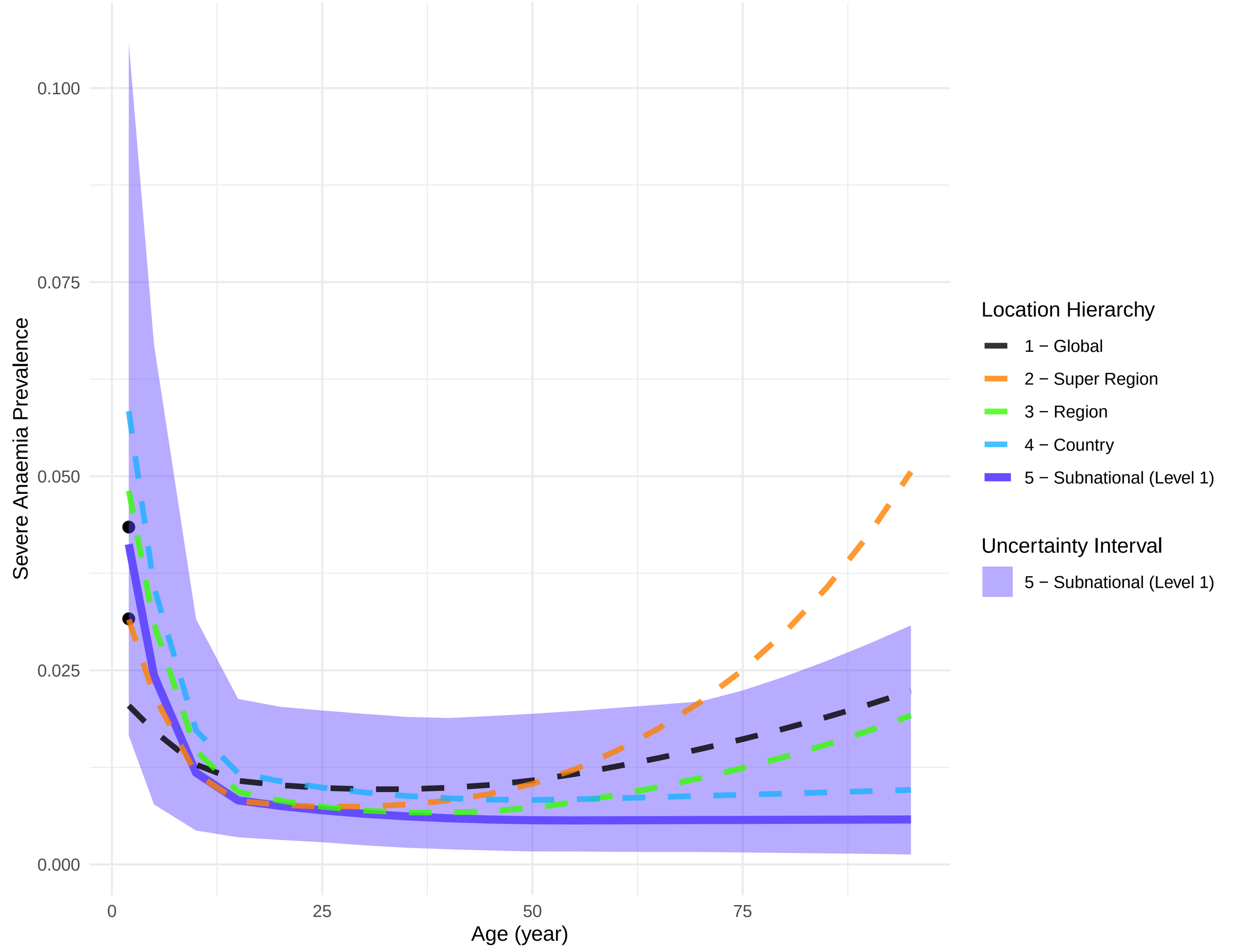

MR−BRT Age/Sex Fits − Males 2+ Years Old − Severe Anaemia Prevalence − Cross River

WHO−2024 Adjustment Method

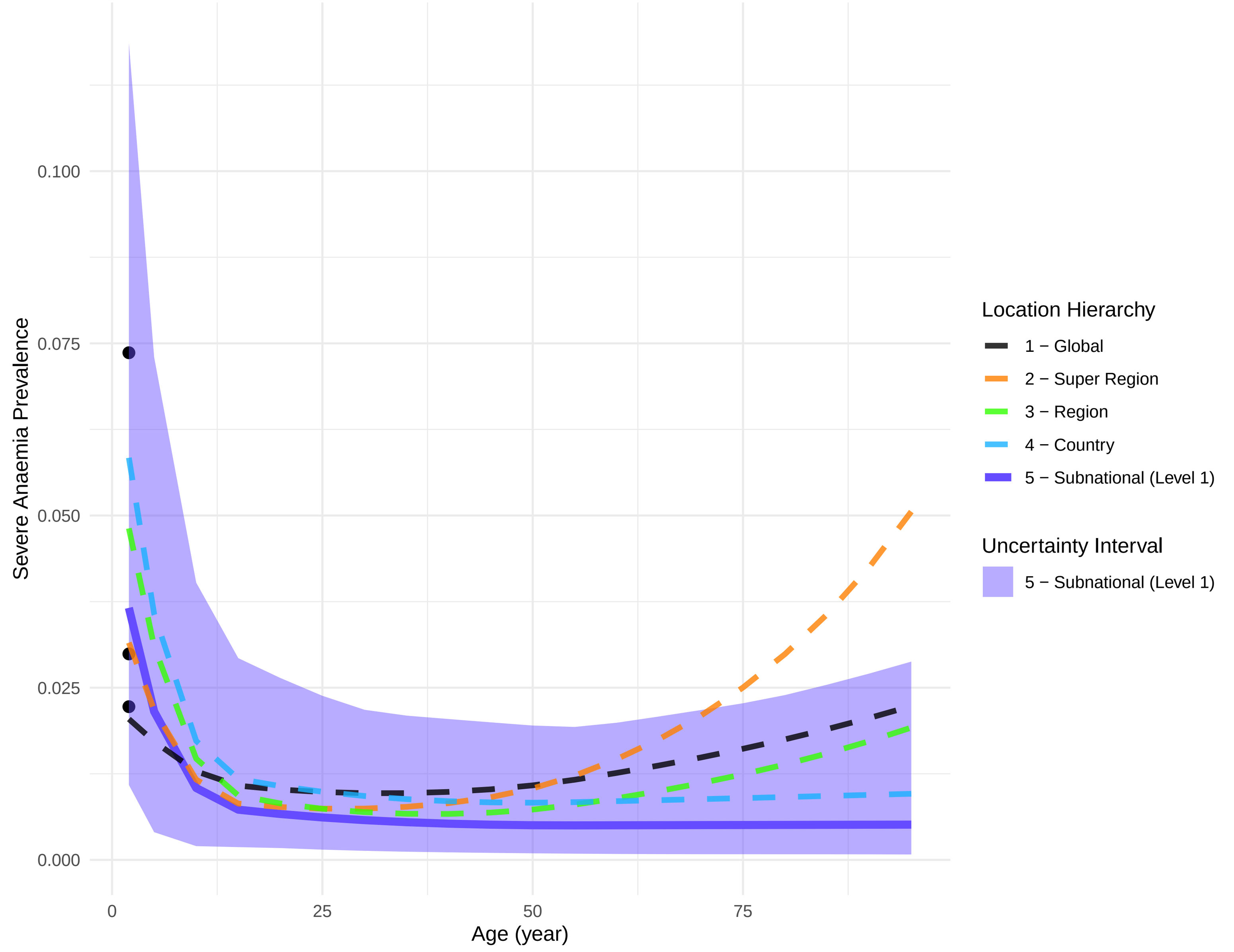

MR−BRT Age/Sex Fits − Males 2+ Years Old − Severe Anaemia Prevalence − Delta

WHO−2024 Adjustment Method

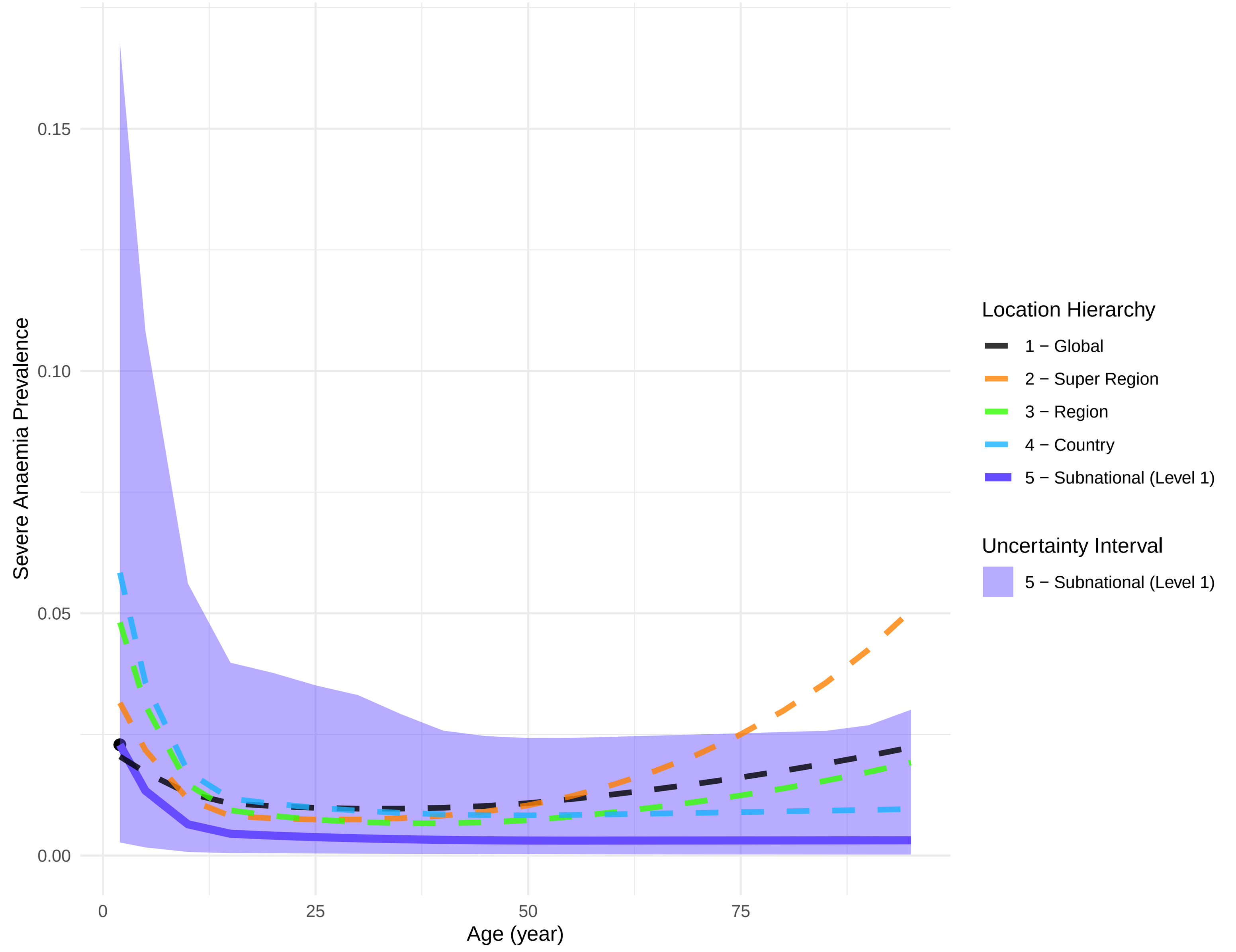

MR−BRT Age/Sex Fits − Males 2+ Years Old − Severe Anaemia Prevalence − Ebonyi

WHO−2024 Adjustment Method

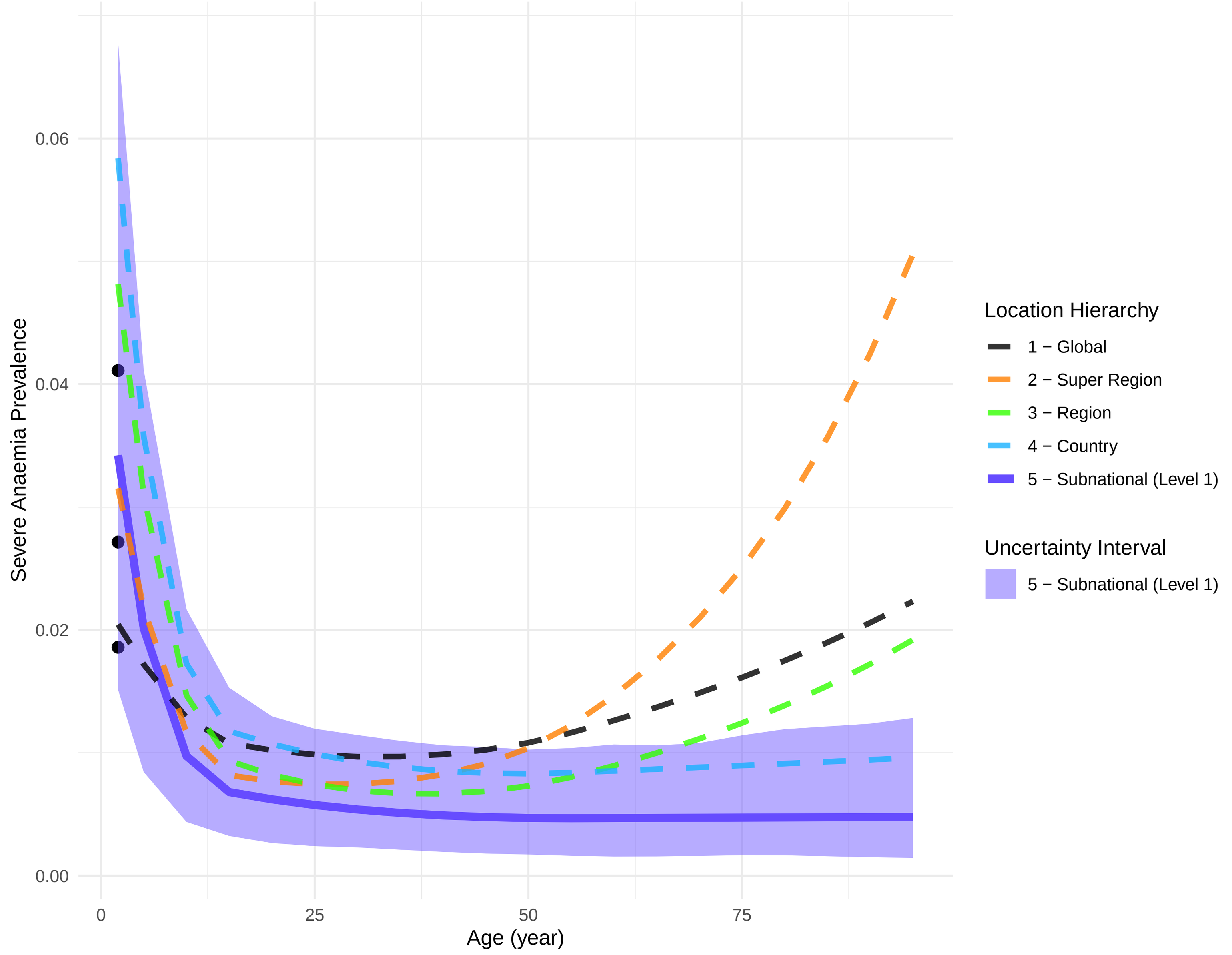

MR−BRT Age/Sex Fits − Males 2+ Years Old − Severe Anaemia Prevalence − Edo

WHO−2024 Adjustment Method

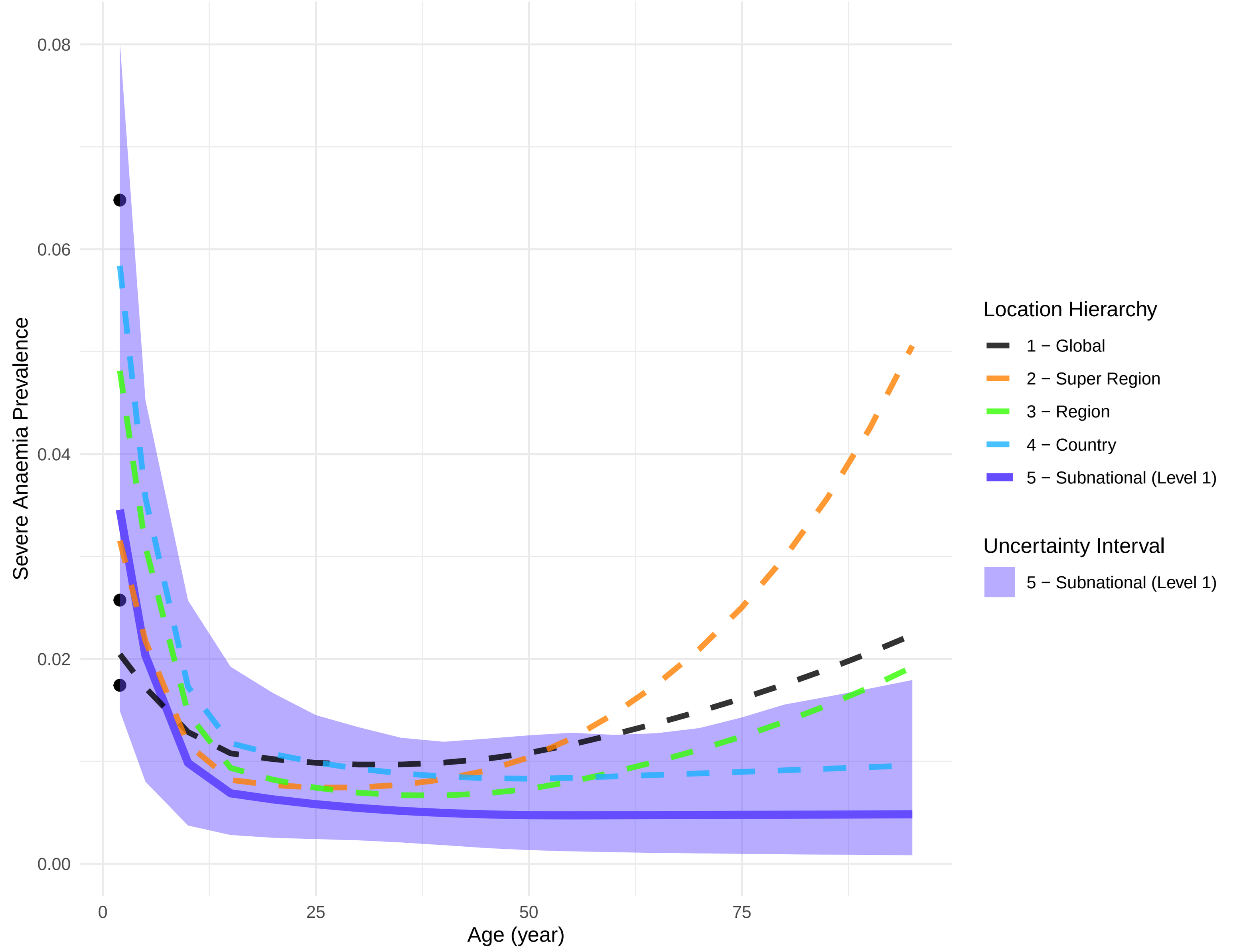

MR−BRT Age/Sex Fits − Males 2+ Years Old − Severe Anaemia Prevalence − Ekiti

WHO−2024 Adjustment Method

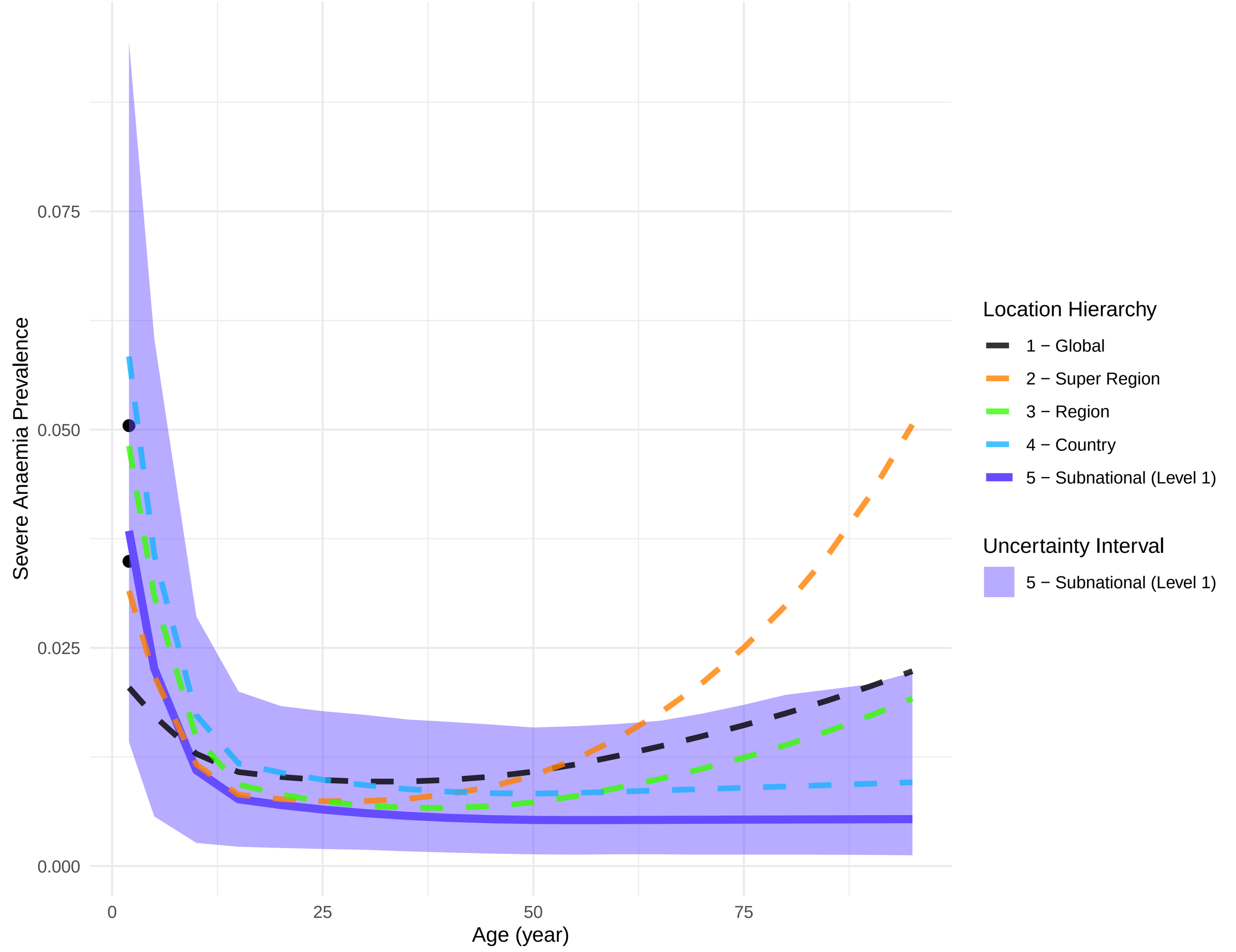

MR−BRT Age/Sex Fits − Males 2+ Years Old − Severe Anaemia Prevalence − Enugu

WHO−2024 Adjustment Method

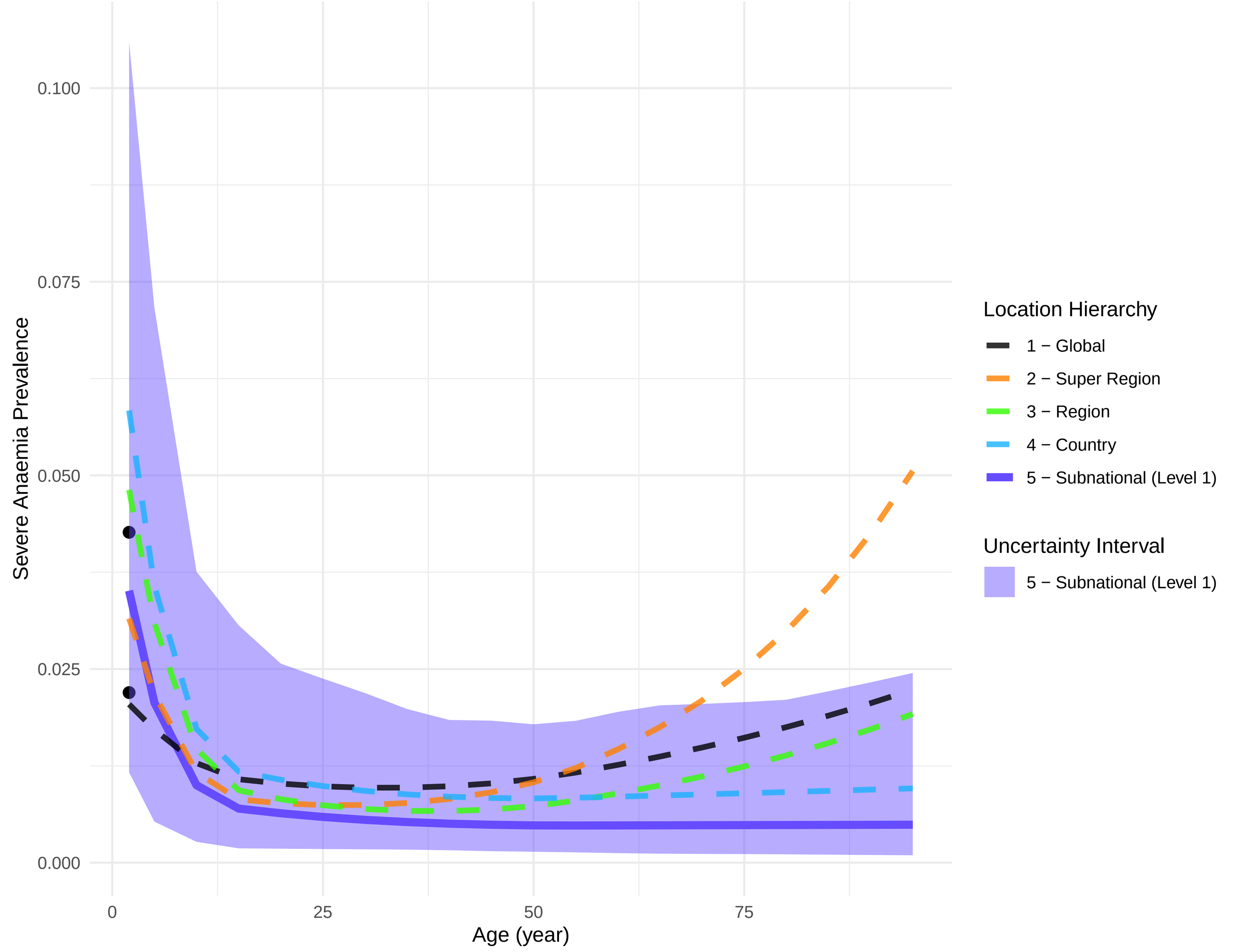

MR−BRT Age/Sex Fits − Males 2+ Years Old − Severe Anaemia Prevalence − FCT (Abuja)

WHO−2024 Adjustment Method

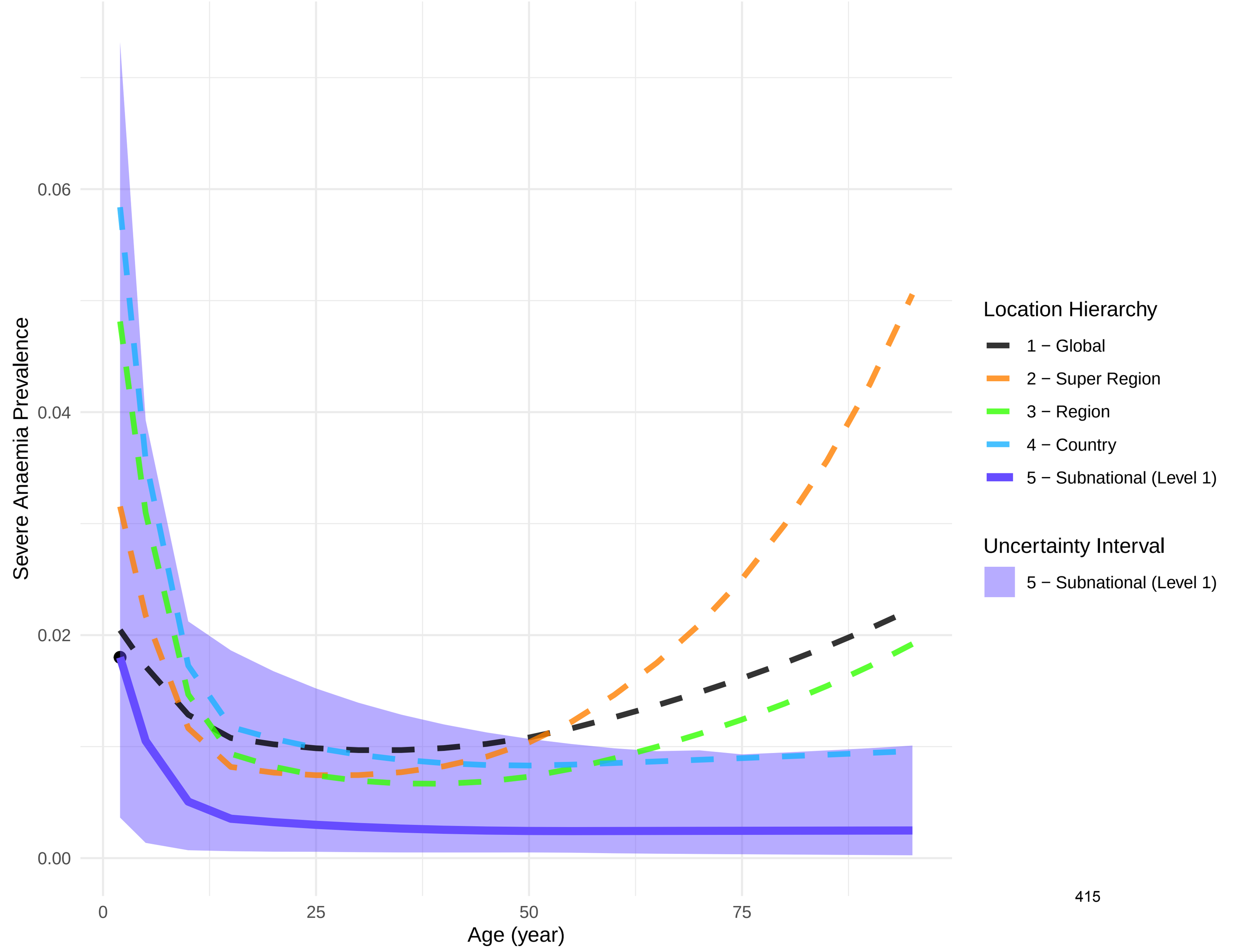

MR−BRT Age/Sex Fits − Males 2+ Years Old − Severe Anaemia Prevalence − Gombe
WHO−2024 Adjustment Method

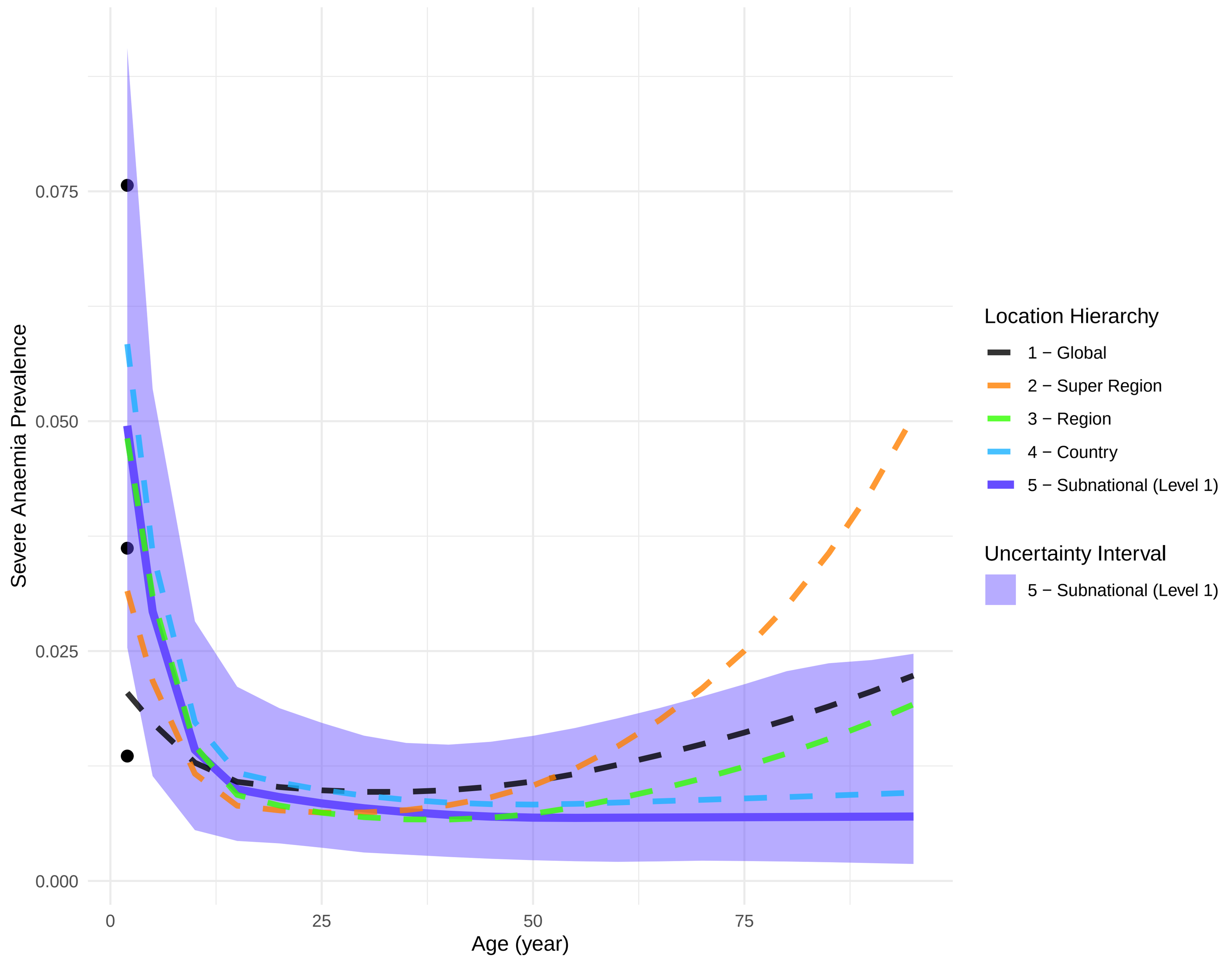

MR−BRT Age/Sex Fits − Males 2+ Years Old − Severe Anaemia Prevalence − Imo
WHO−2024 Adjustment Method

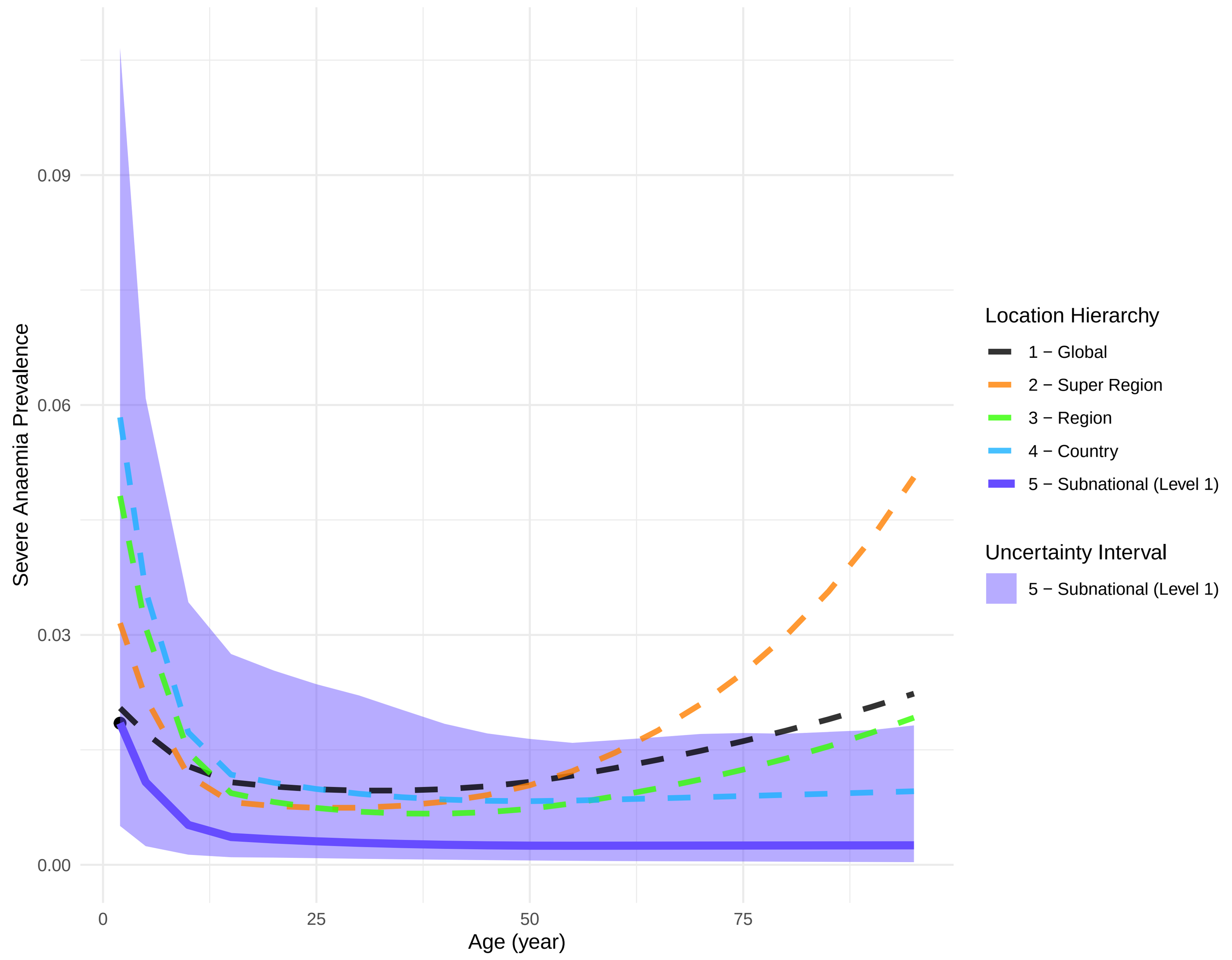

MR−BRT Age/Sex Fits − Males 2+ Years Old − Severe Anaemia Prevalence − Jigawa
WHO−2024 Adjustment Method

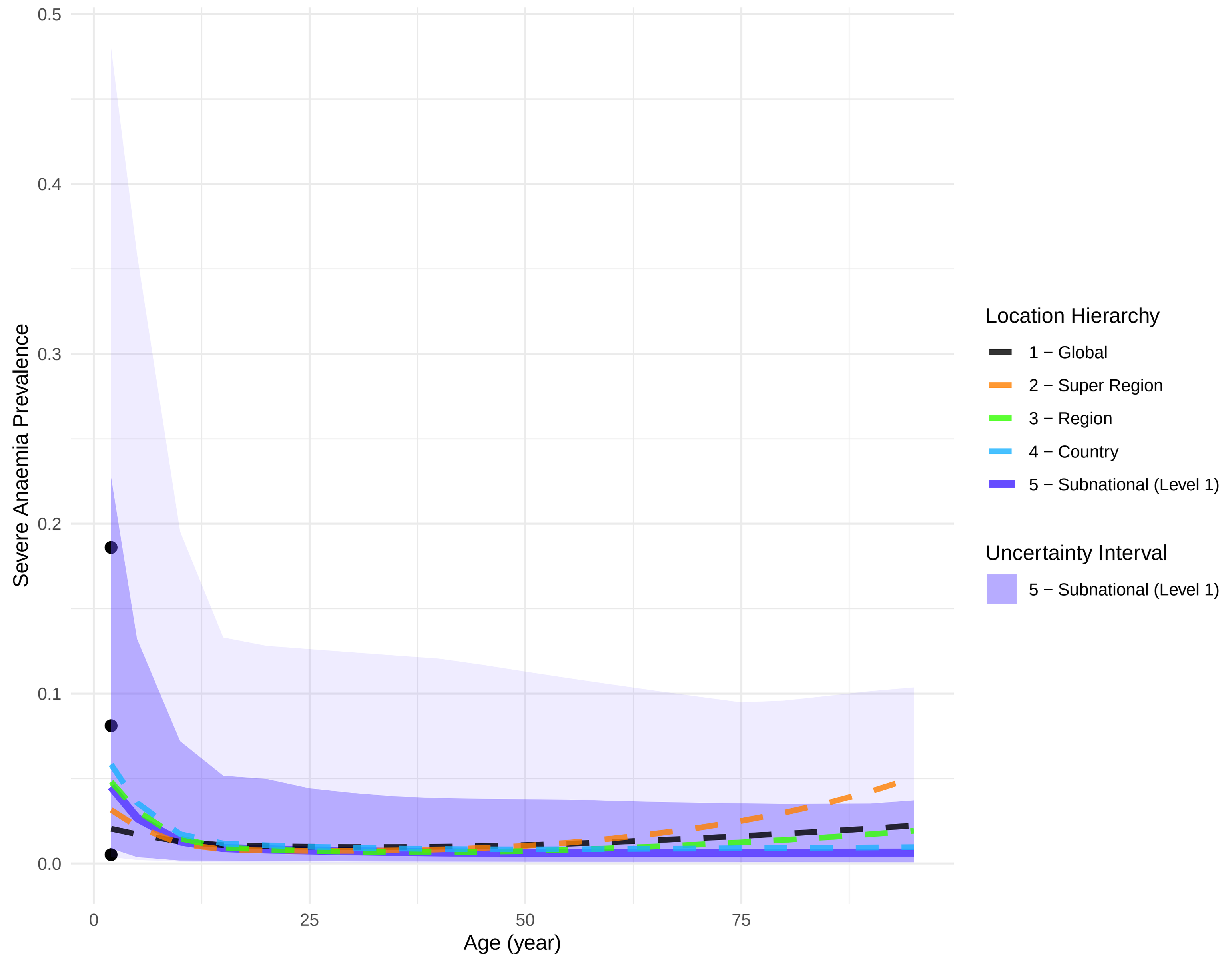

MR−BRT Age/Sex Fits − Males 2+ Years Old − Severe Anaemia Prevalence − Kaduna
WHO−2024 Adjustment Method

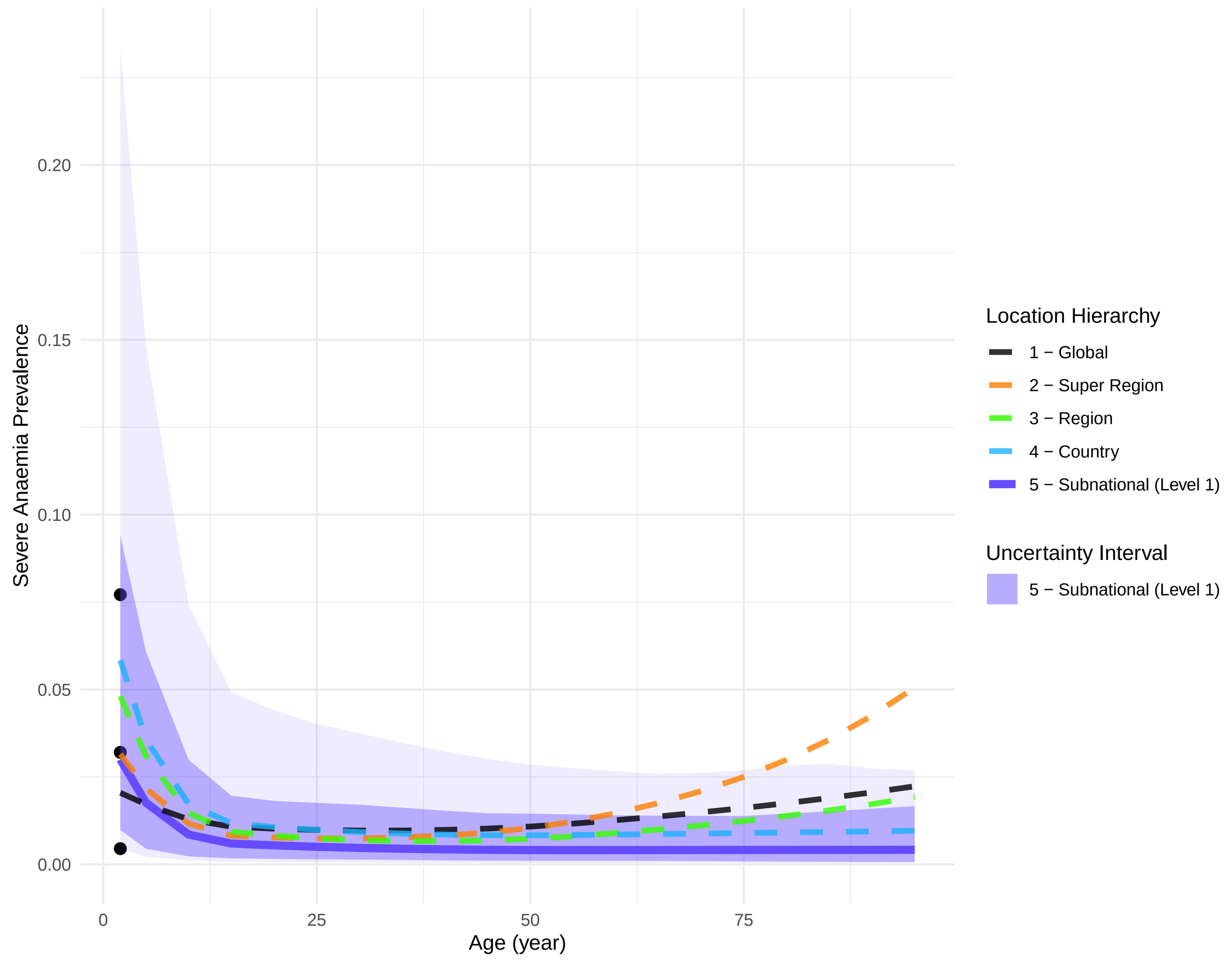

MR−BRT Age/Sex Fits − Males 2+ Years Old − Severe Anaemia Prevalence − Kano
WHO−2024 Adjustment Method

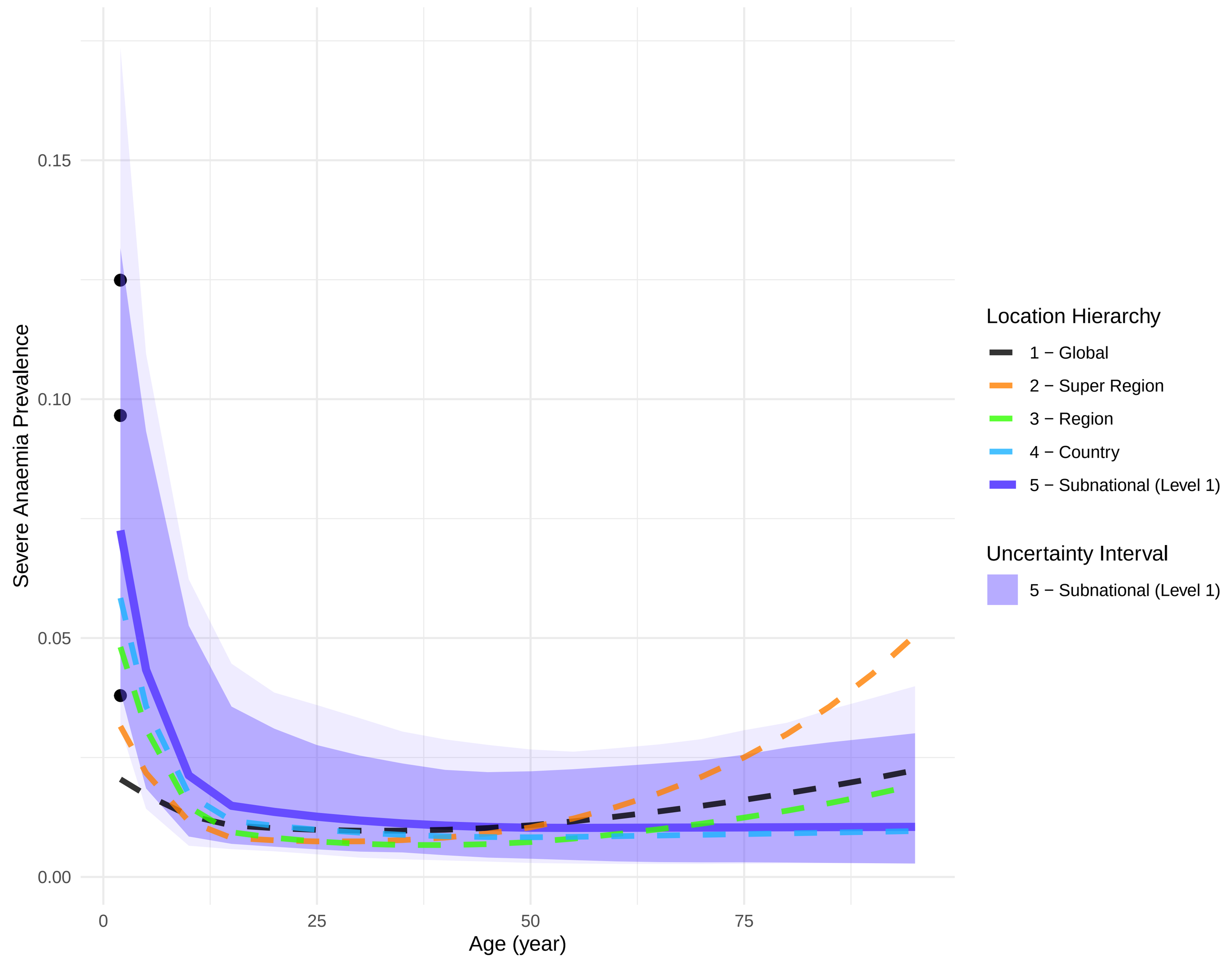

MR−BRT Age/Sex Fits − Males 2+ Years Old − Severe Anaemia Prevalence − Katsina
WHO−2024 Adjustment Method

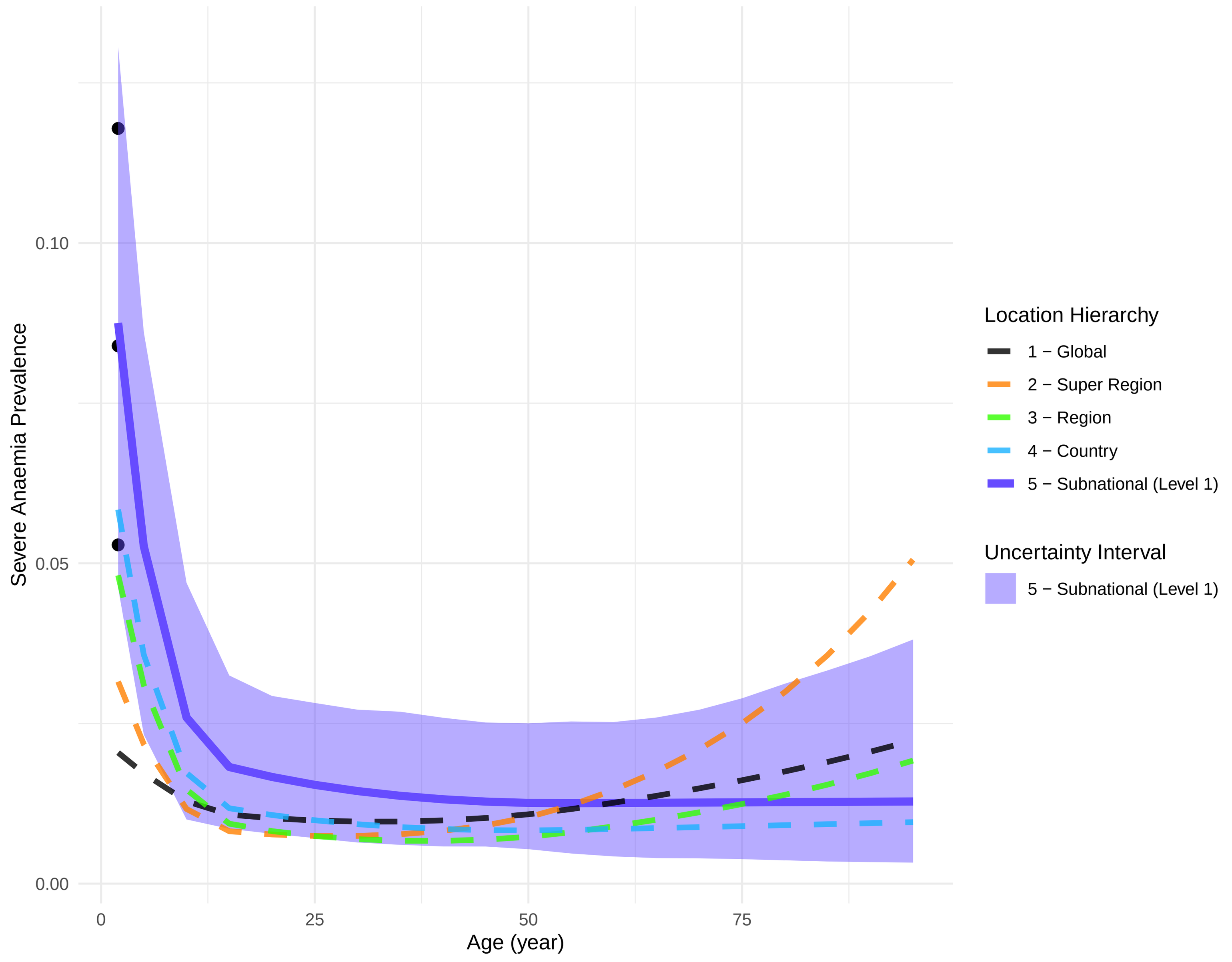

MR−BRT Age/Sex Fits − Males 2+ Years Old − Severe Anaemia Prevalence − Kebbi
WHO−2024 Adjustment Method

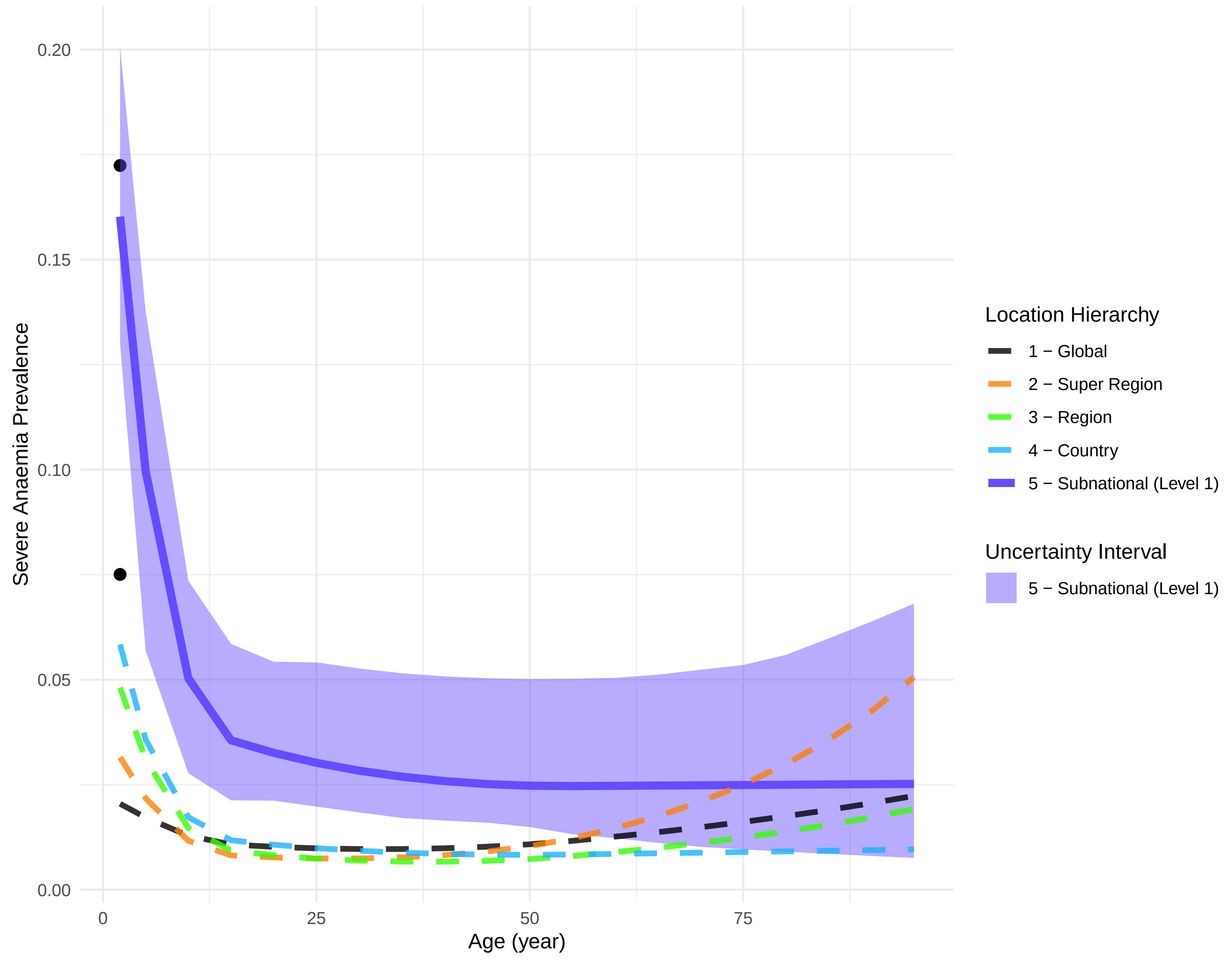

MR−BRT Age/Sex Fits − Males 2+ Years Old − Severe Anaemia Prevalence − Kogi
WHO−2024 Adjustment Method

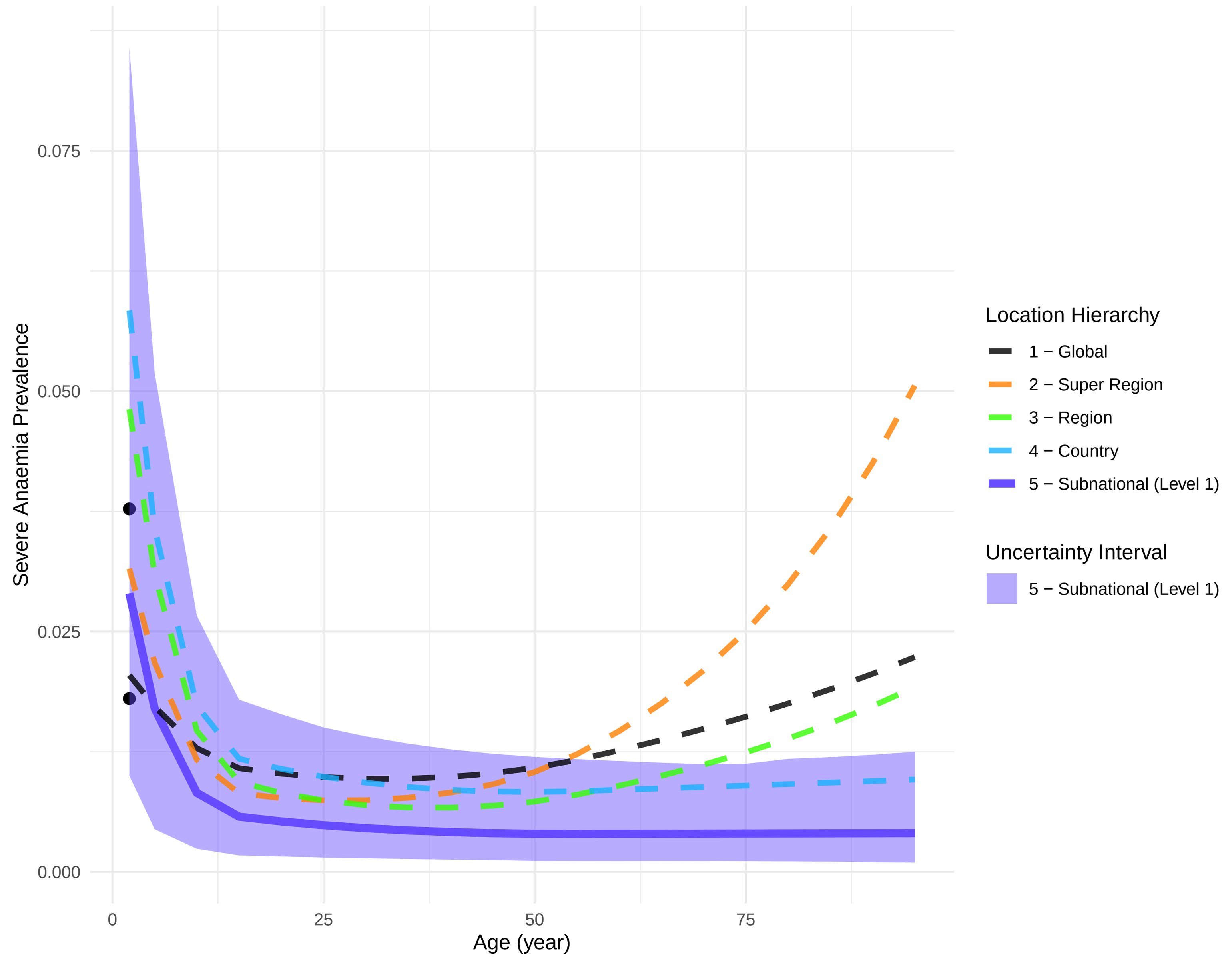

MR−BRT Age/Sex Fits − Males 2+ Years Old − Severe Anaemia Prevalence − Kwara
WHO−2024 Adjustment Method

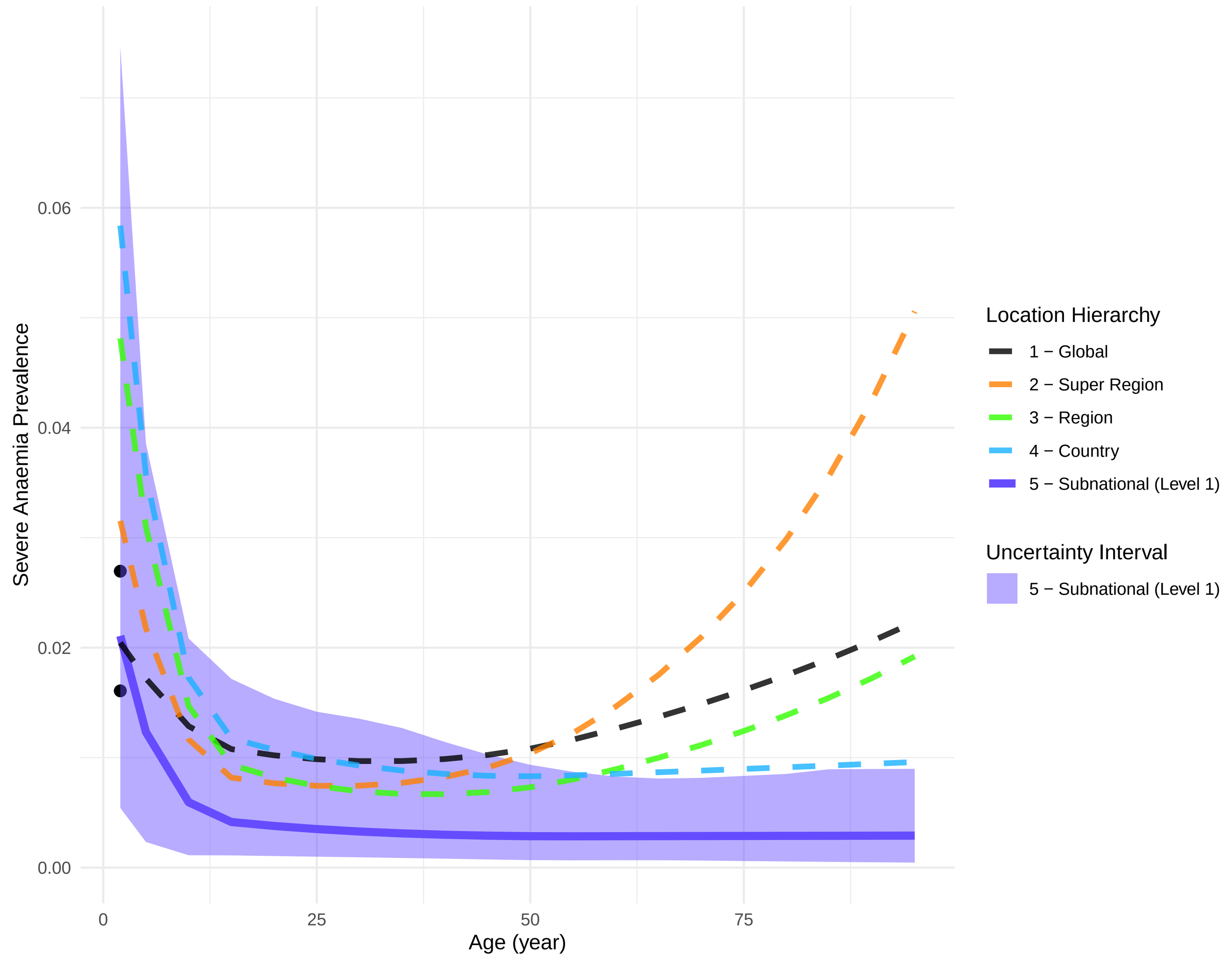

MR−BRT Age/Sex Fits − Males 2+ Years Old − Severe Anaemia Prevalence − Nasarawa
WHO−2024 Adjustment Method

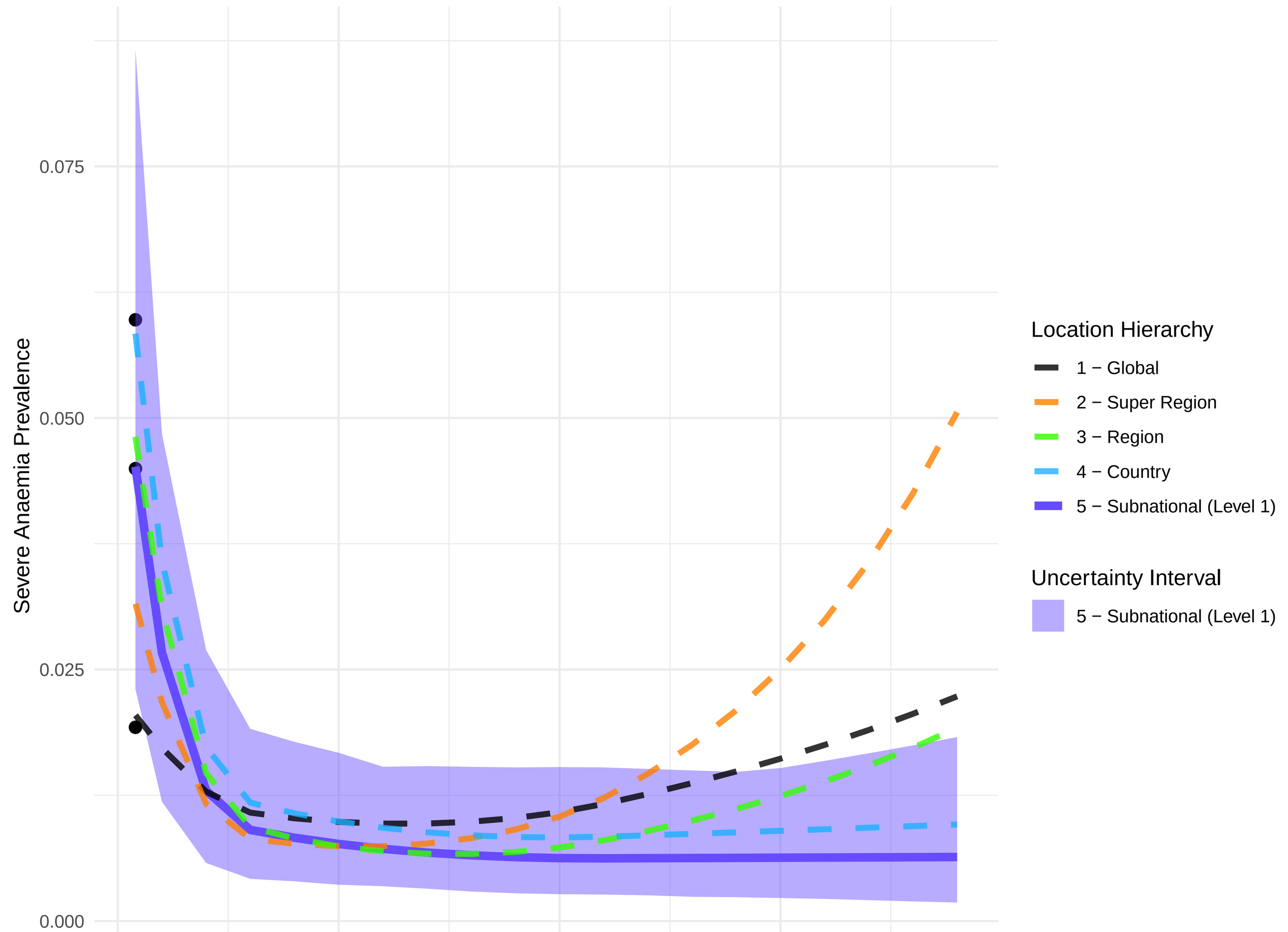

MR−BRT Age/Sex Fits − Males 2+ Years Old − Severe Anaemia Prevalence − Niger
WHO−2024 Adjustment Method

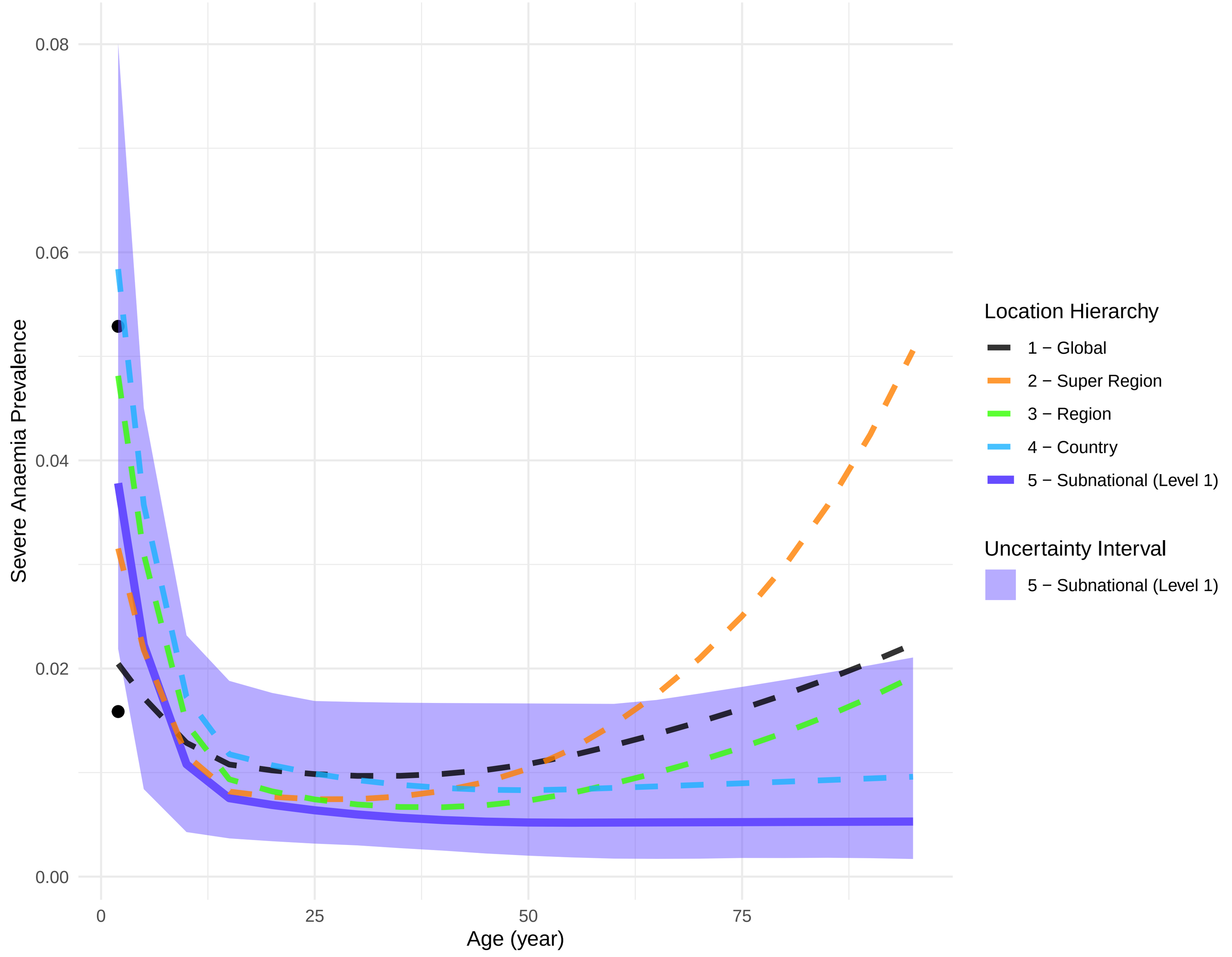

MR−BRT Age/Sex Fits − Males 2+ Years Old − Severe Anaemia Prevalence − Ogun
WHO−2024 Adjustment Method

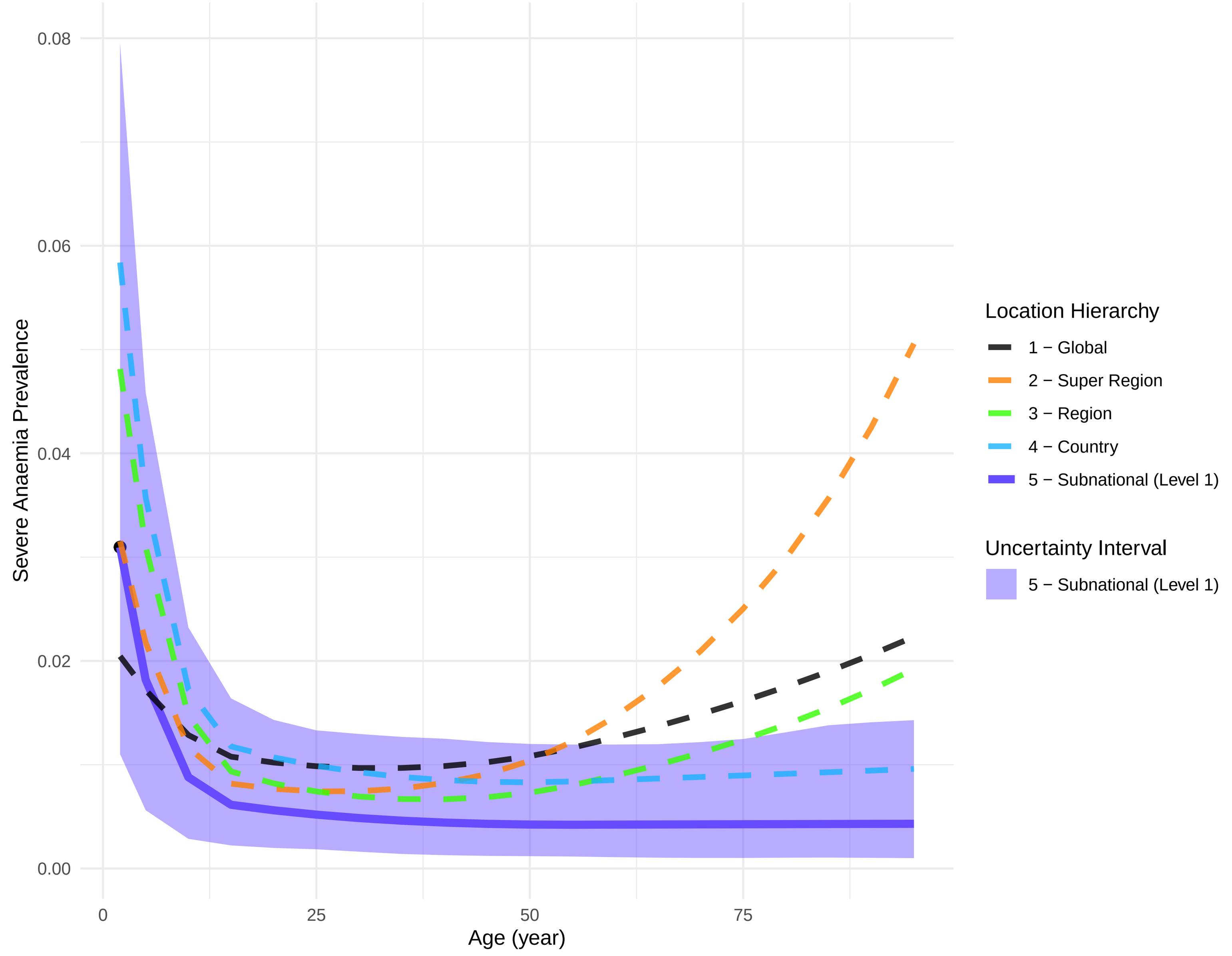

MR−BRT Age/Sex Fits − Males 2+ Years Old − Severe Anaemia Prevalence − Ondo
WHO−2024 Adjustment Method

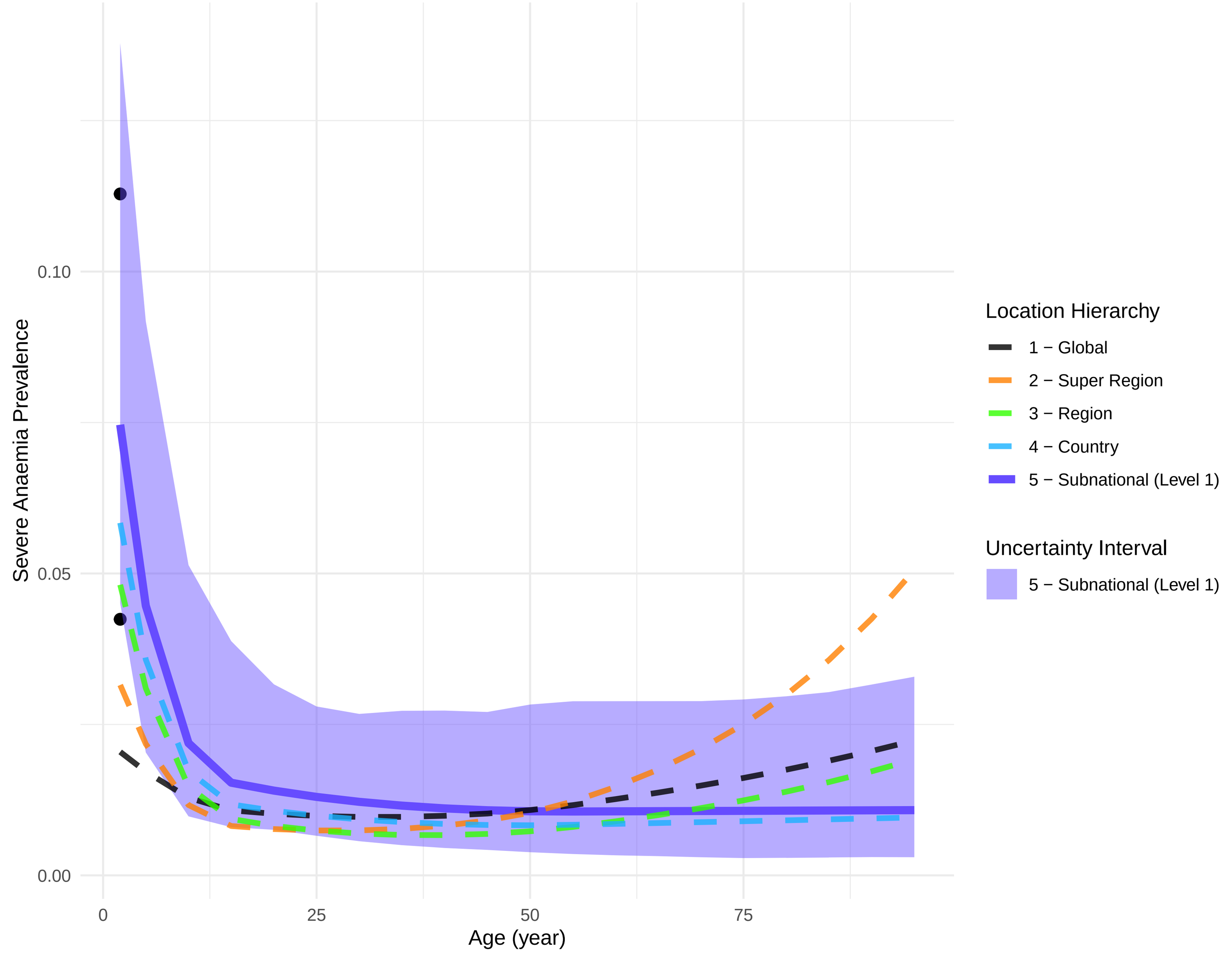

MR−BRT Age/Sex Fits − Males 2+ Years Old − Severe Anaemia Prevalence − Osun
WHO−2024 Adjustment Method

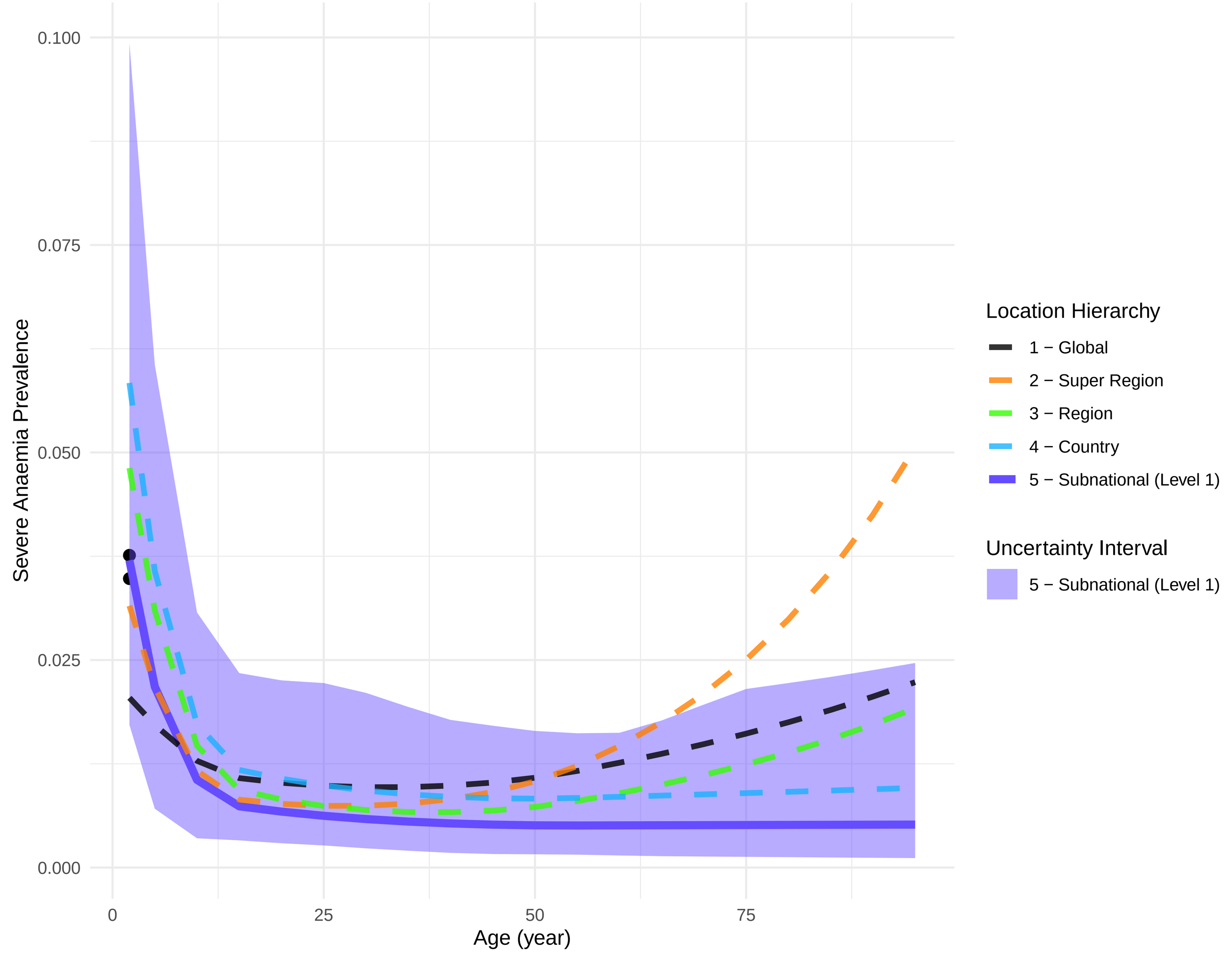

MR−BRT Age/Sex Fits − Males 2+ Years Old − Severe Anaemia Prevalence − Oyo
WHO−2024 Adjustment Method

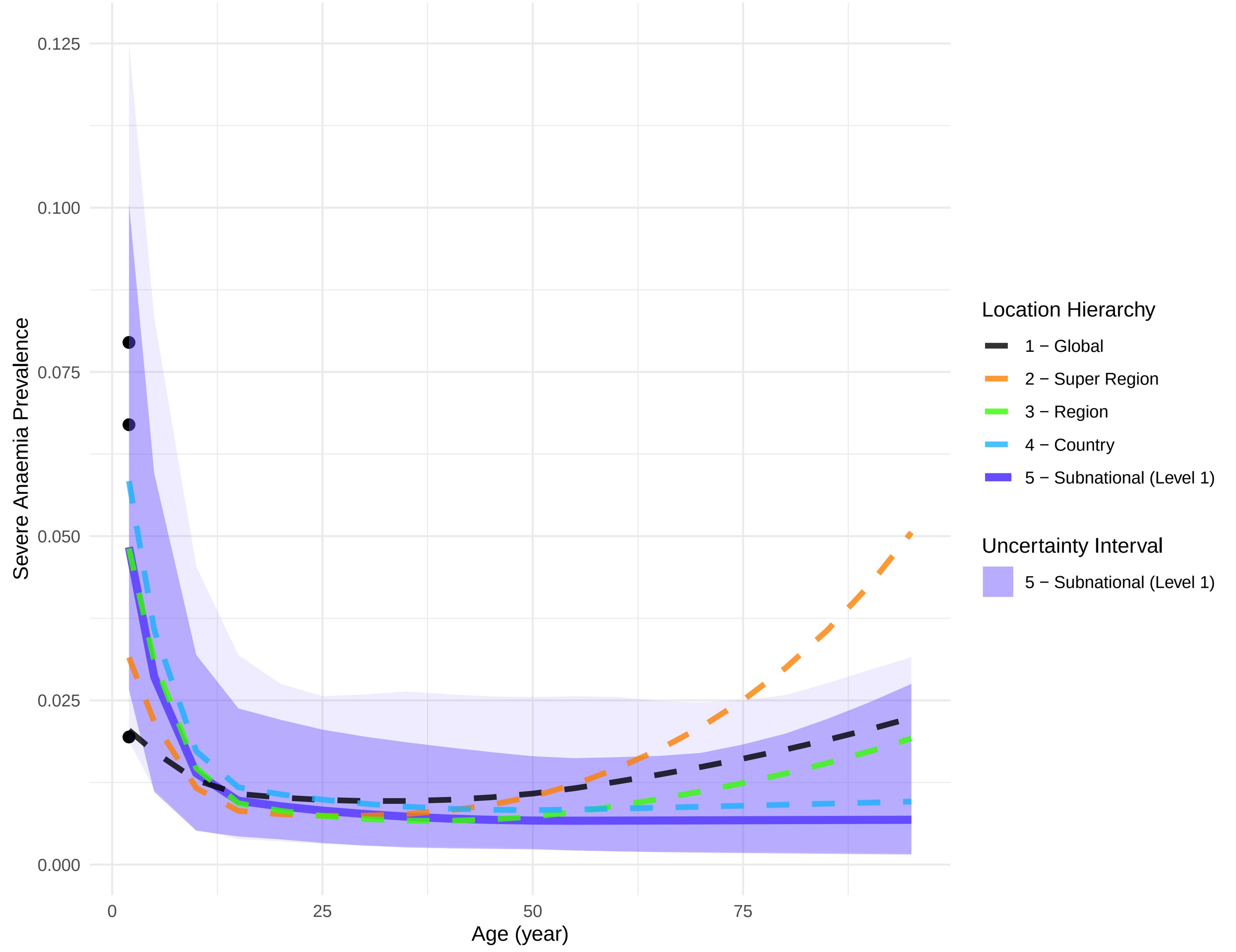

MR−BRT Age/Sex Fits − Males 2+ Years Old − Severe Anaemia Prevalence − Plateau
WHO−2024 Adjustment Method

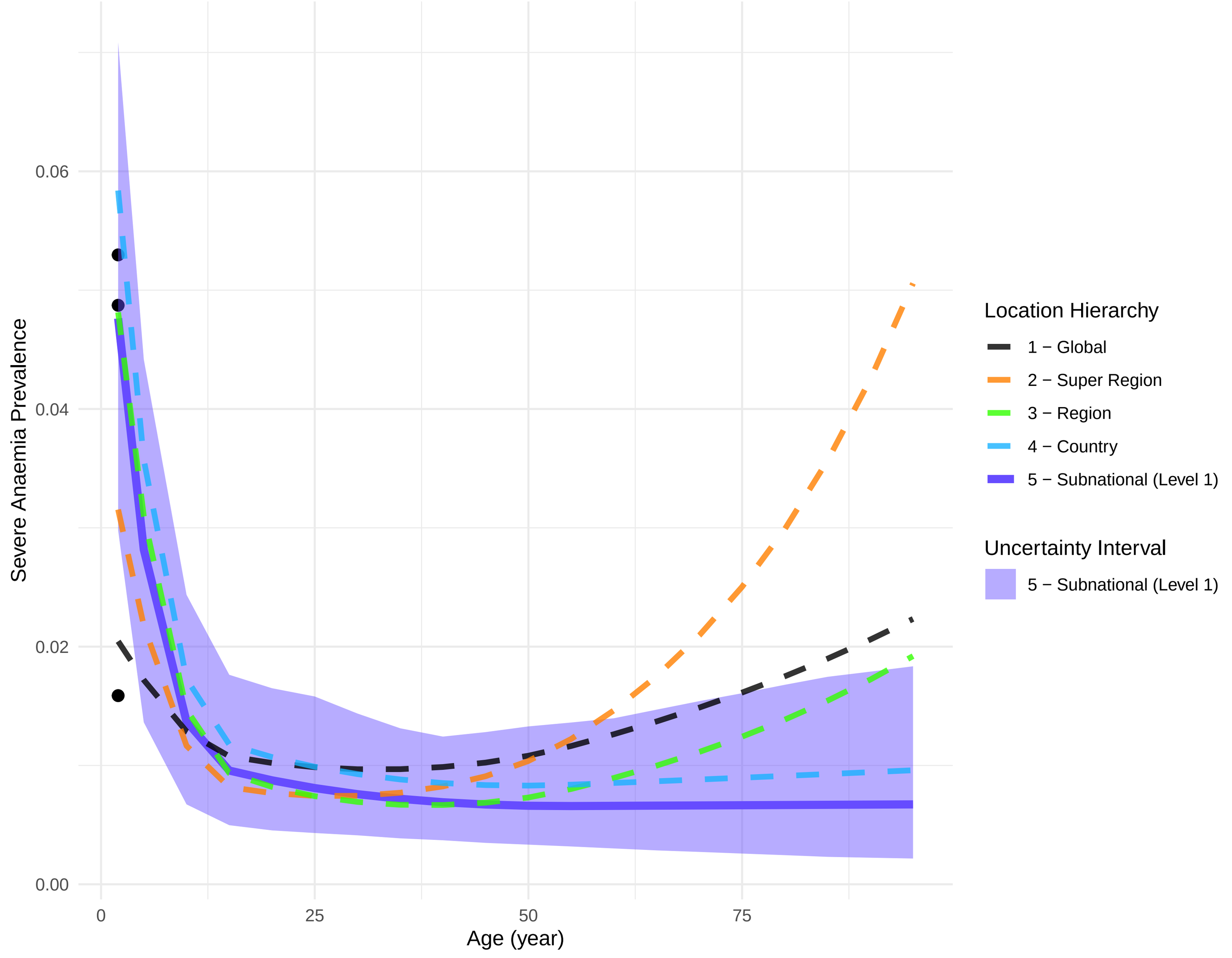

MR−BRT Age/Sex Fits − Males 2+ Years Old − Severe Anaemia Prevalence − Rivers
WHO−2024 Adjustment Method

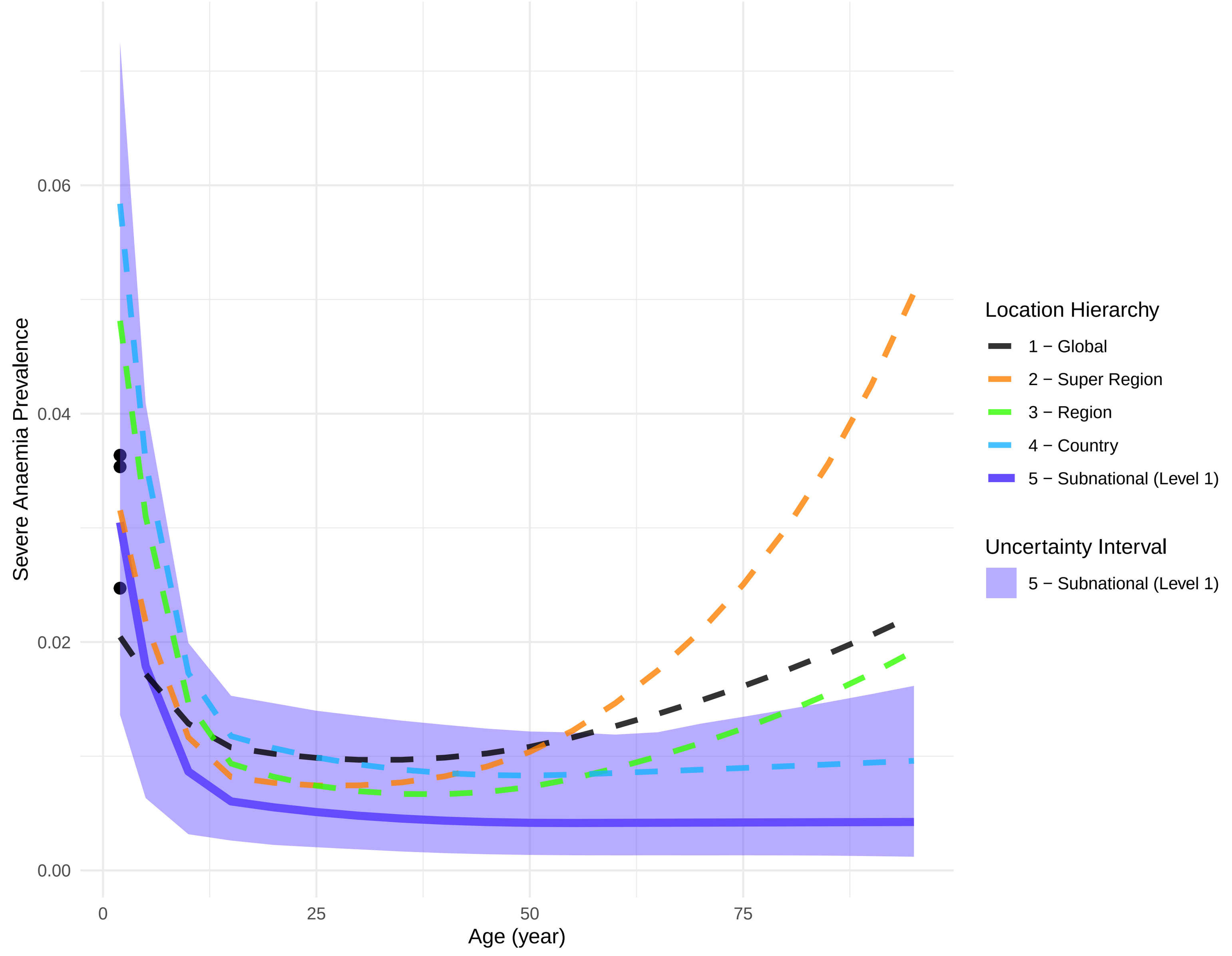

MR−BRT Age/Sex Fits − Males 2+ Years Old − Severe Anaemia Prevalence − Sokoto
WHO−2024 Adjustment Method

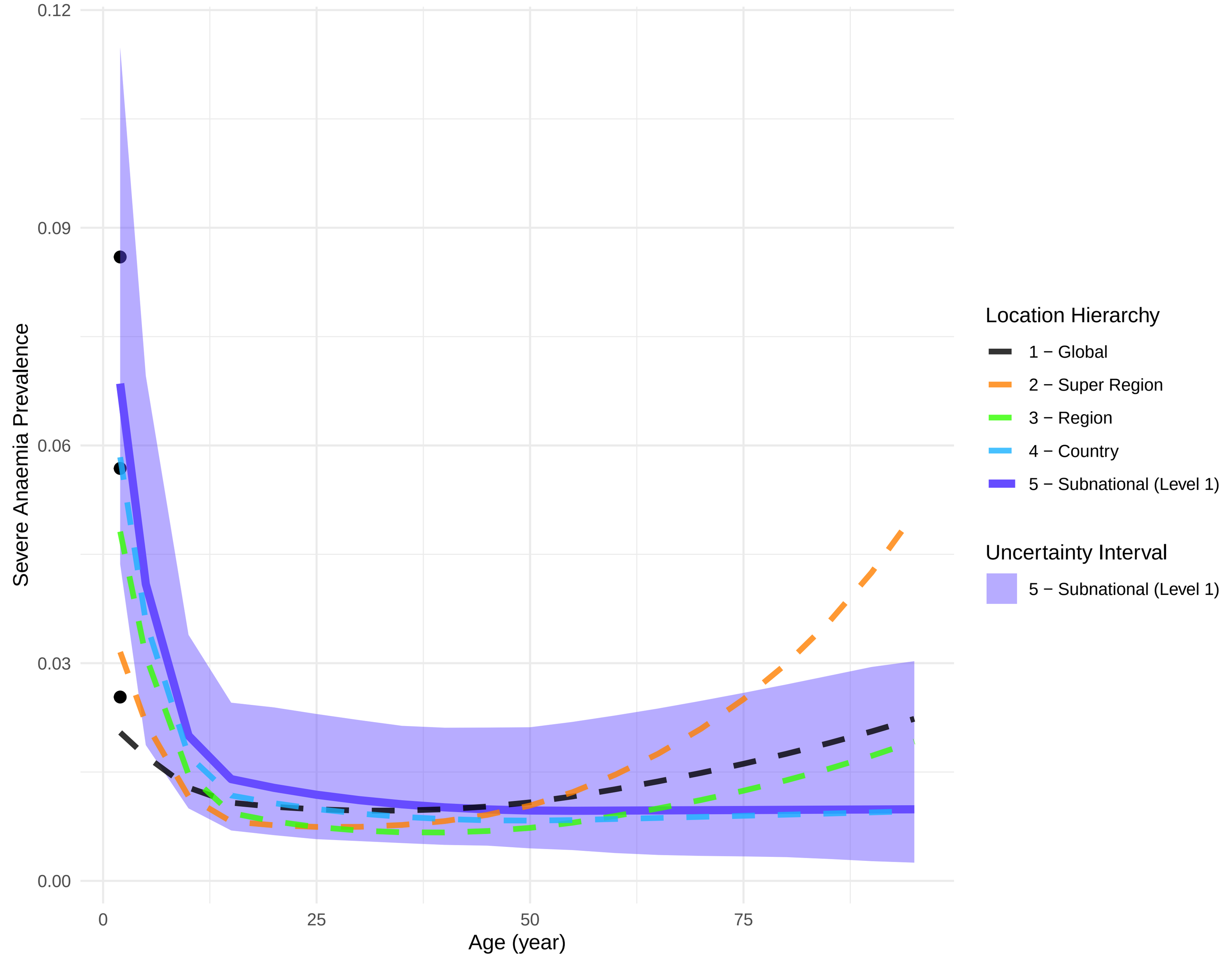

MR−BRT Age/Sex Fits − Males 2+ Years Old − Severe Anaemia Prevalence − Taraba
WHO−2024 Adjustment Method

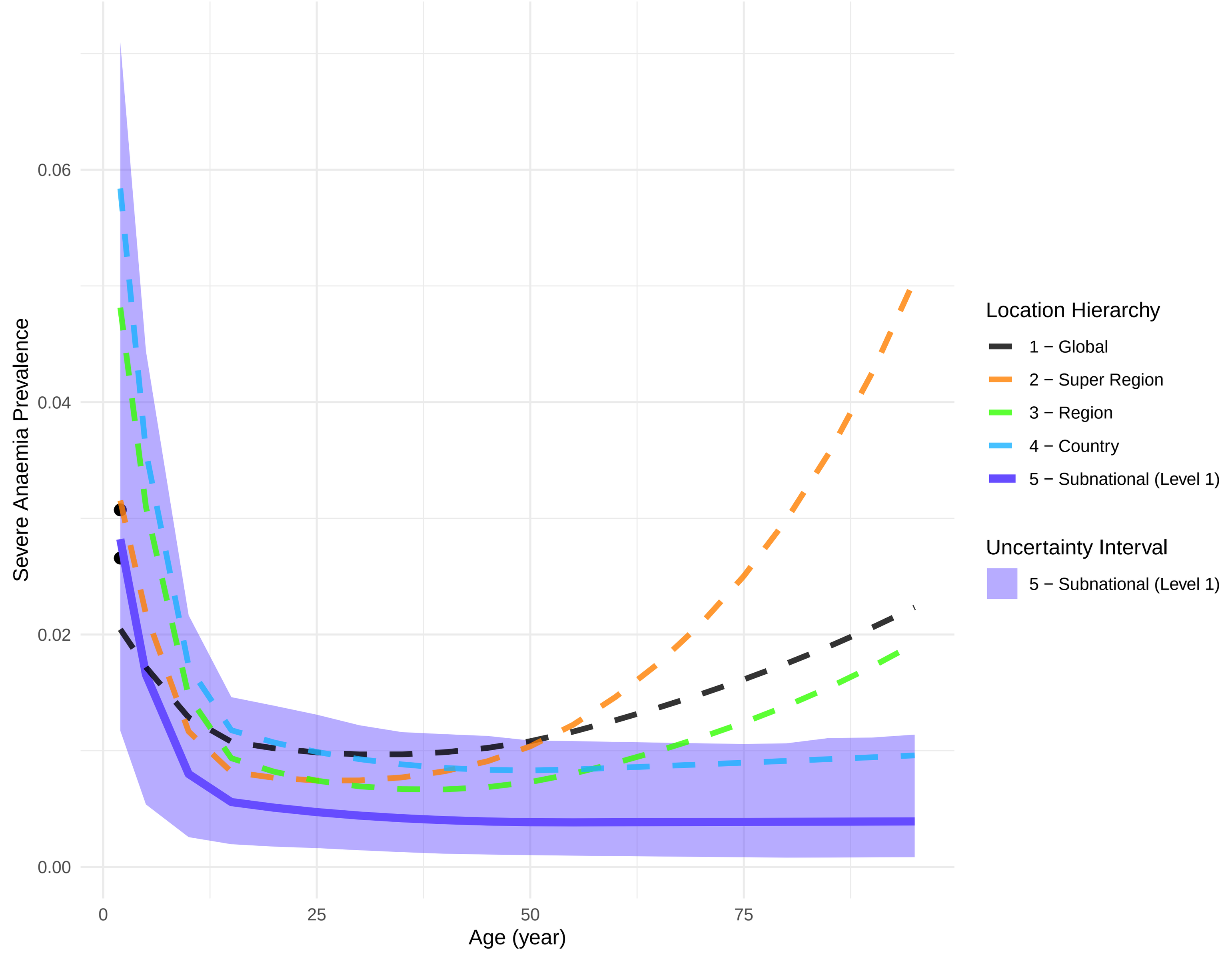

MR−BRT Age/Sex Fits − Males 2+ Years Old − Severe Anaemia Prevalence − Yobe
WHO−2024 Adjustment Method

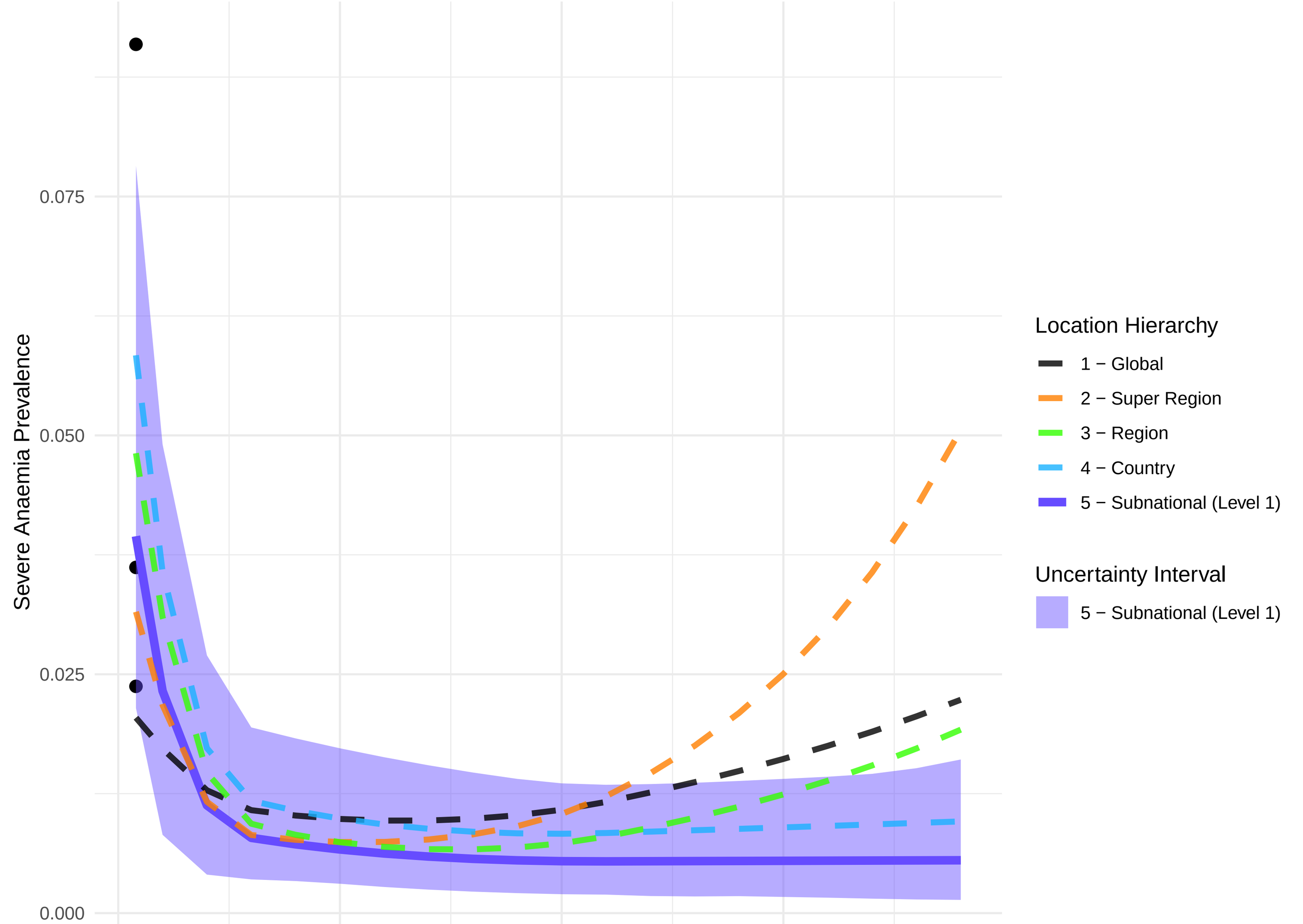

MR−BRT Age/Sex Fits − Males 2+ Years Old − Severe Anaemia Prevalence − Zamfara
WHO−2024 Adjustment Method

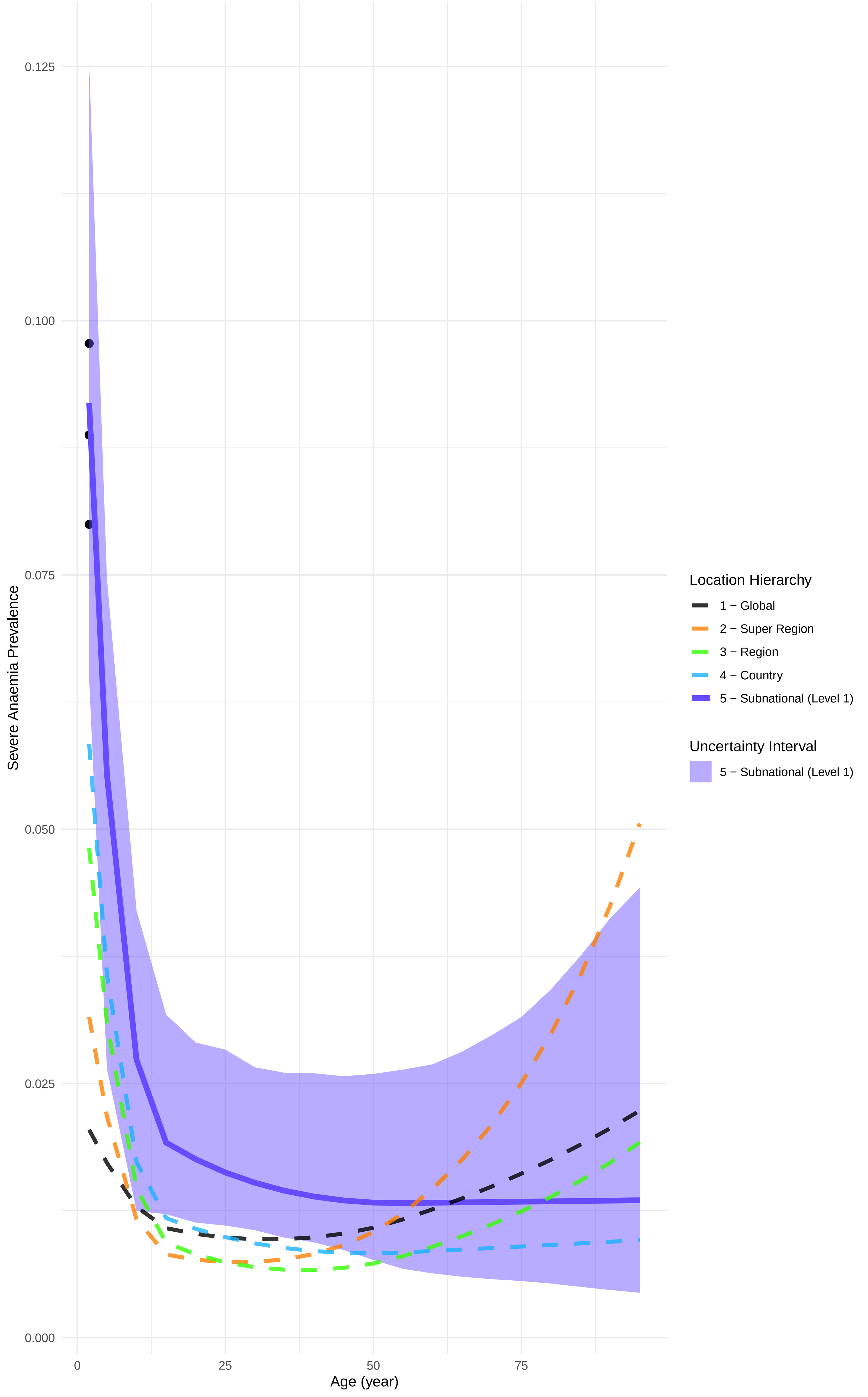

MR−BRT Age/Sex Fits − Males 2+ Years Old − Severe Anaemia Prevalence − Senegal
WHO−2024 Adjustment Method

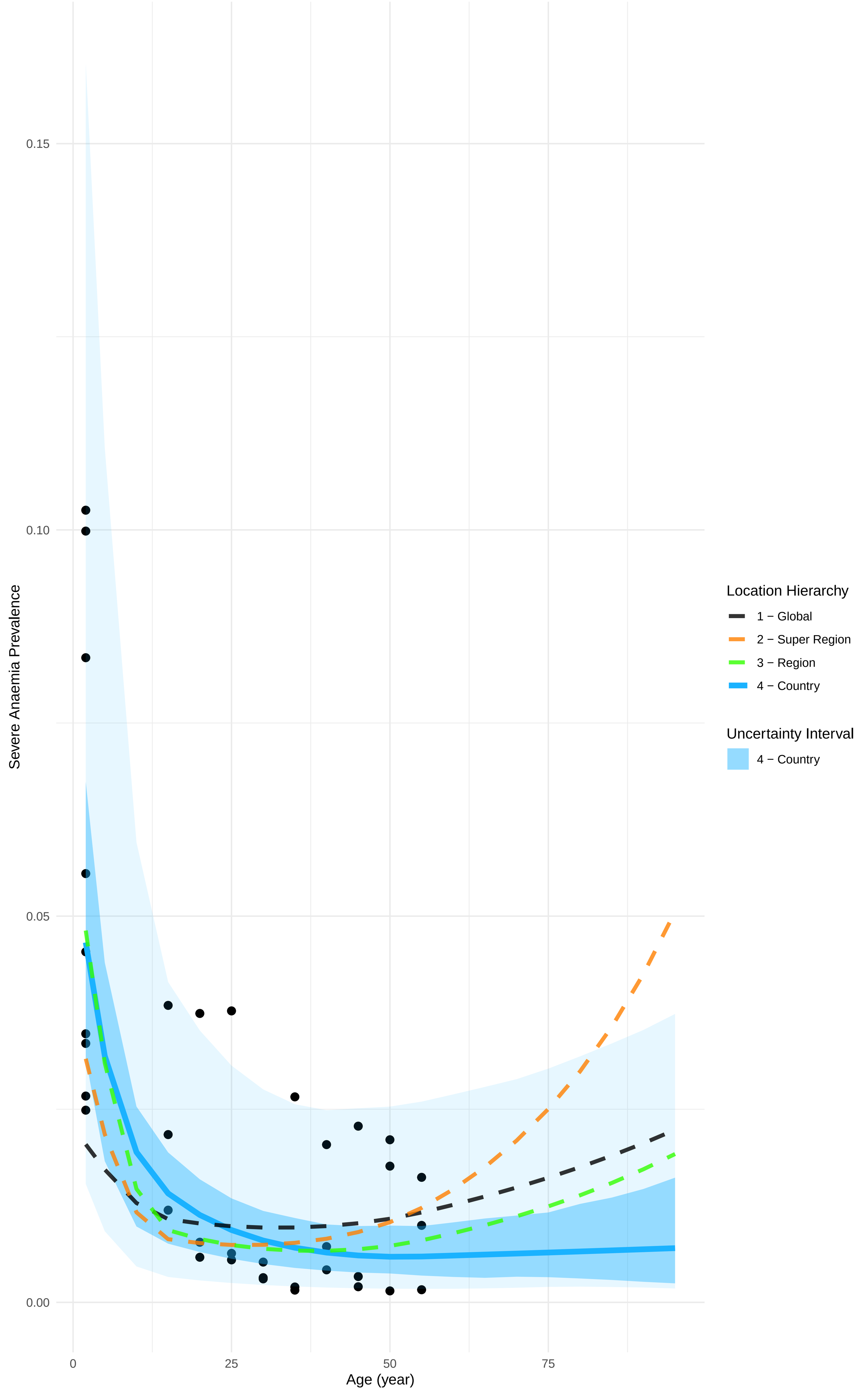

MR−BRT Age/Sex Fits − Males 2+ Years Old − Severe Anaemia Prevalence − Sierra Leone
WHO−2024 Adjustment Method

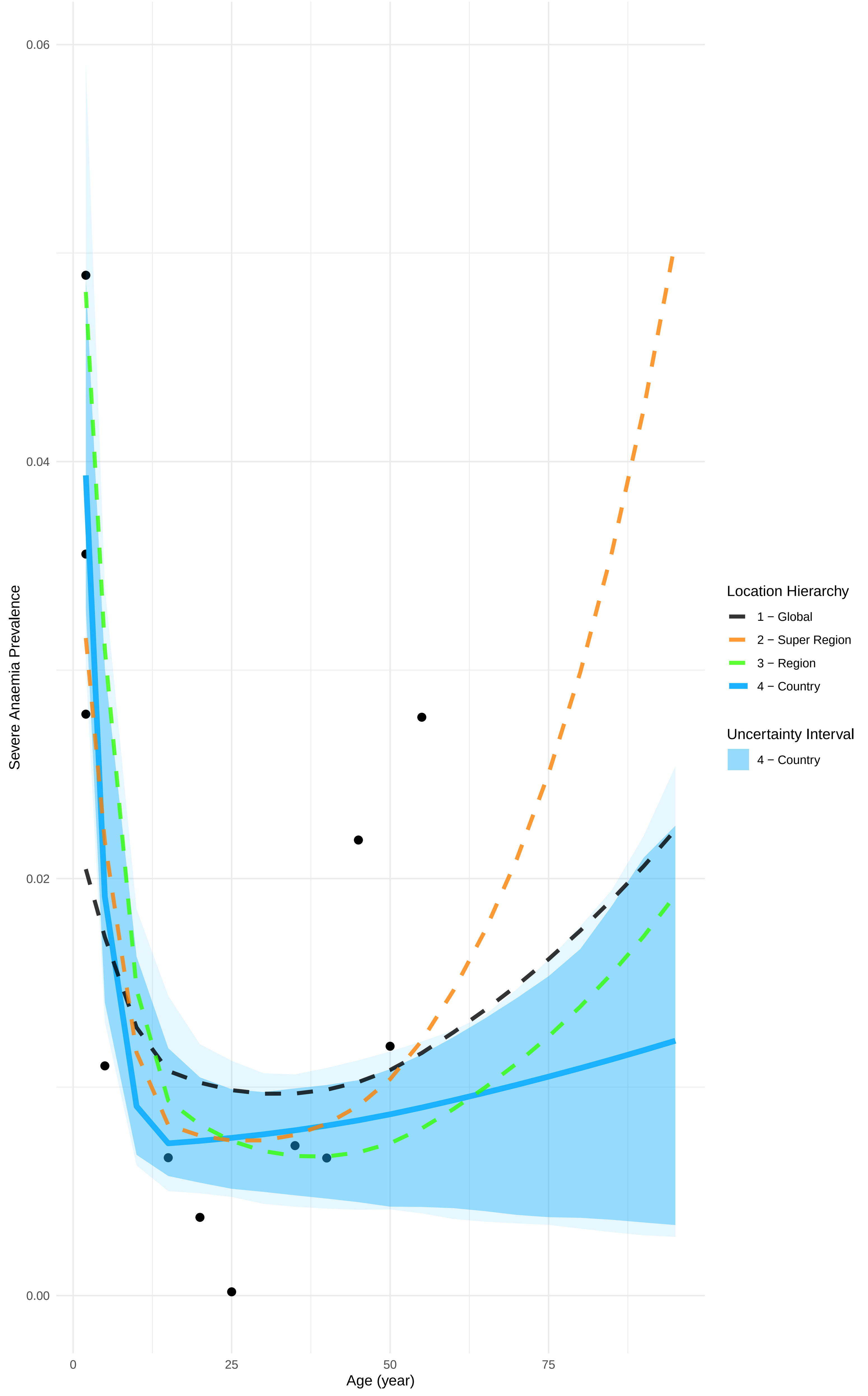

MR−BRT Age/Sex Fits − Males 2+ Years Old − Severe Anaemia Prevalence − Togo
WHO−2024 Adjustment Method

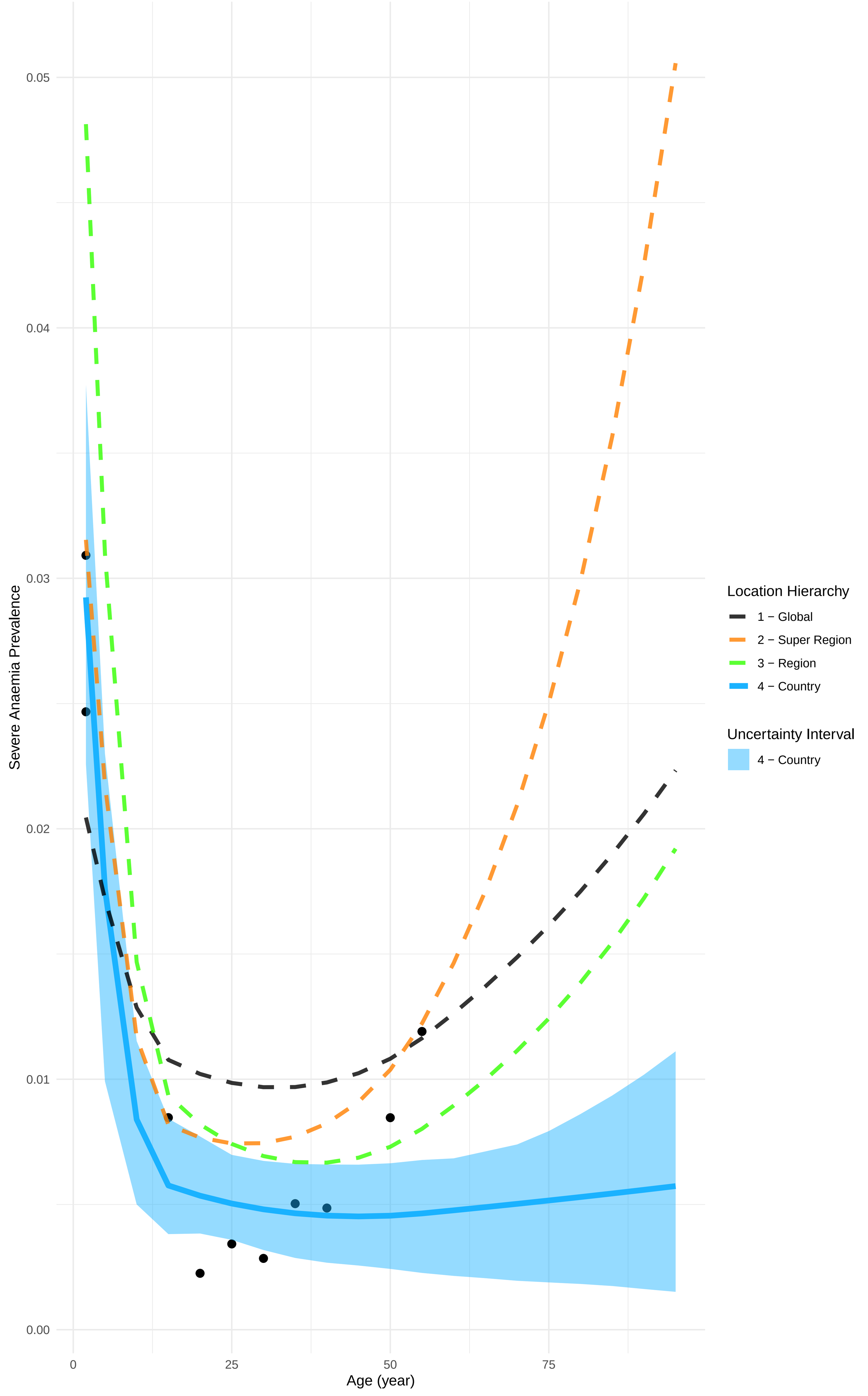

MR−BRT Age/Sex Fits − Non−pregnant Females 2+ Years Old − Severe Anaemia Prevalence − Global
WHO−2024 Adjustment Method

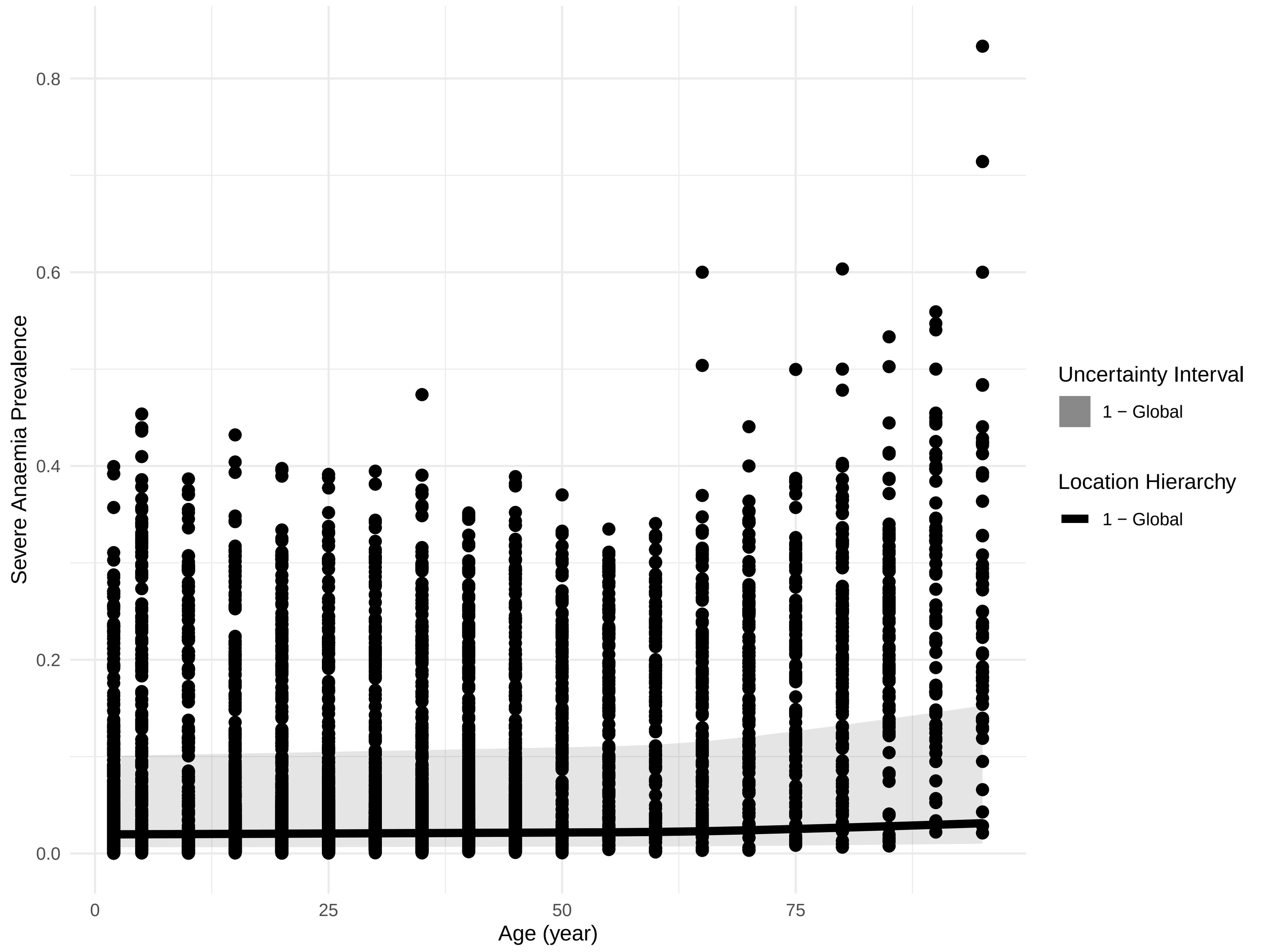

MR−BRT Age/Sex Fits − Non−pregnant Females 2+ Years Old − Severe Anaemia Prevalence − Central Europe, Eastern Europe, and Central Asia
WHO−2024 Adjustment Method

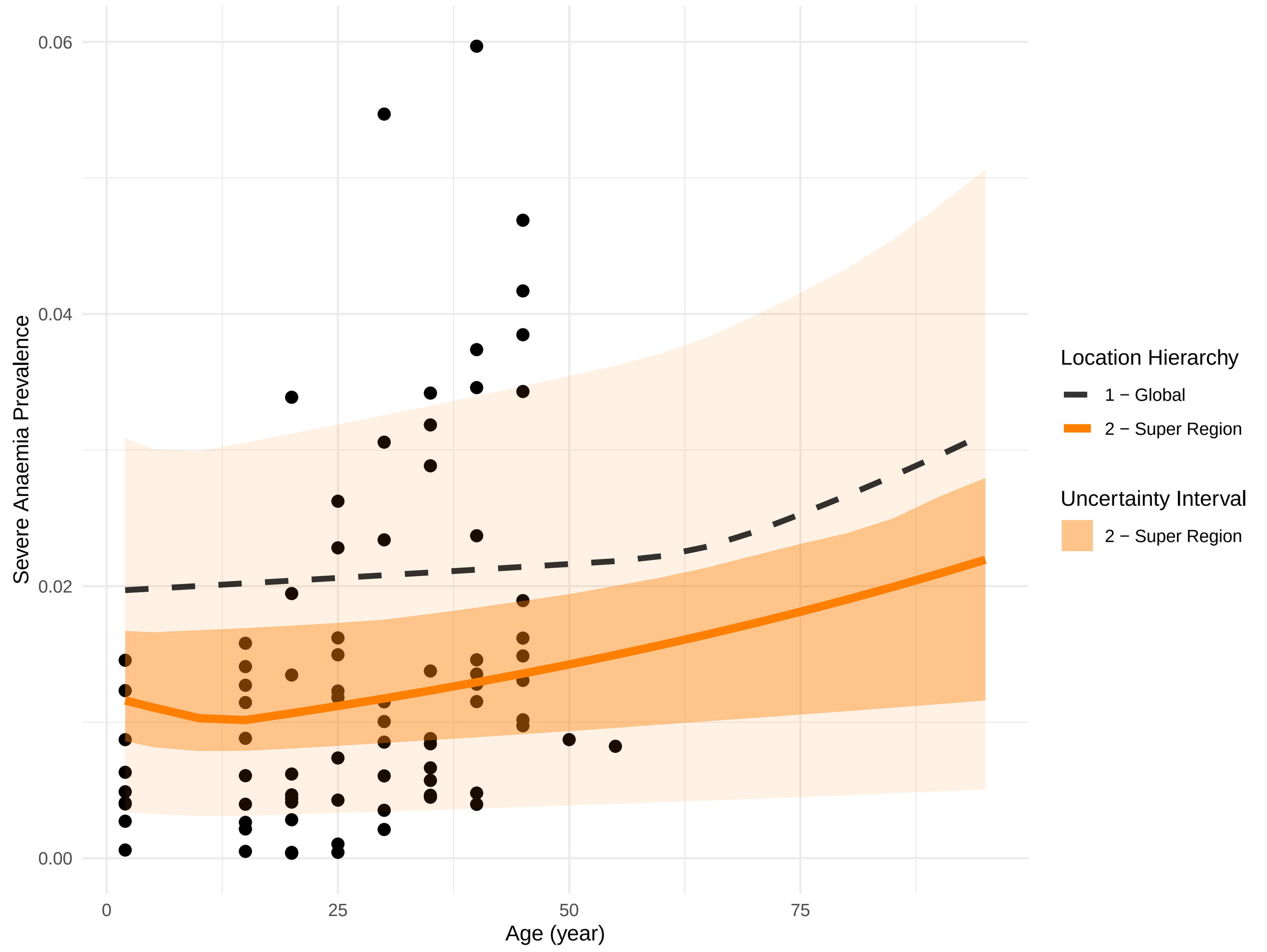

MR−BRT Age/Sex Fits − Non−pregnant Females 2+ Years Old − Severe Anaemia Prevalence − Central Asia
WHO−2024 Adjustment Method

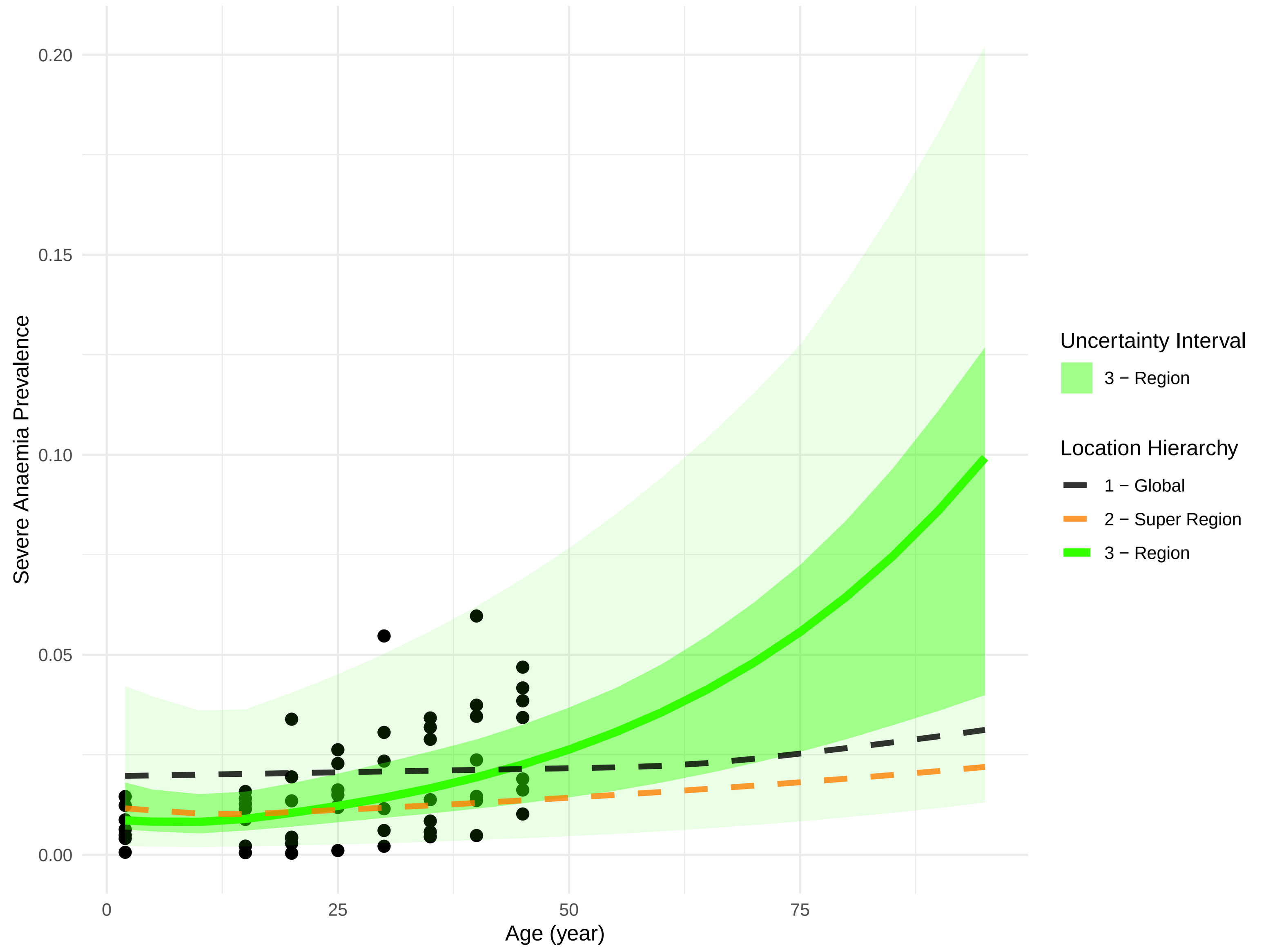

MR−BRT Age/Sex Fits − Non−pregnant Females 2+ Years Old − Severe Anaemia Prevalence − Armenia
WHO−2024 Adjustment Method

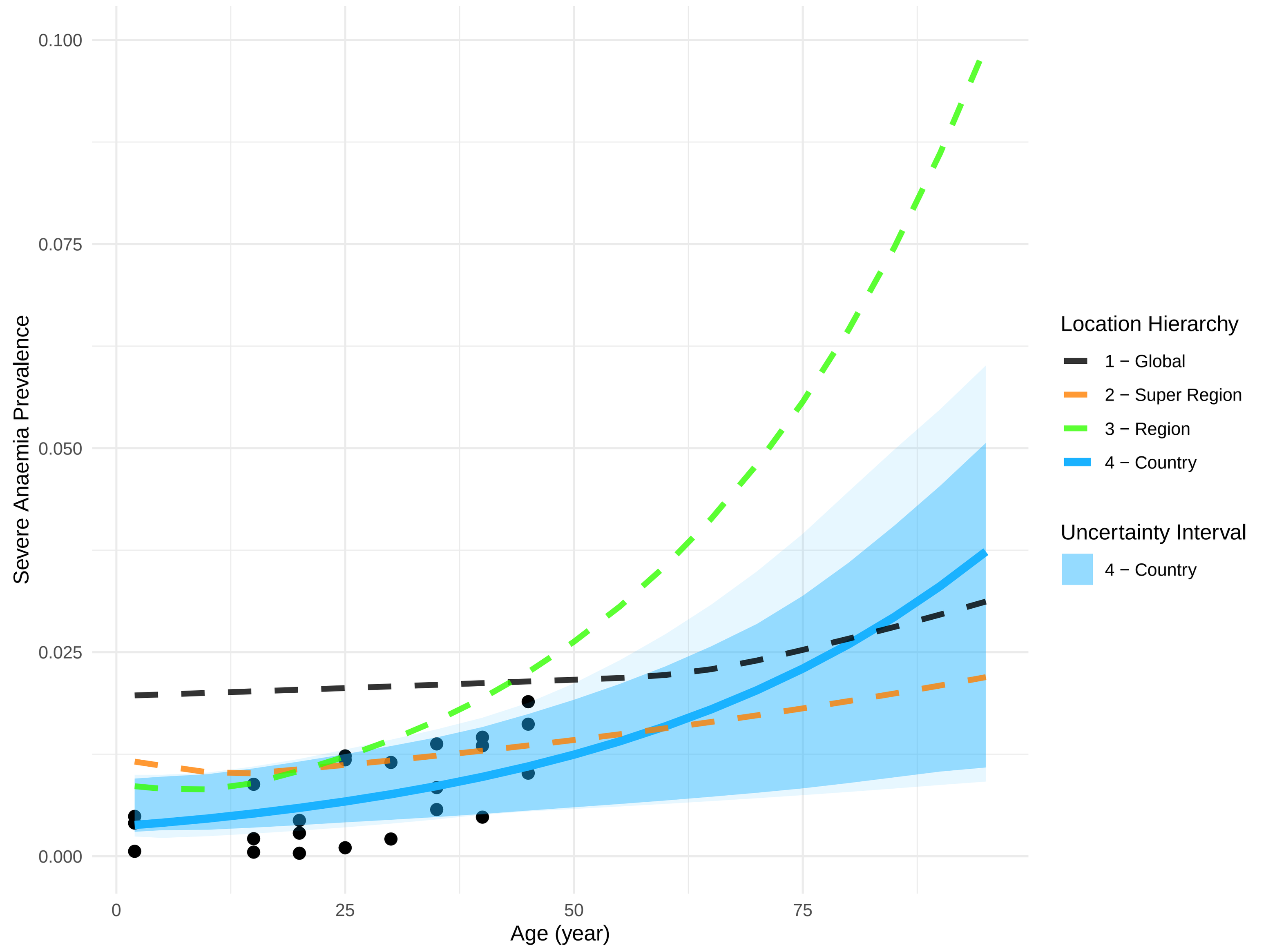

MR−BRT Age/Sex Fits − Non−pregnant Females 2+ Years Old − Severe Anaemia Prevalence − Azerbaijan
WHO−2024 Adjustment Method

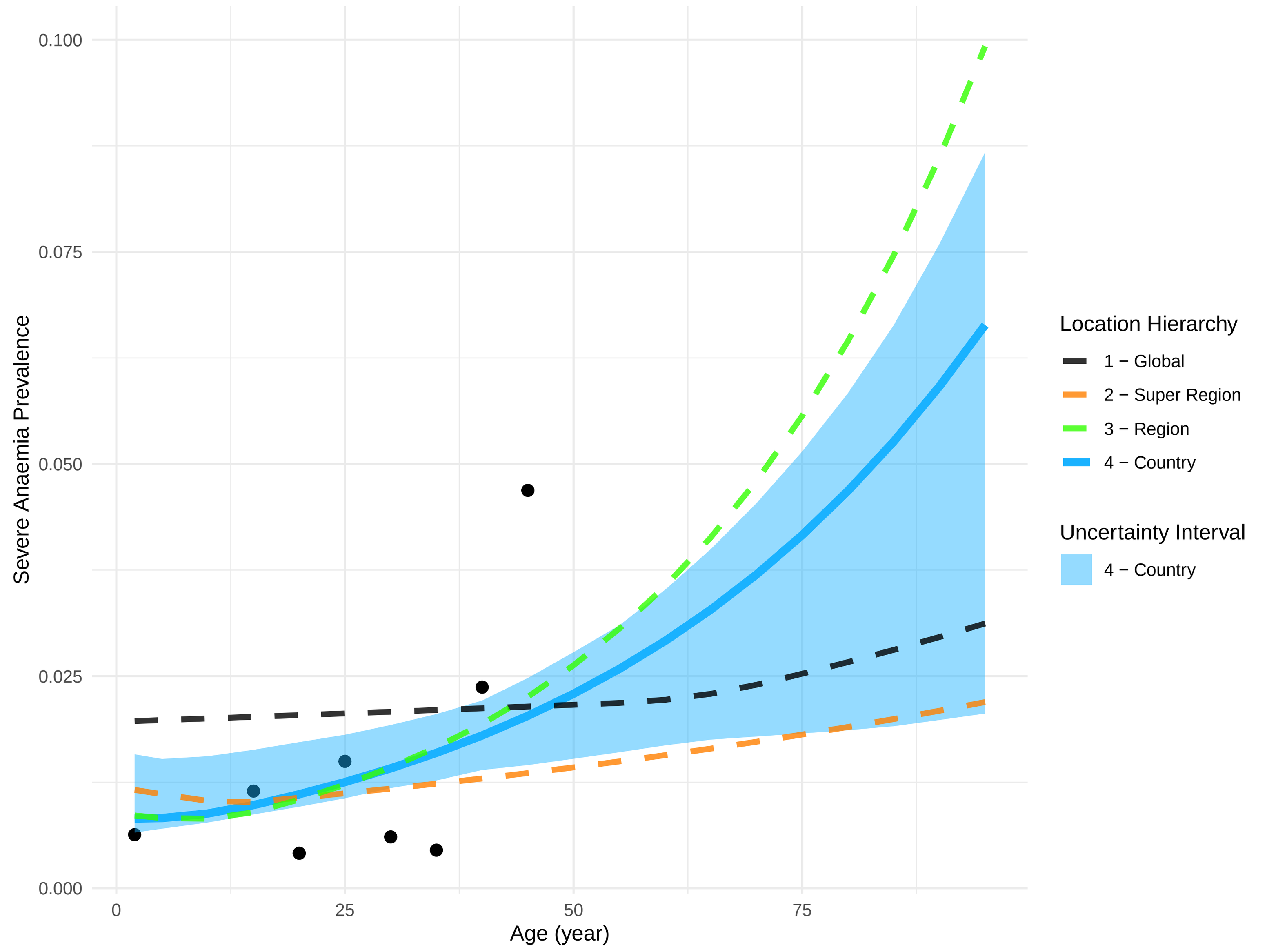

MR−BRT Age/Sex Fits − Non−pregnant Females 2+ Years Old − Severe Anaemia Prevalence − Kyrgyzstan
WHO−2024 Adjustment Method

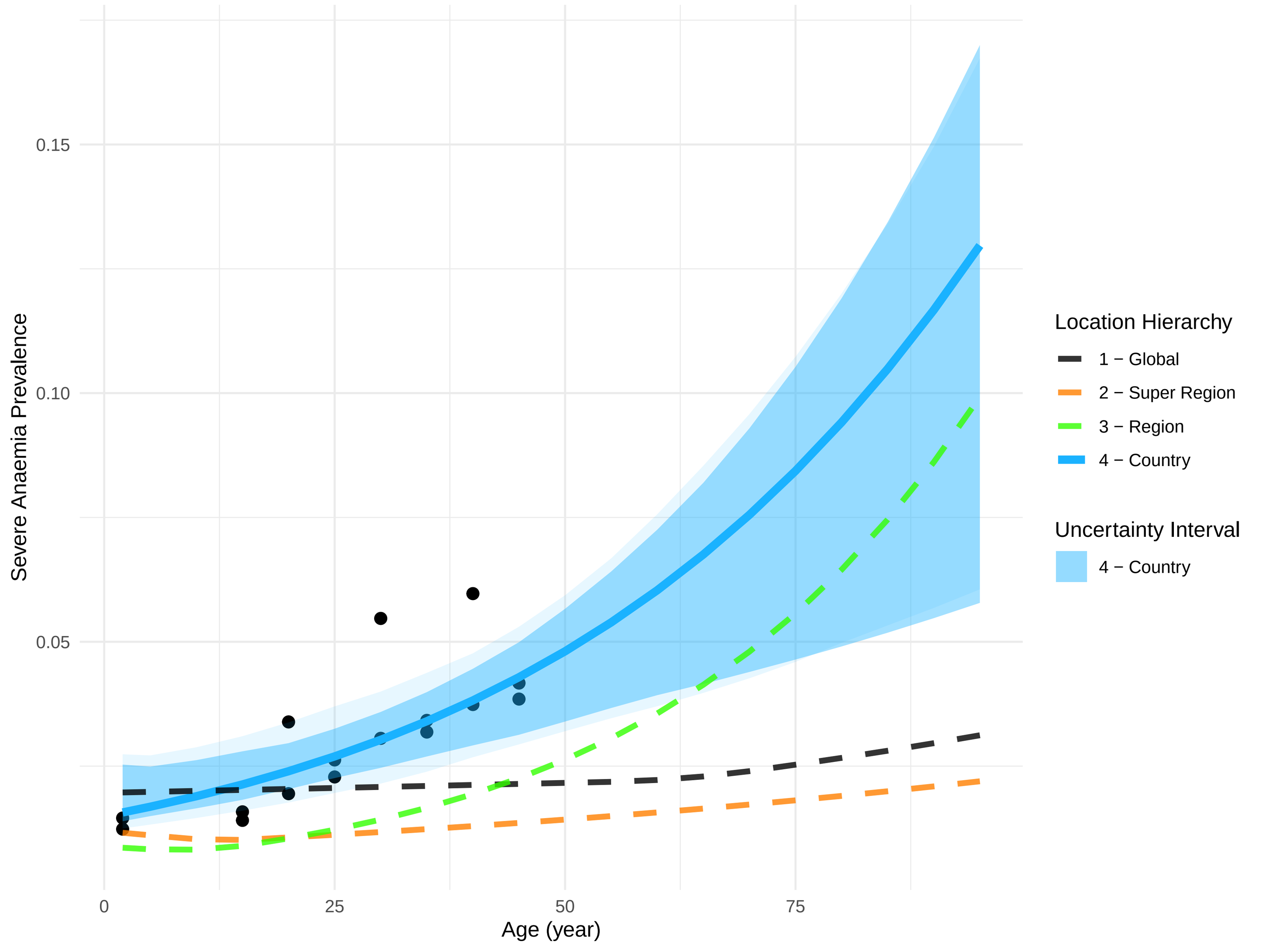

MR−BRT Age/Sex Fits − Non−pregnant Females 2+ Years Old − Severe Anaemia Prevalence − Tajikistan
WHO−2024 Adjustment Method

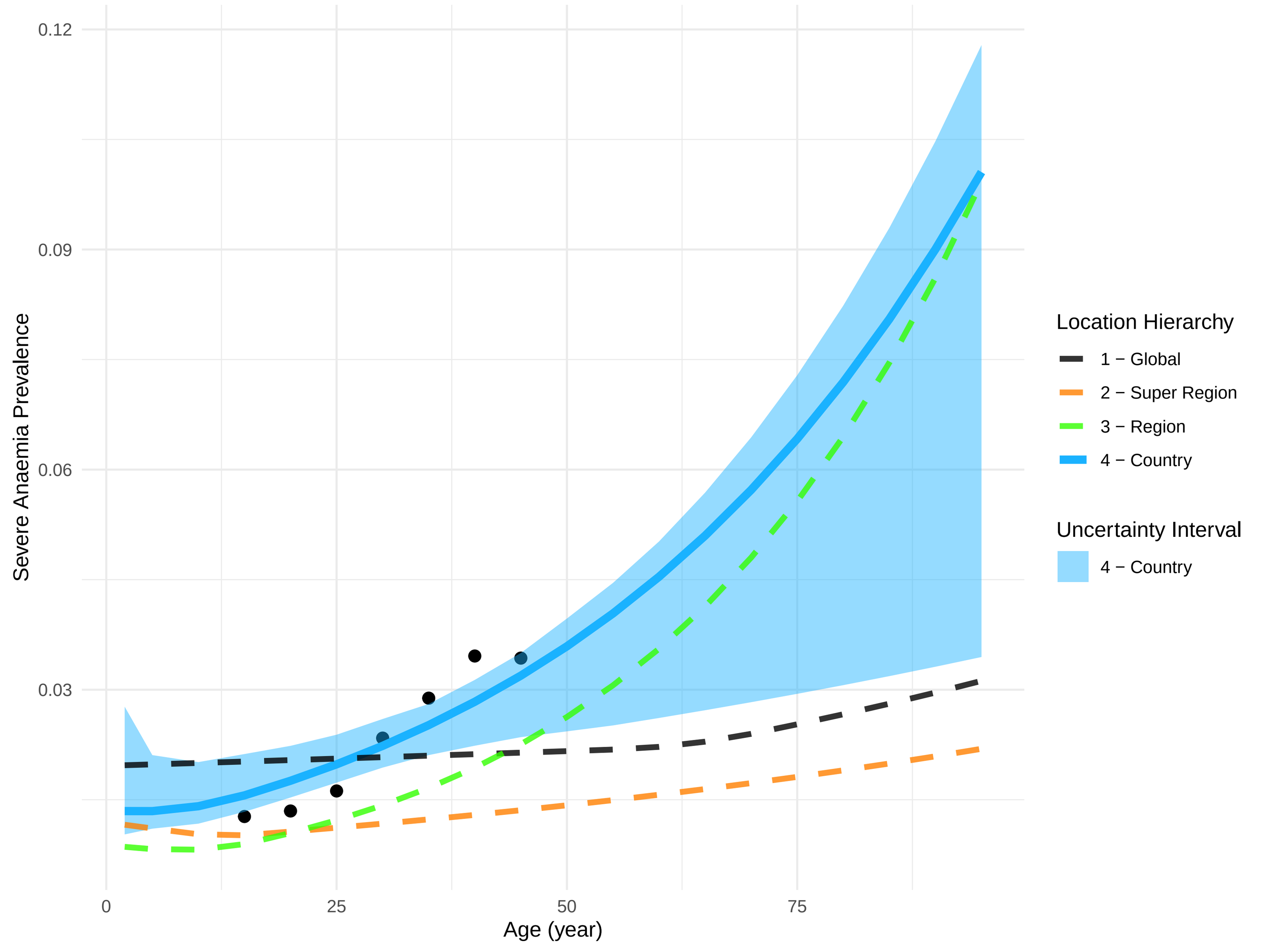

MR−BRT Age/Sex Fits − Non−pregnant Females 2+ Years Old − Severe Anaemia Prevalence − Uzbekistan
WHO−2024 Adjustment Method

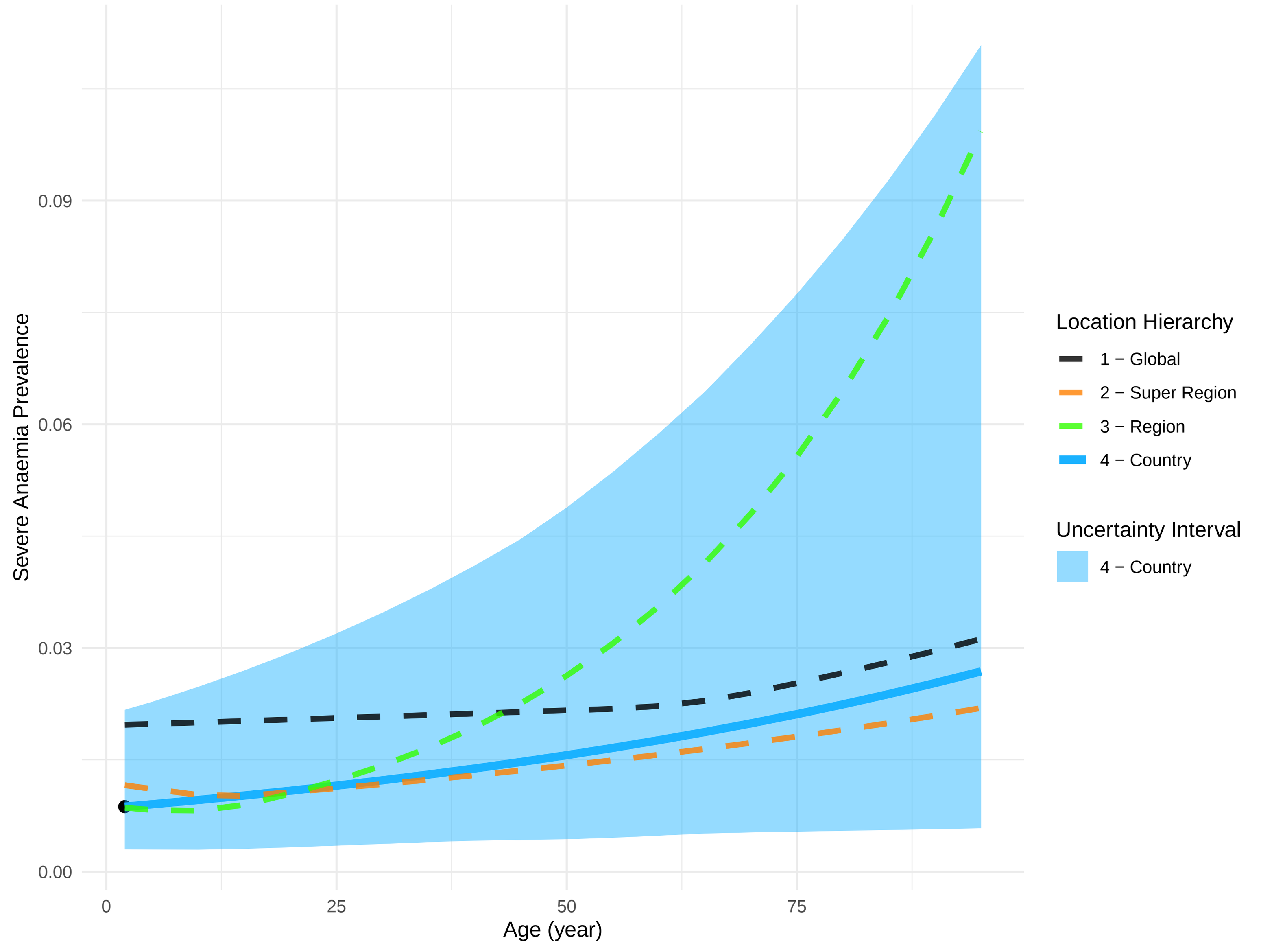

MR−BRT Age/Sex Fits − Non−pregnant Females 2+ Years Old − Severe Anaemia Prevalence − Central Europe
WHO−2024 Adjustment Method

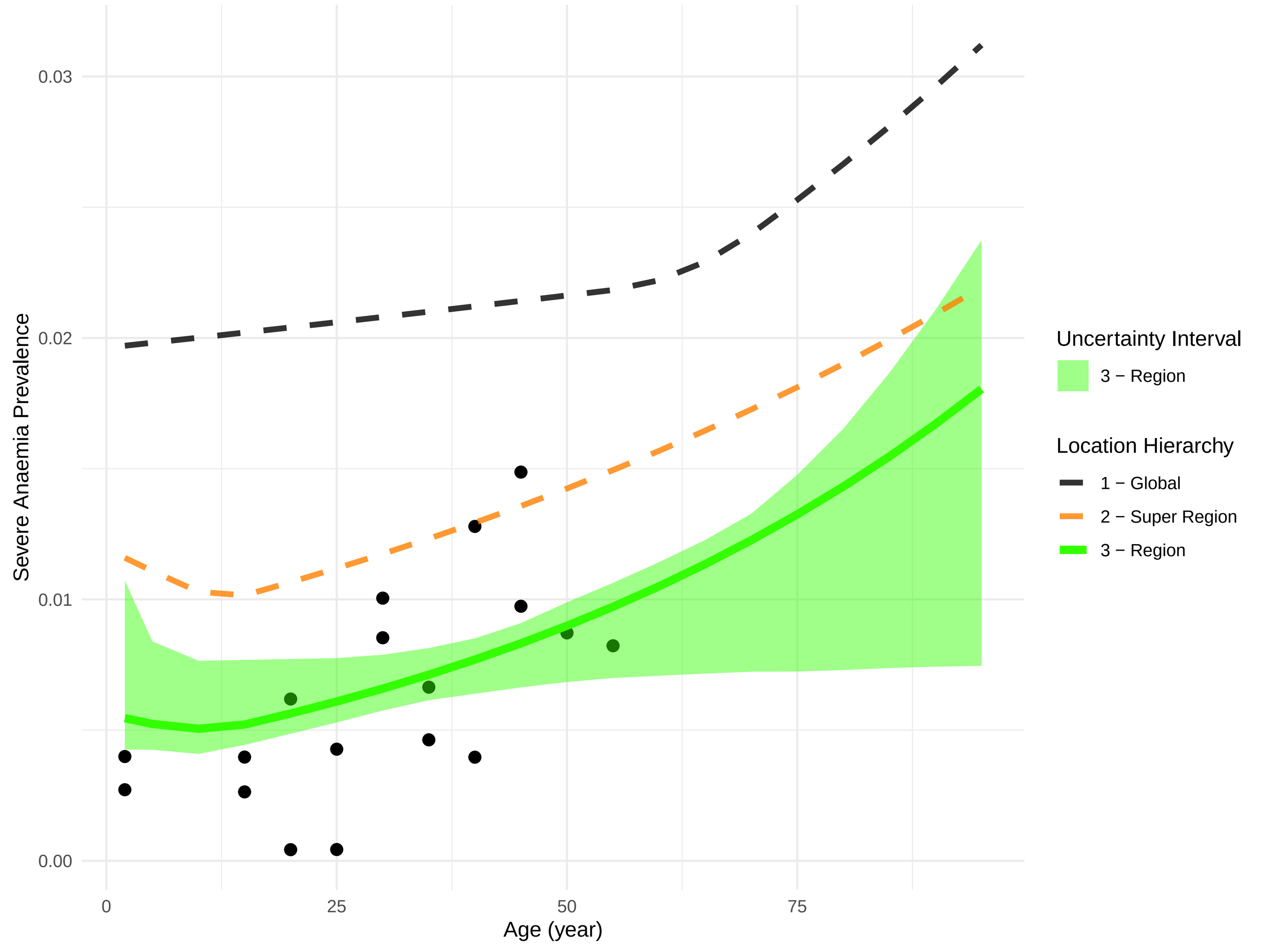

MR−BRT Age/Sex Fits − Non−pregnant Females 2+ Years Old − Severe Anaemia Prevalence − Albania
WHO−2024 Adjustment Method

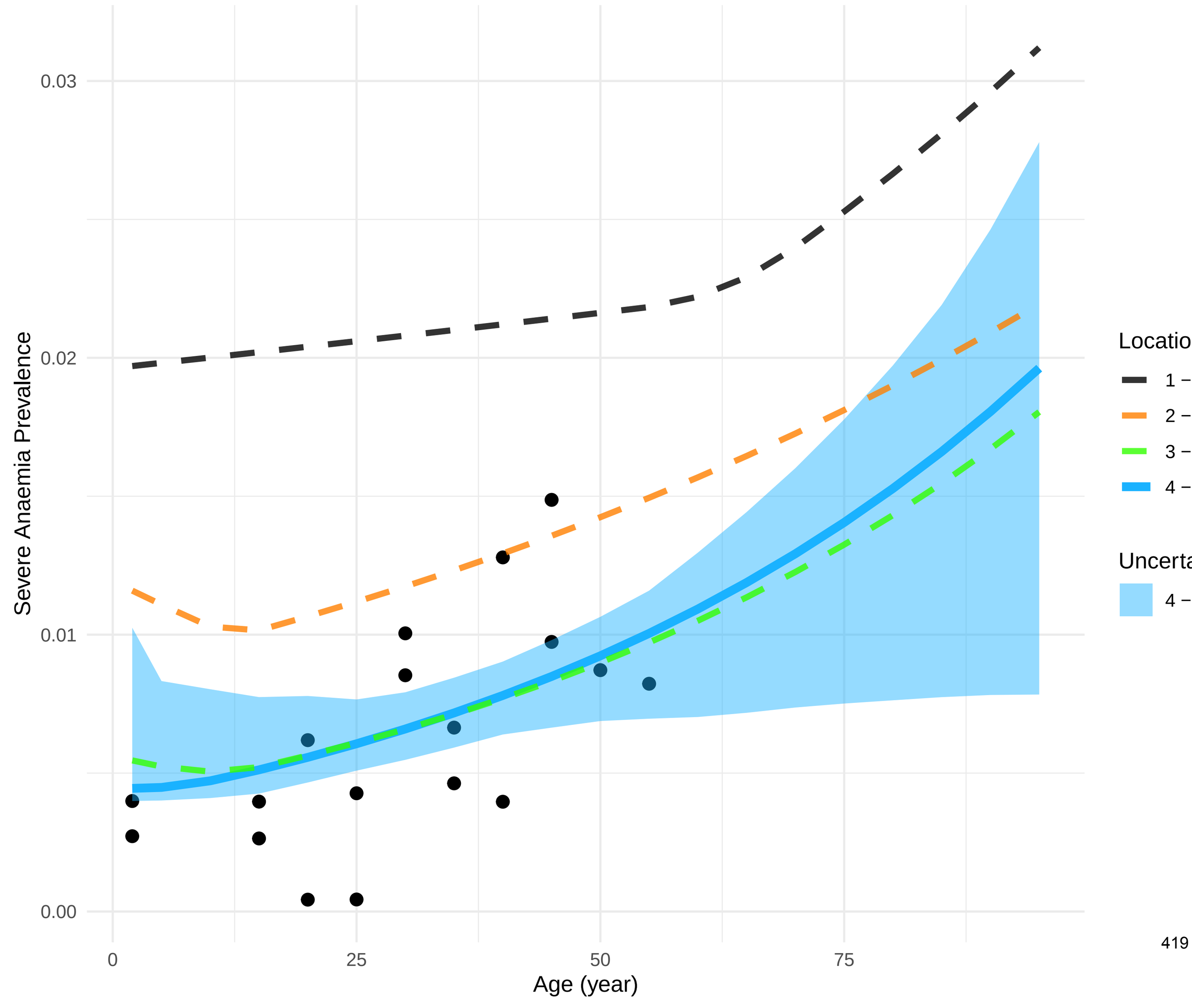

MR−BRT Age/Sex Fits − Non−pregnant Females 2+ Years Old − Severe Anaemia Prevalence − Eastern Europe
WHO−2024 Adjustment Method

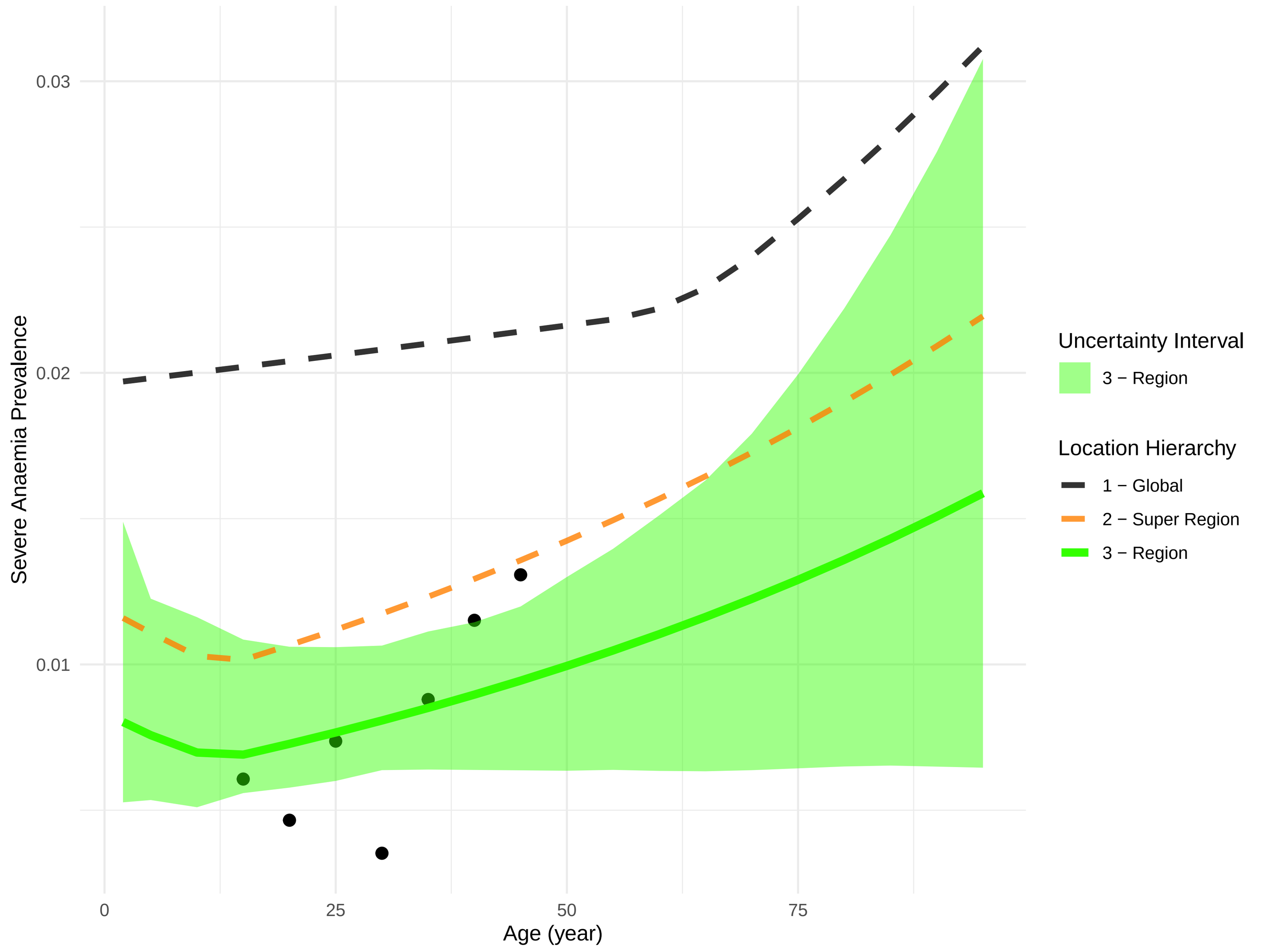

MR−BRT Age/Sex Fits − Non−pregnant Females 2+ Years Old − Severe Anaemia Prevalence − Republic of Moldova
WHO−2024 Adjustment Method

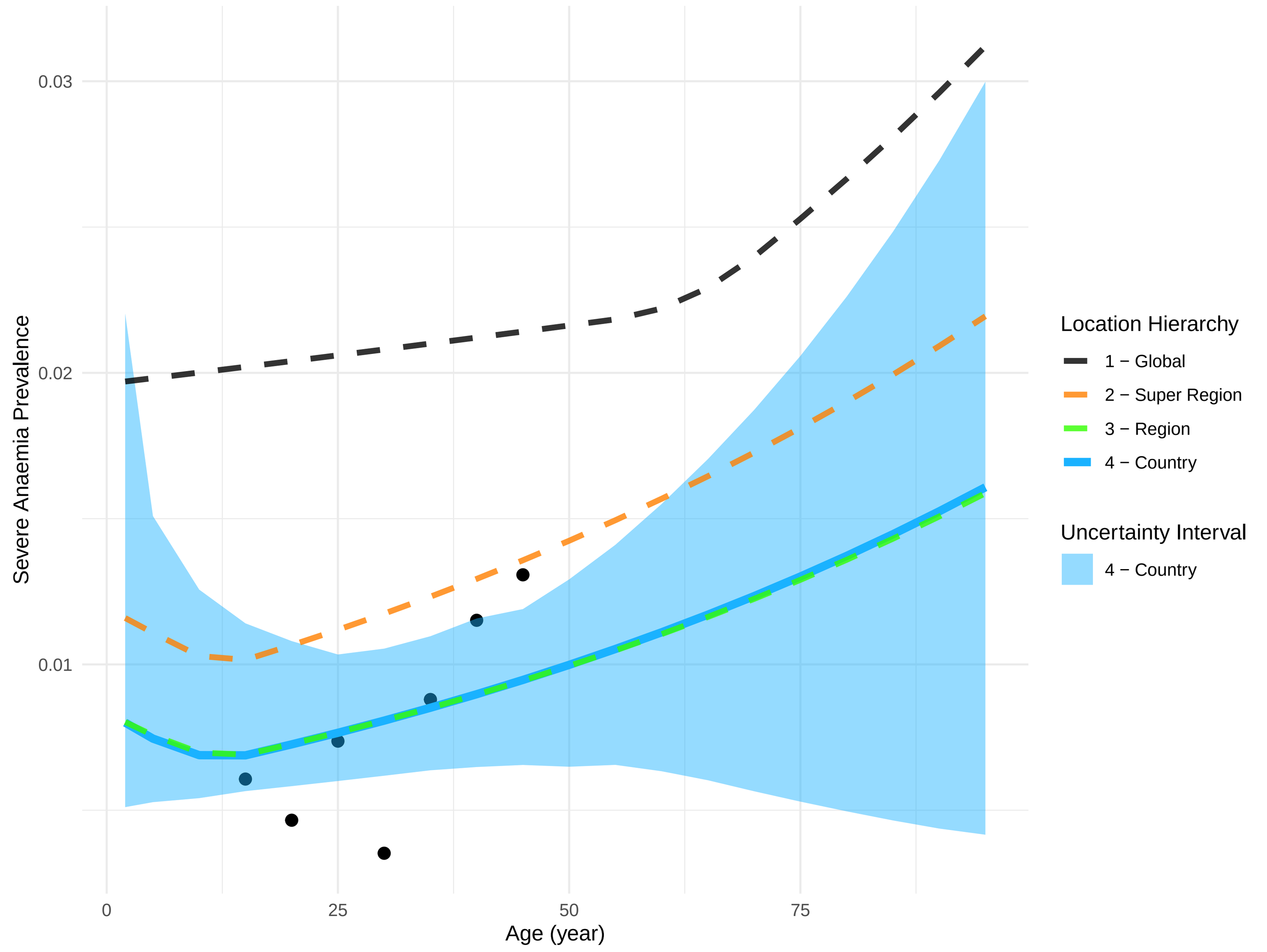

MR−BRT Age/Sex Fits − Non−pregnant Females 2+ Years Old − Severe Anaemia Prevalence − High−income
WHO−2024 Adjustment Method

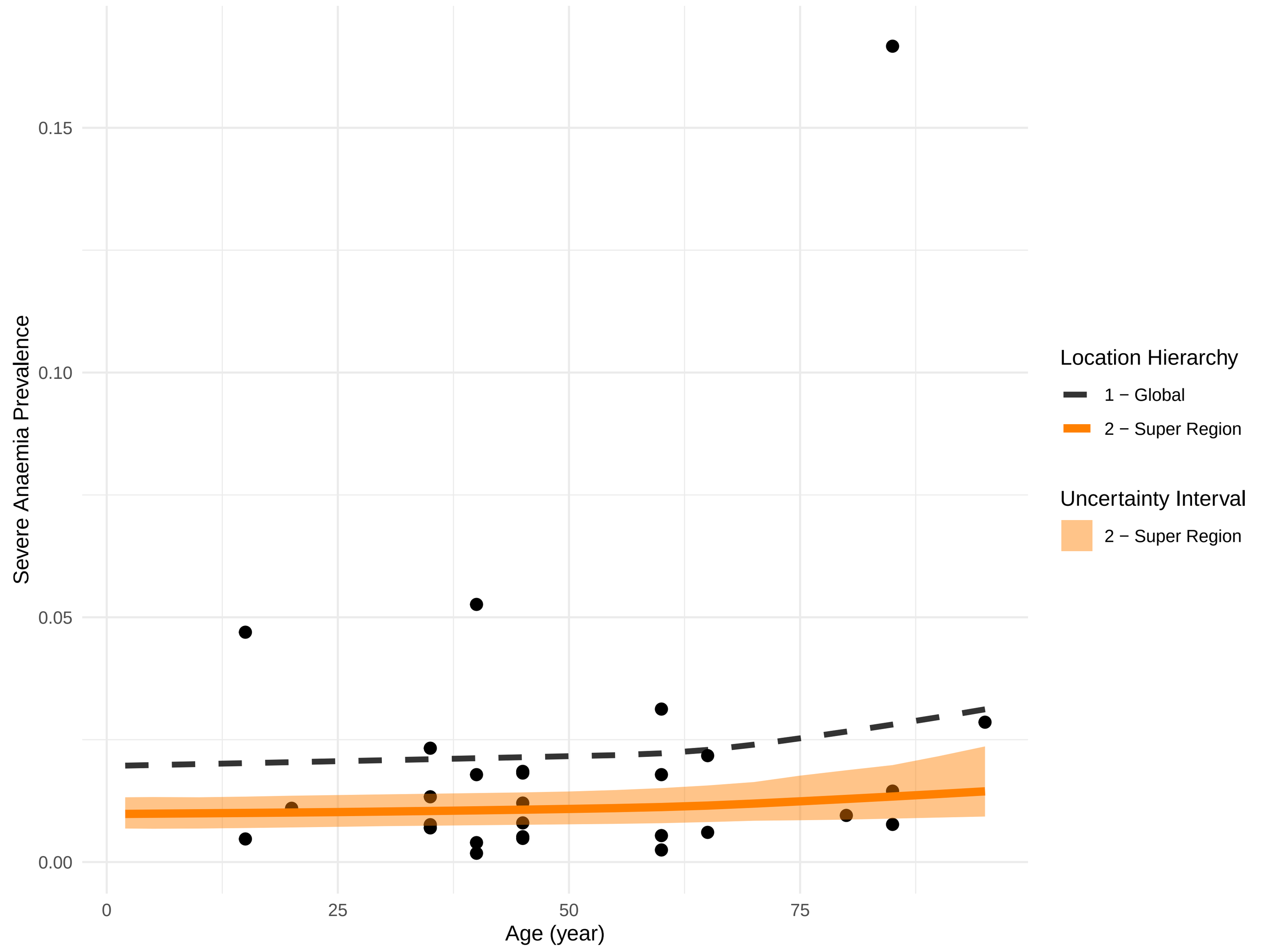

MR−BRT Age/Sex Fits − Non−pregnant Females 2+ Years Old − Severe Anaemia Prevalence − Western Europe
WHO−2024 Adjustment Method

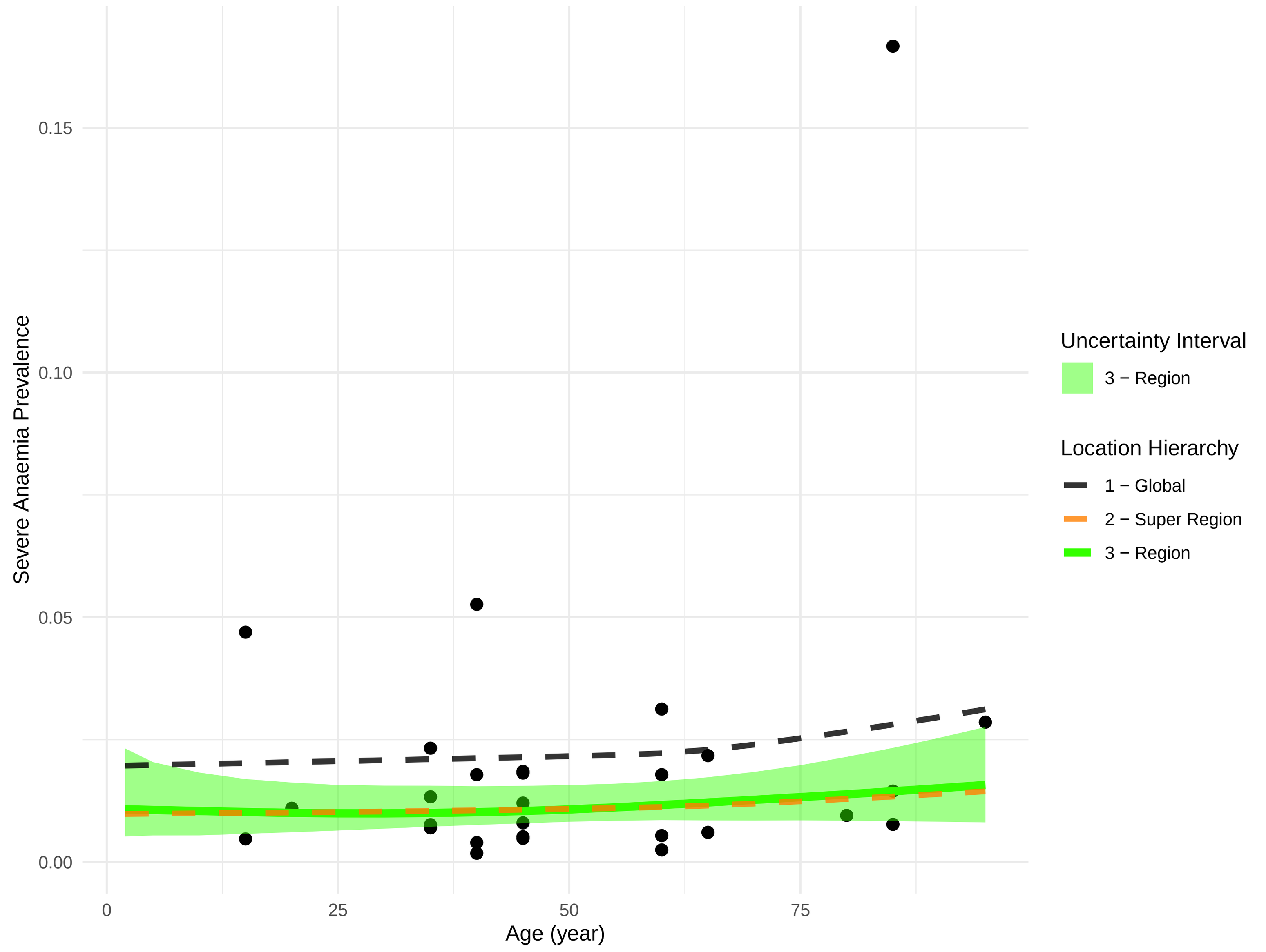

MR−BRT Age/Sex Fits − Non−pregnant Females 2+ Years Old − Severe Anaemia Prevalence − United Kingdom
WHO−2024 Adjustment Method

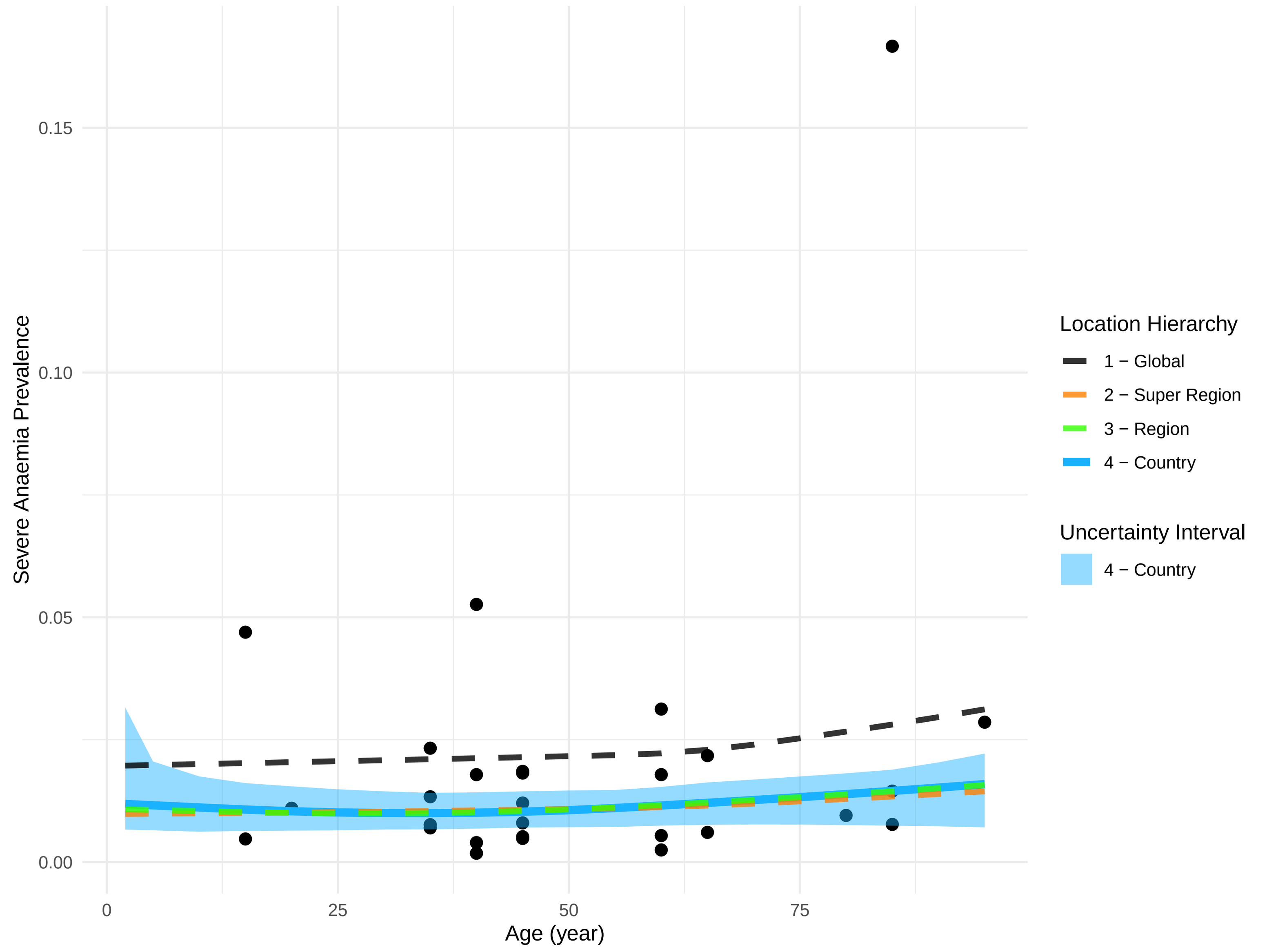

MR−BRT Age/Sex Fits − Non−pregnant Females 2+ Years Old − Severe Anaemia Prevalence − England
WHO−2024 Adjustment Method

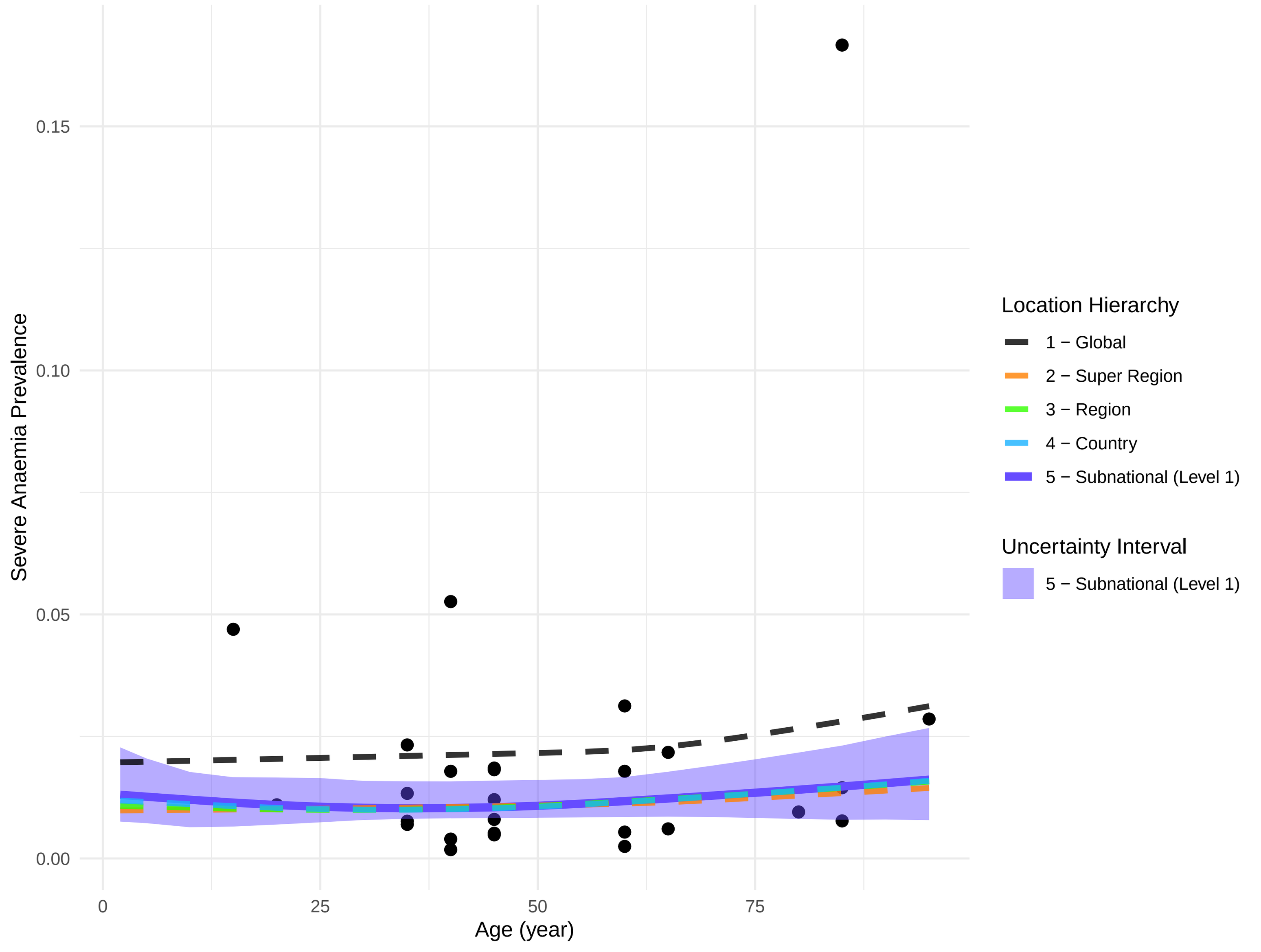

MR−BRT Age/Sex Fits − Non−pregnant Females 2+ Years Old − Severe Anaemia Prevalence − East of England
WHO−2024 Adjustment Method

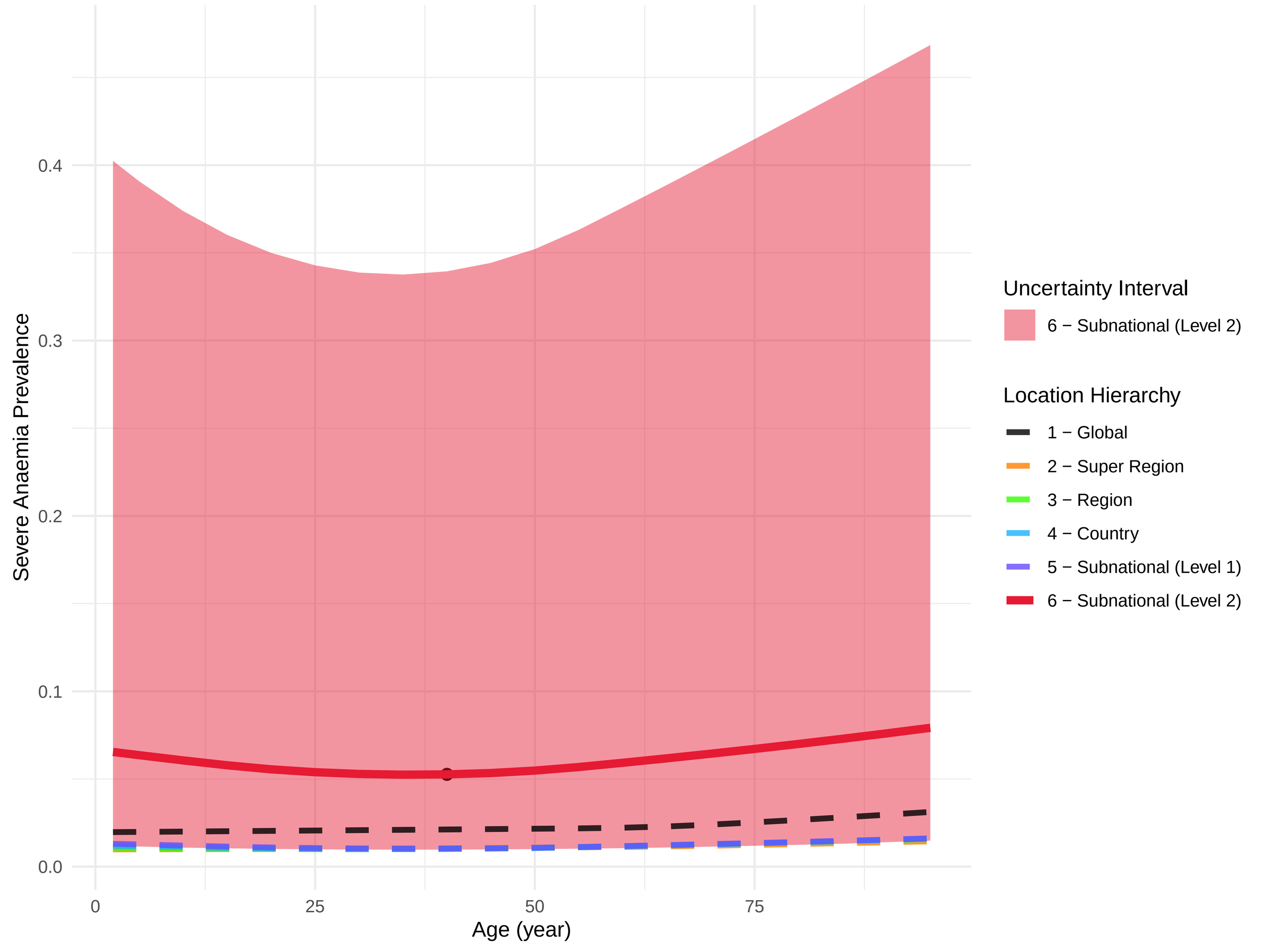

MR−BRT Age/Sex Fits − Non−pregnant Females 2+ Years Old − Severe Anaemia Prevalence − Greater London
WHO−2024 Adjustment Method

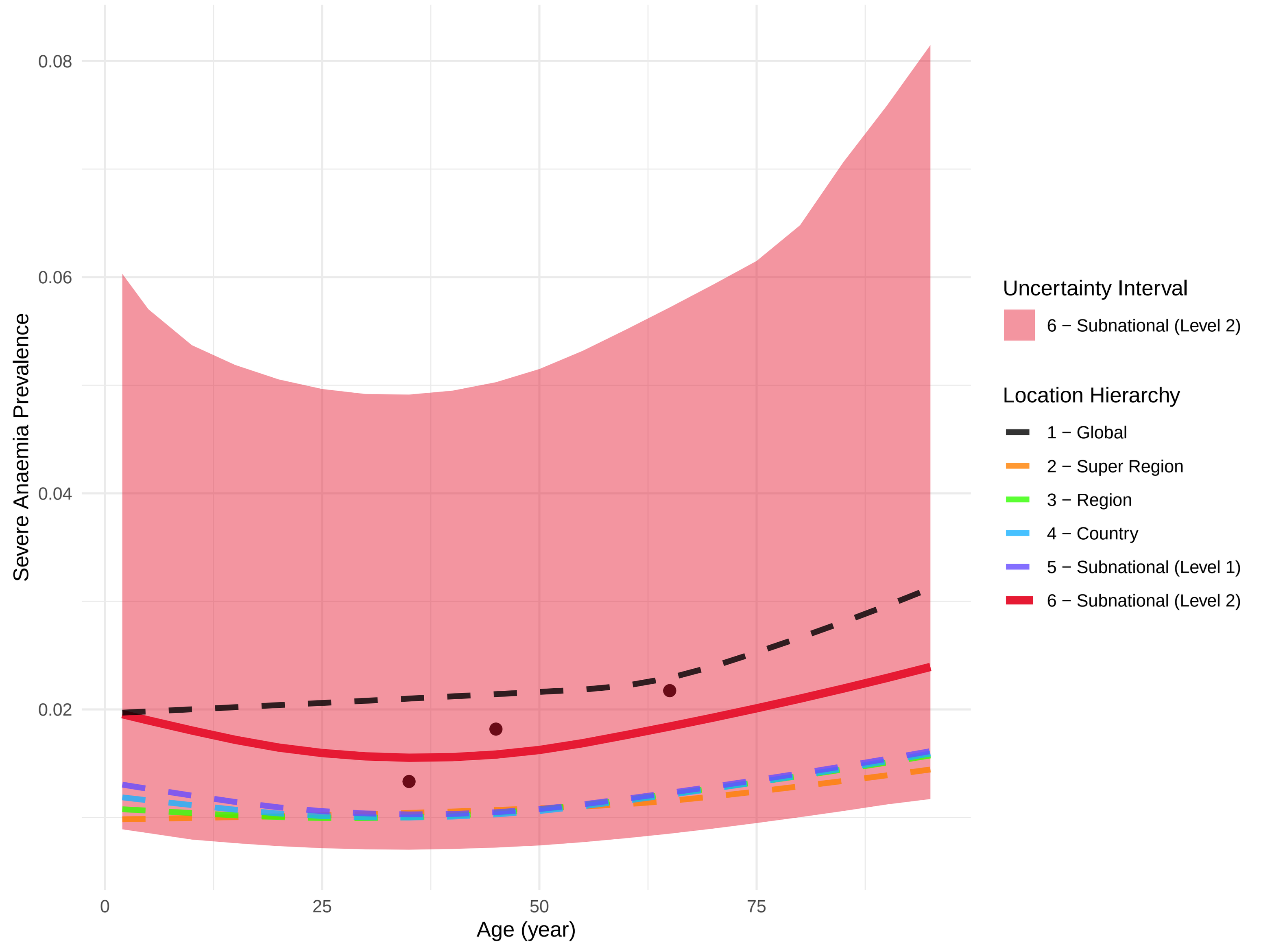

MR−BRT Age/Sex Fits − Non−pregnant Females 2+ Years Old − Severe Anaemia Prevalence − North West England
WHO−2024 Adjustment Method

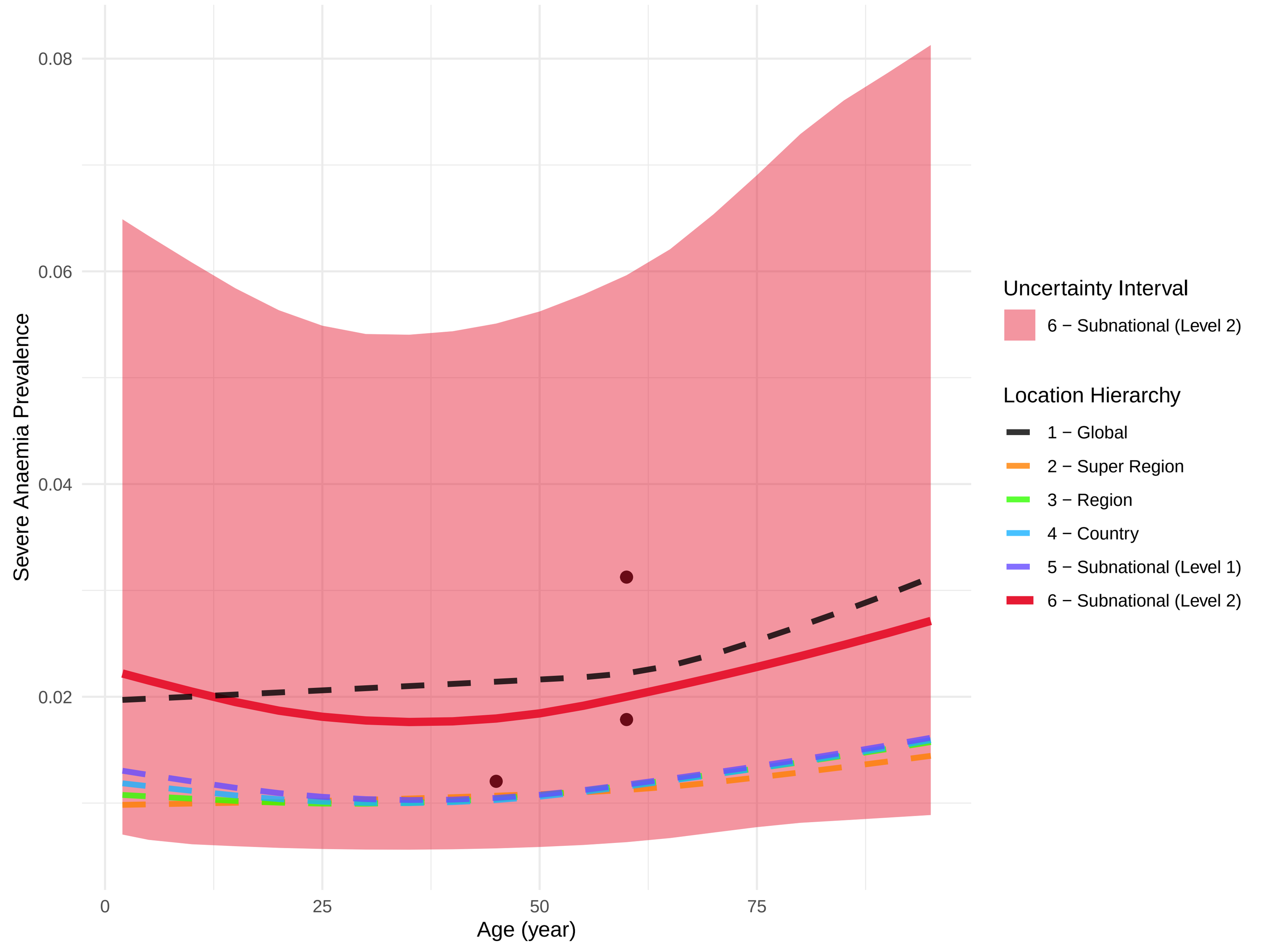

MR−BRT Age/Sex Fits − Non−pregnant Females 2+ Years Old − Severe Anaemia Prevalence − South West England
WHO−2024 Adjustment Method

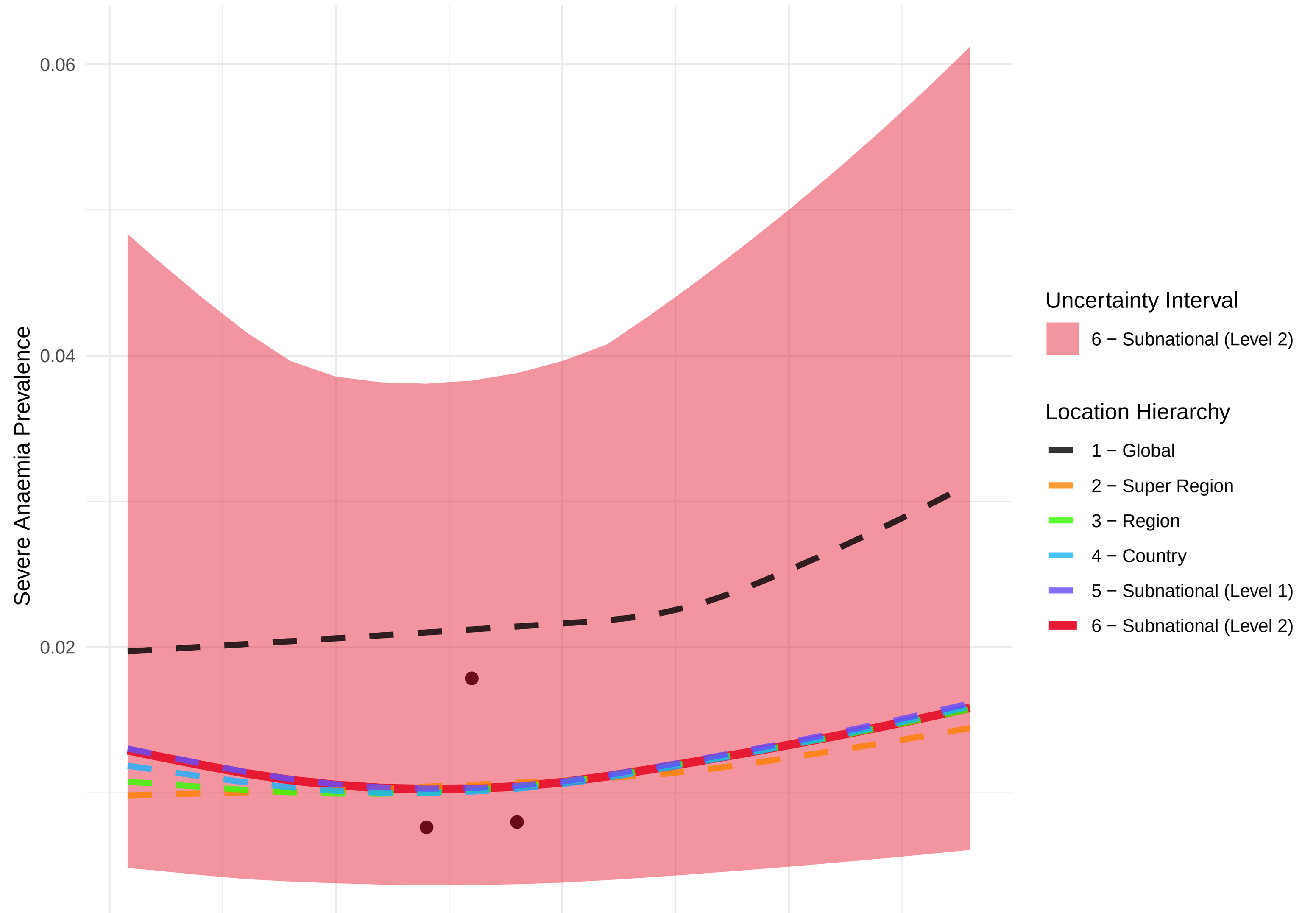

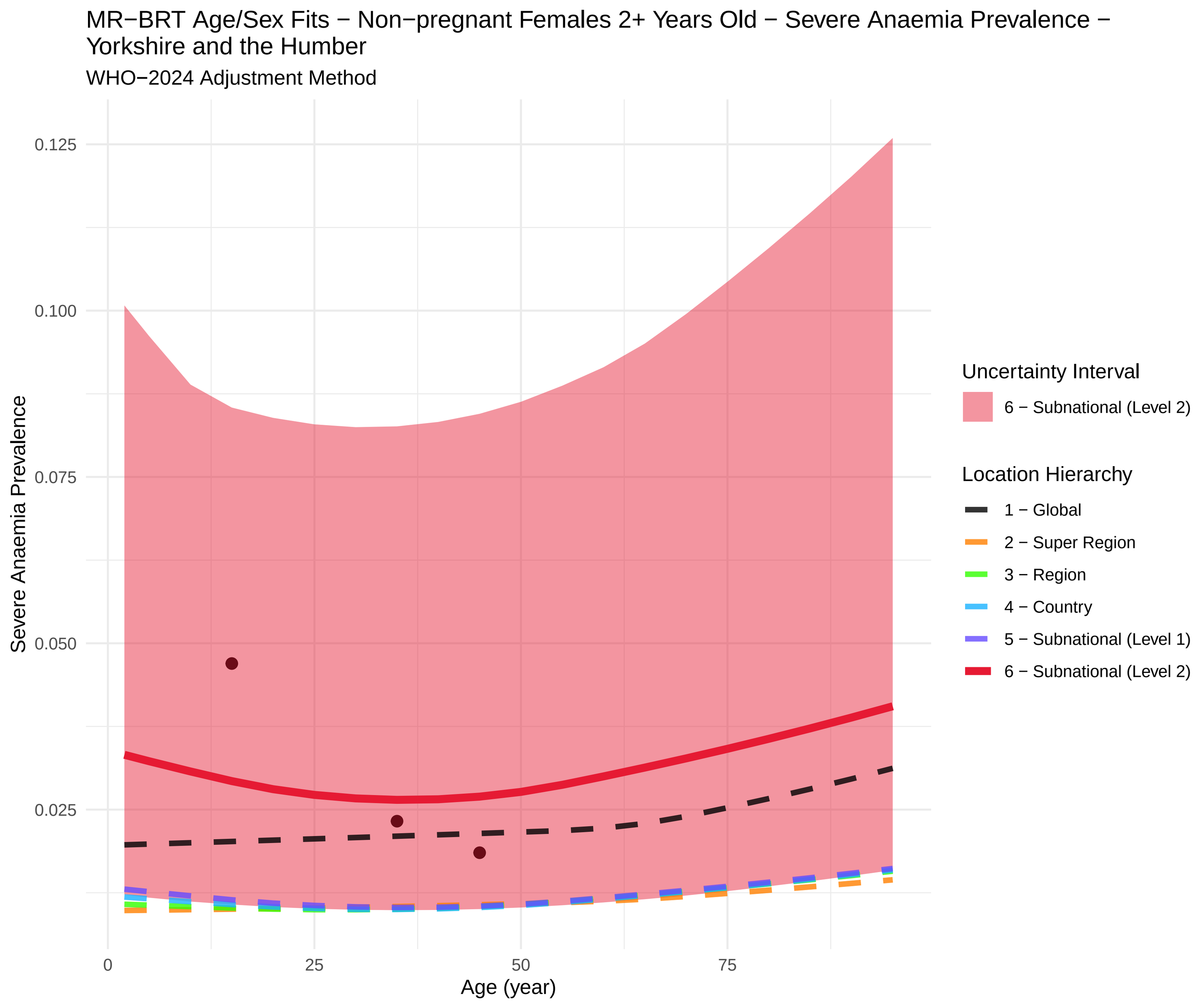
MR−BRT Age/Sex Fits − Non−pregnant Females 2+ Years Old − Severe Anaemia Prevalence − Yorkshire and the Humber
WHO−2024 Adjustment Method
Severe Anaemia Prevalence
Age (year)
Uncertainty Interval
6 − Subnational (Level 2)
Location Hierarchy
1 − Global
2 − Super Region
3 − Region
4 − Country
5 − Subnational (Level 1)
6 − Subnational (Level 2)

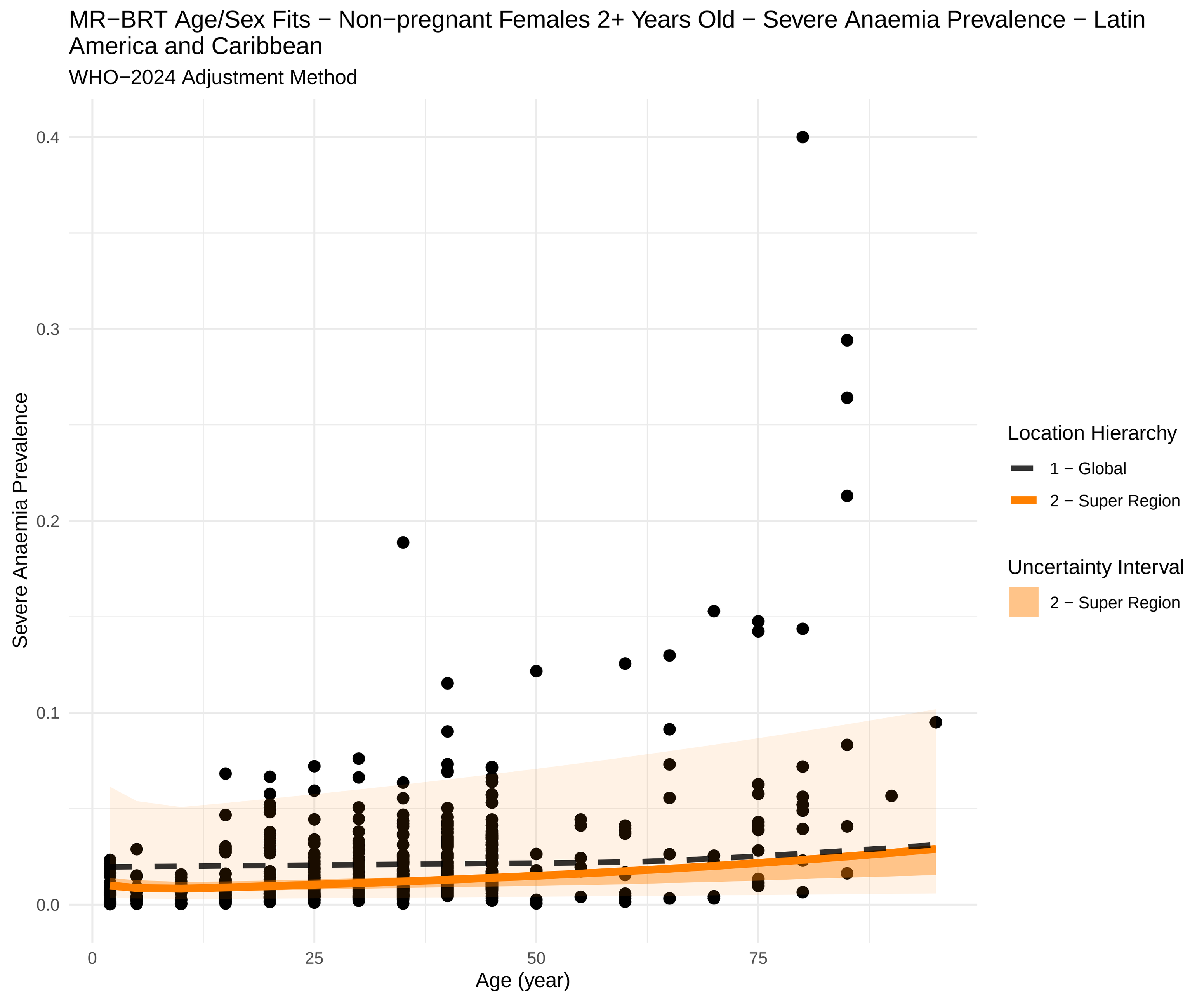
MR−BRT Age/Sex Fits − Non−pregnant Females 2+ Years Old − Severe Anaemia Prevalence − Latin America and Caribbean
WHO−2024 Adjustment Method
Severe Anaemia Prevalence
Age (year)
Location Hierarchy
1 − Global
2 − Super Region
Uncertainty Interval
2 − Super Region

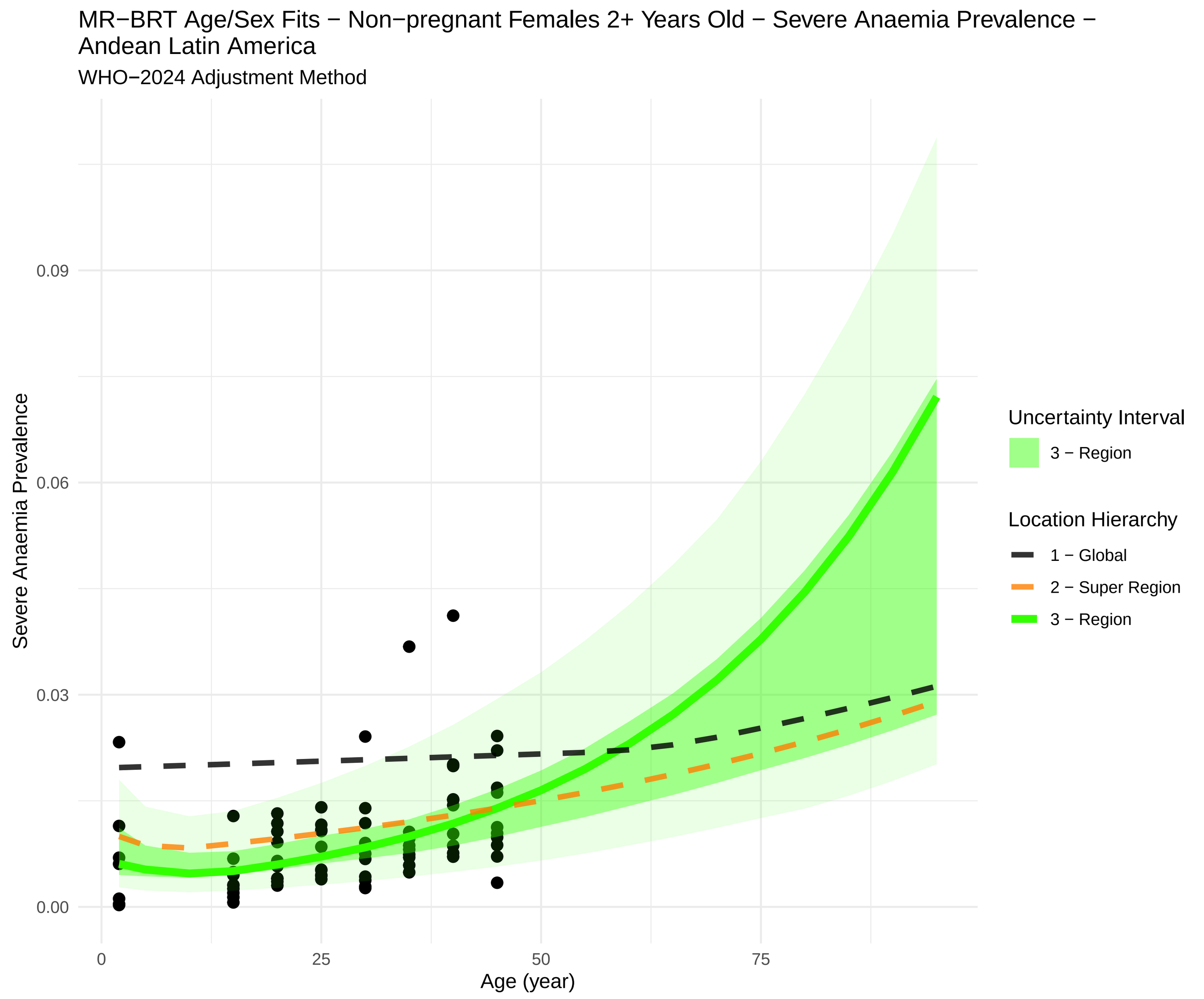
MR−BRT Age/Sex Fits − Non−pregnant Females 2+ Years Old − Severe Anaemia Prevalence − Andean Latin America
WHO−2024 Adjustment Method
Severe Anaemia Prevalence
Age (year)
Uncertainty Interval
3 − Region
Location Hierarchy
1 − Global
2 − Super Region
3 − Region

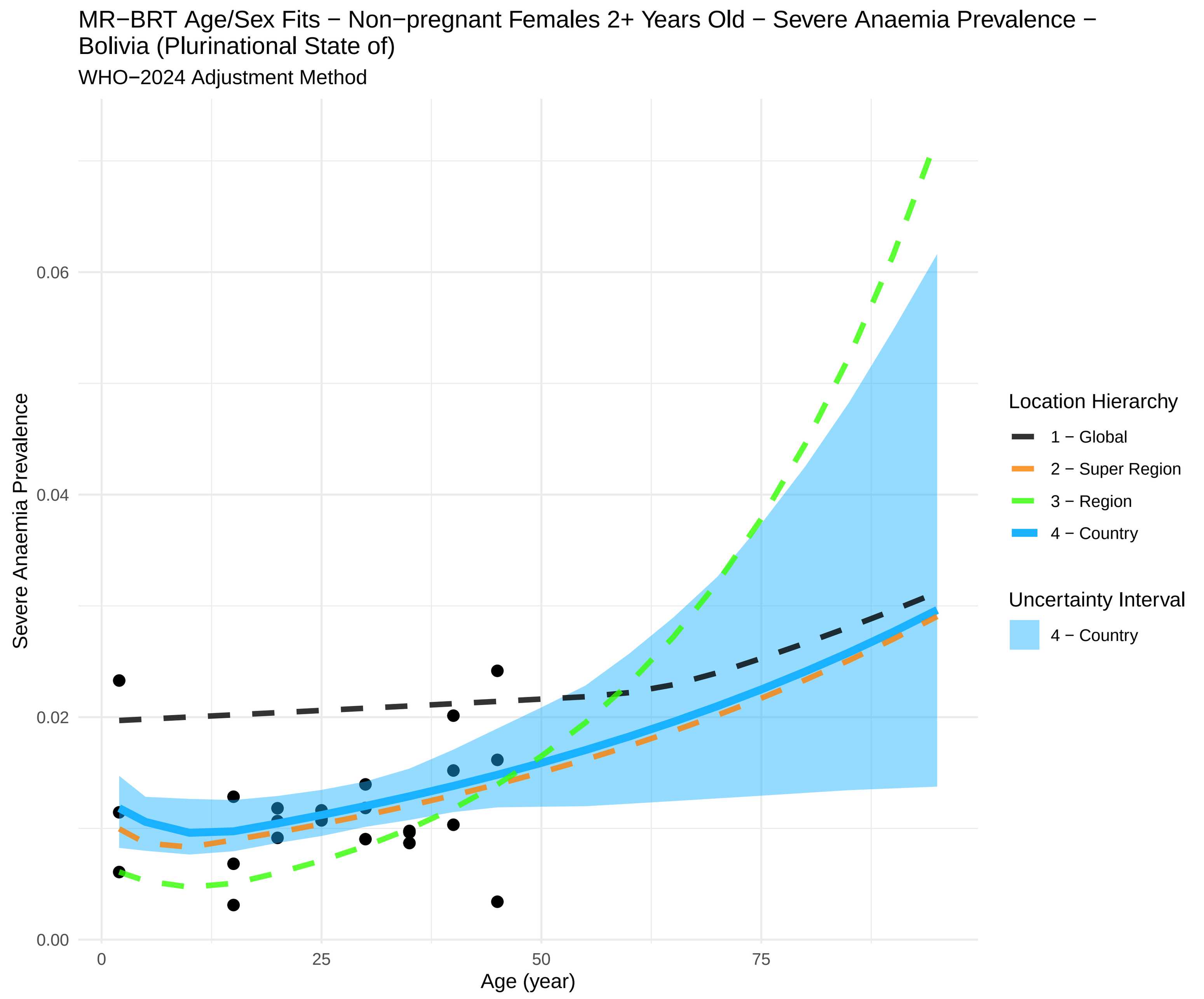
MR−BRT Age/Sex Fits − Non−pregnant Females 2+ Years Old − Severe Anaemia Prevalence − Bolivia (Plurinational State of)
WHO−2024 Adjustment Method
Severe Anaemia Prevalence
Age (year)
Location Hierarchy
1 − Global
2 − Super Region
3 − Region
4 − Country
Uncertainty Interval
4 − Country

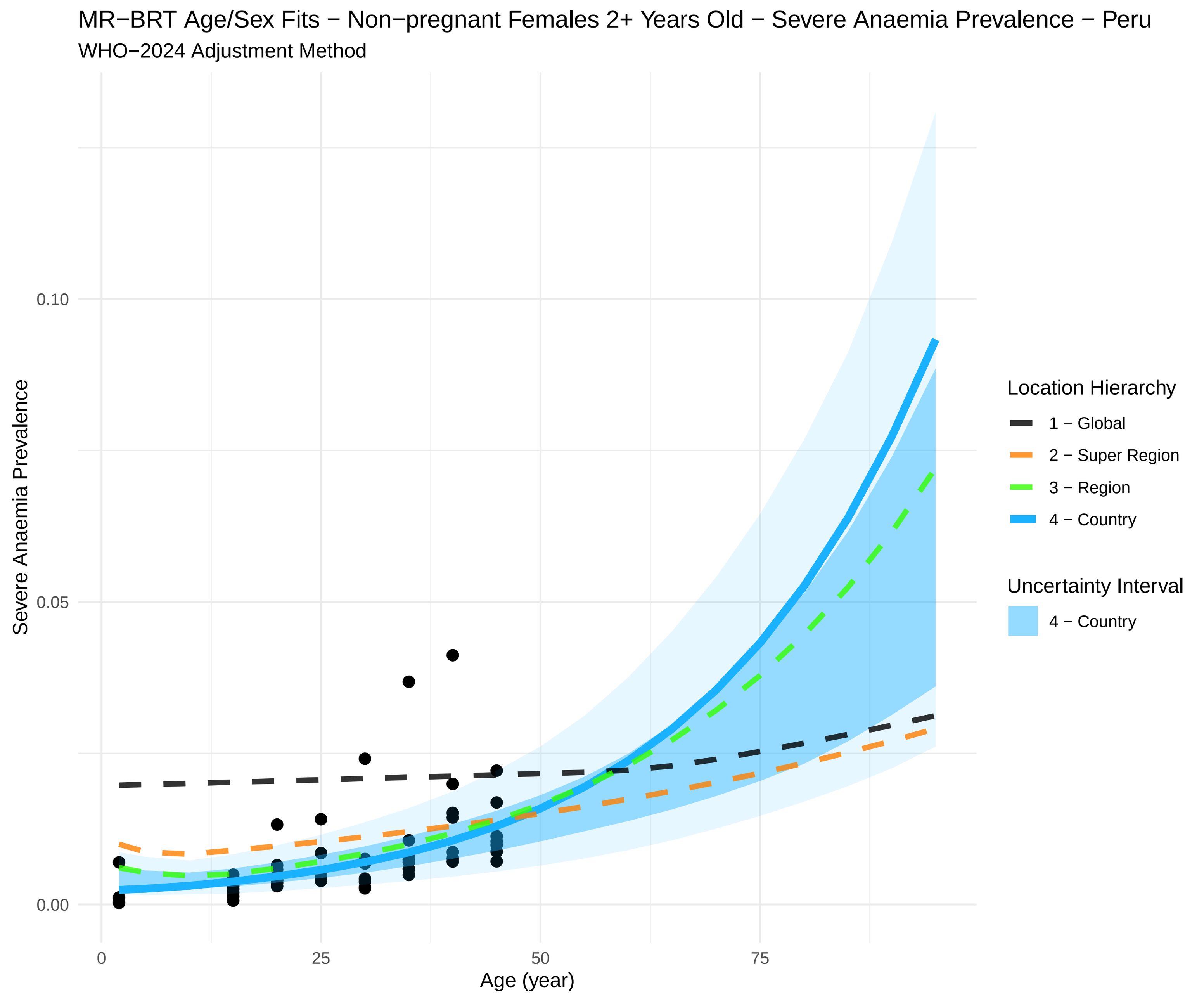
MR−BRT Age/Sex Fits − Non−pregnant Females 2+ Years Old − Severe Anaemia Prevalence − Peru
WHO−2024 Adjustment Method
Severe Anaemia Prevalence
Age (year)
Location Hierarchy
1 − Global
2 − Super Region
3 − Region
4 − Country
Uncertainty Interval
4 − Country

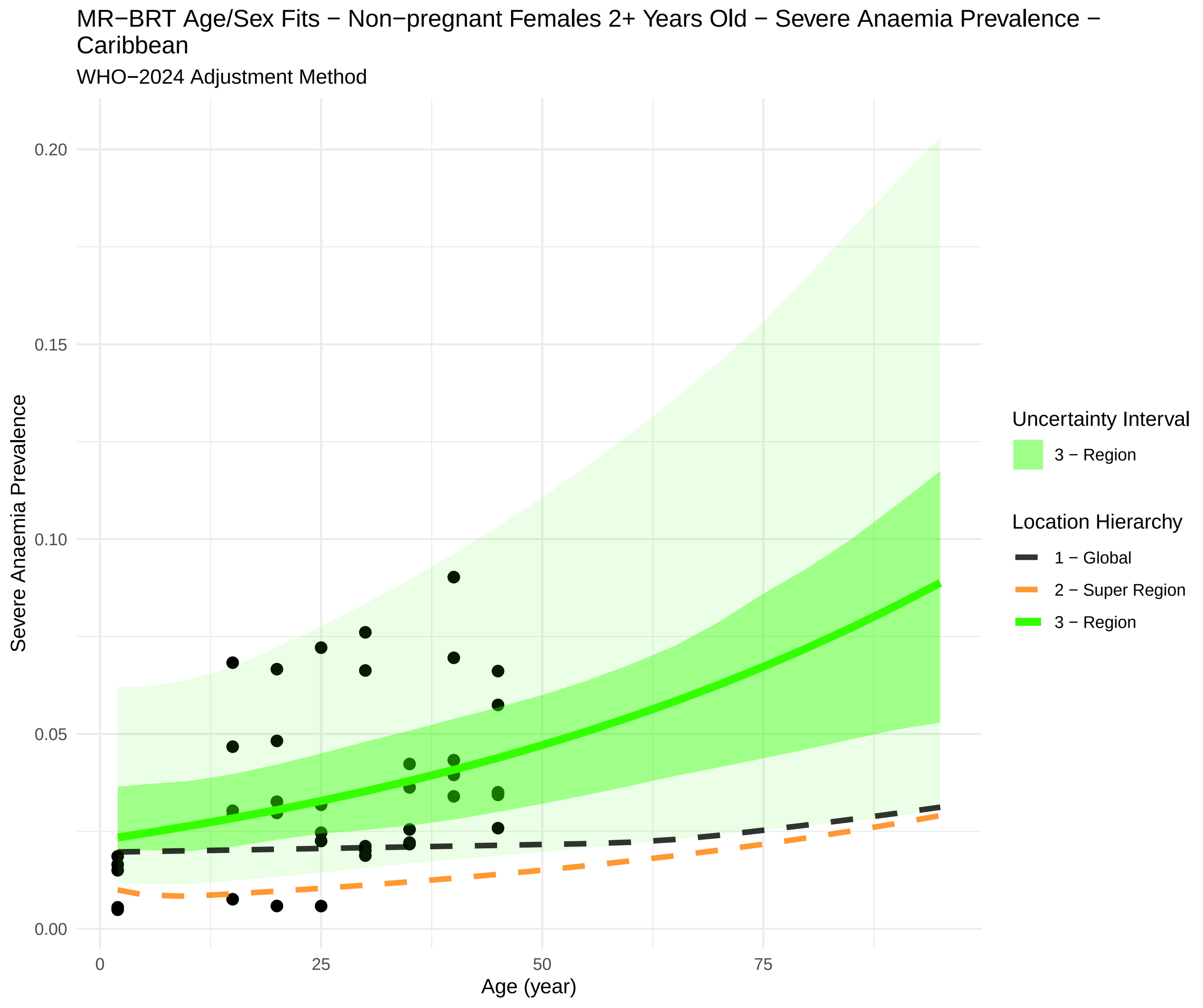
MR−BRT Age/Sex Fits − Non−pregnant Females 2+ Years Old − Severe Anaemia Prevalence − Caribbean
WHO−2024 Adjustment Method
Severe Anaemia Prevalence
Age (year)
Uncertainty Interval
3 − Region
Location Hierarchy
1 − Global
2 − Super Region
3 − Region

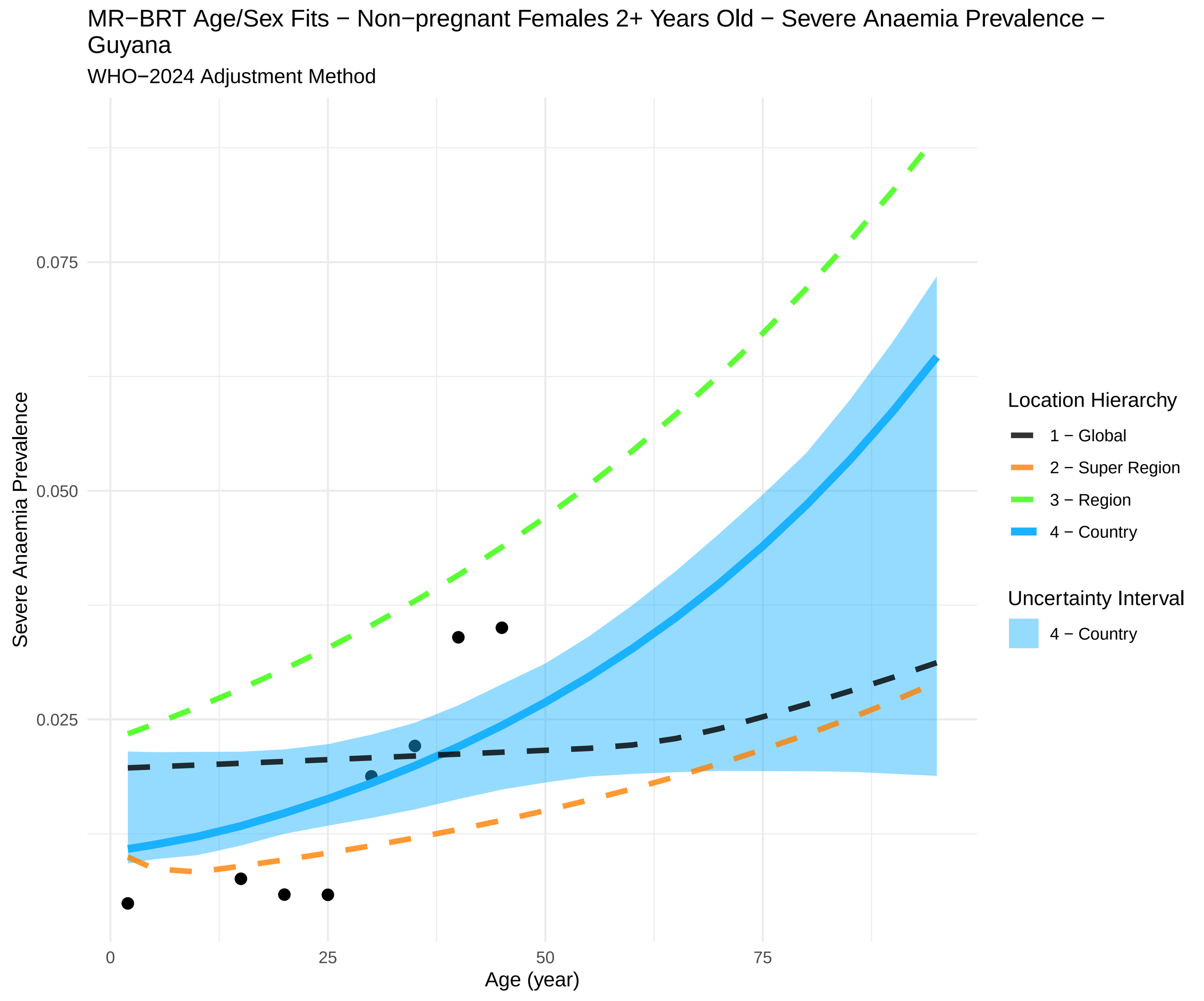
MR−BRT Age/Sex Fits − Non−pregnant Females 2+ Years Old − Severe Anaemia Prevalence − Guyana
WHO−2024 Adjustment Method
Severe Anaemia Prevalence
Age (year)
Location Hierarchy
1 − Global
2 − Super Region
3 − Region
4 − Country
Uncertainty Interval
4 − Country

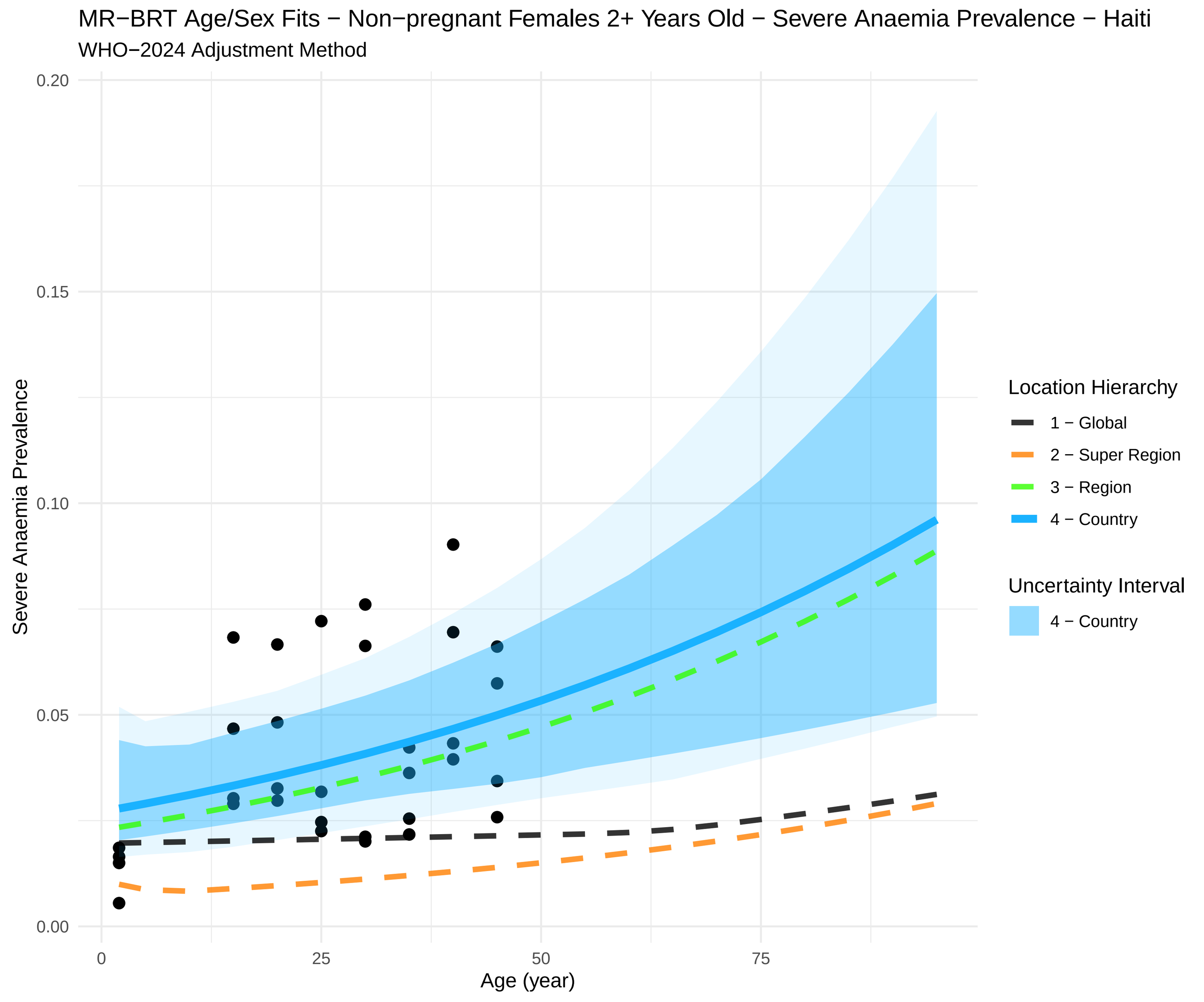
MR−BRT Age/Sex Fits − Non−pregnant Females 2+ Years Old − Severe Anaemia Prevalence − Haiti
WHO−2024 Adjustment Method
Severe Anaemia Prevalence
Age (year)
Location Hierarchy
1 − Global
2 − Super Region
3 − Region
4 − Country
Uncertainty Interval
4 − Country

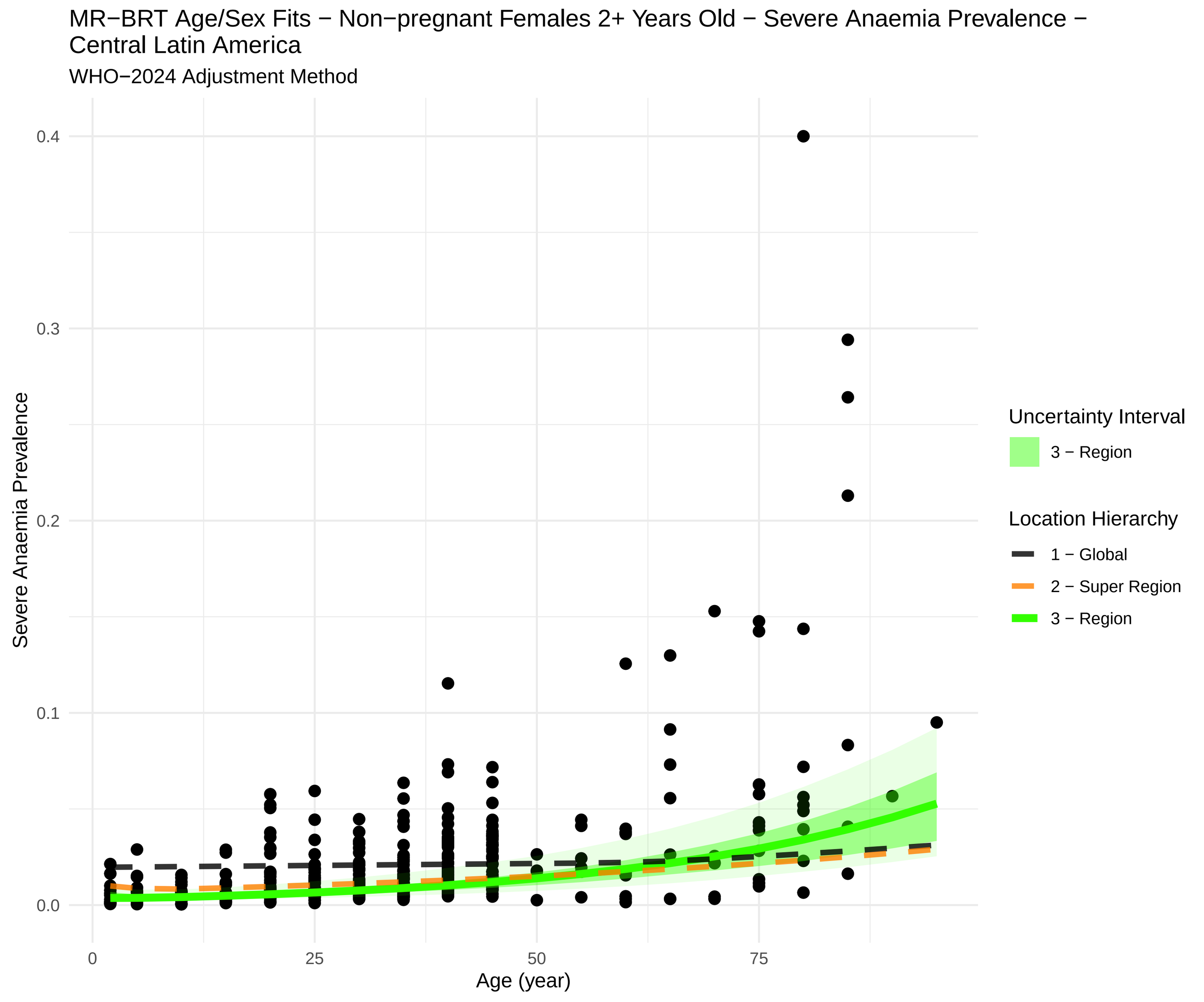
MR−BRT Age/Sex Fits − Non−pregnant Females 2+ Years Old − Severe Anaemia Prevalence − Central Latin America
WHO−2024 Adjustment Method
Severe Anaemia Prevalence
Age (year)
Uncertainty Interval
3 − Region
Location Hierarchy
1 − Global
2 − Super Region
3 − Region

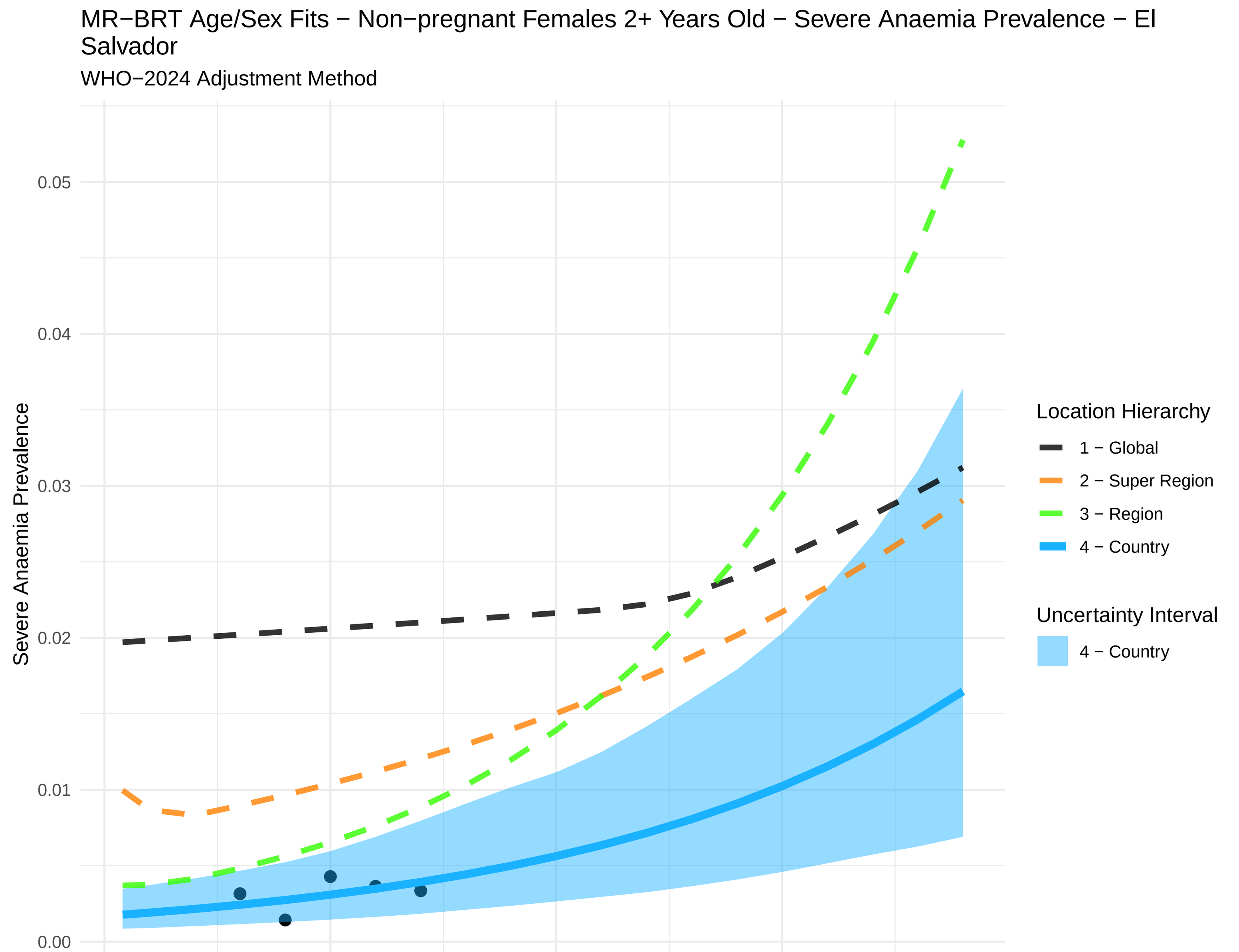
MR−BRT Age/Sex Fits − Non−pregnant Females 2+ Years Old − Severe Anaemia Prevalence − El Salvador
WHO−2024 Adjustment Method
Severe Anaemia Prevalence
Age (year)
Location Hierarchy
1 − Global
2 − Super Region
3 − Region
4 − Country
Uncertainty Interval
4 − Country

MR−BRT Age/Sex Fits − Non-pregnant Females 2+ Years Old − Severe Anaemia Prevalence − Guatemala
WHO−2024 Adjustment Method

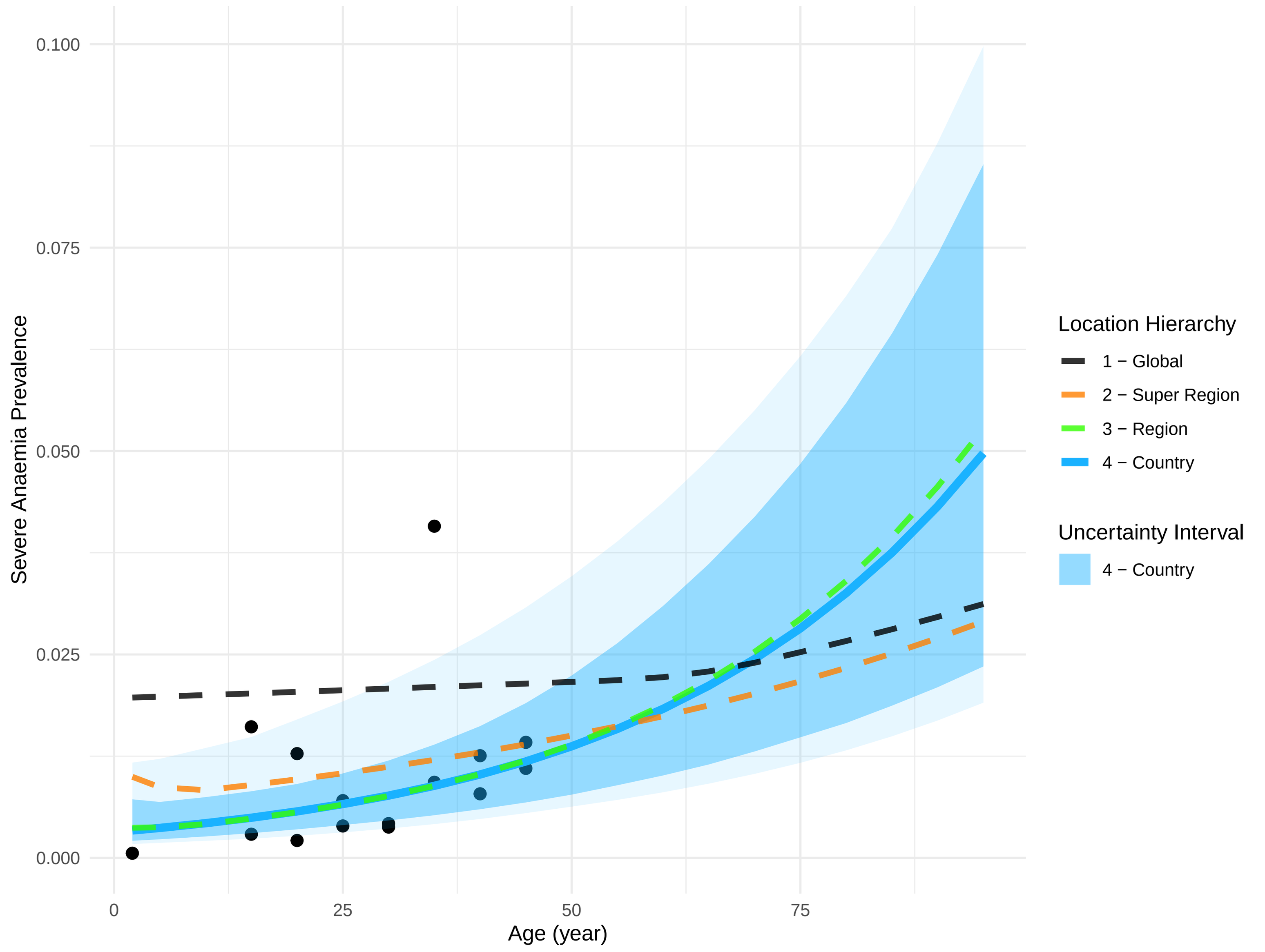

MR−BRT Age/Sex Fits − Non-pregnant Females 2+ Years Old − Severe Anaemia Prevalence − Honduras
WHO−2024 Adjustment Method

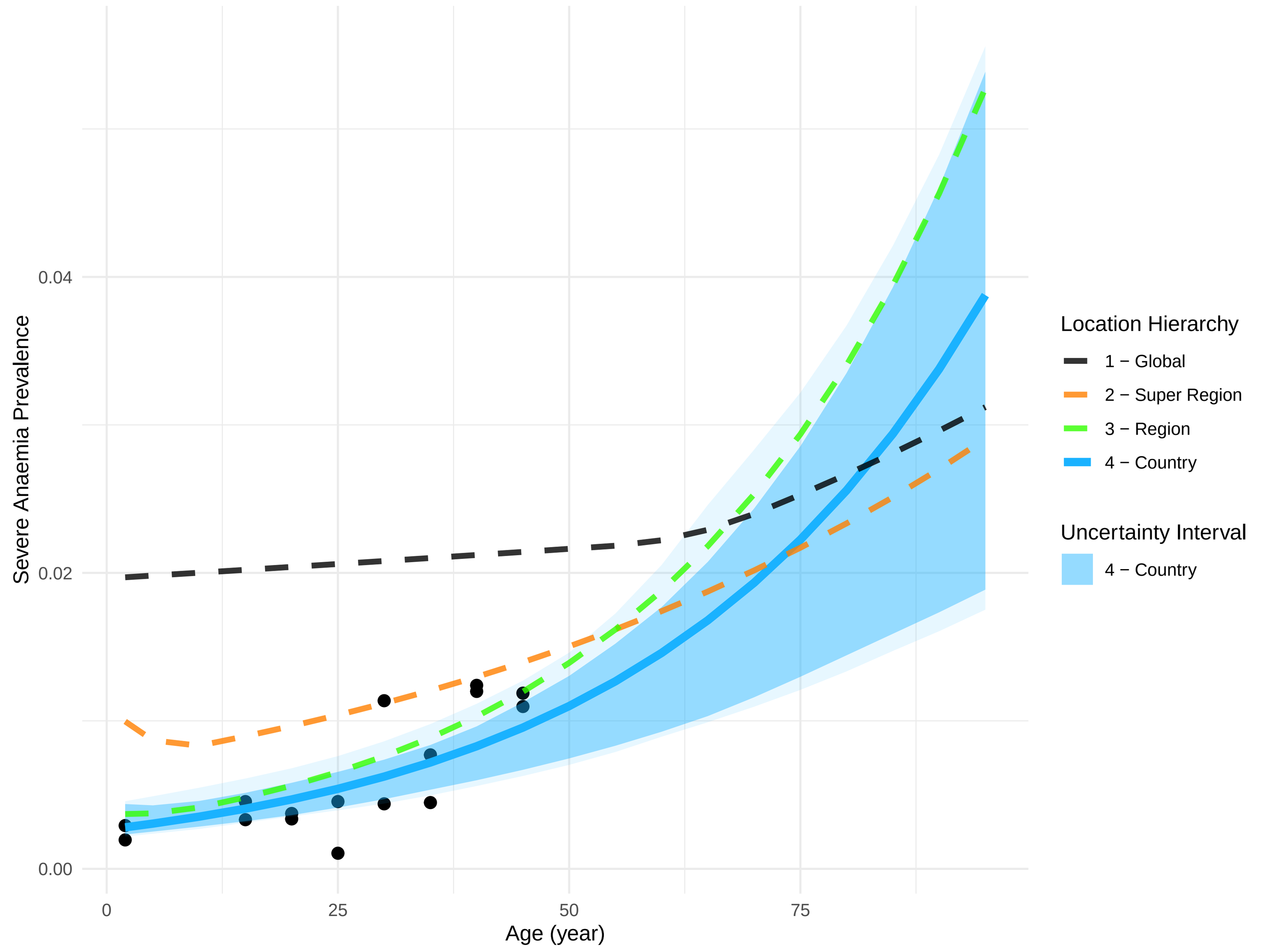

MR−BRT Age/Sex Fits − Non-pregnant Females 2+ Years Old − Severe Anaemia Prevalence − Mexico
WHO−2024 Adjustment Method

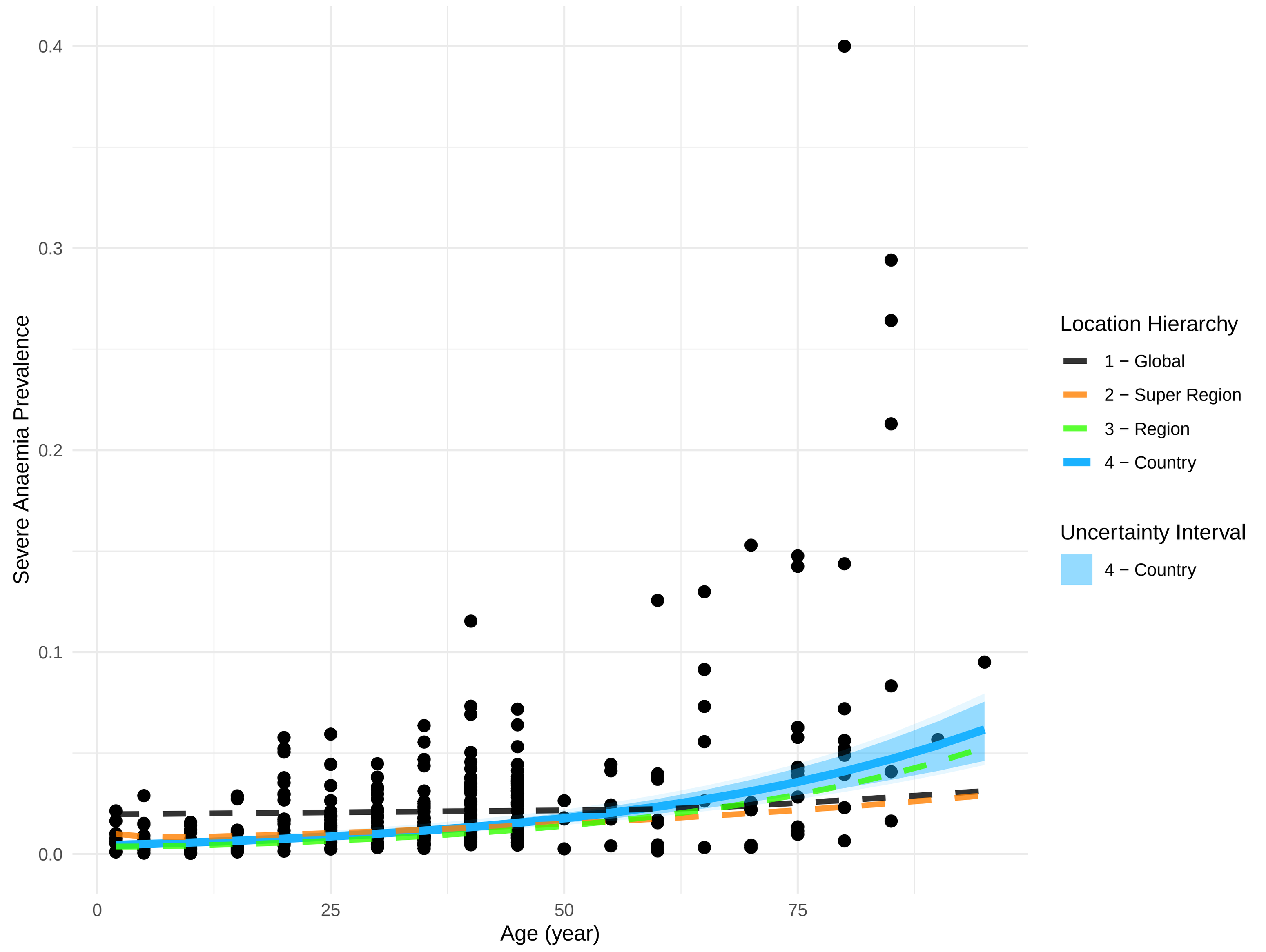

MR−BRT Age/Sex Fits − Non-pregnant Females 2+ Years Old − Severe Anaemia Prevalence − Aguascalientes
WHO−2024 Adjustment Method

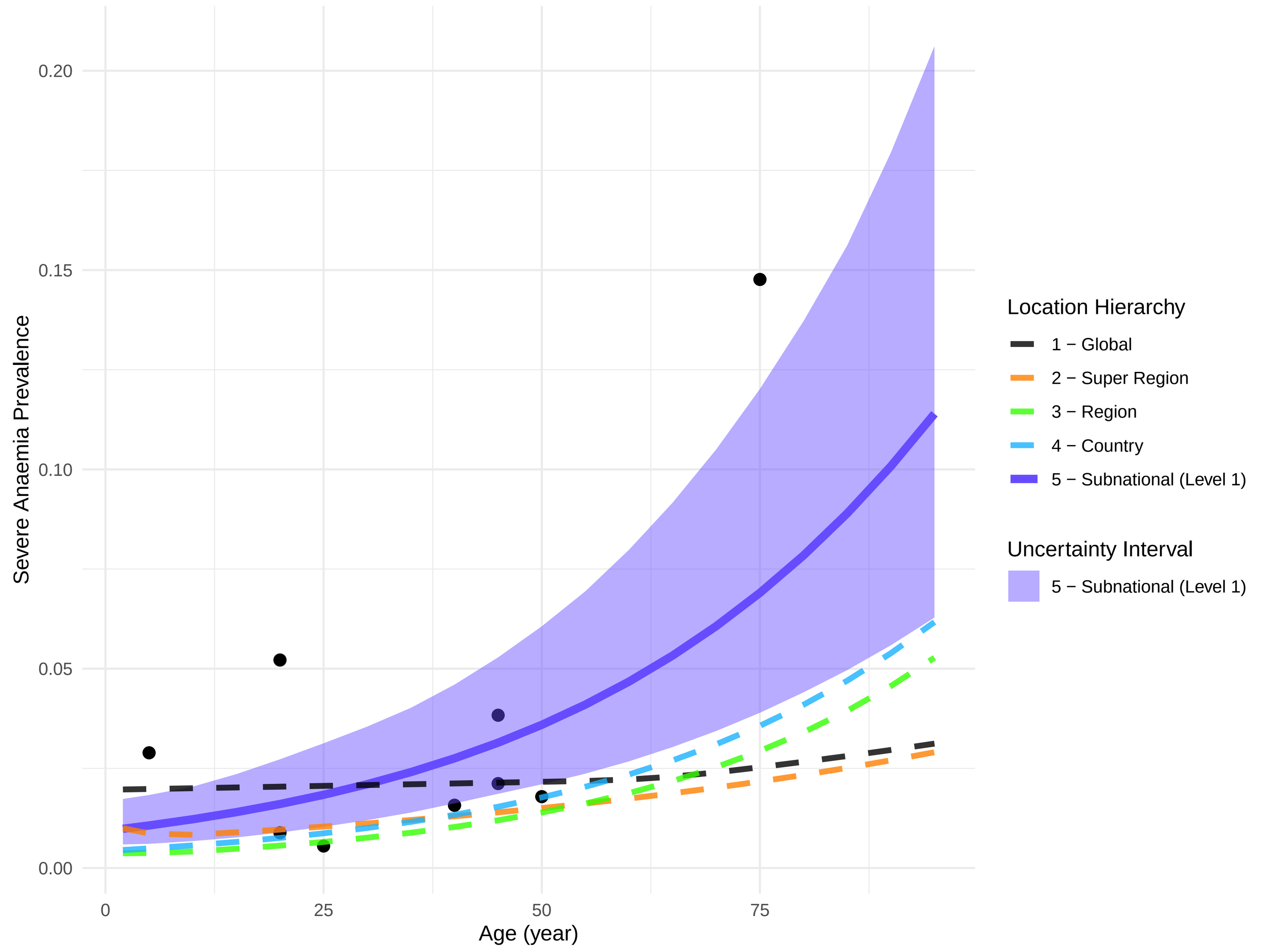

MR−BRT Age/Sex Fits − Non-pregnant Females 2+ Years Old − Severe Anaemia Prevalence − Baja California
WHO−2024 Adjustment Method

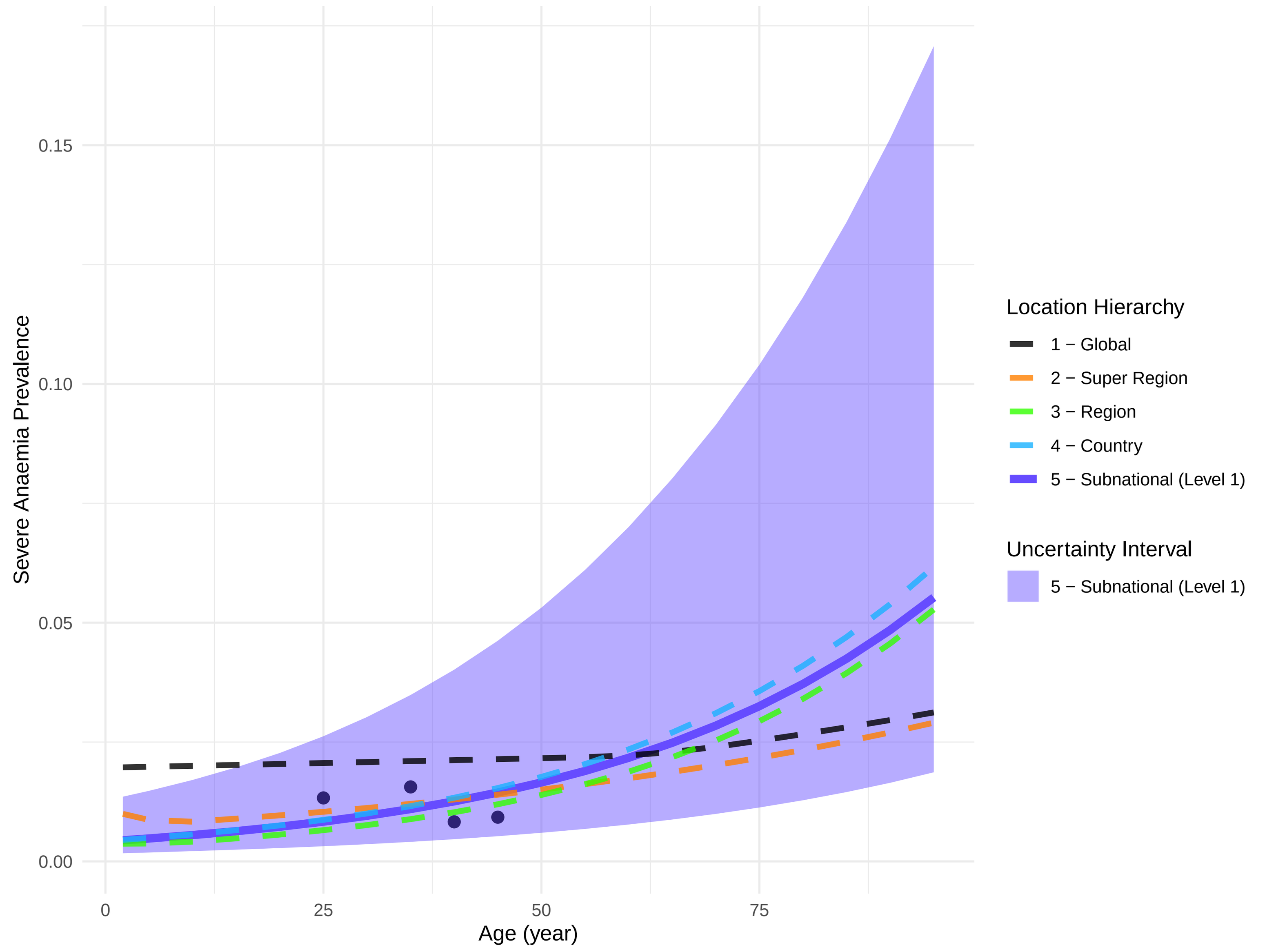

MR−BRT Age/Sex Fits − Non-pregnant Females 2+ Years Old − Severe Anaemia Prevalence − Baja California Sur
WHO−2024 Adjustment Method

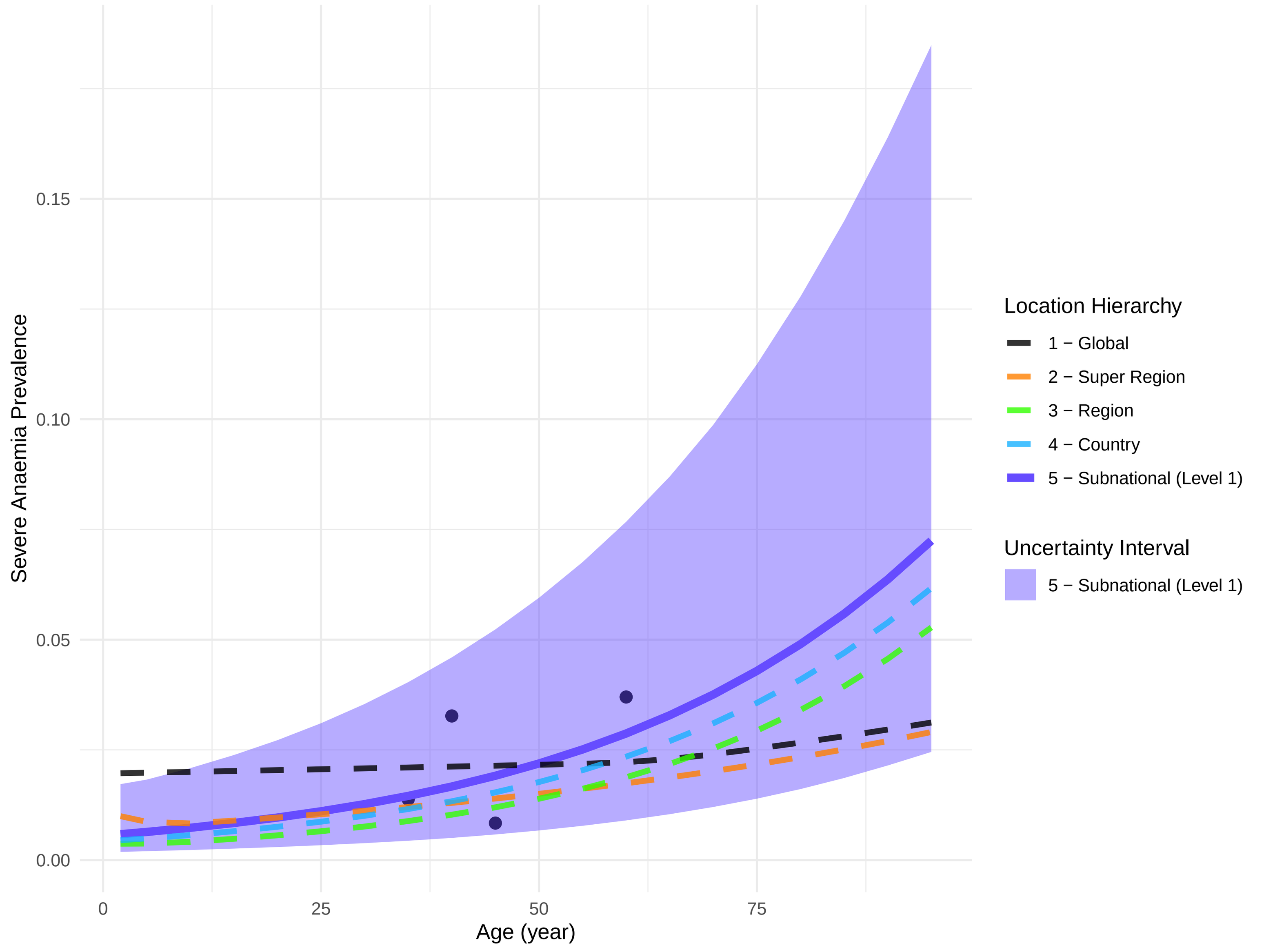

MR−BRT Age/Sex Fits − Non-pregnant Females 2+ Years Old − Severe Anaemia Prevalence − Campeche
WHO−2024 Adjustment Method

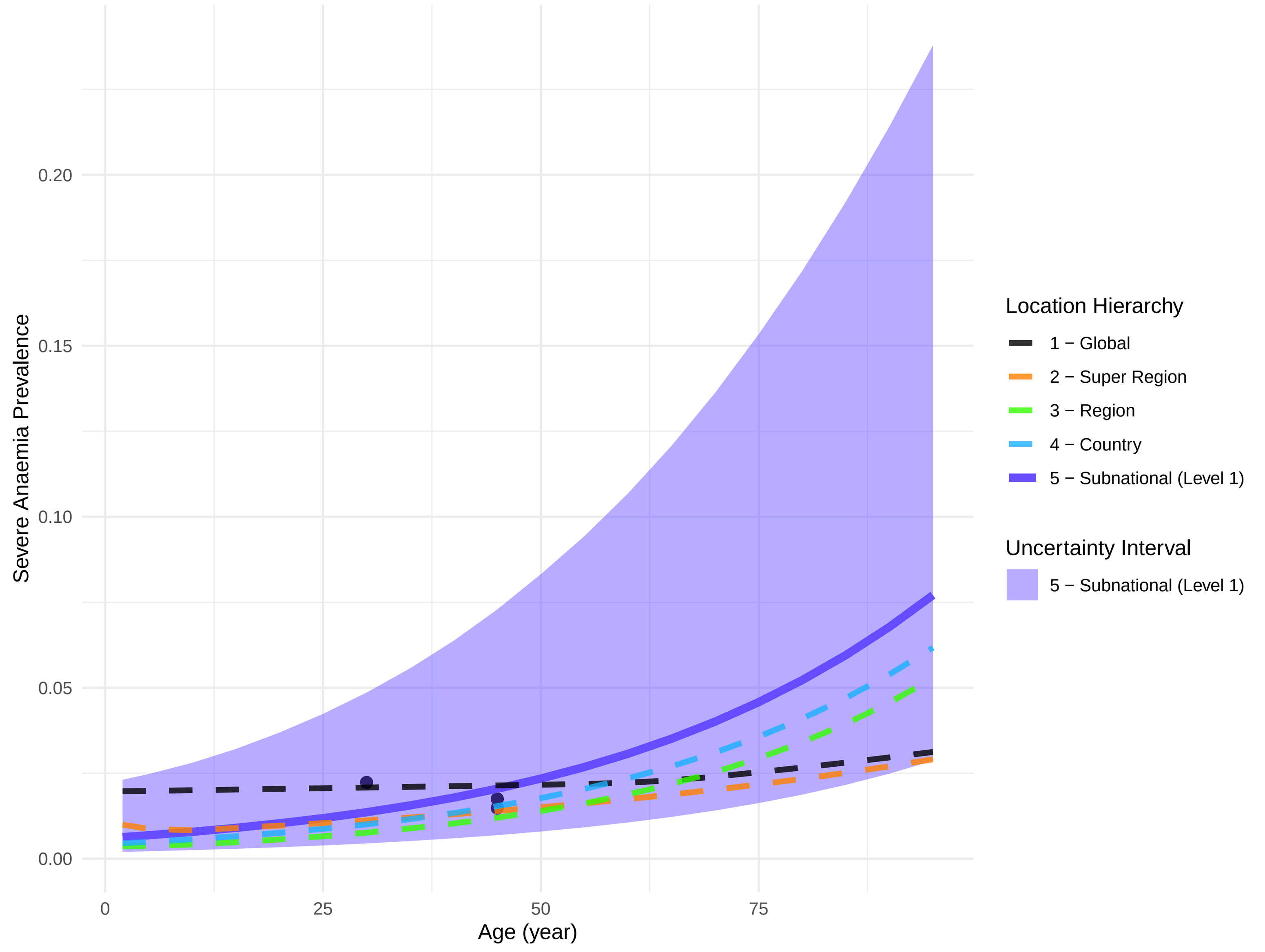

MR−BRT Age/Sex Fits − Non-pregnant Females 2+ Years Old − Severe Anaemia Prevalence − Chiapas
WHO−2024 Adjustment Method

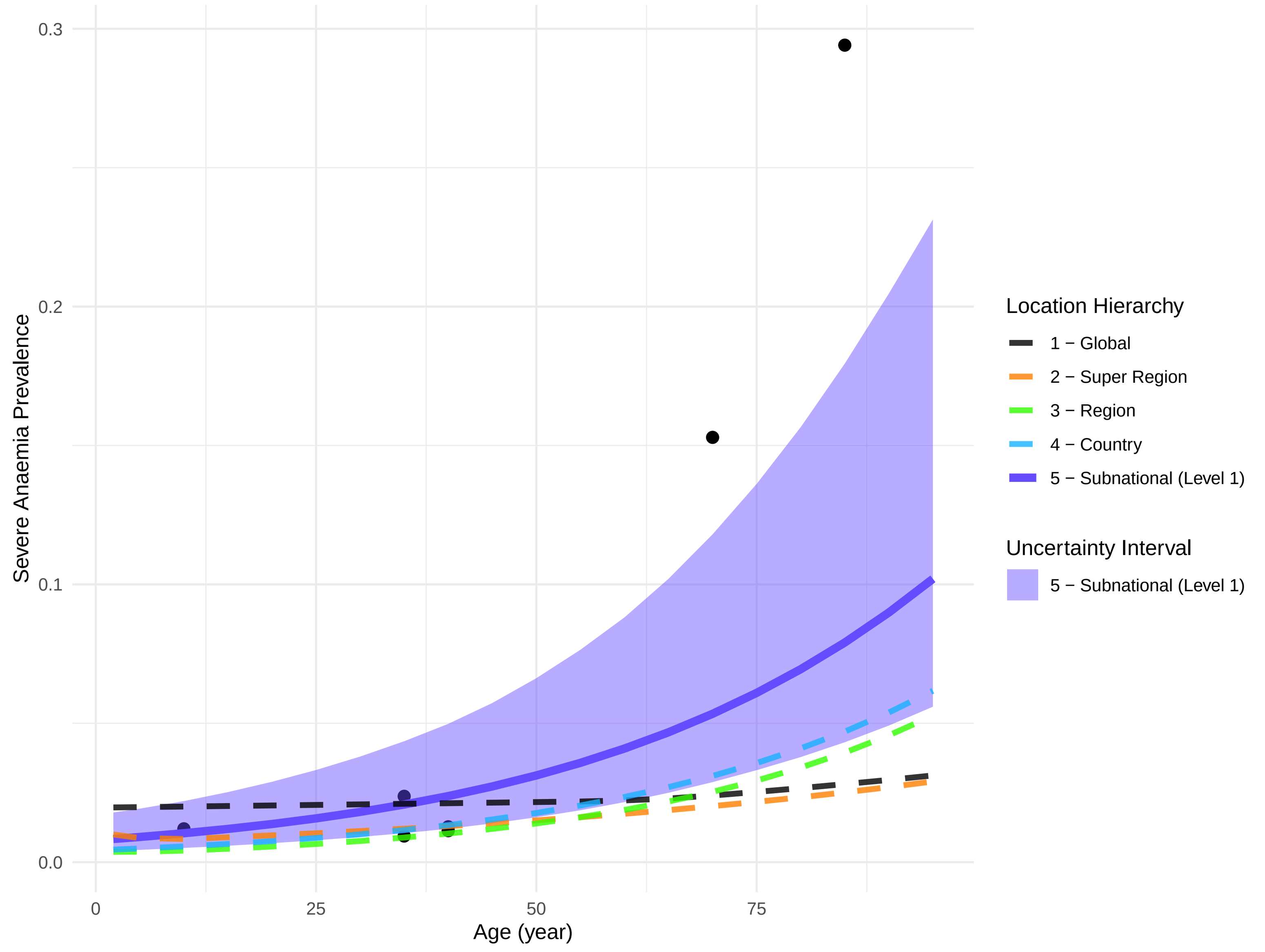

MR−BRT Age/Sex Fits − Non-pregnant Females 2+ Years Old − Severe Anaemia Prevalence − Chihuahua
WHO−2024 Adjustment Method

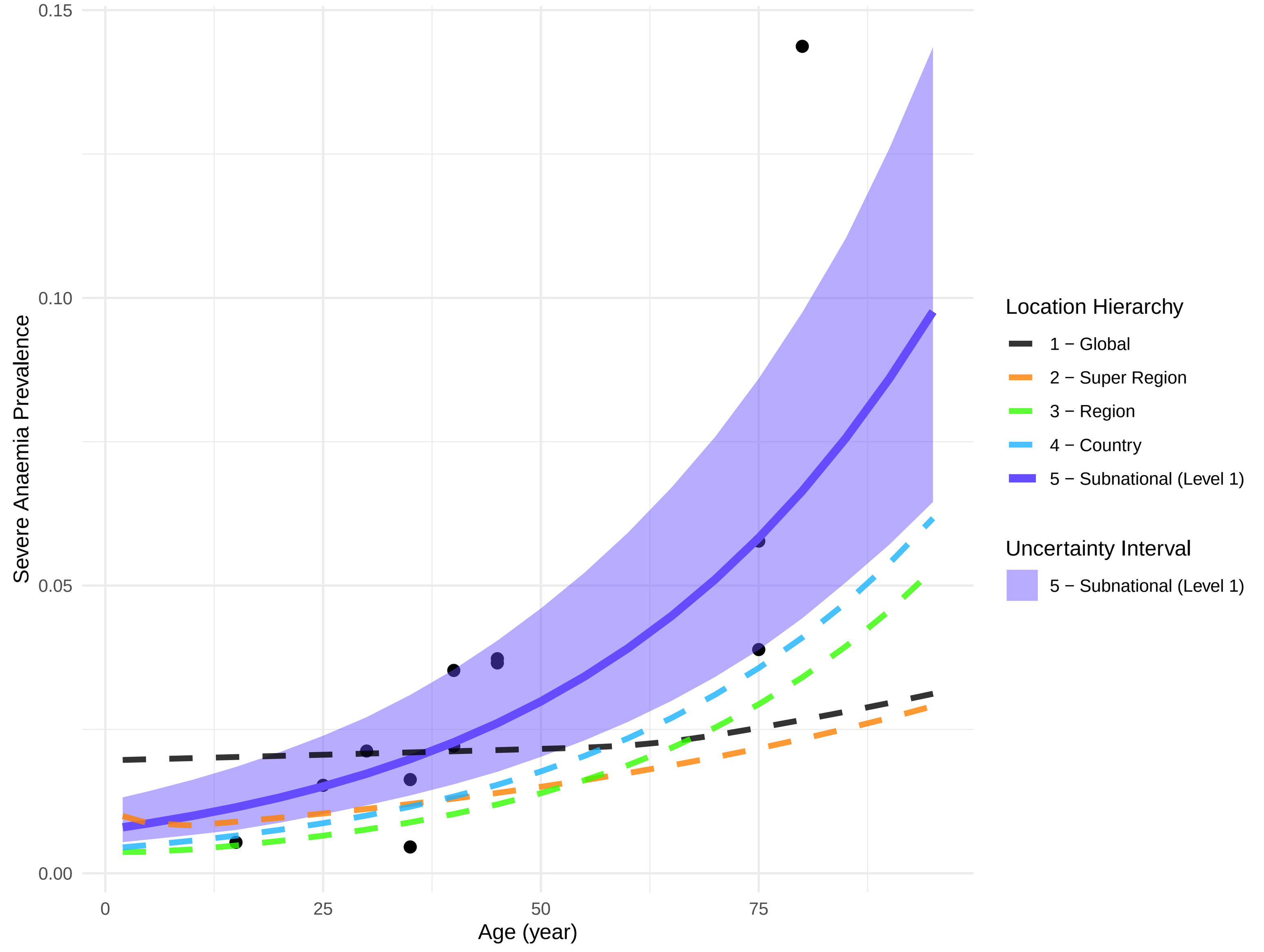

MR−BRT Age/Sex Fits − Non-pregnant Females 2+ Years Old − Severe Anaemia Prevalence − Coahuila
WHO−2024 Adjustment Method

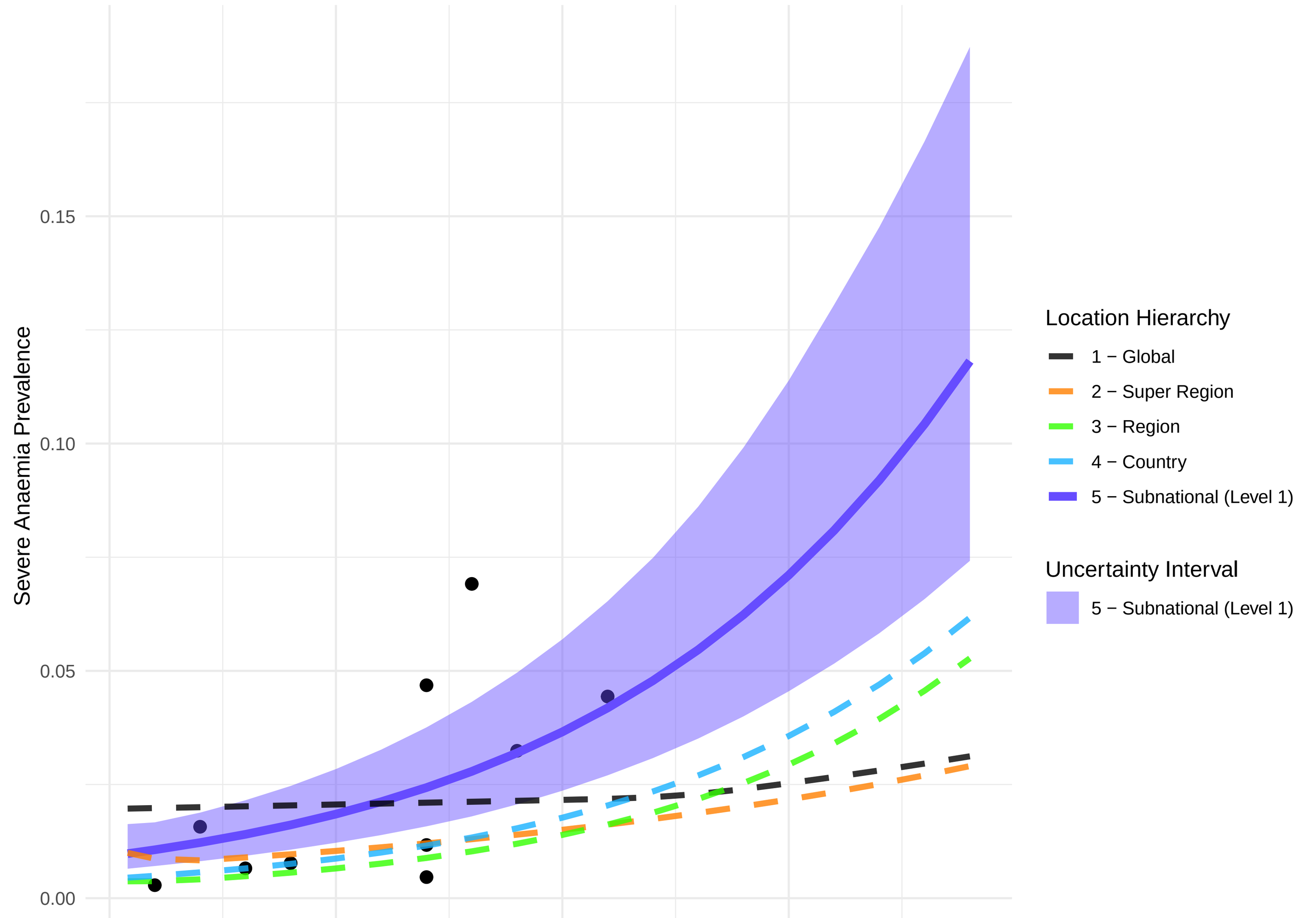

MR−BRT Age/Sex Fits − Non−pregnant Females 2+ Years Old − Severe Anaemia Prevalence − Colima
WHO−2024 Adjustment Method

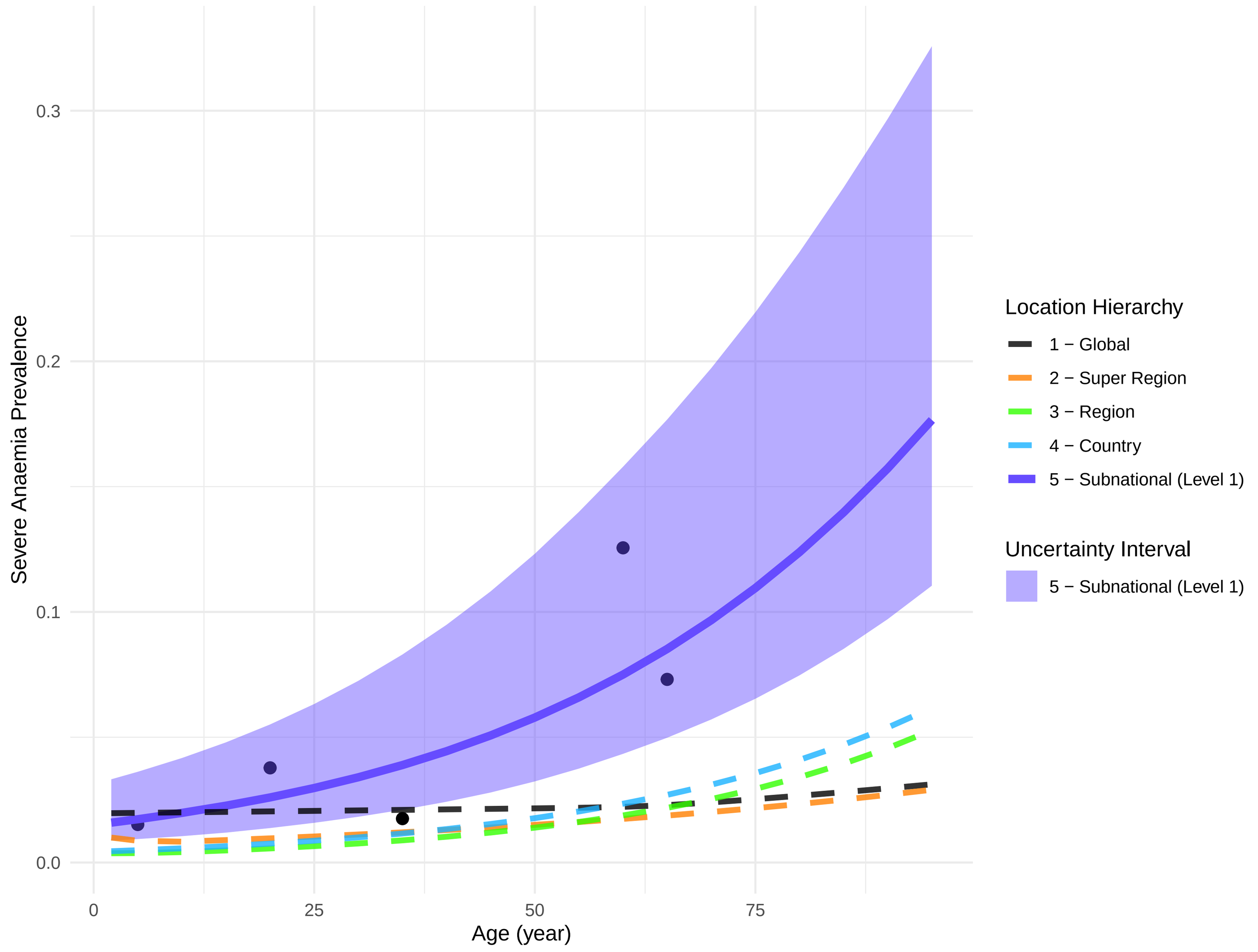

MR−BRT Age/Sex Fits − Non−pregnant Females 2+ Years Old − Severe Anaemia Prevalence − Durango
WHO−2024 Adjustment Method

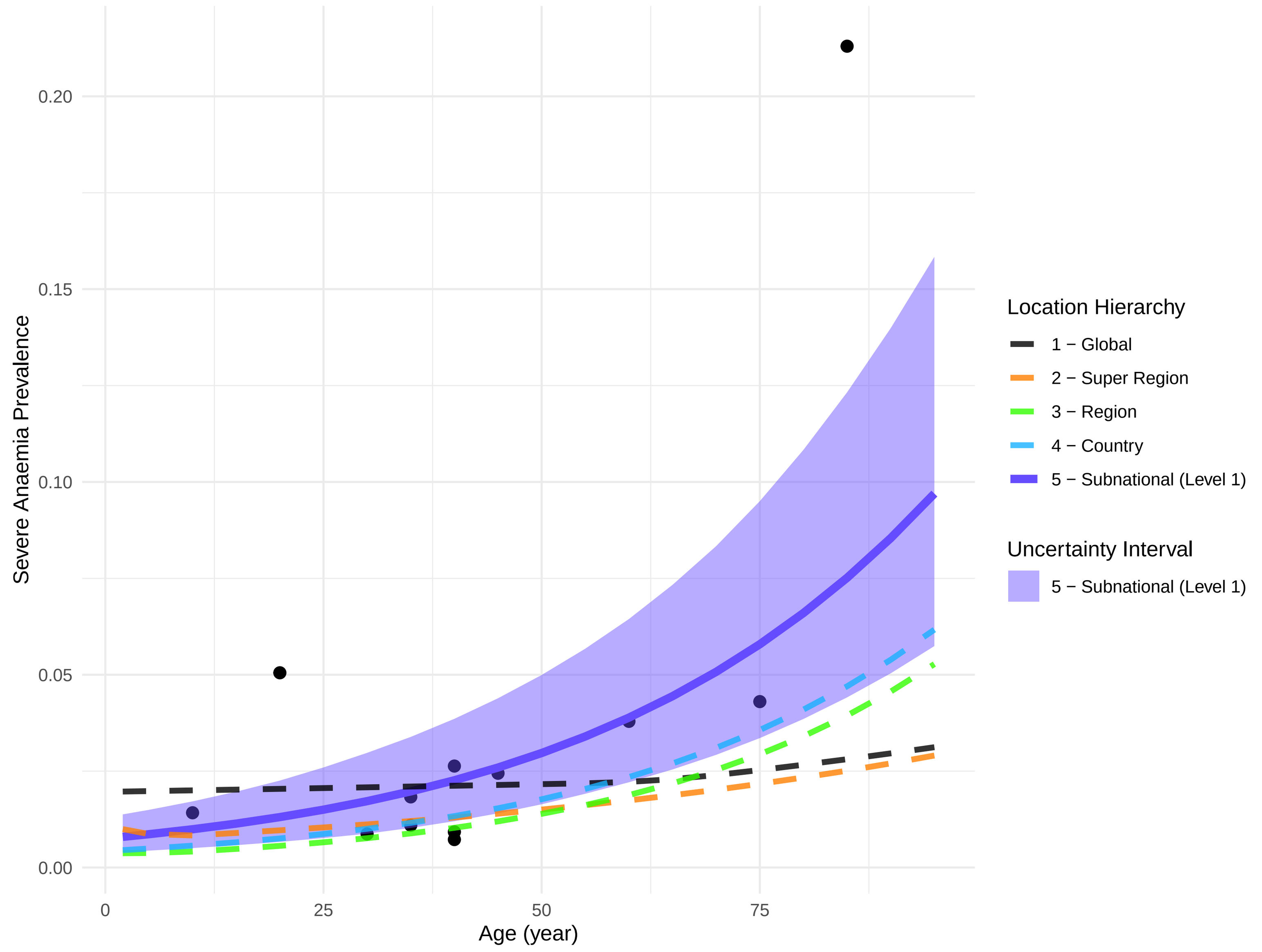

MR−BRT Age/Sex Fits − Non−pregnant Females 2+ Years Old − Severe Anaemia Prevalence − Guanajuato
WHO−2024 Adjustment Method

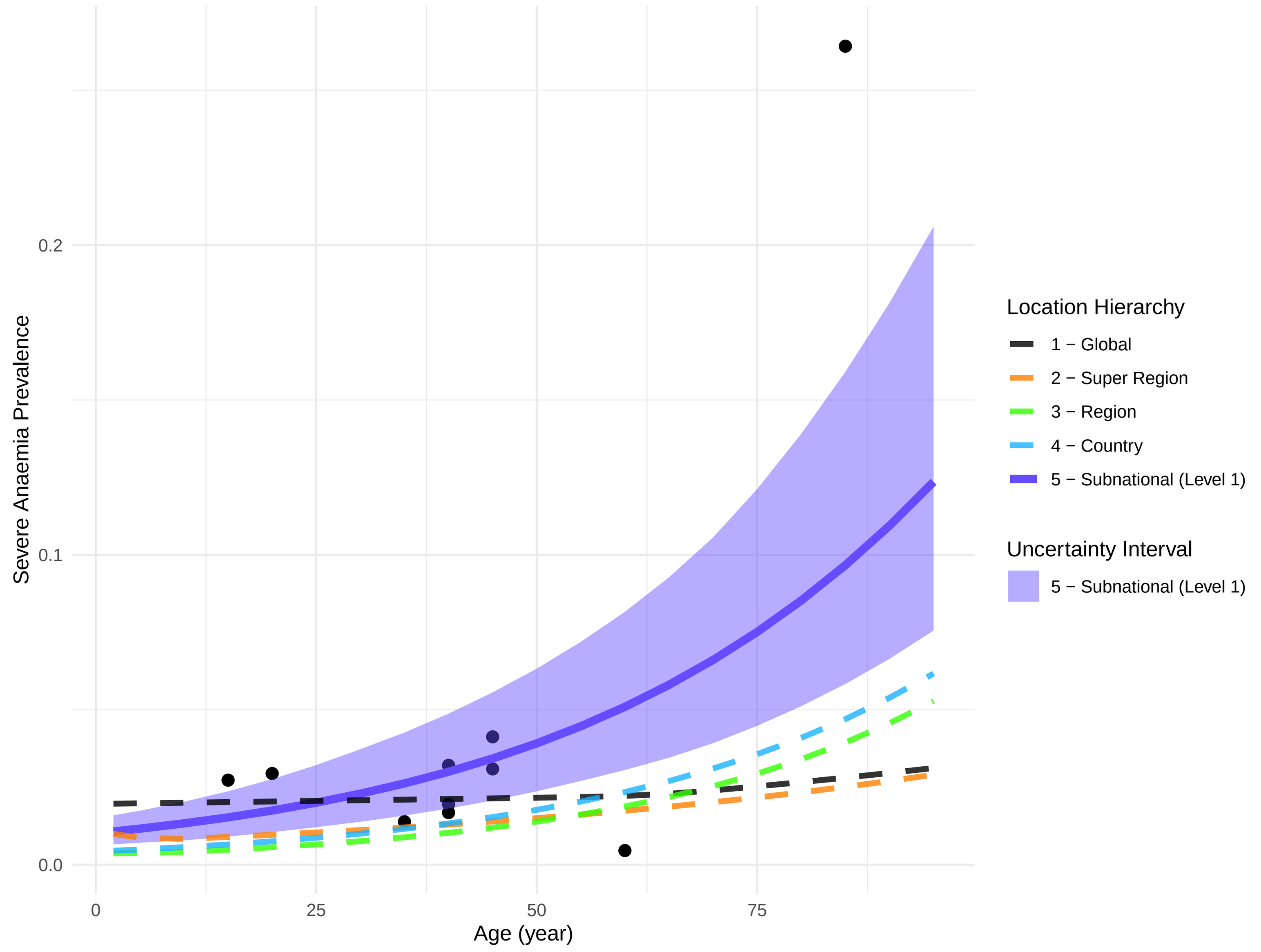

MR−BRT Age/Sex Fits − Non−pregnant Females 2+ Years Old − Severe Anaemia Prevalence − Guerrero
WHO−2024 Adjustment Method

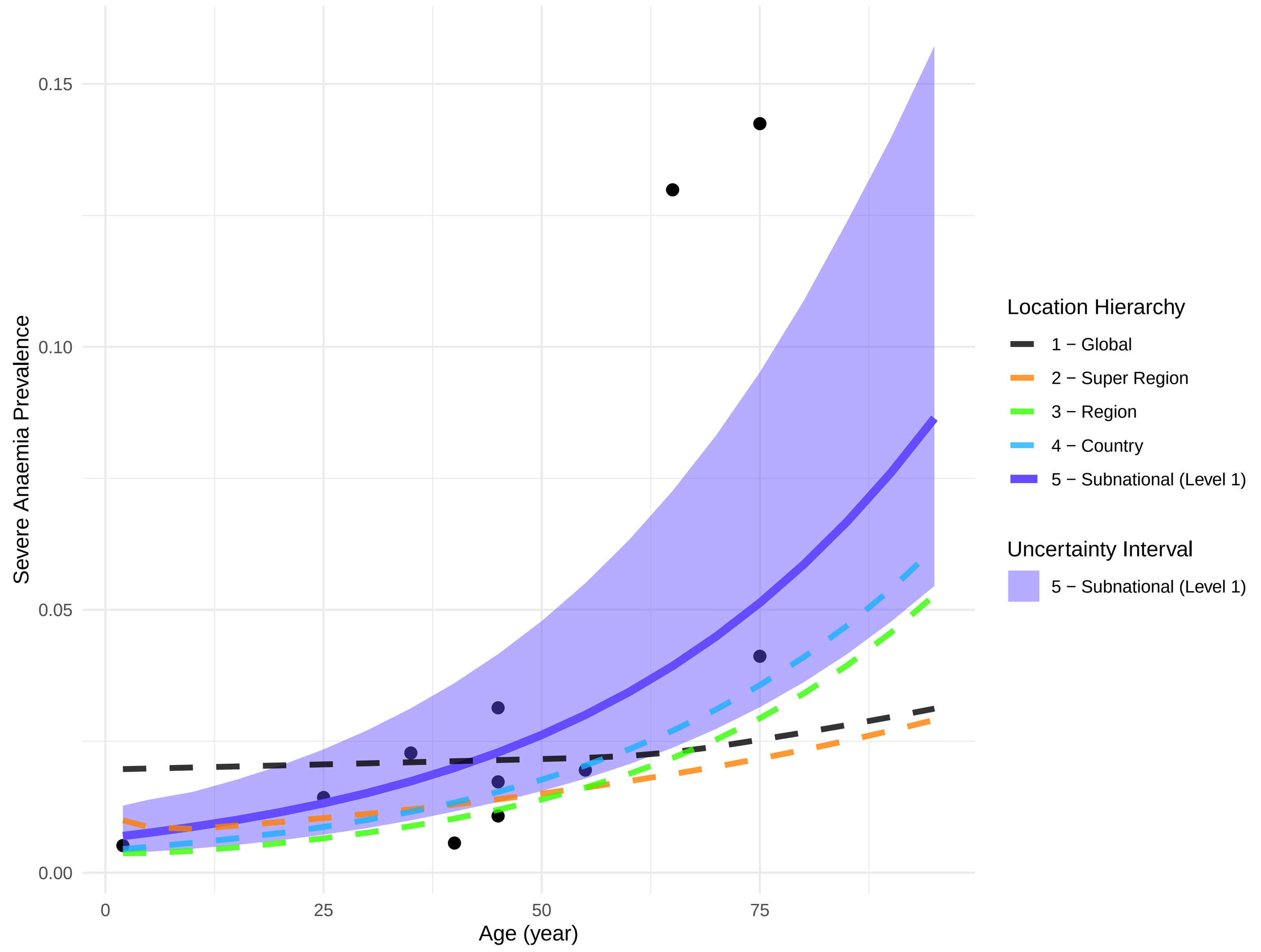

MR−BRT Age/Sex Fits − Non−pregnant Females 2+ Years Old − Severe Anaemia Prevalence − Hidalgo
WHO−2024 Adjustment Method

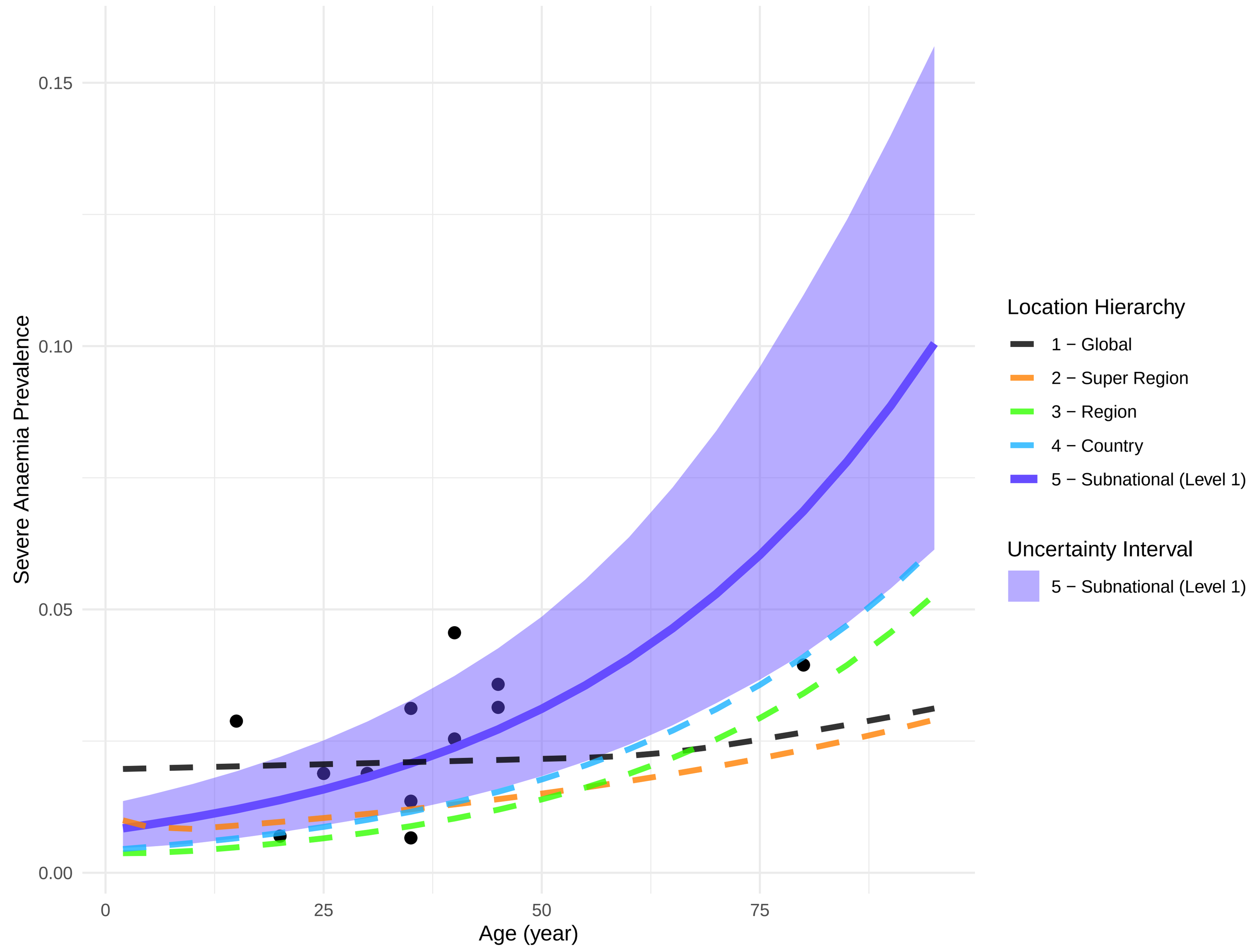

MR−BRT Age/Sex Fits − Non−pregnant Females 2+ Years Old − Severe Anaemia Prevalence − Jalisco
WHO−2024 Adjustment Method

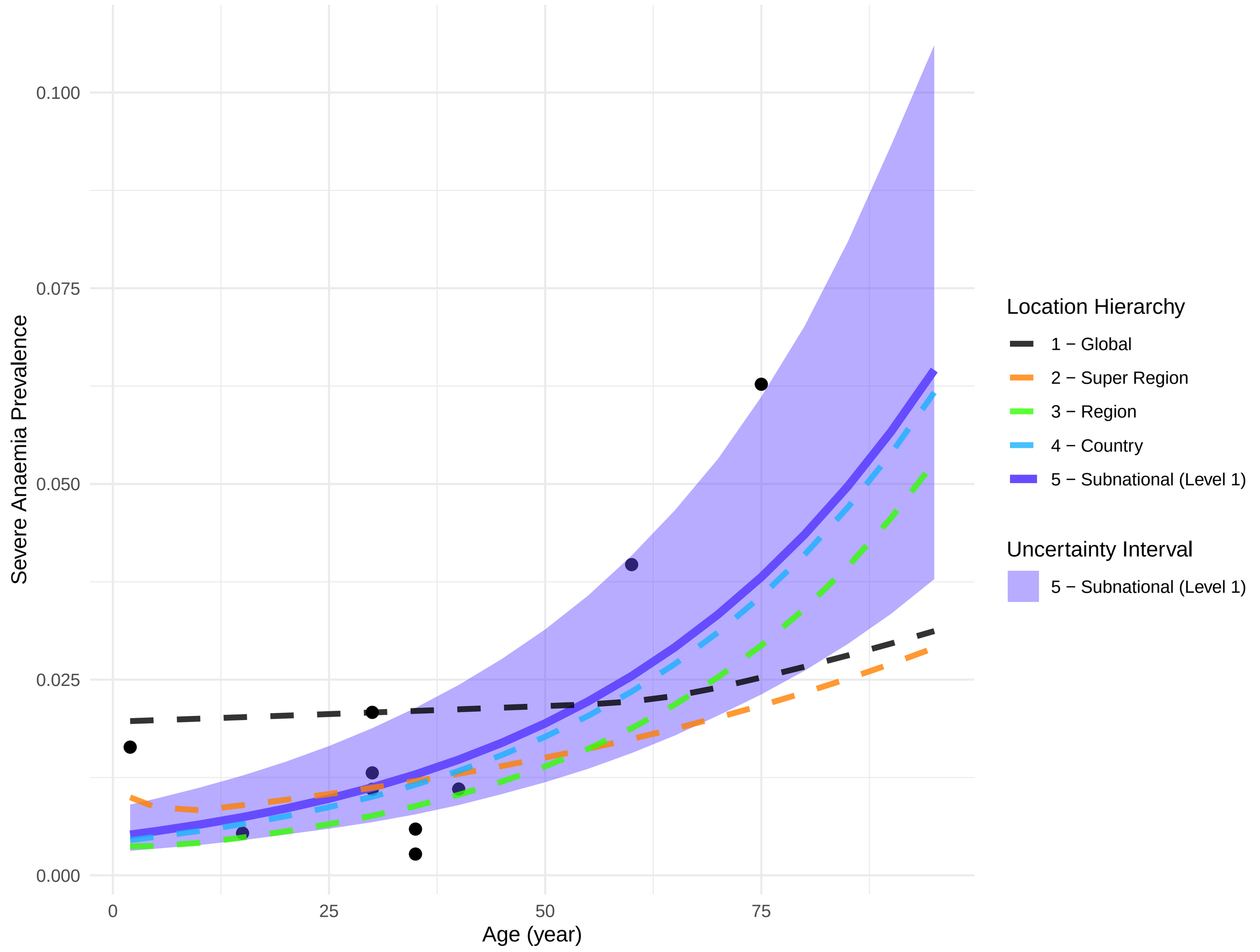

MR−BRT Age/Sex Fits − Non−pregnant Females 2+ Years Old − Severe Anaemia Prevalence − México
WHO−2024 Adjustment Method

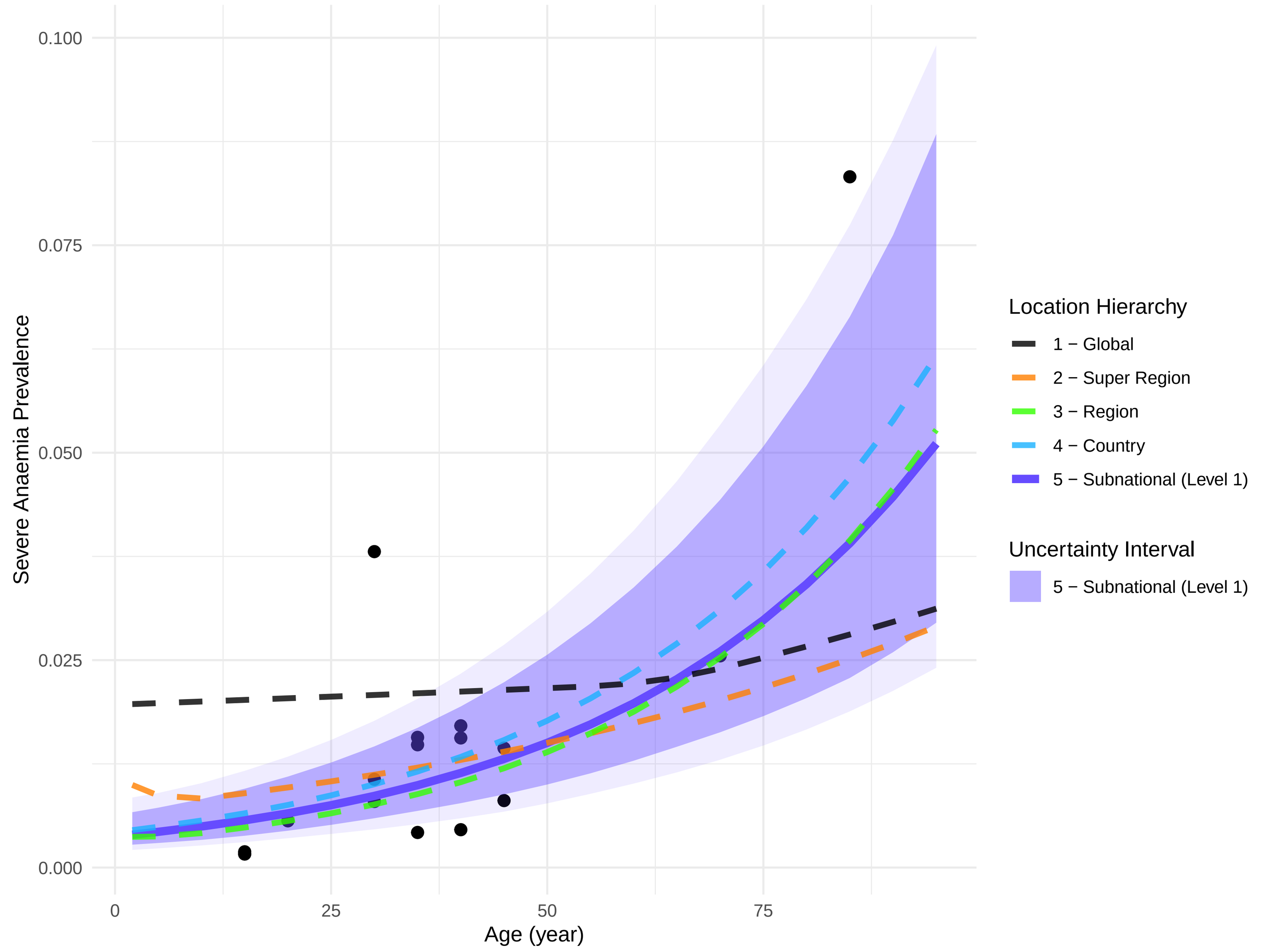

MR−BRT Age/Sex Fits − Non−pregnant Females 2+ Years Old − Severe Anaemia Prevalence − Mexico City
WHO−2024 Adjustment Method

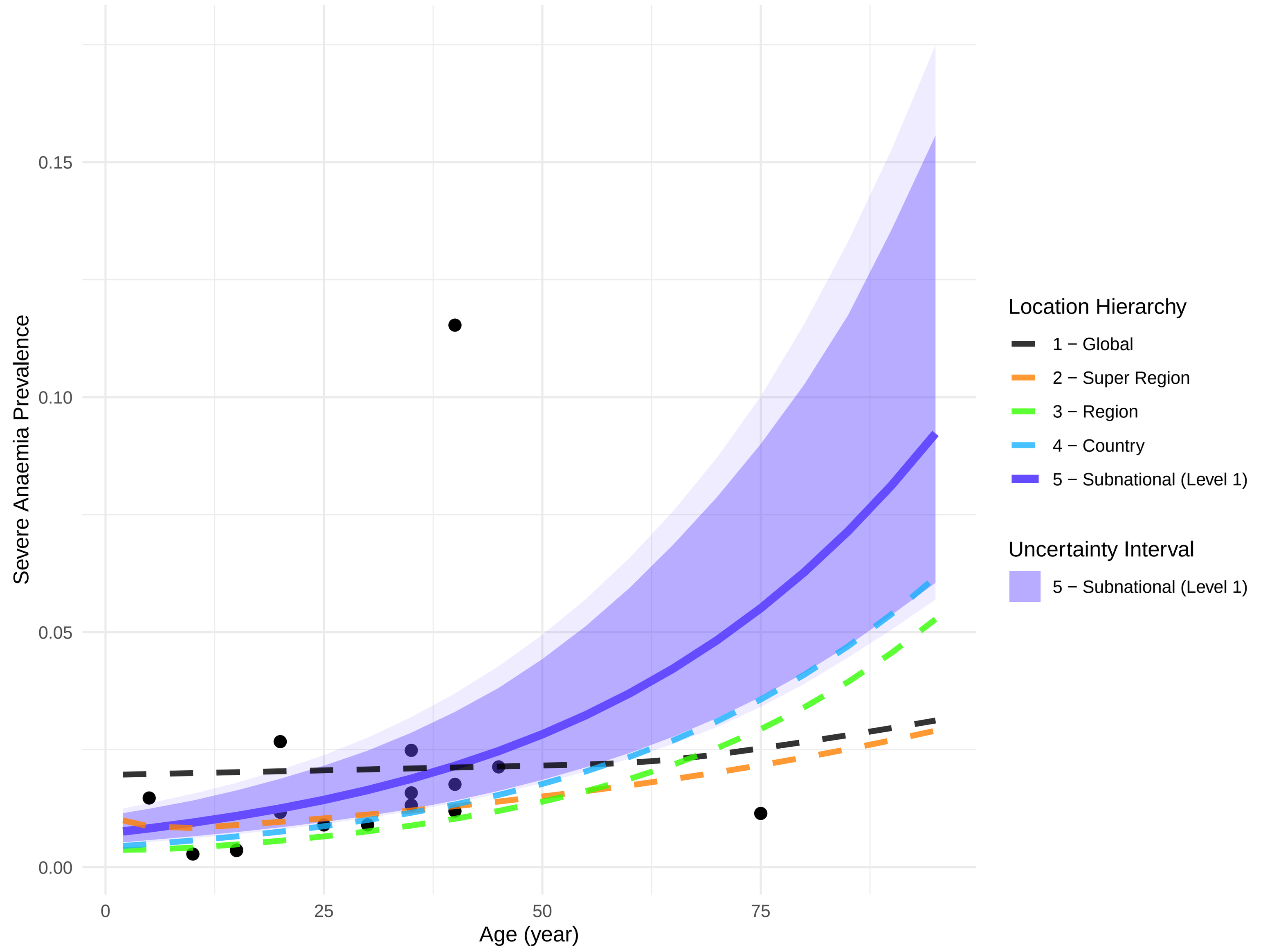

MR−BRT Age/Sex Fits − Non−pregnant Females 2+ Years Old − Severe Anaemia Prevalence − Michoacán de Ocampo
WHO−2024 Adjustment Method

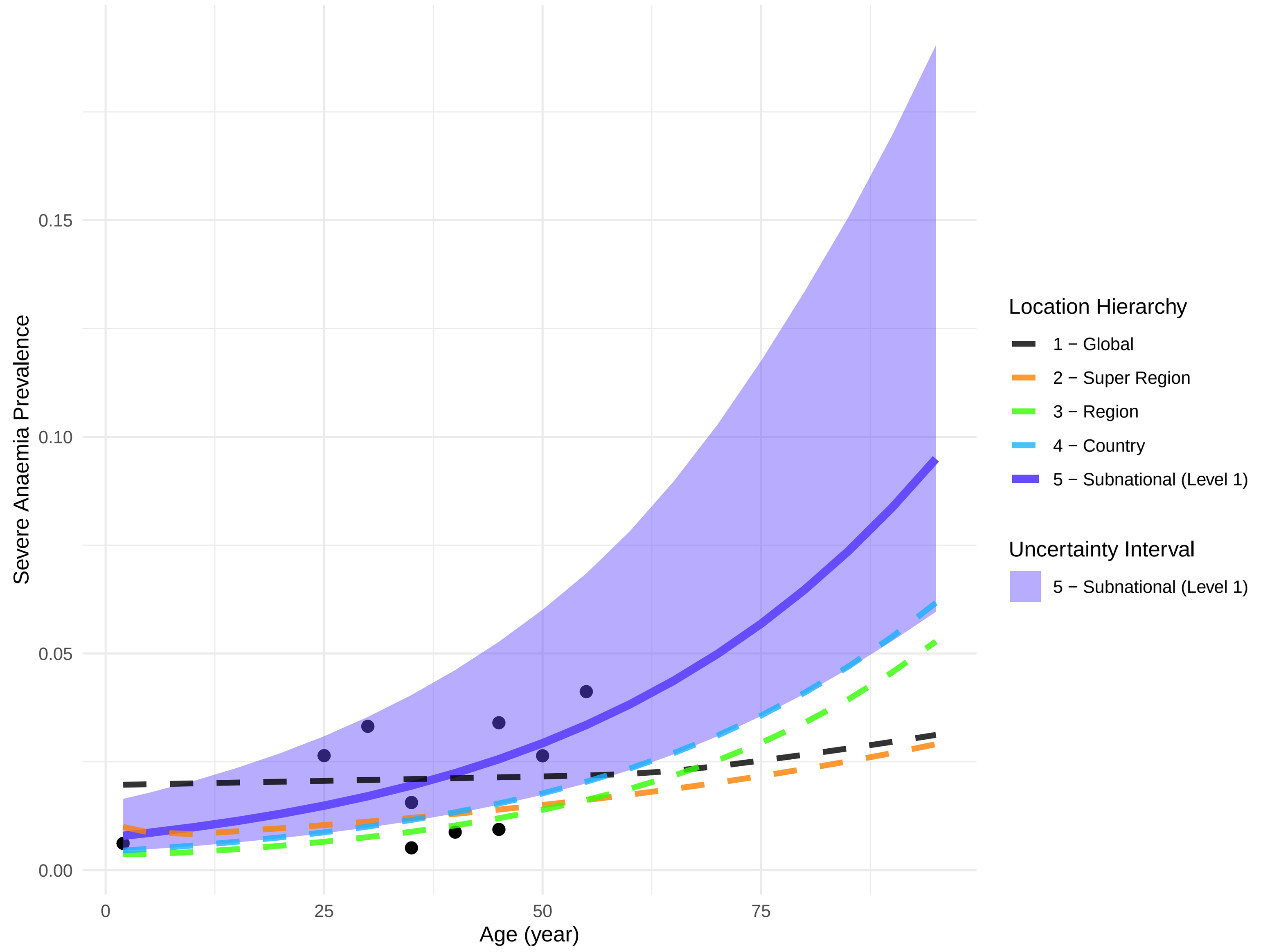

MR−BRT Age/Sex Fits − Non−pregnant Females 2+ Years Old − Severe Anaemia Prevalence − Morelos
WHO−2024 Adjustment Method

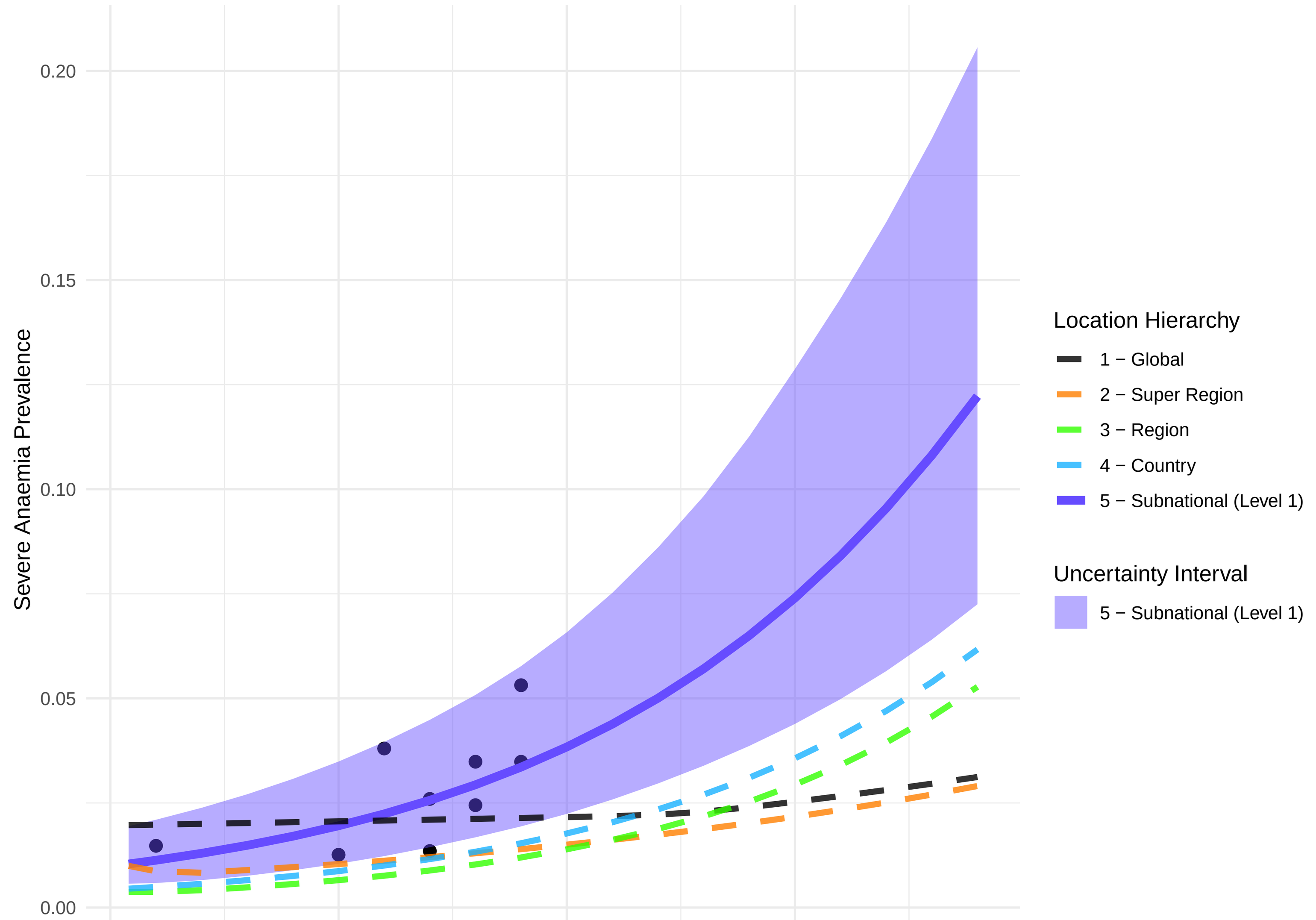

MR−BRT Age/Sex Fits − Non−pregnant Females 2+ Years Old − Severe Anaemia Prevalence − Nayarit
WHO−2024 Adjustment Method

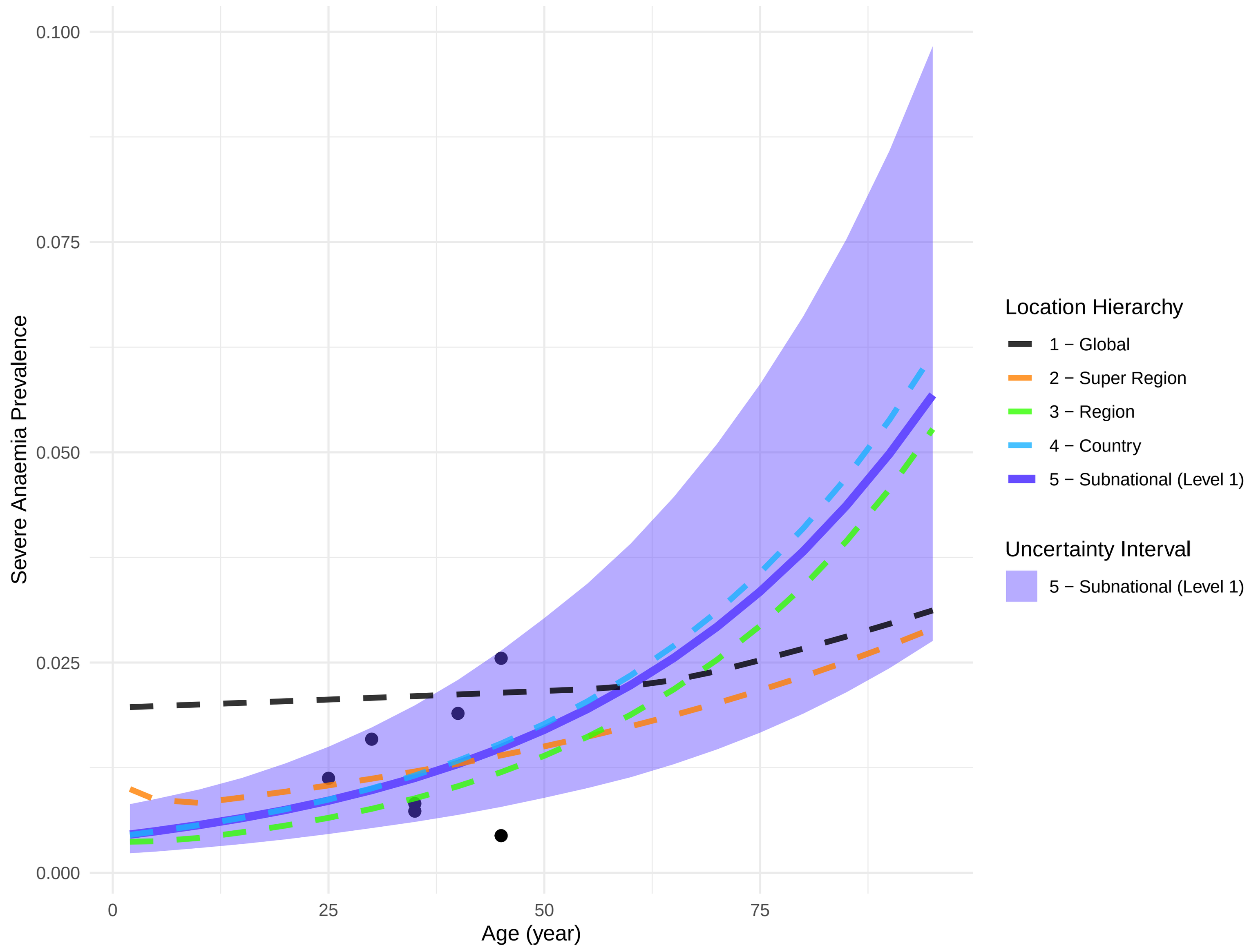

MR−BRT Age/Sex Fits − Non−pregnant Females 2+ Years Old − Severe Anaemia Prevalence − Nuevo León
WHO−2024 Adjustment Method

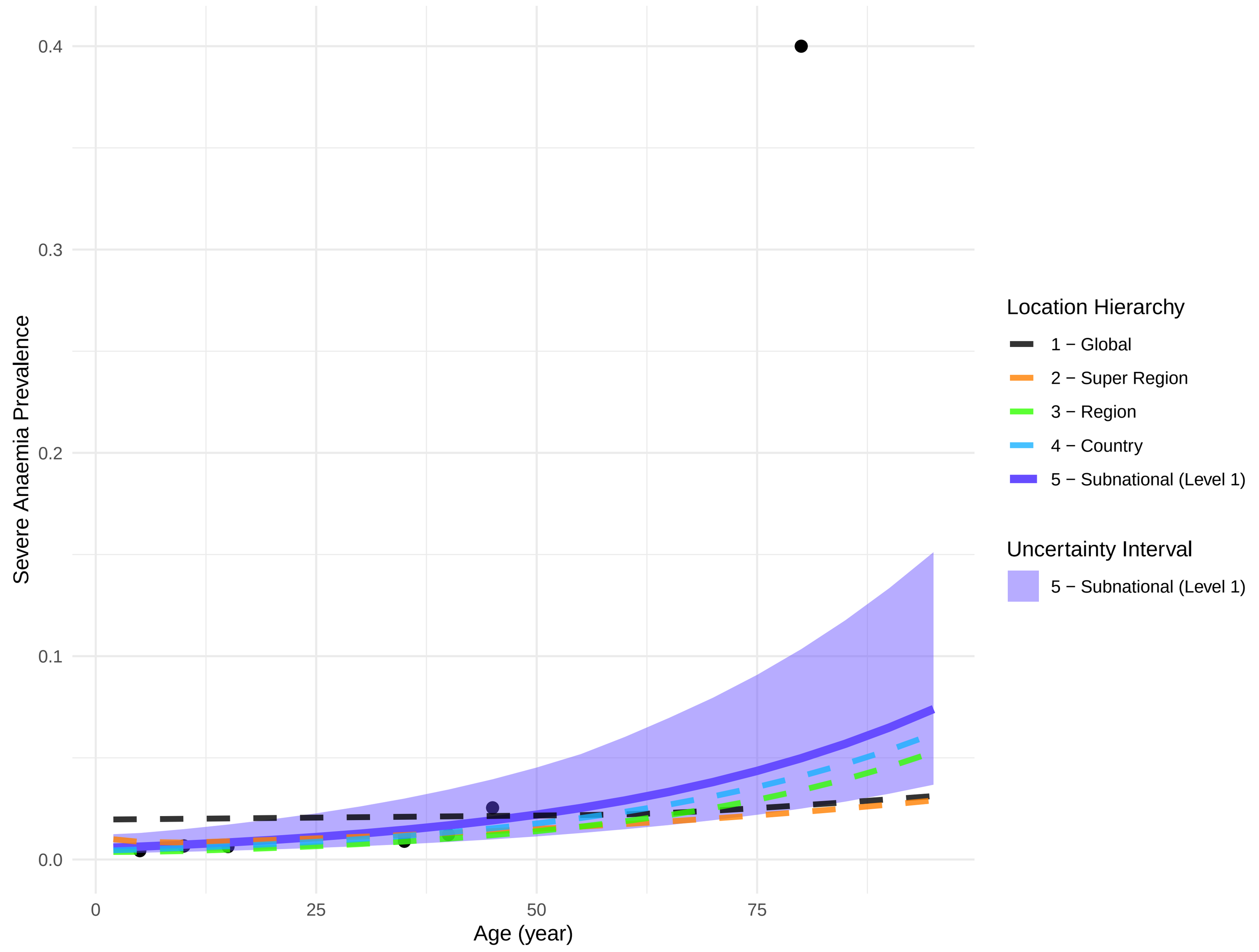

MR−BRT Age/Sex Fits − Non−pregnant Females 2+ Years Old − Severe Anaemia Prevalence − Oaxaca
WHO−2024 Adjustment Method

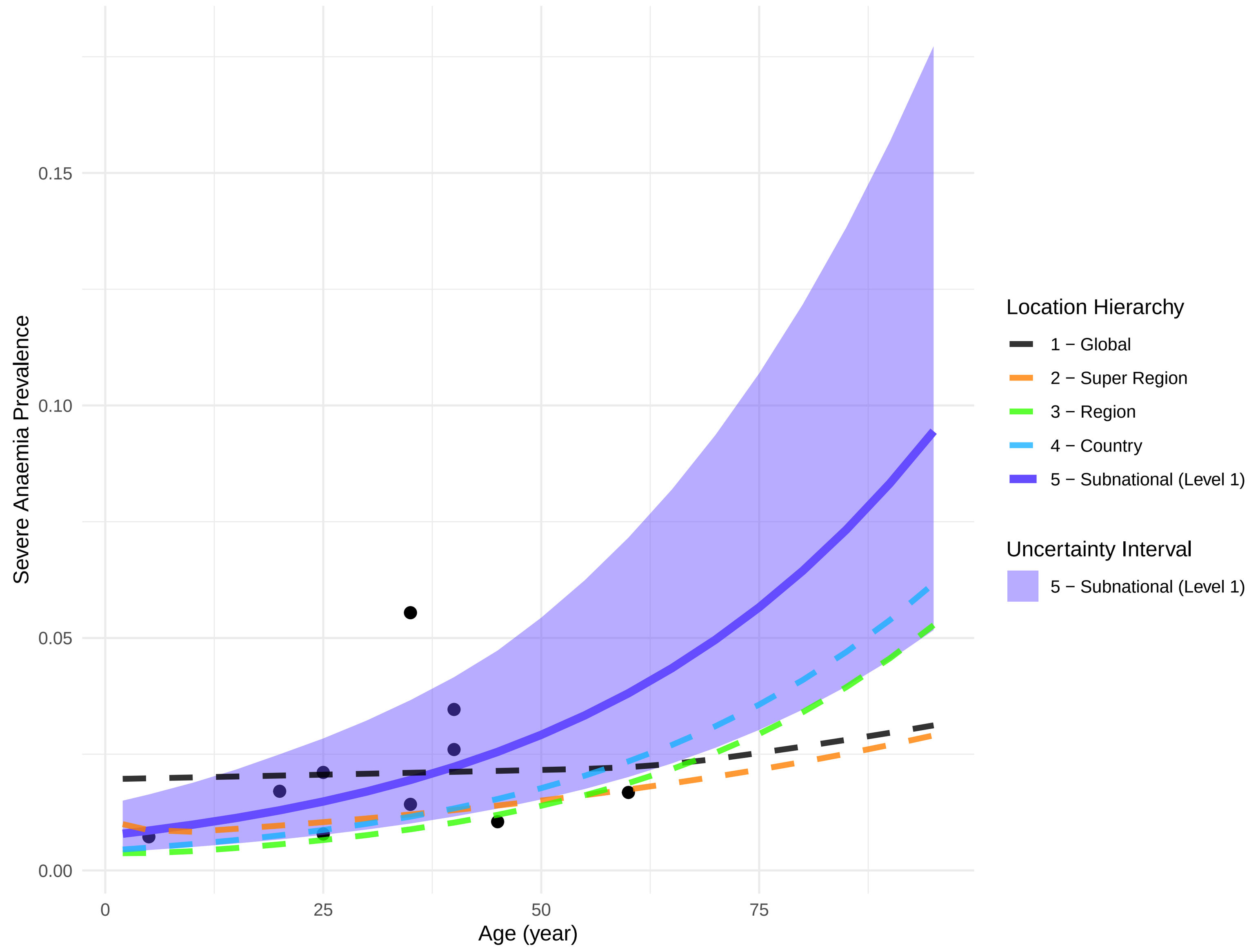

MR−BRT Age/Sex Fits − Non−pregnant Females 2+ Years Old − Severe Anaemia Prevalence − Puebla
WHO−2024 Adjustment Method

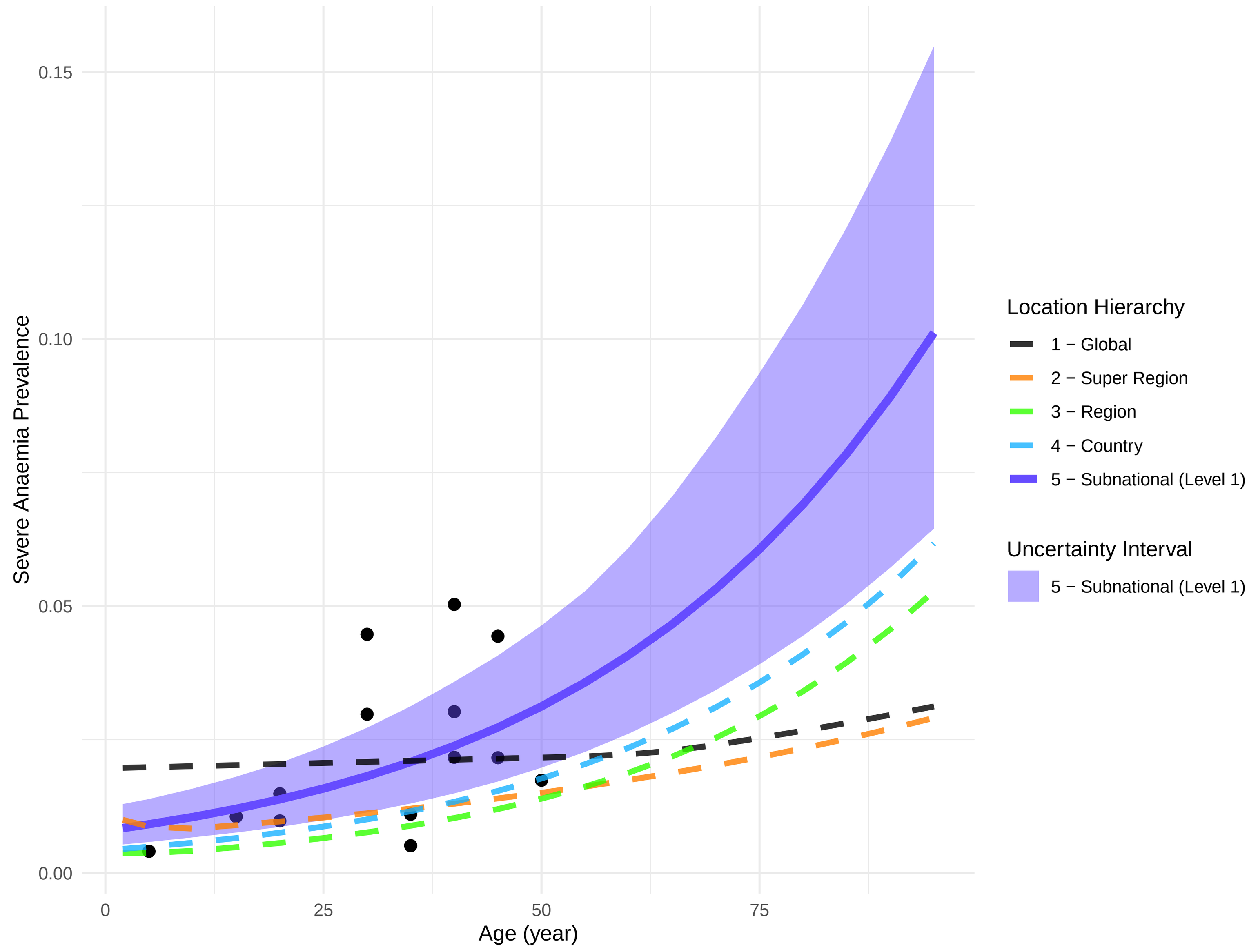

MR−BRT Age/Sex Fits − Non−pregnant Females 2+ Years Old − Severe Anaemia Prevalence − Querétaro
WHO−2024 Adjustment Method

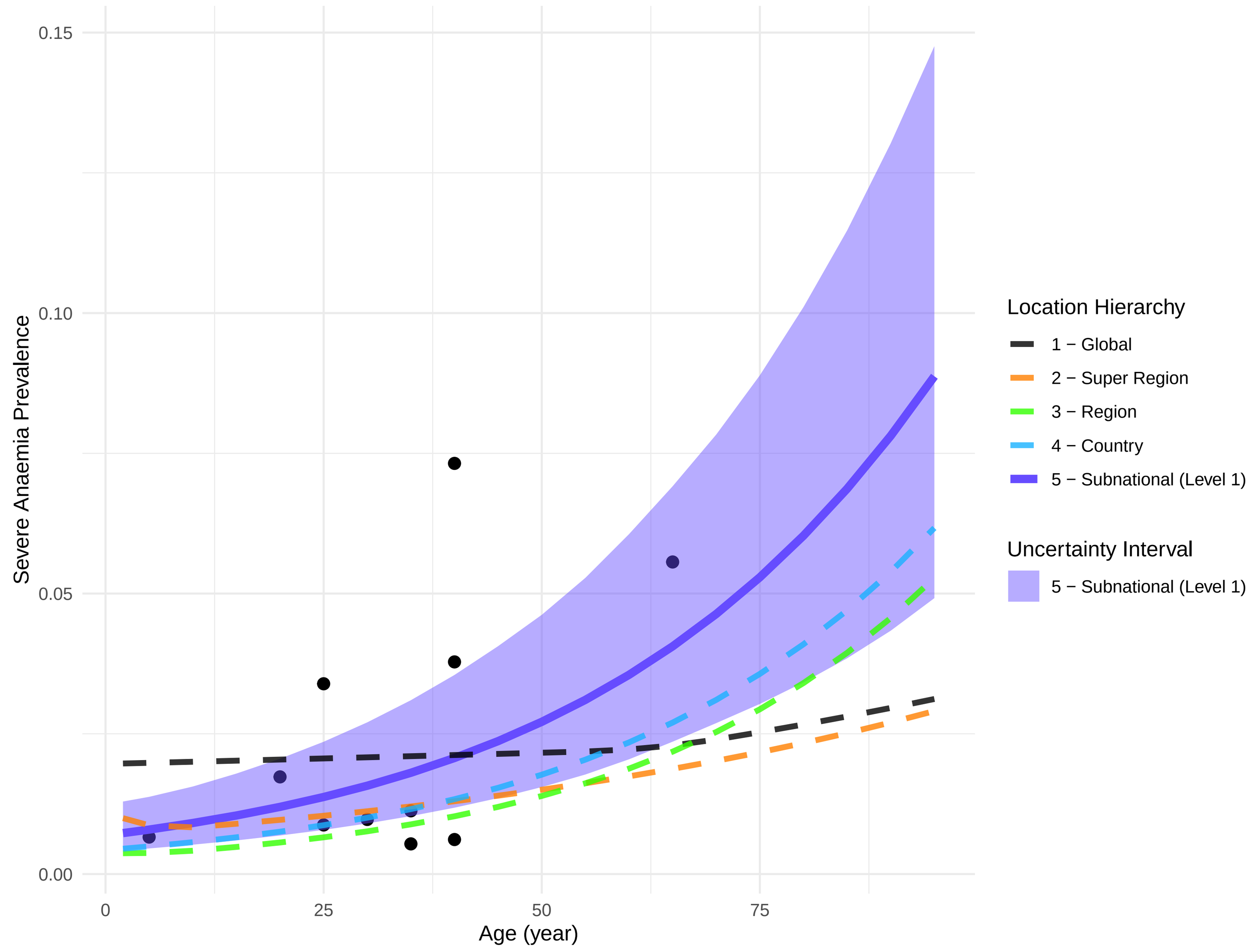

MR−BRT Age/Sex Fits − Non−pregnant Females 2+ Years Old − Severe Anaemia Prevalence − Quintana Roo
WHO−2024 Adjustment Method

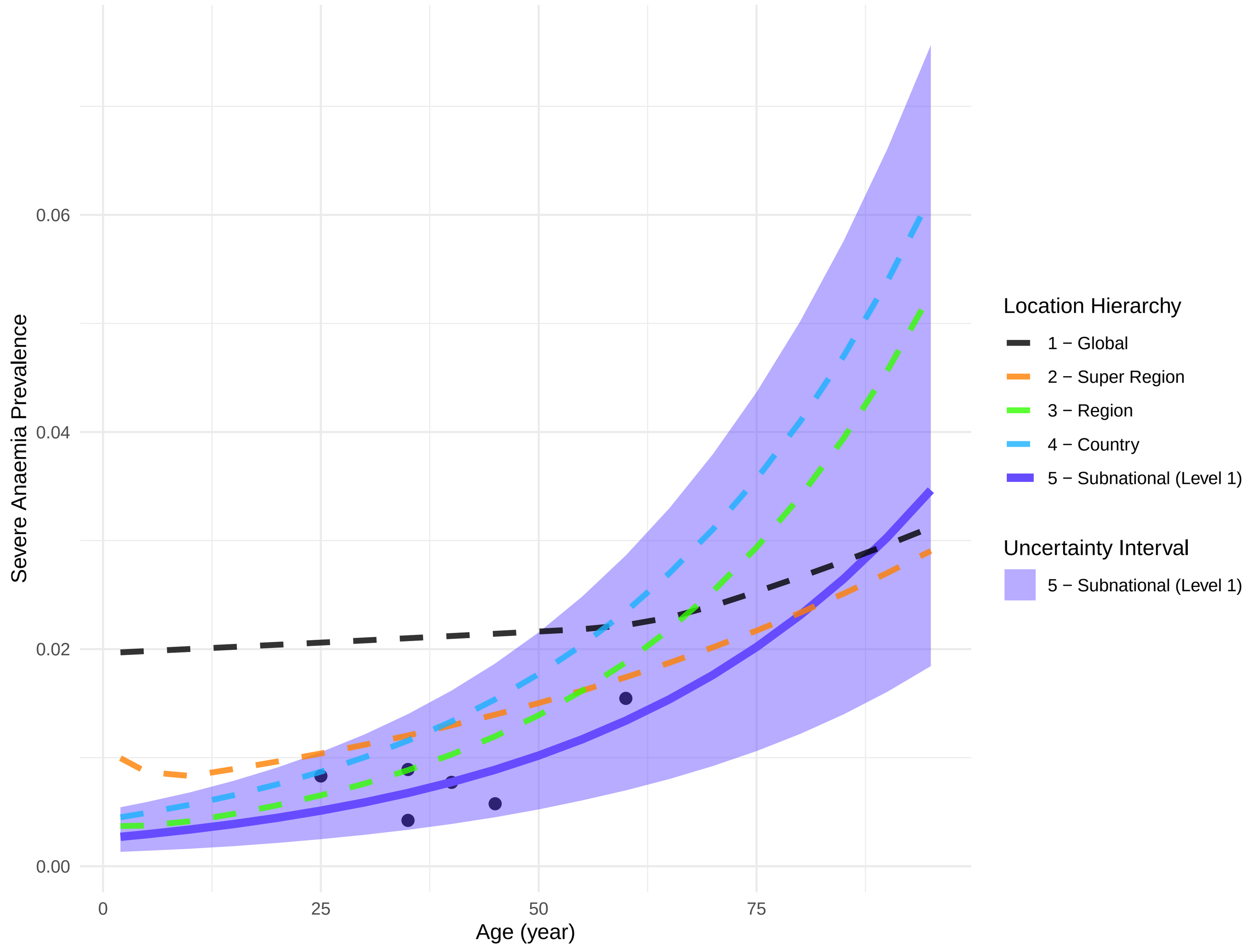

MR−BRT Age/Sex Fits − Non−pregnant Females 2+ Years Old − Severe Anaemia Prevalence − San Luis Potosí
WHO−2024 Adjustment Method

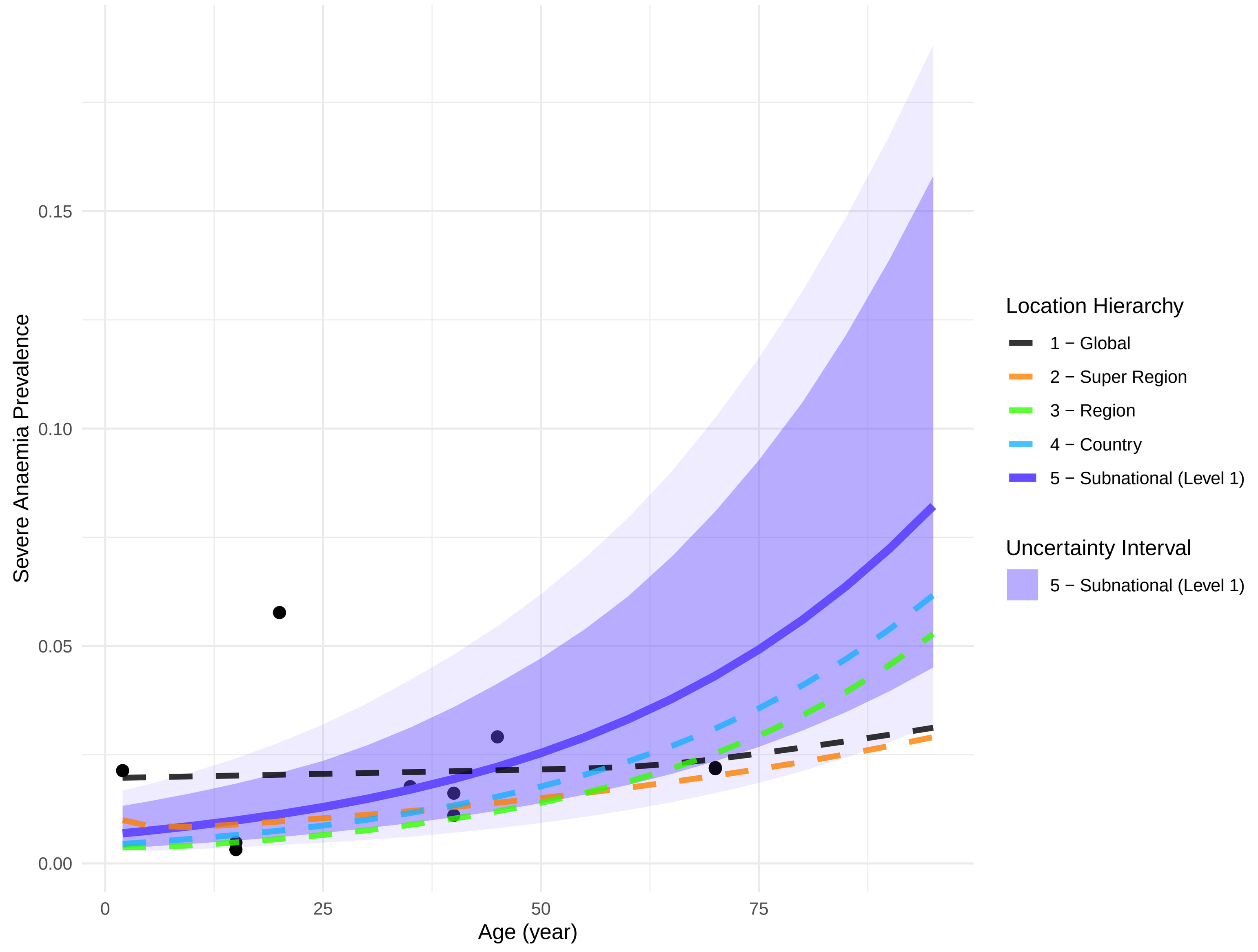

MR−BRT Age/Sex Fits − Non−pregnant Females 2+ Years Old − Severe Anaemia Prevalence − Sinaloa
WHO−2024 Adjustment Method

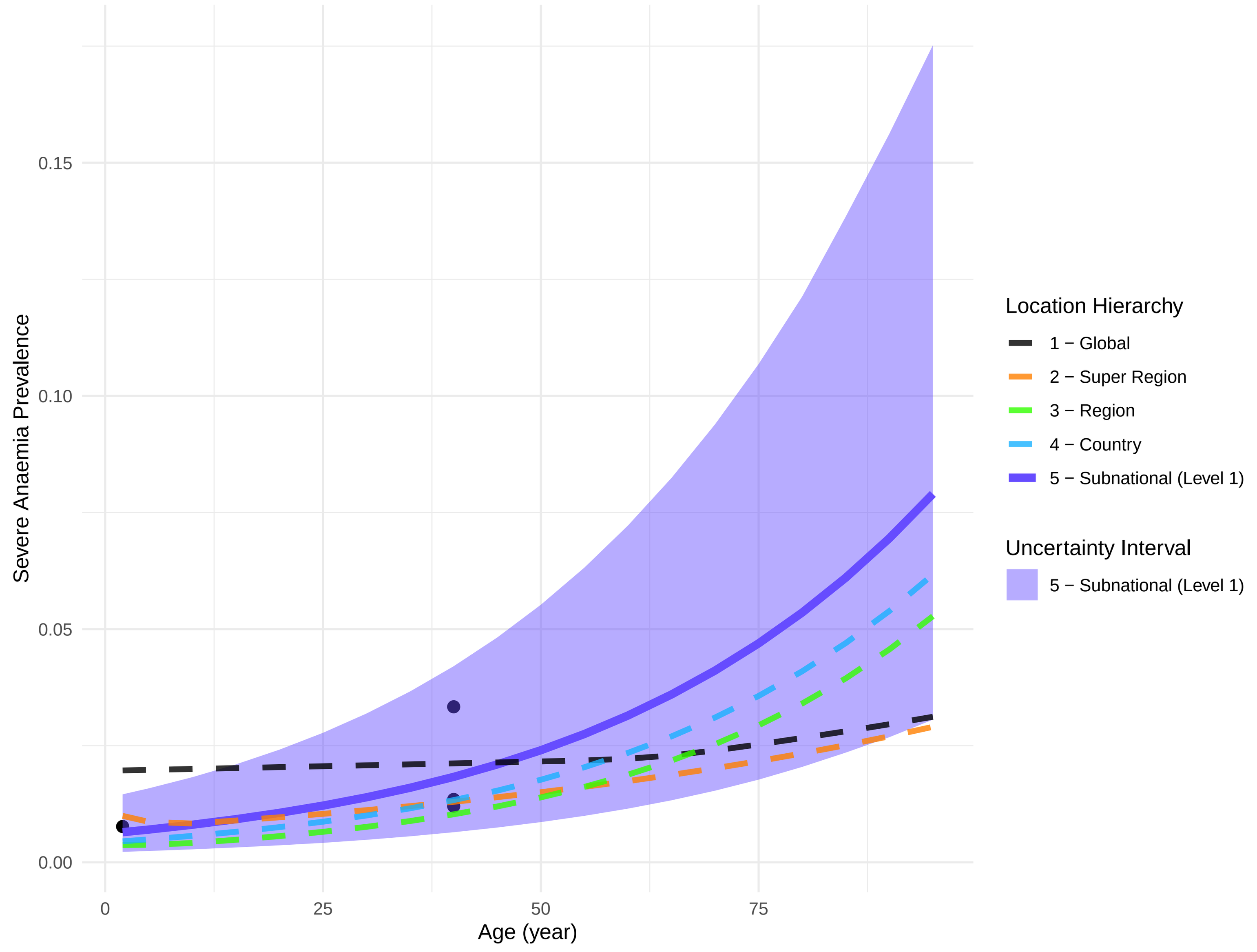

MR−BRT Age/Sex Fits − Non−pregnant Females 2+ Years Old − Severe Anaemia Prevalence − Sonora
WHO−2024 Adjustment Method

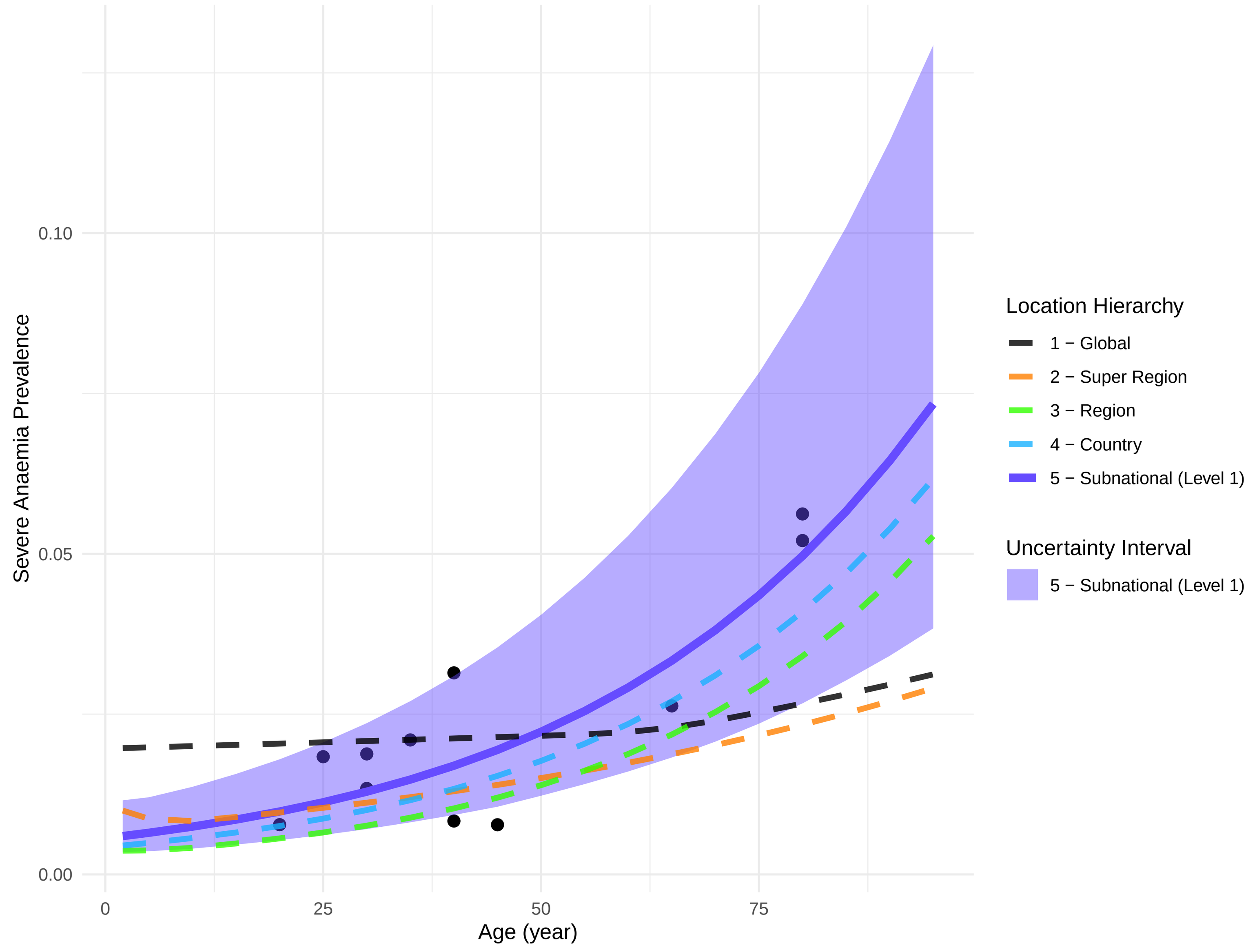

MR−BRT Age/Sex Fits − Non−pregnant Females 2+ Years Old − Severe Anaemia Prevalence − Tabasco
WHO−2024 Adjustment Method

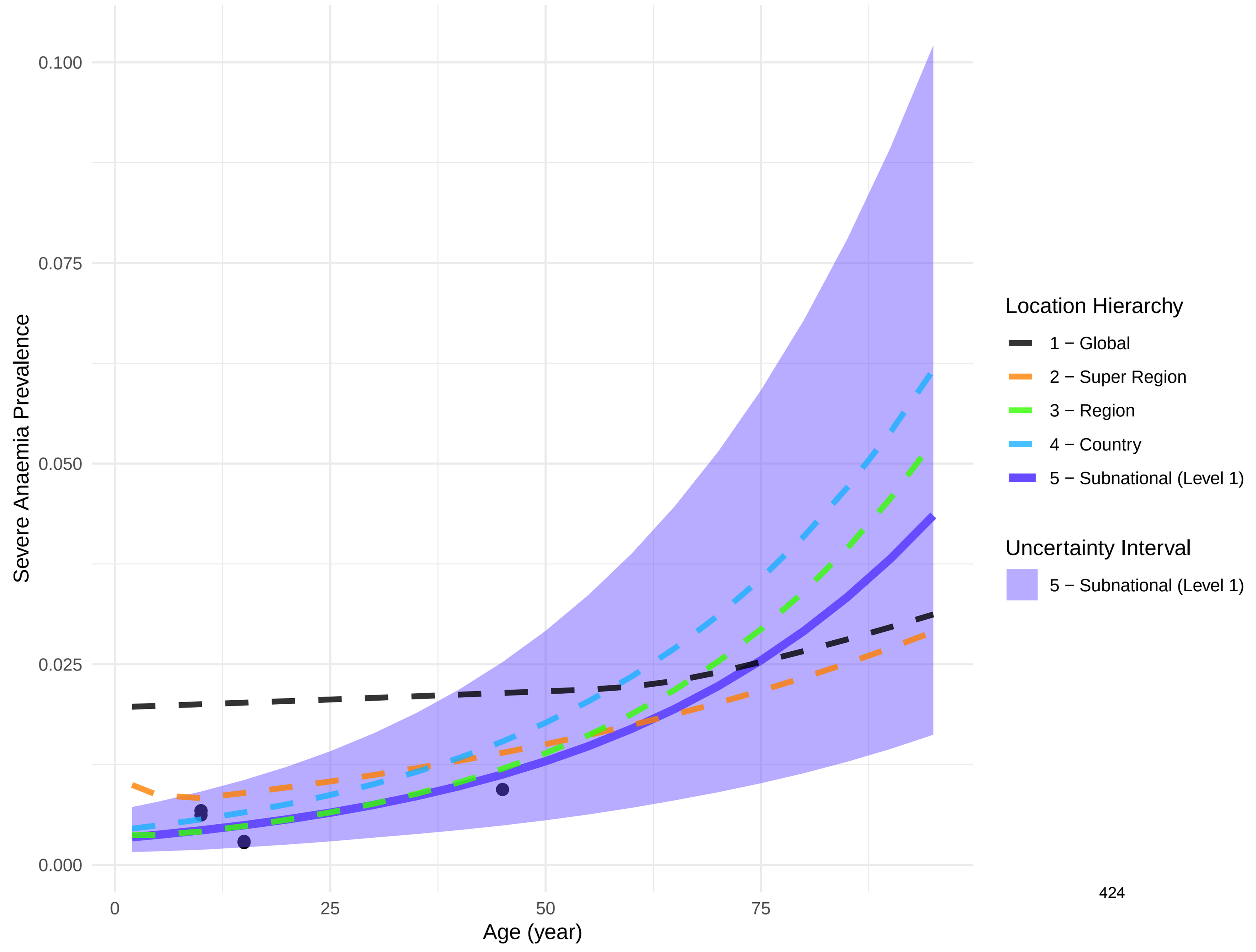

MR−BRT Age/Sex Fits − Non−pregnant Females 2+ Years Old − Severe Anaemia Prevalence − Tamaulipas
WHO−2024 Adjustment Method

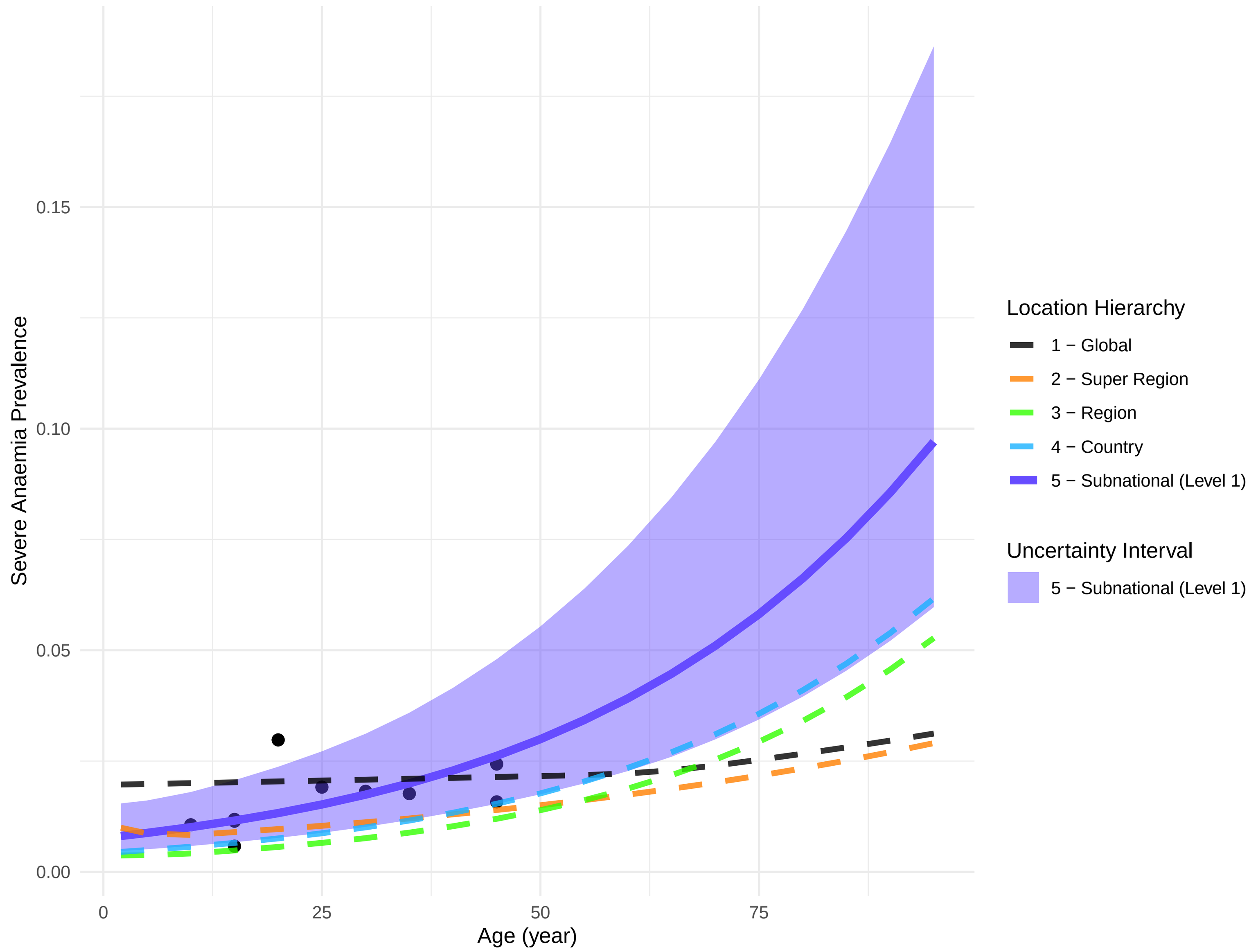

MR−BRT Age/Sex Fits − Non−pregnant Females 2+ Years Old − Severe Anaemia Prevalence − Tlaxcala
WHO−2024 Adjustment Method

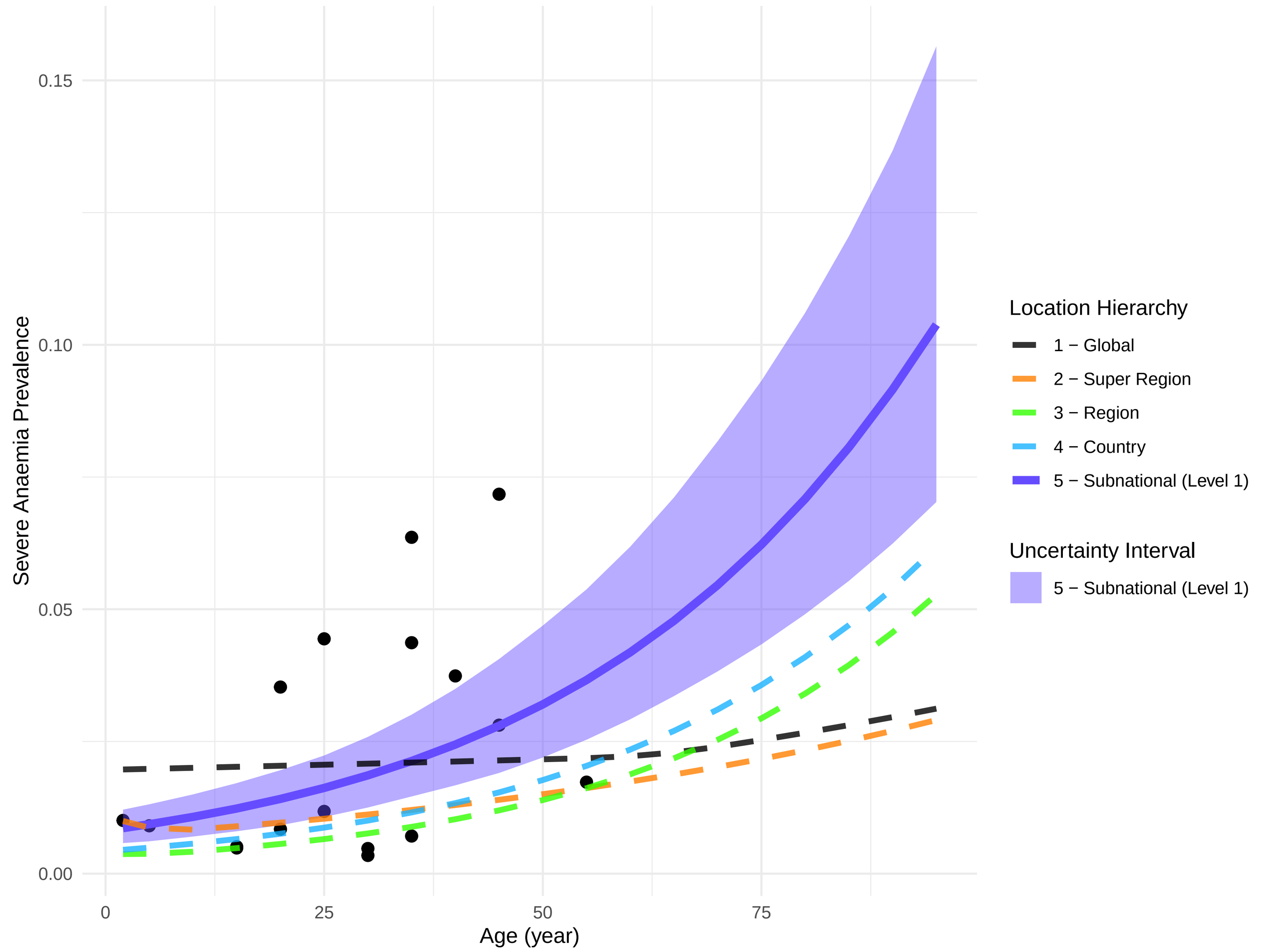

MR−BRT Age/Sex Fits − Non−pregnant Females 2+ Years Old − Severe Anaemia Prevalence − Veracruz de Ignacio de la Llave
WHO−2024 Adjustment Method

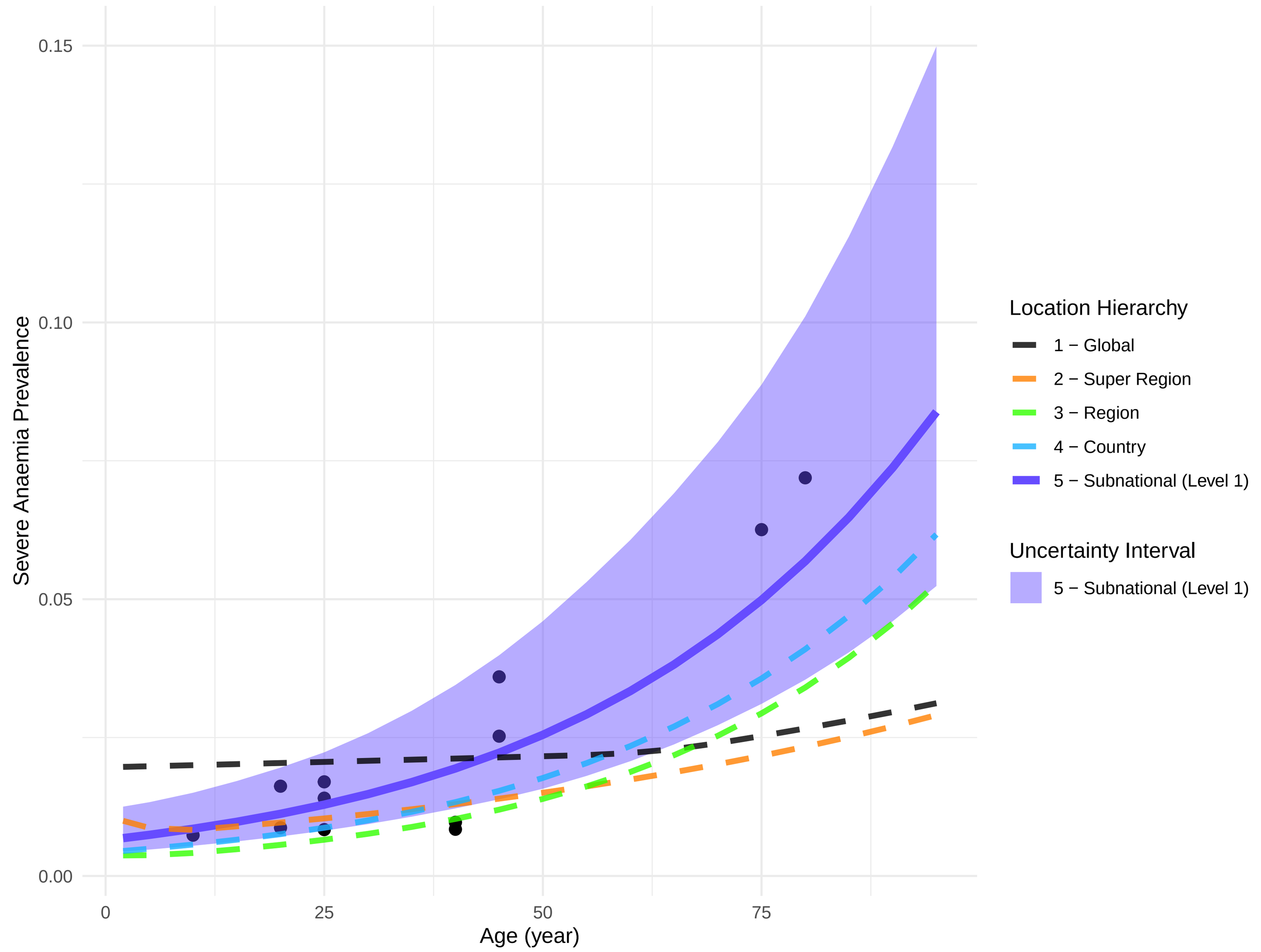

MR−BRT Age/Sex Fits − Non−pregnant Females 2+ Years Old − Severe Anaemia Prevalence − Yucatán
WHO−2024 Adjustment Method

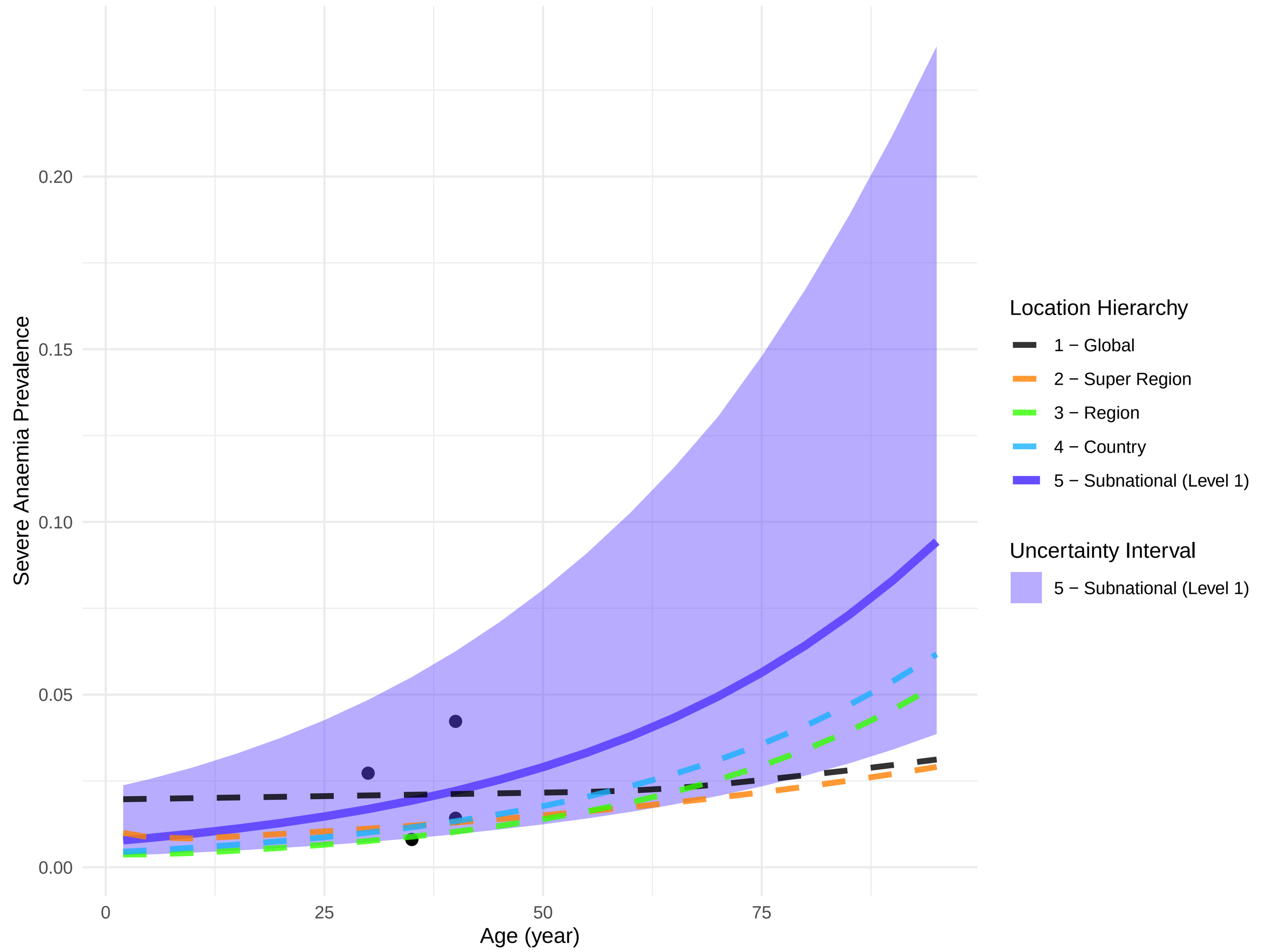

MR−BRT Age/Sex Fits − Non−pregnant Females 2+ Years Old − Severe Anaemia Prevalence − Zacatecas
WHO−2024 Adjustment Method

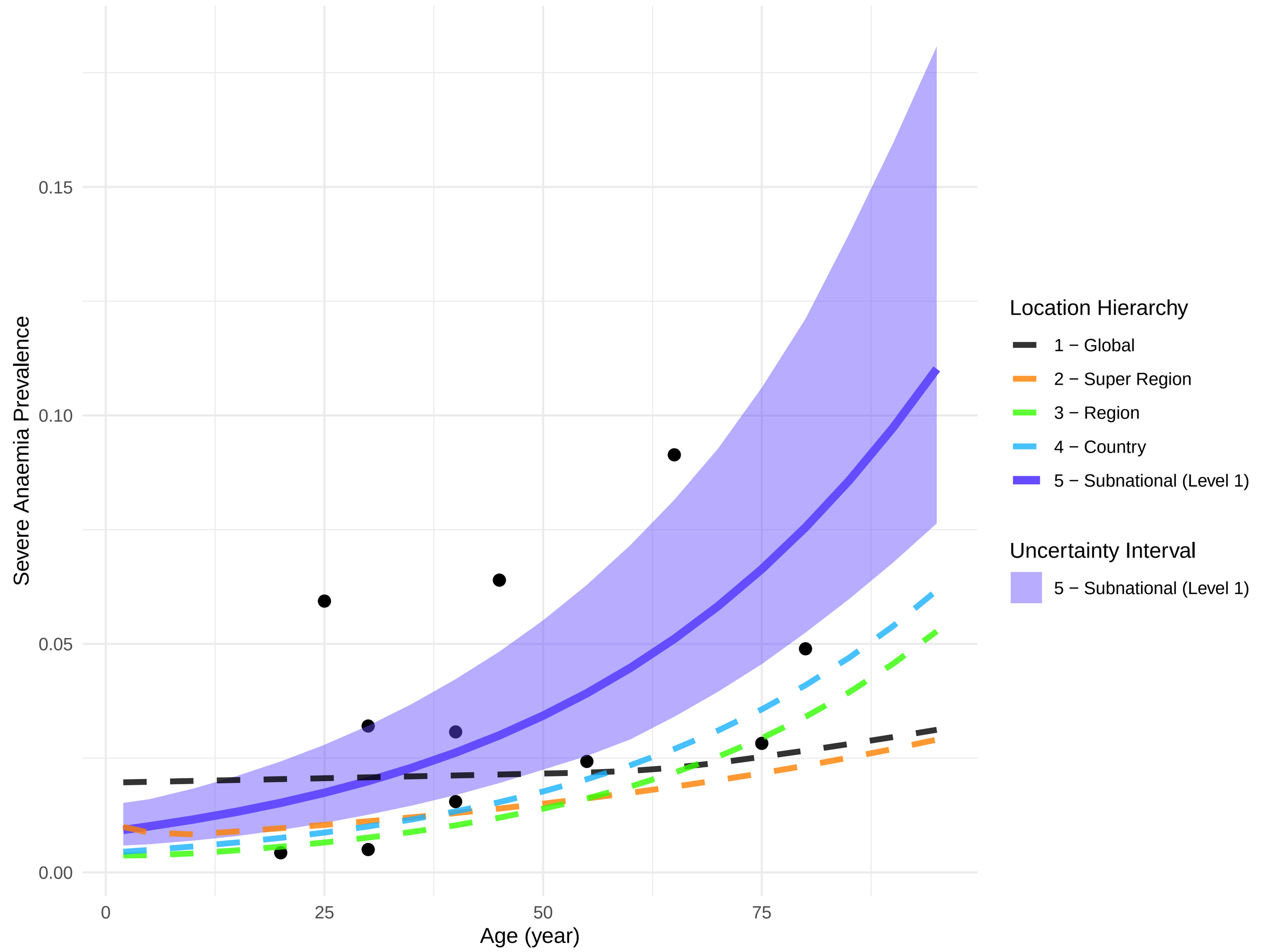

MR−BRT Age/Sex Fits − Non−pregnant Females 2+ Years Old − Severe Anaemia Prevalence − Tropical Latin America
WHO−2024 Adjustment Method

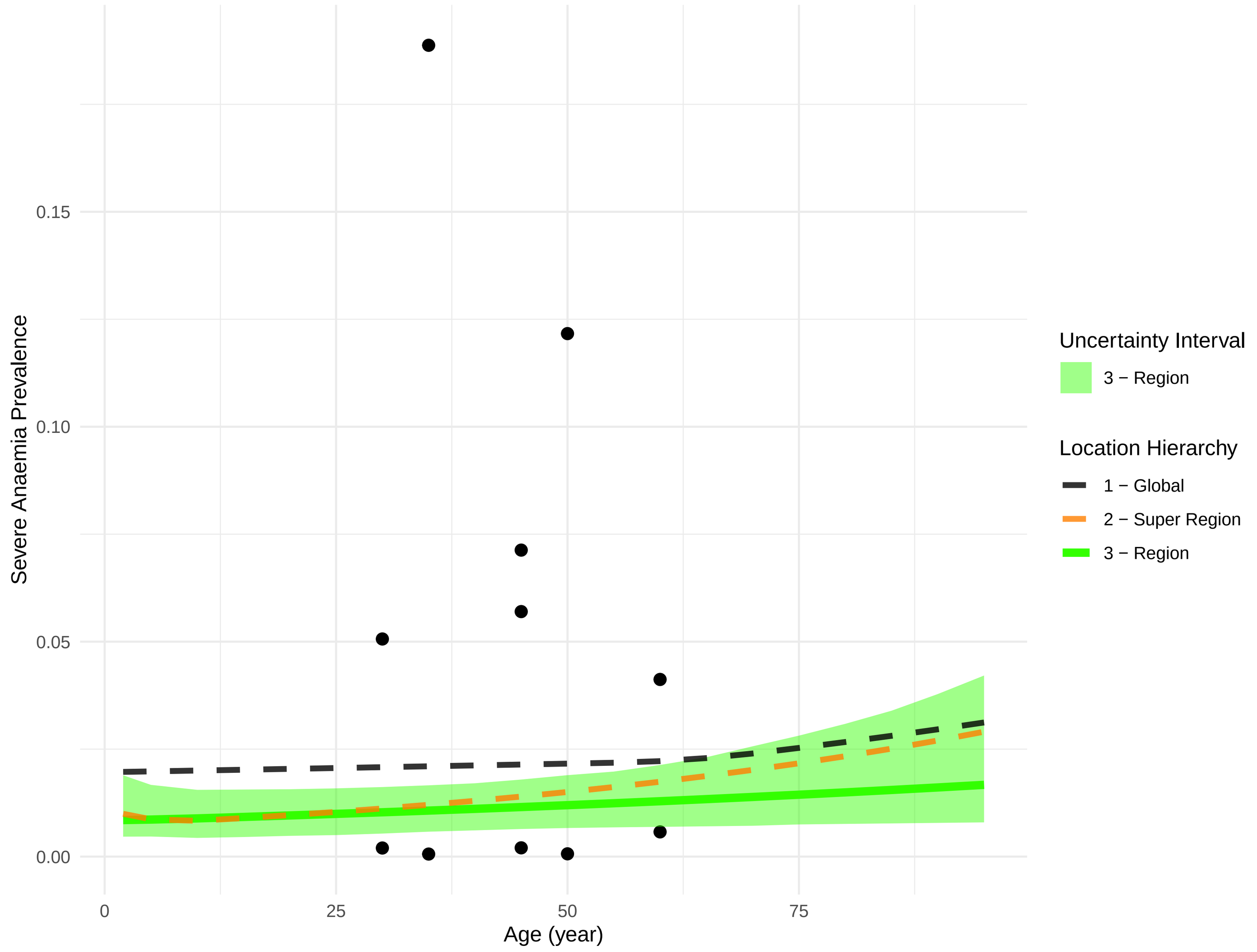

MR−BRT Age/Sex Fits − Non−pregnant Females 2+ Years Old − Severe Anaemia Prevalence − Brazil
WHO−2024 Adjustment Method

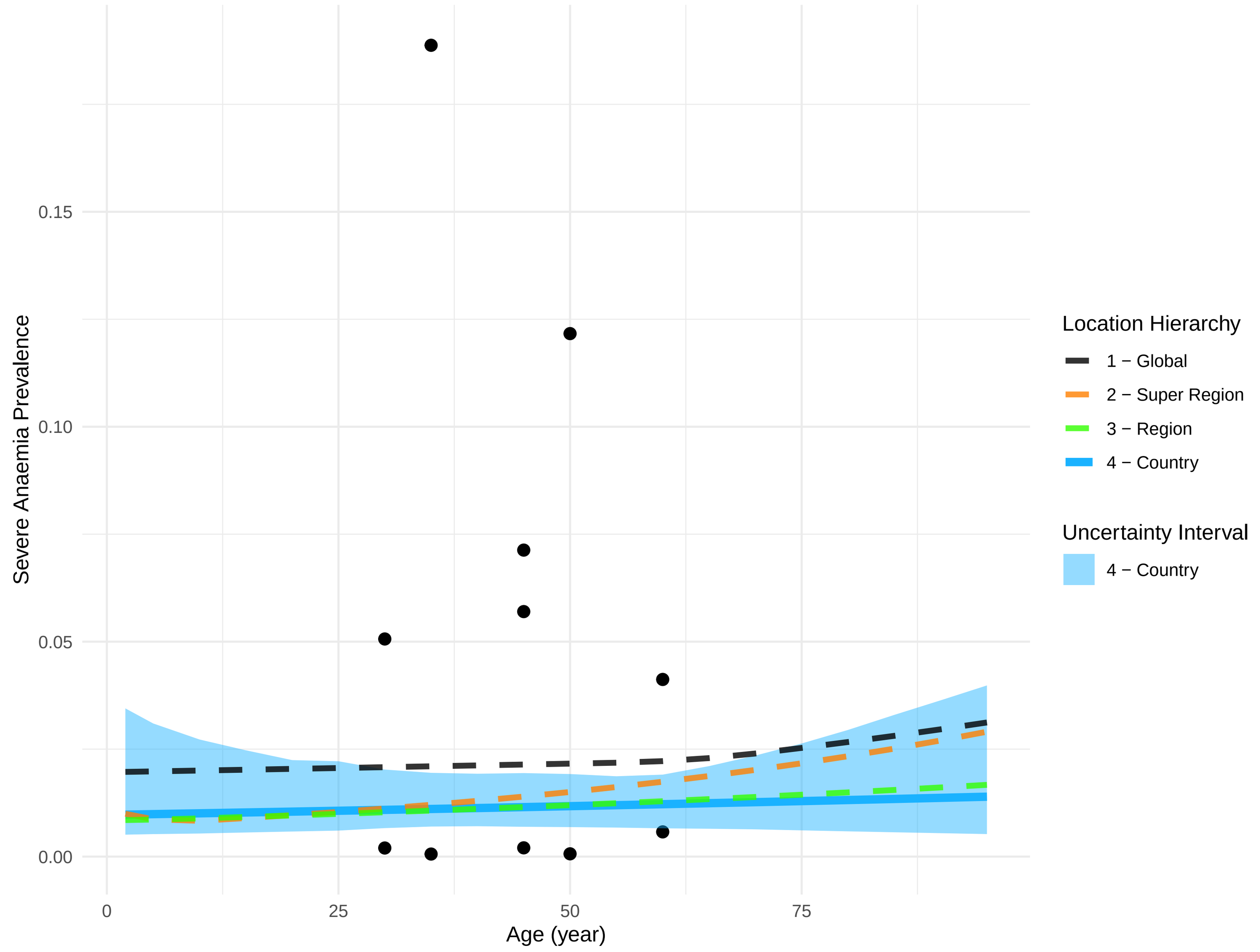

MR−BRT Age/Sex Fits − Non−pregnant Females 2+ Years Old − Severe Anaemia Prevalence − Acre
WHO−2024 Adjustment Method

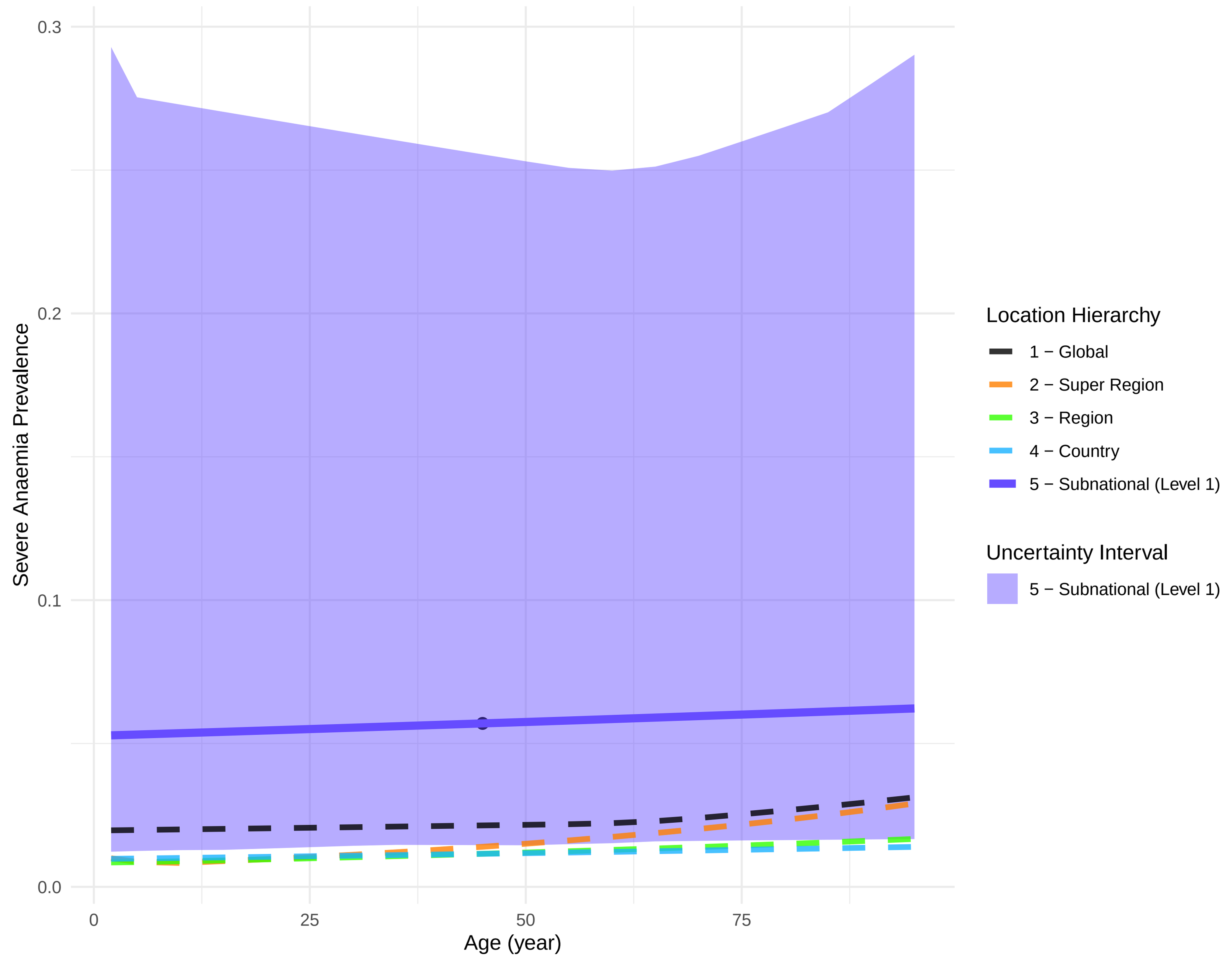

MR−BRT Age/Sex Fits − Non−pregnant Females 2+ Years Old − Severe Anaemia Prevalence − Amazonas
WHO−2024 Adjustment Method

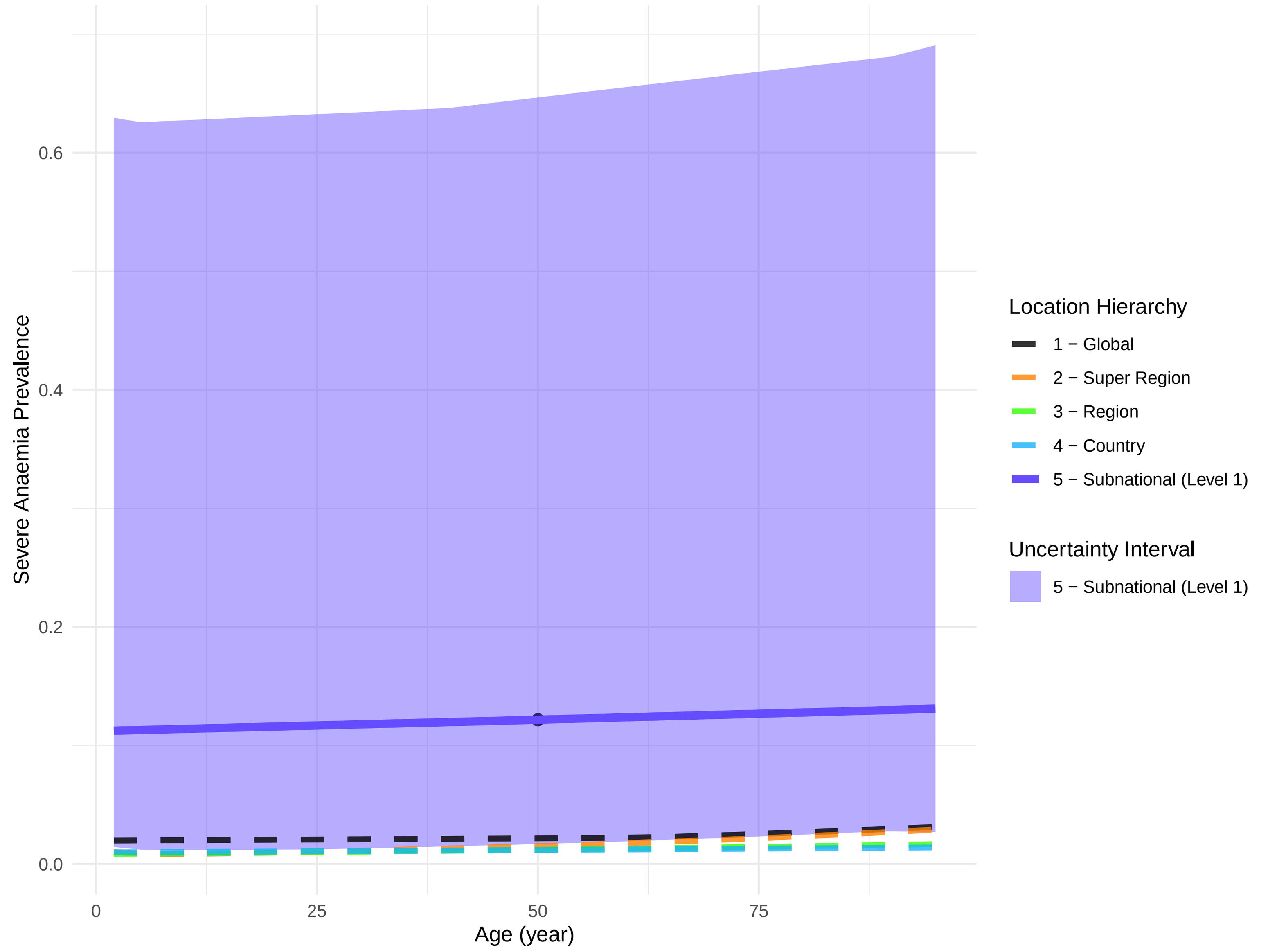

MR−BRT Age/Sex Fits − Non−pregnant Females 2+ Years Old − Severe Anaemia Prevalence − Ceará
WHO−2024 Adjustment Method

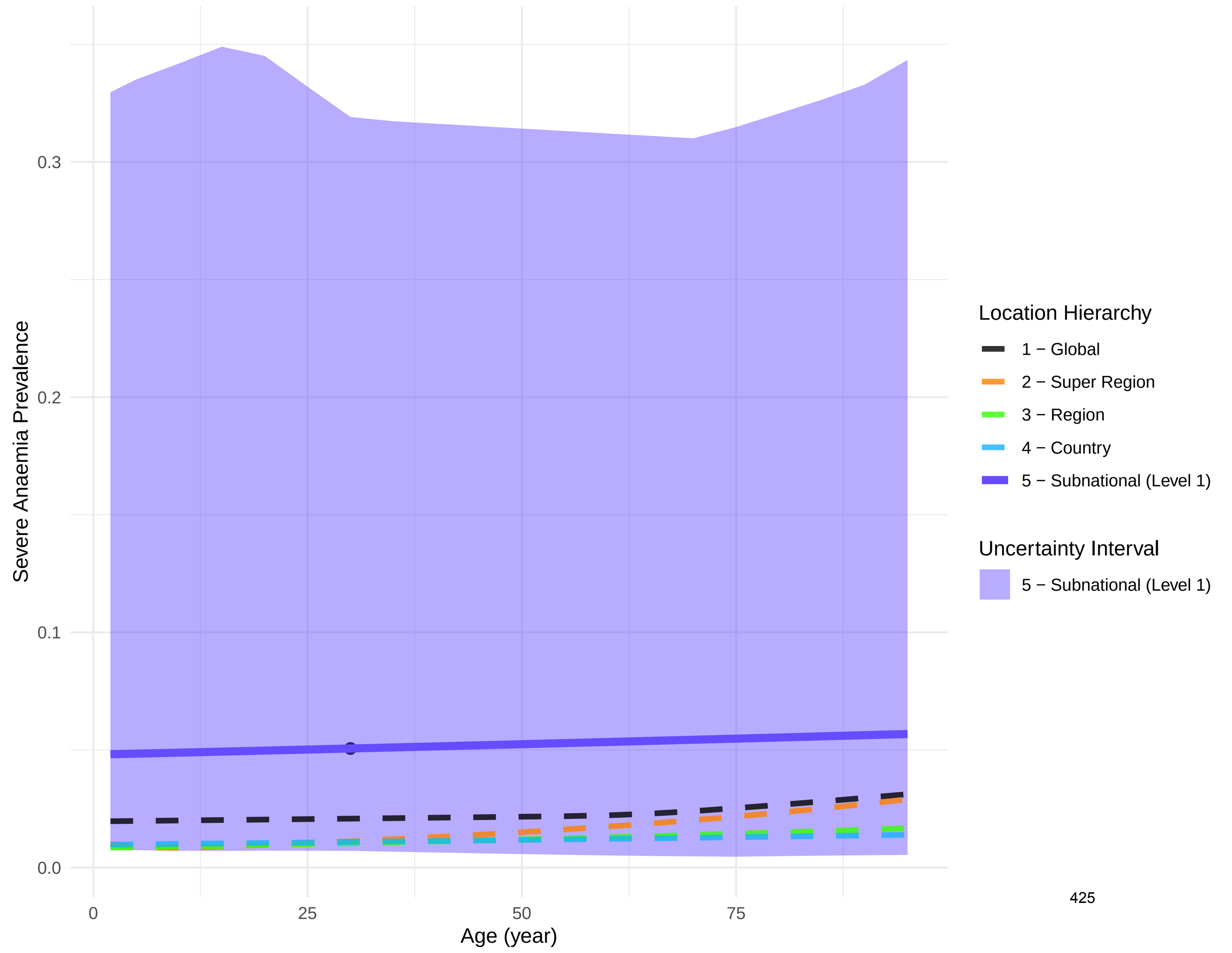

MR−BRT Age/Sex Fits − Non−pregnant Females 2+ Years Old − Severe Anaemia Prevalence − Piaui
WHO−2024 Adjustment Method

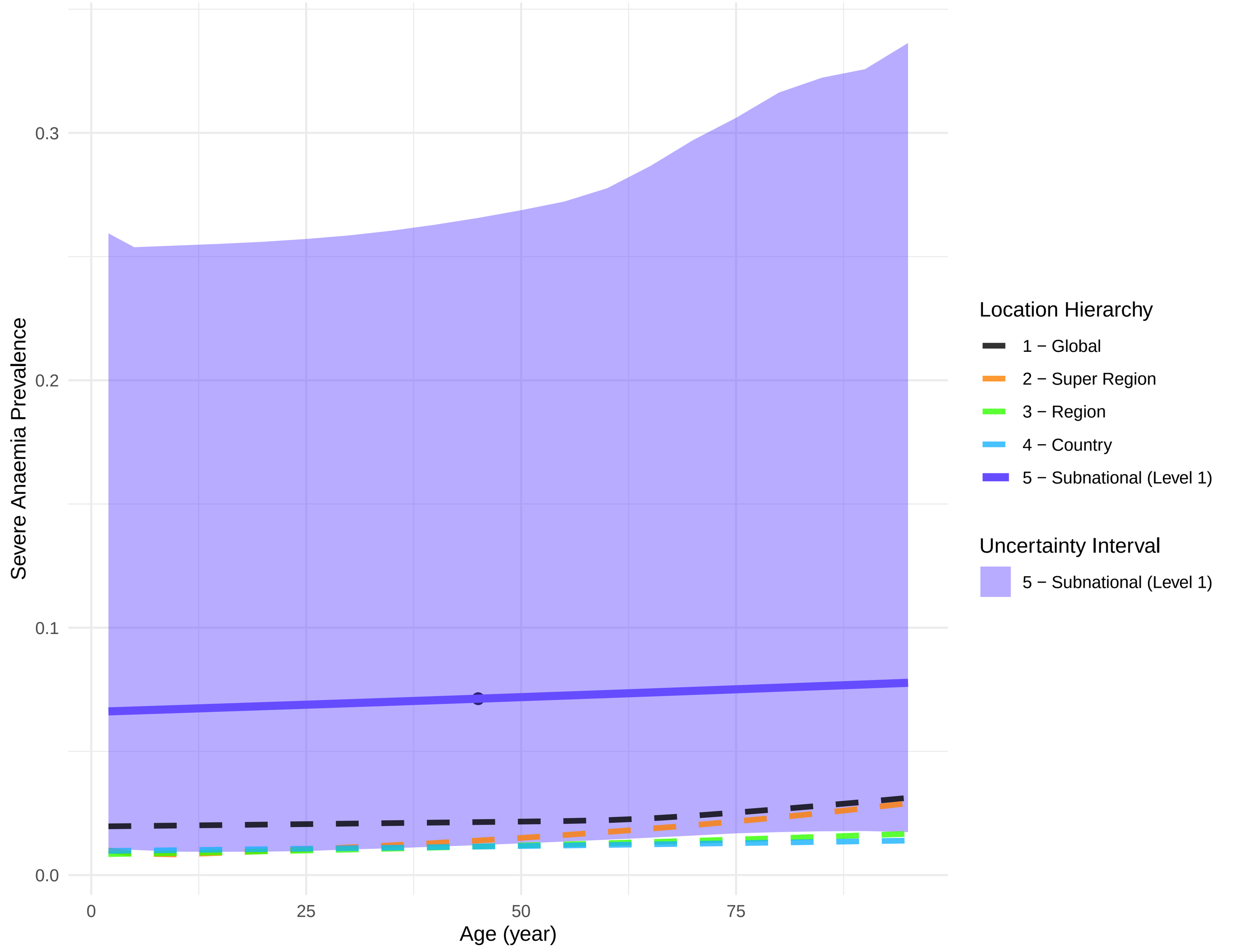

MR−BRT Age/Sex Fits − Non−pregnant Females 2+ Years Old − Severe Anaemia Prevalence − Rio de Janeiro
WHO−2024 Adjustment Method

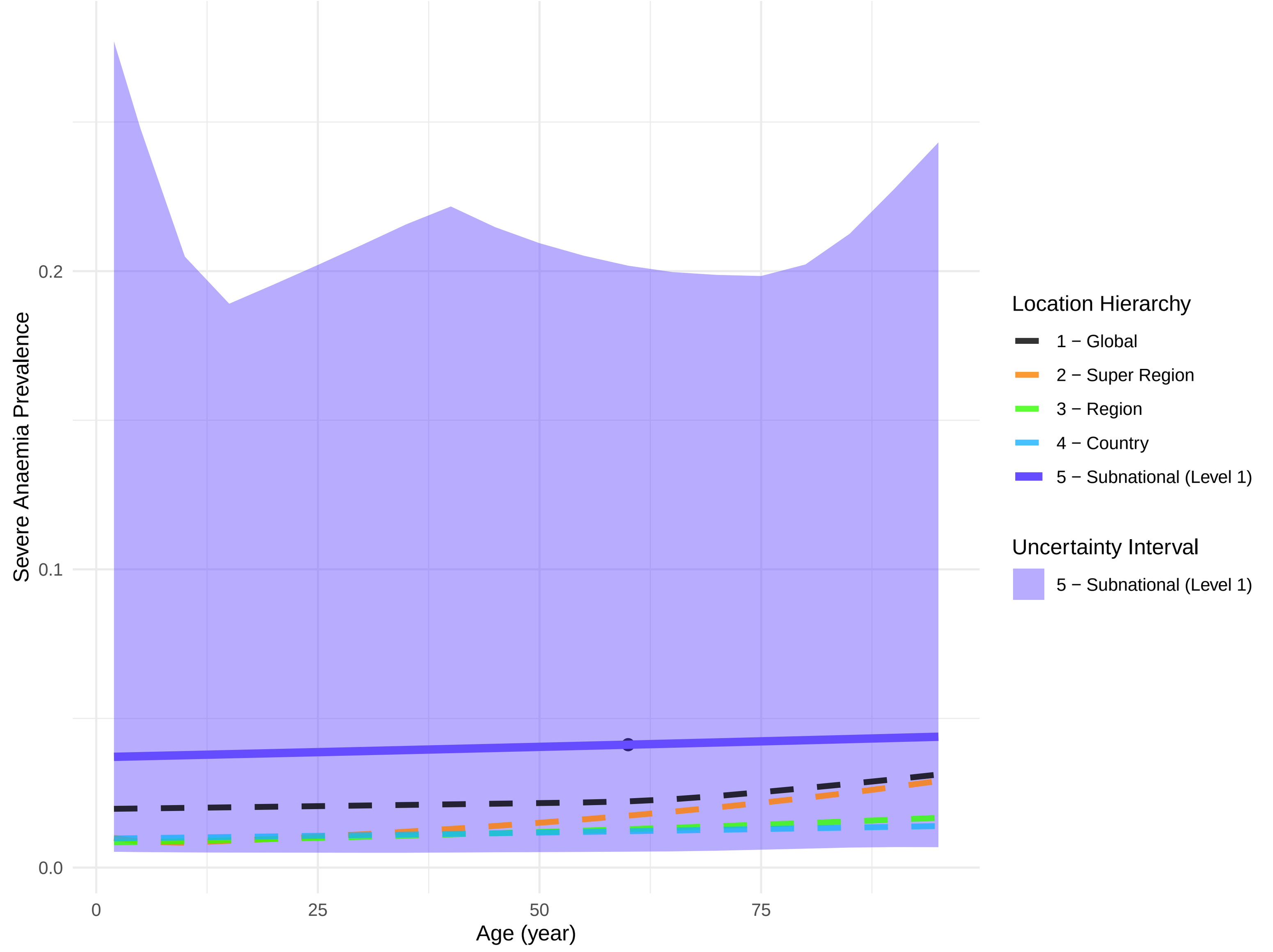

MR−BRT Age/Sex Fits − Non−pregnant Females 2+ Years Old − Severe Anaemia Prevalence − Tocantins
WHO−2024 Adjustment Method

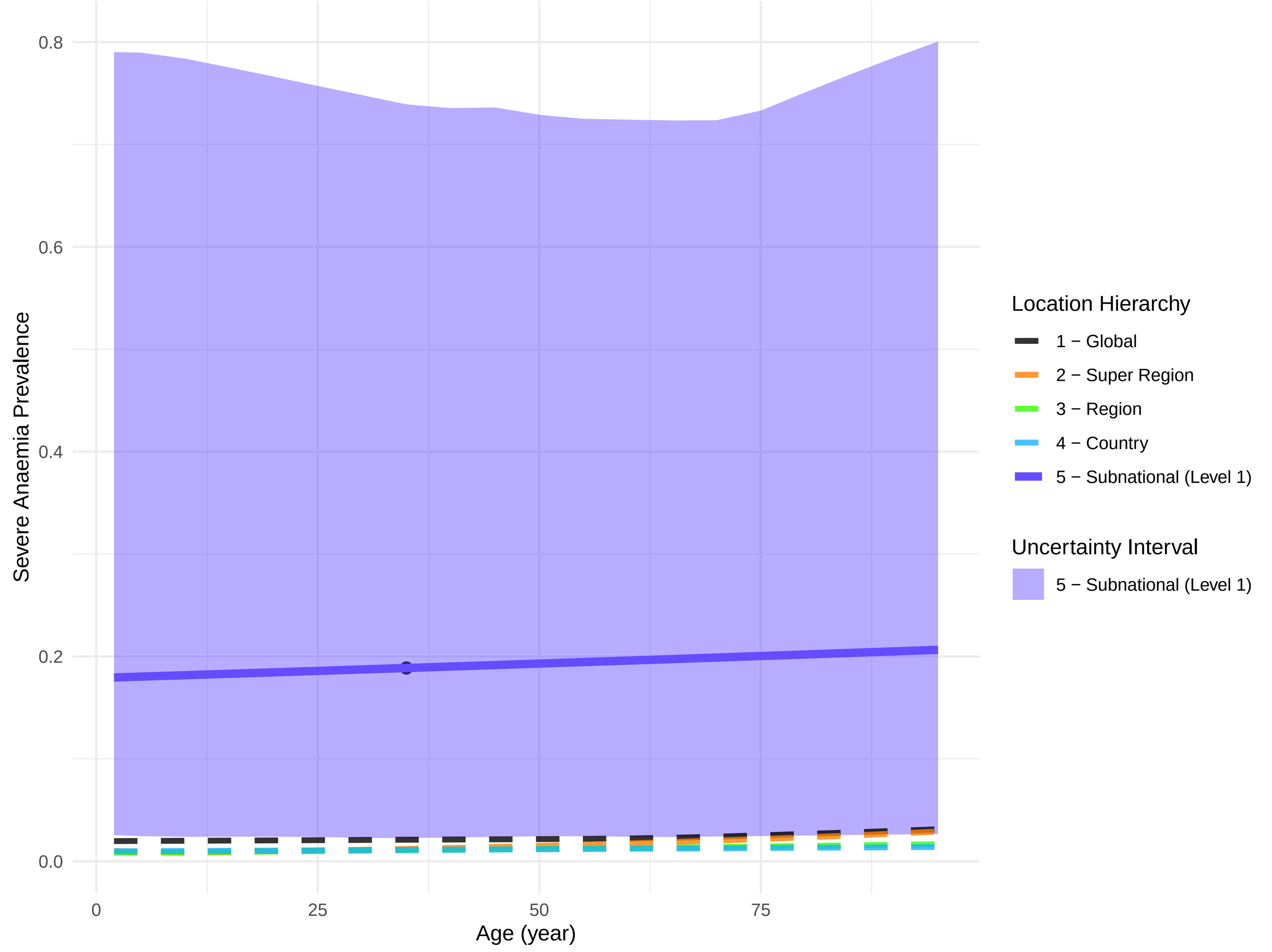

MR−BRT Age/Sex Fits − Non−pregnant Females 2+ Years Old − Severe Anaemia Prevalence − North Africa and Middle East
WHO−2024 Adjustment Method

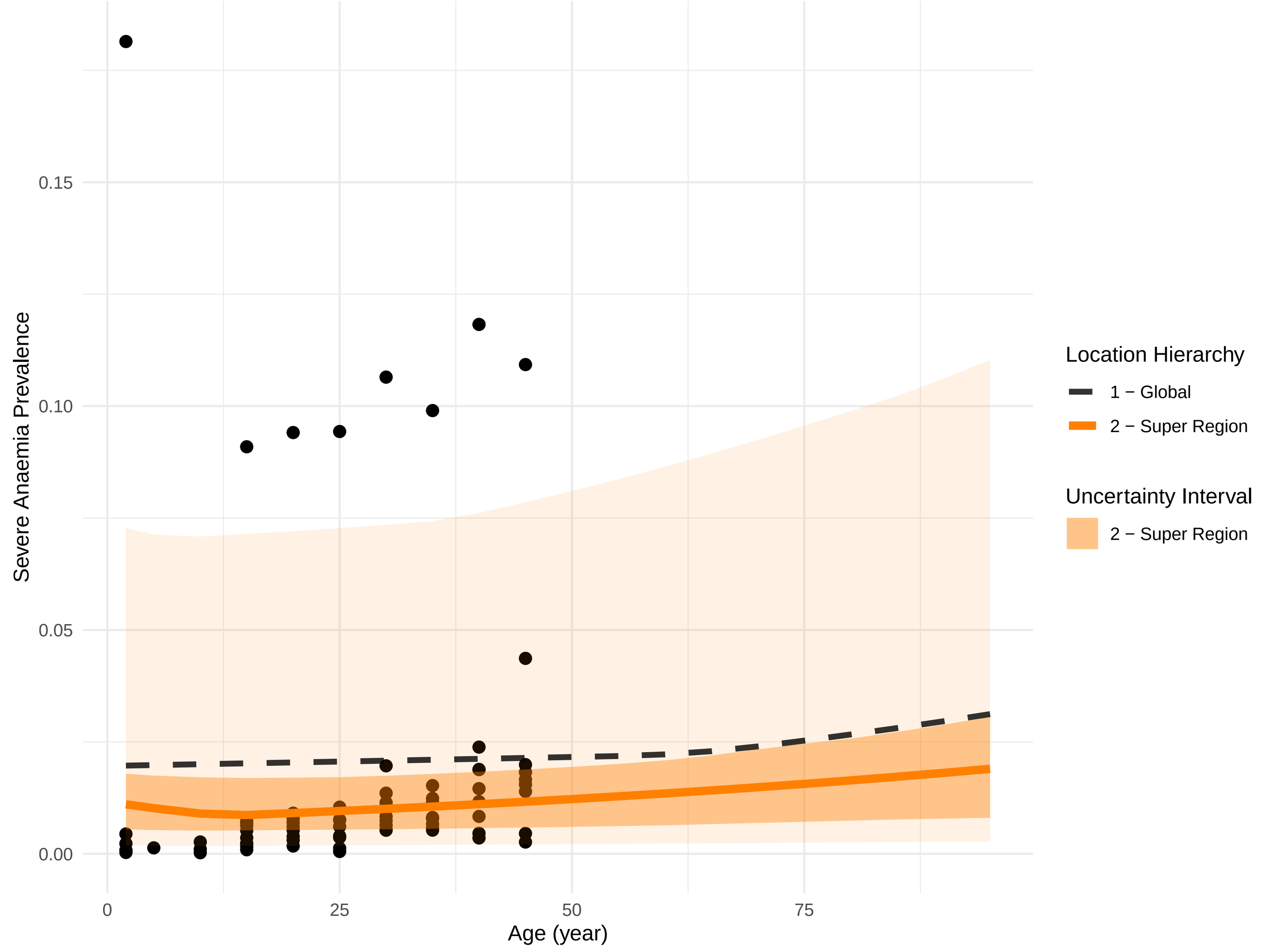

MR−BRT Age/Sex Fits − Non−pregnant Females 2+ Years Old − Severe Anaemia Prevalence − North Africa and Middle East
WHO−2024 Adjustment Method

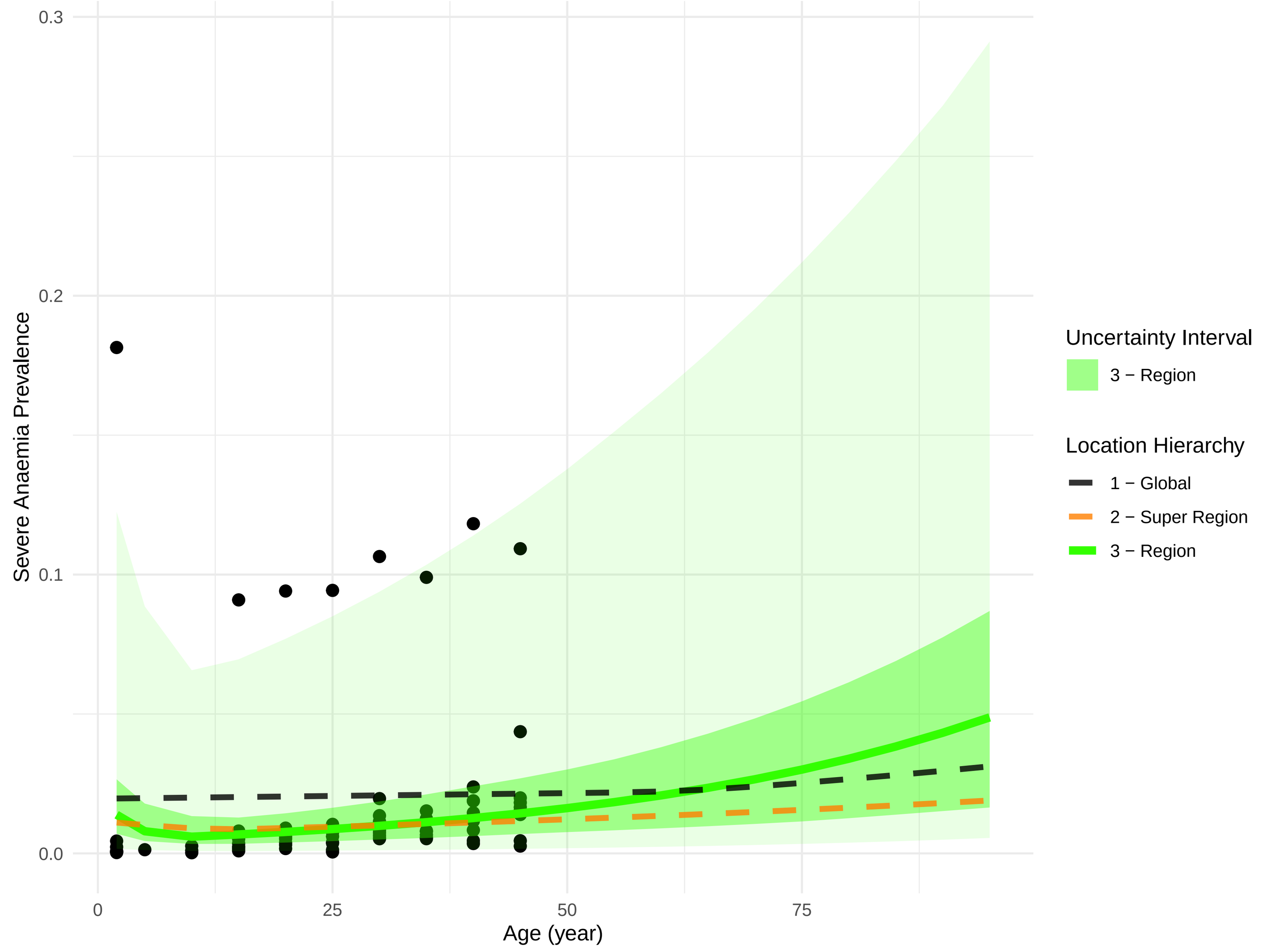

MR−BRT Age/Sex Fits − Non−pregnant Females 2+ Years Old − Severe Anaemia Prevalence − Egypt
WHO−2024 Adjustment Method

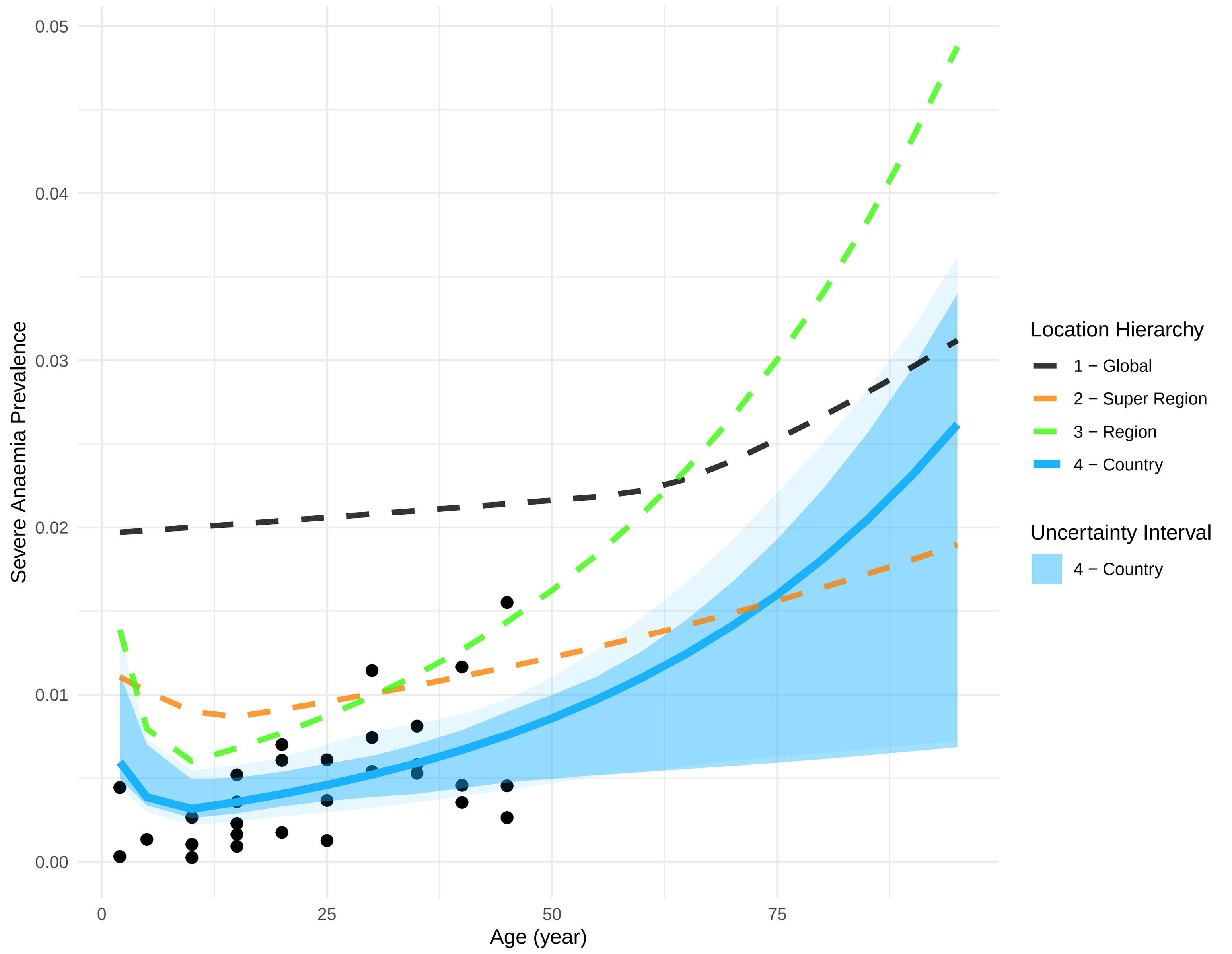

MR−BRT Age/Sex Fits − Non−pregnant Females 2+ Years Old − Severe Anaemia Prevalence − Jordan
WHO−2024 Adjustment Method

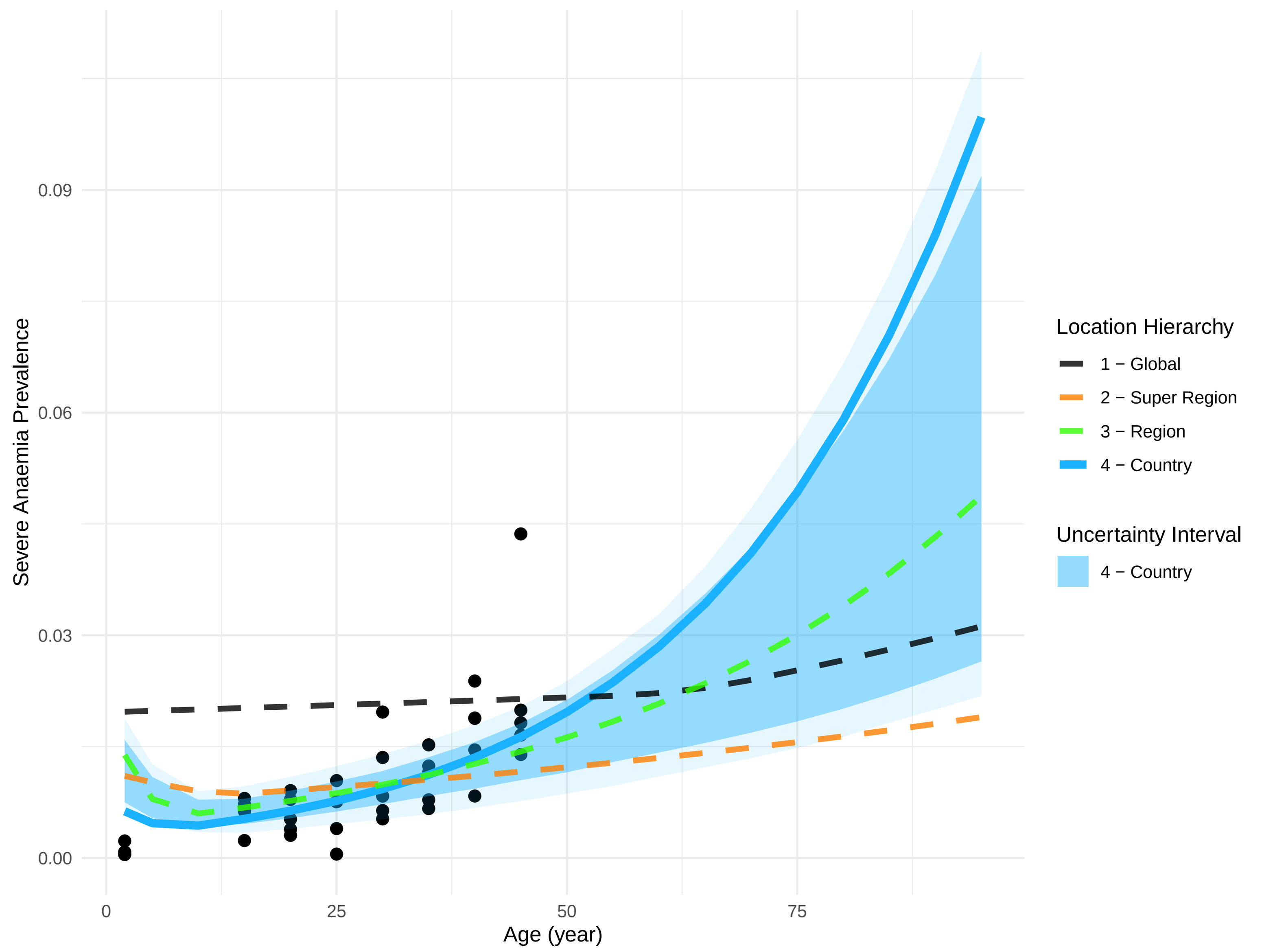

MR−BRT Age/Sex Fits − Non−pregnant Females 2+ Years Old − Severe Anaemia Prevalence − Yemen
WHO−2024 Adjustment Method

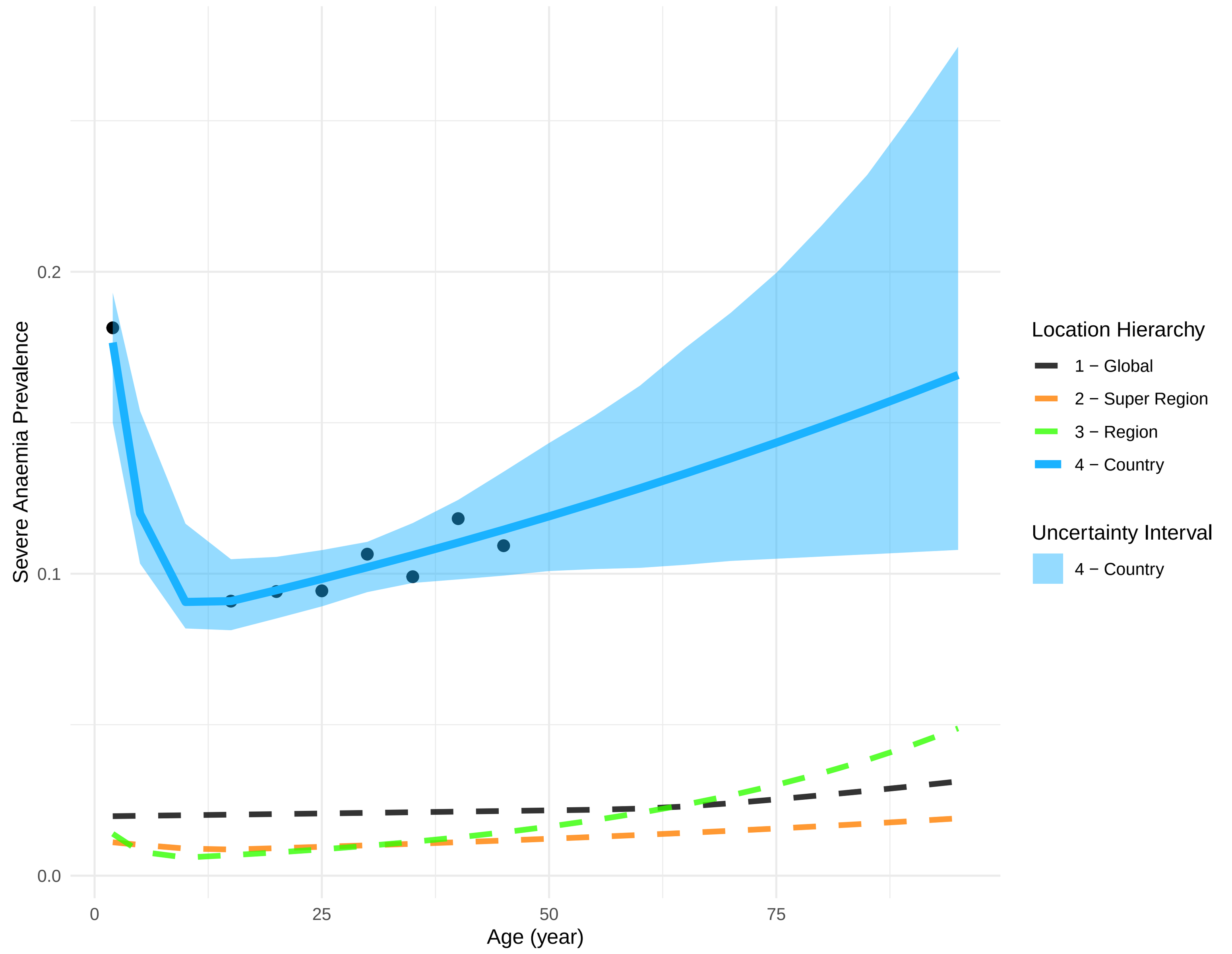

MR−BRT Age/Sex Fits − Non−pregnant Females 2+ Years Old − Severe Anaemia Prevalence − South Asia
WHO−2024 Adjustment Method

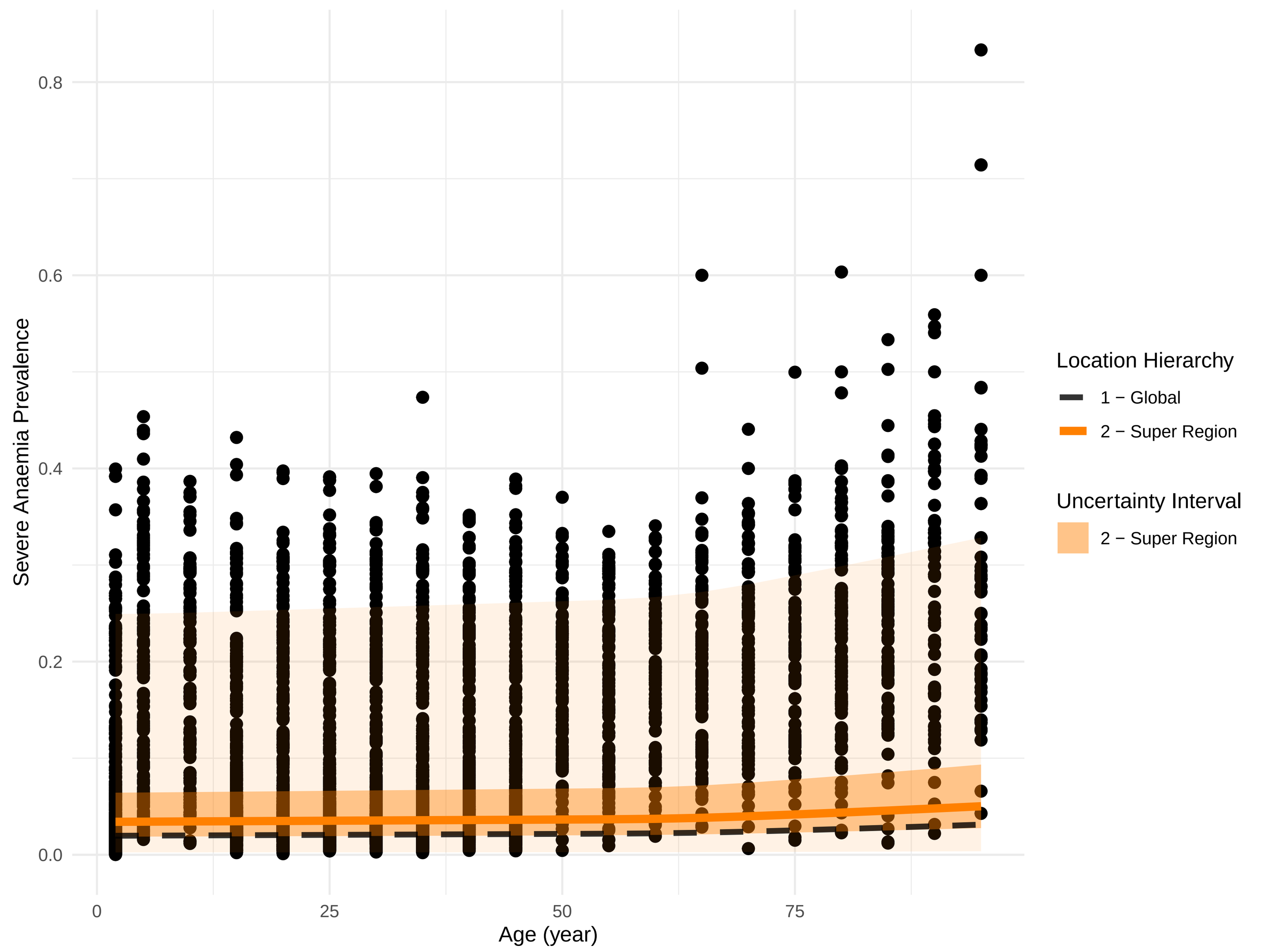

MR−BRT Age/Sex Fits − Non−pregnant Females 2+ Years Old − Severe Anaemia Prevalence − South Asia
WHO−2024 Adjustment Method

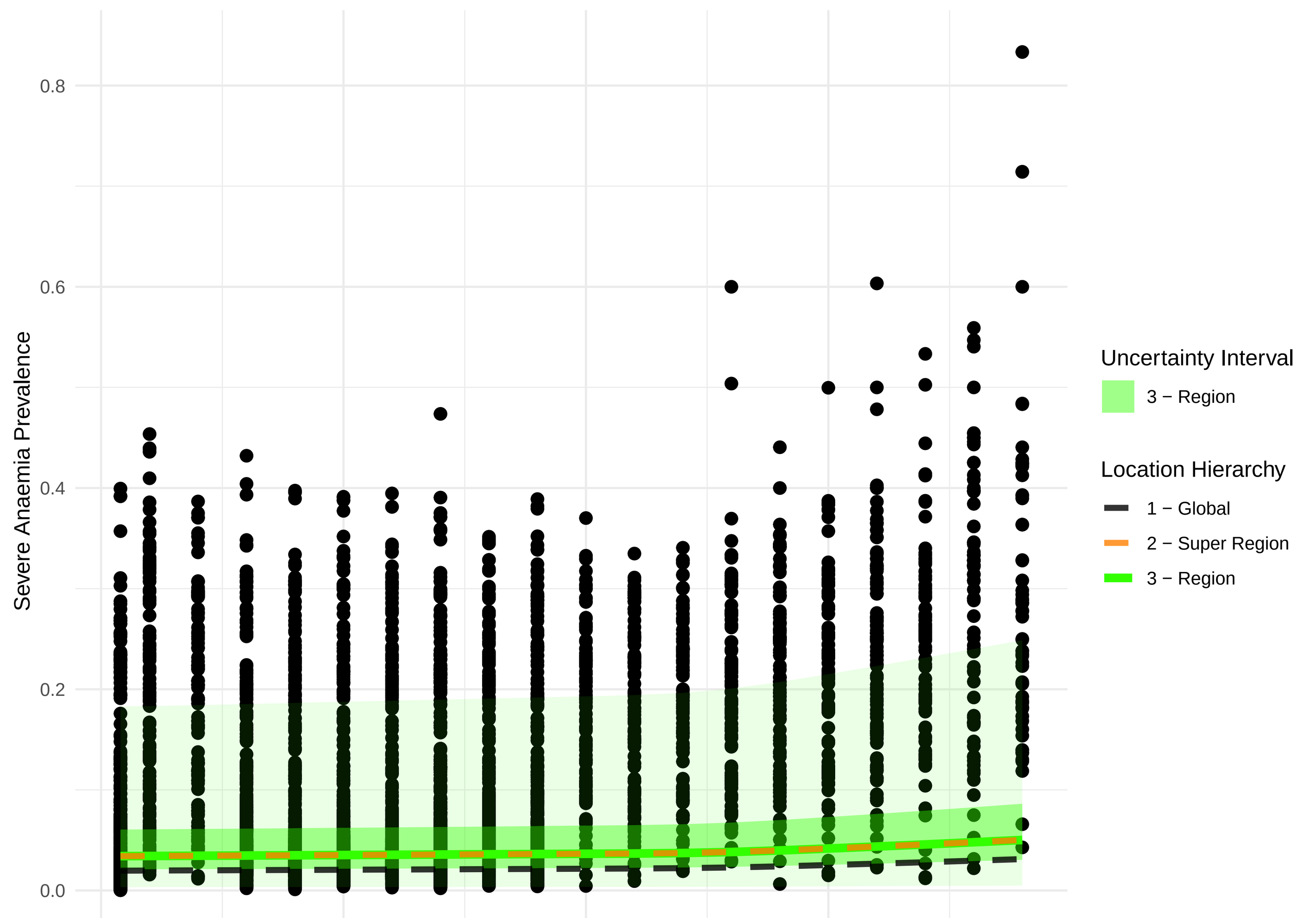

MR−BRT Age/Sex Fits − Non−pregnant Females 2+ Years Old − Severe Anaemia Prevalence − Bangladesh
WHO−2024 Adjustment Method

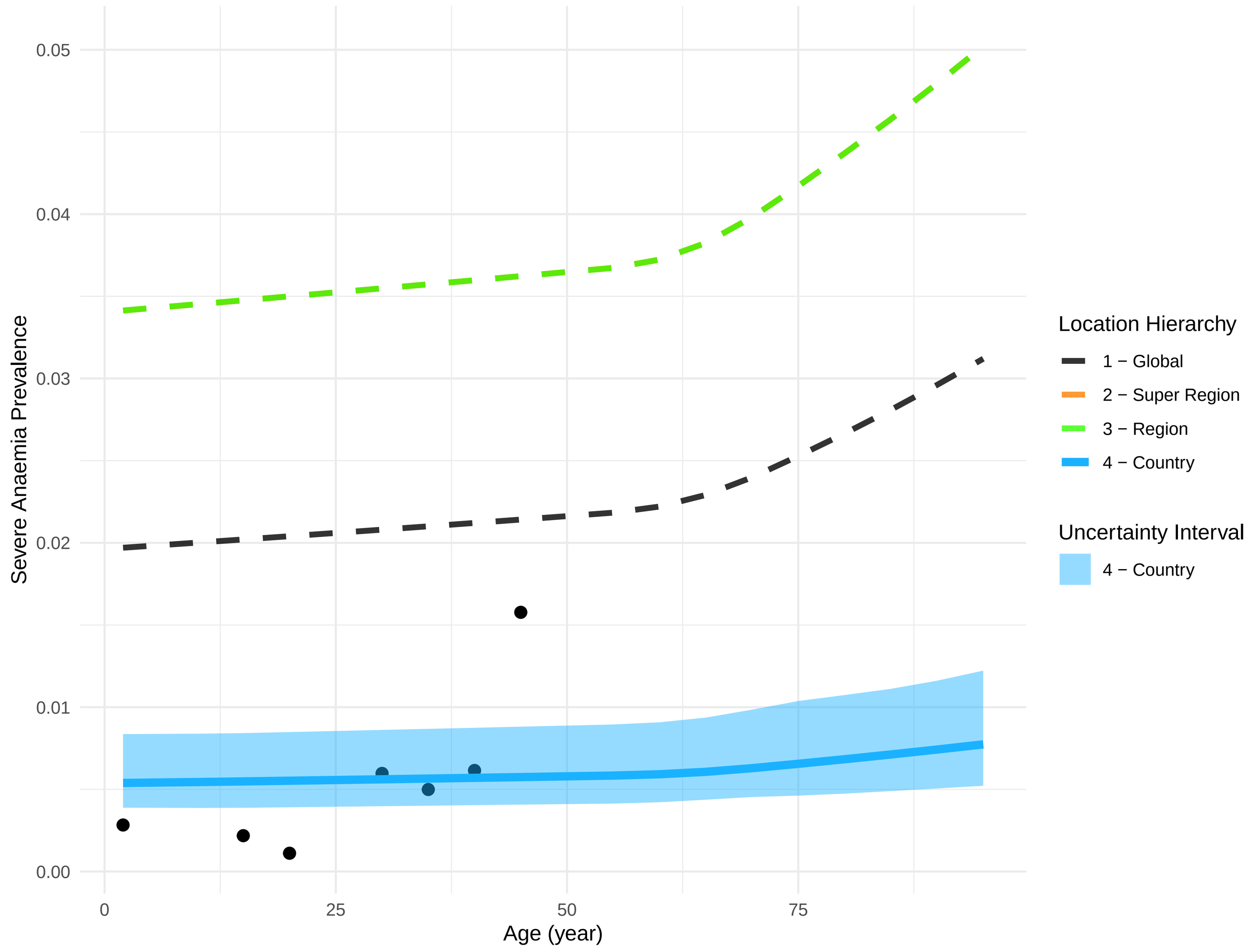

MR−BRT Age/Sex Fits − Non−pregnant Females 2+ Years Old − Severe Anaemia Prevalence − India
WHO−2024 Adjustment Method

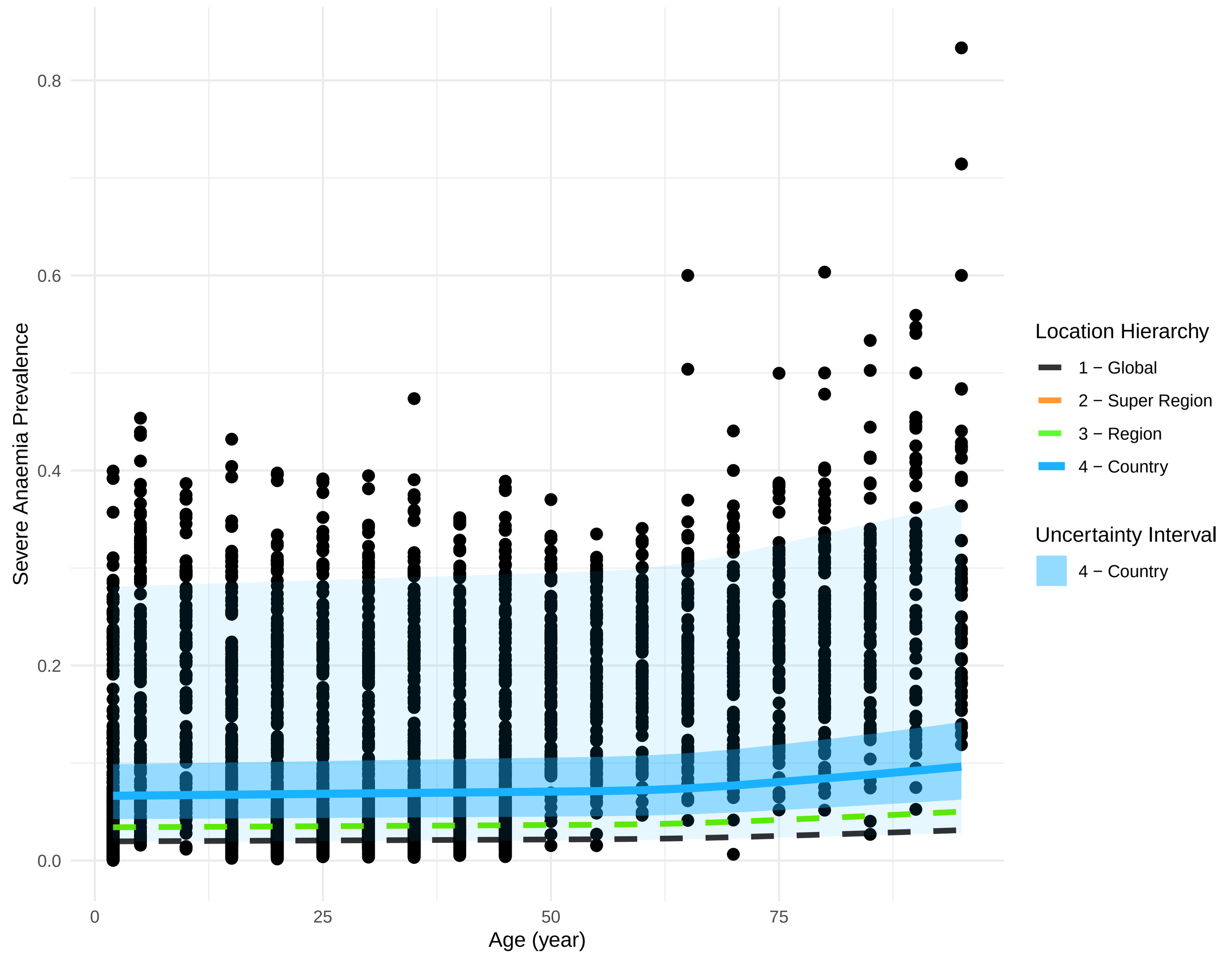

MR−BRT Age/Sex Fits − Non−pregnant Females 2+ Years Old − Severe Anaemia Prevalence − Andhra Pradesh
WHO−2024 Adjustment Method

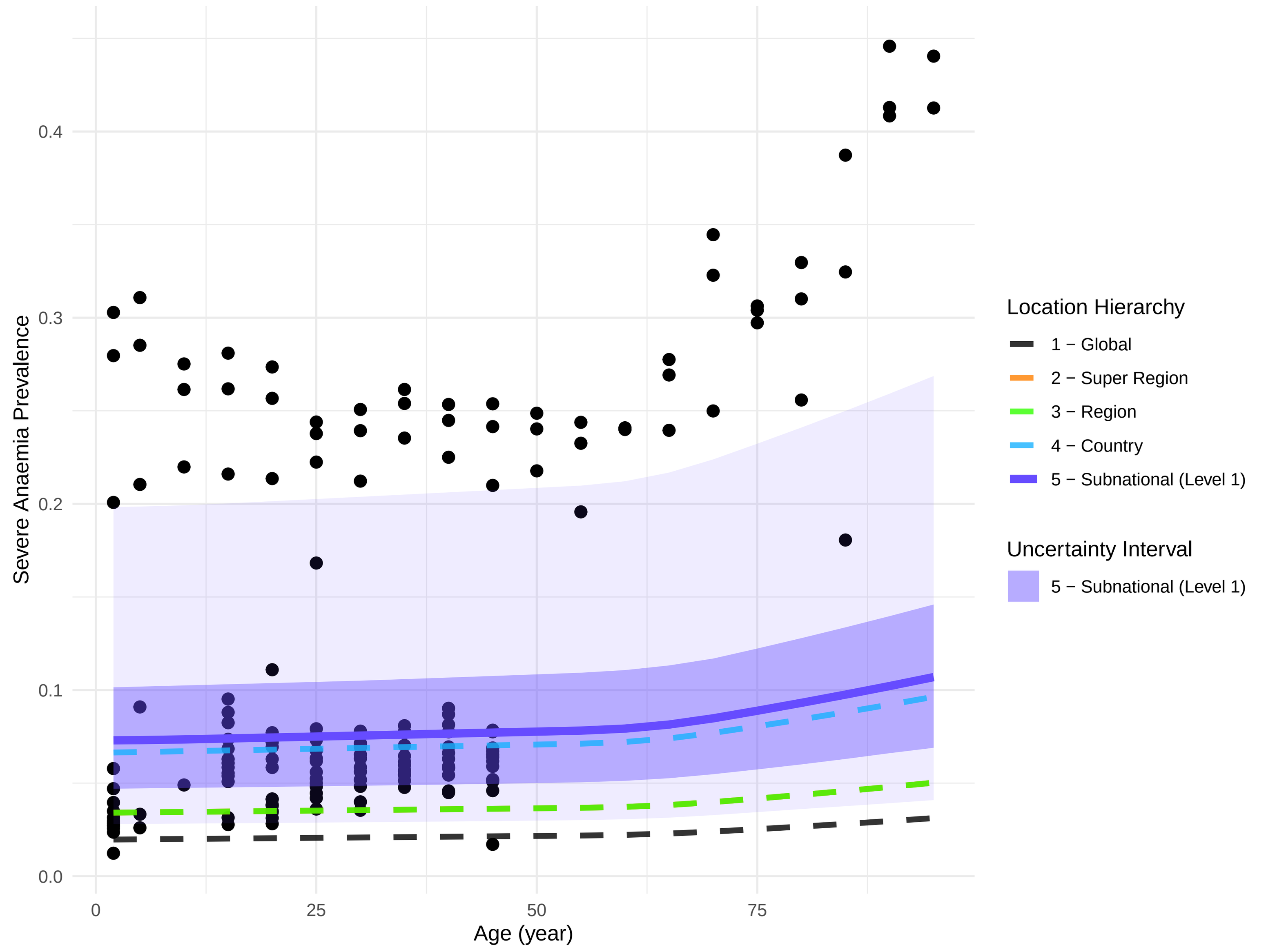

MR−BRT Age/Sex Fits − Non−pregnant Females 2+ Years Old − Severe Anaemia Prevalence − Andhra Pradesh, Rural
WHO−2024 Adjustment Method

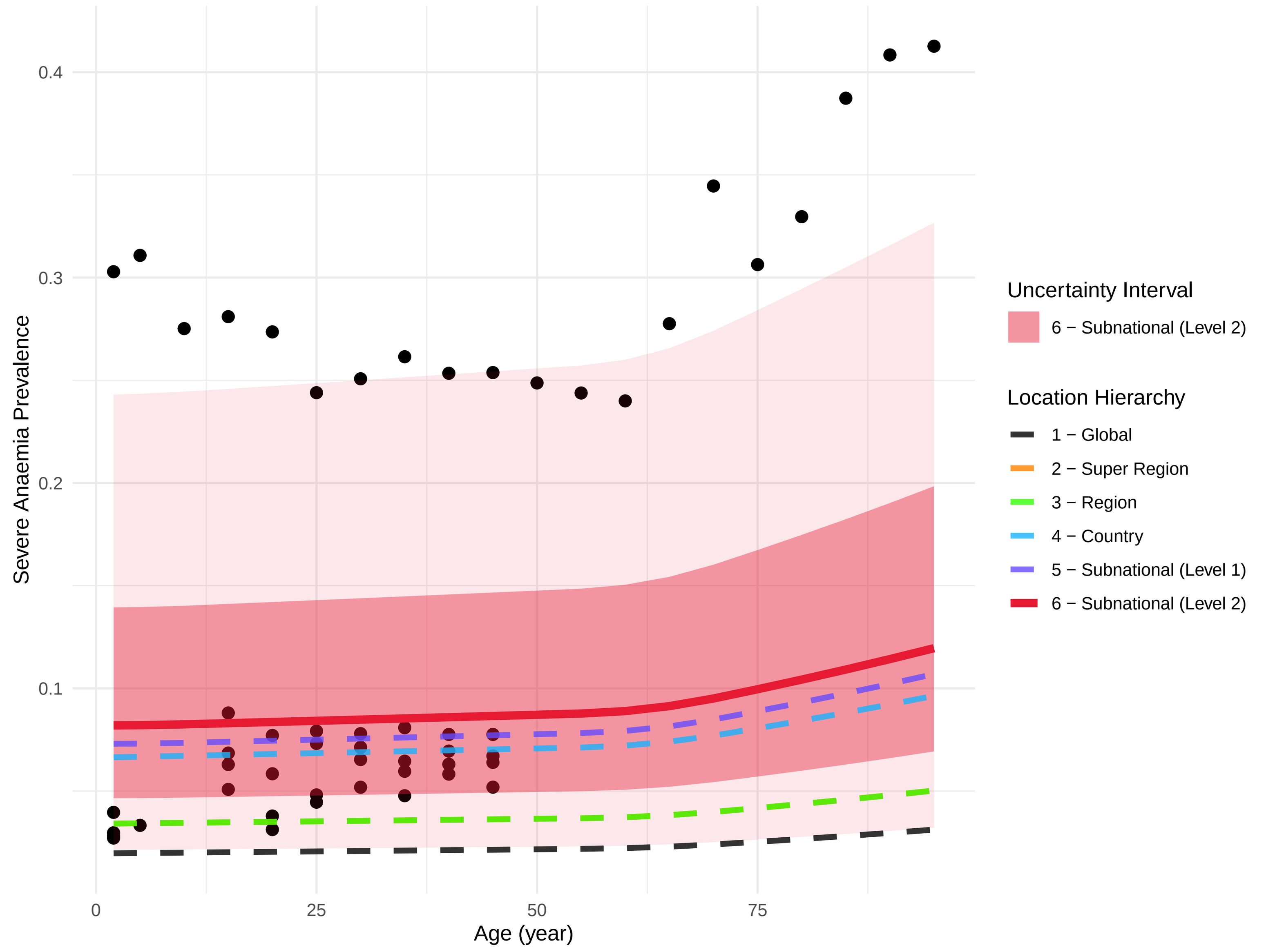

MR−BRT Age/Sex Fits − Non−pregnant Females 2+ Years Old − Severe Anaemia Prevalence − Andhra Pradesh, Urban
WHO−2024 Adjustment Method

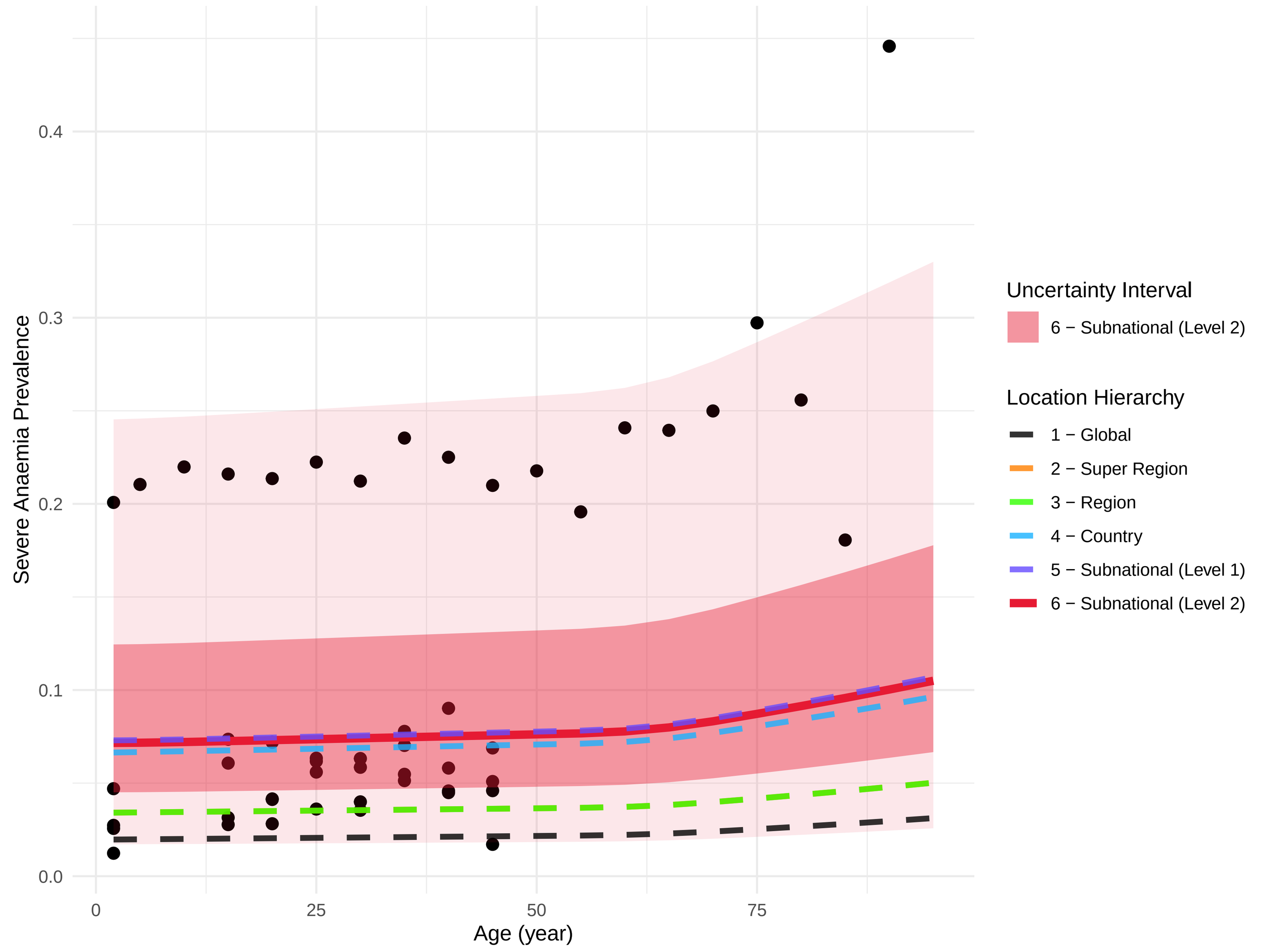

MR−BRT Age/Sex Fits − Non−pregnant Females 2+ Years Old − Severe Anaemia Prevalence − Arunachal Pradesh
WHO−2024 Adjustment Method

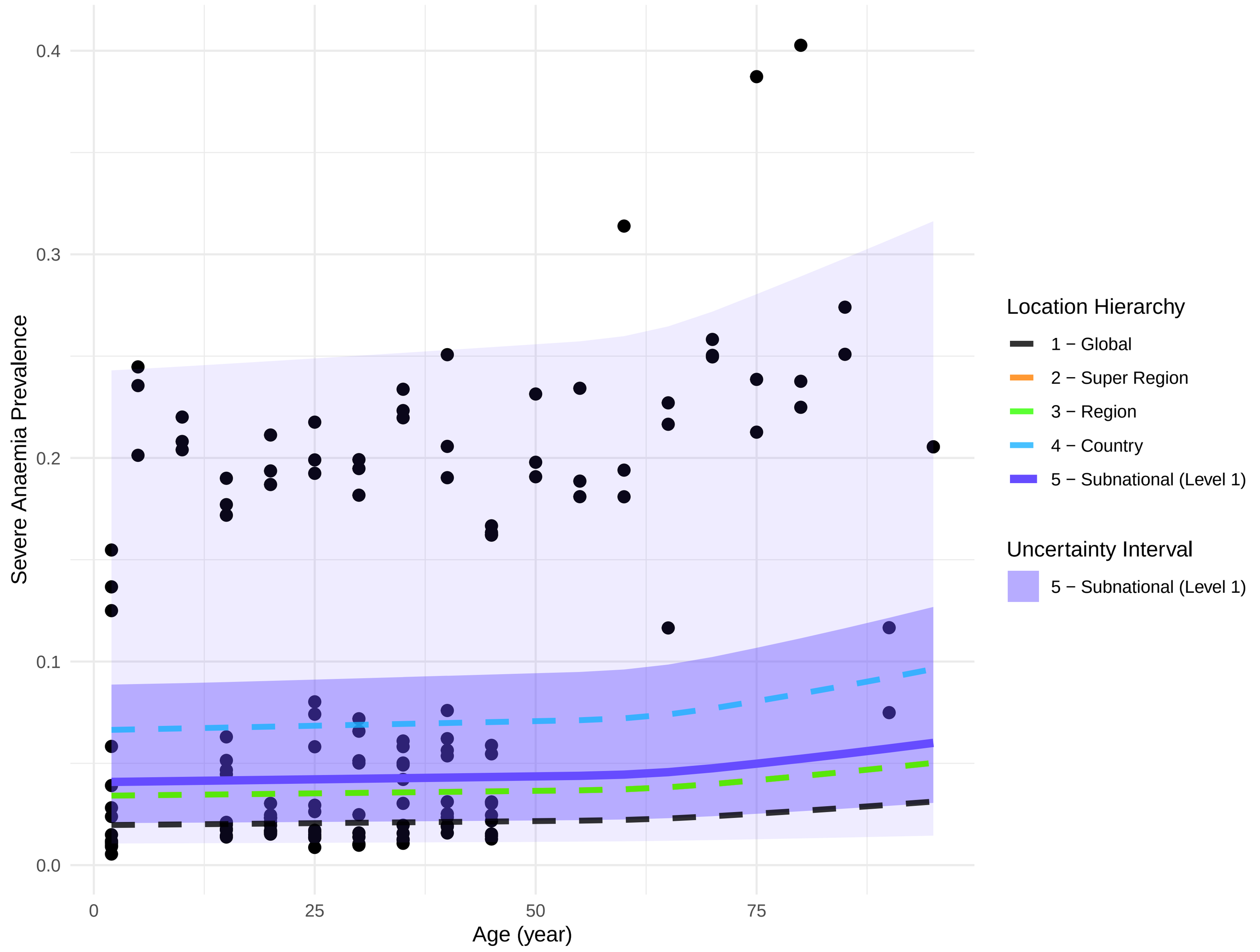

MR−BRT Age/Sex Fits − Non−pregnant Females 2+ Years Old − Severe Anaemia Prevalence − Arunachal Pradesh, Rural
WHO−2024 Adjustment Method

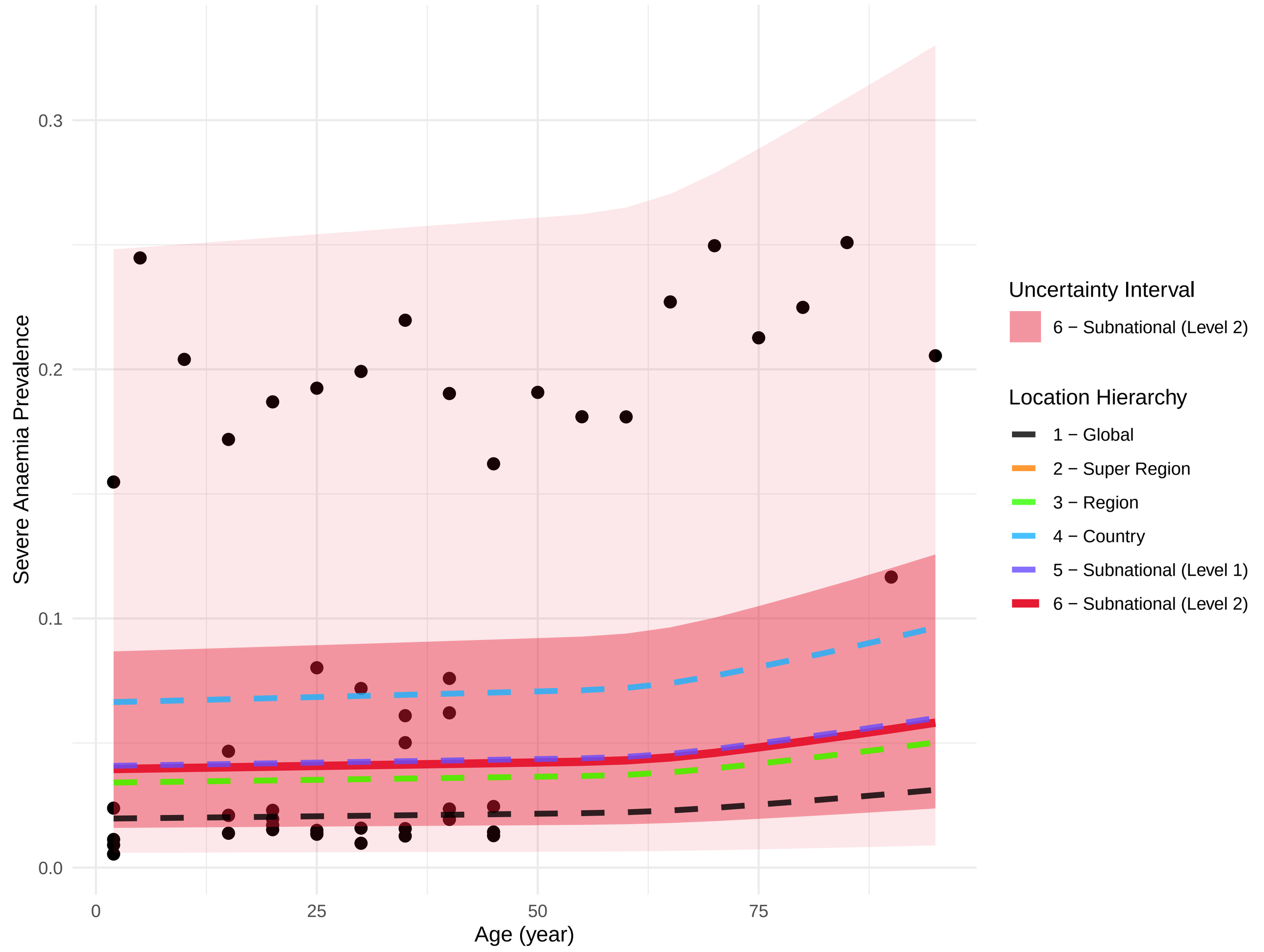

MR−BRT Age/Sex Fits − Non−pregnant Females 2+ Years Old − Severe Anaemia Prevalence − Arunachal Pradesh, Urban
WHO−2024 Adjustment Method

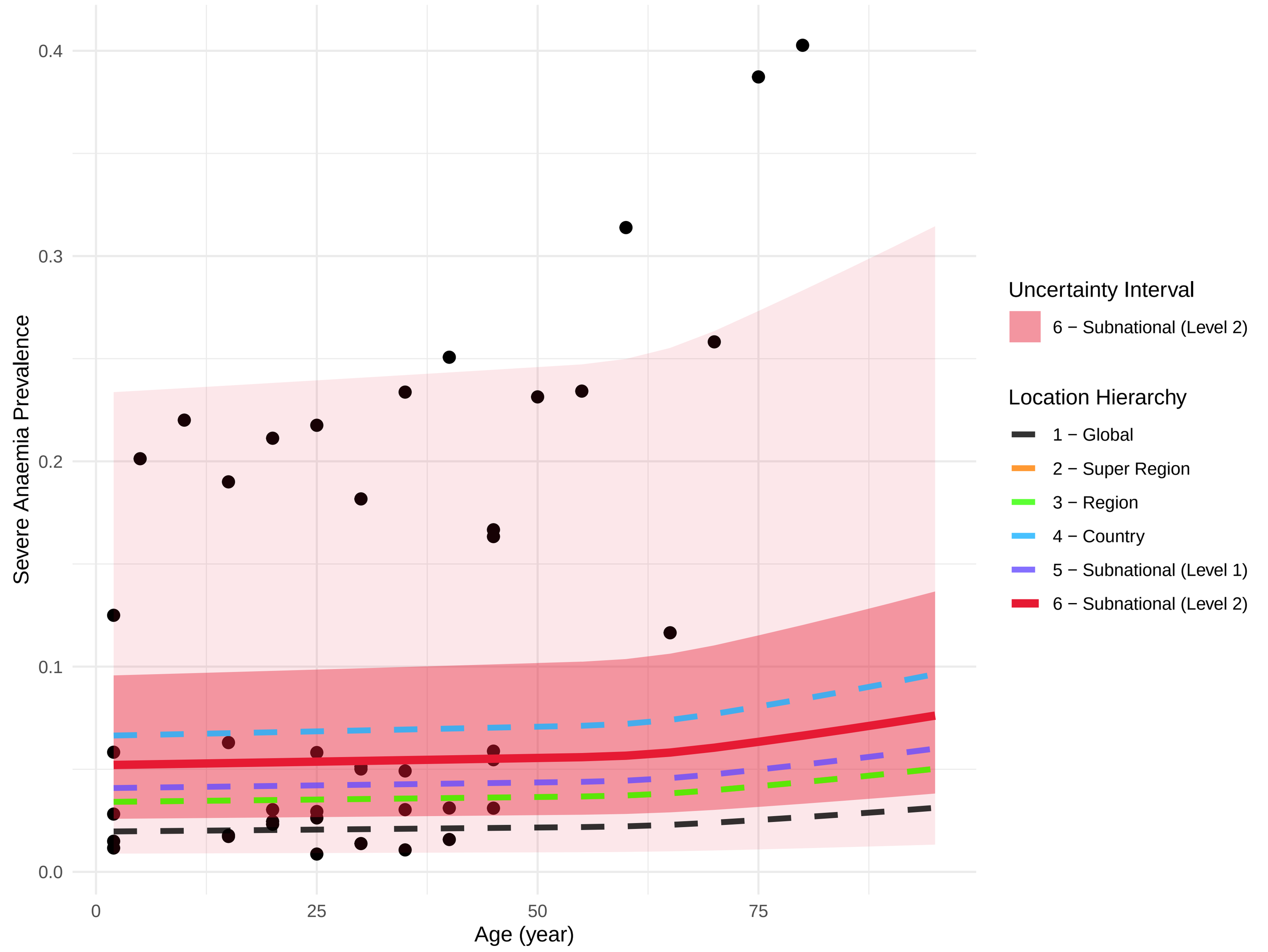

MR−BRT Age/Sex Fits − Non−pregnant Females 2+ Years Old − Severe Anaemia Prevalence − Assam
WHO−2024 Adjustment Method

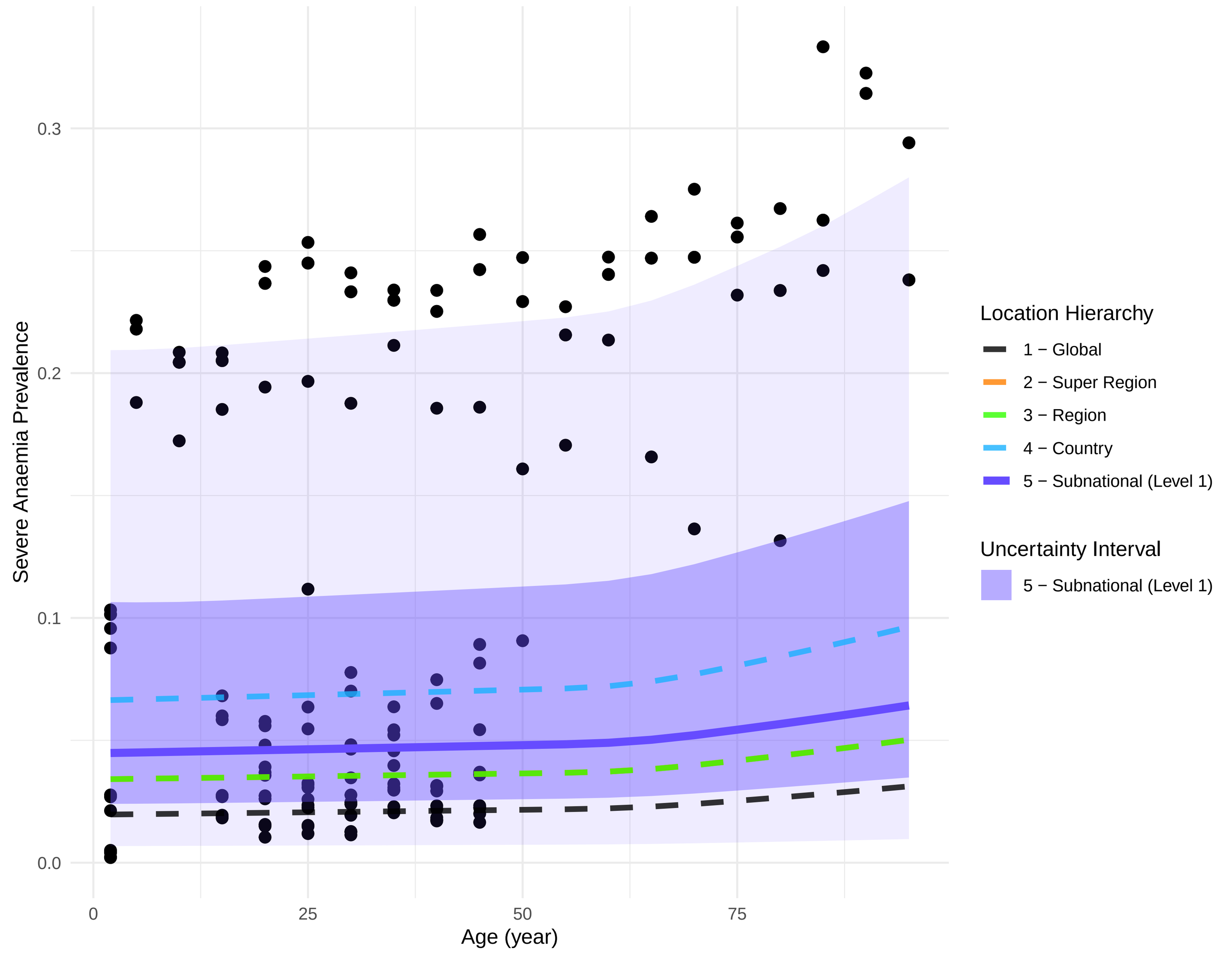

MR−BRT Age/Sex Fits − Non−pregnant Females 2+ Years Old − Severe Anaemia Prevalence − Assam, Rural
WHO−2024 Adjustment Method

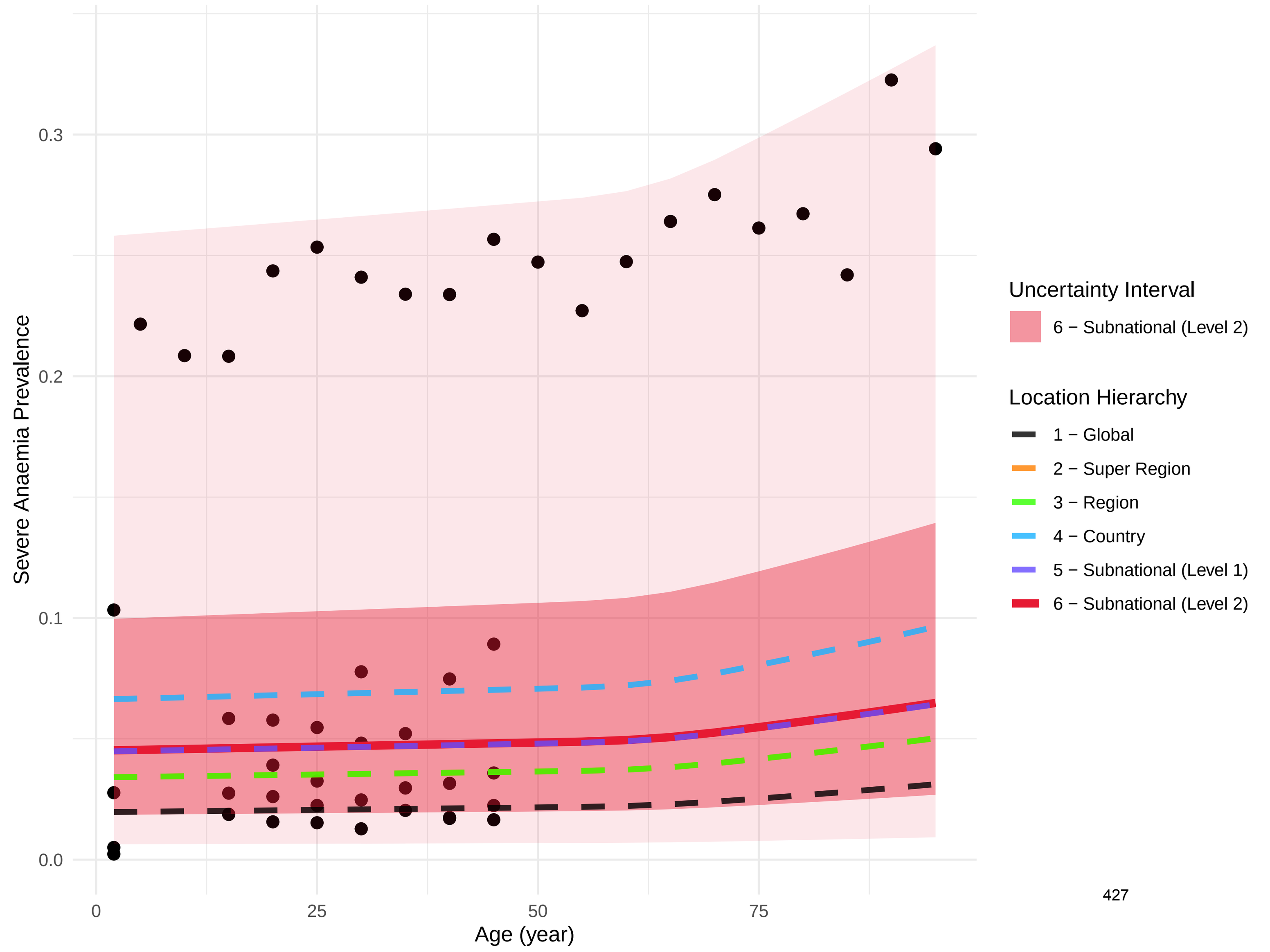

MR−BRT Age/Sex Fits − Non−pregnant Females 2+ Years Old − Severe Anaemia Prevalence − Assam, Urban
WHO−2024 Adjustment Method

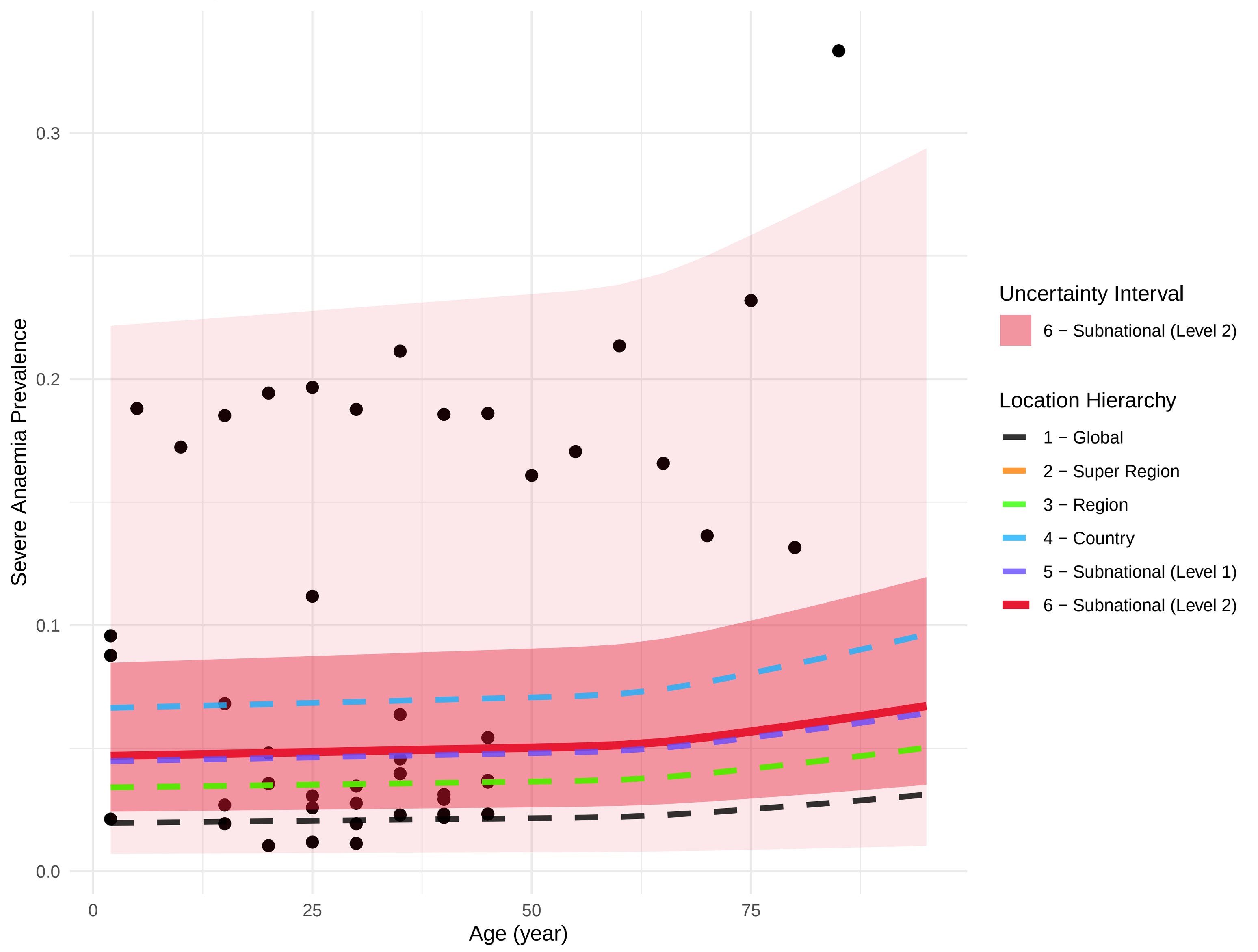

MR−BRT Age/Sex Fits − Non−pregnant Females 2+ Years Old − Severe Anaemia Prevalence − Bihar
WHO−2024 Adjustment Method

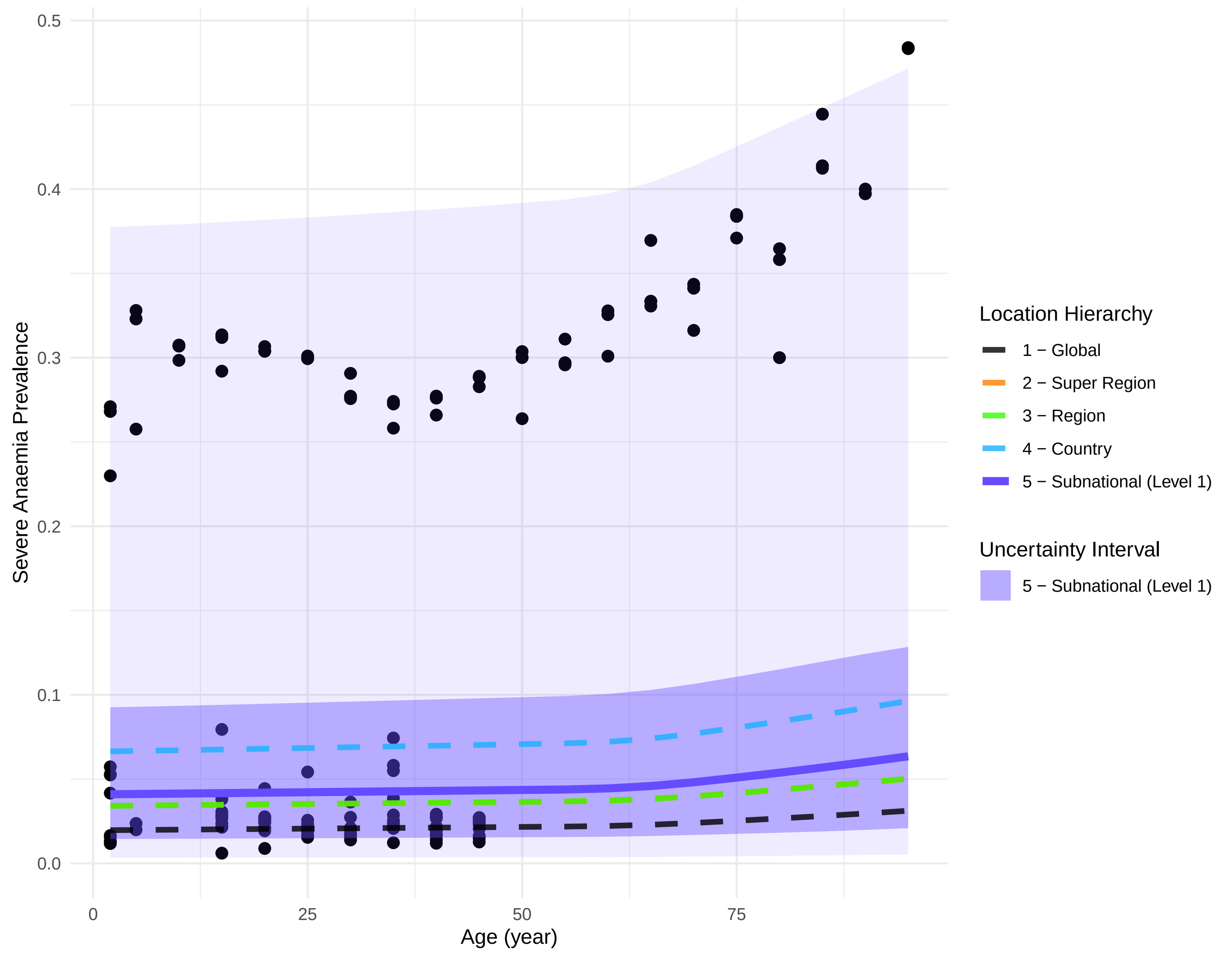

MR−BRT Age/Sex Fits − Non−pregnant Females 2+ Years Old − Severe Anaemia Prevalence − Bihar, Rural
WHO−2024 Adjustment Method

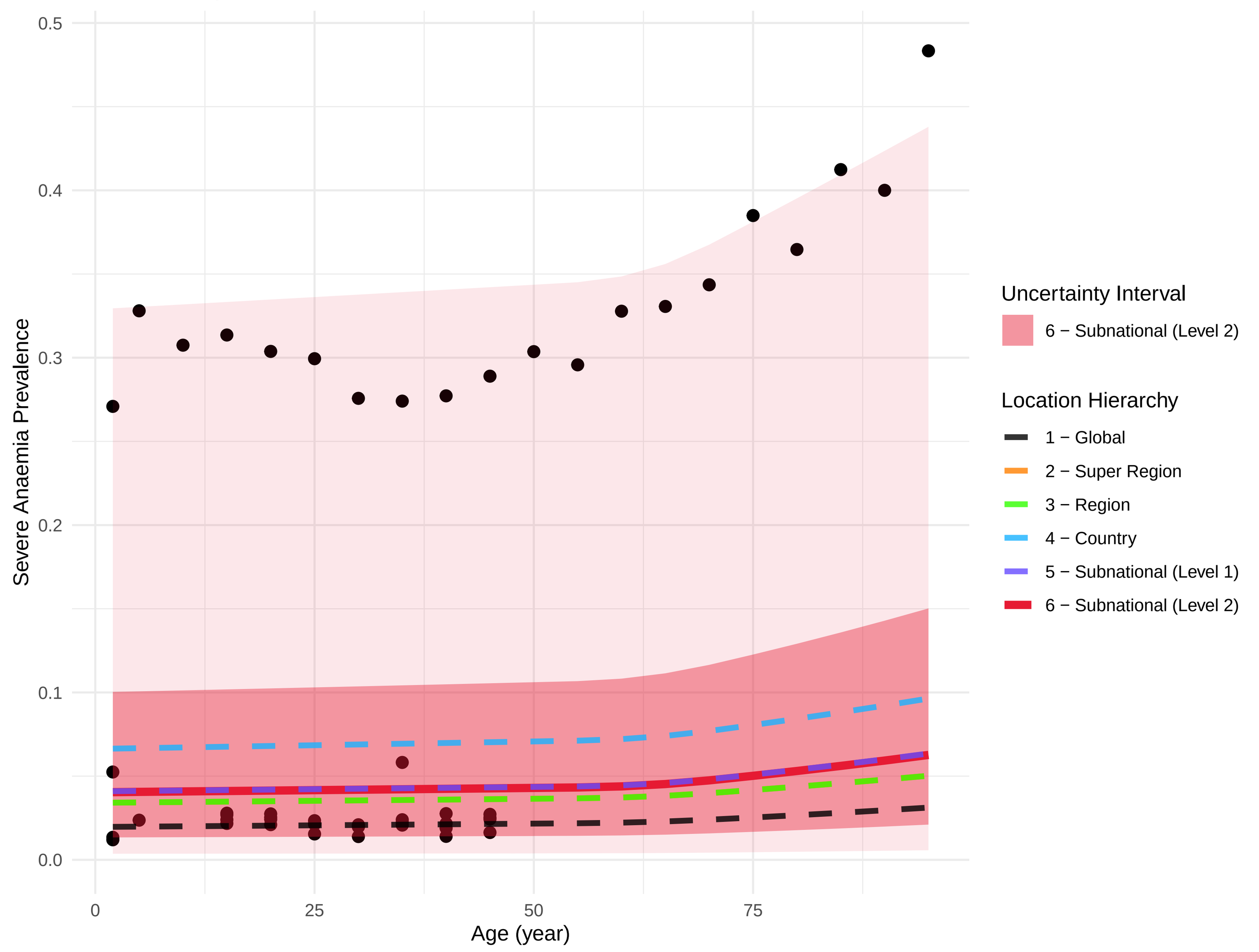

MR−BRT Age/Sex Fits − Non−pregnant Females 2+ Years Old − Severe Anaemia Prevalence − Bihar, Urban
WHO−2024 Adjustment Method

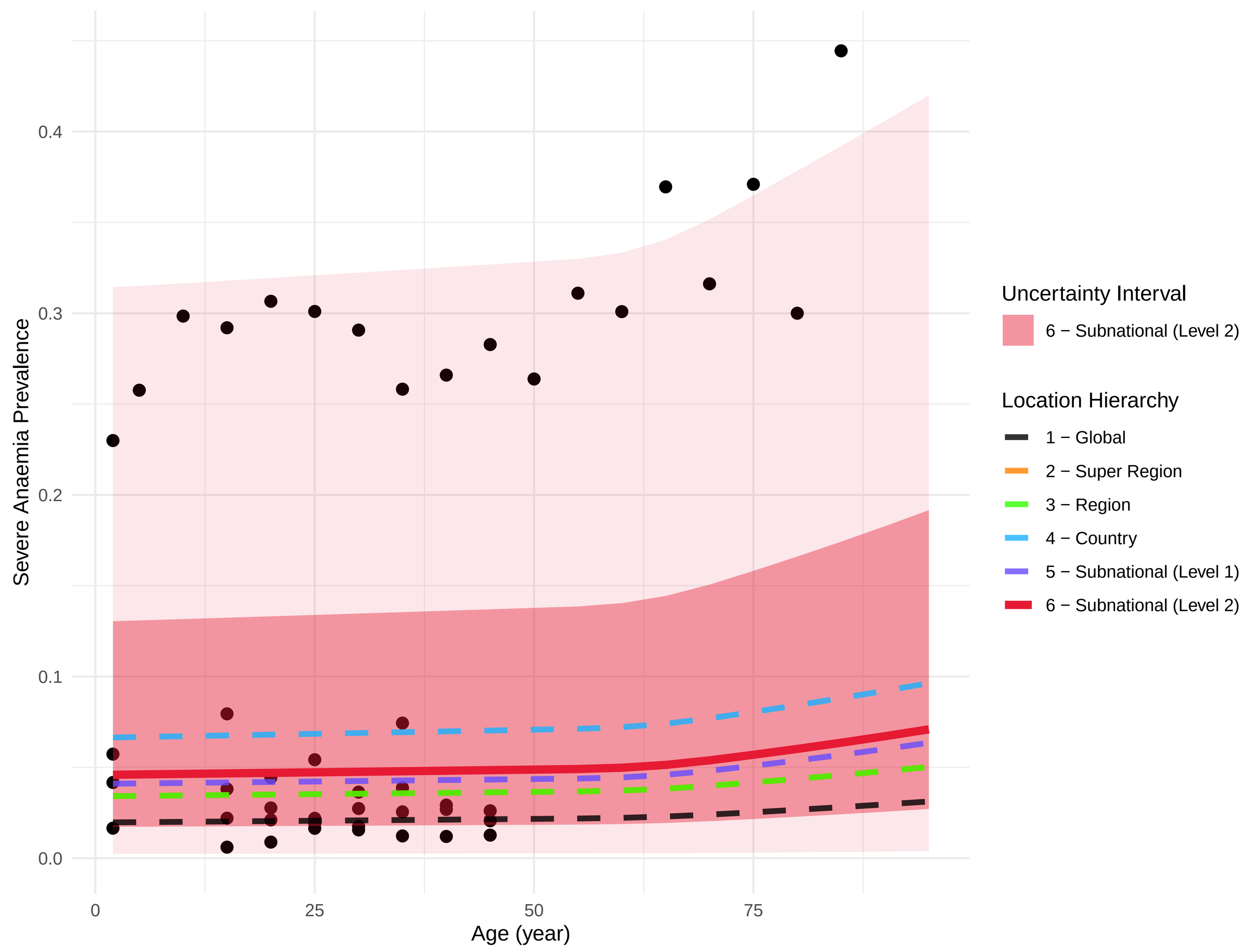

MR−BRT Age/Sex Fits − Non−pregnant Females 2+ Years Old − Severe Anaemia Prevalence − Chhattisgarh
WHO−2024 Adjustment Method

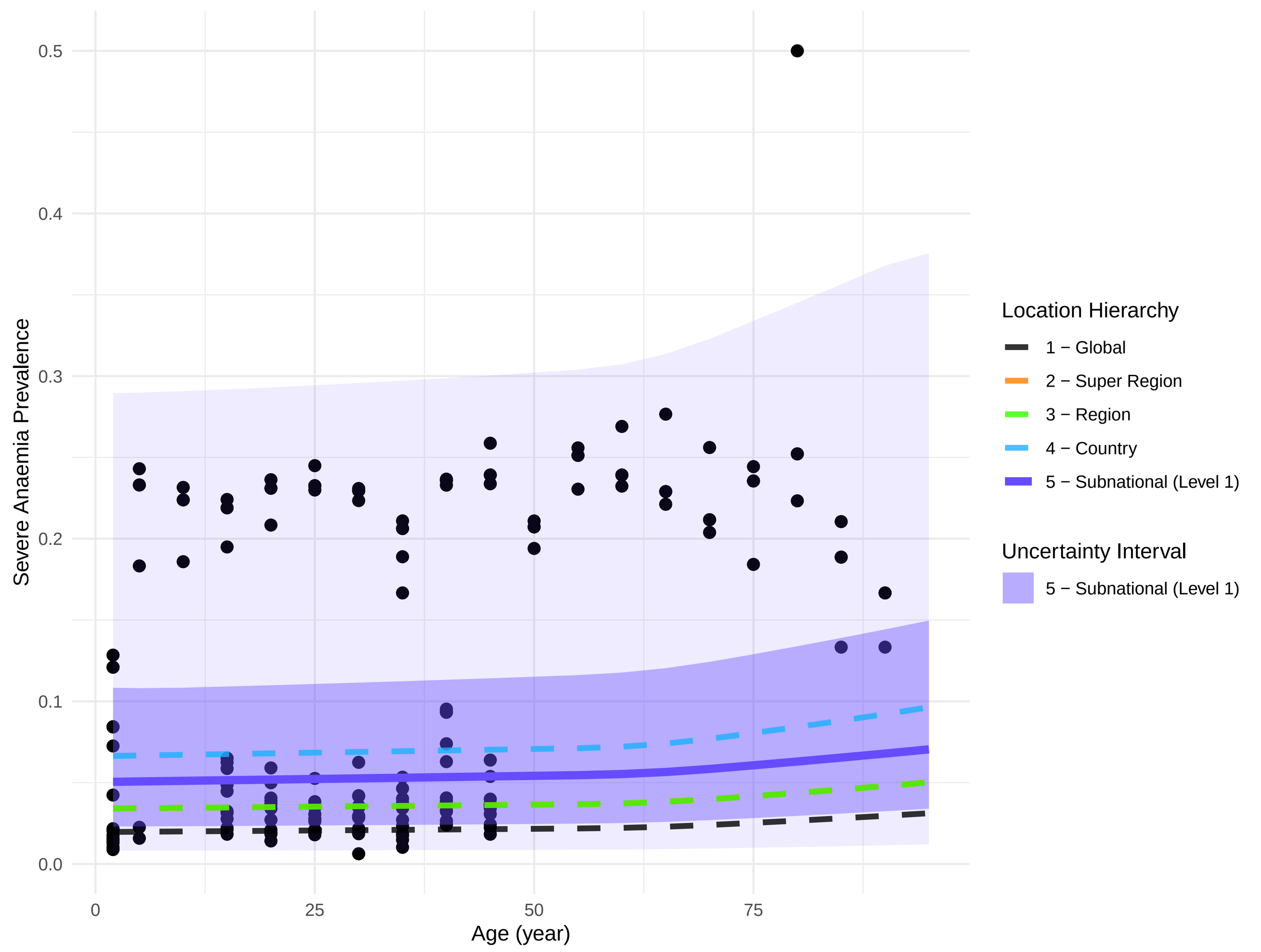

MR−BRT Age/Sex Fits − Non−pregnant Females 2+ Years Old − Severe Anaemia Prevalence − Chhattisgarh, Rural
WHO−2024 Adjustment Method

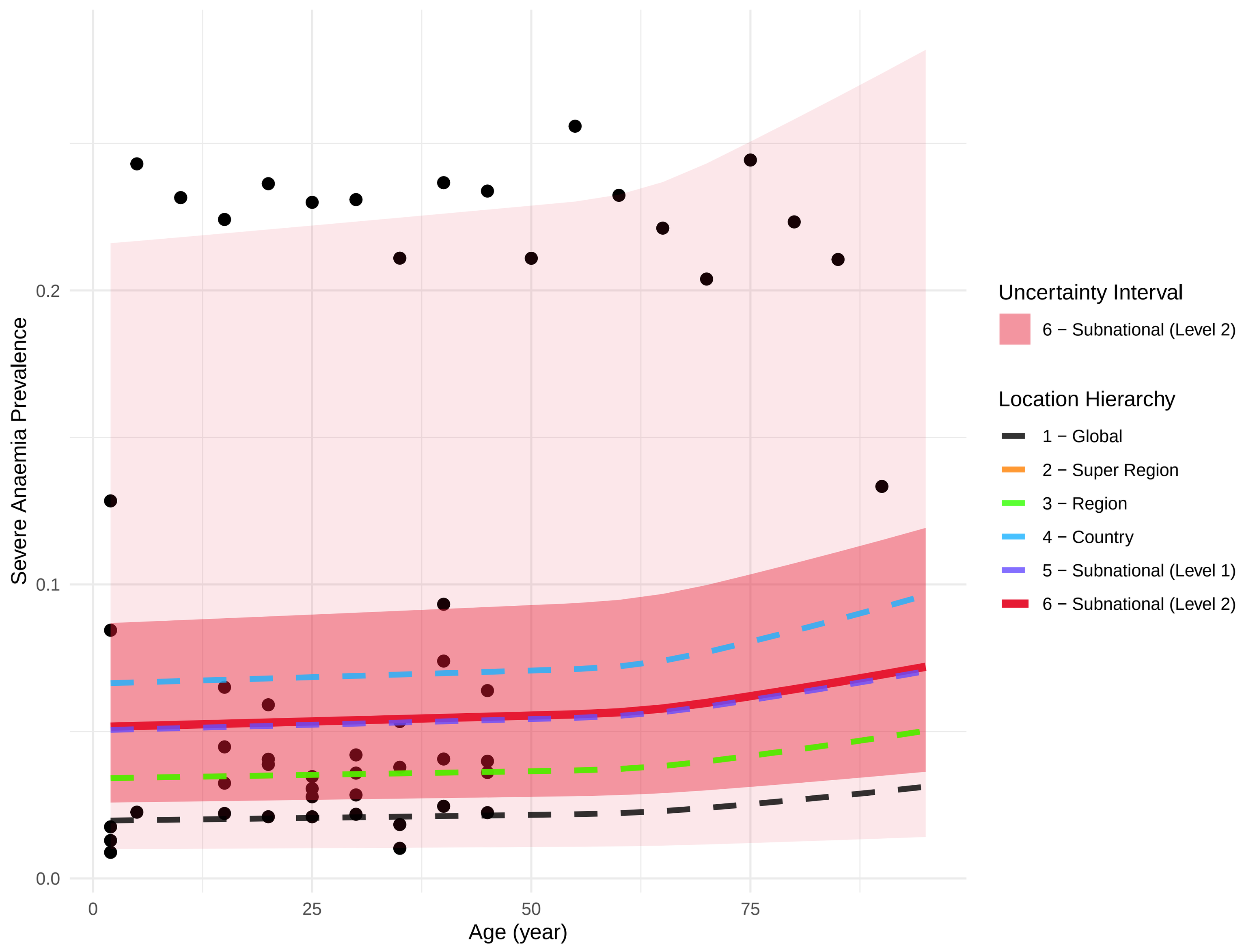

MR−BRT Age/Sex Fits − Non−pregnant Females 2+ Years Old − Severe Anaemia Prevalence − Chhattisgarh, Urban
WHO−2024 Adjustment Method

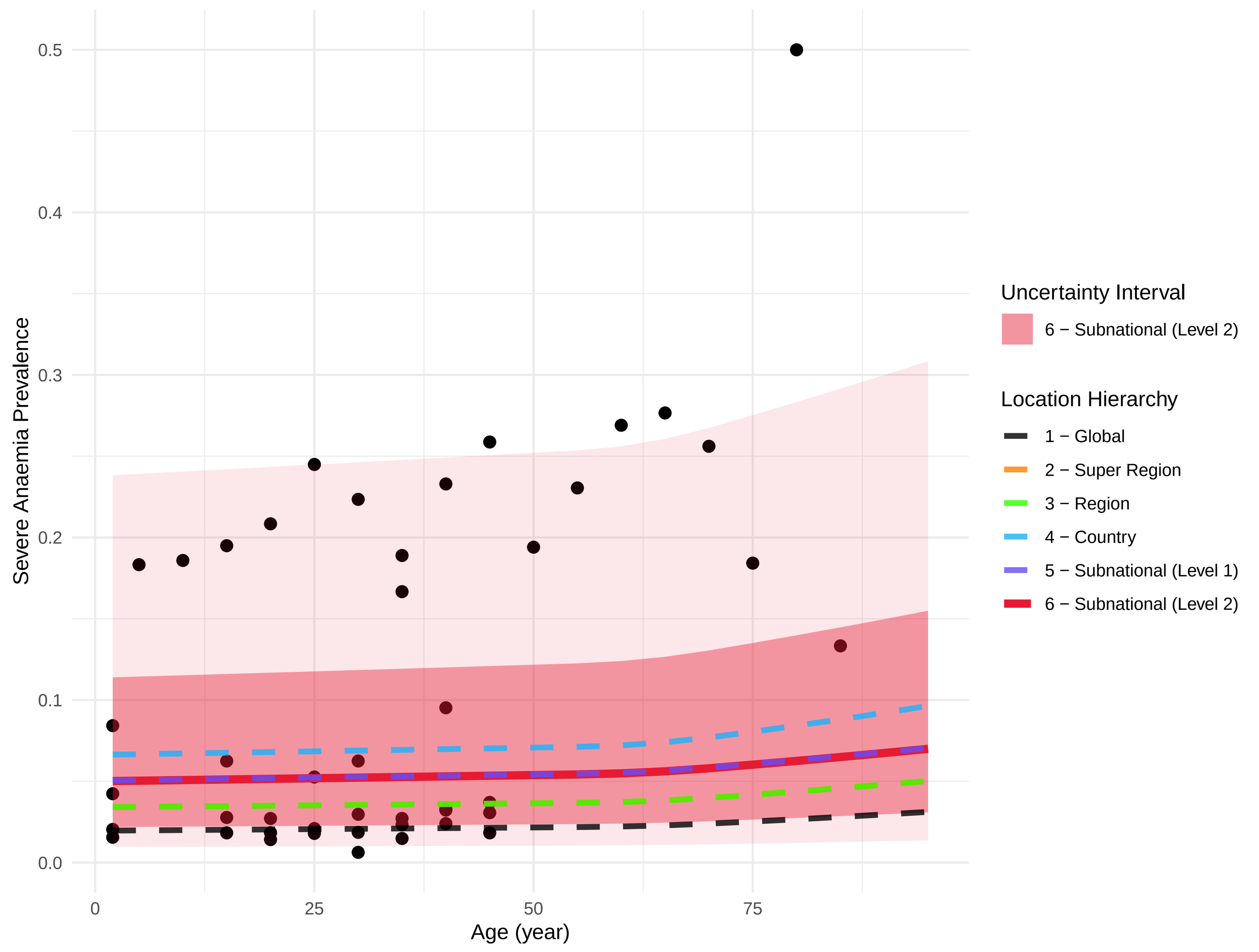

MR−BRT Age/Sex Fits − Non−pregnant Females 2+ Years Old − Severe Anaemia Prevalence − Delhi
WHO−2024 Adjustment Method

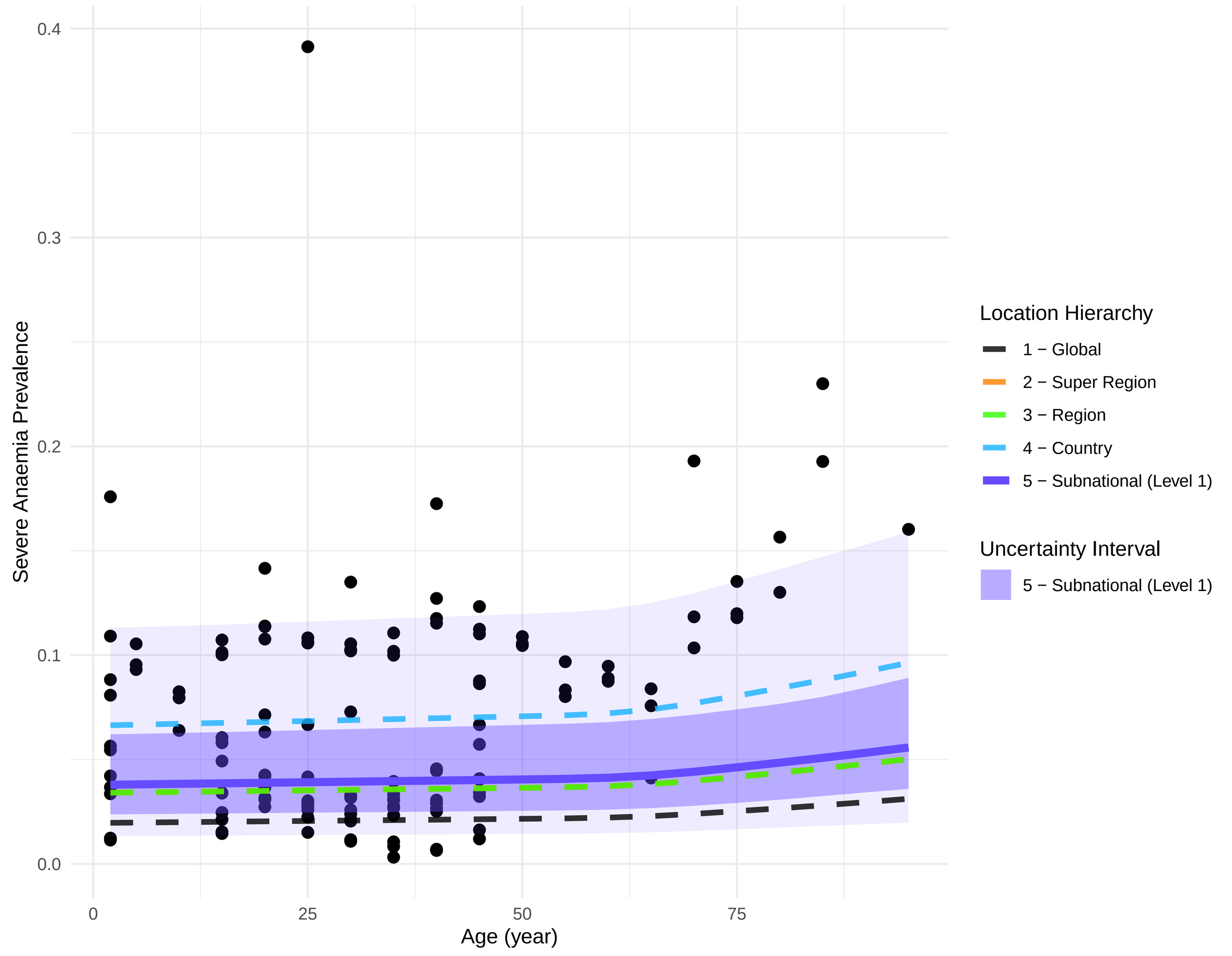

MR−BRT Age/Sex Fits − Non−pregnant Females 2+ Years Old − Severe Anaemia Prevalence − Delhi, Rural
WHO−2024 Adjustment Method

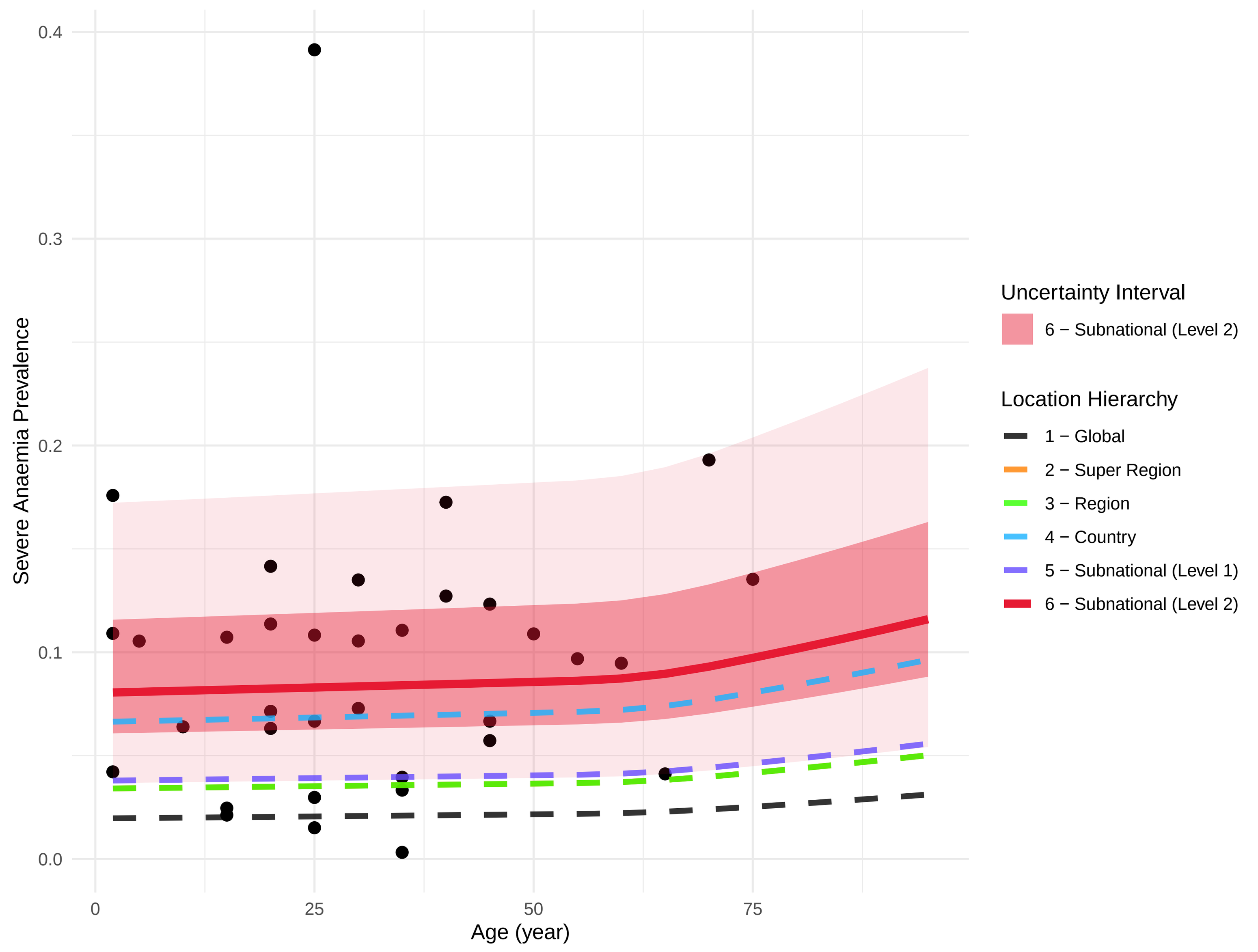

MR−BRT Age/Sex Fits − Non−pregnant Females 2+ Years Old − Severe Anaemia Prevalence − Delhi, Urban
WHO−2024 Adjustment Method

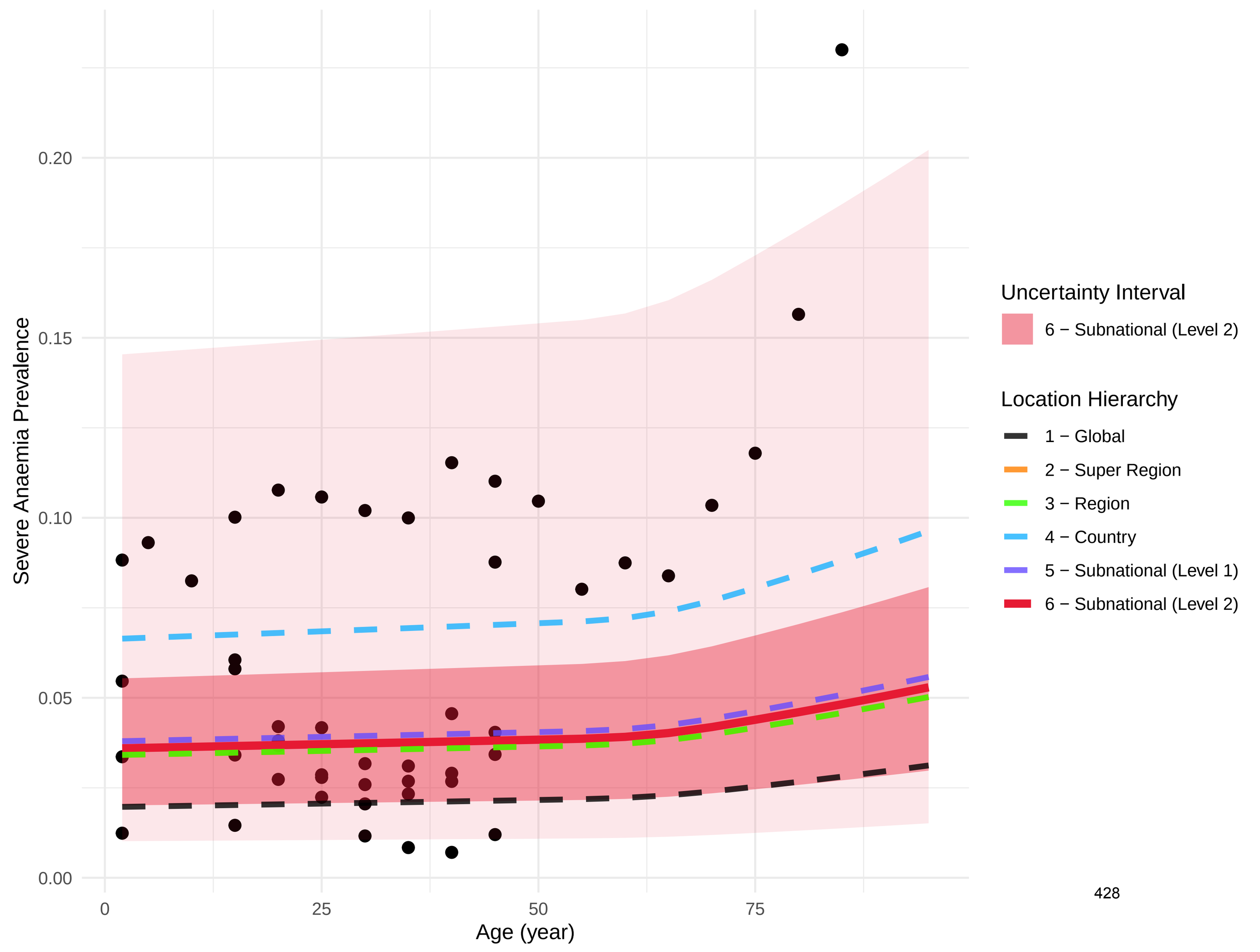

MR−BRT Age/Sex Fits − Non−pregnant Females 2+ Years Old − Severe Anaemia Prevalence − Goa
WHO−2024 Adjustment Method

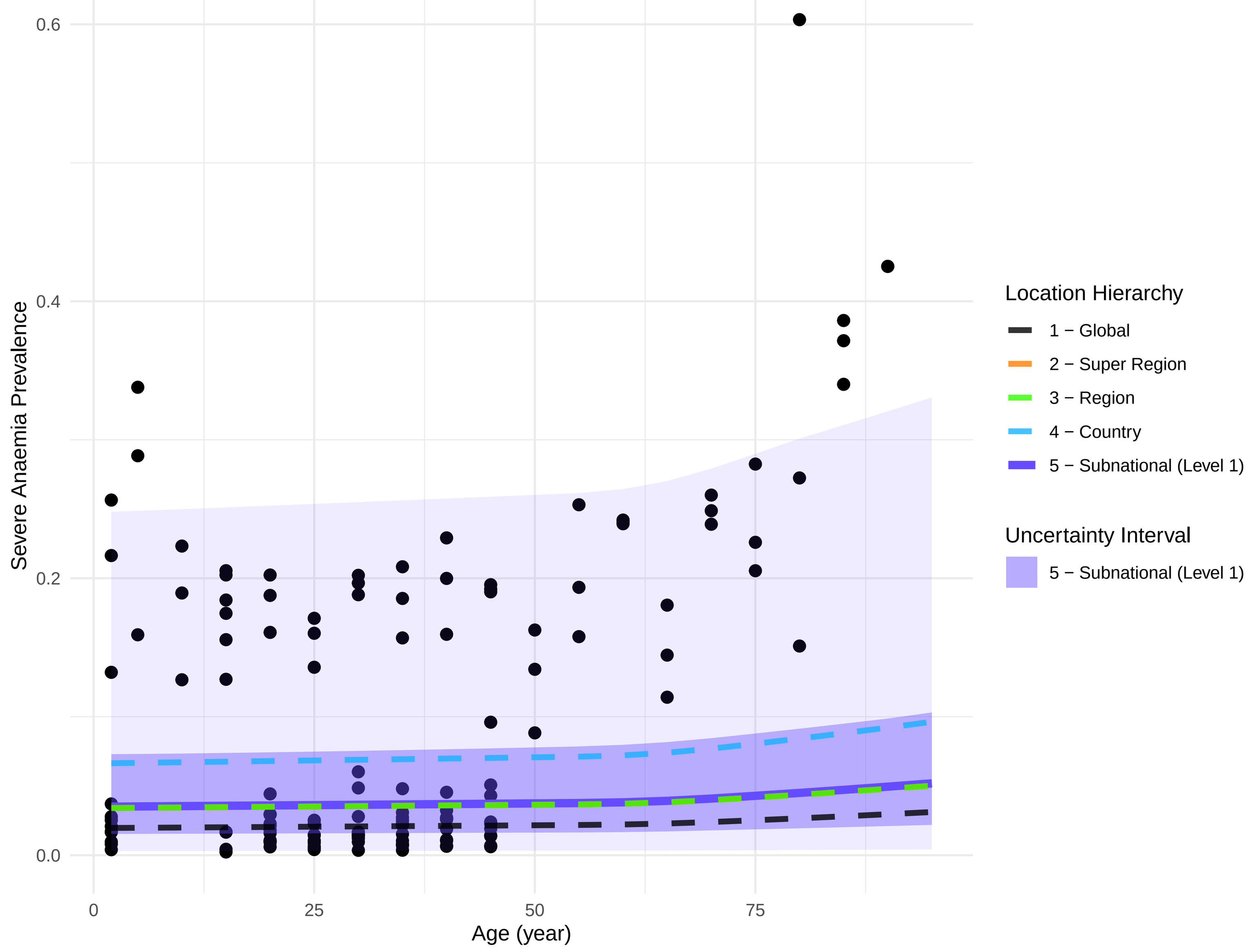

MR−BRT Age/Sex Fits − Non−pregnant Females 2+ Years Old − Severe Anaemia Prevalence − Goa, Rural
WHO−2024 Adjustment Method

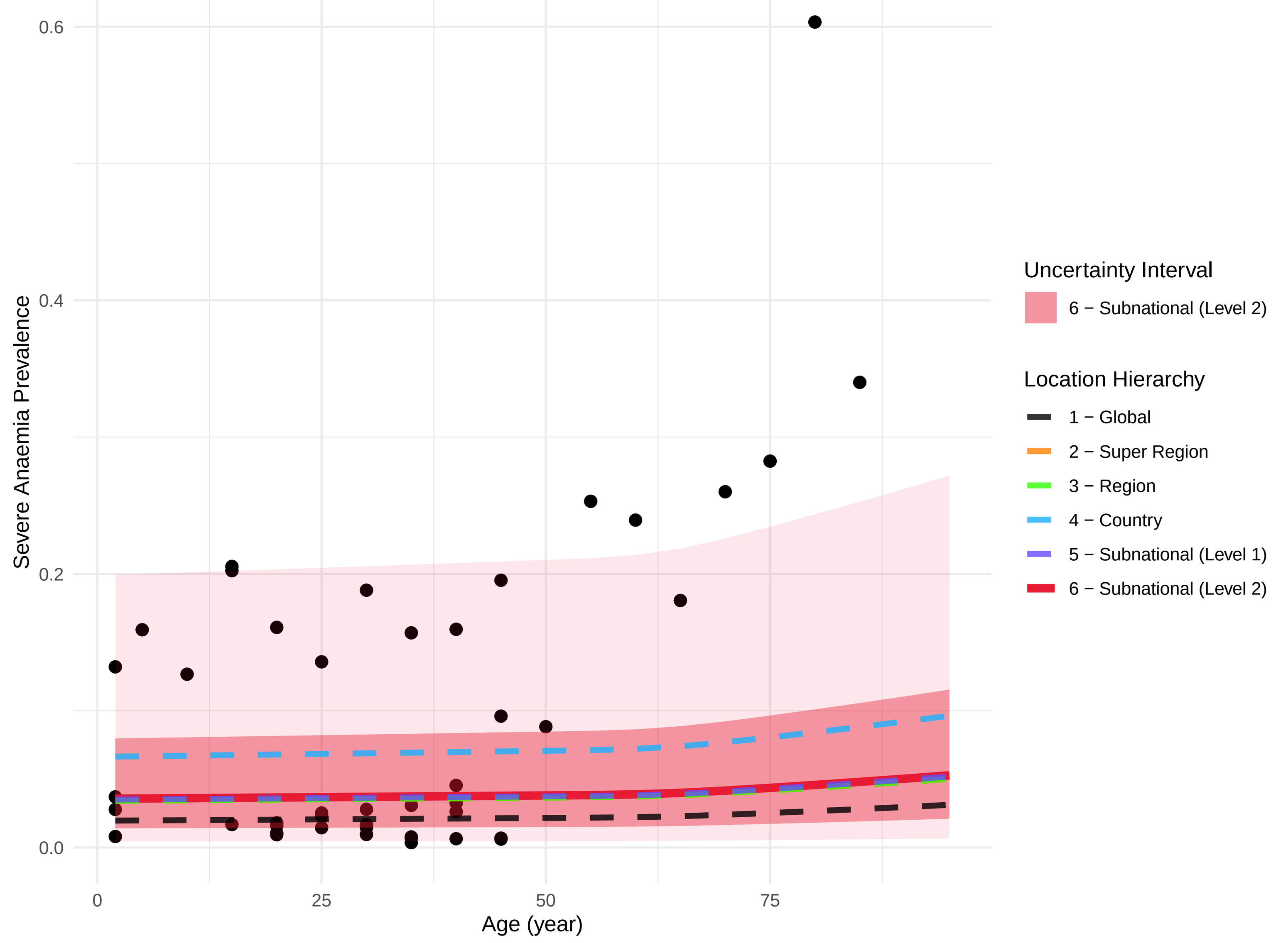

MR−BRT Age/Sex Fits − Non−pregnant Females 2+ Years Old − Severe Anaemia Prevalence − Goa, Urban
WHO−2024 Adjustment Method

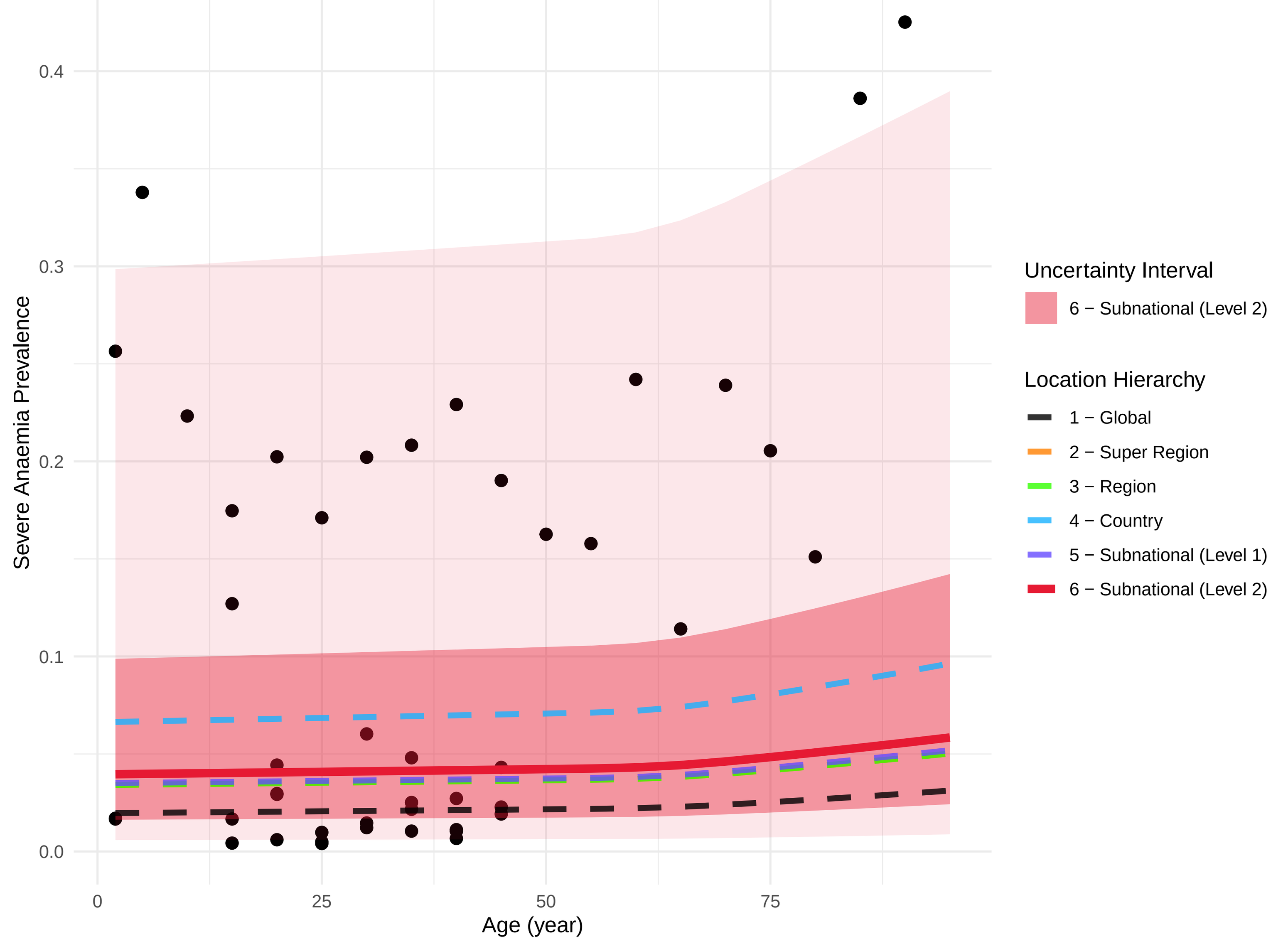

MR−BRT Age/Sex Fits − Non−pregnant Females 2+ Years Old − Severe Anaemia Prevalence − Gujarat
WHO−2024 Adjustment Method

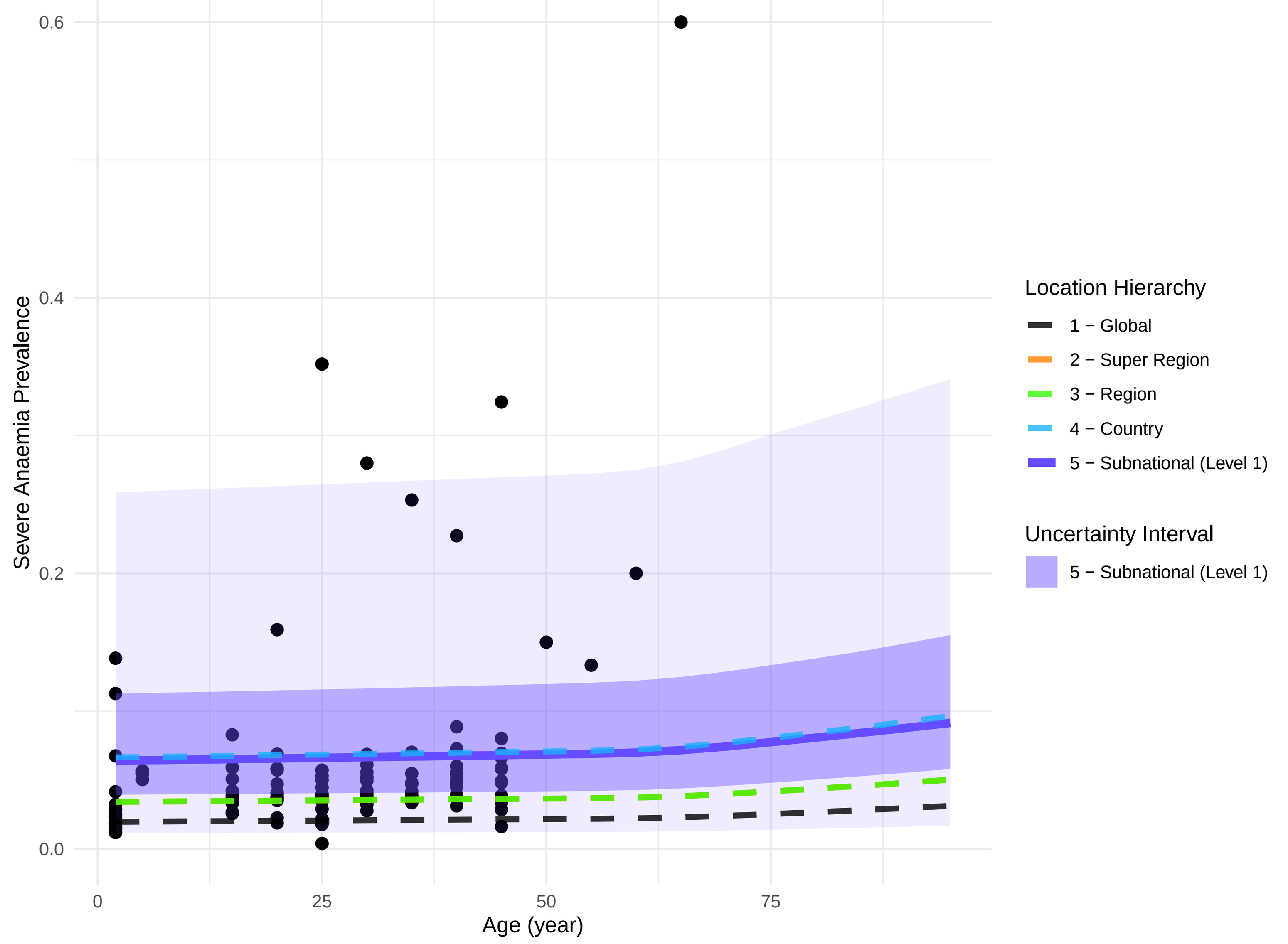

MR−BRT Age/Sex Fits − Non−pregnant Females 2+ Years Old − Severe Anaemia Prevalence − Gujarat, Rural
WHO−2024 Adjustment Method

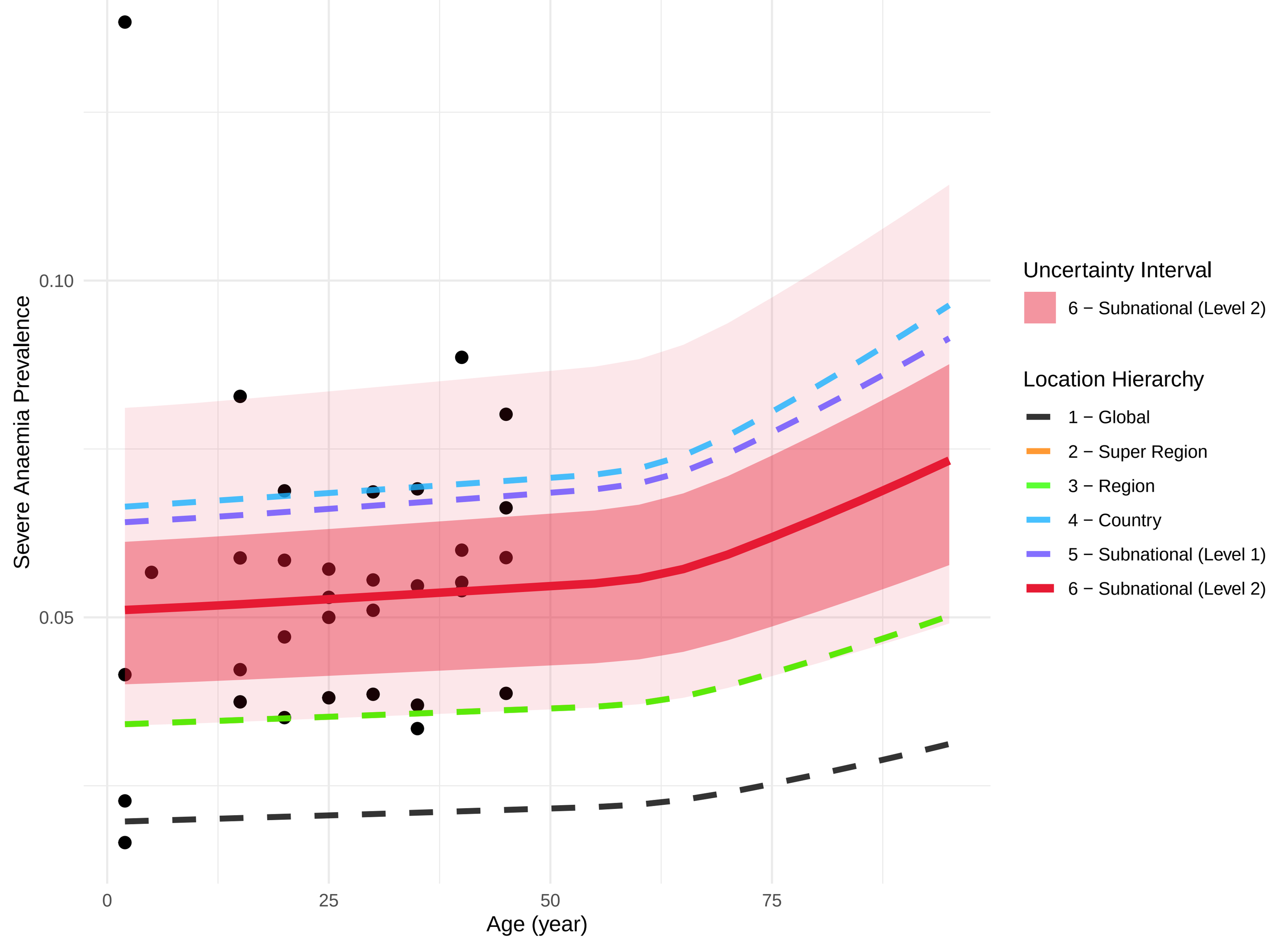

MR−BRT Age/Sex Fits − Non−pregnant Females 2+ Years Old − Severe Anaemia Prevalence − Gujarat, Urban
WHO−2024 Adjustment Method

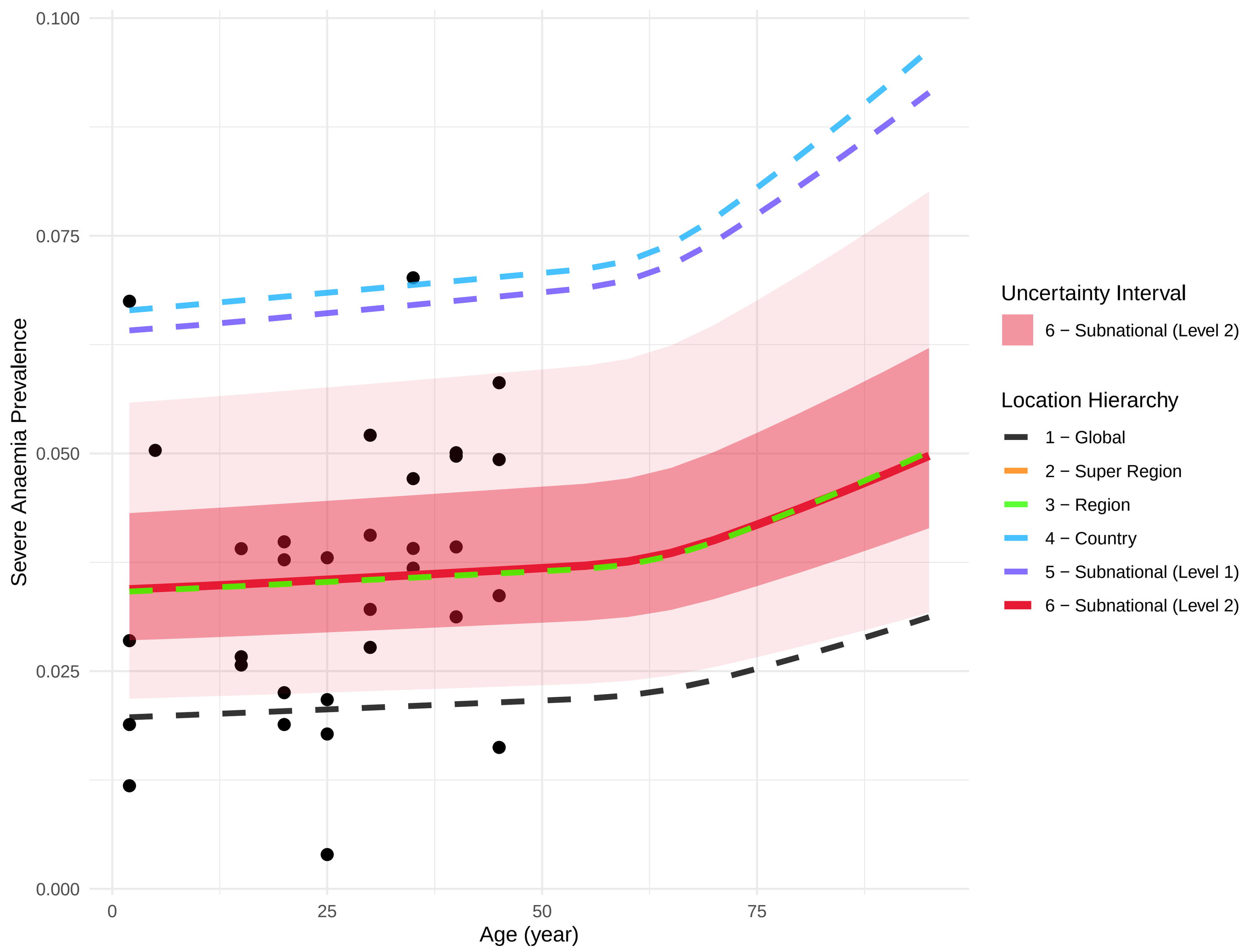

MR−BRT Age/Sex Fits − Non−pregnant Females 2+ Years Old − Severe Anaemia Prevalence − Haryana
WHO−2024 Adjustment Method

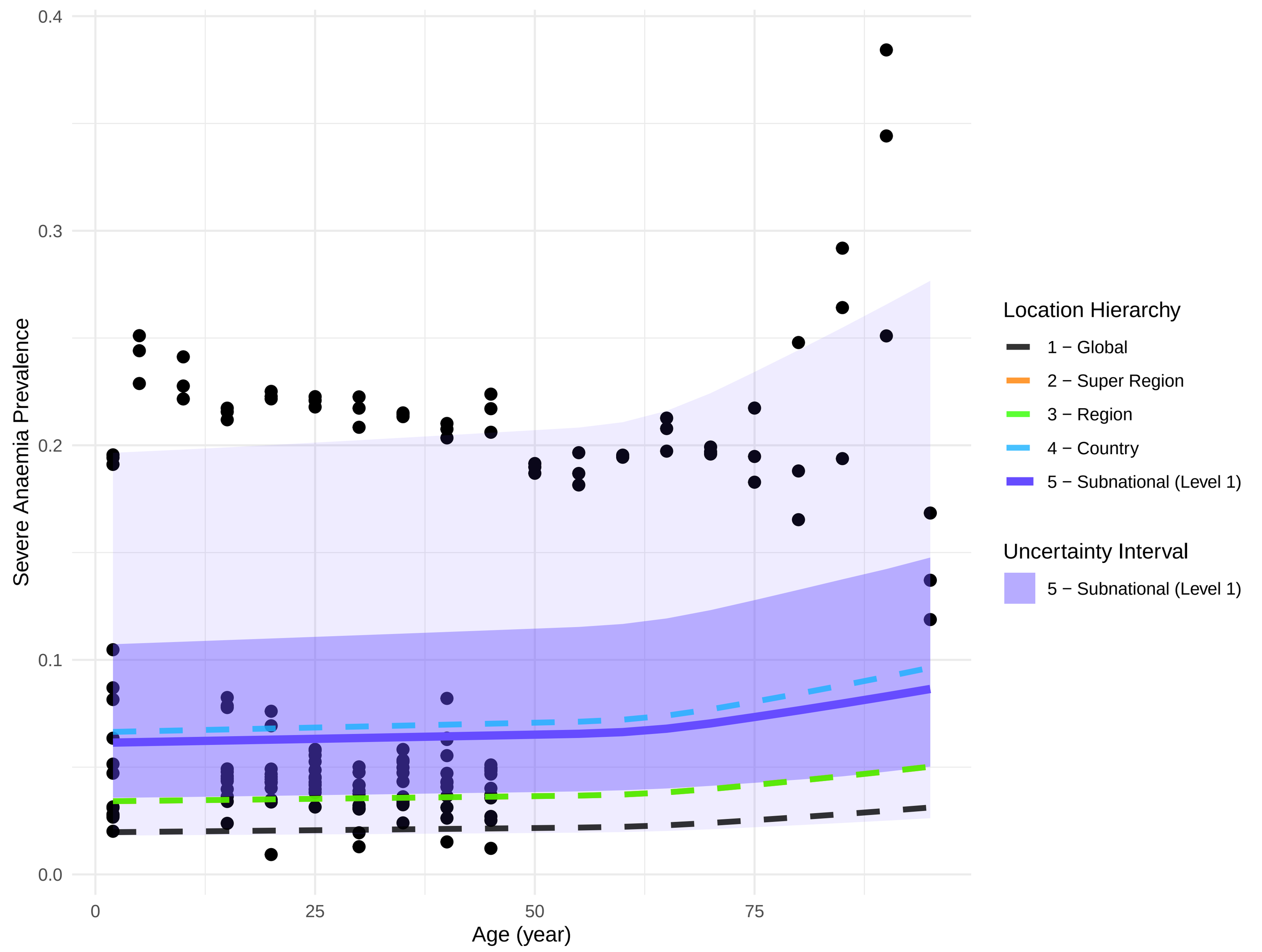

MR−BRT Age/Sex Fits − Non−pregnant Females 2+ Years Old − Severe Anaemia Prevalence − Haryana, Rural
WHO−2024 Adjustment Method

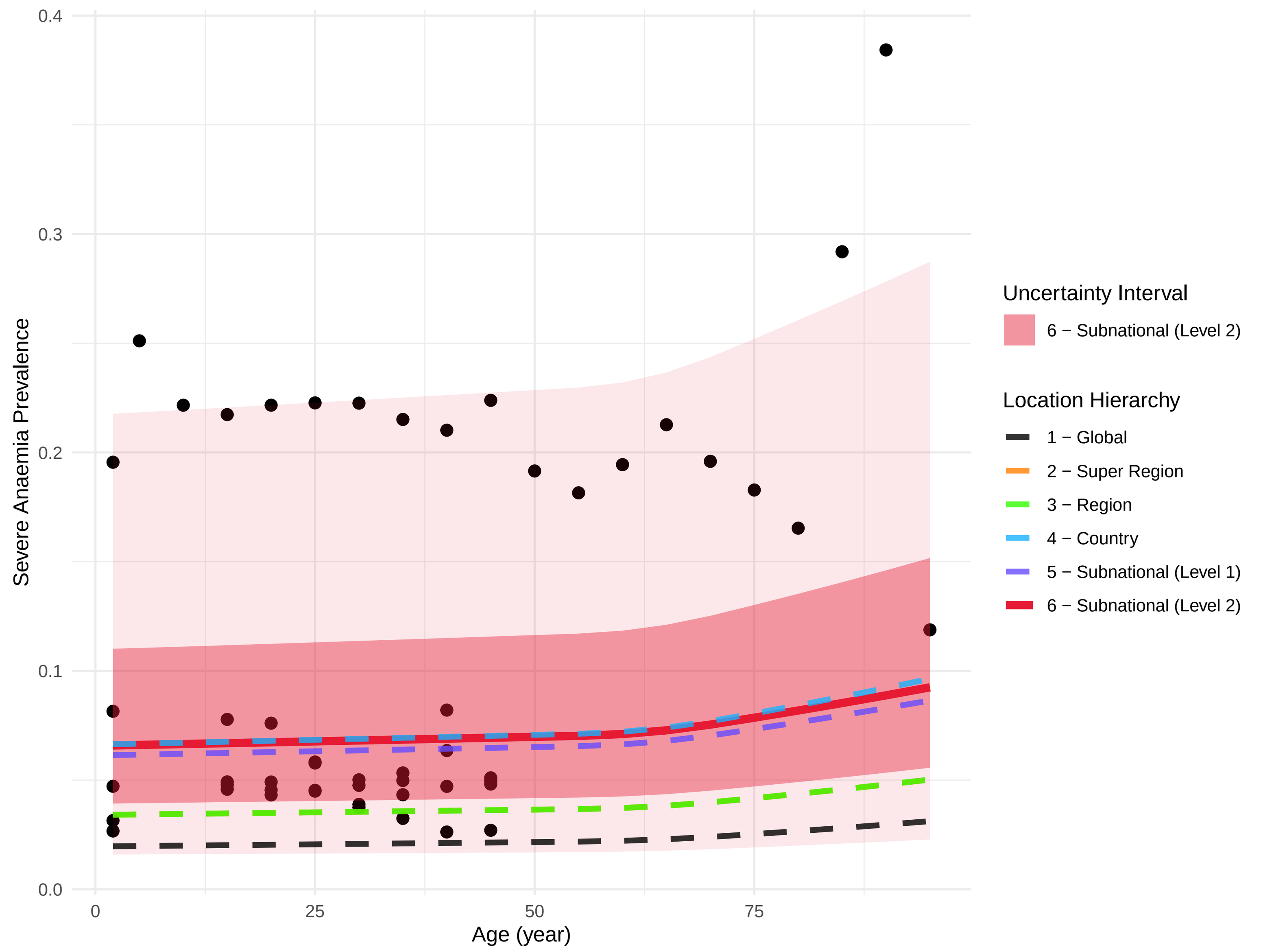

MR−BRT Age/Sex Fits − Non−pregnant Females 2+ Years Old − Severe Anaemia Prevalence − Haryana, Urban
WHO−2024 Adjustment Method

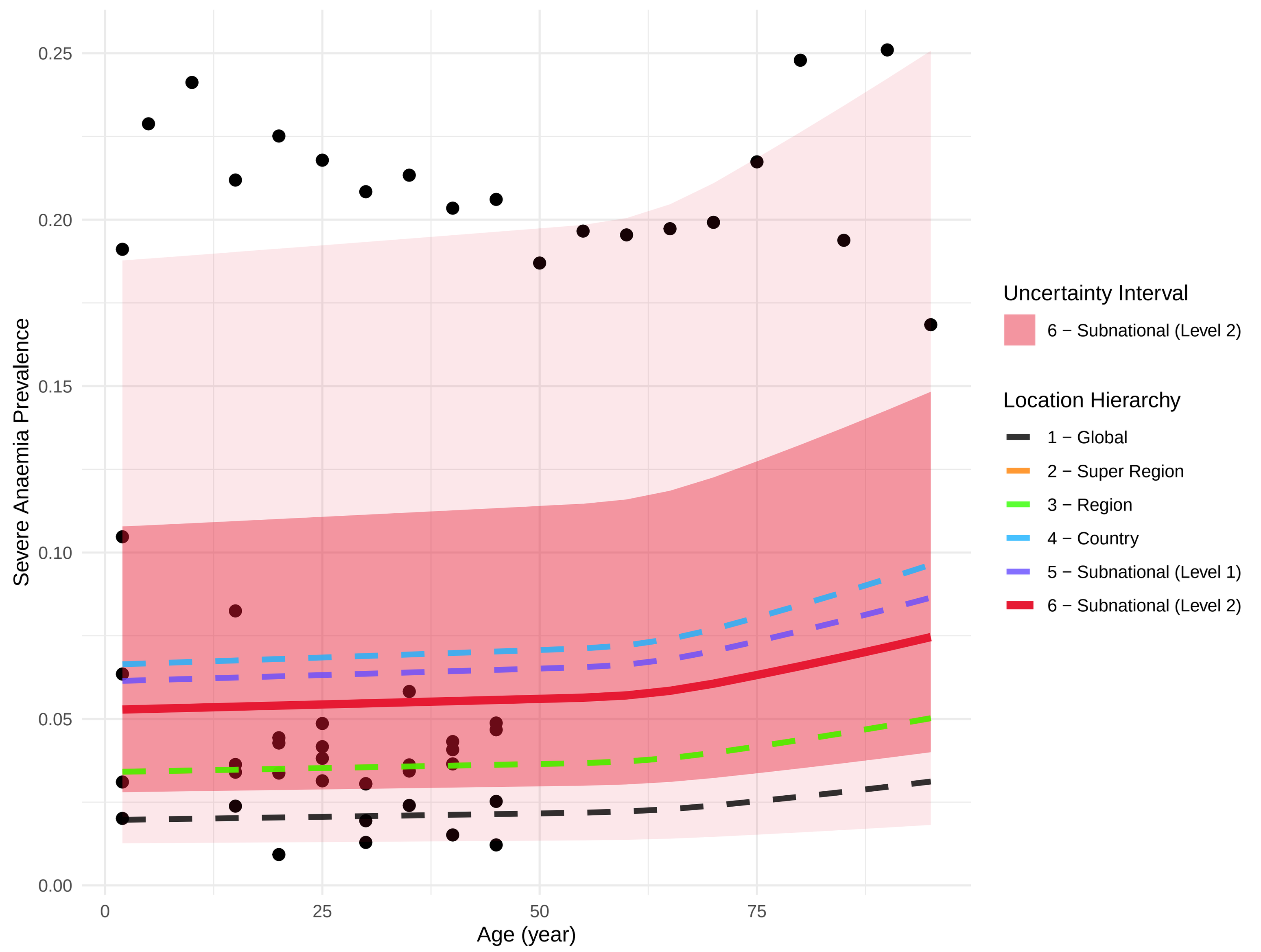

MR−BRT Age/Sex Fits − Non−pregnant Females 2+ Years Old − Severe Anaemia Prevalence − Himachal Pradesh
WHO−2024 Adjustment Method

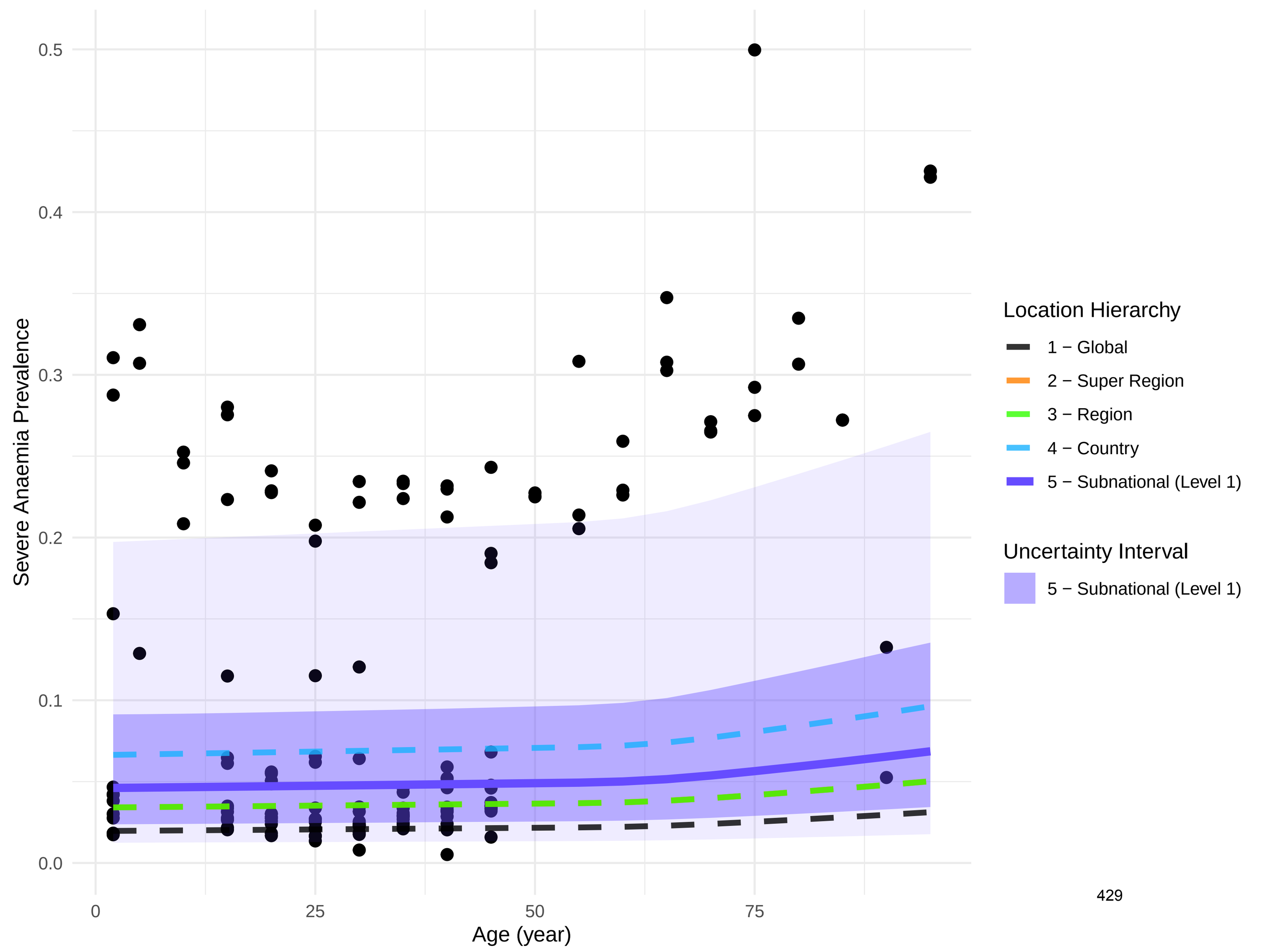

MR−BRT Age/Sex Fits − Non−pregnant Females 2+ Years Old − Severe Anaemia Prevalence − Himachal Pradesh, Rural
WHO−2024 Adjustment Method

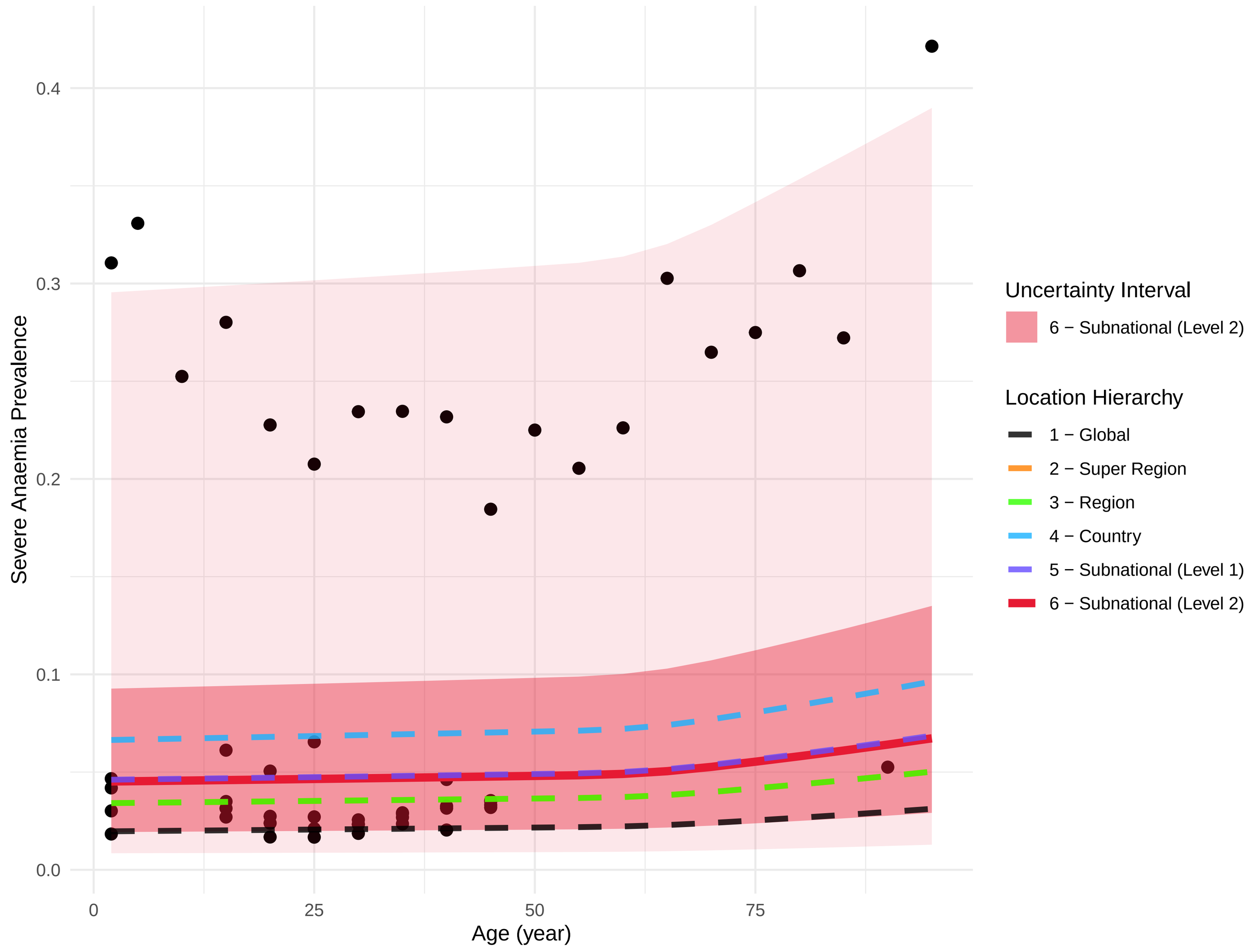

MR−BRT Age/Sex Fits − Non−pregnant Females 2+ Years Old − Severe Anaemia Prevalence − Himachal Pradesh, Urban
WHO−2024 Adjustment Method

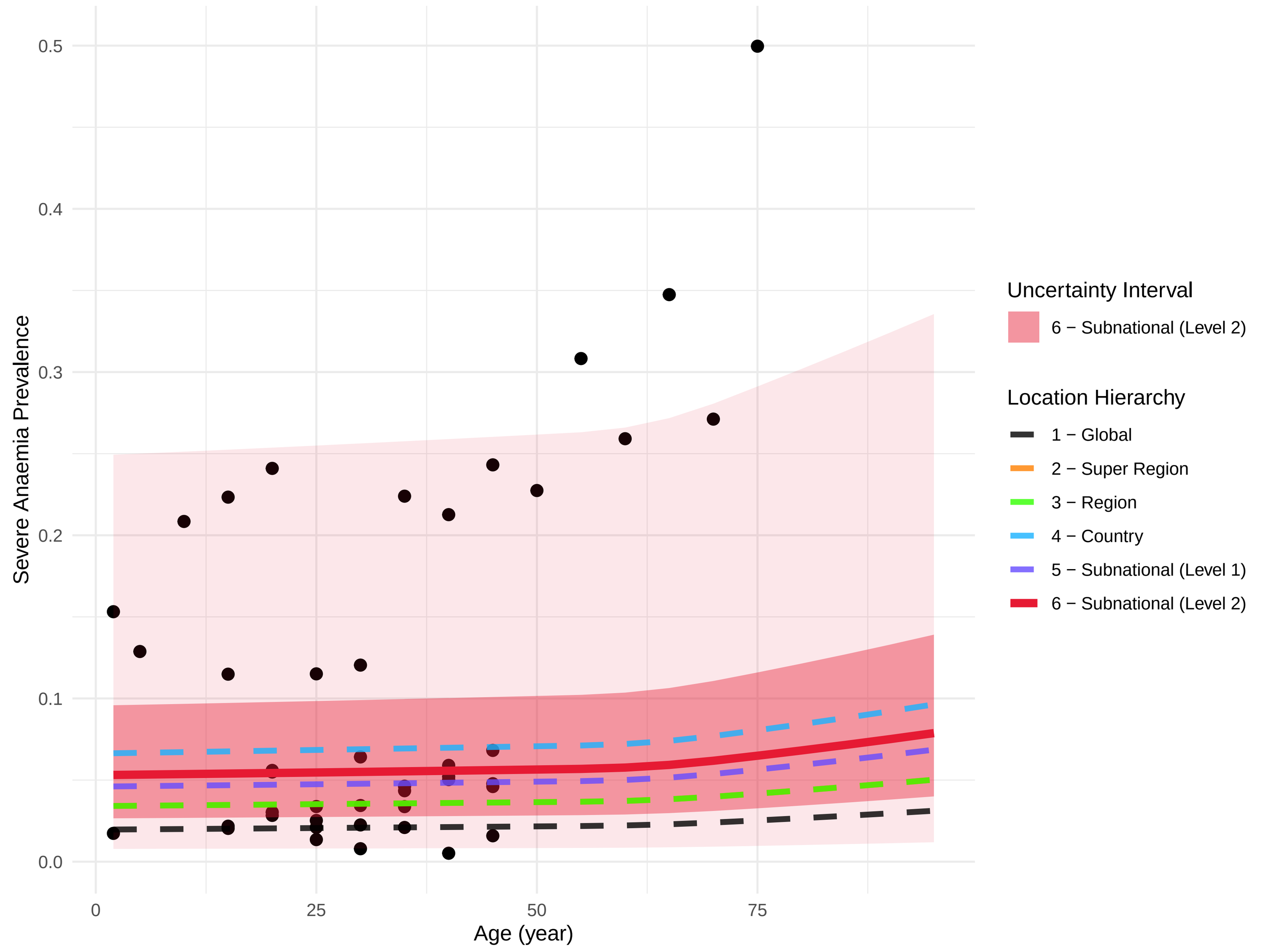

MR−BRT Age/Sex Fits − Non−pregnant Females 2+ Years Old − Severe Anaemia Prevalence − Jammu & Kashmir and Ladakh
WHO−2024 Adjustment Method

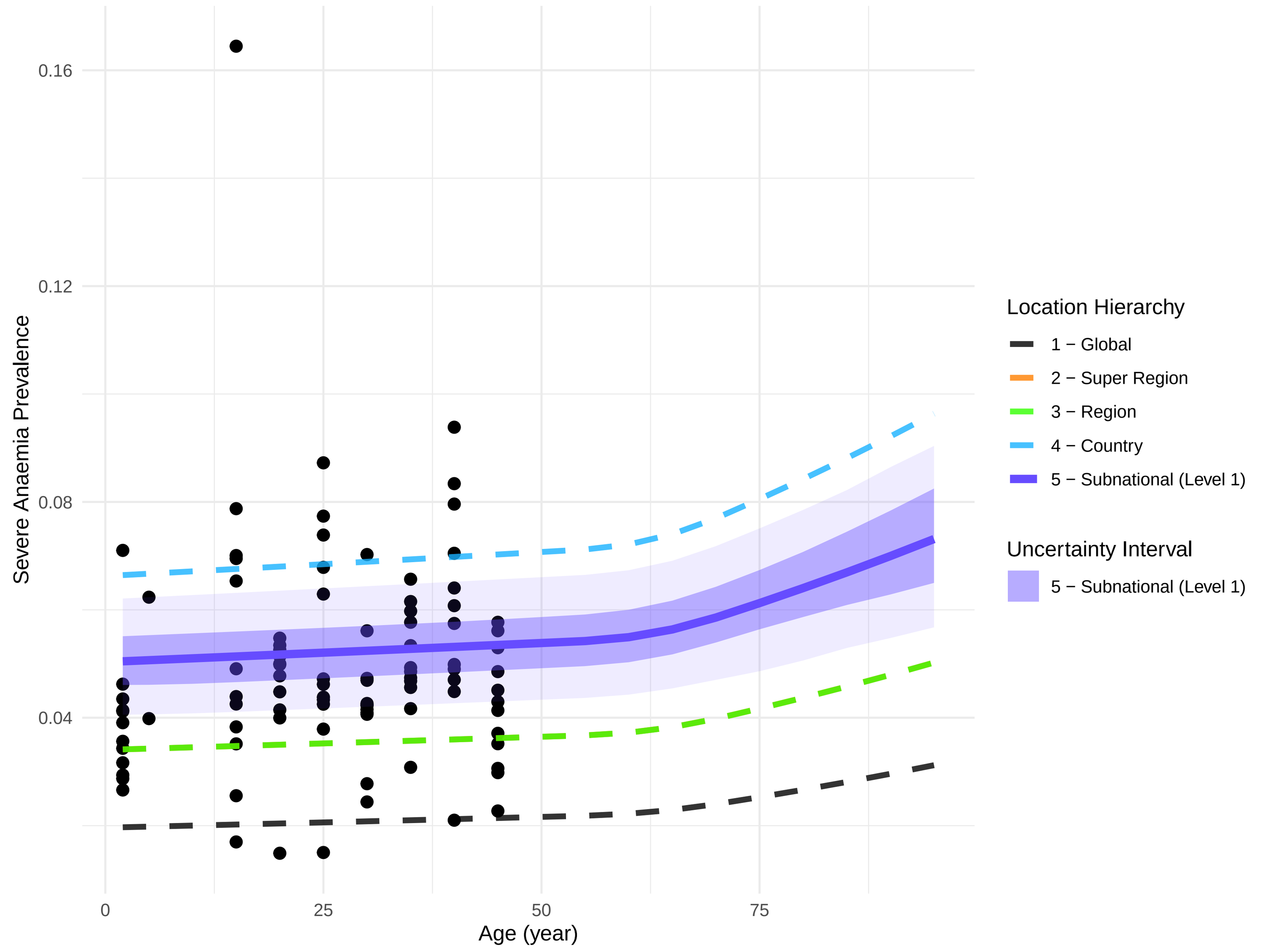

MR−BRT Age/Sex Fits − Non−pregnant Females 2+ Years Old − Severe Anaemia Prevalence − Jammu & Kashmir and Ladakh, Rural
WHO−2024 Adjustment Method

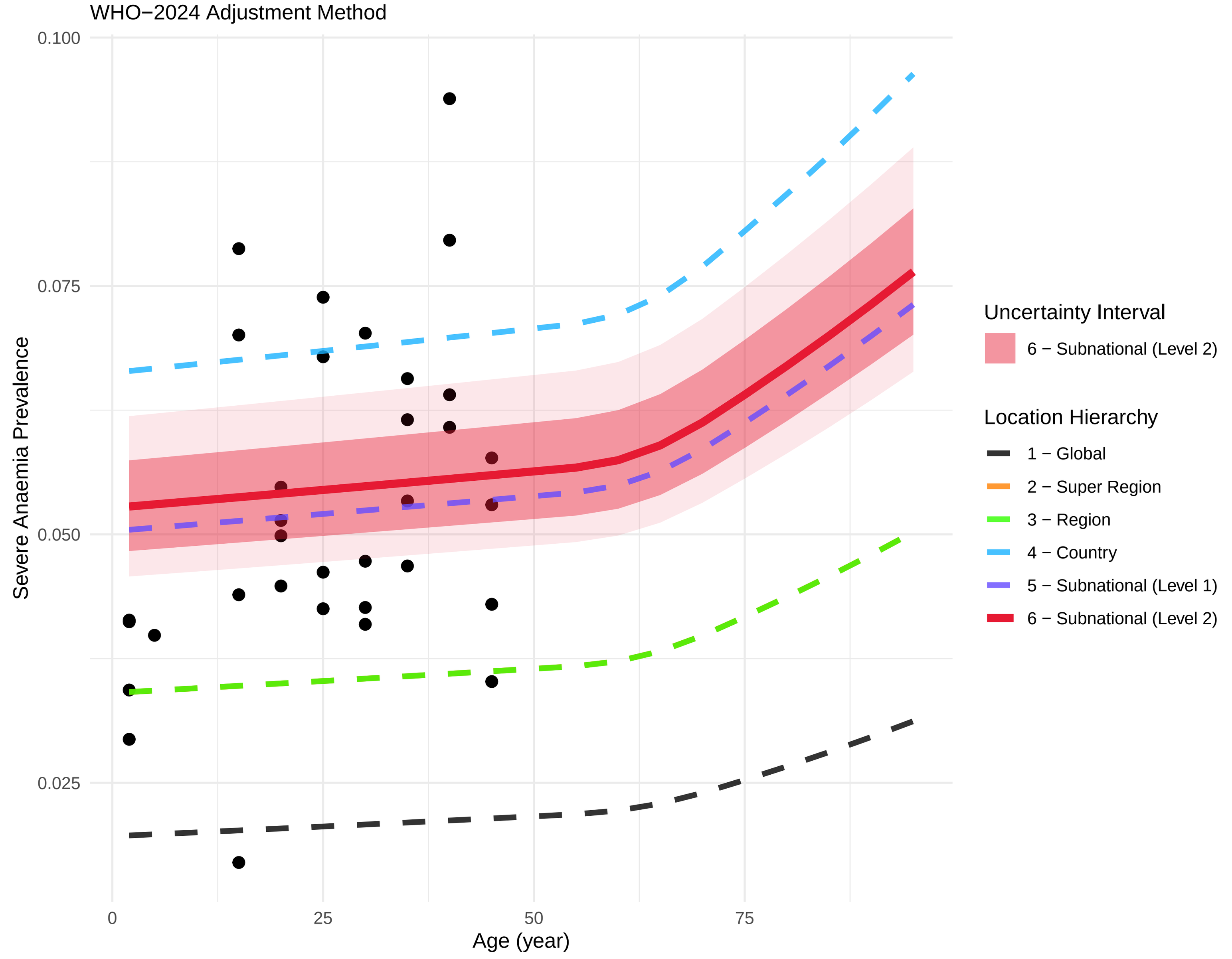

MR−BRT Age/Sex Fits − Non−pregnant Females 2+ Years Old − Severe Anaemia Prevalence − Jammu & Kashmir and Ladakh, Urban
WHO−2024 Adjustment Method

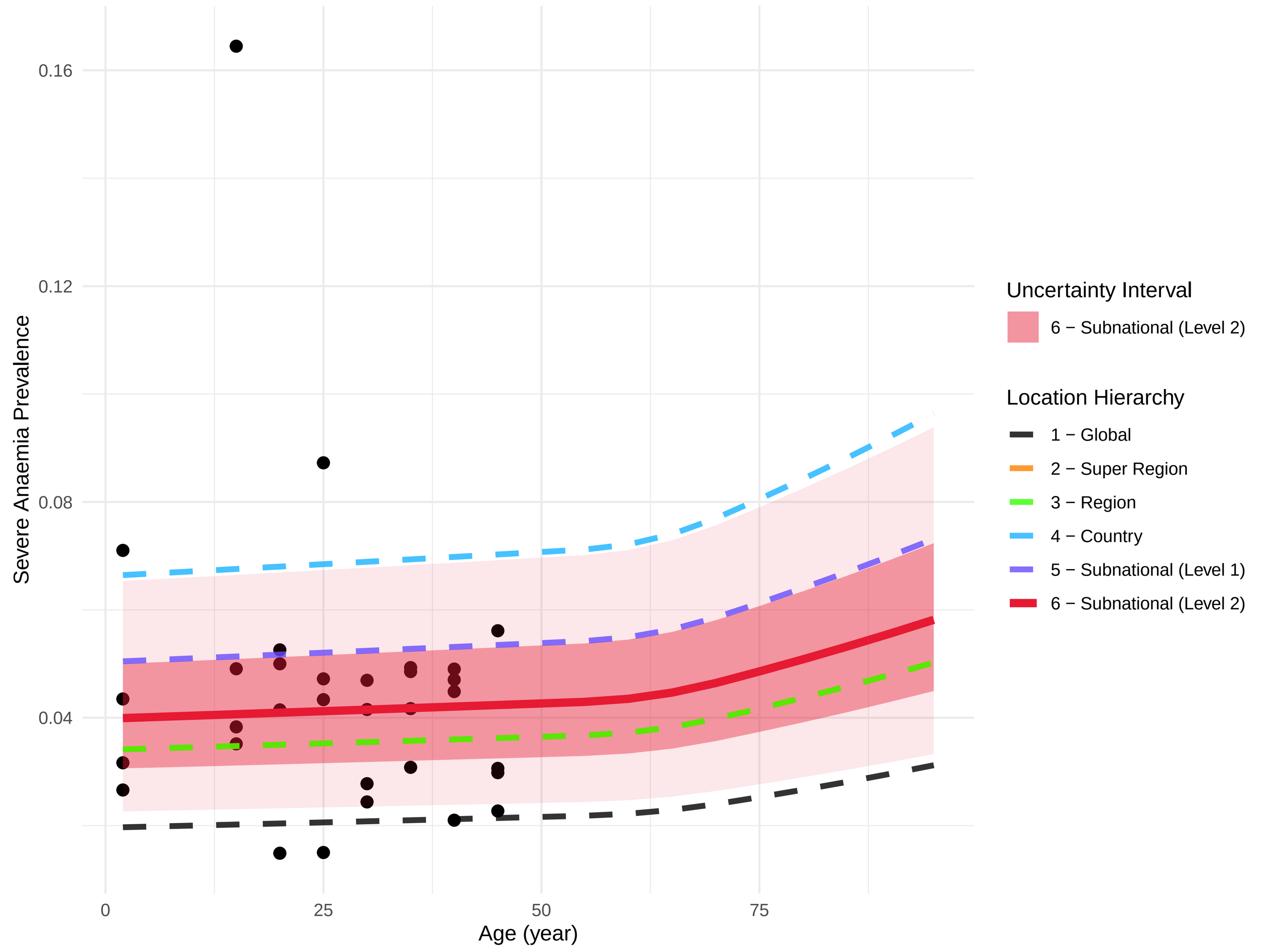

MR−BRT Age/Sex Fits − Non−pregnant Females 2+ Years Old − Severe Anaemia Prevalence − Jharkhand
WHO−2024 Adjustment Method

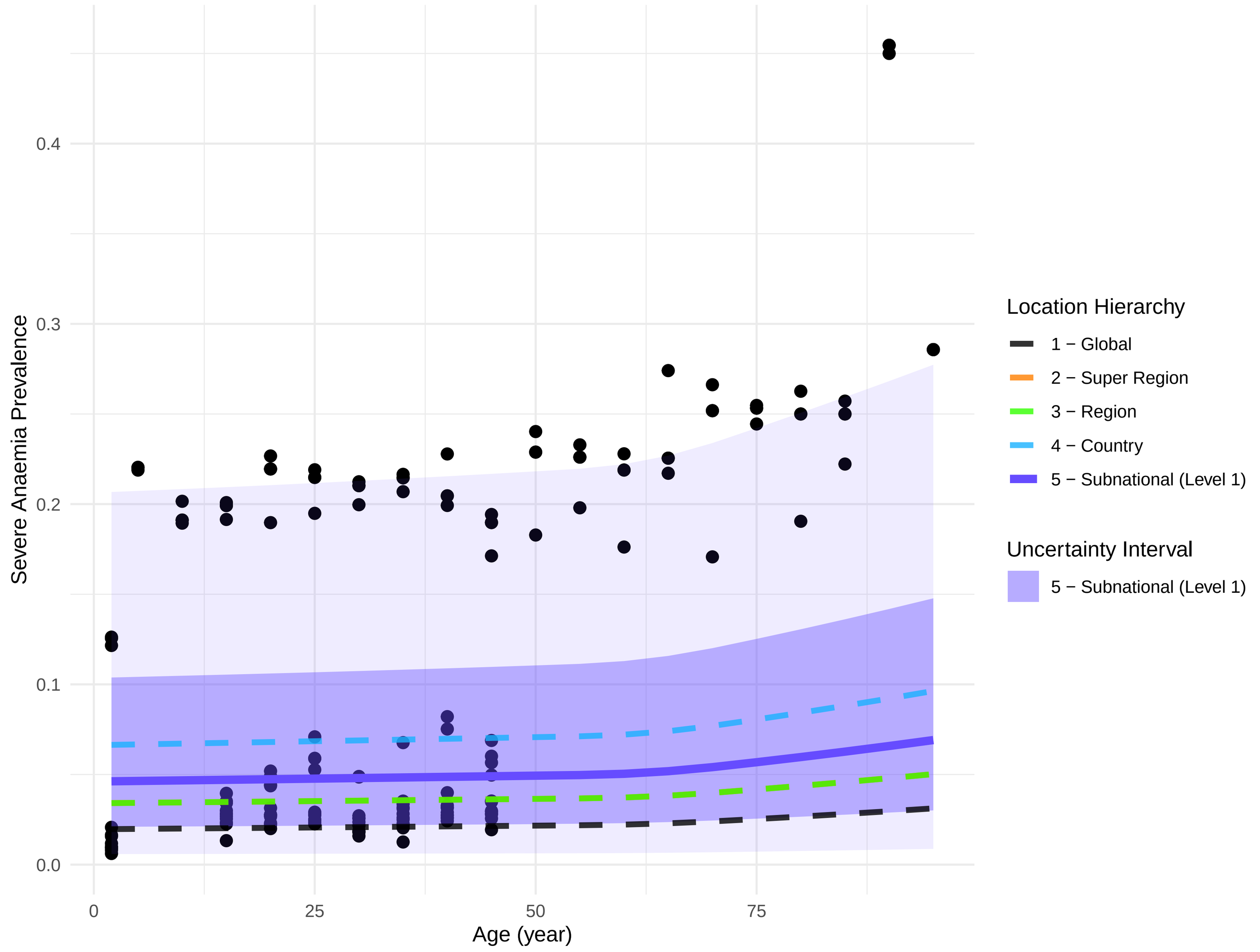

MR−BRT Age/Sex Fits − Non−pregnant Females 2+ Years Old − Severe Anaemia Prevalence − Jharkhand, Rural
WHO−2024 Adjustment Method

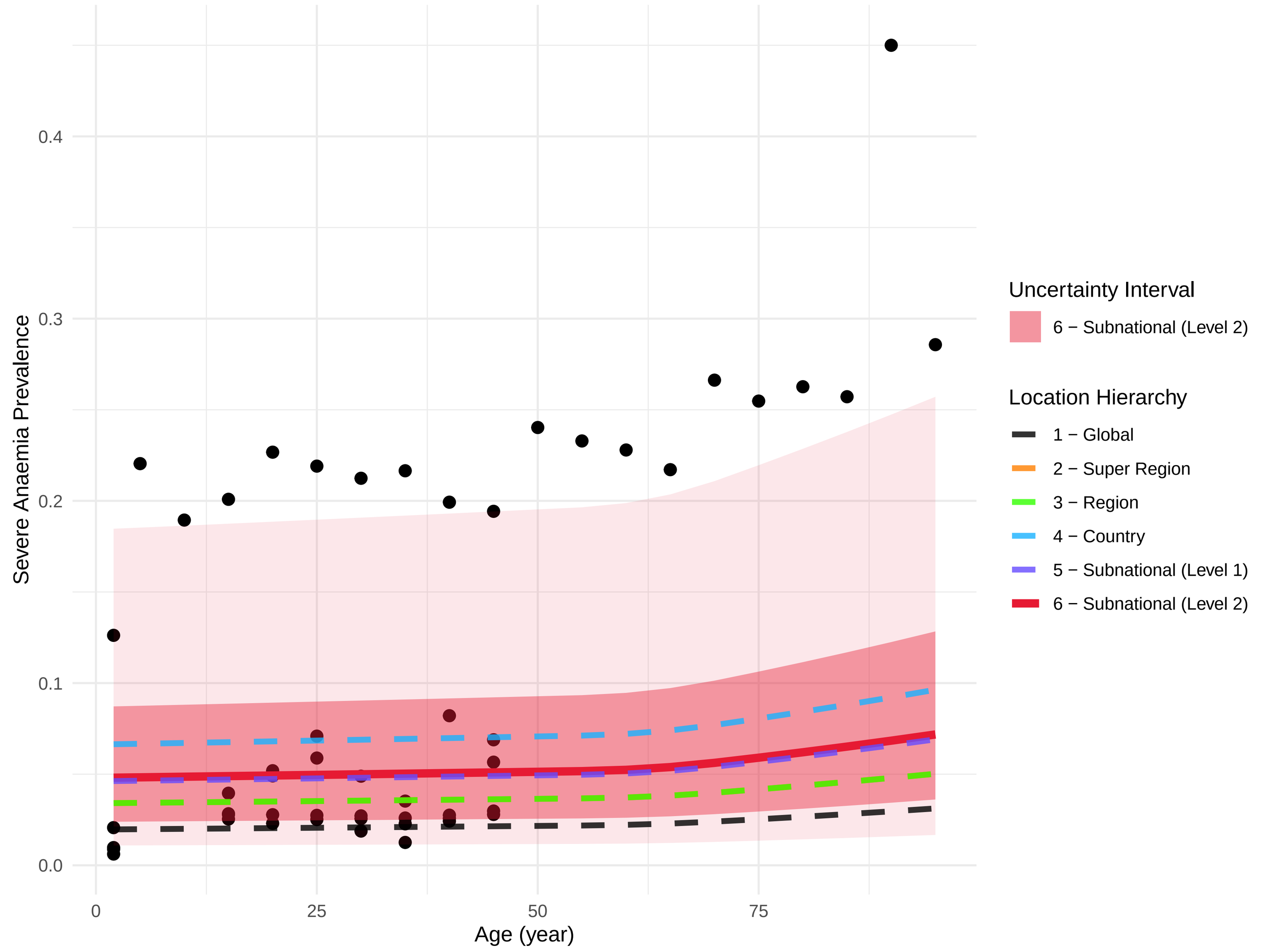

MR−BRT Age/Sex Fits − Non−pregnant Females 2+ Years Old − Severe Anaemia Prevalence − Jharkhand, Urban
WHO−2024 Adjustment Method

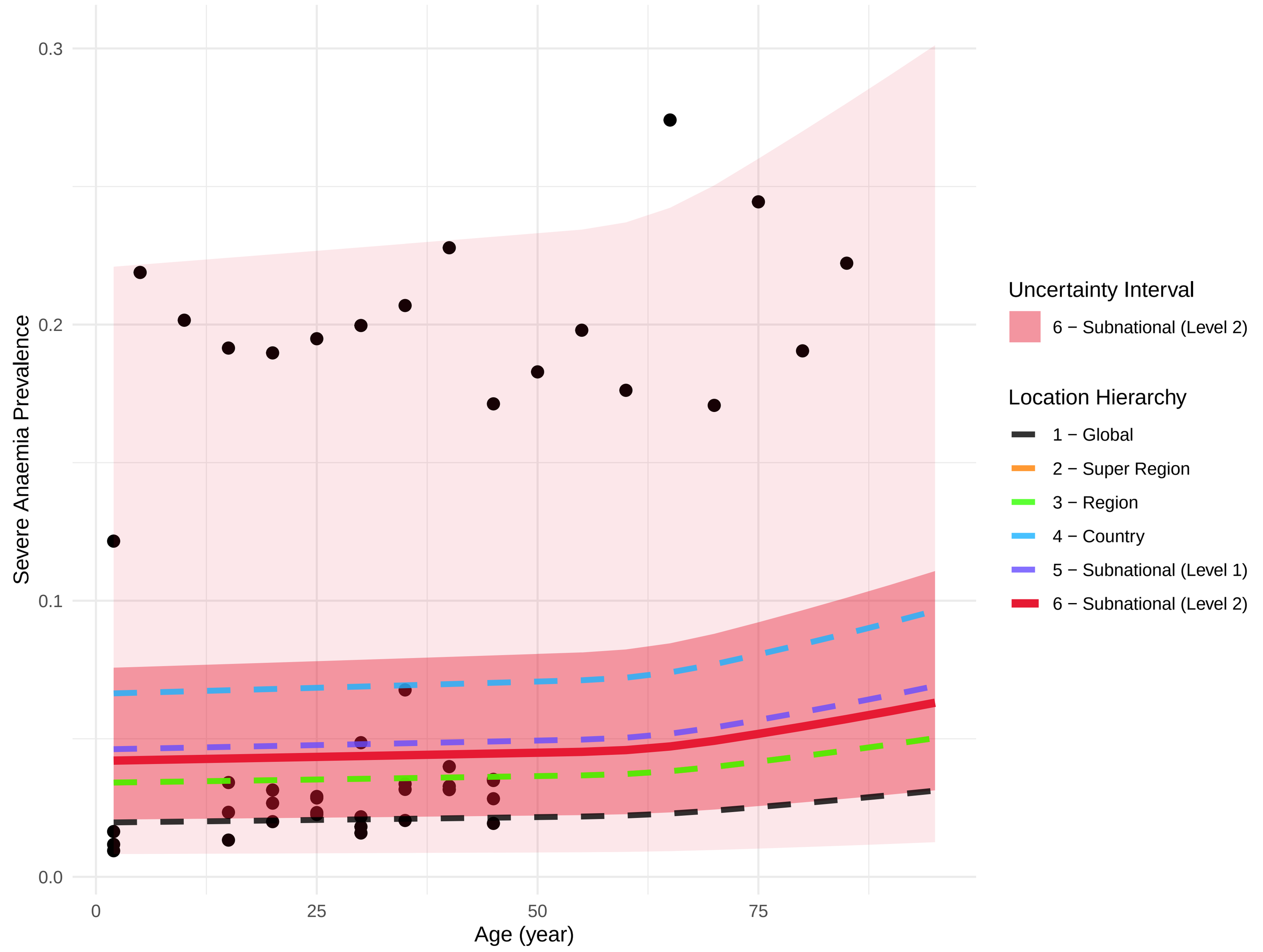

MR−BRT Age/Sex Fits − Non−pregnant Females 2+ Years Old − Severe Anaemia Prevalence − Karnataka
WHO−2024 Adjustment Method

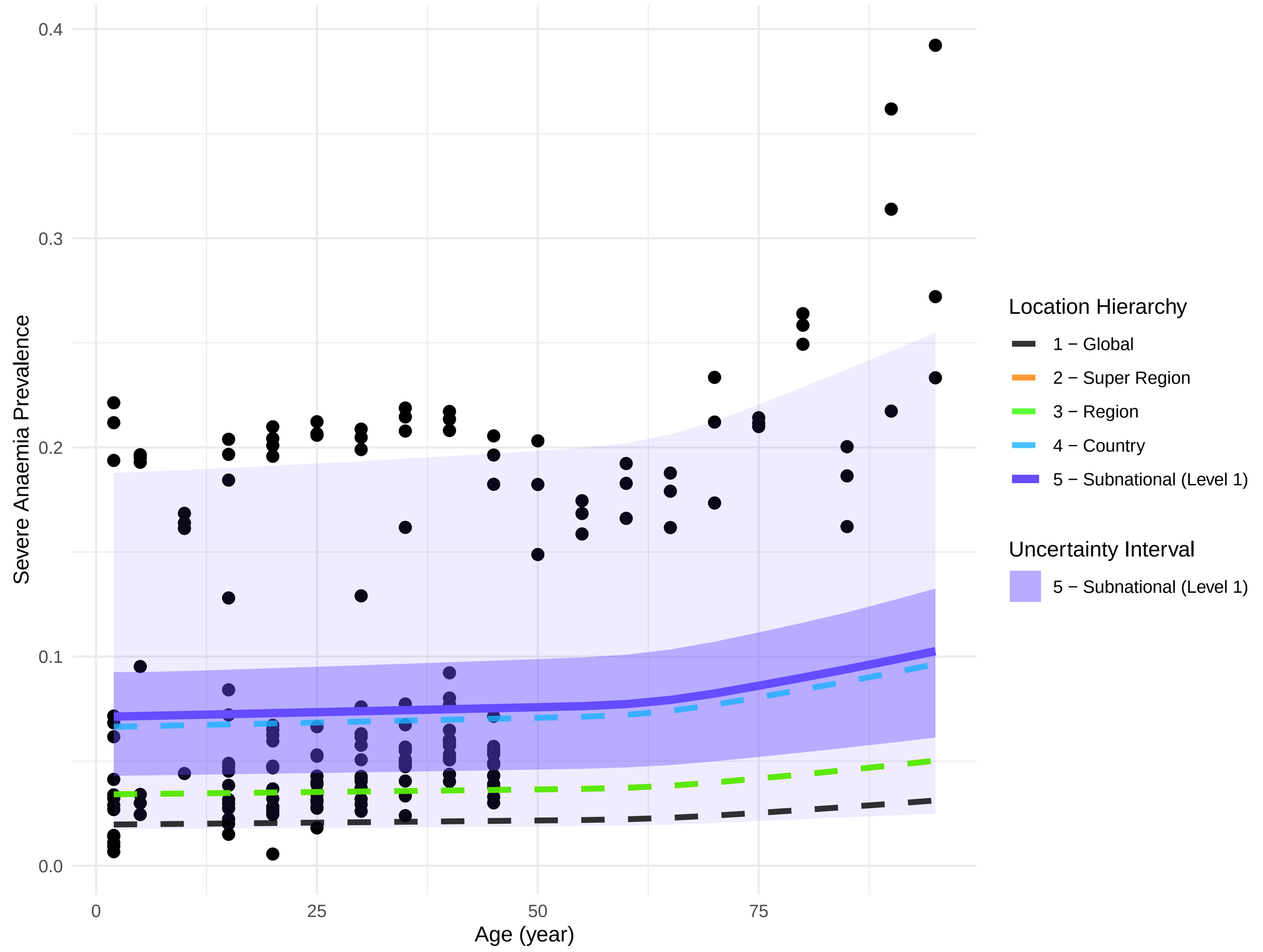

MR−BRT Age/Sex Fits − Non−pregnant Females 2+ Years Old − Severe Anaemia Prevalence − Karnataka, Rural
WHO−2024 Adjustment Method

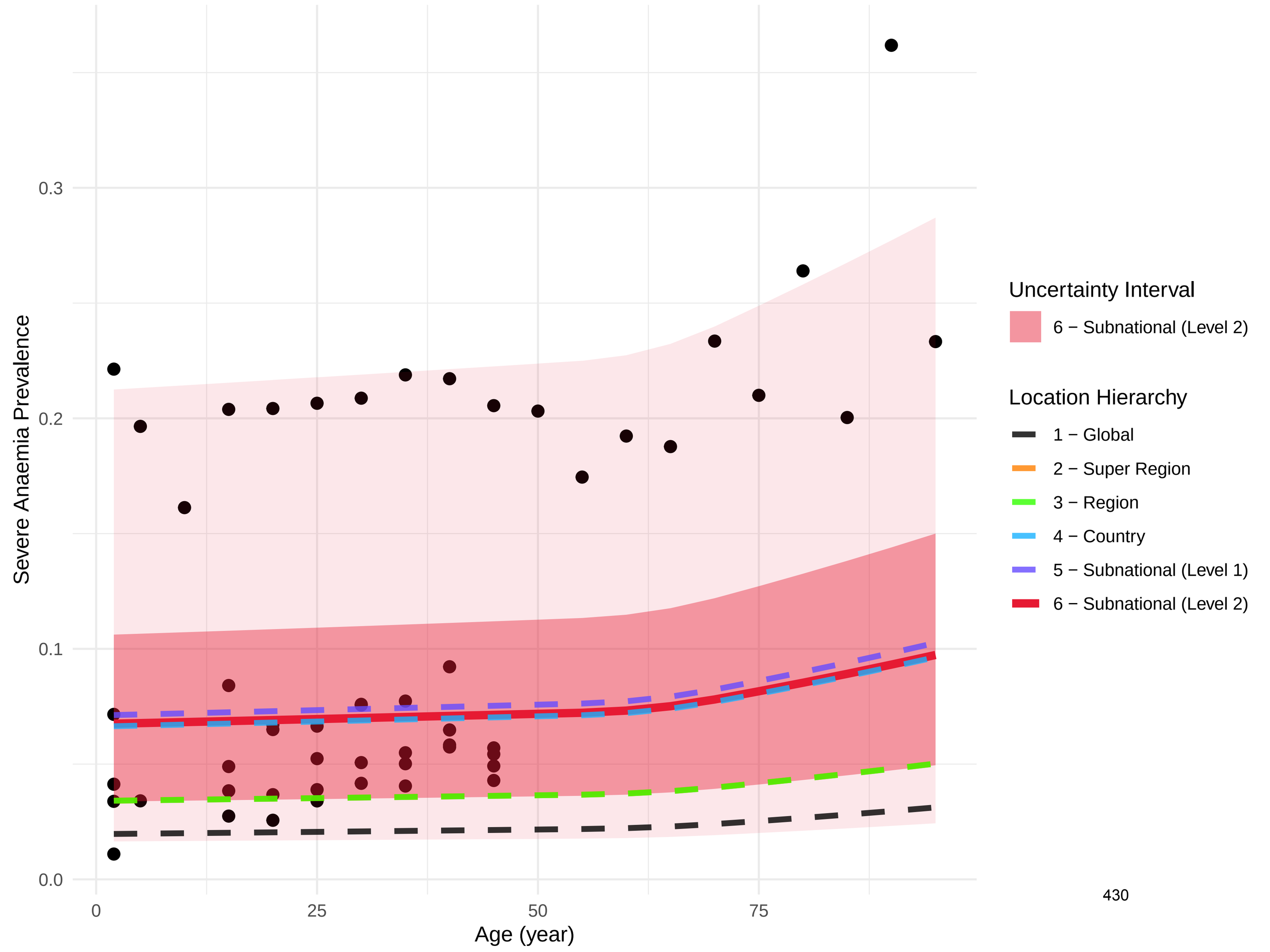

MR−BRT Age/Sex Fits − Non−pregnant Females 2+ Years Old − Severe Anaemia Prevalence − Karnataka, Urban
WHO−2024 Adjustment Method

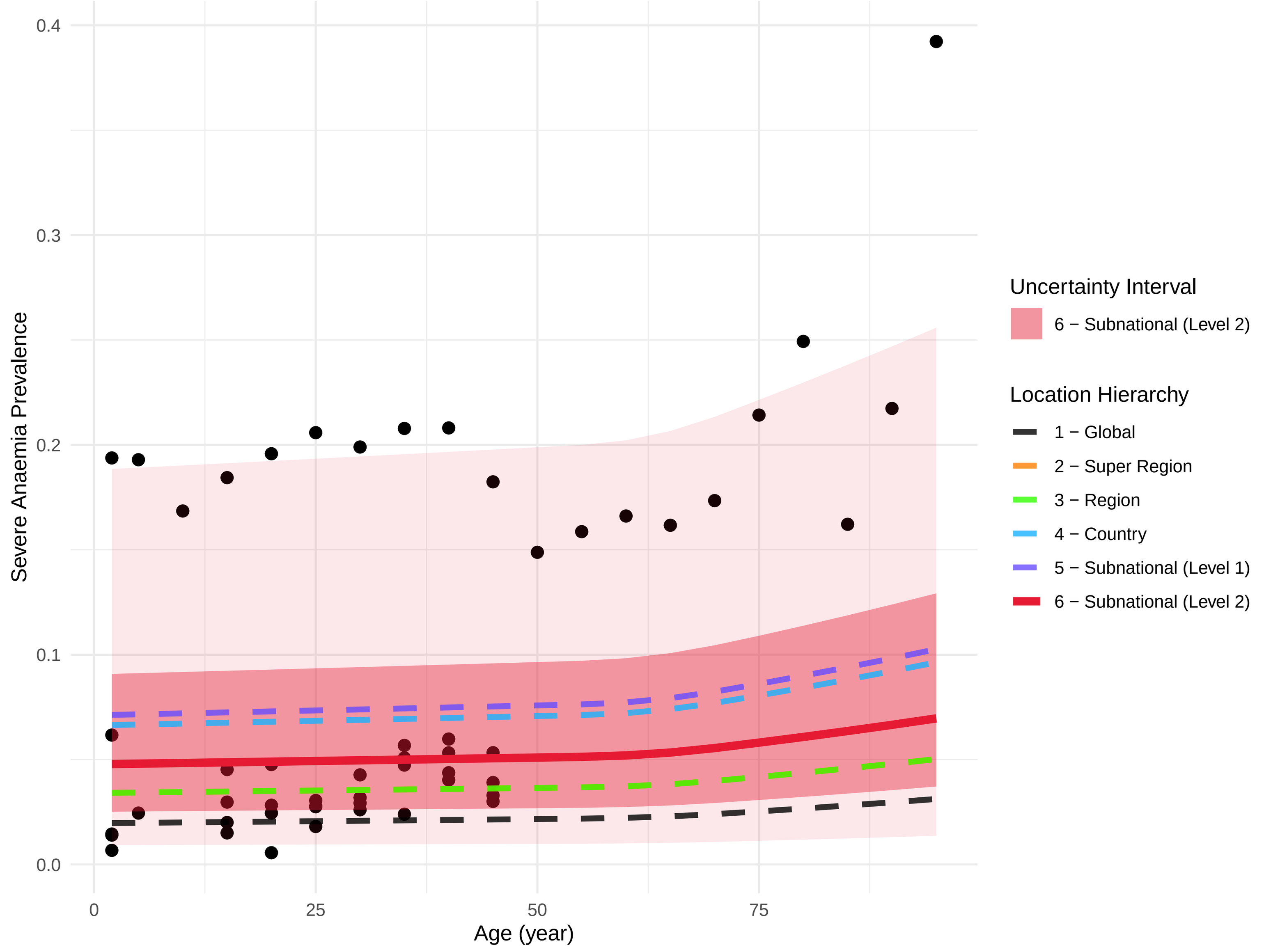

MR−BRT Age/Sex Fits − Non−pregnant Females 2+ Years Old − Severe Anaemia Prevalence − Kerala
WHO−2024 Adjustment Method

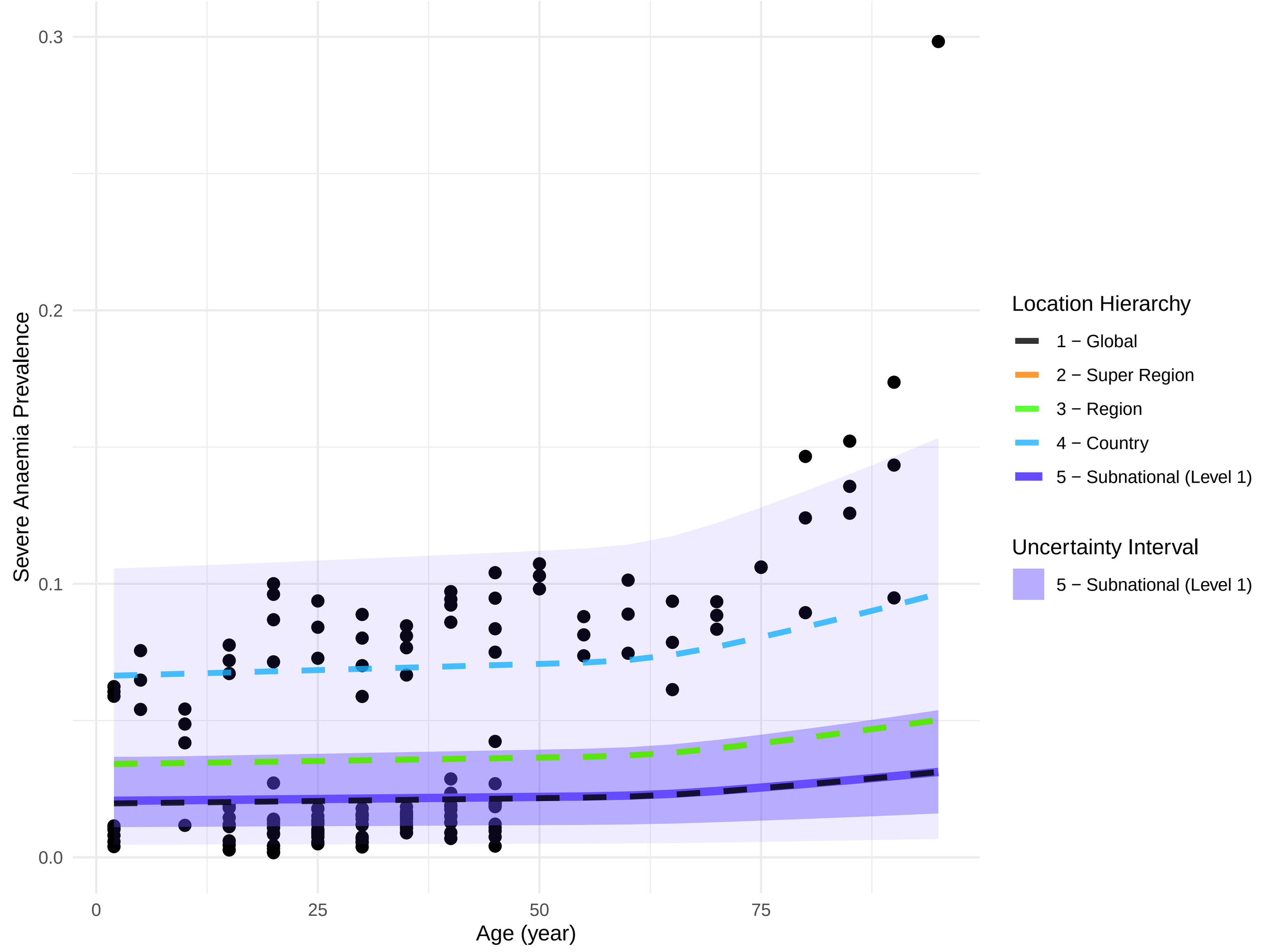

MR−BRT Age/Sex Fits − Non−pregnant Females 2+ Years Old − Severe Anaemia Prevalence − Kerala, Rural
WHO−2024 Adjustment Method

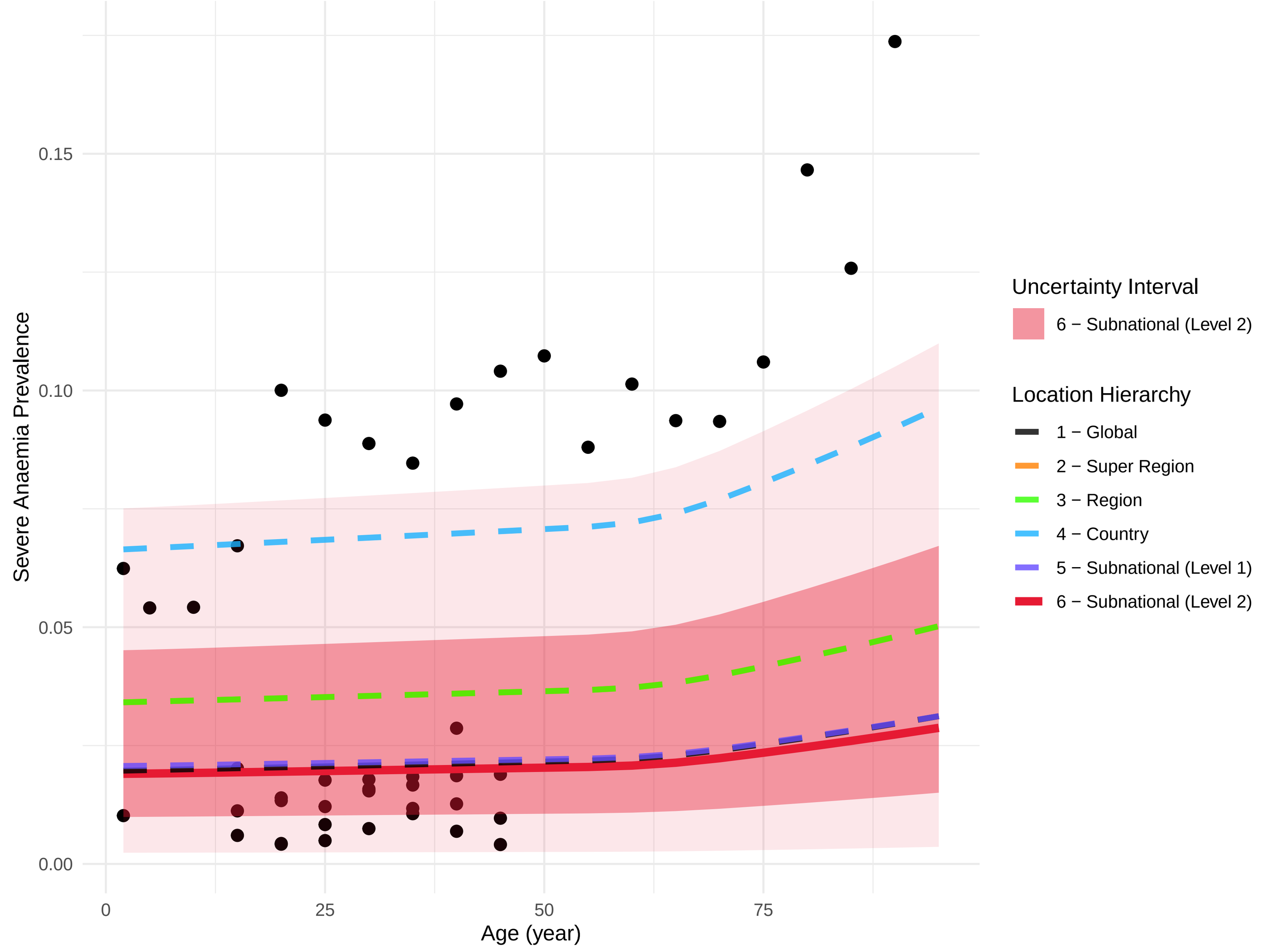

MR−BRT Age/Sex Fits − Non−pregnant Females 2+ Years Old − Severe Anaemia Prevalence − Kerala, Urban
WHO−2024 Adjustment Method

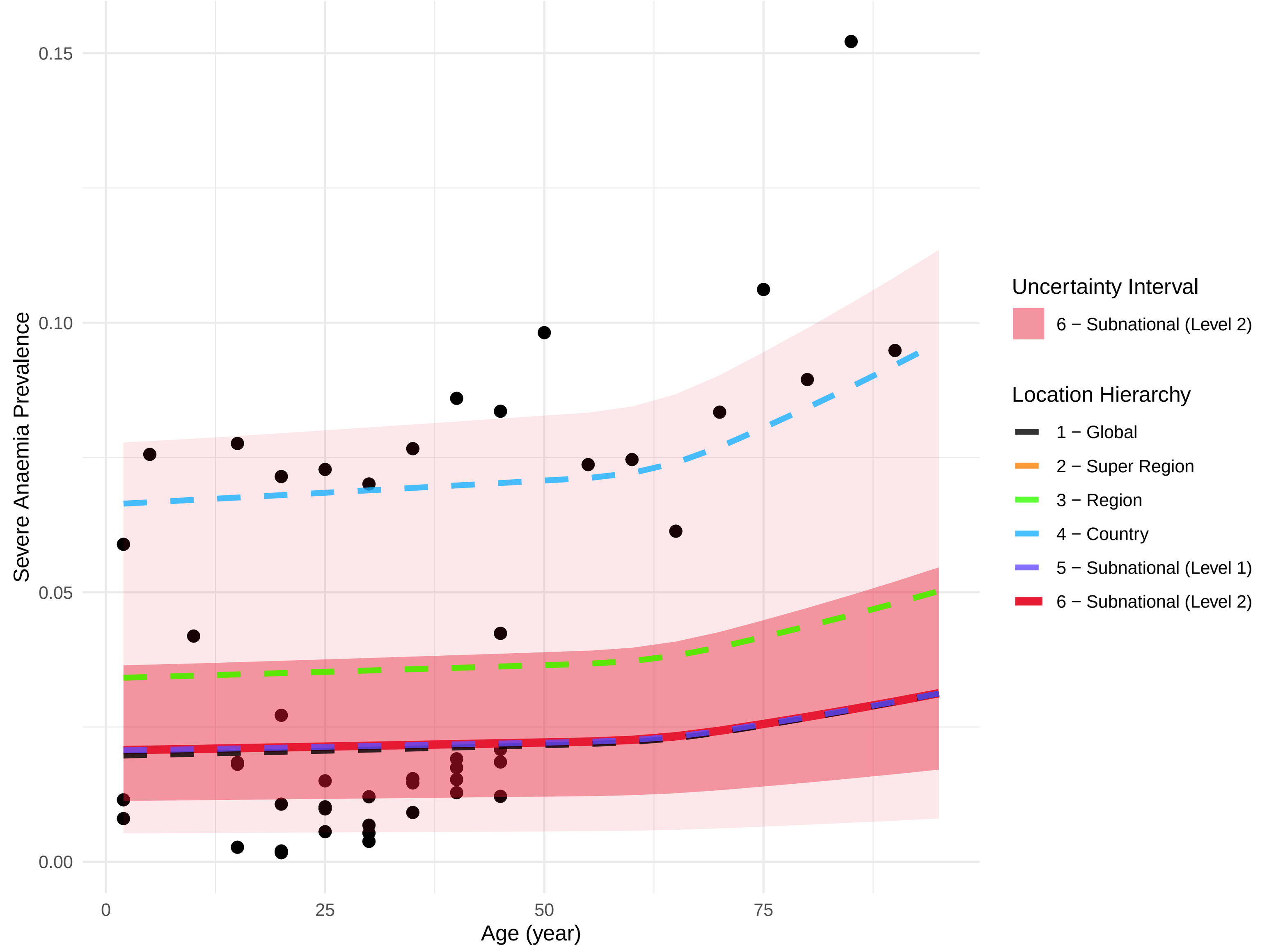

MR−BRT Age/Sex Fits − Non−pregnant Females 2+ Years Old − Severe Anaemia Prevalence − Madhya Pradesh
WHO−2024 Adjustment Method

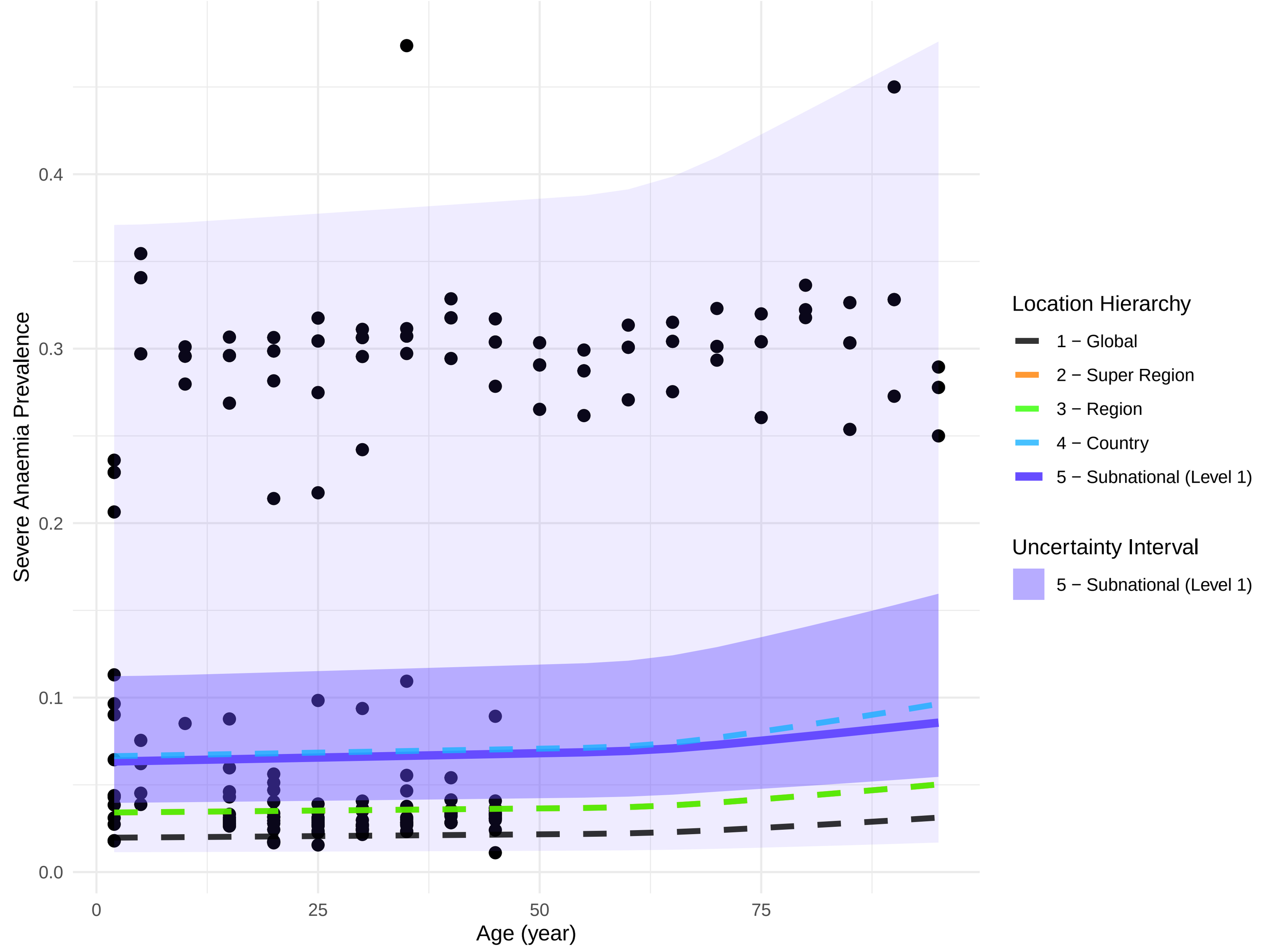

MR−BRT Age/Sex Fits − Non−pregnant Females 2+ Years Old − Severe Anaemia Prevalence − Madhya Pradesh, Rural
WHO−2024 Adjustment Method

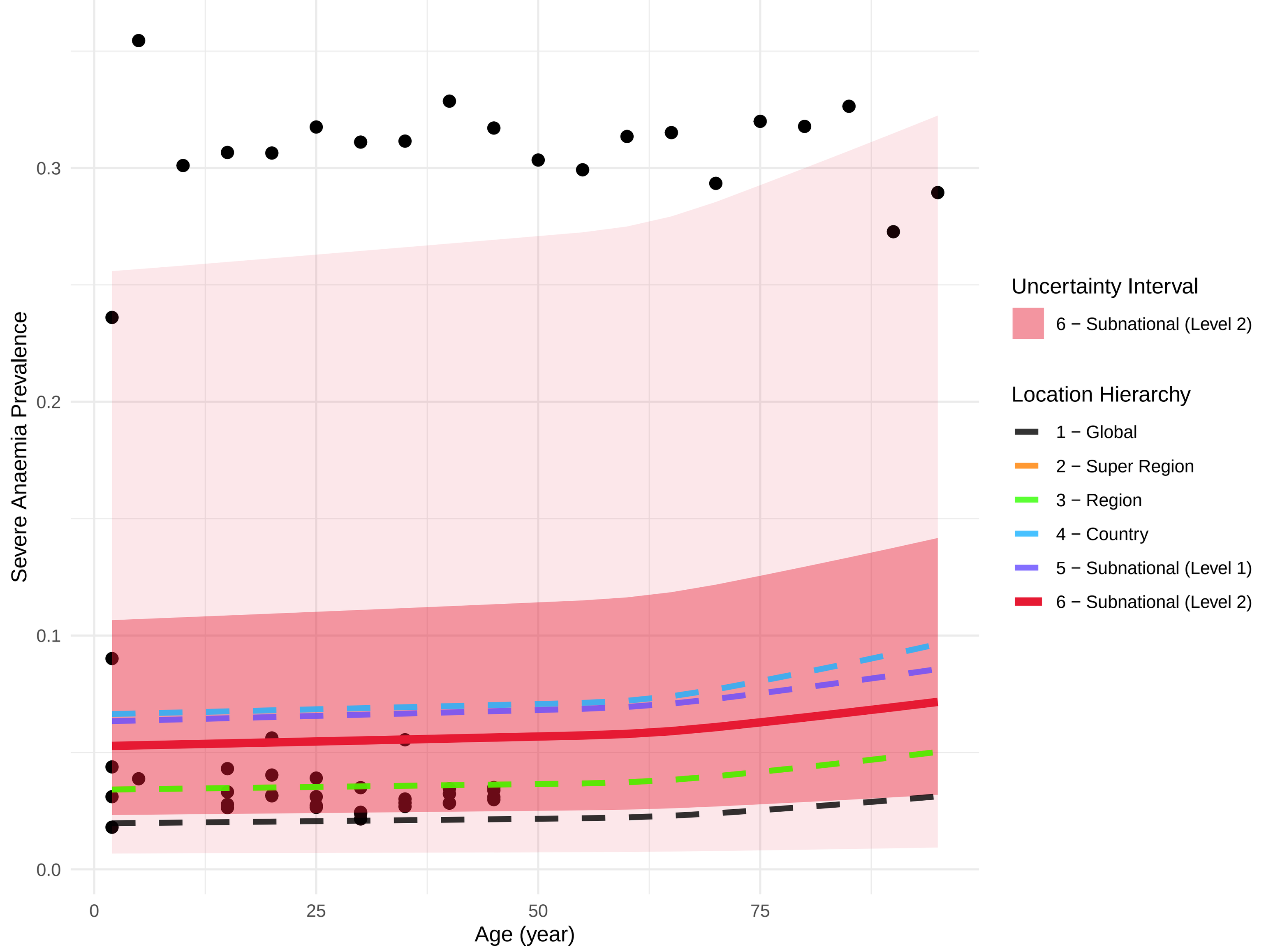

MR−BRT Age/Sex Fits − Non−pregnant Females 2+ Years Old − Severe Anaemia Prevalence − Madhya Pradesh, Urban
WHO−2024 Adjustment Method

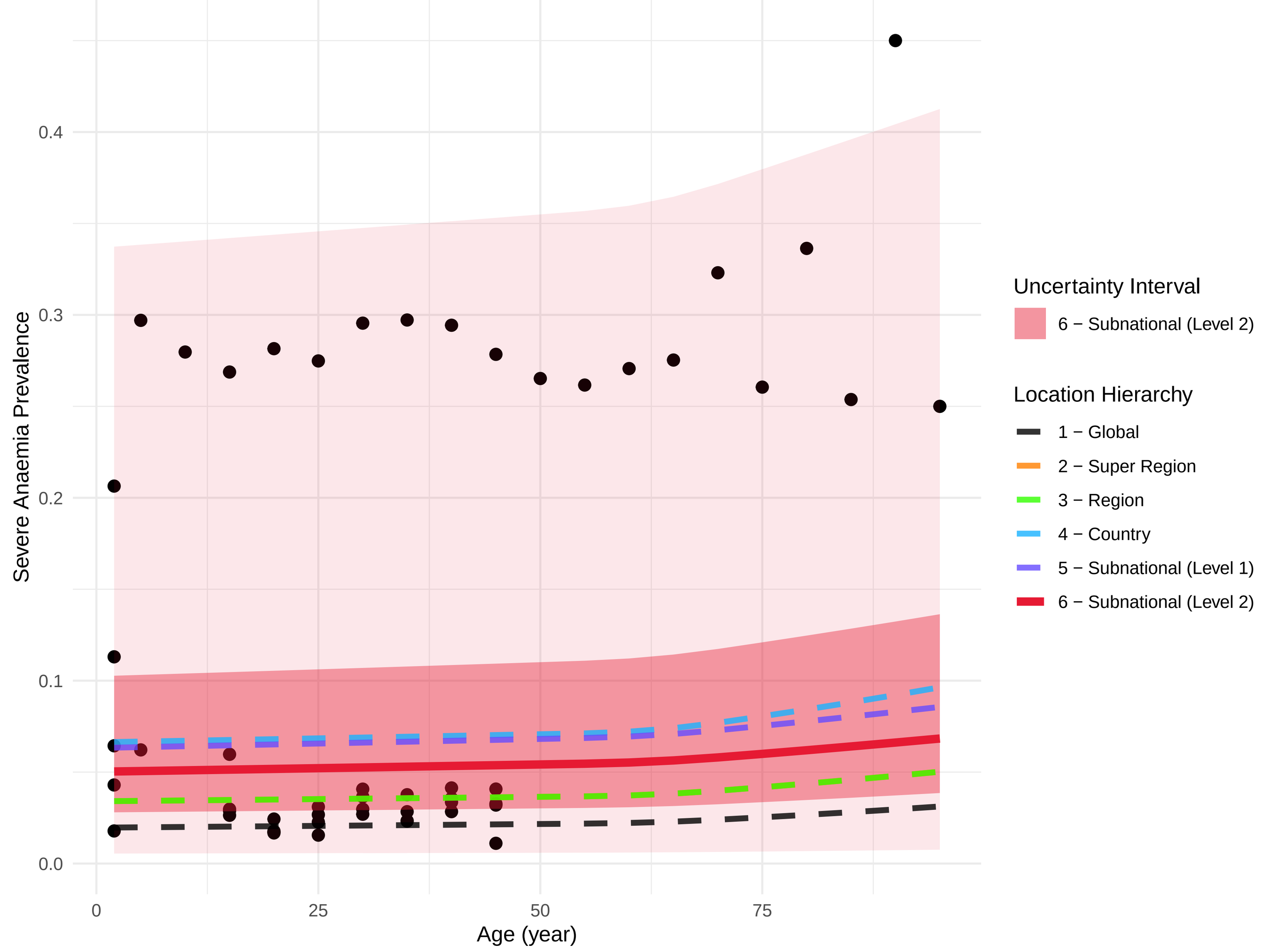

MR−BRT Age/Sex Fits − Non−pregnant Females 2+ Years Old − Severe Anaemia Prevalence − Maharashtra
WHO−2024 Adjustment Method

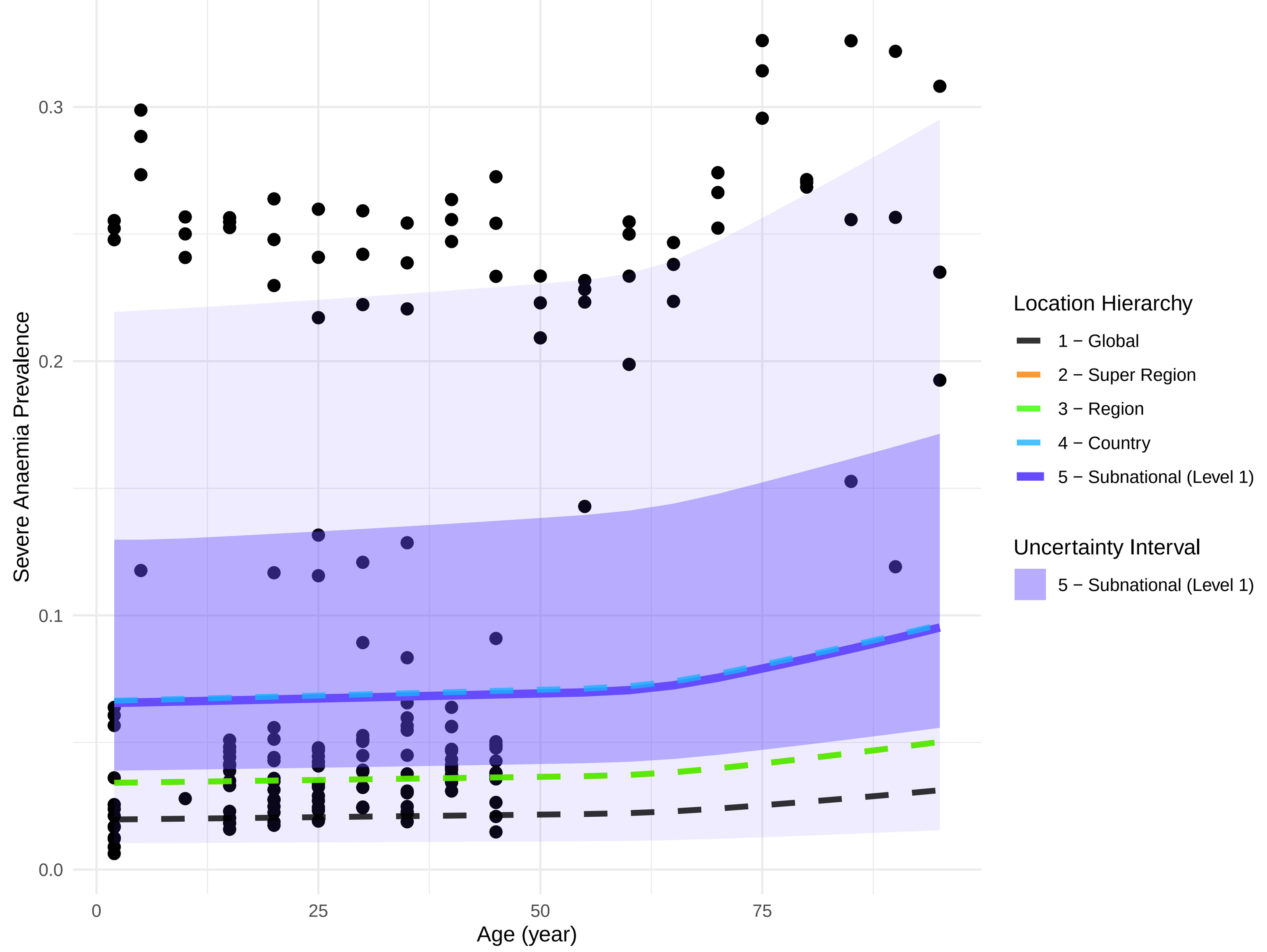

MR−BRT Age/Sex Fits − Non−pregnant Females 2+ Years Old − Severe Anaemia Prevalence − Maharashtra, Rural
WHO−2024 Adjustment Method

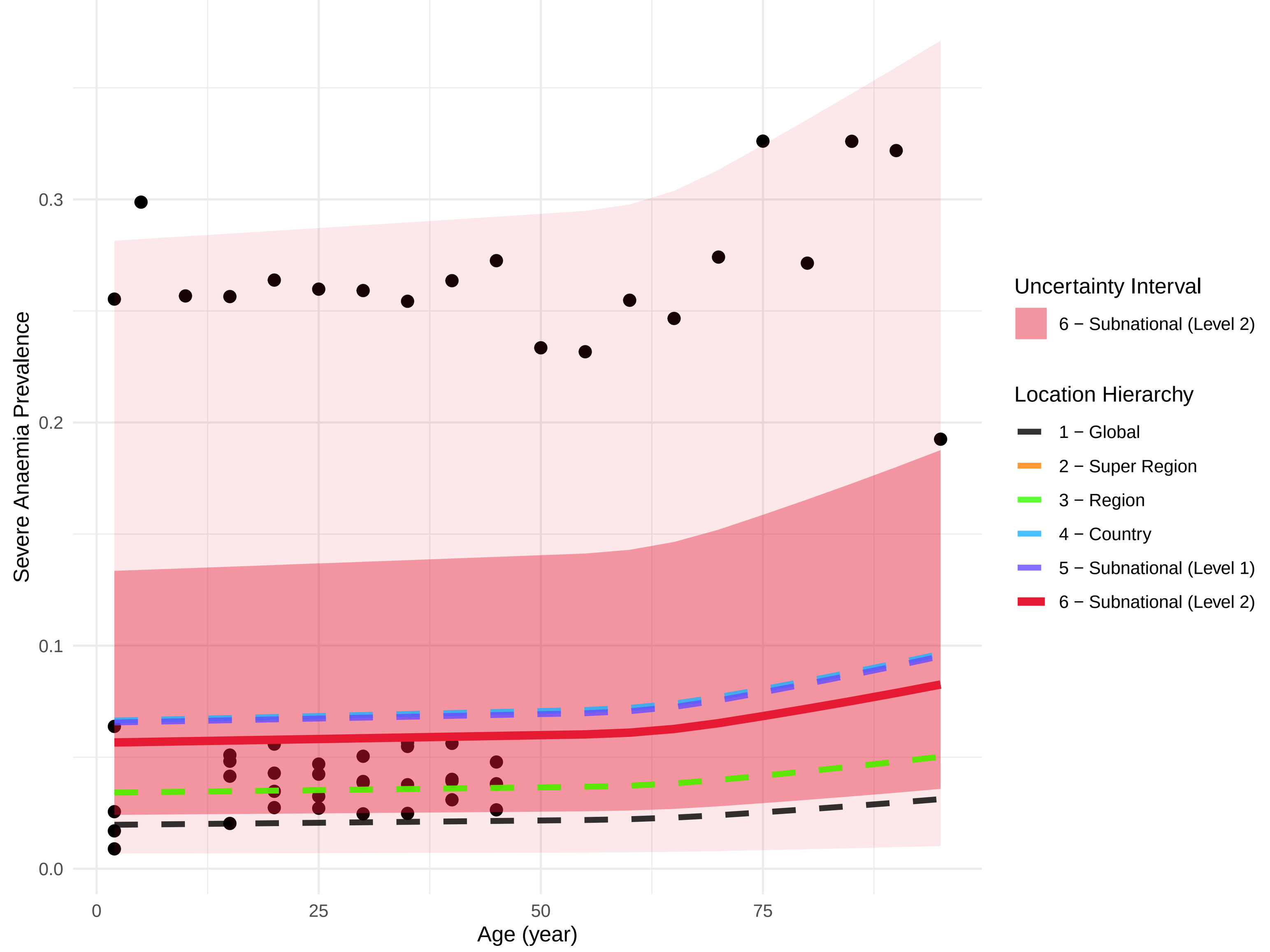

MR−BRT Age/Sex Fits − Non−pregnant Females 2+ Years Old − Severe Anaemia Prevalence − Maharashtra, Urban
WHO−2024 Adjustment Method

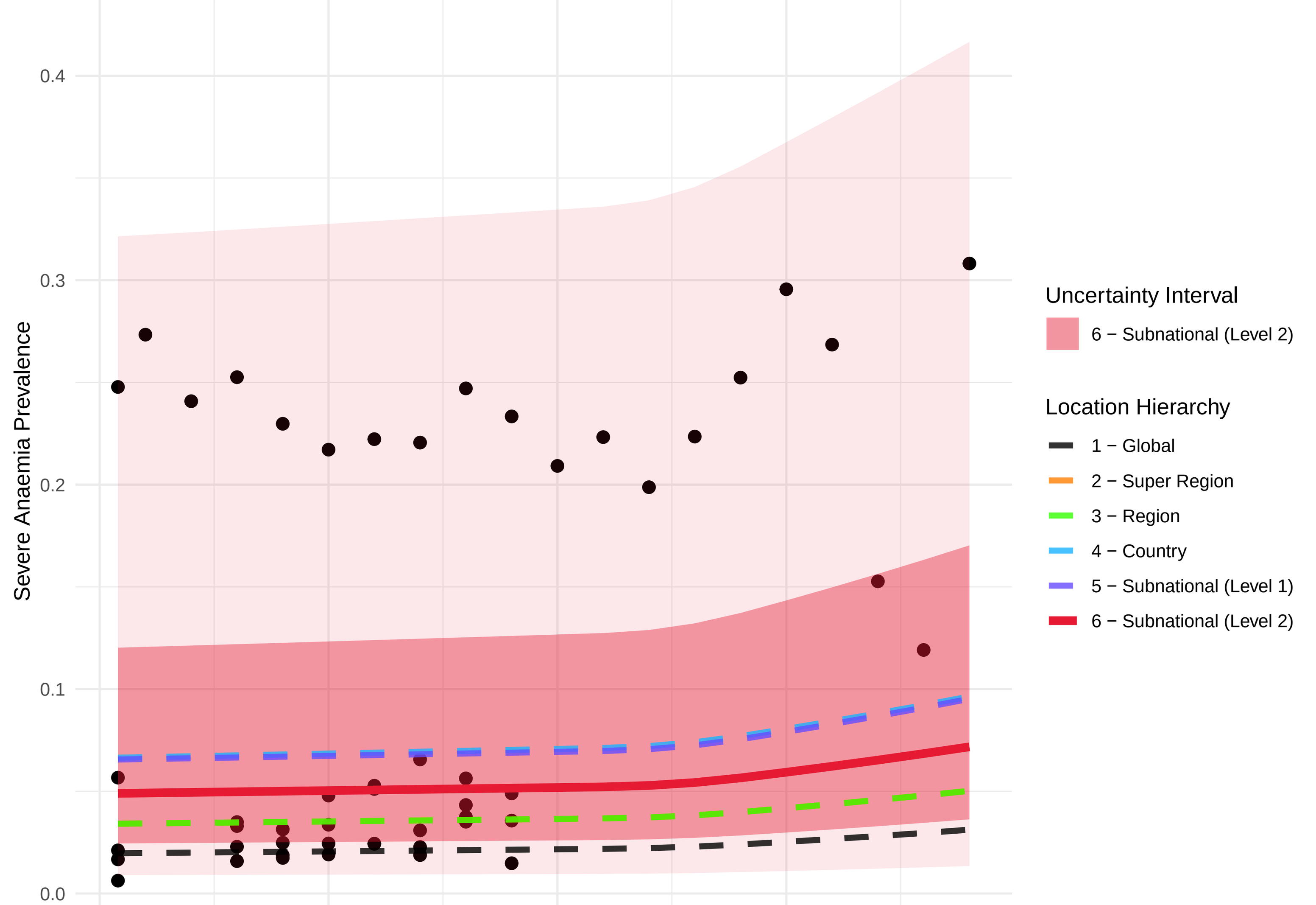

MR−BRT Age/Sex Fits − Non−pregnant Females 2+ Years Old − Severe Anaemia Prevalence − Manipur
WHO−2024 Adjustment Method

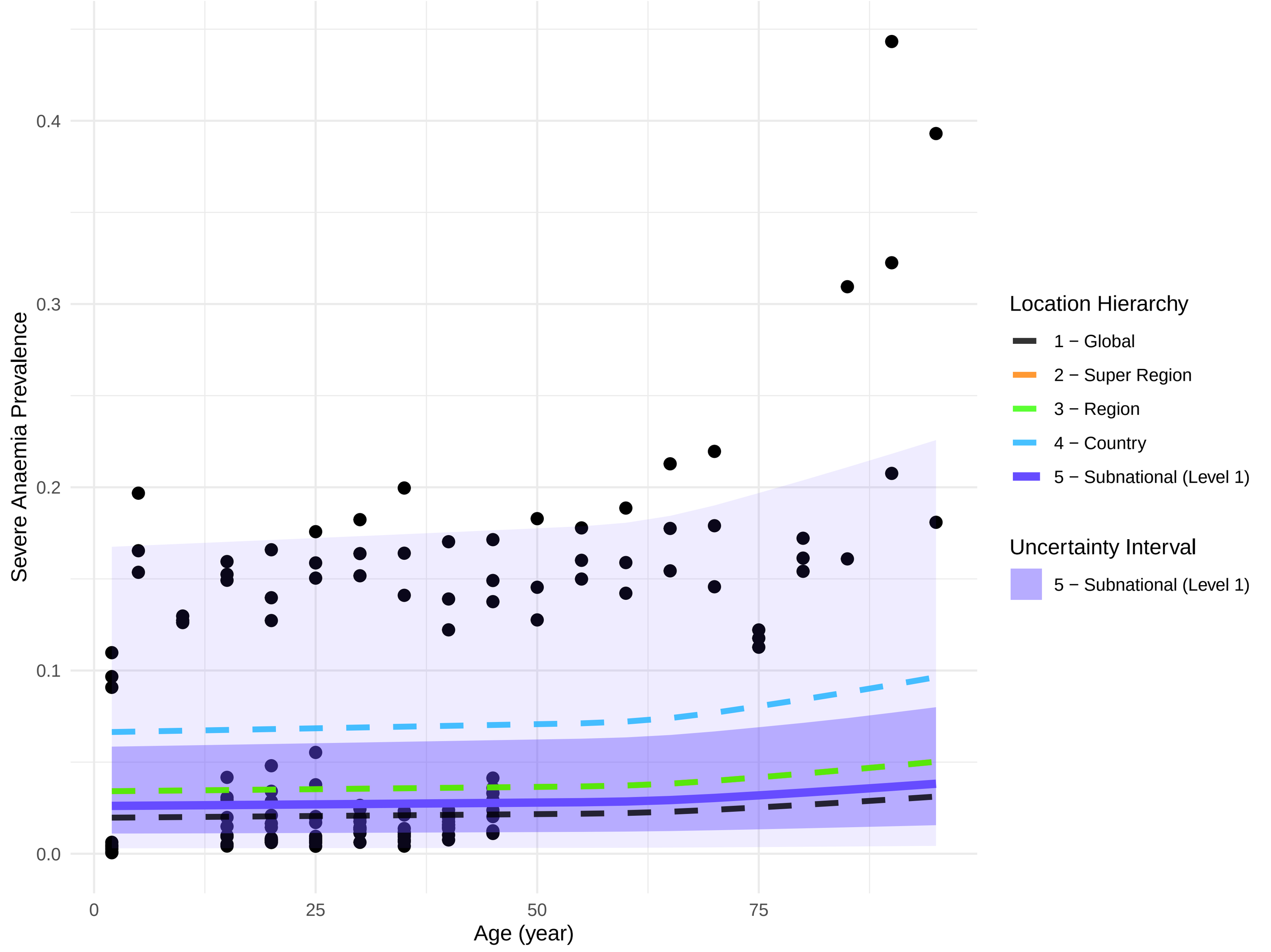

MR−BRT Age/Sex Fits − Non−pregnant Females 2+ Years Old − Severe Anaemia Prevalence − Manipur, Rural
WHO−2024 Adjustment Method

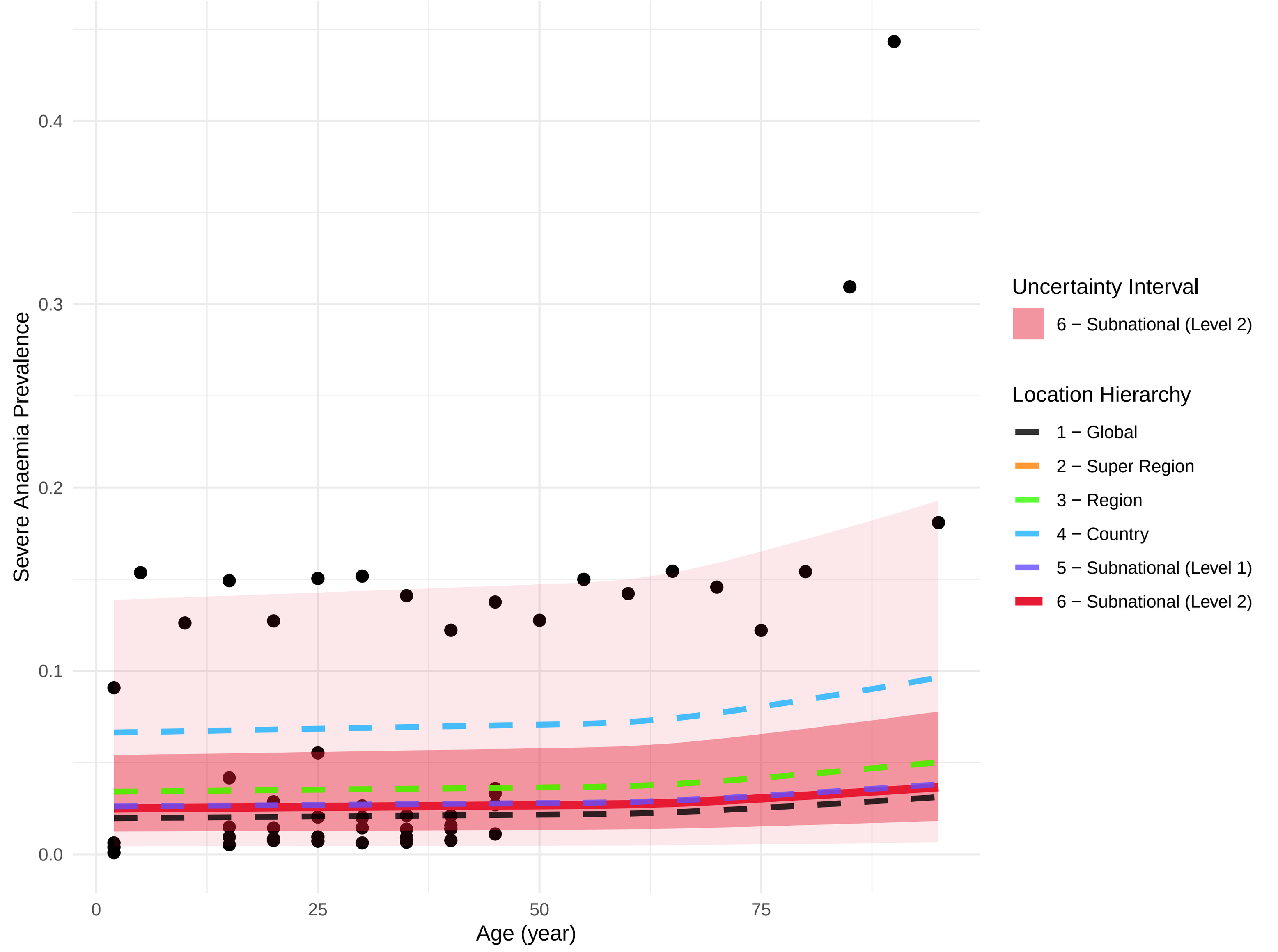

MR−BRT Age/Sex Fits − Non−pregnant Females 2+ Years Old − Severe Anaemia Prevalence − Manipur, Urban
WHO−2024 Adjustment Method

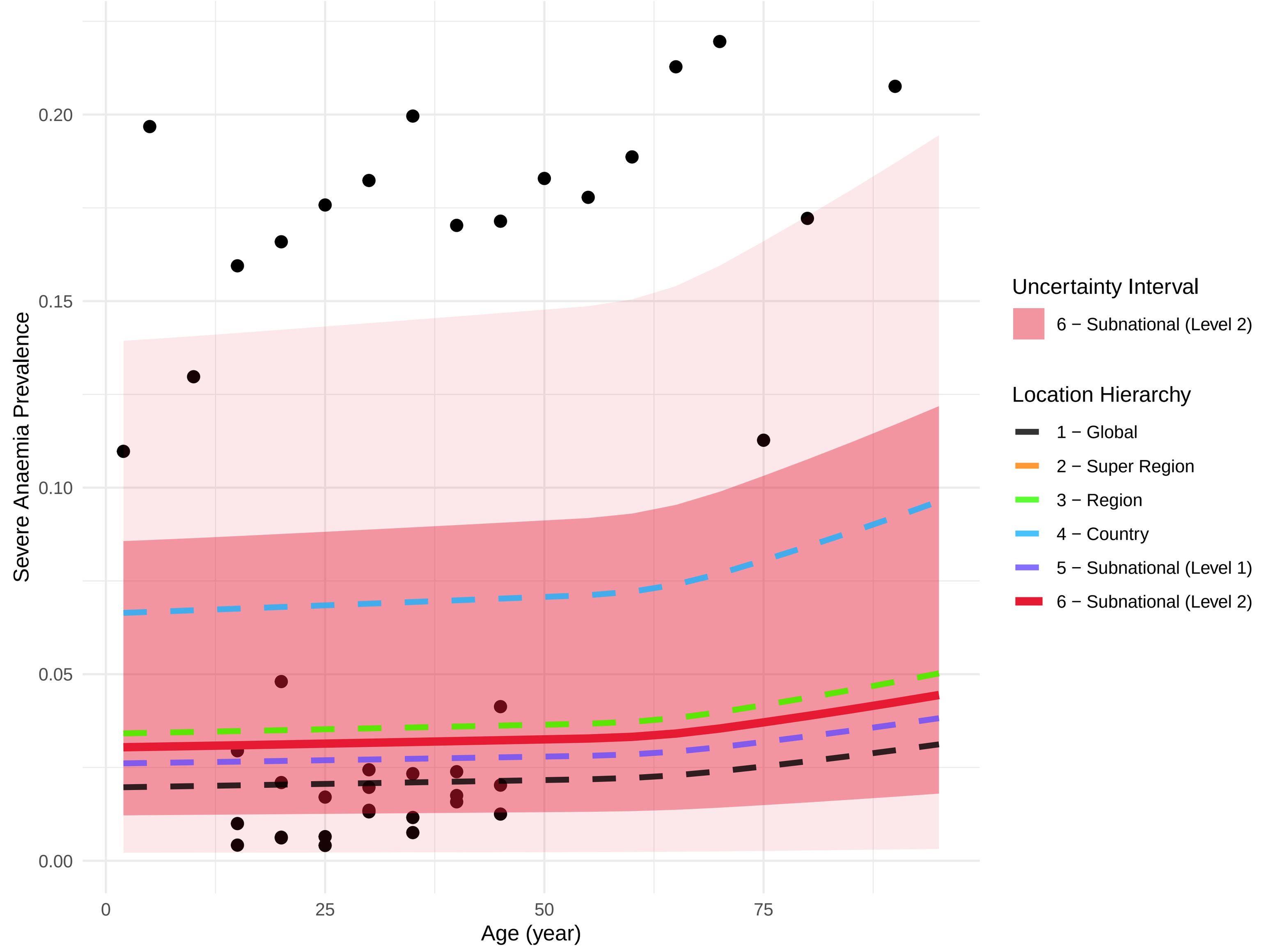

MR−BRT Age/Sex Fits − Non−pregnant Females 2+ Years Old − Severe Anaemia Prevalence − Meghalaya
WHO−2024 Adjustment Method

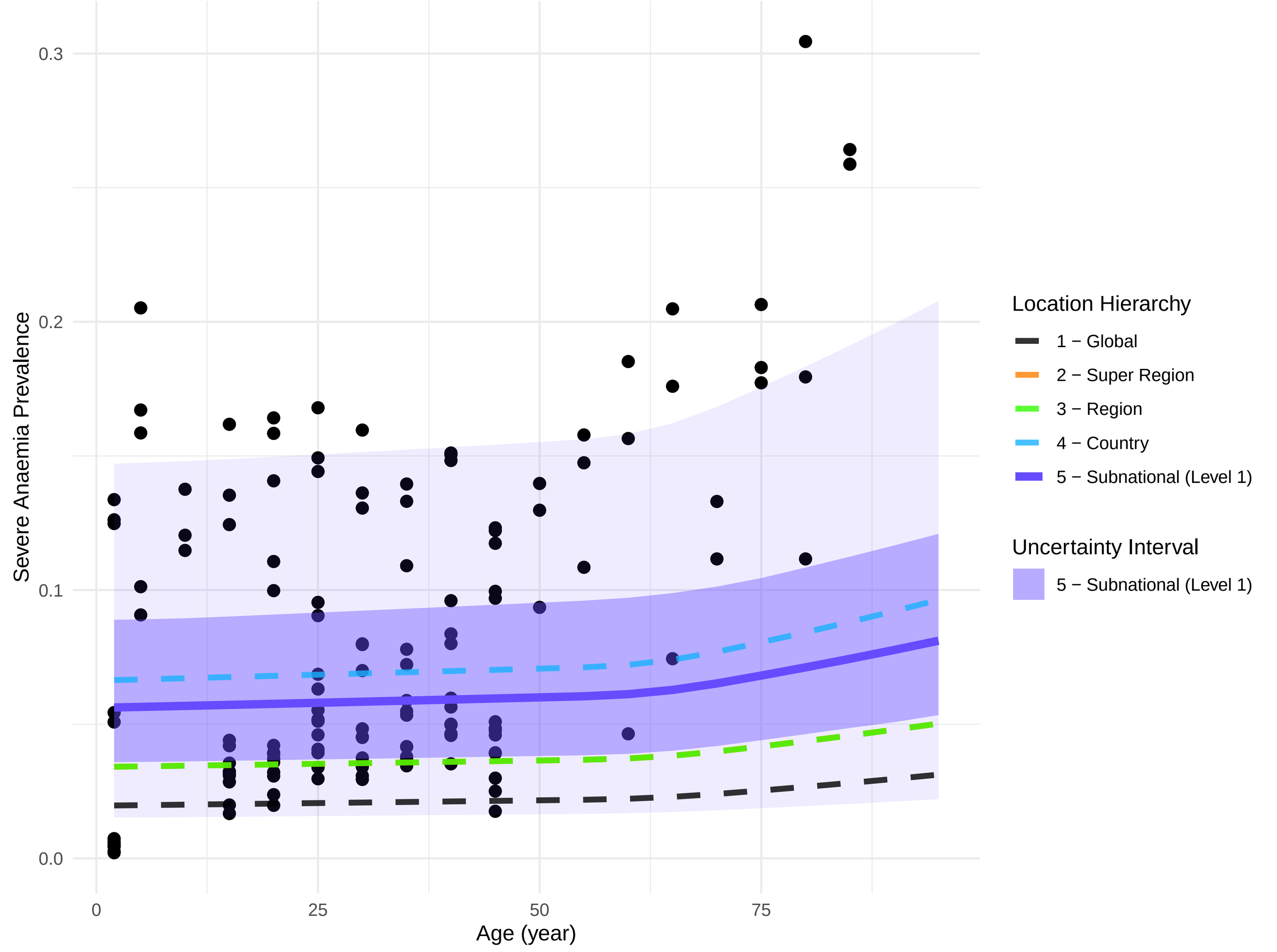

MR−BRT Age/Sex Fits − Non−pregnant Females 2+ Years Old − Severe Anaemia Prevalence − Meghalaya, Rural
WHO−2024 Adjustment Method

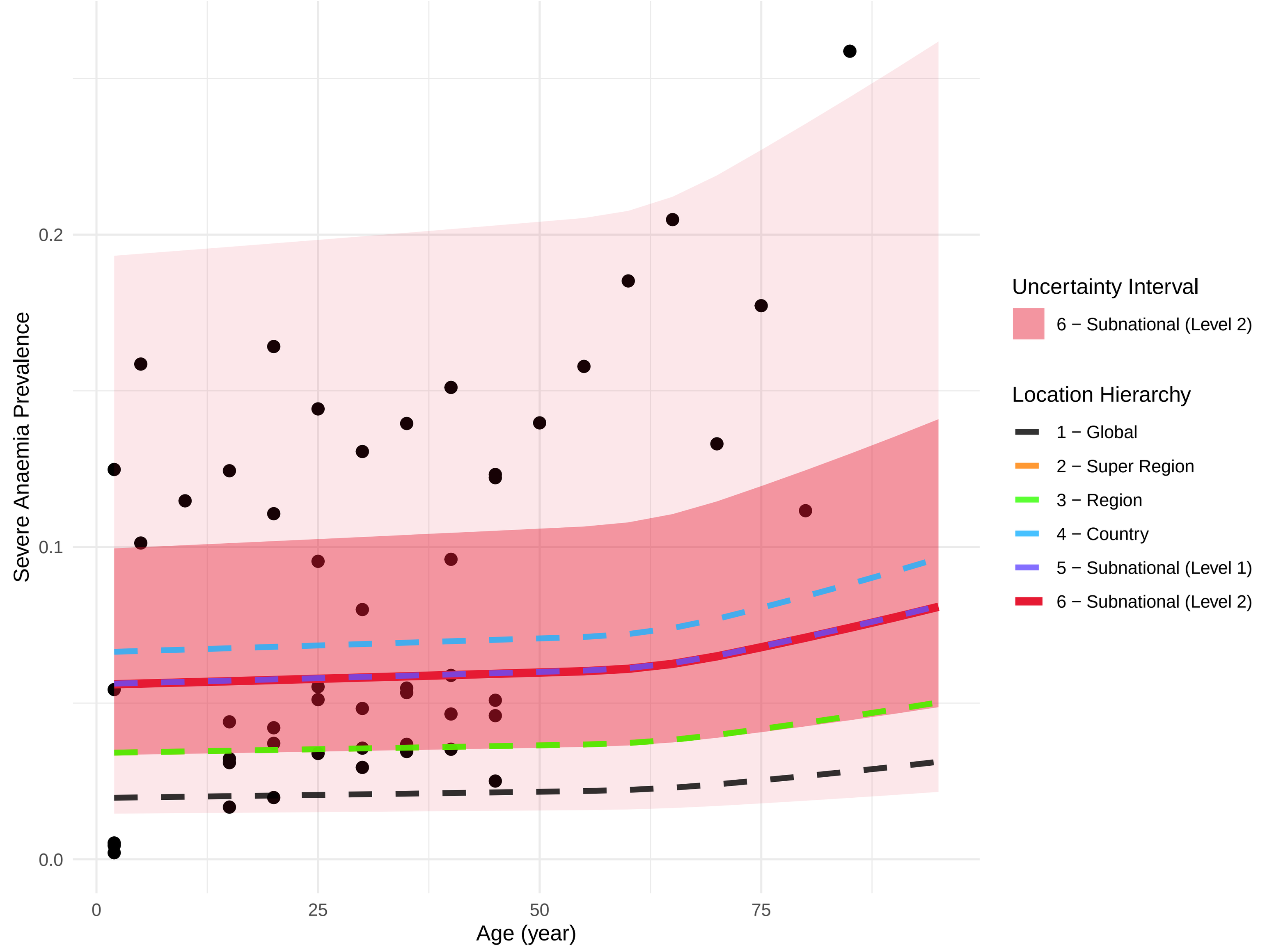

MR−BRT Age/Sex Fits − Non−pregnant Females 2+ Years Old − Severe Anaemia Prevalence − Meghalaya, Urban
WHO−2024 Adjustment Method

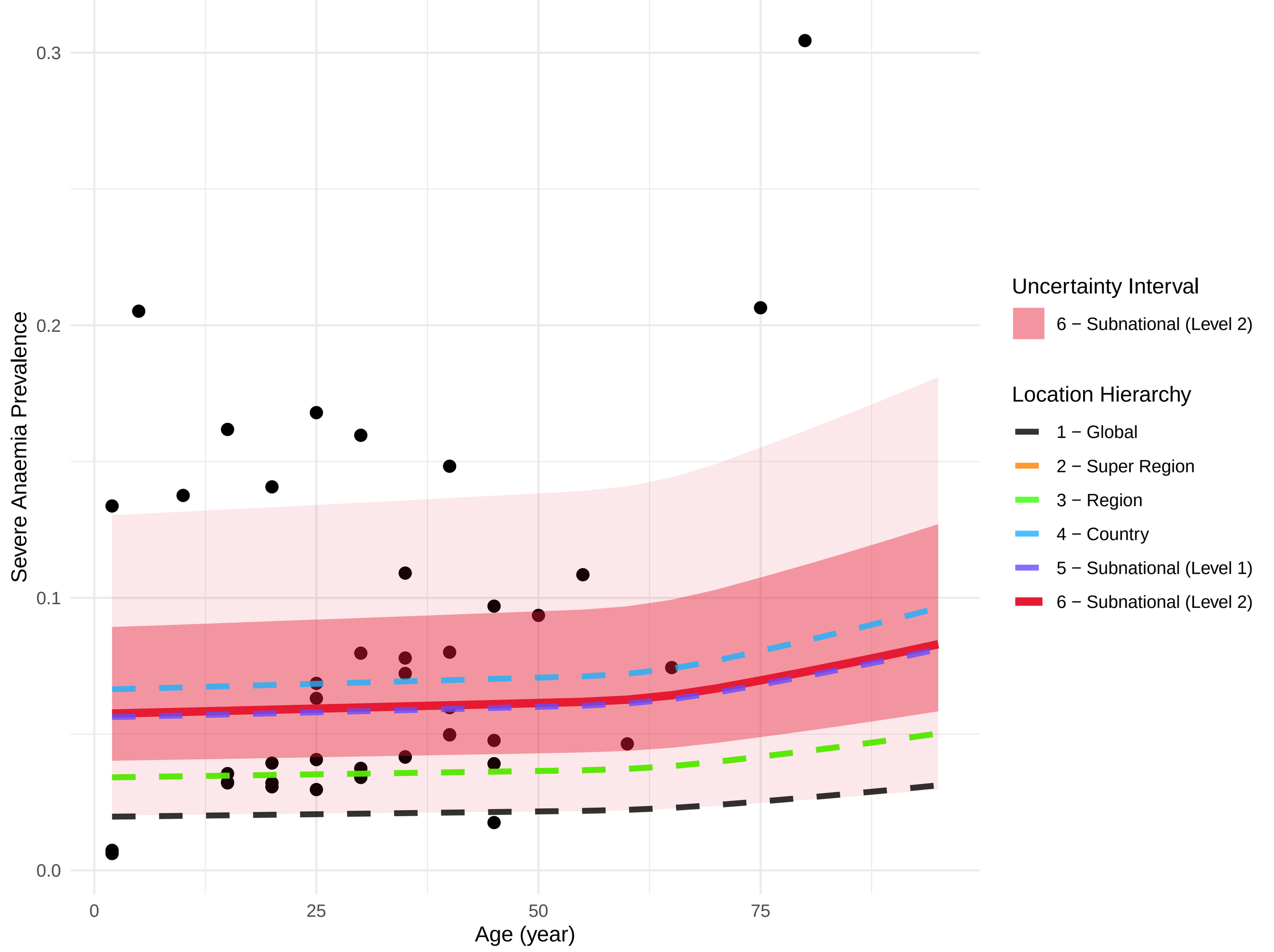

MR−BRT Age/Sex Fits − Non−pregnant Females 2+ Years Old − Severe Anaemia Prevalence − Mizoram
WHO−2024 Adjustment Method

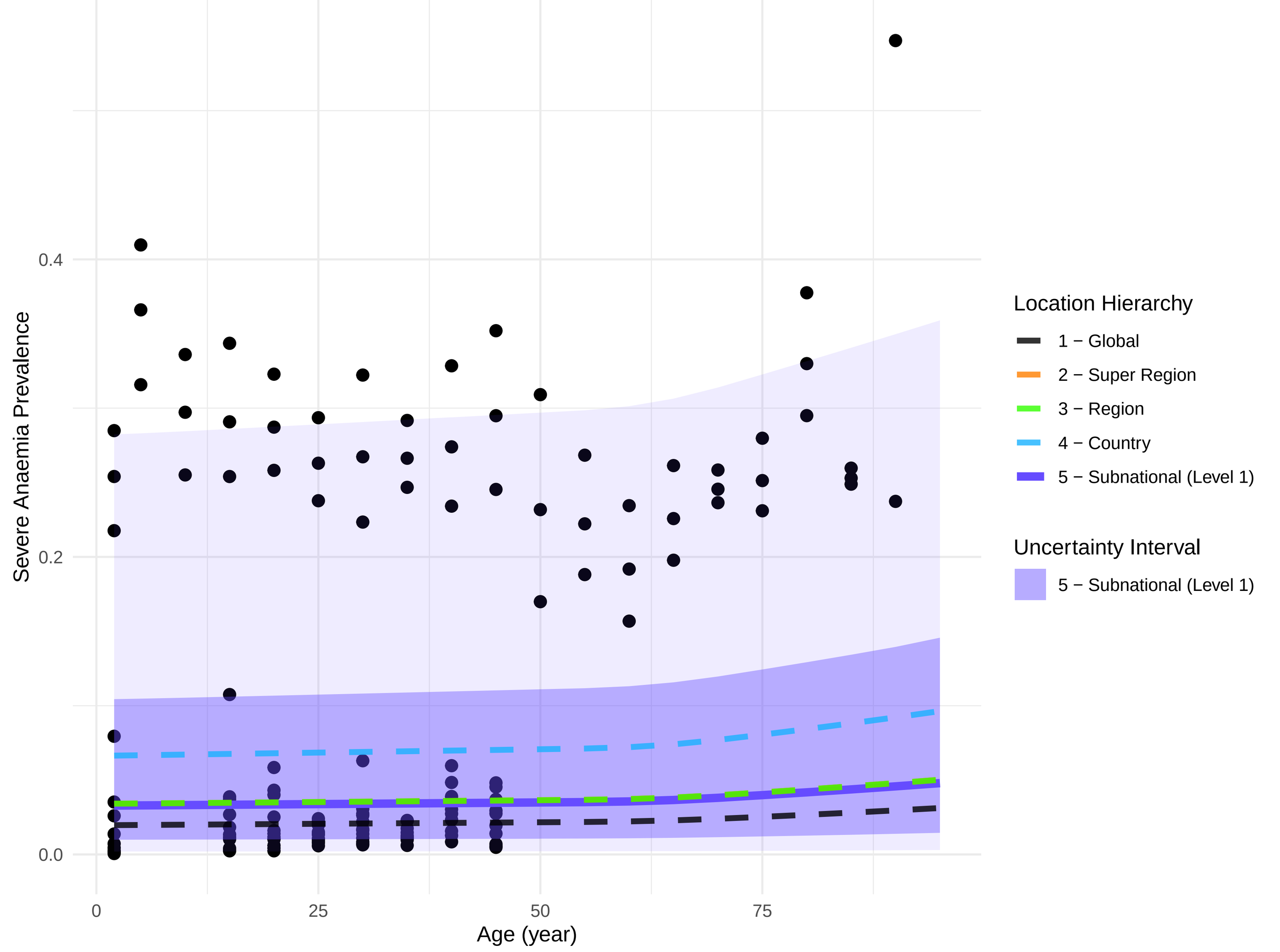

MR−BRT Age/Sex Fits − Non−pregnant Females 2+ Years Old − Severe Anaemia Prevalence − Mizoram, Rural
WHO−2024 Adjustment Method

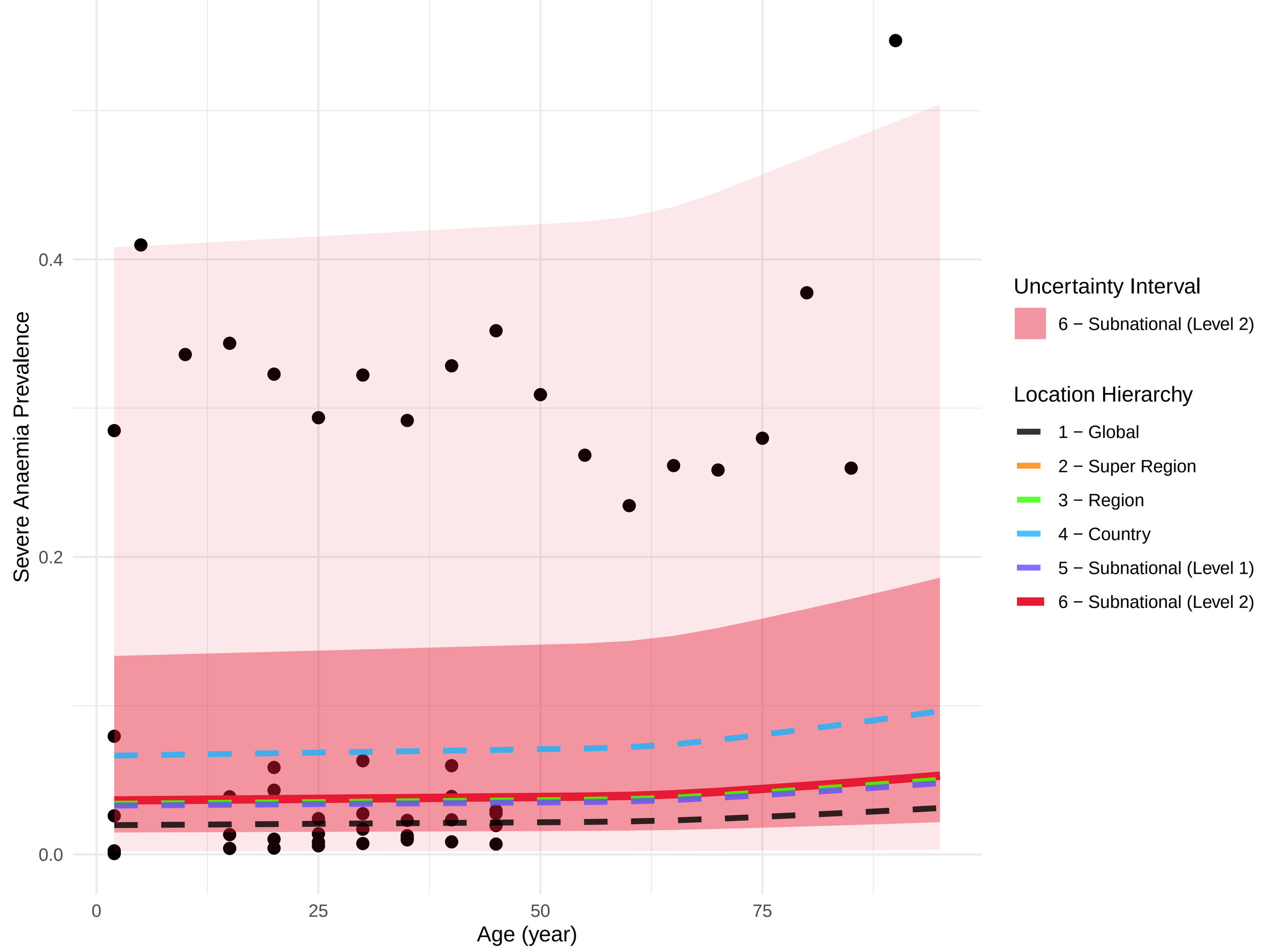

MR−BRT Age/Sex Fits − Non−pregnant Females 2+ Years Old − Severe Anaemia Prevalence − Mizoram, Urban
WHO−2024 Adjustment Method

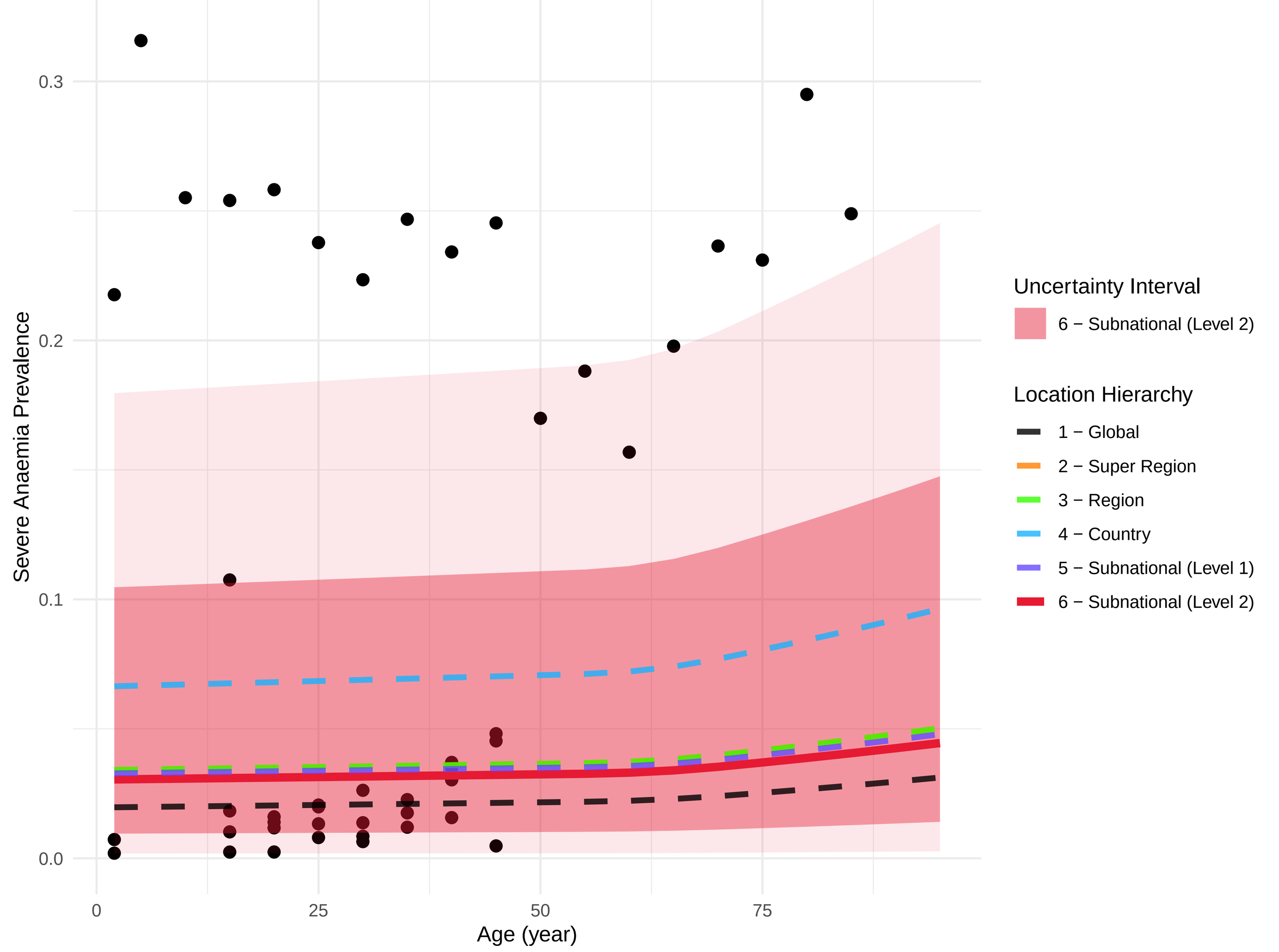

MR−BRT Age/Sex Fits − Non−pregnant Females 2+ Years Old − Severe Anaemia Prevalence − Nagaland
WHO−2024 Adjustment Method

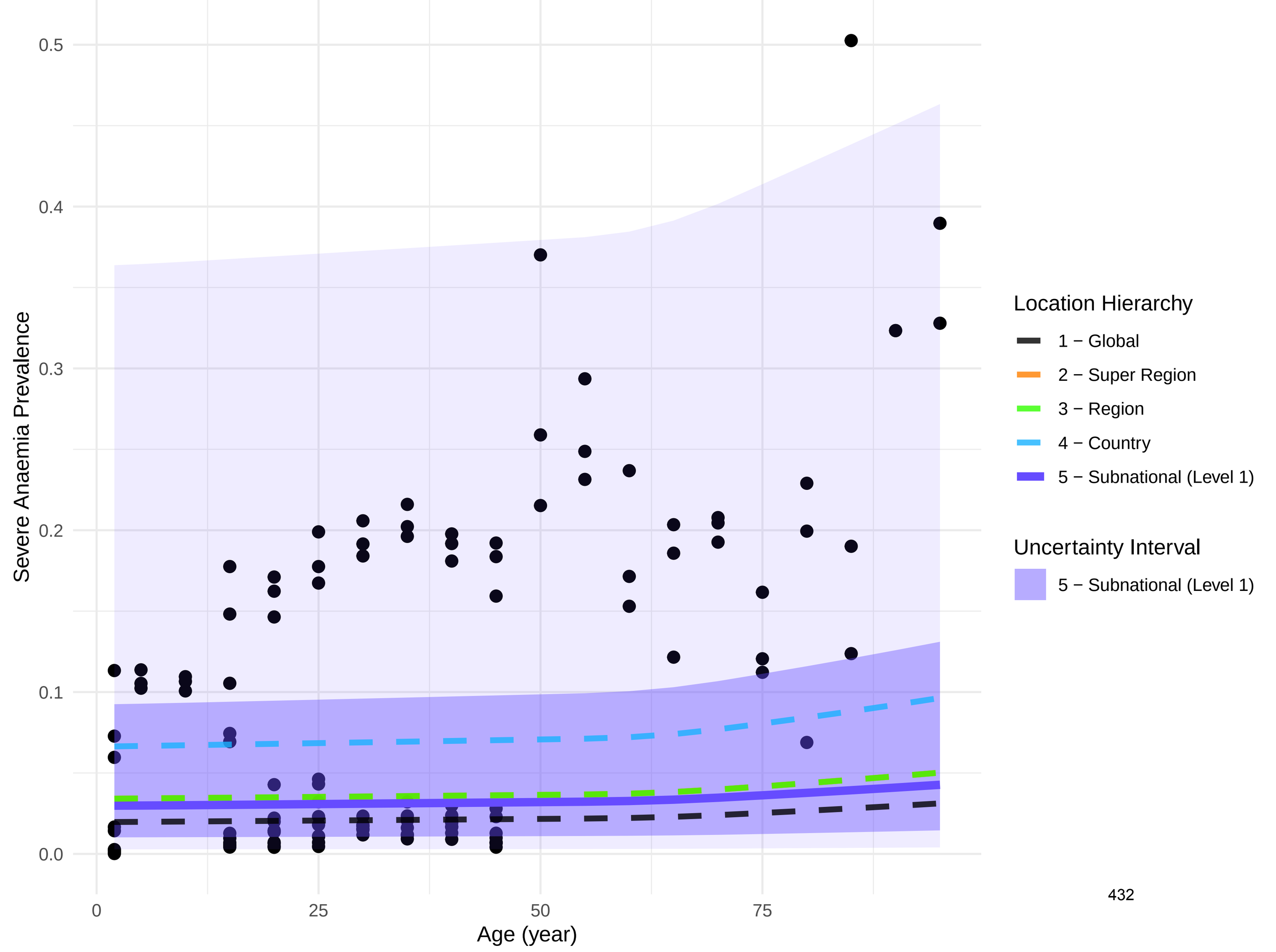

MR−BRT Age/Sex Fits − Non−pregnant Females 2+ Years Old − Severe Anaemia Prevalence − Nagaland, Rural
WHO−2024 Adjustment Method

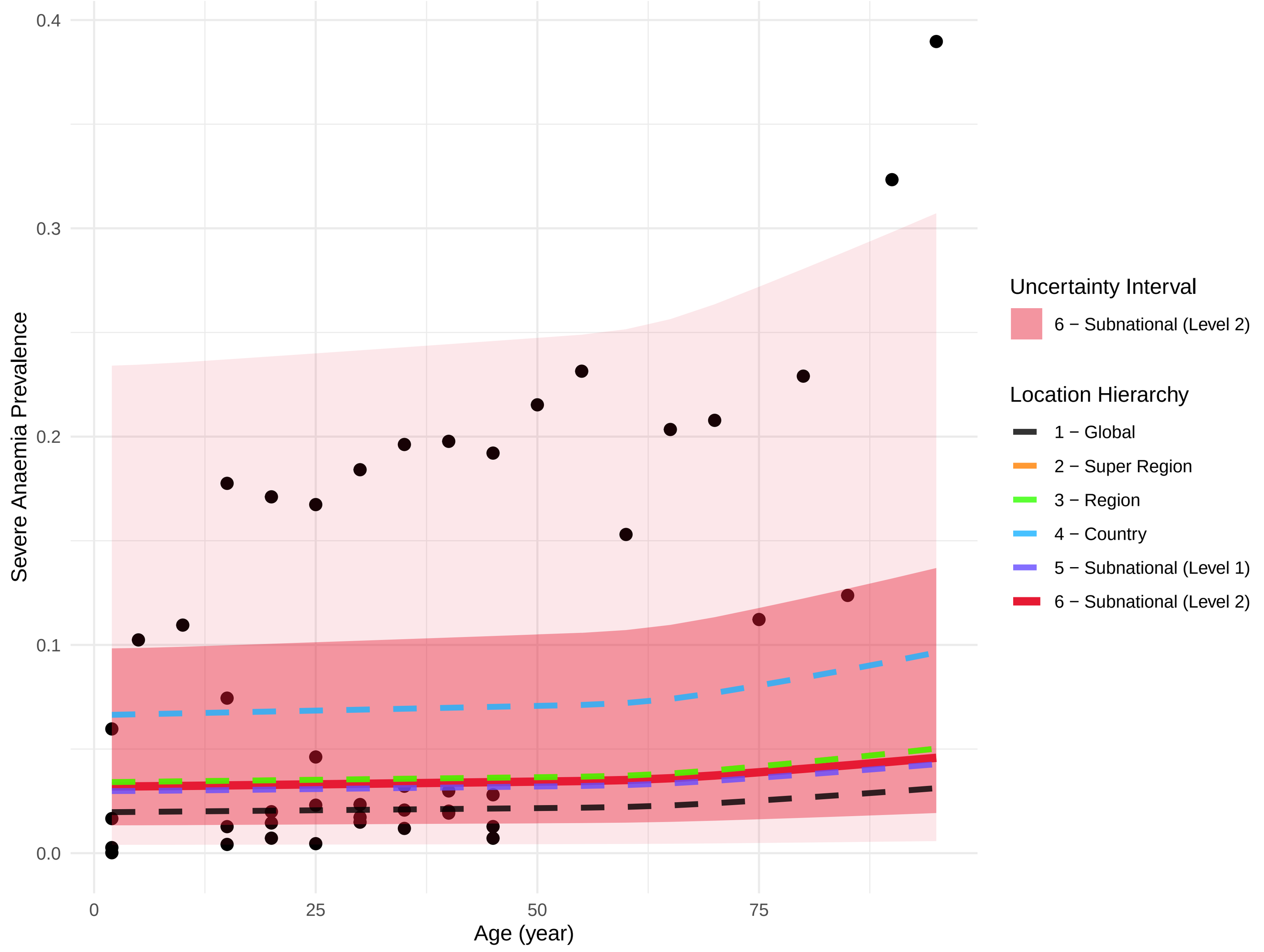

MR−BRT Age/Sex Fits − Non−pregnant Females 2+ Years Old − Severe Anaemia Prevalence − Nagaland, Urban
WHO−2024 Adjustment Method

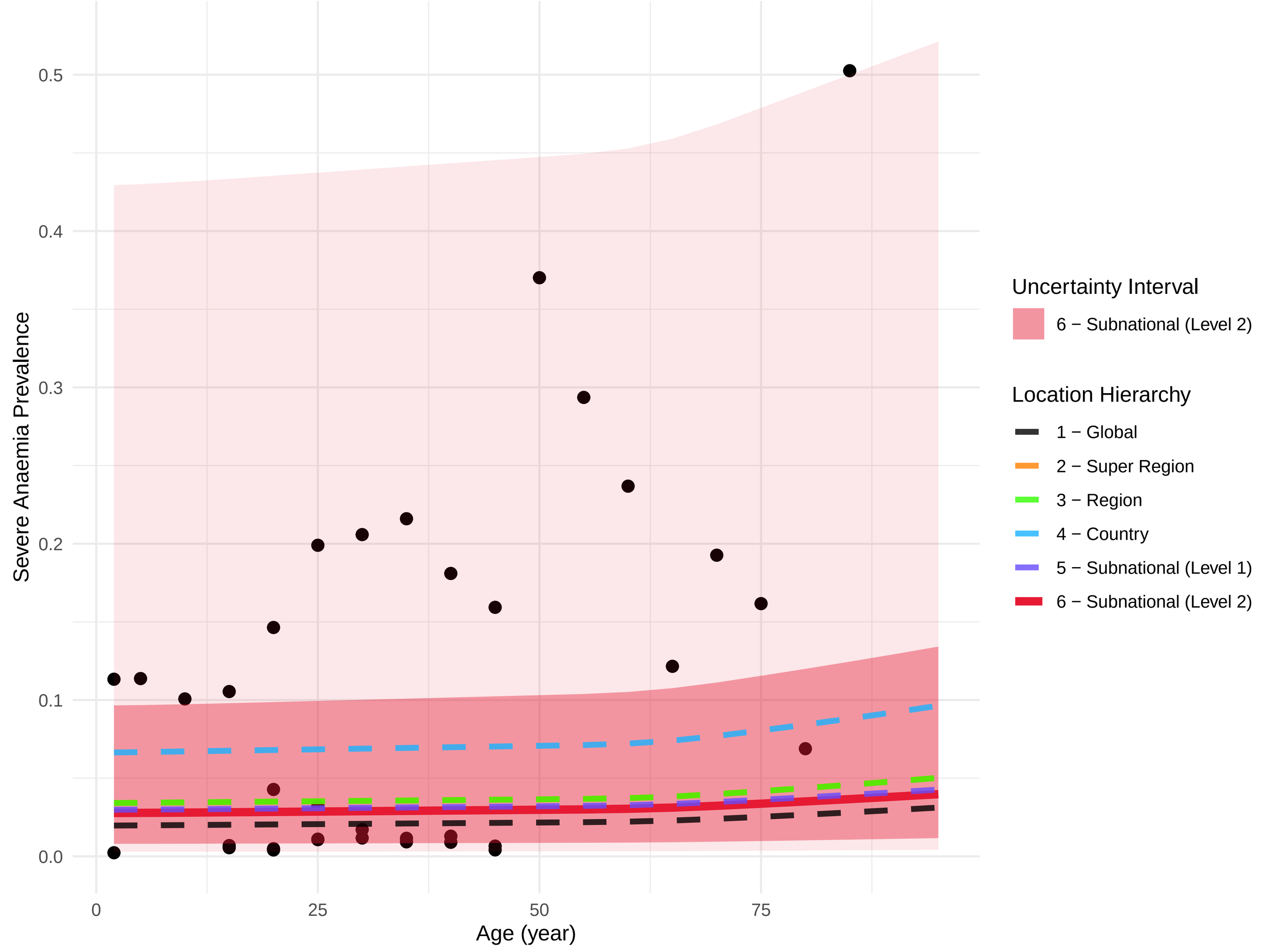

MR−BRT Age/Sex Fits − Non−pregnant Females 2+ Years Old − Severe Anaemia Prevalence − Odisha
WHO−2024 Adjustment Method

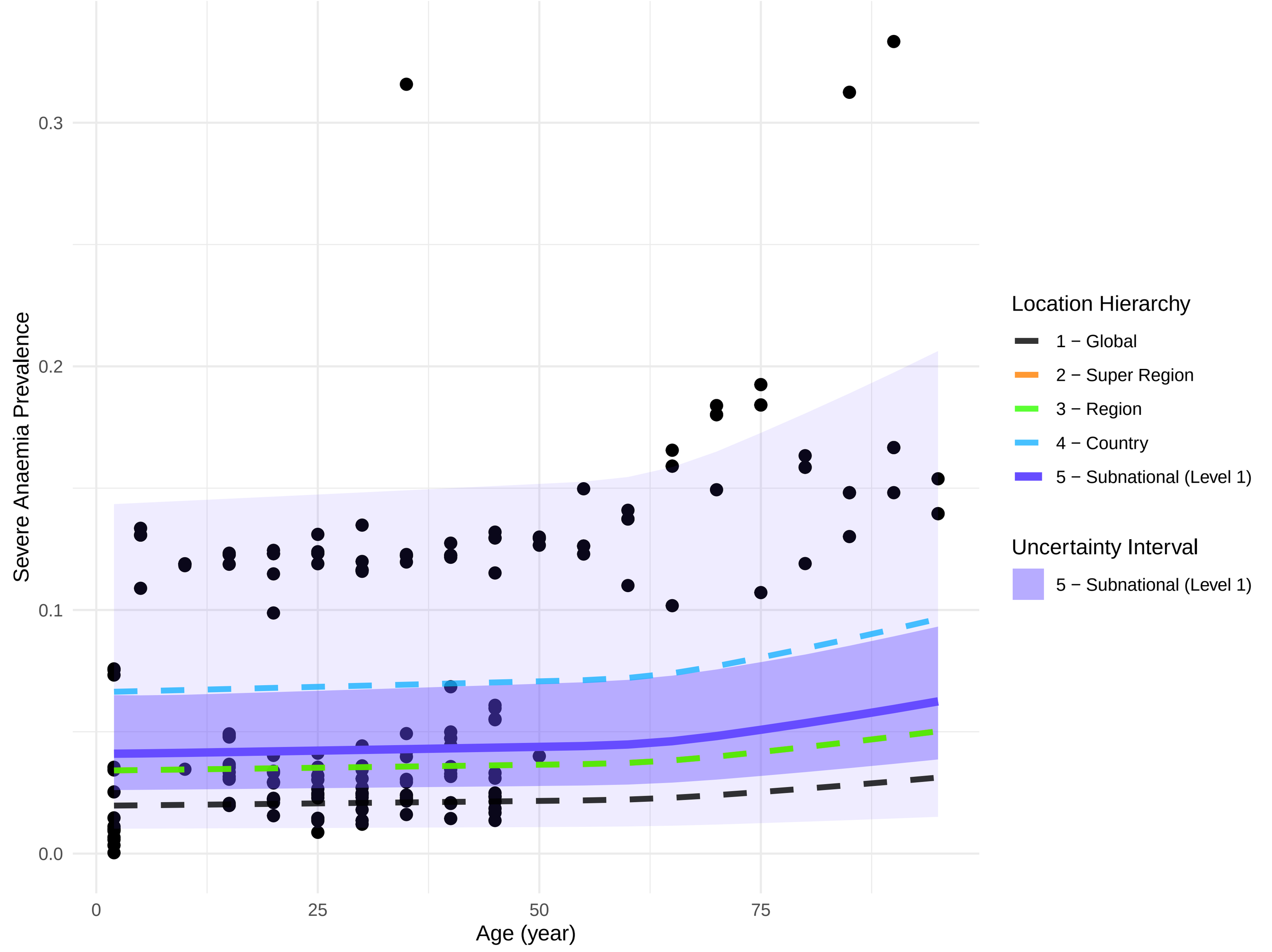

MR−BRT Age/Sex Fits − Non−pregnant Females 2+ Years Old − Severe Anaemia Prevalence − Odisha, Rural
WHO−2024 Adjustment Method

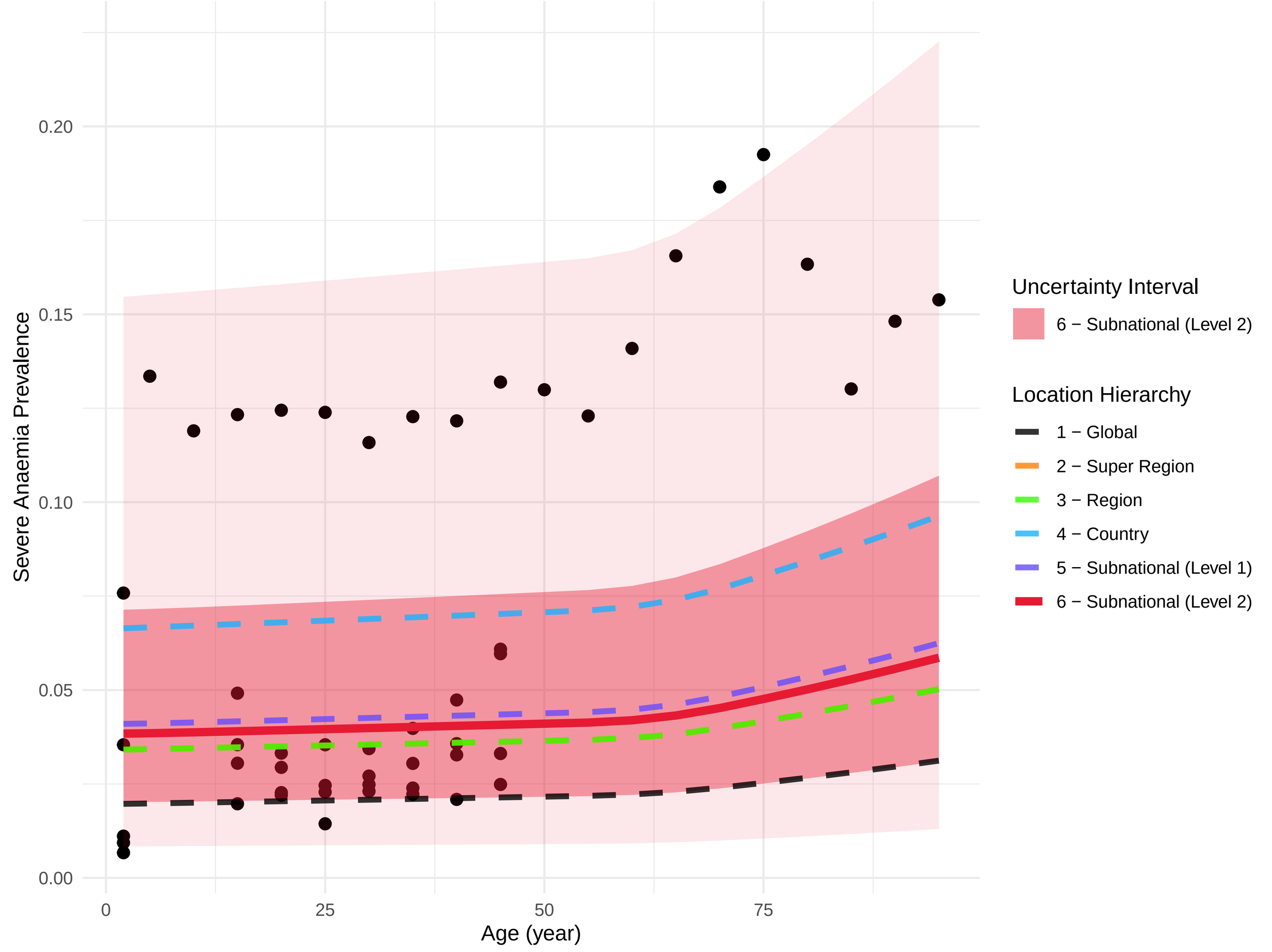

MR−BRT Age/Sex Fits − Non−pregnant Females 2+ Years Old − Severe Anaemia Prevalence − Odisha, Urban
WHO−2024 Adjustment Method

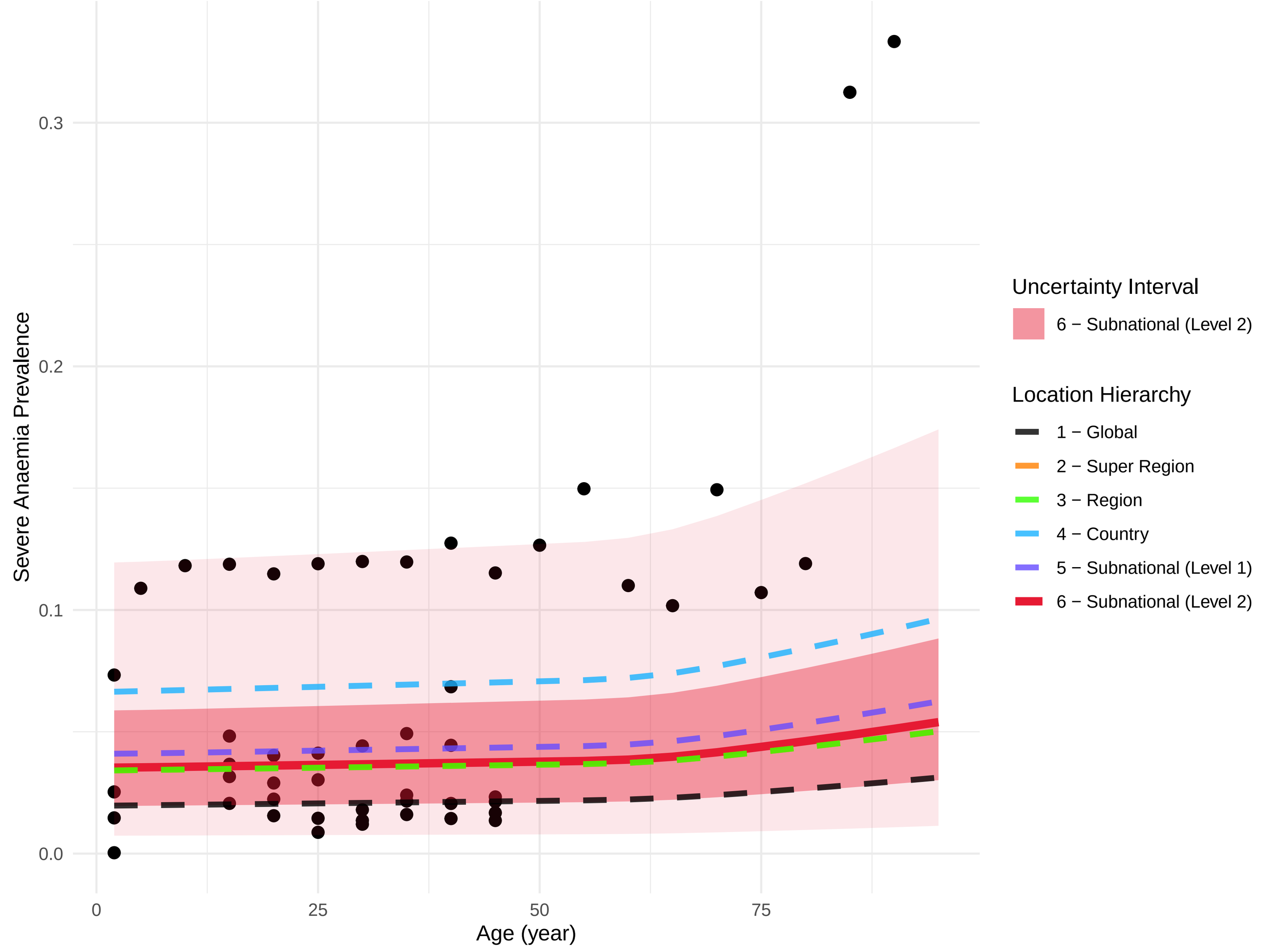

MR−BRT Age/Sex Fits − Non−pregnant Females 2+ Years Old − Severe Anaemia Prevalence − Other Union Territories
WHO−2024 Adjustment Method

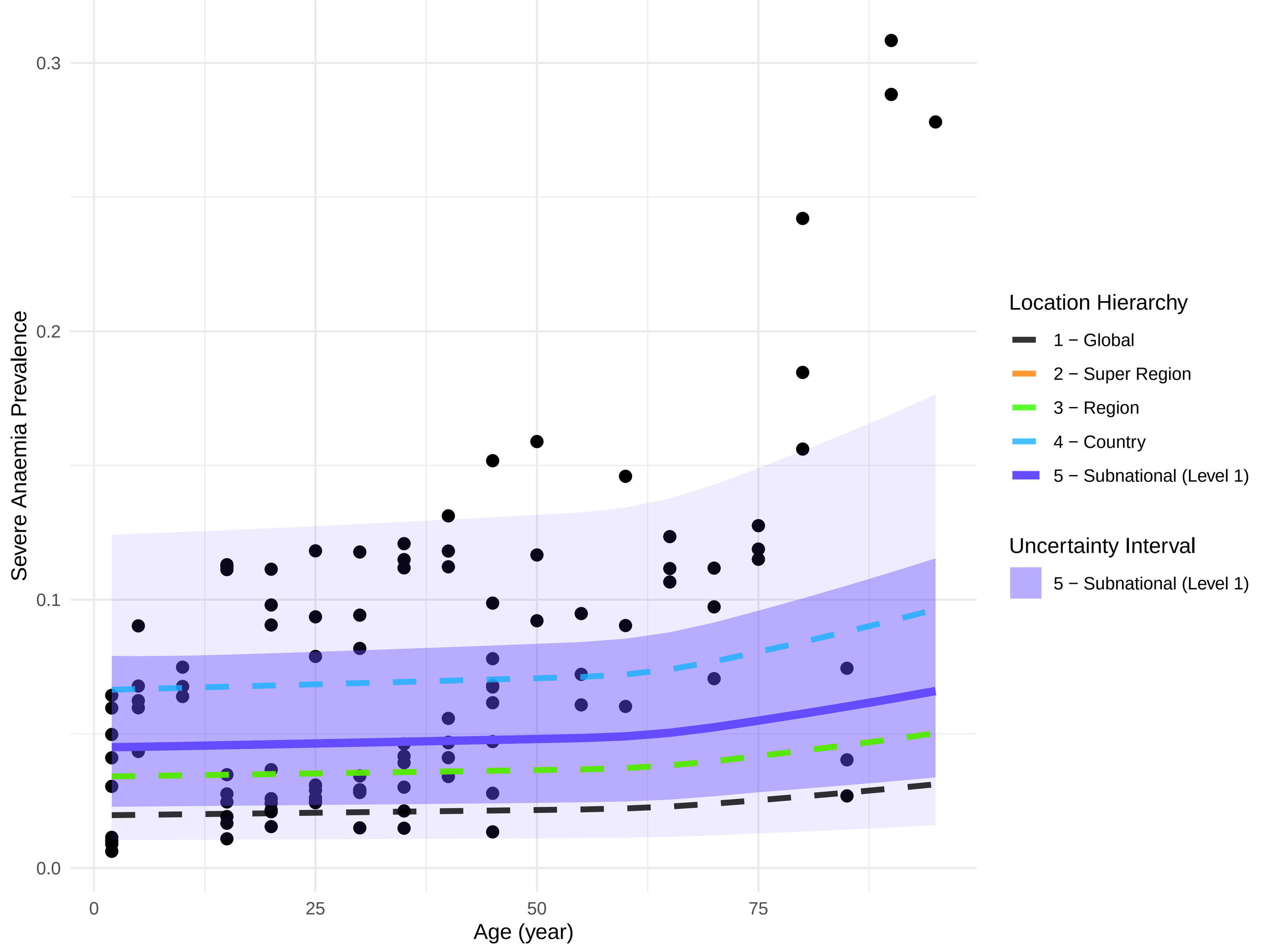

MR−BRT Age/Sex Fits − Non−pregnant Females 2+ Years Old − Severe Anaemia Prevalence − Other Union Territories, Rural
WHO−2024 Adjustment Method

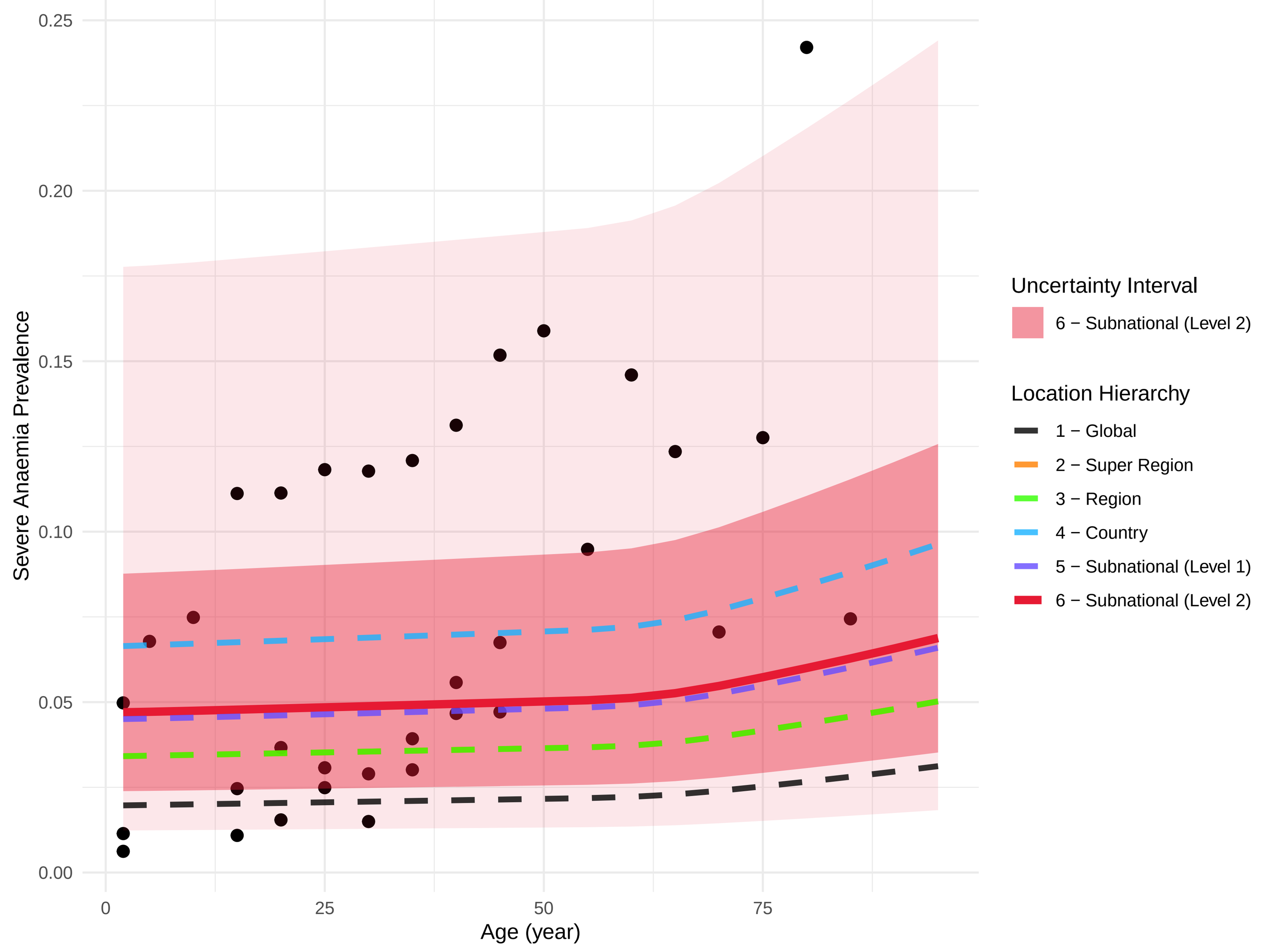

MR−BRT Age/Sex Fits − Non−pregnant Females 2+ Years Old − Severe Anaemia Prevalence − Other Union Territories, Urban
WHO−2024 Adjustment Method

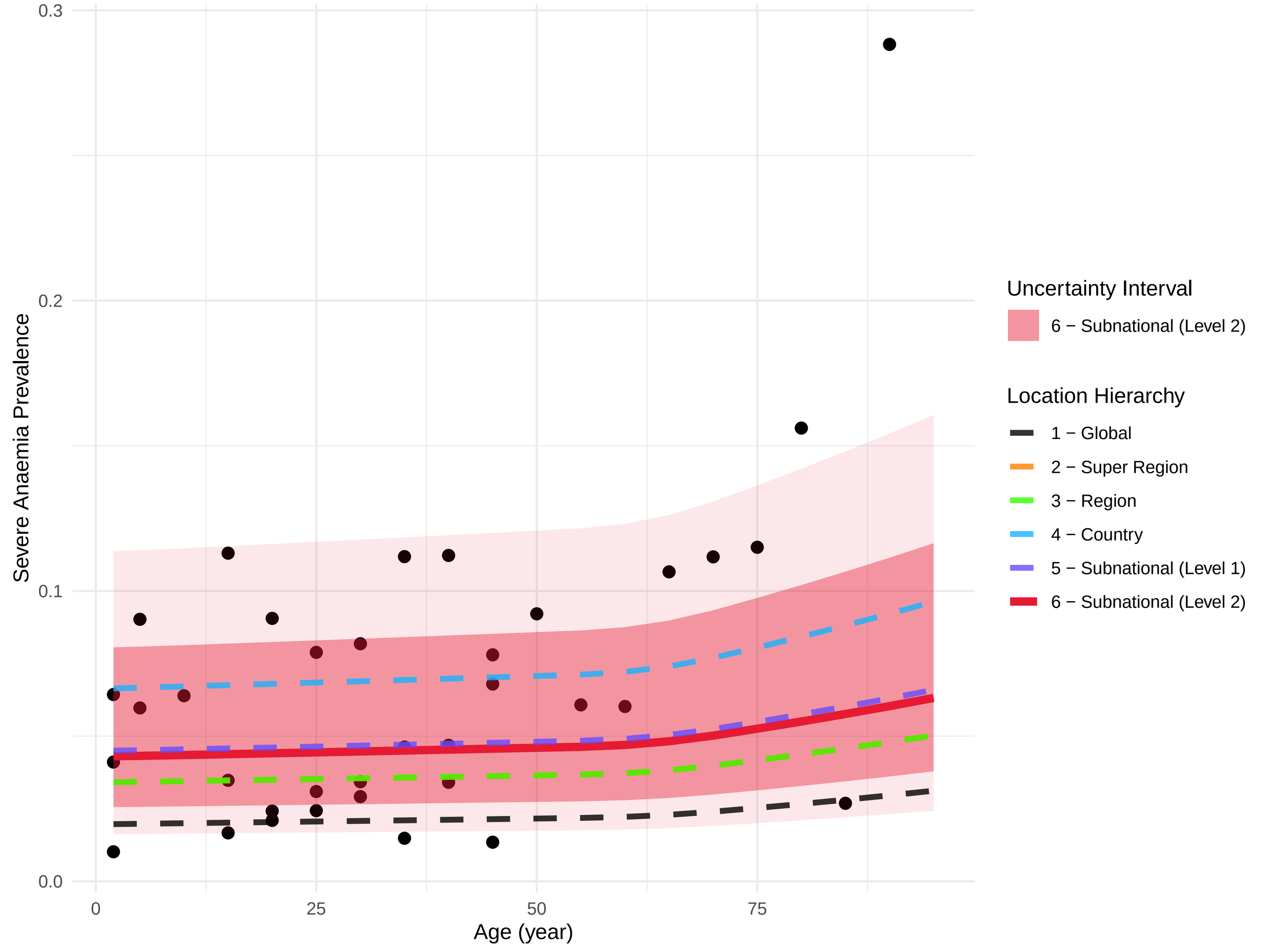

MR−BRT Age/Sex Fits − Non−pregnant Females 2+ Years Old − Severe Anaemia Prevalence − Punjab
WHO−2024 Adjustment Method

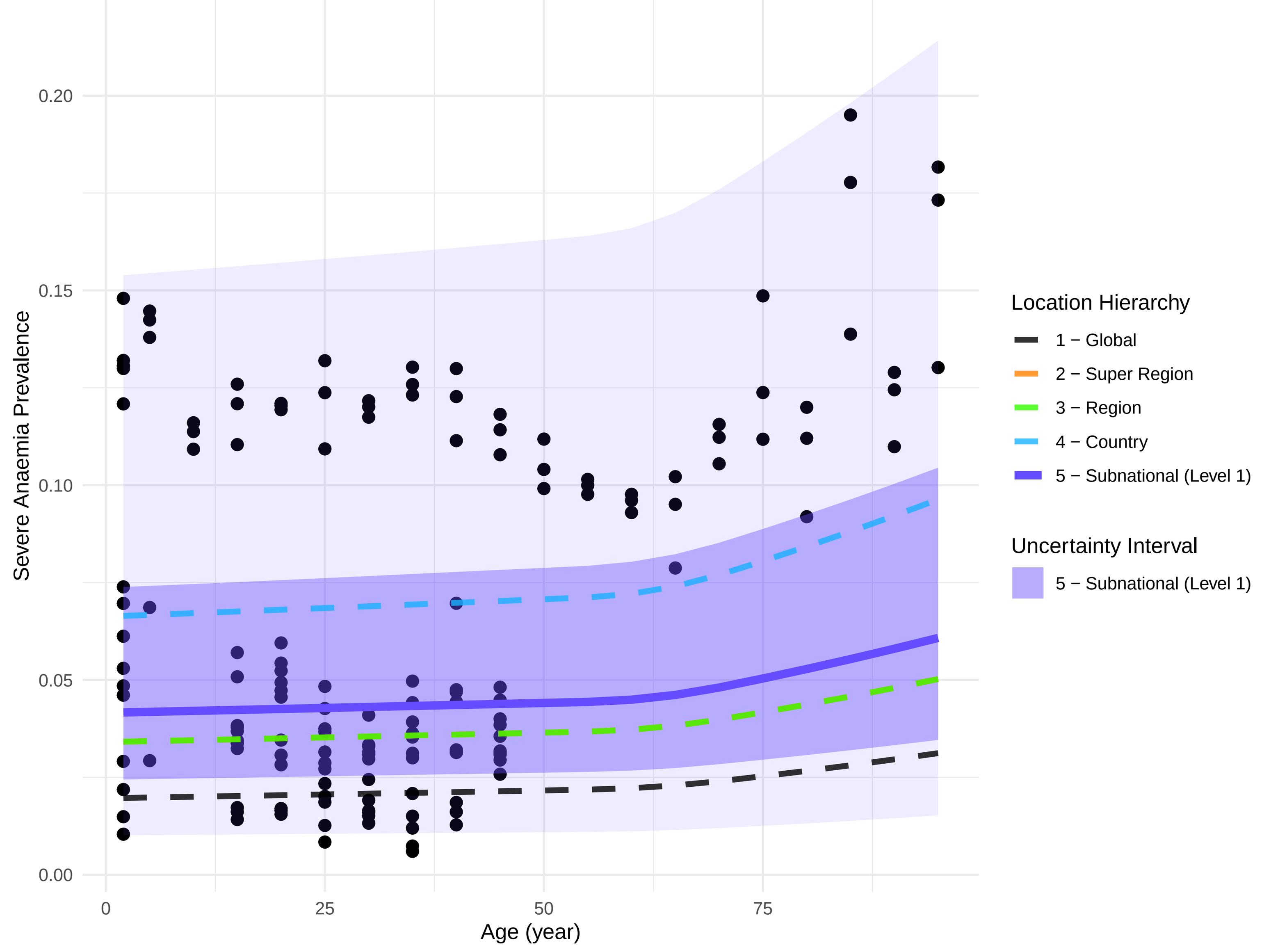

MR−BRT Age/Sex Fits − Non−pregnant Females 2+ Years Old − Severe Anaemia Prevalence − Punjab, Rural
WHO−2024 Adjustment Method

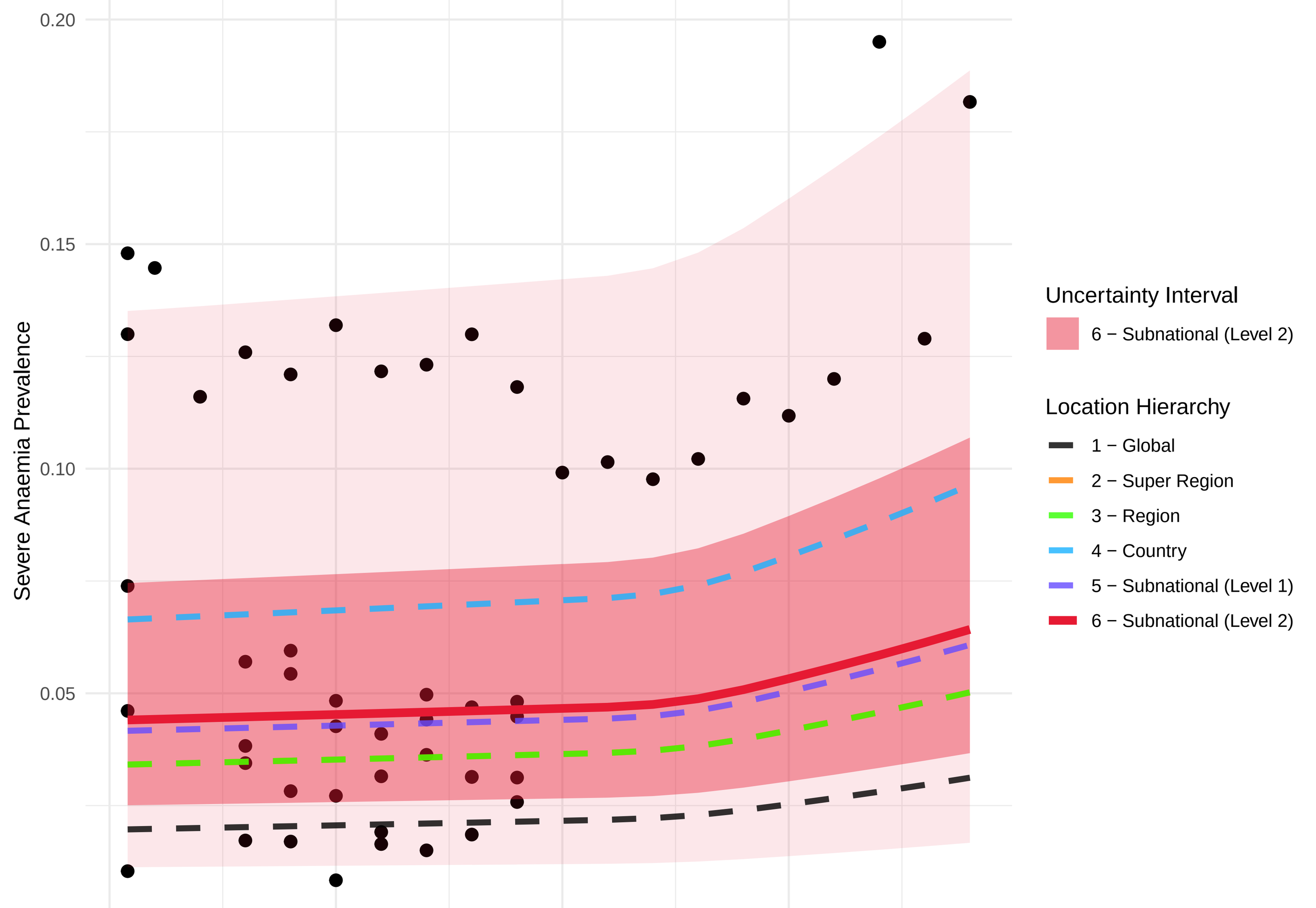

MR−BRT Age/Sex Fits − Non−pregnant Females 2+ Years Old − Severe Anaemia Prevalence − Punjab, Urban
WHO−2024 Adjustment Method

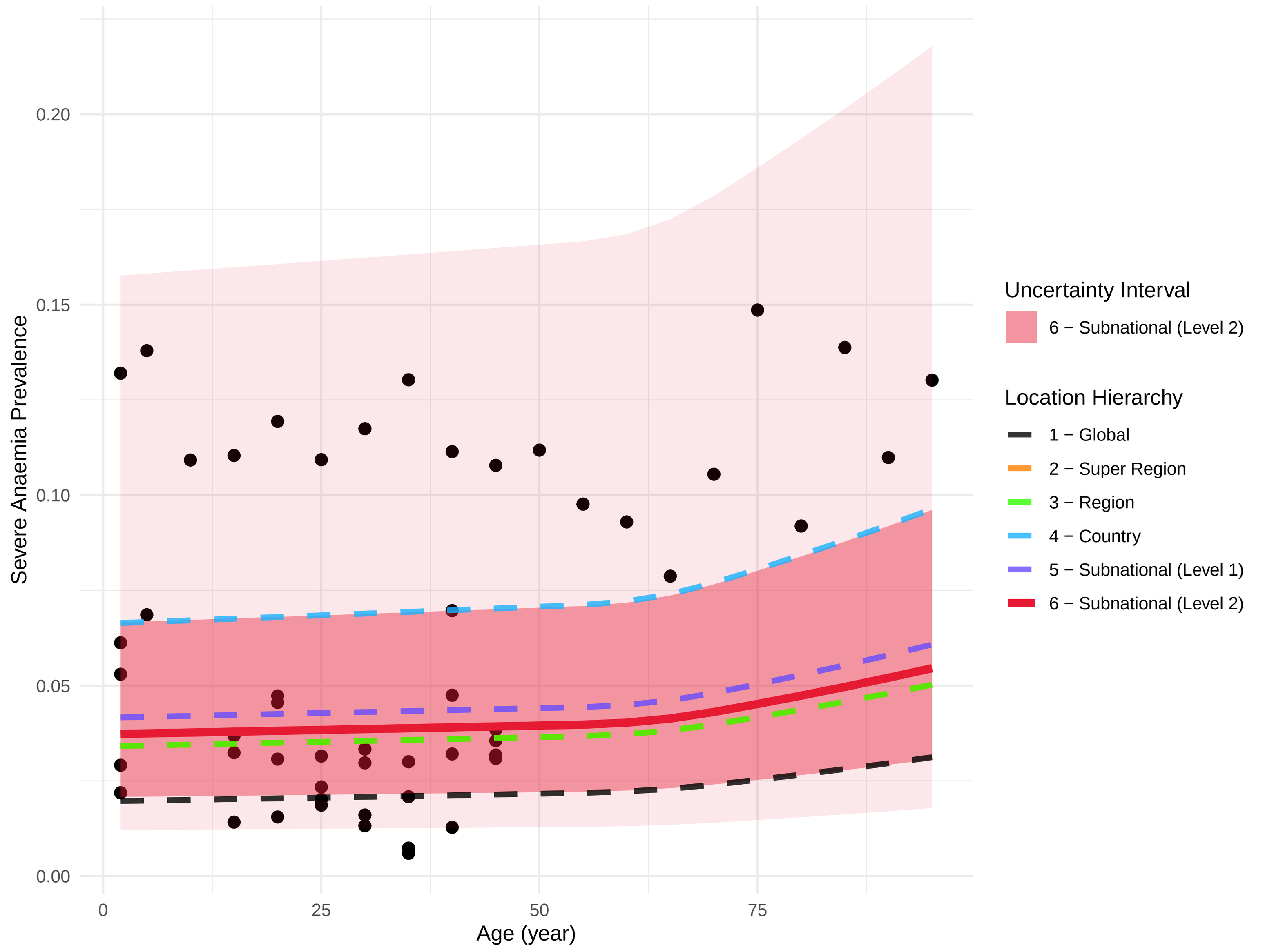

MR−BRT Age/Sex Fits − Non−pregnant Females 2+ Years Old − Severe Anaemia Prevalence − Rajasthan
WHO−2024 Adjustment Method

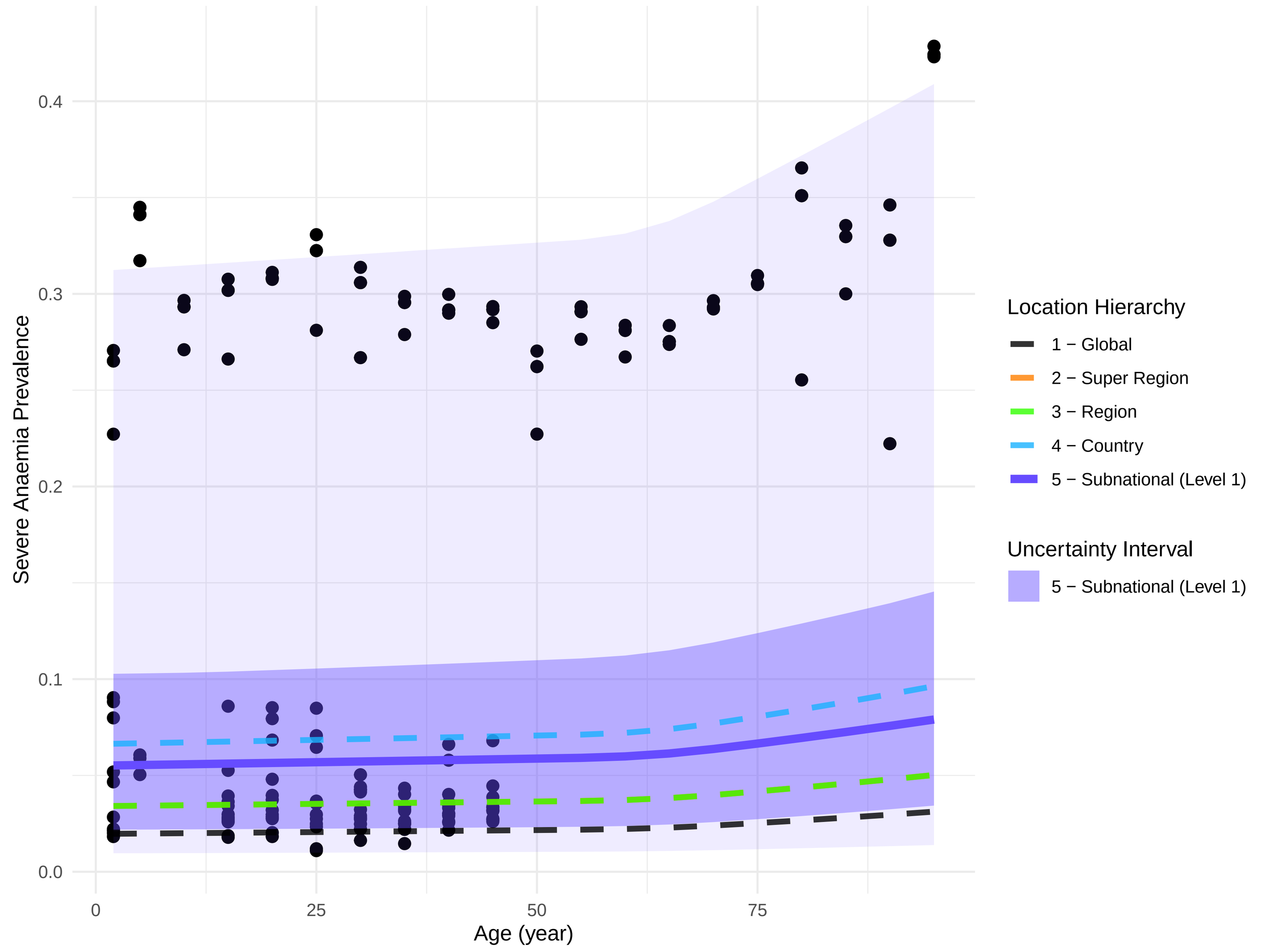

MR−BRT Age/Sex Fits − Non−pregnant Females 2+ Years Old − Severe Anaemia Prevalence − Rajasthan, Rural
WHO−2024 Adjustment Method

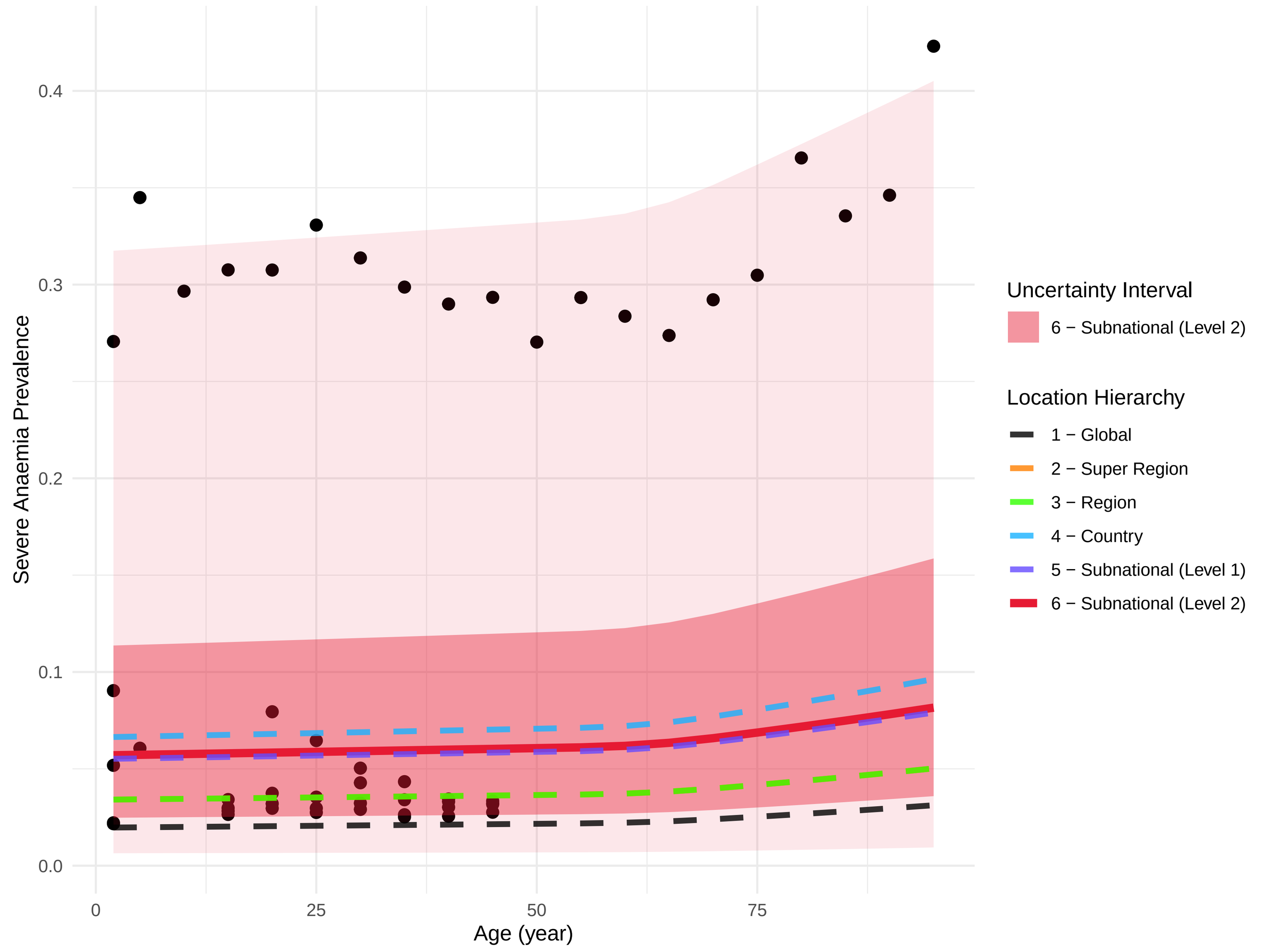

MR−BRT Age/Sex Fits − Non−pregnant Females 2+ Years Old − Severe Anaemia Prevalence − Rajasthan, Urban
WHO−2024 Adjustment Method

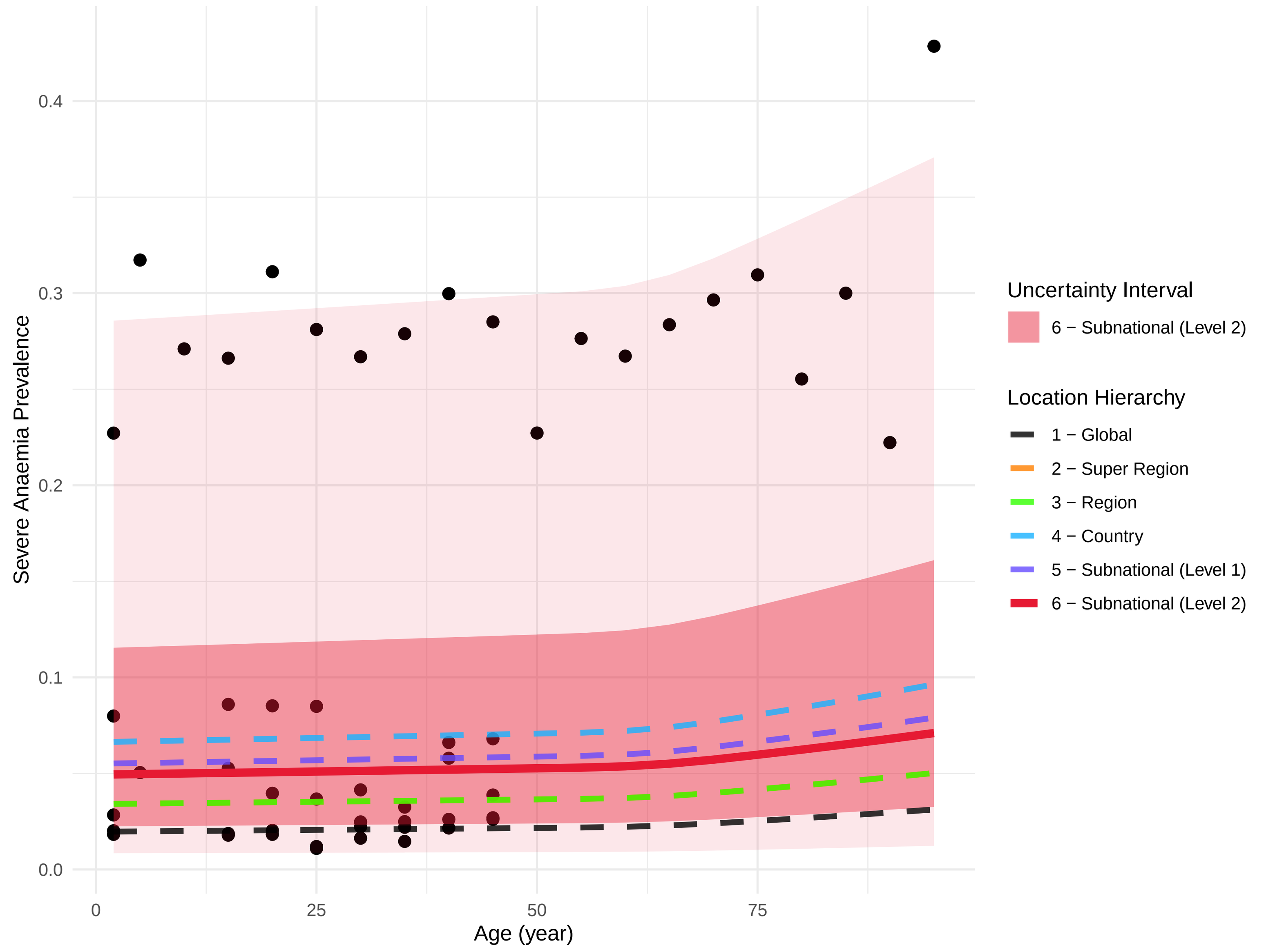

MR−BRT Age/Sex Fits − Non−pregnant Females 2+ Years Old − Severe Anaemia Prevalence − Sikkim
WHO−2024 Adjustment Method

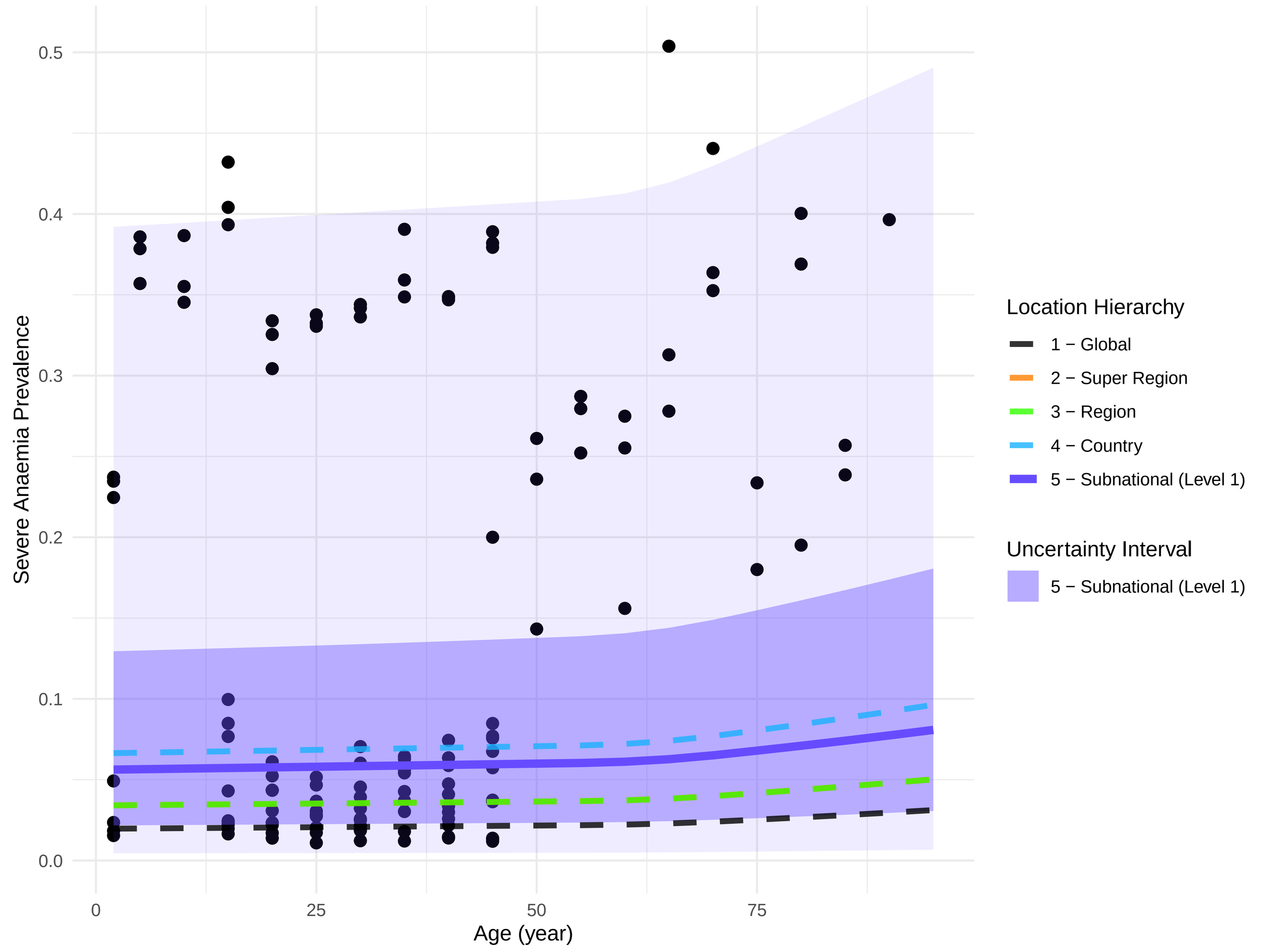

MR−BRT Age/Sex Fits − Non−pregnant Females 2+ Years Old − Severe Anaemia Prevalence − Sikkim, Rural
WHO−2024 Adjustment Method

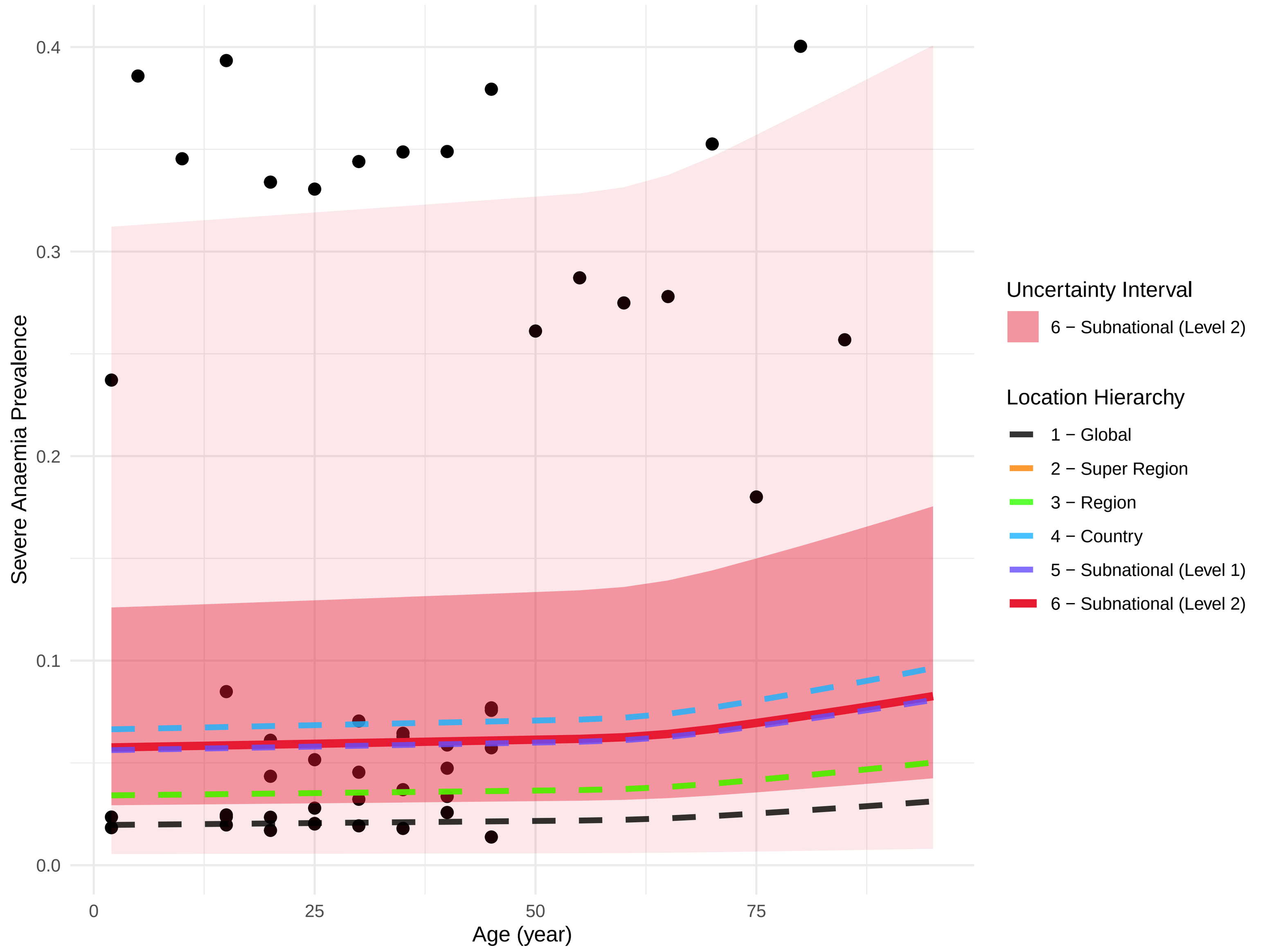

MR−BRT Age/Sex Fits − Non−pregnant Females 2+ Years Old − Severe Anaemia Prevalence − Sikkim, Urban
WHO−2024 Adjustment Method

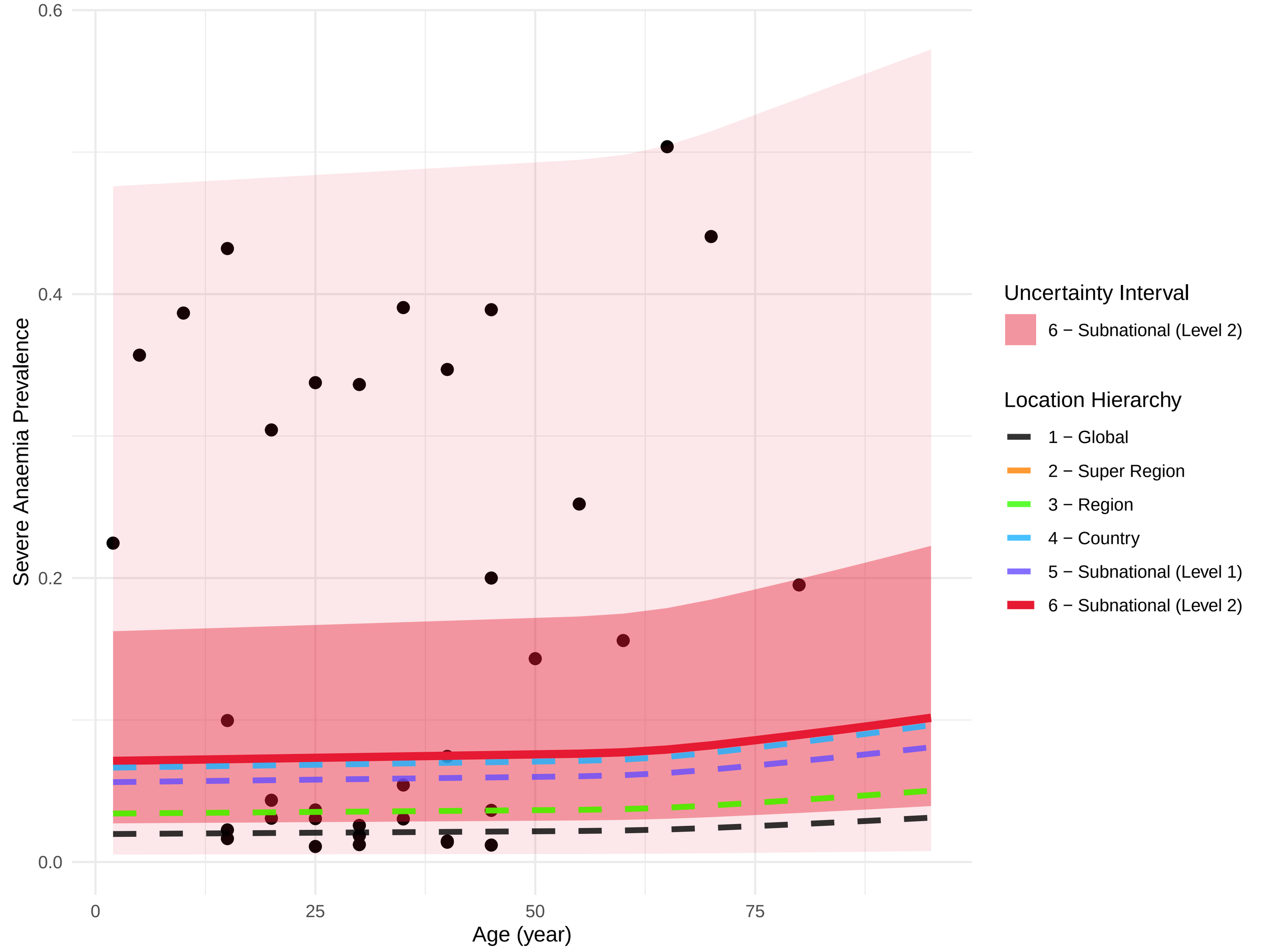

MR−BRT Age/Sex Fits − Non−pregnant Females 2+ Years Old − Severe Anaemia Prevalence − Tamil Nadu
WHO−2024 Adjustment Method

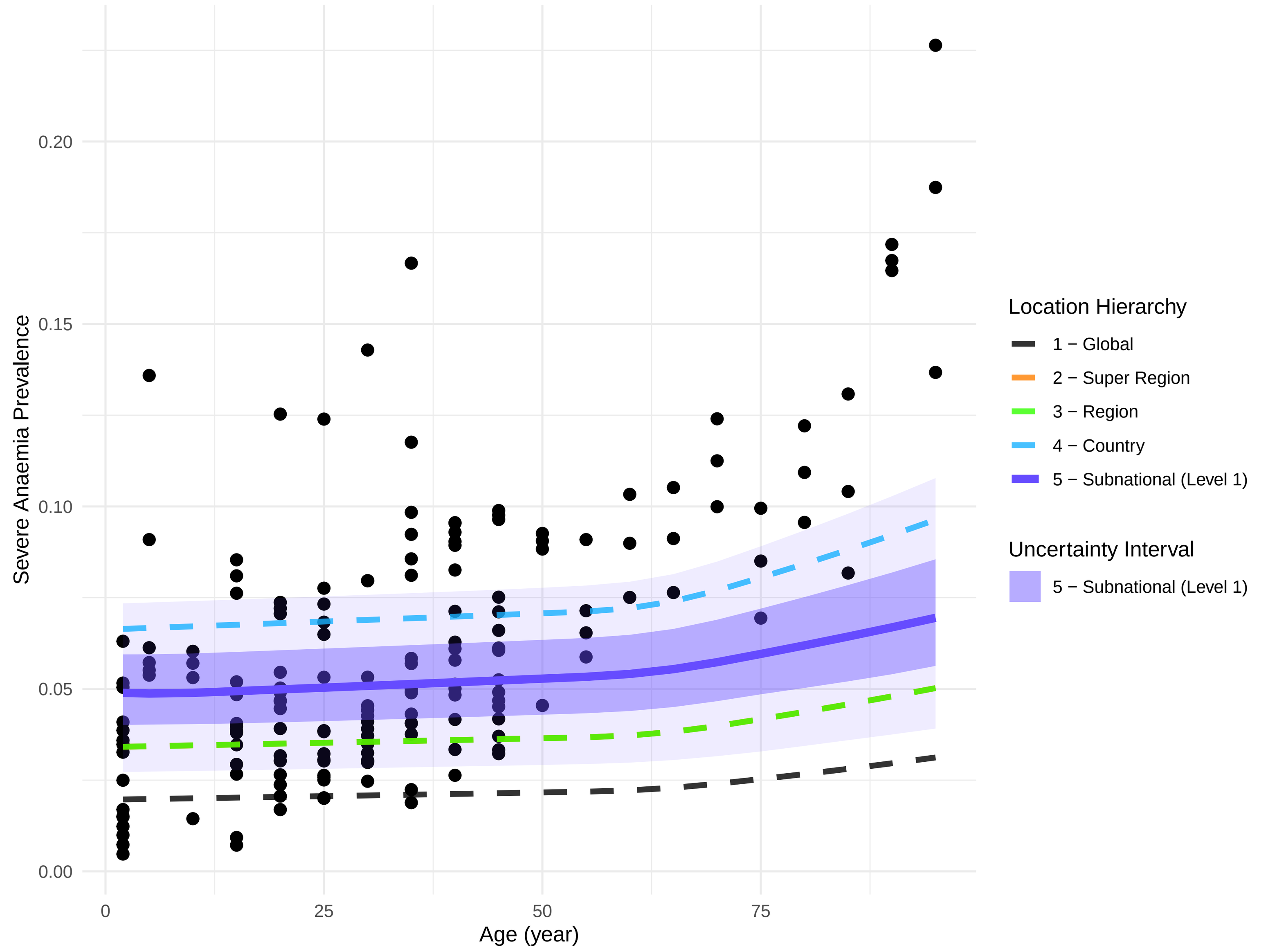

MR−BRT Age/Sex Fits − Non−pregnant Females 2+ Years Old − Severe Anaemia Prevalence − Tamil Nadu, Rural
WHO−2024 Adjustment Method

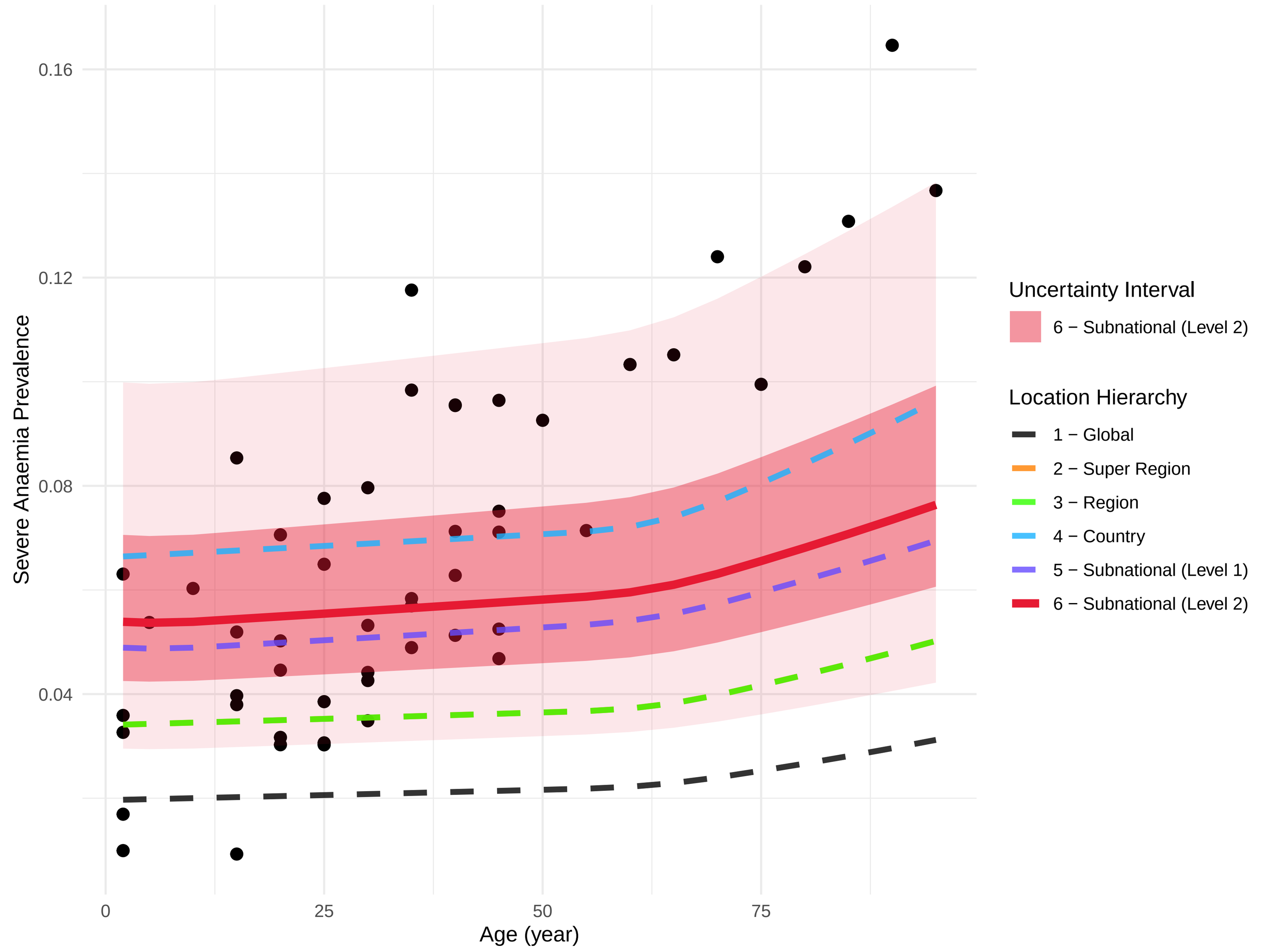

MR−BRT Age/Sex Fits − Non−pregnant Females 2+ Years Old − Severe Anaemia Prevalence − Tamil Nadu, Urban
WHO−2024 Adjustment Method

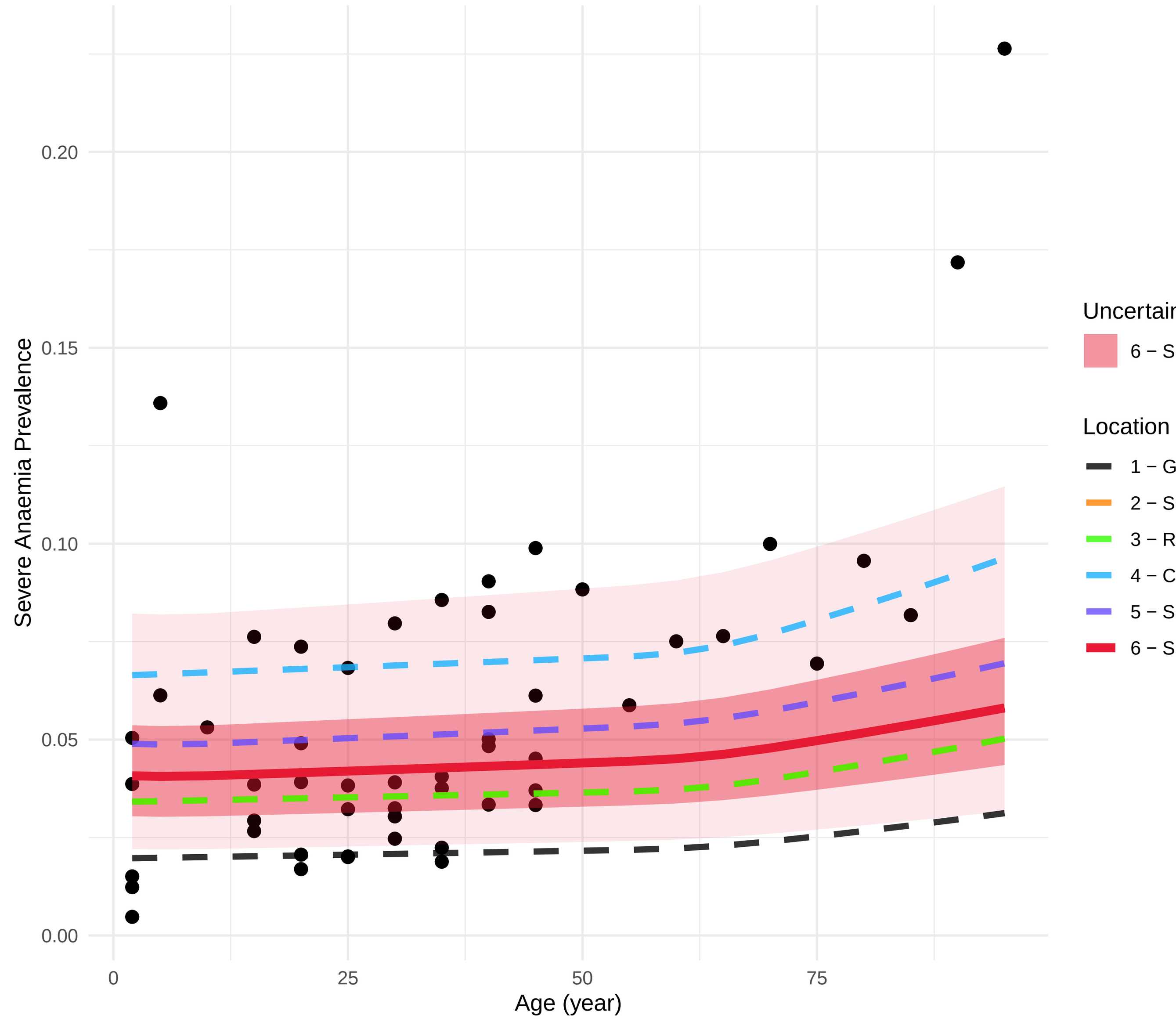

MR−BRT Age/Sex Fits − Non-pregnant Females 2+ Years Old − Severe Anaemia Prevalence − Telangana
WHO−2024 Adjustment Method

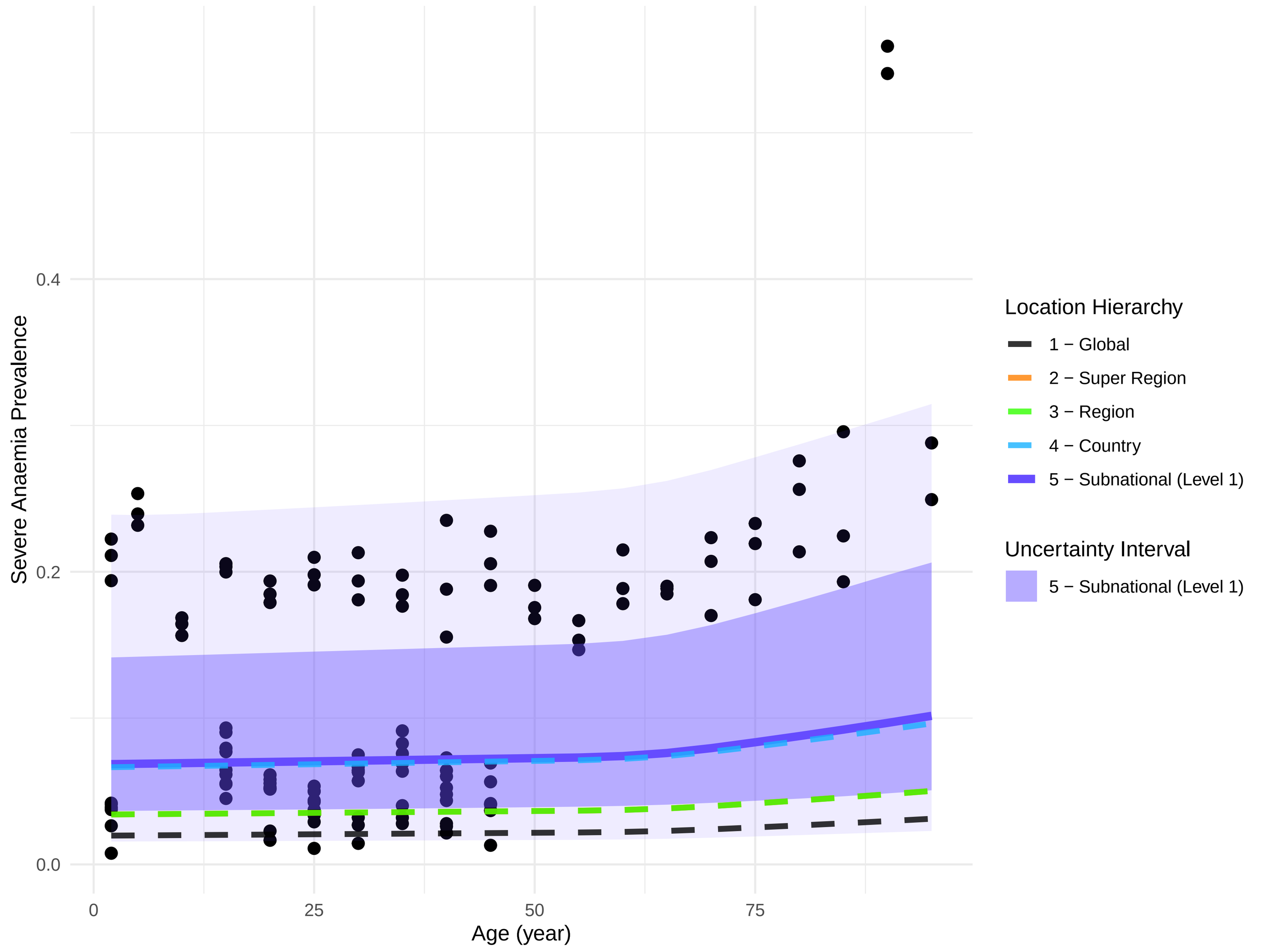

MR−BRT Age/Sex Fits − Non-pregnant Females 2+ Years Old − Severe Anaemia Prevalence − Telangana, Rural
WHO−2024 Adjustment Method

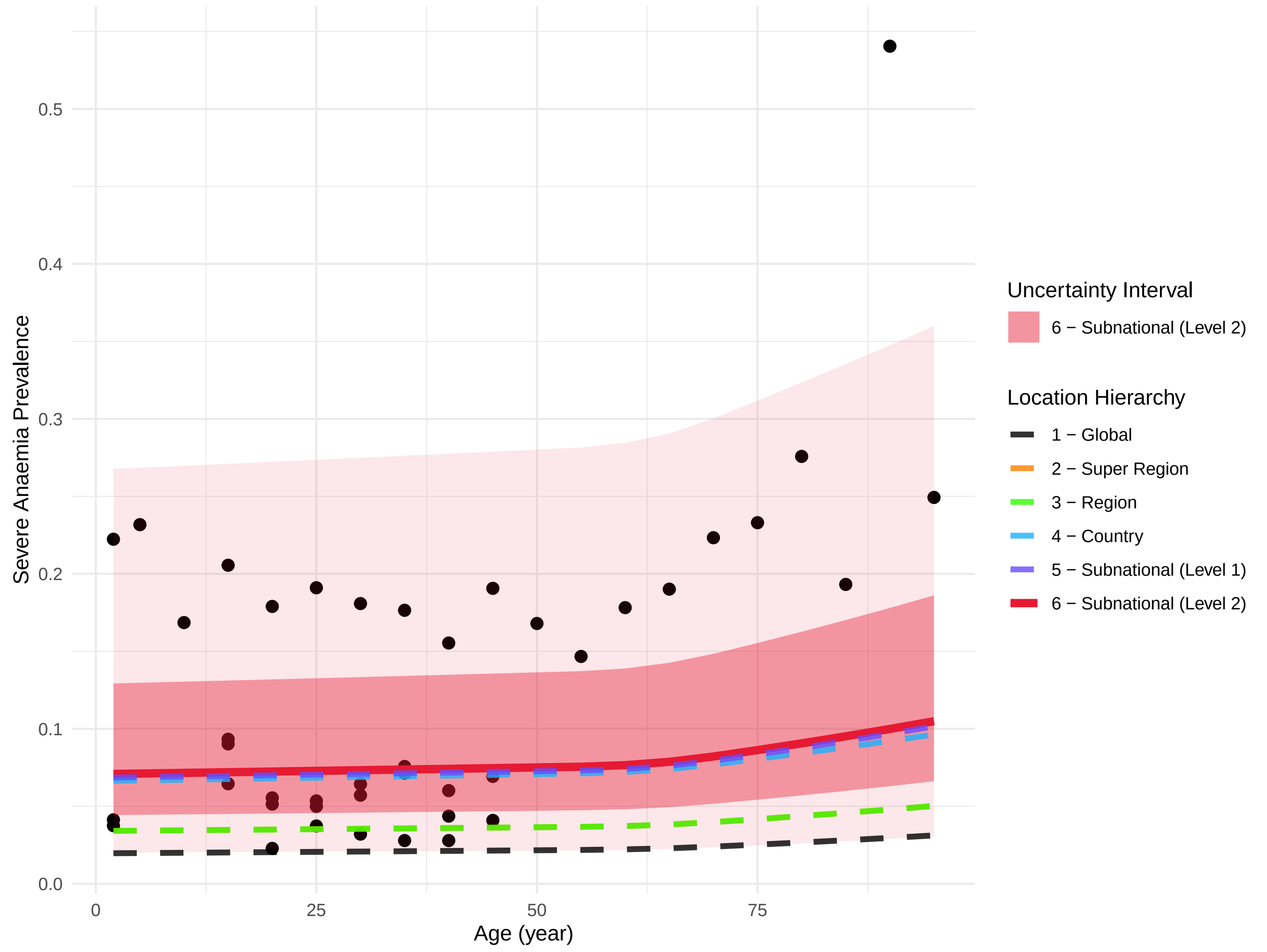

MR−BRT Age/Sex Fits − Non-pregnant Females 2+ Years Old − Severe Anaemia Prevalence − Telangana, Urban
WHO−2024 Adjustment Method

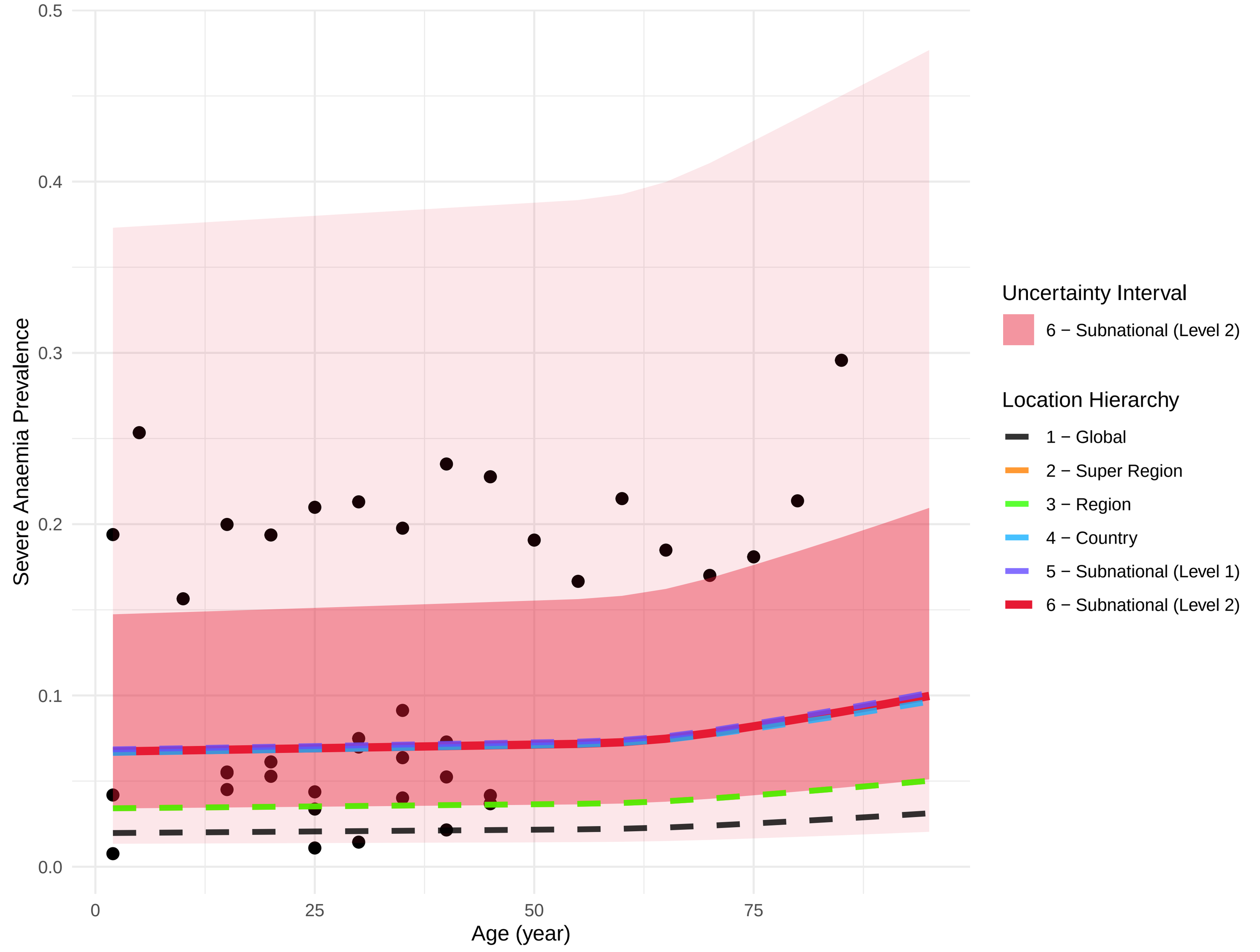

MR−BRT Age/Sex Fits − Non-pregnant Females 2+ Years Old − Severe Anaemia Prevalence − Tripura
WHO−2024 Adjustment Method

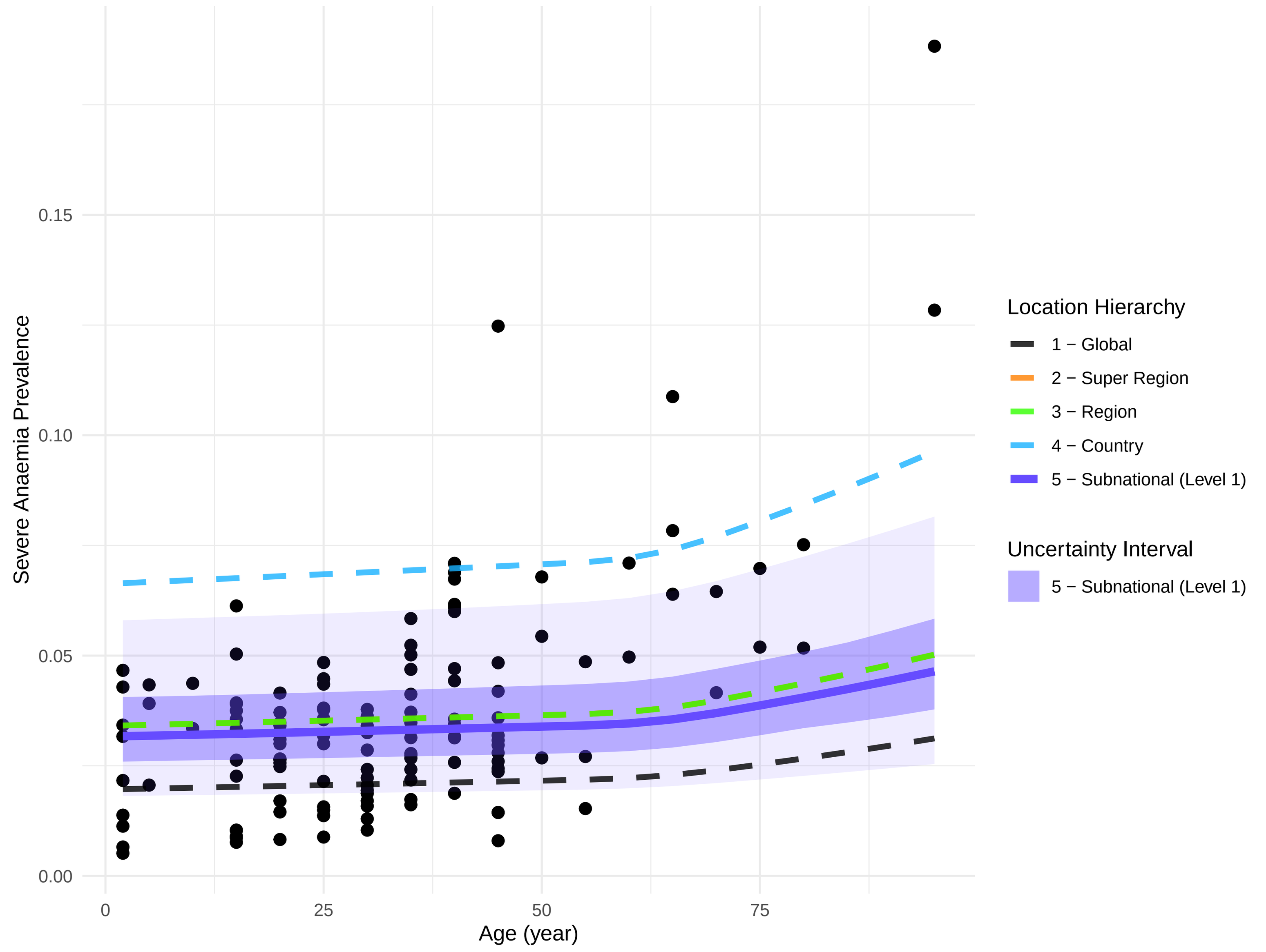

MR−BRT Age/Sex Fits − Non-pregnant Females 2+ Years Old − Severe Anaemia Prevalence − Tripura, Rural
WHO−2024 Adjustment Method

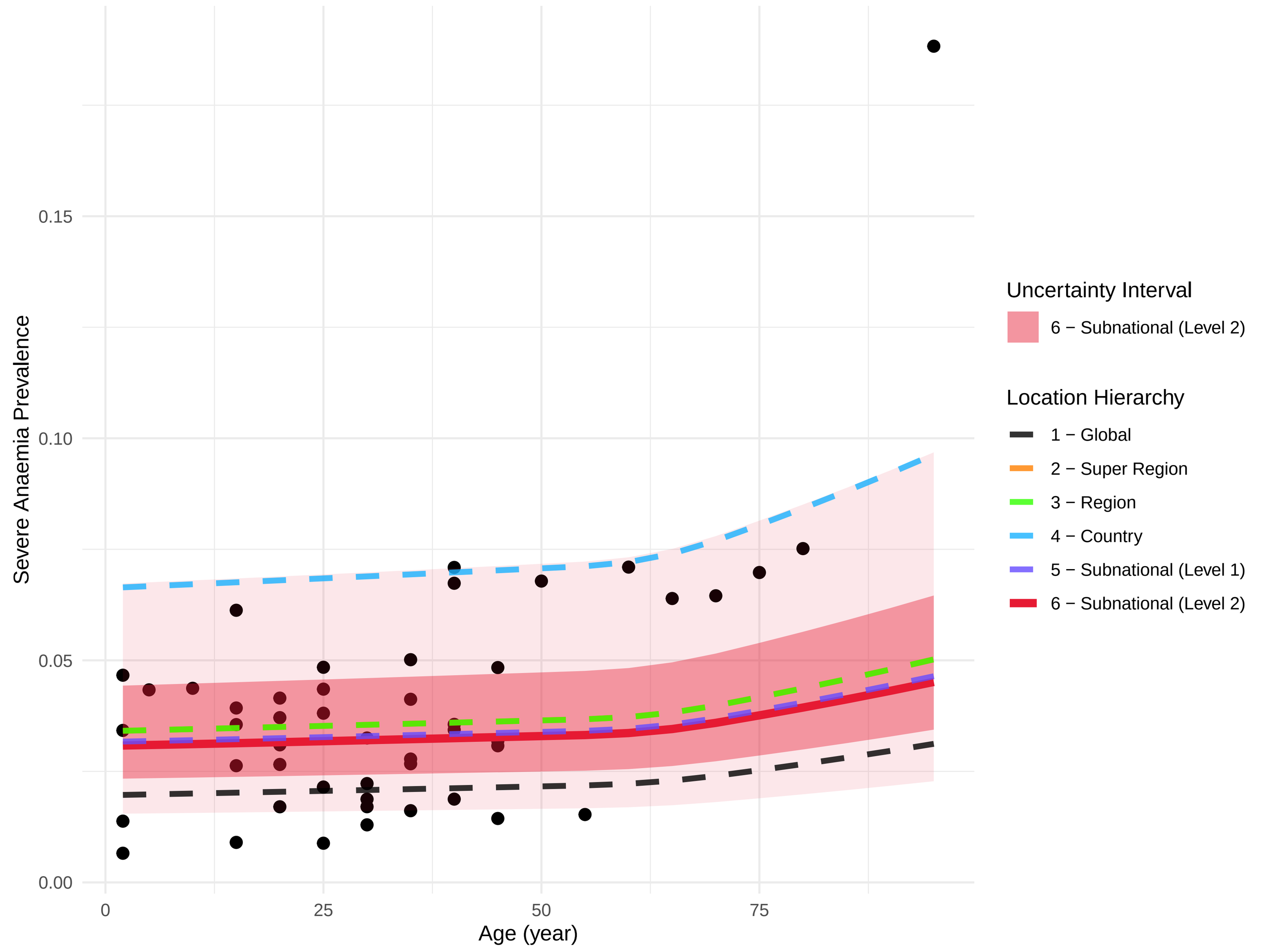

MR−BRT Age/Sex Fits − Non-pregnant Females 2+ Years Old − Severe Anaemia Prevalence − Tripura, Urban
WHO−2024 Adjustment Method

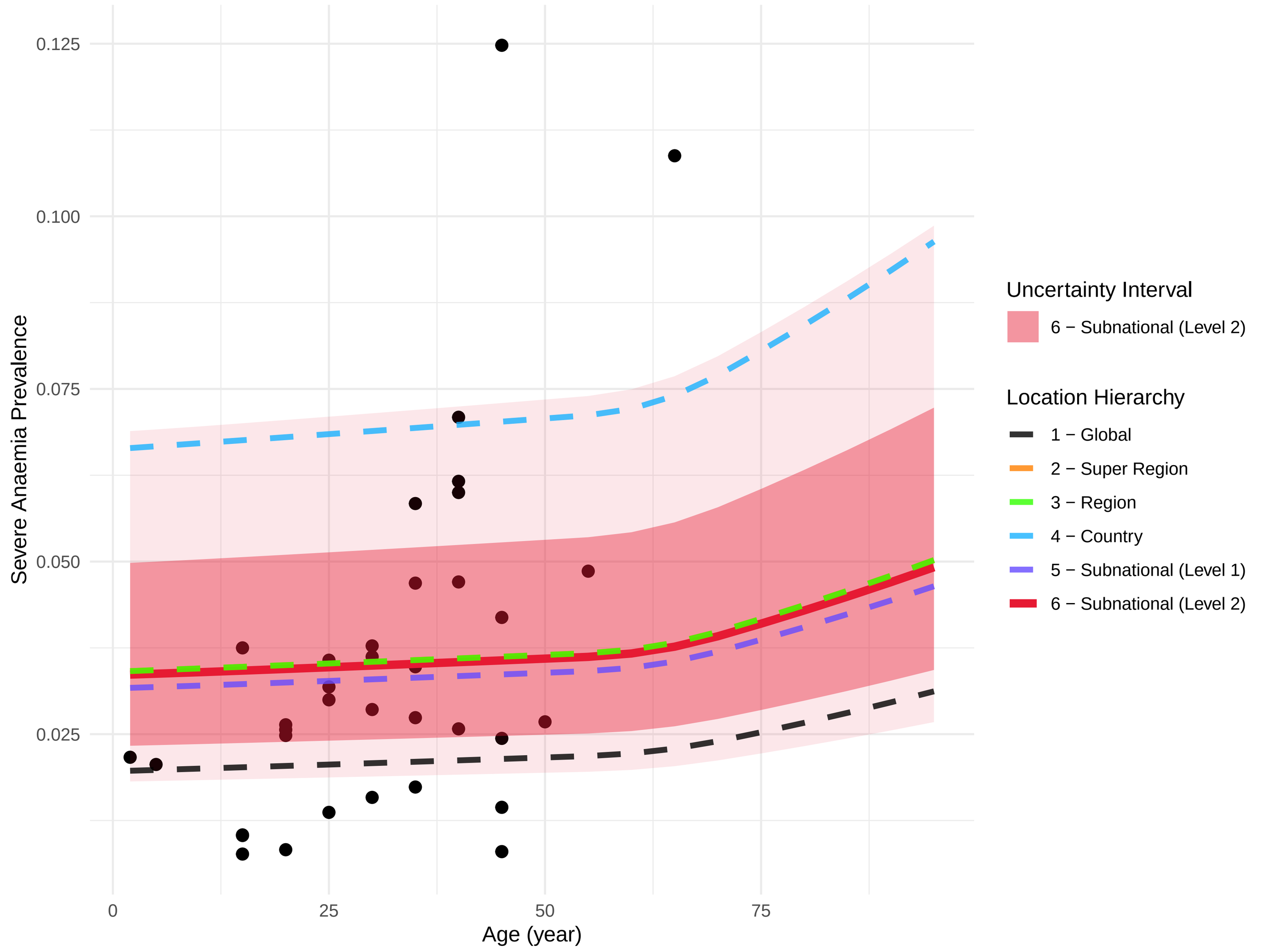

MR−BRT Age/Sex Fits − Non-pregnant Females 2+ Years Old − Severe Anaemia Prevalence − Uttar Pradesh
WHO−2024 Adjustment Method

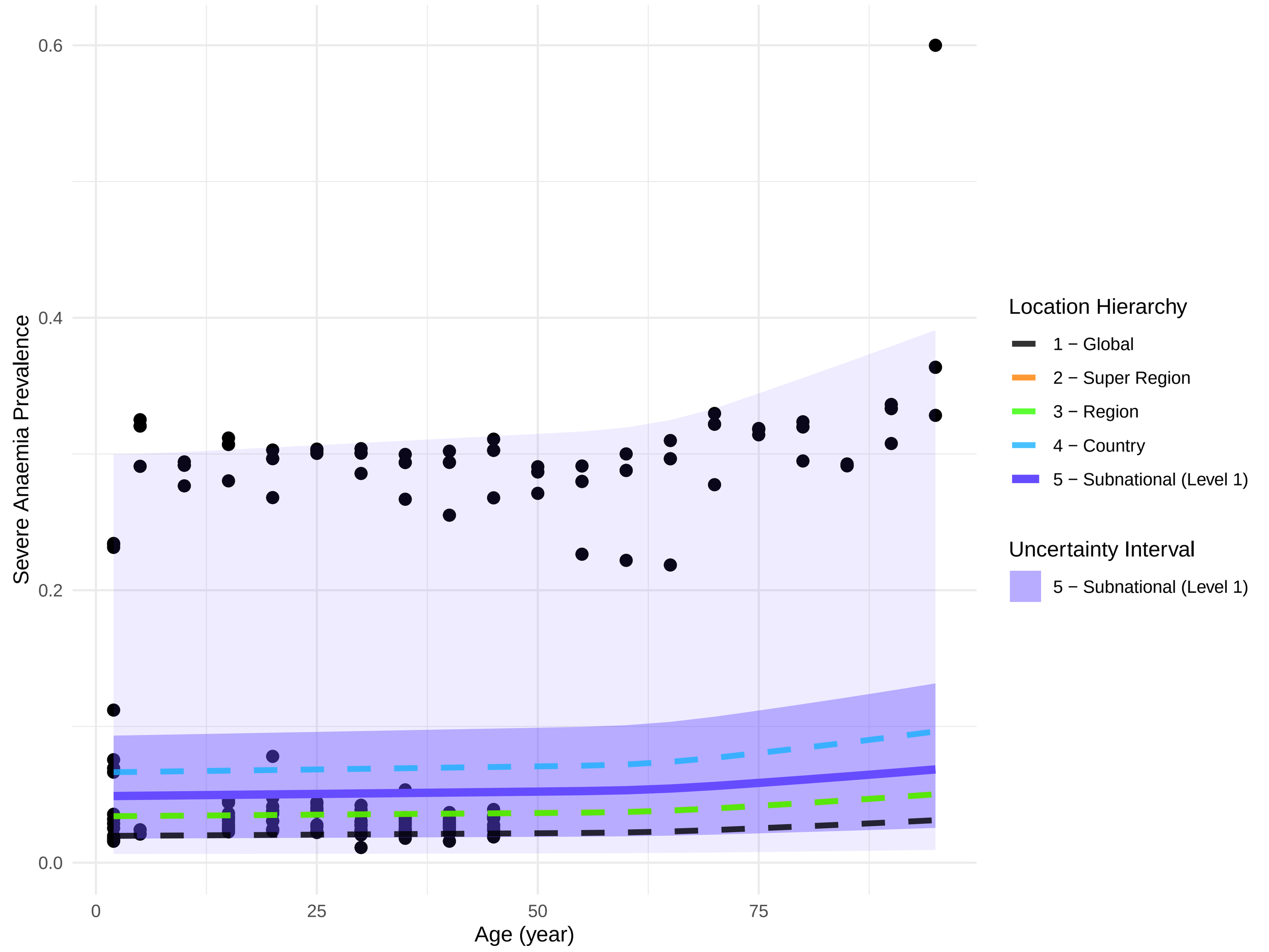

MR−BRT Age/Sex Fits − Non-pregnant Females 2+ Years Old − Severe Anaemia Prevalence − Uttar Pradesh, Rural
WHO−2024 Adjustment Method

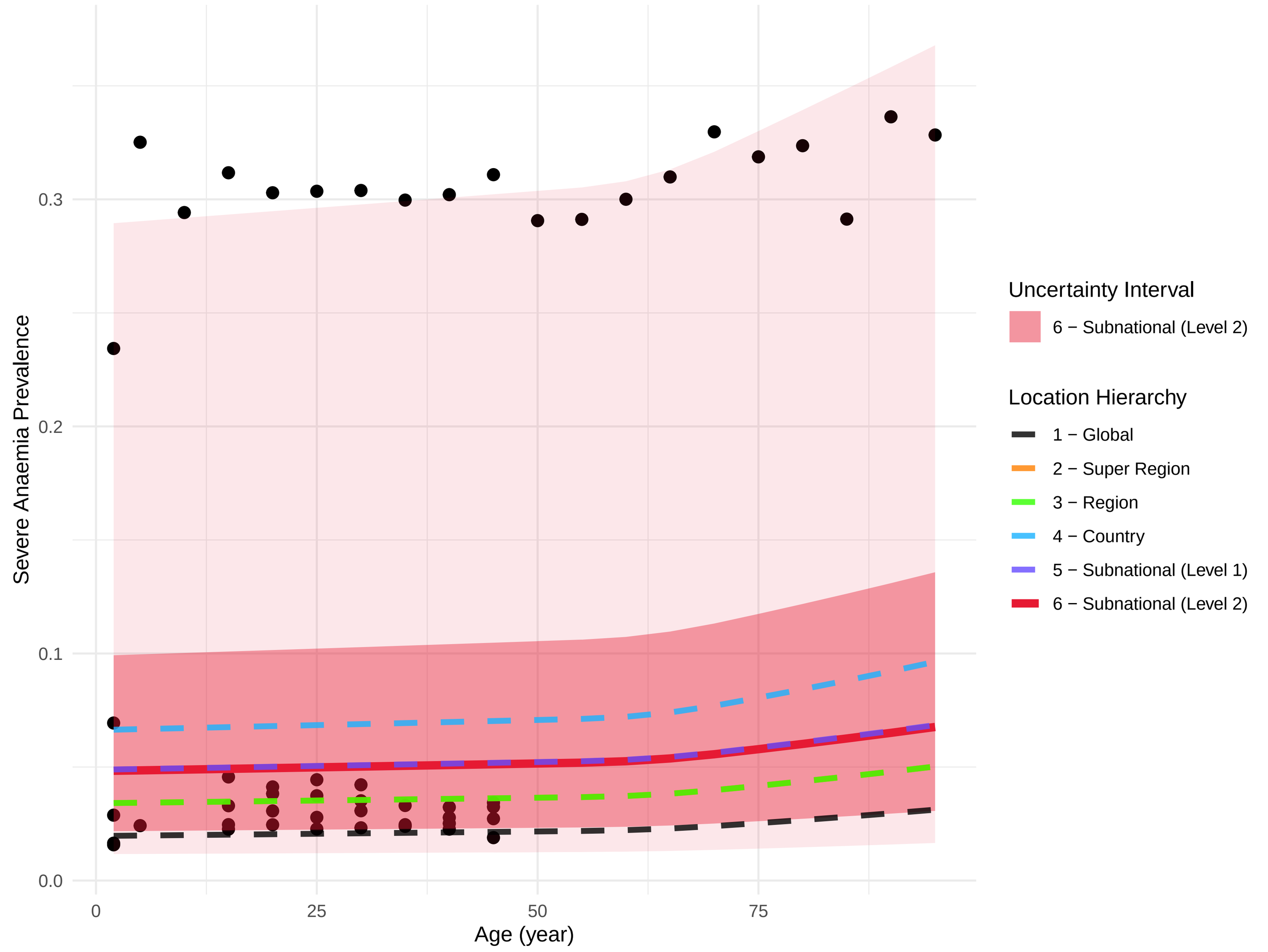

MR−BRT Age/Sex Fits − Non-pregnant Females 2+ Years Old − Severe Anaemia Prevalence − Uttar Pradesh, Urban
WHO−2024 Adjustment Method

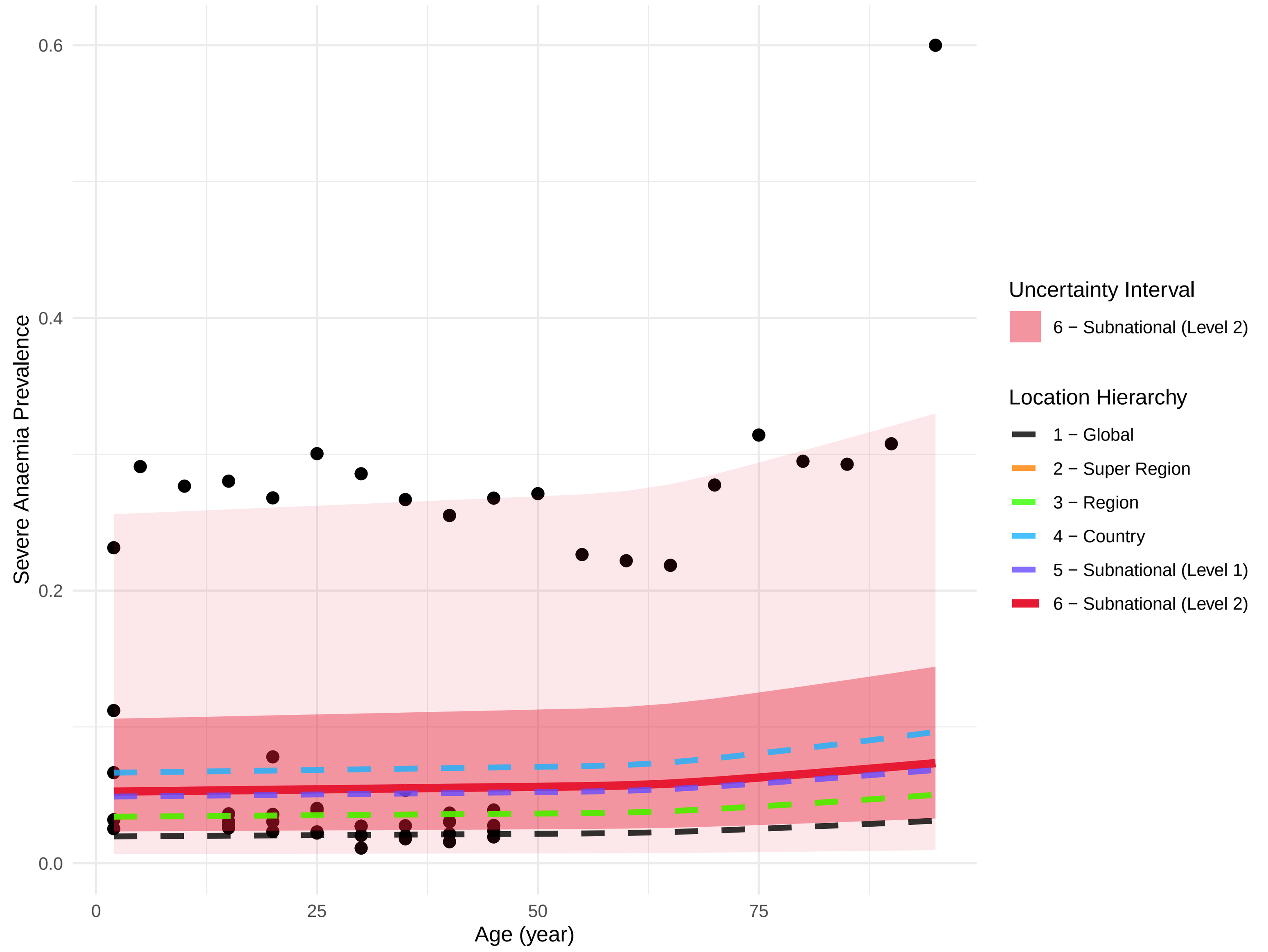

MR−BRT Age/Sex Fits − Non-pregnant Females 2+ Years Old − Severe Anaemia Prevalence − Uttarakhand
WHO−2024 Adjustment Method

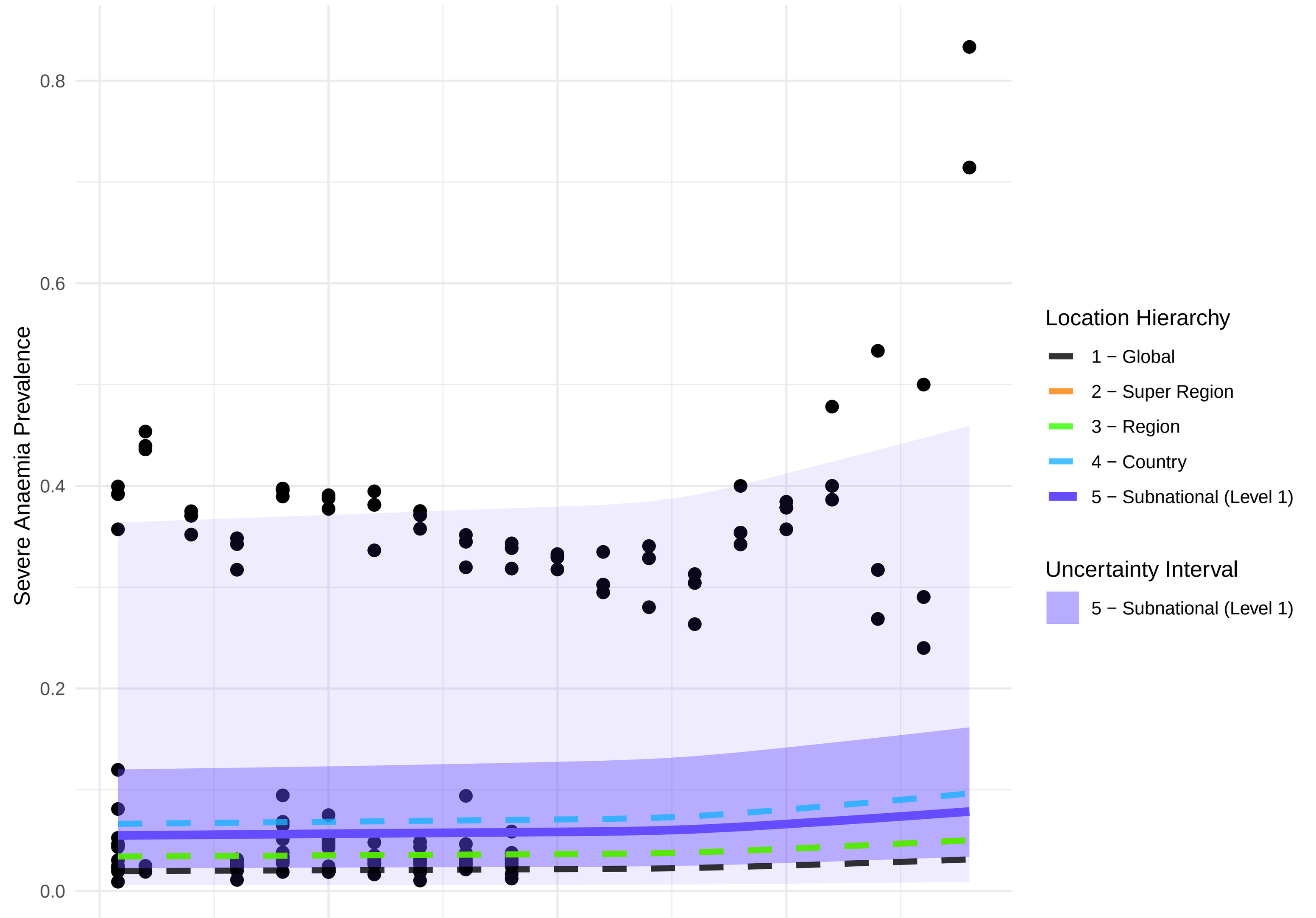

MR−BRT Age/Sex Fits − Non−pregnant Females 2+ Years Old − Severe Anaemia Prevalence − Uttarakhand, Rural
WHO−2024 Adjustment Method

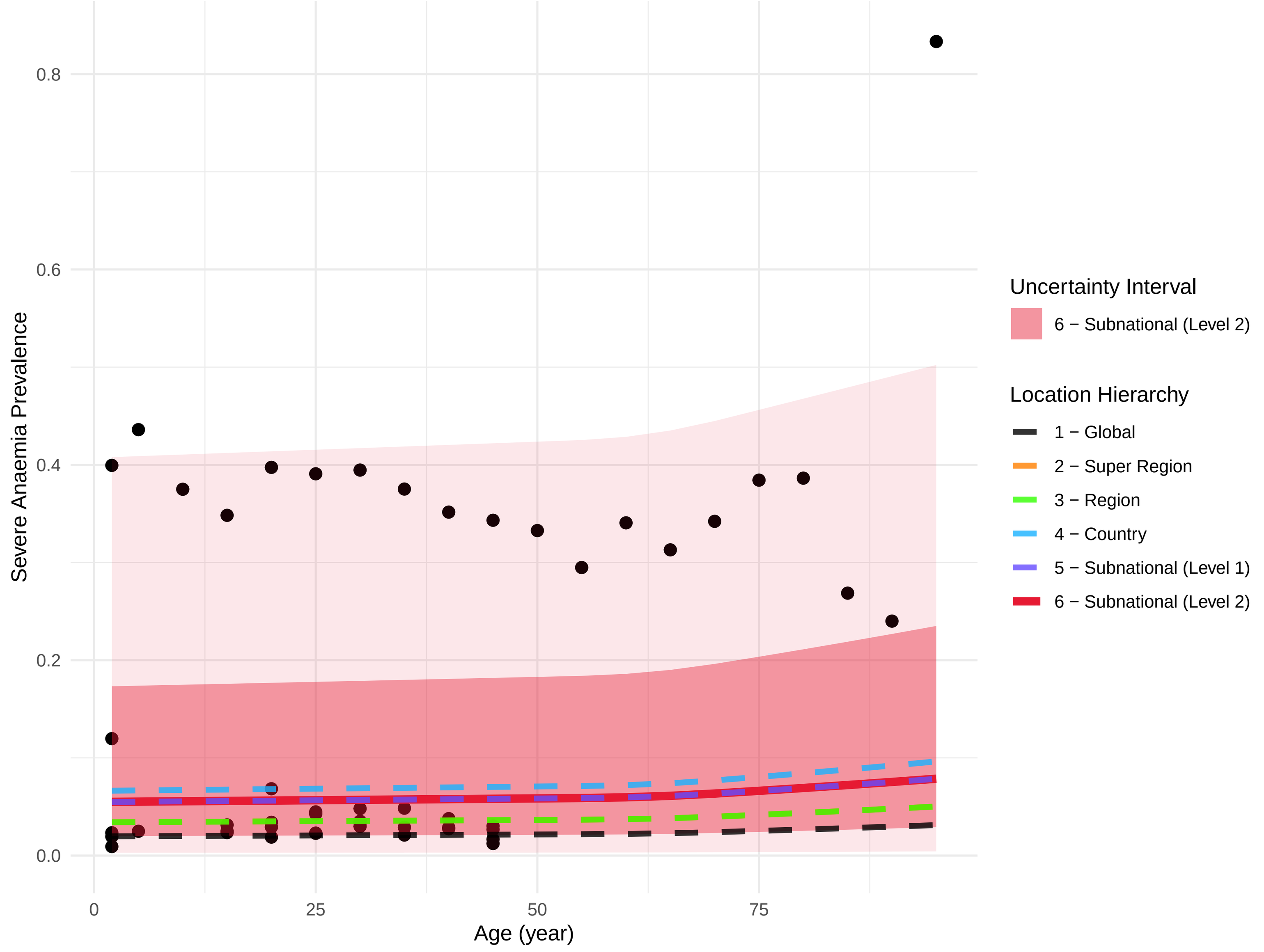

MR−BRT Age/Sex Fits − Non−pregnant Females 2+ Years Old − Severe Anaemia Prevalence − Uttarakhand, Urban
WHO−2024 Adjustment Method

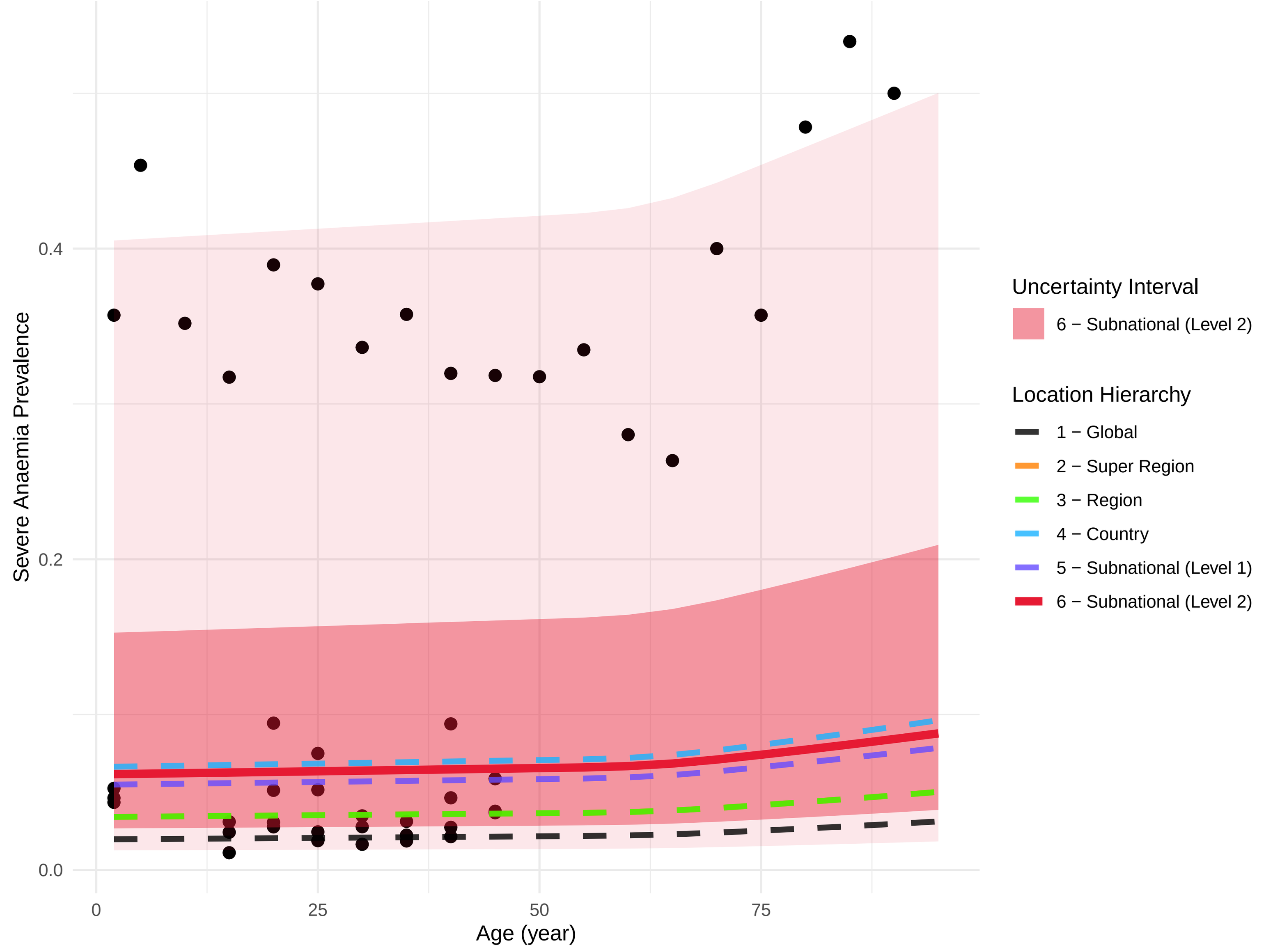

MR−BRT Age/Sex Fits − Non−pregnant Females 2+ Years Old − Severe Anaemia Prevalence − West Bengal
WHO−2024 Adjustment Method

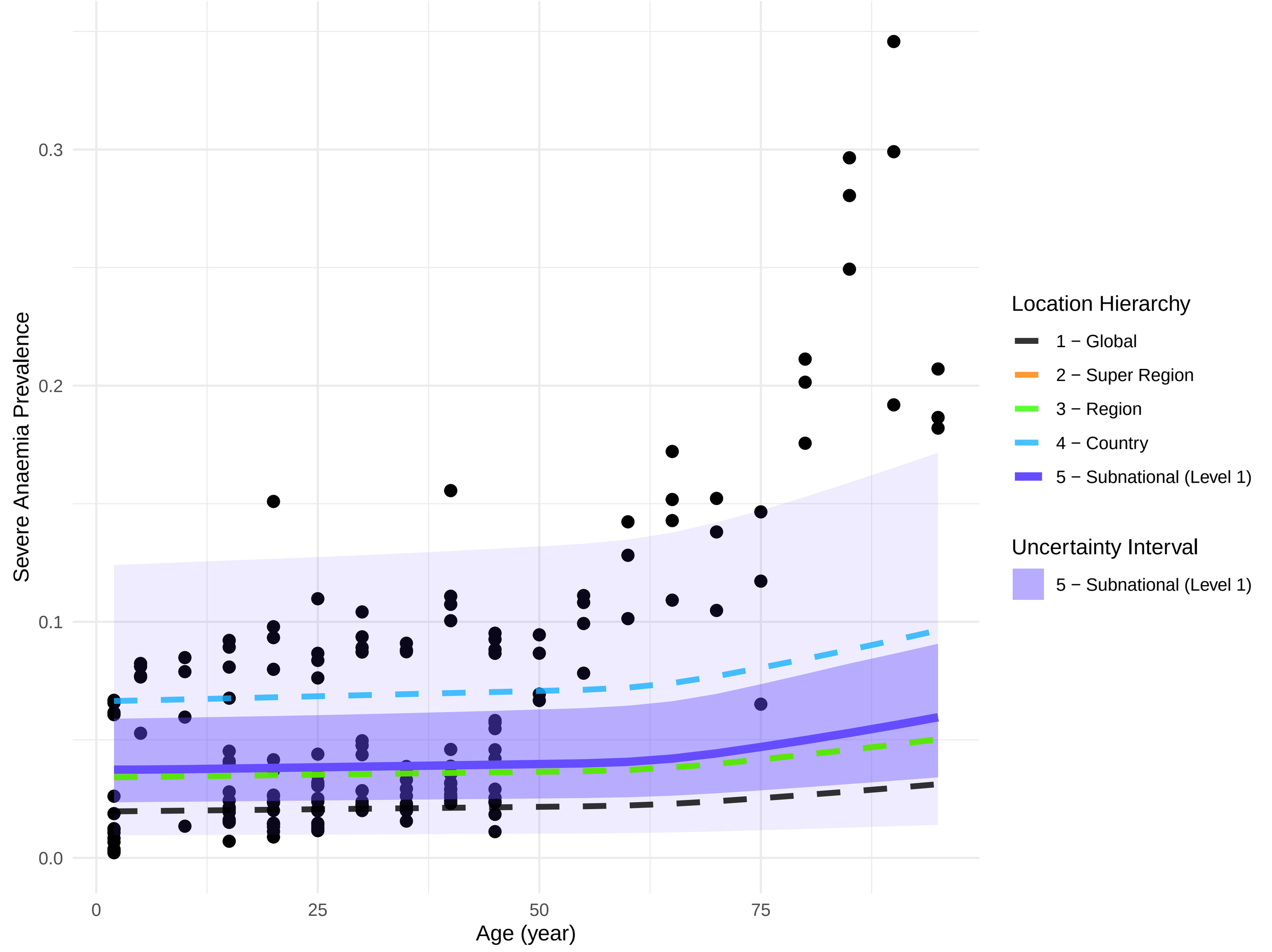

MR−BRT Age/Sex Fits − Non−pregnant Females 2+ Years Old − Severe Anaemia Prevalence − West Bengal, Rural
WHO−2024 Adjustment Method

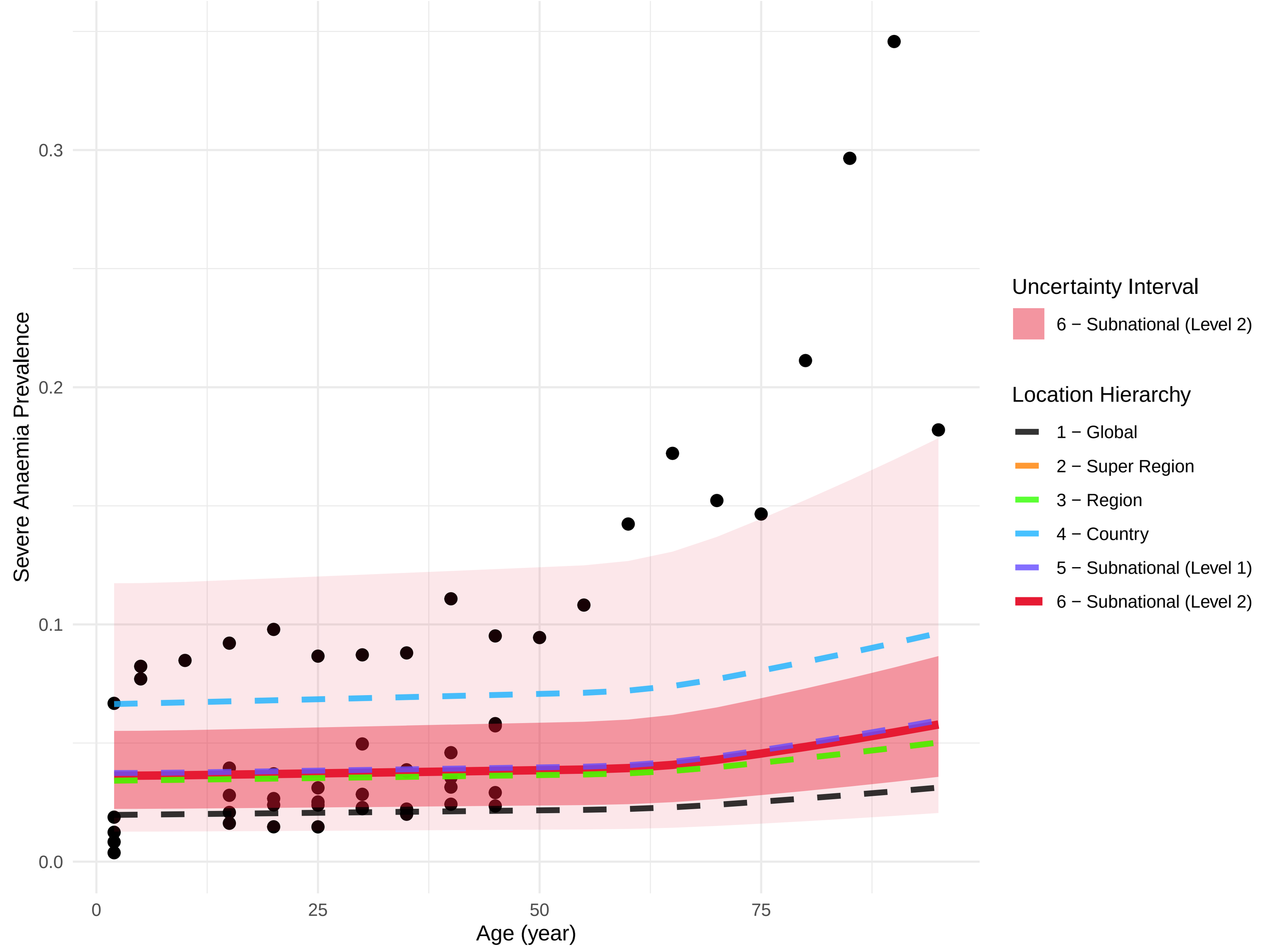

MR−BRT Age/Sex Fits − Non−pregnant Females 2+ Years Old − Severe Anaemia Prevalence − West Bengal, Urban
WHO−2024 Adjustment Method

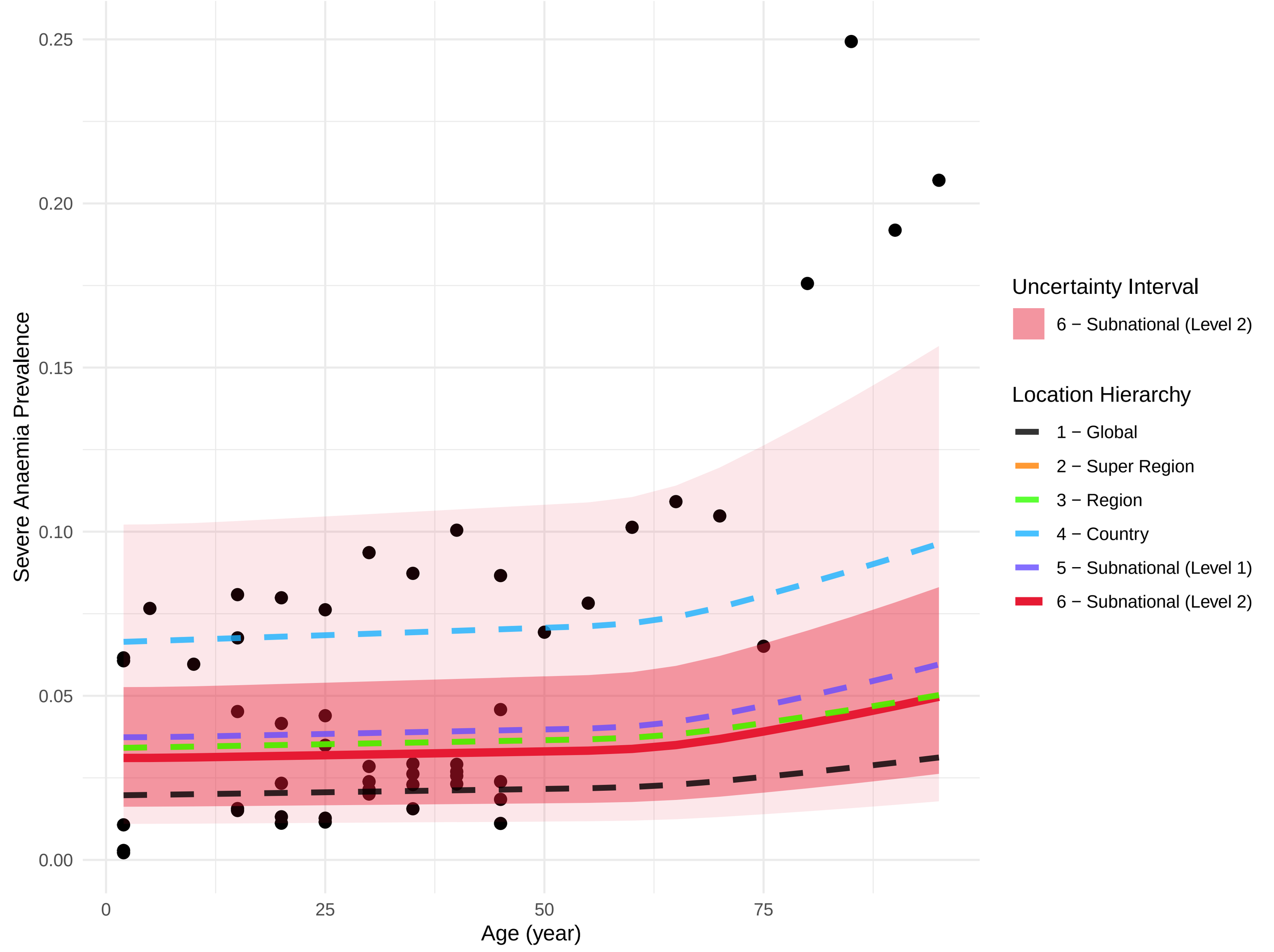

MR−BRT Age/Sex Fits − Non−pregnant Females 2+ Years Old − Severe Anaemia Prevalence − Nepal
WHO−2024 Adjustment Method

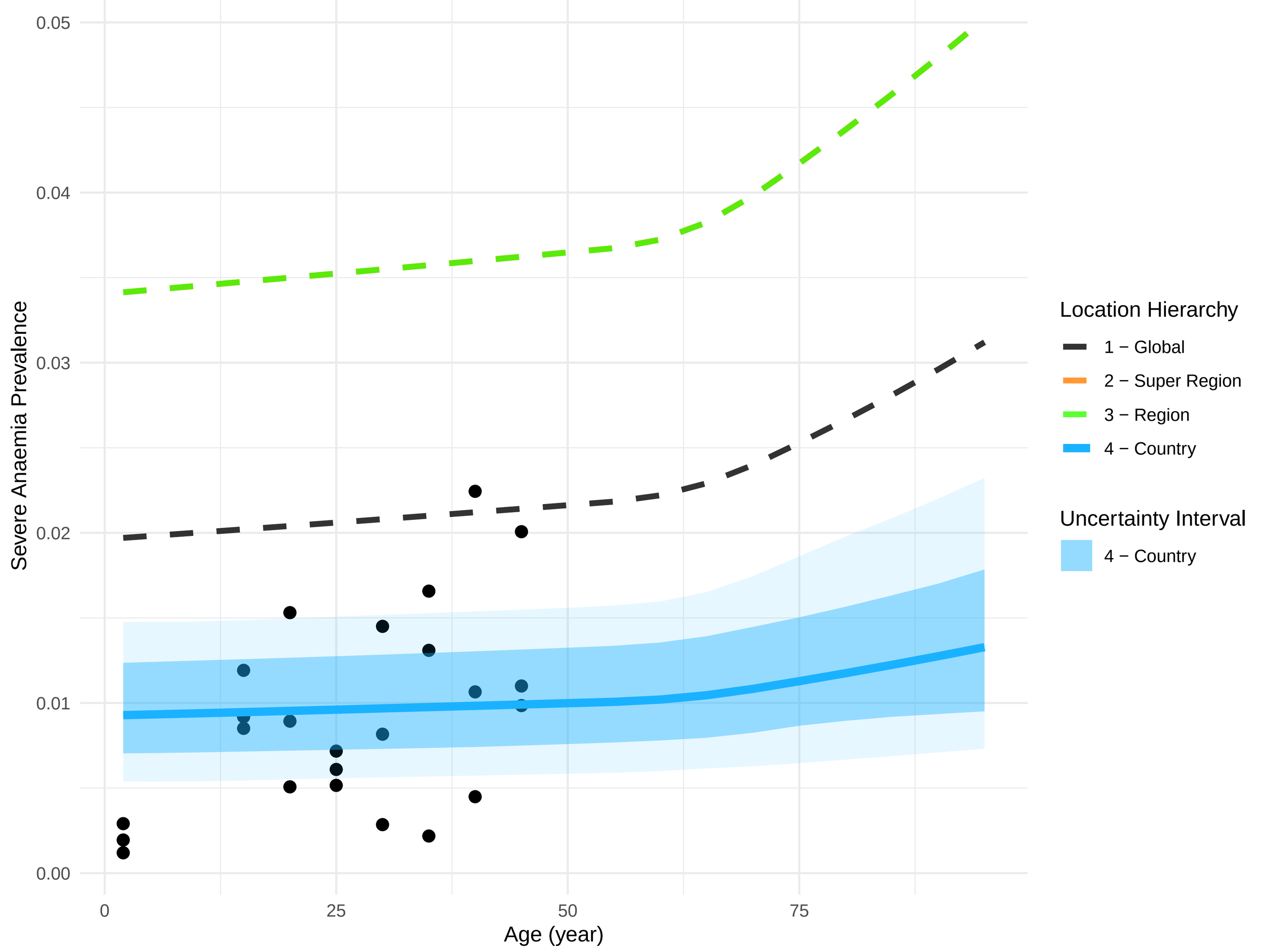

MR−BRT Age/Sex Fits − Non−pregnant Females 2+ Years Old − Severe Anaemia Prevalence − Pakistan
WHO−2024 Adjustment Method

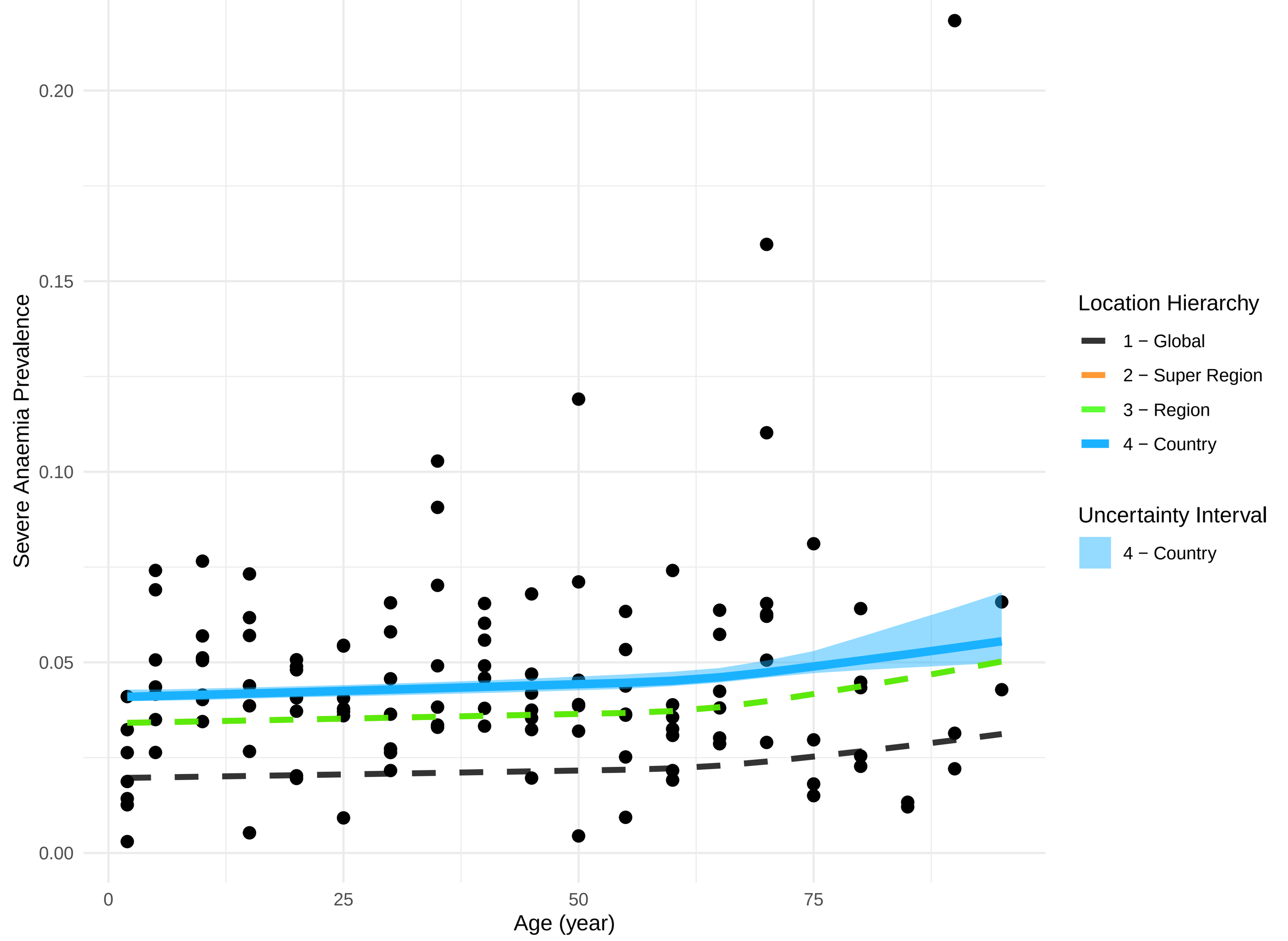

MR−BRT Age/Sex Fits − Non−pregnant Females 2+ Years Old − Severe Anaemia Prevalence − Azad Jammu & Kashmir
WHO−2024 Adjustment Method

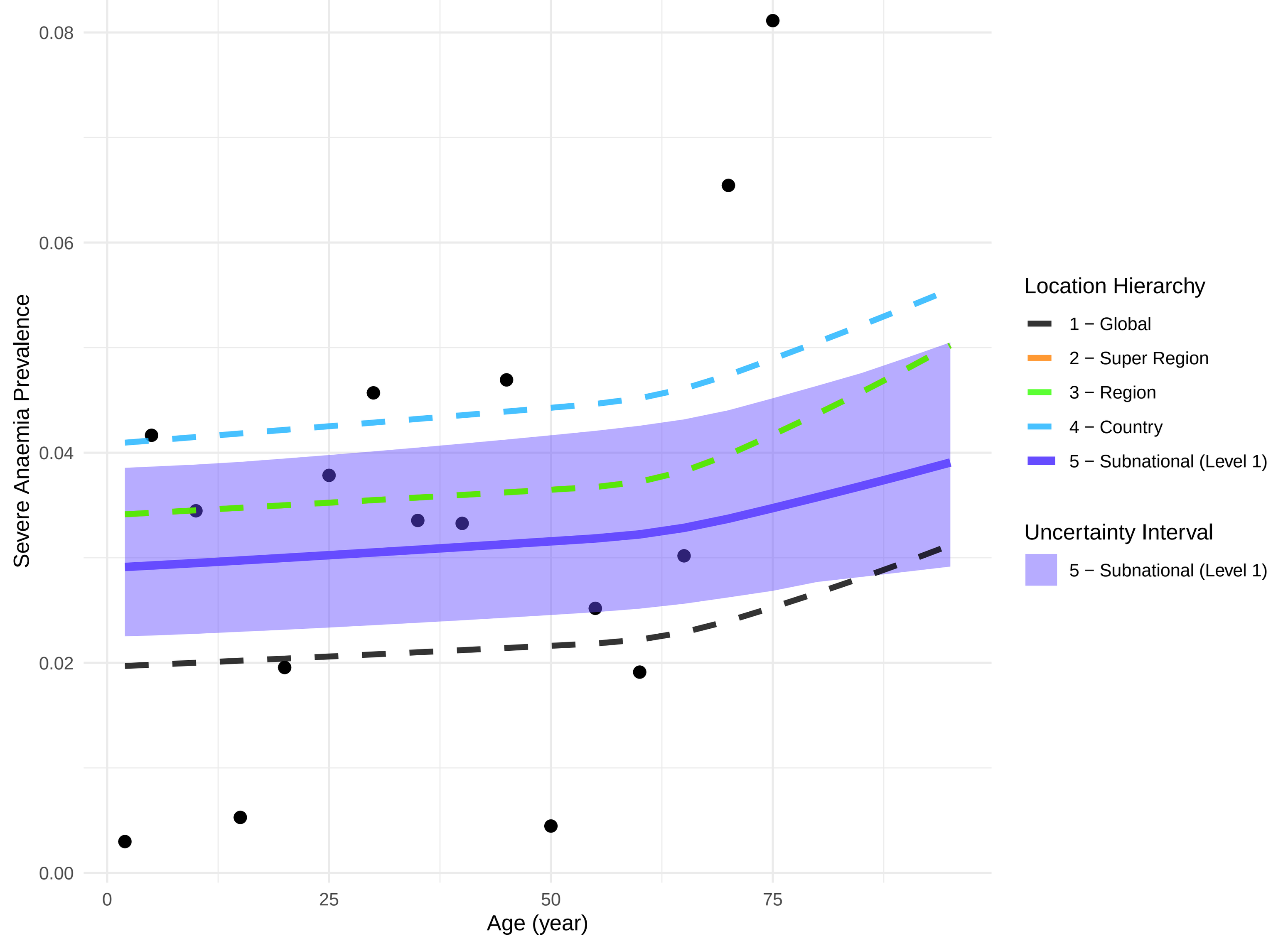

MR−BRT Age/Sex Fits − Non−pregnant Females 2+ Years Old − Severe Anaemia Prevalence − Balochistan
WHO−2024 Adjustment Method

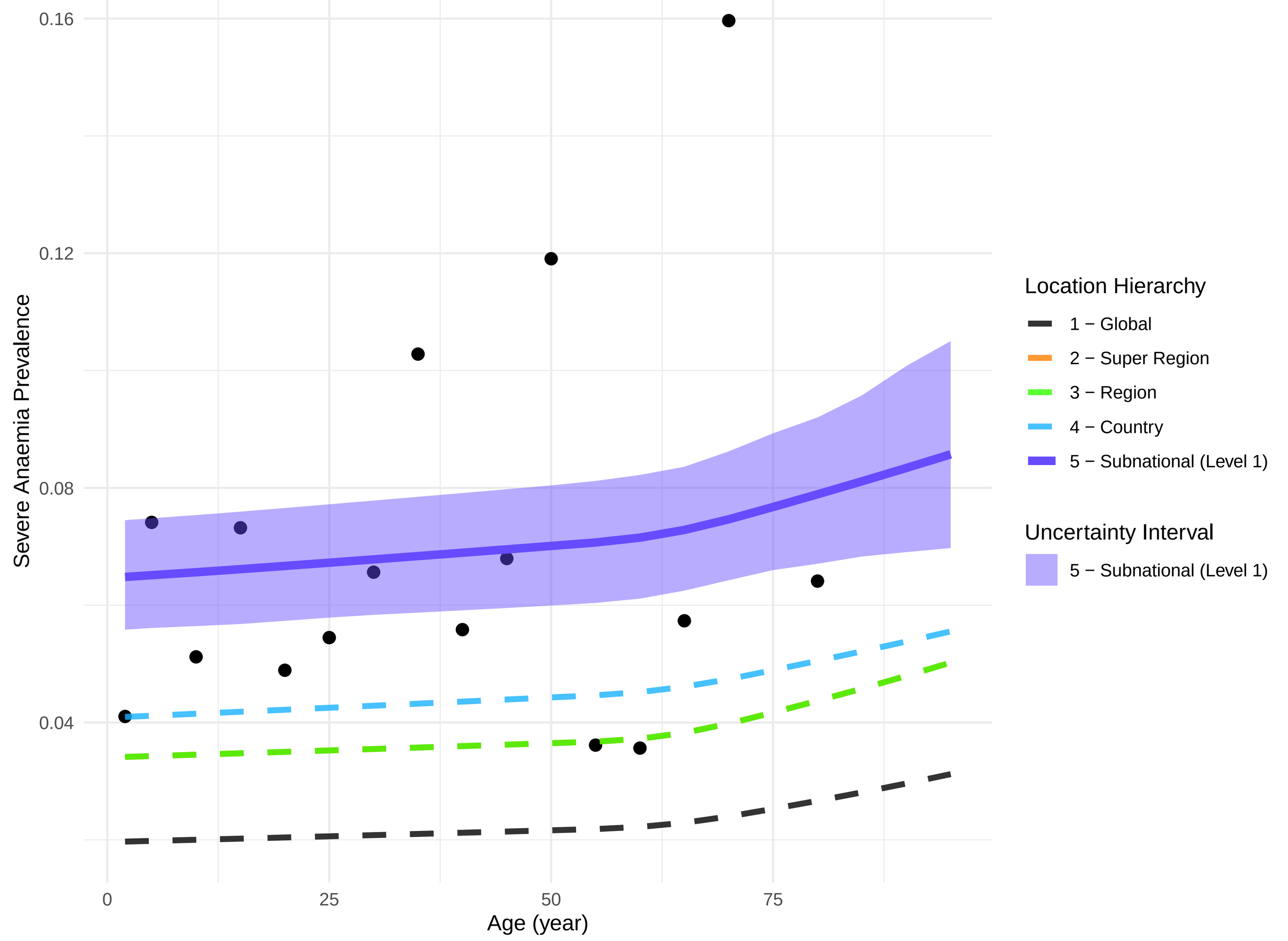

MR−BRT Age/Sex Fits − Non−pregnant Females 2+ Years Old − Severe Anaemia Prevalence − Gilgit−Baltistan
WHO−2024 Adjustment Method

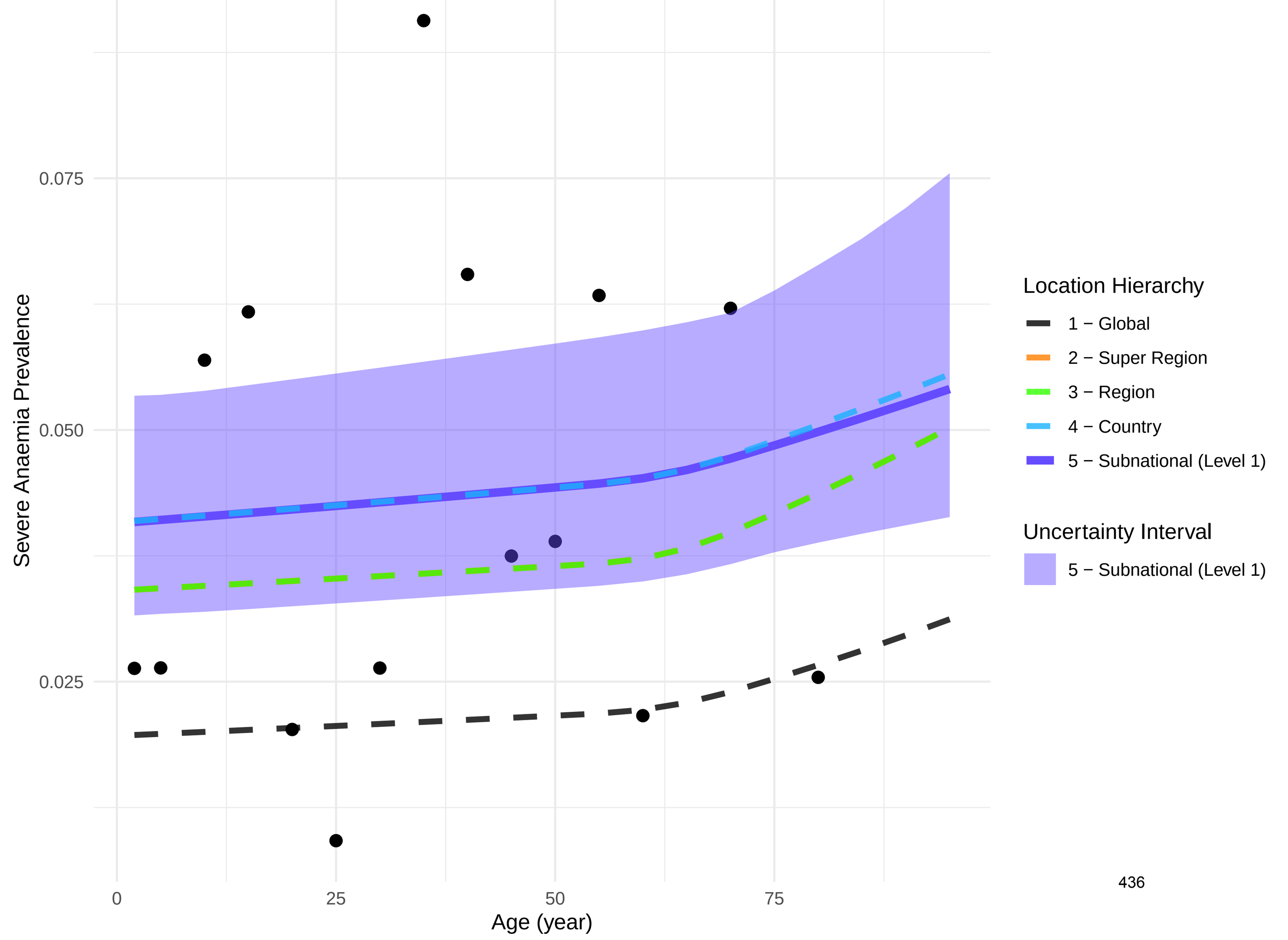

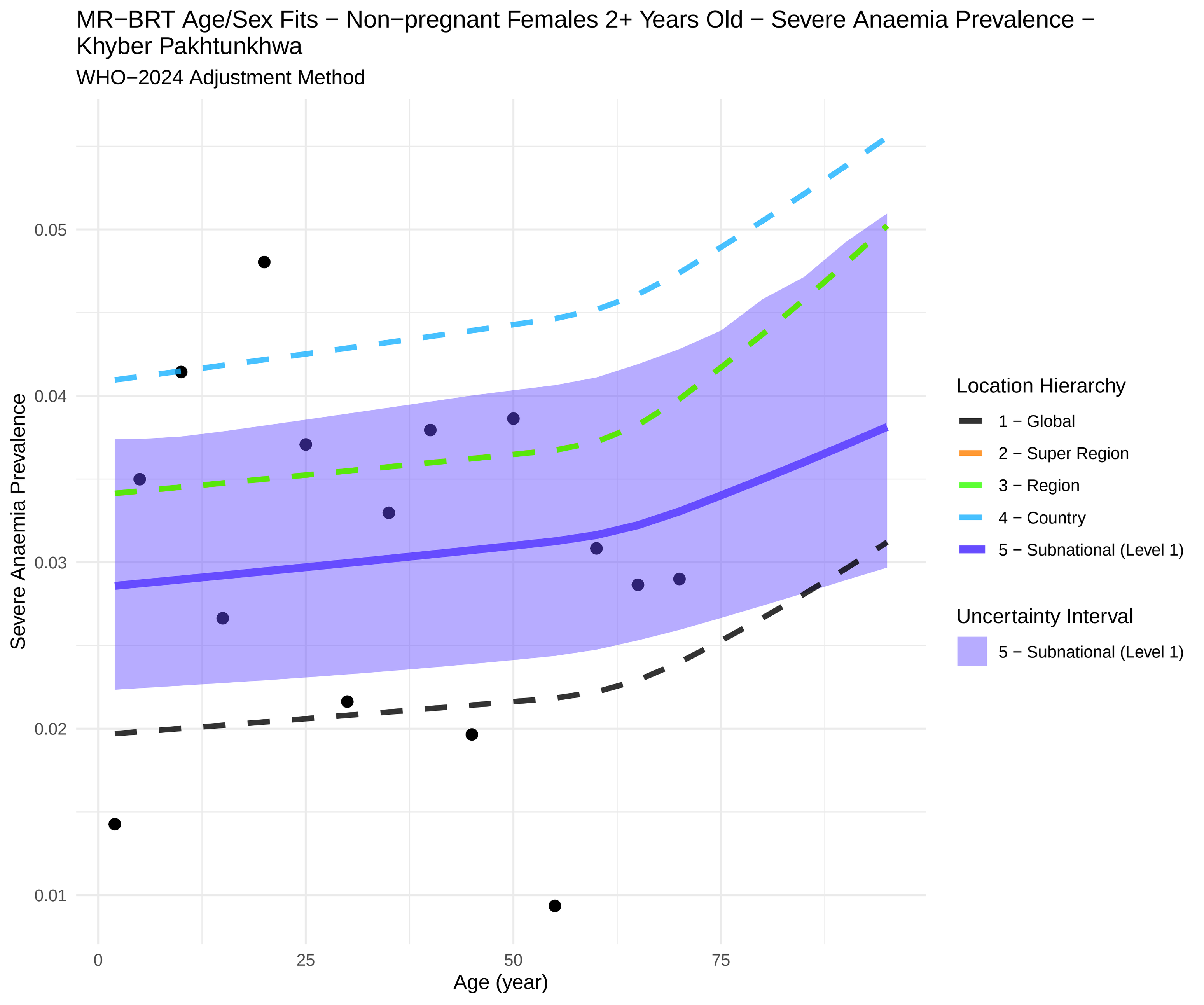
MR−BRT Age/Sex Fits − Non−pregnant Females 2+ Years Old − Severe Anaemia Prevalence − Khyber Pakhtunkhwa
WHO−2024 Adjustment Method
Severe Anaemia Prevalence
Age (year)
Location Hierarchy
1 − Global
2 − Super Region
3 − Region
4 − Country
5 − Subnational (Level 1)
Uncertainty Interval
5 − Subnational (Level 1)

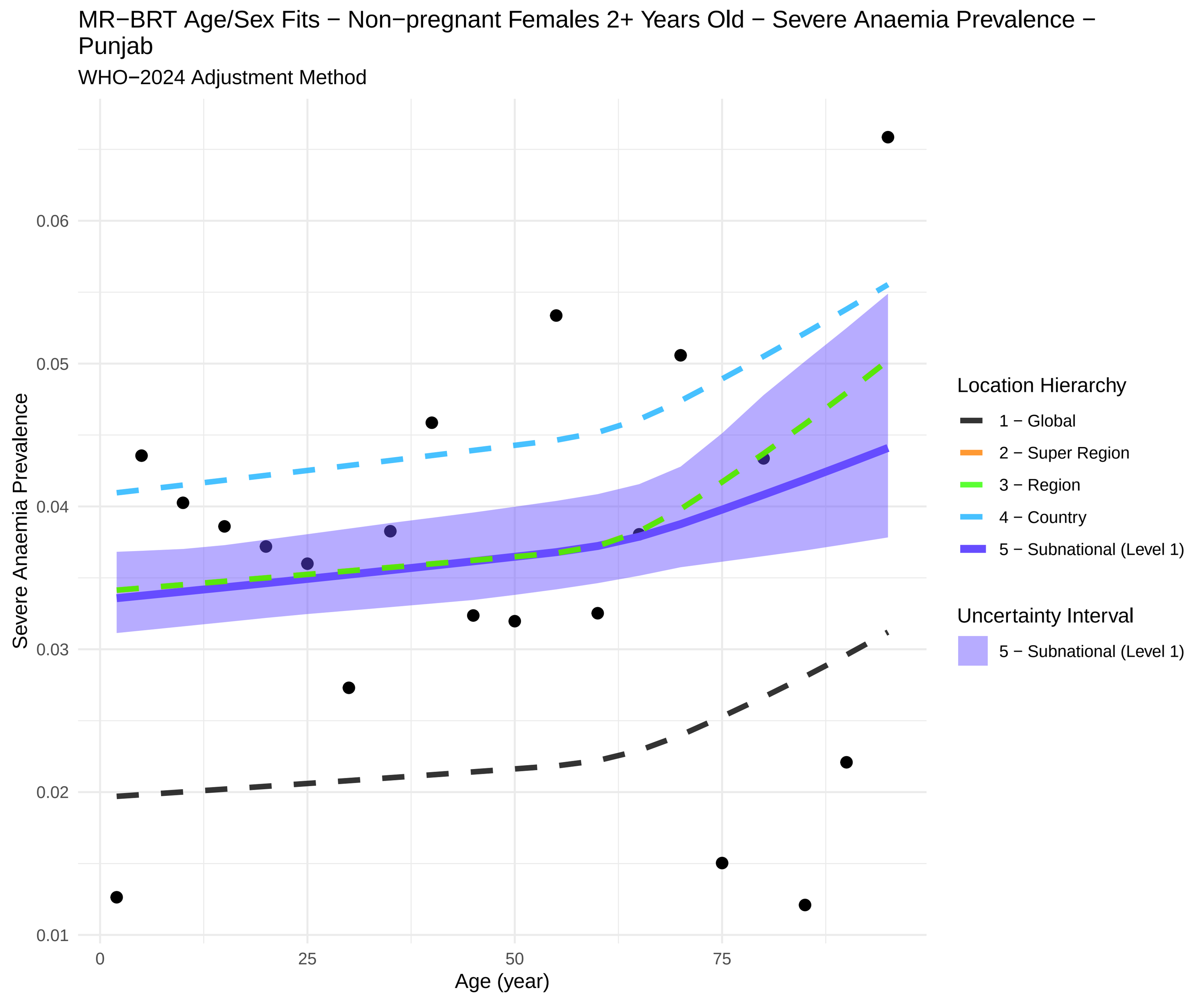
MR−BRT Age/Sex Fits − Non−pregnant Females 2+ Years Old − Severe Anaemia Prevalence − Punjab
WHO−2024 Adjustment Method
Severe Anaemia Prevalence
Age (year)
Location Hierarchy
1 − Global
2 − Super Region
3 − Region
4 − Country
5 − Subnational (Level 1)
Uncertainty Interval
5 − Subnational (Level 1)

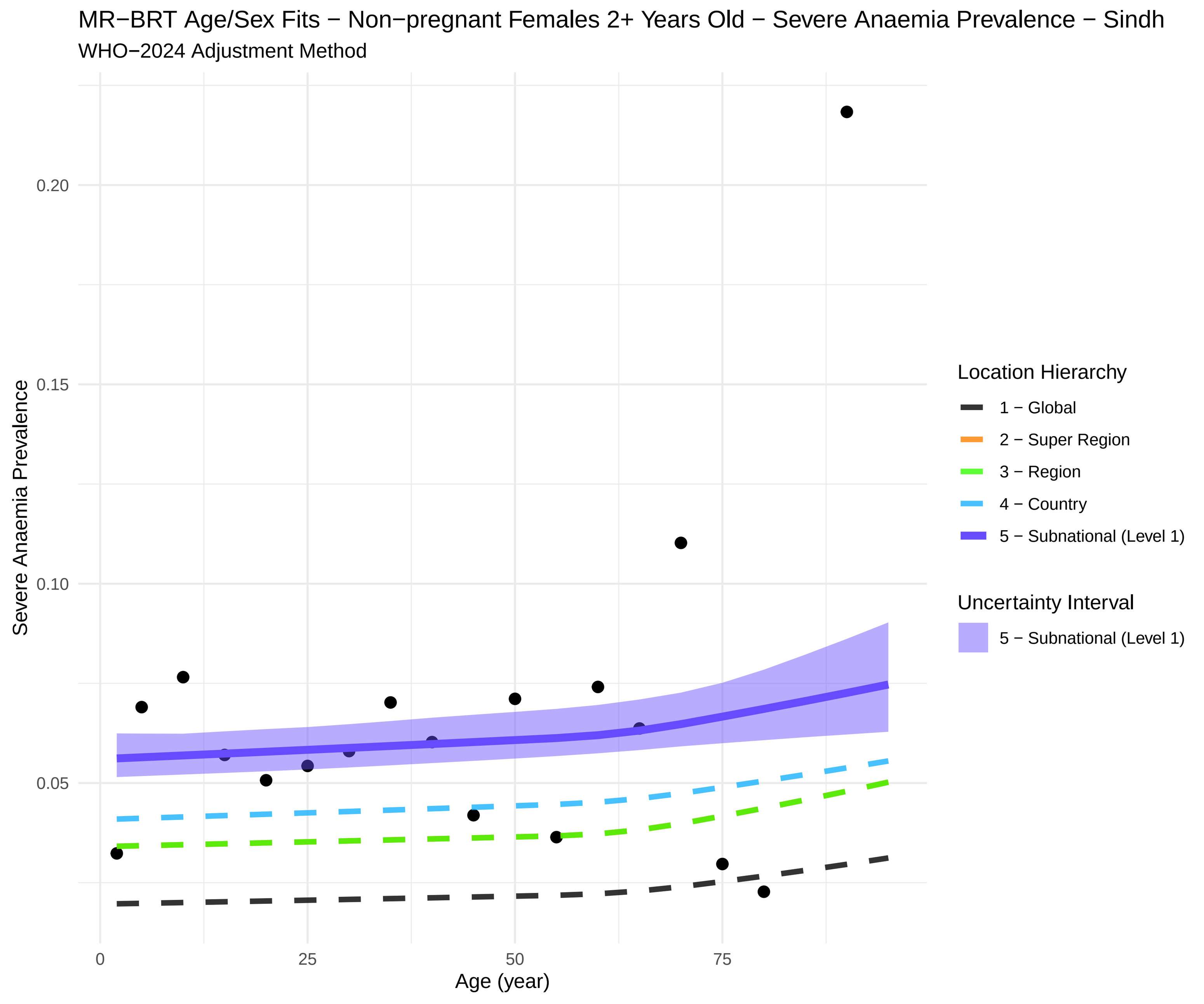
MR−BRT Age/Sex Fits − Non−pregnant Females 2+ Years Old − Severe Anaemia Prevalence − Sindh
WHO−2024 Adjustment Method
Severe Anaemia Prevalence
Age (year)
Location Hierarchy
1 − Global
2 − Super Region
3 − Region
4 − Country
5 − Subnational (Level 1)
Uncertainty Interval
5 − Subnational (Level 1)

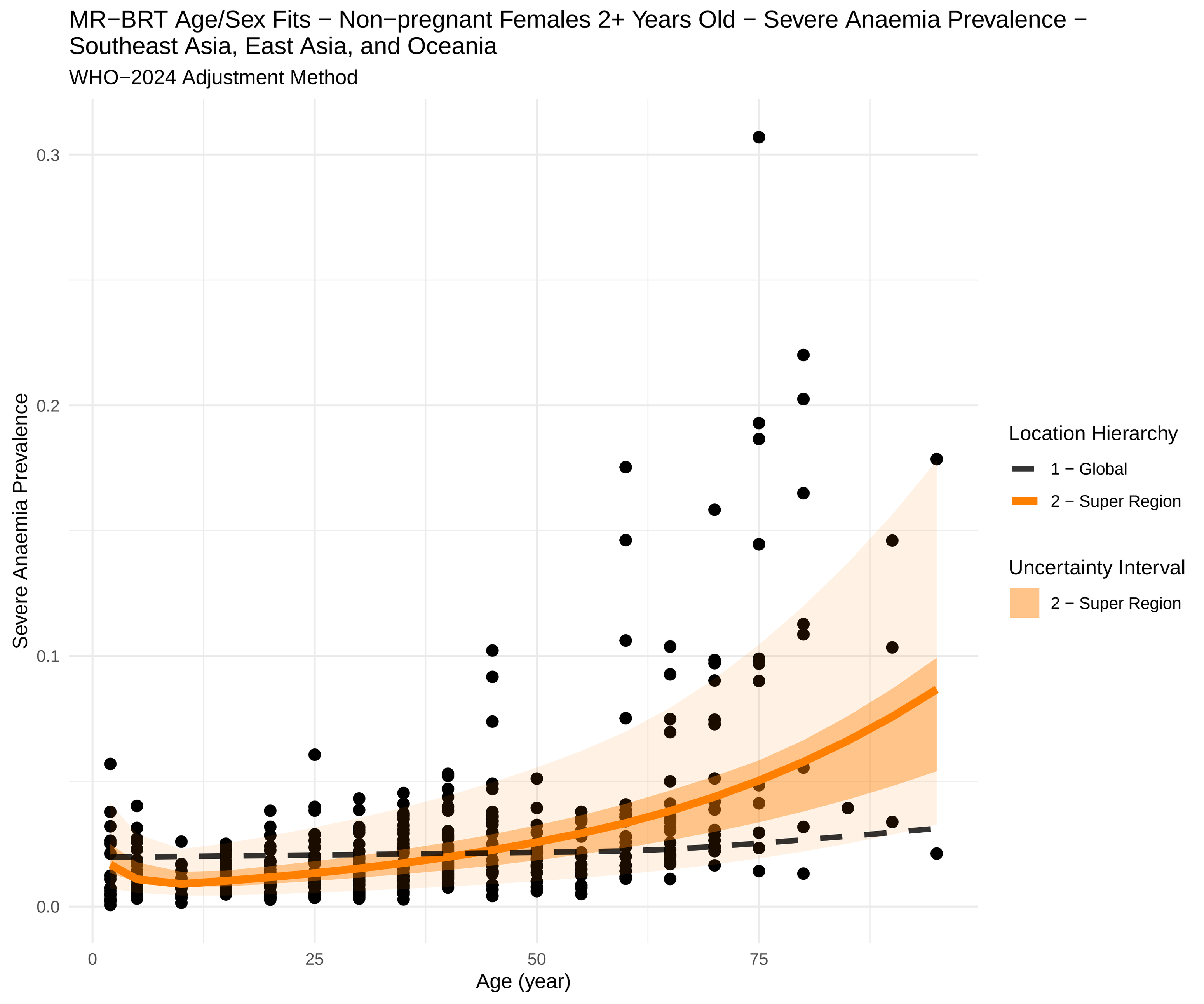
MR−BRT Age/Sex Fits − Non−pregnant Females 2+ Years Old − Severe Anaemia Prevalence − Southeast Asia, East Asia, and Oceania
WHO−2024 Adjustment Method
Severe Anaemia Prevalence
Age (year)
Location Hierarchy
1 − Global
2 − Super Region
Uncertainty Interval
2 − Super Region

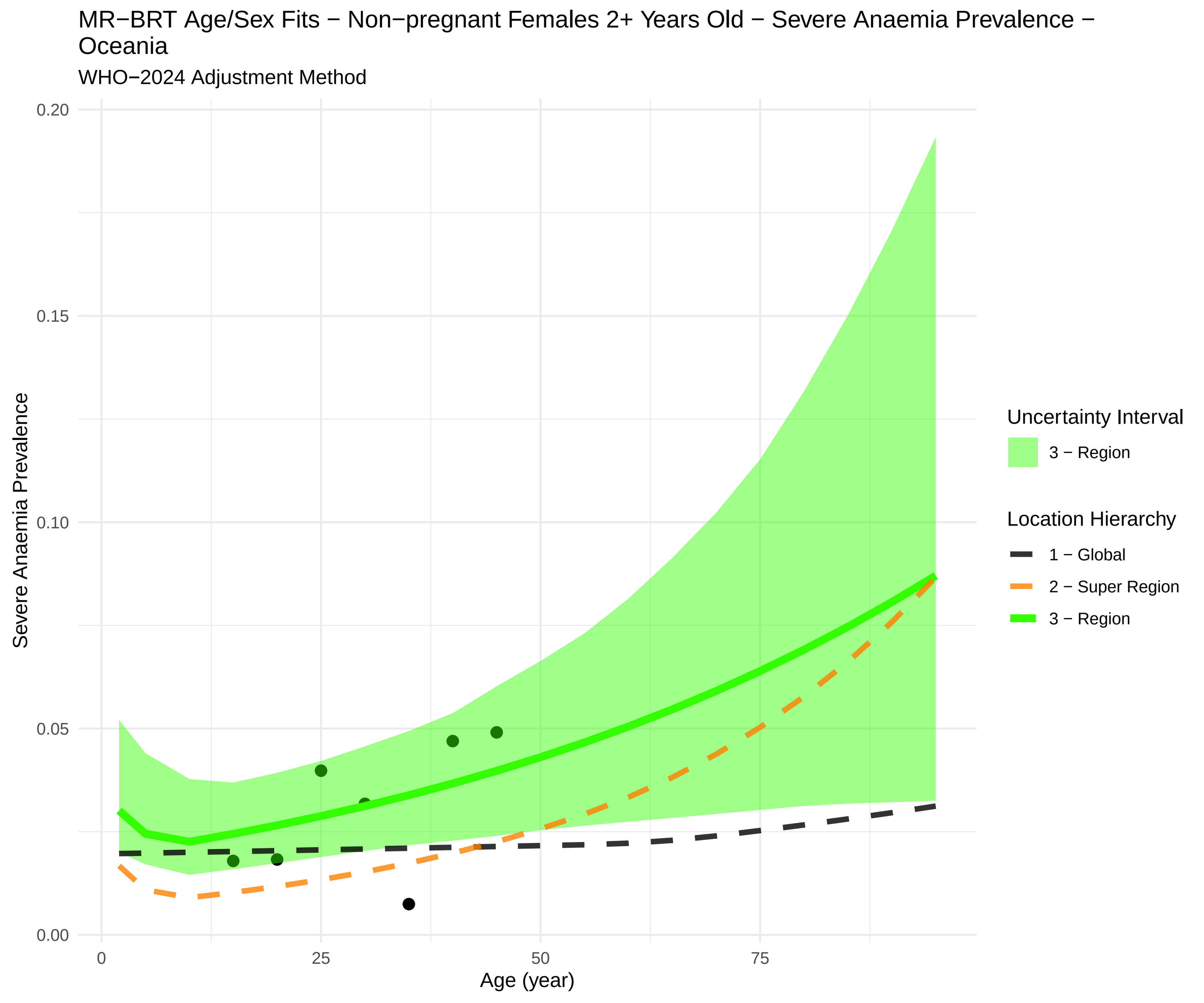
MR−BRT Age/Sex Fits − Non−pregnant Females 2+ Years Old − Severe Anaemia Prevalence − Oceania
WHO−2024 Adjustment Method
Severe Anaemia Prevalence
Age (year)
Uncertainty Interval
3 − Region
Location Hierarchy
1 − Global
2 − Super Region
3 − Region

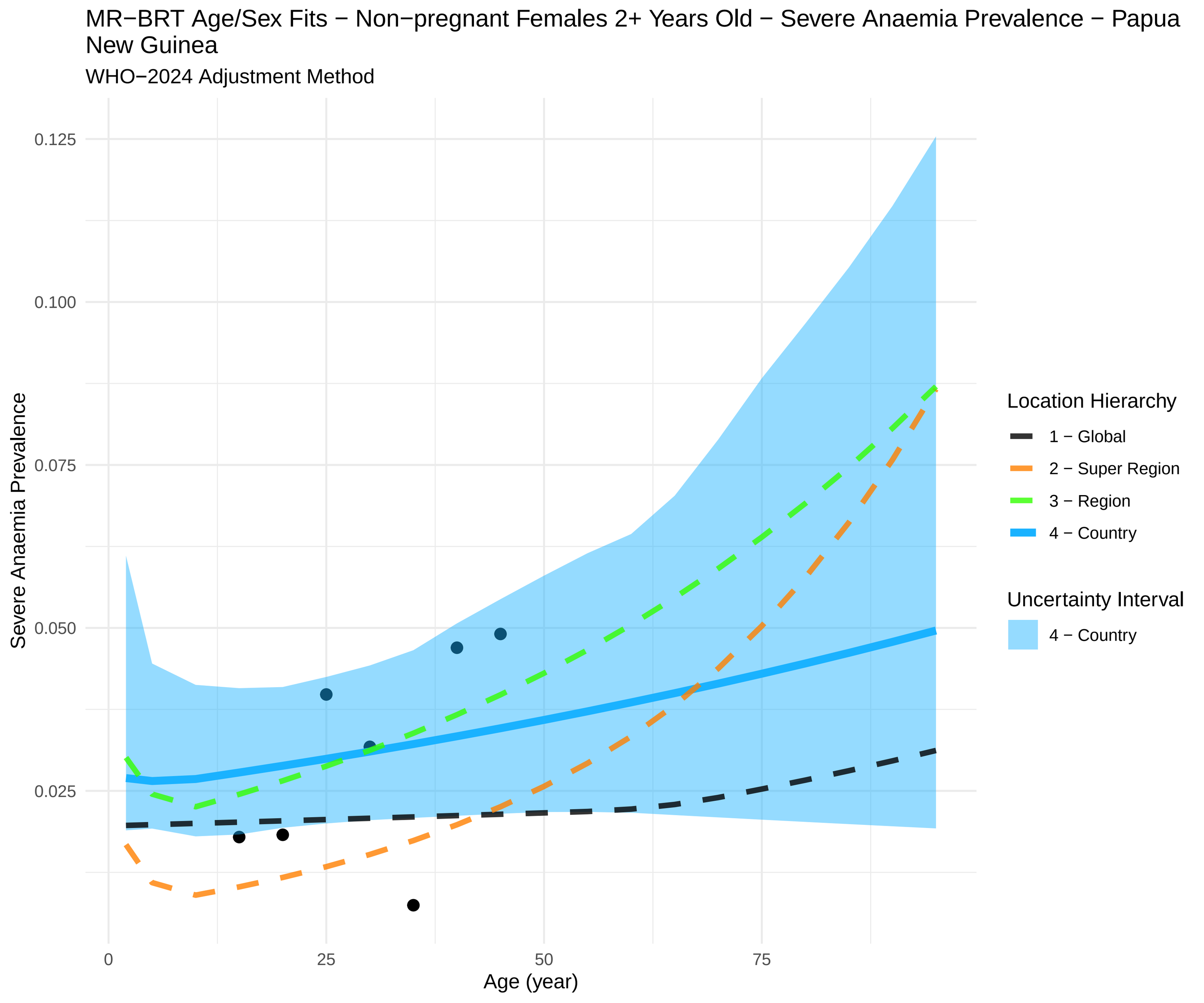
MR−BRT Age/Sex Fits − Non−pregnant Females 2+ Years Old − Severe Anaemia Prevalence − Papua New Guinea
WHO−2024 Adjustment Method
Severe Anaemia Prevalence
Age (year)
Location Hierarchy
1 − Global
2 − Super Region
3 − Region
4 − Country
Uncertainty Interval
4 − Country

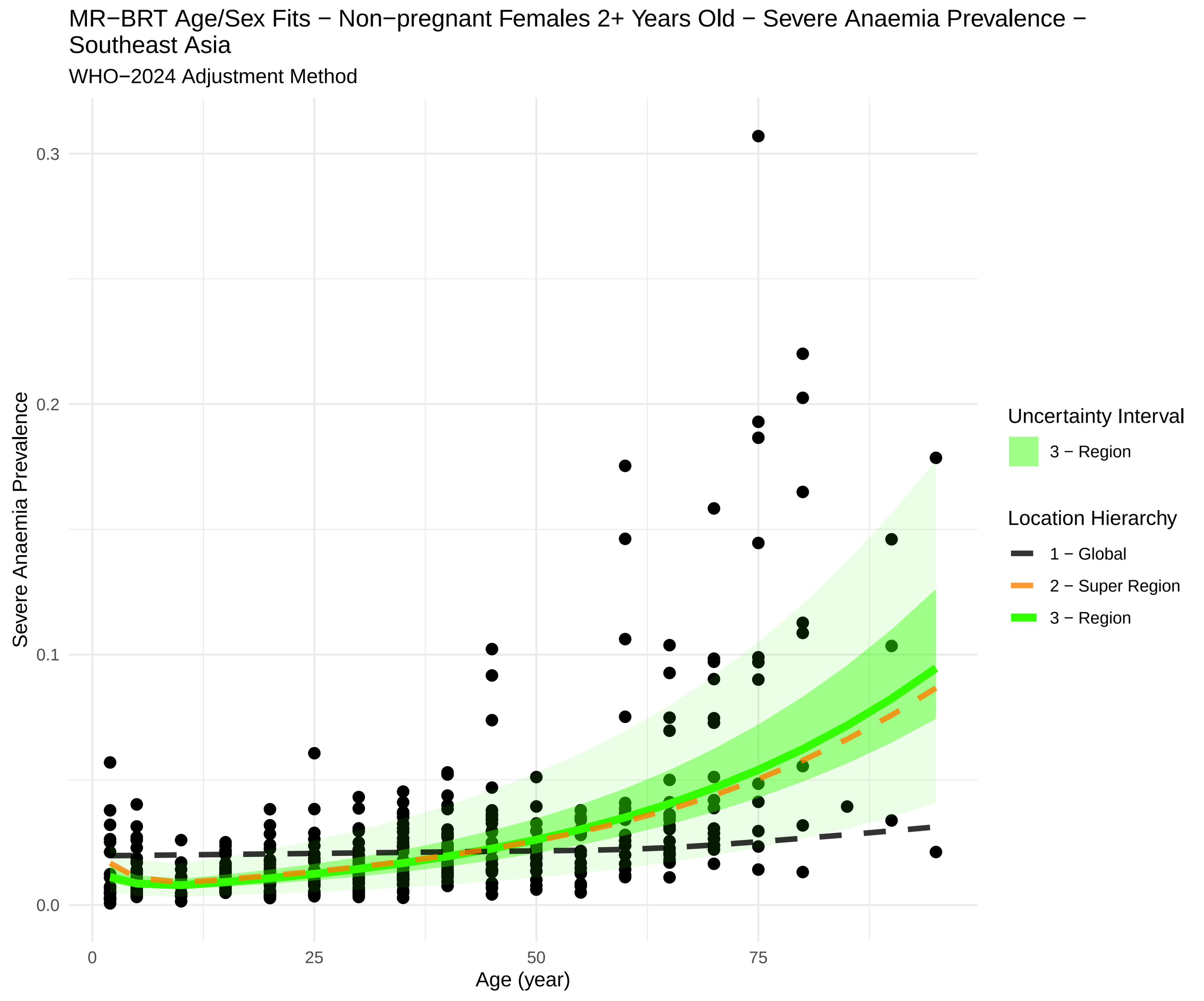
MR−BRT Age/Sex Fits − Non−pregnant Females 2+ Years Old − Severe Anaemia Prevalence − Southeast Asia
WHO−2024 Adjustment Method
Severe Anaemia Prevalence
Age (year)
Uncertainty Interval
3 − Region
Location Hierarchy
1 − Global
2 − Super Region
3 − Region

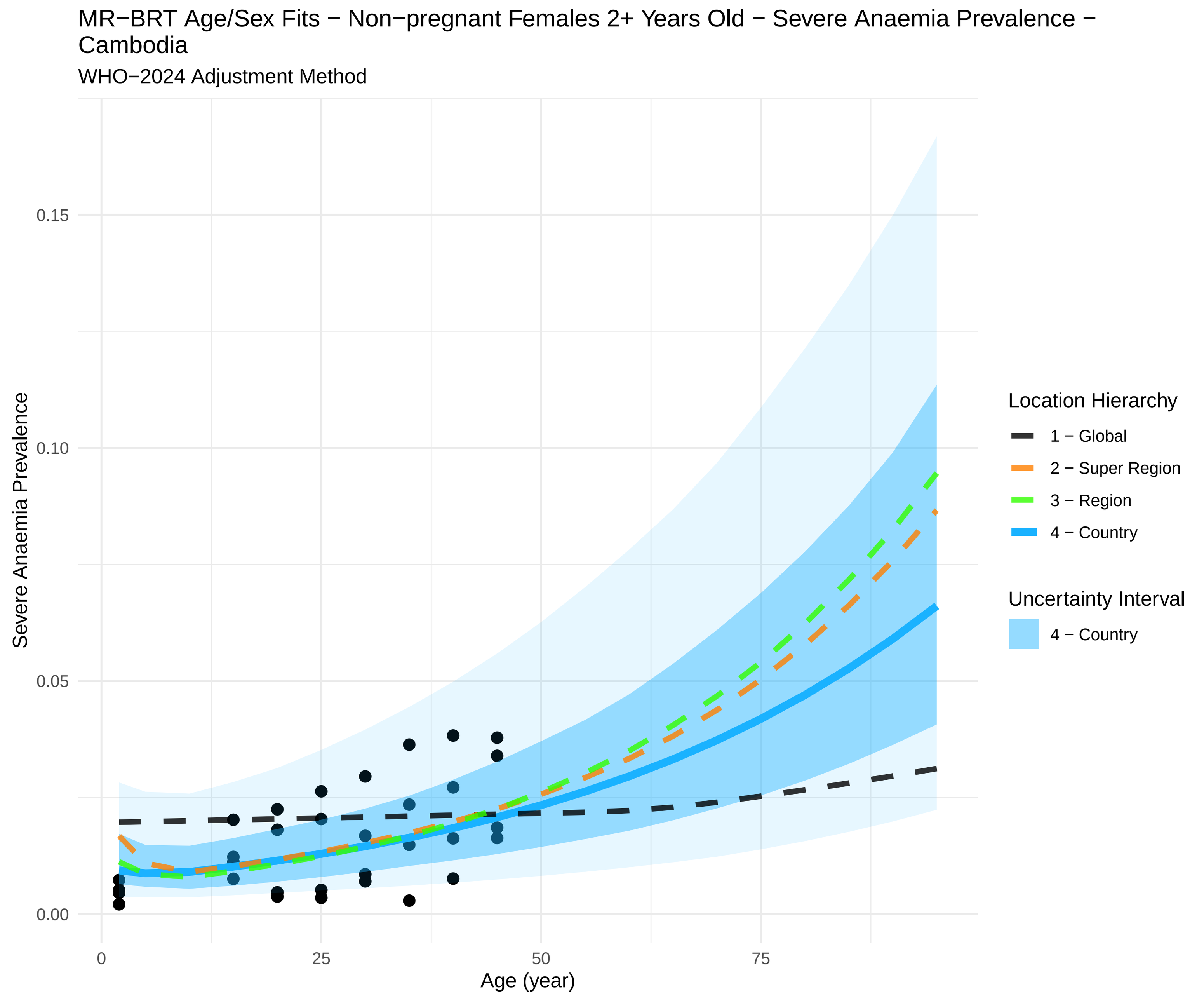
MR−BRT Age/Sex Fits − Non−pregnant Females 2+ Years Old − Severe Anaemia Prevalence − Cambodia
WHO−2024 Adjustment Method
Severe Anaemia Prevalence
Age (year)
Location Hierarchy
1 − Global
2 − Super Region
3 − Region
4 − Country
Uncertainty Interval
4 − Country

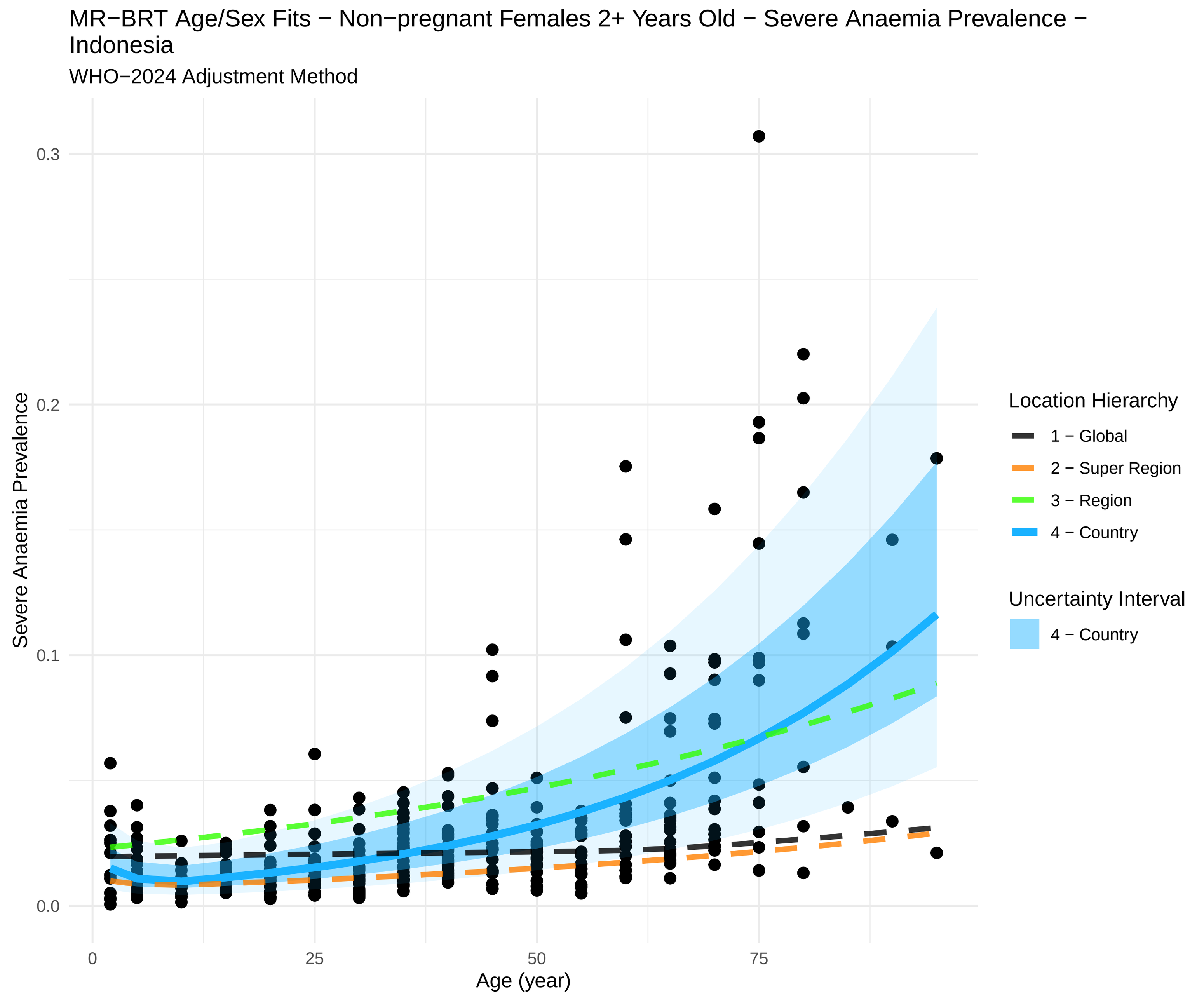
MR−BRT Age/Sex Fits − Non−pregnant Females 2+ Years Old − Severe Anaemia Prevalence − Indonesia
WHO−2024 Adjustment Method
Severe Anaemia Prevalence
Age (year)
Location Hierarchy
1 − Global
2 − Super Region
3 − Region
4 − Country
Uncertainty Interval
4 − Country

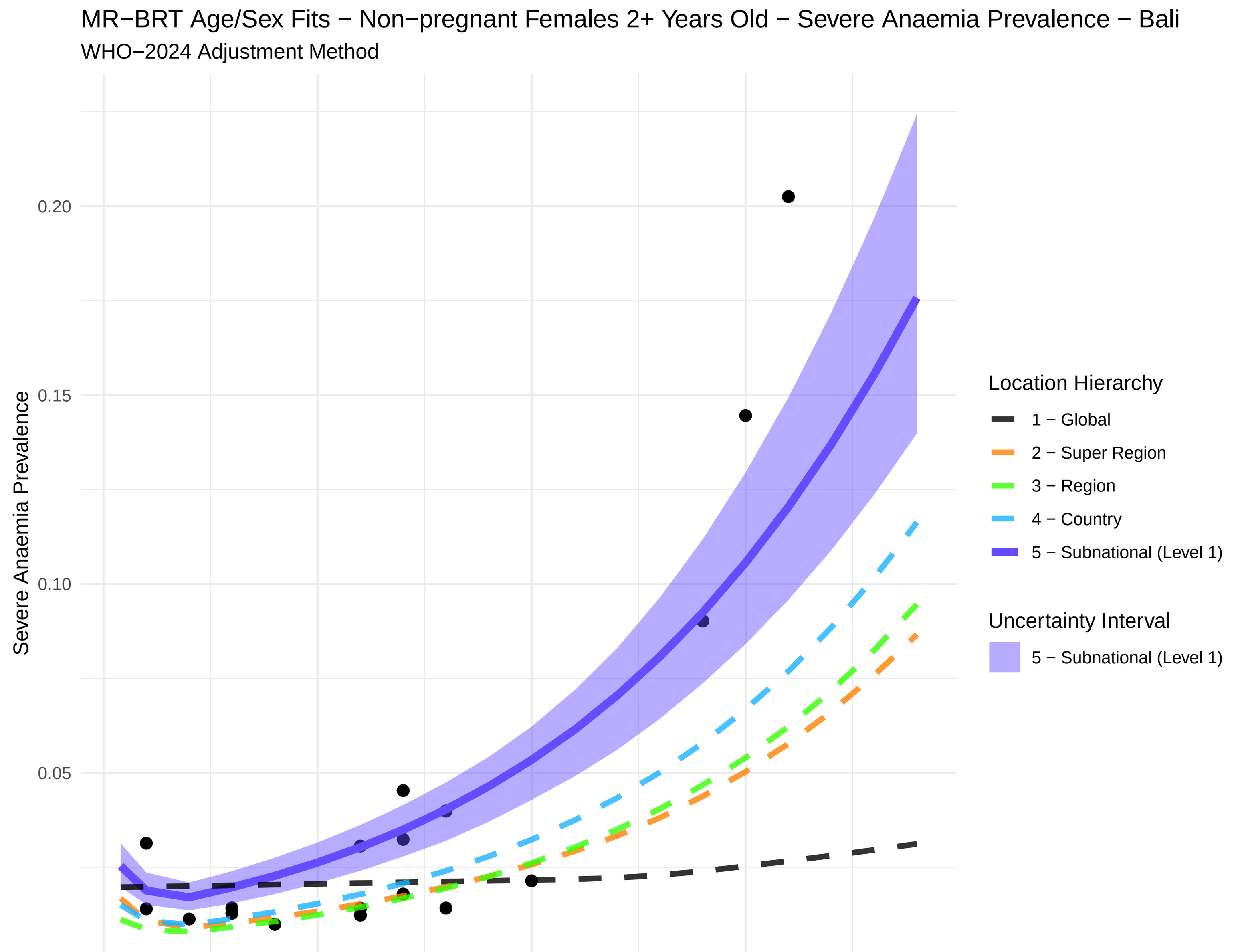
MR−BRT Age/Sex Fits − Non−pregnant Females 2+ Years Old − Severe Anaemia Prevalence − Bali
WHO−2024 Adjustment Method
Severe Anaemia Prevalence
Age (year)
Location Hierarchy
1 − Global
2 − Super Region
3 − Region
4 − Country
5 − Subnational (Level 1)
Uncertainty Interval
5 − Subnational (Level 1)

MR−BRT Age/Sex Fits − Non−pregnant Females 2+ Years Old − Severe Anaemia Prevalence − Bangka−Belitung Islands
WHO−2024 Adjustment Method

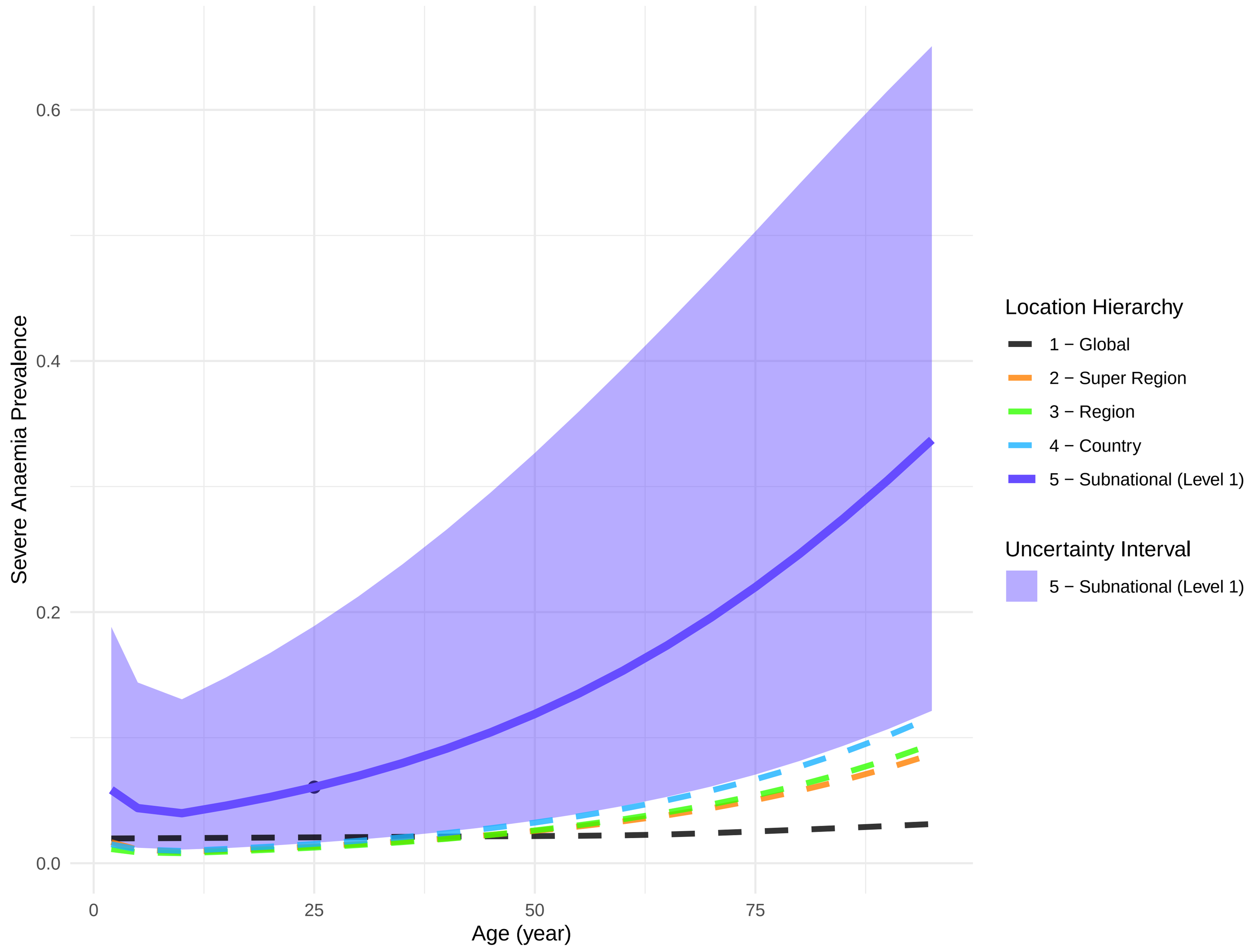

MR−BRT Age/Sex Fits − Non−pregnant Females 2+ Years Old − Severe Anaemia Prevalence − Banten
WHO−2024 Adjustment Method

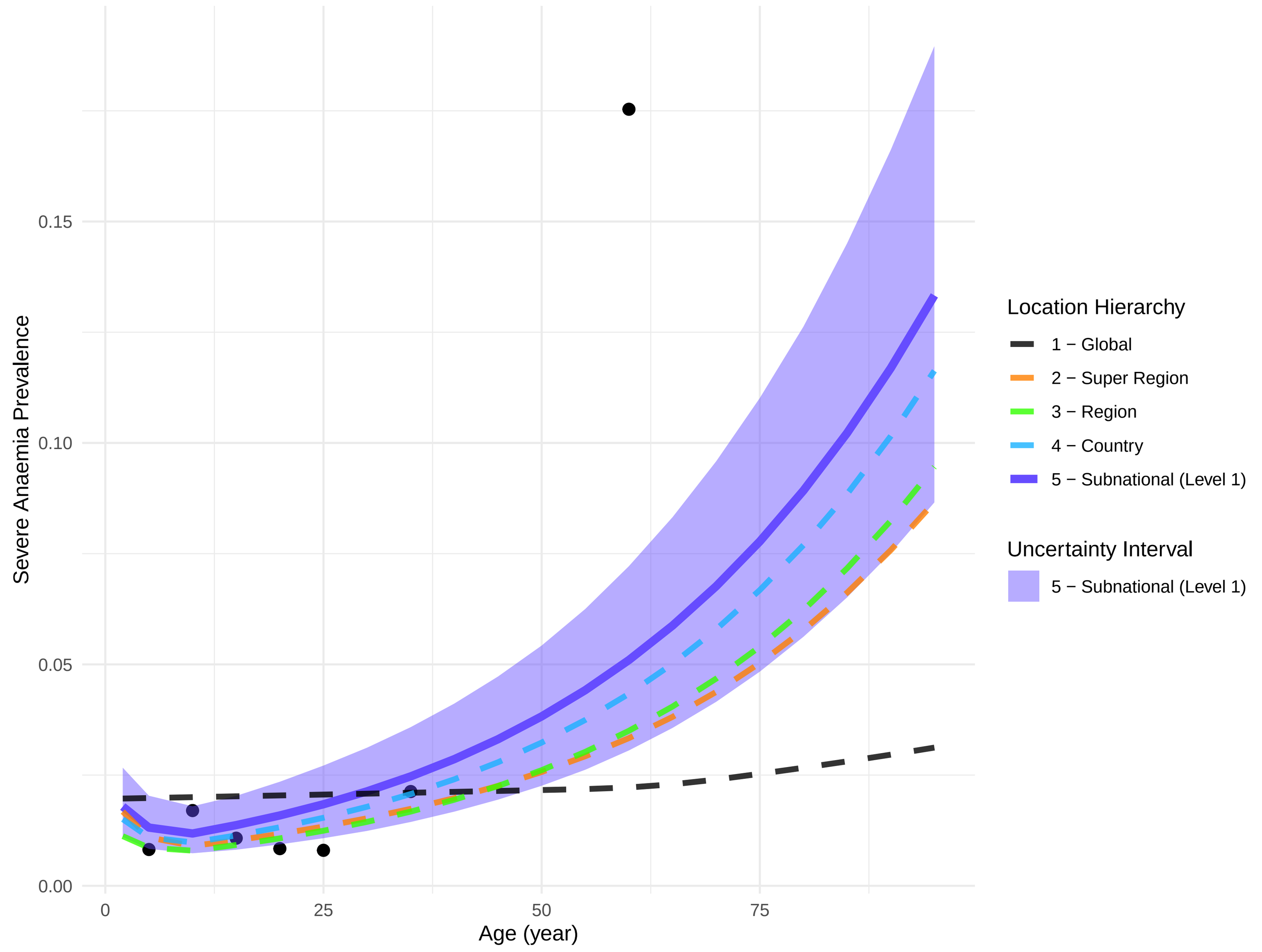

MR−BRT Age/Sex Fits − Non−pregnant Females 2+ Years Old − Severe Anaemia Prevalence − Jakarta
WHO−2024 Adjustment Method

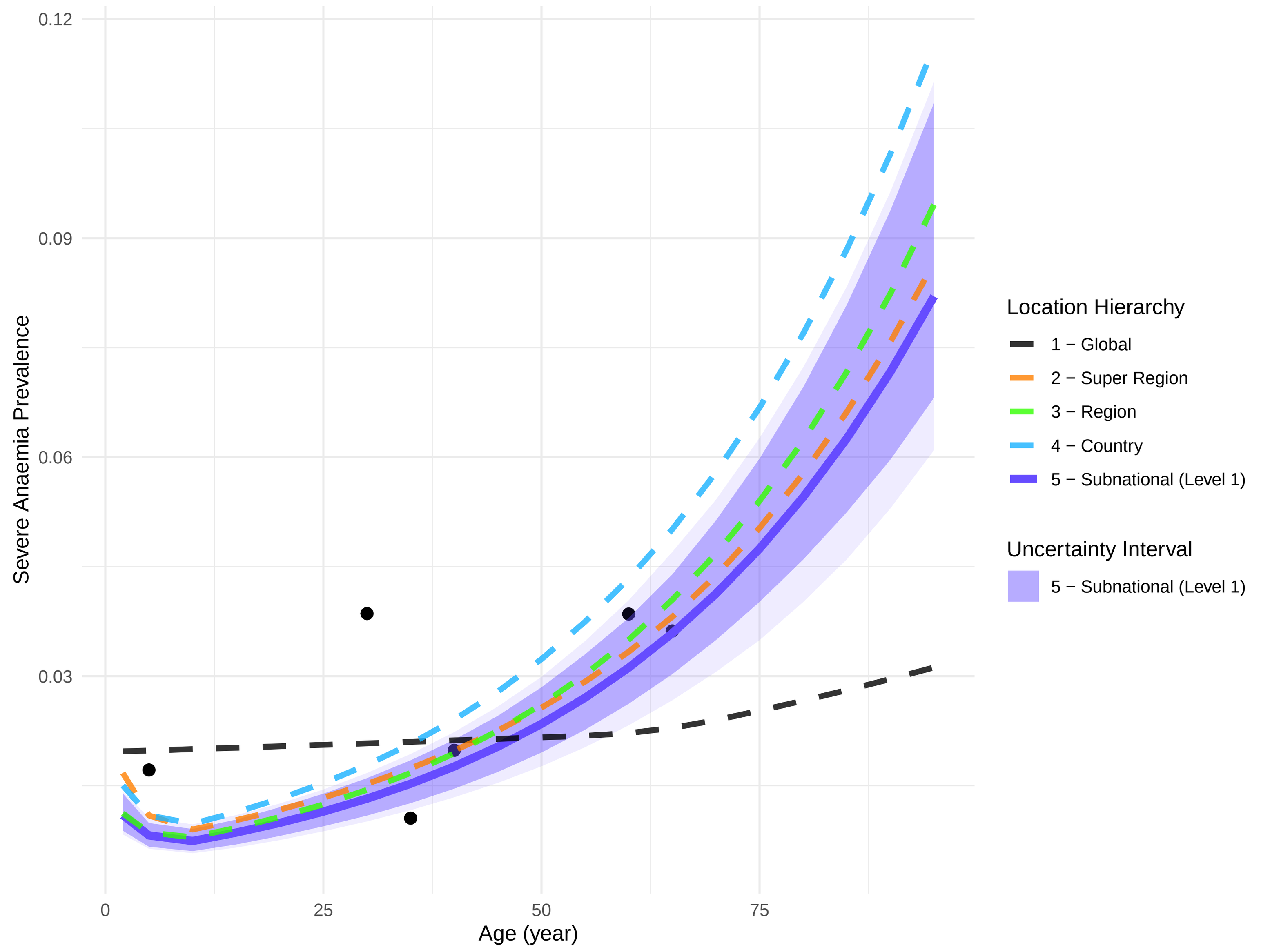

MR−BRT Age/Sex Fits − Non−pregnant Females 2+ Years Old − Severe Anaemia Prevalence − West Java
WHO−2024 Adjustment Method

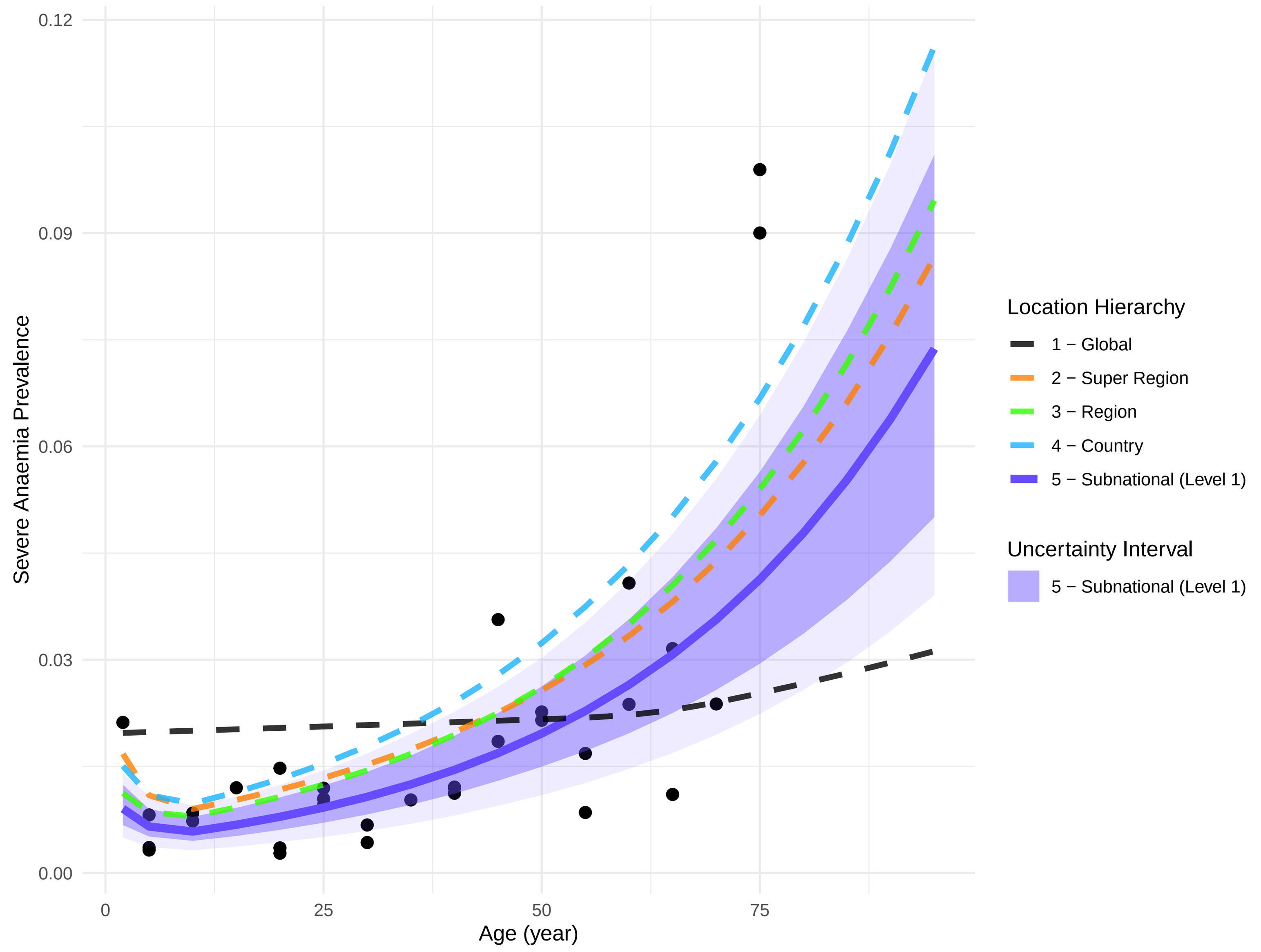

MR−BRT Age/Sex Fits − Non−pregnant Females 2+ Years Old − Severe Anaemia Prevalence − Central Java
WHO−2024 Adjustment Method

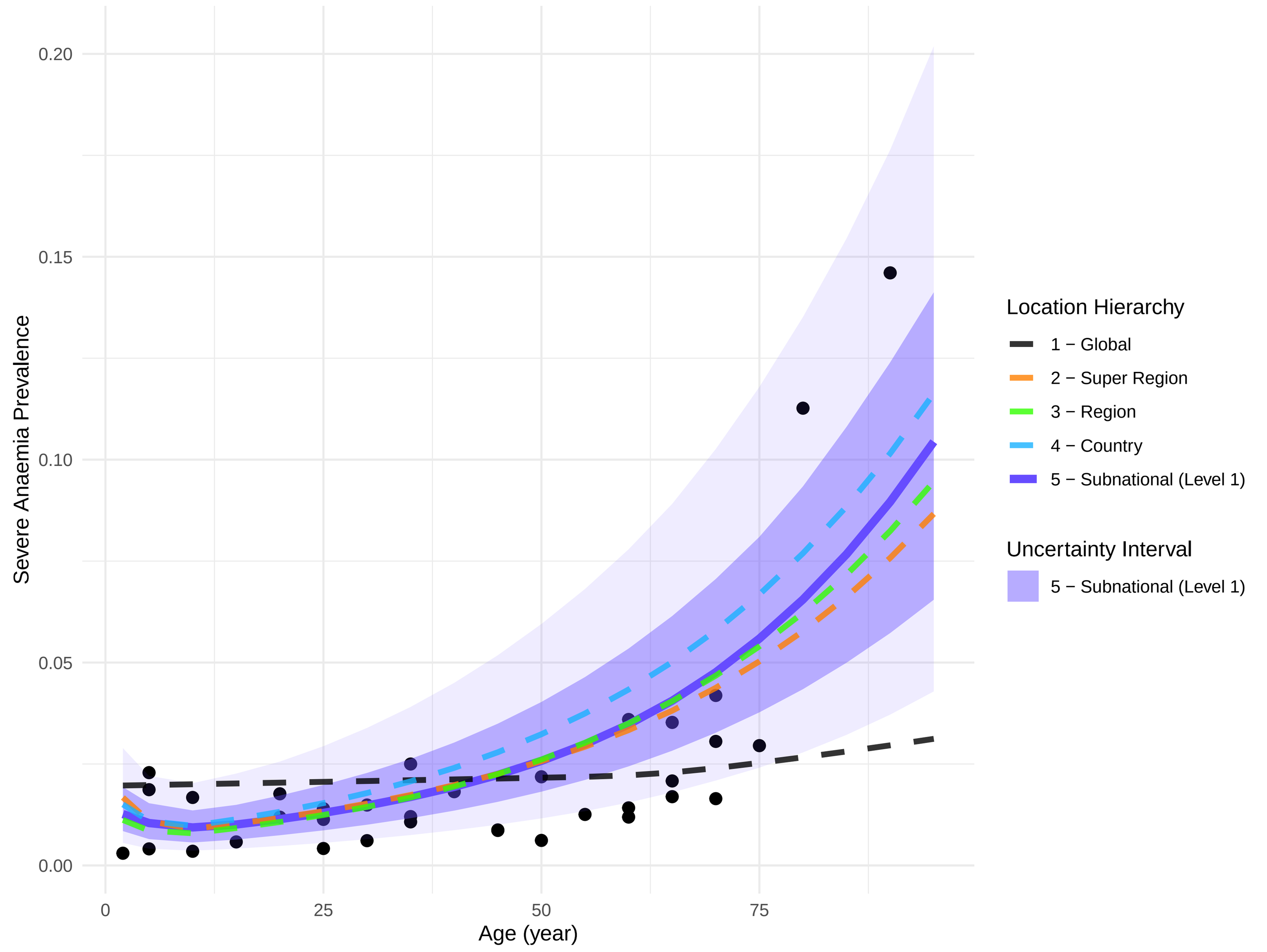

MR−BRT Age/Sex Fits − Non−pregnant Females 2+ Years Old − Severe Anaemia Prevalence − East Java
WHO−2024 Adjustment Method

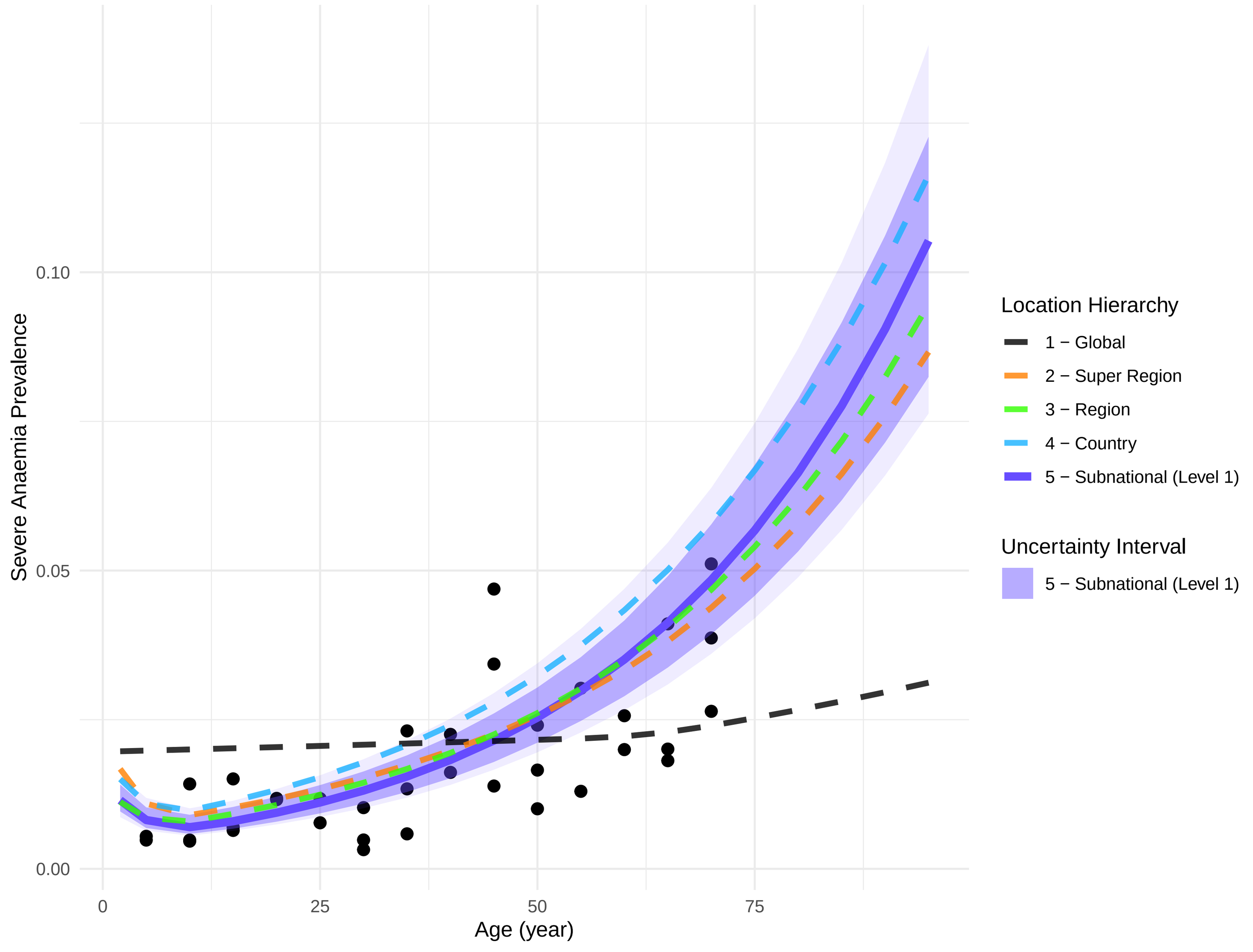

MR−BRT Age/Sex Fits − Non−pregnant Females 2+ Years Old − Severe Anaemia Prevalence − South Kalimantan
WHO−2024 Adjustment Method

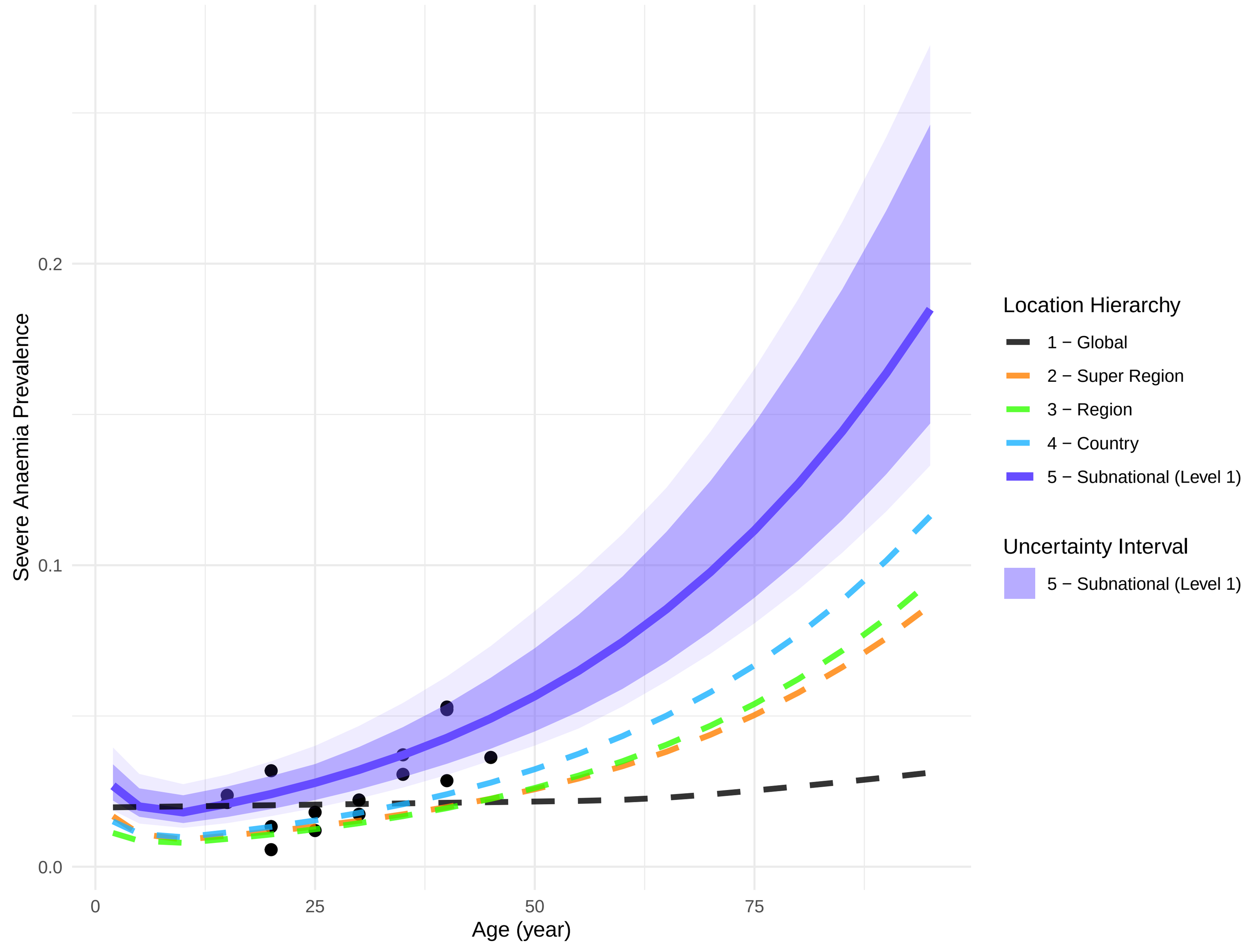

MR−BRT Age/Sex Fits − Non−pregnant Females 2+ Years Old − Severe Anaemia Prevalence − Lampung
WHO−2024 Adjustment Method

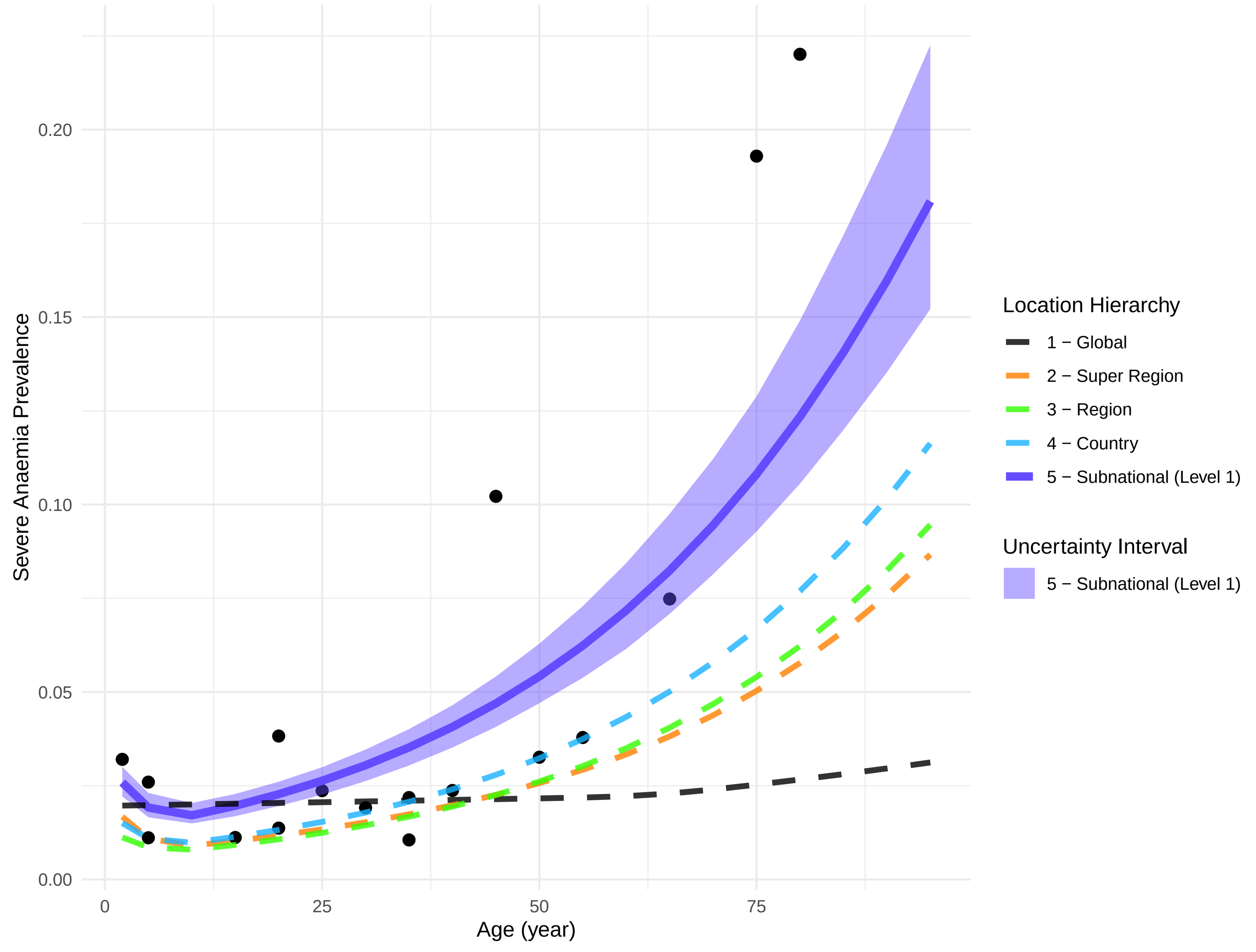

MR−BRT Age/Sex Fits − Non−pregnant Females 2+ Years Old − Severe Anaemia Prevalence − West Nusa Tenggara
WHO−2024 Adjustment Method

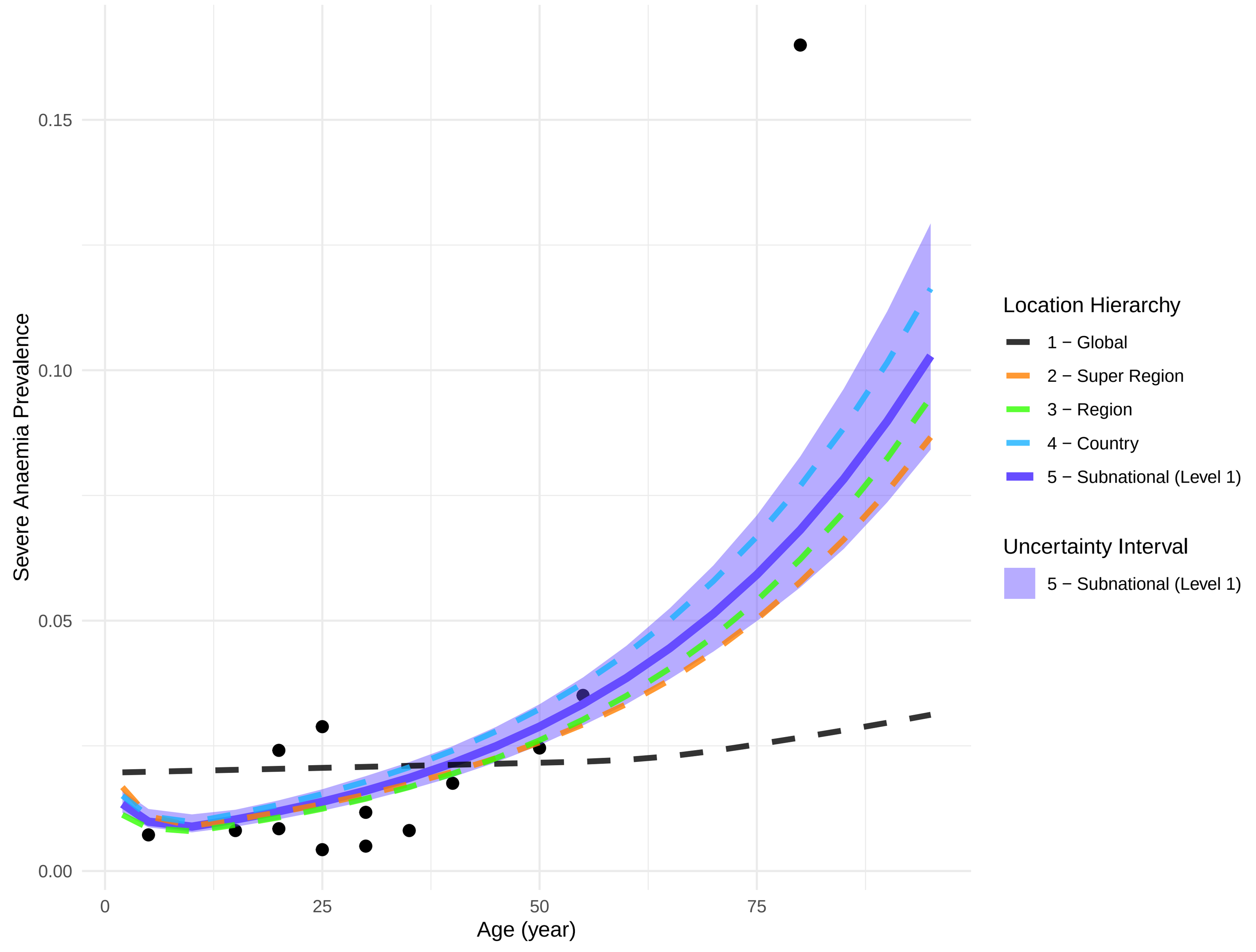

MR−BRT Age/Sex Fits − Non−pregnant Females 2+ Years Old − Severe Anaemia Prevalence − South Sulawesi
WHO−2024 Adjustment Method

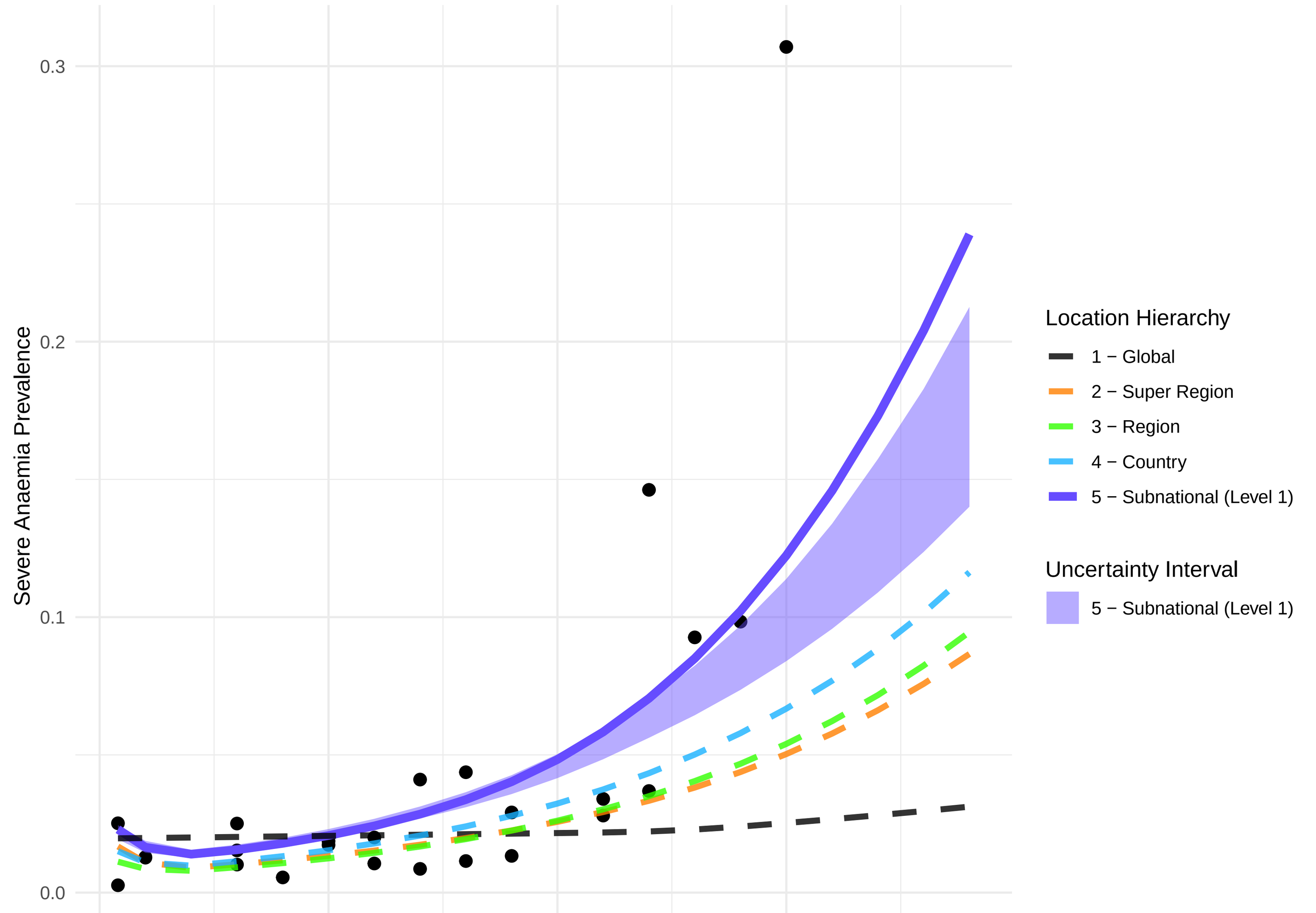

MR−BRT Age/Sex Fits − Non-pregnant Females 2+ Years Old − Severe Anaemia Prevalence − West Sumatra
WHO−2024 Adjustment Method

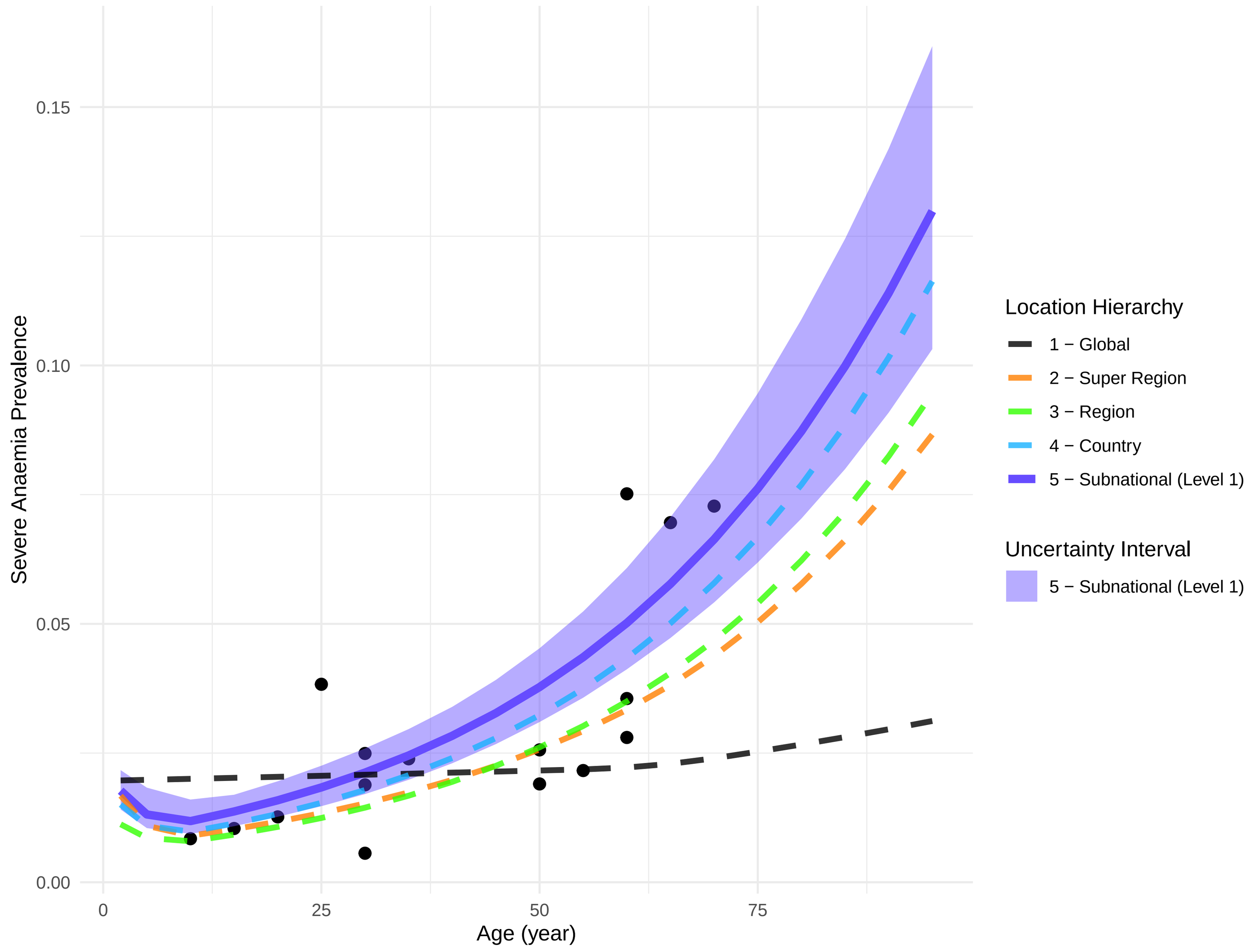

MR−BRT Age/Sex Fits − Non-pregnant Females 2+ Years Old − Severe Anaemia Prevalence − South Sumatra
WHO−2024 Adjustment Method

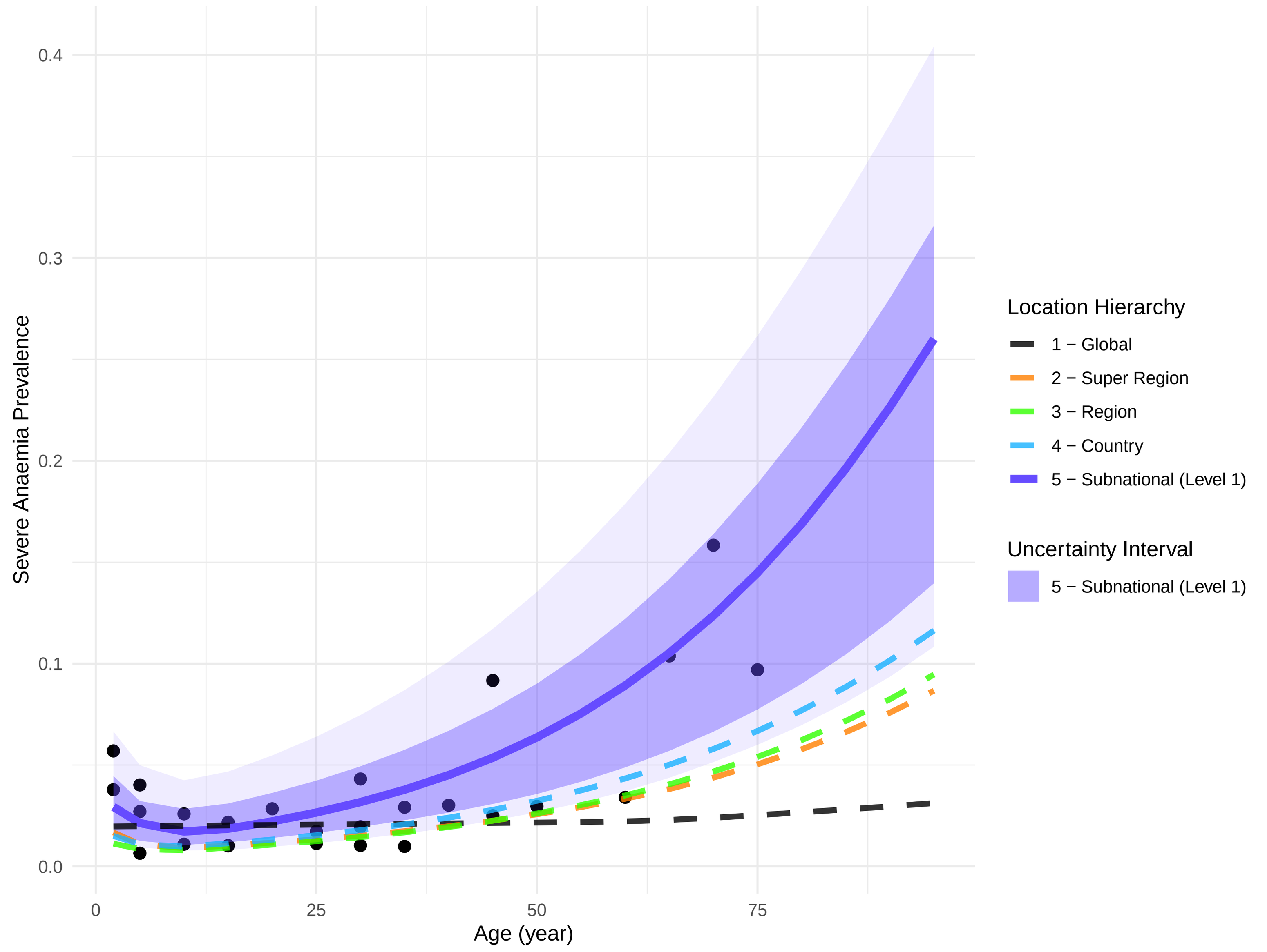

MR−BRT Age/Sex Fits − Non-pregnant Females 2+ Years Old − Severe Anaemia Prevalence − North Sumatra
WHO−2024 Adjustment Method

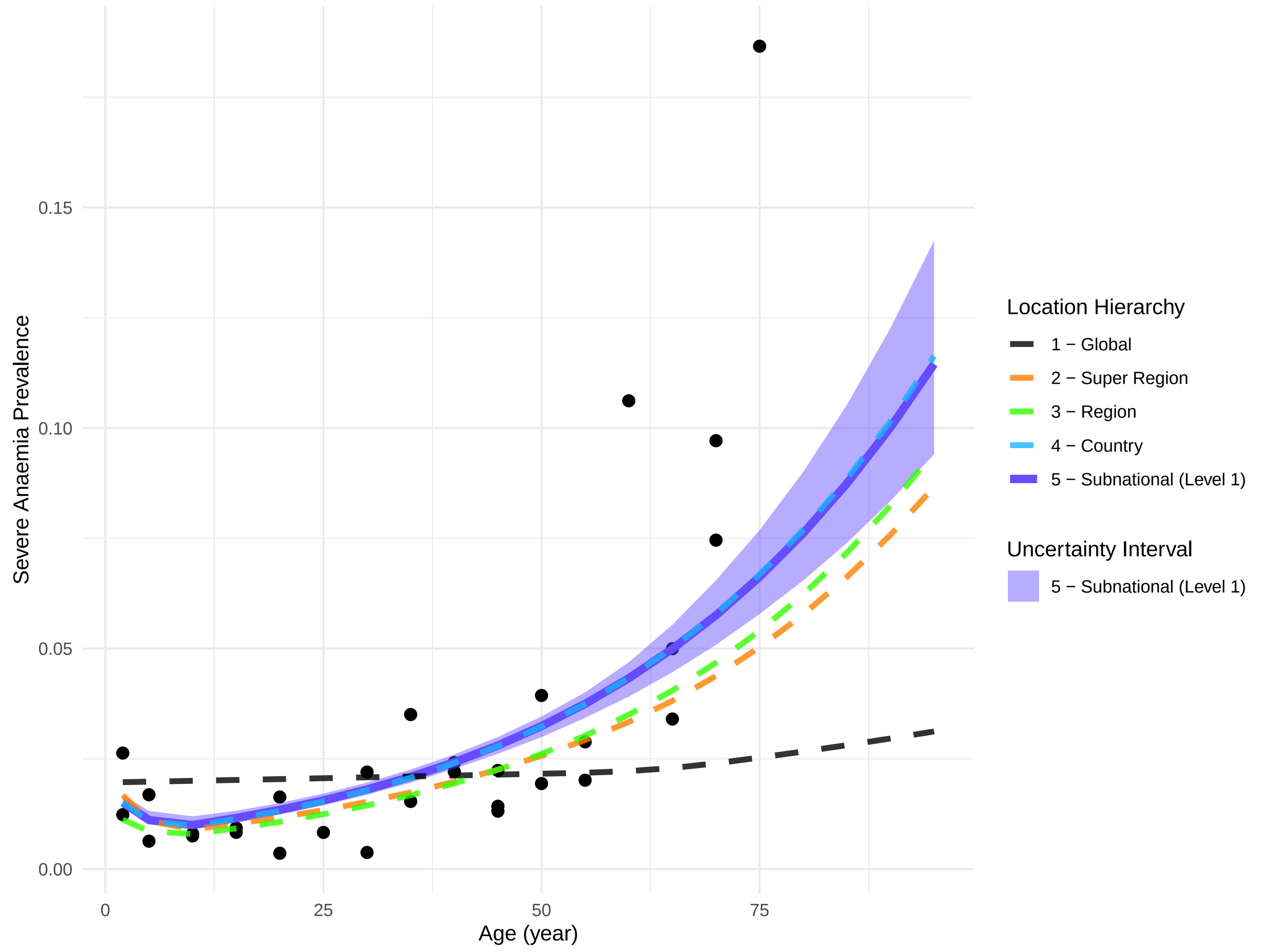

MR−BRT Age/Sex Fits − Non-pregnant Females 2+ Years Old − Severe Anaemia Prevalence − Yogyakarta
WHO−2024 Adjustment Method

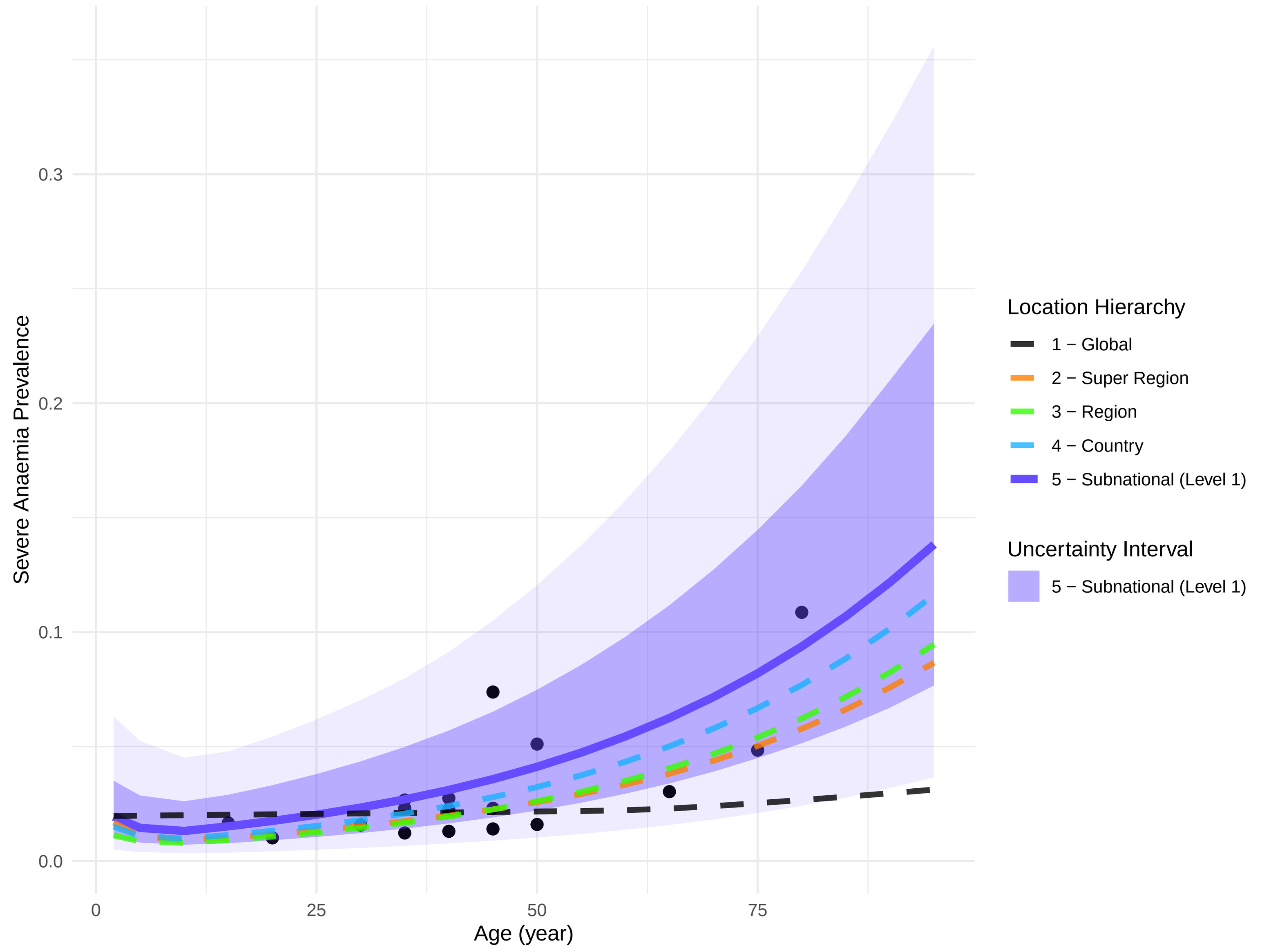

MR−BRT Age/Sex Fits − Non-pregnant Females 2+ Years Old − Severe Anaemia Prevalence − Myanmar
WHO−2024 Adjustment Method

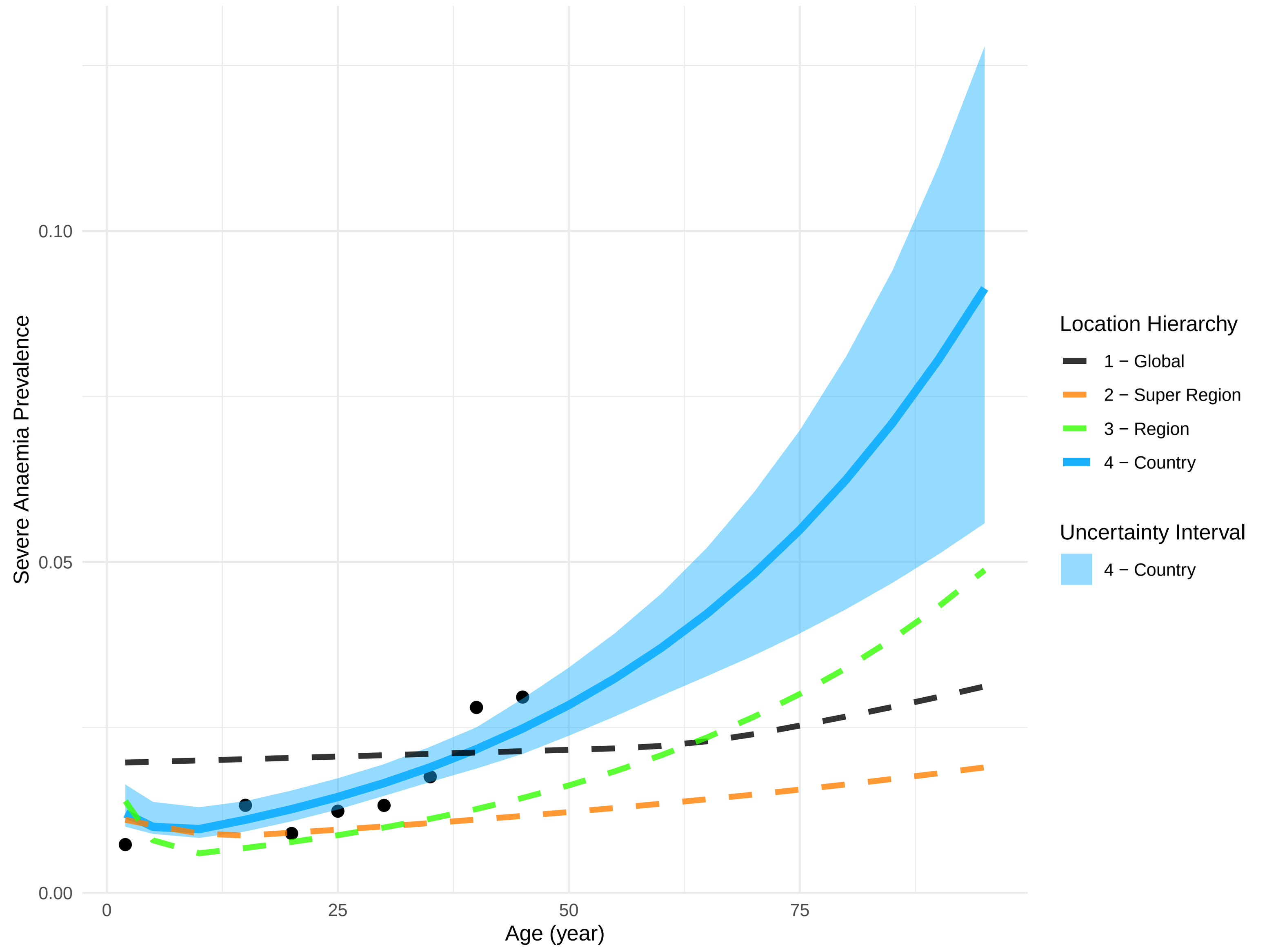

MR−BRT Age/Sex Fits − Non-pregnant Females 2+ Years Old − Severe Anaemia Prevalence − Timor−Leste
WHO−2024 Adjustment Method

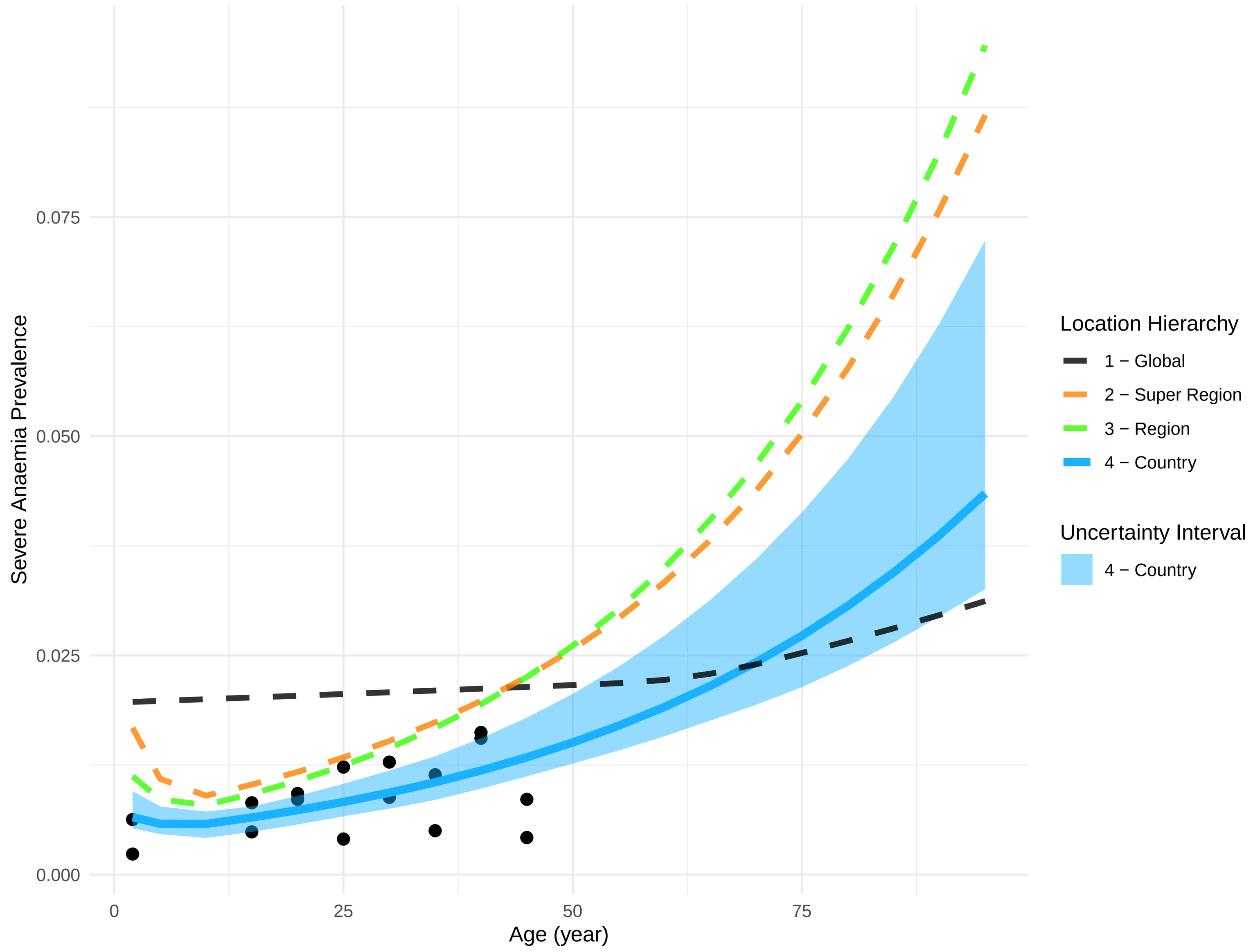

MR−BRT Age/Sex Fits − Non-pregnant Females 2+ Years Old − Severe Anaemia Prevalence − Sub-Saharan Africa
WHO−2024 Adjustment Method

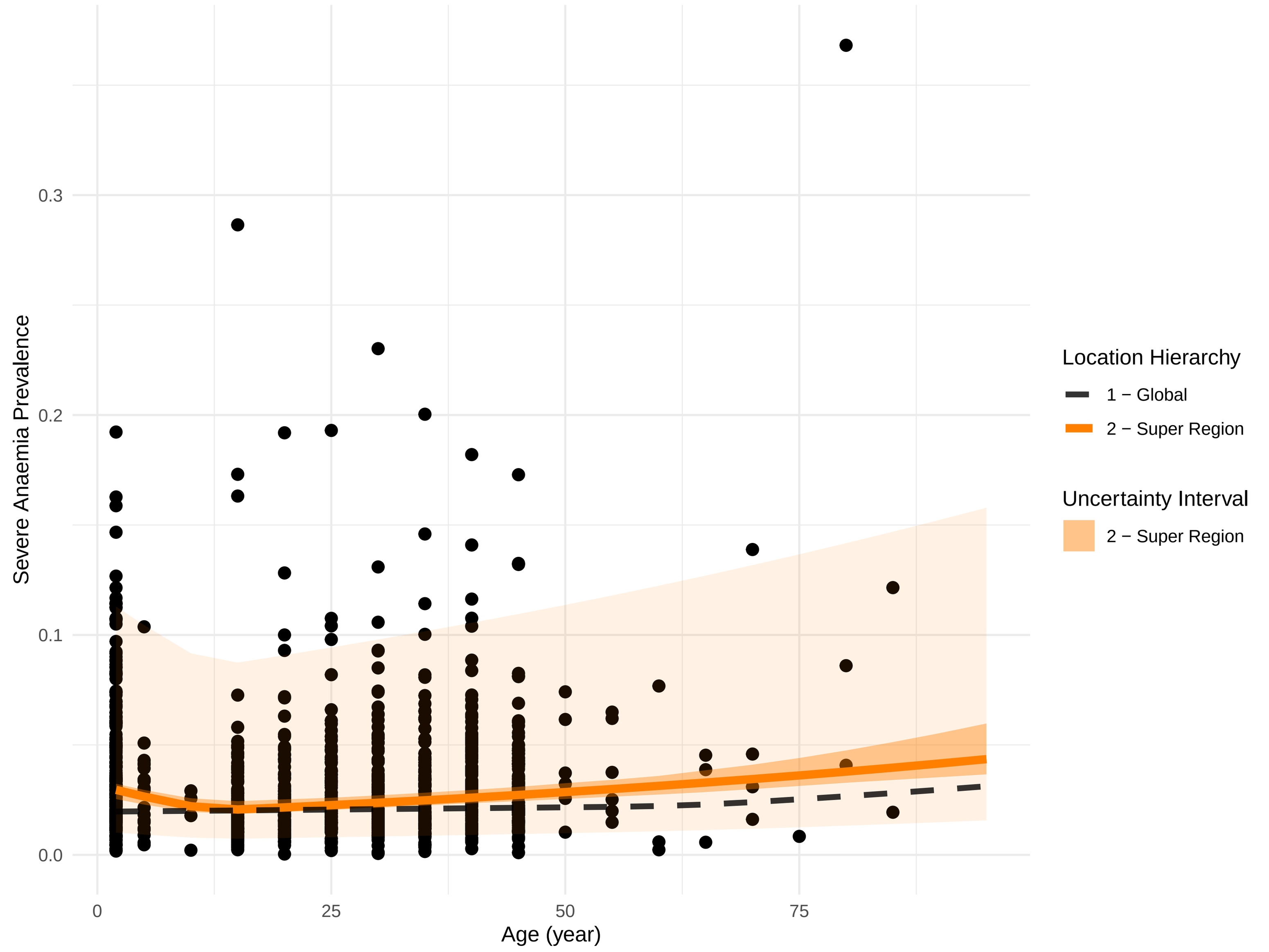

MR−BRT Age/Sex Fits − Non-pregnant Females 2+ Years Old − Severe Anaemia Prevalence − Central Sub-Saharan Africa
WHO−2024 Adjustment Method

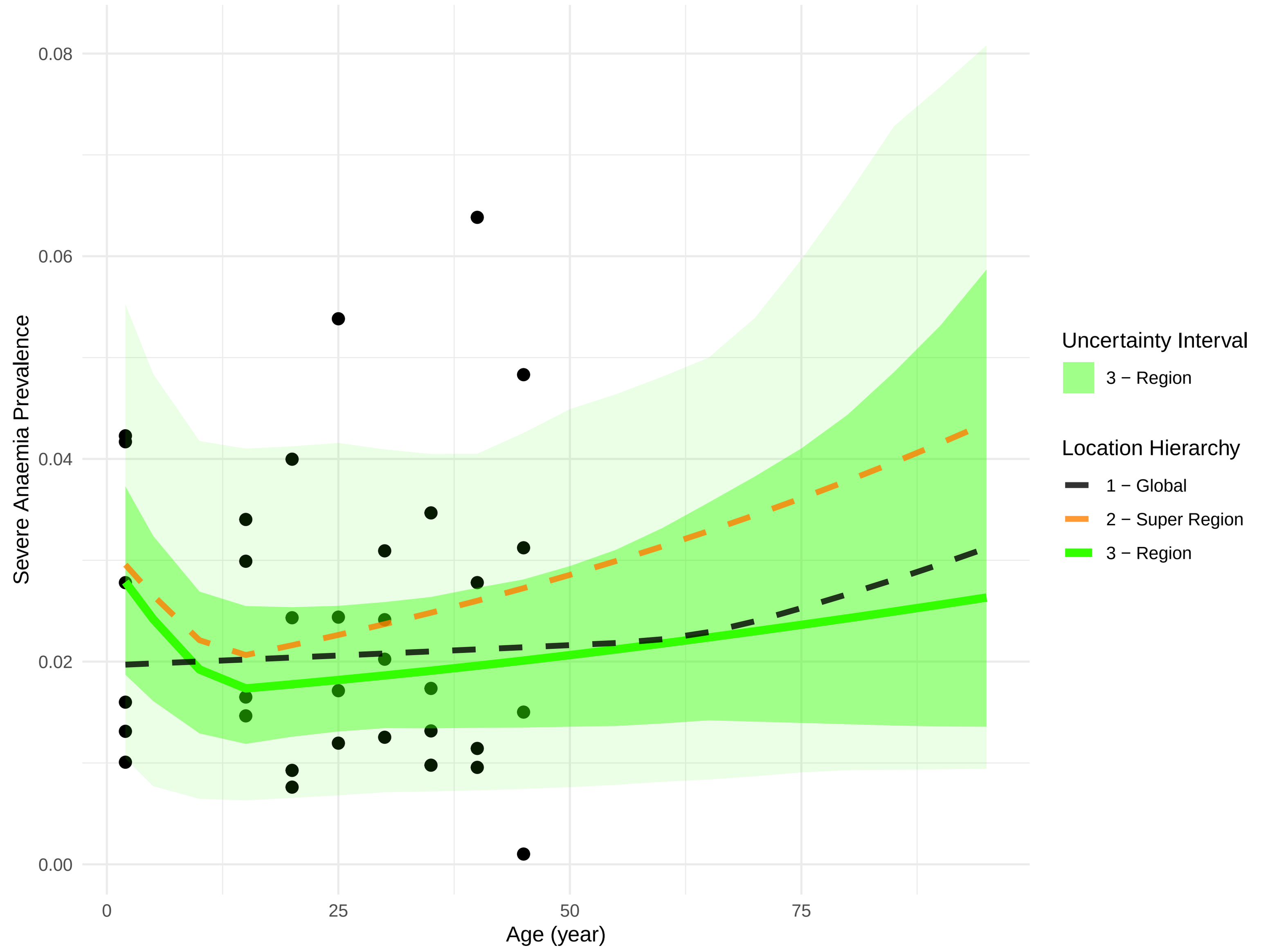

MR−BRT Age/Sex Fits − Non-pregnant Females 2+ Years Old − Severe Anaemia Prevalence − Angola
WHO−2024 Adjustment Method

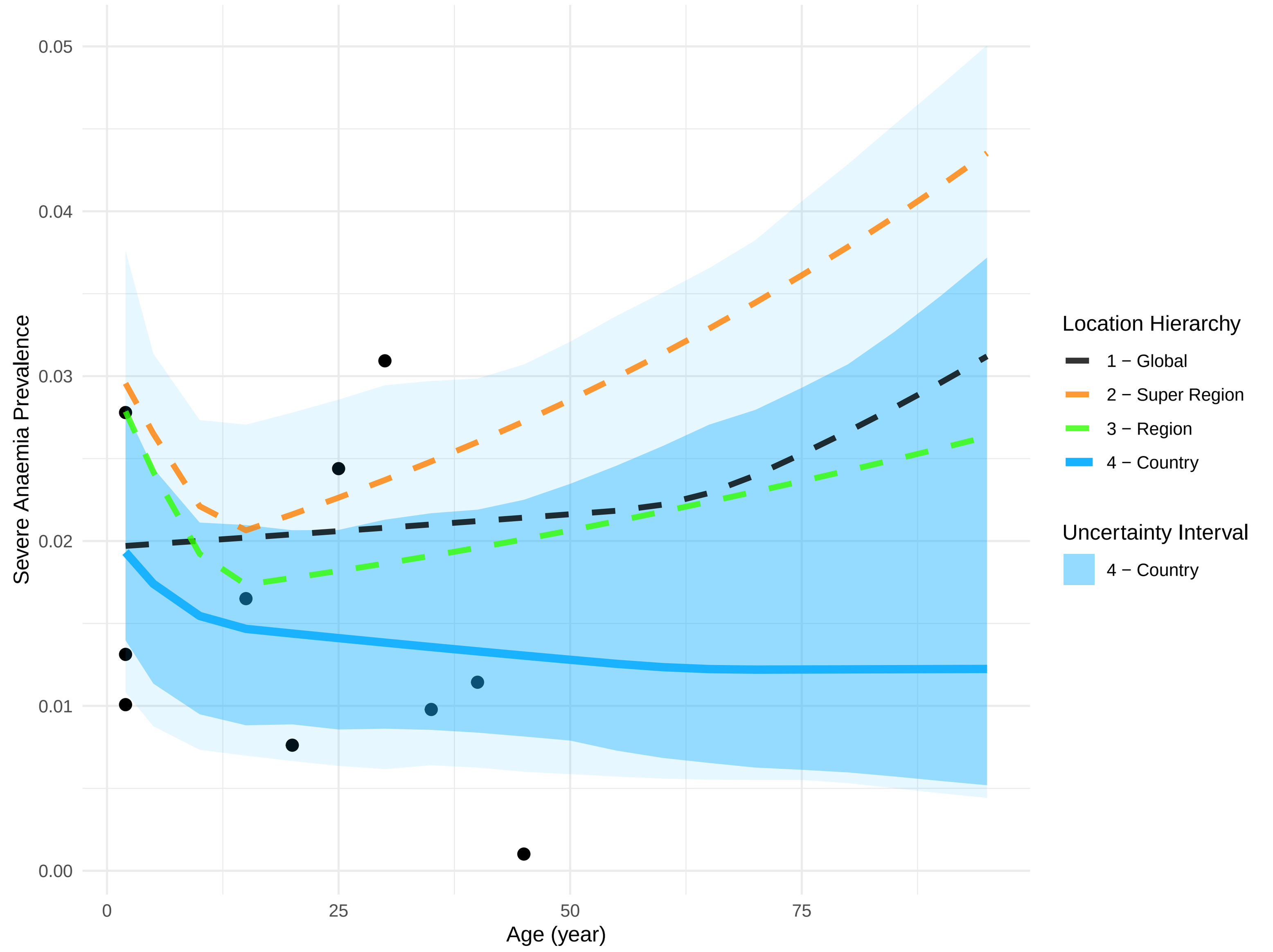

MR−BRT Age/Sex Fits − Non-pregnant Females 2+ Years Old − Severe Anaemia Prevalence − Democratic Republic of the Congo
WHO−2024 Adjustment Method

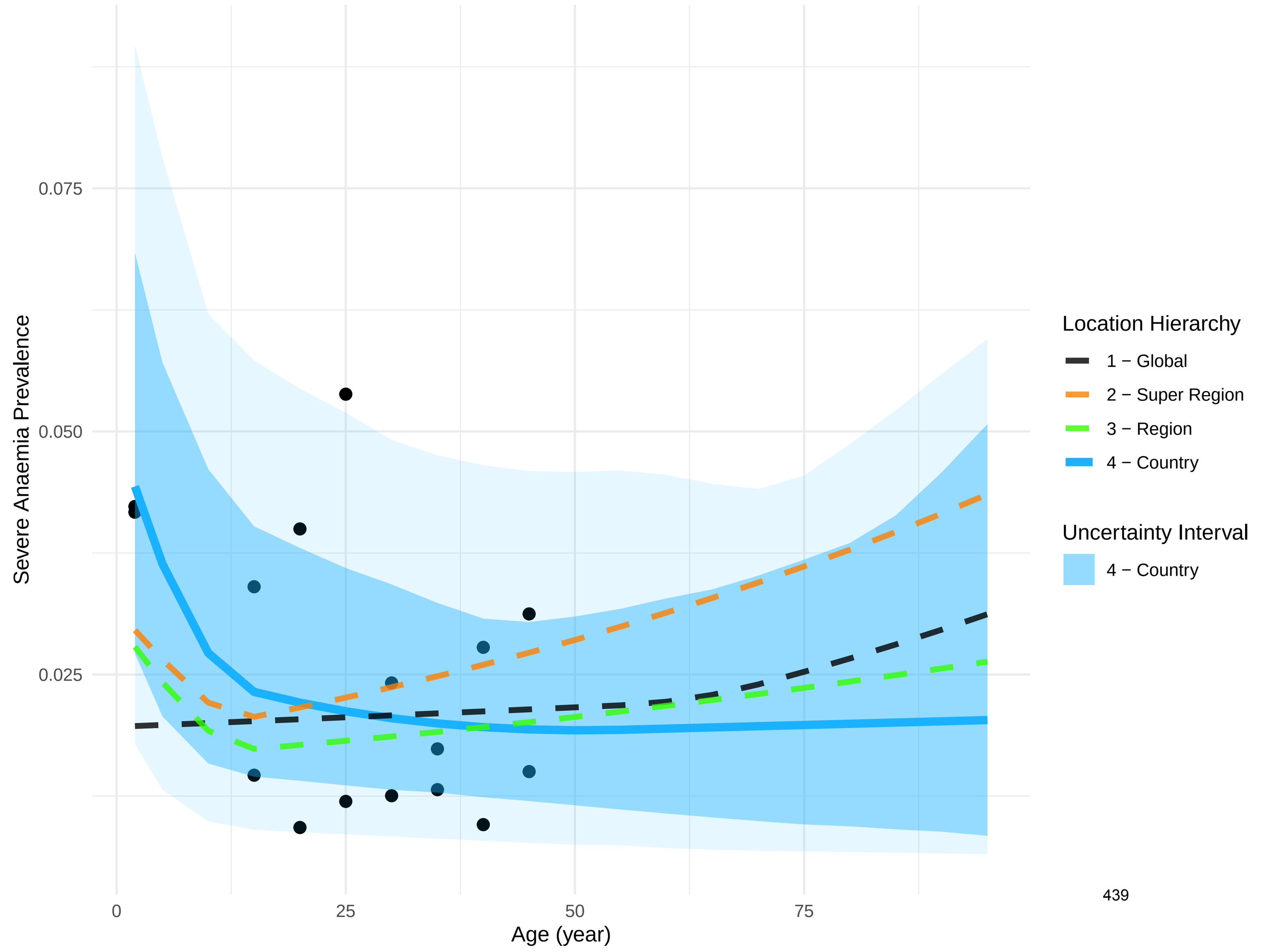

MR−BRT Age/Sex Fits − Non−pregnant Females 2+ Years Old − Severe Anaemia Prevalence − Gabon
WHO−2024 Adjustment Method

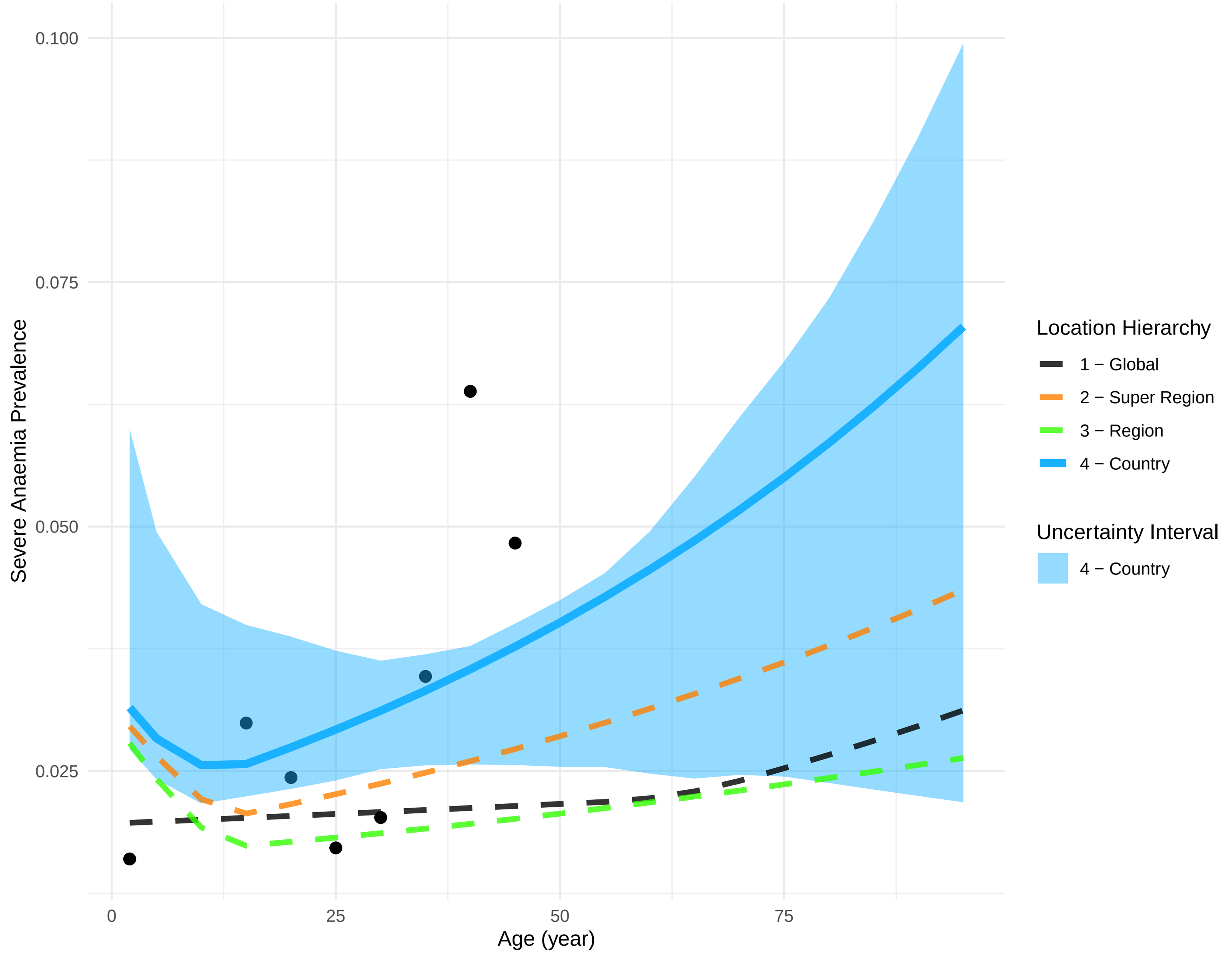

MR−BRT Age/Sex Fits − Non−pregnant Females 2+ Years Old − Severe Anaemia Prevalence − Eastern Sub−Saharan Africa
WHO−2024 Adjustment Method

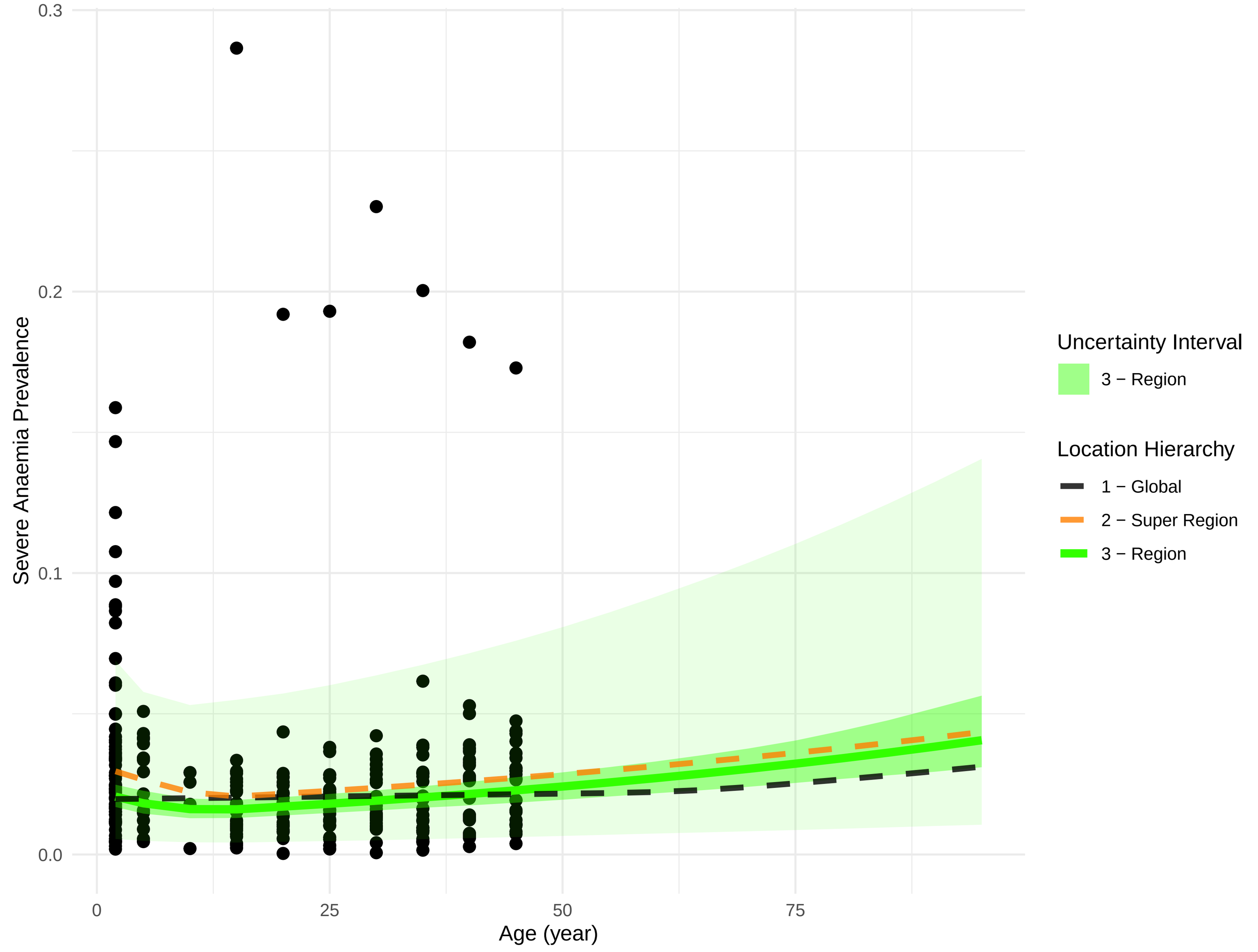

MR−BRT Age/Sex Fits − Non−pregnant Females 2+ Years Old − Severe Anaemia Prevalence − Burundi
WHO−2024 Adjustment Method

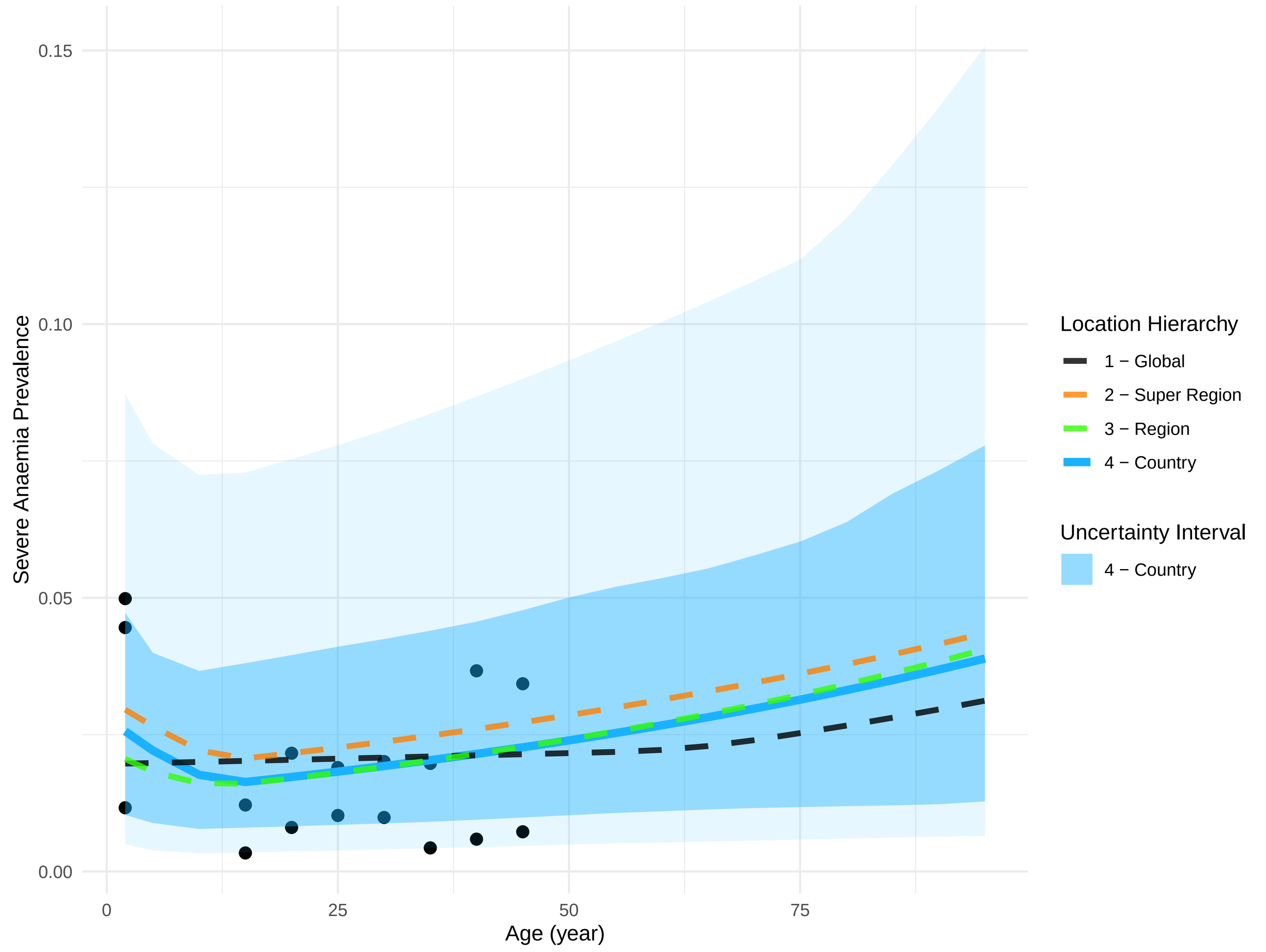

MR−BRT Age/Sex Fits − Non−pregnant Females 2+ Years Old − Severe Anaemia Prevalence − Ethiopia
WHO−2024 Adjustment Method

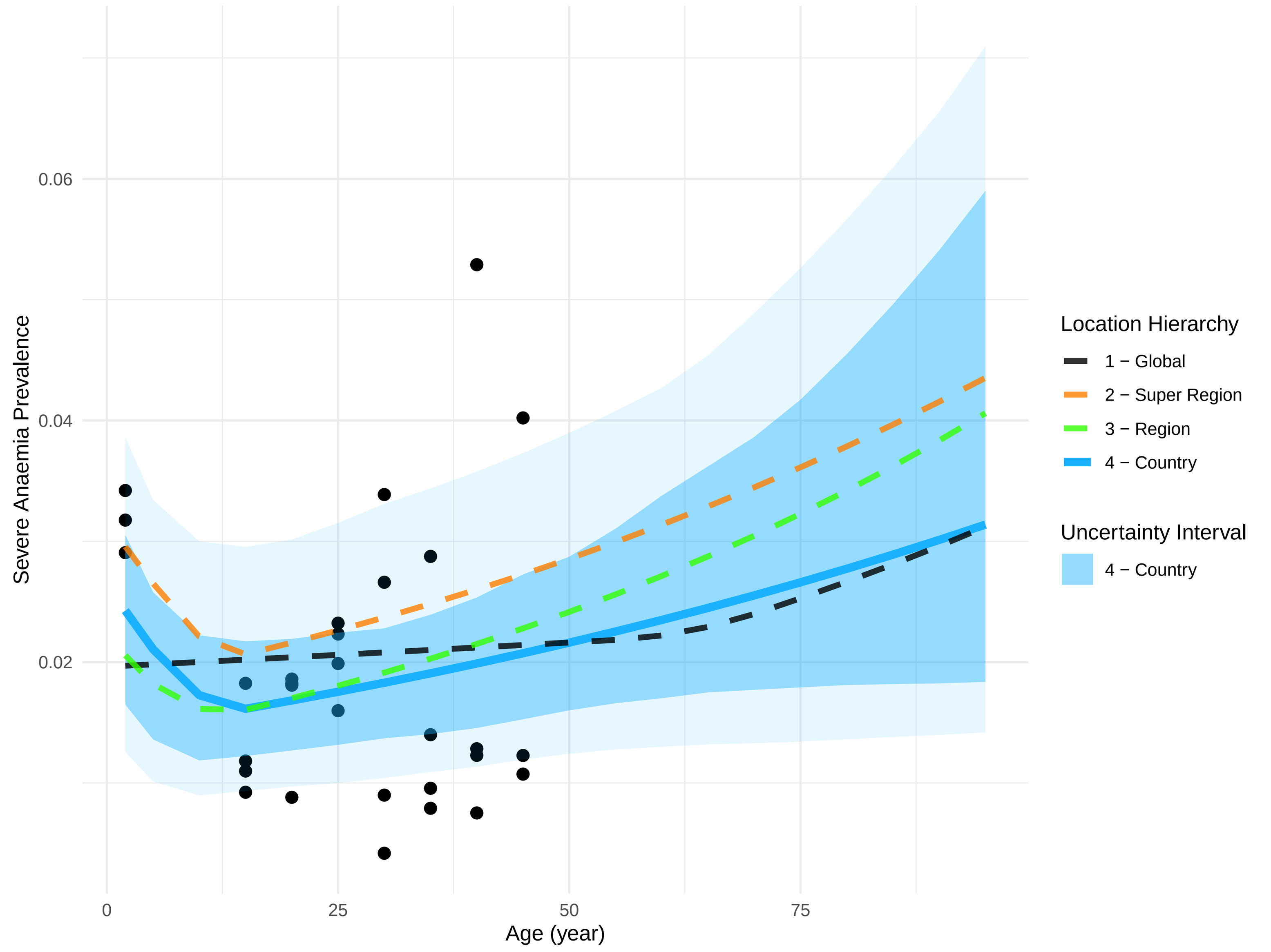

MR−BRT Age/Sex Fits − Non−pregnant Females 2+ Years Old − Severe Anaemia Prevalence − Kenya
WHO−2024 Adjustment Method

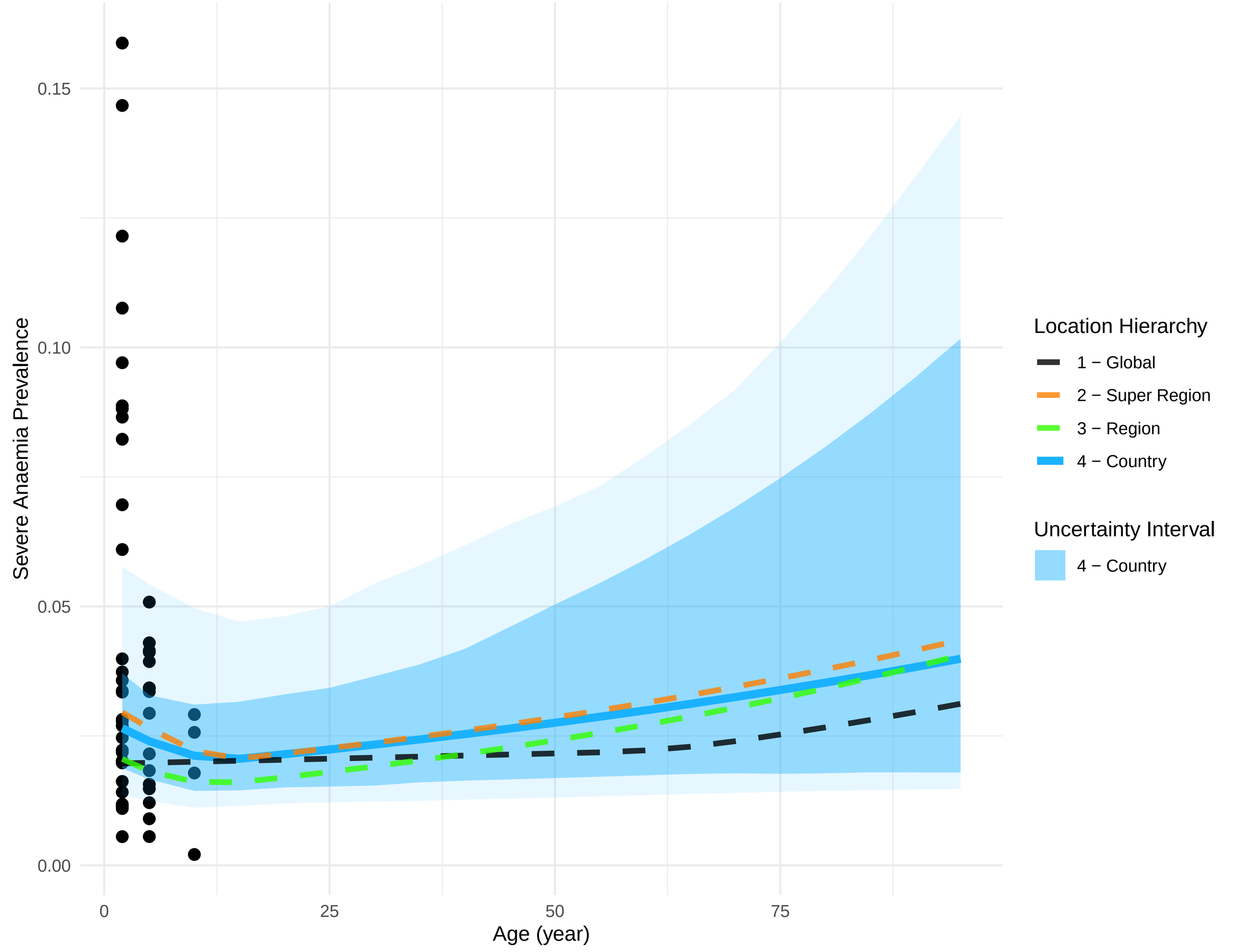

MR−BRT Age/Sex Fits − Non−pregnant Females 2+ Years Old − Severe Anaemia Prevalence − Bungoma
WHO−2024 Adjustment Method

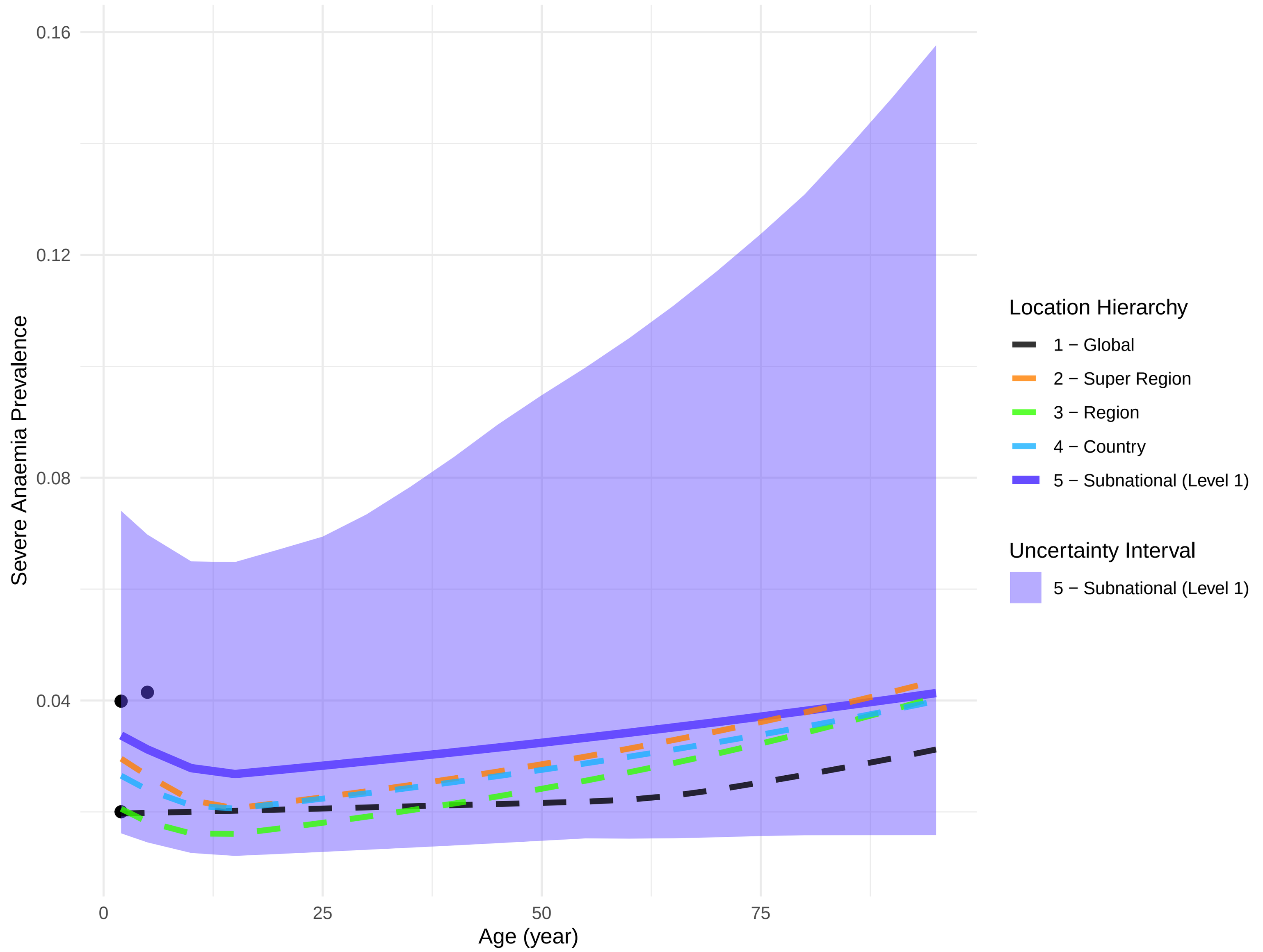

MR−BRT Age/Sex Fits − Non−pregnant Females 2+ Years Old − Severe Anaemia Prevalence − Busia
WHO−2024 Adjustment Method

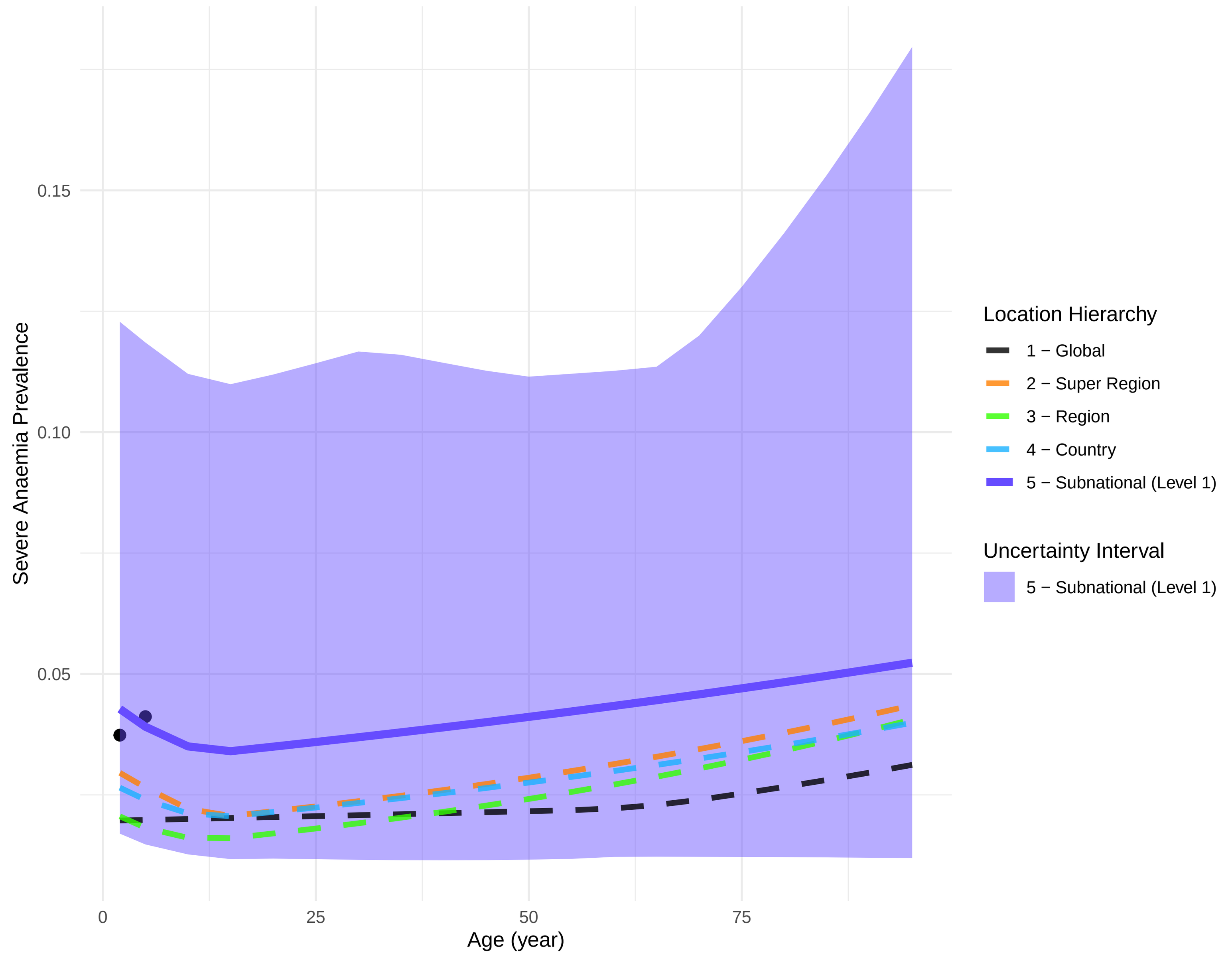

MR−BRT Age/Sex Fits − Non−pregnant Females 2+ Years Old − Severe Anaemia Prevalence − Elgeyo−Marakwet
WHO−2024 Adjustment Method

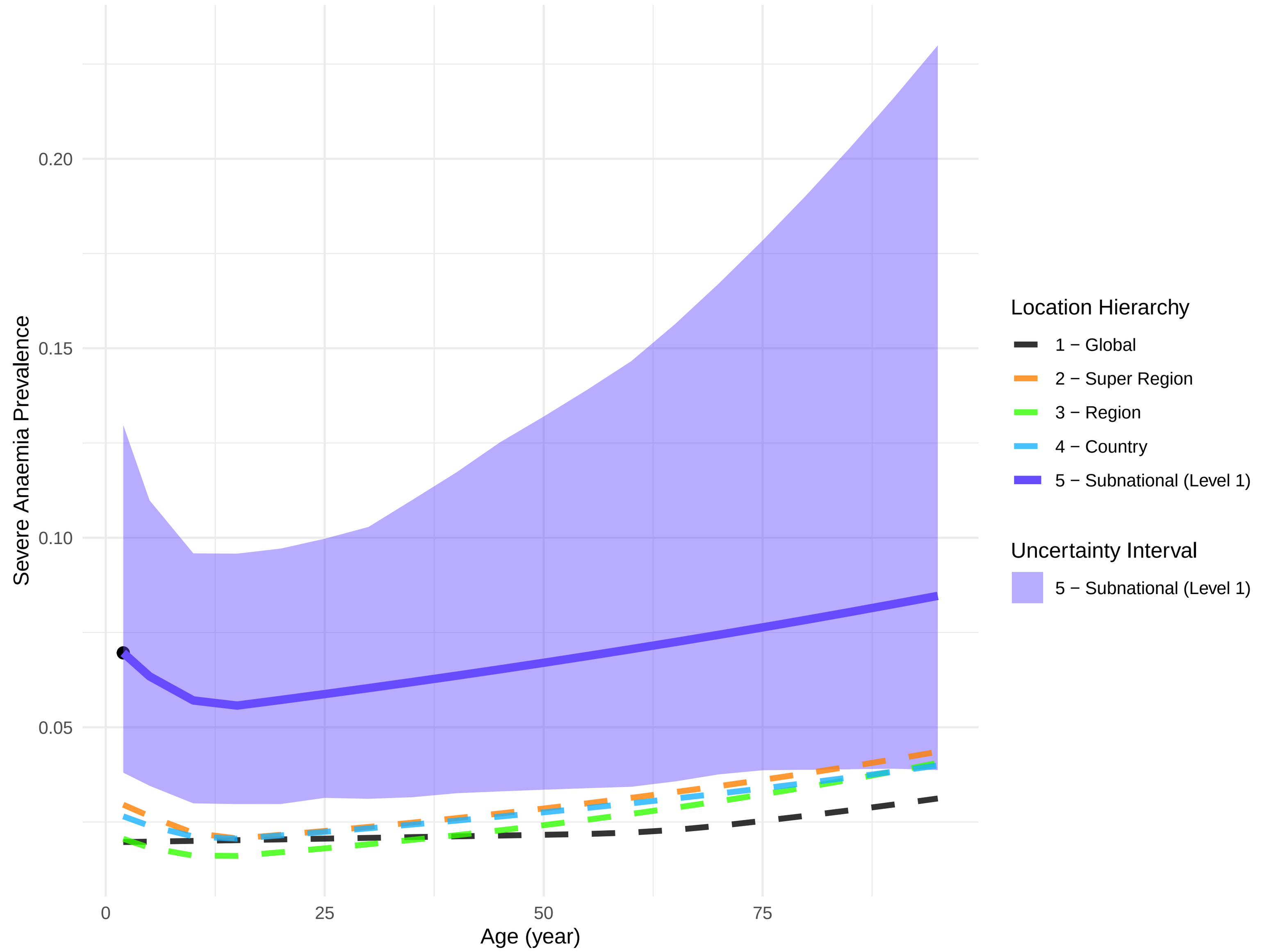

MR−BRT Age/Sex Fits − Non−pregnant Females 2+ Years Old − Severe Anaemia Prevalence − Garissa
WHO−2024 Adjustment Method

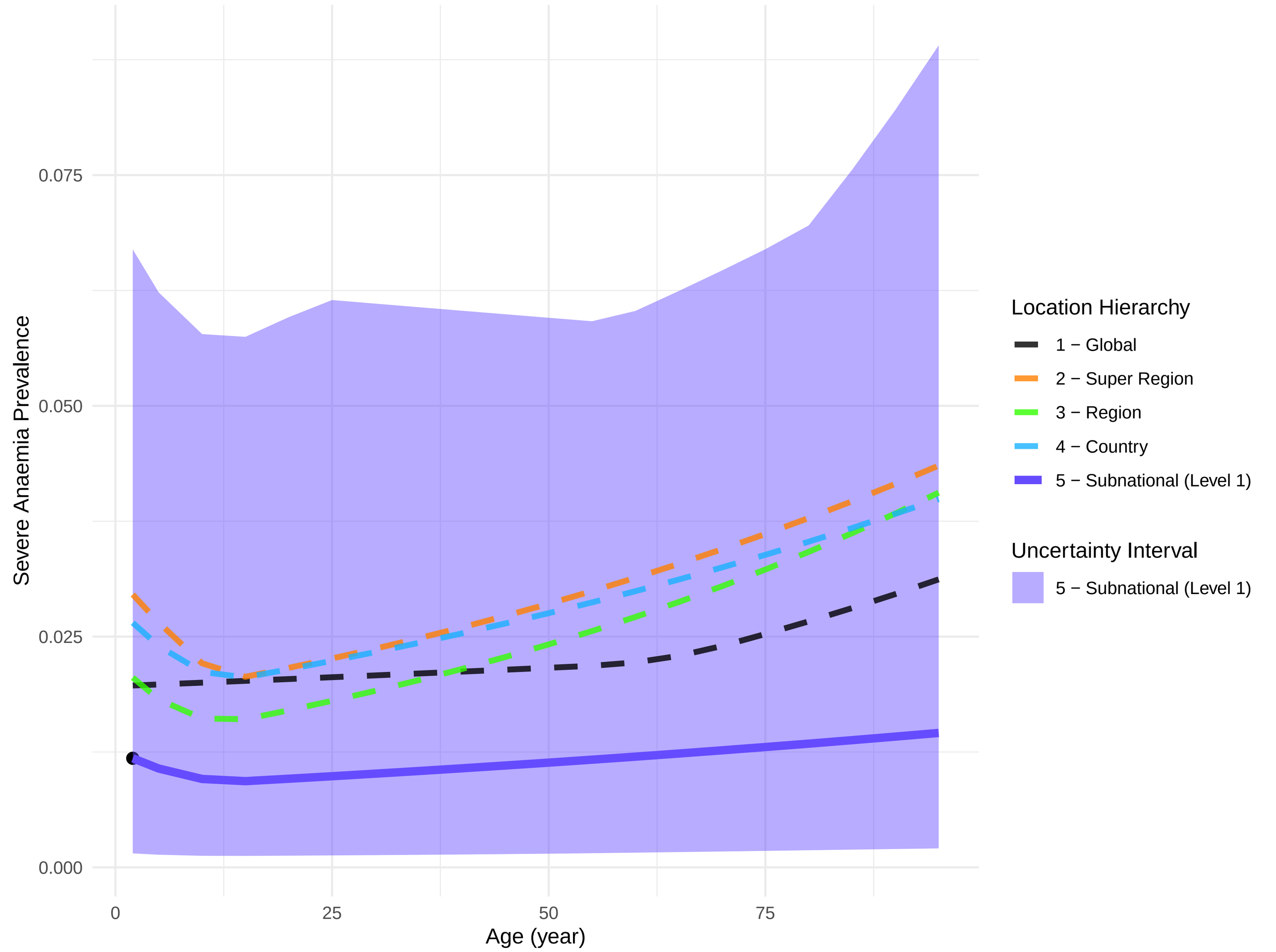

MR−BRT Age/Sex Fits − Non−pregnant Females 2+ Years Old − Severe Anaemia Prevalence − Homa Bay
WHO−2024 Adjustment Method

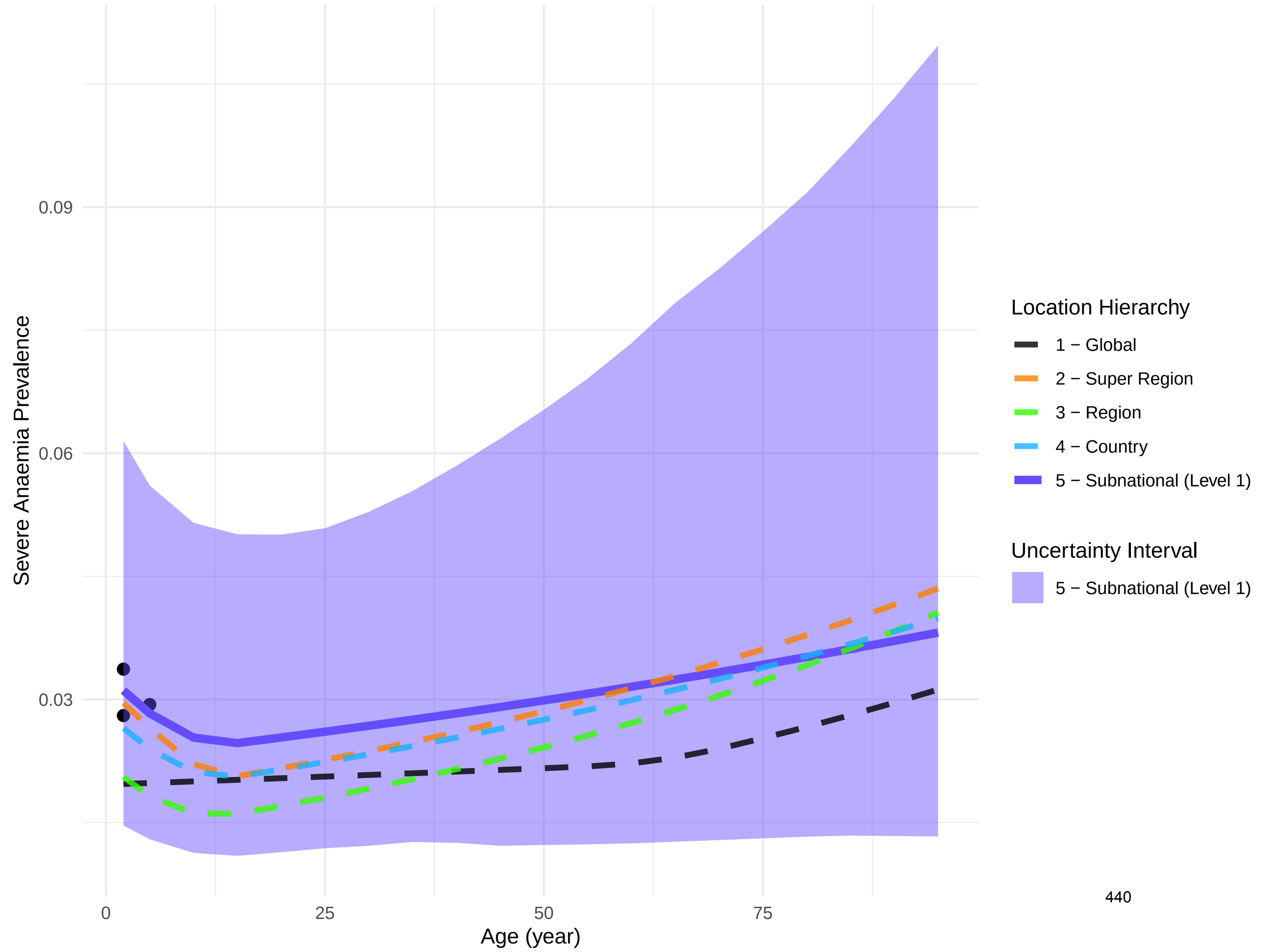

MR−BRT Age/Sex Fits − Non−pregnant Females 2+ Years Old − Severe Anaemia Prevalence − Isiolo
WHO−2024 Adjustment Method

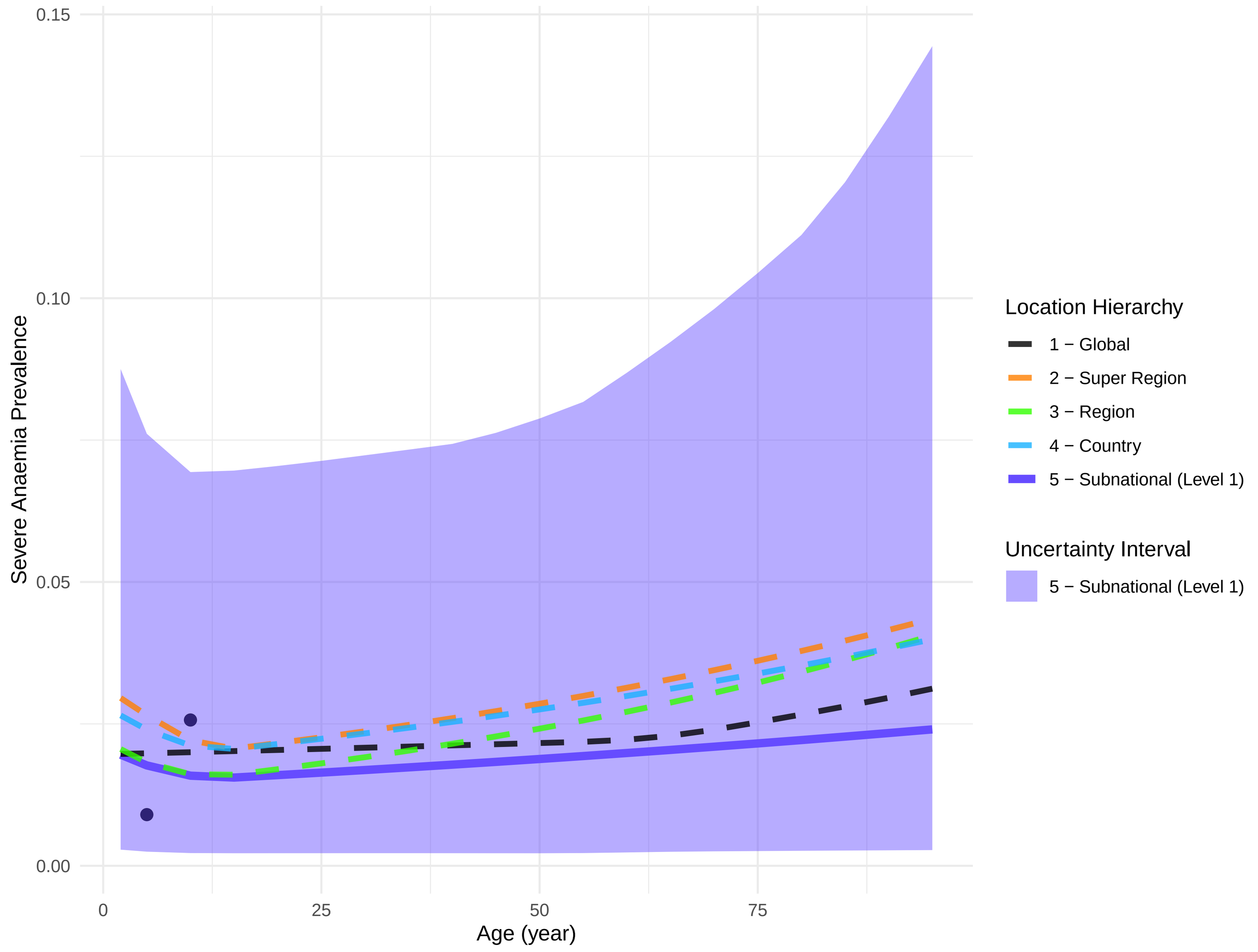

MR−BRT Age/Sex Fits − Non−pregnant Females 2+ Years Old − Severe Anaemia Prevalence − Kajiado
WHO−2024 Adjustment Method

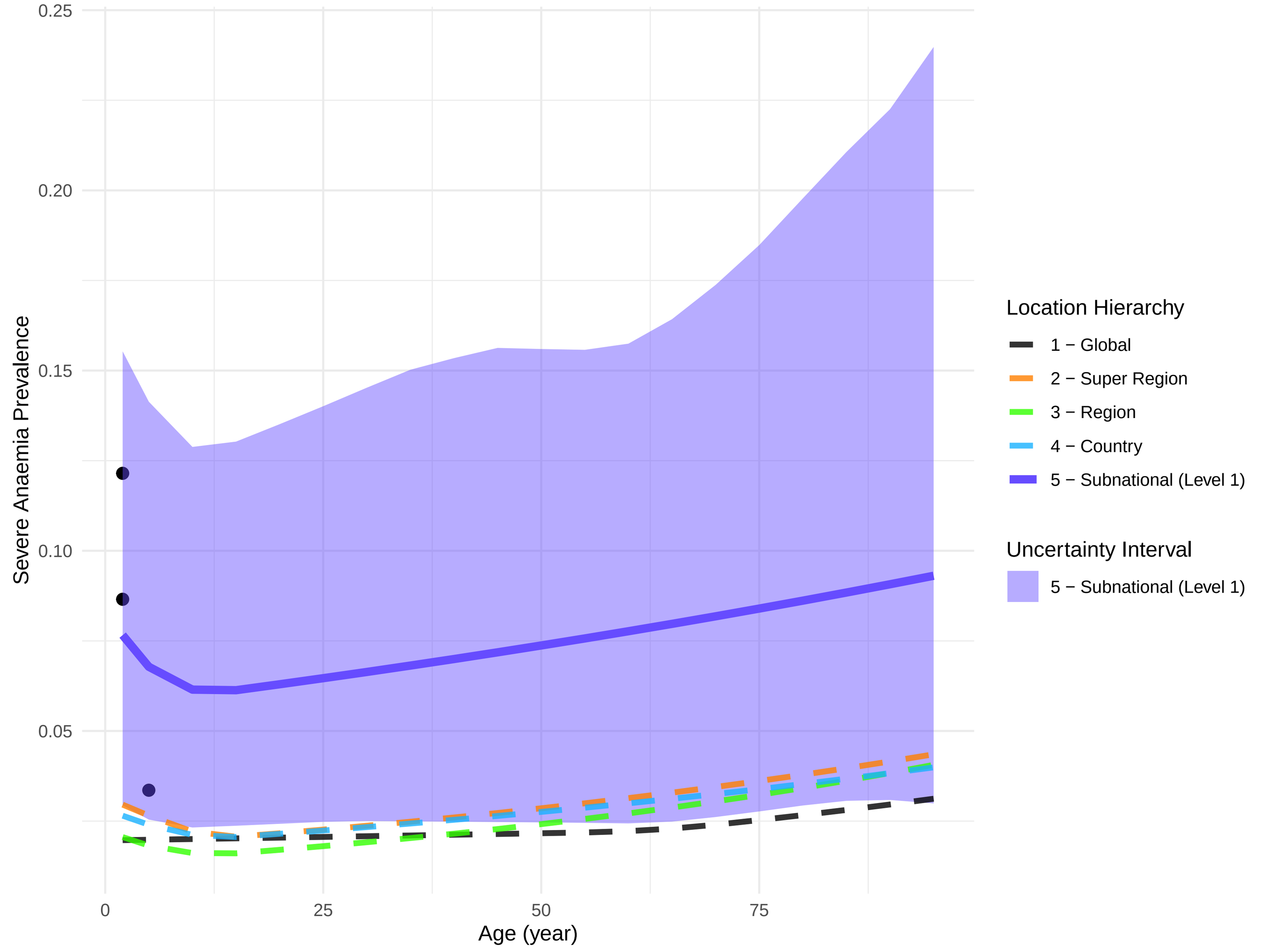

MR−BRT Age/Sex Fits − Non−pregnant Females 2+ Years Old − Severe Anaemia Prevalence − Kakamega
WHO−2024 Adjustment Method

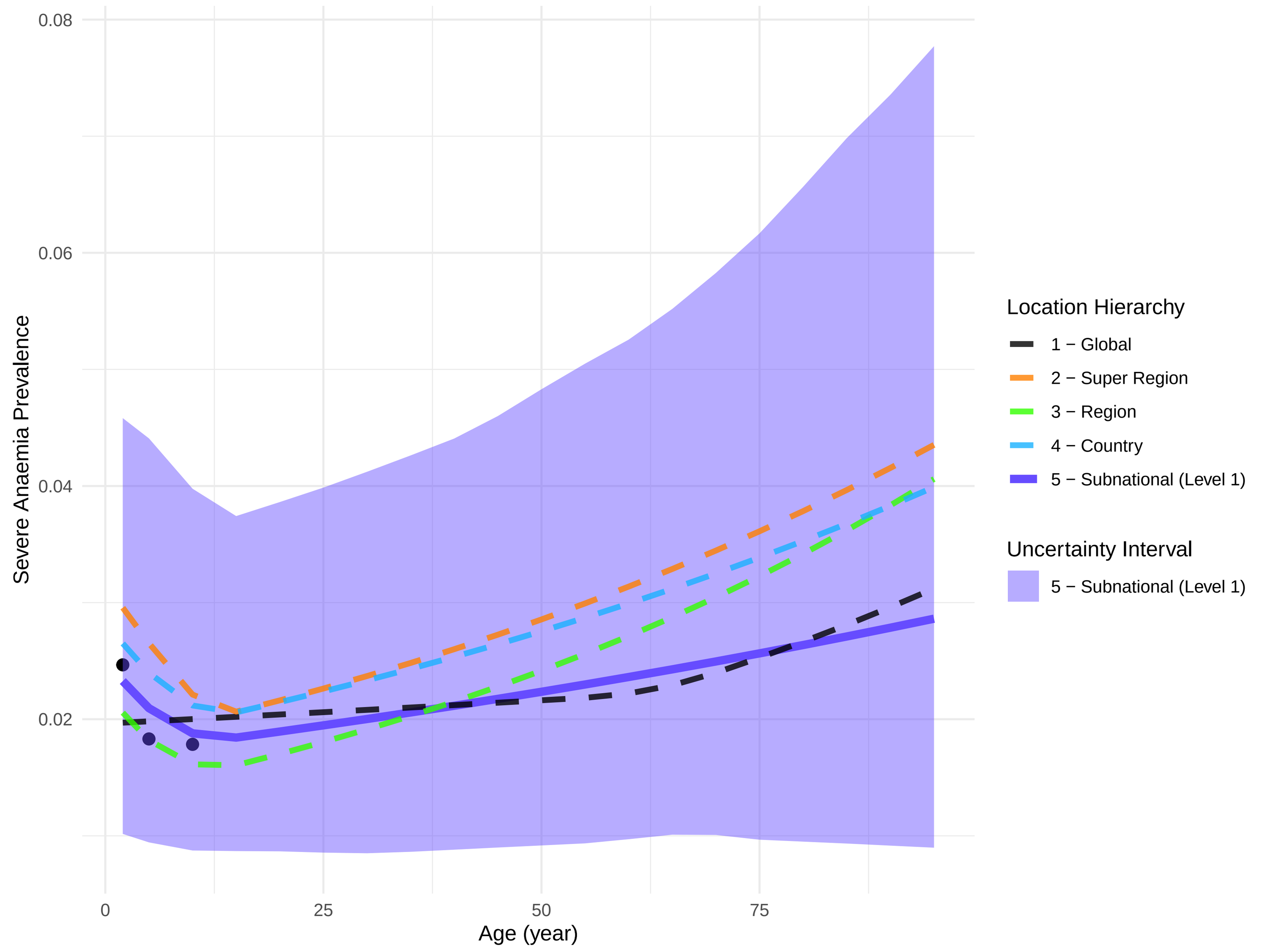

MR−BRT Age/Sex Fits − Non−pregnant Females 2+ Years Old − Severe Anaemia Prevalence − Kilifi
WHO−2024 Adjustment Method

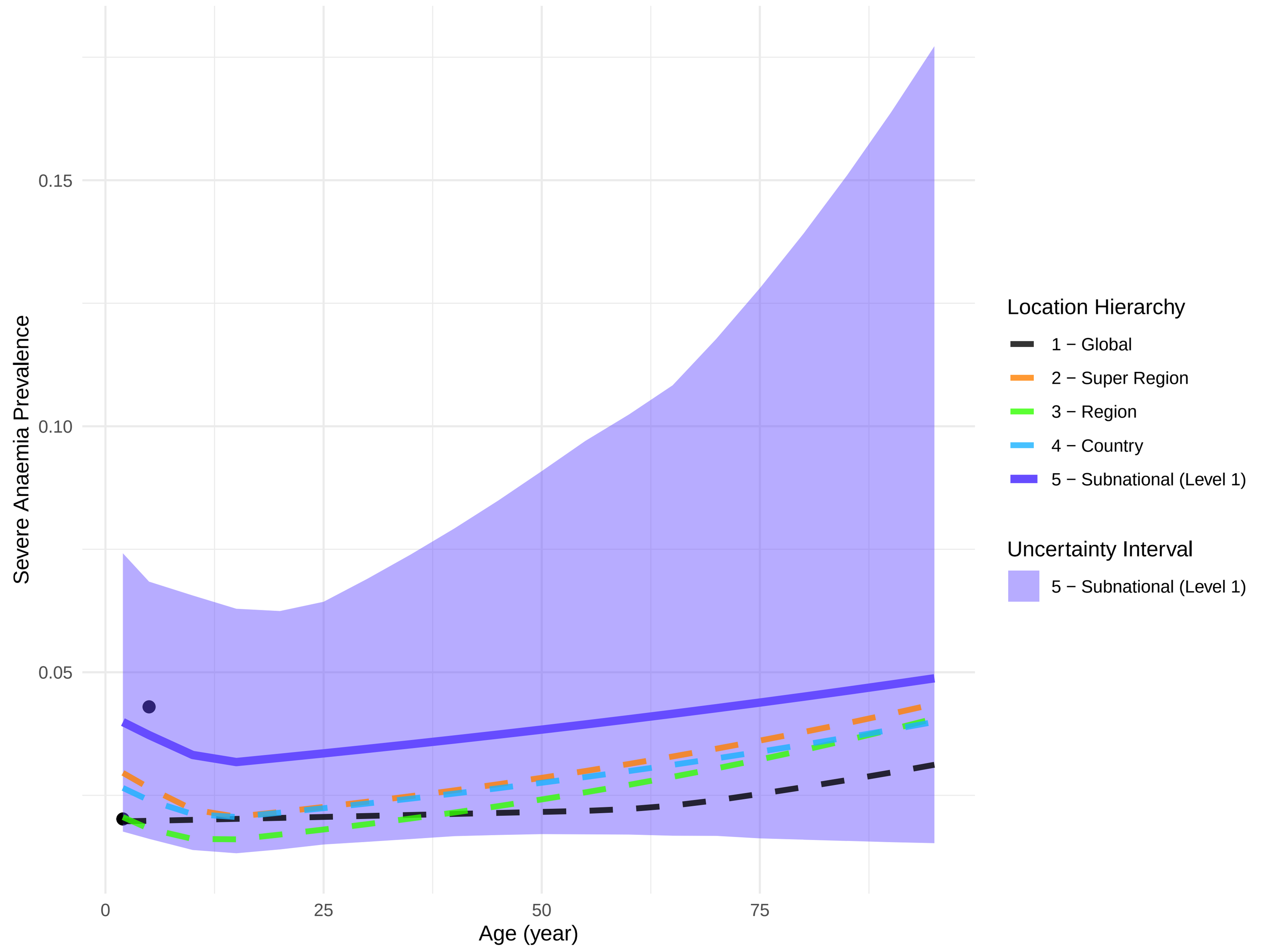

MR−BRT Age/Sex Fits − Non−pregnant Females 2+ Years Old − Severe Anaemia Prevalence − Kisii
WHO−2024 Adjustment Method

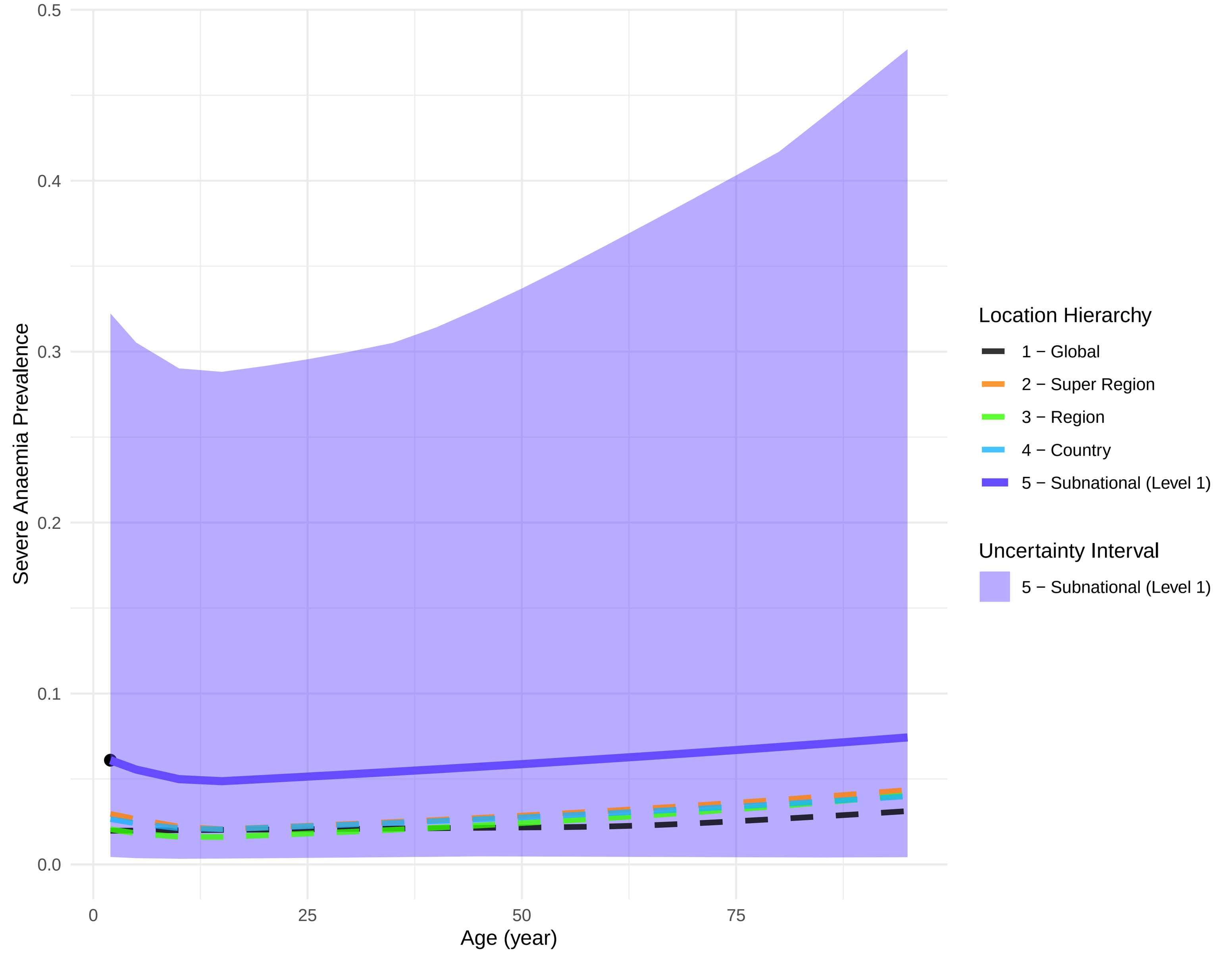

MR−BRT Age/Sex Fits − Non−pregnant Females 2+ Years Old − Severe Anaemia Prevalence − Kisumu
WHO−2024 Adjustment Method

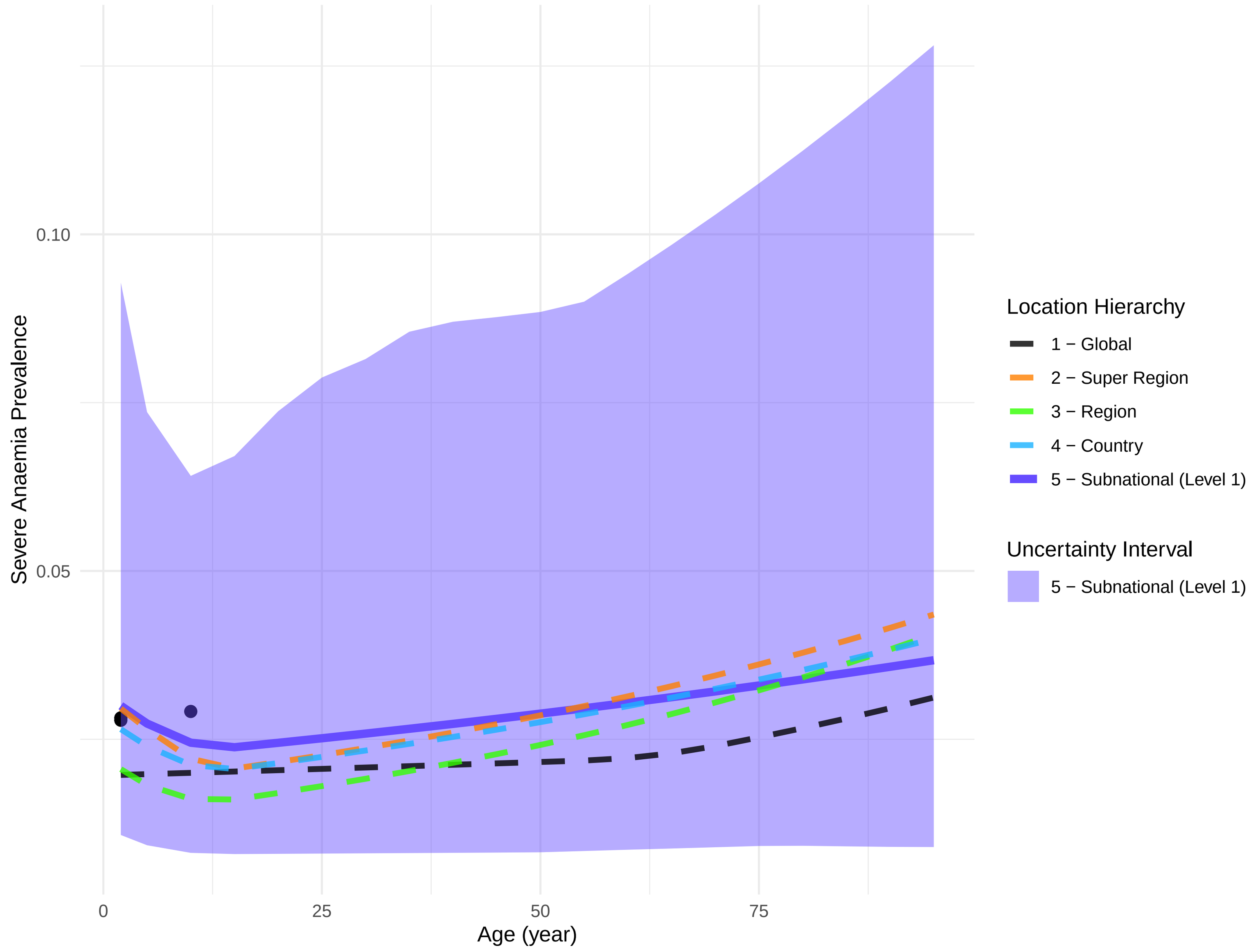

MR−BRT Age/Sex Fits − Non−pregnant Females 2+ Years Old − Severe Anaemia Prevalence − Kwale
WHO−2024 Adjustment Method

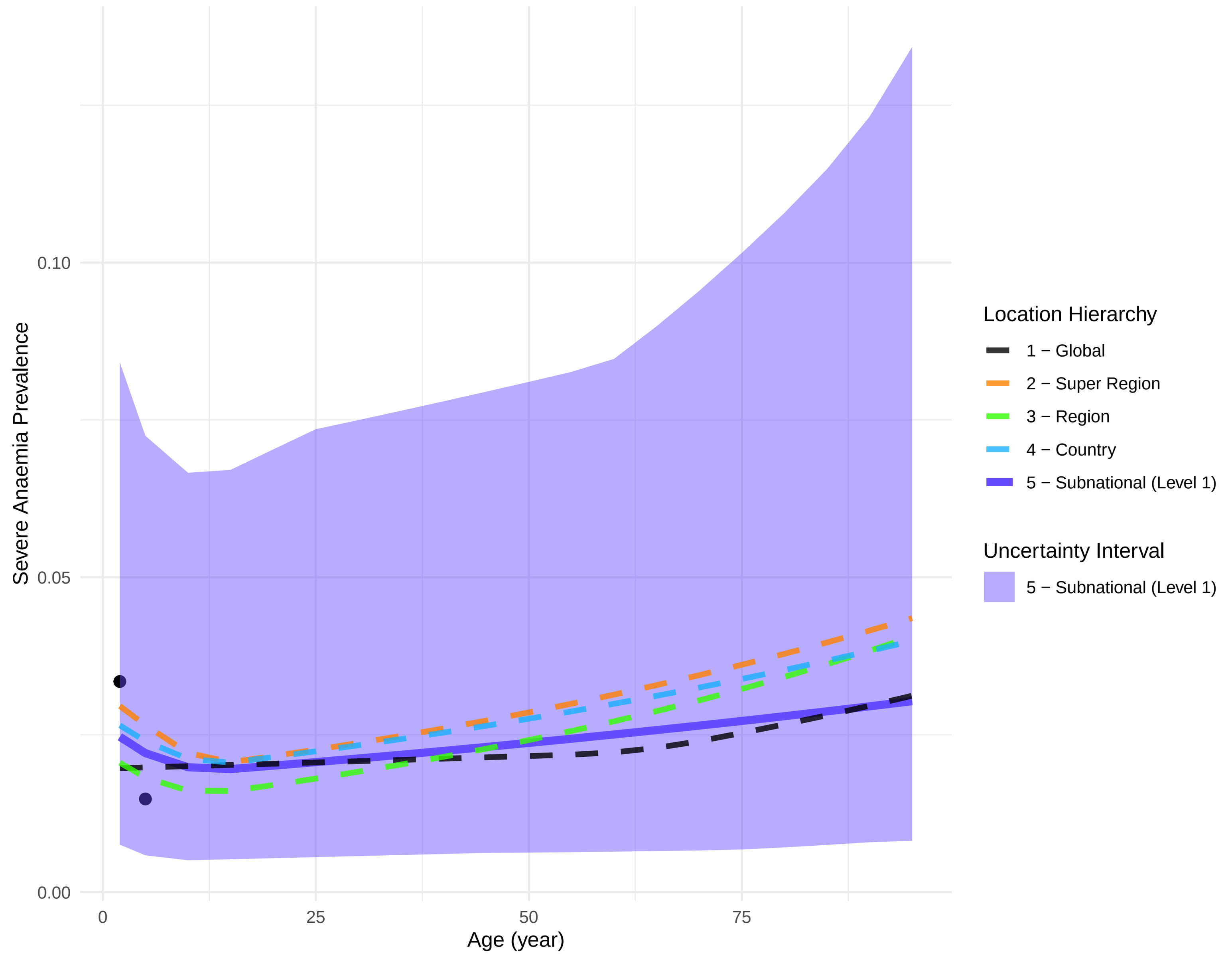

MR−BRT Age/Sex Fits − Non−pregnant Females 2+ Years Old − Severe Anaemia Prevalence − Lamu
WHO−2024 Adjustment Method

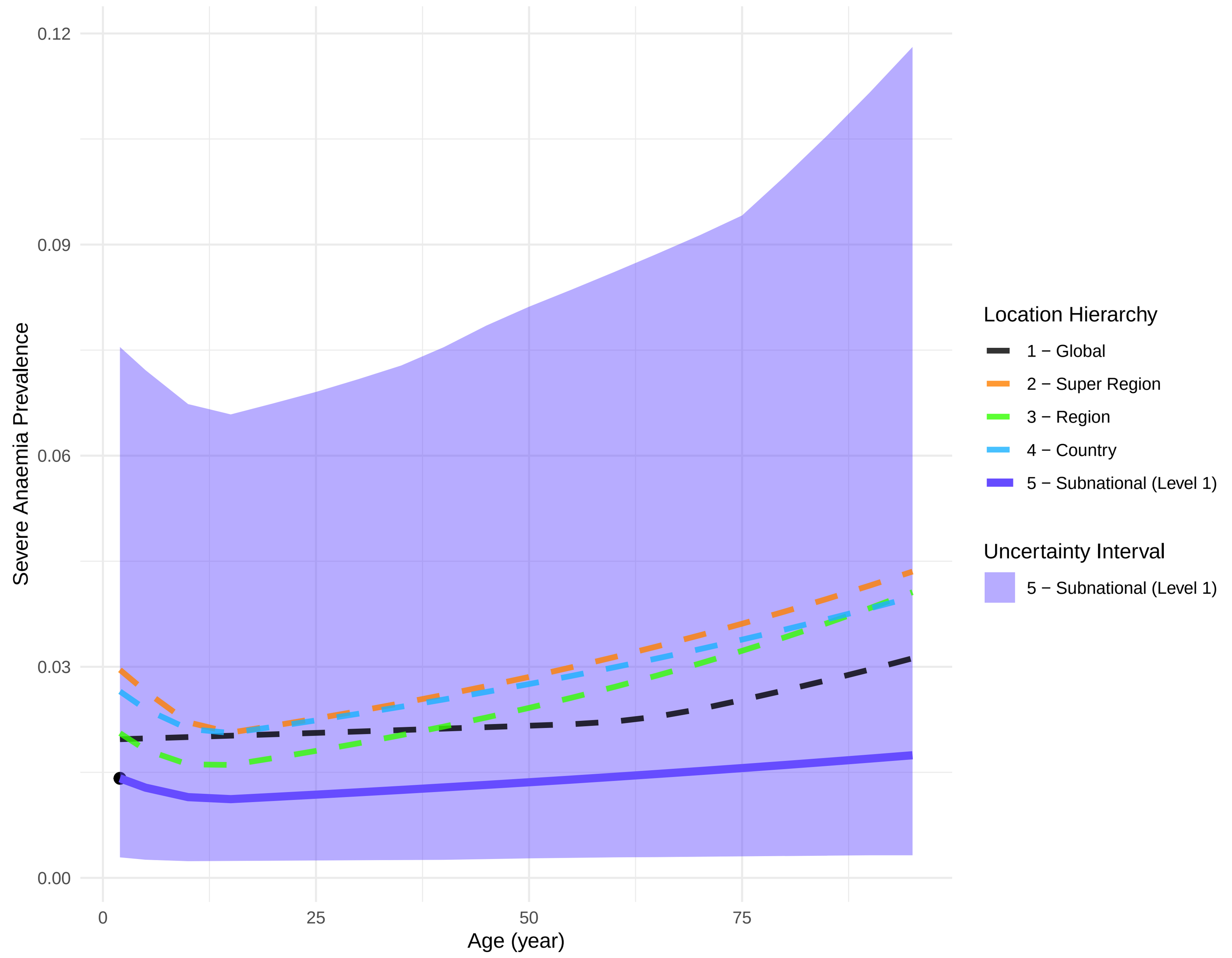

MR−BRT Age/Sex Fits − Non−pregnant Females 2+ Years Old − Severe Anaemia Prevalence − Machakos
WHO−2024 Adjustment Method

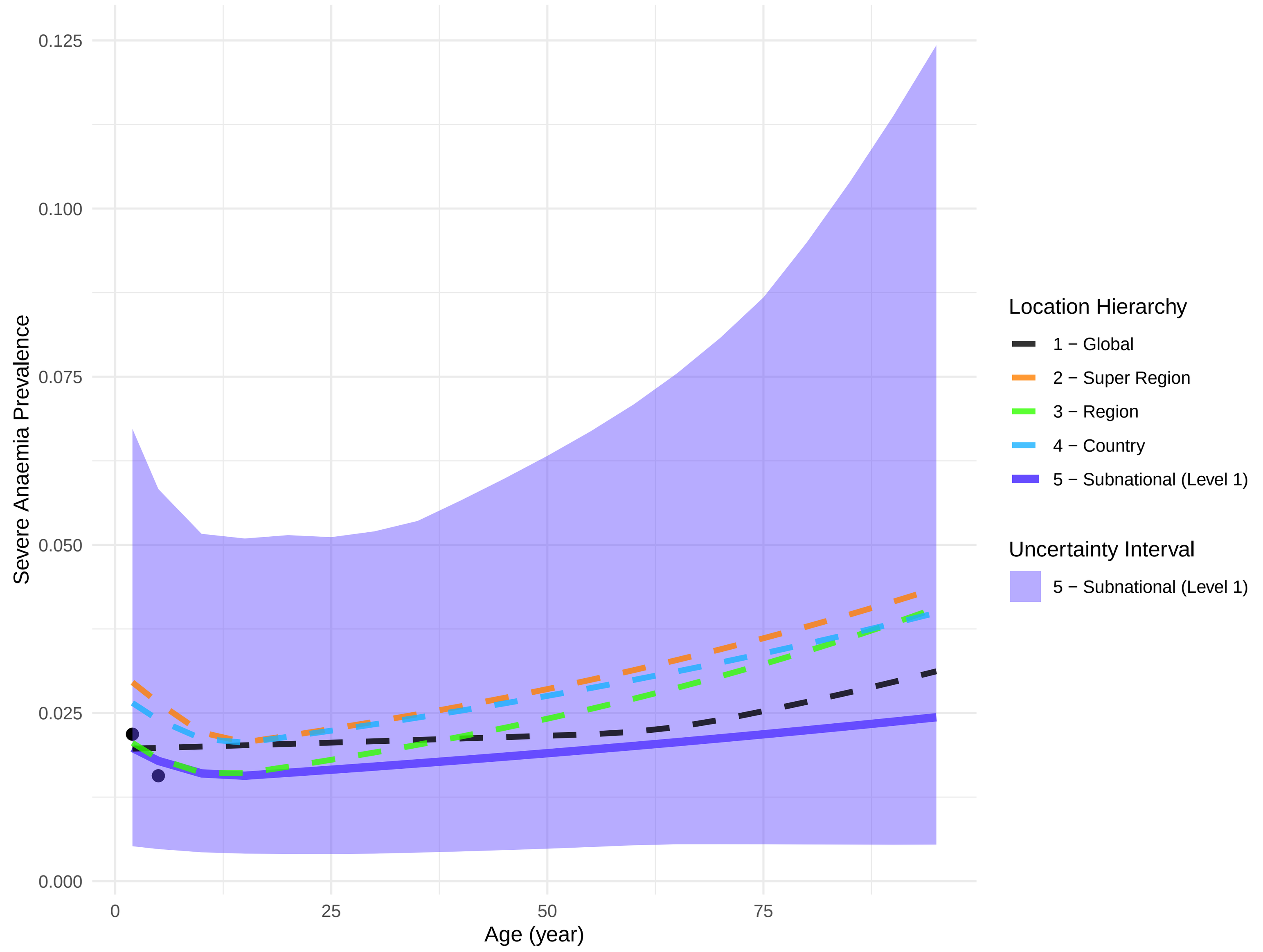

MR−BRT Age/Sex Fits − Non−pregnant Females 2+ Years Old − Severe Anaemia Prevalence − Mandera
WHO−2024 Adjustment Method

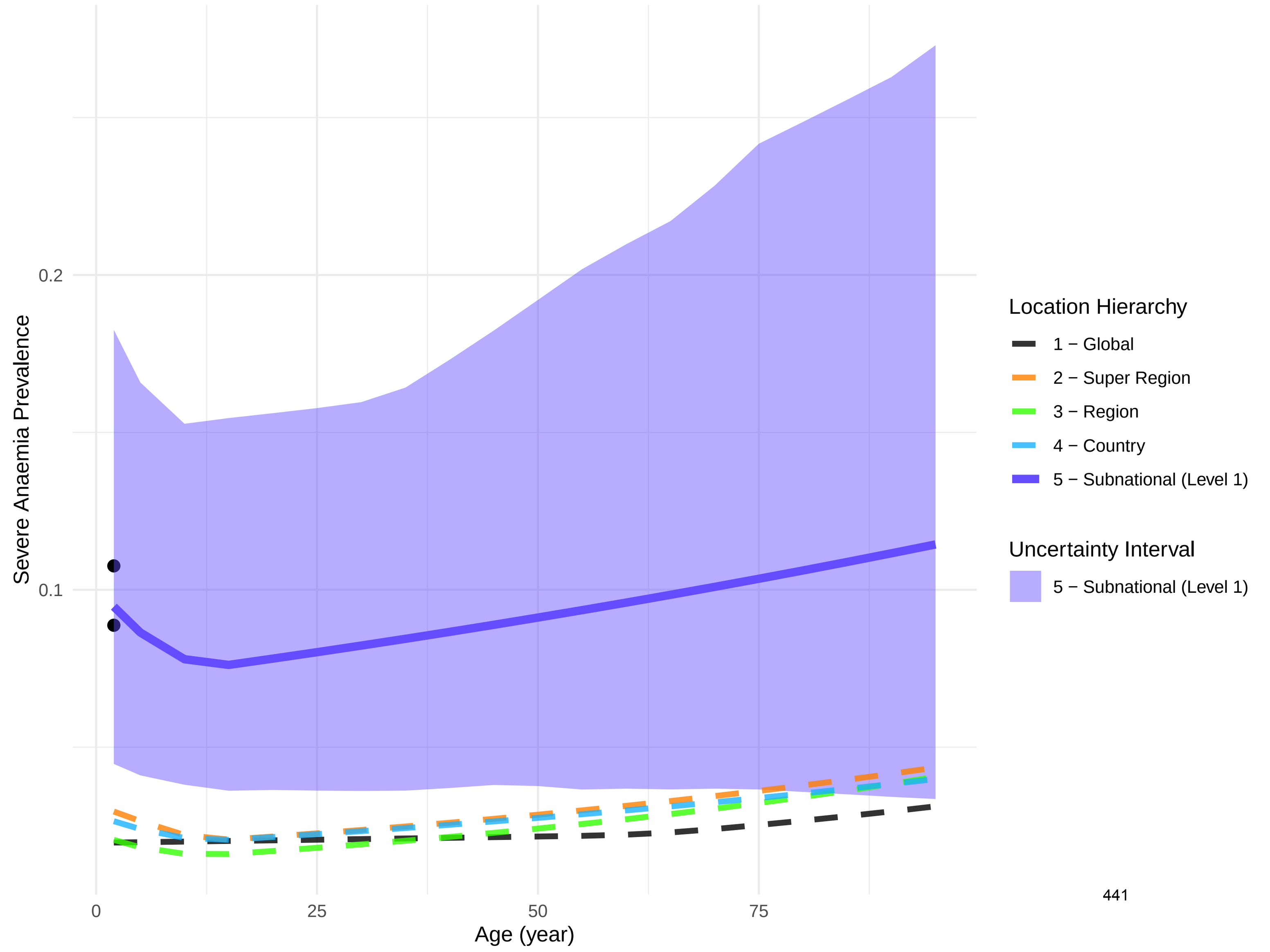

MR−BRT Age/Sex Fits − Non−pregnant Females 2+ Years Old − Severe Anaemia Prevalence − Marsabit
WHO−2024 Adjustment Method

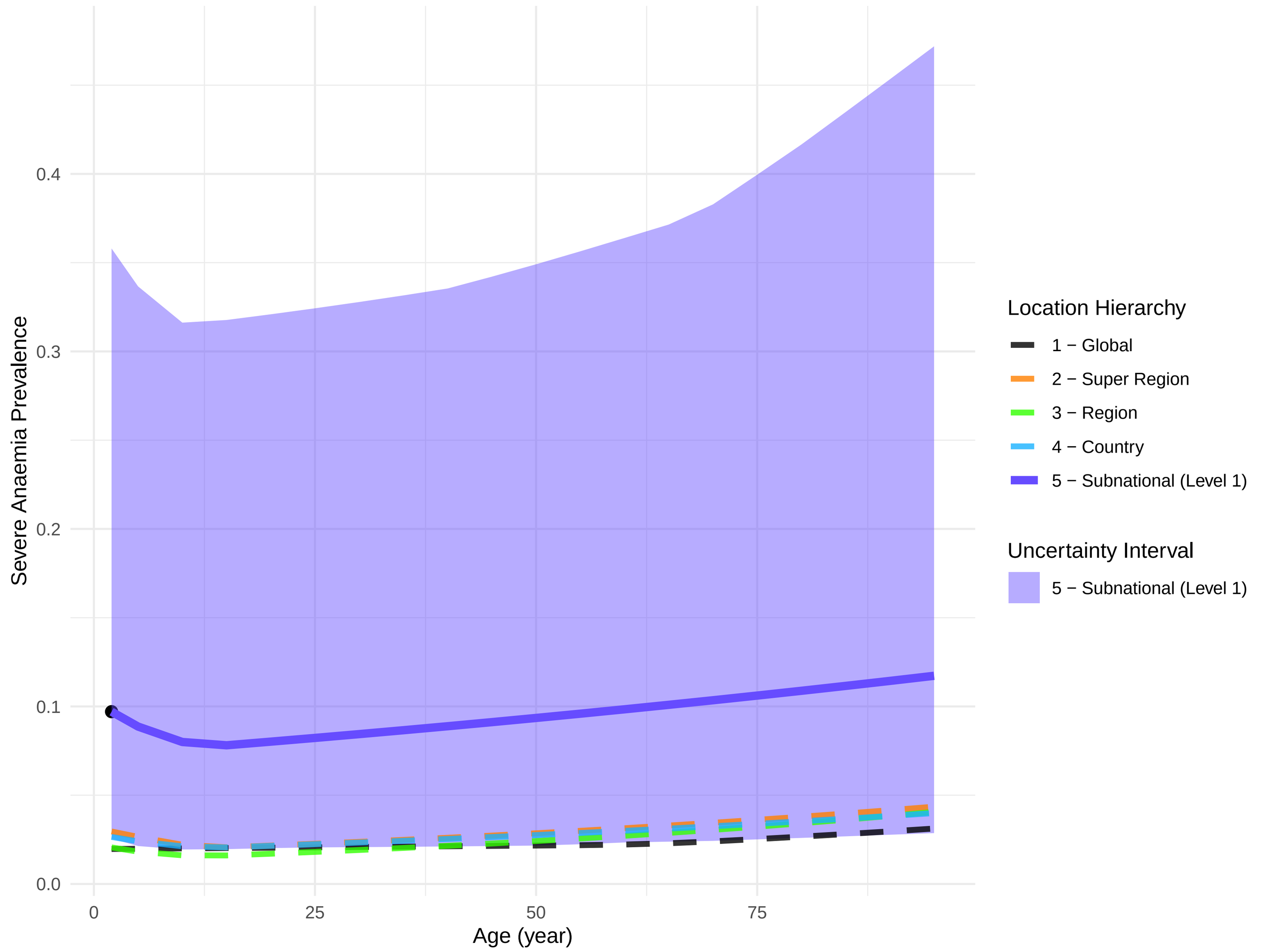

MR−BRT Age/Sex Fits − Non−pregnant Females 2+ Years Old − Severe Anaemia Prevalence − Migori
WHO−2024 Adjustment Method

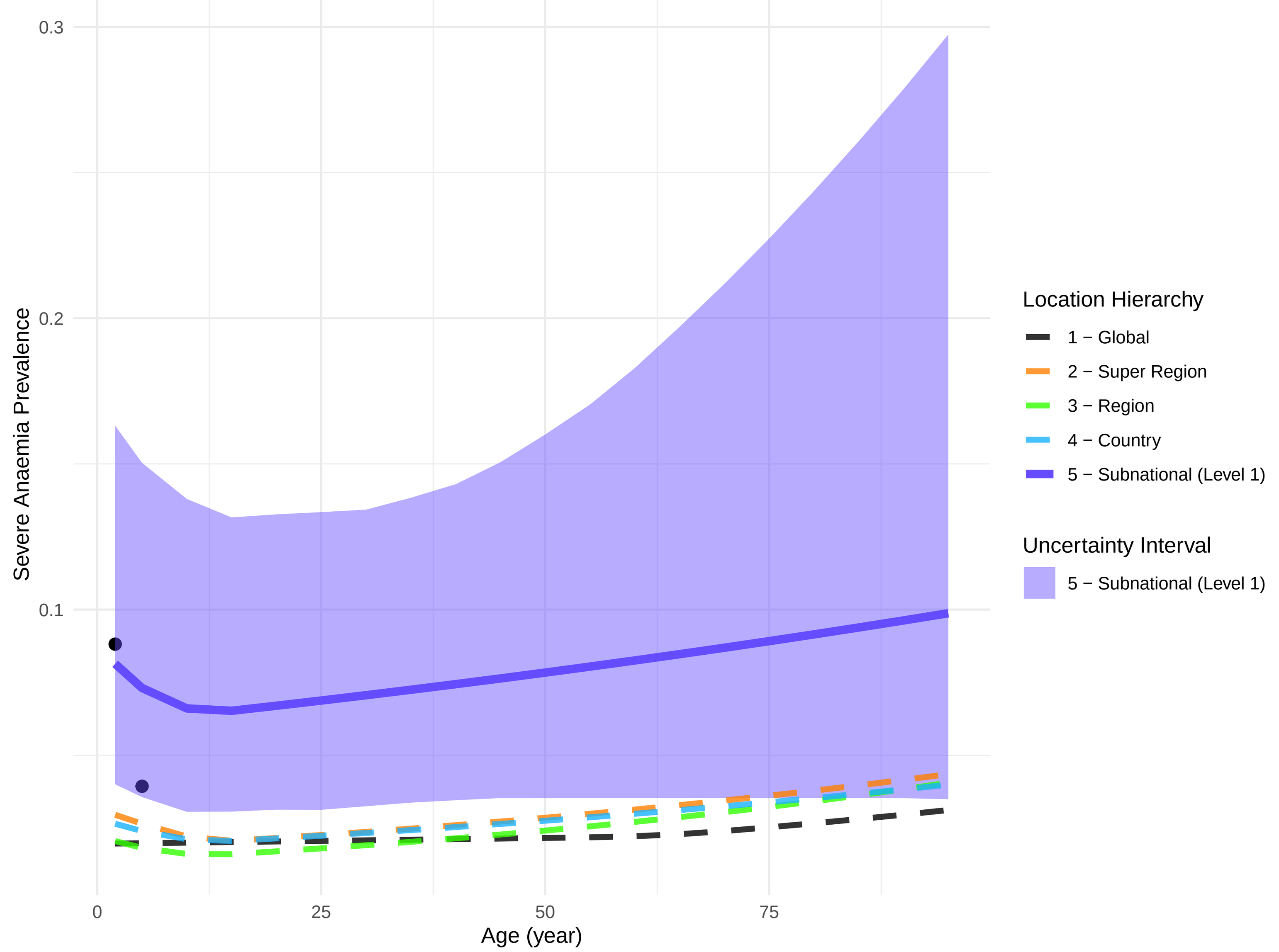

MR−BRT Age/Sex Fits − Non−pregnant Females 2+ Years Old − Severe Anaemia Prevalence − Mombasa
WHO−2024 Adjustment Method

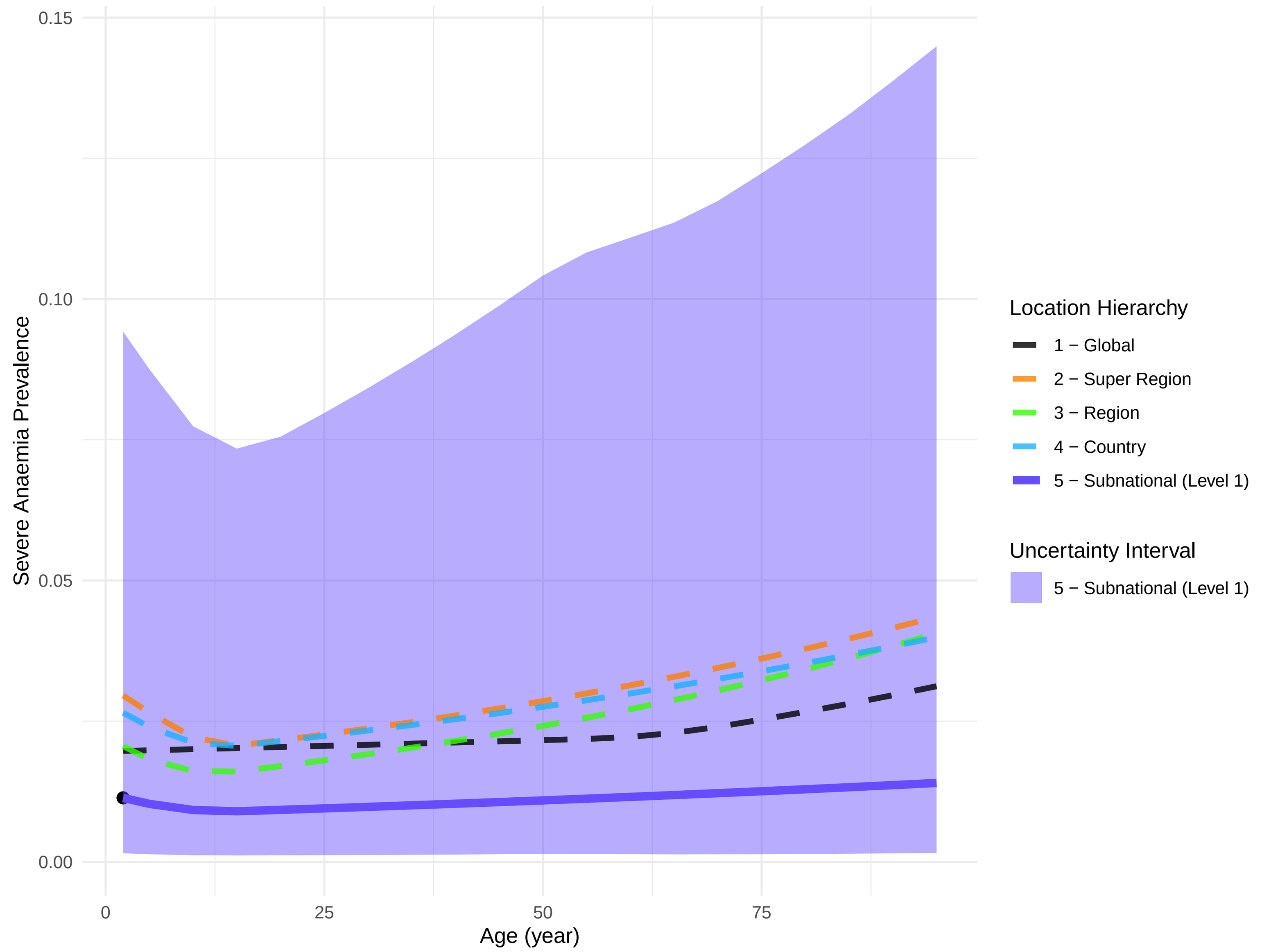

MR−BRT Age/Sex Fits − Non−pregnant Females 2+ Years Old − Severe Anaemia Prevalence − Nairobi
WHO−2024 Adjustment Method

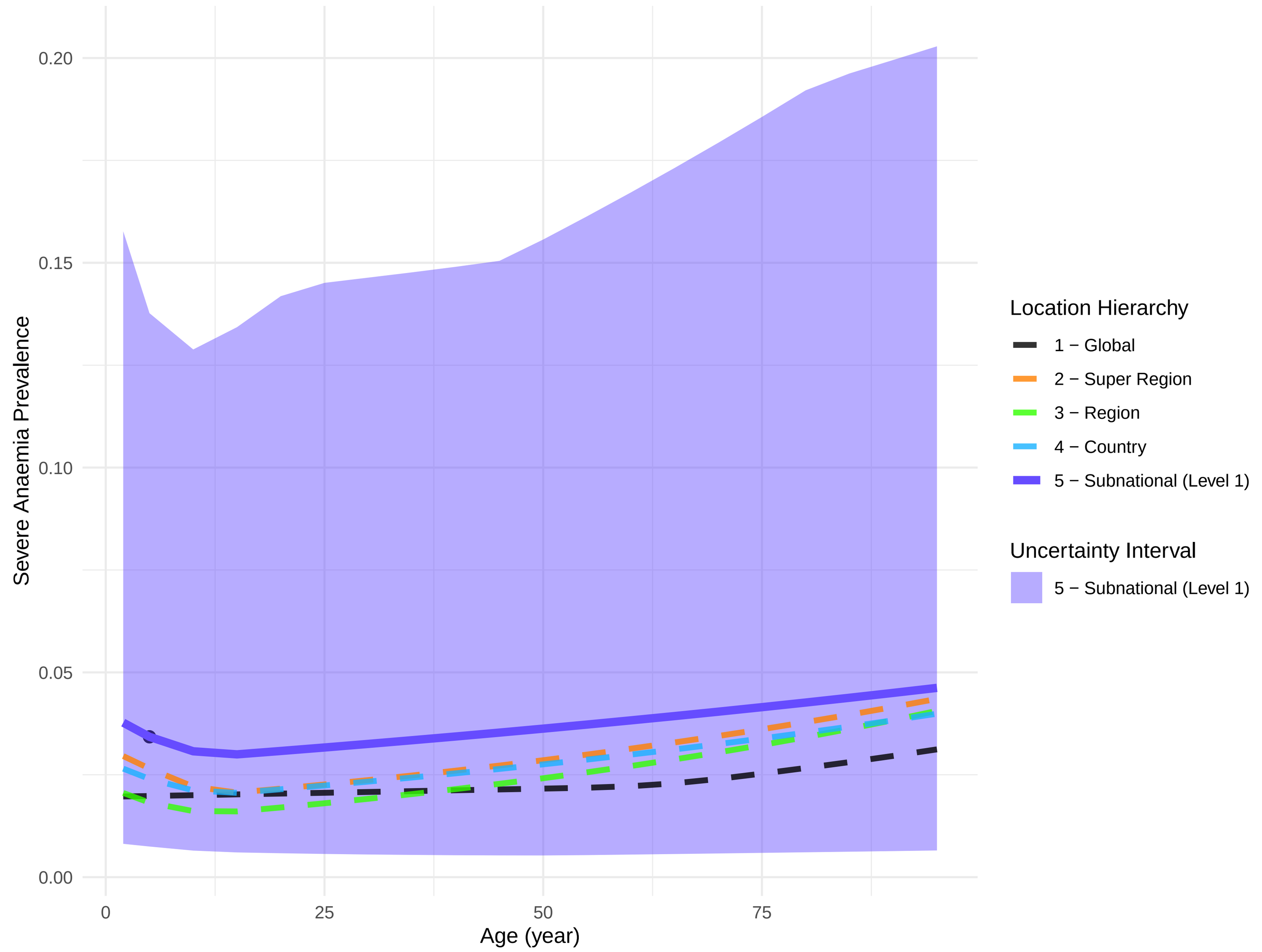

MR−BRT Age/Sex Fits − Non−pregnant Females 2+ Years Old − Severe Anaemia Prevalence − Siaya
WHO−2024 Adjustment Method

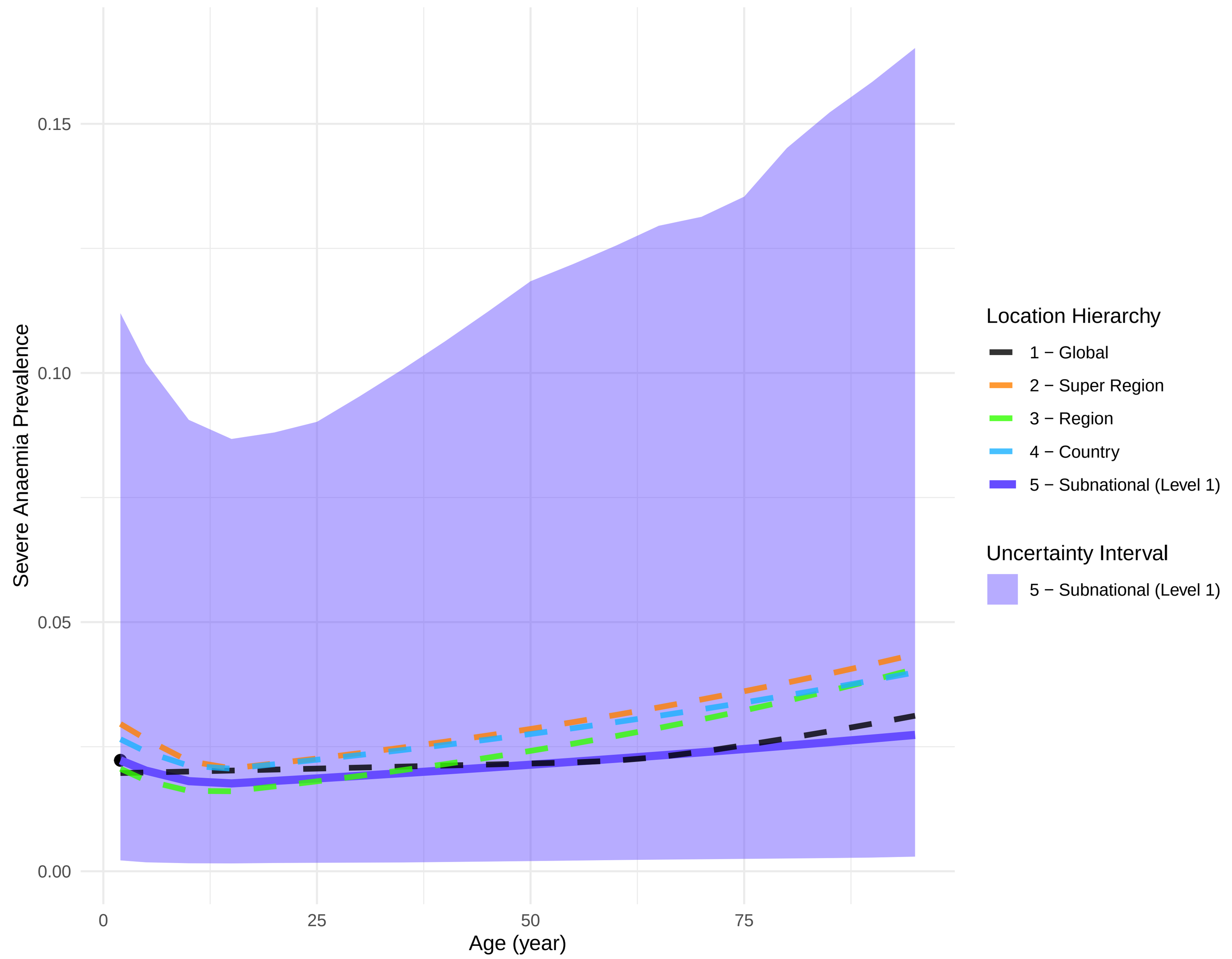

MR−BRT Age/Sex Fits − Non−pregnant Females 2+ Years Old − Severe Anaemia Prevalence − Tana River
WHO−2024 Adjustment Method

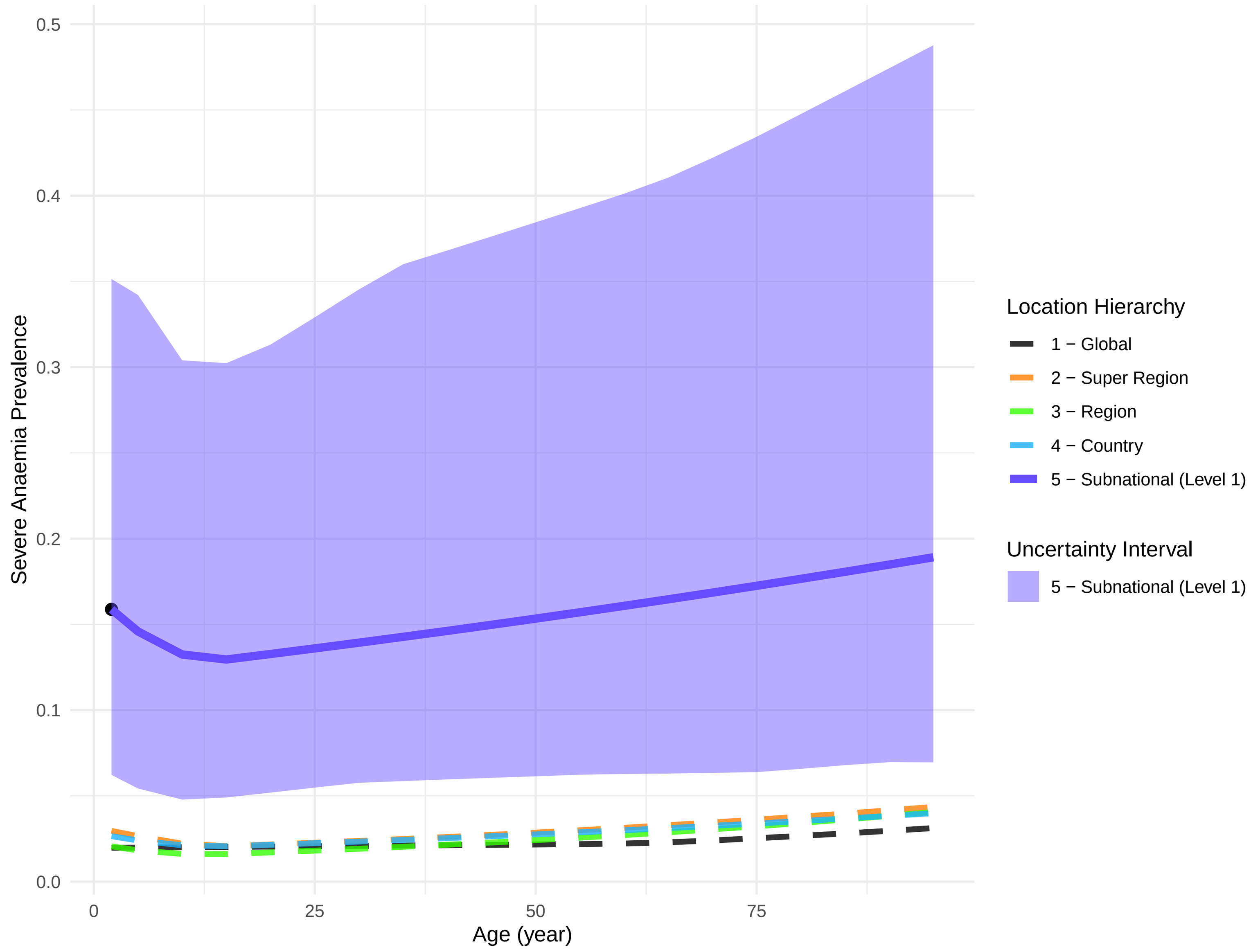

MR−BRT Age/Sex Fits − Non−pregnant Females 2+ Years Old − Severe Anaemia Prevalence − Trans Nzoia
WHO−2024 Adjustment Method

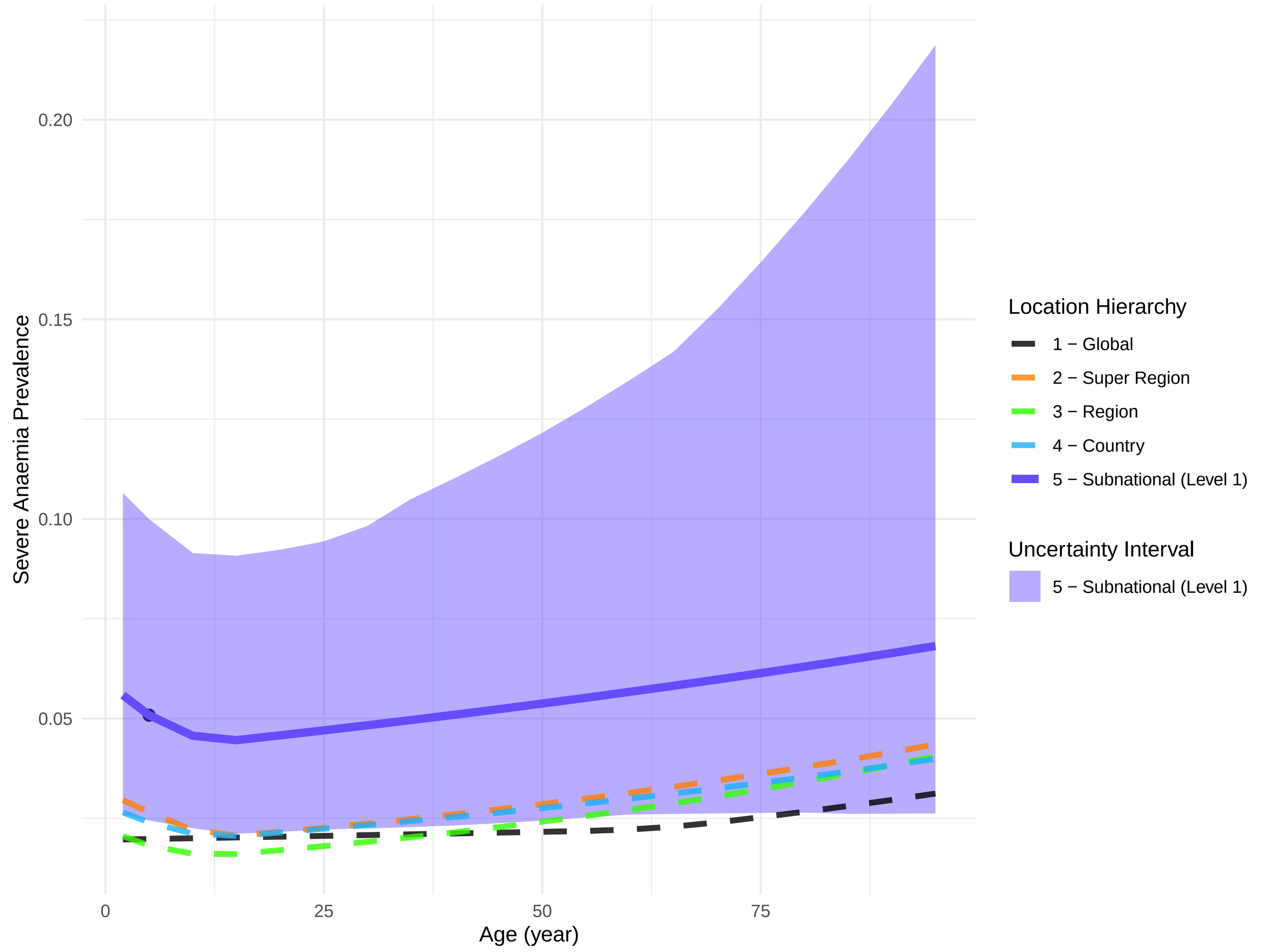

MR−BRT Age/Sex Fits − Non−pregnant Females 2+ Years Old − Severe Anaemia Prevalence − Turkana
WHO−2024 Adjustment Method

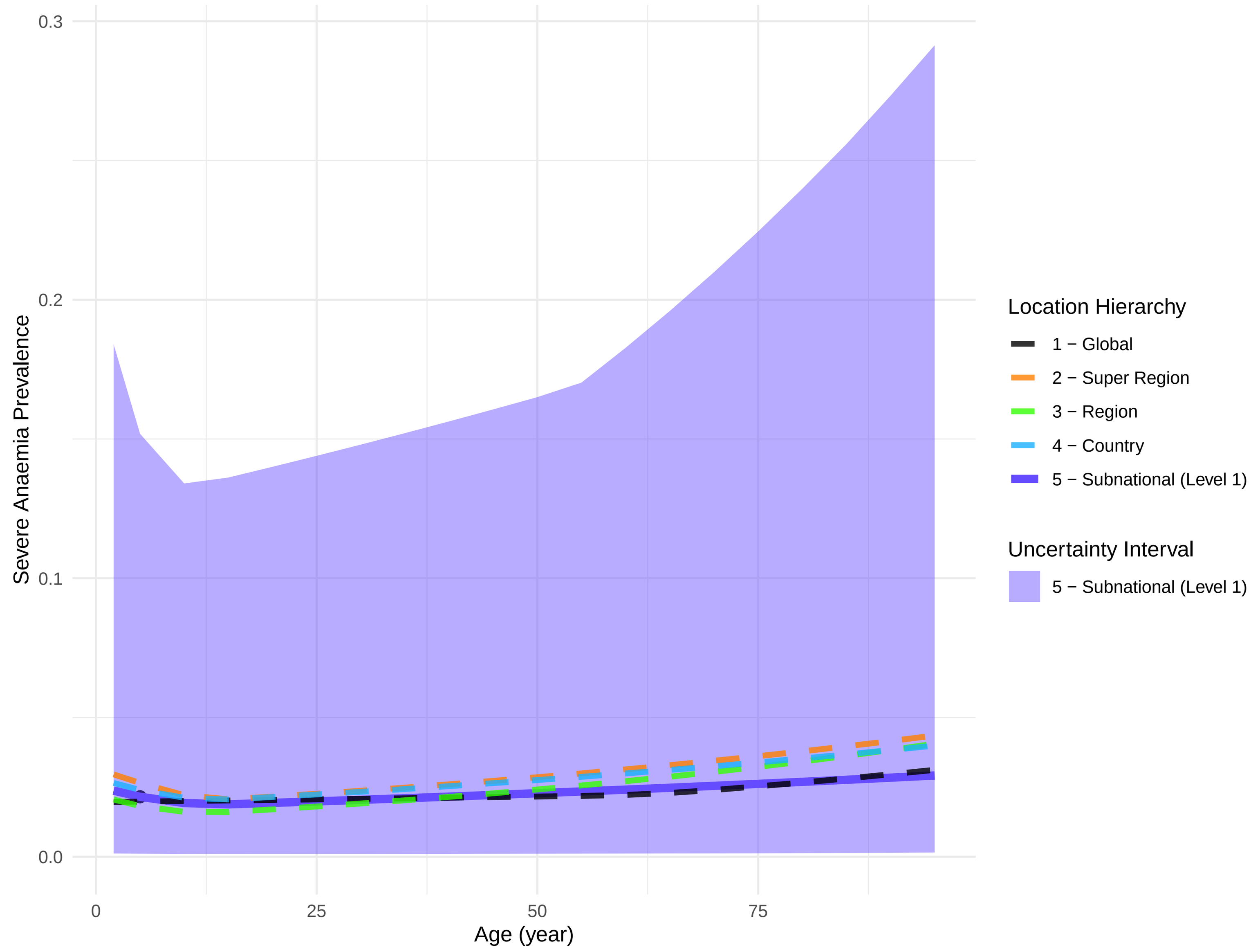

MR−BRT Age/Sex Fits − Non−pregnant Females 2+ Years Old − Severe Anaemia Prevalence − Vihiga
WHO−2024 Adjustment Method

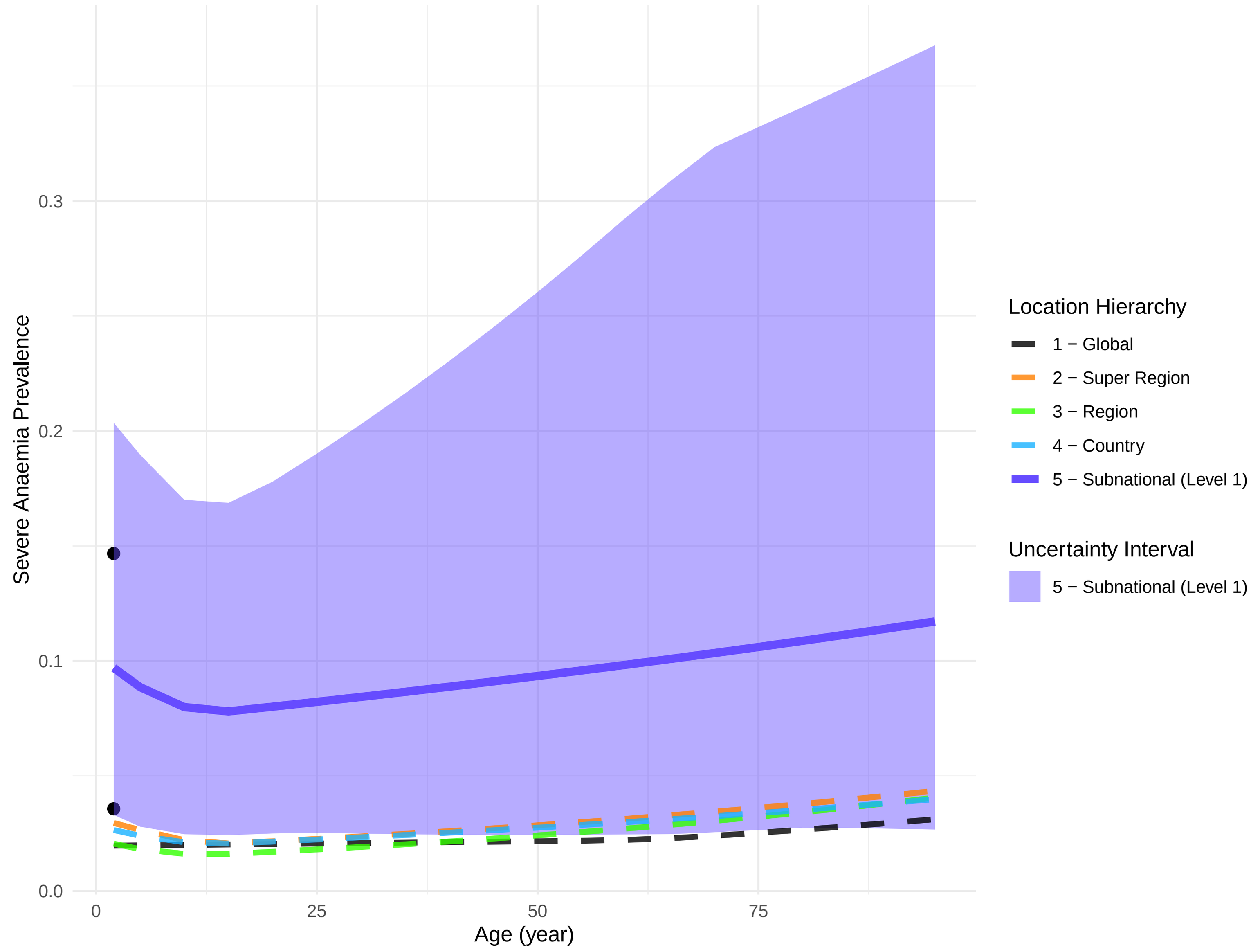

MR−BRT Age/Sex Fits − Non−pregnant Females 2+ Years Old − Severe Anaemia Prevalence − Wajir
WHO−2024 Adjustment Method

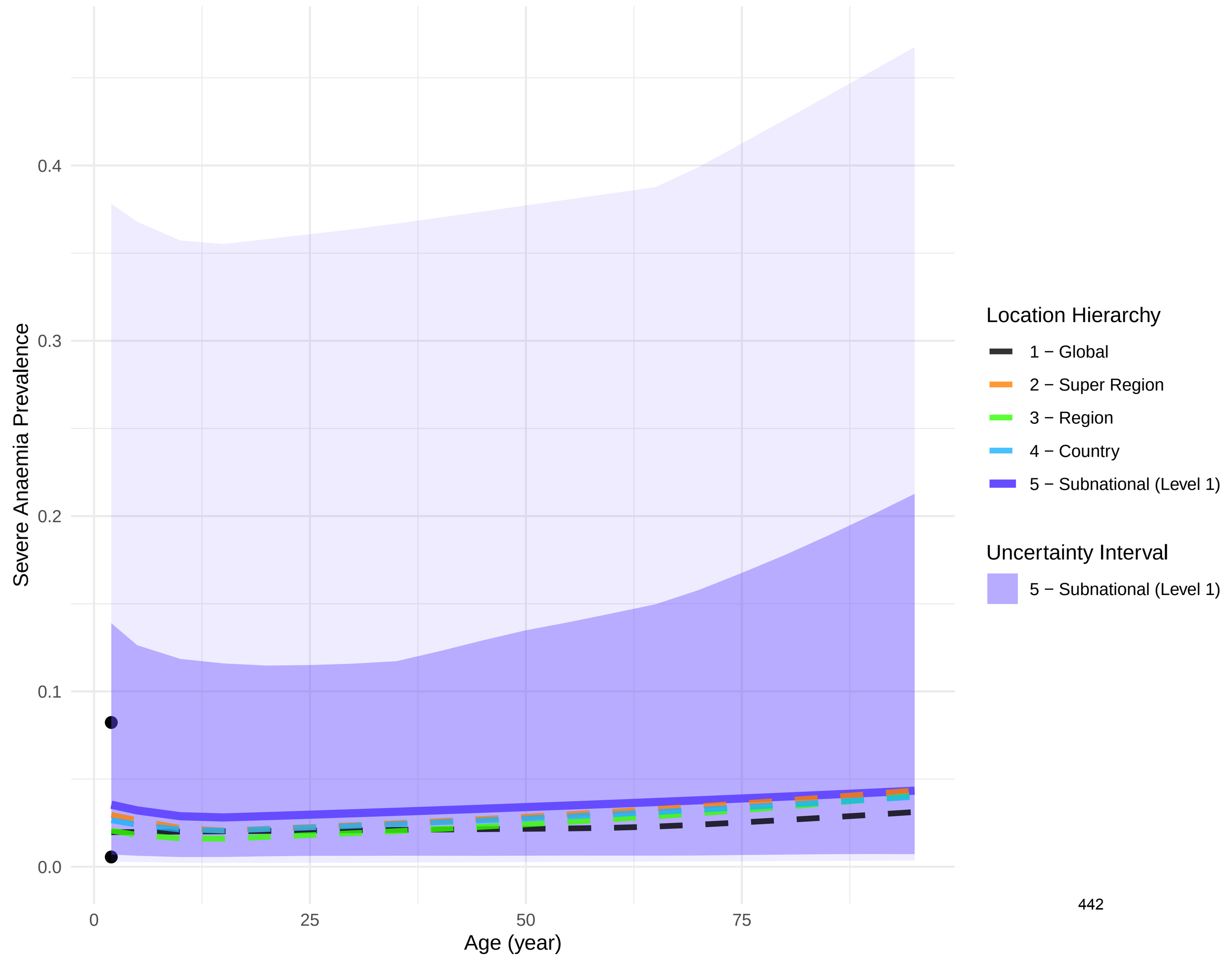

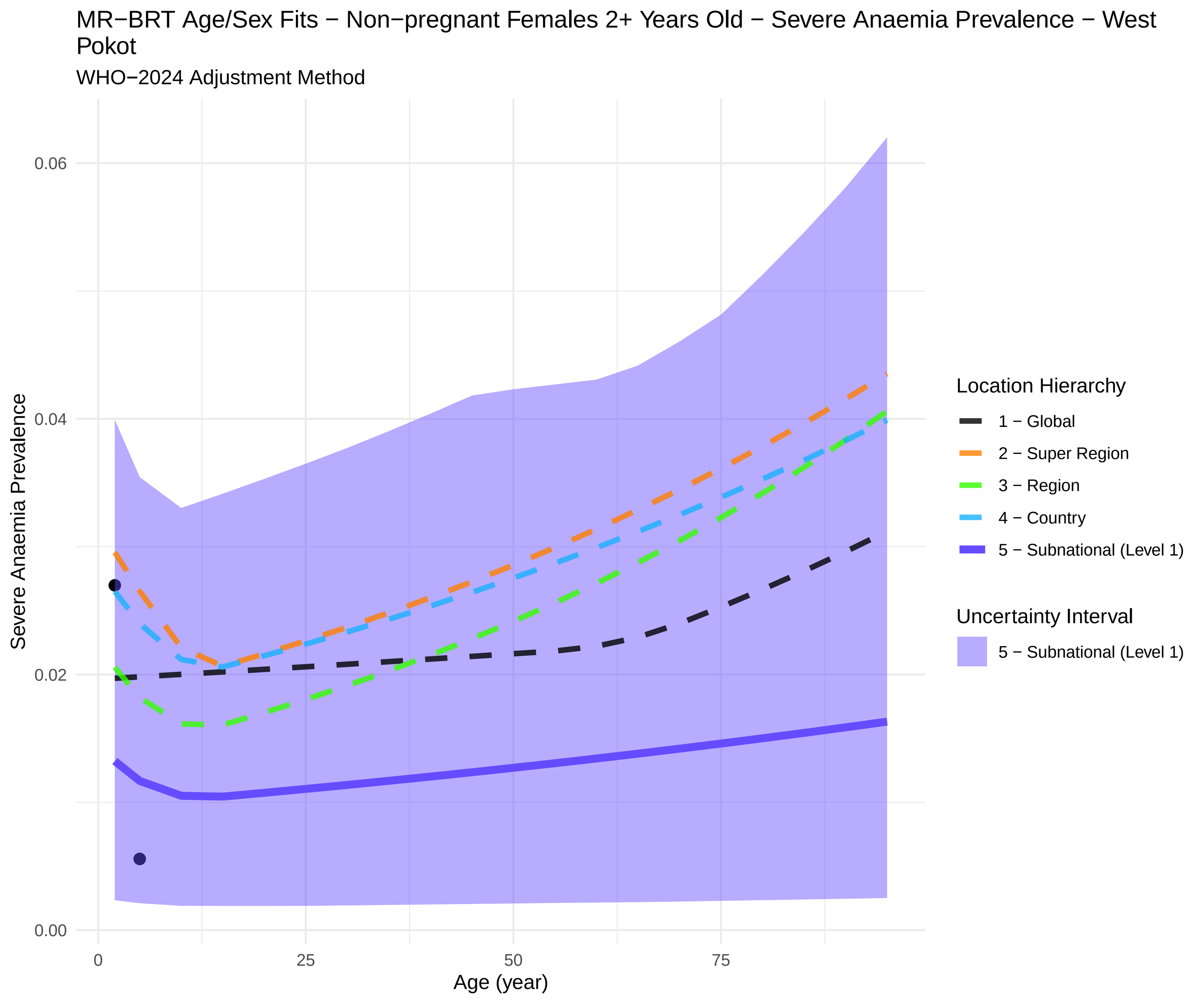
MR−BRT Age/Sex Fits − Non−pregnant Females 2+ Years Old − Severe Anaemia Prevalence − West Pokot
WHO−2024 Adjustment Method
Severe Anaemia Prevalence
Age (year)
Location Hierarchy
1 − Global
2 − Super Region
3 − Region
4 − Country
5 − Subnational (Level 1)
Uncertainty Interval
5 − Subnational (Level 1)

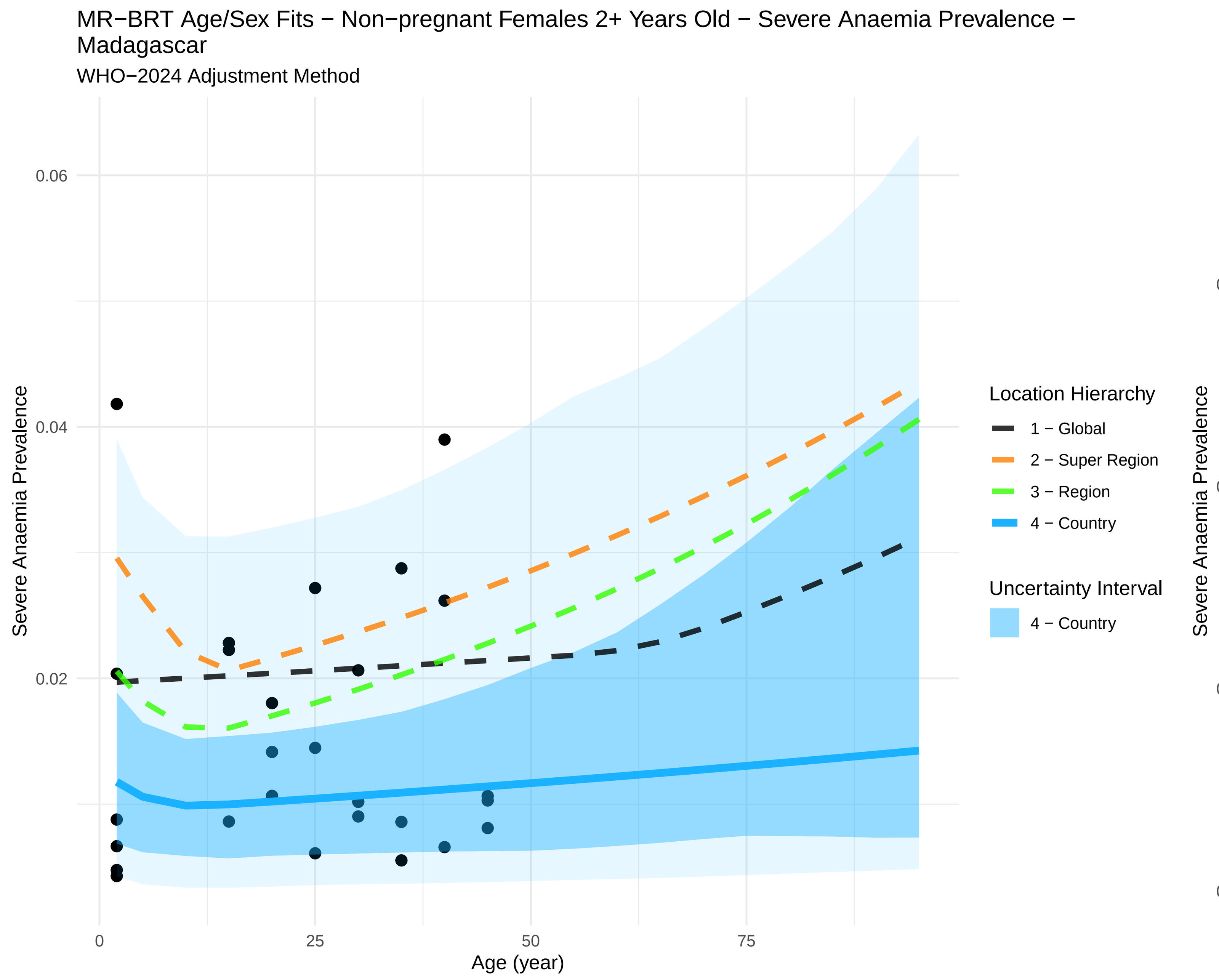
MR−BRT Age/Sex Fits − Non−pregnant Females 2+ Years Old − Severe Anaemia Prevalence − Madagascar
WHO−2024 Adjustment Method
Severe Anaemia Prevalence
Age (year)
Location Hierarchy
1 − Global
2 − Super Region
3 − Region
4 − Country
Uncertainty Interval
4 − Country

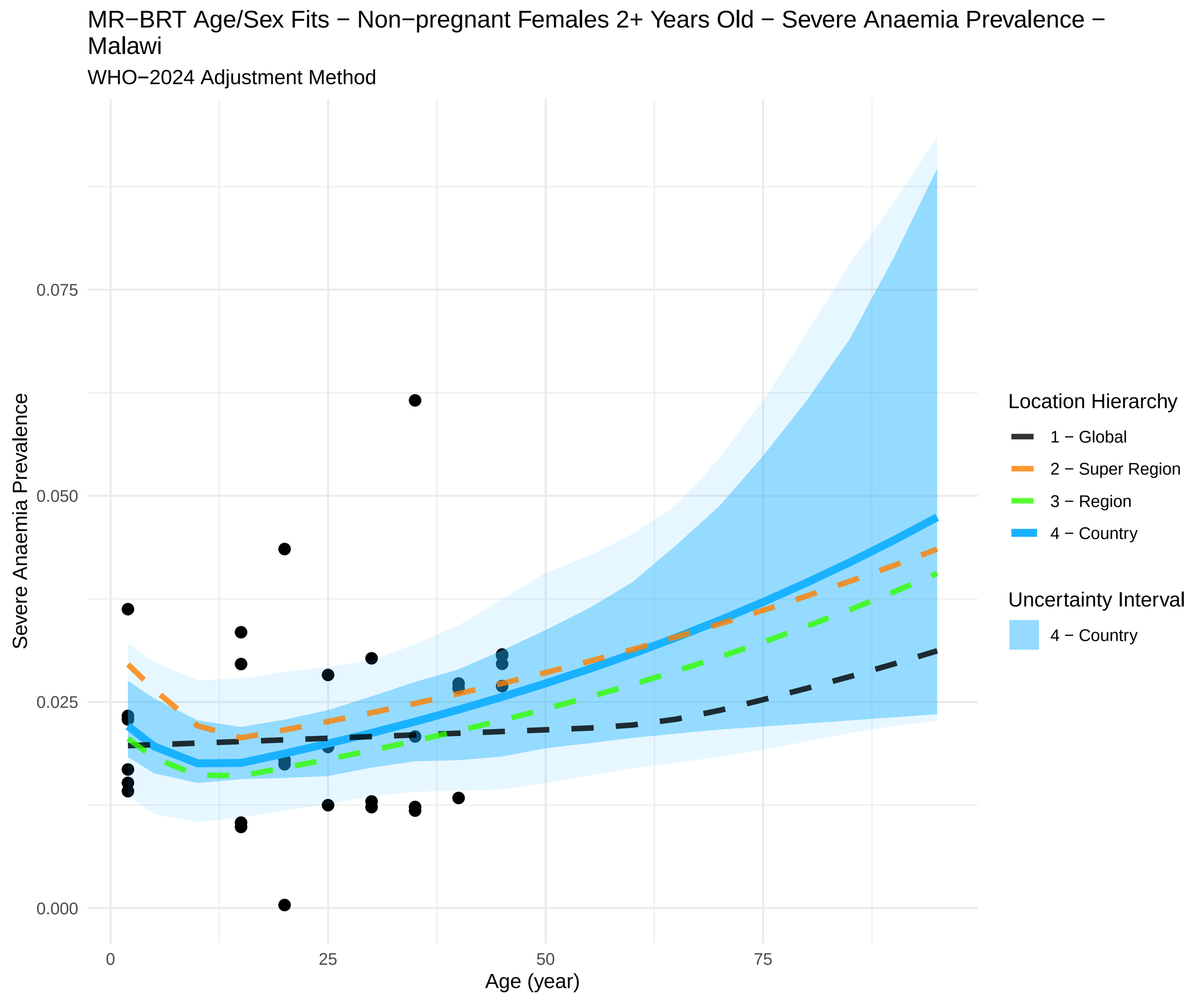
MR−BRT Age/Sex Fits − Non−pregnant Females 2+ Years Old − Severe Anaemia Prevalence − Malawi
WHO−2024 Adjustment Method
Severe Anaemia Prevalence
Age (year)
Location Hierarchy
1 − Global
2 − Super Region
3 − Region
4 − Country
Uncertainty Interval
4 − Country

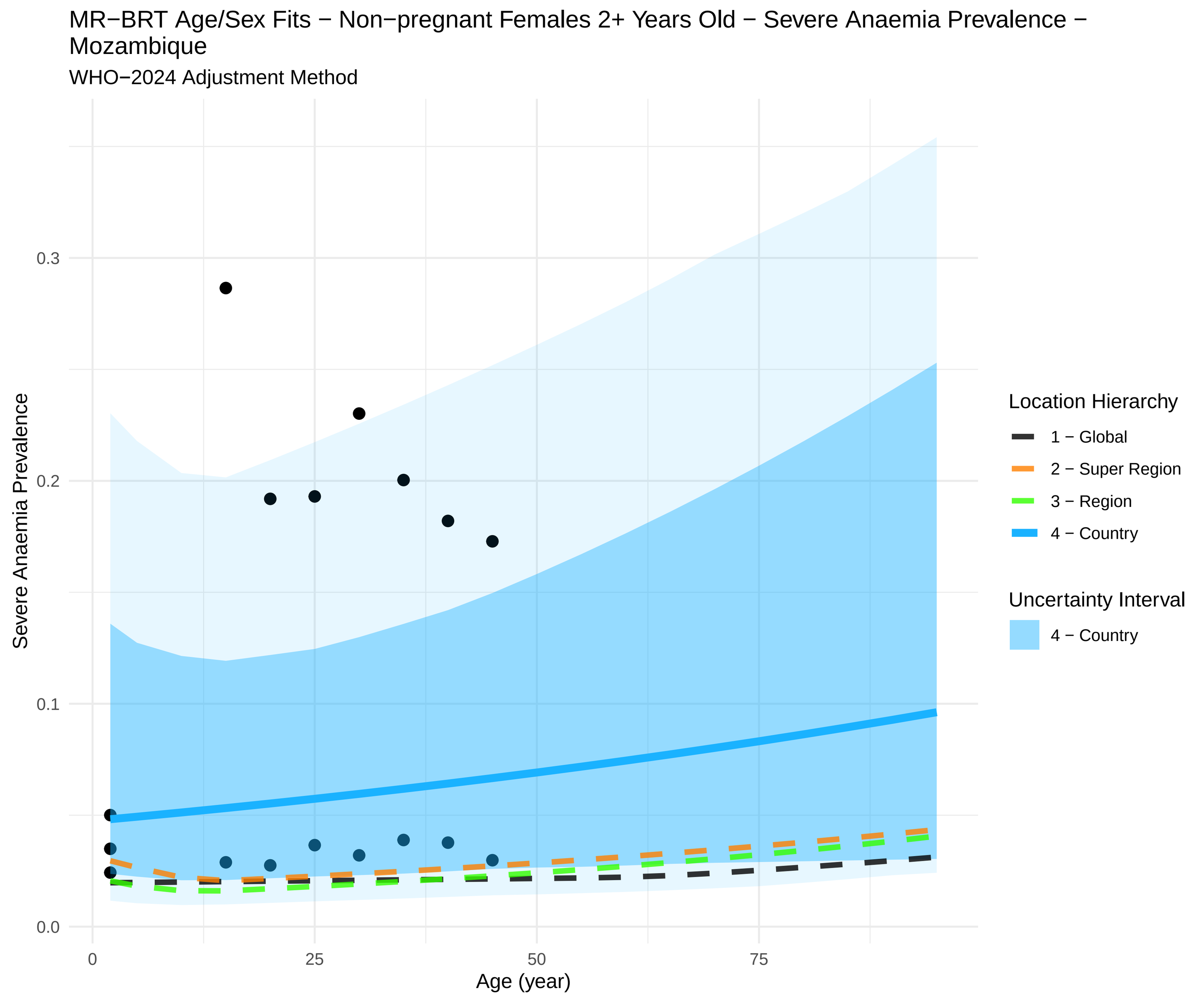
MR−BRT Age/Sex Fits − Non−pregnant Females 2+ Years Old − Severe Anaemia Prevalence − Mozambique
WHO−2024 Adjustment Method
Severe Anaemia Prevalence
Age (year)
Location Hierarchy
1 − Global
2 − Super Region
3 − Region
4 − Country
Uncertainty Interval
4 − Country

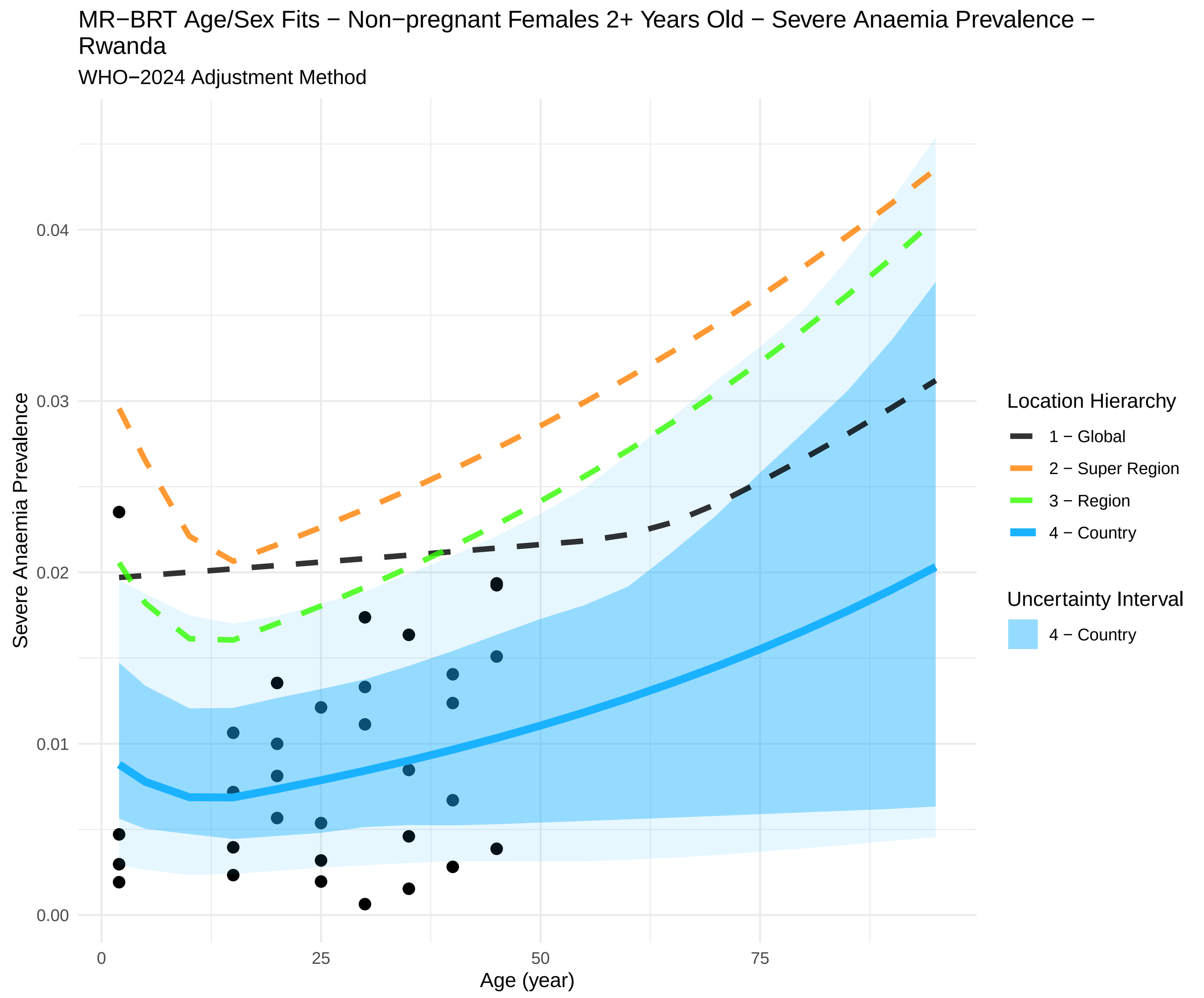
MR−BRT Age/Sex Fits − Non−pregnant Females 2+ Years Old − Severe Anaemia Prevalence − Rwanda
WHO−2024 Adjustment Method
Severe Anaemia Prevalence
Age (year)
Location Hierarchy
1 − Global
2 − Super Region
3 − Region
4 − Country
Uncertainty Interval
4 − Country

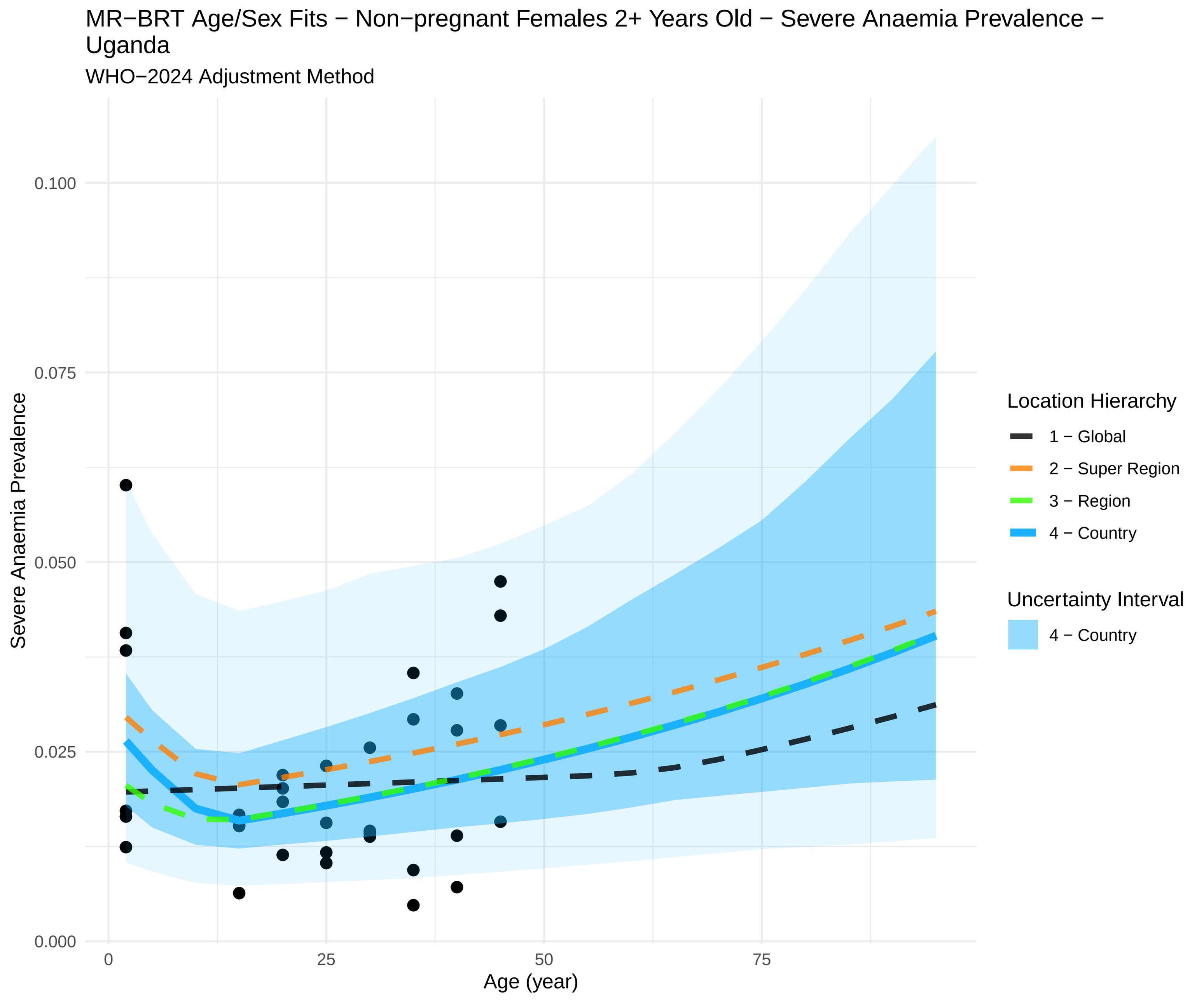
MR−BRT Age/Sex Fits − Non−pregnant Females 2+ Years Old − Severe Anaemia Prevalence − Uganda
WHO−2024 Adjustment Method
Severe Anaemia Prevalence
Age (year)
Location Hierarchy
1 − Global
2 − Super Region
3 − Region
4 − Country
Uncertainty Interval
4 − Country

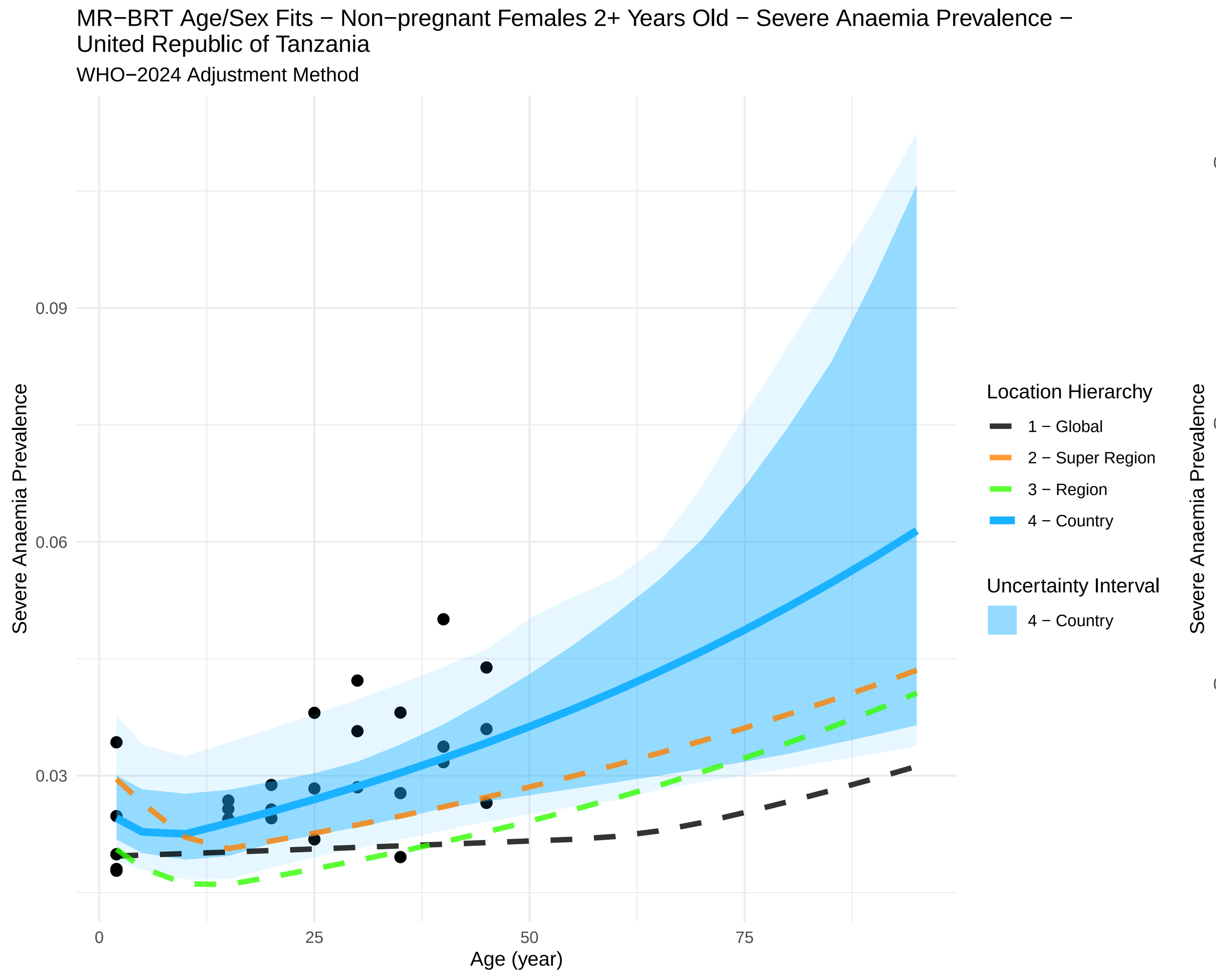
MR−BRT Age/Sex Fits − Non−pregnant Females 2+ Years Old − Severe Anaemia Prevalence − United Republic of Tanzania
WHO−2024 Adjustment Method
Severe Anaemia Prevalence
Age (year)
Location Hierarchy
1 − Global
2 − Super Region
3 − Region
4 − Country
Uncertainty Interval
4 − Country

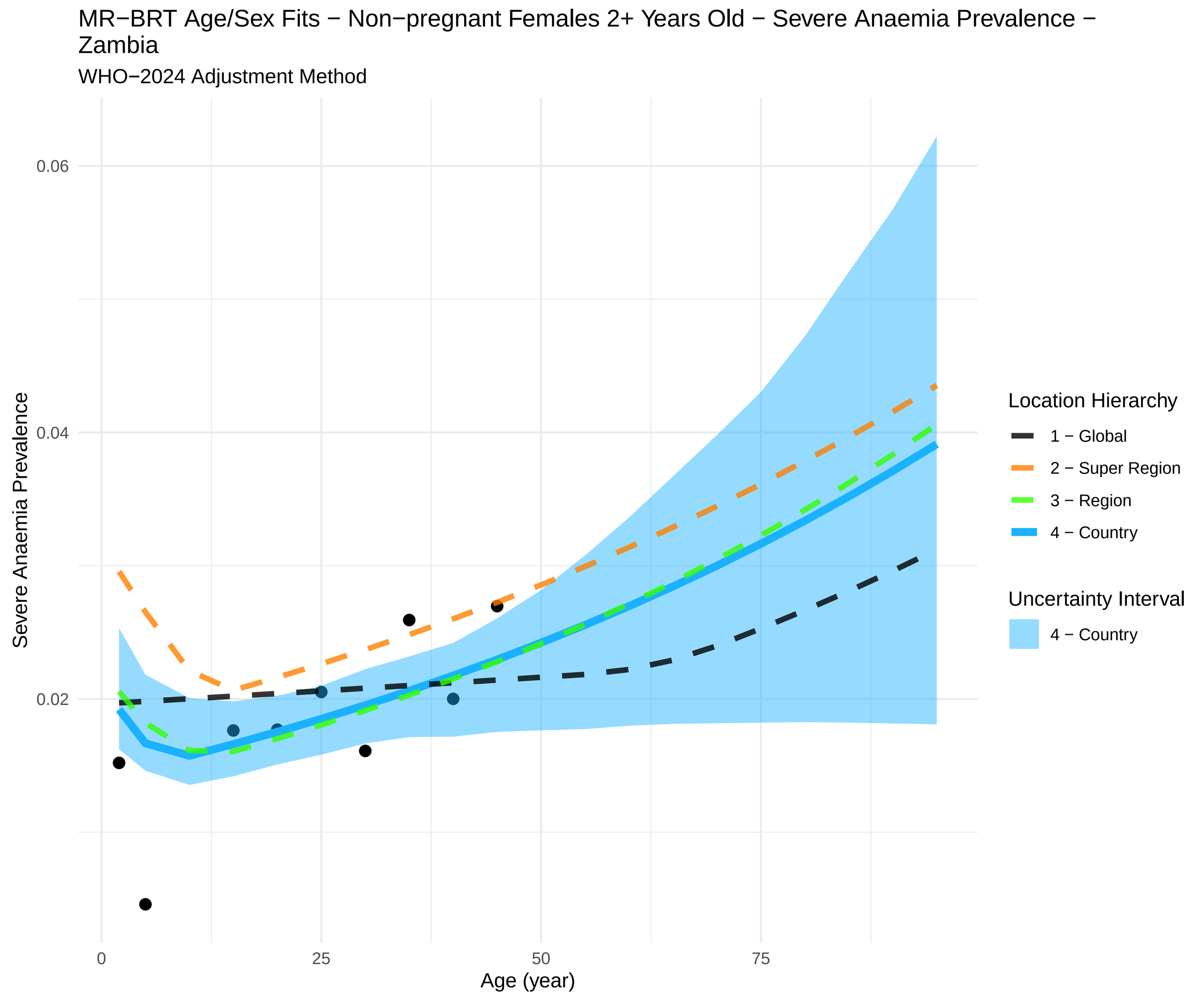
MR−BRT Age/Sex Fits − Non−pregnant Females 2+ Years Old − Severe Anaemia Prevalence − Zambia
WHO−2024 Adjustment Method
Severe Anaemia Prevalence
Age (year)
Location Hierarchy
1 − Global
2 − Super Region
3 − Region
4 − Country
Uncertainty Interval
4 − Country

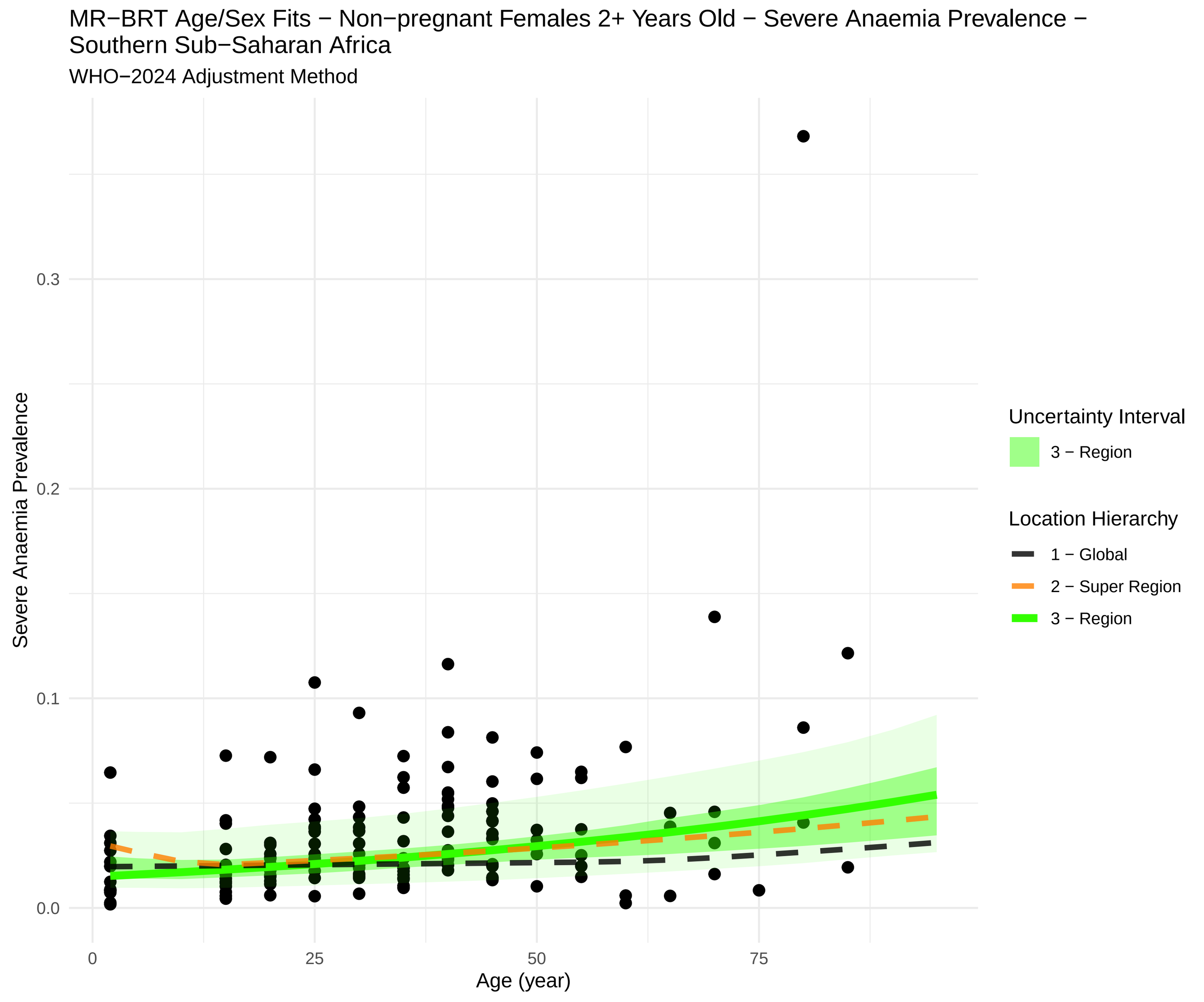
MR−BRT Age/Sex Fits − Non−pregnant Females 2+ Years Old − Severe Anaemia Prevalence − Southern Sub−Saharan Africa
WHO−2024 Adjustment Method
Severe Anaemia Prevalence
Age (year)
Uncertainty Interval
3 − Region
Location Hierarchy
1 − Global
2 − Super Region
3 − Region

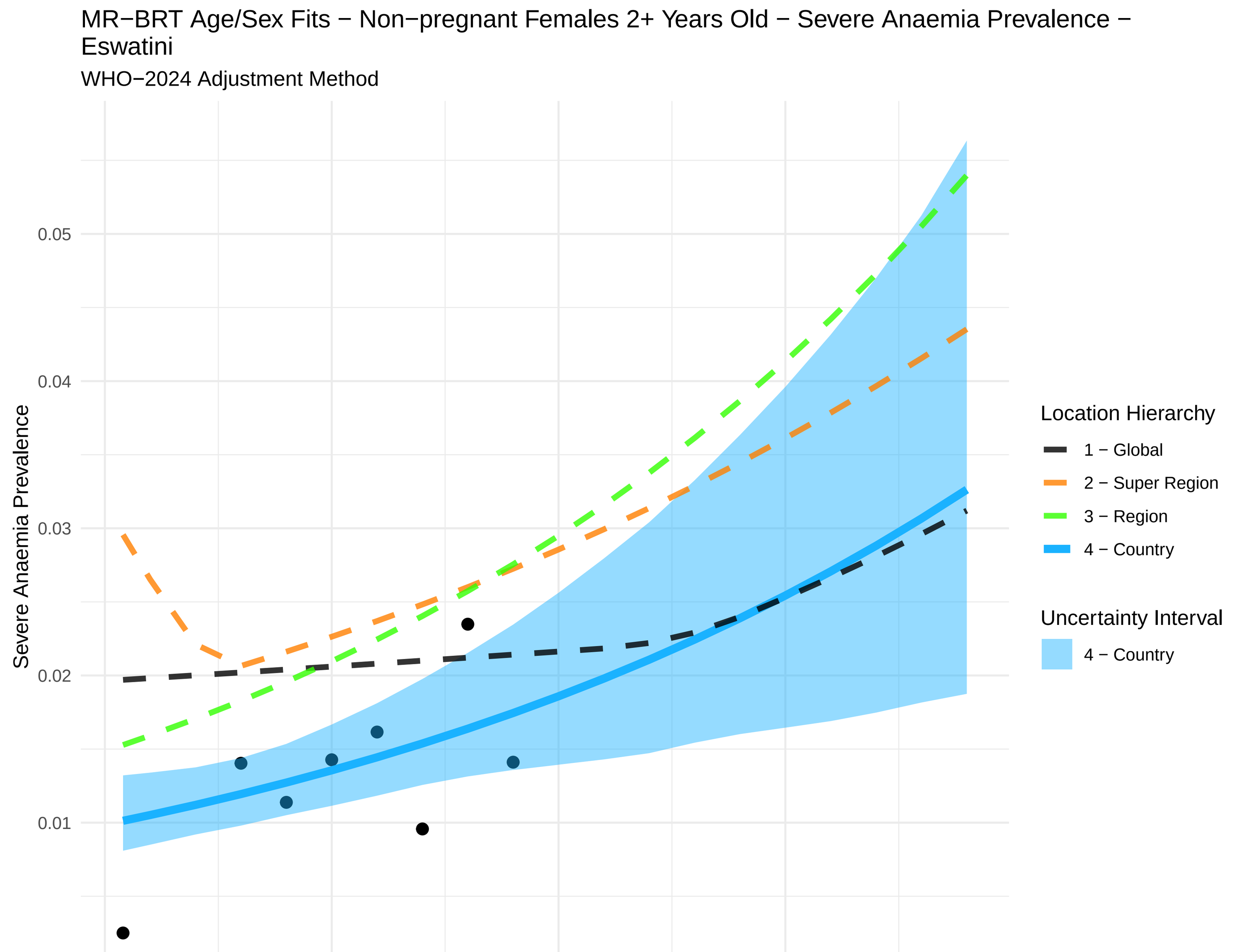
MR−BRT Age/Sex Fits − Non−pregnant Females 2+ Years Old − Severe Anaemia Prevalence − Eswatini
WHO−2024 Adjustment Method
Severe Anaemia Prevalence
Age (year)
Location Hierarchy
1 − Global
2 − Super Region
3 − Region
4 − Country
Uncertainty Interval
4 − Country

MR−BRT Age/Sex Fits − Non-pregnant Females 2+ Years Old − Severe Anaemia Prevalence − Lesotho
WHO−2024 Adjustment Method

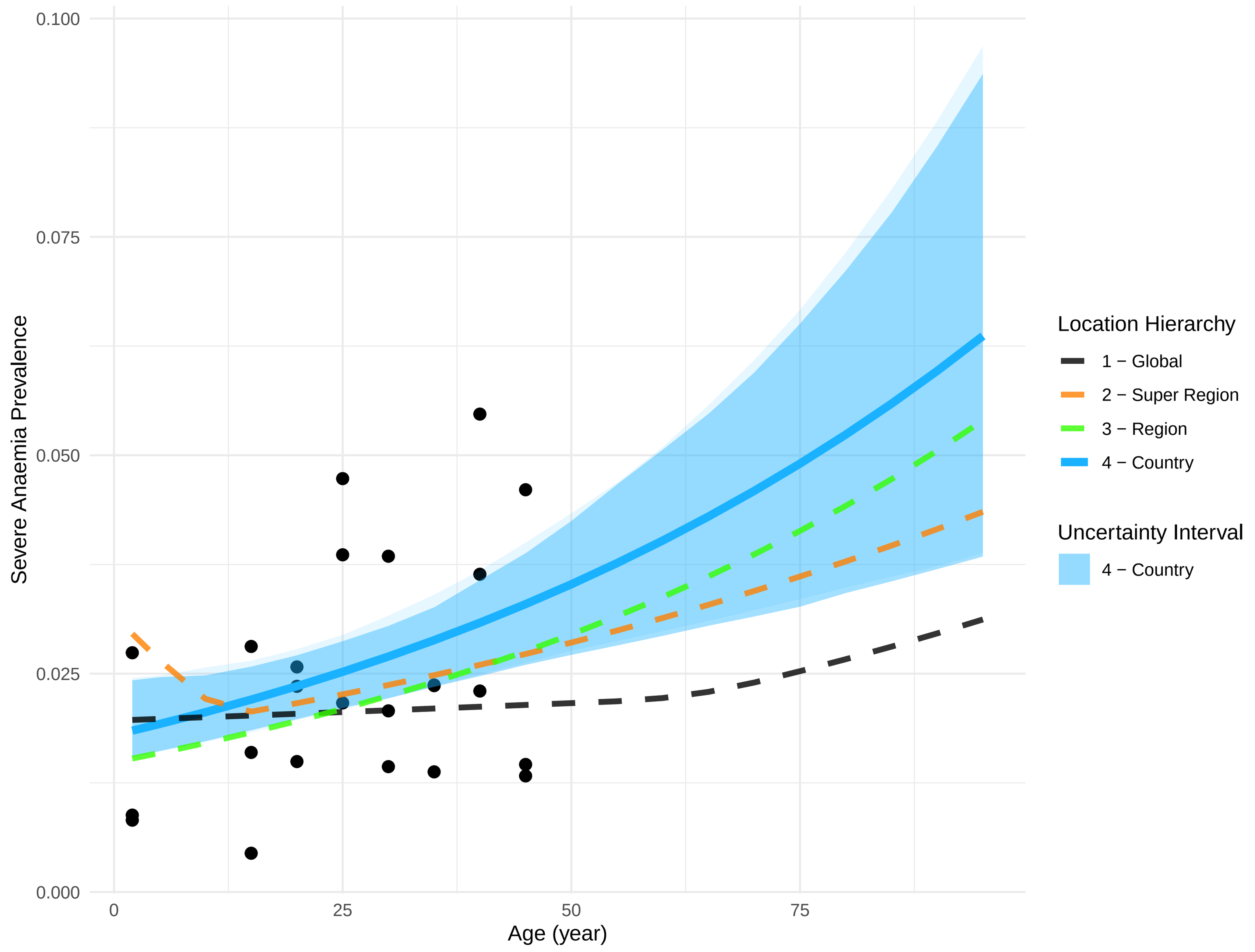

MR−BRT Age/Sex Fits − Non-pregnant Females 2+ Years Old − Severe Anaemia Prevalence − Namibia
WHO−2024 Adjustment Method

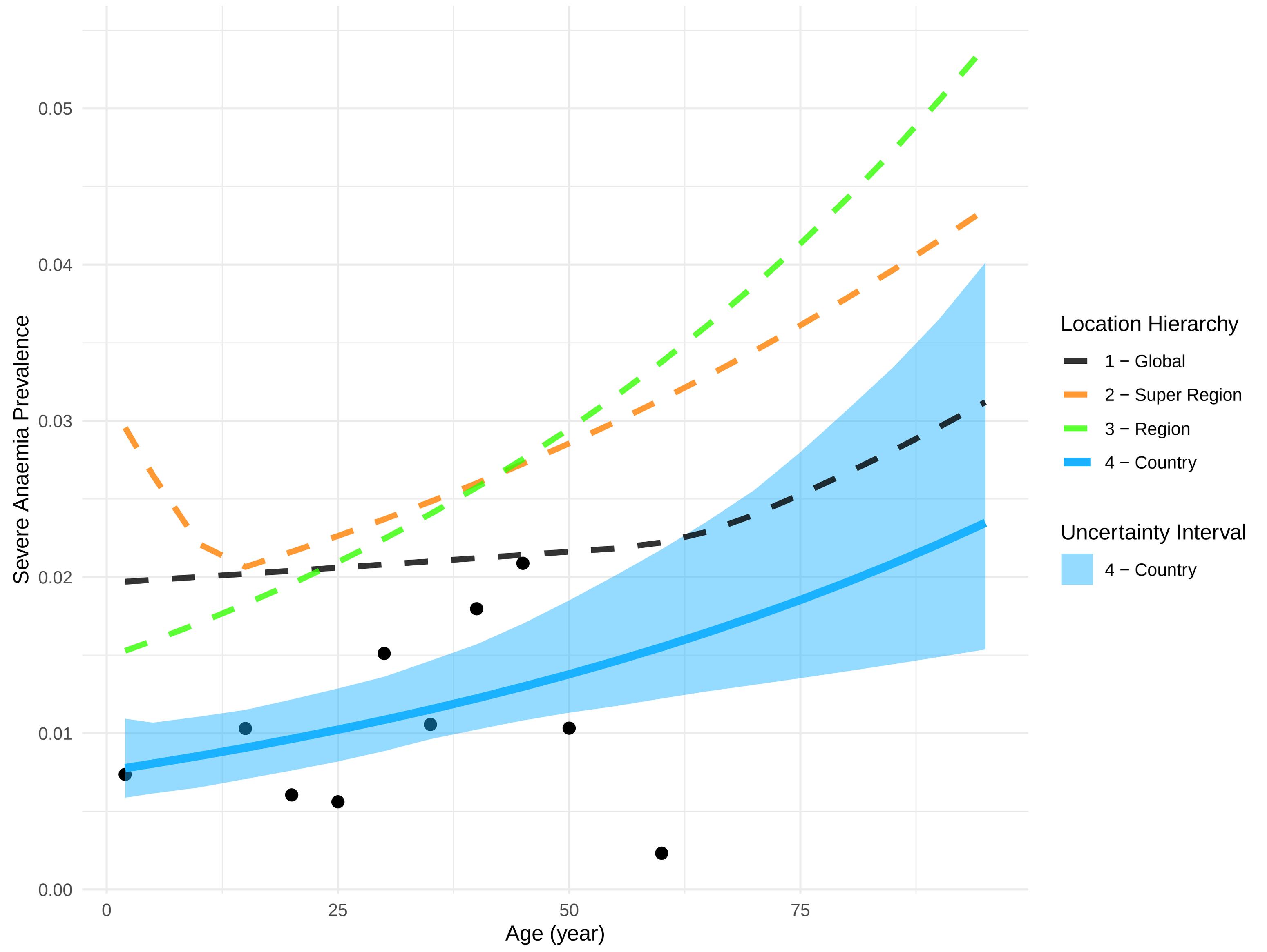

MR−BRT Age/Sex Fits − Non-pregnant Females 2+ Years Old − Severe Anaemia Prevalence − South Africa
WHO−2024 Adjustment Method

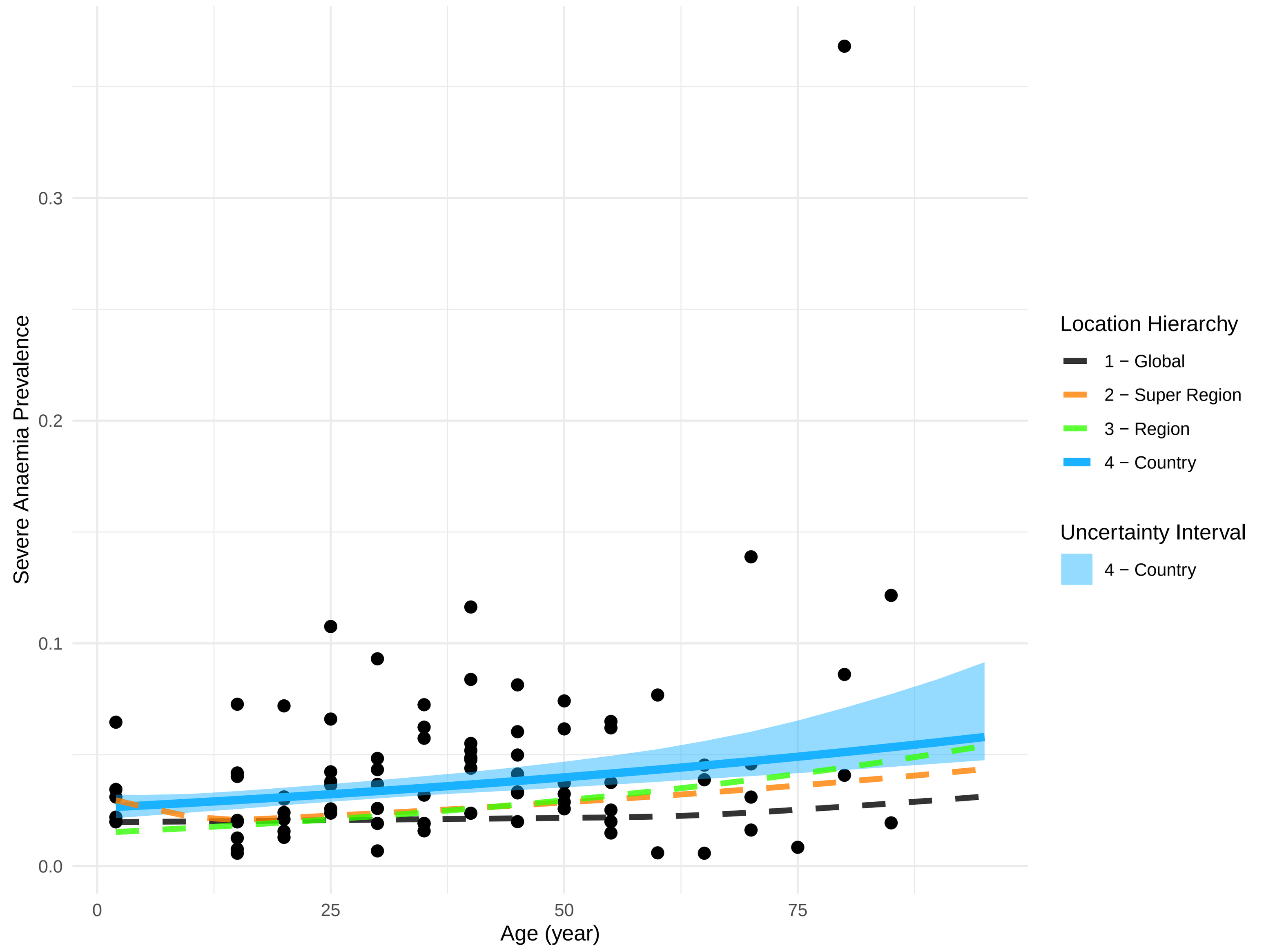

MR−BRT Age/Sex Fits − Non-pregnant Females 2+ Years Old − Severe Anaemia Prevalence − Eastern Cape
WHO−2024 Adjustment Method

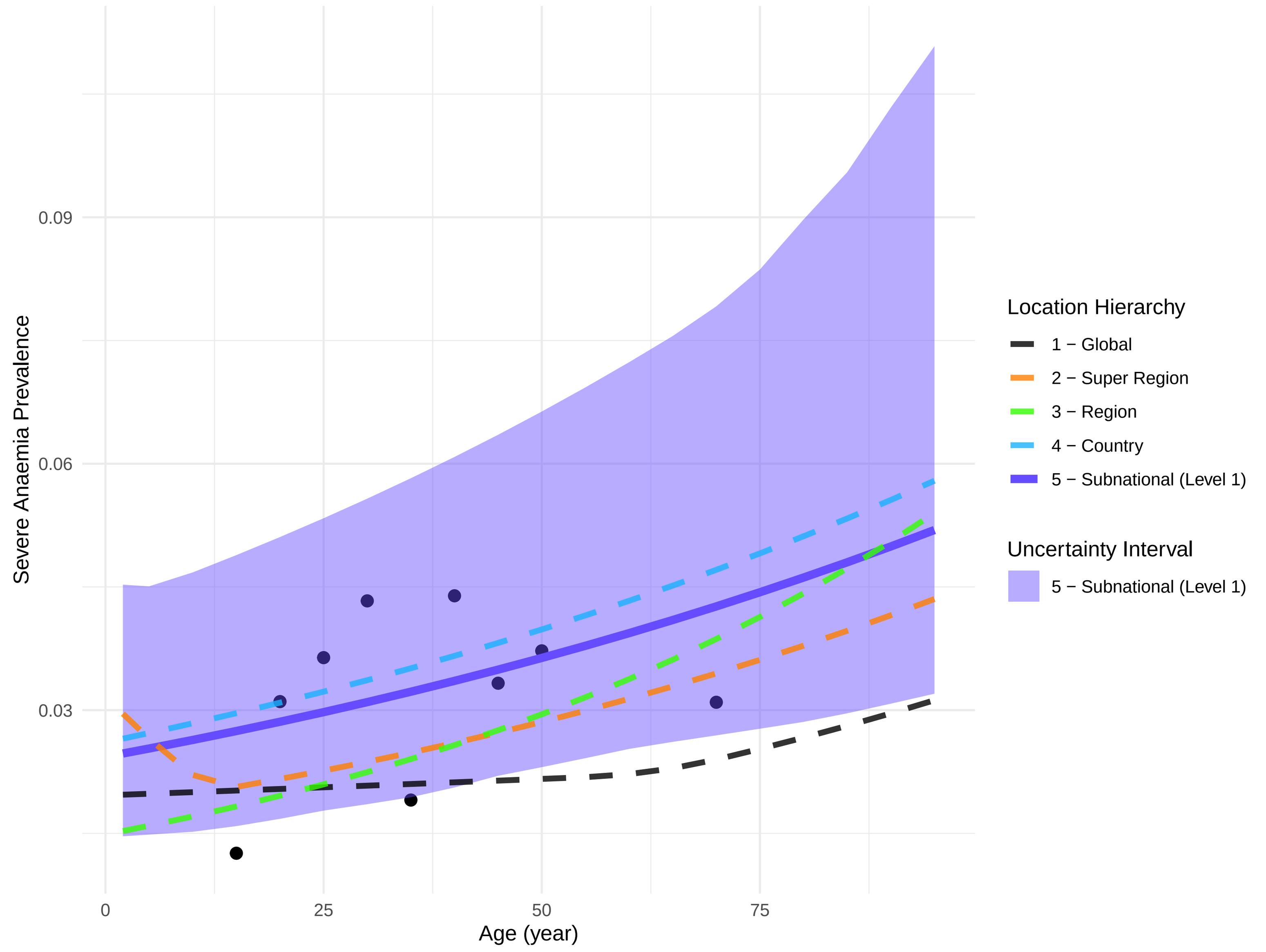

MR−BRT Age/Sex Fits − Non-pregnant Females 2+ Years Old − Severe Anaemia Prevalence − Free State
WHO−2024 Adjustment Method

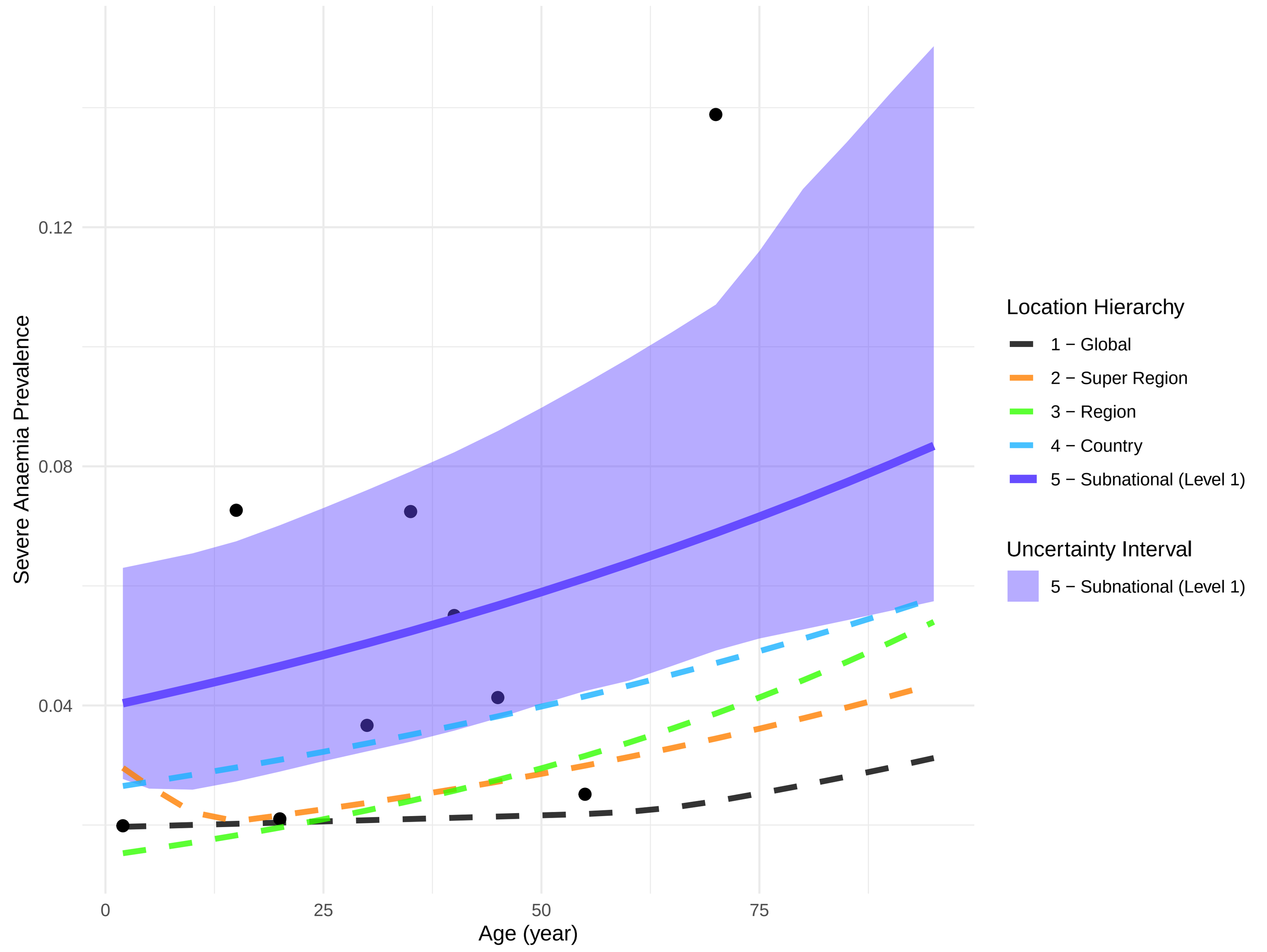

MR−BRT Age/Sex Fits − Non-pregnant Females 2+ Years Old − Severe Anaemia Prevalence − Gauteng
WHO−2024 Adjustment Method

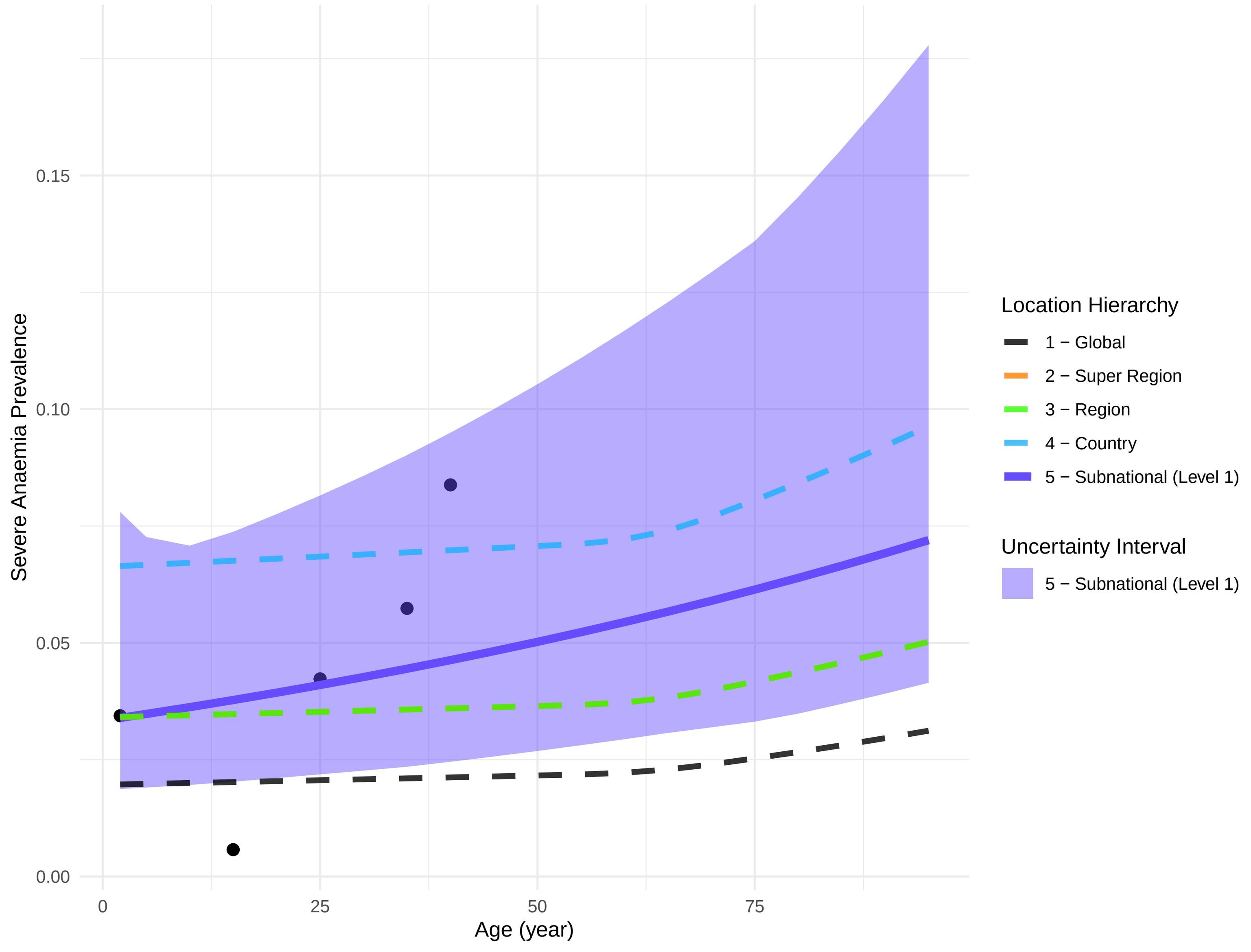

MR−BRT Age/Sex Fits − Non-pregnant Females 2+ Years Old − Severe Anaemia Prevalence − KwaZulu−Natal
WHO−2024 Adjustment Method

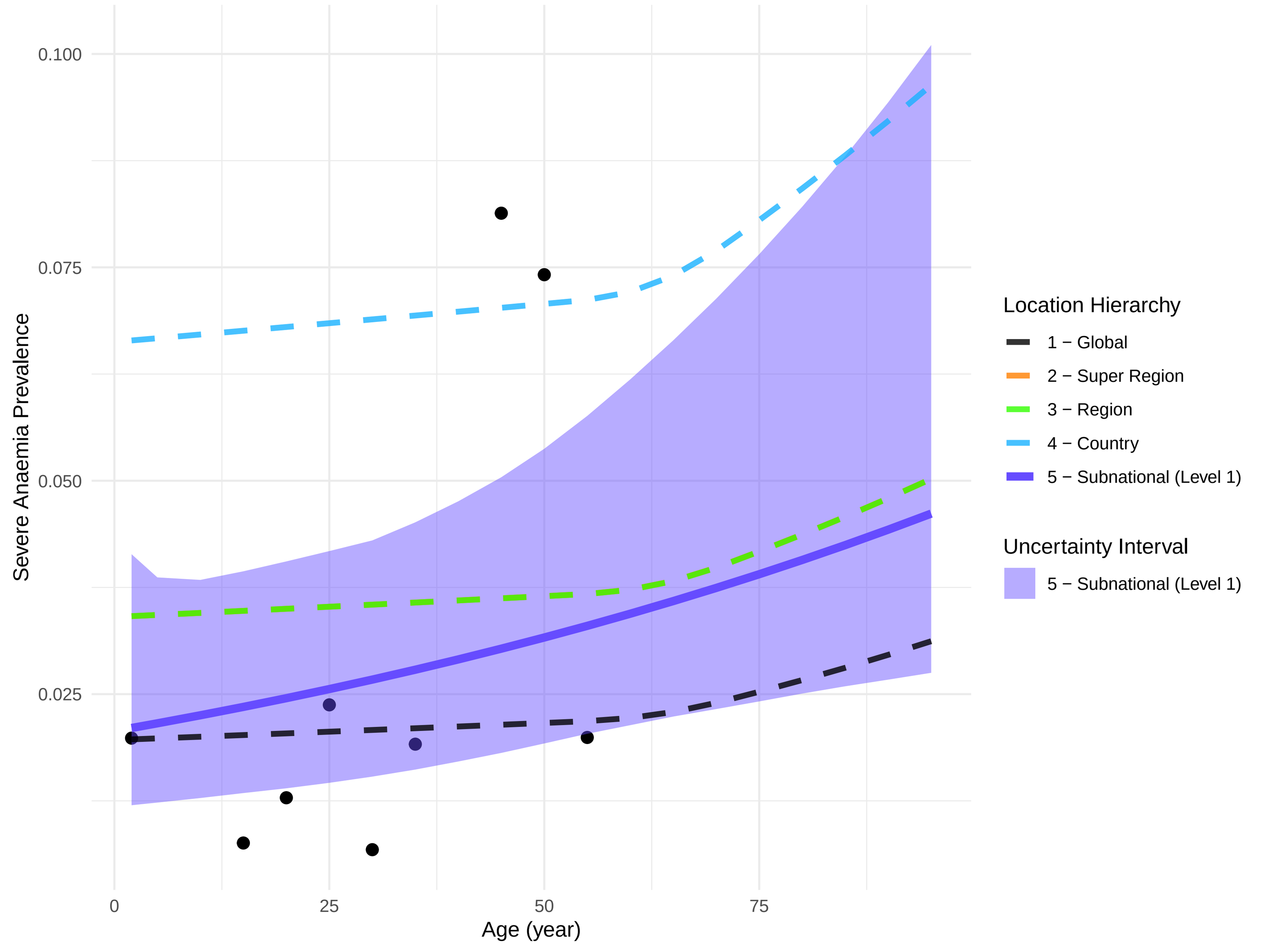

MR−BRT Age/Sex Fits − Non-pregnant Females 2+ Years Old − Severe Anaemia Prevalence − Limpopo
WHO−2024 Adjustment Method

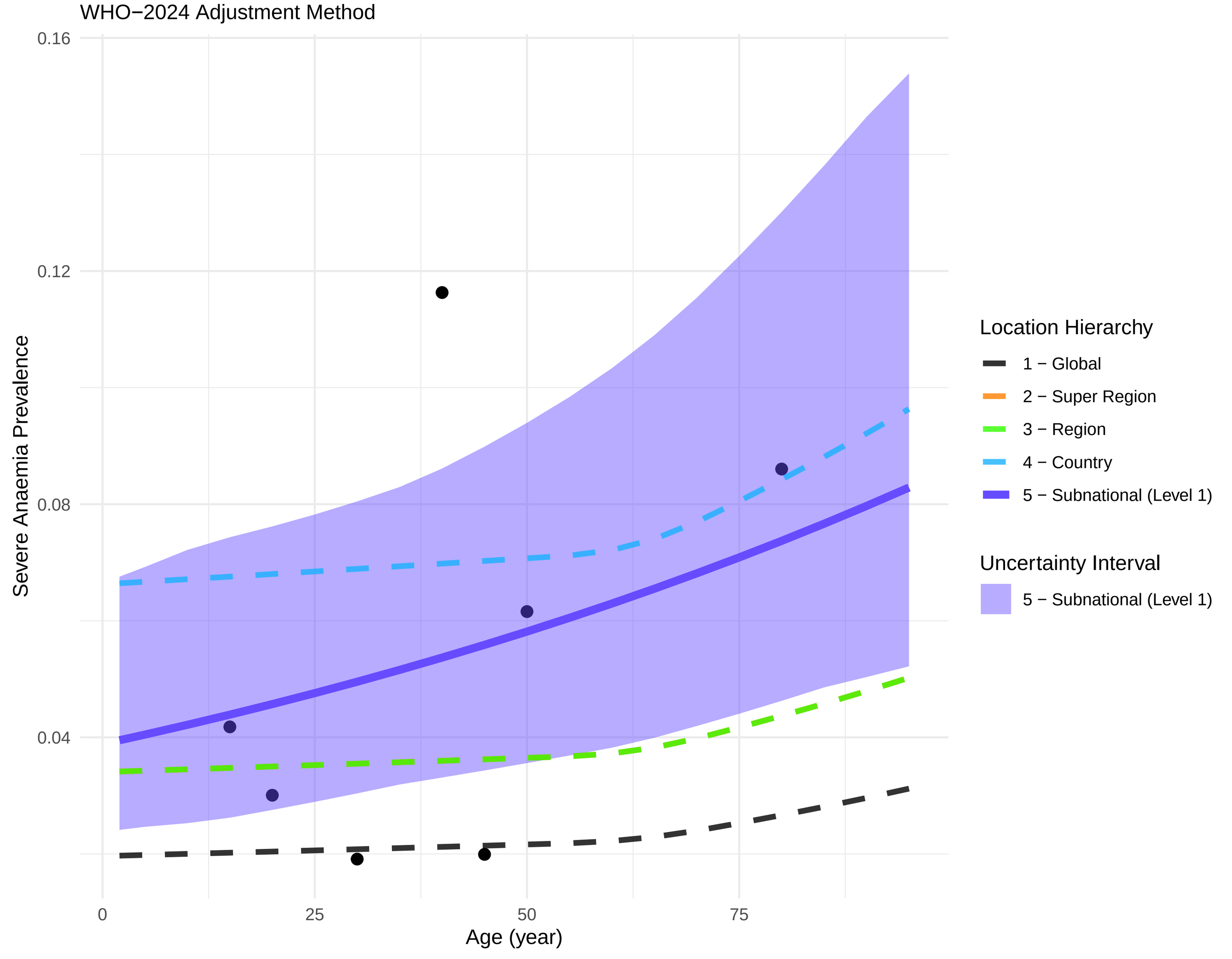

MR−BRT Age/Sex Fits − Non-pregnant Females 2+ Years Old − Severe Anaemia Prevalence − Mpumalanga
WHO−2024 Adjustment Method

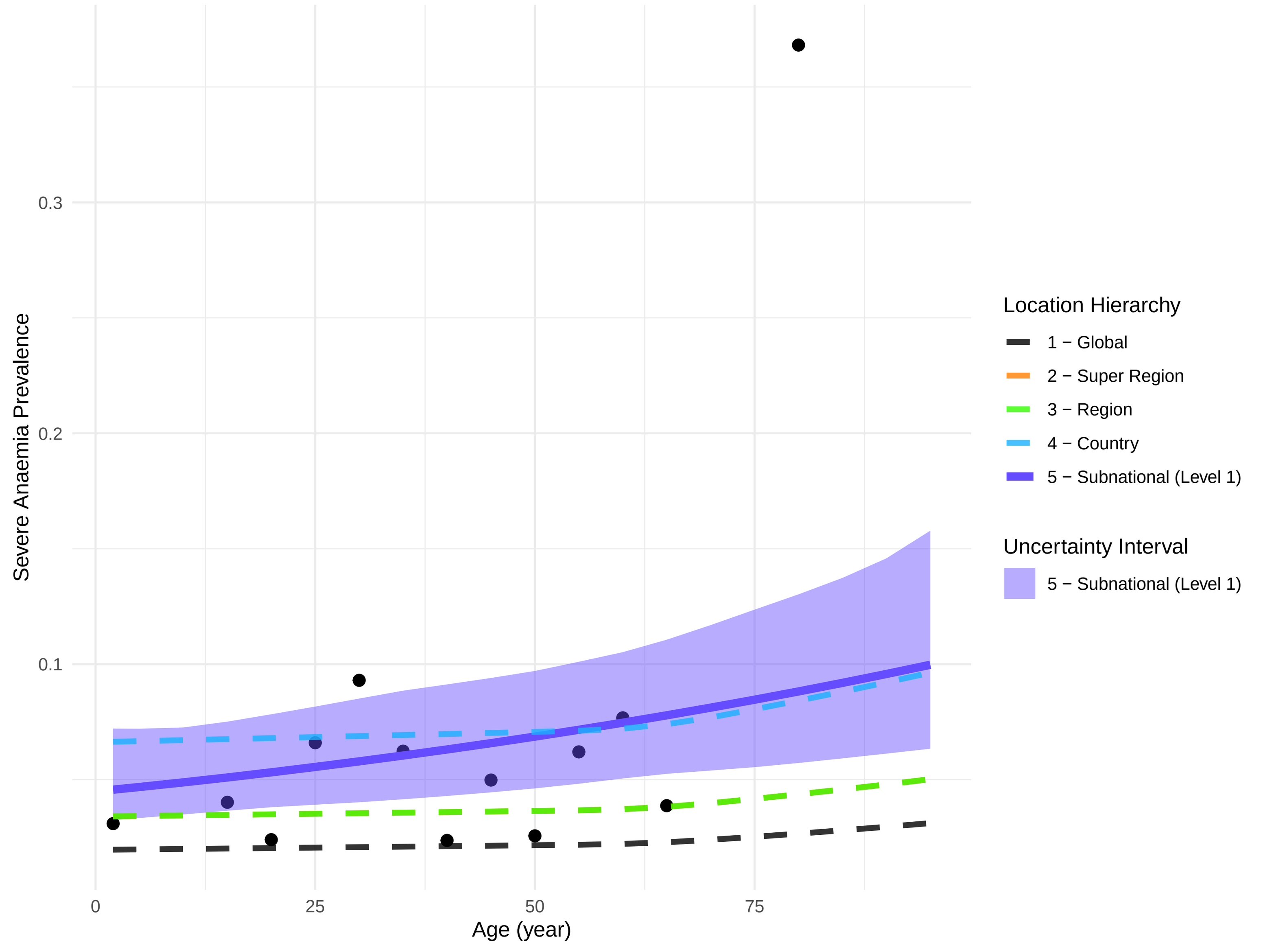

MR−BRT Age/Sex Fits − Non-pregnant Females 2+ Years Old − Severe Anaemia Prevalence − North−West
WHO−2024 Adjustment Method

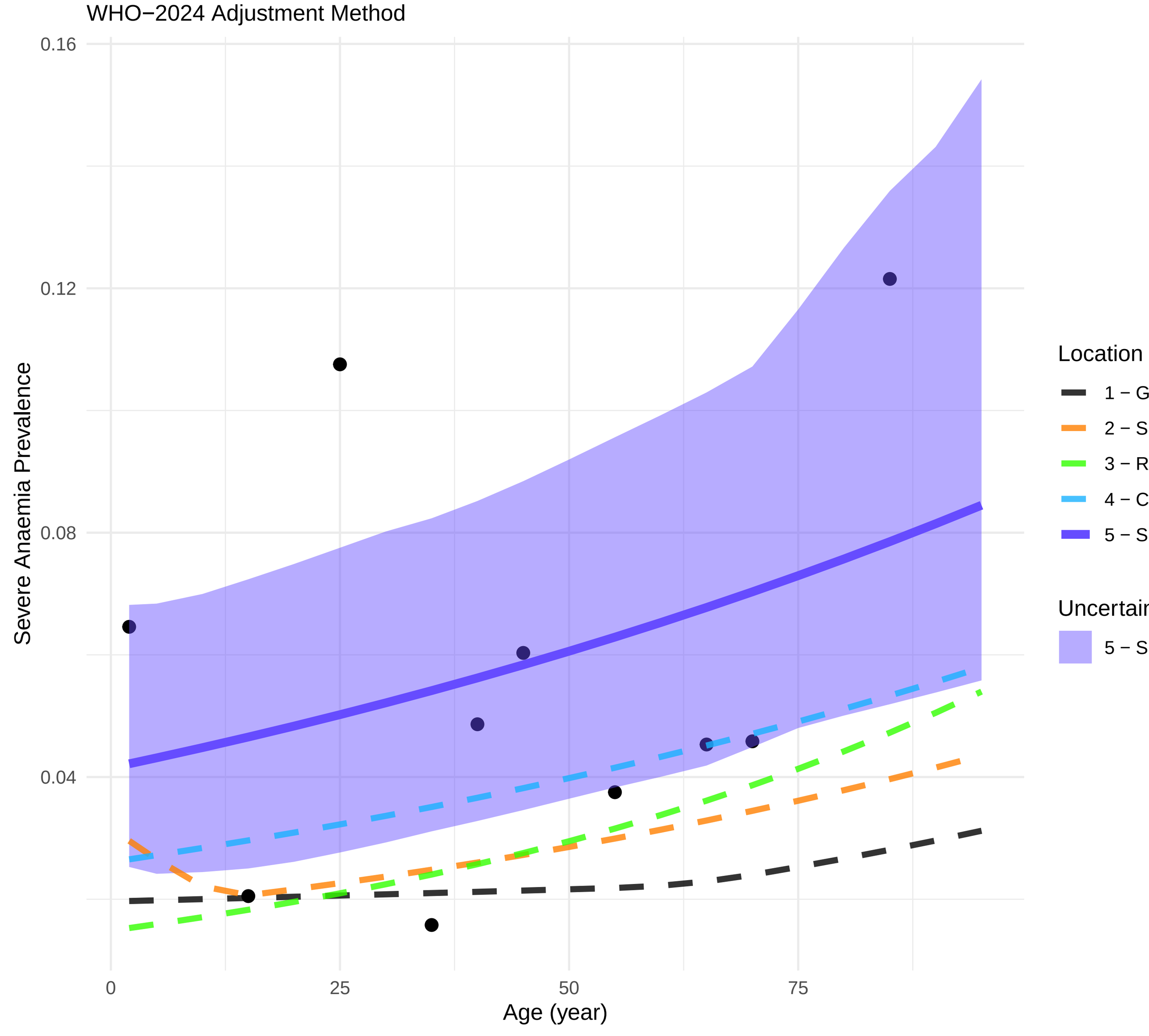

MR−BRT Age/Sex Fits − Non−pregnant Females 2+ Years Old − Severe Anaemia Prevalence − Northern Cape
WHO−2024 Adjustment Method

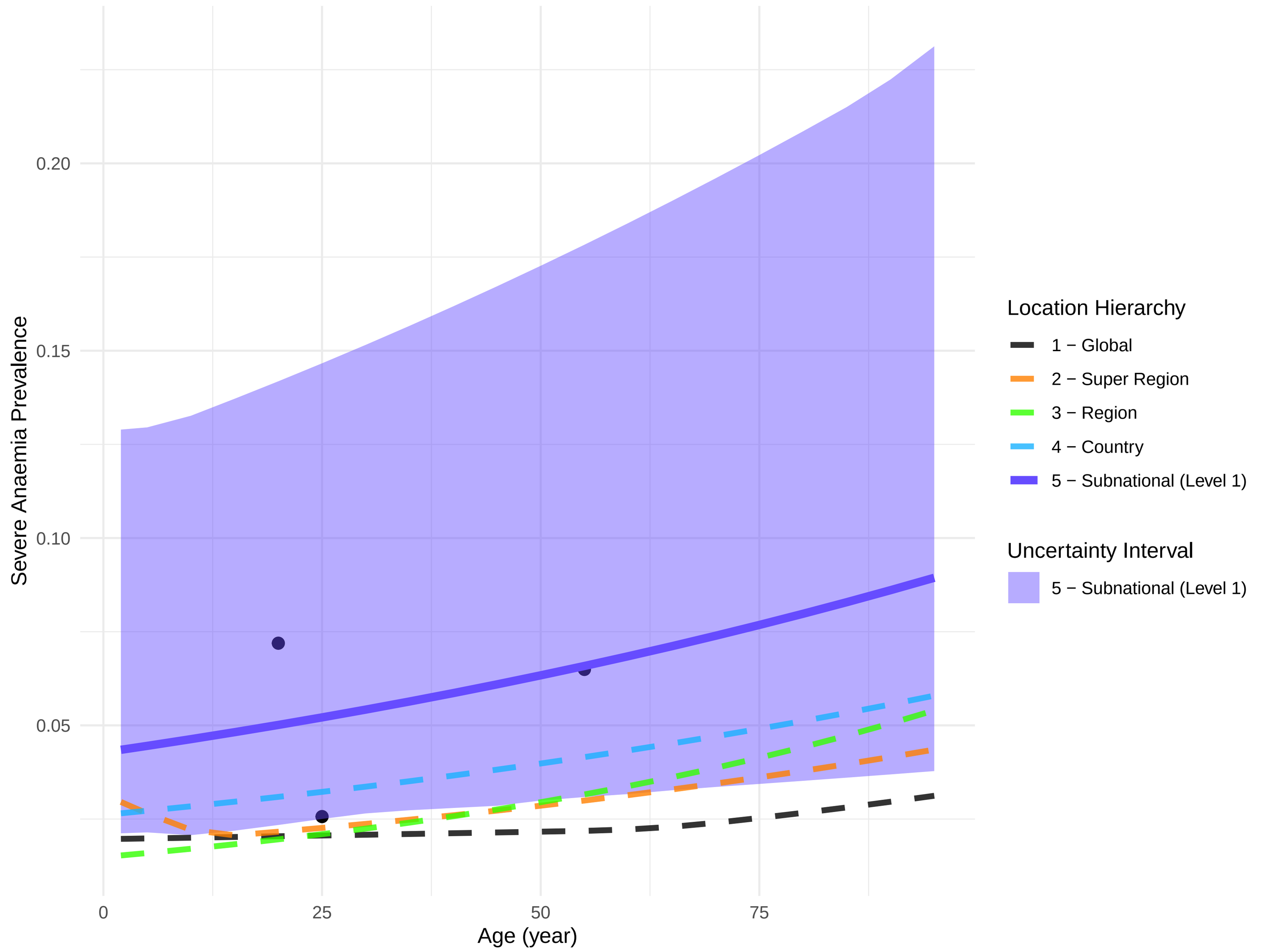

MR−BRT Age/Sex Fits − Non−pregnant Females 2+ Years Old − Severe Anaemia Prevalence − Western Cape
WHO−2024 Adjustment Method

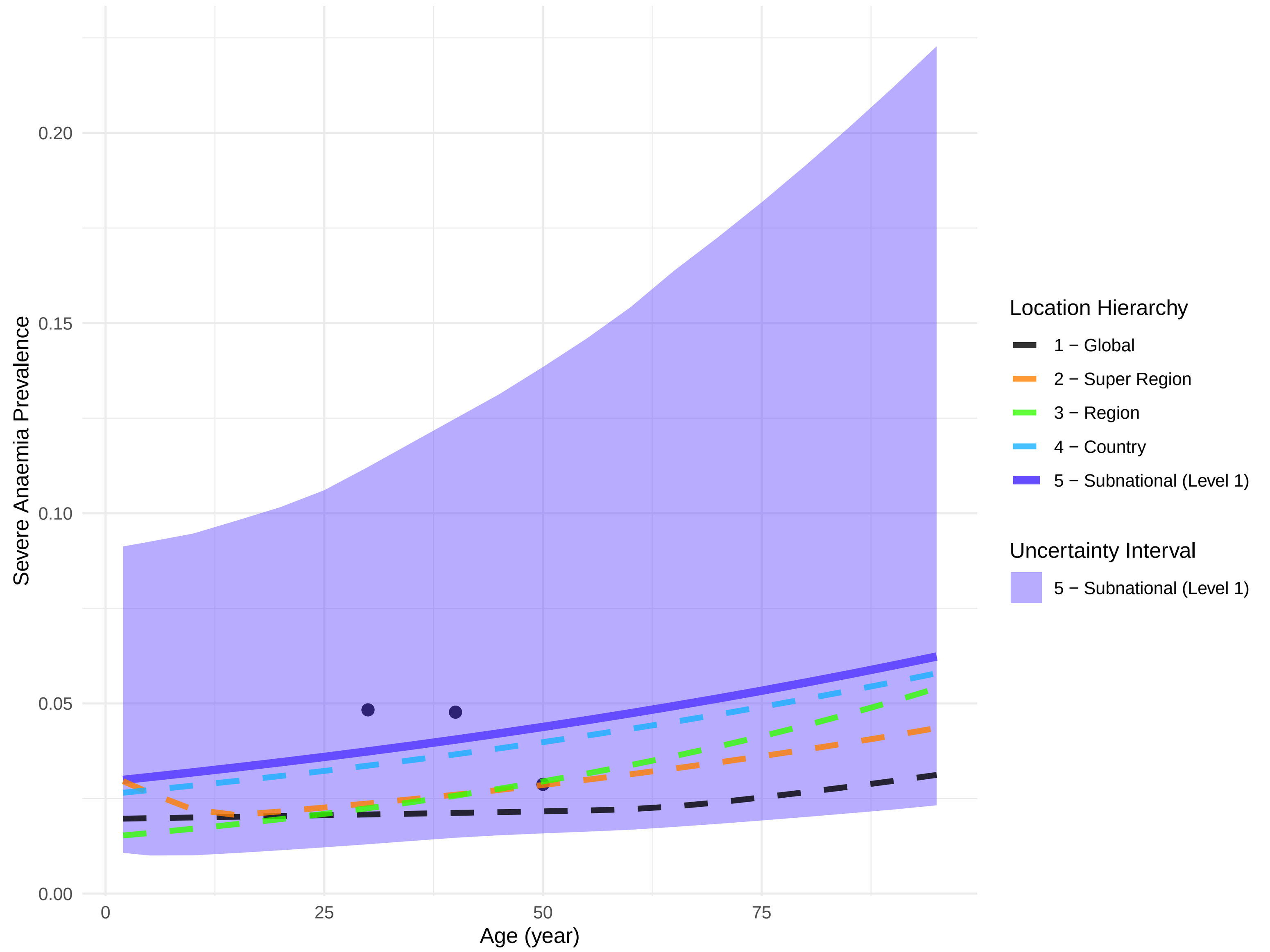

MR−BRT Age/Sex Fits − Non−pregnant Females 2+ Years Old − Severe Anaemia Prevalence − Zimbabwe
WHO−2024 Adjustment Method

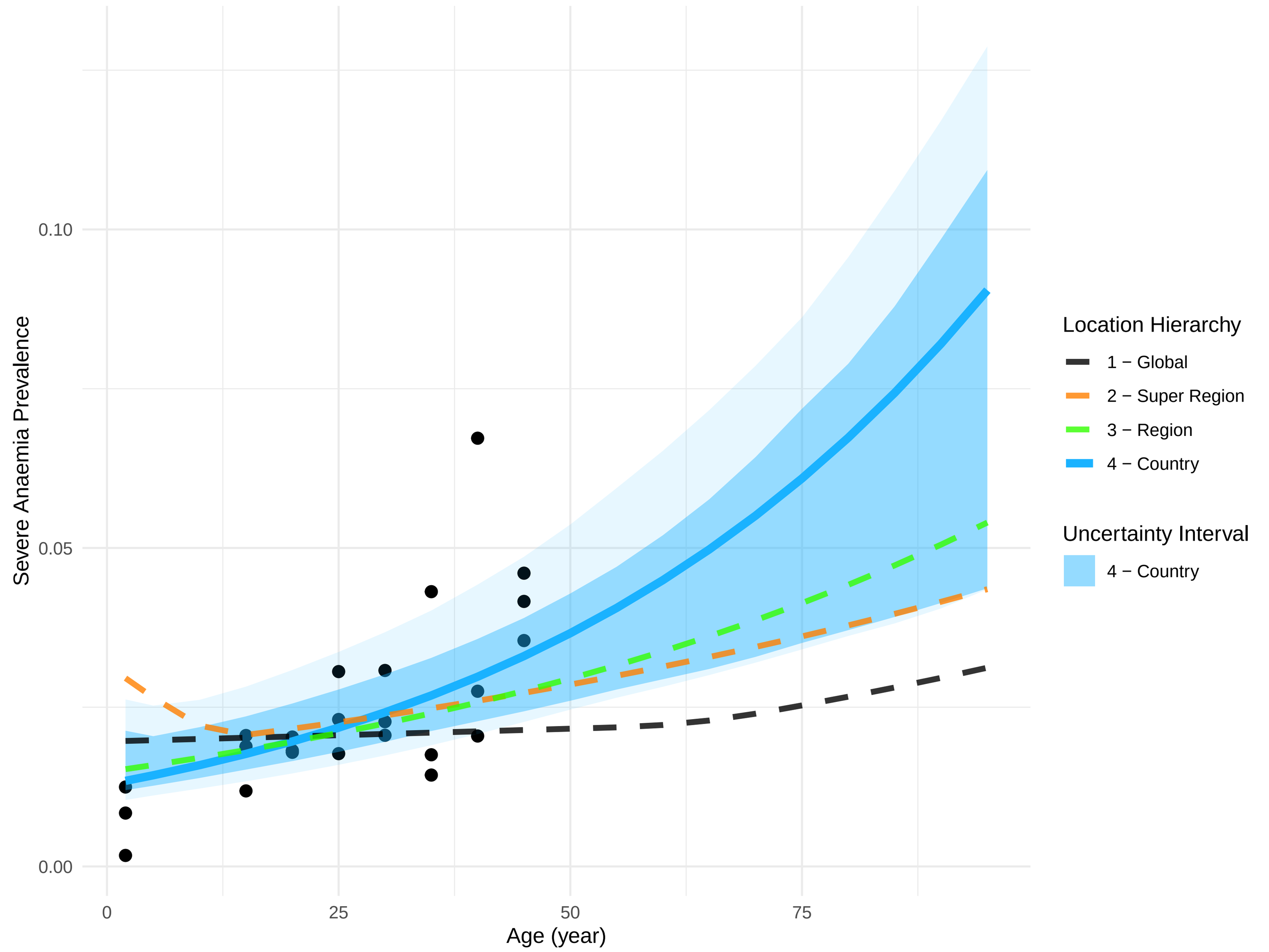

MR−BRT Age/Sex Fits − Non−pregnant Females 2+ Years Old − Severe Anaemia Prevalence − Western Sub−Saharan Africa
WHO−2024 Adjustment Method

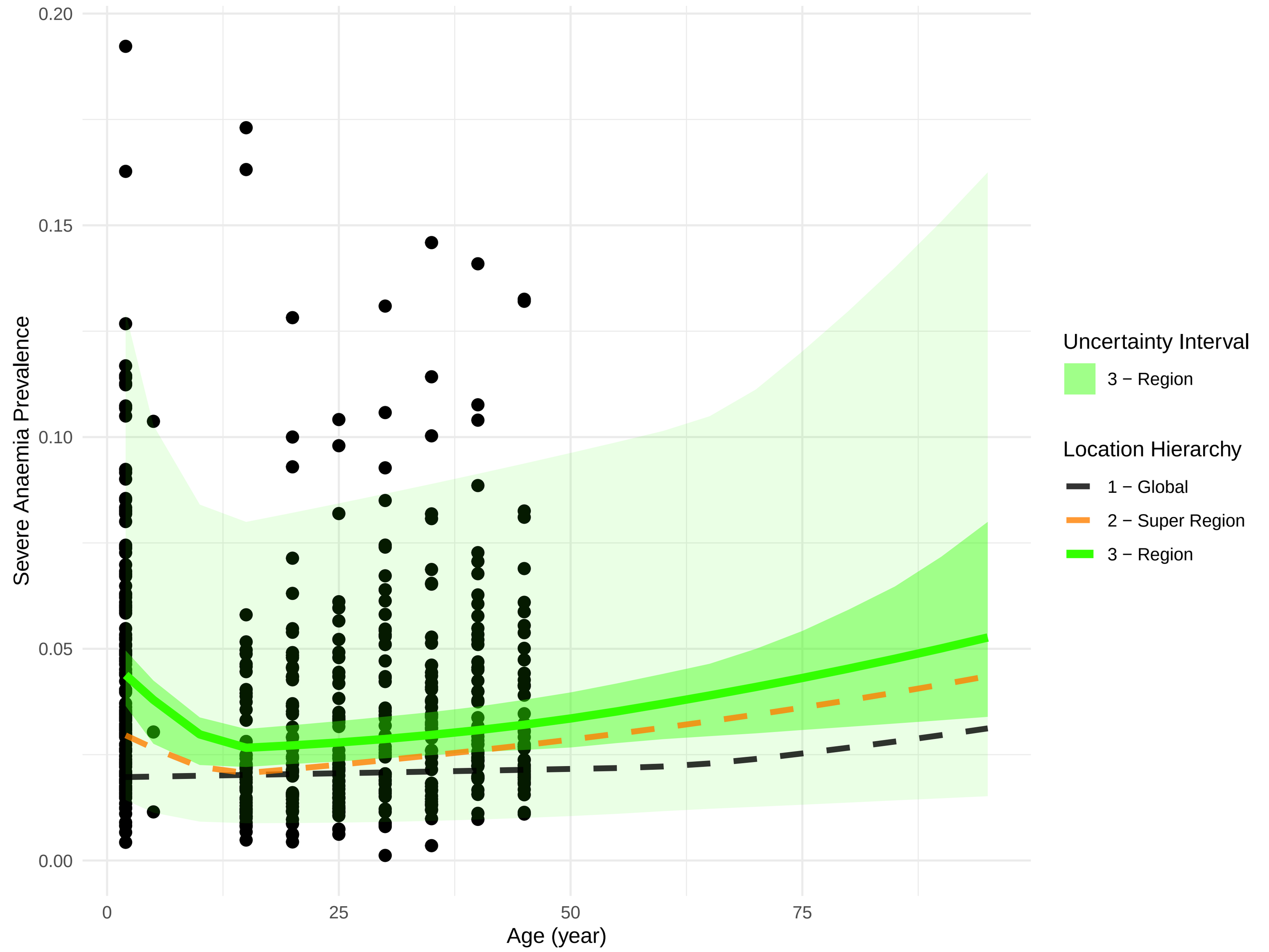

MR−BRT Age/Sex Fits − Non−pregnant Females 2+ Years Old − Severe Anaemia Prevalence − Benin
WHO−2024 Adjustment Method

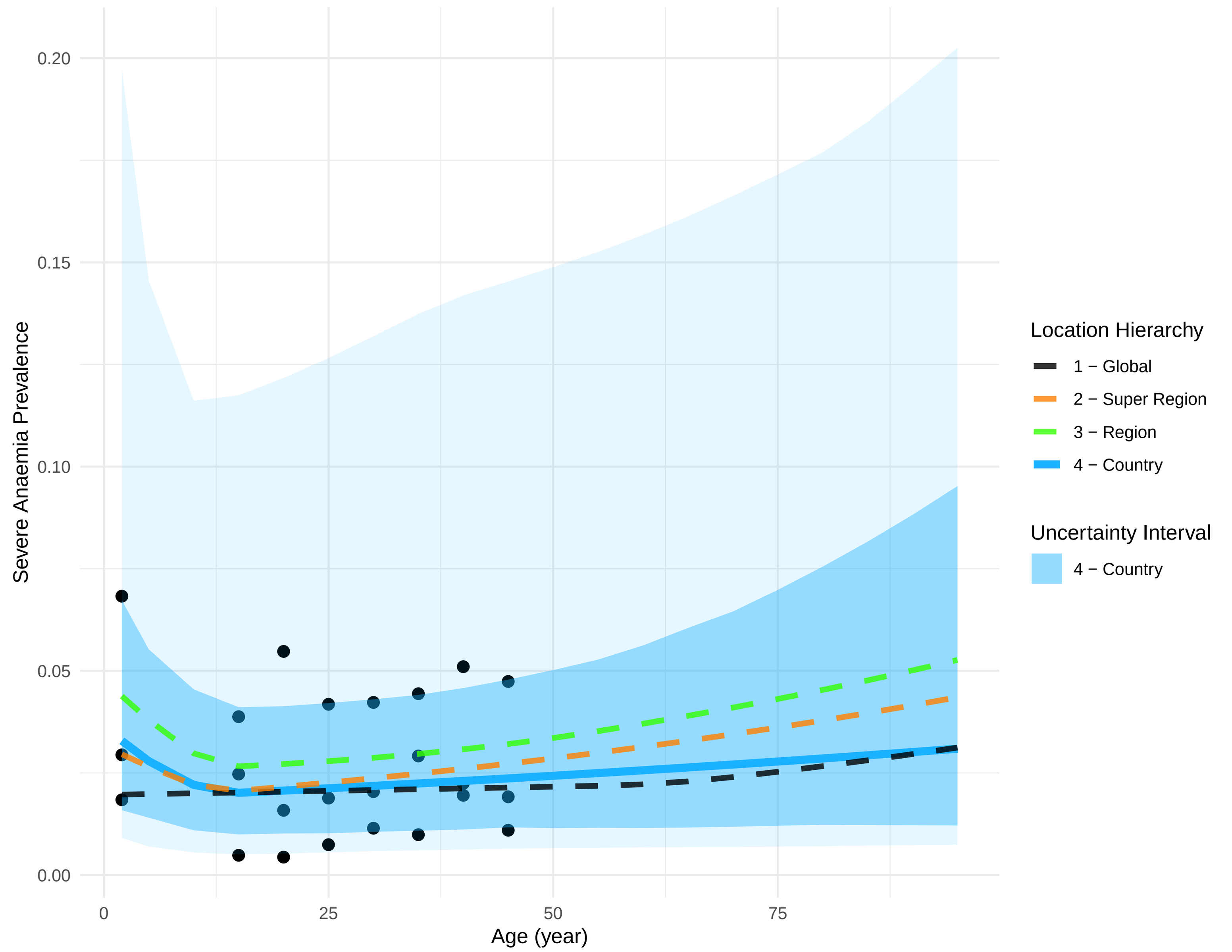

MR−BRT Age/Sex Fits − Non−pregnant Females 2+ Years Old − Severe Anaemia Prevalence − Burkina Faso
WHO−2024 Adjustment Method

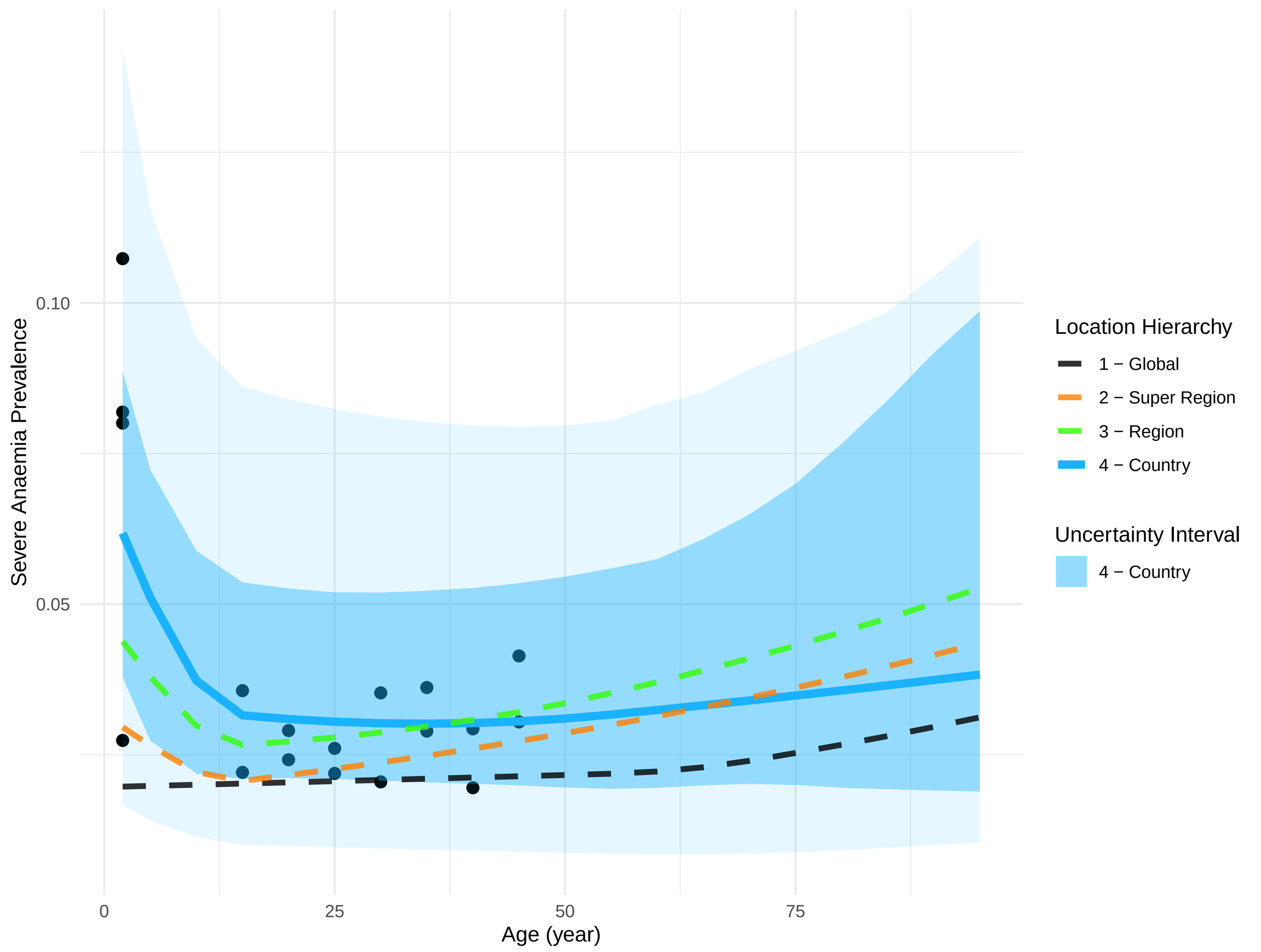

MR−BRT Age/Sex Fits − Non−pregnant Females 2+ Years Old − Severe Anaemia Prevalence − Cameroon
WHO−2024 Adjustment Method

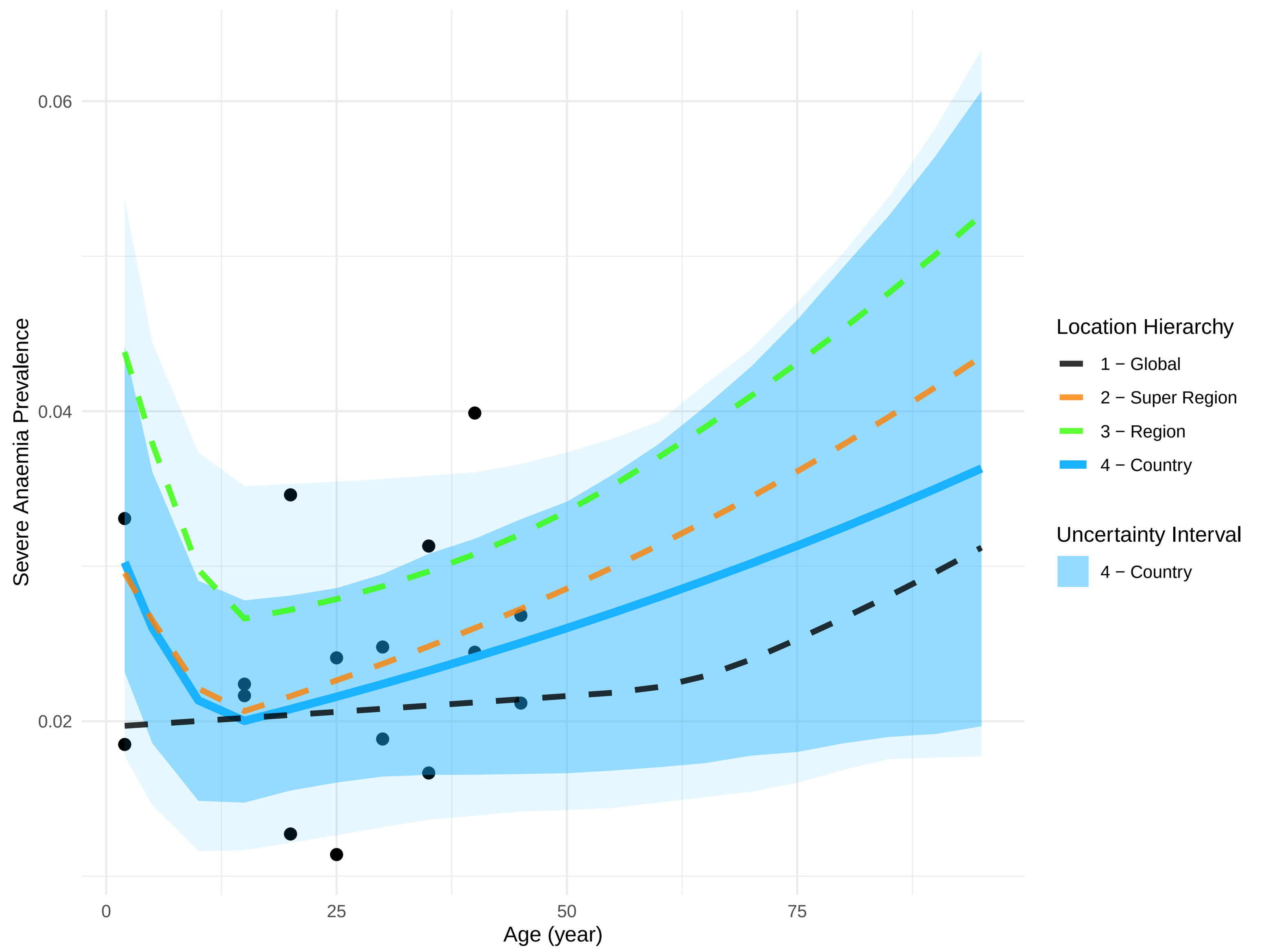

MR−BRT Age/Sex Fits − Non−pregnant Females 2+ Years Old − Severe Anaemia Prevalence − Côte d'Ivoire
WHO−2024 Adjustment Method

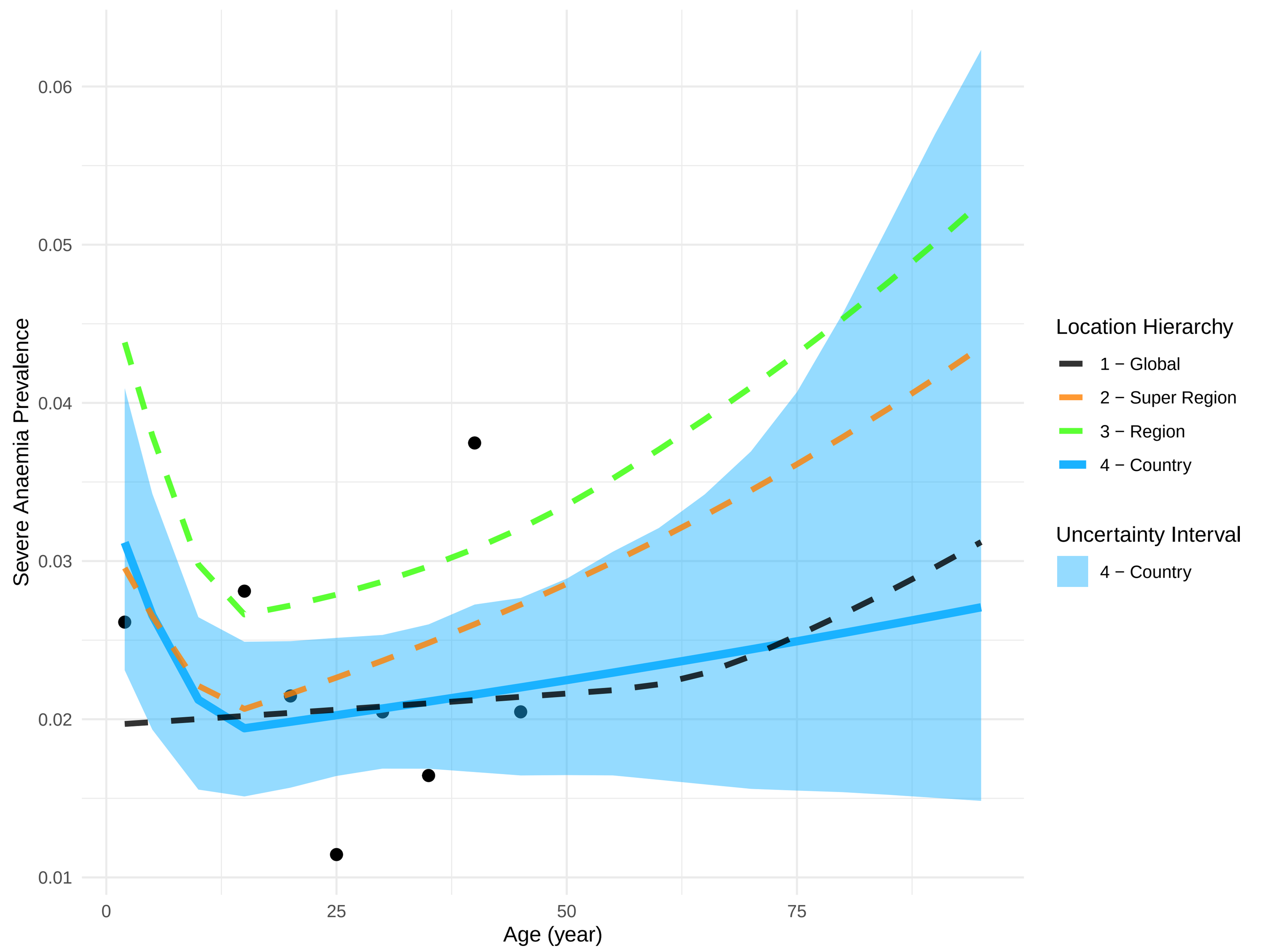

MR−BRT Age/Sex Fits − Non−pregnant Females 2+ Years Old − Severe Anaemia Prevalence − Gambia
WHO−2024 Adjustment Method

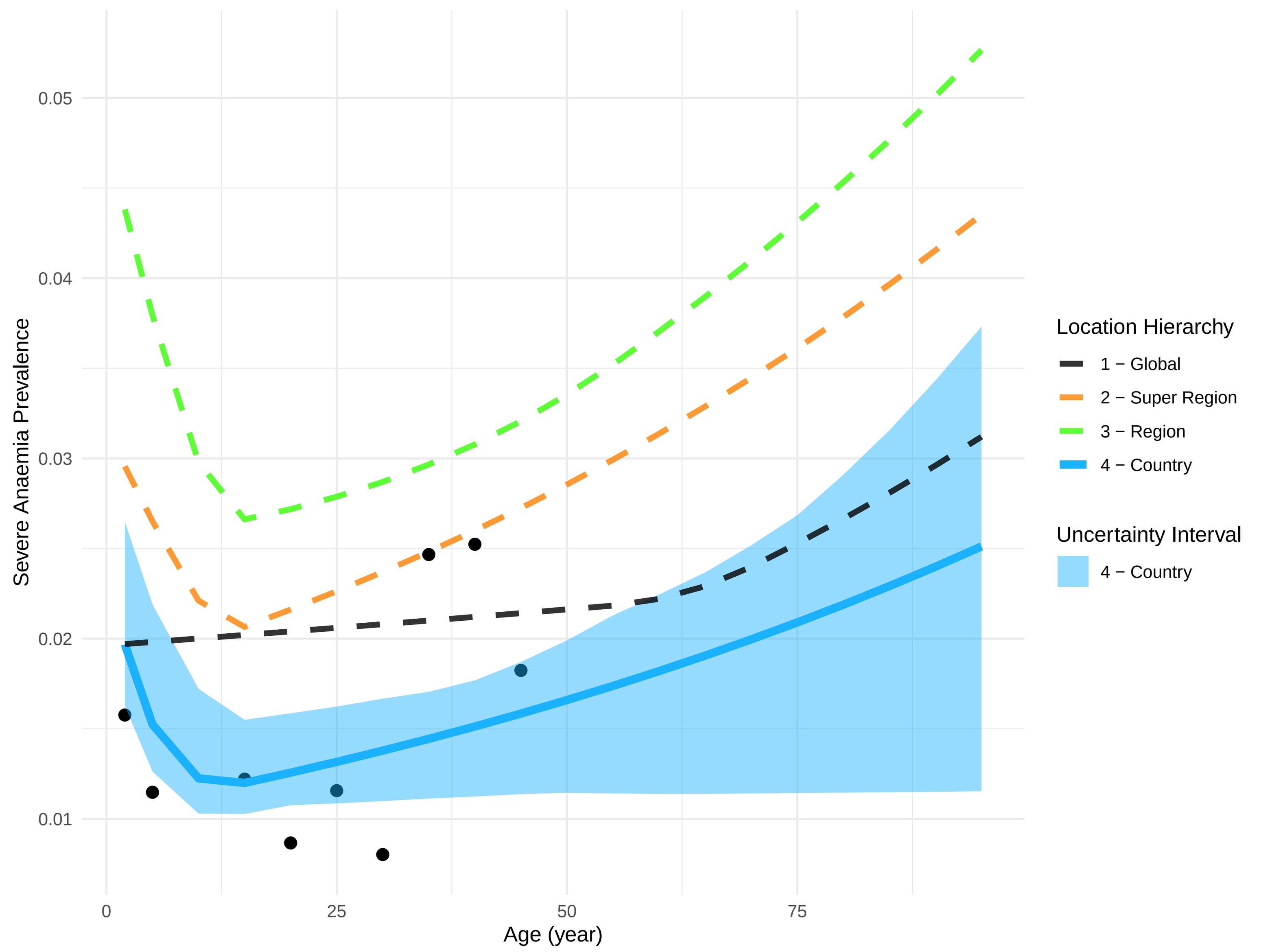

MR−BRT Age/Sex Fits − Non−pregnant Females 2+ Years Old − Severe Anaemia Prevalence − Ghana
WHO−2024 Adjustment Method

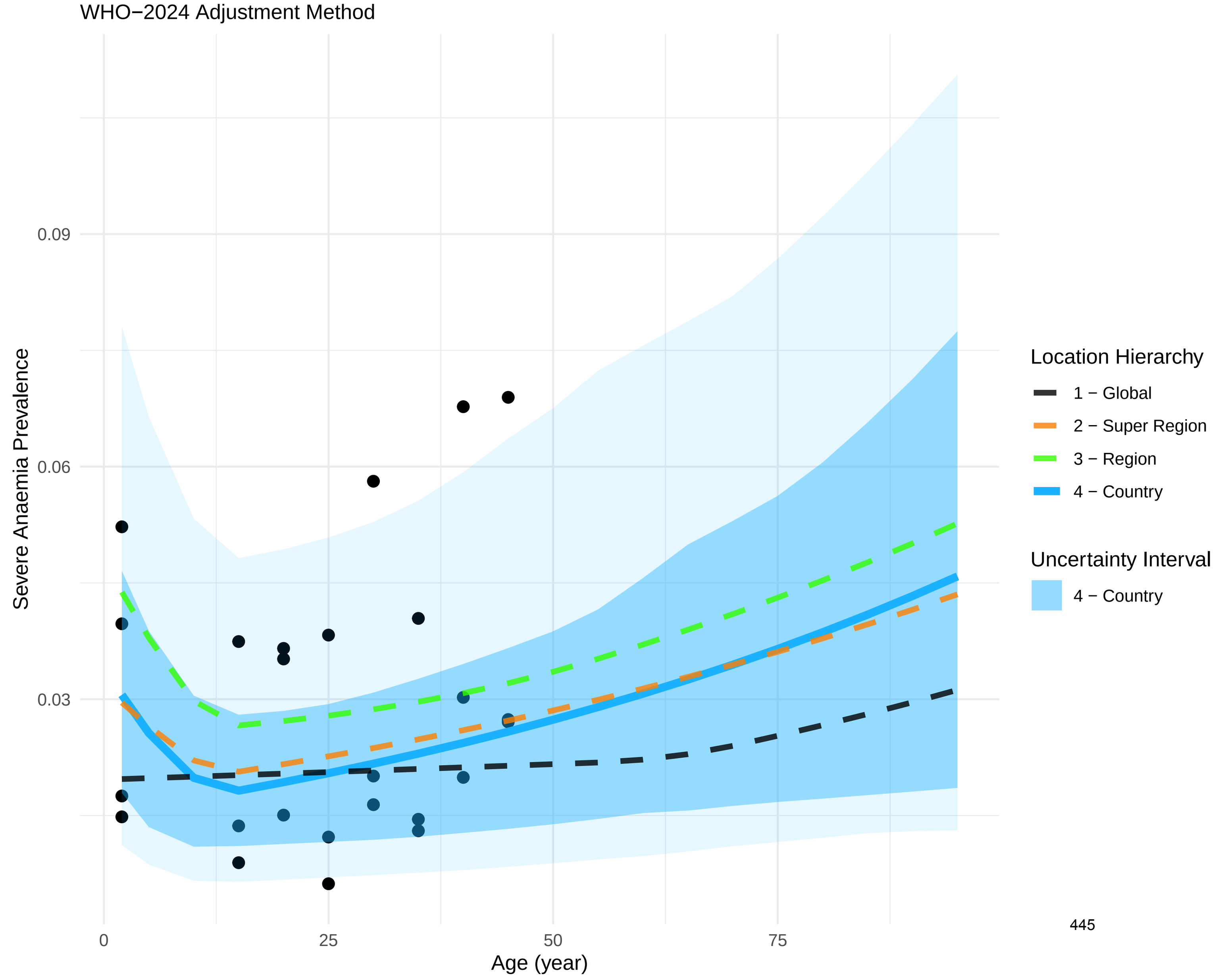

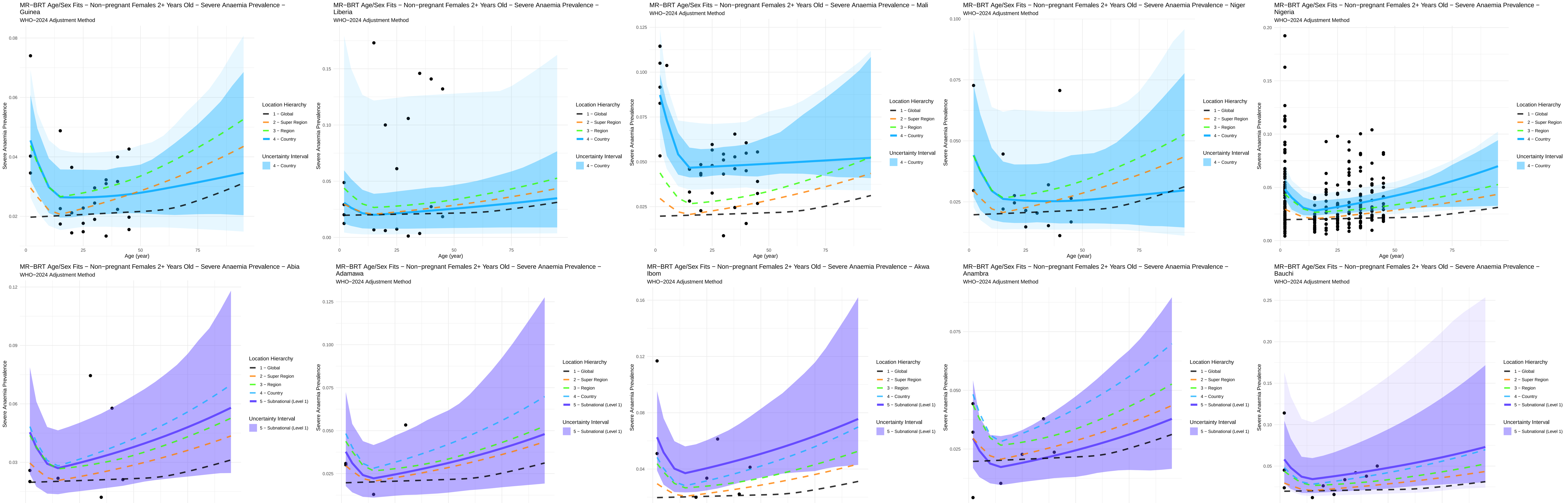
MR−BRT Age/Sex Fits − Non−pregnant Females 2+ Years Old − Severe Anaemia Prevalence − Guinea
WHO−2024 Adjustment Method
MR−BRT Age/Sex Fits − Non−pregnant Females 2+ Years Old − Severe Anaemia Prevalence − Liberia
WHO−2024 Adjustment Method
MR−BRT Age/Sex Fits − Non−pregnant Females 2+ Years Old − Severe Anaemia Prevalence − Mali
WHO−2024 Adjustment Method
MR−BRT Age/Sex Fits − Non−pregnant Females 2+ Years Old − Severe Anaemia Prevalence − Niger
WHO−2024 Adjustment Method
MR−BRT Age/Sex Fits − Non−pregnant Females 2+ Years Old − Severe Anaemia Prevalence − Nigeria
WHO−2024 Adjustment Method
Location Hierarchy
1 − Global
2 − Super Region
3 − Region
4 − Country
Uncertainty Interval
4 − Country
MR−BRT Age/Sex Fits − Non−pregnant Females 2+ Years Old − Severe Anaemia Prevalence − Abia
WHO−2024 Adjustment Method
MR−BRT Age/Sex Fits − Non−pregnant Females 2+ Years Old − Severe Anaemia Prevalence − Adamawa
WHO−2024 Adjustment Method
MR−BRT Age/Sex Fits − Non−pregnant Females 2+ Years Old − Severe Anaemia Prevalence − Akwa Ibom
WHO−2024 Adjustment Method
MR−BRT Age/Sex Fits − Non−pregnant Females 2+ Years Old − Severe Anaemia Prevalence − Anambra
WHO−2024 Adjustment Method
MR−BRT Age/Sex Fits − Non−pregnant Females 2+ Years Old − Severe Anaemia Prevalence − Bauchi
WHO−2024 Adjustment Method
Location Hierarchy
1 − Global
2 − Super Region
3 − Region
4 − Country
5 − Subnational (Level 1)
Uncertainty Interval
5 − Subnational (Level 1)
Severe Anaemia Prevalence
Age (year)

MR−BRT Age/Sex Fits − Non−pregnant Females 2+ Years Old − Severe Anaemia Prevalence − Bayelsa
WHO−2024 Adjustment Method

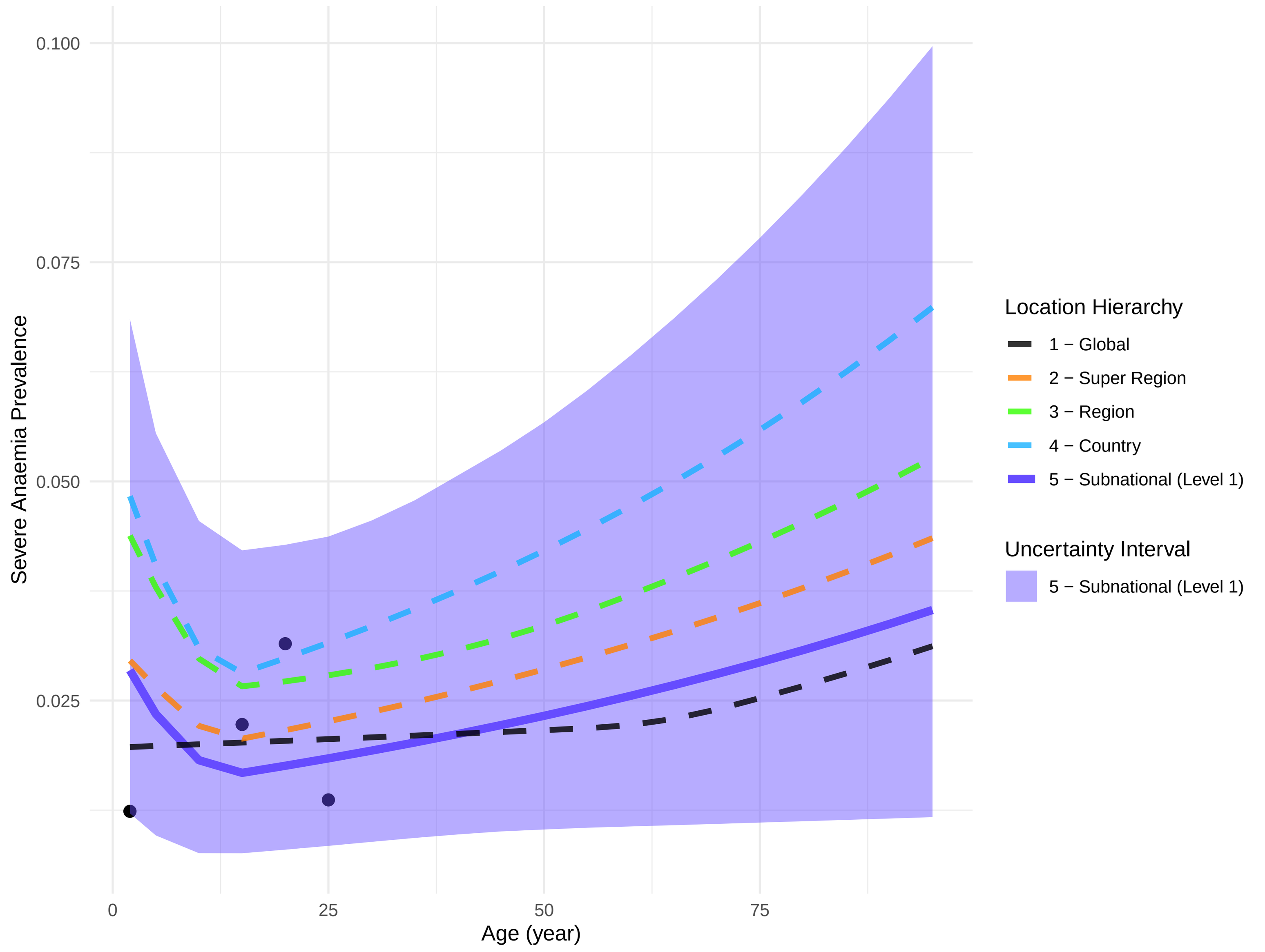

MR−BRT Age/Sex Fits − Non−pregnant Females 2+ Years Old − Severe Anaemia Prevalence − Benue
WHO−2024 Adjustment Method

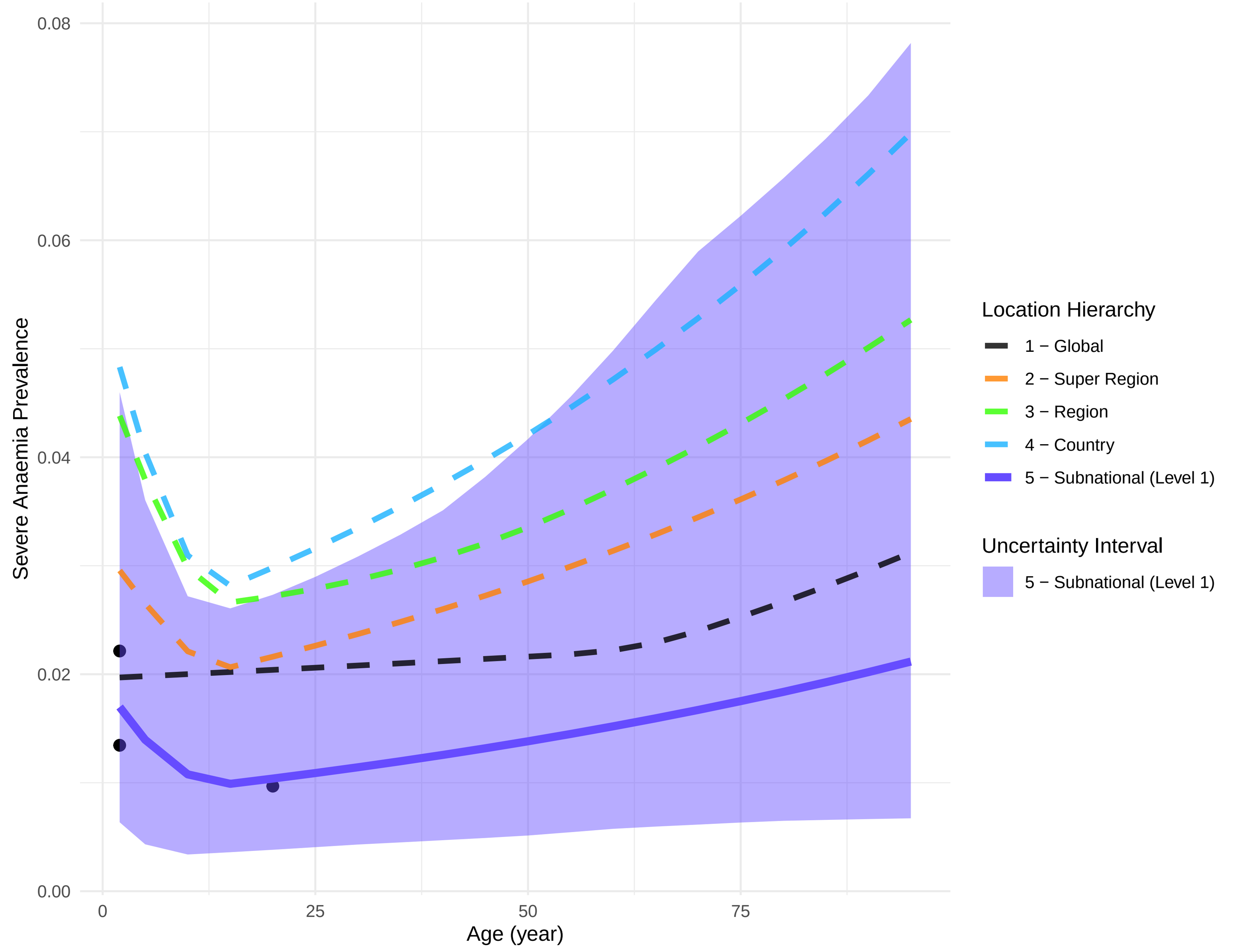

MR−BRT Age/Sex Fits − Non−pregnant Females 2+ Years Old − Severe Anaemia Prevalence − Borno
WHO−2024 Adjustment Method

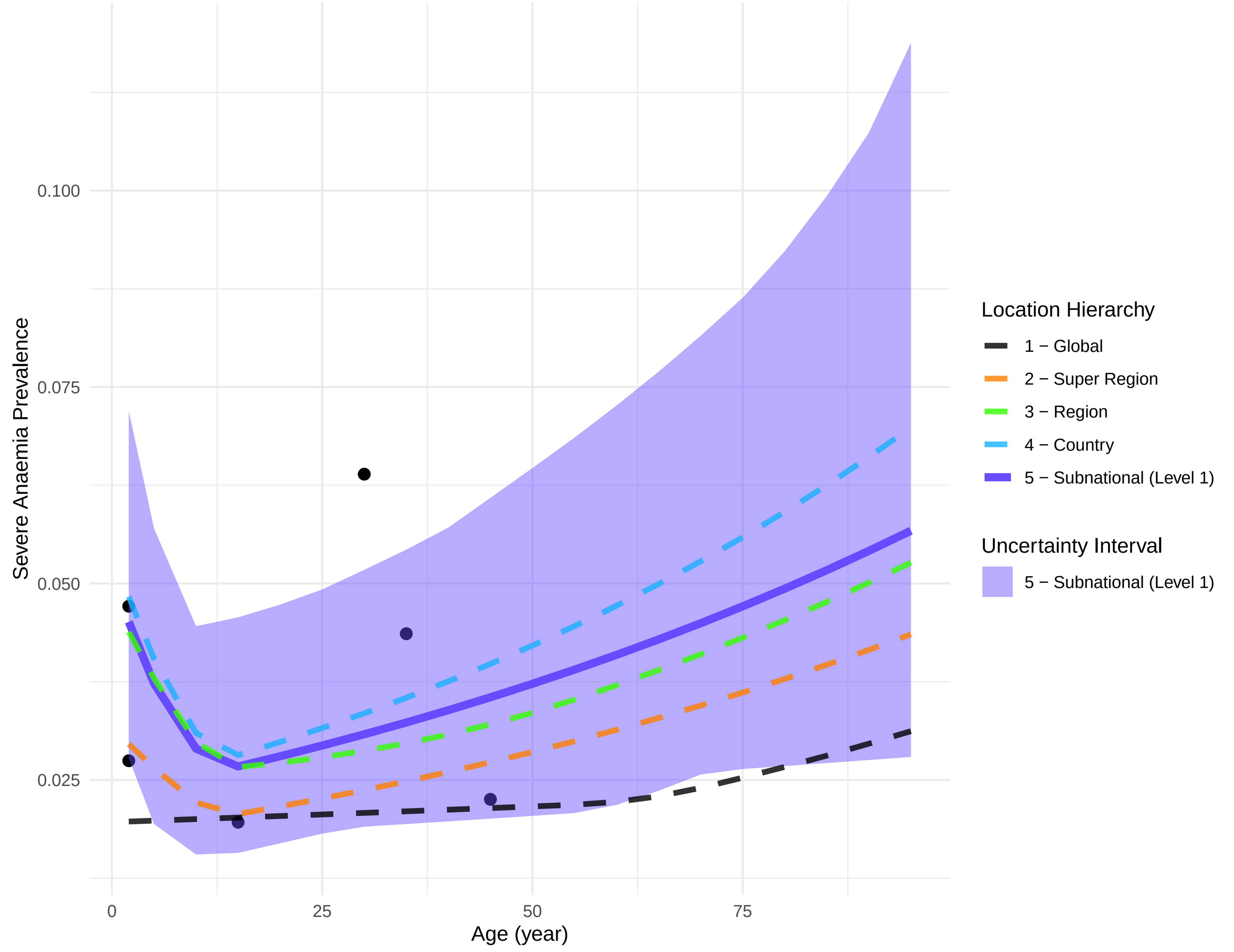

MR−BRT Age/Sex Fits − Non−pregnant Females 2+ Years Old − Severe Anaemia Prevalence − Cross River
WHO−2024 Adjustment Method

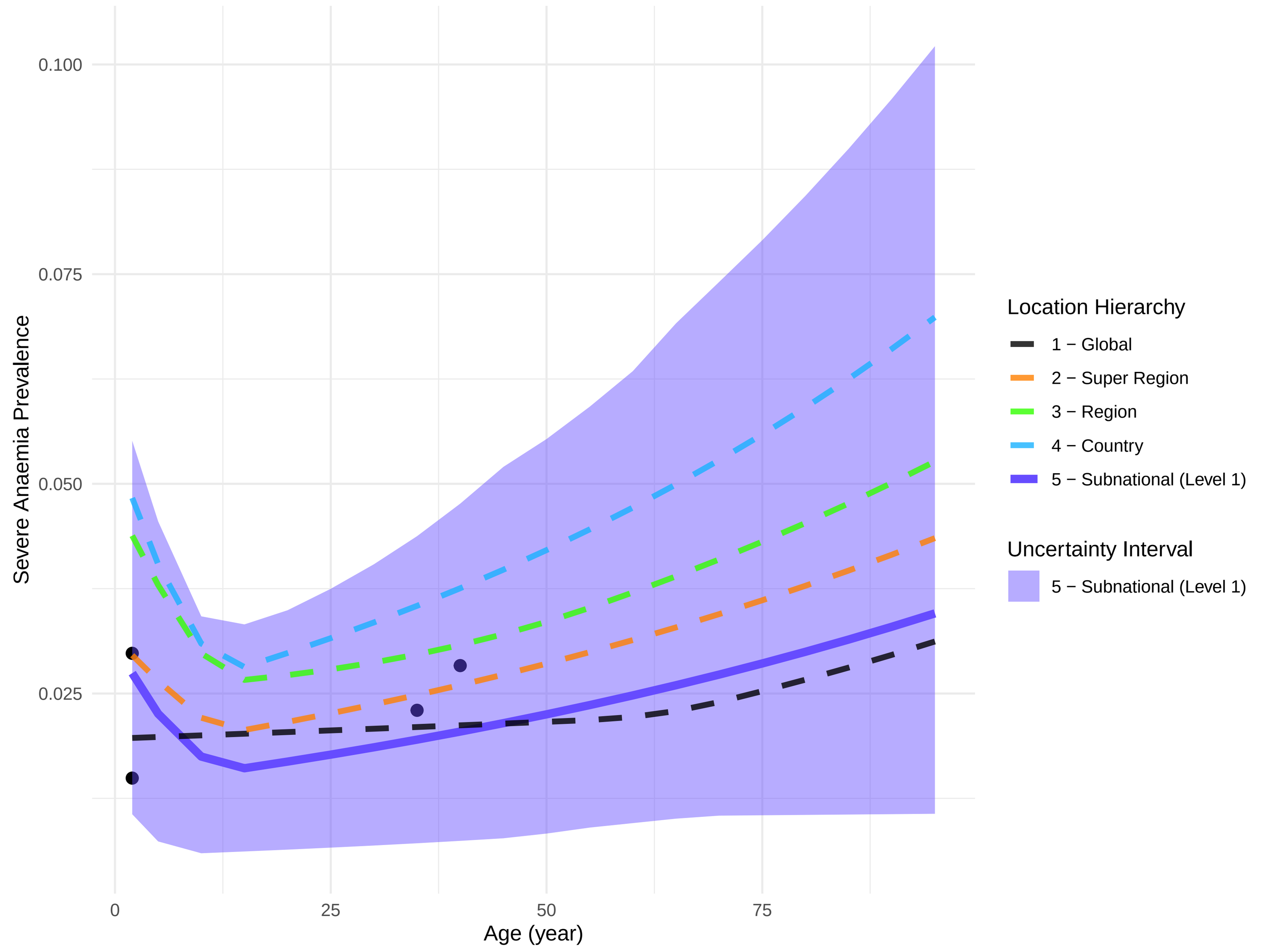

MR−BRT Age/Sex Fits − Non−pregnant Females 2+ Years Old − Severe Anaemia Prevalence − Delta
WHO−2024 Adjustment Method

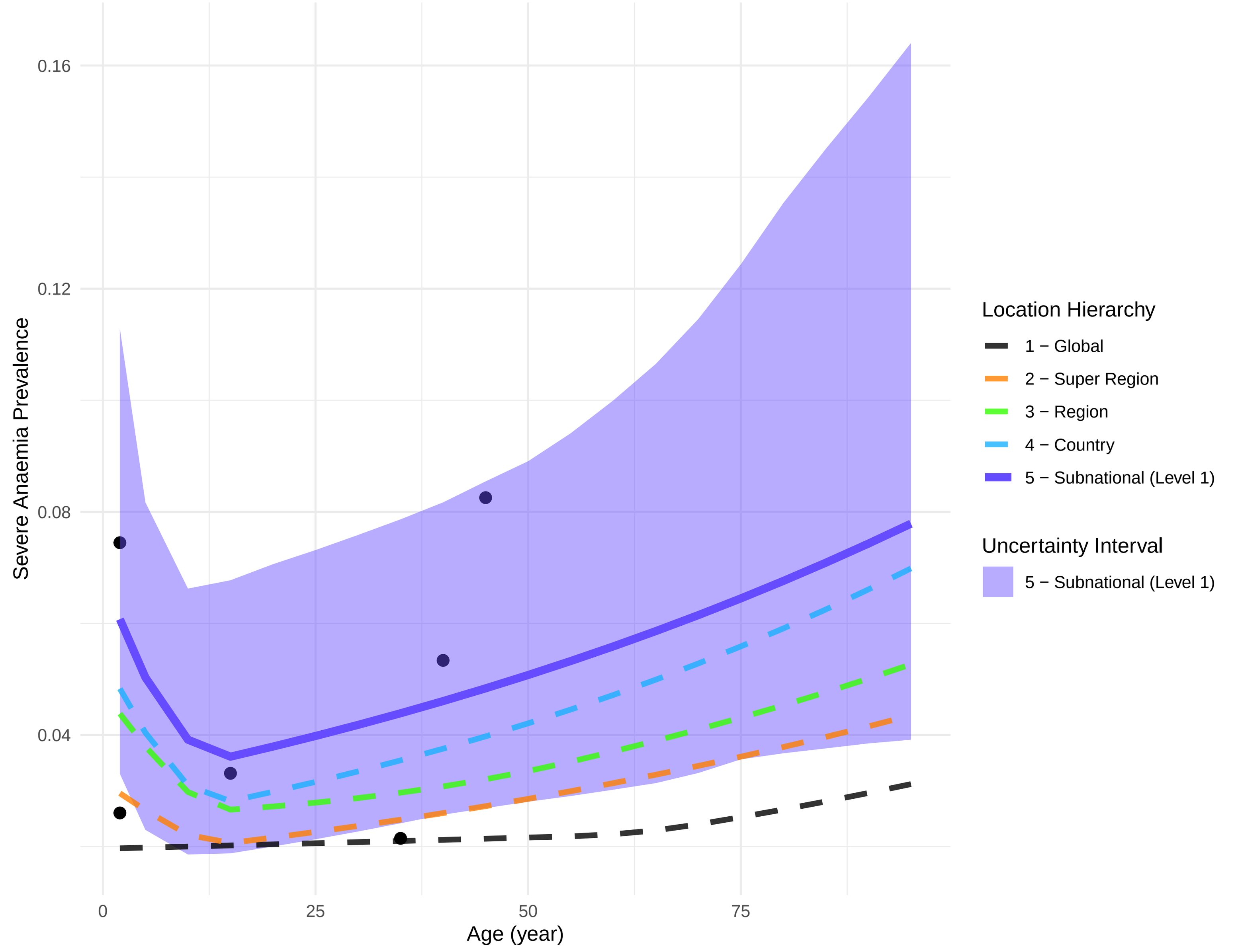

MR−BRT Age/Sex Fits − Non−pregnant Females 2+ Years Old − Severe Anaemia Prevalence − Ebonyi
WHO−2024 Adjustment Method

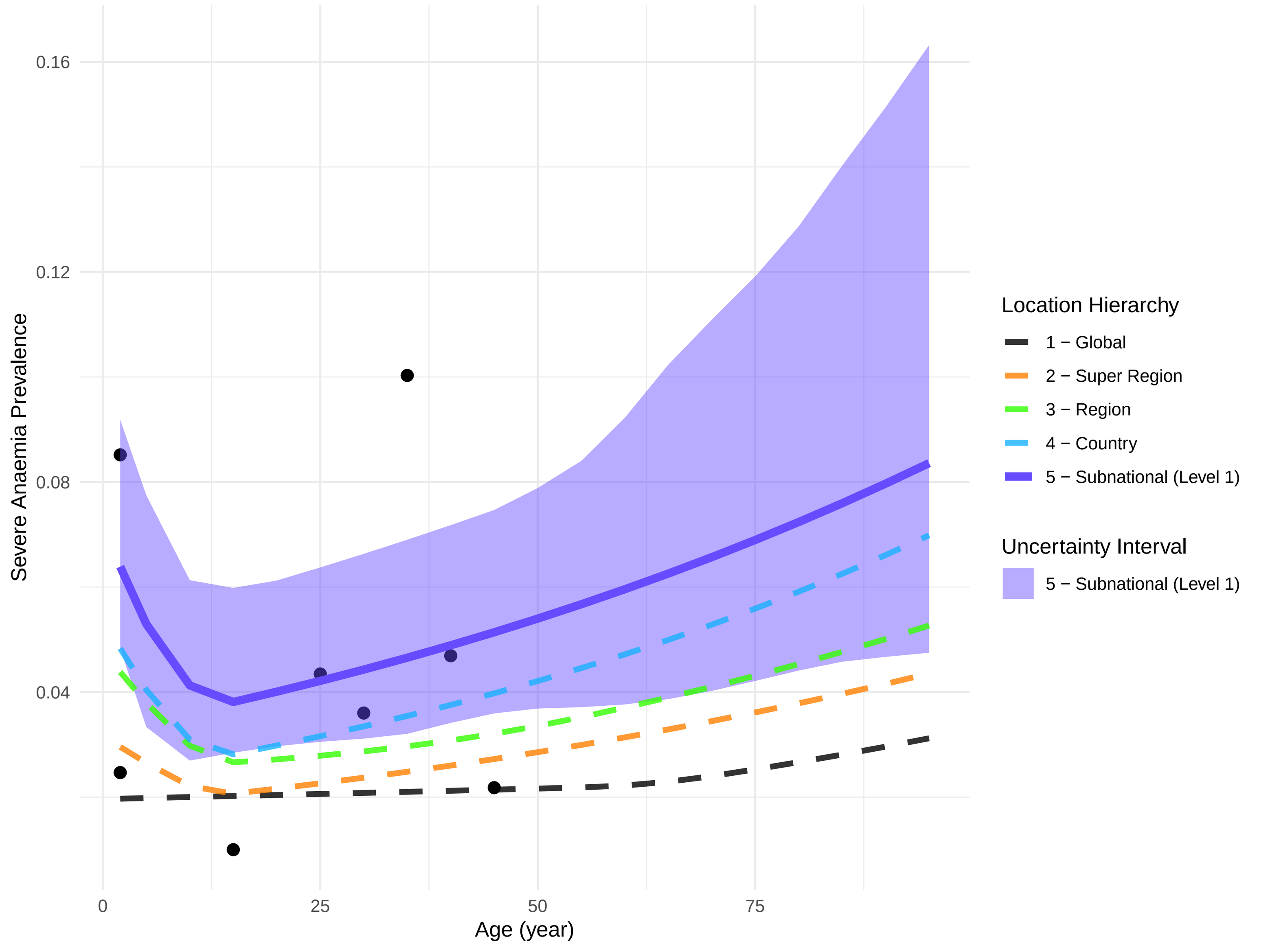

MR−BRT Age/Sex Fits − Non−pregnant Females 2+ Years Old − Severe Anaemia Prevalence − Edo
WHO−2024 Adjustment Method

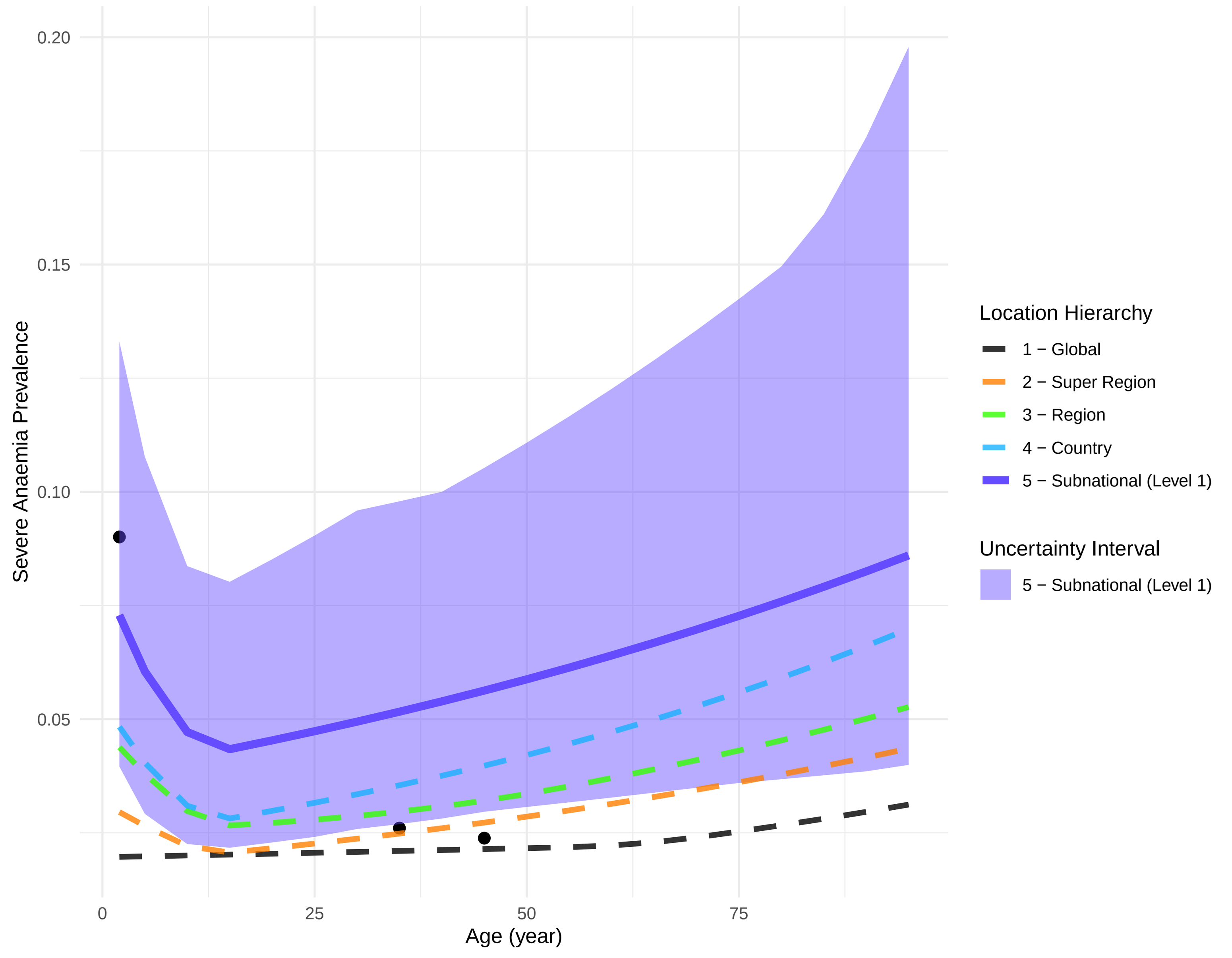

MR−BRT Age/Sex Fits − Non−pregnant Females 2+ Years Old − Severe Anaemia Prevalence − Ekiti
WHO−2024 Adjustment Method

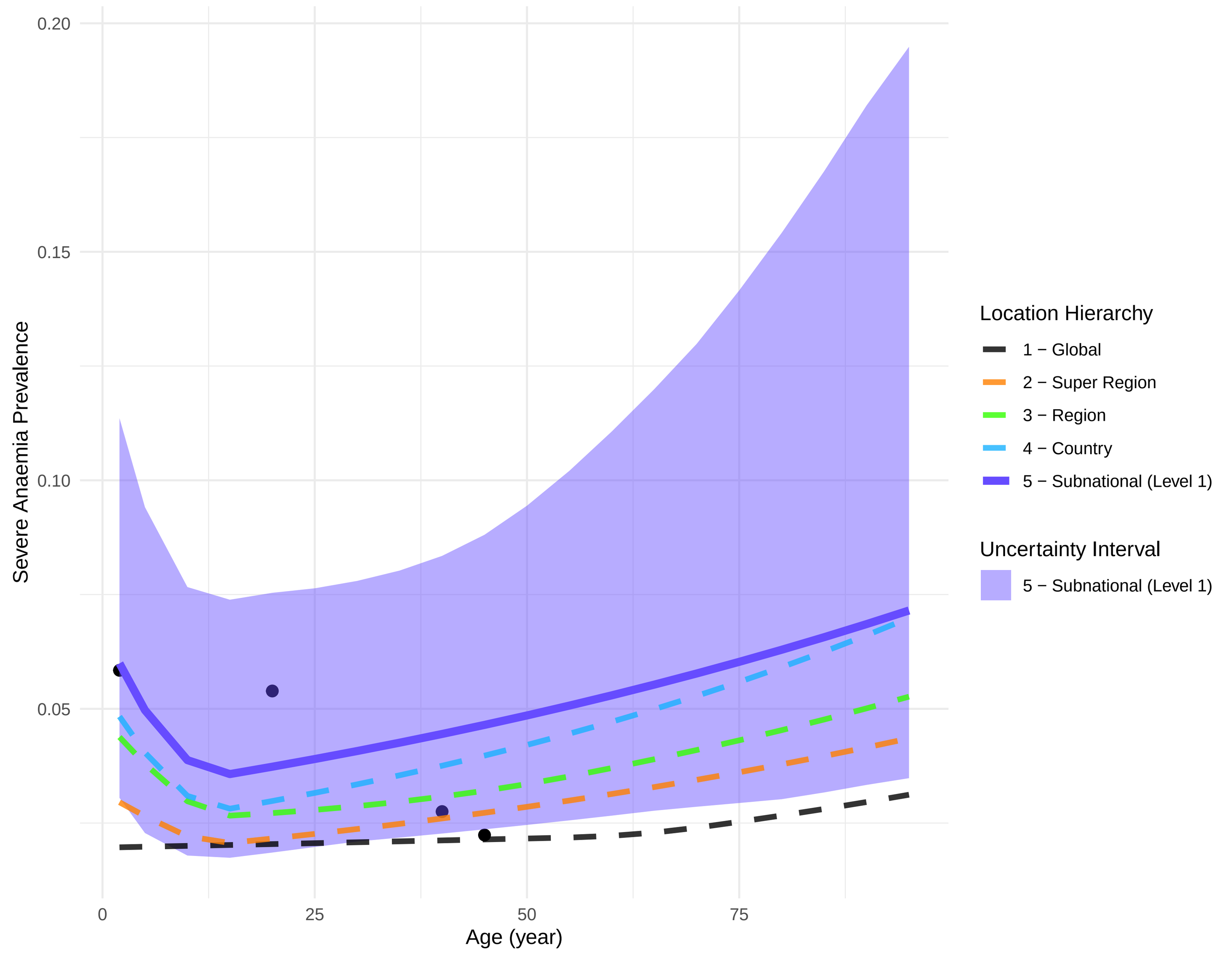

MR−BRT Age/Sex Fits − Non−pregnant Females 2+ Years Old − Severe Anaemia Prevalence − Enugu
WHO−2024 Adjustment Method

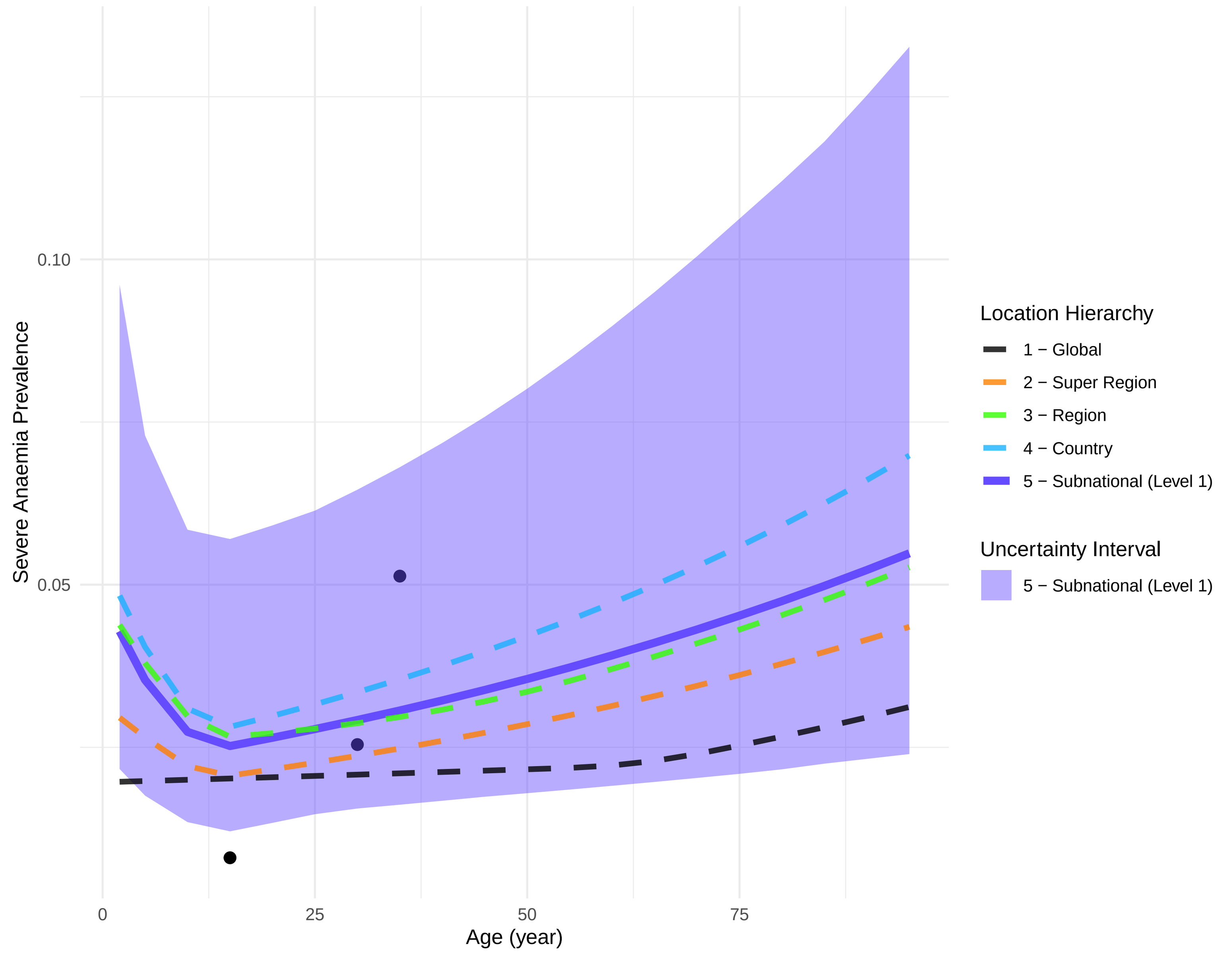

MR−BRT Age/Sex Fits − Non−pregnant Females 2+ Years Old − Severe Anaemia Prevalence − FCT (Abuja)
WHO−2024 Adjustment Method

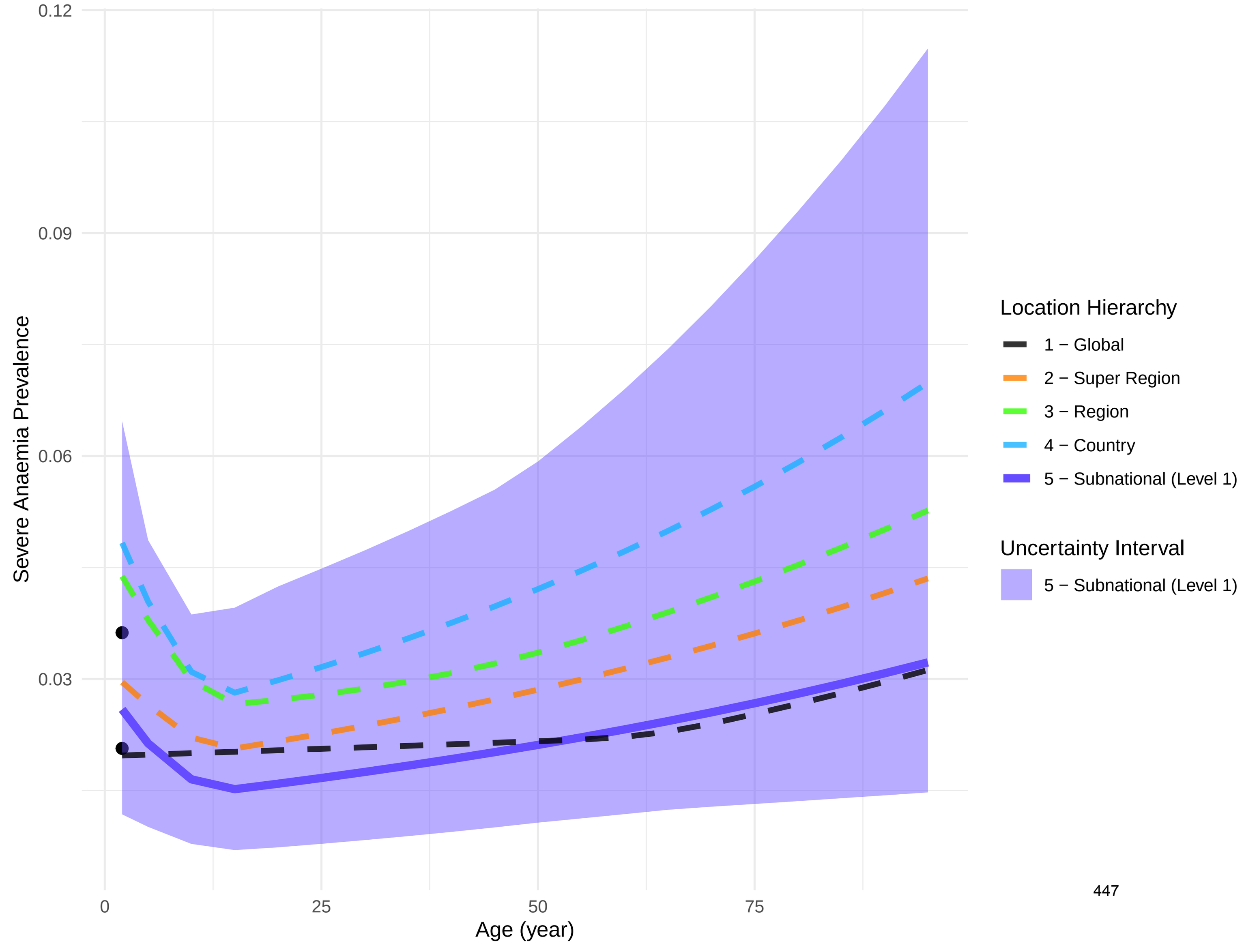

MR−BRT Age/Sex Fits − Non−pregnant Females 2+ Years Old − Severe Anaemia Prevalence − Gombe
WHO−2024 Adjustment Method

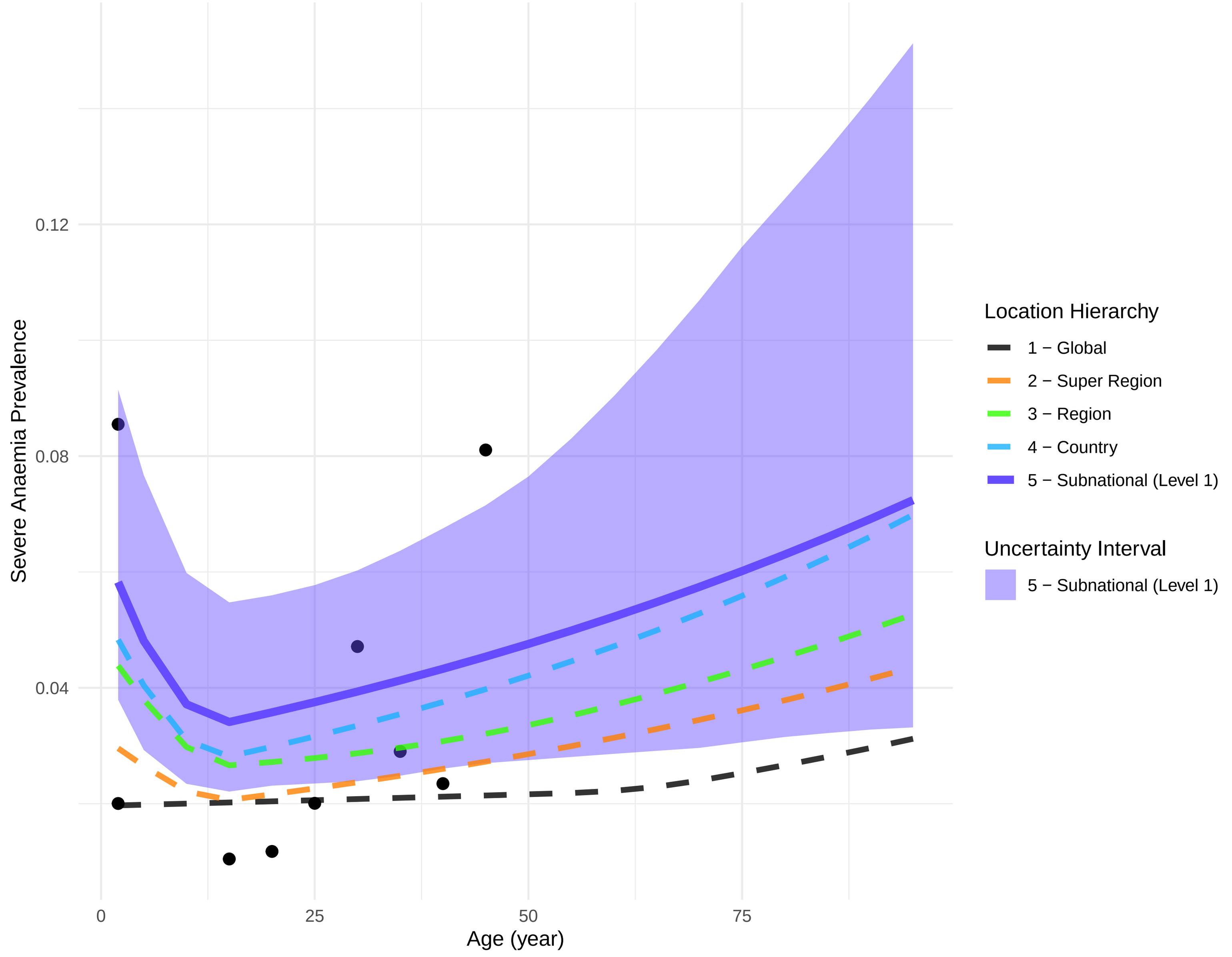

MR−BRT Age/Sex Fits − Non−pregnant Females 2+ Years Old − Severe Anaemia Prevalence − Imo
WHO−2024 Adjustment Method

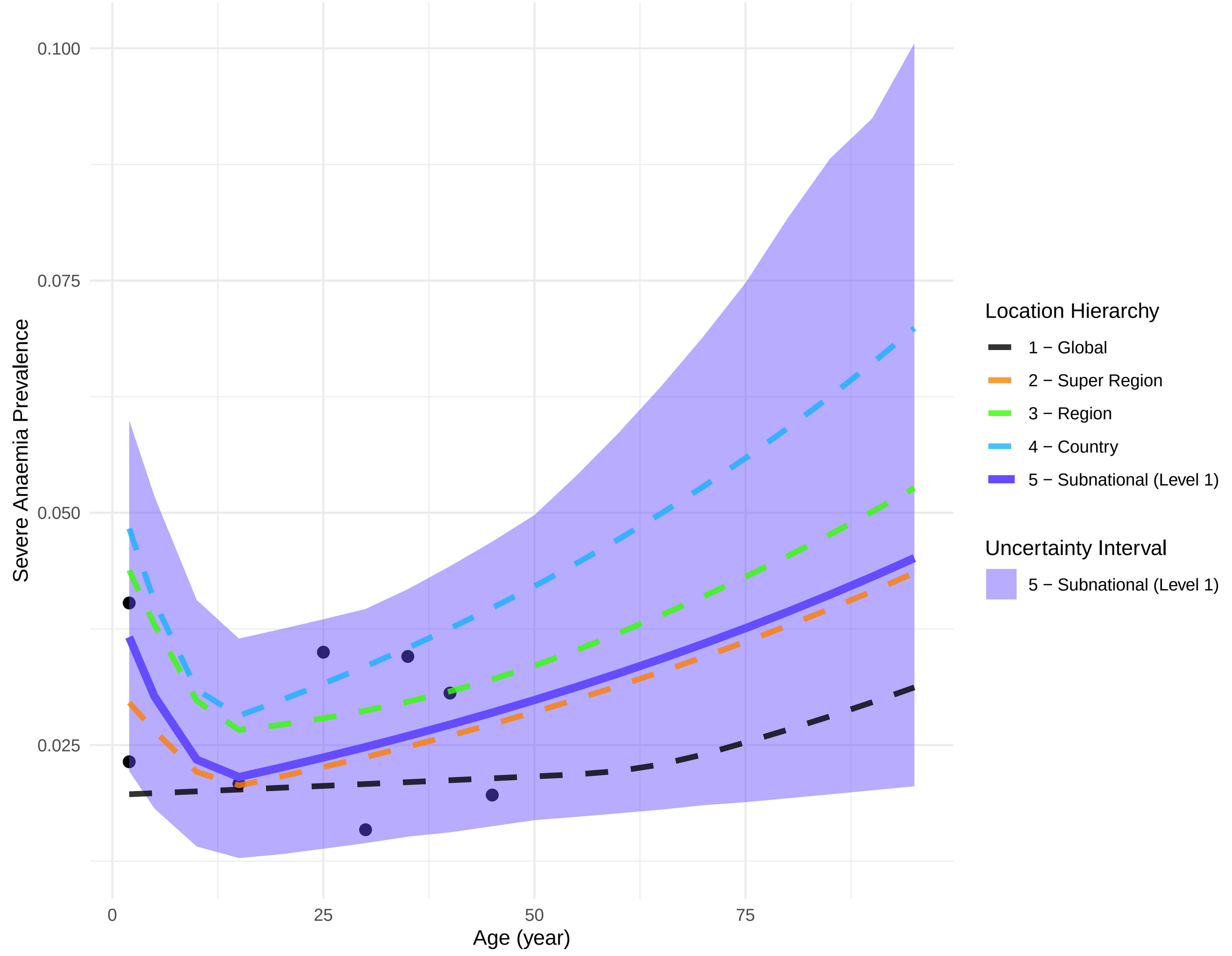

MR−BRT Age/Sex Fits − Non−pregnant Females 2+ Years Old − Severe Anaemia Prevalence − Jigawa
WHO−2024 Adjustment Method

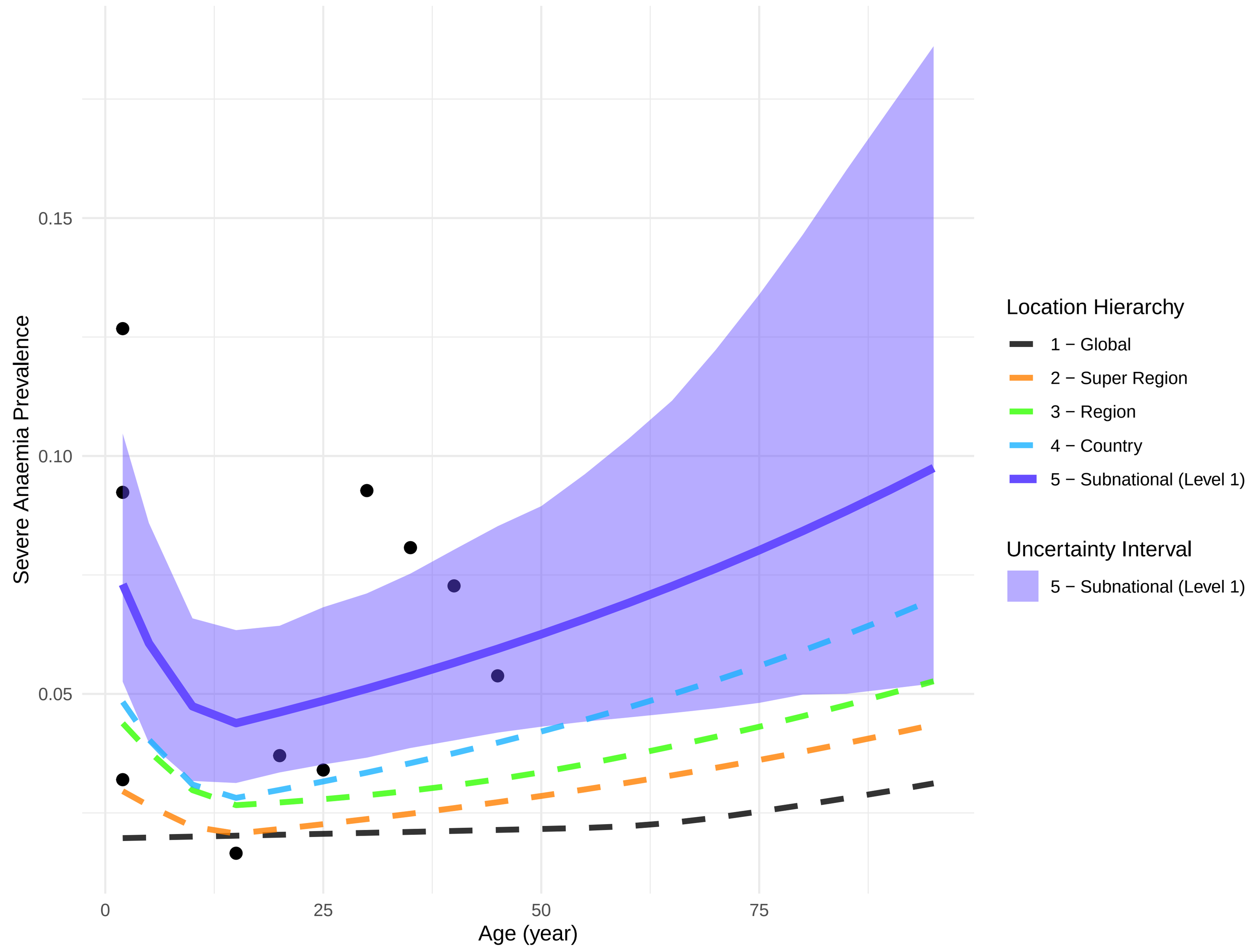

MR−BRT Age/Sex Fits − Non−pregnant Females 2+ Years Old − Severe Anaemia Prevalence − Kaduna
WHO−2024 Adjustment Method

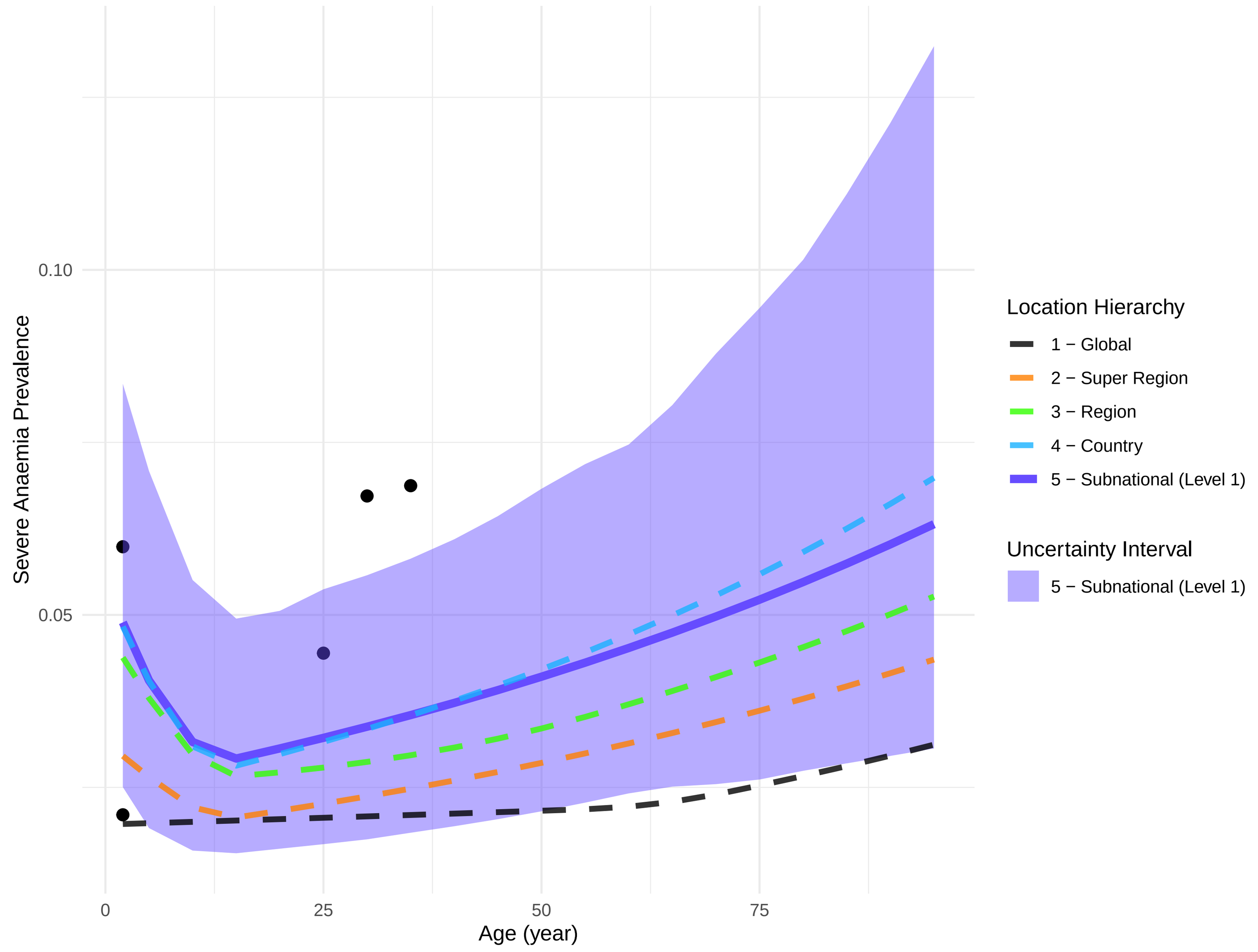

MR−BRT Age/Sex Fits − Non−pregnant Females 2+ Years Old − Severe Anaemia Prevalence − Kano
WHO−2024 Adjustment Method

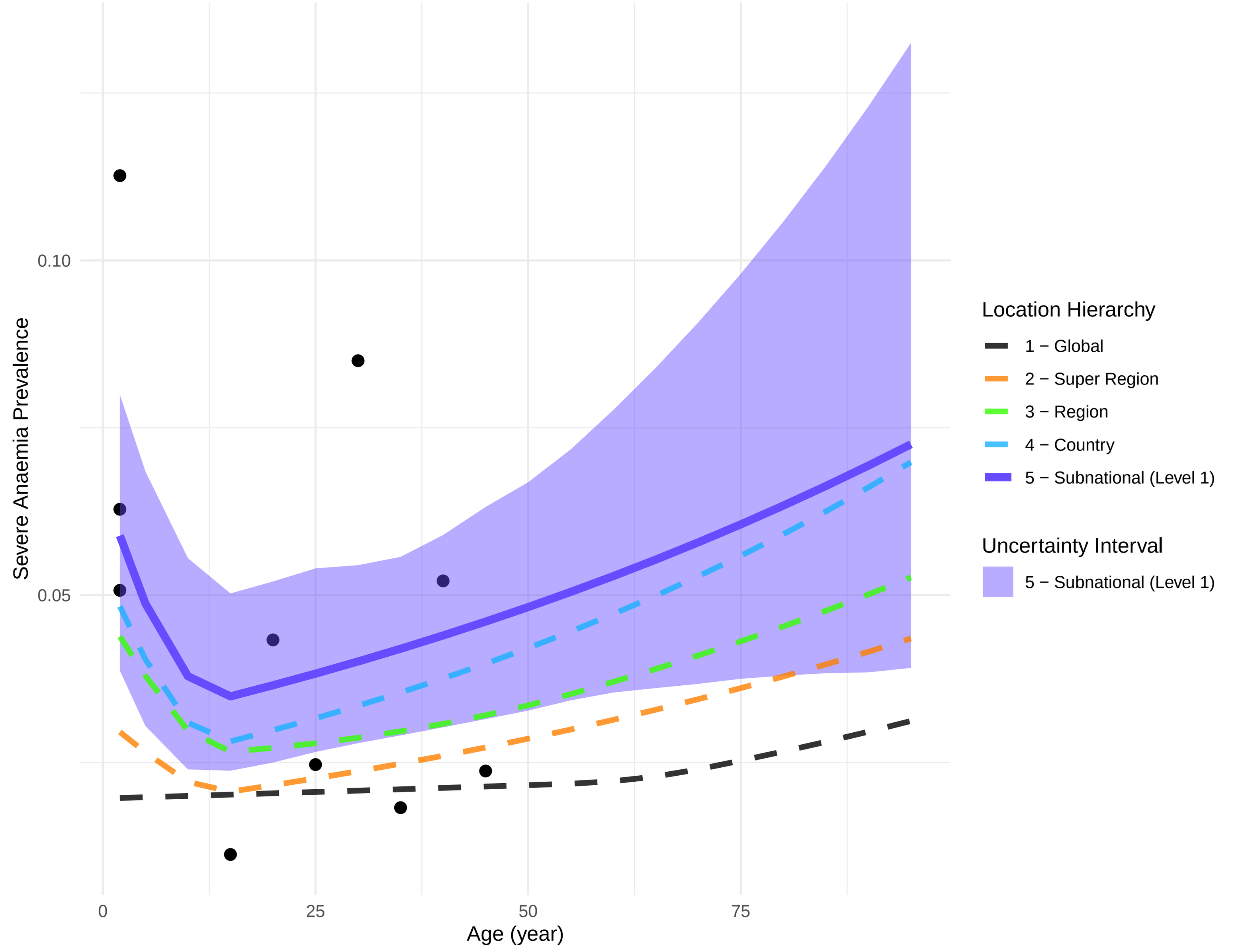

MR−BRT Age/Sex Fits − Non−pregnant Females 2+ Years Old − Severe Anaemia Prevalence − Katsina
WHO−2024 Adjustment Method

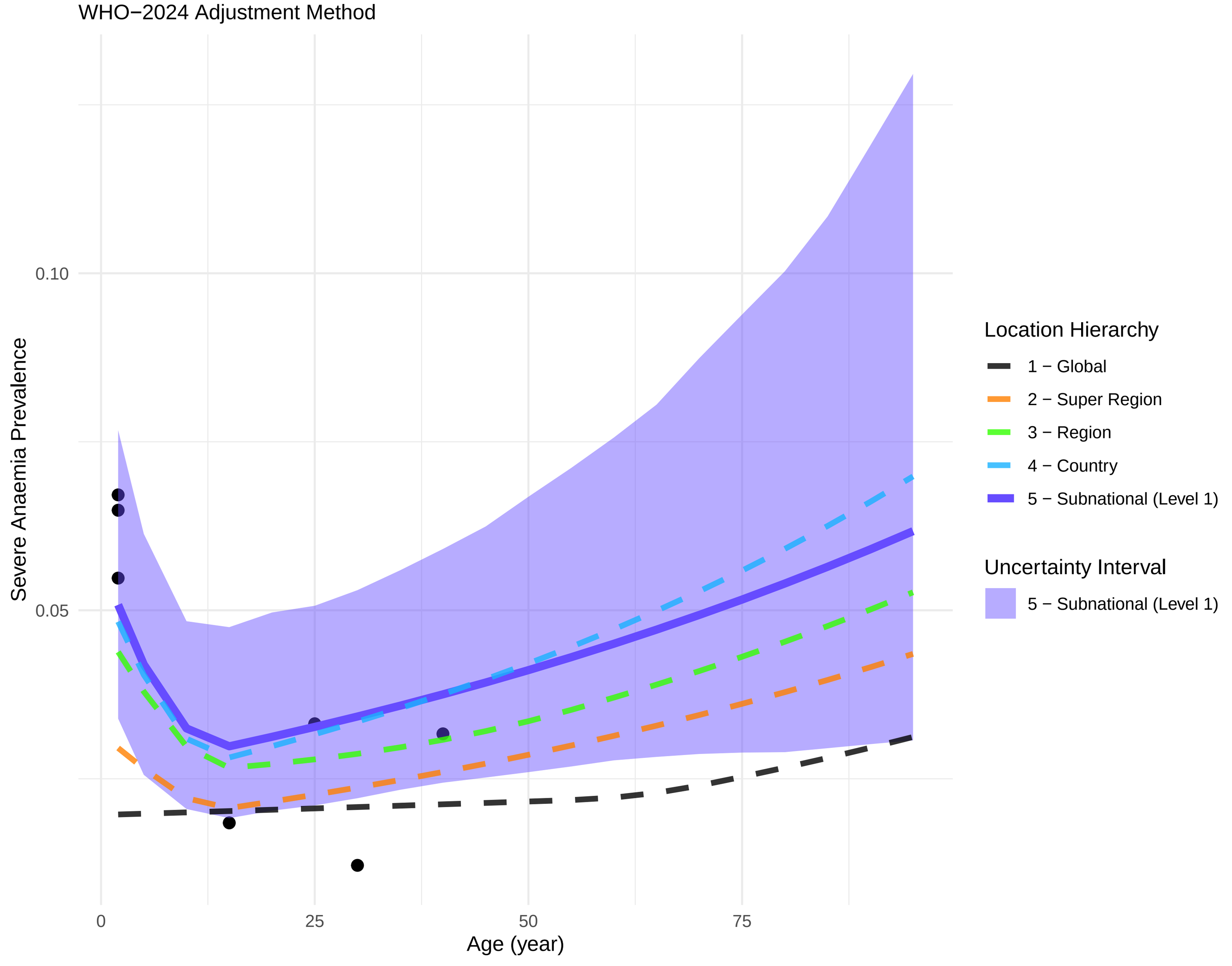

MR−BRT Age/Sex Fits − Non−pregnant Females 2+ Years Old − Severe Anaemia Prevalence − Kebbi
WHO−2024 Adjustment Method

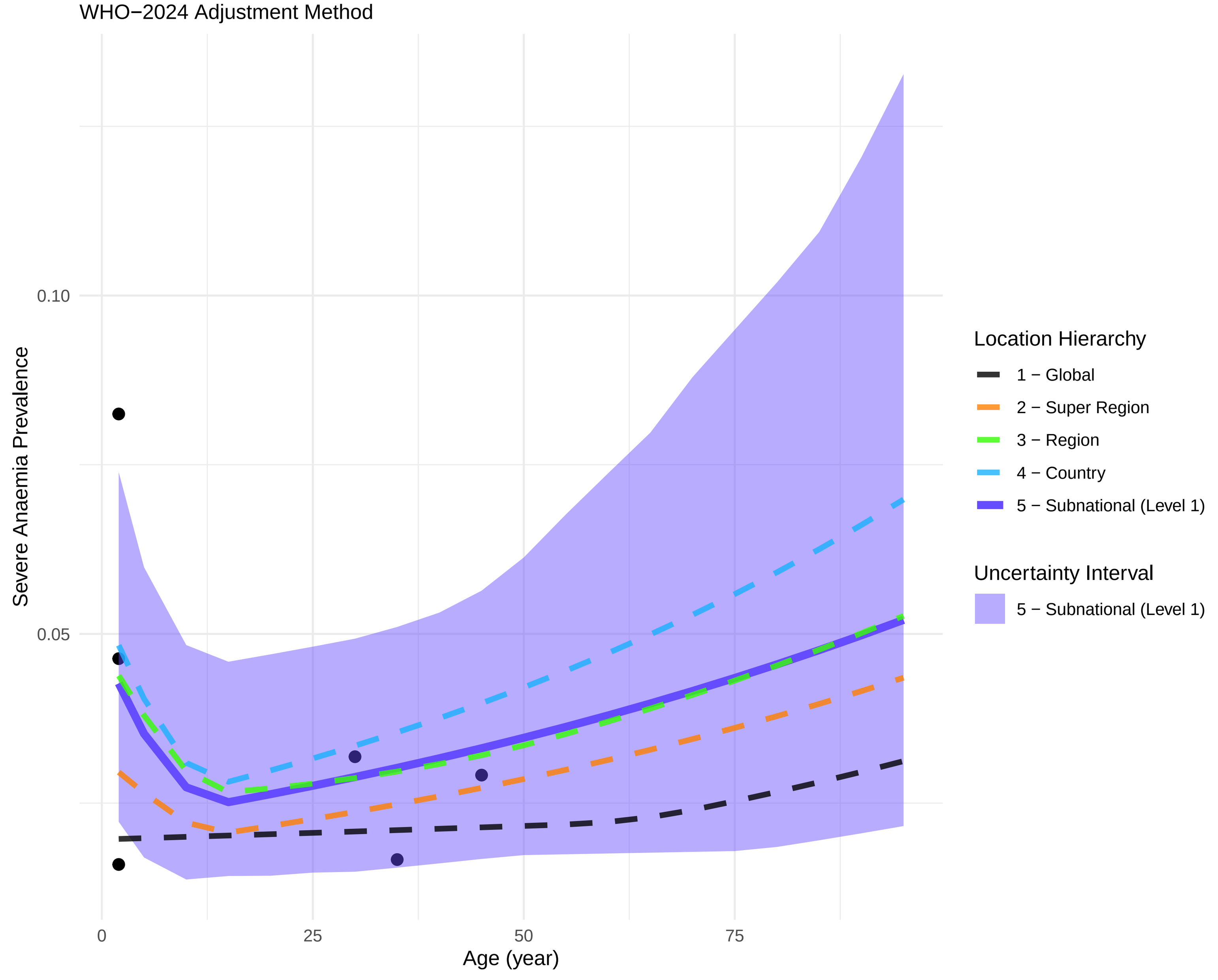

MR−BRT Age/Sex Fits − Non−pregnant Females 2+ Years Old − Severe Anaemia Prevalence − Kogi
WHO−2024 Adjustment Method

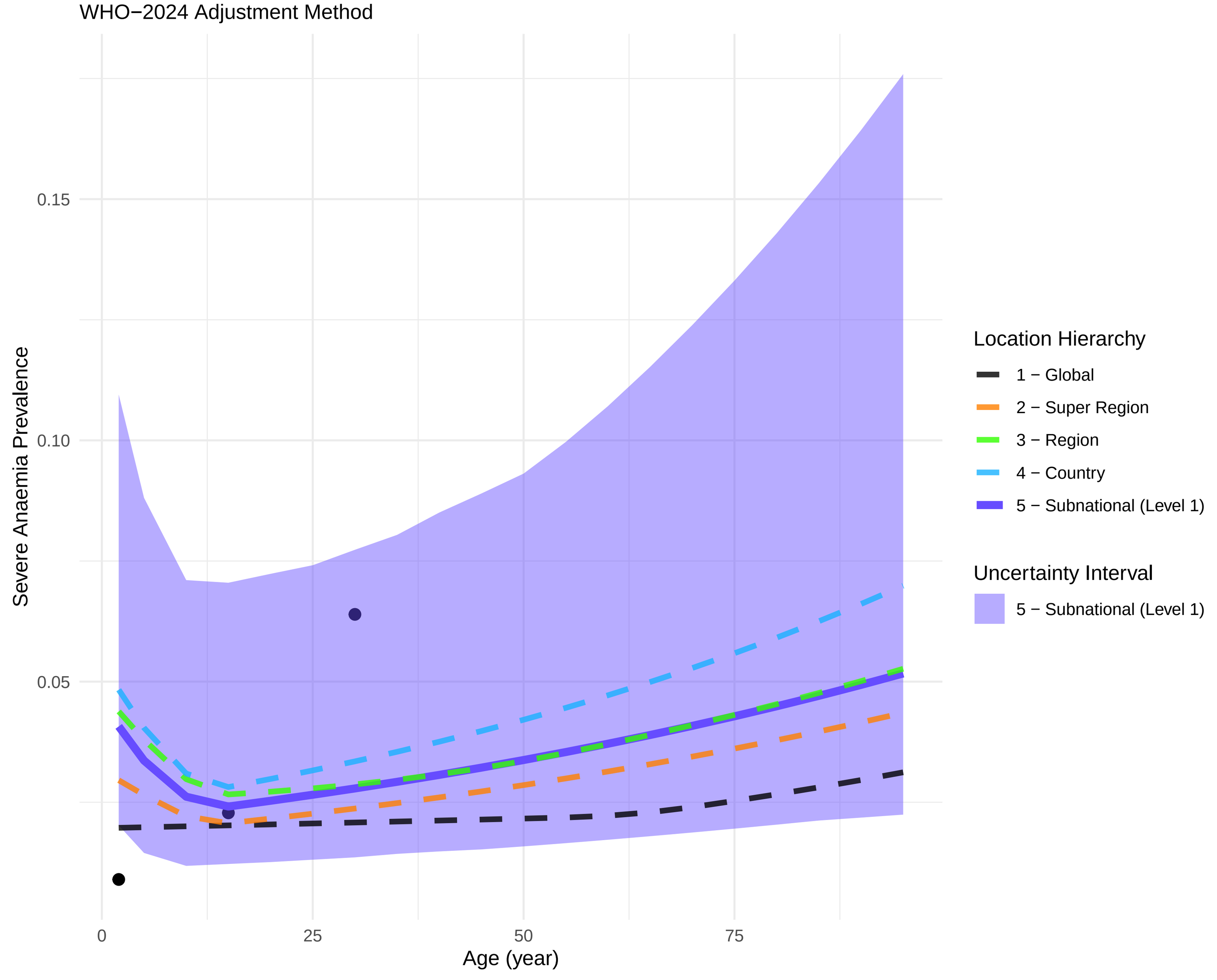

MR−BRT Age/Sex Fits − Non−pregnant Females 2+ Years Old − Severe Anaemia Prevalence − Kwara
WHO−2024 Adjustment Method

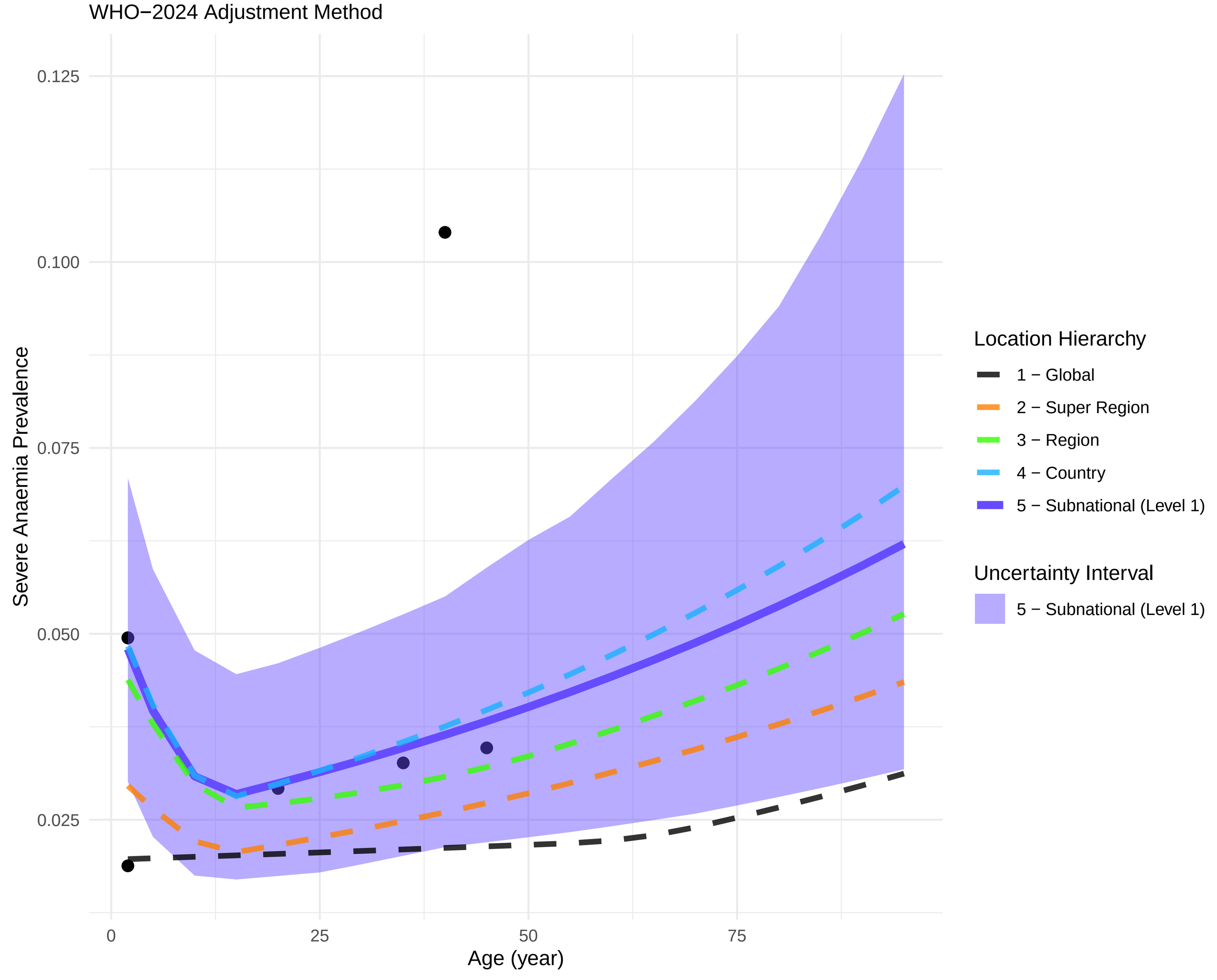

MR−BRT Age/Sex Fits − Non−pregnant Females 2+ Years Old − Severe Anaemia Prevalence − Lagos
WHO−2024 Adjustment Method

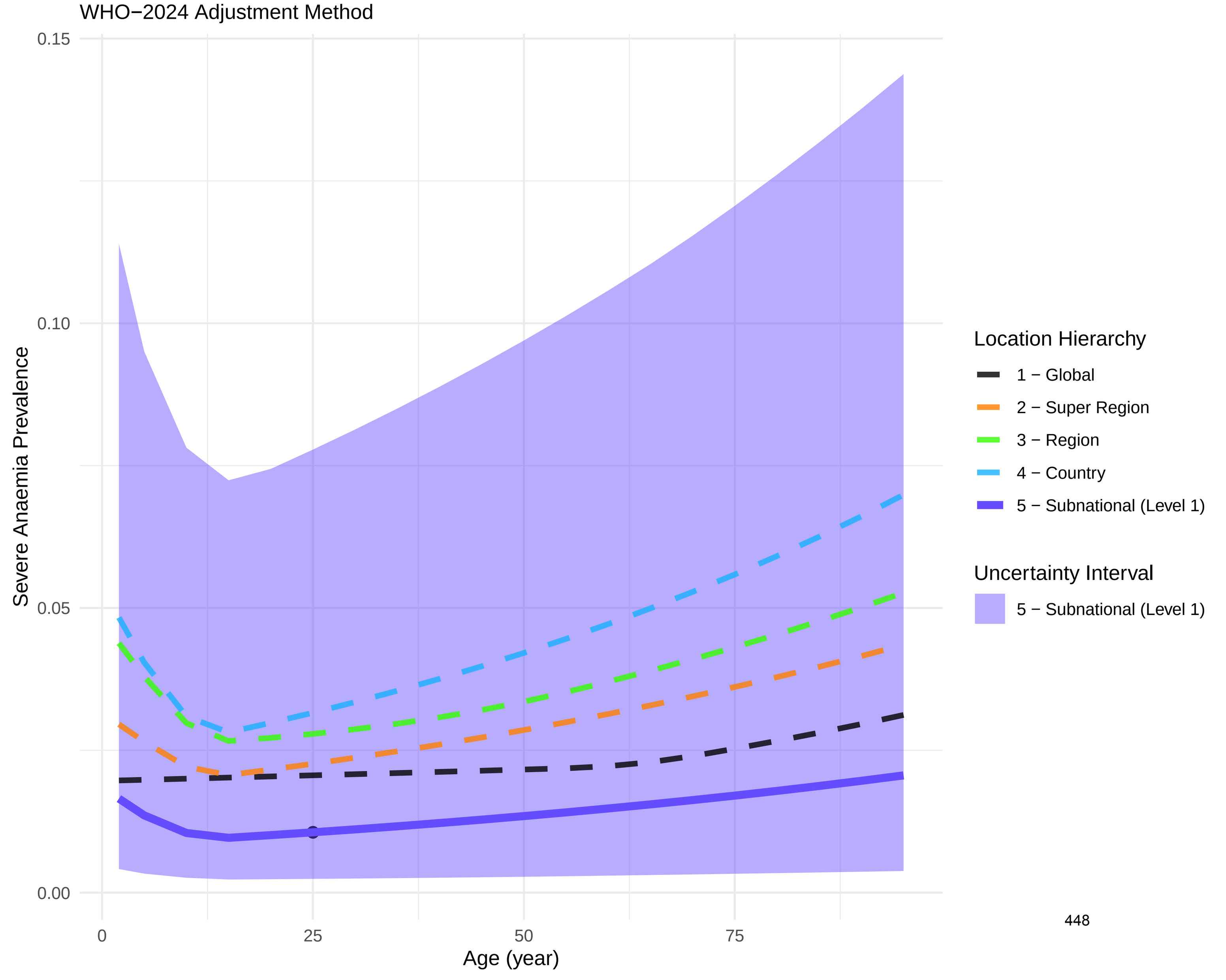

MR−BRT Age/Sex Fits − Non-pregnant Females 2+ Years Old − Severe Anaemia Prevalence − Nasarawa
WHO−2024 Adjustment Method

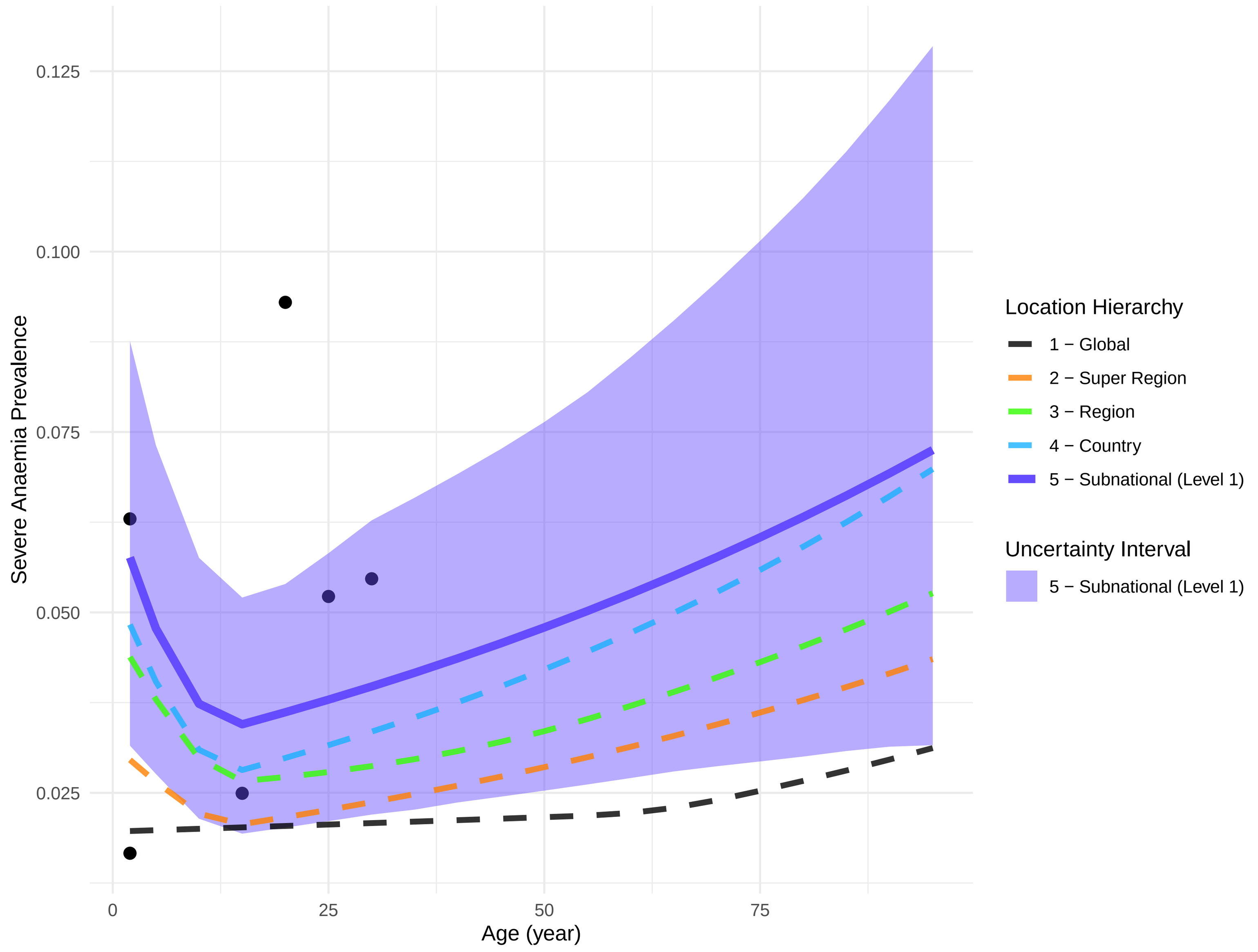

MR−BRT Age/Sex Fits − Non-pregnant Females 2+ Years Old − Severe Anaemia Prevalence − Niger
WHO−2024 Adjustment Method

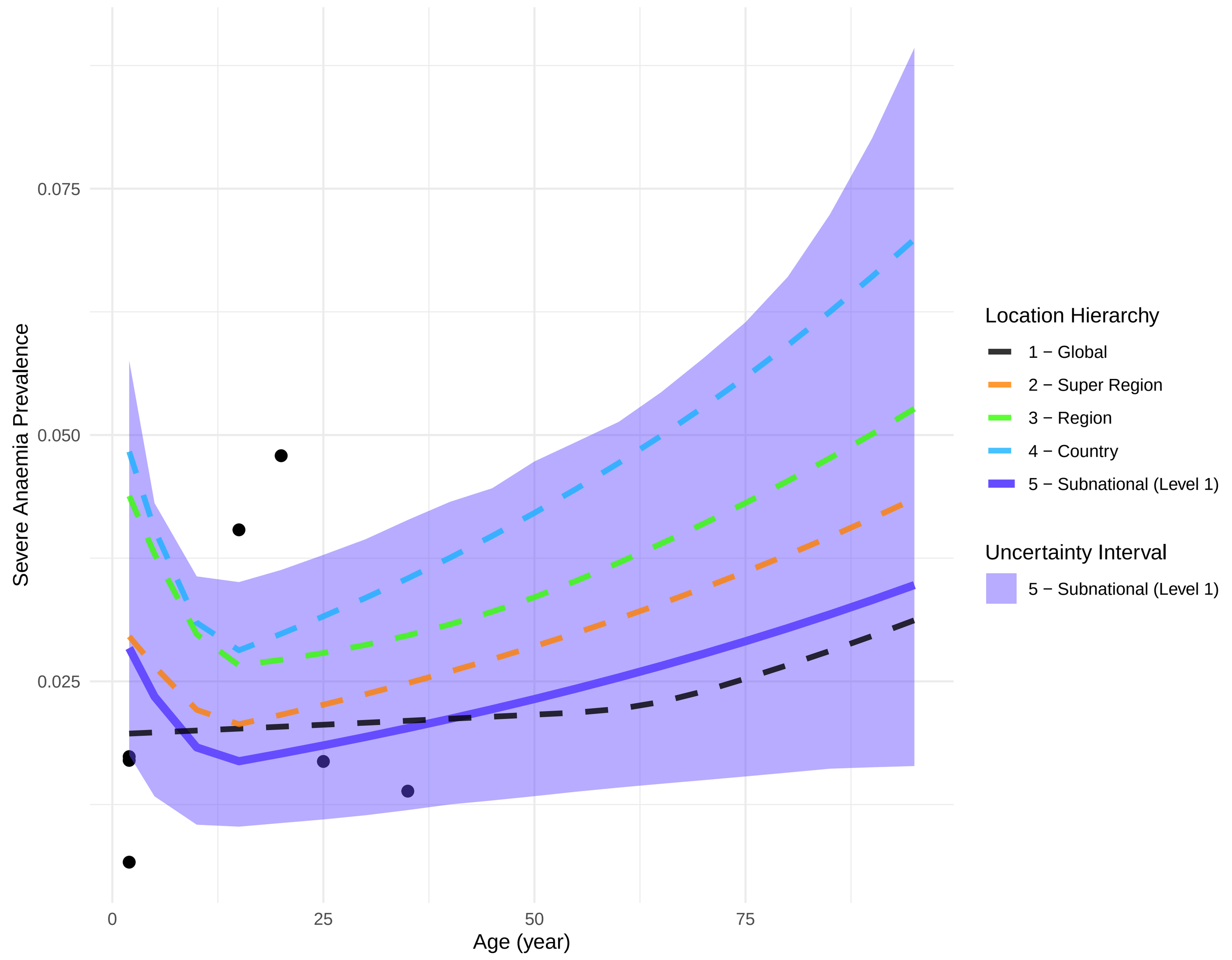

MR−BRT Age/Sex Fits − Non-pregnant Females 2+ Years Old − Severe Anaemia Prevalence − Ogun
WHO−2024 Adjustment Method

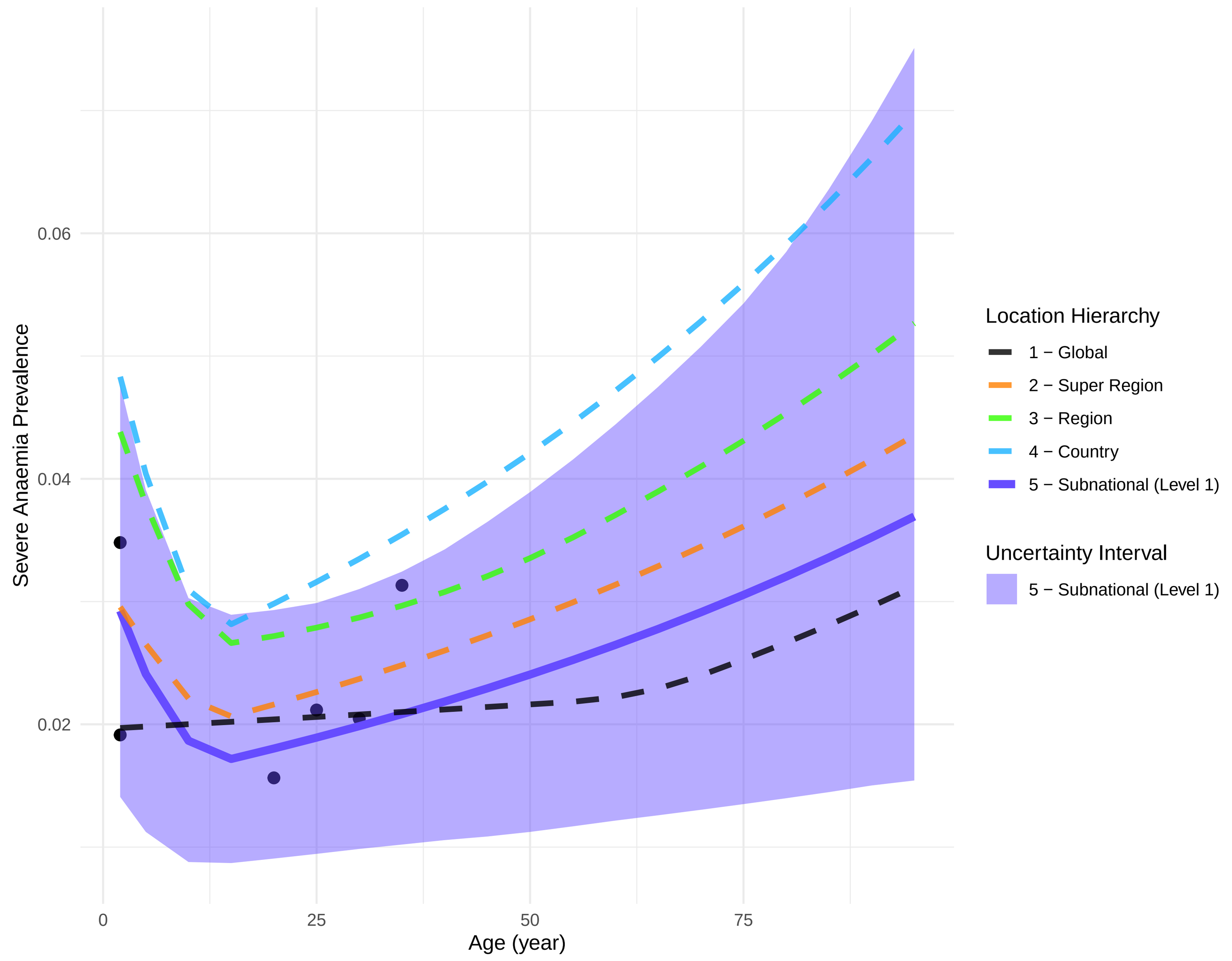

MR−BRT Age/Sex Fits − Non-pregnant Females 2+ Years Old − Severe Anaemia Prevalence − Ondo
WHO−2024 Adjustment Method

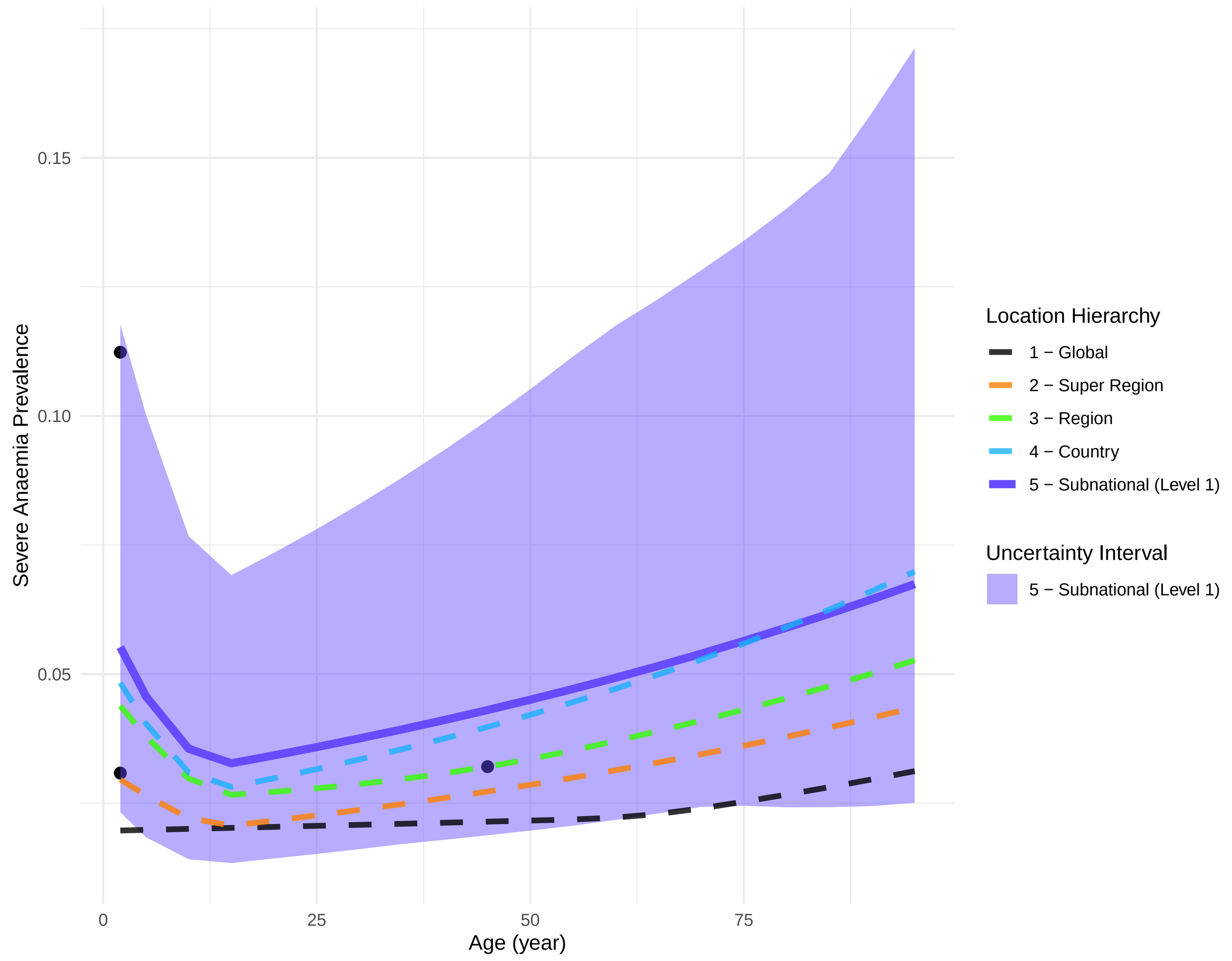

MR−BRT Age/Sex Fits − Non-pregnant Females 2+ Years Old − Severe Anaemia Prevalence − Osun
WHO−2024 Adjustment Method

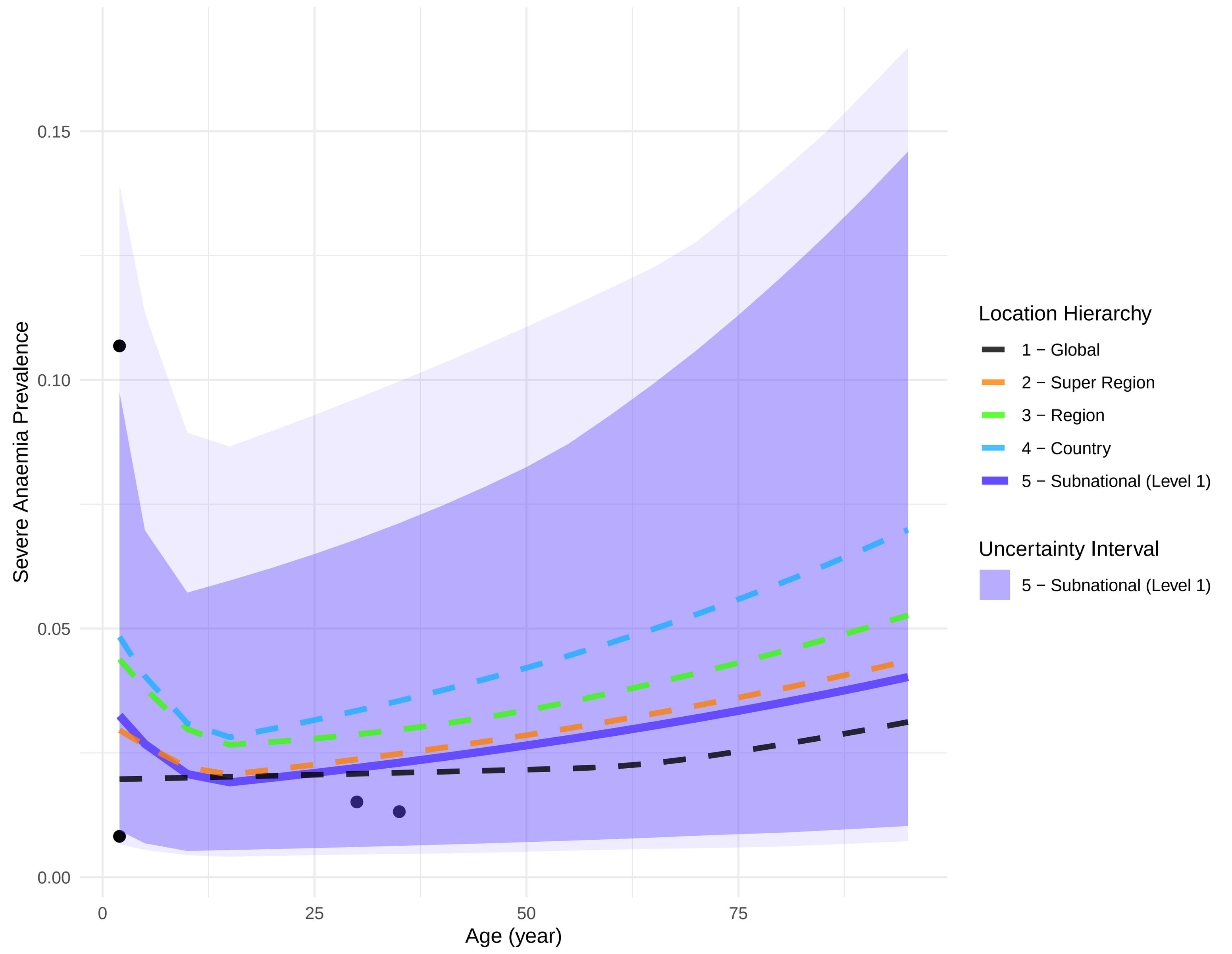

MR−BRT Age/Sex Fits − Non-pregnant Females 2+ Years Old − Severe Anaemia Prevalence − Oyo
WHO−2024 Adjustment Method

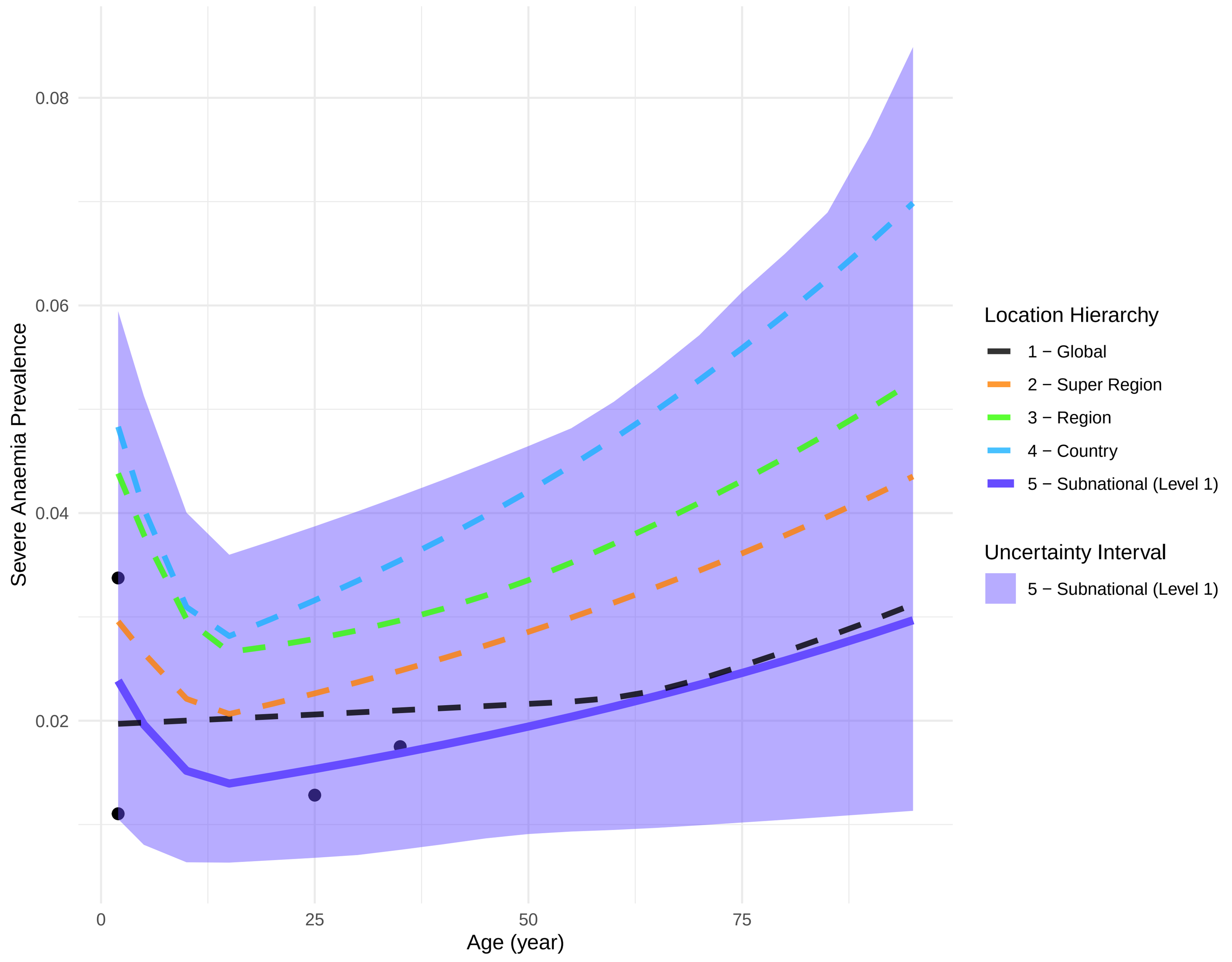

MR−BRT Age/Sex Fits − Non-pregnant Females 2+ Years Old − Severe Anaemia Prevalence − Plateau
WHO−2024 Adjustment Method

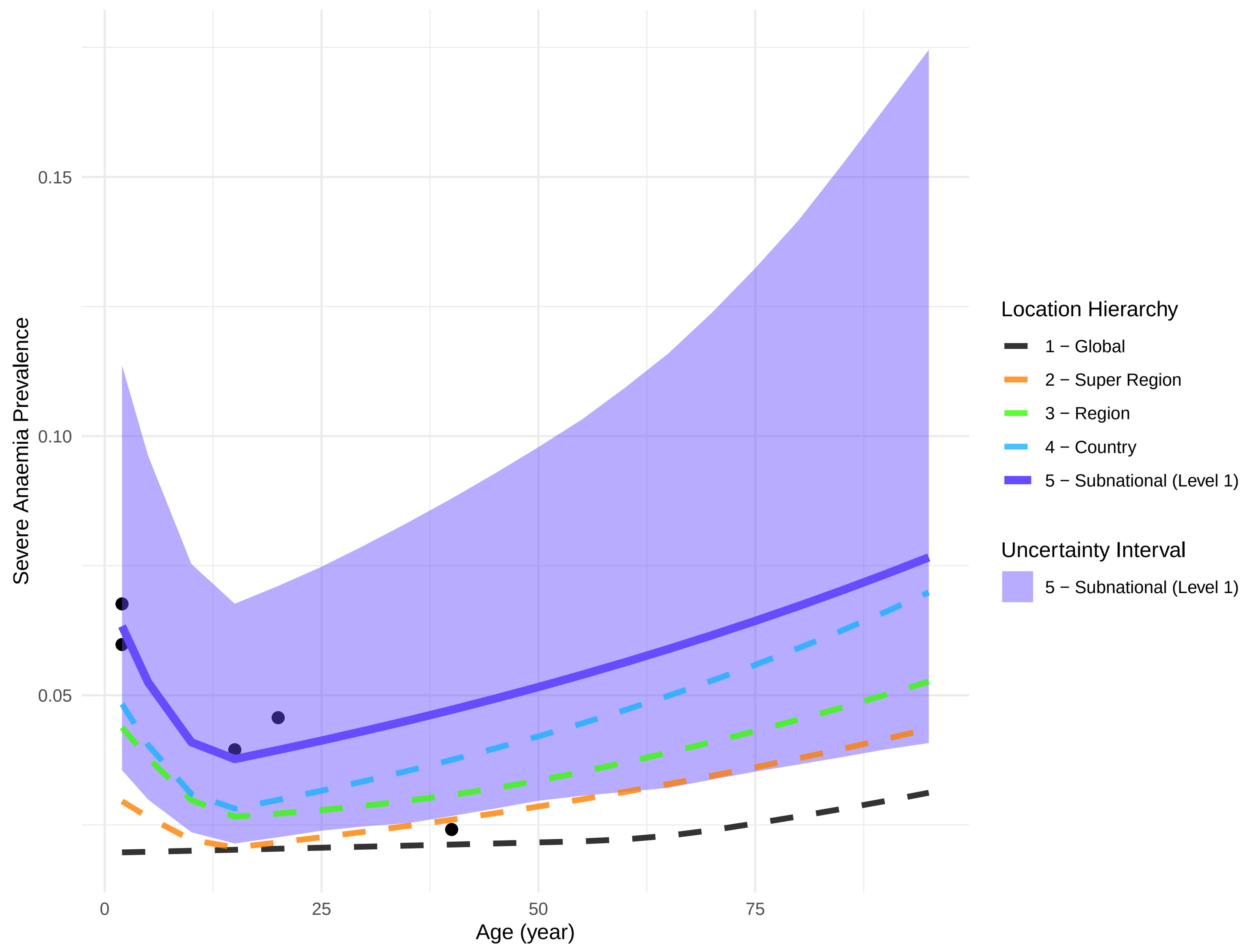

MR−BRT Age/Sex Fits − Non-pregnant Females 2+ Years Old − Severe Anaemia Prevalence − Rivers
WHO−2024 Adjustment Method

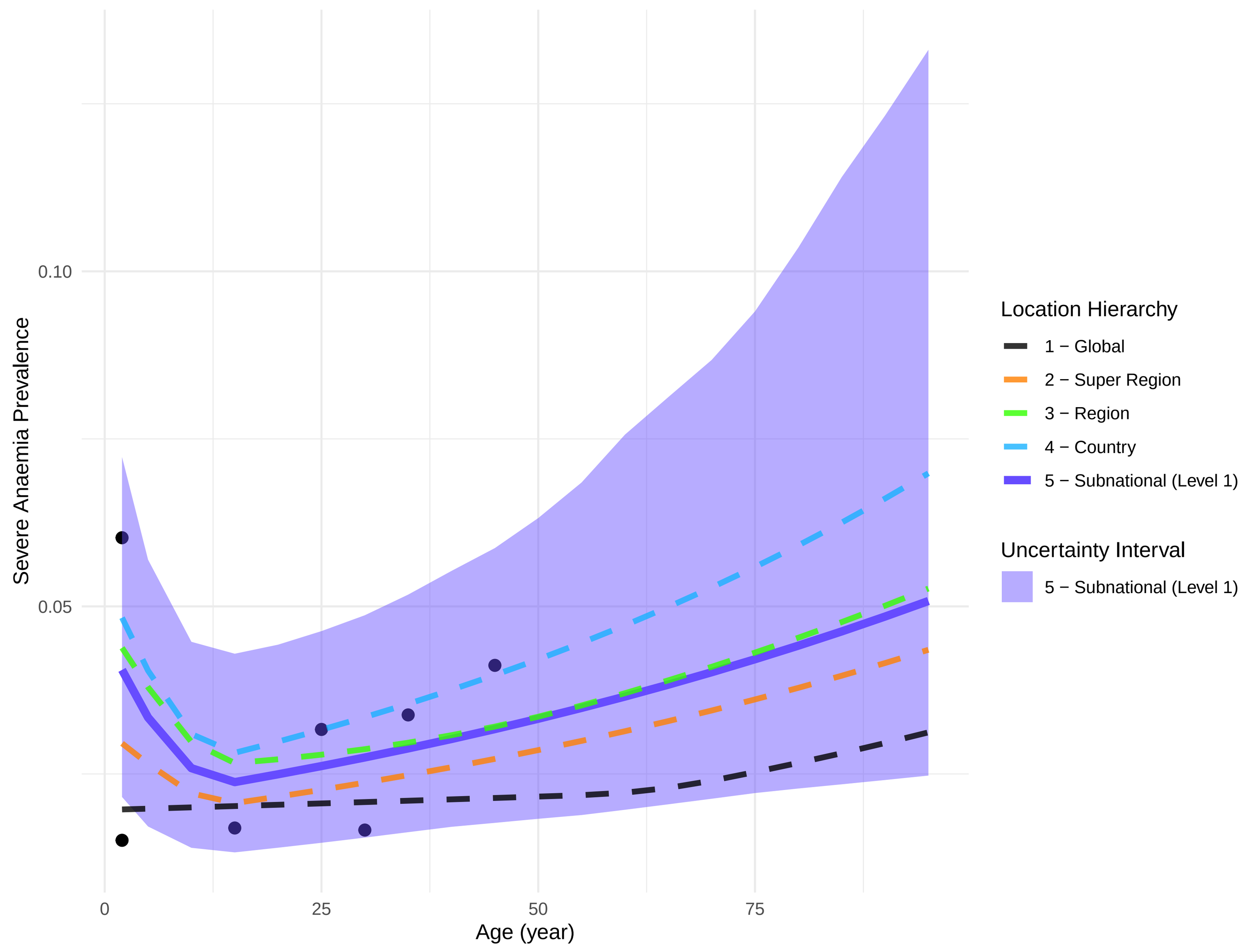

MR−BRT Age/Sex Fits − Non-pregnant Females 2+ Years Old − Severe Anaemia Prevalence − Sokoto
WHO−2024 Adjustment Method

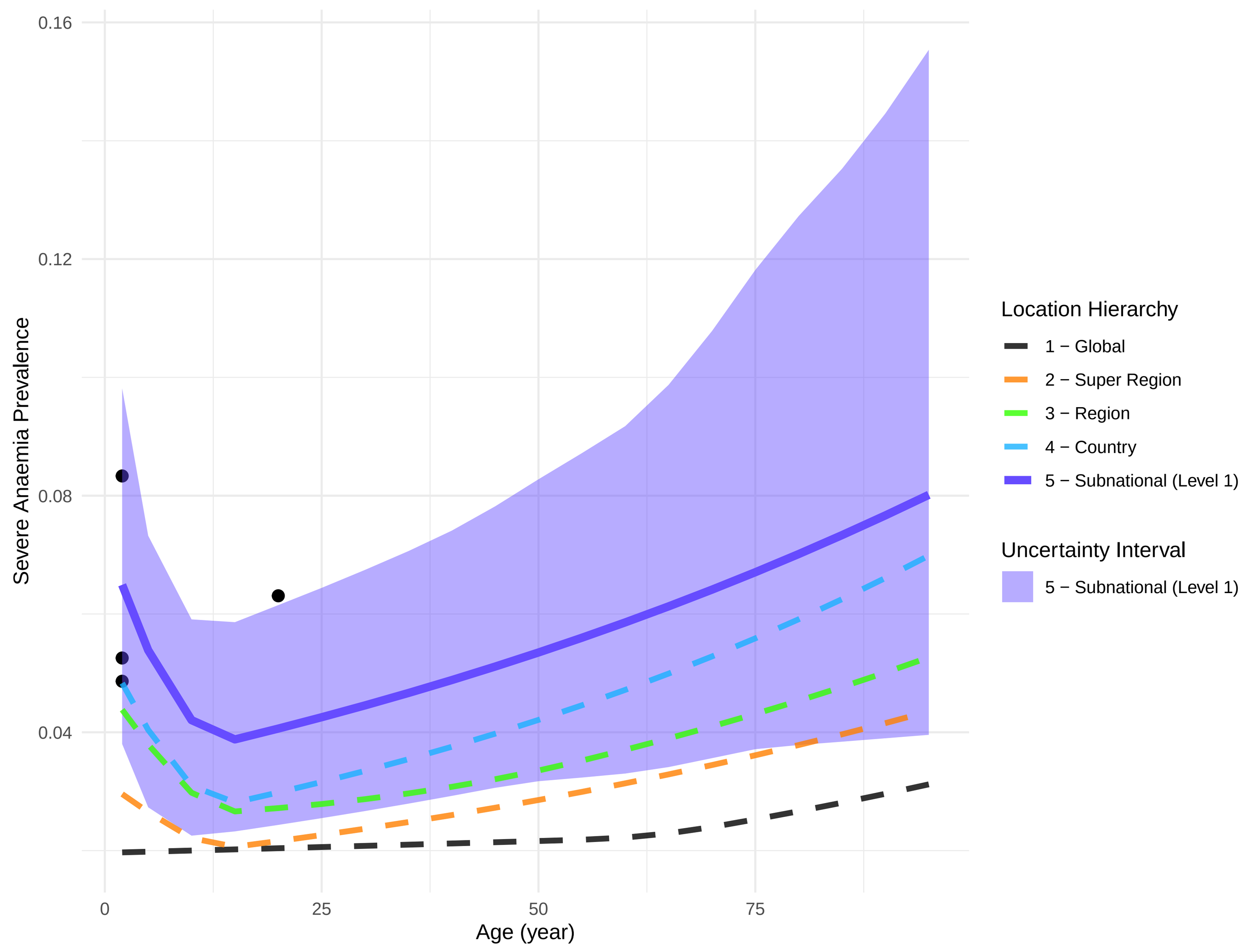

MR−BRT Age/Sex Fits − Non-pregnant Females 2+ Years Old − Severe Anaemia Prevalence − Taraba
WHO−2024 Adjustment Method

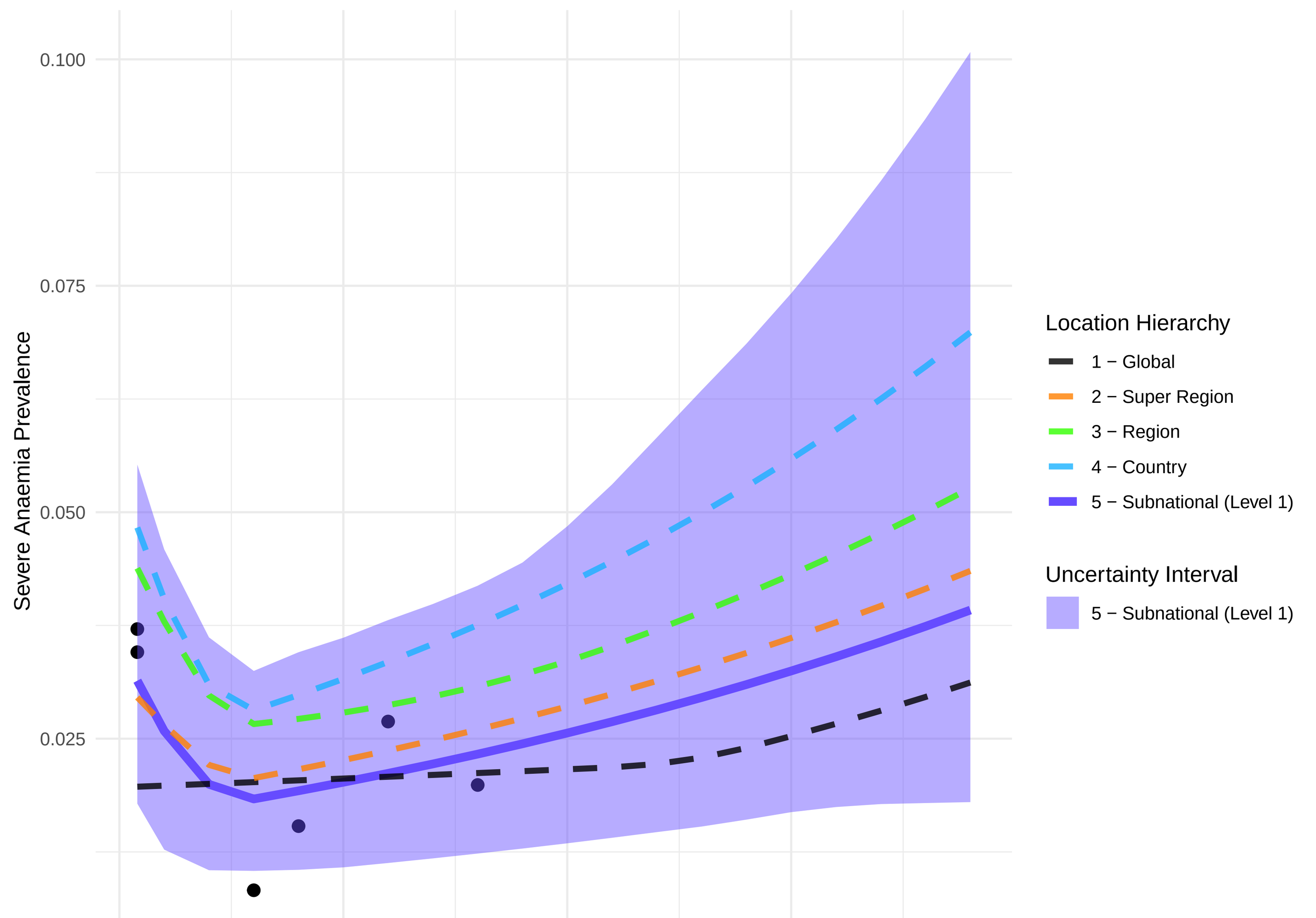

MR−BRT Age/Sex Fits − Non−pregnant Females 2+ Years Old − Severe Anaemia Prevalence − Yobe
WHO−2024 Adjustment Method

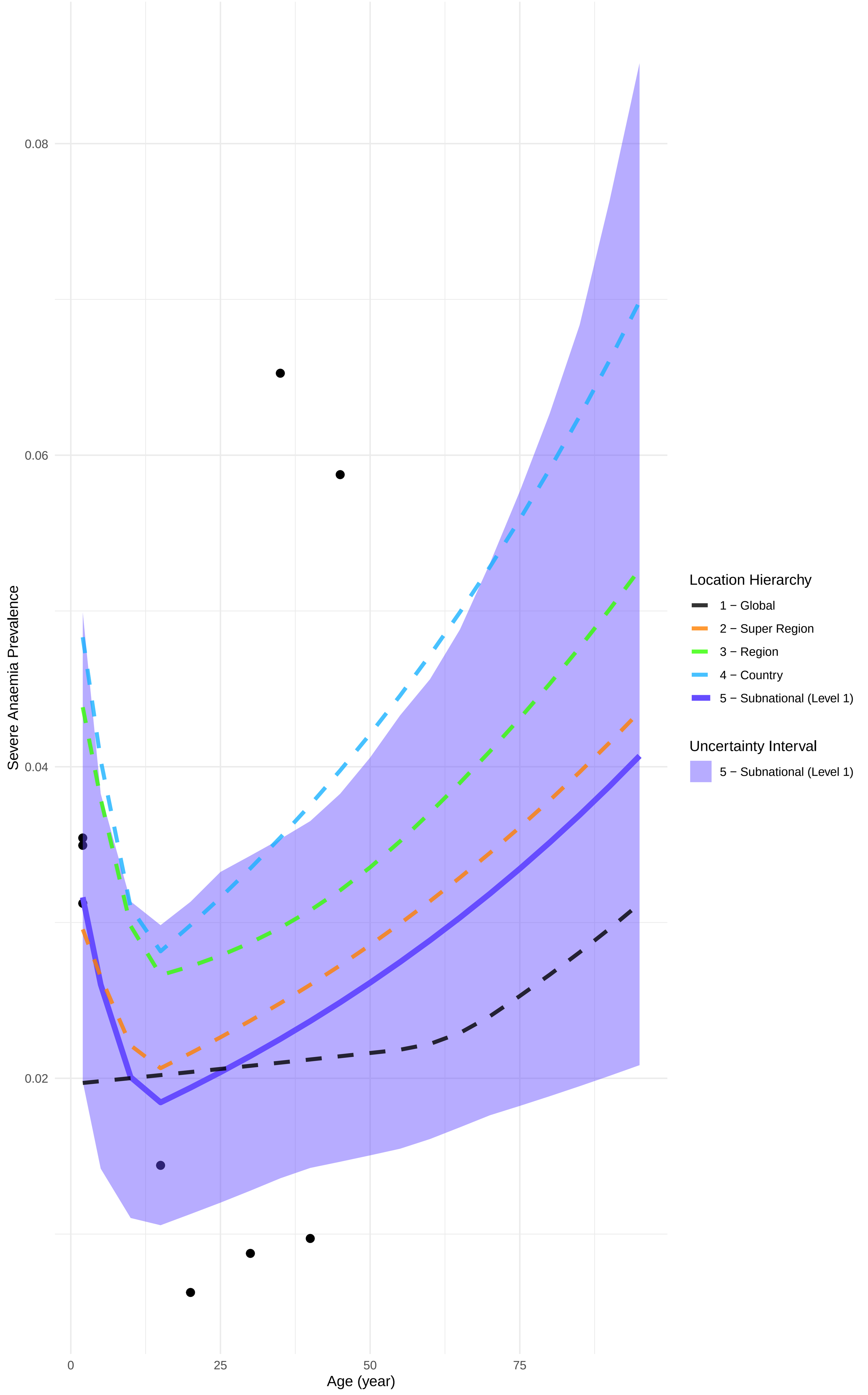

MR−BRT Age/Sex Fits − Non−pregnant Females 2+ Years Old − Severe Anaemia Prevalence − Zamfara
WHO−2024 Adjustment Method

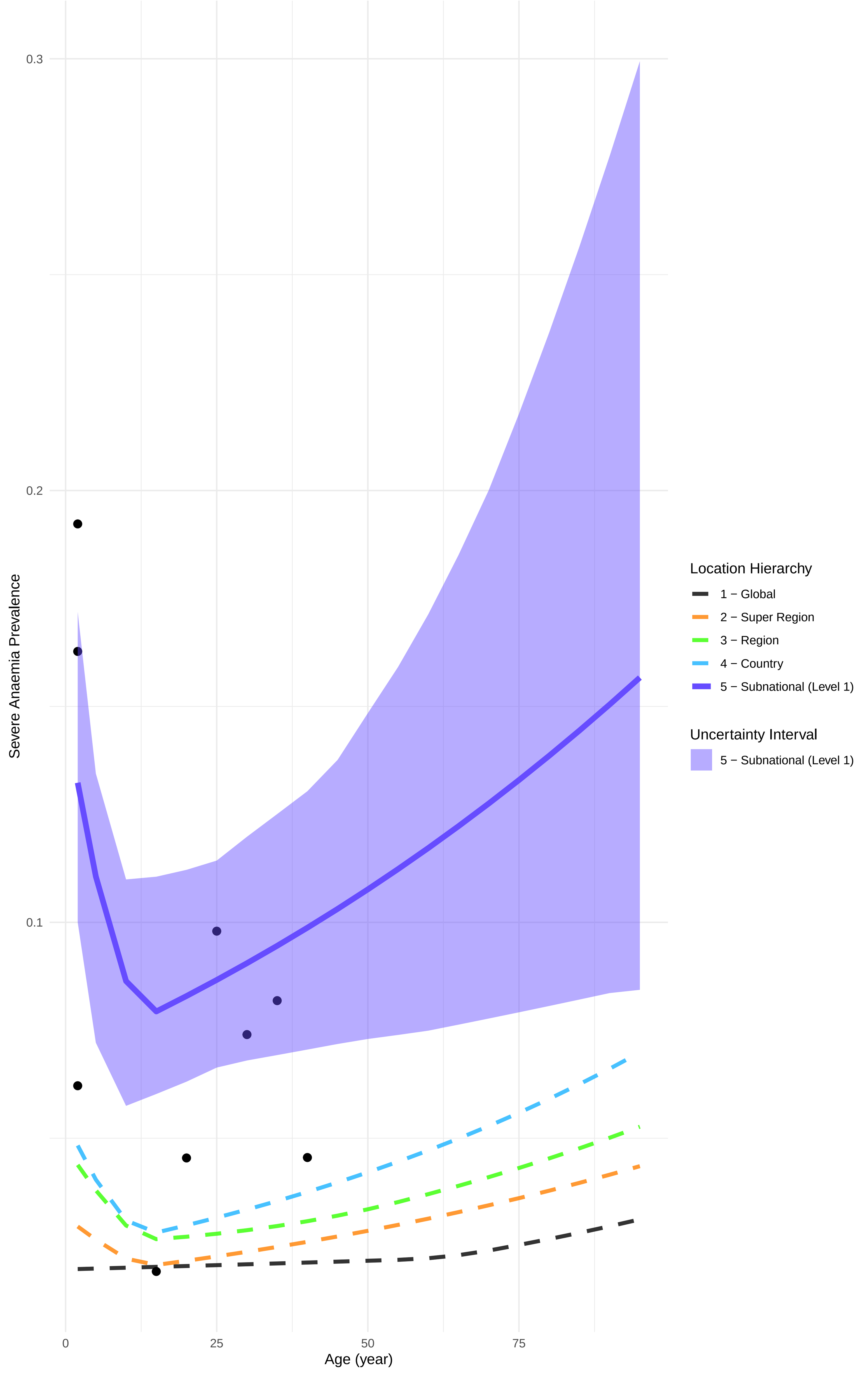

MR−BRT Age/Sex Fits − Non−pregnant Females 2+ Years Old − Severe Anaemia Prevalence − Senegal
WHO−2024 Adjustment Method

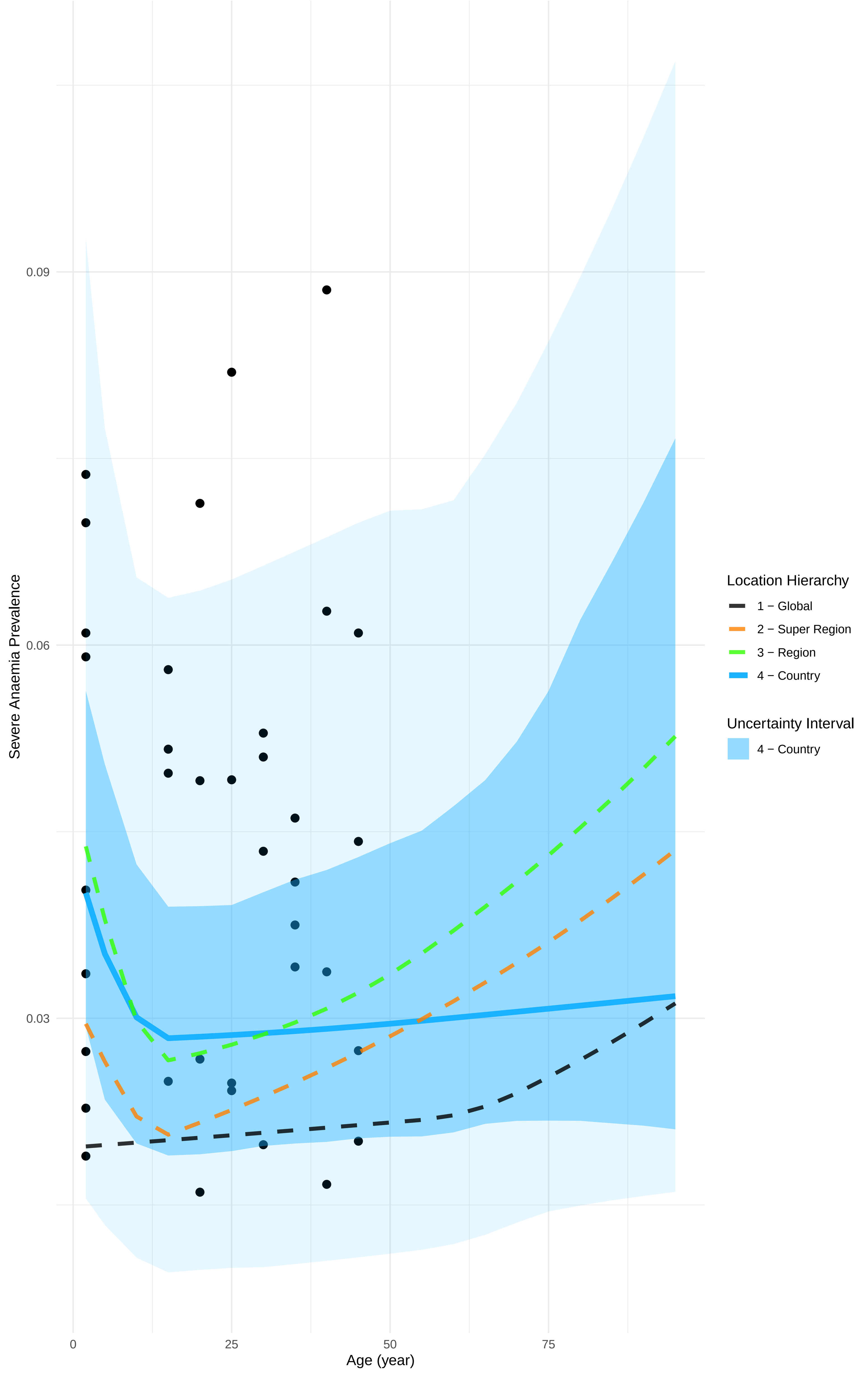

MR−BRT Age/Sex Fits − Non−pregnant Females 2+ Years Old − Severe Anaemia Prevalence − Sierra Leone
WHO−2024 Adjustment Method

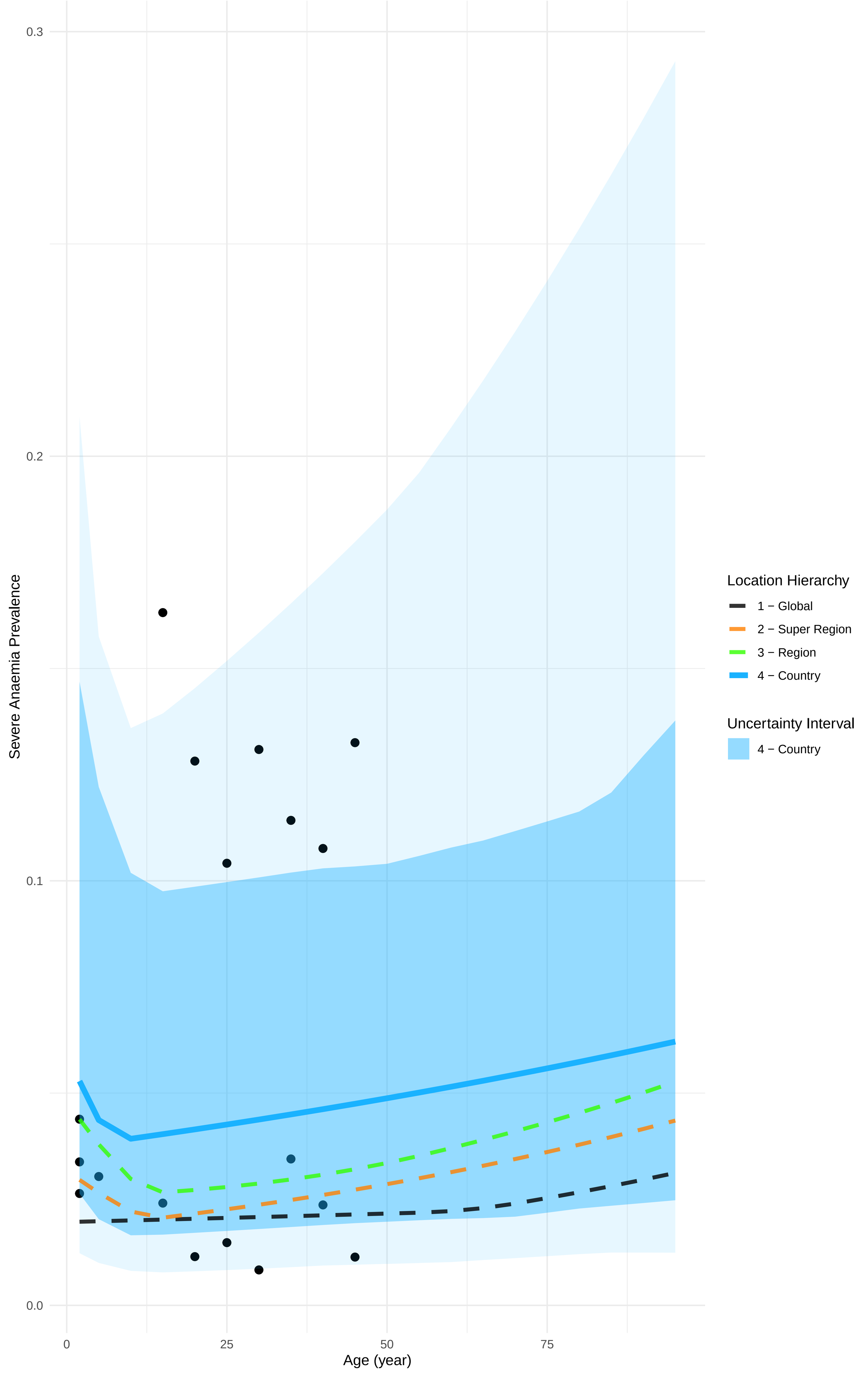

MR−BRT Age/Sex Fits − Non−pregnant Females 2+ Years Old − Severe Anaemia Prevalence − Togo
WHO−2024 Adjustment Method

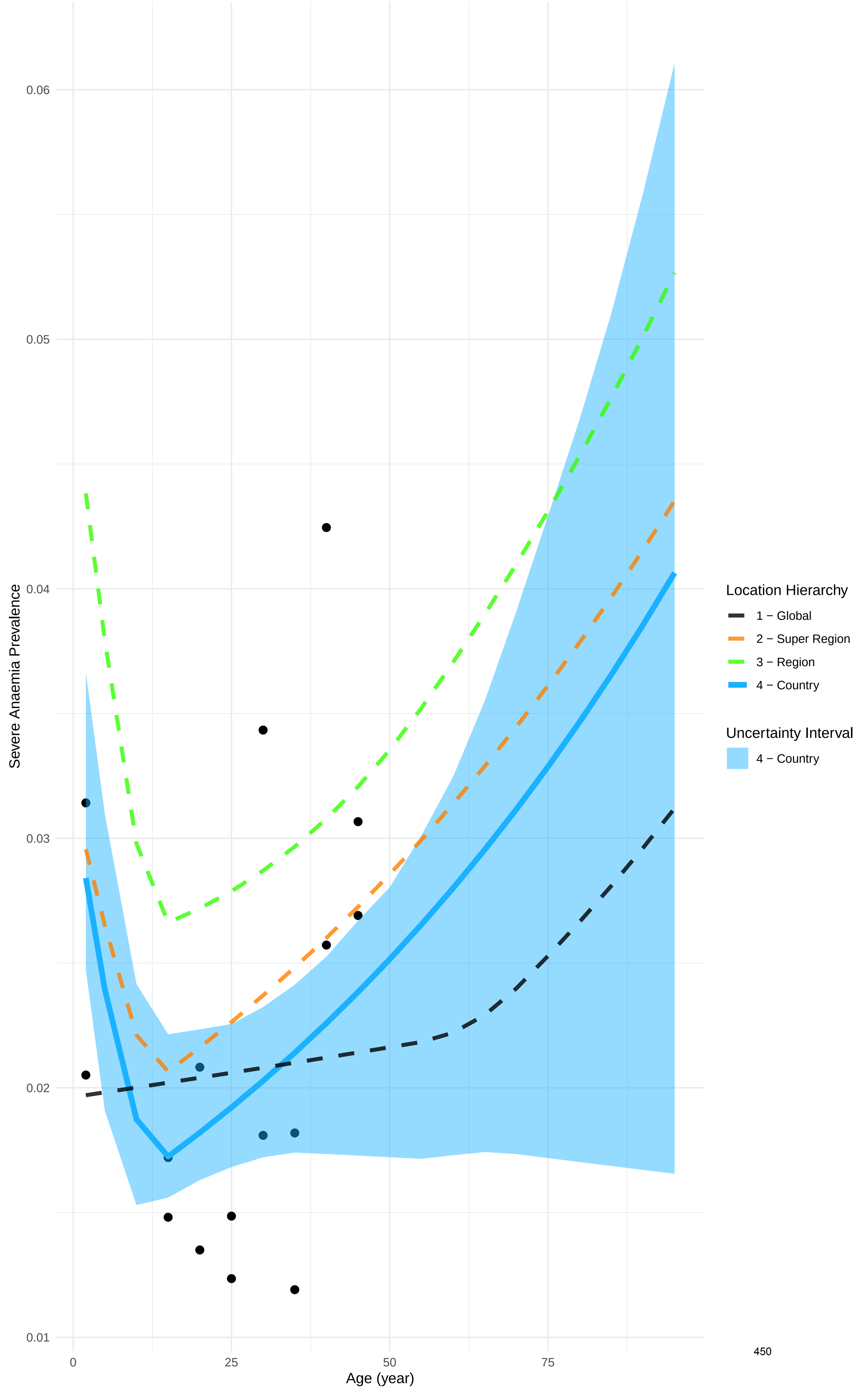

MR−BRT Age/Sex Fits − Males 2+ Years Old − Severe Anaemia Prevalence − Global
WHO−2001 Adjustment Method

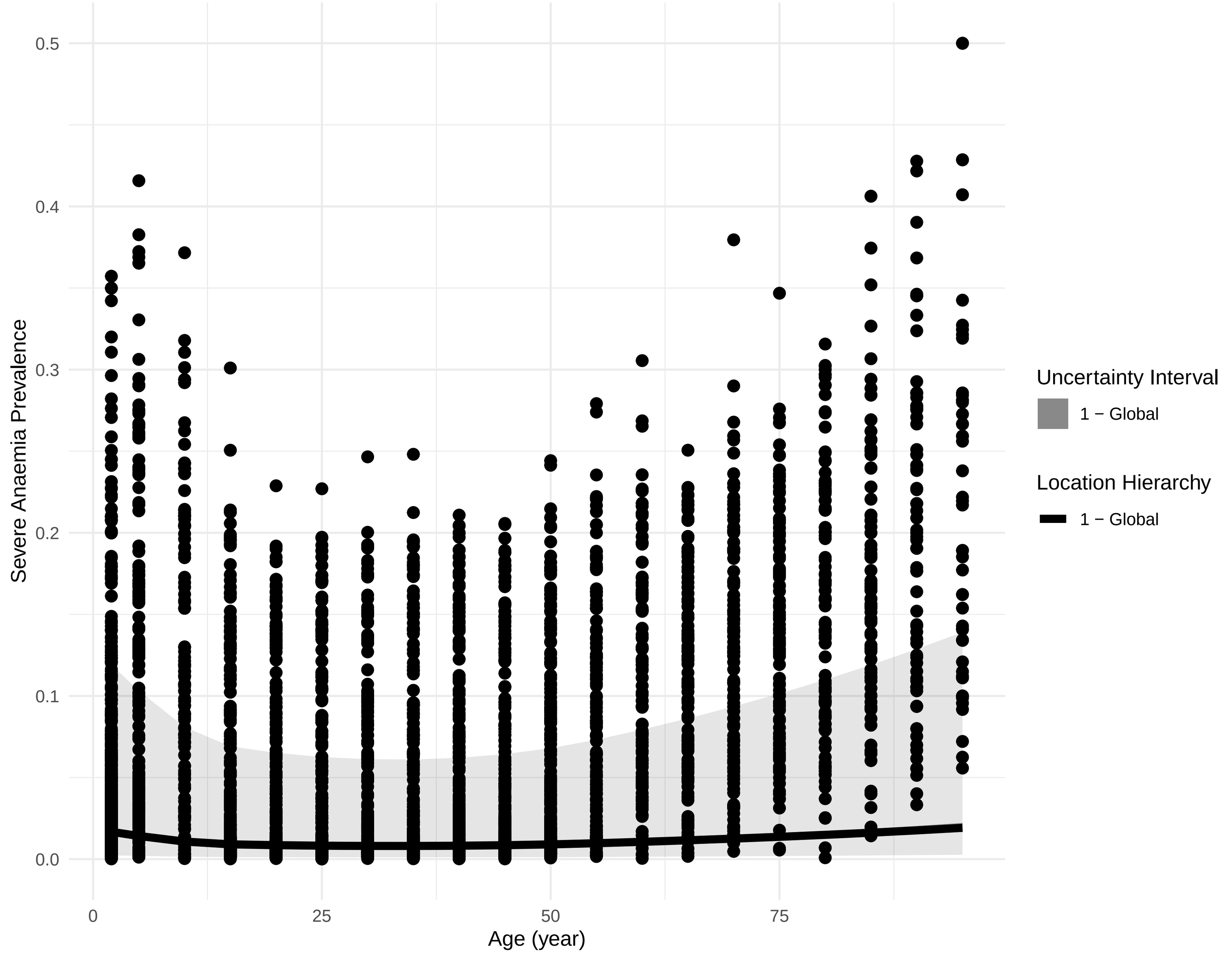

MR−BRT Age/Sex Fits − Males 2+ Years Old − Severe Anaemia Prevalence − Central Europe, Eastern Europe, and Central Asia
WHO−2001 Adjustment Method

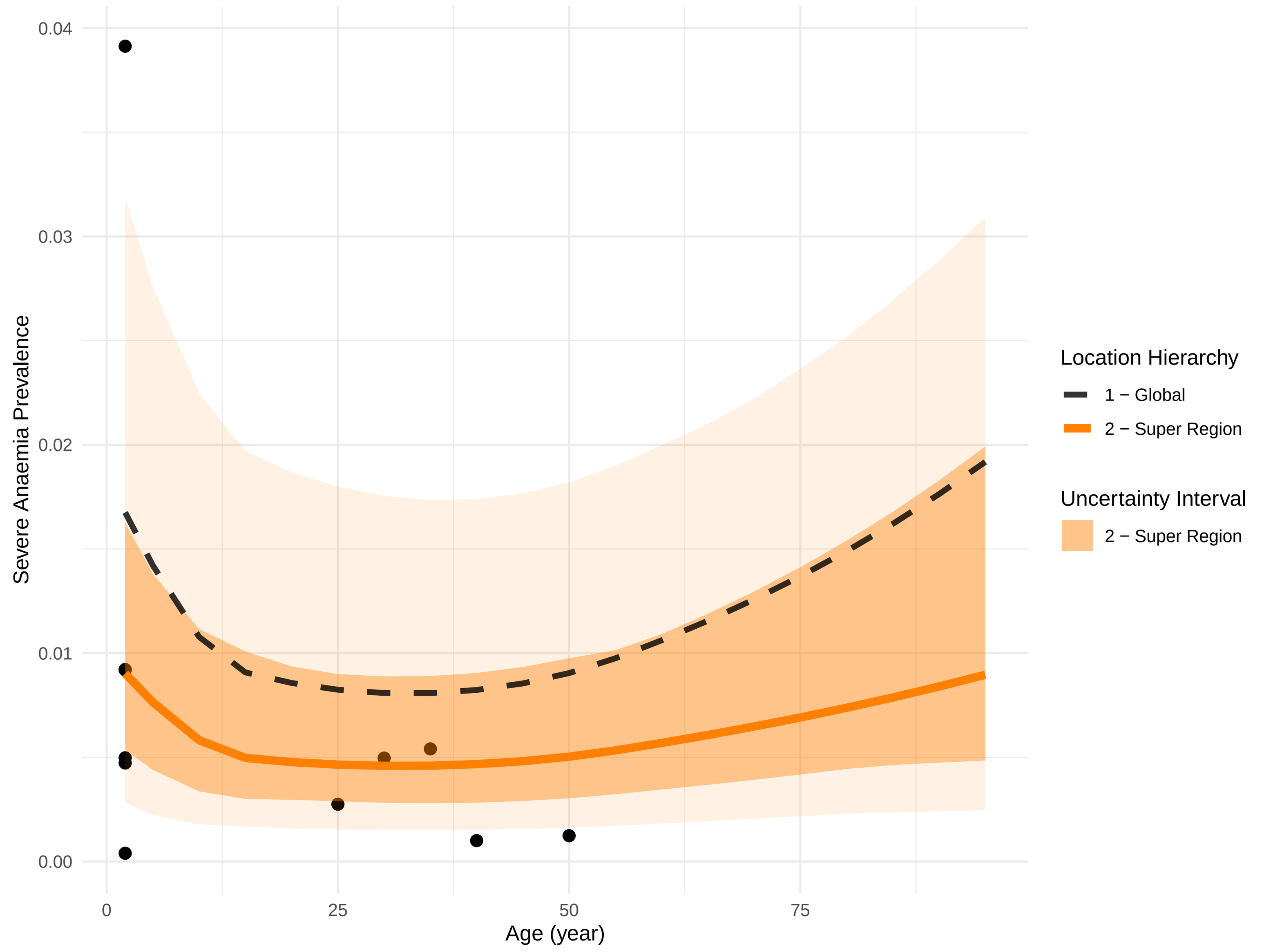

MR−BRT Age/Sex Fits − Males 2+ Years Old − Severe Anaemia Prevalence − Central Asia
WHO−2001 Adjustment Method

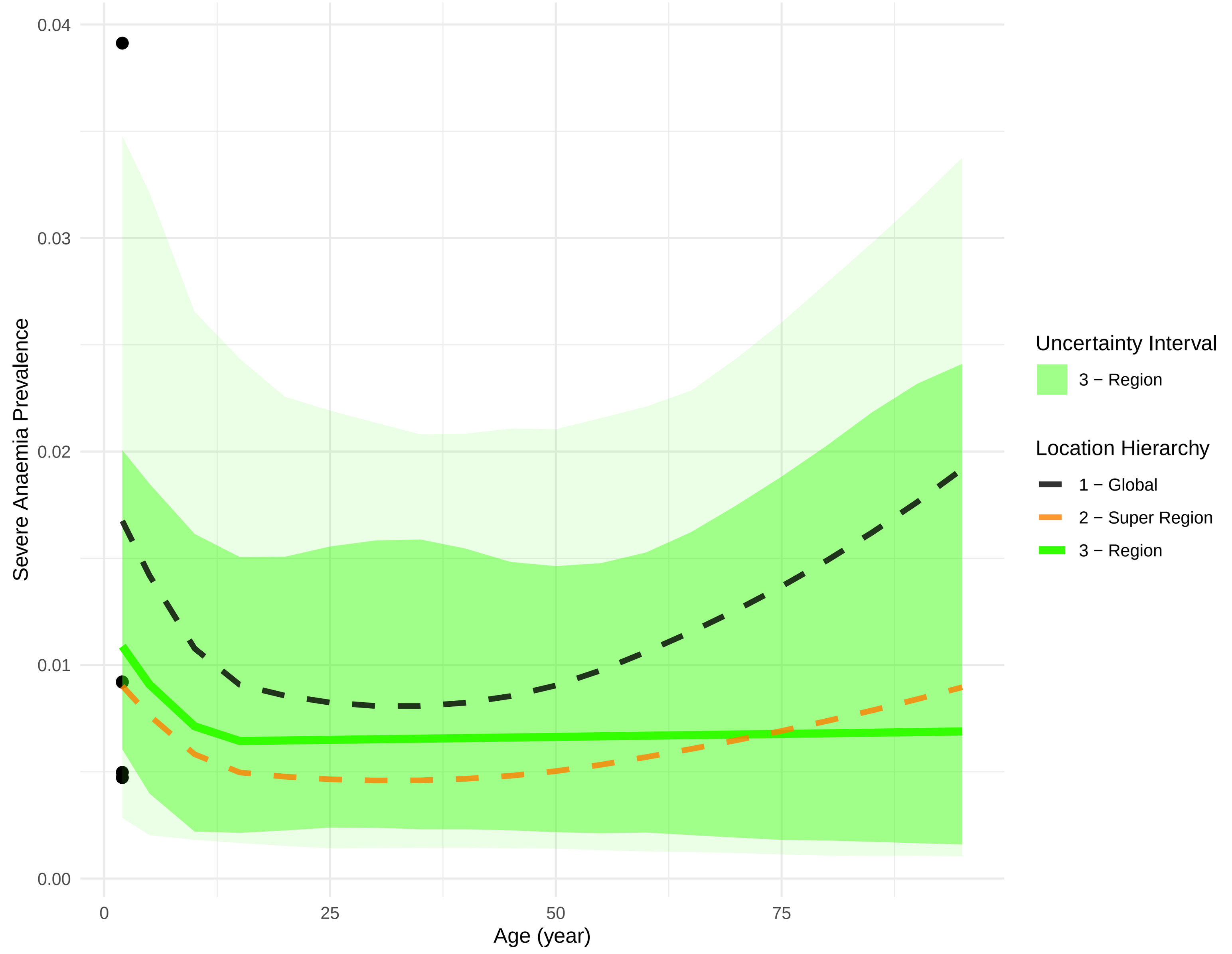

MR−BRT Age/Sex Fits − Males 2+ Years Old − Severe Anaemia Prevalence − Armenia
WHO−2001 Adjustment Method

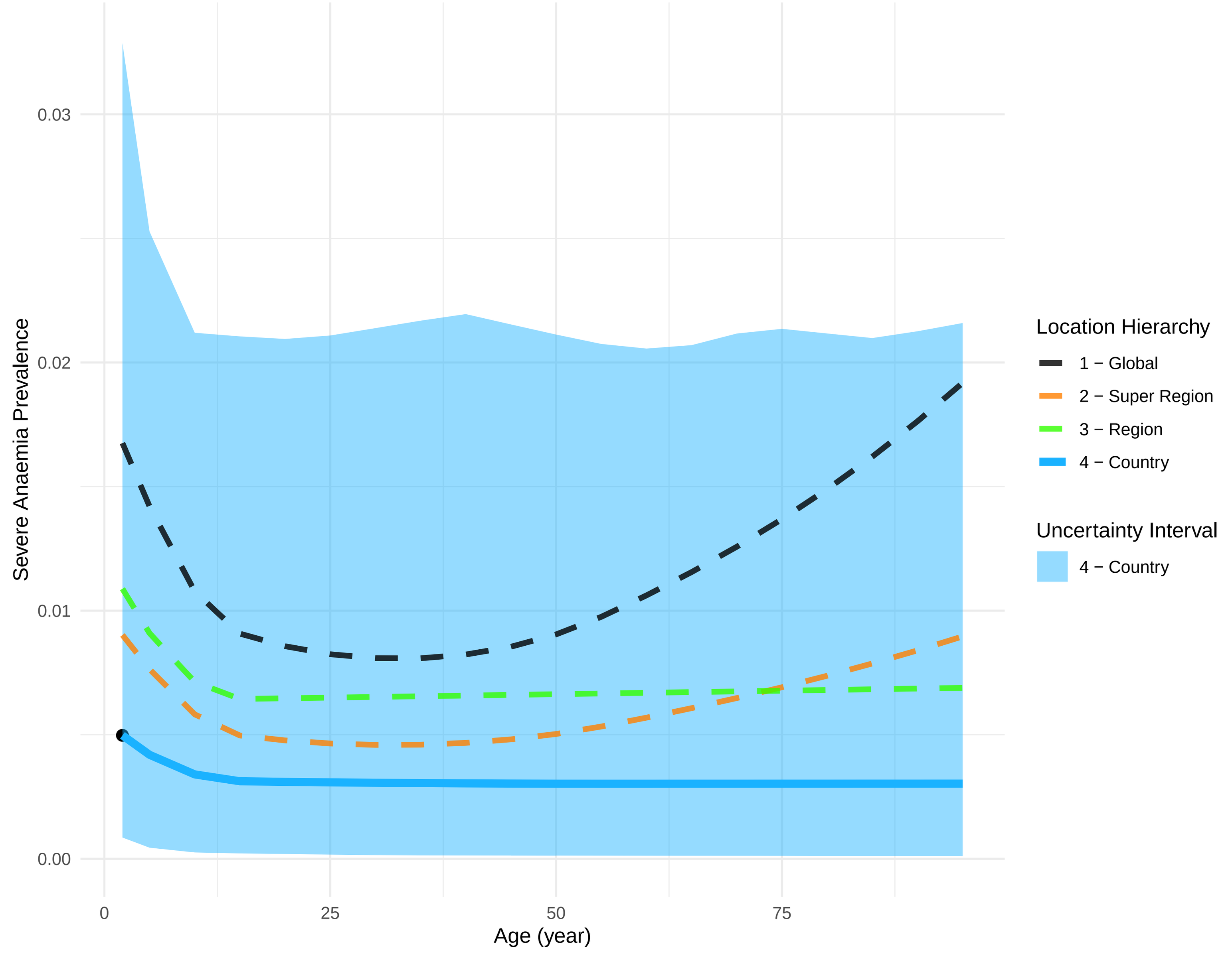

MR−BRT Age/Sex Fits − Males 2+ Years Old − Severe Anaemia Prevalence − Azerbaijan
WHO−2001 Adjustment Method

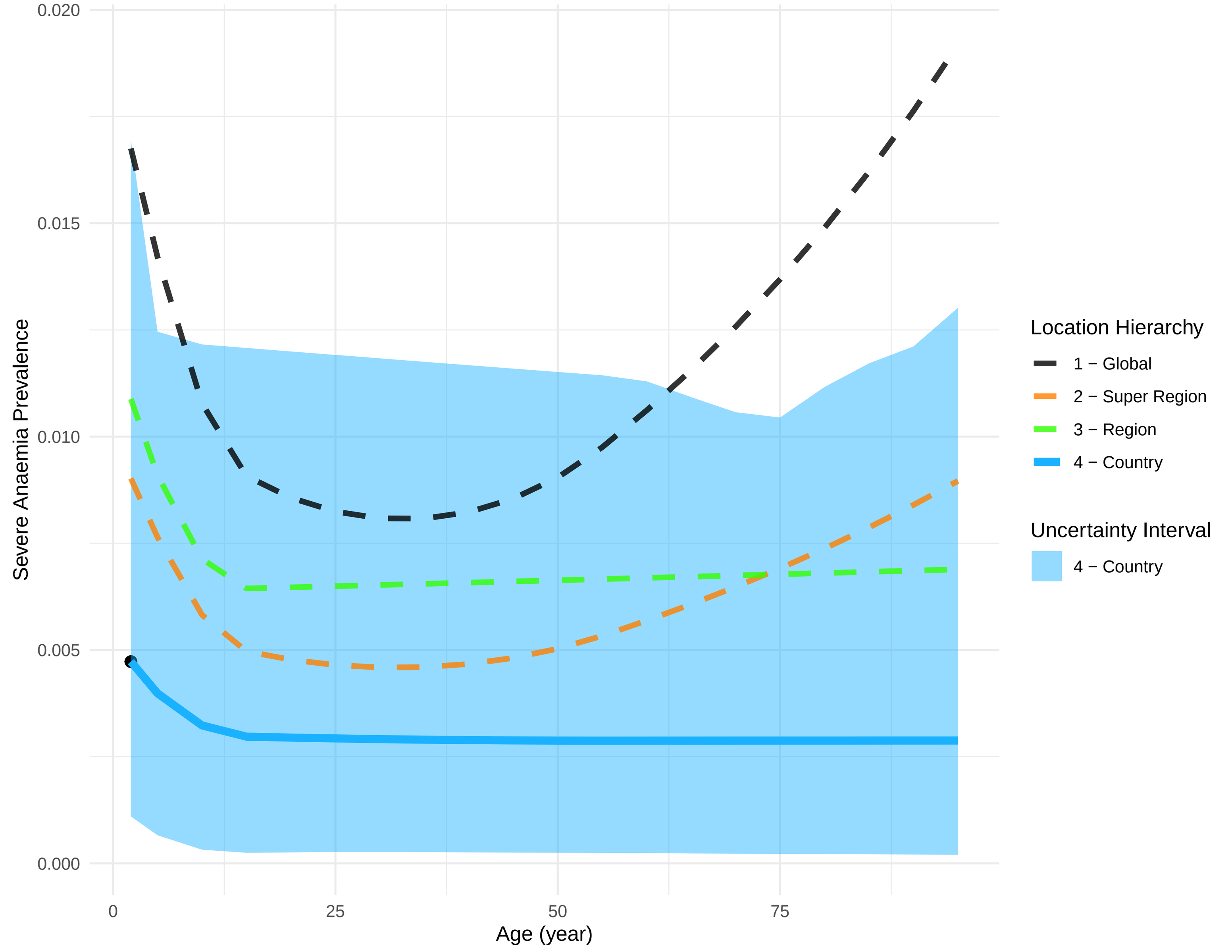

MR−BRT Age/Sex Fits − Males 2+ Years Old − Severe Anaemia Prevalence − Kyrgyzstan
WHO−2001 Adjustment Method

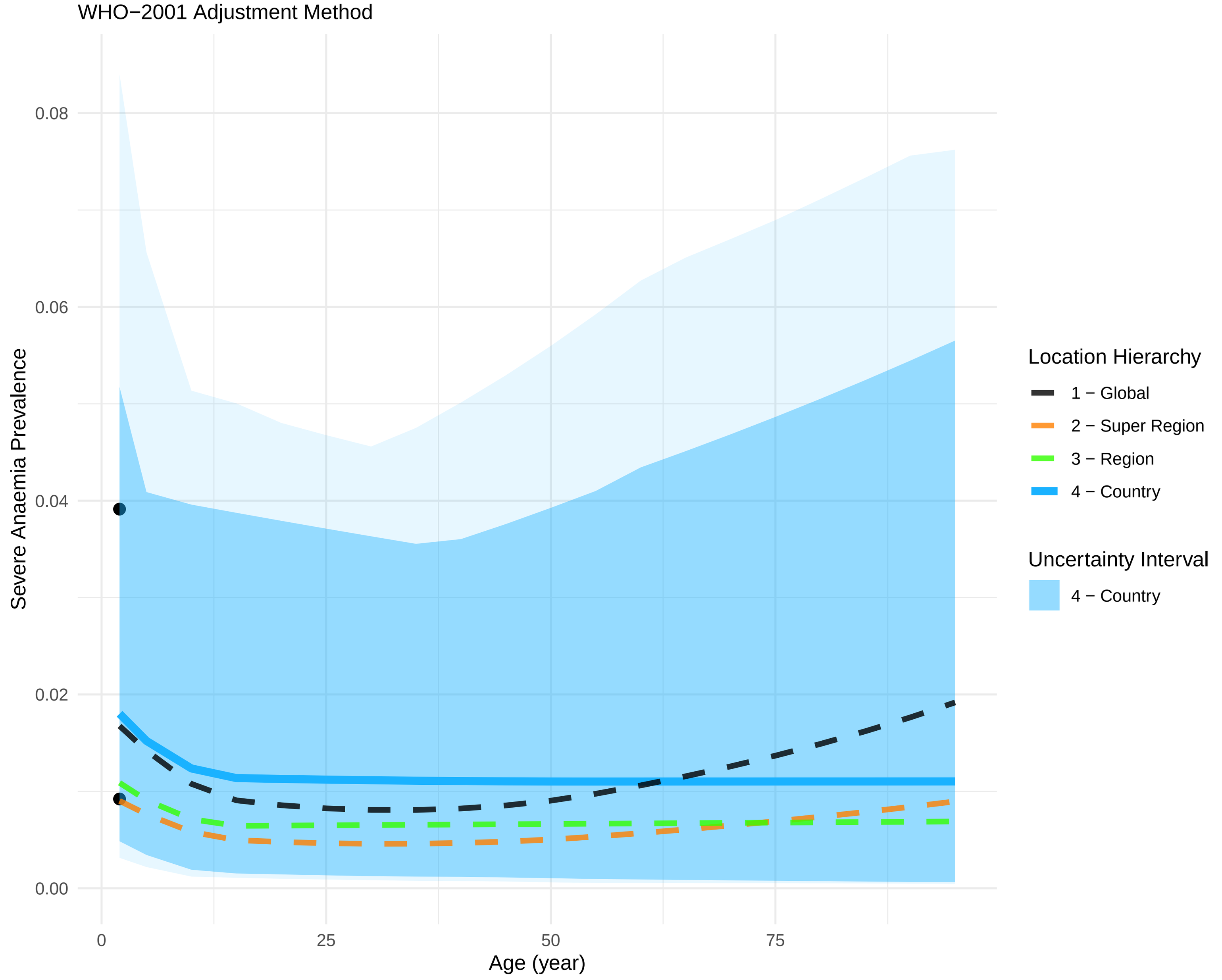

MR−BRT Age/Sex Fits − Males 2+ Years Old − Severe Anaemia Prevalence − Uzbekistan
WHO−2001 Adjustment Method

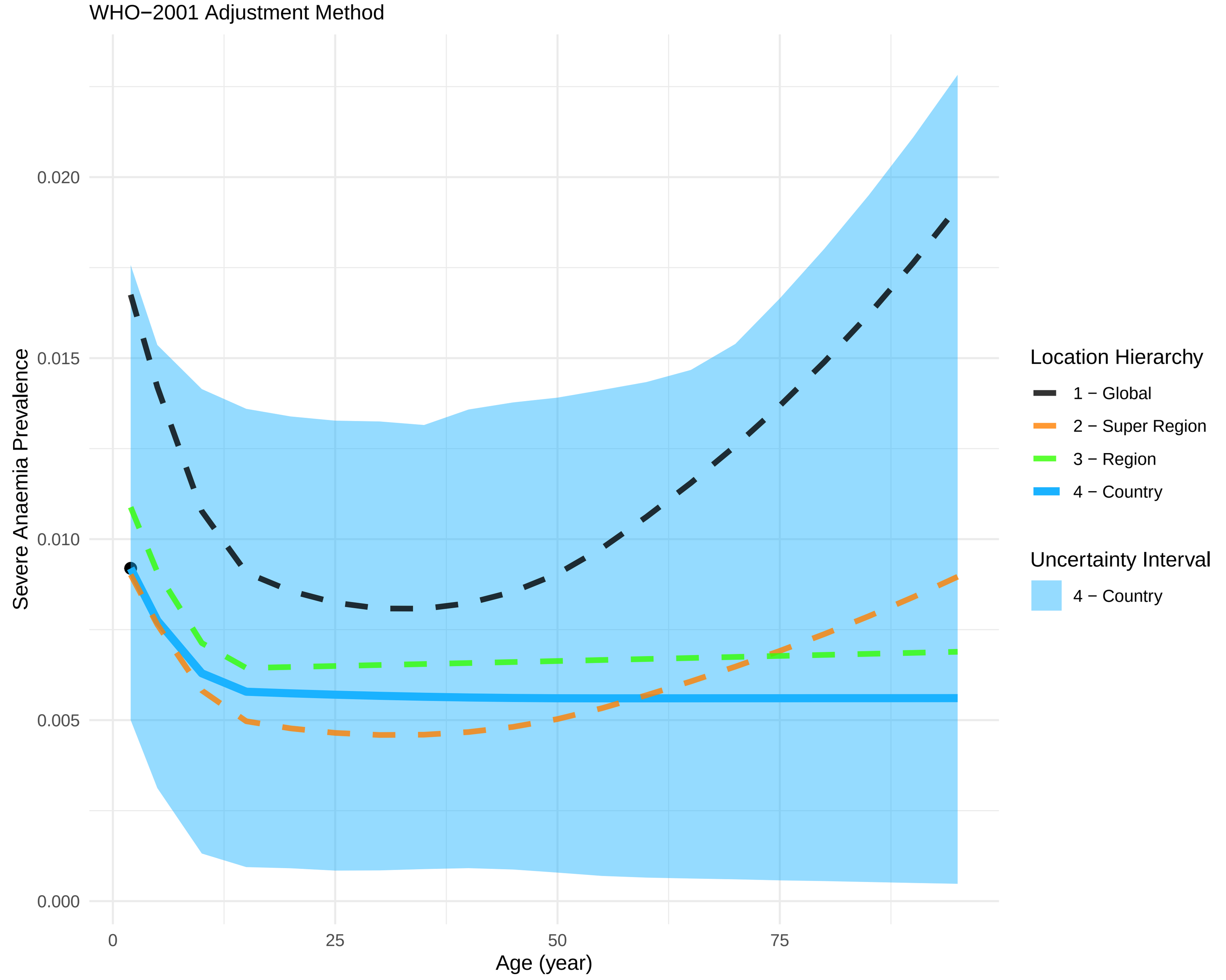

MR−BRT Age/Sex Fits − Males 2+ Years Old − Severe Anaemia Prevalence − Central Europe
WHO−2001 Adjustment Method

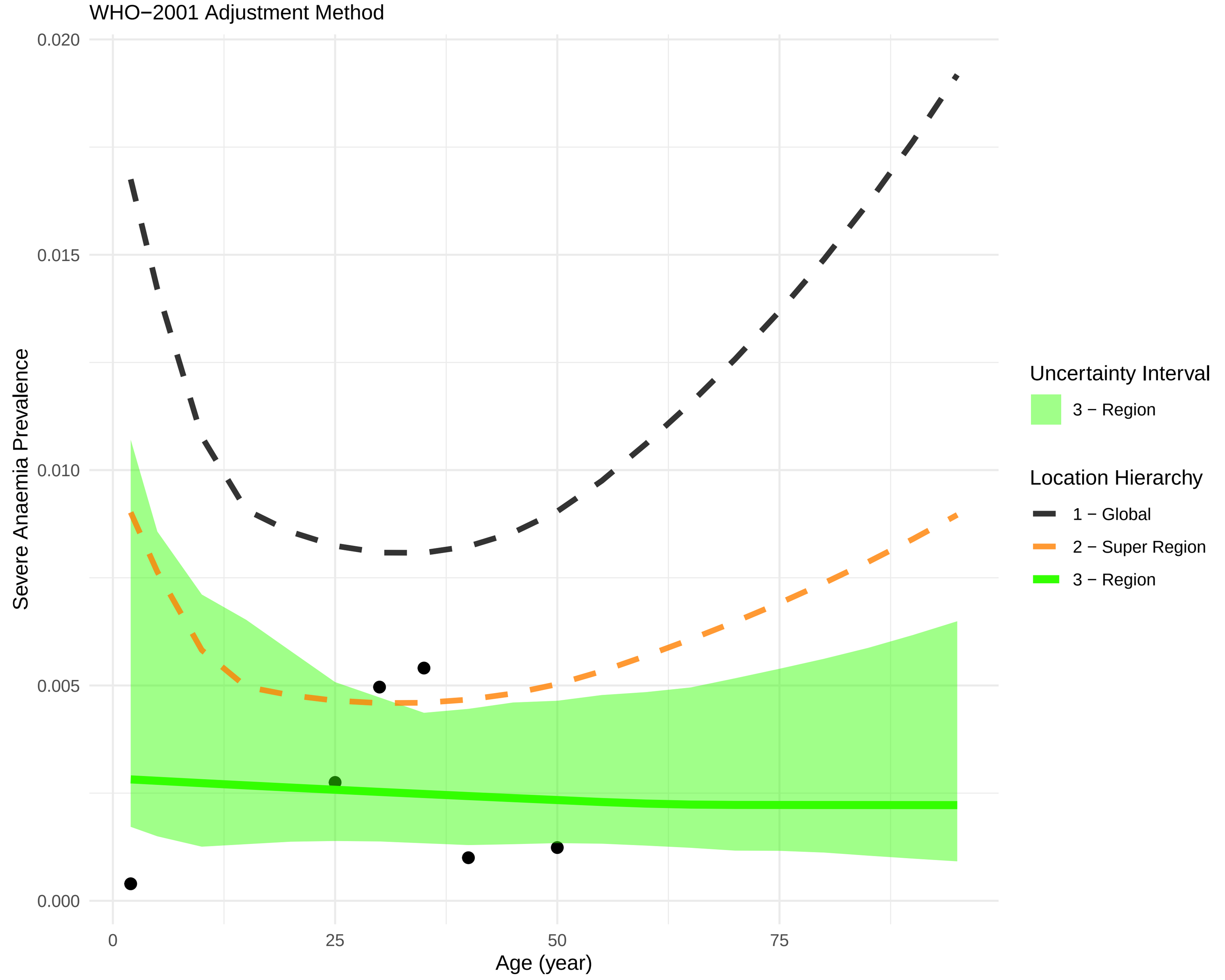

MR−BRT Age/Sex Fits − Males 2+ Years Old − Severe Anaemia Prevalence − Albania
WHO−2001 Adjustment Method

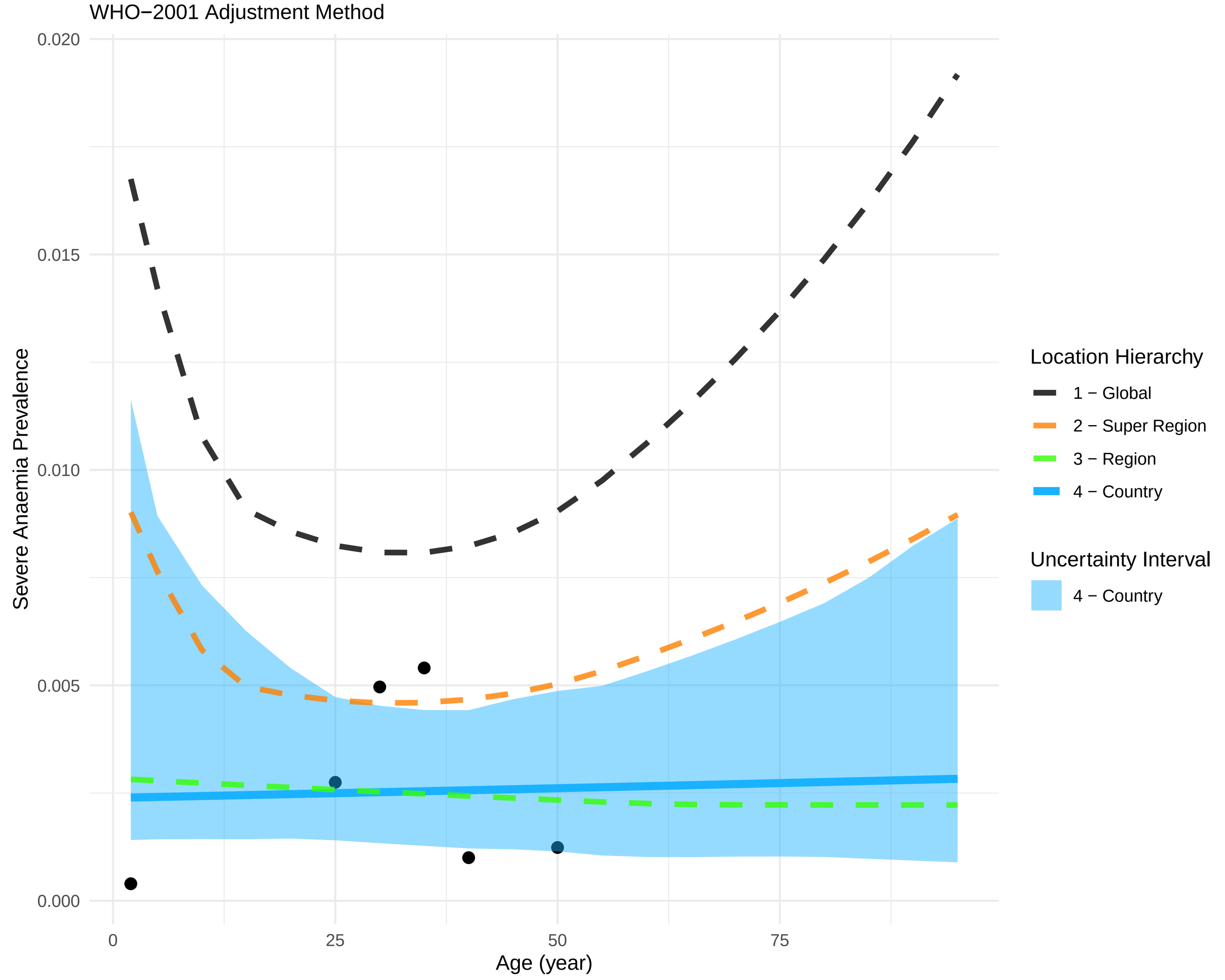

MR−BRT Age/Sex Fits − Males 2+ Years Old − Severe Anaemia Prevalence − High−income
WHO−2001 Adjustment Method

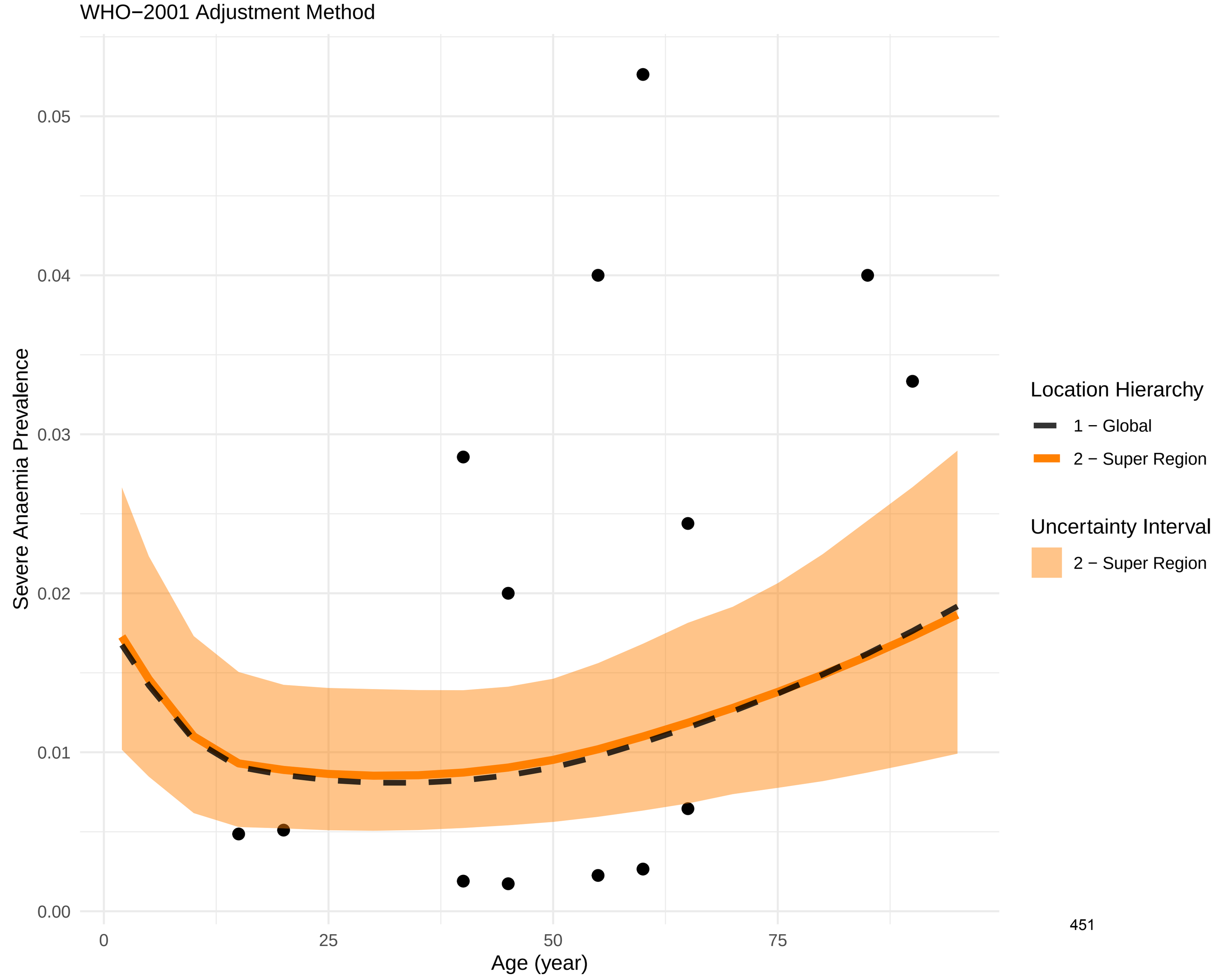

MR−BRT Age/Sex Fits − Males 2+ Years Old − Severe Anaemia Prevalence − Western Europe
WHO−2001 Adjustment Method

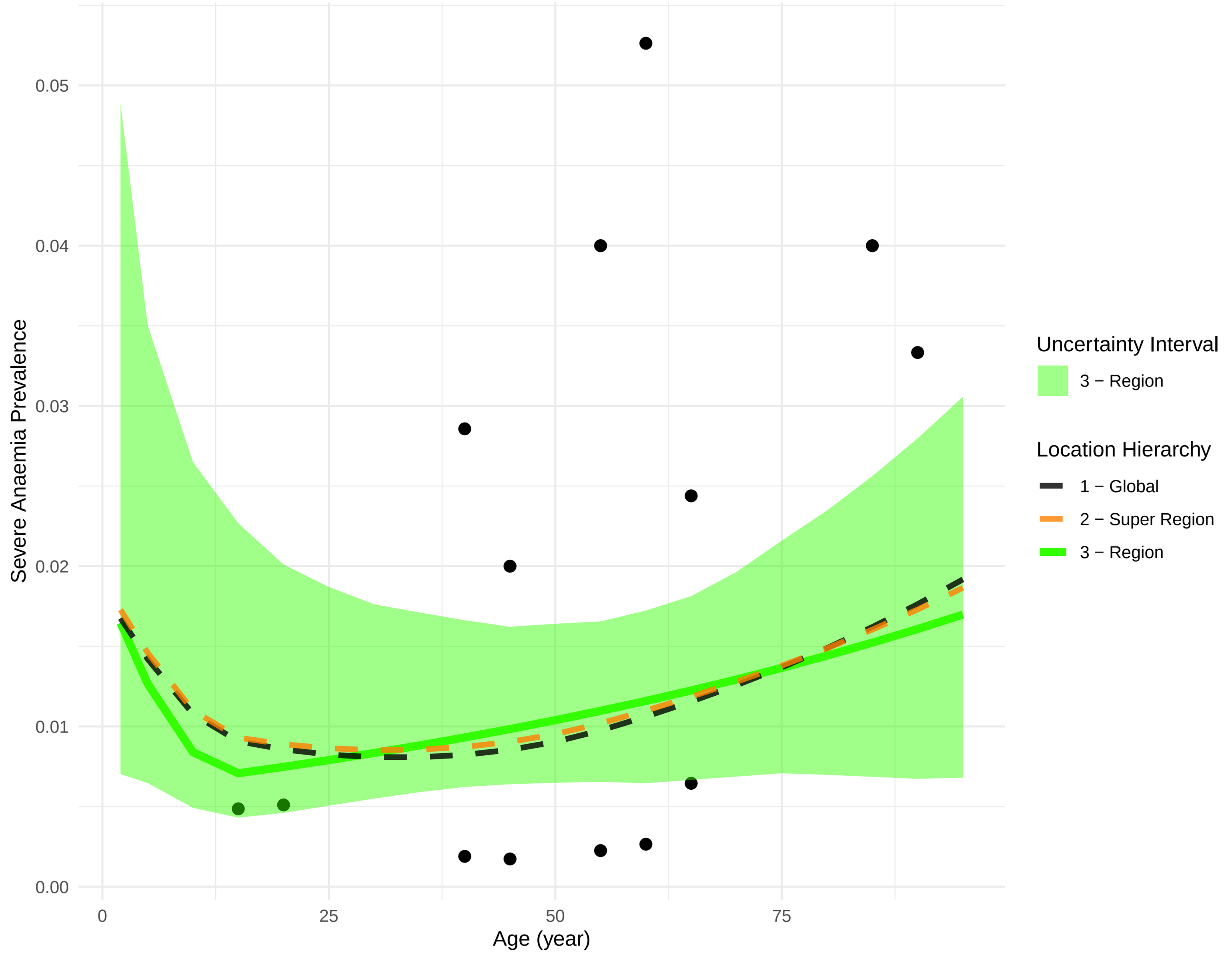

MR−BRT Age/Sex Fits − Males 2+ Years Old − Severe Anaemia Prevalence − United Kingdom
WHO−2001 Adjustment Method

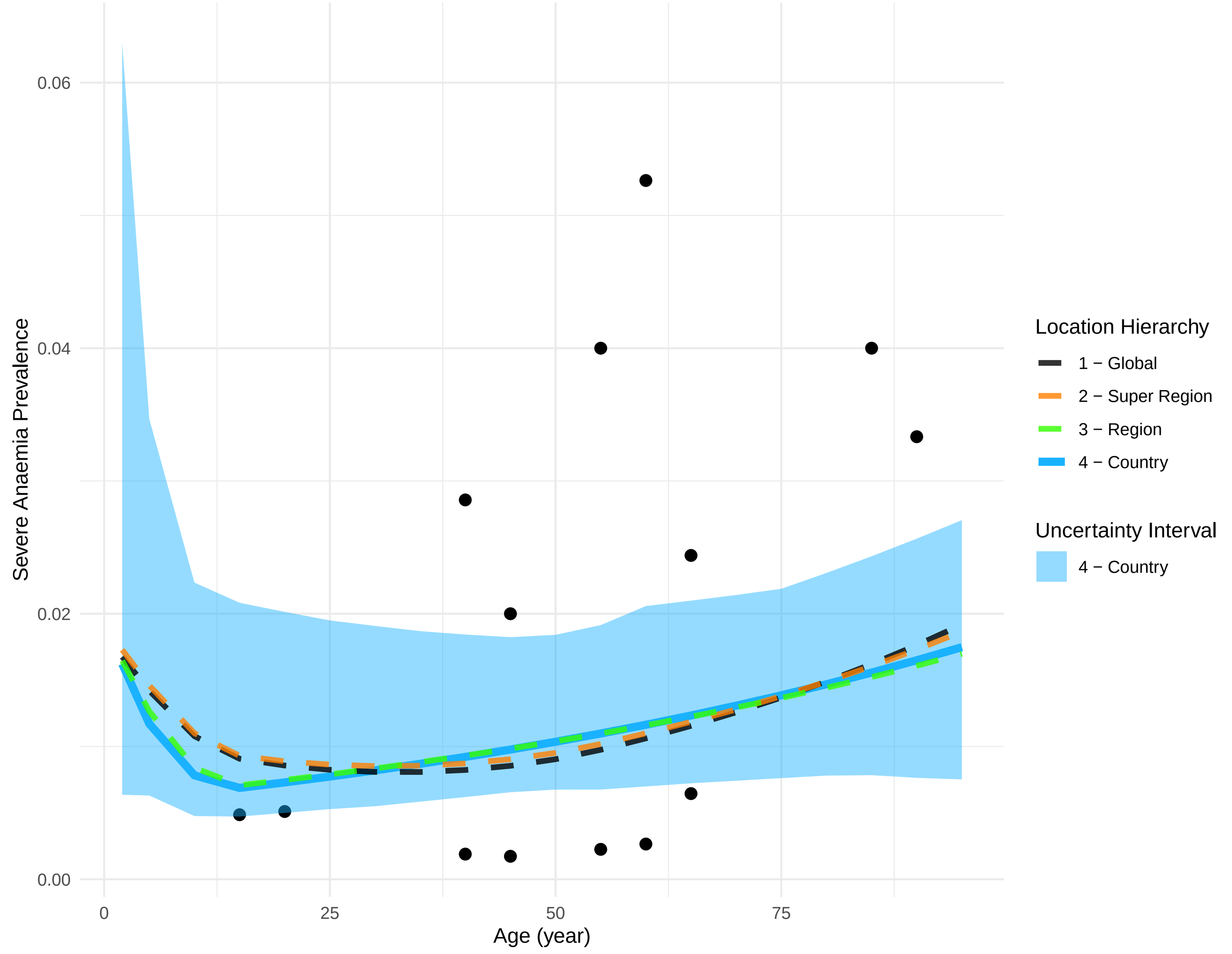

MR−BRT Age/Sex Fits − Males 2+ Years Old − Severe Anaemia Prevalence − England
WHO−2001 Adjustment Method

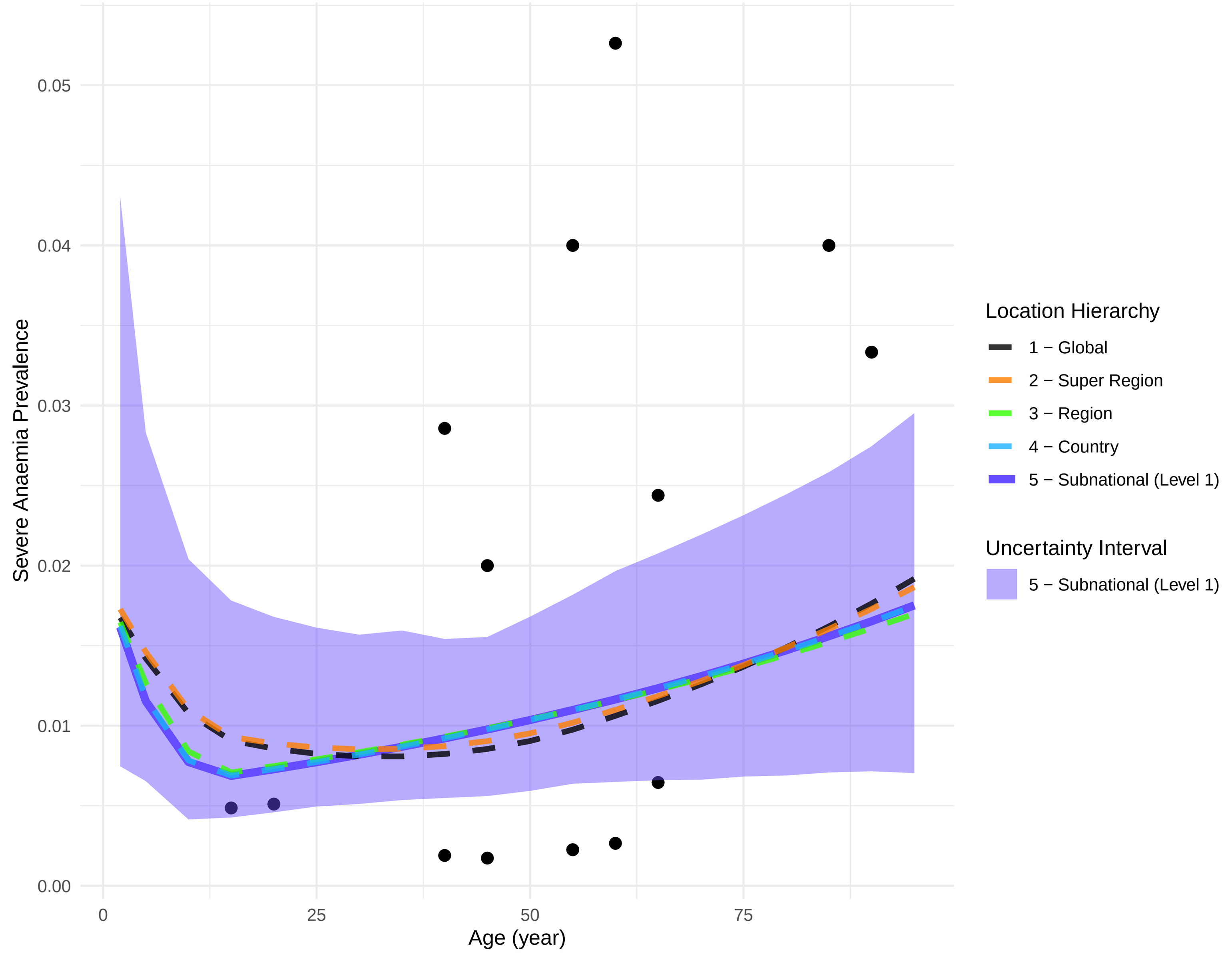

MR−BRT Age/Sex Fits − Males 2+ Years Old − Severe Anaemia Prevalence − East of England
WHO−2001 Adjustment Method

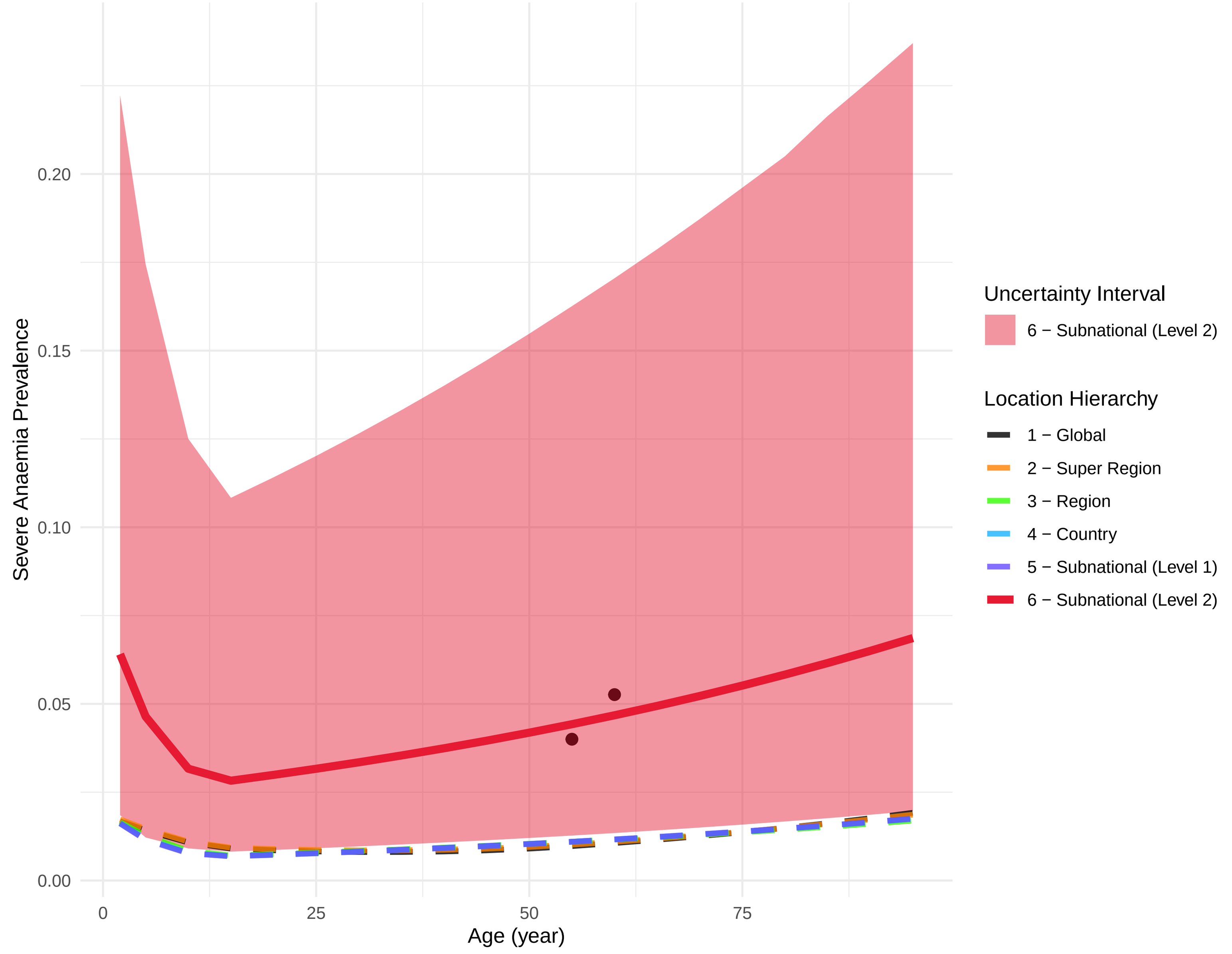

MR−BRT Age/Sex Fits − Males 2+ Years Old − Severe Anaemia Prevalence − South West England
WHO−2001 Adjustment Method

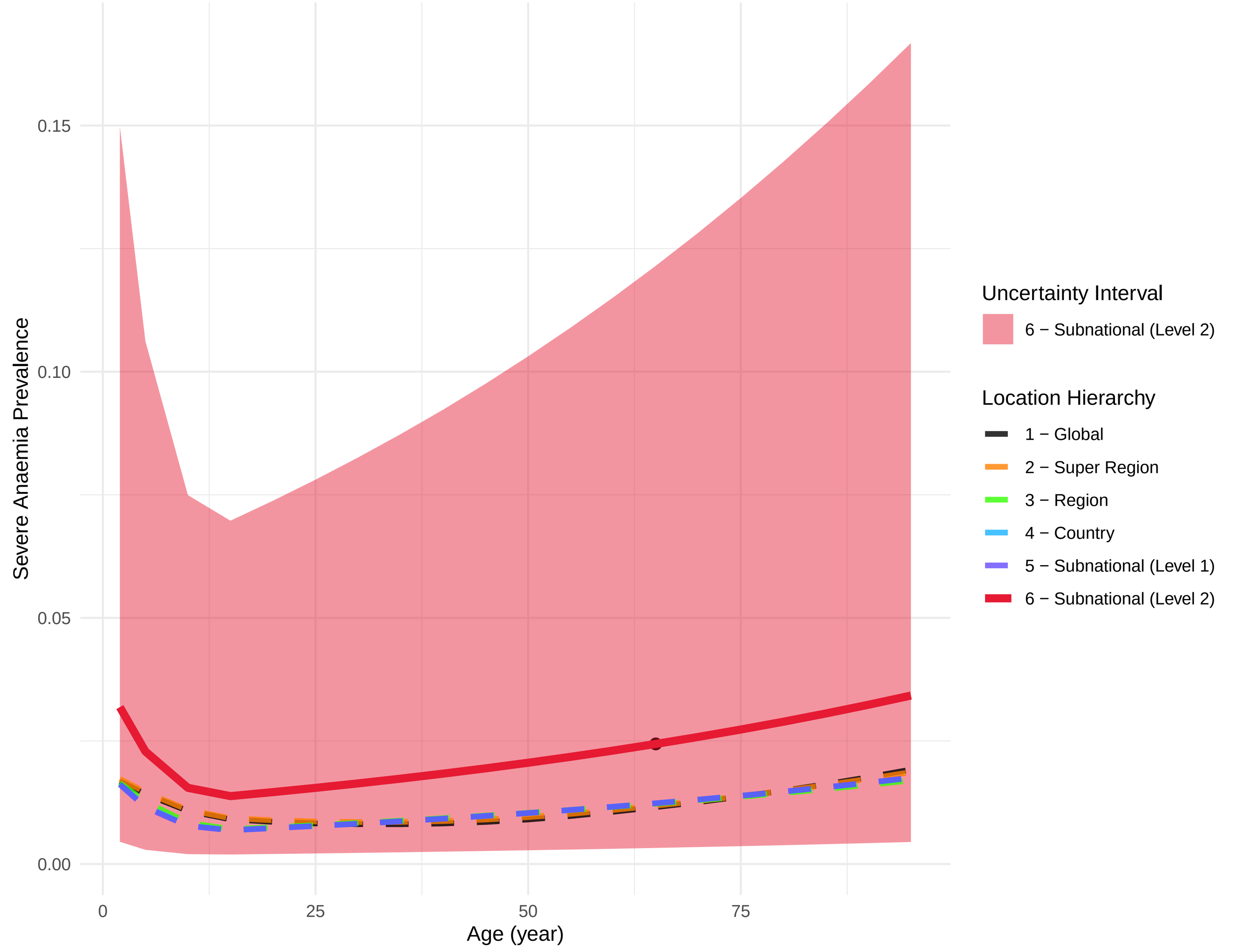

MR−BRT Age/Sex Fits − Males 2+ Years Old − Severe Anaemia Prevalence − West Midlands
WHO−2001 Adjustment Method

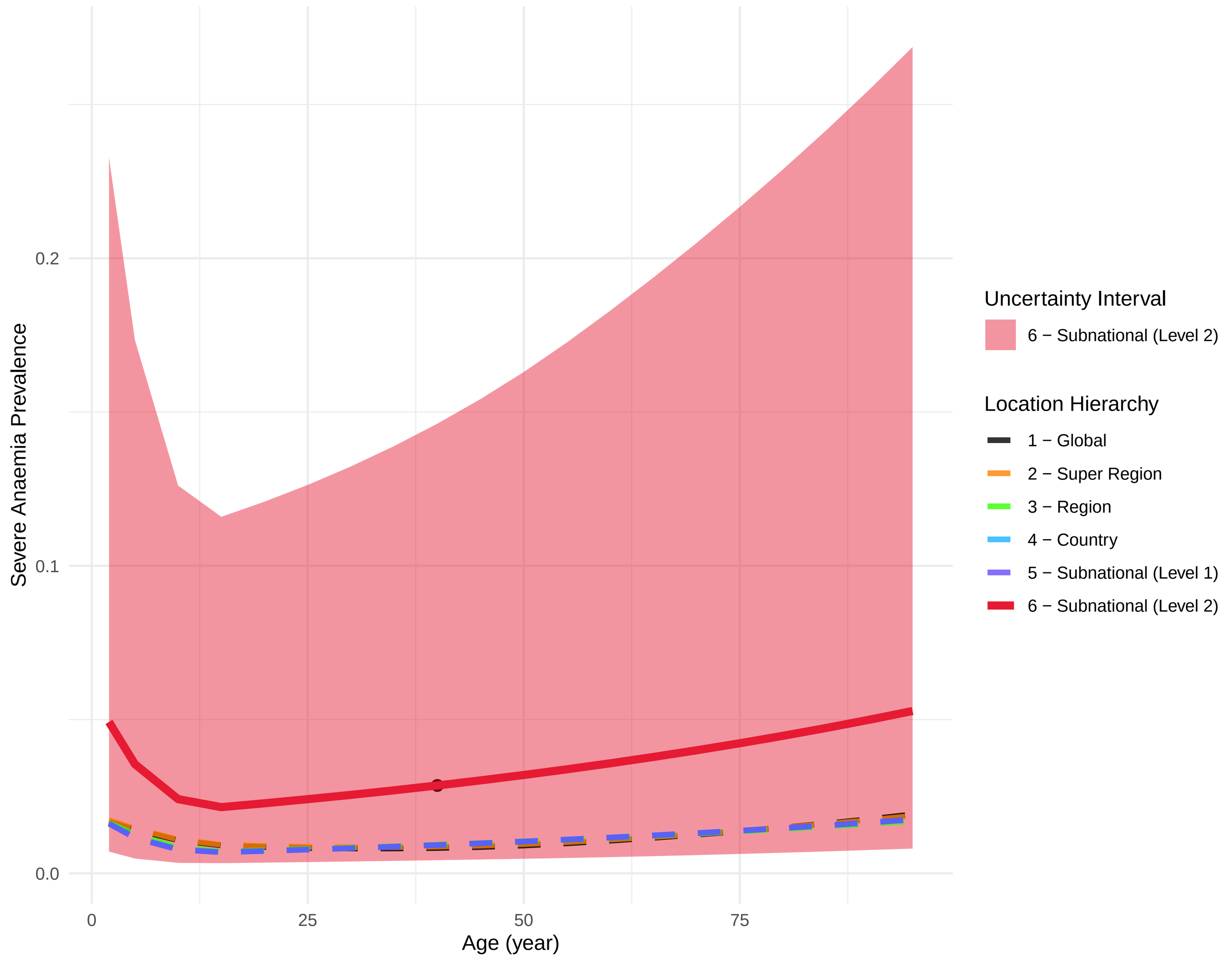

MR−BRT Age/Sex Fits − Males 2+ Years Old − Severe Anaemia Prevalence − Yorkshire and the Humber
WHO−2001 Adjustment Method

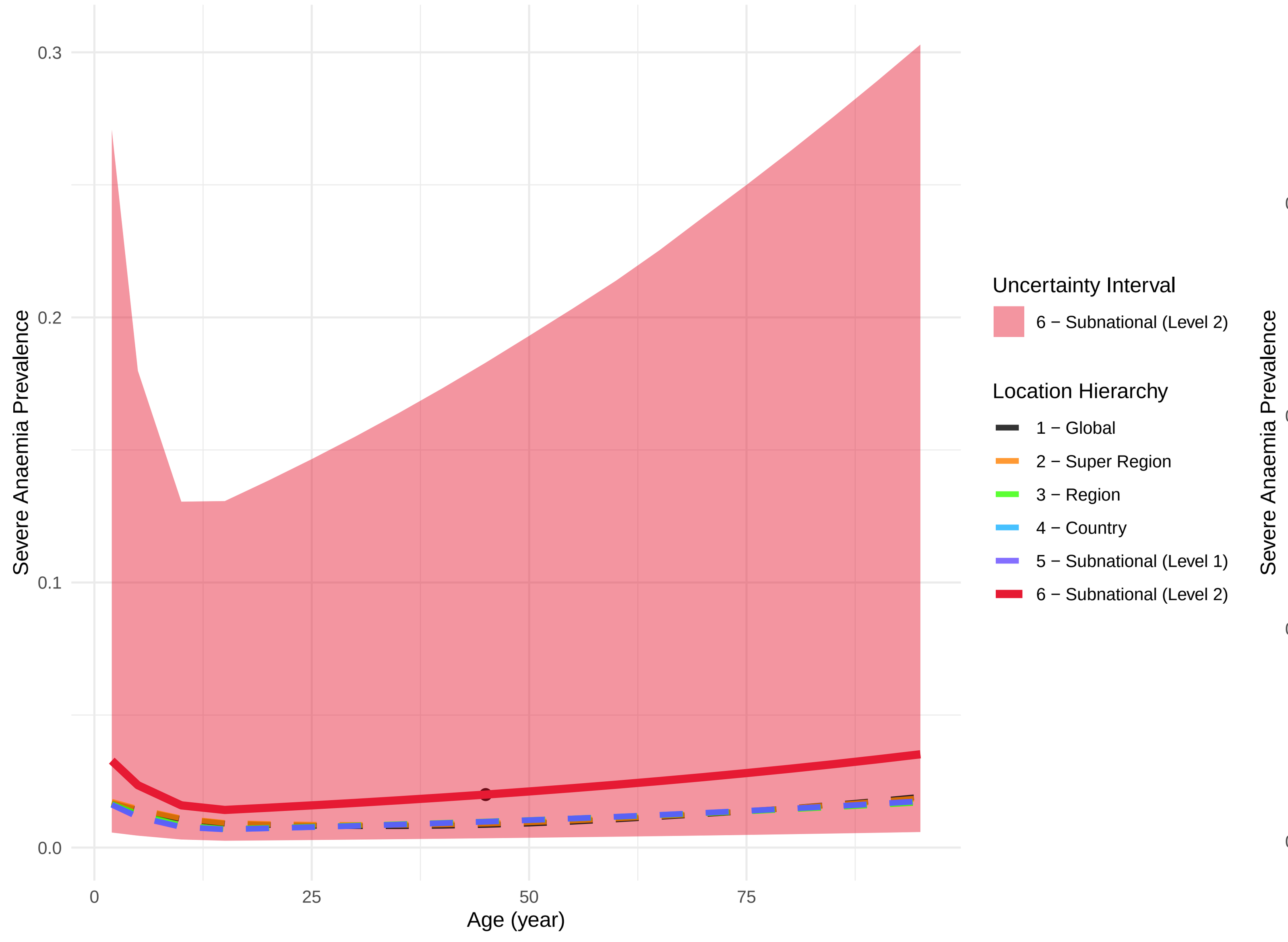

MR−BRT Age/Sex Fits − Males 2+ Years Old − Severe Anaemia Prevalence − Latin America and Caribbean
WHO−2001 Adjustment Method

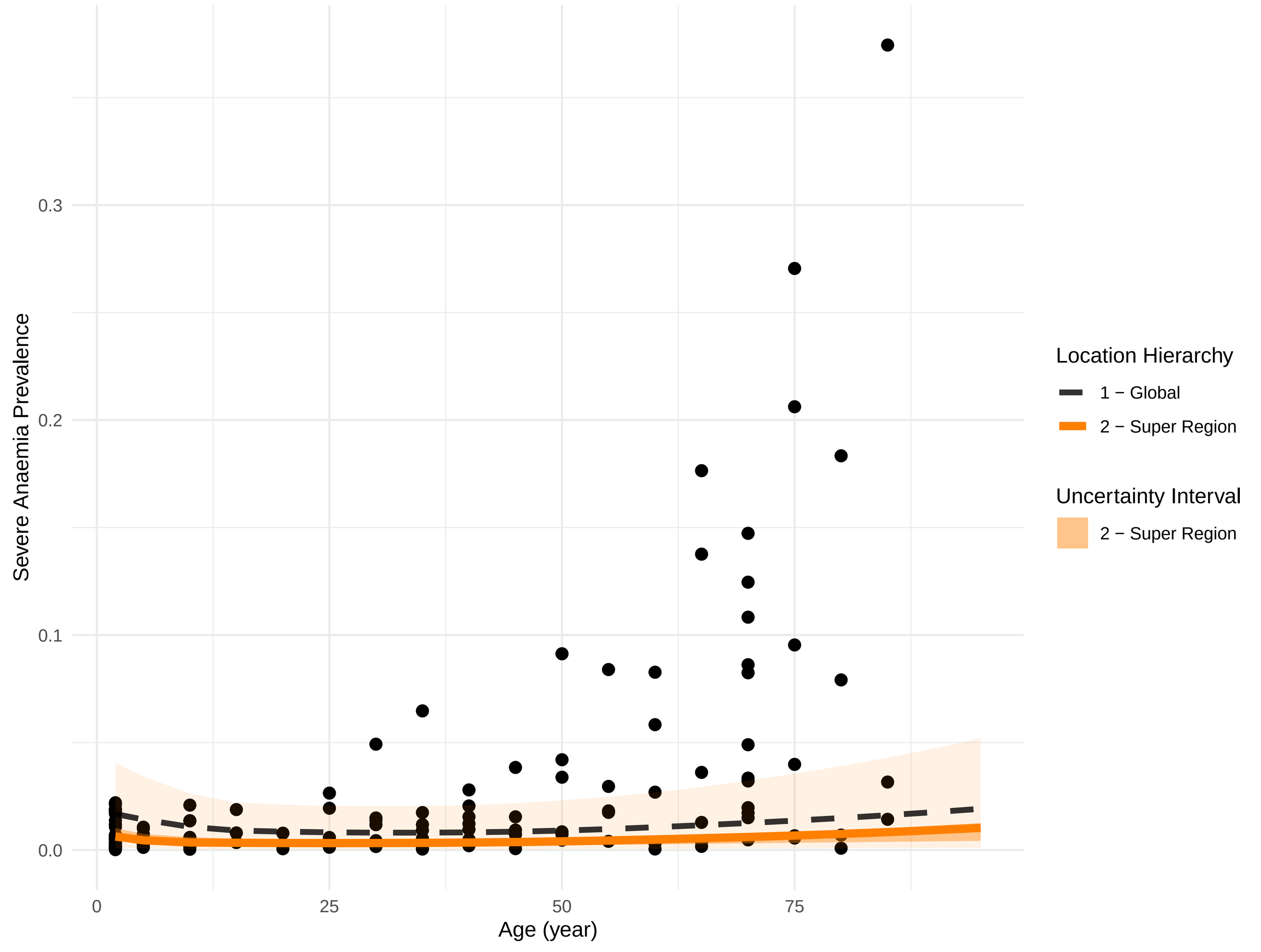

MR−BRT Age/Sex Fits − Males 2+ Years Old − Severe Anaemia Prevalence − Andean Latin America
WHO−2001 Adjustment Method

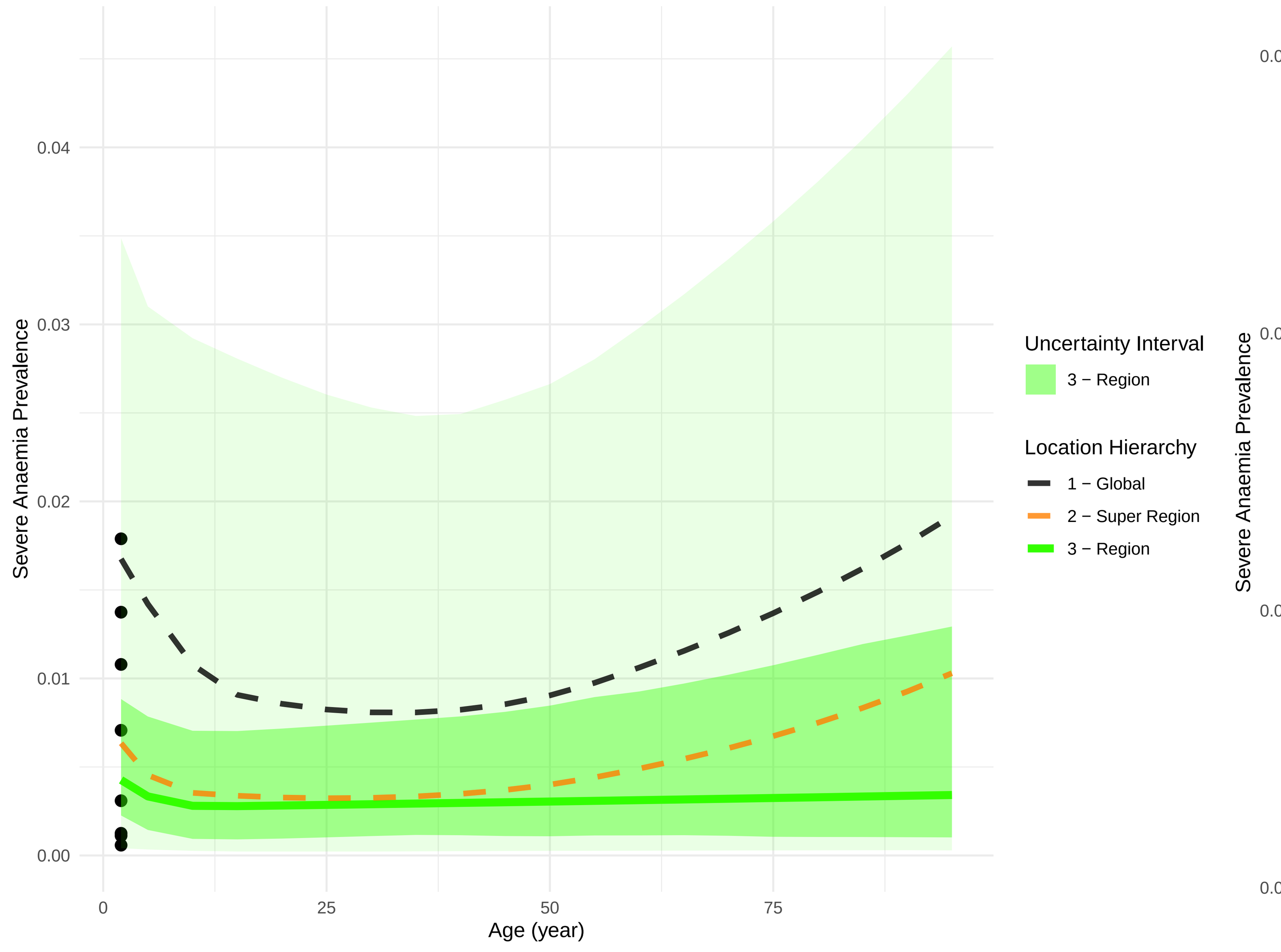

MR−BRT Age/Sex Fits − Males 2+ Years Old − Severe Anaemia Prevalence − Bolivia (Plurinational State of)
WHO−2001 Adjustment Method

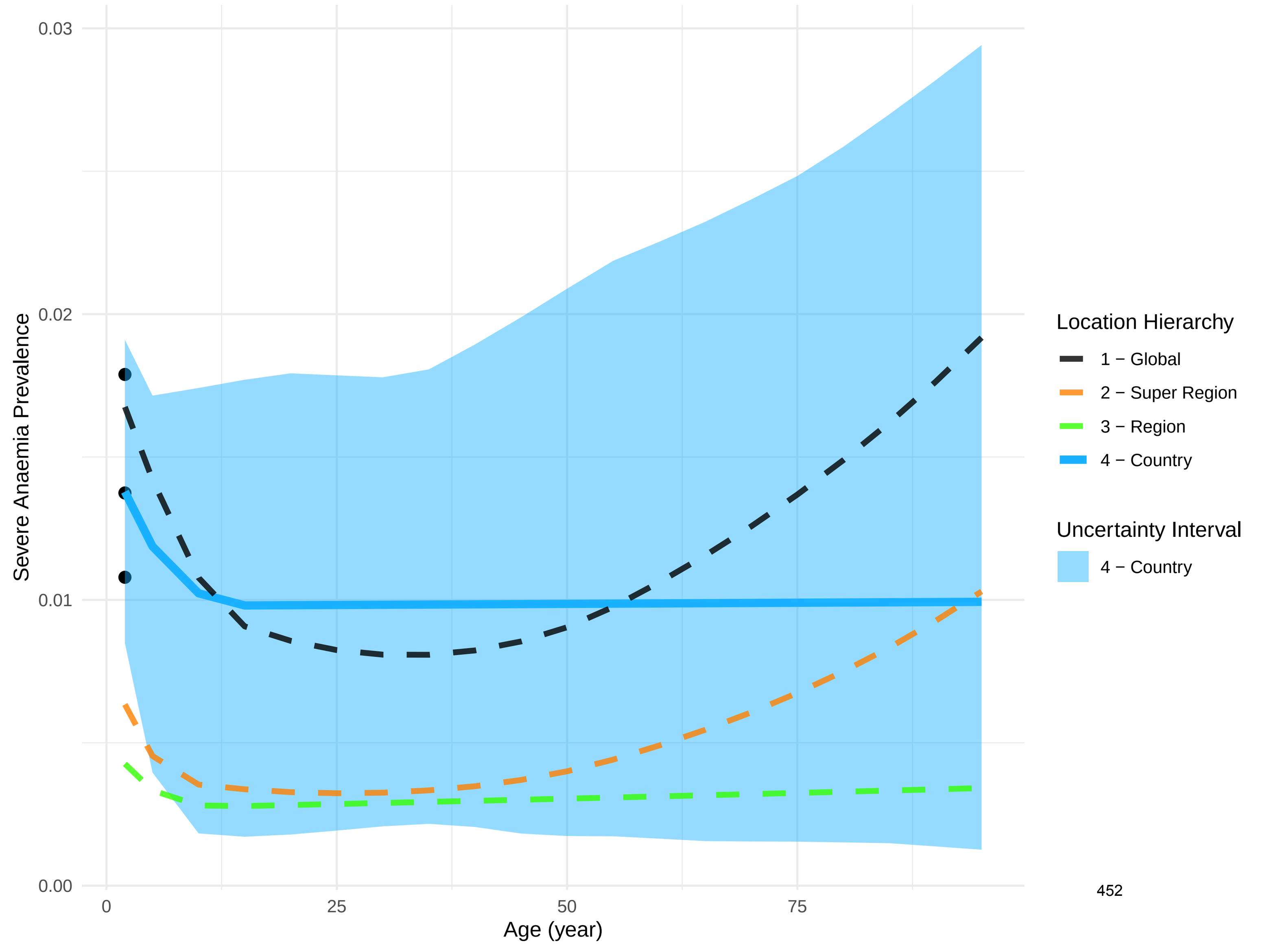

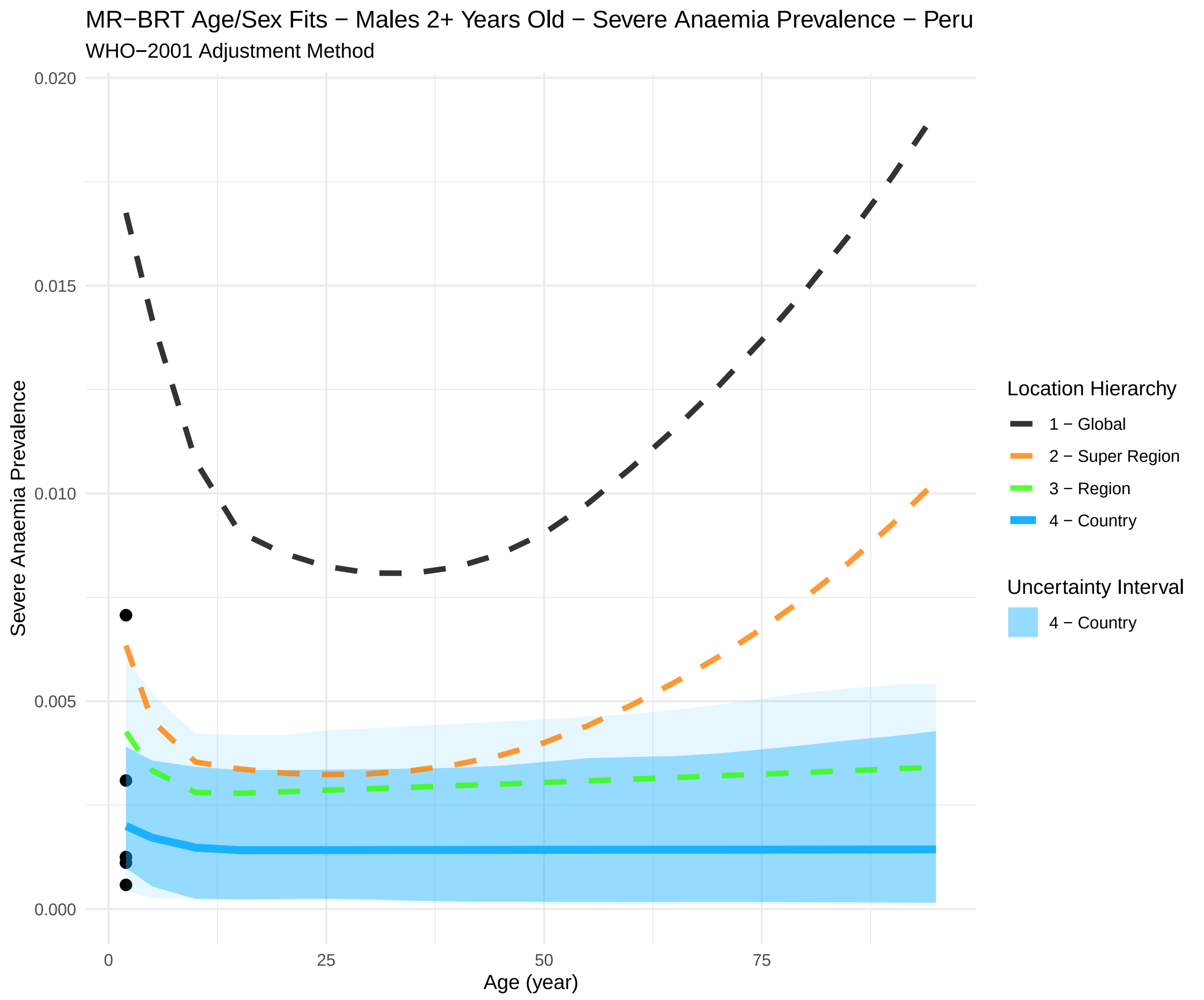
MR−BRT Age/Sex Fits − Males 2+ Years Old − Severe Anaemia Prevalence − Peru
WHO−2001 Adjustment Method
Severe Anaemia Prevalence
Age (year)
Location Hierarchy
1 − Global
2 − Super Region
3 − Region
4 − Country
Uncertainty Interval
4 − Country

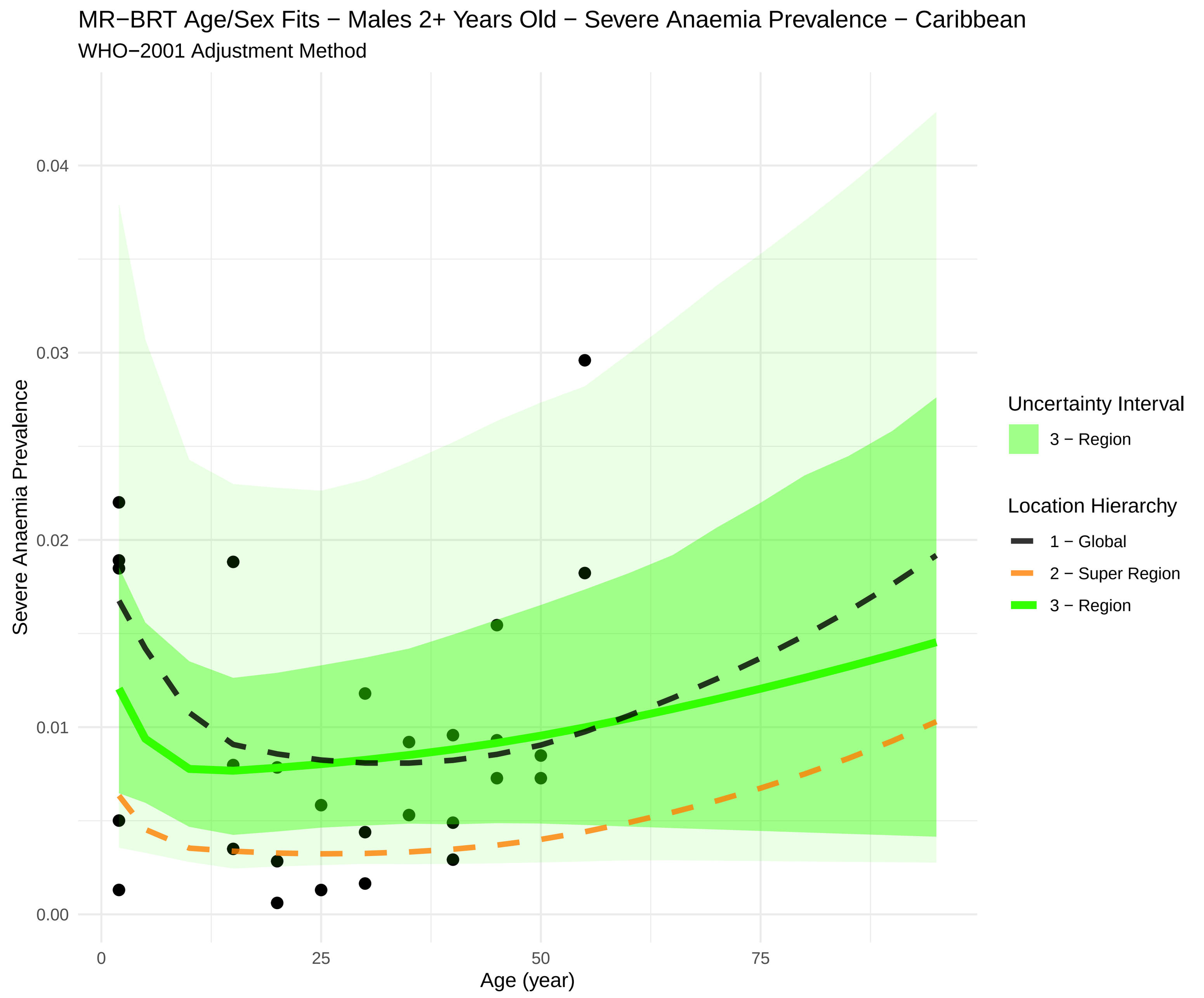
MR−BRT Age/Sex Fits − Males 2+ Years Old − Severe Anaemia Prevalence − Caribbean
WHO−2001 Adjustment Method
Severe Anaemia Prevalence
Age (year)
Uncertainty Interval
3 − Region
Location Hierarchy
1 − Global
2 − Super Region
3 − Region

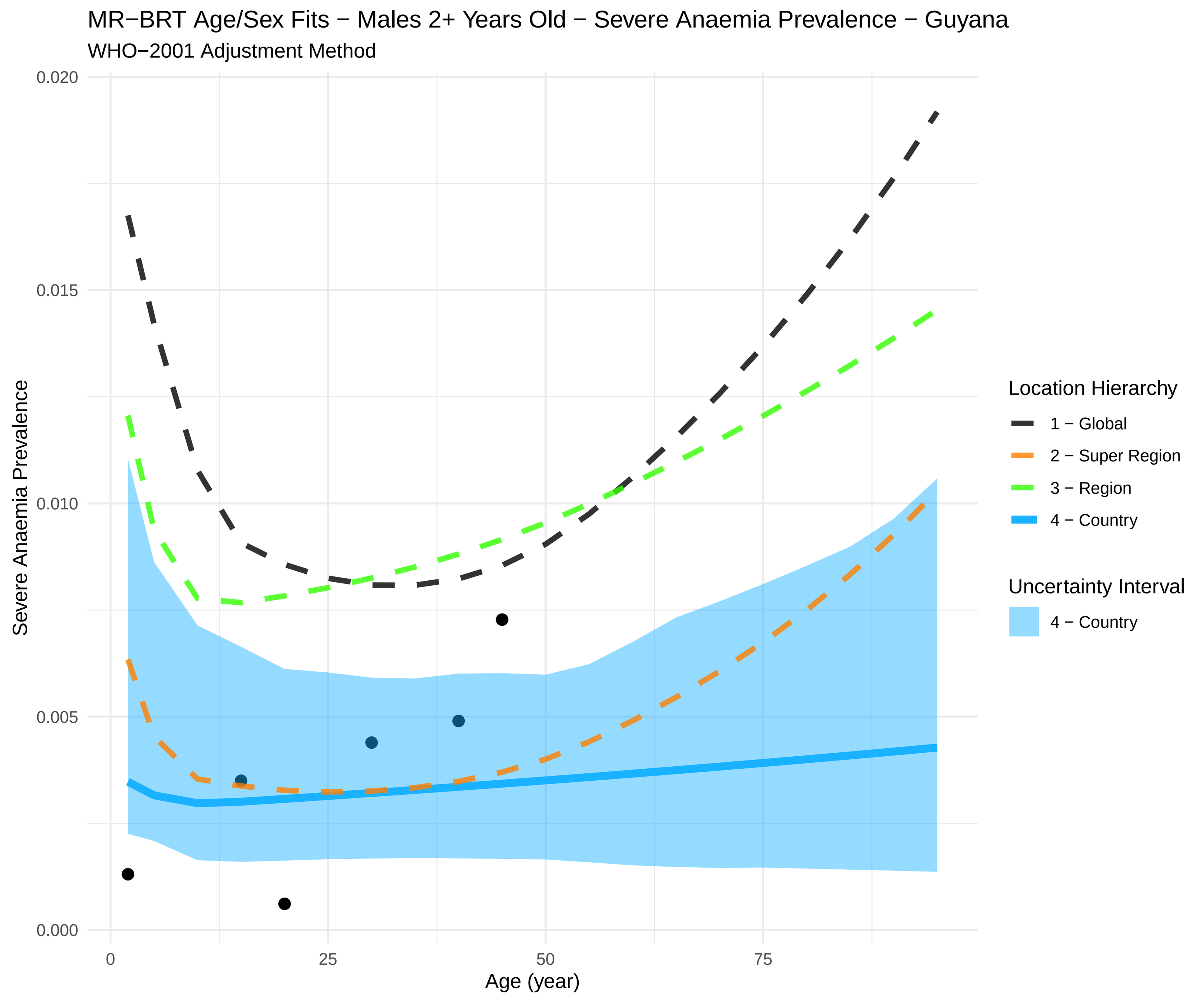
MR−BRT Age/Sex Fits − Males 2+ Years Old − Severe Anaemia Prevalence − Guyana
WHO−2001 Adjustment Method
Severe Anaemia Prevalence
Age (year)
Location Hierarchy
1 − Global
2 − Super Region
3 − Region
4 − Country
Uncertainty Interval
4 − Country

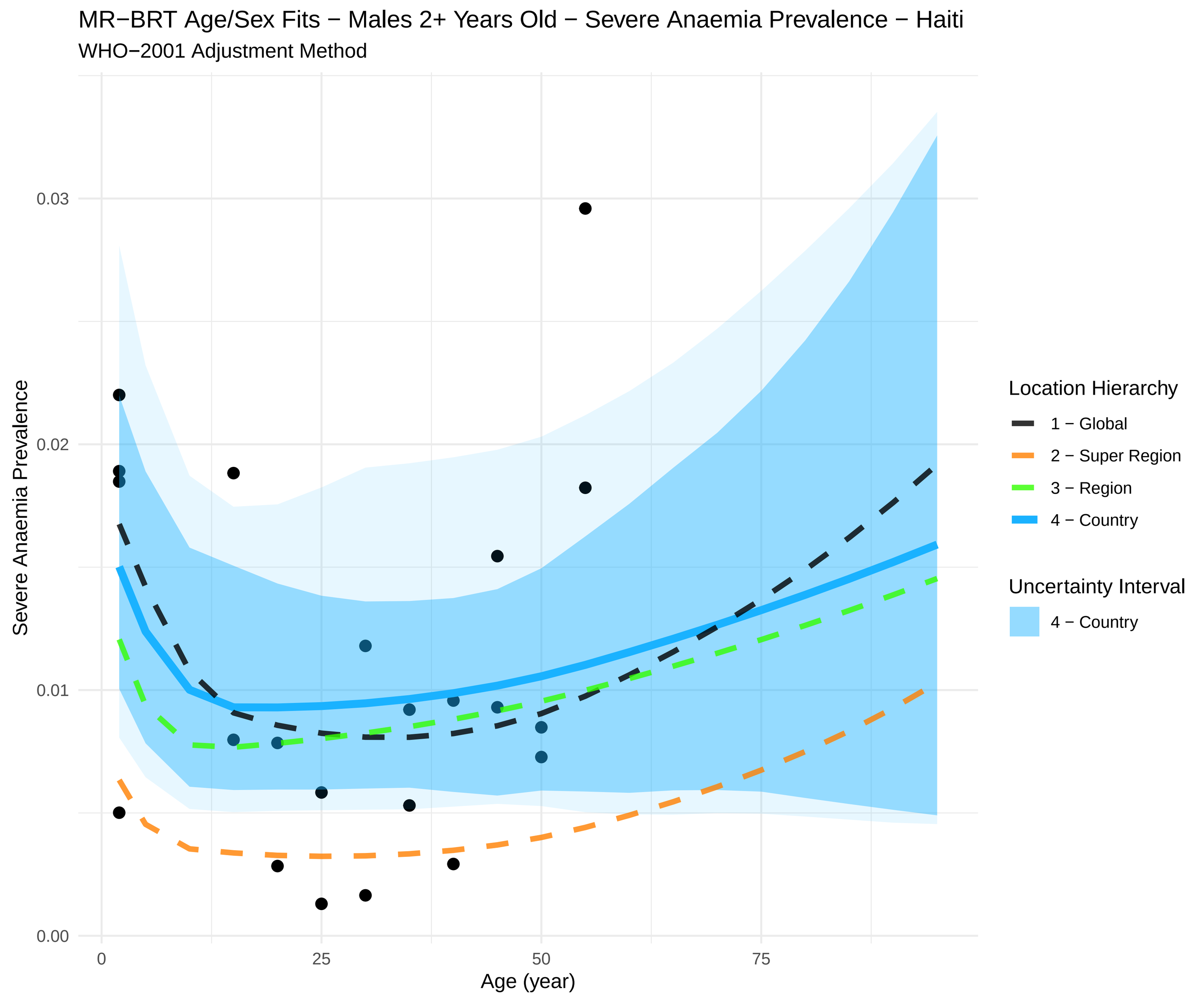
MR−BRT Age/Sex Fits − Males 2+ Years Old − Severe Anaemia Prevalence − Haiti
WHO−2001 Adjustment Method
Severe Anaemia Prevalence
Age (year)
Location Hierarchy
1 − Global
2 − Super Region
3 − Region
4 − Country
Uncertainty Interval
4 − Country

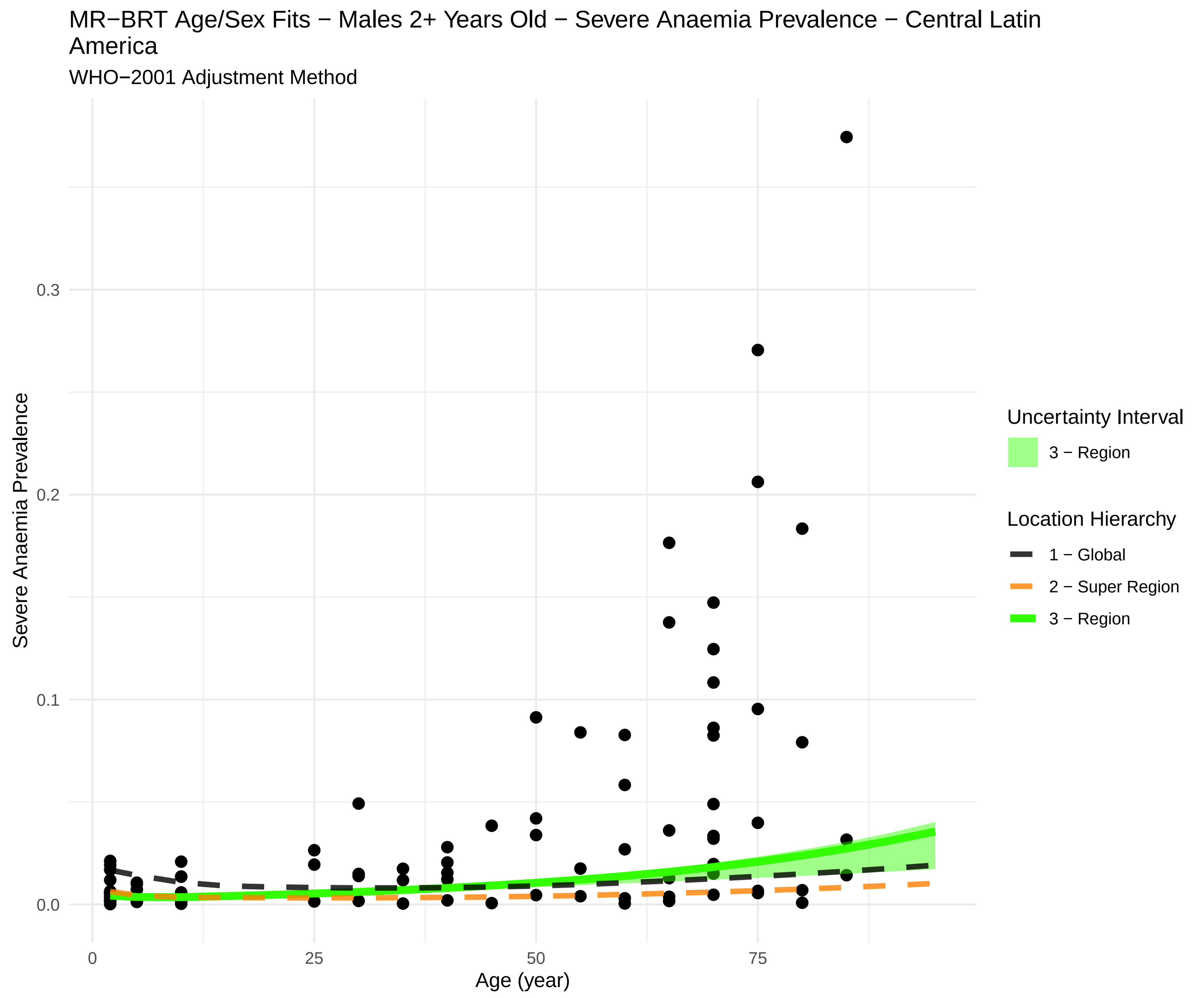
MR−BRT Age/Sex Fits − Males 2+ Years Old − Severe Anaemia Prevalence − Central Latin America
WHO−2001 Adjustment Method
Severe Anaemia Prevalence
Age (year)
Uncertainty Interval
3 − Region
Location Hierarchy
1 − Global
2 − Super Region
3 − Region

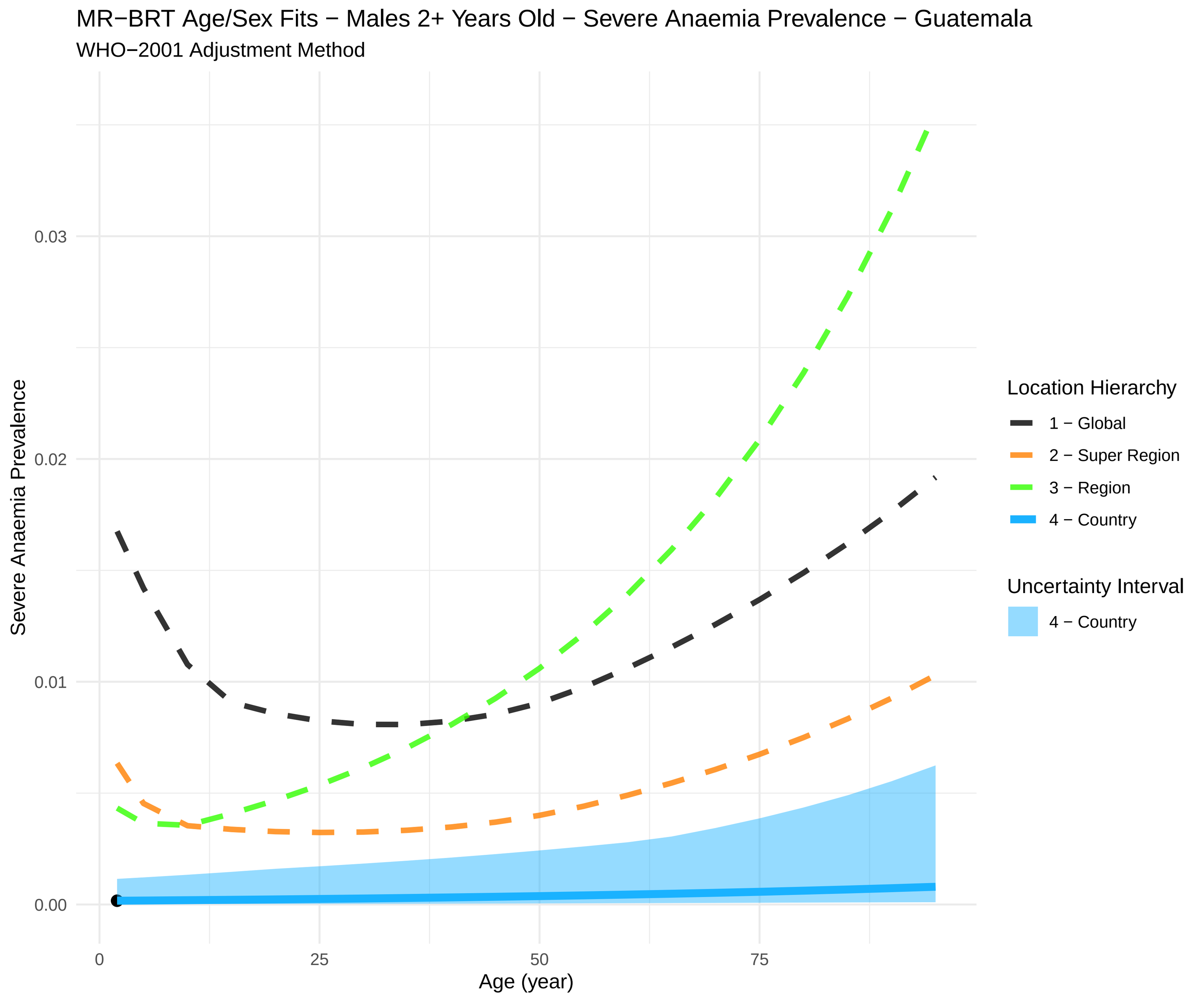
MR−BRT Age/Sex Fits − Males 2+ Years Old − Severe Anaemia Prevalence − Guatemala
WHO−2001 Adjustment Method
Severe Anaemia Prevalence
Age (year)
Location Hierarchy
1 − Global
2 − Super Region
3 − Region
4 − Country
Uncertainty Interval
4 − Country

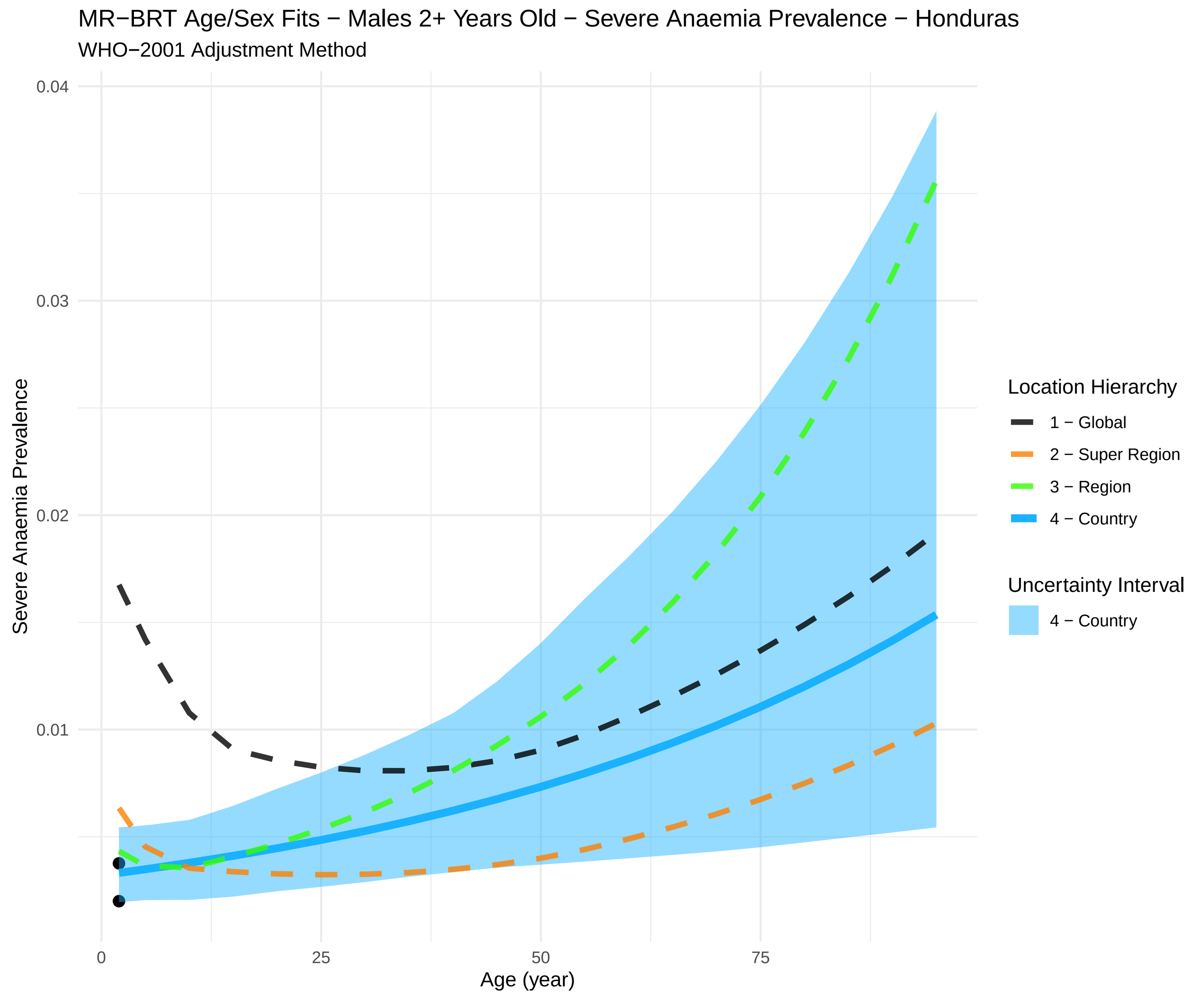
MR−BRT Age/Sex Fits − Males 2+ Years Old − Severe Anaemia Prevalence − Honduras
WHO−2001 Adjustment Method
Severe Anaemia Prevalence
Age (year)
Location Hierarchy
1 − Global
2 − Super Region
3 − Region
4 − Country
Uncertainty Interval
4 − Country

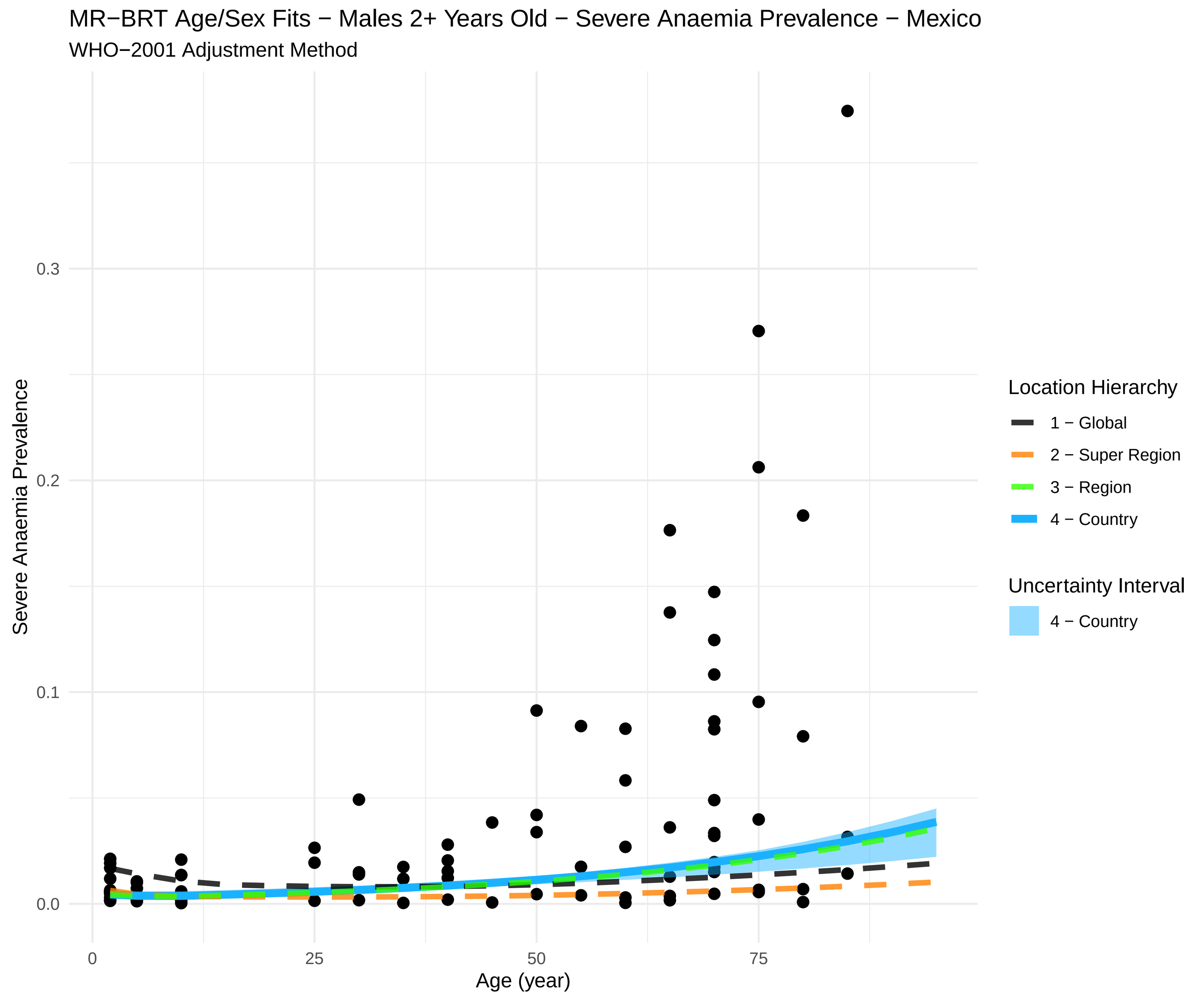
MR−BRT Age/Sex Fits − Males 2+ Years Old − Severe Anaemia Prevalence − Mexico
WHO−2001 Adjustment Method
Severe Anaemia Prevalence
Age (year)
Location Hierarchy
1 − Global
2 − Super Region
3 − Region
4 − Country
Uncertainty Interval
4 − Country

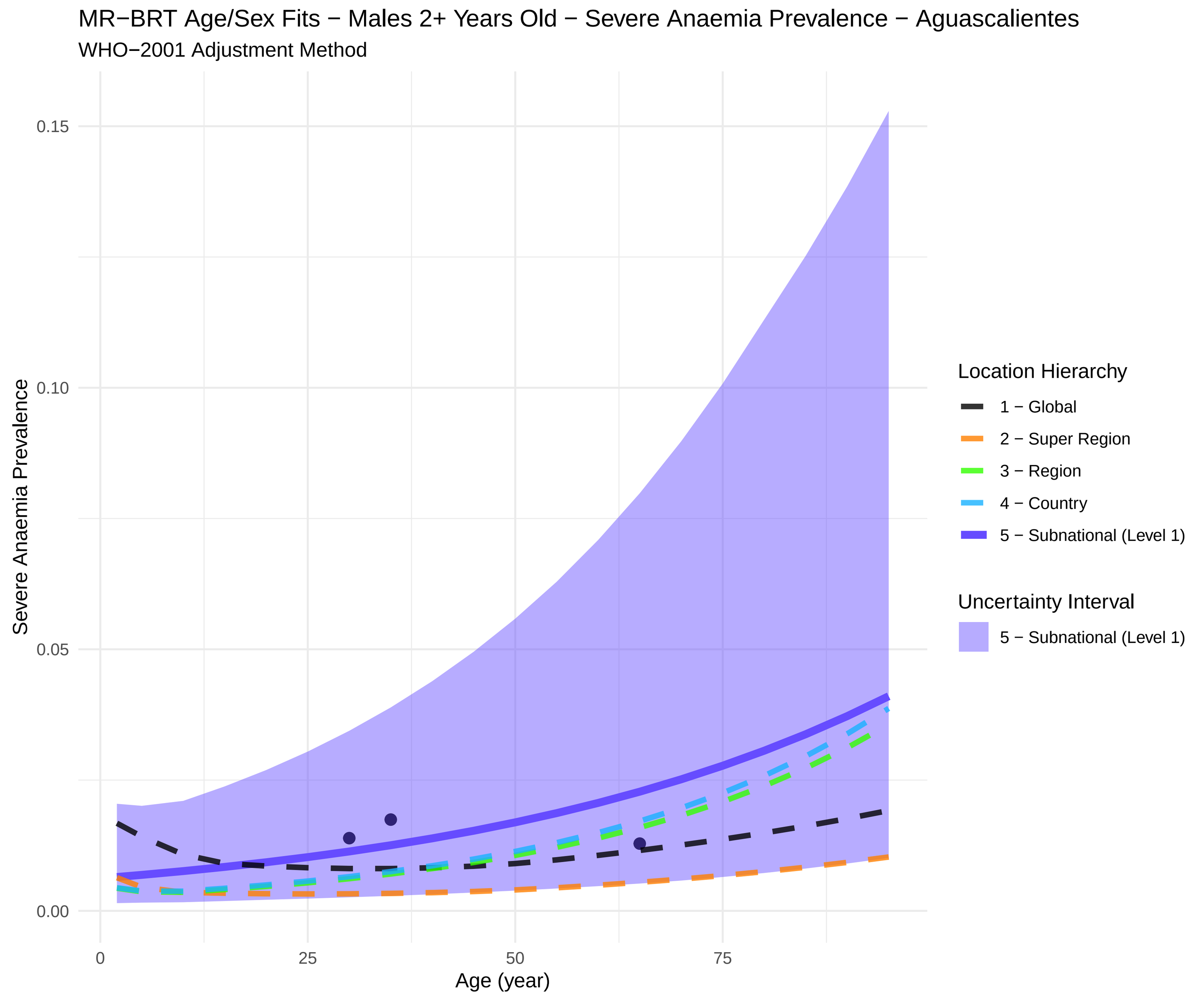
MR−BRT Age/Sex Fits − Males 2+ Years Old − Severe Anaemia Prevalence − Aguascalientes
WHO−2001 Adjustment Method
Severe Anaemia Prevalence
Age (year)
Location Hierarchy
1 − Global
2 − Super Region
3 − Region
4 − Country
5 − Subnational (Level 1)
Uncertainty Interval
5 − Subnational (Level 1)

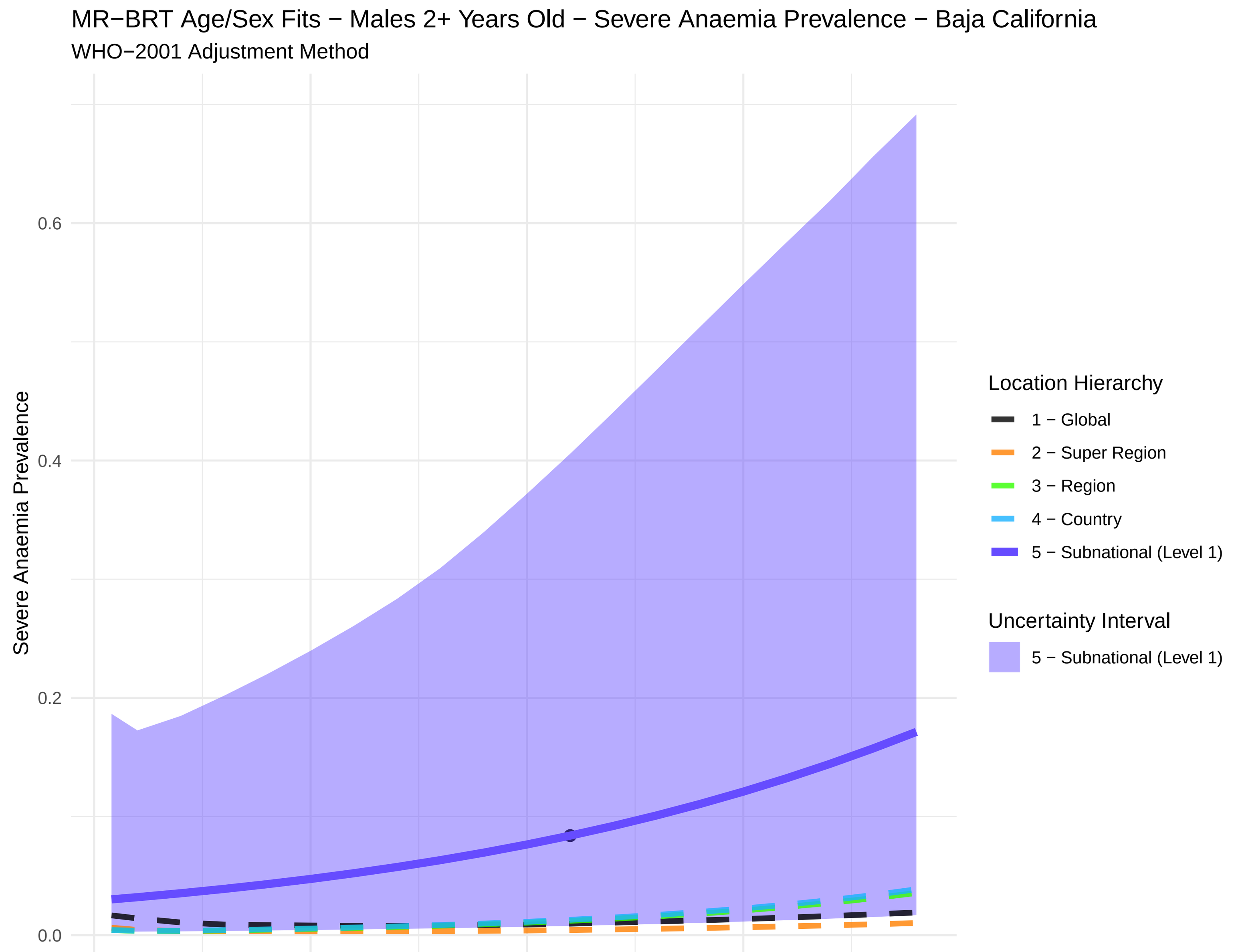
MR−BRT Age/Sex Fits − Males 2+ Years Old − Severe Anaemia Prevalence − Baja California
WHO−2001 Adjustment Method
Severe Anaemia Prevalence
Age (year)
Location Hierarchy
1 − Global
2 − Super Region
3 − Region
4 − Country
5 − Subnational (Level 1)
Uncertainty Interval
5 − Subnational (Level 1)

MR−BRT Age/Sex Fits − Males 2+ Years Old − Severe Anaemia Prevalence − Campeche
WHO−2001 Adjustment Method

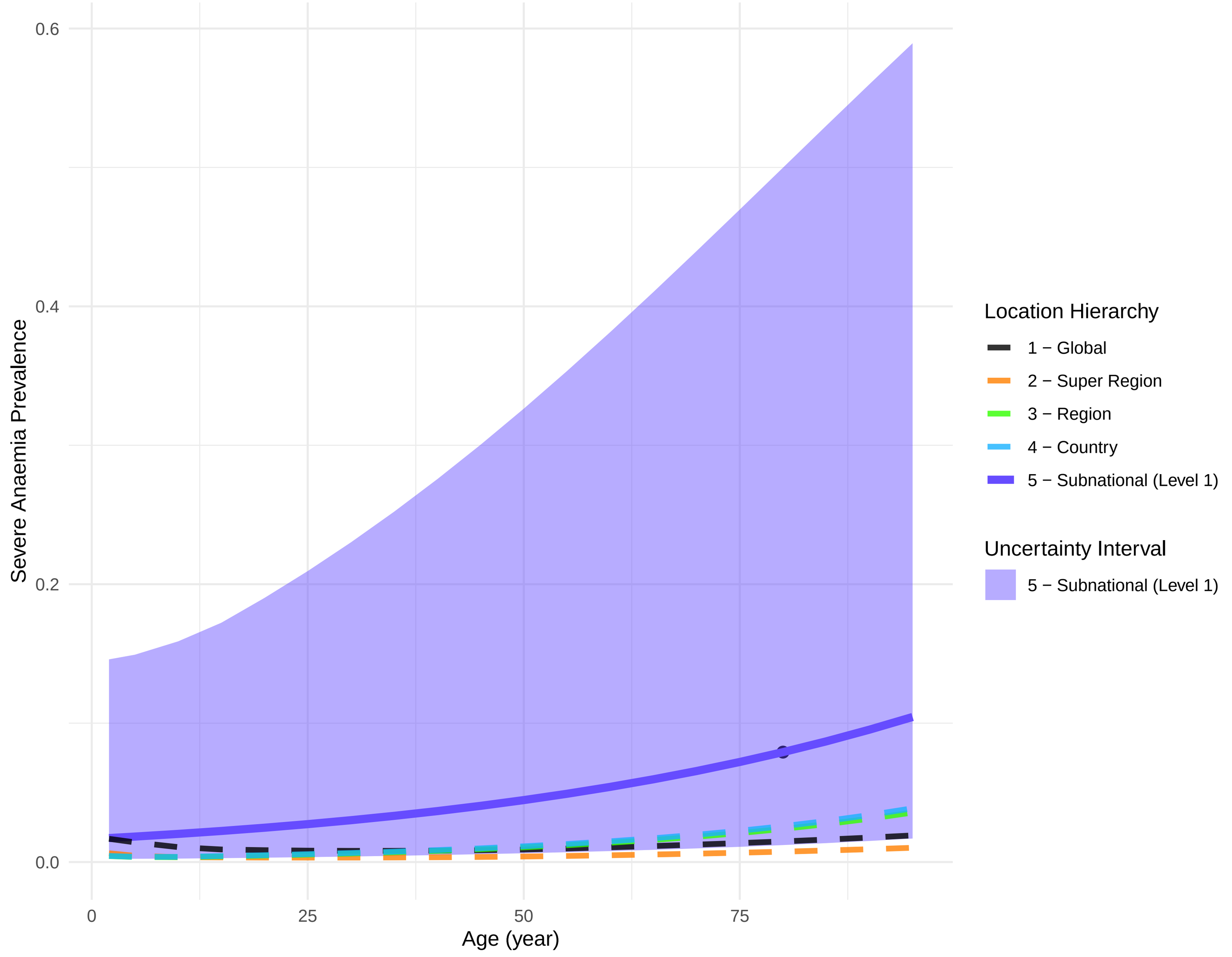

MR−BRT Age/Sex Fits − Males 2+ Years Old − Severe Anaemia Prevalence − Chiapas
WHO−2001 Adjustment Method

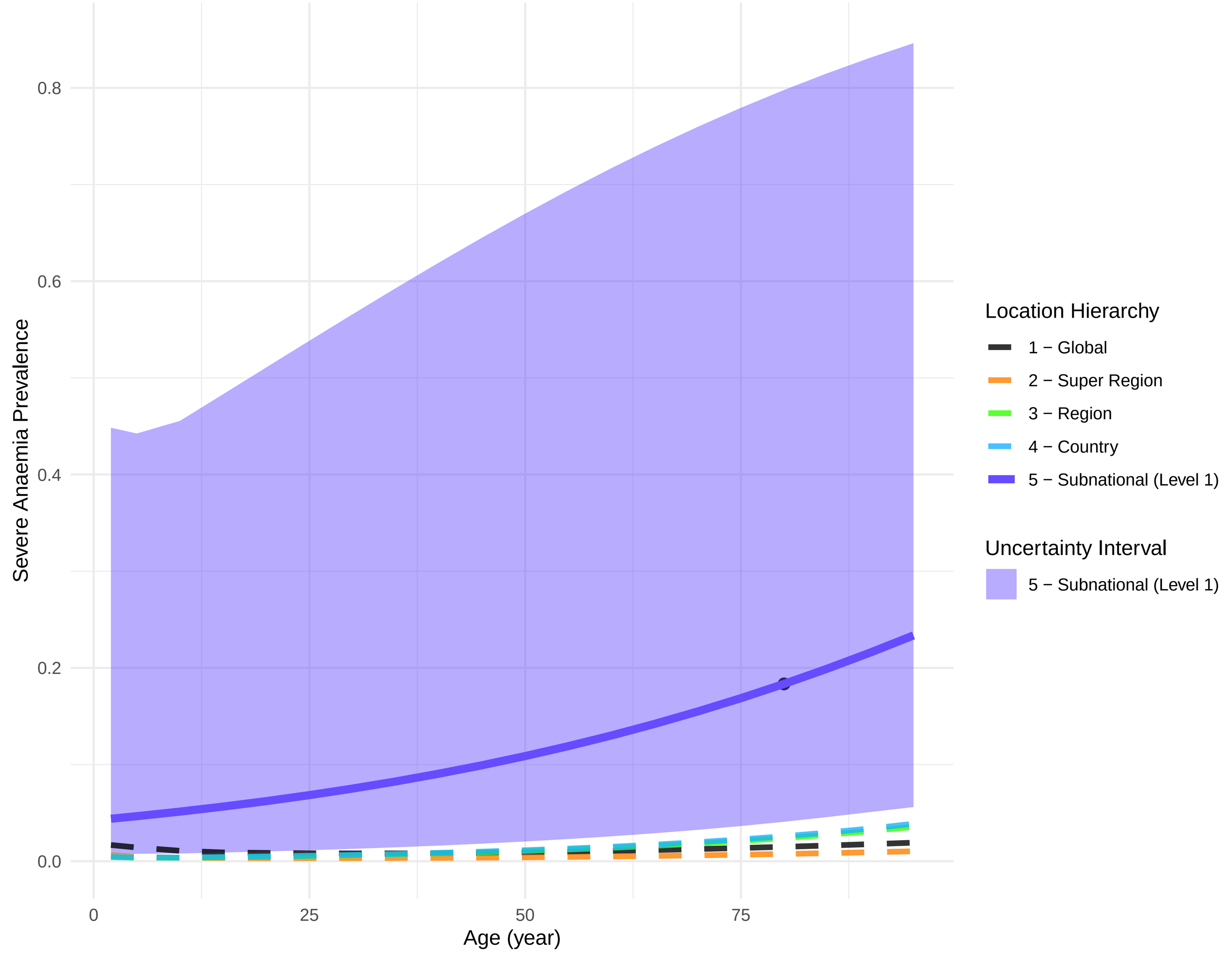

MR−BRT Age/Sex Fits − Males 2+ Years Old − Severe Anaemia Prevalence − Chihuahua
WHO−2001 Adjustment Method

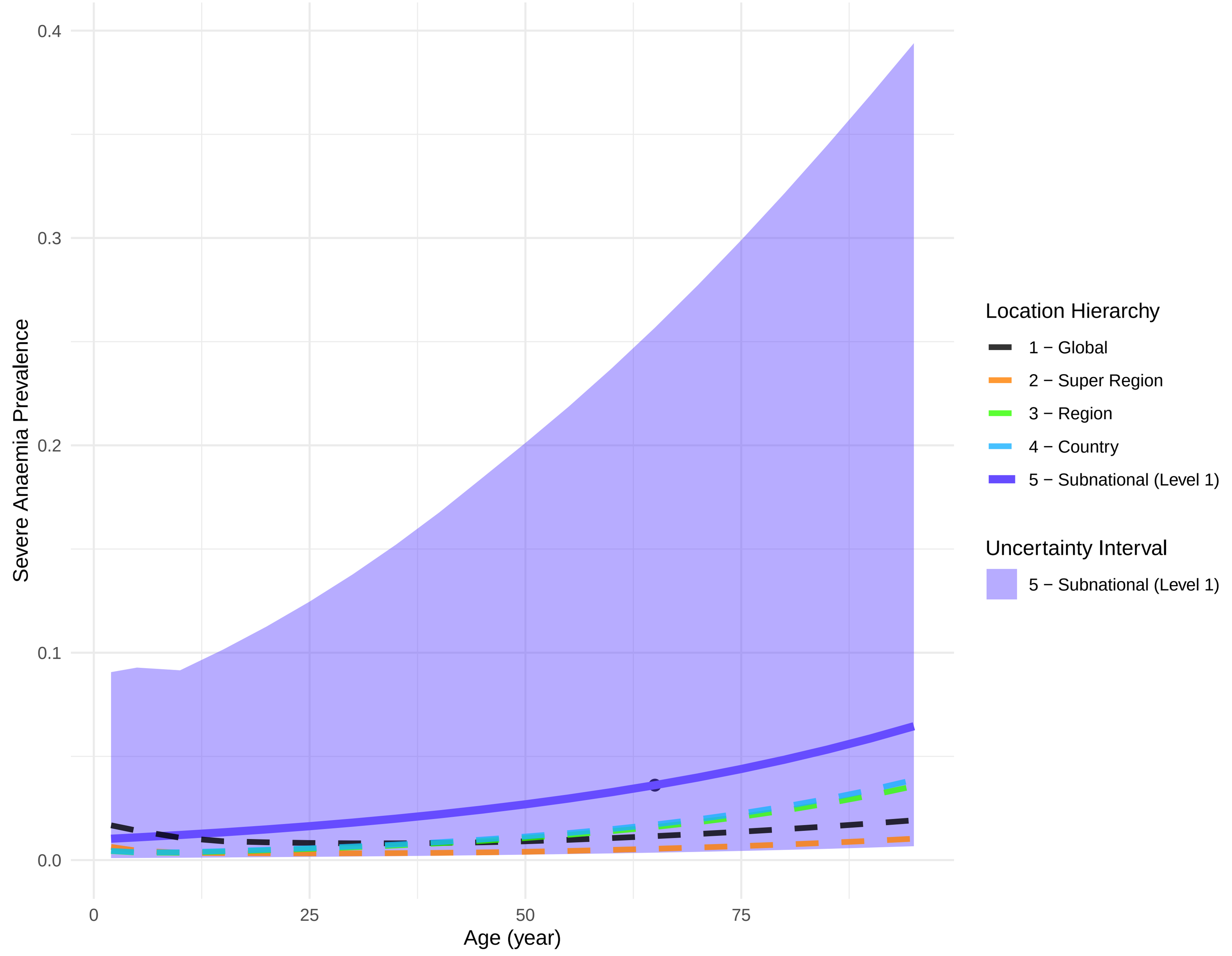

MR−BRT Age/Sex Fits − Males 2+ Years Old − Severe Anaemia Prevalence − Coahuila
WHO−2001 Adjustment Method

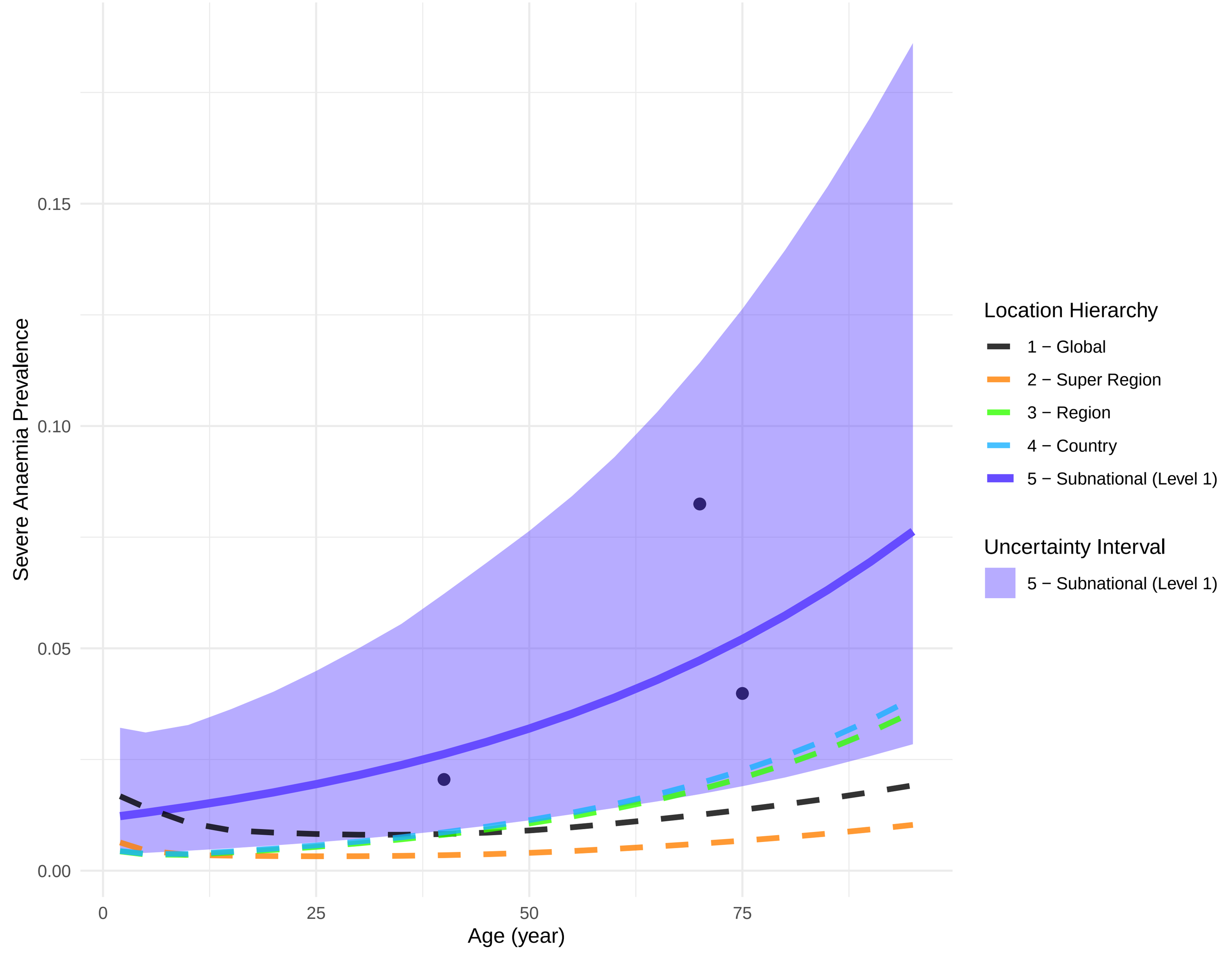

MR−BRT Age/Sex Fits − Males 2+ Years Old − Severe Anaemia Prevalence − Colima
WHO−2001 Adjustment Method

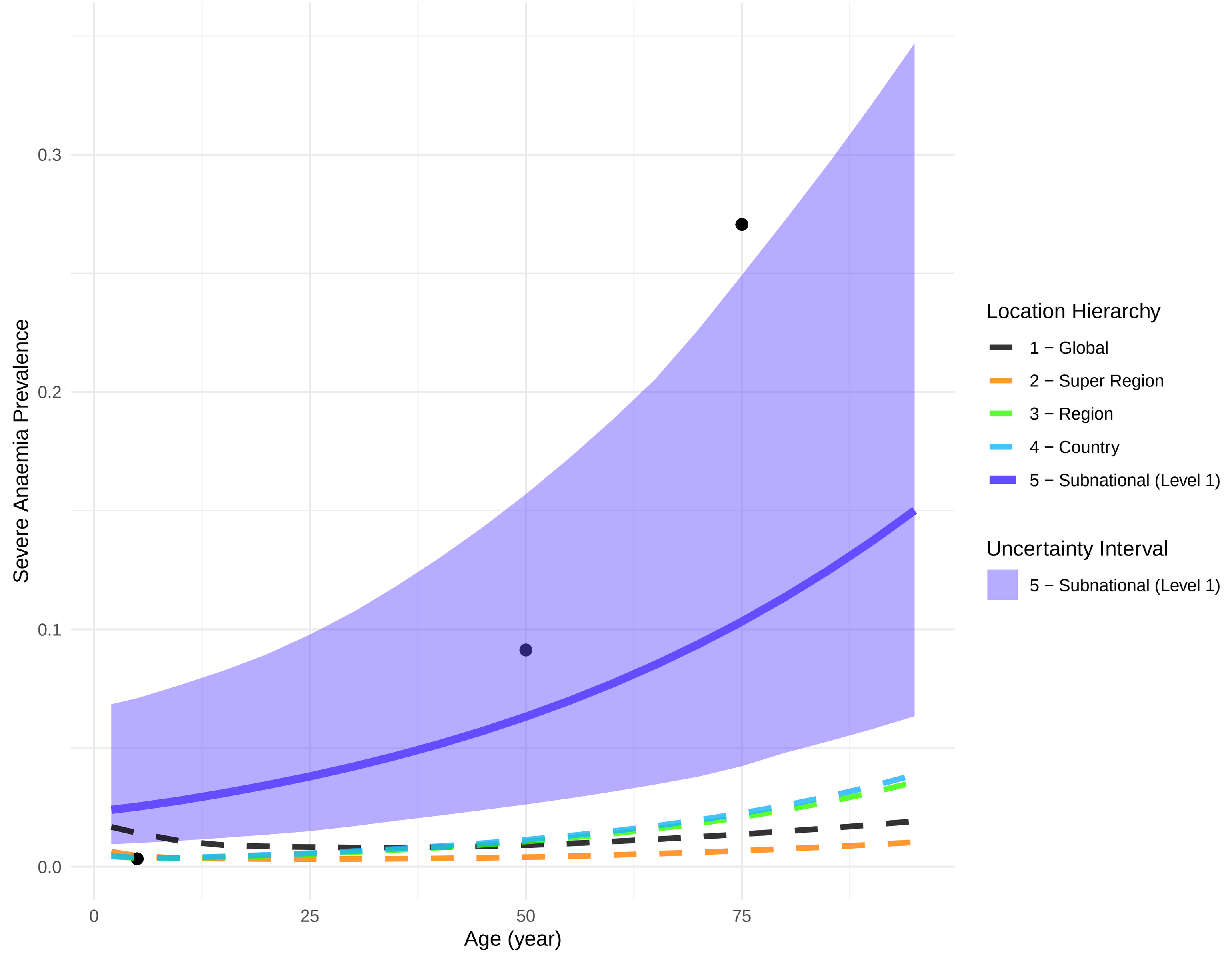

MR−BRT Age/Sex Fits − Males 2+ Years Old − Severe Anaemia Prevalence − Durango
WHO−2001 Adjustment Method

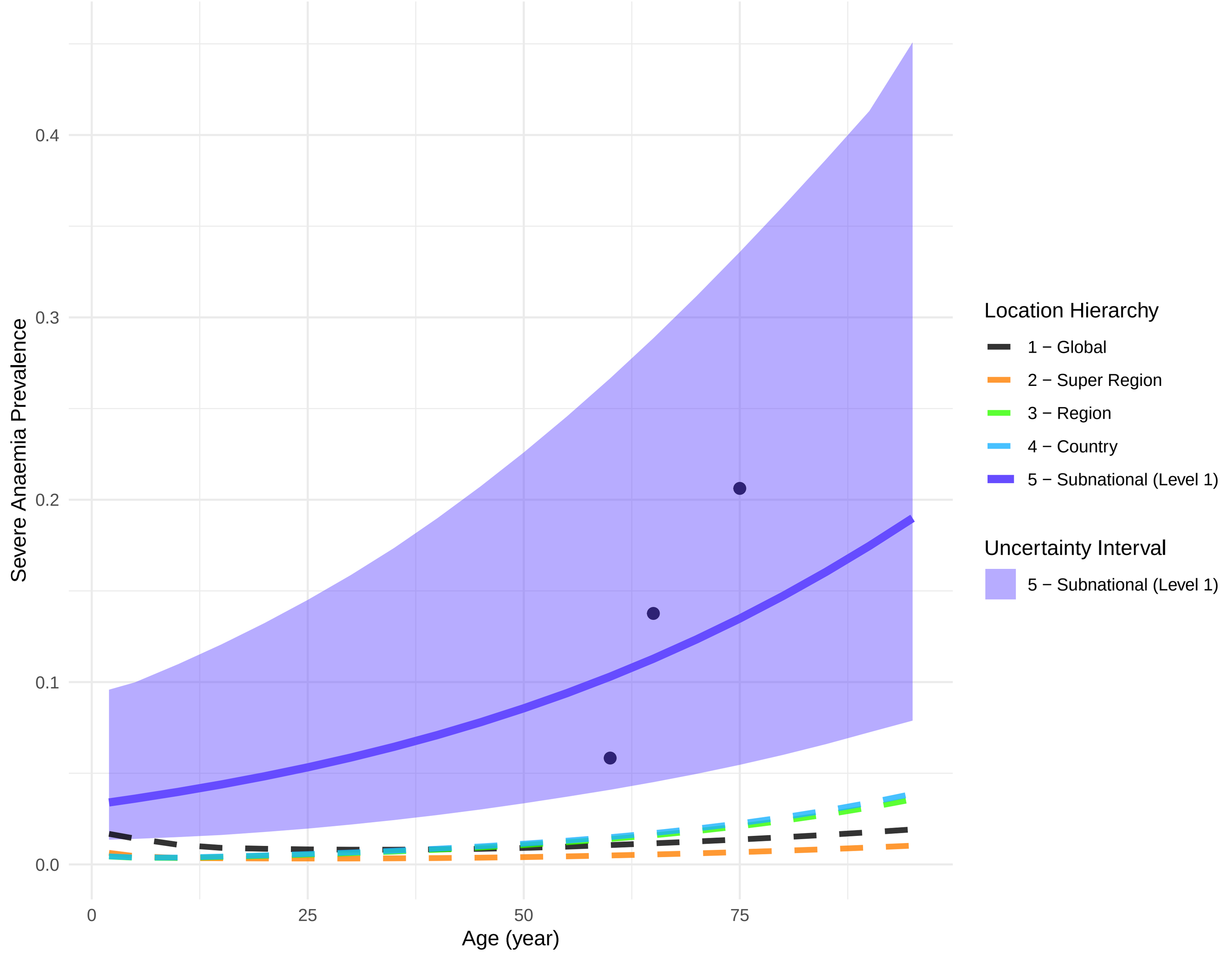

MR−BRT Age/Sex Fits − Males 2+ Years Old − Severe Anaemia Prevalence − Guanajuato
WHO−2001 Adjustment Method

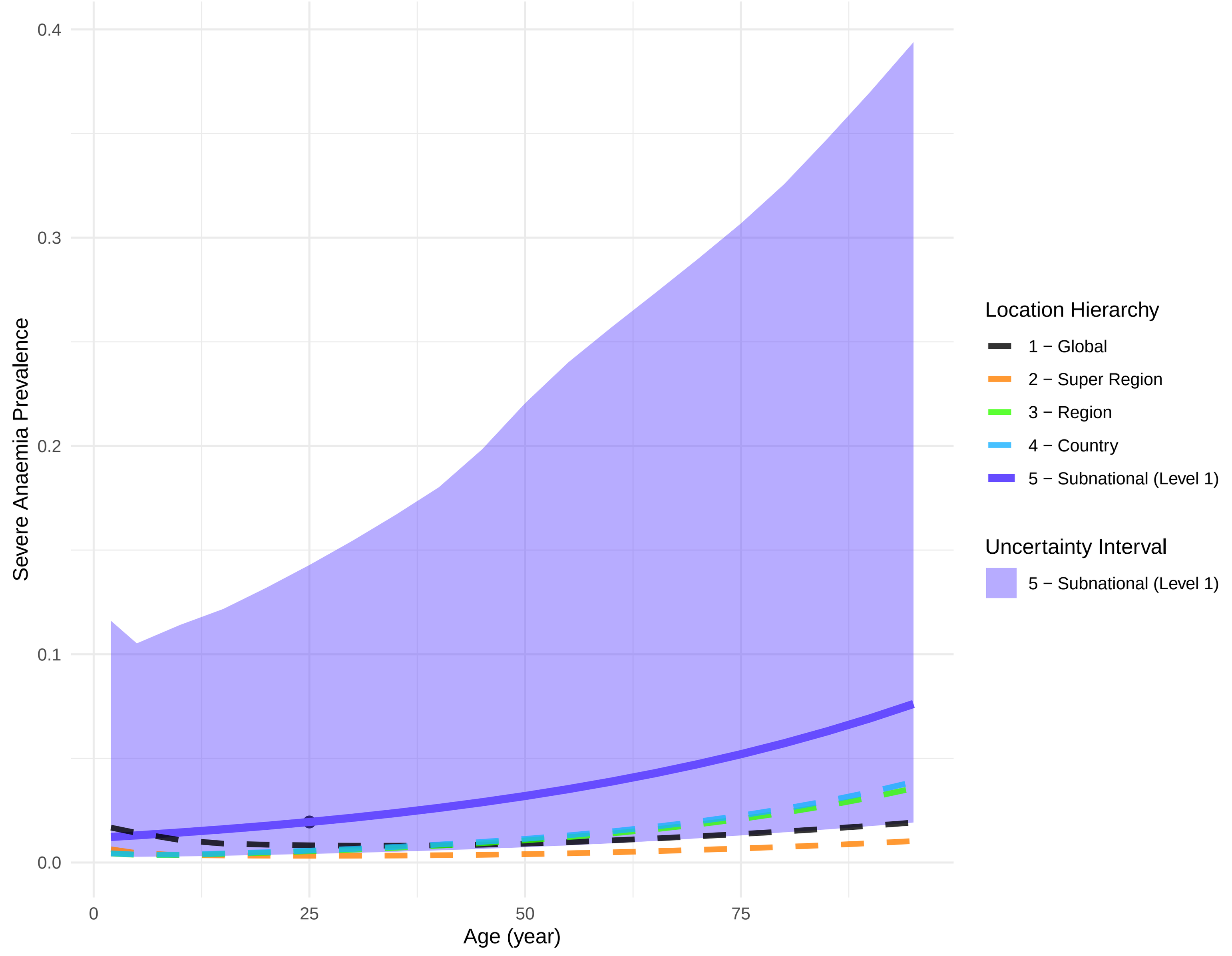

MR−BRT Age/Sex Fits − Males 2+ Years Old − Severe Anaemia Prevalence − Guerrero
WHO−2001 Adjustment Method

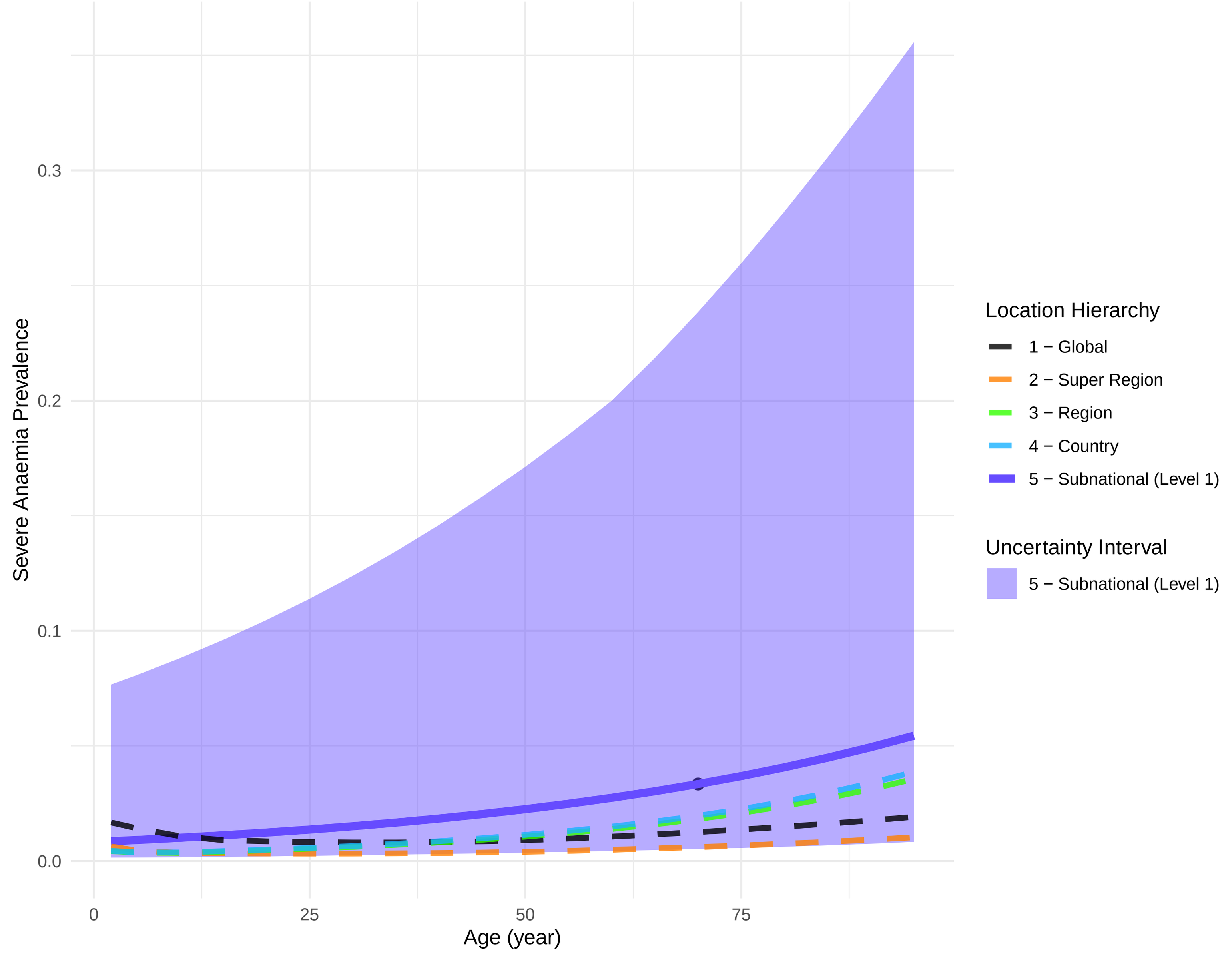

MR−BRT Age/Sex Fits − Males 2+ Years Old − Severe Anaemia Prevalence − Hidalgo
WHO−2001 Adjustment Method

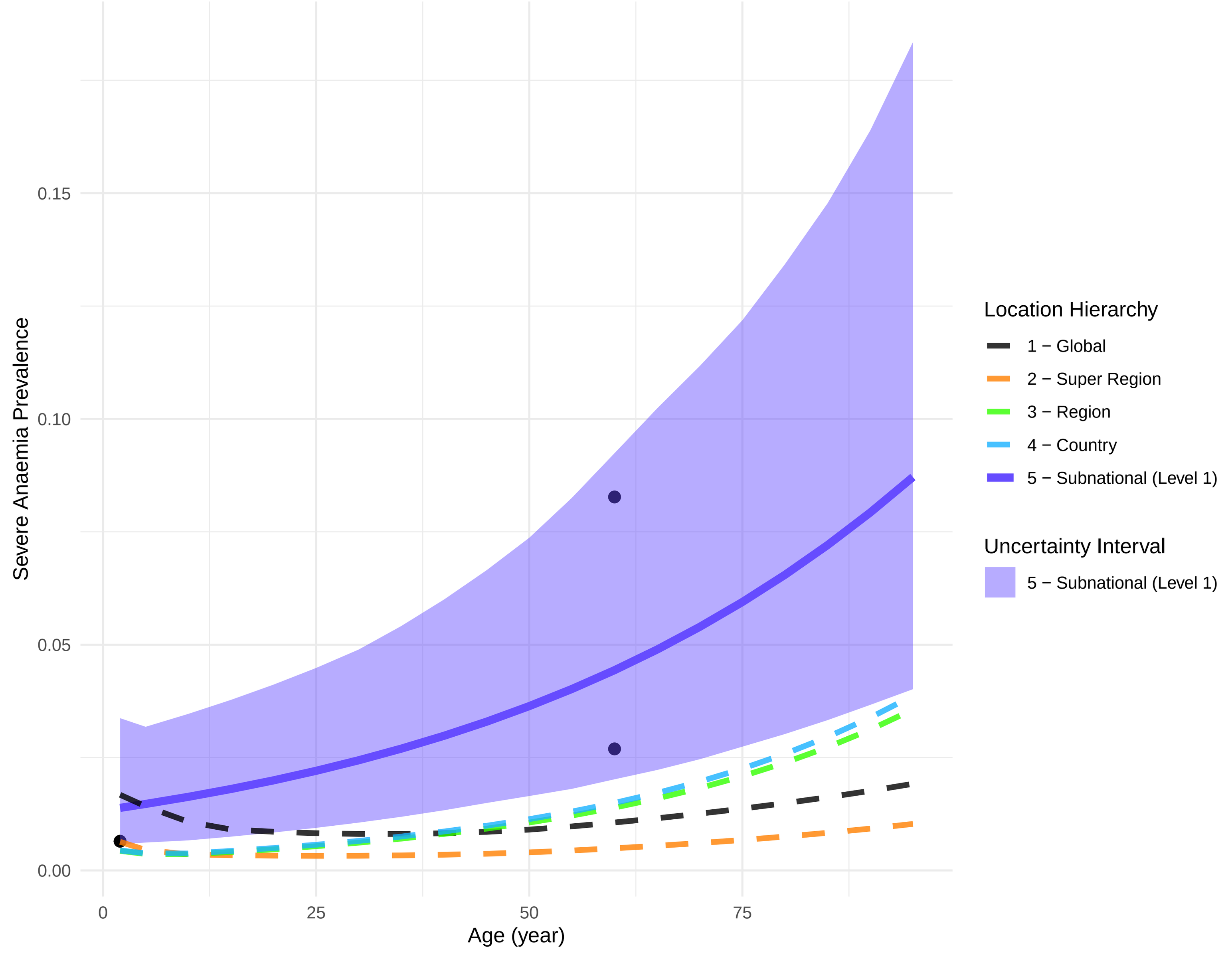

MR−BRT Age/Sex Fits − Males 2+ Years Old − Severe Anaemia Prevalence − México
WHO−2001 Adjustment Method

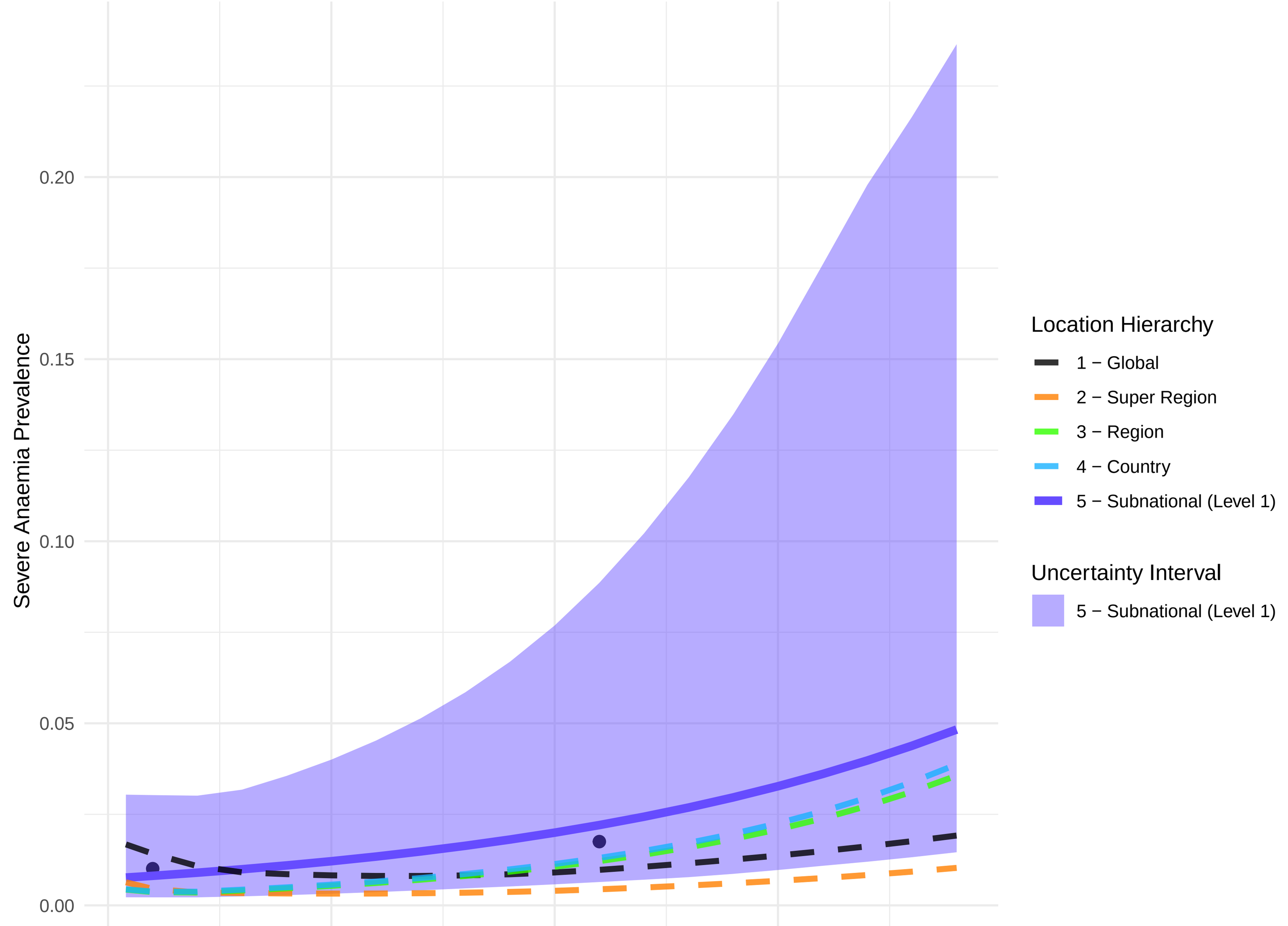

MR−BRT Age/Sex Fits − Males 2+ Years Old − Severe Anaemia Prevalence − Mexico City
WHO−2001 Adjustment Method

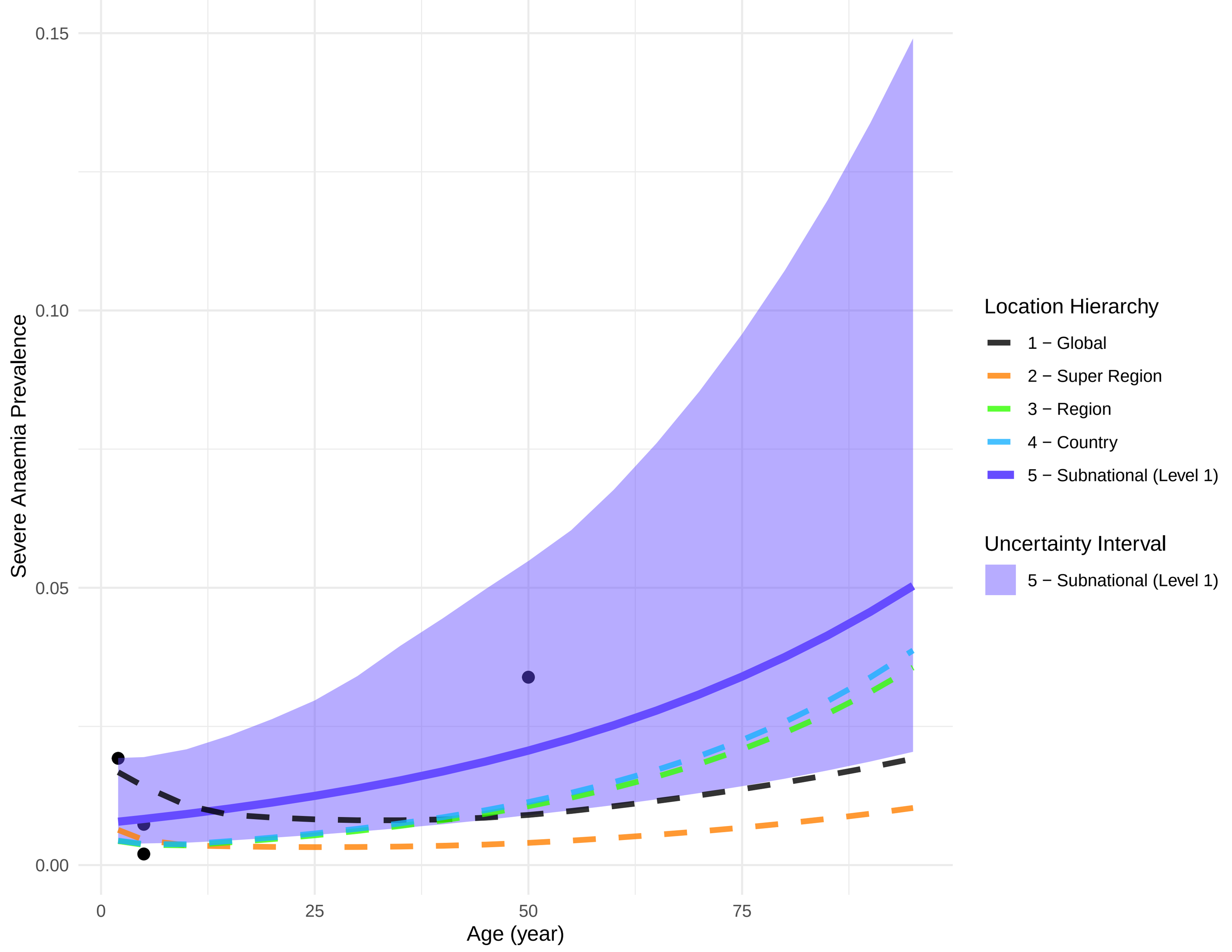

MR−BRT Age/Sex Fits − Males 2+ Years Old − Severe Anaemia Prevalence − Michoacán de Ocampo
WHO−2001 Adjustment Method

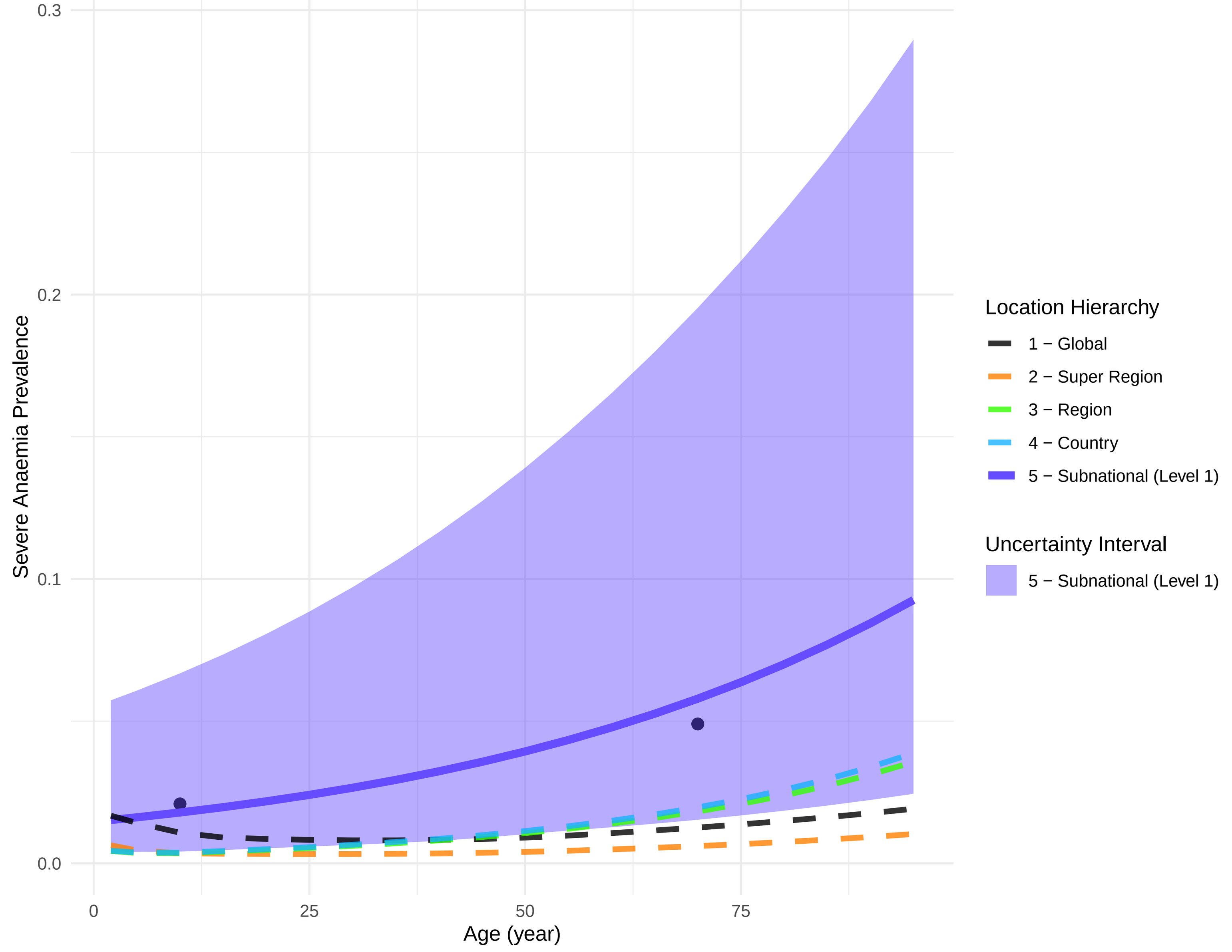

MR−BRT Age/Sex Fits − Males 2+ Years Old − Severe Anaemia Prevalence − Morelos
WHO−2001 Adjustment Method

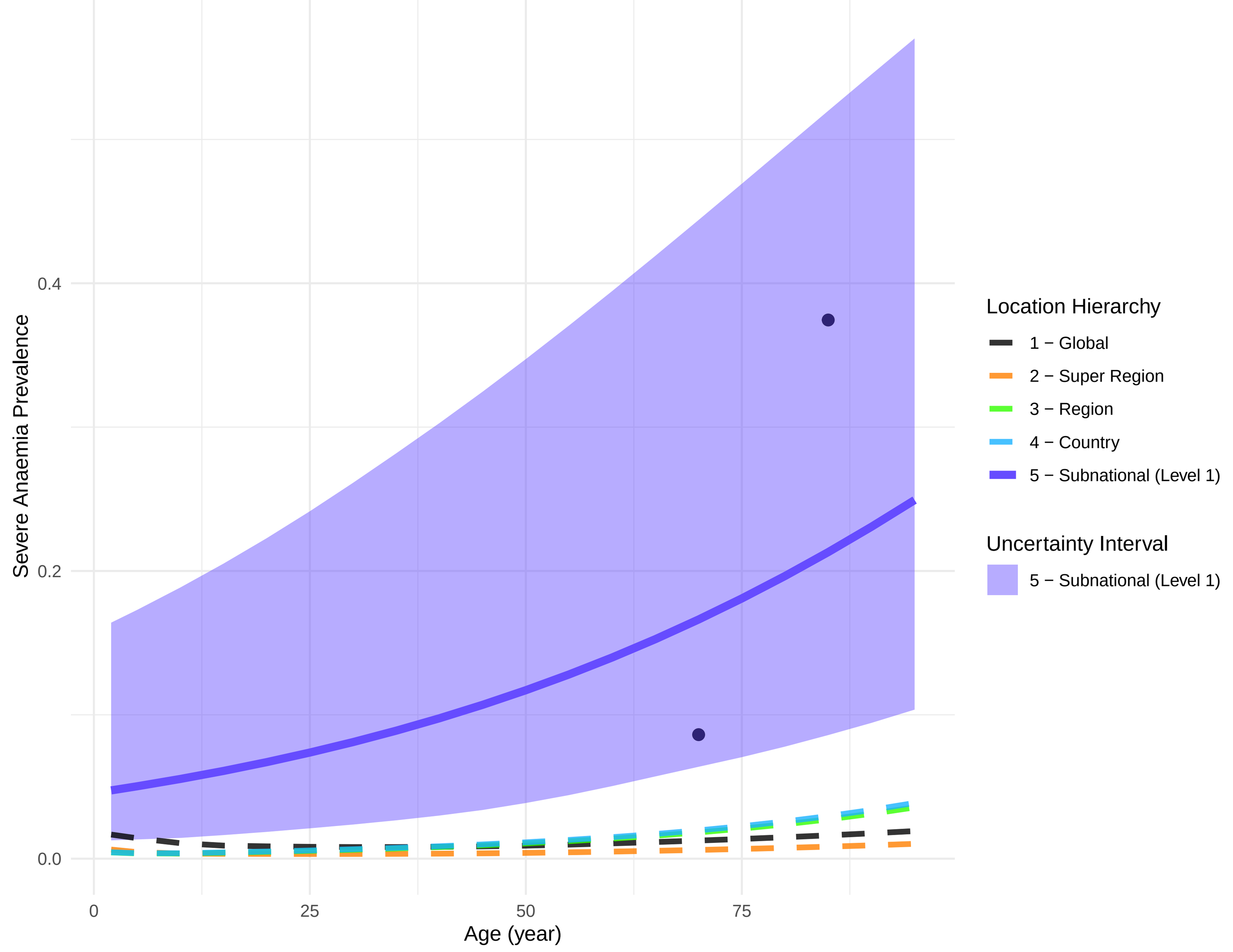

MR−BRT Age/Sex Fits − Males 2+ Years Old − Severe Anaemia Prevalence − Nayarit
WHO−2001 Adjustment Method

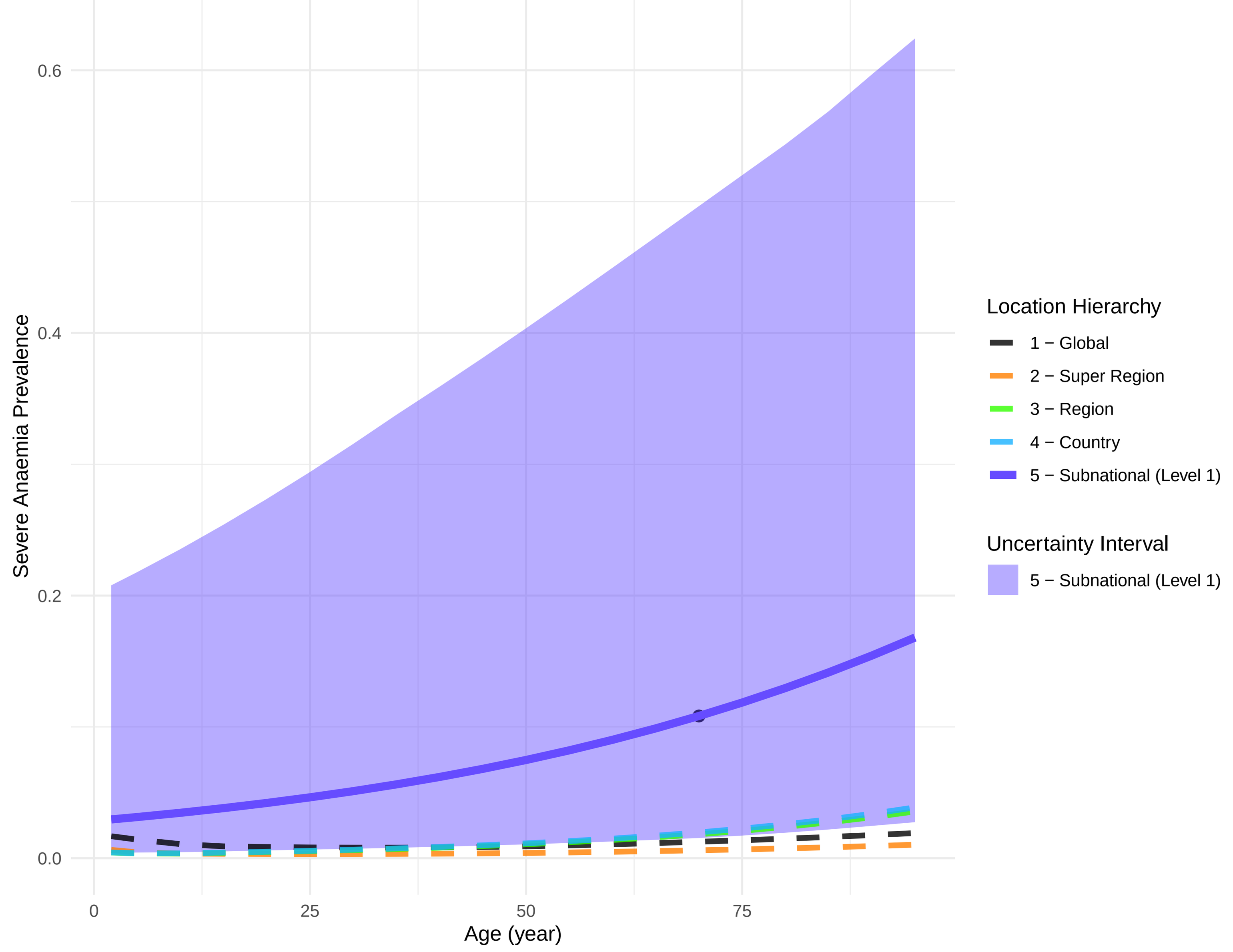

MR−BRT Age/Sex Fits − Males 2+ Years Old − Severe Anaemia Prevalence − Nuevo León
WHO−2001 Adjustment Method

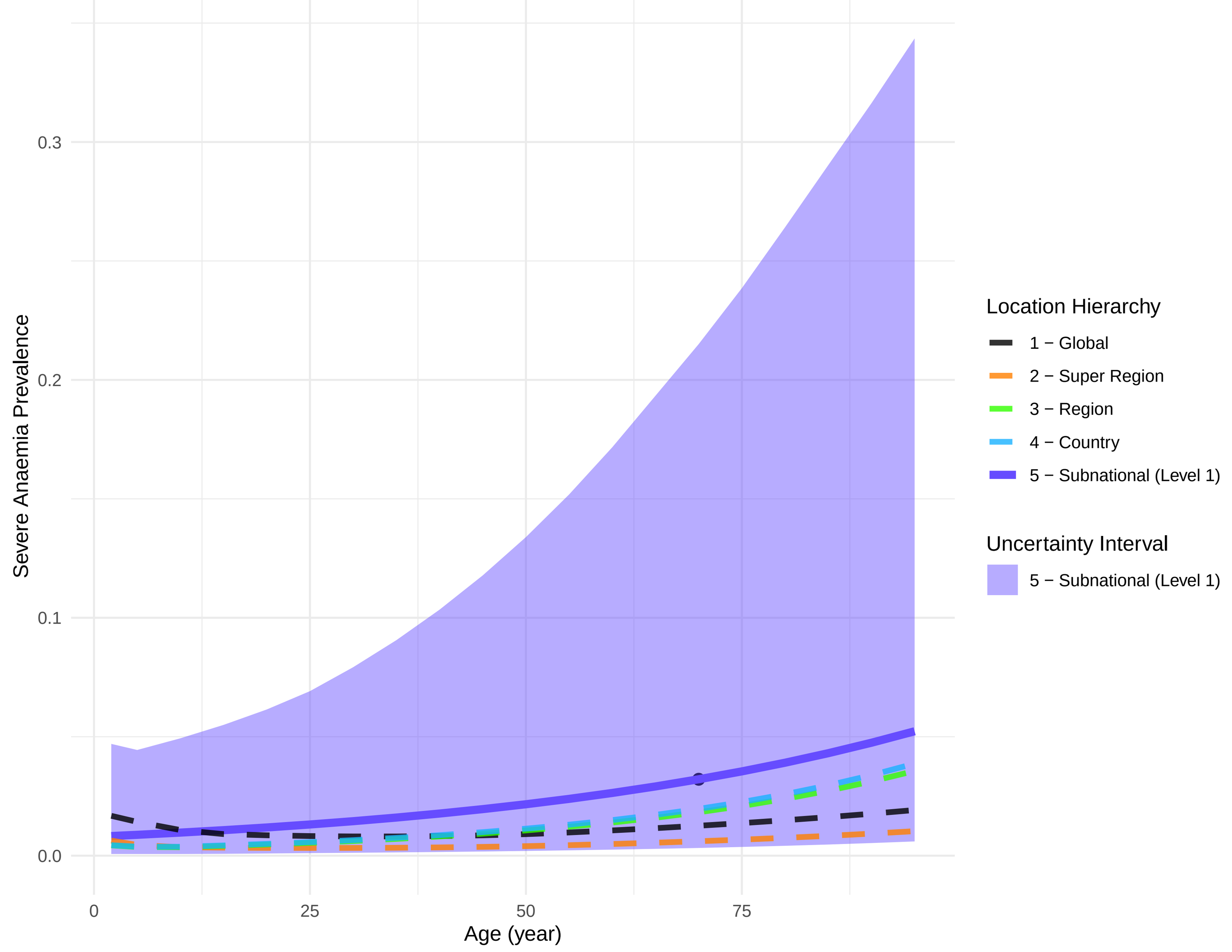

MR−BRT Age/Sex Fits − Males 2+ Years Old − Severe Anaemia Prevalence − Oaxaca
WHO−2001 Adjustment Method

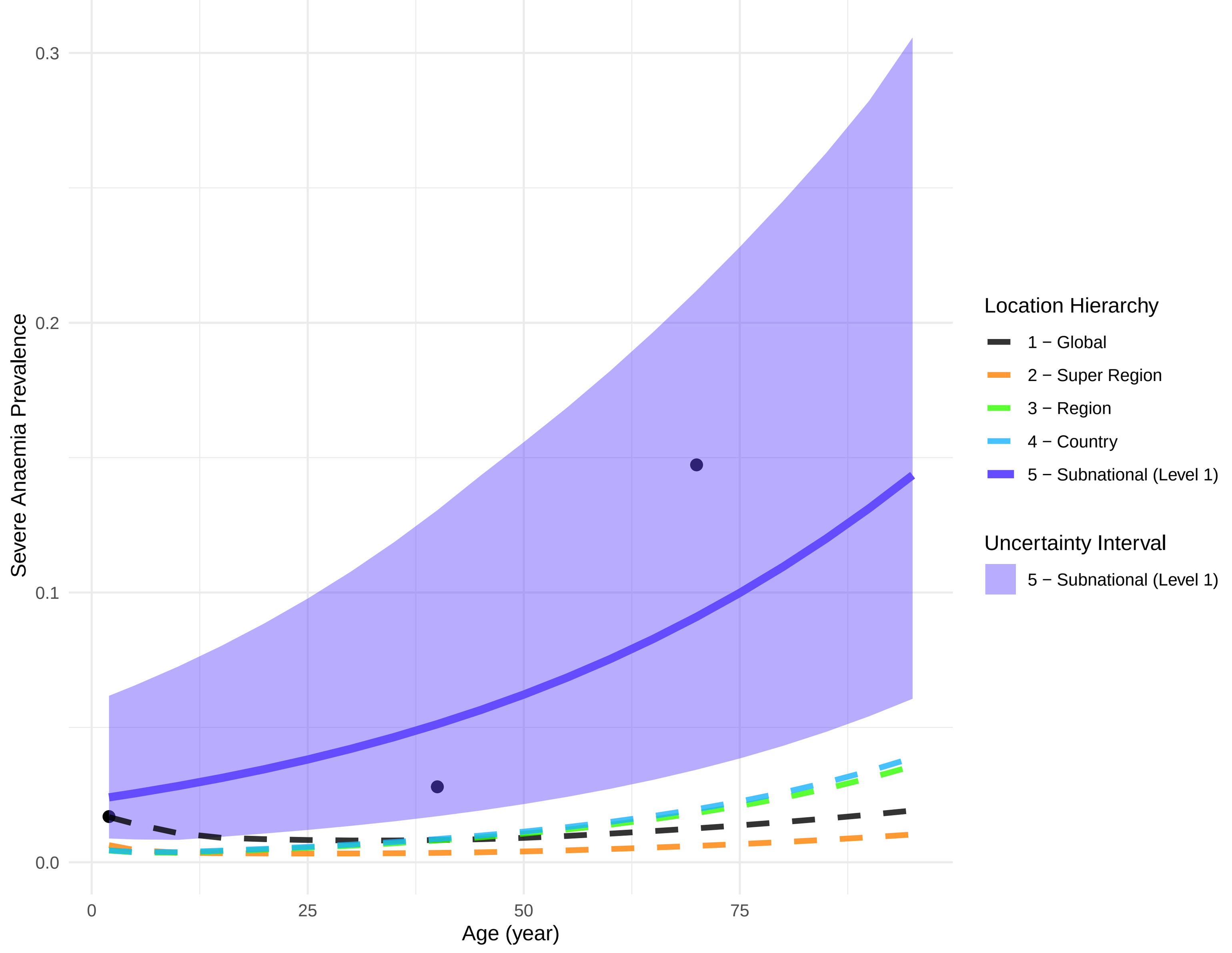

MR−BRT Age/Sex Fits − Males 2+ Years Old − Severe Anaemia Prevalence − Puebla
WHO−2001 Adjustment Method

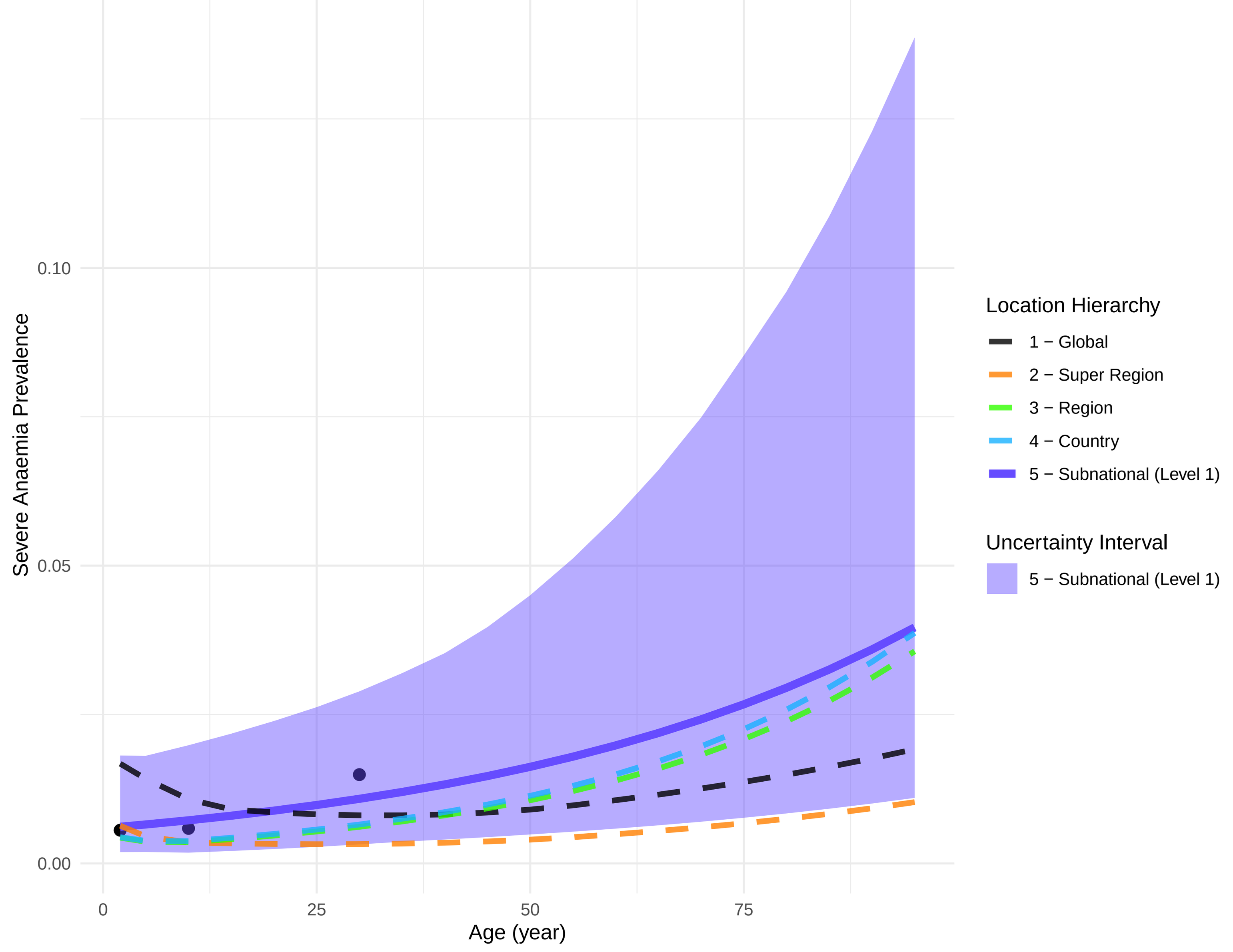

MR−BRT Age/Sex Fits − Males 2+ Years Old − Severe Anaemia Prevalence − Querétaro
WHO−2001 Adjustment Method

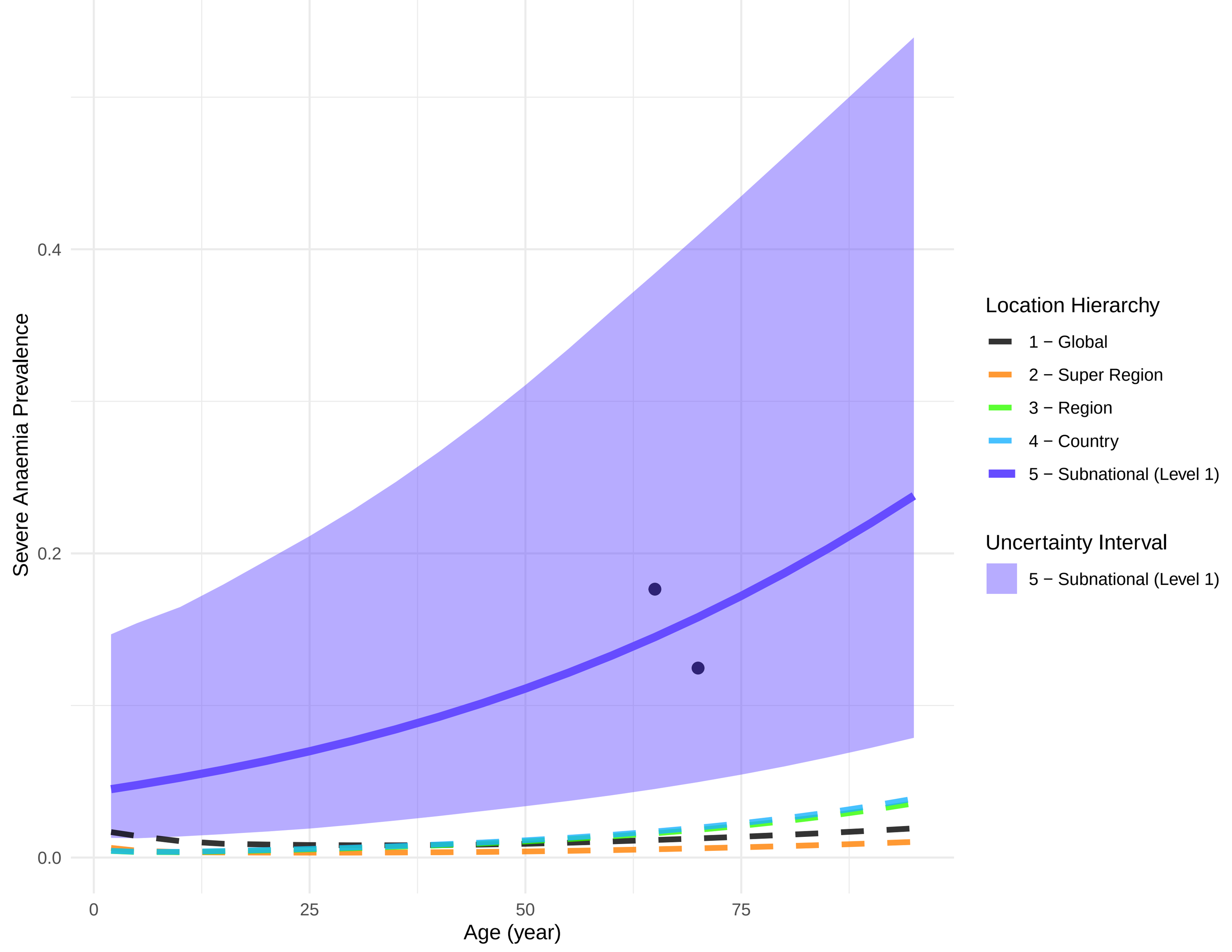

MR−BRT Age/Sex Fits − Males 2+ Years Old − Severe Anaemia Prevalence − Quintana Roo
WHO−2001 Adjustment Method

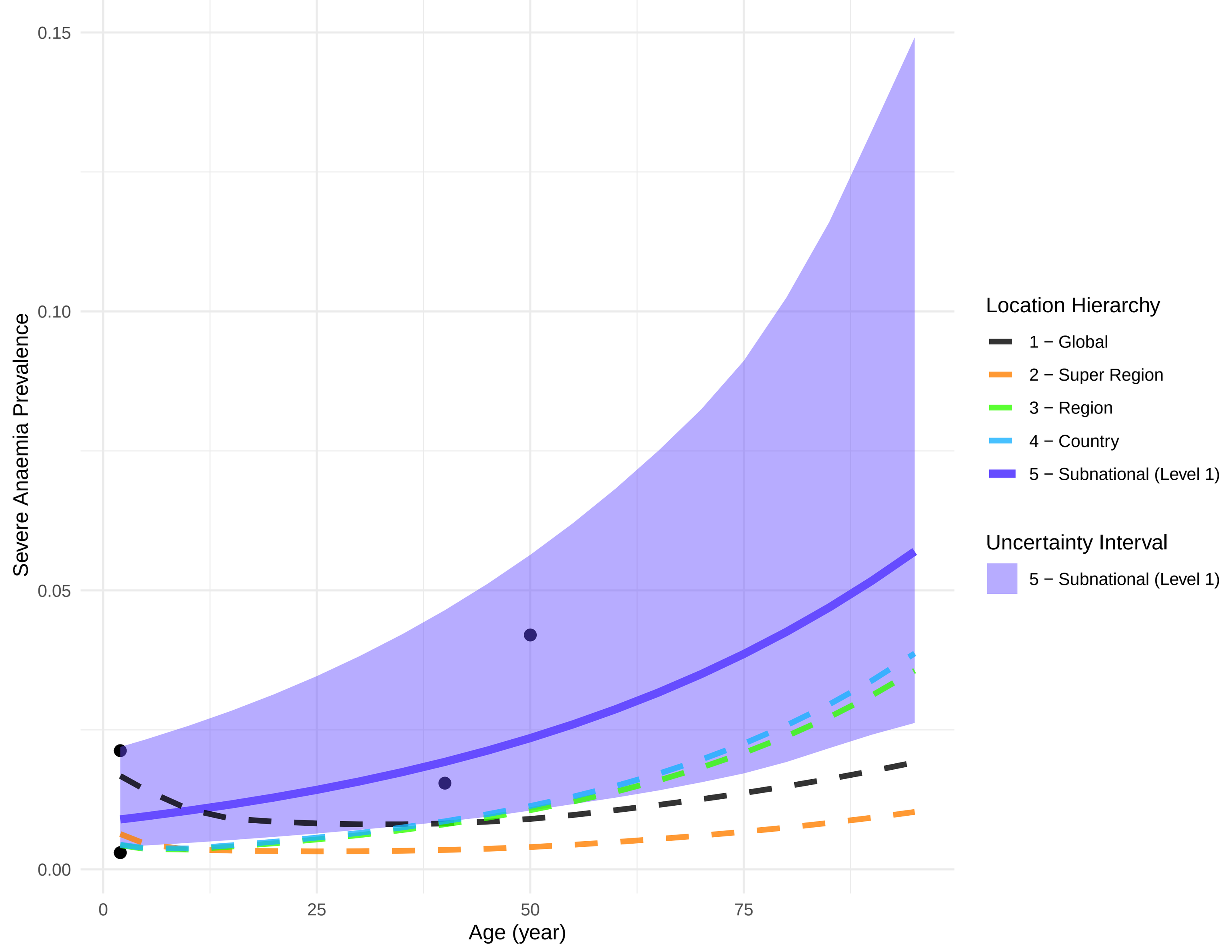

MR−BRT Age/Sex Fits − Males 2+ Years Old − Severe Anaemia Prevalence − San Luis Potosí
WHO−2001 Adjustment Method

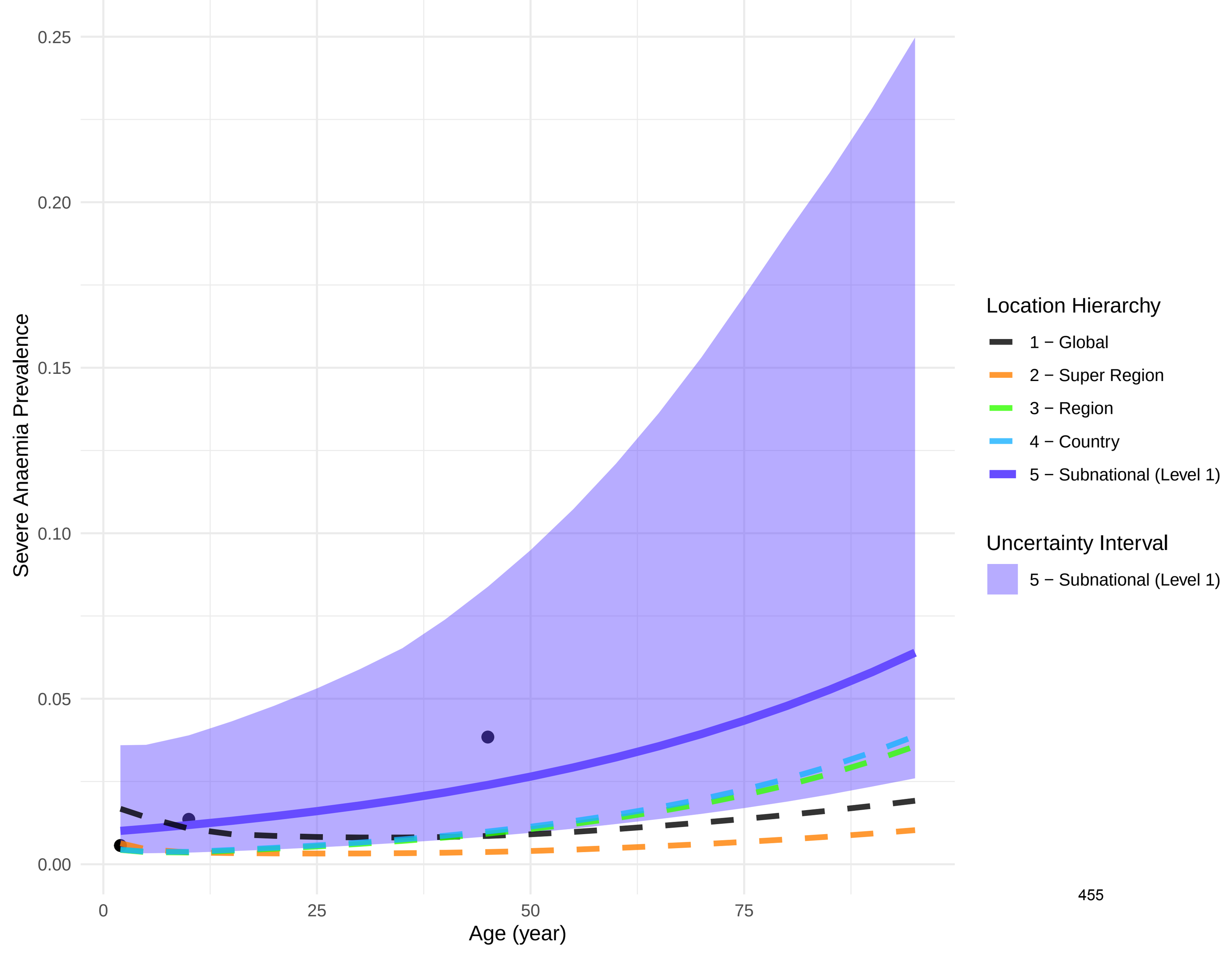

MR−BRT Age/Sex Fits − Males 2+ Years Old − Severe Anaemia Prevalence − Sinaloa
WHO−2001 Adjustment Method

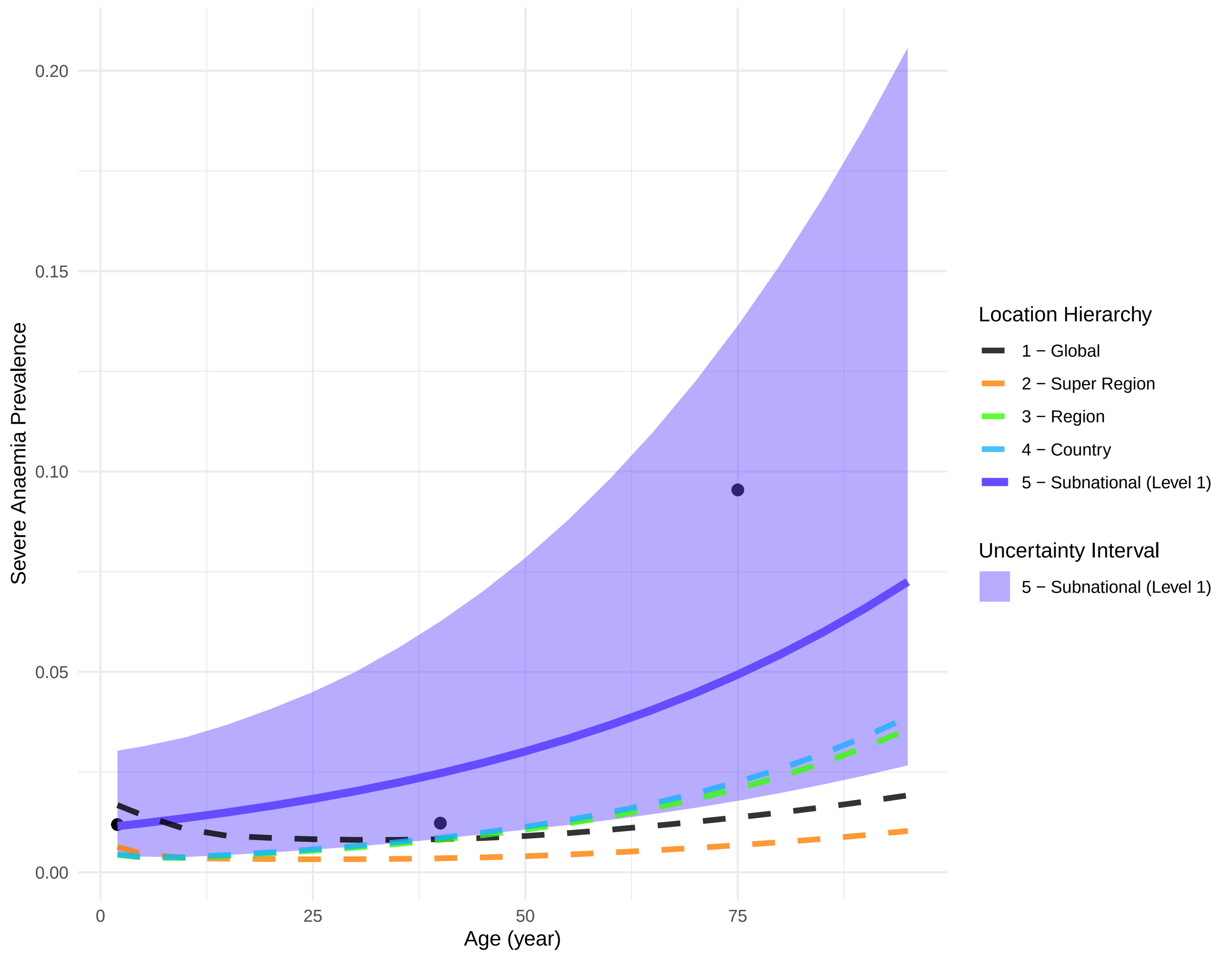

MR−BRT Age/Sex Fits − Males 2+ Years Old − Severe Anaemia Prevalence − Tabasco
WHO−2001 Adjustment Method

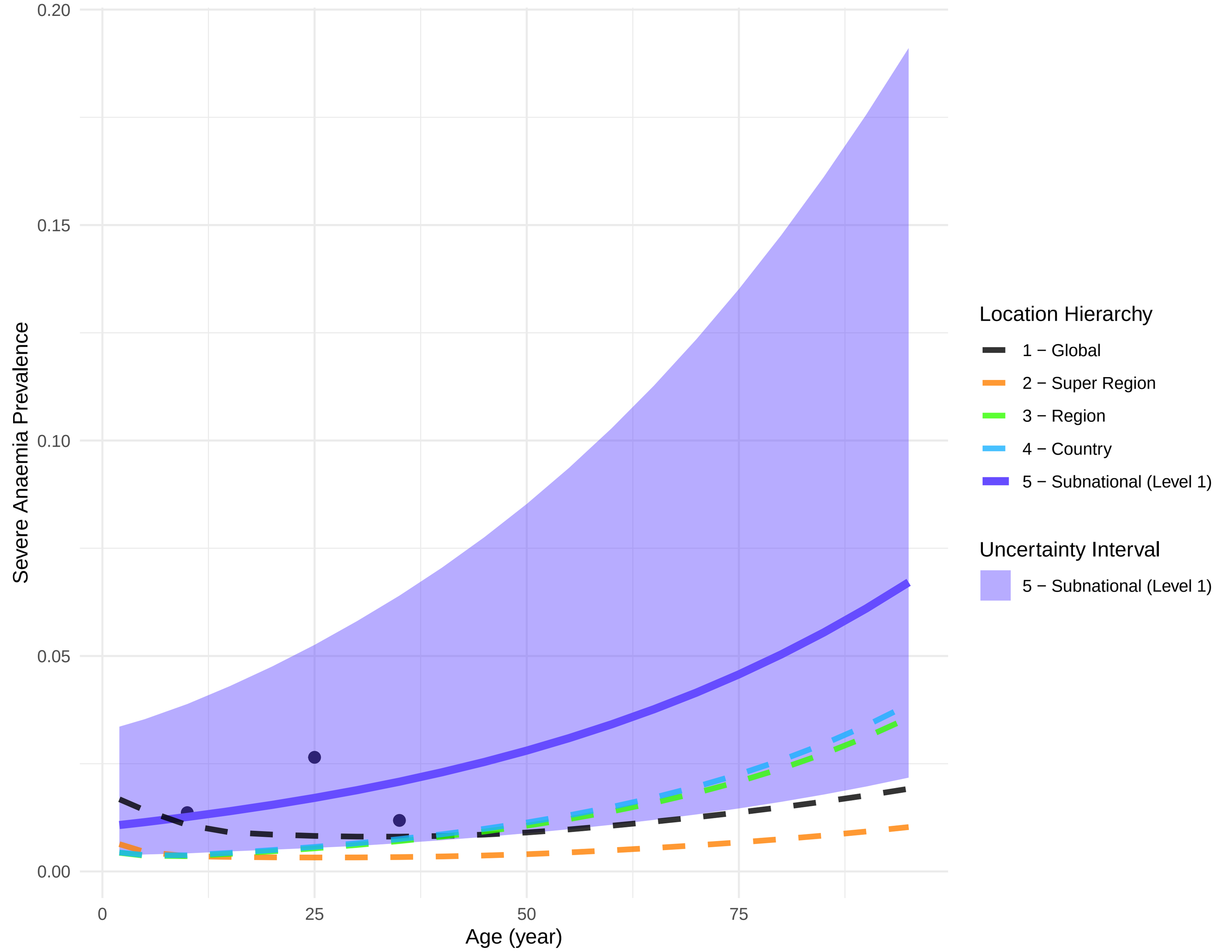

MR−BRT Age/Sex Fits − Males 2+ Years Old − Severe Anaemia Prevalence − Tamaulipas
WHO−2001 Adjustment Method

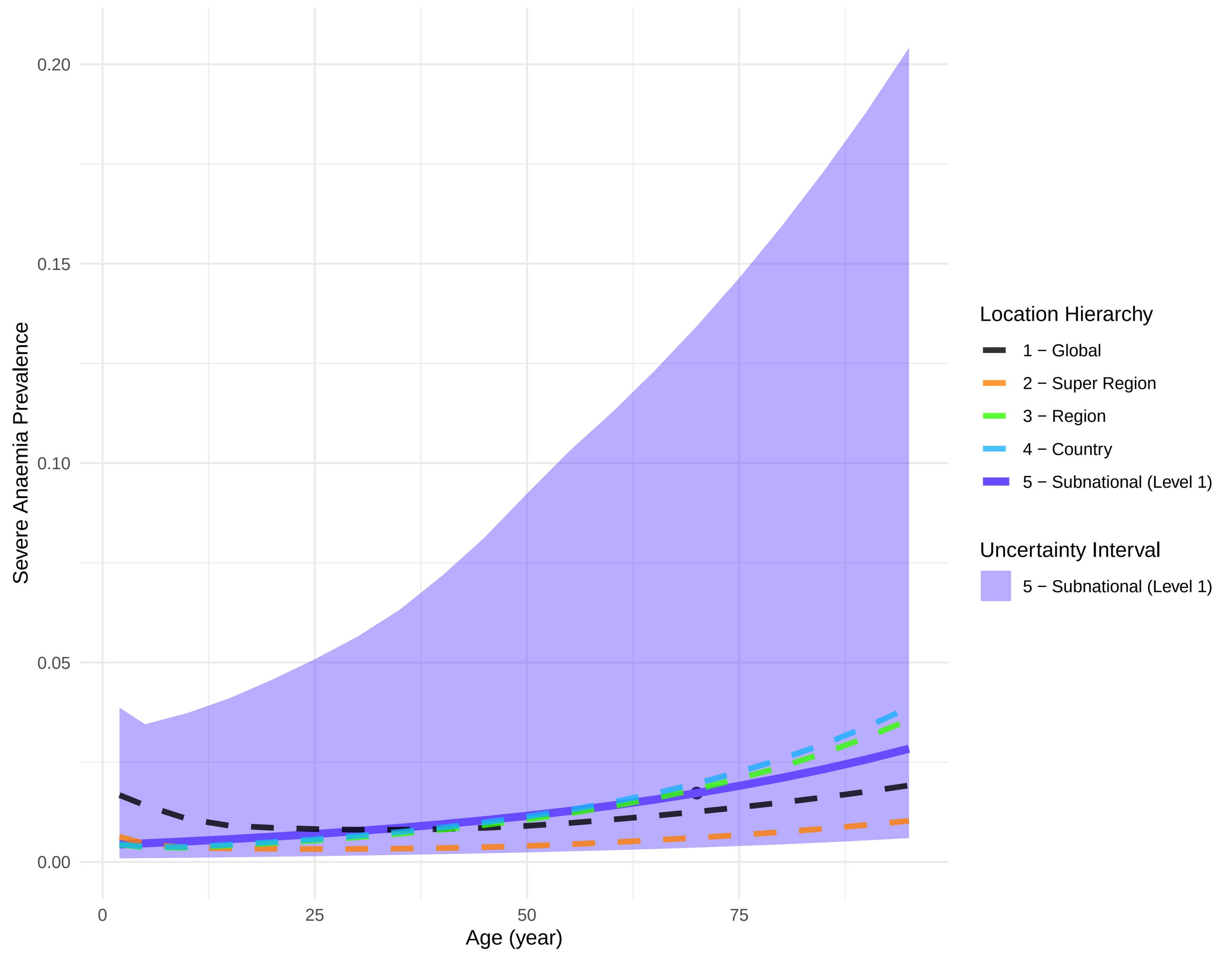

MR−BRT Age/Sex Fits − Males 2+ Years Old − Severe Anaemia Prevalence − Tlaxcala
WHO−2001 Adjustment Method

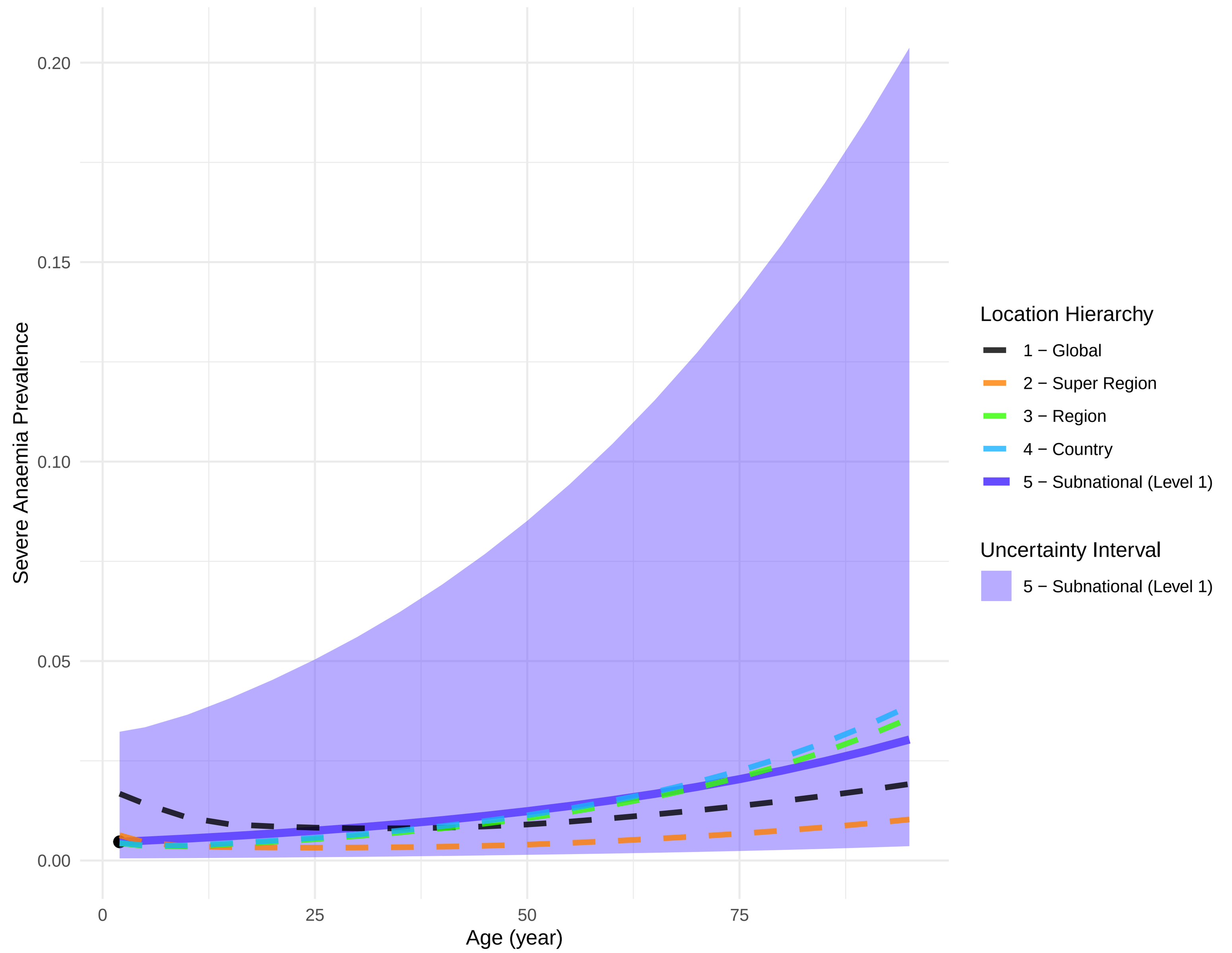

MR−BRT Age/Sex Fits − Males 2+ Years Old − Severe Anaemia Prevalence − Veracruz de Ignacio de la Llave
WHO−2001 Adjustment Method

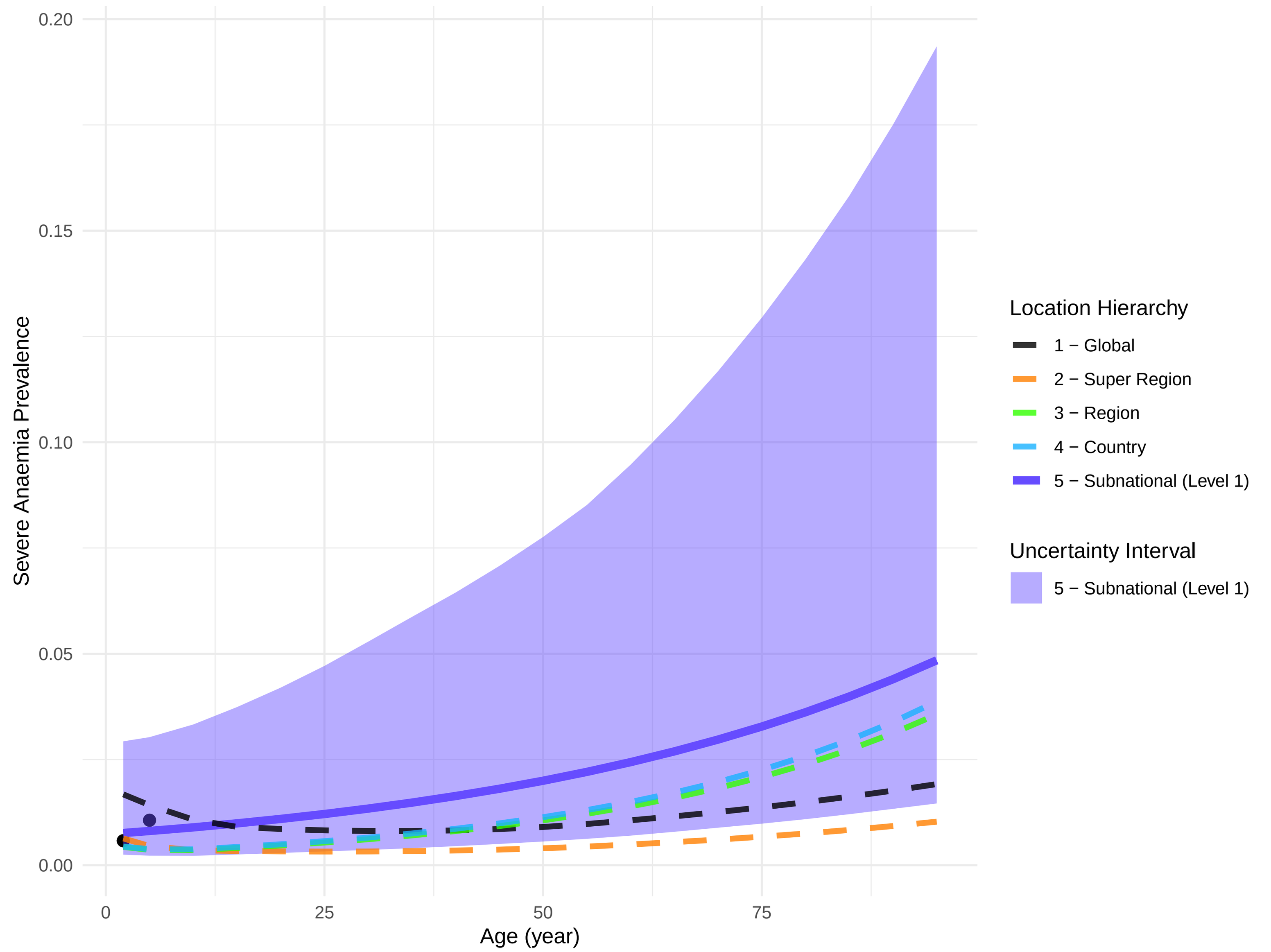

MR−BRT Age/Sex Fits − Males 2+ Years Old − Severe Anaemia Prevalence − Yucatán
WHO−2001 Adjustment Method

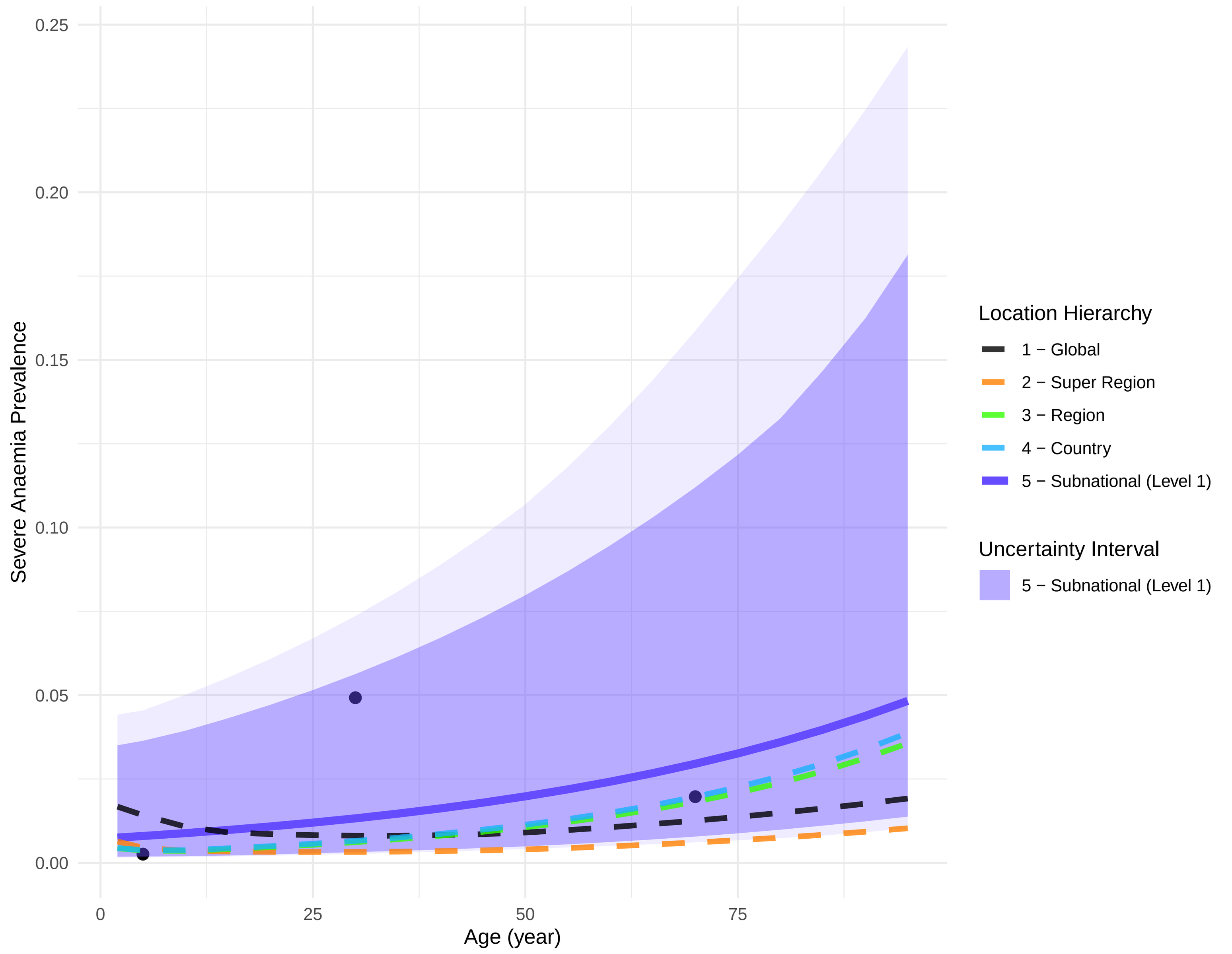

MR−BRT Age/Sex Fits − Males 2+ Years Old − Severe Anaemia Prevalence − Tropical Latin America
WHO−2001 Adjustment Method

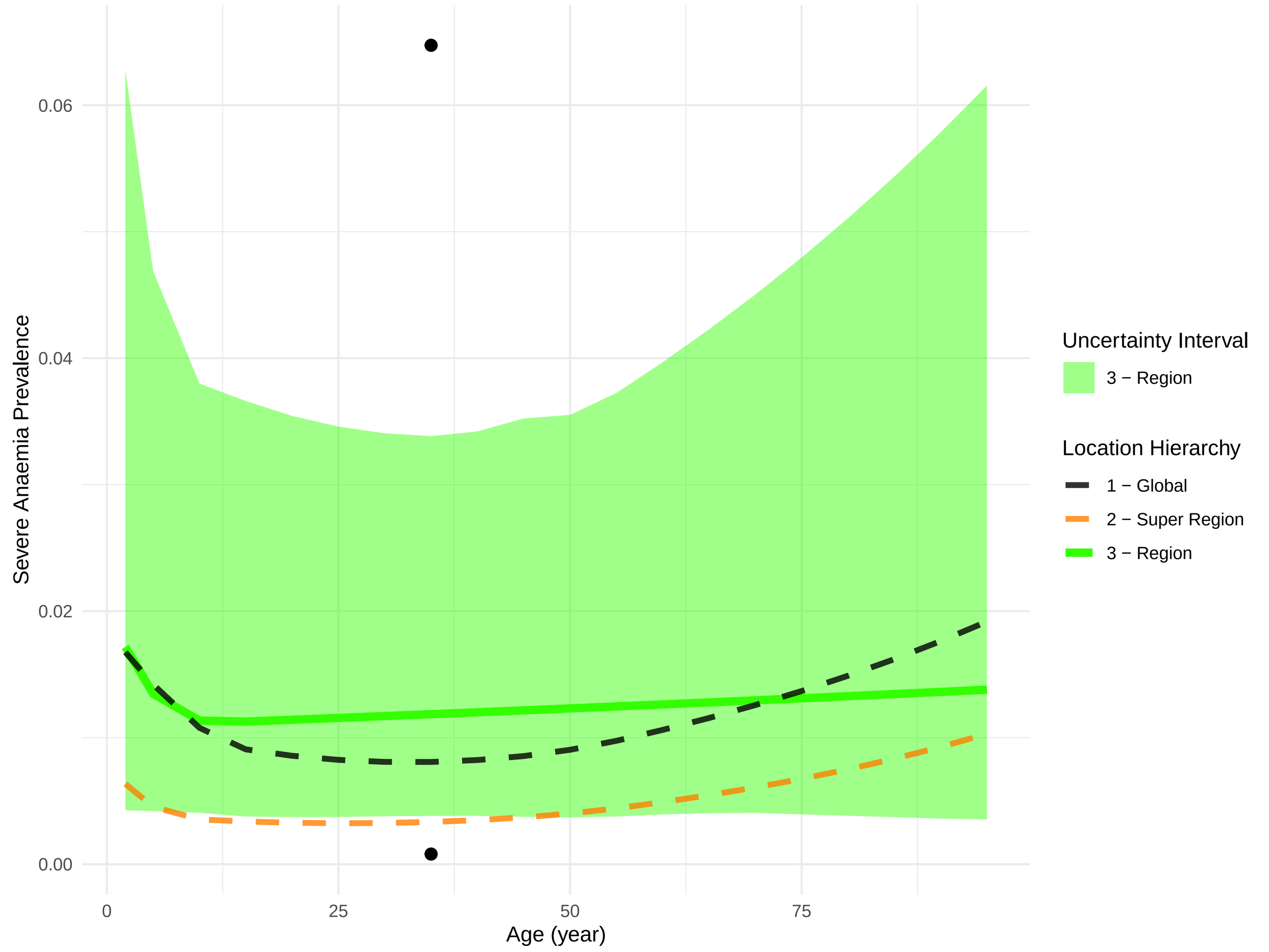

MR−BRT Age/Sex Fits − Males 2+ Years Old − Severe Anaemia Prevalence − Brazil
WHO−2001 Adjustment Method

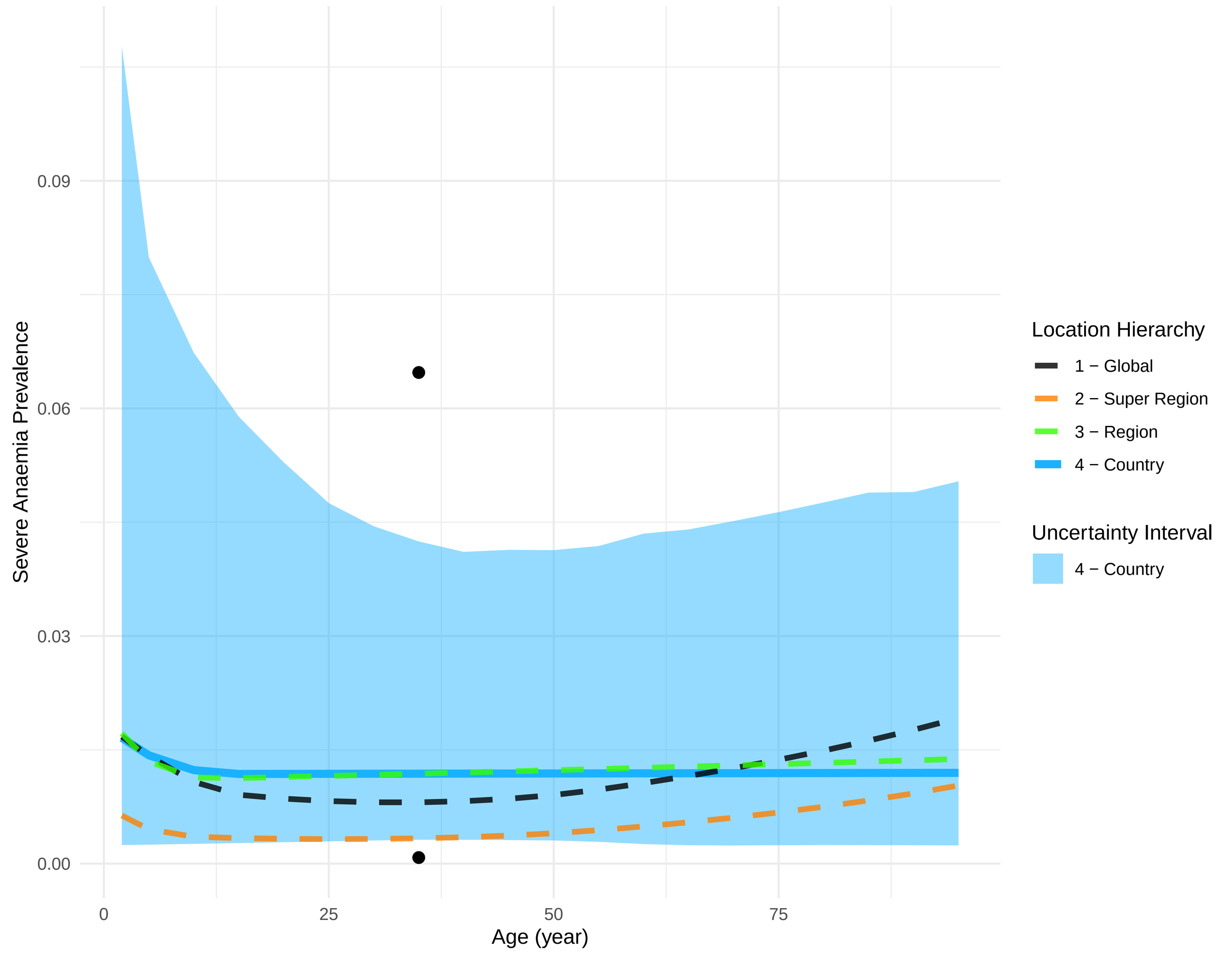

MR−BRT Age/Sex Fits − Males 2+ Years Old − Severe Anaemia Prevalence − Amazonas
WHO−2001 Adjustment Method

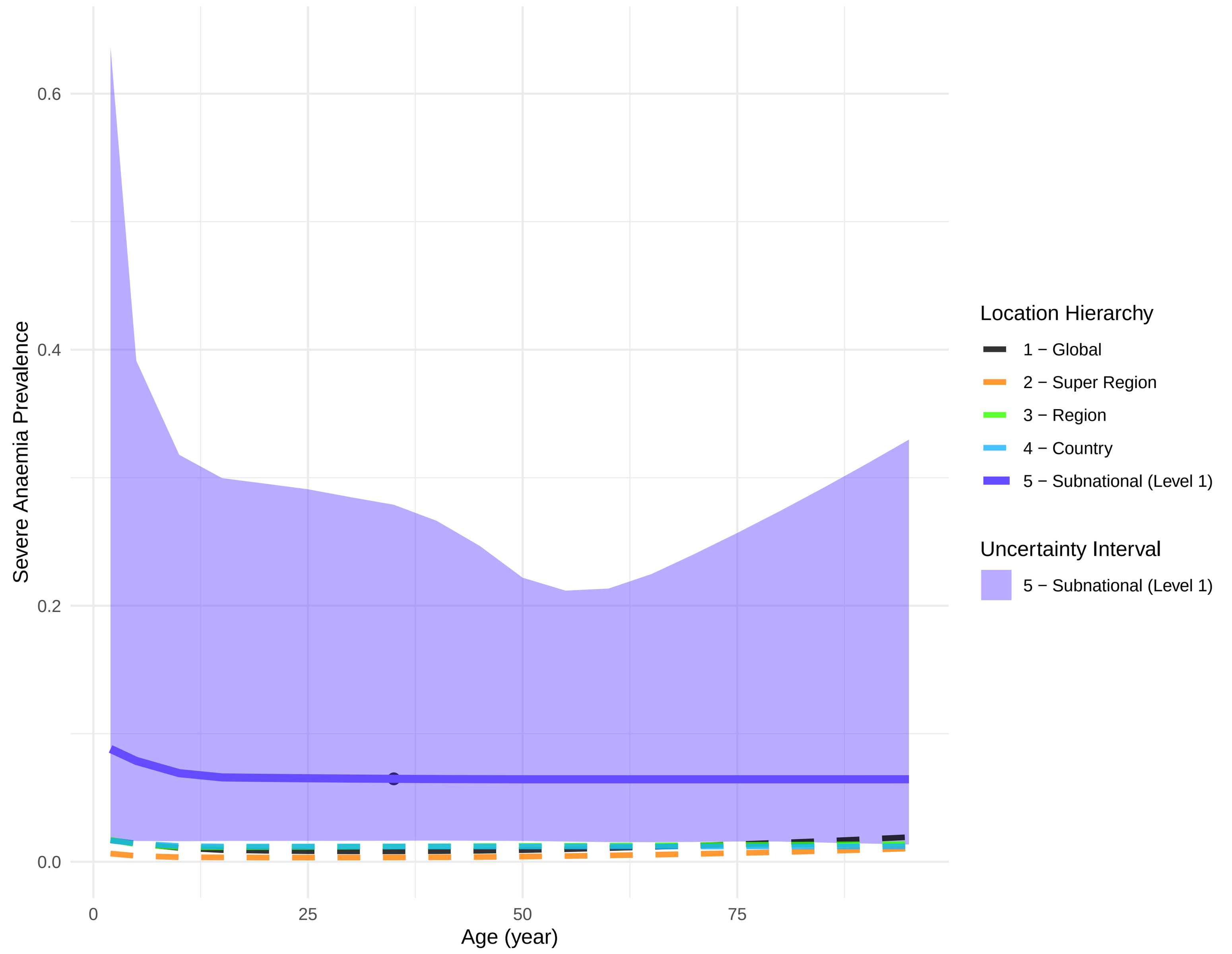

MR−BRT Age/Sex Fits − Males 2+ Years Old − Severe Anaemia Prevalence − North Africa and Middle East
WHO−2001 Adjustment Method

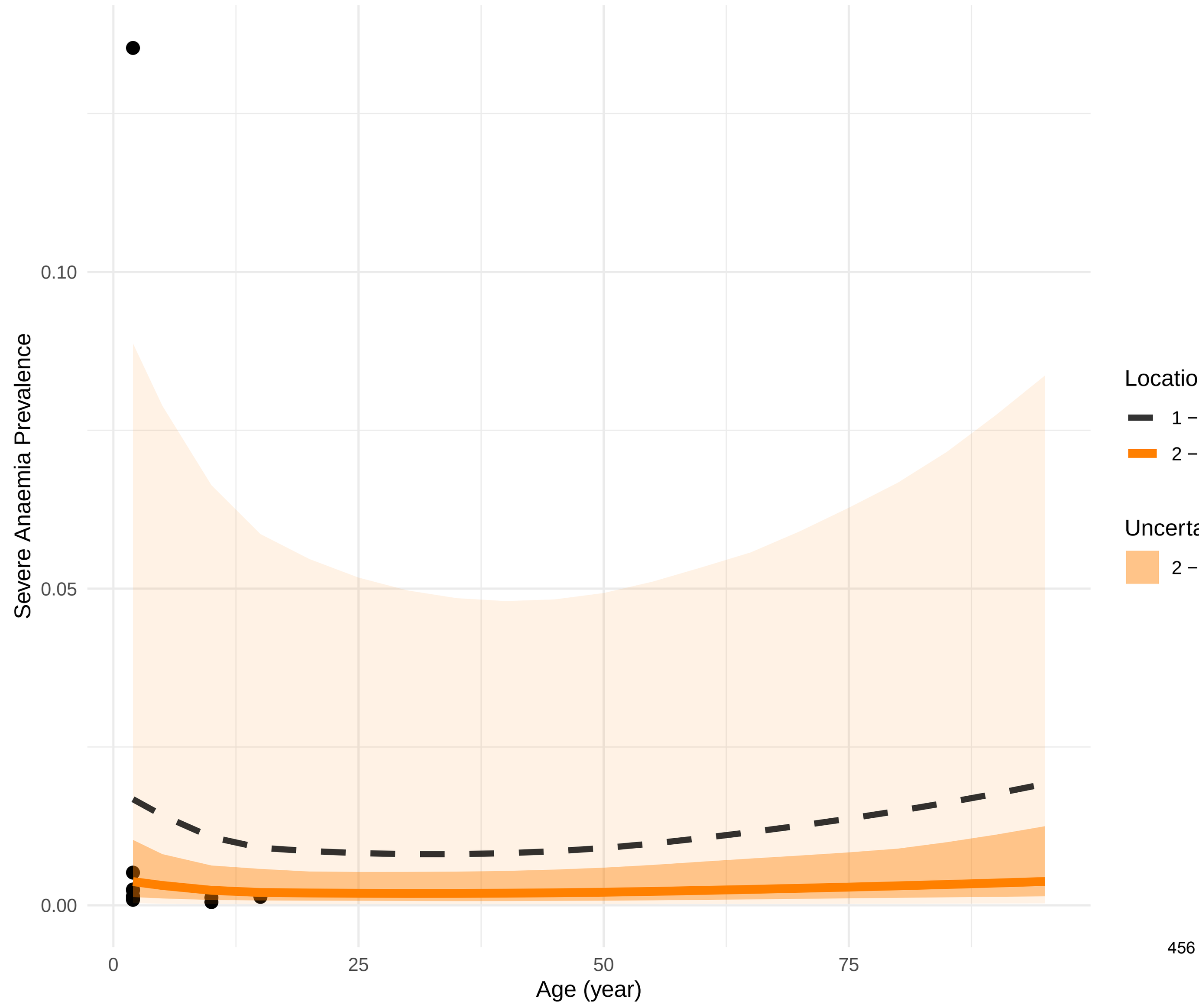

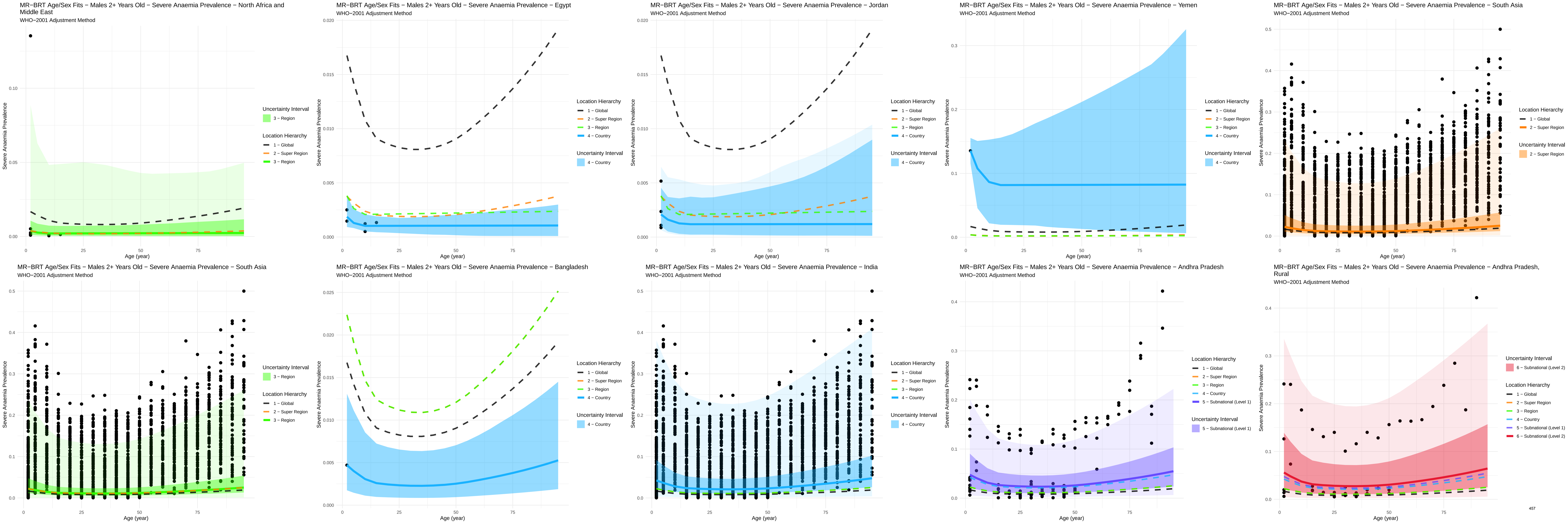
MR−BRT Age/Sex Fits − Males 2+ Years Old − Severe Anaemia Prevalence − North Africa and Middle East
WHO−2001 Adjustment Method
Severe Anaemia Prevalence
Age (year)
Uncertainty Interval
3 − Region
Location Hierarchy
1 − Global
2 − Super Region
3 − Region
MR−BRT Age/Sex Fits − Males 2+ Years Old − Severe Anaemia Prevalence − Egypt
WHO−2001 Adjustment Method
Location Hierarchy
1 − Global
2 − Super Region
3 − Region
4 − Country
Uncertainty Interval
4 − Country
MR−BRT Age/Sex Fits − Males 2+ Years Old − Severe Anaemia Prevalence − Jordan
WHO−2001 Adjustment Method
MR−BRT Age/Sex Fits − Males 2+ Years Old − Severe Anaemia Prevalence − Yemen
WHO−2001 Adjustment Method
MR−BRT Age/Sex Fits − Males 2+ Years Old − Severe Anaemia Prevalence − South Asia
WHO−2001 Adjustment Method
Location Hierarchy
1 − Global
2 − Super Region
Uncertainty Interval
2 − Super Region
MR−BRT Age/Sex Fits − Males 2+ Years Old − Severe Anaemia Prevalence − South Asia
WHO−2001 Adjustment Method
Uncertainty Interval
3 − Region
Location Hierarchy
1 − Global
2 − Super Region
3 − Region
MR−BRT Age/Sex Fits − Males 2+ Years Old − Severe Anaemia Prevalence − Bangladesh
WHO−2001 Adjustment Method
MR−BRT Age/Sex Fits − Males 2+ Years Old − Severe Anaemia Prevalence − India
WHO−2001 Adjustment Method
MR−BRT Age/Sex Fits − Males 2+ Years Old − Severe Anaemia Prevalence − Andhra Pradesh
WHO−2001 Adjustment Method
Location Hierarchy
1 − Global
2 − Super Region
3 − Region
4 − Country
5 − Subnational (Level 1)
Uncertainty Interval
5 − Subnational (Level 1)
MR−BRT Age/Sex Fits − Males 2+ Years Old − Severe Anaemia Prevalence − Andhra Pradesh, Rural
WHO−2001 Adjustment Method
Uncertainty Interval
6 − Subnational (Level 2)
Location Hierarchy
1 − Global
2 − Super Region
3 − Region
4 − Country
5 − Subnational (Level 1)
6 − Subnational (Level 2)

MR−BRT Age/Sex Fits − Males 2+ Years Old − Severe Anaemia Prevalence − Andhra Pradesh, Urban
WHO−2001 Adjustment Method

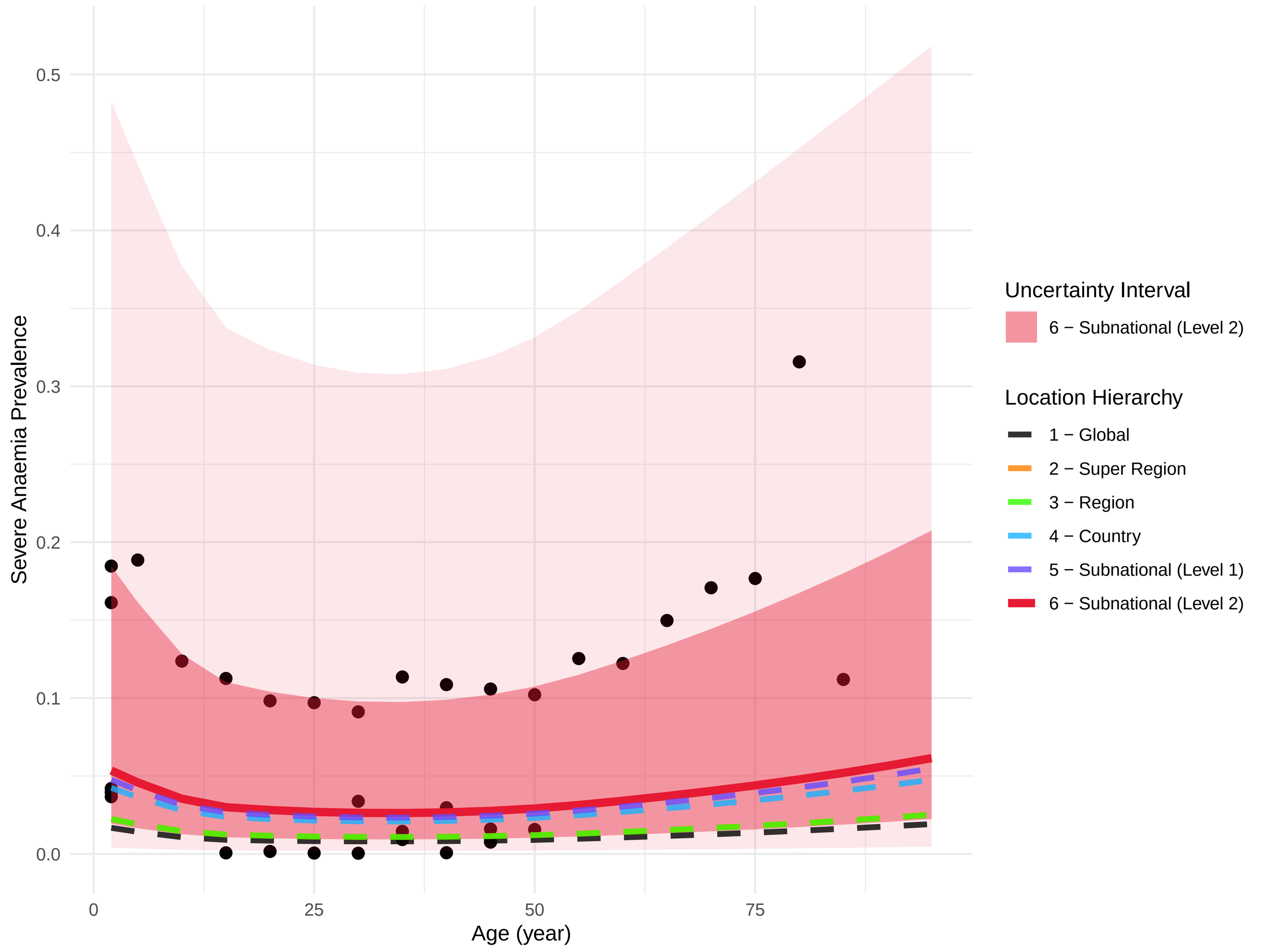

MR−BRT Age/Sex Fits − Males 2+ Years Old − Severe Anaemia Prevalence − Arunachal Pradesh
WHO−2001 Adjustment Method

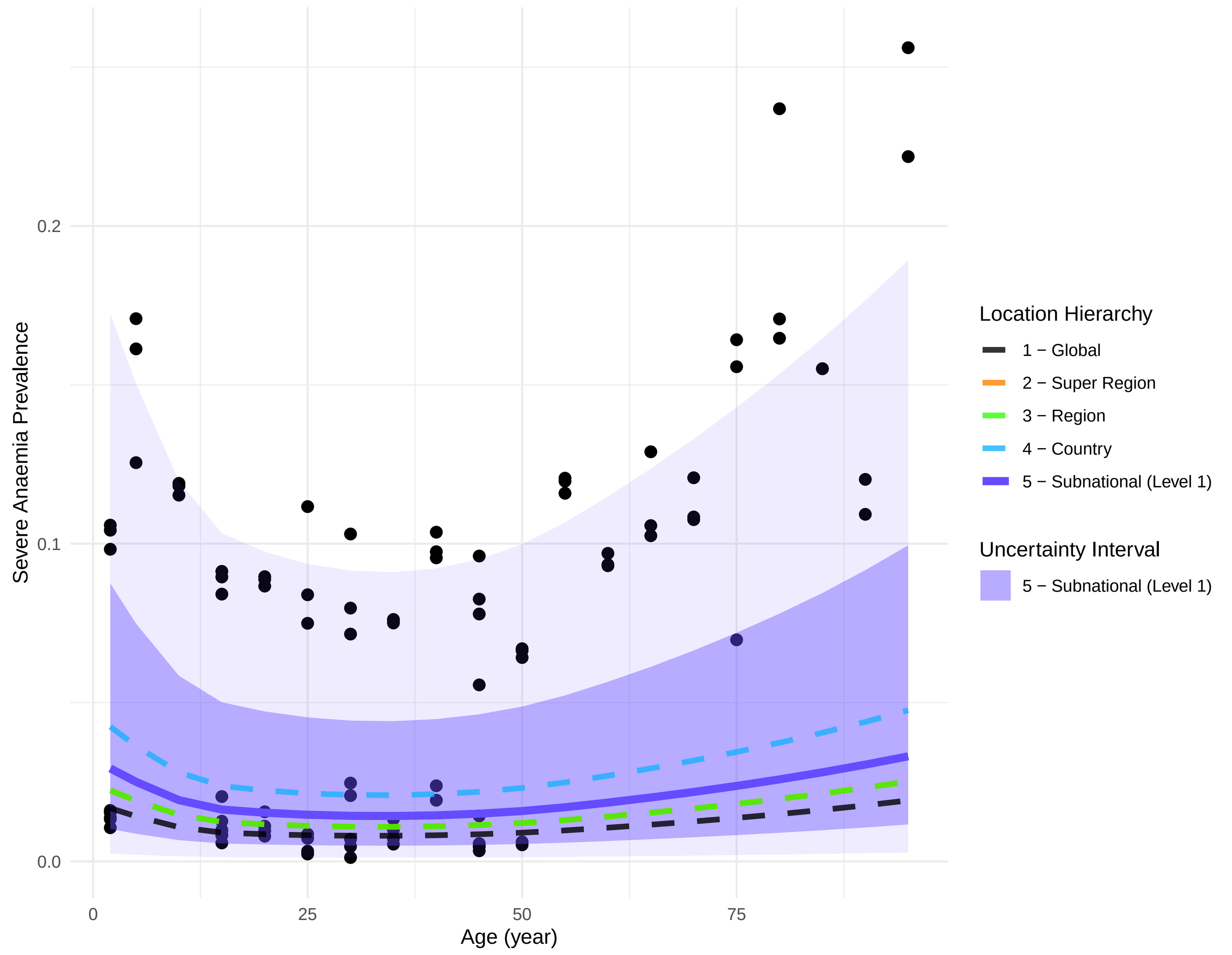

MR−BRT Age/Sex Fits − Males 2+ Years Old − Severe Anaemia Prevalence − Arunachal Pradesh, Rural
WHO−2001 Adjustment Method

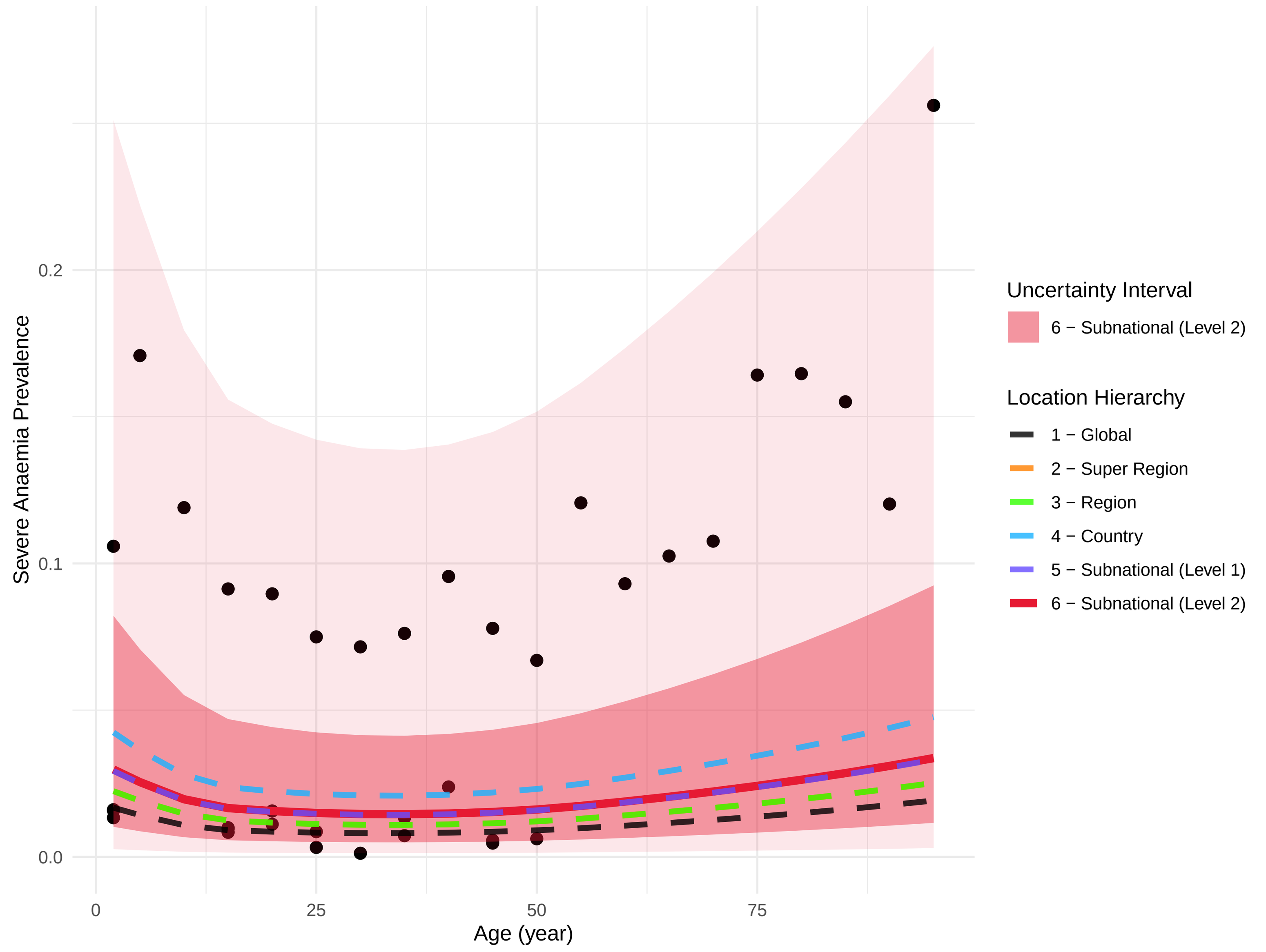

MR−BRT Age/Sex Fits − Males 2+ Years Old − Severe Anaemia Prevalence − Arunachal Pradesh, Urban
WHO−2001 Adjustment Method

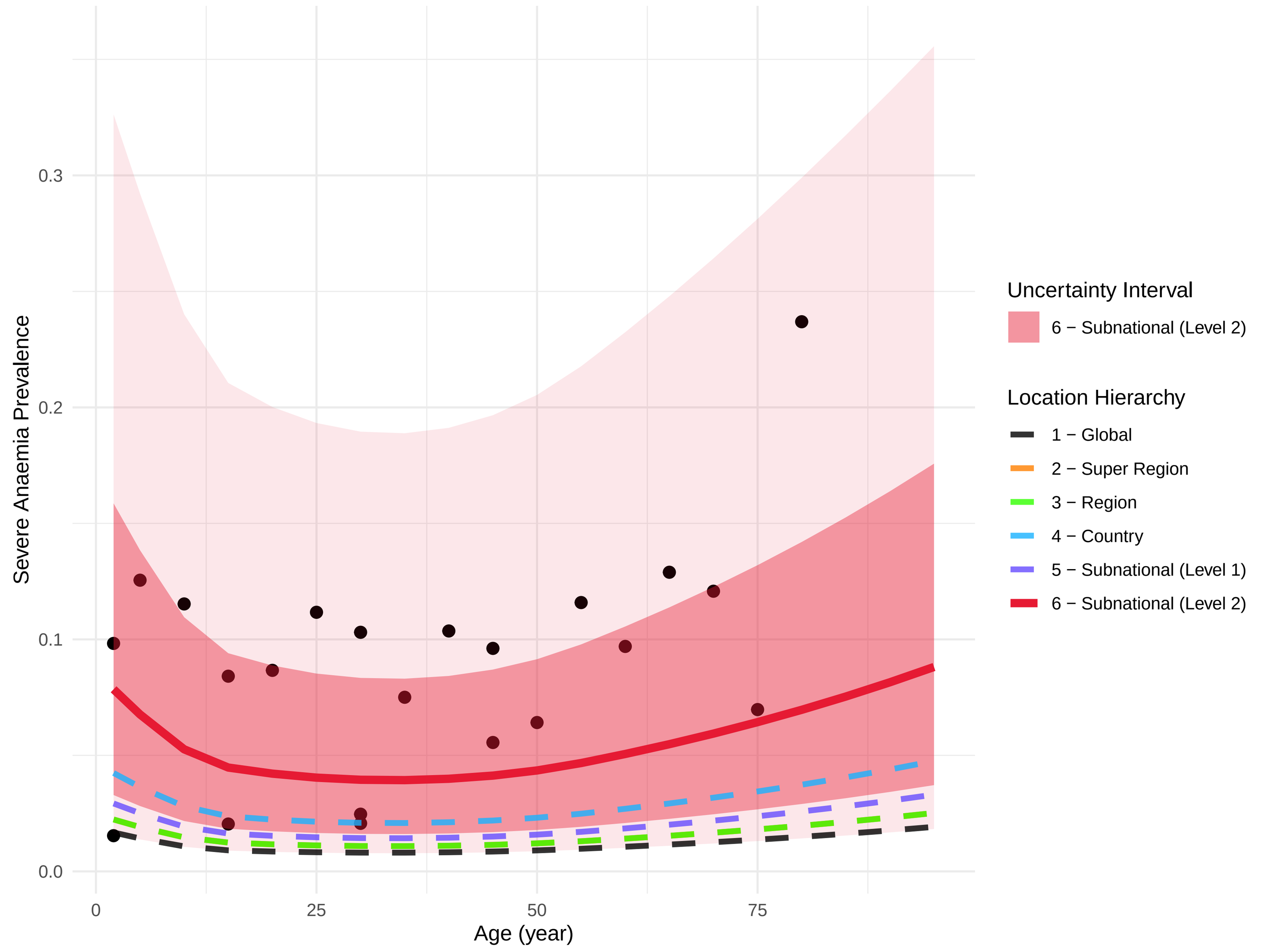

MR−BRT Age/Sex Fits − Males 2+ Years Old − Severe Anaemia Prevalence − Assam
WHO−2001 Adjustment Method

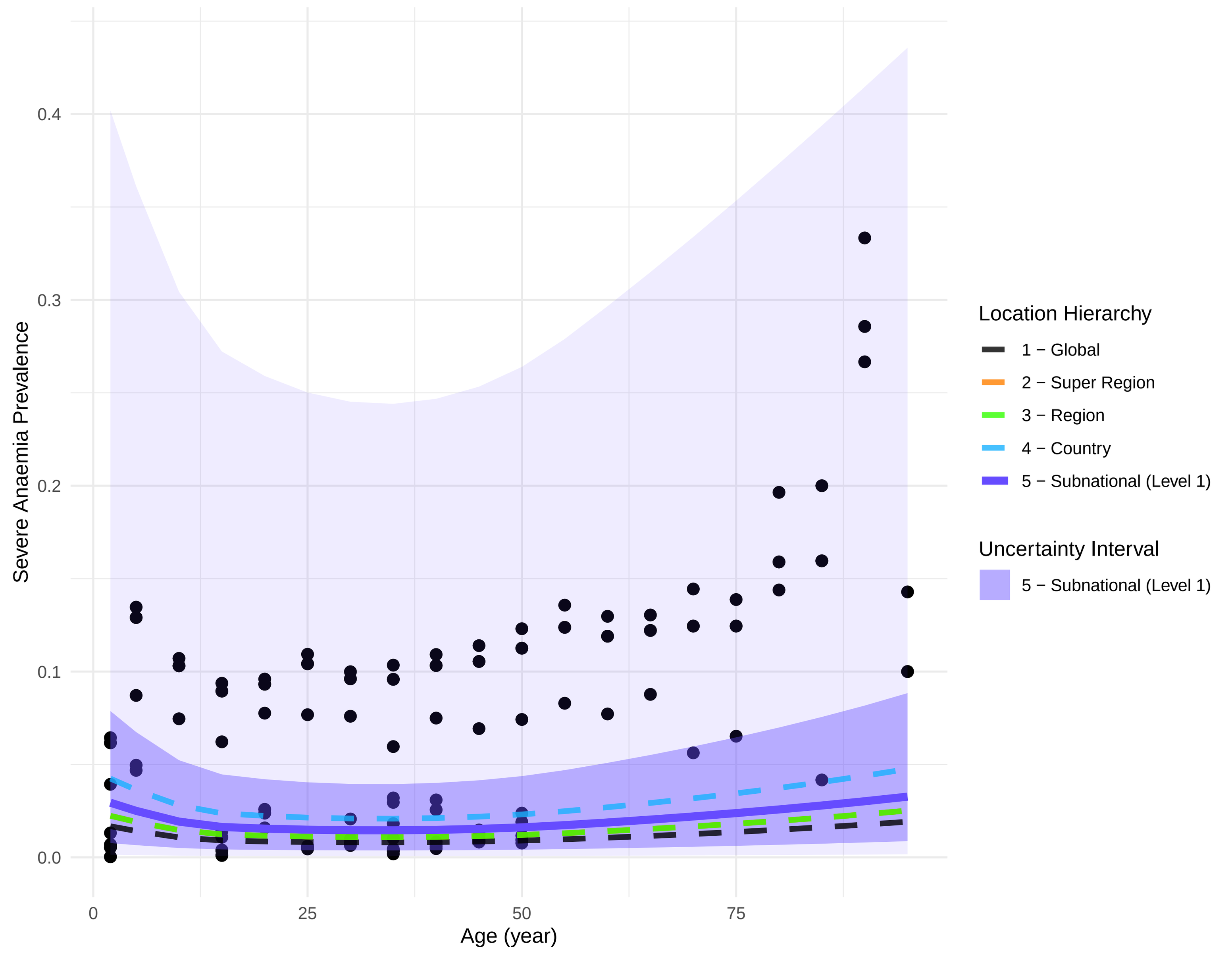

MR−BRT Age/Sex Fits − Males 2+ Years Old − Severe Anaemia Prevalence − Assam, Rural
WHO−2001 Adjustment Method

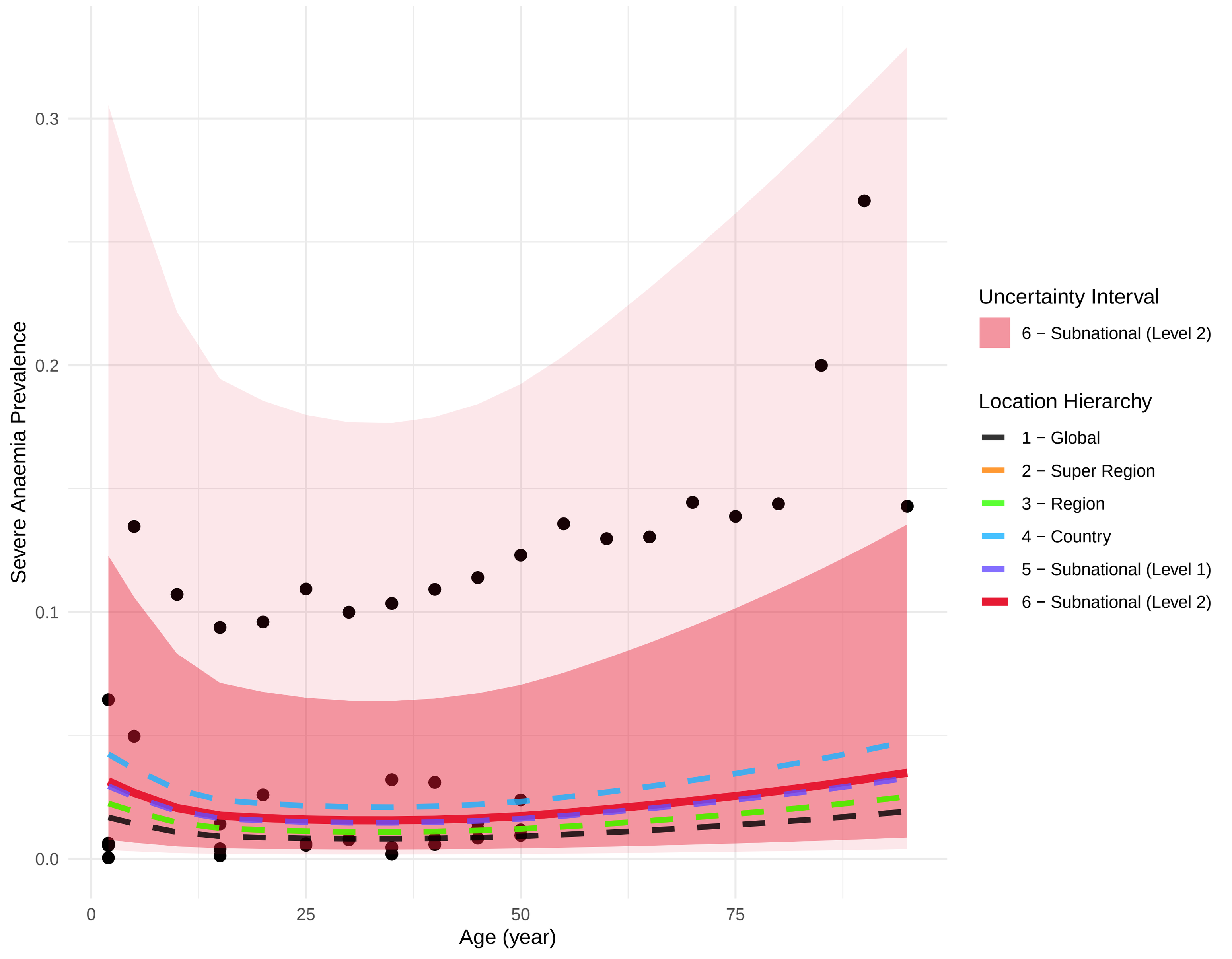

MR−BRT Age/Sex Fits − Males 2+ Years Old − Severe Anaemia Prevalence − Assam, Urban
WHO−2001 Adjustment Method

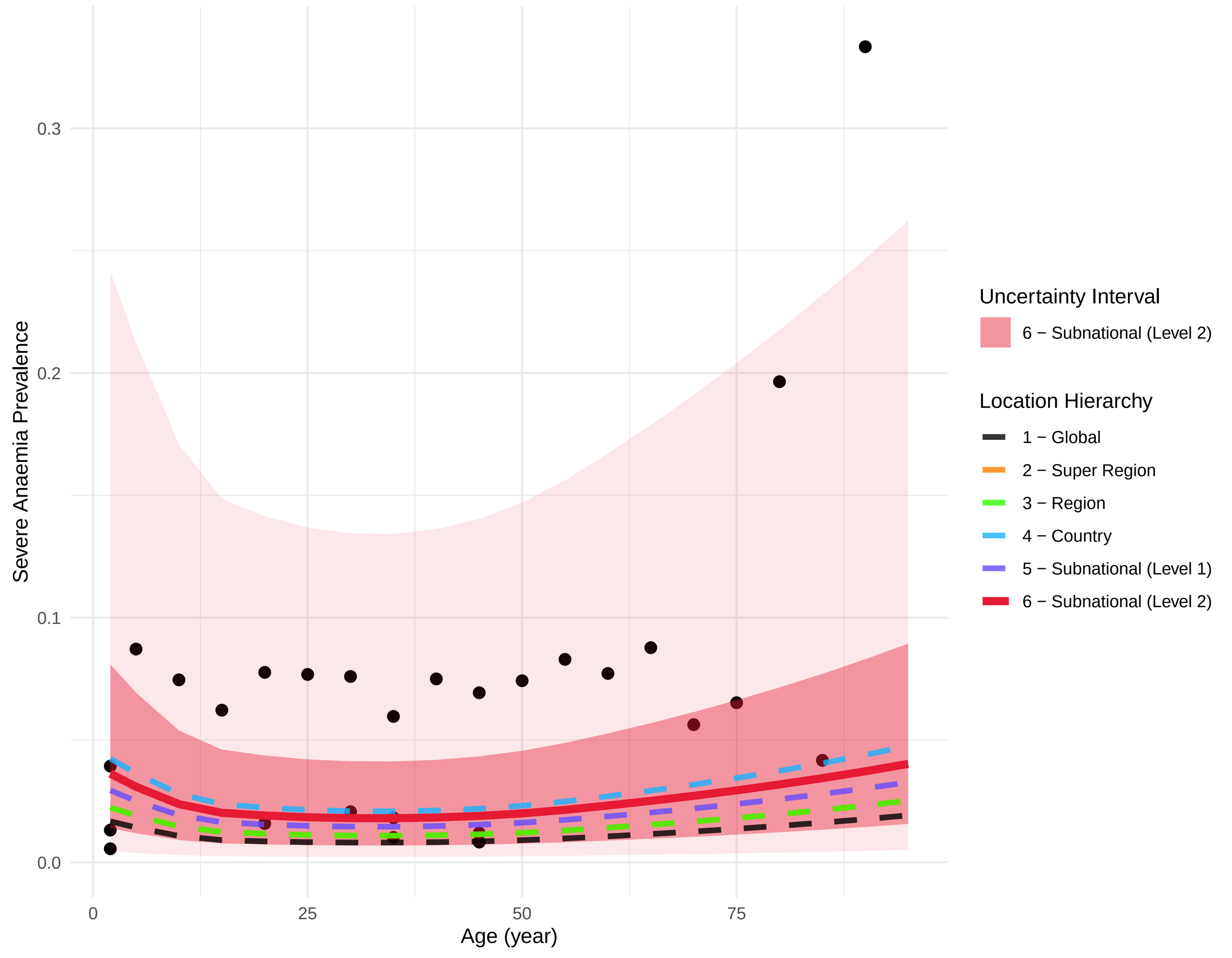

MR−BRT Age/Sex Fits − Males 2+ Years Old − Severe Anaemia Prevalence − Bihar
WHO−2001 Adjustment Method

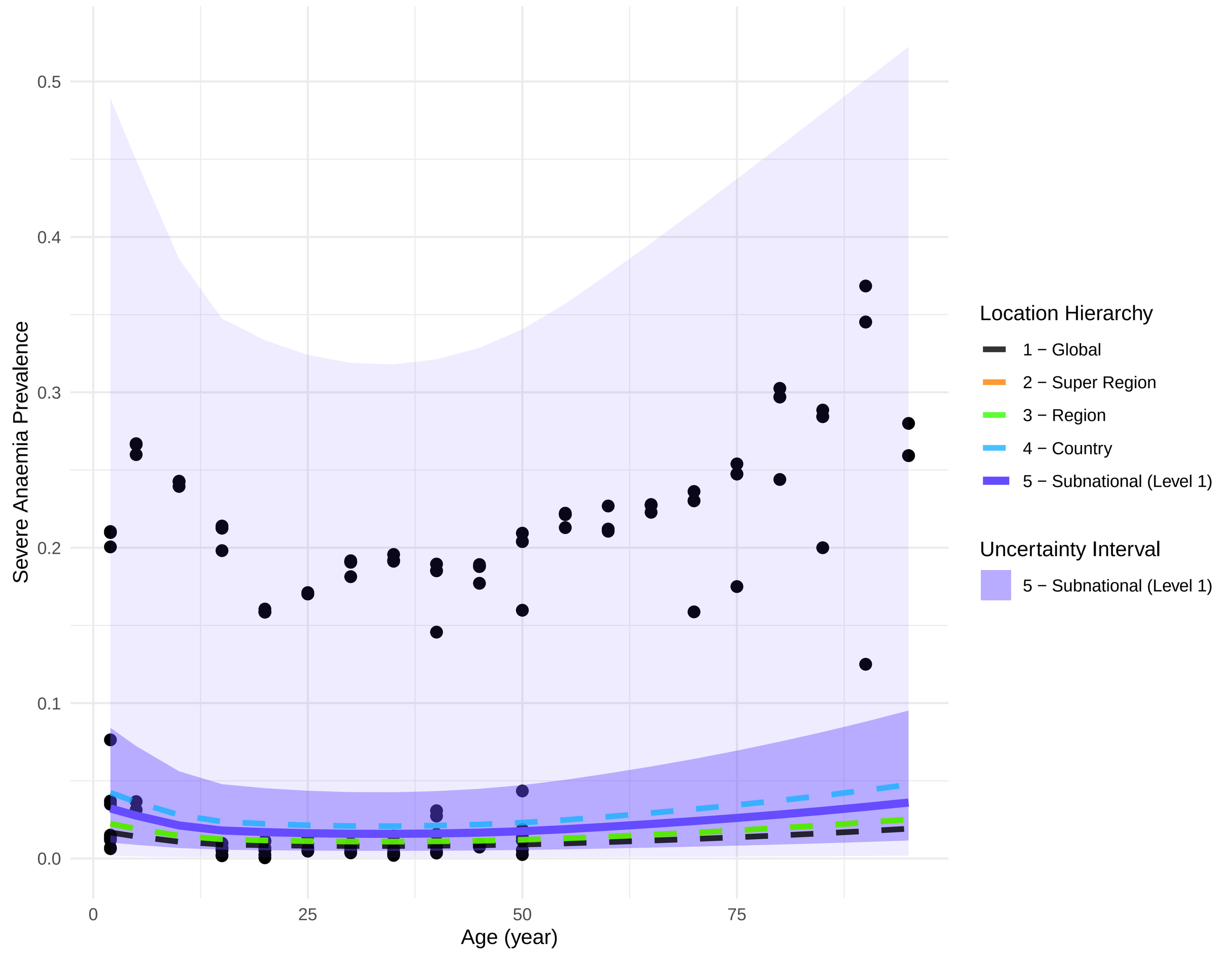

MR−BRT Age/Sex Fits − Males 2+ Years Old − Severe Anaemia Prevalence − Bihar, Rural
WHO−2001 Adjustment Method

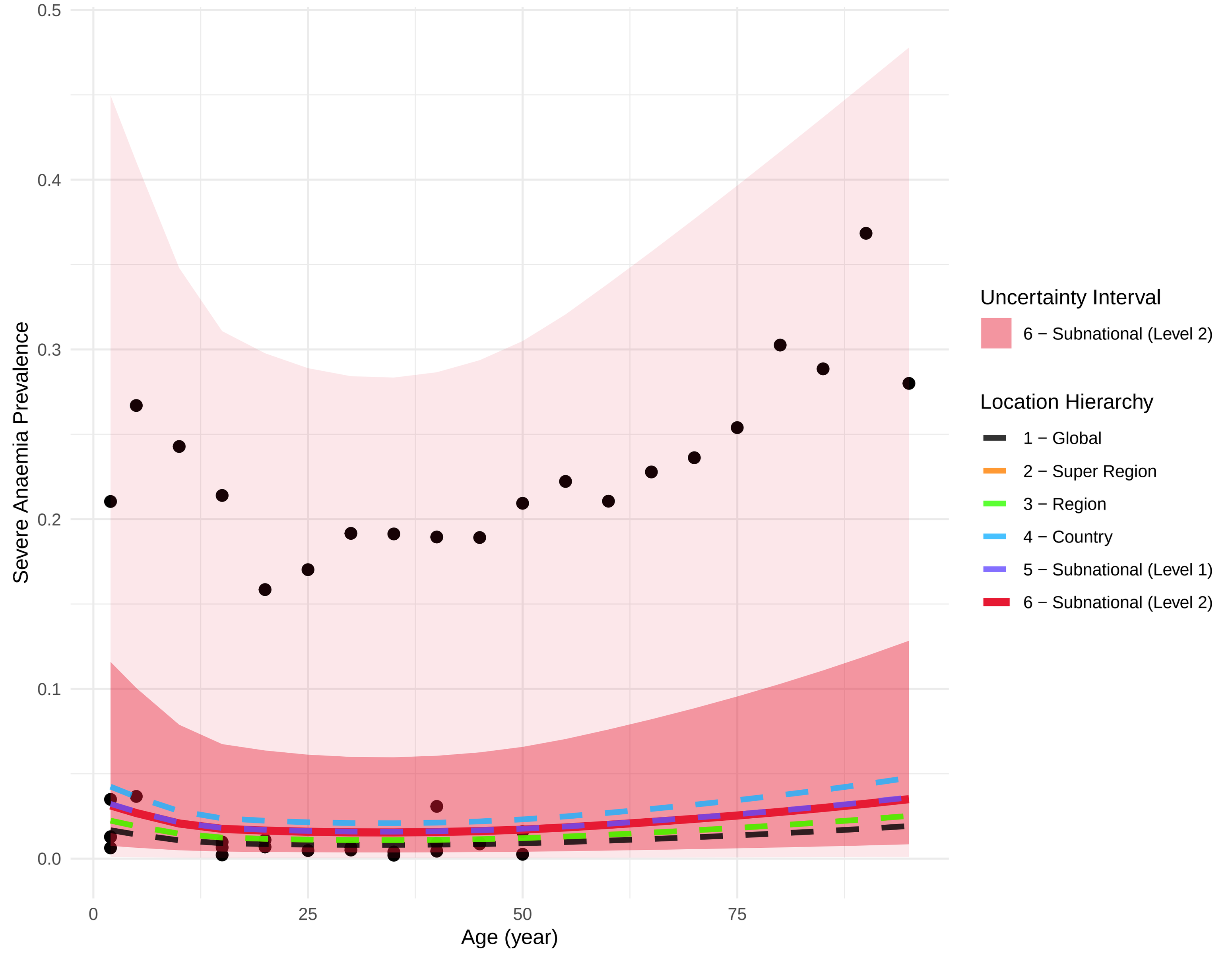

MR−BRT Age/Sex Fits − Males 2+ Years Old − Severe Anaemia Prevalence − Bihar, Urban
WHO−2001 Adjustment Method

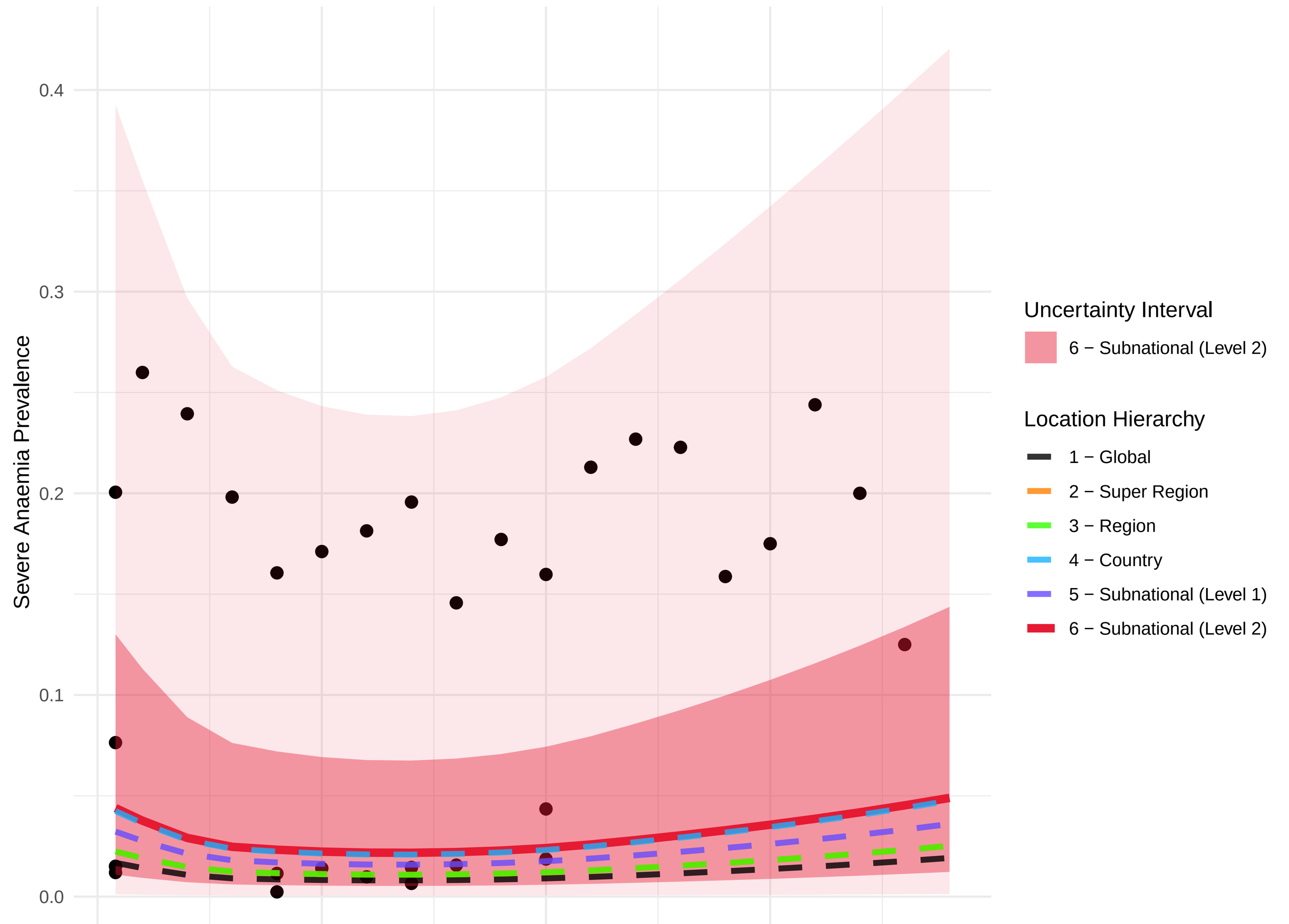

MR−BRT Age/Sex Fits − Males 2+ Years Old − Severe Anaemia Prevalence − Chhattisgarh
WHO−2001 Adjustment Method

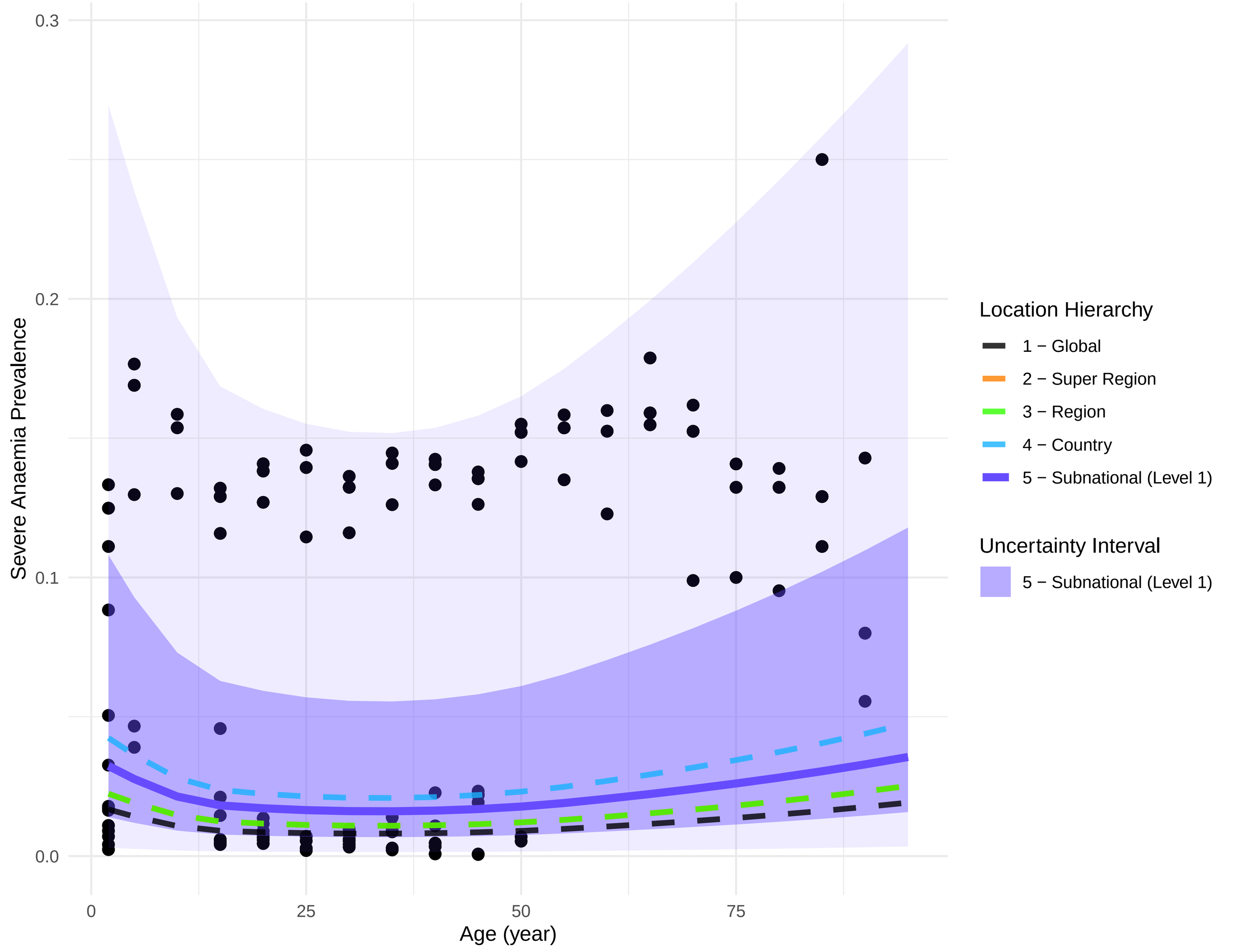

MR−BRT Age/Sex Fits − Males 2+ Years Old − Severe Anaemia Prevalence − Chhattisgarh, Rural
WHO−2001 Adjustment Method

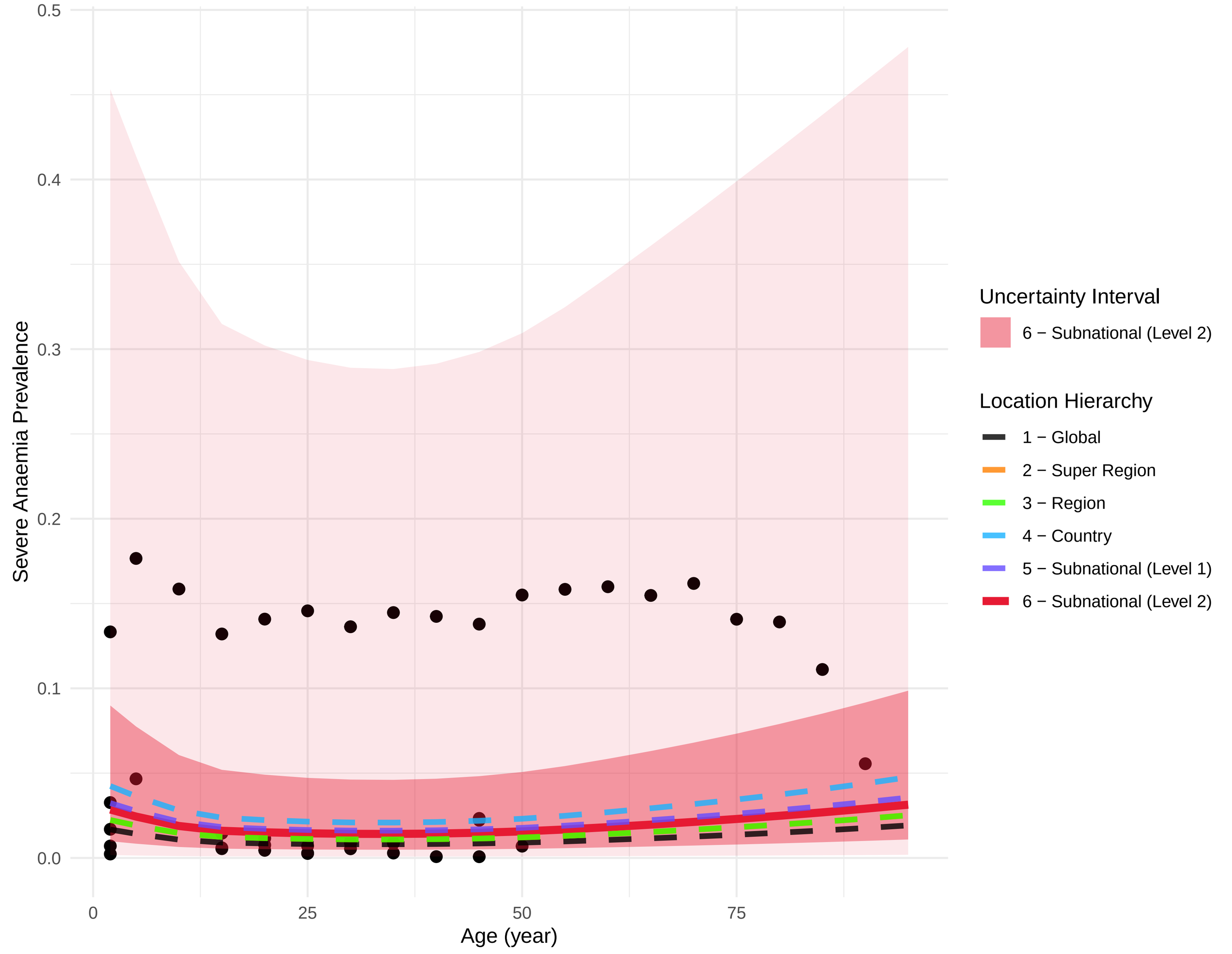

MR−BRT Age/Sex Fits − Males 2+ Years Old − Severe Anaemia Prevalence − Chhattisgarh, Urban
WHO−2001 Adjustment Method

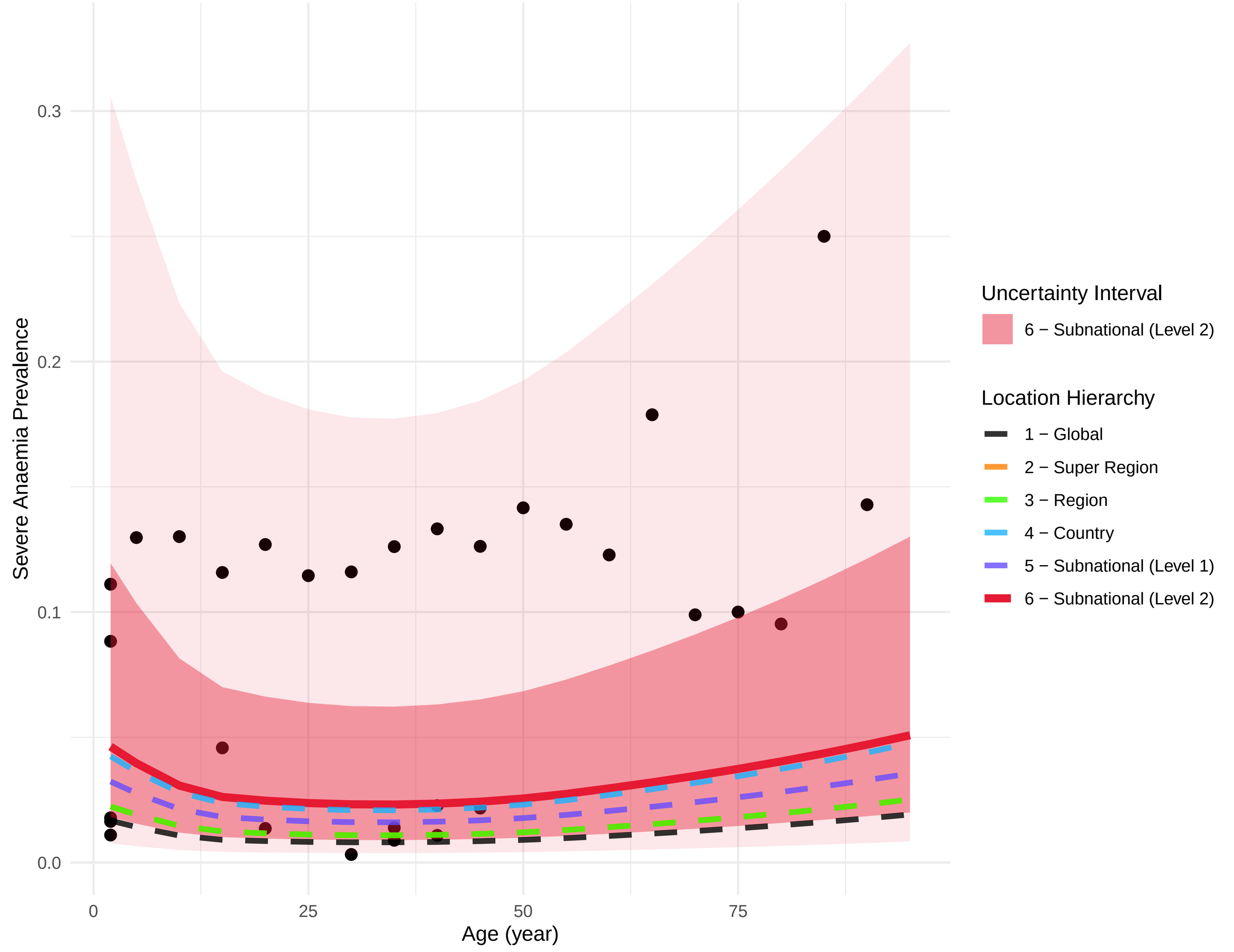

MR−BRT Age/Sex Fits − Males 2+ Years Old − Severe Anaemia Prevalence − Delhi
WHO−2001 Adjustment Method

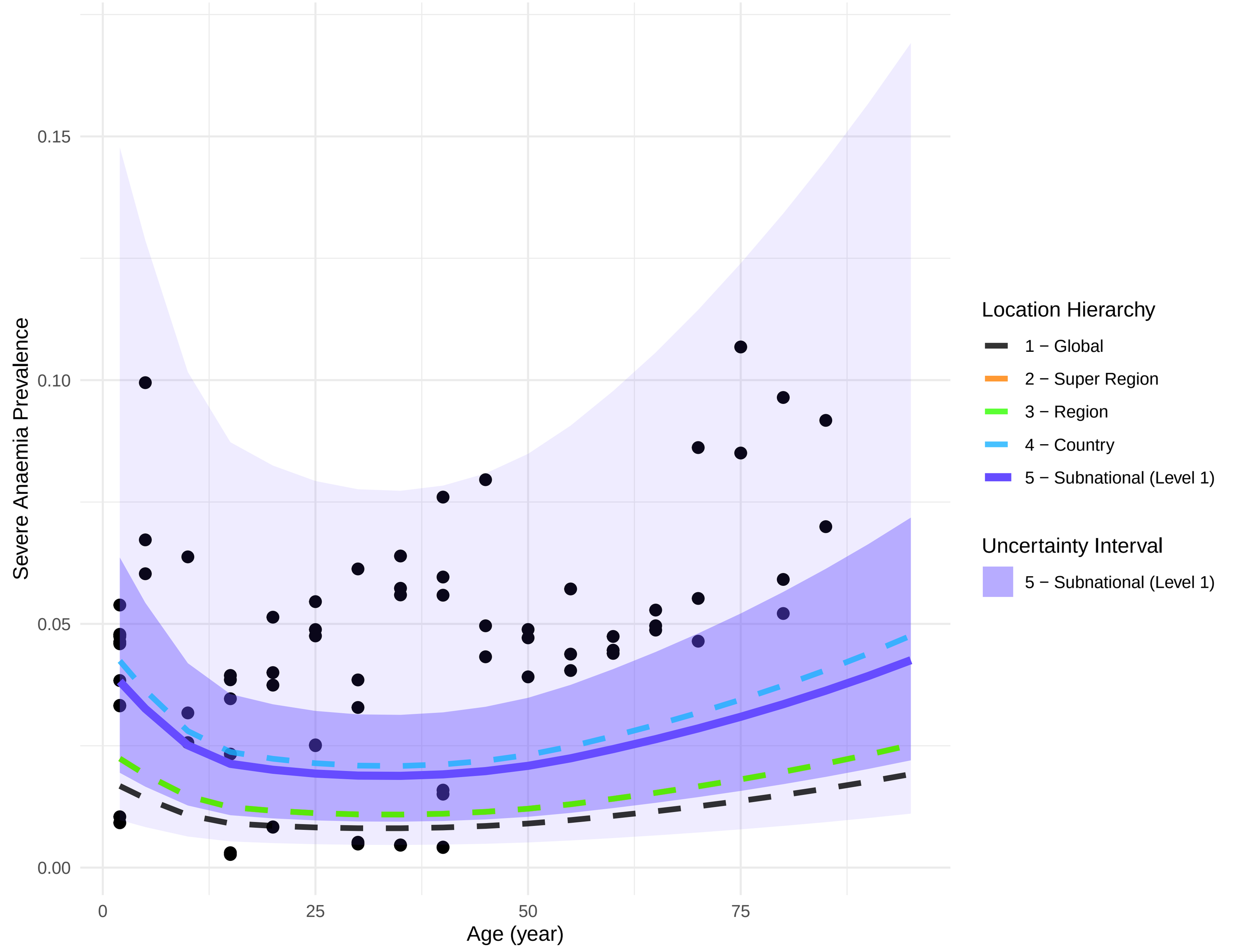

MR−BRT Age/Sex Fits − Males 2+ Years Old − Severe Anaemia Prevalence − Delhi, Rural
WHO−2001 Adjustment Method

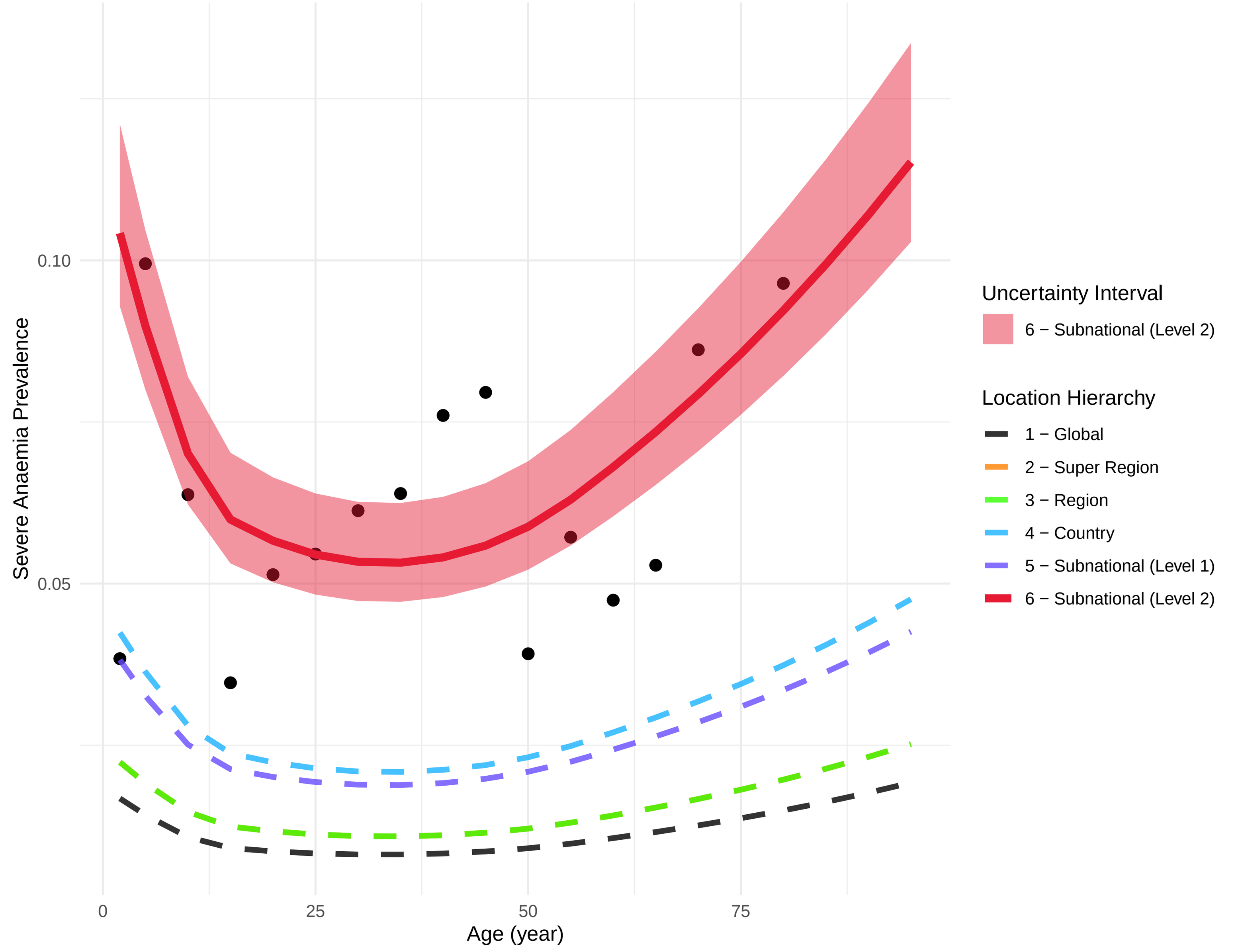

MR−BRT Age/Sex Fits − Males 2+ Years Old − Severe Anaemia Prevalence − Delhi, Urban
WHO−2001 Adjustment Method

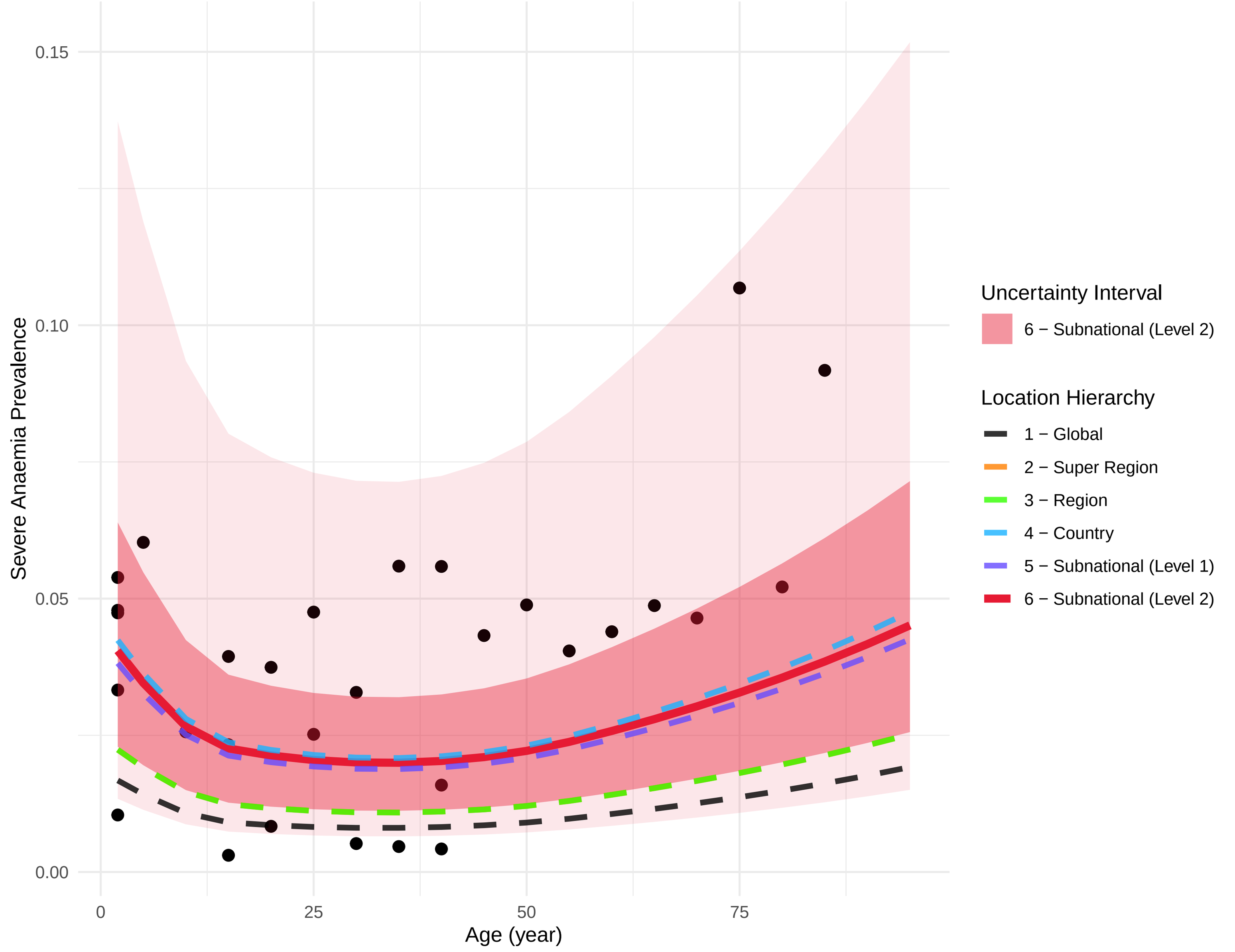

MR−BRT Age/Sex Fits − Males 2+ Years Old − Severe Anaemia Prevalence − Goa
WHO−2001 Adjustment Method

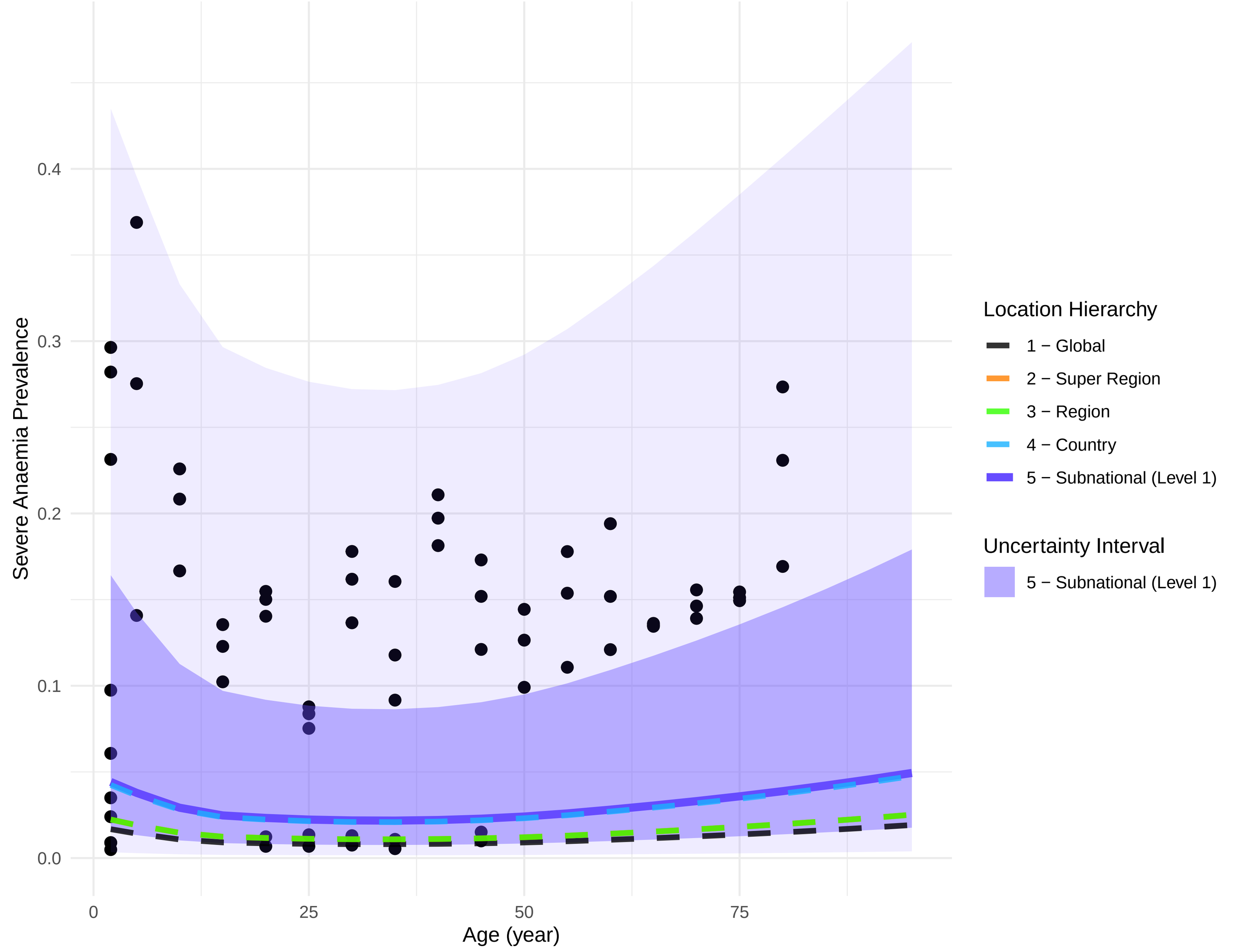

MR−BRT Age/Sex Fits − Males 2+ Years Old − Severe Anaemia Prevalence − Goa, Rural
WHO−2001 Adjustment Method

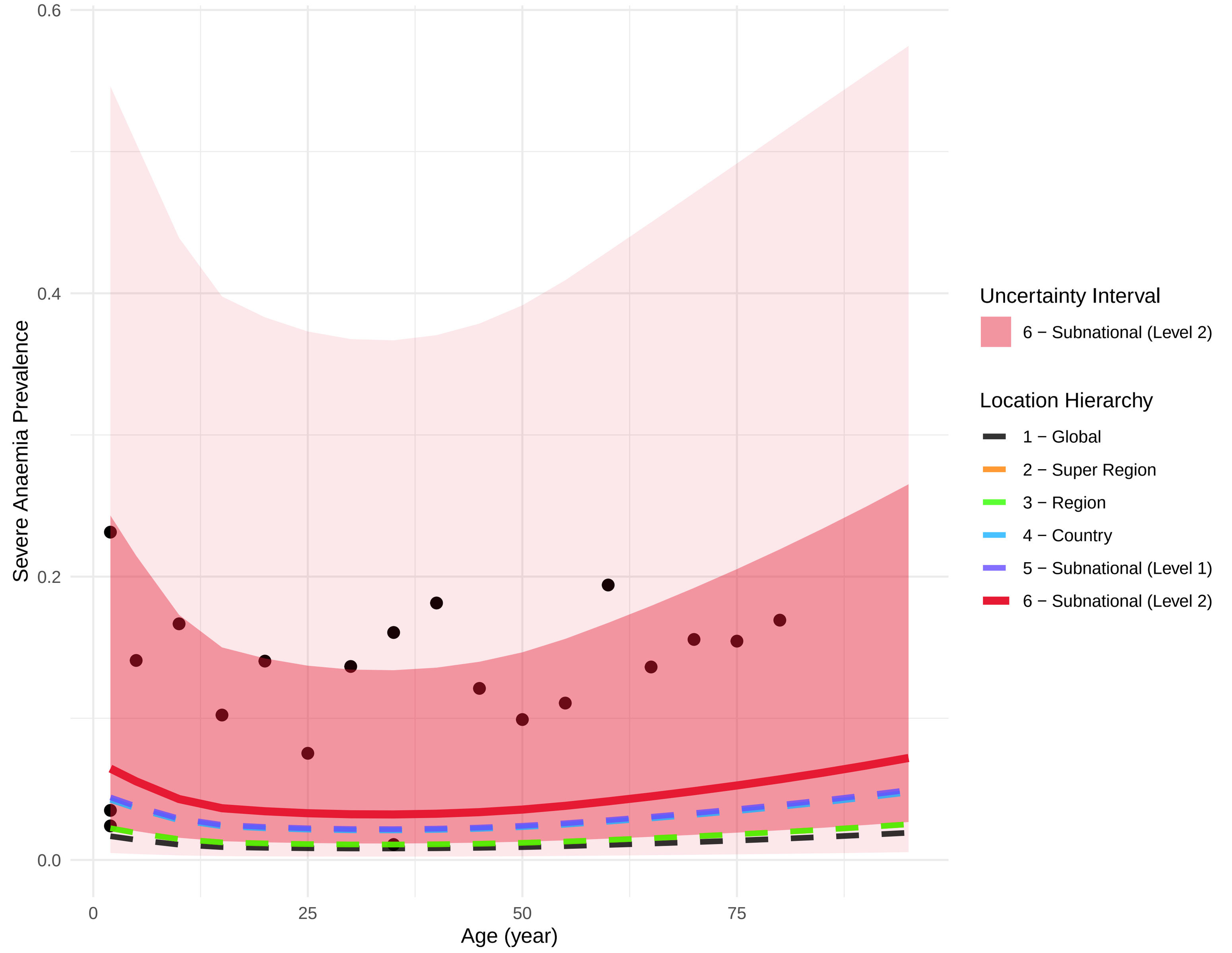

MR−BRT Age/Sex Fits − Males 2+ Years Old − Severe Anaemia Prevalence − Goa, Urban
WHO−2001 Adjustment Method

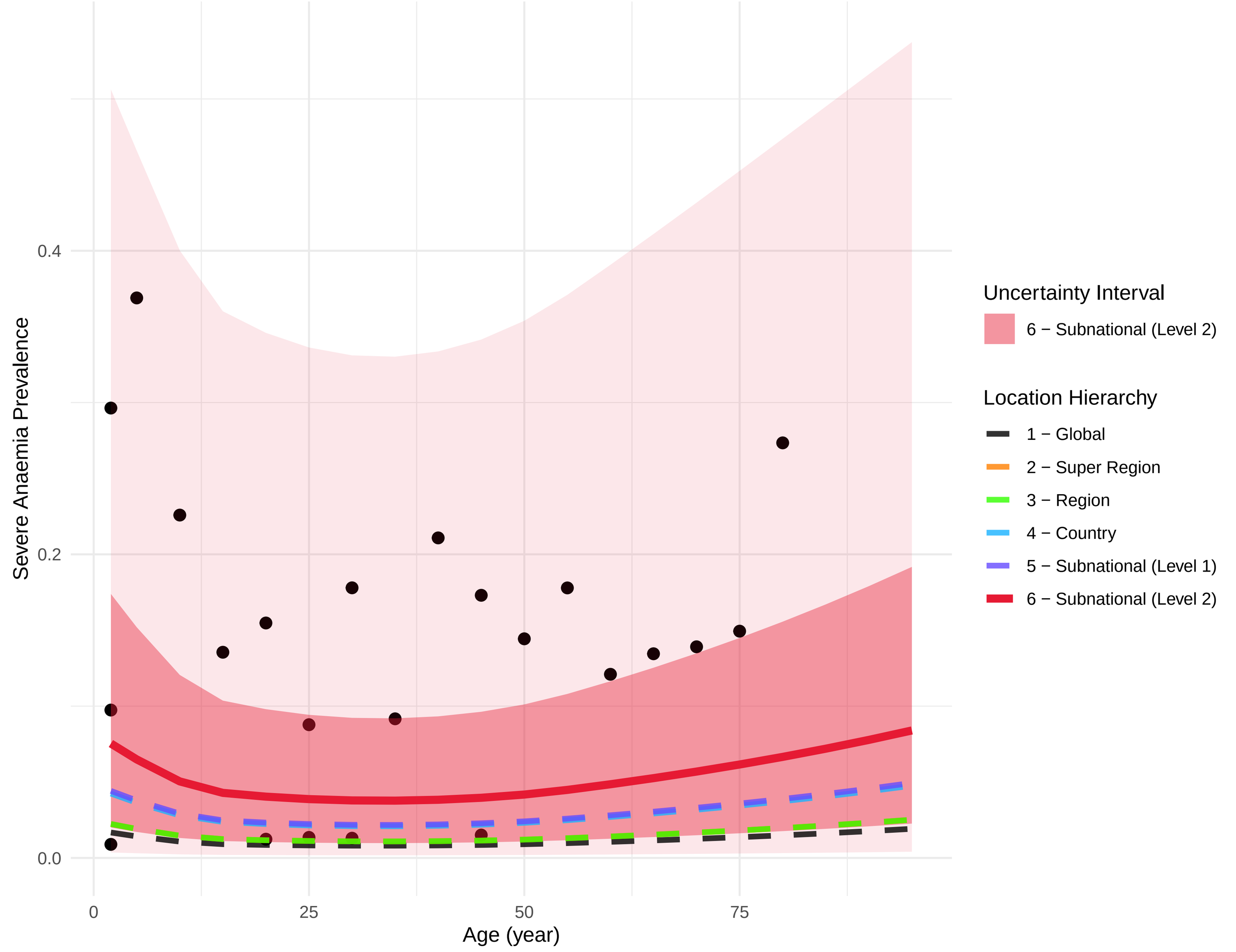

MR−BRT Age/Sex Fits − Males 2+ Years Old − Severe Anaemia Prevalence − Gujarat
WHO−2001 Adjustment Method

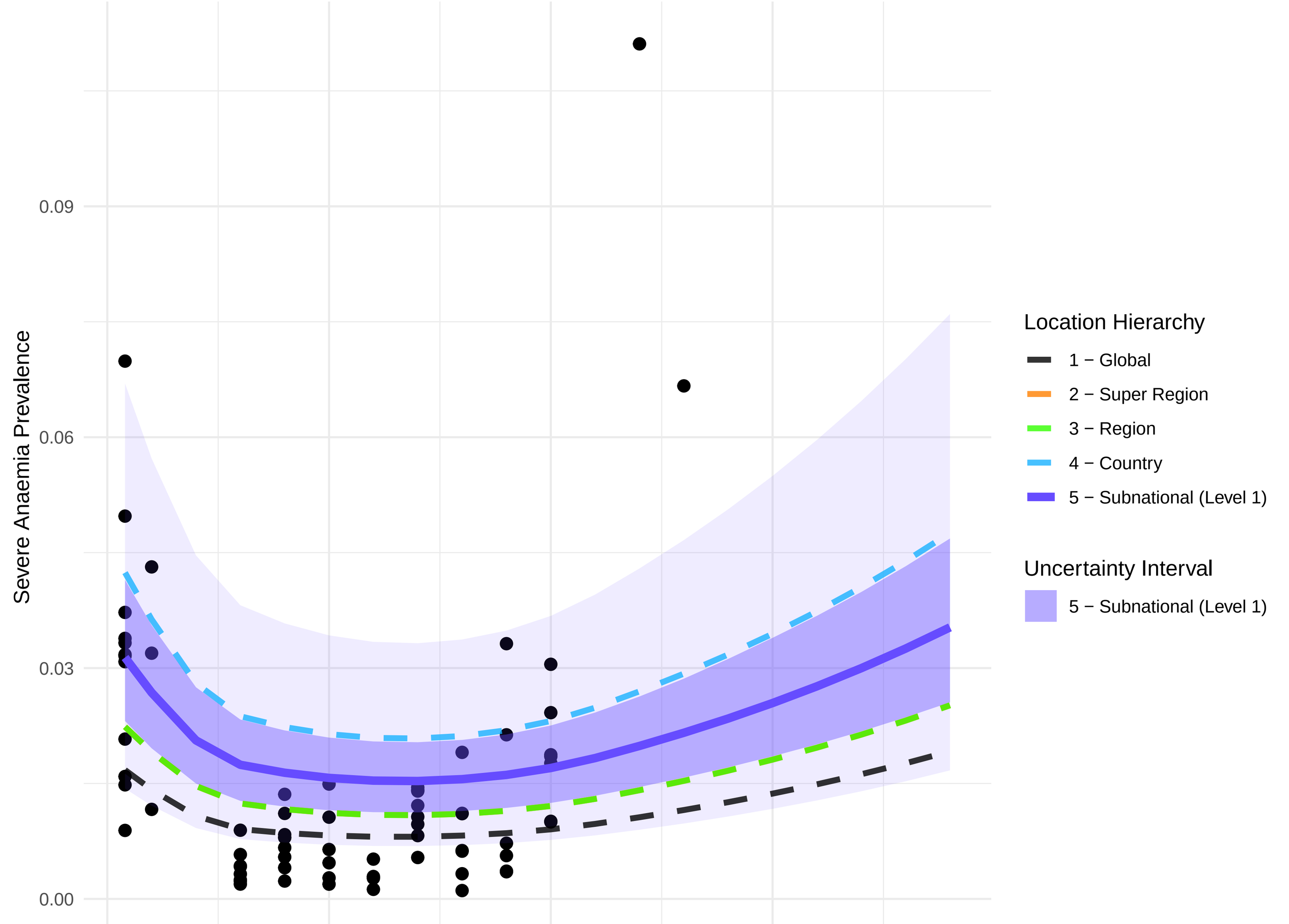

MR−BRT Age/Sex Fits − Males 2+ Years Old − Severe Anaemia Prevalence − Gujarat, Rural
WHO−2001 Adjustment Method

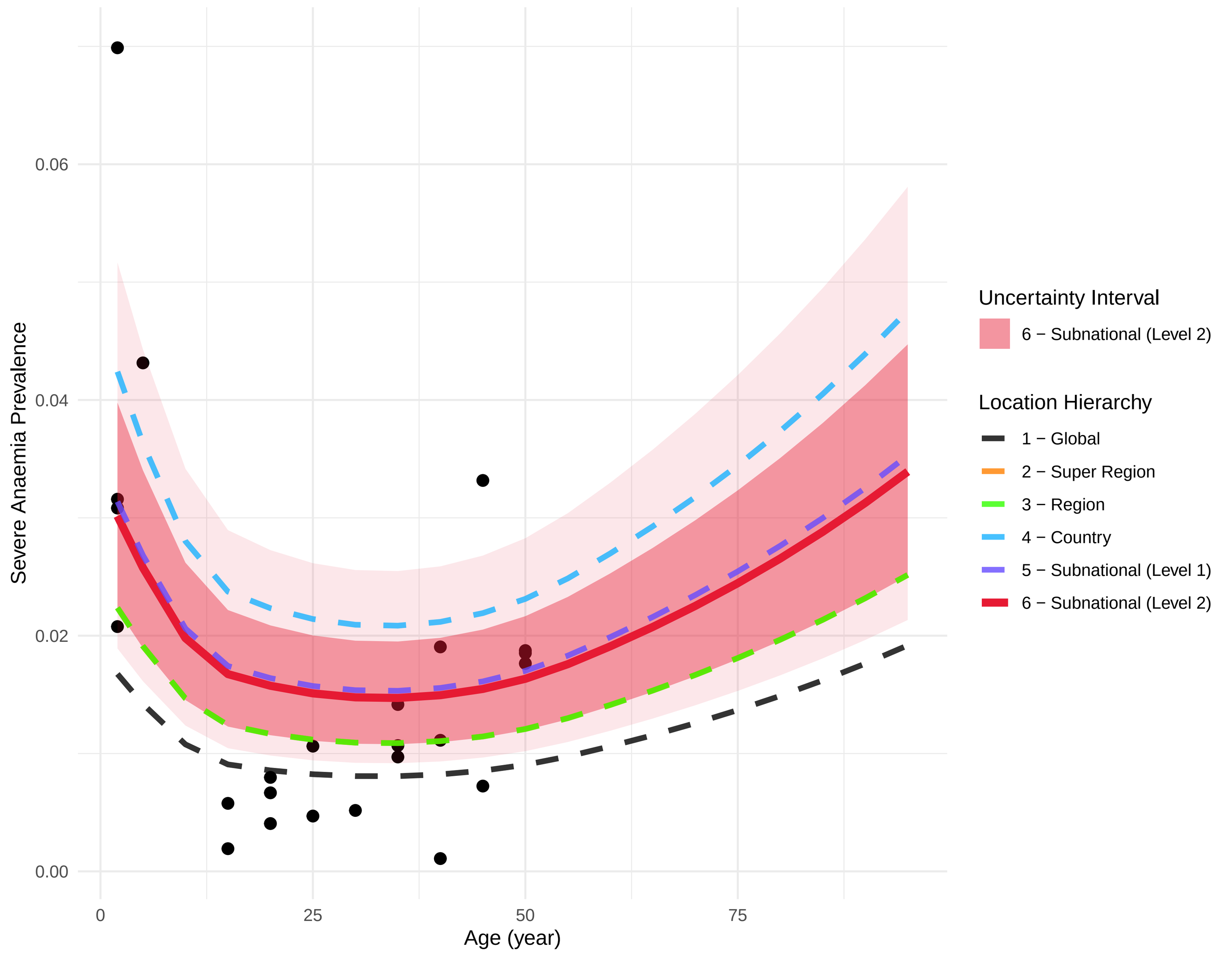

MR−BRT Age/Sex Fits − Males 2+ Years Old − Severe Anaemia Prevalence − Gujarat, Urban
WHO−2001 Adjustment Method

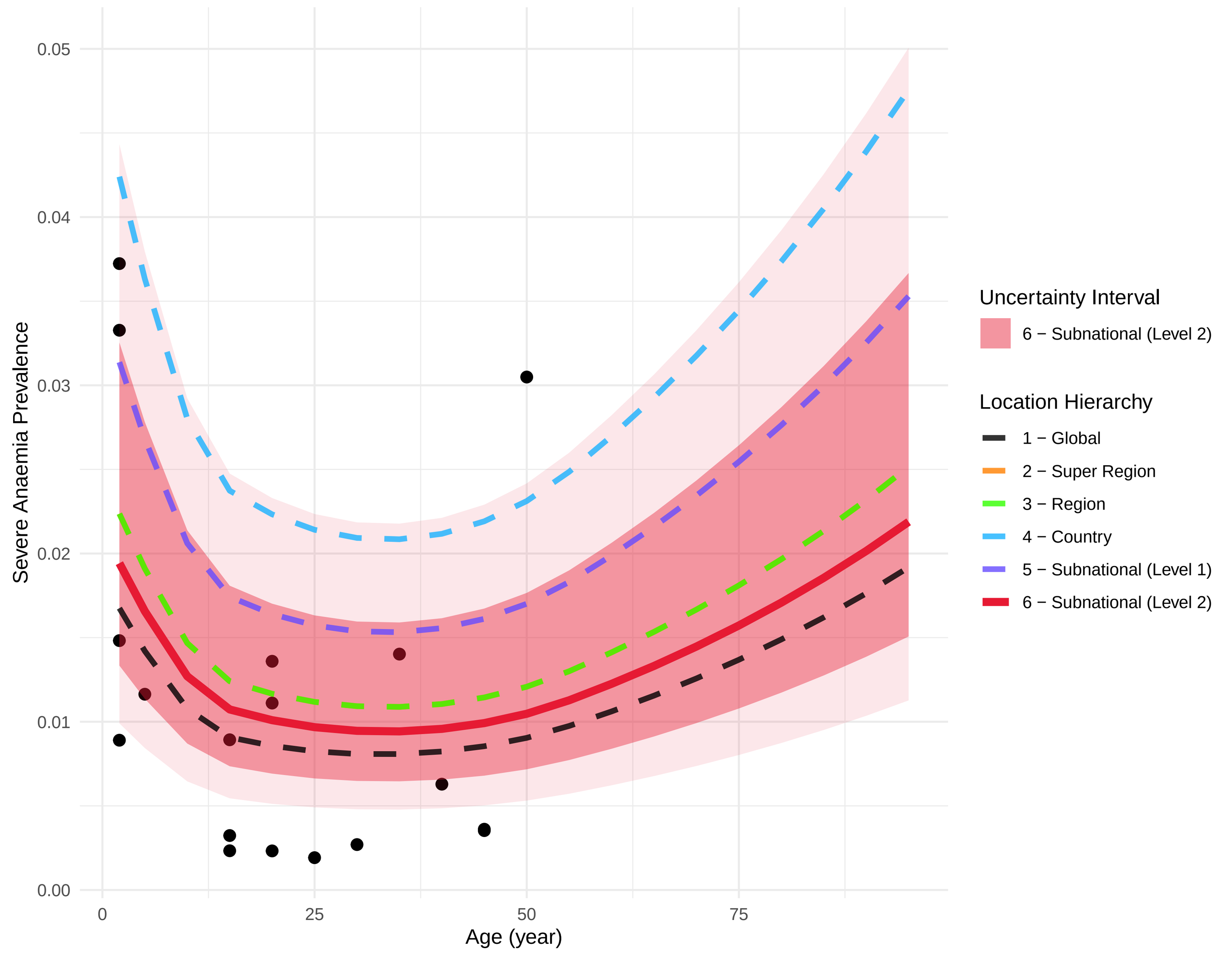

MR−BRT Age/Sex Fits − Males 2+ Years Old − Severe Anaemia Prevalence − Haryana
WHO−2001 Adjustment Method

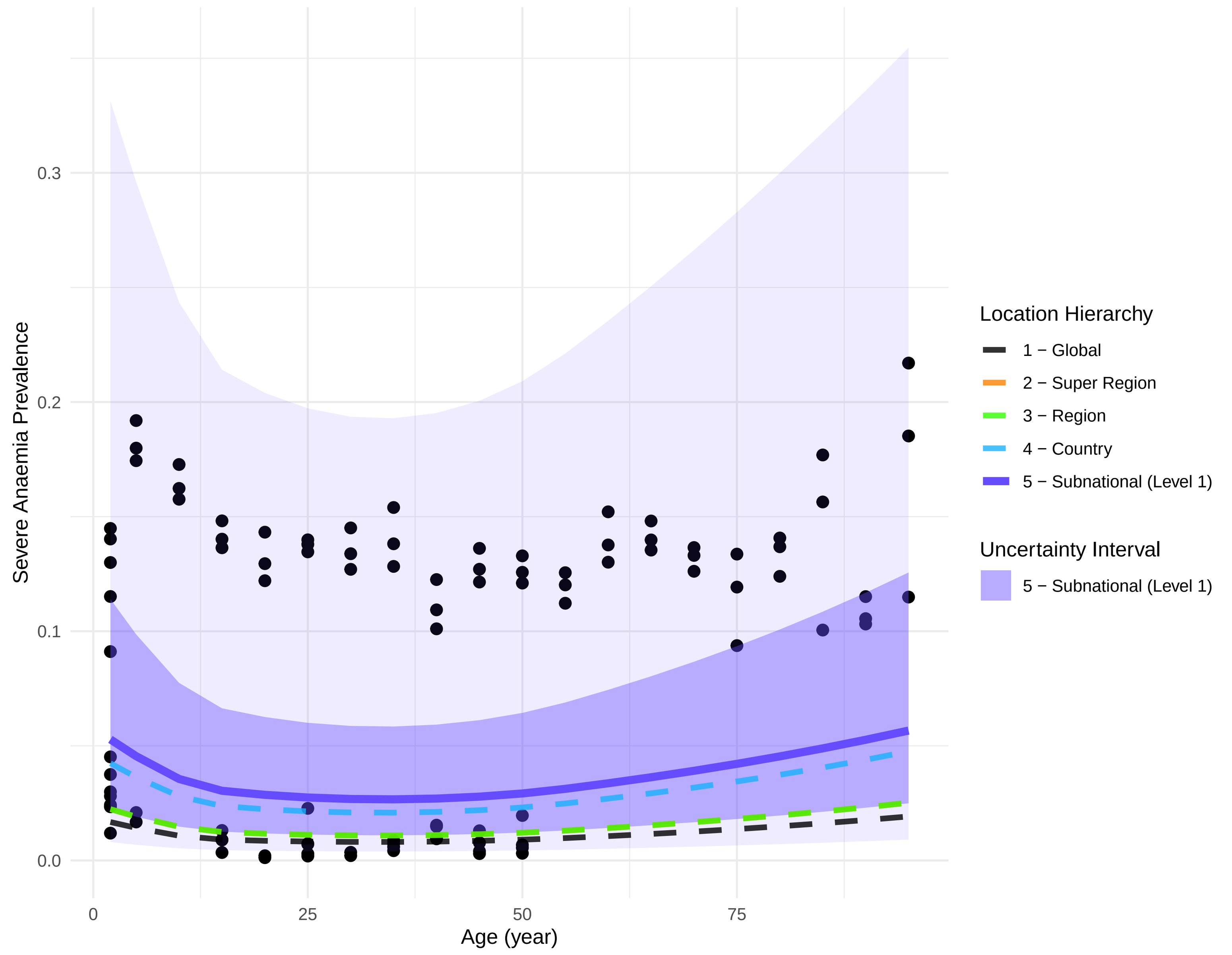

MR−BRT Age/Sex Fits − Males 2+ Years Old − Severe Anaemia Prevalence − Haryana, Rural
WHO−2001 Adjustment Method

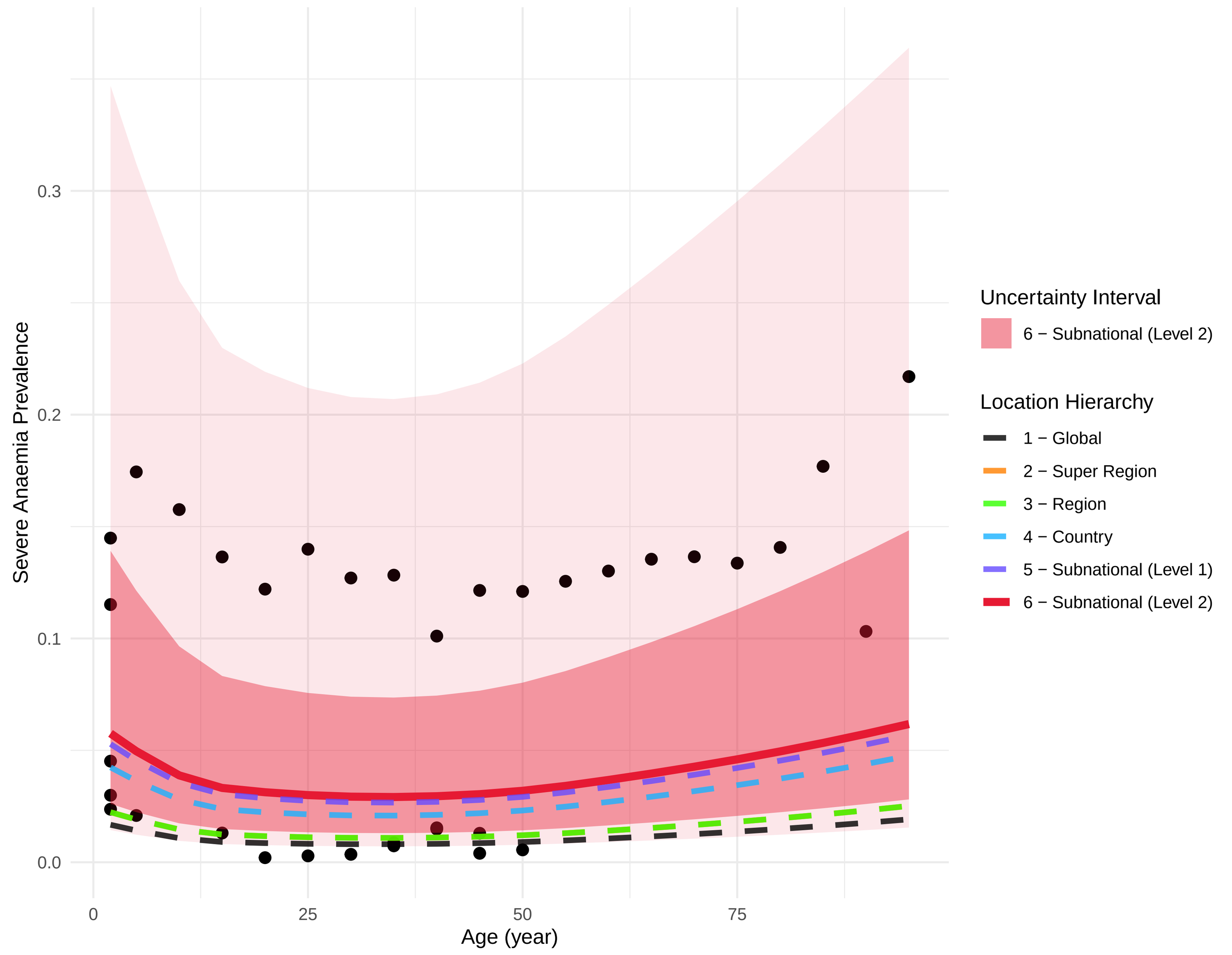

MR−BRT Age/Sex Fits − Males 2+ Years Old − Severe Anaemia Prevalence − Haryana, Urban
WHO−2001 Adjustment Method

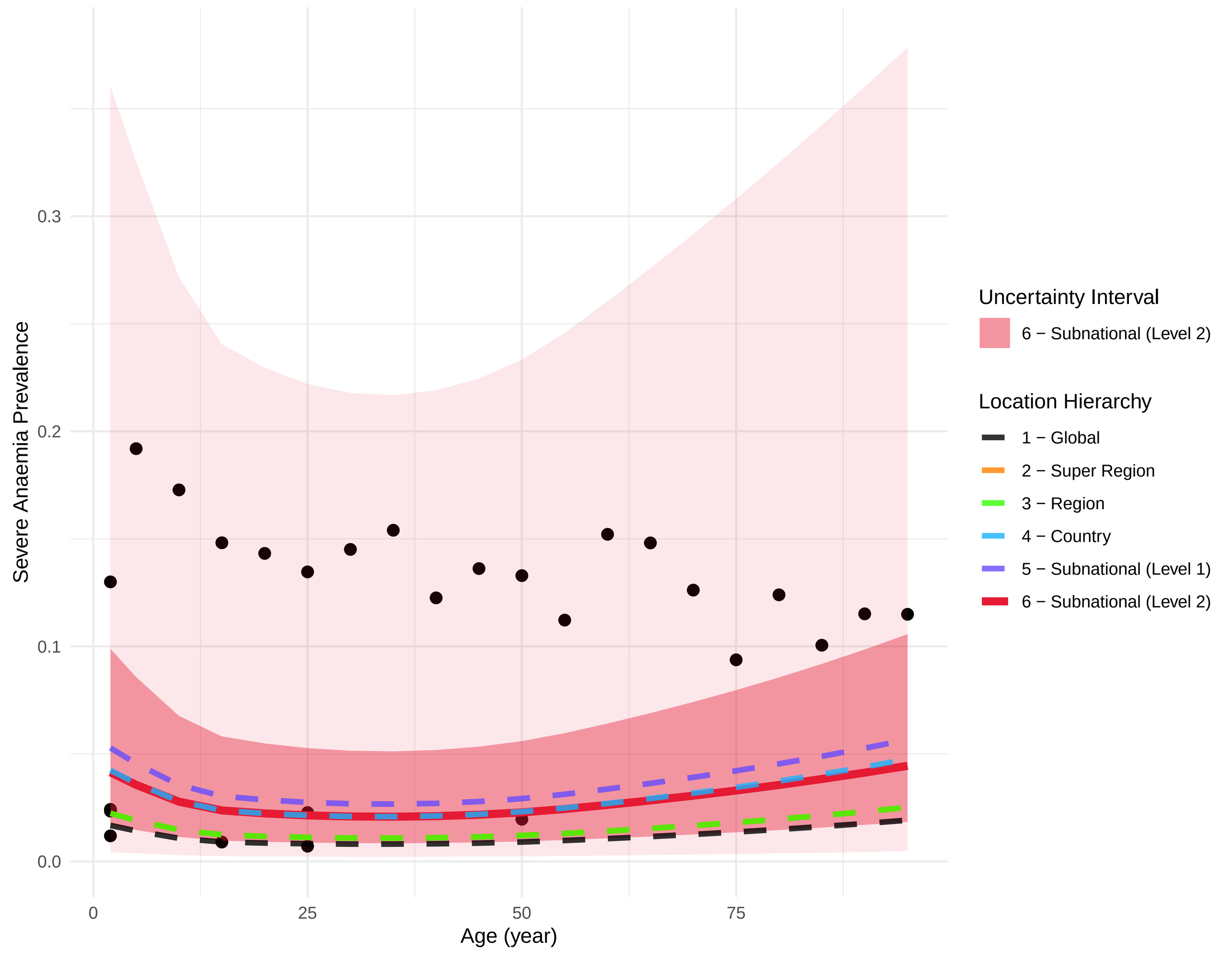

MR−BRT Age/Sex Fits − Males 2+ Years Old − Severe Anaemia Prevalence − Himachal Pradesh
WHO−2001 Adjustment Method

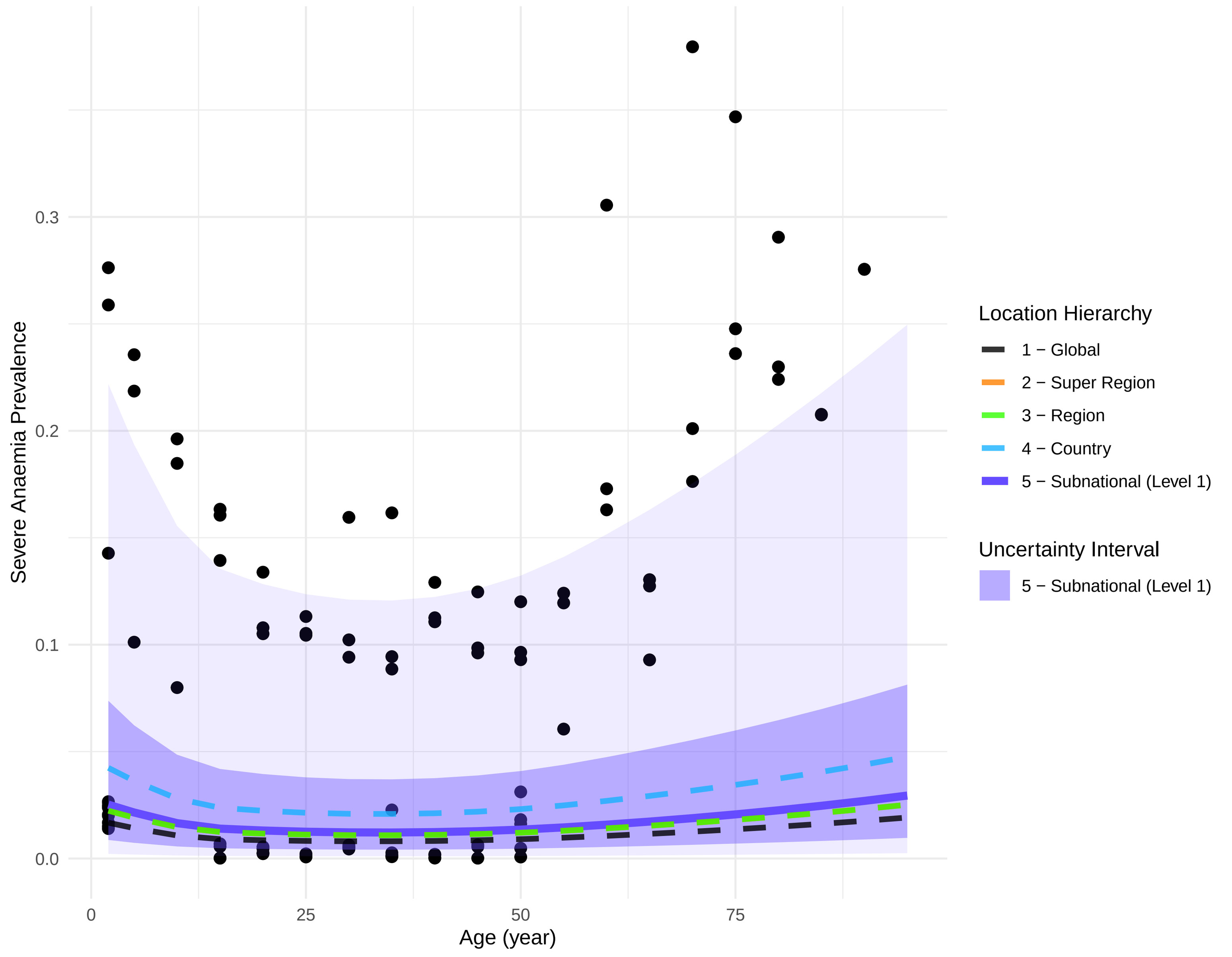

MR−BRT Age/Sex Fits − Males 2+ Years Old − Severe Anaemia Prevalence − Himachal Pradesh, Rural
WHO−2001 Adjustment Method

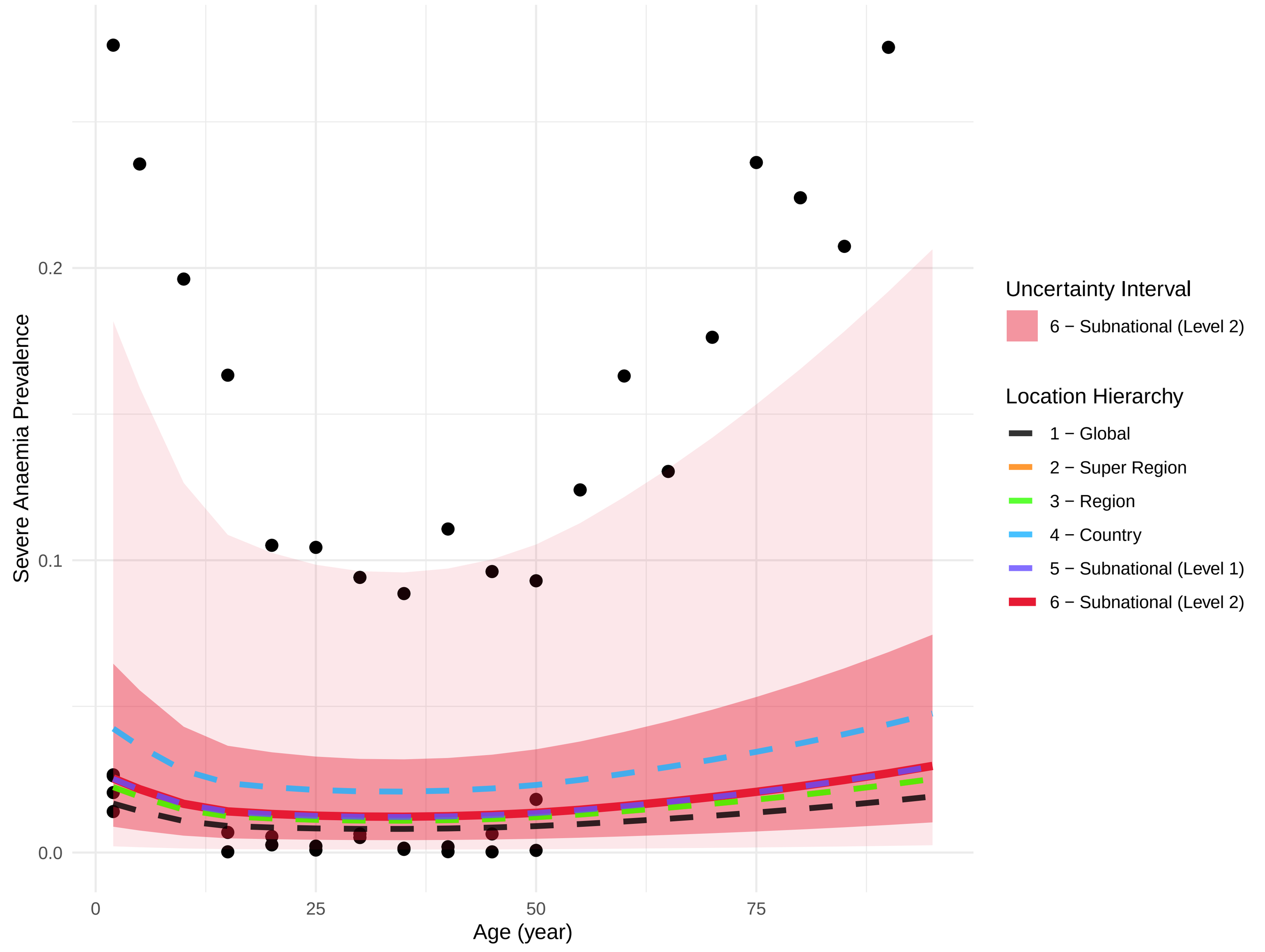

MR−BRT Age/Sex Fits − Males 2+ Years Old − Severe Anaemia Prevalence − Himachal Pradesh, Urban
WHO−2001 Adjustment Method

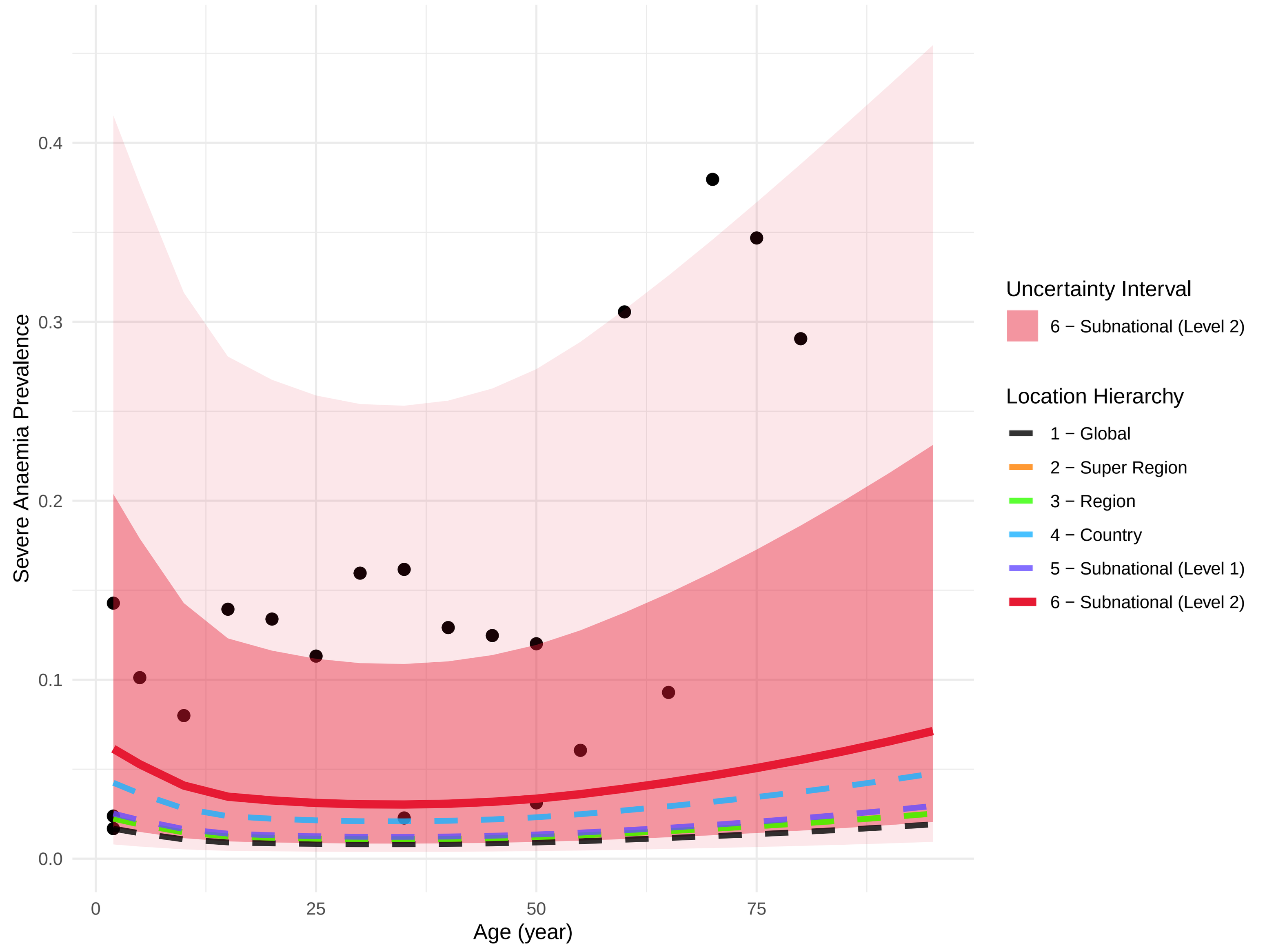

MR−BRT Age/Sex Fits − Males 2+ Years Old − Severe Anaemia Prevalence − Jammu & Kashmir and Ladakh
WHO−2001 Adjustment Method

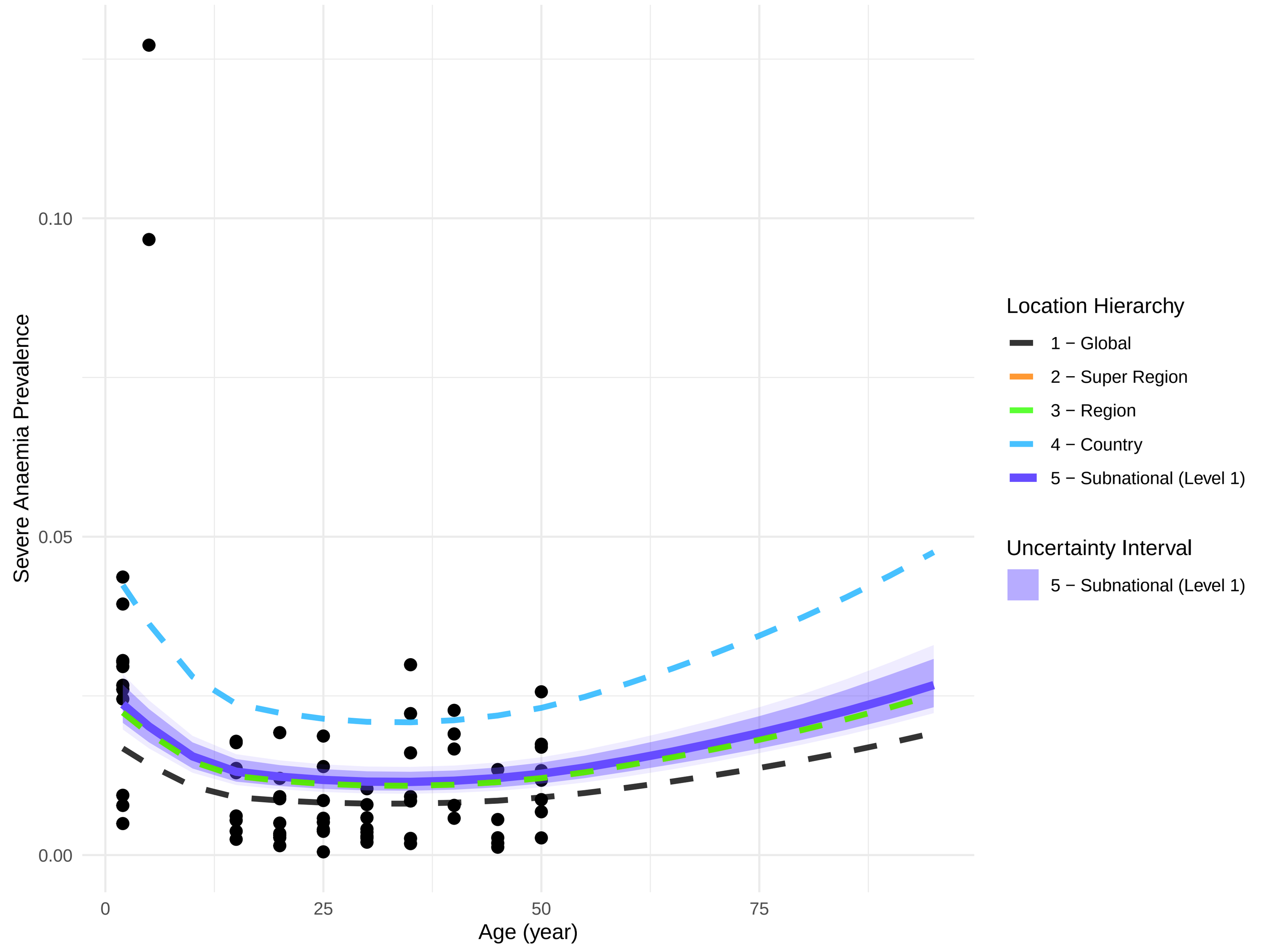

MR−BRT Age/Sex Fits − Males 2+ Years Old − Severe Anaemia Prevalence − Jammu & Kashmir and Ladakh, Rural
WHO−2001 Adjustment Method

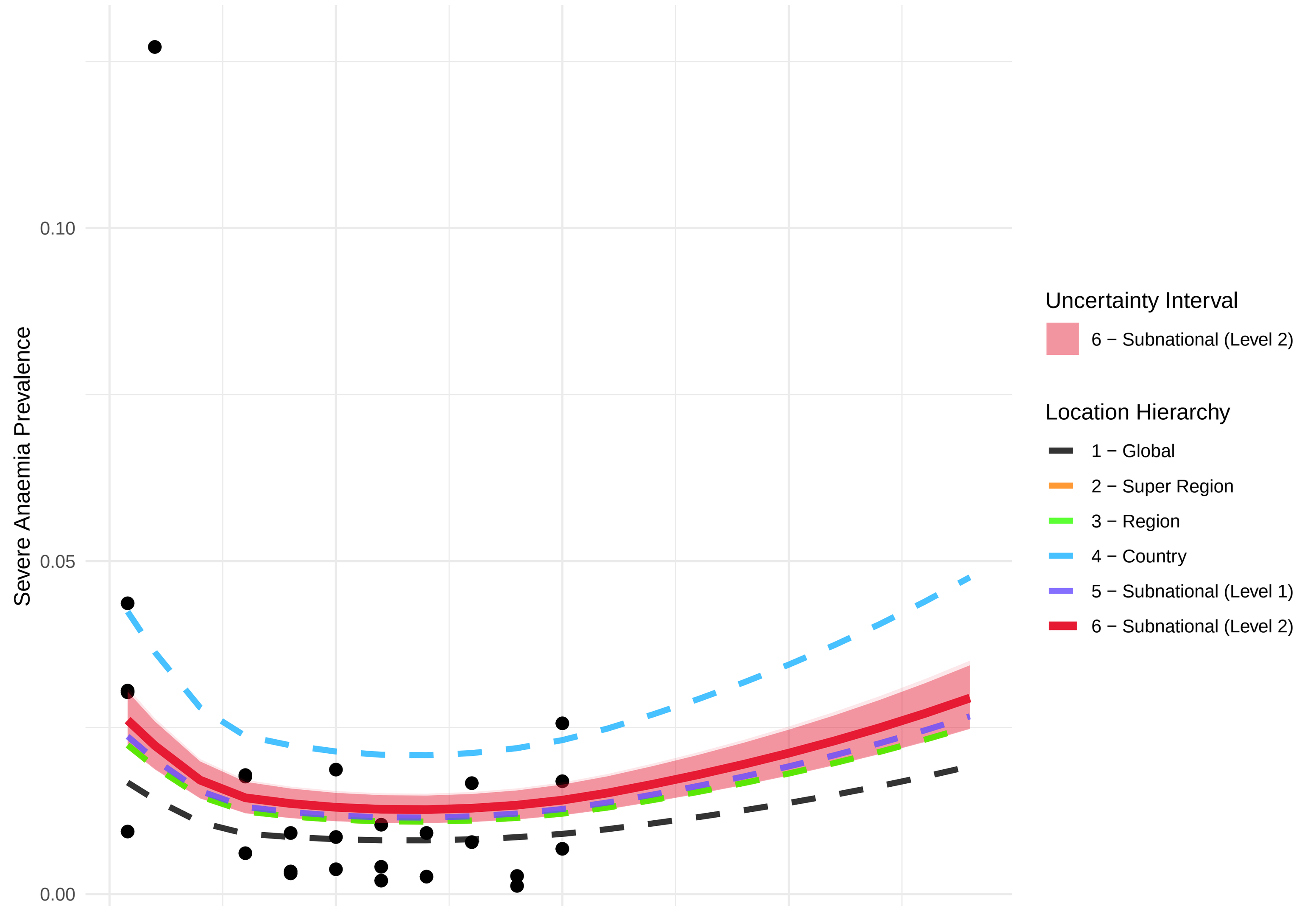

MR−BRT Age/Sex Fits − Males 2+ Years Old − Severe Anaemia Prevalence − Jammu & Kashmir and Ladakh, Urban
WHO−2001 Adjustment Method

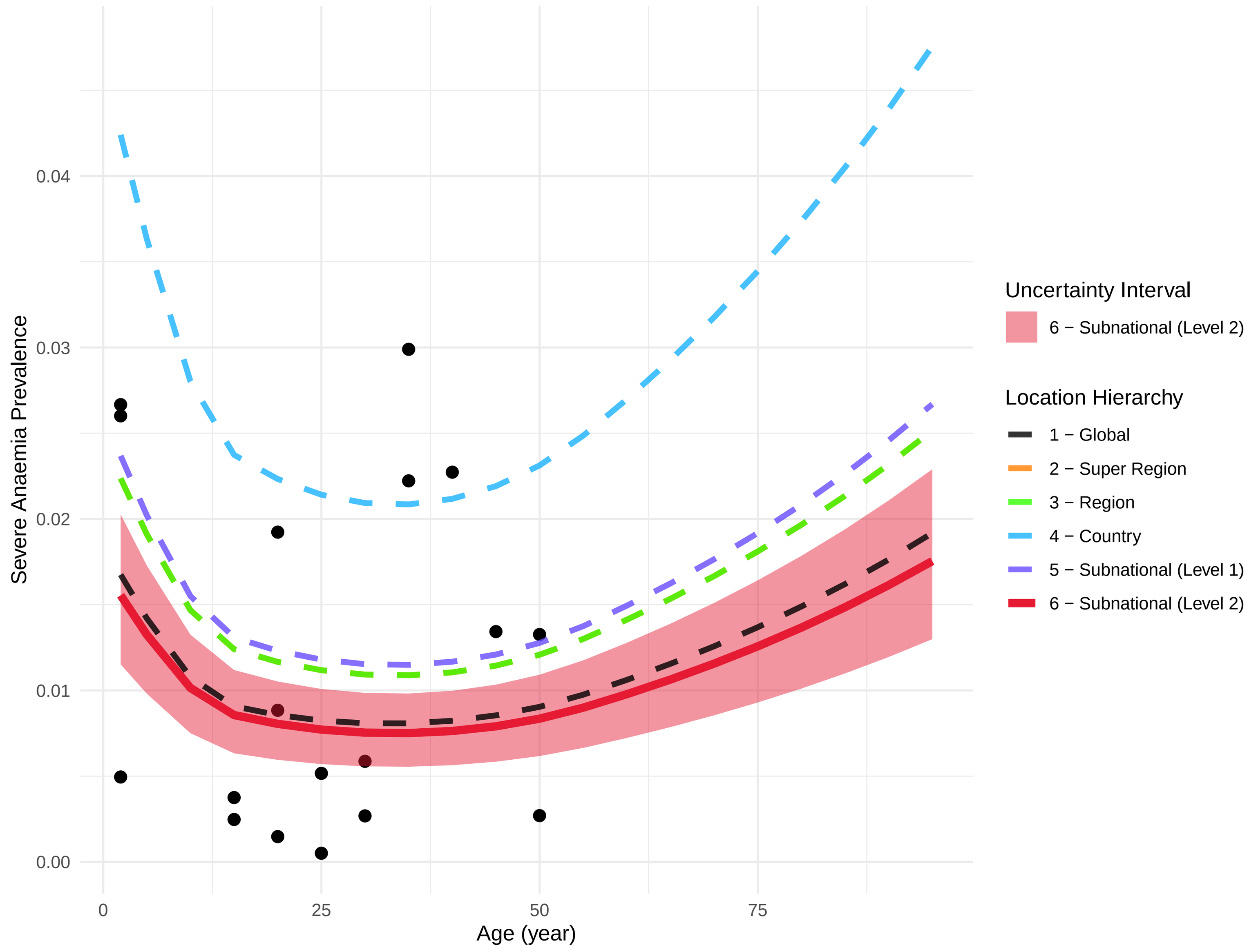

MR−BRT Age/Sex Fits − Males 2+ Years Old − Severe Anaemia Prevalence − Jharkhand
WHO−2001 Adjustment Method

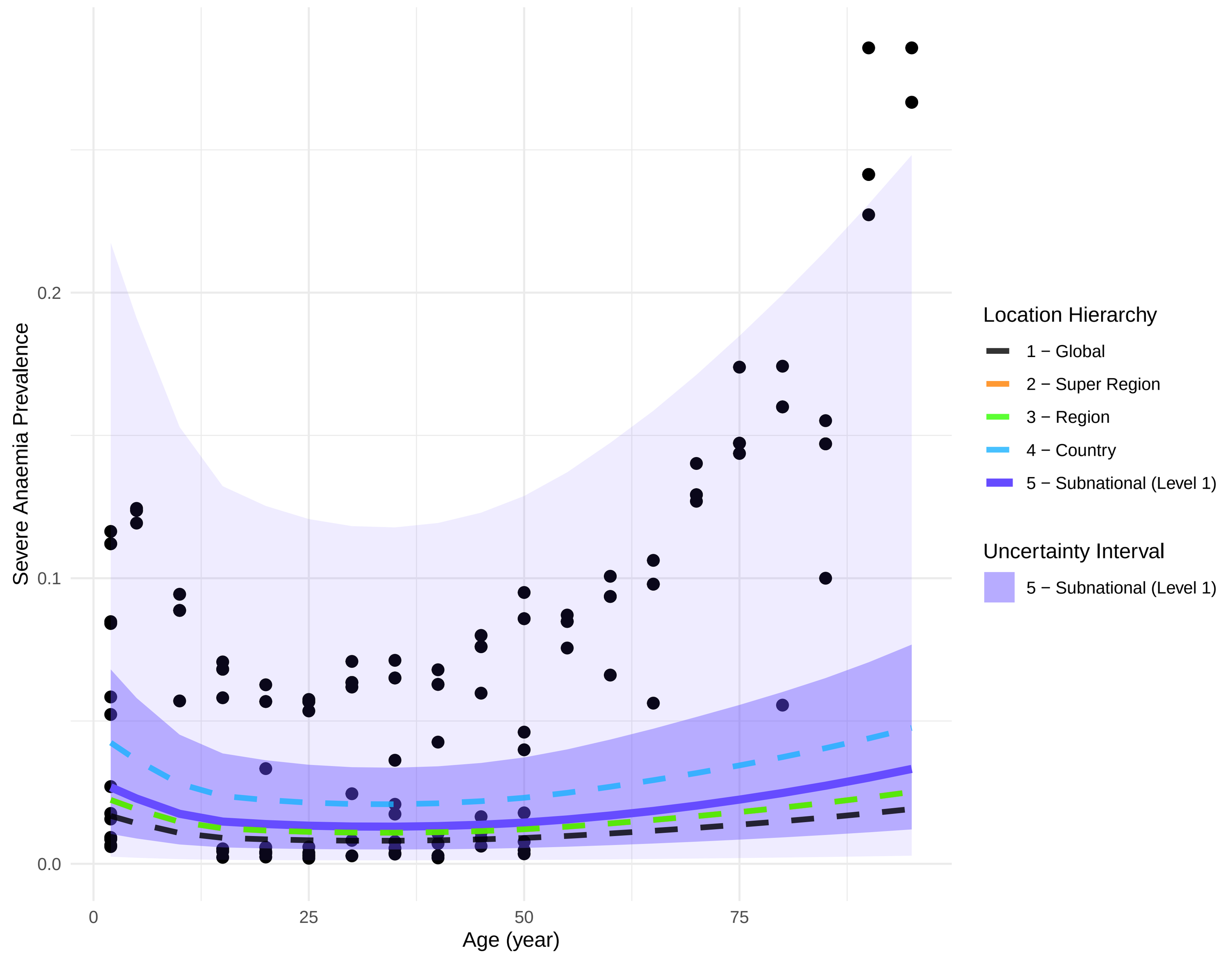

MR−BRT Age/Sex Fits − Males 2+ Years Old − Severe Anaemia Prevalence − Jharkhand, Rural
WHO−2001 Adjustment Method

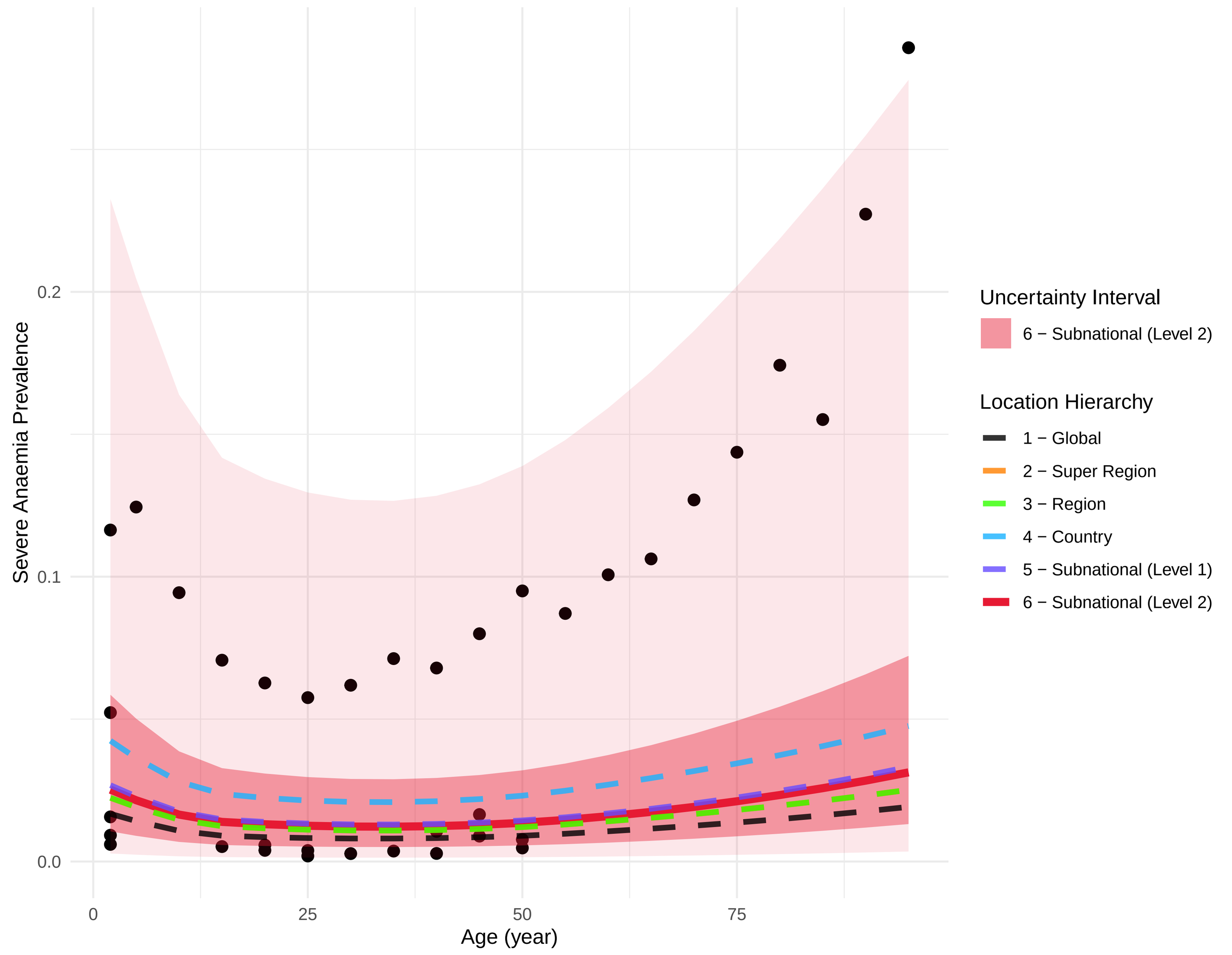

MR−BRT Age/Sex Fits − Males 2+ Years Old − Severe Anaemia Prevalence − Jharkhand, Urban
WHO−2001 Adjustment Method

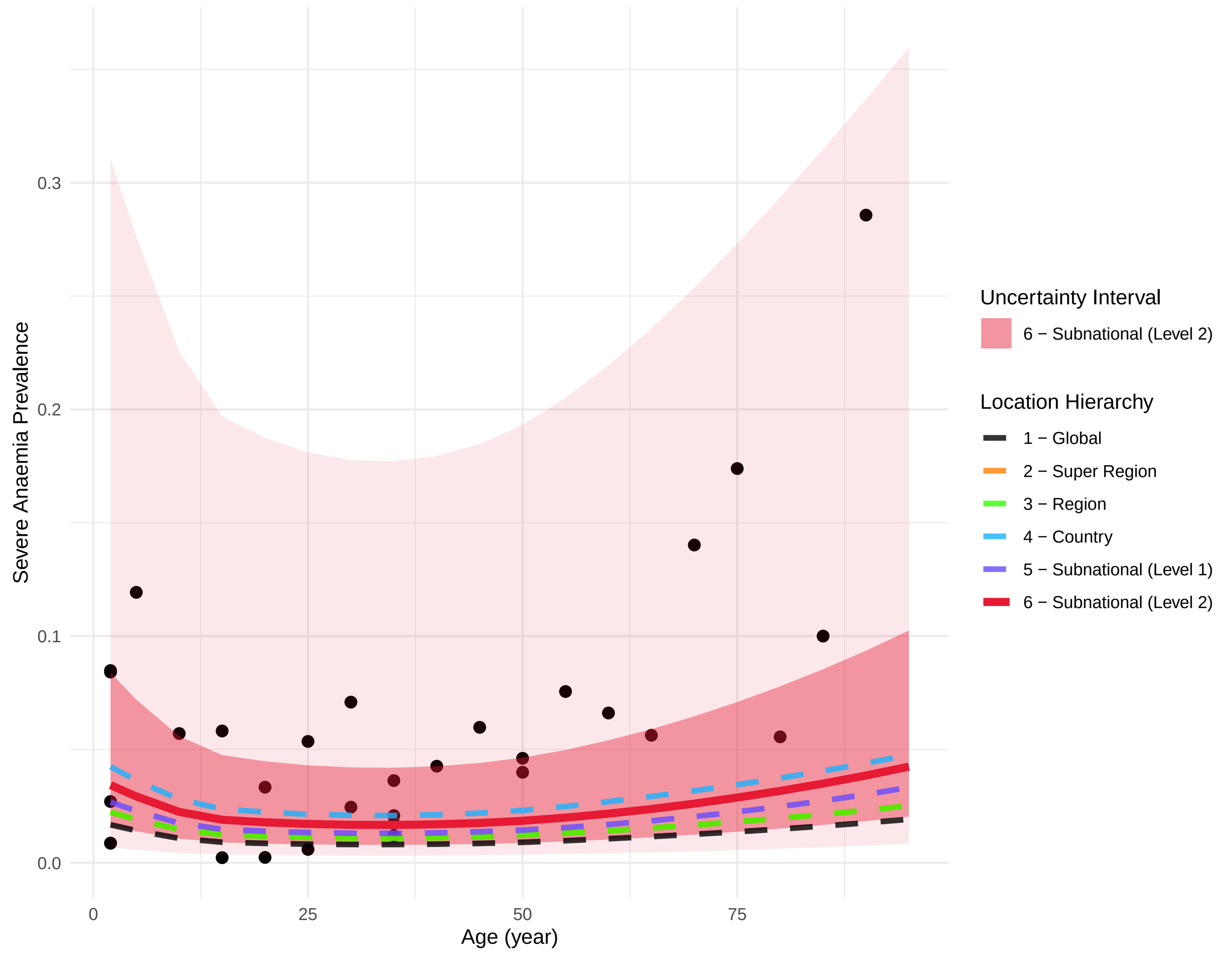

MR−BRT Age/Sex Fits − Males 2+ Years Old − Severe Anaemia Prevalence − Karnataka
WHO−2001 Adjustment Method

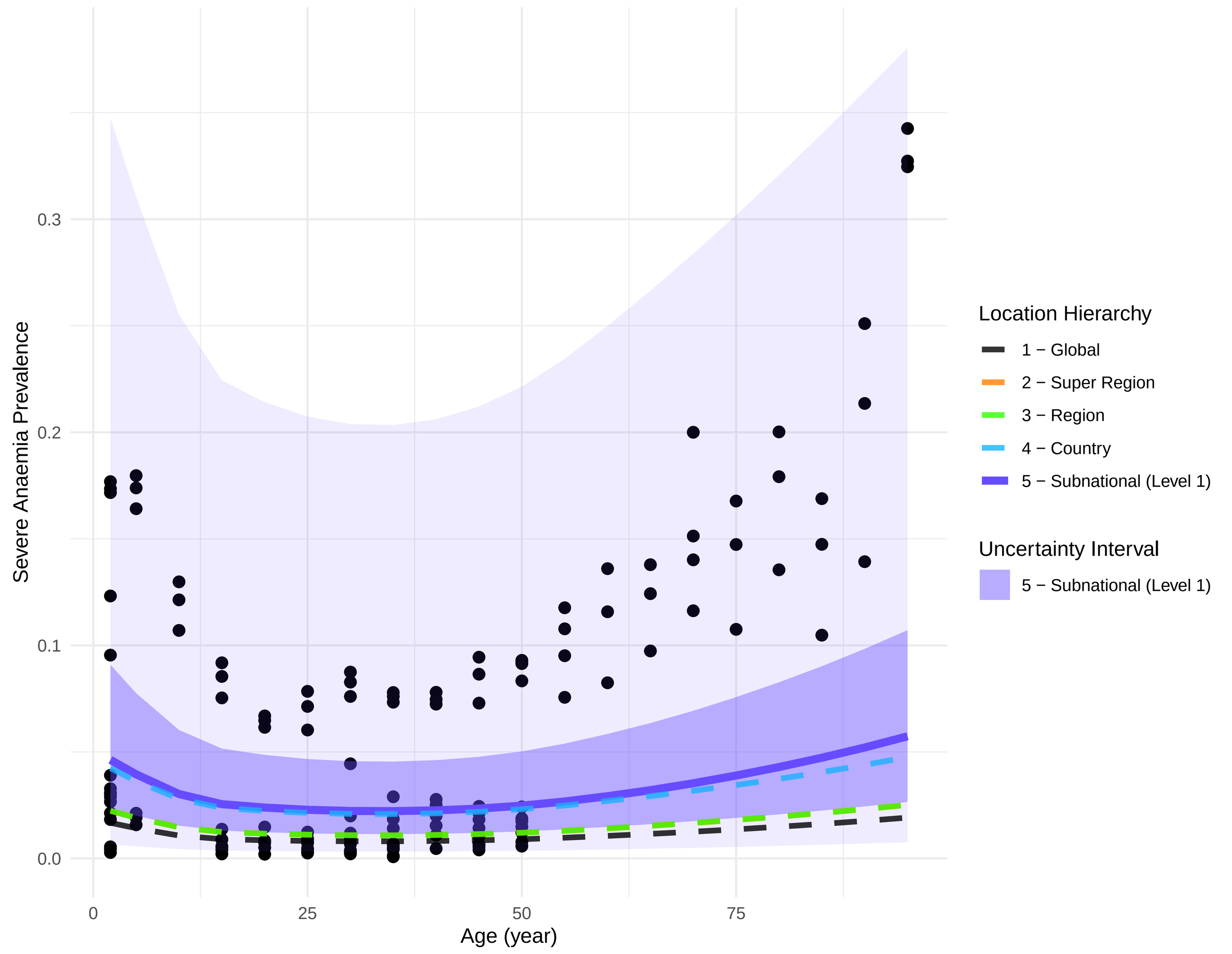

MR−BRT Age/Sex Fits − Males 2+ Years Old − Severe Anaemia Prevalence − Karnataka, Rural
WHO−2001 Adjustment Method

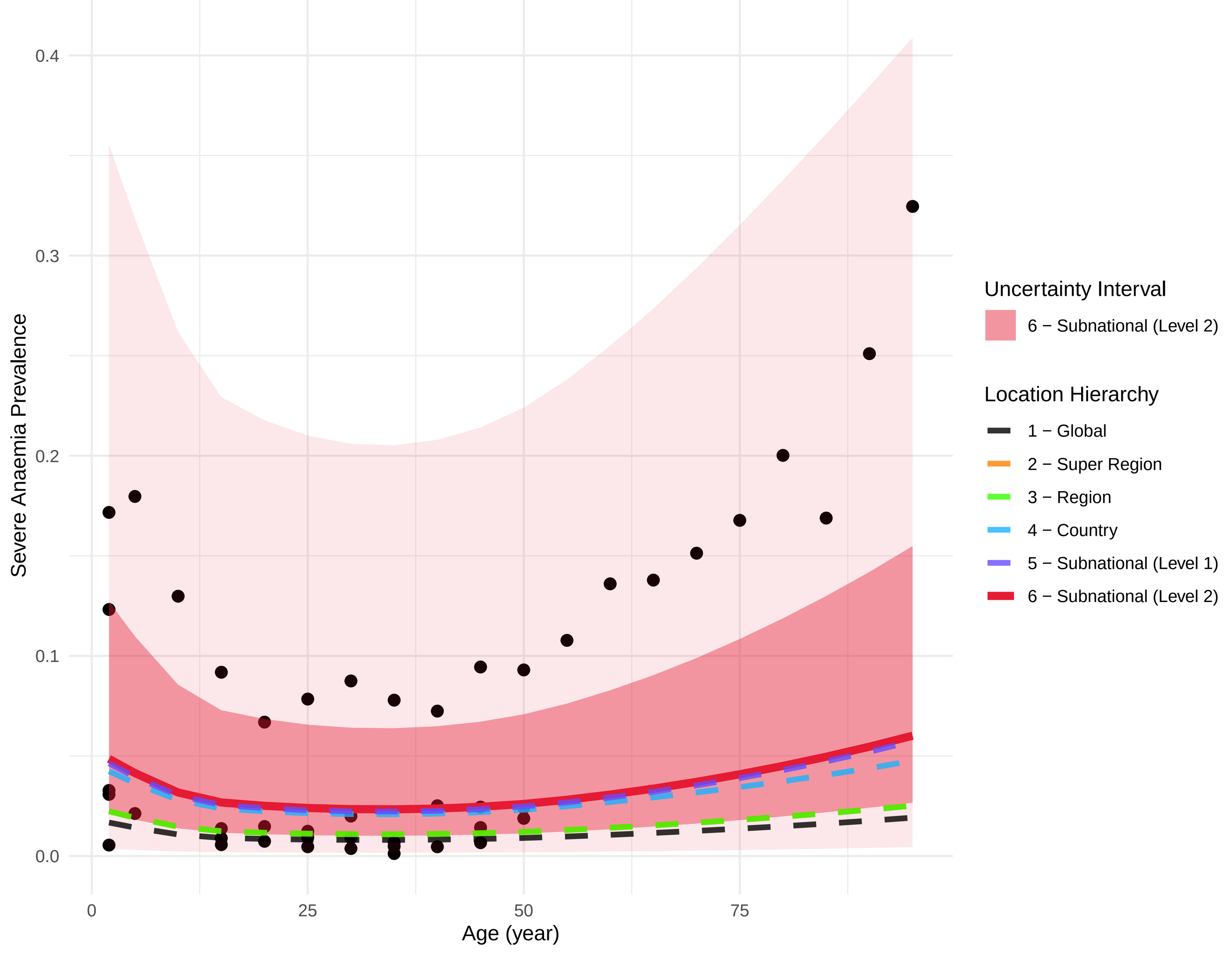

MR−BRT Age/Sex Fits − Males 2+ Years Old − Severe Anaemia Prevalence − Karnataka, Urban
WHO−2001 Adjustment Method

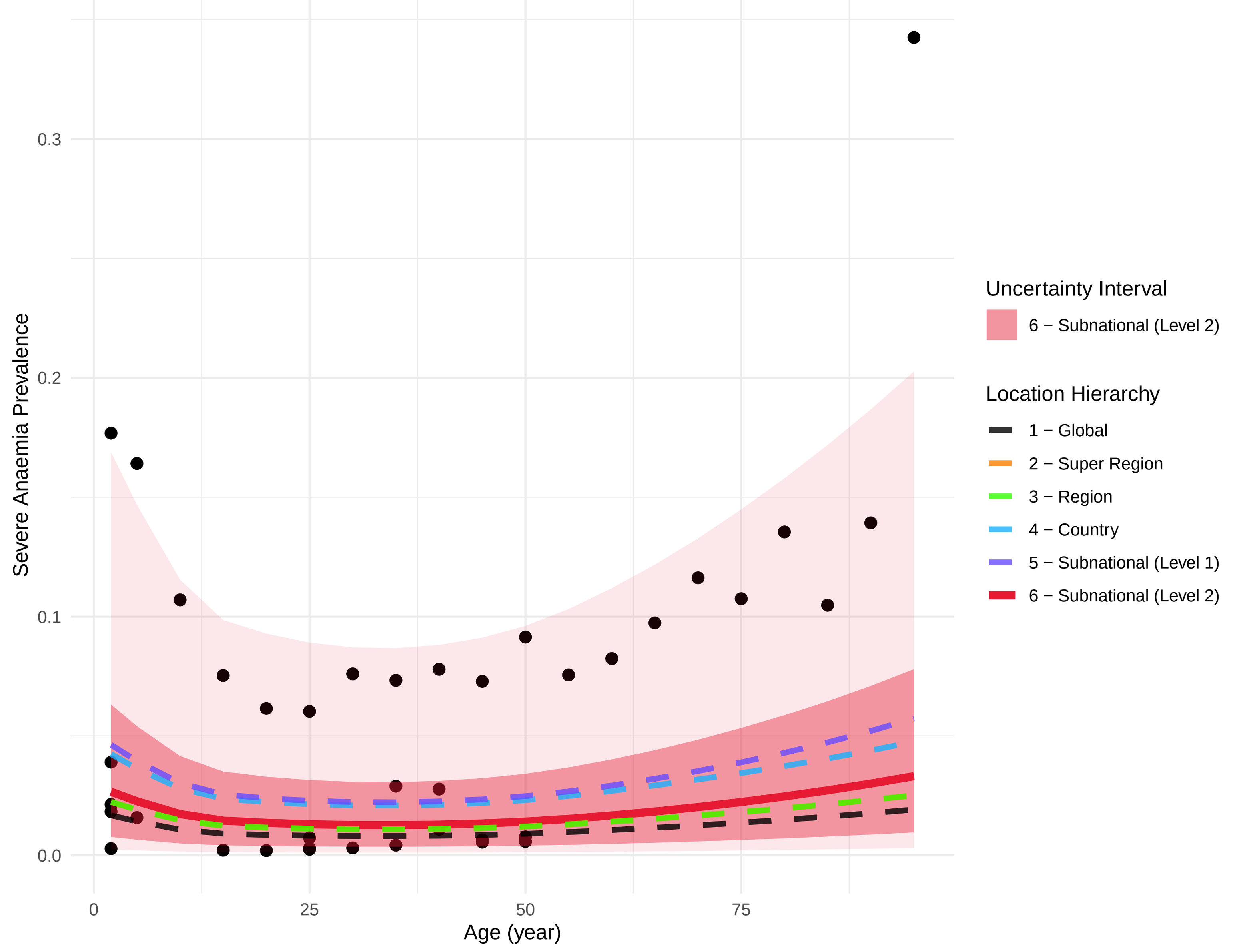

MR−BRT Age/Sex Fits − Males 2+ Years Old − Severe Anaemia Prevalence − Kerala
WHO−2001 Adjustment Method

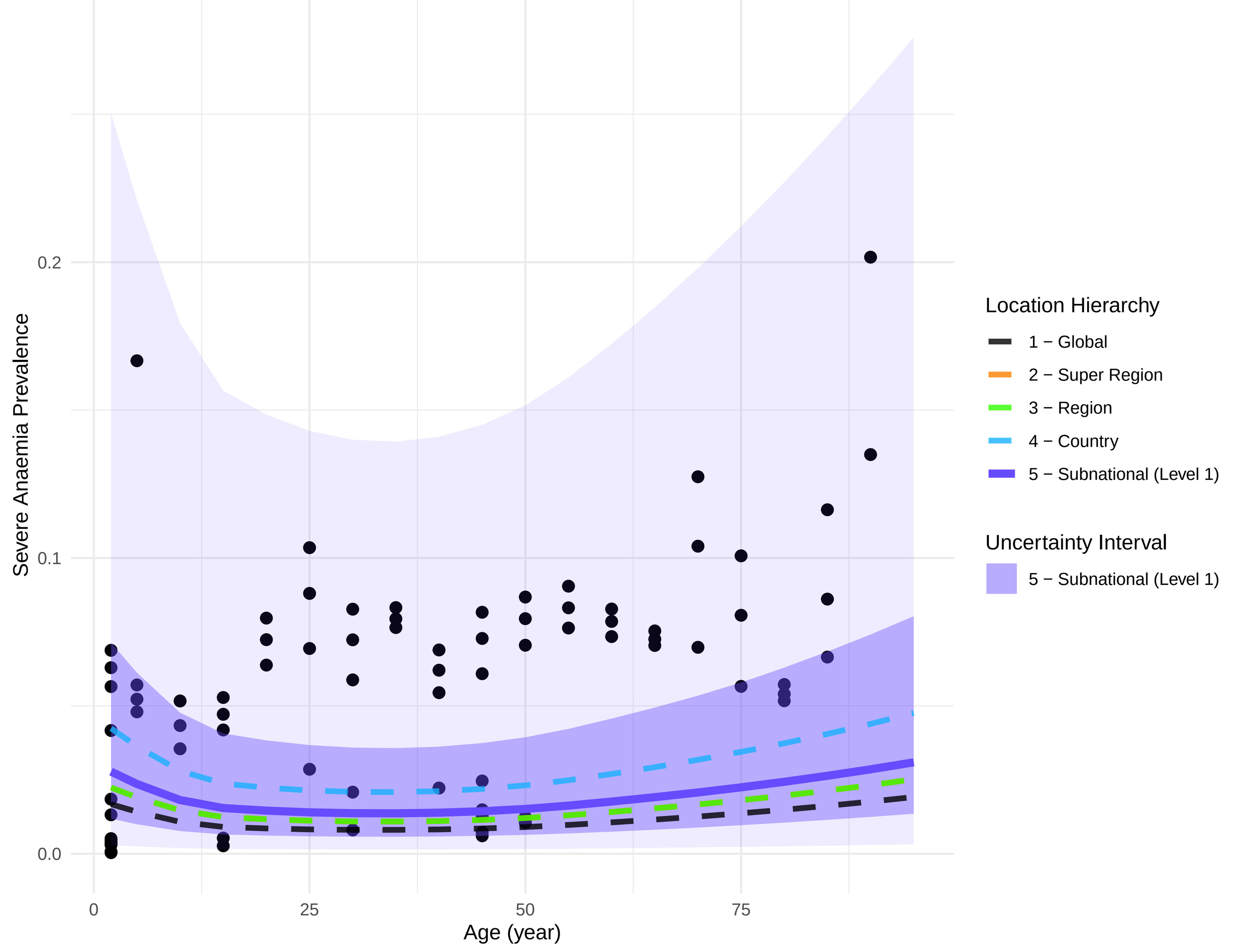

MR−BRT Age/Sex Fits − Males 2+ Years Old − Severe Anaemia Prevalence − Kerala, Rural
WHO−2001 Adjustment Method

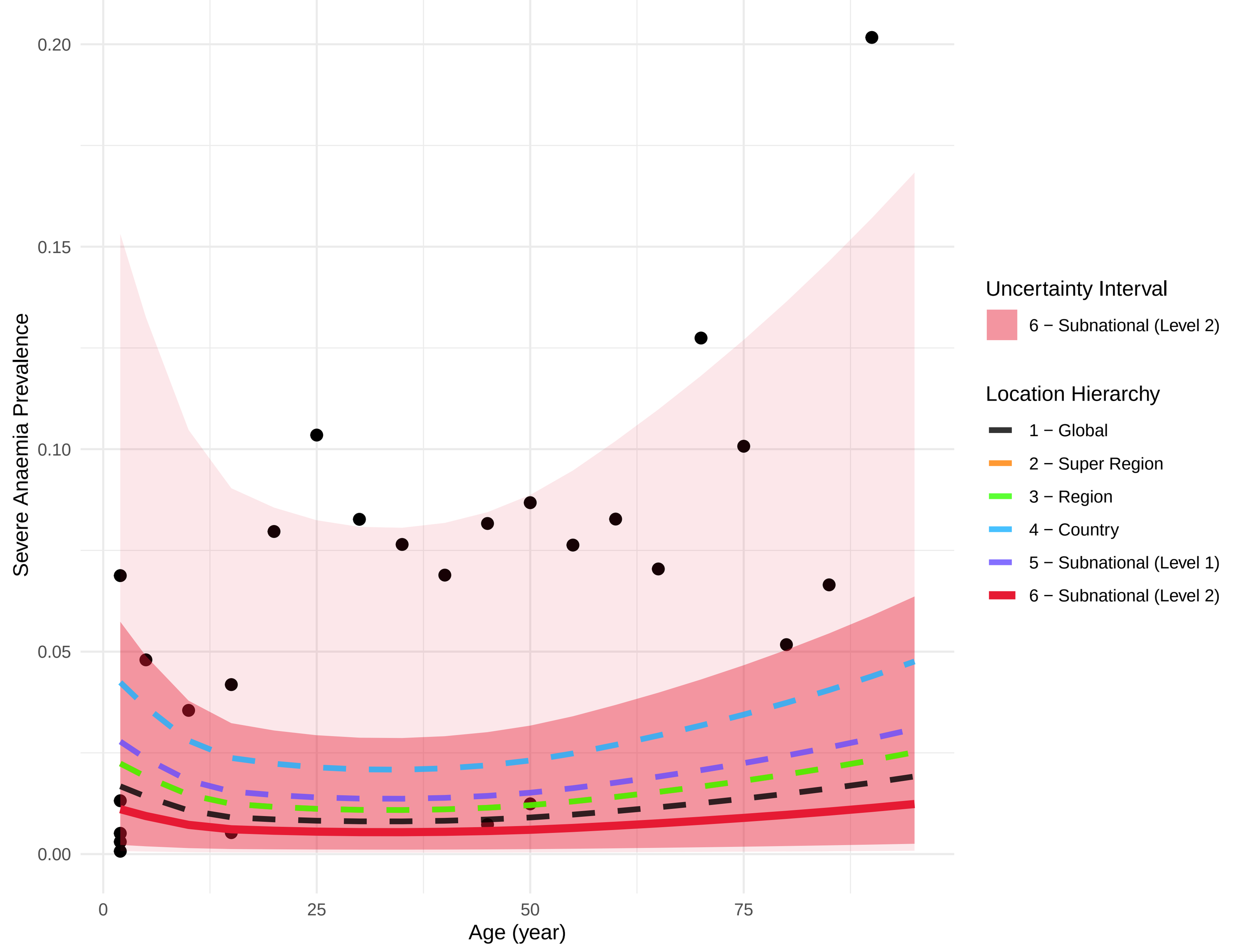

MR−BRT Age/Sex Fits − Males 2+ Years Old − Severe Anaemia Prevalence − Kerala, Urban
WHO−2001 Adjustment Method

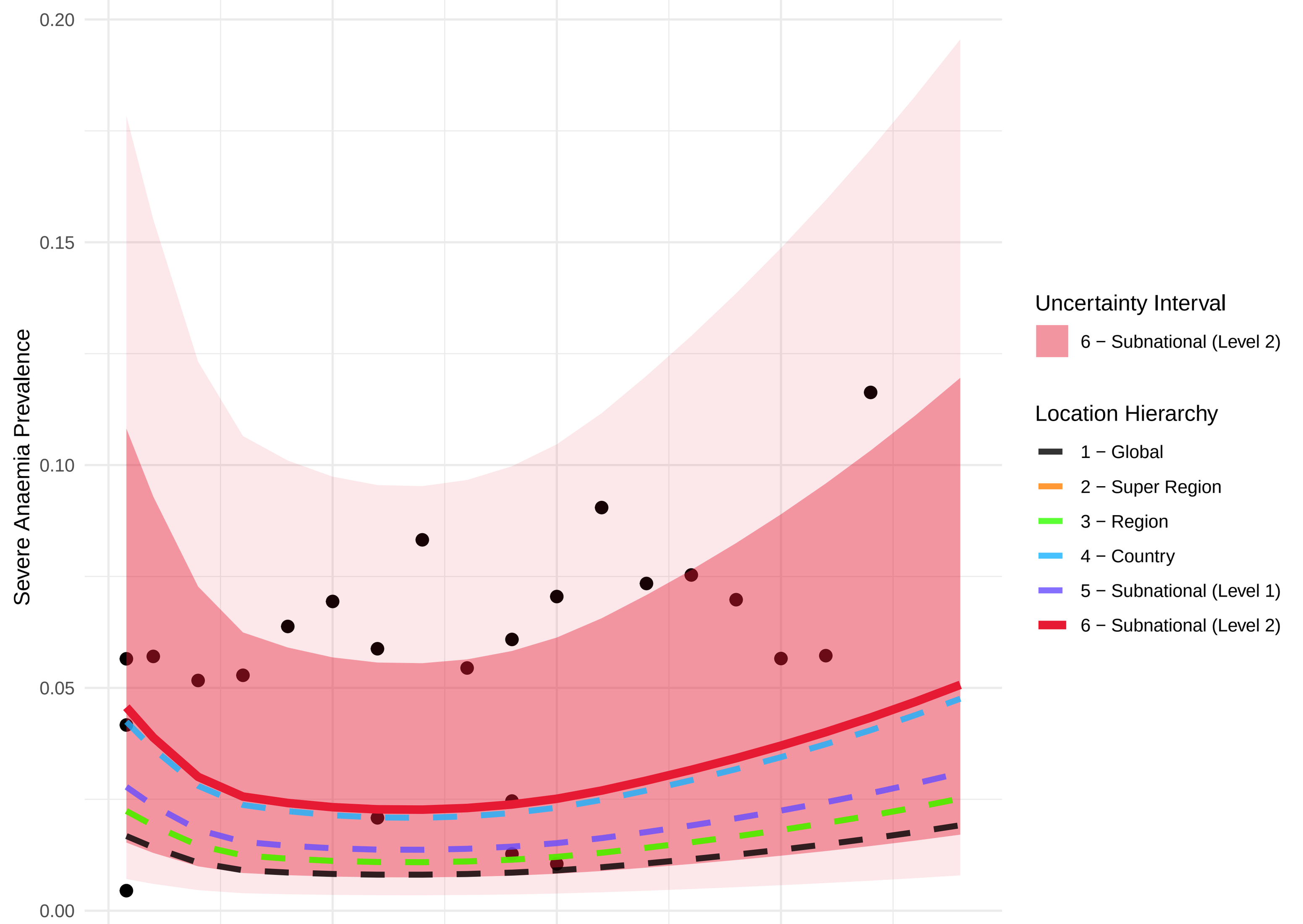

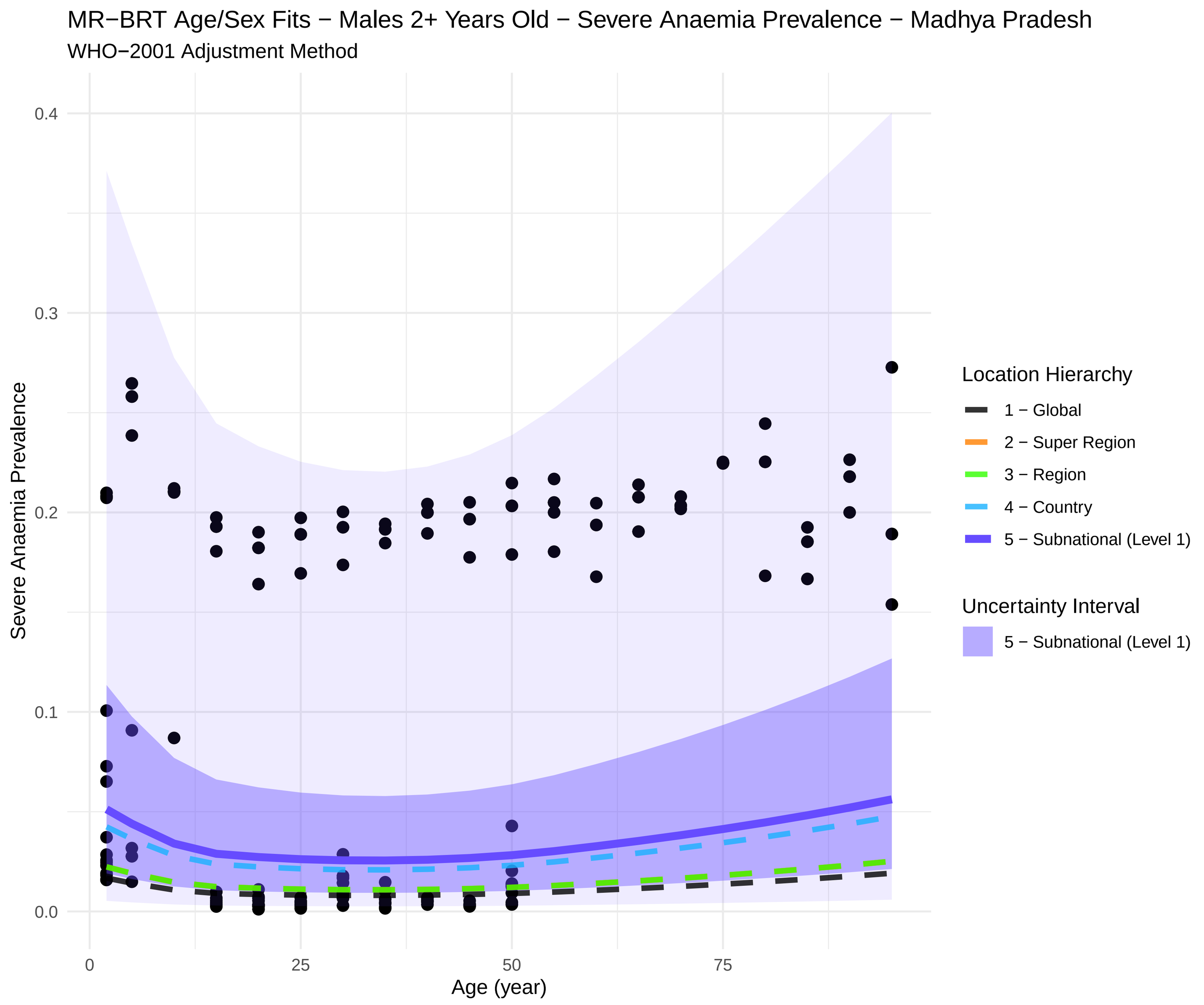
MR−BRT Age/Sex Fits − Males 2+ Years Old − Severe Anaemia Prevalence − Madhya Pradesh
WHO−2001 Adjustment Method
Severe Anaemia Prevalence
Age (year)
Location Hierarchy
1 − Global
2 − Super Region
3 − Region
4 − Country
5 − Subnational (Level 1)
Uncertainty Interval
5 − Subnational (Level 1)

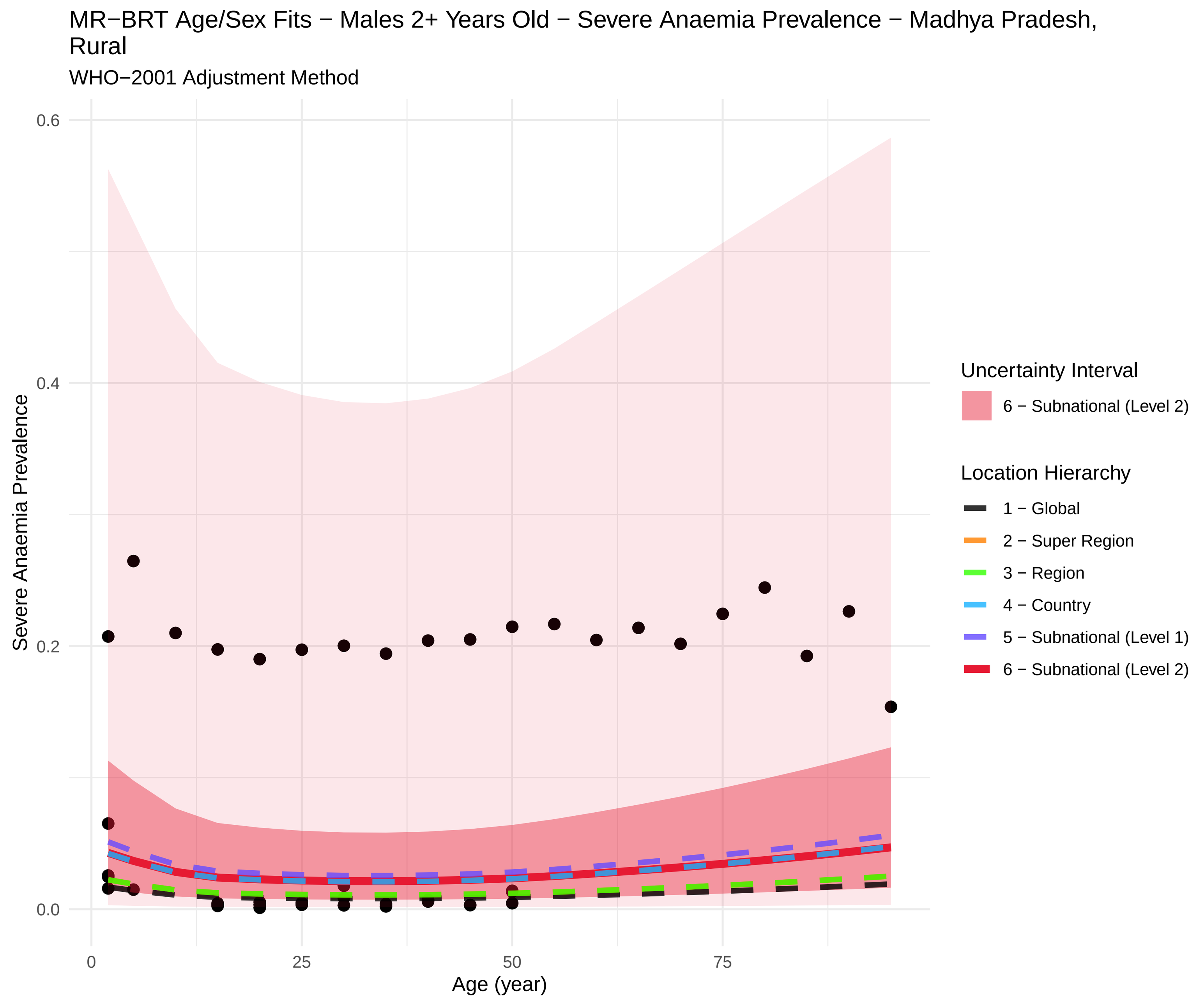
MR−BRT Age/Sex Fits − Males 2+ Years Old − Severe Anaemia Prevalence − Madhya Pradesh, Rural
WHO−2001 Adjustment Method
Severe Anaemia Prevalence
Age (year)
Uncertainty Interval
6 − Subnational (Level 2)
Location Hierarchy
1 − Global
2 − Super Region
3 − Region
4 − Country
5 − Subnational (Level 1)
6 − Subnational (Level 2)

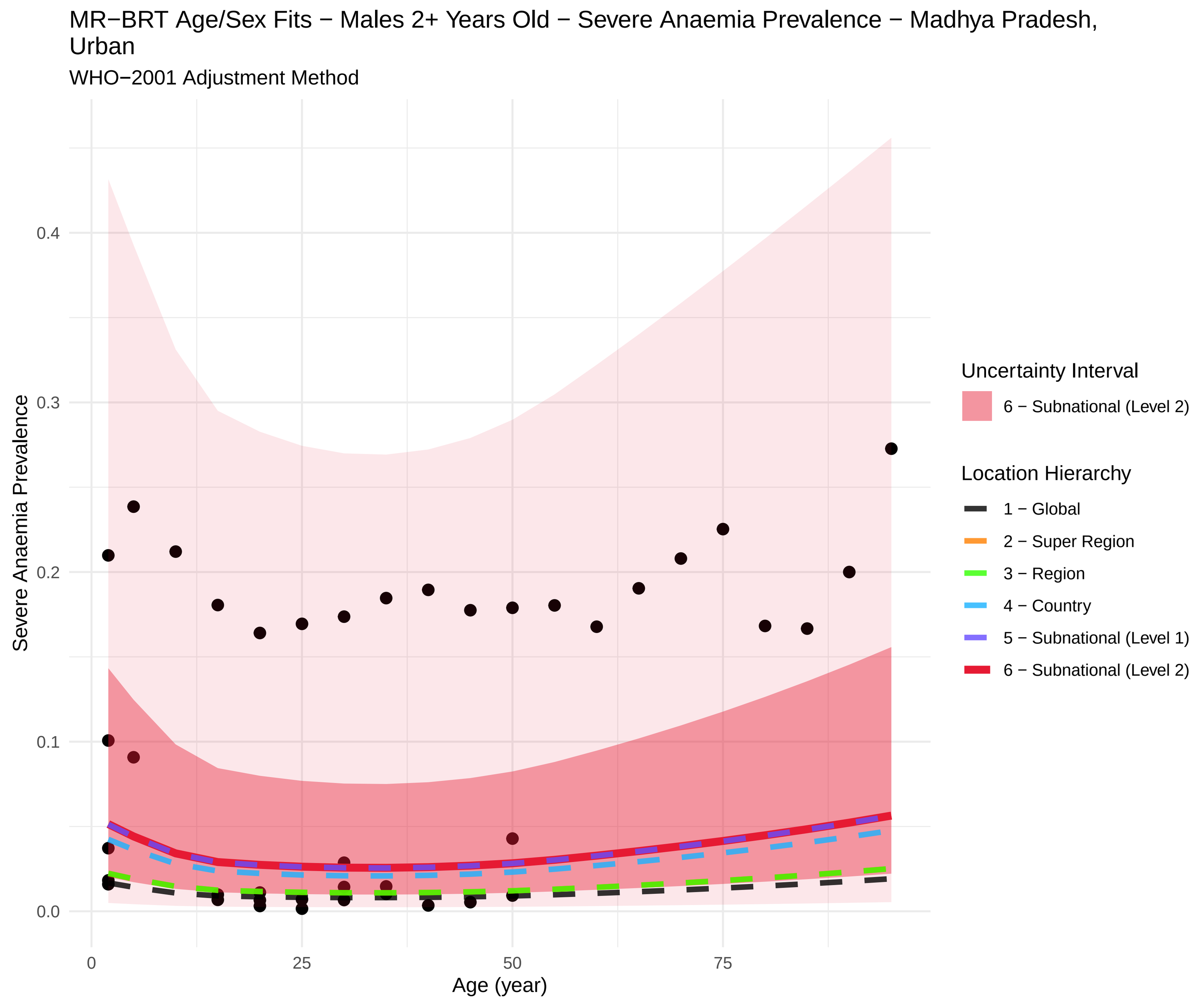
MR−BRT Age/Sex Fits − Males 2+ Years Old − Severe Anaemia Prevalence − Madhya Pradesh, Urban
WHO−2001 Adjustment Method
Severe Anaemia Prevalence
Age (year)
Uncertainty Interval
6 − Subnational (Level 2)
Location Hierarchy
1 − Global
2 − Super Region
3 − Region
4 − Country
5 − Subnational (Level 1)
6 − Subnational (Level 2)

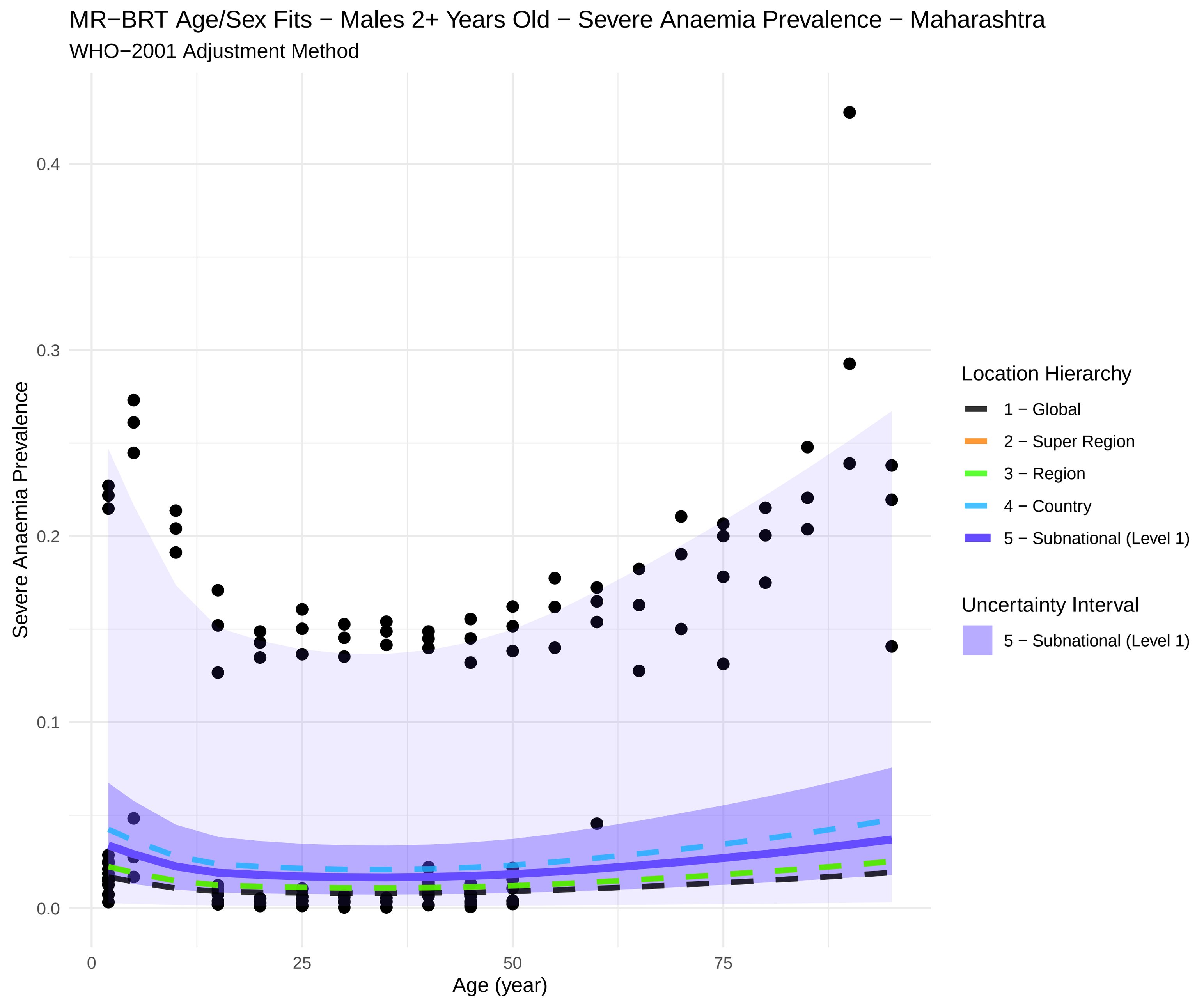
MR−BRT Age/Sex Fits − Males 2+ Years Old − Severe Anaemia Prevalence − Maharashtra
WHO−2001 Adjustment Method
Severe Anaemia Prevalence
Age (year)
Location Hierarchy
1 − Global
2 − Super Region
3 − Region
4 − Country
5 − Subnational (Level 1)
Uncertainty Interval
5 − Subnational (Level 1)

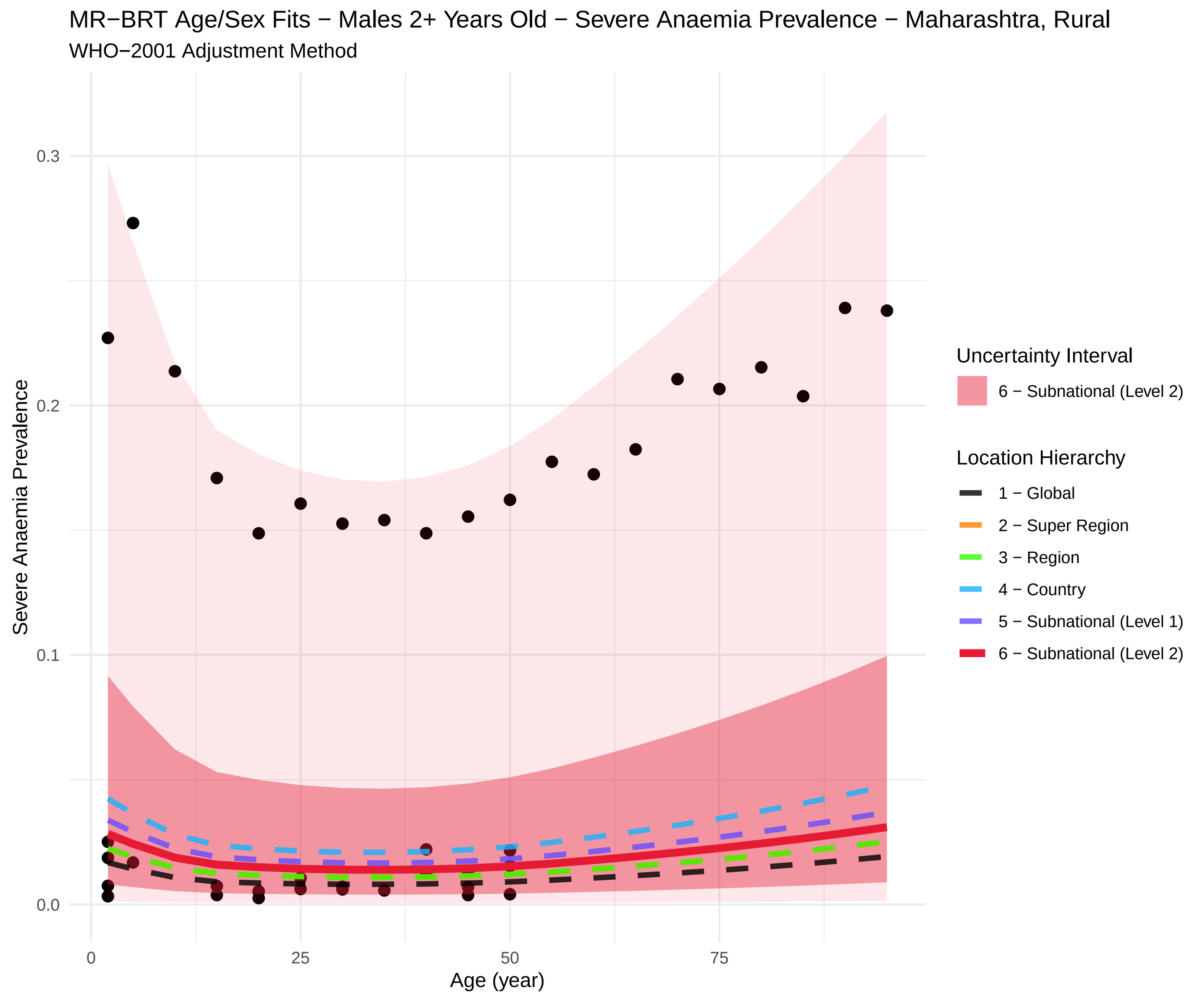
MR−BRT Age/Sex Fits − Males 2+ Years Old − Severe Anaemia Prevalence − Maharashtra, Rural
WHO−2001 Adjustment Method
Severe Anaemia Prevalence
Age (year)
Uncertainty Interval
6 − Subnational (Level 2)
Location Hierarchy
1 − Global
2 − Super Region
3 − Region
4 − Country
5 − Subnational (Level 1)
6 − Subnational (Level 2)

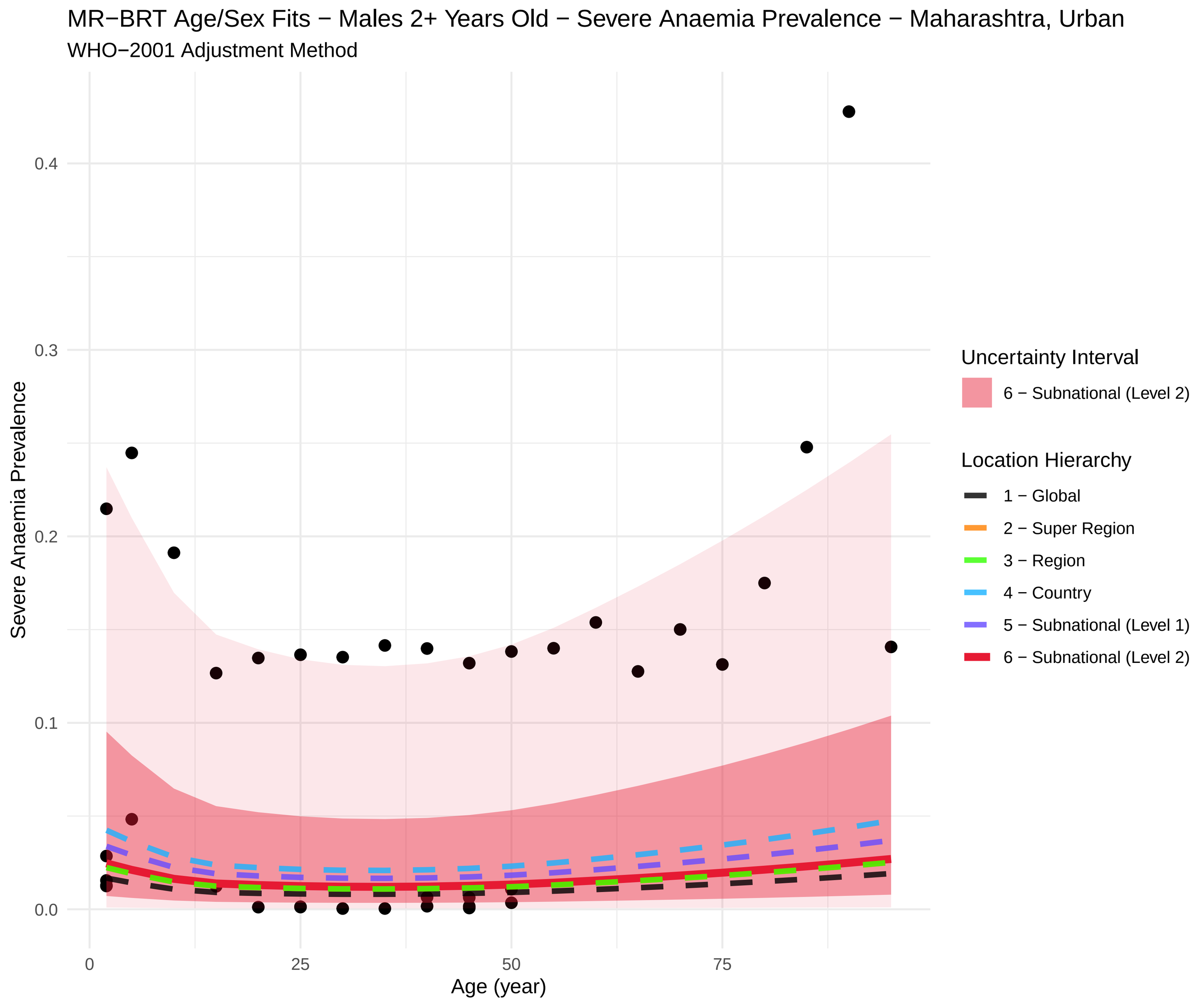
MR−BRT Age/Sex Fits − Males 2+ Years Old − Severe Anaemia Prevalence − Maharashtra, Urban
WHO−2001 Adjustment Method
Severe Anaemia Prevalence
Age (year)
Uncertainty Interval
6 − Subnational (Level 2)
Location Hierarchy
1 − Global
2 − Super Region
3 − Region
4 − Country
5 − Subnational (Level 1)
6 − Subnational (Level 2)

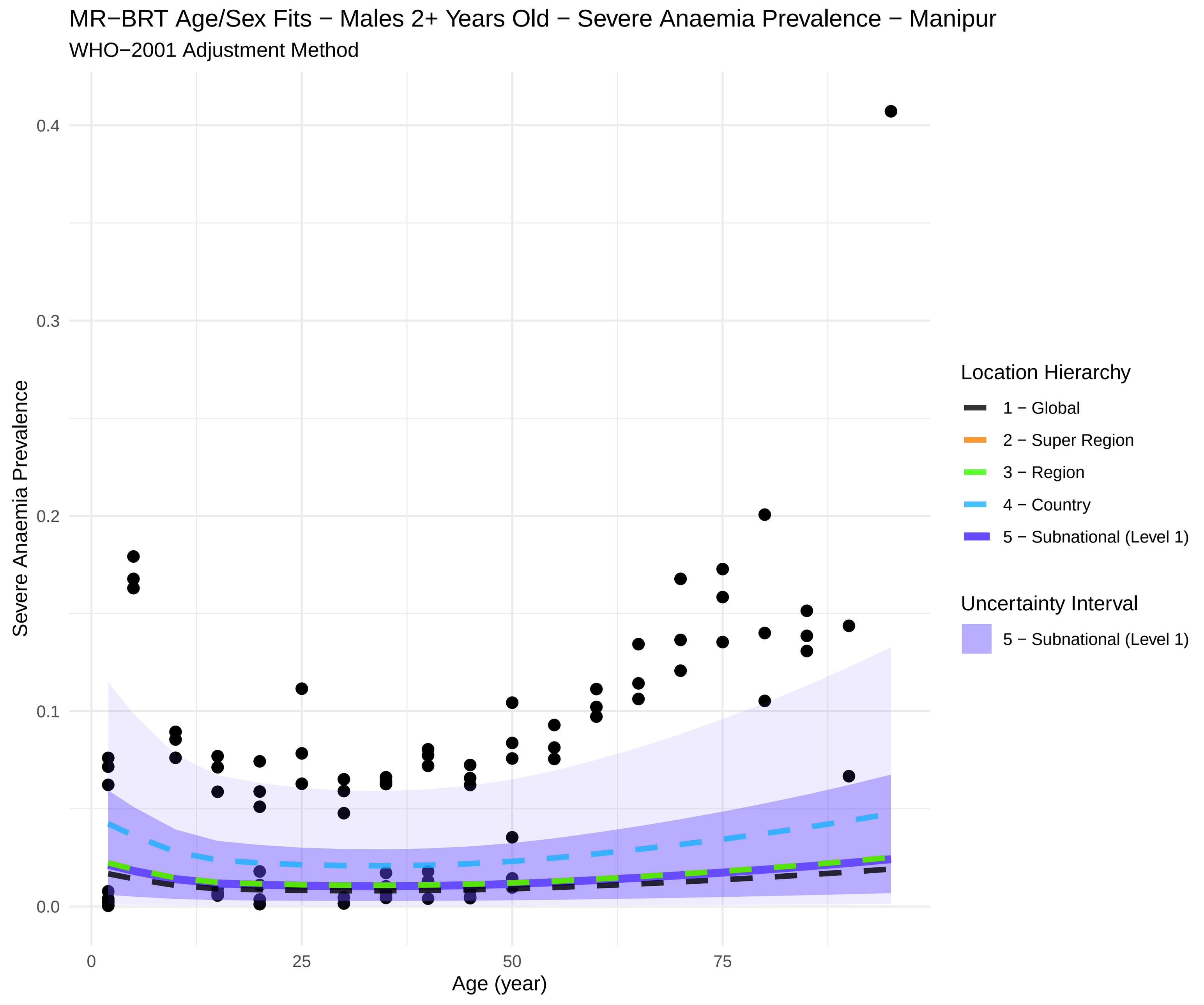
MR−BRT Age/Sex Fits − Males 2+ Years Old − Severe Anaemia Prevalence − Manipur
WHO−2001 Adjustment Method
Severe Anaemia Prevalence
Age (year)
Location Hierarchy
1 − Global
2 − Super Region
3 − Region
4 − Country
5 − Subnational (Level 1)
Uncertainty Interval
5 − Subnational (Level 1)

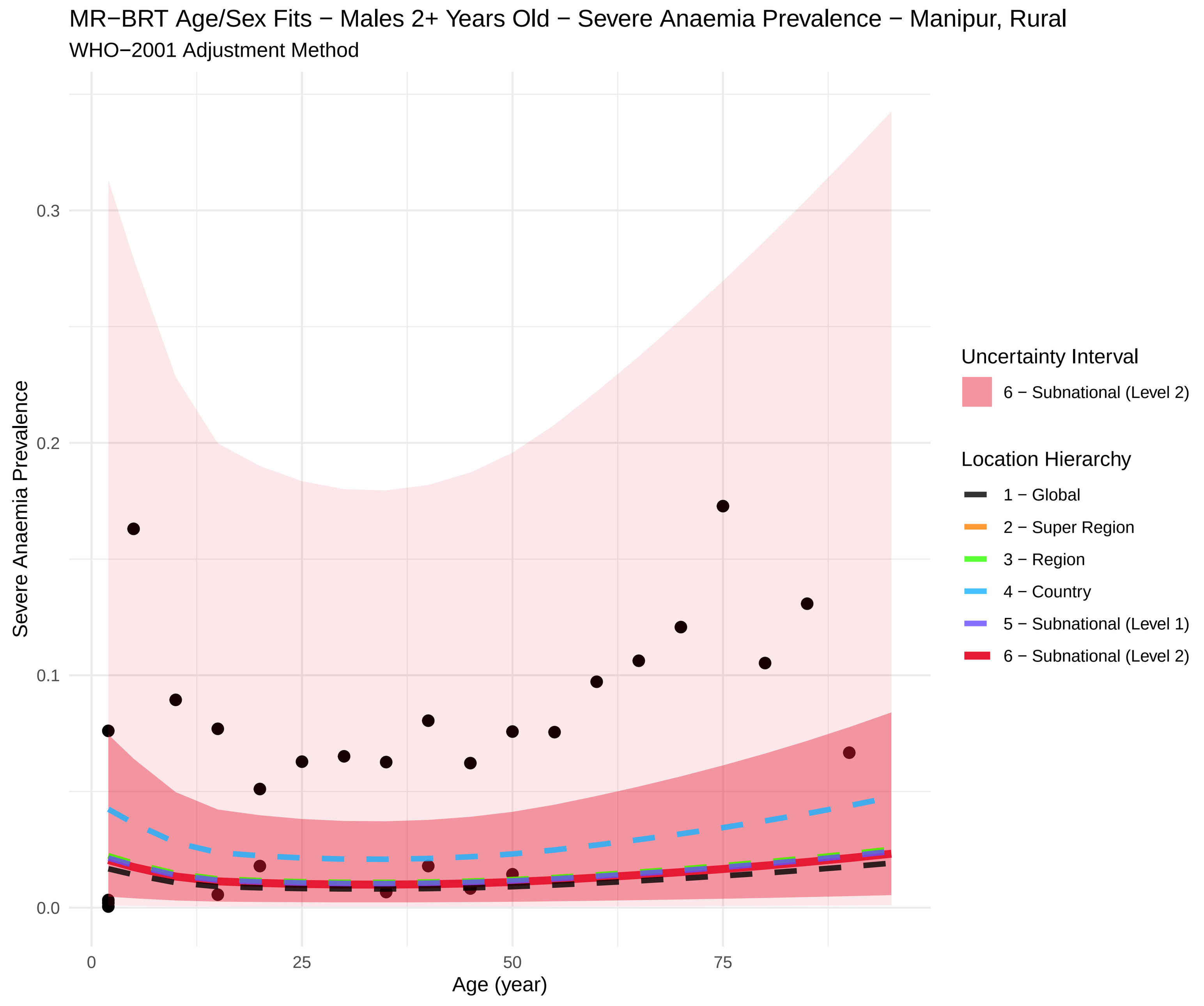
MR−BRT Age/Sex Fits − Males 2+ Years Old − Severe Anaemia Prevalence − Manipur, Rural
WHO−2001 Adjustment Method
Severe Anaemia Prevalence
Age (year)
Uncertainty Interval
6 − Subnational (Level 2)
Location Hierarchy
1 − Global
2 − Super Region
3 − Region
4 − Country
5 − Subnational (Level 1)
6 − Subnational (Level 2)

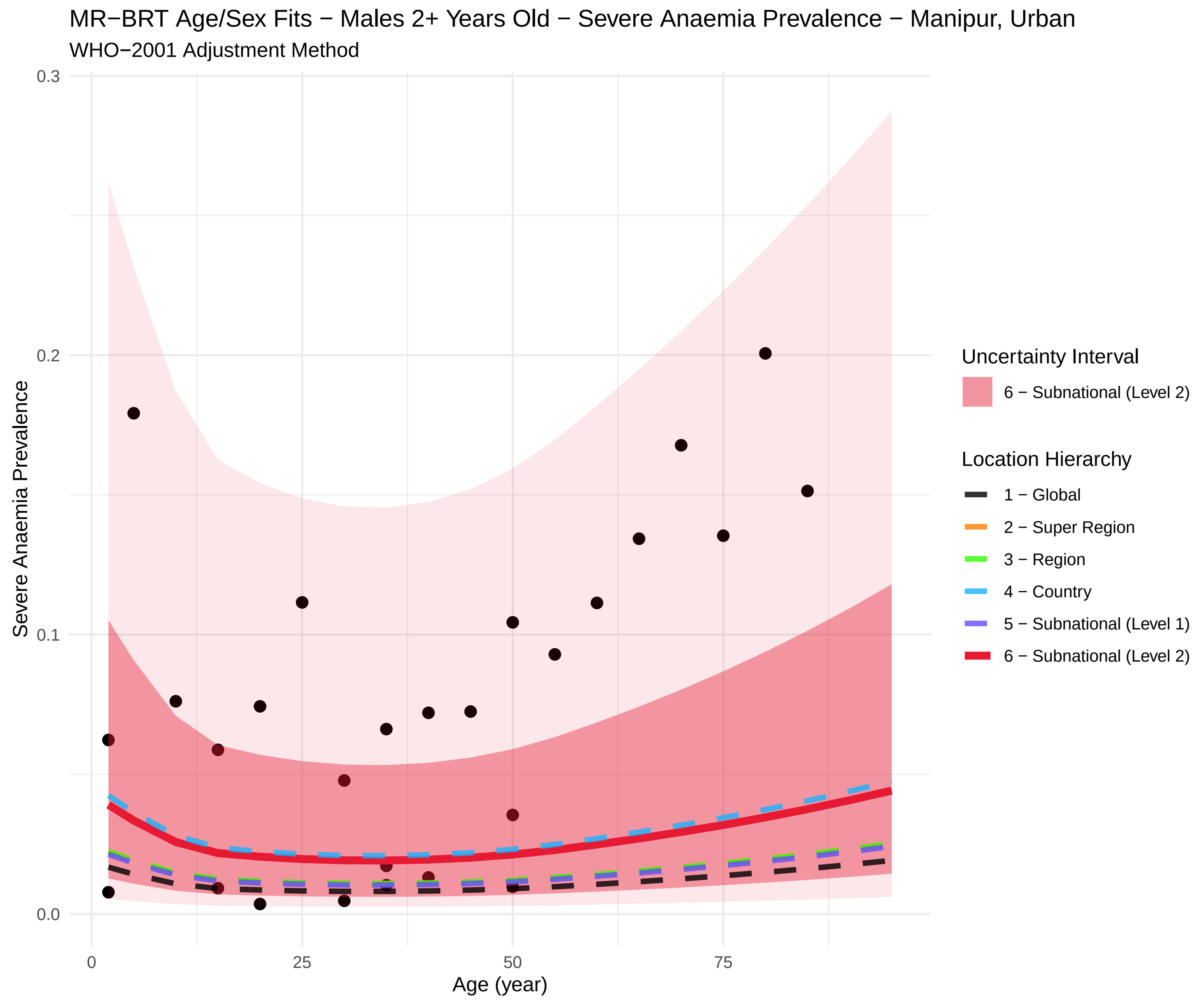
MR−BRT Age/Sex Fits − Males 2+ Years Old − Severe Anaemia Prevalence − Manipur, Urban
WHO−2001 Adjustment Method
Severe Anaemia Prevalence
Age (year)
Uncertainty Interval
6 − Subnational (Level 2)
Location Hierarchy
1 − Global
2 − Super Region
3 − Region
4 − Country
5 − Subnational (Level 1)
6 − Subnational (Level 2)

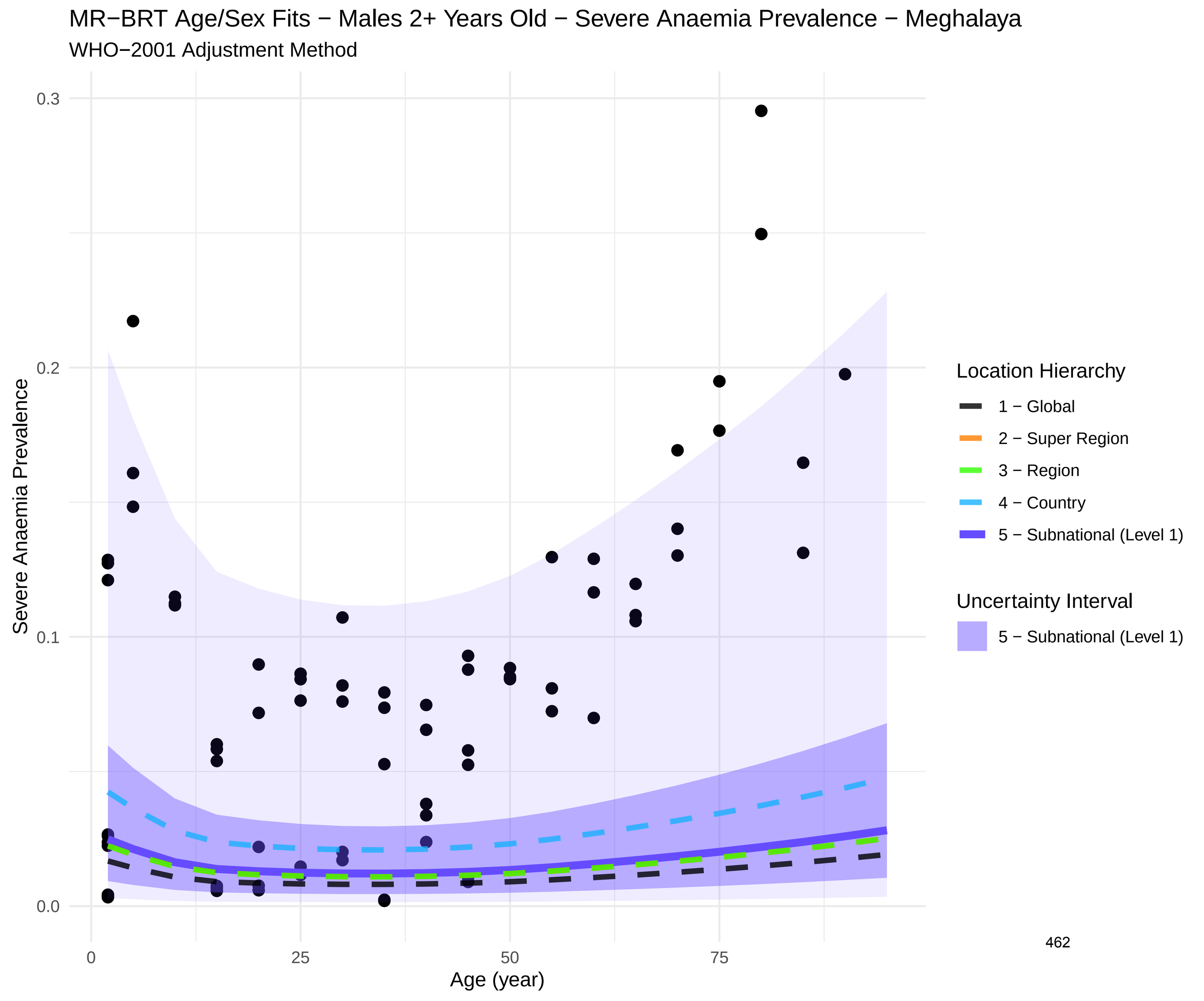
MR−BRT Age/Sex Fits − Males 2+ Years Old − Severe Anaemia Prevalence − Meghalaya
WHO−2001 Adjustment Method
Severe Anaemia Prevalence
Age (year)
Location Hierarchy
1 − Global
2 − Super Region
3 − Region
4 − Country
5 − Subnational (Level 1)
Uncertainty Interval
5 − Subnational (Level 1)

MR−BRT Age/Sex Fits − Males 2+ Years Old − Severe Anaemia Prevalence − Meghalaya, Rural
WHO−2001 Adjustment Method

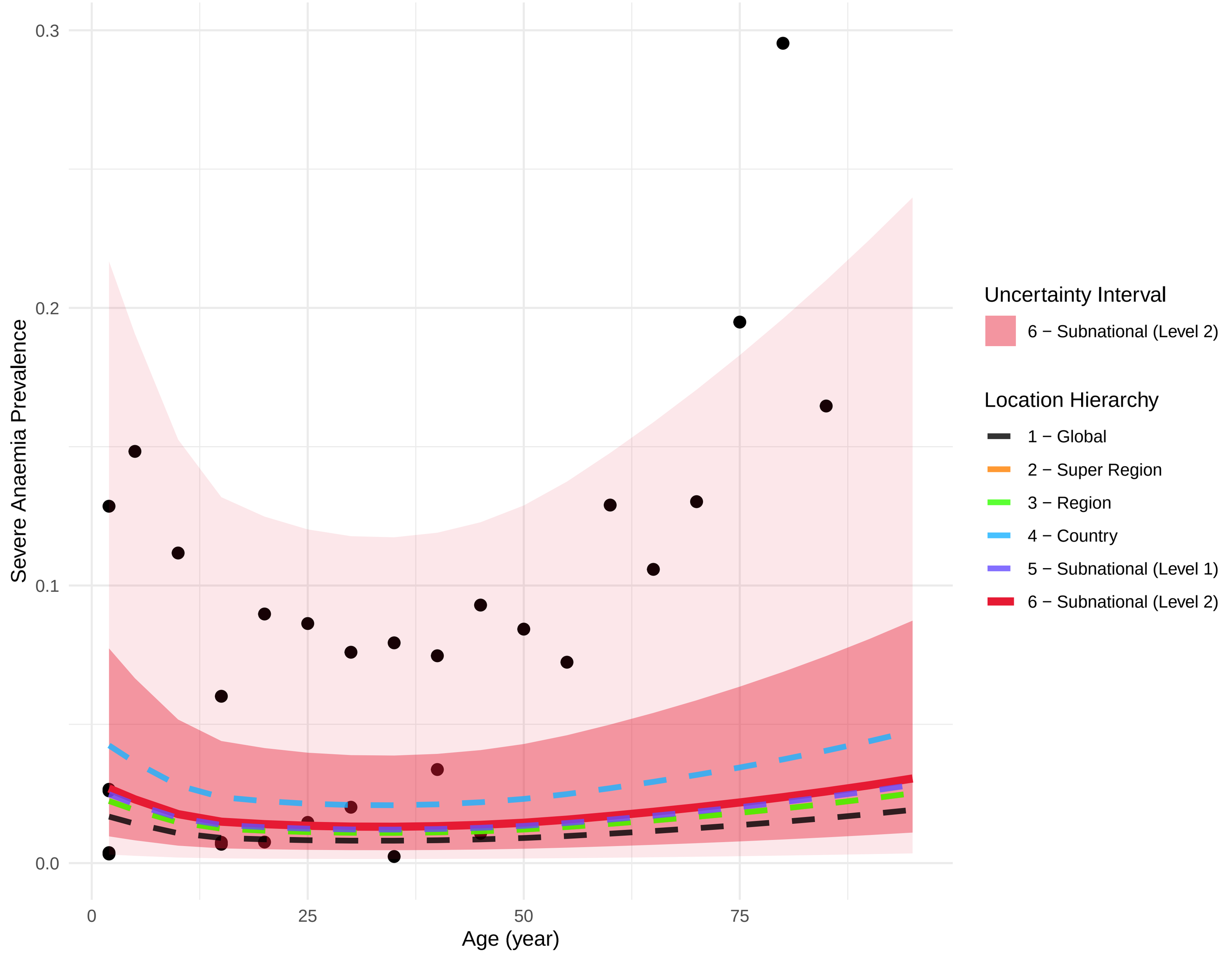

MR−BRT Age/Sex Fits − Males 2+ Years Old − Severe Anaemia Prevalence − Meghalaya, Urban
WHO−2001 Adjustment Method

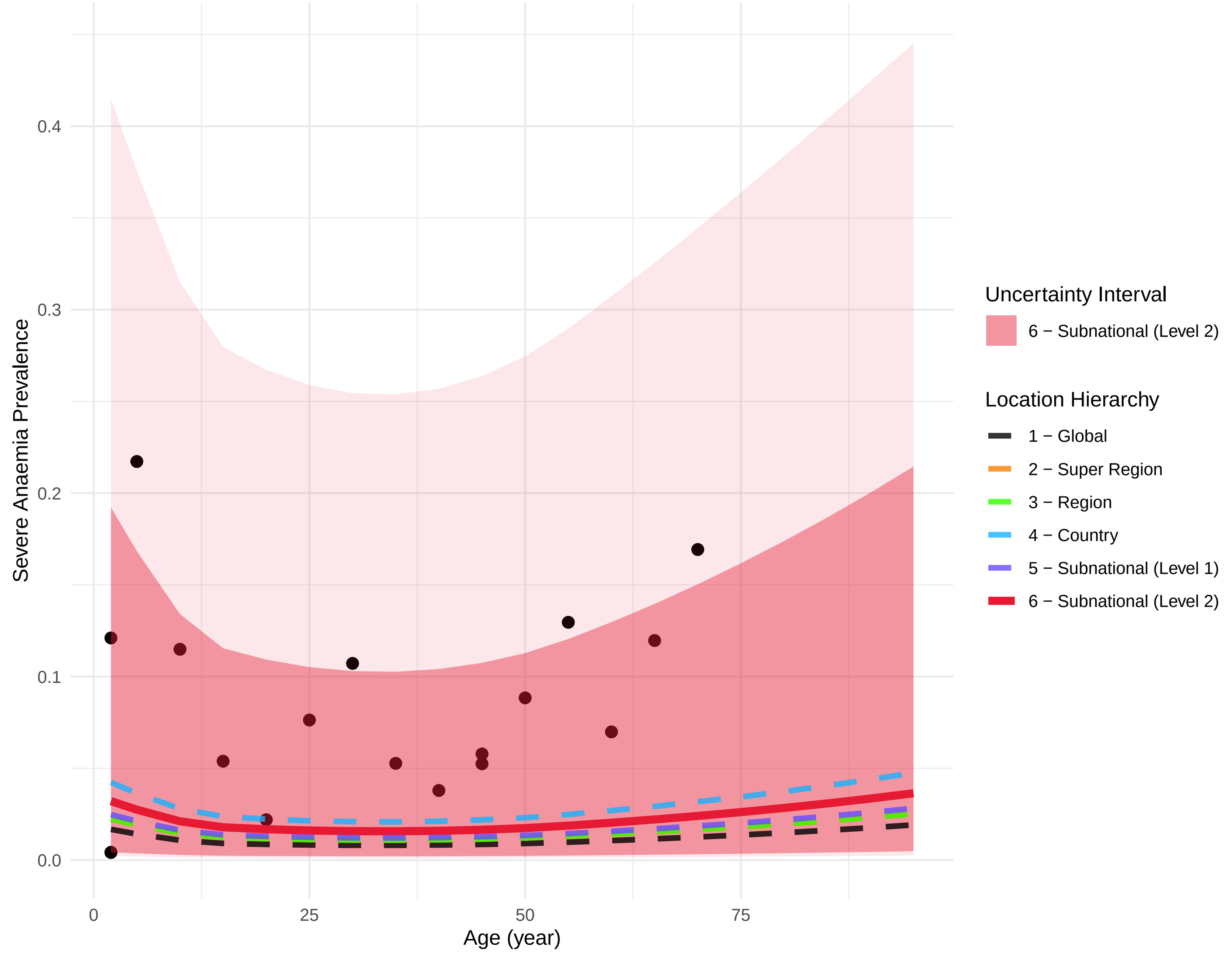

MR−BRT Age/Sex Fits − Males 2+ Years Old − Severe Anaemia Prevalence − Mizoram
WHO−2001 Adjustment Method

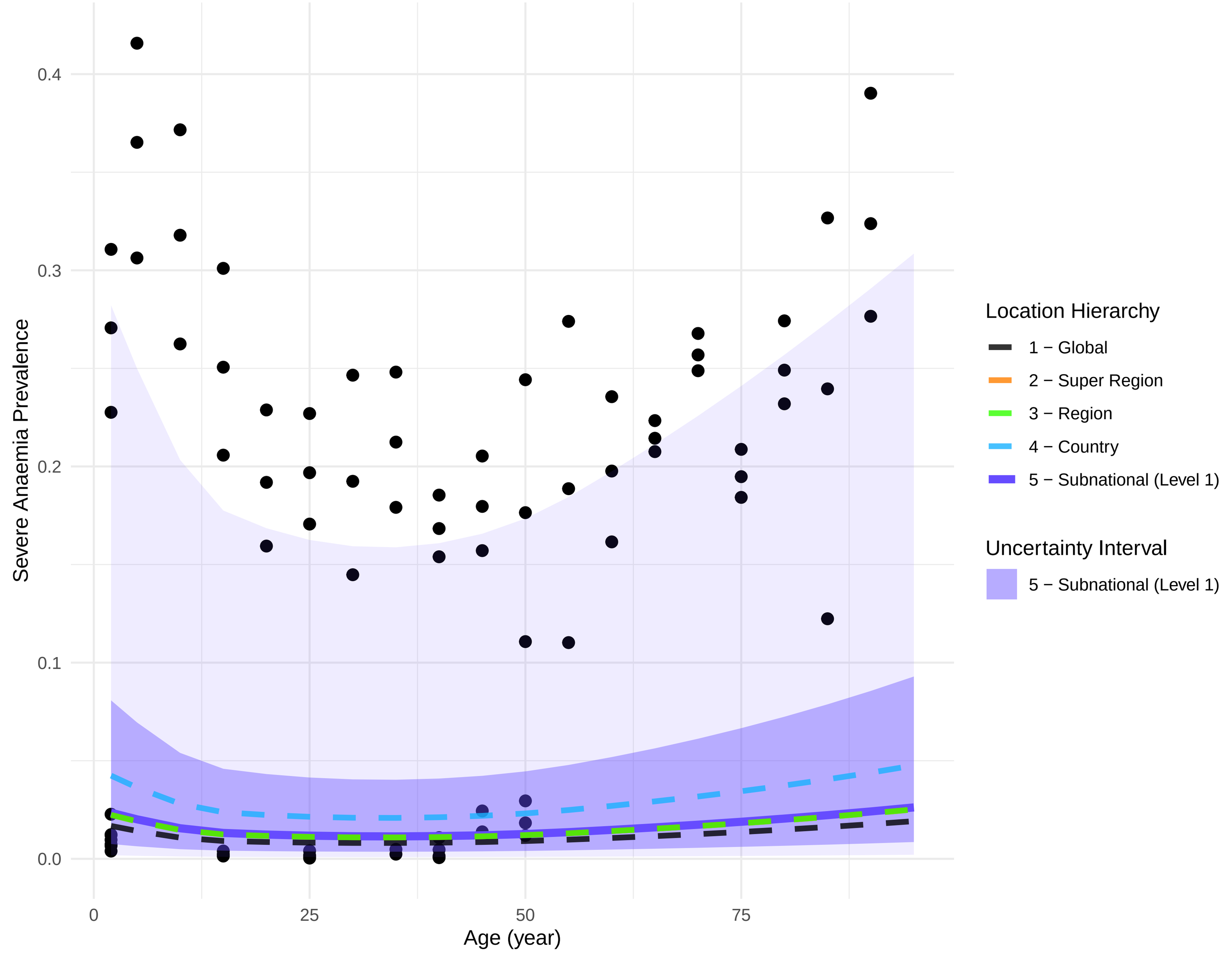

MR−BRT Age/Sex Fits − Males 2+ Years Old − Severe Anaemia Prevalence − Mizoram, Rural
WHO−2001 Adjustment Method

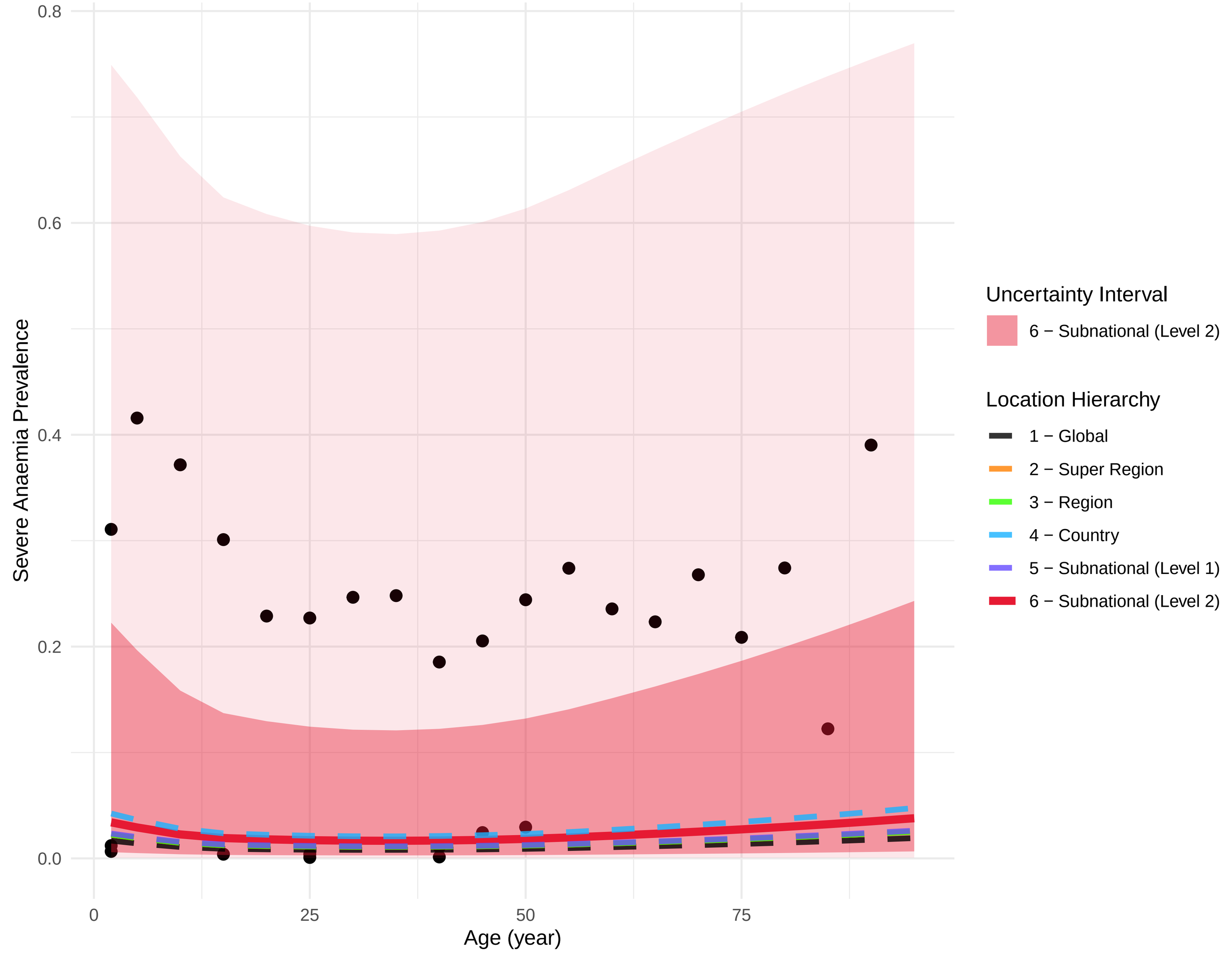

MR−BRT Age/Sex Fits − Males 2+ Years Old − Severe Anaemia Prevalence − Mizoram, Urban
WHO−2001 Adjustment Method

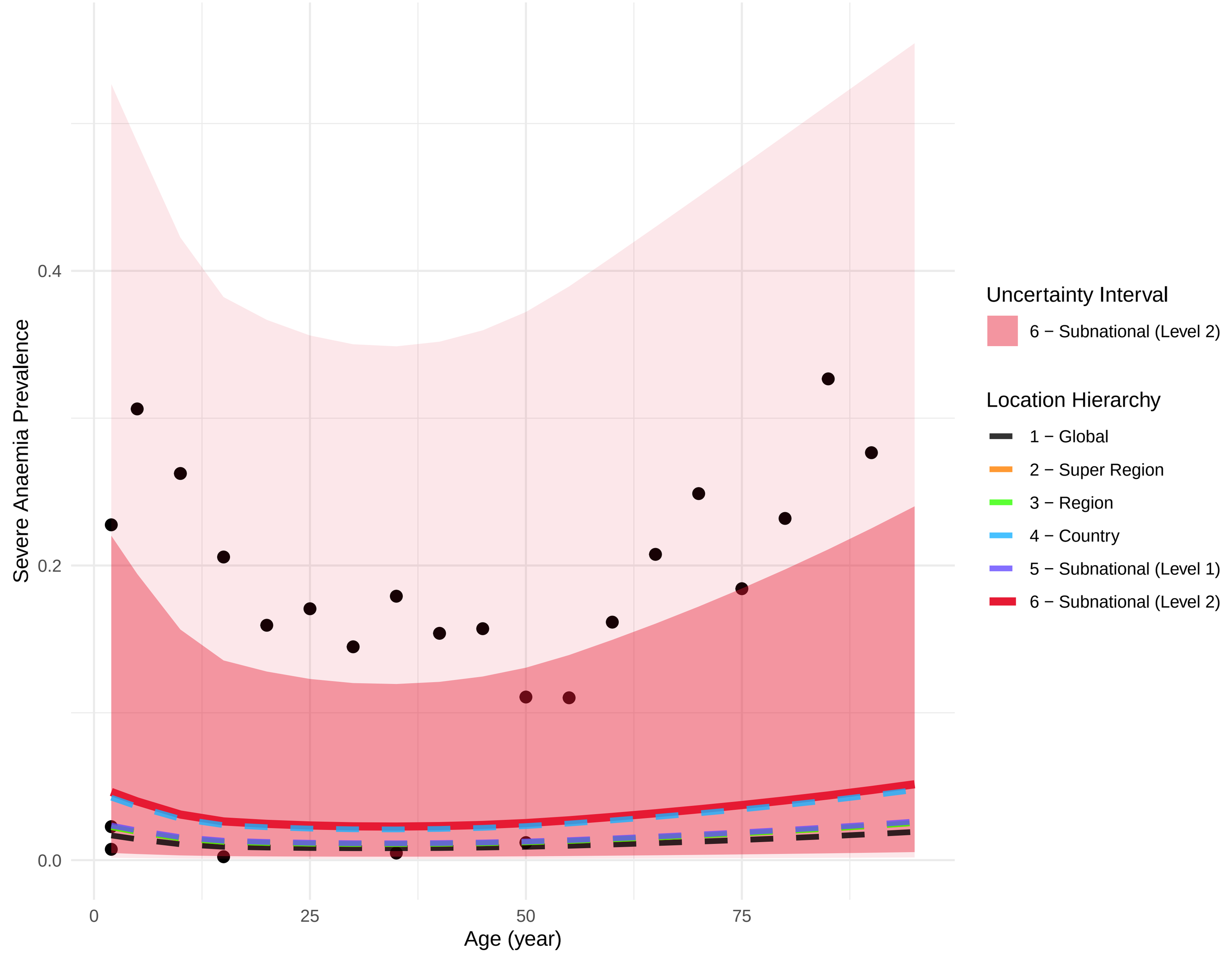

MR−BRT Age/Sex Fits − Males 2+ Years Old − Severe Anaemia Prevalence − Nagaland
WHO−2001 Adjustment Method

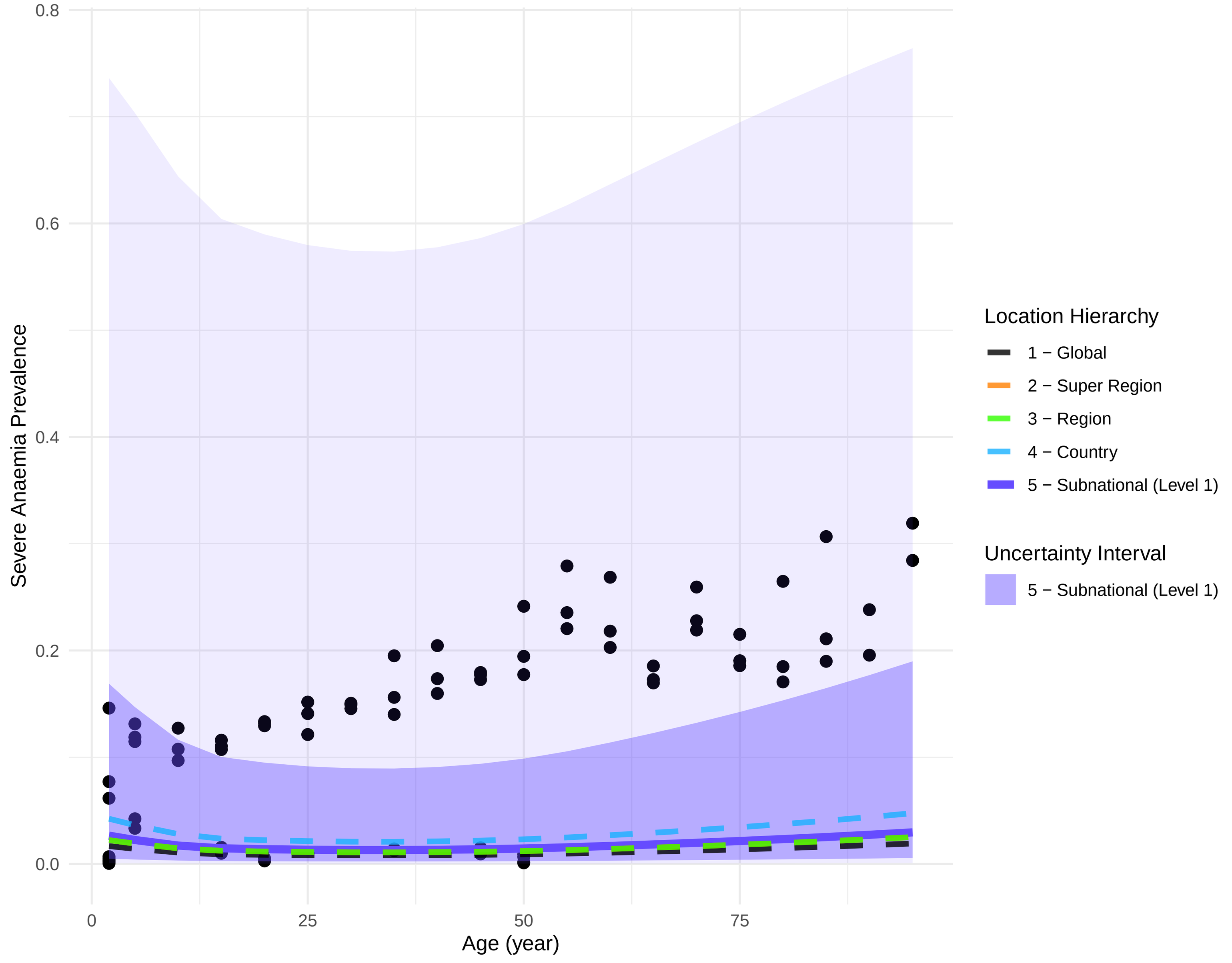

MR−BRT Age/Sex Fits − Males 2+ Years Old − Severe Anaemia Prevalence − Nagaland, Rural
WHO−2001 Adjustment Method

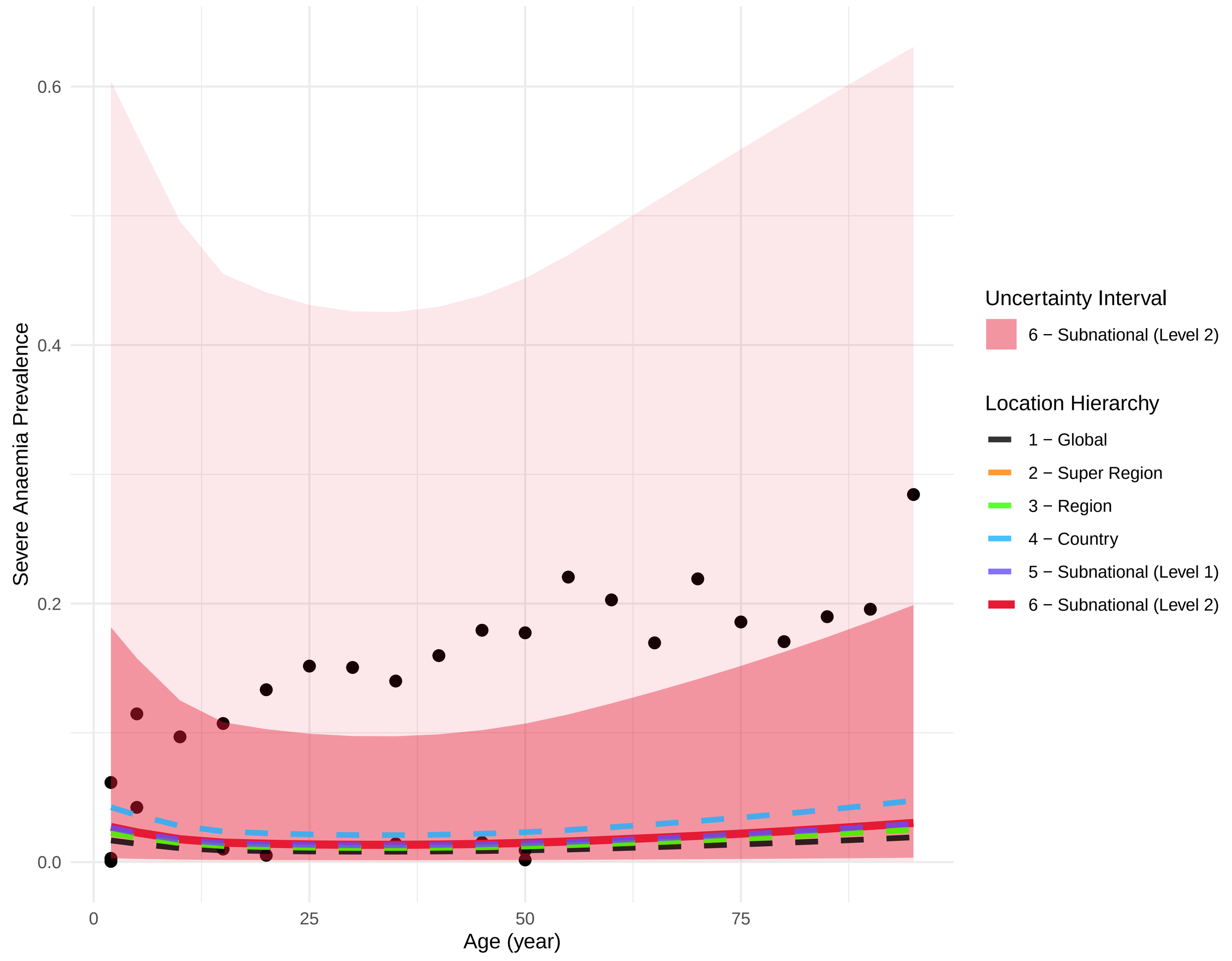

MR−BRT Age/Sex Fits − Males 2+ Years Old − Severe Anaemia Prevalence − Nagaland, Urban
WHO−2001 Adjustment Method

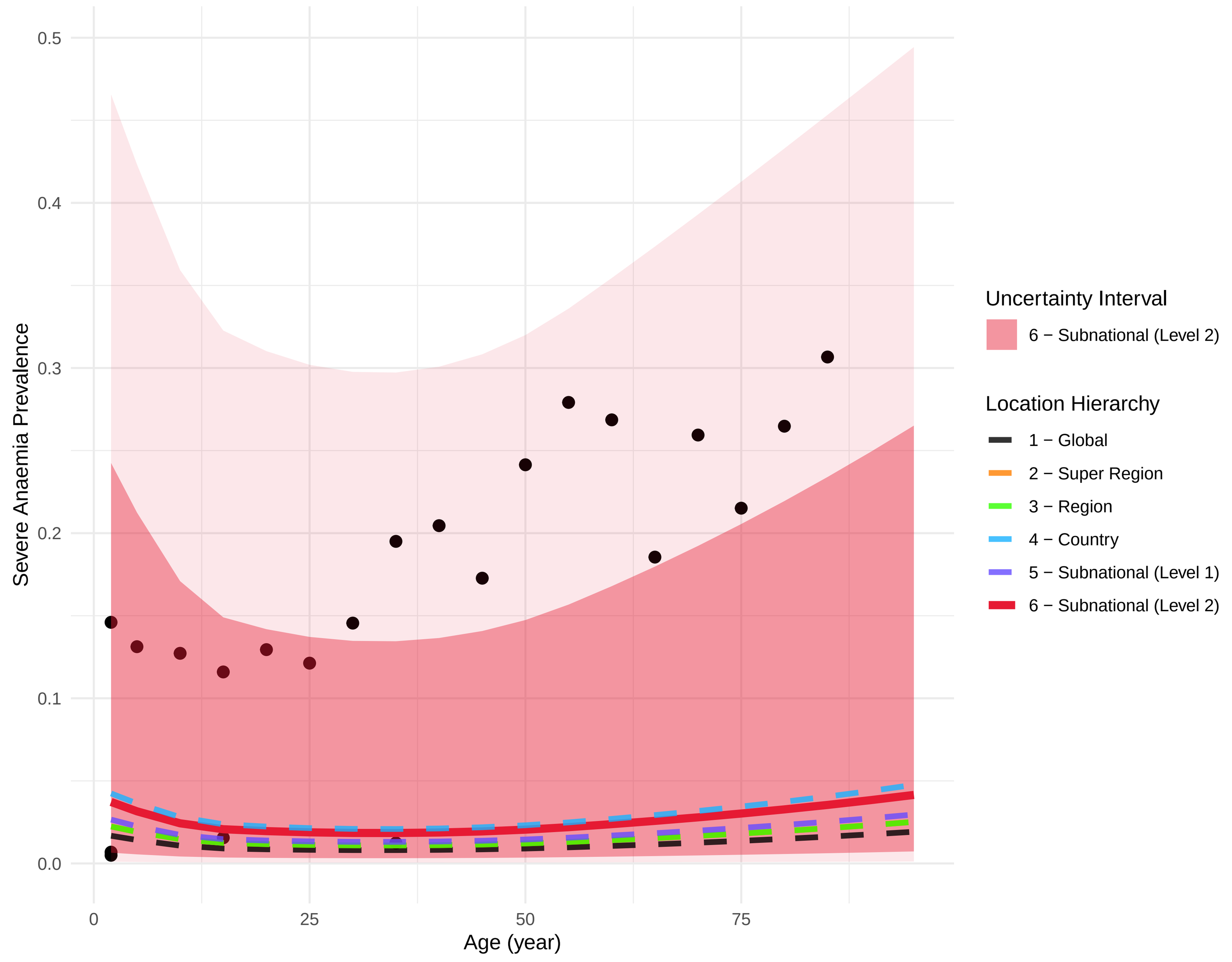

MR−BRT Age/Sex Fits − Males 2+ Years Old − Severe Anaemia Prevalence − Odisha
WHO−2001 Adjustment Method

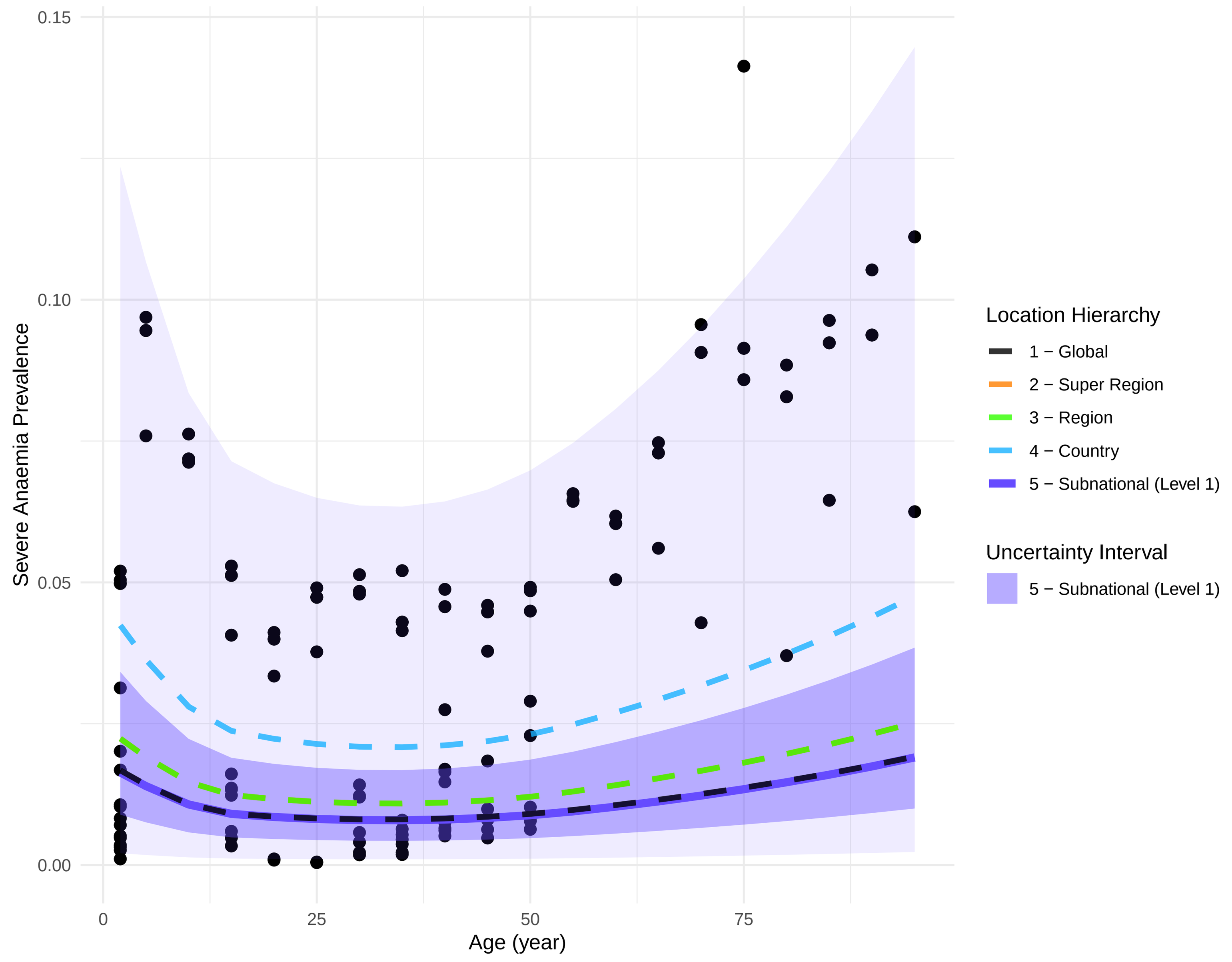

MR−BRT Age/Sex Fits − Males 2+ Years Old − Severe Anaemia Prevalence − Odisha, Rural
WHO−2001 Adjustment Method

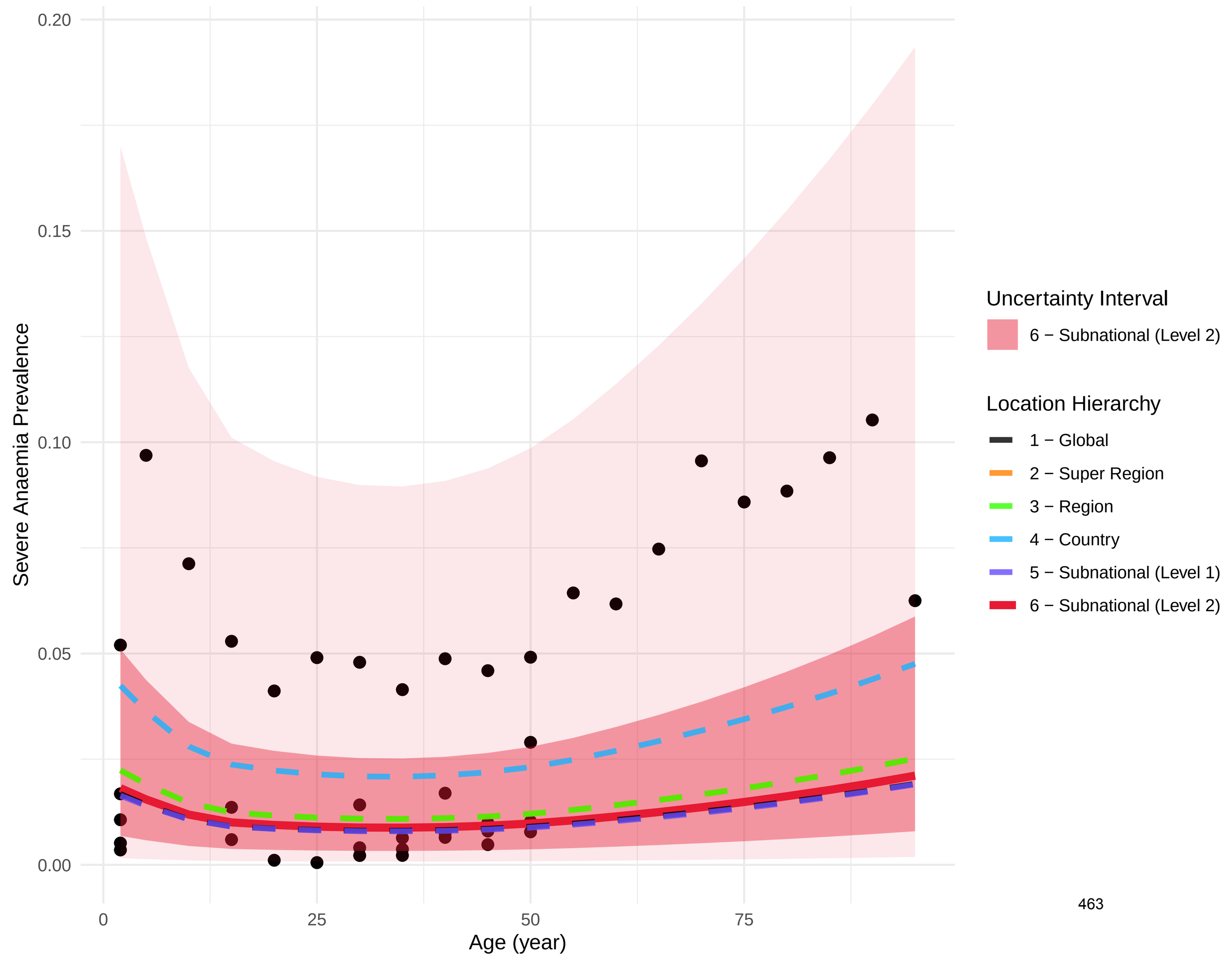

MR−BRT Age/Sex Fits − Males 2+ Years Old − Severe Anaemia Prevalence − Odisha, Urban
WHO−2001 Adjustment Method

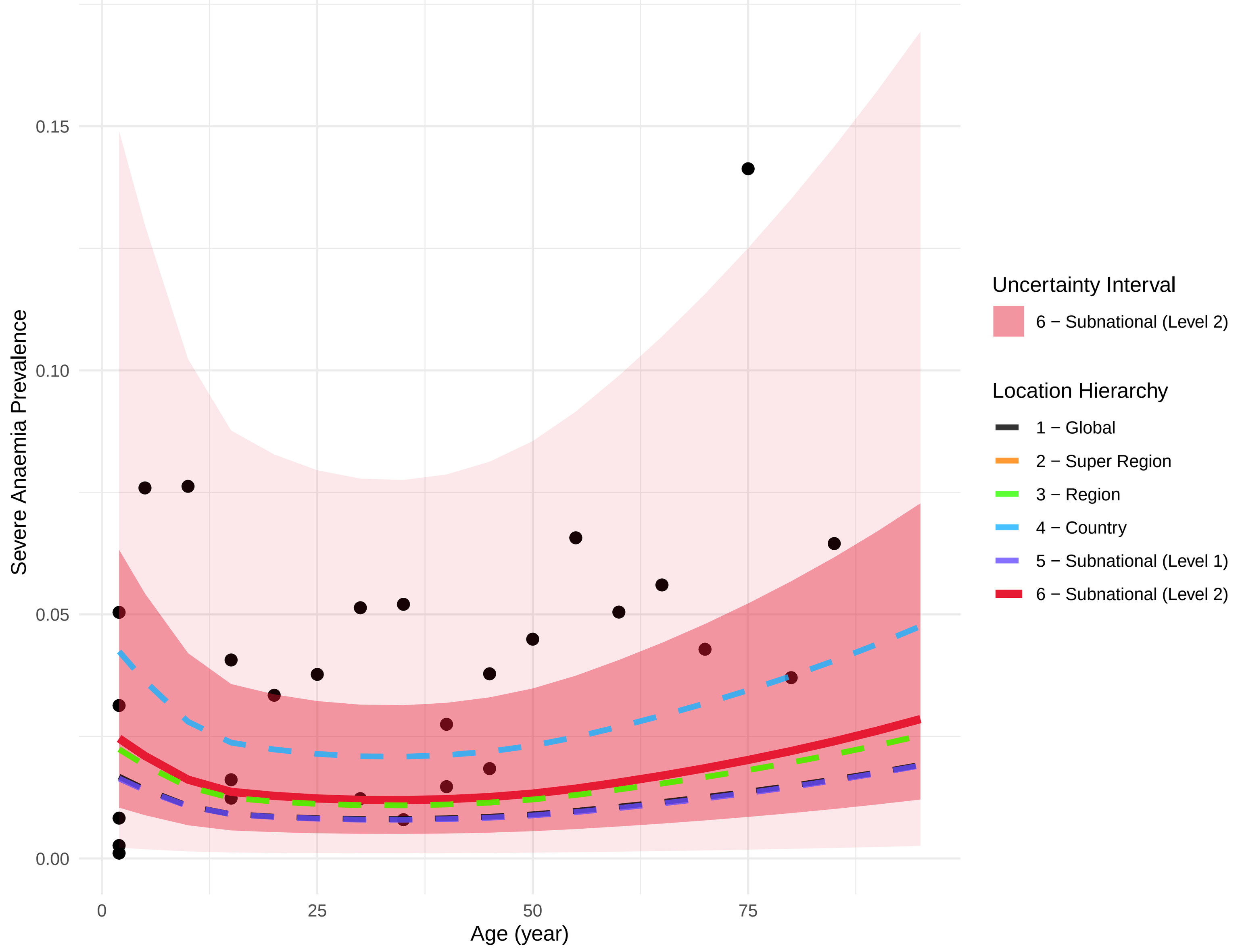

MR−BRT Age/Sex Fits − Males 2+ Years Old − Severe Anaemia Prevalence − Other Union Territories
WHO−2001 Adjustment Method

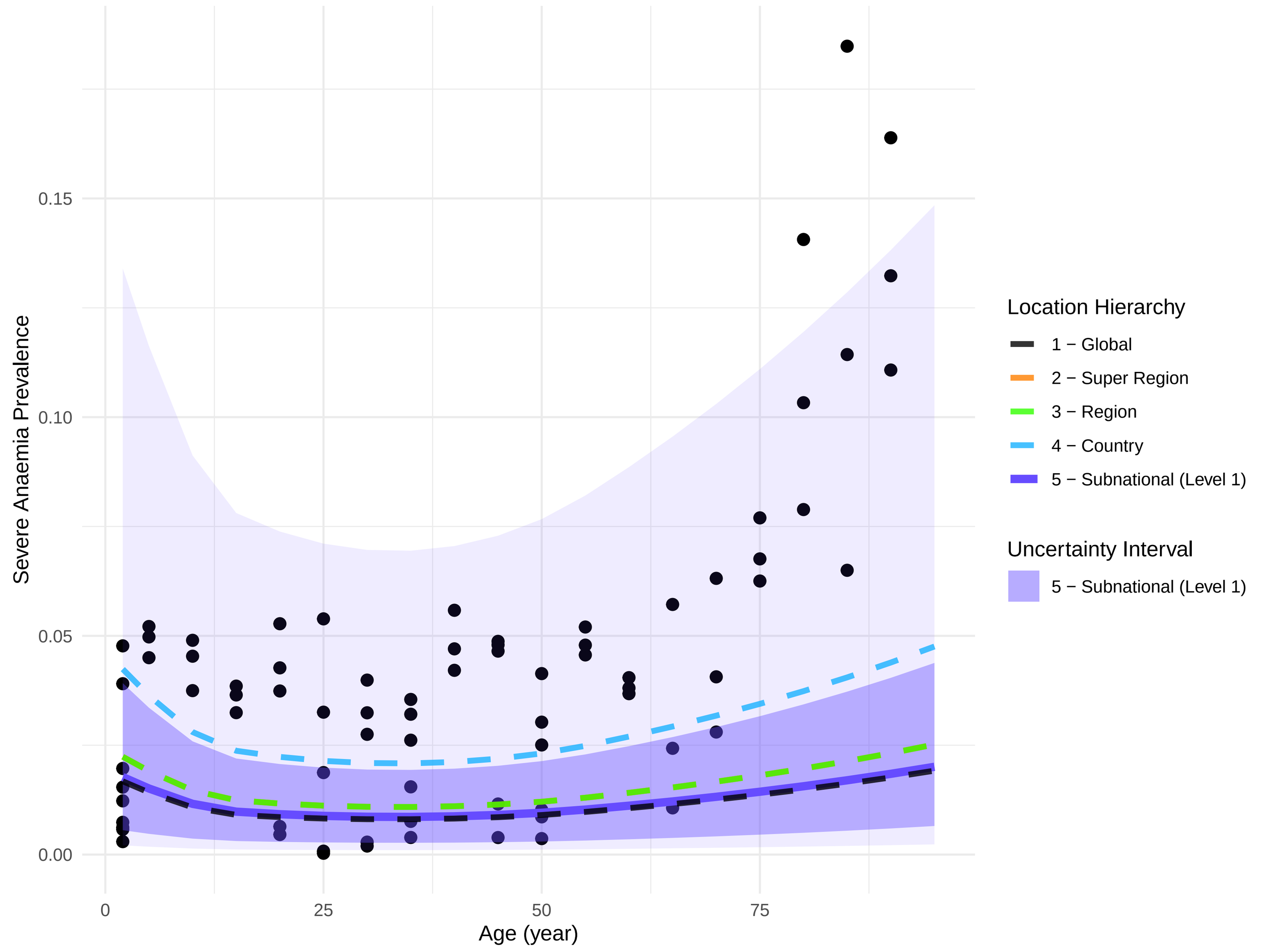

MR−BRT Age/Sex Fits − Males 2+ Years Old − Severe Anaemia Prevalence − Other Union Territories, Rural
WHO−2001 Adjustment Method

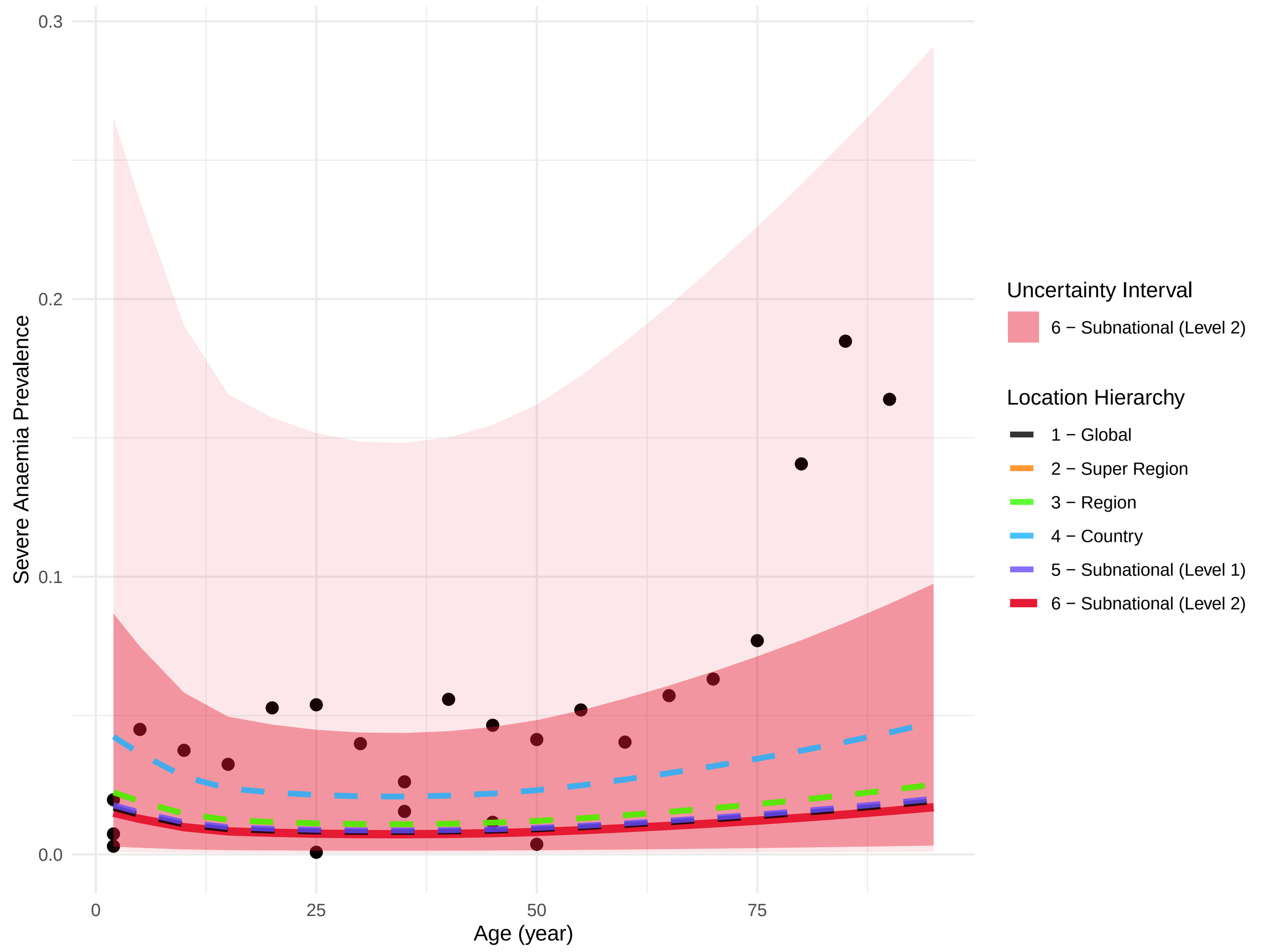

MR−BRT Age/Sex Fits − Males 2+ Years Old − Severe Anaemia Prevalence − Other Union Territories, Urban
WHO−2001 Adjustment Method

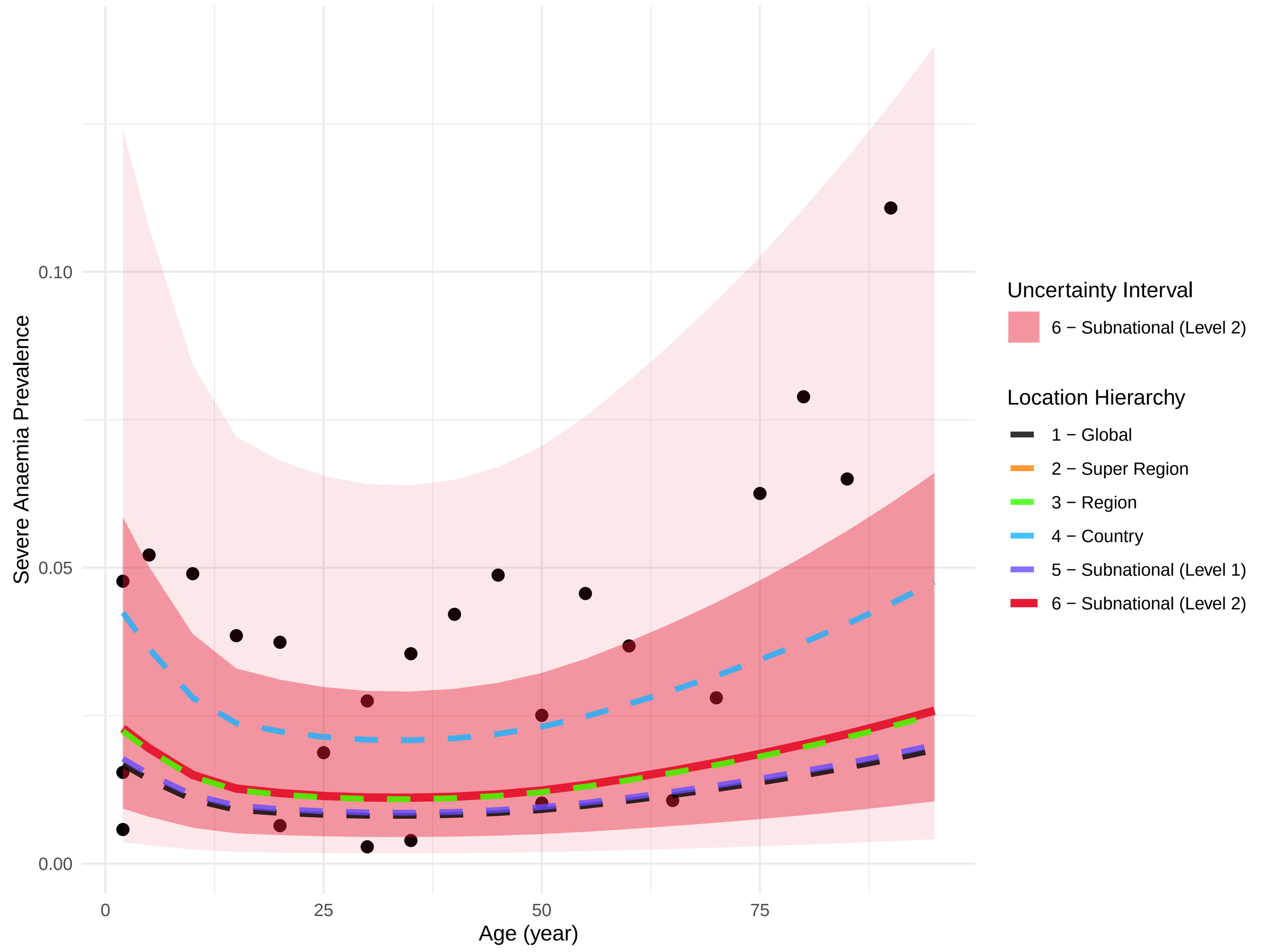

MR−BRT Age/Sex Fits − Males 2+ Years Old − Severe Anaemia Prevalence − Punjab
WHO−2001 Adjustment Method

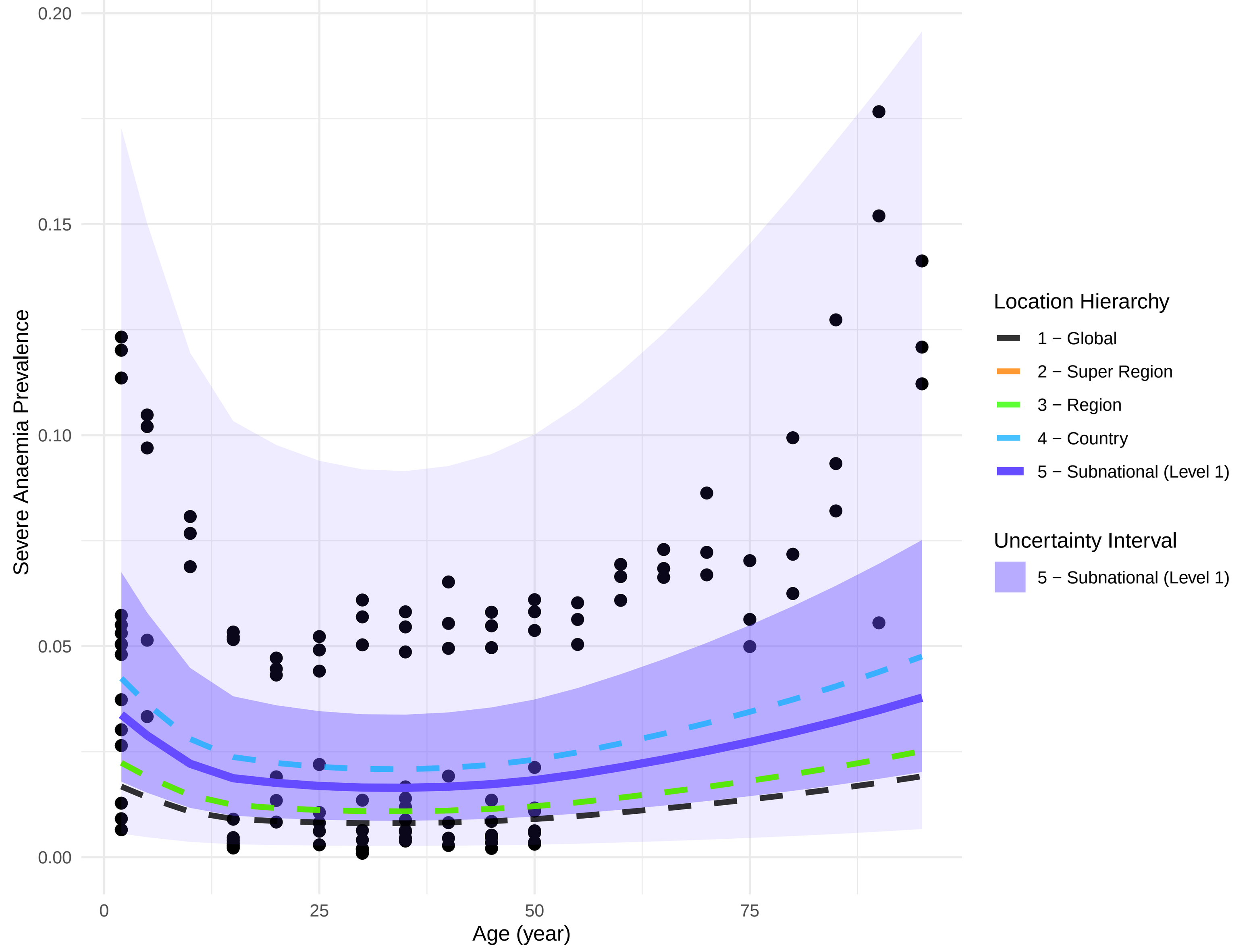

MR−BRT Age/Sex Fits − Males 2+ Years Old − Severe Anaemia Prevalence − Punjab, Rural
WHO−2001 Adjustment Method

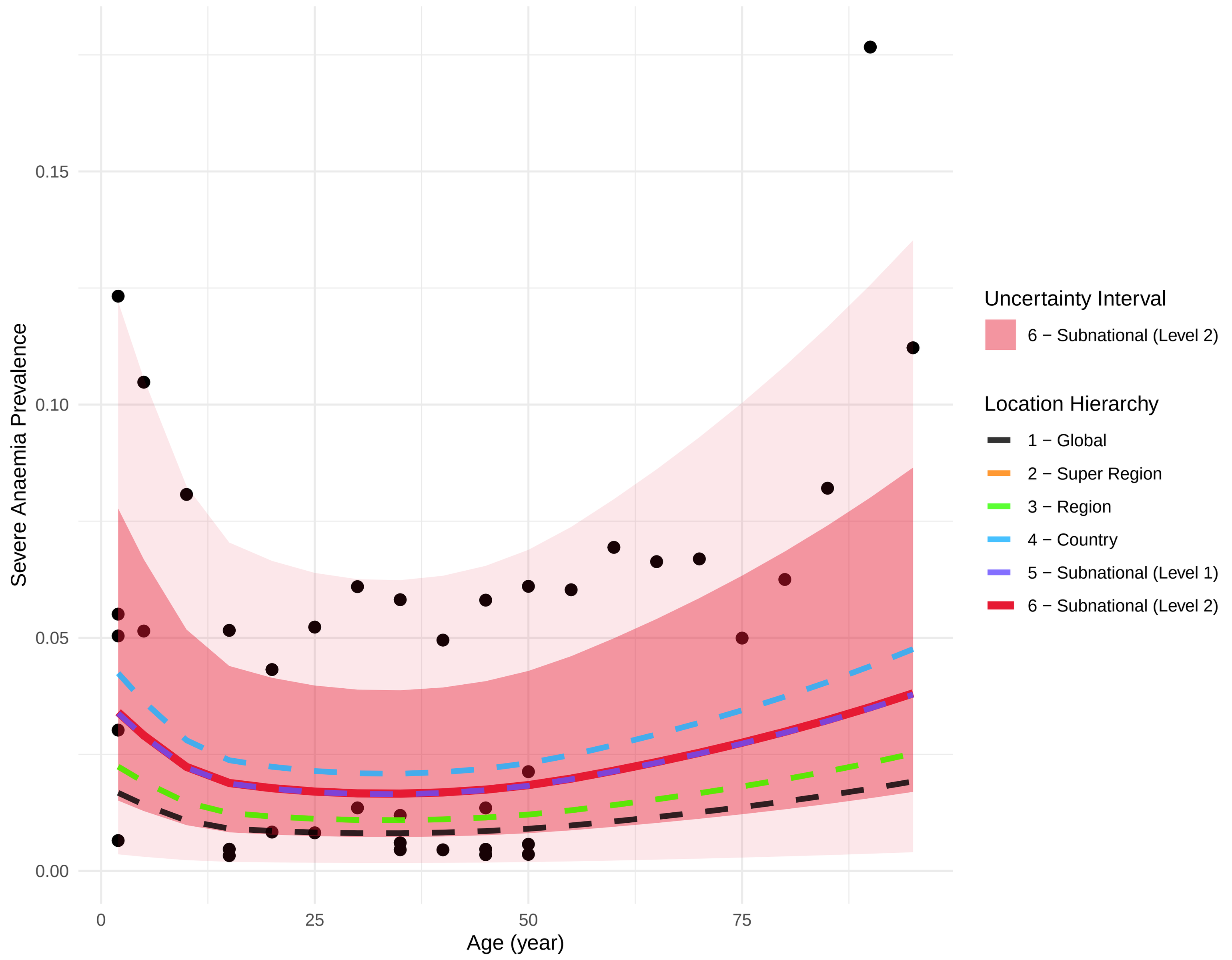

MR−BRT Age/Sex Fits − Males 2+ Years Old − Severe Anaemia Prevalence − Punjab, Urban
WHO−2001 Adjustment Method

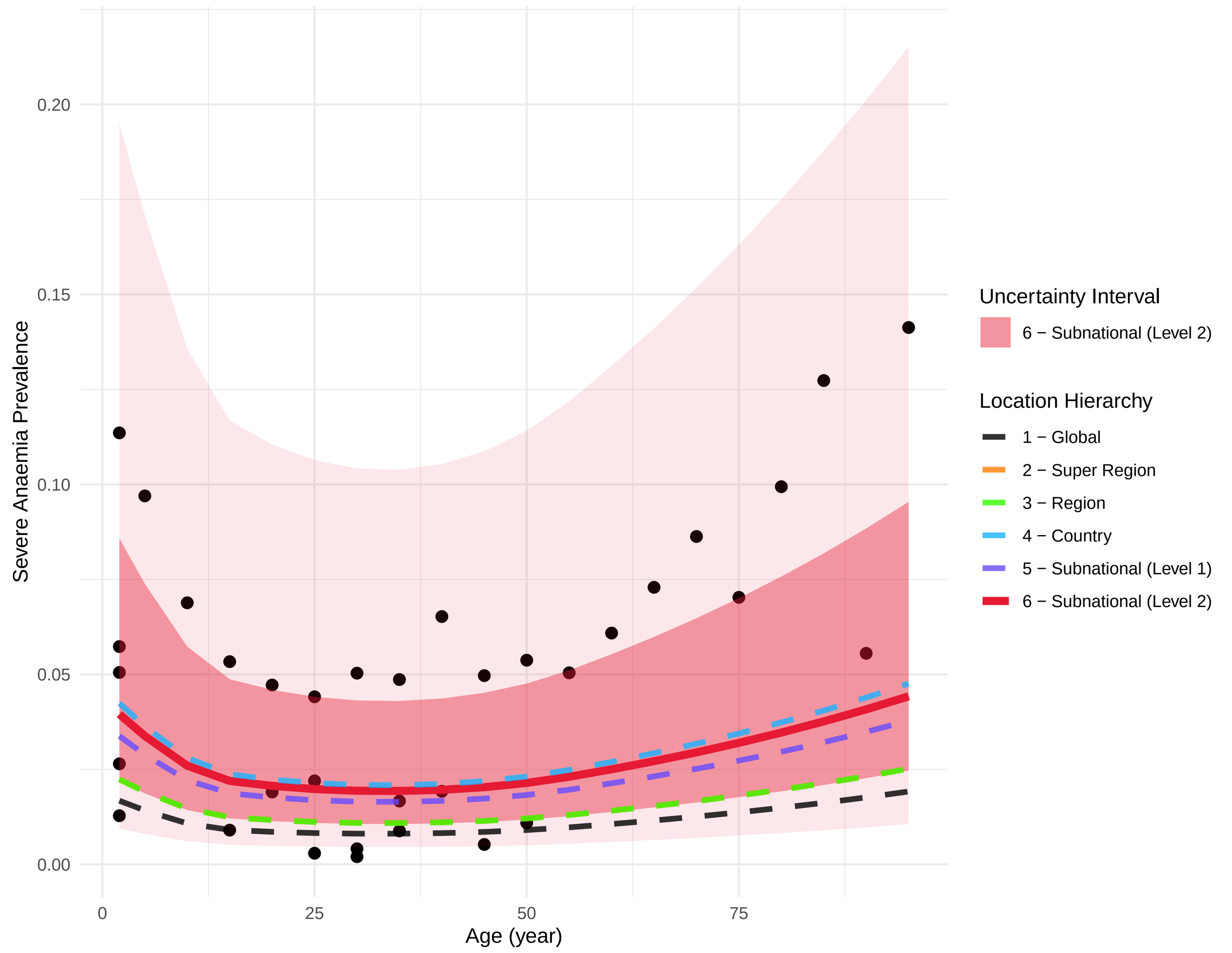

MR−BRT Age/Sex Fits − Males 2+ Years Old − Severe Anaemia Prevalence − Rajasthan
WHO−2001 Adjustment Method

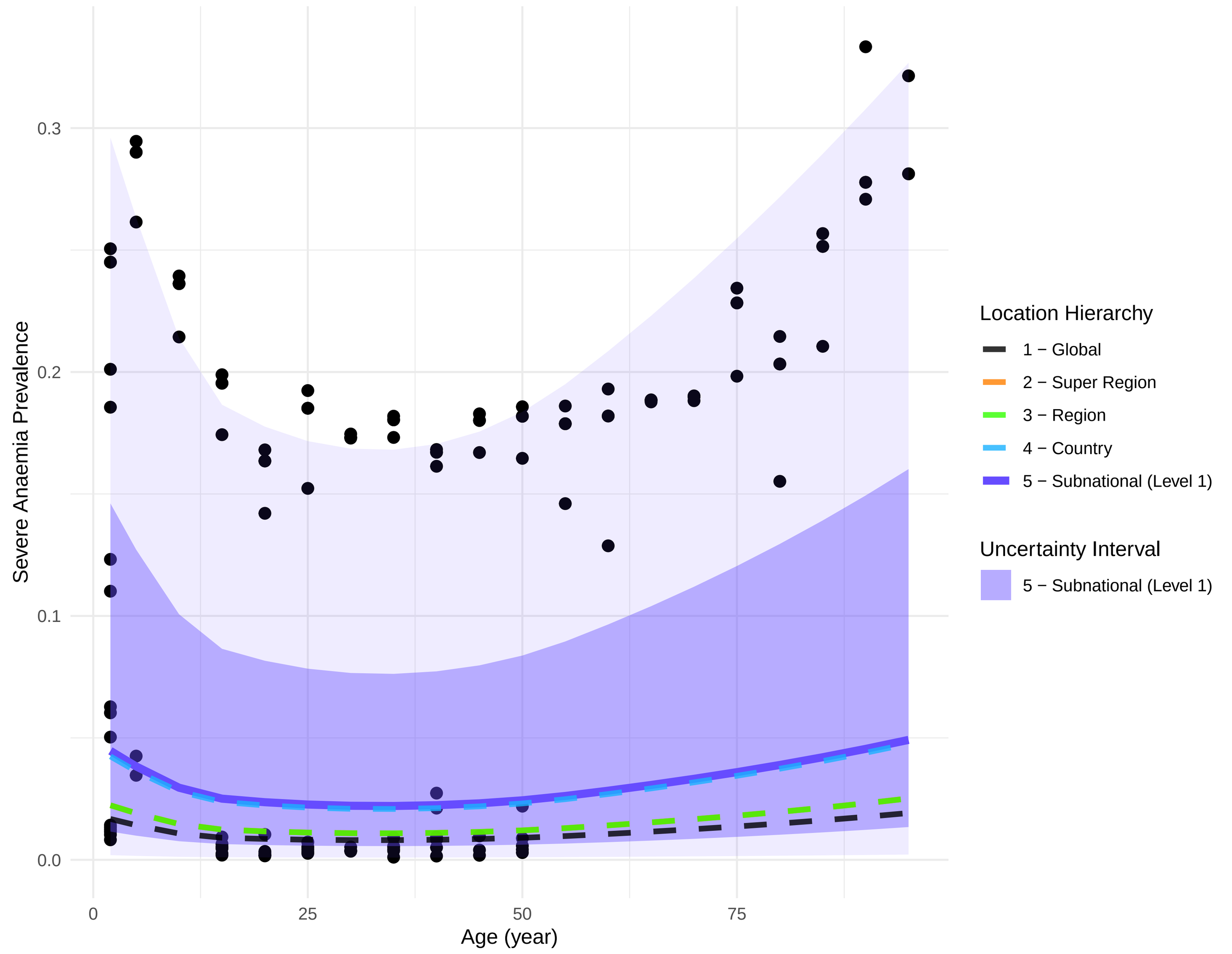

MR−BRT Age/Sex Fits − Males 2+ Years Old − Severe Anaemia Prevalence − Rajasthan, Rural
WHO−2001 Adjustment Method

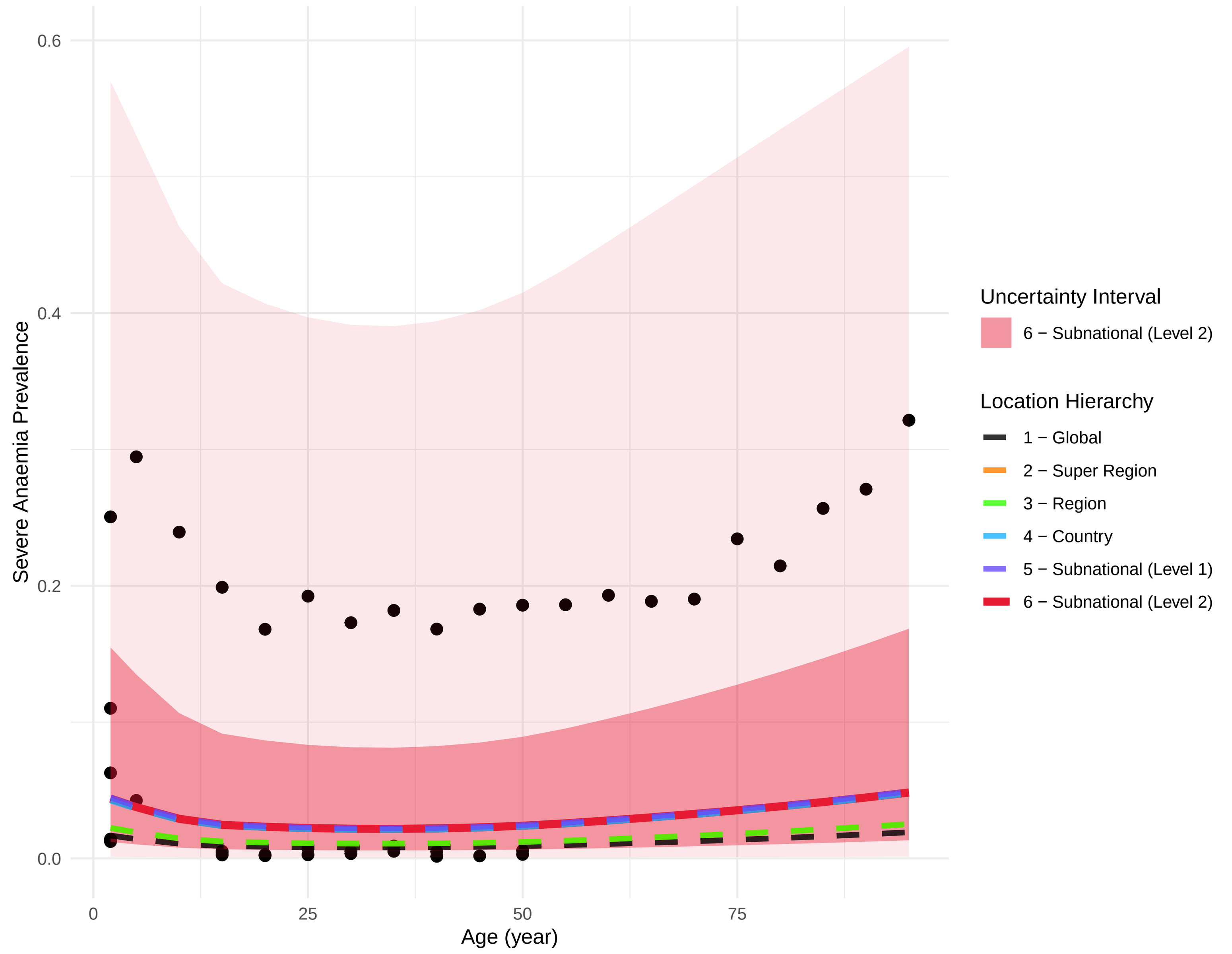

MR−BRT Age/Sex Fits − Males 2+ Years Old − Severe Anaemia Prevalence − Rajasthan, Urban
WHO−2001 Adjustment Method

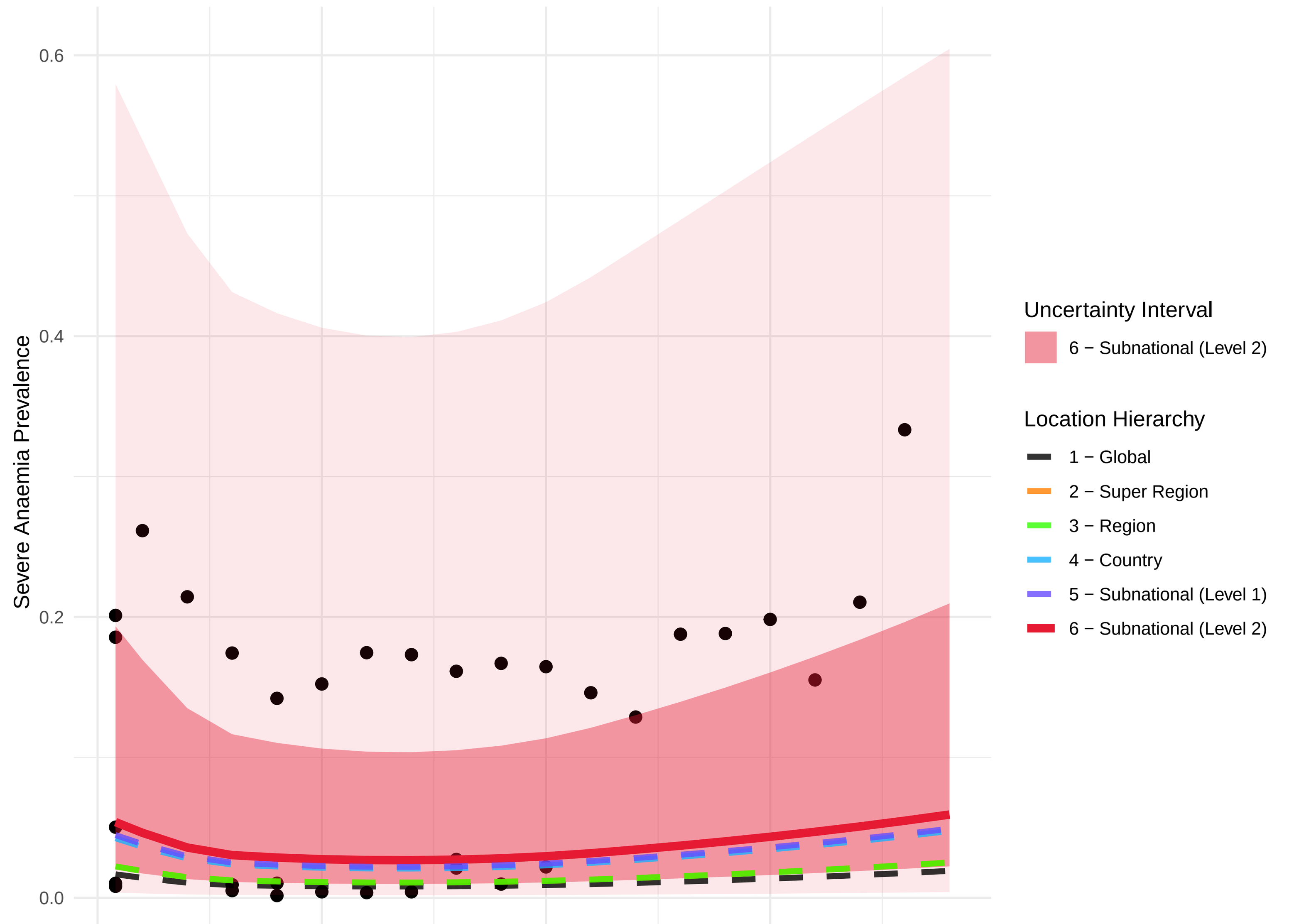

MR−BRT Age/Sex Fits − Males 2+ Years Old − Severe Anaemia Prevalence − Sikkim
WHO−2001 Adjustment Method

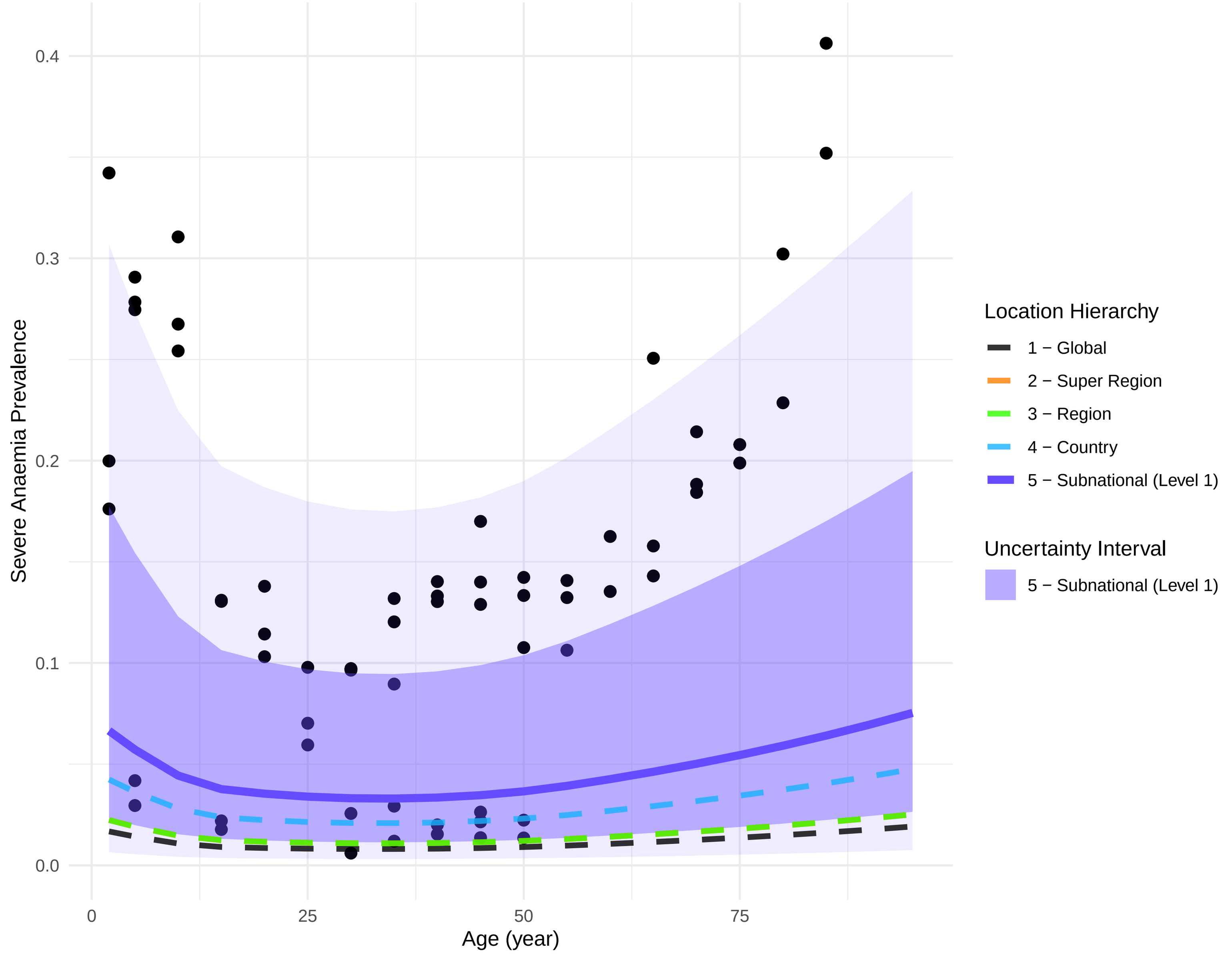

MR−BRT Age/Sex Fits − Males 2+ Years Old − Severe Anaemia Prevalence − Sikkim, Rural
WHO−2001 Adjustment Method

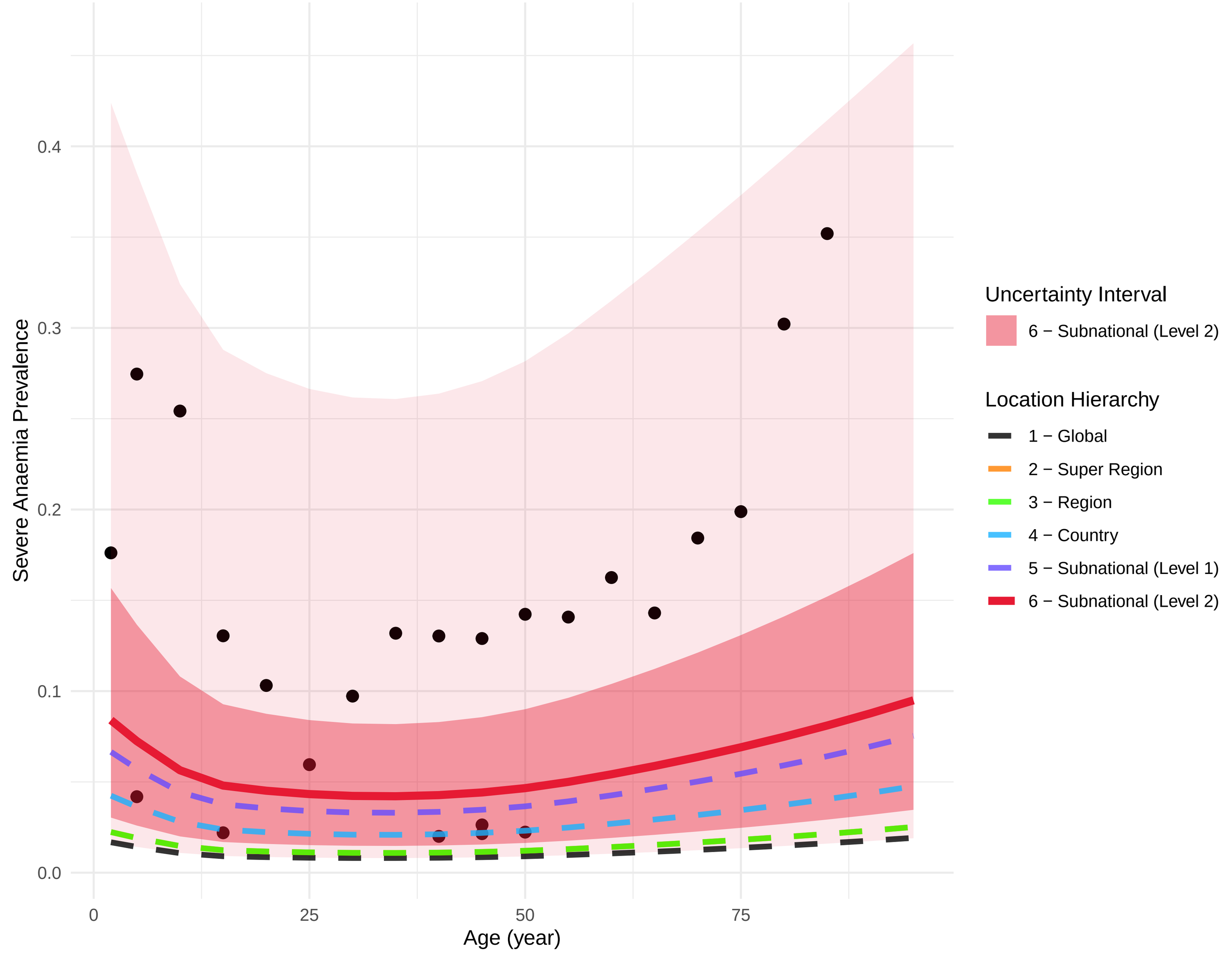

MR−BRT Age/Sex Fits − Males 2+ Years Old − Severe Anaemia Prevalence − Sikkim, Urban
WHO−2001 Adjustment Method

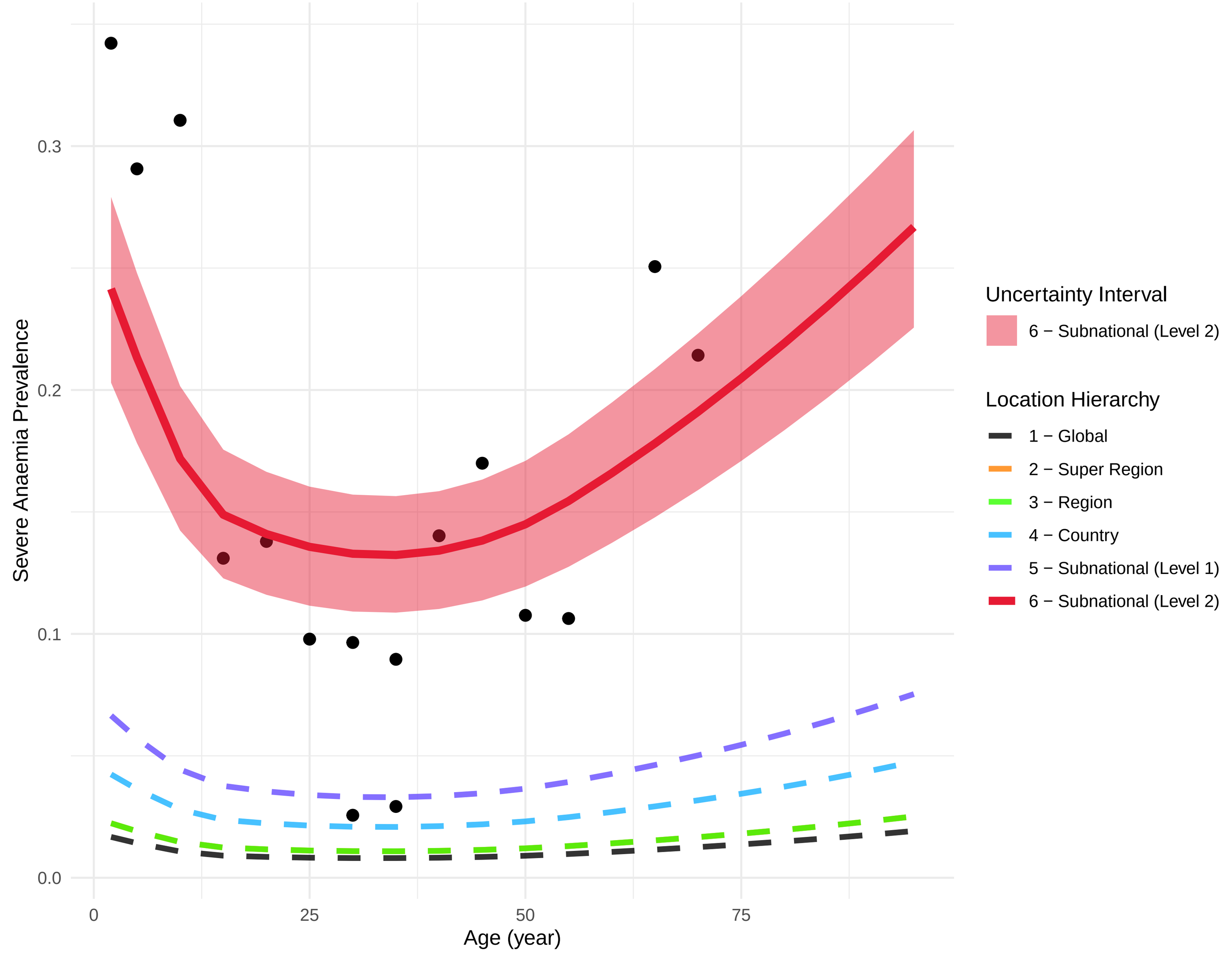

MR−BRT Age/Sex Fits − Males 2+ Years Old − Severe Anaemia Prevalence − Tamil Nadu
WHO−2001 Adjustment Method

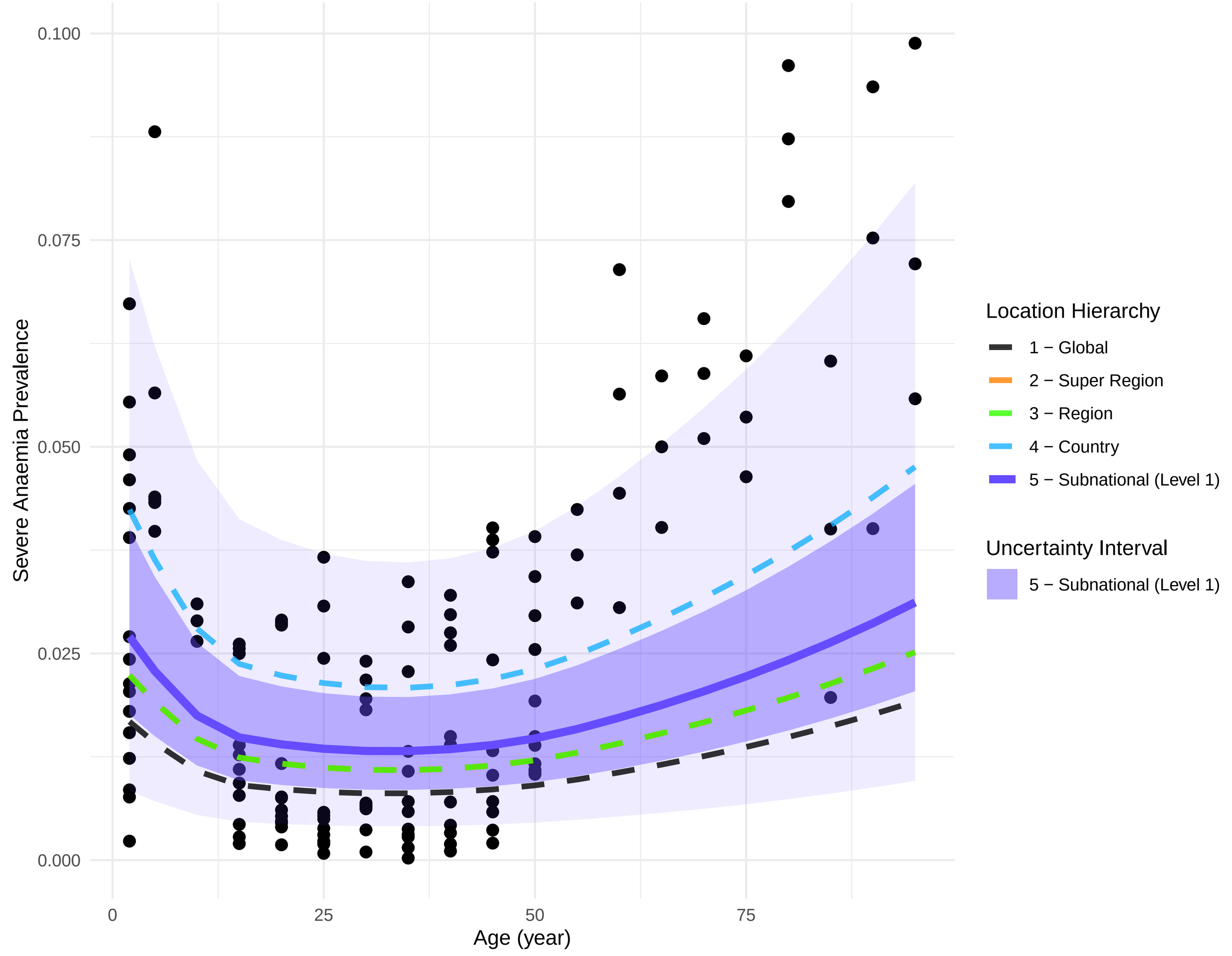

MR−BRT Age/Sex Fits − Males 2+ Years Old − Severe Anaemia Prevalence − Tamil Nadu, Rural
WHO−2001 Adjustment Method

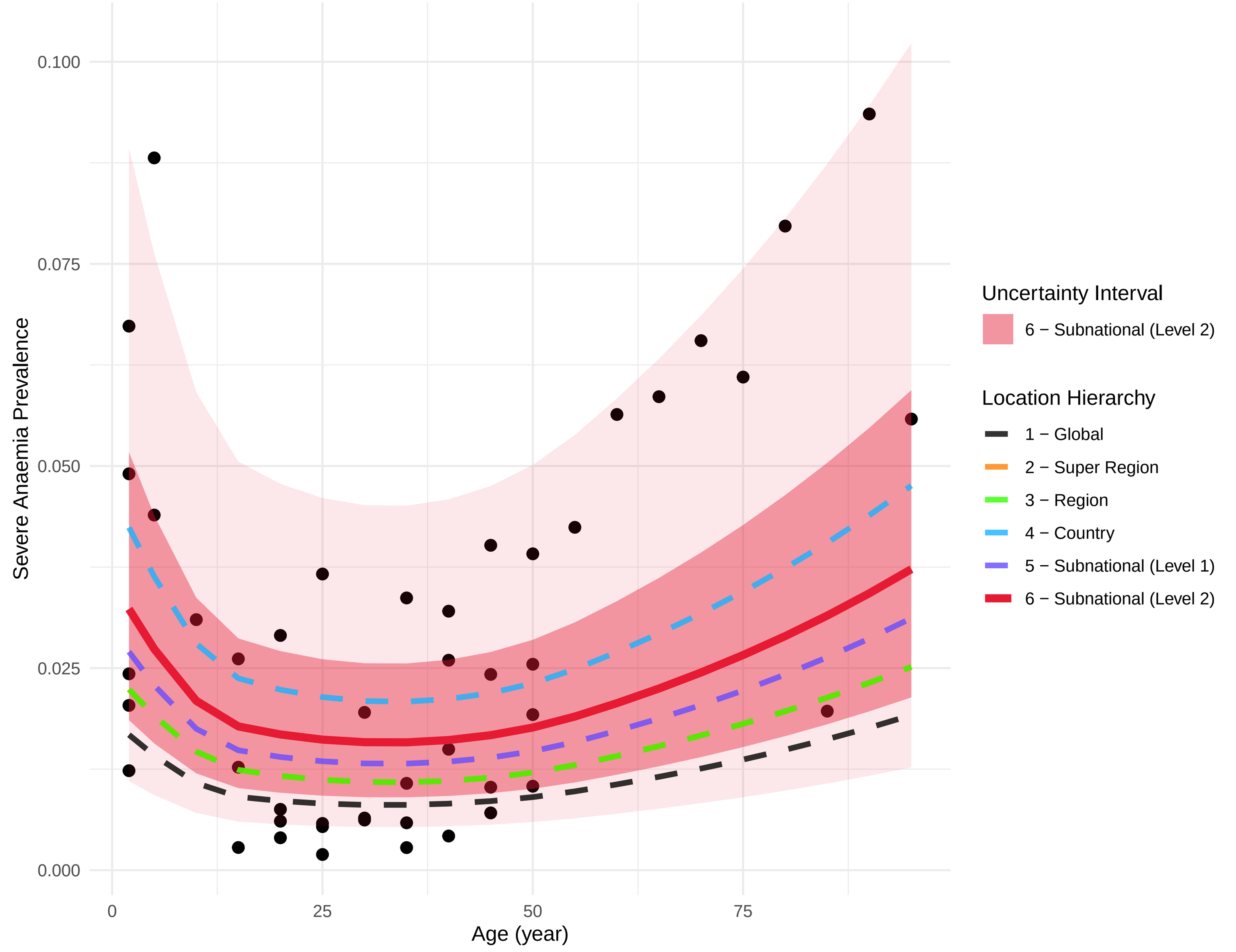

MR−BRT Age/Sex Fits − Males 2+ Years Old − Severe Anaemia Prevalence − Tamil Nadu, Urban
WHO−2001 Adjustment Method

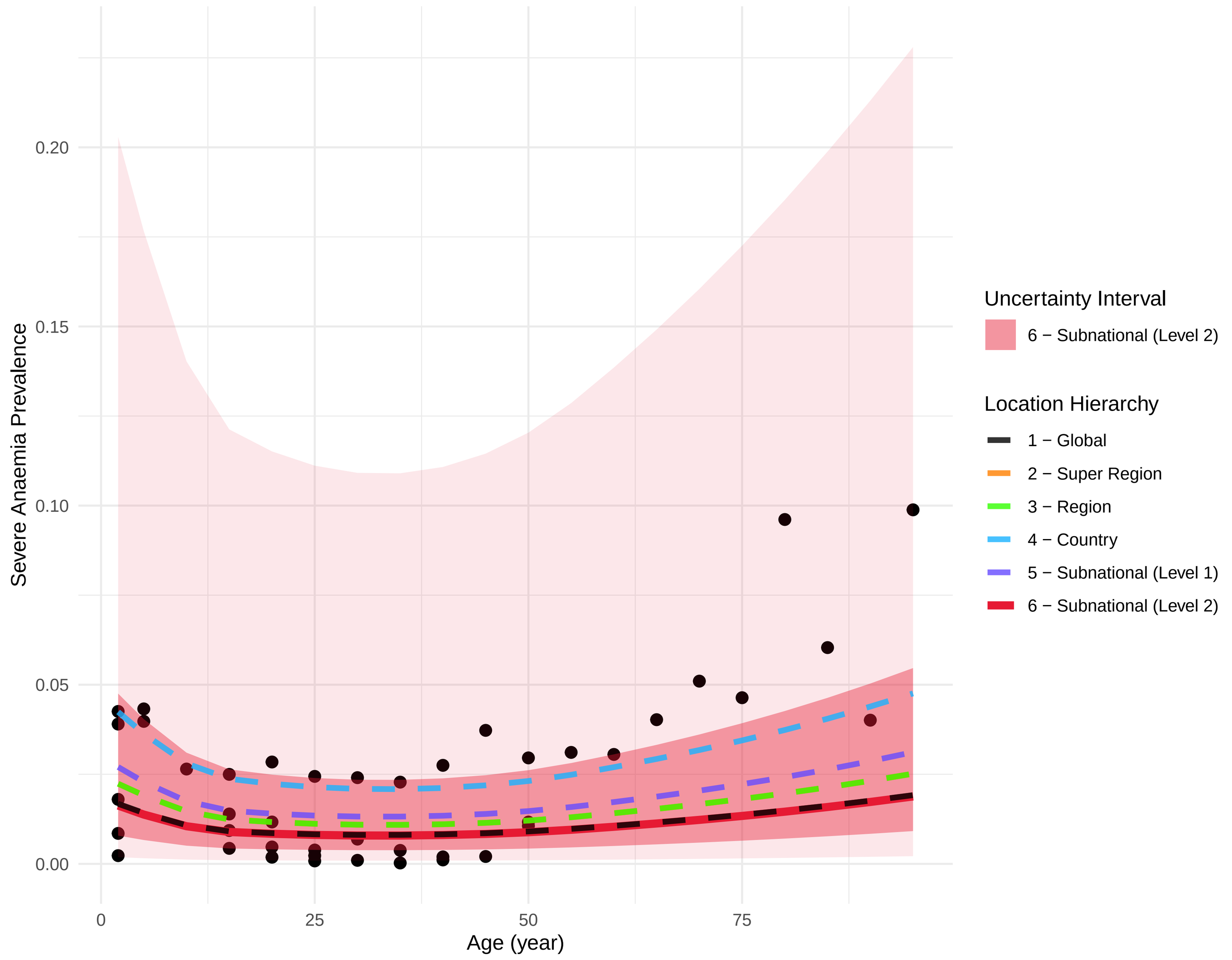

MR−BRT Age/Sex Fits − Males 2+ Years Old − Severe Anaemia Prevalence − Telangana
WHO−2001 Adjustment Method

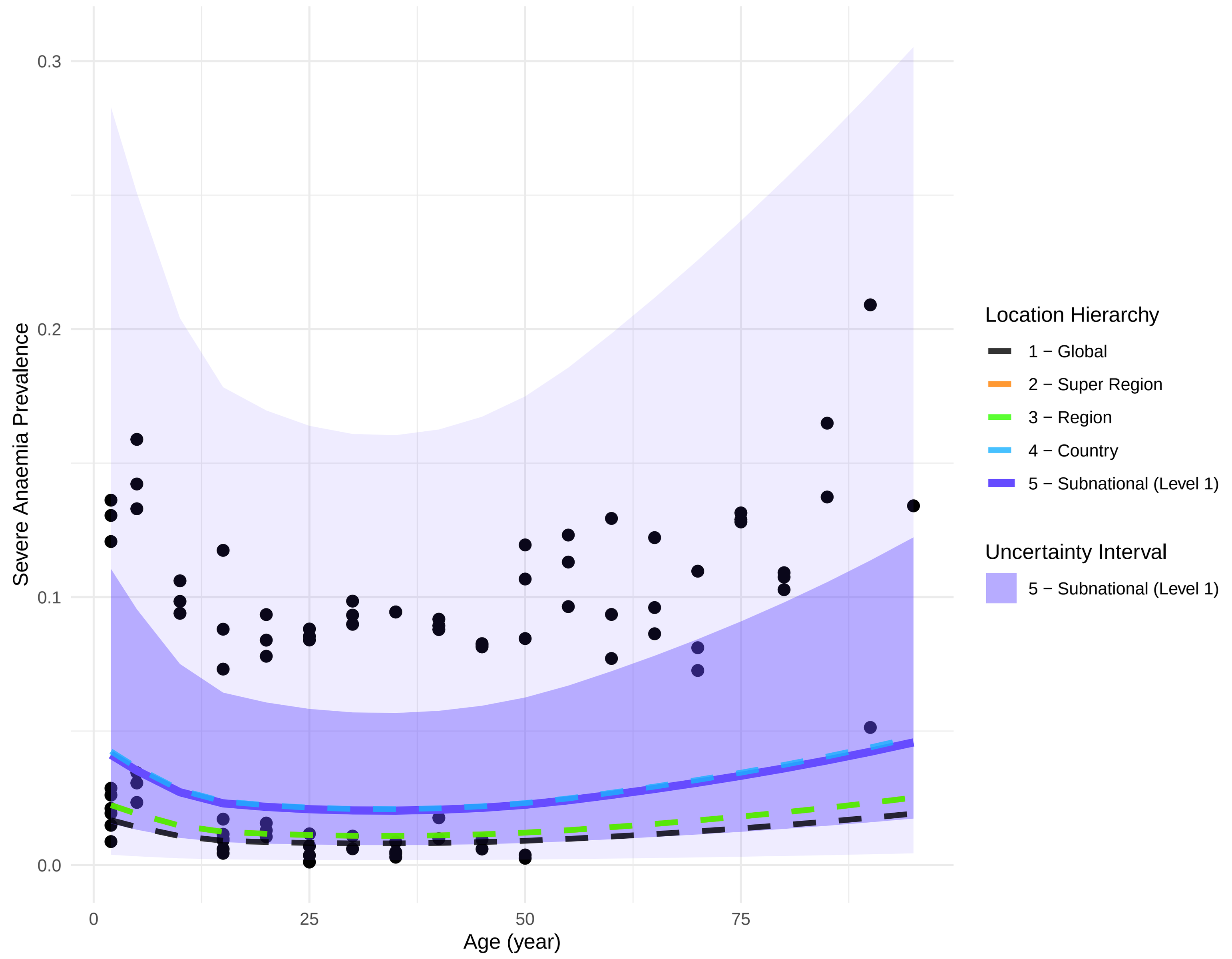

MR−BRT Age/Sex Fits − Males 2+ Years Old − Severe Anaemia Prevalence − Telangana, Rural
WHO−2001 Adjustment Method

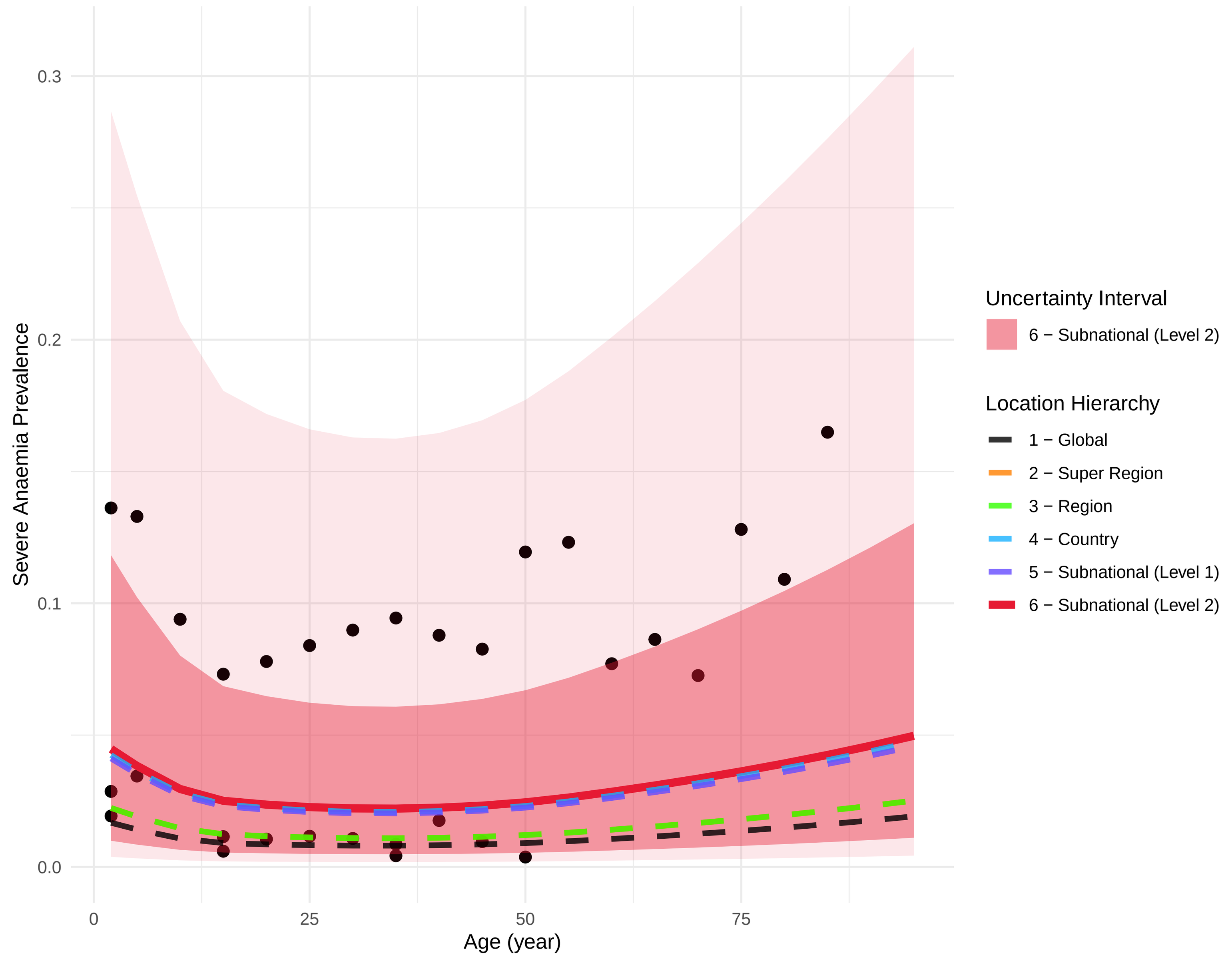

MR−BRT Age/Sex Fits − Males 2+ Years Old − Severe Anaemia Prevalence − Telangana, Urban
WHO−2001 Adjustment Method

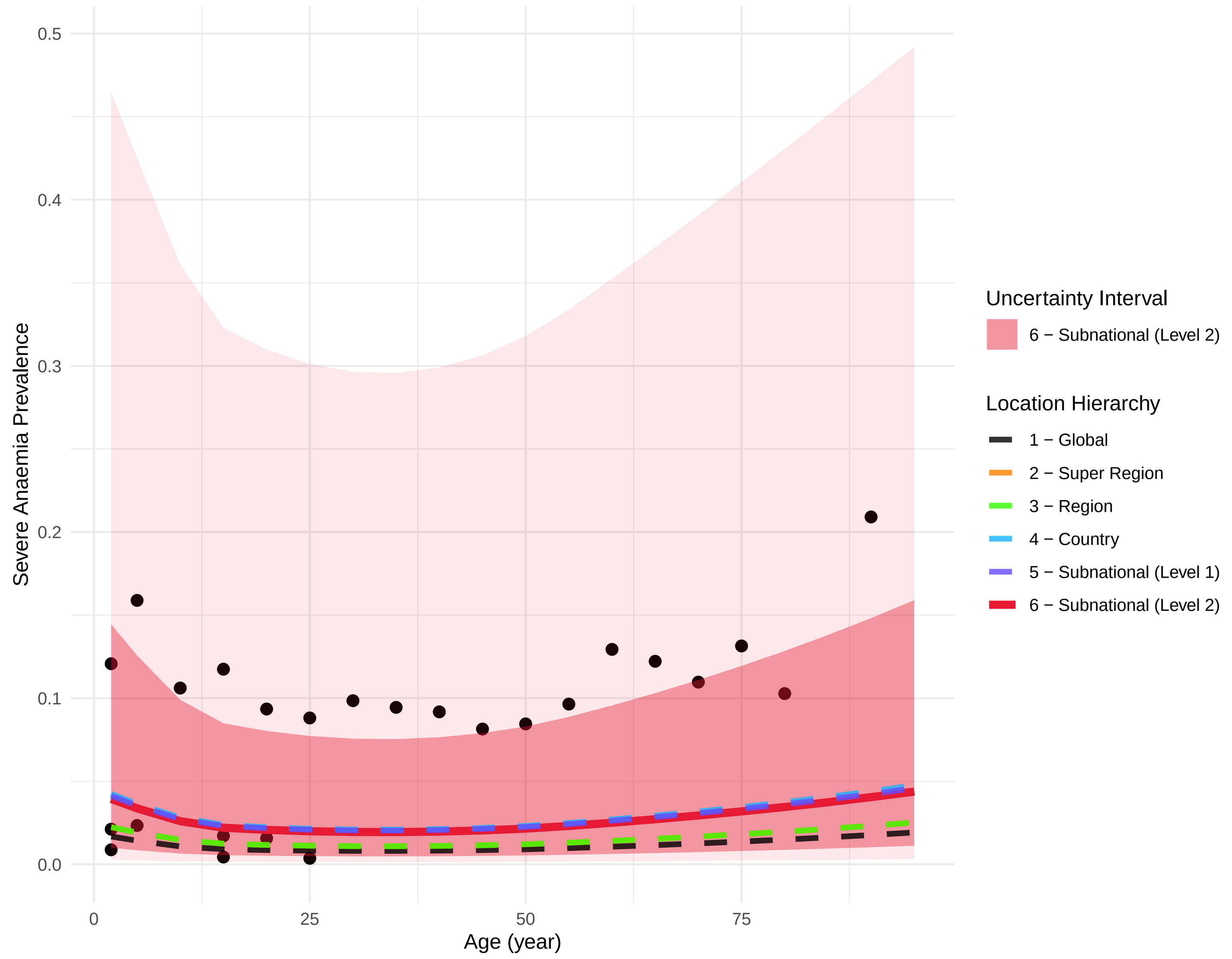

MR−BRT Age/Sex Fits − Males 2+ Years Old − Severe Anaemia Prevalence − Tripura
WHO−2001 Adjustment Method

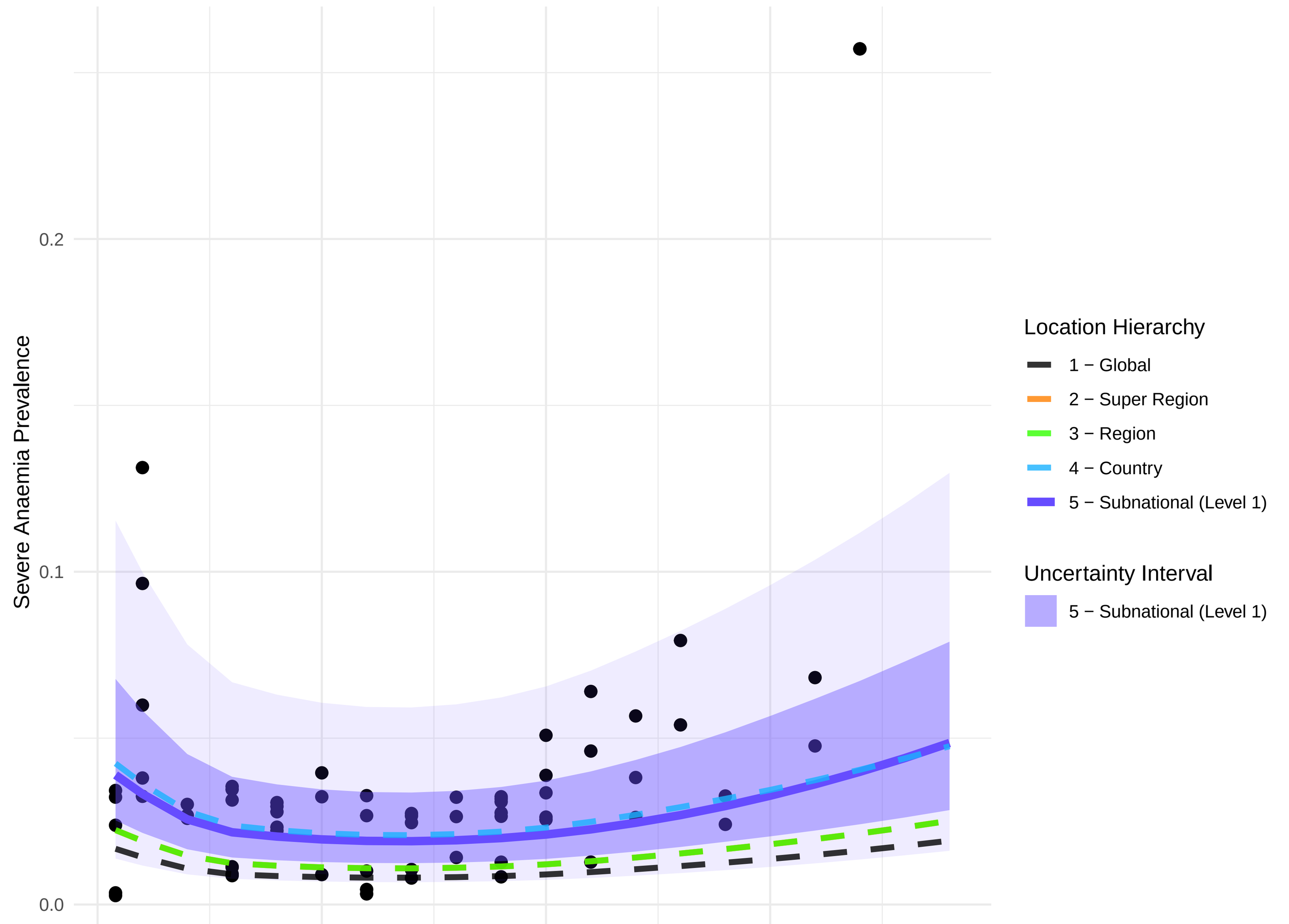

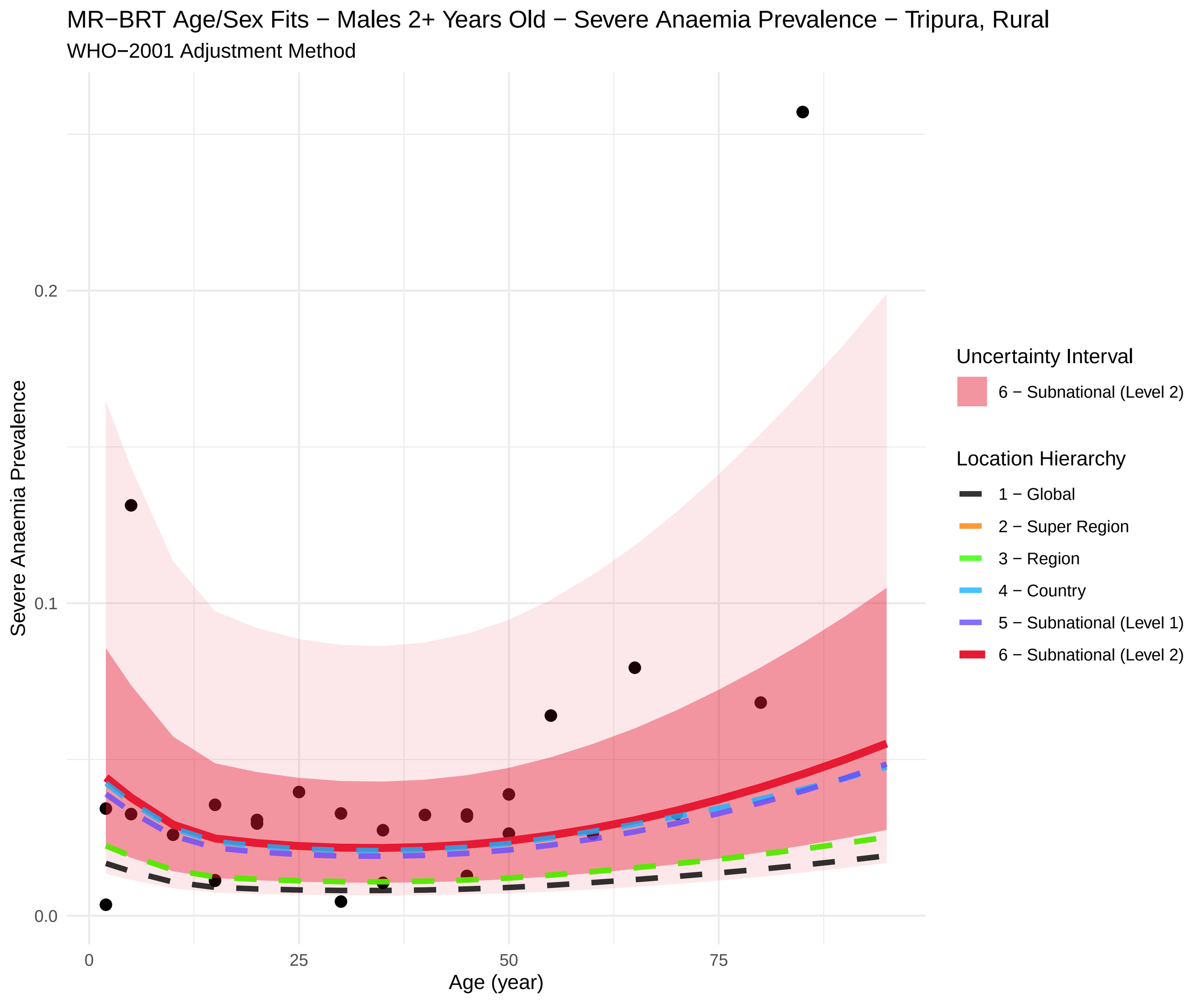
MR−BRT Age/Sex Fits − Males 2+ Years Old − Severe Anaemia Prevalence − Tripura, Rural
WHO−2001 Adjustment Method
Severe Anaemia Prevalence
Age (year)
Uncertainty Interval
6 − Subnational (Level 2)
Location Hierarchy
1 − Global
2 − Super Region
3 − Region
4 − Country
5 − Subnational (Level 1)
6 − Subnational (Level 2)

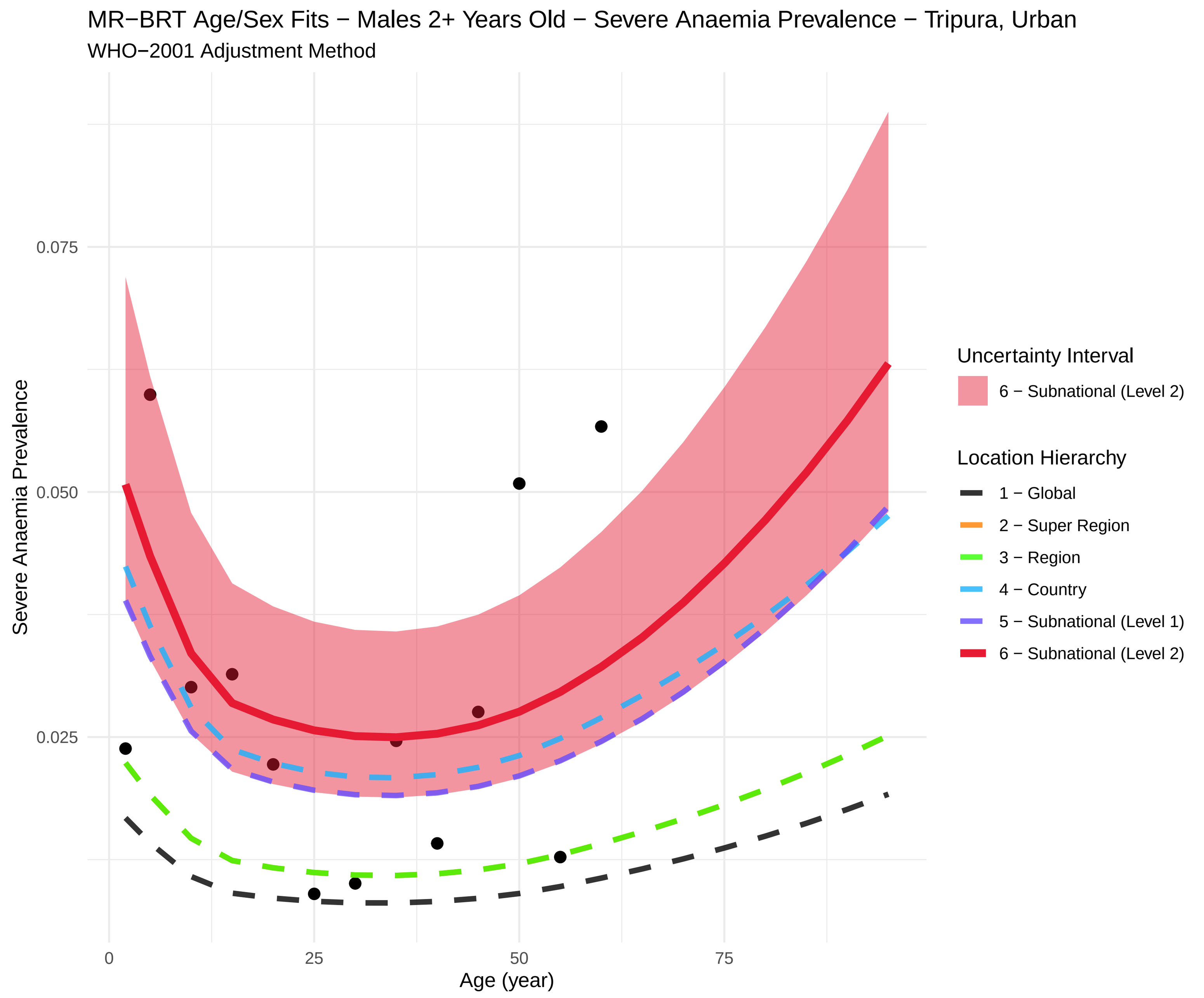
MR−BRT Age/Sex Fits − Males 2+ Years Old − Severe Anaemia Prevalence − Tripura, Urban
WHO−2001 Adjustment Method
Severe Anaemia Prevalence
Age (year)
Uncertainty Interval
6 − Subnational (Level 2)
Location Hierarchy
1 − Global
2 − Super Region
3 − Region
4 − Country
5 − Subnational (Level 1)
6 − Subnational (Level 2)

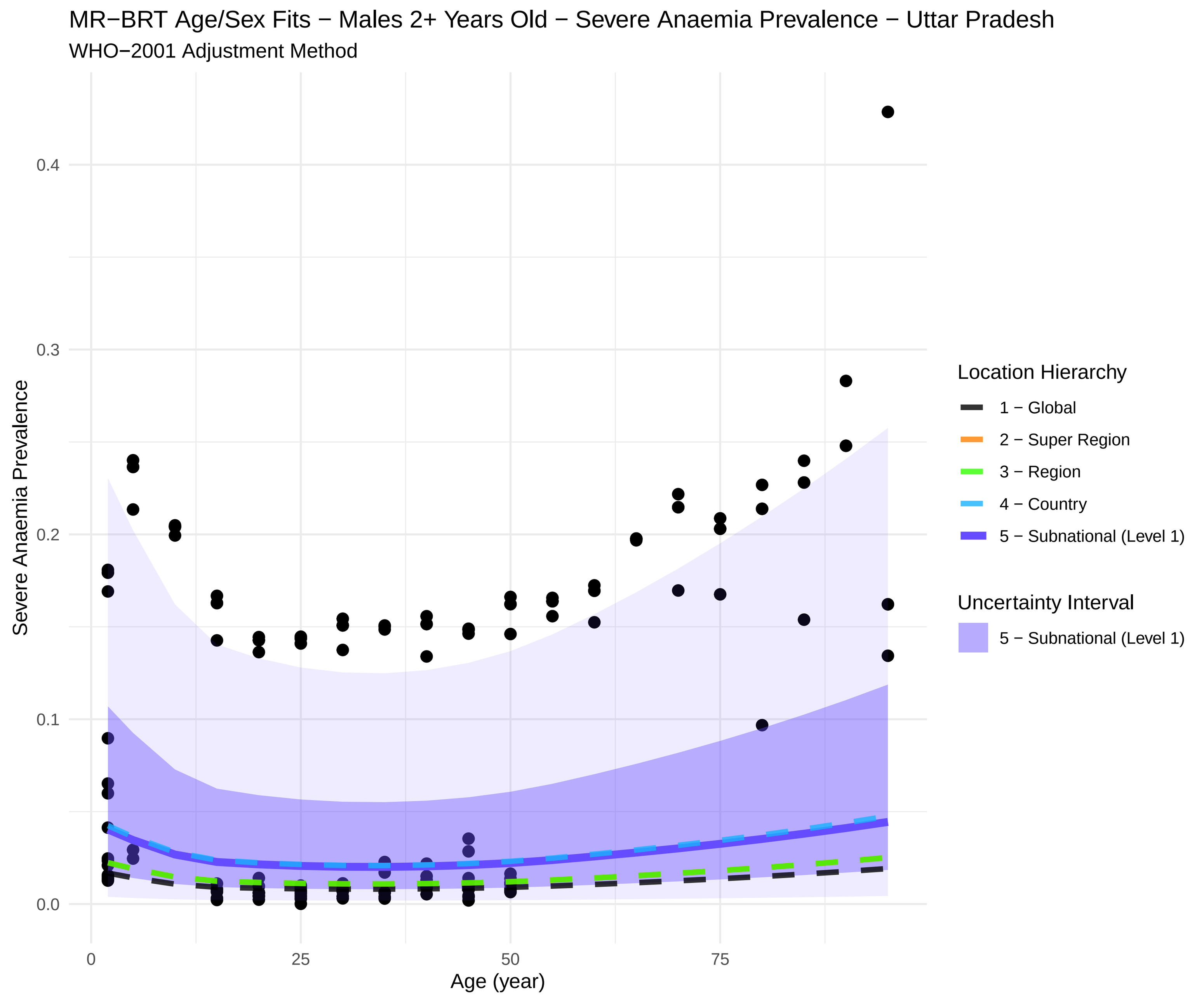
MR−BRT Age/Sex Fits − Males 2+ Years Old − Severe Anaemia Prevalence − Uttar Pradesh
WHO−2001 Adjustment Method
Severe Anaemia Prevalence
Age (year)
Location Hierarchy
1 − Global
2 − Super Region
3 − Region
4 − Country
5 − Subnational (Level 1)
Uncertainty Interval
5 − Subnational (Level 1)

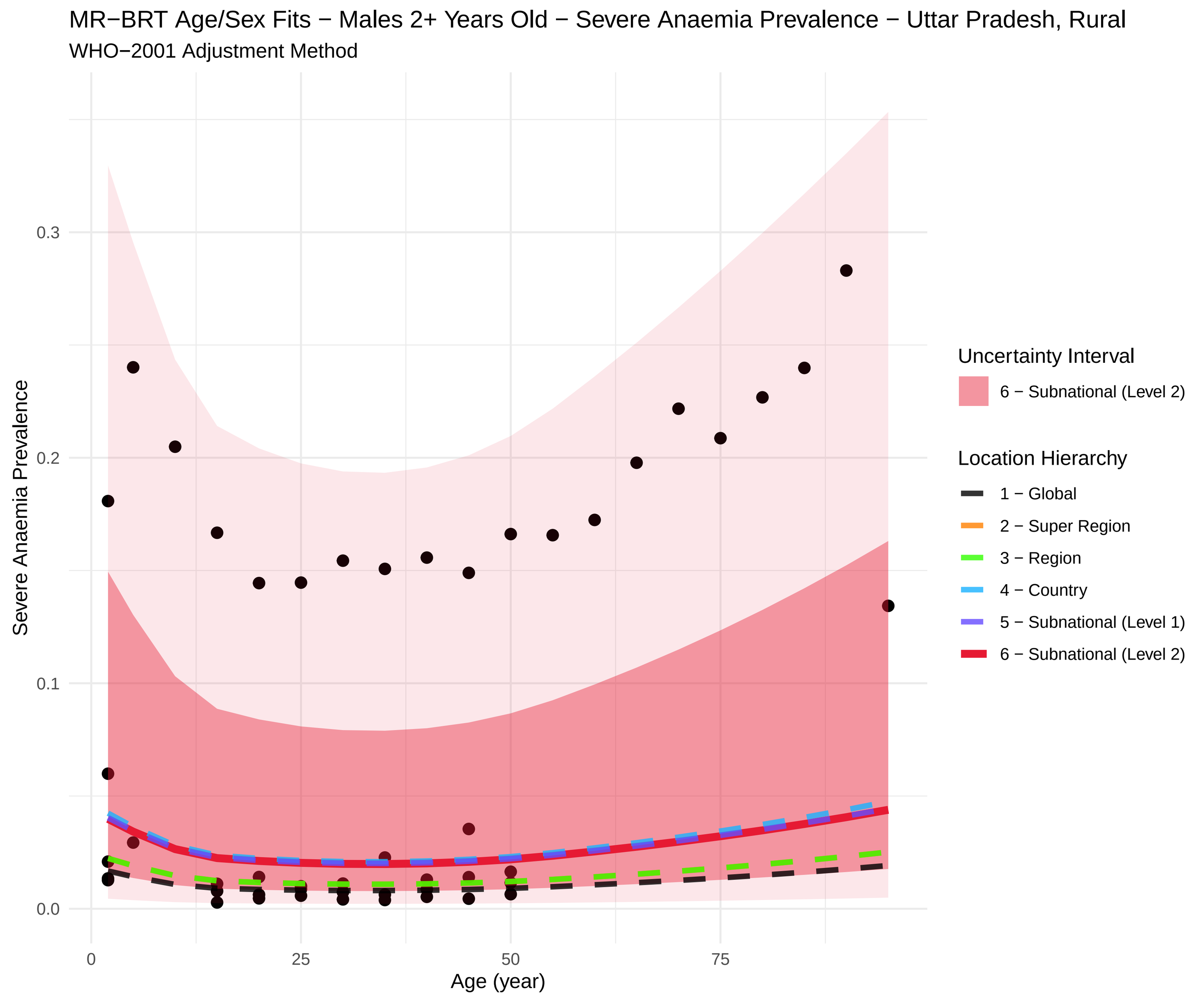
MR−BRT Age/Sex Fits − Males 2+ Years Old − Severe Anaemia Prevalence − Uttar Pradesh, Rural
WHO−2001 Adjustment Method
Severe Anaemia Prevalence
Age (year)
Uncertainty Interval
6 − Subnational (Level 2)
Location Hierarchy
1 − Global
2 − Super Region
3 − Region
4 − Country
5 − Subnational (Level 1)
6 − Subnational (Level 2)

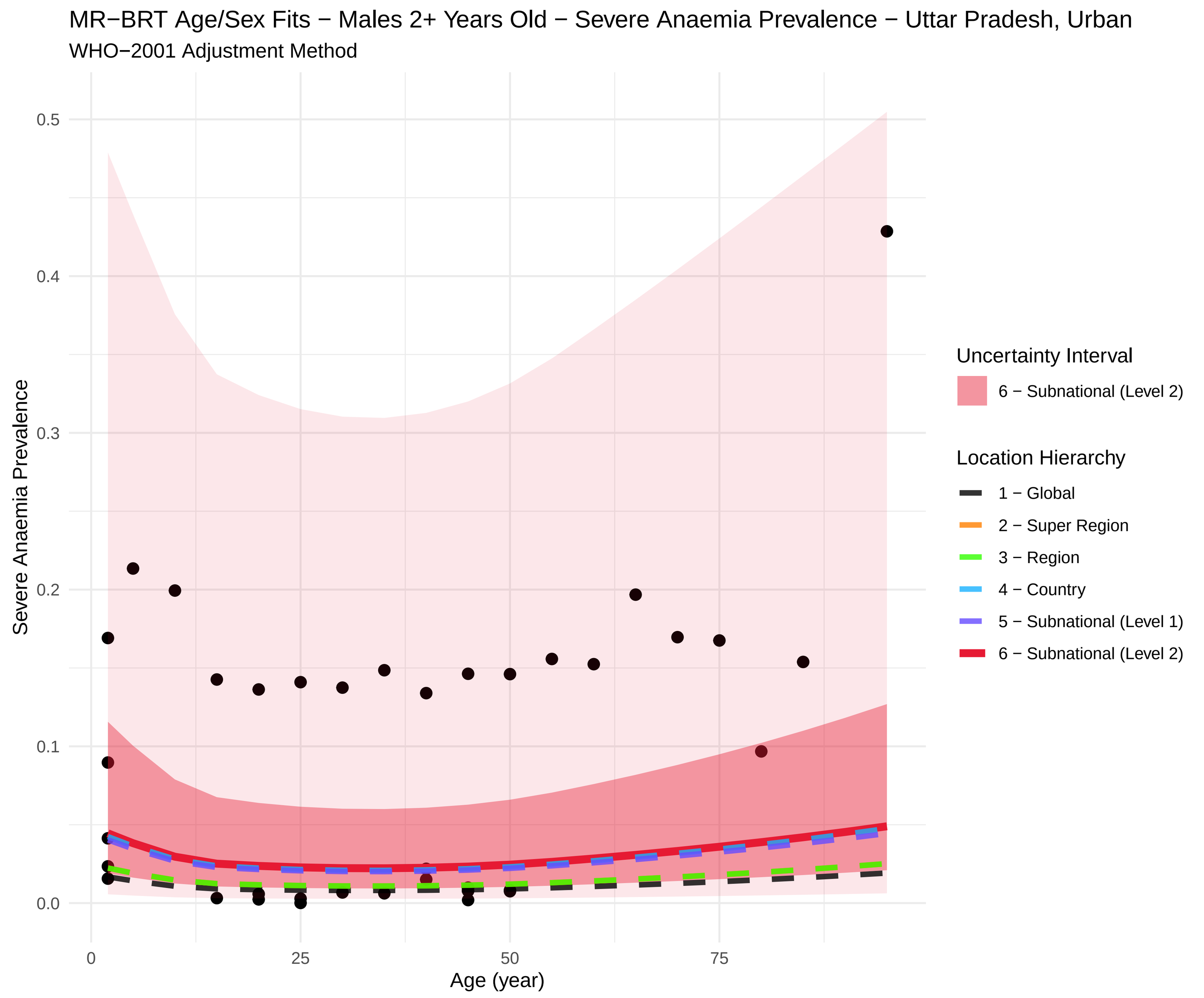
MR−BRT Age/Sex Fits − Males 2+ Years Old − Severe Anaemia Prevalence − Uttar Pradesh, Urban
WHO−2001 Adjustment Method
Severe Anaemia Prevalence
Age (year)
Uncertainty Interval
6 − Subnational (Level 2)
Location Hierarchy
1 − Global
2 − Super Region
3 − Region
4 − Country
5 − Subnational (Level 1)
6 − Subnational (Level 2)

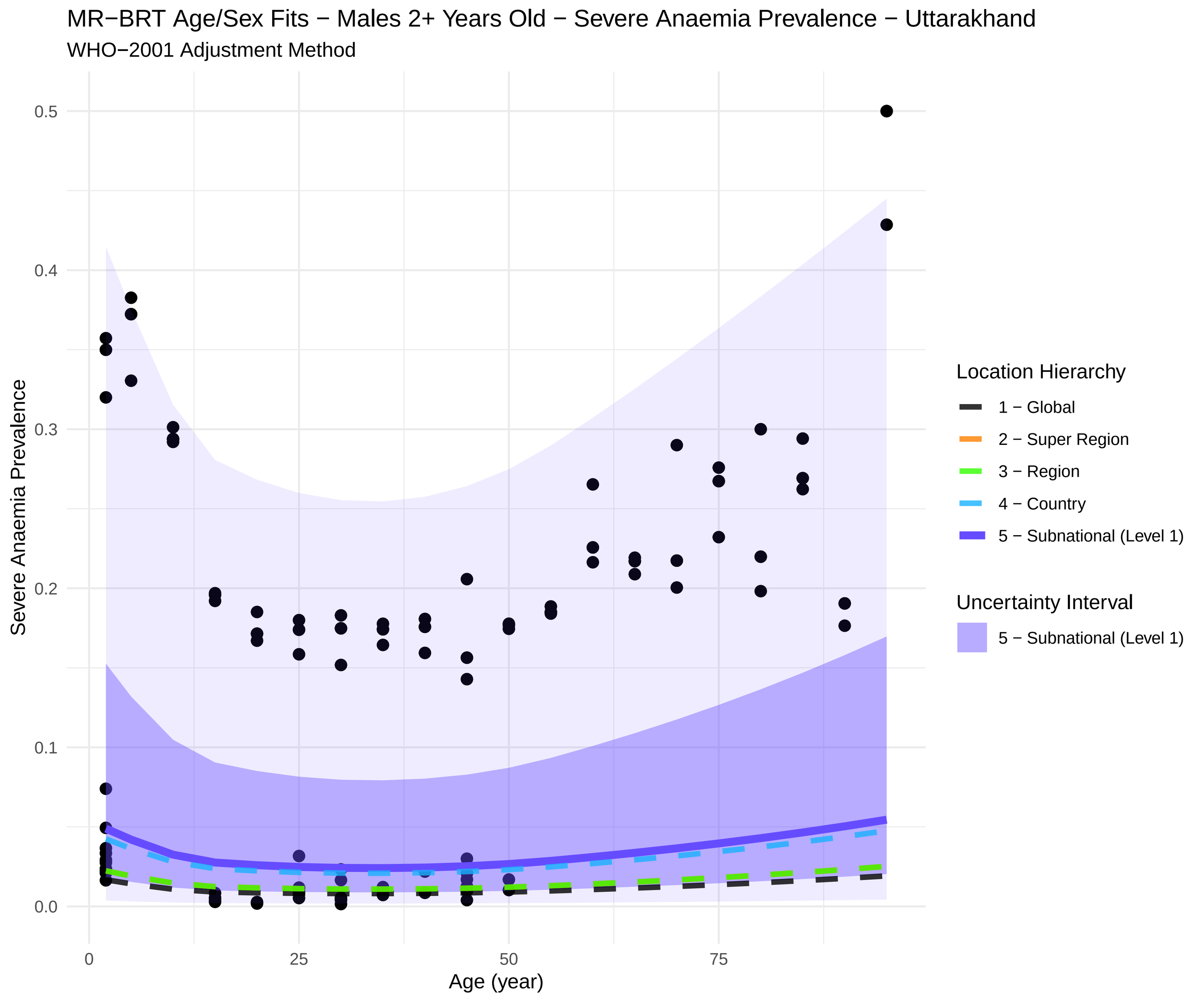
MR−BRT Age/Sex Fits − Males 2+ Years Old − Severe Anaemia Prevalence − Uttarakhand
WHO−2001 Adjustment Method
Severe Anaemia Prevalence
Age (year)
Location Hierarchy
1 − Global
2 − Super Region
3 − Region
4 − Country
5 − Subnational (Level 1)
Uncertainty Interval
5 − Subnational (Level 1)

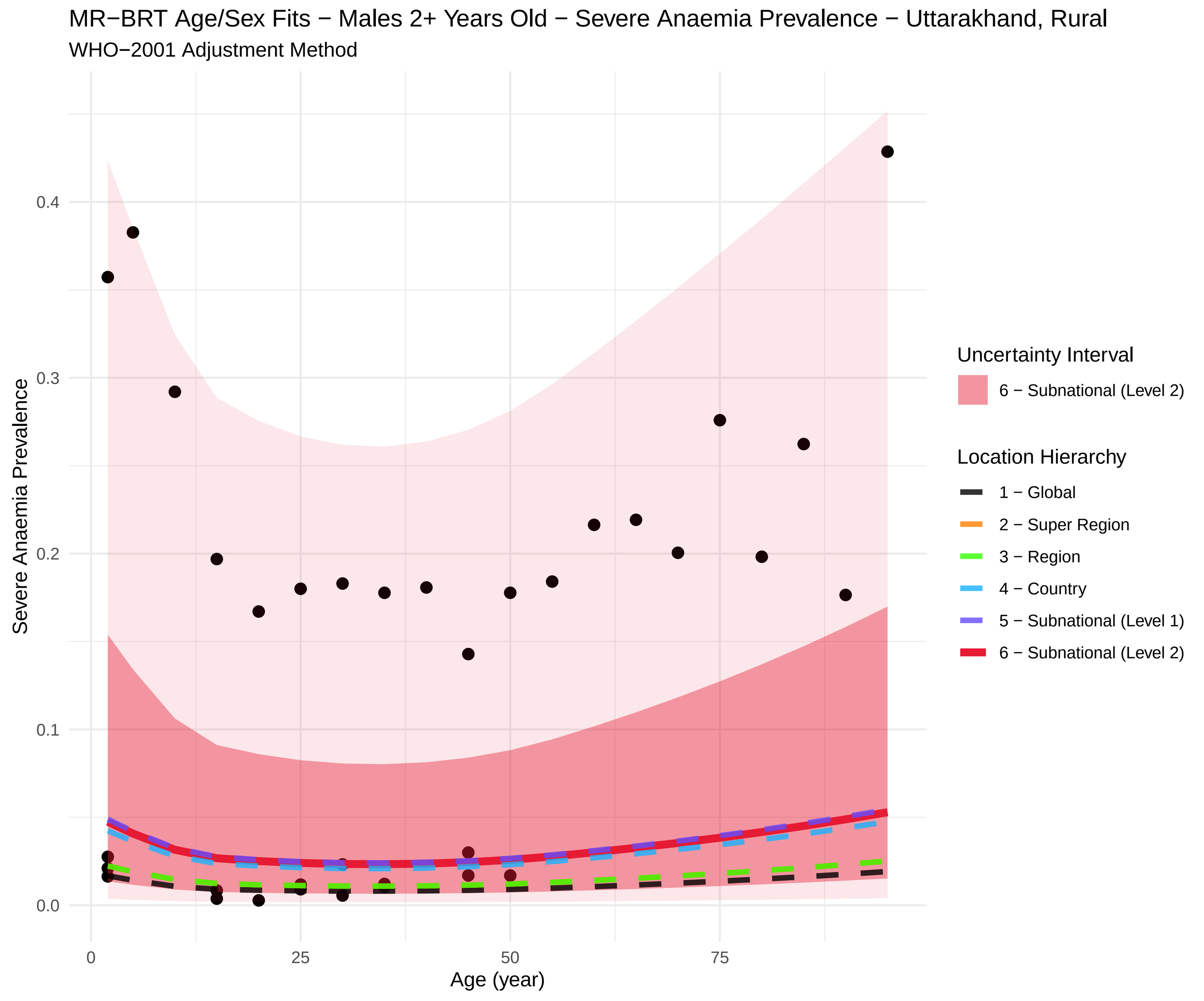
MR−BRT Age/Sex Fits − Males 2+ Years Old − Severe Anaemia Prevalence − Uttarakhand, Rural
WHO−2001 Adjustment Method
Severe Anaemia Prevalence
Age (year)
Uncertainty Interval
6 − Subnational (Level 2)
Location Hierarchy
1 − Global
2 − Super Region
3 − Region
4 − Country
5 − Subnational (Level 1)
6 − Subnational (Level 2)

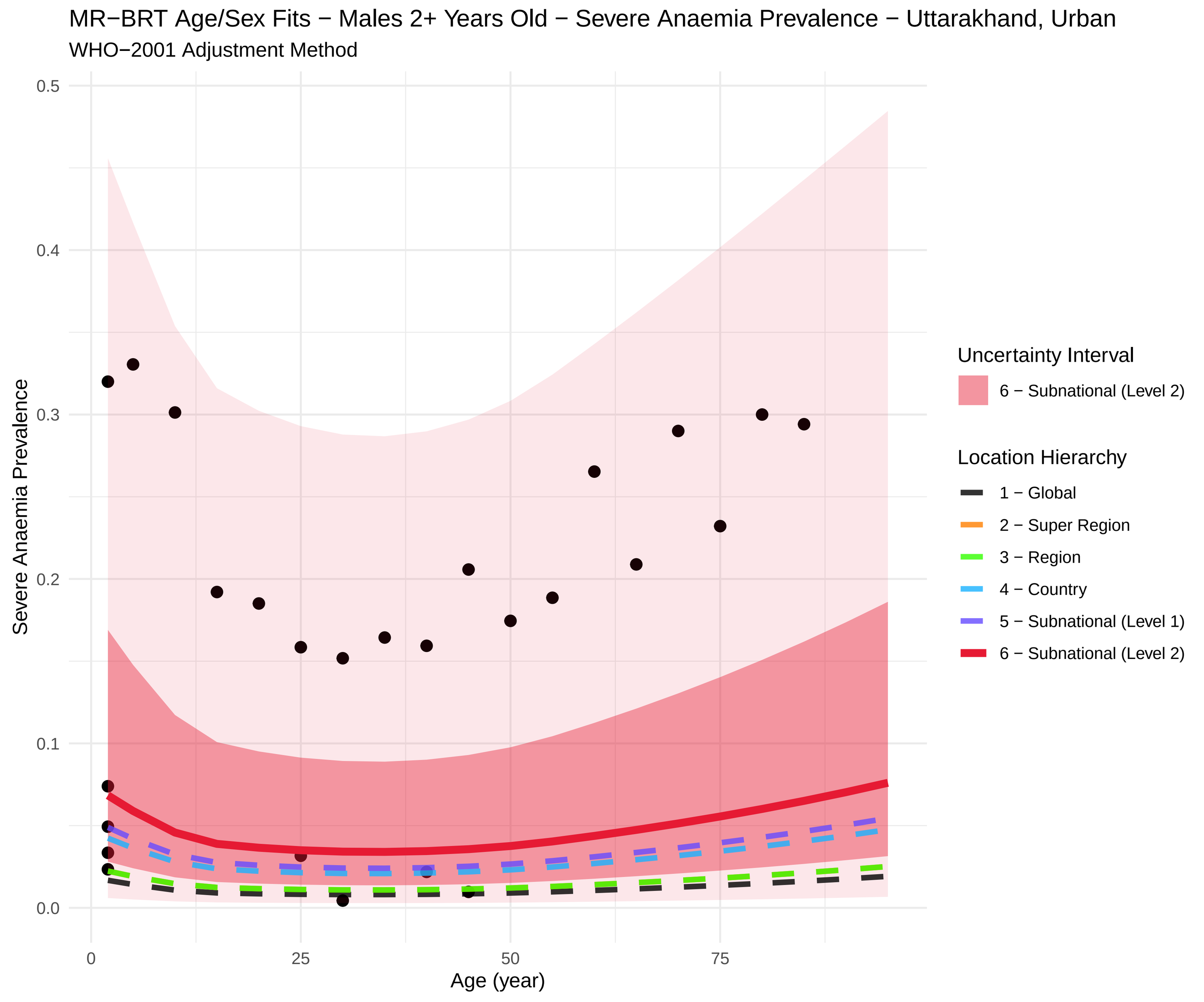
MR−BRT Age/Sex Fits − Males 2+ Years Old − Severe Anaemia Prevalence − Uttarakhand, Urban
WHO−2001 Adjustment Method
Severe Anaemia Prevalence
Age (year)
Uncertainty Interval
6 − Subnational (Level 2)
Location Hierarchy
1 − Global
2 − Super Region
3 − Region
4 − Country
5 − Subnational (Level 1)
6 − Subnational (Level 2)

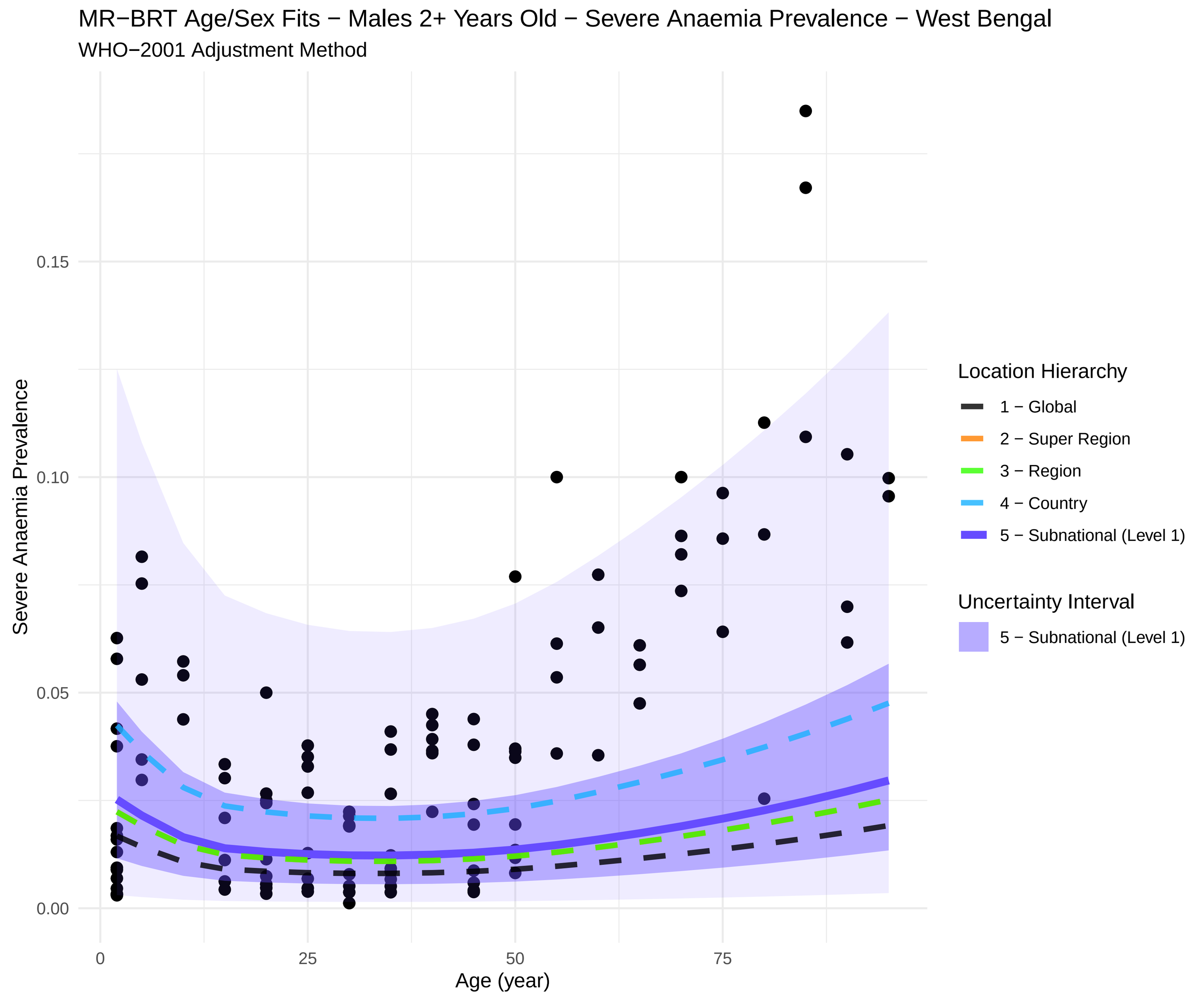
MR−BRT Age/Sex Fits − Males 2+ Years Old − Severe Anaemia Prevalence − West Bengal
WHO−2001 Adjustment Method
Severe Anaemia Prevalence
Age (year)
Location Hierarchy
1 − Global
2 − Super Region
3 − Region
4 − Country
5 − Subnational (Level 1)
Uncertainty Interval
5 − Subnational (Level 1)

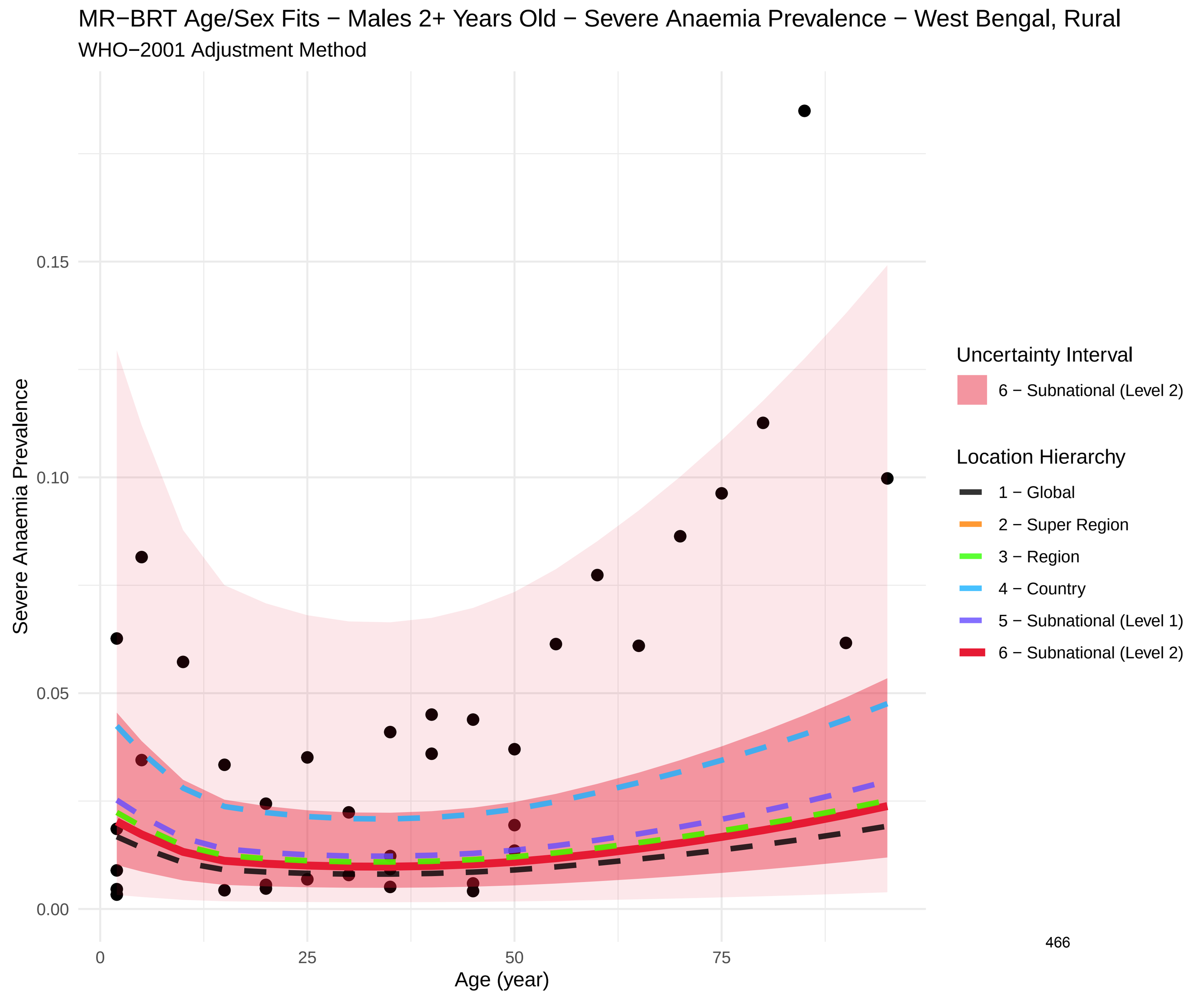
MR−BRT Age/Sex Fits − Males 2+ Years Old − Severe Anaemia Prevalence − West Bengal, Rural
WHO−2001 Adjustment Method
Severe Anaemia Prevalence
Age (year)
Uncertainty Interval
6 − Subnational (Level 2)
Location Hierarchy
1 − Global
2 − Super Region
3 − Region
4 − Country
5 − Subnational (Level 1)
6 − Subnational (Level 2)

MR−BRT Age/Sex Fits − Males 2+ Years Old − Severe Anaemia Prevalence − West Bengal, Urban
WHO−2001 Adjustment Method

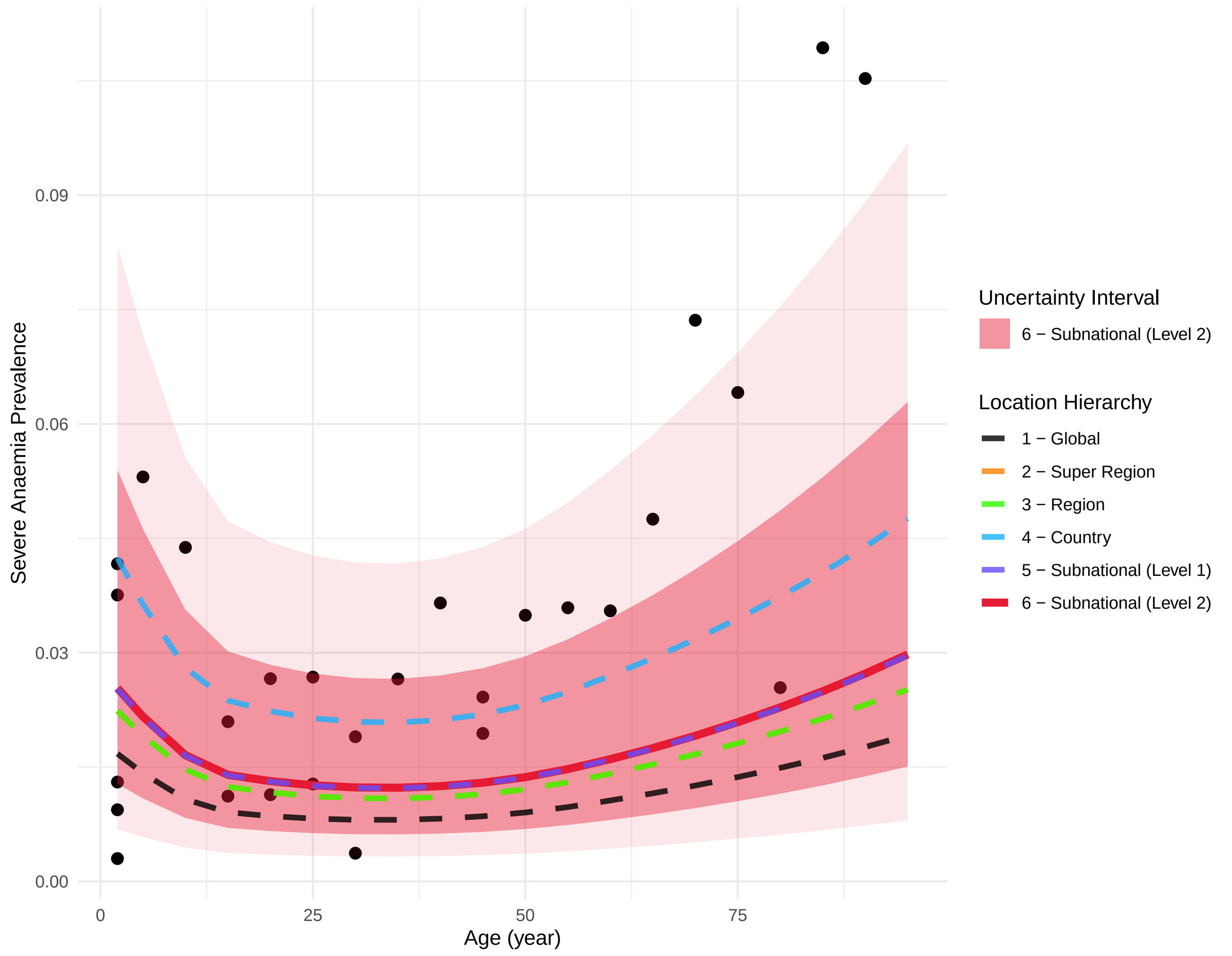

MR−BRT Age/Sex Fits − Males 2+ Years Old − Severe Anaemia Prevalence − Nepal
WHO−2001 Adjustment Method

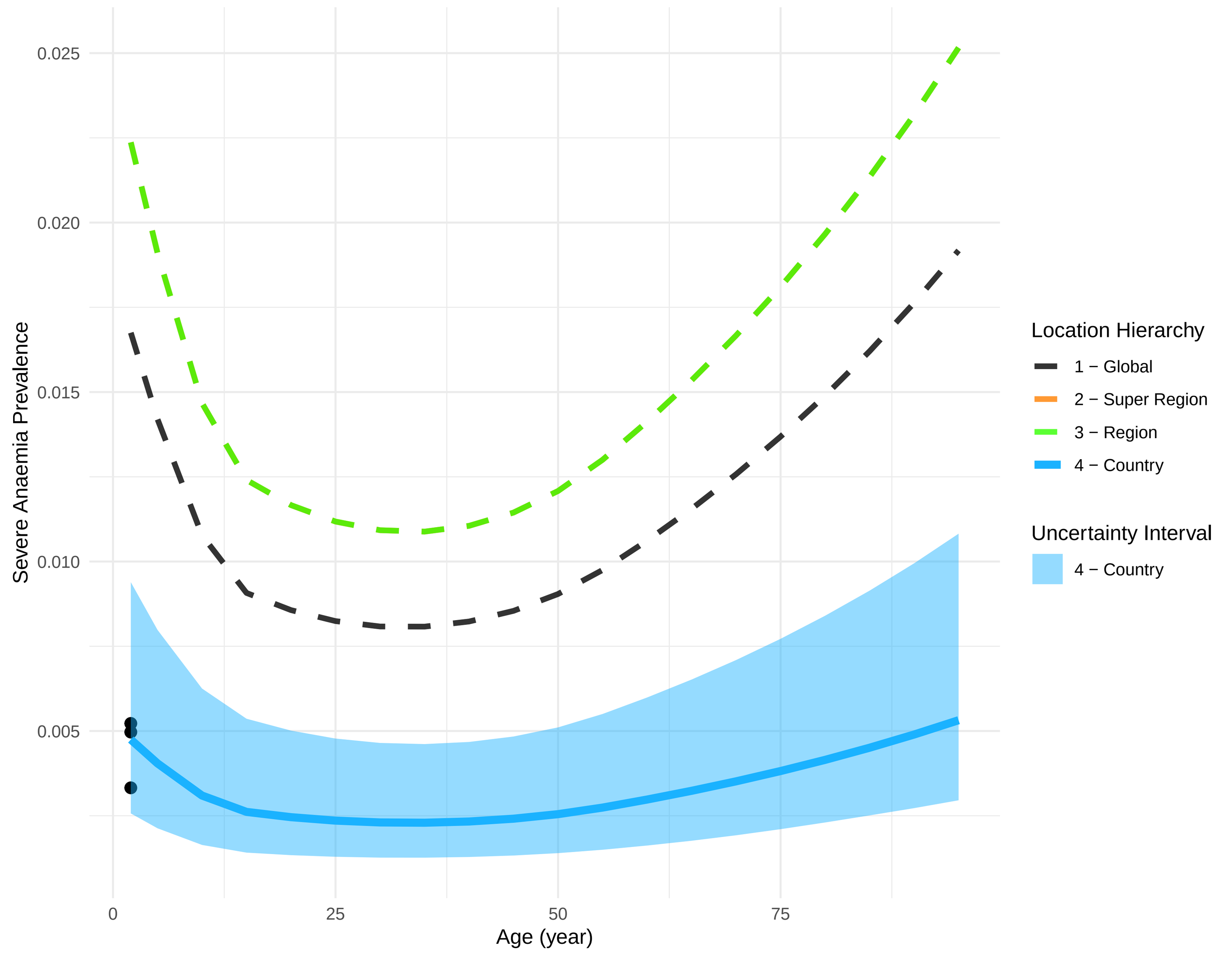

MR−BRT Age/Sex Fits − Males 2+ Years Old − Severe Anaemia Prevalence − Southeast Asia, East Asia, and Oceania
WHO−2001 Adjustment Method

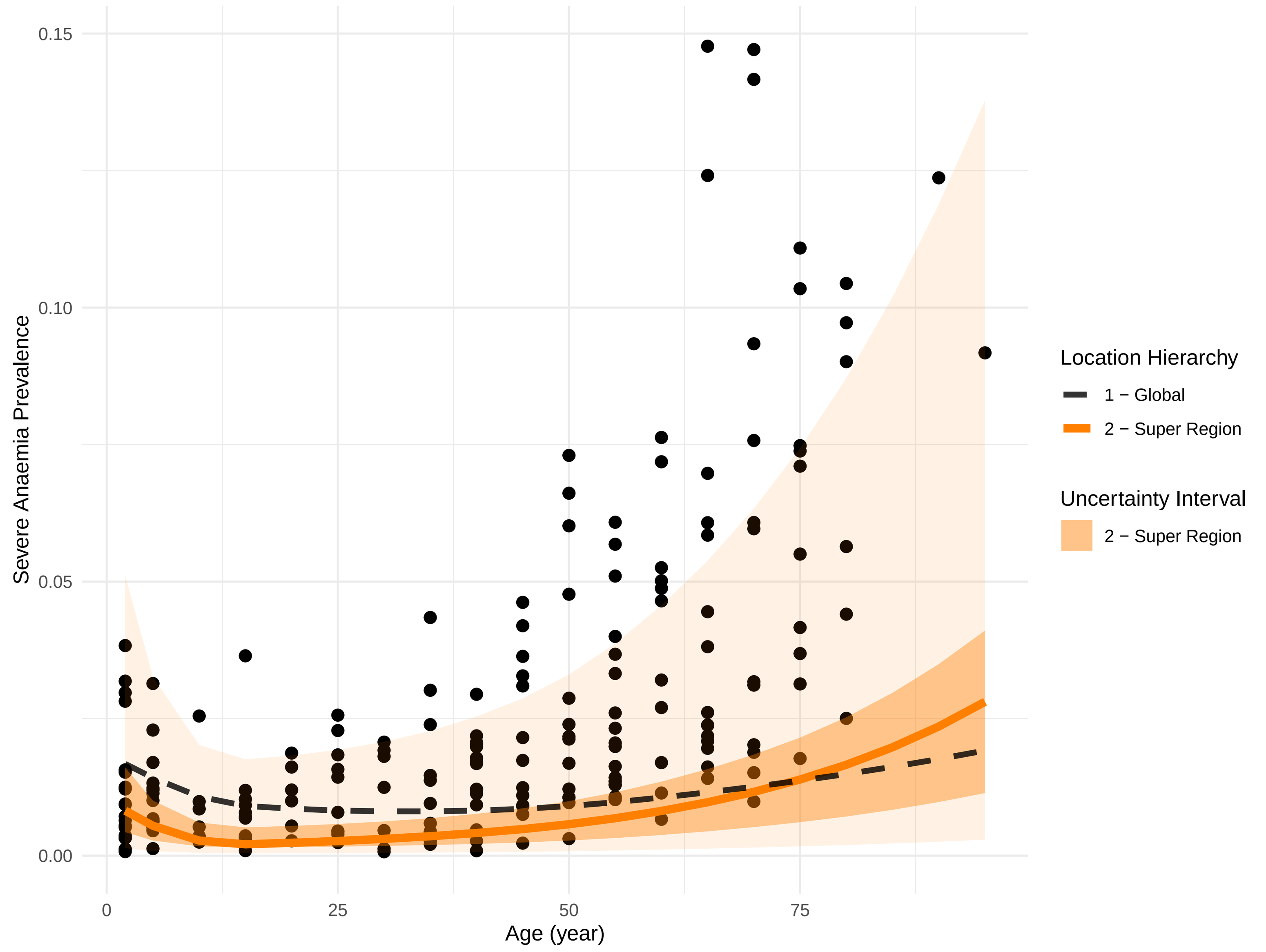

MR−BRT Age/Sex Fits − Males 2+ Years Old − Severe Anaemia Prevalence − Southeast Asia
WHO−2001 Adjustment Method

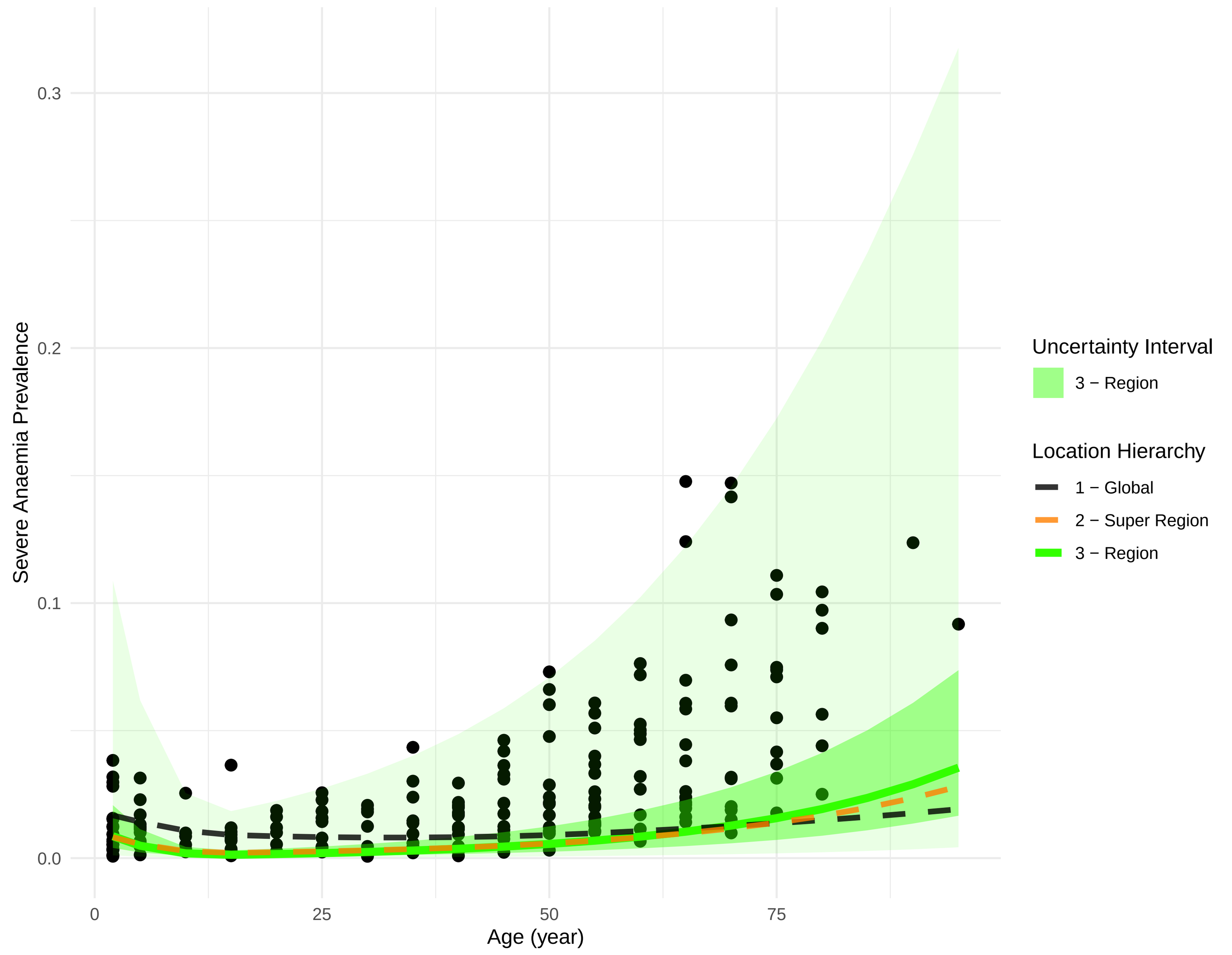

MR−BRT Age/Sex Fits − Males 2+ Years Old − Severe Anaemia Prevalence − Cambodia
WHO−2001 Adjustment Method

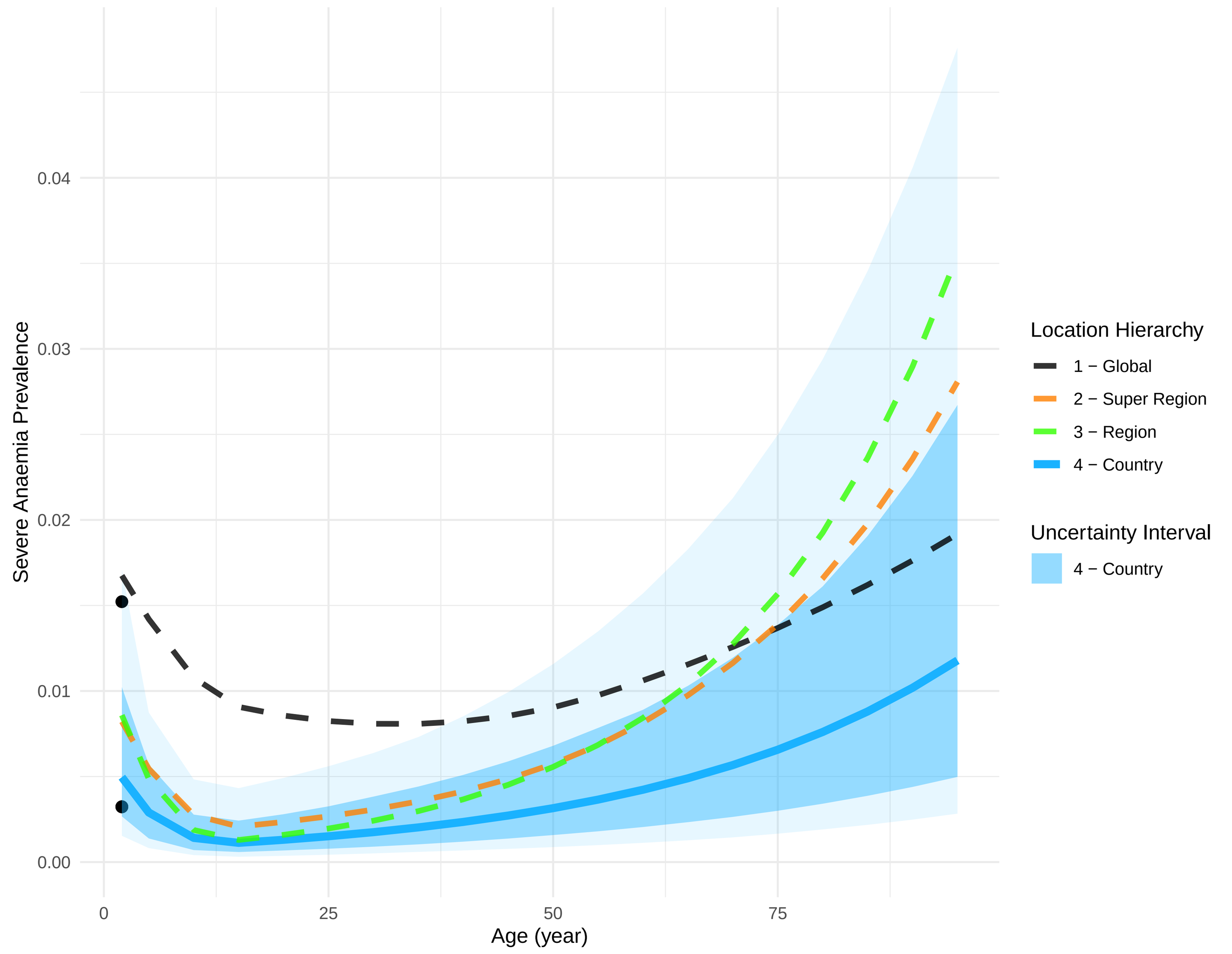

MR−BRT Age/Sex Fits − Males 2+ Years Old − Severe Anaemia Prevalence − Indonesia
WHO−2001 Adjustment Method

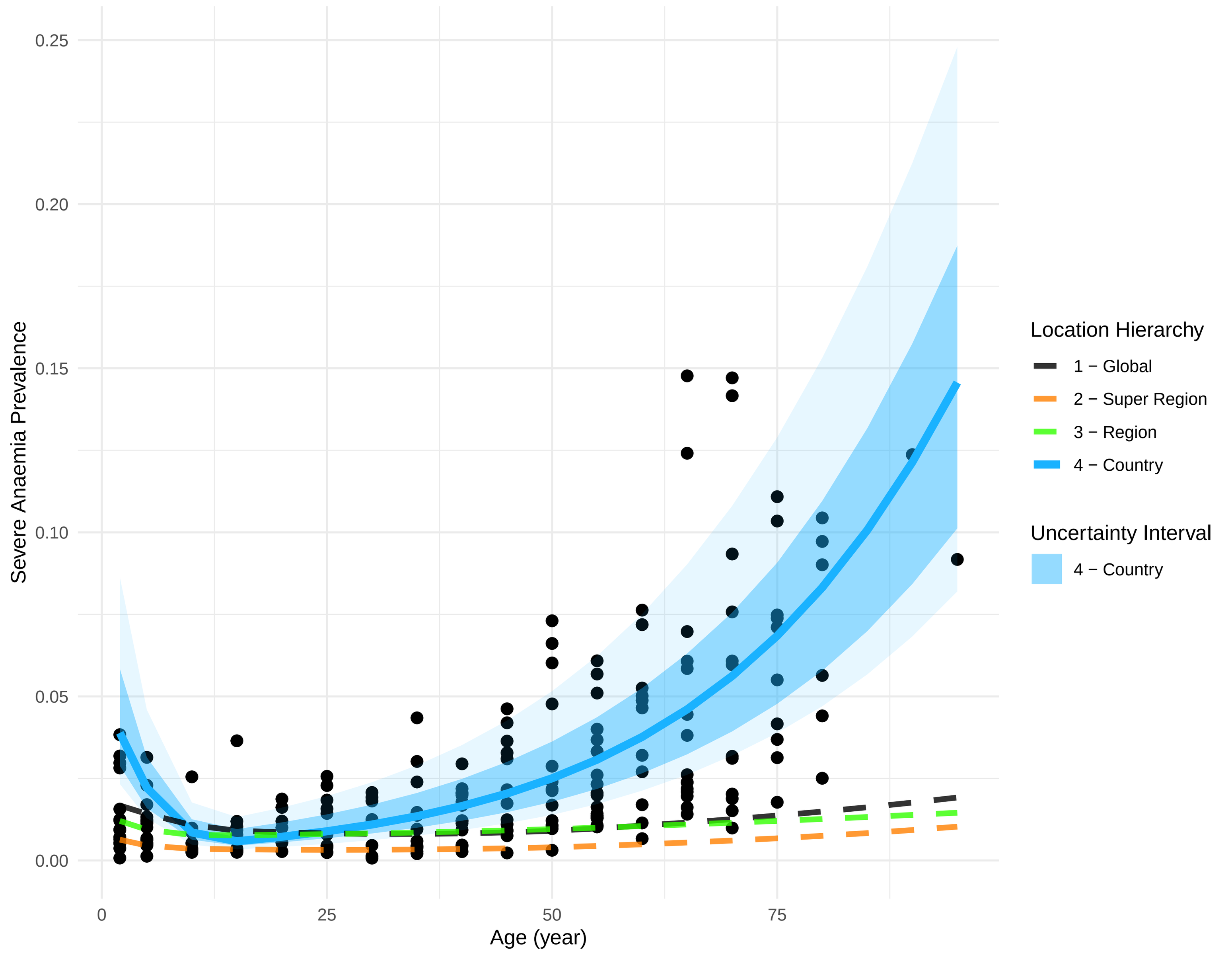

MR−BRT Age/Sex Fits − Males 2+ Years Old − Severe Anaemia Prevalence − Bali
WHO−2001 Adjustment Method

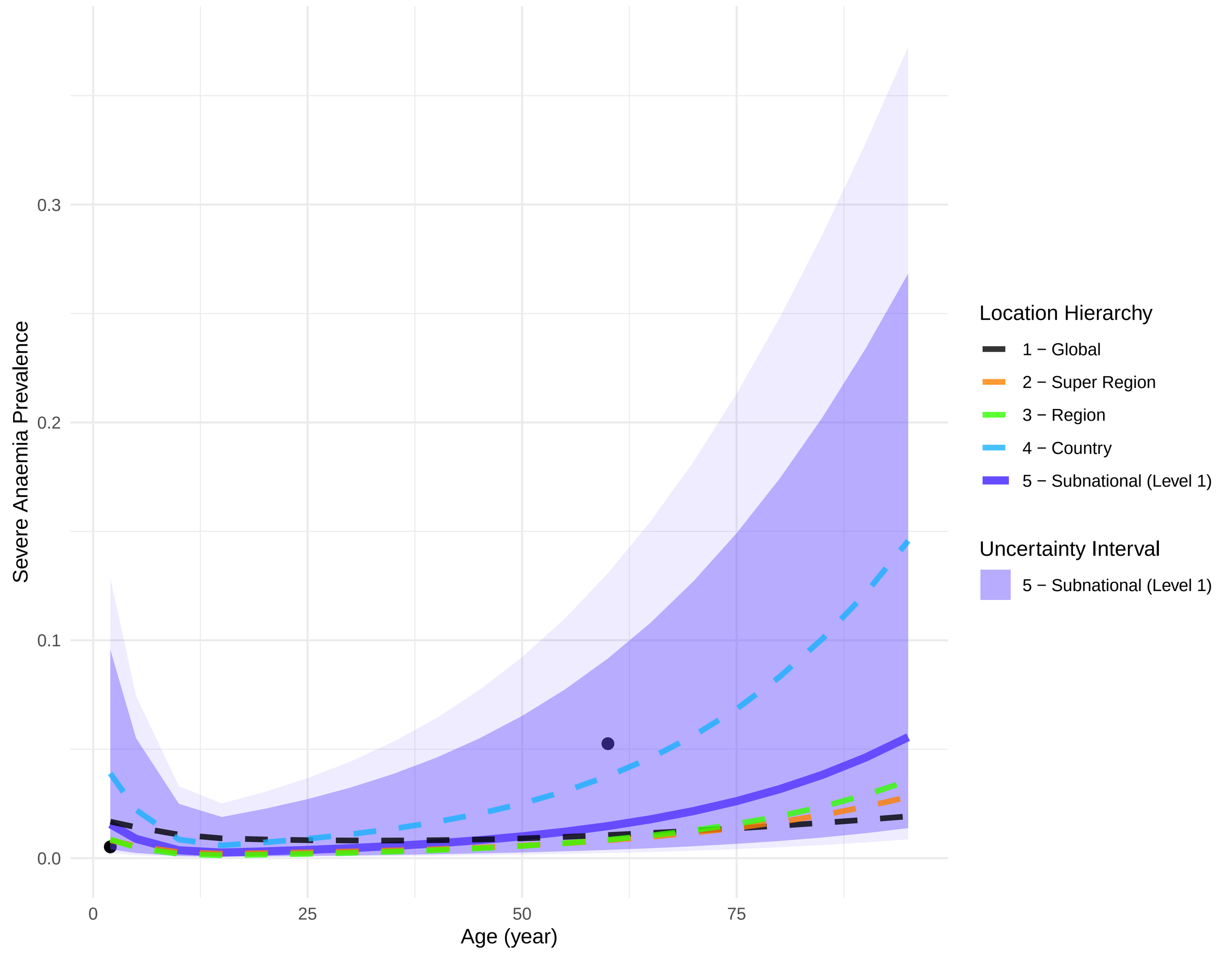

MR−BRT Age/Sex Fits − Males 2+ Years Old − Severe Anaemia Prevalence − Banten
WHO−2001 Adjustment Method

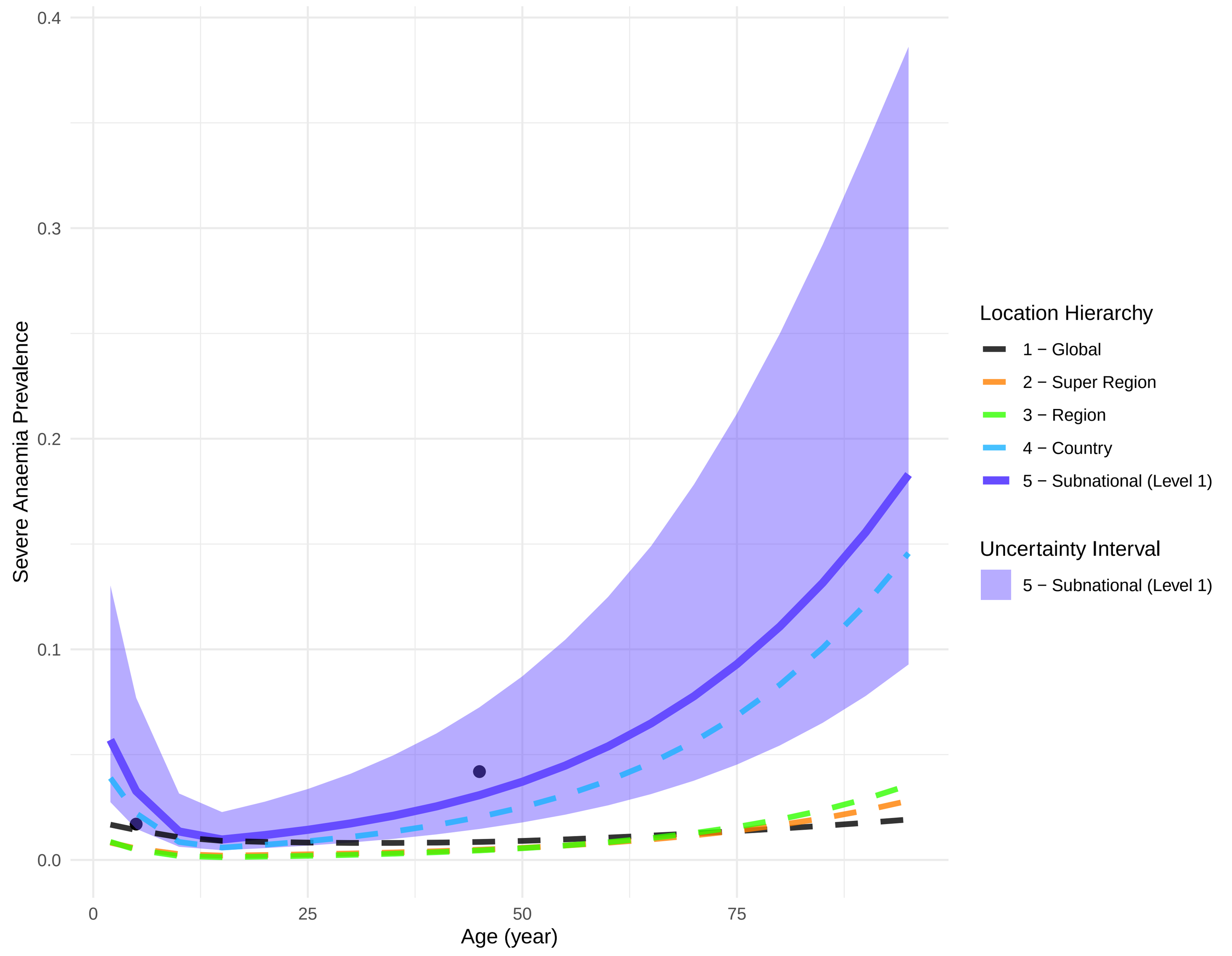

MR−BRT Age/Sex Fits − Males 2+ Years Old − Severe Anaemia Prevalence − Jakarta
WHO−2001 Adjustment Method

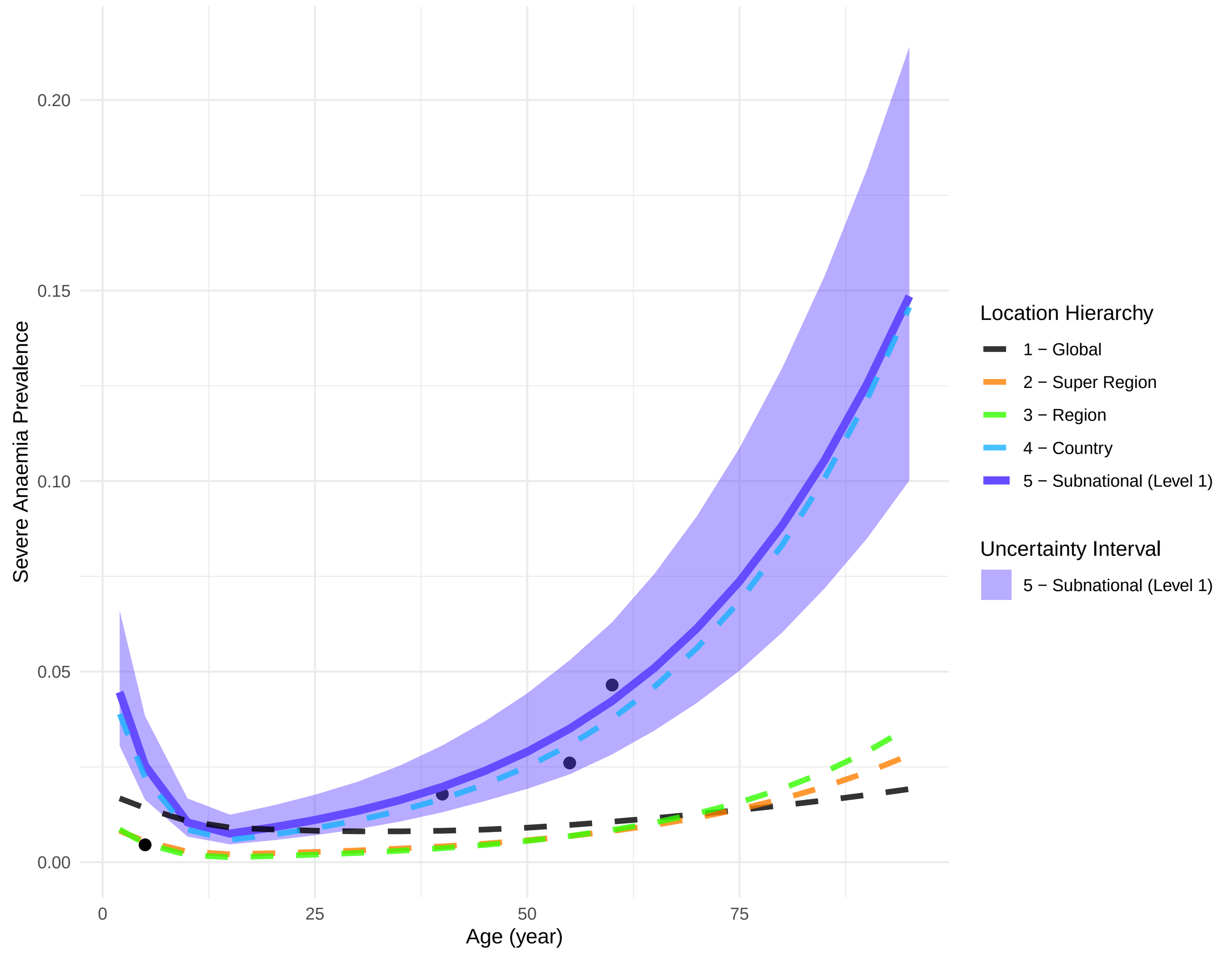

MR−BRT Age/Sex Fits − Males 2+ Years Old − Severe Anaemia Prevalence − West Java
WHO−2001 Adjustment Method

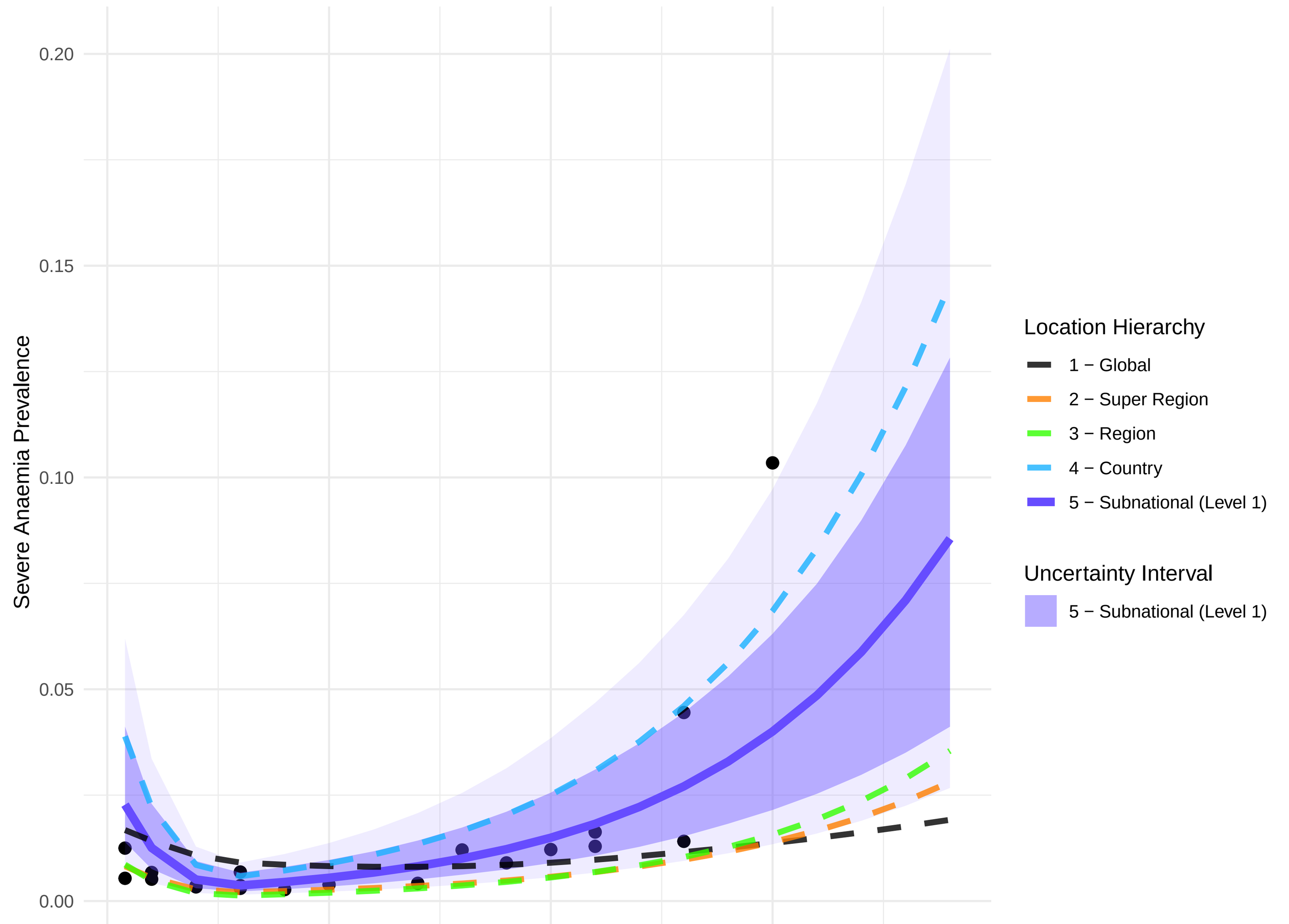

MR−BRT Age/Sex Fits − Males 2+ Years Old − Severe Anaemia Prevalence − Central Java
WHO−2001 Adjustment Method

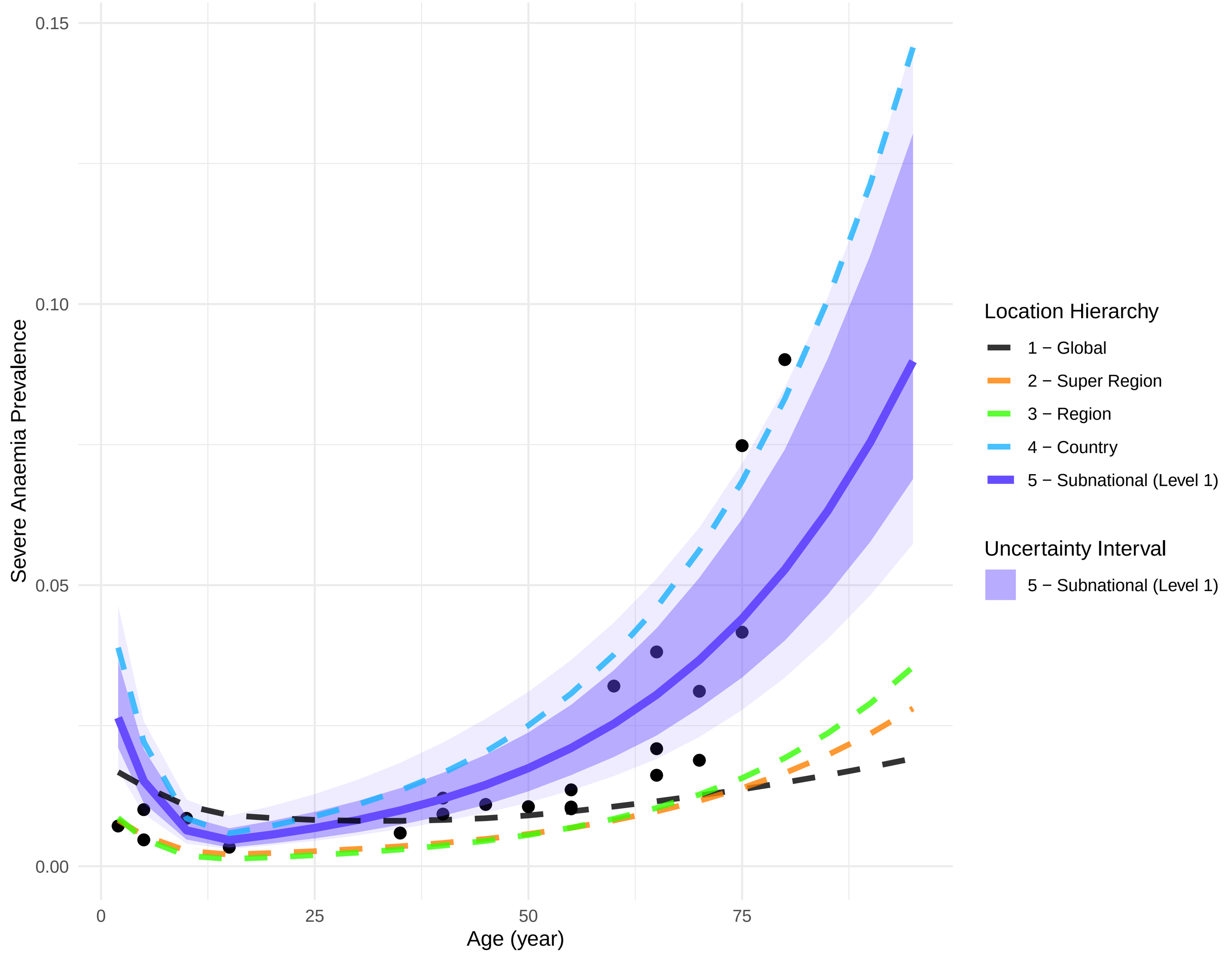

MR−BRT Age/Sex Fits − Males 2+ Years Old − Severe Anaemia Prevalence − East Java
WHO−2001 Adjustment Method

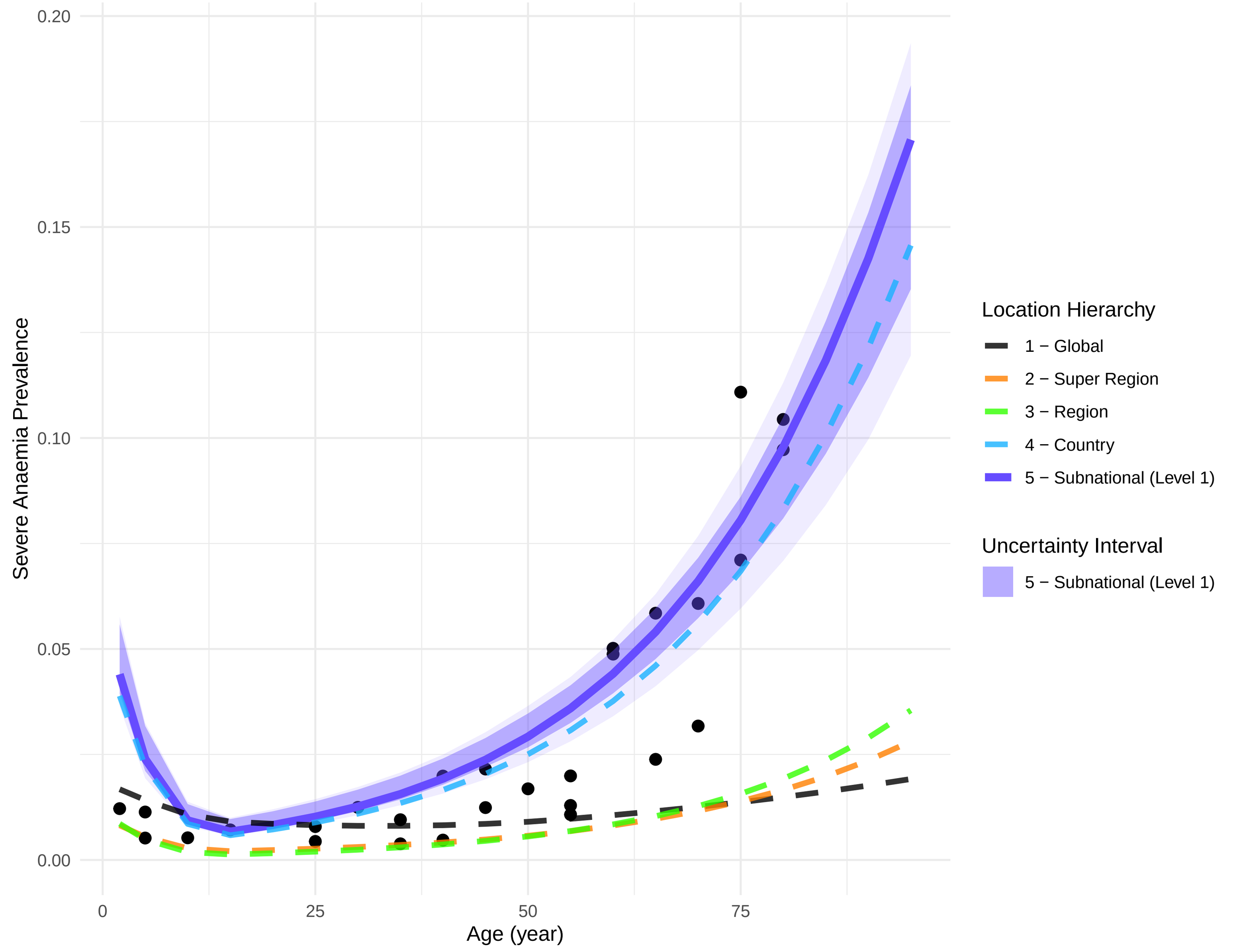

MR−BRT Age/Sex Fits − Males 2+ Years Old − Severe Anaemia Prevalence − South Kalimantan
WHO−2001 Adjustment Method

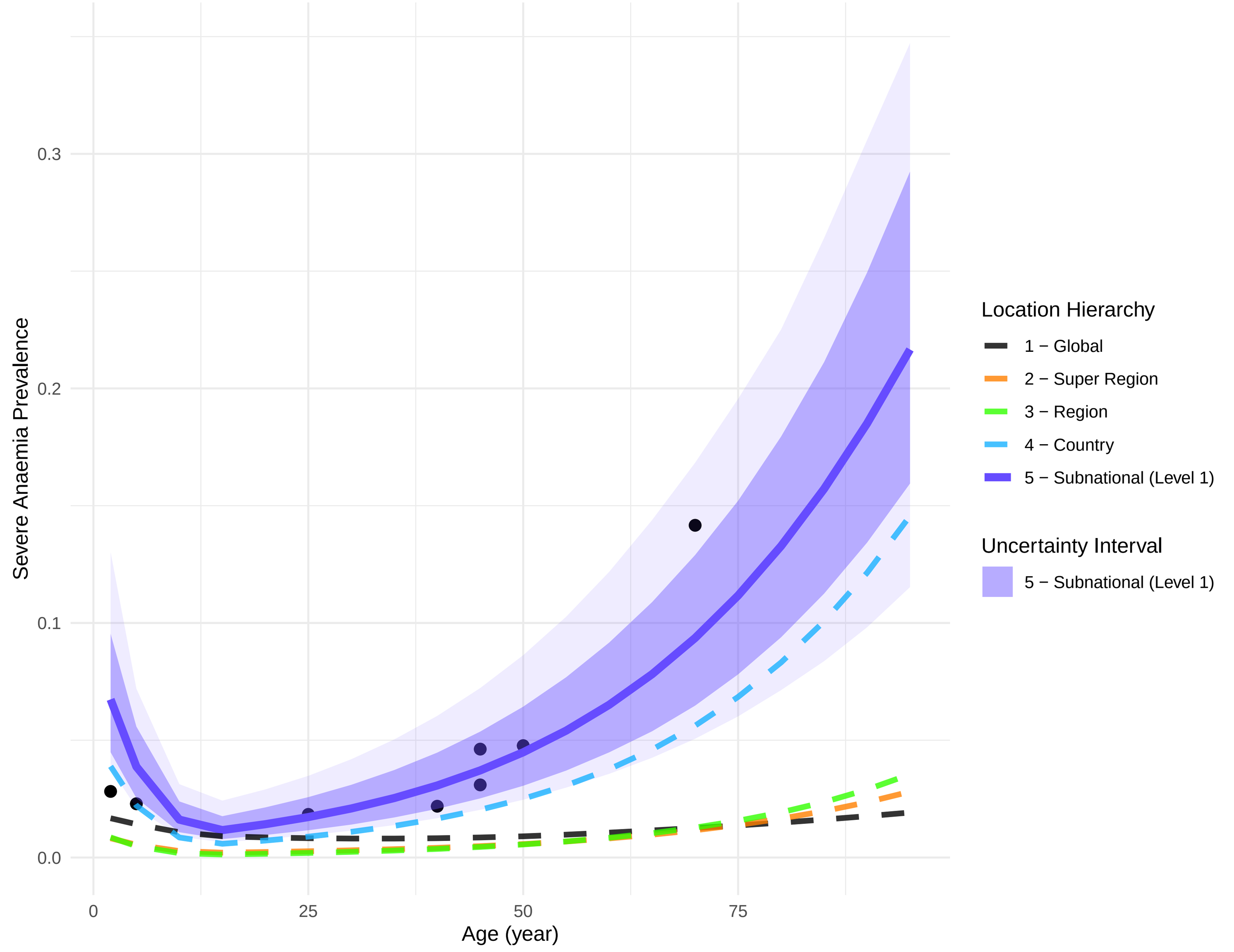

MR−BRT Age/Sex Fits − Males 2+ Years Old − Severe Anaemia Prevalence − Lampung
WHO−2001 Adjustment Method

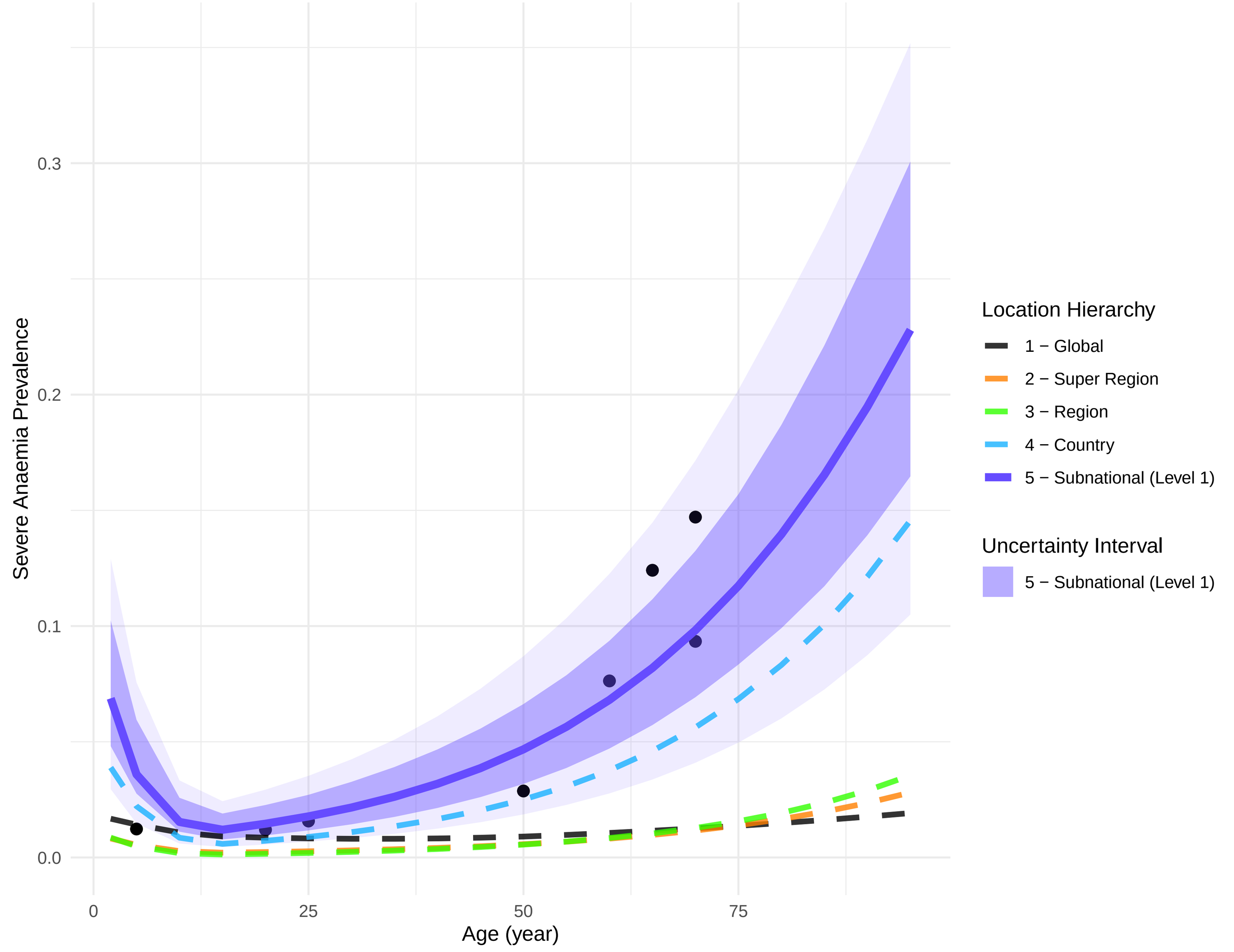

MR−BRT Age/Sex Fits − Males 2+ Years Old − Severe Anaemia Prevalence − West Nusa Tenggara
WHO−2001 Adjustment Method

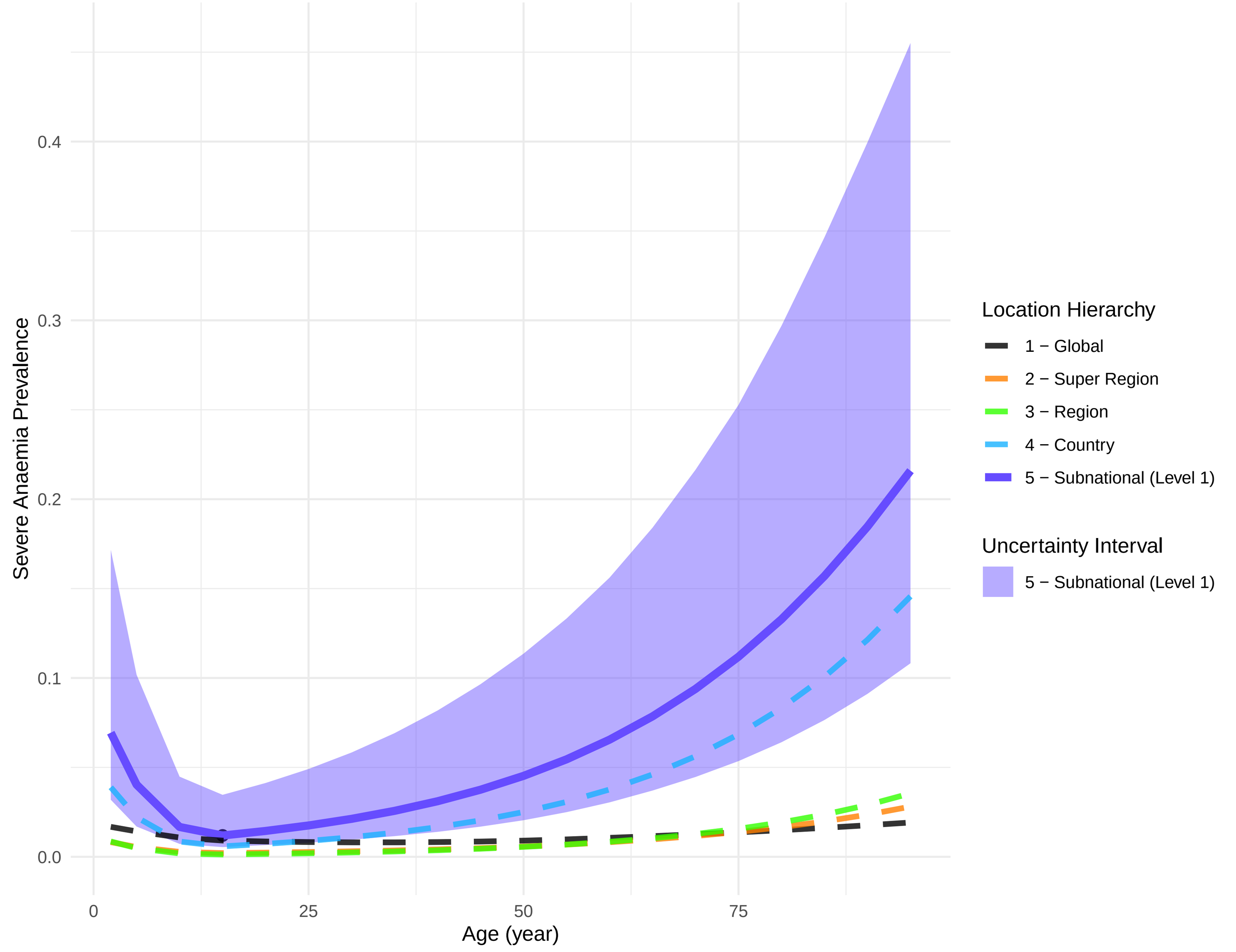

MR−BRT Age/Sex Fits − Males 2+ Years Old − Severe Anaemia Prevalence − South Sulawesi
WHO−2001 Adjustment Method

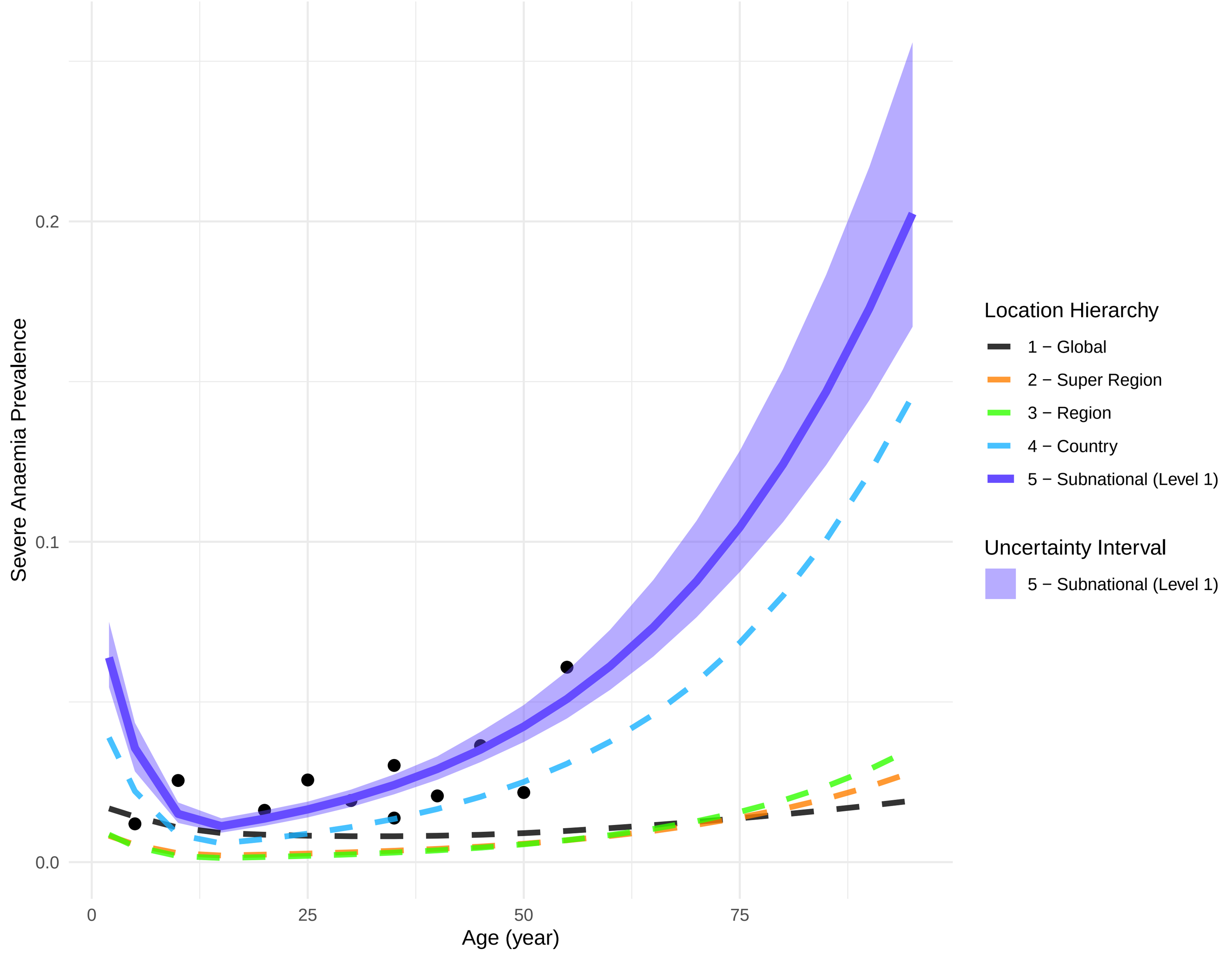

MR−BRT Age/Sex Fits − Males 2+ Years Old − Severe Anaemia Prevalence − West Sumatra
WHO−2001 Adjustment Method

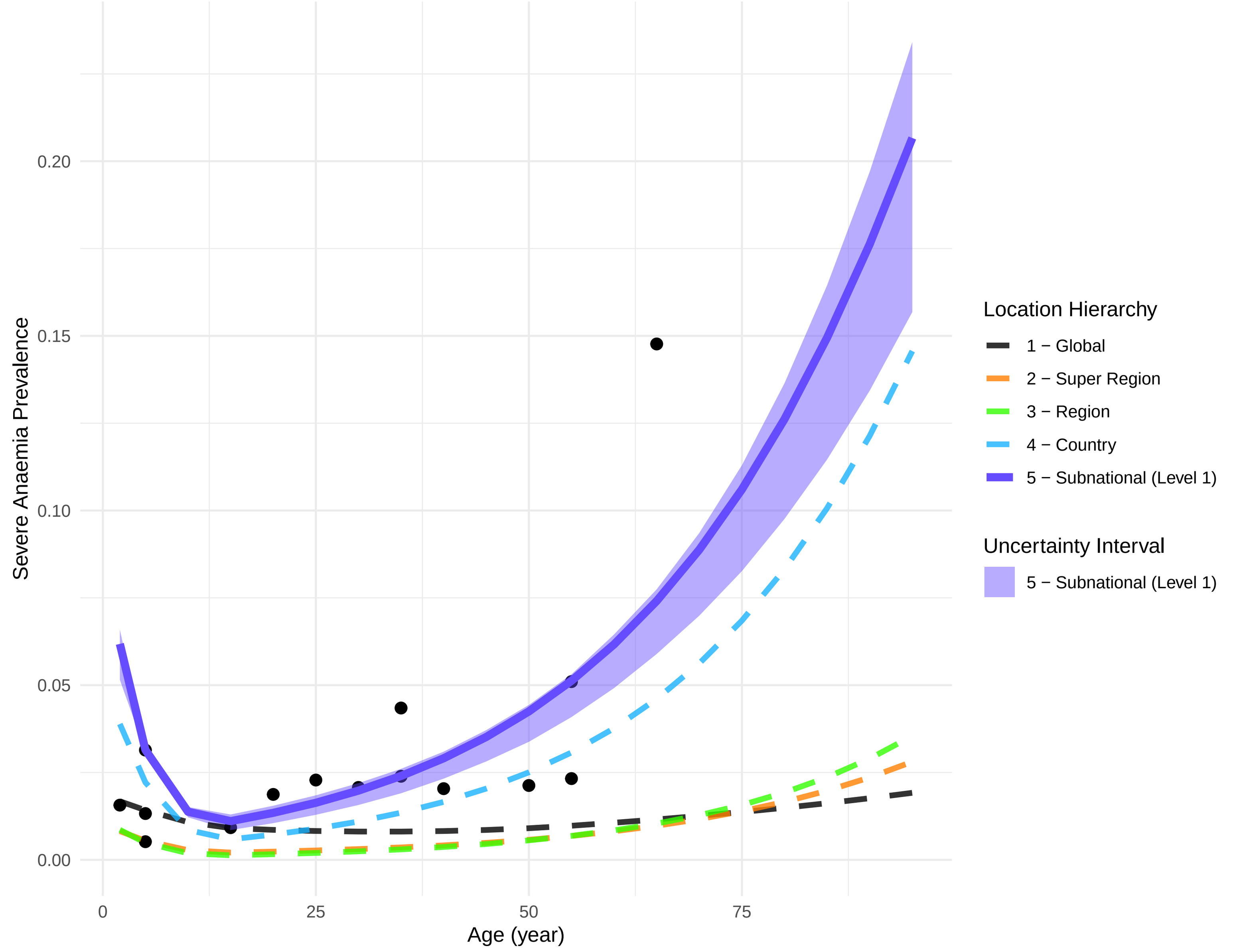

MR−BRT Age/Sex Fits − Males 2+ Years Old − Severe Anaemia Prevalence − South Sumatra
WHO−2001 Adjustment Method

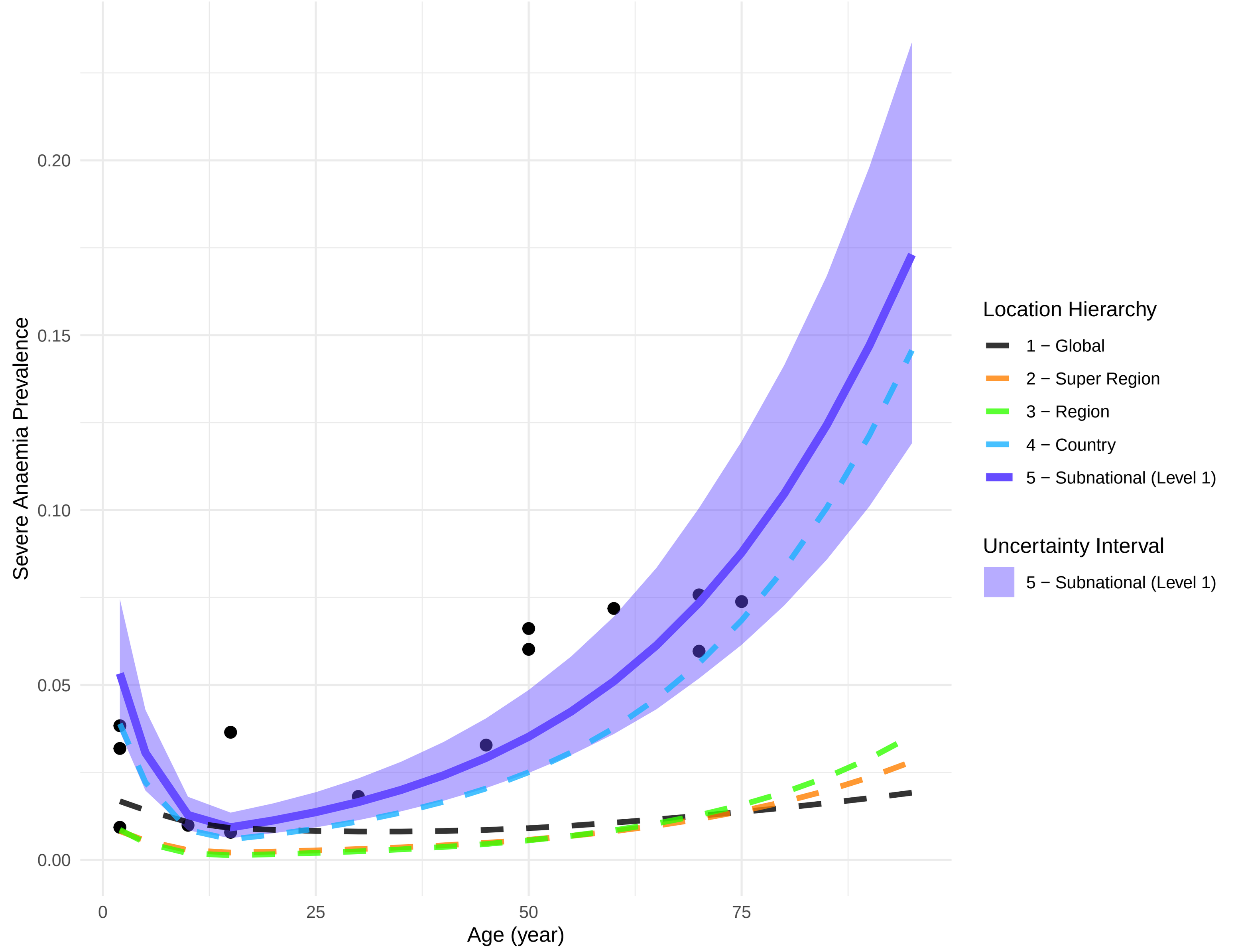

MR−BRT Age/Sex Fits − Males 2+ Years Old − Severe Anaemia Prevalence − North Sumatra
WHO−2001 Adjustment Method

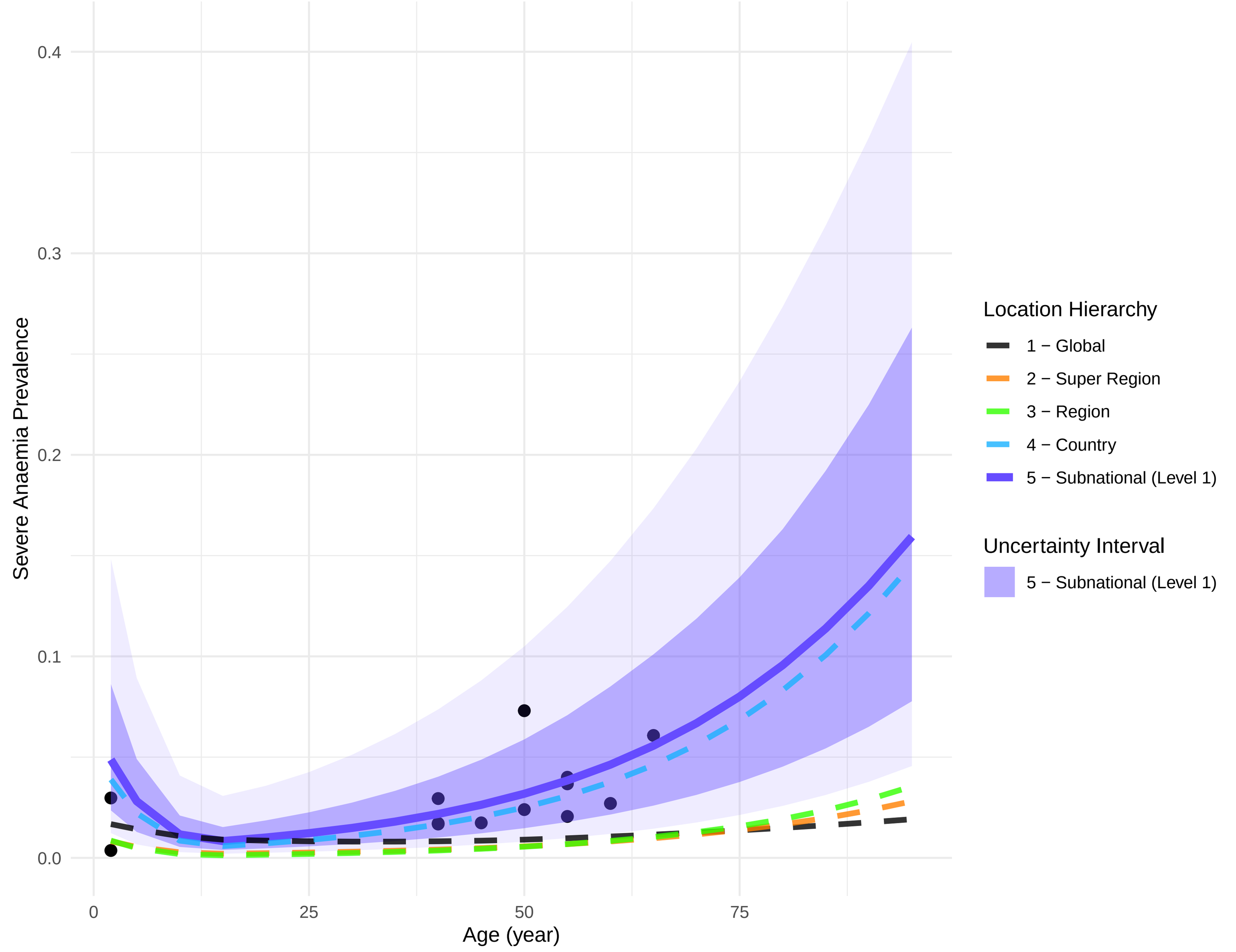

MR−BRT Age/Sex Fits − Males 2+ Years Old − Severe Anaemia Prevalence − Yogyakarta
WHO−2001 Adjustment Method

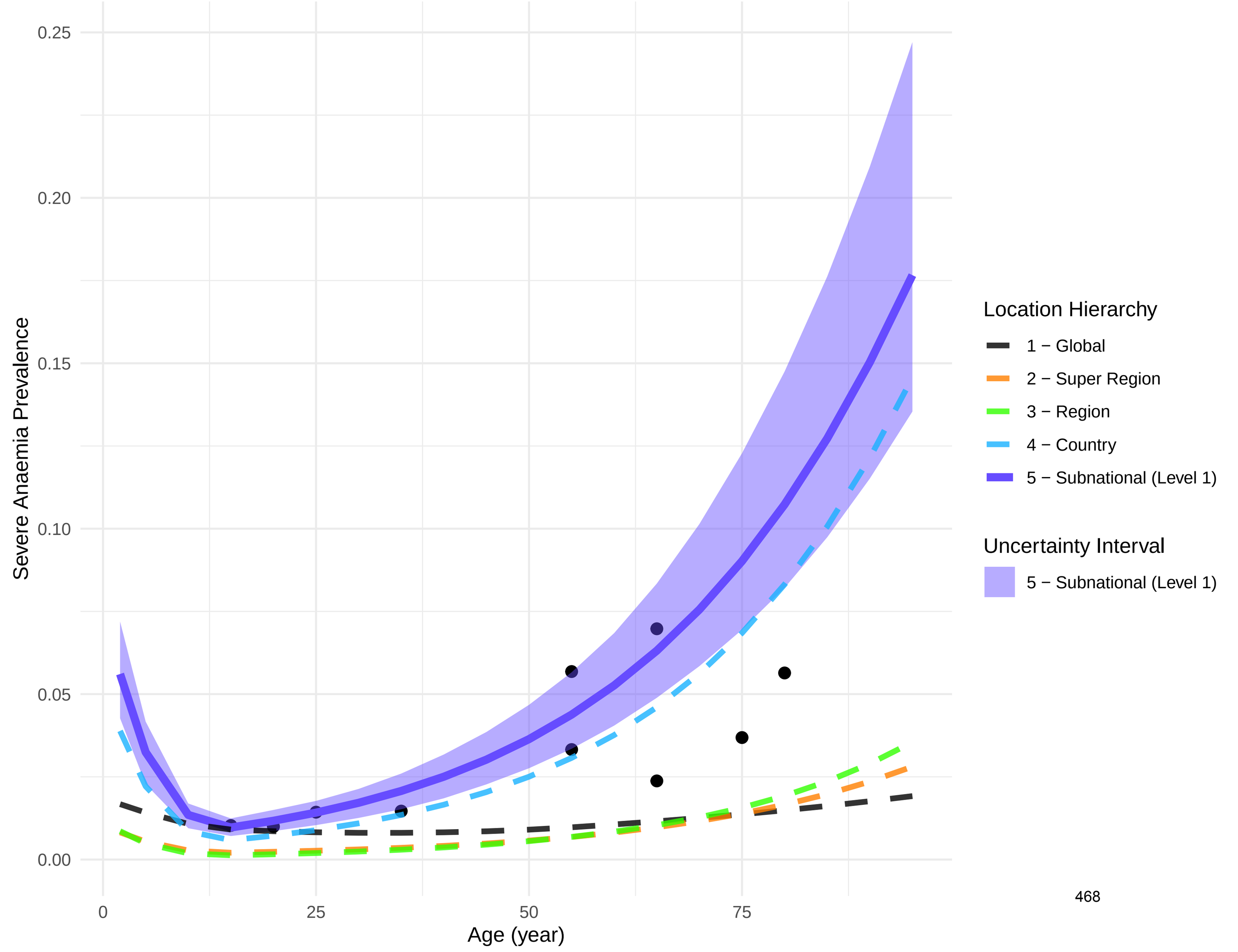

MR−BRT Age/Sex Fits − Males 2+ Years Old − Severe Anaemia Prevalence − Myanmar
WHO−2001 Adjustment Method

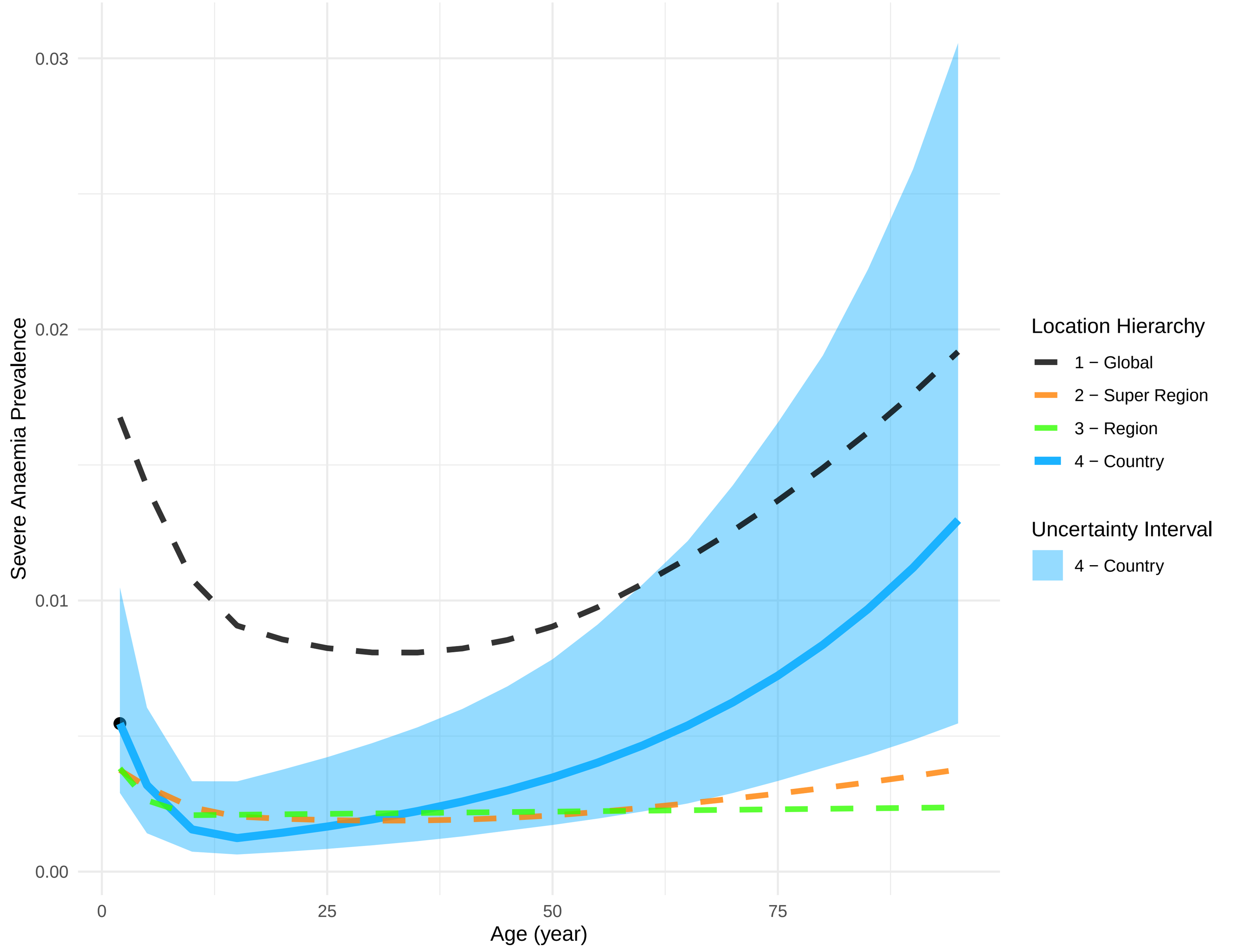

MR−BRT Age/Sex Fits − Males 2+ Years Old − Severe Anaemia Prevalence − Timor−Leste
WHO−2001 Adjustment Method

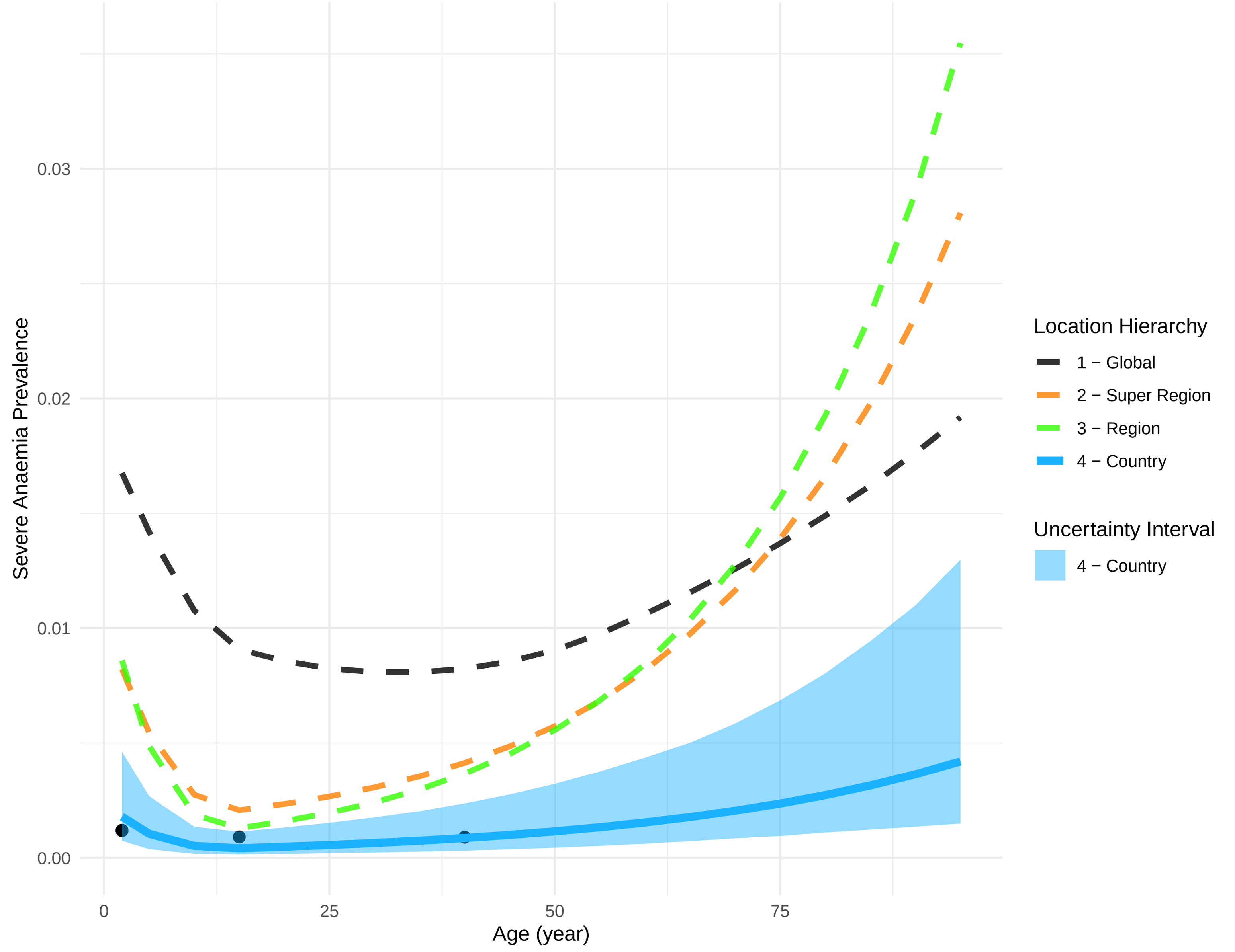

MR−BRT Age/Sex Fits − Males 2+ Years Old − Severe Anaemia Prevalence − Sub−Saharan Africa
WHO−2001 Adjustment Method

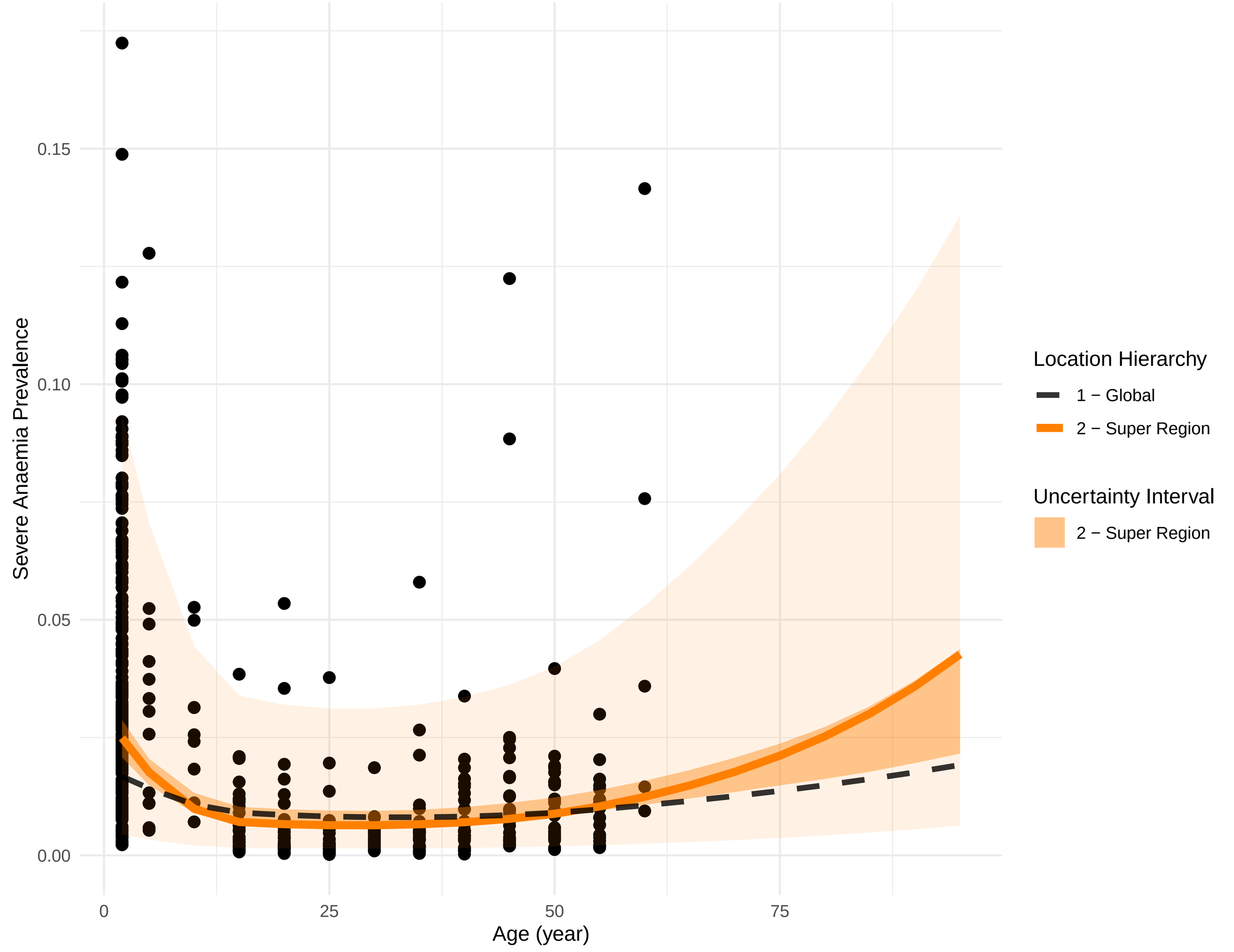

MR−BRT Age/Sex Fits − Males 2+ Years Old − Severe Anaemia Prevalence − Central Sub−Saharan Africa
WHO−2001 Adjustment Method

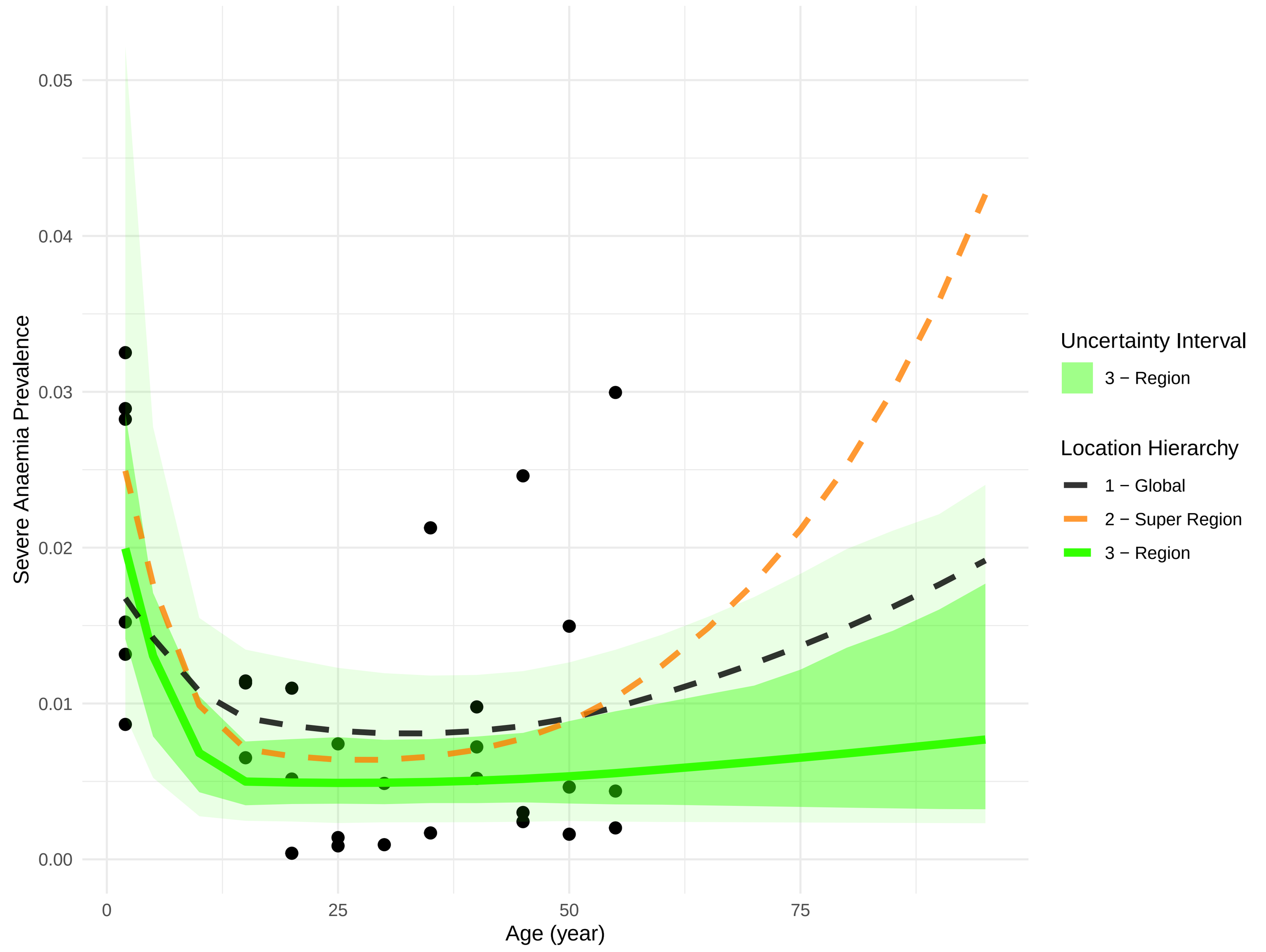

MR−BRT Age/Sex Fits − Males 2+ Years Old − Severe Anaemia Prevalence − Angola
WHO−2001 Adjustment Method

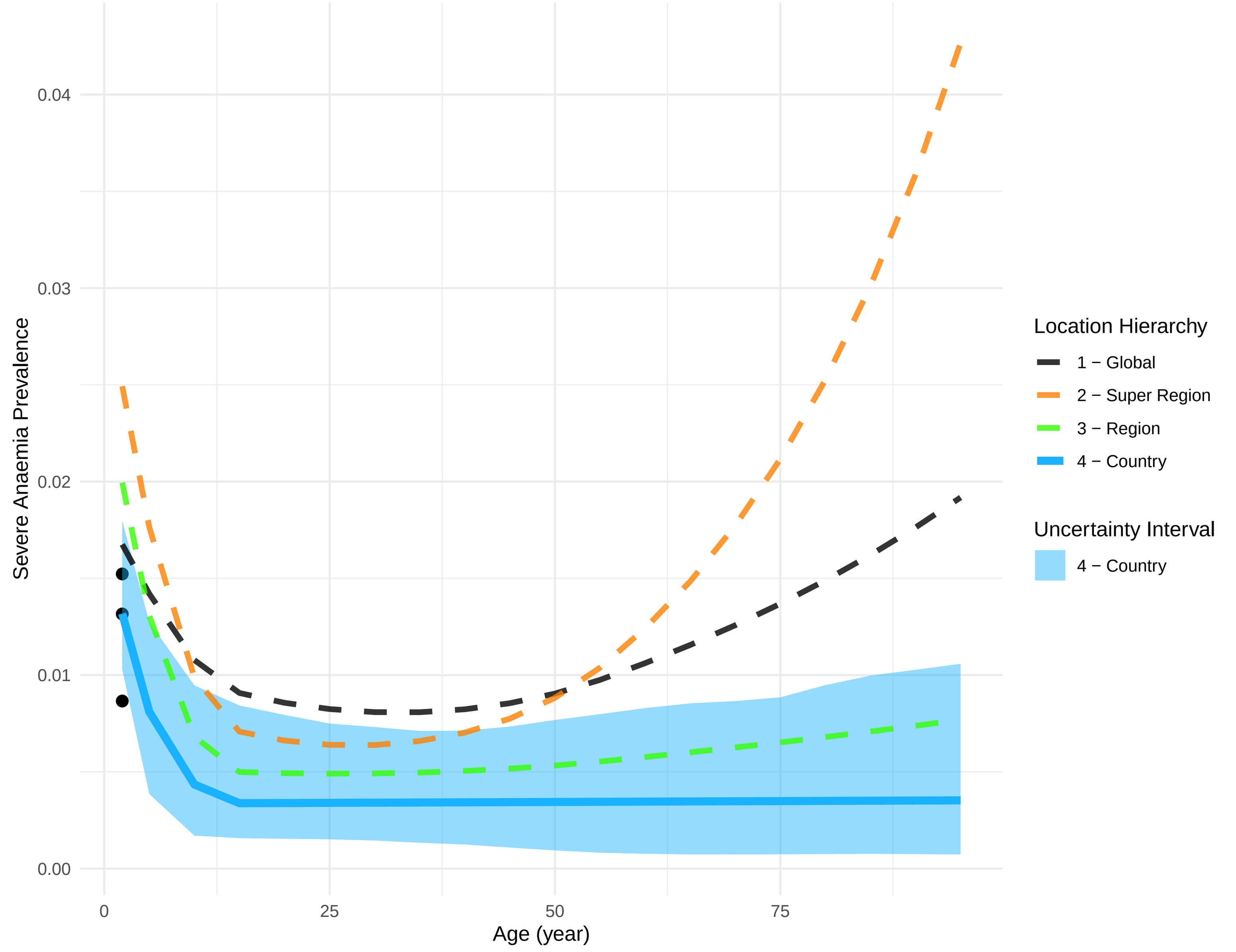

MR−BRT Age/Sex Fits − Males 2+ Years Old − Severe Anaemia Prevalence − Democratic Republic of the Congo
WHO−2001 Adjustment Method

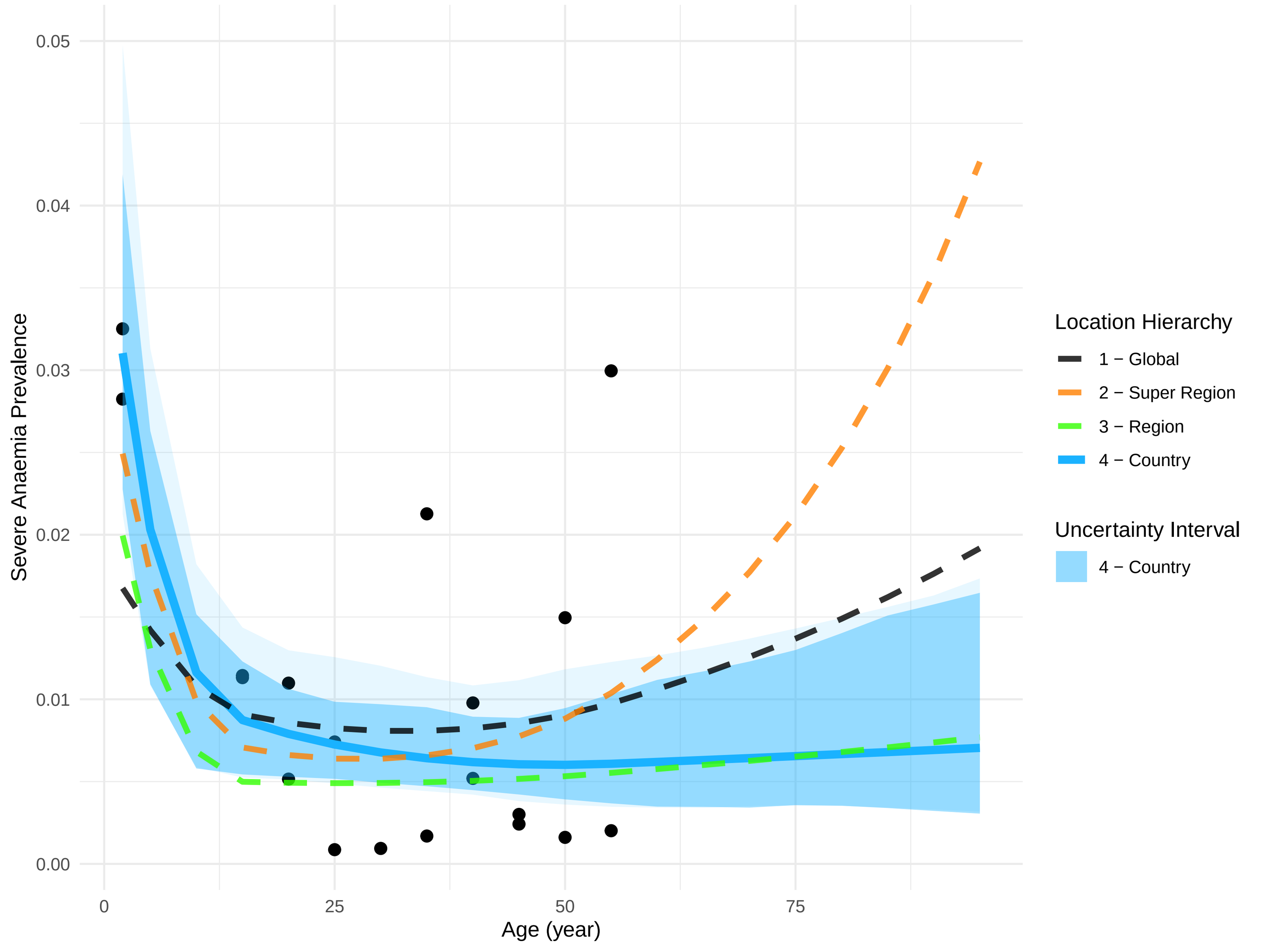

MR−BRT Age/Sex Fits − Males 2+ Years Old − Severe Anaemia Prevalence − Gabon
WHO−2001 Adjustment Method

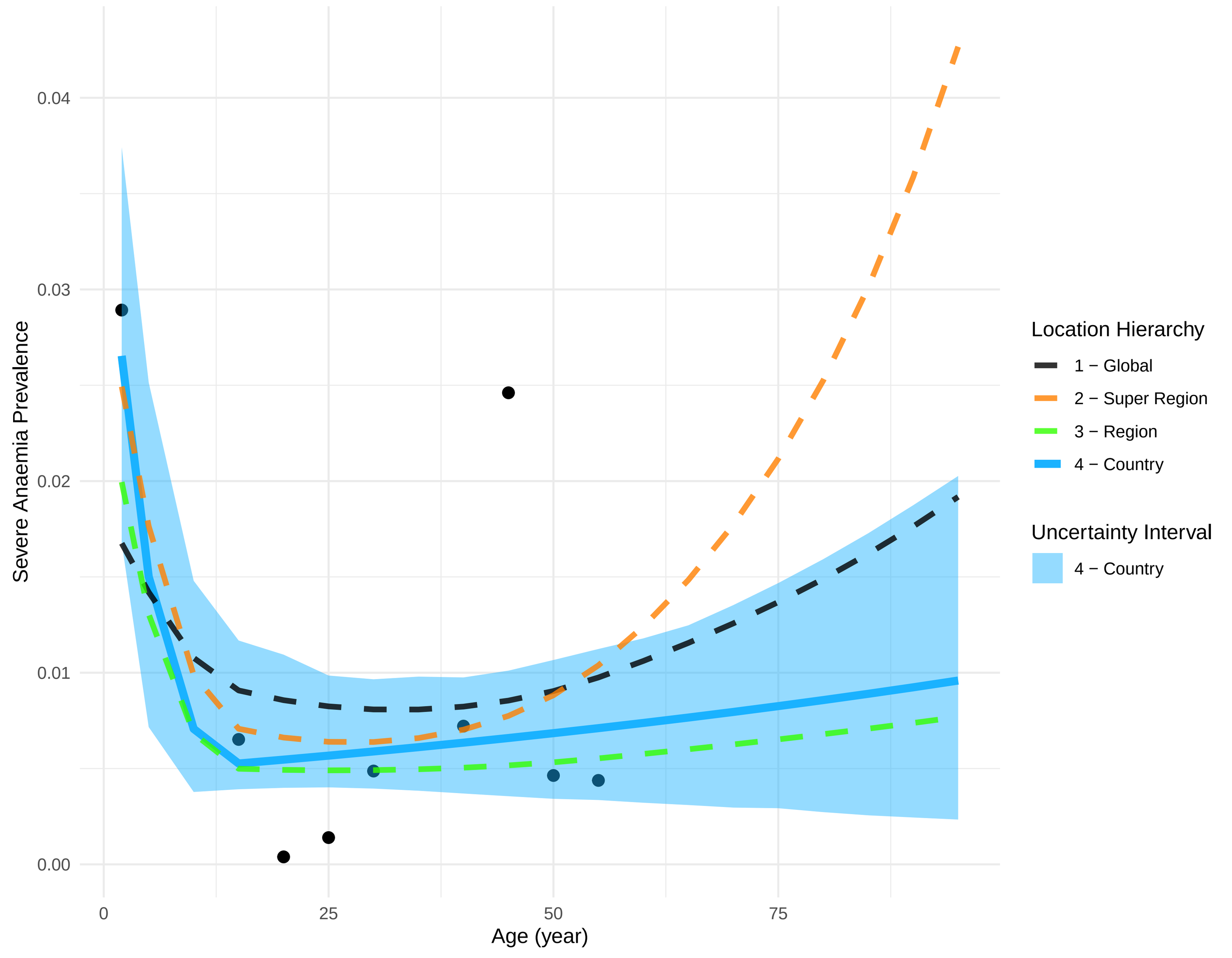

MR−BRT Age/Sex Fits − Males 2+ Years Old − Severe Anaemia Prevalence − Eastern Sub−Saharan Africa
WHO−2001 Adjustment Method

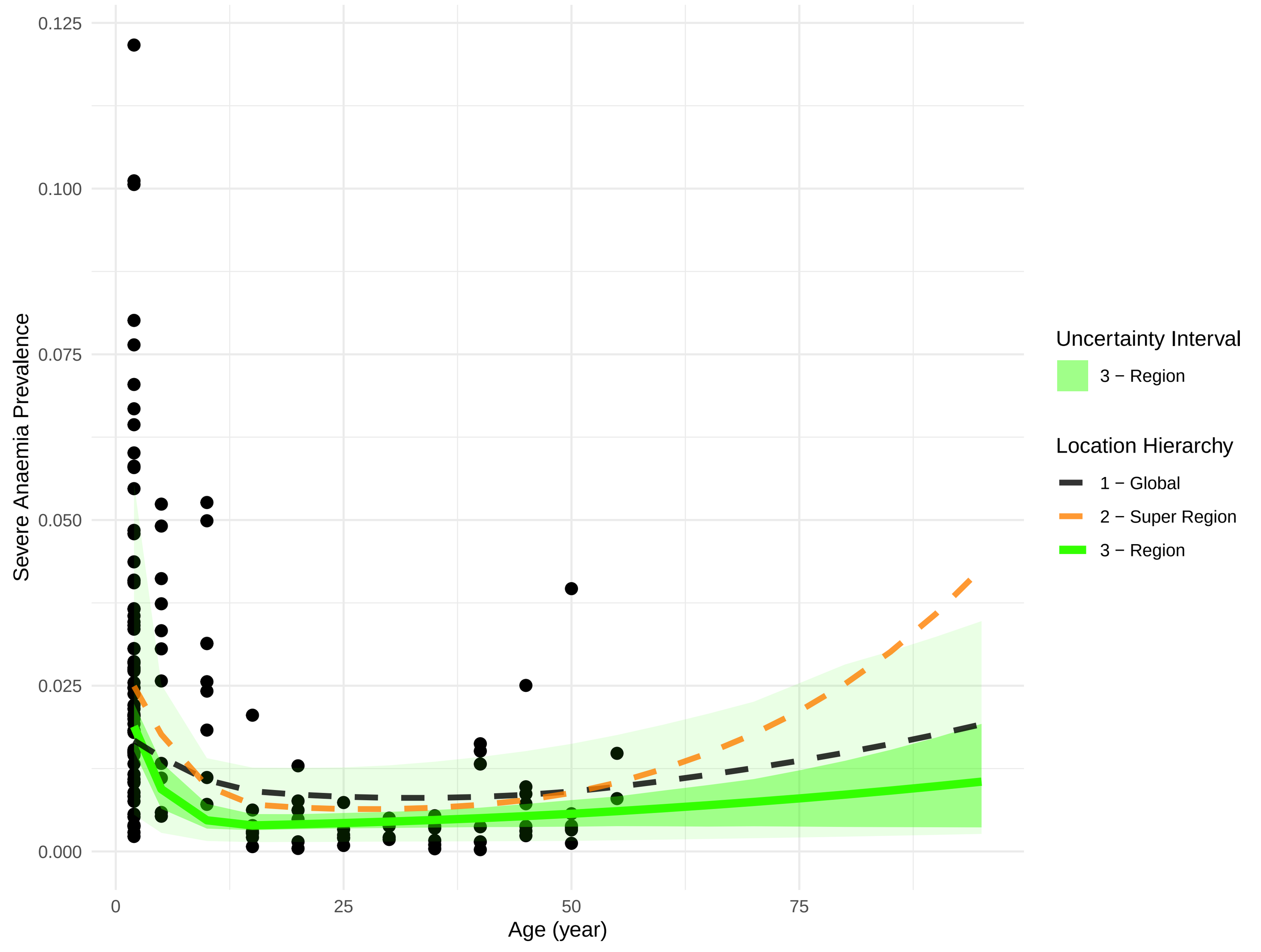

MR−BRT Age/Sex Fits − Males 2+ Years Old − Severe Anaemia Prevalence − Burundi
WHO−2001 Adjustment Method

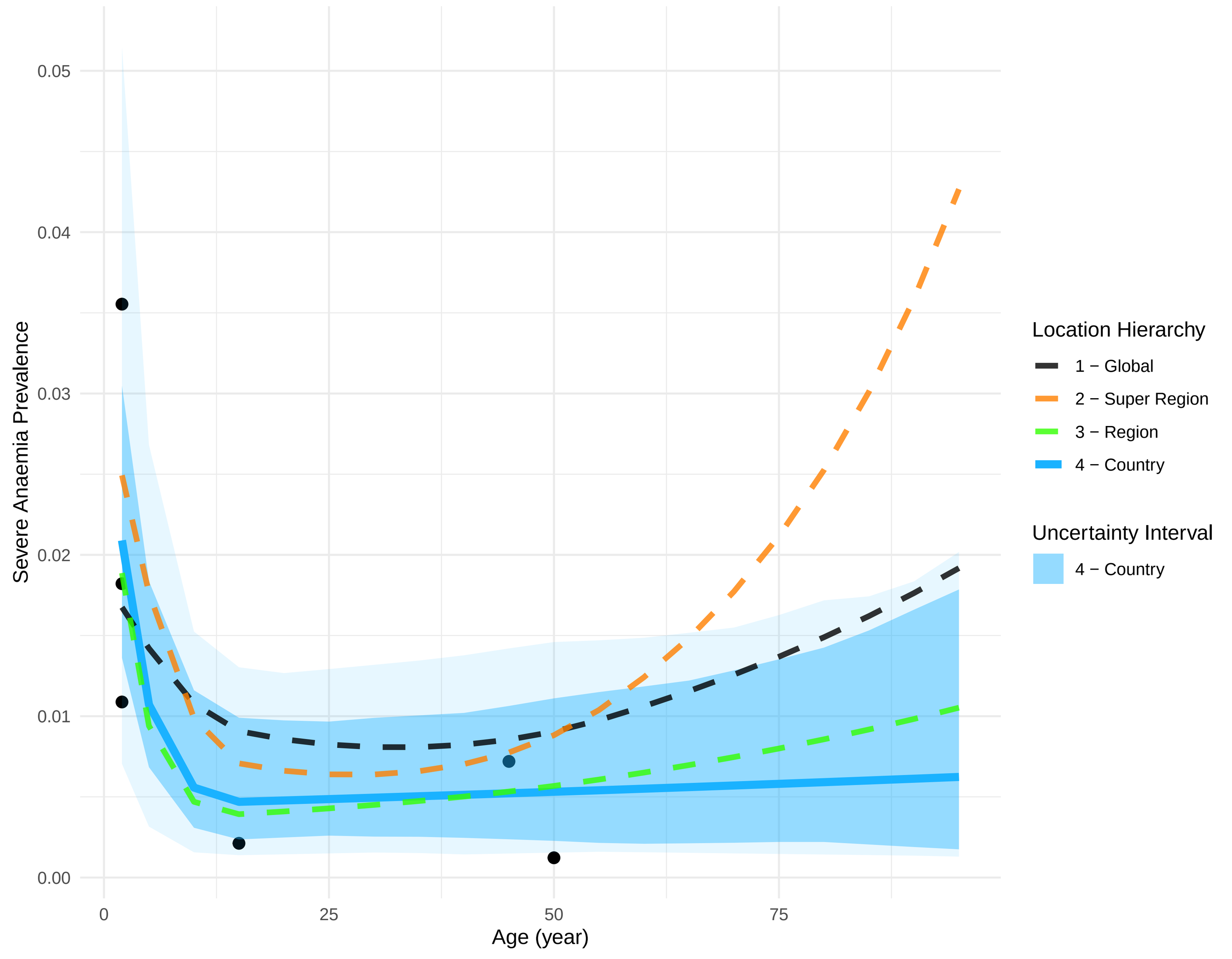

MR−BRT Age/Sex Fits − Males 2+ Years Old − Severe Anaemia Prevalence − Ethiopia
WHO−2001 Adjustment Method

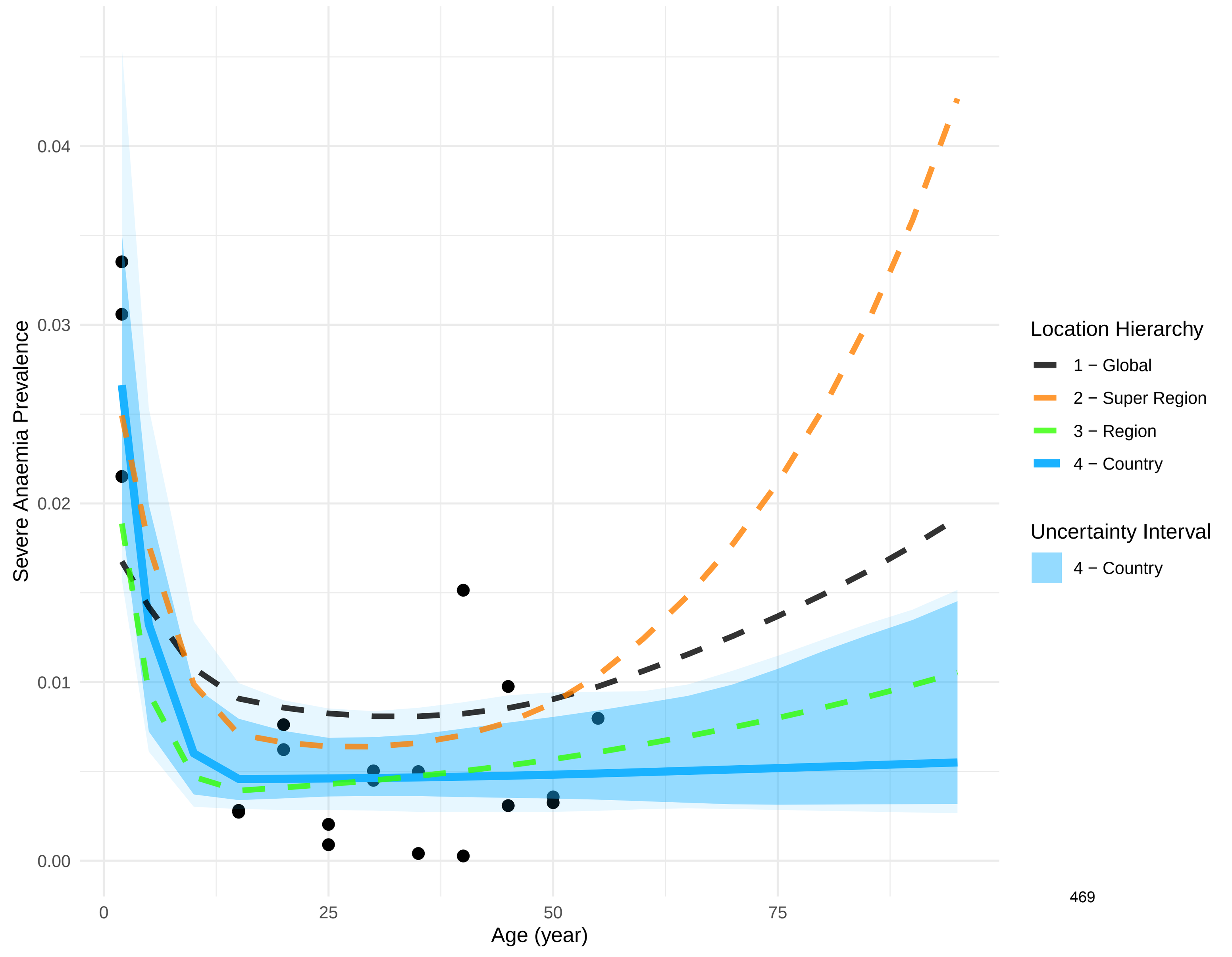

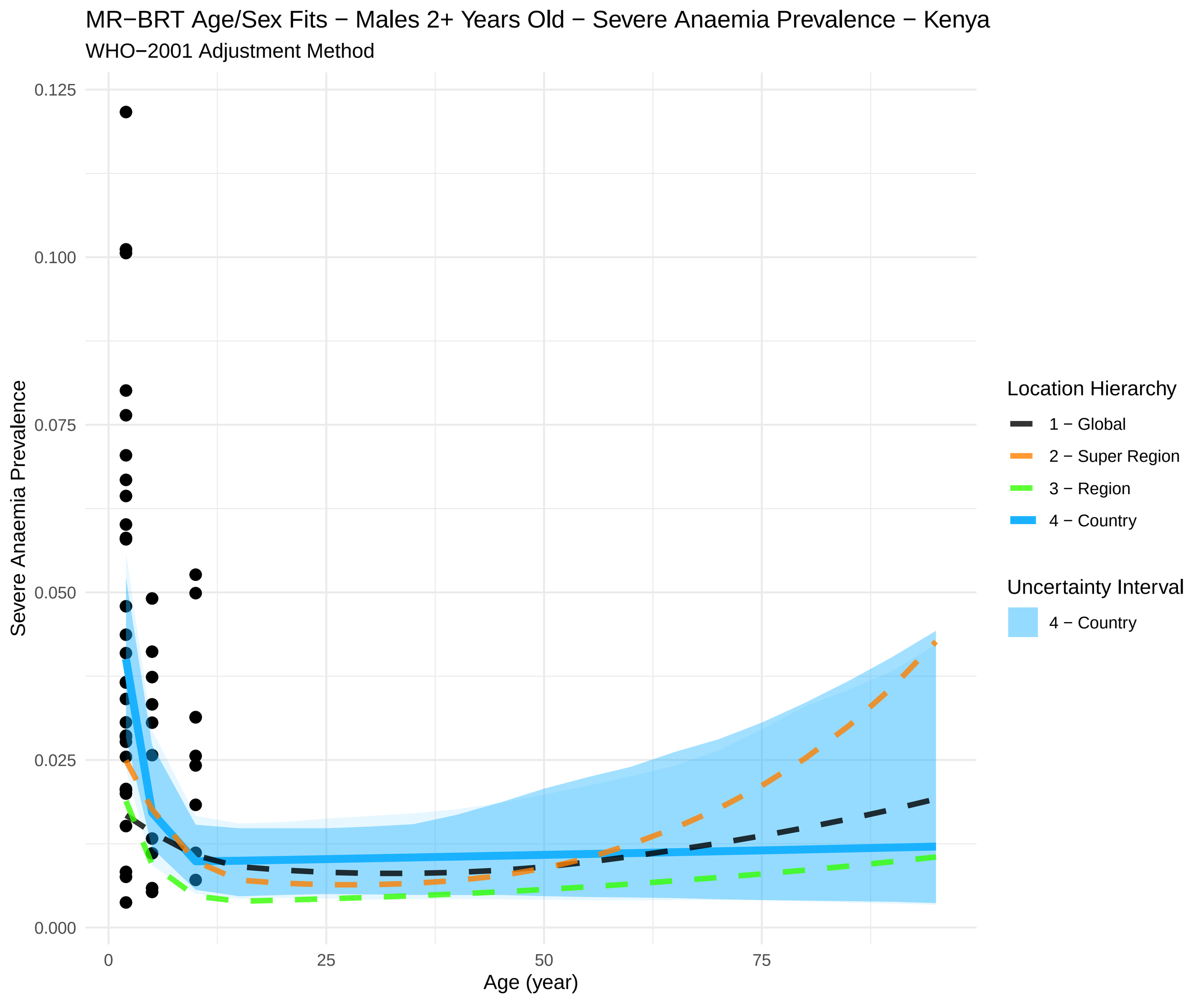
MR−BRT Age/Sex Fits − Males 2+ Years Old − Severe Anaemia Prevalence − Kenya
WHO−2001 Adjustment Method
Severe Anaemia Prevalence
0.000
0.025
0.050
0.075
0.100
0.125
0
25
50
75
Age (year)
Location Hierarchy
1 − Global
2 − Super Region
3 − Region
4 − Country
Uncertainty Interval
4 − Country

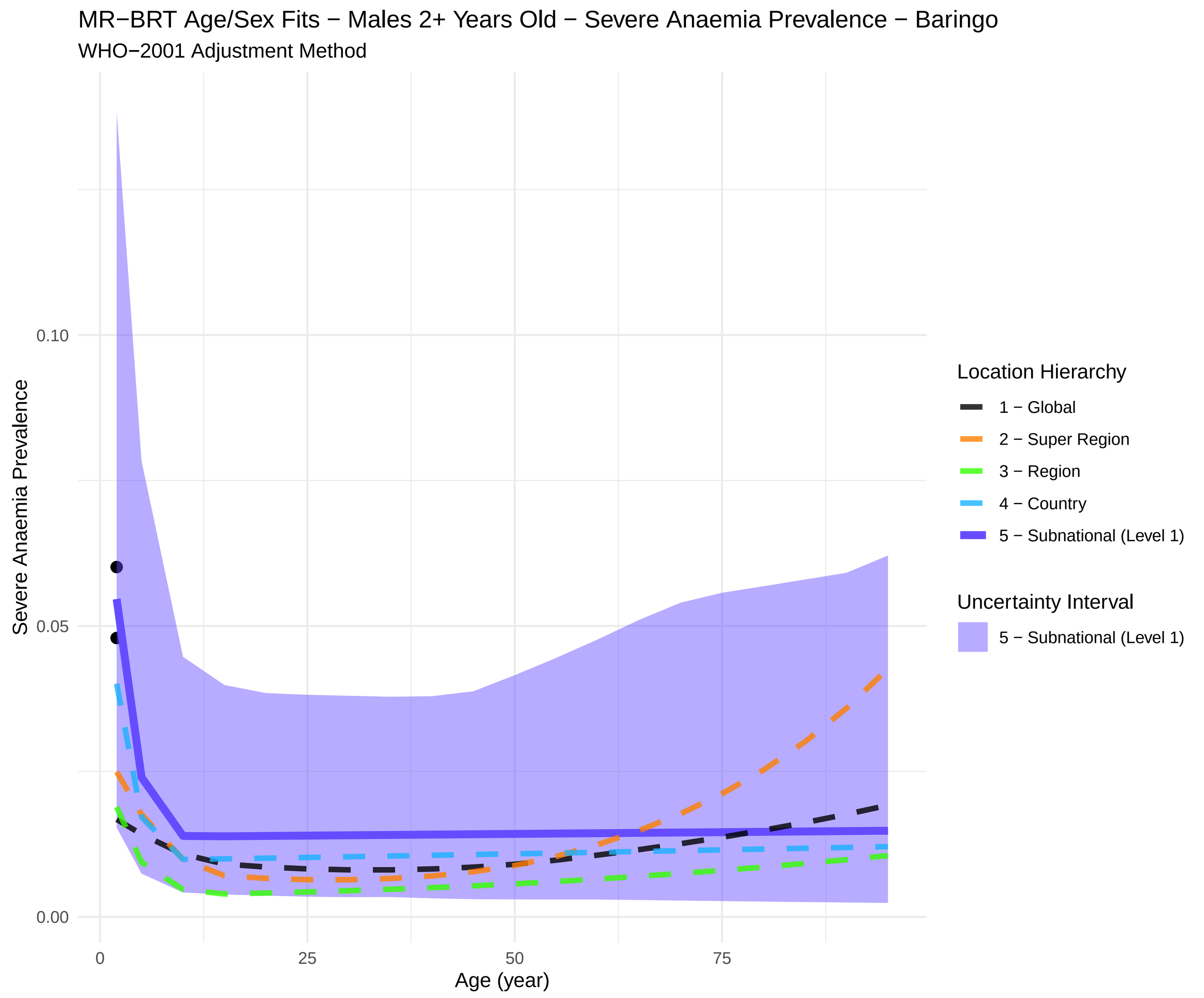
MR−BRT Age/Sex Fits − Males 2+ Years Old − Severe Anaemia Prevalence − Baringo
WHO−2001 Adjustment Method
Severe Anaemia Prevalence
0.00
0.05
0.10
0
25
50
75
Age (year)
Location Hierarchy
1 − Global
2 − Super Region
3 − Region
4 − Country
5 − Subnational (Level 1)
Uncertainty Interval
5 − Subnational (Level 1)

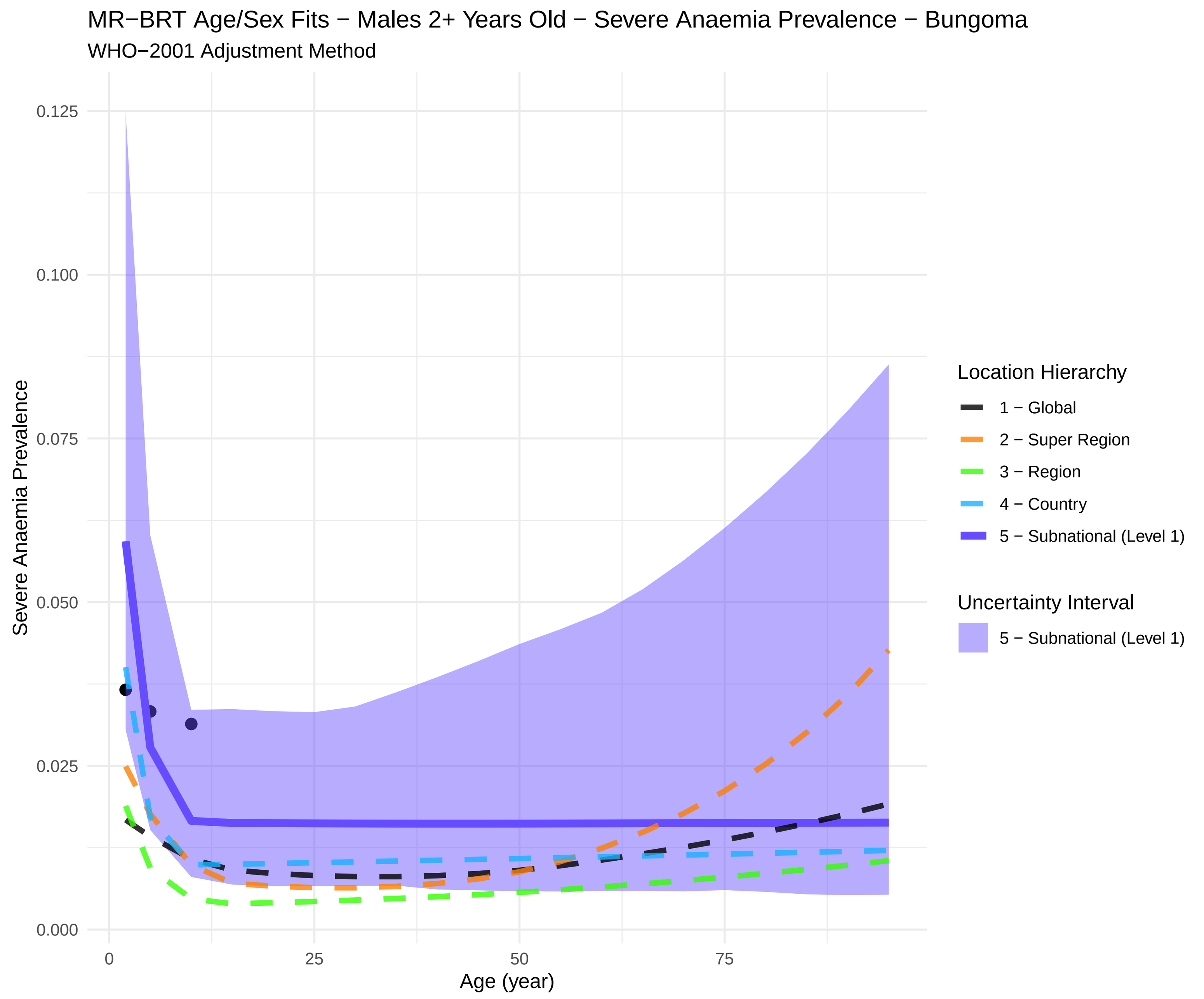
MR−BRT Age/Sex Fits − Males 2+ Years Old − Severe Anaemia Prevalence − Bungoma
WHO−2001 Adjustment Method
Severe Anaemia Prevalence
0.000
0.025
0.050
0.075
0.100
0.125
0
25
50
75
Age (year)
Location Hierarchy
1 − Global
2 − Super Region
3 − Region
4 − Country
5 − Subnational (Level 1)
Uncertainty Interval
5 − Subnational (Level 1)

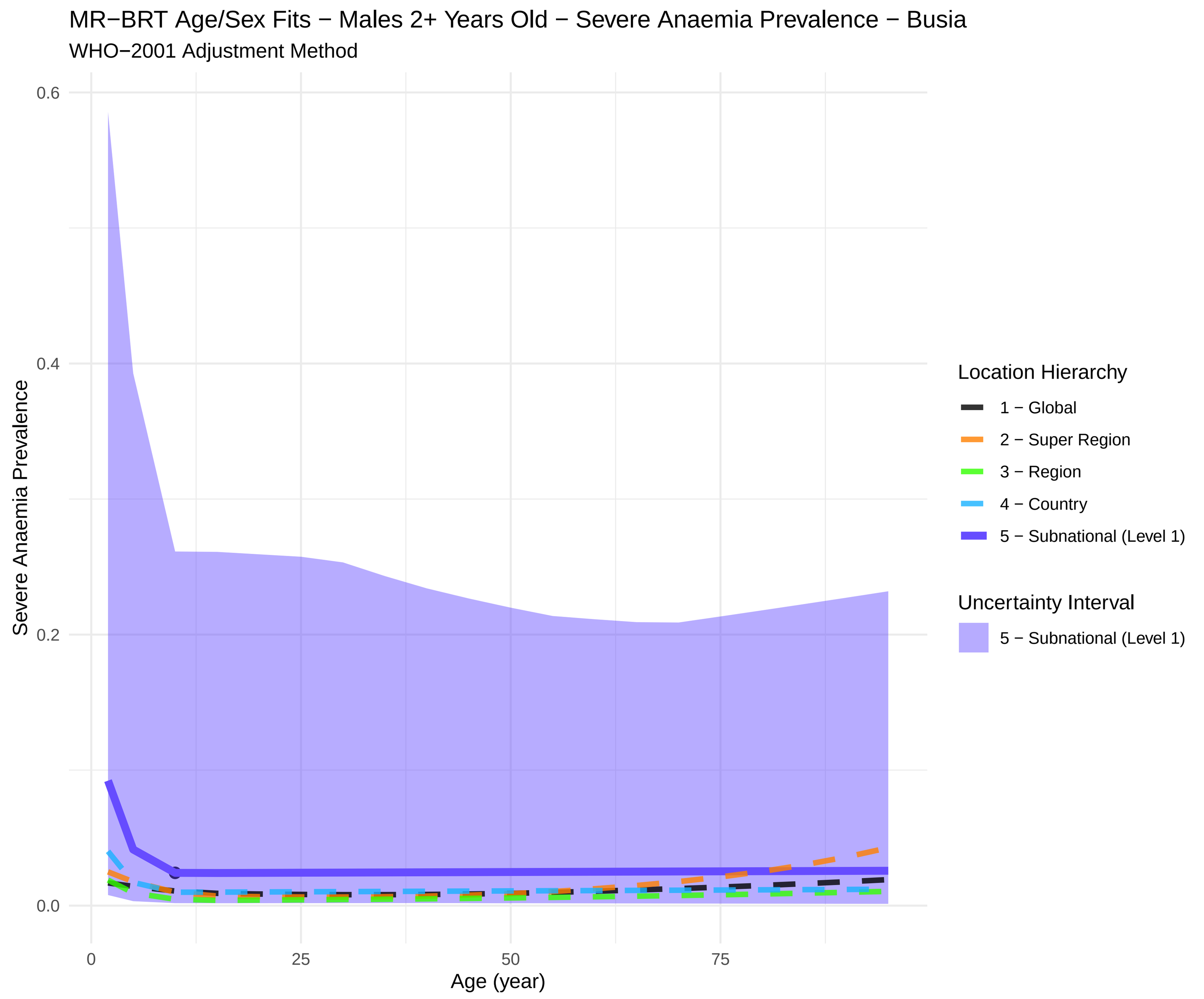
MR−BRT Age/Sex Fits − Males 2+ Years Old − Severe Anaemia Prevalence − Busia
WHO−2001 Adjustment Method
Severe Anaemia Prevalence
0.0
0.2
0.4
0.6
0
25
50
75
Age (year)
Location Hierarchy
1 − Global
2 − Super Region
3 − Region
4 − Country
5 − Subnational (Level 1)
Uncertainty Interval
5 − Subnational (Level 1)

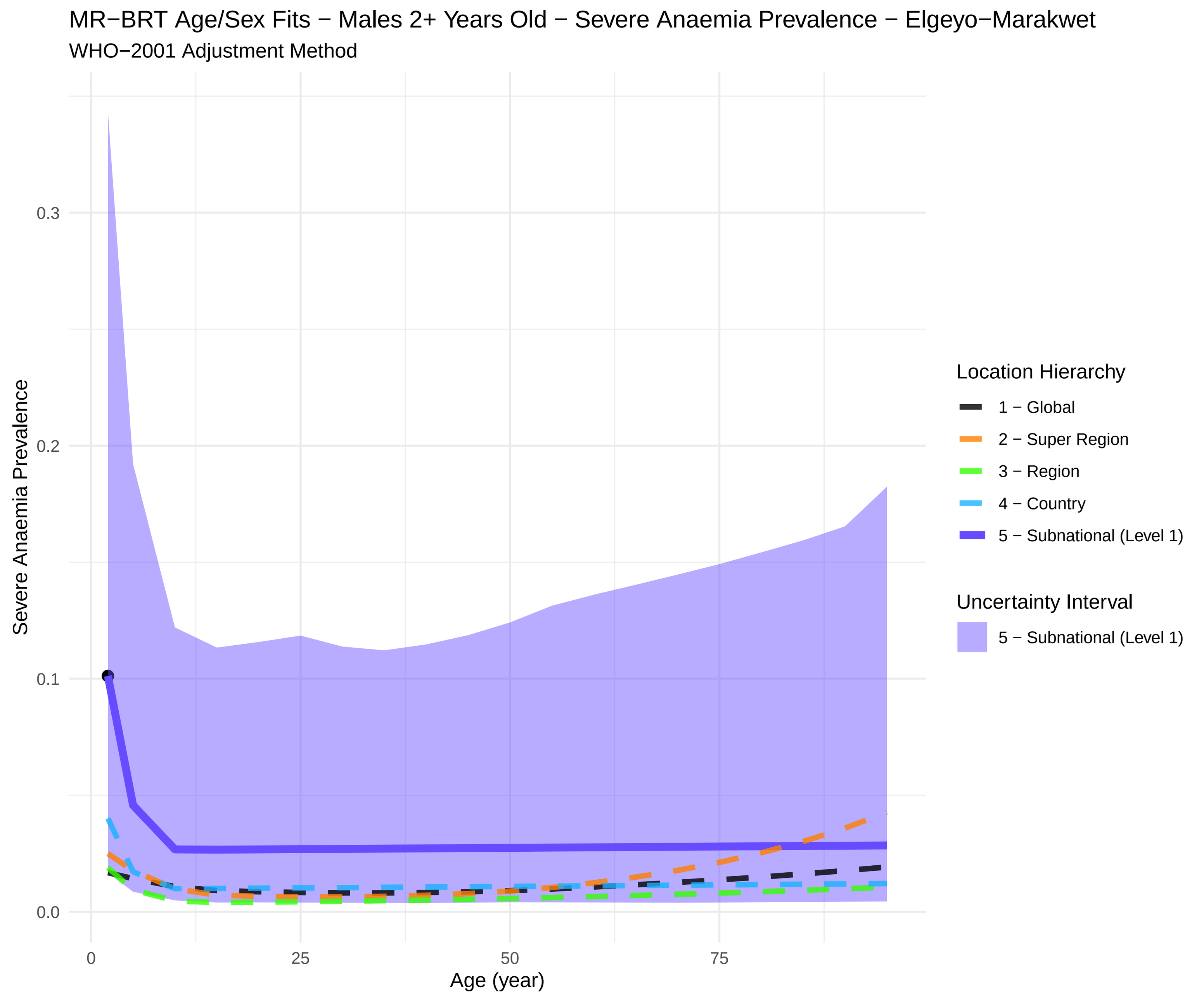
MR−BRT Age/Sex Fits − Males 2+ Years Old − Severe Anaemia Prevalence − Elgeyo−Marakwet
WHO−2001 Adjustment Method
Severe Anaemia Prevalence
0.0
0.1
0.2
0.3
0
25
50
75
Age (year)
Location Hierarchy
1 − Global
2 − Super Region
3 − Region
4 − Country
5 − Subnational (Level 1)
Uncertainty Interval
5 − Subnational (Level 1)

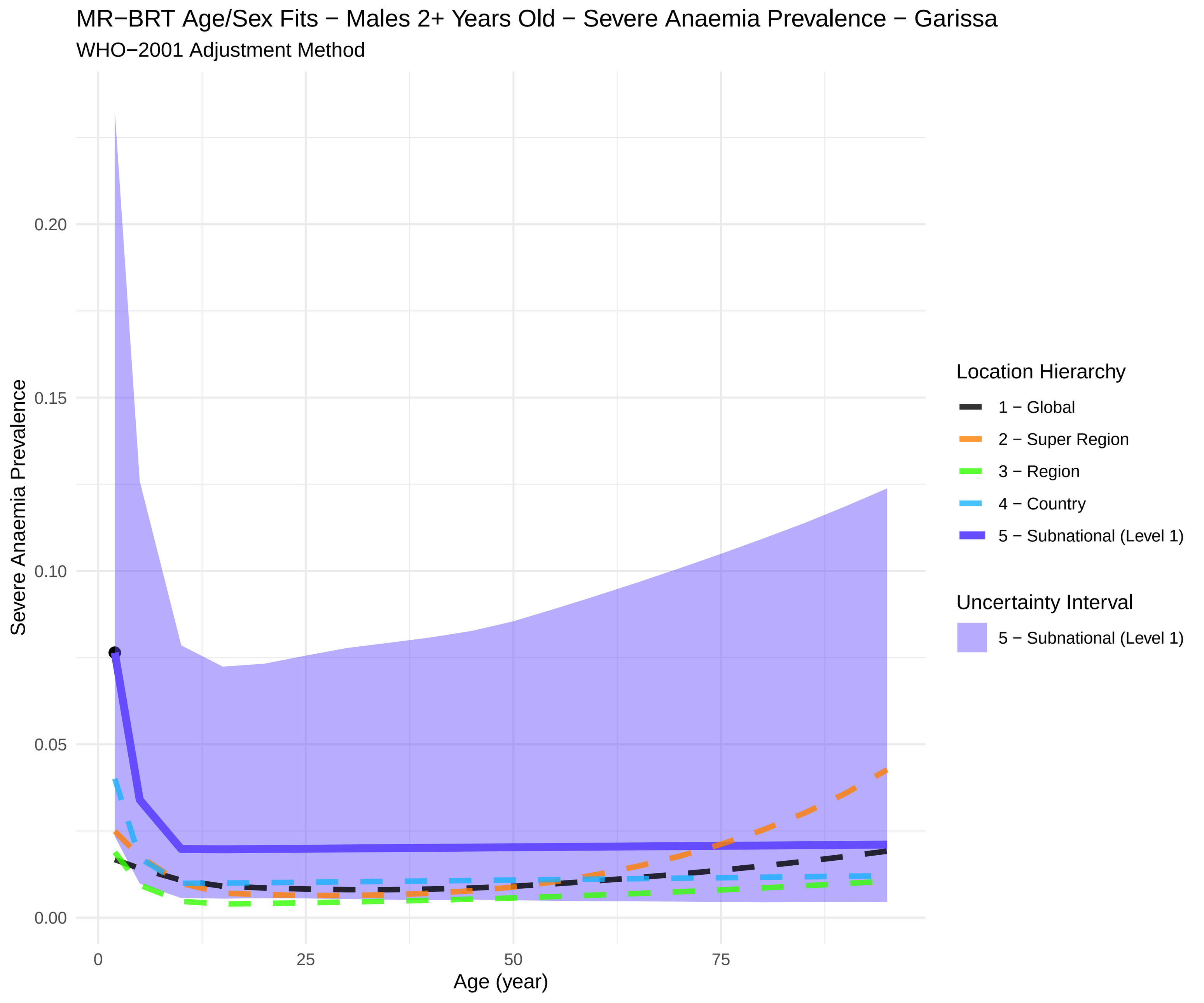
MR−BRT Age/Sex Fits − Males 2+ Years Old − Severe Anaemia Prevalence − Garissa
WHO−2001 Adjustment Method
Severe Anaemia Prevalence
0.00
0.05
0.10
0.15
0.20
0
25
50
75
Age (year)
Location Hierarchy
1 − Global
2 − Super Region
3 − Region
4 − Country
5 − Subnational (Level 1)
Uncertainty Interval
5 − Subnational (Level 1)

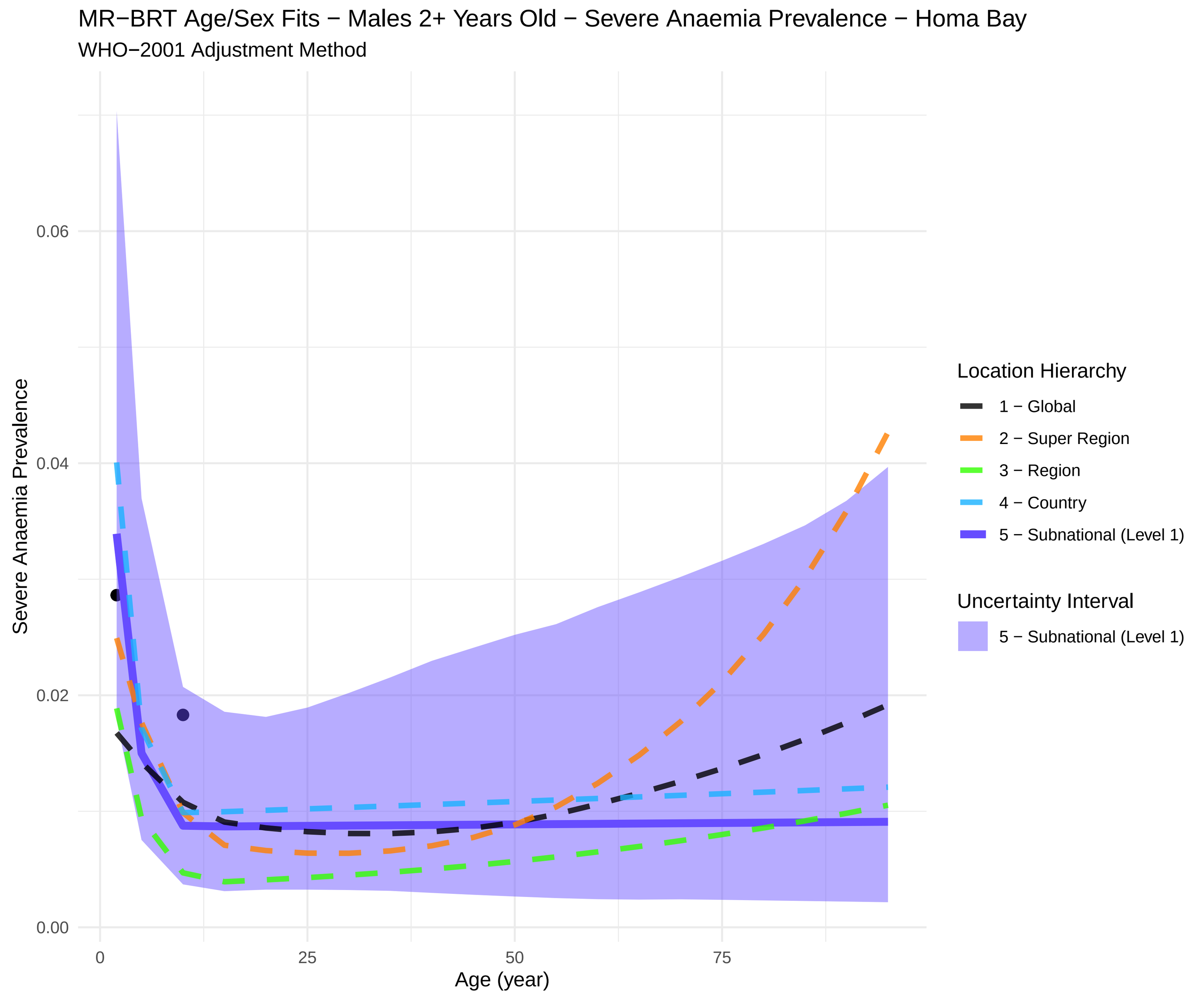
MR−BRT Age/Sex Fits − Males 2+ Years Old − Severe Anaemia Prevalence − Homa Bay
WHO−2001 Adjustment Method
Severe Anaemia Prevalence
0.00
0.02
0.04
0.06
0
25
50
75
Age (year)
Location Hierarchy
1 − Global
2 − Super Region
3 − Region
4 − Country
5 − Subnational (Level 1)
Uncertainty Interval
5 − Subnational (Level 1)

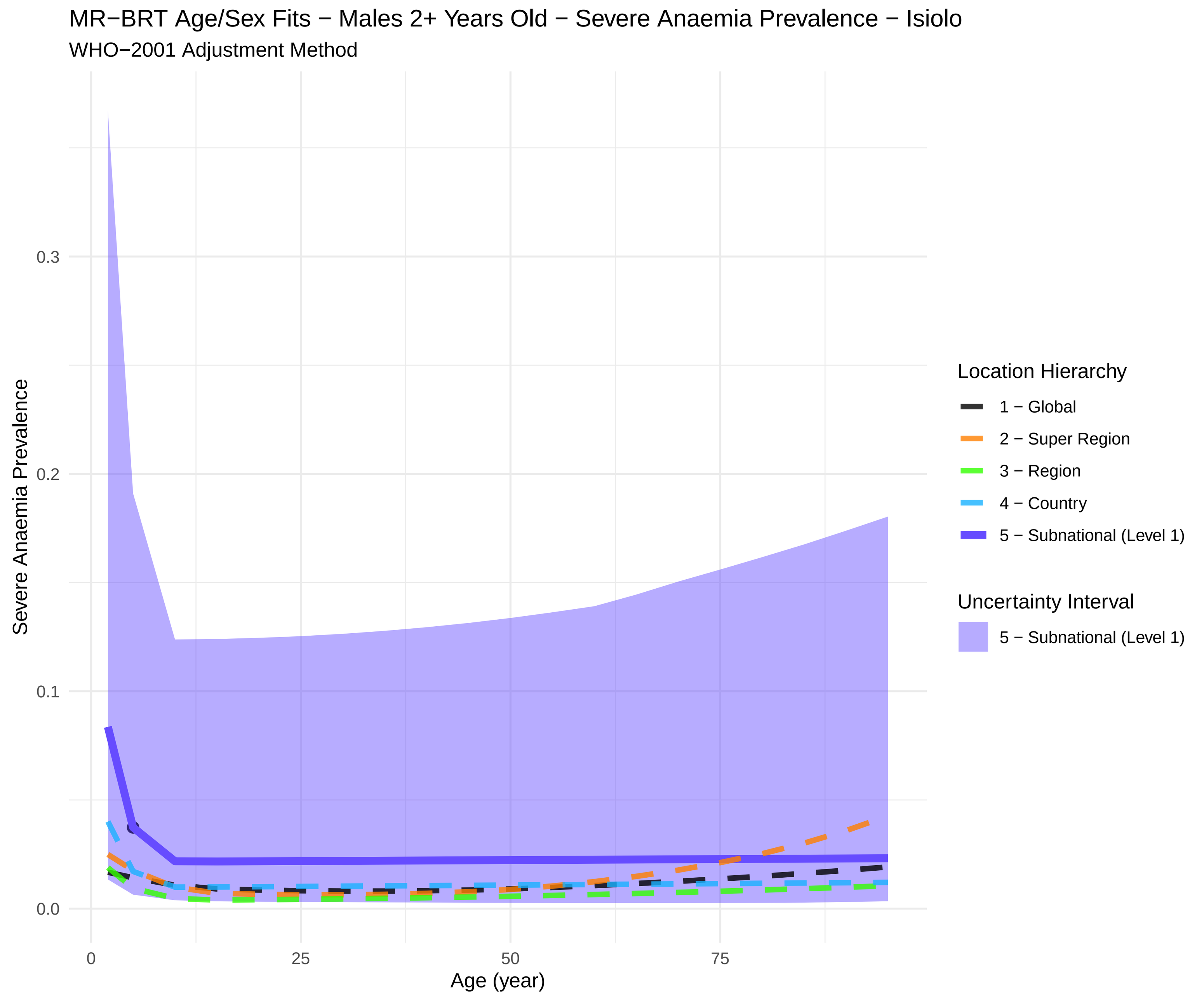
MR−BRT Age/Sex Fits − Males 2+ Years Old − Severe Anaemia Prevalence − Isiolo
WHO−2001 Adjustment Method
Severe Anaemia Prevalence
0.0
0.1
0.2
0.3
0
25
50
75
Age (year)
Location Hierarchy
1 − Global
2 − Super Region
3 − Region
4 − Country
5 − Subnational (Level 1)
Uncertainty Interval
5 − Subnational (Level 1)

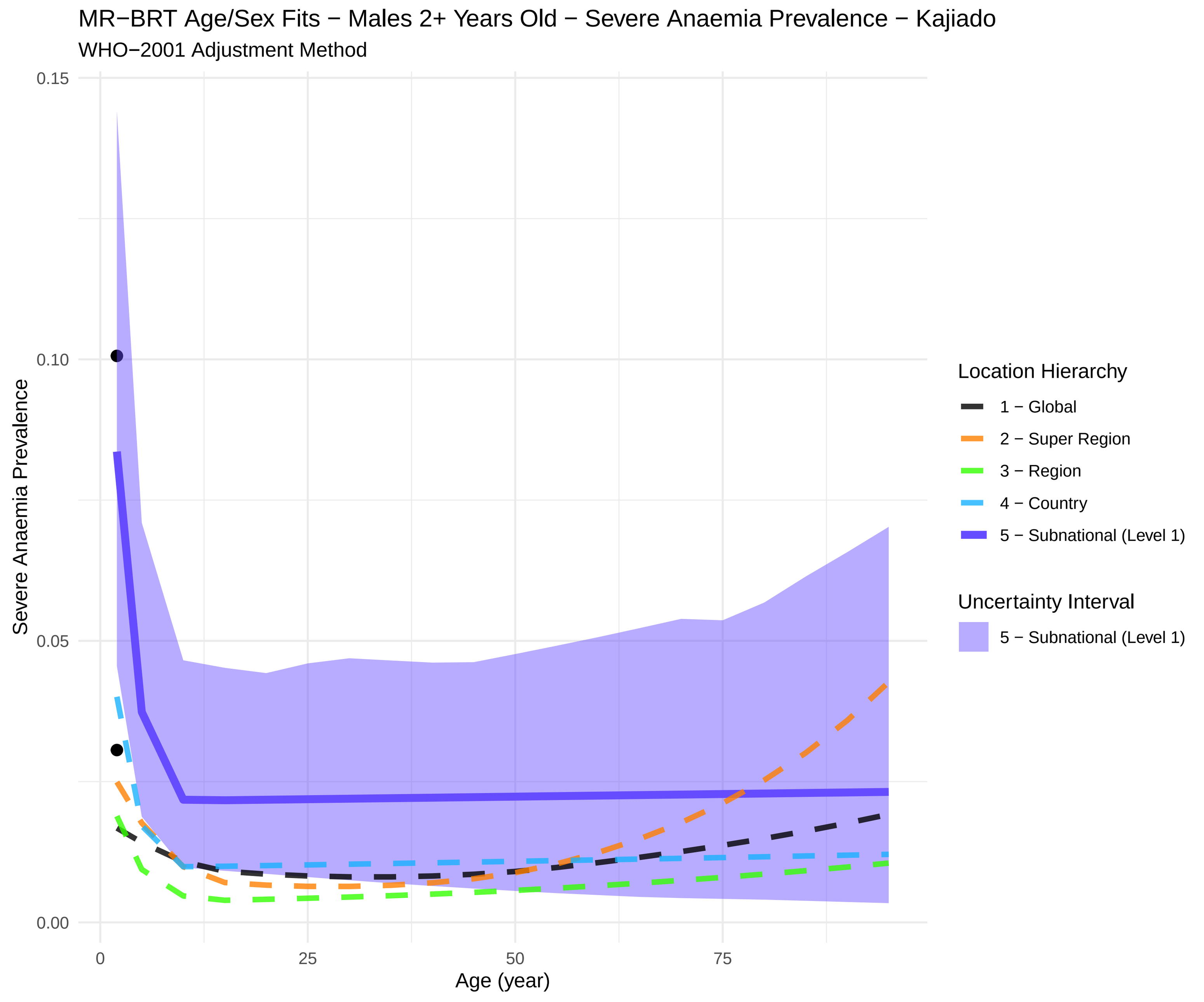
MR−BRT Age/Sex Fits − Males 2+ Years Old − Severe Anaemia Prevalence − Kajiado
WHO−2001 Adjustment Method
Severe Anaemia Prevalence
0.00
0.05
0.10
0.15
0
25
50
75
Age (year)
Location Hierarchy
1 − Global
2 − Super Region
3 − Region
4 − Country
5 − Subnational (Level 1)
Uncertainty Interval
5 − Subnational (Level 1)

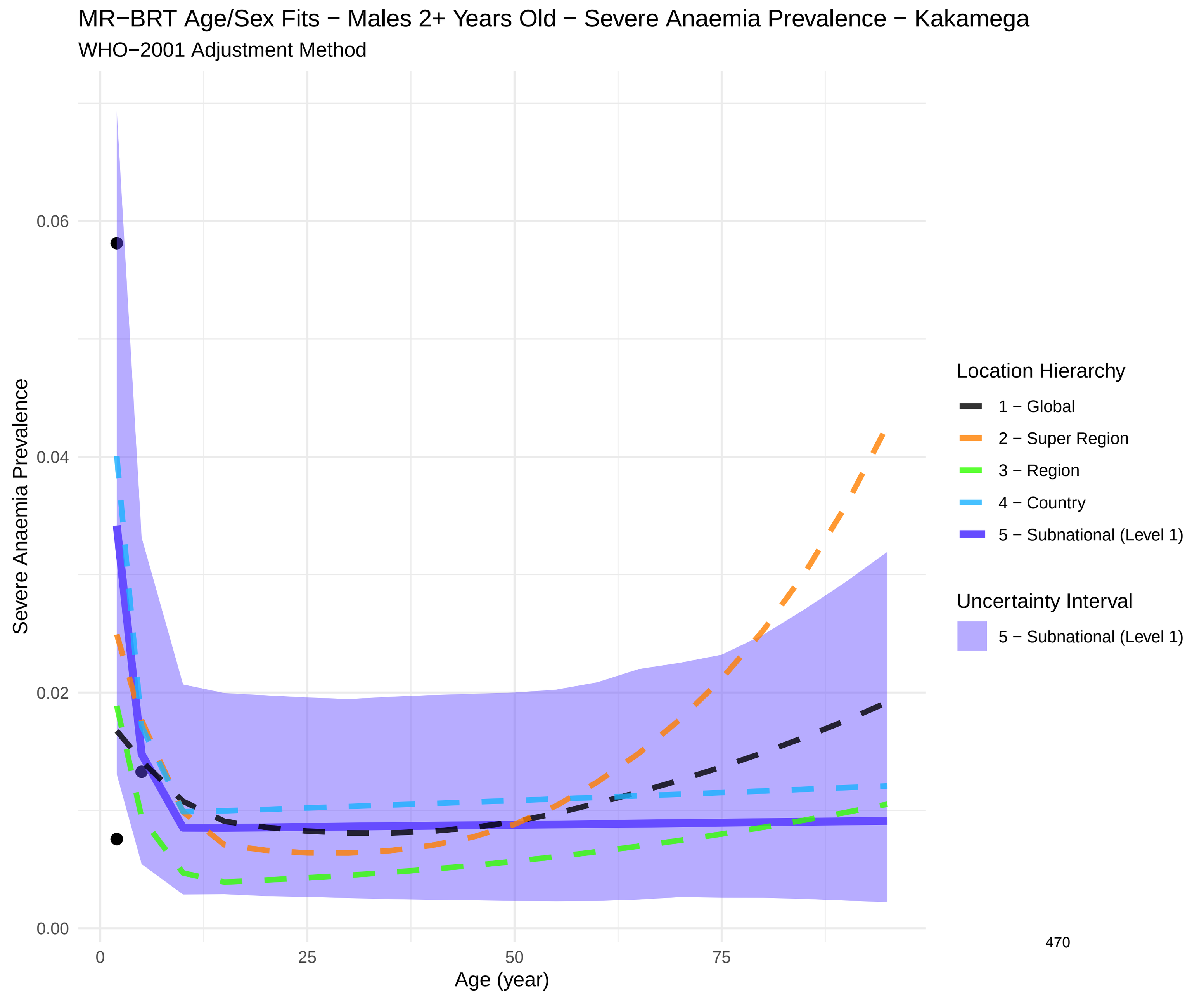
MR−BRT Age/Sex Fits − Males 2+ Years Old − Severe Anaemia Prevalence − Kakamega
WHO−2001 Adjustment Method
Severe Anaemia Prevalence
0.00
0.02
0.04
0.06
0
25
50
75
Age (year)
Location Hierarchy
1 − Global
2 − Super Region
3 − Region
4 − Country
5 − Subnational (Level 1)
Uncertainty Interval
5 − Subnational (Level 1)

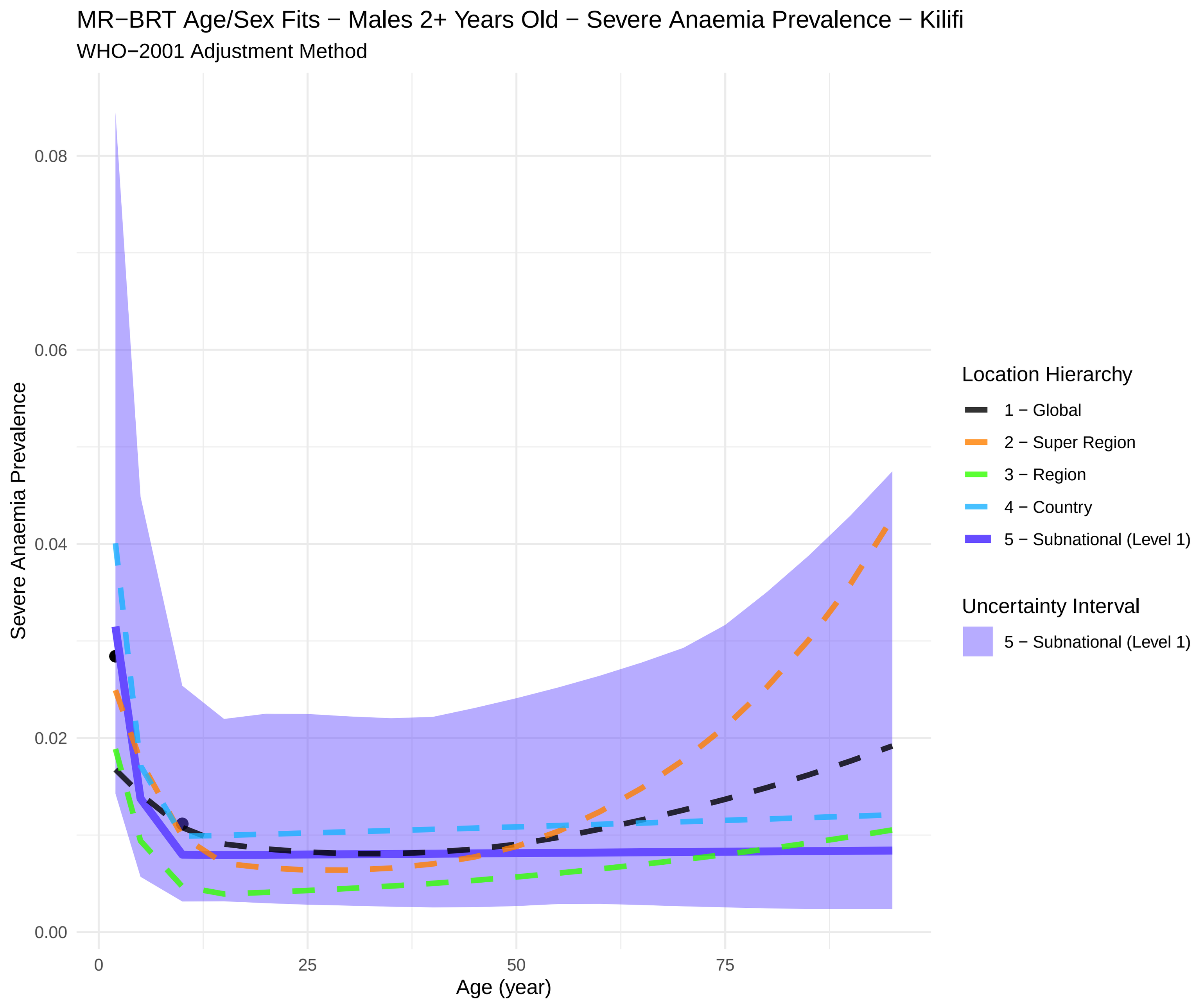
MR−BRT Age/Sex Fits − Males 2+ Years Old − Severe Anaemia Prevalence − Kilifi
WHO−2001 Adjustment Method
Severe Anaemia Prevalence
0.00
0.02
0.04
0.06
0.08
0
25
50
75
Age (year)
Location Hierarchy
1 − Global
2 − Super Region
3 − Region
4 − Country
5 − Subnational (Level 1)
Uncertainty Interval
5 − Subnational (Level 1)

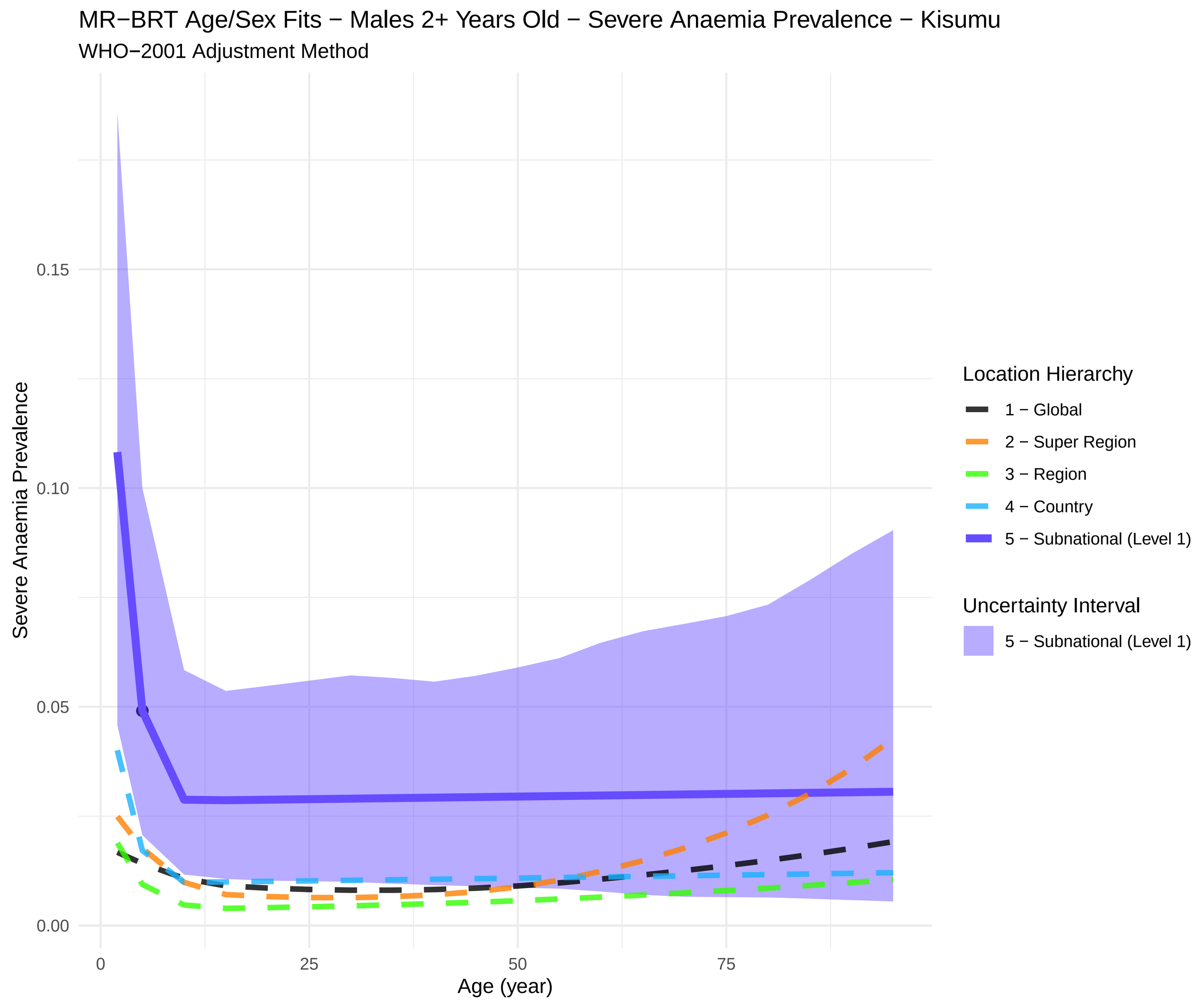
MR−BRT Age/Sex Fits − Males 2+ Years Old − Severe Anaemia Prevalence − Kisumu
WHO−2001 Adjustment Method
Severe Anaemia Prevalence
0.00
0.05
0.10
0.15
0
25
50
75
Age (year)
Location Hierarchy
1 − Global
2 − Super Region
3 − Region
4 − Country
5 − Subnational (Level 1)
Uncertainty Interval
5 − Subnational (Level 1)

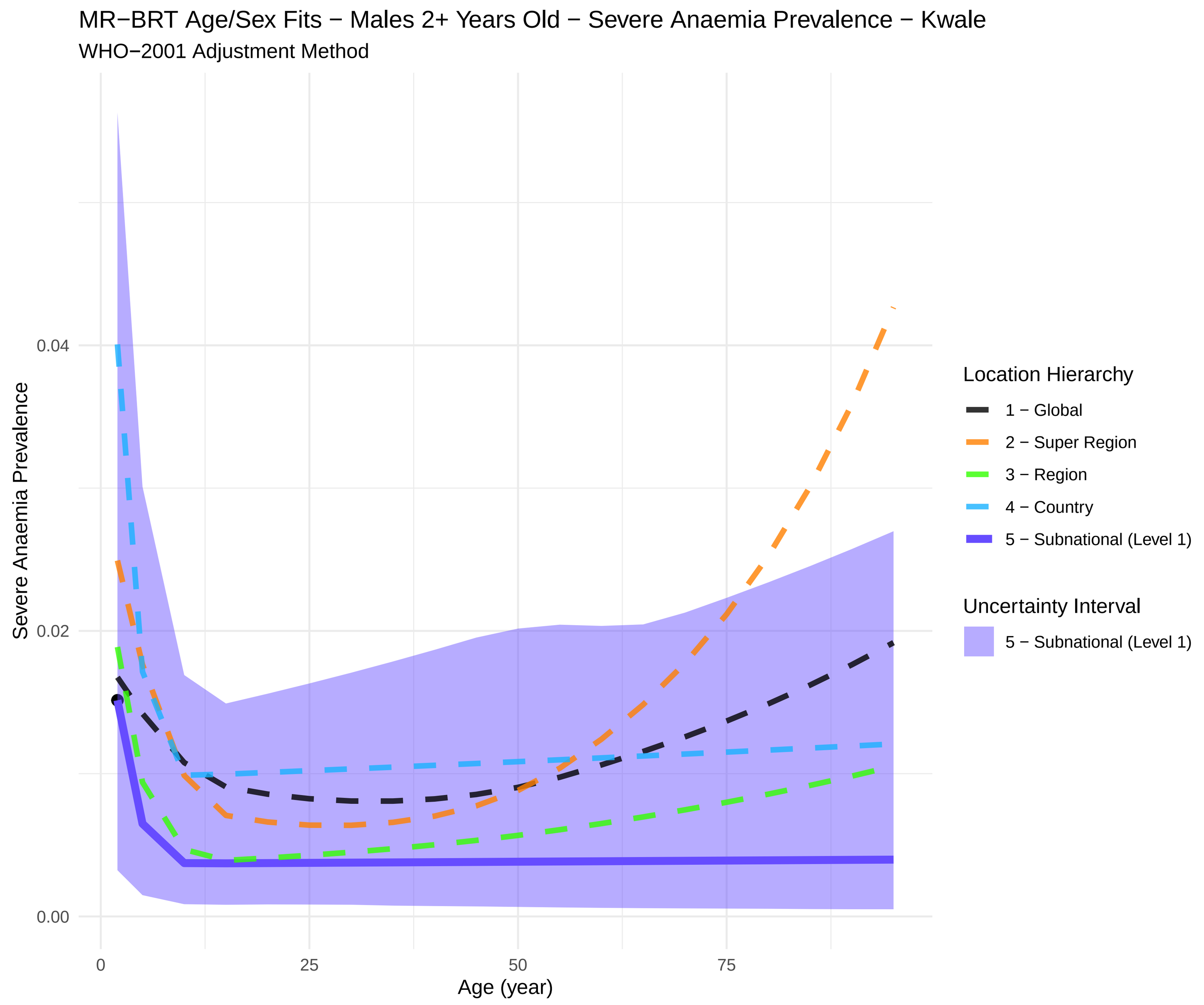
MR−BRT Age/Sex Fits − Males 2+ Years Old − Severe Anaemia Prevalence − Kwale
WHO−2001 Adjustment Method
Severe Anaemia Prevalence
0.00
0.02
0.04
0
25
50
75
Age (year)
Location Hierarchy
1 − Global
2 − Super Region
3 − Region
4 − Country
5 − Subnational (Level 1)
Uncertainty Interval
5 − Subnational (Level 1)

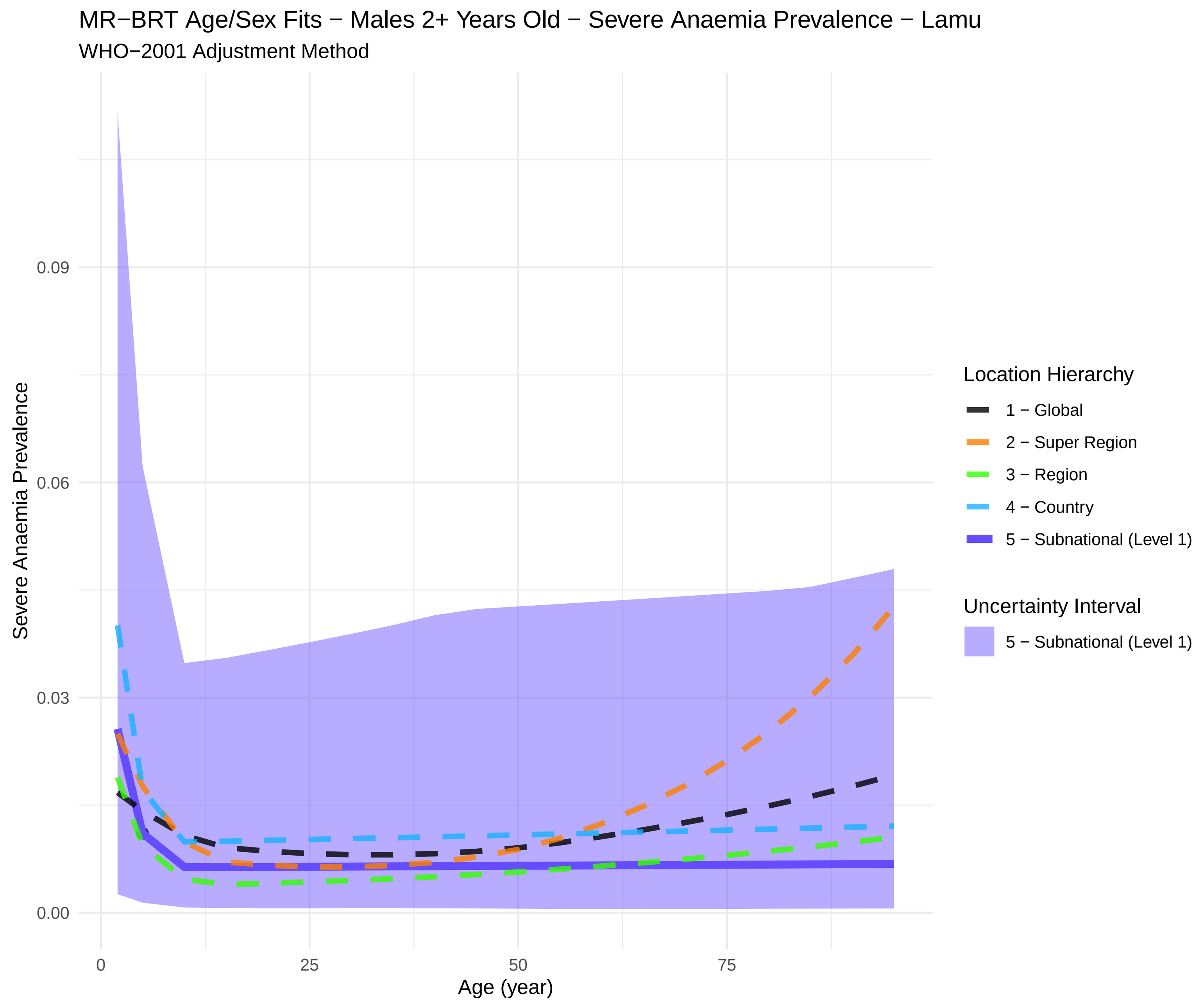
MR−BRT Age/Sex Fits − Males 2+ Years Old − Severe Anaemia Prevalence − Lamu
WHO−2001 Adjustment Method
Severe Anaemia Prevalence
0.00
0.03
0.06
0.09
0
25
50
75
Age (year)
Location Hierarchy
1 − Global
2 − Super Region
3 − Region
4 − Country
5 − Subnational (Level 1)
Uncertainty Interval
5 − Subnational (Level 1)

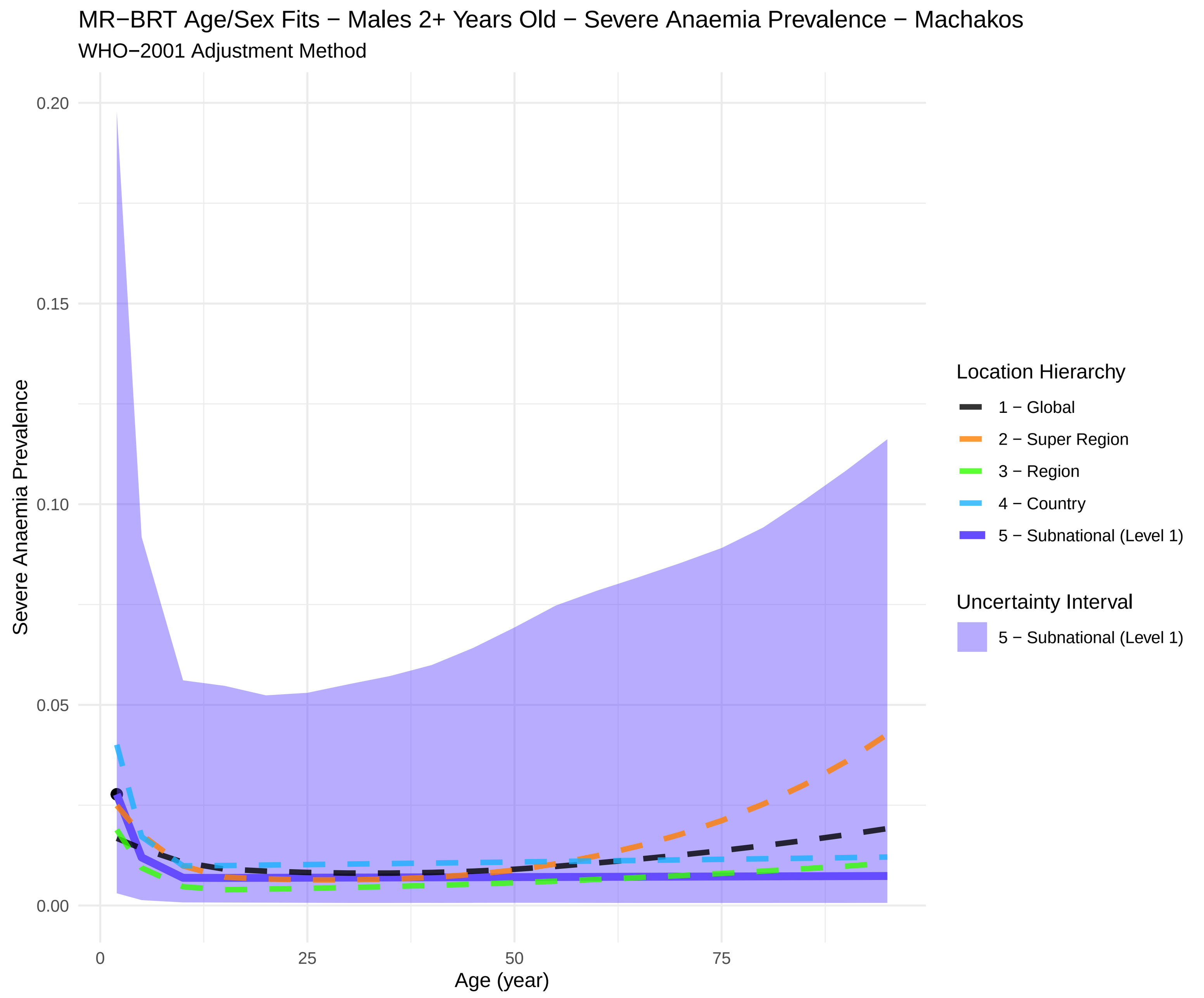
MR−BRT Age/Sex Fits − Males 2+ Years Old − Severe Anaemia Prevalence − Machakos
WHO−2001 Adjustment Method
Severe Anaemia Prevalence
0.00
0.05
0.10
0.15
0.20
0
25
50
75
Age (year)
Location Hierarchy
1 − Global
2 − Super Region
3 − Region
4 − Country
5 − Subnational (Level 1)
Uncertainty Interval
5 − Subnational (Level 1)

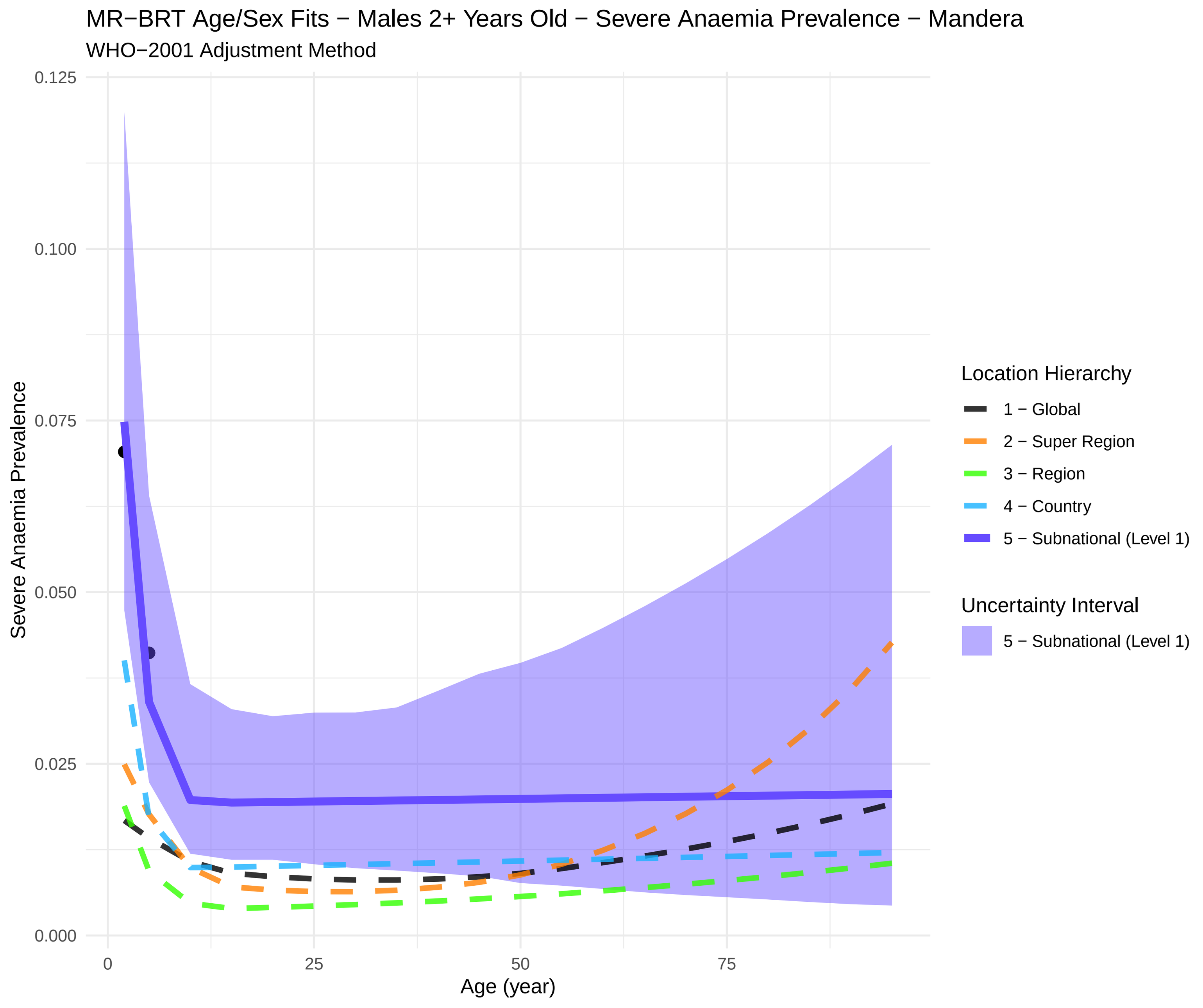
MR−BRT Age/Sex Fits − Males 2+ Years Old − Severe Anaemia Prevalence − Mandera
WHO−2001 Adjustment Method
Severe Anaemia Prevalence
0.000
0.025
0.050
0.075
0.100
0.125
0
25
50
75
Age (year)
Location Hierarchy
1 − Global
2 − Super Region
3 − Region
4 − Country
5 − Subnational (Level 1)
Uncertainty Interval
5 − Subnational (Level 1)

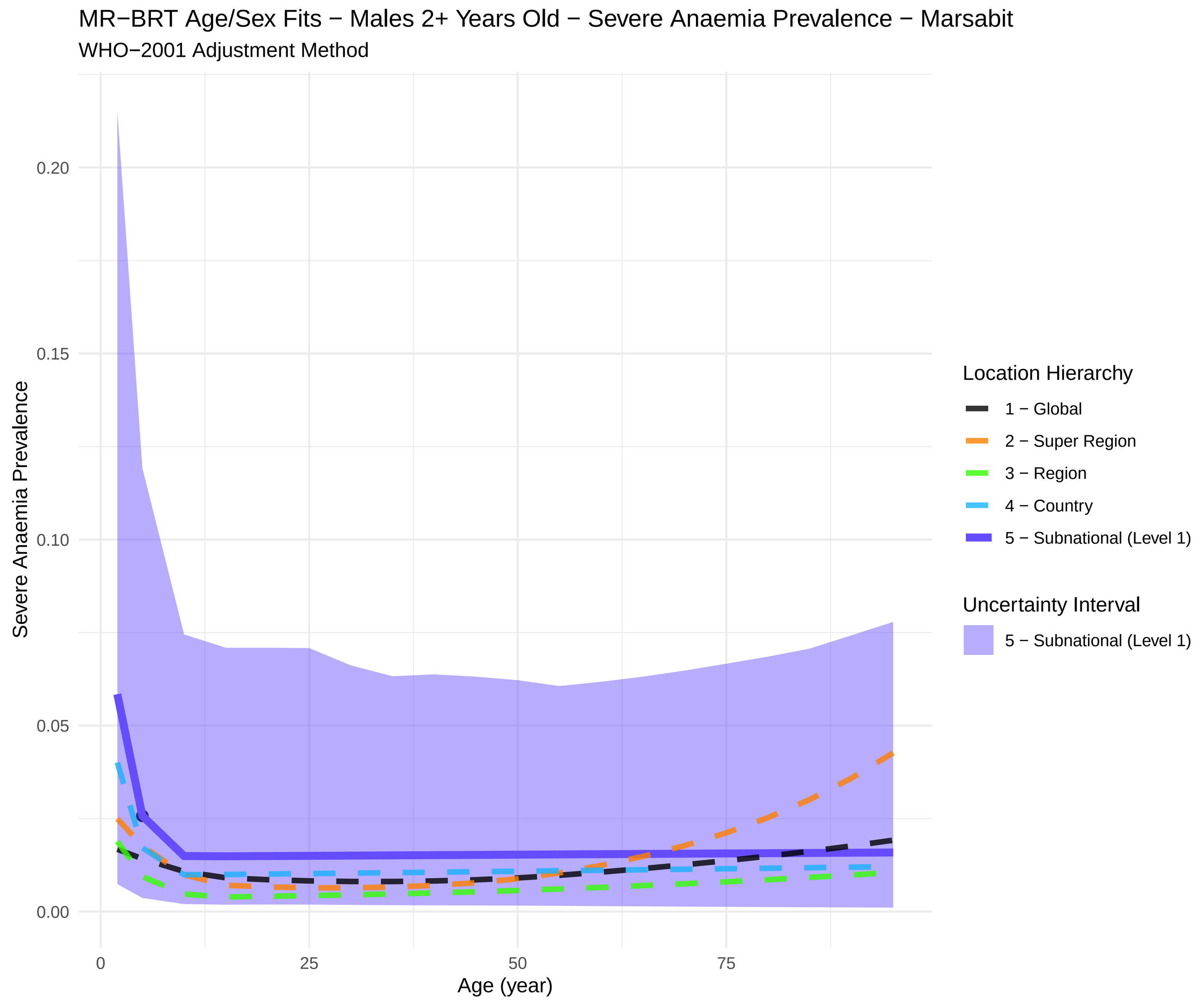
MR−BRT Age/Sex Fits − Males 2+ Years Old − Severe Anaemia Prevalence − Marsabit
WHO−2001 Adjustment Method
Severe Anaemia Prevalence
0.00
0.05
0.10
0.15
0.20
0
25
50
75
Age (year)
Location Hierarchy
1 − Global
2 − Super Region
3 − Region
4 − Country
5 − Subnational (Level 1)
Uncertainty Interval
5 − Subnational (Level 1)

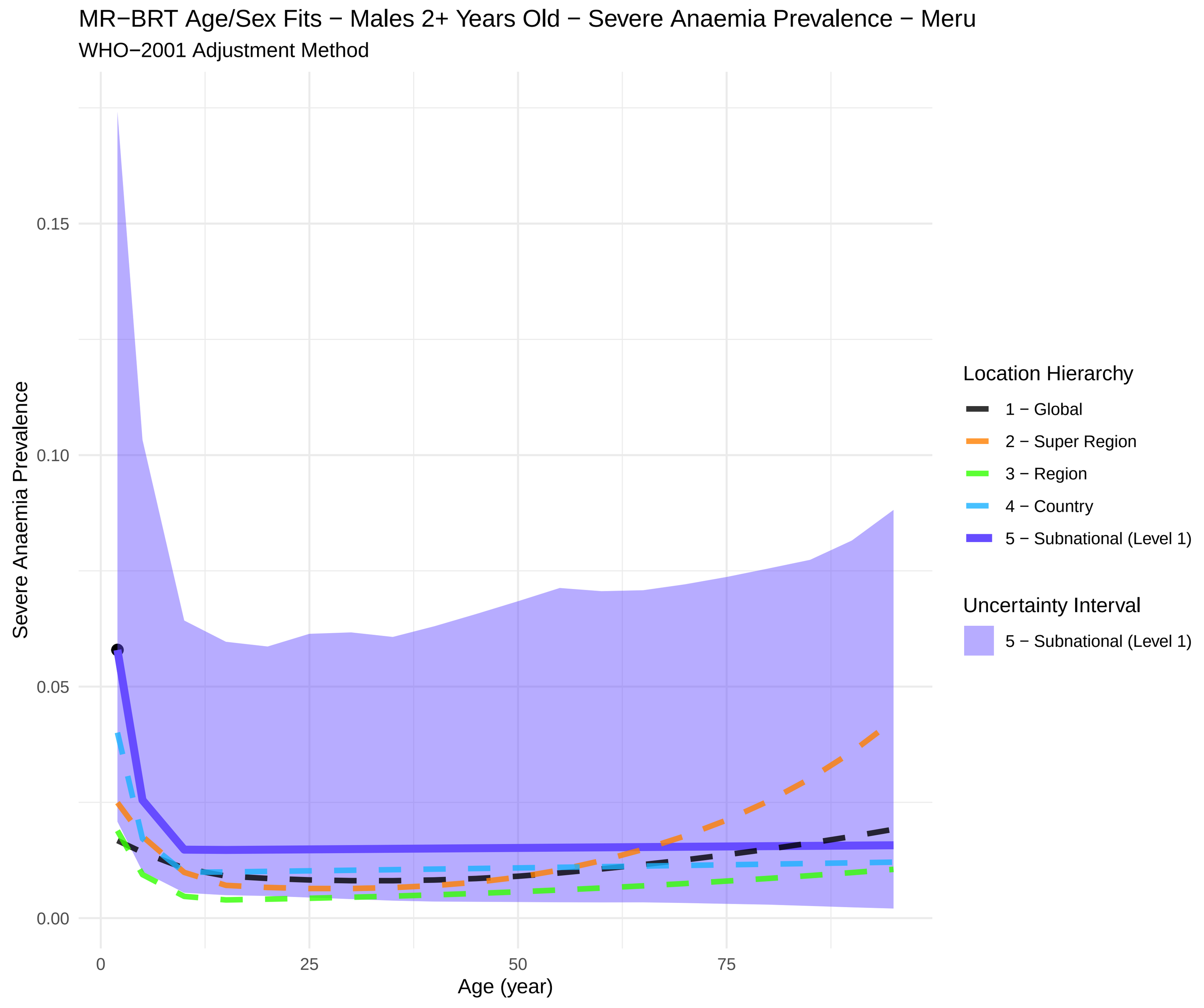
MR−BRT Age/Sex Fits − Males 2+ Years Old − Severe Anaemia Prevalence − Meru
WHO−2001 Adjustment Method
Severe Anaemia Prevalence
0.00
0.05
0.10
0.15
0
25
50
75
Age (year)
Location Hierarchy
1 − Global
2 − Super Region
3 − Region
4 − Country
5 − Subnational (Level 1)
Uncertainty Interval
5 − Subnational (Level 1)

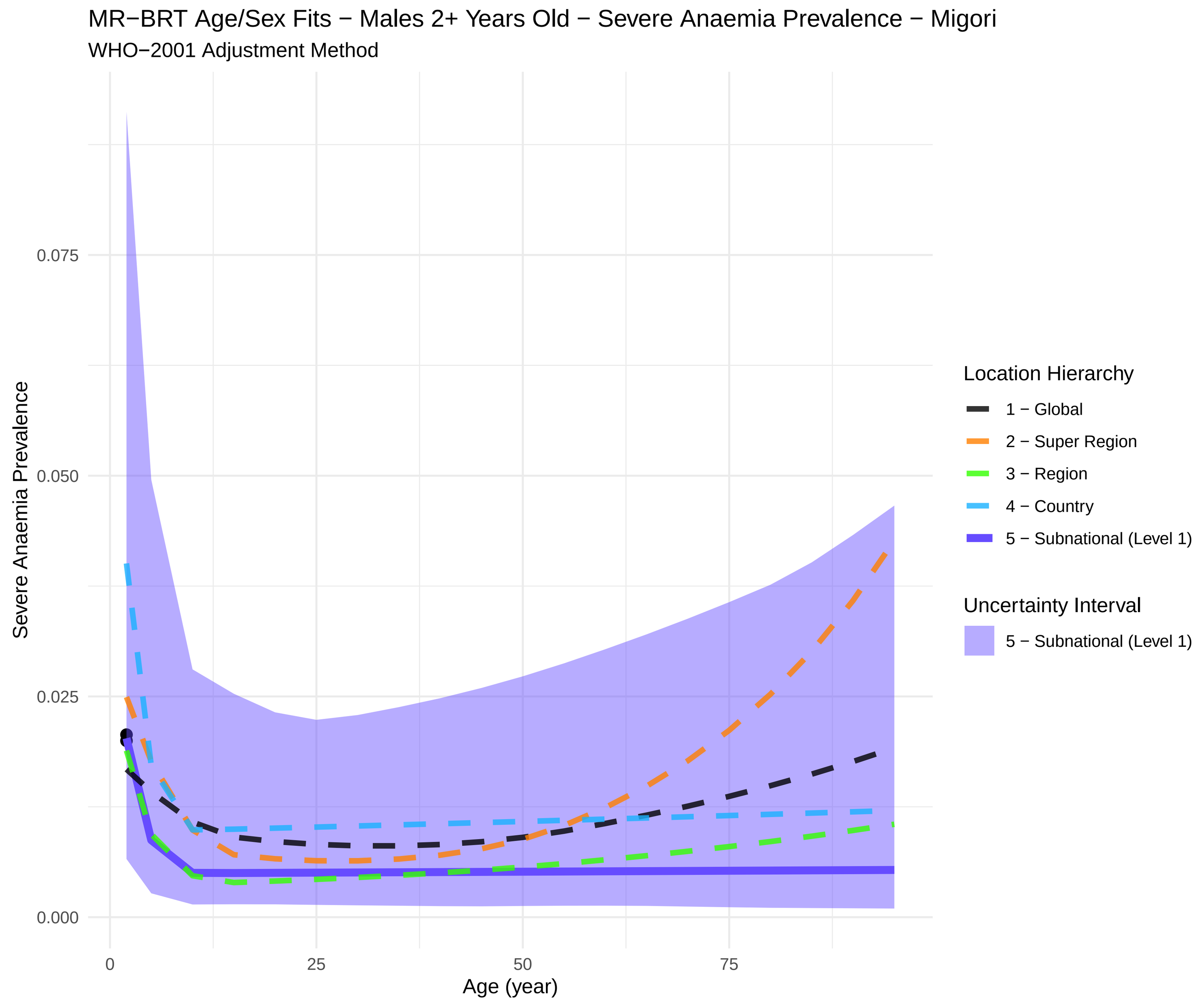
MR−BRT Age/Sex Fits − Males 2+ Years Old − Severe Anaemia Prevalence − Migori
WHO−2001 Adjustment Method
Severe Anaemia Prevalence
0.000
0.025
0.050
0.075
0
25
50
75
Age (year)
Location Hierarchy
1 − Global
2 − Super Region
3 − Region
4 − Country
5 − Subnational (Level 1)
Uncertainty Interval
5 − Subnational (Level 1)

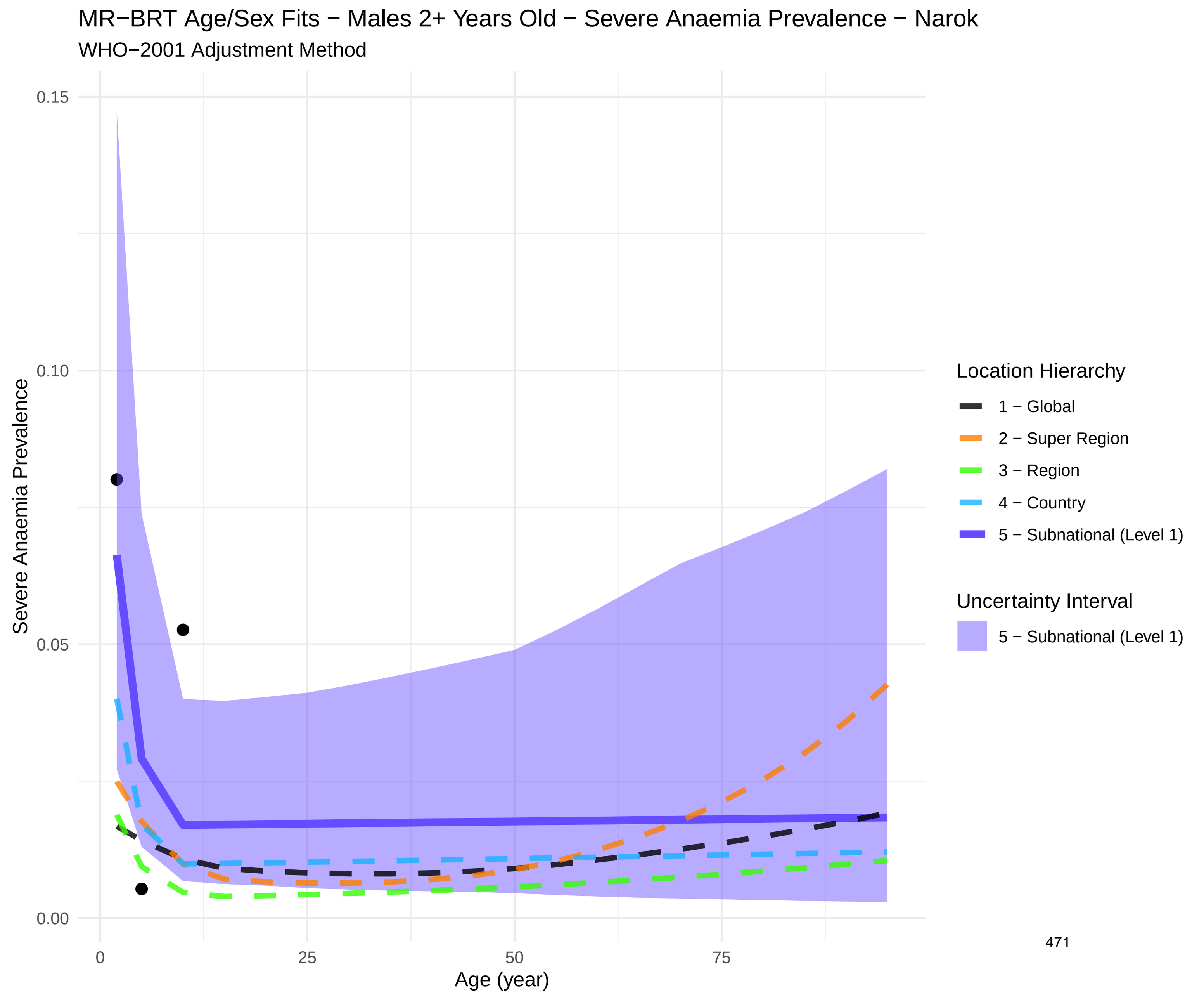
MR−BRT Age/Sex Fits − Males 2+ Years Old − Severe Anaemia Prevalence − Narok
WHO−2001 Adjustment Method
Severe Anaemia Prevalence
0.00
0.05
0.10
0.15
0
25
50
75
Age (year)
Location Hierarchy
1 − Global
2 − Super Region
3 − Region
4 − Country
5 − Subnational (Level 1)
Uncertainty Interval
5 − Subnational (Level 1)

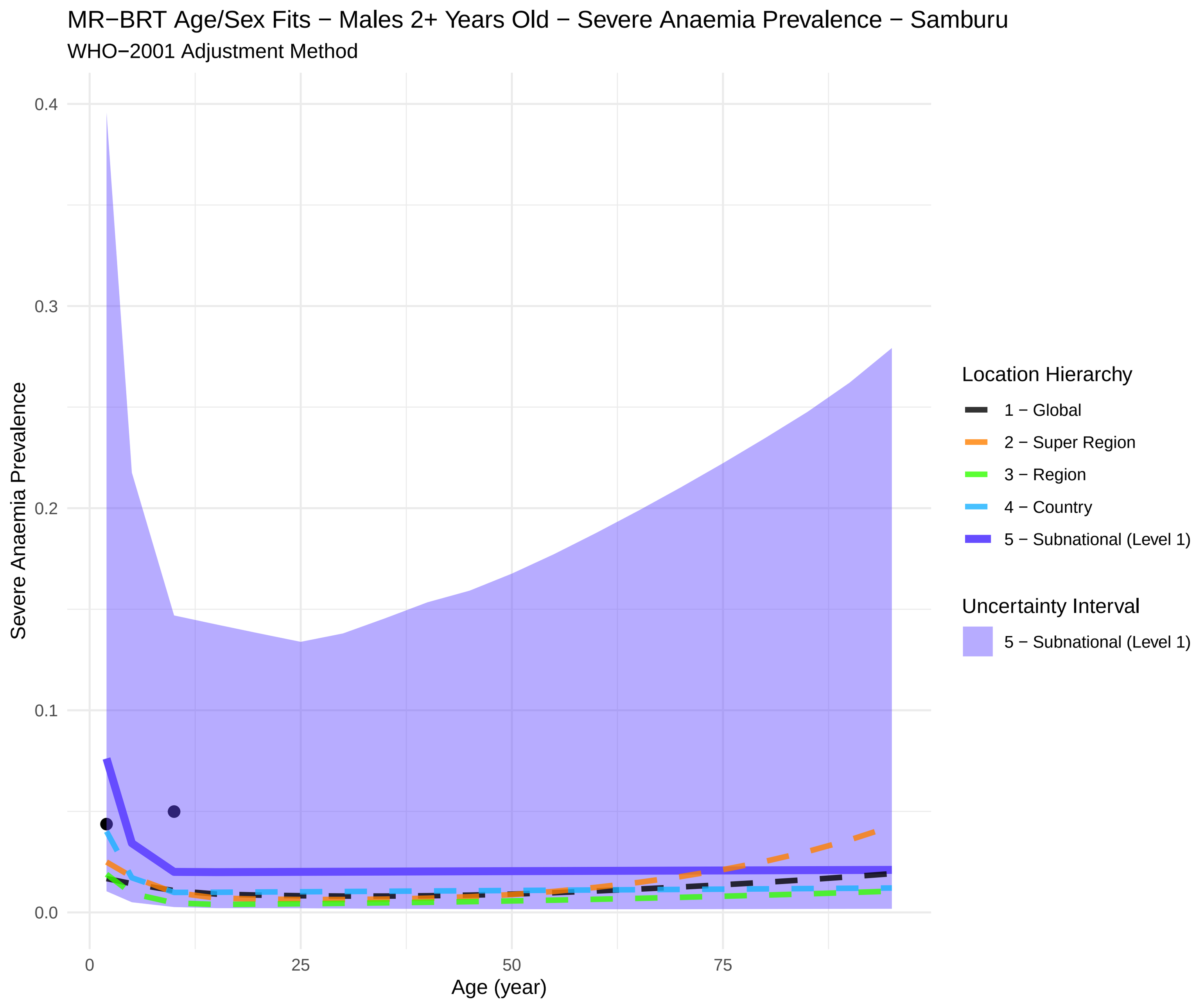
MR−BRT Age/Sex Fits − Males 2+ Years Old − Severe Anaemia Prevalence − Samburu
WHO−2001 Adjustment Method
Severe Anaemia Prevalence
Age (year)
0.0
0.1
0.2
0.3
0.4
0
25
50
75
Location Hierarchy
1 − Global
2 − Super Region
3 − Region
4 − Country
5 − Subnational (Level 1)
Uncertainty Interval
5 − Subnational (Level 1)

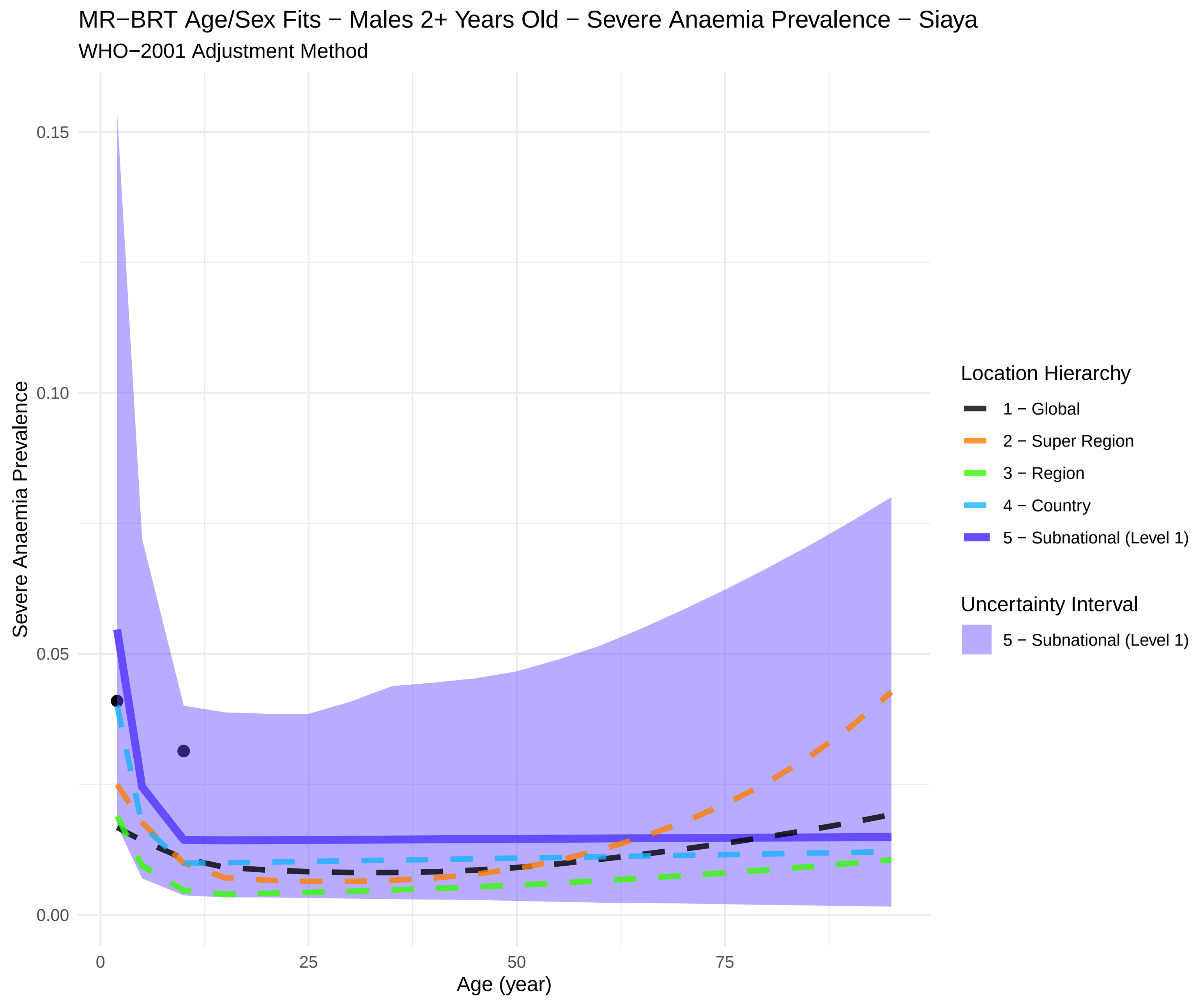
MR−BRT Age/Sex Fits − Males 2+ Years Old − Severe Anaemia Prevalence − Siaya
WHO−2001 Adjustment Method
Severe Anaemia Prevalence
Age (year)
0.00
0.05
0.10
0.15
0
25
50
75
Location Hierarchy
1 − Global
2 − Super Region
3 − Region
4 − Country
5 − Subnational (Level 1)
Uncertainty Interval
5 − Subnational (Level 1)

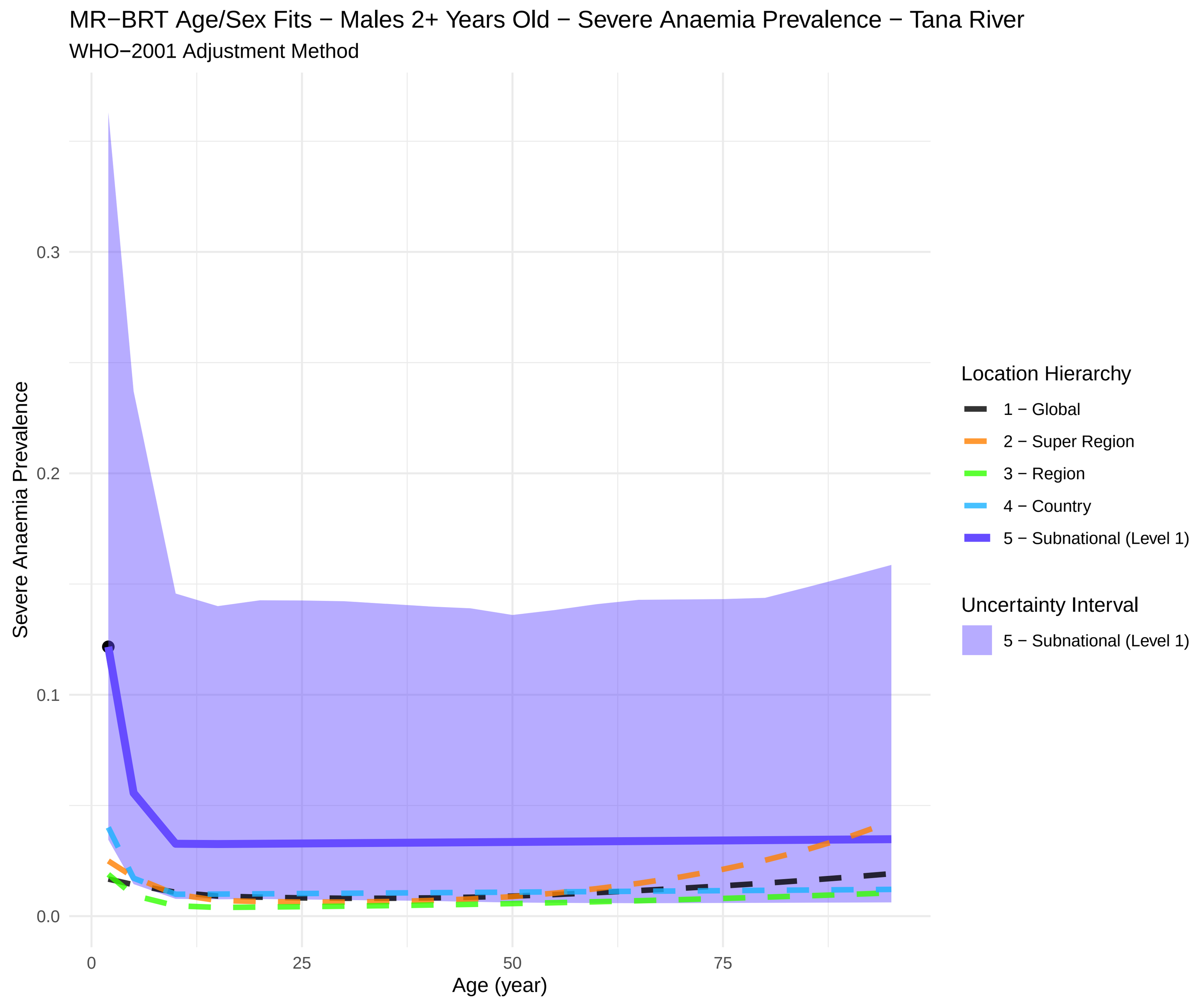
MR−BRT Age/Sex Fits − Males 2+ Years Old − Severe Anaemia Prevalence − Tana River
WHO−2001 Adjustment Method
Severe Anaemia Prevalence
Age (year)
0.0
0.1
0.2
0.3
0
25
50
75
Location Hierarchy
1 − Global
2 − Super Region
3 − Region
4 − Country
5 − Subnational (Level 1)
Uncertainty Interval
5 − Subnational (Level 1)

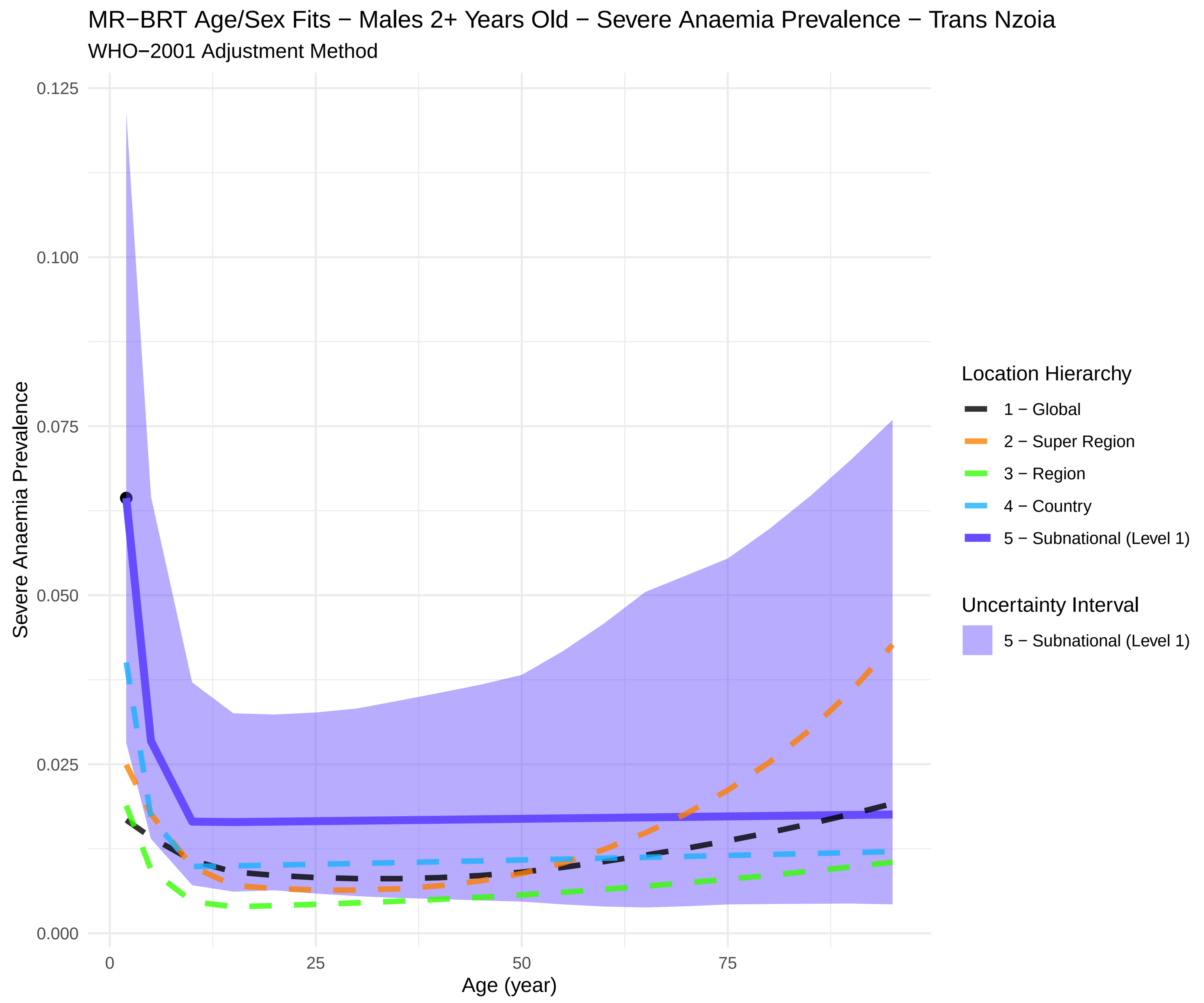
MR−BRT Age/Sex Fits − Males 2+ Years Old − Severe Anaemia Prevalence − Trans Nzoia
WHO−2001 Adjustment Method
Severe Anaemia Prevalence
Age (year)
0.000
0.025
0.050
0.075
0.100
0.125
0
25
50
75
Location Hierarchy
1 − Global
2 − Super Region
3 − Region
4 − Country
5 − Subnational (Level 1)
Uncertainty Interval
5 − Subnational (Level 1)

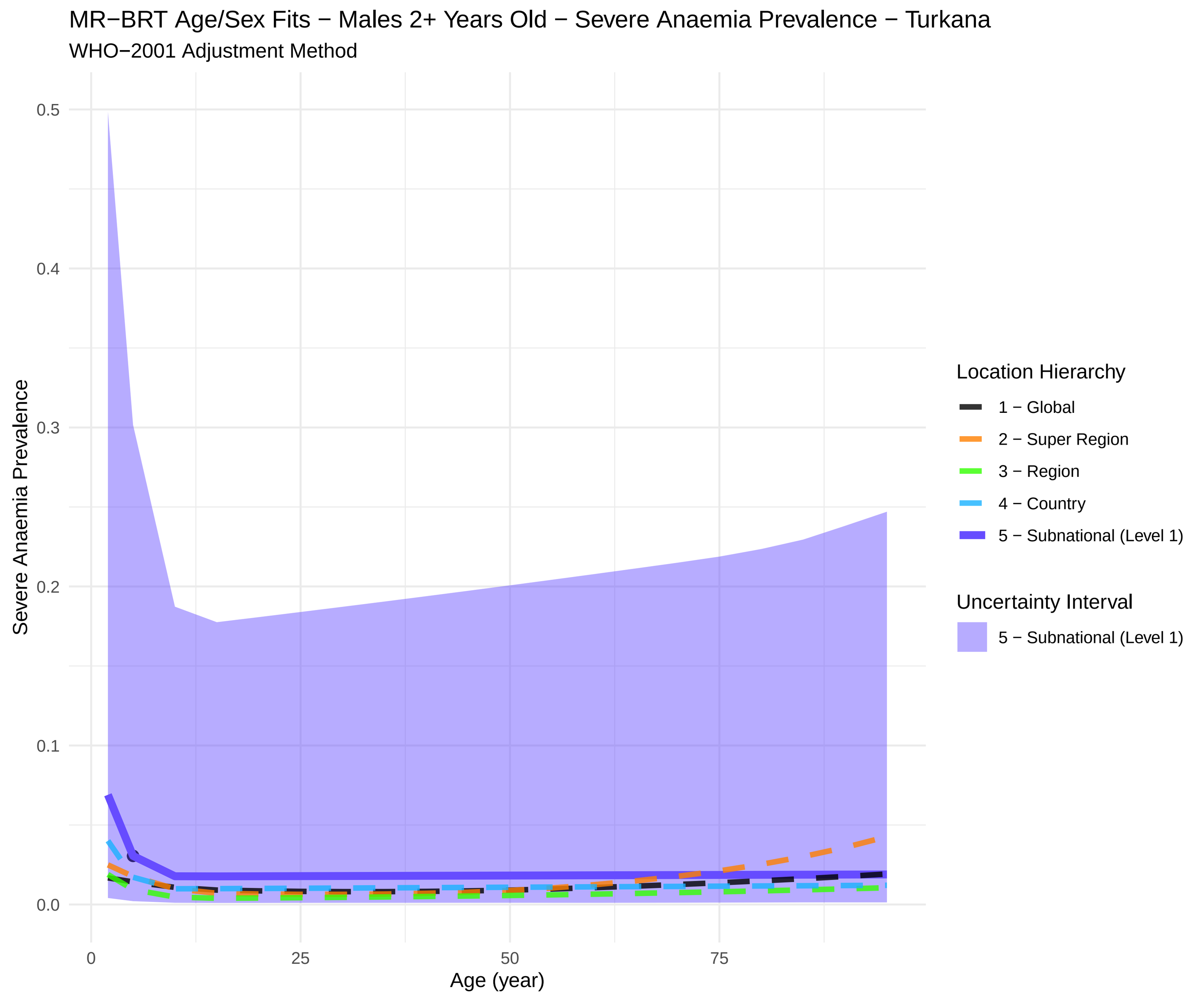
MR−BRT Age/Sex Fits − Males 2+ Years Old − Severe Anaemia Prevalence − Turkana
WHO−2001 Adjustment Method
Severe Anaemia Prevalence
Age (year)
0.0
0.1
0.2
0.3
0.4
0.5
0
25
50
75
Location Hierarchy
1 − Global
2 − Super Region
3 − Region
4 − Country
5 − Subnational (Level 1)
Uncertainty Interval
5 − Subnational (Level 1)

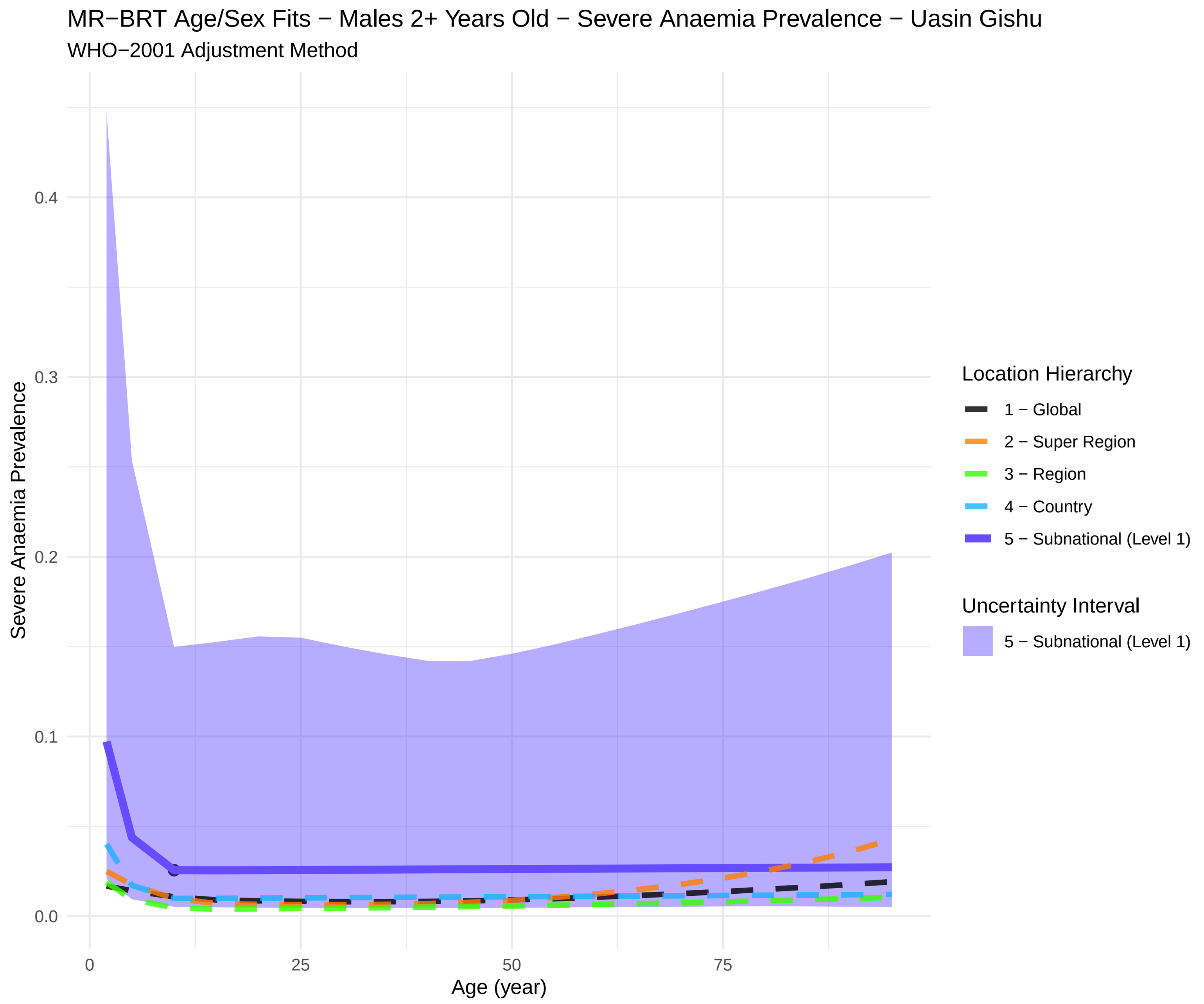
MR−BRT Age/Sex Fits − Males 2+ Years Old − Severe Anaemia Prevalence − Uasin Gishu
WHO−2001 Adjustment Method
Severe Anaemia Prevalence
Age (year)
0.0
0.1
0.2
0.3
0.4
0
25
50
75
Location Hierarchy
1 − Global
2 − Super Region
3 − Region
4 − Country
5 − Subnational (Level 1)
Uncertainty Interval
5 − Subnational (Level 1)

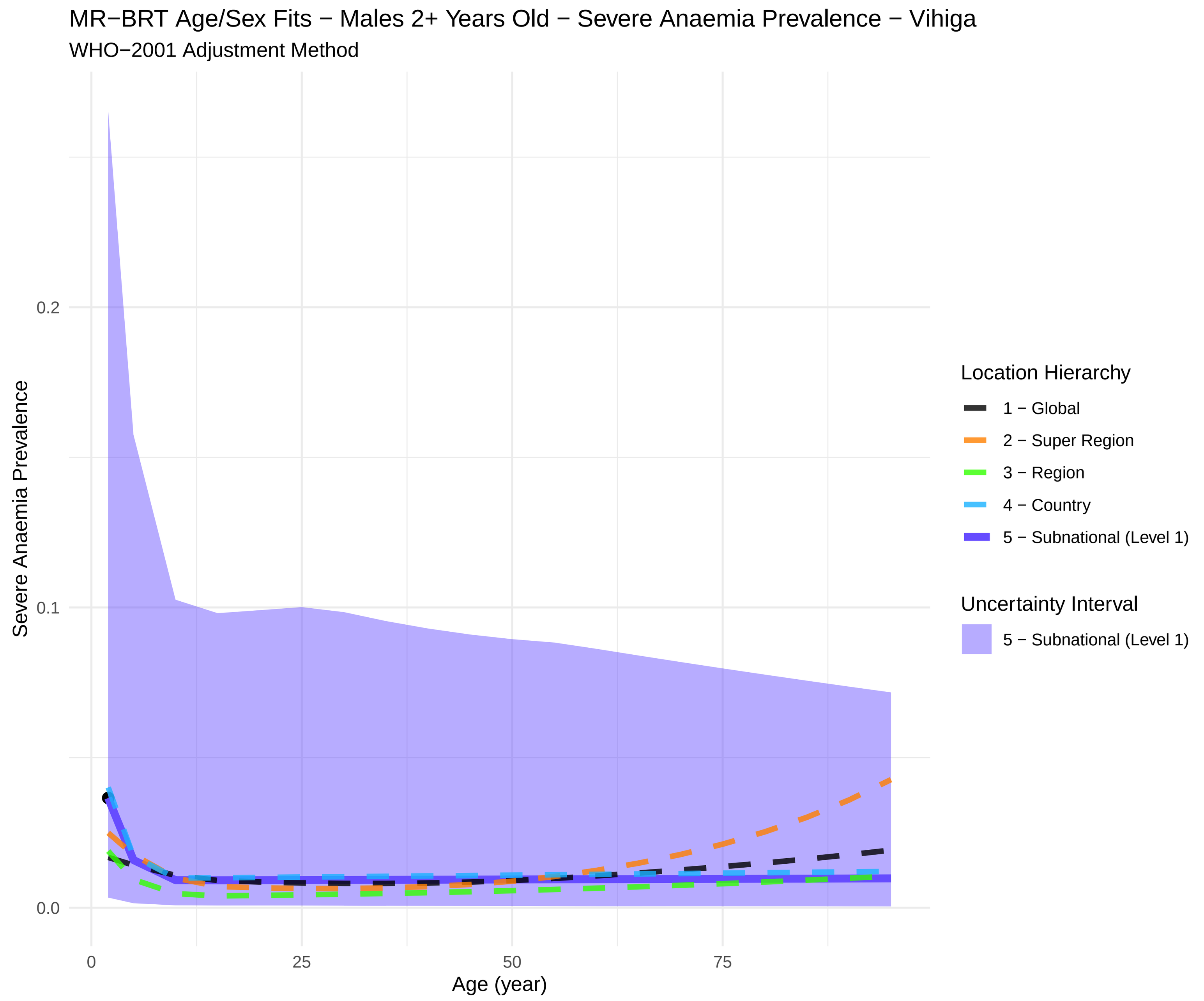
MR−BRT Age/Sex Fits − Males 2+ Years Old − Severe Anaemia Prevalence − Vihiga
WHO−2001 Adjustment Method
Severe Anaemia Prevalence
Age (year)
0.0
0.1
0.2
0
25
50
75
Location Hierarchy
1 − Global
2 − Super Region
3 − Region
4 − Country
5 − Subnational (Level 1)
Uncertainty Interval
5 − Subnational (Level 1)

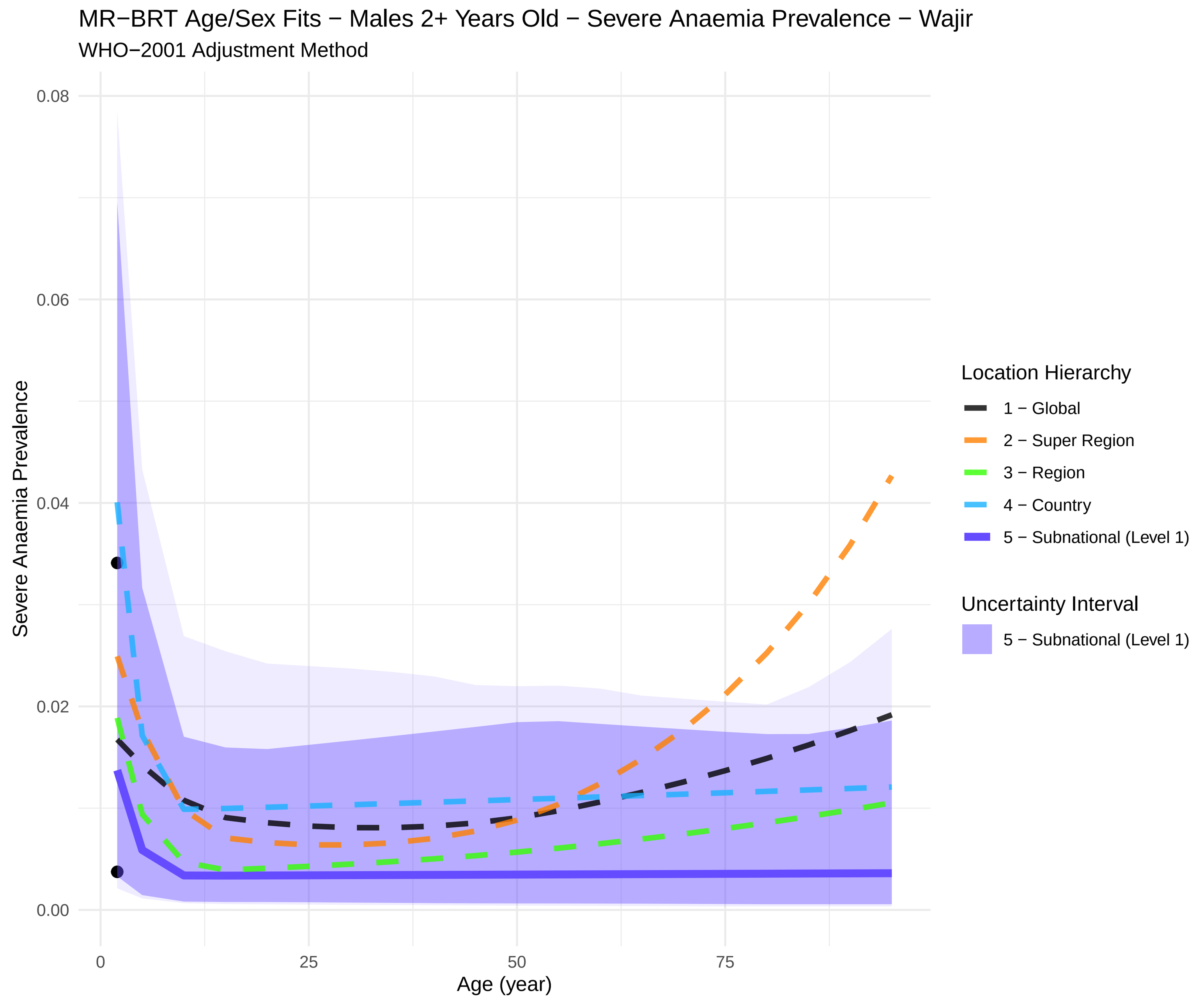
MR−BRT Age/Sex Fits − Males 2+ Years Old − Severe Anaemia Prevalence − Wajir
WHO−2001 Adjustment Method
Severe Anaemia Prevalence
Age (year)
0.00
0.02
0.04
0.06
0.08
0
25
50
75
Location Hierarchy
1 − Global
2 − Super Region
3 − Region
4 − Country
5 − Subnational (Level 1)
Uncertainty Interval
5 − Subnational (Level 1)

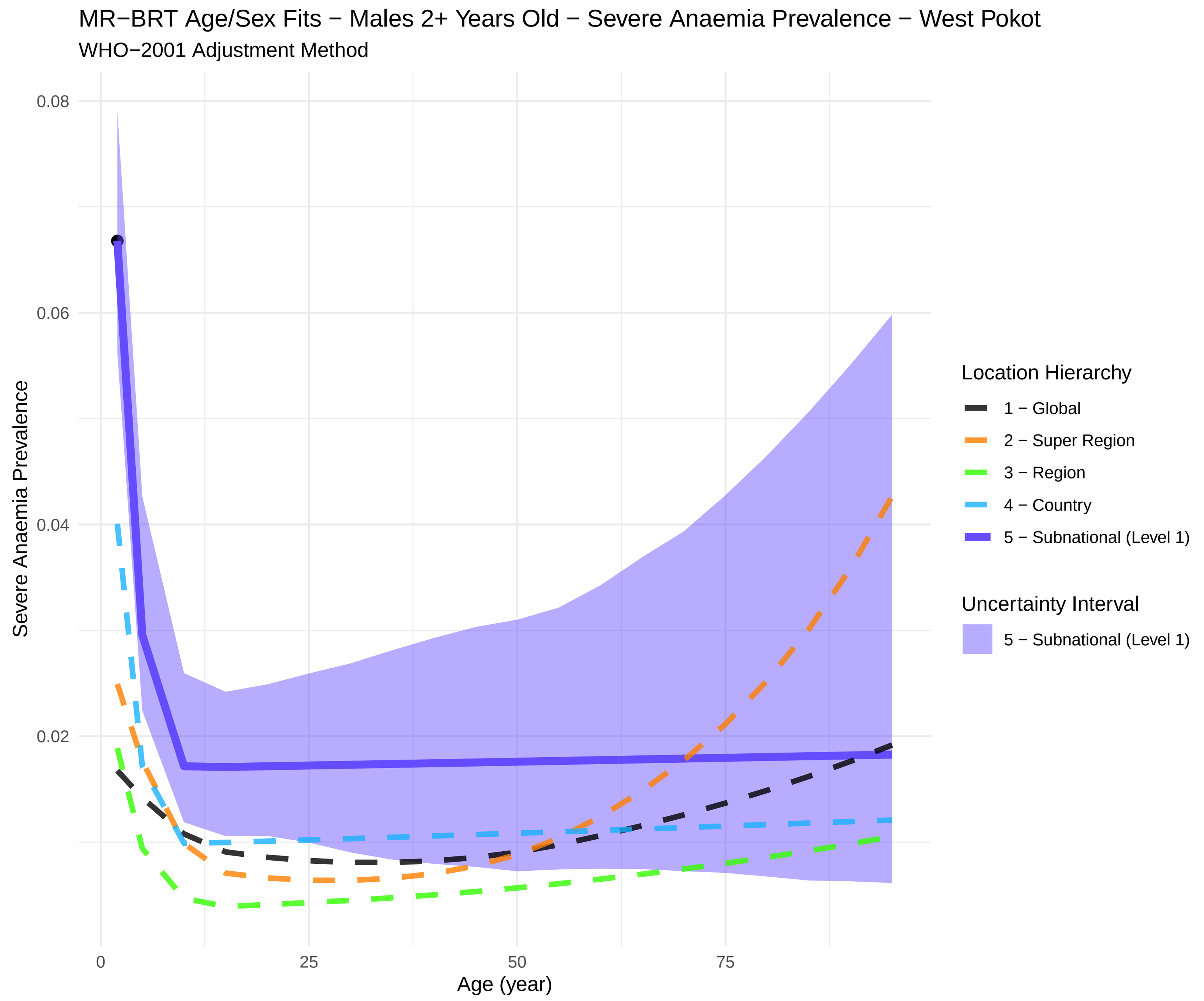
MR−BRT Age/Sex Fits − Males 2+ Years Old − Severe Anaemia Prevalence − West Pokot
WHO−2001 Adjustment Method
Severe Anaemia Prevalence
Age (year)
0.02
0.04
0.06
0.08
0
25
50
75
Location Hierarchy
1 − Global
2 − Super Region
3 − Region
4 − Country
5 − Subnational (Level 1)
Uncertainty Interval
5 − Subnational (Level 1)

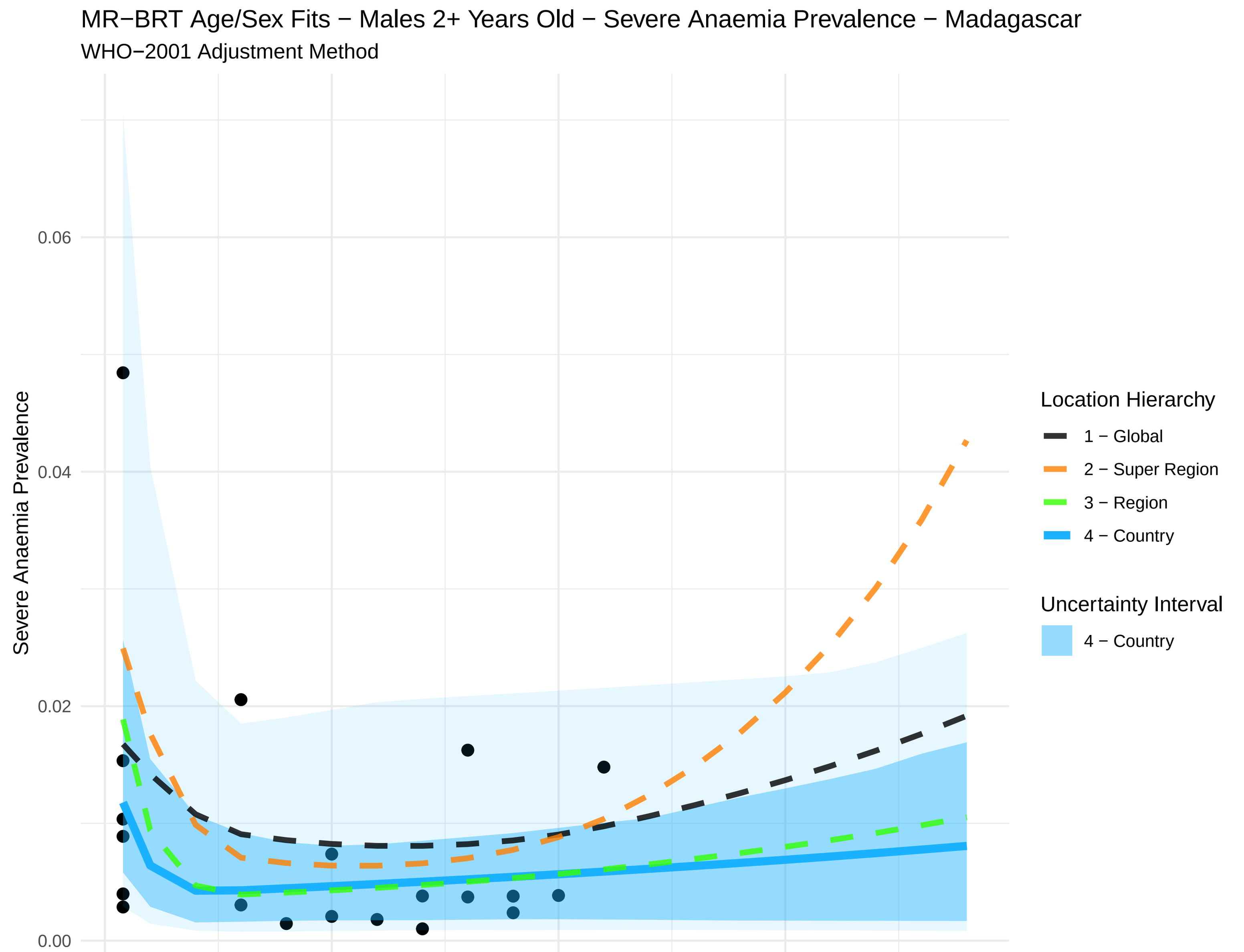
MR−BRT Age/Sex Fits − Males 2+ Years Old − Severe Anaemia Prevalence − Madagascar
WHO−2001 Adjustment Method
Severe Anaemia Prevalence
Age (year)
0.00
0.02
0.04
0.06
0
25
50
75
Location Hierarchy
1 − Global
2 − Super Region
3 − Region
4 − Country
Uncertainty Interval
4 − Country

MR−BRT Age/Sex Fits − Males 2+ Years Old − Severe Anaemia Prevalence − Malawi
WHO−2001 Adjustment Method

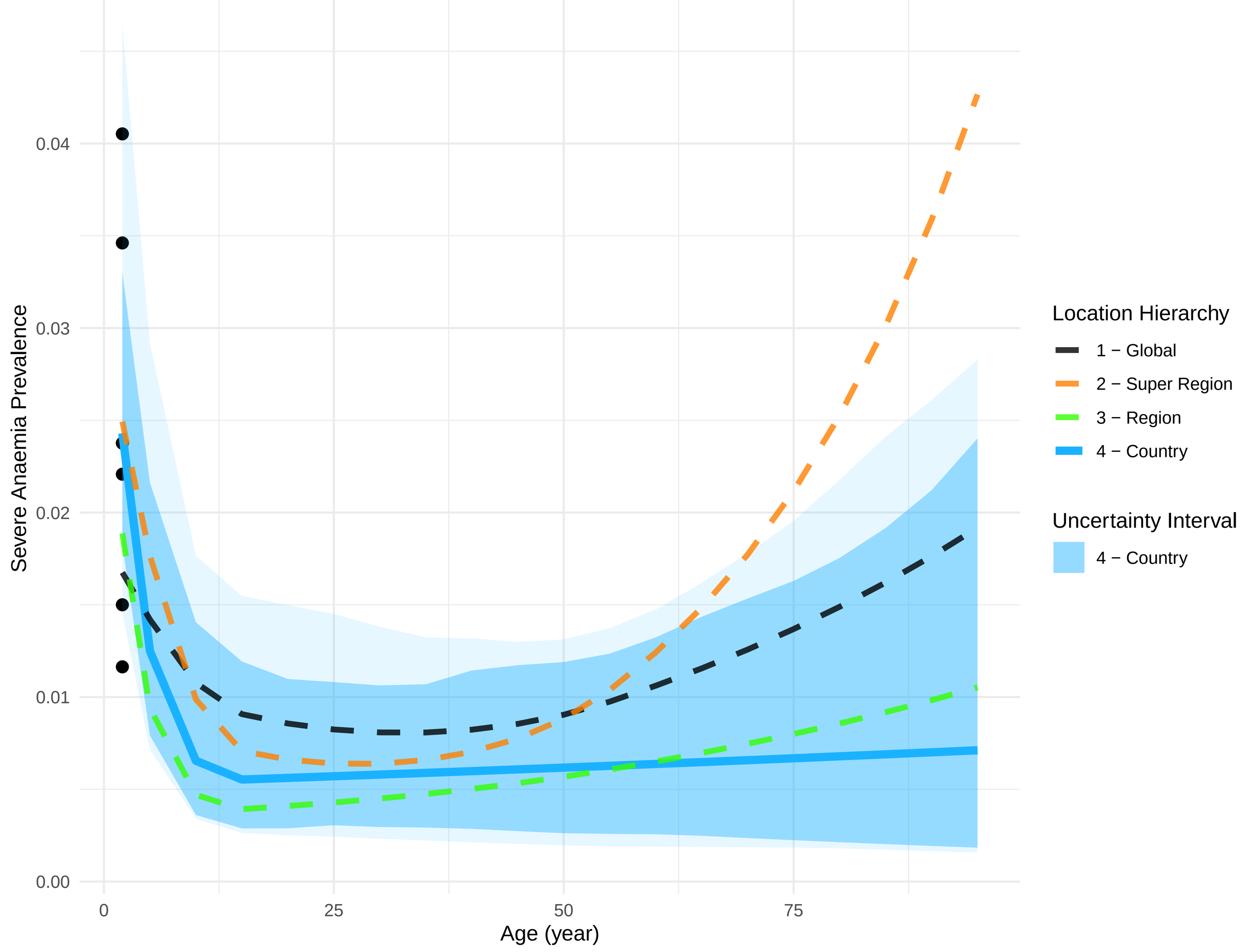

MR−BRT Age/Sex Fits − Males 2+ Years Old − Severe Anaemia Prevalence − Mozambique
WHO−2001 Adjustment Method

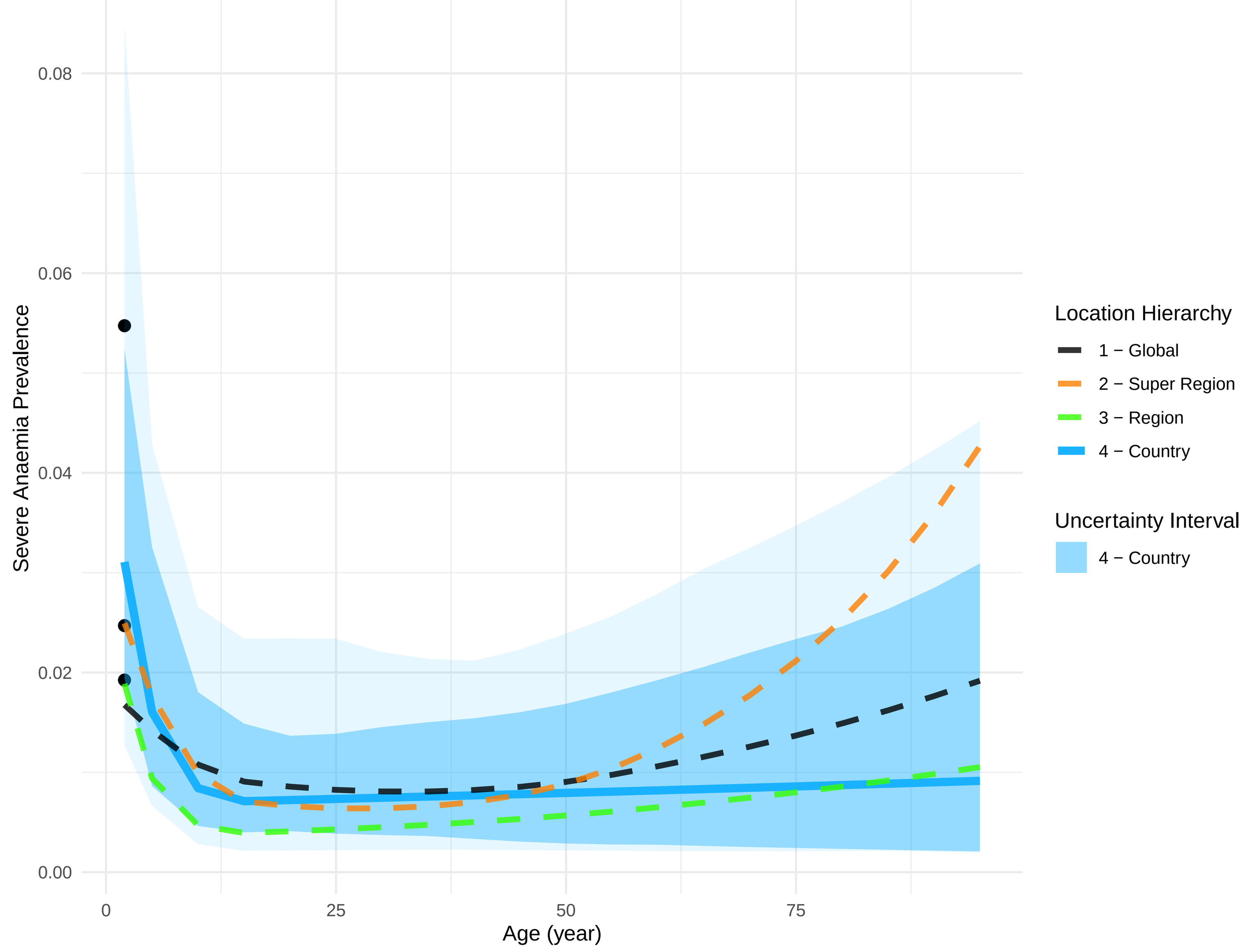

MR−BRT Age/Sex Fits − Males 2+ Years Old − Severe Anaemia Prevalence − Rwanda
WHO−2001 Adjustment Method

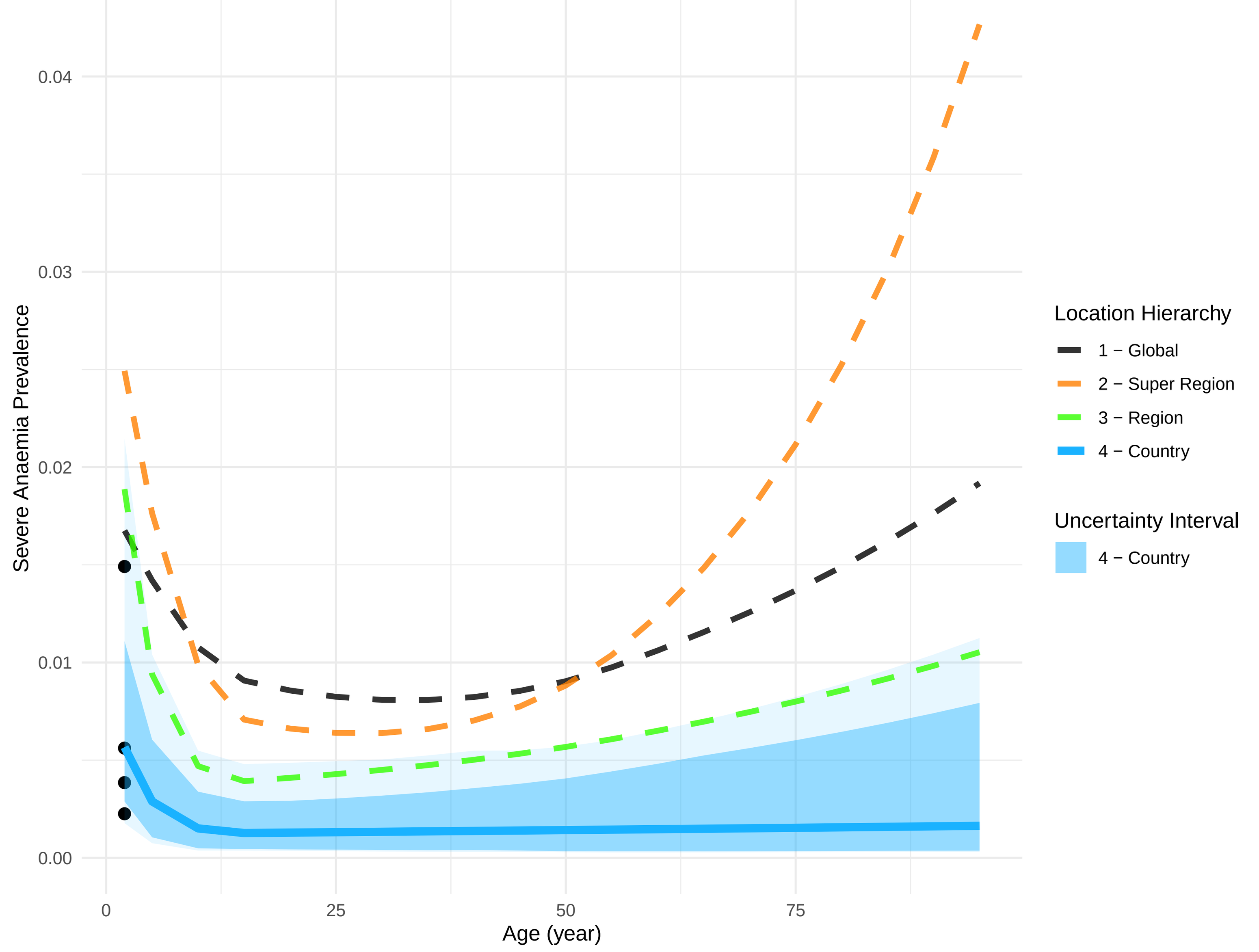

MR−BRT Age/Sex Fits − Males 2+ Years Old − Severe Anaemia Prevalence − Uganda
WHO−2001 Adjustment Method

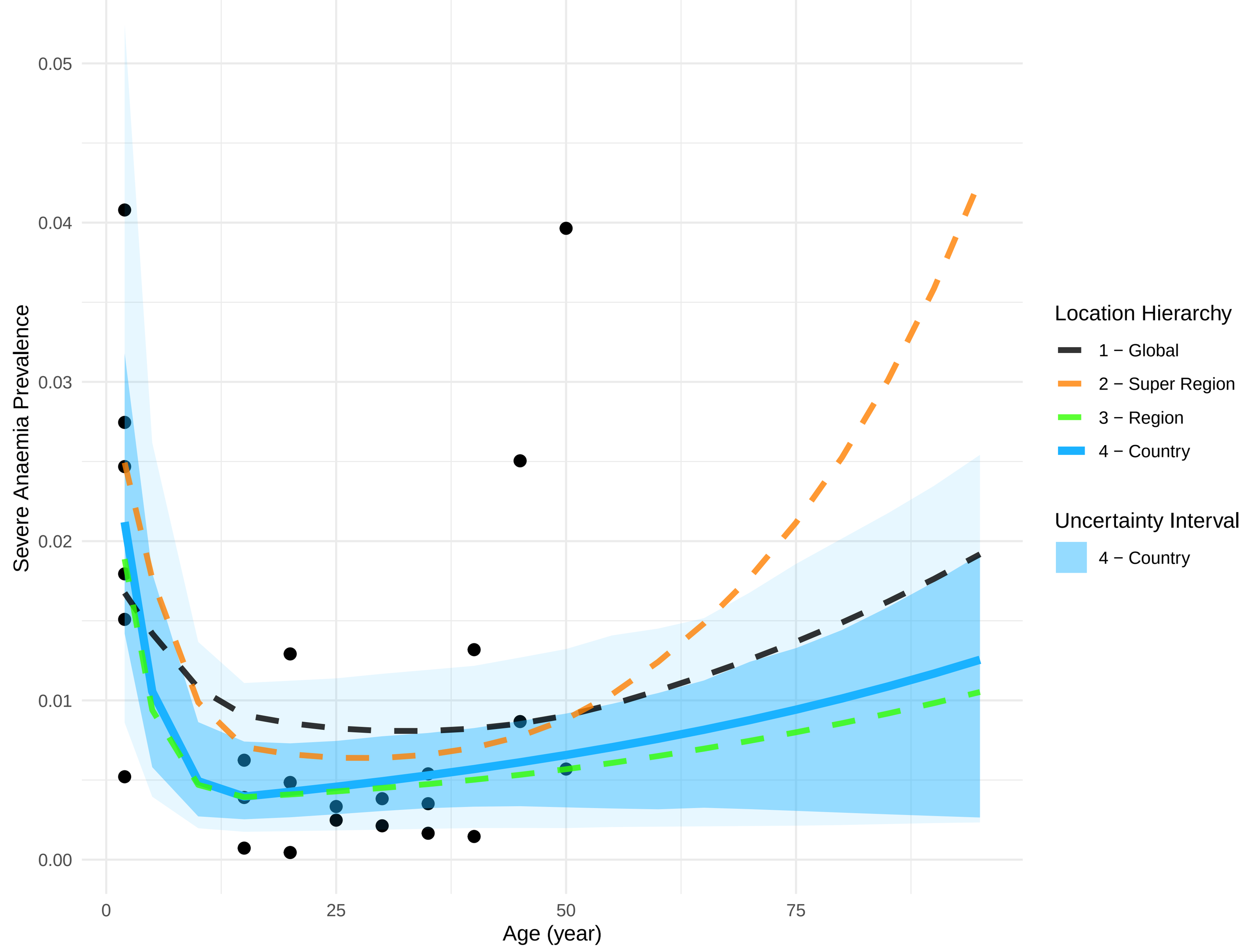

MR−BRT Age/Sex Fits − Males 2+ Years Old − Severe Anaemia Prevalence − United Republic of Tanzania
WHO−2001 Adjustment Method

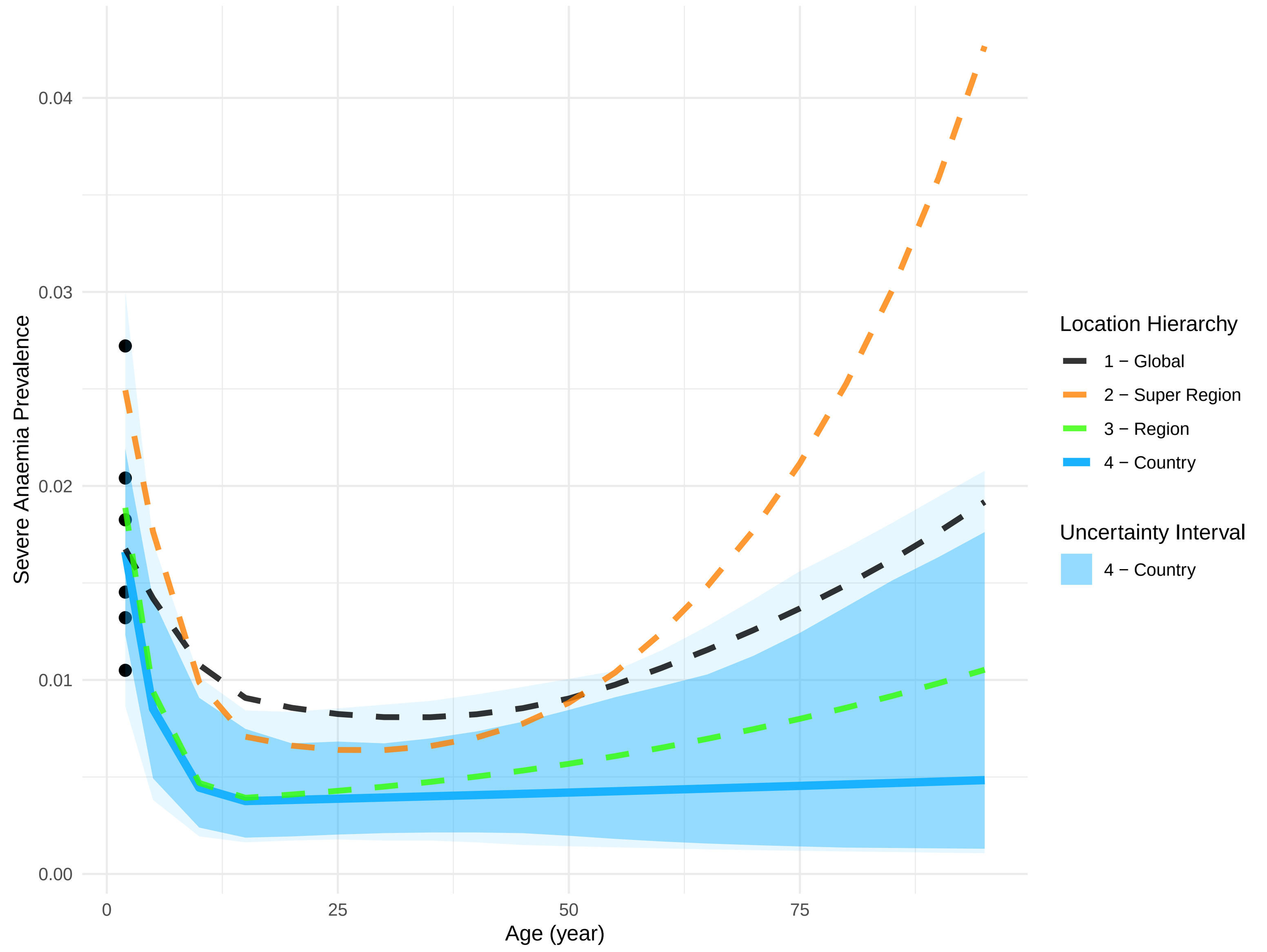

MR−BRT Age/Sex Fits − Males 2+ Years Old − Severe Anaemia Prevalence − Zambia
WHO−2001 Adjustment Method

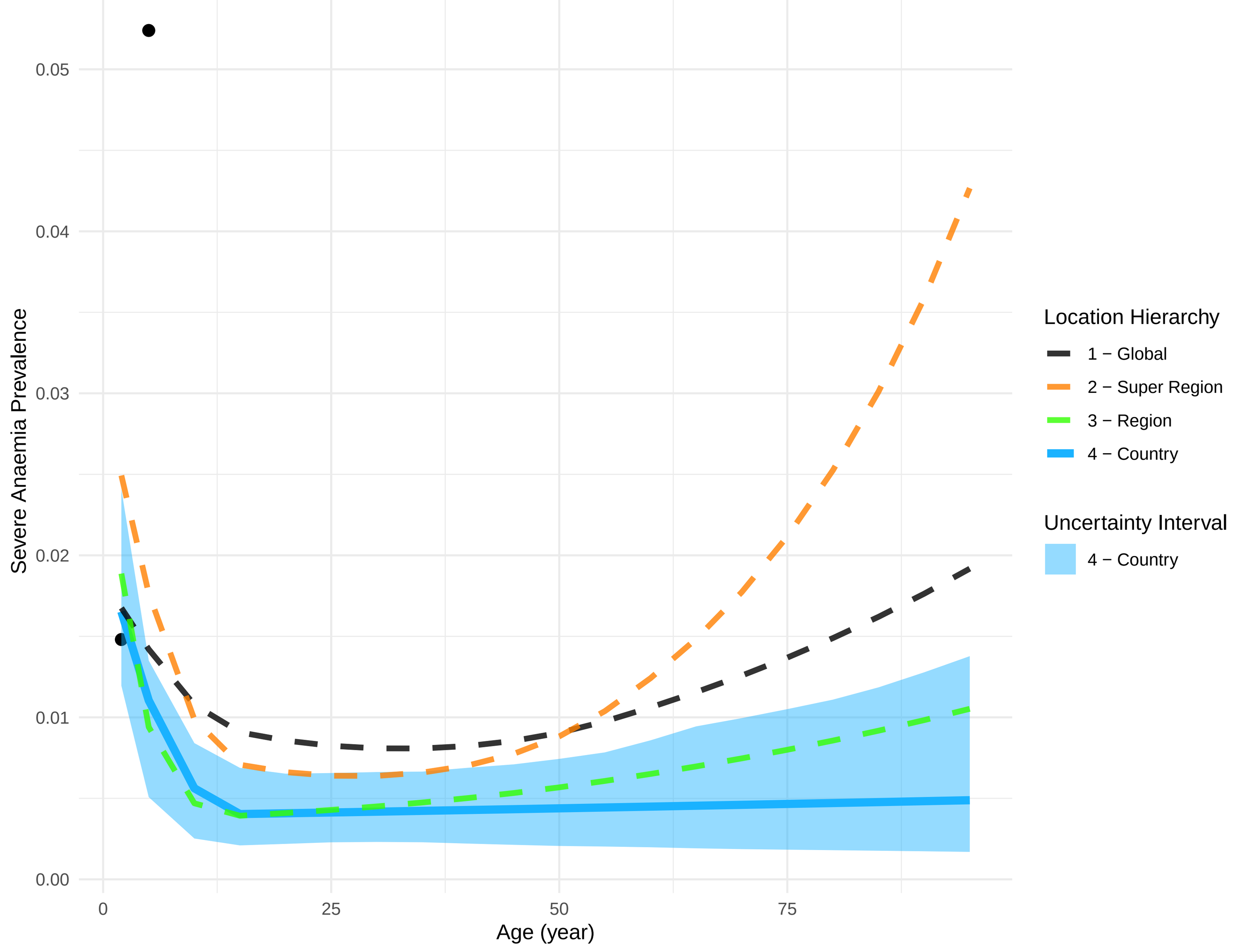

MR−BRT Age/Sex Fits − Males 2+ Years Old − Severe Anaemia Prevalence − Southern Sub−Saharan Africa
WHO−2001 Adjustment Method

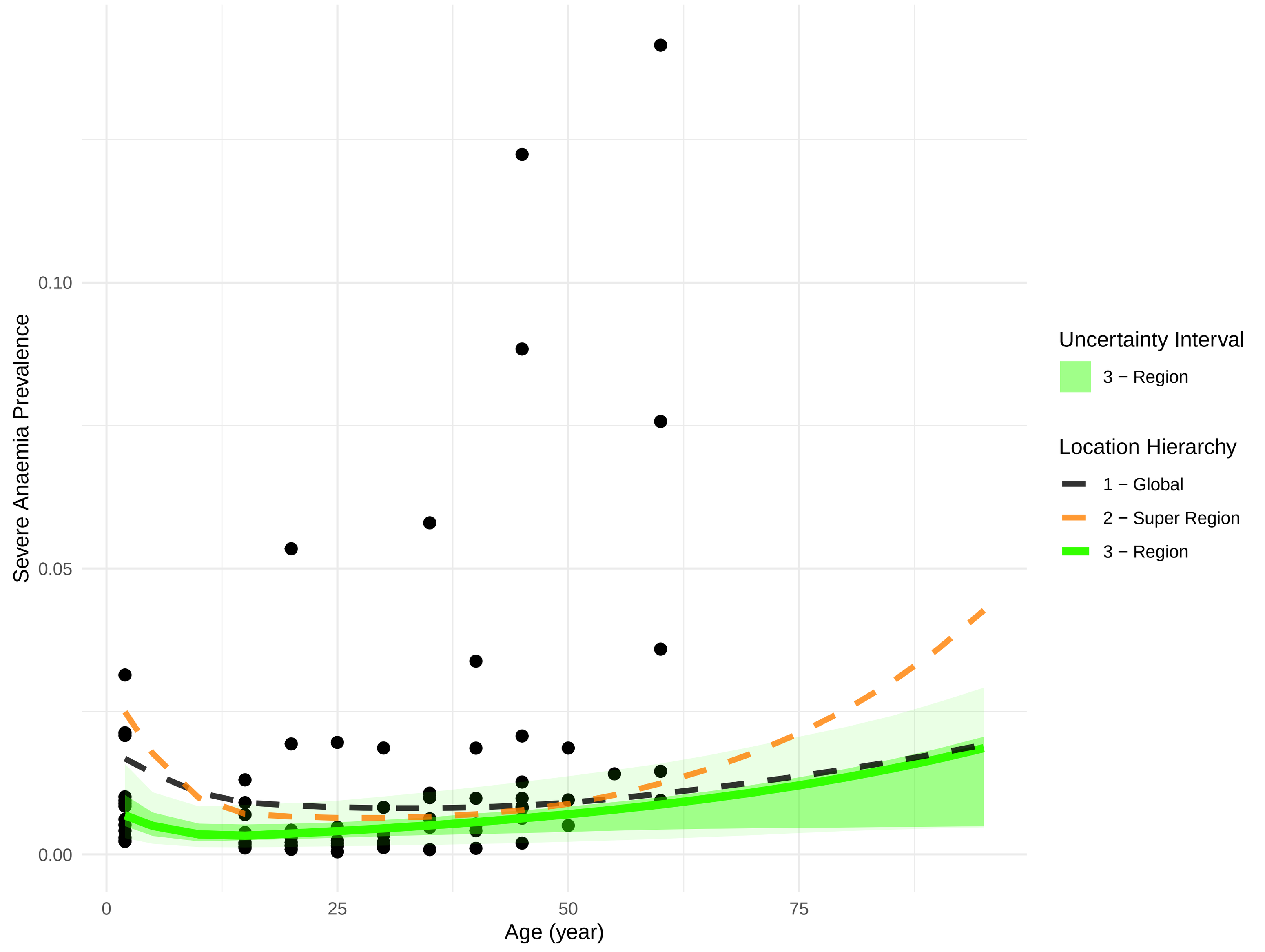

MR−BRT Age/Sex Fits − Males 2+ Years Old − Severe Anaemia Prevalence − Eswatini
WHO−2001 Adjustment Method

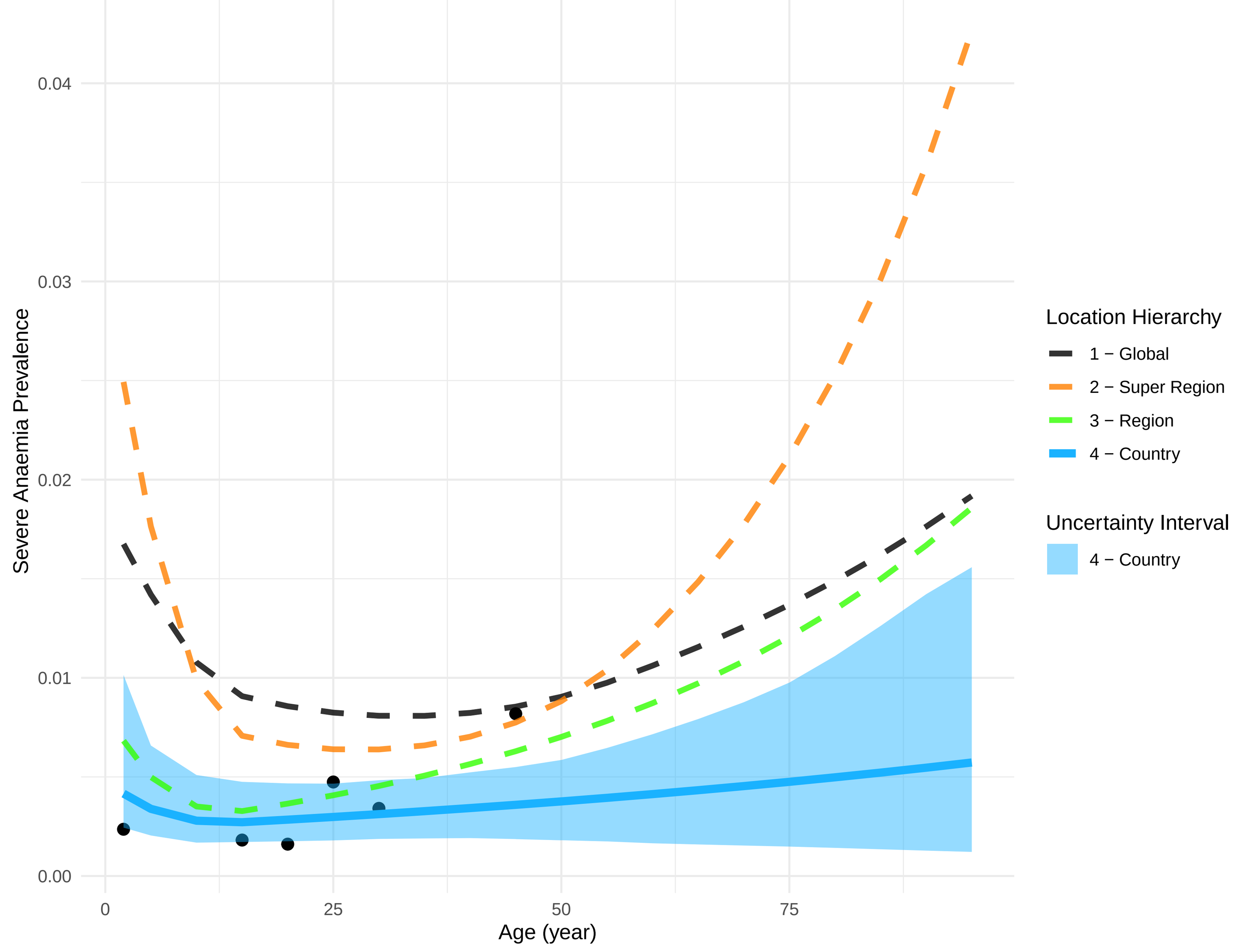

MR−BRT Age/Sex Fits − Males 2+ Years Old − Severe Anaemia Prevalence − Lesotho
WHO−2001 Adjustment Method

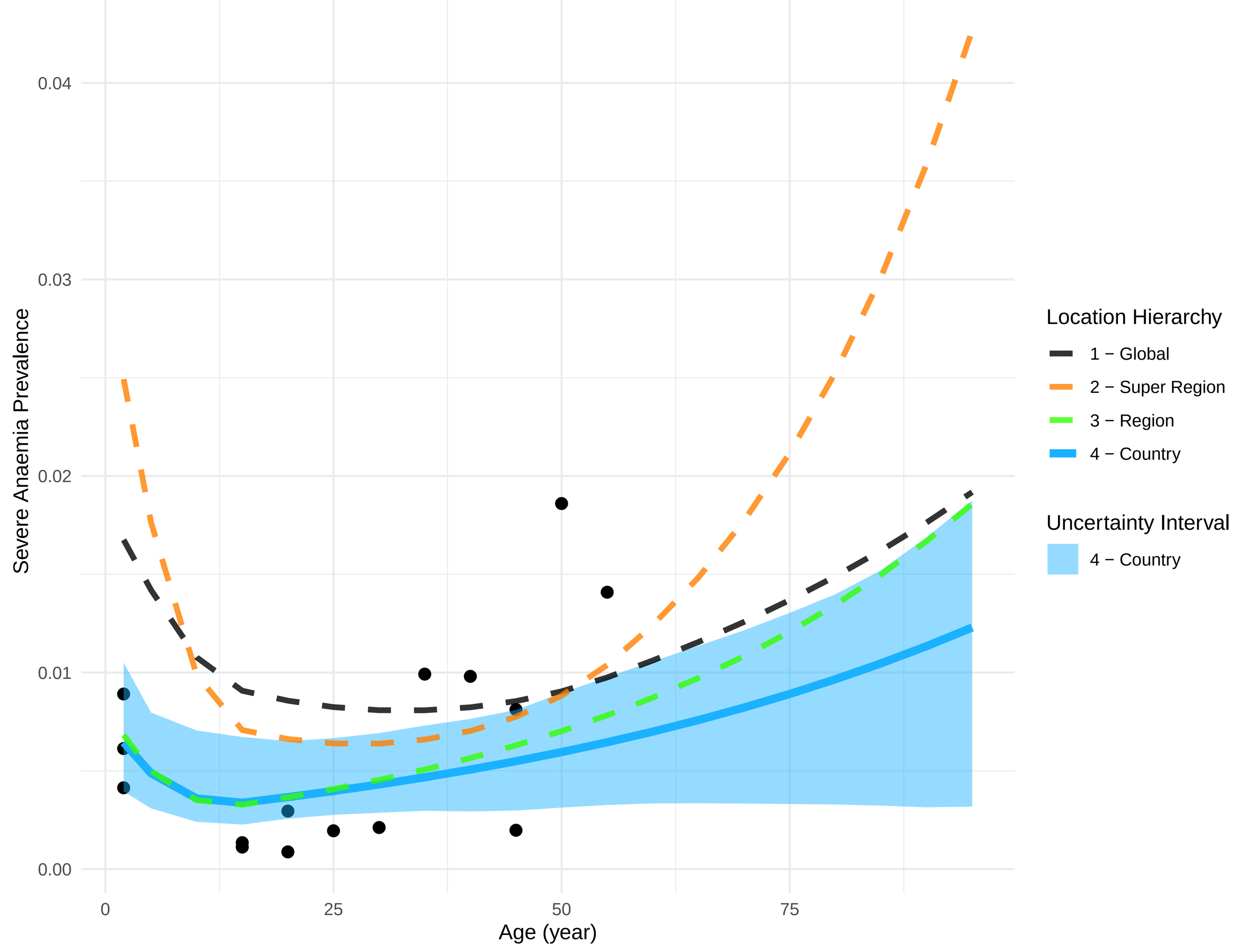

MR−BRT Age/Sex Fits − Males 2+ Years Old − Severe Anaemia Prevalence − Namibia
WHO−2001 Adjustment Method

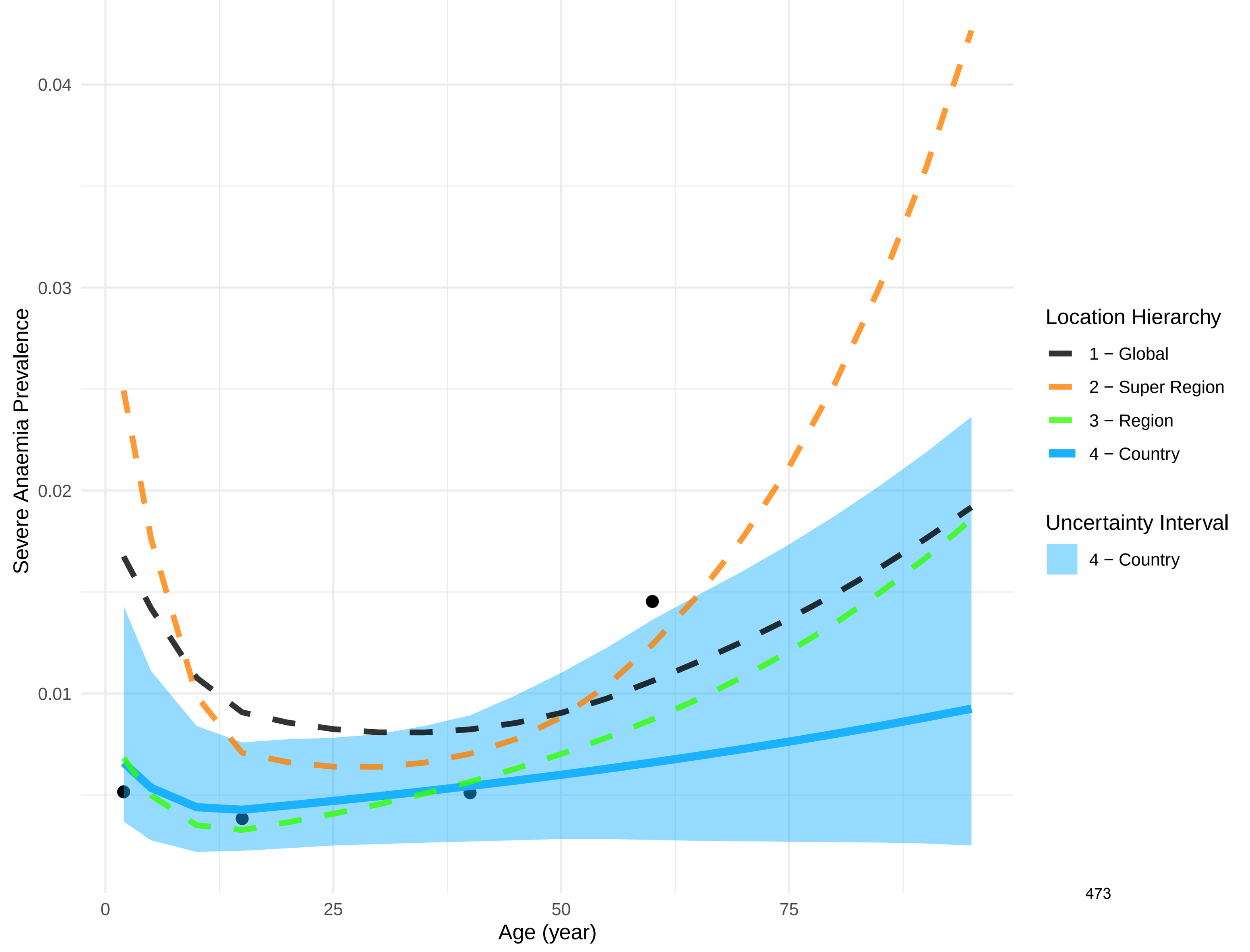

MR−BRT Age/Sex Fits − Males 2+ Years Old − Severe Anaemia Prevalence − South Africa
WHO−2001 Adjustment Method

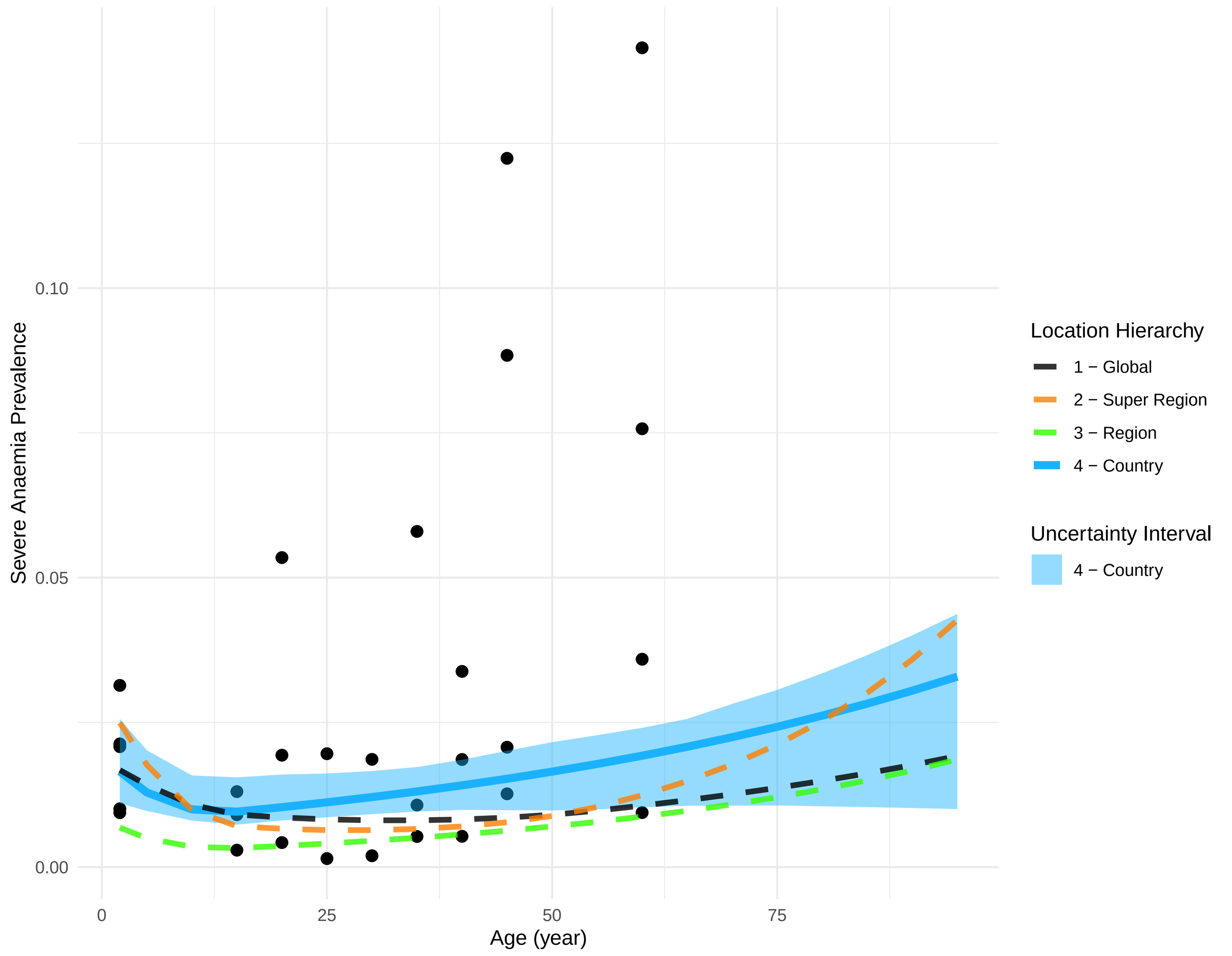

MR−BRT Age/Sex Fits − Males 2+ Years Old − Severe Anaemia Prevalence − Eastern Cape
WHO−2001 Adjustment Method

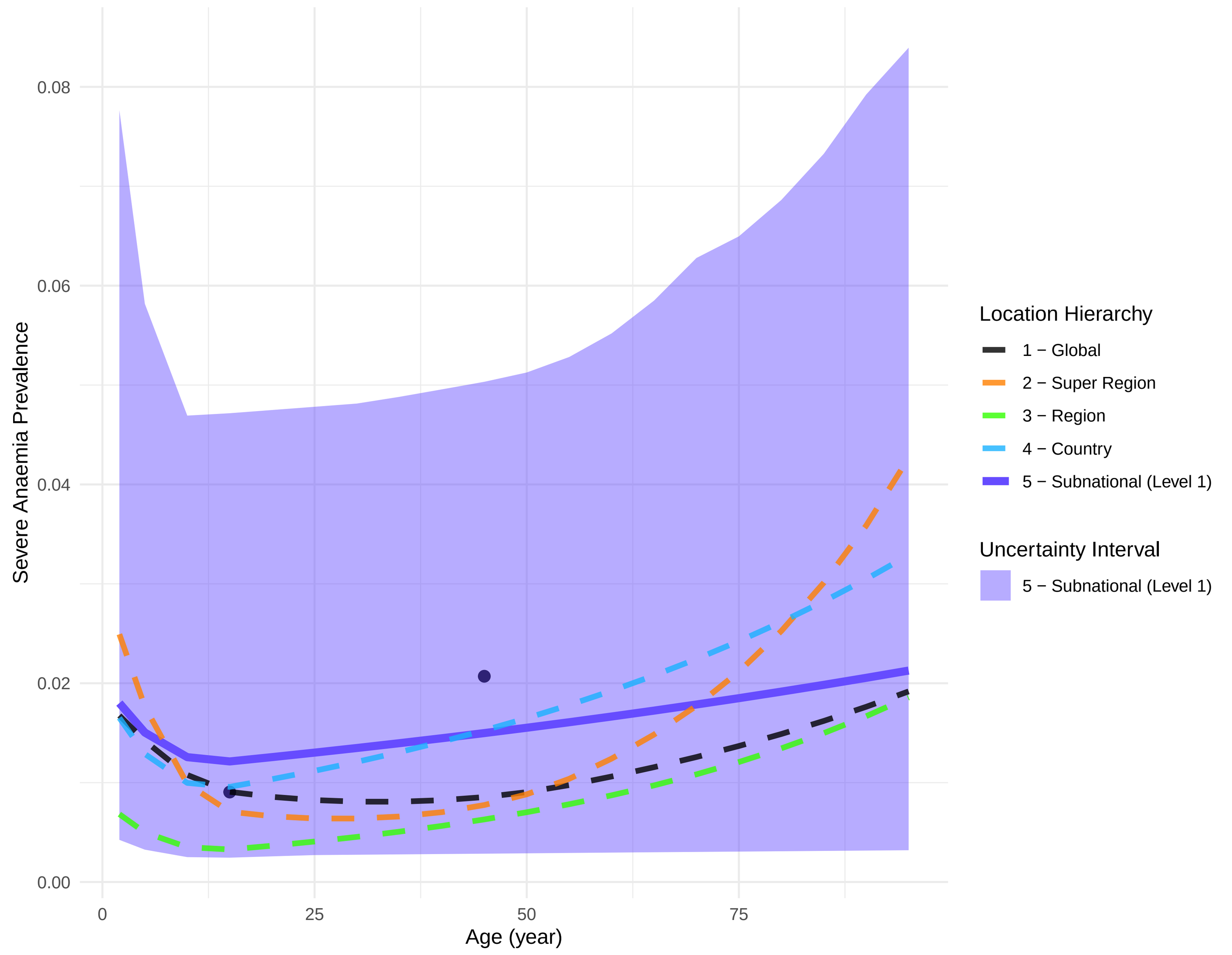

MR−BRT Age/Sex Fits − Males 2+ Years Old − Severe Anaemia Prevalence − Free State
WHO−2001 Adjustment Method

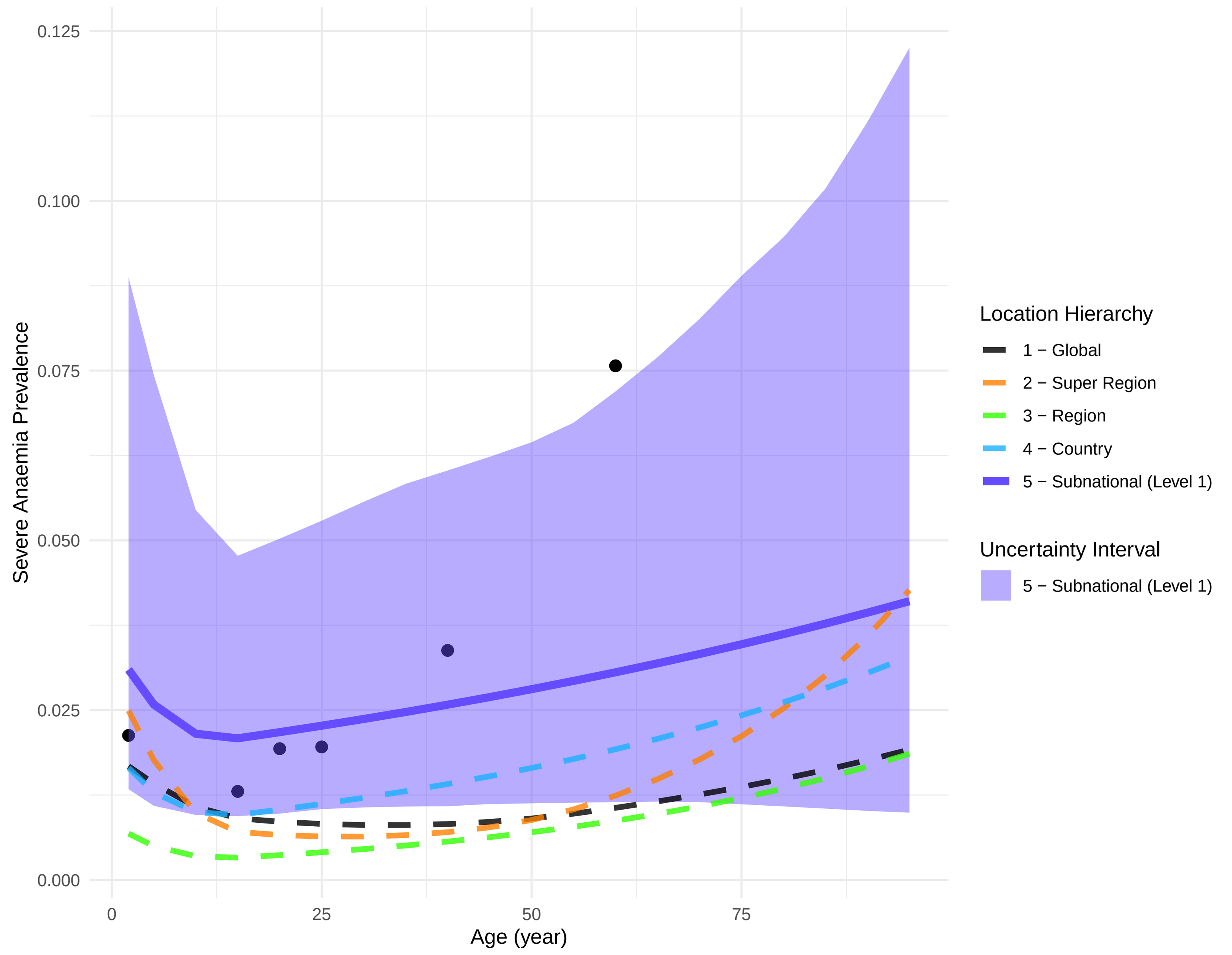

MR−BRT Age/Sex Fits − Males 2+ Years Old − Severe Anaemia Prevalence − Gauteng
WHO−2001 Adjustment Method

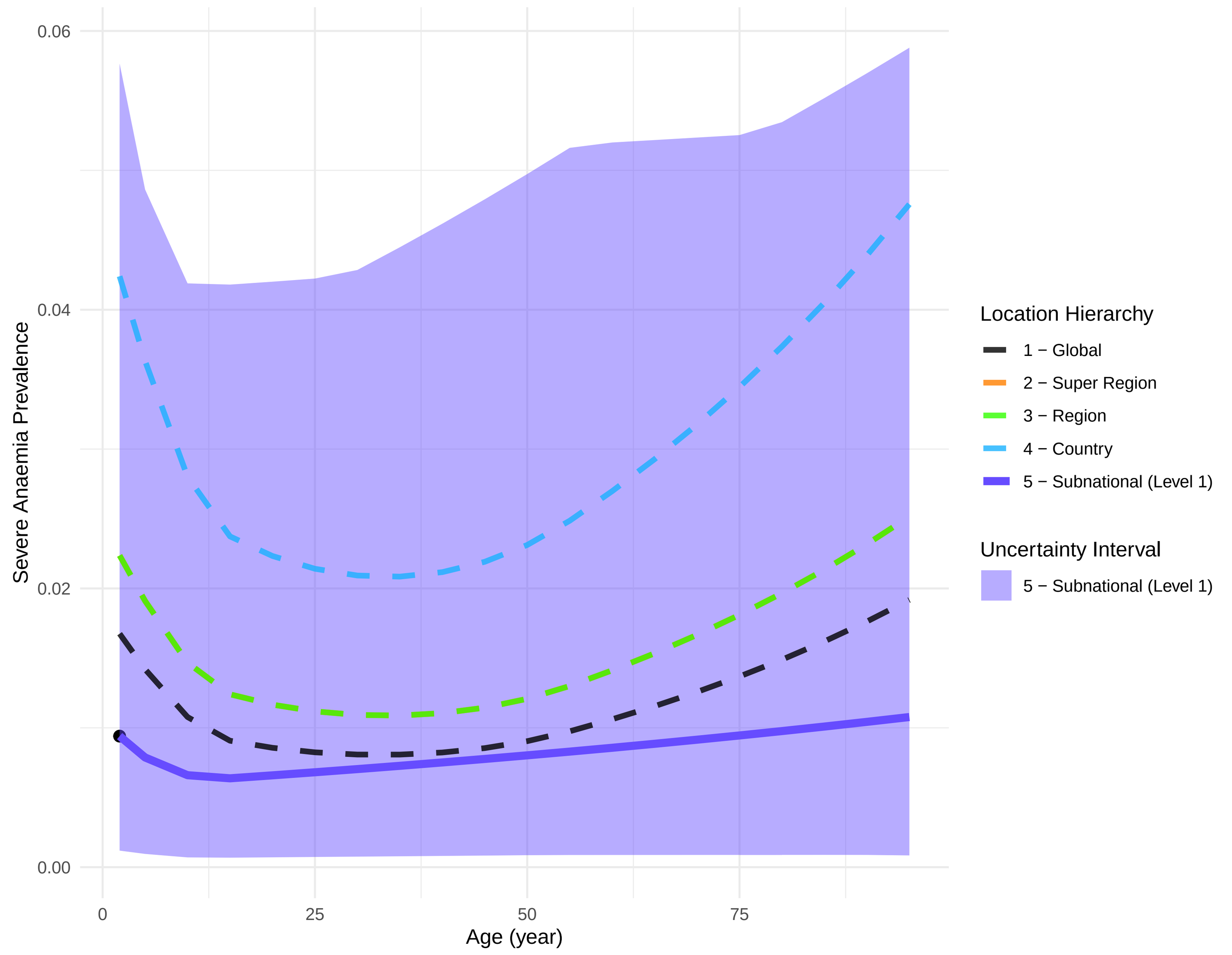

MR−BRT Age/Sex Fits − Males 2+ Years Old − Severe Anaemia Prevalence − Limpopo
WHO−2001 Adjustment Method

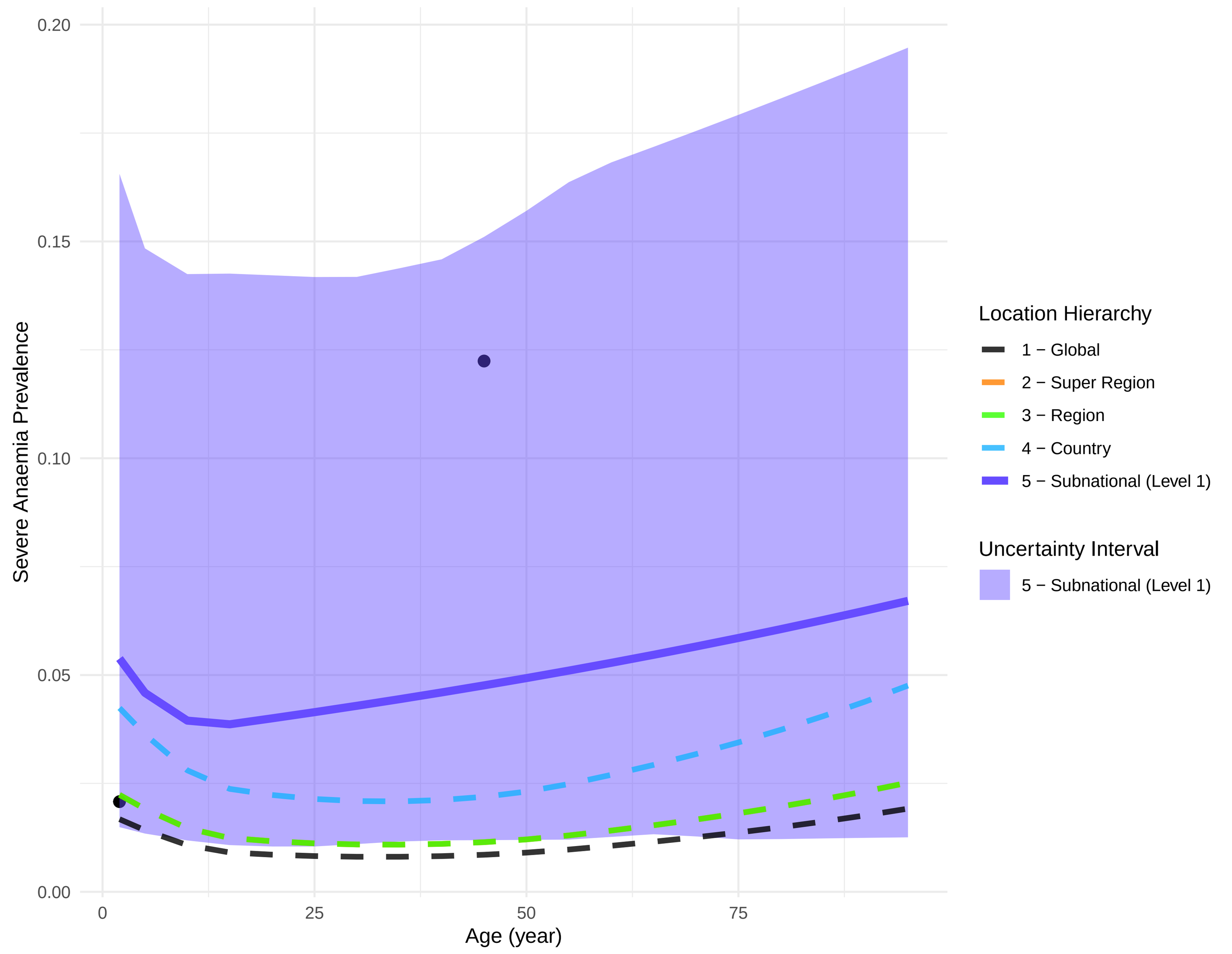

MR−BRT Age/Sex Fits − Males 2+ Years Old − Severe Anaemia Prevalence − Mpumalanga
WHO−2001 Adjustment Method

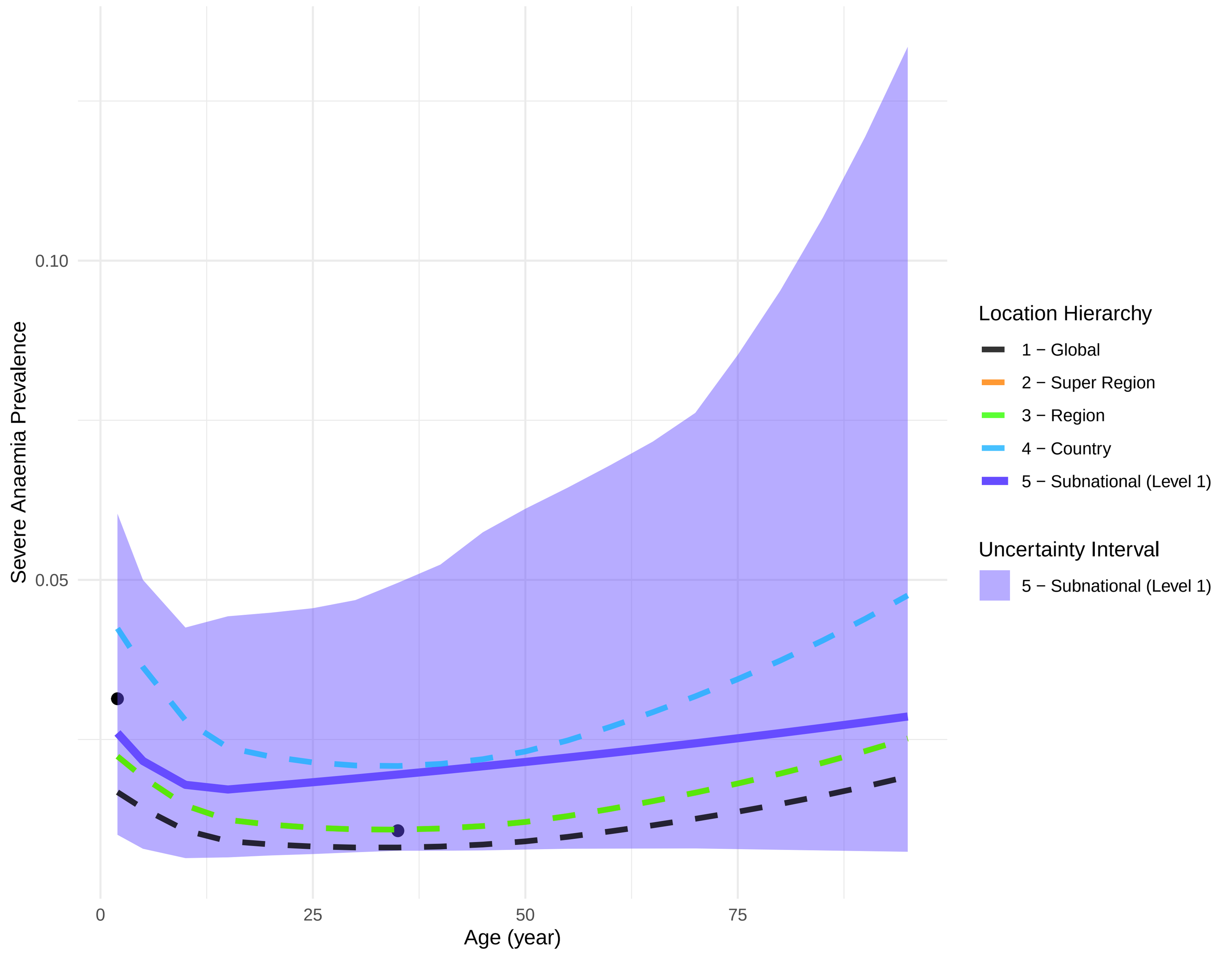

MR−BRT Age/Sex Fits − Males 2+ Years Old − Severe Anaemia Prevalence − North−West
WHO−2001 Adjustment Method

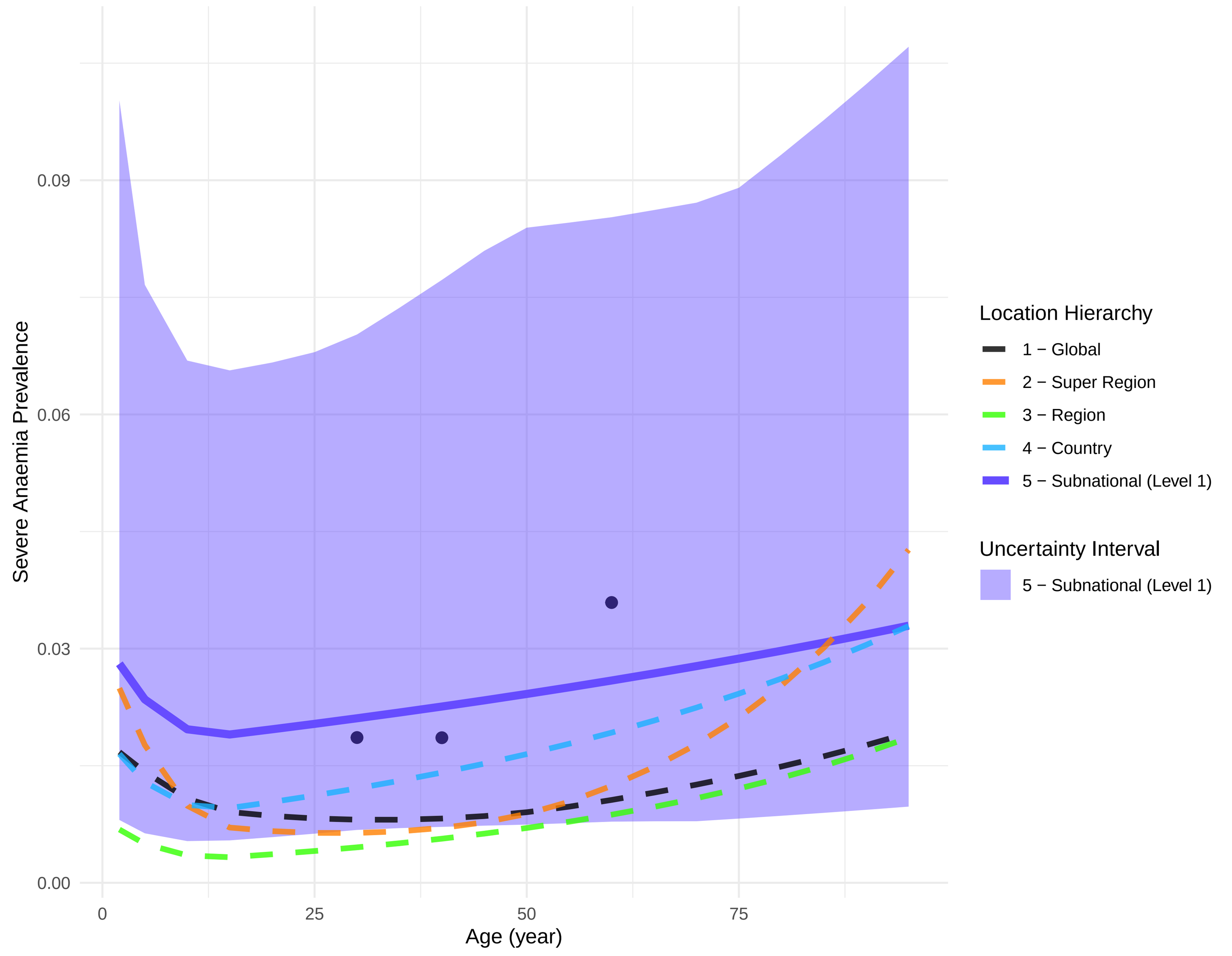

MR−BRT Age/Sex Fits − Males 2+ Years Old − Severe Anaemia Prevalence − Northern Cape
WHO−2001 Adjustment Method

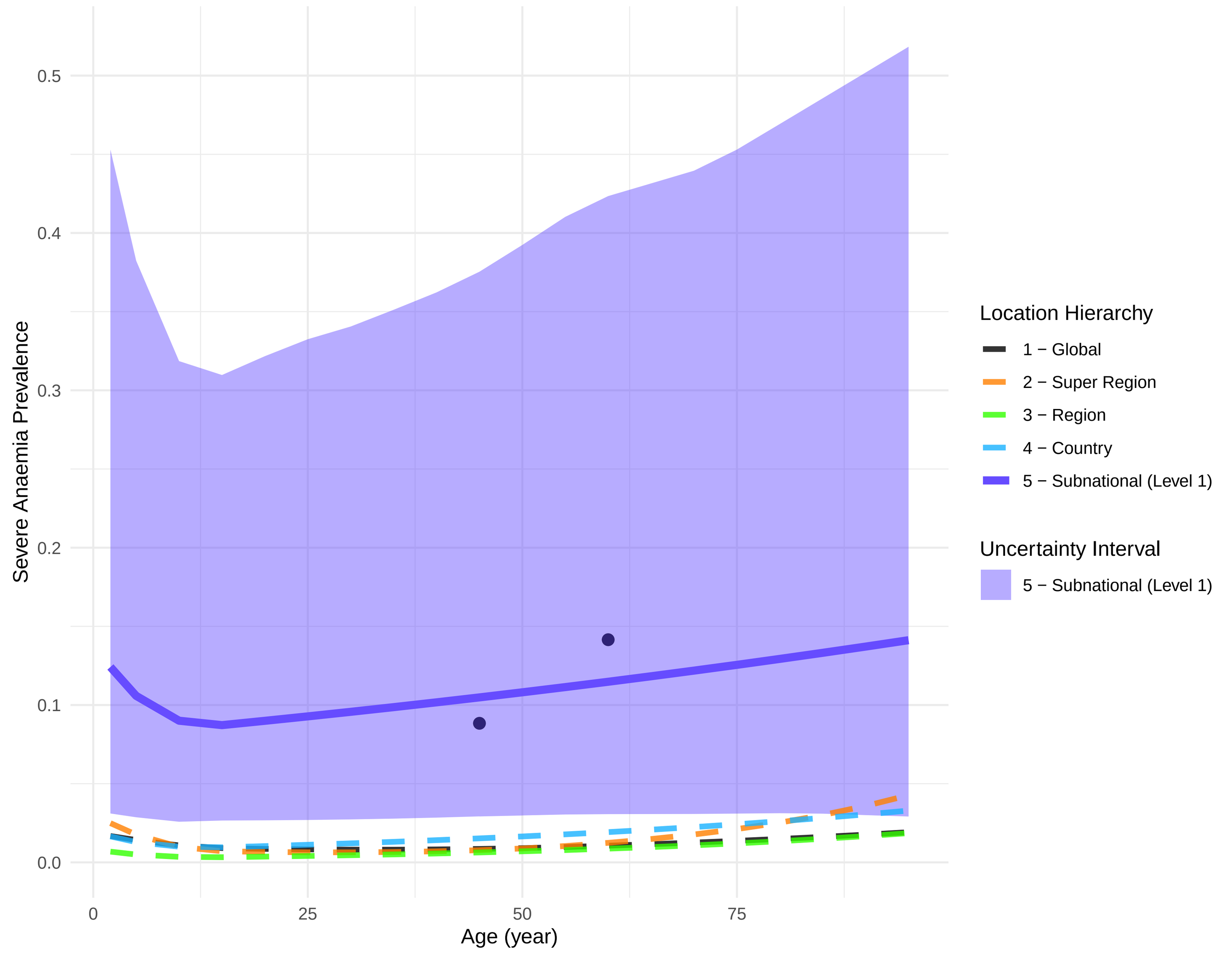

MR−BRT Age/Sex Fits − Males 2+ Years Old − Severe Anaemia Prevalence − Western Cape
WHO−2001 Adjustment Method

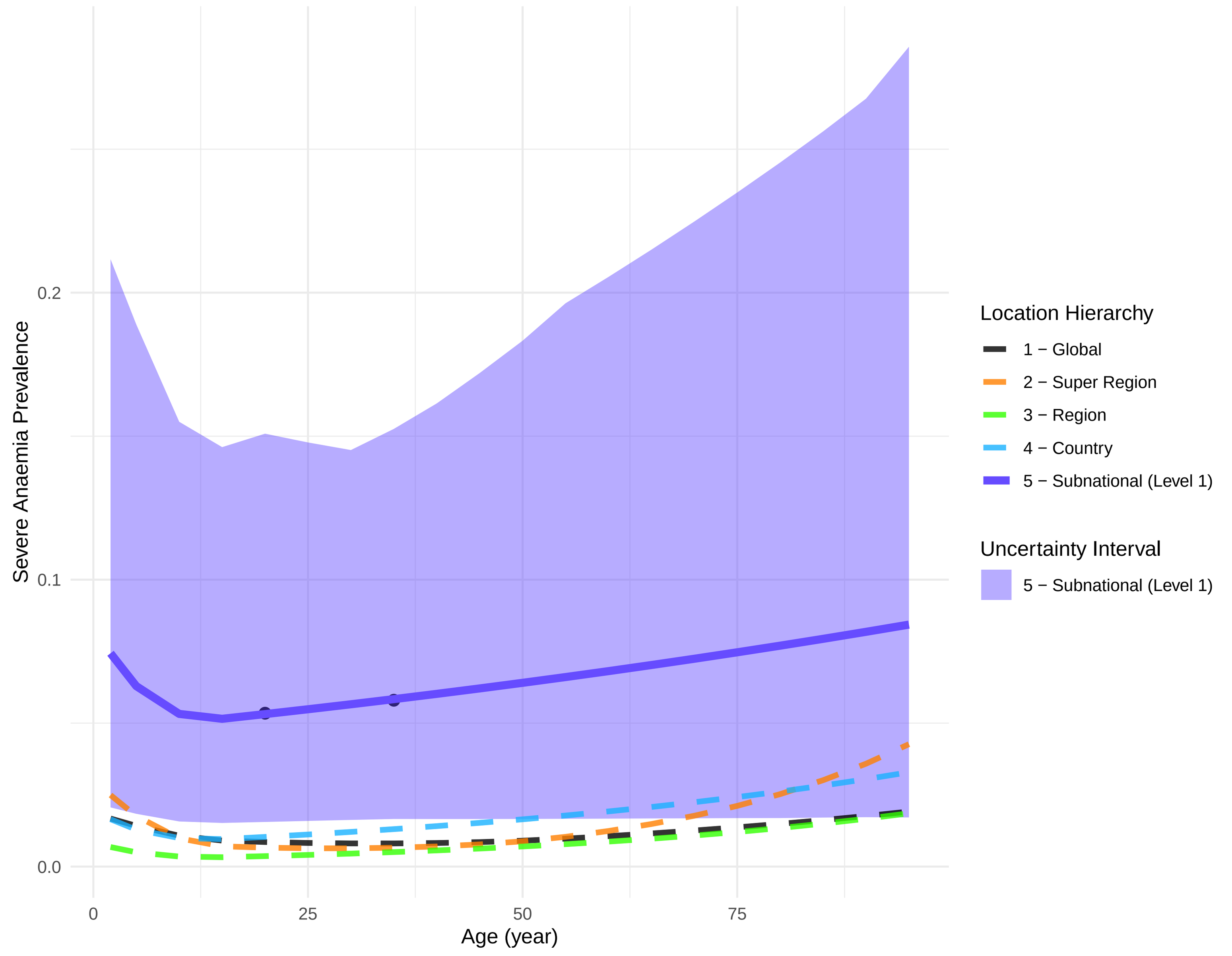

MR−BRT Age/Sex Fits − Males 2+ Years Old − Severe Anaemia Prevalence − Zimbabwe
WHO−2001 Adjustment Method

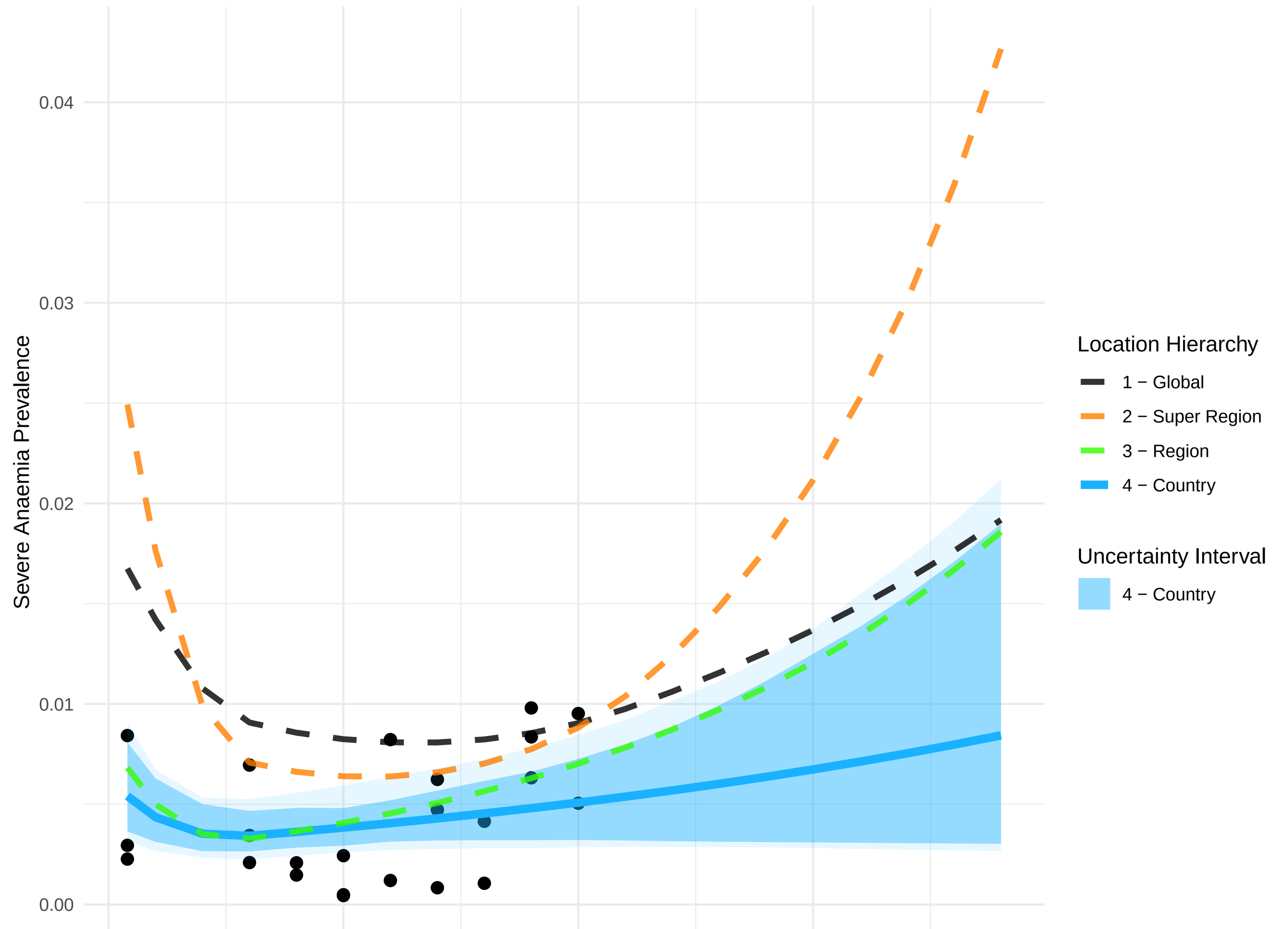

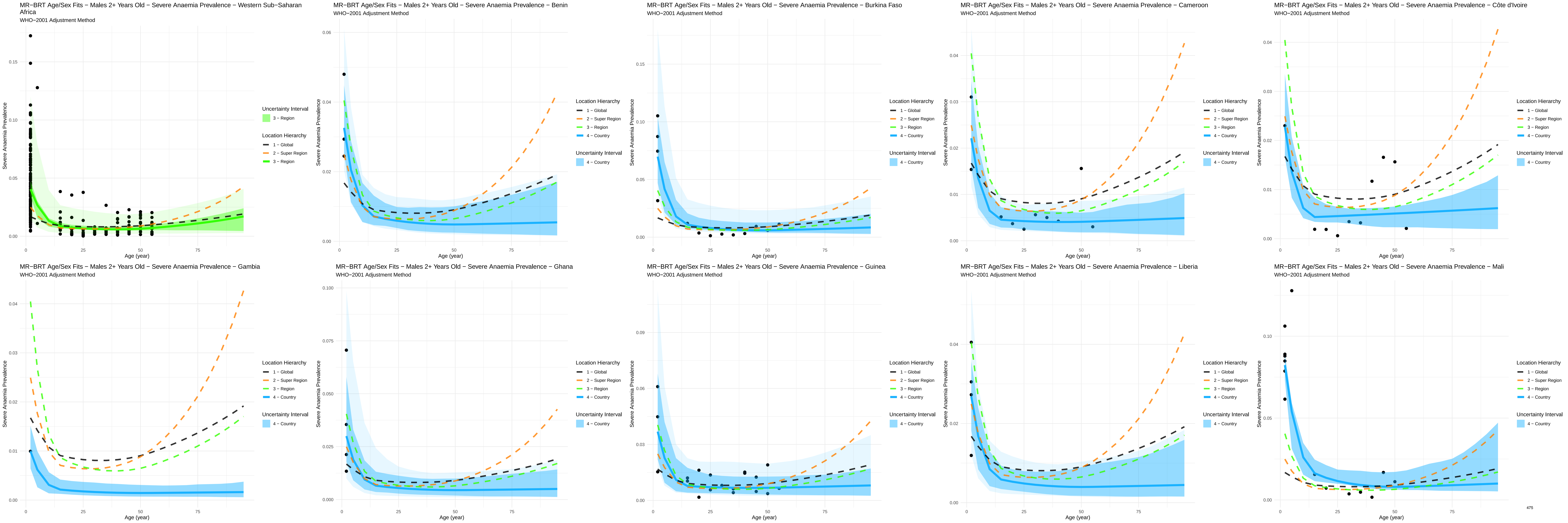
MR−BRT Age/Sex Fits − Males 2+ Years Old − Severe Anaemia Prevalence − Western Sub−Saharan Africa
WHO−2001 Adjustment Method
Severe Anaemia Prevalence
Age (year)
Uncertainty Interval
3 − Region
Location Hierarchy
1 − Global
2 − Super Region
3 − Region
MR−BRT Age/Sex Fits − Males 2+ Years Old − Severe Anaemia Prevalence − Benin
WHO−2001 Adjustment Method
MR−BRT Age/Sex Fits − Males 2+ Years Old − Severe Anaemia Prevalence − Burkina Faso
WHO−2001 Adjustment Method
MR−BRT Age/Sex Fits − Males 2+ Years Old − Severe Anaemia Prevalence − Cameroon
WHO−2001 Adjustment Method
MR−BRT Age/Sex Fits − Males 2+ Years Old − Severe Anaemia Prevalence − Côte d'Ivoire
WHO−2001 Adjustment Method
MR−BRT Age/Sex Fits − Males 2+ Years Old − Severe Anaemia Prevalence − Gambia
WHO−2001 Adjustment Method
MR−BRT Age/Sex Fits − Males 2+ Years Old − Severe Anaemia Prevalence − Ghana
WHO−2001 Adjustment Method
MR−BRT Age/Sex Fits − Males 2+ Years Old − Severe Anaemia Prevalence − Guinea
WHO−2001 Adjustment Method
MR−BRT Age/Sex Fits − Males 2+ Years Old − Severe Anaemia Prevalence − Liberia
WHO−2001 Adjustment Method
MR−BRT Age/Sex Fits − Males 2+ Years Old − Severe Anaemia Prevalence − Mali
WHO−2001 Adjustment Method
Location Hierarchy
1 − Global
2 − Super Region
3 − Region
4 − Country
Uncertainty Interval
4 − Country

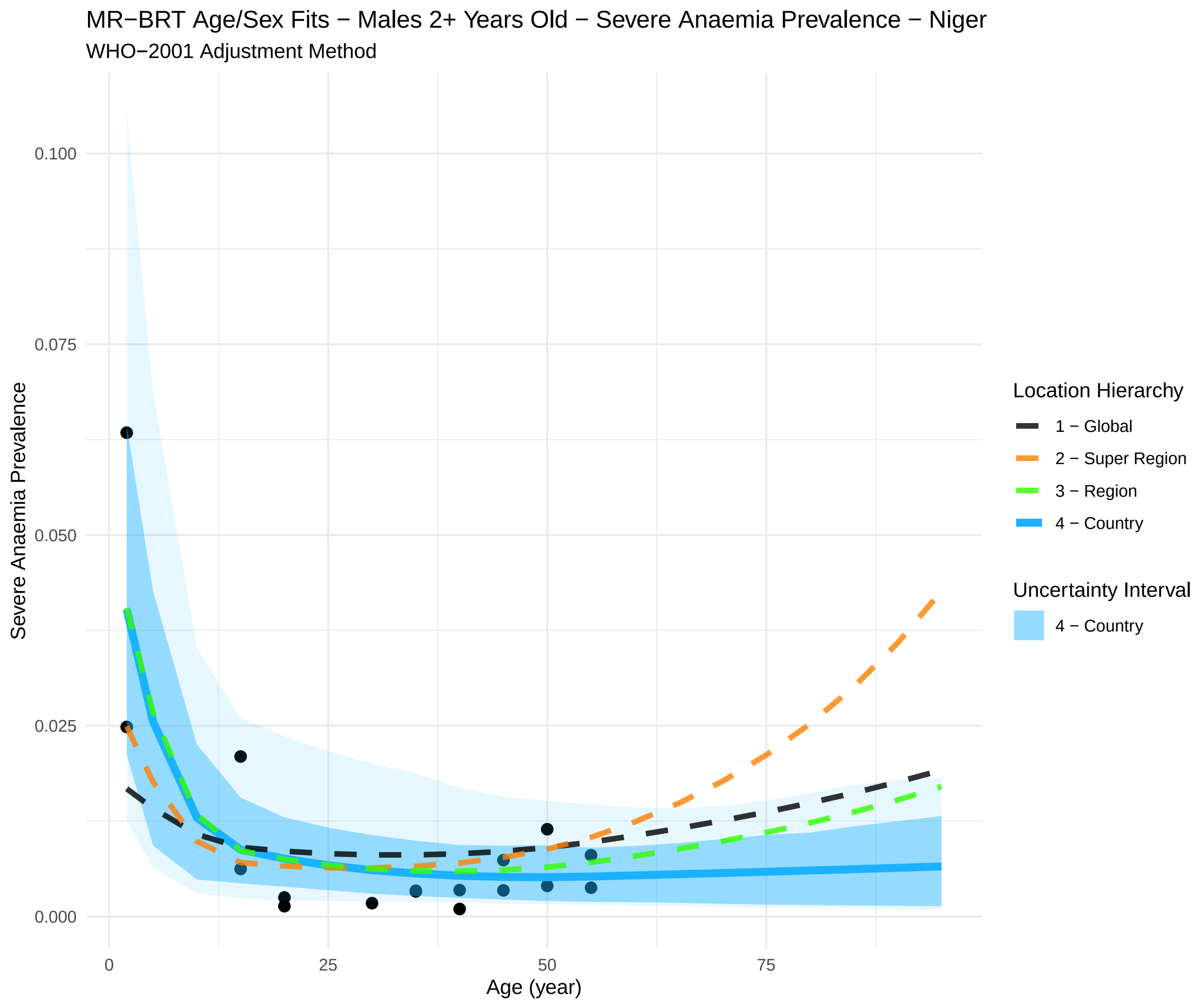
MR−BRT Age/Sex Fits − Males 2+ Years Old − Severe Anaemia Prevalence − Niger
WHO−2001 Adjustment Method
Severe Anaemia Prevalence
Age (year)
Location Hierarchy
1 − Global
2 − Super Region
3 − Region
4 − Country
Uncertainty Interval
4 − Country

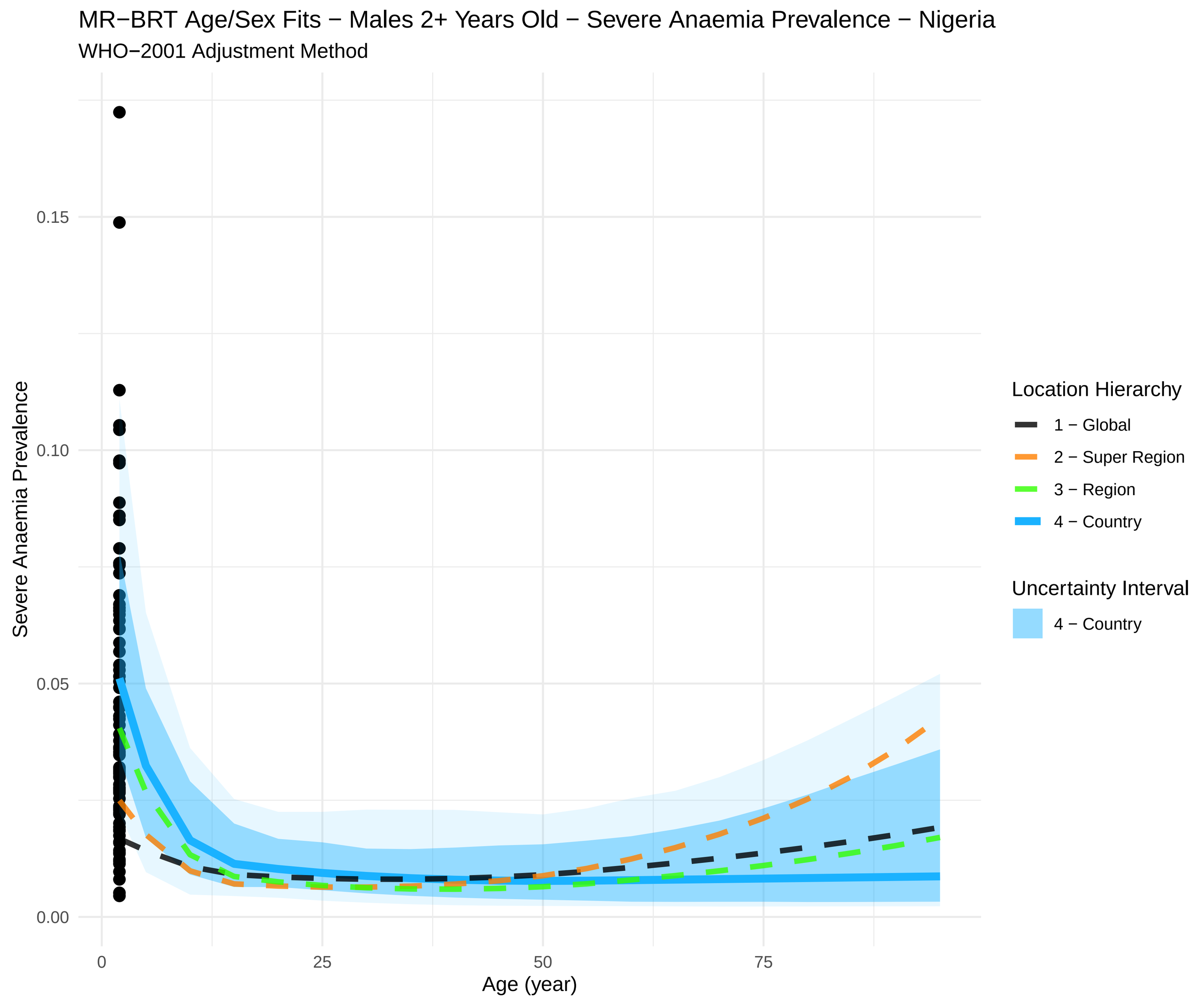
MR−BRT Age/Sex Fits − Males 2+ Years Old − Severe Anaemia Prevalence − Nigeria
WHO−2001 Adjustment Method
Severe Anaemia Prevalence
Age (year)
Location Hierarchy
1 − Global
2 − Super Region
3 − Region
4 − Country
Uncertainty Interval
4 − Country

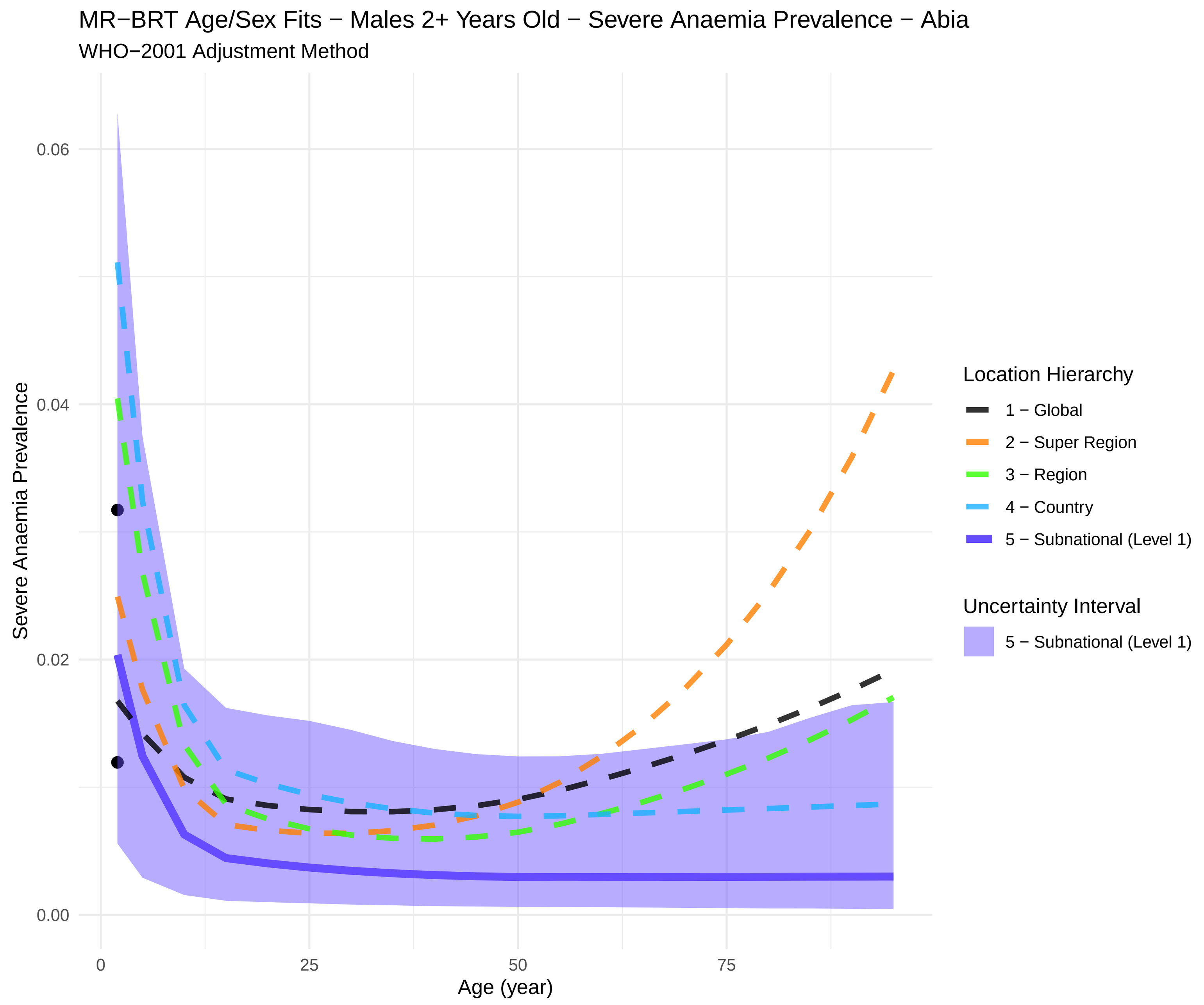
MR−BRT Age/Sex Fits − Males 2+ Years Old − Severe Anaemia Prevalence − Abia
WHO−2001 Adjustment Method
Severe Anaemia Prevalence
Age (year)
Location Hierarchy
1 − Global
2 − Super Region
3 − Region
4 − Country
5 − Subnational (Level 1)
Uncertainty Interval
5 − Subnational (Level 1)

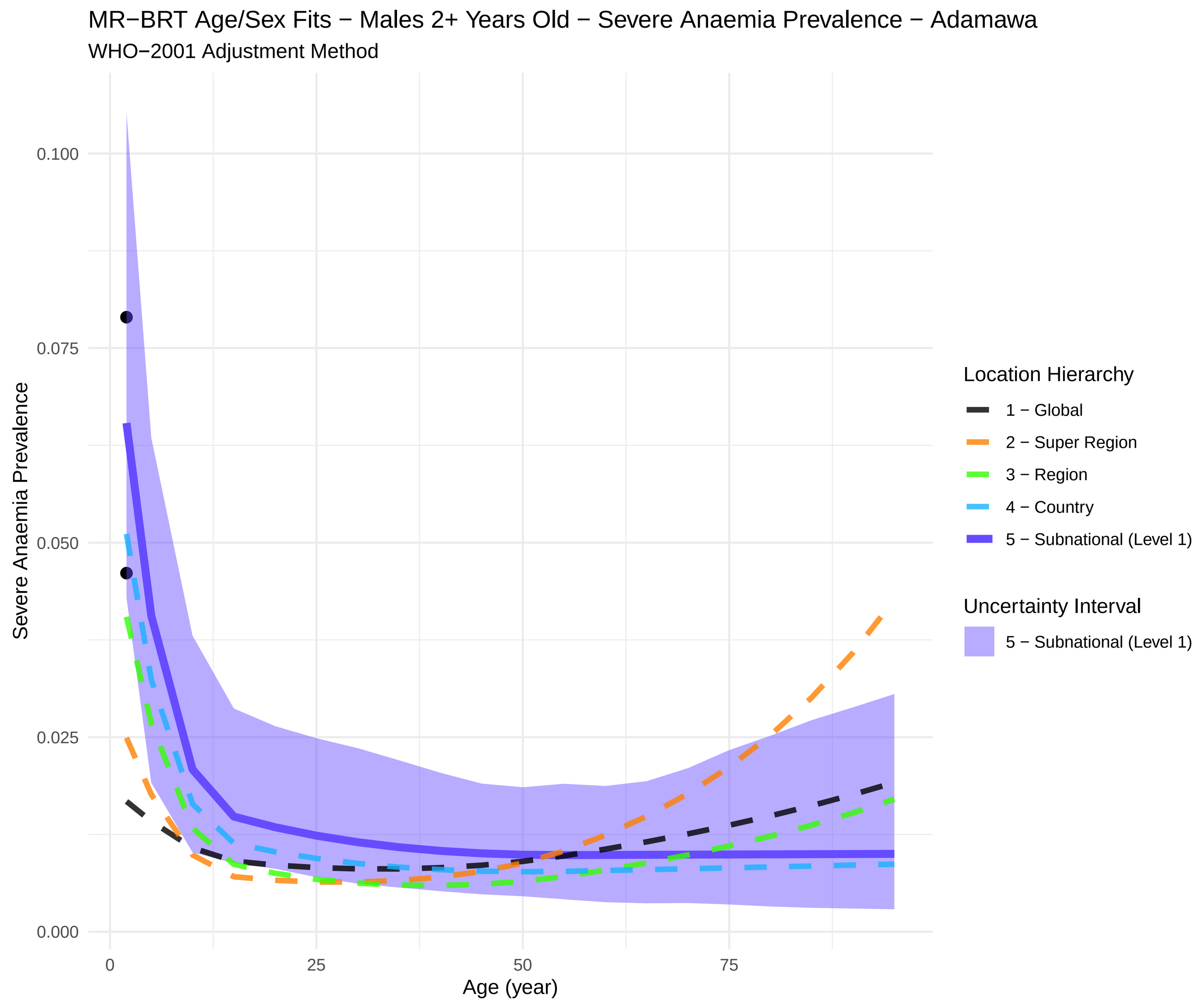
MR−BRT Age/Sex Fits − Males 2+ Years Old − Severe Anaemia Prevalence − Adamawa
WHO−2001 Adjustment Method
Severe Anaemia Prevalence
Age (year)
Location Hierarchy
1 − Global
2 − Super Region
3 − Region
4 − Country
5 − Subnational (Level 1)
Uncertainty Interval
5 − Subnational (Level 1)

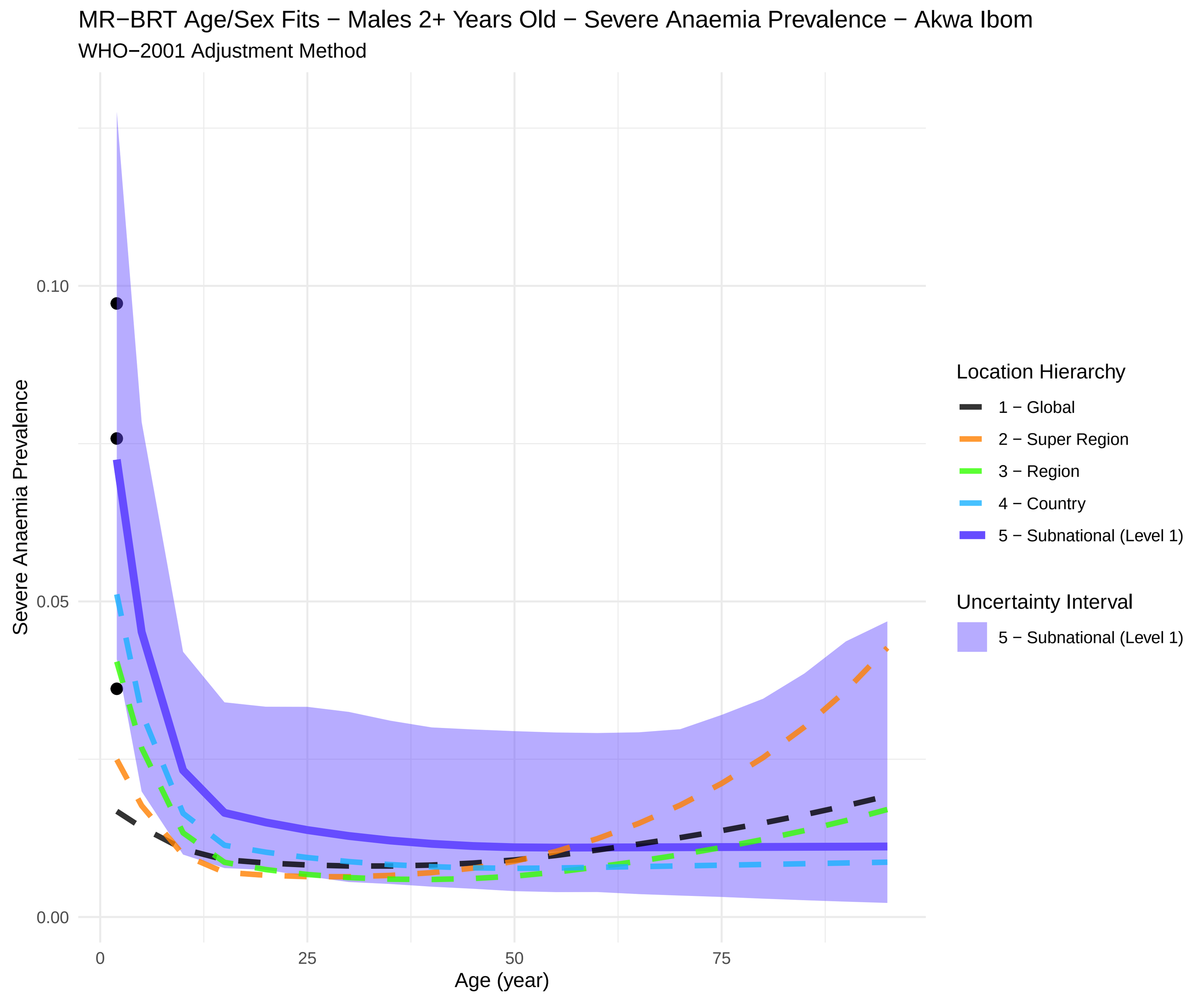
MR−BRT Age/Sex Fits − Males 2+ Years Old − Severe Anaemia Prevalence − Akwa Ibom
WHO−2001 Adjustment Method
Severe Anaemia Prevalence
Age (year)
Location Hierarchy
1 − Global
2 − Super Region
3 − Region
4 − Country
5 − Subnational (Level 1)
Uncertainty Interval
5 − Subnational (Level 1)

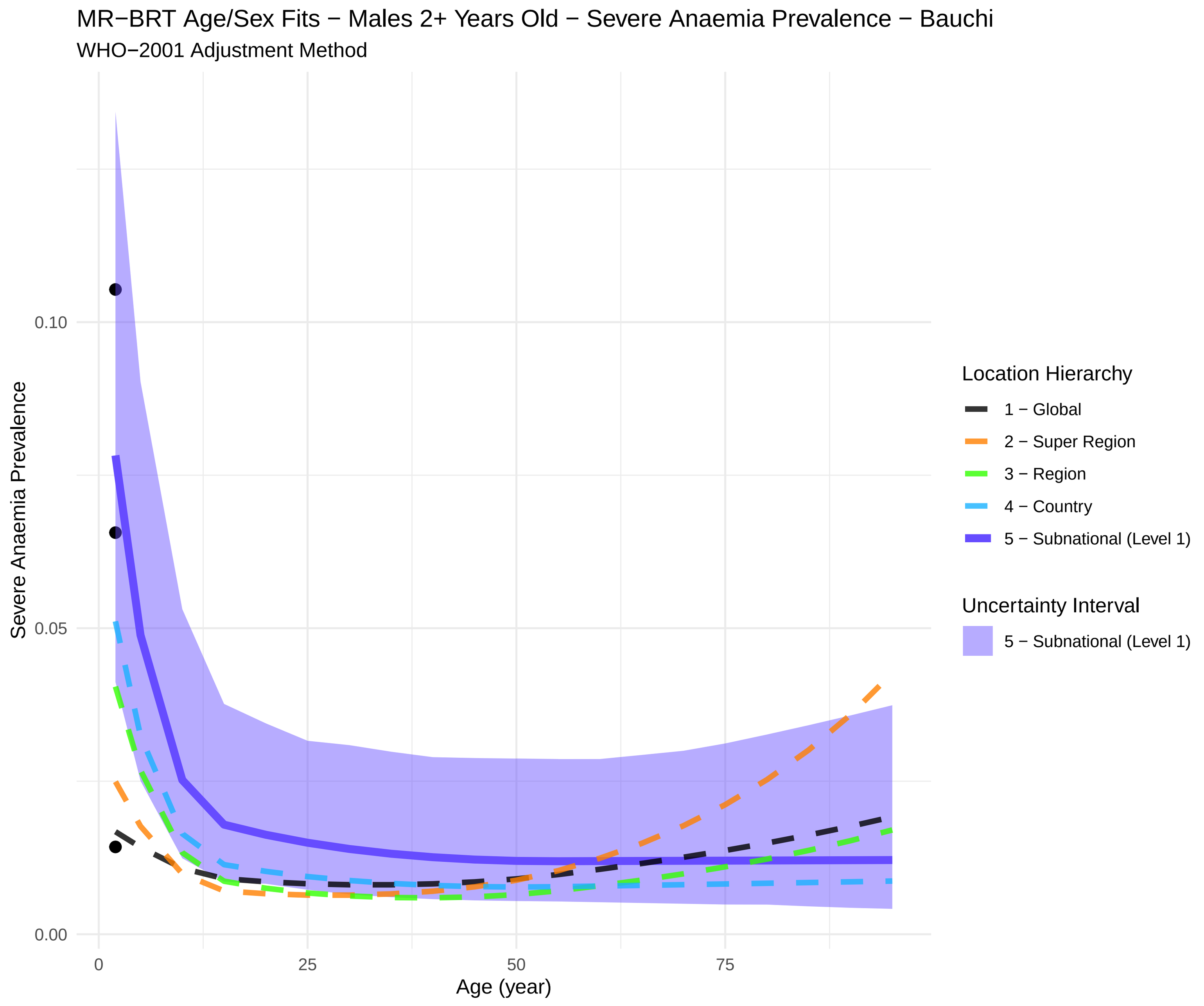
MR−BRT Age/Sex Fits − Males 2+ Years Old − Severe Anaemia Prevalence − Bauchi
WHO−2001 Adjustment Method
Severe Anaemia Prevalence
Age (year)
Location Hierarchy
1 − Global
2 − Super Region
3 − Region
4 − Country
5 − Subnational (Level 1)
Uncertainty Interval
5 − Subnational (Level 1)

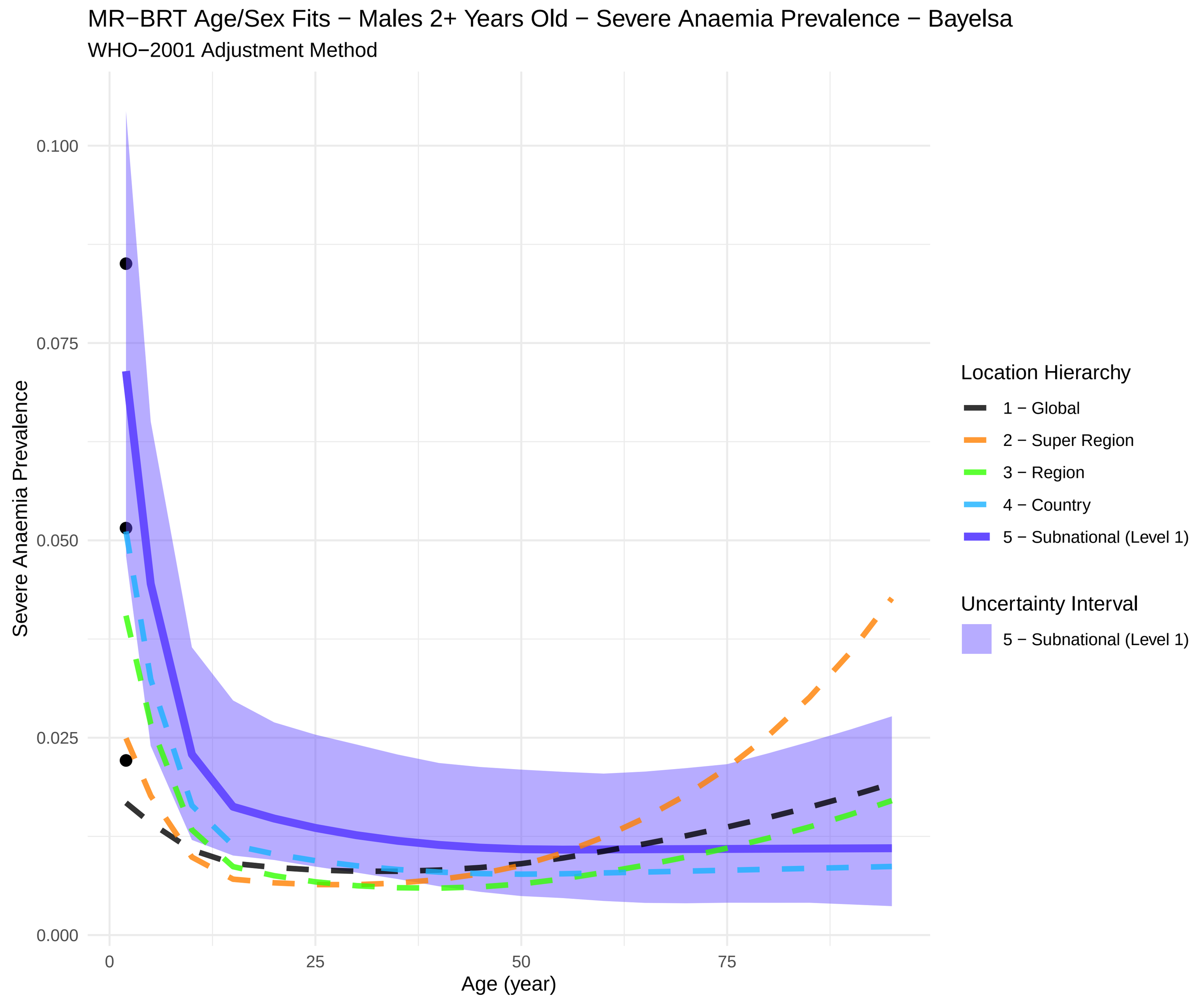
MR−BRT Age/Sex Fits − Males 2+ Years Old − Severe Anaemia Prevalence − Bayelsa
WHO−2001 Adjustment Method
Severe Anaemia Prevalence
Age (year)
Location Hierarchy
1 − Global
2 − Super Region
3 − Region
4 − Country
5 − Subnational (Level 1)
Uncertainty Interval
5 − Subnational (Level 1)

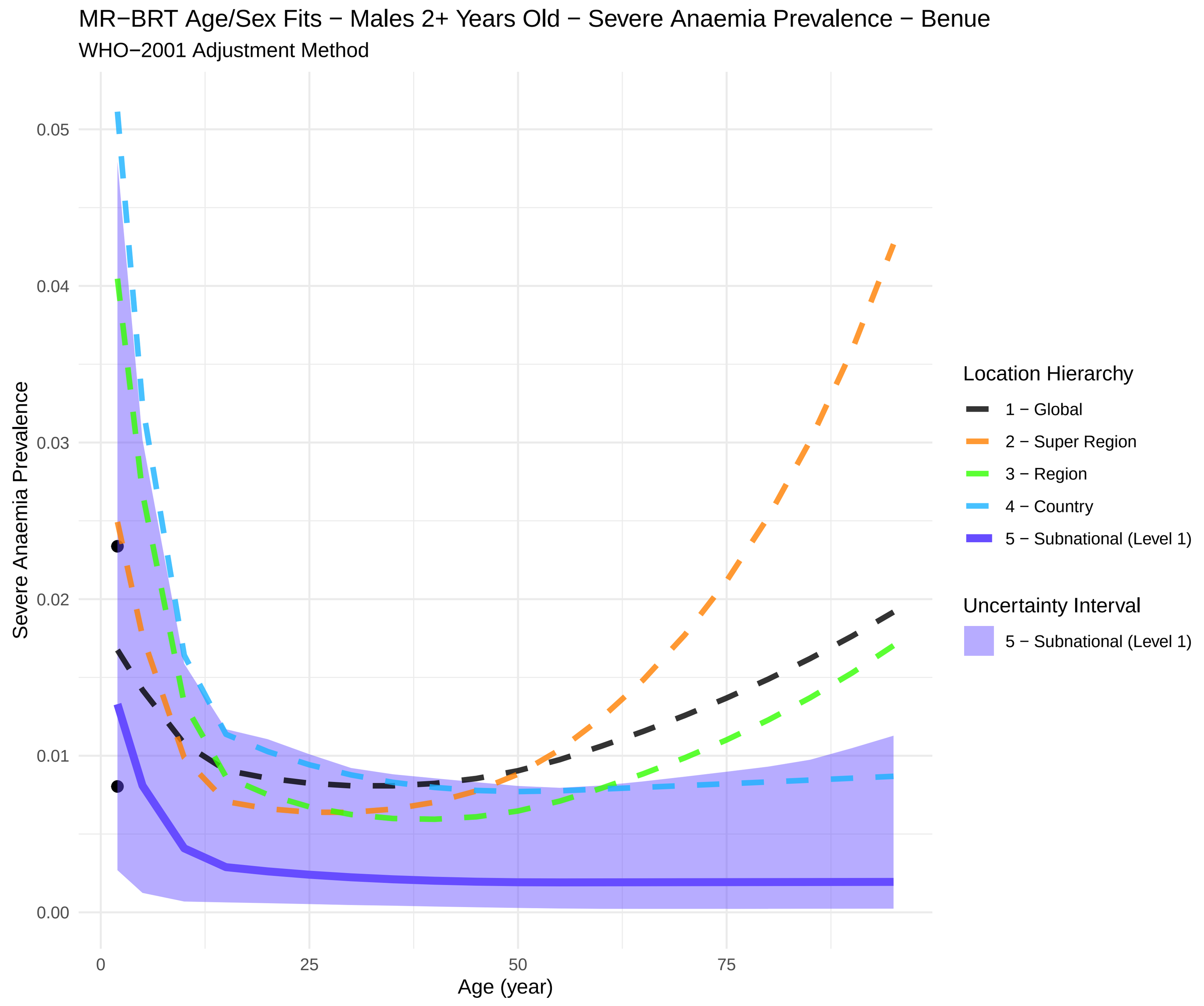
MR−BRT Age/Sex Fits − Males 2+ Years Old − Severe Anaemia Prevalence − Benue
WHO−2001 Adjustment Method
Severe Anaemia Prevalence
Age (year)
Location Hierarchy
1 − Global
2 − Super Region
3 − Region
4 − Country
5 − Subnational (Level 1)
Uncertainty Interval
5 − Subnational (Level 1)

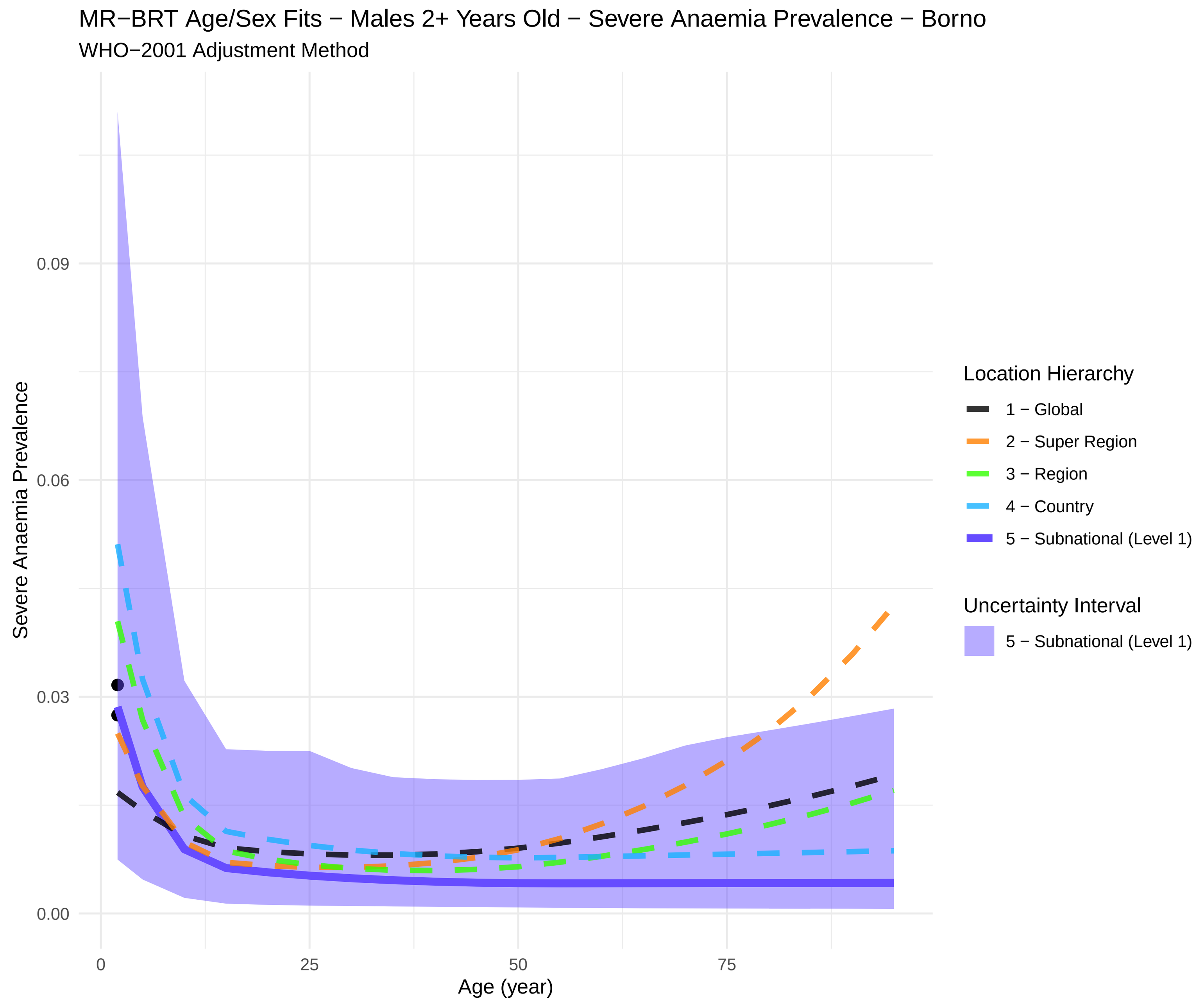
MR−BRT Age/Sex Fits − Males 2+ Years Old − Severe Anaemia Prevalence − Borno
WHO−2001 Adjustment Method
Severe Anaemia Prevalence
Age (year)
Location Hierarchy
1 − Global
2 − Super Region
3 − Region
4 − Country
5 − Subnational (Level 1)
Uncertainty Interval
5 − Subnational (Level 1)

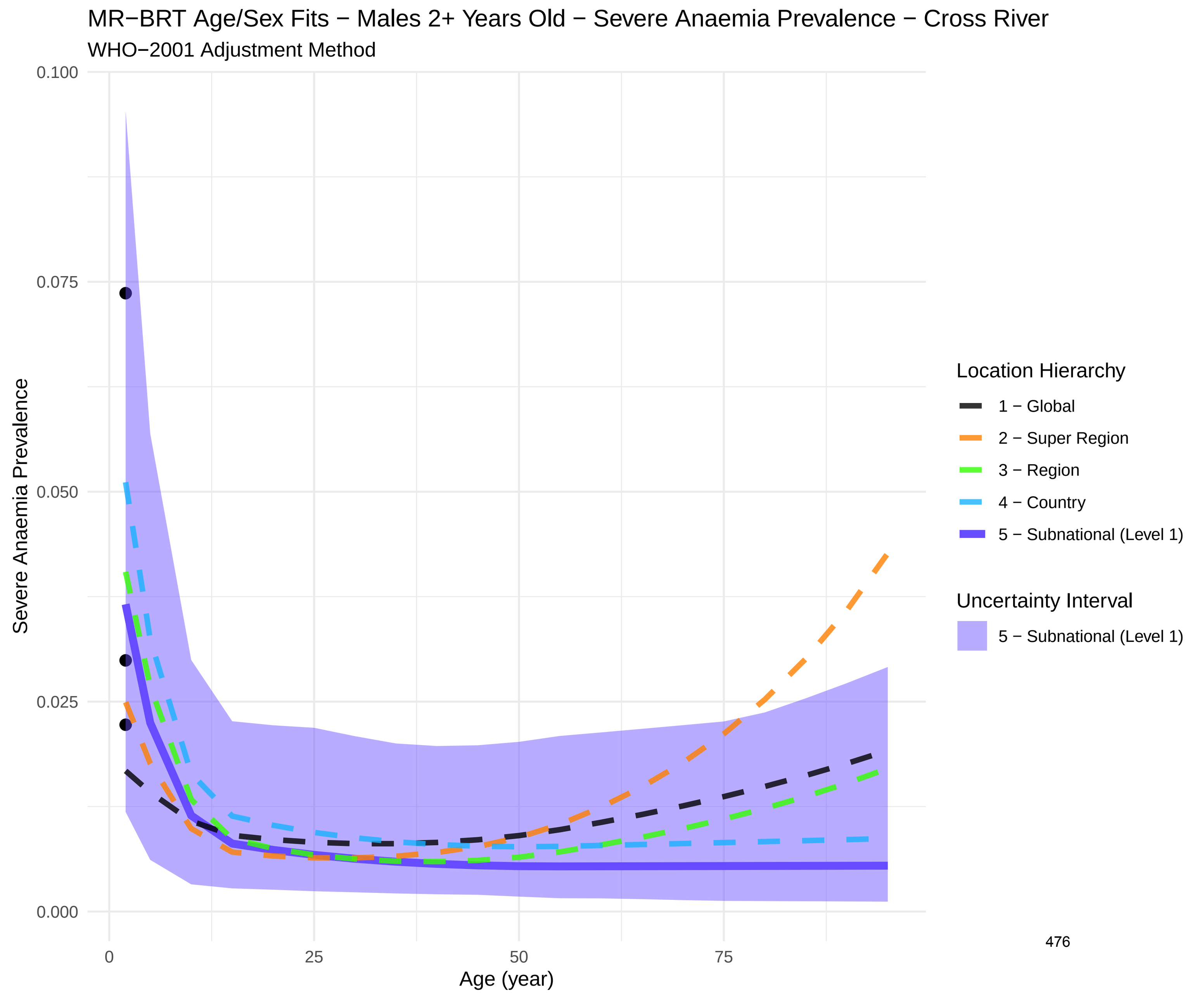
MR−BRT Age/Sex Fits − Males 2+ Years Old − Severe Anaemia Prevalence − Cross River
WHO−2001 Adjustment Method
Severe Anaemia Prevalence
Age (year)
Location Hierarchy
1 − Global
2 − Super Region
3 − Region
4 − Country
5 − Subnational (Level 1)
Uncertainty Interval
5 − Subnational (Level 1)

MR−BRT Age/Sex Fits − Males 2+ Years Old − Severe Anaemia Prevalence − Delta
WHO−2001 Adjustment Method

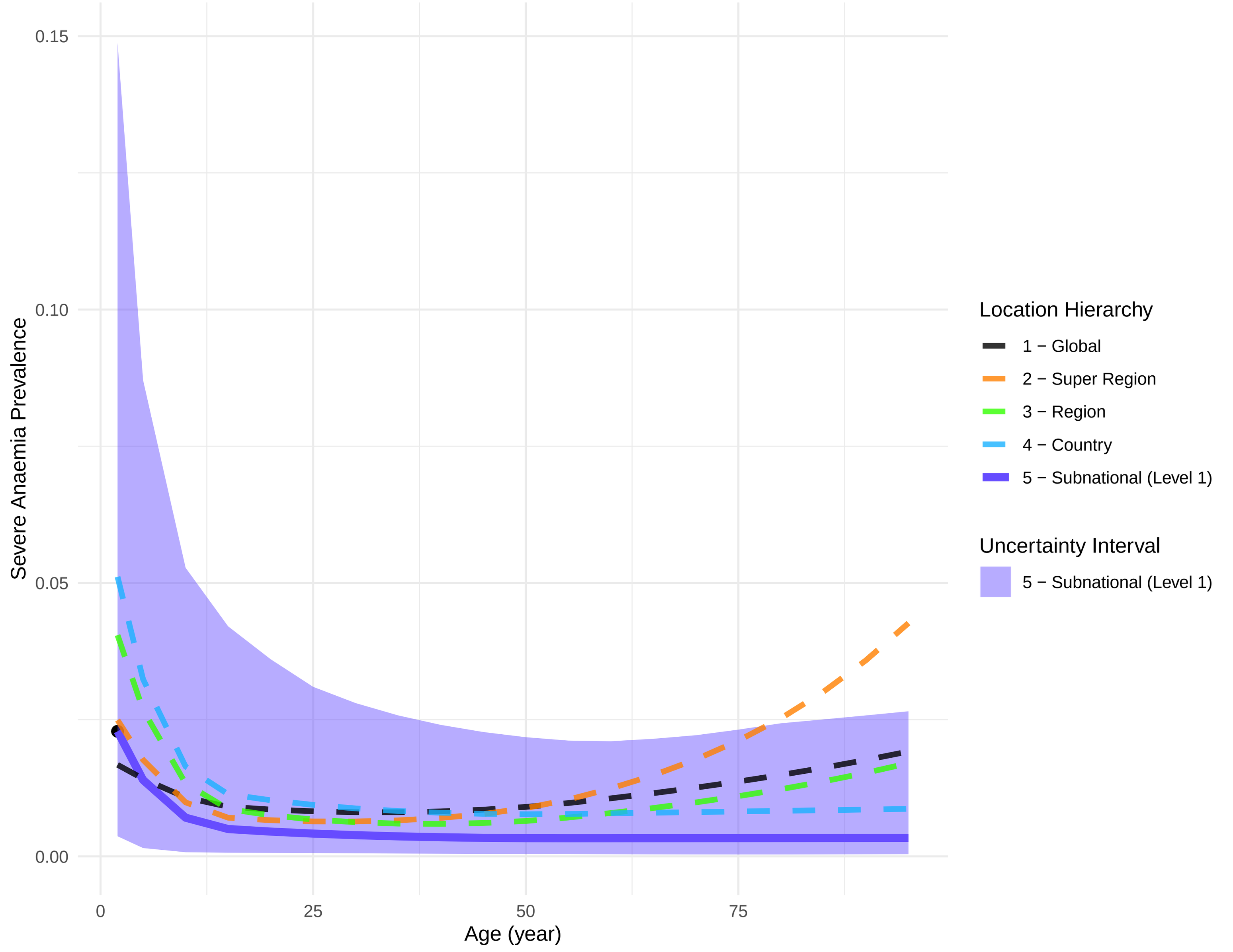

MR−BRT Age/Sex Fits − Males 2+ Years Old − Severe Anaemia Prevalence − Ebonyi
WHO−2001 Adjustment Method

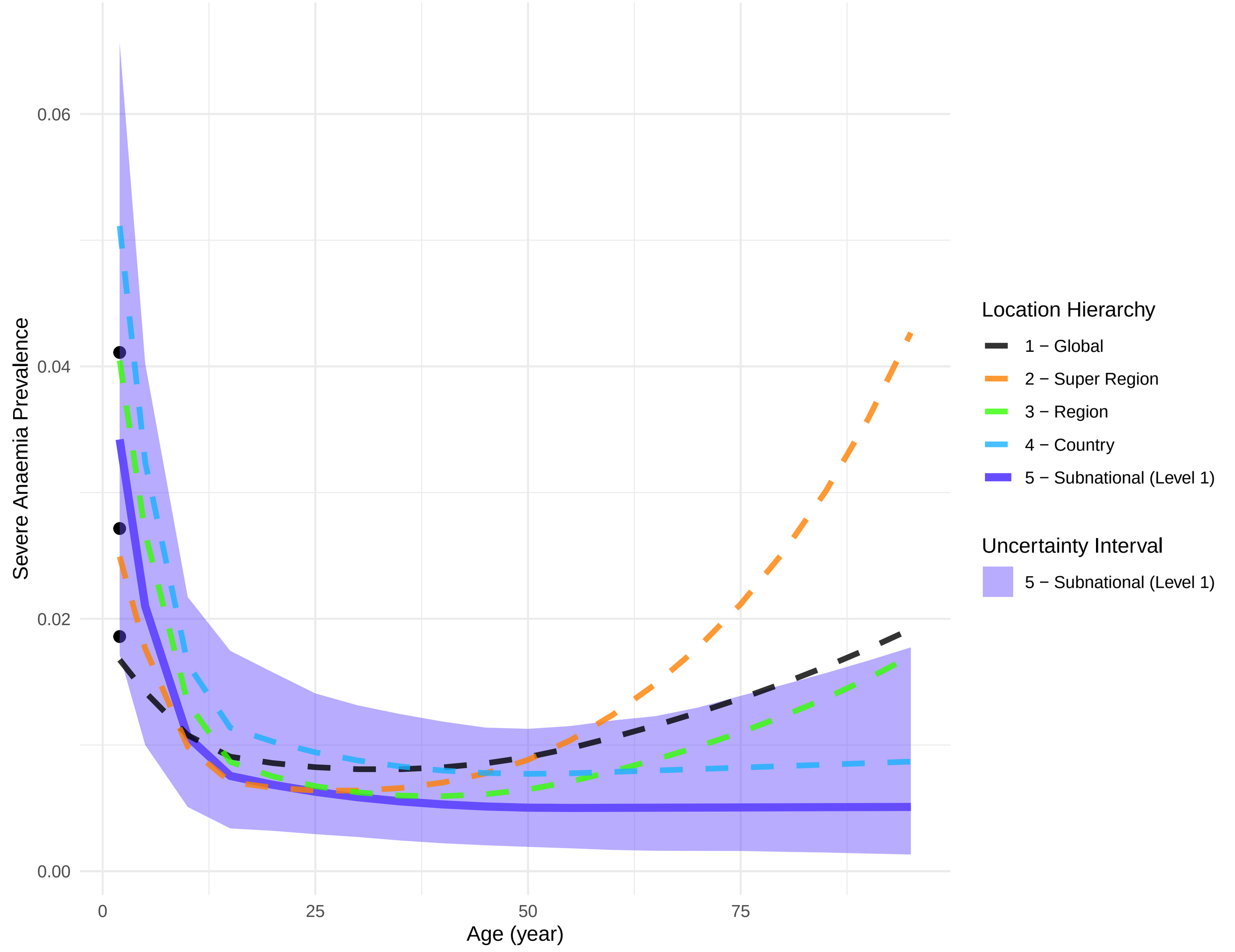

MR−BRT Age/Sex Fits − Males 2+ Years Old − Severe Anaemia Prevalence − Edo
WHO−2001 Adjustment Method

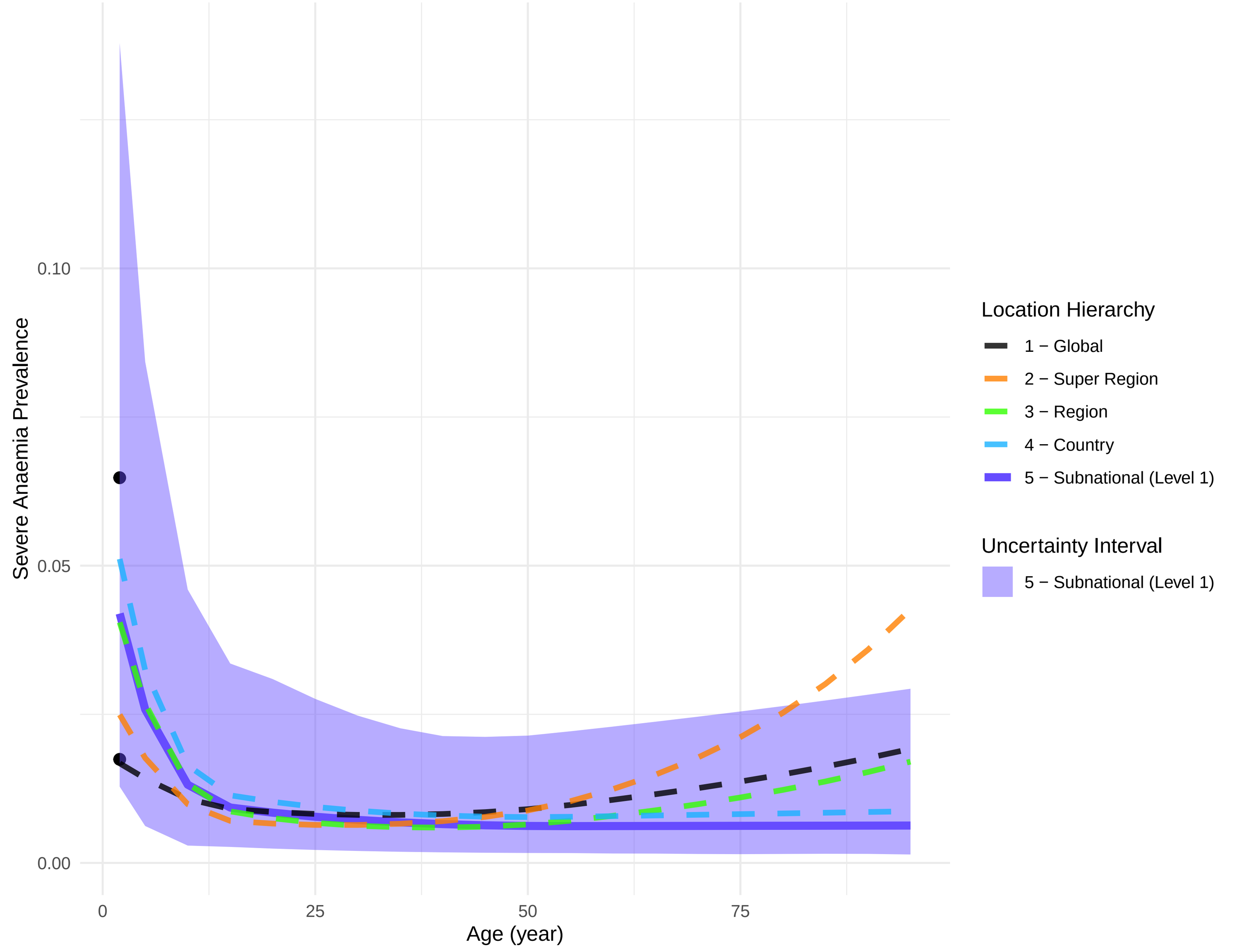

MR−BRT Age/Sex Fits − Males 2+ Years Old − Severe Anaemia Prevalence − Ekiti
WHO−2001 Adjustment Method

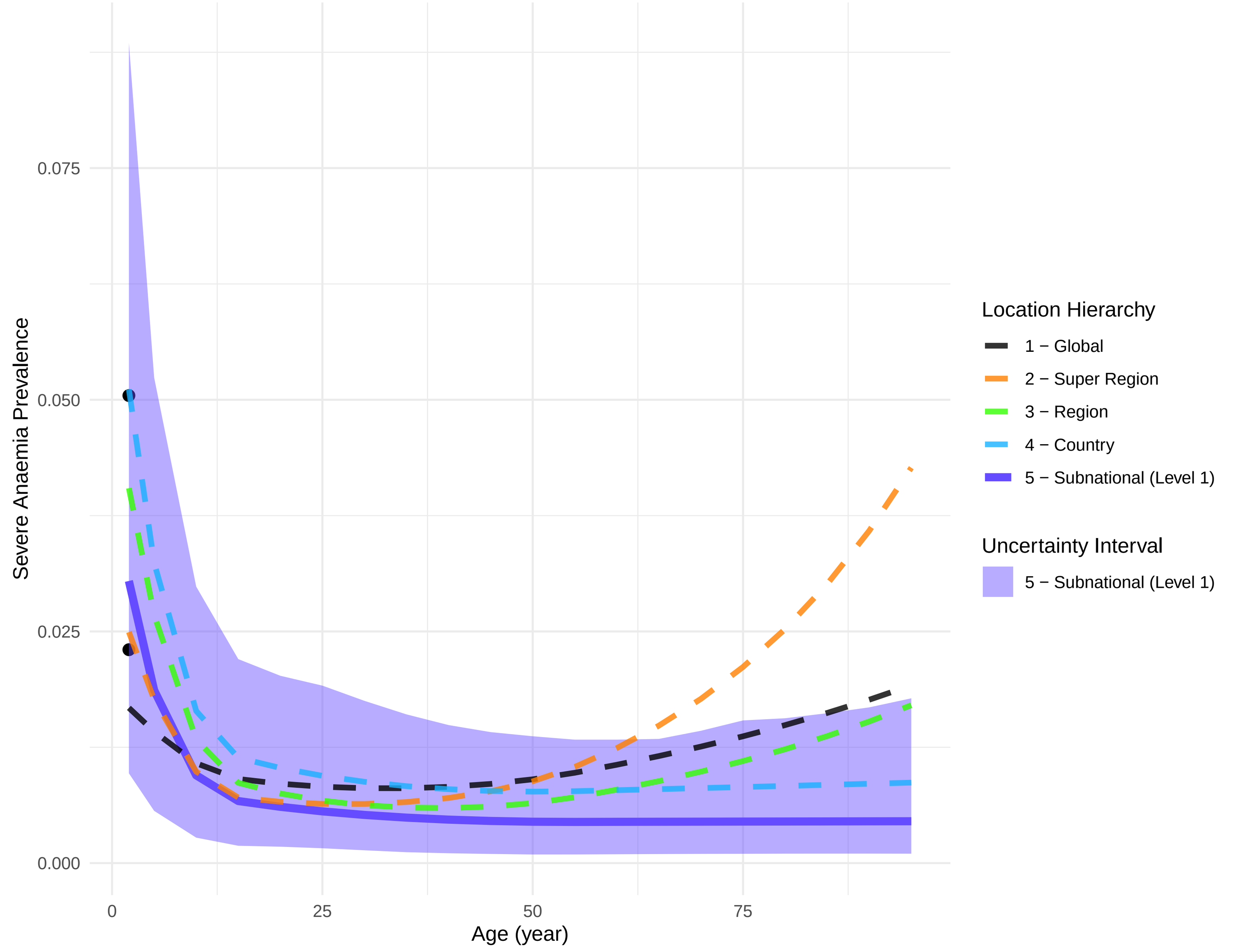

MR−BRT Age/Sex Fits − Males 2+ Years Old − Severe Anaemia Prevalence − Enugu
WHO−2001 Adjustment Method

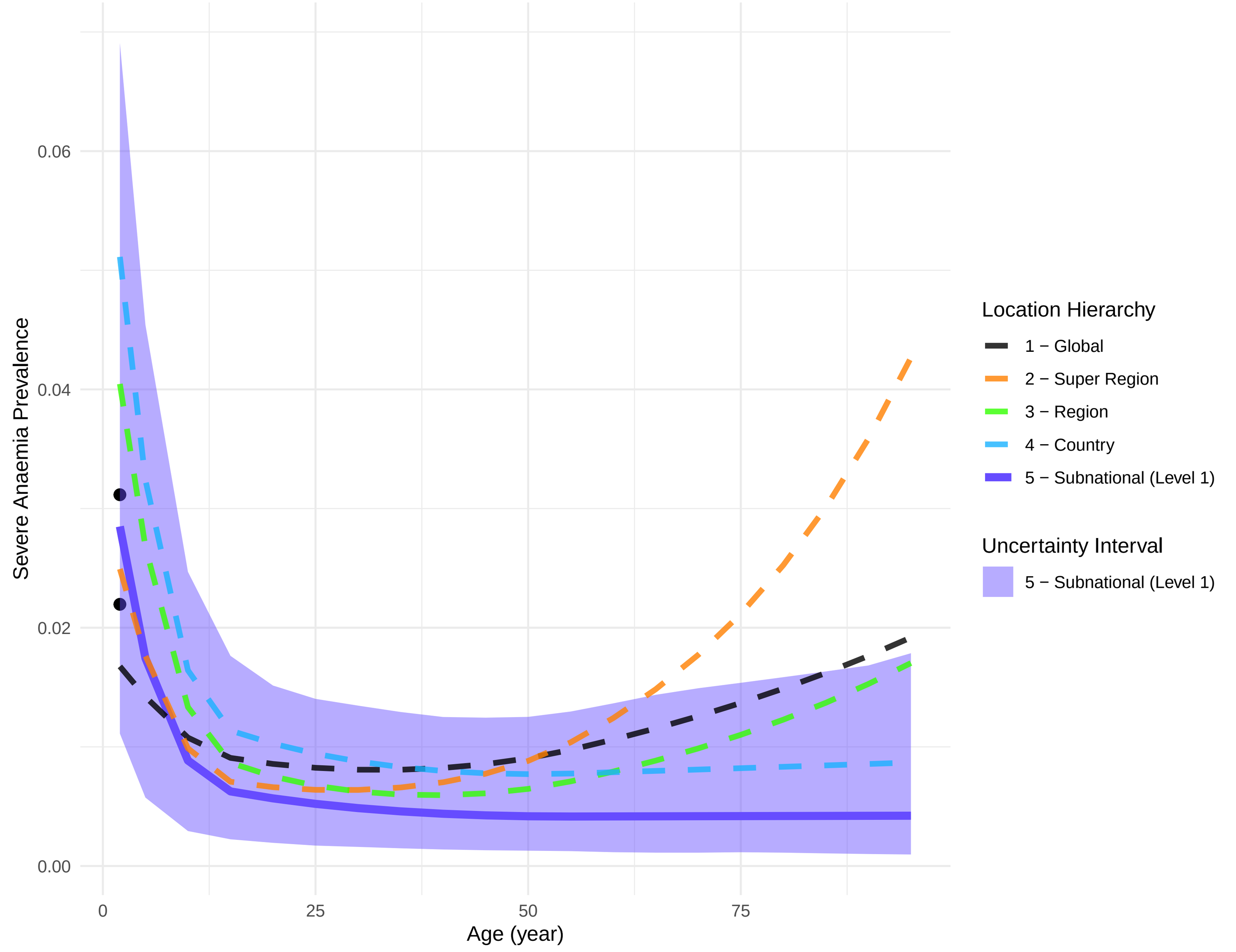

MR−BRT Age/Sex Fits − Males 2+ Years Old − Severe Anaemia Prevalence − FCT (Abuja)
WHO−2001 Adjustment Method

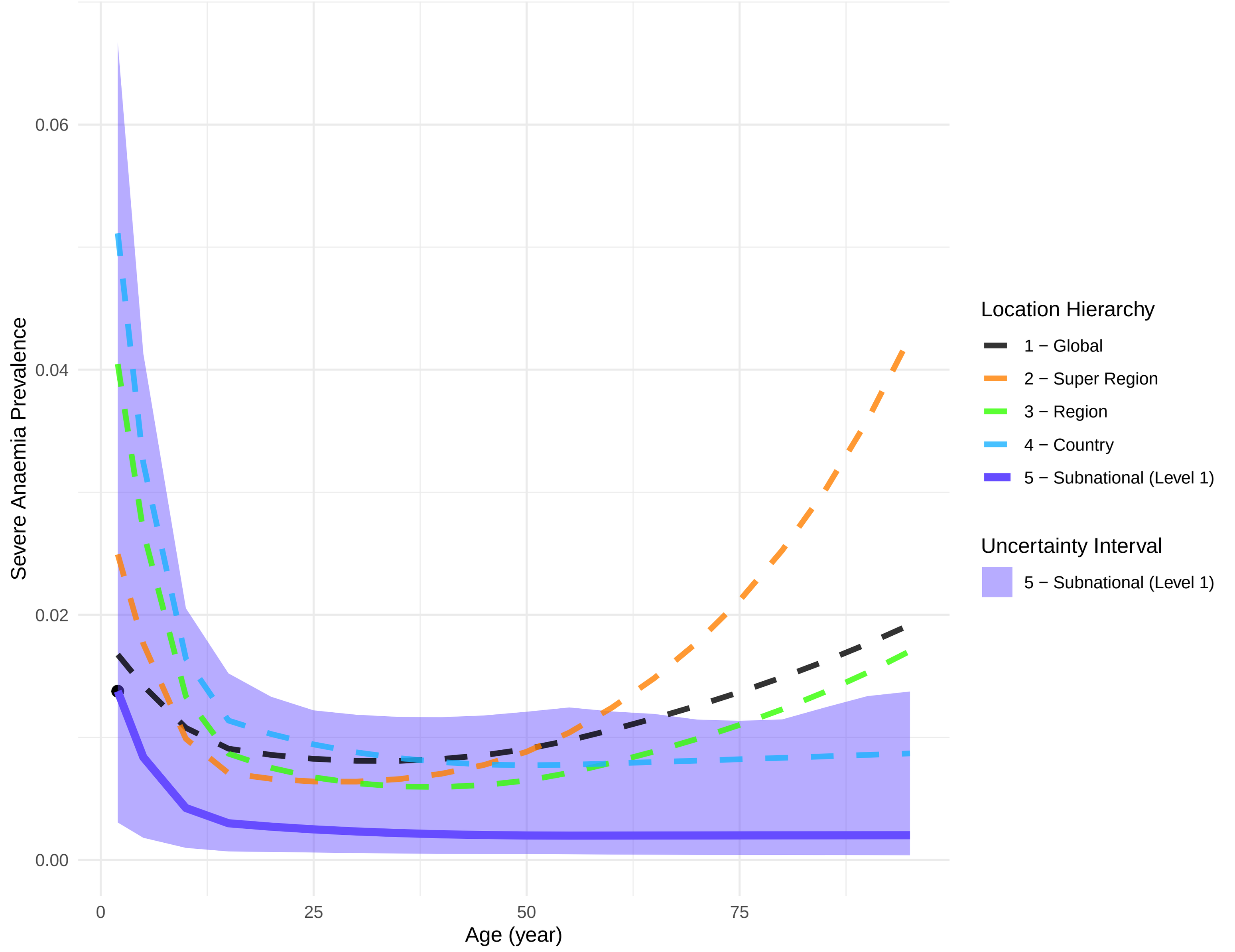

MR−BRT Age/Sex Fits − Males 2+ Years Old − Severe Anaemia Prevalence − Gombe
WHO−2001 Adjustment Method

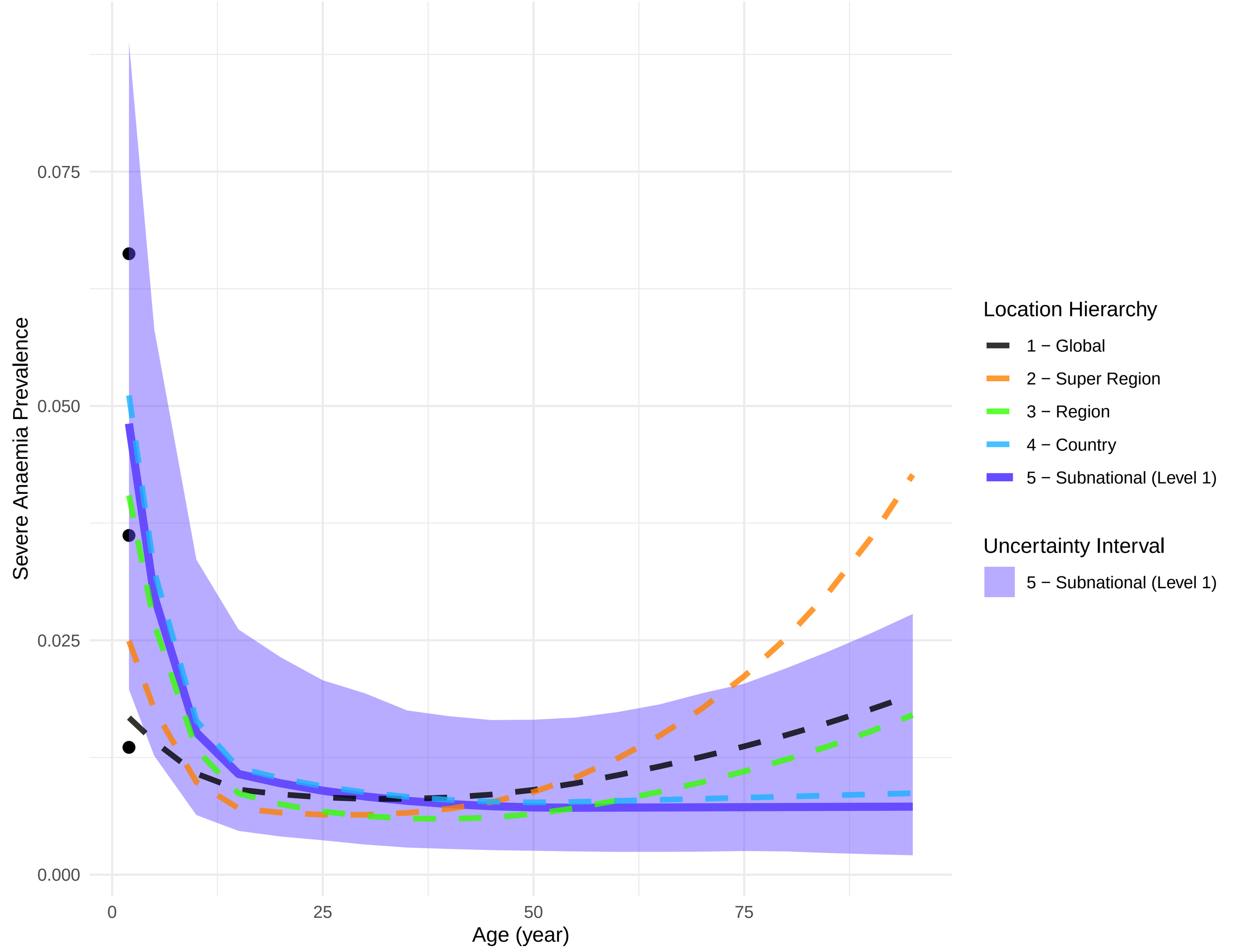

MR−BRT Age/Sex Fits − Males 2+ Years Old − Severe Anaemia Prevalence − Imo
WHO−2001 Adjustment Method

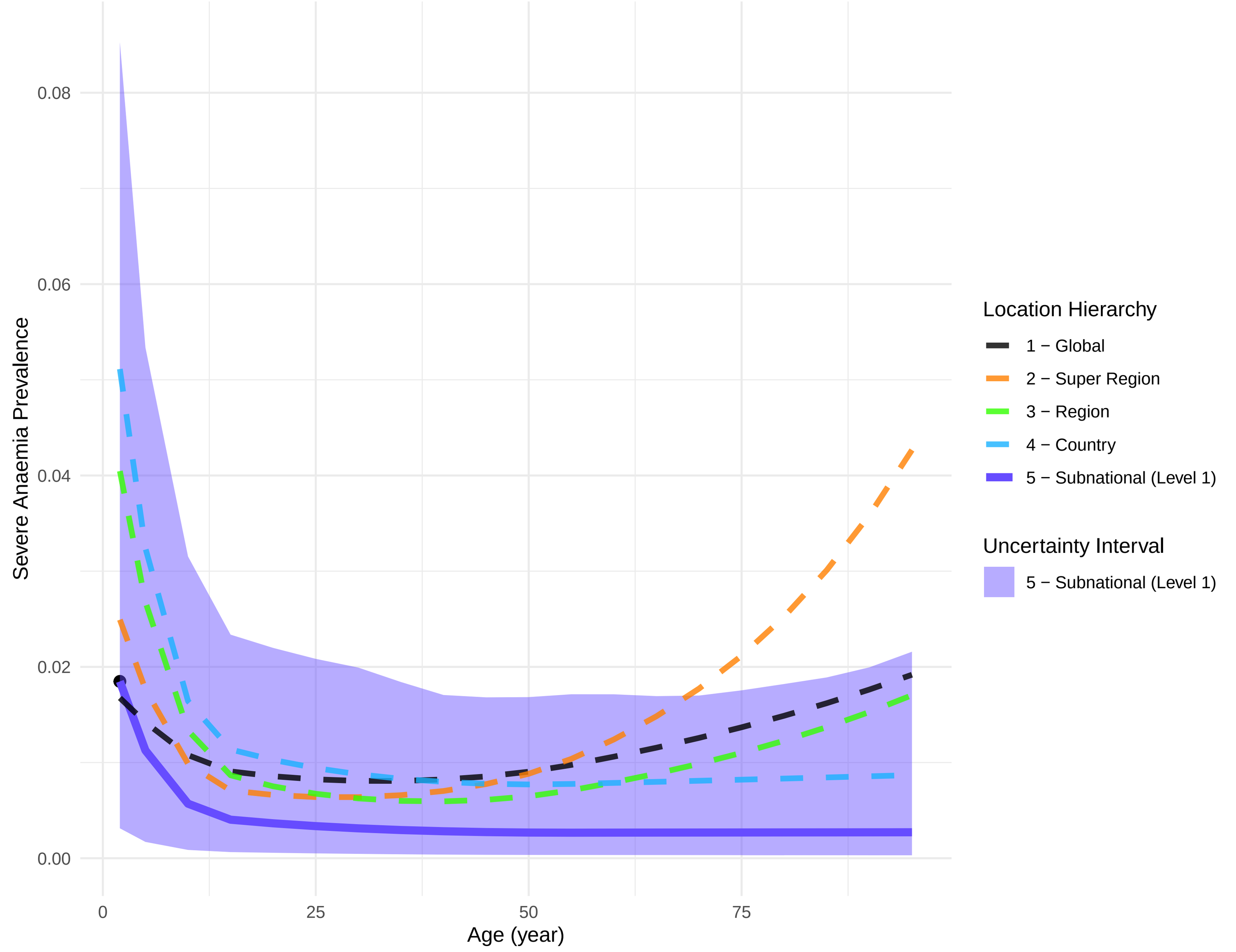

MR−BRT Age/Sex Fits − Males 2+ Years Old − Severe Anaemia Prevalence − Jigawa
WHO−2001 Adjustment Method

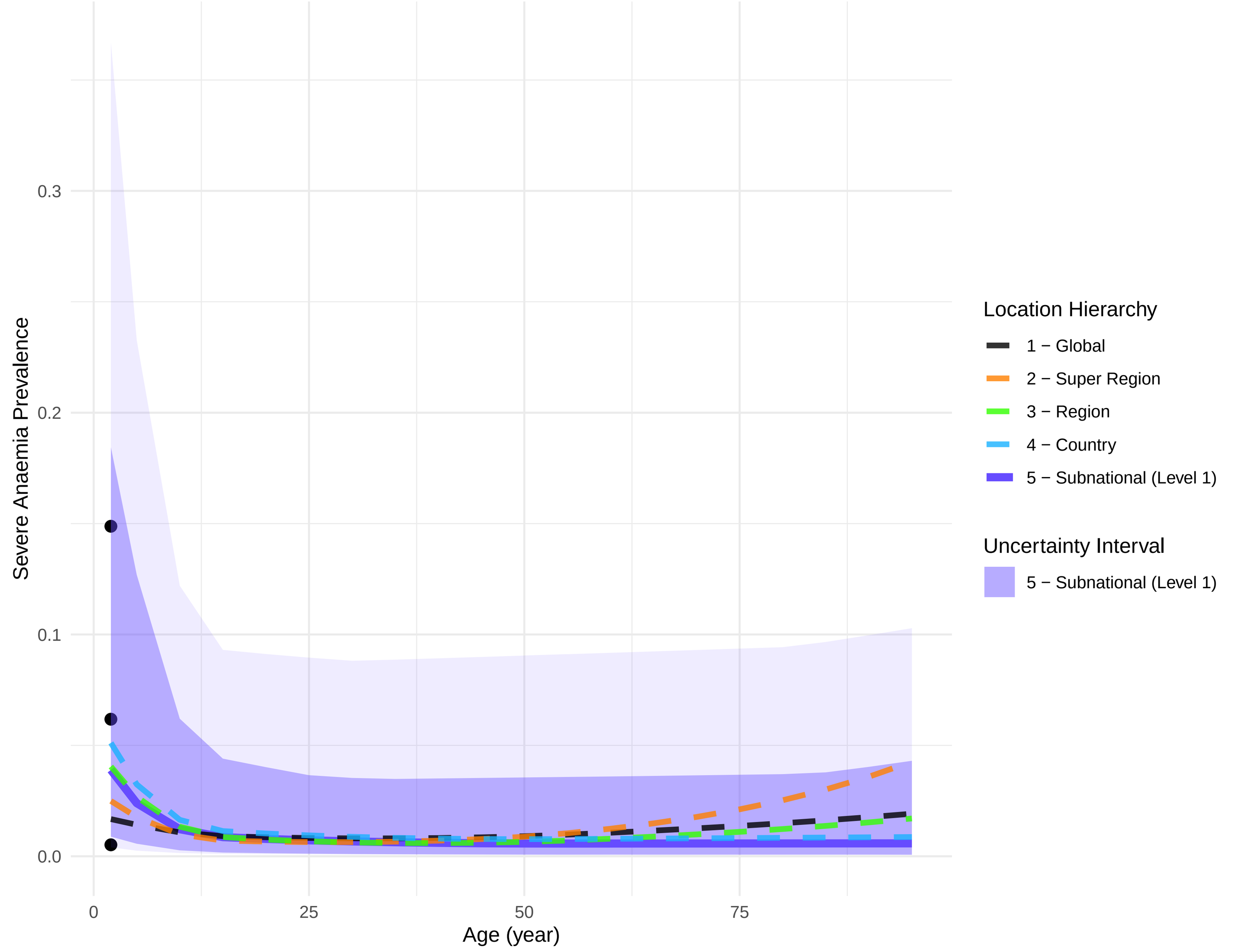

MR−BRT Age/Sex Fits − Males 2+ Years Old − Severe Anaemia Prevalence − Kaduna
WHO−2001 Adjustment Method

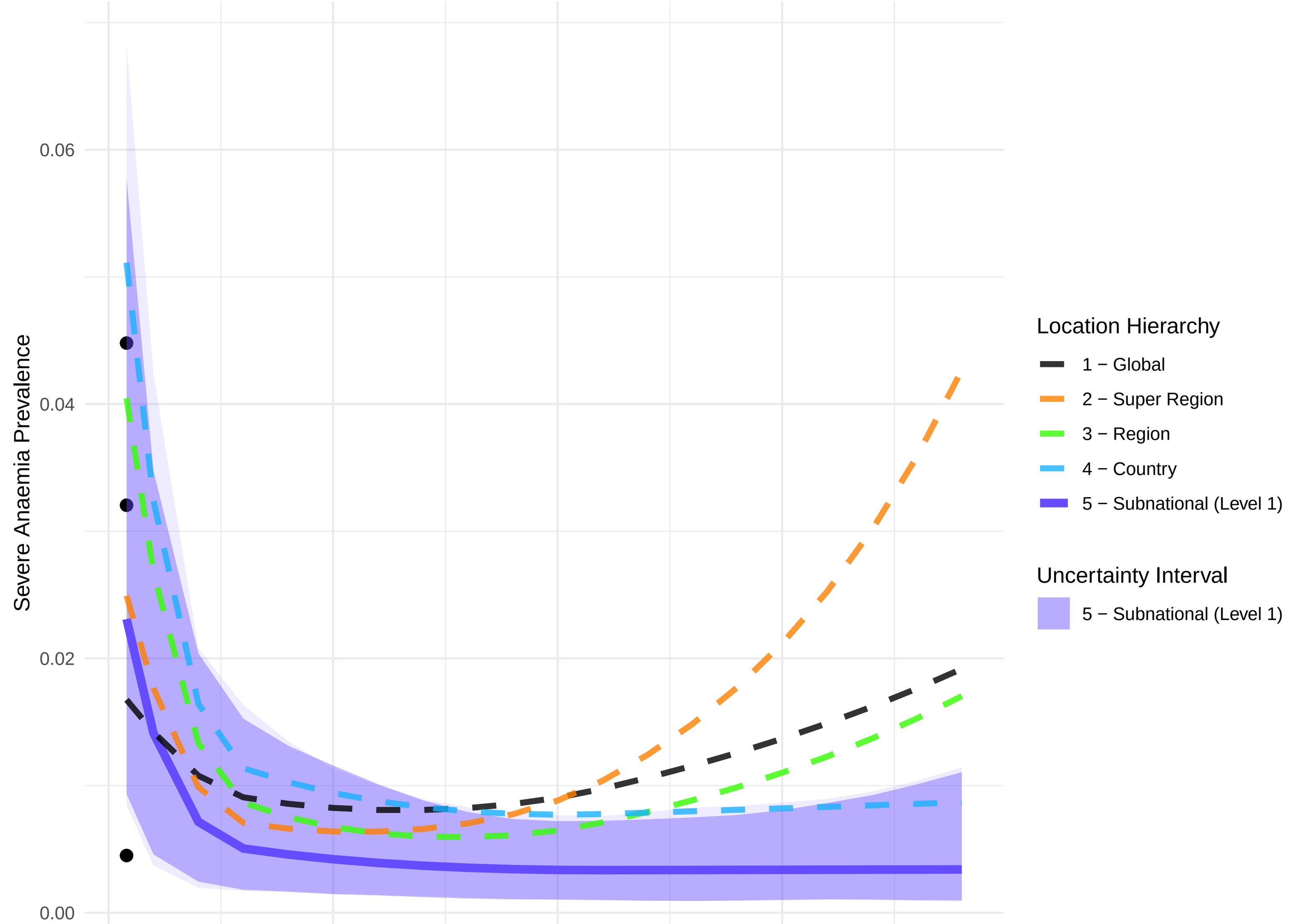

MR−BRT Age/Sex Fits − Males 2+ Years Old − Severe Anaemia Prevalence − Kano
WHO−2001 Adjustment Method

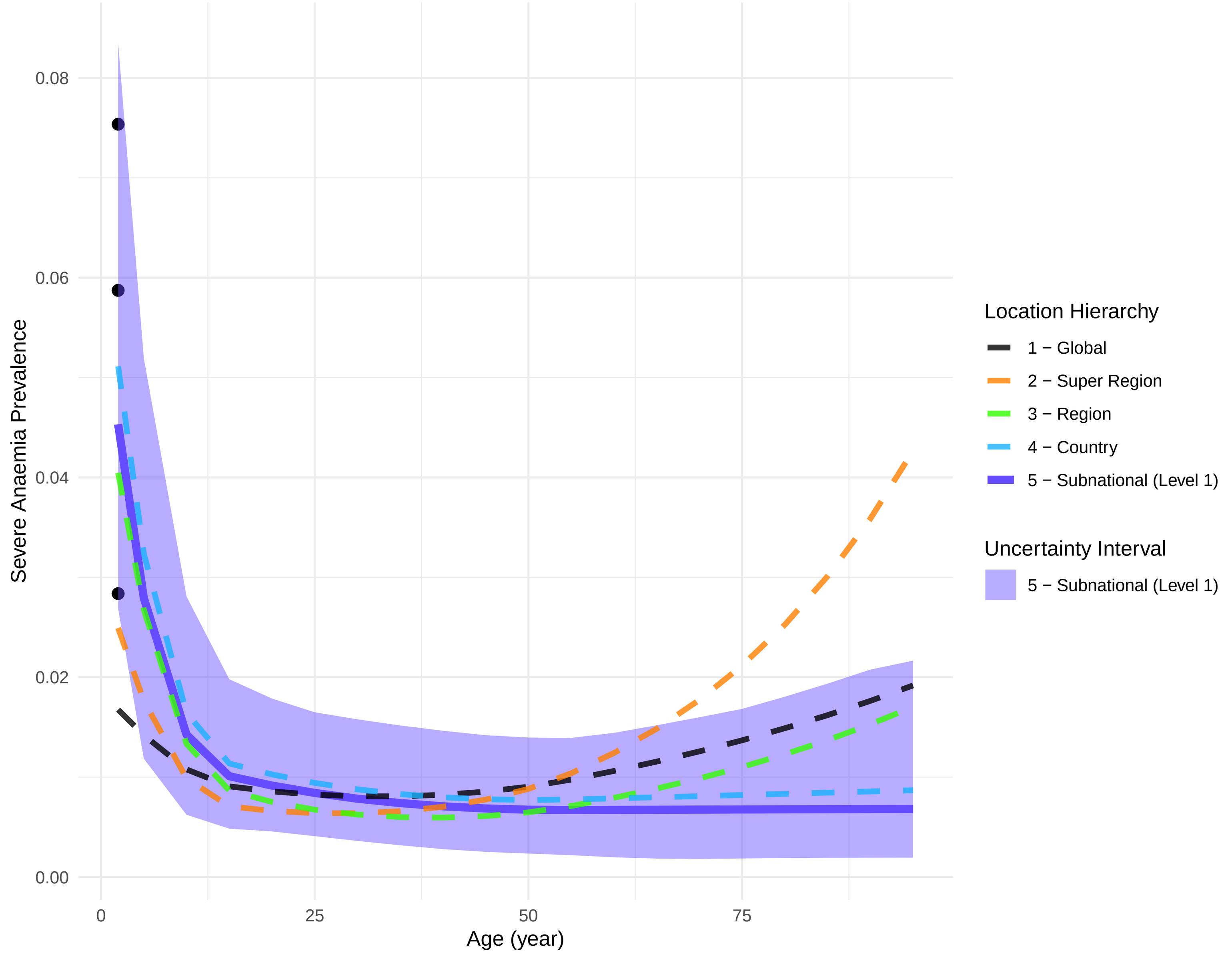

MR−BRT Age/Sex Fits − Males 2+ Years Old − Severe Anaemia Prevalence − Katsina
WHO−2001 Adjustment Method

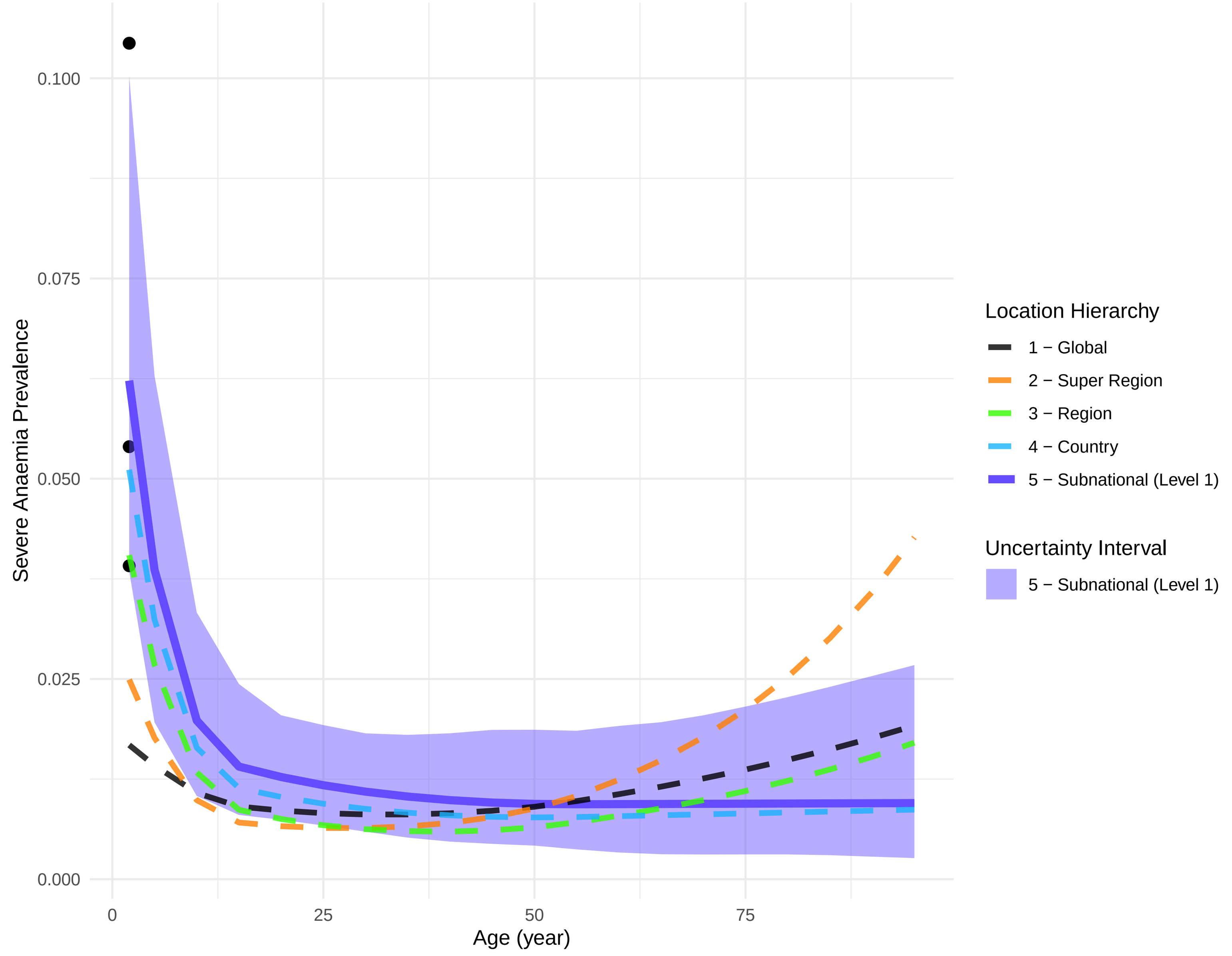

MR−BRT Age/Sex Fits − Males 2+ Years Old − Severe Anaemia Prevalence − Kebbi
WHO−2001 Adjustment Method

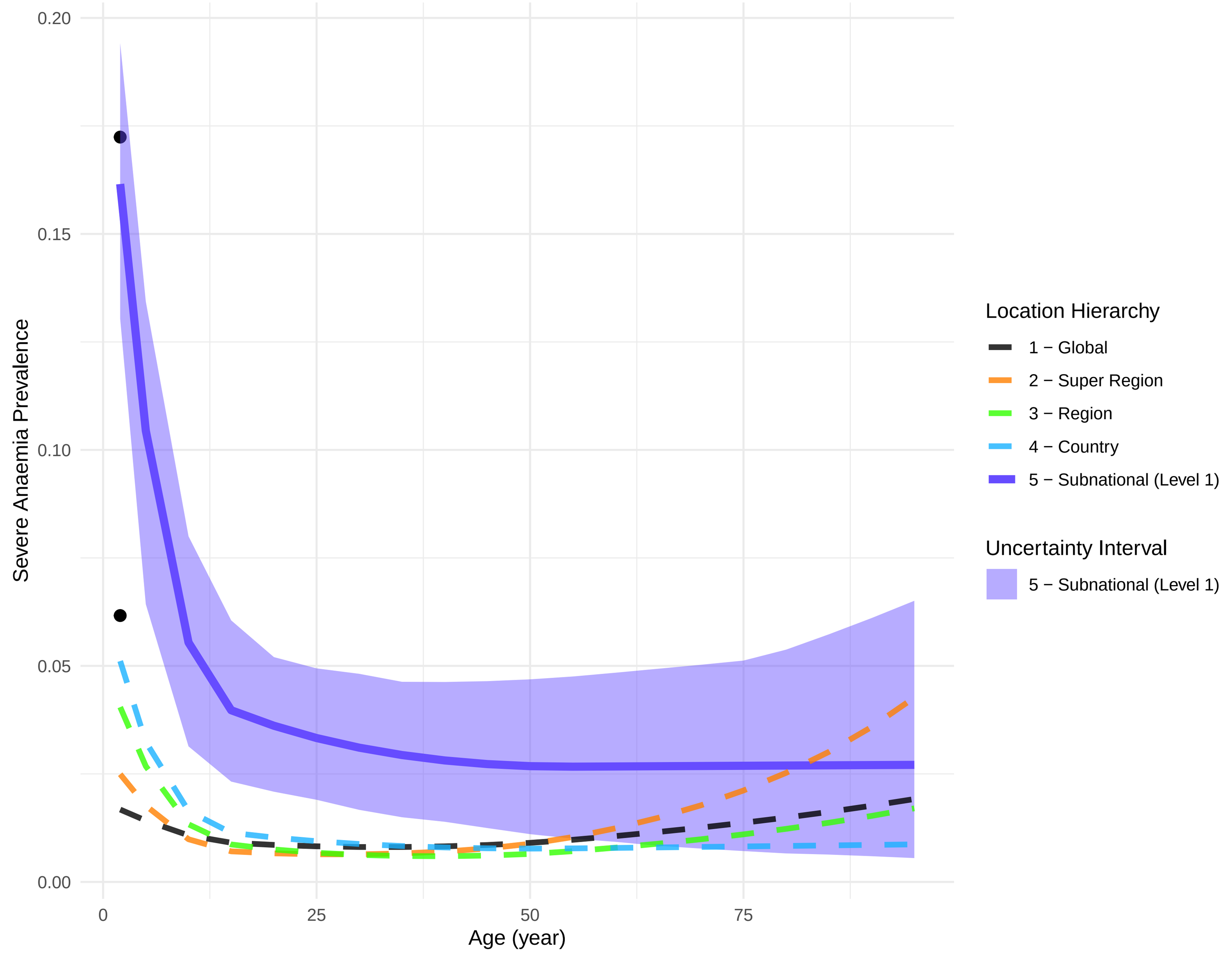

MR−BRT Age/Sex Fits − Males 2+ Years Old − Severe Anaemia Prevalence − Kogi
WHO−2001 Adjustment Method

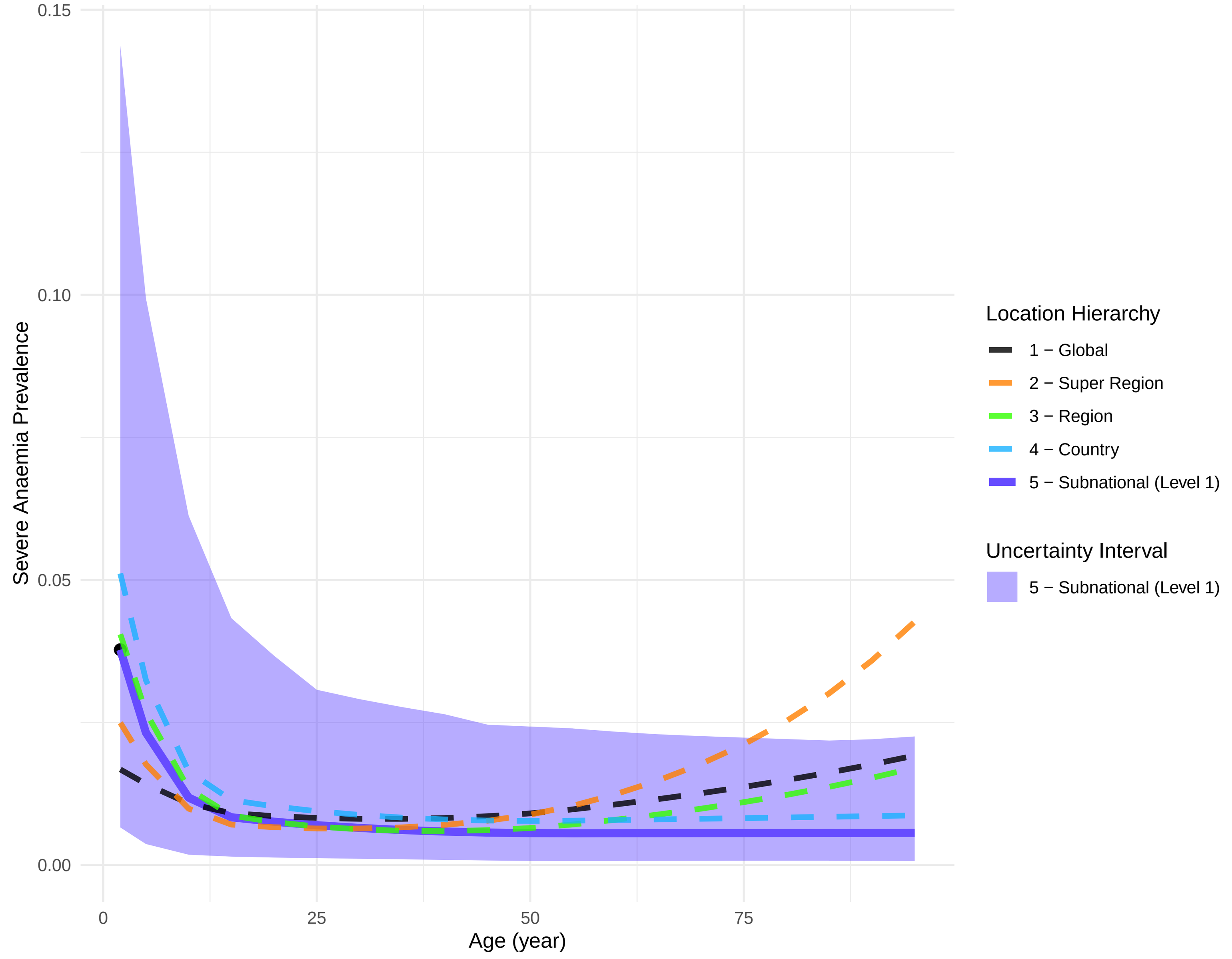

MR−BRT Age/Sex Fits − Males 2+ Years Old − Severe Anaemia Prevalence − Kwara
WHO−2001 Adjustment Method

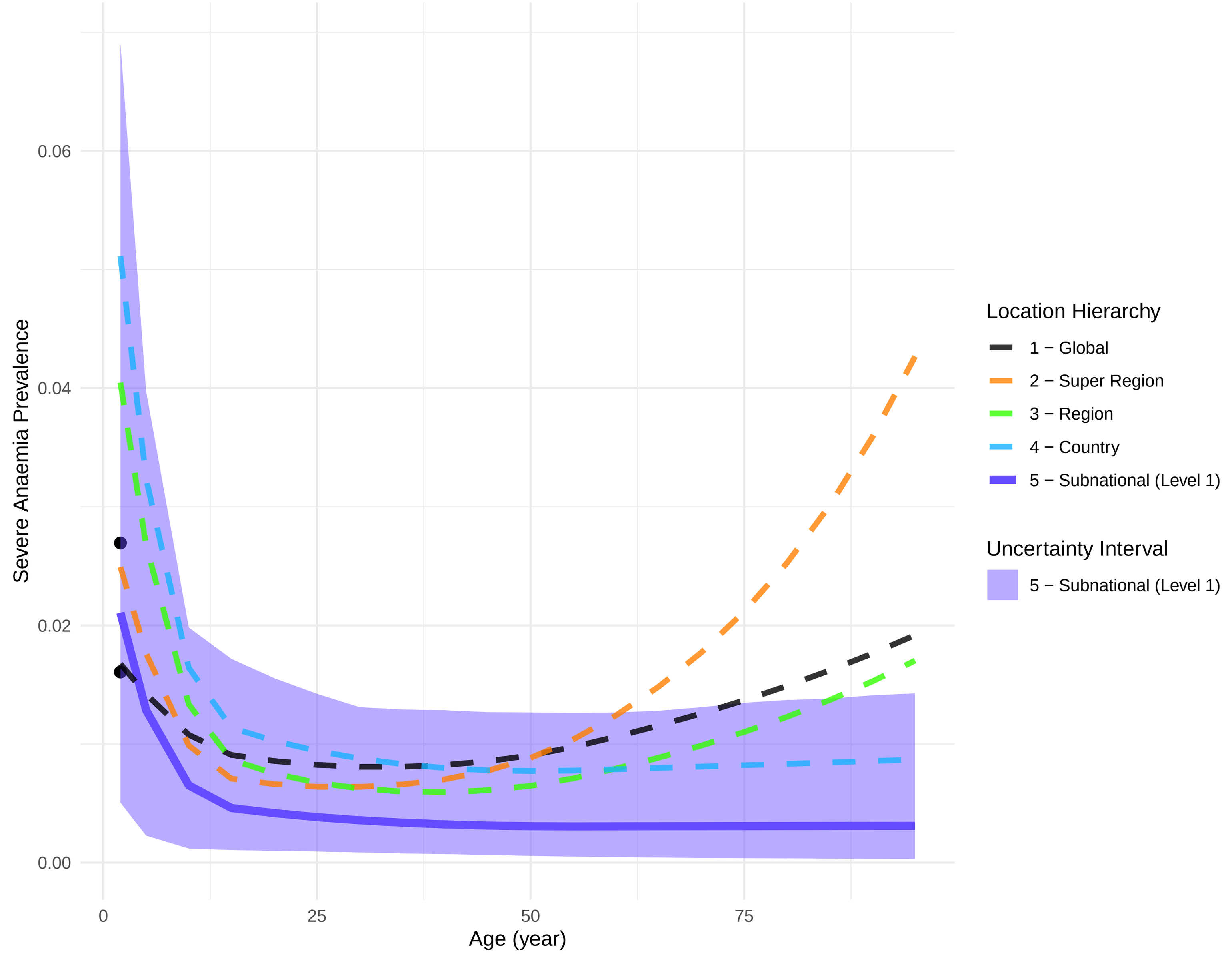

MR−BRT Age/Sex Fits − Males 2+ Years Old − Severe Anaemia Prevalence − Nasarawa
WHO−2001 Adjustment Method

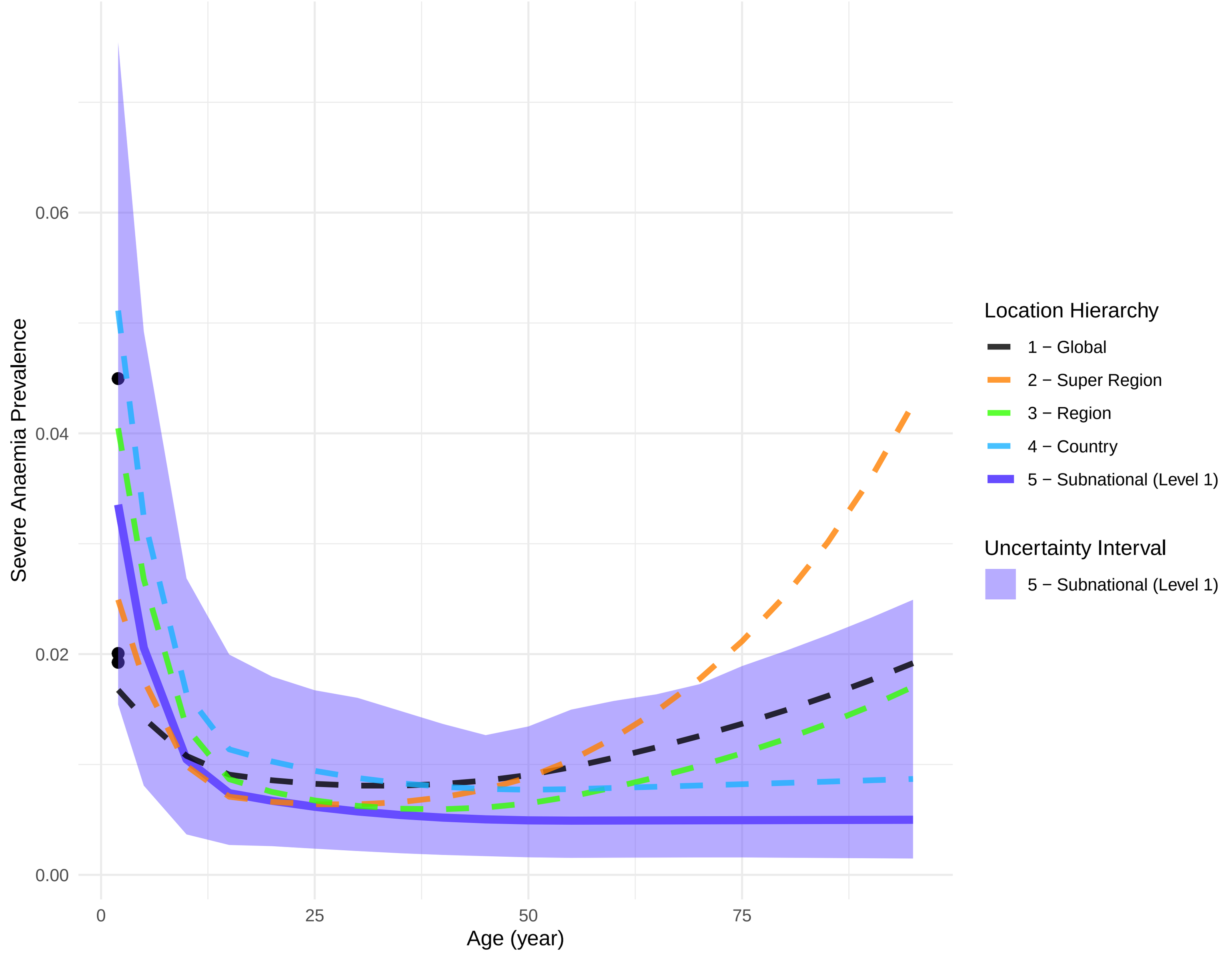

MR−BRT Age/Sex Fits − Males 2+ Years Old − Severe Anaemia Prevalence − Niger
WHO−2001 Adjustment Method

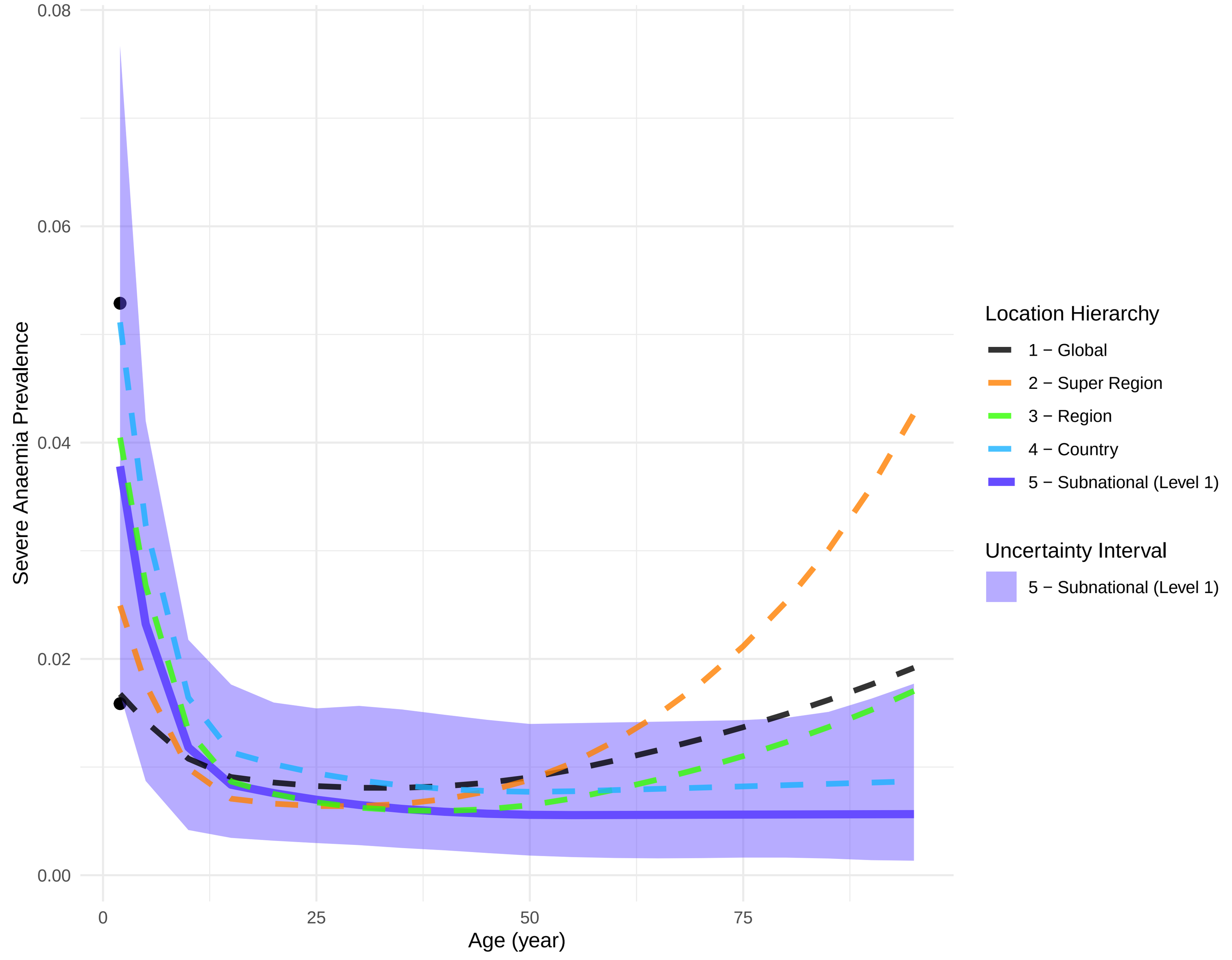

MR−BRT Age/Sex Fits − Males 2+ Years Old − Severe Anaemia Prevalence − Ogun
WHO−2001 Adjustment Method

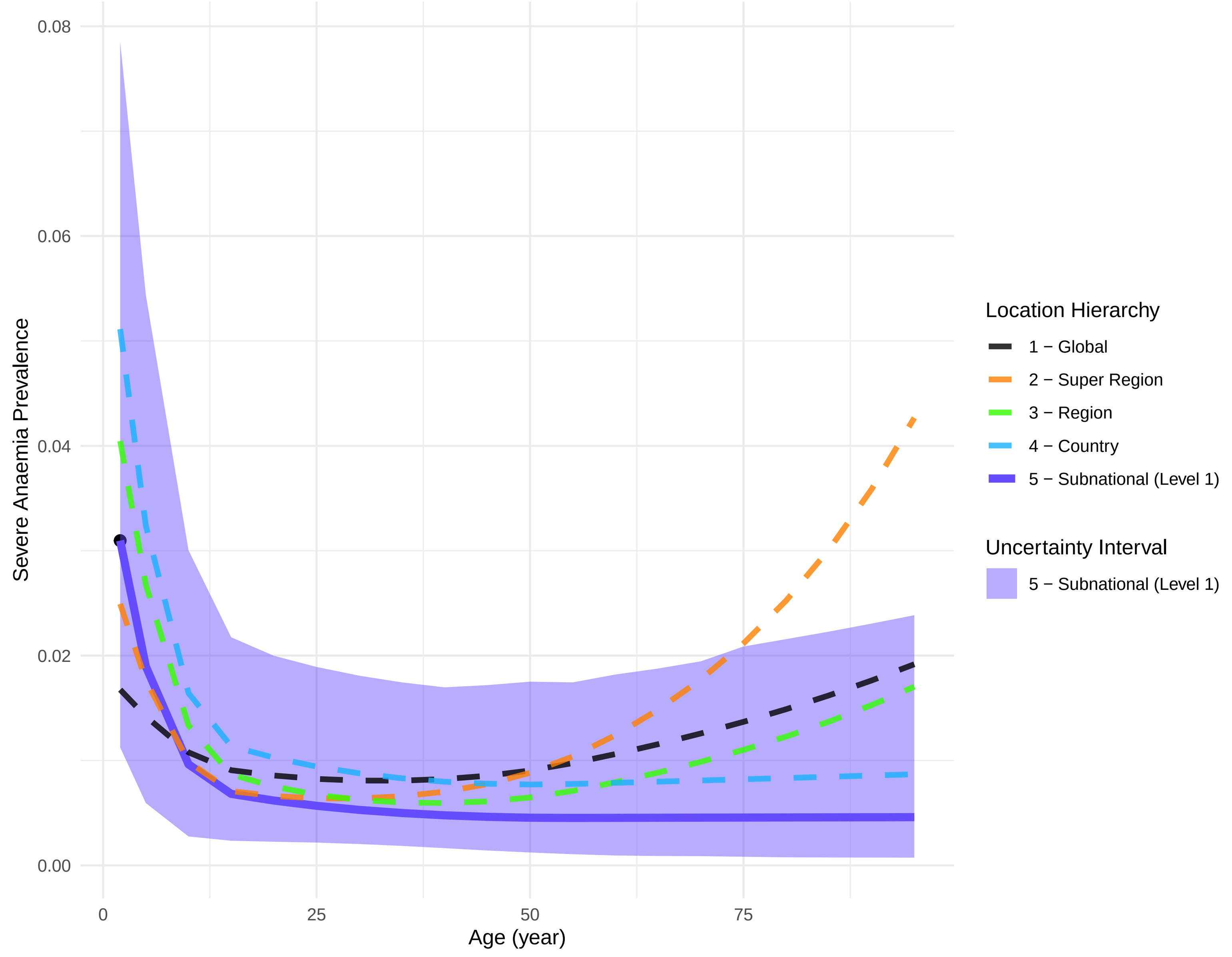

MR−BRT Age/Sex Fits − Males 2+ Years Old − Severe Anaemia Prevalence − Ondo
WHO−2001 Adjustment Method

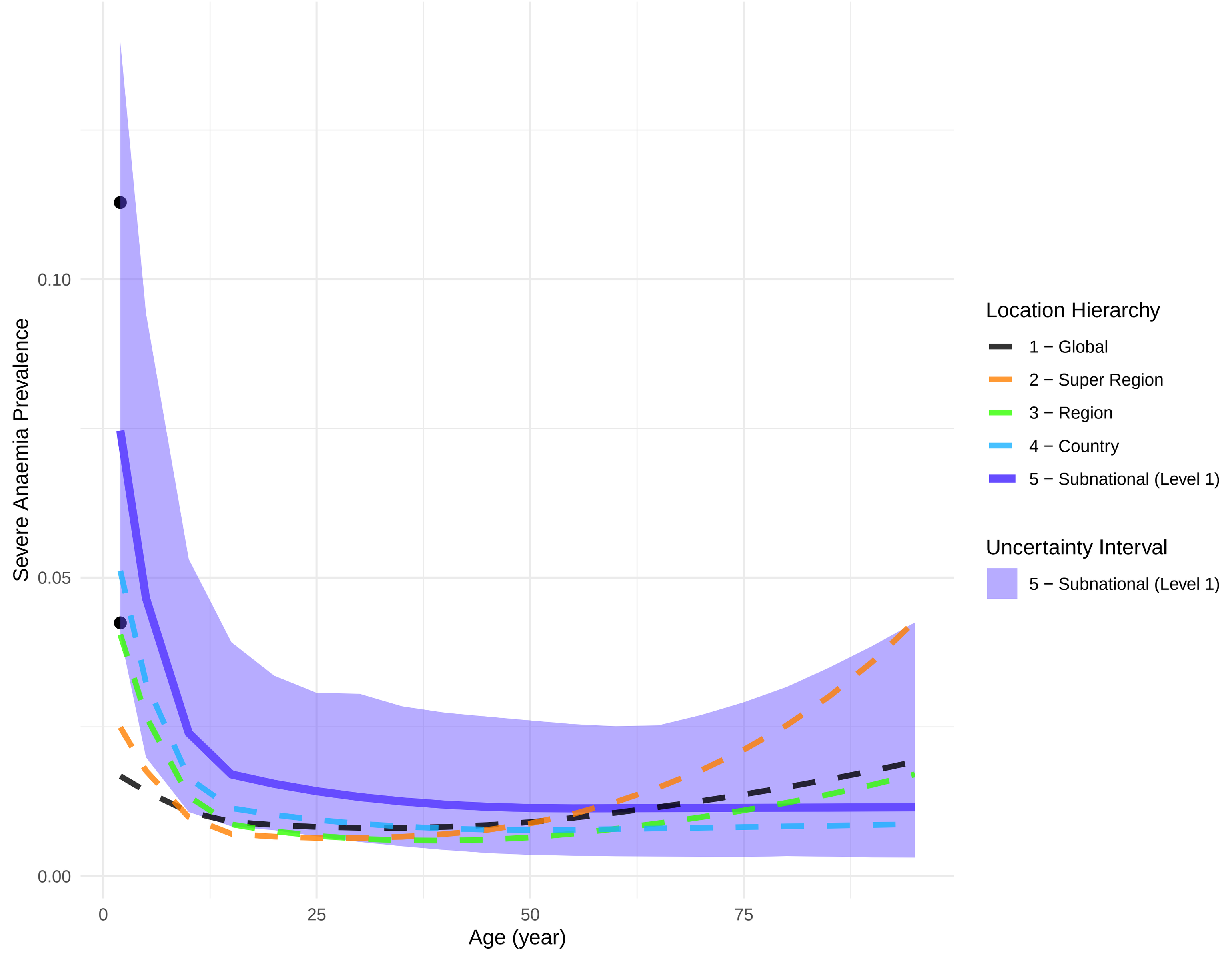

MR−BRT Age/Sex Fits − Males 2+ Years Old − Severe Anaemia Prevalence − Osun
WHO−2001 Adjustment Method

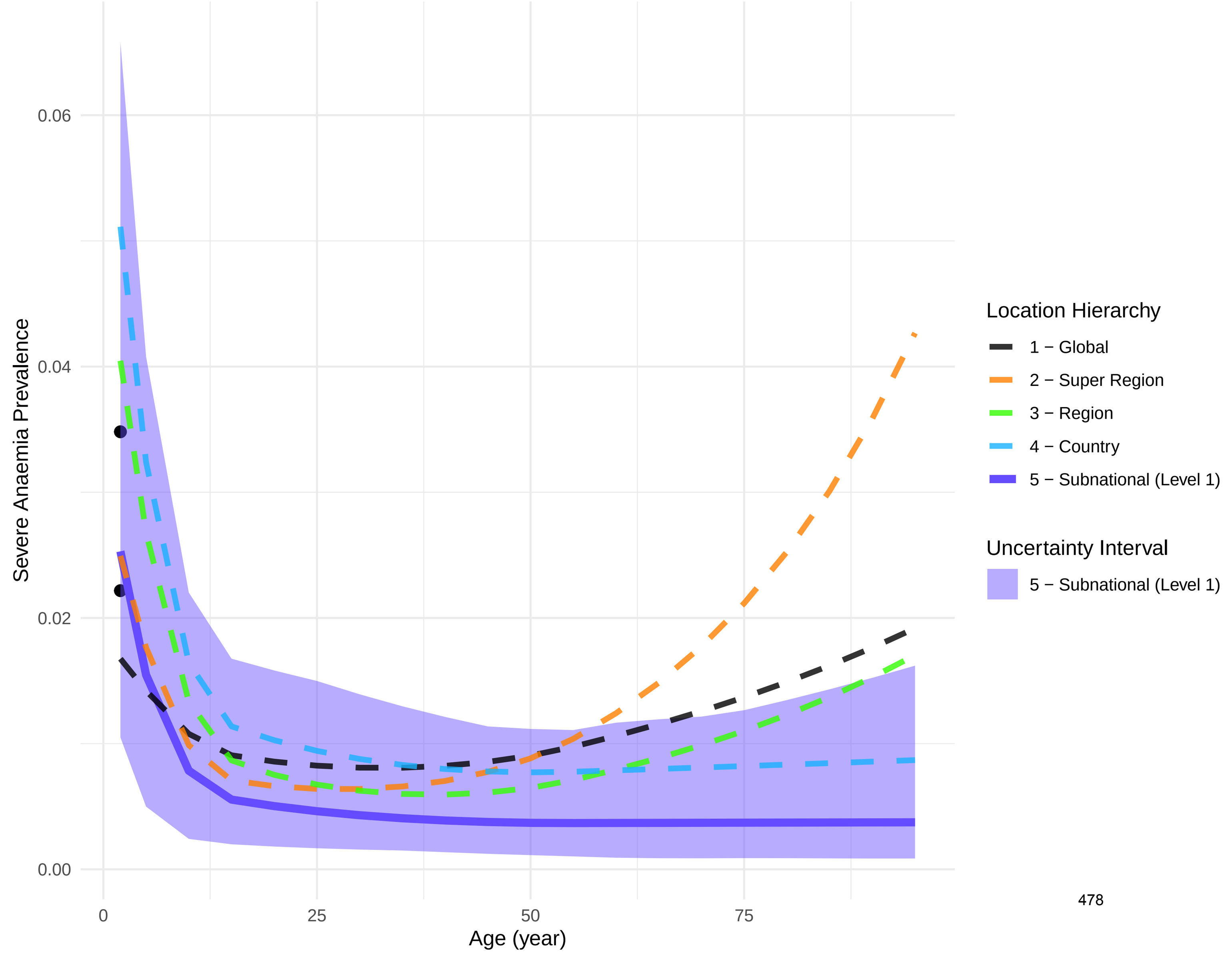

MR−BRT Age/Sex Fits − Males 2+ Years Old − Severe Anaemia Prevalence − Oyo
WHO−2001 Adjustment Method

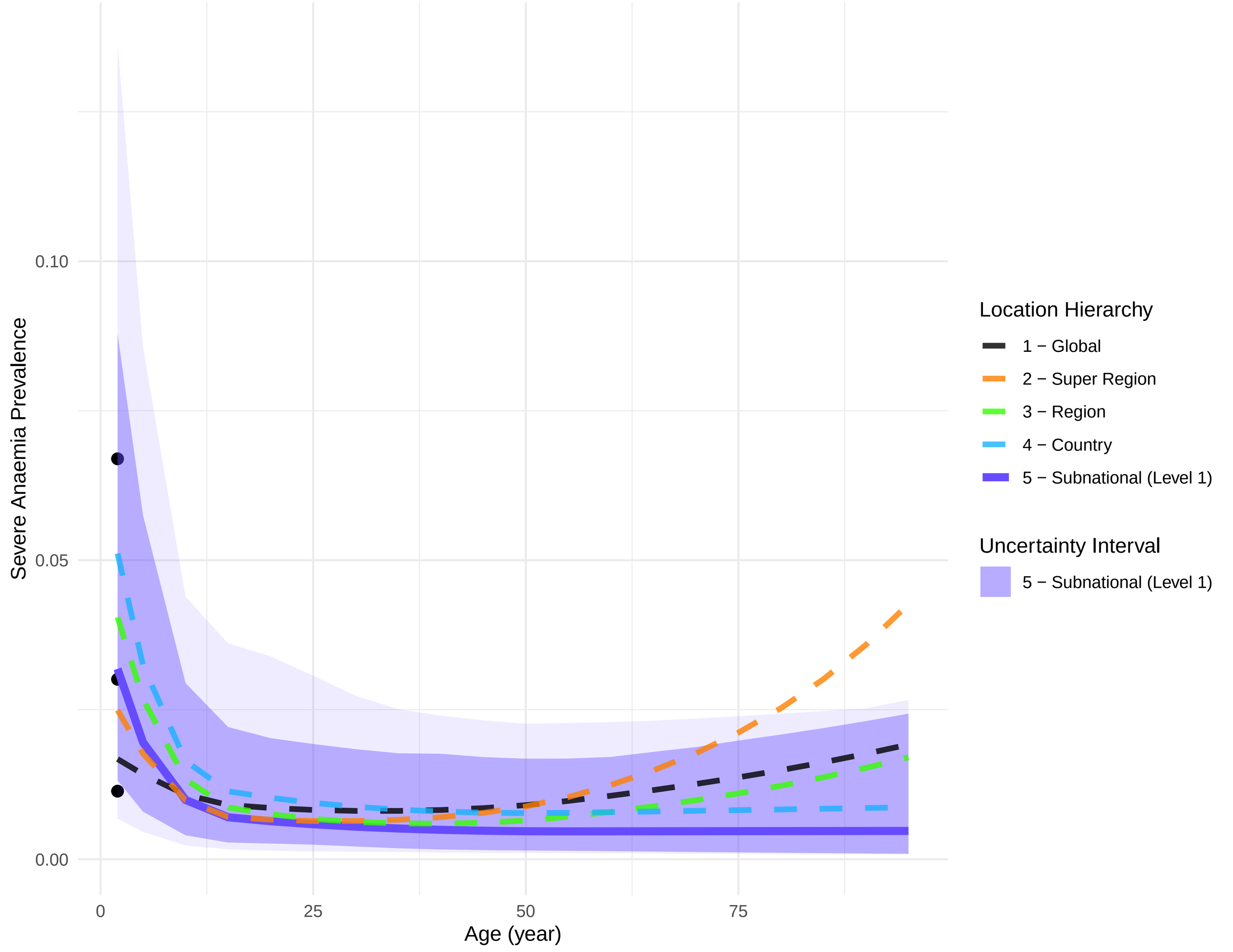

MR−BRT Age/Sex Fits − Males 2+ Years Old − Severe Anaemia Prevalence − Plateau
WHO−2001 Adjustment Method

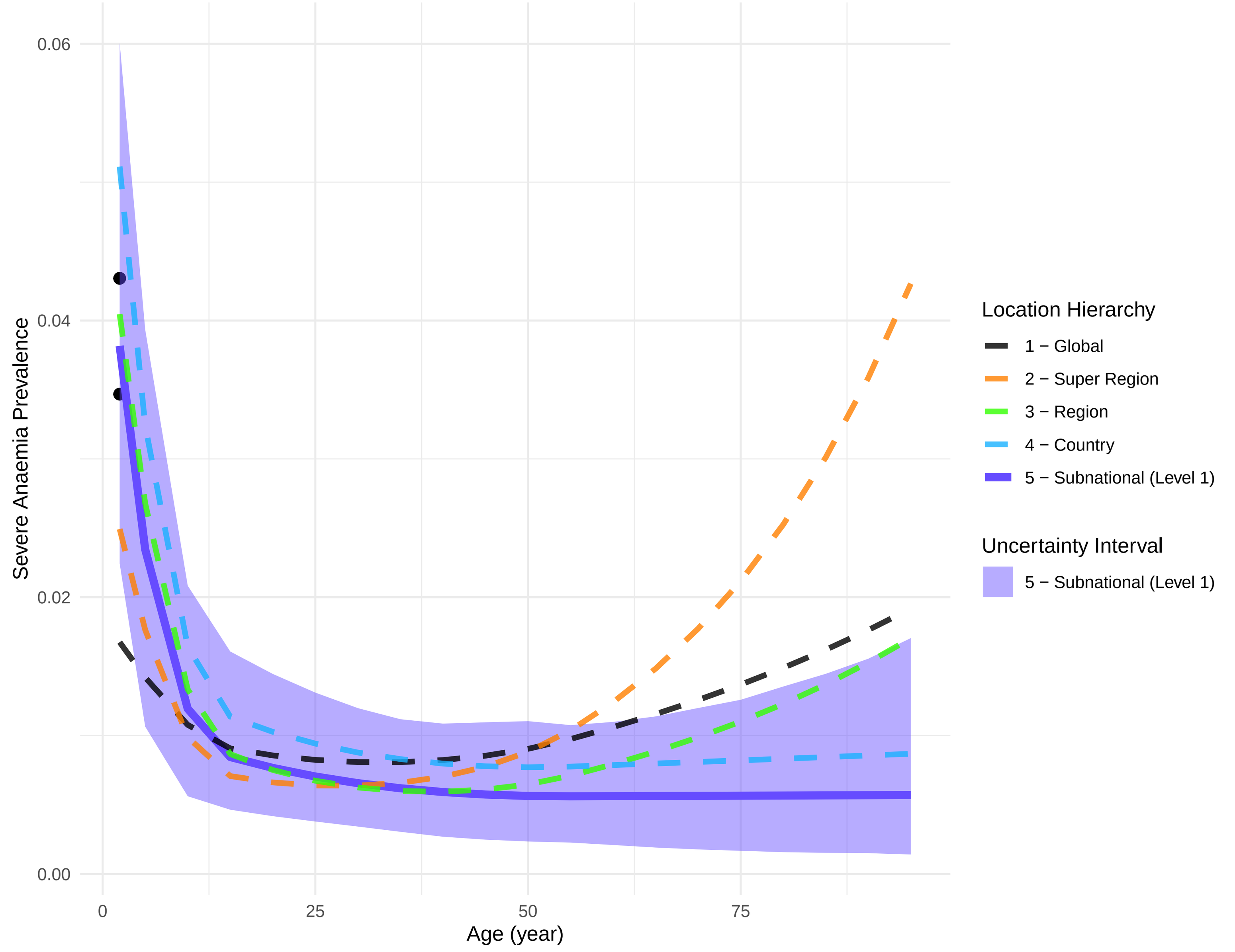

MR−BRT Age/Sex Fits − Males 2+ Years Old − Severe Anaemia Prevalence − Rivers
WHO−2001 Adjustment Method

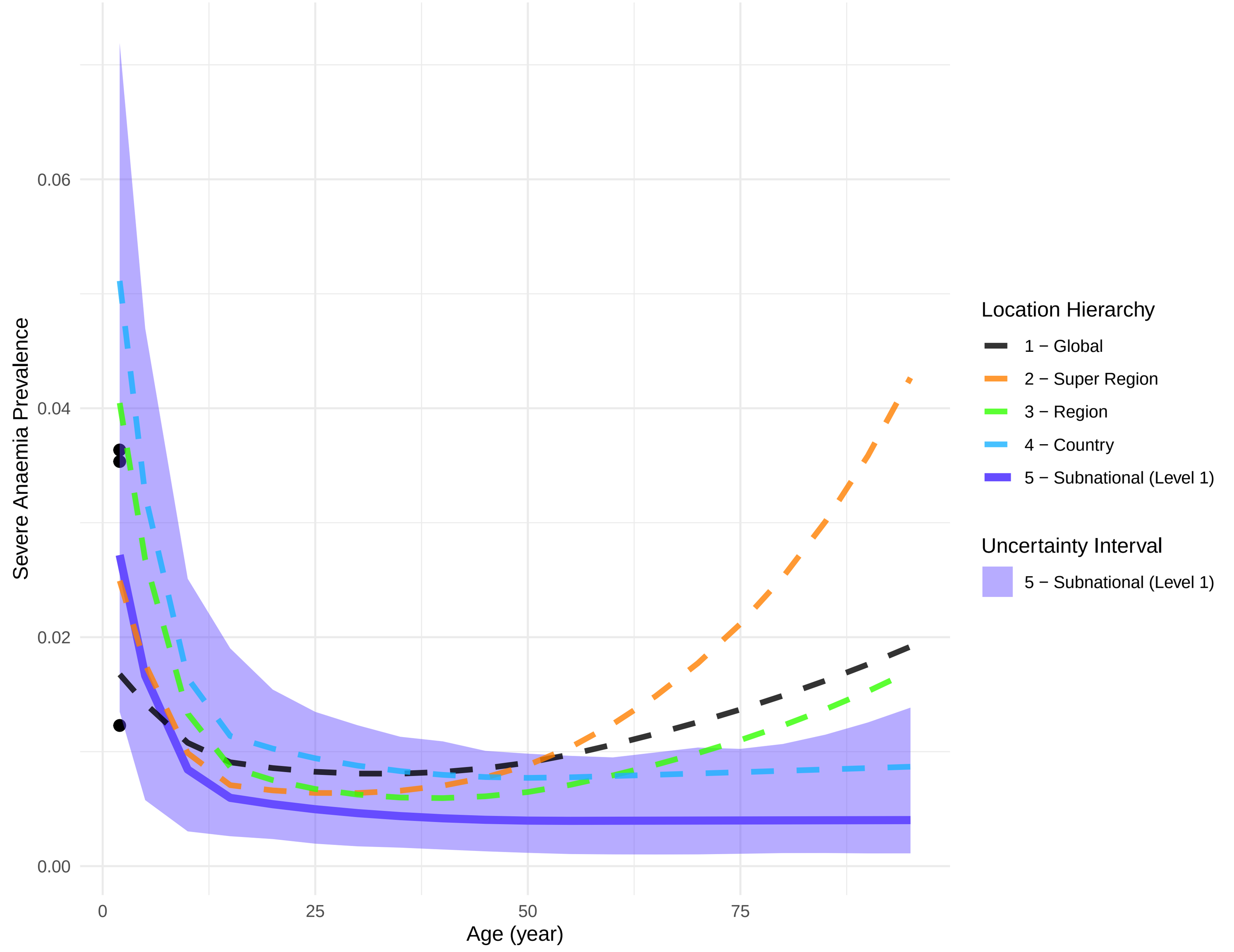

MR−BRT Age/Sex Fits − Males 2+ Years Old − Severe Anaemia Prevalence − Sokoto
WHO−2001 Adjustment Method

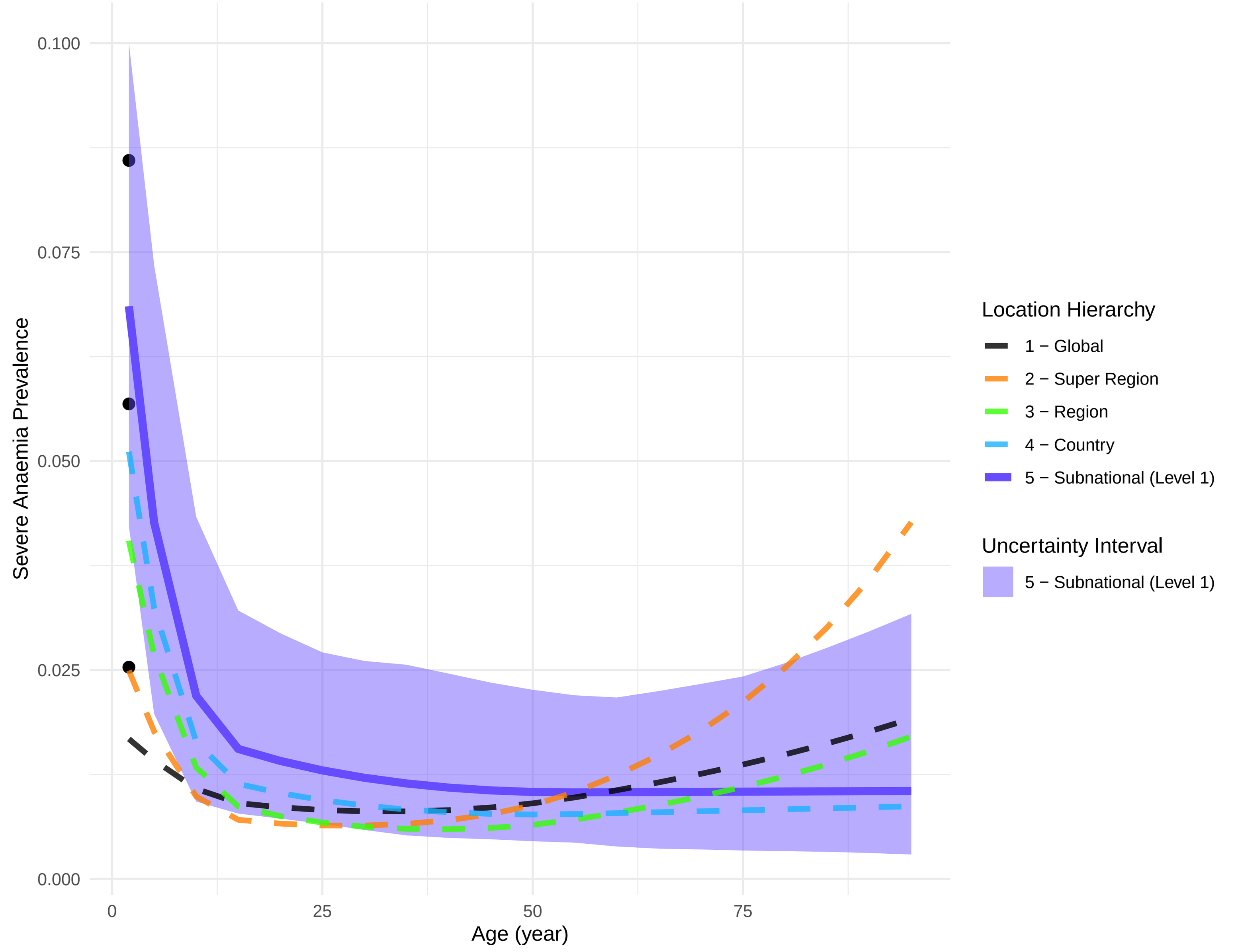

MR−BRT Age/Sex Fits − Males 2+ Years Old − Severe Anaemia Prevalence − Taraba
WHO−2001 Adjustment Method

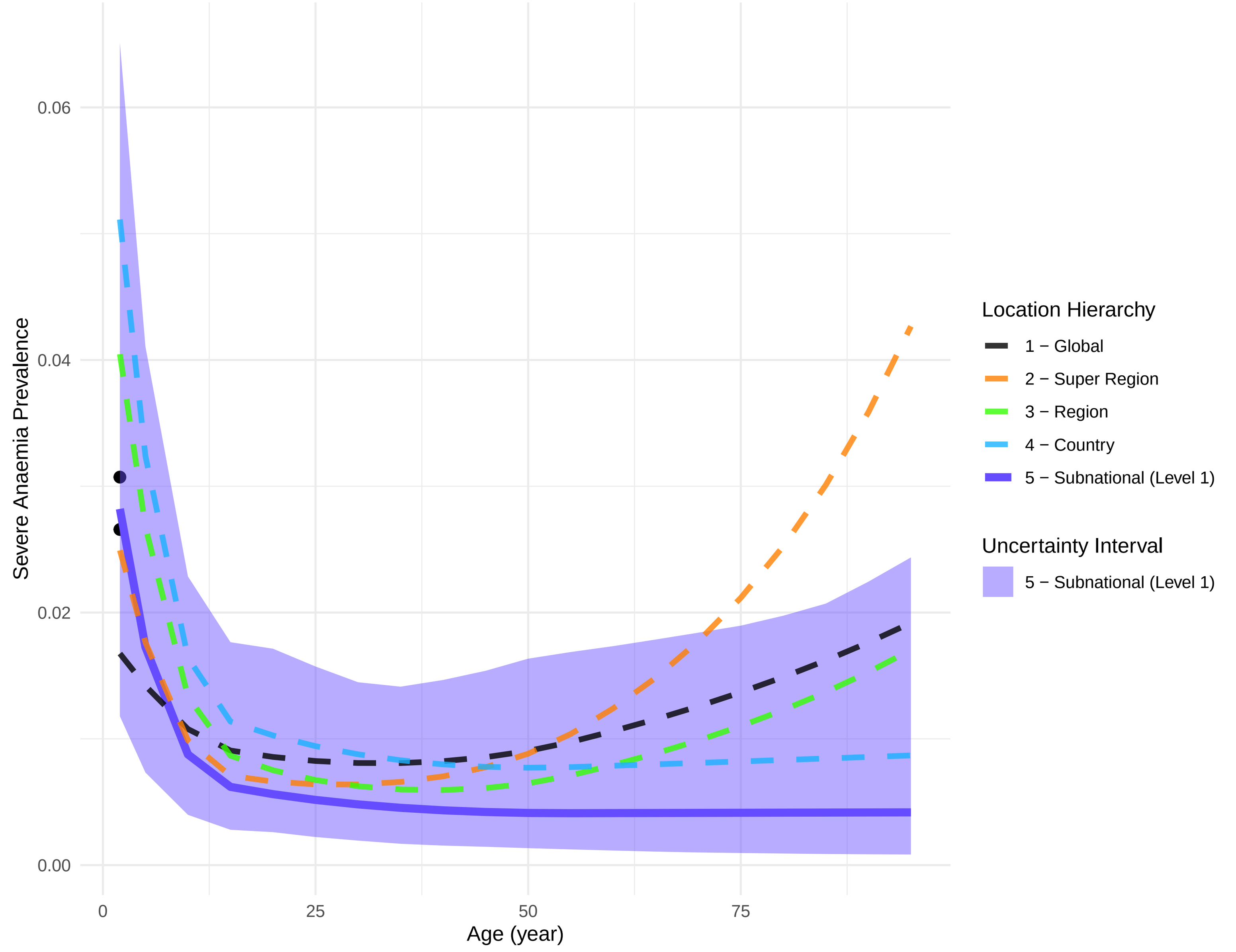

MR−BRT Age/Sex Fits − Males 2+ Years Old − Severe Anaemia Prevalence − Yobe
WHO−2001 Adjustment Method

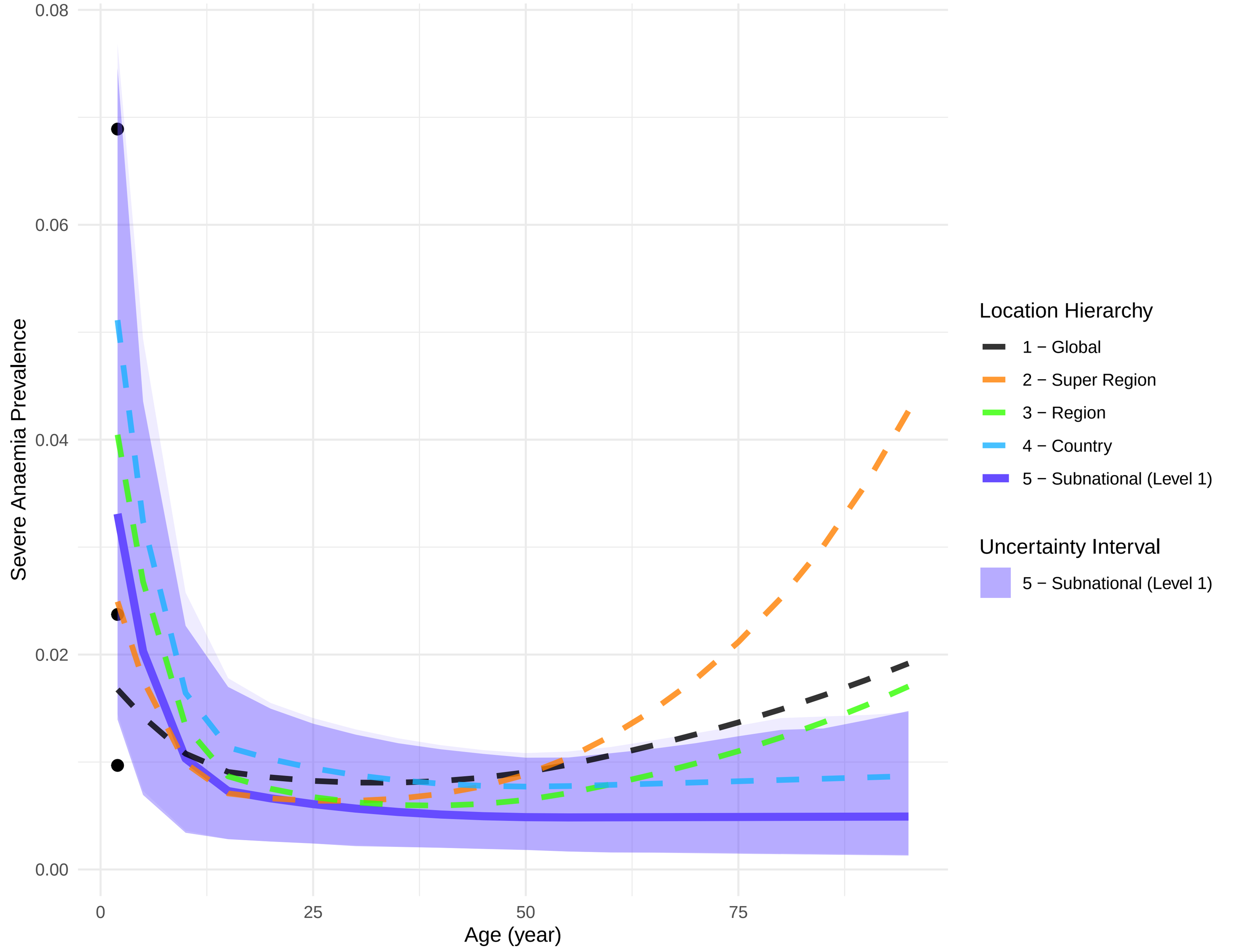

MR−BRT Age/Sex Fits − Males 2+ Years Old − Severe Anaemia Prevalence − Zamfara
WHO−2001 Adjustment Method

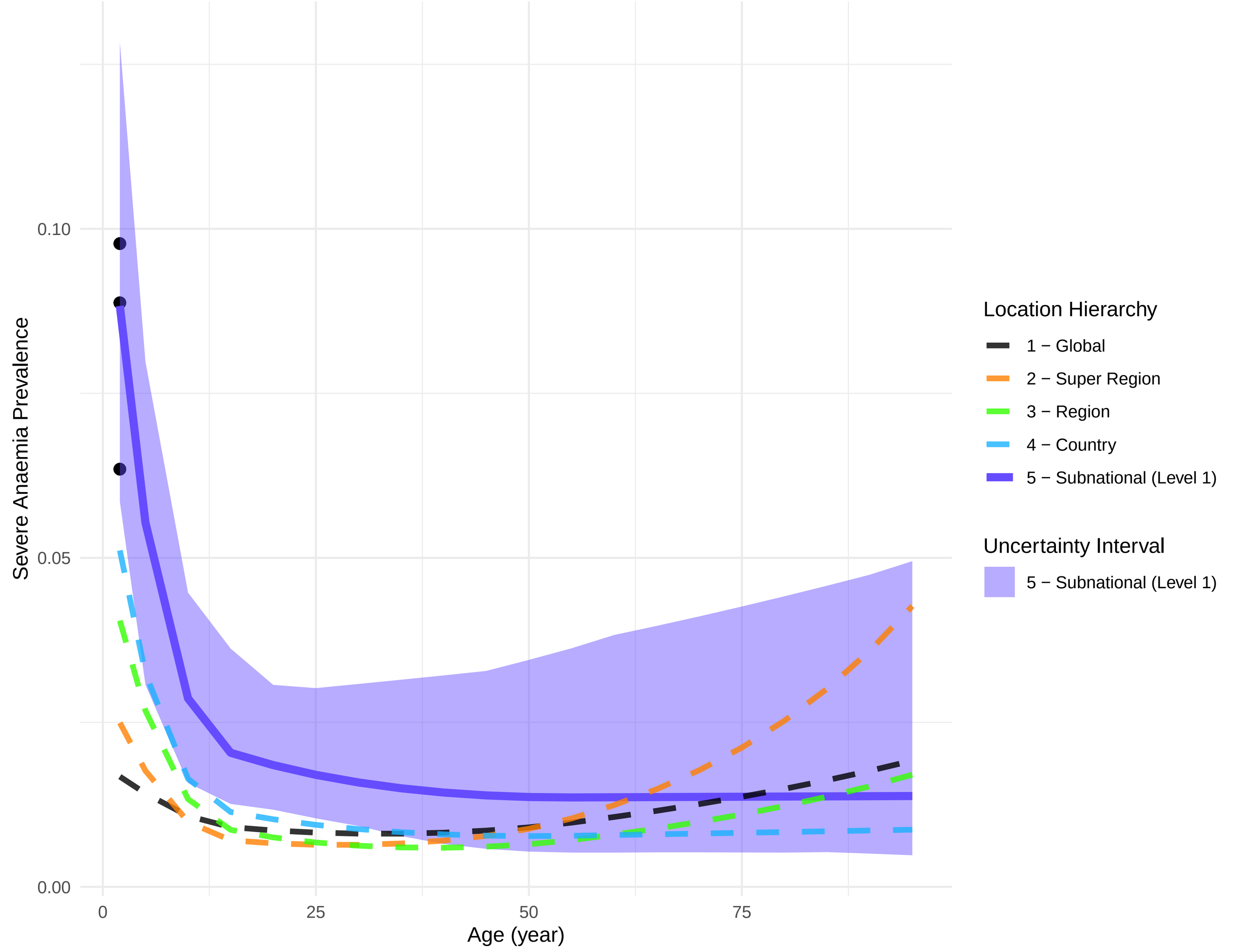

MR−BRT Age/Sex Fits − Males 2+ Years Old − Severe Anaemia Prevalence − Senegal
WHO−2001 Adjustment Method

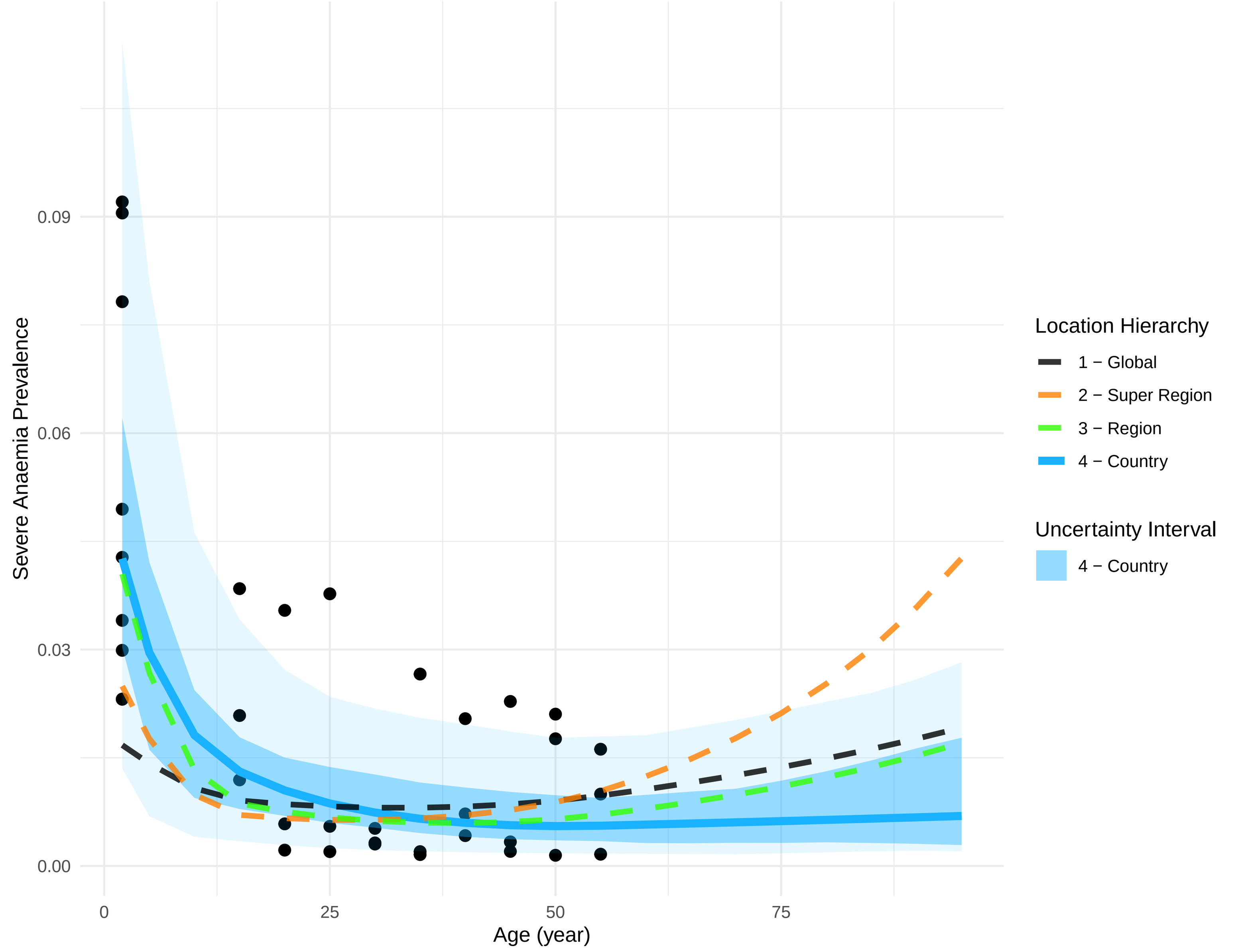

MR−BRT Age/Sex Fits − Males 2+ Years Old − Severe Anaemia Prevalence − Sierra Leone
WHO−2001 Adjustment Method

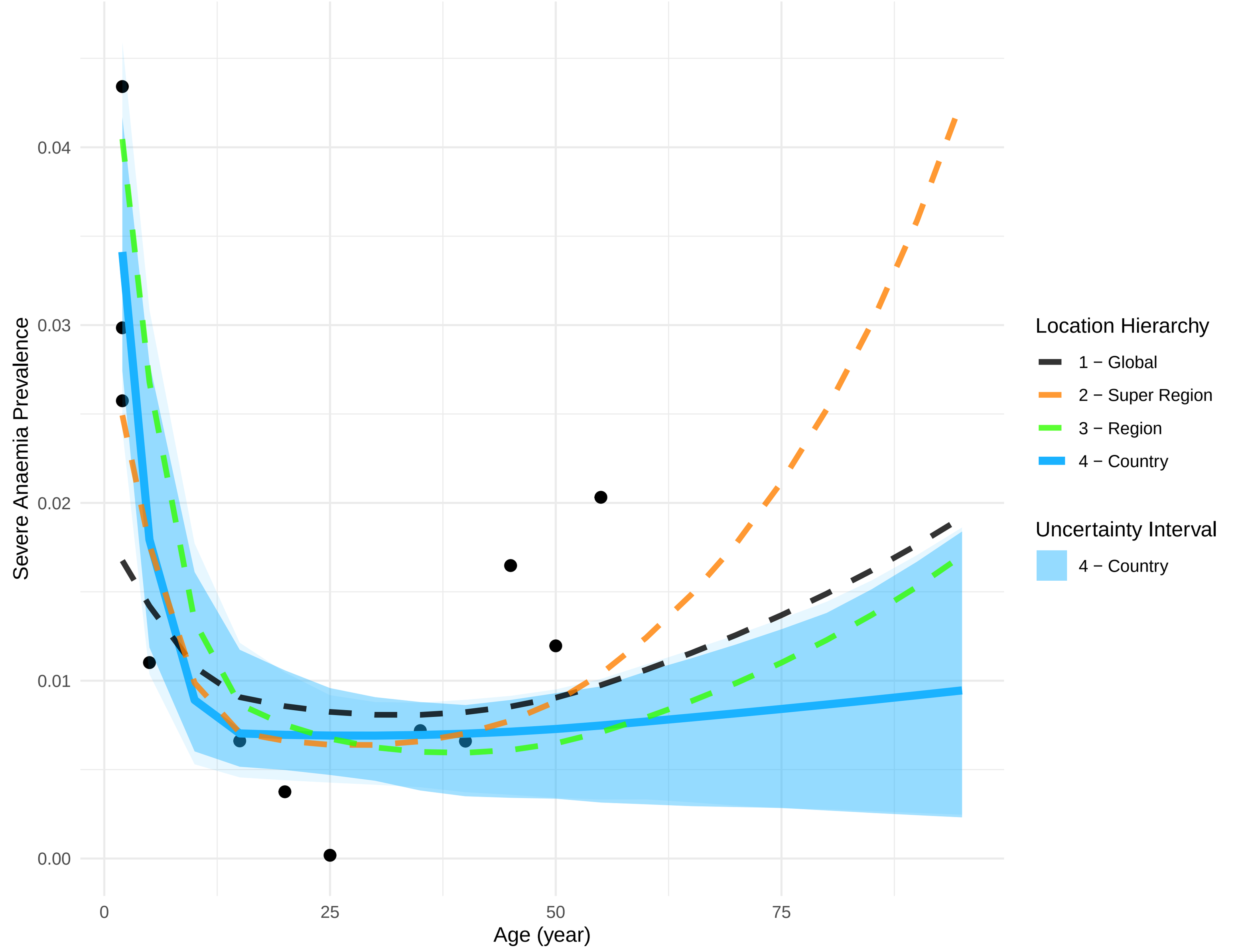

MR−BRT Age/Sex Fits − Males 2+ Years Old − Severe Anaemia Prevalence − Togo
WHO−2001 Adjustment Method

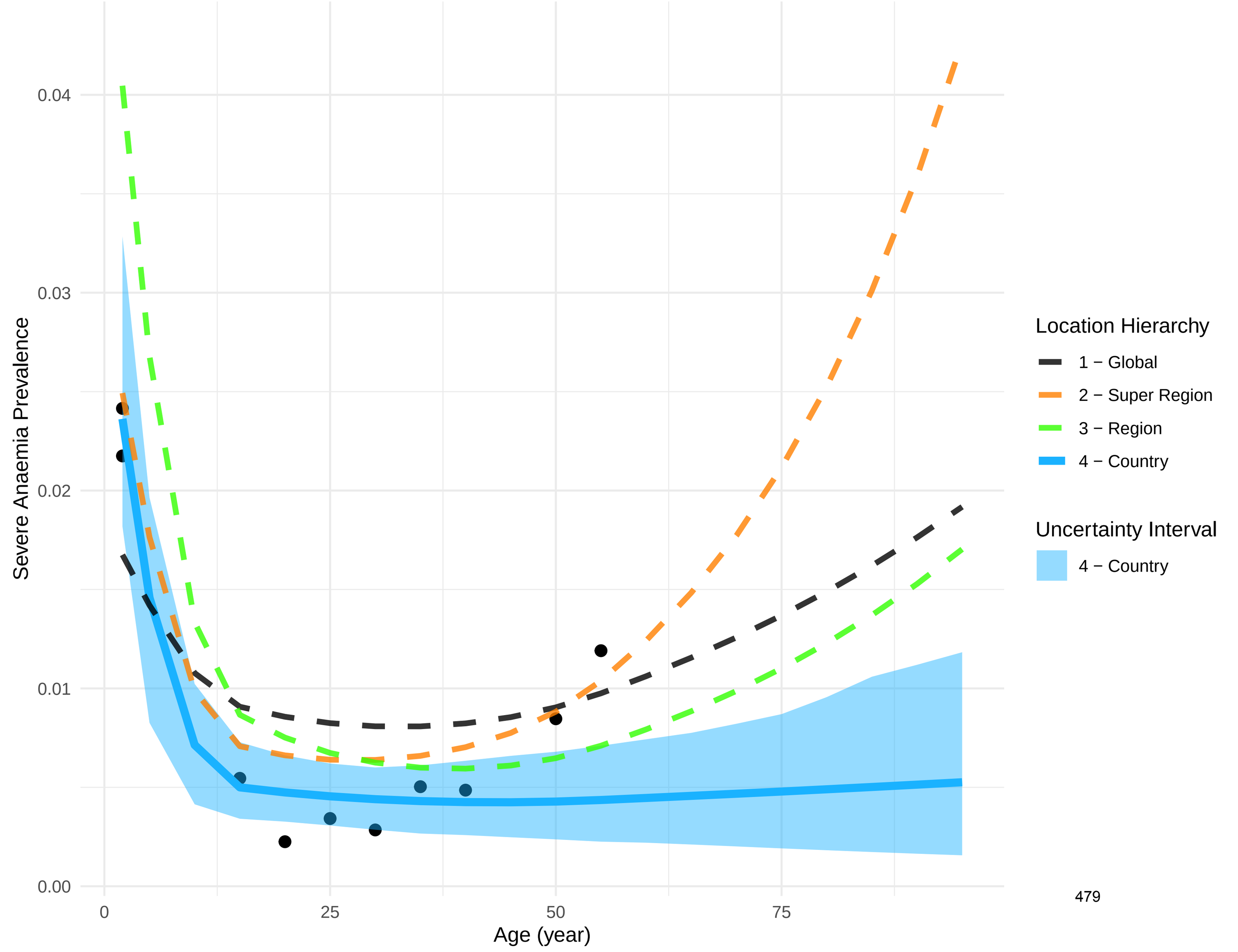

MR−BRT Age/Sex Fits − Non-pregnant Females 2+ Years Old − Severe Anaemia Prevalence − Global
WHO−2001 Adjustment Method

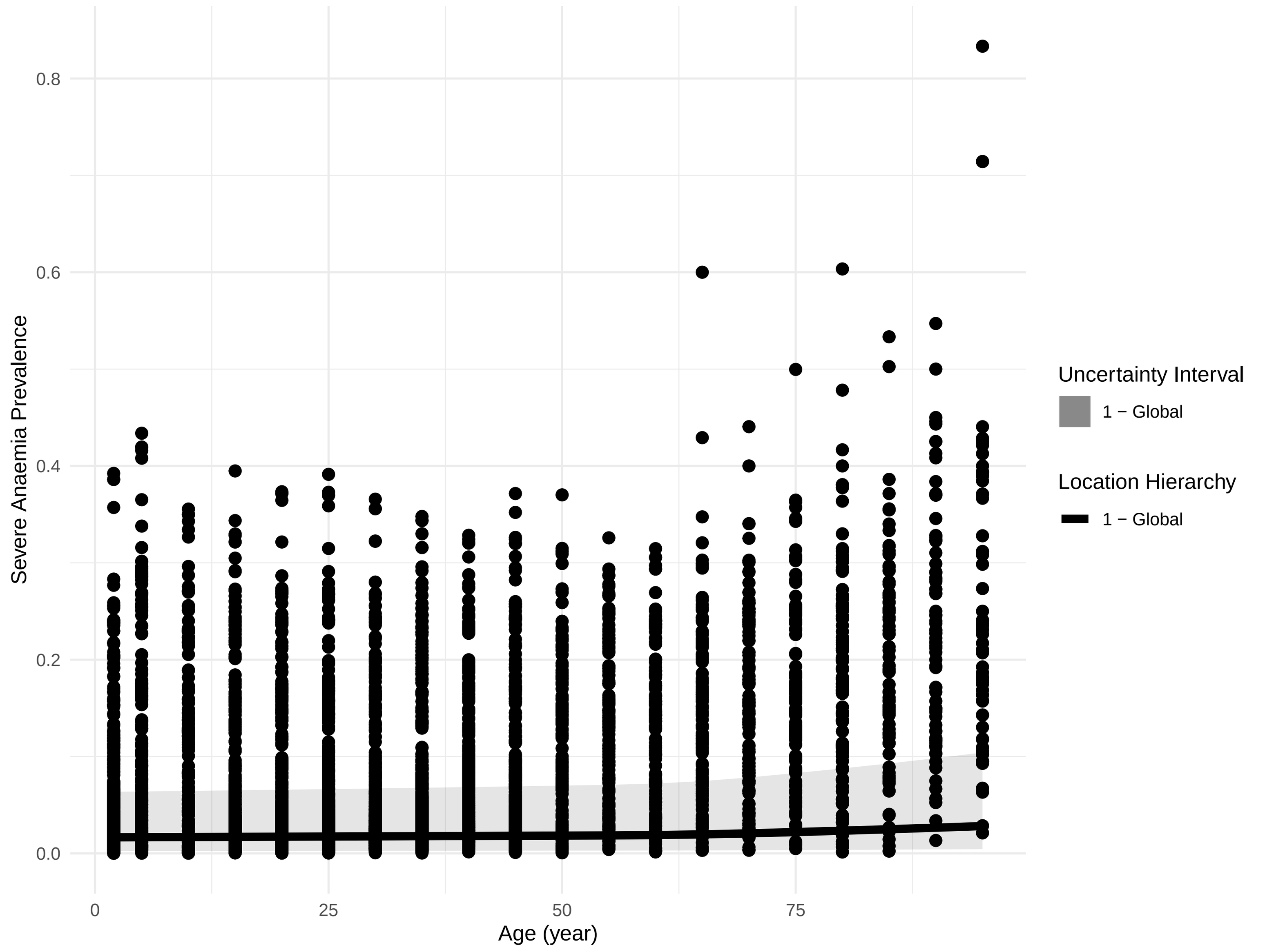

MR−BRT Age/Sex Fits − Non-pregnant Females 2+ Years Old − Severe Anaemia Prevalence − Central Europe, Eastern Europe, and Central Asia
WHO−2001 Adjustment Method

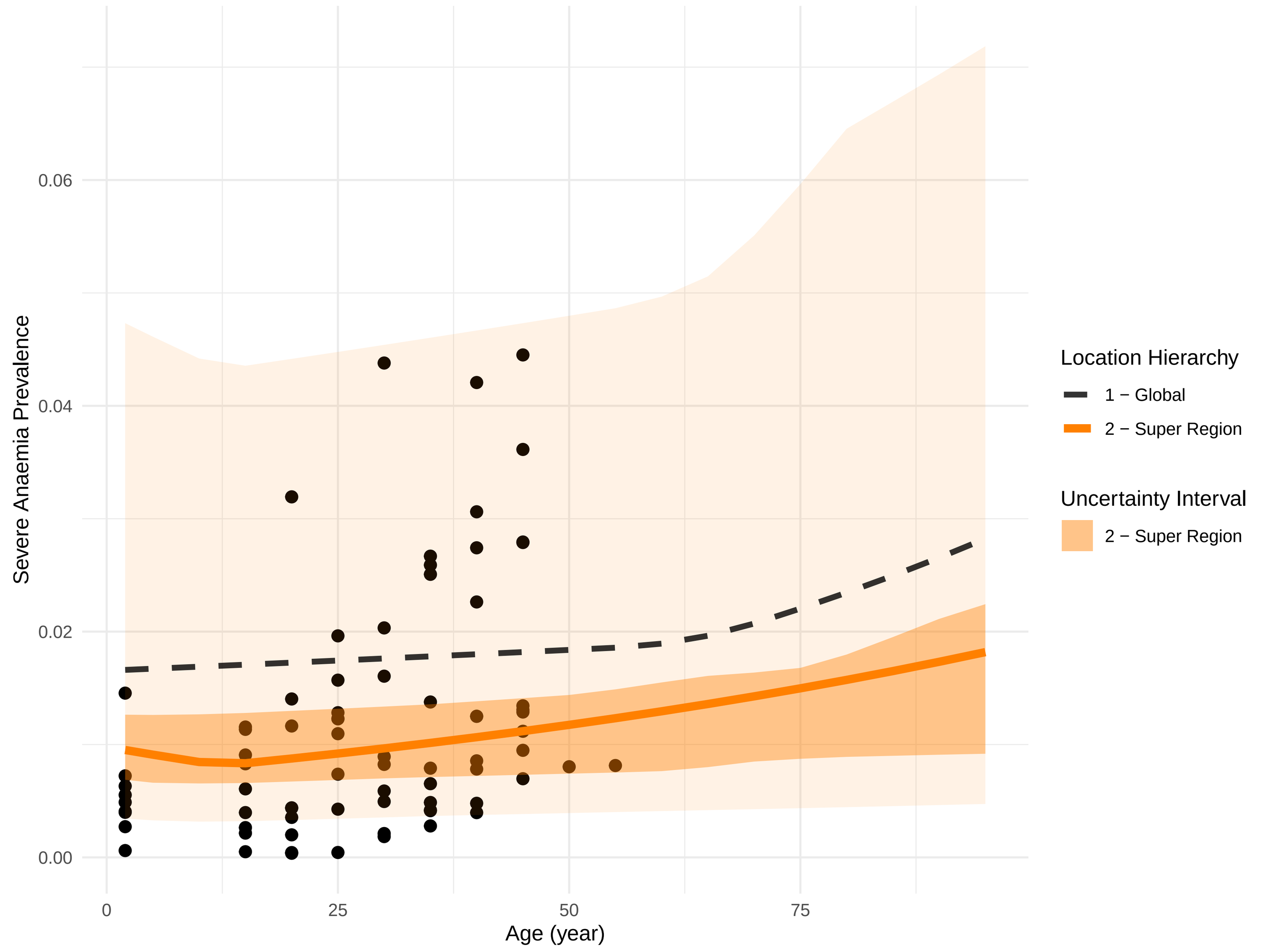

MR−BRT Age/Sex Fits − Non-pregnant Females 2+ Years Old − Severe Anaemia Prevalence − Central Asia
WHO−2001 Adjustment Method

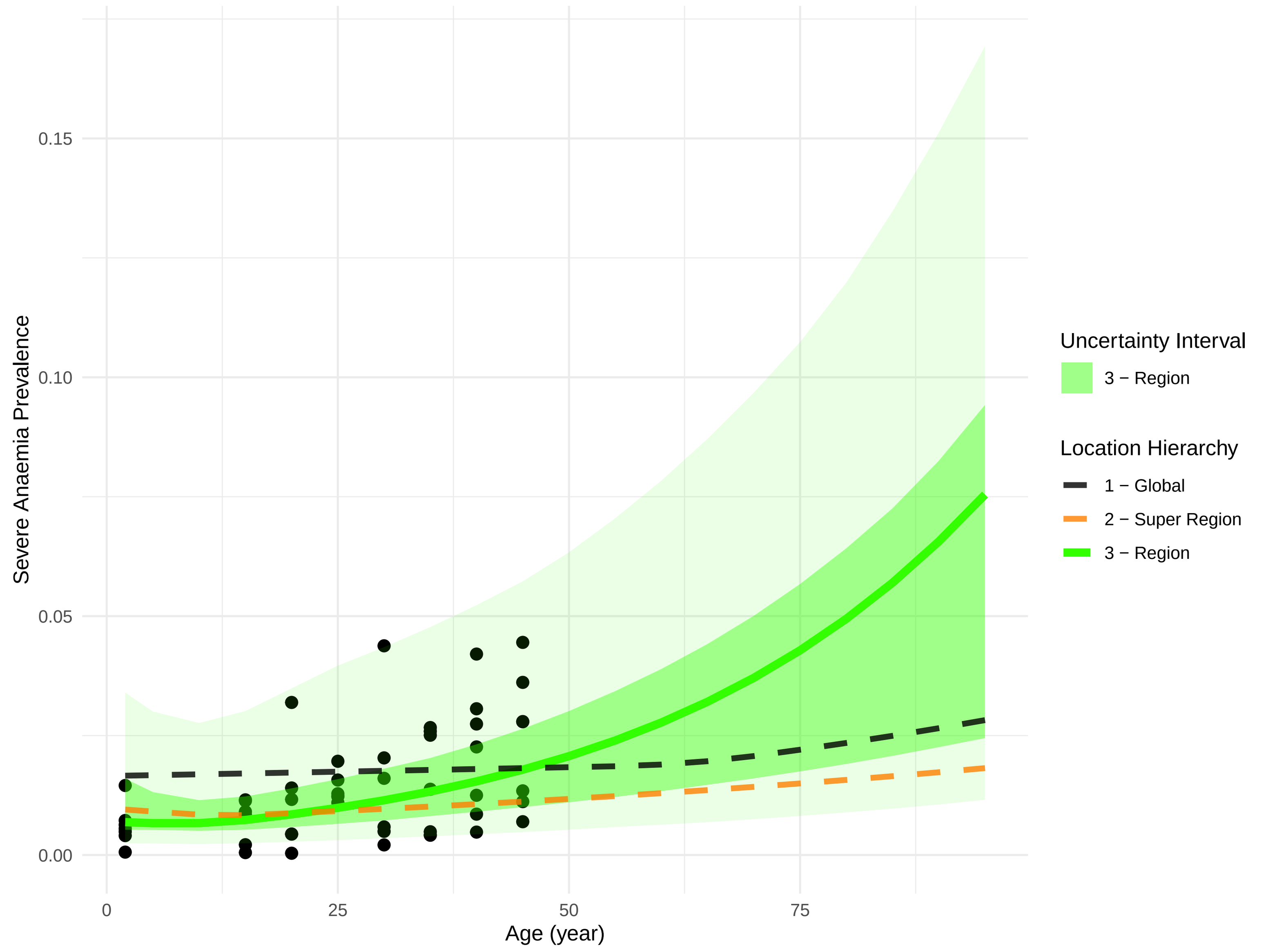

MR−BRT Age/Sex Fits − Non-pregnant Females 2+ Years Old − Severe Anaemia Prevalence − Armenia
WHO−2001 Adjustment Method

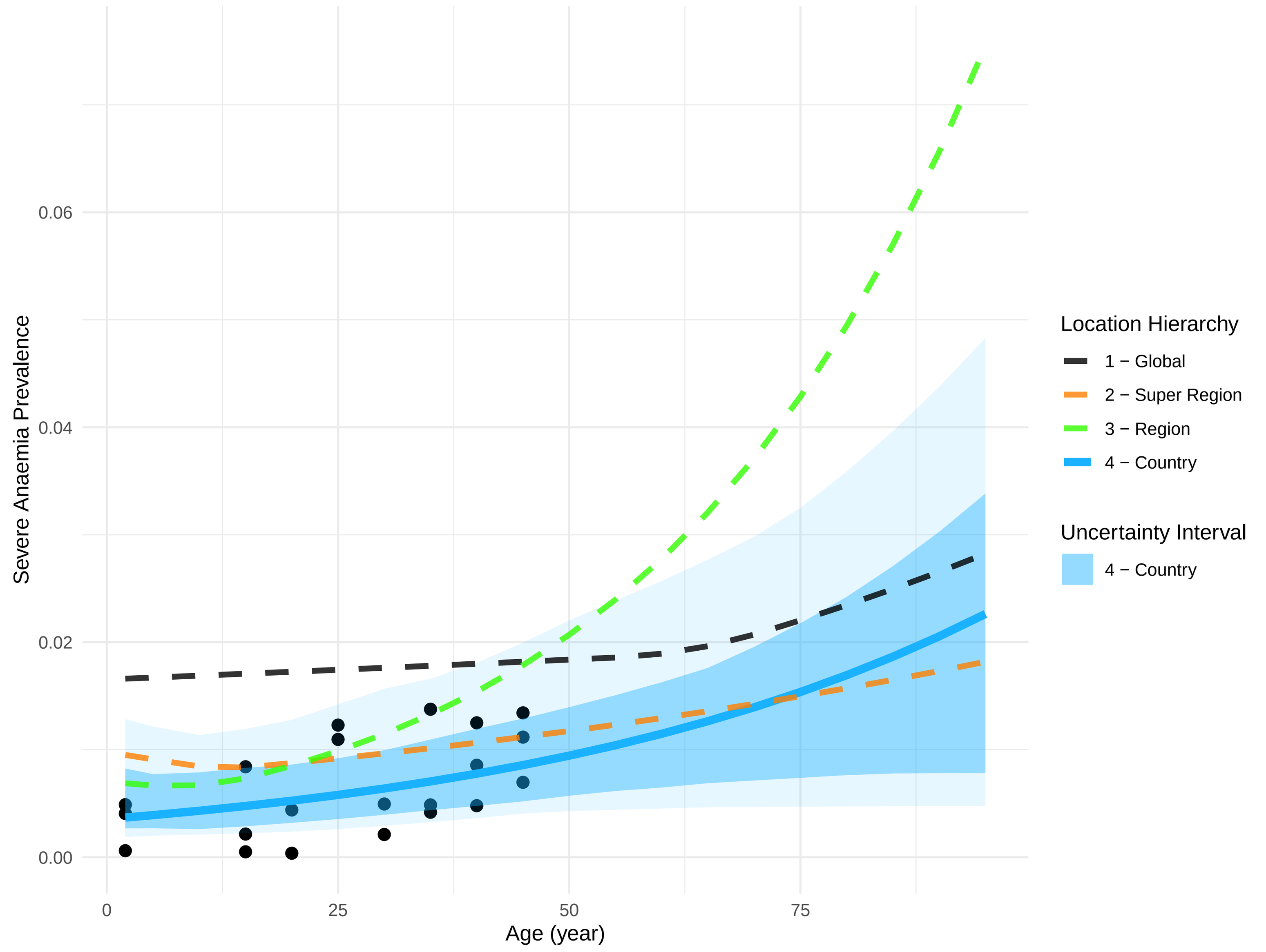

MR−BRT Age/Sex Fits − Non-pregnant Females 2+ Years Old − Severe Anaemia Prevalence − Azerbaijan
WHO−2001 Adjustment Method

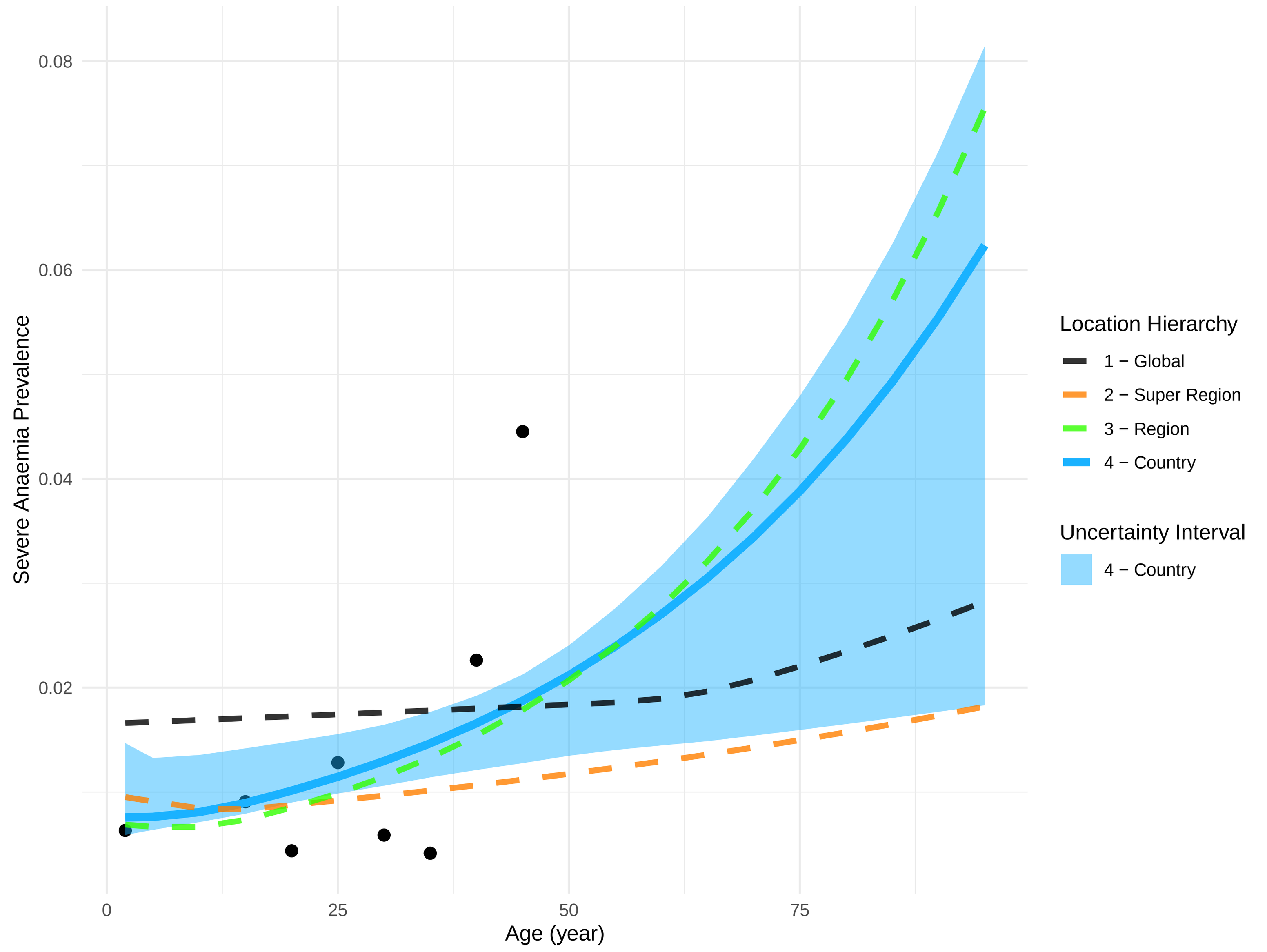

MR−BRT Age/Sex Fits − Non-pregnant Females 2+ Years Old − Severe Anaemia Prevalence − Kyrgyzstan
WHO−2001 Adjustment Method

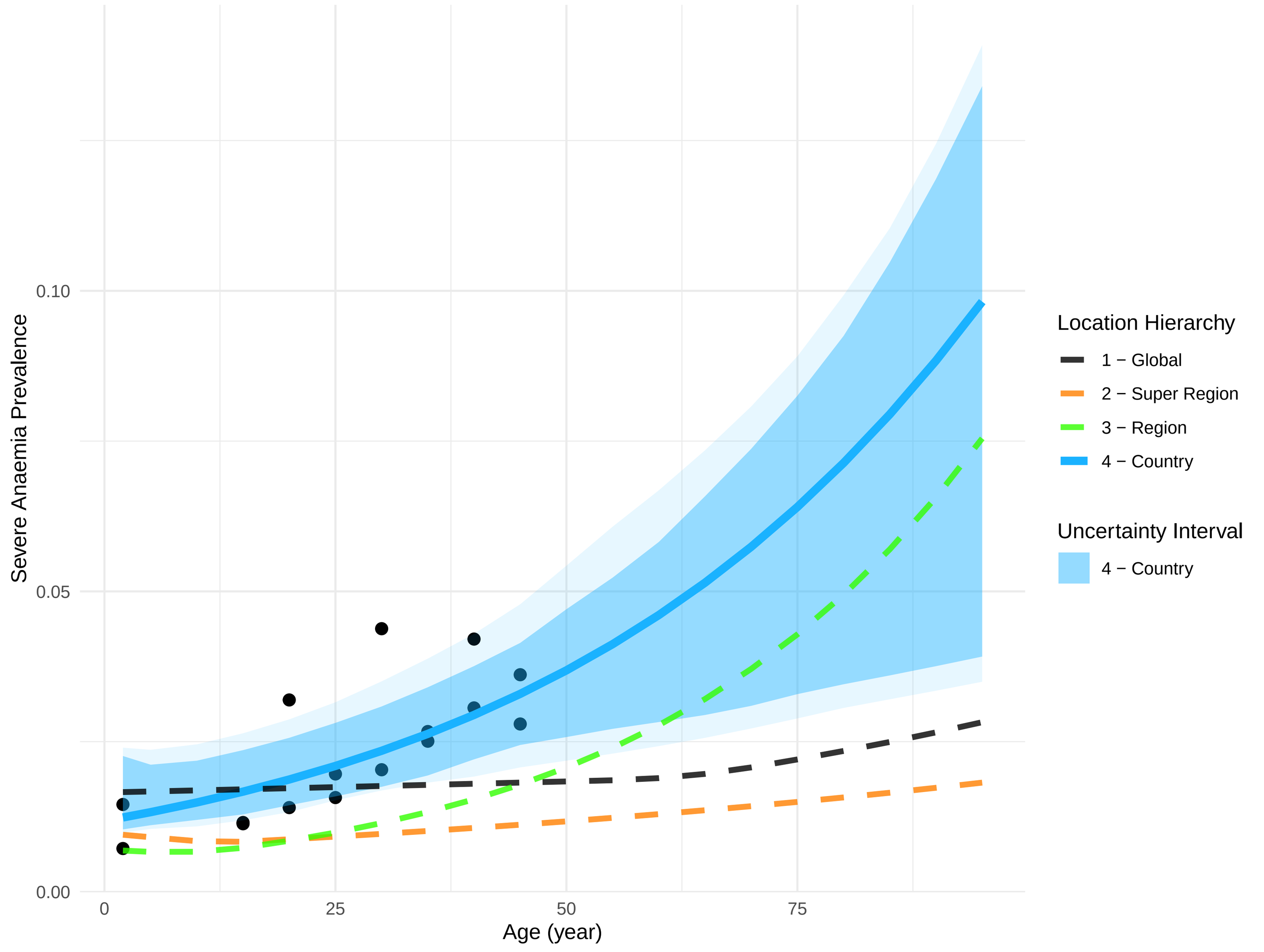

MR−BRT Age/Sex Fits − Non-pregnant Females 2+ Years Old − Severe Anaemia Prevalence − Tajikistan
WHO−2001 Adjustment Method

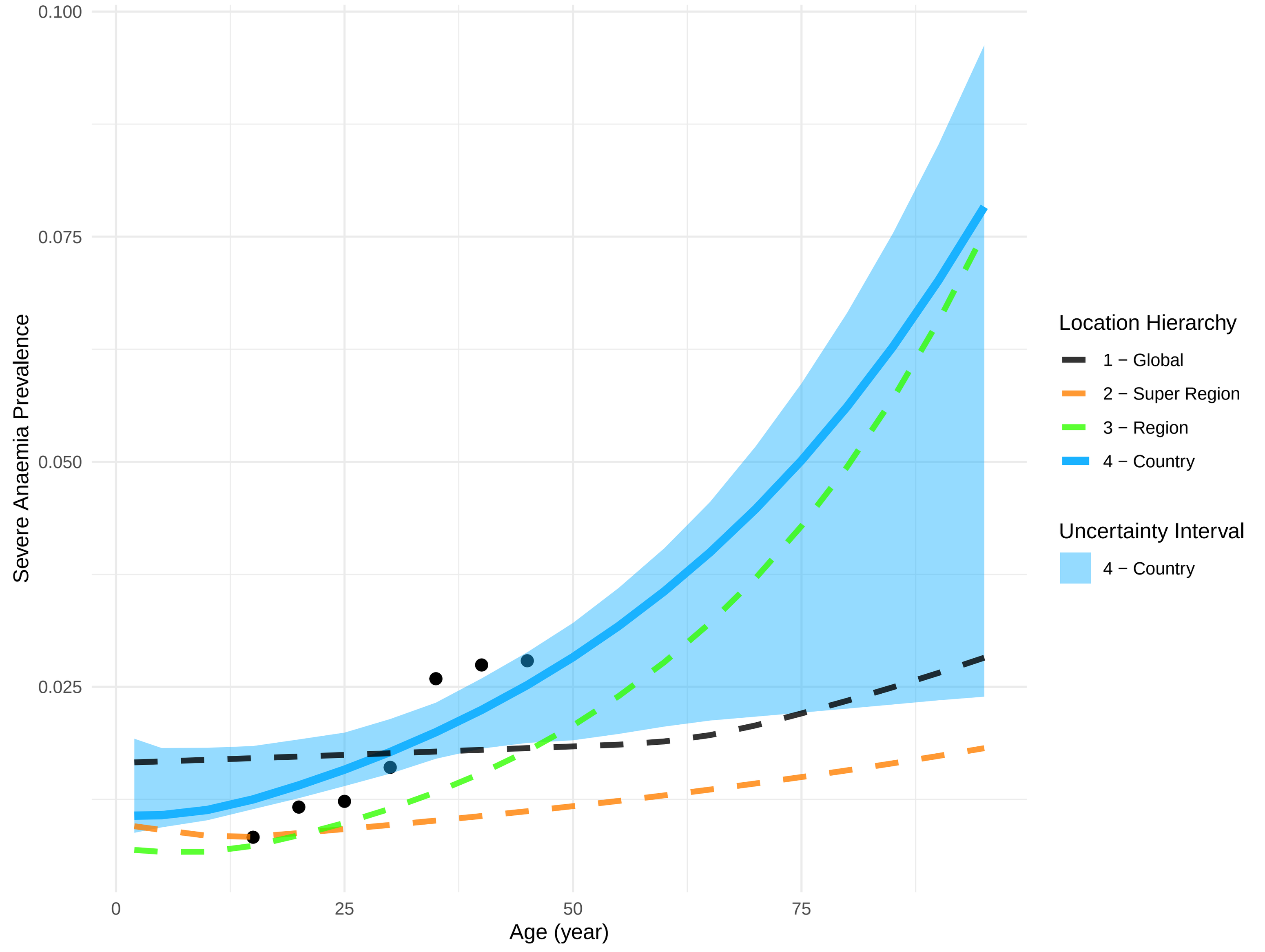

MR−BRT Age/Sex Fits − Non-pregnant Females 2+ Years Old − Severe Anaemia Prevalence − Uzbekistan
WHO−2001 Adjustment Method

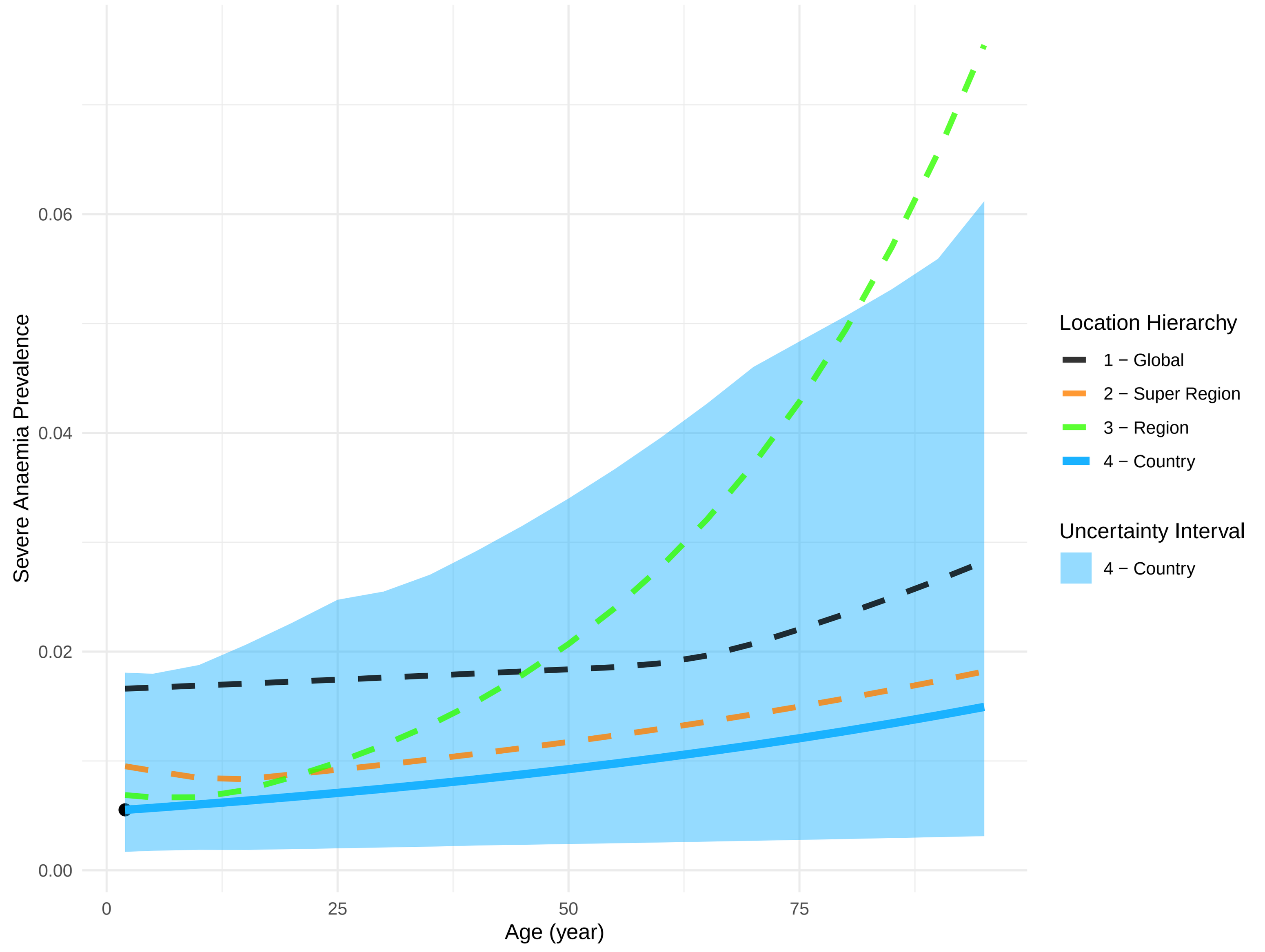

MR−BRT Age/Sex Fits − Non-pregnant Females 2+ Years Old − Severe Anaemia Prevalence − Central Europe
WHO−2001 Adjustment Method

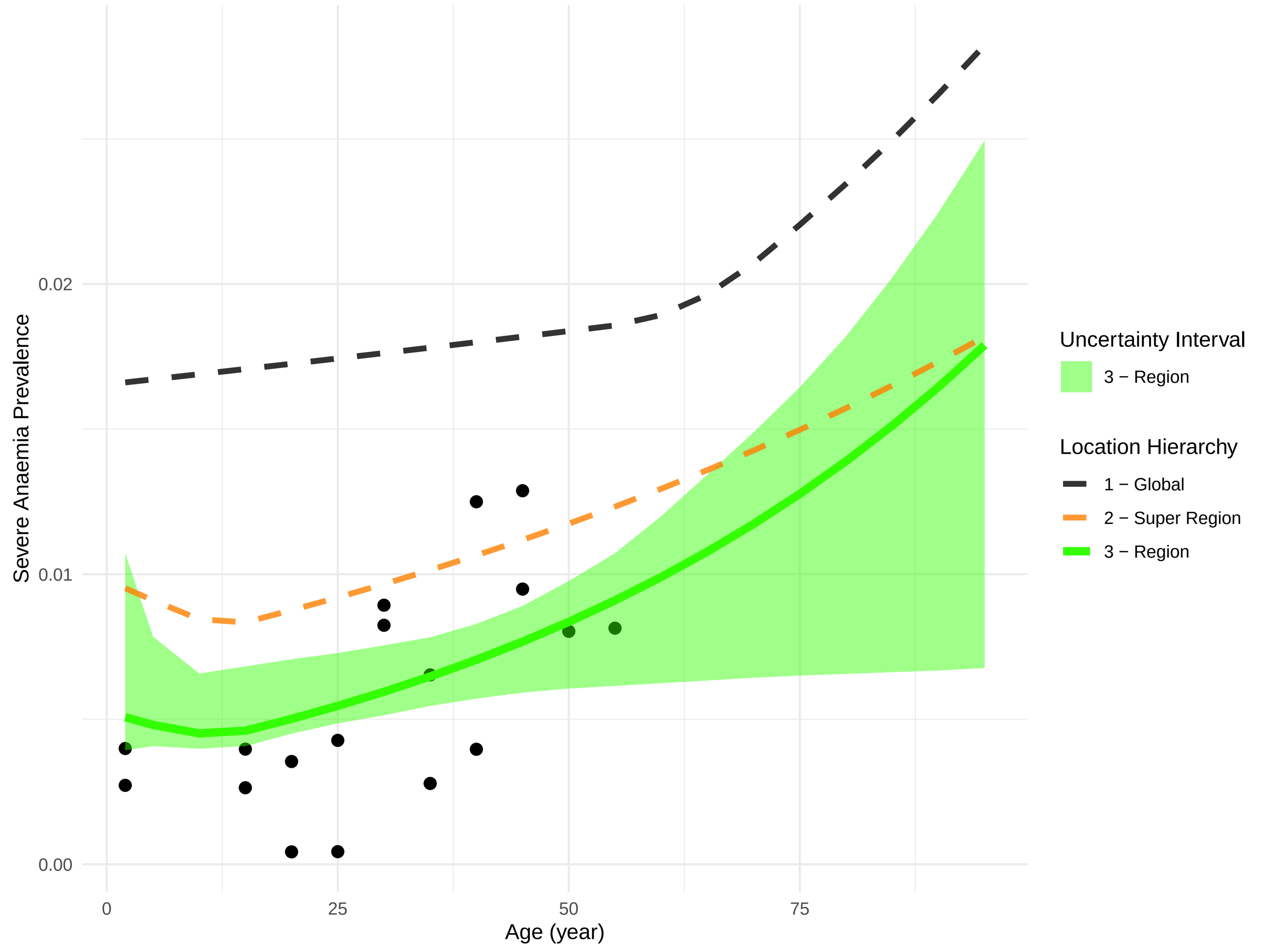

MR−BRT Age/Sex Fits − Non-pregnant Females 2+ Years Old − Severe Anaemia Prevalence − Albania
WHO−2001 Adjustment Method

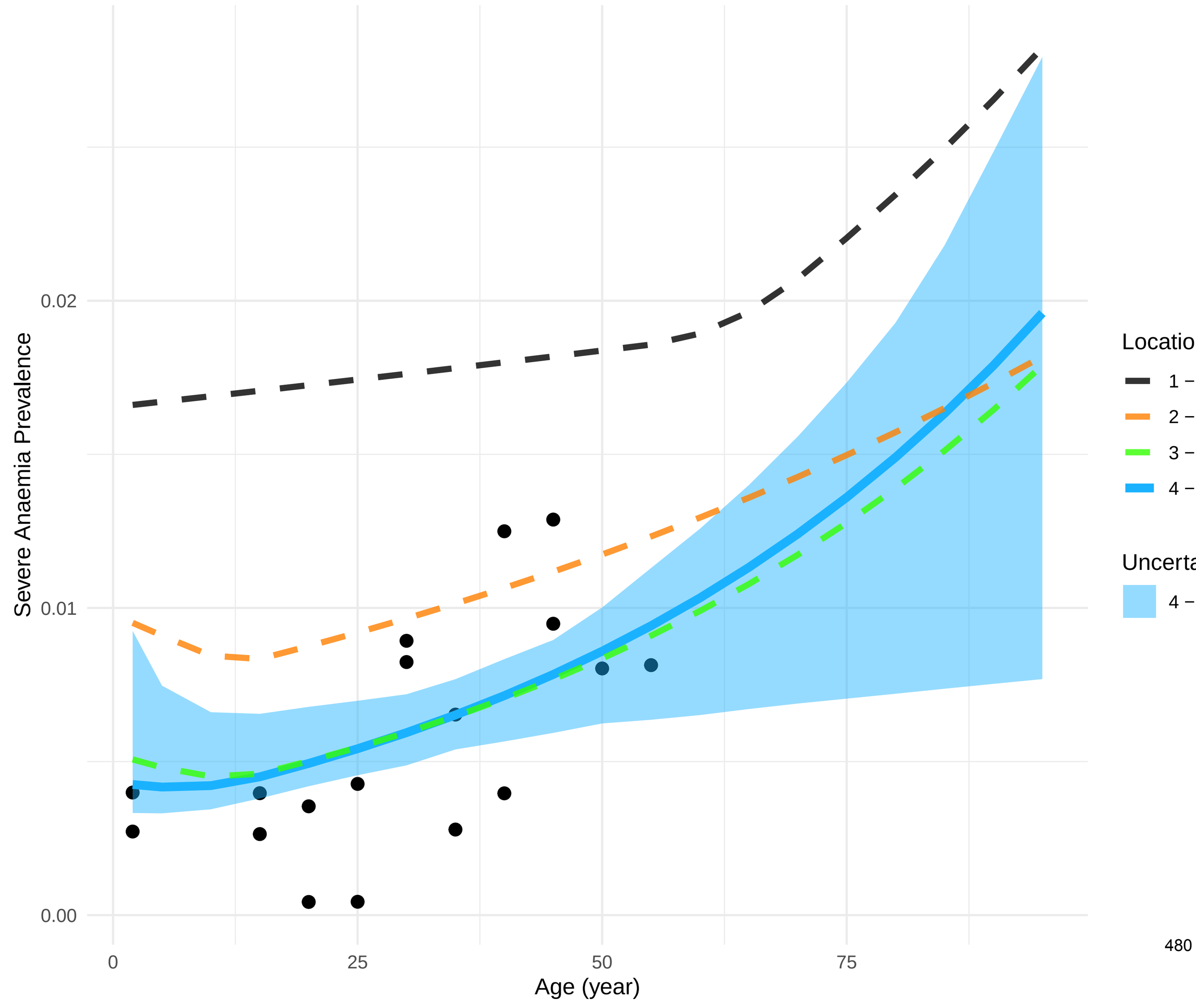

MR−BRT Age/Sex Fits − Non−pregnant Females 2+ Years Old − Severe Anaemia Prevalence − Eastern Europe
WHO−2001 Adjustment Method

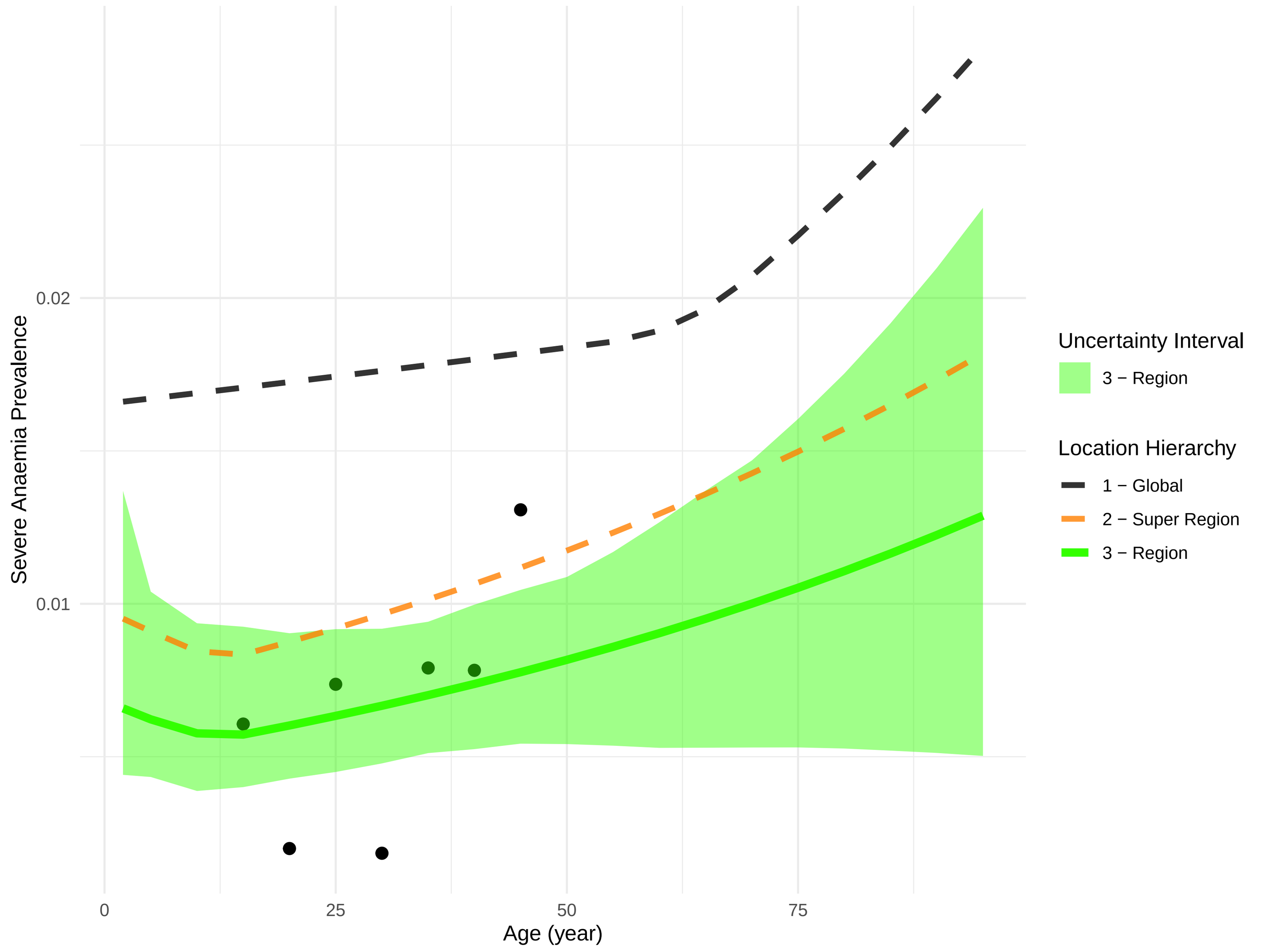

MR−BRT Age/Sex Fits − Non−pregnant Females 2+ Years Old − Severe Anaemia Prevalence − Republic of Moldova
WHO−2001 Adjustment Method

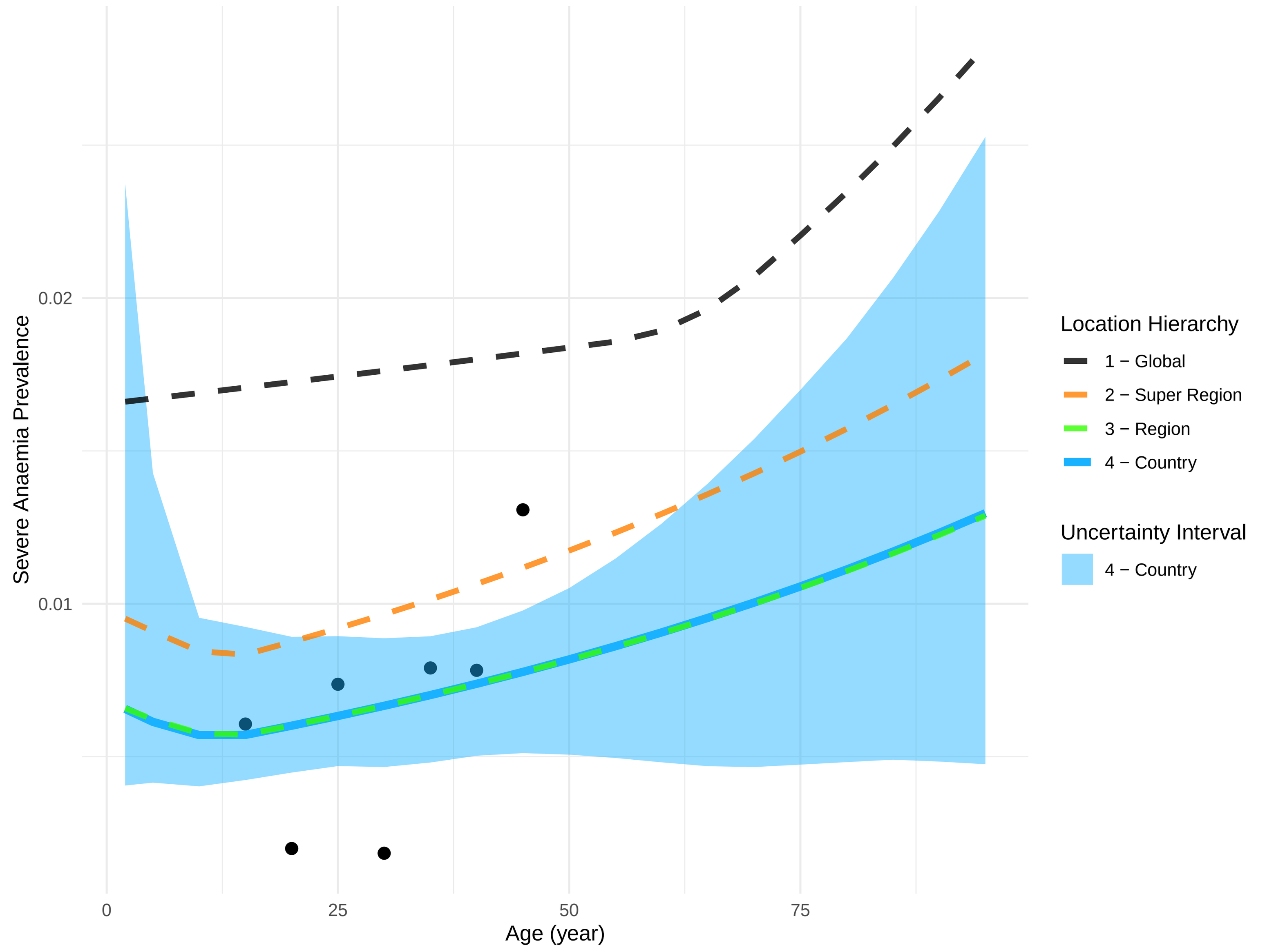

MR−BRT Age/Sex Fits − Non−pregnant Females 2+ Years Old − Severe Anaemia Prevalence − High−income
WHO−2001 Adjustment Method

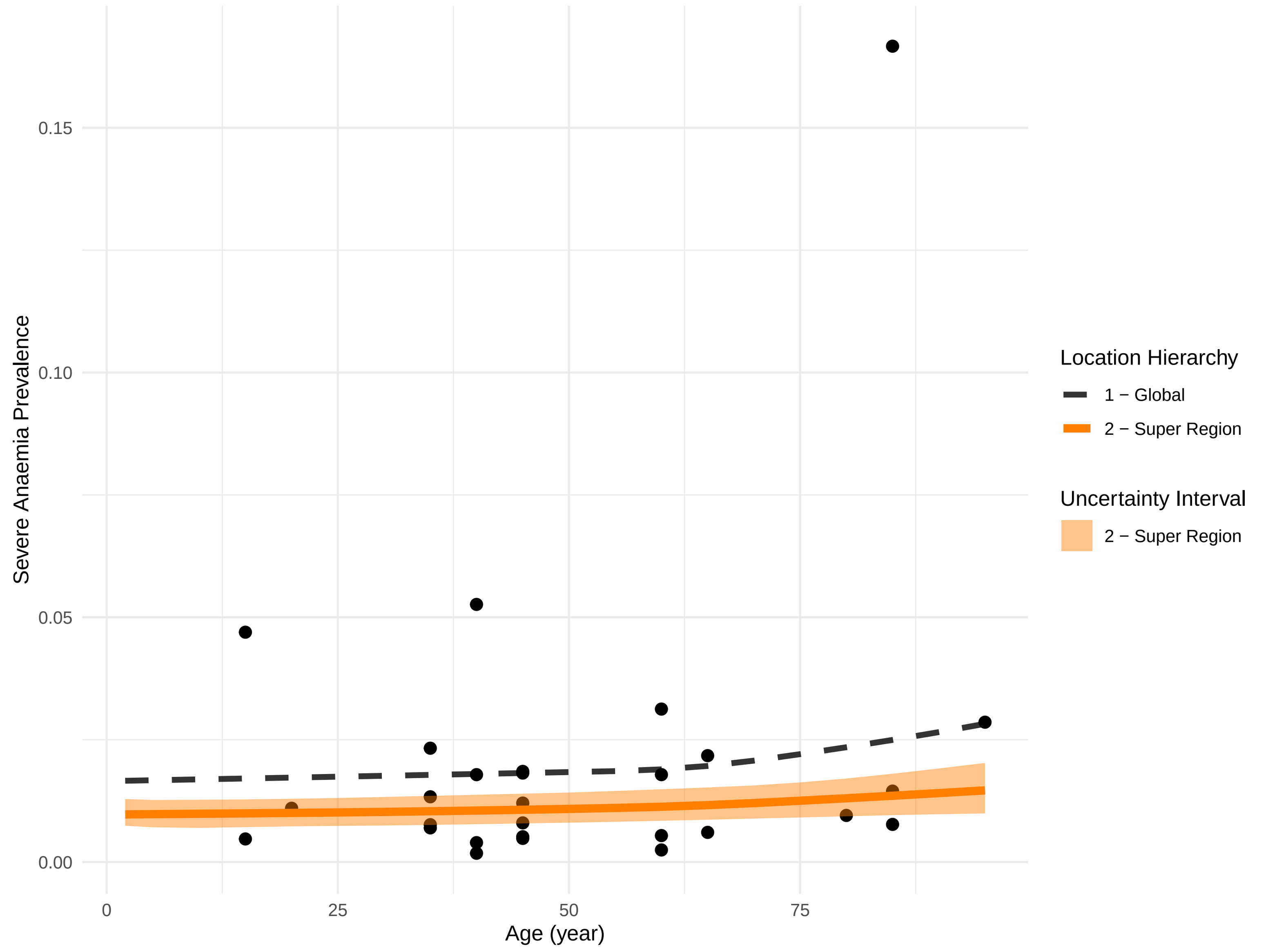

MR−BRT Age/Sex Fits − Non−pregnant Females 2+ Years Old − Severe Anaemia Prevalence − Western Europe
WHO−2001 Adjustment Method

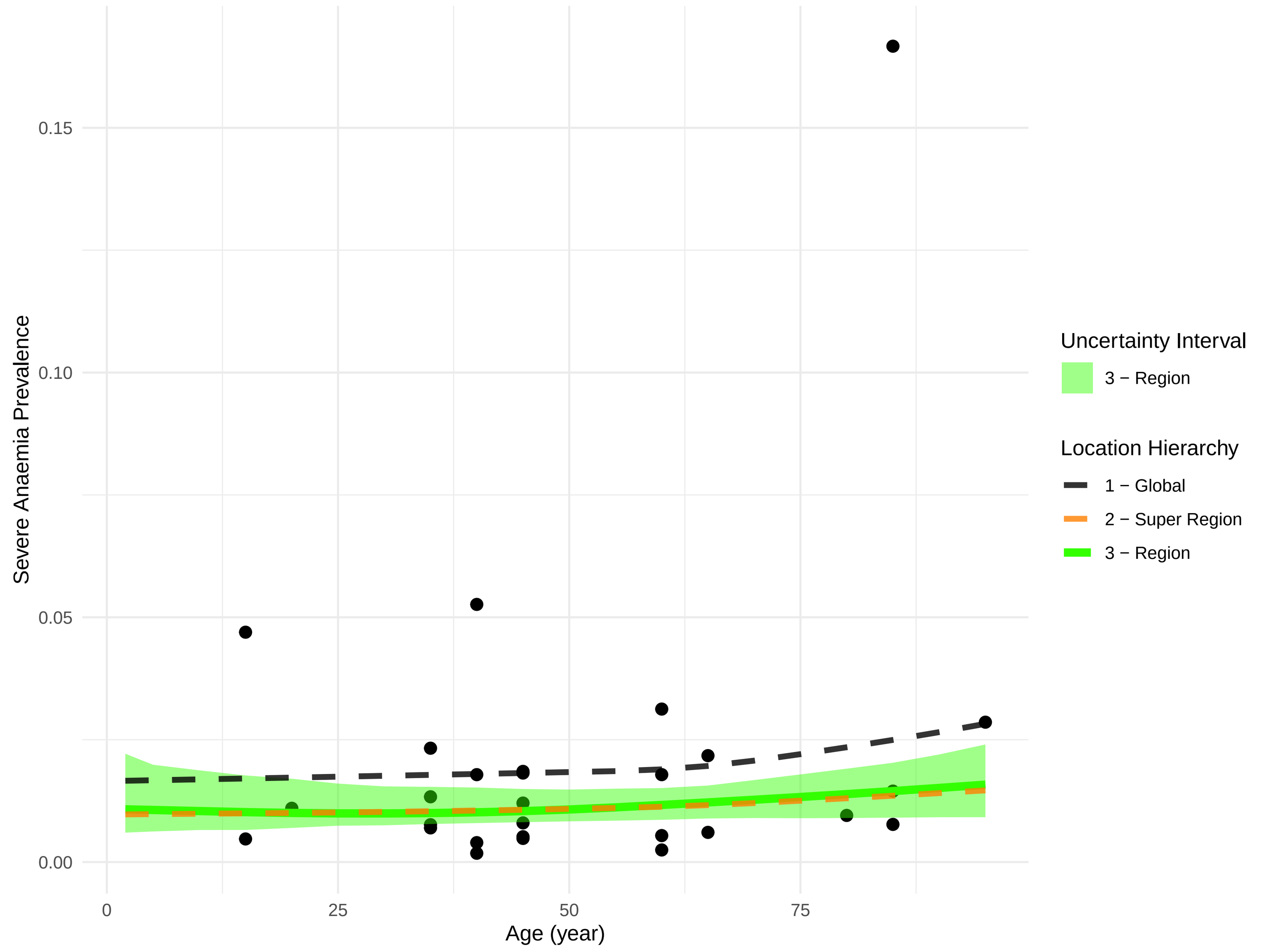

MR−BRT Age/Sex Fits − Non−pregnant Females 2+ Years Old − Severe Anaemia Prevalence − United Kingdom
WHO−2001 Adjustment Method

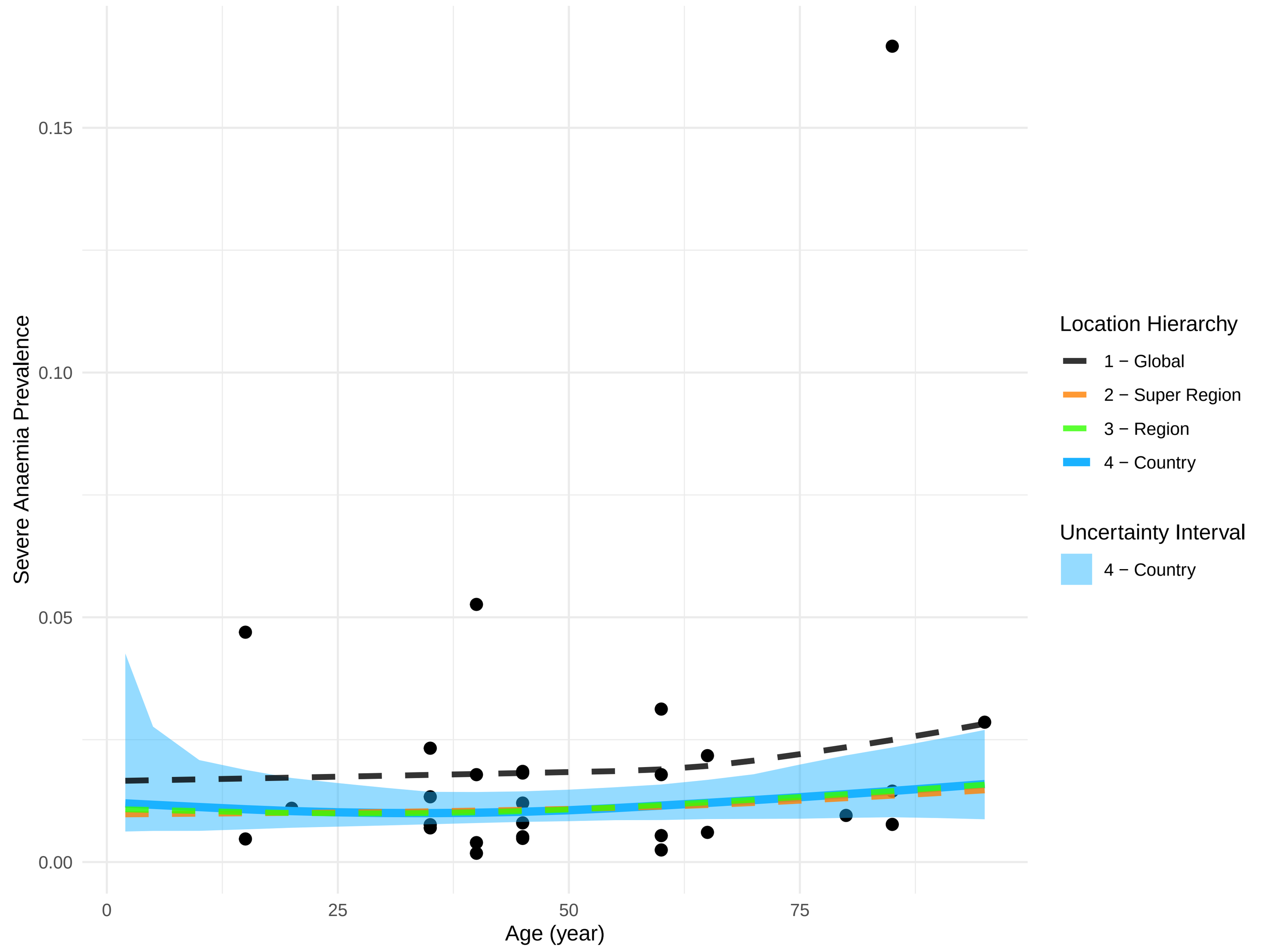

MR−BRT Age/Sex Fits − Non−pregnant Females 2+ Years Old − Severe Anaemia Prevalence − England
WHO−2001 Adjustment Method

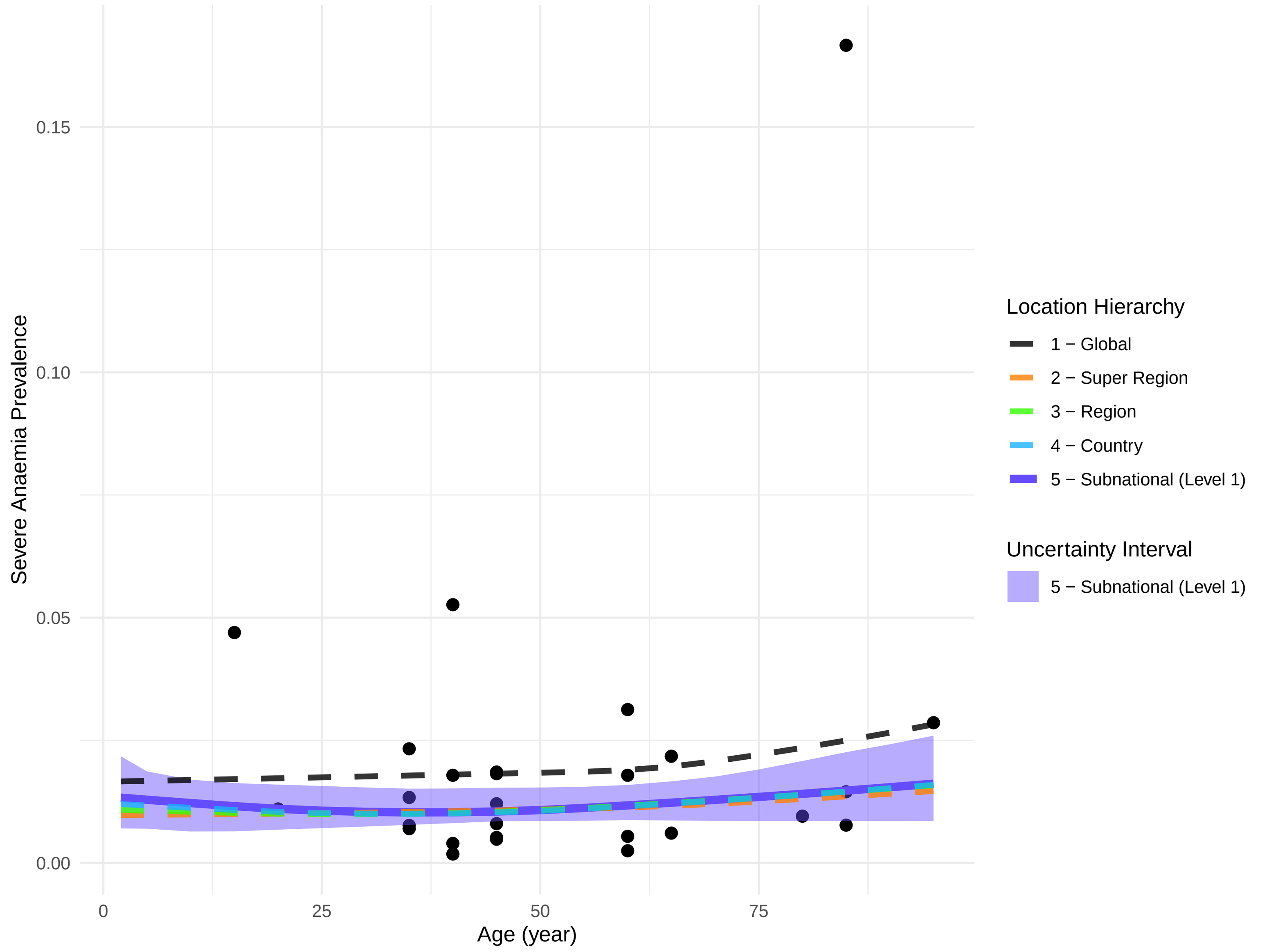

MR−BRT Age/Sex Fits − Non−pregnant Females 2+ Years Old − Severe Anaemia Prevalence − East of England
WHO−2001 Adjustment Method

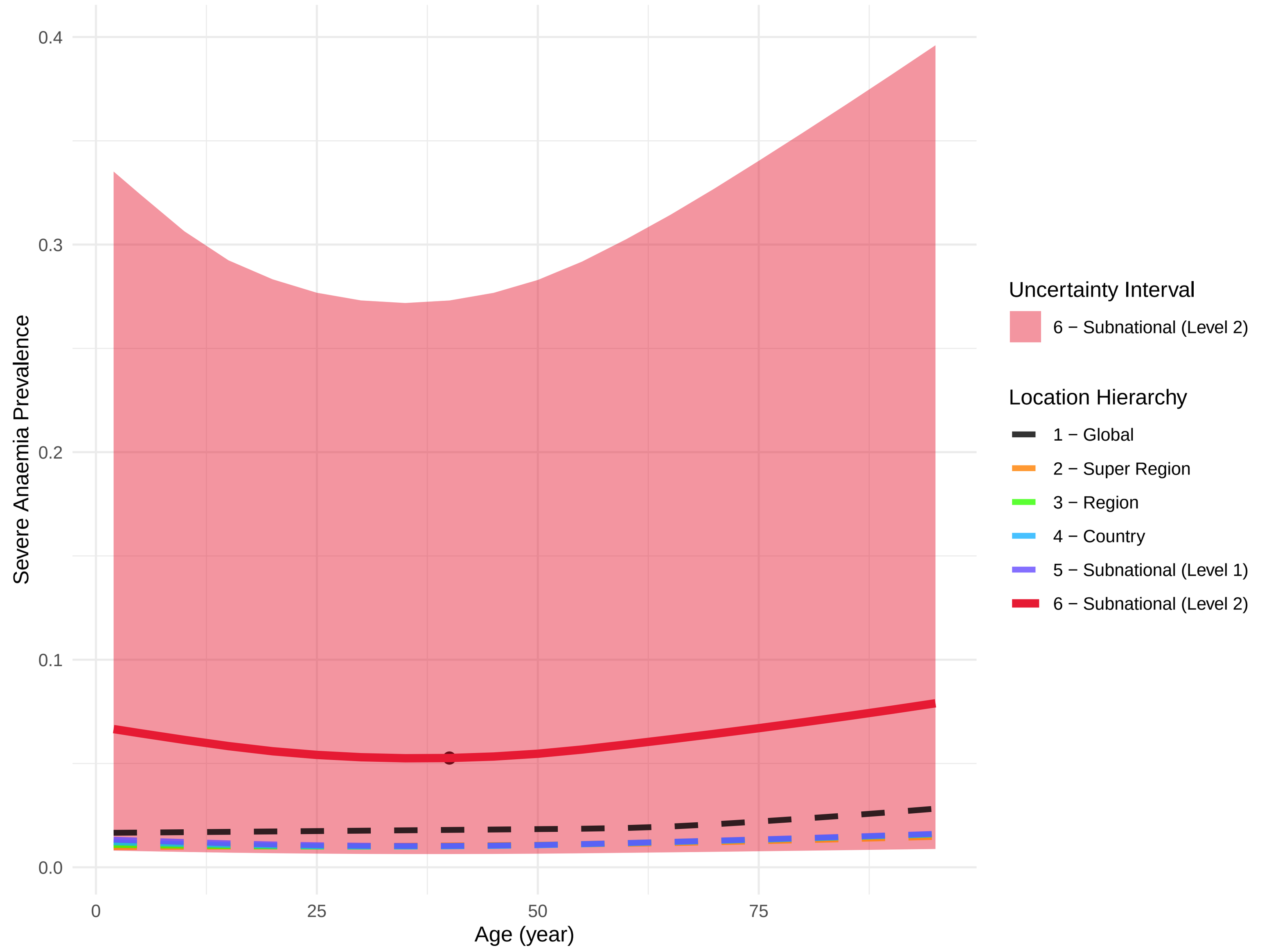

MR−BRT Age/Sex Fits − Non−pregnant Females 2+ Years Old − Severe Anaemia Prevalence − Greater London
WHO−2001 Adjustment Method

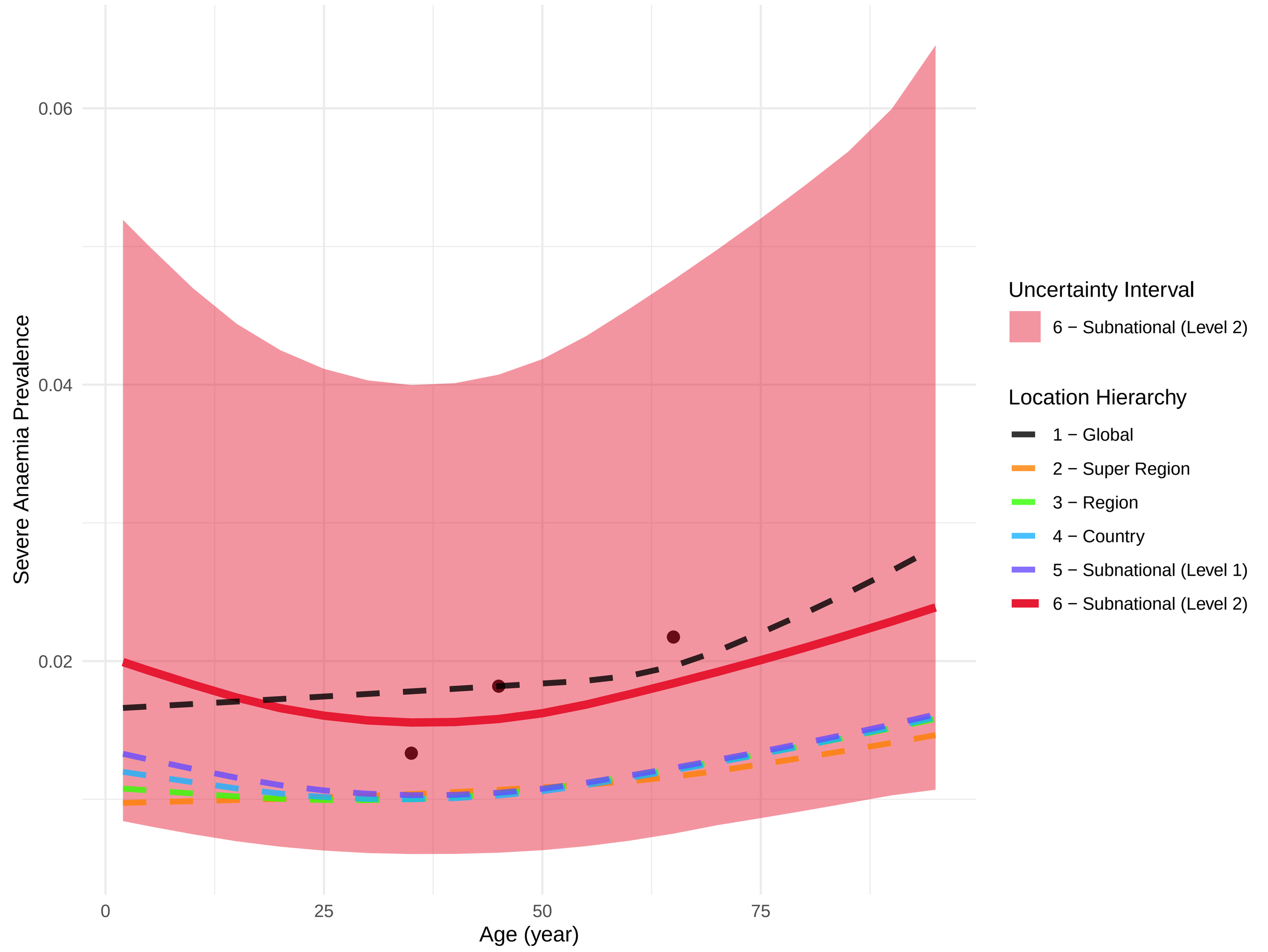

MR−BRT Age/Sex Fits − Non−pregnant Females 2+ Years Old − Severe Anaemia Prevalence − North West England
WHO−2001 Adjustment Method

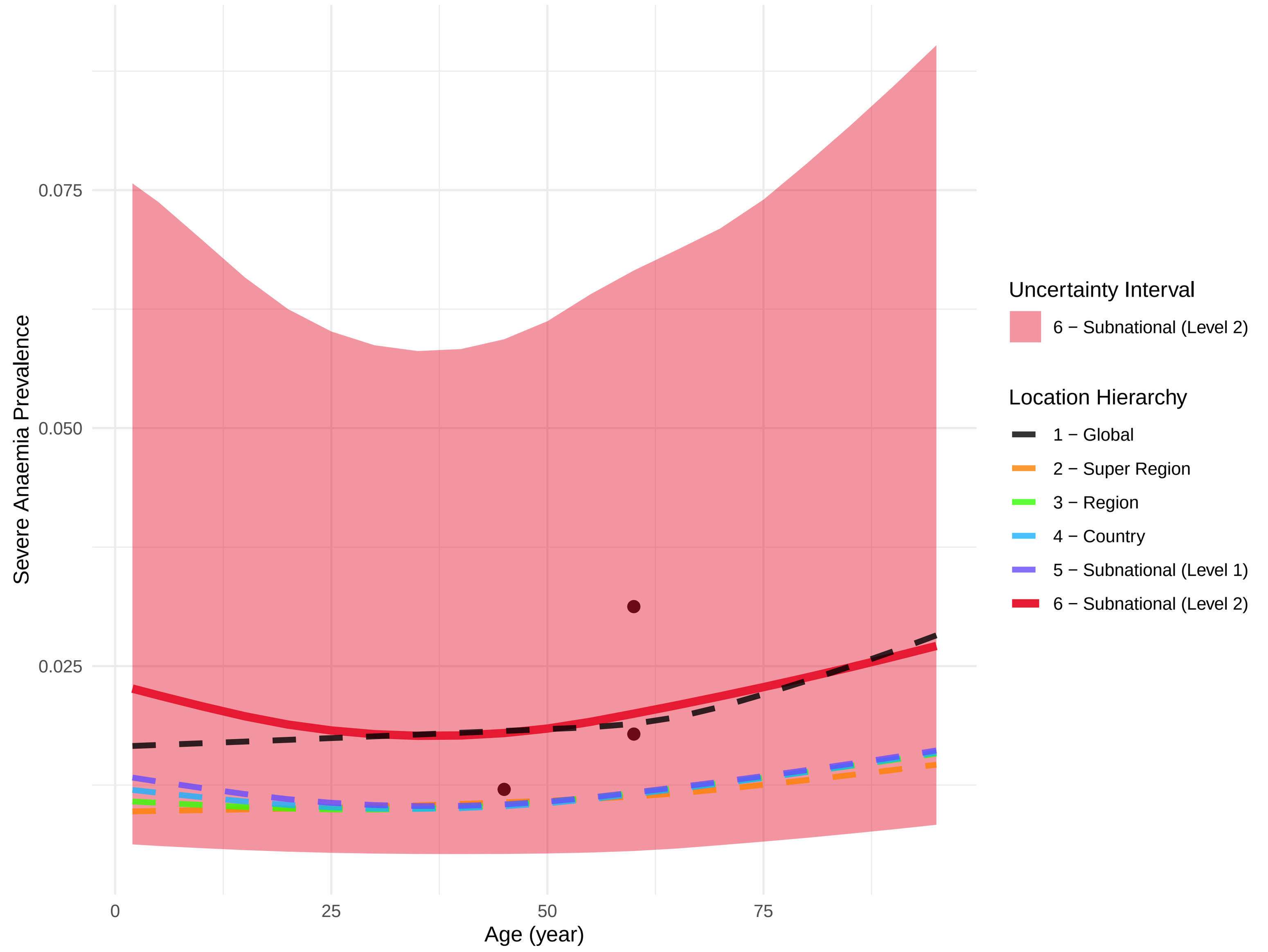

MR−BRT Age/Sex Fits − Non−pregnant Females 2+ Years Old − Severe Anaemia Prevalence − South West England
WHO−2001 Adjustment Method

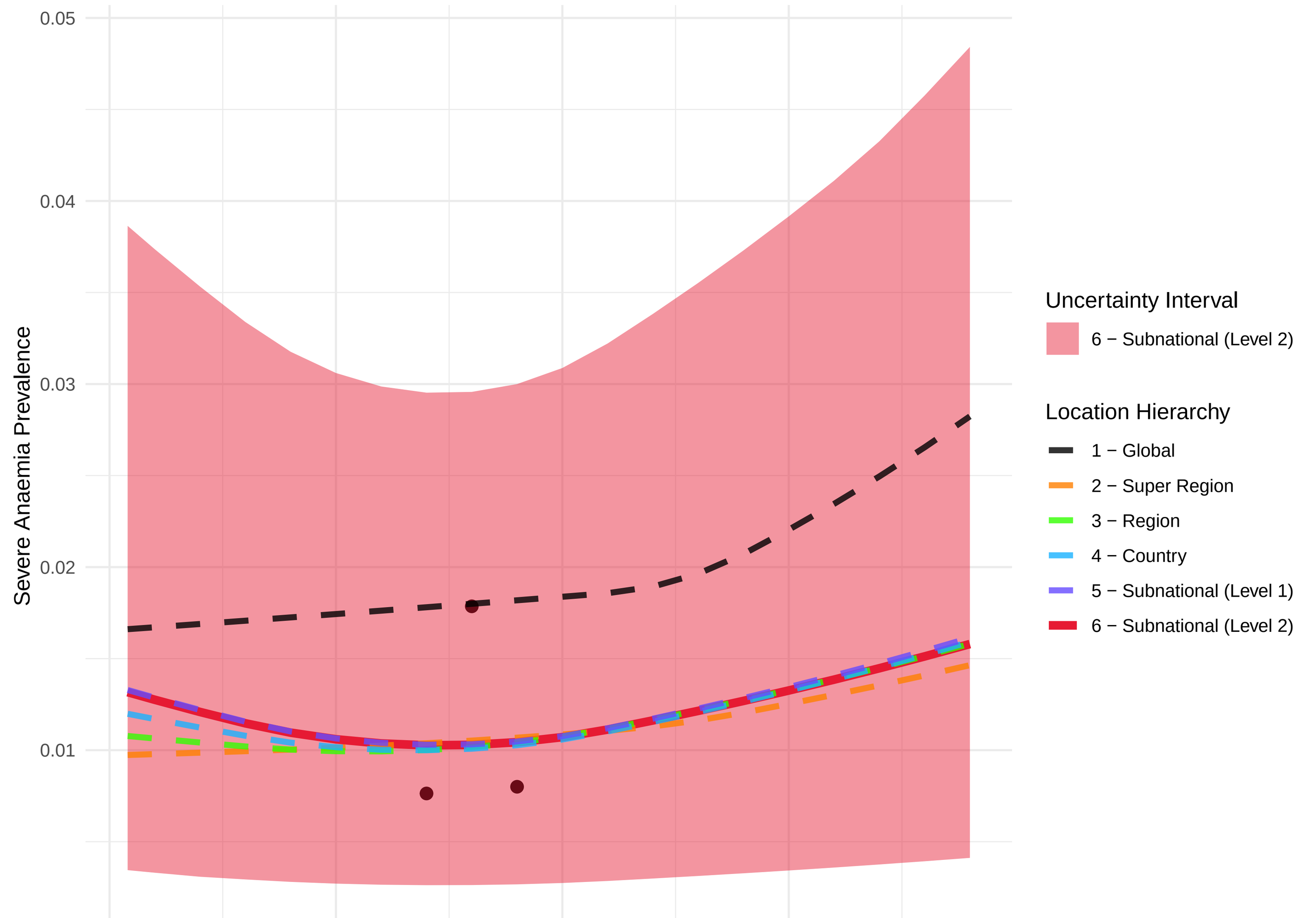

MR−BRT Age/Sex Fits − Non-pregnant Females 2+ Years Old − Severe Anaemia Prevalence − Yorkshire and the Humber
WHO−2001 Adjustment Method

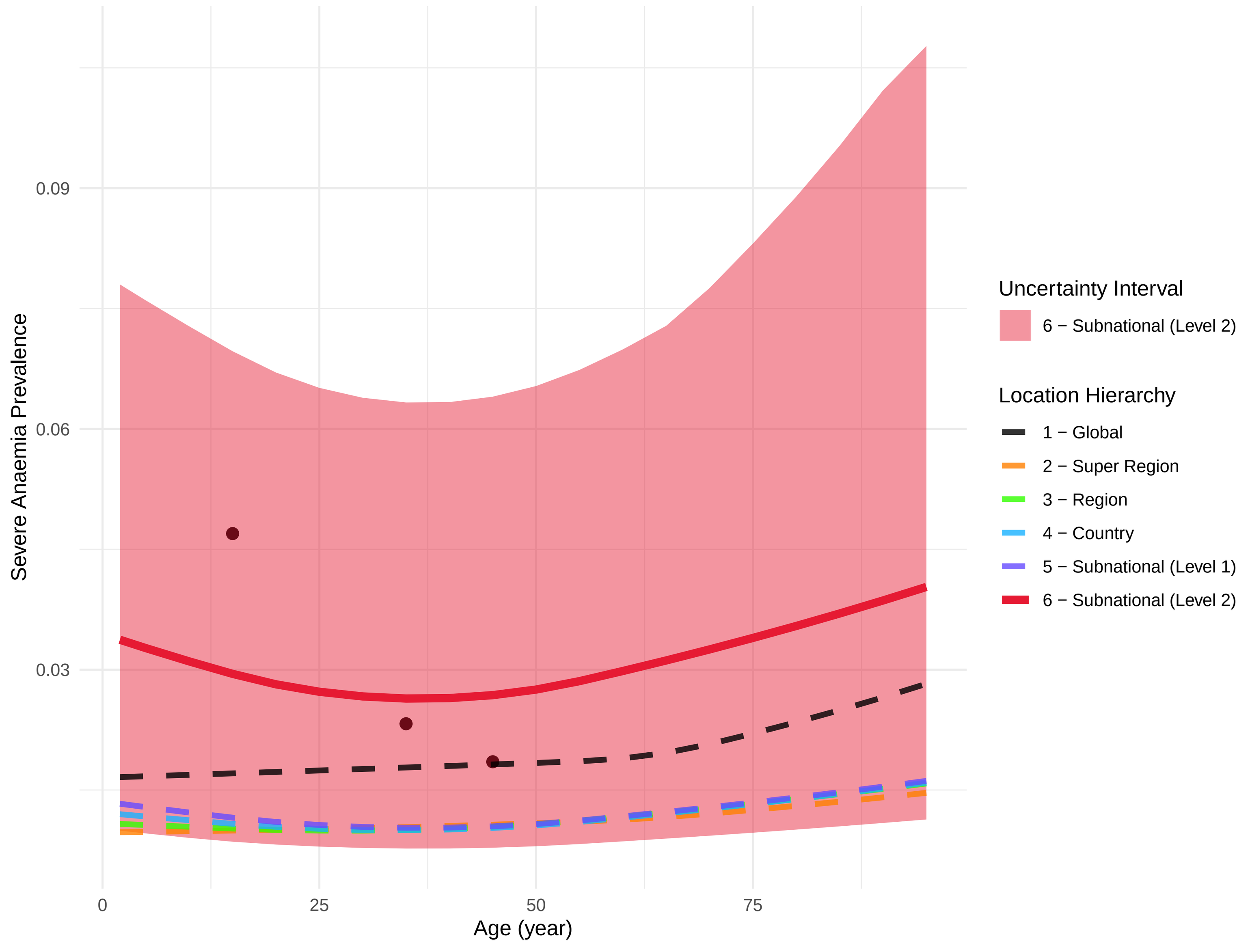

MR−BRT Age/Sex Fits − Non-pregnant Females 2+ Years Old − Severe Anaemia Prevalence − Latin America and Caribbean
WHO−2001 Adjustment Method

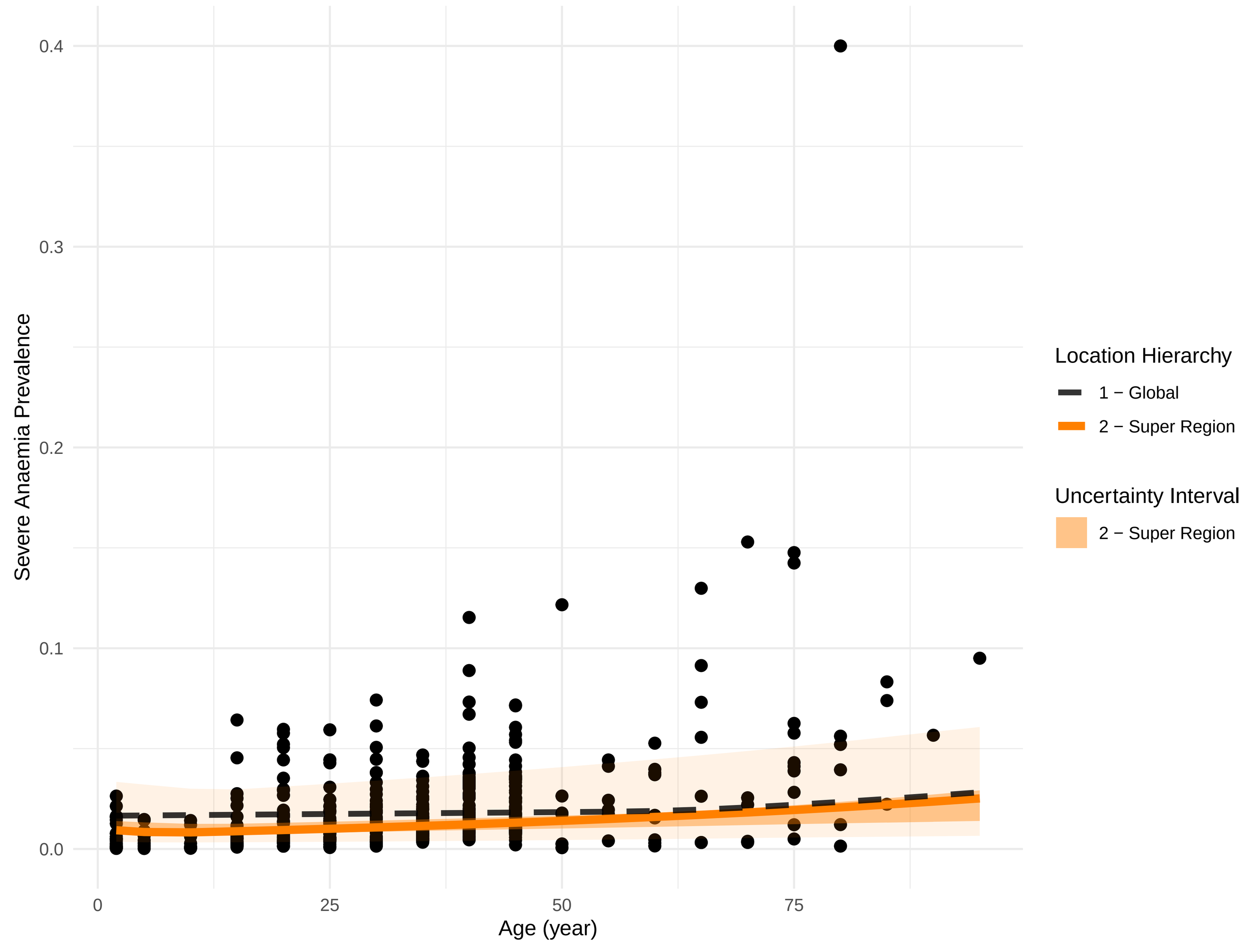

MR−BRT Age/Sex Fits − Non-pregnant Females 2+ Years Old − Severe Anaemia Prevalence − Andean Latin America
WHO−2001 Adjustment Method

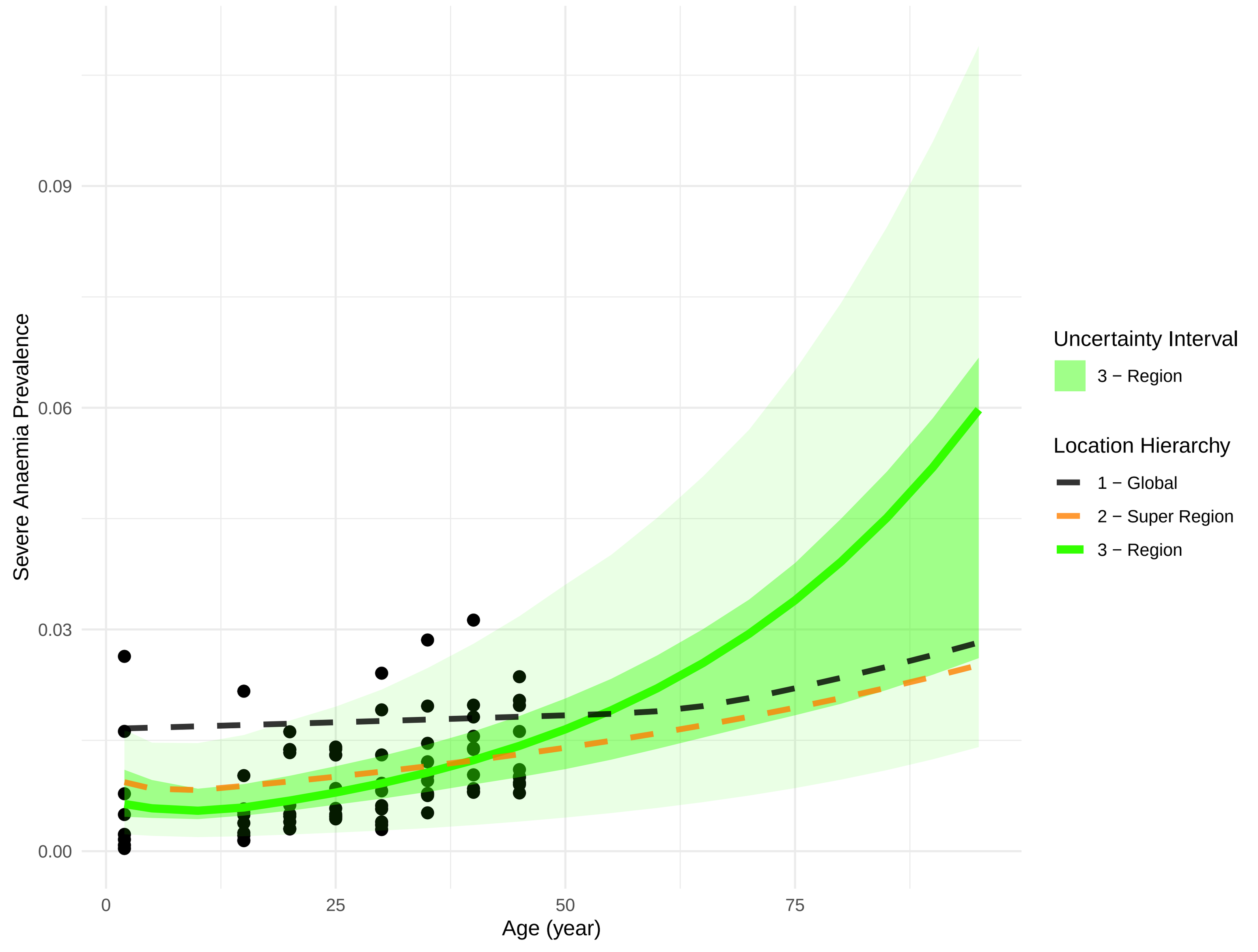

MR−BRT Age/Sex Fits − Non-pregnant Females 2+ Years Old − Severe Anaemia Prevalence − Bolivia (Plurinational State of)
WHO−2001 Adjustment Method

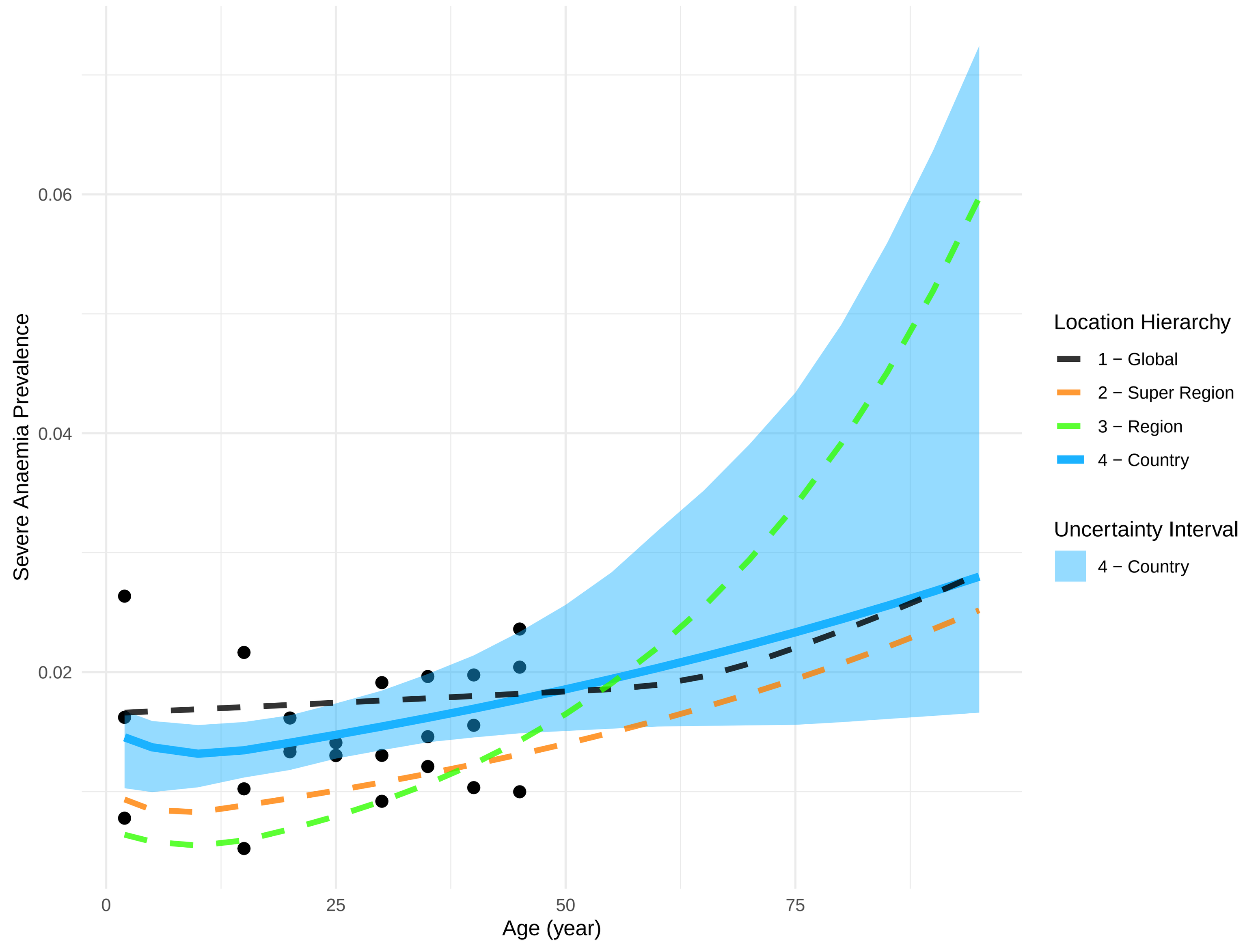

MR−BRT Age/Sex Fits − Non-pregnant Females 2+ Years Old − Severe Anaemia Prevalence − Peru
WHO−2001 Adjustment Method

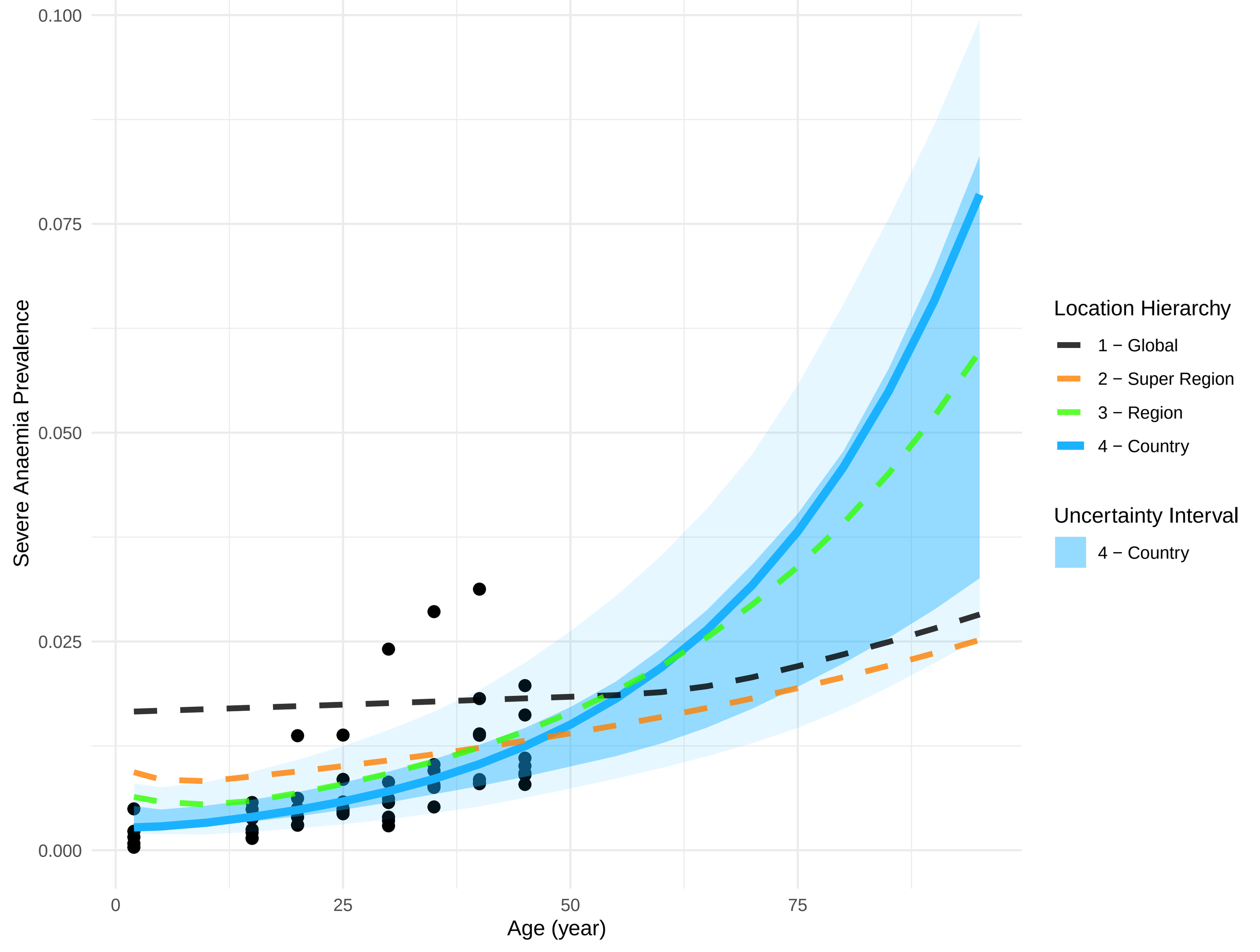

MR−BRT Age/Sex Fits − Non-pregnant Females 2+ Years Old − Severe Anaemia Prevalence − Caribbean
WHO−2001 Adjustment Method

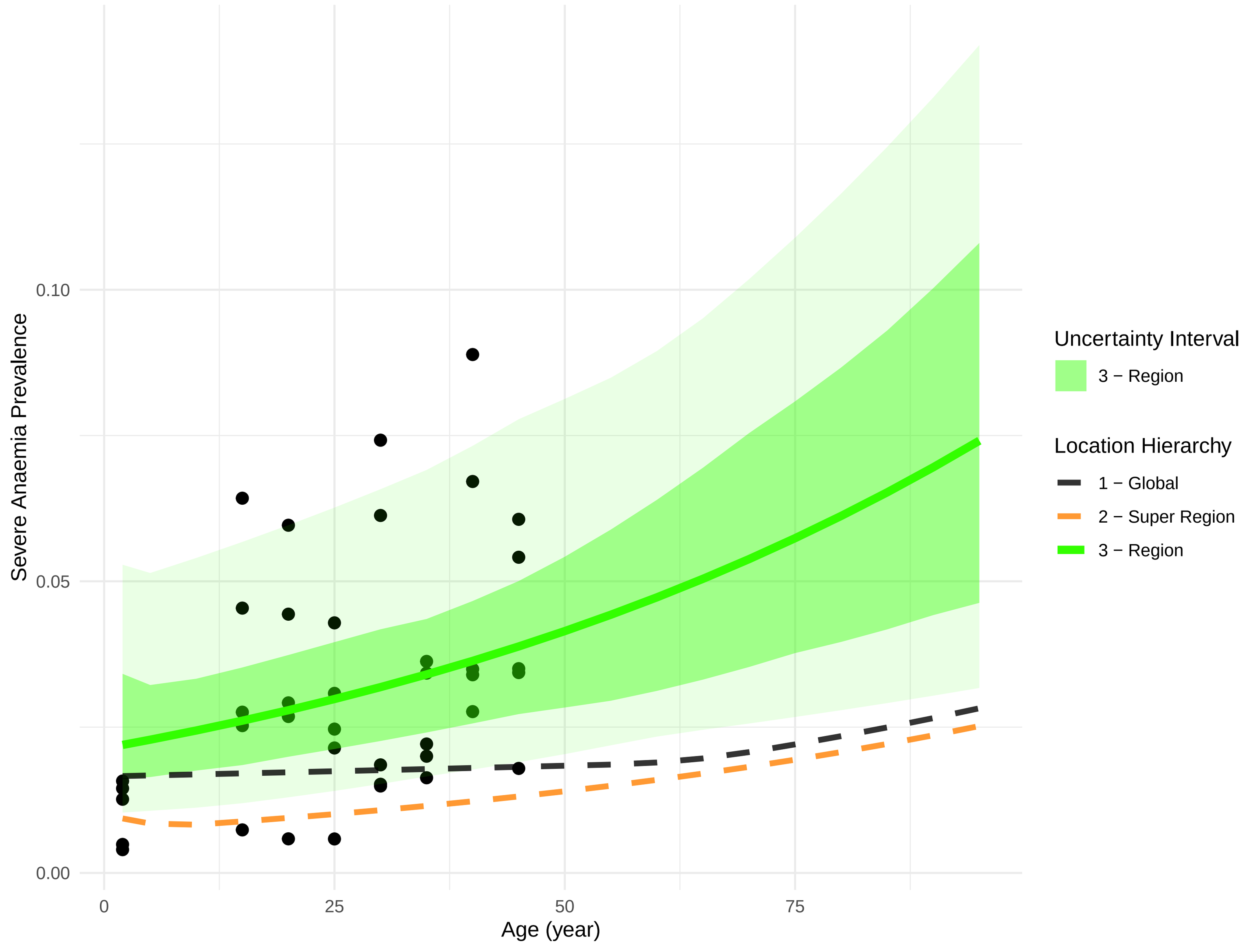

MR−BRT Age/Sex Fits − Non-pregnant Females 2+ Years Old − Severe Anaemia Prevalence − Guyana
WHO−2001 Adjustment Method

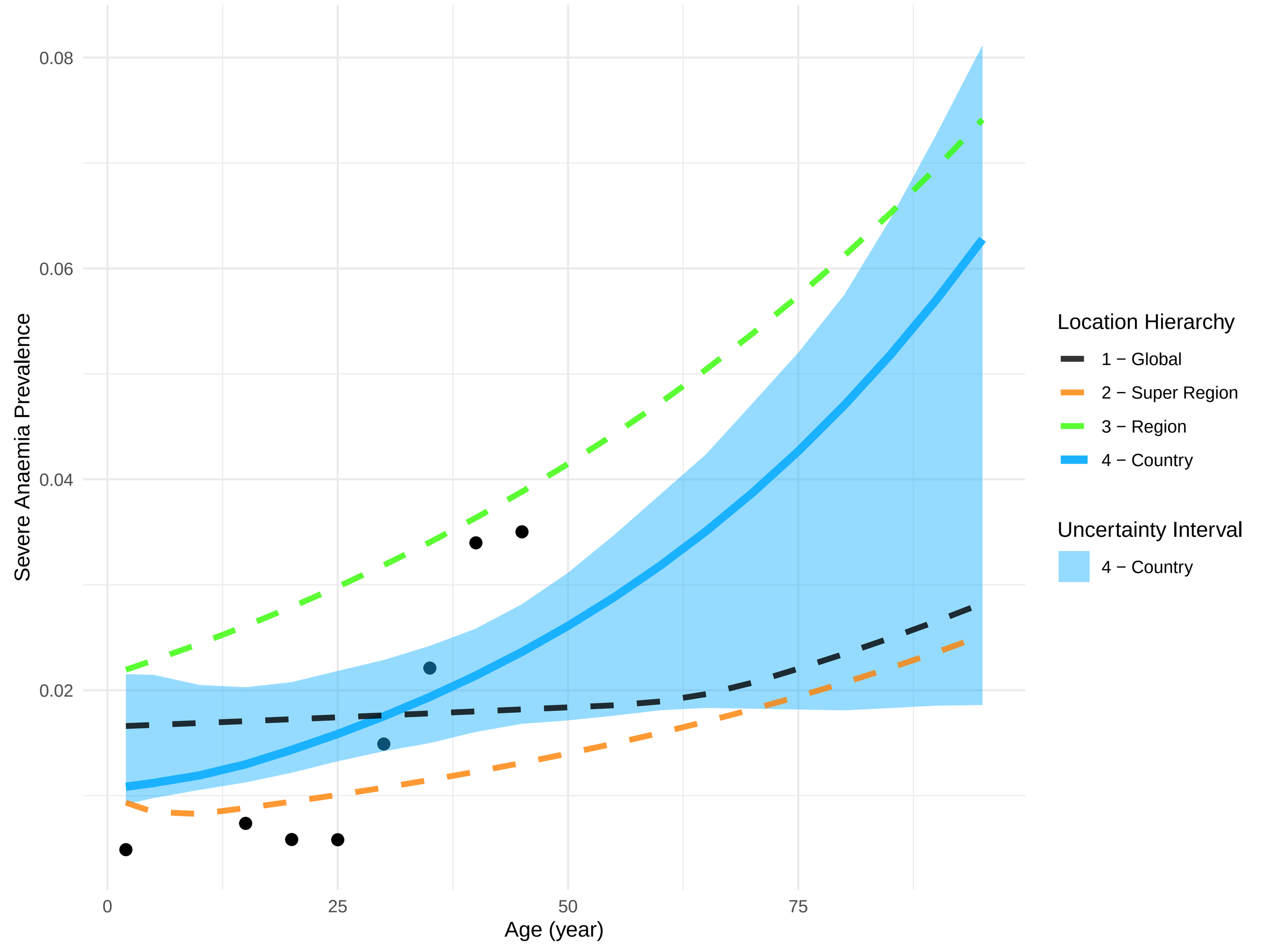

MR−BRT Age/Sex Fits − Non-pregnant Females 2+ Years Old − Severe Anaemia Prevalence − Haiti
WHO−2001 Adjustment Method

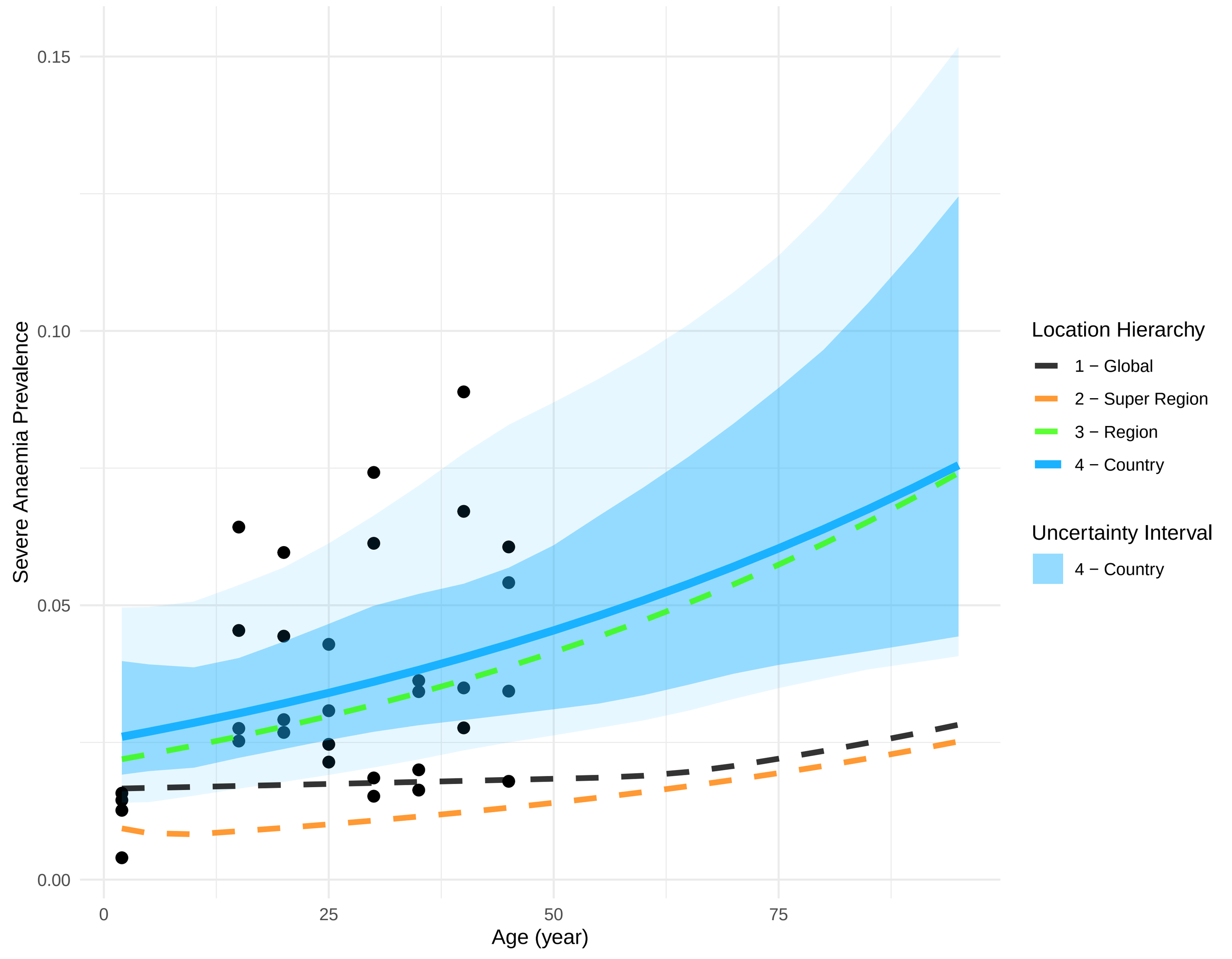

MR−BRT Age/Sex Fits − Non-pregnant Females 2+ Years Old − Severe Anaemia Prevalence − Central Latin America
WHO−2001 Adjustment Method

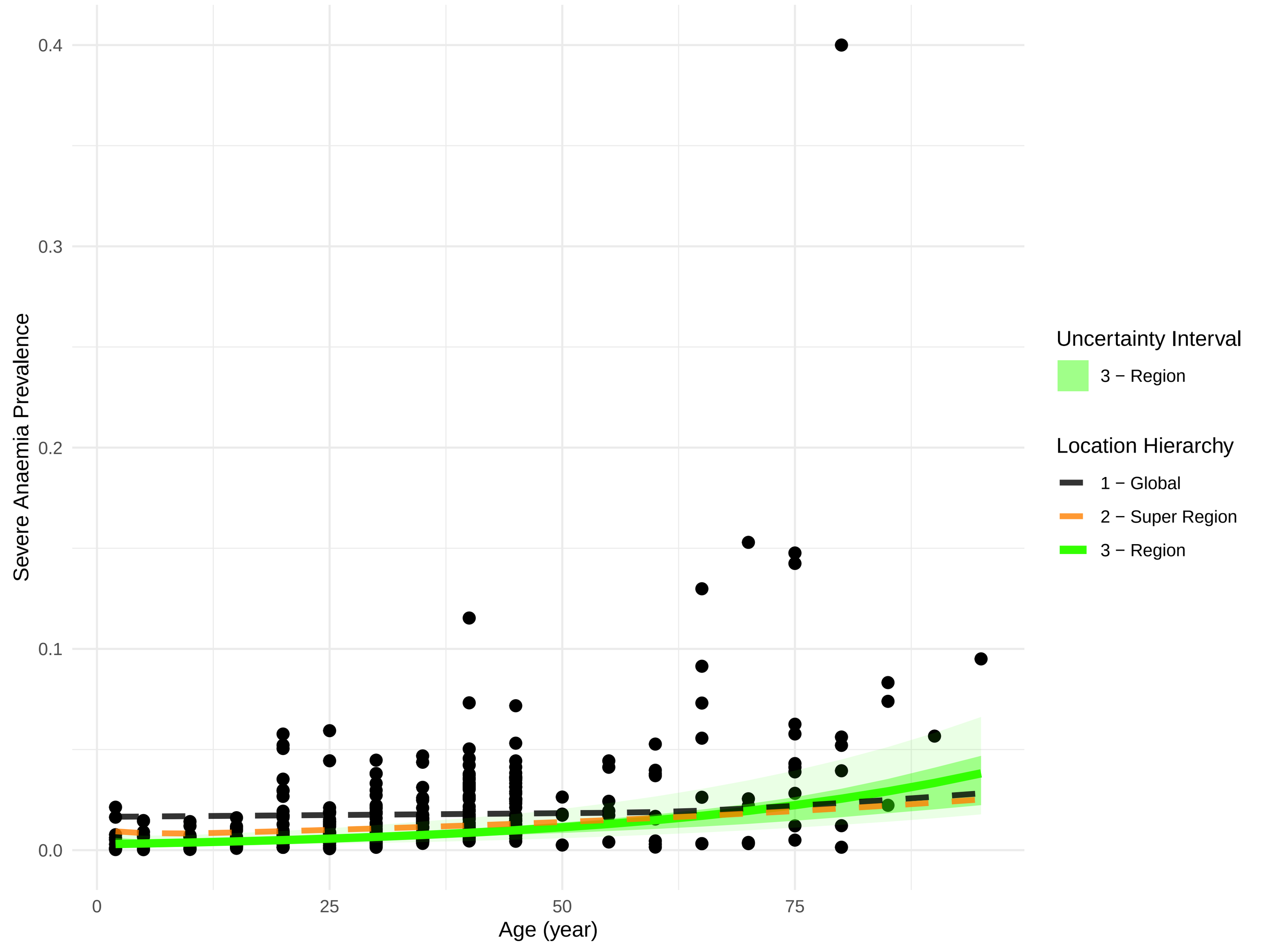

MR−BRT Age/Sex Fits − Non-pregnant Females 2+ Years Old − Severe Anaemia Prevalence − El Salvador
WHO−2001 Adjustment Method

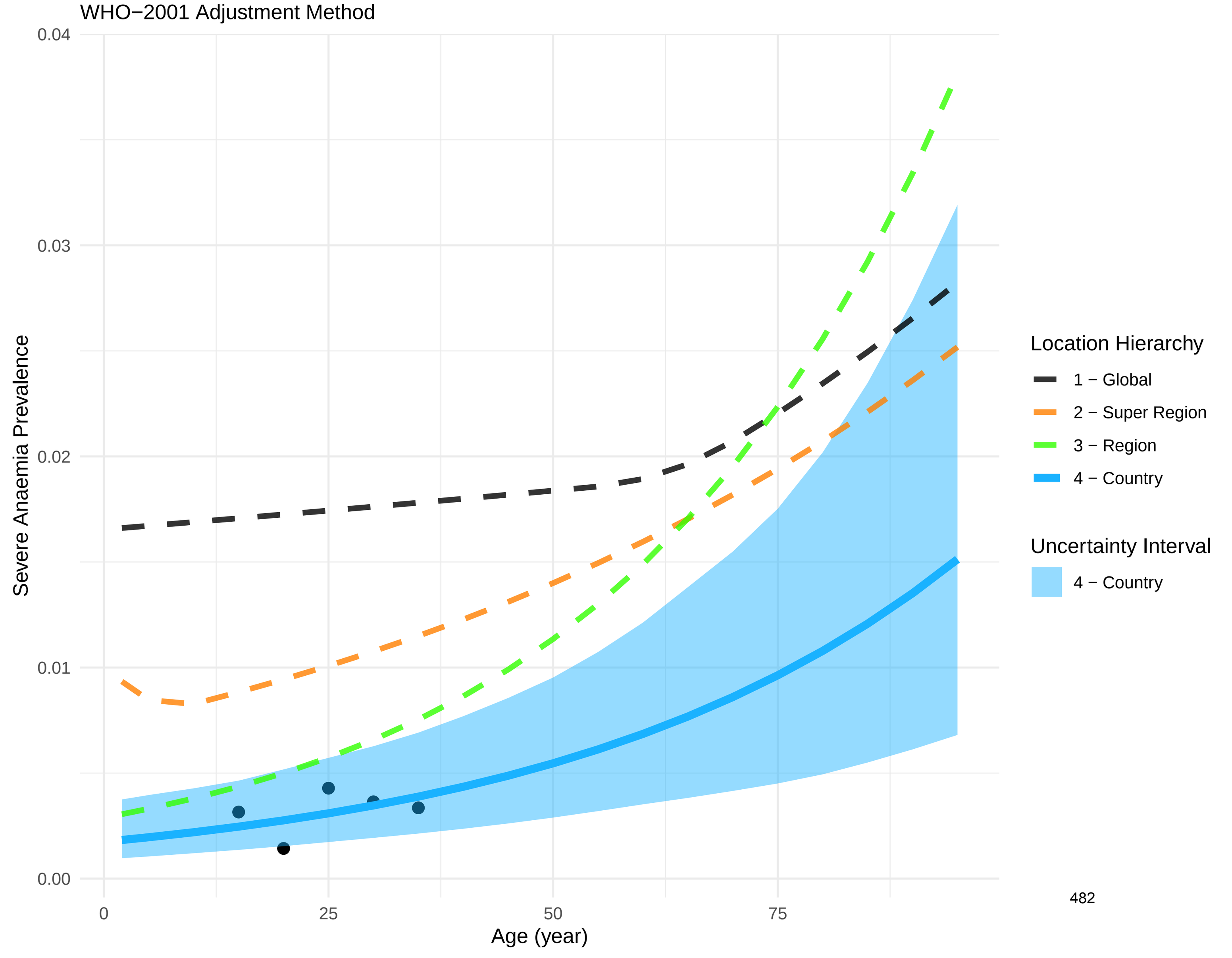

MR−BRT Age/Sex Fits − Non−pregnant Females 2+ Years Old − Severe Anaemia Prevalence − Guatemala
WHO−2001 Adjustment Method

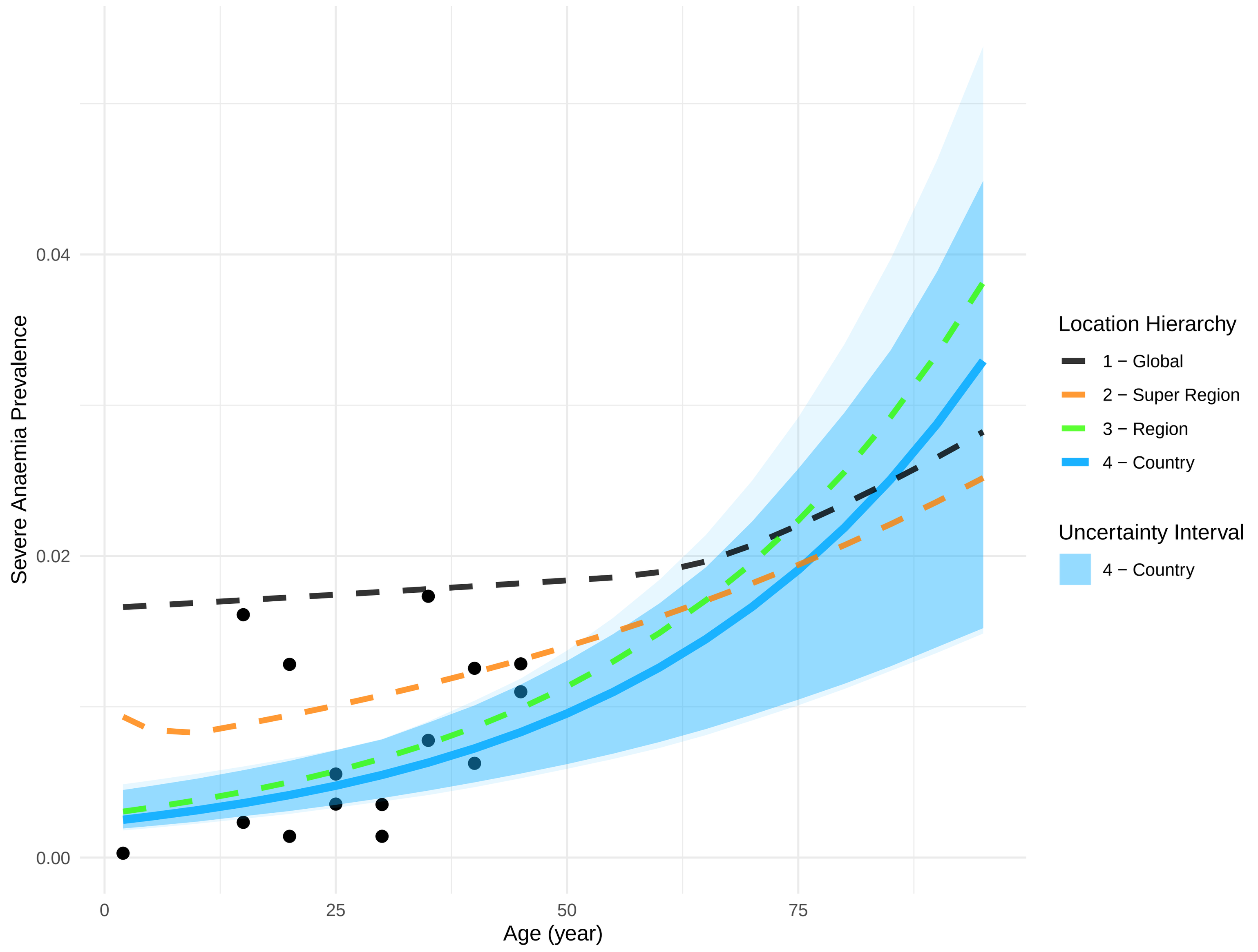

MR−BRT Age/Sex Fits − Non−pregnant Females 2+ Years Old − Severe Anaemia Prevalence − Honduras
WHO−2001 Adjustment Method

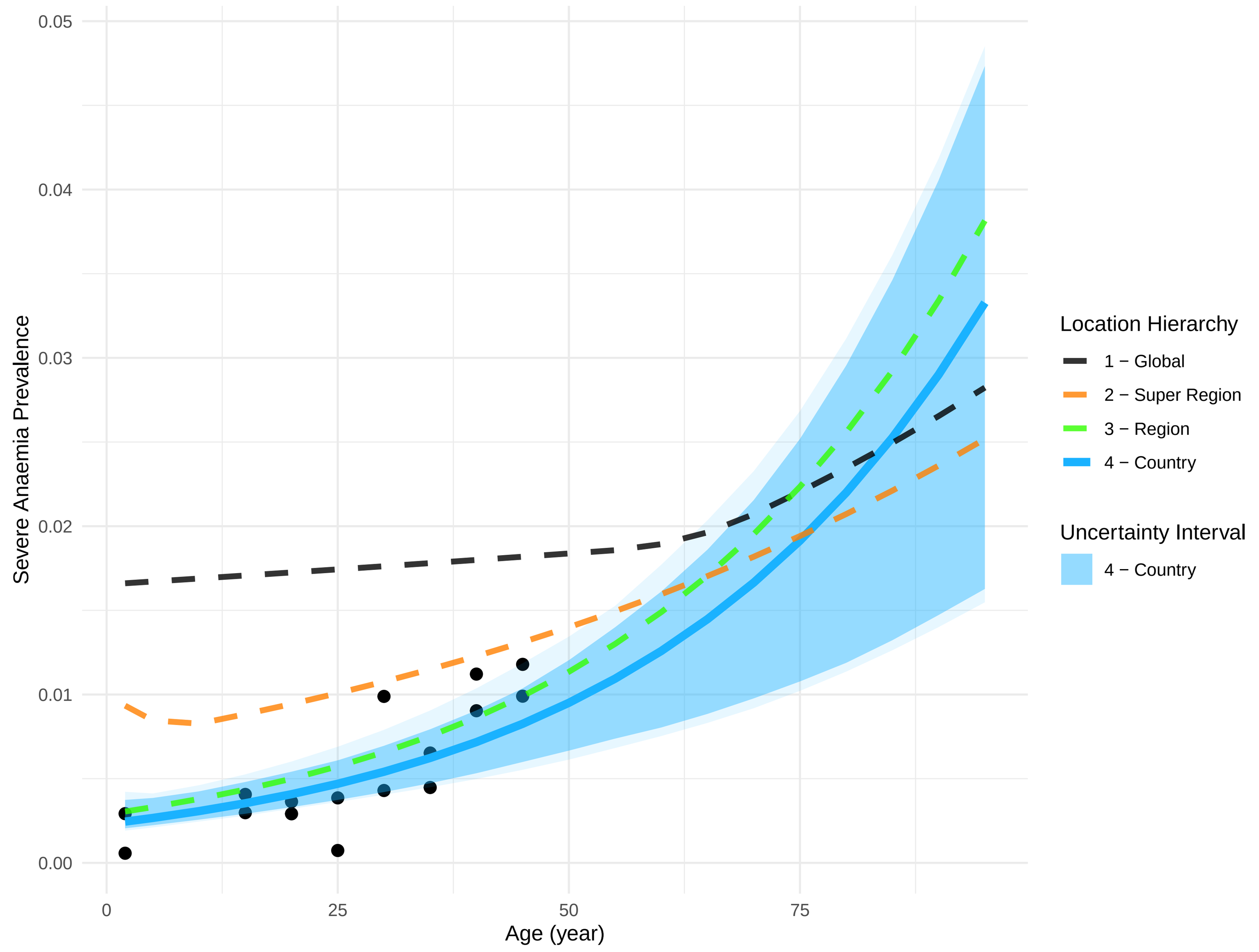

MR−BRT Age/Sex Fits − Non−pregnant Females 2+ Years Old − Severe Anaemia Prevalence − Mexico
WHO−2001 Adjustment Method

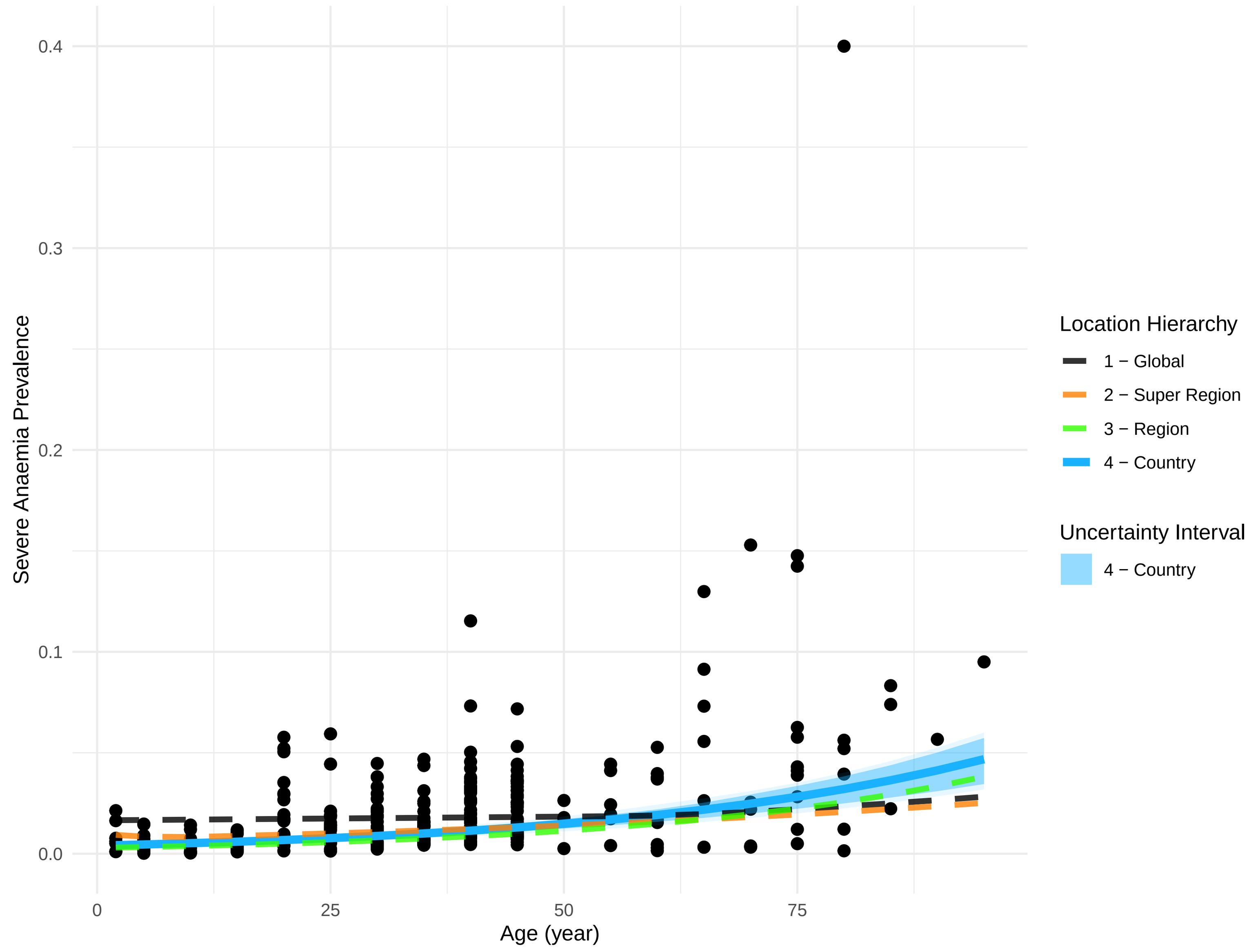

MR−BRT Age/Sex Fits − Non−pregnant Females 2+ Years Old − Severe Anaemia Prevalence − Aguascalientes
WHO−2001 Adjustment Method

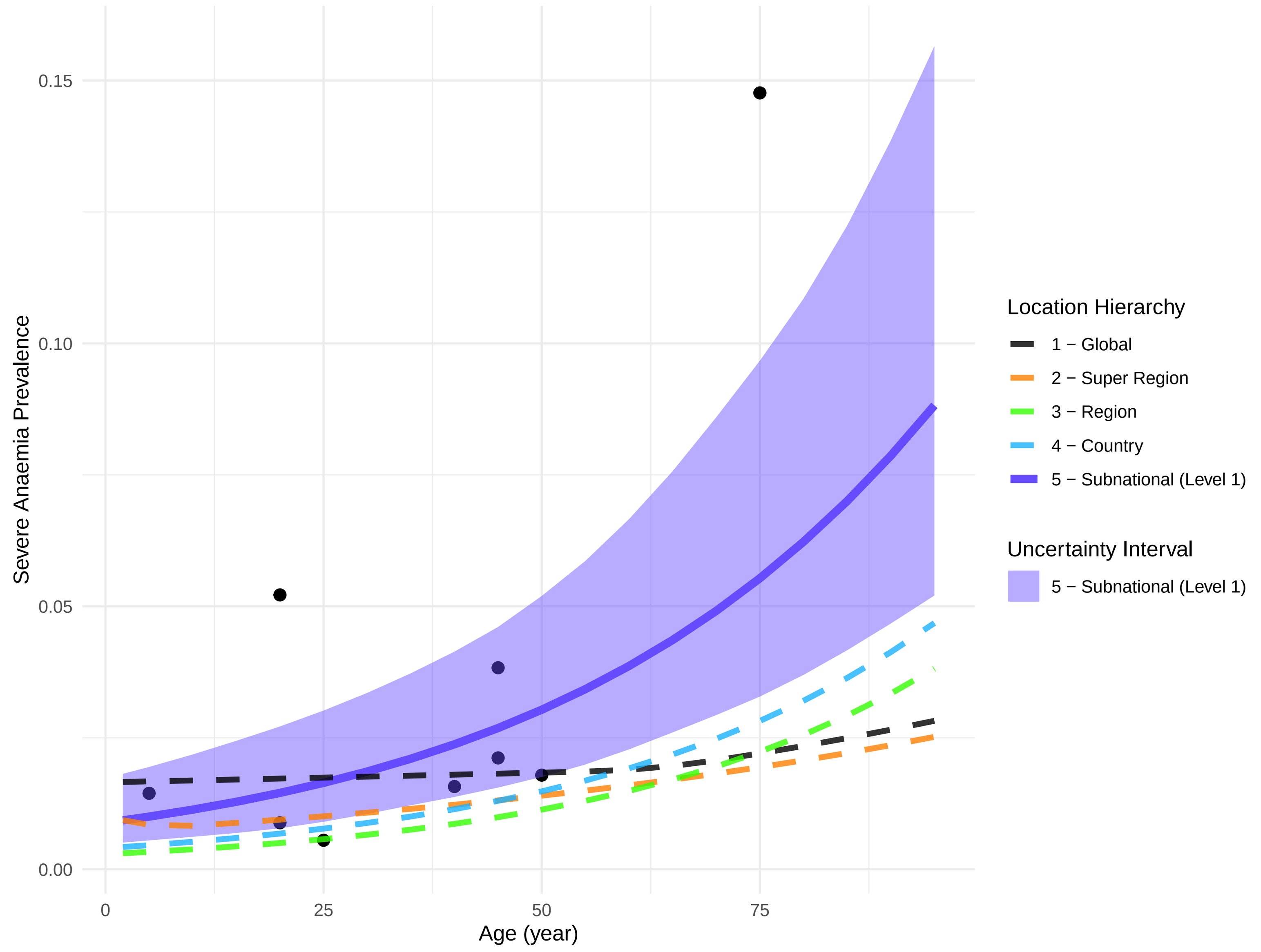

MR−BRT Age/Sex Fits − Non−pregnant Females 2+ Years Old − Severe Anaemia Prevalence − Baja California
WHO−2001 Adjustment Method

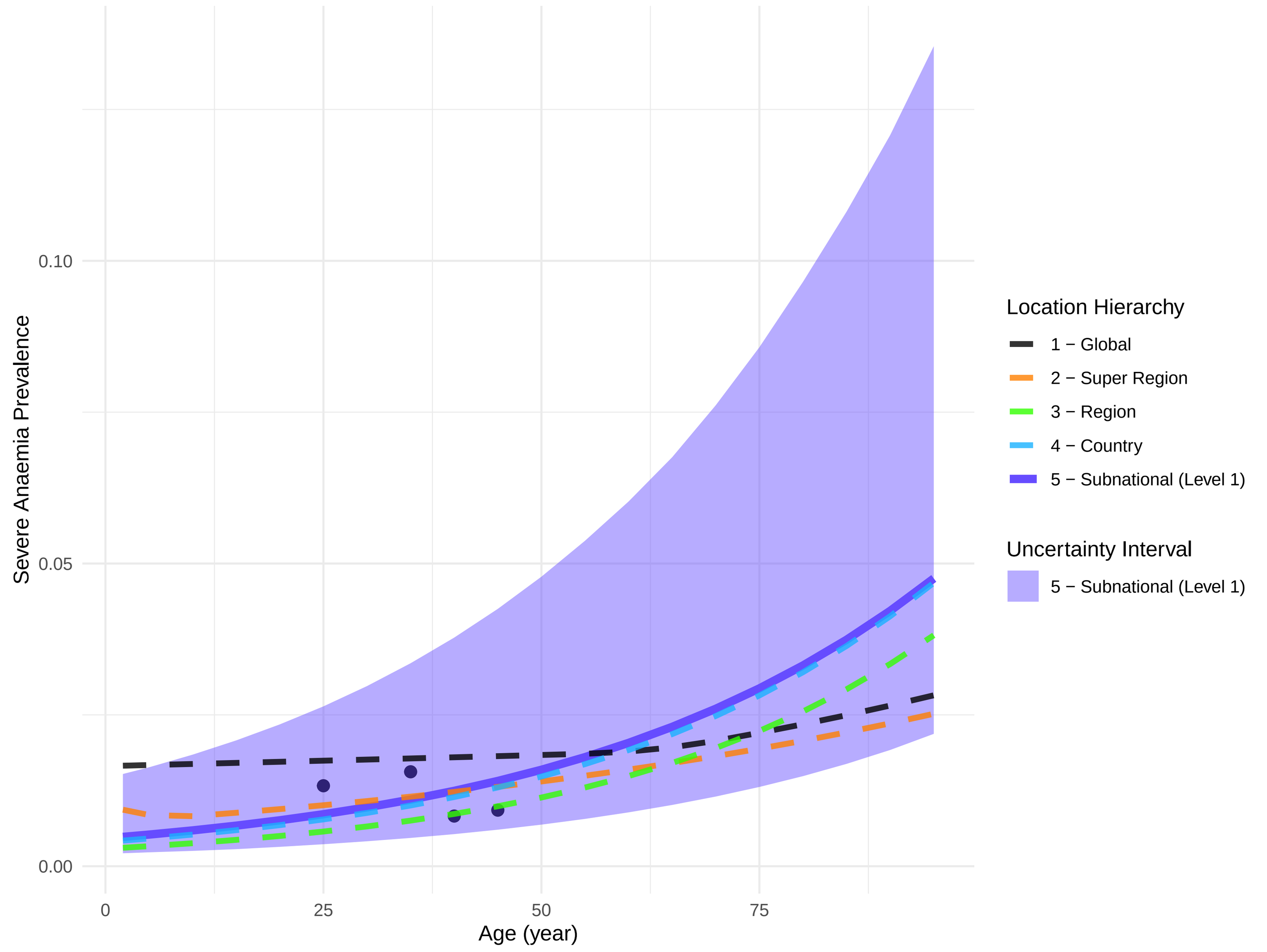

MR−BRT Age/Sex Fits − Non−pregnant Females 2+ Years Old − Severe Anaemia Prevalence − Baja California Sur
WHO−2001 Adjustment Method

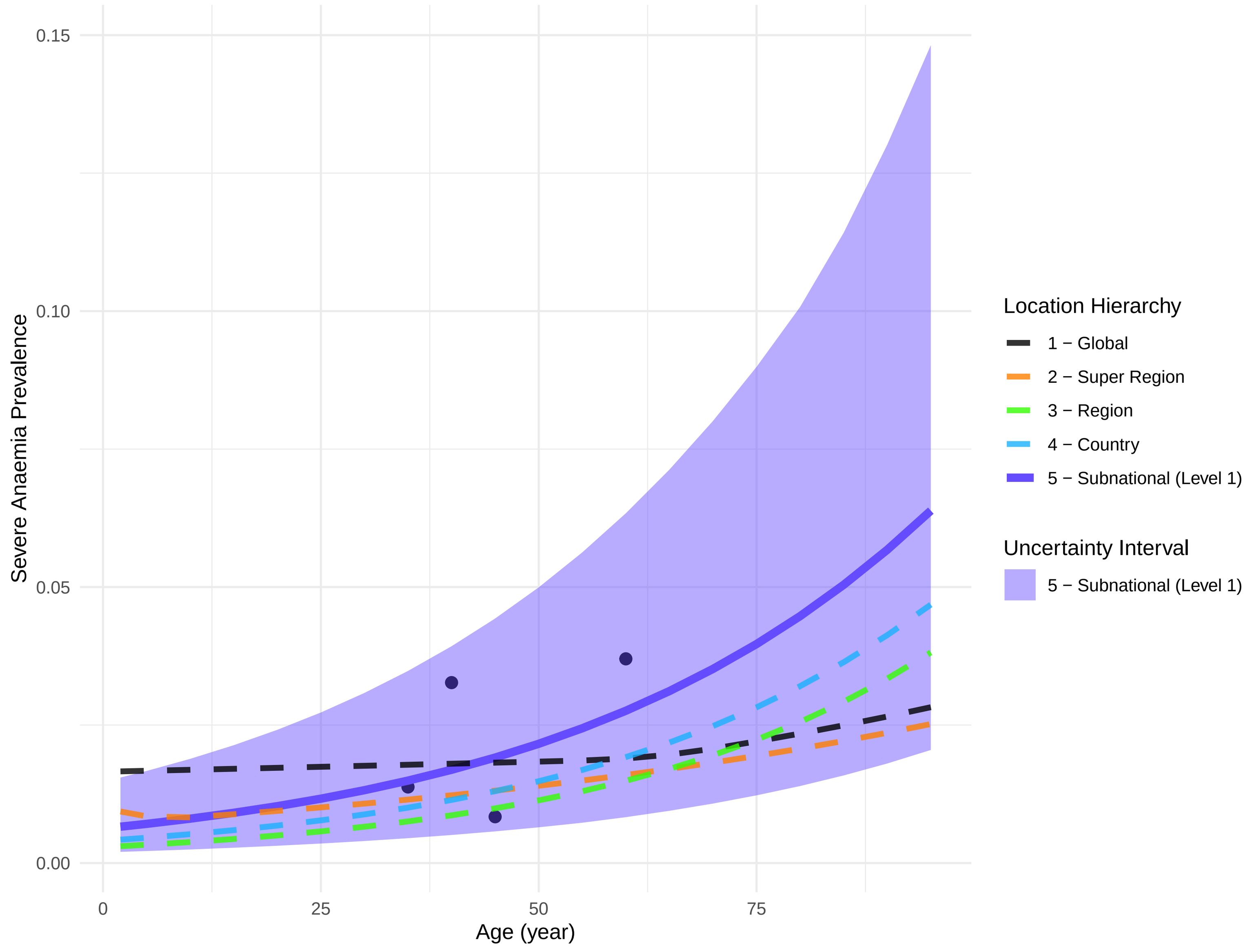

MR−BRT Age/Sex Fits − Non−pregnant Females 2+ Years Old − Severe Anaemia Prevalence − Campeche
WHO−2001 Adjustment Method

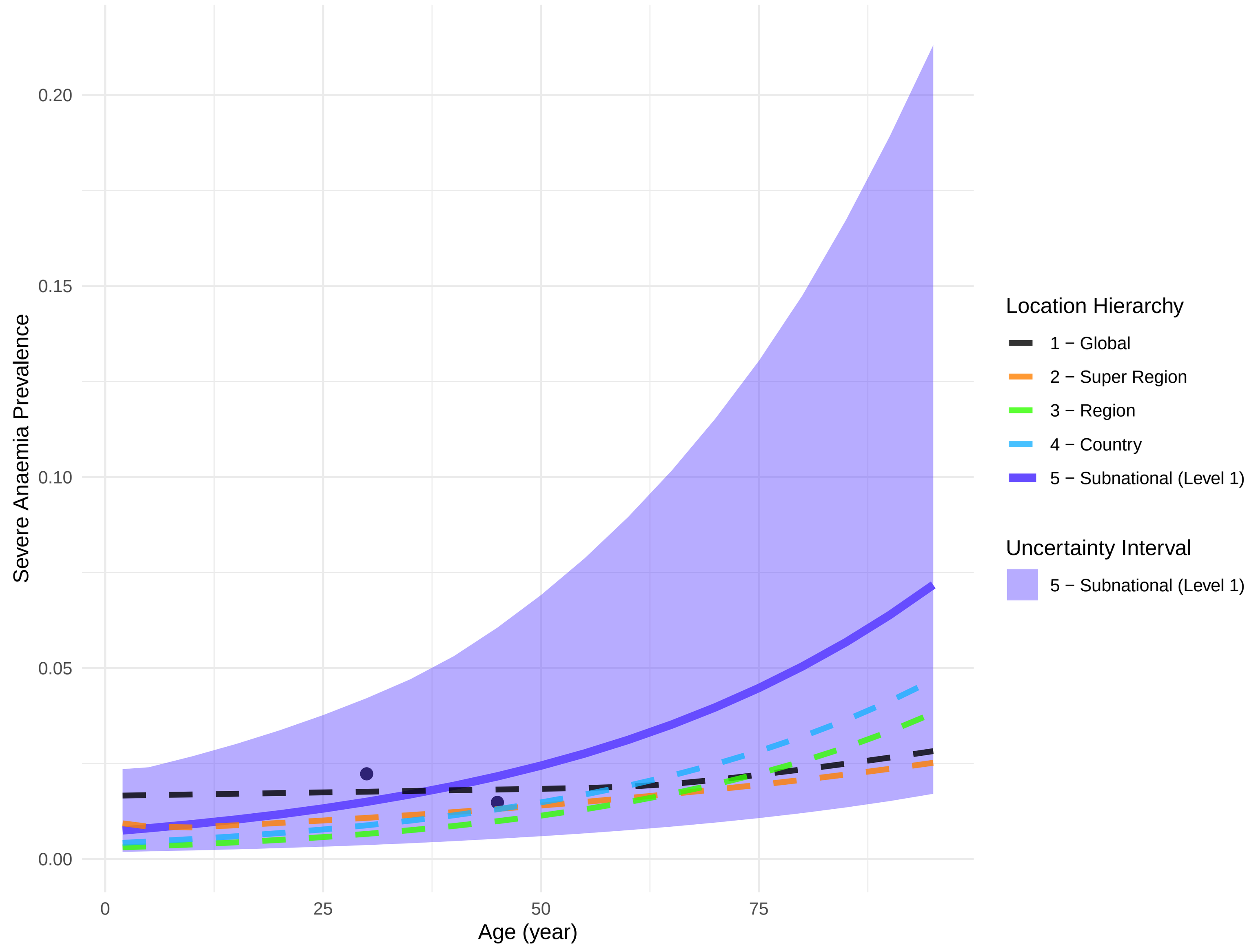

MR−BRT Age/Sex Fits − Non−pregnant Females 2+ Years Old − Severe Anaemia Prevalence − Chiapas
WHO−2001 Adjustment Method

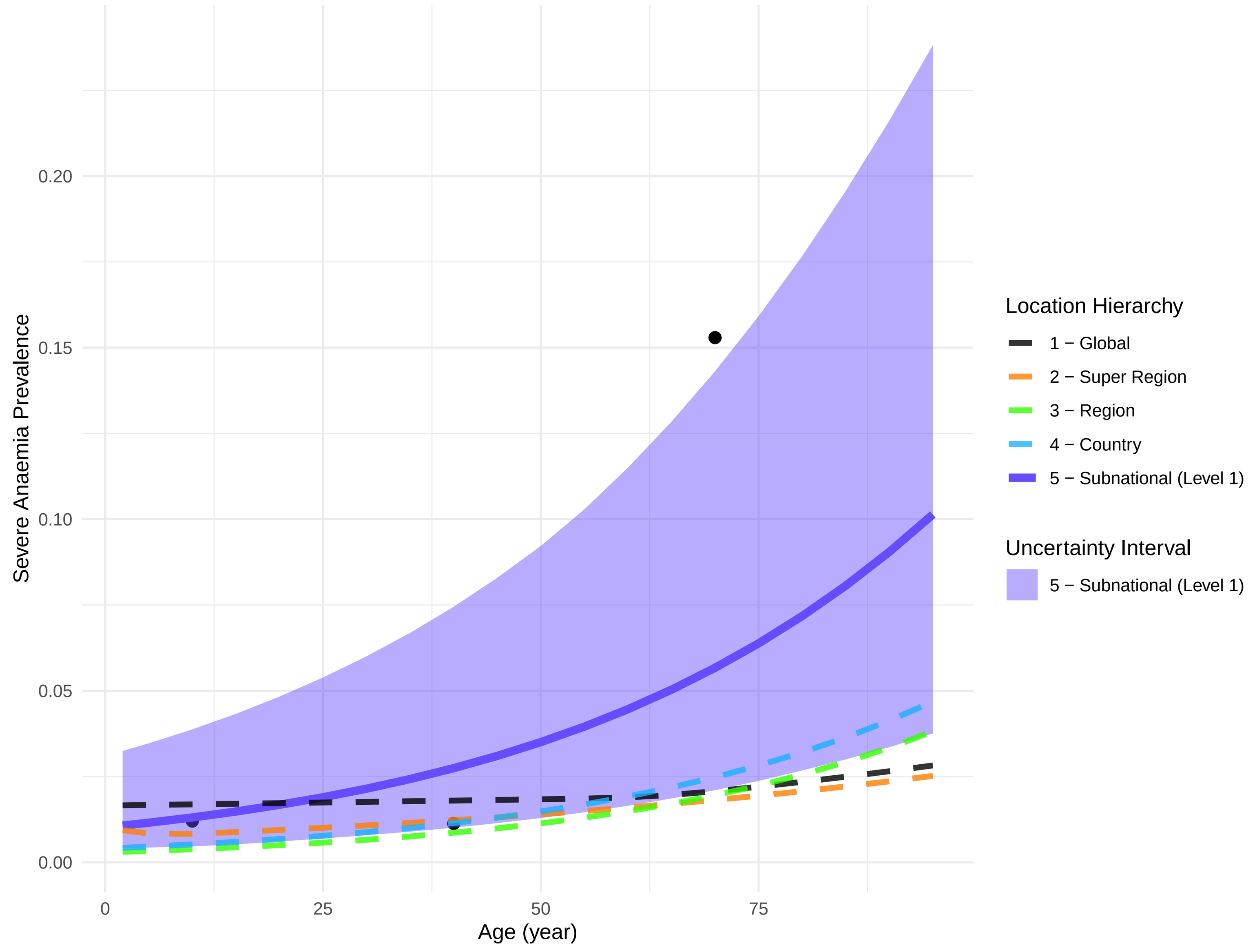

MR−BRT Age/Sex Fits − Non−pregnant Females 2+ Years Old − Severe Anaemia Prevalence − Chihuahua
WHO−2001 Adjustment Method

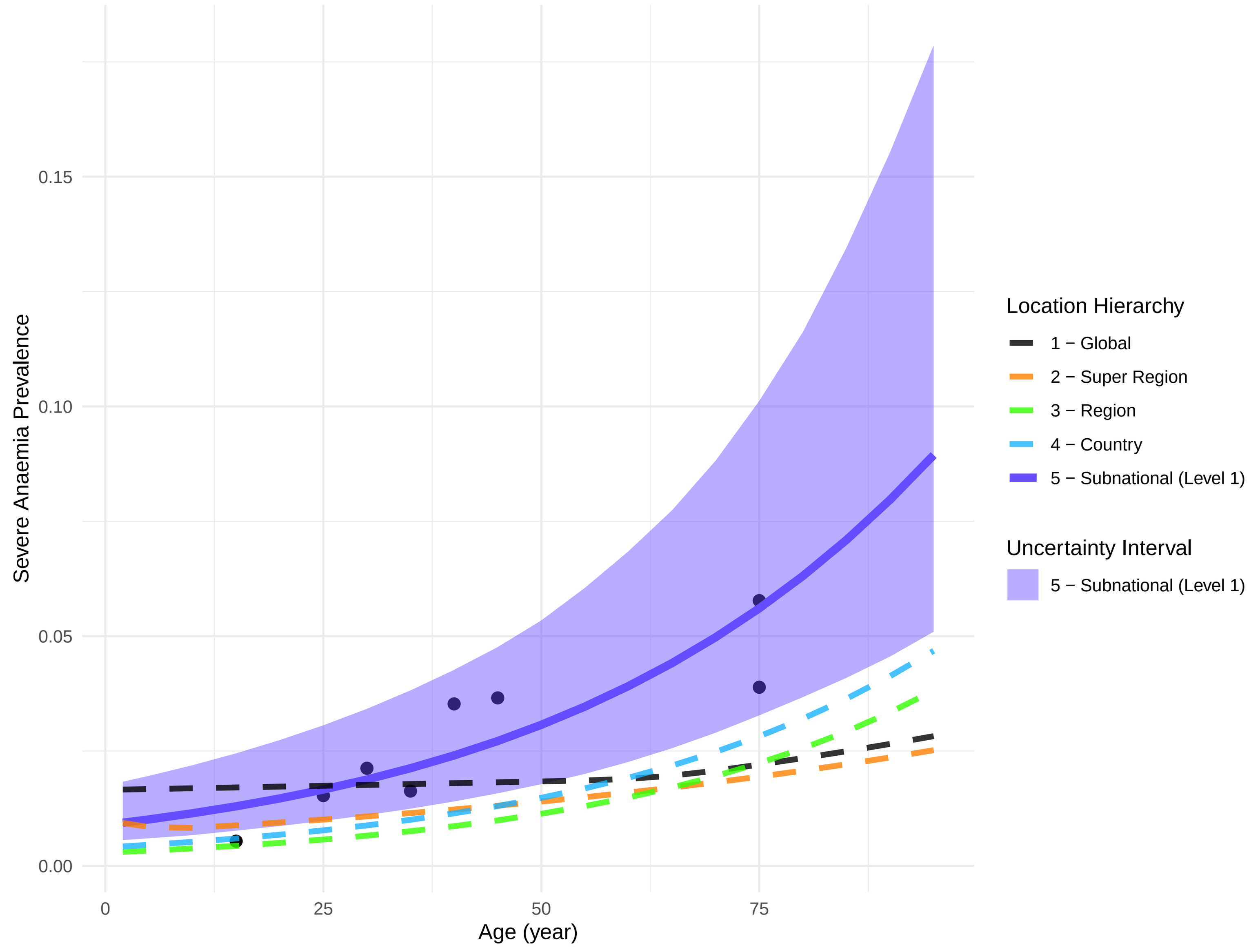

MR−BRT Age/Sex Fits − Non−pregnant Females 2+ Years Old − Severe Anaemia Prevalence − Coahuila
WHO−2001 Adjustment Method

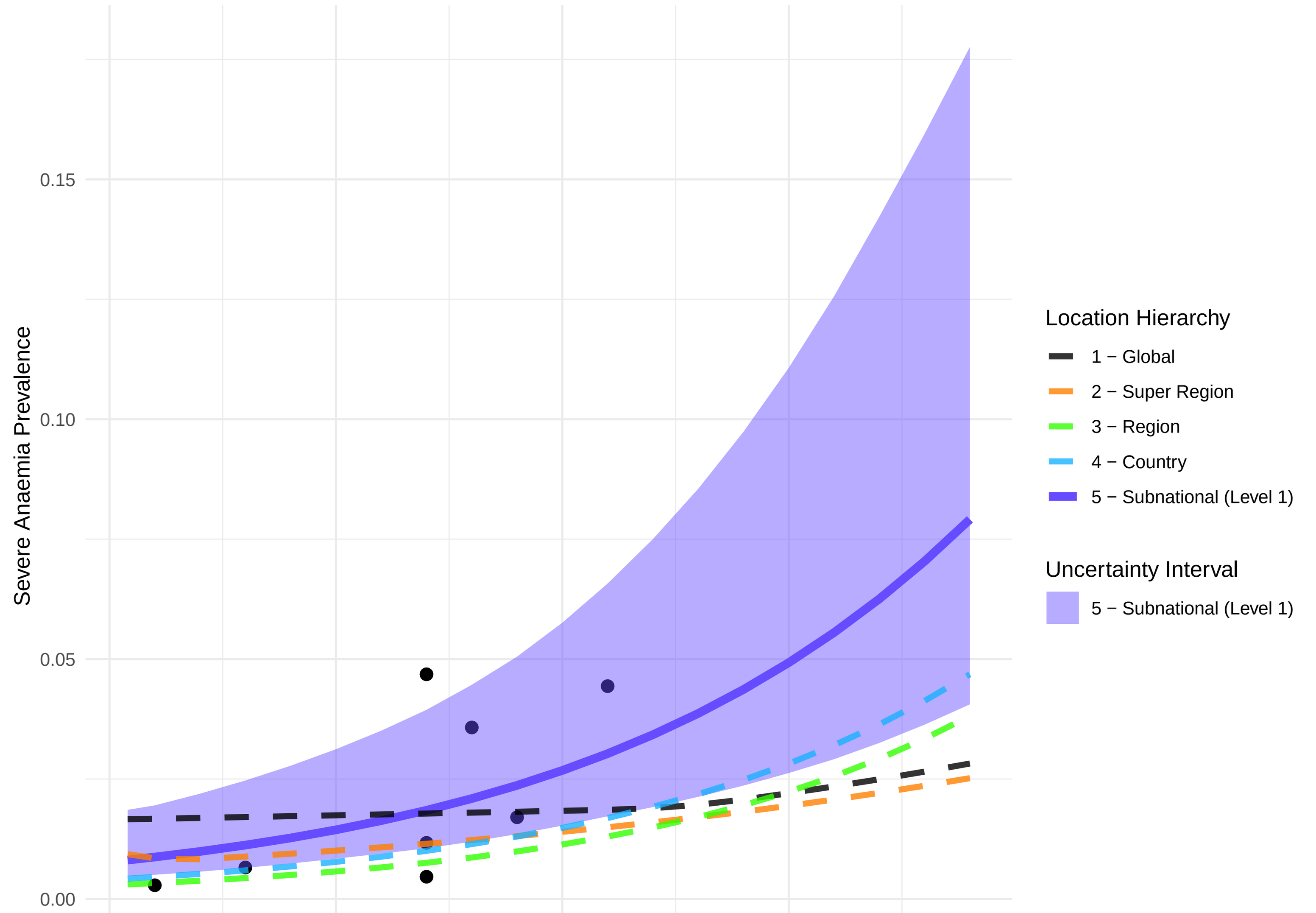

MR−BRT Age/Sex Fits − Non−pregnant Females 2+ Years Old − Severe Anaemia Prevalence − Colima
WHO−2001 Adjustment Method

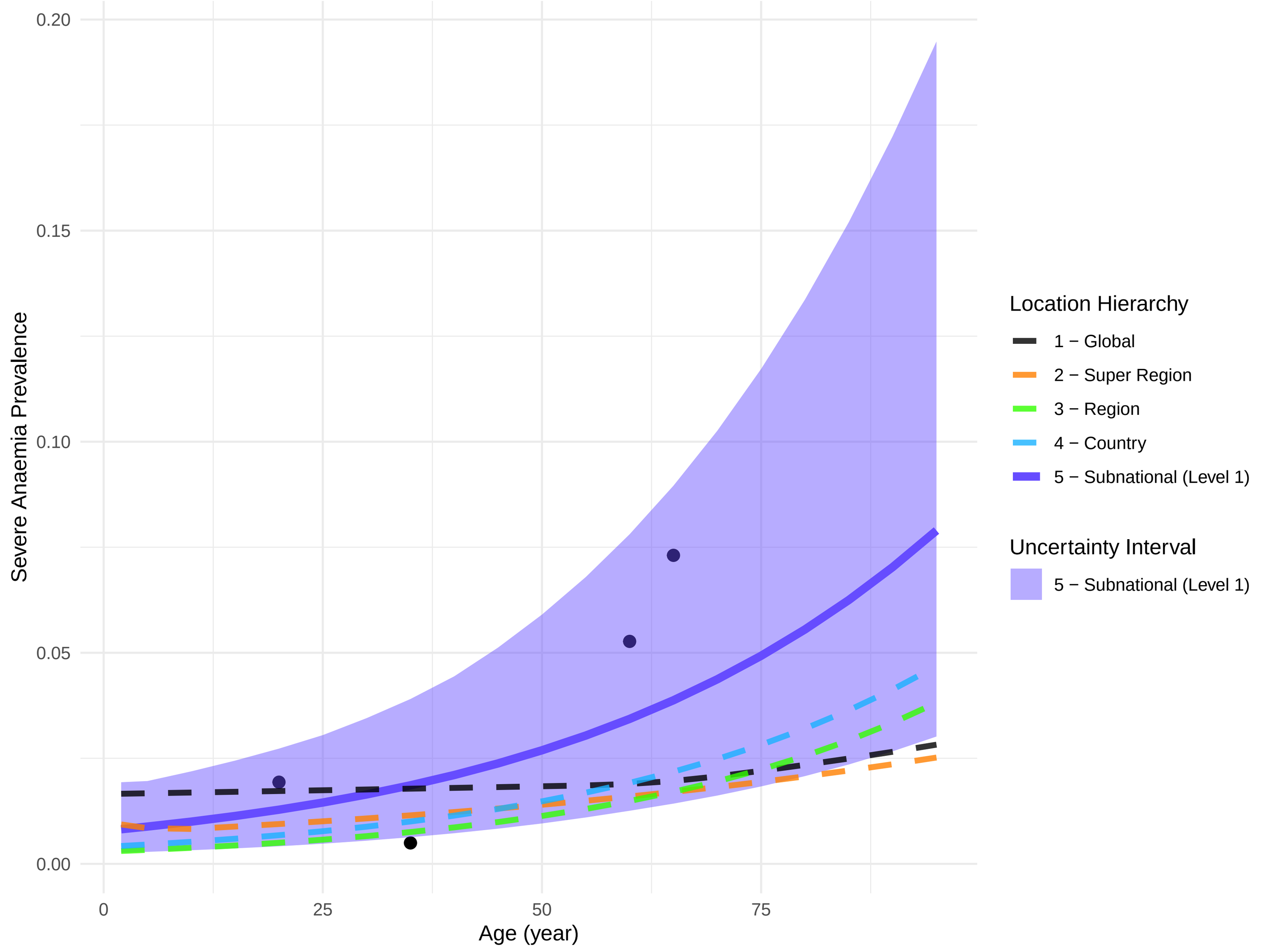

MR−BRT Age/Sex Fits − Non−pregnant Females 2+ Years Old − Severe Anaemia Prevalence − Durango
WHO−2001 Adjustment Method

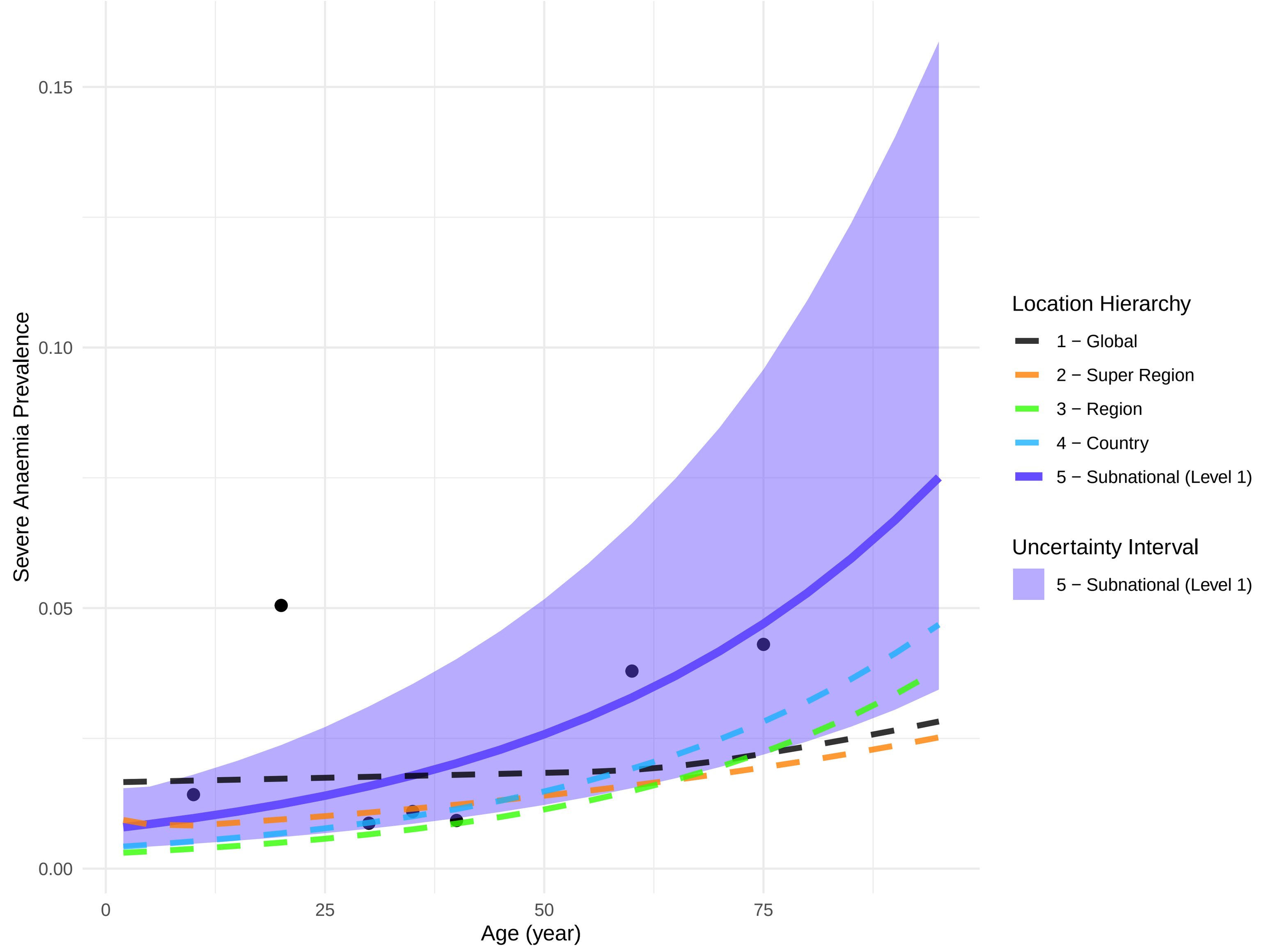

MR−BRT Age/Sex Fits − Non−pregnant Females 2+ Years Old − Severe Anaemia Prevalence − Guanajuato
WHO−2001 Adjustment Method

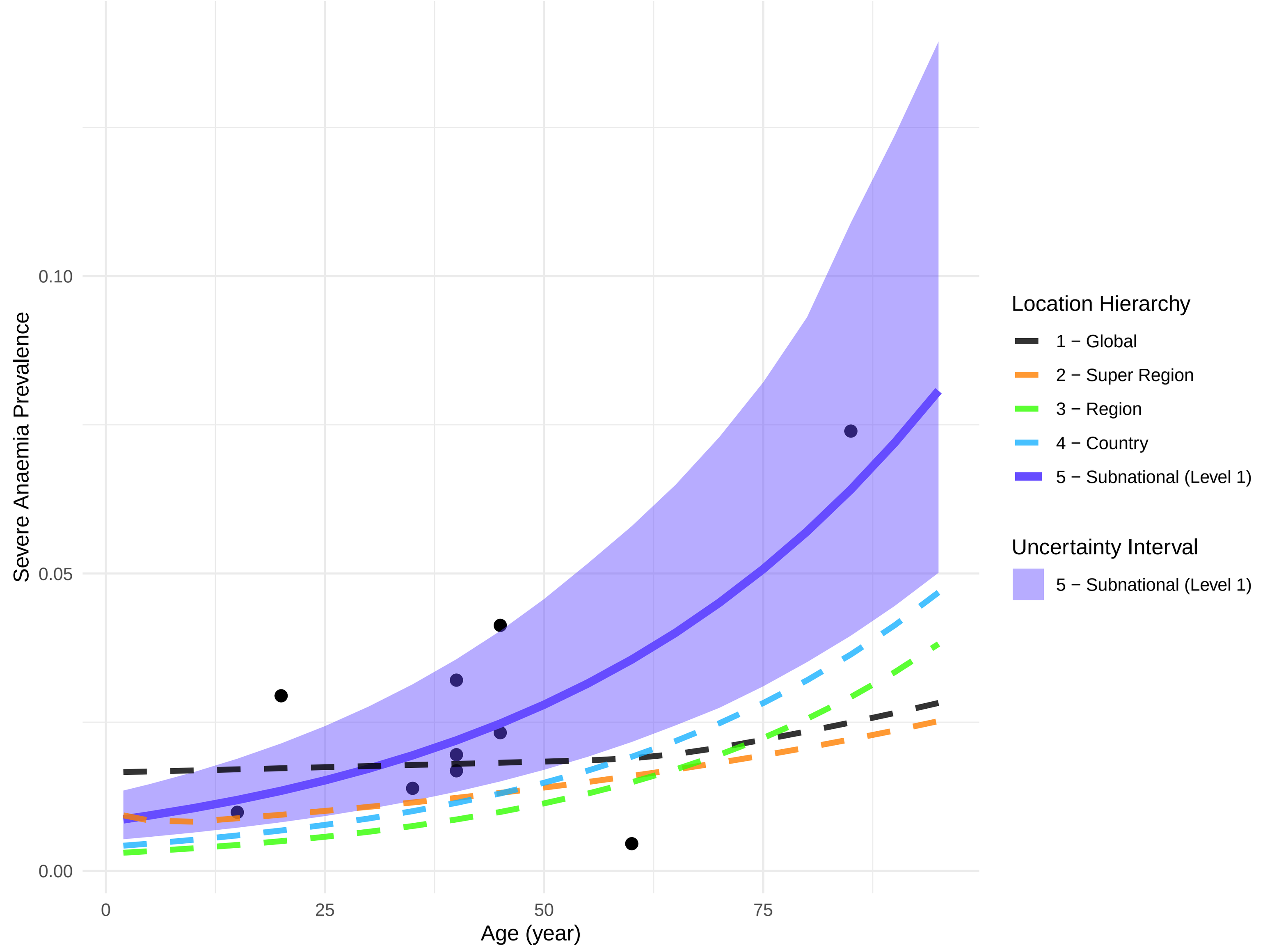

MR−BRT Age/Sex Fits − Non−pregnant Females 2+ Years Old − Severe Anaemia Prevalence − Guerrero
WHO−2001 Adjustment Method

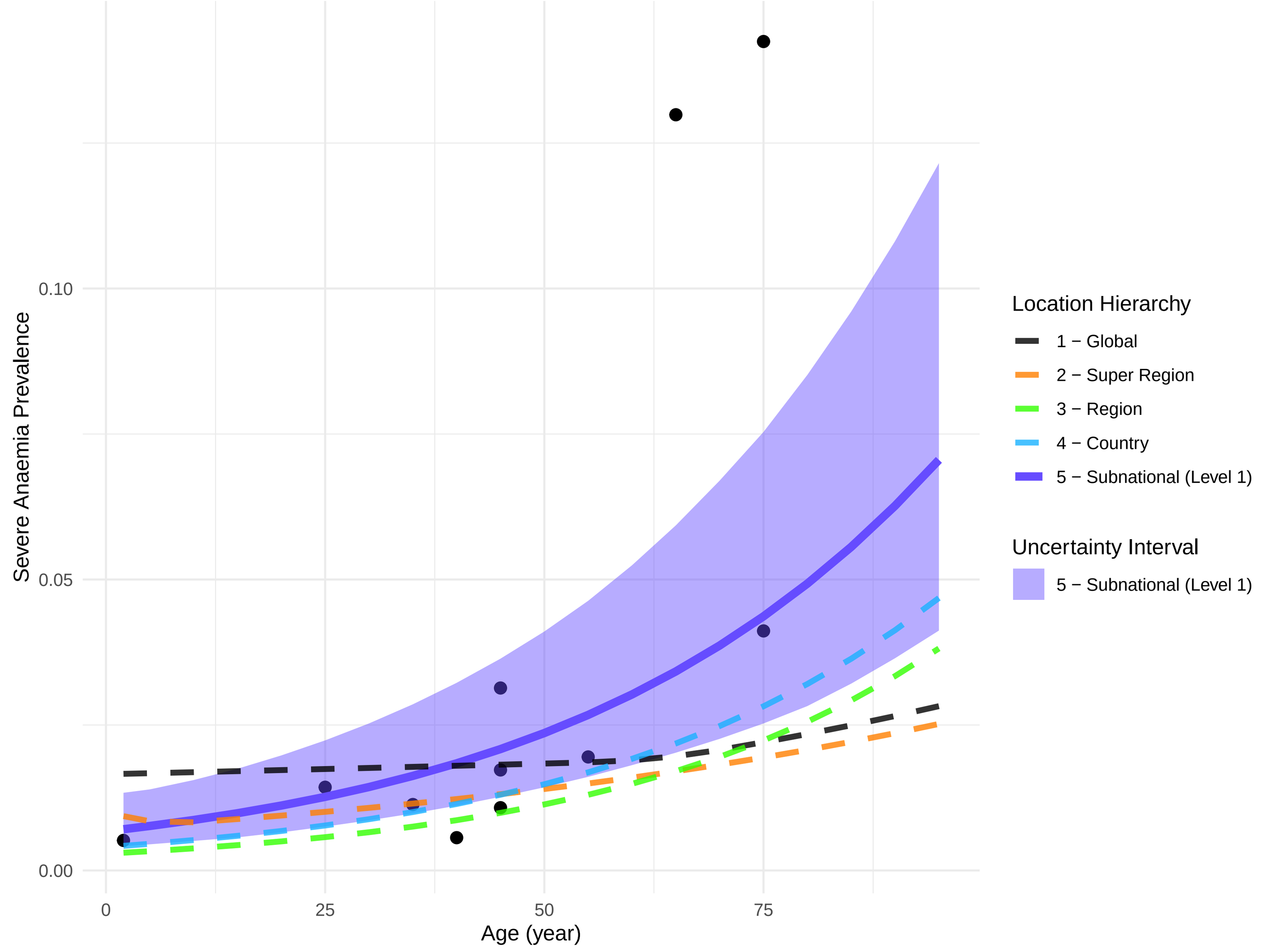

MR−BRT Age/Sex Fits − Non−pregnant Females 2+ Years Old − Severe Anaemia Prevalence − Hidalgo
WHO−2001 Adjustment Method

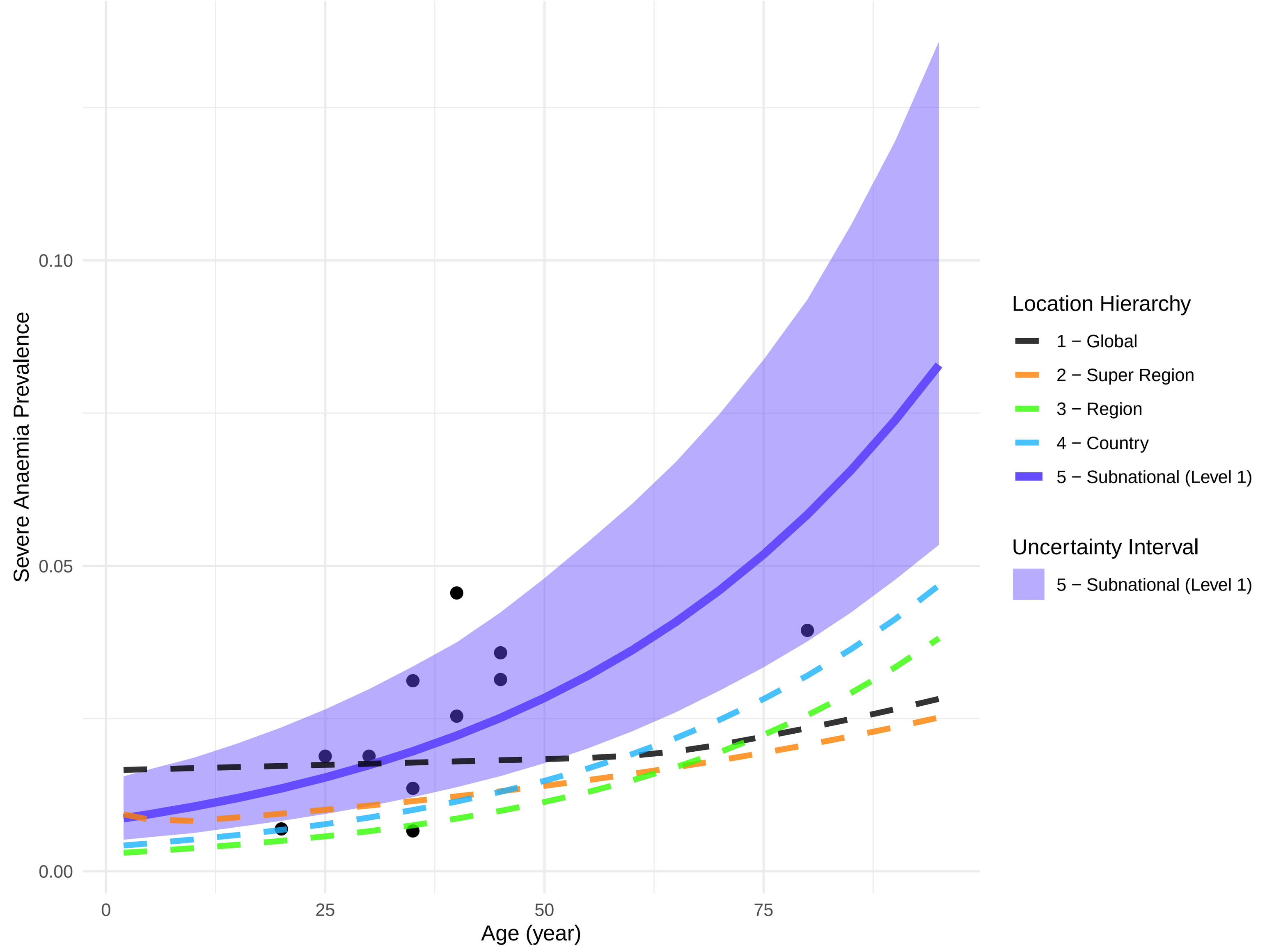

MR−BRT Age/Sex Fits − Non−pregnant Females 2+ Years Old − Severe Anaemia Prevalence − Jalisco
WHO−2001 Adjustment Method

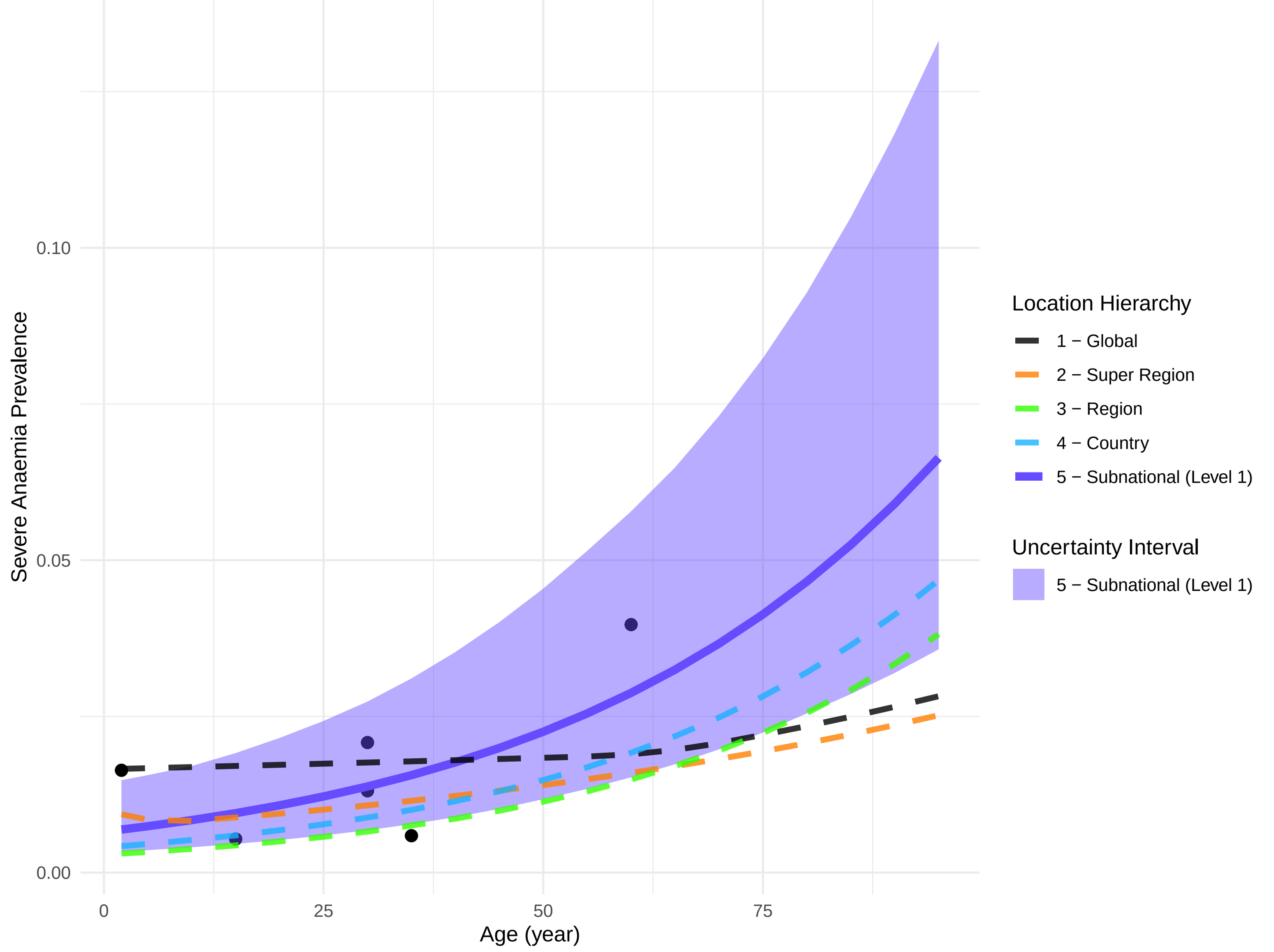

MR−BRT Age/Sex Fits − Non−pregnant Females 2+ Years Old − Severe Anaemia Prevalence − México
WHO−2001 Adjustment Method

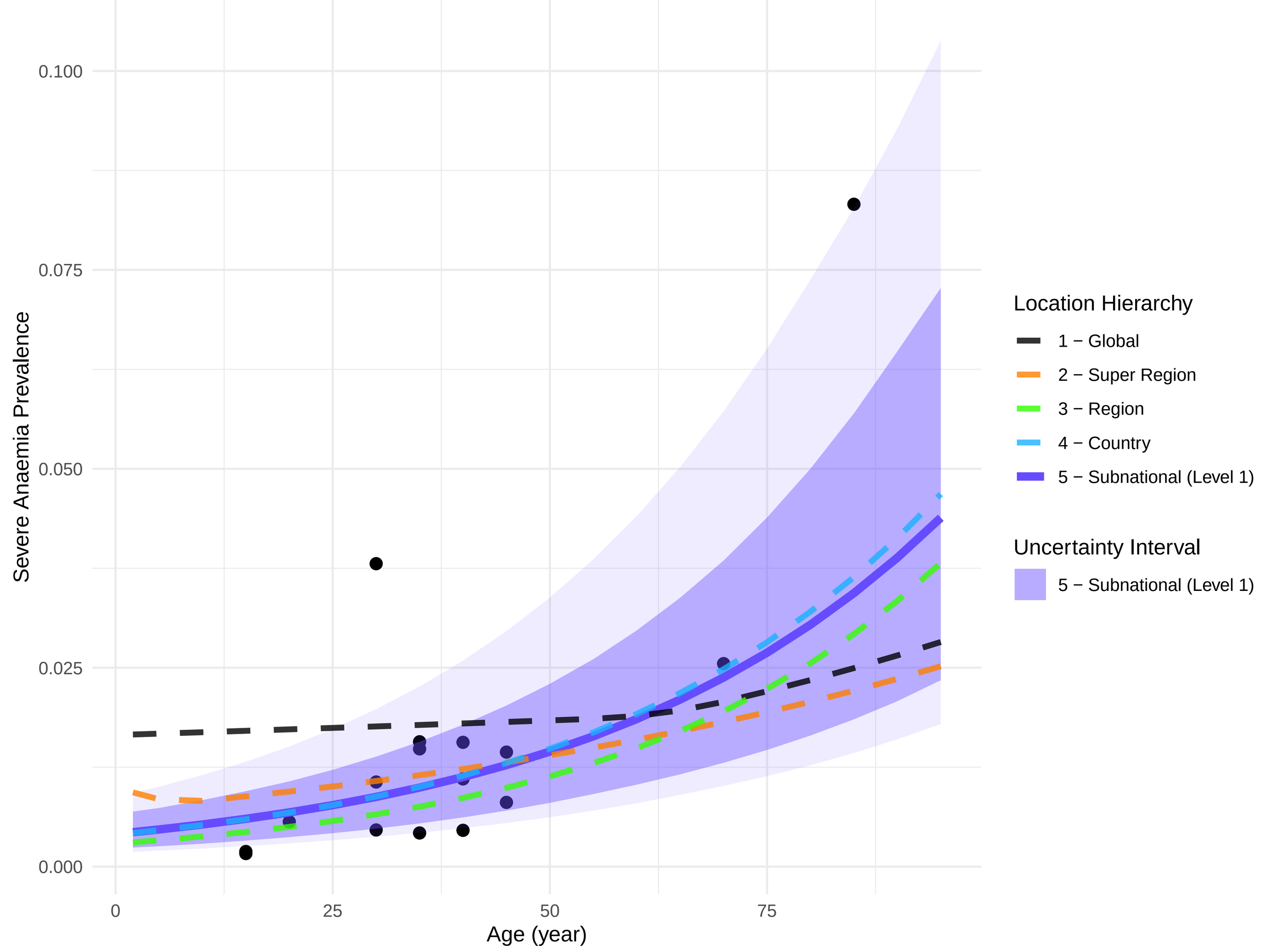

MR−BRT Age/Sex Fits − Non−pregnant Females 2+ Years Old − Severe Anaemia Prevalence − Mexico City
WHO−2001 Adjustment Method

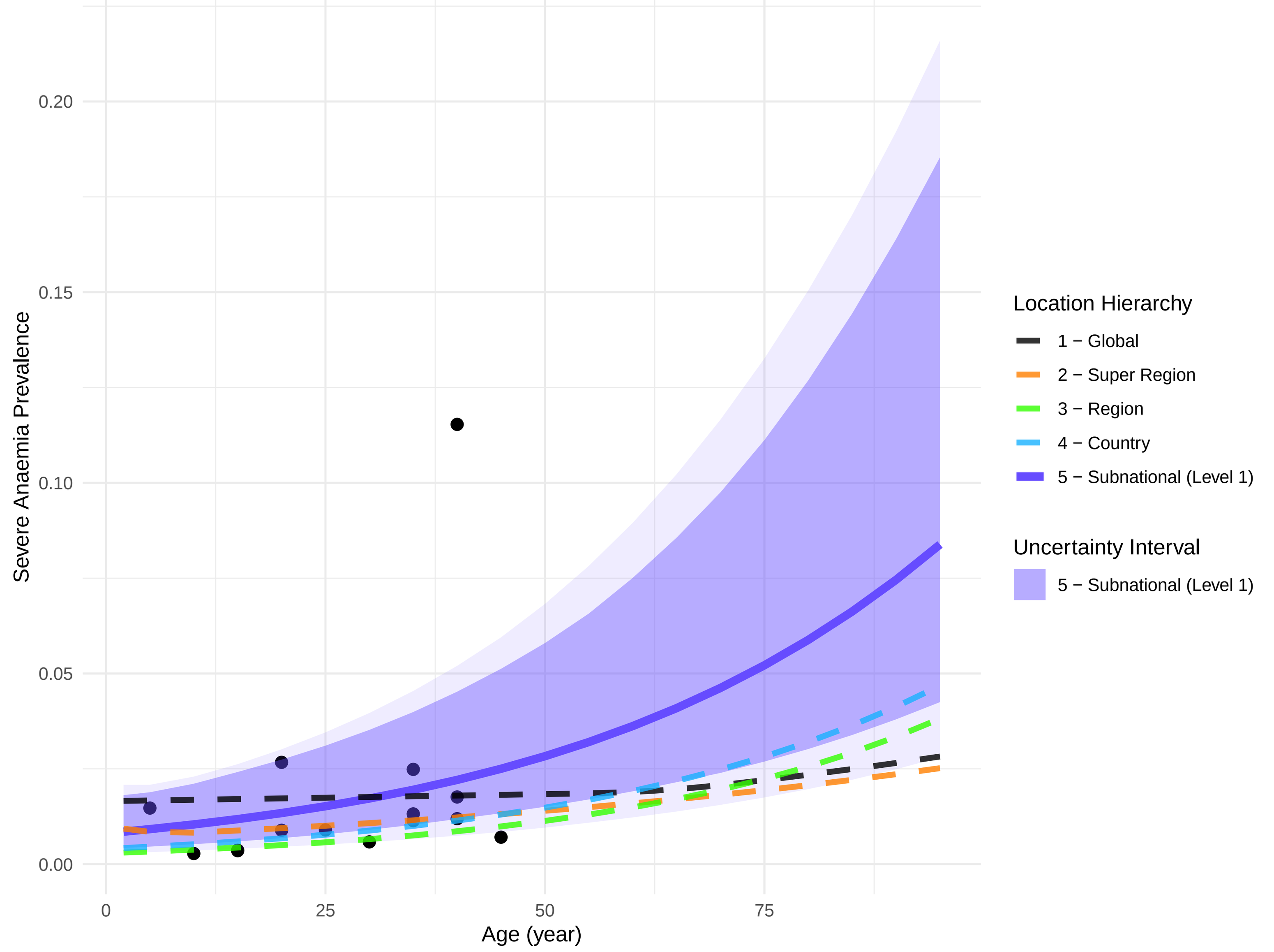

MR−BRT Age/Sex Fits − Non−pregnant Females 2+ Years Old − Severe Anaemia Prevalence − Michoacán de Ocampo
WHO−2001 Adjustment Method

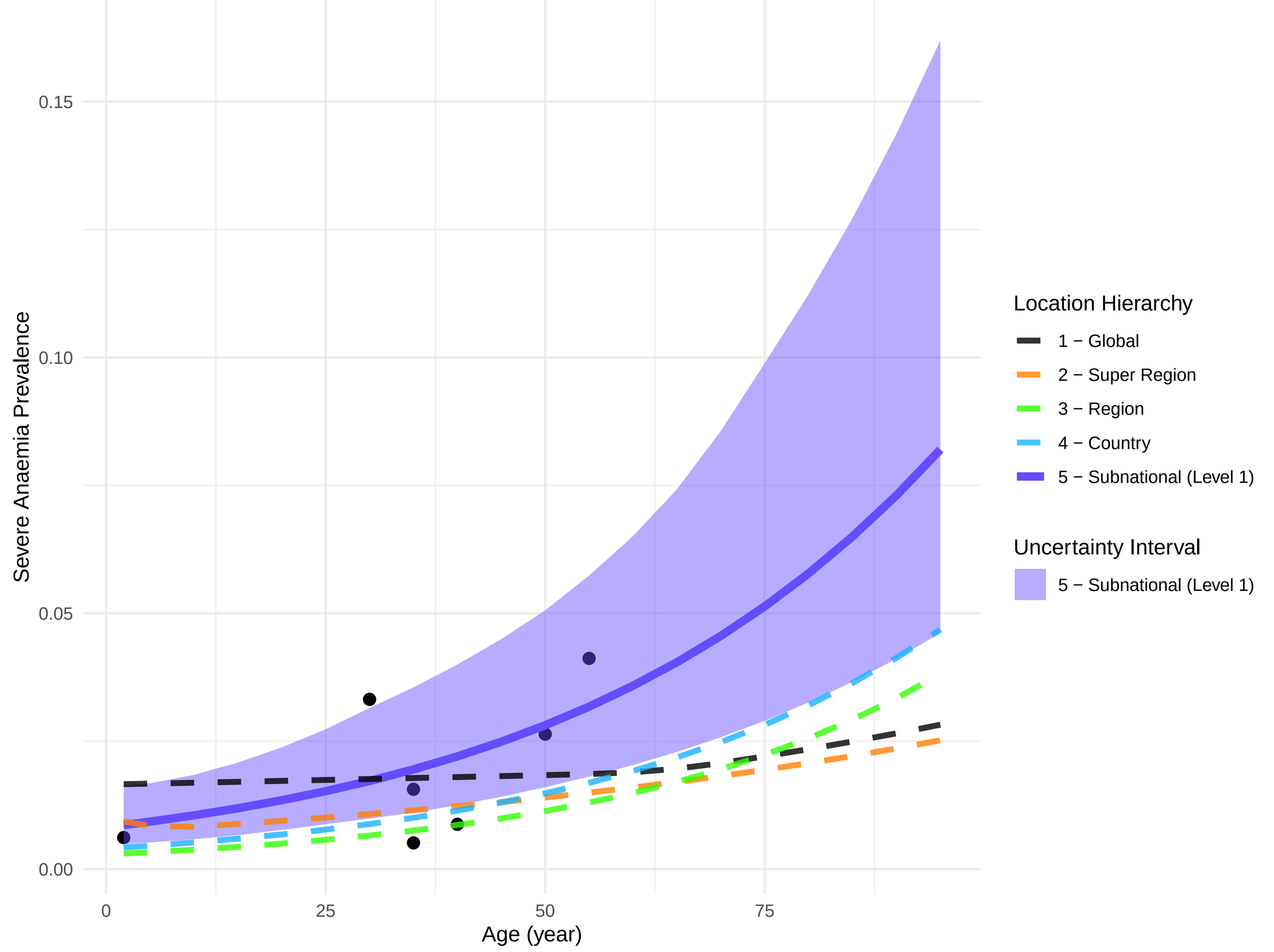

MR−BRT Age/Sex Fits − Non−pregnant Females 2+ Years Old − Severe Anaemia Prevalence − Morelos
WHO−2001 Adjustment Method

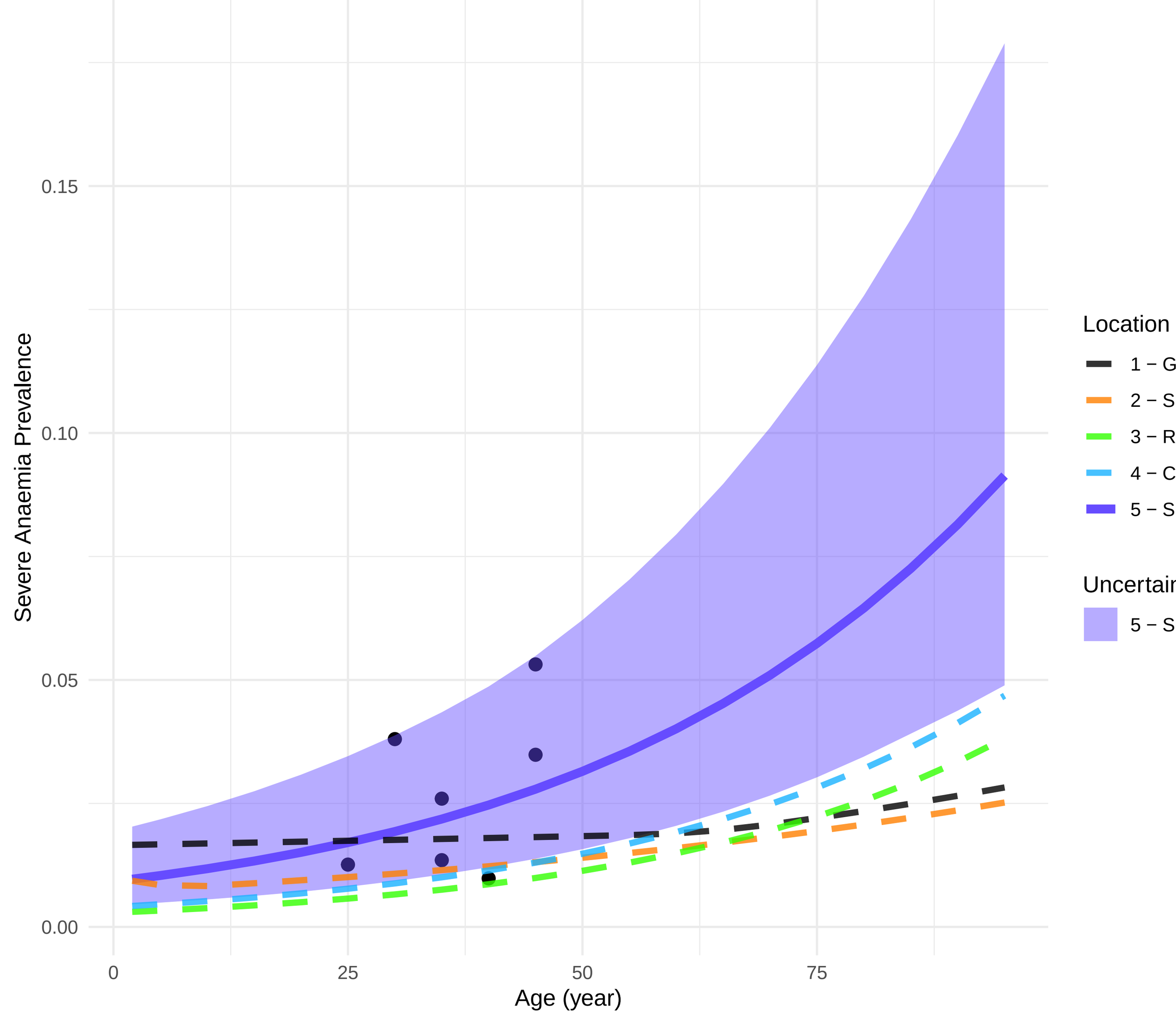

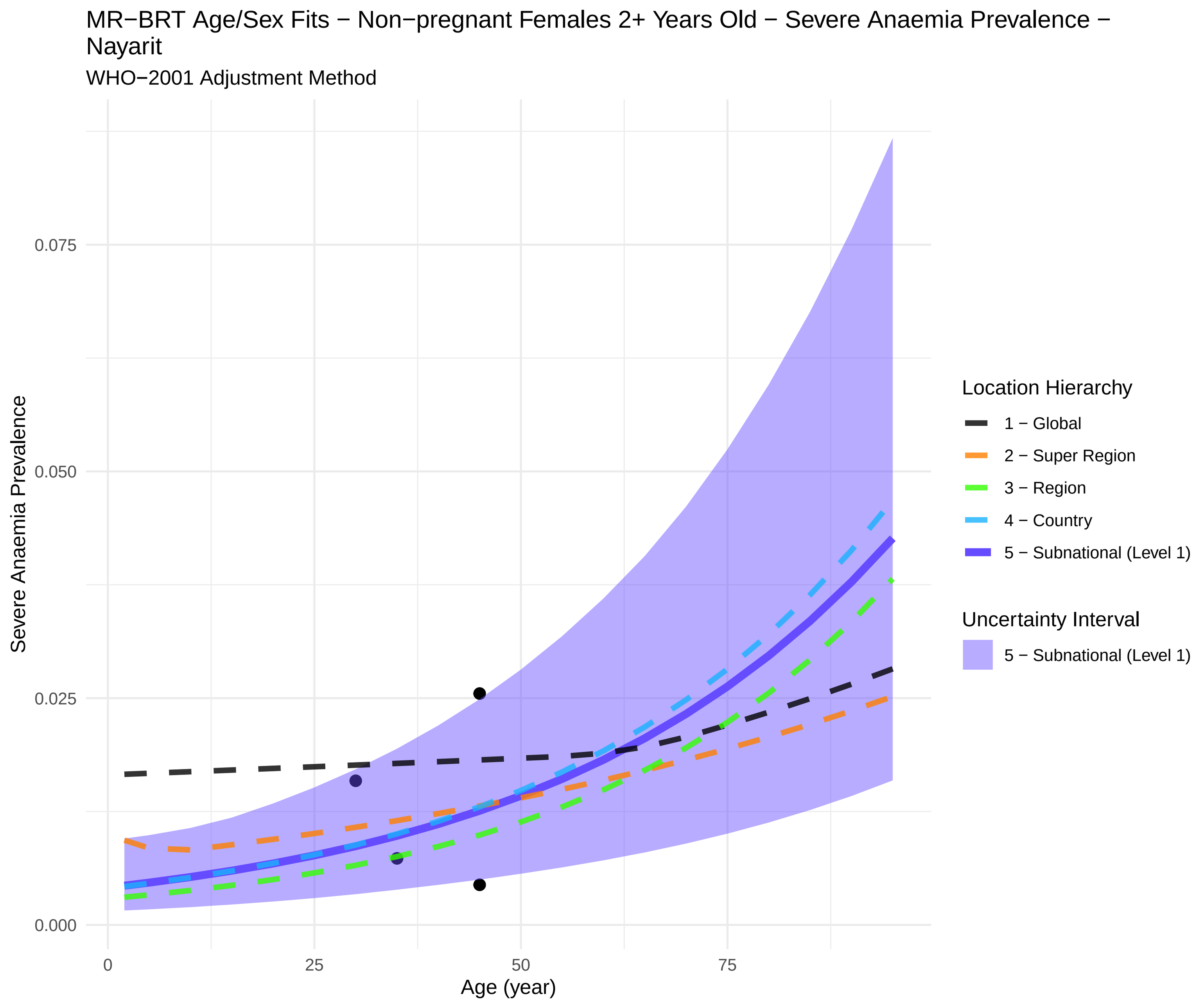
MR−BRT Age/Sex Fits − Non−pregnant Females 2+ Years Old − Severe Anaemia Prevalence − Nayarit
WHO−2001 Adjustment Method
Severe Anaemia Prevalence
Age (year)
Location Hierarchy
1 − Global
2 − Super Region
3 − Region
4 − Country
5 − Subnational (Level 1)
Uncertainty Interval
5 − Subnational (Level 1)

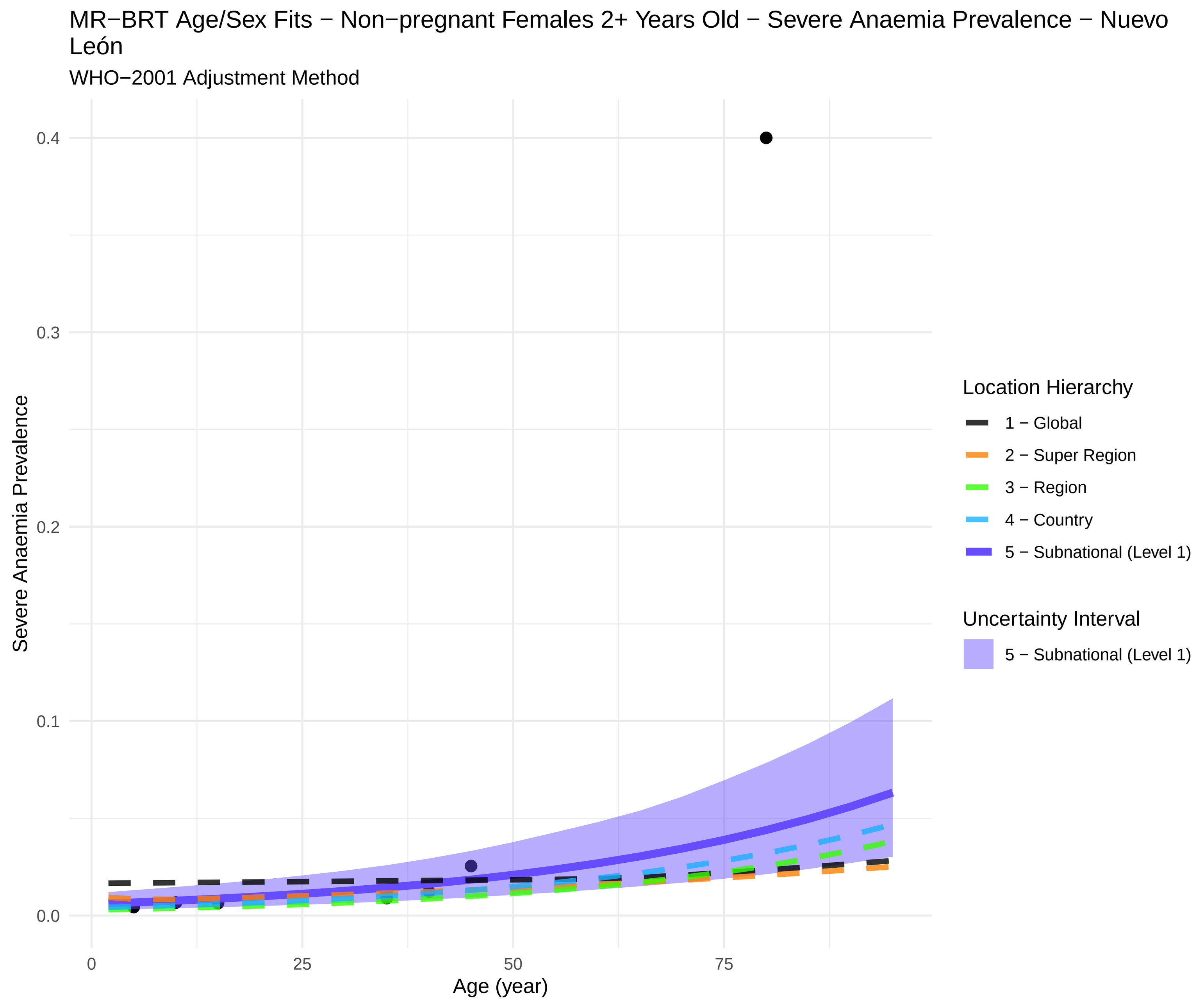
MR−BRT Age/Sex Fits − Non−pregnant Females 2+ Years Old − Severe Anaemia Prevalence − Nuevo León
WHO−2001 Adjustment Method
Severe Anaemia Prevalence
Age (year)
Location Hierarchy
1 − Global
2 − Super Region
3 − Region
4 − Country
5 − Subnational (Level 1)
Uncertainty Interval
5 − Subnational (Level 1)

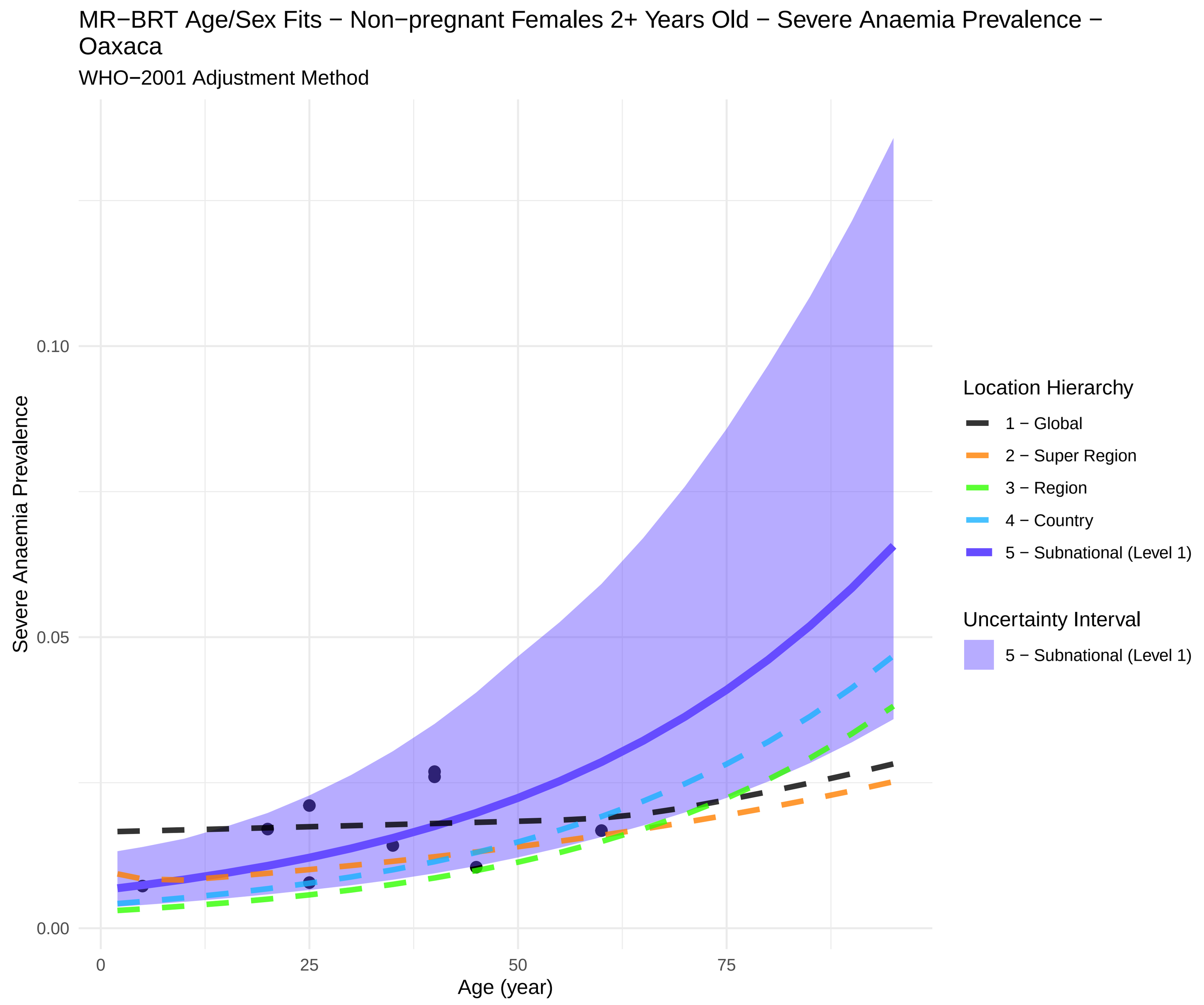
MR−BRT Age/Sex Fits − Non−pregnant Females 2+ Years Old − Severe Anaemia Prevalence − Oaxaca
WHO−2001 Adjustment Method
Severe Anaemia Prevalence
Age (year)
Location Hierarchy
1 − Global
2 − Super Region
3 − Region
4 − Country
5 − Subnational (Level 1)
Uncertainty Interval
5 − Subnational (Level 1)

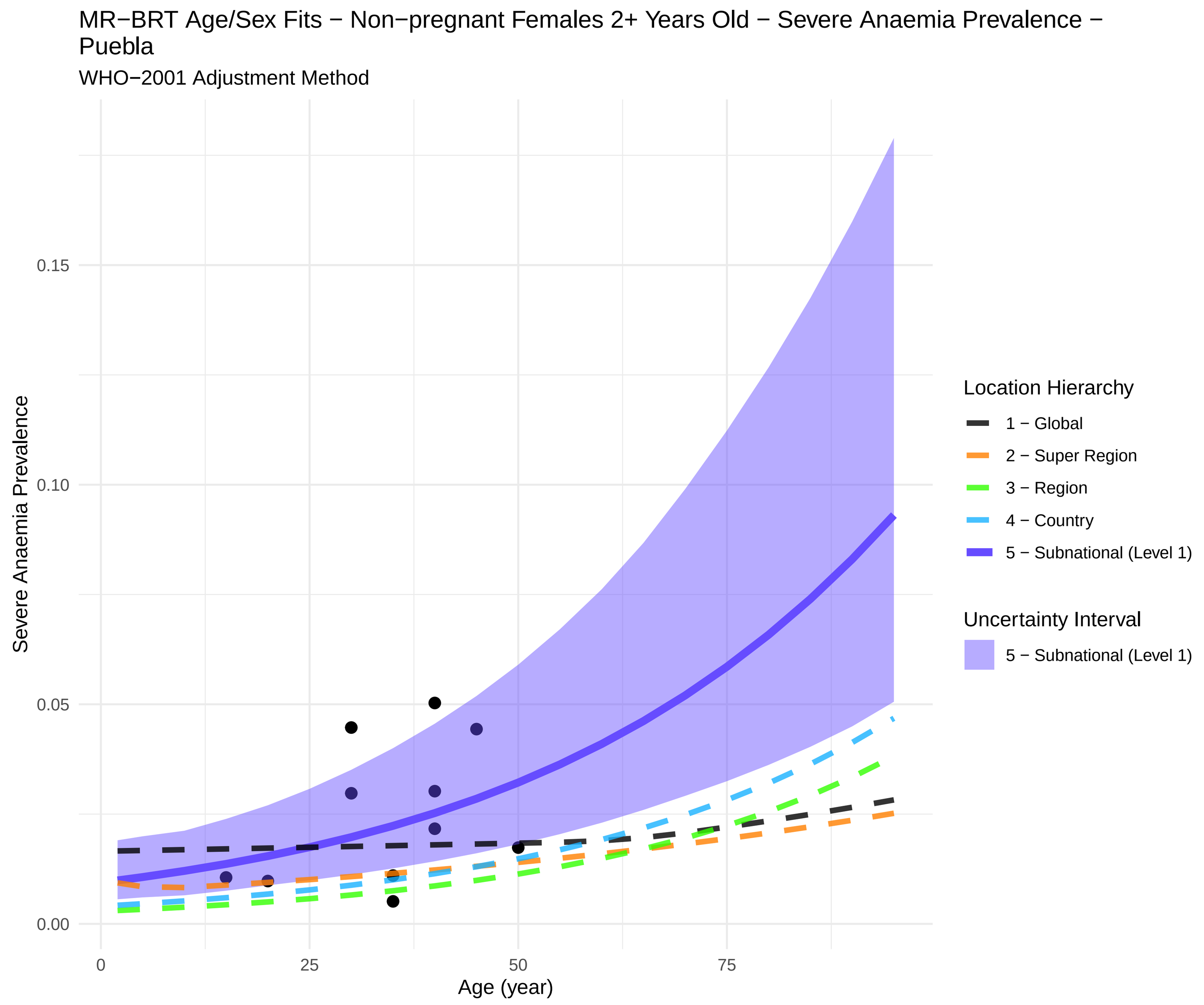
MR−BRT Age/Sex Fits − Non−pregnant Females 2+ Years Old − Severe Anaemia Prevalence − Puebla
WHO−2001 Adjustment Method
Severe Anaemia Prevalence
Age (year)
Location Hierarchy
1 − Global
2 − Super Region
3 − Region
4 − Country
5 − Subnational (Level 1)
Uncertainty Interval
5 − Subnational (Level 1)

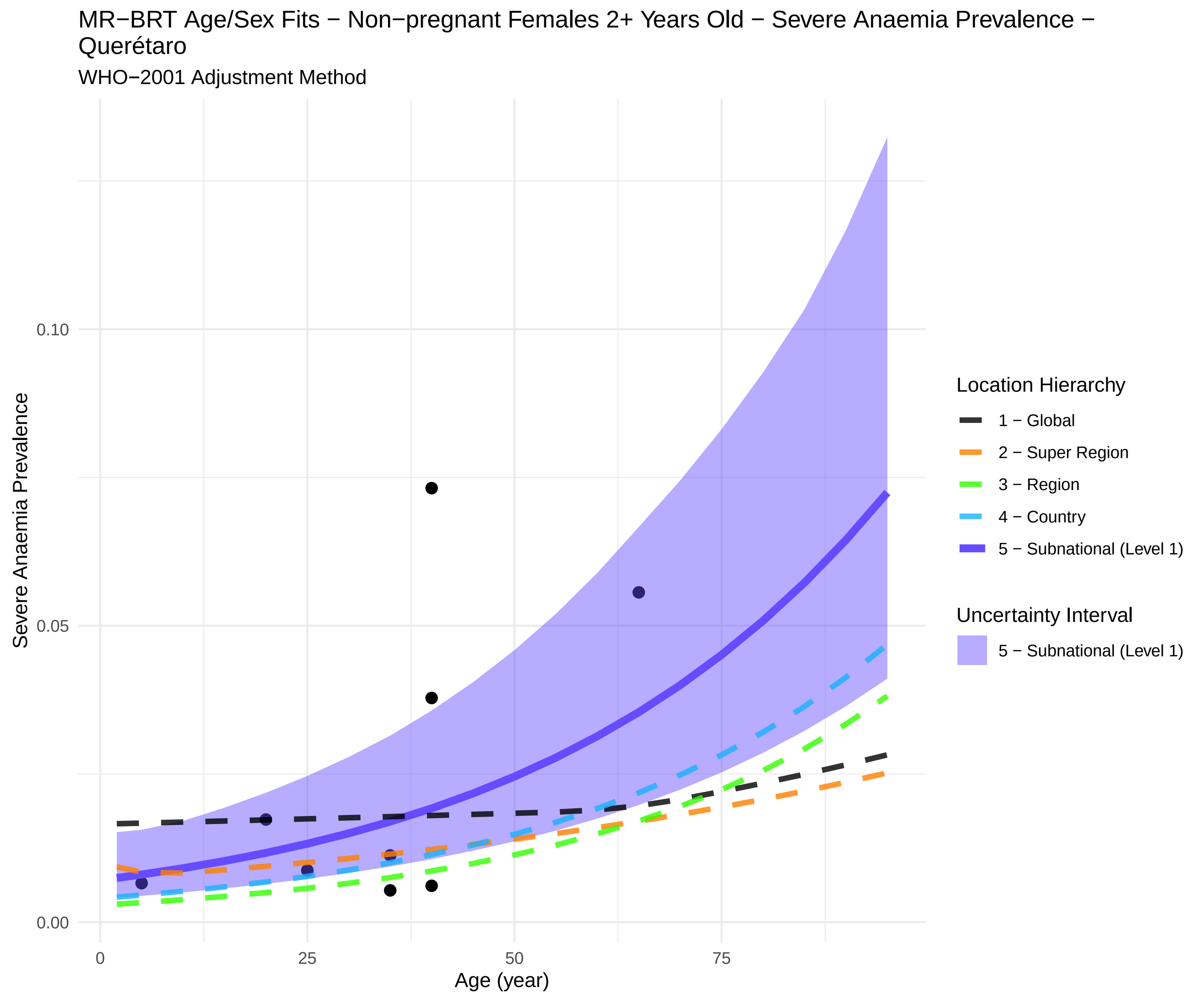
MR−BRT Age/Sex Fits − Non−pregnant Females 2+ Years Old − Severe Anaemia Prevalence − Querétaro
WHO−2001 Adjustment Method
Severe Anaemia Prevalence
Age (year)
Location Hierarchy
1 − Global
2 − Super Region
3 − Region
4 − Country
5 − Subnational (Level 1)
Uncertainty Interval
5 − Subnational (Level 1)

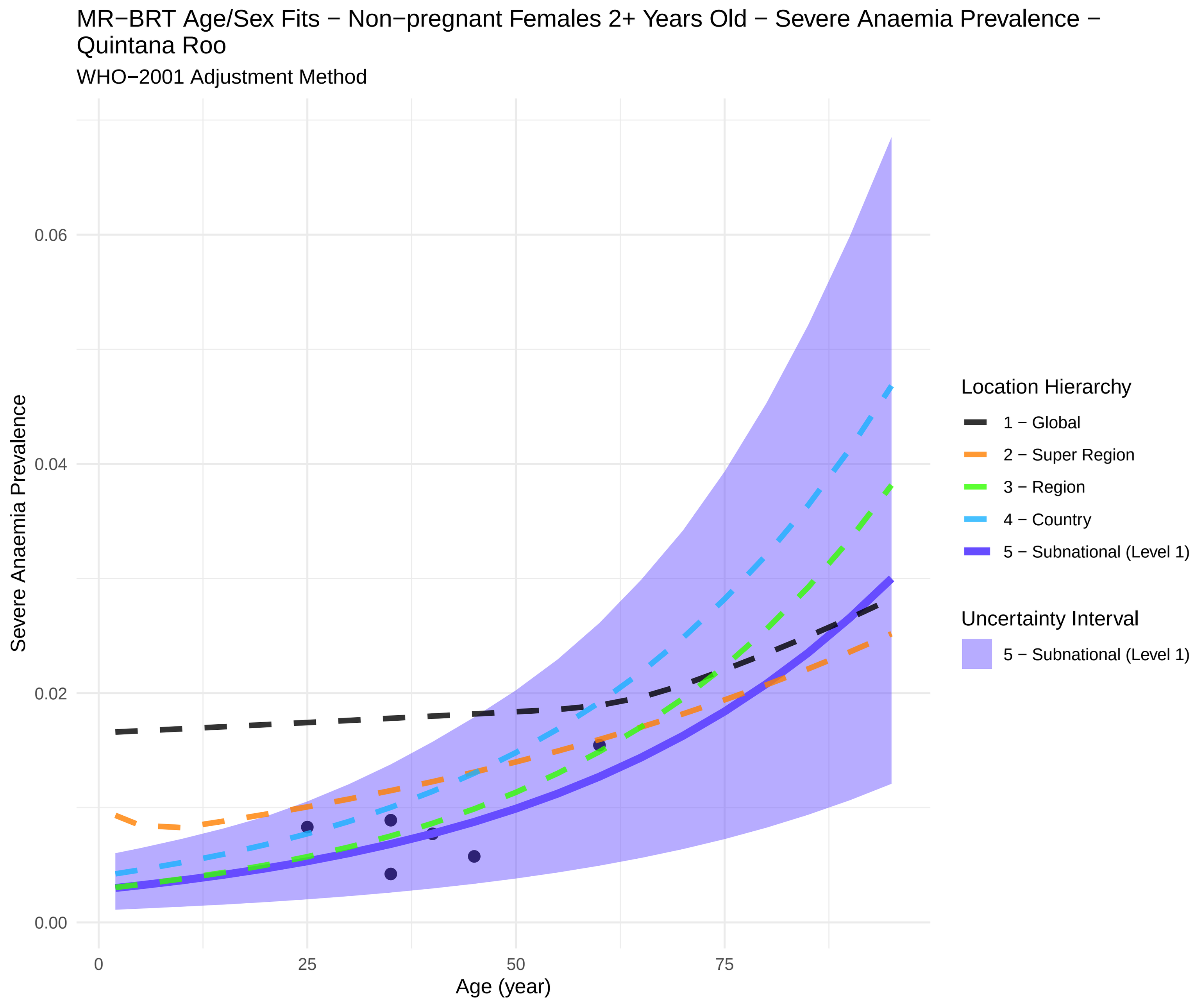
MR−BRT Age/Sex Fits − Non−pregnant Females 2+ Years Old − Severe Anaemia Prevalence − Quintana Roo
WHO−2001 Adjustment Method
Severe Anaemia Prevalence
Age (year)
Location Hierarchy
1 − Global
2 − Super Region
3 − Region
4 − Country
5 − Subnational (Level 1)
Uncertainty Interval
5 − Subnational (Level 1)

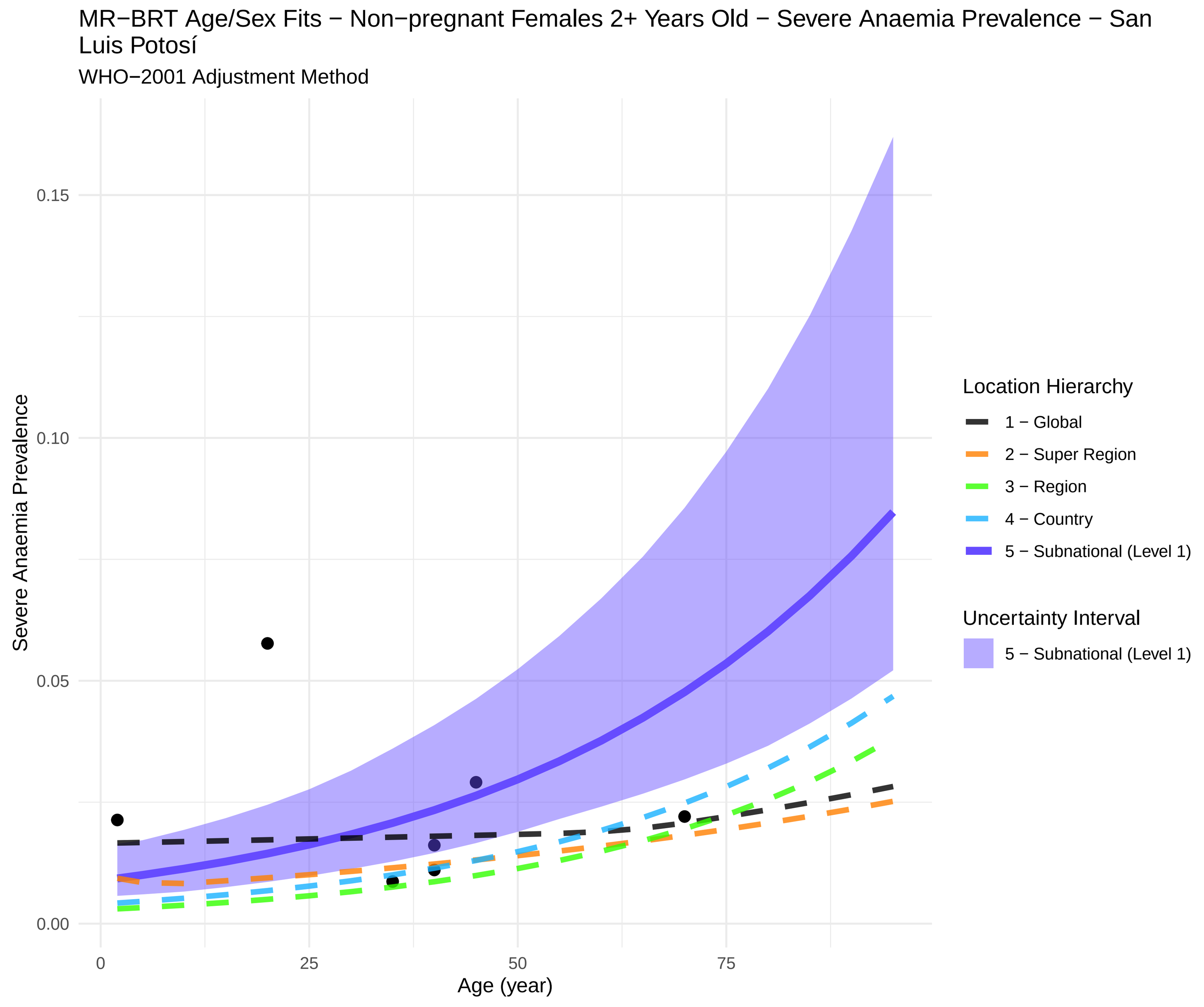
MR−BRT Age/Sex Fits − Non−pregnant Females 2+ Years Old − Severe Anaemia Prevalence − San Luis Potosí
WHO−2001 Adjustment Method
Severe Anaemia Prevalence
Age (year)
Location Hierarchy
1 − Global
2 − Super Region
3 − Region
4 − Country
5 − Subnational (Level 1)
Uncertainty Interval
5 − Subnational (Level 1)

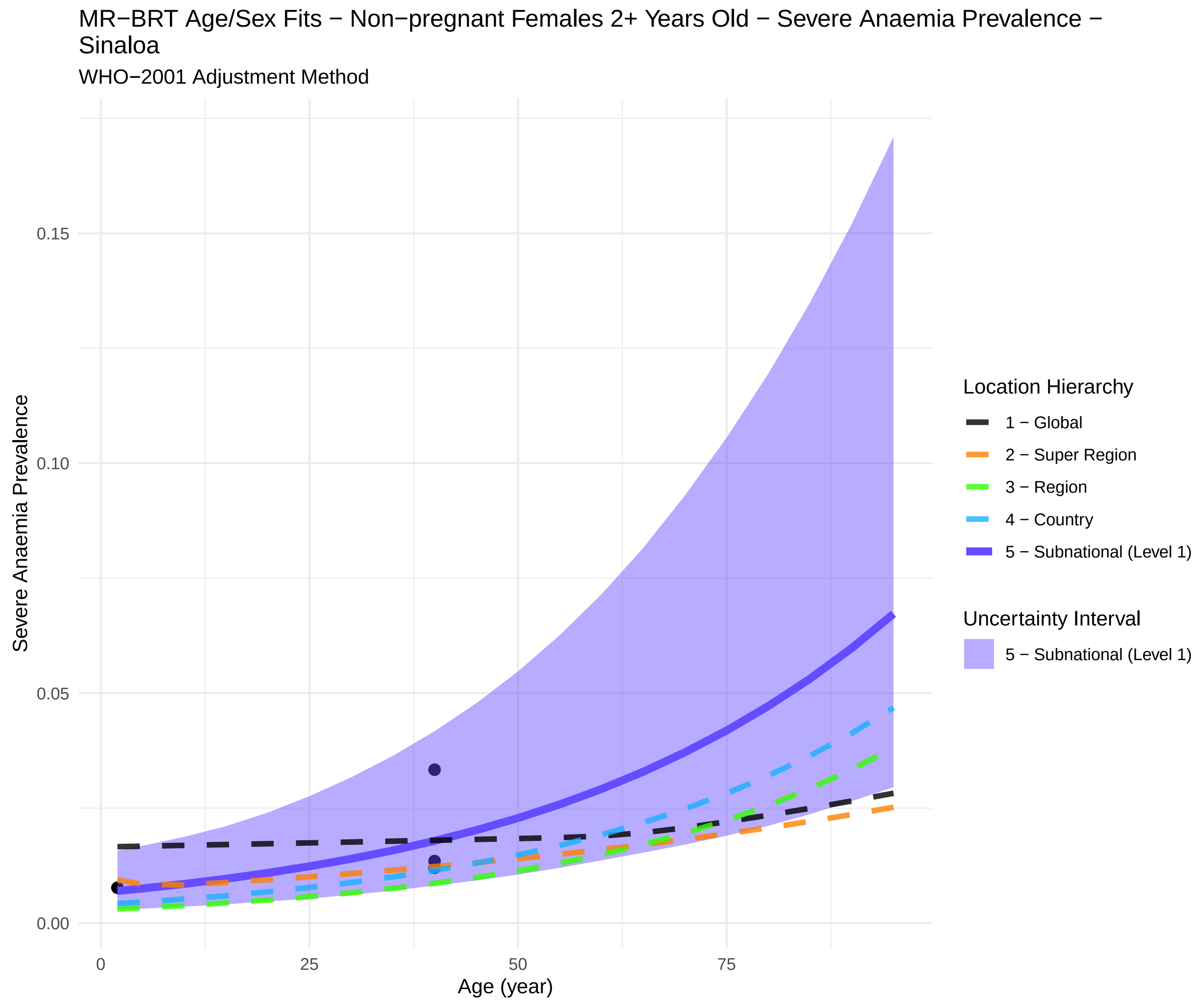
MR−BRT Age/Sex Fits − Non−pregnant Females 2+ Years Old − Severe Anaemia Prevalence − Sinaloa
WHO−2001 Adjustment Method
Severe Anaemia Prevalence
Age (year)
Location Hierarchy
1 − Global
2 − Super Region
3 − Region
4 − Country
5 − Subnational (Level 1)
Uncertainty Interval
5 − Subnational (Level 1)

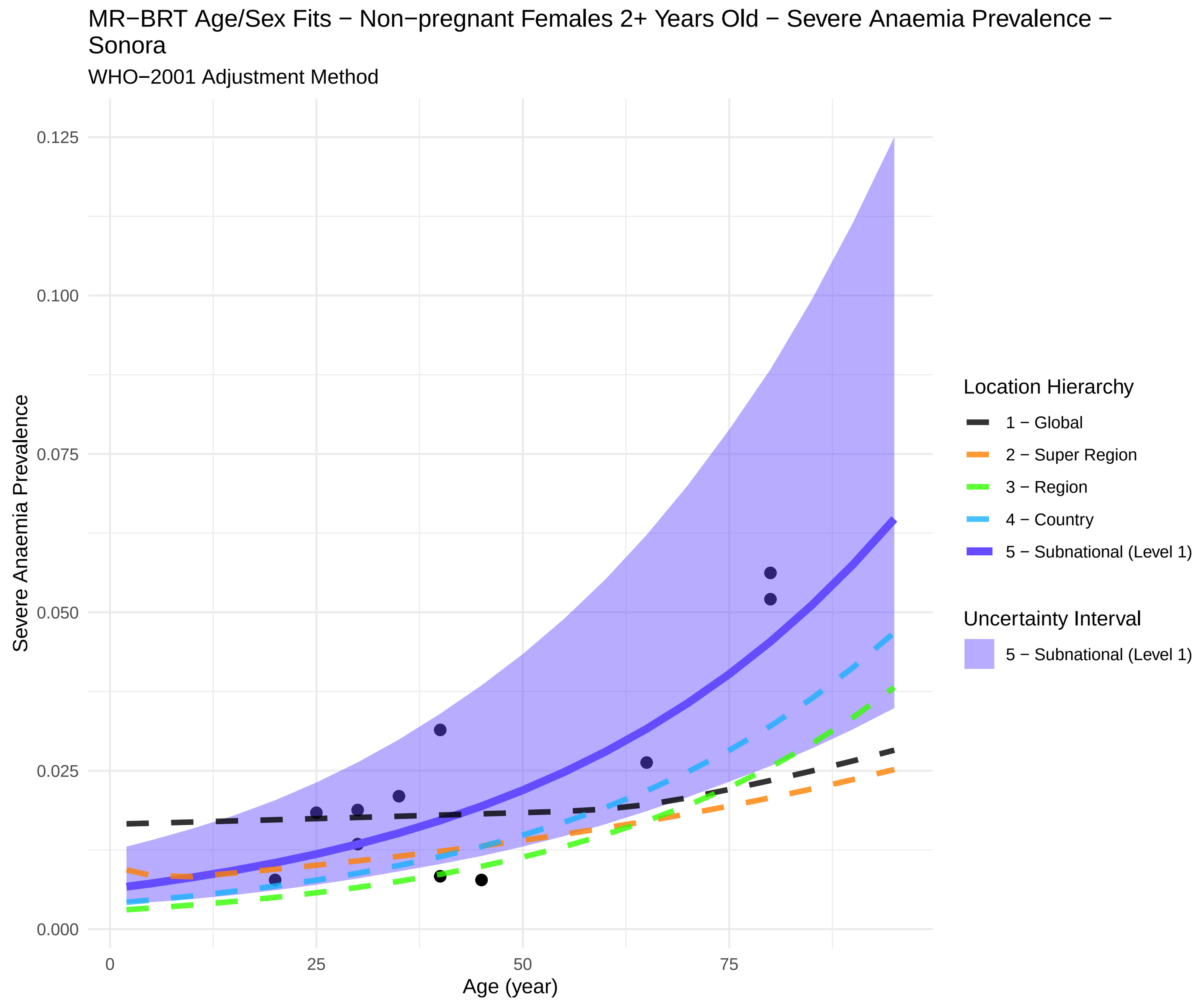
MR−BRT Age/Sex Fits − Non−pregnant Females 2+ Years Old − Severe Anaemia Prevalence − Sonora
WHO−2001 Adjustment Method
Severe Anaemia Prevalence
Age (year)
Location Hierarchy
1 − Global
2 − Super Region
3 − Region
4 − Country
5 − Subnational (Level 1)
Uncertainty Interval
5 − Subnational (Level 1)

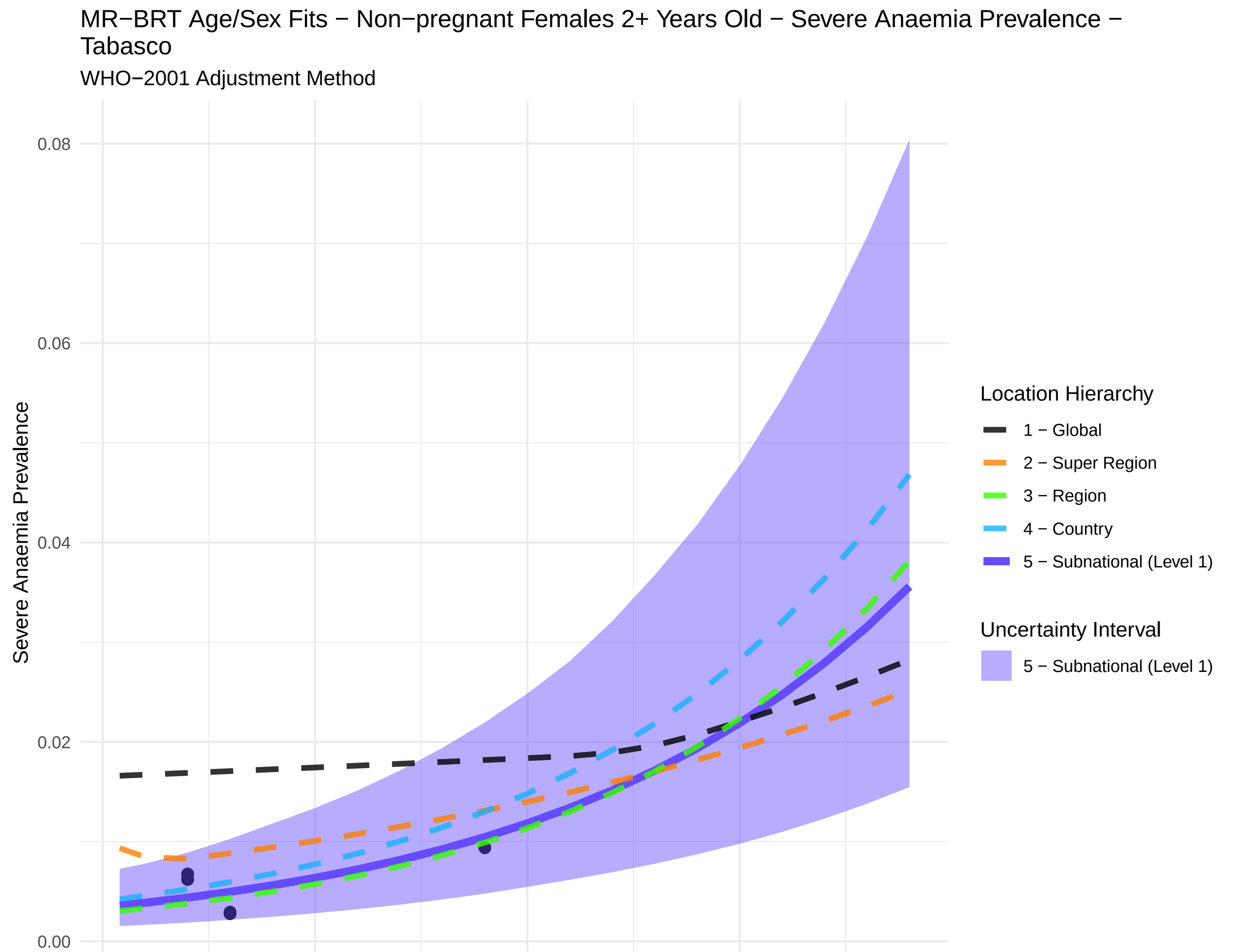
MR−BRT Age/Sex Fits − Non−pregnant Females 2+ Years Old − Severe Anaemia Prevalence − Tabasco
WHO−2001 Adjustment Method
Severe Anaemia Prevalence
Age (year)
Location Hierarchy
1 − Global
2 − Super Region
3 − Region
4 − Country
5 − Subnational (Level 1)
Uncertainty Interval
5 − Subnational (Level 1)

MR−BRT Age/Sex Fits − Non−pregnant Females 2+ Years Old − Severe Anaemia Prevalence − Tamaulipas
WHO−2001 Adjustment Method

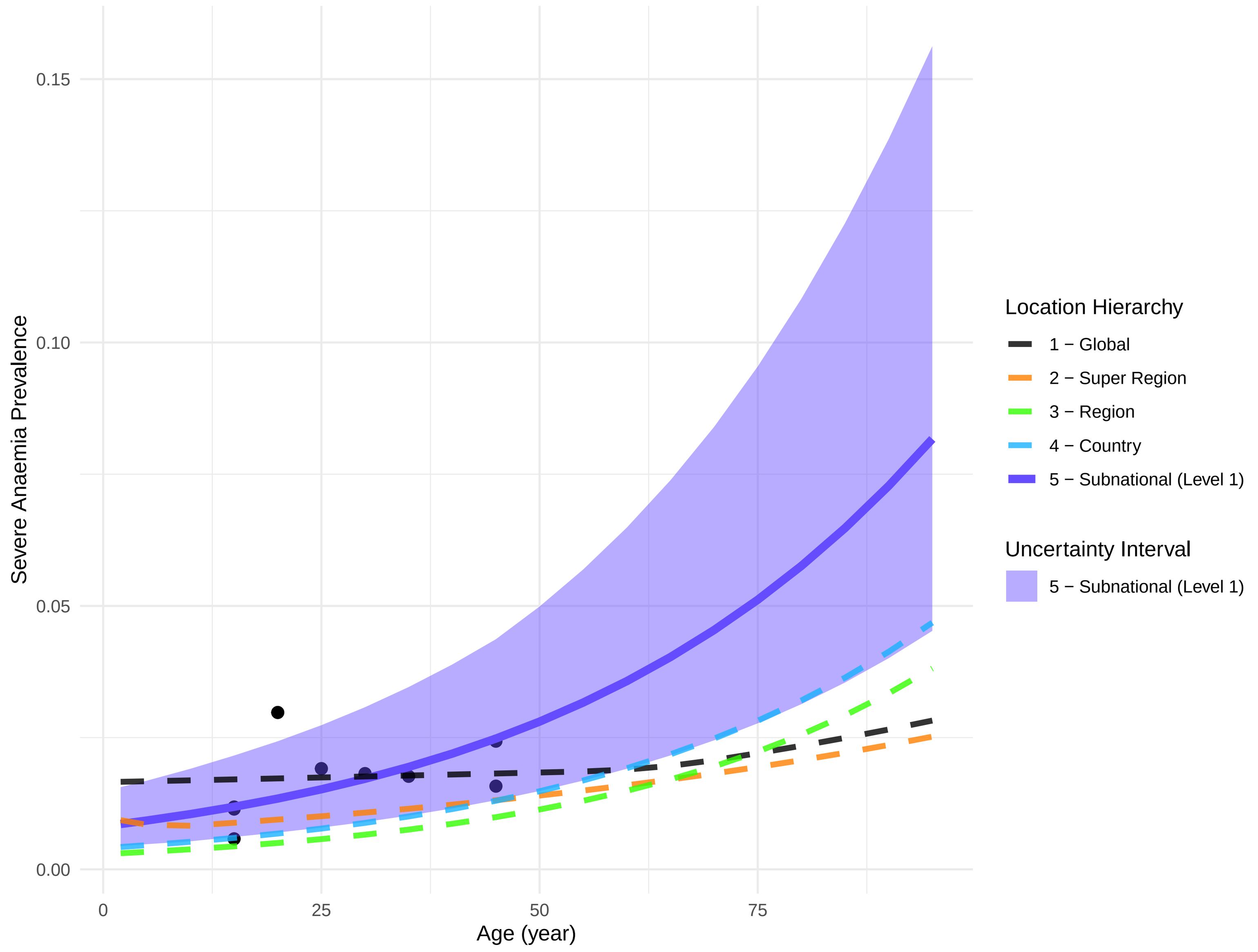

MR−BRT Age/Sex Fits − Non−pregnant Females 2+ Years Old − Severe Anaemia Prevalence − Tlaxcala
WHO−2001 Adjustment Method

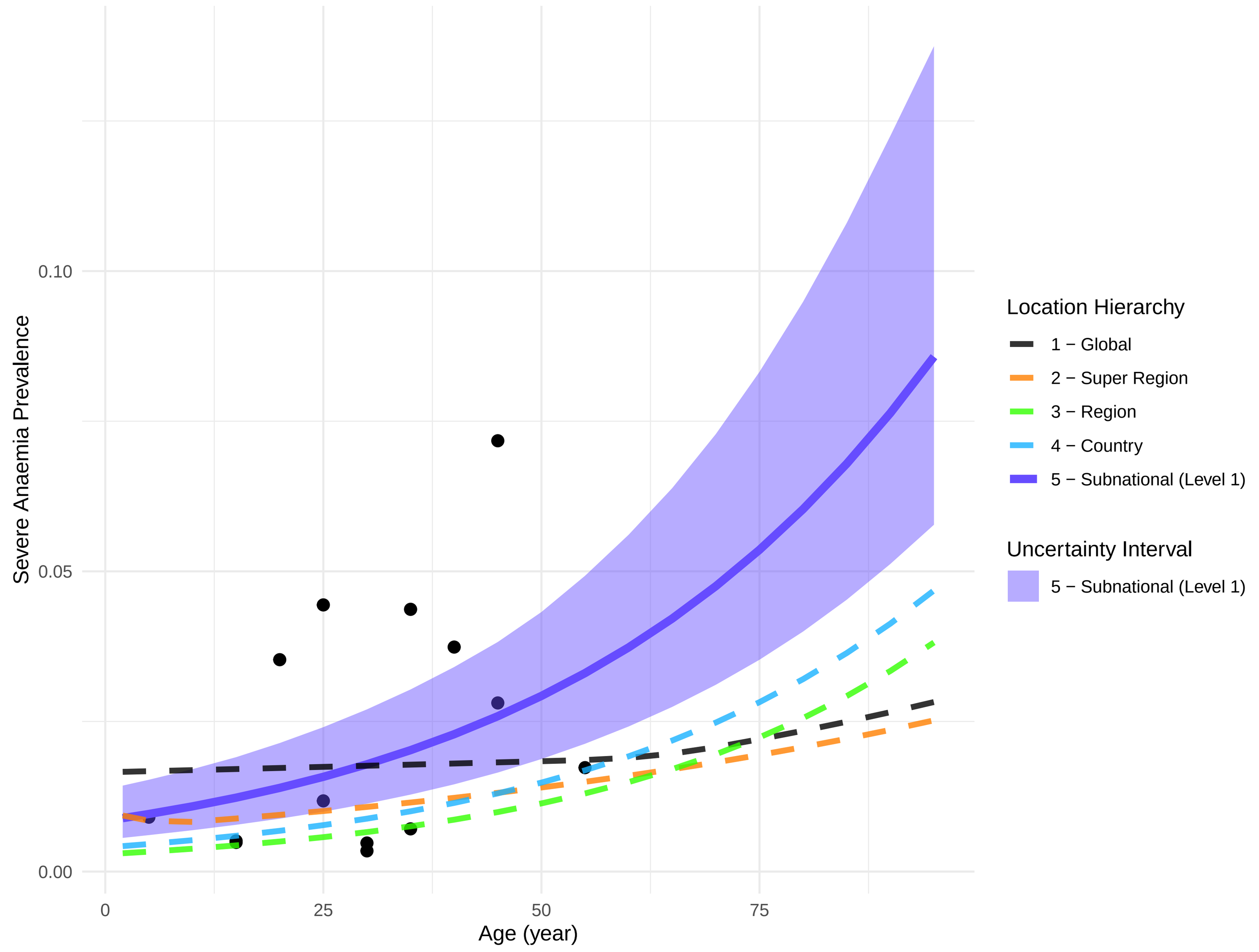

MR−BRT Age/Sex Fits − Non−pregnant Females 2+ Years Old − Severe Anaemia Prevalence − Veracruz de Ignacio de la Llave
WHO−2001 Adjustment Method

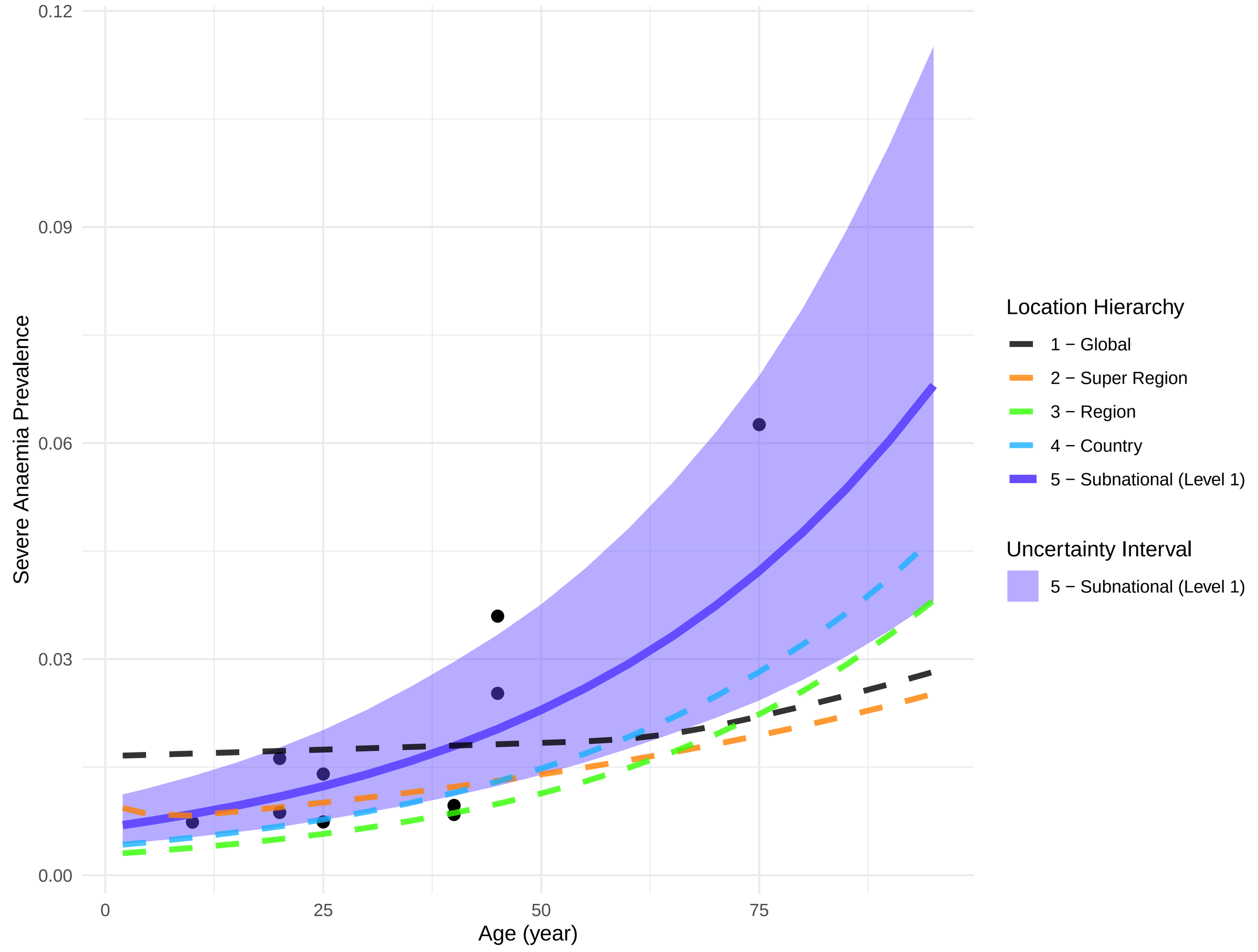

MR−BRT Age/Sex Fits − Non−pregnant Females 2+ Years Old − Severe Anaemia Prevalence − Yucatán
WHO−2001 Adjustment Method

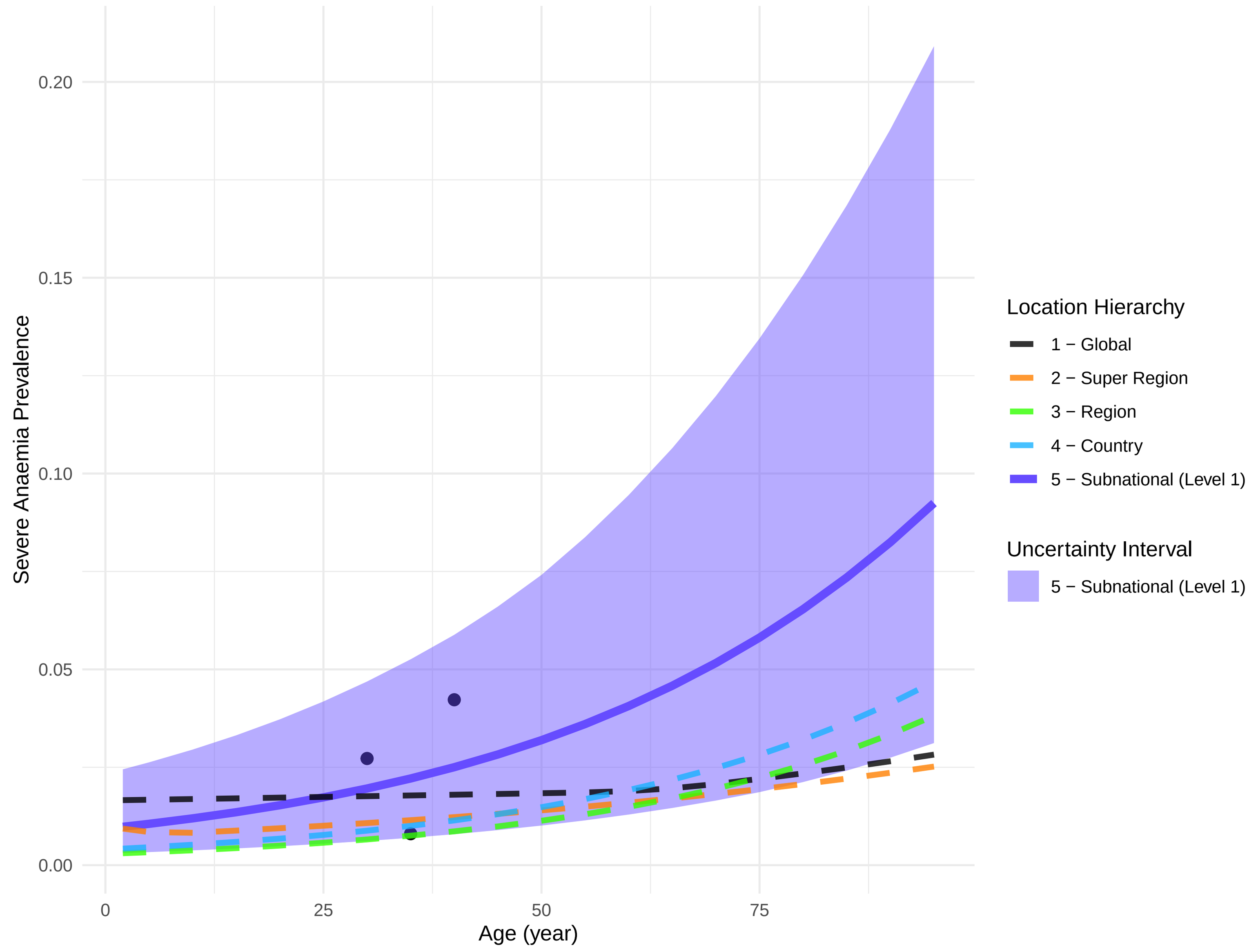

MR−BRT Age/Sex Fits − Non−pregnant Females 2+ Years Old − Severe Anaemia Prevalence − Zacatecas
WHO−2001 Adjustment Method

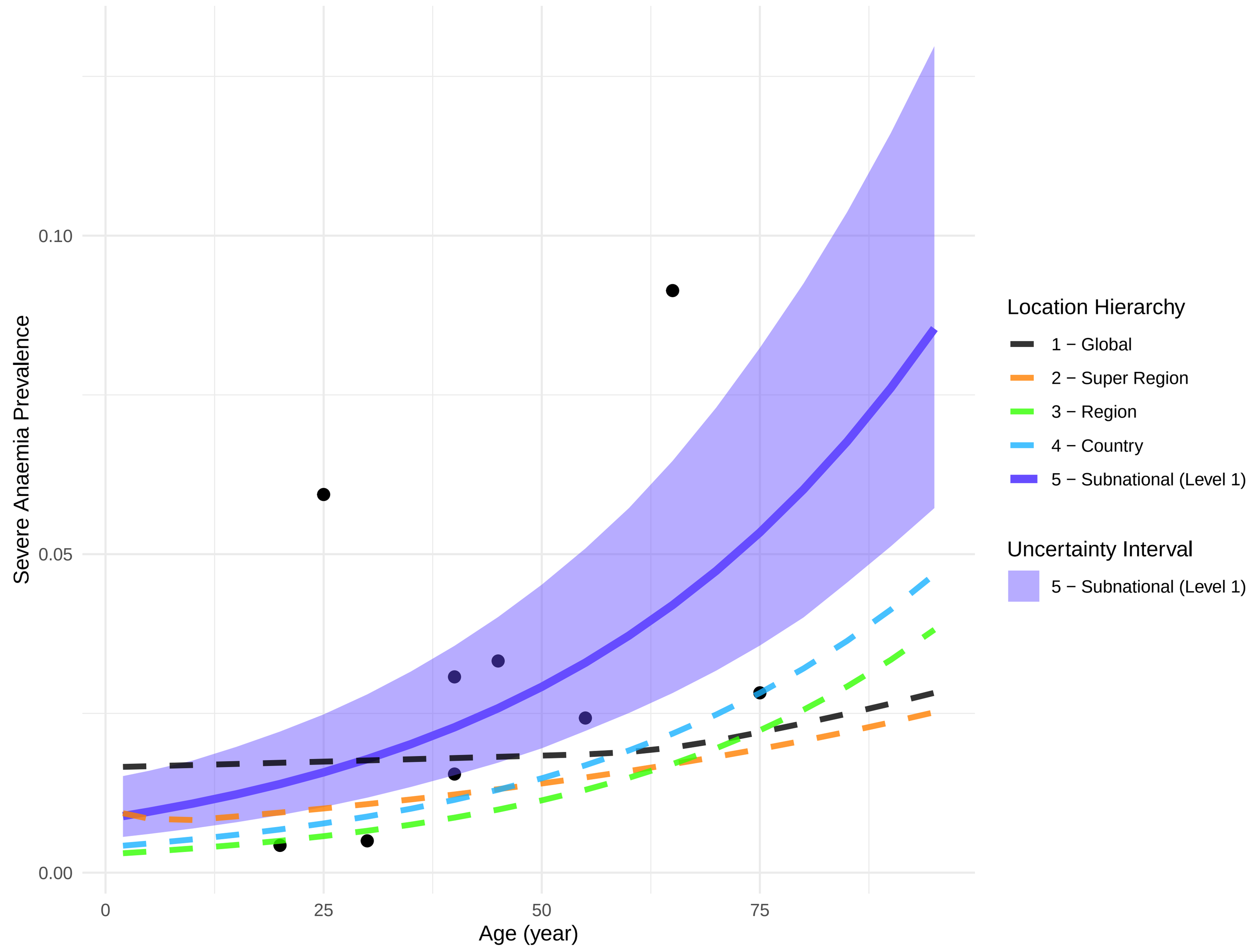

MR−BRT Age/Sex Fits − Non−pregnant Females 2+ Years Old − Severe Anaemia Prevalence − Tropical Latin America
WHO−2001 Adjustment Method

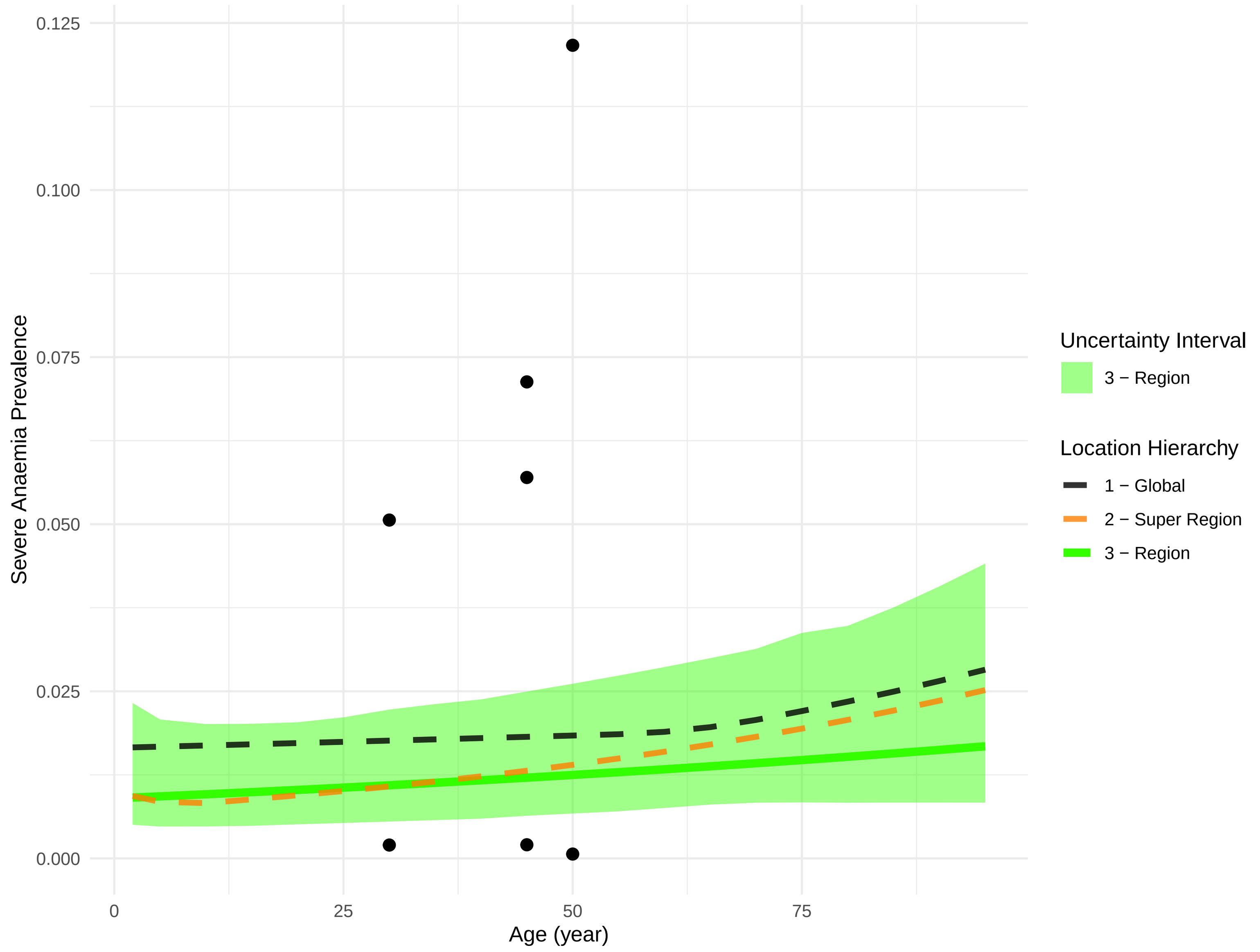

MR−BRT Age/Sex Fits − Non−pregnant Females 2+ Years Old − Severe Anaemia Prevalence − Brazil
WHO−2001 Adjustment Method

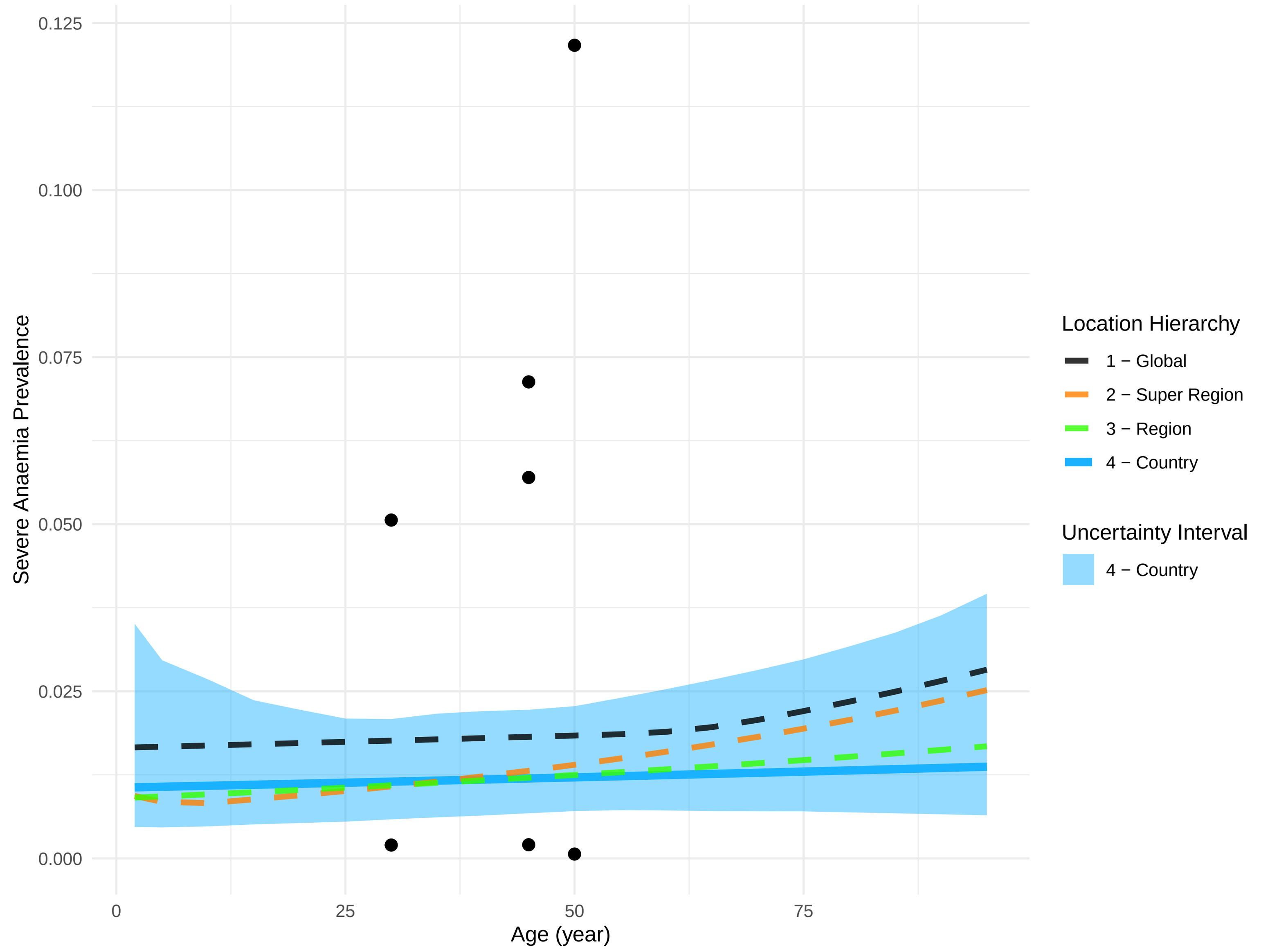

MR−BRT Age/Sex Fits − Non−pregnant Females 2+ Years Old − Severe Anaemia Prevalence − Acre
WHO−2001 Adjustment Method

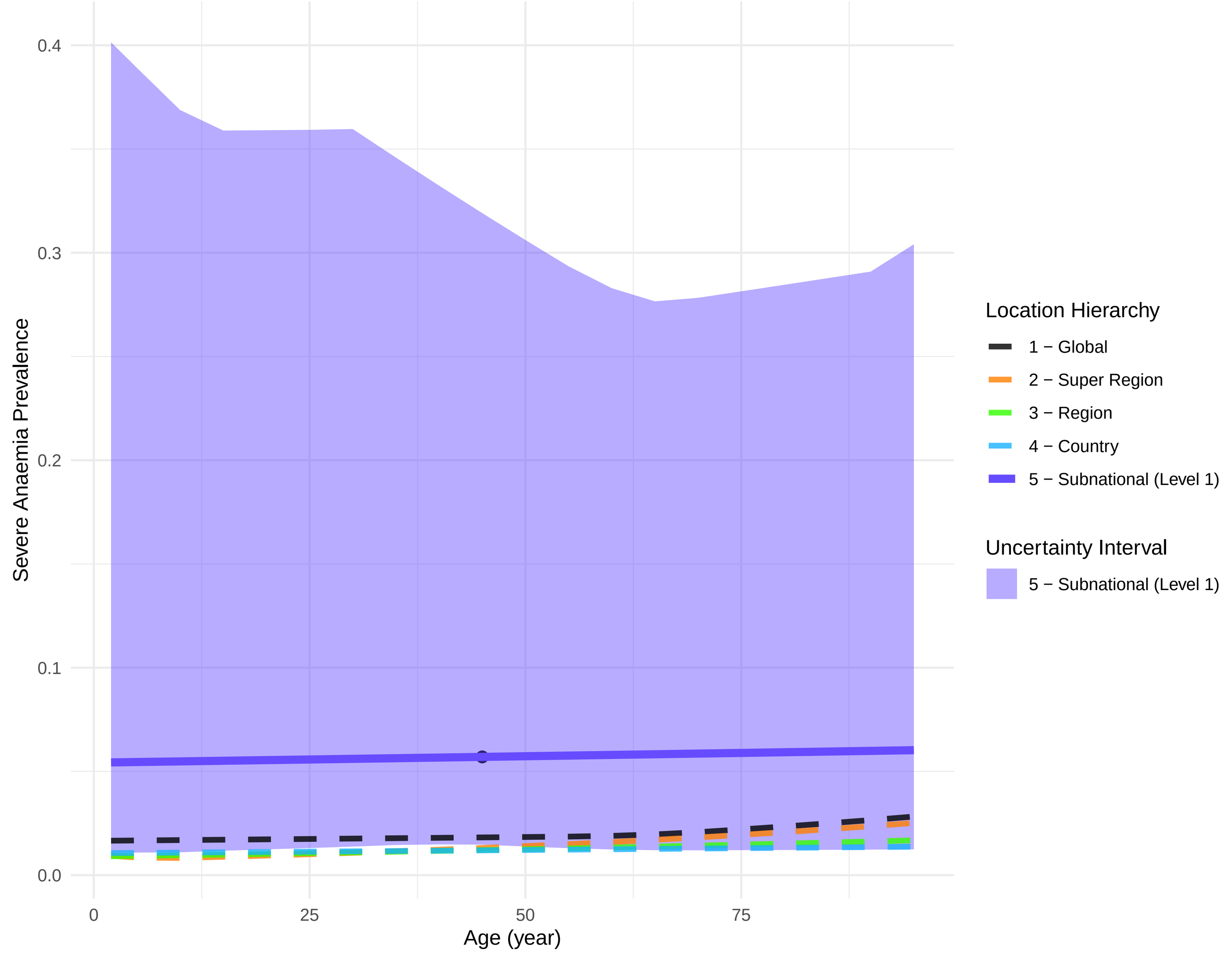

MR−BRT Age/Sex Fits − Non−pregnant Females 2+ Years Old − Severe Anaemia Prevalence − Amazonas
WHO−2001 Adjustment Method

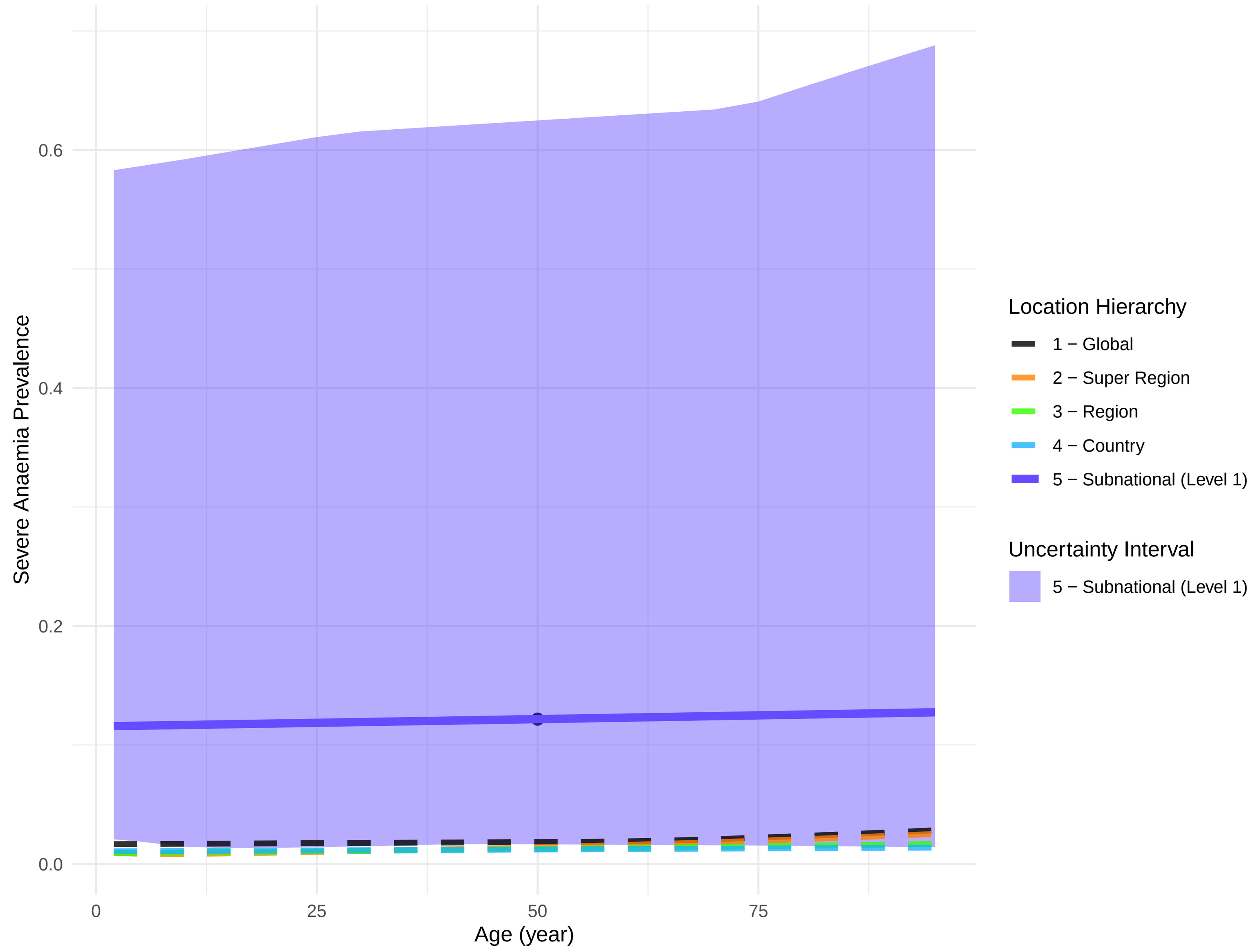

MR−BRT Age/Sex Fits − Non−pregnant Females 2+ Years Old − Severe Anaemia Prevalence − Ceará
WHO−2001 Adjustment Method

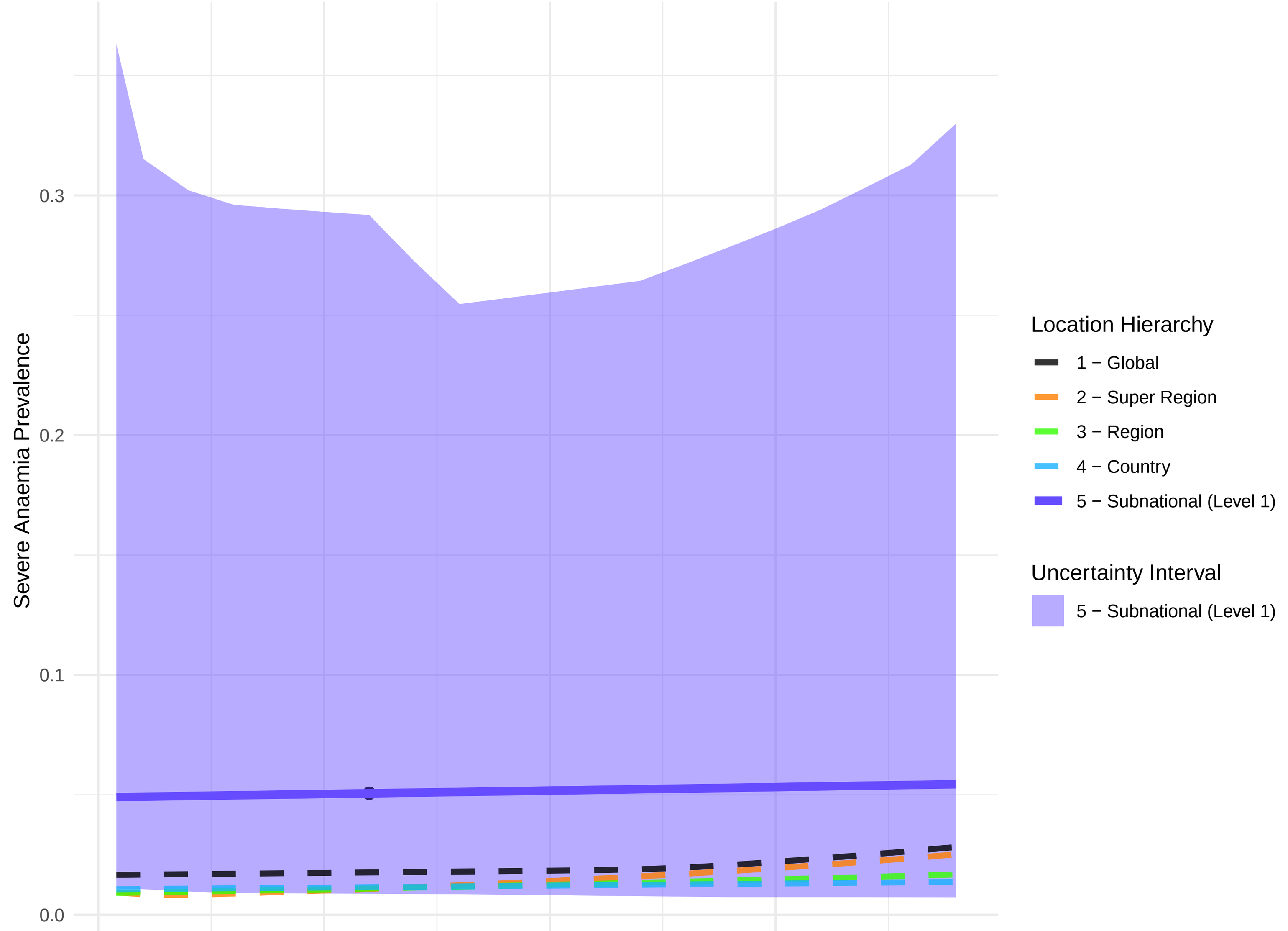

MR−BRT Age/Sex Fits − Non−pregnant Females 2+ Years Old − Severe Anaemia Prevalence − Piaui
WHO−2001 Adjustment Method

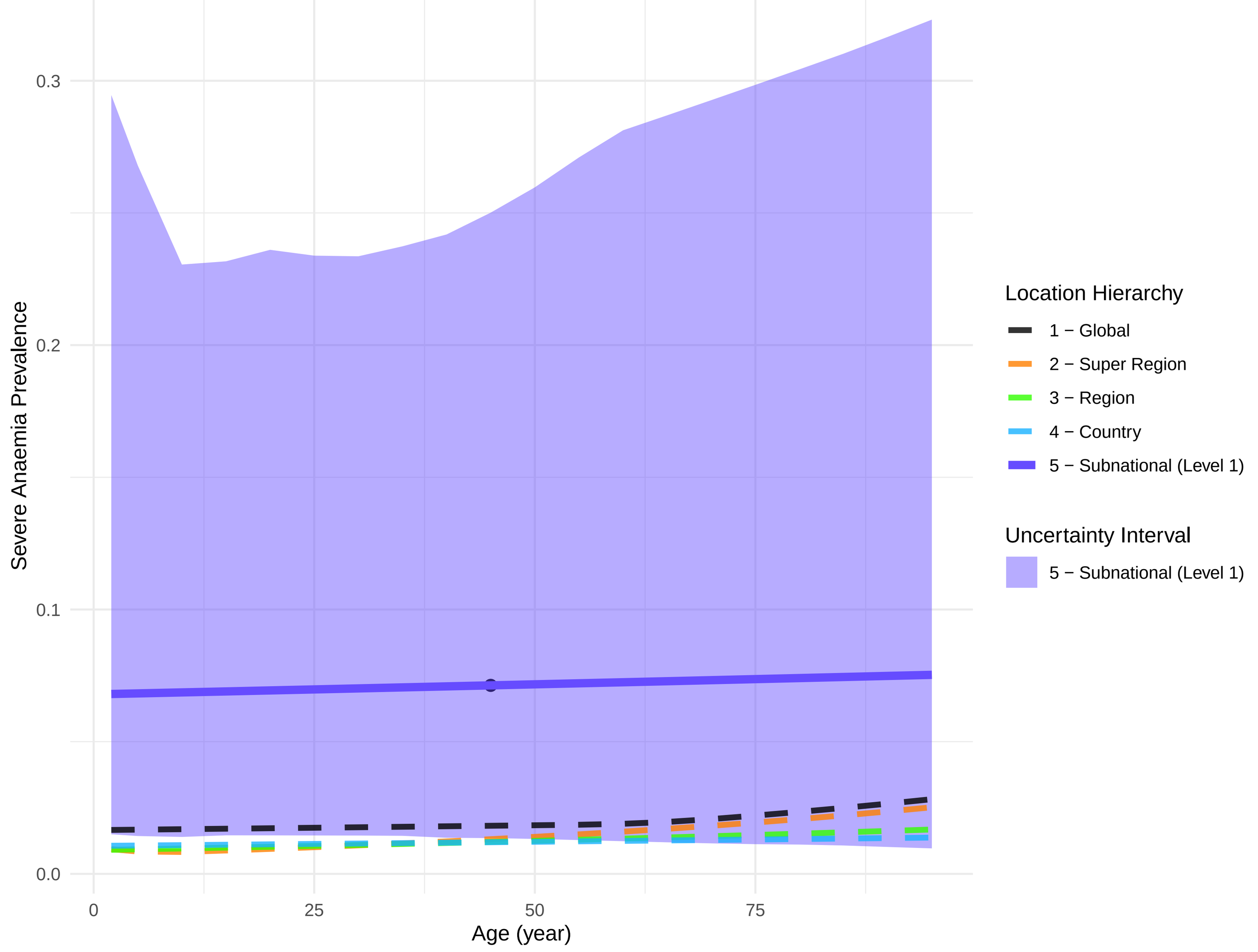

MR−BRT Age/Sex Fits − Non−pregnant Females 2+ Years Old − Severe Anaemia Prevalence − North Africa and Middle East
WHO−2001 Adjustment Method

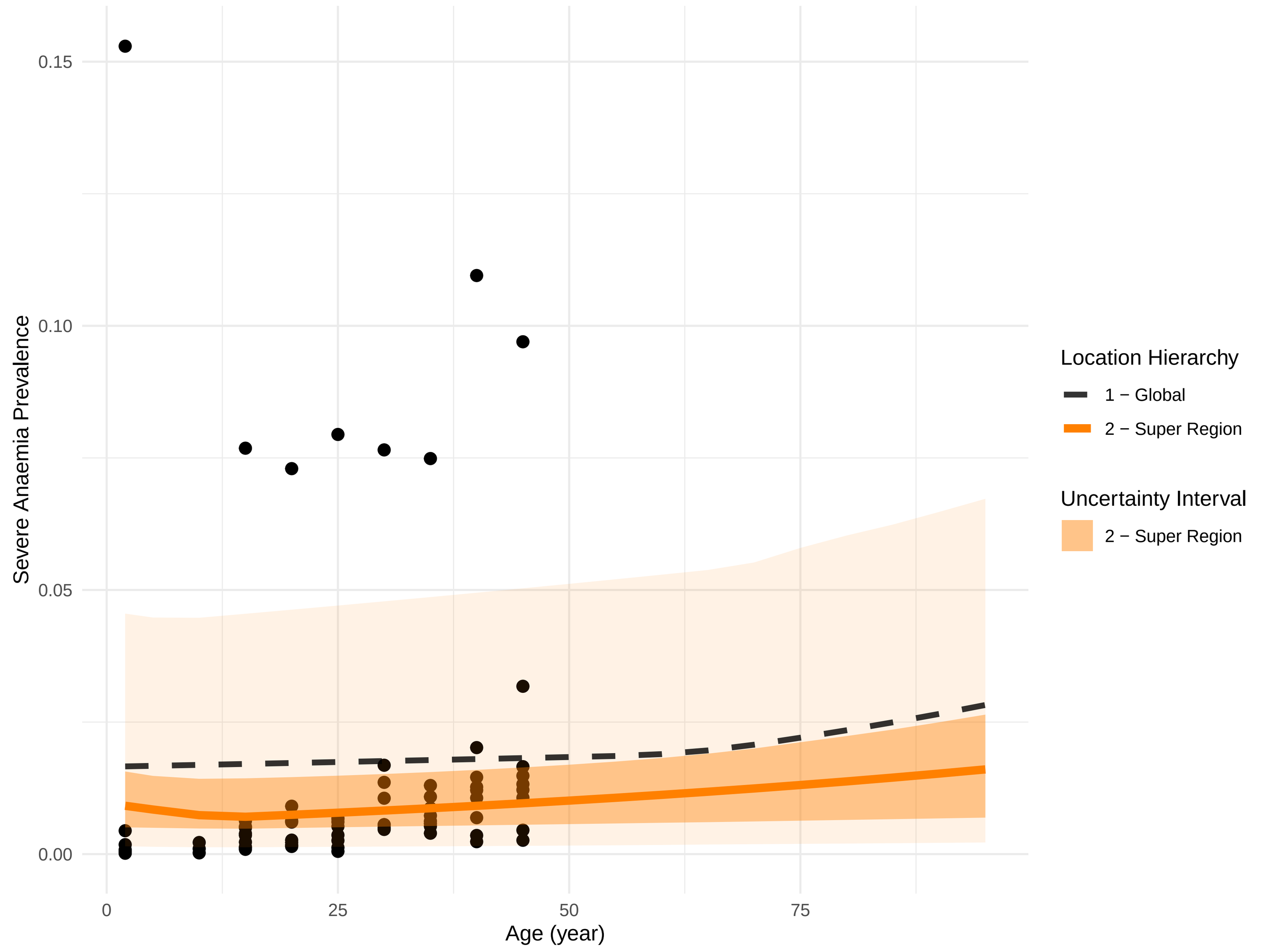

MR−BRT Age/Sex Fits − Non−pregnant Females 2+ Years Old − Severe Anaemia Prevalence − North Africa and Middle East
WHO−2001 Adjustment Method

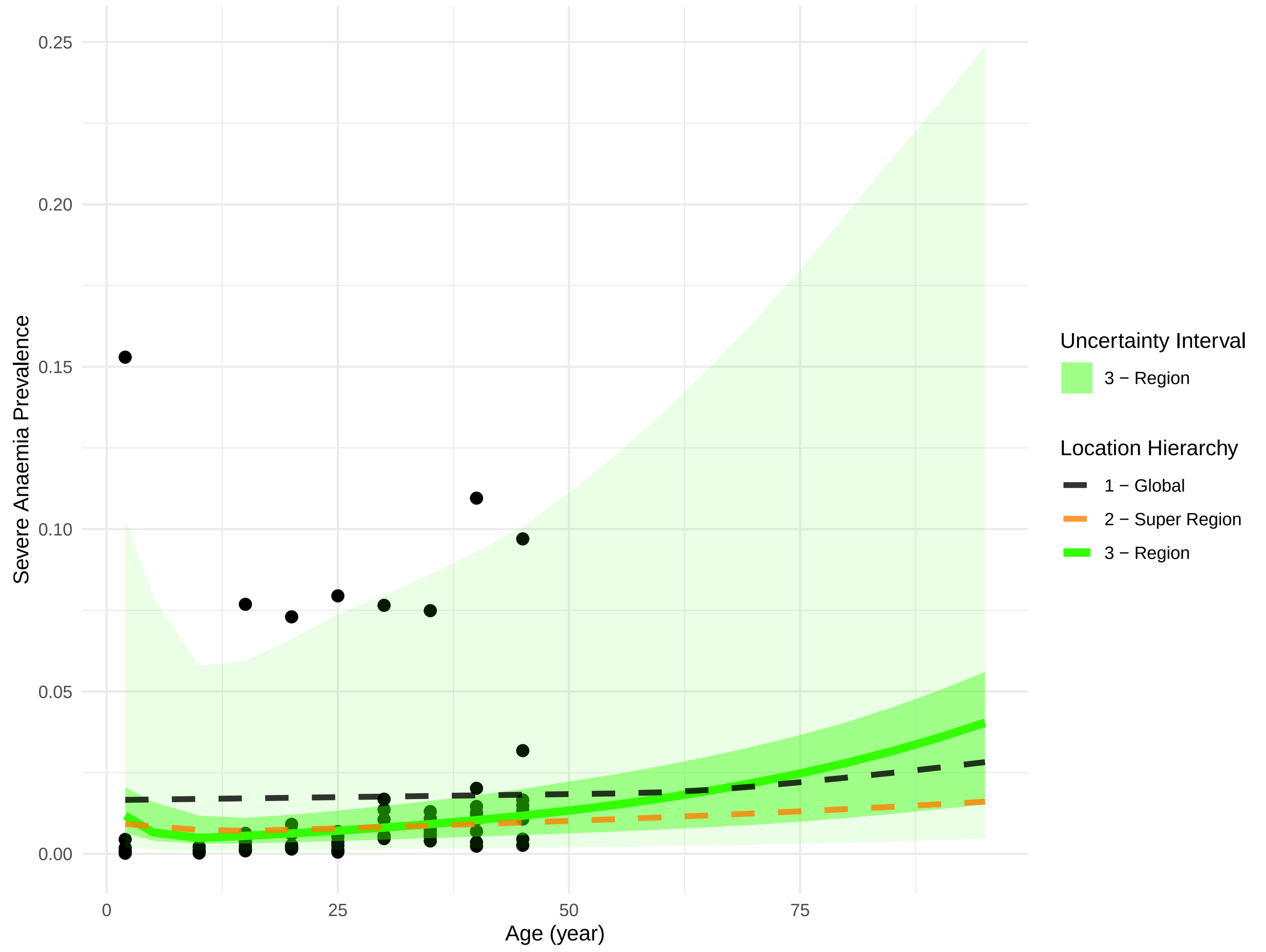

MR−BRT Age/Sex Fits − Non−pregnant Females 2+ Years Old − Severe Anaemia Prevalence − Egypt
WHO−2001 Adjustment Method

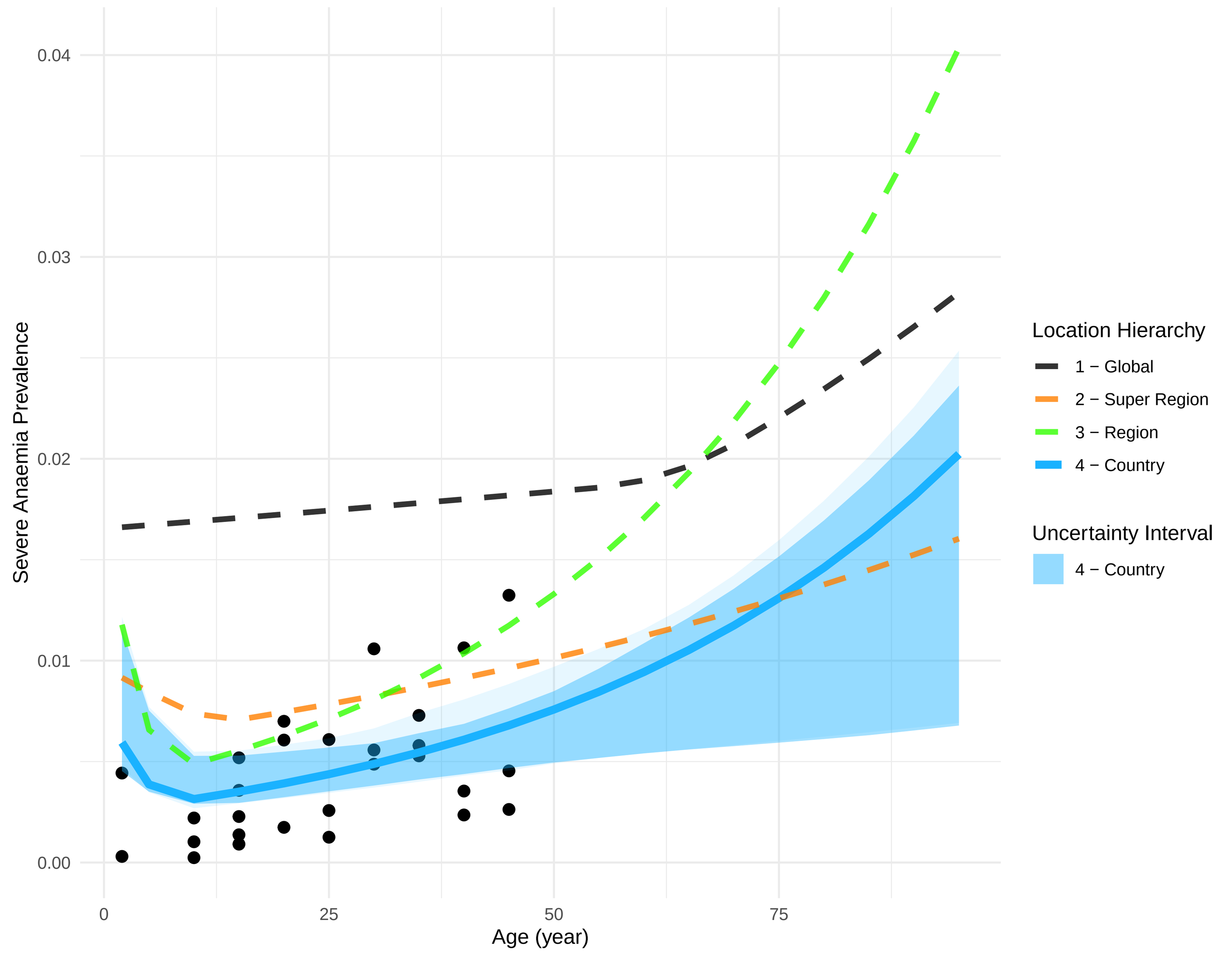

MR−BRT Age/Sex Fits − Non−pregnant Females 2+ Years Old − Severe Anaemia Prevalence − Jordan
WHO−2001 Adjustment Method

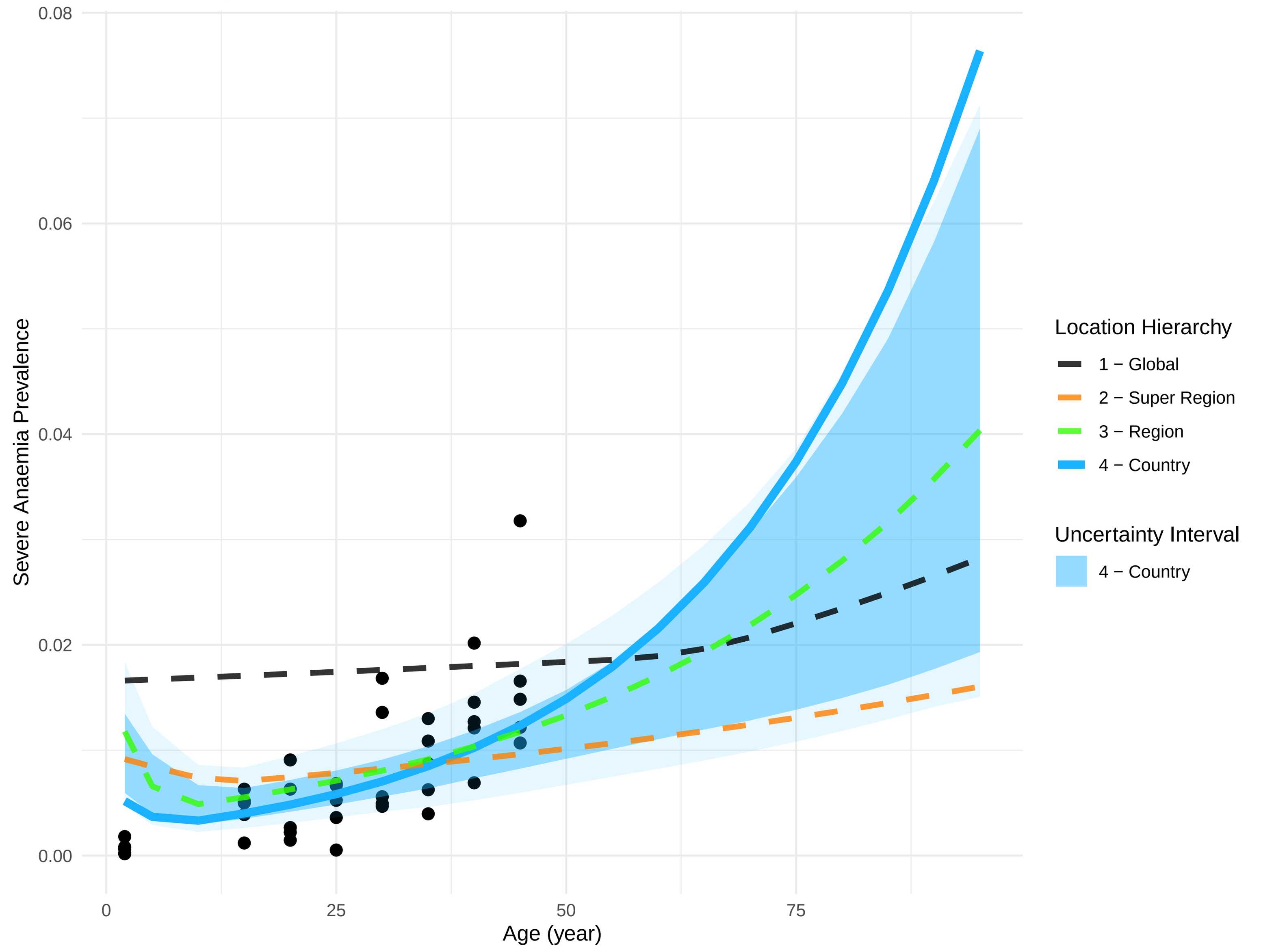

MR−BRT Age/Sex Fits − Non−pregnant Females 2+ Years Old − Severe Anaemia Prevalence − Yemen
WHO−2001 Adjustment Method

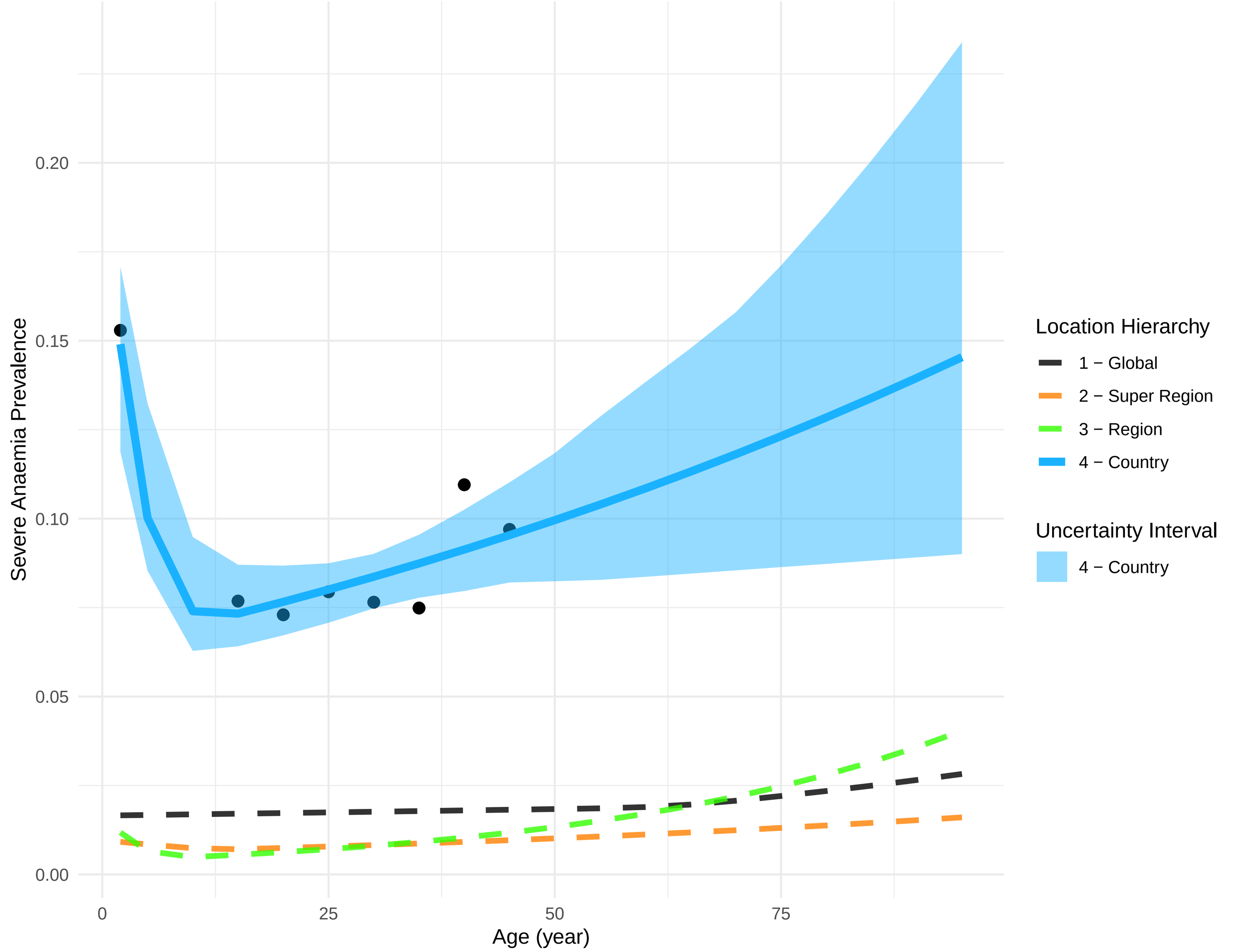

MR−BRT Age/Sex Fits − Non−pregnant Females 2+ Years Old − Severe Anaemia Prevalence − South Asia
WHO−2001 Adjustment Method

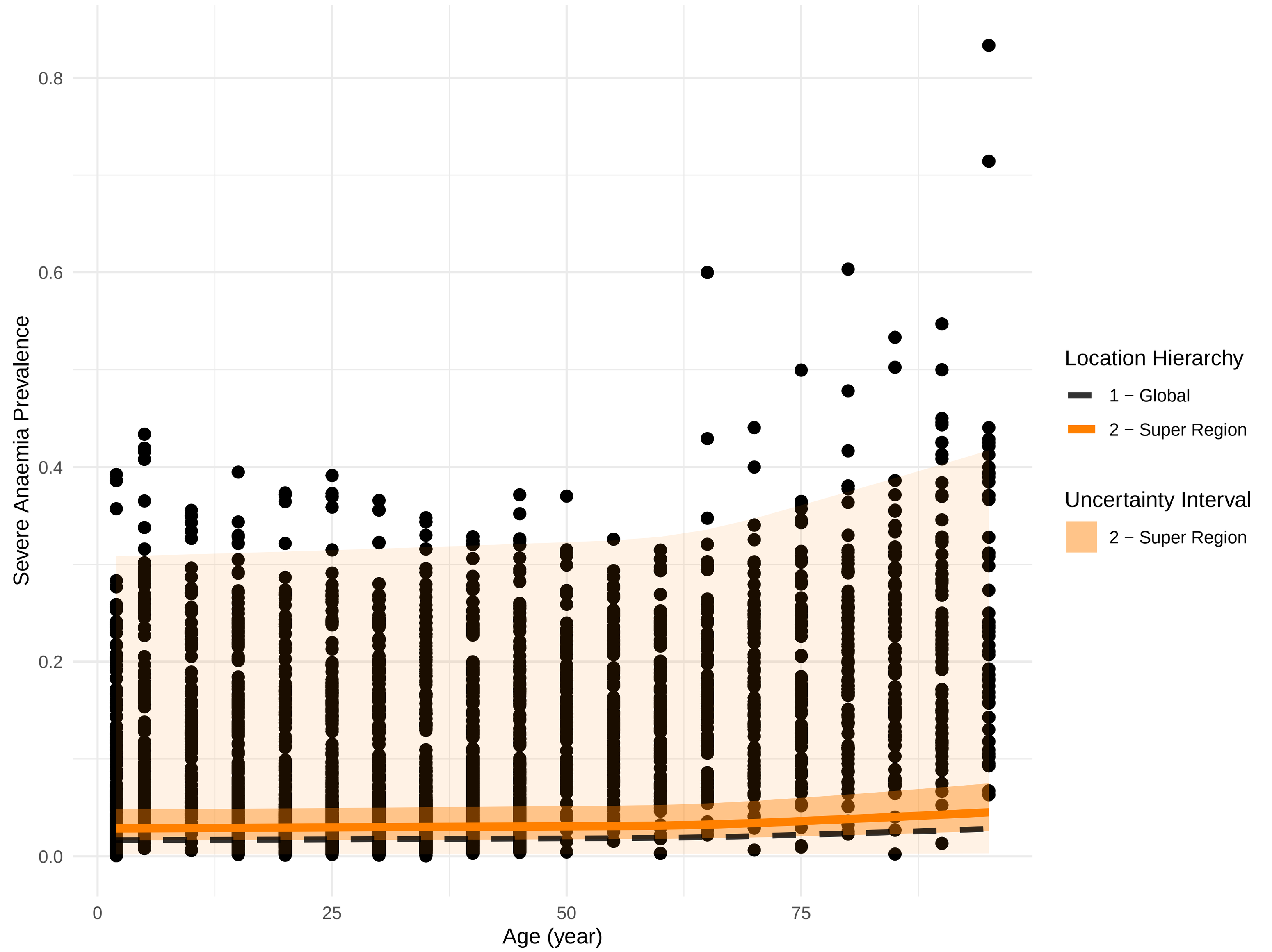

MR−BRT Age/Sex Fits − Non−pregnant Females 2+ Years Old − Severe Anaemia Prevalence − South Asia
WHO−2001 Adjustment Method

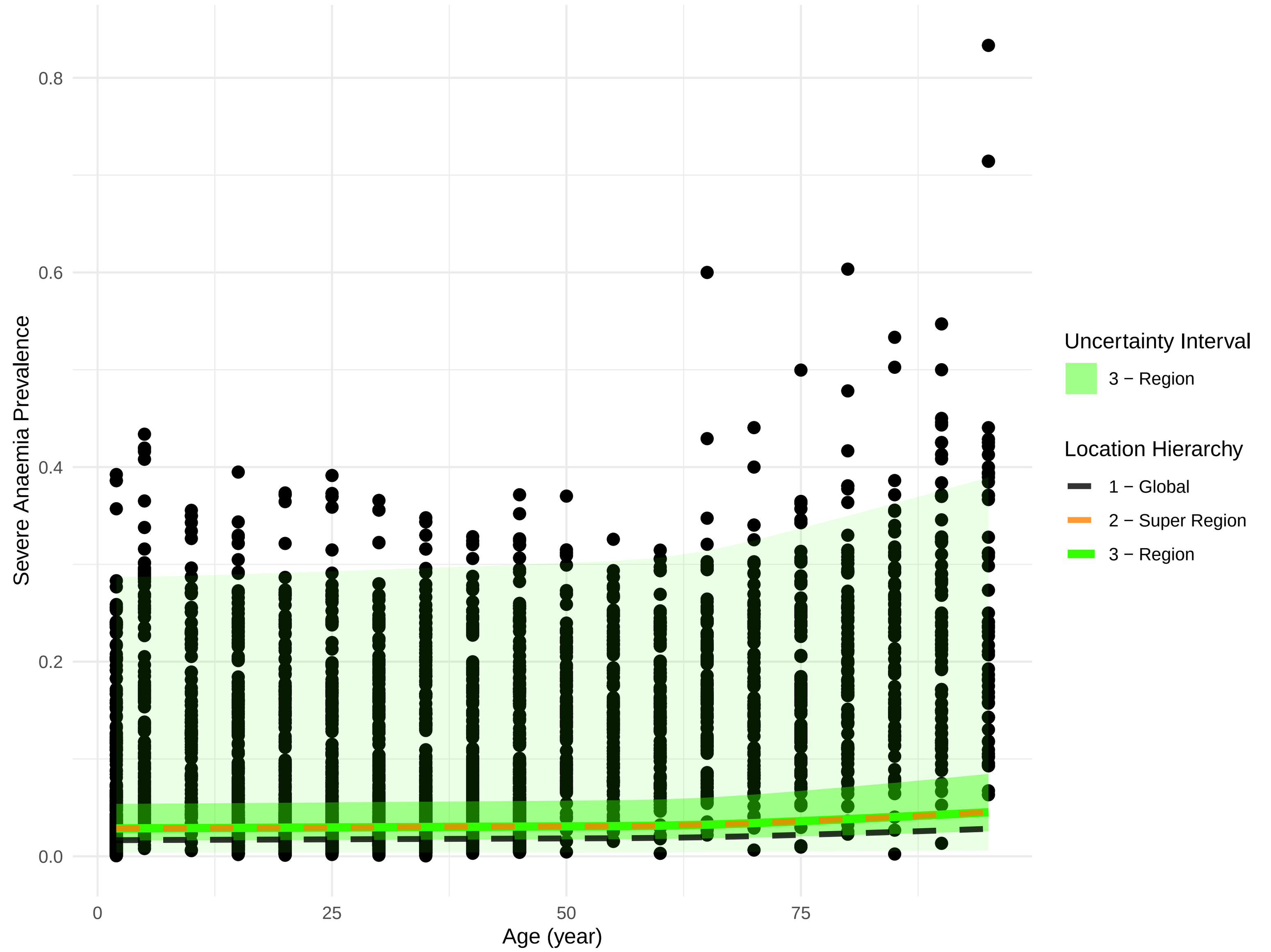

MR−BRT Age/Sex Fits − Non−pregnant Females 2+ Years Old − Severe Anaemia Prevalence − Bangladesh
WHO−2001 Adjustment Method

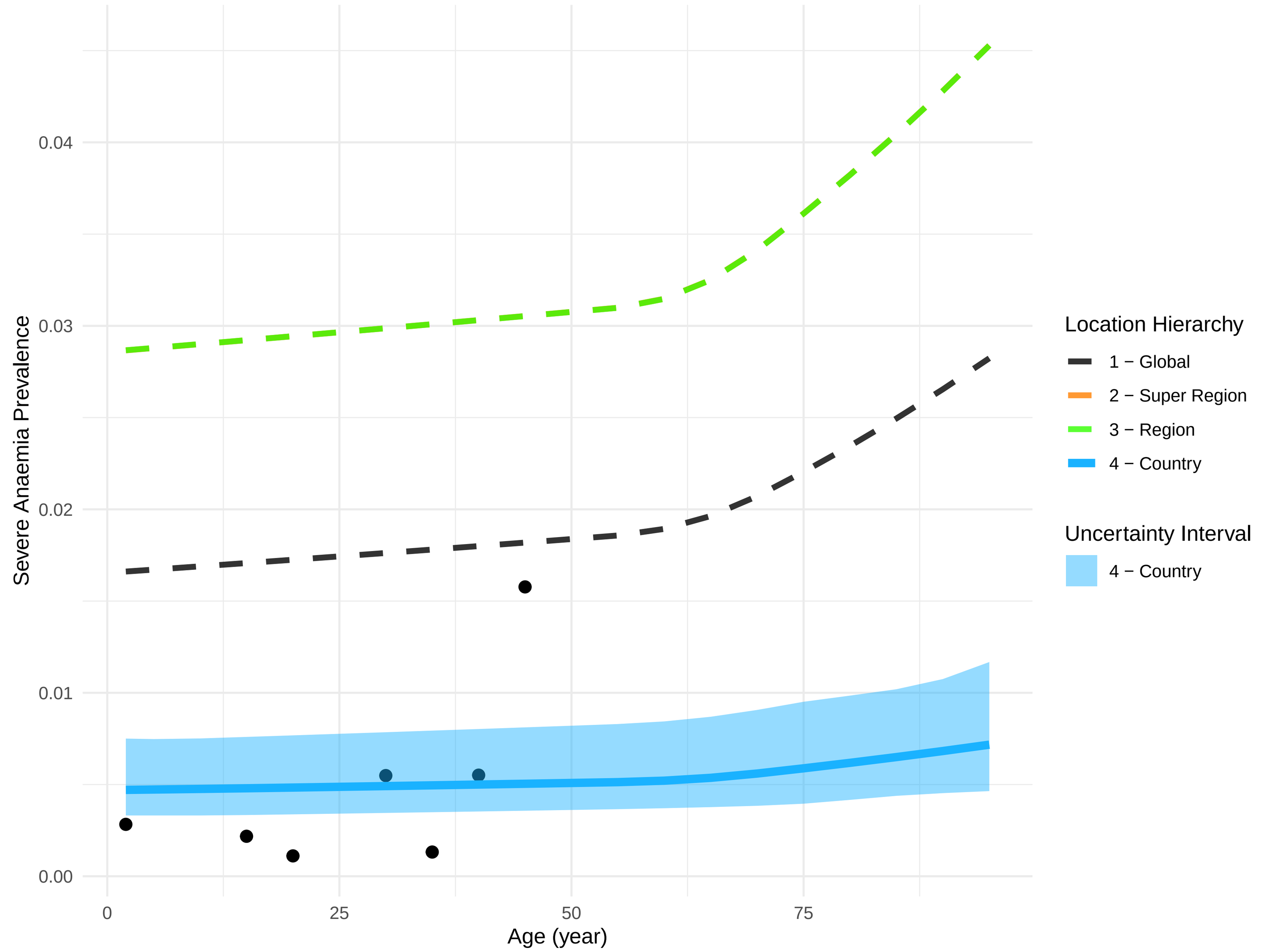

MR−BRT Age/Sex Fits − Non−pregnant Females 2+ Years Old − Severe Anaemia Prevalence − India
WHO−2001 Adjustment Method

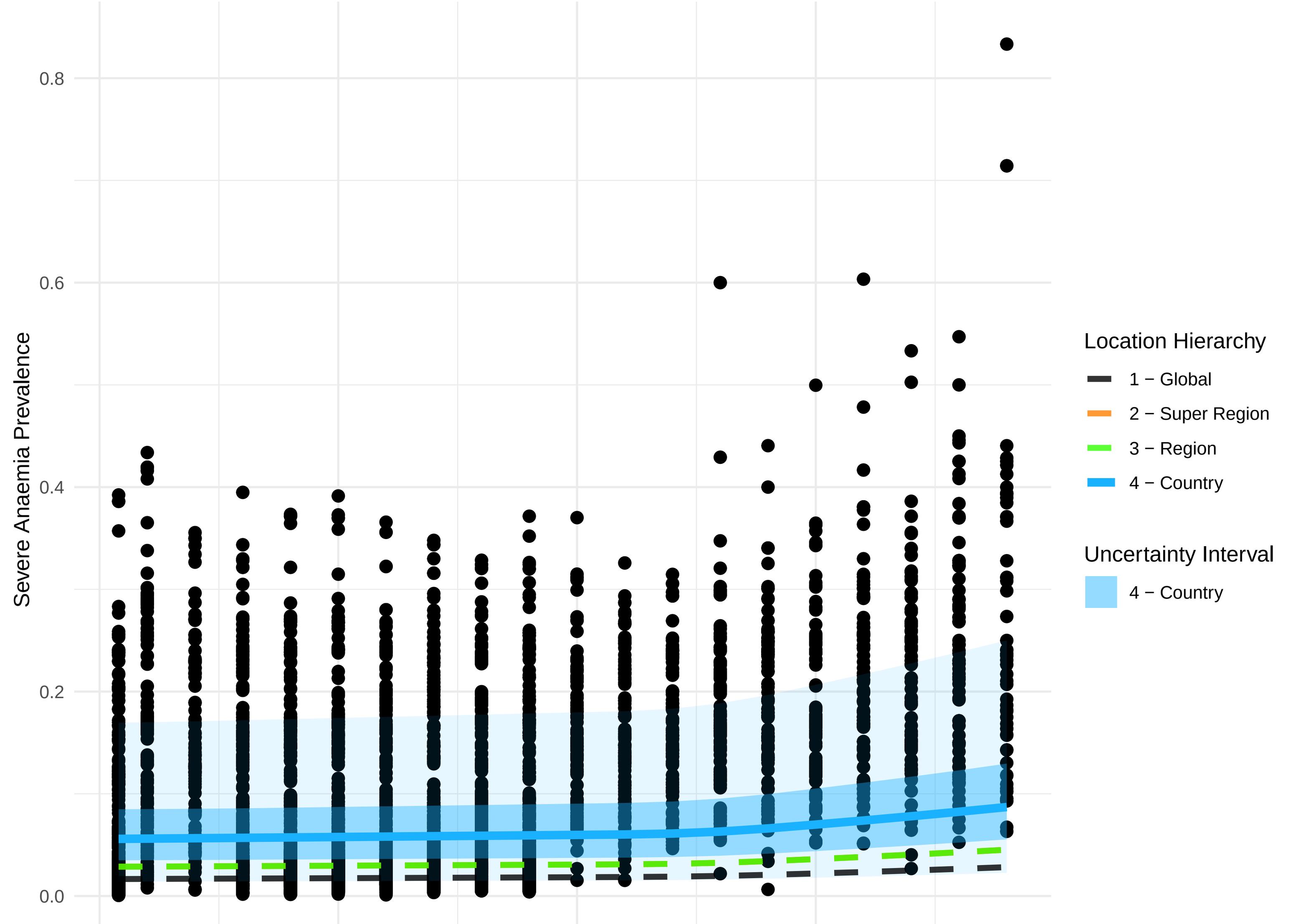

MR−BRT Age/Sex Fits − Non−pregnant Females 2+ Years Old − Severe Anaemia Prevalence − Andhra Pradesh
WHO−2001 Adjustment Method

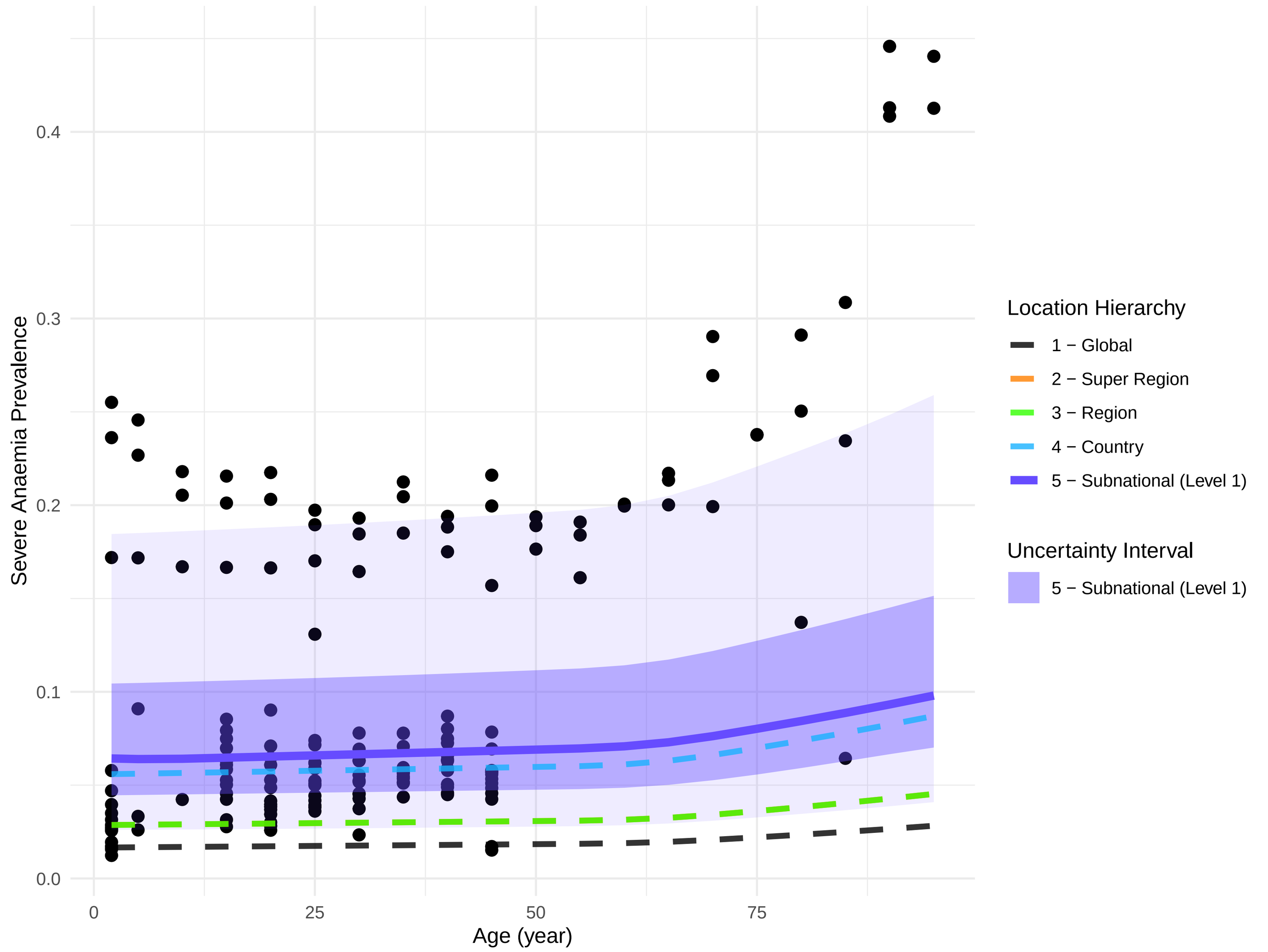

MR−BRT Age/Sex Fits − Non−pregnant Females 2+ Years Old − Severe Anaemia Prevalence − Andhra Pradesh, Rural
WHO−2001 Adjustment Method

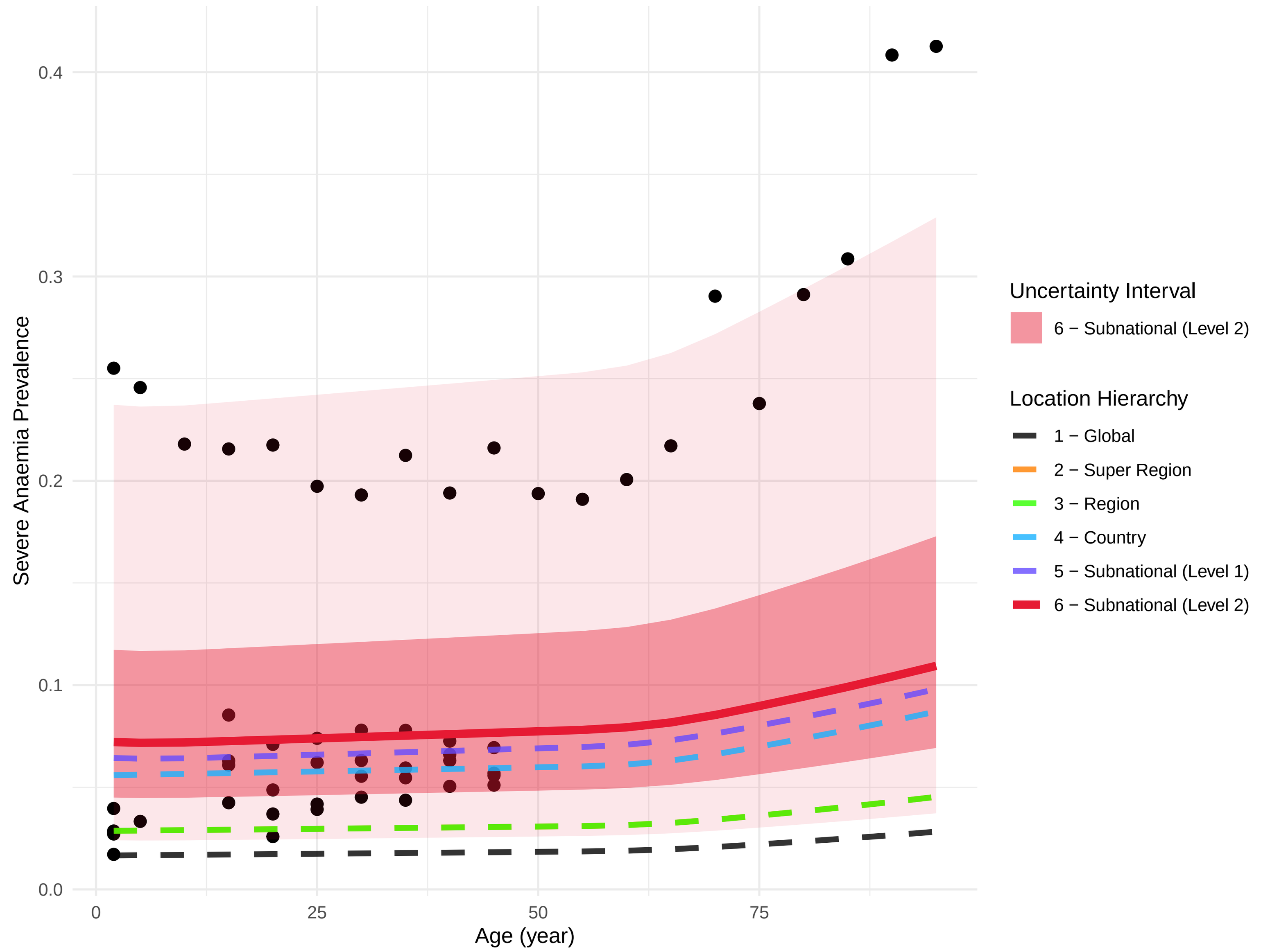

MR−BRT Age/Sex Fits − Non−pregnant Females 2+ Years Old − Severe Anaemia Prevalence − Andhra Pradesh, Urban
WHO−2001 Adjustment Method

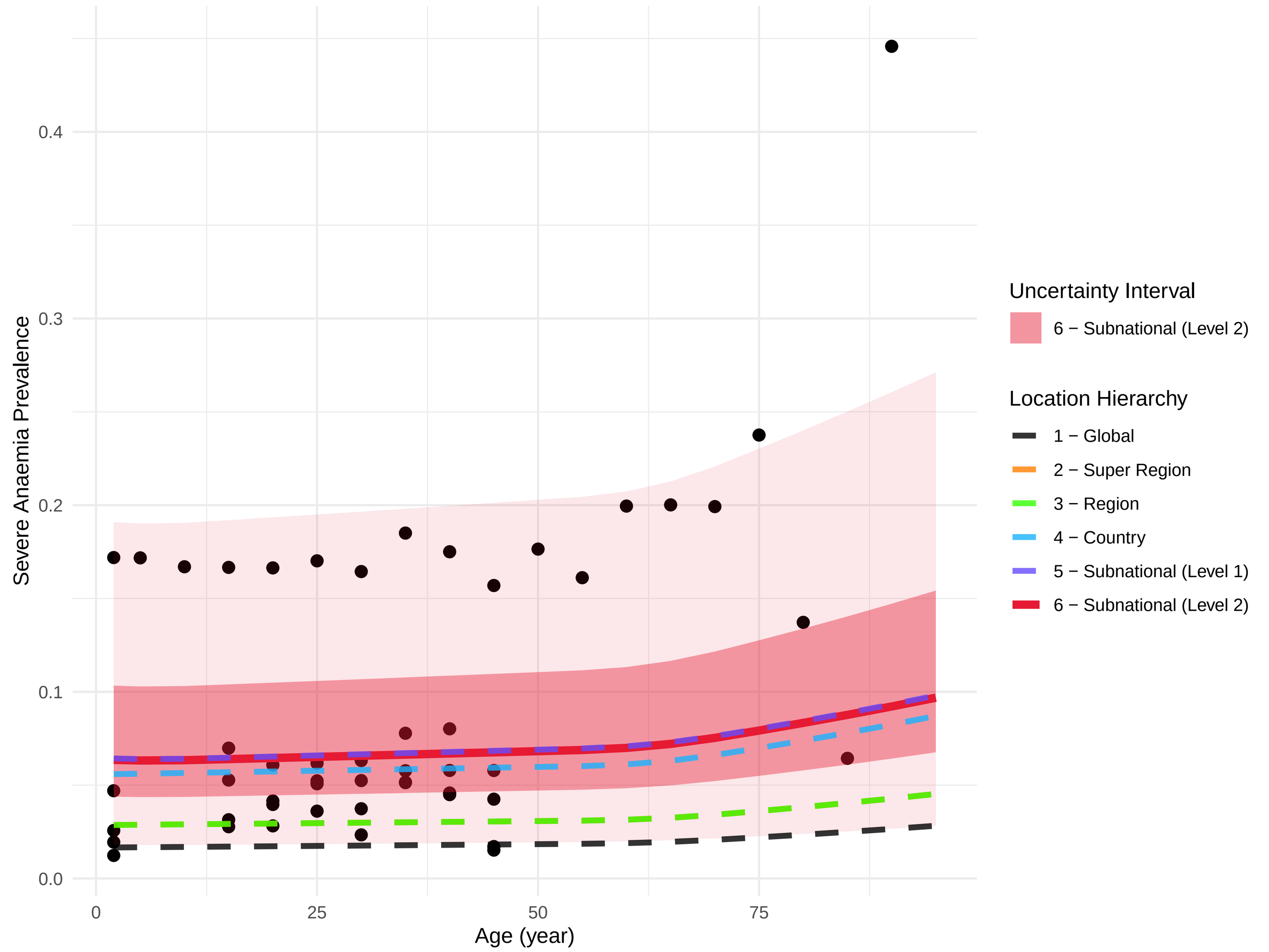

MR−BRT Age/Sex Fits − Non−pregnant Females 2+ Years Old − Severe Anaemia Prevalence − Arunachal Pradesh
WHO−2001 Adjustment Method

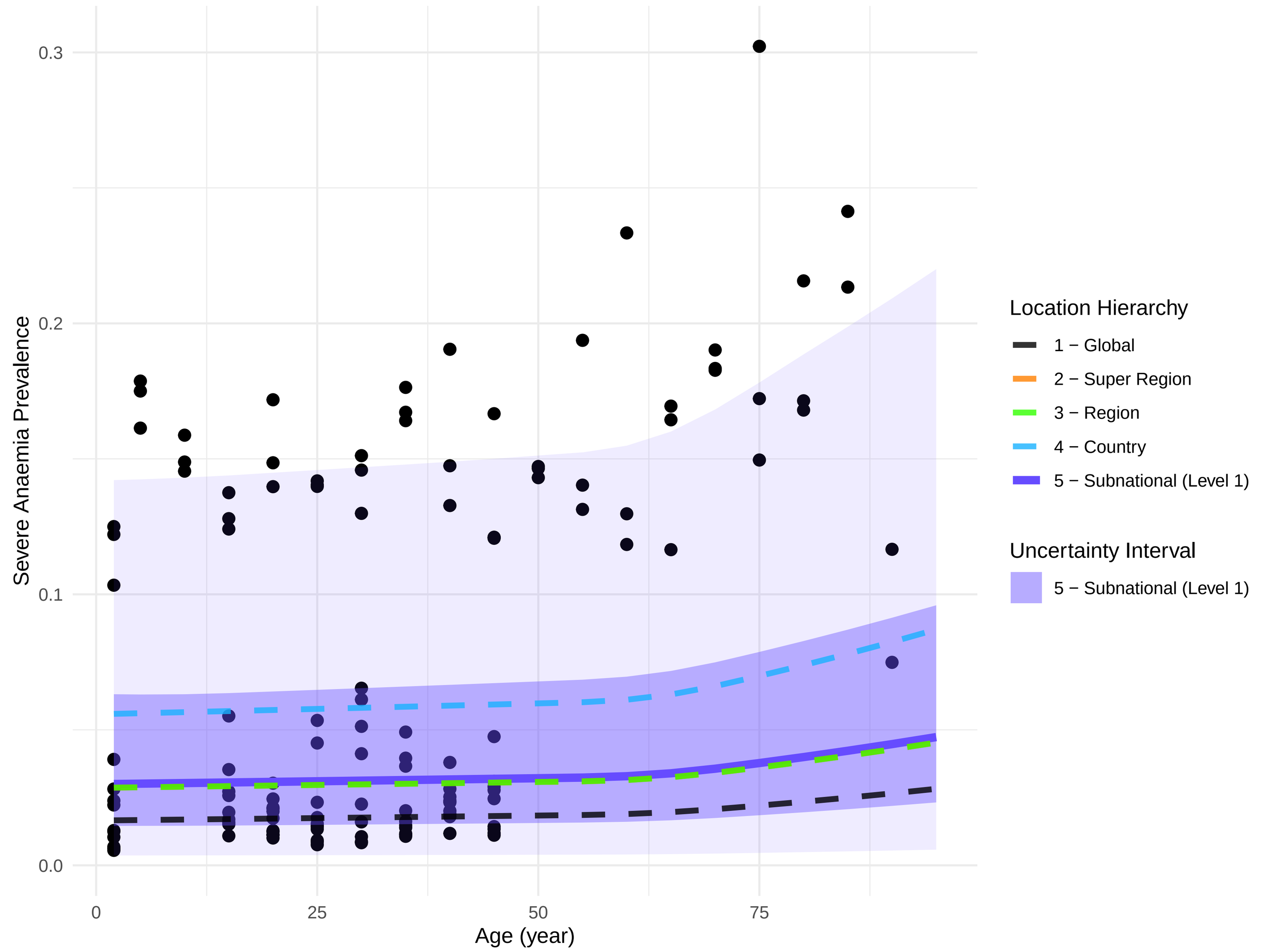

MR−BRT Age/Sex Fits − Non−pregnant Females 2+ Years Old − Severe Anaemia Prevalence − Arunachal Pradesh, Rural
WHO−2001 Adjustment Method

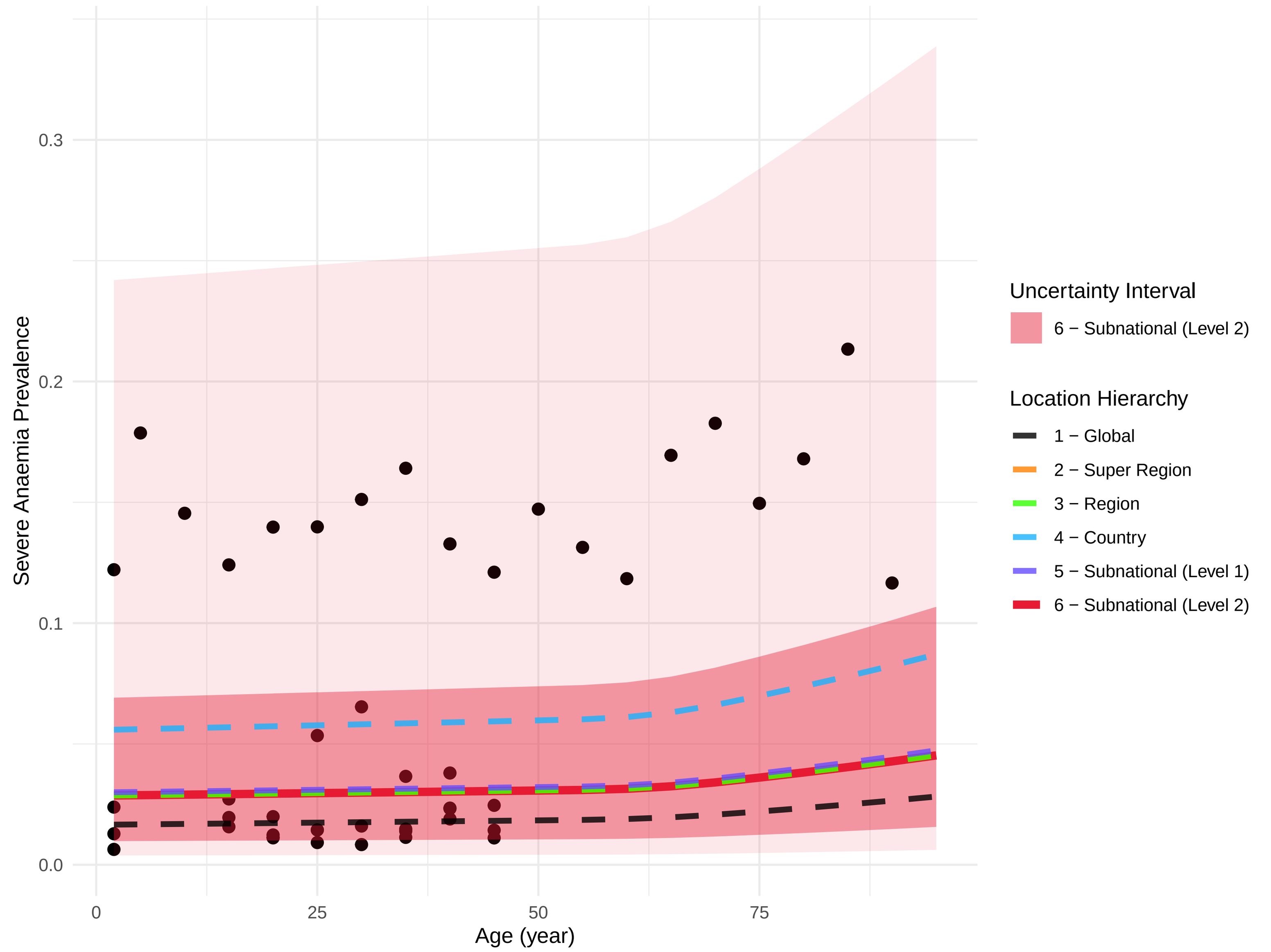

MR−BRT Age/Sex Fits − Non−pregnant Females 2+ Years Old − Severe Anaemia Prevalence − Arunachal Pradesh, Urban
WHO−2001 Adjustment Method

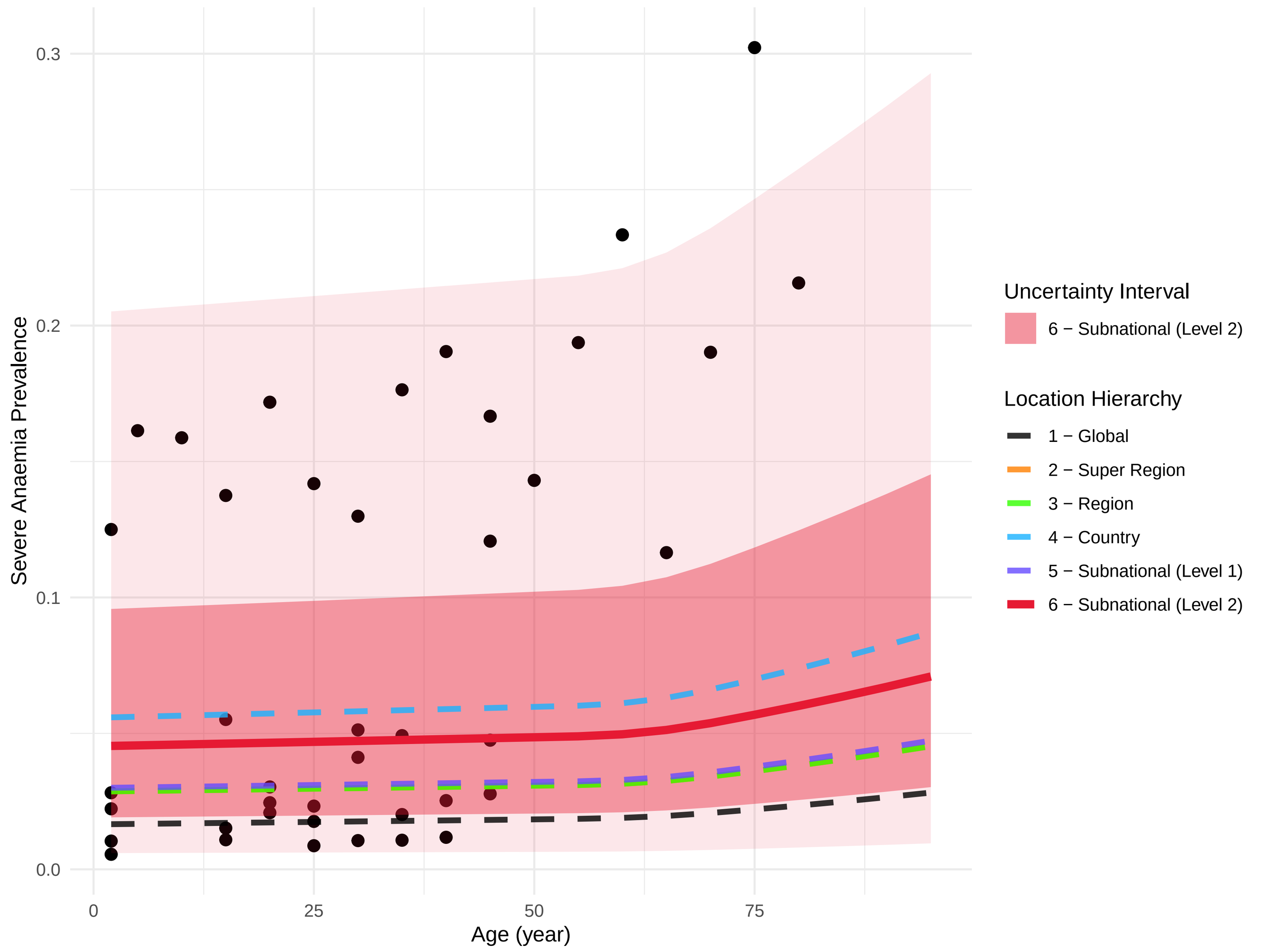

MR−BRT Age/Sex Fits − Non−pregnant Females 2+ Years Old − Severe Anaemia Prevalence − Assam
WHO−2001 Adjustment Method

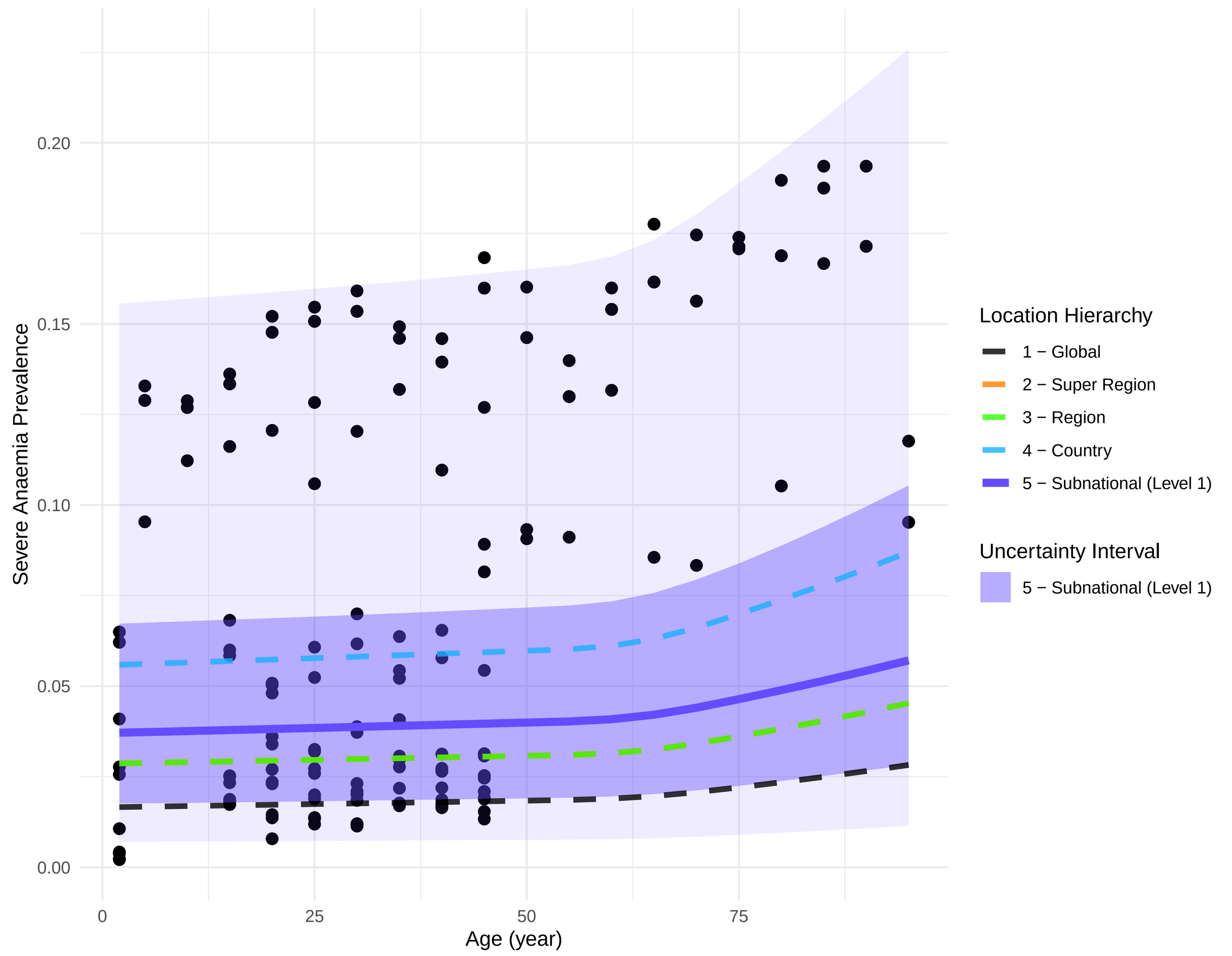

MR−BRT Age/Sex Fits − Non−pregnant Females 2+ Years Old − Severe Anaemia Prevalence − Assam, Rural
WHO−2001 Adjustment Method

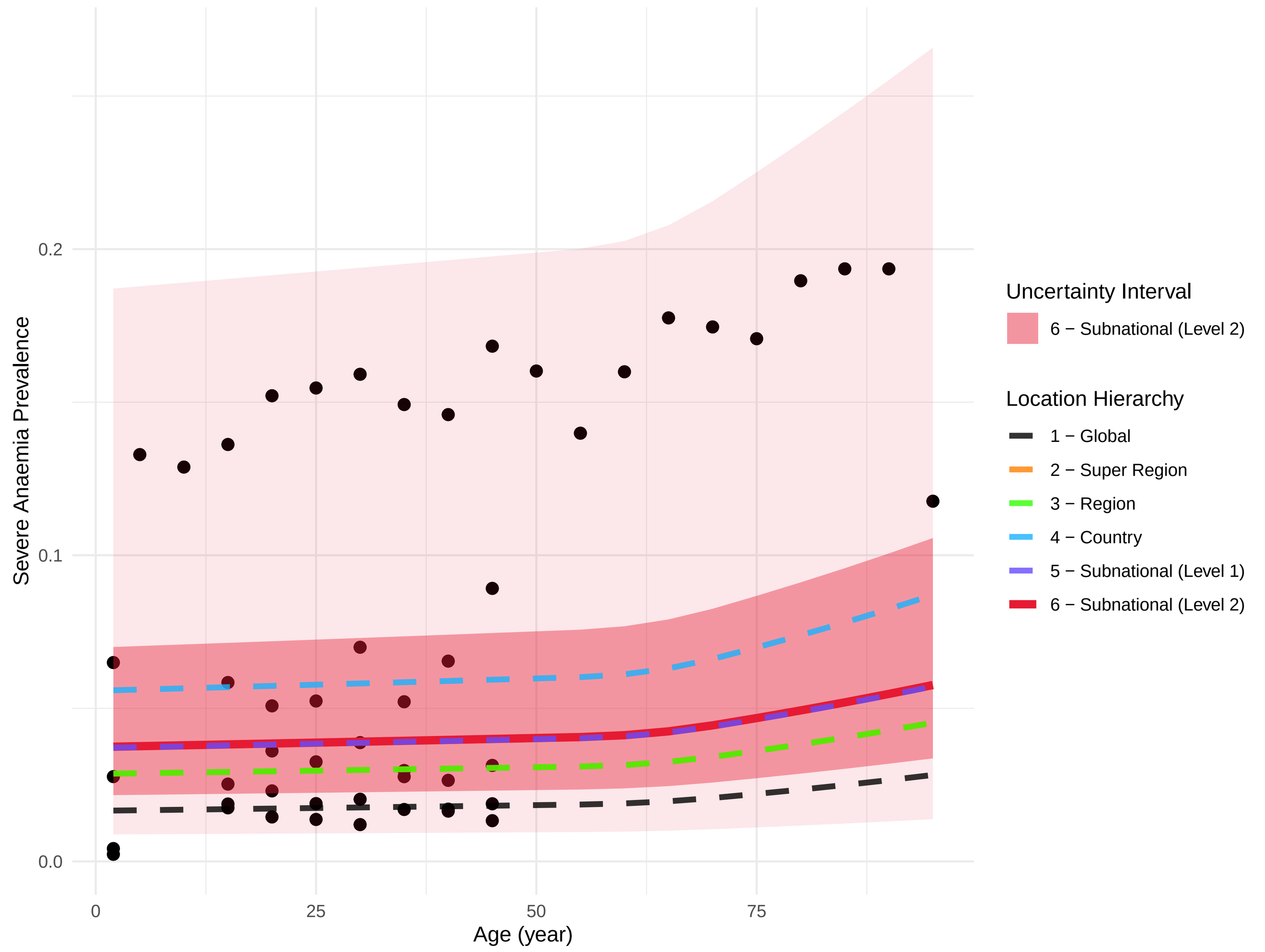

MR−BRT Age/Sex Fits − Non−pregnant Females 2+ Years Old − Severe Anaemia Prevalence − Assam, Urban
WHO−2001 Adjustment Method

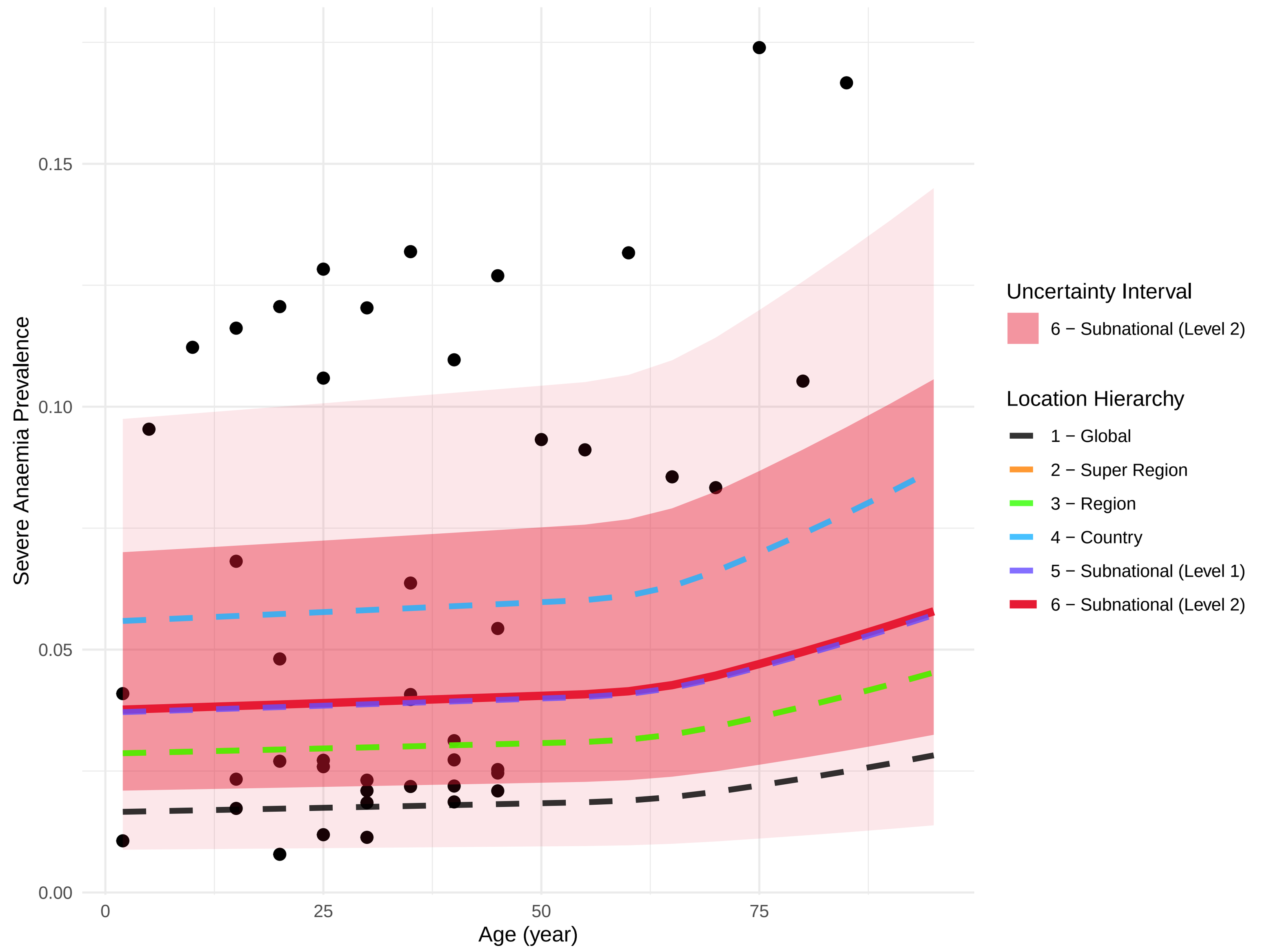

MR−BRT Age/Sex Fits − Non−pregnant Females 2+ Years Old − Severe Anaemia Prevalence − Bihar
WHO−2001 Adjustment Method

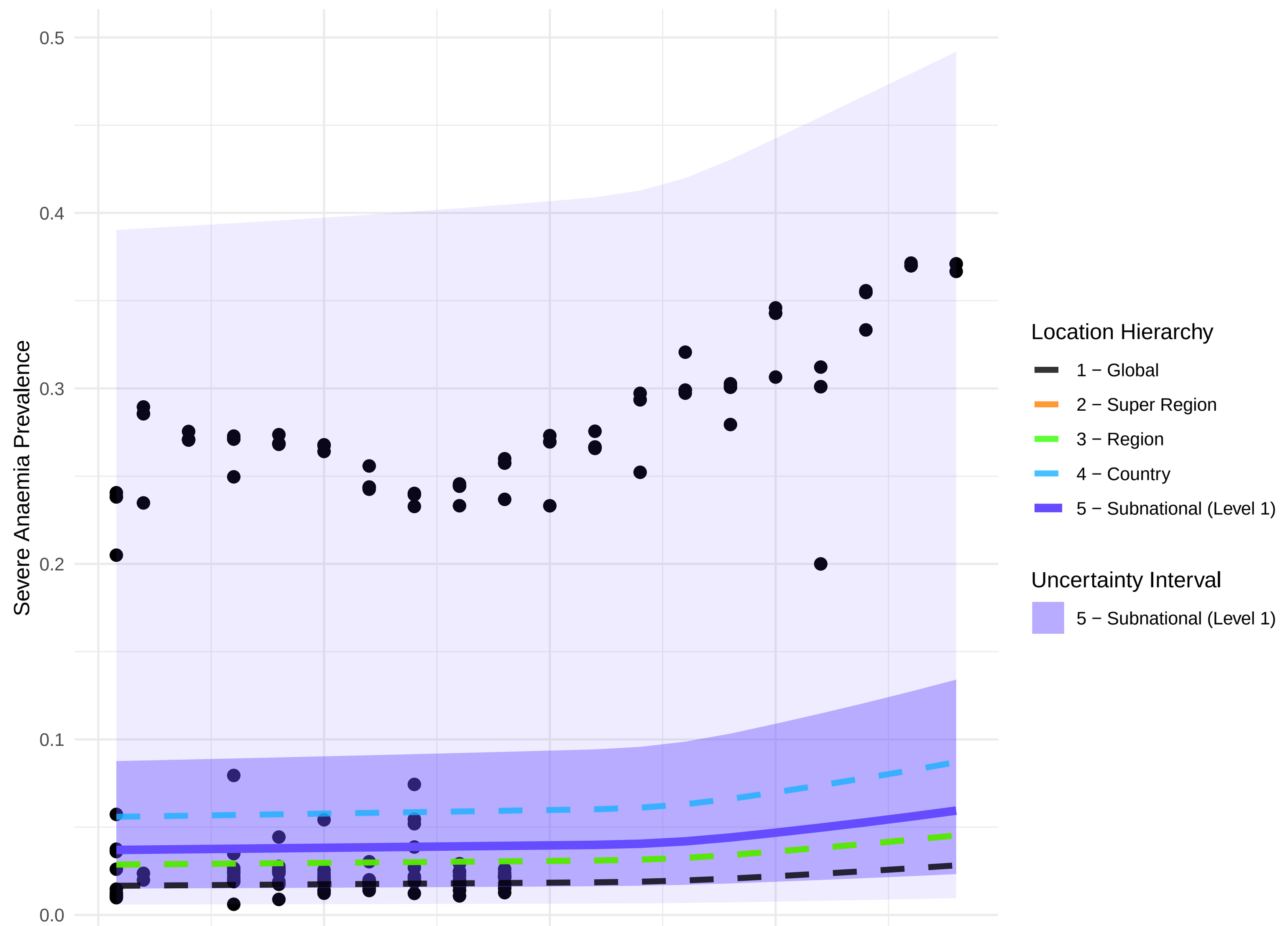

MR−BRT Age/Sex Fits − Non−pregnant Females 2+ Years Old − Severe Anaemia Prevalence − Bihar, Rural
WHO−2001 Adjustment Method

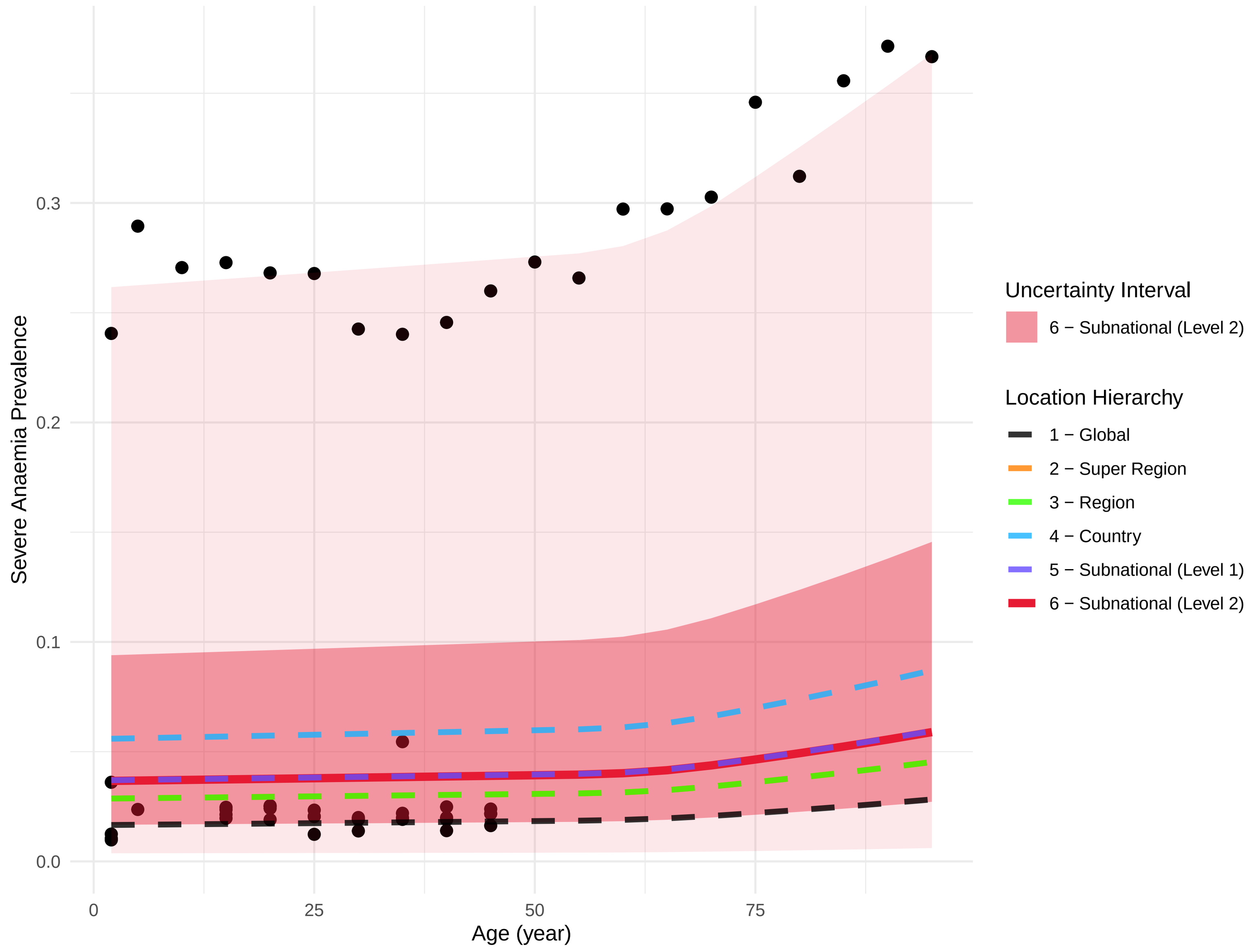

MR−BRT Age/Sex Fits − Non−pregnant Females 2+ Years Old − Severe Anaemia Prevalence − Bihar, Urban
WHO−2001 Adjustment Method

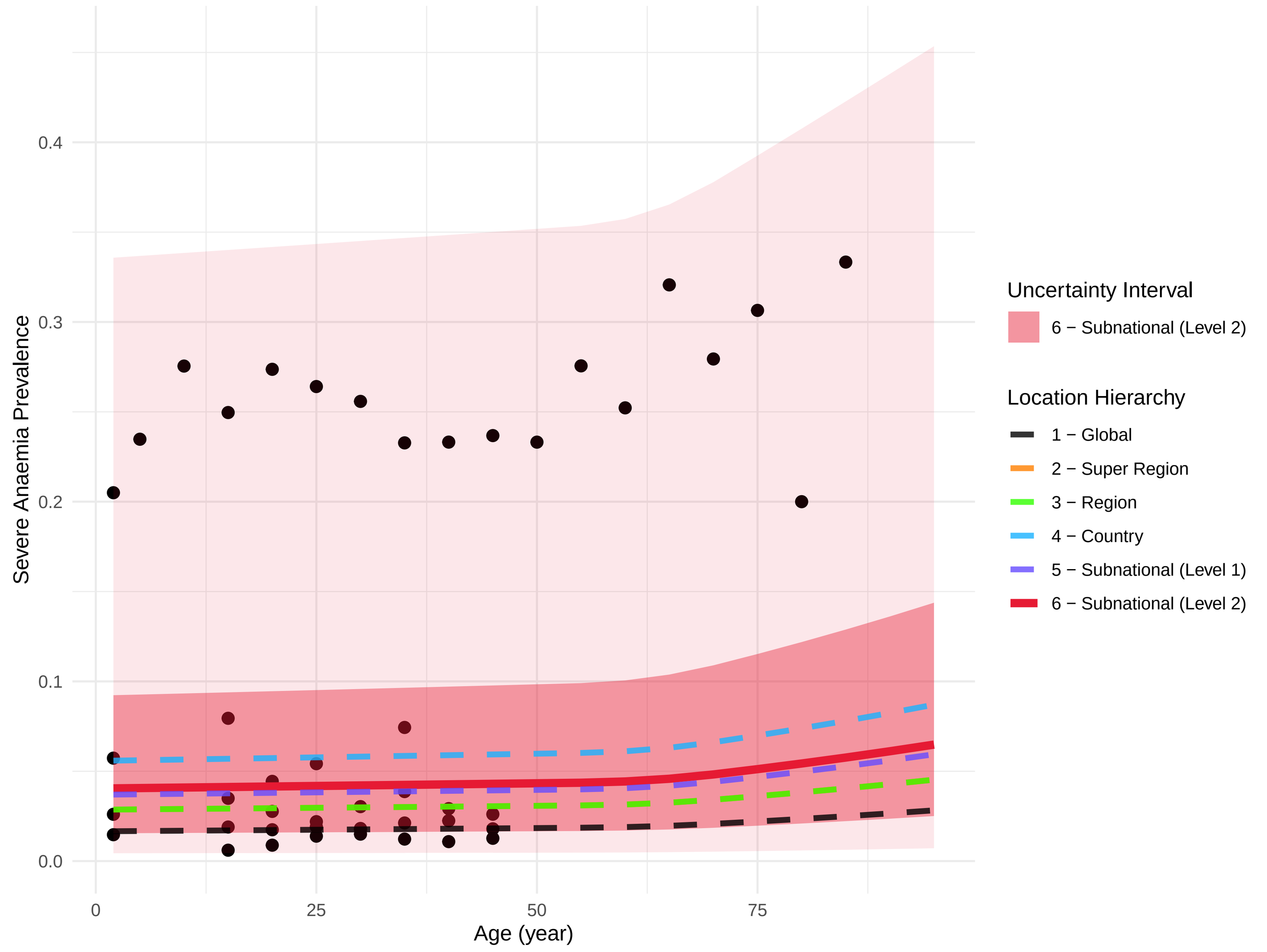

MR−BRT Age/Sex Fits − Non−pregnant Females 2+ Years Old − Severe Anaemia Prevalence − Chhattisgarh
WHO−2001 Adjustment Method

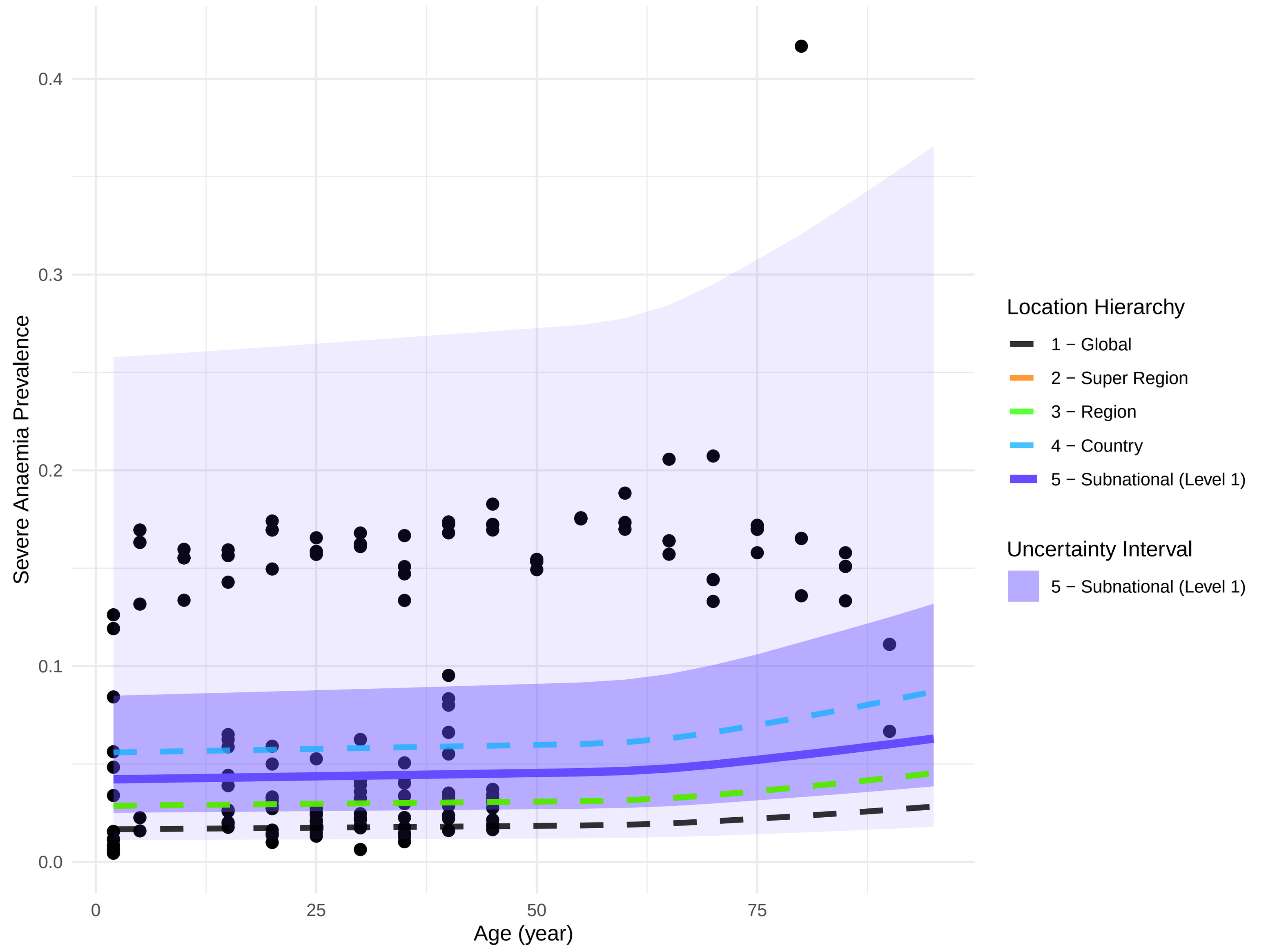

MR−BRT Age/Sex Fits − Non−pregnant Females 2+ Years Old − Severe Anaemia Prevalence − Chhattisgarh, Rural
WHO−2001 Adjustment Method

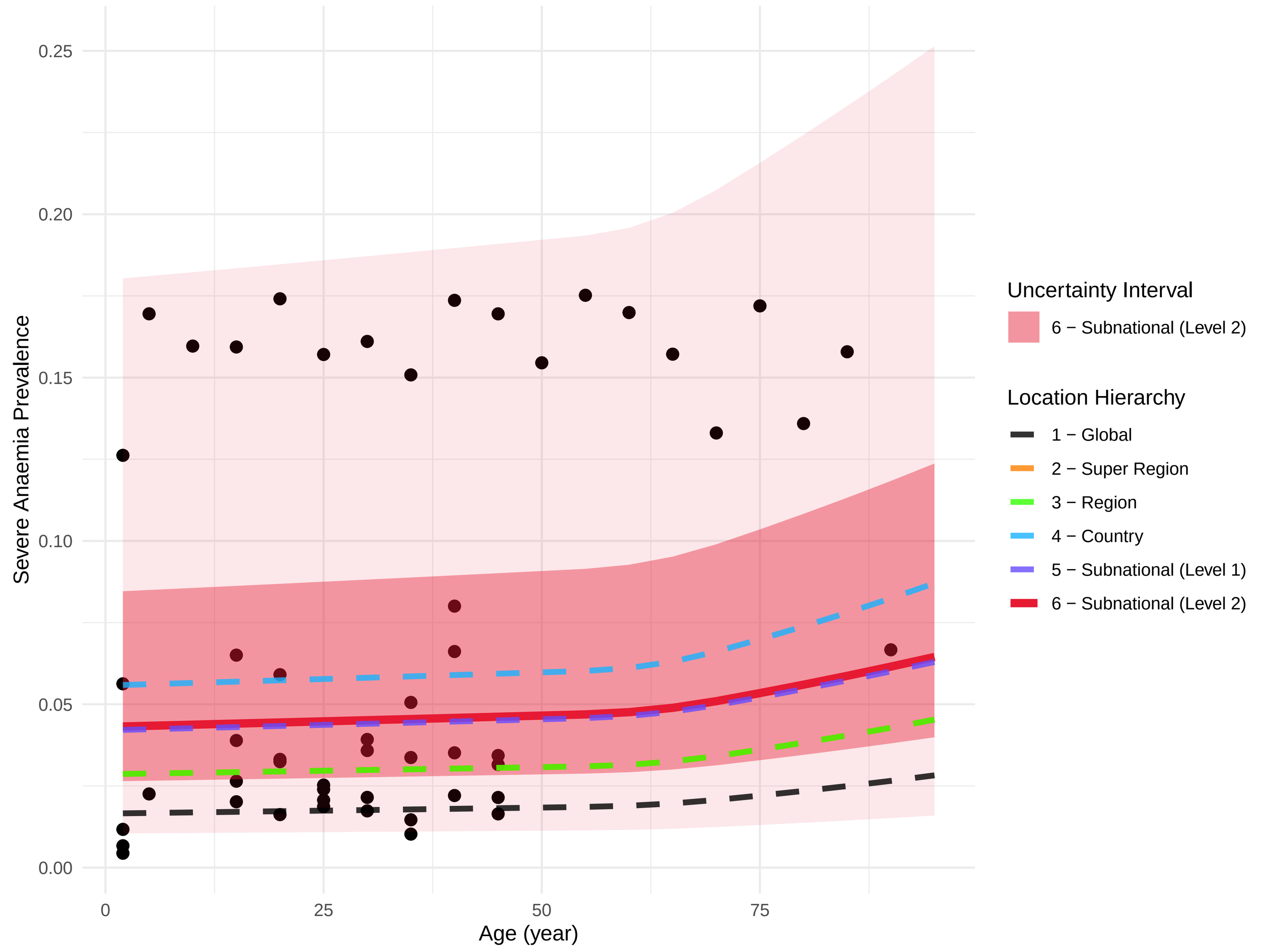

MR−BRT Age/Sex Fits − Non−pregnant Females 2+ Years Old − Severe Anaemia Prevalence − Chhattisgarh, Urban
WHO−2001 Adjustment Method

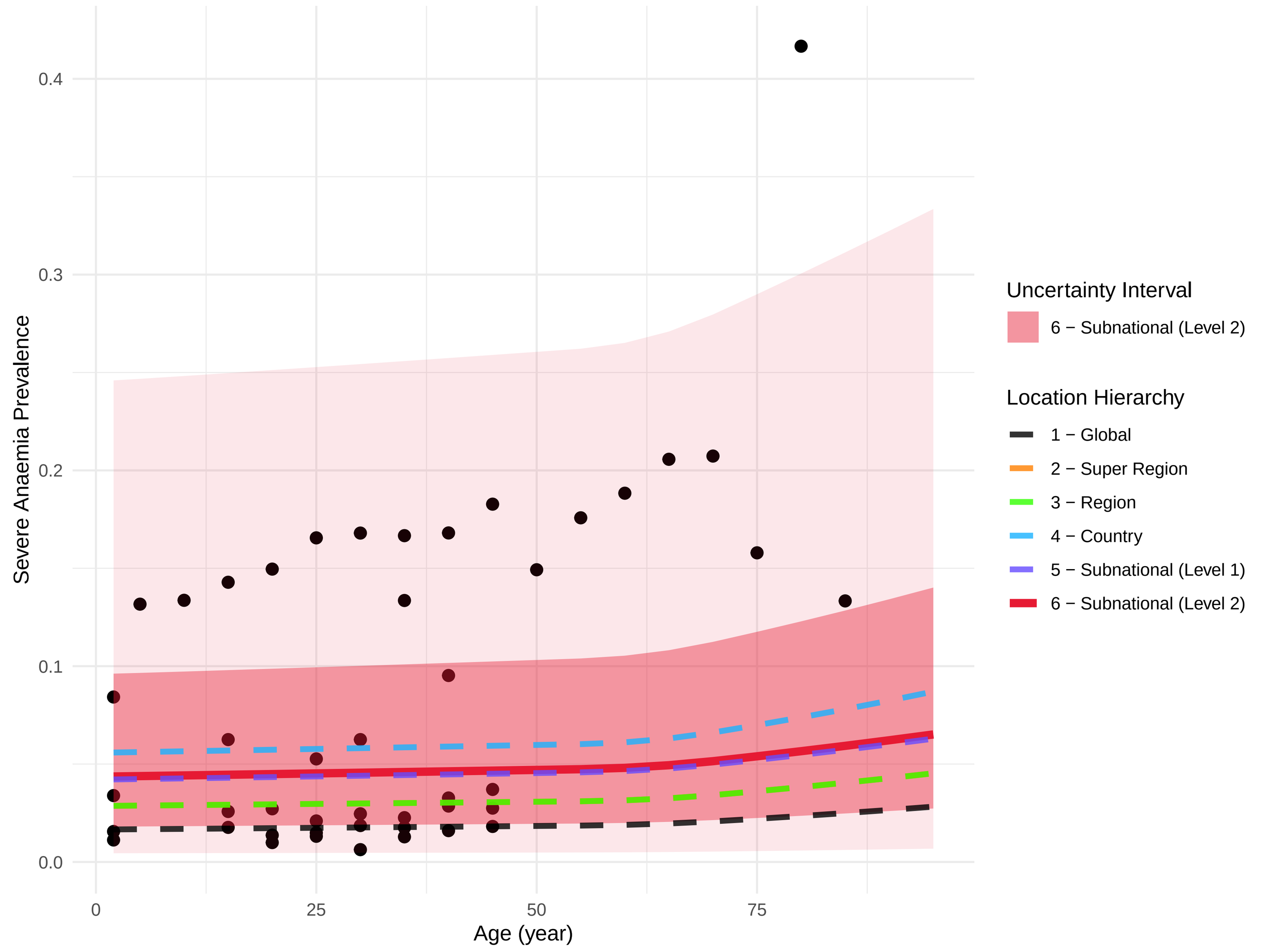

MR−BRT Age/Sex Fits − Non−pregnant Females 2+ Years Old − Severe Anaemia Prevalence − Delhi
WHO−2001 Adjustment Method

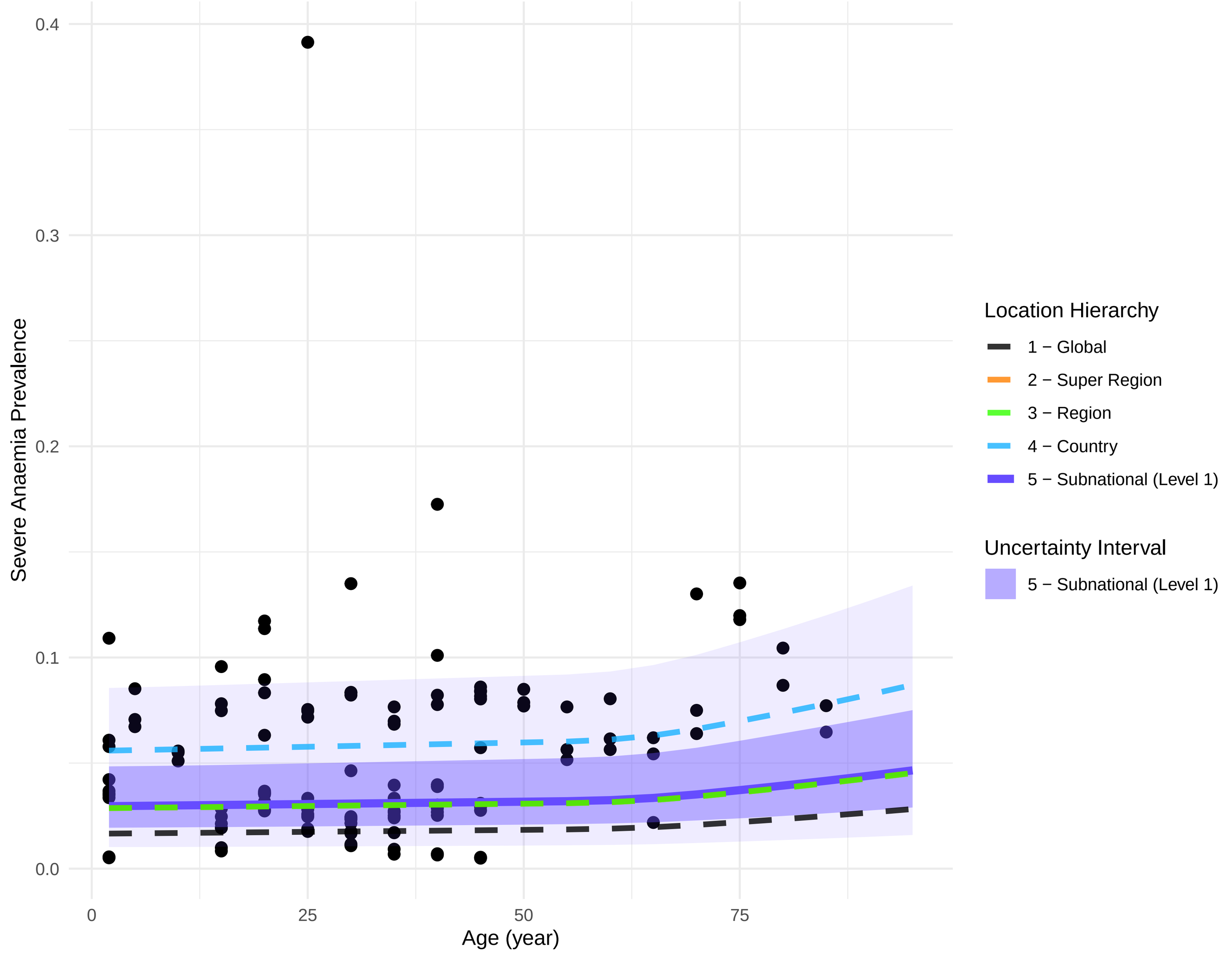

MR−BRT Age/Sex Fits − Non−pregnant Females 2+ Years Old − Severe Anaemia Prevalence − Delhi, Rural
WHO−2001 Adjustment Method

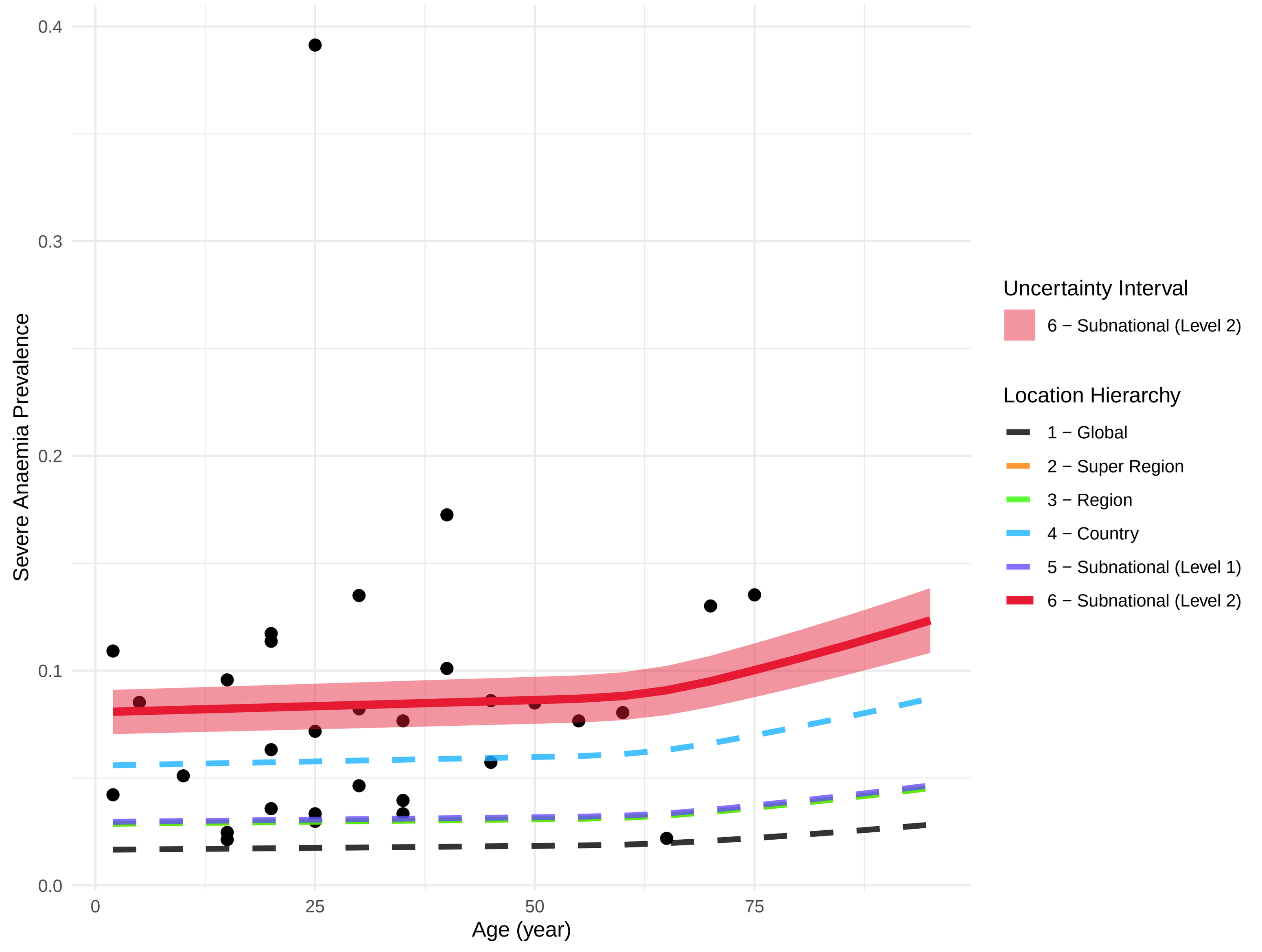

MR−BRT Age/Sex Fits − Non−pregnant Females 2+ Years Old − Severe Anaemia Prevalence − Delhi, Urban
WHO−2001 Adjustment Method

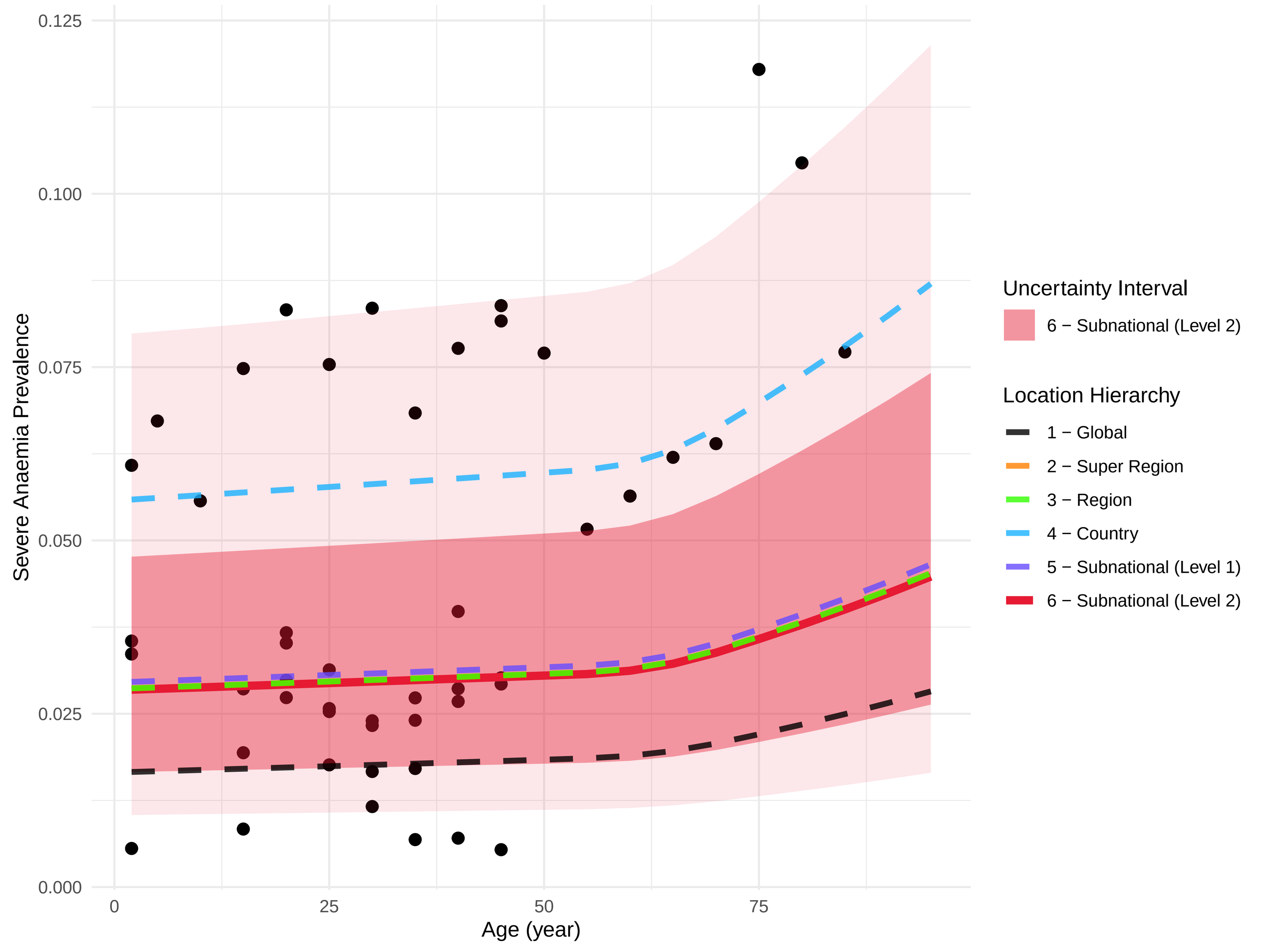

MR−BRT Age/Sex Fits − Non−pregnant Females 2+ Years Old − Severe Anaemia Prevalence − Goa
WHO−2001 Adjustment Method

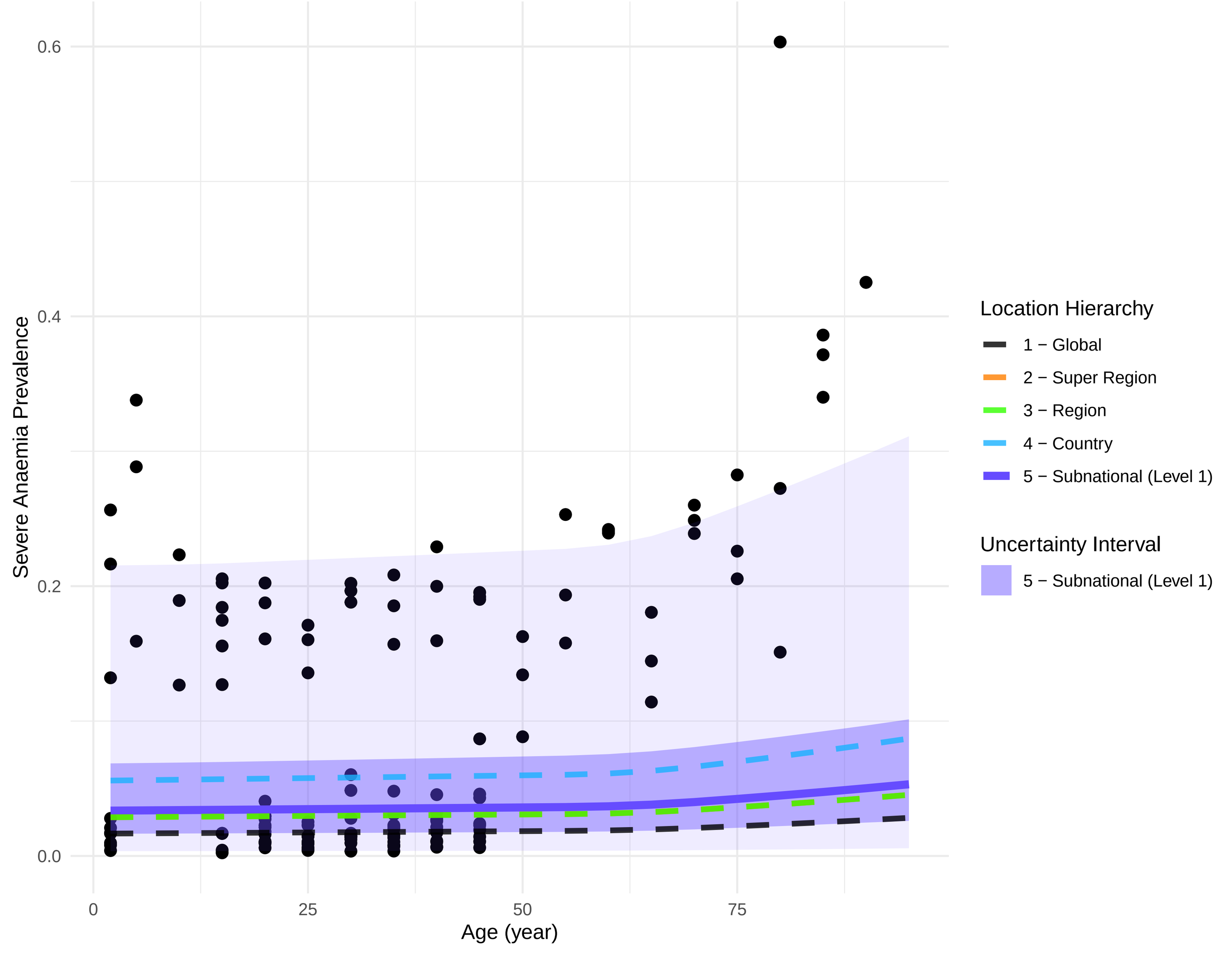

MR−BRT Age/Sex Fits − Non−pregnant Females 2+ Years Old − Severe Anaemia Prevalence − Goa, Rural
WHO−2001 Adjustment Method

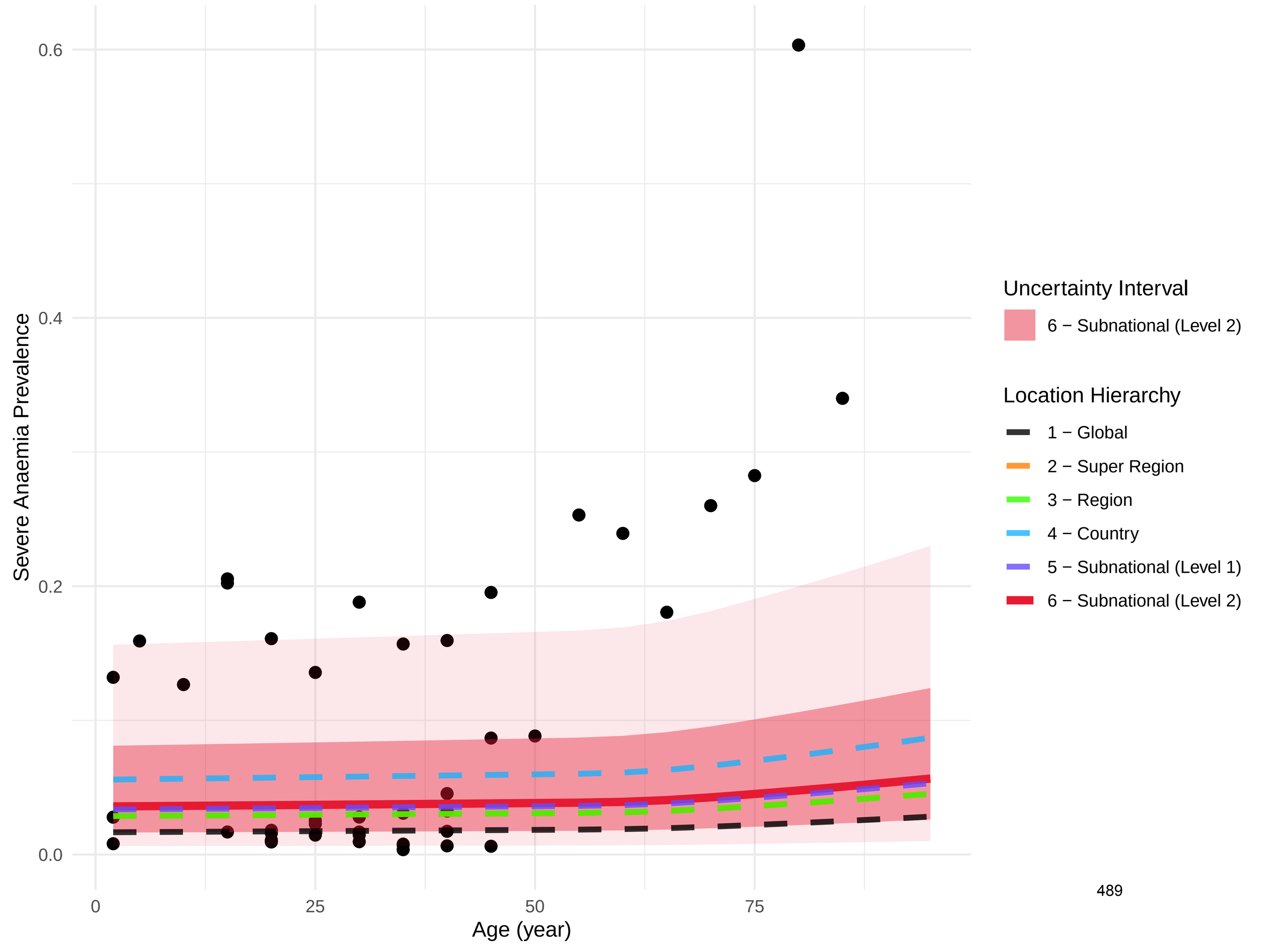

MR−BRT Age/Sex Fits − Non−pregnant Females 2+ Years Old − Severe Anaemia Prevalence − Goa, Urban
WHO−2001 Adjustment Method

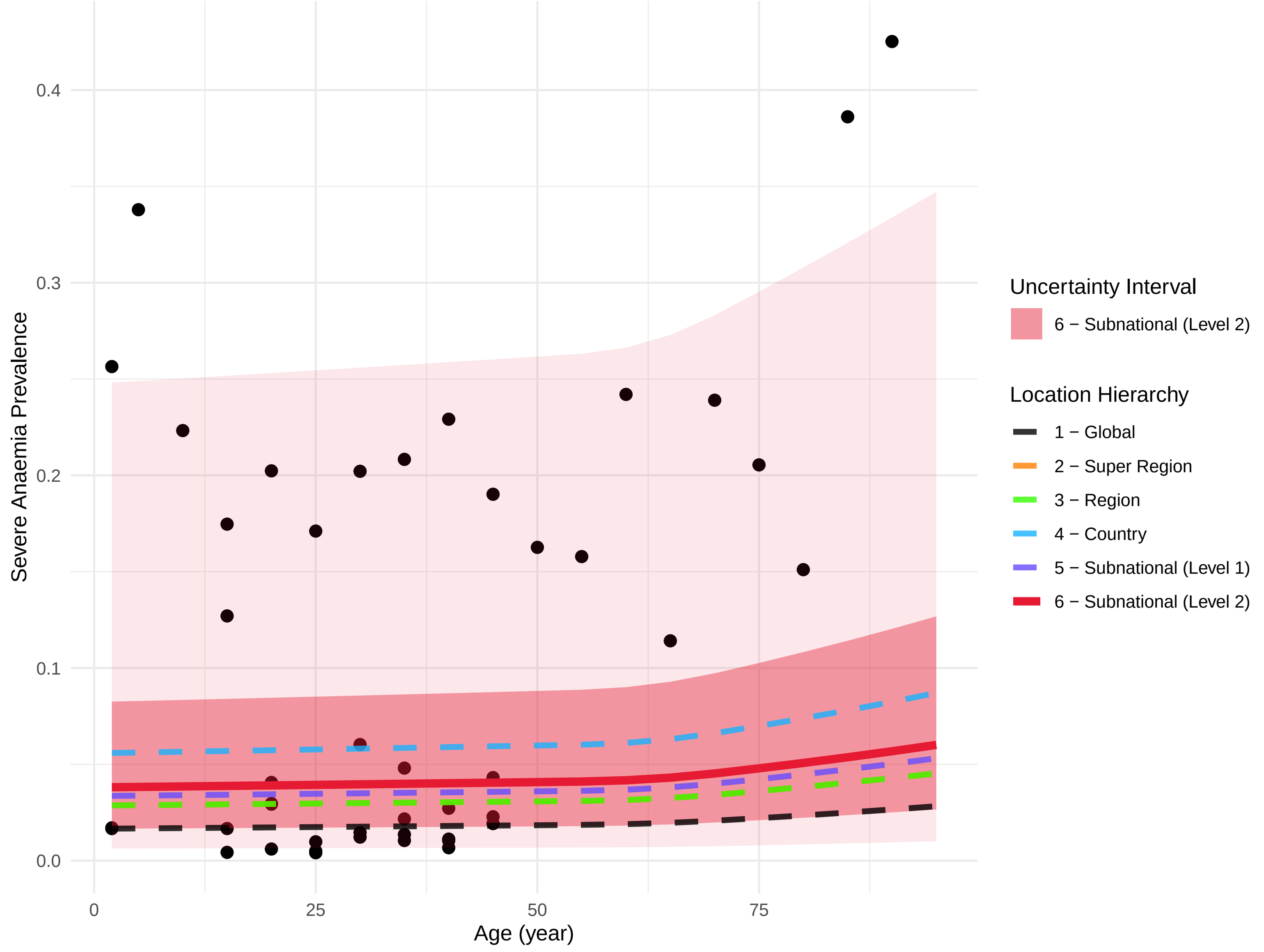

MR−BRT Age/Sex Fits − Non−pregnant Females 2+ Years Old − Severe Anaemia Prevalence − Gujarat
WHO−2001 Adjustment Method

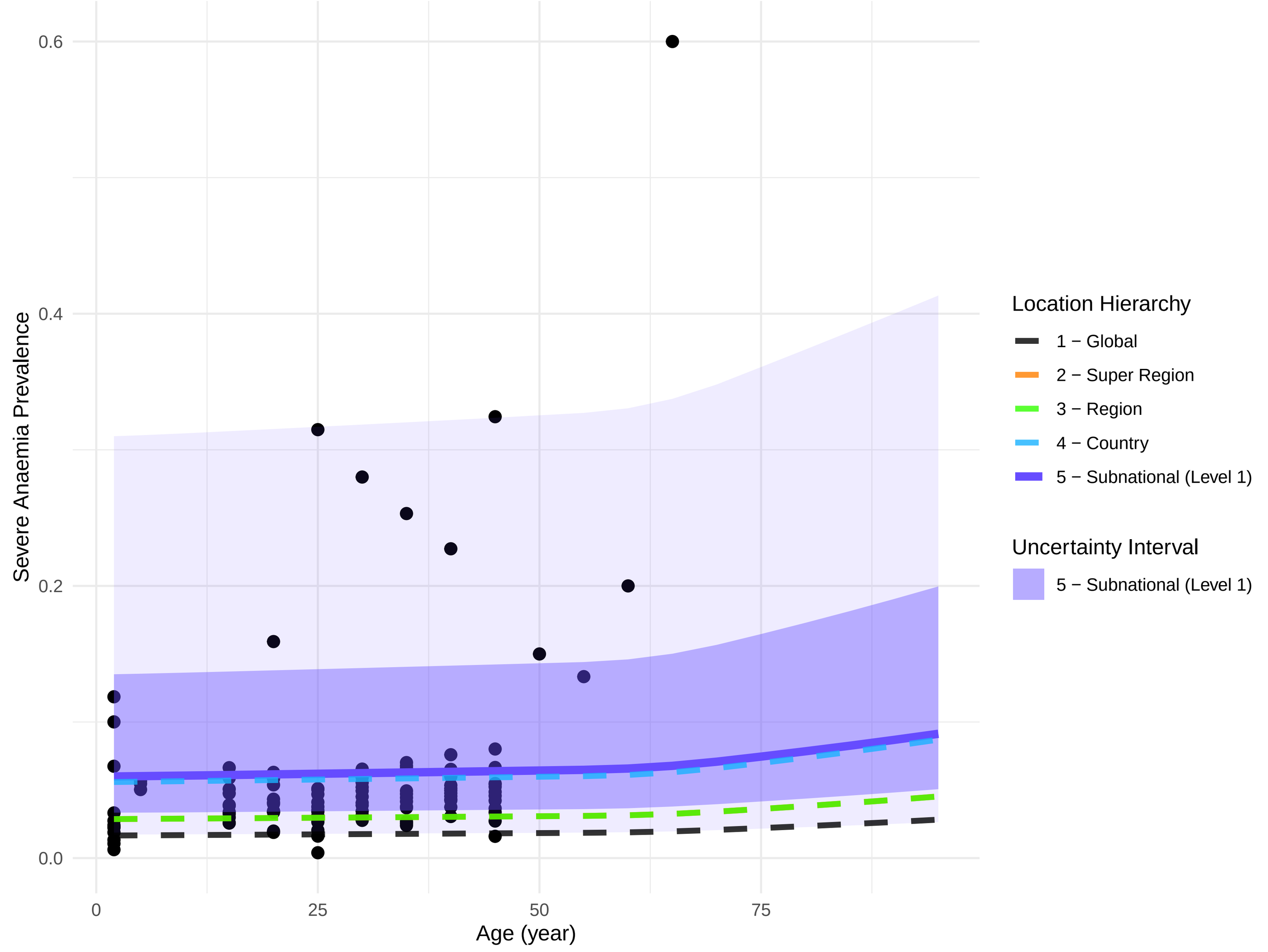

MR−BRT Age/Sex Fits − Non−pregnant Females 2+ Years Old − Severe Anaemia Prevalence − Gujarat, Rural
WHO−2001 Adjustment Method

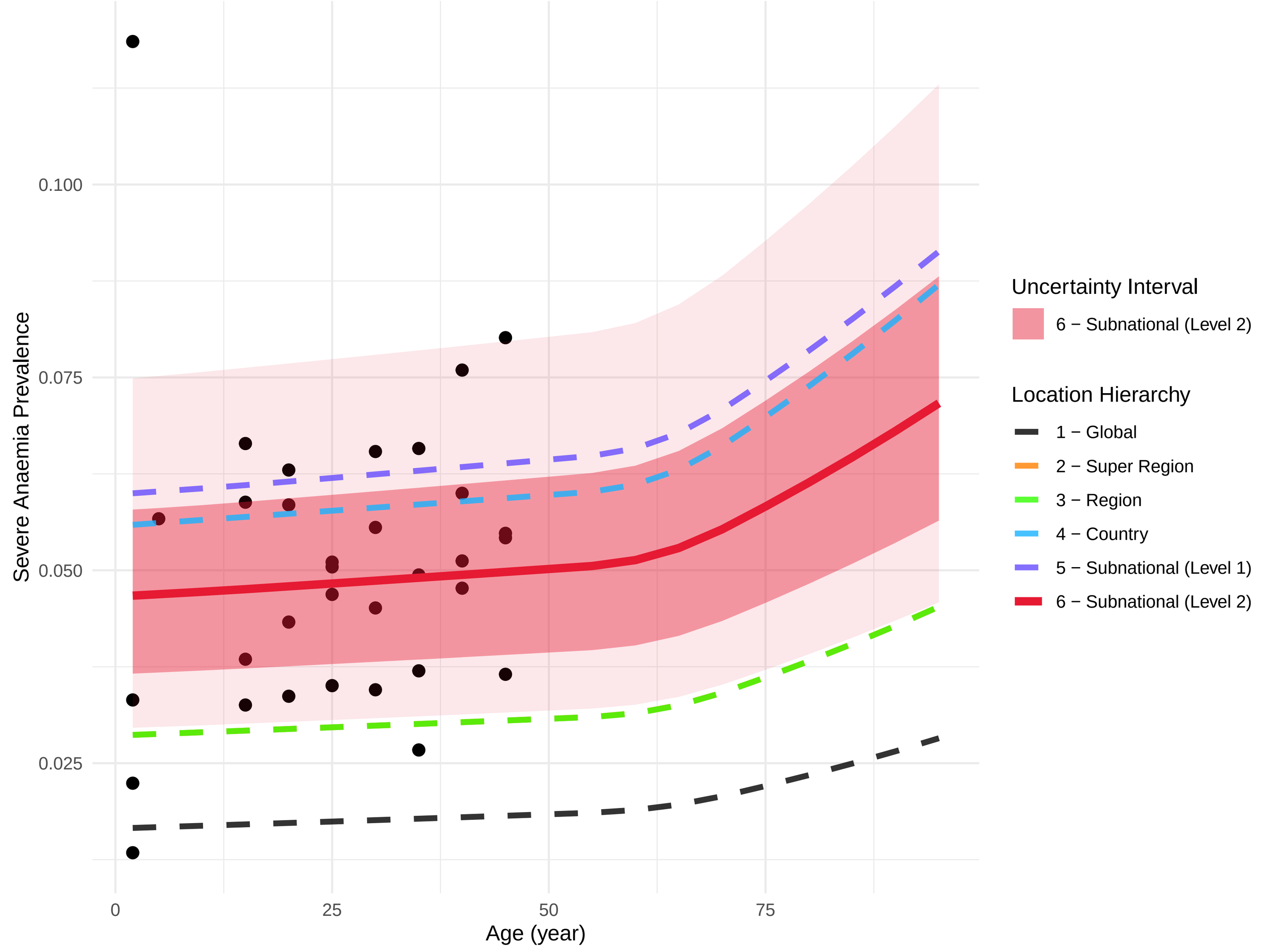

MR−BRT Age/Sex Fits − Non−pregnant Females 2+ Years Old − Severe Anaemia Prevalence − Gujarat, Urban
WHO−2001 Adjustment Method

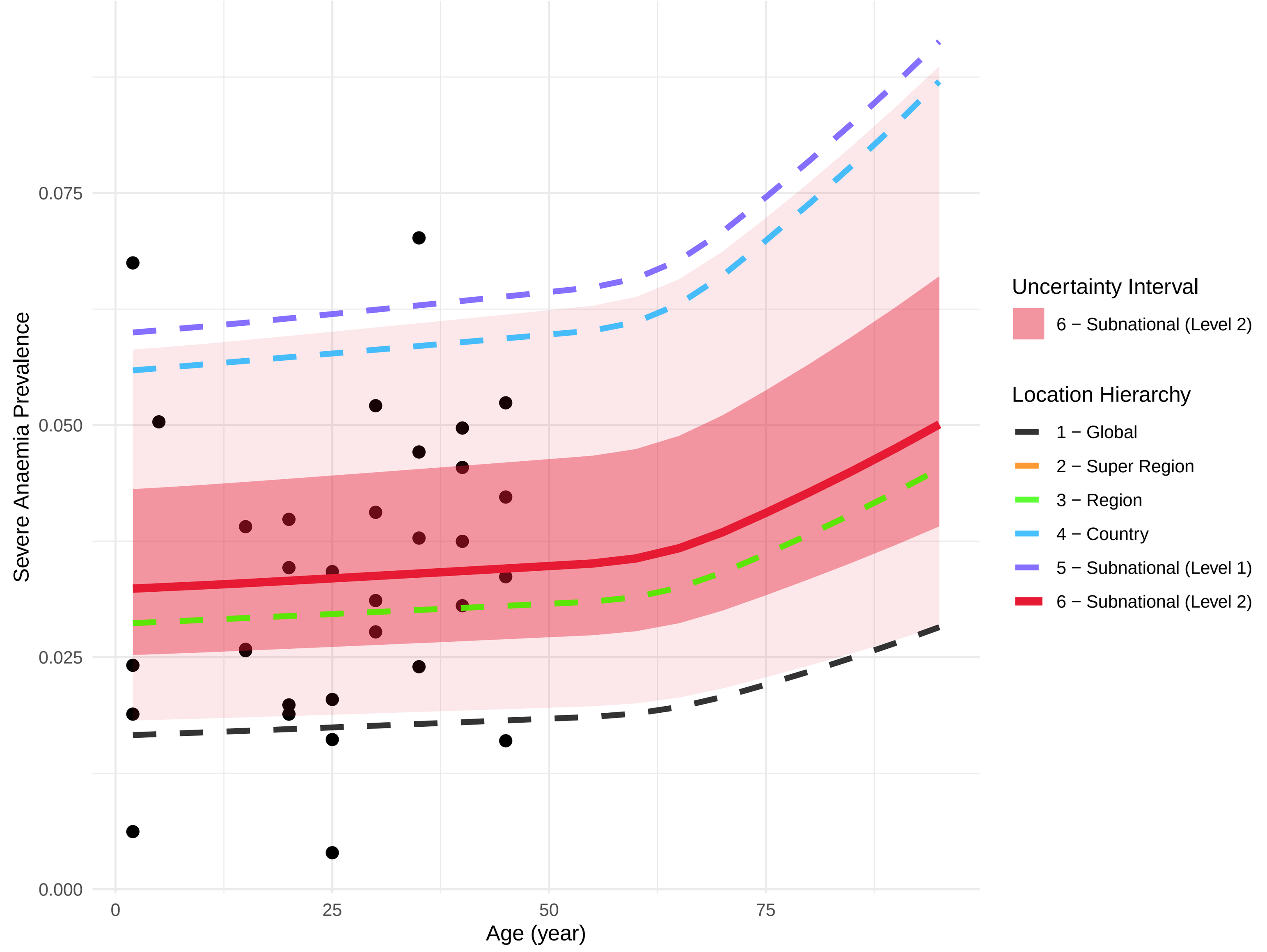

MR−BRT Age/Sex Fits − Non−pregnant Females 2+ Years Old − Severe Anaemia Prevalence − Haryana
WHO−2001 Adjustment Method

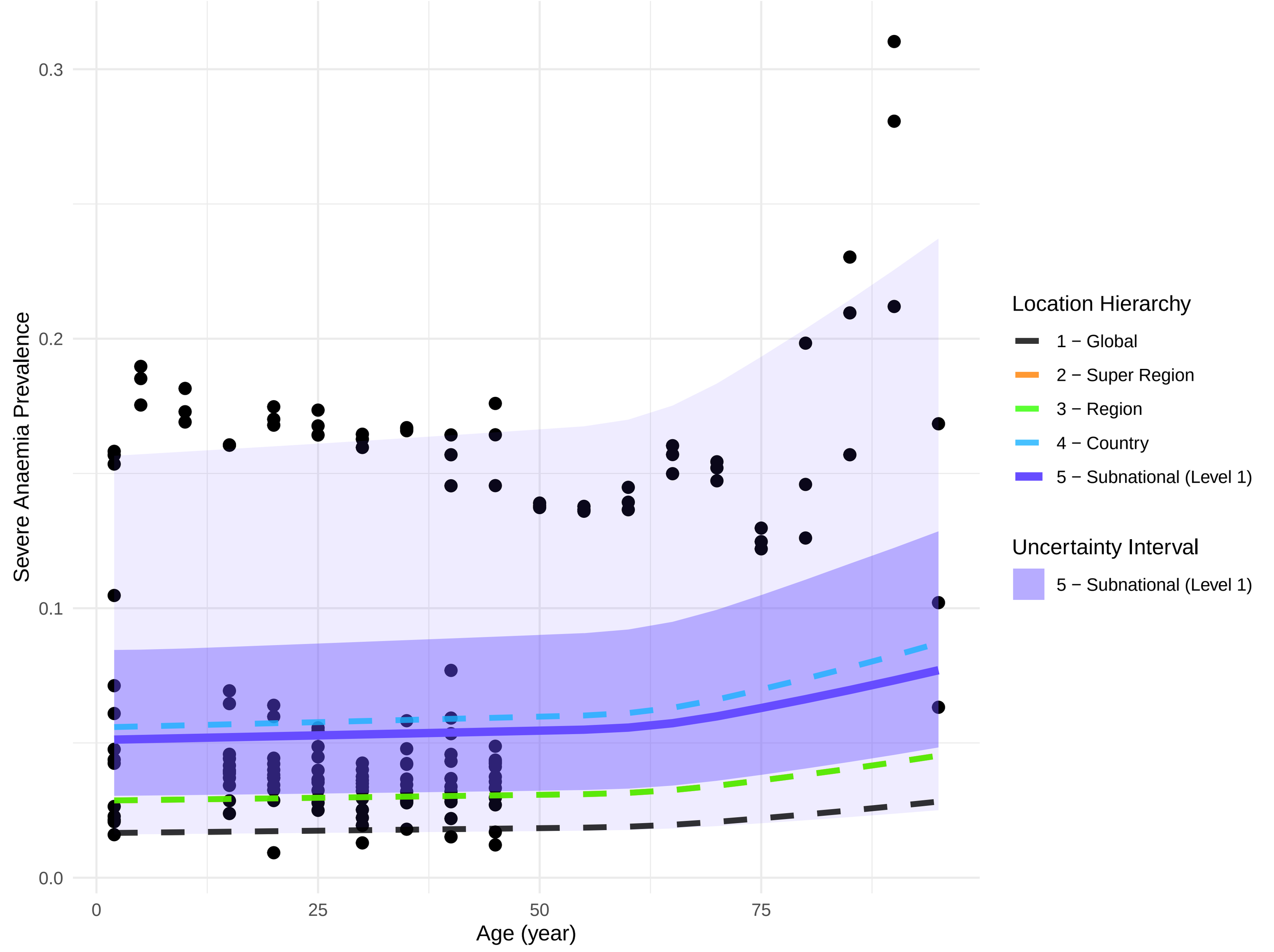

MR−BRT Age/Sex Fits − Non−pregnant Females 2+ Years Old − Severe Anaemia Prevalence − Haryana, Rural
WHO−2001 Adjustment Method

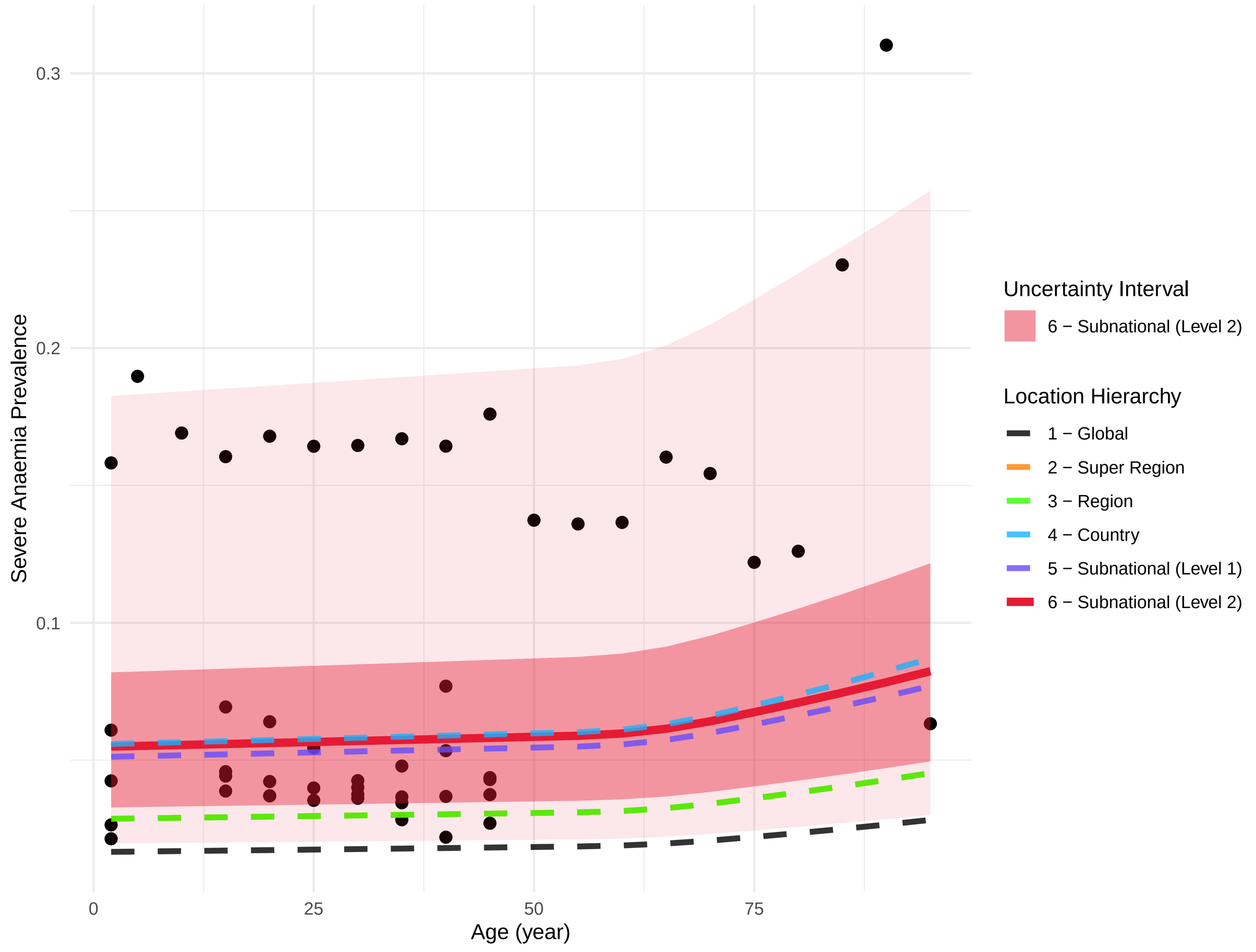

MR−BRT Age/Sex Fits − Non−pregnant Females 2+ Years Old − Severe Anaemia Prevalence − Haryana, Urban
WHO−2001 Adjustment Method

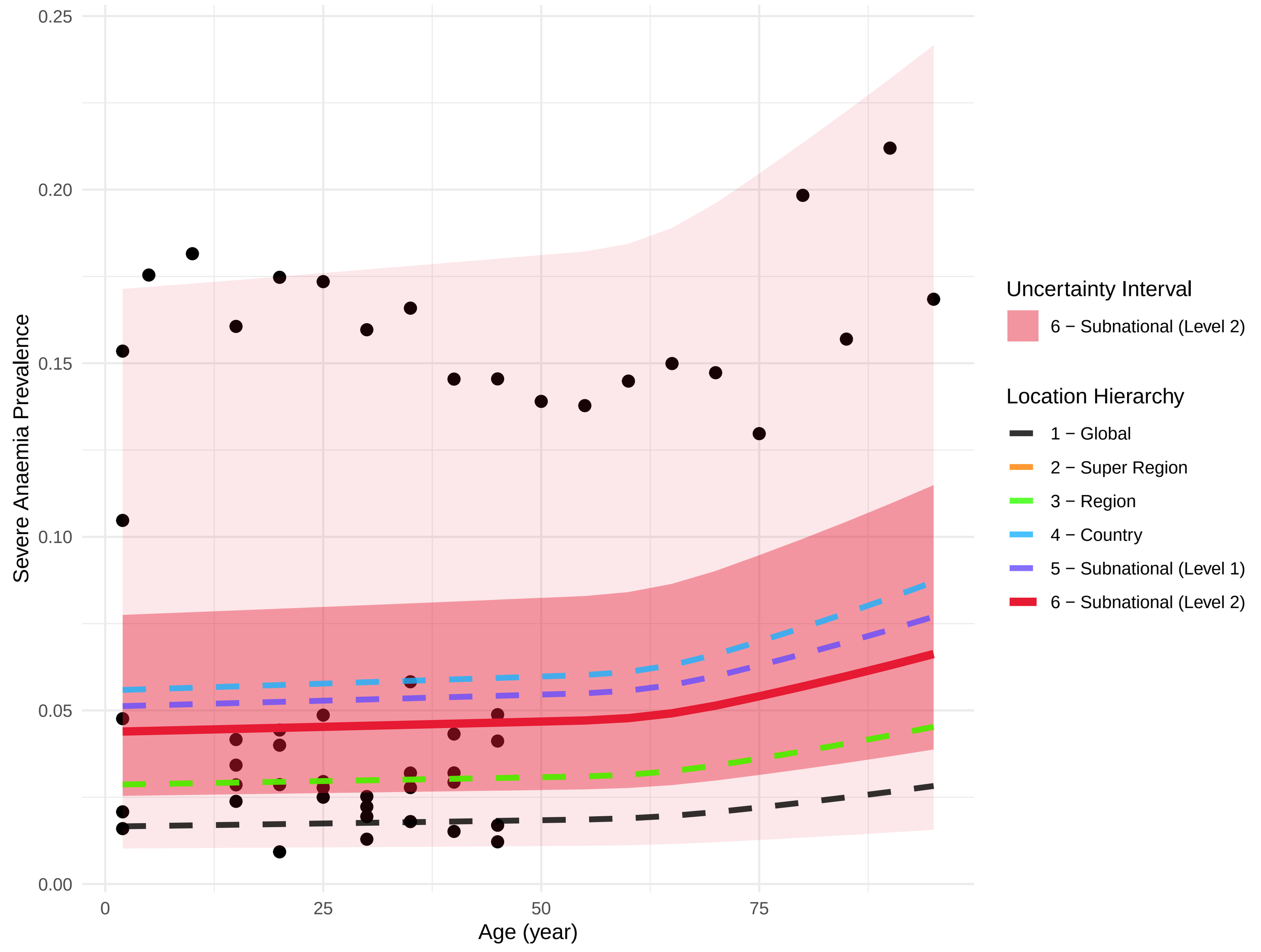

MR−BRT Age/Sex Fits − Non−pregnant Females 2+ Years Old − Severe Anaemia Prevalence − Himachal Pradesh
WHO−2001 Adjustment Method

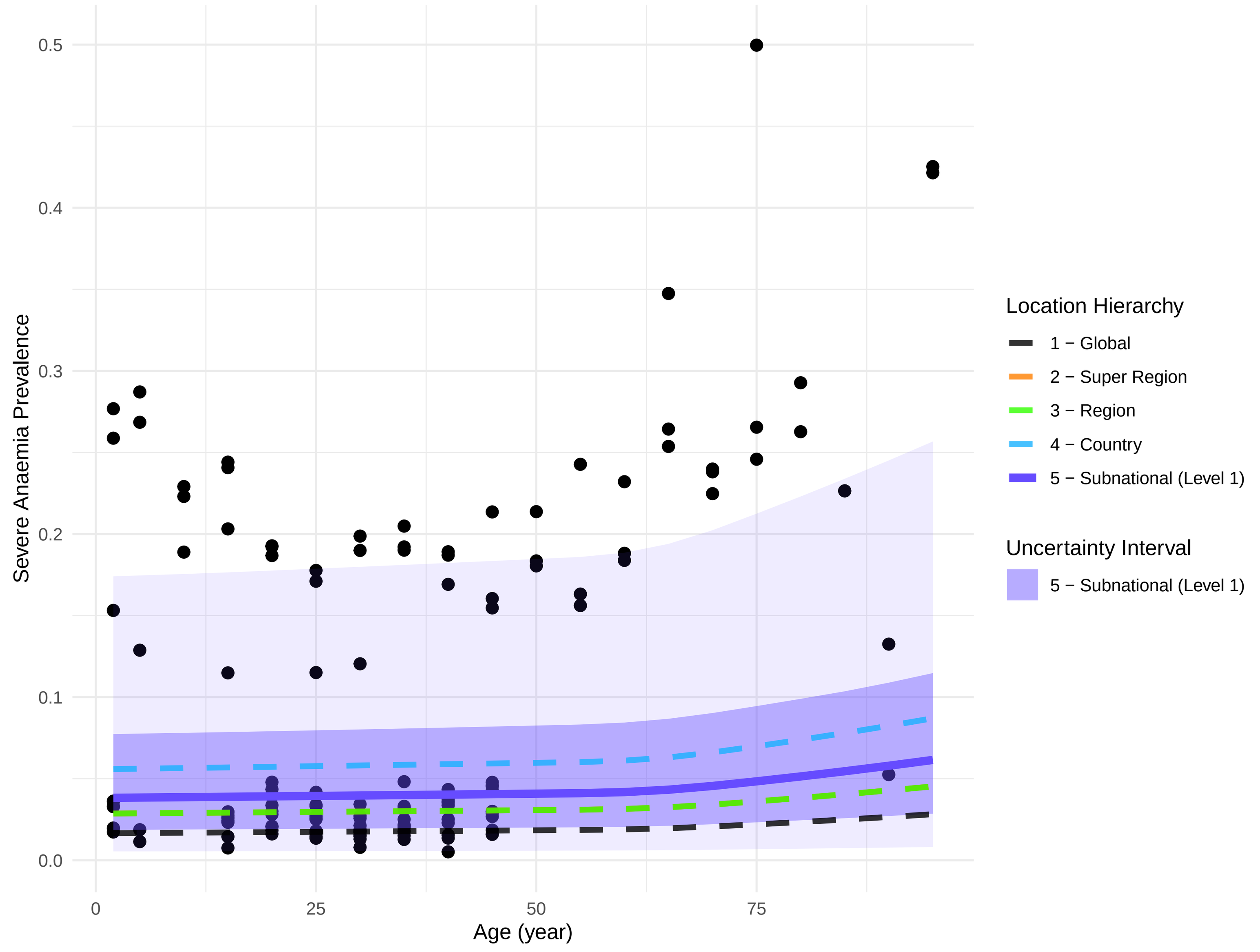

MR−BRT Age/Sex Fits − Non−pregnant Females 2+ Years Old − Severe Anaemia Prevalence − Himachal Pradesh, Rural
WHO−2001 Adjustment Method

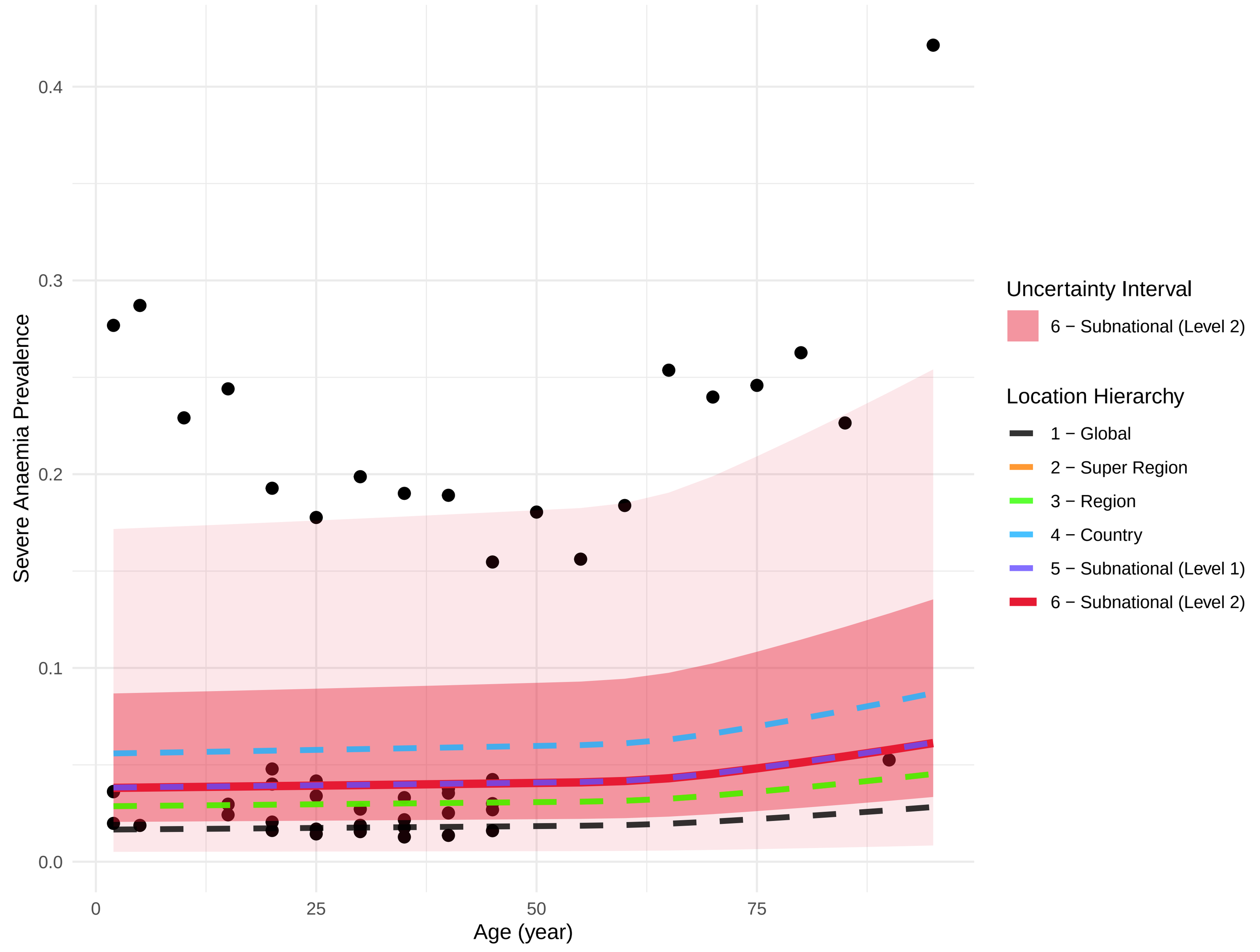

MR−BRT Age/Sex Fits − Non−pregnant Females 2+ Years Old − Severe Anaemia Prevalence − Himachal Pradesh, Urban
WHO−2001 Adjustment Method

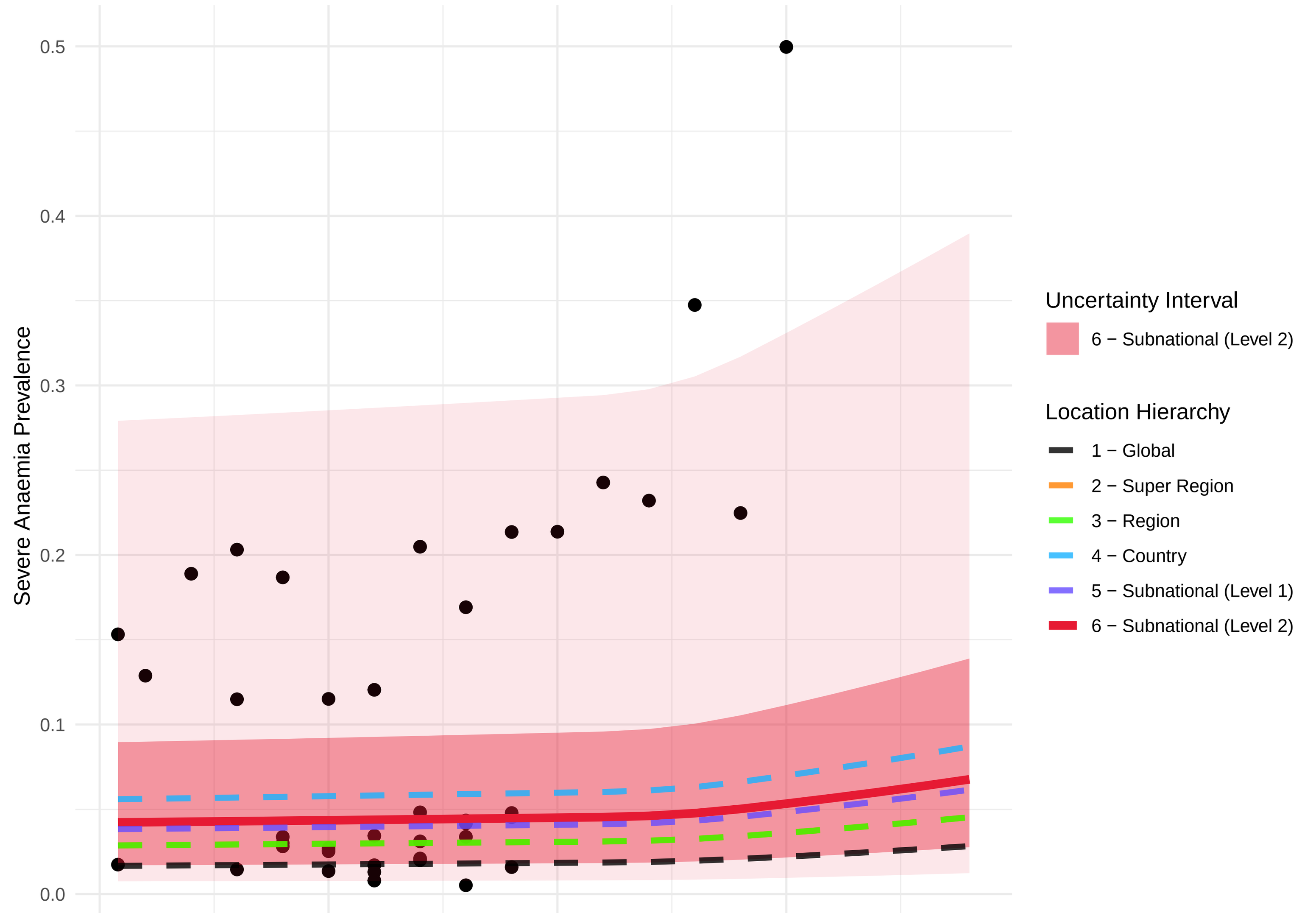

MR−BRT Age/Sex Fits − Non−pregnant Females 2+ Years Old − Severe Anaemia Prevalence − Jammu & Kashmir and Ladakh
WHO−2001 Adjustment Method

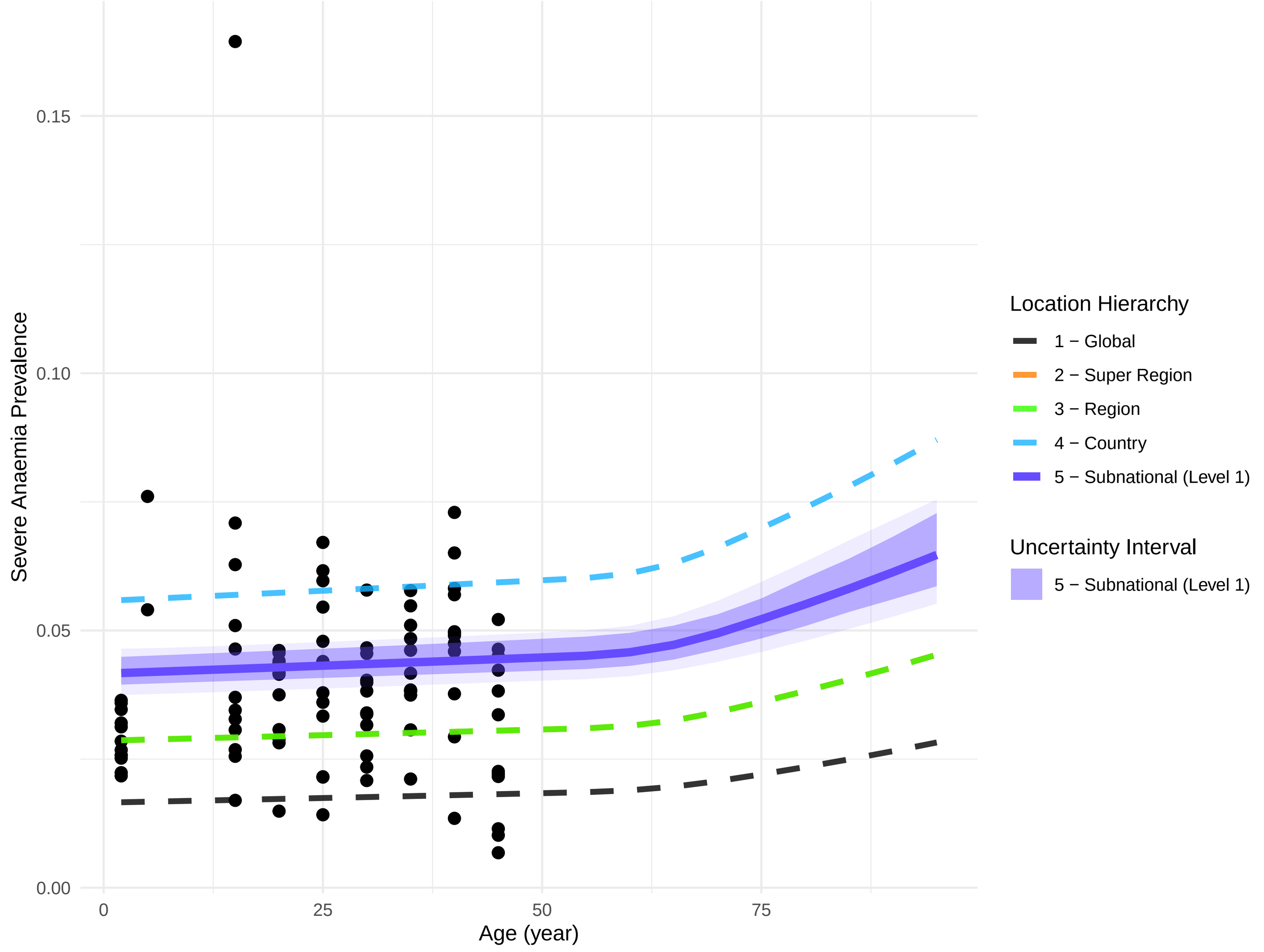

MR−BRT Age/Sex Fits − Non−pregnant Females 2+ Years Old − Severe Anaemia Prevalence − Jammu & Kashmir and Ladakh, Rural
WHO−2001 Adjustment Method

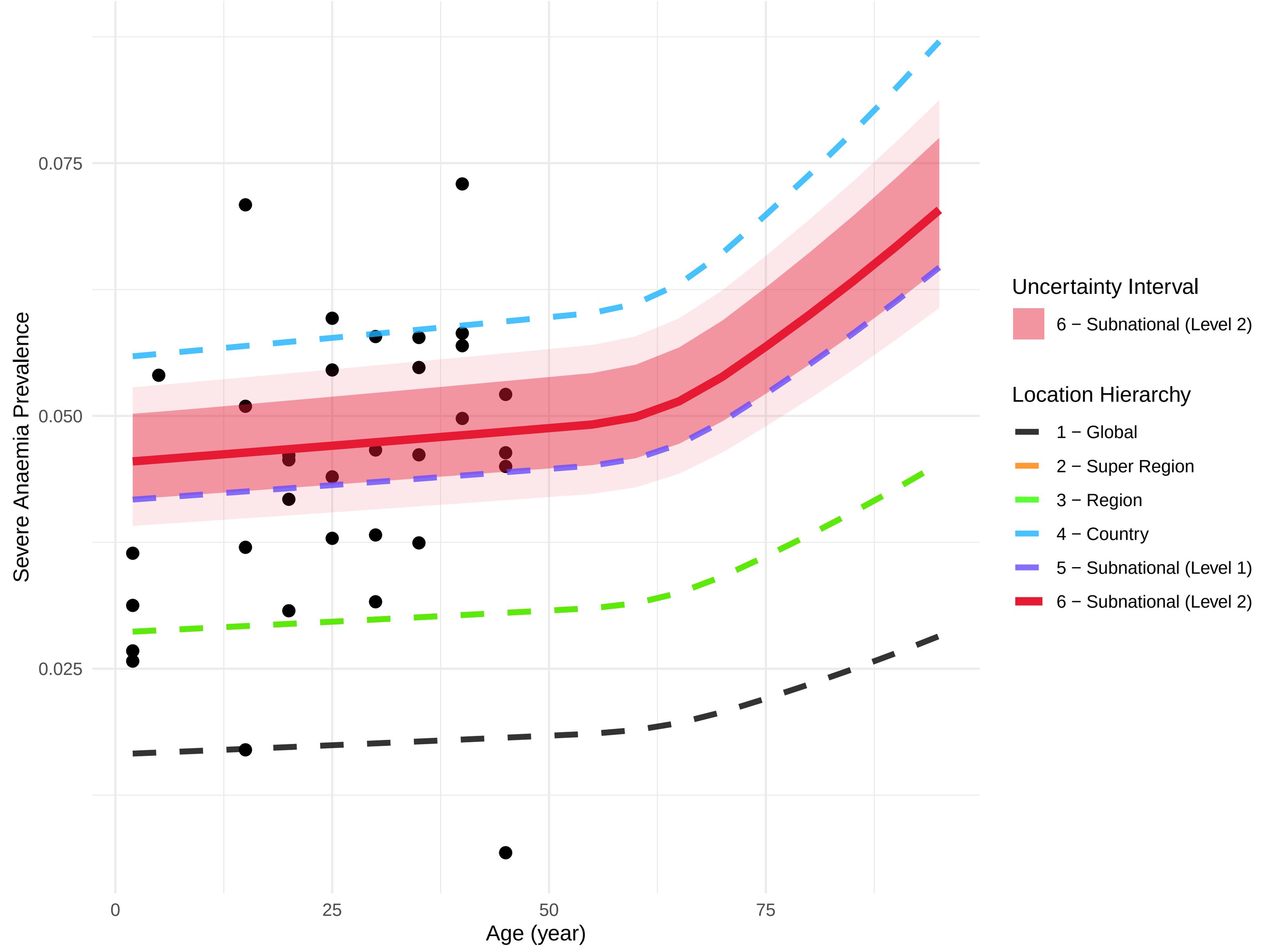

MR−BRT Age/Sex Fits − Non−pregnant Females 2+ Years Old − Severe Anaemia Prevalence − Jammu & Kashmir and Ladakh, Urban
WHO−2001 Adjustment Method

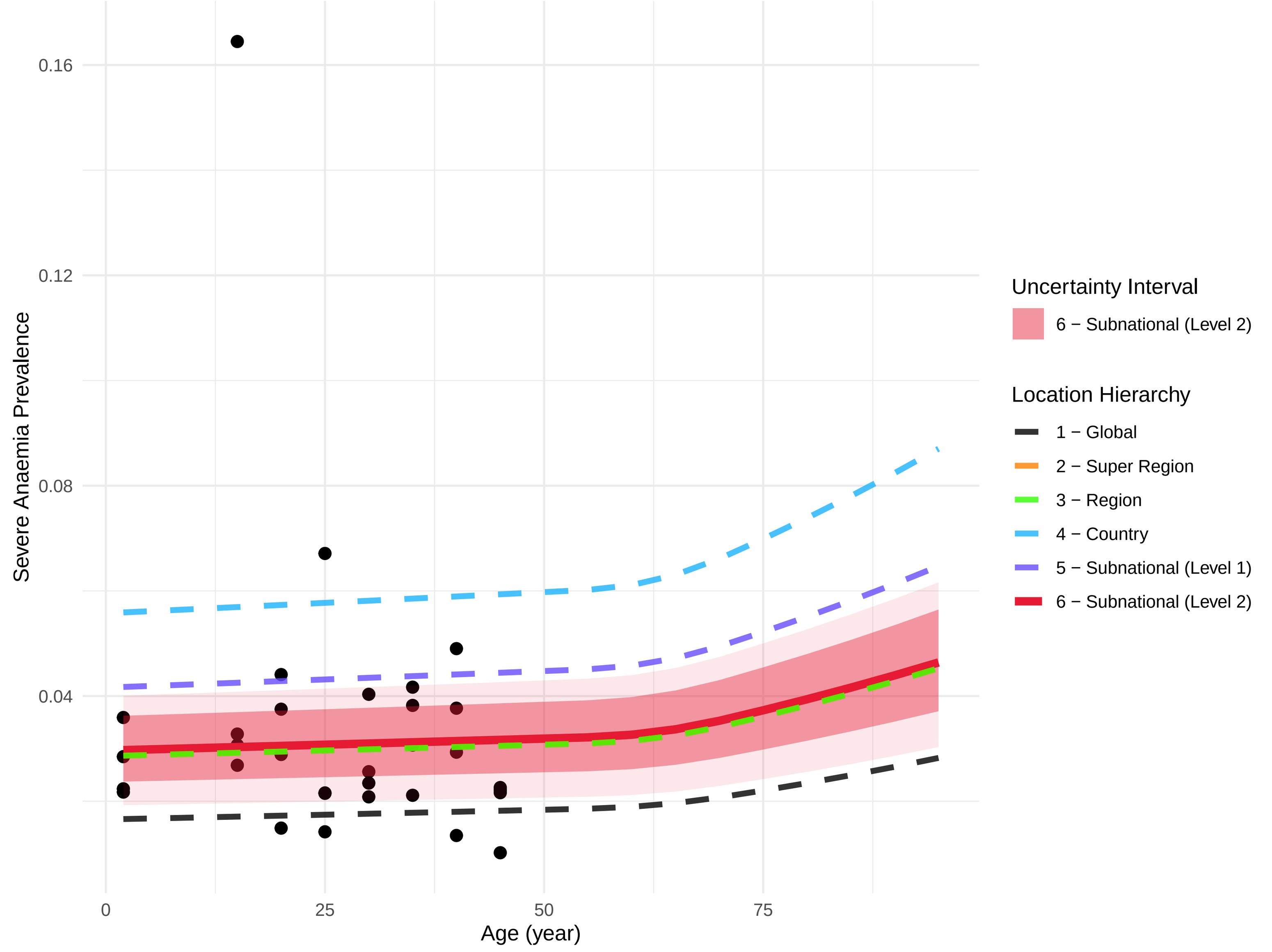

MR−BRT Age/Sex Fits − Non−pregnant Females 2+ Years Old − Severe Anaemia Prevalence − Jharkhand
WHO−2001 Adjustment Method

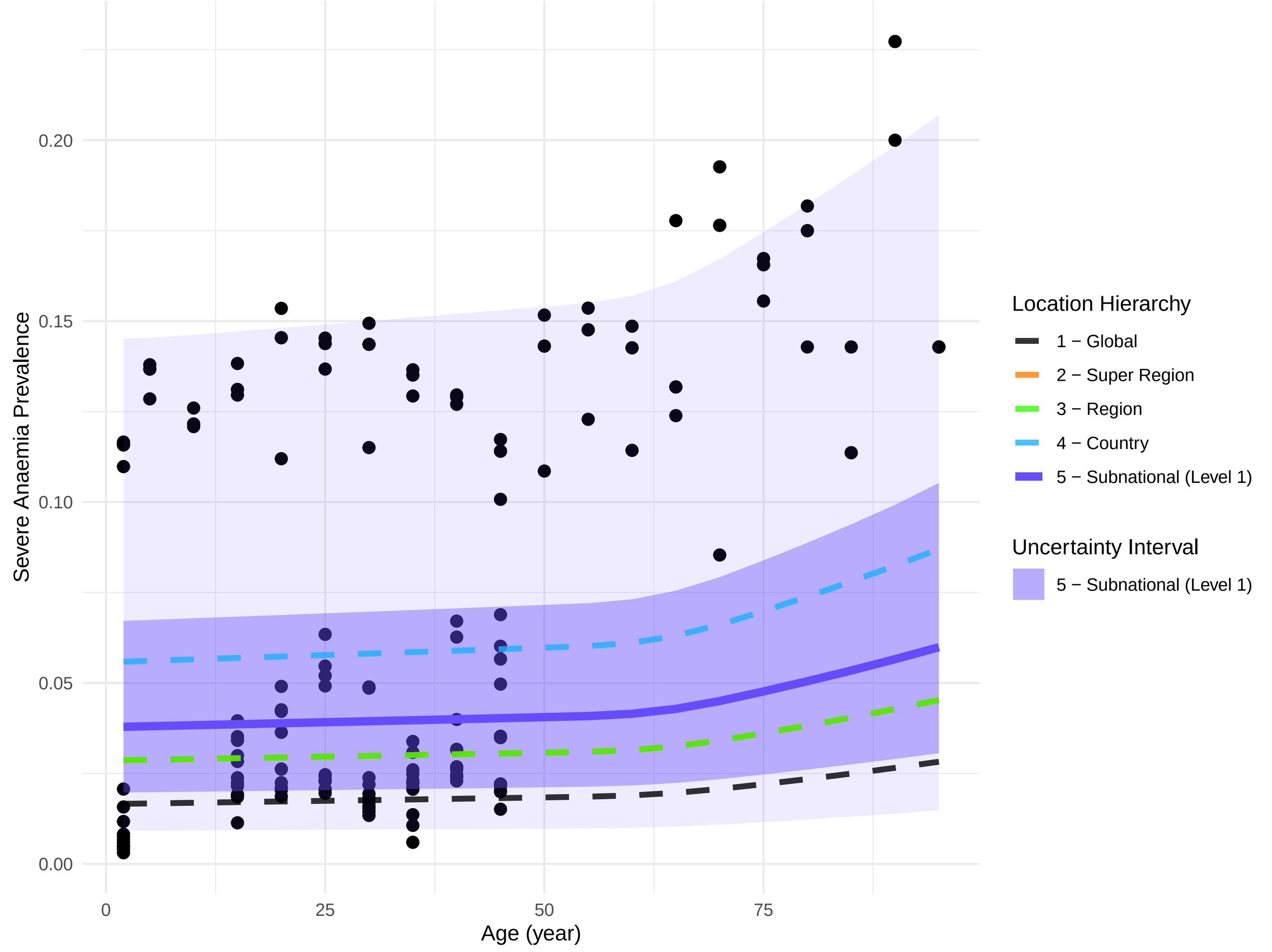

MR−BRT Age/Sex Fits − Non−pregnant Females 2+ Years Old − Severe Anaemia Prevalence − Jharkhand, Rural
WHO−2001 Adjustment Method

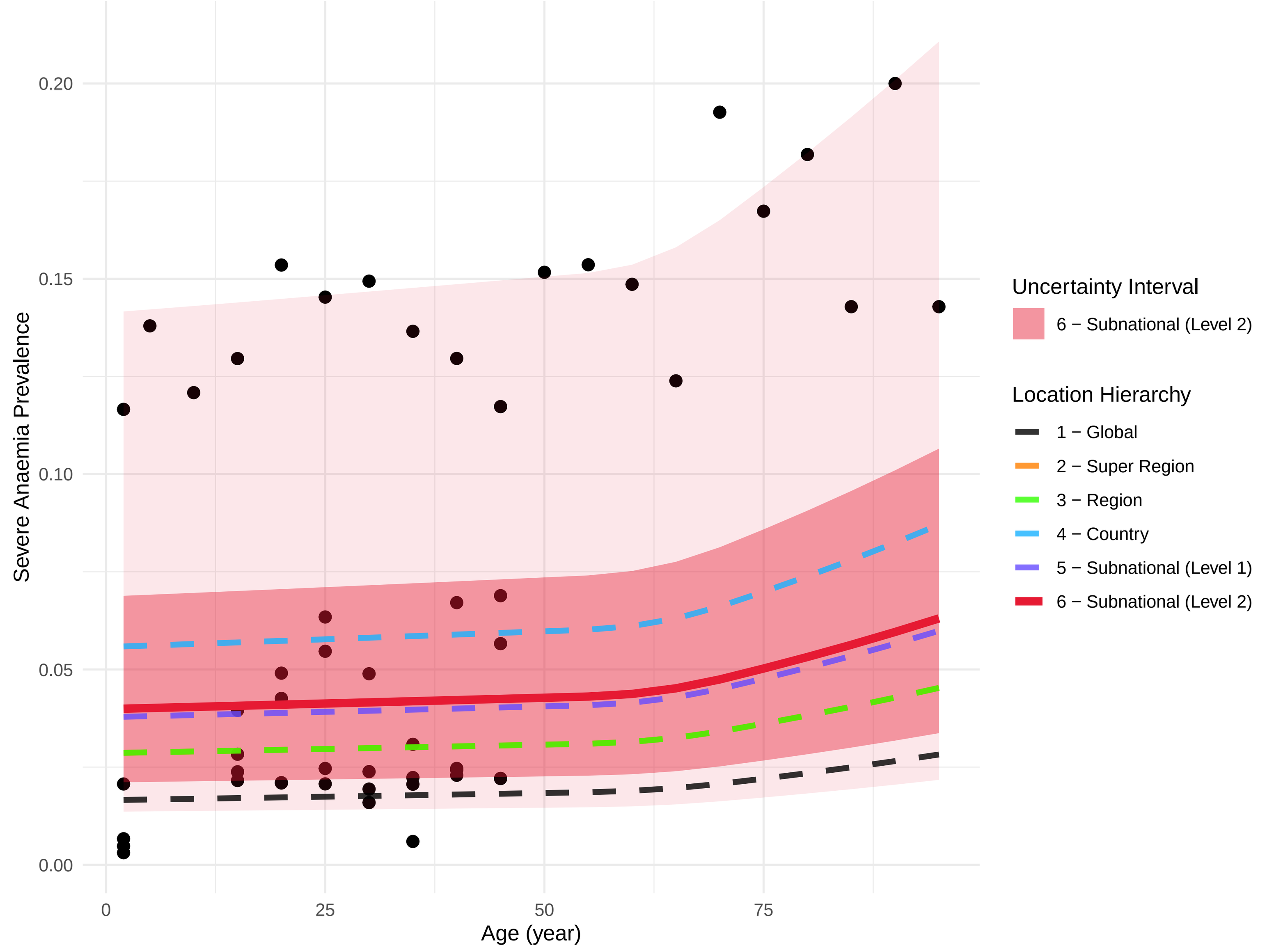

MR−BRT Age/Sex Fits − Non−pregnant Females 2+ Years Old − Severe Anaemia Prevalence − Jharkhand, Urban
WHO−2001 Adjustment Method

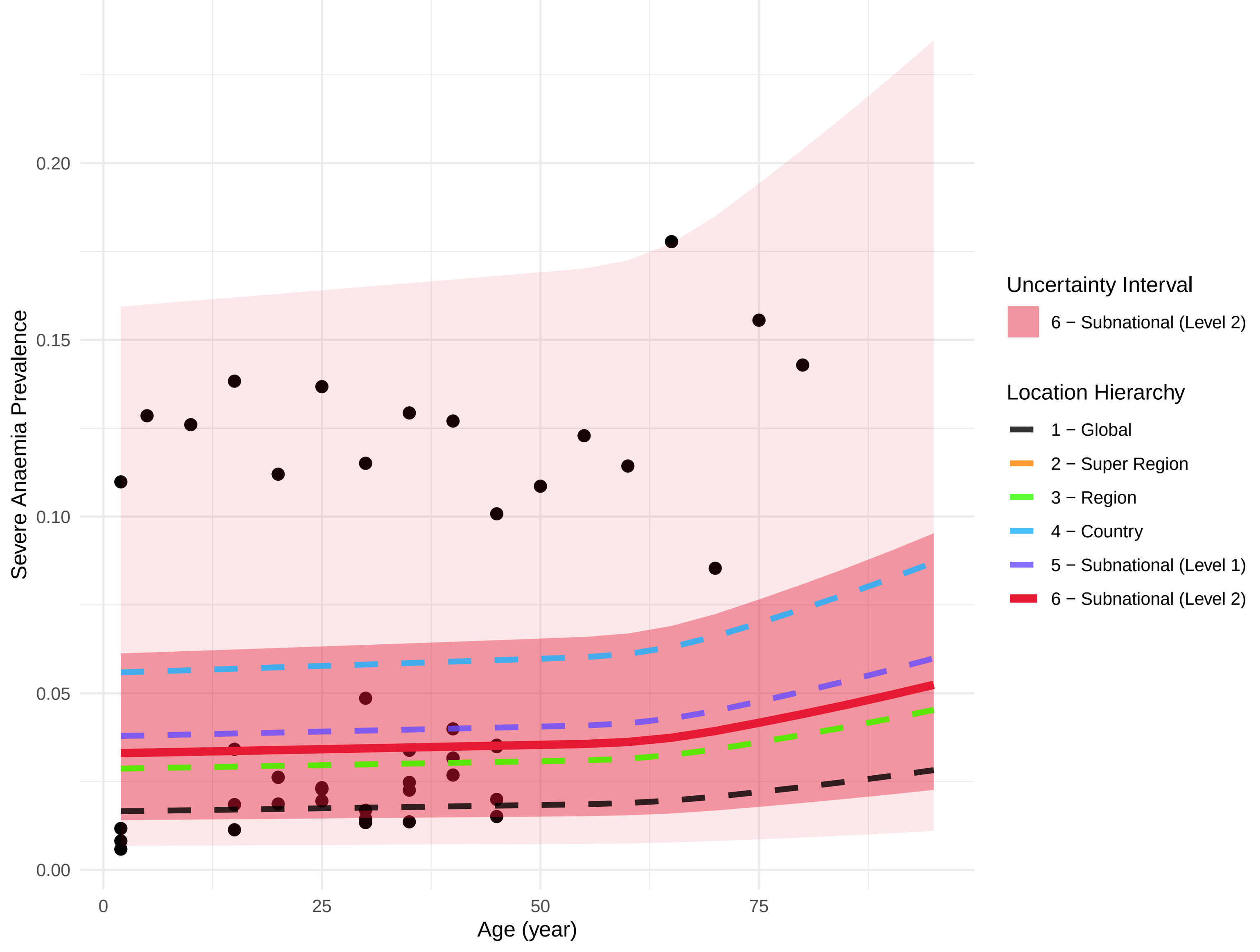

MR−BRT Age/Sex Fits − Non−pregnant Females 2+ Years Old − Severe Anaemia Prevalence − Karnataka
WHO−2001 Adjustment Method

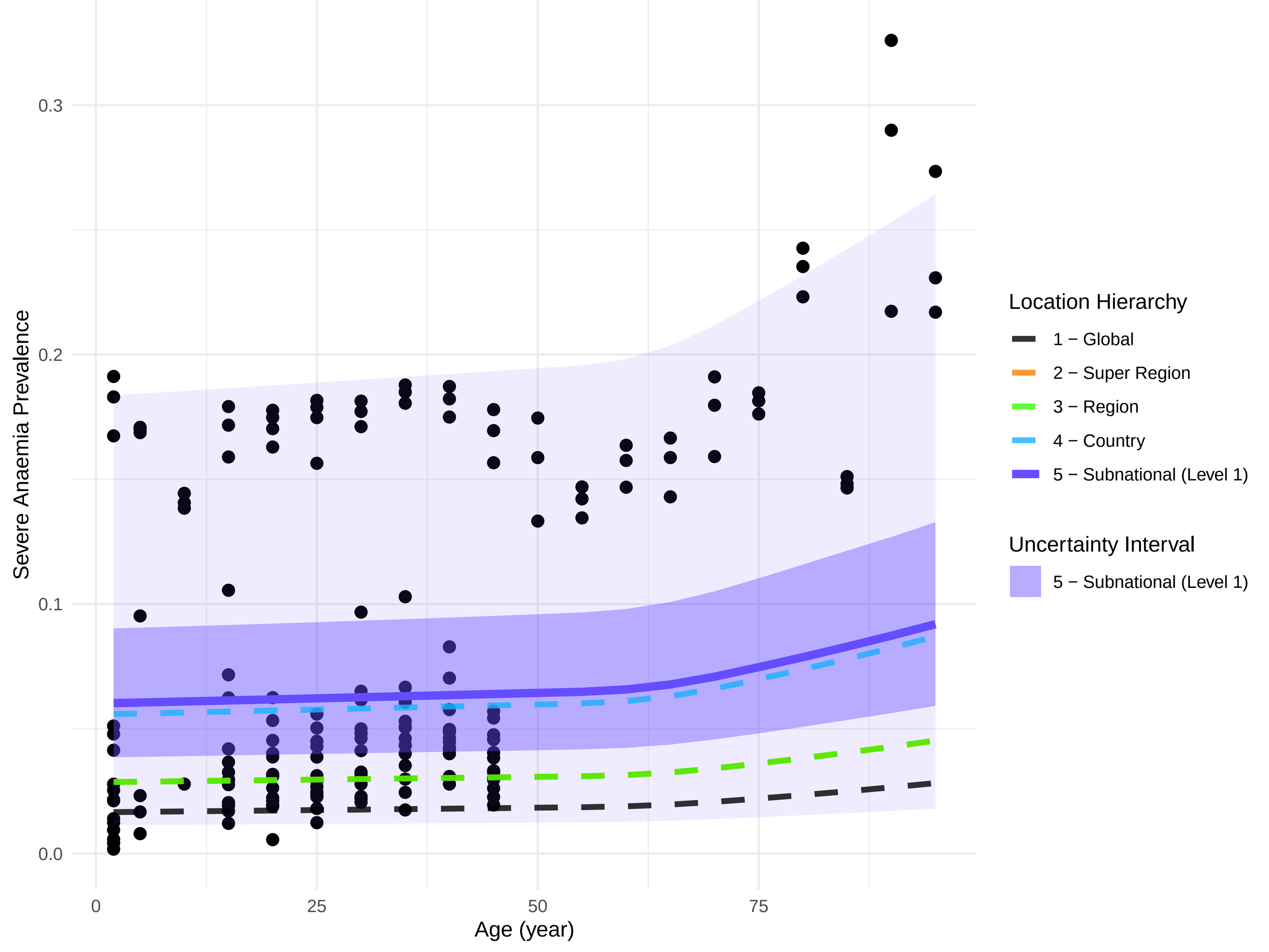

MR−BRT Age/Sex Fits − Non−pregnant Females 2+ Years Old − Severe Anaemia Prevalence − Karnataka, Rural
WHO−2001 Adjustment Method

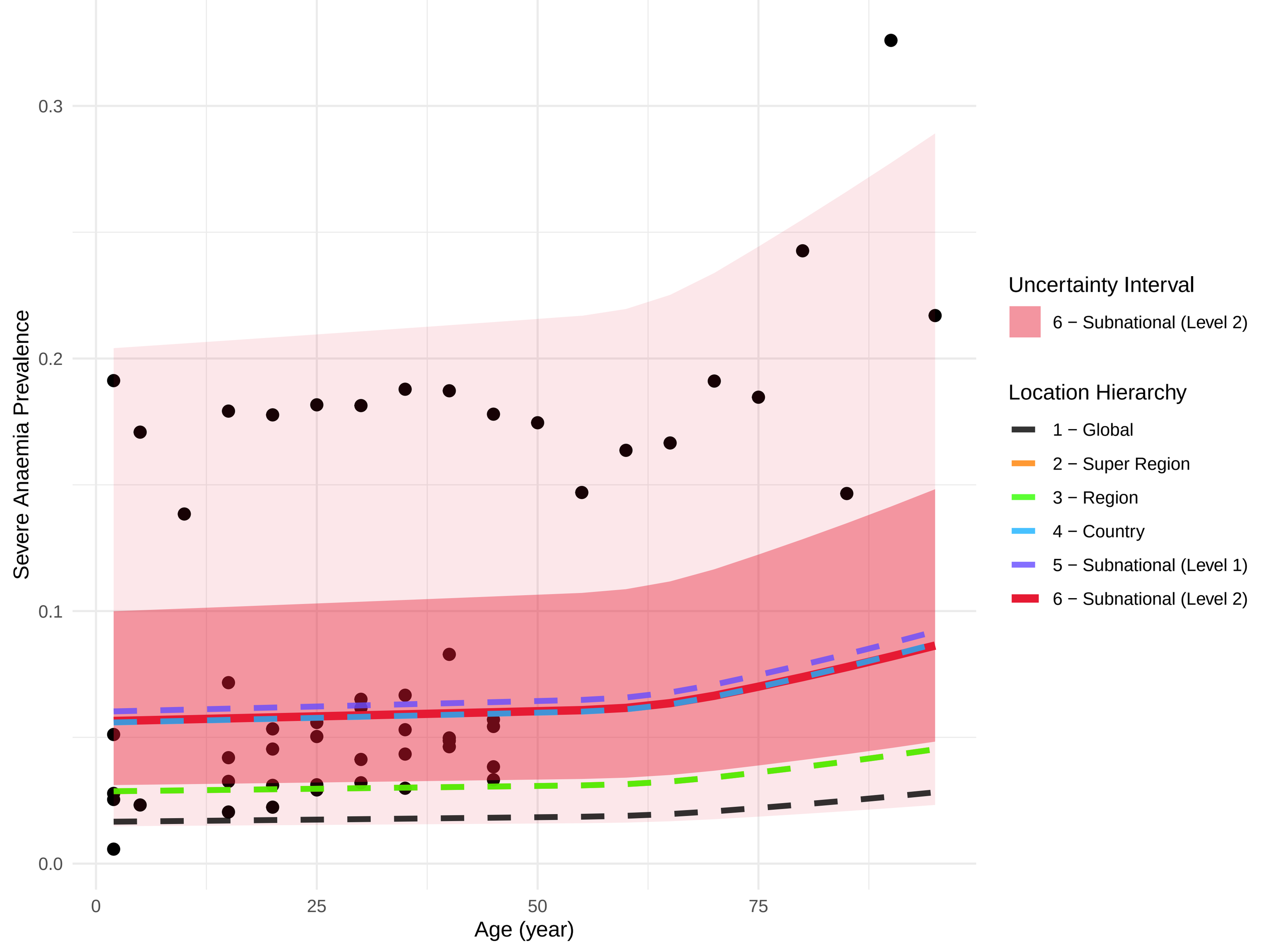

MR−BRT Age/Sex Fits − Non−pregnant Females 2+ Years Old − Severe Anaemia Prevalence − Karnataka, Urban
WHO−2001 Adjustment Method

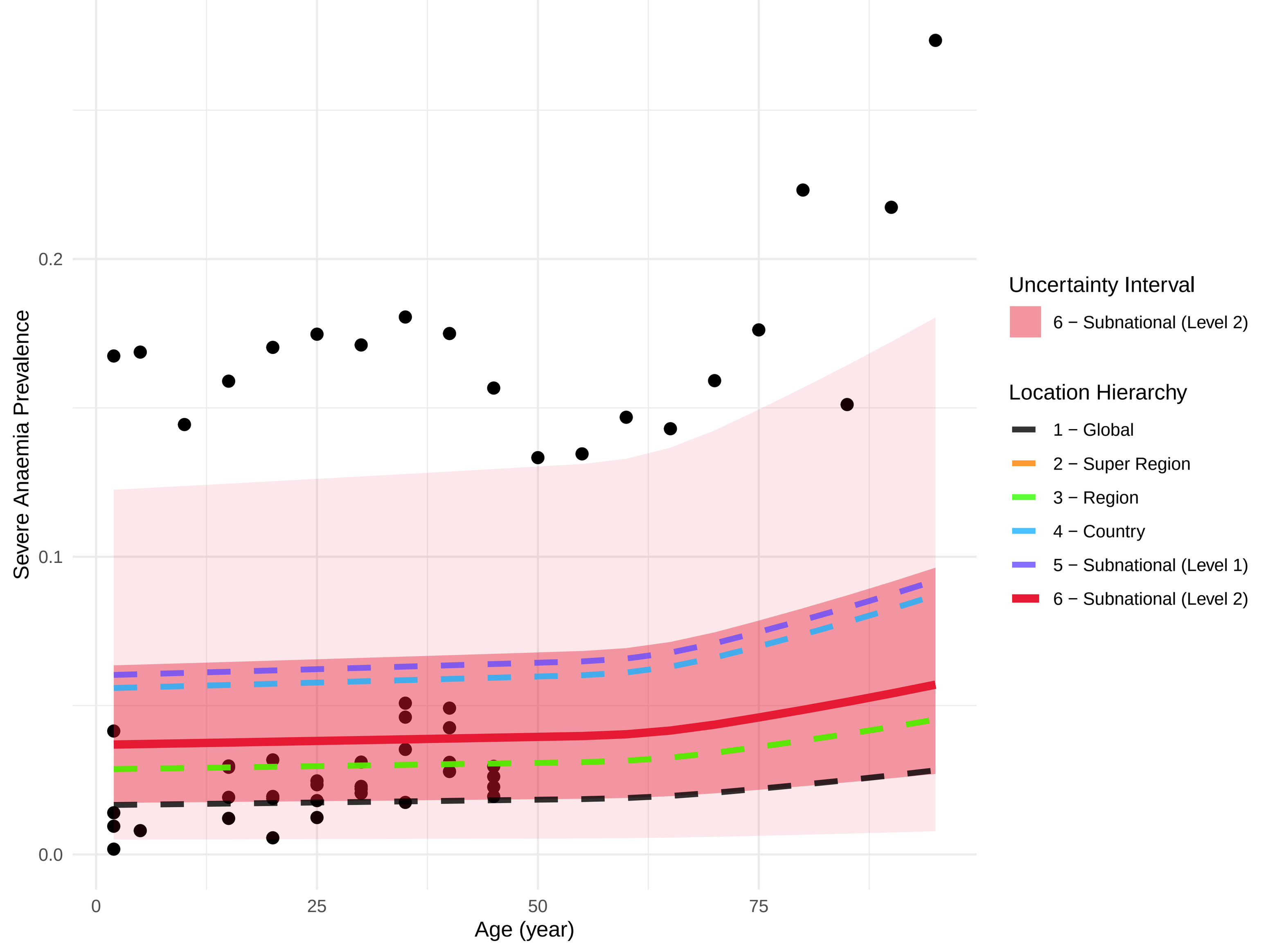

MR−BRT Age/Sex Fits − Non−pregnant Females 2+ Years Old − Severe Anaemia Prevalence − Kerala
WHO−2001 Adjustment Method

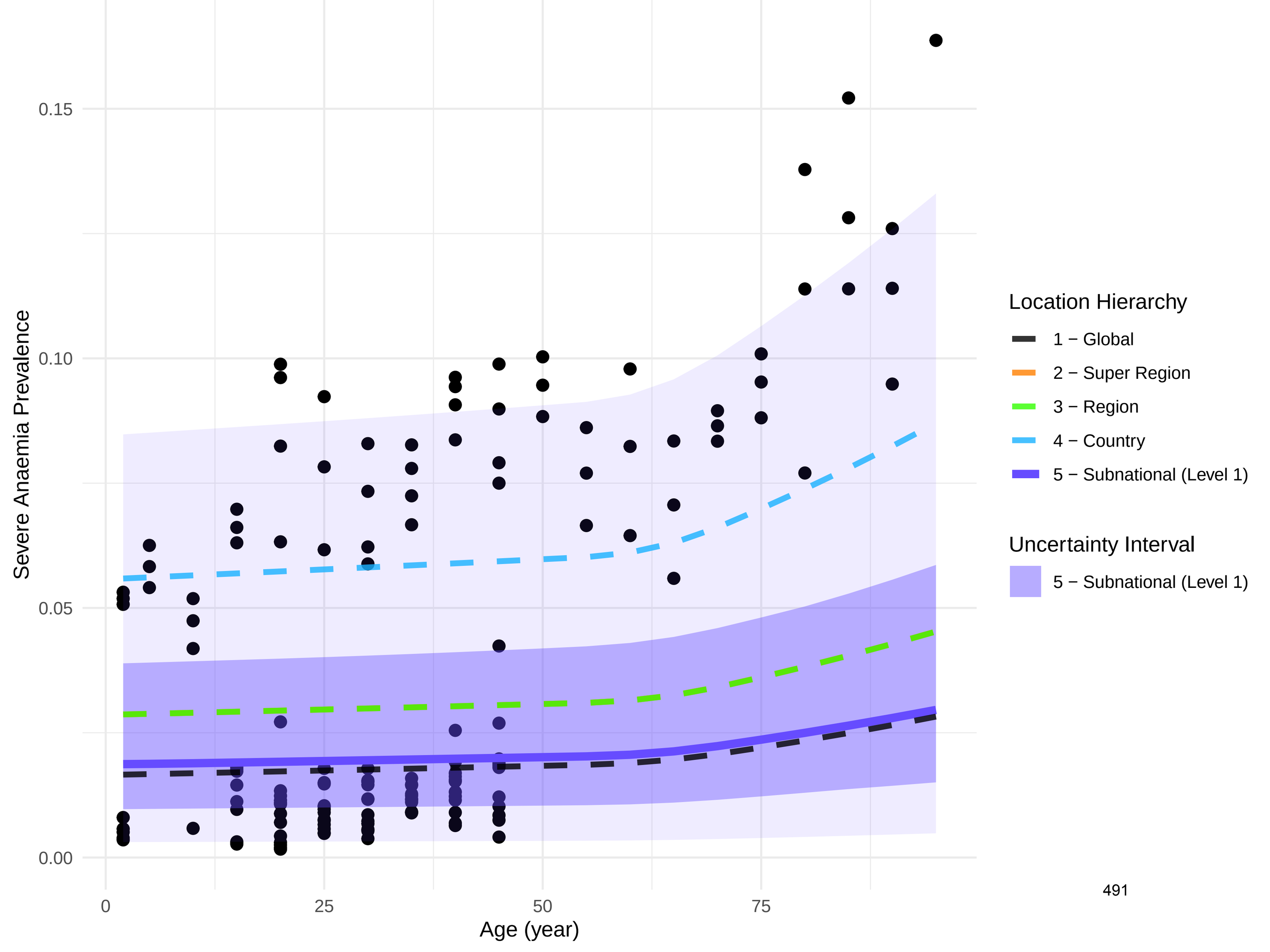

MR−BRT Age/Sex Fits − Non−pregnant Females 2+ Years Old − Severe Anaemia Prevalence − Kerala, Rural
WHO−2001 Adjustment Method

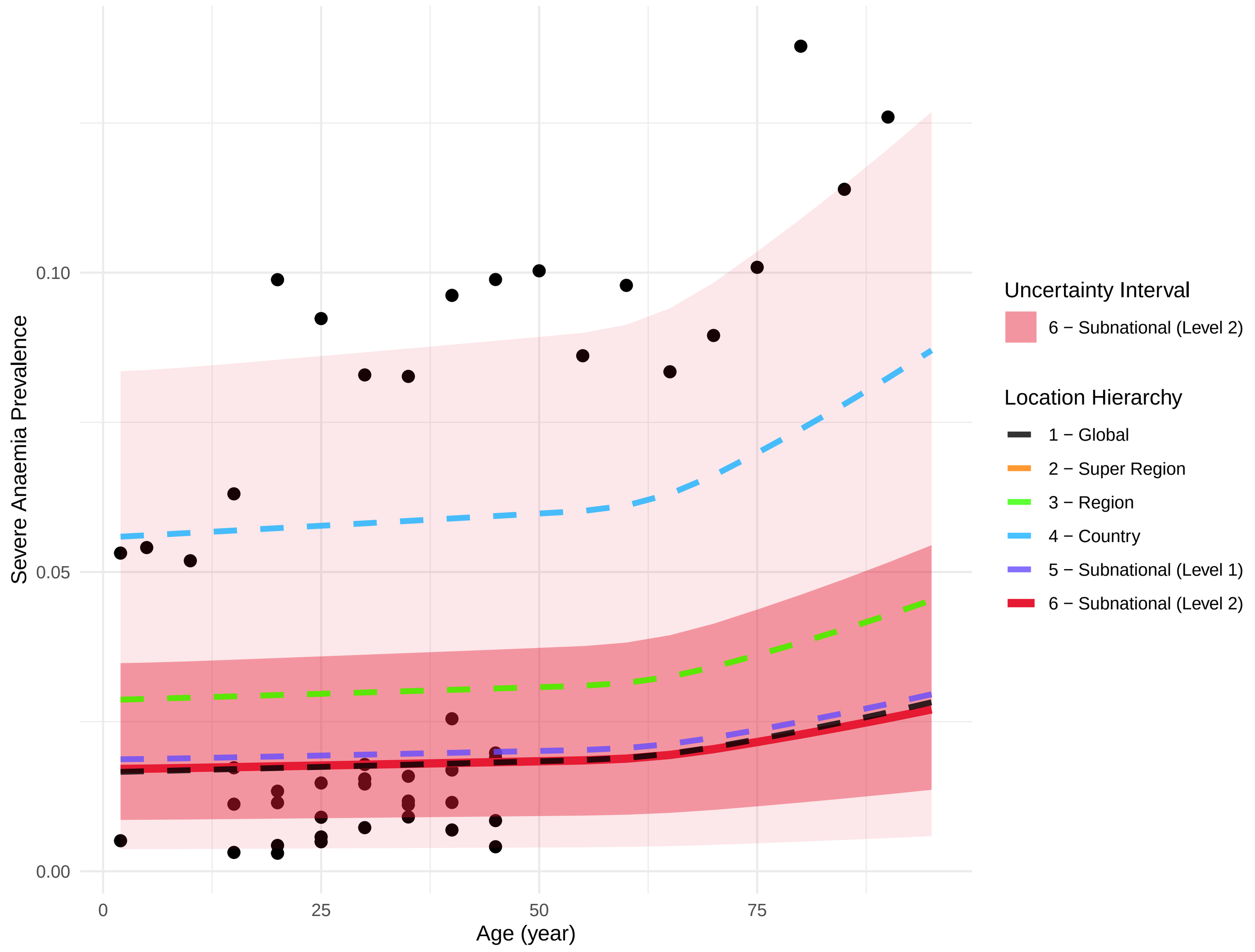

MR−BRT Age/Sex Fits − Non−pregnant Females 2+ Years Old − Severe Anaemia Prevalence − Kerala, Urban
WHO−2001 Adjustment Method

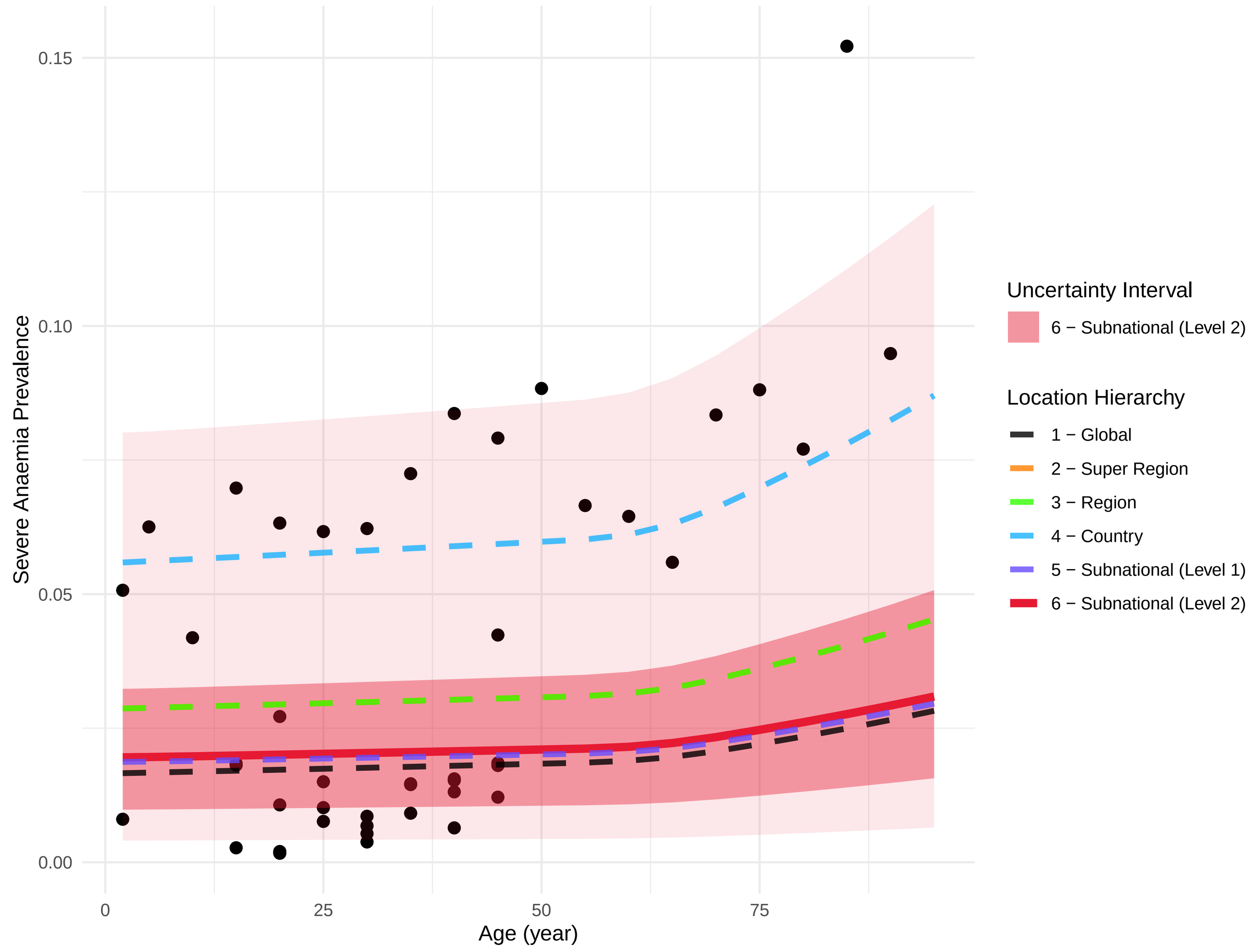

MR−BRT Age/Sex Fits − Non−pregnant Females 2+ Years Old − Severe Anaemia Prevalence − Madhya Pradesh
WHO−2001 Adjustment Method

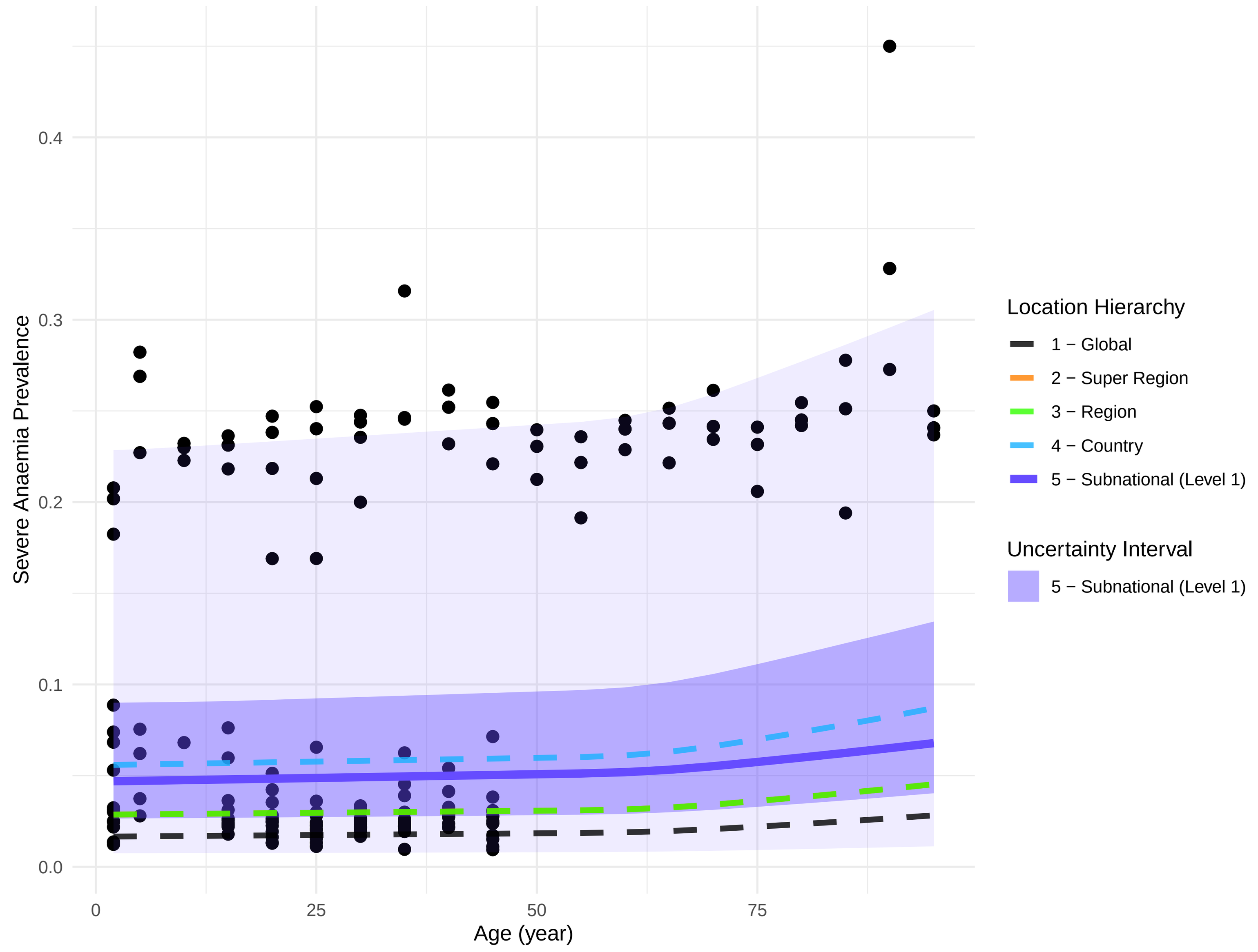

MR−BRT Age/Sex Fits − Non−pregnant Females 2+ Years Old − Severe Anaemia Prevalence − Madhya Pradesh, Rural
WHO−2001 Adjustment Method

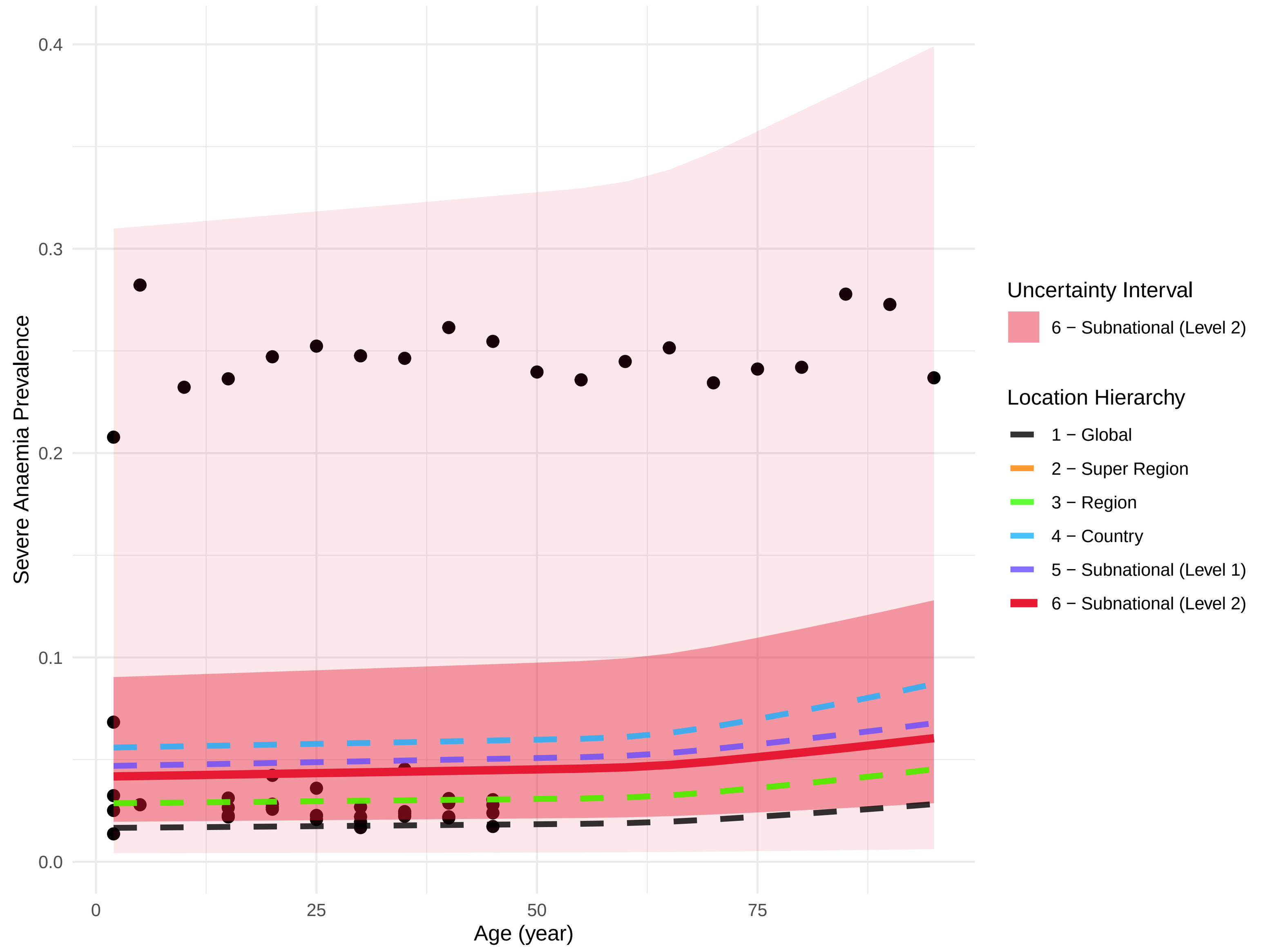

MR−BRT Age/Sex Fits − Non−pregnant Females 2+ Years Old − Severe Anaemia Prevalence − Madhya Pradesh, Urban
WHO−2001 Adjustment Method

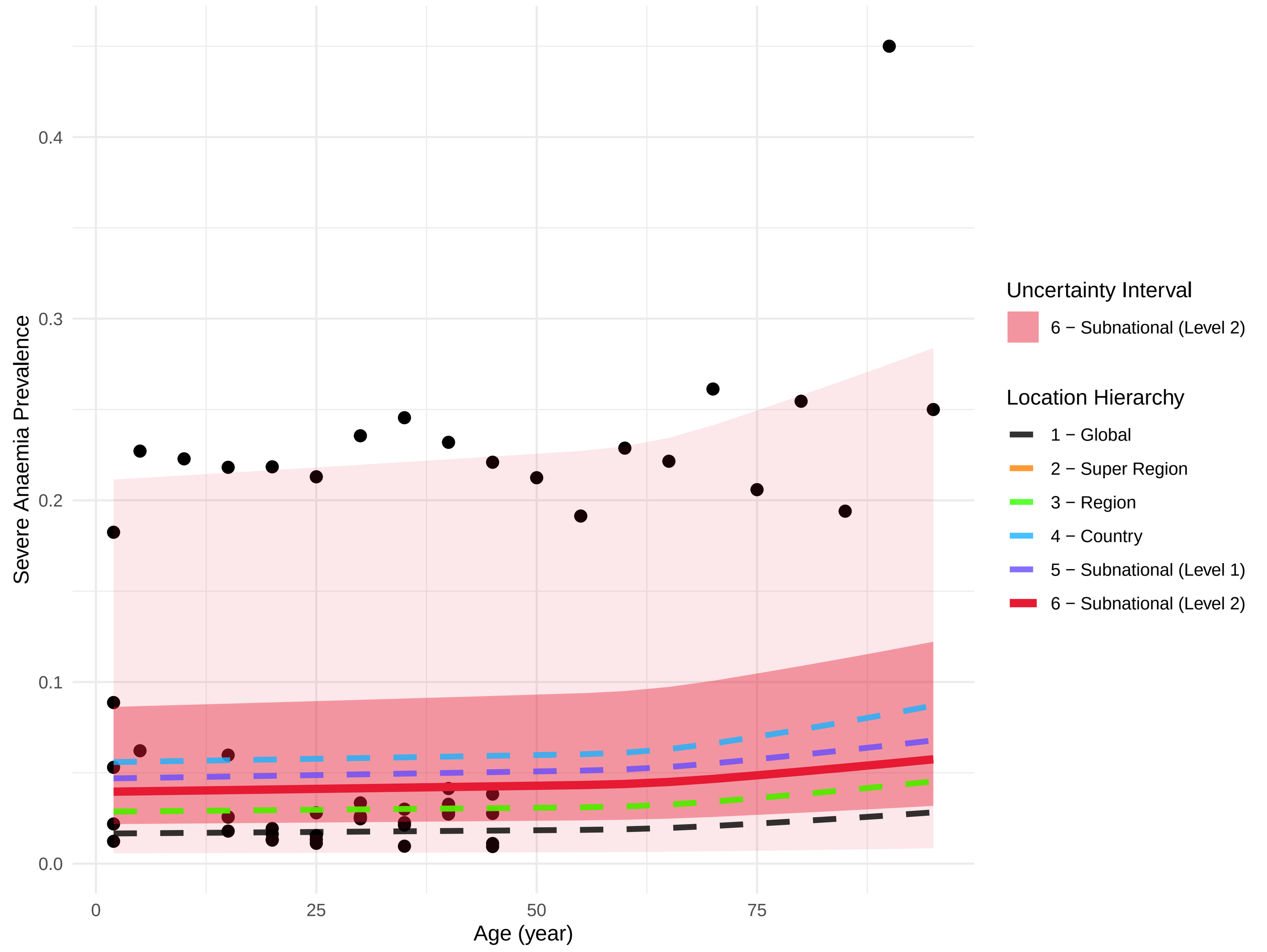

MR−BRT Age/Sex Fits − Non−pregnant Females 2+ Years Old − Severe Anaemia Prevalence − Maharashtra
WHO−2001 Adjustment Method

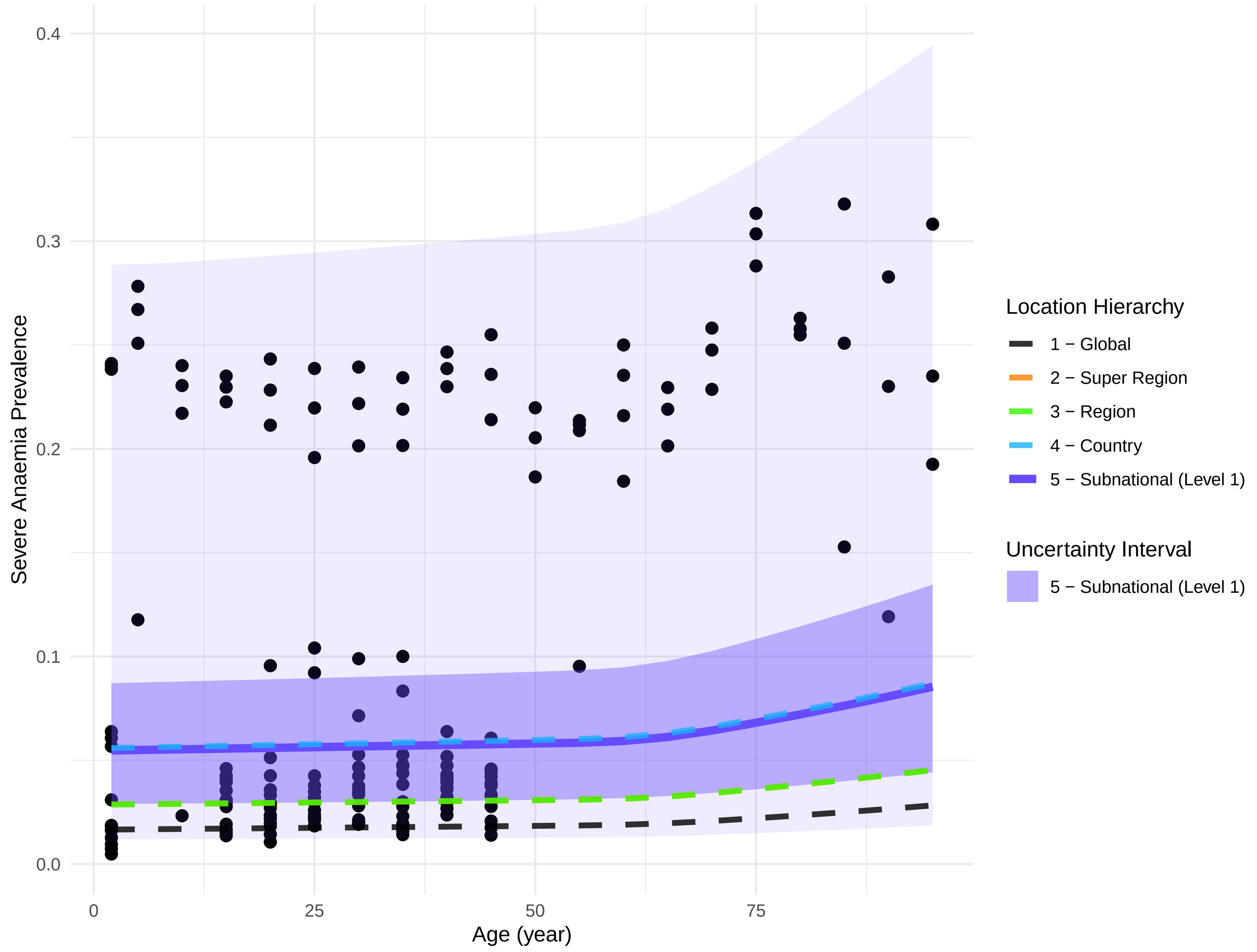

MR−BRT Age/Sex Fits − Non−pregnant Females 2+ Years Old − Severe Anaemia Prevalence − Maharashtra, Rural
WHO−2001 Adjustment Method

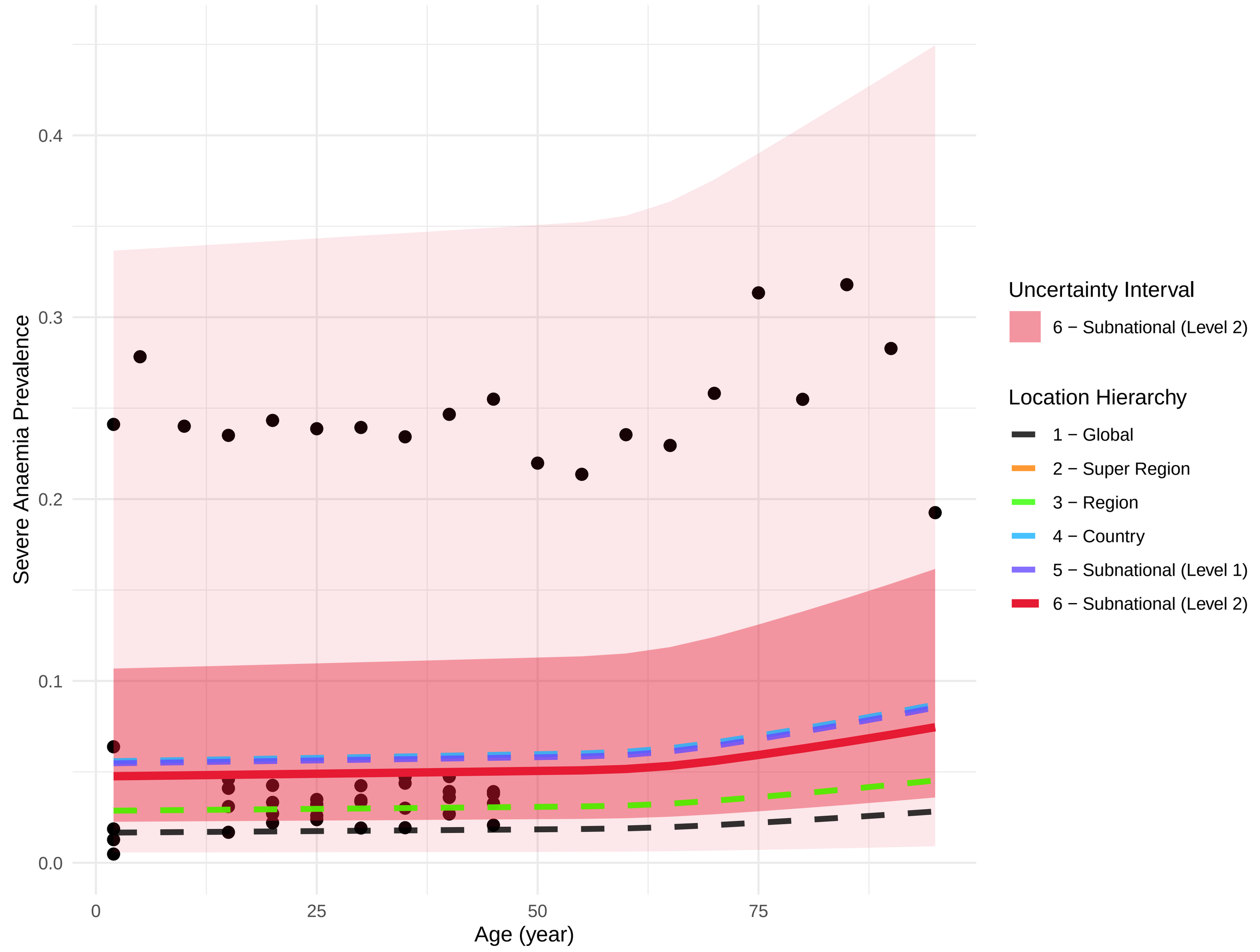

MR−BRT Age/Sex Fits − Non−pregnant Females 2+ Years Old − Severe Anaemia Prevalence − Maharashtra, Urban
WHO−2001 Adjustment Method

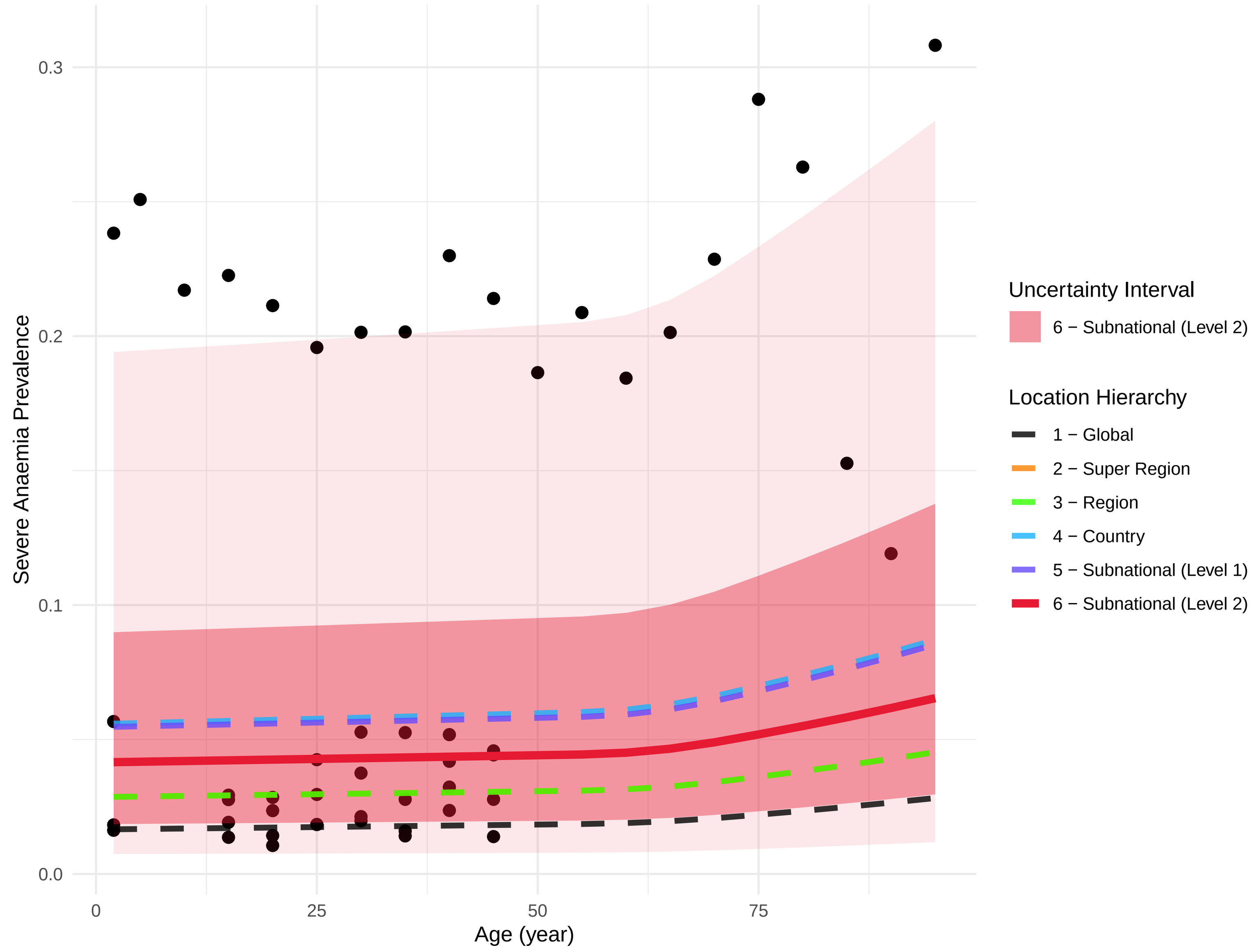

MR−BRT Age/Sex Fits − Non−pregnant Females 2+ Years Old − Severe Anaemia Prevalence − Manipur
WHO−2001 Adjustment Method

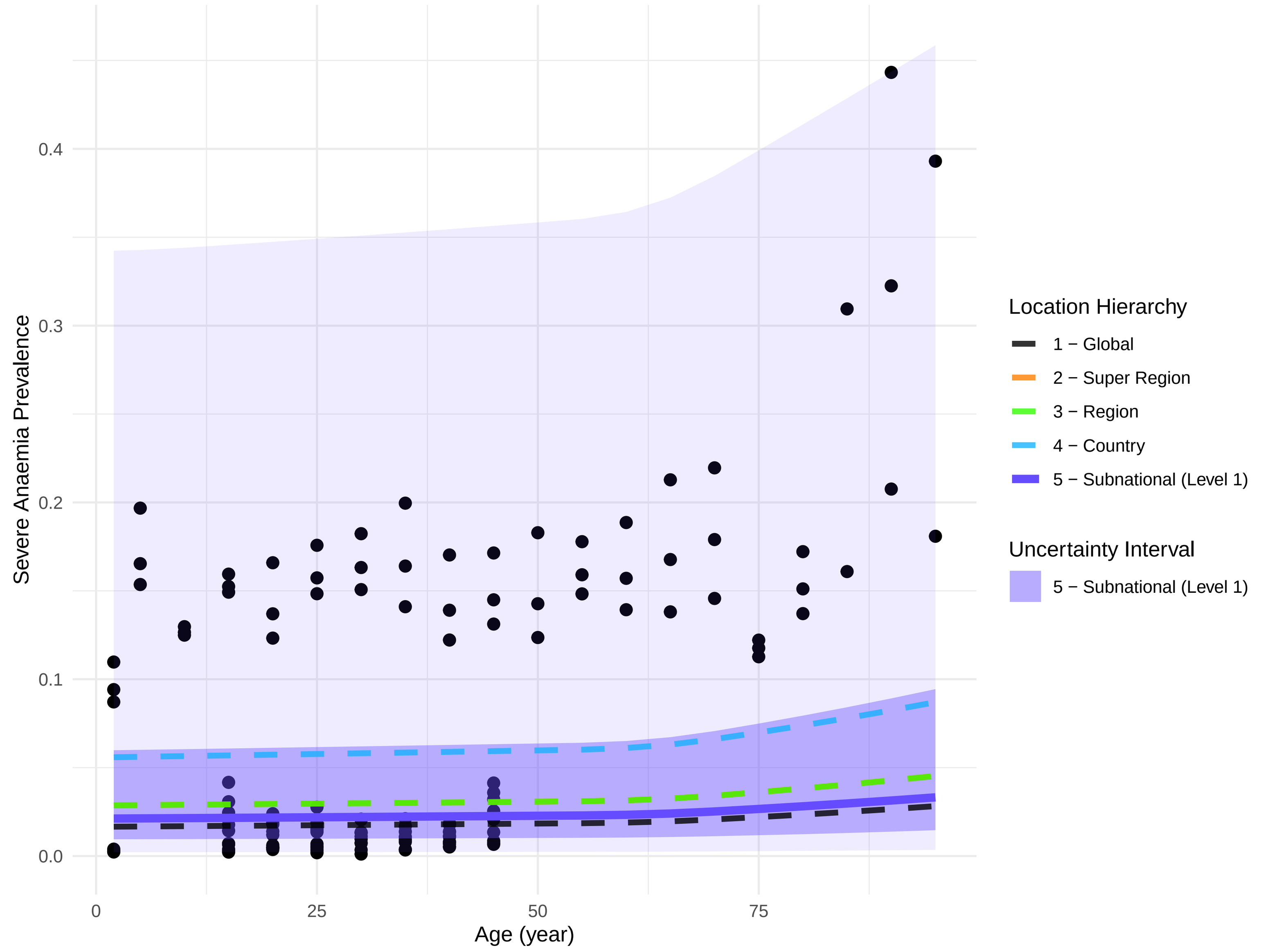

MR−BRT Age/Sex Fits − Non−pregnant Females 2+ Years Old − Severe Anaemia Prevalence − Manipur, Rural
WHO−2001 Adjustment Method

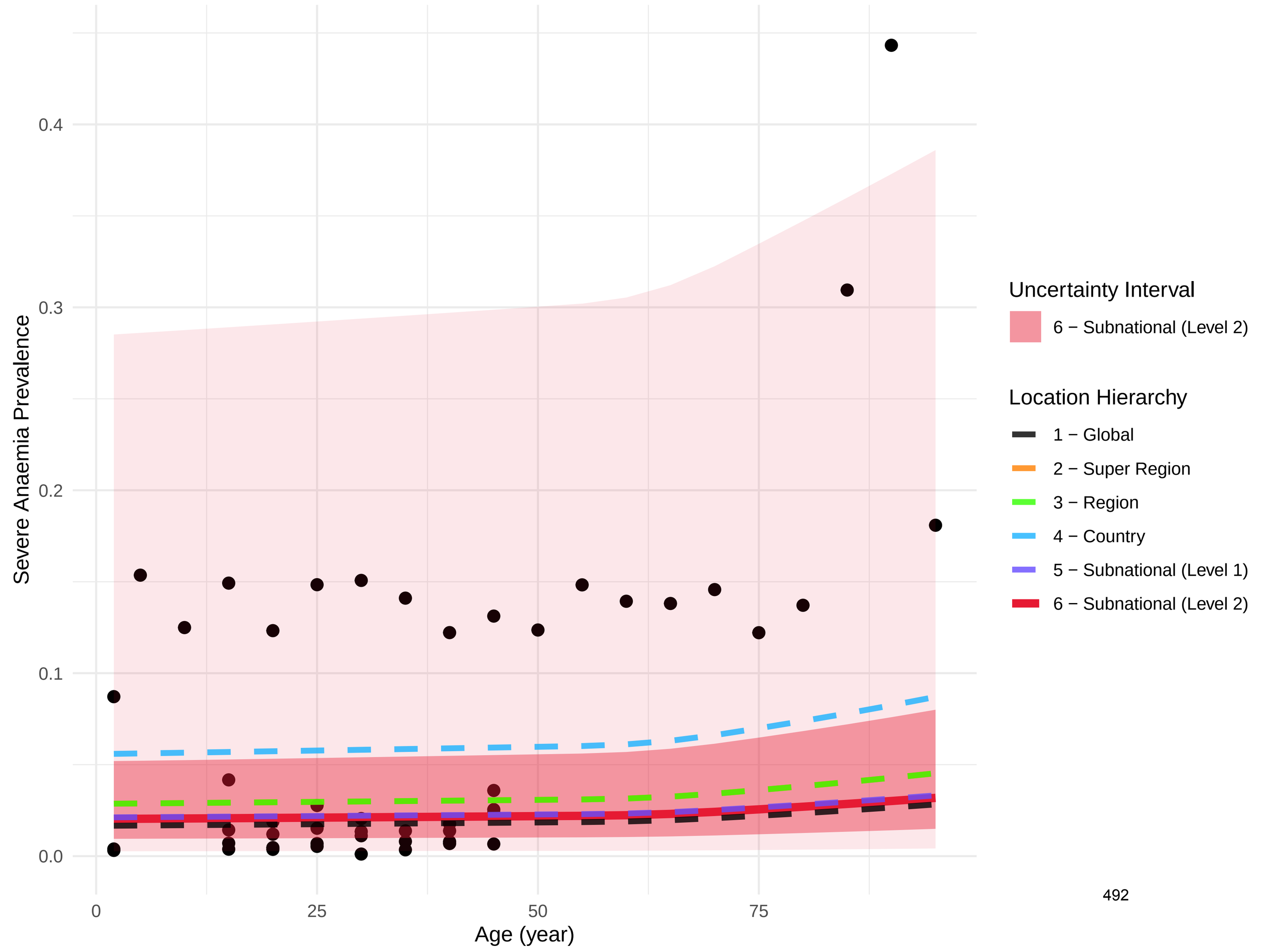

MR−BRT Age/Sex Fits − Non−pregnant Females 2+ Years Old − Severe Anaemia Prevalence − Manipur, Urban
WHO−2001 Adjustment Method

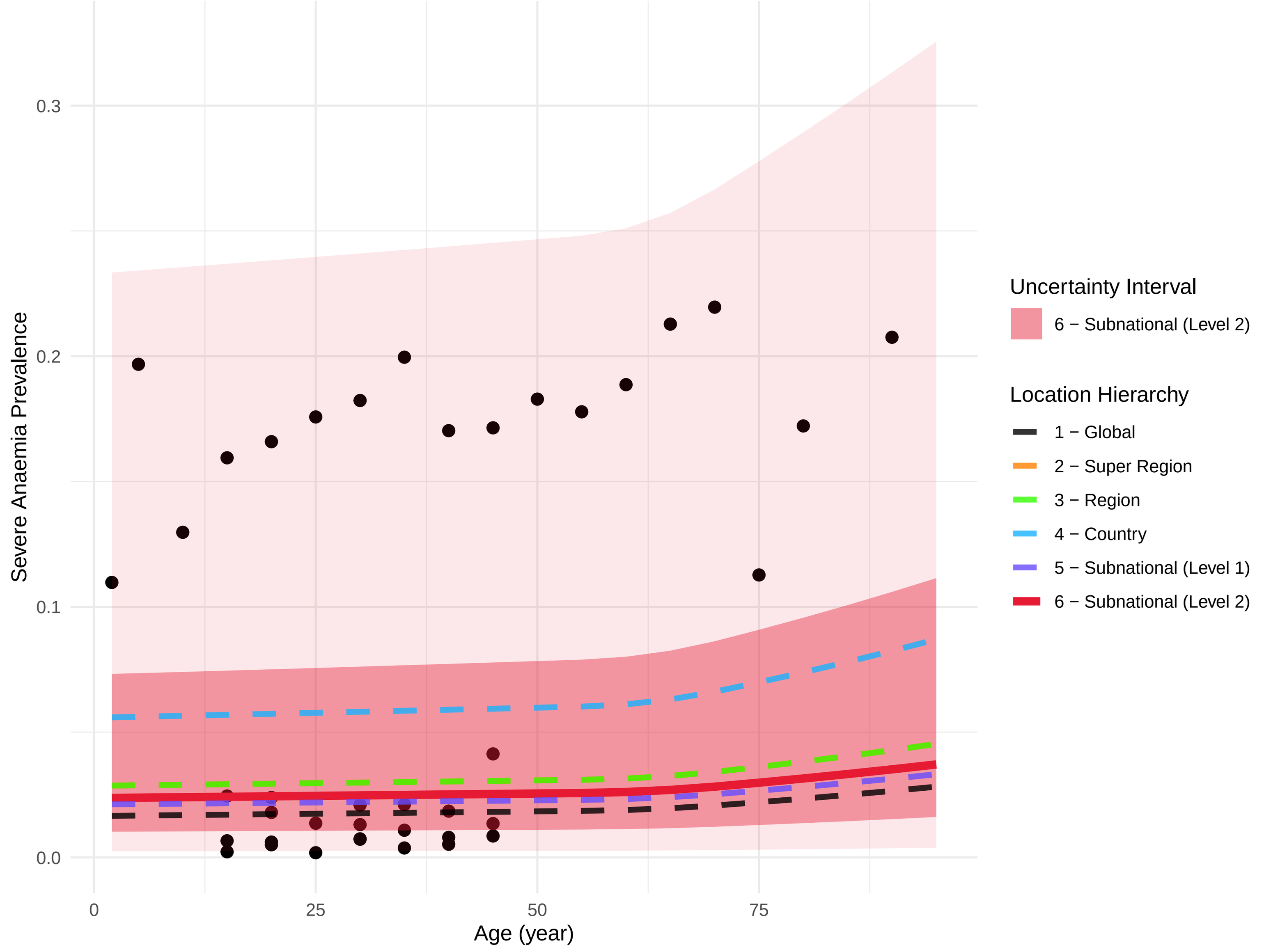

MR−BRT Age/Sex Fits − Non−pregnant Females 2+ Years Old − Severe Anaemia Prevalence − Meghalaya
WHO−2001 Adjustment Method

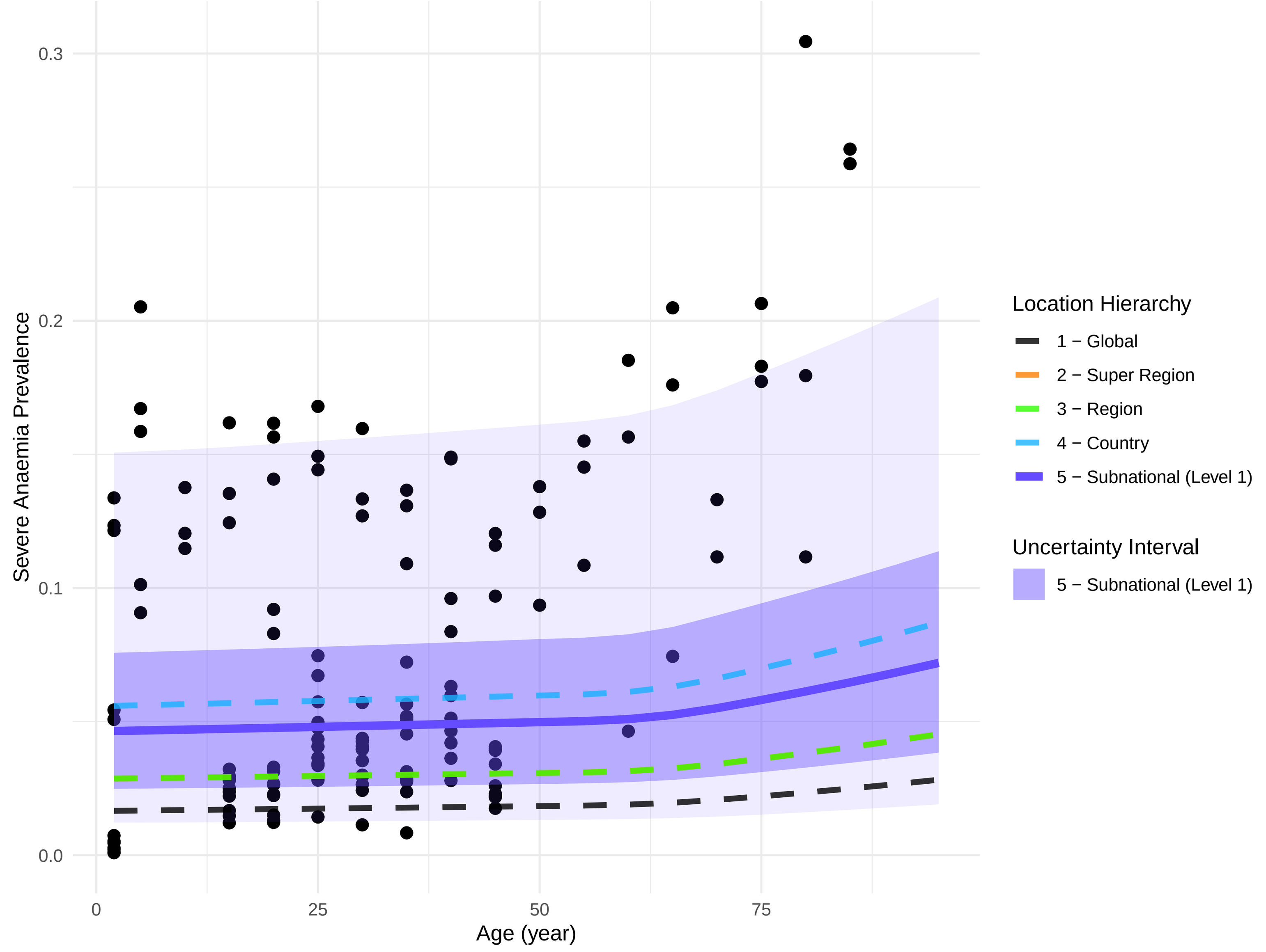

MR−BRT Age/Sex Fits − Non−pregnant Females 2+ Years Old − Severe Anaemia Prevalence − Meghalaya, Rural
WHO−2001 Adjustment Method

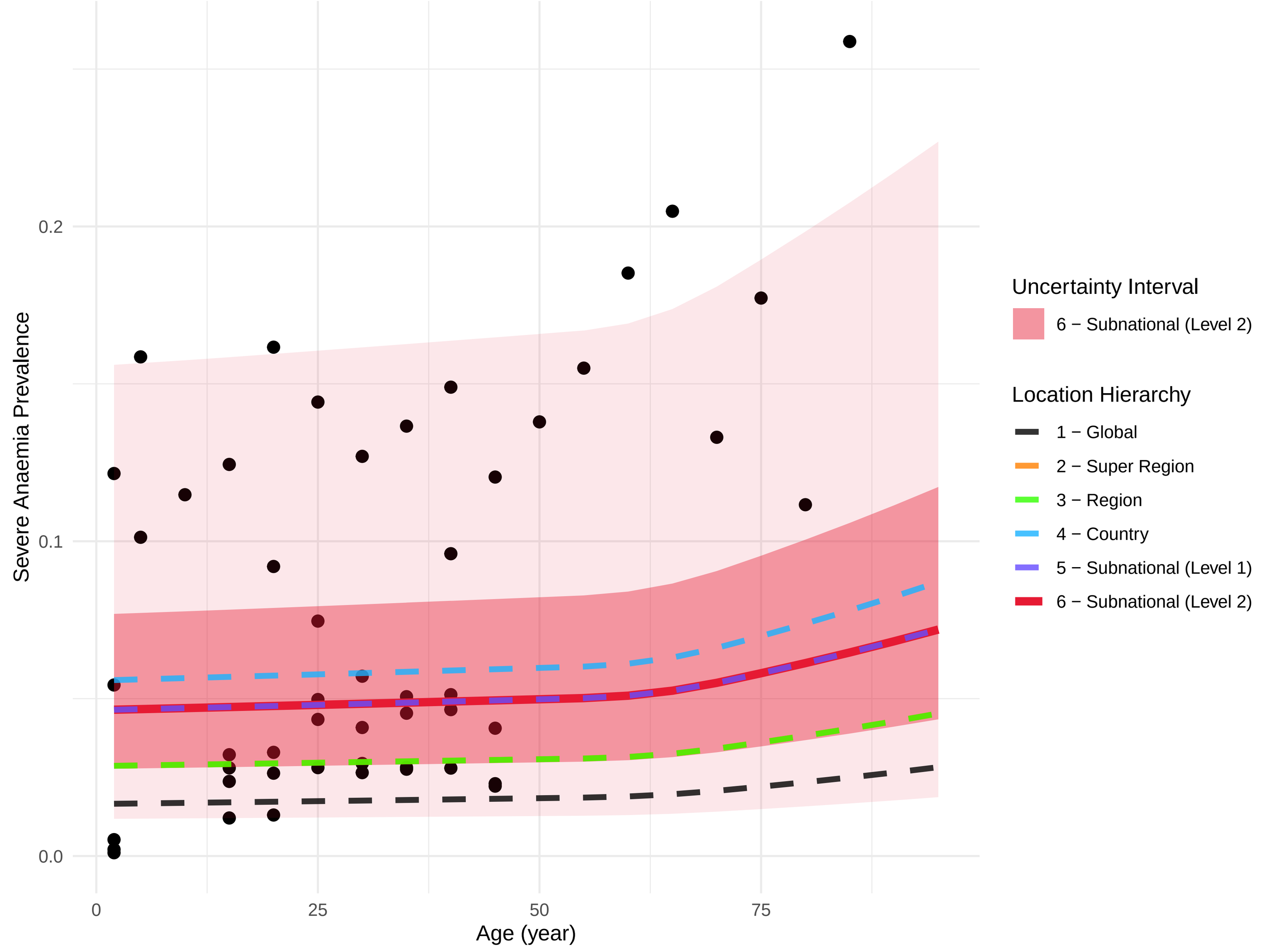

MR−BRT Age/Sex Fits − Non−pregnant Females 2+ Years Old − Severe Anaemia Prevalence − Meghalaya, Urban
WHO−2001 Adjustment Method

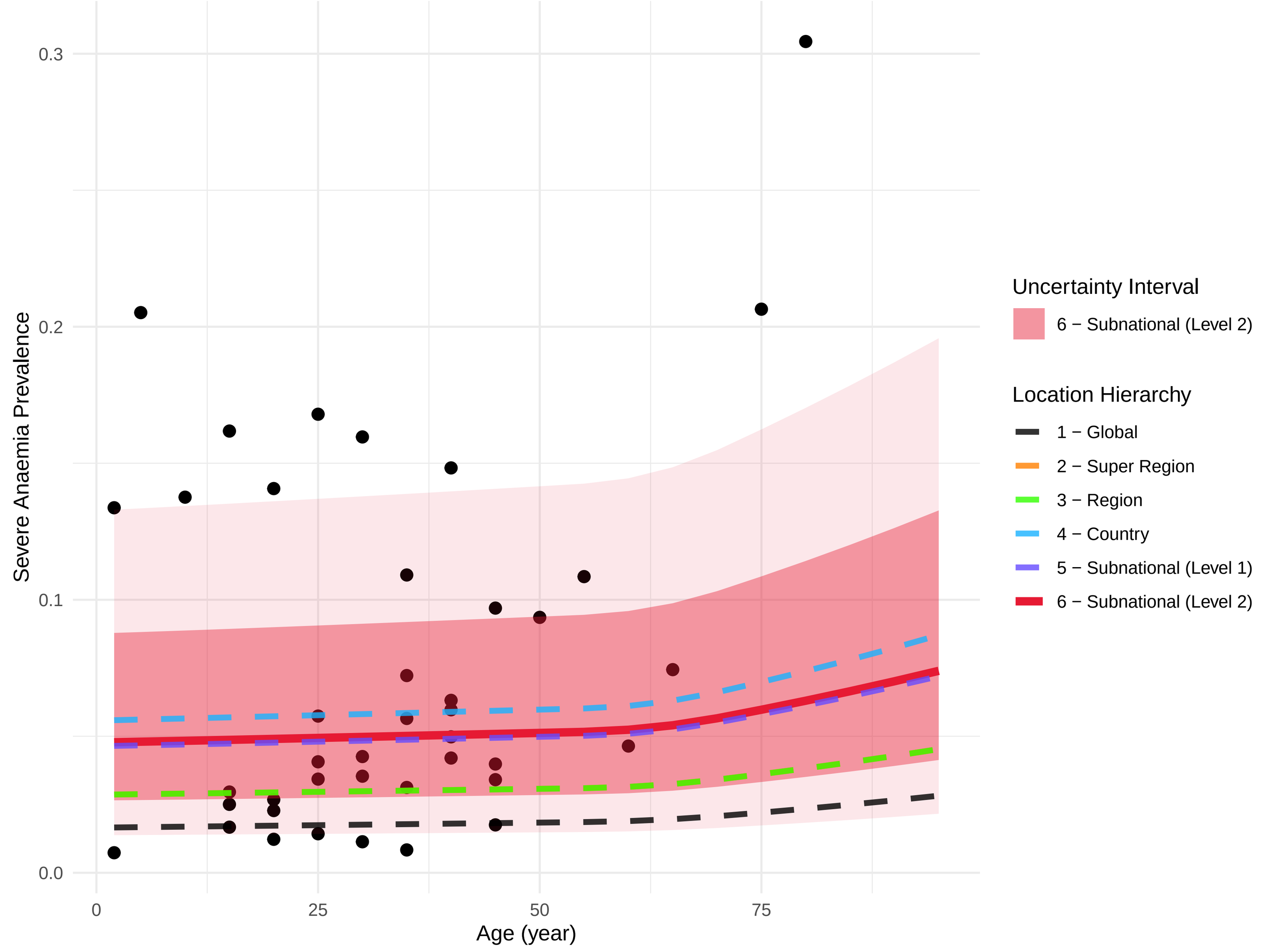

MR−BRT Age/Sex Fits − Non−pregnant Females 2+ Years Old − Severe Anaemia Prevalence − Mizoram
WHO−2001 Adjustment Method

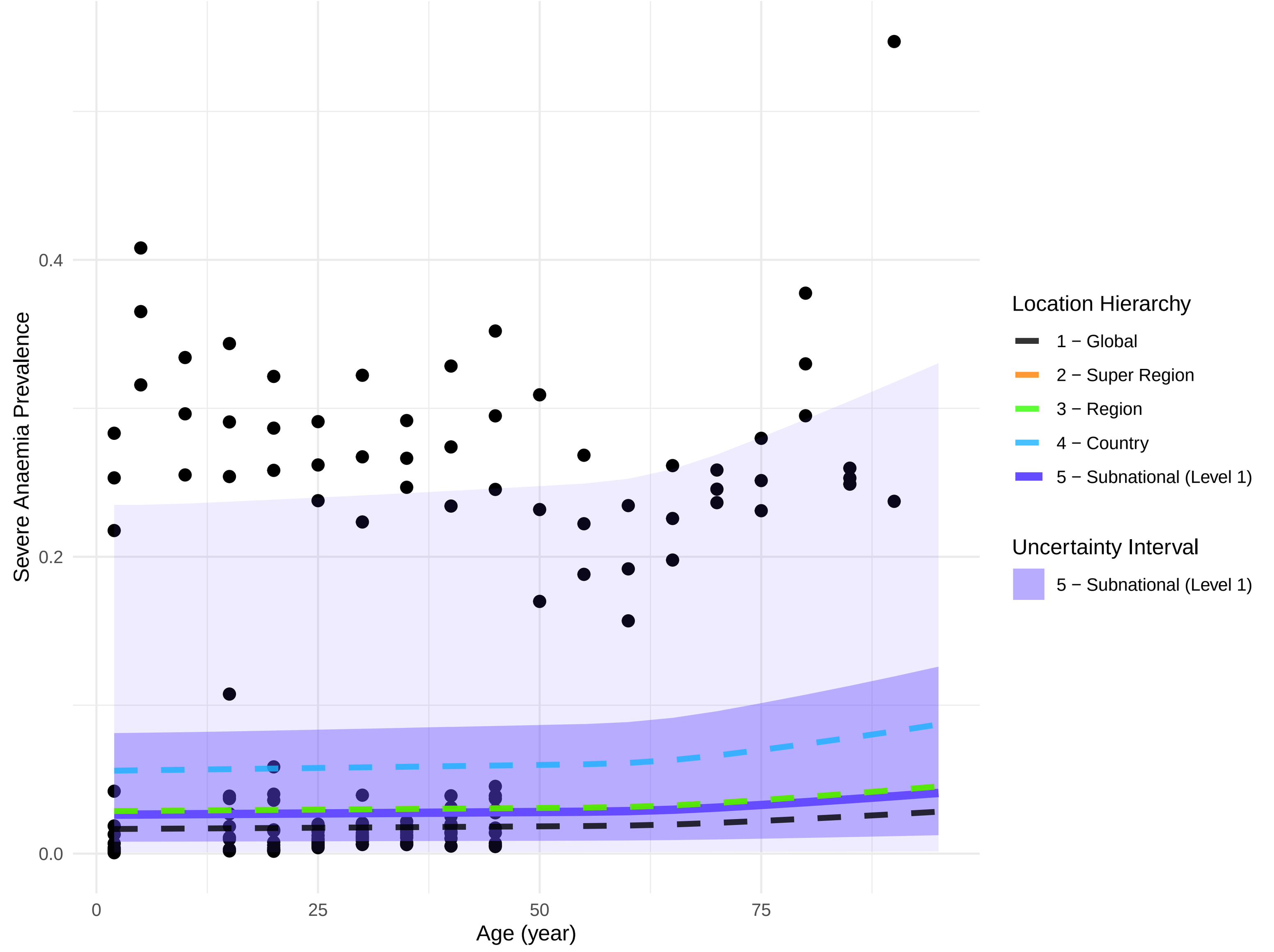

MR−BRT Age/Sex Fits − Non−pregnant Females 2+ Years Old − Severe Anaemia Prevalence − Mizoram, Rural
WHO−2001 Adjustment Method

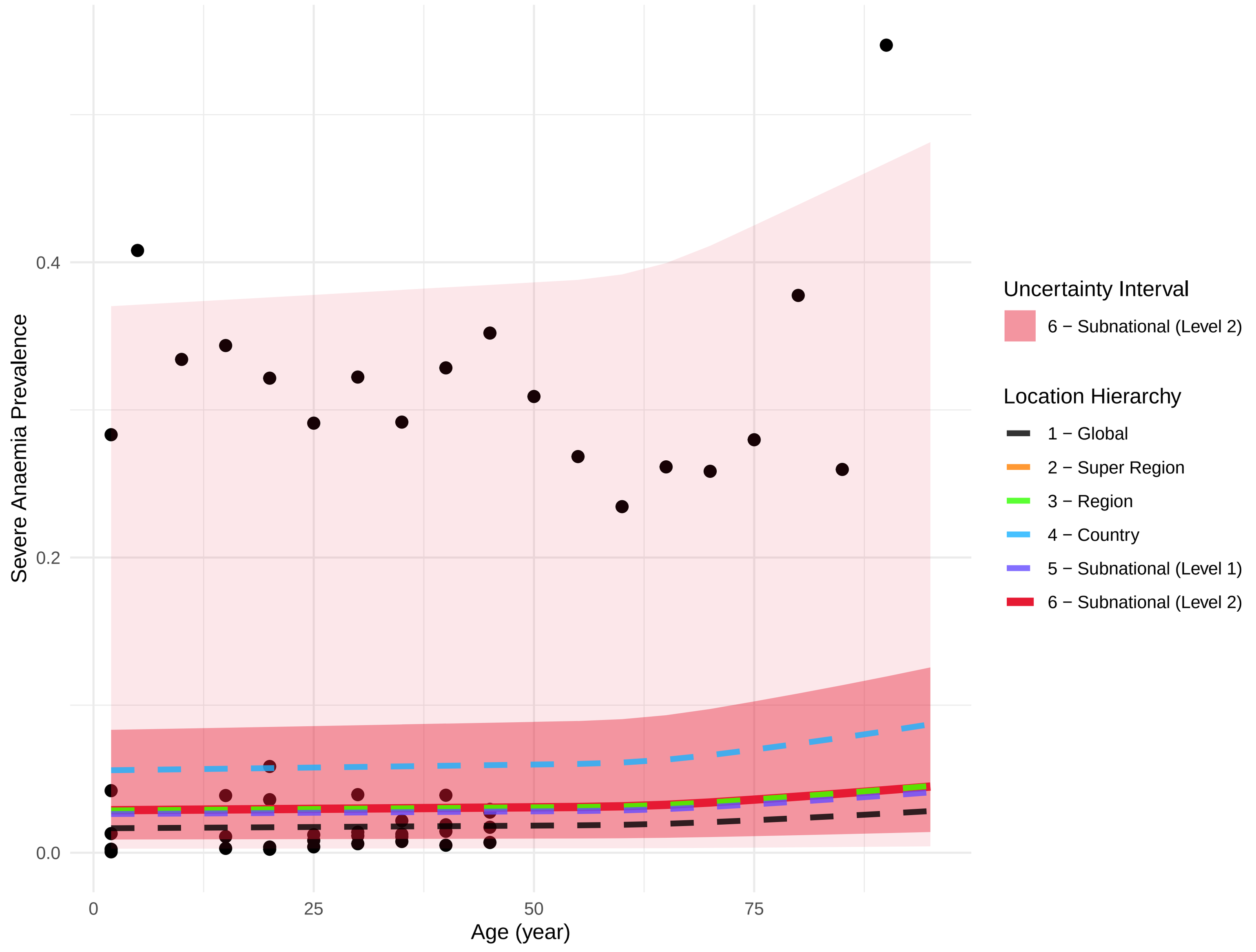

MR−BRT Age/Sex Fits − Non−pregnant Females 2+ Years Old − Severe Anaemia Prevalence − Mizoram, Urban
WHO−2001 Adjustment Method

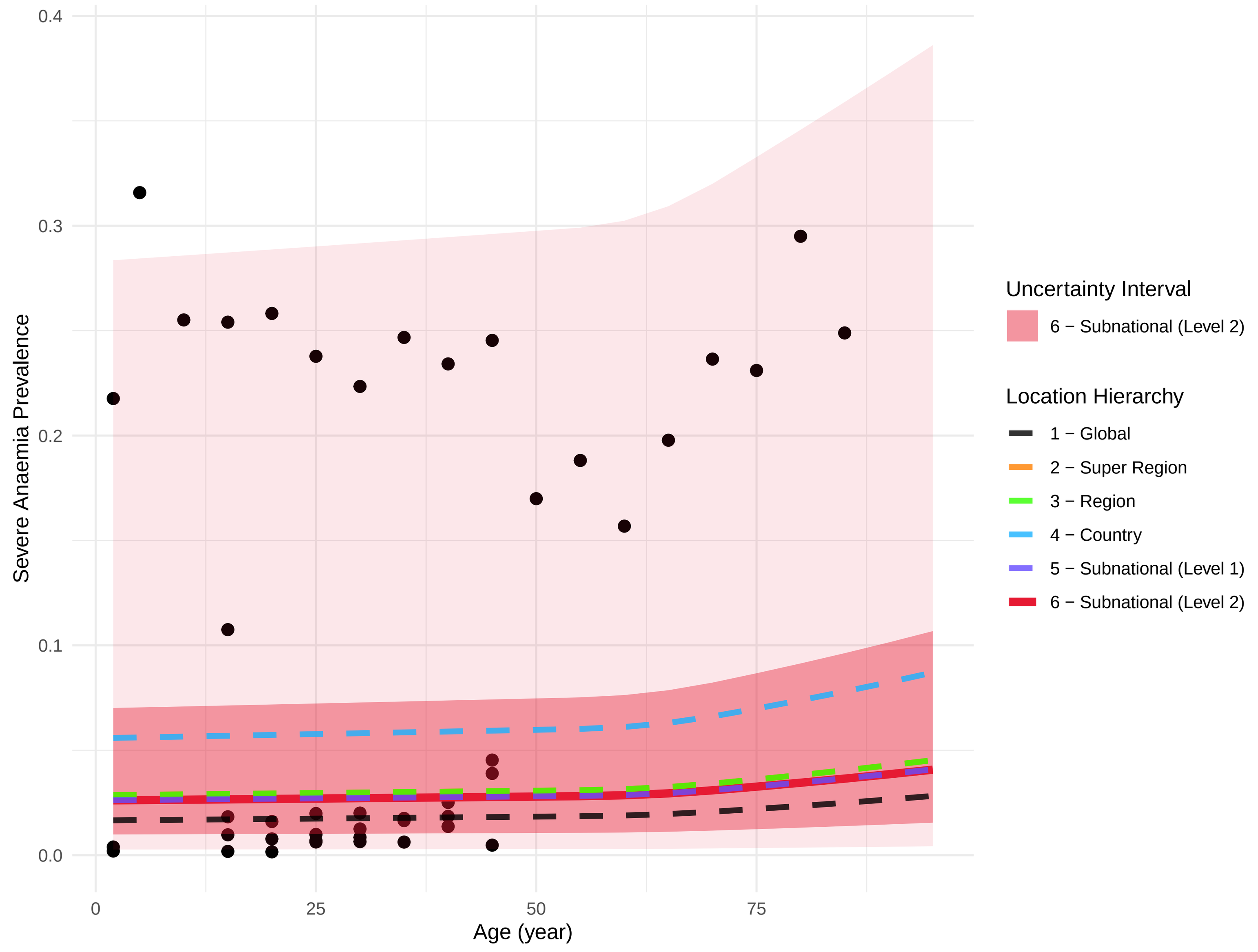

MR−BRT Age/Sex Fits − Non−pregnant Females 2+ Years Old − Severe Anaemia Prevalence − Nagaland
WHO−2001 Adjustment Method

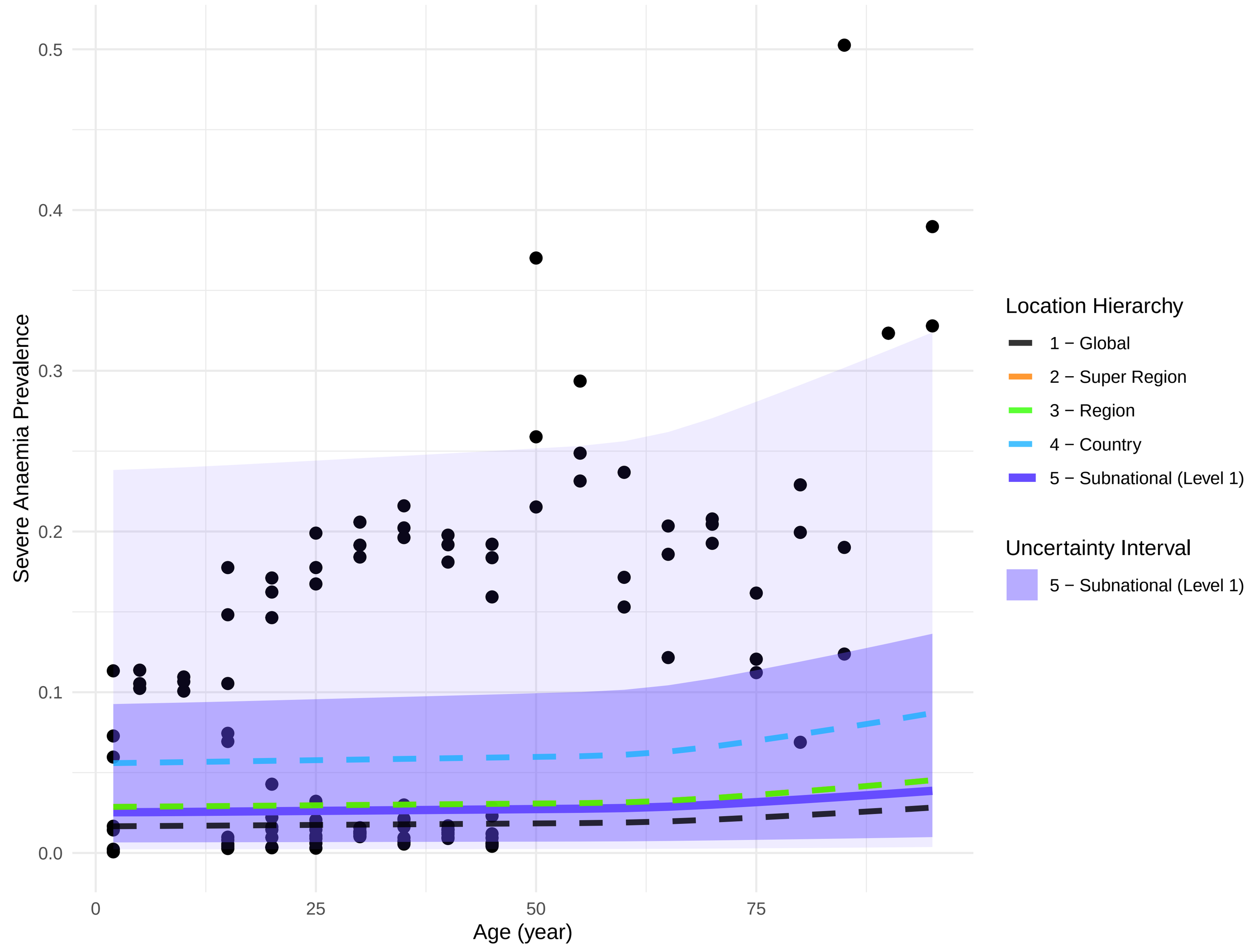

MR−BRT Age/Sex Fits − Non−pregnant Females 2+ Years Old − Severe Anaemia Prevalence − Nagaland, Rural
WHO−2001 Adjustment Method

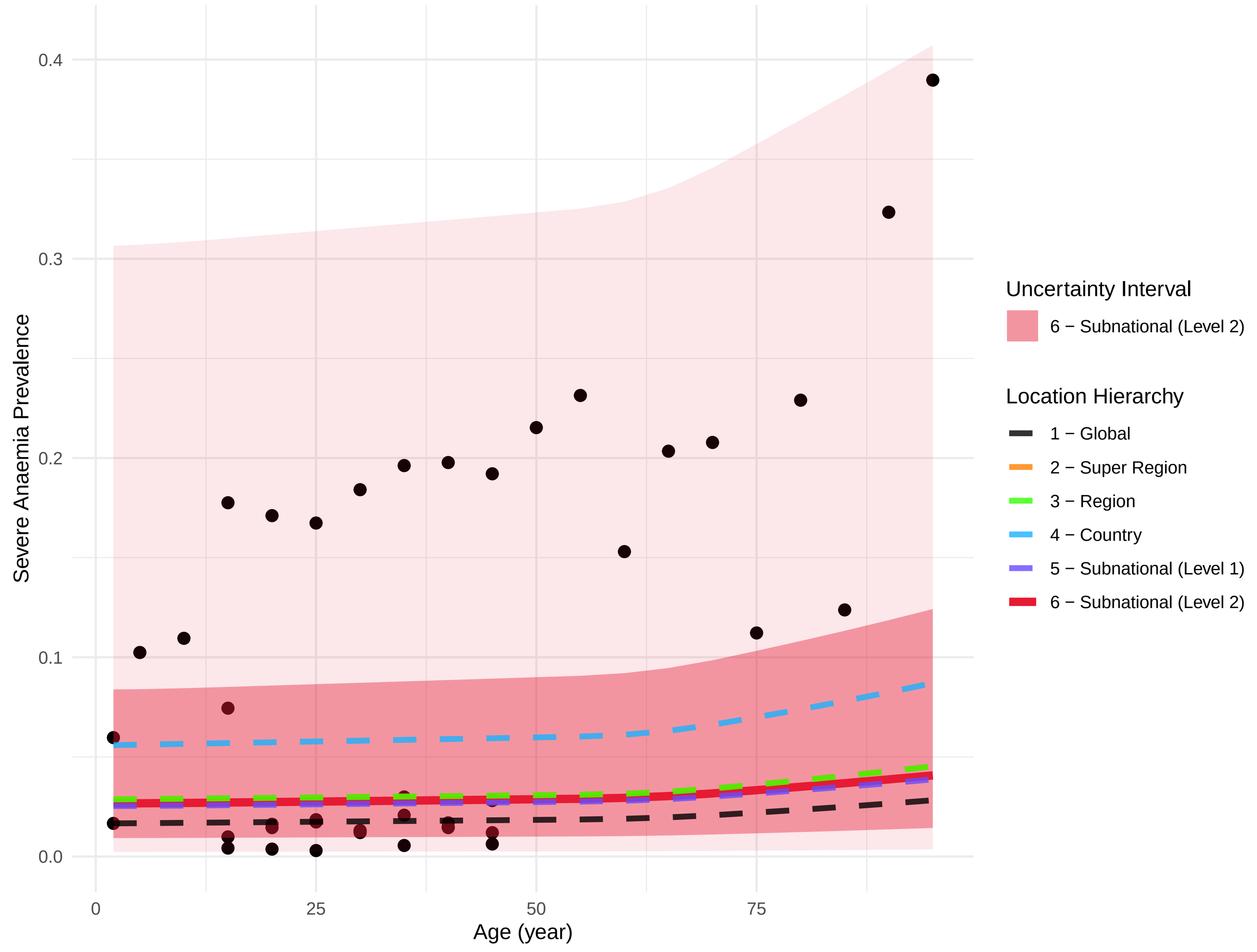

MR−BRT Age/Sex Fits − Non−pregnant Females 2+ Years Old − Severe Anaemia Prevalence − Nagaland, Urban
WHO−2001 Adjustment Method

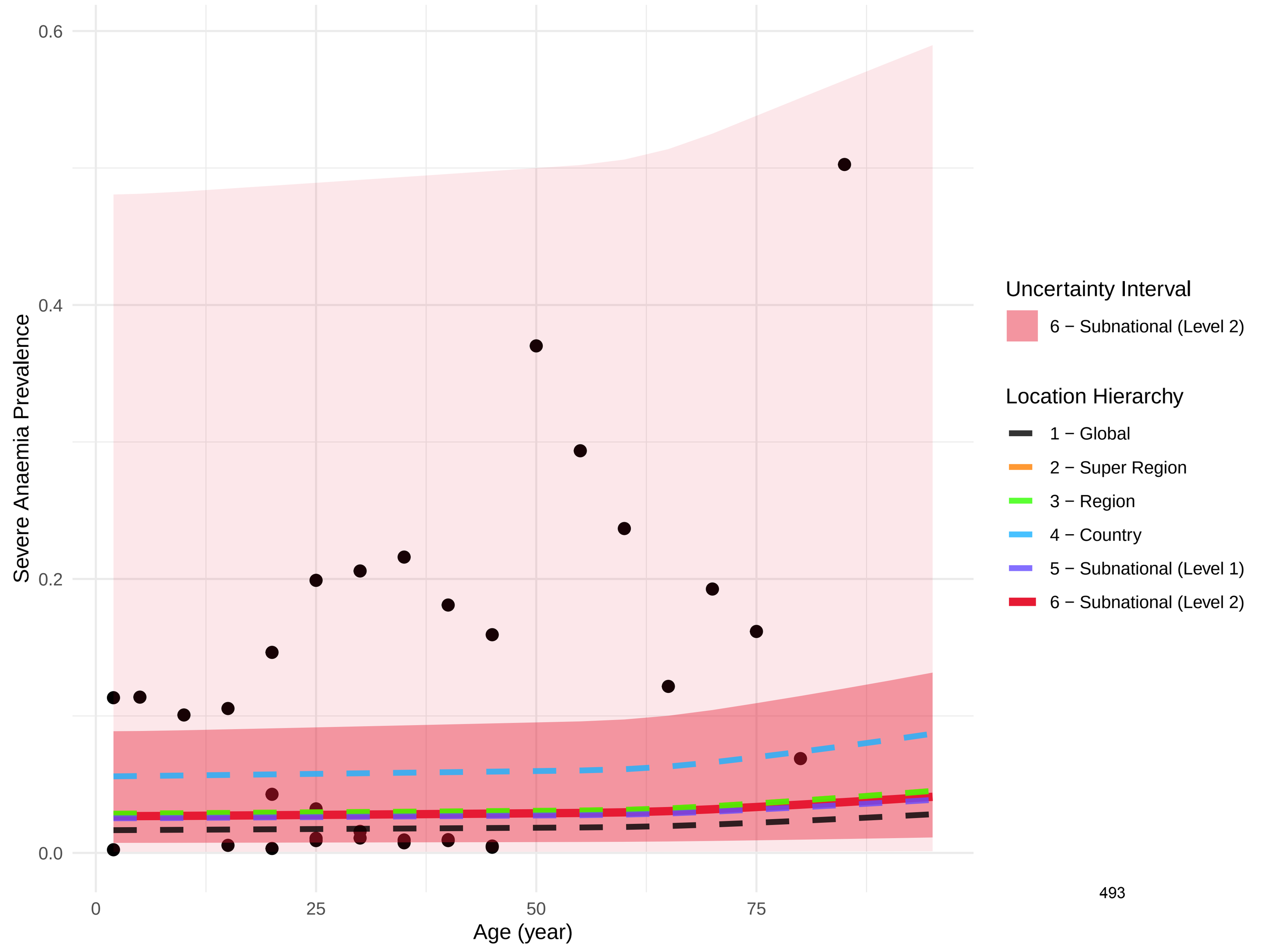

MR−BRT Age/Sex Fits − Non−pregnant Females 2+ Years Old − Severe Anaemia Prevalence − Odisha
WHO−2001 Adjustment Method

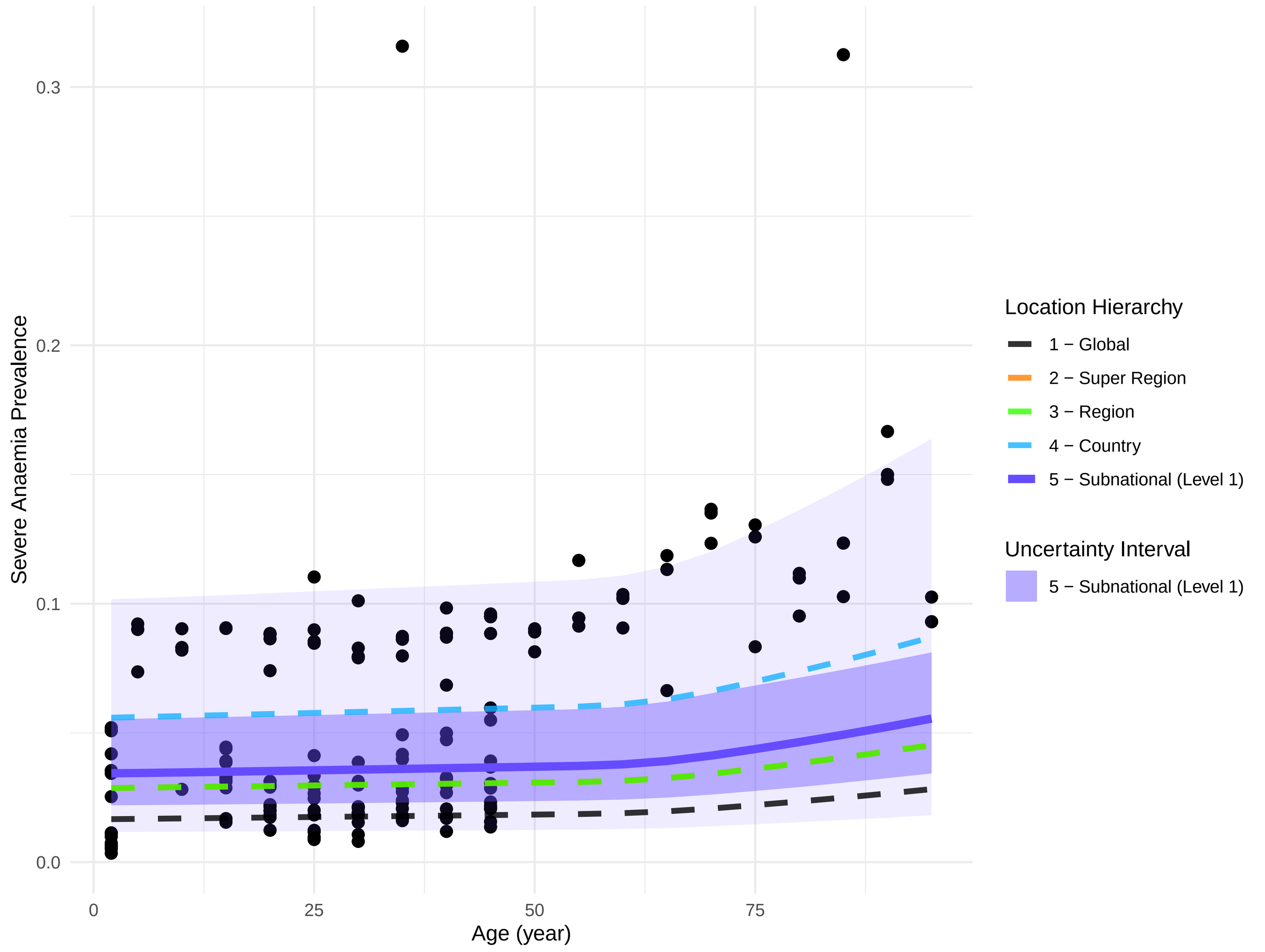

MR−BRT Age/Sex Fits − Non−pregnant Females 2+ Years Old − Severe Anaemia Prevalence − Odisha, Rural
WHO−2001 Adjustment Method

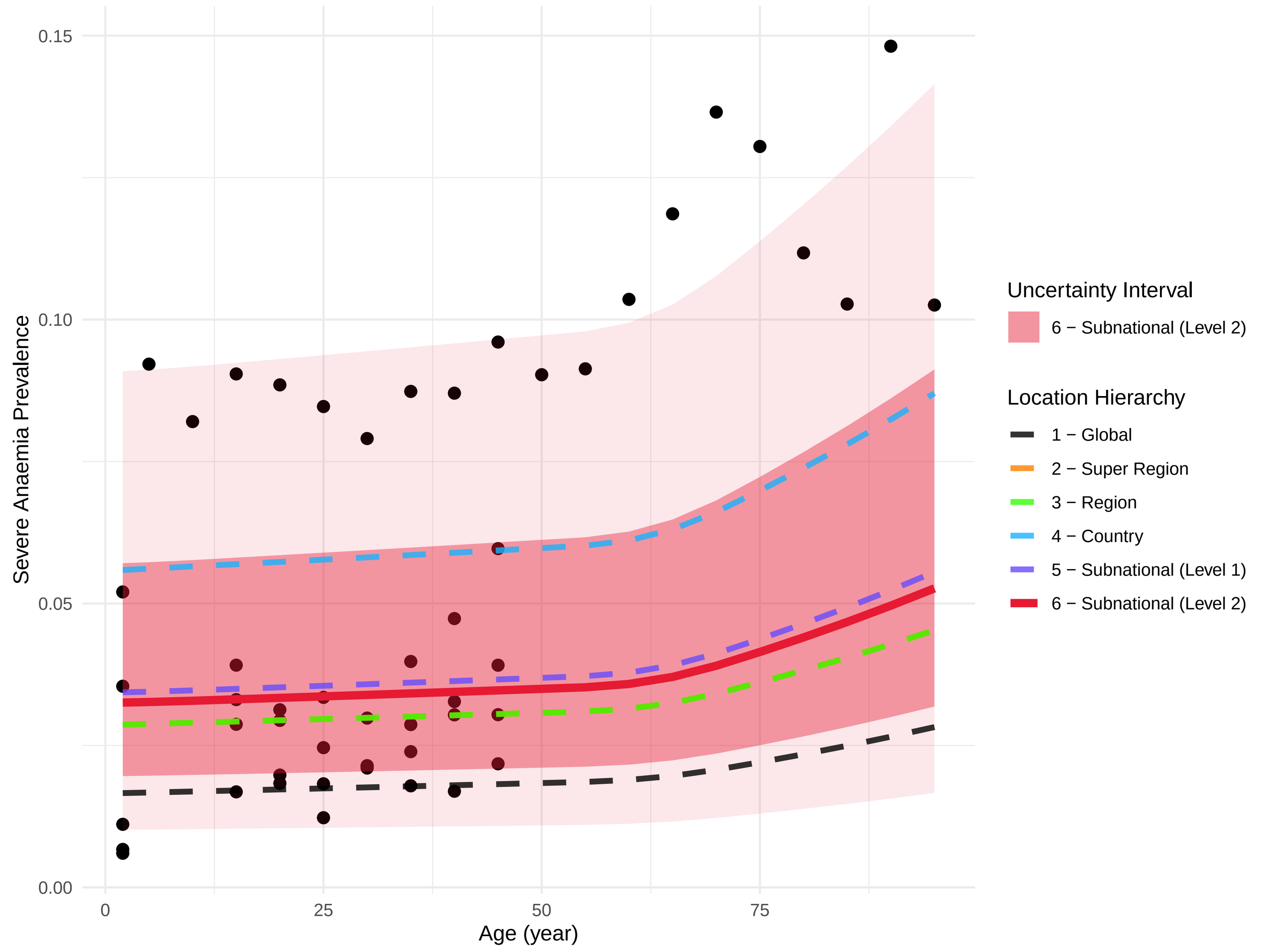

MR−BRT Age/Sex Fits − Non−pregnant Females 2+ Years Old − Severe Anaemia Prevalence − Odisha, Urban
WHO−2001 Adjustment Method

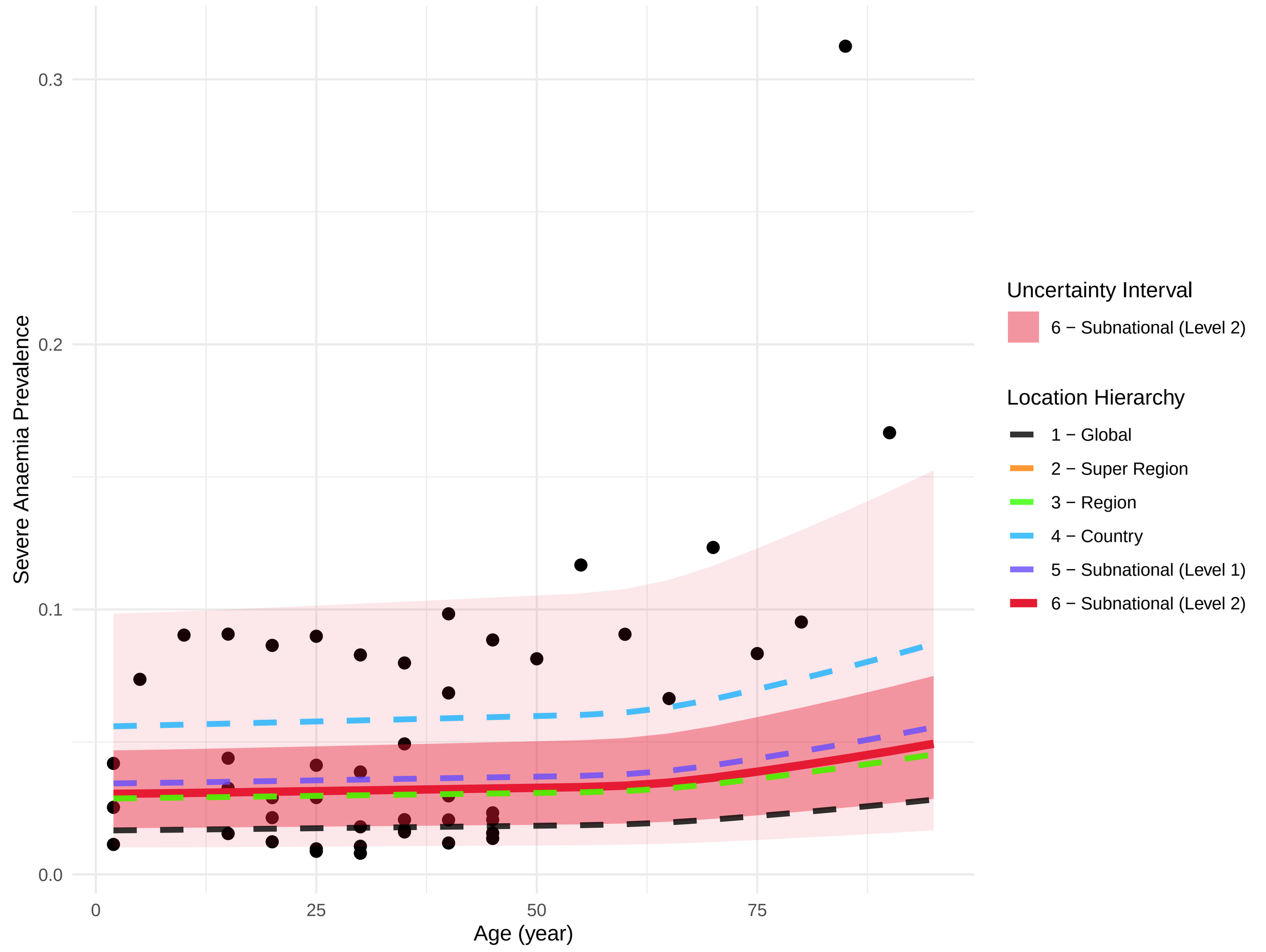

MR−BRT Age/Sex Fits − Non−pregnant Females 2+ Years Old − Severe Anaemia Prevalence − Other Union Territories
WHO−2001 Adjustment Method

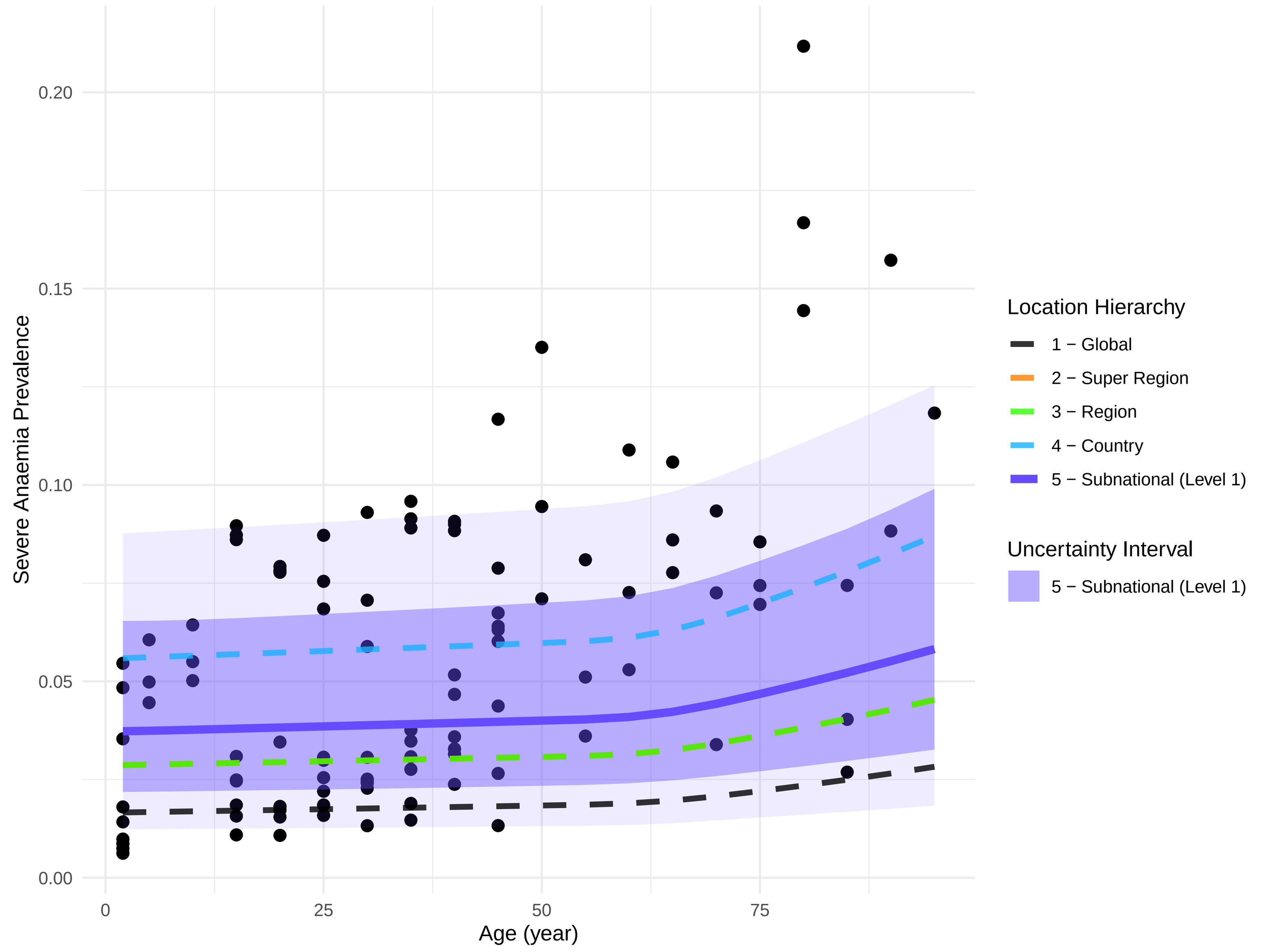

MR−BRT Age/Sex Fits − Non−pregnant Females 2+ Years Old − Severe Anaemia Prevalence − Other Union Territories, Rural
WHO−2001 Adjustment Method

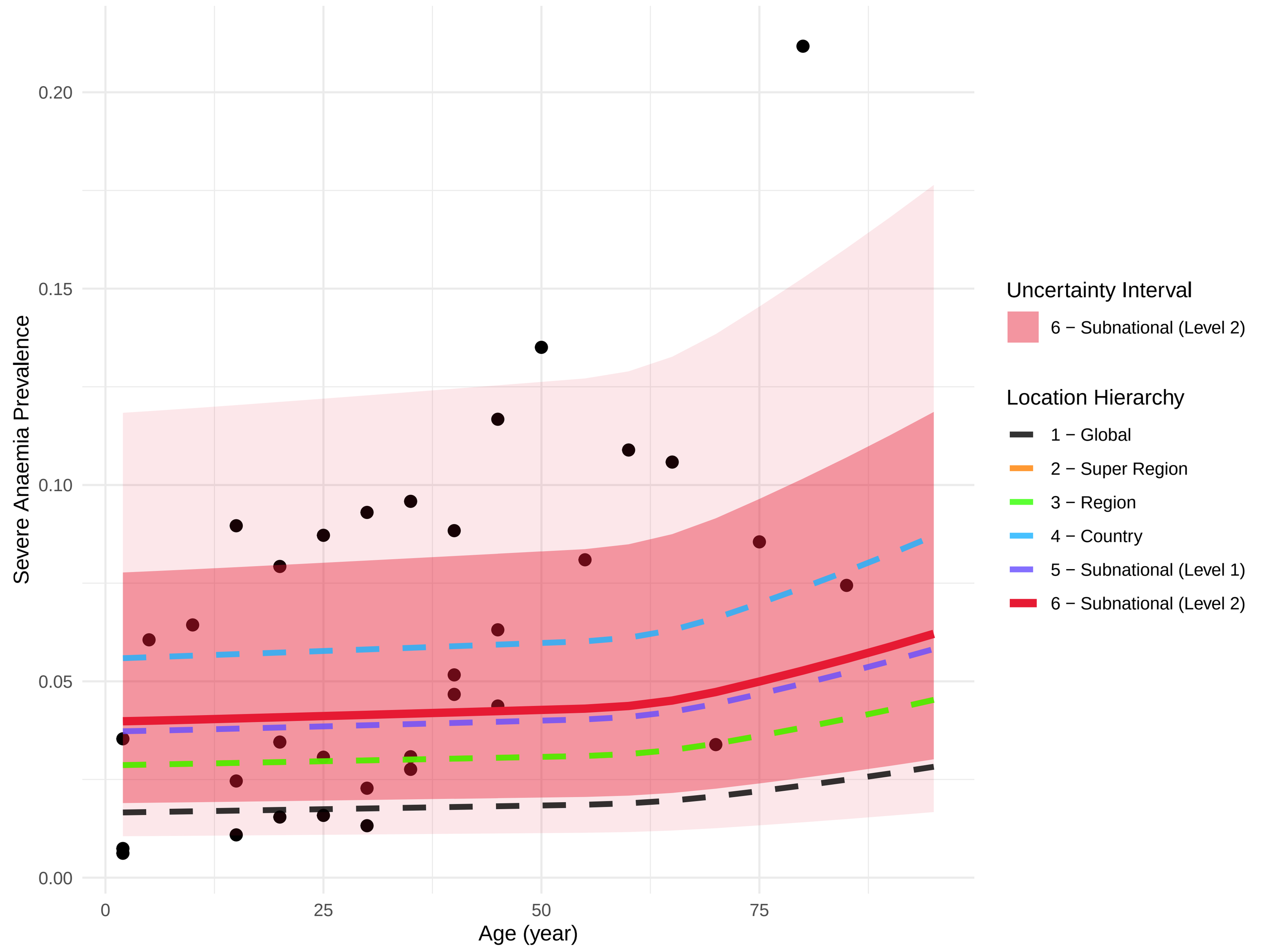

MR−BRT Age/Sex Fits − Non−pregnant Females 2+ Years Old − Severe Anaemia Prevalence − Other Union Territories, Urban
WHO−2001 Adjustment Method

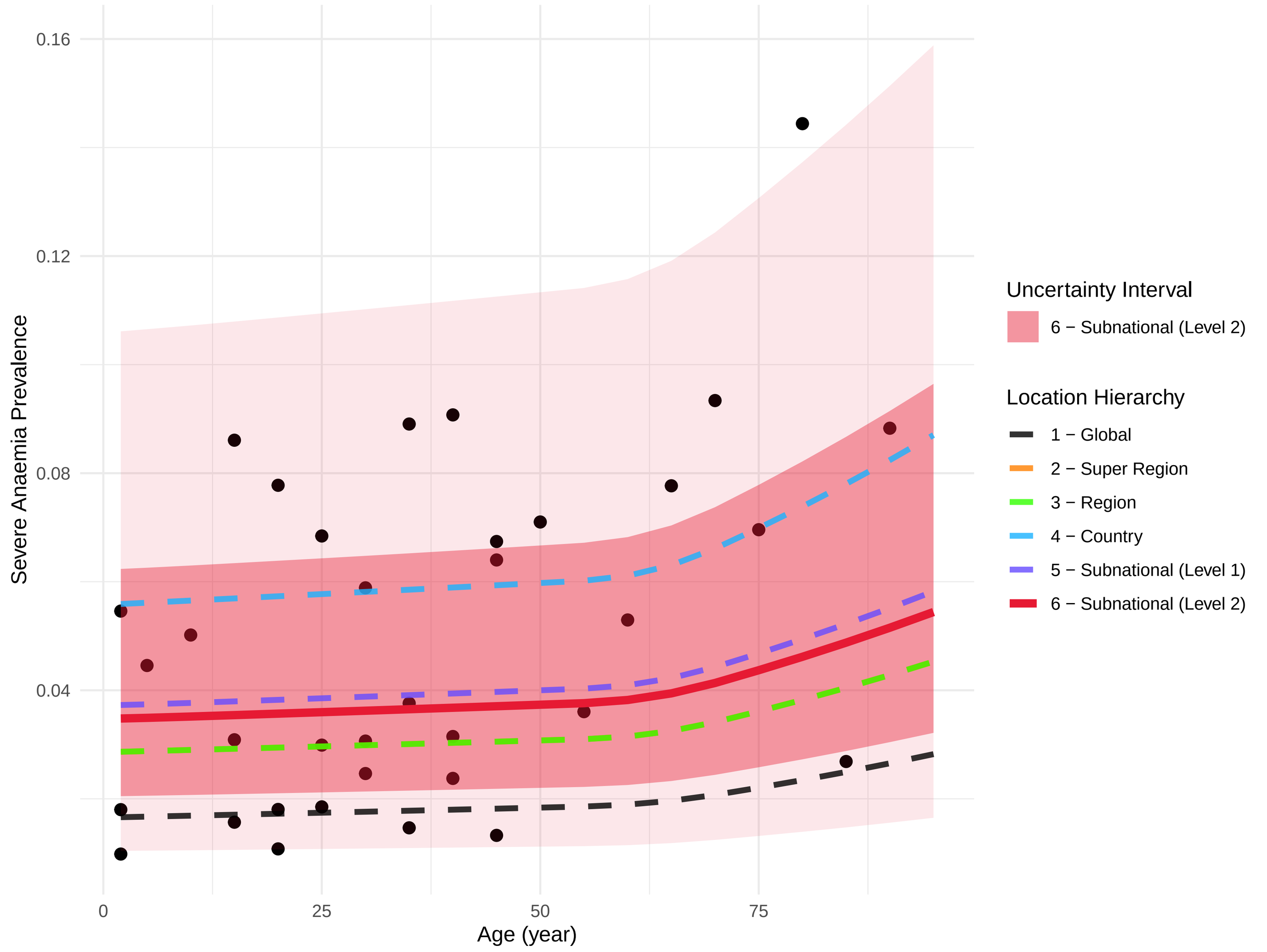

MR−BRT Age/Sex Fits − Non−pregnant Females 2+ Years Old − Severe Anaemia Prevalence − Punjab
WHO−2001 Adjustment Method

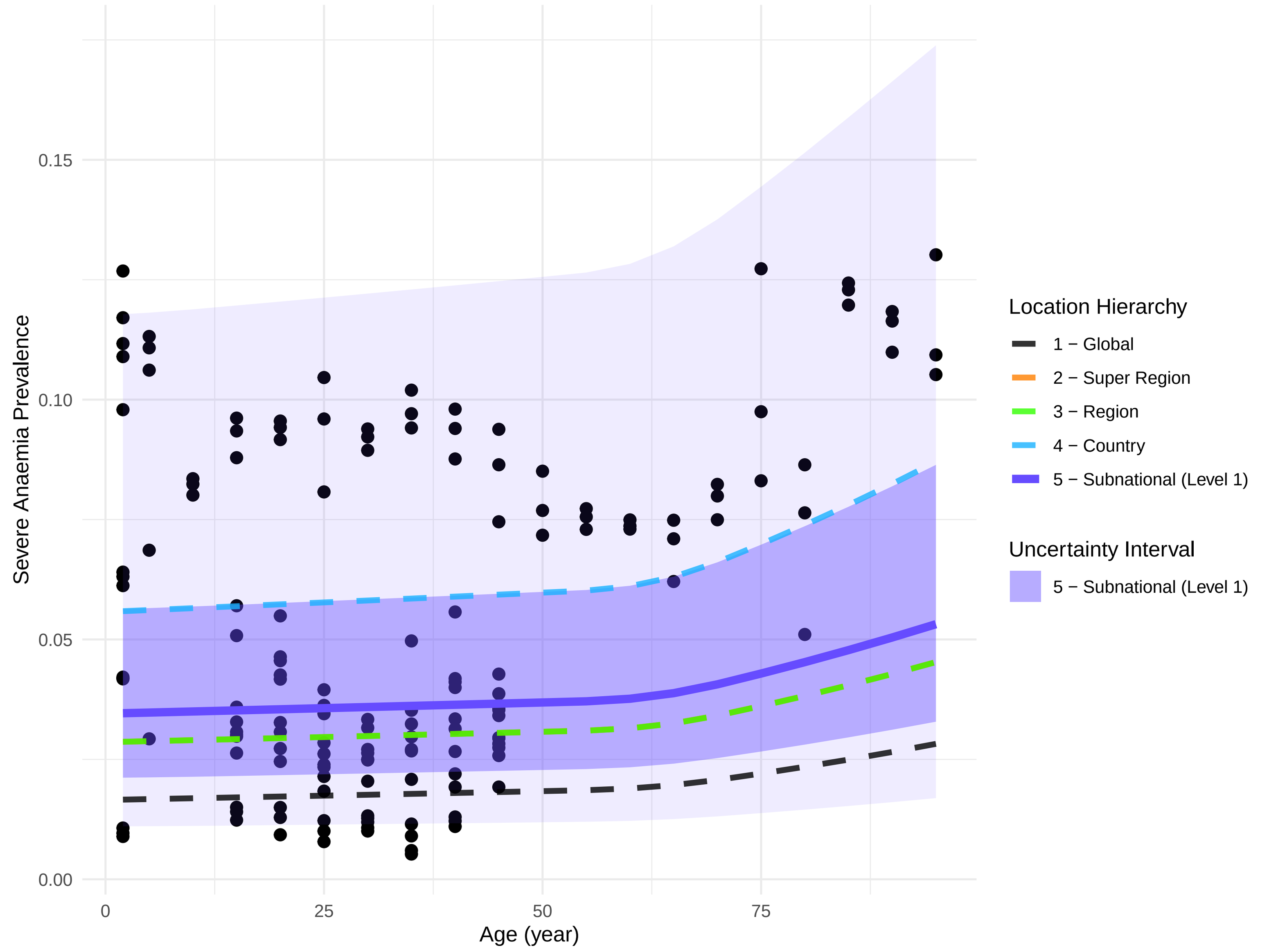

MR−BRT Age/Sex Fits − Non−pregnant Females 2+ Years Old − Severe Anaemia Prevalence − Punjab, Rural
WHO−2001 Adjustment Method

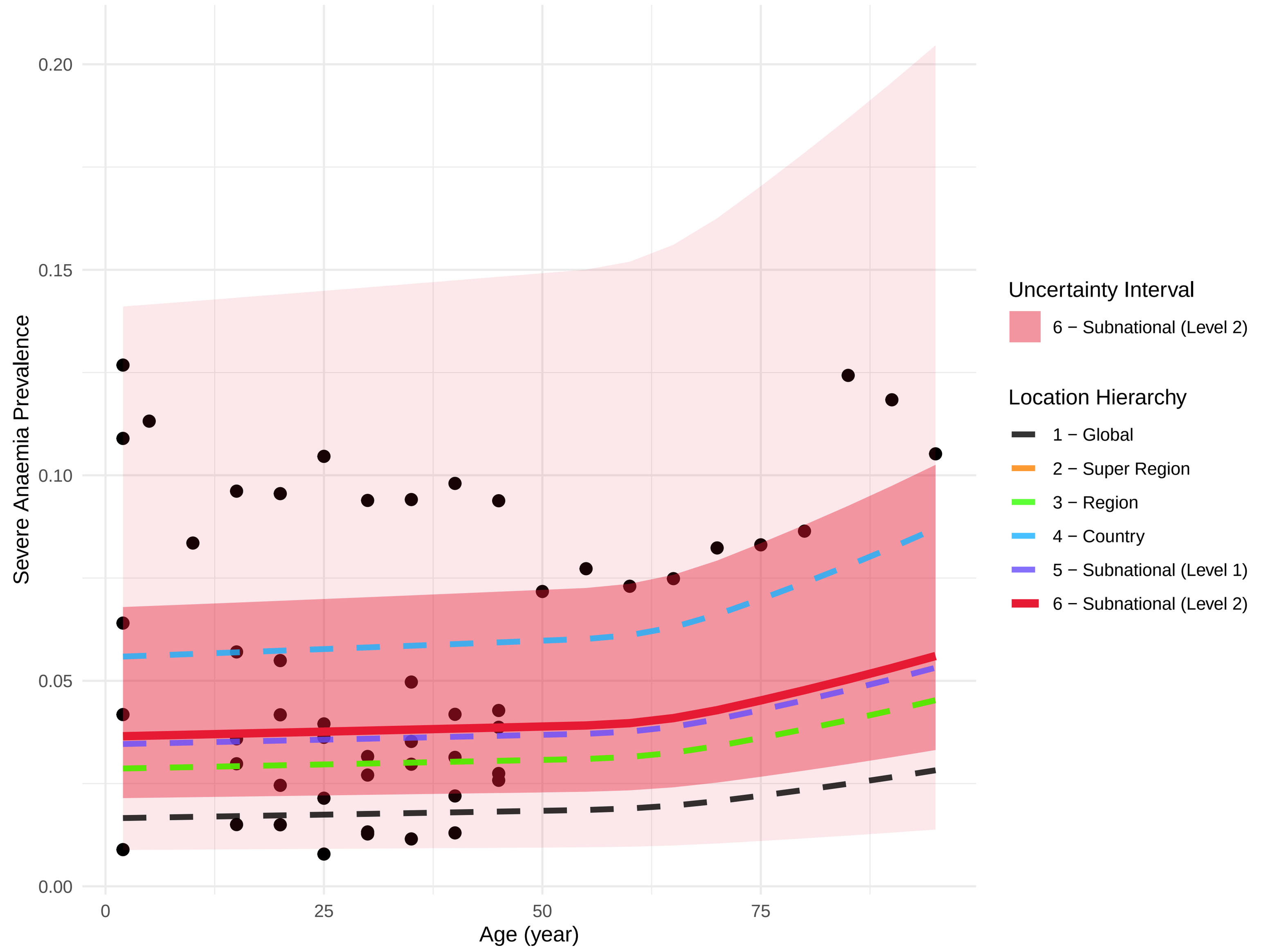

MR−BRT Age/Sex Fits − Non−pregnant Females 2+ Years Old − Severe Anaemia Prevalence − Punjab, Urban
WHO−2001 Adjustment Method

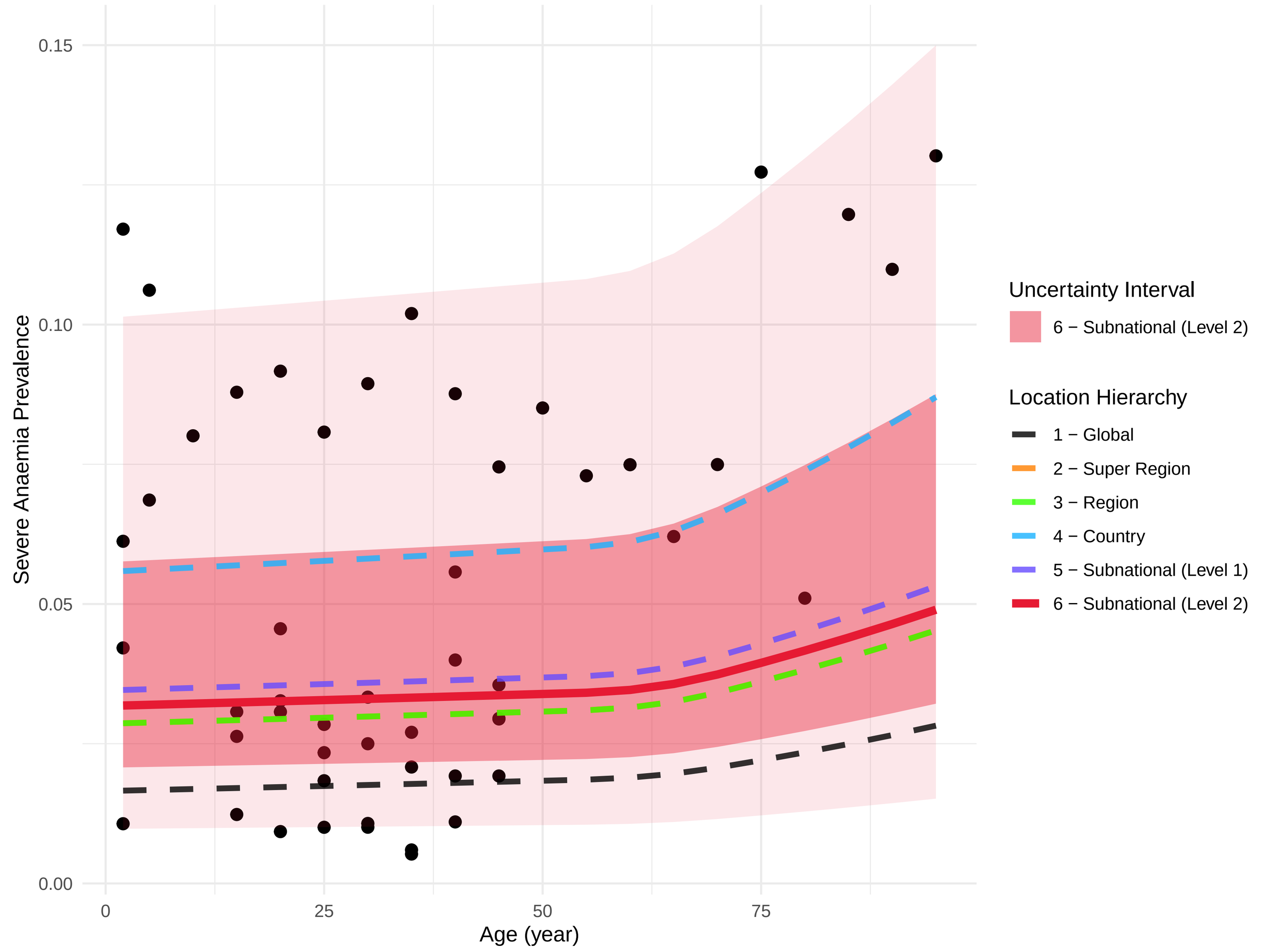

MR−BRT Age/Sex Fits − Non−pregnant Females 2+ Years Old − Severe Anaemia Prevalence − Rajasthan
WHO−2001 Adjustment Method

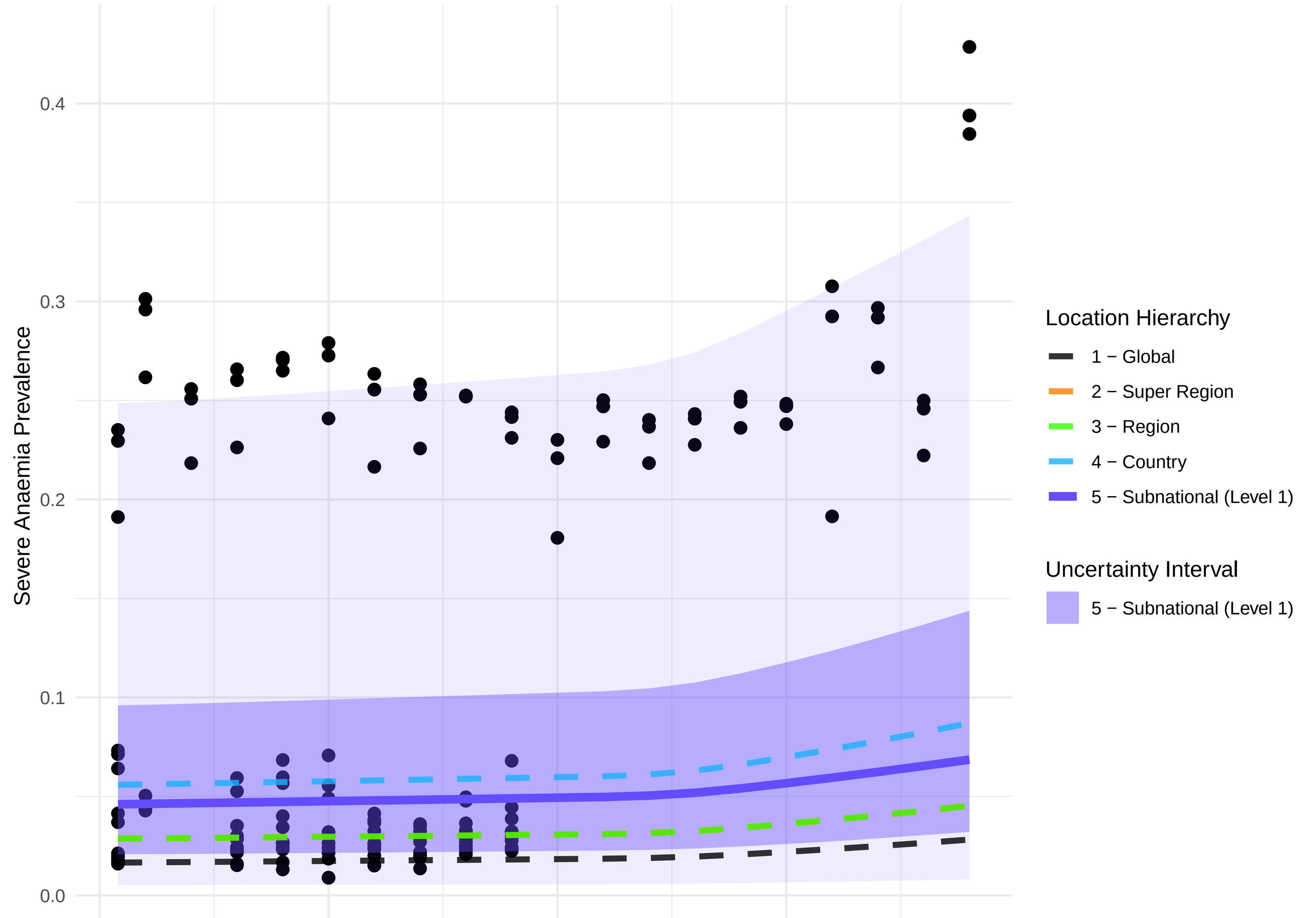

MR−BRT Age/Sex Fits − Non−pregnant Females 2+ Years Old − Severe Anaemia Prevalence − Rajasthan, Rural
WHO−2001 Adjustment Method

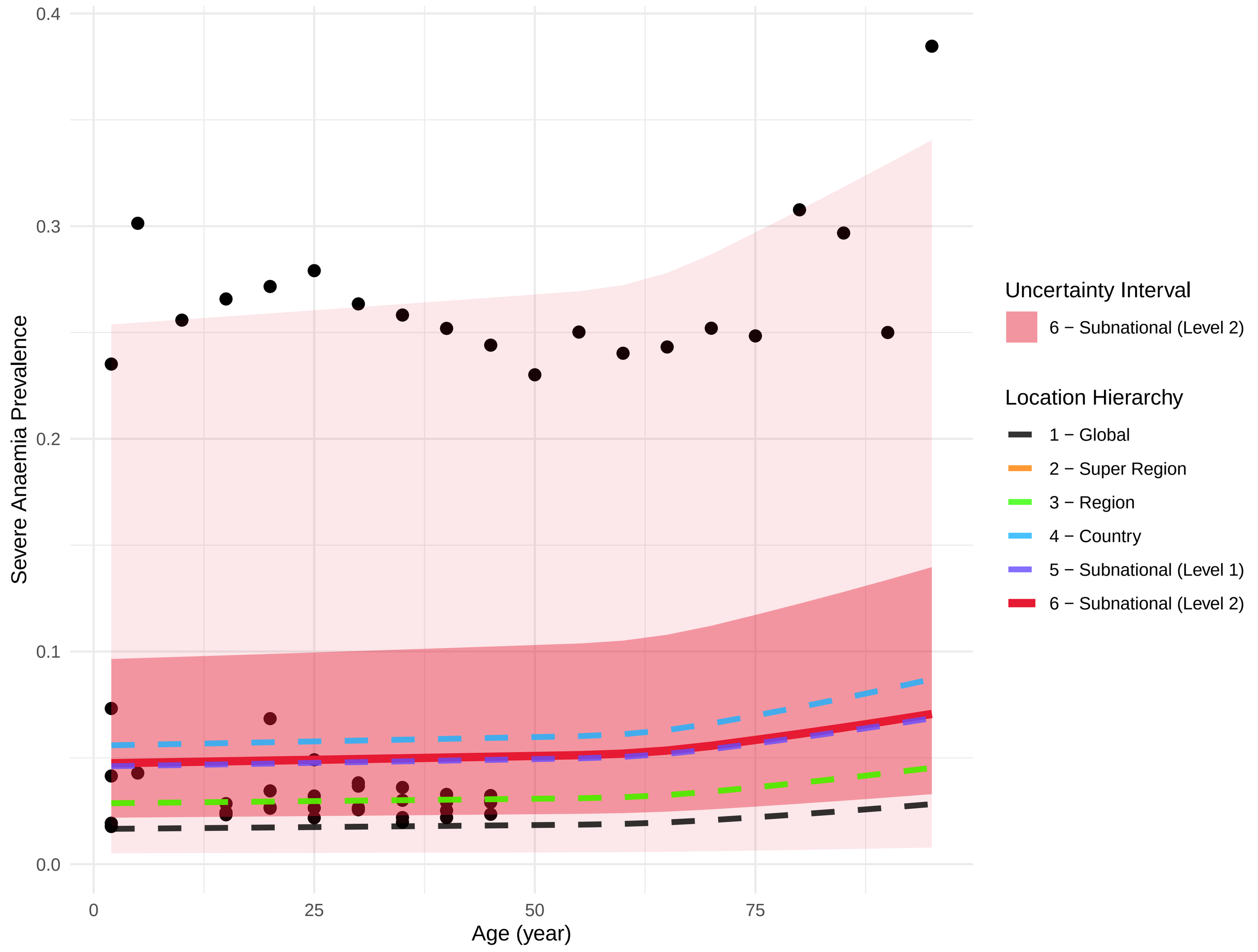

MR−BRT Age/Sex Fits − Non−pregnant Females 2+ Years Old − Severe Anaemia Prevalence − Rajasthan, Urban
WHO−2001 Adjustment Method

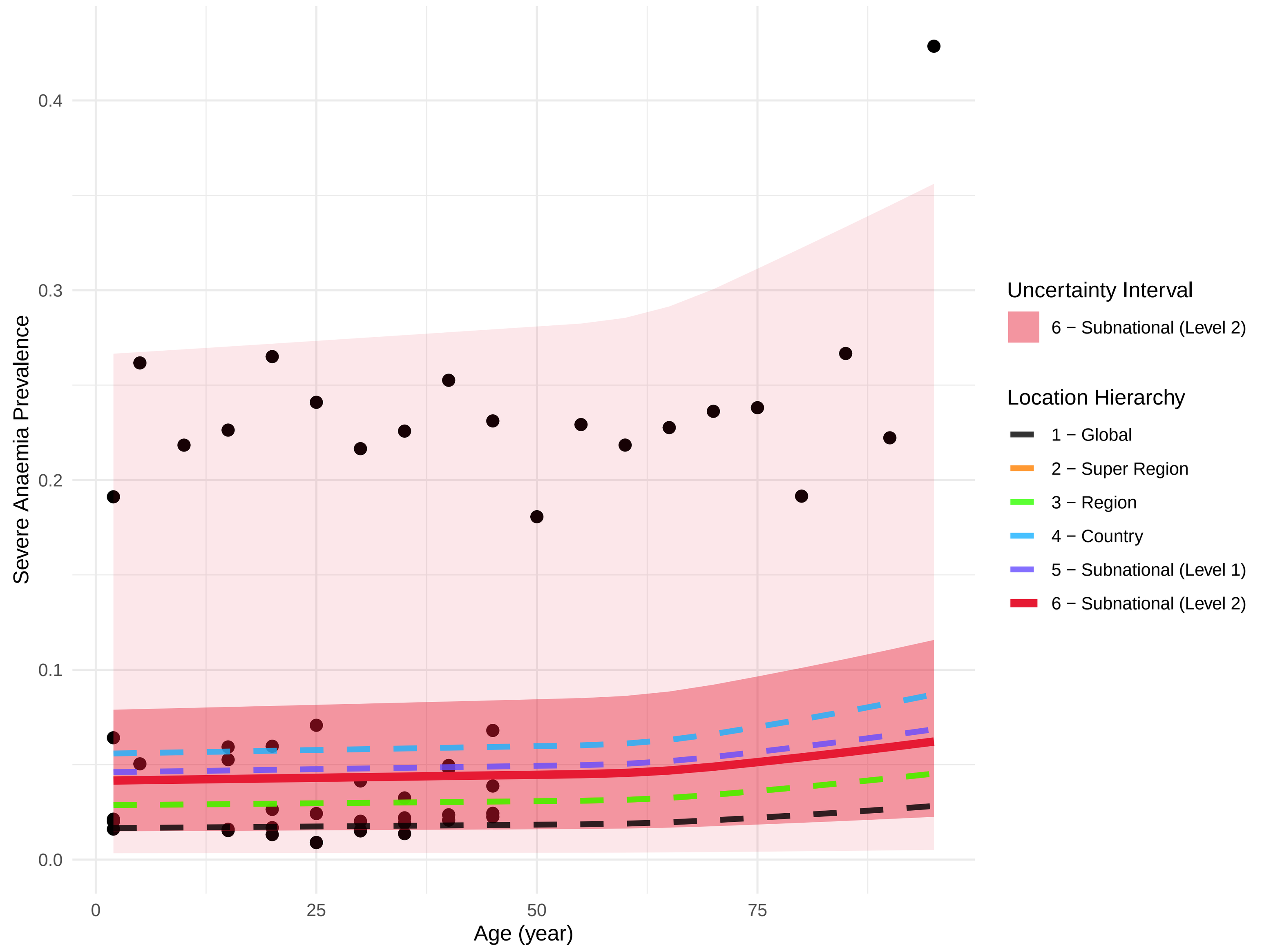

MR−BRT Age/Sex Fits − Non−pregnant Females 2+ Years Old − Severe Anaemia Prevalence − Sikkim
WHO−2001 Adjustment Method

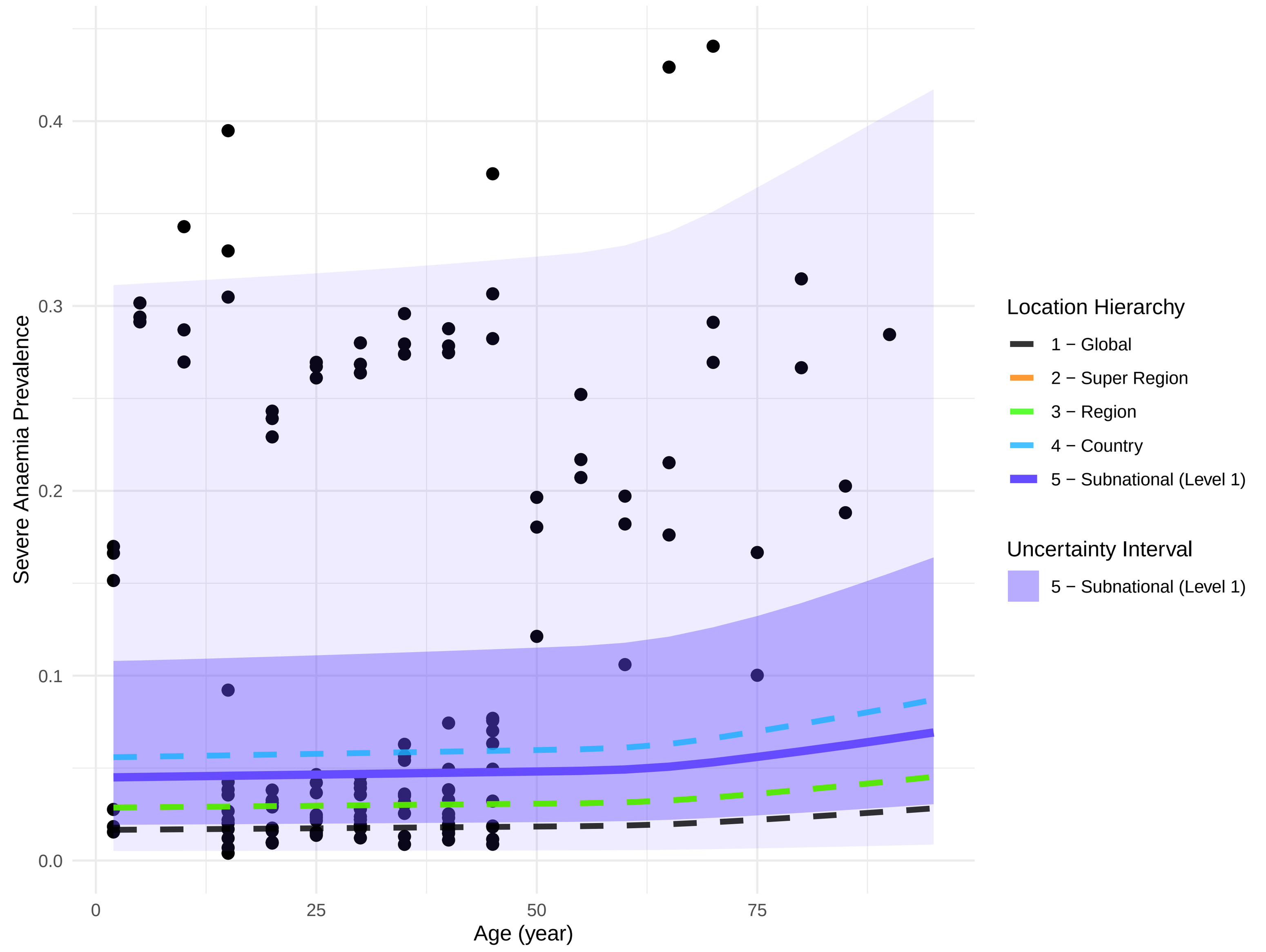

MR−BRT Age/Sex Fits − Non−pregnant Females 2+ Years Old − Severe Anaemia Prevalence − Sikkim, Rural
WHO−2001 Adjustment Method

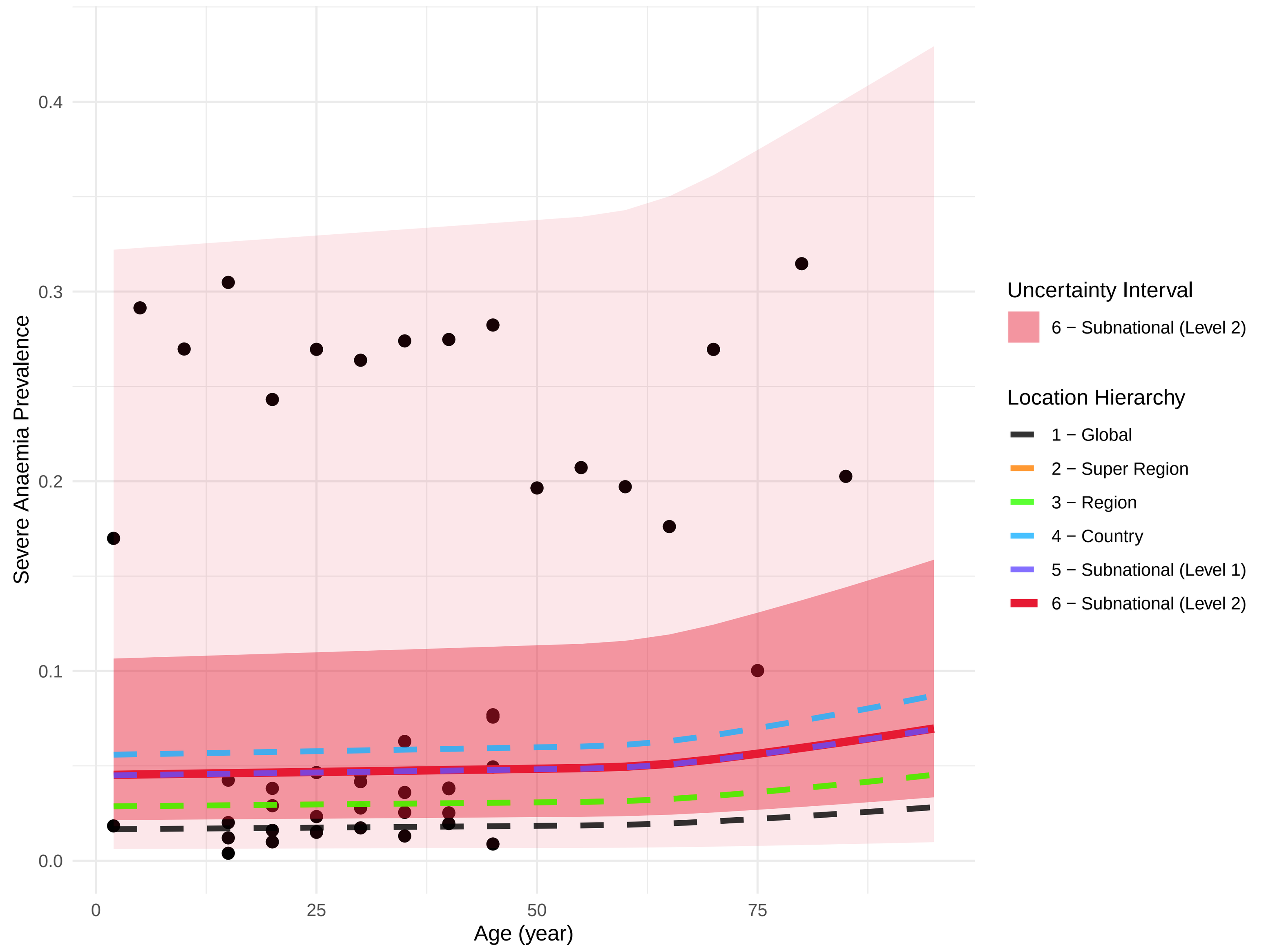

MR−BRT Age/Sex Fits − Non−pregnant Females 2+ Years Old − Severe Anaemia Prevalence − Sikkim, Urban
WHO−2001 Adjustment Method

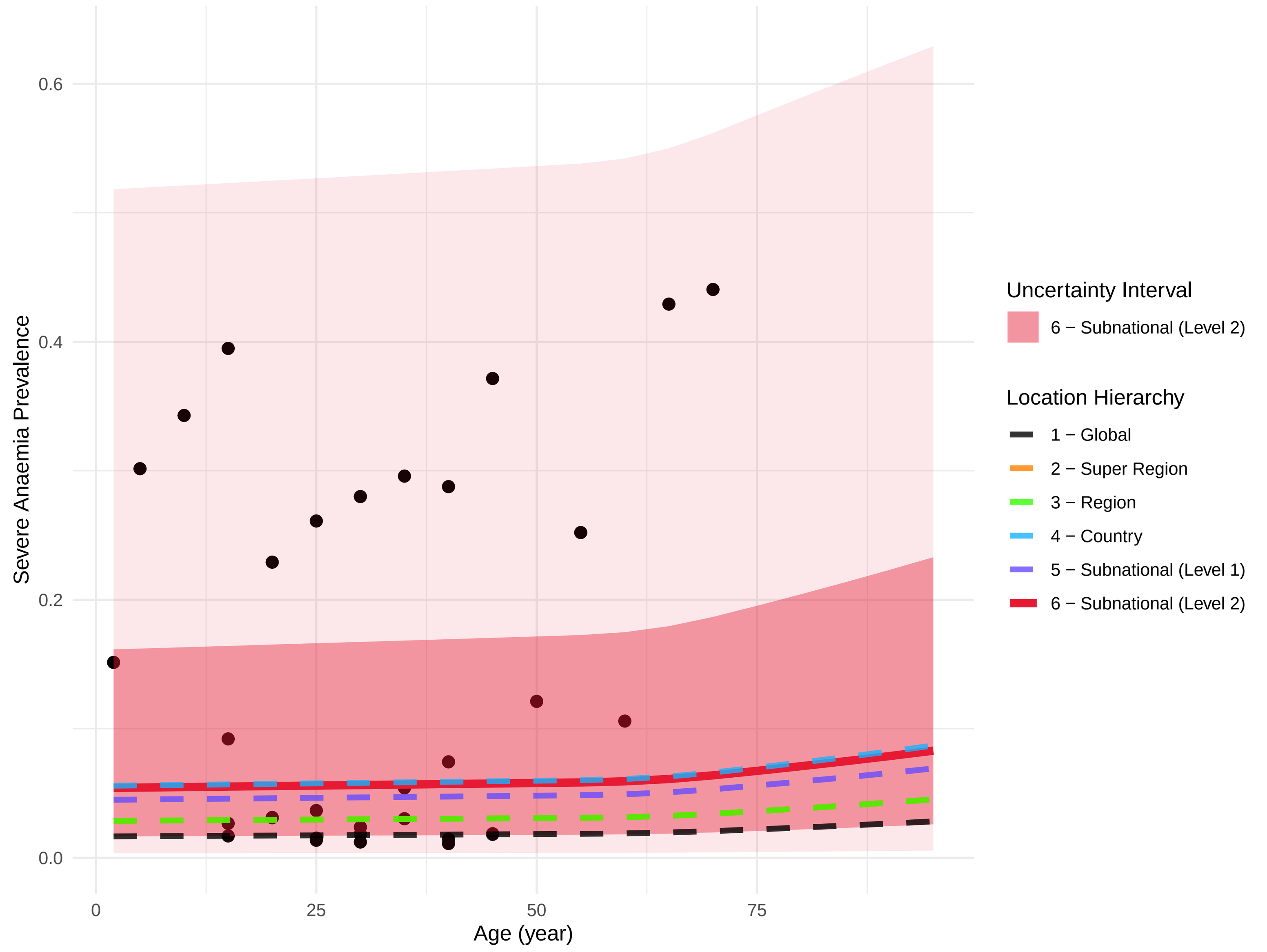

MR−BRT Age/Sex Fits − Non−pregnant Females 2+ Years Old − Severe Anaemia Prevalence − Tamil Nadu
WHO−2001 Adjustment Method

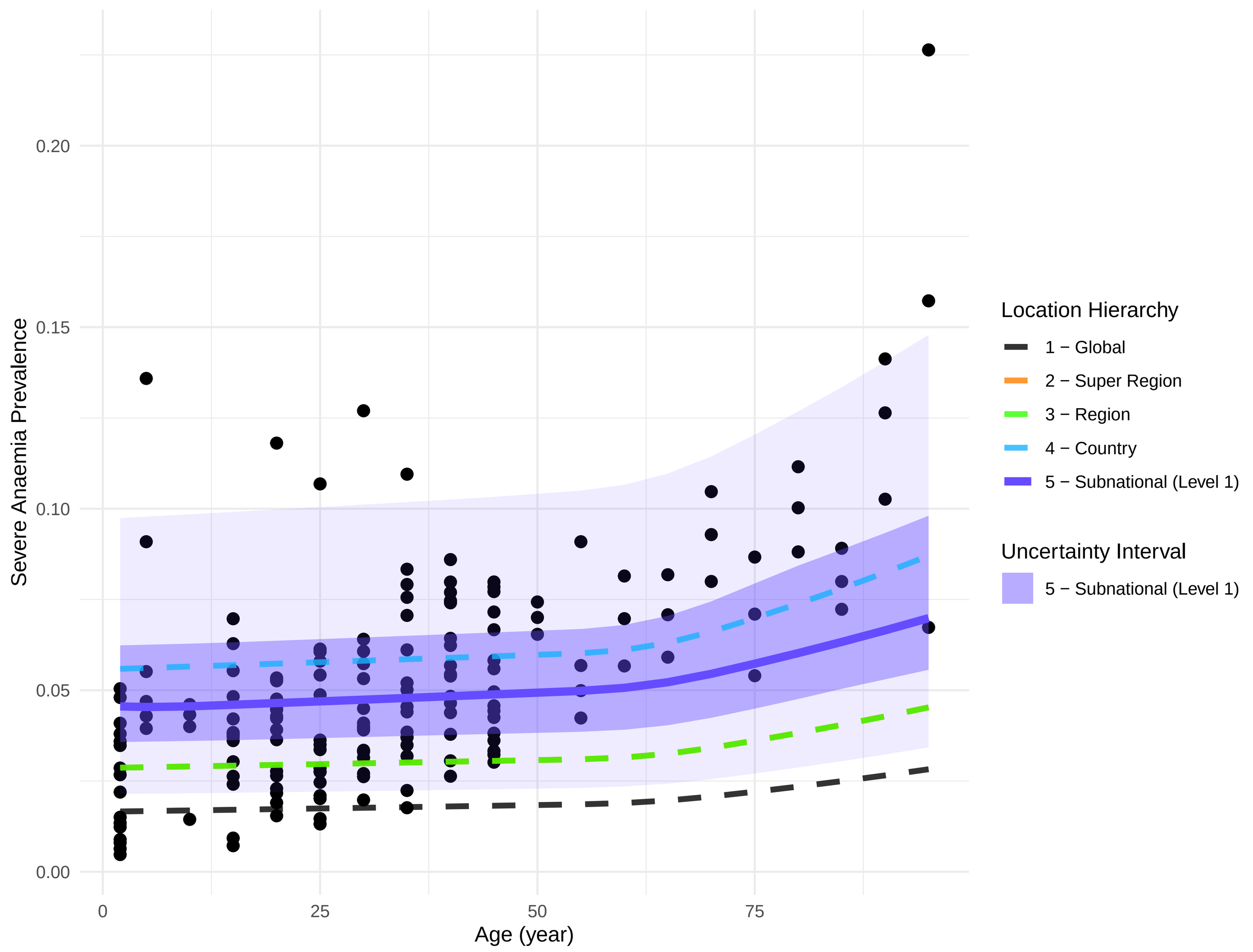

MR−BRT Age/Sex Fits − Non−pregnant Females 2+ Years Old − Severe Anaemia Prevalence − Tamil Nadu, Rural
WHO−2001 Adjustment Method

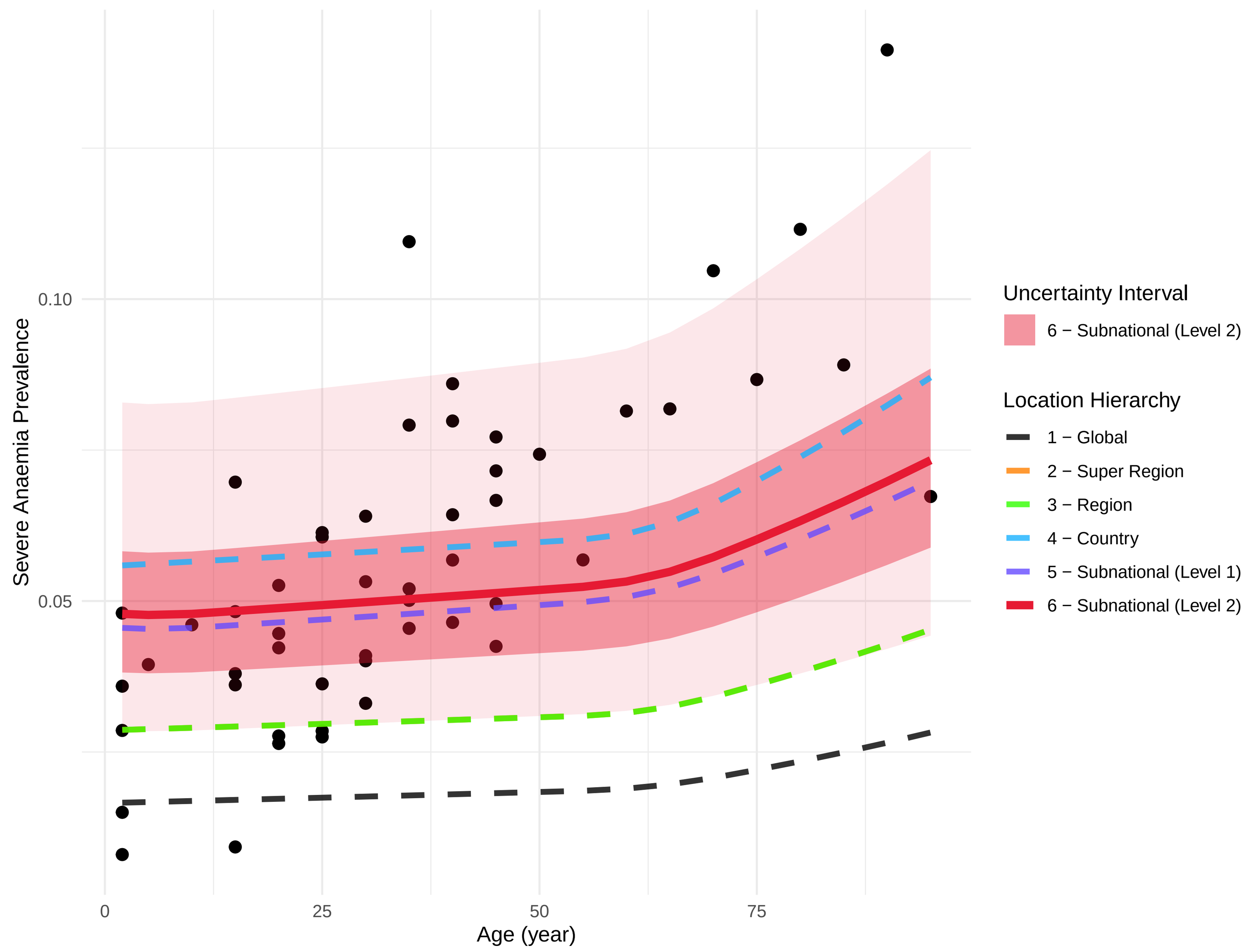

MR−BRT Age/Sex Fits − Non−pregnant Females 2+ Years Old − Severe Anaemia Prevalence − Tamil Nadu, Urban
WHO−2001 Adjustment Method

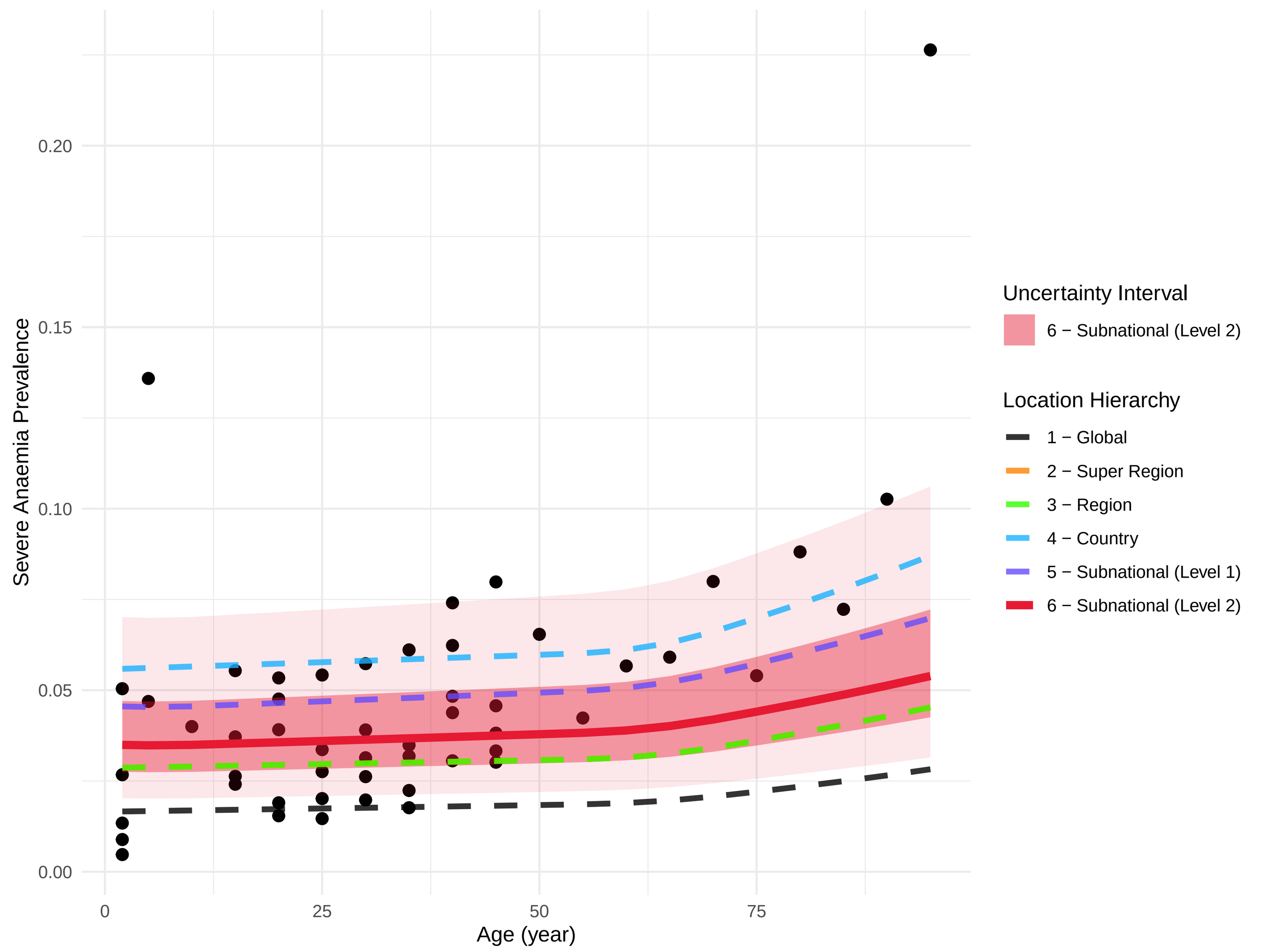

MR−BRT Age/Sex Fits − Non−pregnant Females 2+ Years Old − Severe Anaemia Prevalence − Telangana
WHO−2001 Adjustment Method

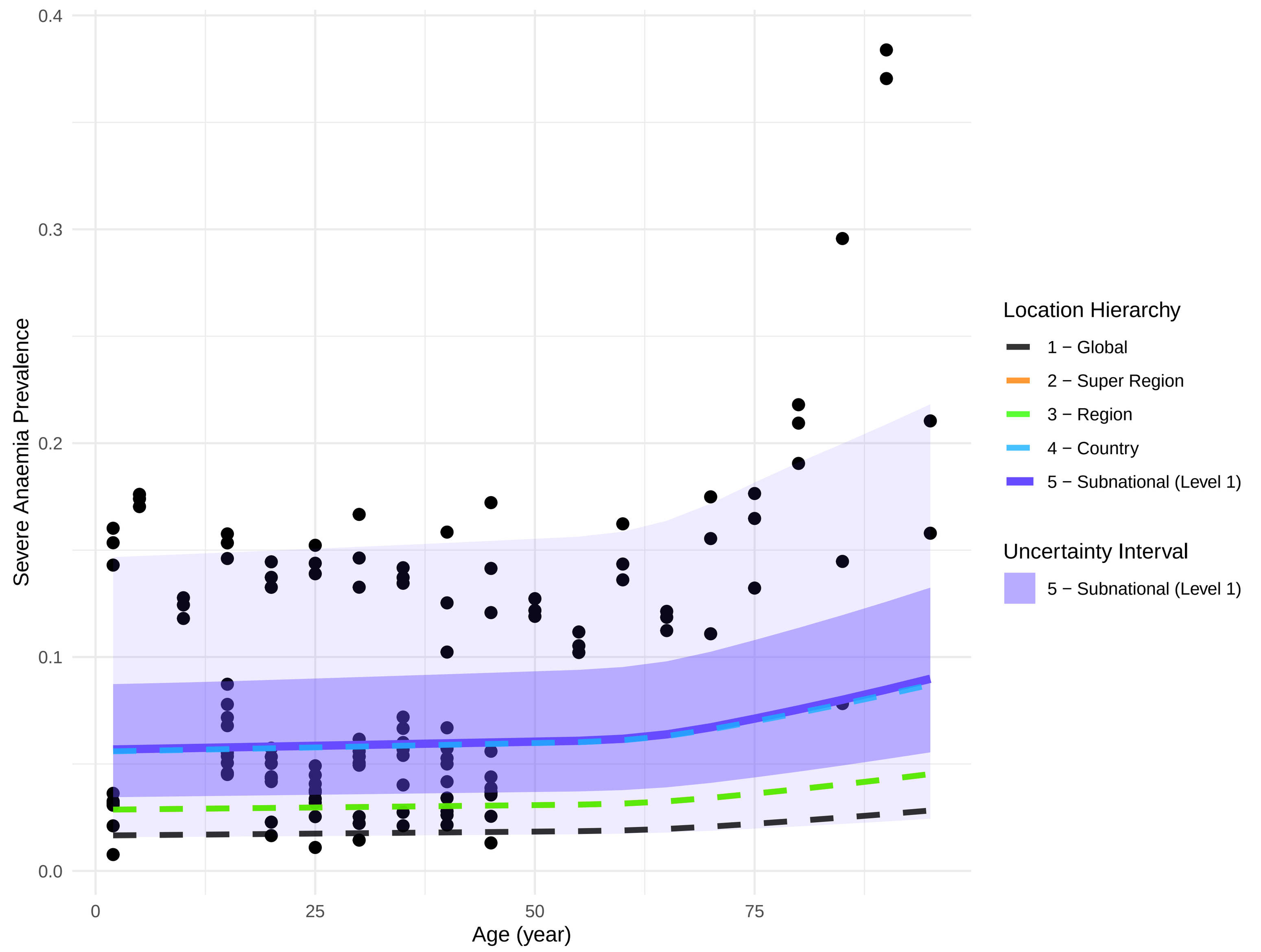

MR−BRT Age/Sex Fits − Non−pregnant Females 2+ Years Old − Severe Anaemia Prevalence − Telangana, Rural
WHO−2001 Adjustment Method

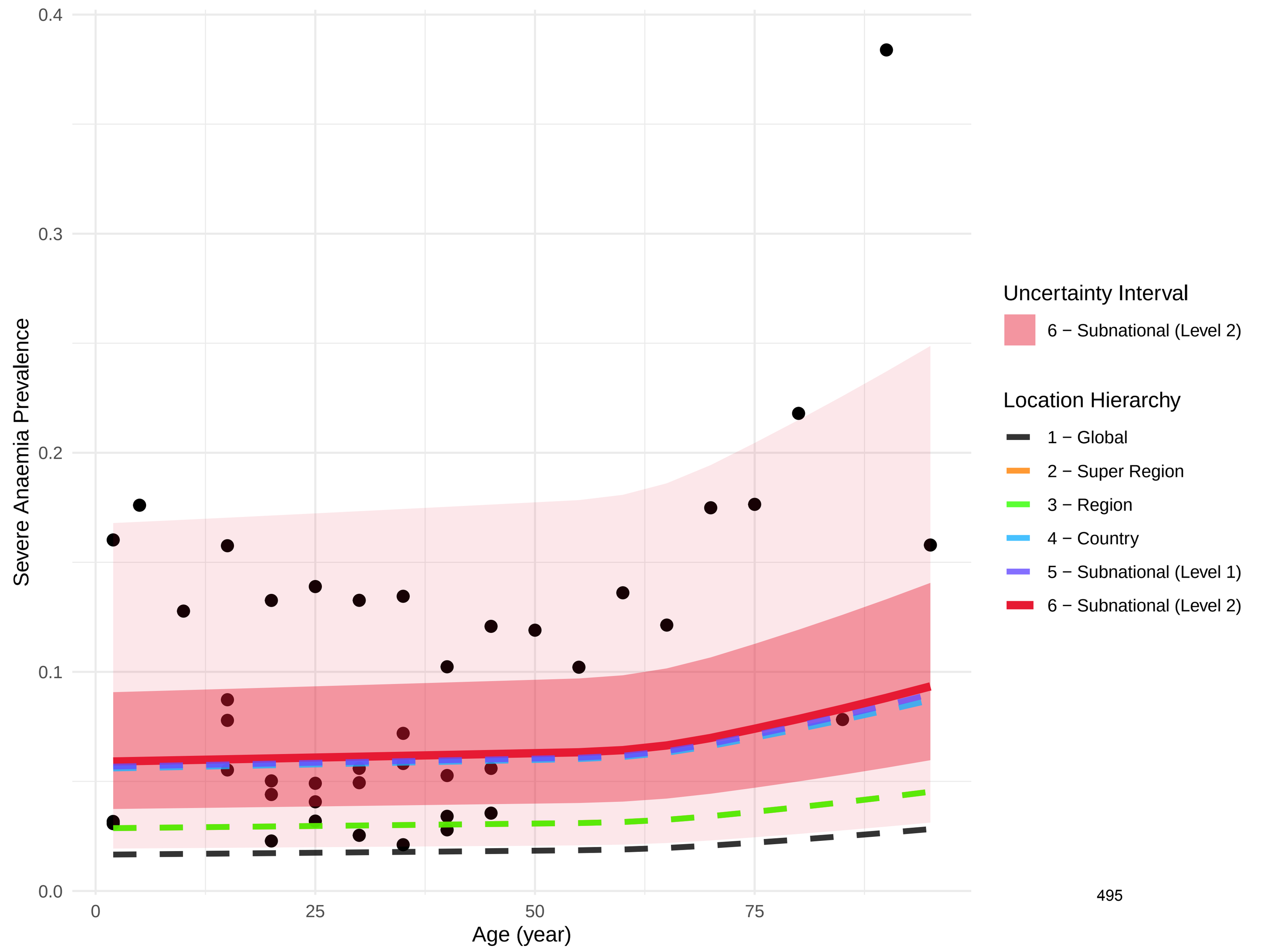

MR−BRT Age/Sex Fits − Non−pregnant Females 2+ Years Old − Severe Anaemia Prevalence − Telangana, Urban
WHO−2001 Adjustment Method

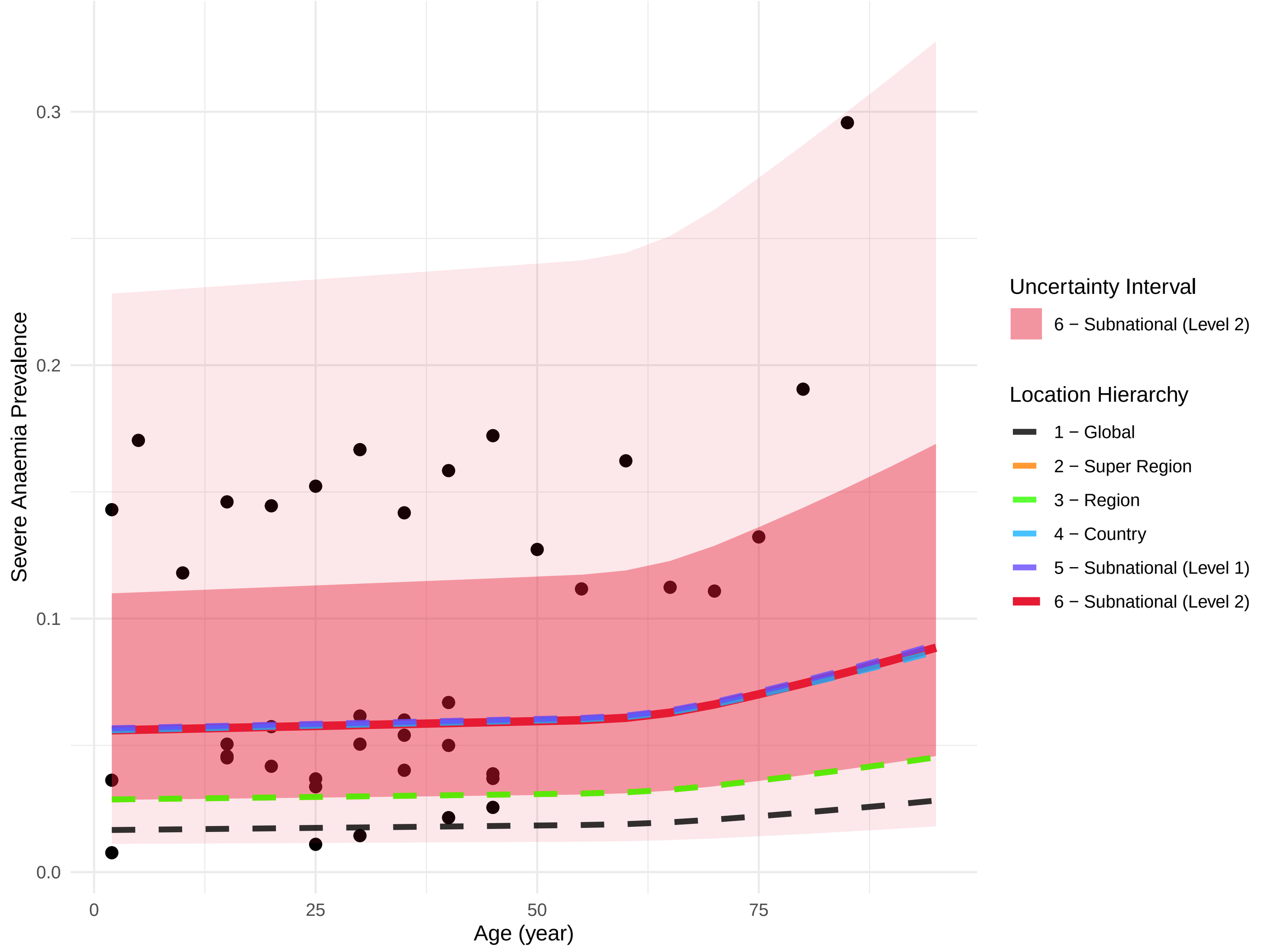

MR−BRT Age/Sex Fits − Non−pregnant Females 2+ Years Old − Severe Anaemia Prevalence − Tripura
WHO−2001 Adjustment Method

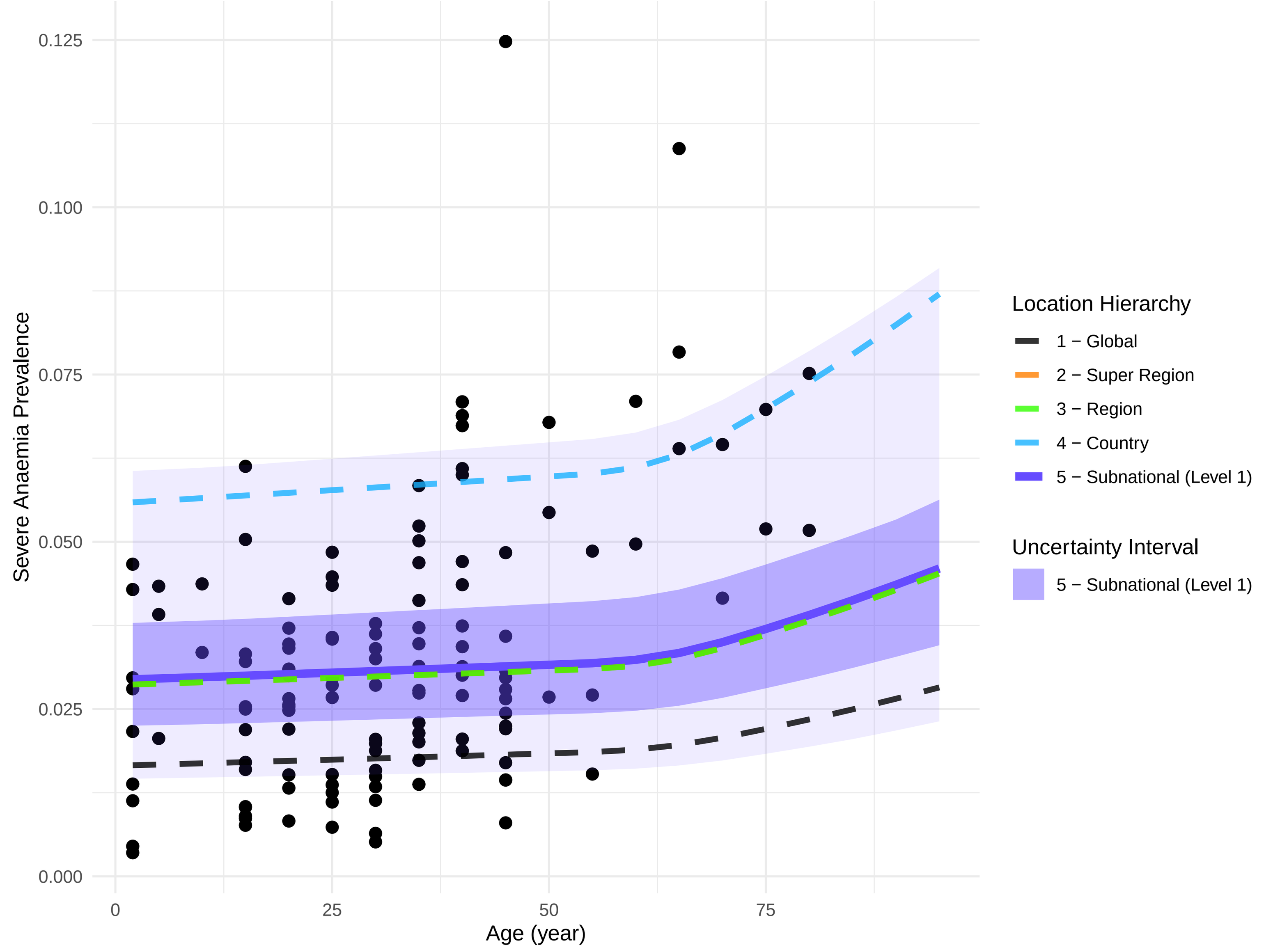

MR−BRT Age/Sex Fits − Non−pregnant Females 2+ Years Old − Severe Anaemia Prevalence − Tripura, Rural
WHO−2001 Adjustment Method

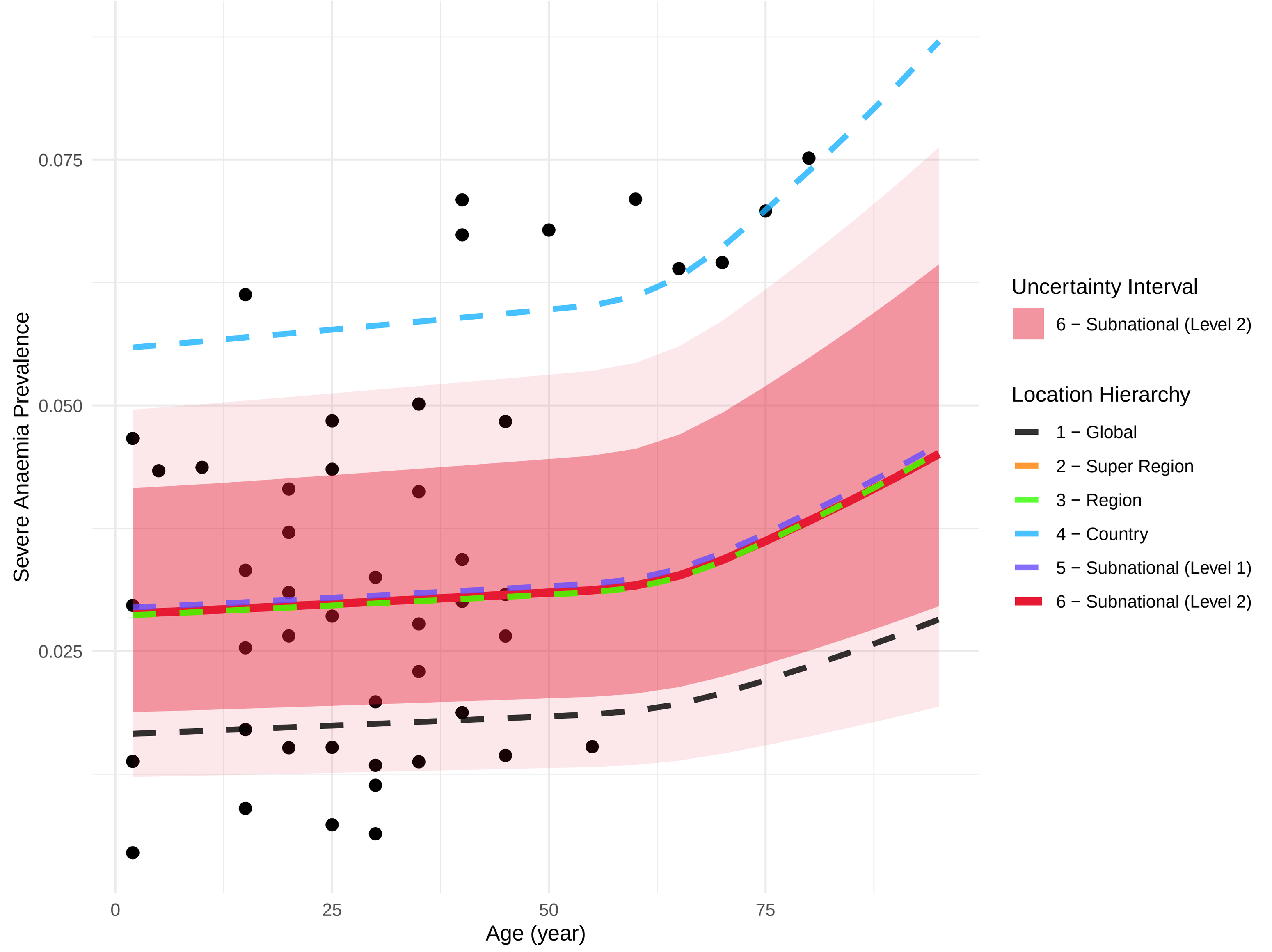

MR−BRT Age/Sex Fits − Non−pregnant Females 2+ Years Old − Severe Anaemia Prevalence − Tripura, Urban
WHO−2001 Adjustment Method

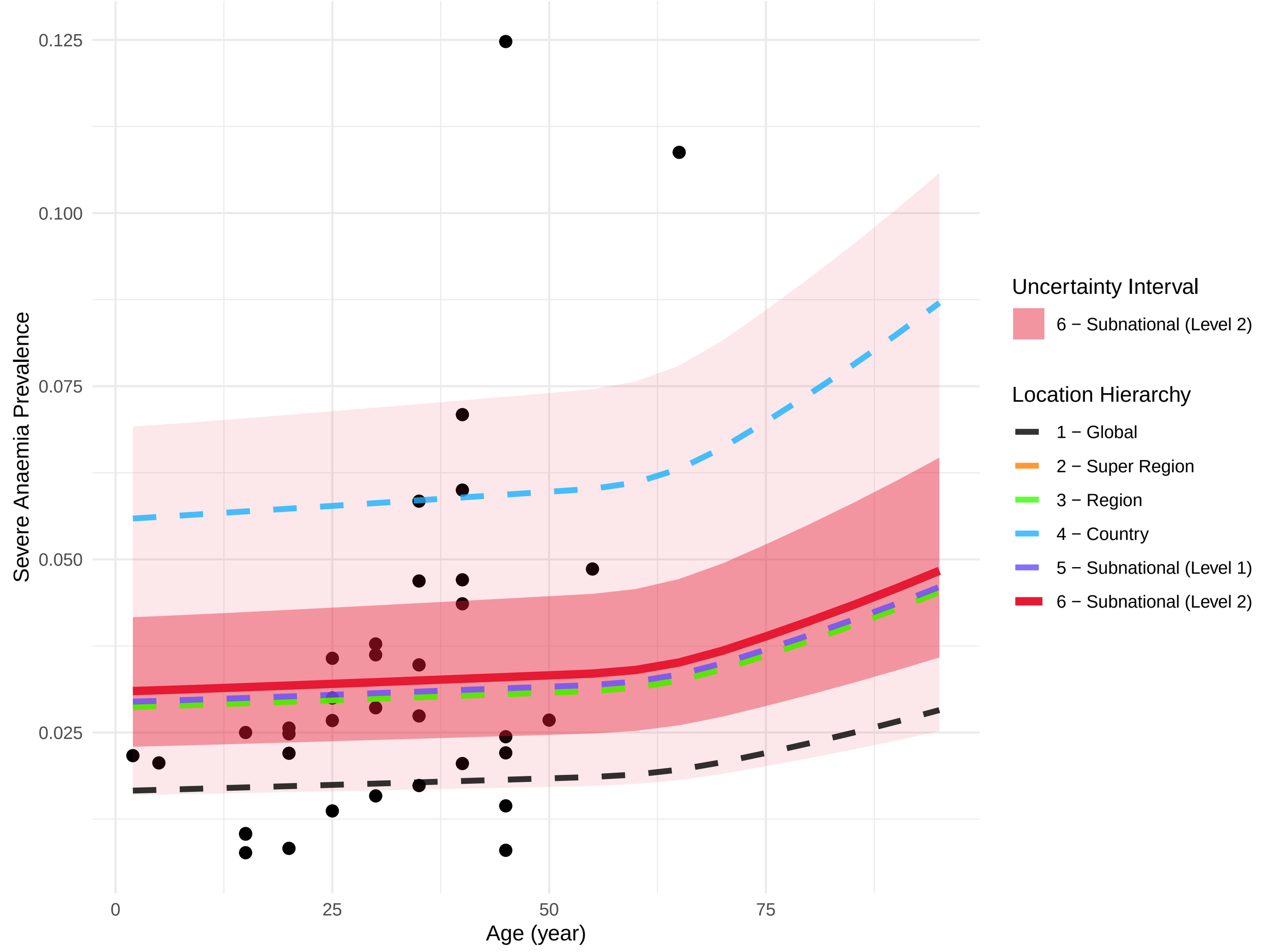

MR−BRT Age/Sex Fits − Non−pregnant Females 2+ Years Old − Severe Anaemia Prevalence − Uttar Pradesh
WHO−2001 Adjustment Method

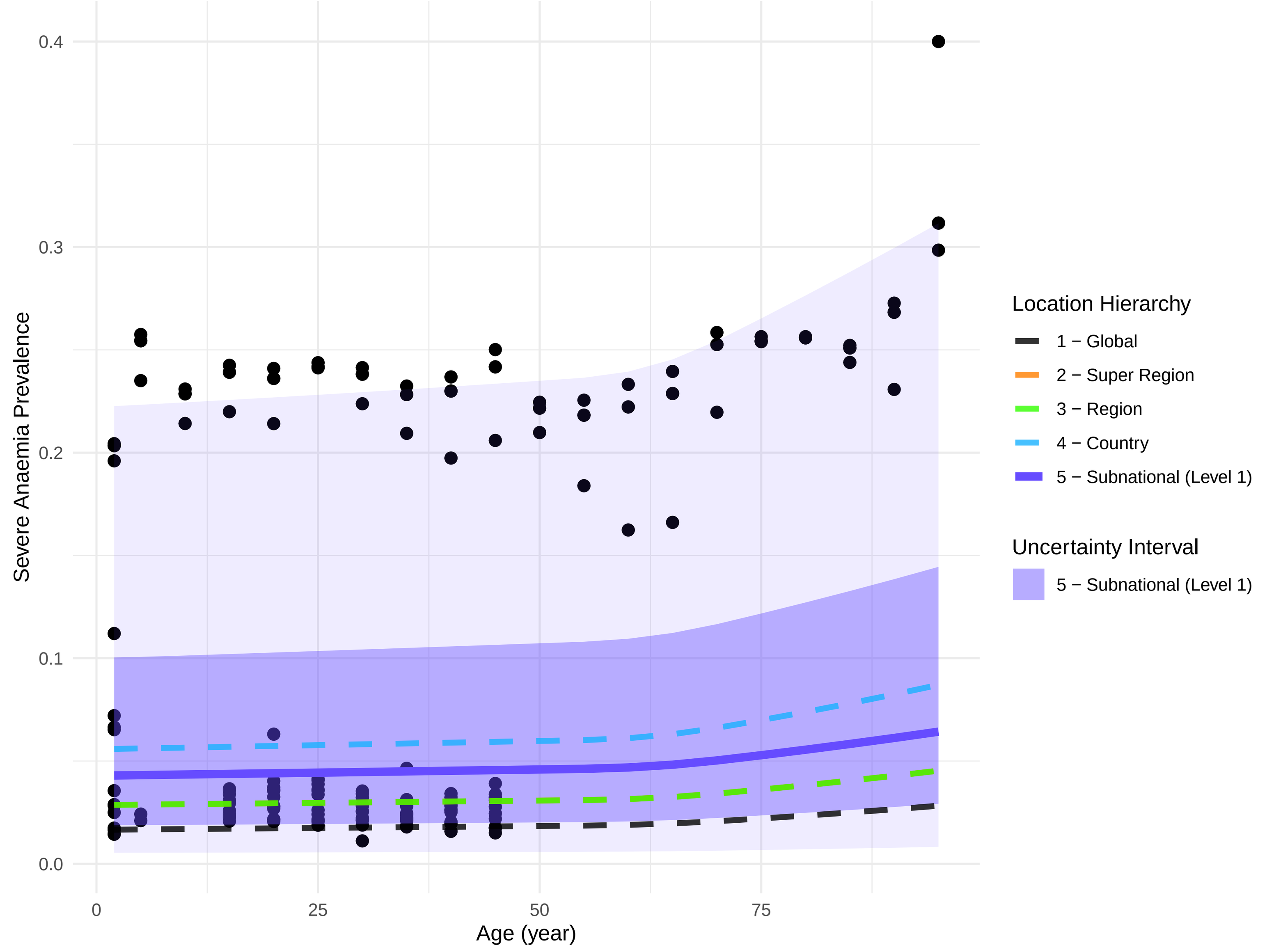

MR−BRT Age/Sex Fits − Non−pregnant Females 2+ Years Old − Severe Anaemia Prevalence − Uttar Pradesh, Rural
WHO−2001 Adjustment Method

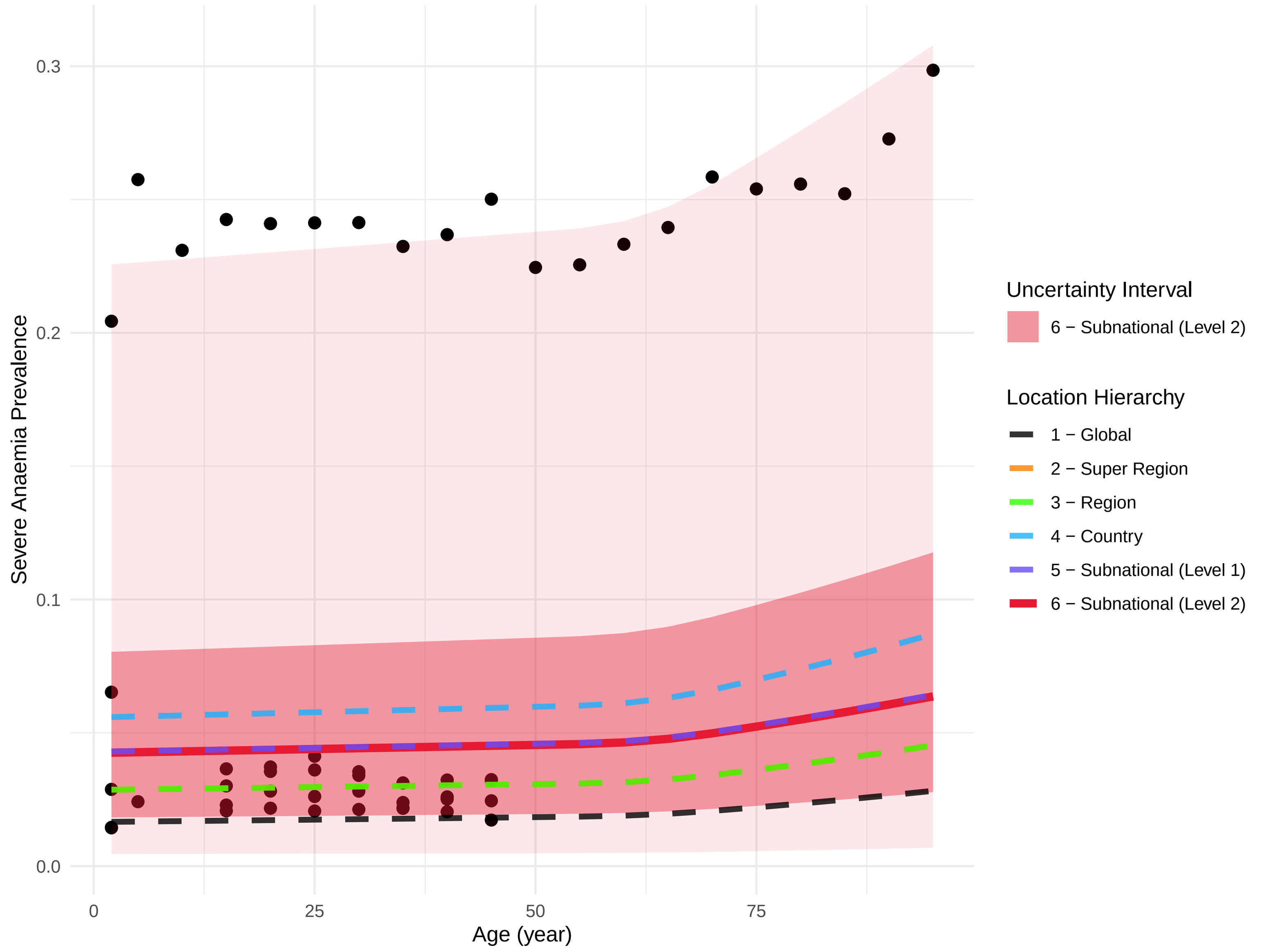

MR−BRT Age/Sex Fits − Non−pregnant Females 2+ Years Old − Severe Anaemia Prevalence − Uttar Pradesh, Urban
WHO−2001 Adjustment Method

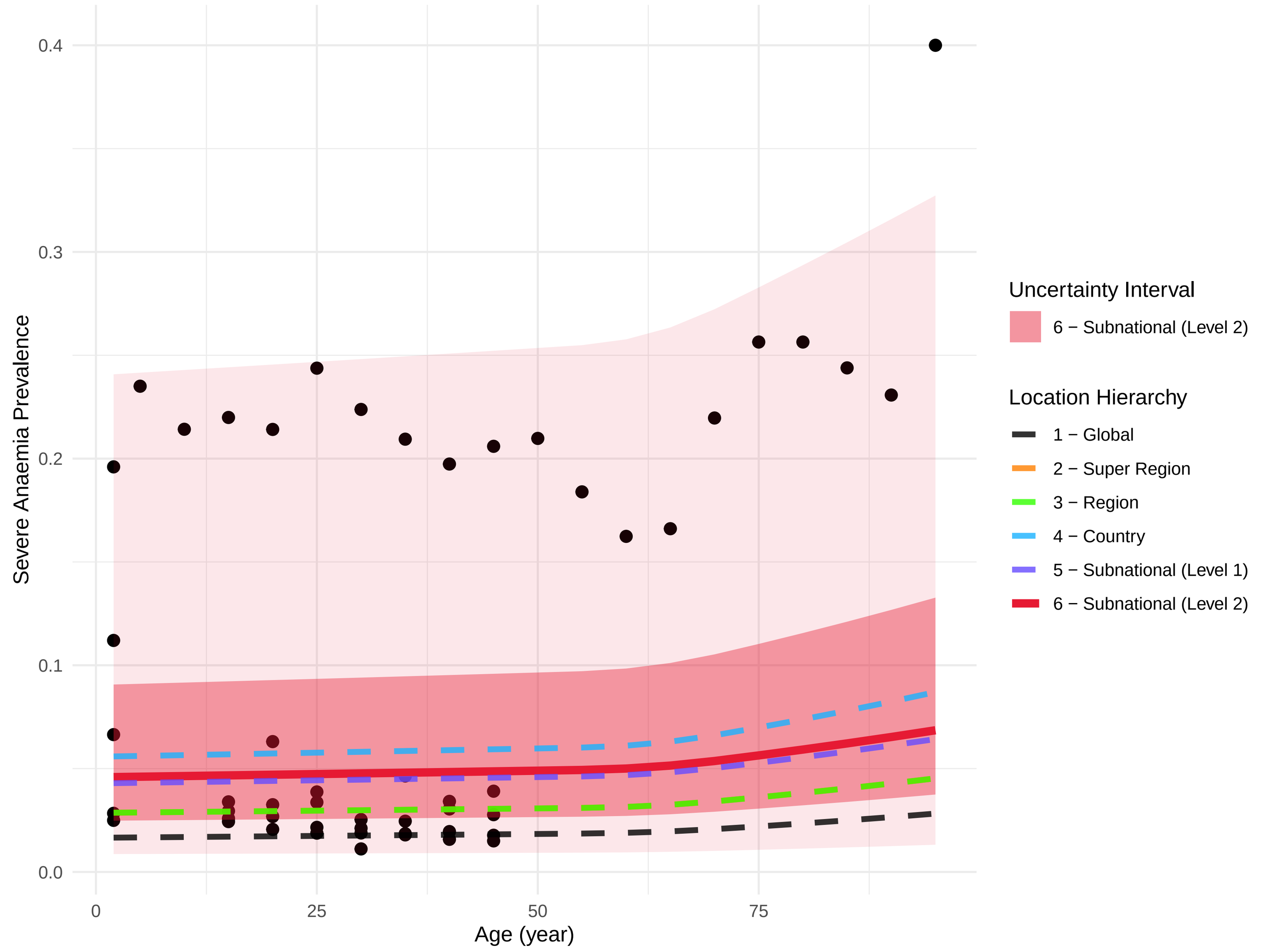

MR−BRT Age/Sex Fits − Non−pregnant Females 2+ Years Old − Severe Anaemia Prevalence − Uttarakhand
WHO−2001 Adjustment Method

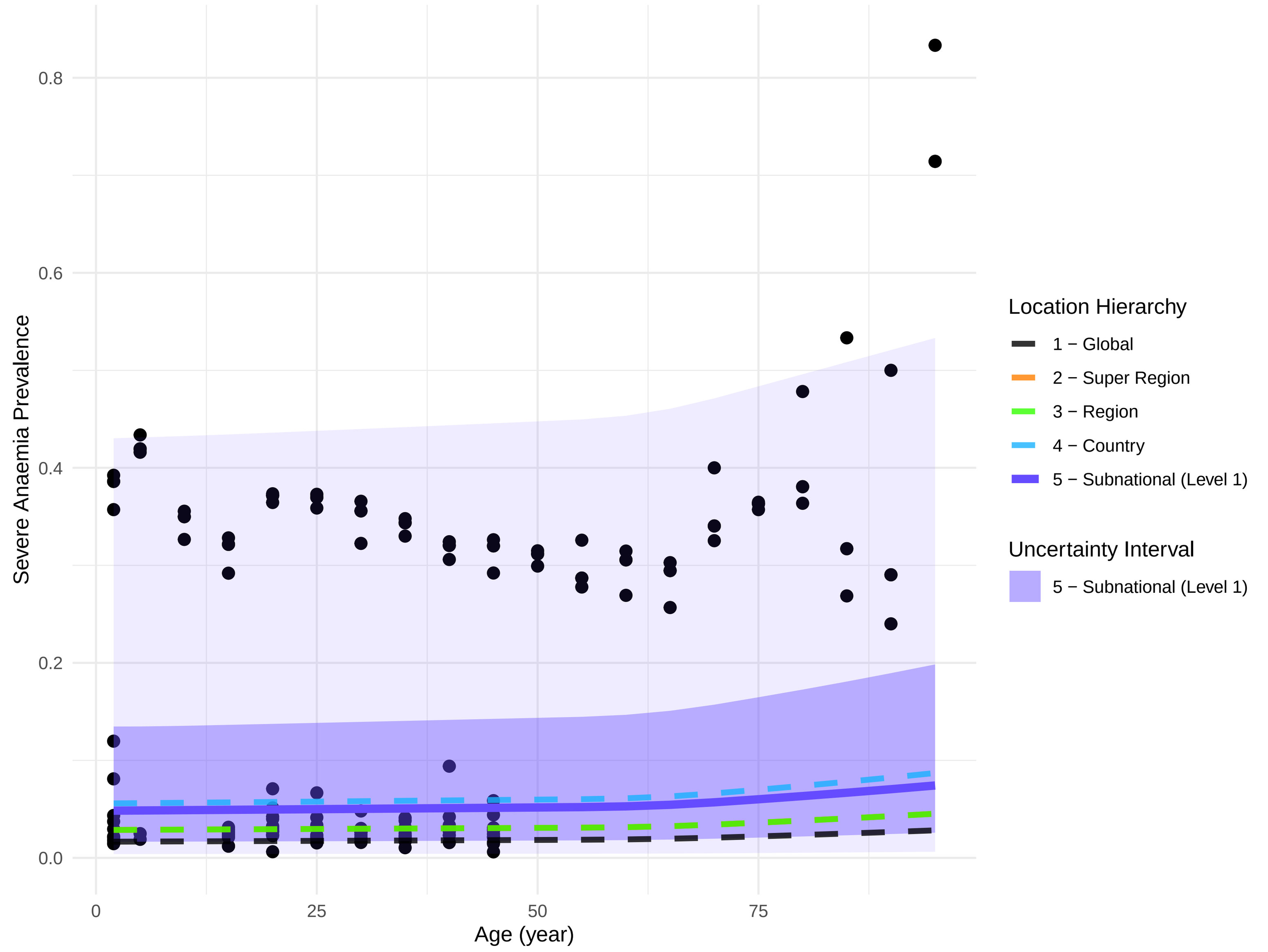

MR−BRT Age/Sex Fits − Non−pregnant Females 2+ Years Old − Severe Anaemia Prevalence − Uttarakhand, Rural
WHO−2001 Adjustment Method

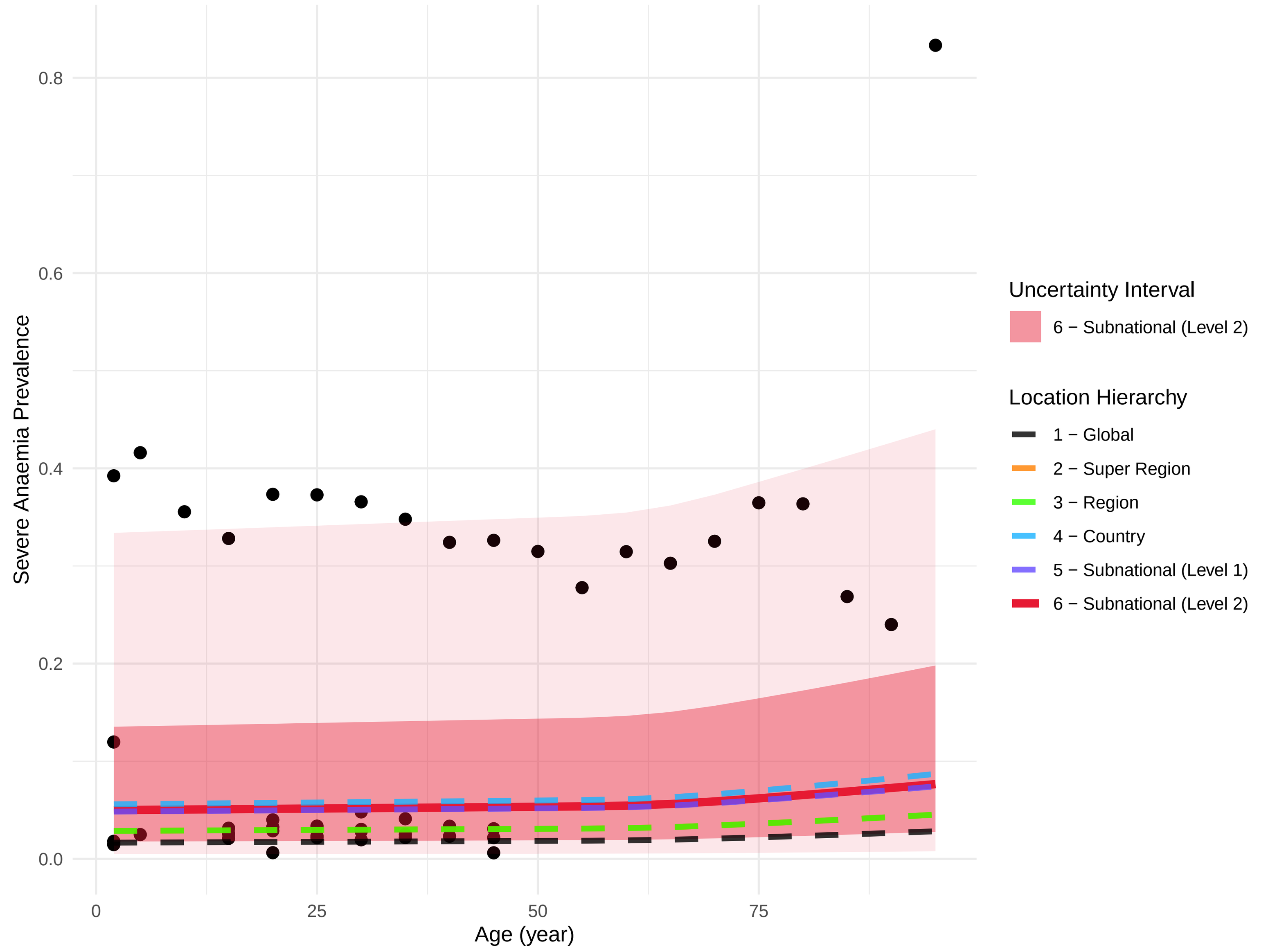

MR−BRT Age/Sex Fits − Non−pregnant Females 2+ Years Old − Severe Anaemia Prevalence − Uttarakhand, Urban
WHO−2001 Adjustment Method

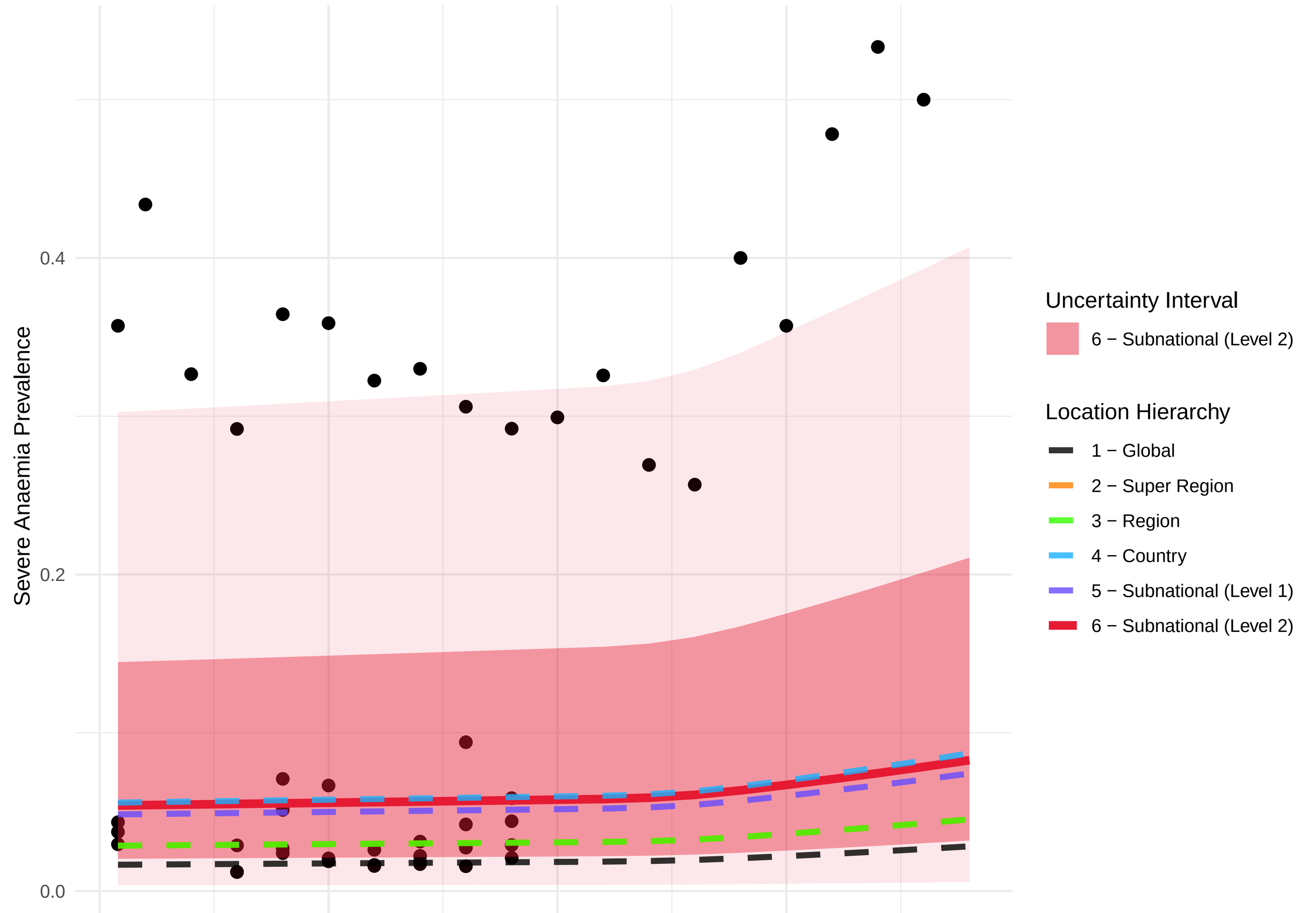

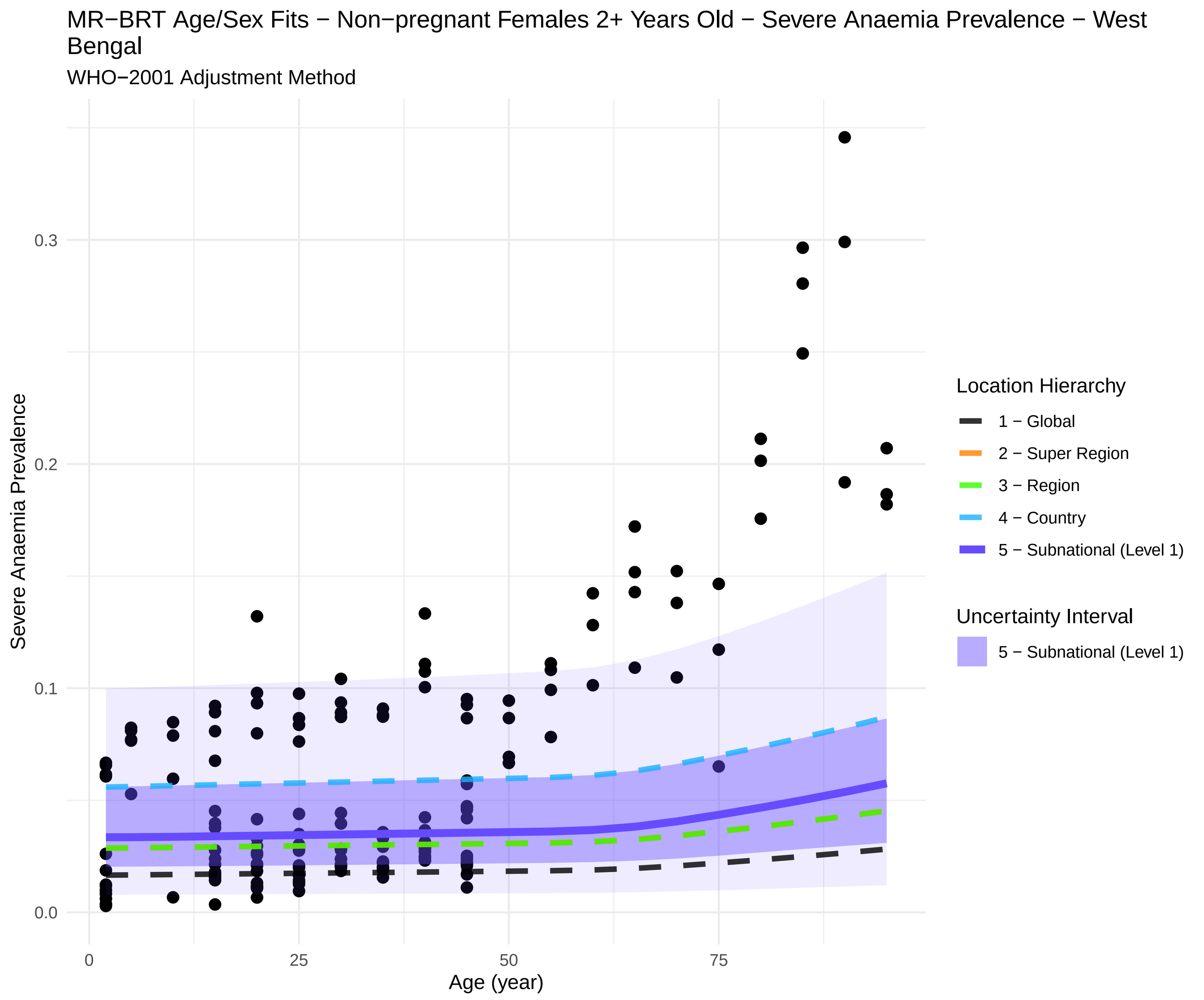
MR−BRT Age/Sex Fits − Non−pregnant Females 2+ Years Old − Severe Anaemia Prevalence − West Bengal
WHO−2001 Adjustment Method
Severe Anaemia Prevalence
Age (year)
Location Hierarchy
1 − Global
2 − Super Region
3 − Region
4 − Country
5 − Subnational (Level 1)
Uncertainty Interval
5 − Subnational (Level 1)

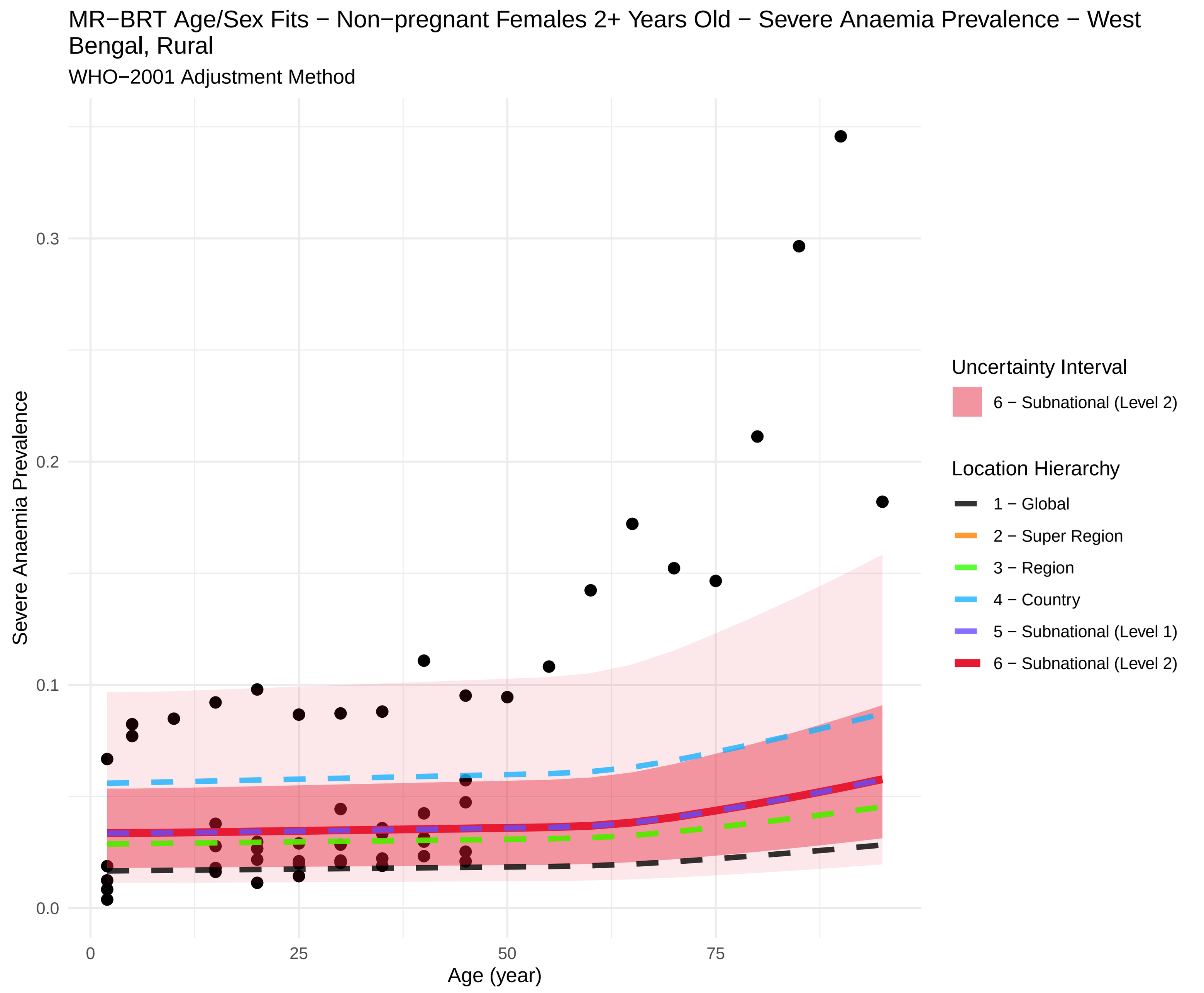
MR−BRT Age/Sex Fits − Non−pregnant Females 2+ Years Old − Severe Anaemia Prevalence − West Bengal, Rural
WHO−2001 Adjustment Method
Severe Anaemia Prevalence
Age (year)
Uncertainty Interval
6 − Subnational (Level 2)
Location Hierarchy
1 − Global
2 − Super Region
3 − Region
4 − Country
5 − Subnational (Level 1)
6 − Subnational (Level 2)

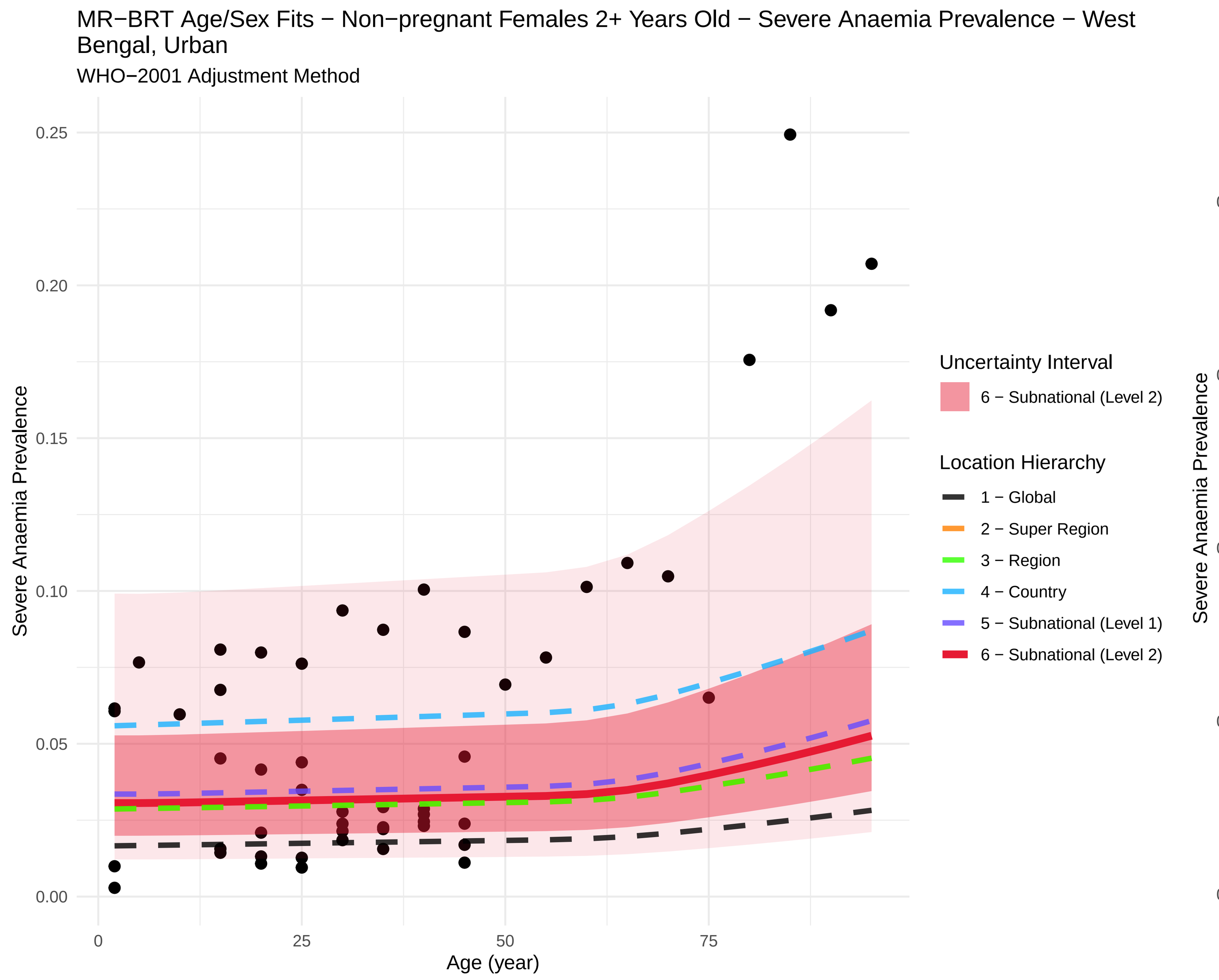
MR−BRT Age/Sex Fits − Non−pregnant Females 2+ Years Old − Severe Anaemia Prevalence − West Bengal, Urban
WHO−2001 Adjustment Method
Severe Anaemia Prevalence
Age (year)
Uncertainty Interval
6 − Subnational (Level 2)
Location Hierarchy
1 − Global
2 − Super Region
3 − Region
4 − Country
5 − Subnational (Level 1)
6 − Subnational (Level 2)

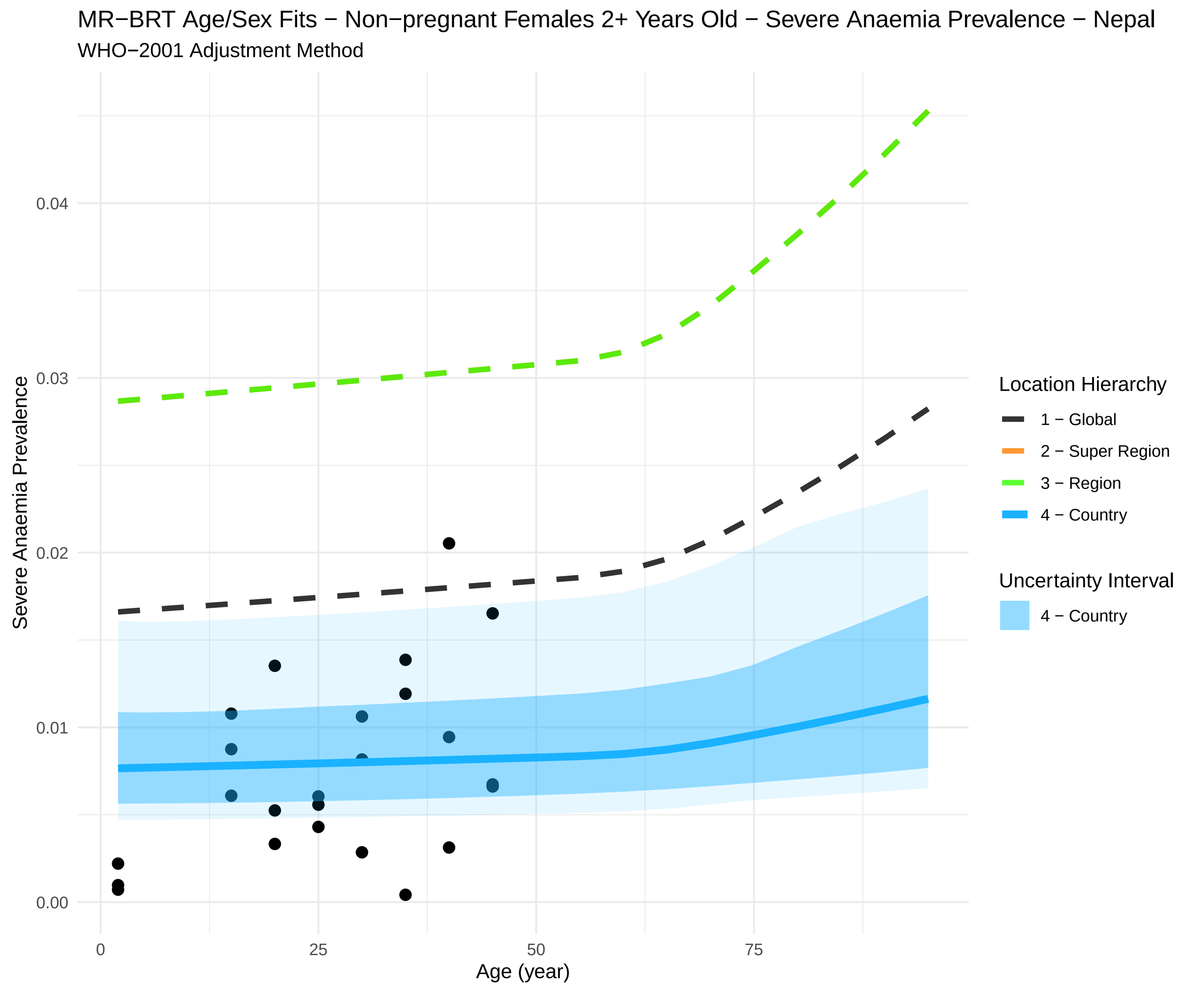
MR−BRT Age/Sex Fits − Non−pregnant Females 2+ Years Old − Severe Anaemia Prevalence − Nepal
WHO−2001 Adjustment Method
Severe Anaemia Prevalence
Age (year)
Location Hierarchy
1 − Global
2 − Super Region
3 − Region
4 − Country
Uncertainty Interval
4 − Country

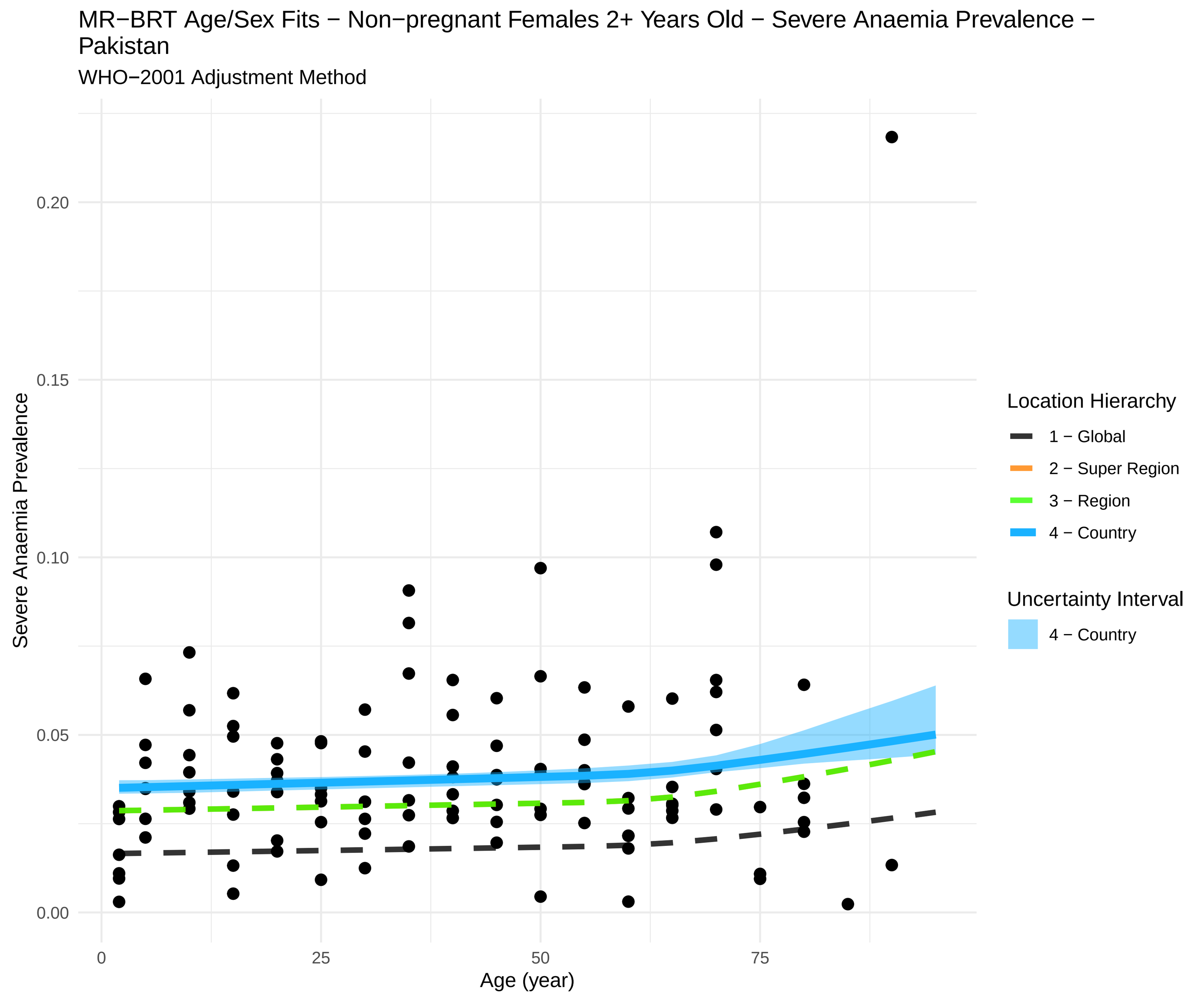
MR−BRT Age/Sex Fits − Non−pregnant Females 2+ Years Old − Severe Anaemia Prevalence − Pakistan
WHO−2001 Adjustment Method
Severe Anaemia Prevalence
Age (year)
Location Hierarchy
1 − Global
2 − Super Region
3 − Region
4 − Country
Uncertainty Interval
4 − Country

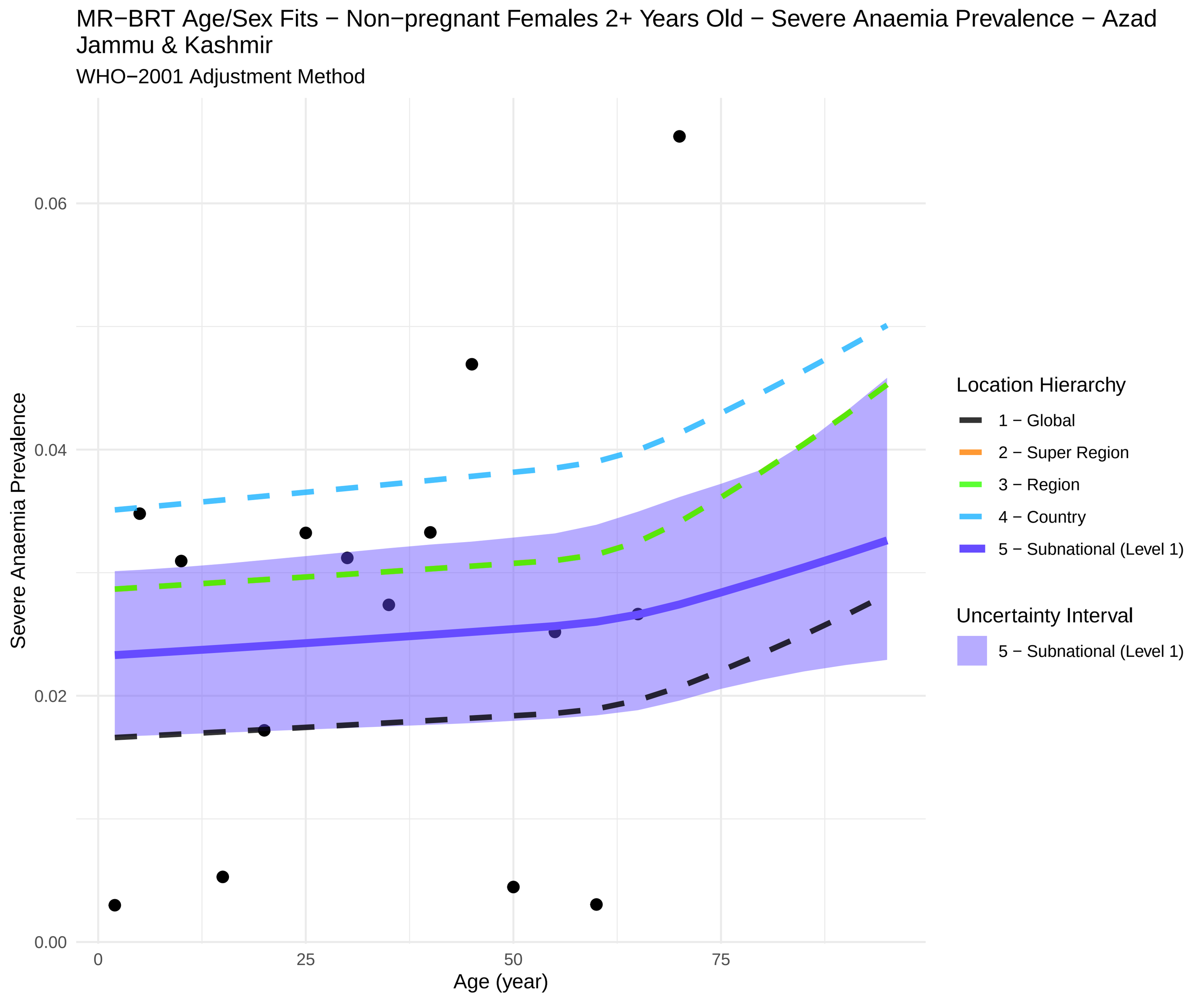
MR−BRT Age/Sex Fits − Non−pregnant Females 2+ Years Old − Severe Anaemia Prevalence − Azad Jammu & Kashmir
WHO−2001 Adjustment Method
Severe Anaemia Prevalence
Age (year)
Location Hierarchy
1 − Global
2 − Super Region
3 − Region
4 − Country
5 − Subnational (Level 1)
Uncertainty Interval
5 − Subnational (Level 1)

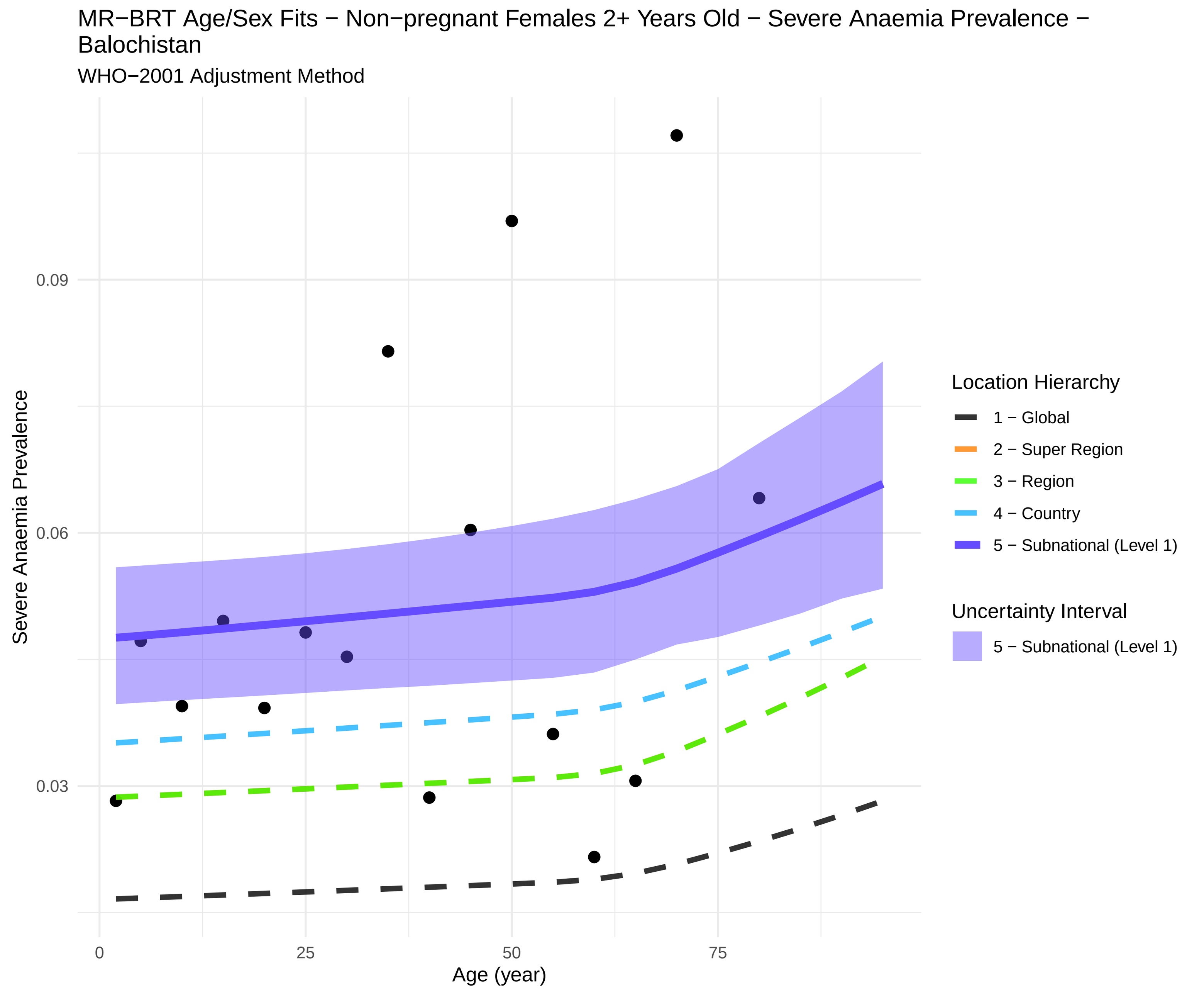
MR−BRT Age/Sex Fits − Non−pregnant Females 2+ Years Old − Severe Anaemia Prevalence − Balochistan
WHO−2001 Adjustment Method
Severe Anaemia Prevalence
Age (year)
Location Hierarchy
1 − Global
2 − Super Region
3 − Region
4 − Country
5 − Subnational (Level 1)
Uncertainty Interval
5 − Subnational (Level 1)

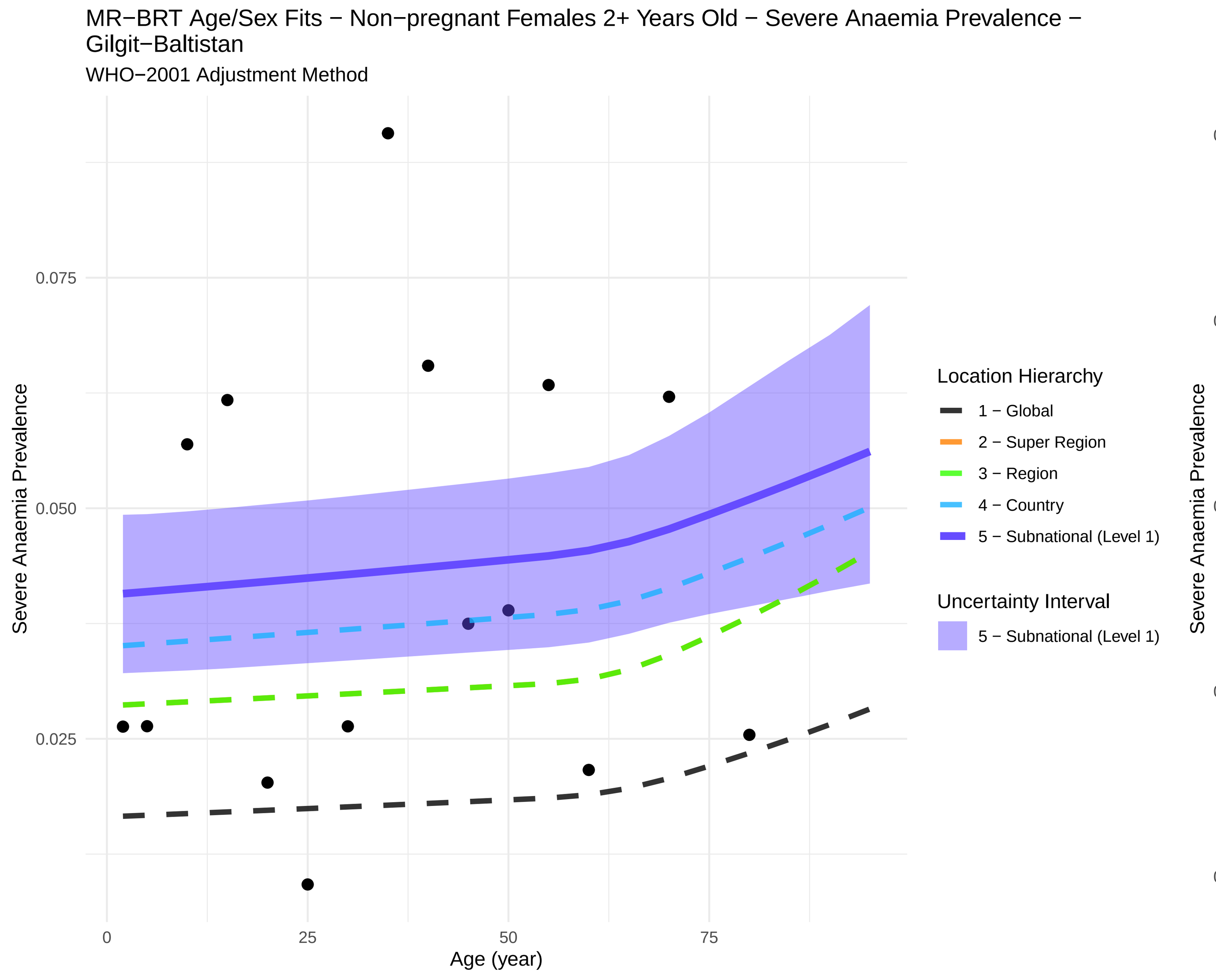
MR−BRT Age/Sex Fits − Non−pregnant Females 2+ Years Old − Severe Anaemia Prevalence − Gilgit−Baltistan
WHO−2001 Adjustment Method
Severe Anaemia Prevalence
Age (year)
Location Hierarchy
1 − Global
2 − Super Region
3 − Region
4 − Country
5 − Subnational (Level 1)
Uncertainty Interval
5 − Subnational (Level 1)

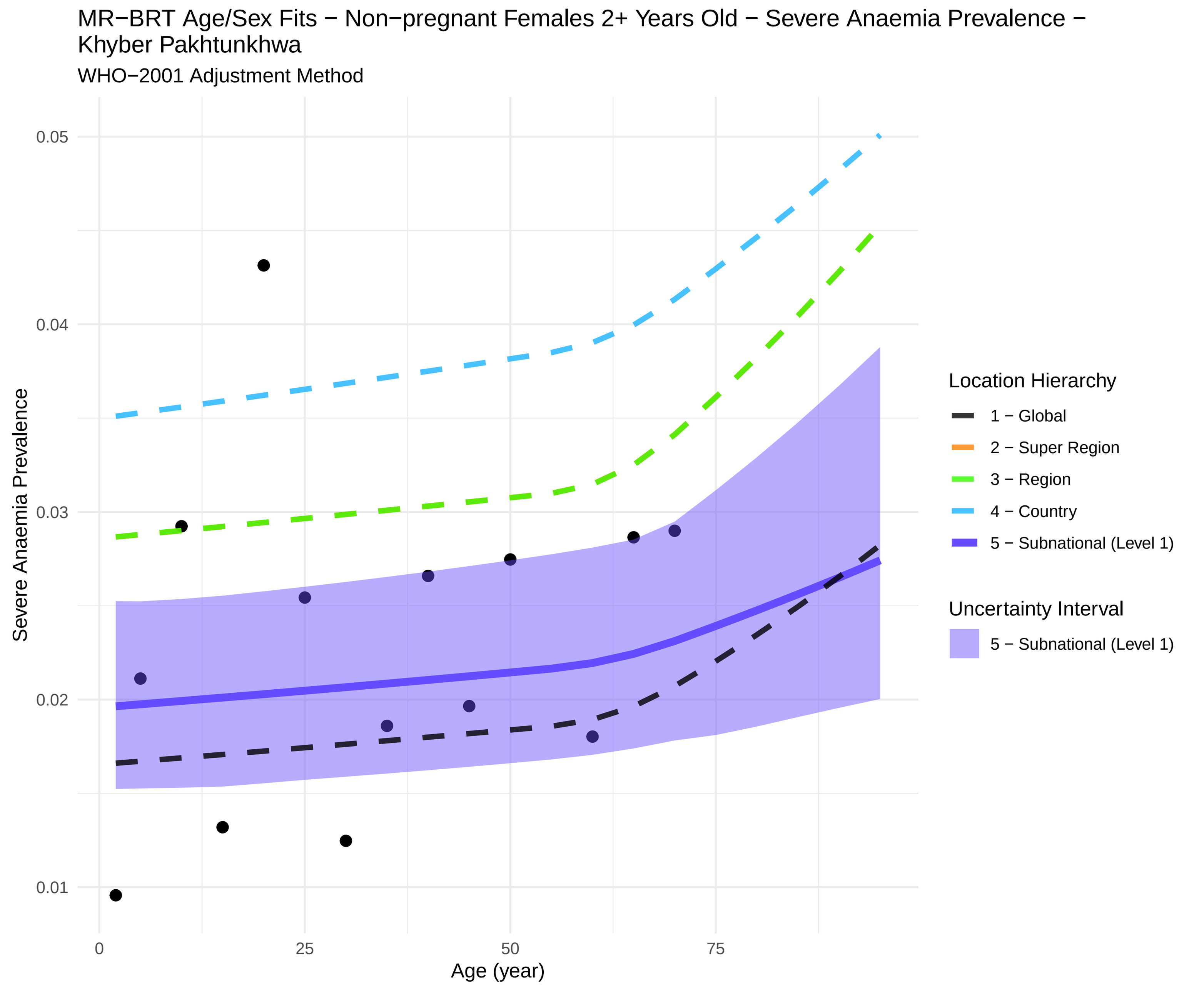
MR−BRT Age/Sex Fits − Non−pregnant Females 2+ Years Old − Severe Anaemia Prevalence − Khyber Pakhtunkhwa
WHO−2001 Adjustment Method
Severe Anaemia Prevalence
Age (year)
Location Hierarchy
1 − Global
2 − Super Region
3 − Region
4 − Country
5 − Subnational (Level 1)
Uncertainty Interval
5 − Subnational (Level 1)

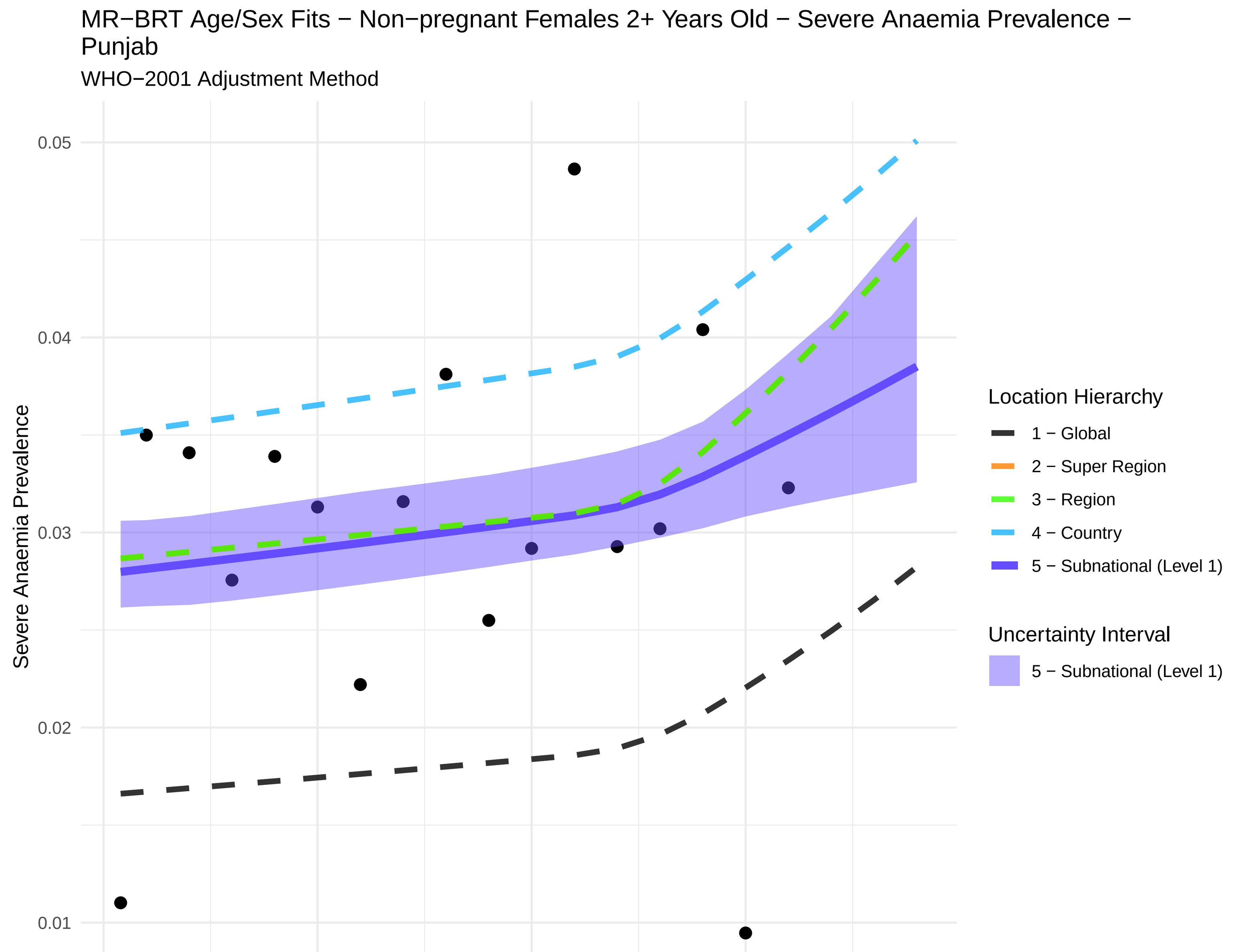
MR−BRT Age/Sex Fits − Non−pregnant Females 2+ Years Old − Severe Anaemia Prevalence − Punjab
WHO−2001 Adjustment Method
Severe Anaemia Prevalence
Age (year)
Location Hierarchy
1 − Global
2 − Super Region
3 − Region
4 − Country
5 − Subnational (Level 1)
Uncertainty Interval
5 − Subnational (Level 1)

MR−BRT Age/Sex Fits − Non−pregnant Females 2+ Years Old − Severe Anaemia Prevalence − Sindh
WHO−2001 Adjustment Method

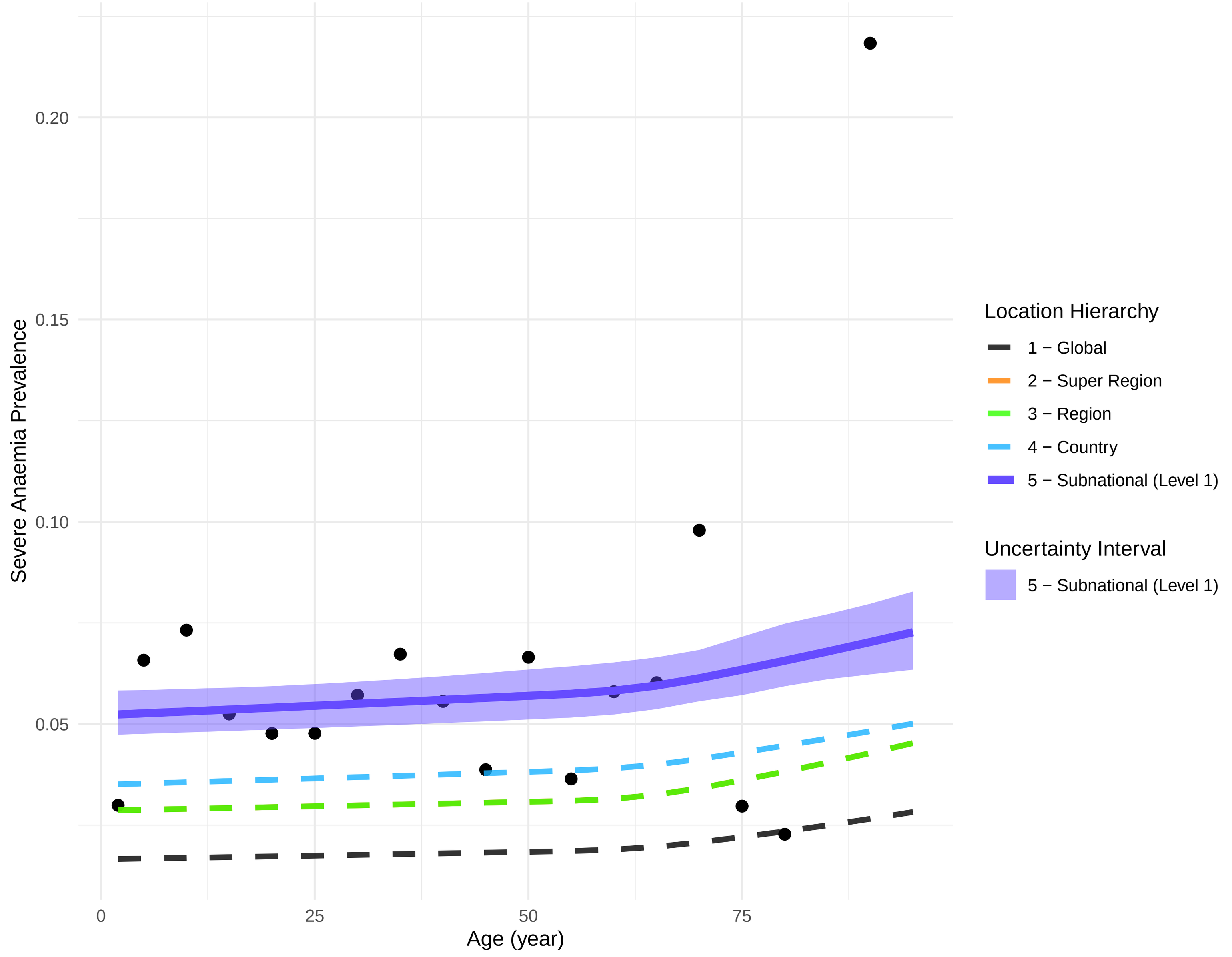

MR−BRT Age/Sex Fits − Non−pregnant Females 2+ Years Old − Severe Anaemia Prevalence − Southeast Asia, East Asia, and Oceania
WHO−2001 Adjustment Method

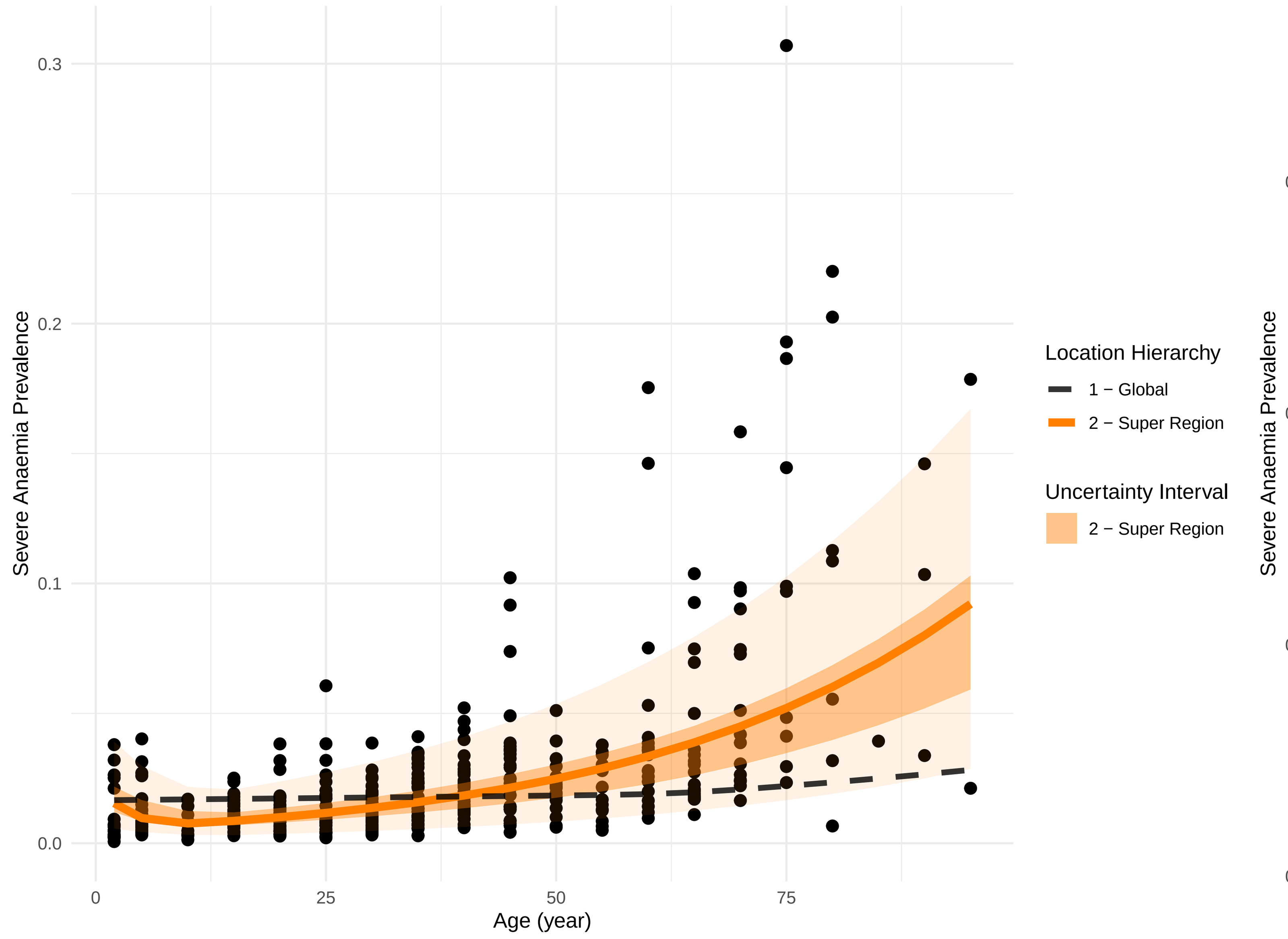

MR−BRT Age/Sex Fits − Non−pregnant Females 2+ Years Old − Severe Anaemia Prevalence − Oceania
WHO−2001 Adjustment Method

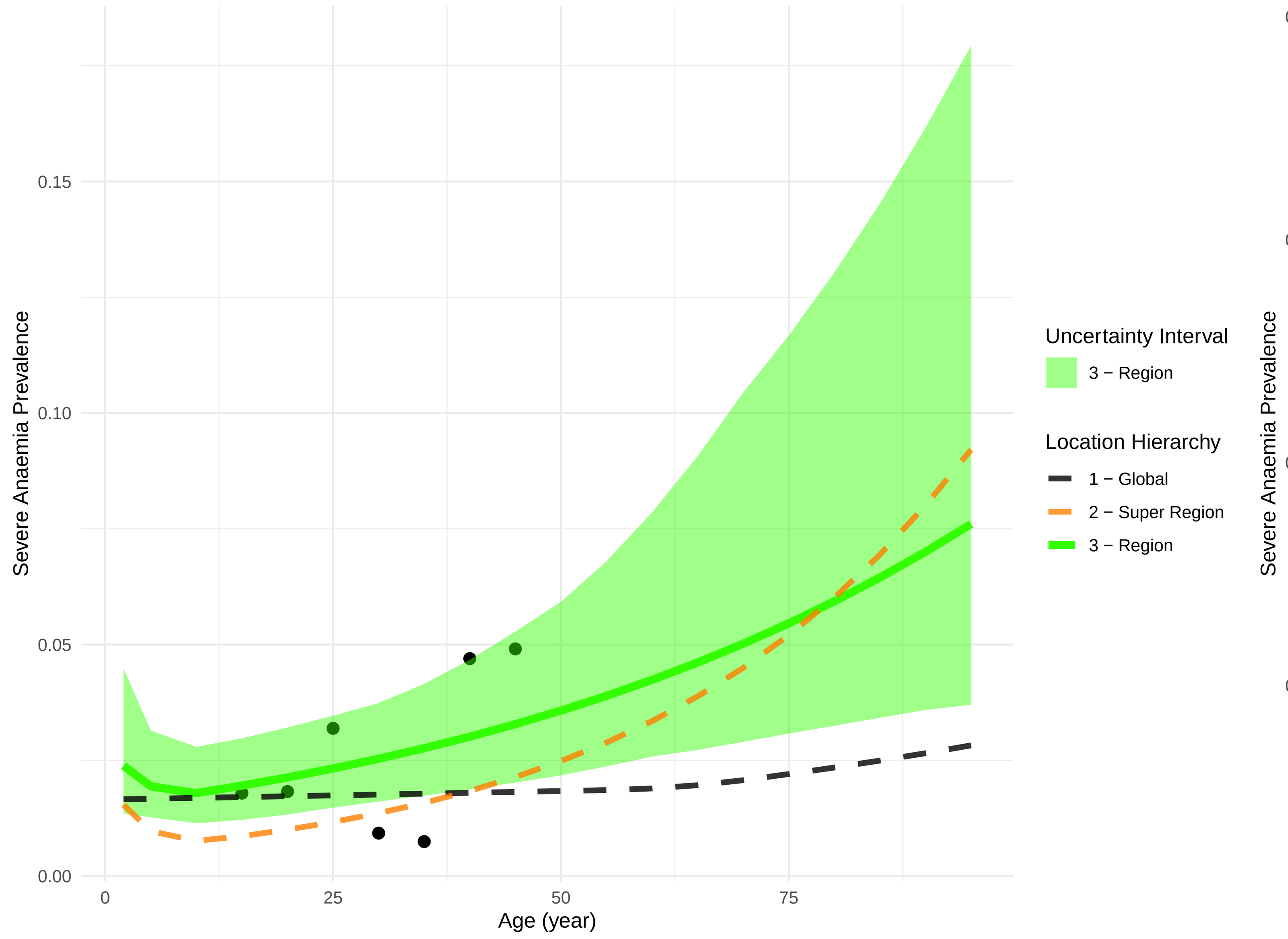

MR−BRT Age/Sex Fits − Non−pregnant Females 2+ Years Old − Severe Anaemia Prevalence − Papua New Guinea
WHO−2001 Adjustment Method

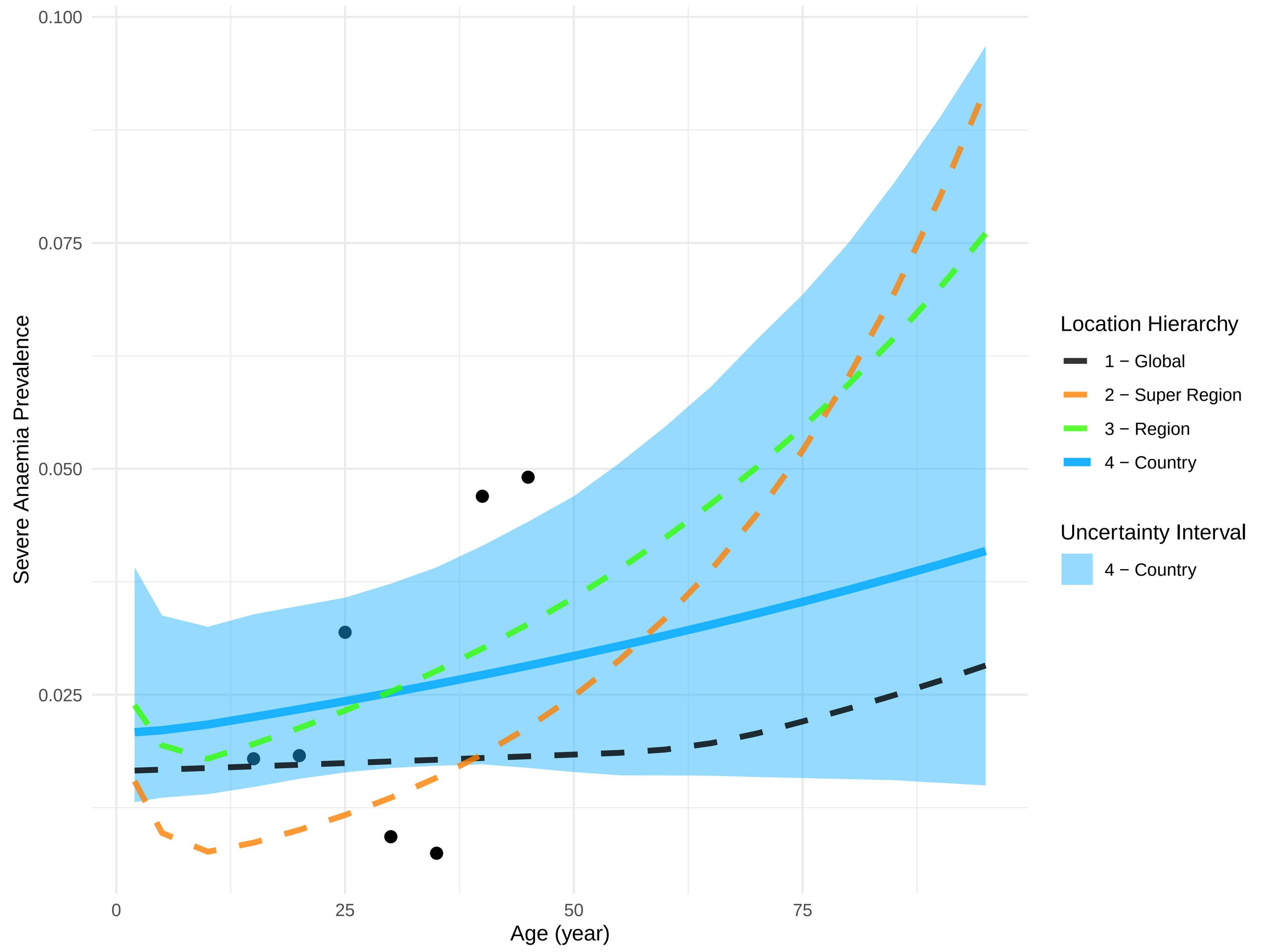

MR−BRT Age/Sex Fits − Non−pregnant Females 2+ Years Old − Severe Anaemia Prevalence − Southeast Asia
WHO−2001 Adjustment Method

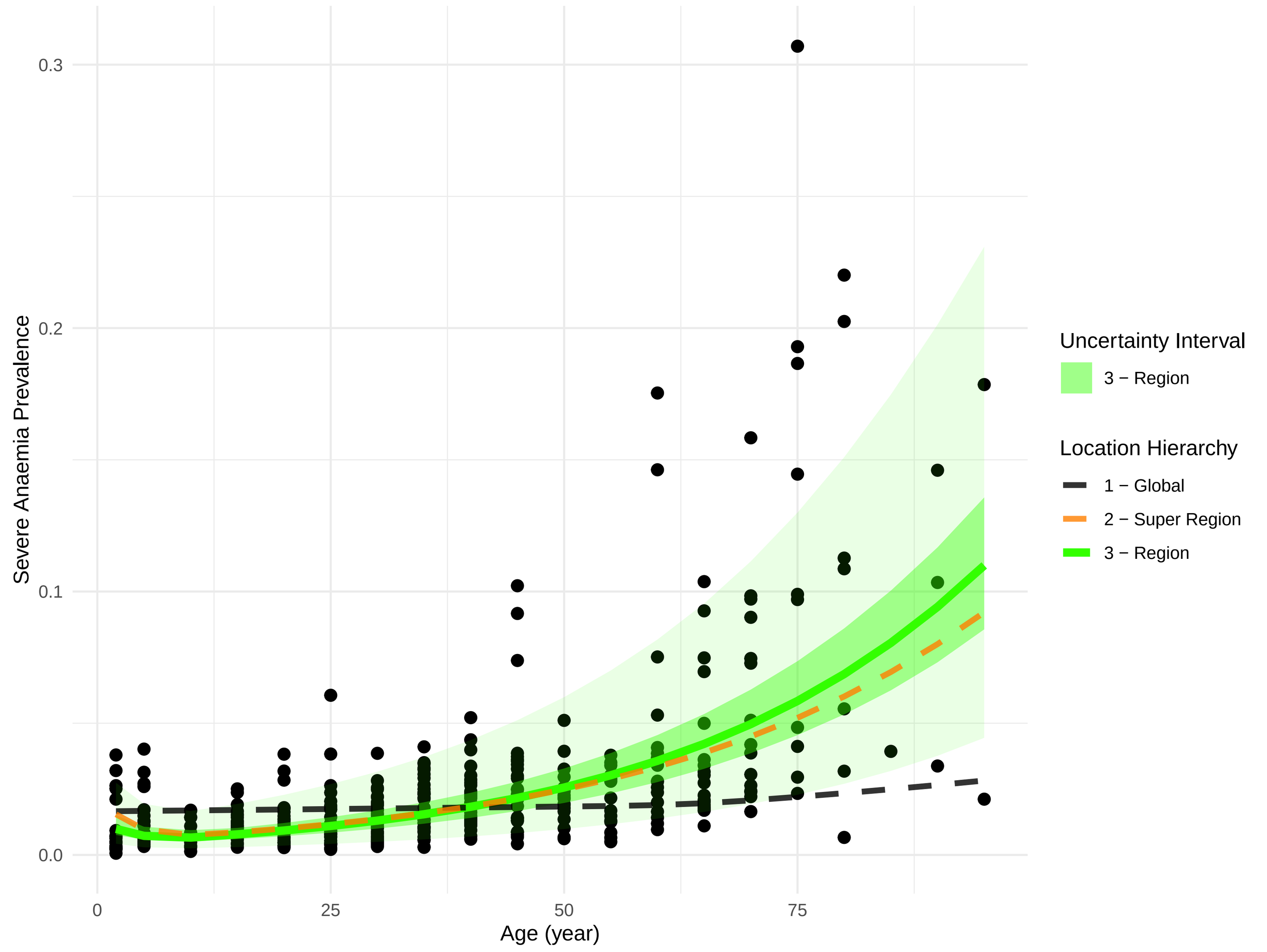

MR−BRT Age/Sex Fits − Non−pregnant Females 2+ Years Old − Severe Anaemia Prevalence − Cambodia
WHO−2001 Adjustment Method

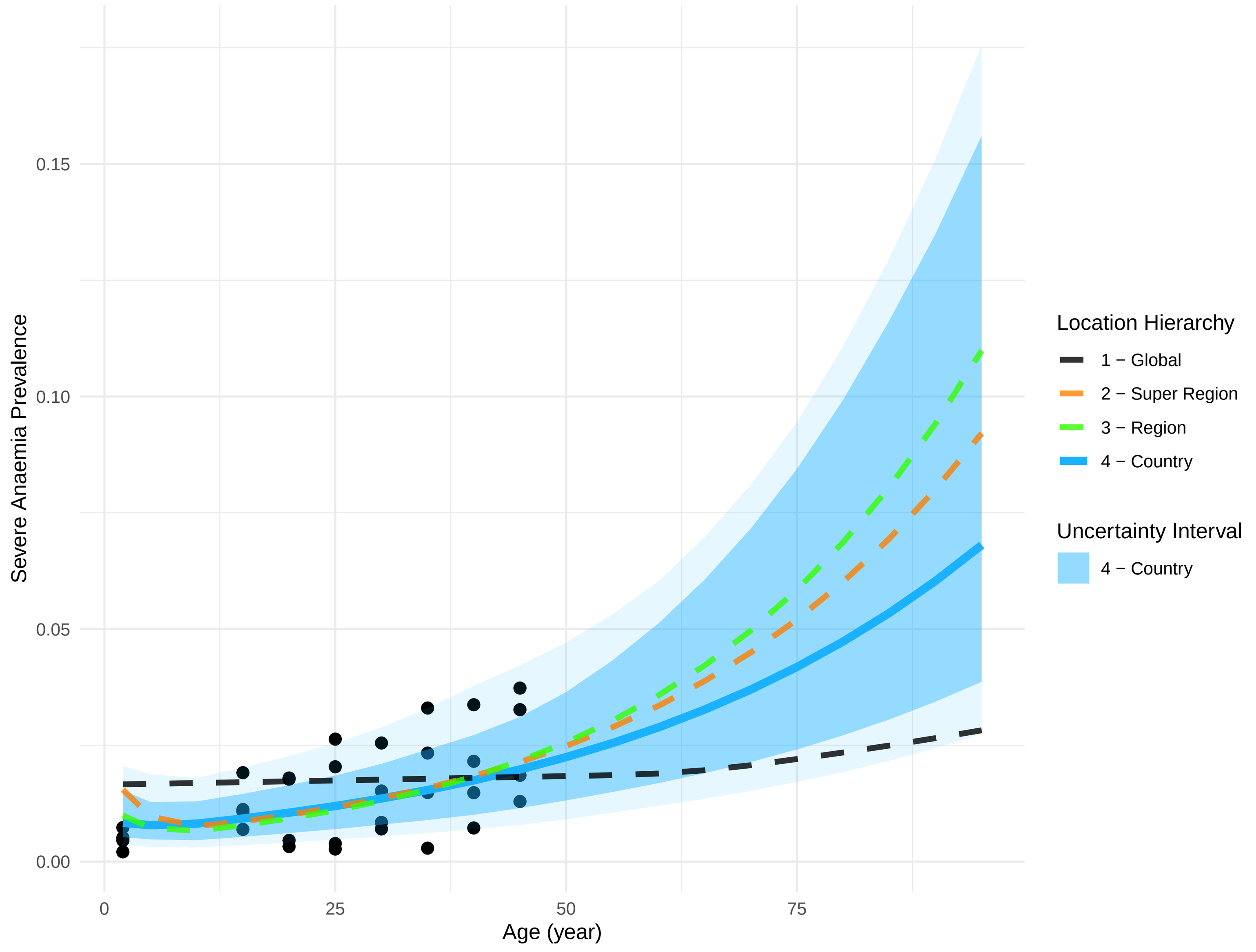

MR−BRT Age/Sex Fits − Non−pregnant Females 2+ Years Old − Severe Anaemia Prevalence − Indonesia
WHO−2001 Adjustment Method

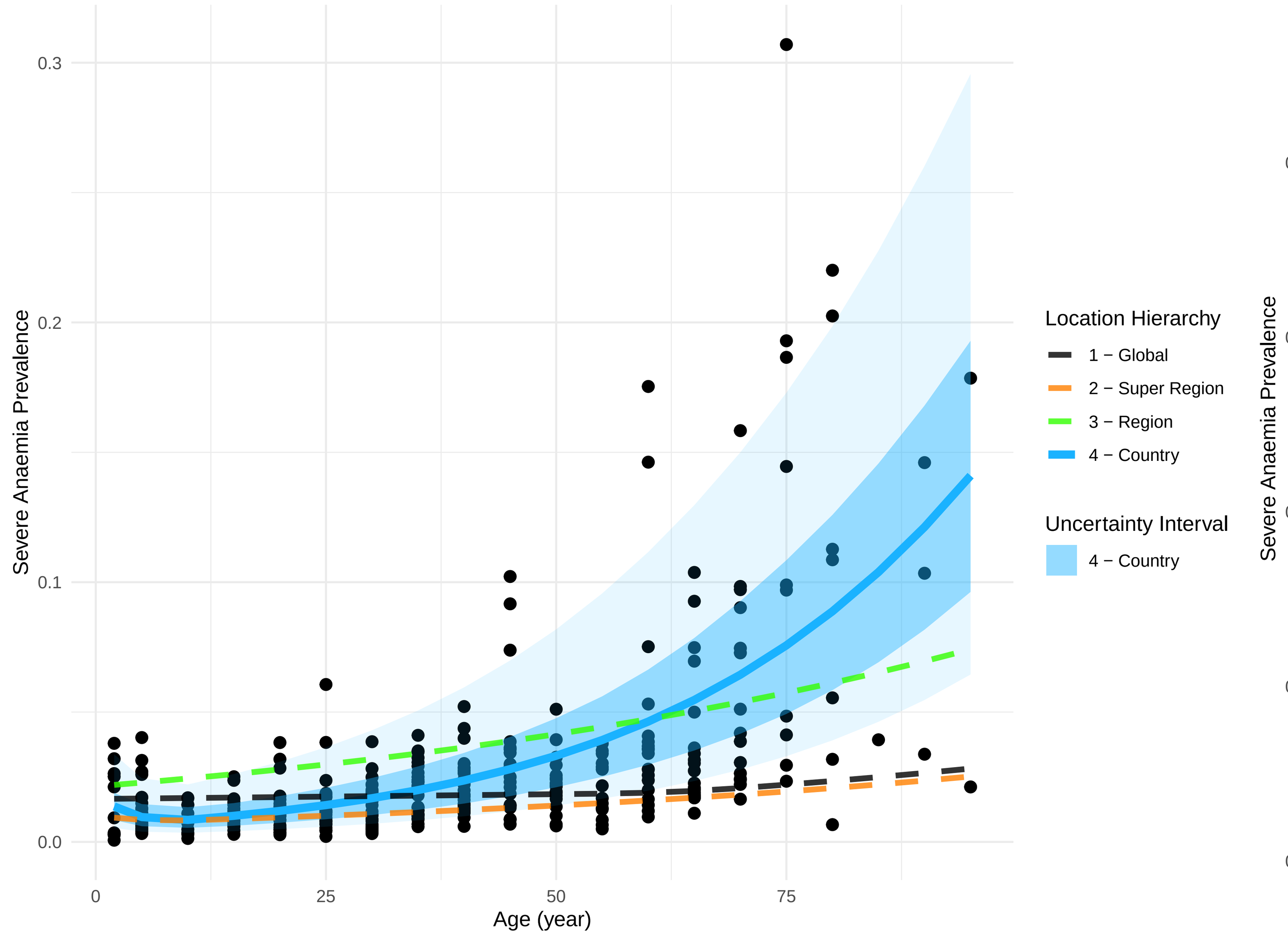

MR−BRT Age/Sex Fits − Non−pregnant Females 2+ Years Old − Severe Anaemia Prevalence − Bali
WHO−2001 Adjustment Method

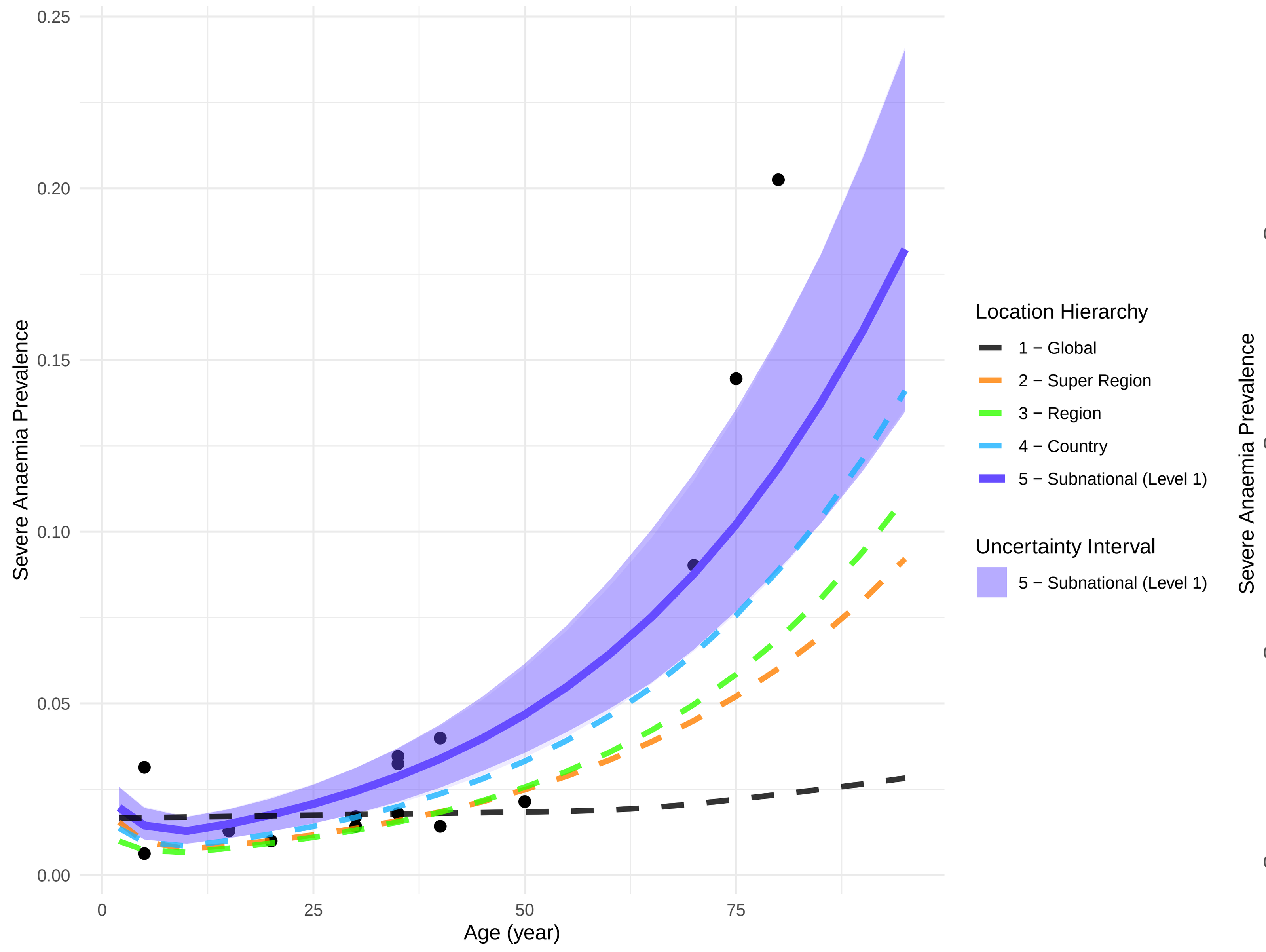

MR−BRT Age/Sex Fits − Non−pregnant Females 2+ Years Old − Severe Anaemia Prevalence − Bangka−Belitung Islands
WHO−2001 Adjustment Method

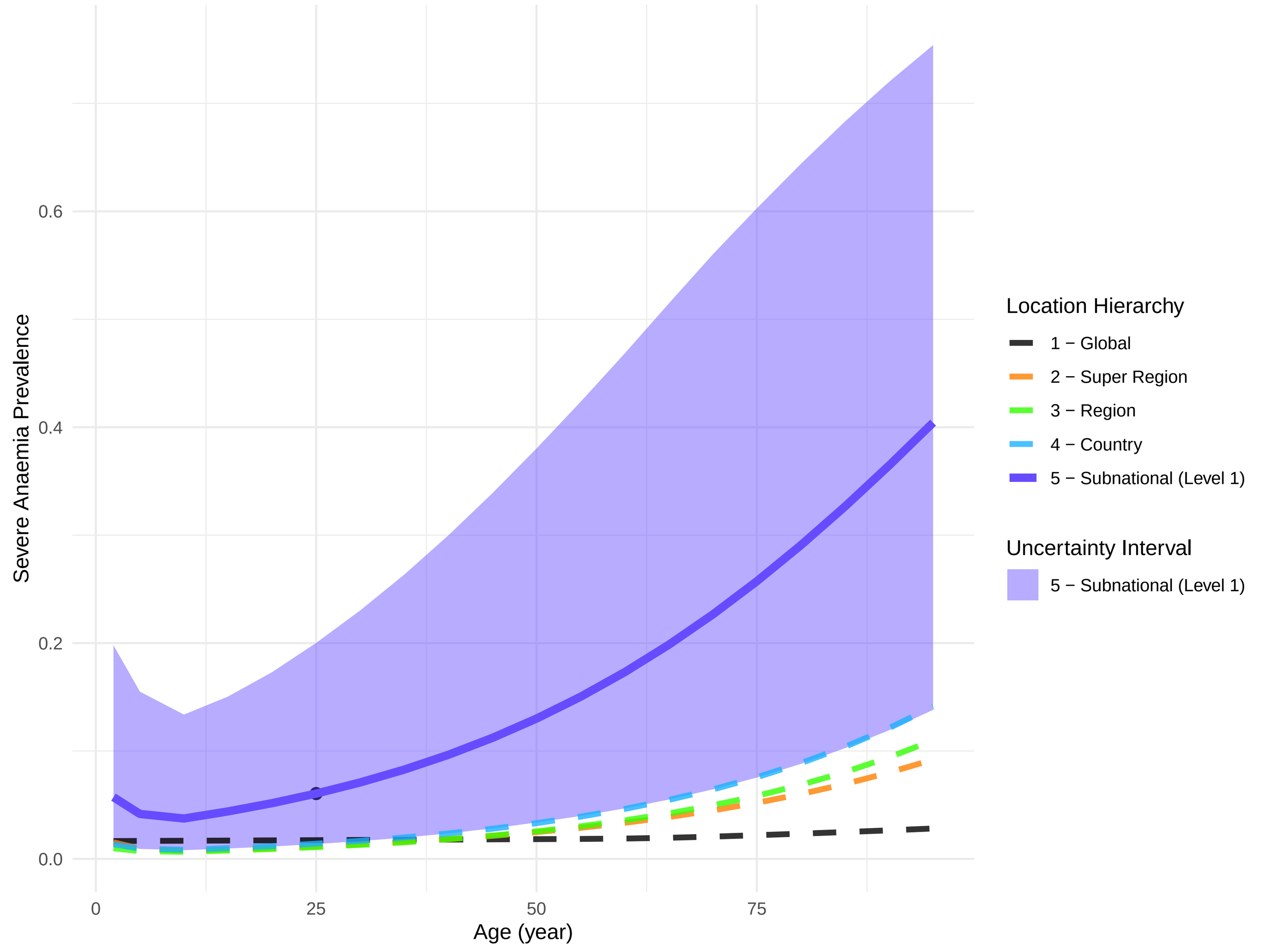

MR−BRT Age/Sex Fits − Non−pregnant Females 2+ Years Old − Severe Anaemia Prevalence − Banten
WHO−2001 Adjustment Method

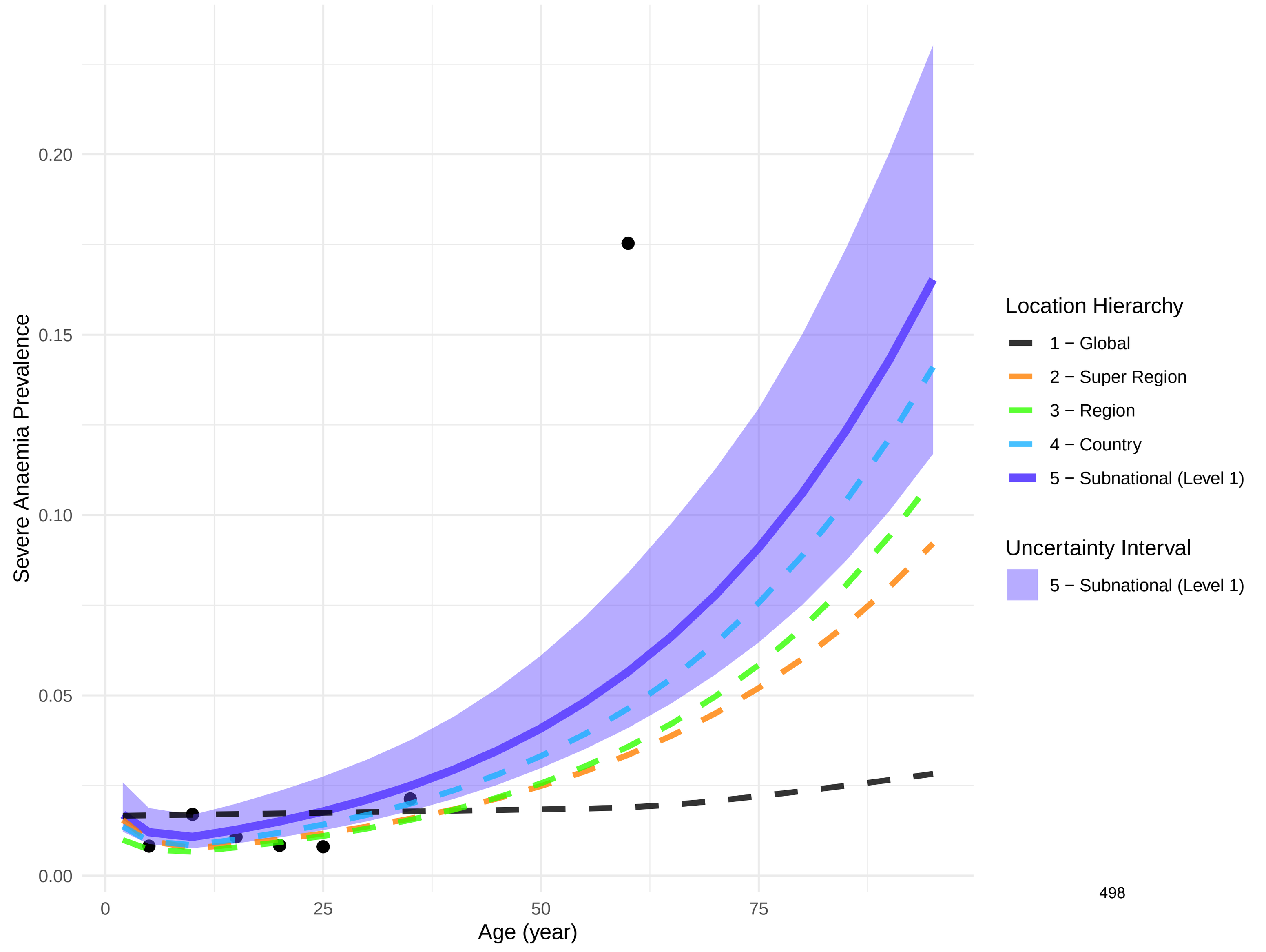

MR−BRT Age/Sex Fits − Non−pregnant Females 2+ Years Old − Severe Anaemia Prevalence − Jakarta
WHO−2001 Adjustment Method

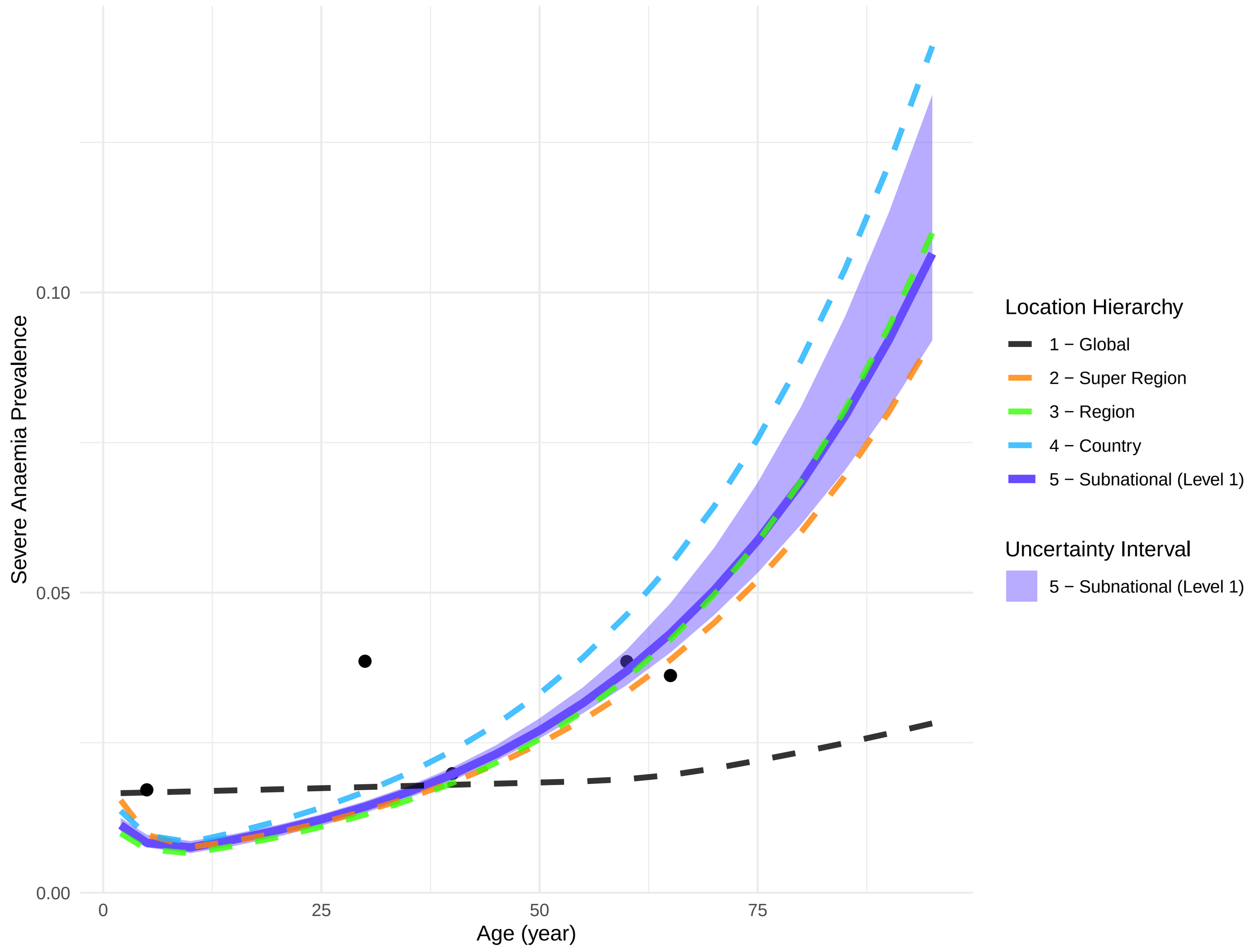

MR−BRT Age/Sex Fits − Non−pregnant Females 2+ Years Old − Severe Anaemia Prevalence − West Java
WHO−2001 Adjustment Method

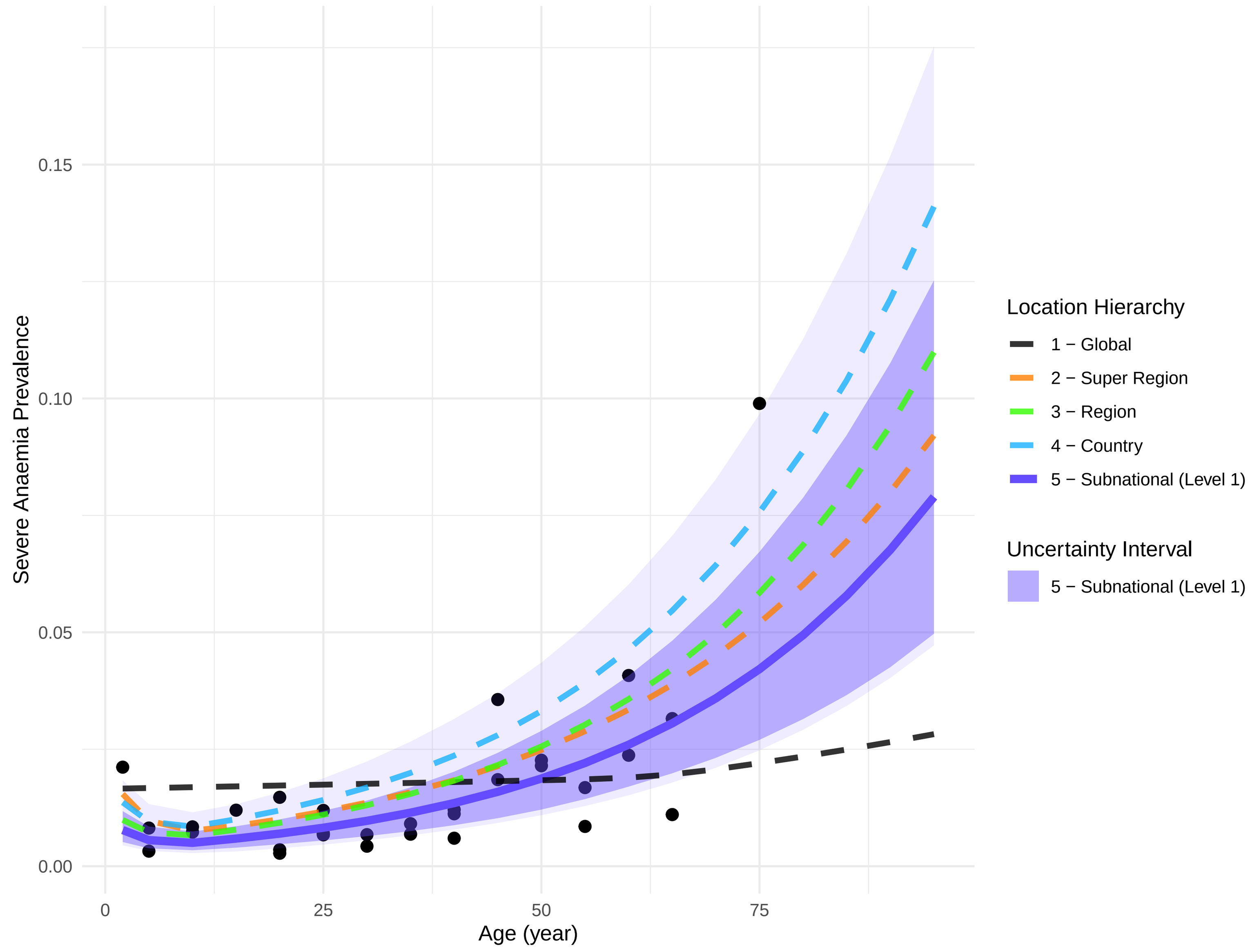

MR−BRT Age/Sex Fits − Non−pregnant Females 2+ Years Old − Severe Anaemia Prevalence − Central Java
WHO−2001 Adjustment Method

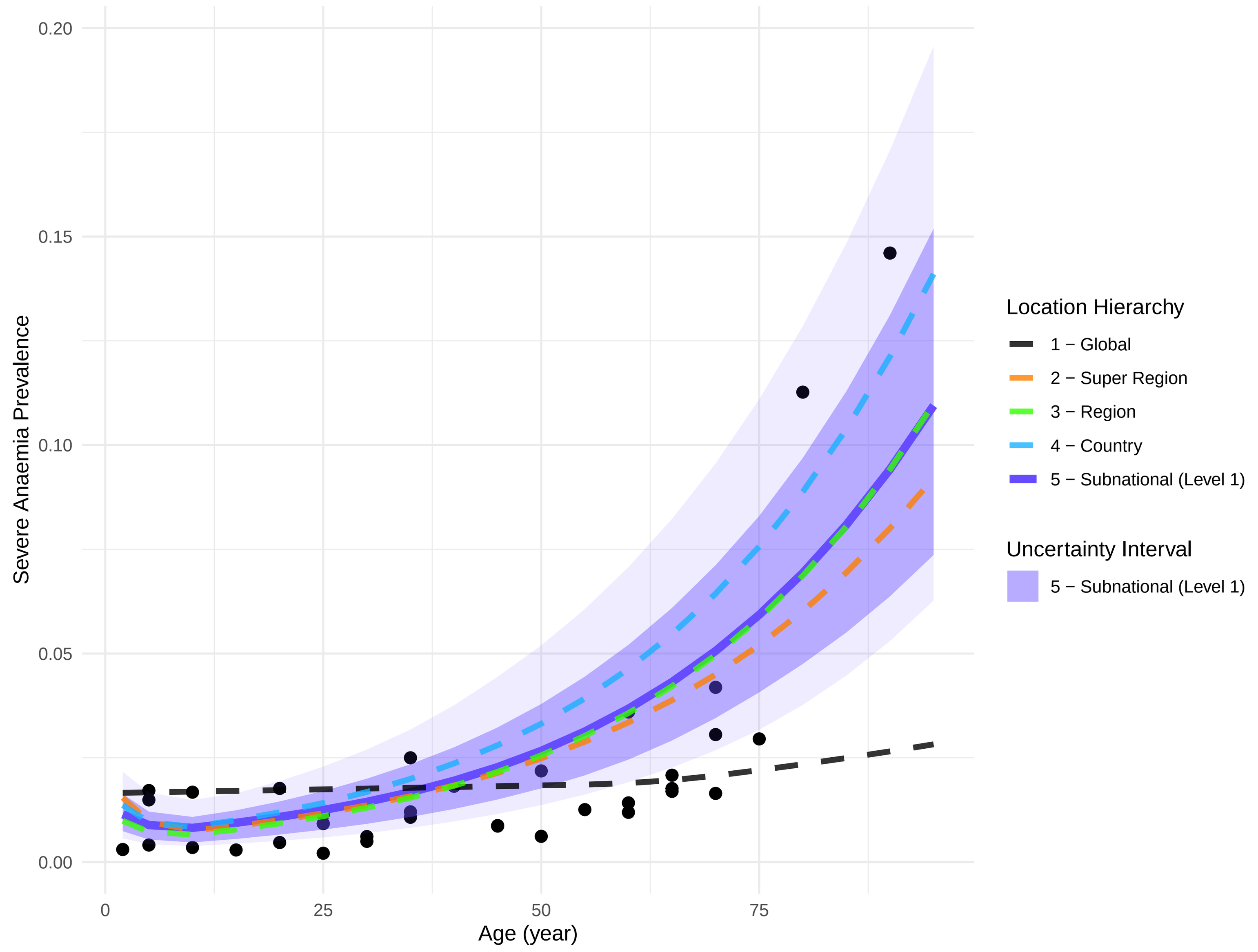

MR−BRT Age/Sex Fits − Non−pregnant Females 2+ Years Old − Severe Anaemia Prevalence − East Java
WHO−2001 Adjustment Method

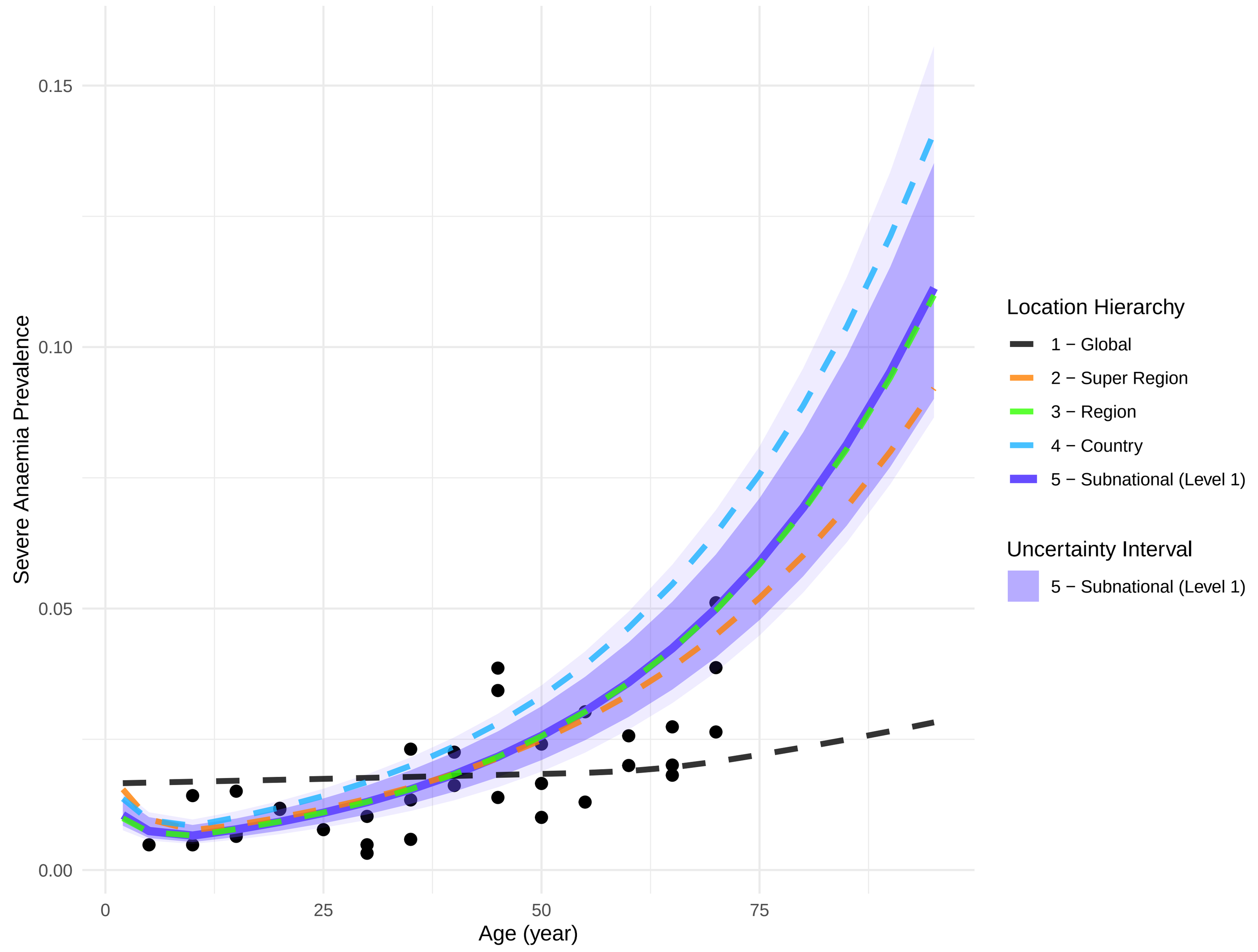

MR−BRT Age/Sex Fits − Non−pregnant Females 2+ Years Old − Severe Anaemia Prevalence − South Kalimantan
WHO−2001 Adjustment Method

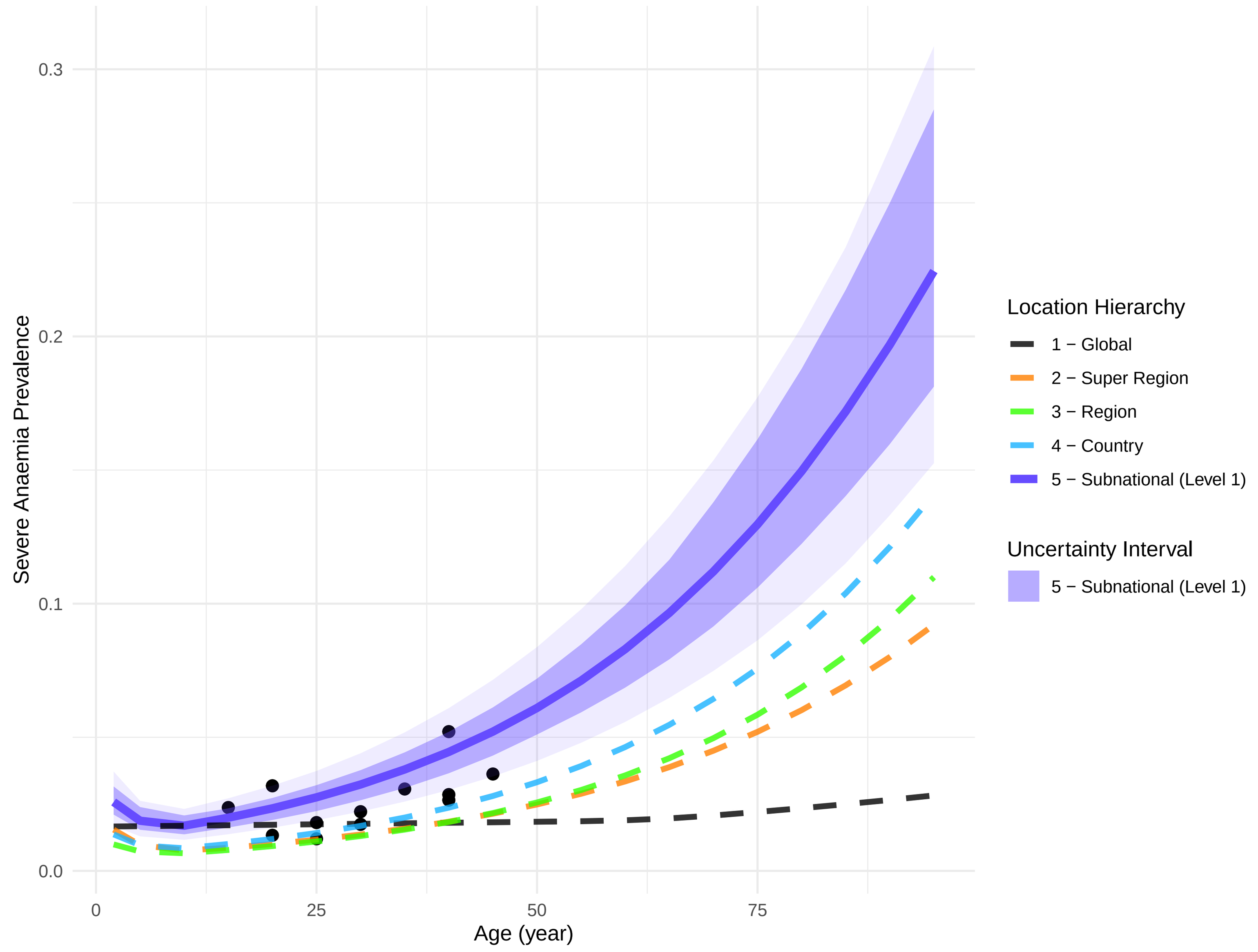

MR−BRT Age/Sex Fits − Non−pregnant Females 2+ Years Old − Severe Anaemia Prevalence − Lampung
WHO−2001 Adjustment Method

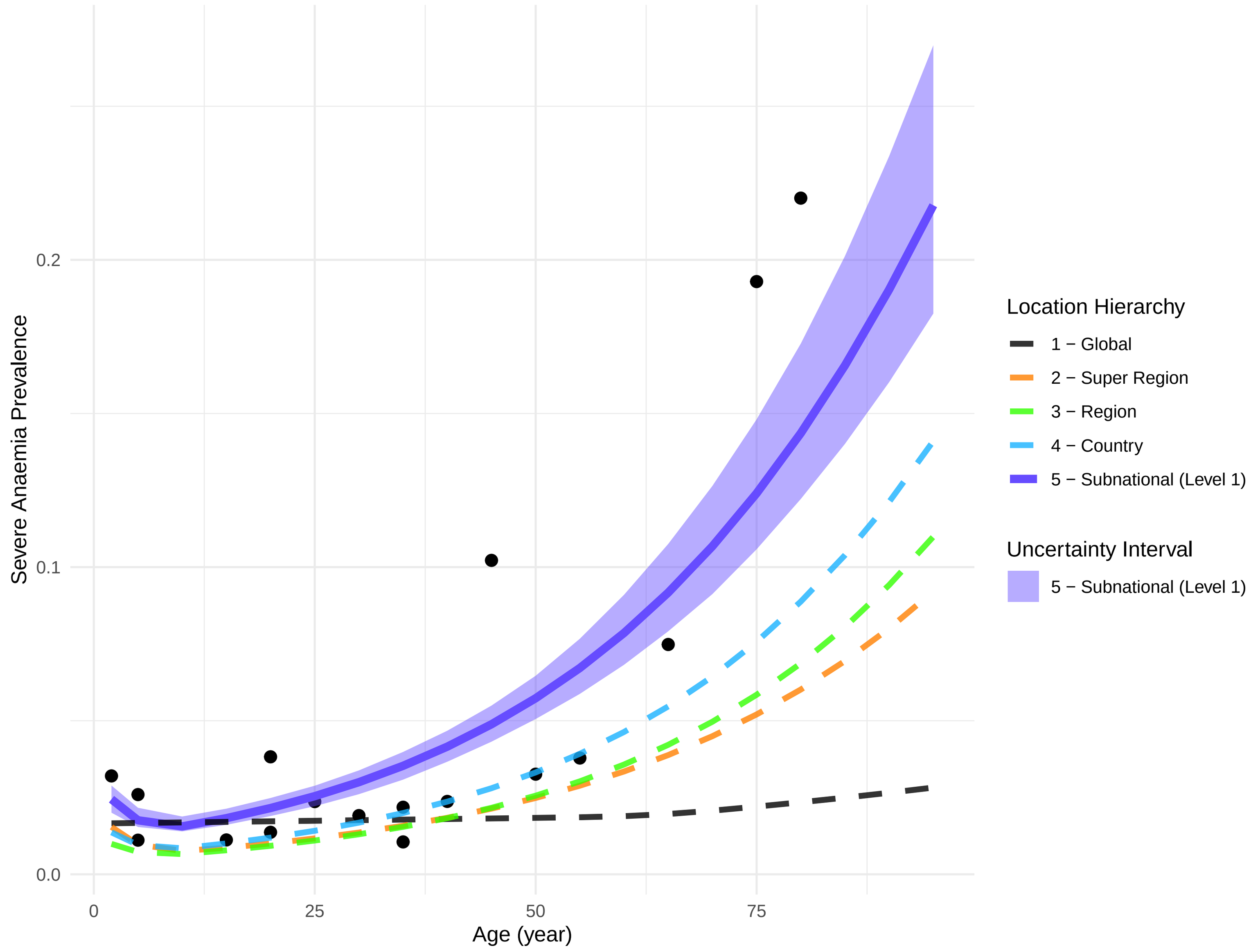

MR−BRT Age/Sex Fits − Non−pregnant Females 2+ Years Old − Severe Anaemia Prevalence − West Nusa Tenggara
WHO−2001 Adjustment Method

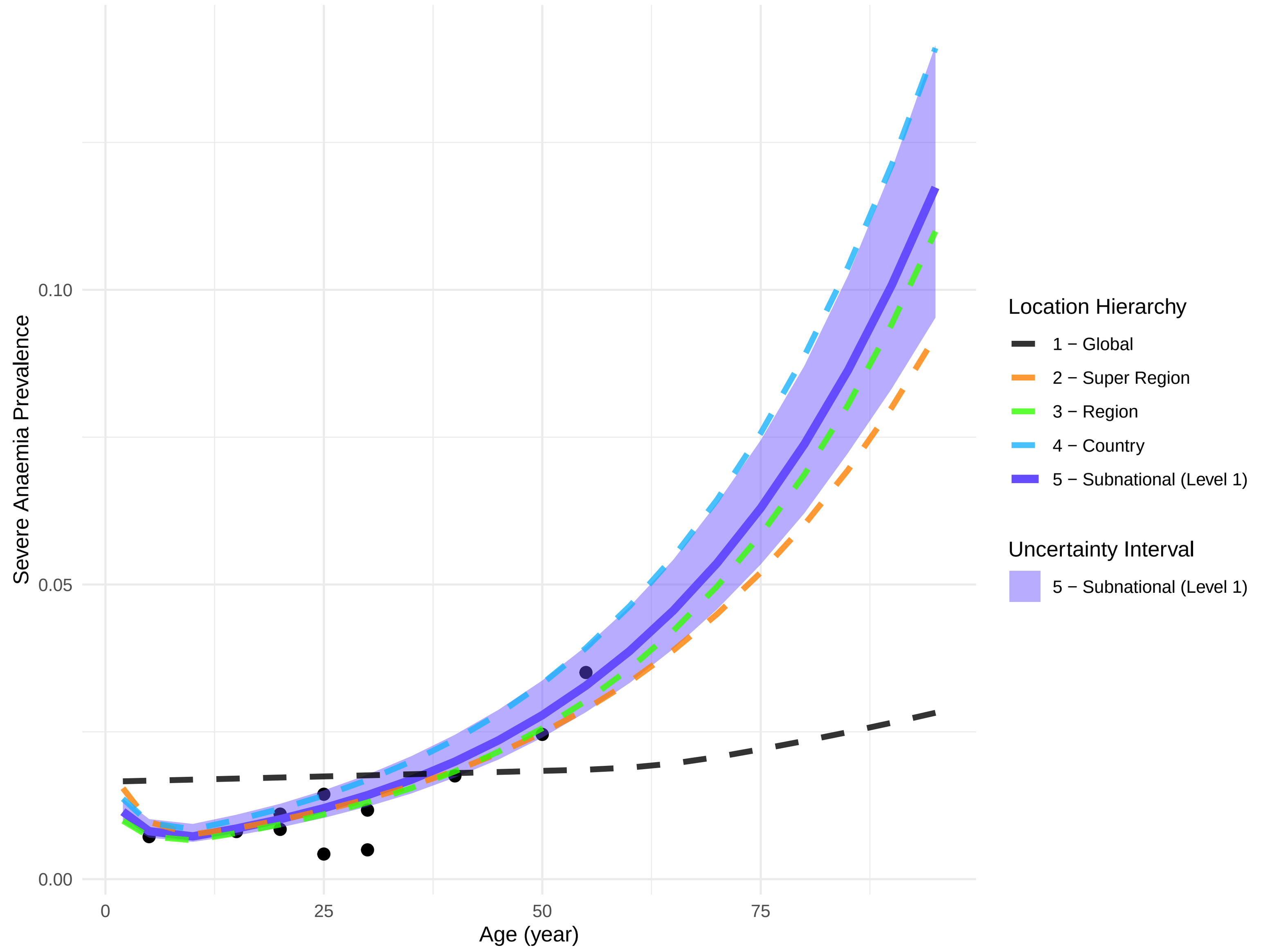

MR−BRT Age/Sex Fits − Non−pregnant Females 2+ Years Old − Severe Anaemia Prevalence − South Sulawesi
WHO−2001 Adjustment Method

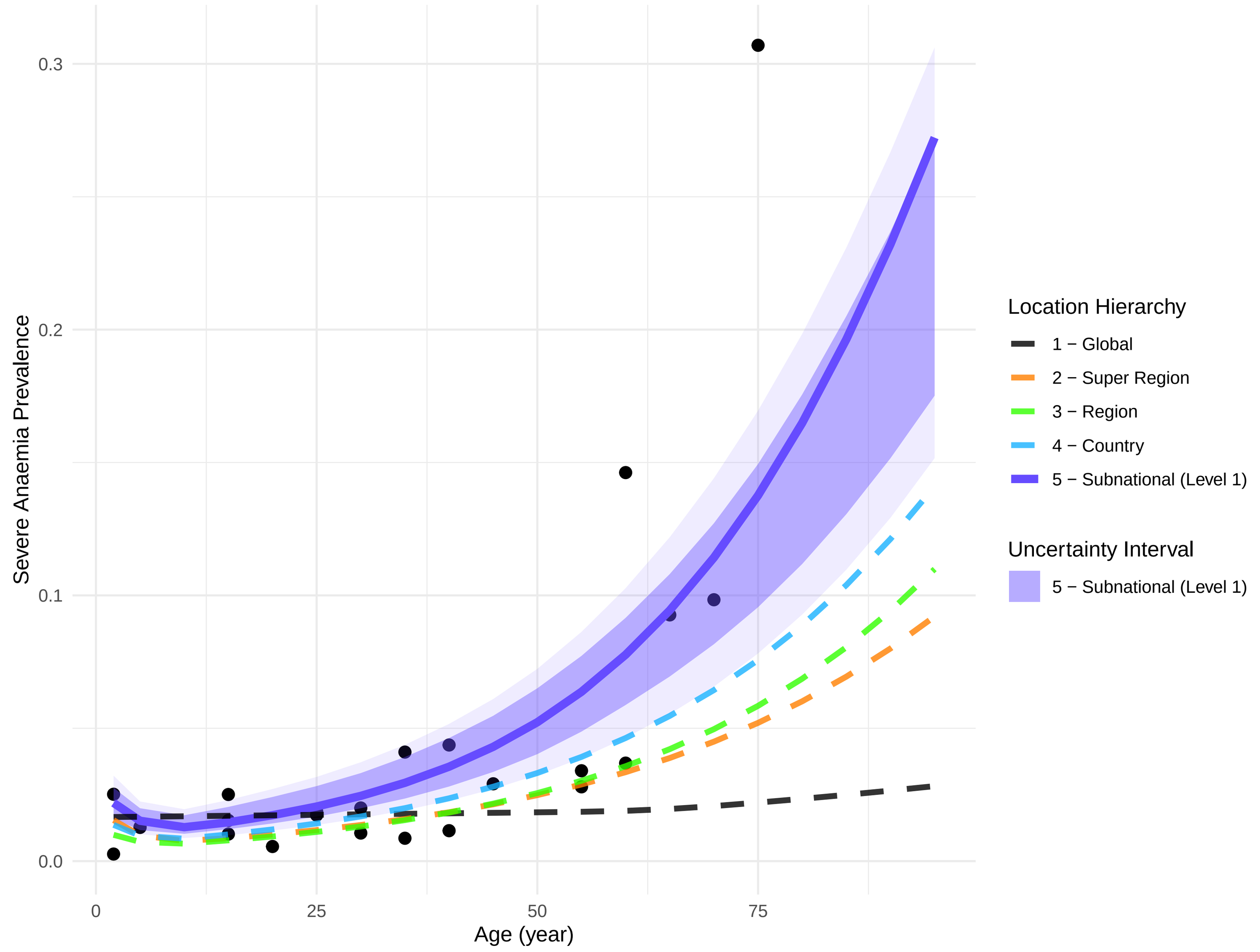

MR−BRT Age/Sex Fits − Non−pregnant Females 2+ Years Old − Severe Anaemia Prevalence − West Sumatra
WHO−2001 Adjustment Method

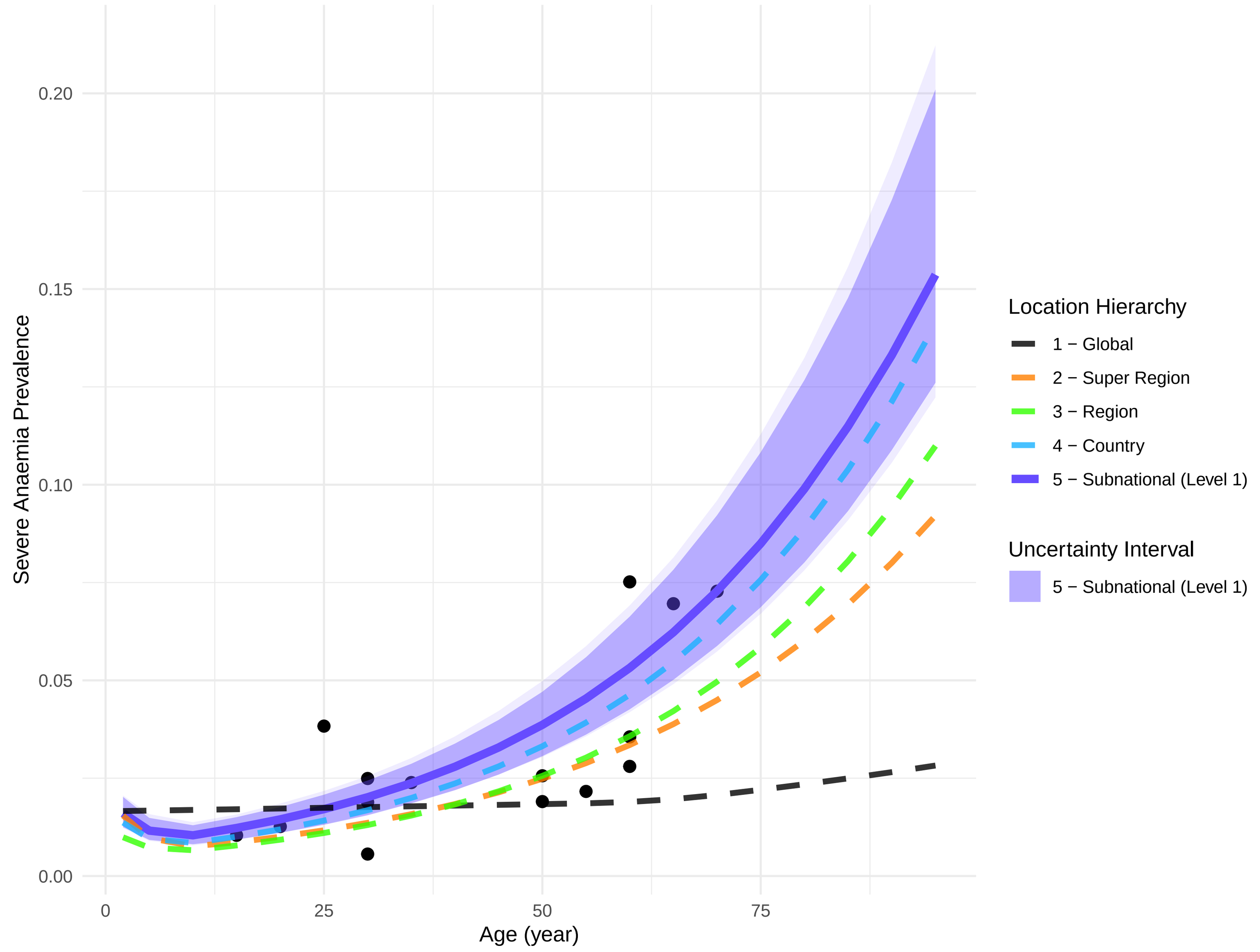

MR−BRT Age/Sex Fits − Non−pregnant Females 2+ Years Old − Severe Anaemia Prevalence − South Sumatra
WHO−2001 Adjustment Method

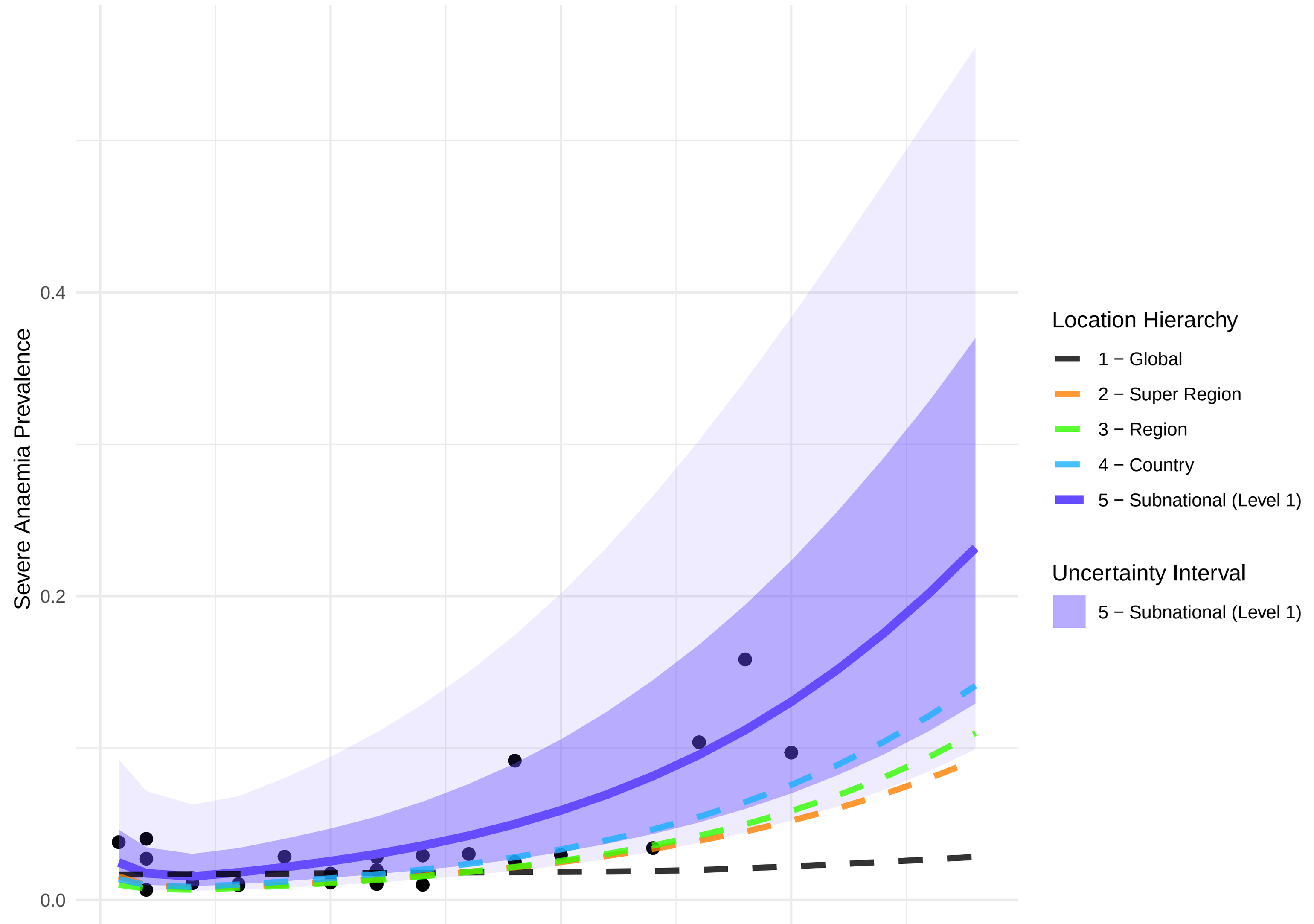

MR−BRT Age/Sex Fits − Non−pregnant Females 2+ Years Old − Severe Anaemia Prevalence − North Sumatra
WHO−2001 Adjustment Method

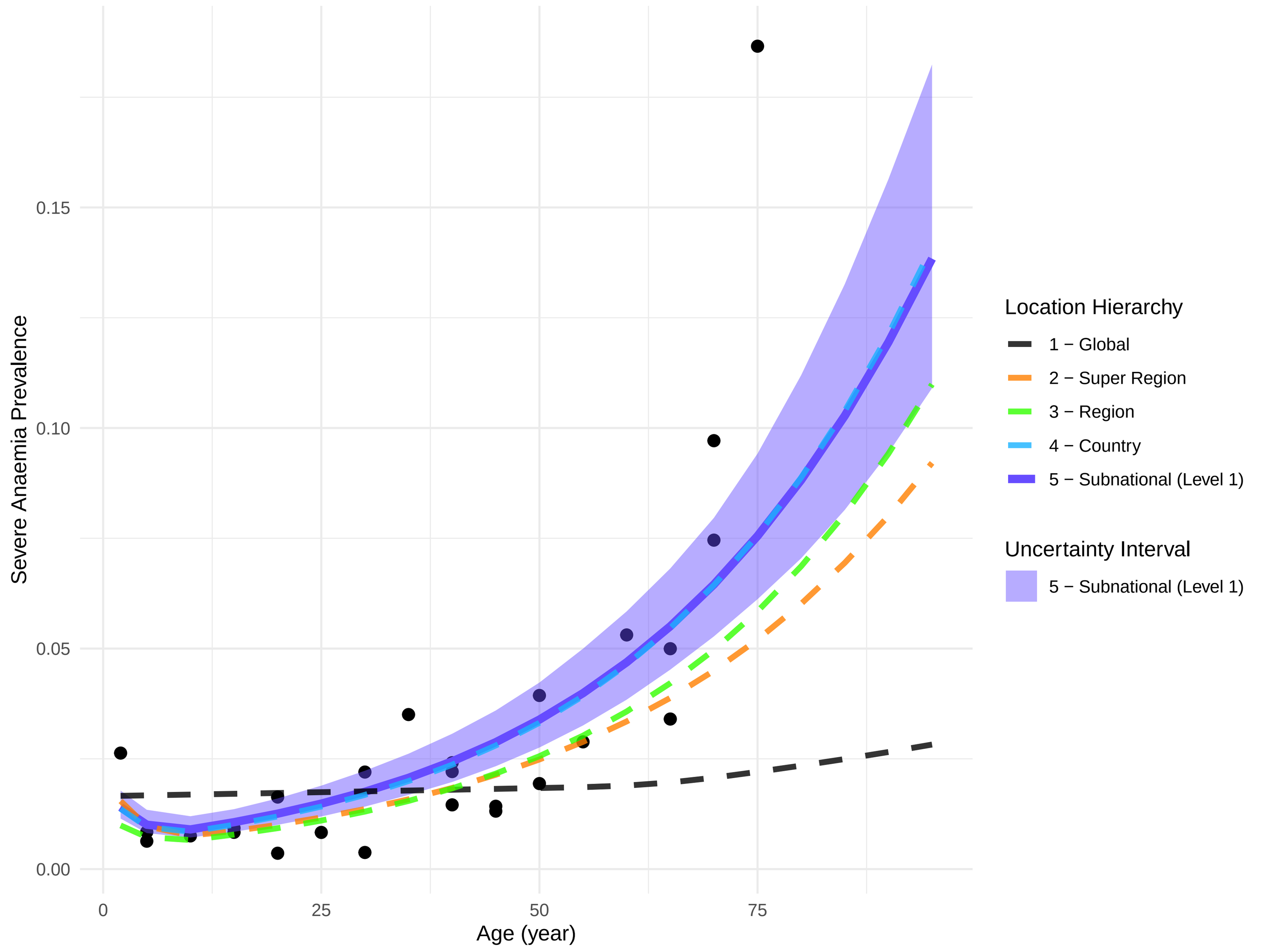

MR−BRT Age/Sex Fits − Non−pregnant Females 2+ Years Old − Severe Anaemia Prevalence − Yogyakarta
WHO−2001 Adjustment Method

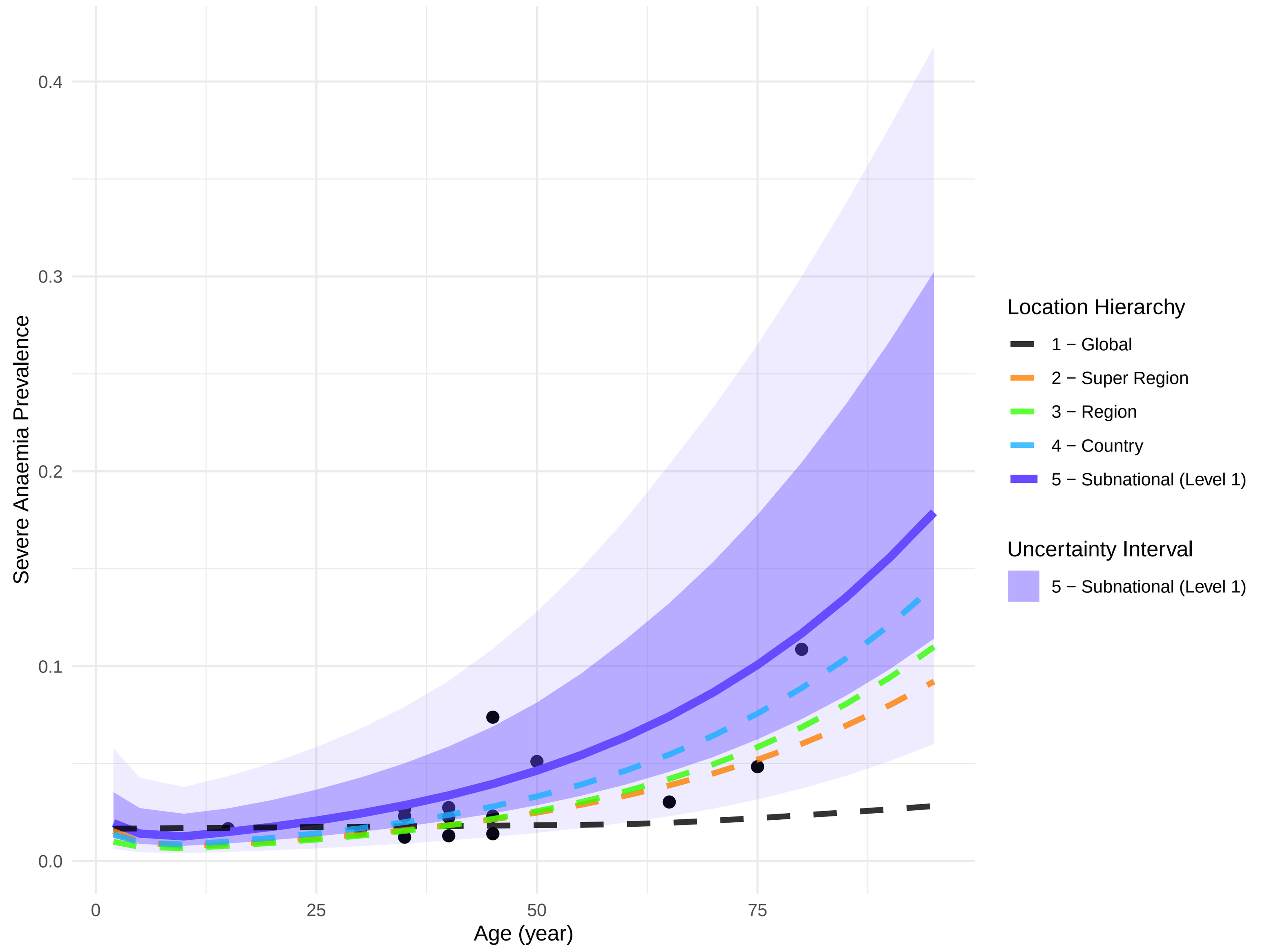

MR−BRT Age/Sex Fits − Non−pregnant Females 2+ Years Old − Severe Anaemia Prevalence − Myanmar
WHO−2001 Adjustment Method

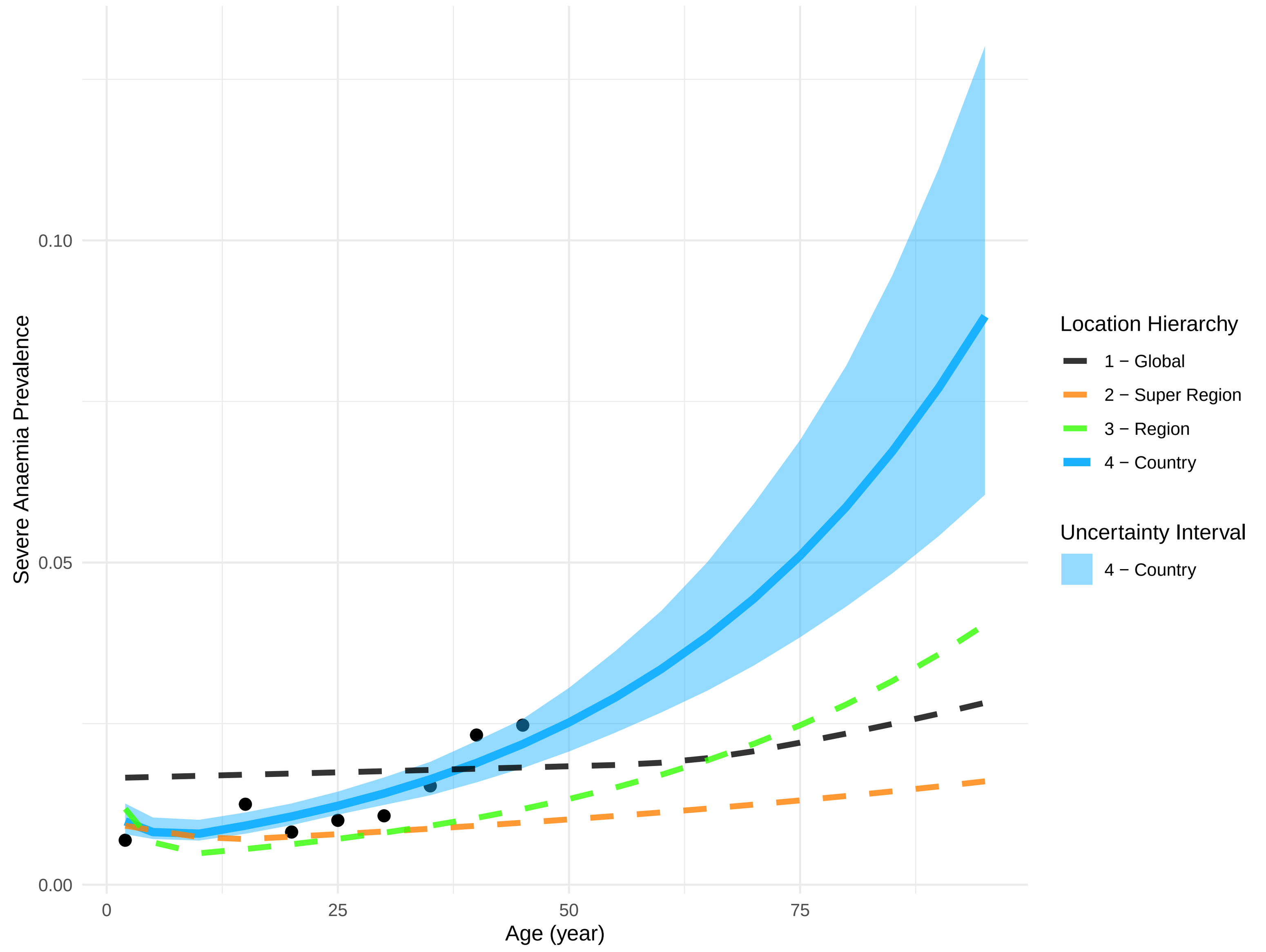

MR−BRT Age/Sex Fits − Non−pregnant Females 2+ Years Old − Severe Anaemia Prevalence − Timor−Leste
WHO−2001 Adjustment Method

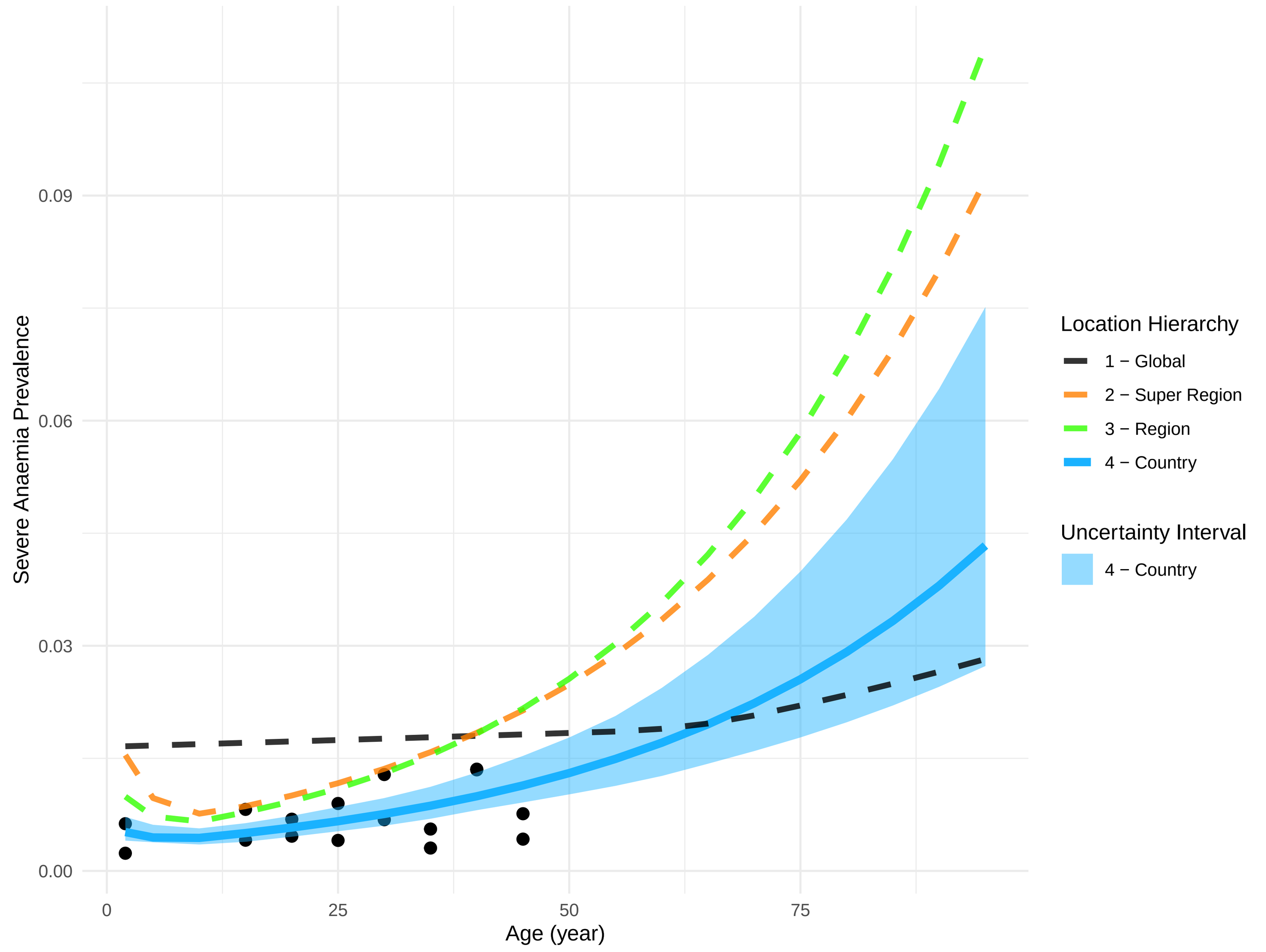

MR−BRT Age/Sex Fits − Non−pregnant Females 2+ Years Old − Severe Anaemia Prevalence − Sub−Saharan Africa
WHO−2001 Adjustment Method

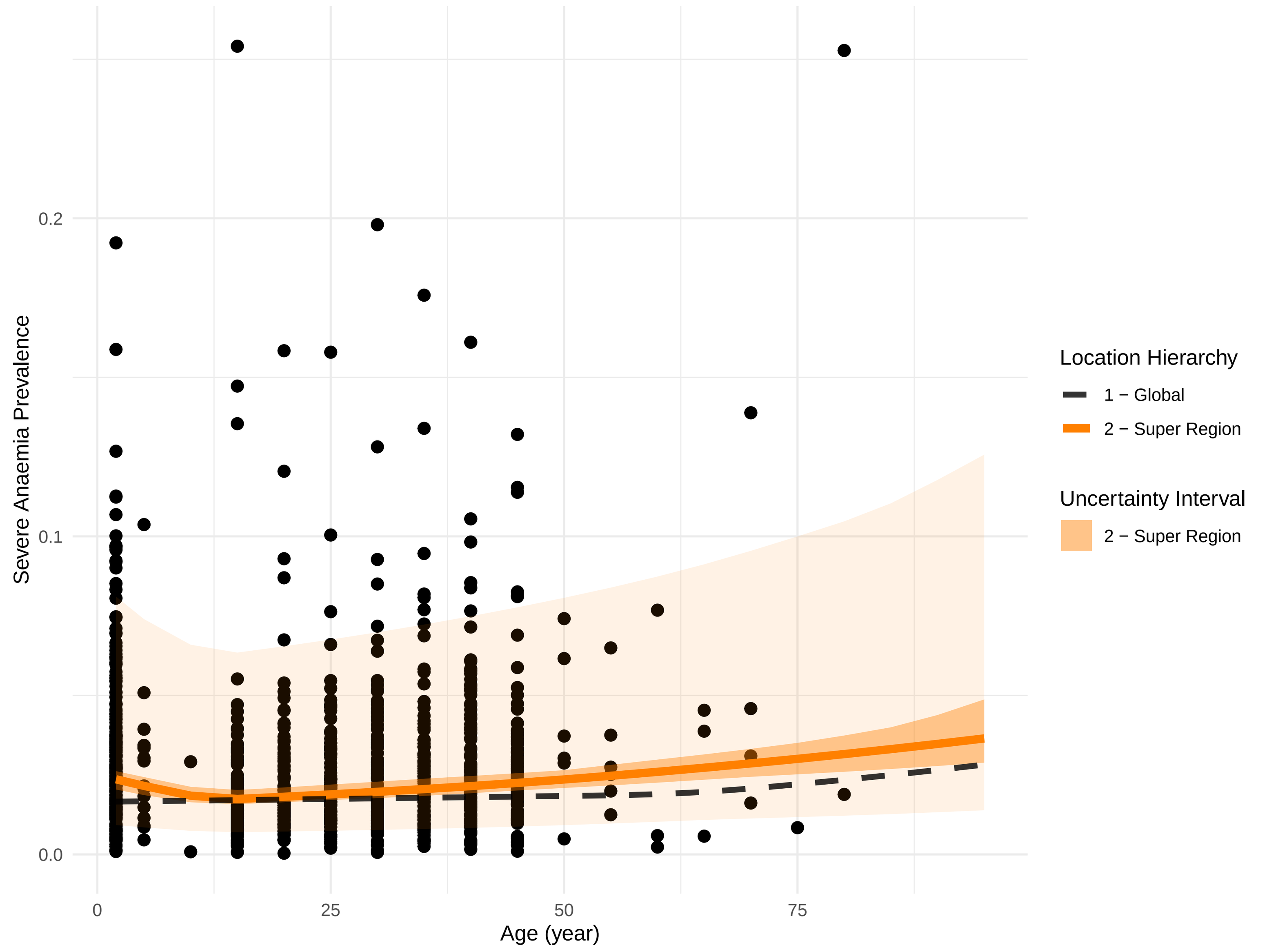

MR−BRT Age/Sex Fits − Non−pregnant Females 2+ Years Old − Severe Anaemia Prevalence − Central Sub−Saharan Africa
WHO−2001 Adjustment Method

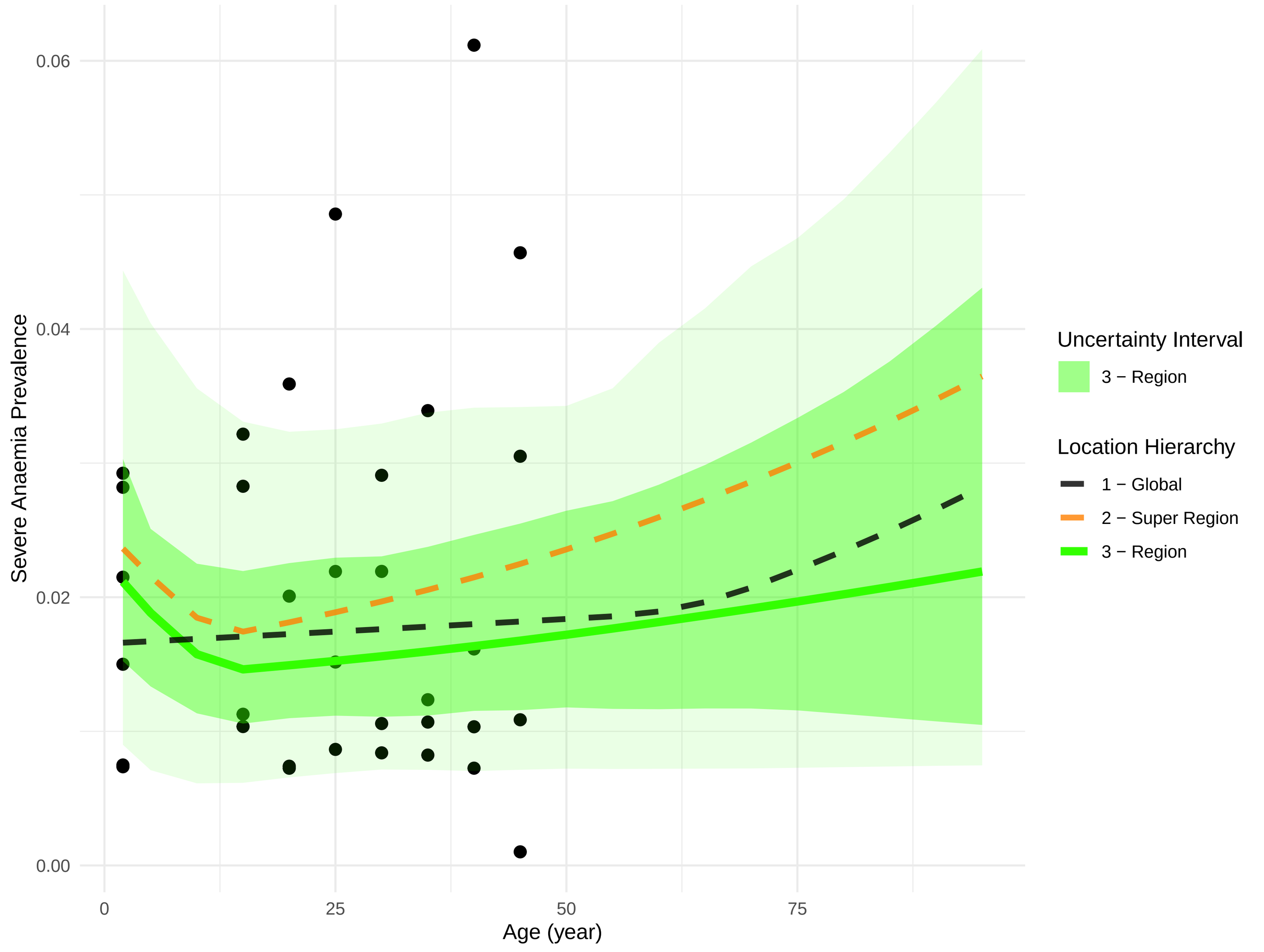

MR−BRT Age/Sex Fits − Non−pregnant Females 2+ Years Old − Severe Anaemia Prevalence − Angola
WHO−2001 Adjustment Method

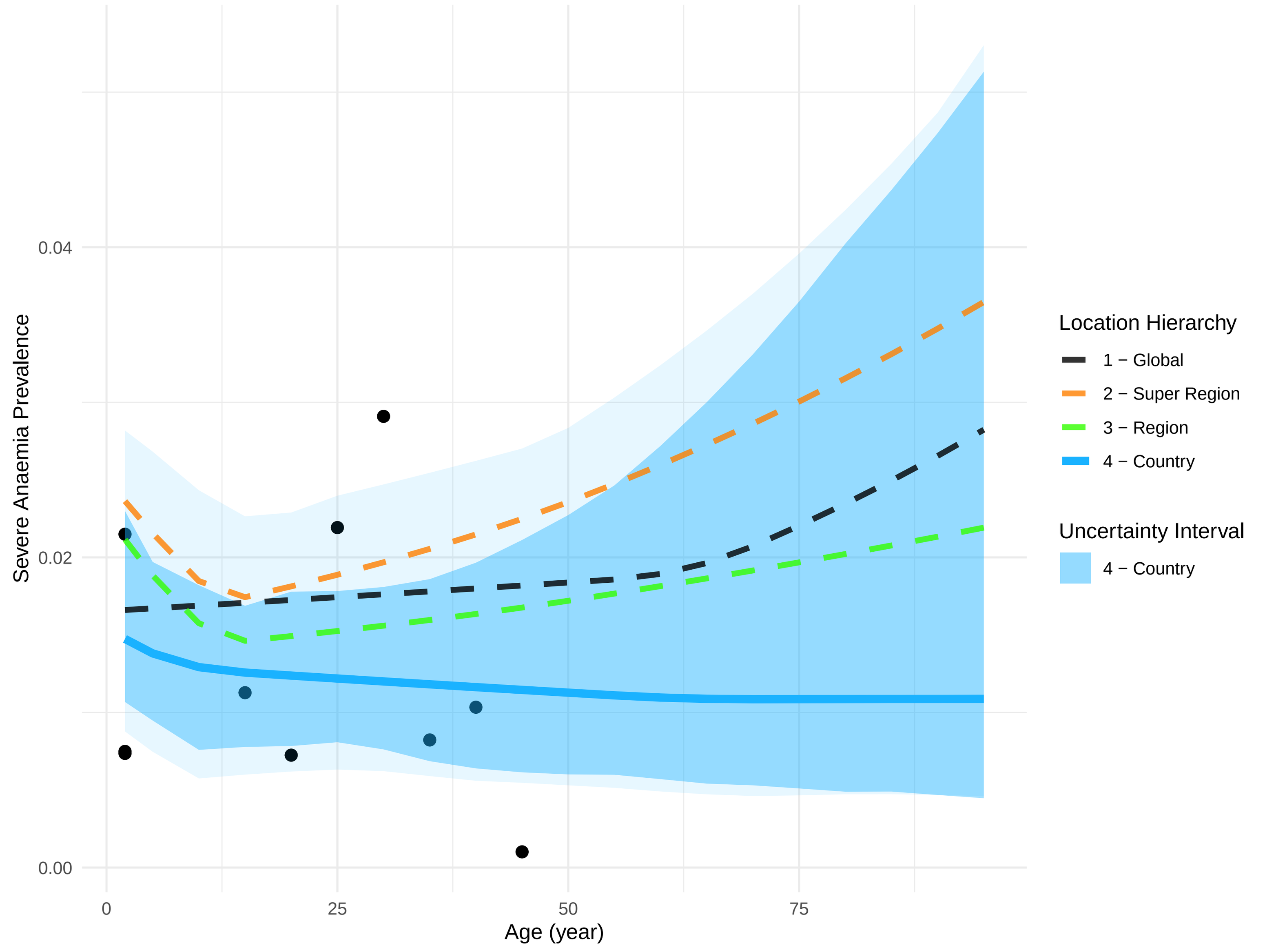

MR−BRT Age/Sex Fits − Non−pregnant Females 2+ Years Old − Severe Anaemia Prevalence − Democratic Republic of the Congo
WHO−2001 Adjustment Method

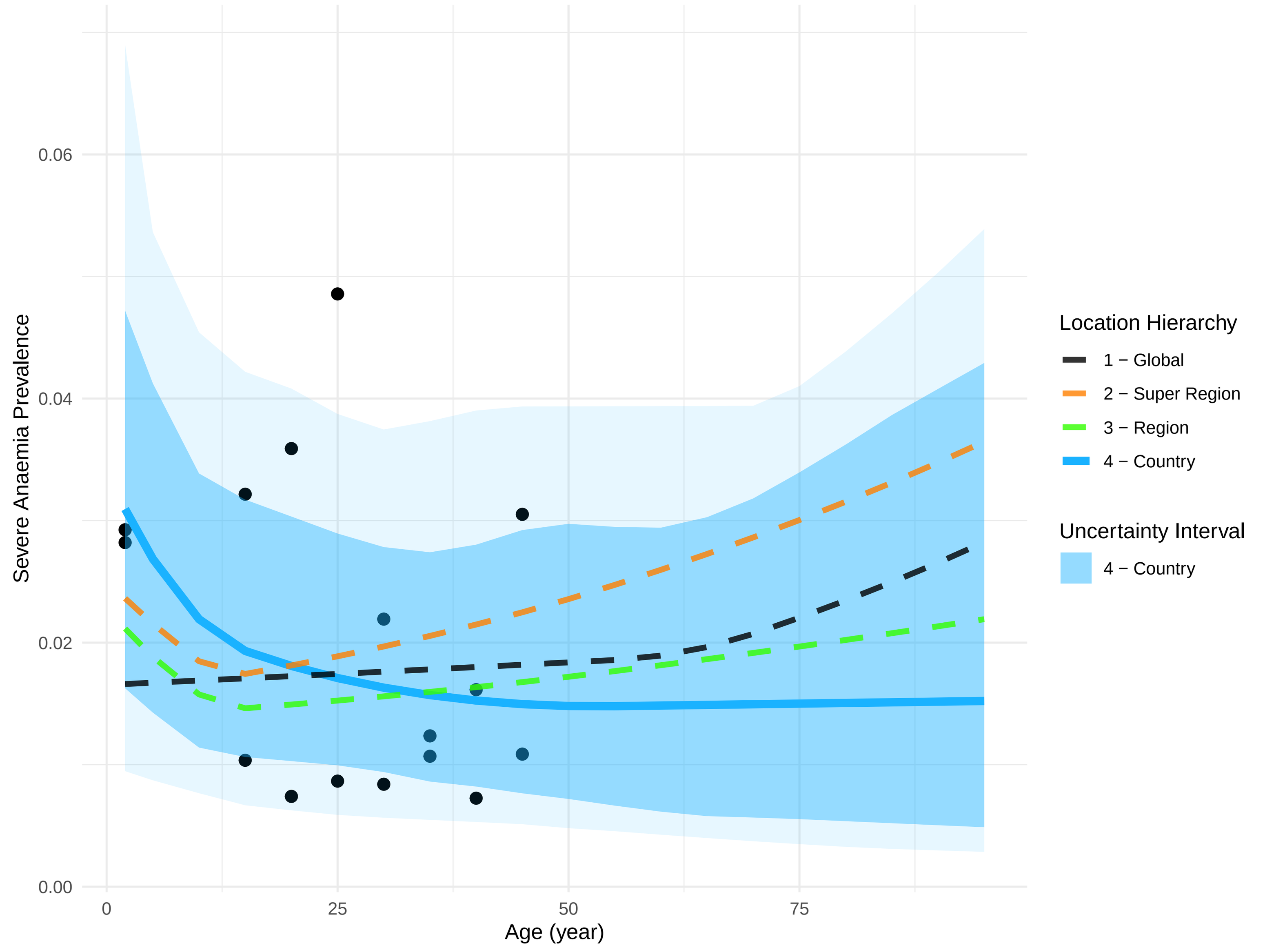

MR−BRT Age/Sex Fits − Non−pregnant Females 2+ Years Old − Severe Anaemia Prevalence − Gabon
WHO−2001 Adjustment Method

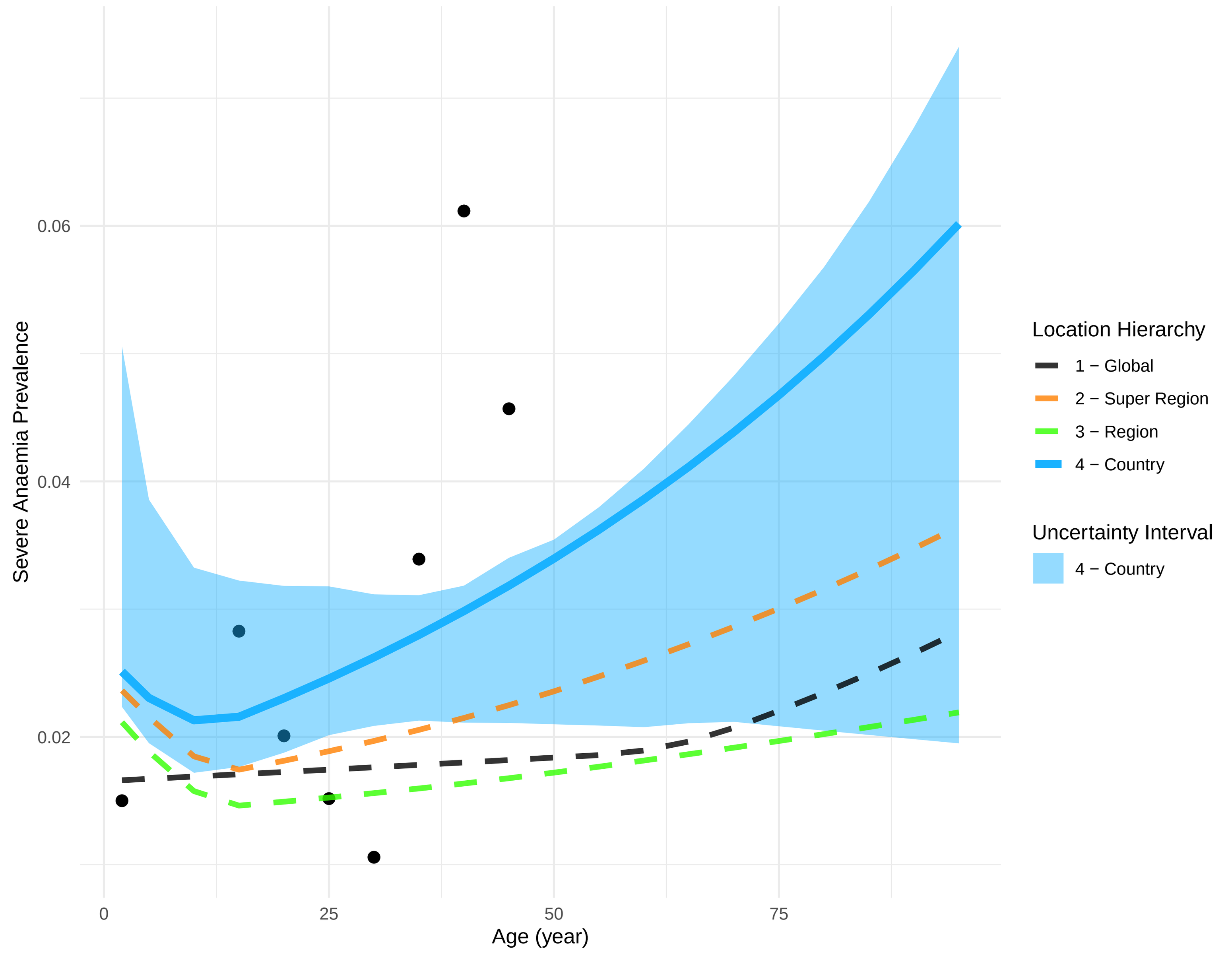

MR−BRT Age/Sex Fits − Non−pregnant Females 2+ Years Old − Severe Anaemia Prevalence − Eastern Sub−Saharan Africa
WHO−2001 Adjustment Method

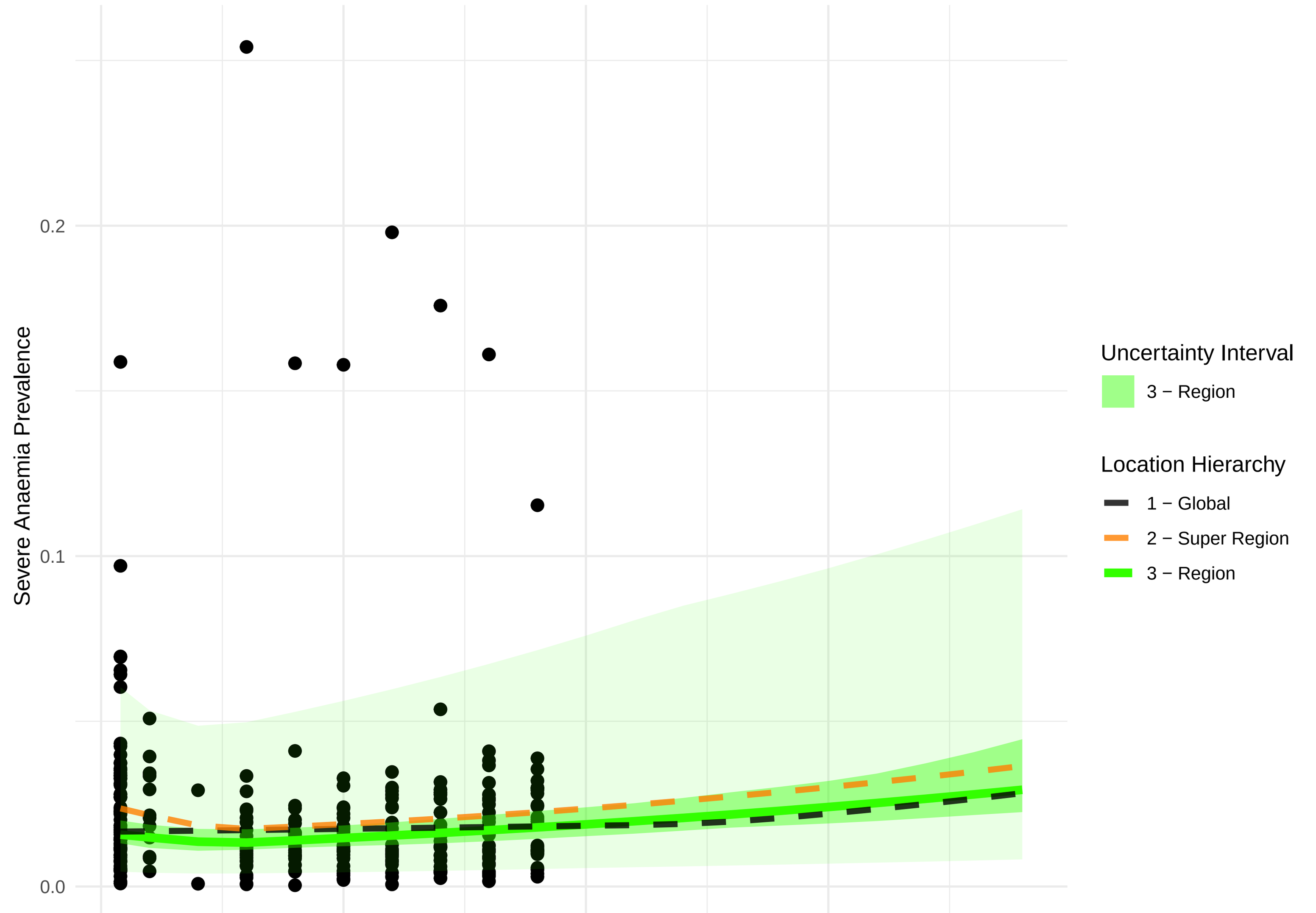

MR−BRT Age/Sex Fits − Non−pregnant Females 2+ Years Old − Severe Anaemia Prevalence − Burundi
WHO−2001 Adjustment Method

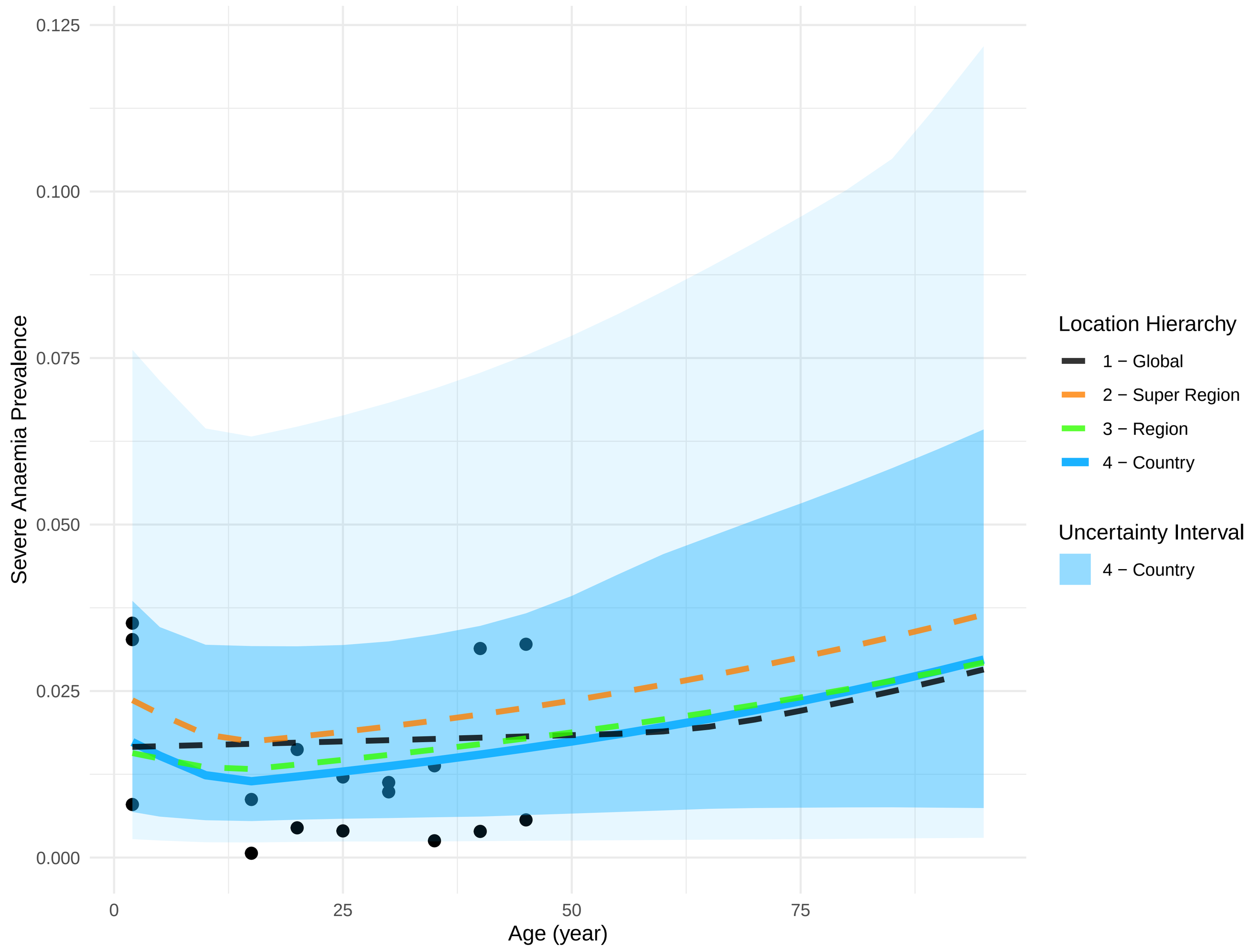

MR−BRT Age/Sex Fits − Non−pregnant Females 2+ Years Old − Severe Anaemia Prevalence − Ethiopia
WHO−2001 Adjustment Method

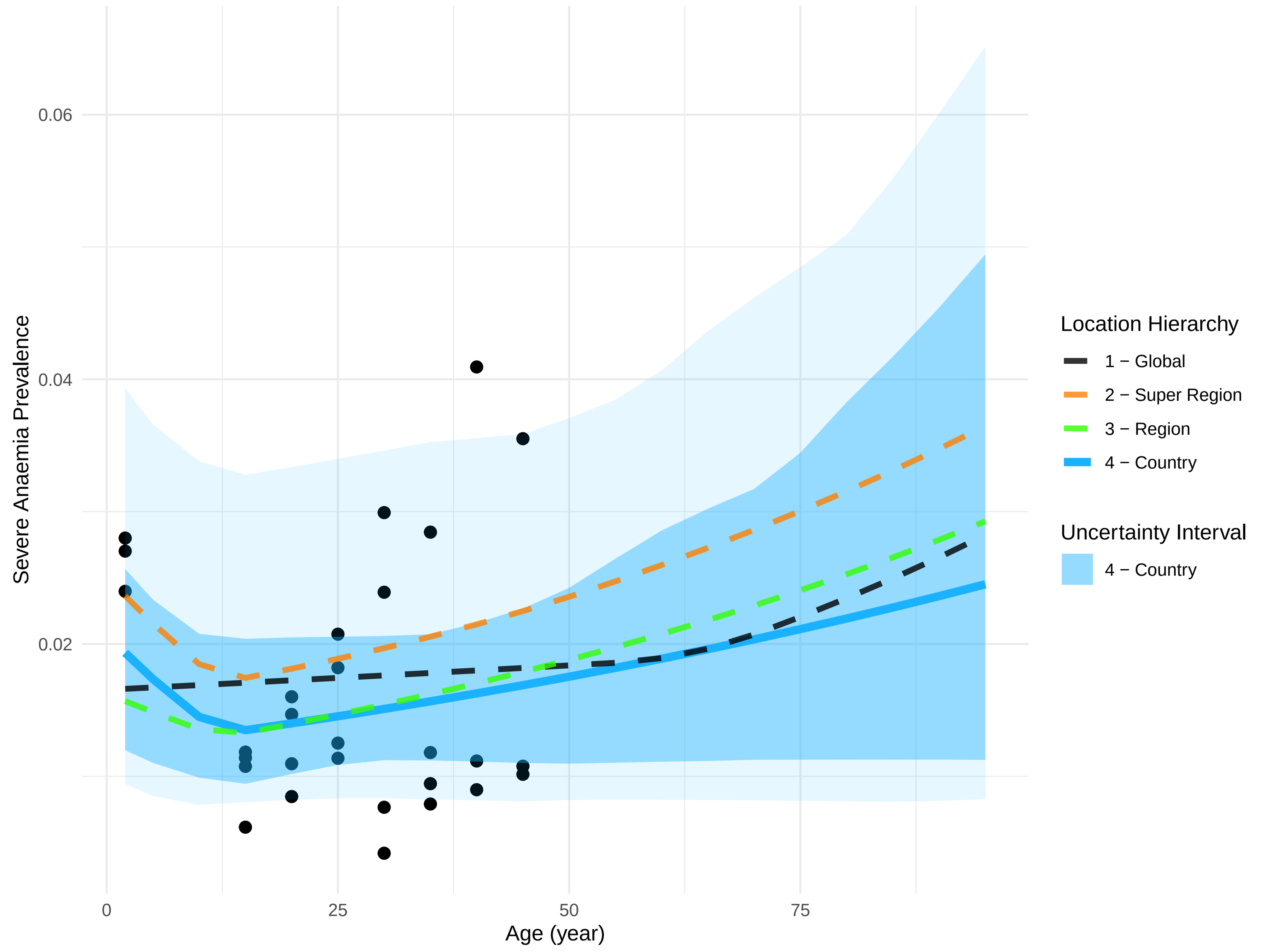

MR−BRT Age/Sex Fits − Non−pregnant Females 2+ Years Old − Severe Anaemia Prevalence − Kenya
WHO−2001 Adjustment Method

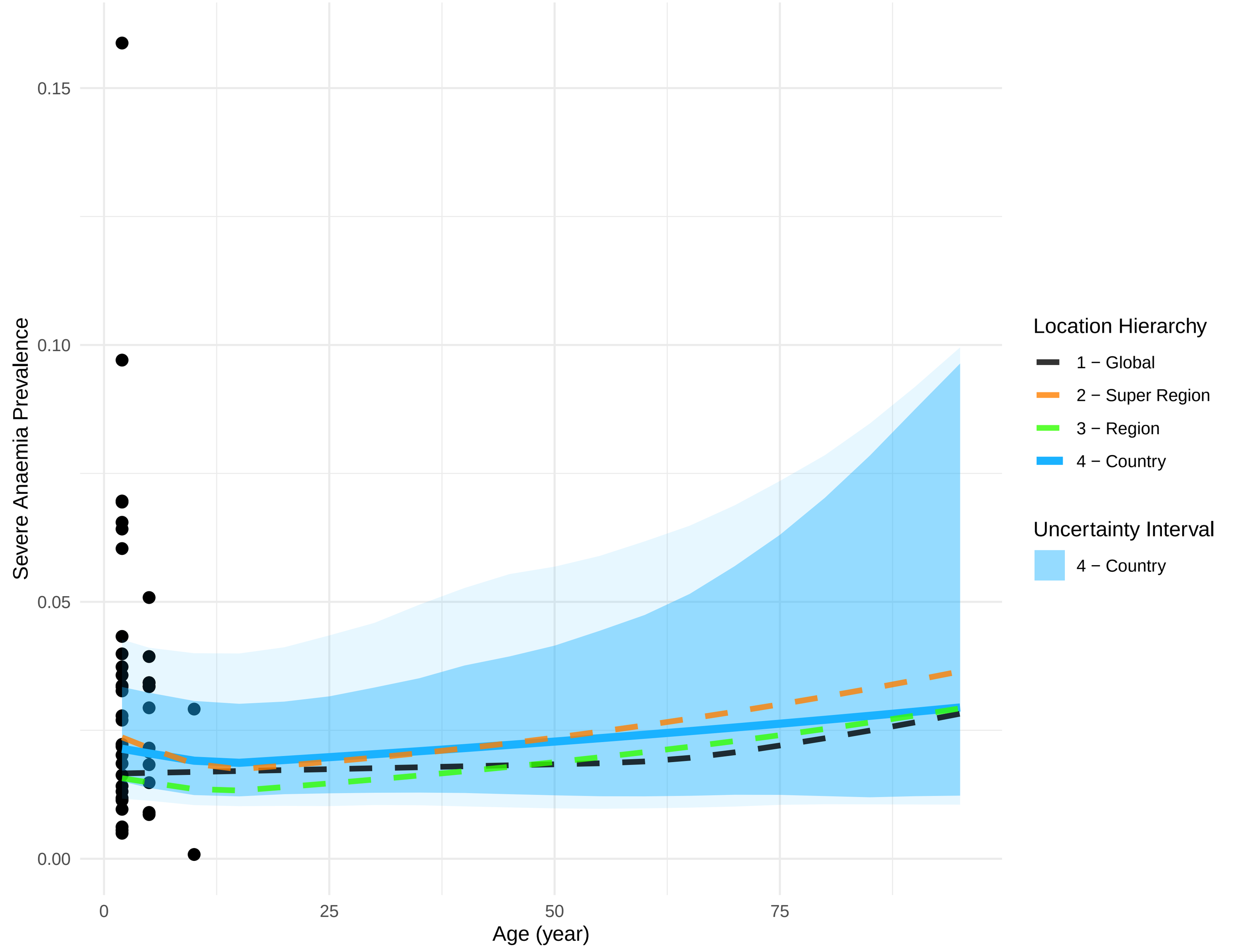

MR−BRT Age/Sex Fits − Non−pregnant Females 2+ Years Old − Severe Anaemia Prevalence − Bungoma
WHO−2001 Adjustment Method

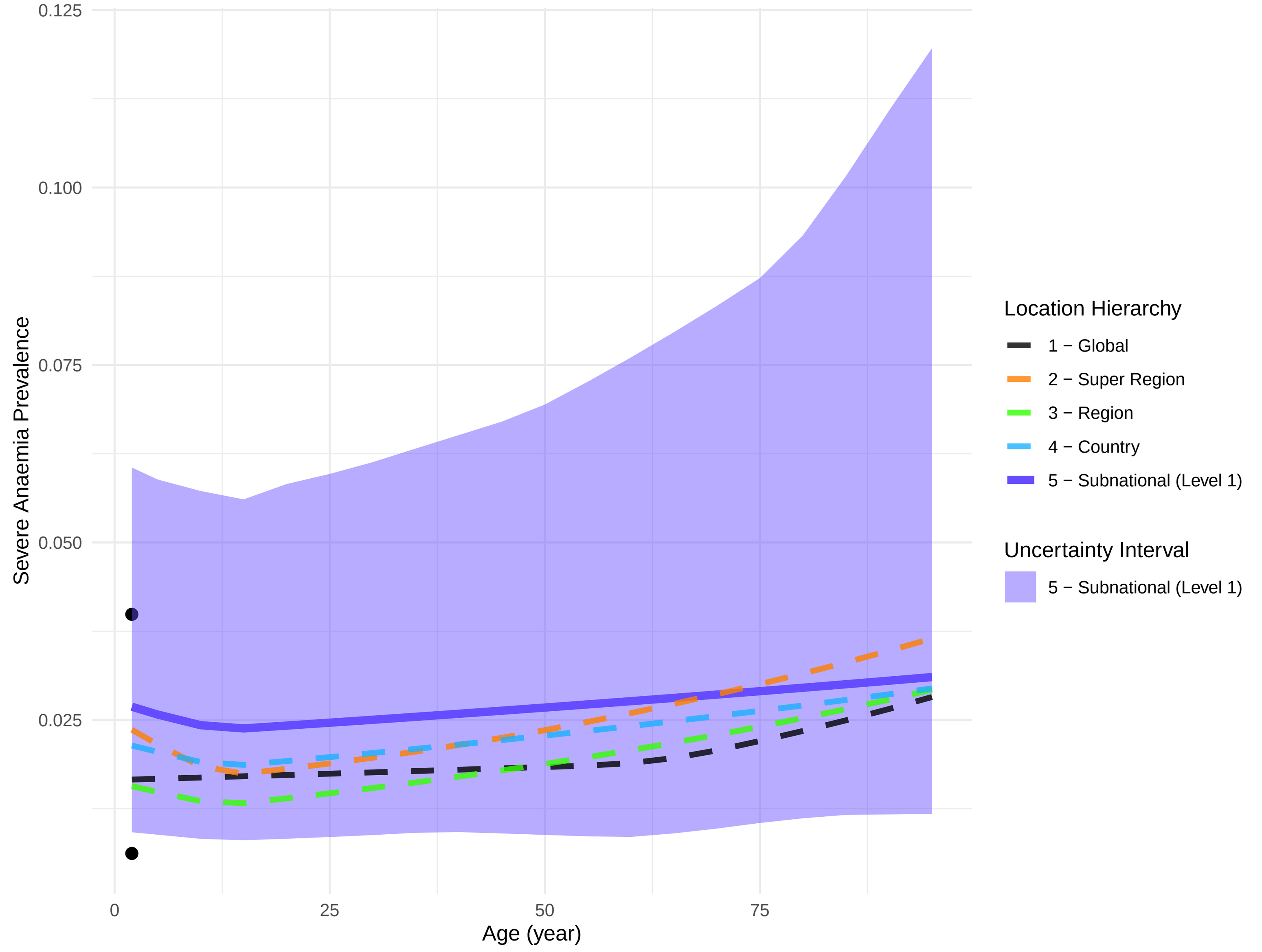

MR−BRT Age/Sex Fits − Non−pregnant Females 2+ Years Old − Severe Anaemia Prevalence − Busia
WHO−2001 Adjustment Method

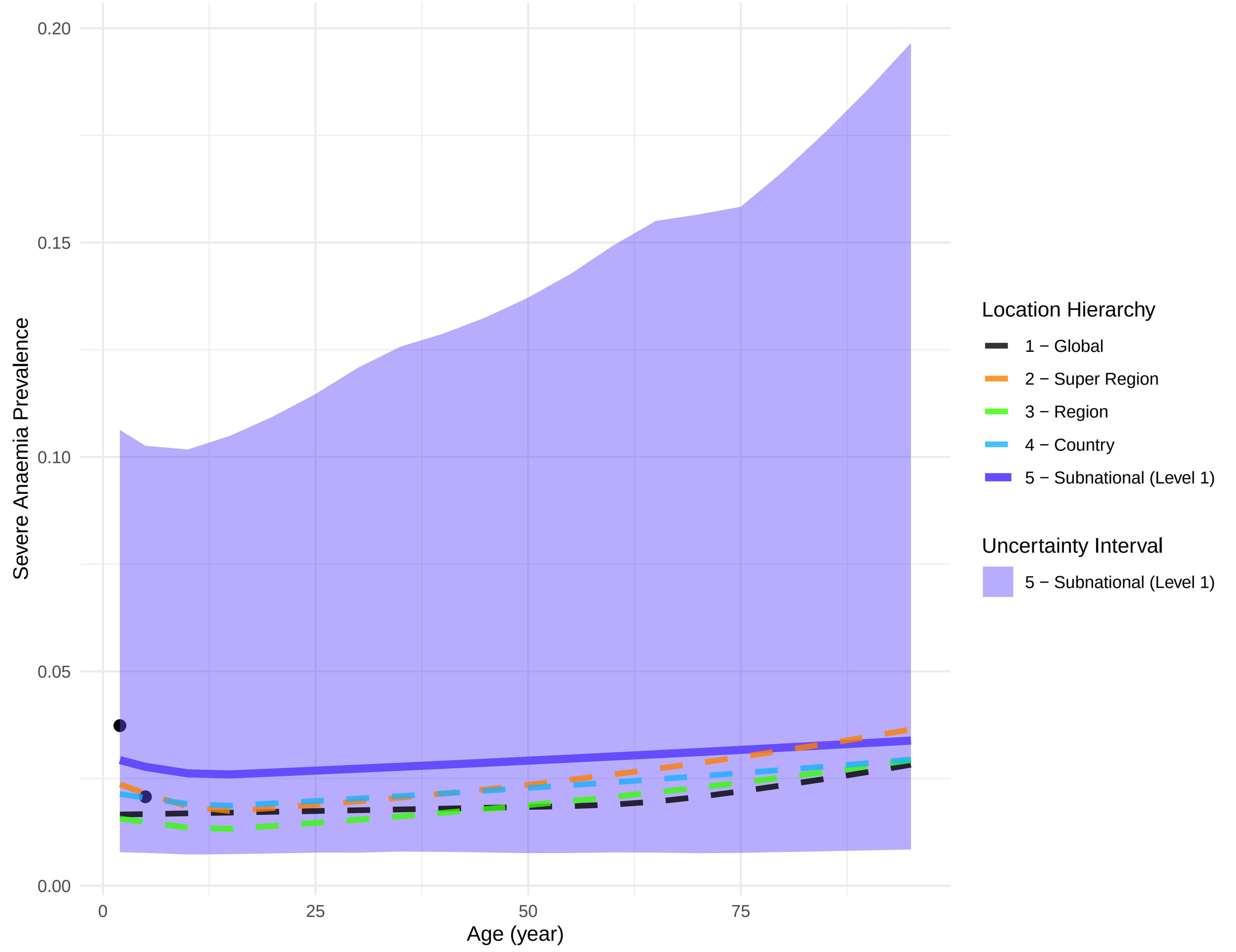

MR−BRT Age/Sex Fits − Non−pregnant Females 2+ Years Old − Severe Anaemia Prevalence − Elgeyo−Marakwet
WHO−2001 Adjustment Method

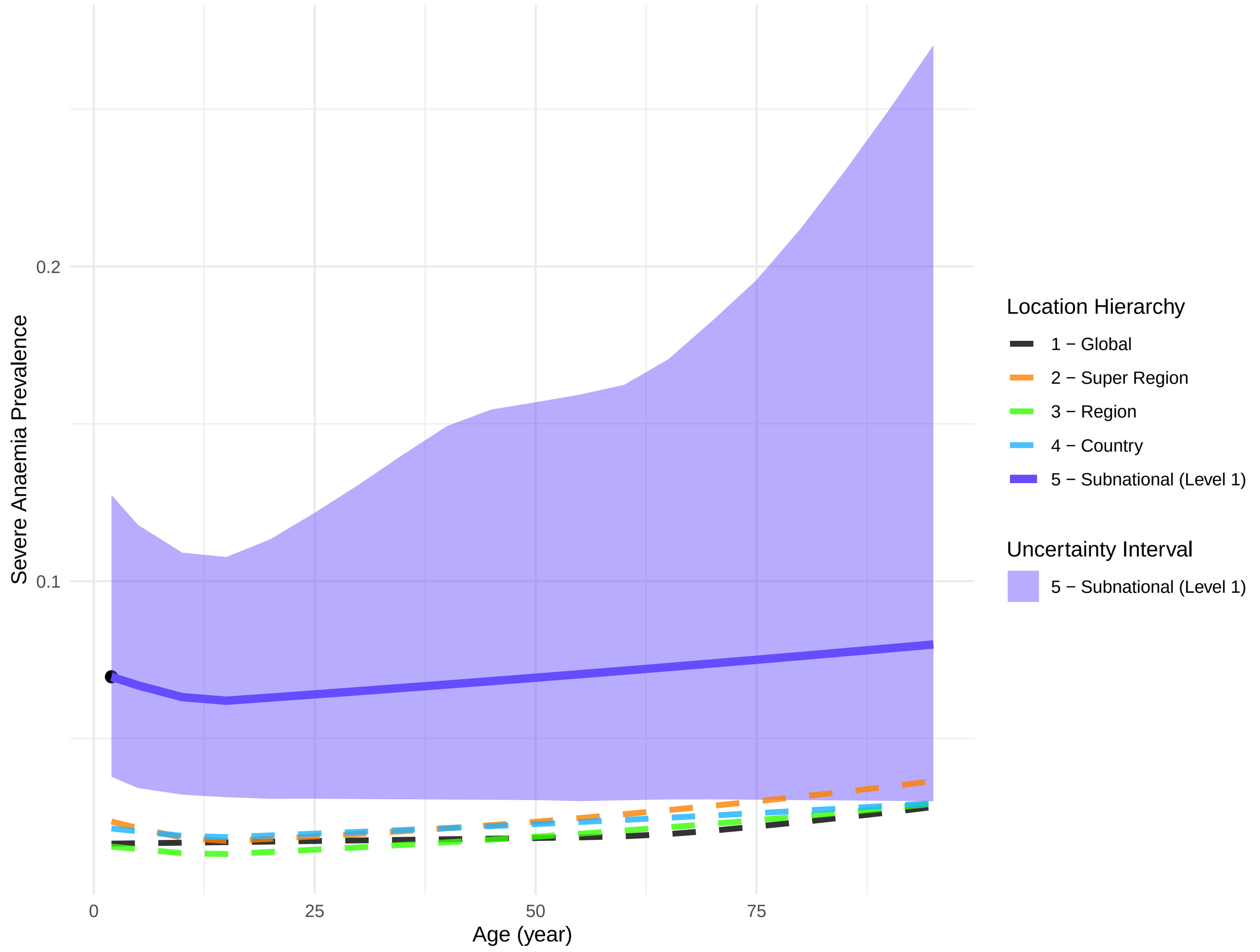

MR−BRT Age/Sex Fits − Non−pregnant Females 2+ Years Old − Severe Anaemia Prevalence − Garissa
WHO−2001 Adjustment Method

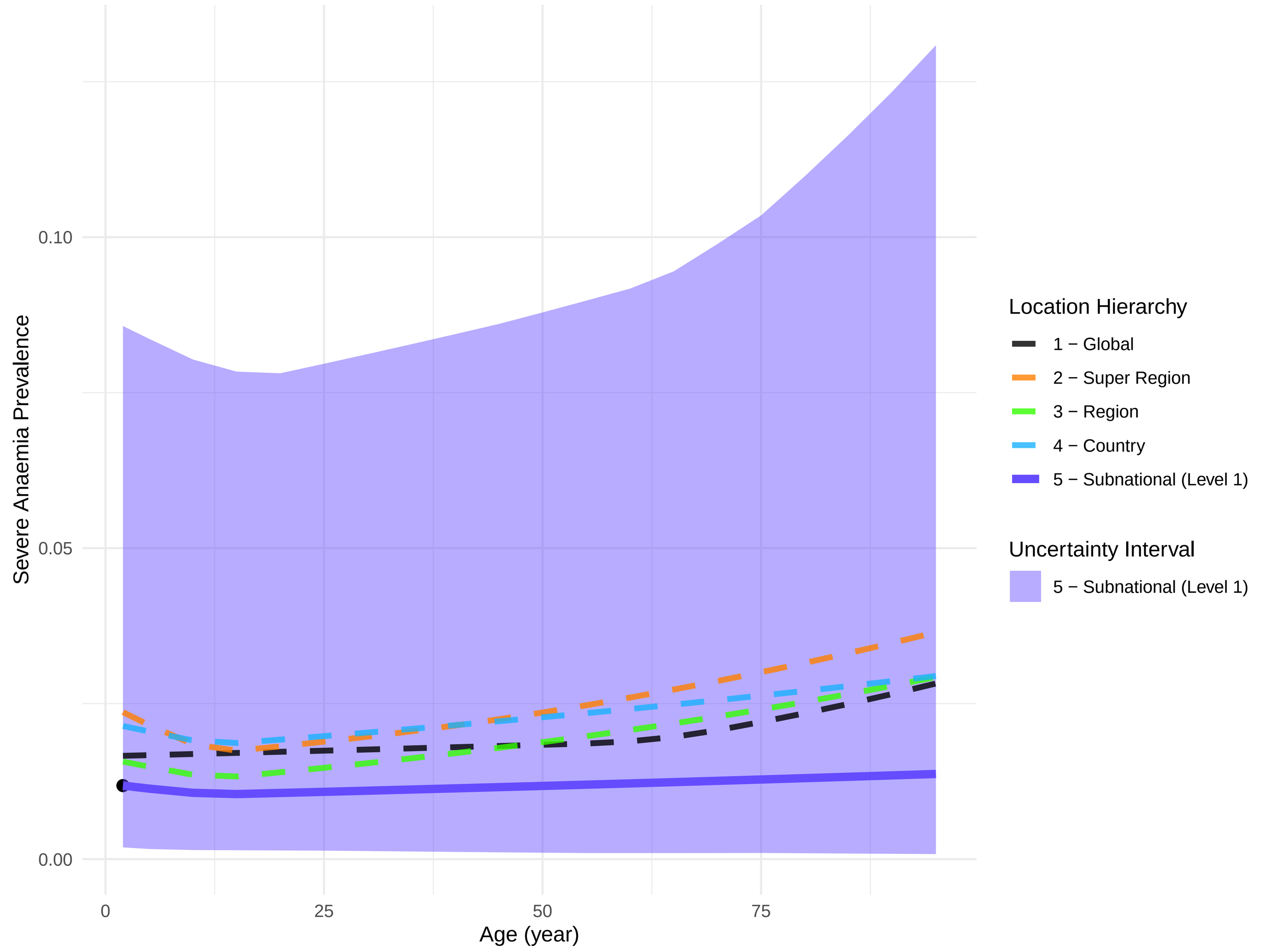

MR−BRT Age/Sex Fits − Non−pregnant Females 2+ Years Old − Severe Anaemia Prevalence − Homa Bay
WHO−2001 Adjustment Method

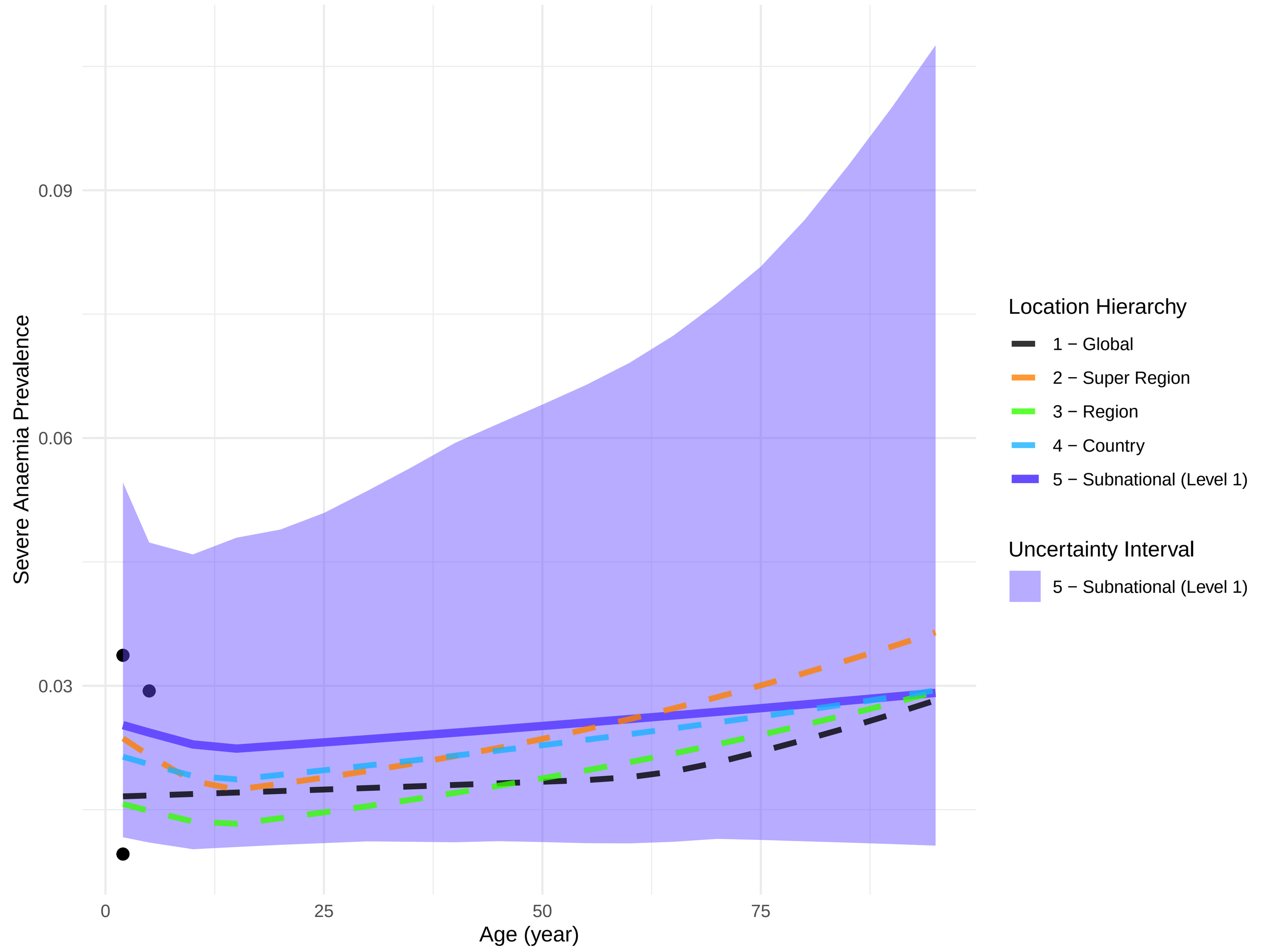

MR−BRT Age/Sex Fits − Non−pregnant Females 2+ Years Old − Severe Anaemia Prevalence − Isiolo
WHO−2001 Adjustment Method

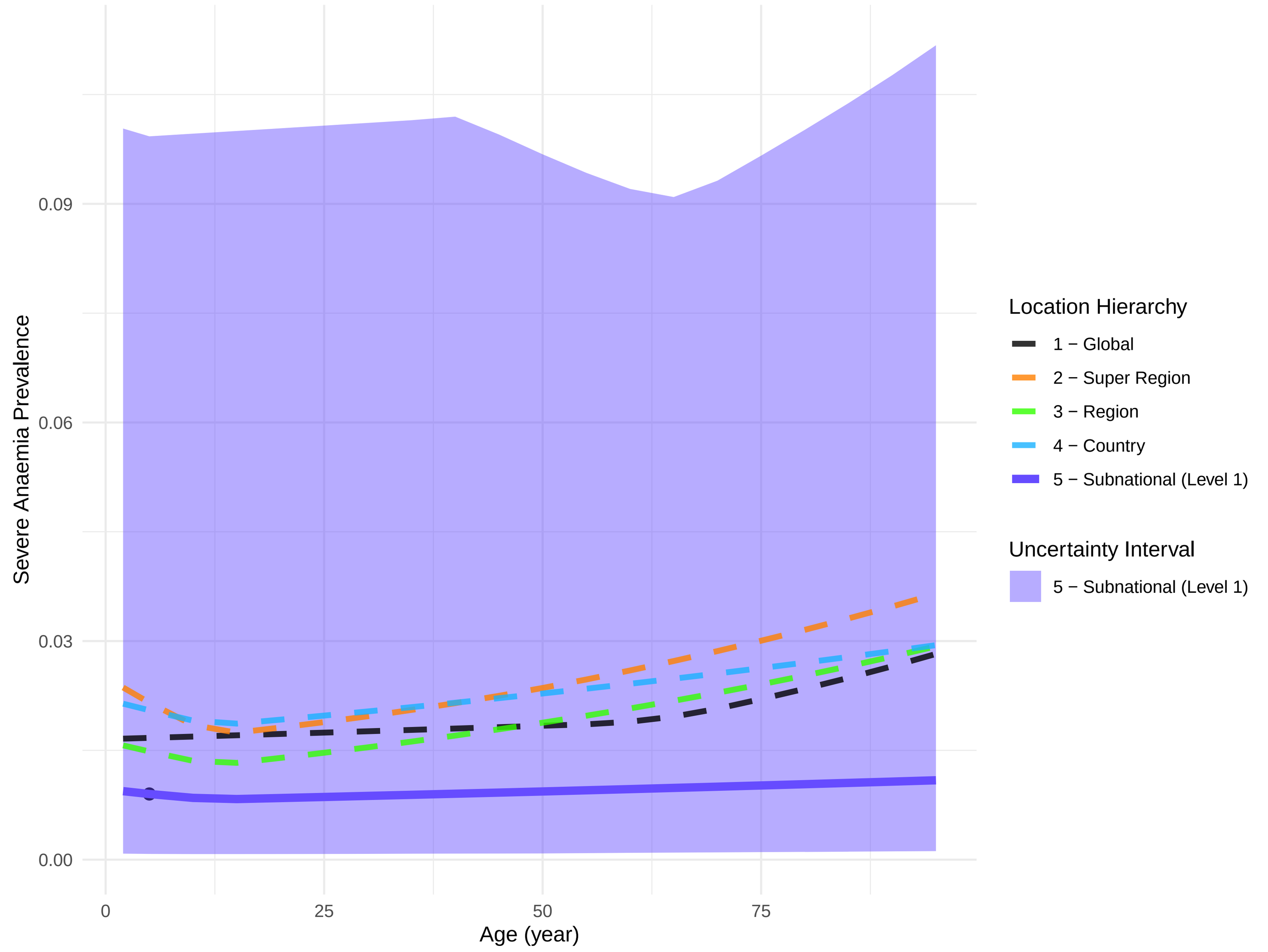

MR−BRT Age/Sex Fits − Non−pregnant Females 2+ Years Old − Severe Anaemia Prevalence − Kajiado
WHO−2001 Adjustment Method

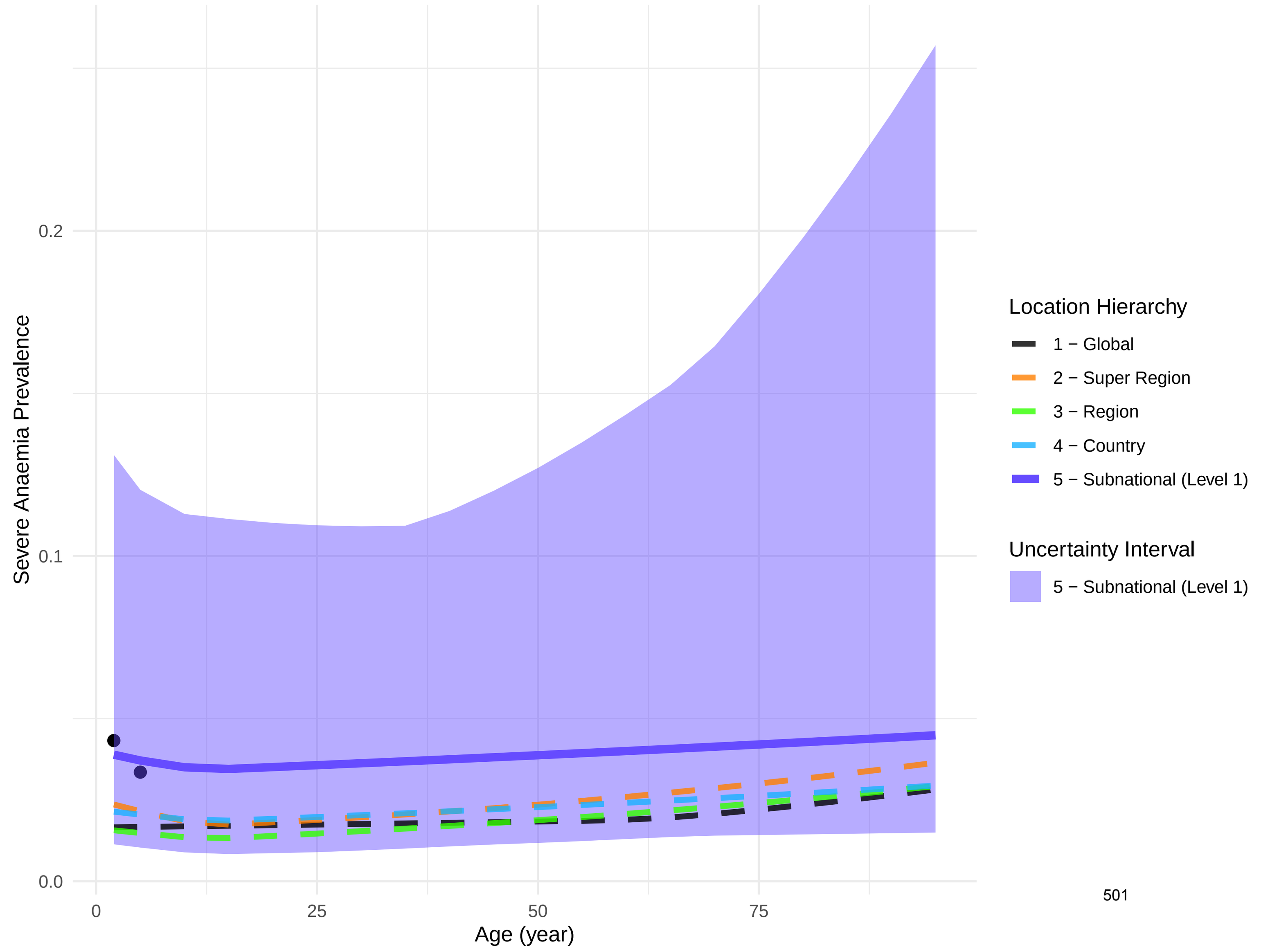

MR−BRT Age/Sex Fits − Non−pregnant Females 2+ Years Old − Severe Anaemia Prevalence − Kakamega
WHO−2001 Adjustment Method

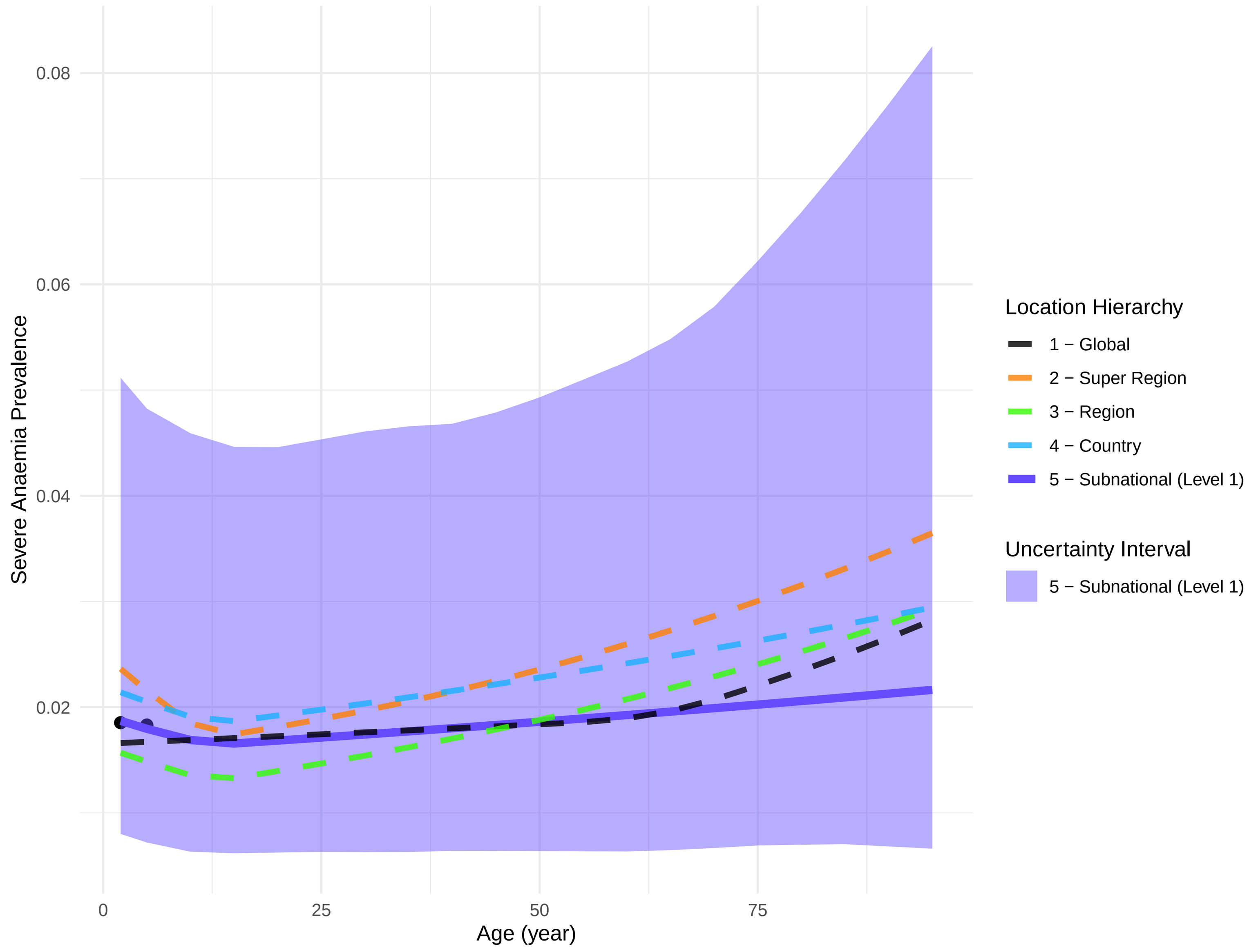

MR−BRT Age/Sex Fits − Non−pregnant Females 2+ Years Old − Severe Anaemia Prevalence − Kilifi
WHO−2001 Adjustment Method

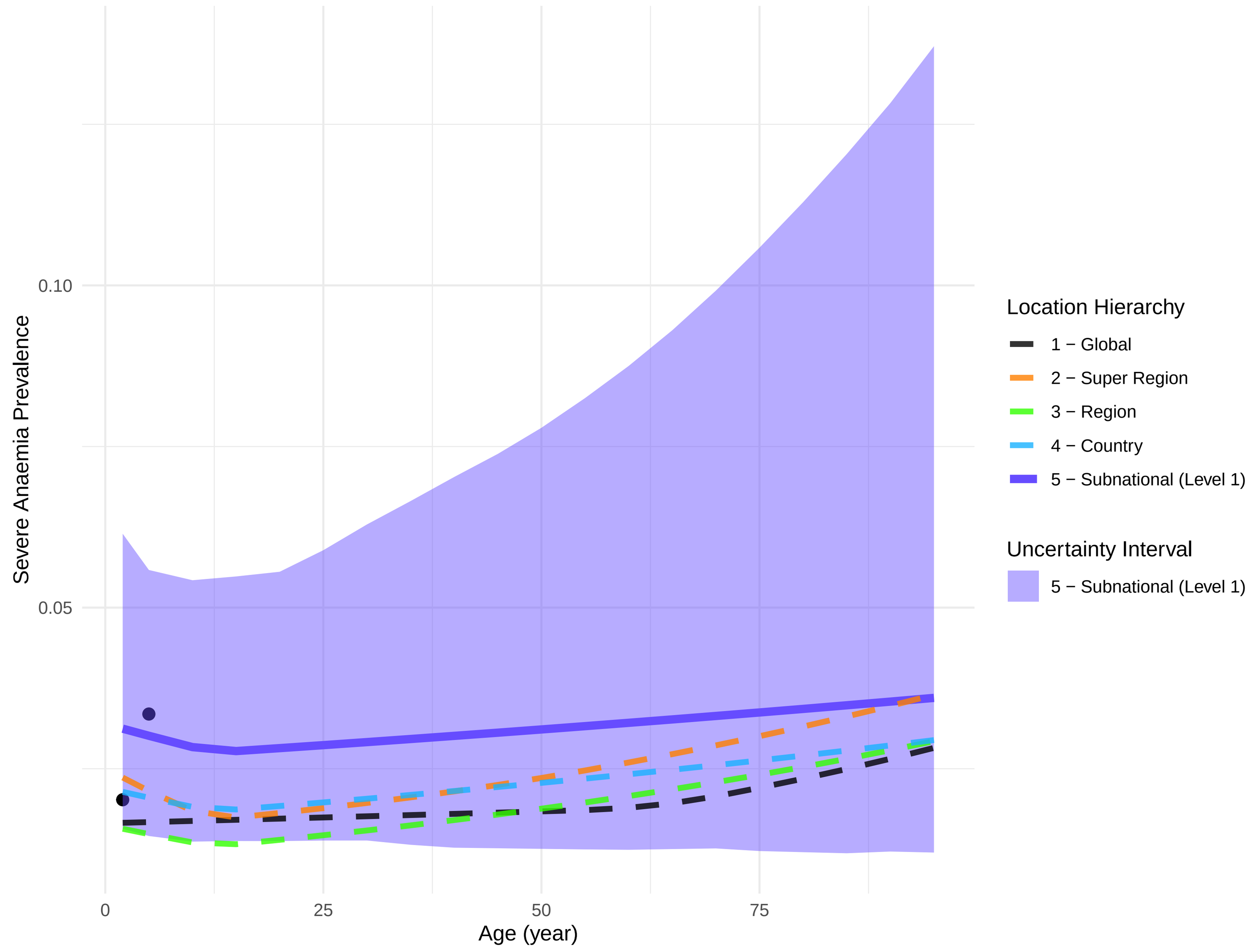

MR−BRT Age/Sex Fits − Non−pregnant Females 2+ Years Old − Severe Anaemia Prevalence − Kisumu
WHO−2001 Adjustment Method

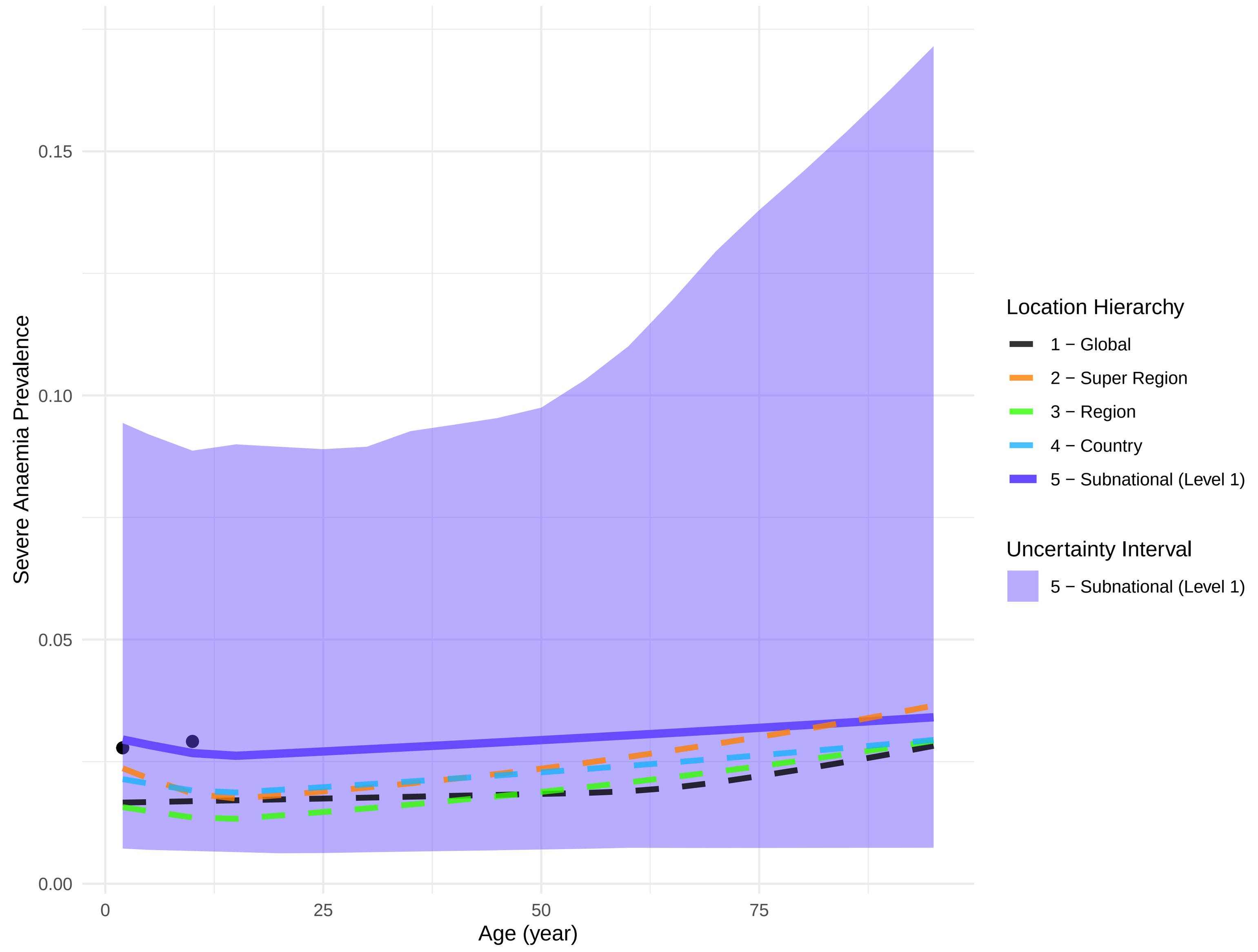

MR−BRT Age/Sex Fits − Non−pregnant Females 2+ Years Old − Severe Anaemia Prevalence − Kwale
WHO−2001 Adjustment Method

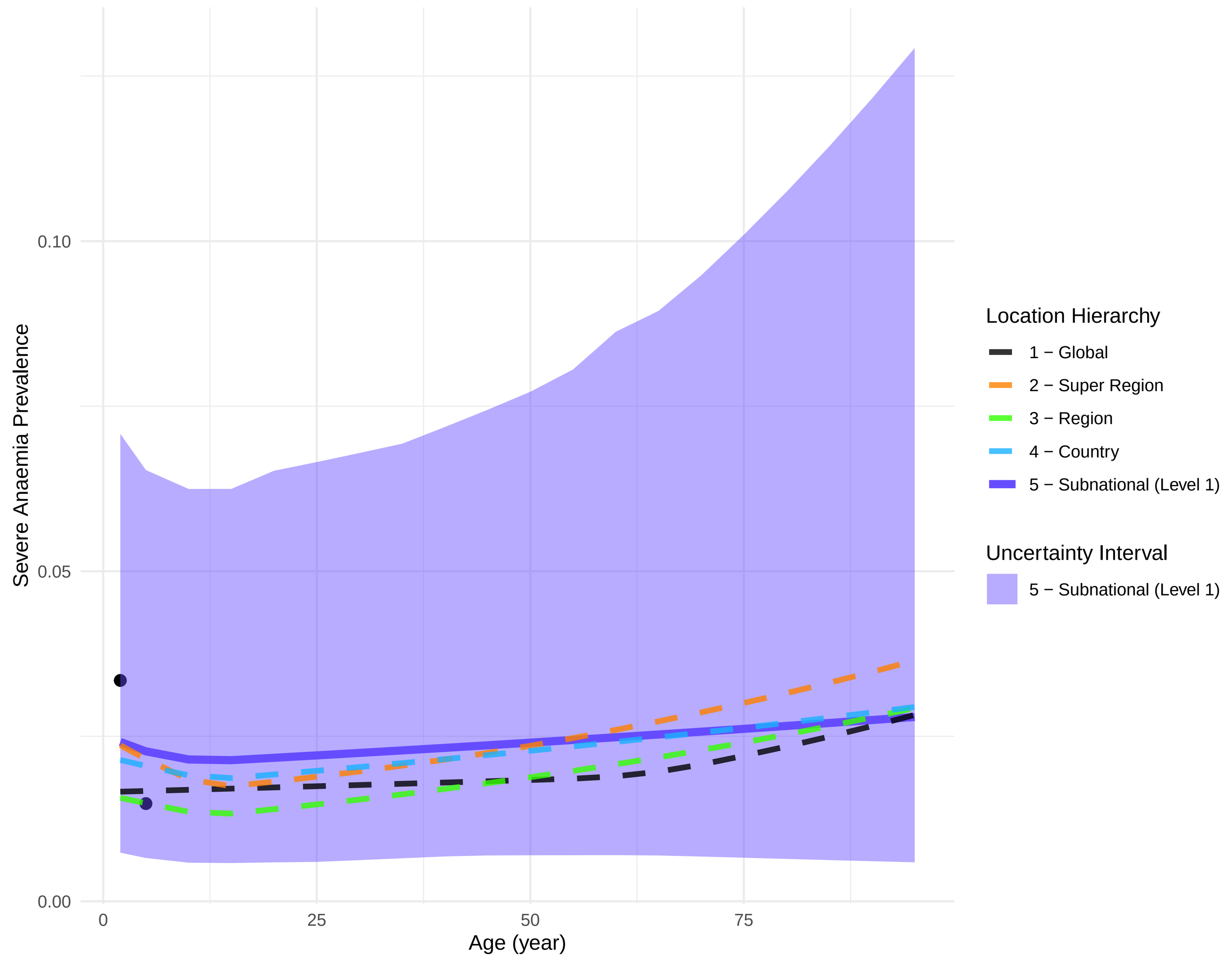

MR−BRT Age/Sex Fits − Non−pregnant Females 2+ Years Old − Severe Anaemia Prevalence − Lamu
WHO−2001 Adjustment Method

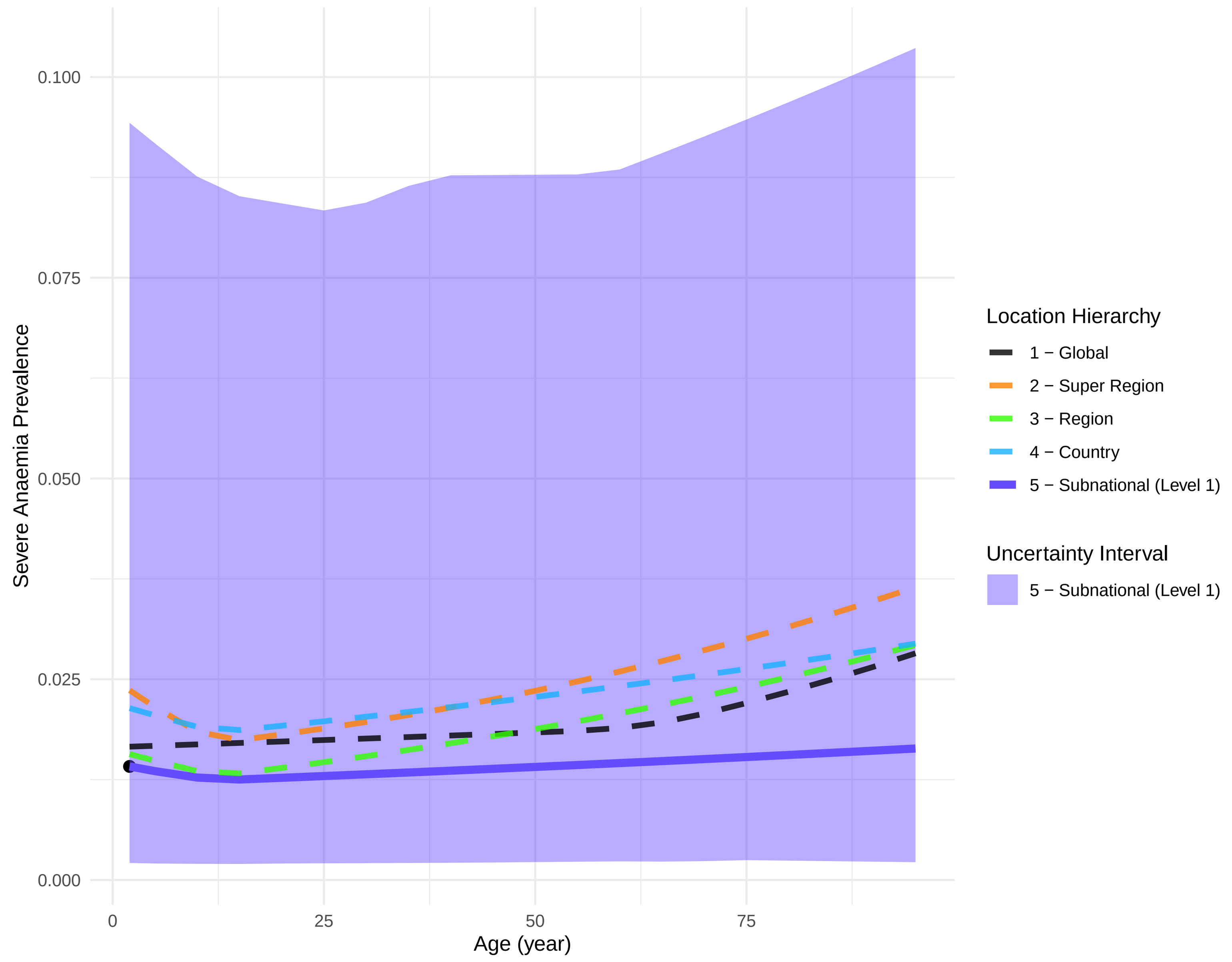

MR−BRT Age/Sex Fits − Non−pregnant Females 2+ Years Old − Severe Anaemia Prevalence − Machakos
WHO−2001 Adjustment Method

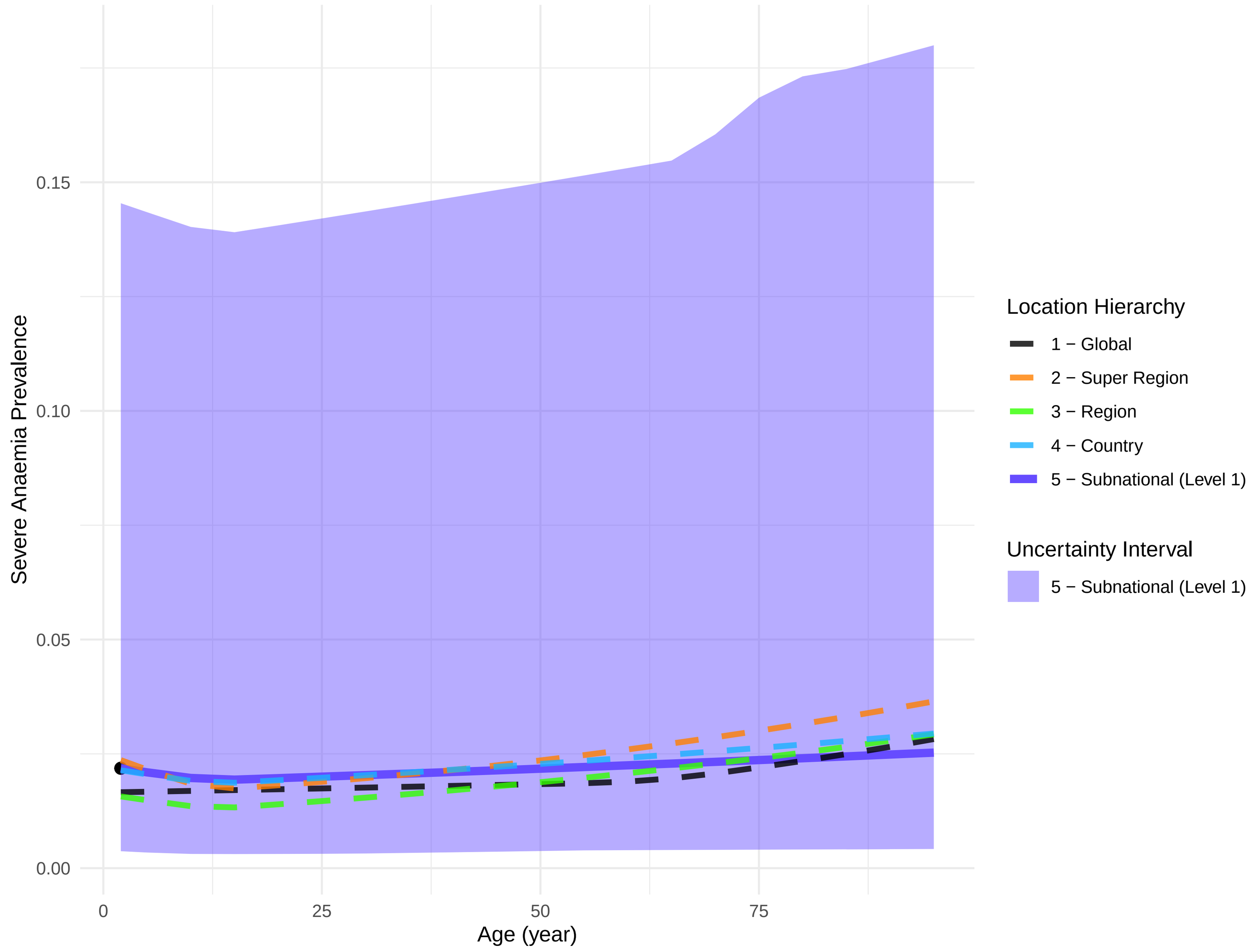

MR−BRT Age/Sex Fits − Non−pregnant Females 2+ Years Old − Severe Anaemia Prevalence − Mandera
WHO−2001 Adjustment Method

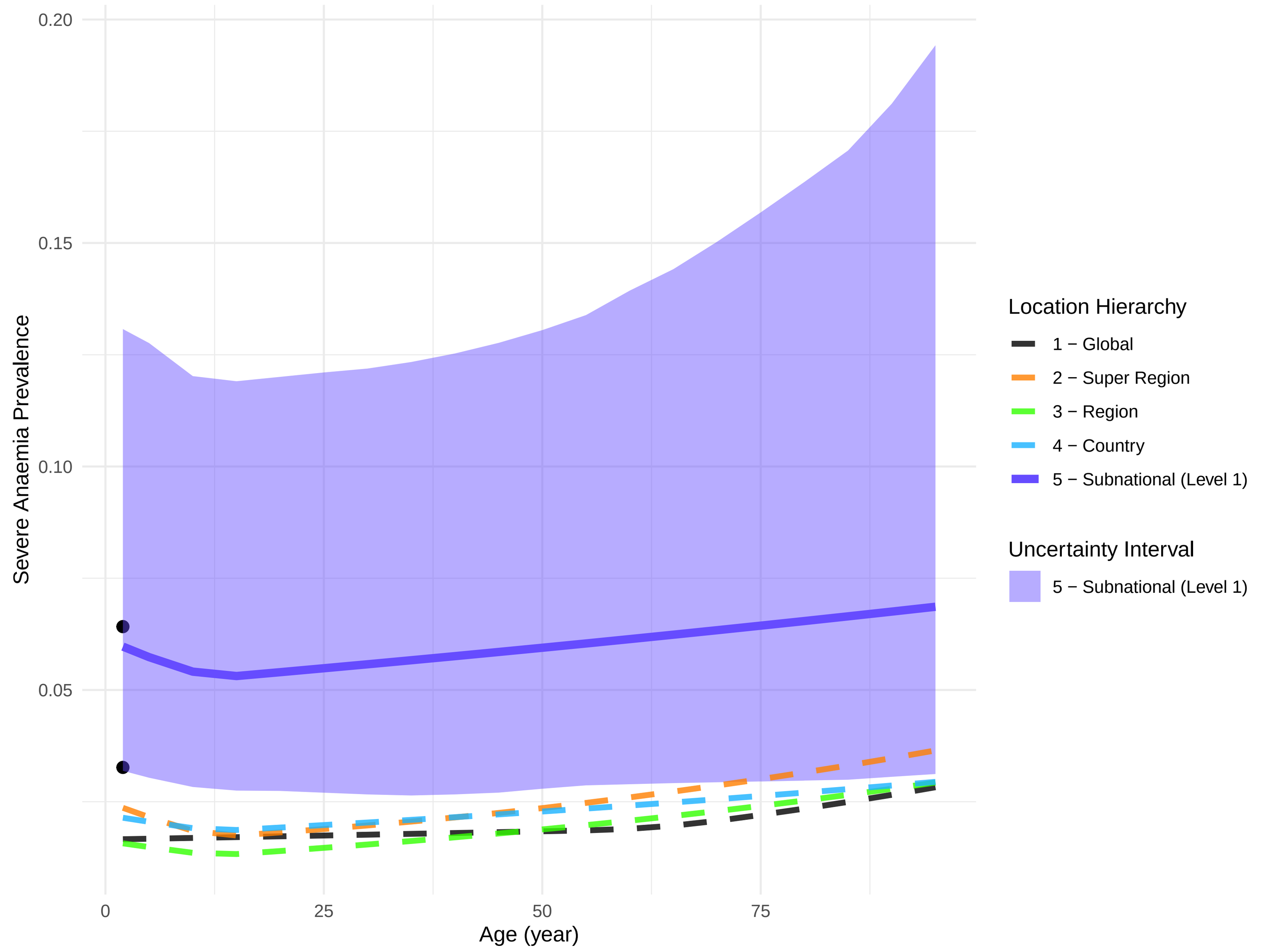

MR−BRT Age/Sex Fits − Non−pregnant Females 2+ Years Old − Severe Anaemia Prevalence − Marsabit
WHO−2001 Adjustment Method

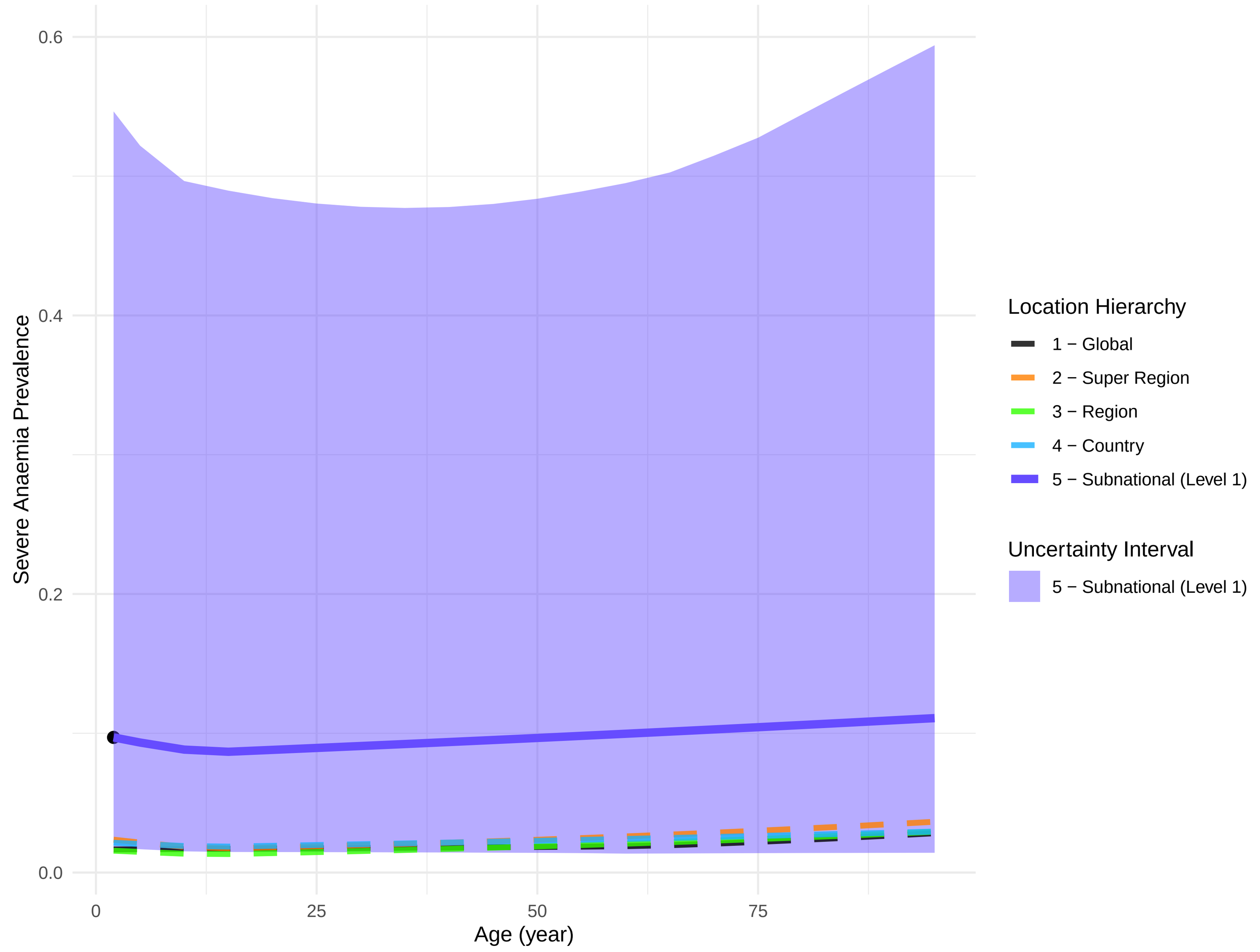

MR−BRT Age/Sex Fits − Non−pregnant Females 2+ Years Old − Severe Anaemia Prevalence − Migori
WHO−2001 Adjustment Method

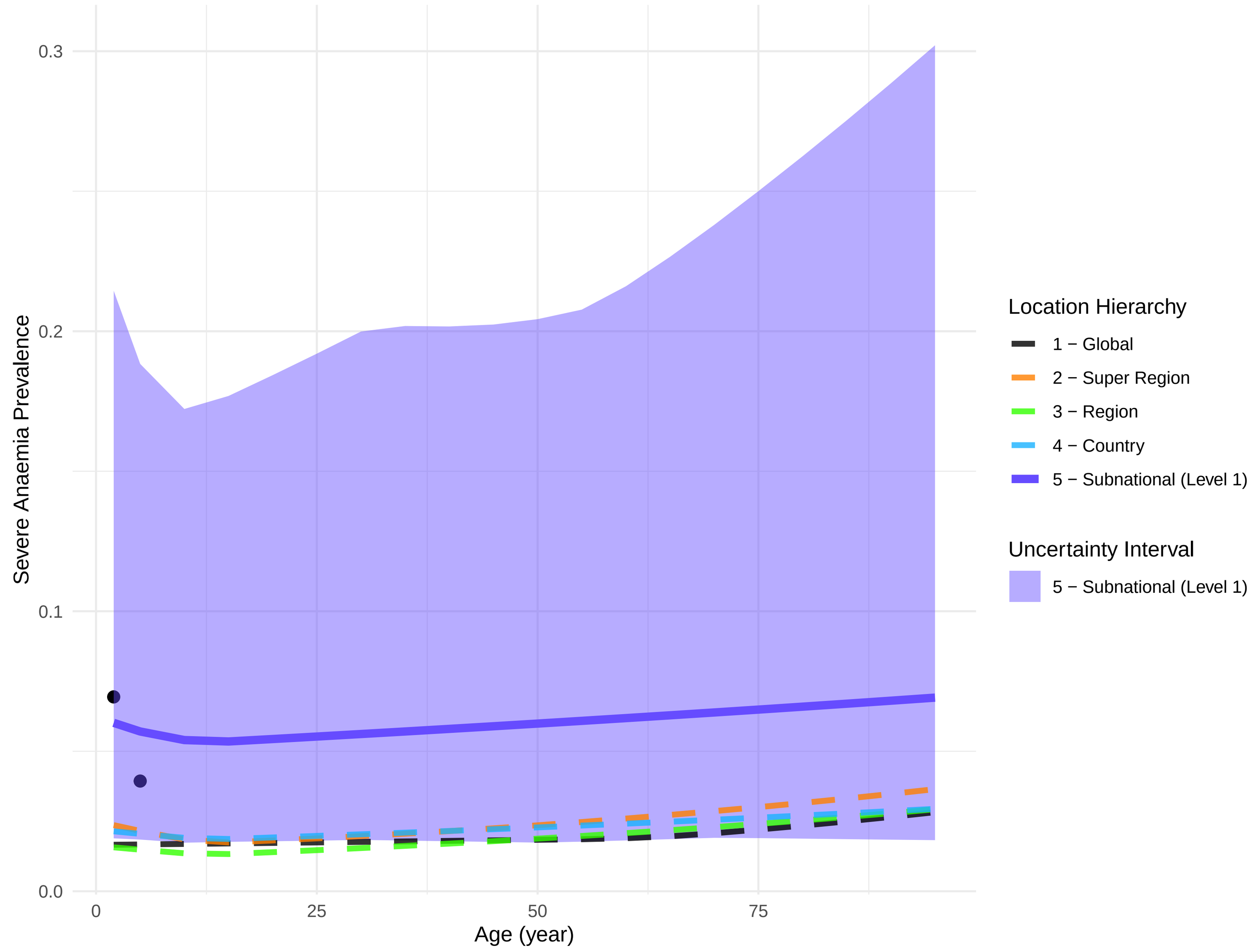

MR−BRT Age/Sex Fits − Non−pregnant Females 2+ Years Old − Severe Anaemia Prevalence − Mombasa
WHO−2001 Adjustment Method

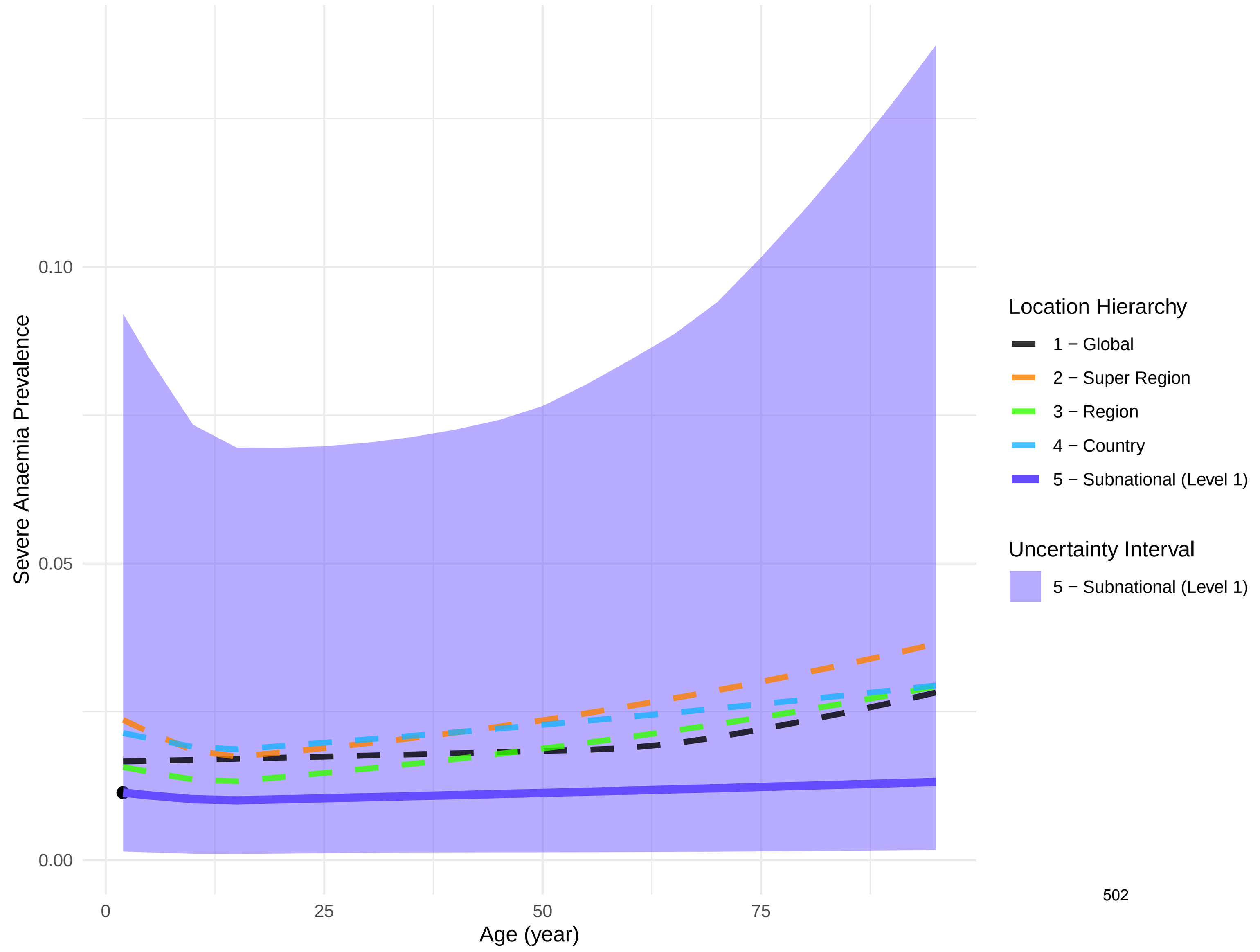

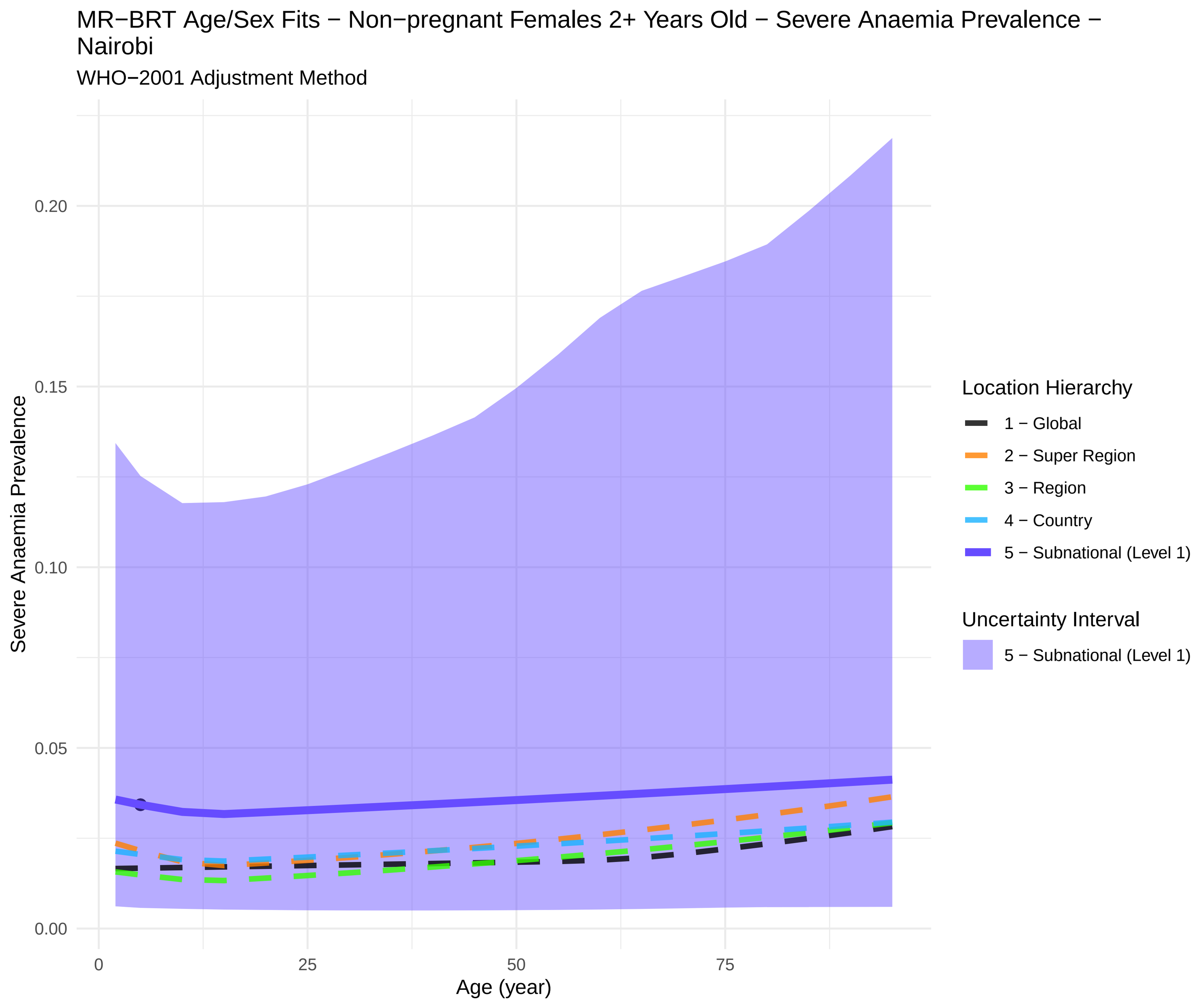
MR−BRT Age/Sex Fits − Non−pregnant Females 2+ Years Old − Severe Anaemia Prevalence − Nairobi
WHO−2001 Adjustment Method
Severe Anaemia Prevalence
Age (year)
Location Hierarchy
1 − Global
2 − Super Region
3 − Region
4 − Country
5 − Subnational (Level 1)
Uncertainty Interval
5 − Subnational (Level 1)

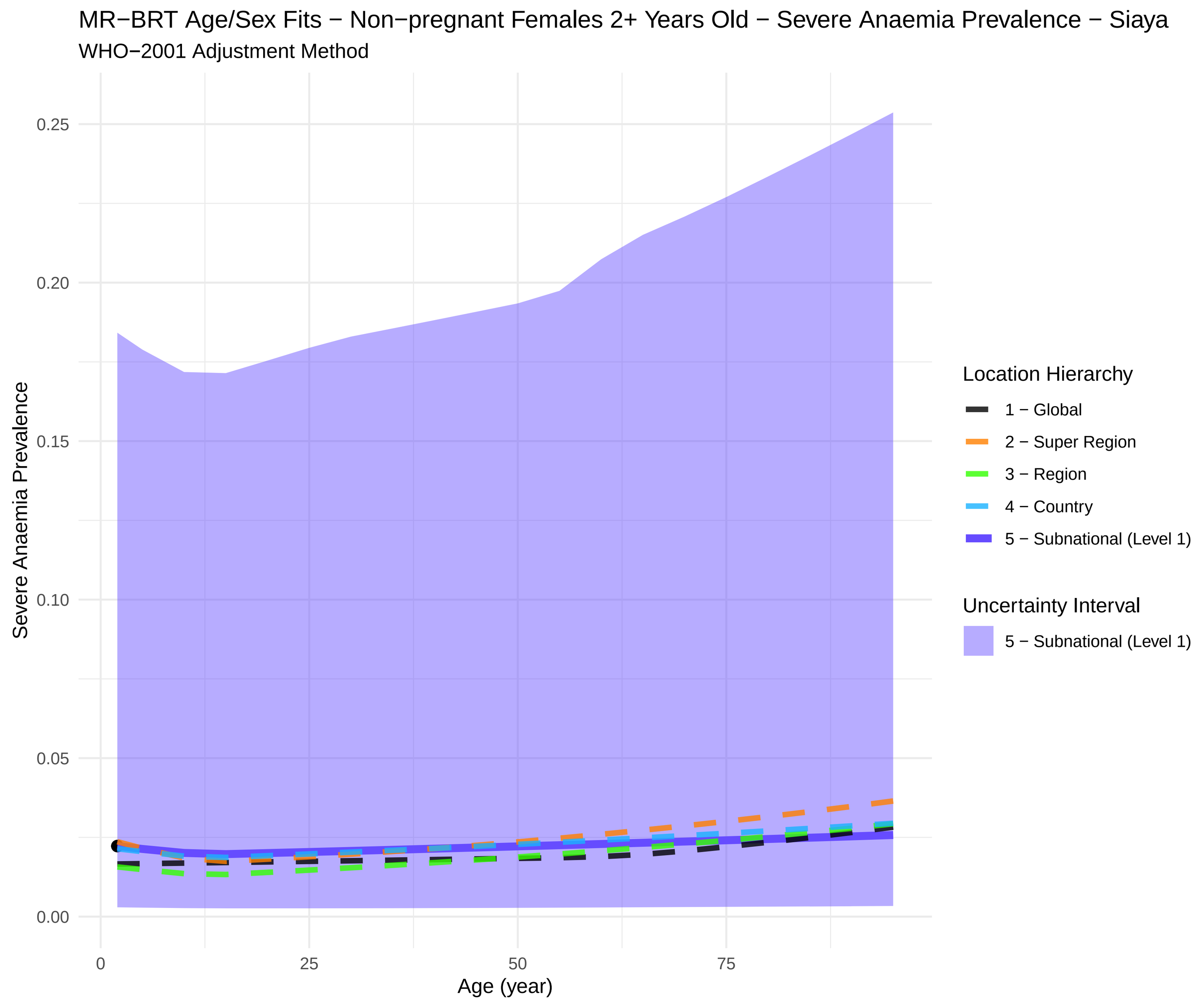
MR−BRT Age/Sex Fits − Non−pregnant Females 2+ Years Old − Severe Anaemia Prevalence − Siaya
WHO−2001 Adjustment Method
Severe Anaemia Prevalence
Age (year)
Location Hierarchy
1 − Global
2 − Super Region
3 − Region
4 − Country
5 − Subnational (Level 1)
Uncertainty Interval
5 − Subnational (Level 1)

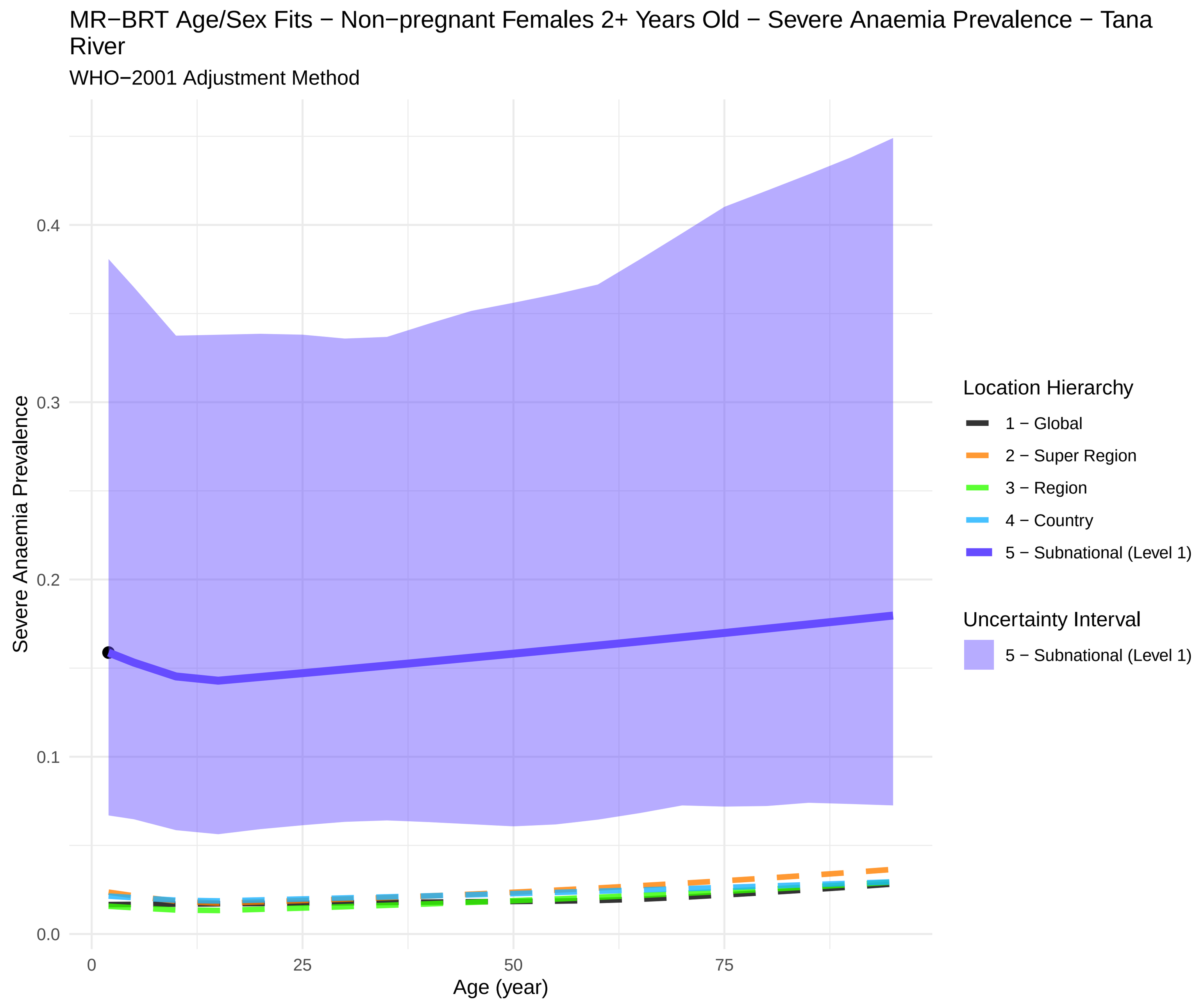
MR−BRT Age/Sex Fits − Non−pregnant Females 2+ Years Old − Severe Anaemia Prevalence − Tana River
WHO−2001 Adjustment Method
Severe Anaemia Prevalence
Age (year)
Location Hierarchy
1 − Global
2 − Super Region
3 − Region
4 − Country
5 − Subnational (Level 1)
Uncertainty Interval
5 − Subnational (Level 1)

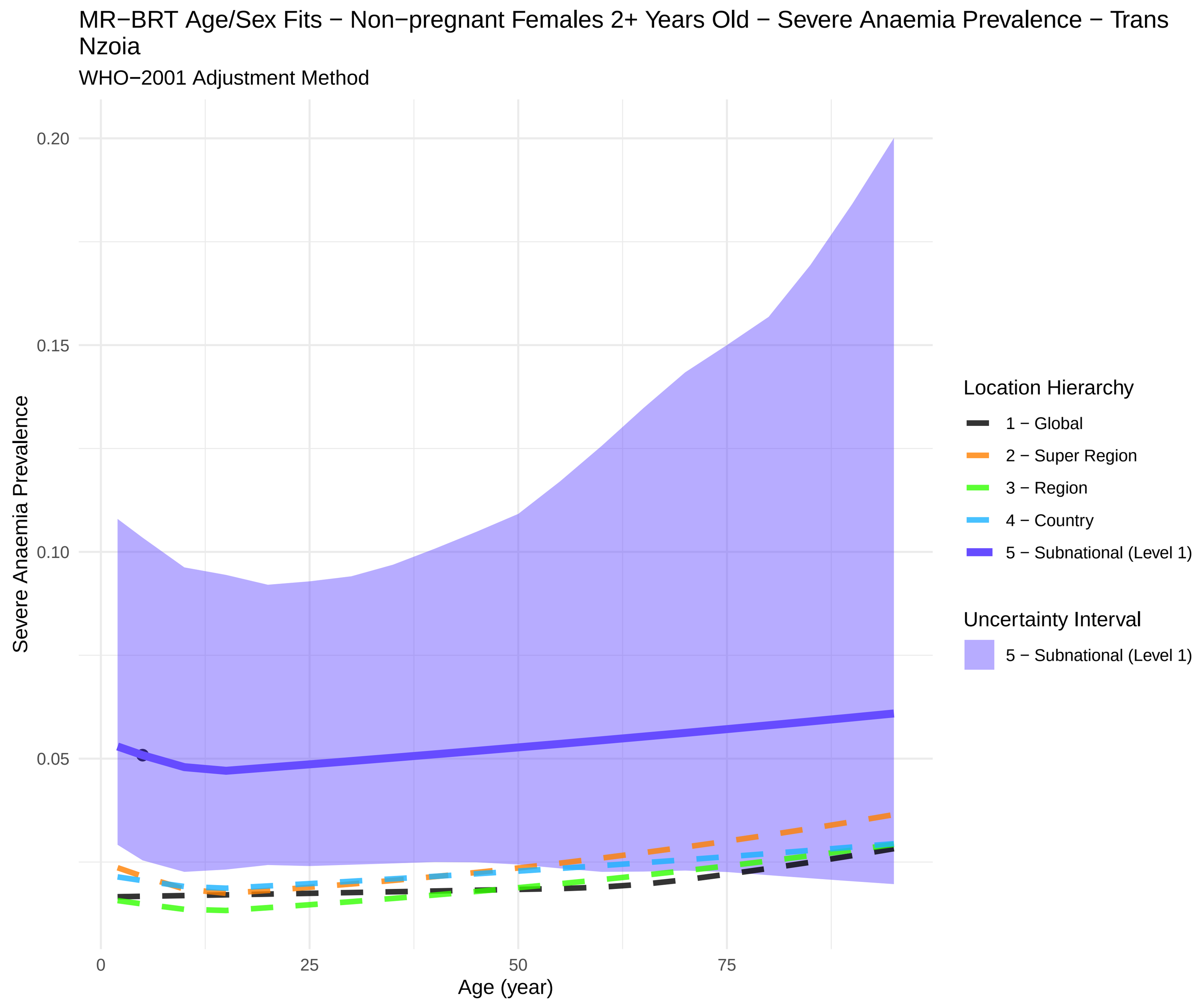
MR−BRT Age/Sex Fits − Non−pregnant Females 2+ Years Old − Severe Anaemia Prevalence − Trans Nzoia
WHO−2001 Adjustment Method
Severe Anaemia Prevalence
Age (year)
Location Hierarchy
1 − Global
2 − Super Region
3 − Region
4 − Country
5 − Subnational (Level 1)
Uncertainty Interval
5 − Subnational (Level 1)

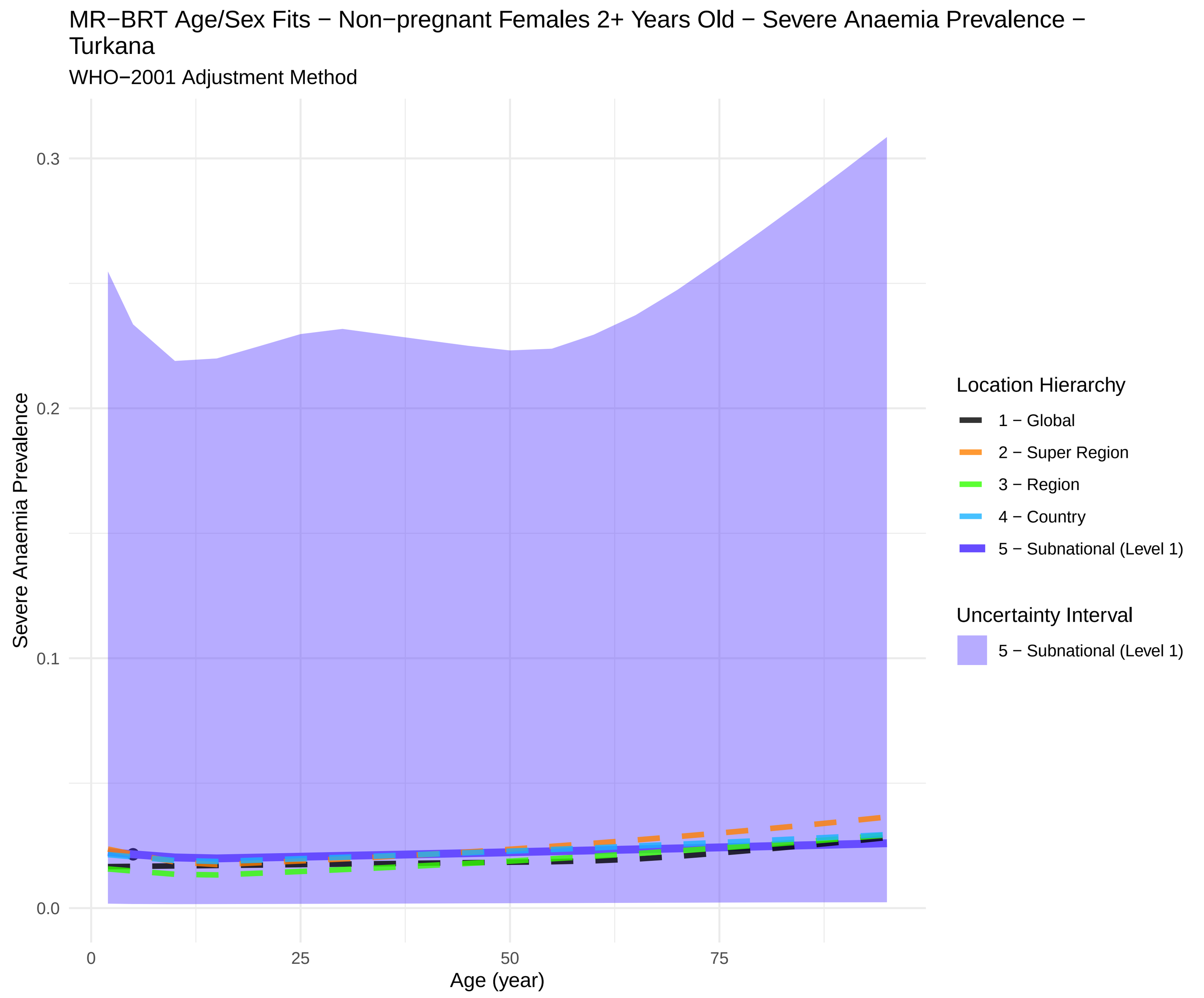
MR−BRT Age/Sex Fits − Non−pregnant Females 2+ Years Old − Severe Anaemia Prevalence − Turkana
WHO−2001 Adjustment Method
Severe Anaemia Prevalence
Age (year)
Location Hierarchy
1 − Global
2 − Super Region
3 − Region
4 − Country
5 − Subnational (Level 1)
Uncertainty Interval
5 − Subnational (Level 1)

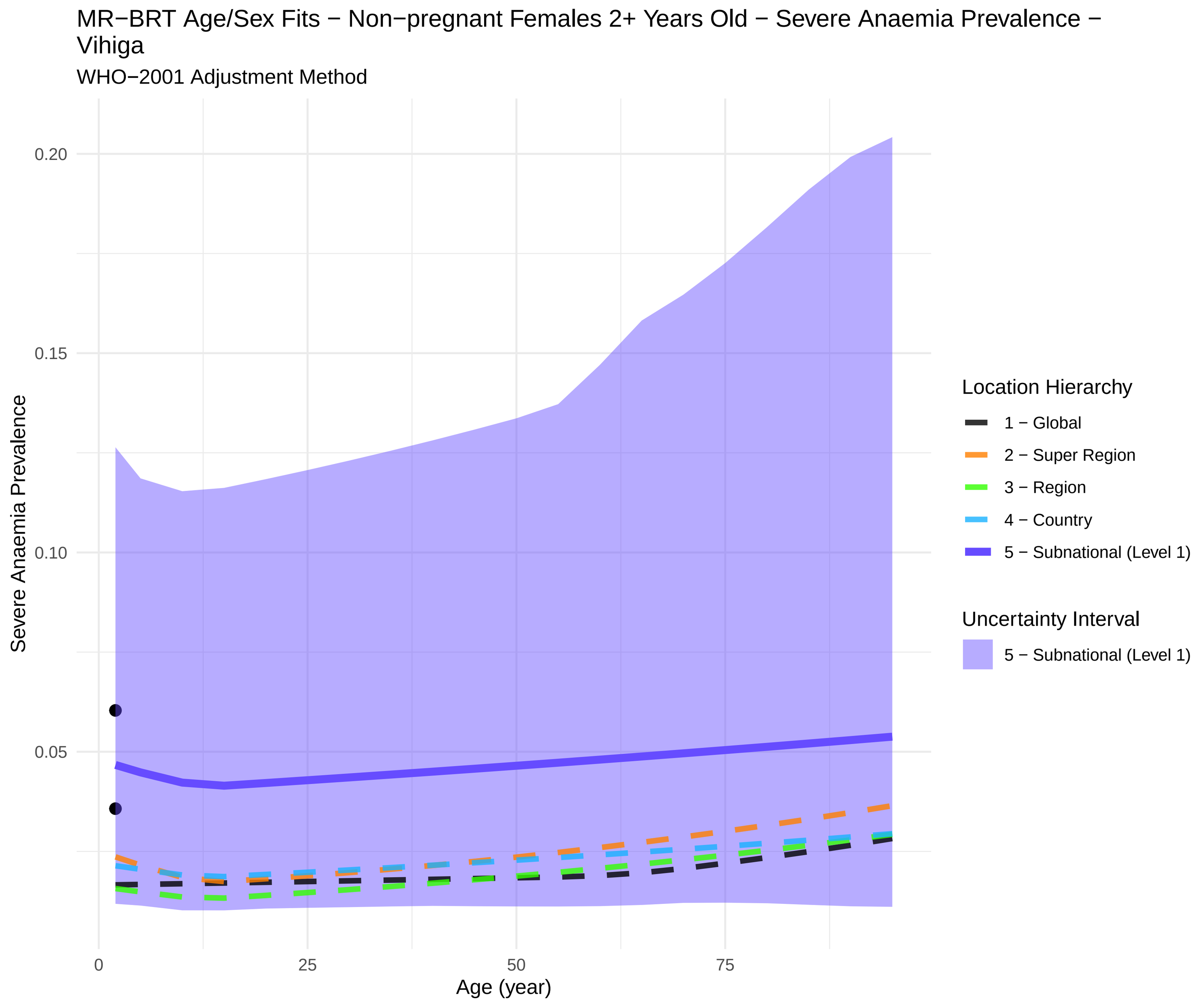
MR−BRT Age/Sex Fits − Non−pregnant Females 2+ Years Old − Severe Anaemia Prevalence − Vihiga
WHO−2001 Adjustment Method
Severe Anaemia Prevalence
Age (year)
Location Hierarchy
1 − Global
2 − Super Region
3 − Region
4 − Country
5 − Subnational (Level 1)
Uncertainty Interval
5 − Subnational (Level 1)

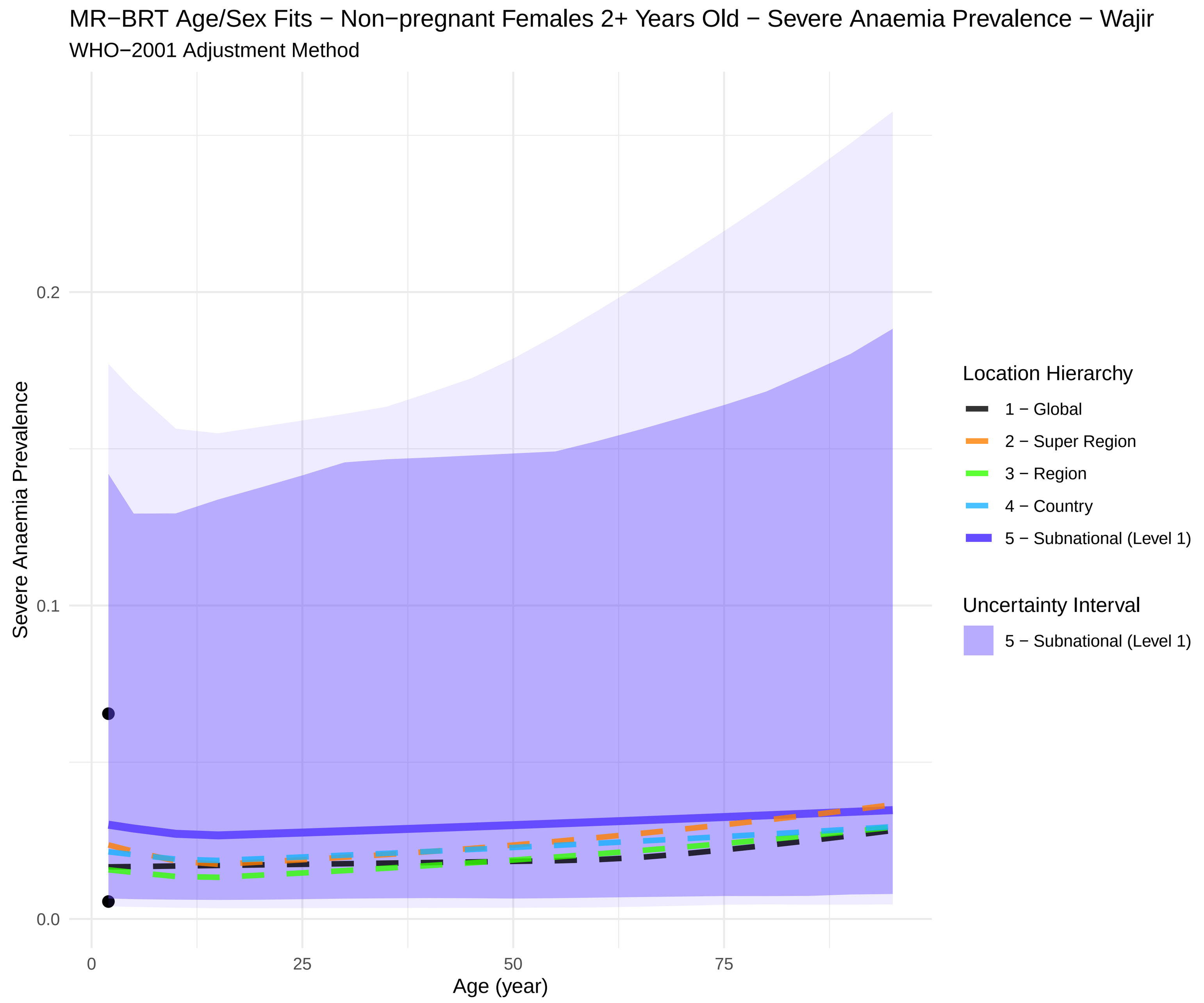
MR−BRT Age/Sex Fits − Non−pregnant Females 2+ Years Old − Severe Anaemia Prevalence − Wajir
WHO−2001 Adjustment Method
Severe Anaemia Prevalence
Age (year)
Location Hierarchy
1 − Global
2 − Super Region
3 − Region
4 − Country
5 − Subnational (Level 1)
Uncertainty Interval
5 − Subnational (Level 1)

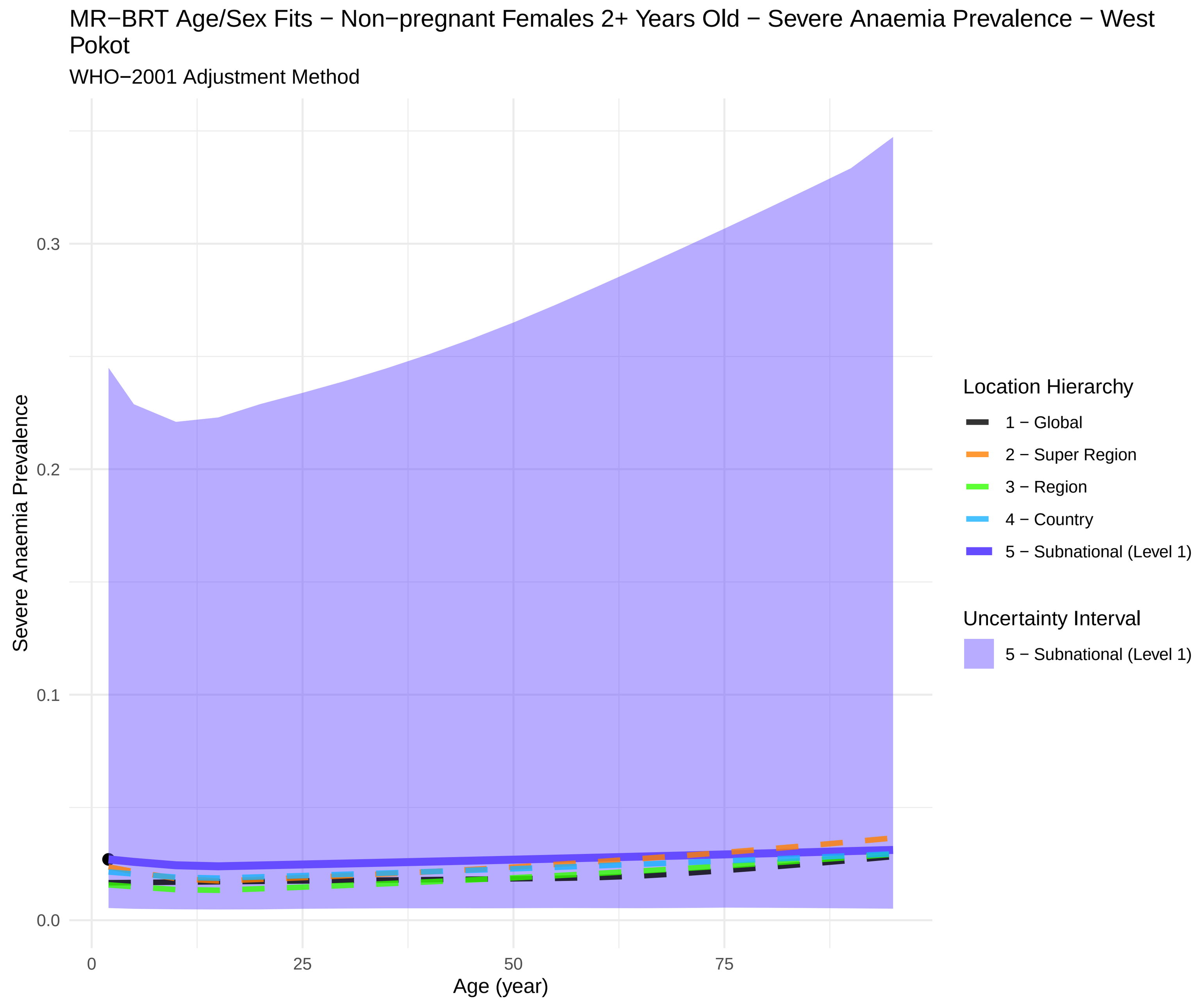
MR−BRT Age/Sex Fits − Non−pregnant Females 2+ Years Old − Severe Anaemia Prevalence − West Pokot
WHO−2001 Adjustment Method
Severe Anaemia Prevalence
Age (year)
Location Hierarchy
1 − Global
2 − Super Region
3 − Region
4 − Country
5 − Subnational (Level 1)
Uncertainty Interval
5 − Subnational (Level 1)

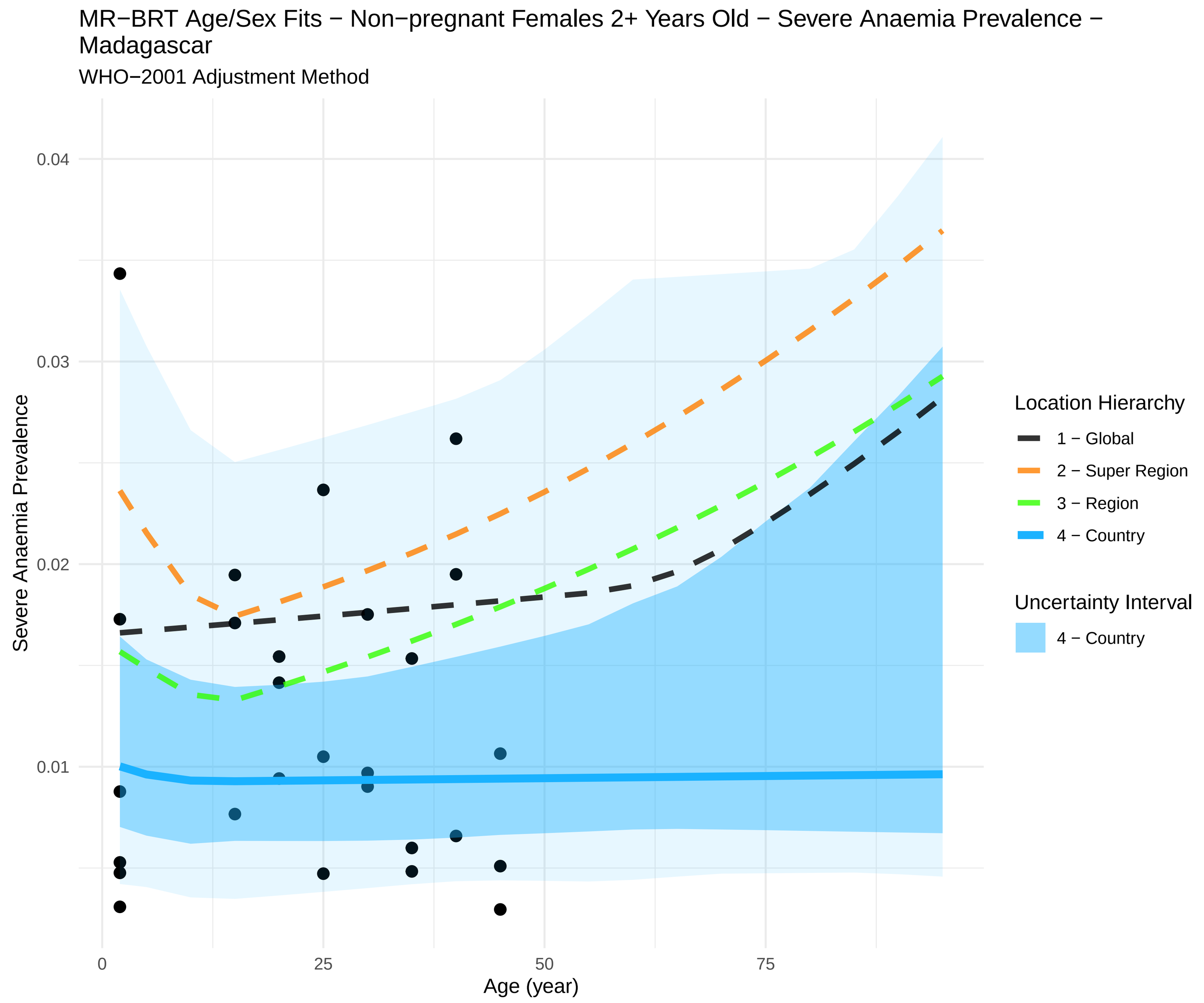
MR−BRT Age/Sex Fits − Non−pregnant Females 2+ Years Old − Severe Anaemia Prevalence − Madagascar
WHO−2001 Adjustment Method
Severe Anaemia Prevalence
Age (year)
Location Hierarchy
1 − Global
2 − Super Region
3 − Region
4 − Country
Uncertainty Interval
4 − Country

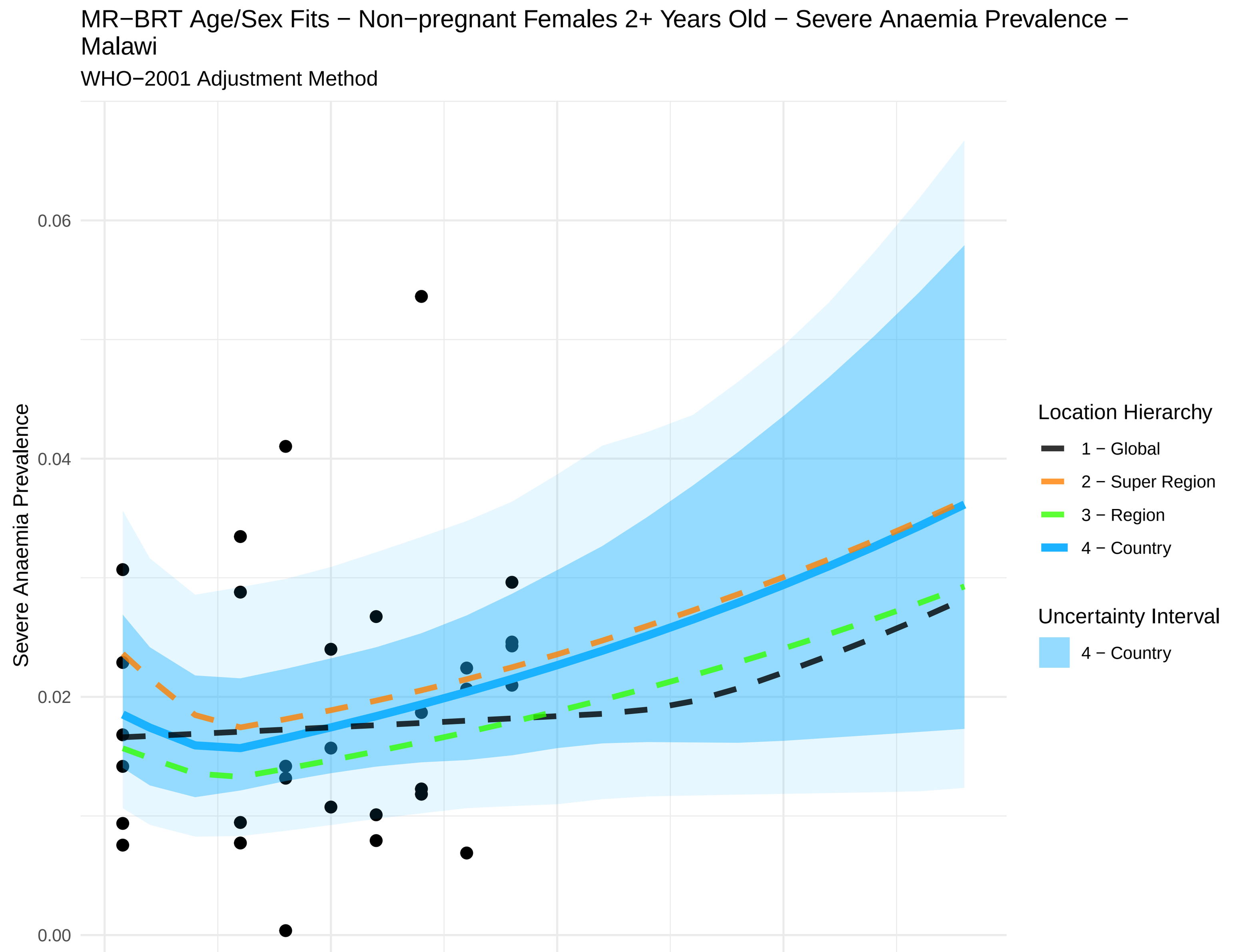
MR−BRT Age/Sex Fits − Non−pregnant Females 2+ Years Old − Severe Anaemia Prevalence − Malawi
WHO−2001 Adjustment Method
Severe Anaemia Prevalence
Age (year)
Location Hierarchy
1 − Global
2 − Super Region
3 − Region
4 − Country
Uncertainty Interval
4 − Country

MR−BRT Age/Sex Fits − Non−pregnant Females 2+ Years Old − Severe Anaemia Prevalence − Mozambique
WHO−2001 Adjustment Method

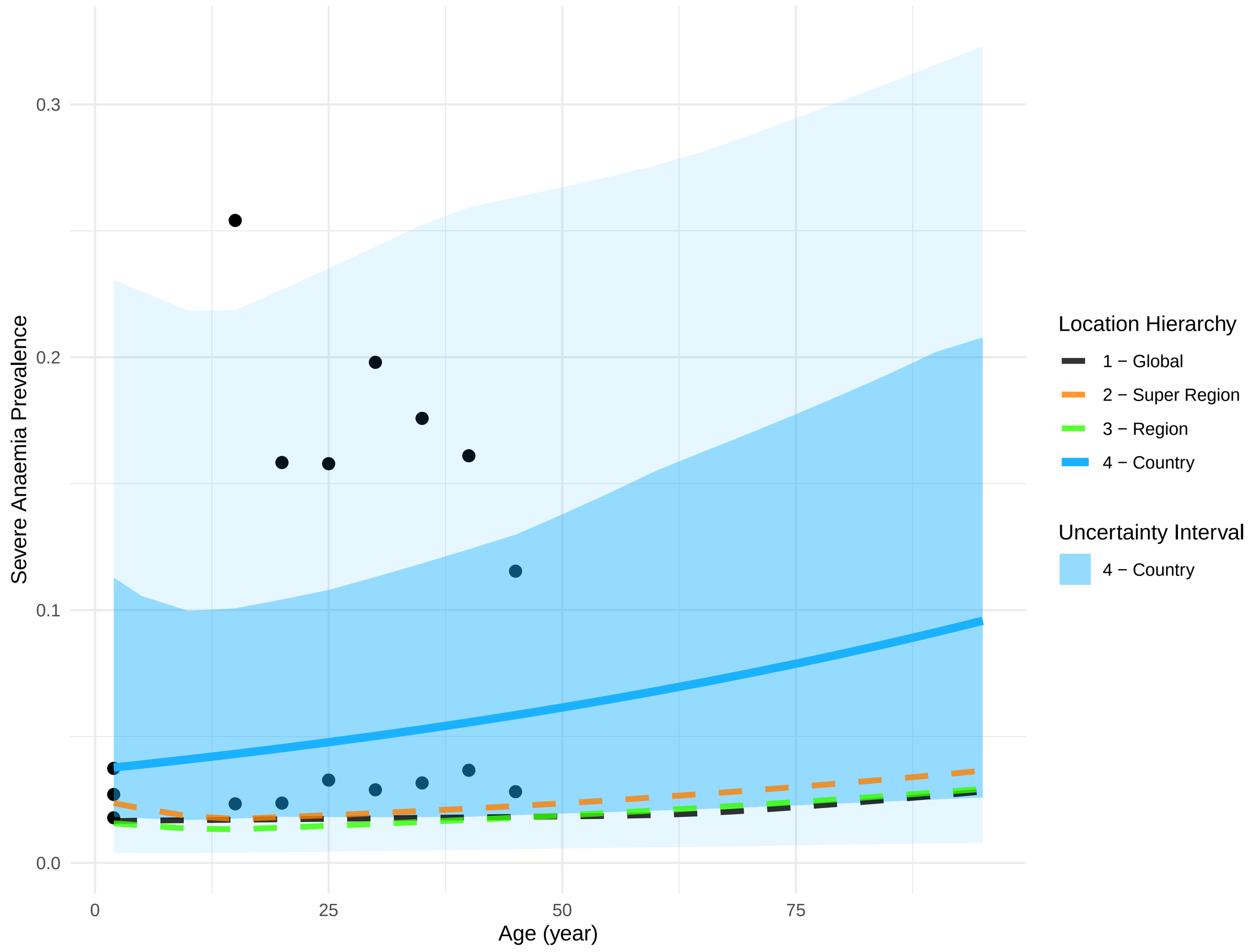

MR−BRT Age/Sex Fits − Non−pregnant Females 2+ Years Old − Severe Anaemia Prevalence − Rwanda
WHO−2001 Adjustment Method

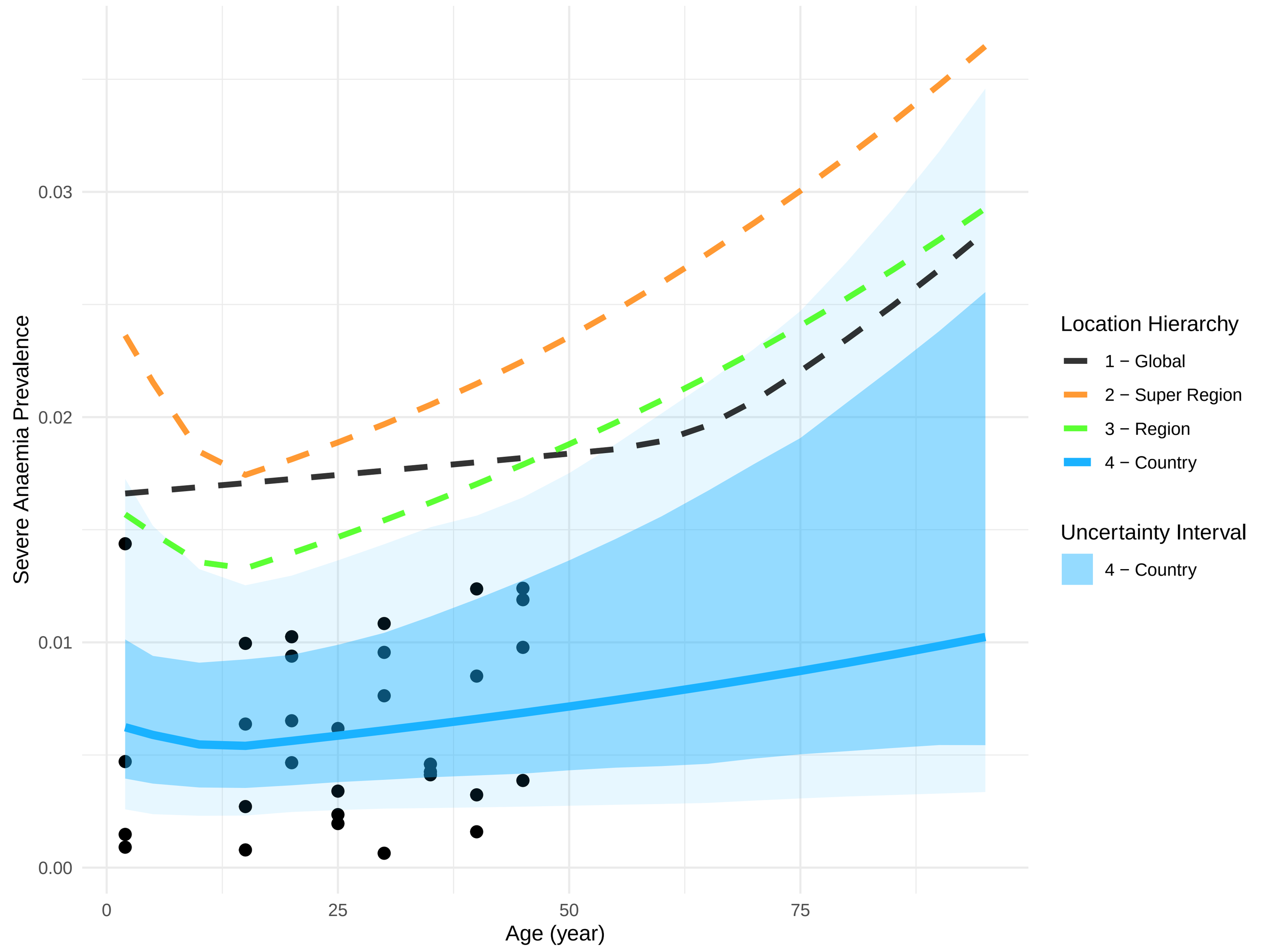

MR−BRT Age/Sex Fits − Non−pregnant Females 2+ Years Old − Severe Anaemia Prevalence − Uganda
WHO−2001 Adjustment Method

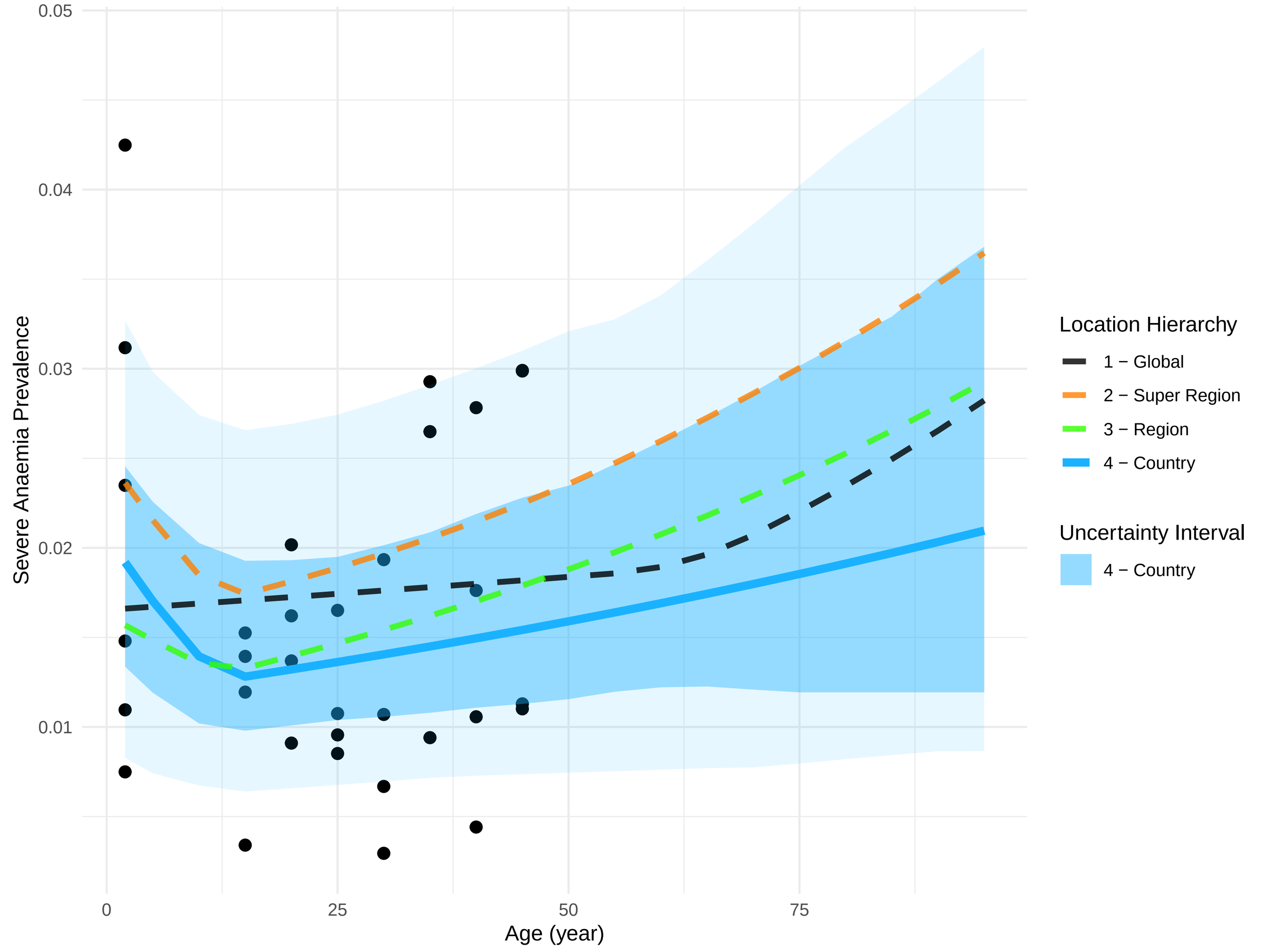

MR−BRT Age/Sex Fits − Non−pregnant Females 2+ Years Old − Severe Anaemia Prevalence − United Republic of Tanzania
WHO−2001 Adjustment Method

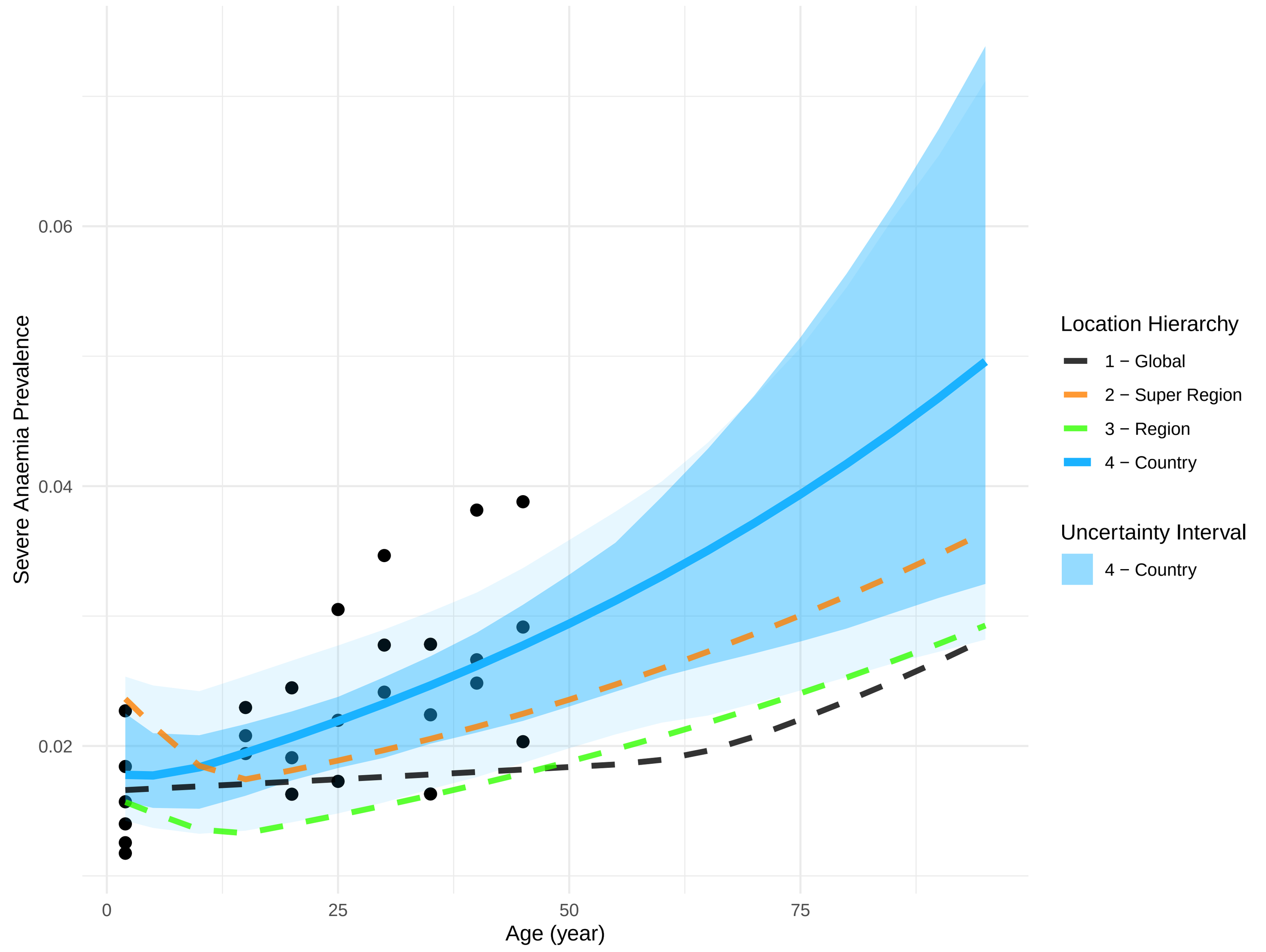

MR−BRT Age/Sex Fits − Non−pregnant Females 2+ Years Old − Severe Anaemia Prevalence − Zambia
WHO−2001 Adjustment Method

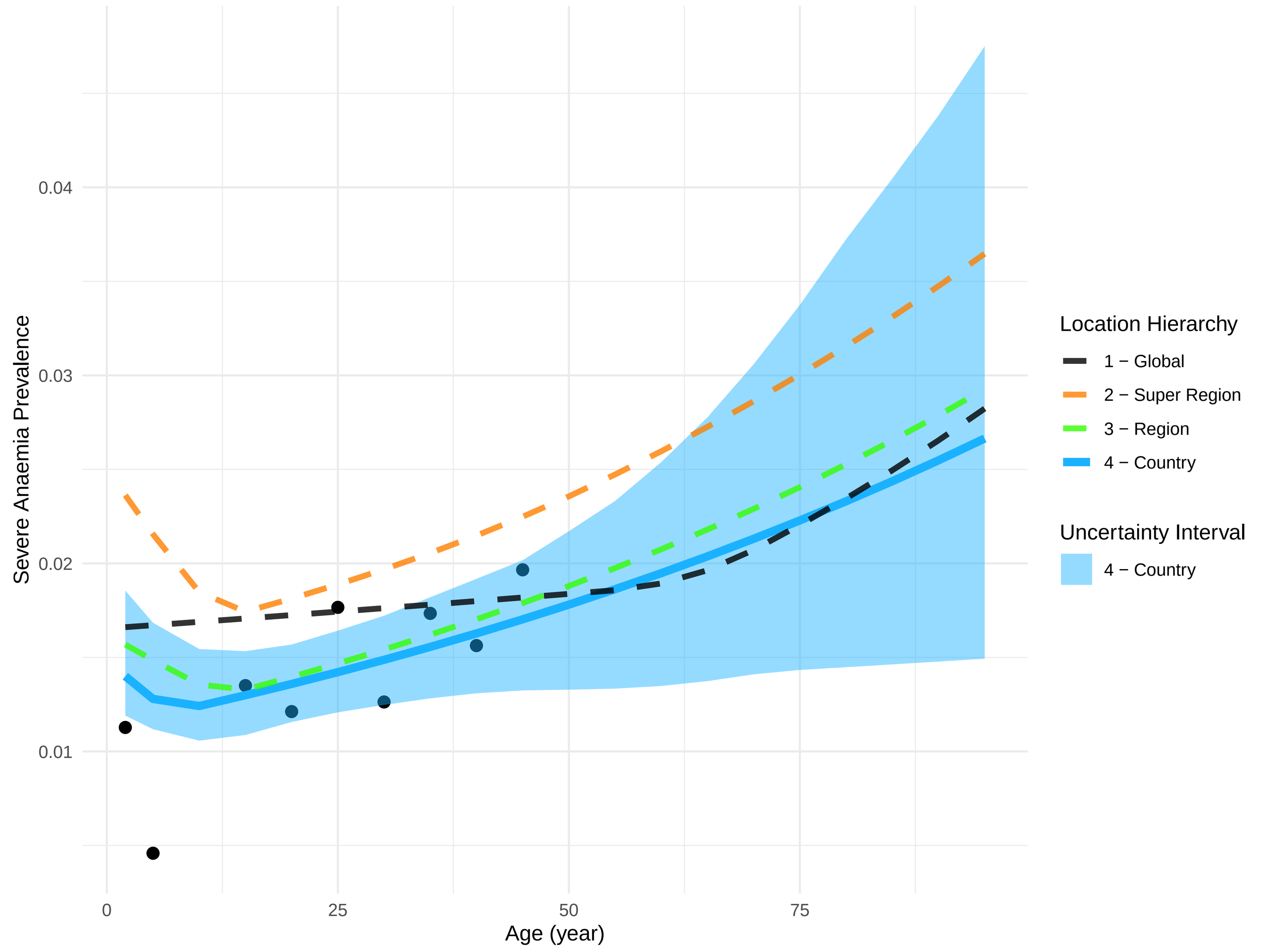

MR−BRT Age/Sex Fits − Non−pregnant Females 2+ Years Old − Severe Anaemia Prevalence − Southern Sub−Saharan Africa
WHO−2001 Adjustment Method

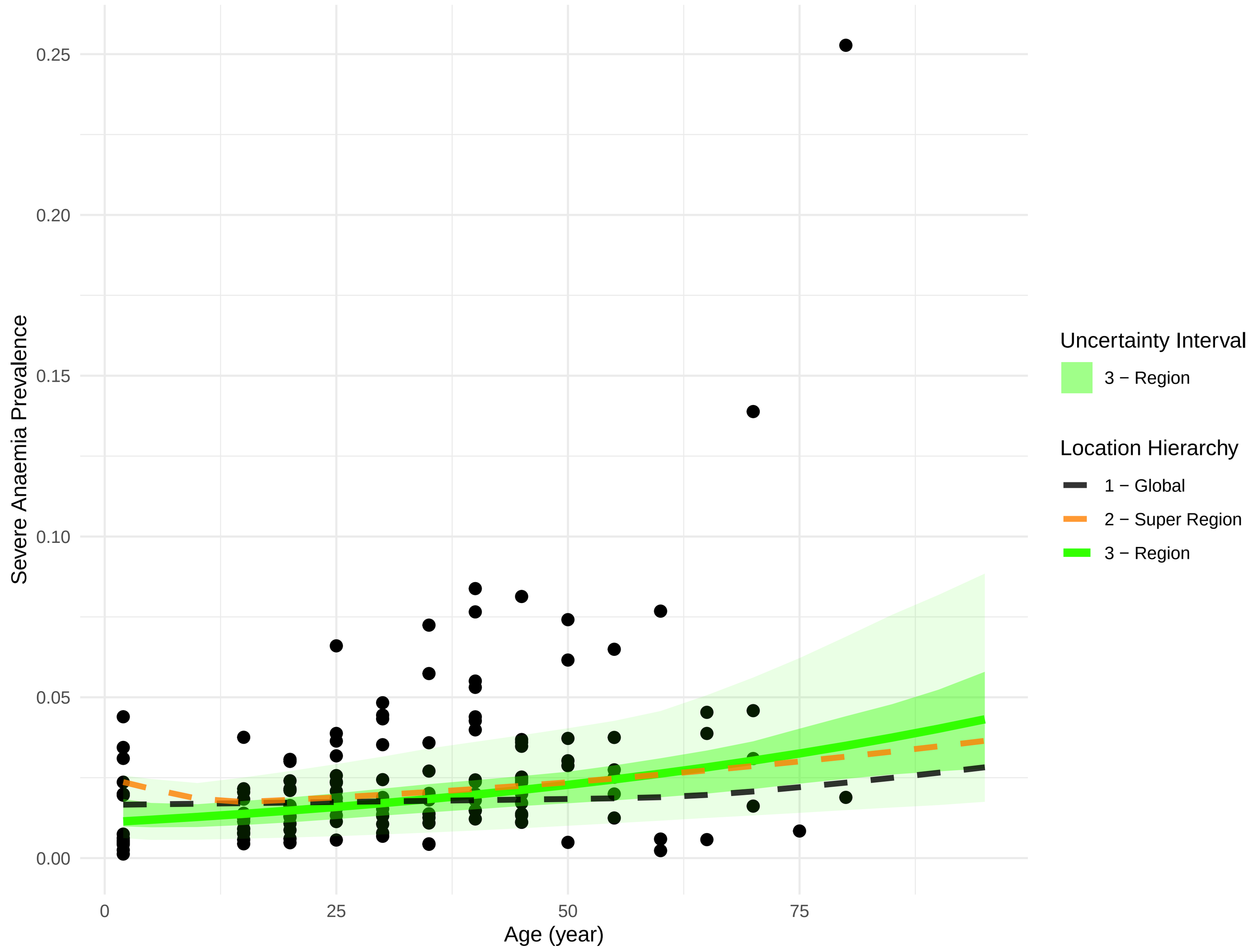

MR−BRT Age/Sex Fits − Non−pregnant Females 2+ Years Old − Severe Anaemia Prevalence − Eswatini
WHO−2001 Adjustment Method

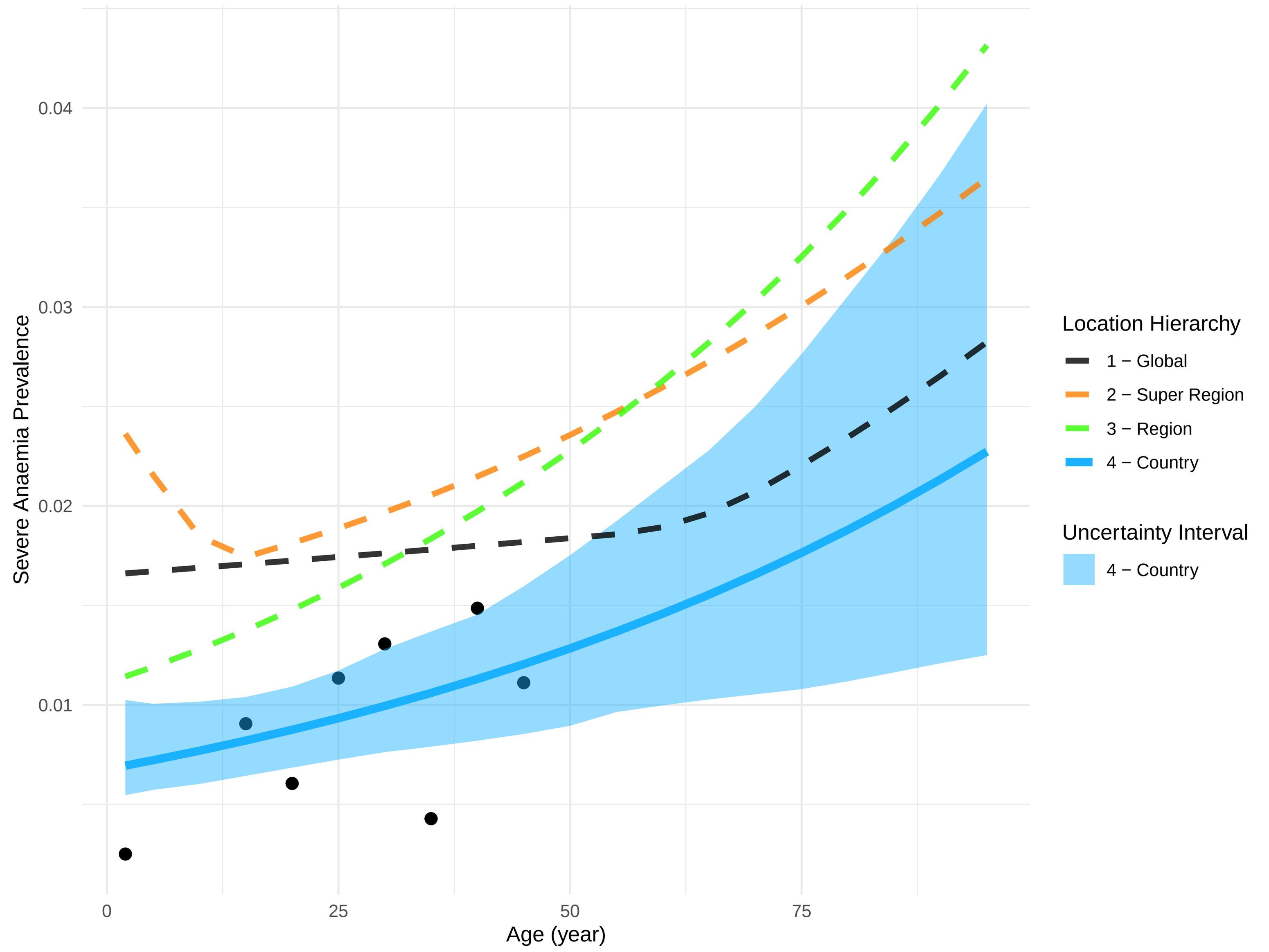

MR−BRT Age/Sex Fits − Non−pregnant Females 2+ Years Old − Severe Anaemia Prevalence − Lesotho
WHO−2001 Adjustment Method

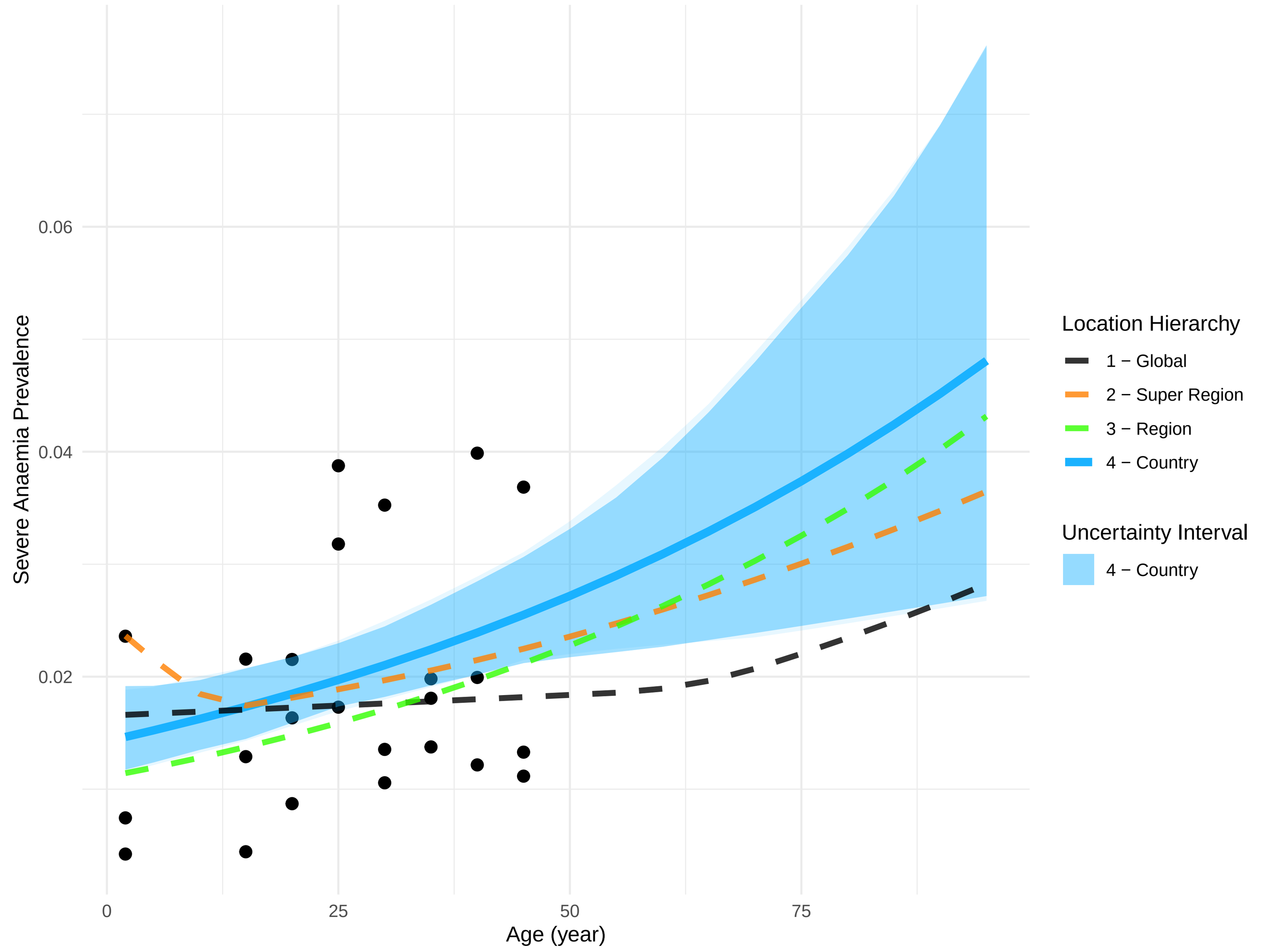

MR−BRT Age/Sex Fits − Non−pregnant Females 2+ Years Old − Severe Anaemia Prevalence − Namibia
WHO−2001 Adjustment Method

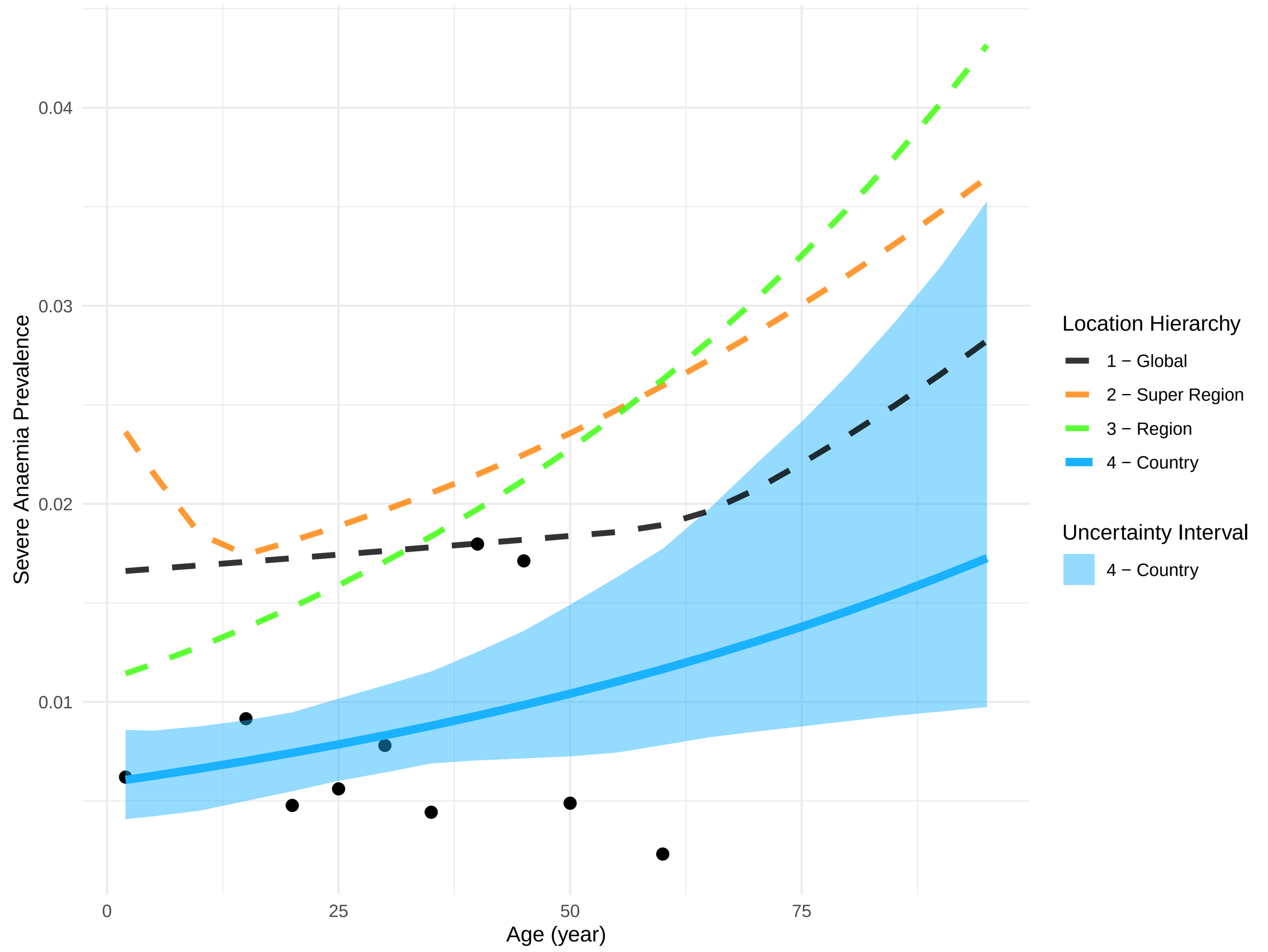

MR−BRT Age/Sex Fits − Non−pregnant Females 2+ Years Old − Severe Anaemia Prevalence − South Africa
WHO−2001 Adjustment Method

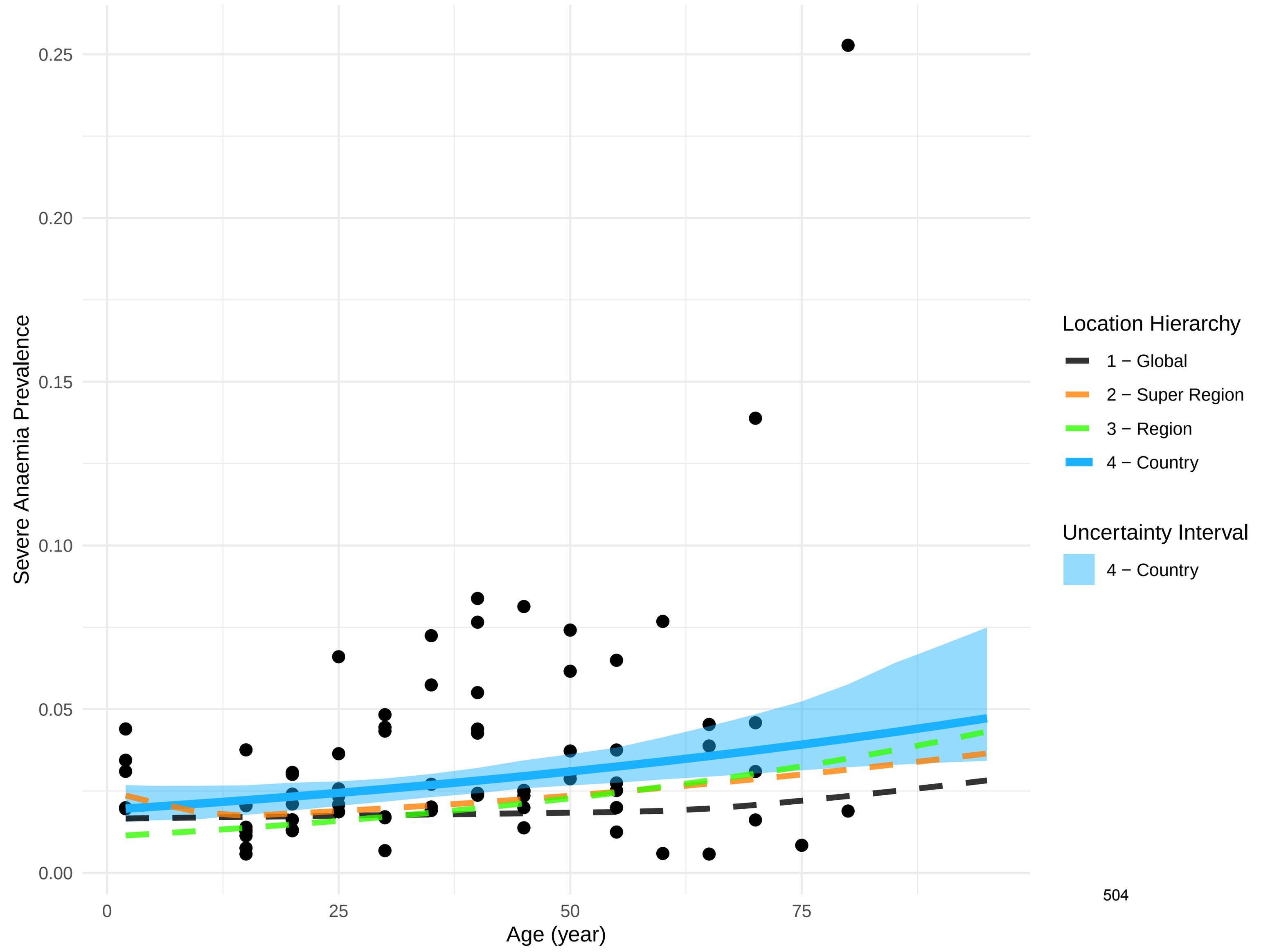

MR−BRT Age/Sex Fits − Non−pregnant Females 2+ Years Old − Severe Anaemia Prevalence − Eastern Cape
WHO−2001 Adjustment Method

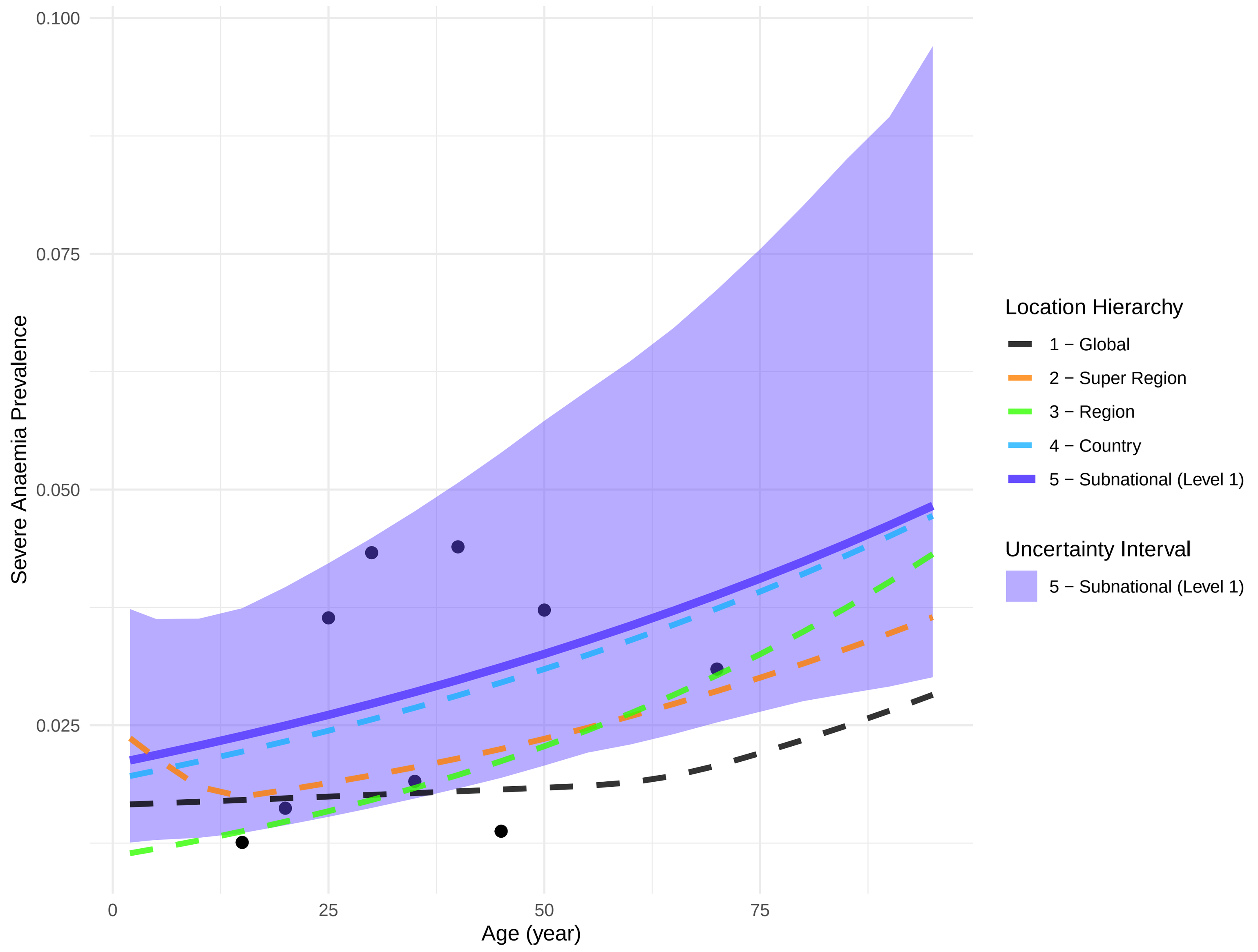

MR−BRT Age/Sex Fits − Non−pregnant Females 2+ Years Old − Severe Anaemia Prevalence − Free State
WHO−2001 Adjustment Method

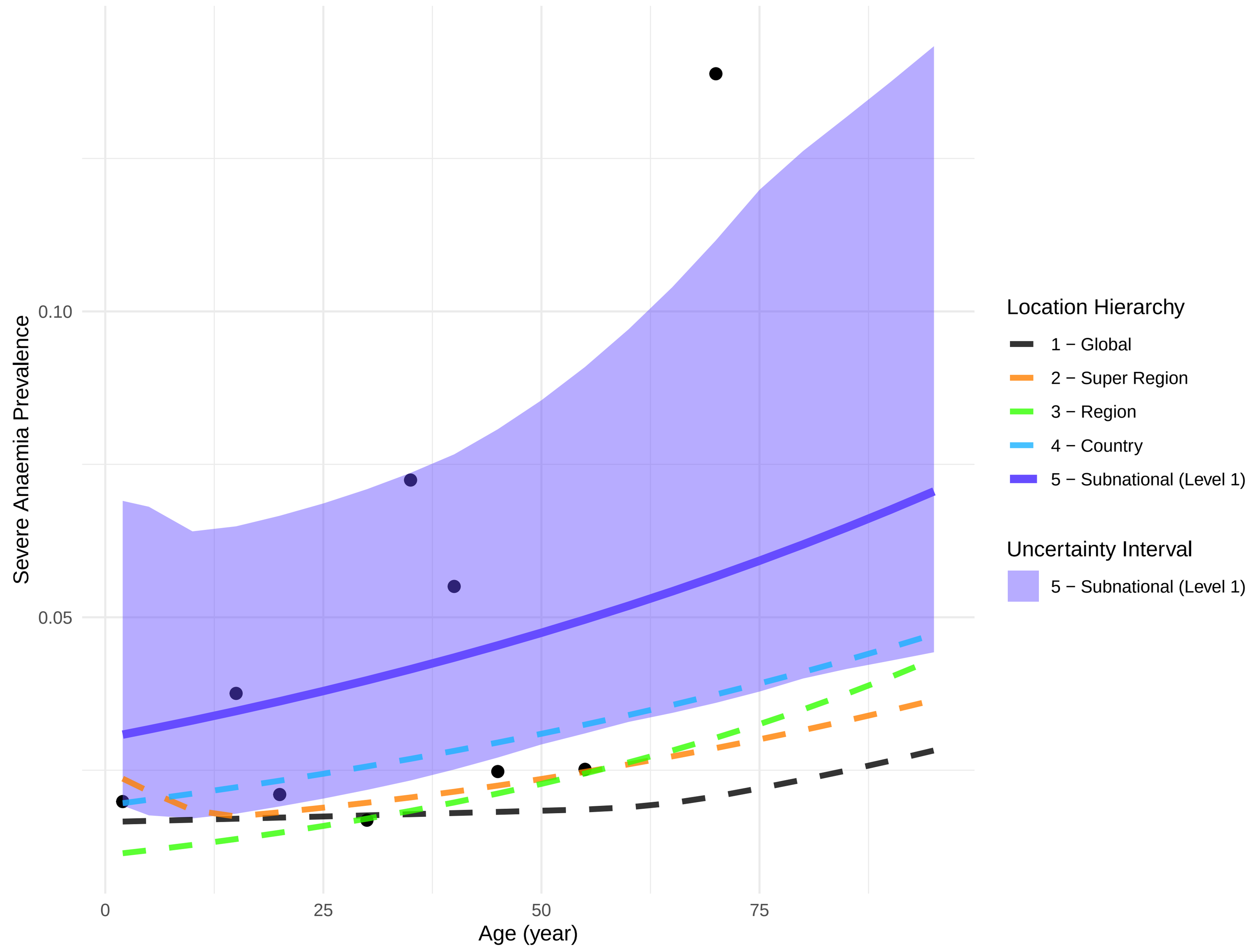

MR−BRT Age/Sex Fits − Non−pregnant Females 2+ Years Old − Severe Anaemia Prevalence − Gauteng
WHO−2001 Adjustment Method

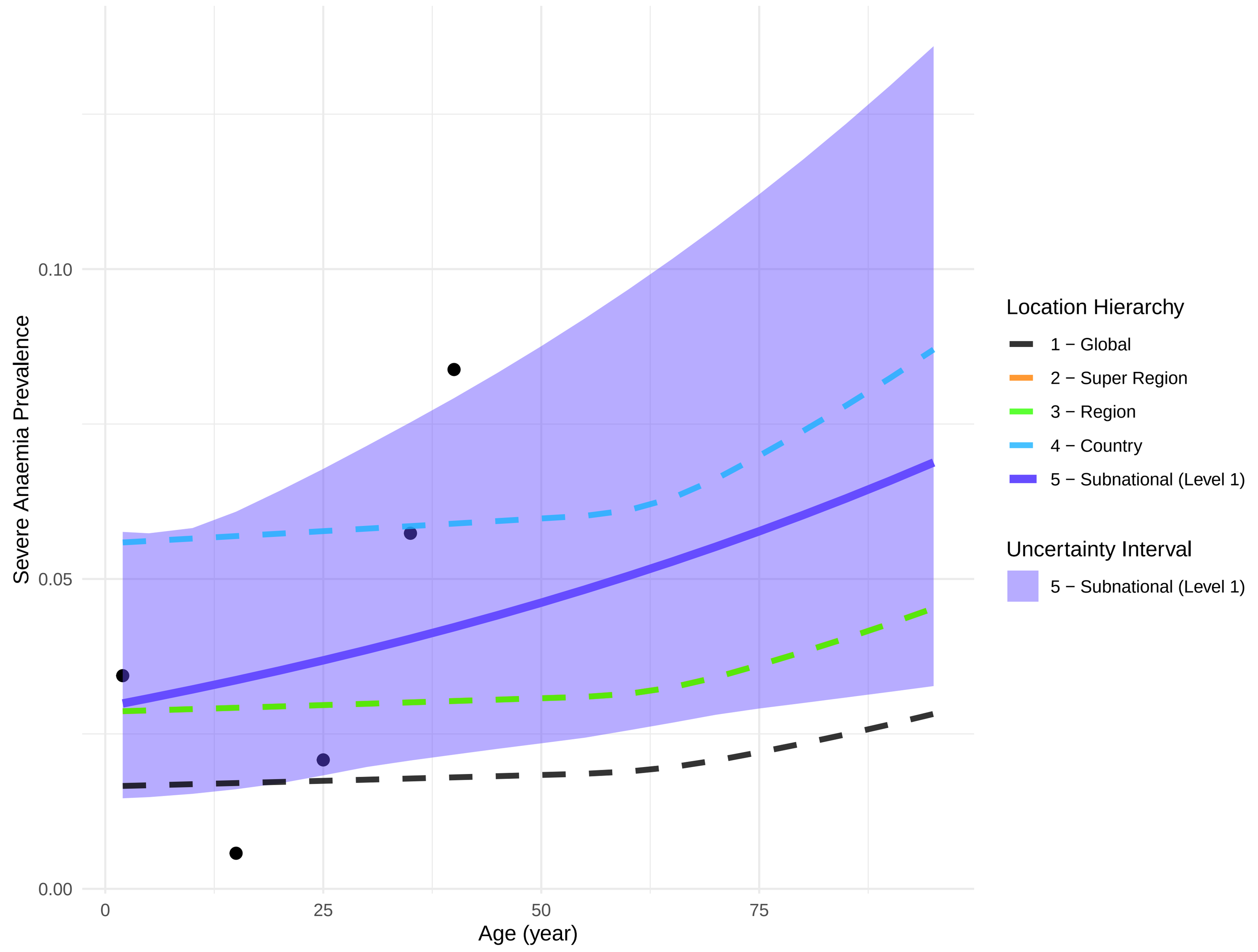

MR−BRT Age/Sex Fits − Non−pregnant Females 2+ Years Old − Severe Anaemia Prevalence − KwaZulu−Natal
WHO−2001 Adjustment Method

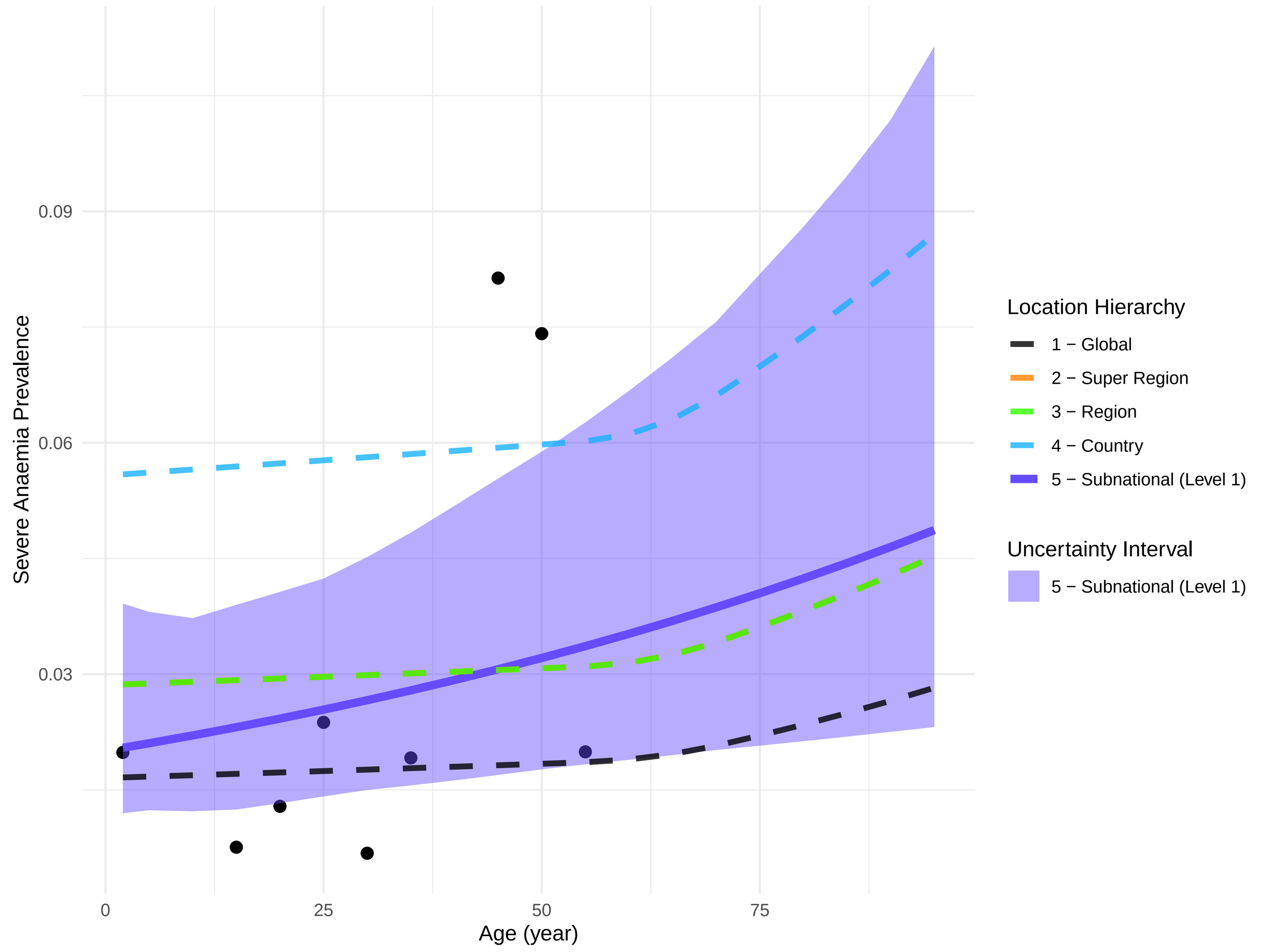

MR−BRT Age/Sex Fits − Non−pregnant Females 2+ Years Old − Severe Anaemia Prevalence − Limpopo
WHO−2001 Adjustment Method

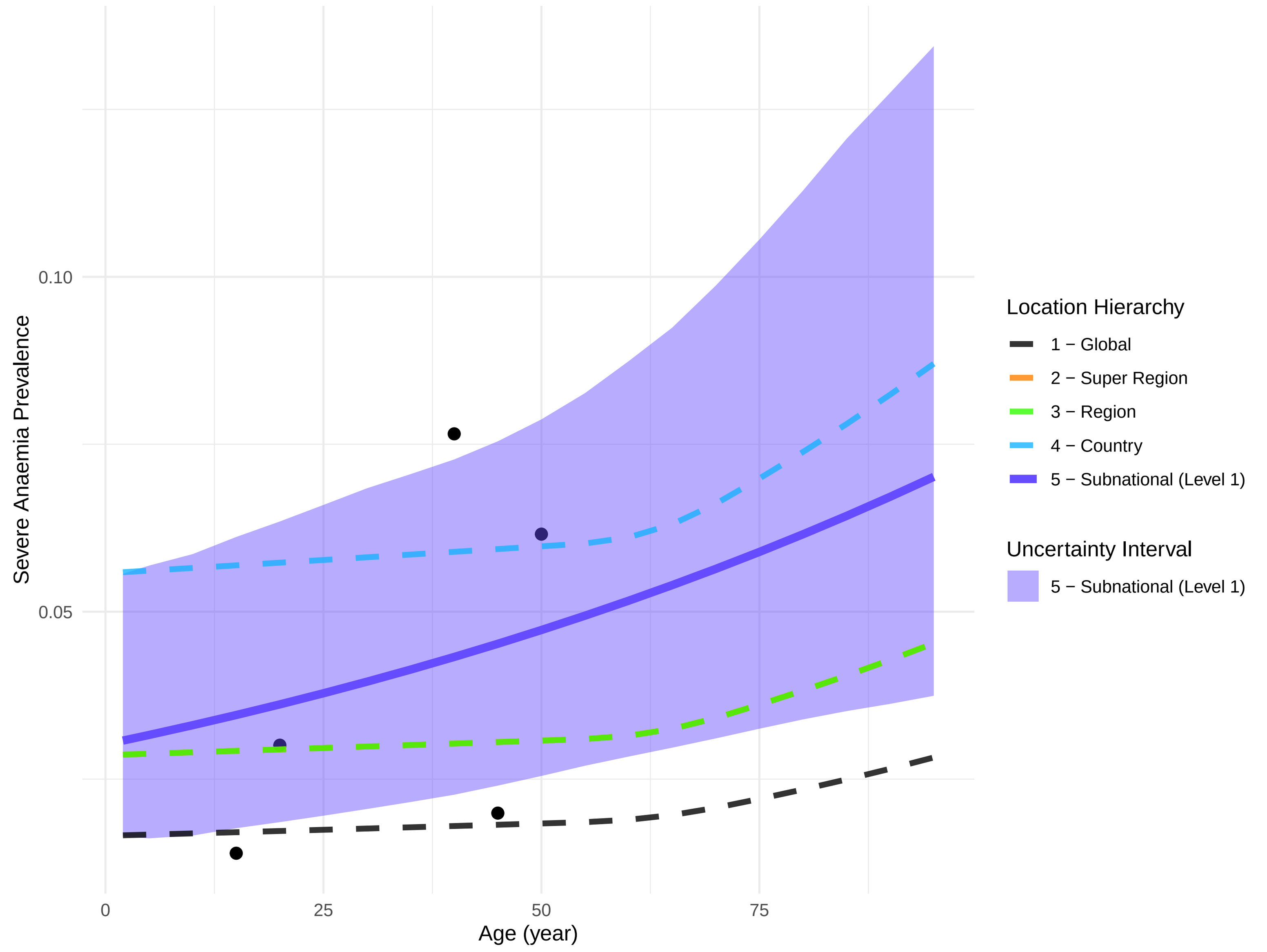

MR−BRT Age/Sex Fits − Non−pregnant Females 2+ Years Old − Severe Anaemia Prevalence − Mpumalanga
WHO−2001 Adjustment Method

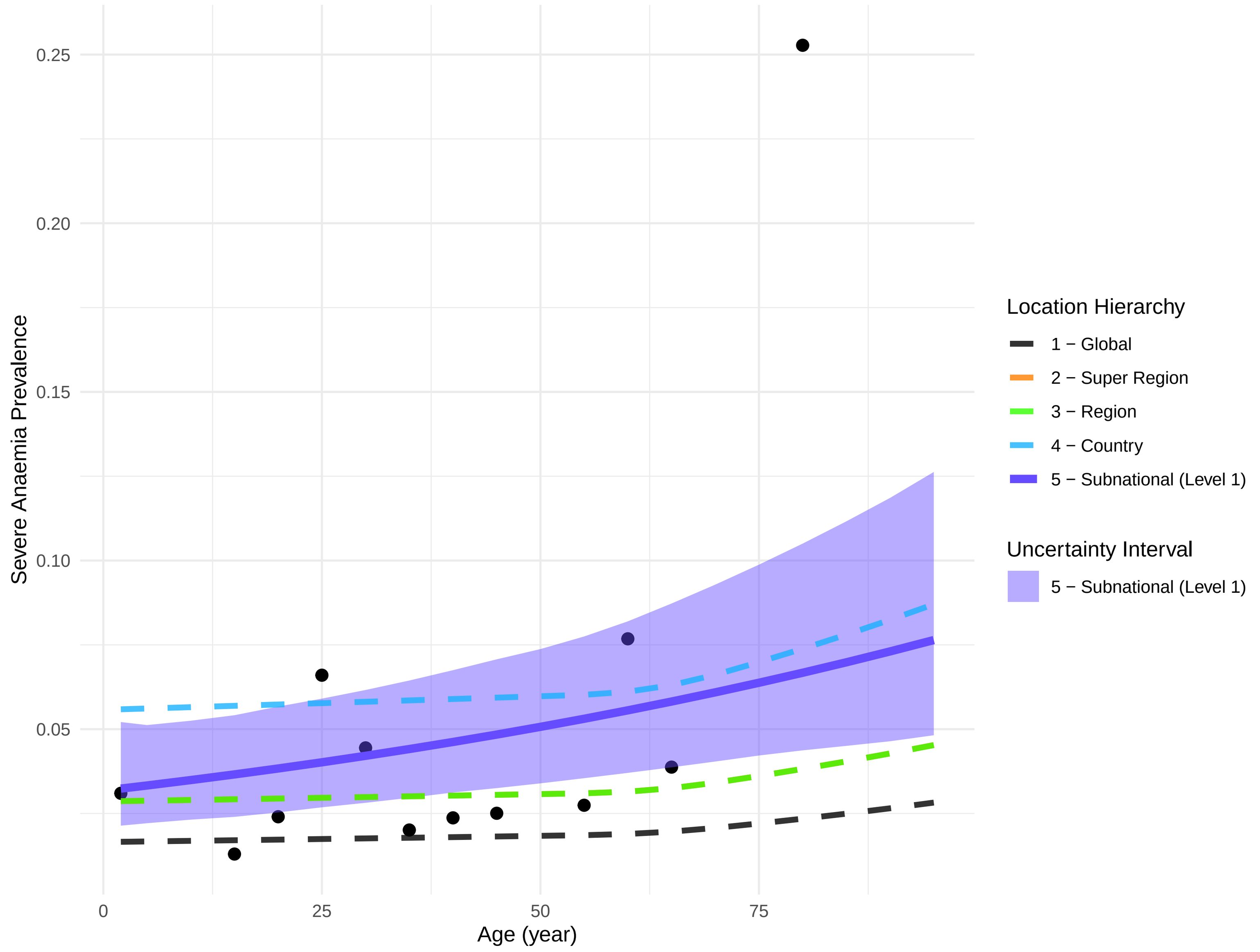

MR−BRT Age/Sex Fits − Non−pregnant Females 2+ Years Old − Severe Anaemia Prevalence − North−West
WHO−2001 Adjustment Method

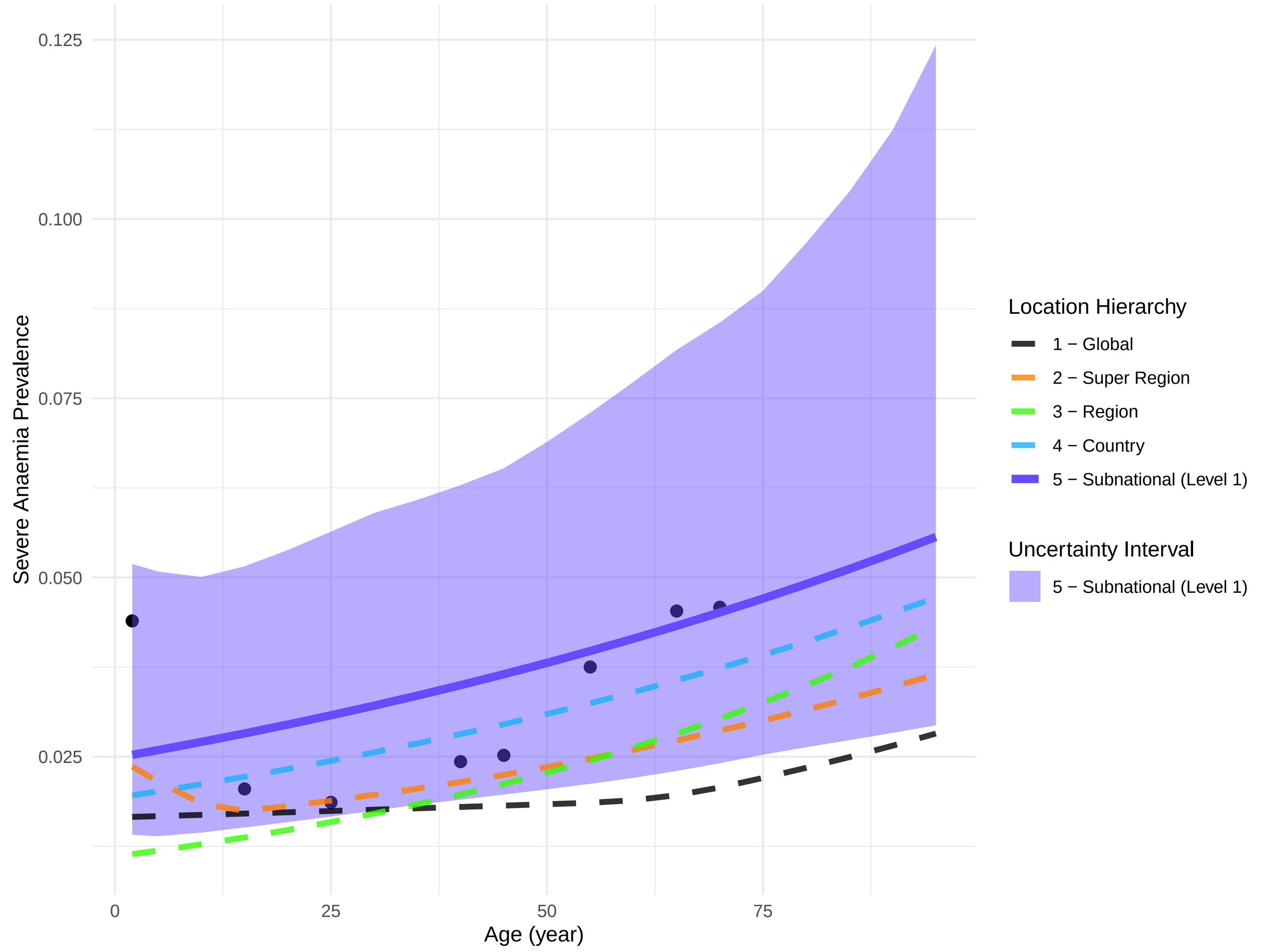

MR−BRT Age/Sex Fits − Non−pregnant Females 2+ Years Old − Severe Anaemia Prevalence − Northern Cape
WHO−2001 Adjustment Method

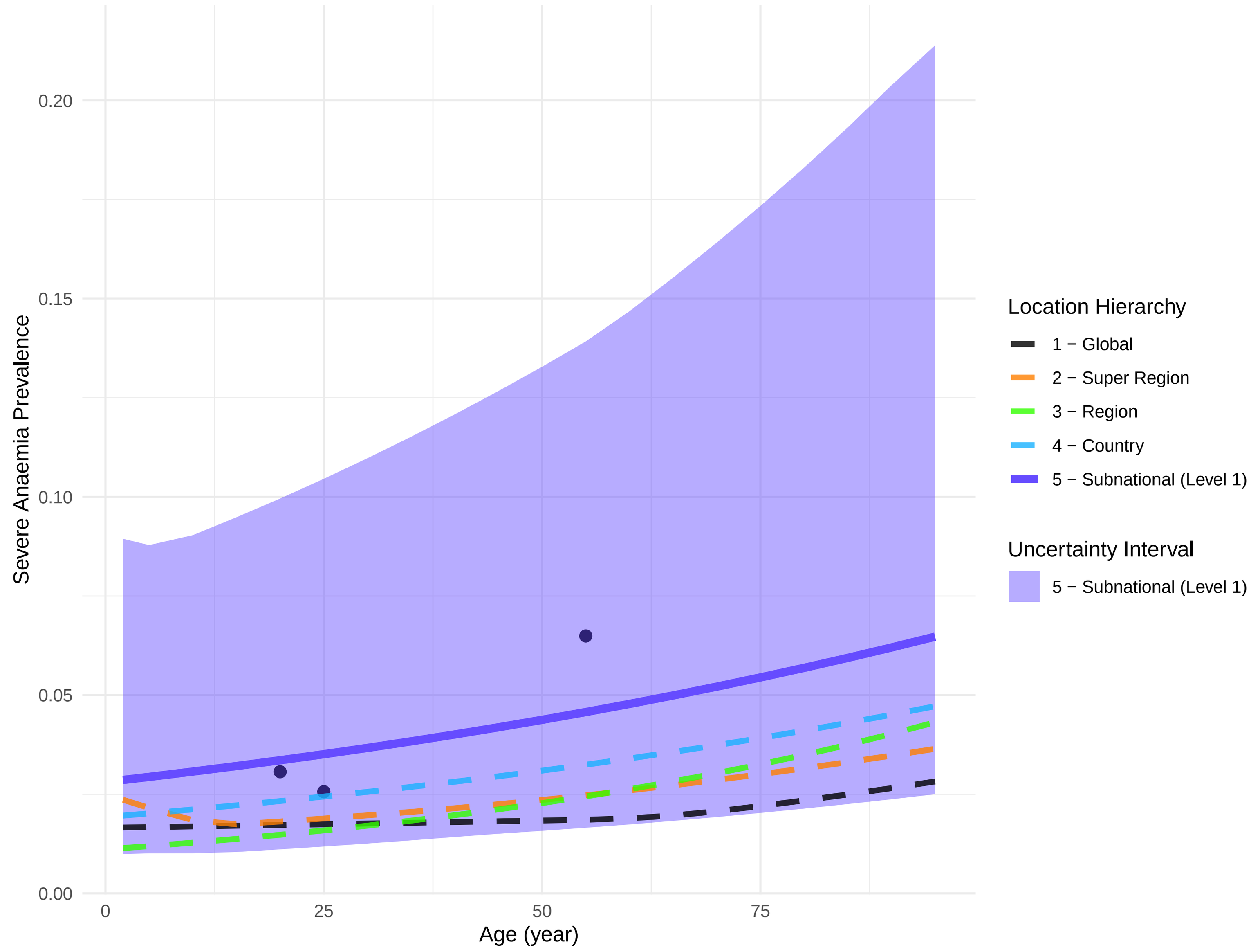

MR−BRT Age/Sex Fits − Non−pregnant Females 2+ Years Old − Severe Anaemia Prevalence − Western Cape
WHO−2001 Adjustment Method

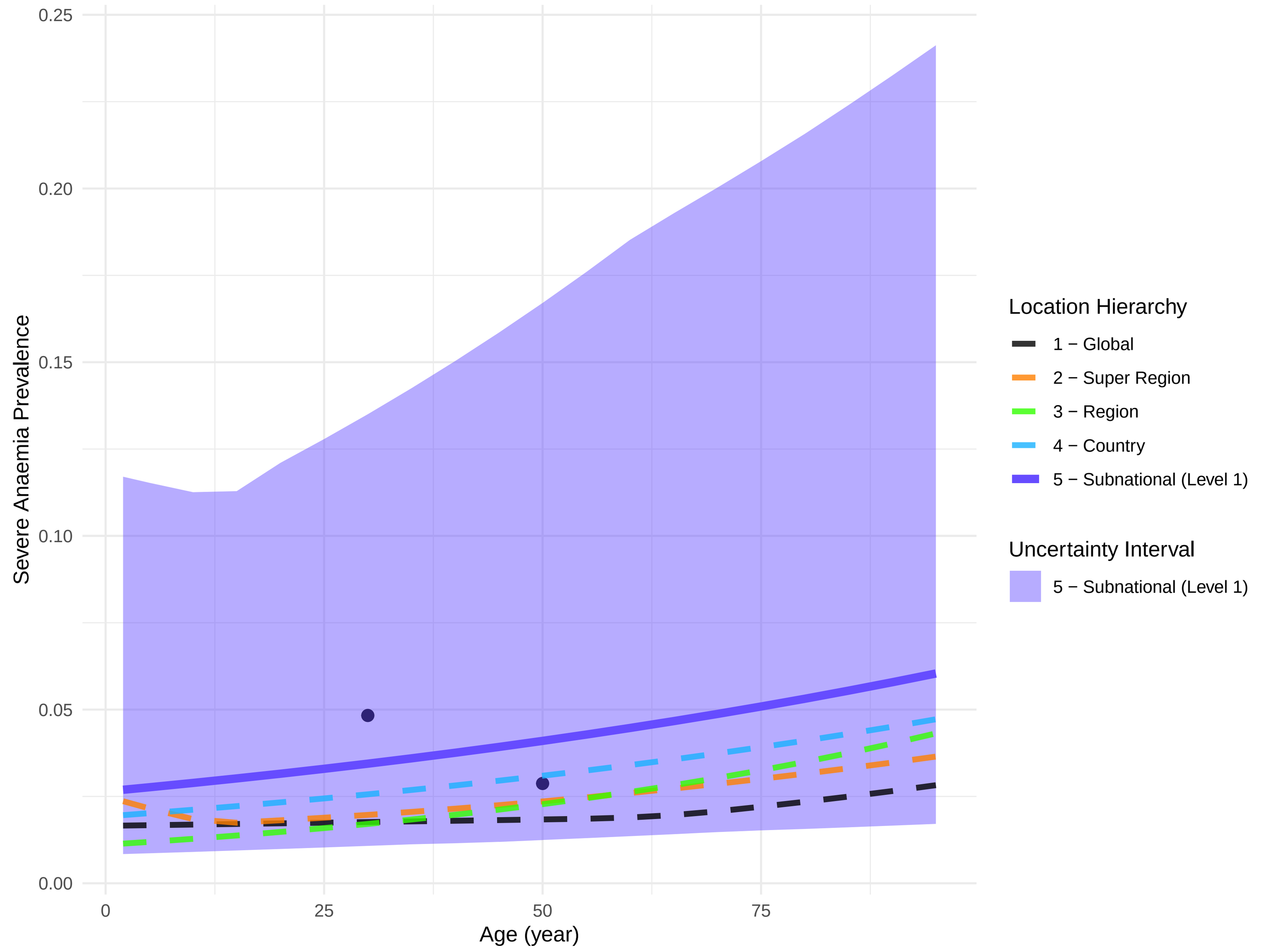

MR−BRT Age/Sex Fits − Non−pregnant Females 2+ Years Old − Severe Anaemia Prevalence − Zimbabwe
WHO−2001 Adjustment Method

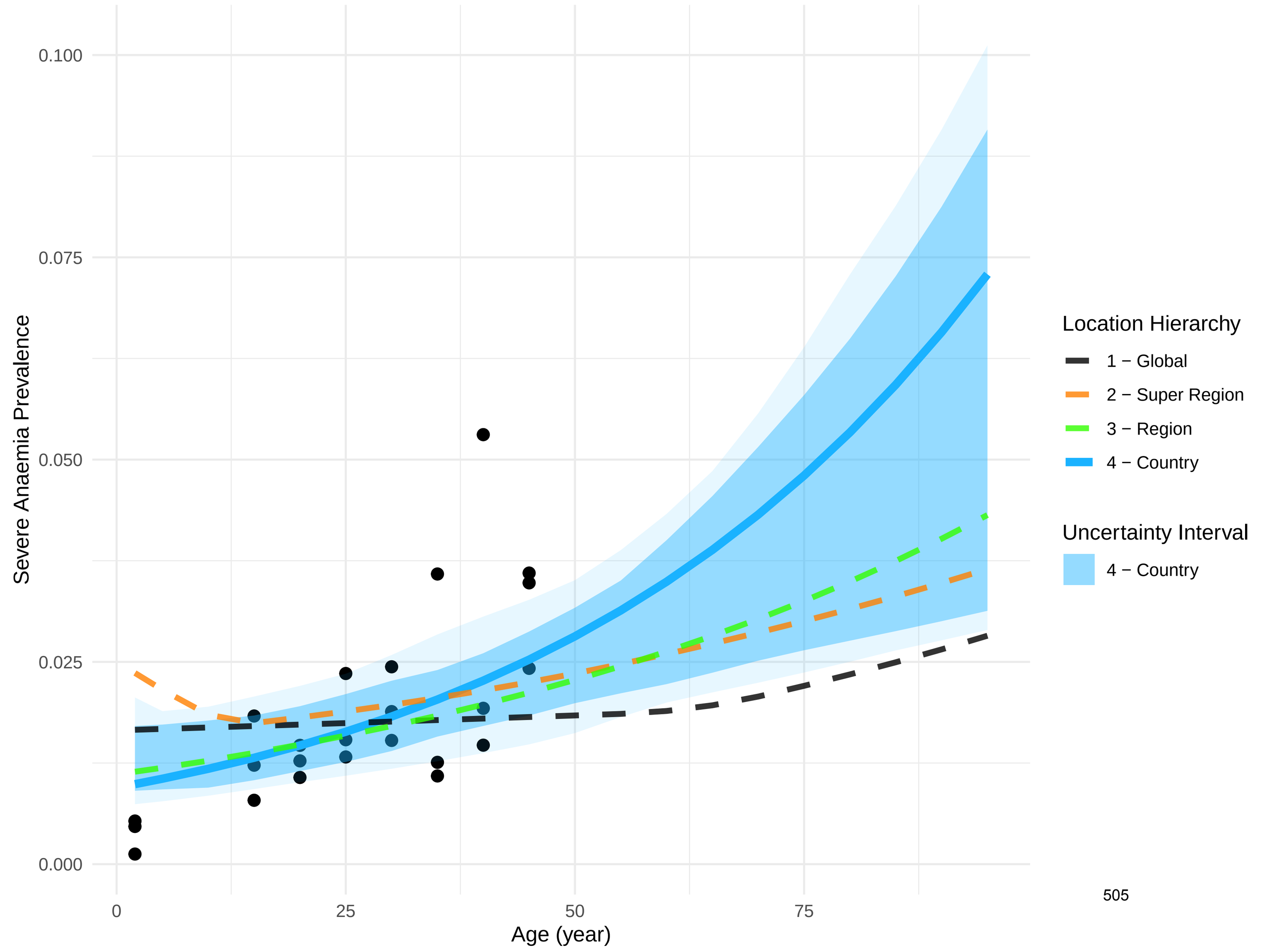

MR−BRT Age/Sex Fits − Non−pregnant Females 2+ Years Old − Severe Anaemia Prevalence − Western Sub−Saharan Africa
WHO−2001 Adjustment Method

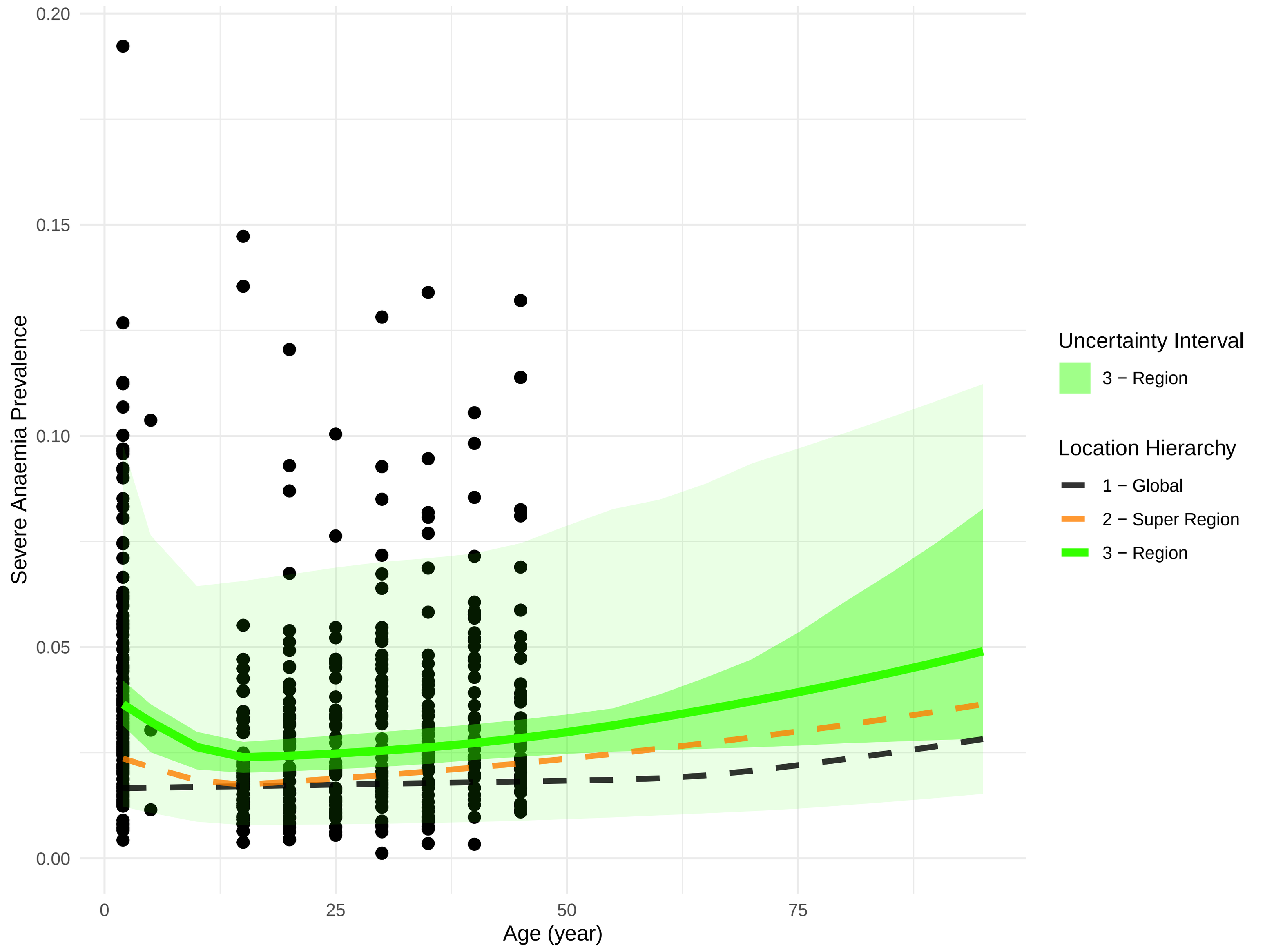

MR−BRT Age/Sex Fits − Non−pregnant Females 2+ Years Old − Severe Anaemia Prevalence − Benin
WHO−2001 Adjustment Method

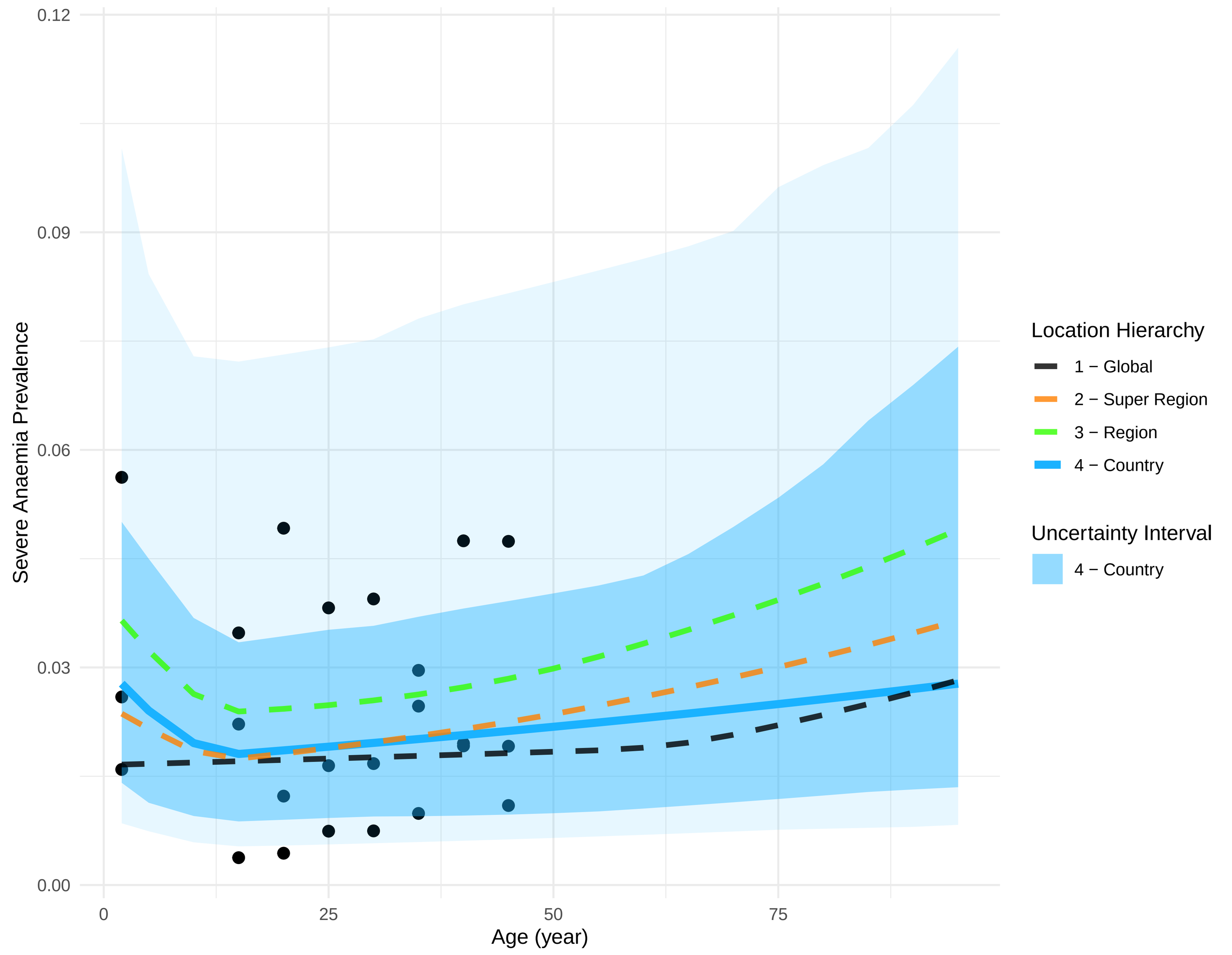

MR−BRT Age/Sex Fits − Non−pregnant Females 2+ Years Old − Severe Anaemia Prevalence − Burkina Faso
WHO−2001 Adjustment Method

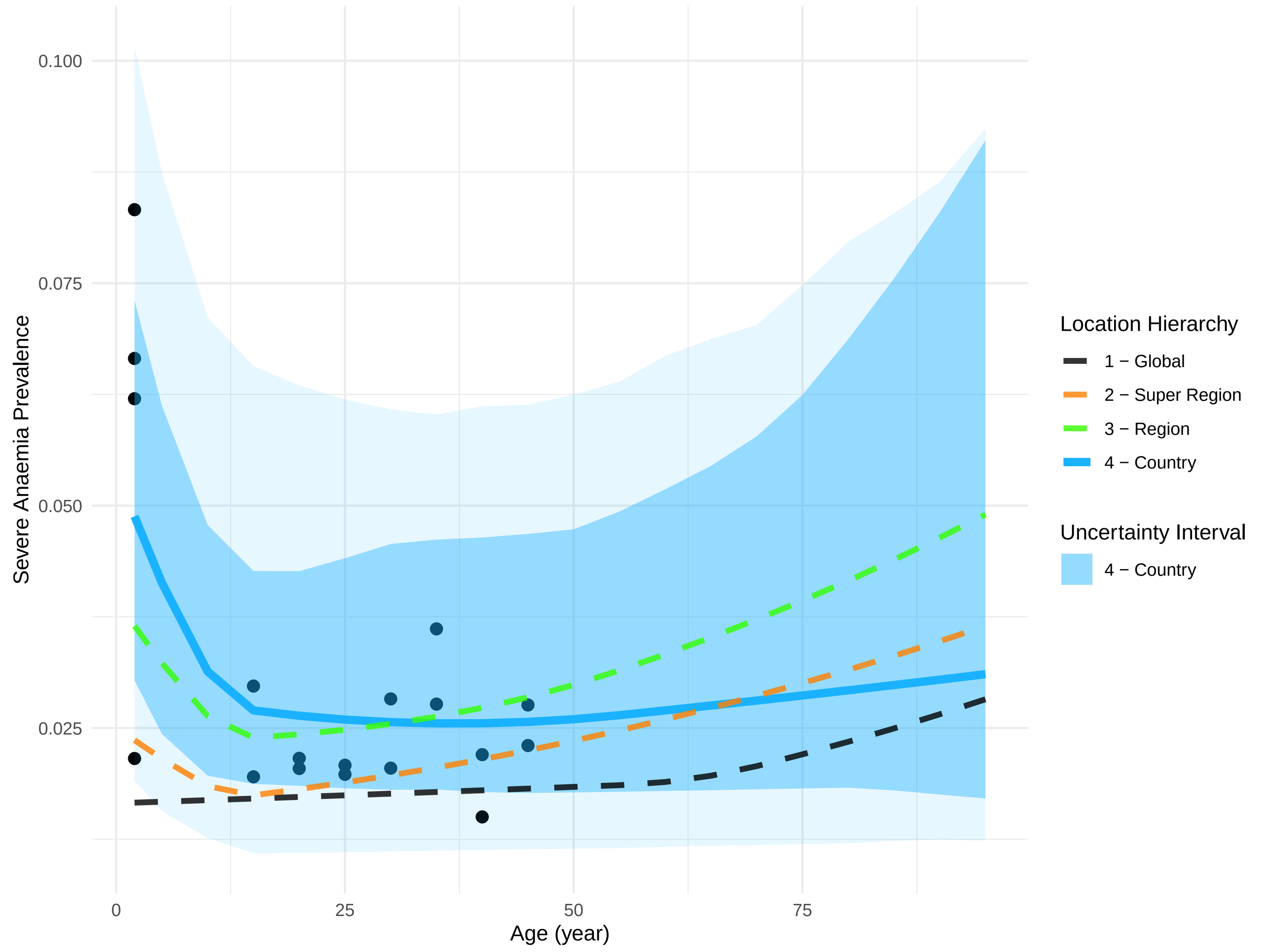

MR−BRT Age/Sex Fits − Non−pregnant Females 2+ Years Old − Severe Anaemia Prevalence − Cameroon
WHO−2001 Adjustment Method

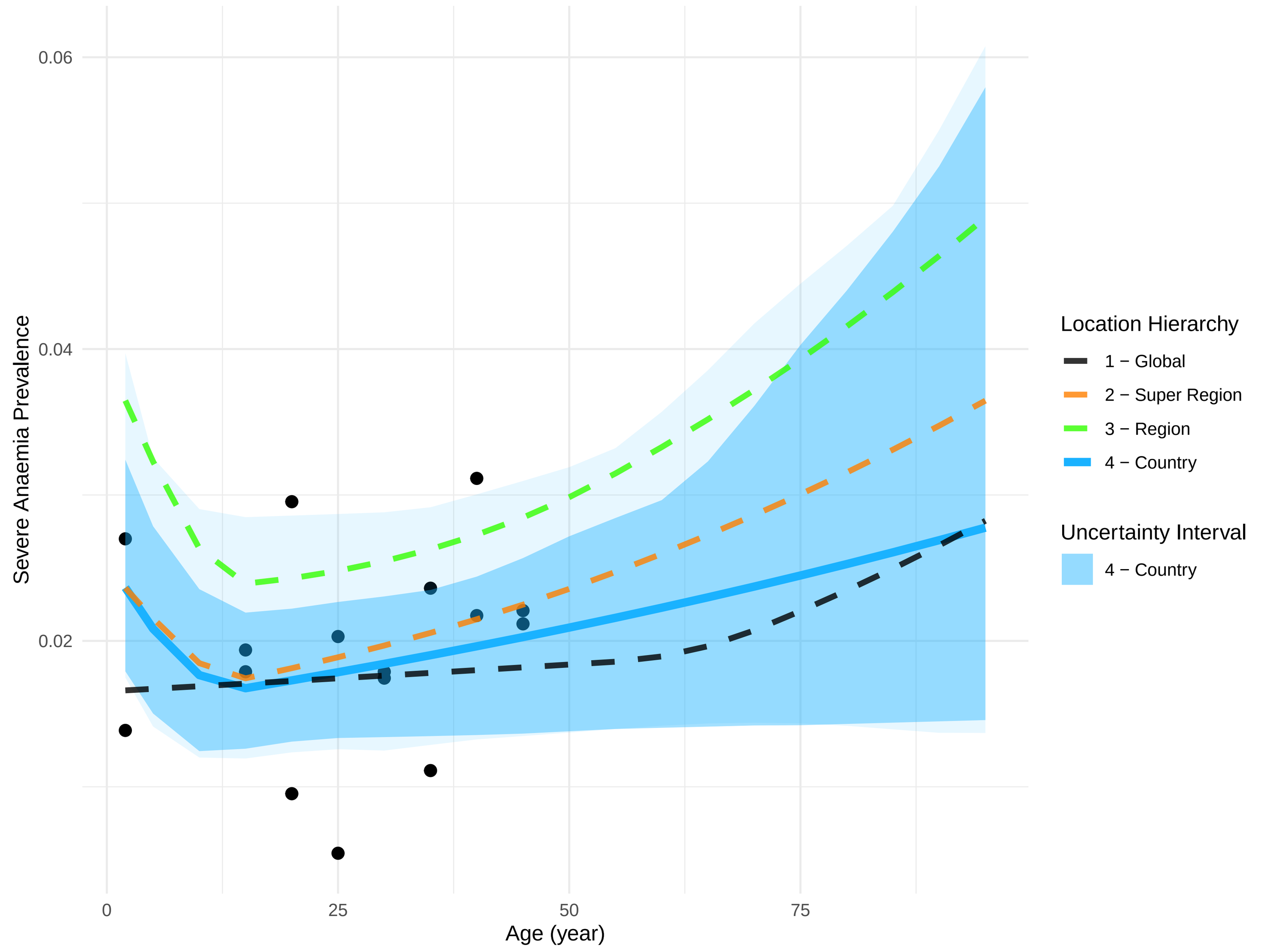

MR−BRT Age/Sex Fits − Non−pregnant Females 2+ Years Old − Severe Anaemia Prevalence − Côte d'Ivoire
WHO−2001 Adjustment Method

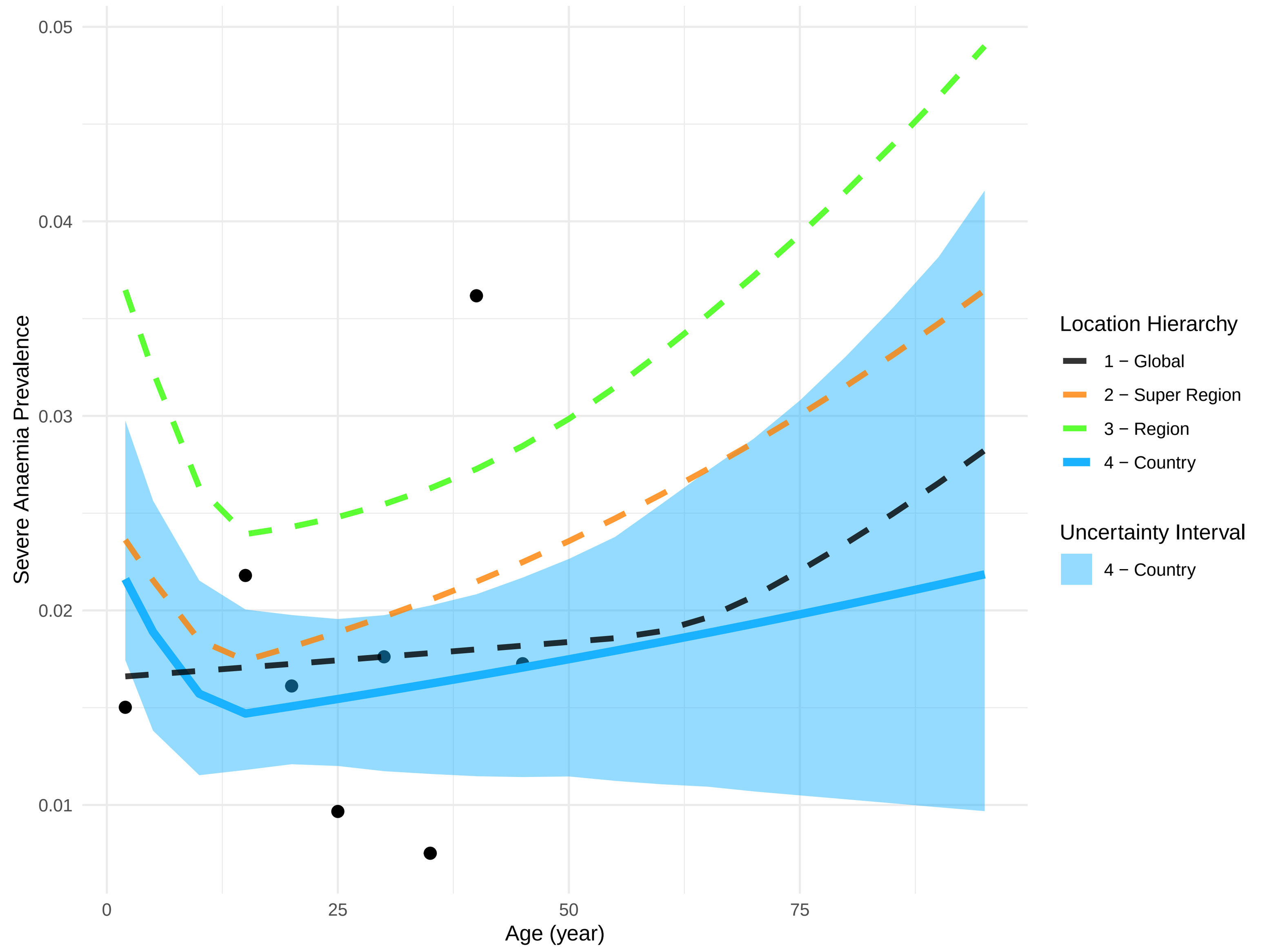

MR−BRT Age/Sex Fits − Non−pregnant Females 2+ Years Old − Severe Anaemia Prevalence − Gambia
WHO−2001 Adjustment Method

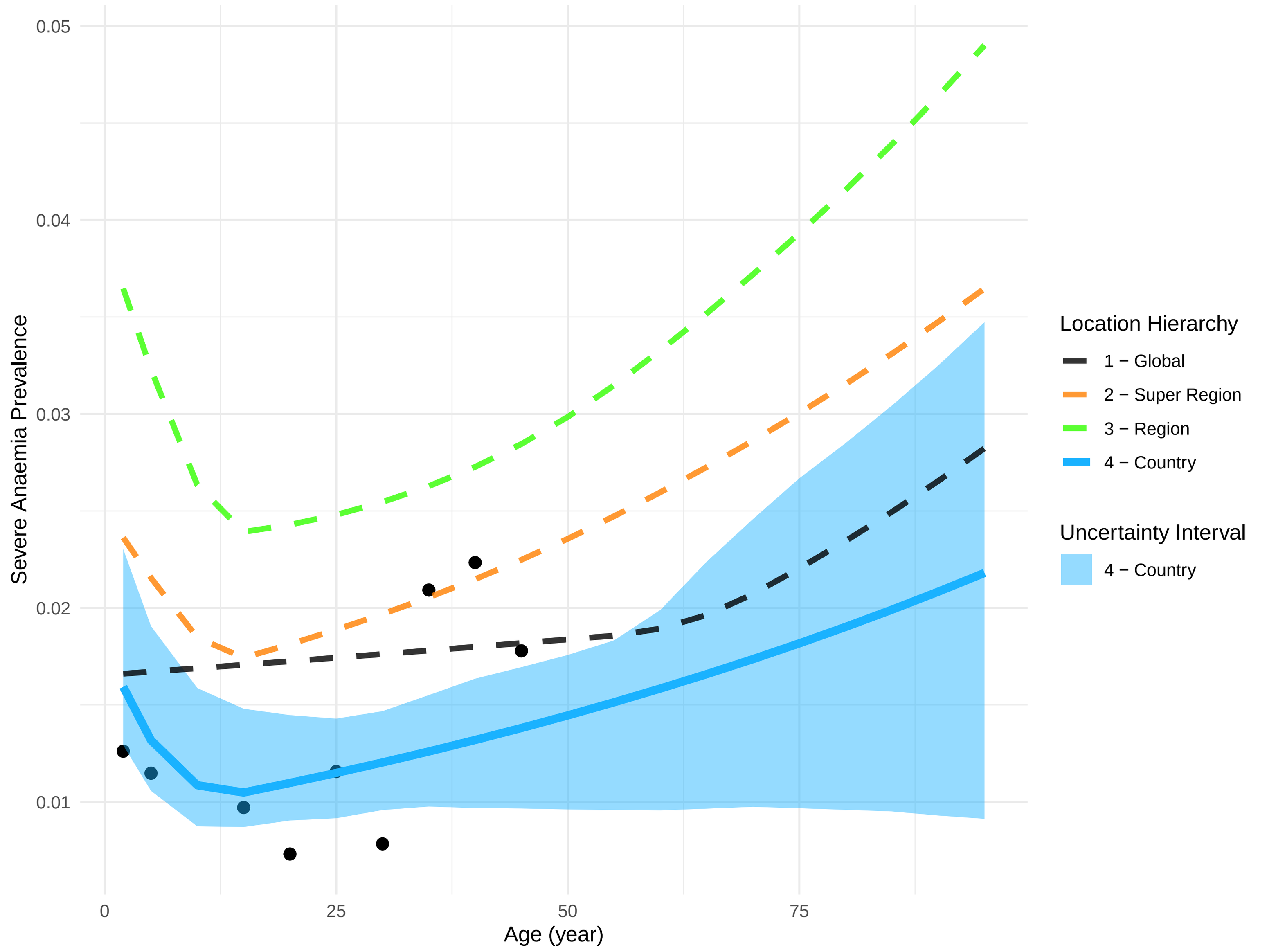

MR−BRT Age/Sex Fits − Non−pregnant Females 2+ Years Old − Severe Anaemia Prevalence − Ghana
WHO−2001 Adjustment Method

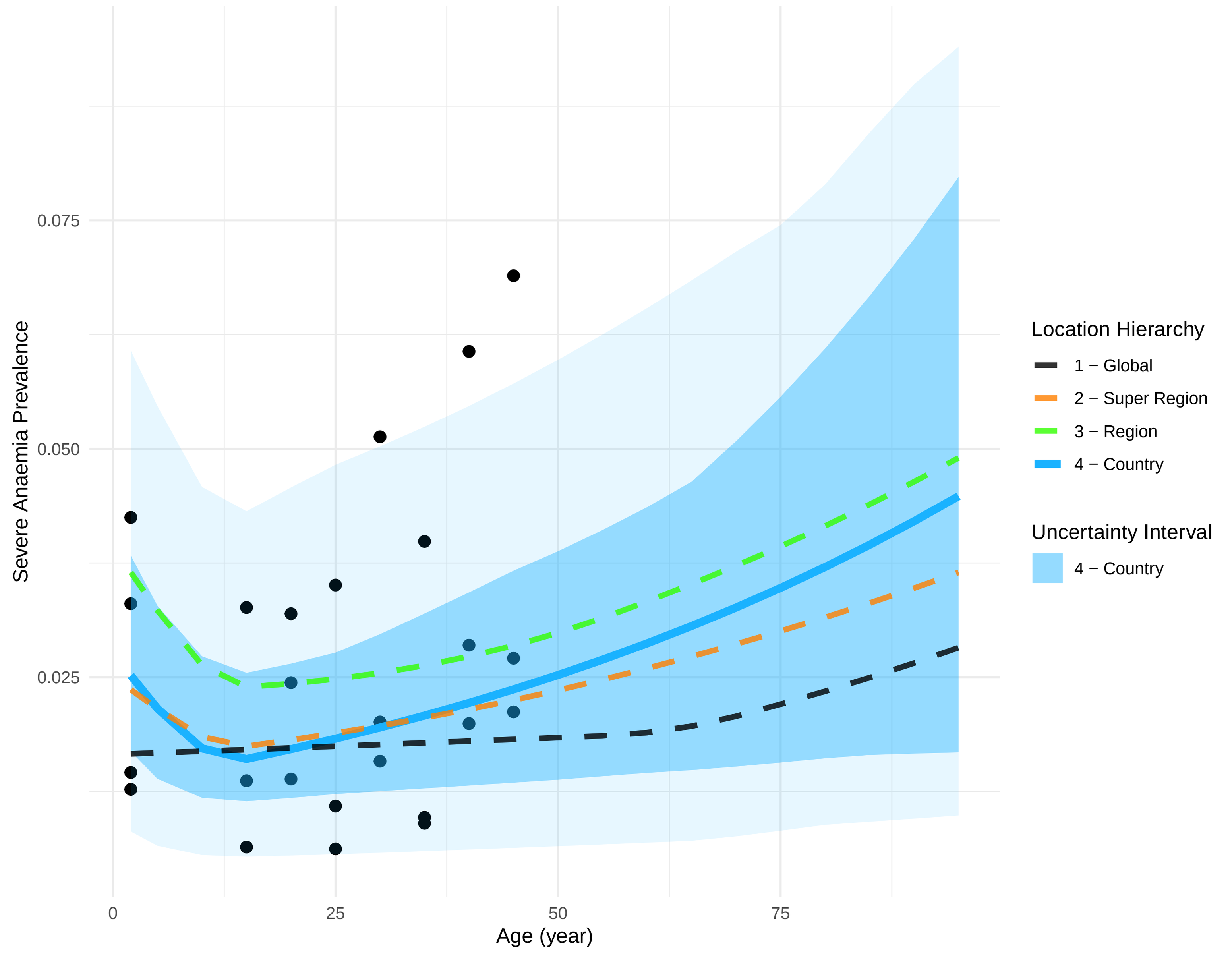

MR−BRT Age/Sex Fits − Non−pregnant Females 2+ Years Old − Severe Anaemia Prevalence − Guinea
WHO−2001 Adjustment Method

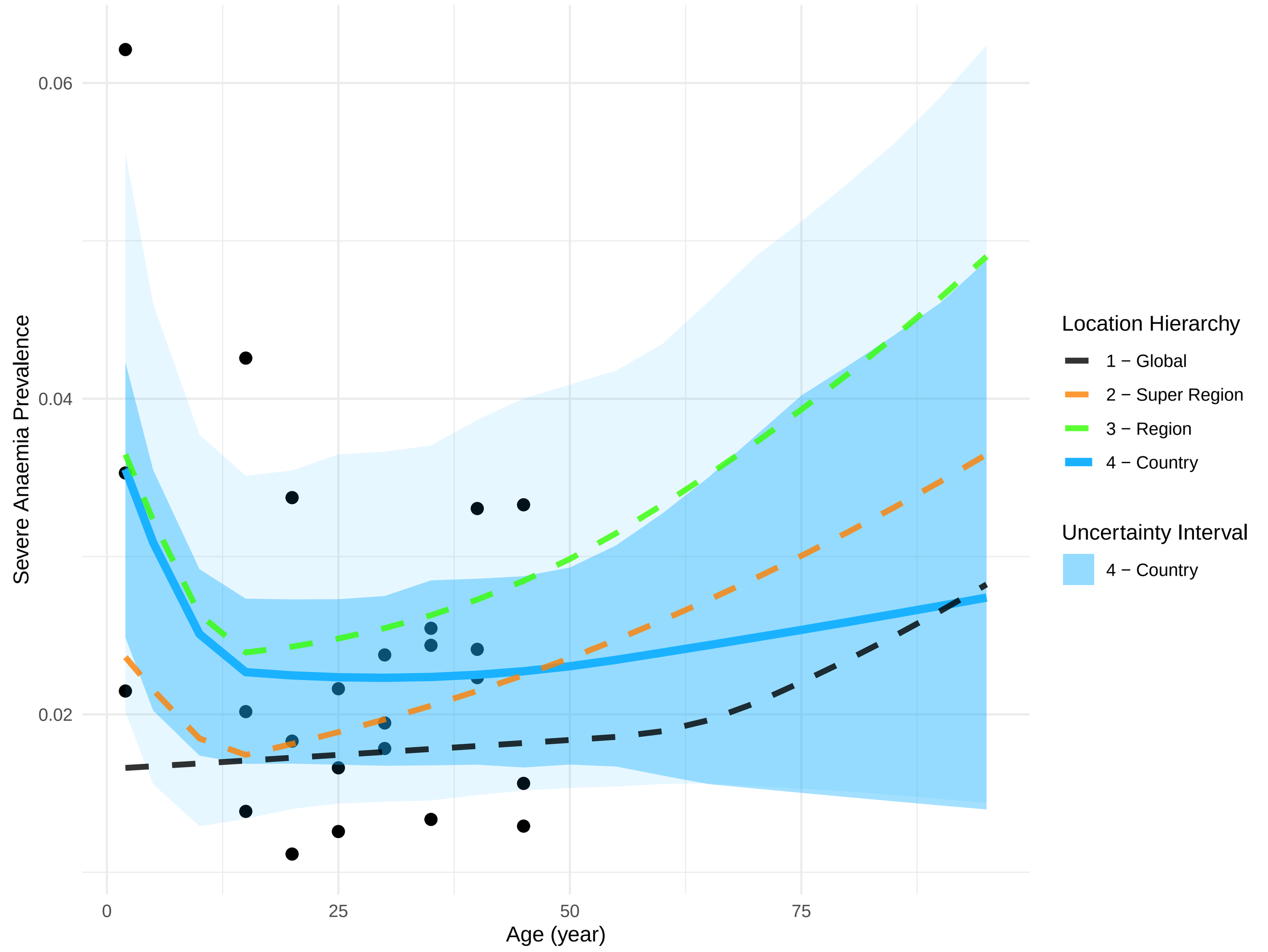

MR−BRT Age/Sex Fits − Non−pregnant Females 2+ Years Old − Severe Anaemia Prevalence − Liberia
WHO−2001 Adjustment Method

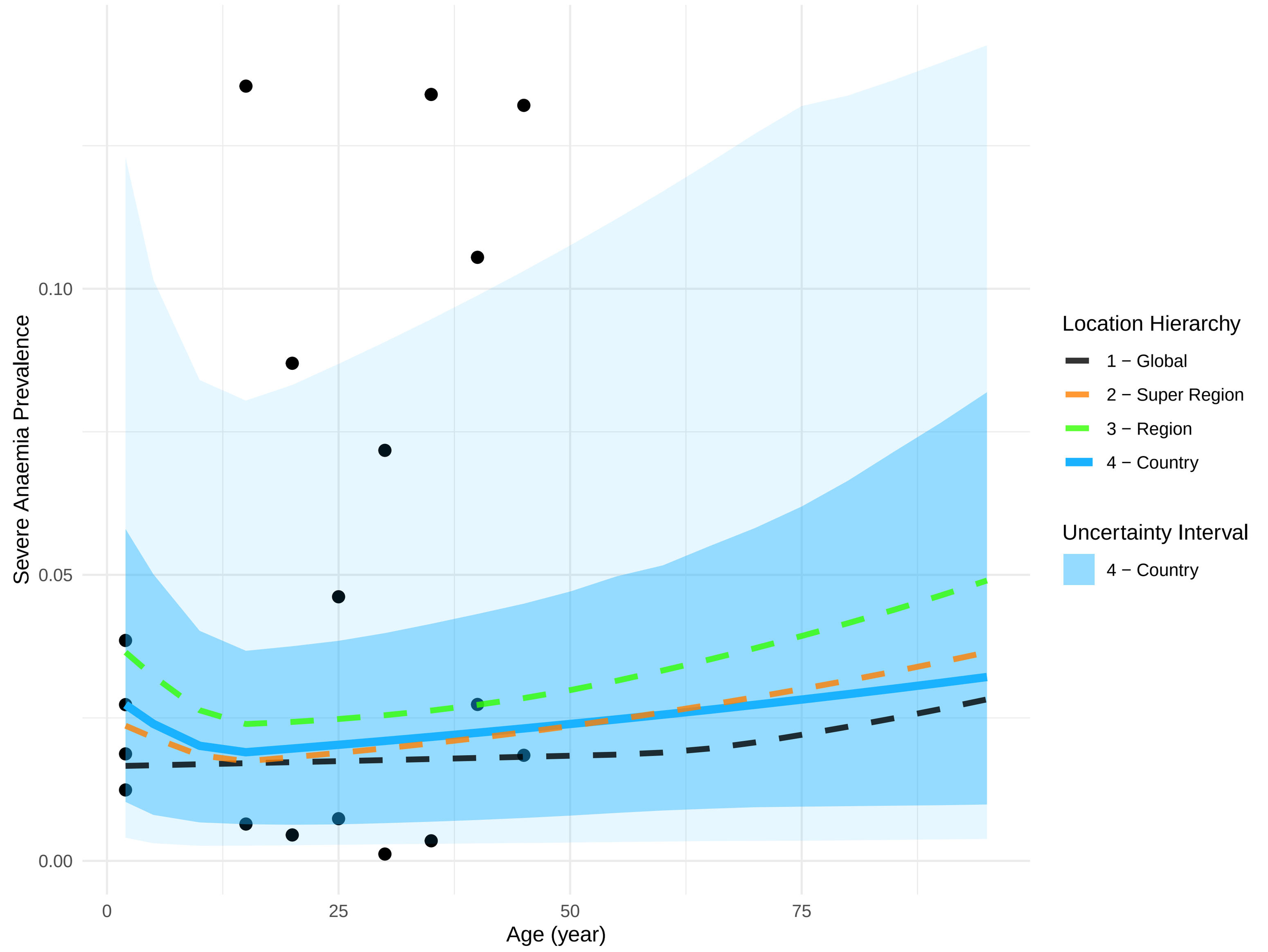

MR−BRT Age/Sex Fits − Non−pregnant Females 2+ Years Old − Severe Anaemia Prevalence − Mali
WHO−2001 Adjustment Method

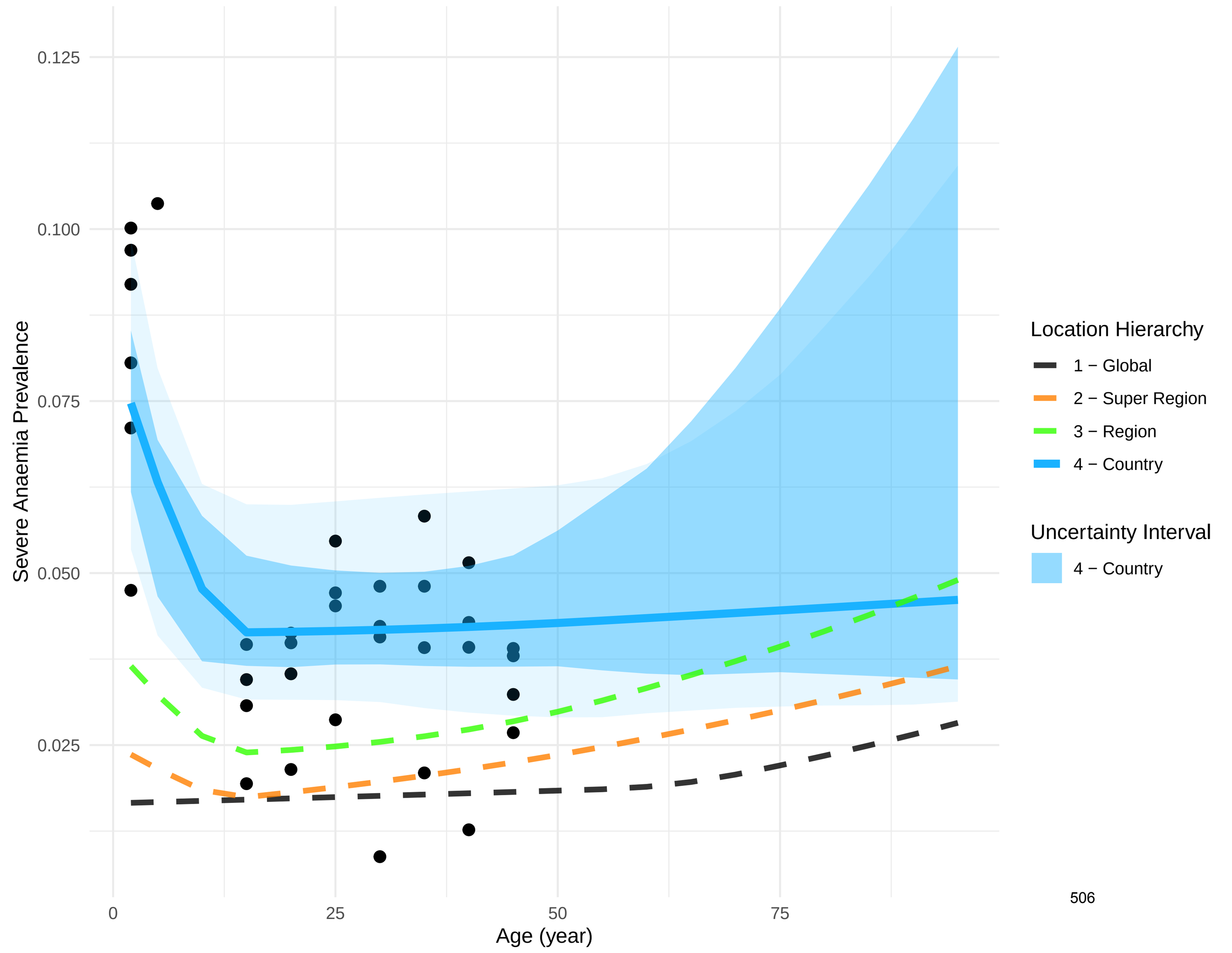

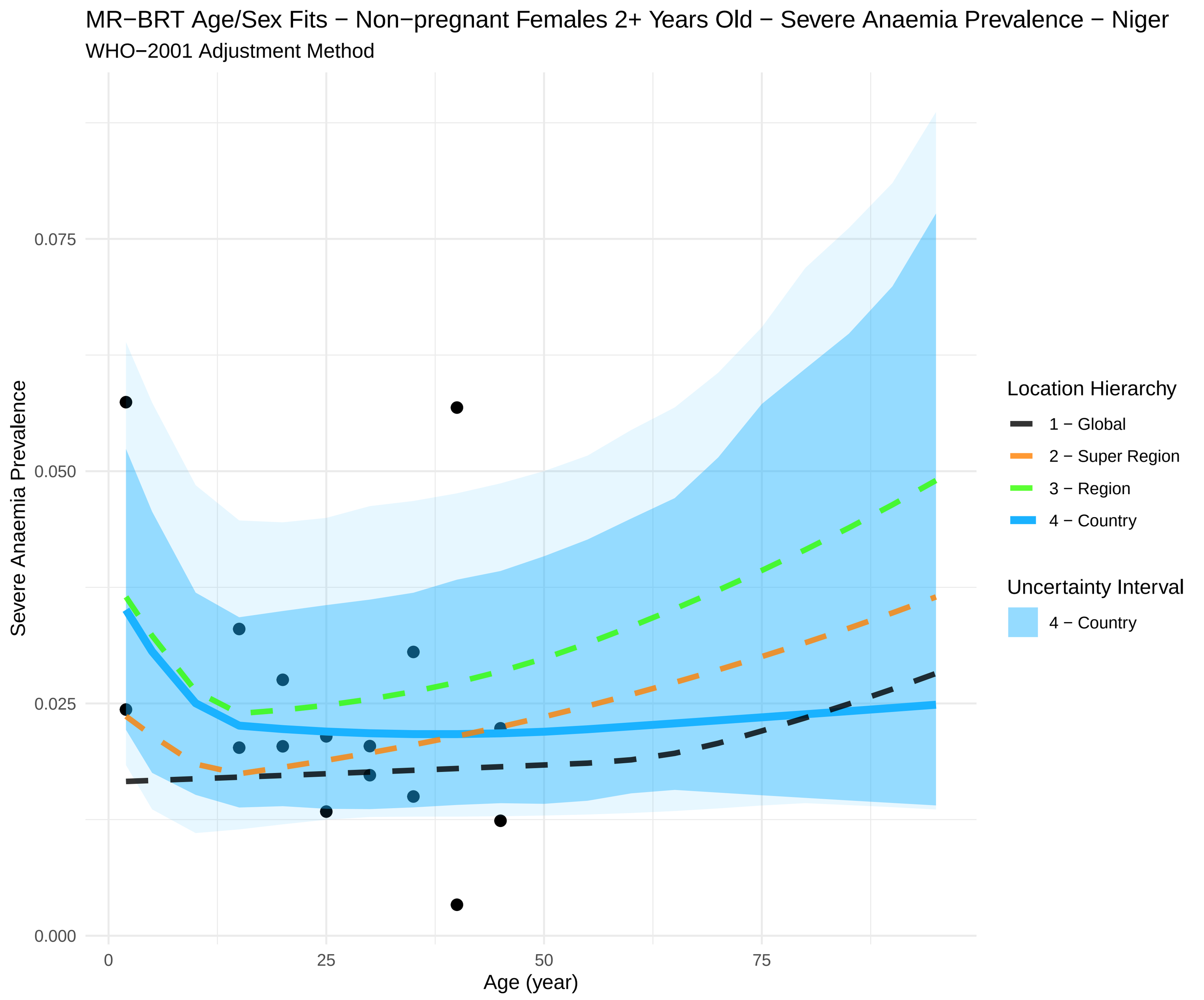
MR−BRT Age/Sex Fits − Non−pregnant Females 2+ Years Old − Severe Anaemia Prevalence − Niger
WHO−2001 Adjustment Method
Severe Anaemia Prevalence
Age (year)
Location Hierarchy
1 − Global
2 − Super Region
3 − Region
4 − Country
Uncertainty Interval
4 − Country

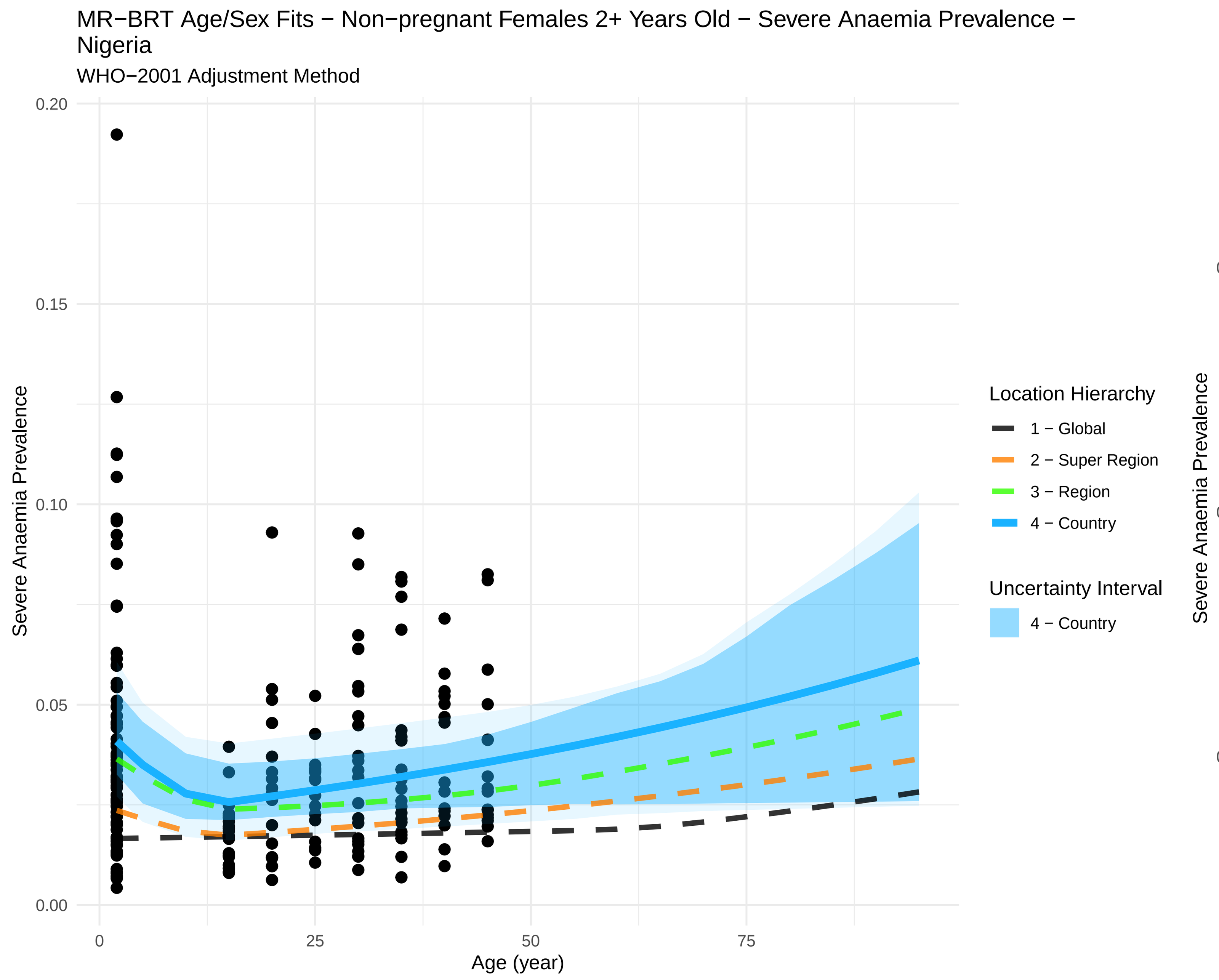
MR−BRT Age/Sex Fits − Non−pregnant Females 2+ Years Old − Severe Anaemia Prevalence − Nigeria
WHO−2001 Adjustment Method
Severe Anaemia Prevalence
Age (year)
Location Hierarchy
1 − Global
2 − Super Region
3 − Region
4 − Country
Uncertainty Interval
4 − Country

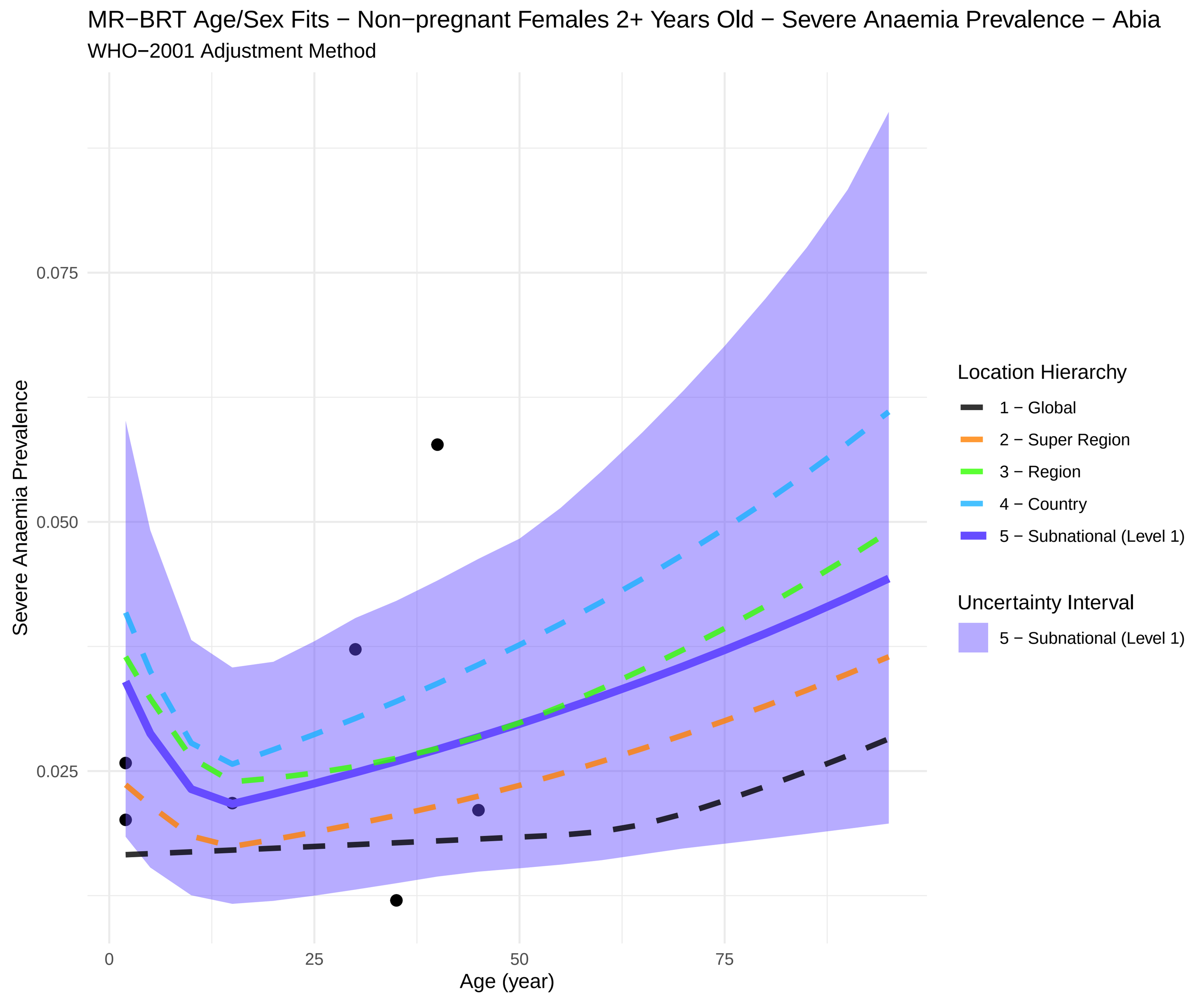
MR−BRT Age/Sex Fits − Non−pregnant Females 2+ Years Old − Severe Anaemia Prevalence − Abia
WHO−2001 Adjustment Method
Severe Anaemia Prevalence
Age (year)
Location Hierarchy
1 − Global
2 − Super Region
3 − Region
4 − Country
5 − Subnational (Level 1)
Uncertainty Interval
5 − Subnational (Level 1)

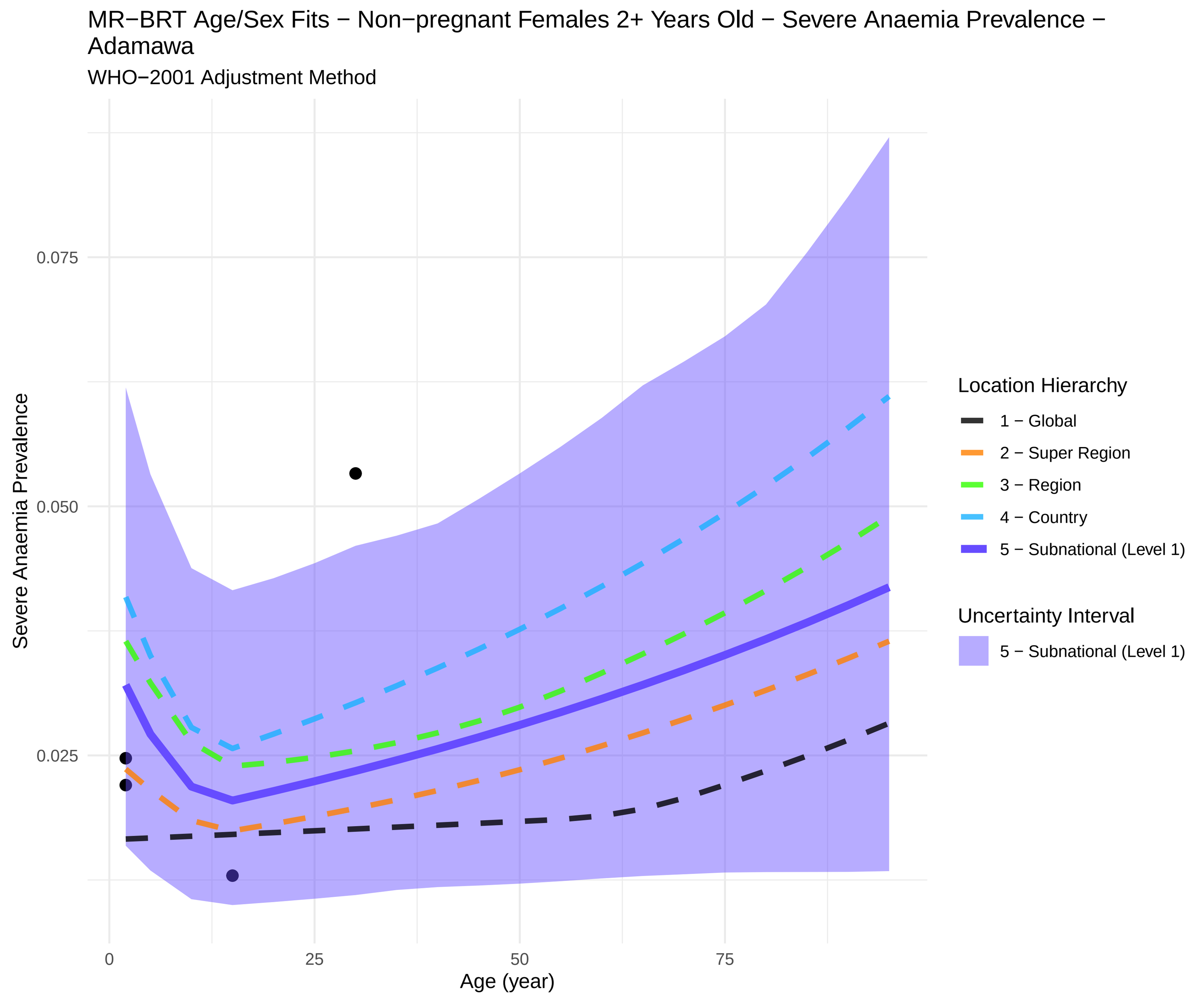
MR−BRT Age/Sex Fits − Non−pregnant Females 2+ Years Old − Severe Anaemia Prevalence − Adamawa
WHO−2001 Adjustment Method
Severe Anaemia Prevalence
Age (year)
Location Hierarchy
1 − Global
2 − Super Region
3 − Region
4 − Country
5 − Subnational (Level 1)
Uncertainty Interval
5 − Subnational (Level 1)

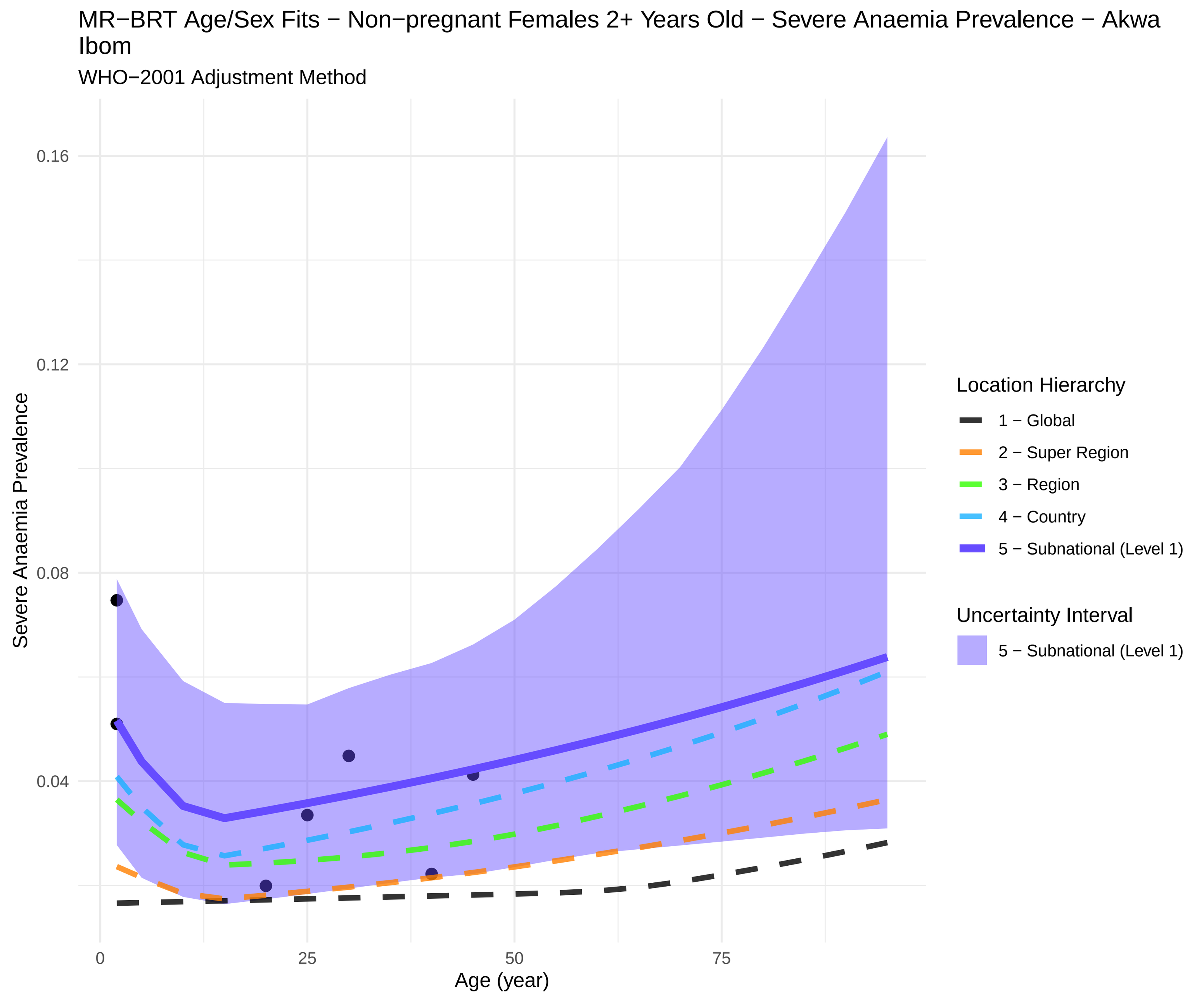
MR−BRT Age/Sex Fits − Non−pregnant Females 2+ Years Old − Severe Anaemia Prevalence − Akwa Ibom
WHO−2001 Adjustment Method
Severe Anaemia Prevalence
Age (year)
Location Hierarchy
1 − Global
2 − Super Region
3 − Region
4 − Country
5 − Subnational (Level 1)
Uncertainty Interval
5 − Subnational (Level 1)

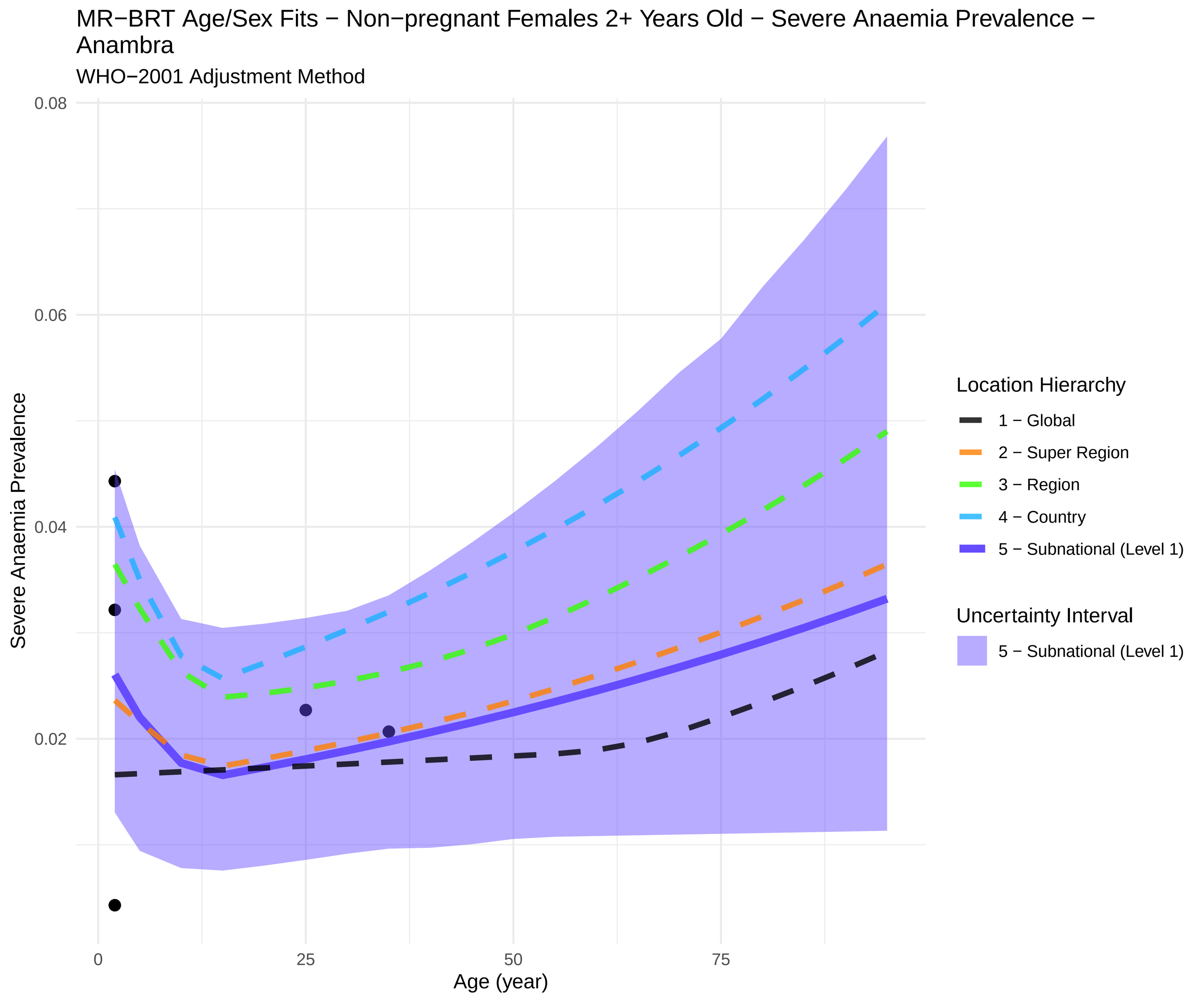
MR−BRT Age/Sex Fits − Non−pregnant Females 2+ Years Old − Severe Anaemia Prevalence − Anambra
WHO−2001 Adjustment Method
Severe Anaemia Prevalence
Age (year)
Location Hierarchy
1 − Global
2 − Super Region
3 − Region
4 − Country
5 − Subnational (Level 1)
Uncertainty Interval
5 − Subnational (Level 1)

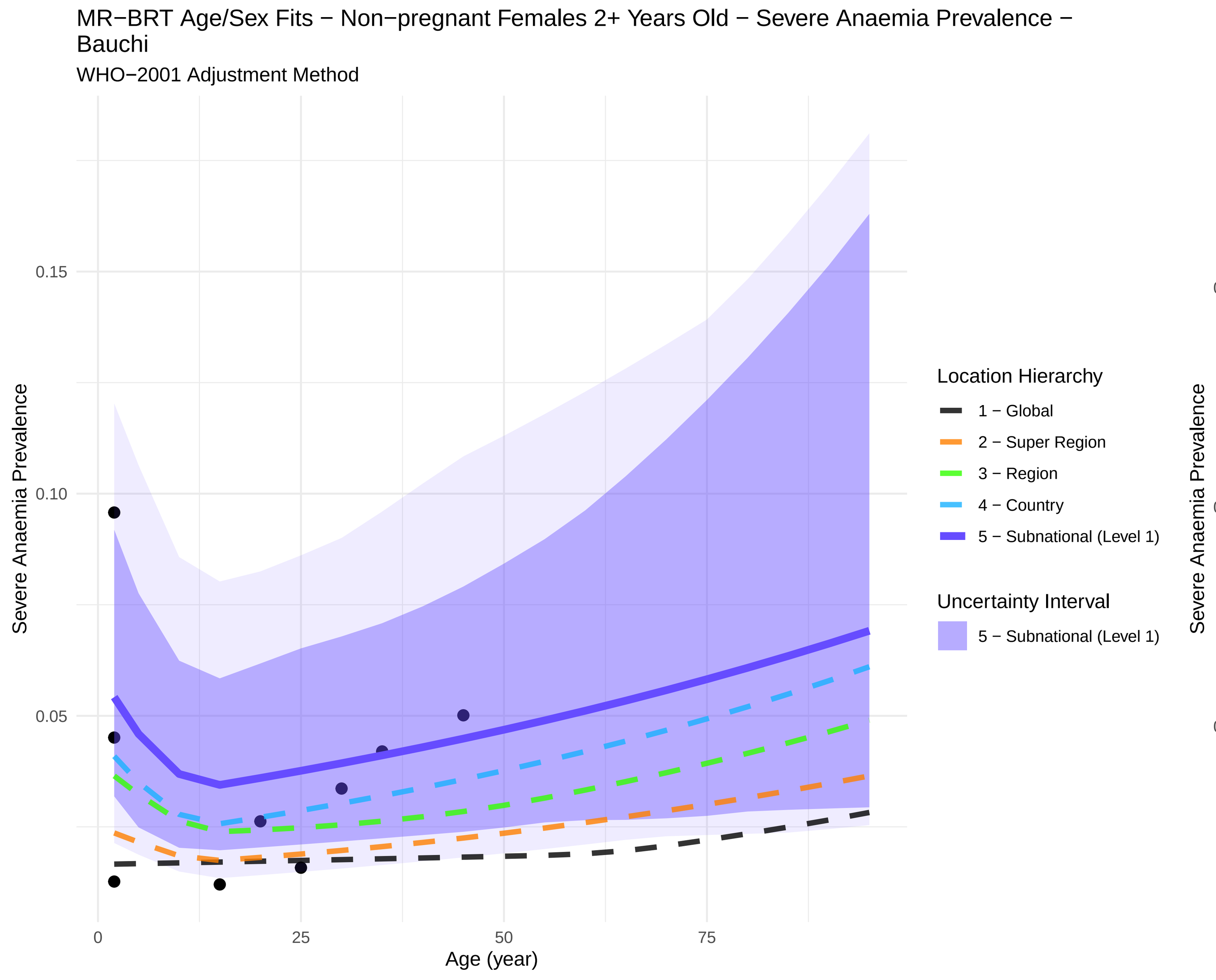
MR−BRT Age/Sex Fits − Non−pregnant Females 2+ Years Old − Severe Anaemia Prevalence − Bauchi
WHO−2001 Adjustment Method
Severe Anaemia Prevalence
Age (year)
Location Hierarchy
1 − Global
2 − Super Region
3 − Region
4 − Country
5 − Subnational (Level 1)
Uncertainty Interval
5 − Subnational (Level 1)

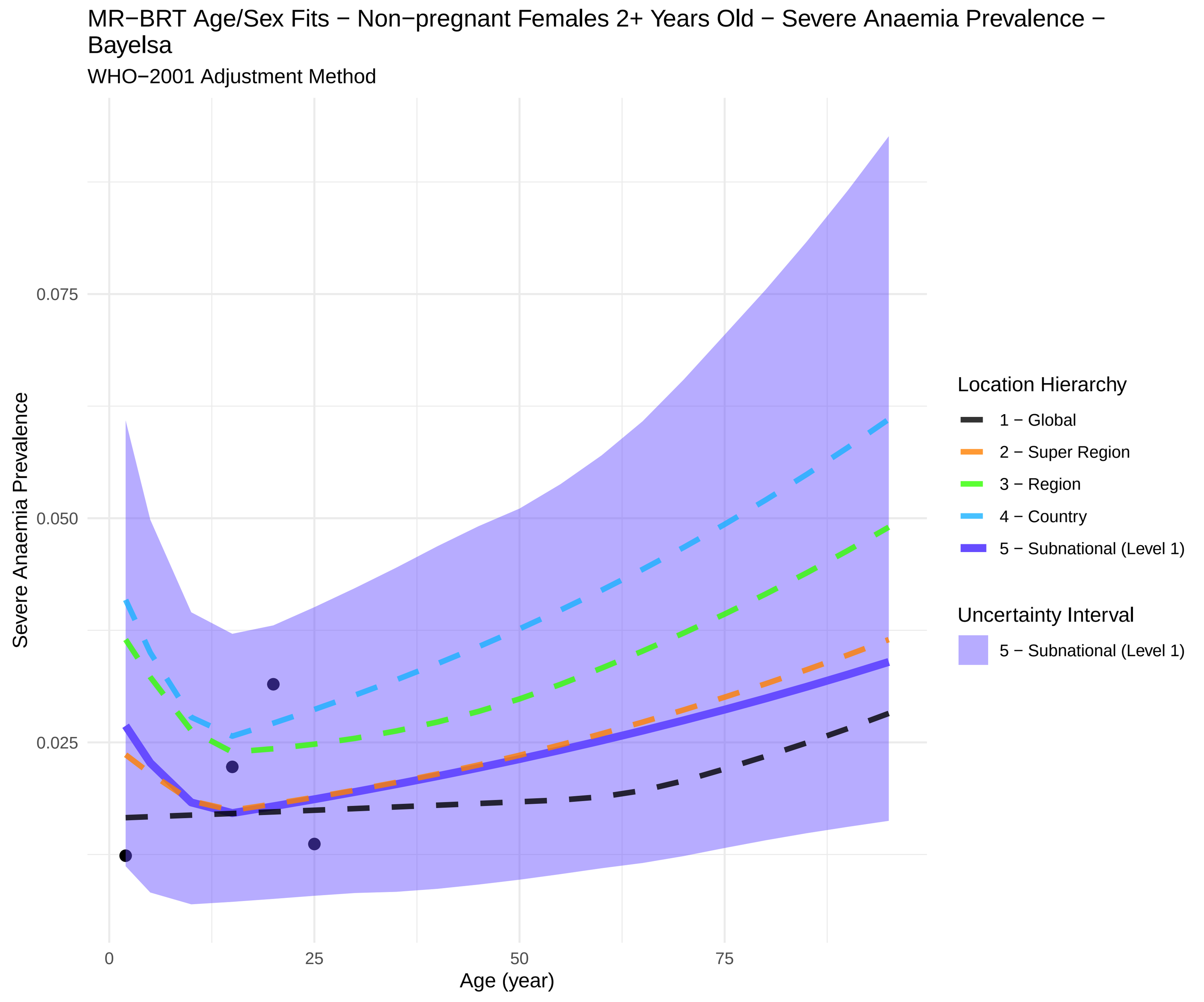
MR−BRT Age/Sex Fits − Non−pregnant Females 2+ Years Old − Severe Anaemia Prevalence − Bayelsa
WHO−2001 Adjustment Method
Severe Anaemia Prevalence
Age (year)
Location Hierarchy
1 − Global
2 − Super Region
3 − Region
4 − Country
5 − Subnational (Level 1)
Uncertainty Interval
5 − Subnational (Level 1)

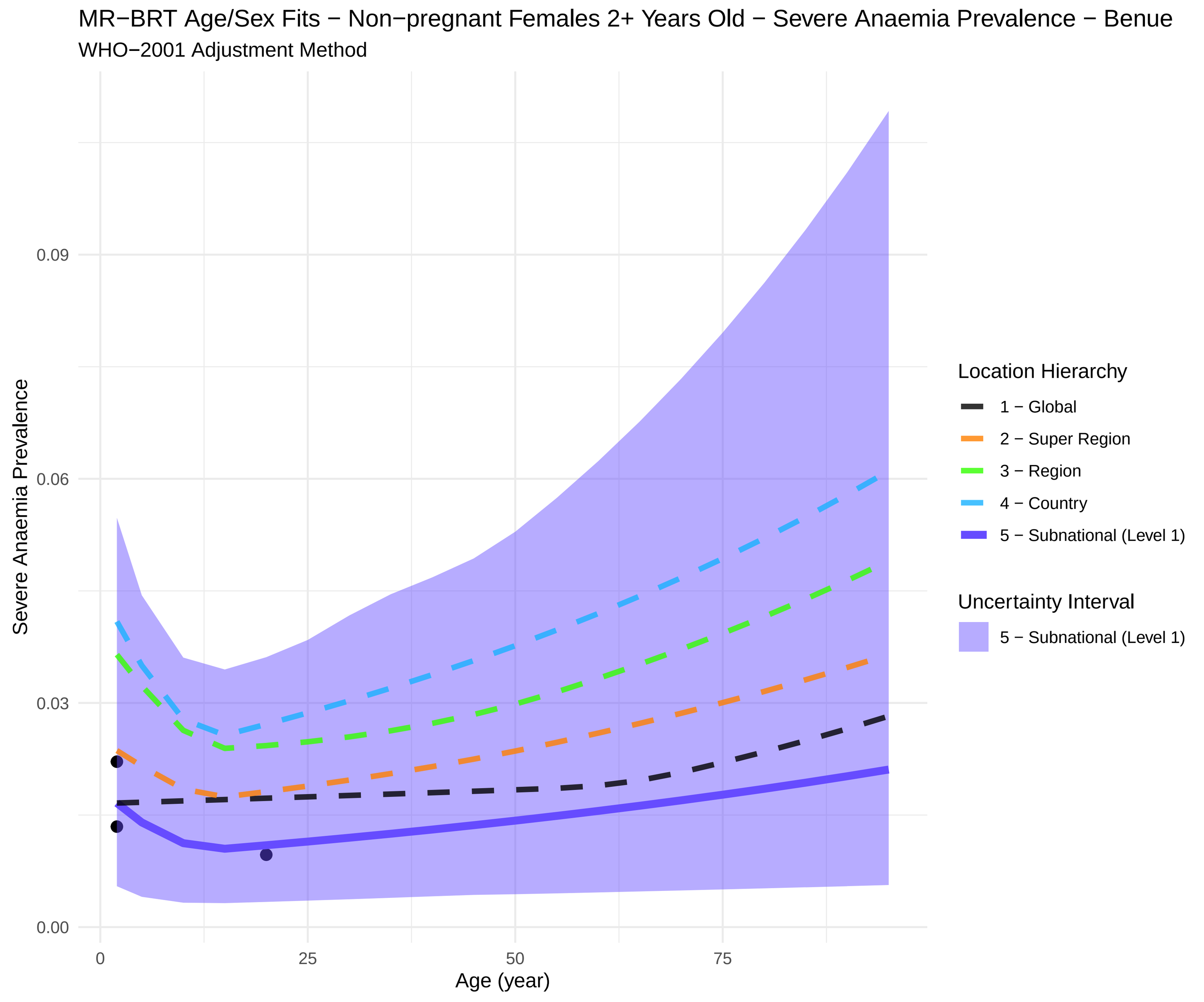
MR−BRT Age/Sex Fits − Non−pregnant Females 2+ Years Old − Severe Anaemia Prevalence − Benue
WHO−2001 Adjustment Method
Severe Anaemia Prevalence
Age (year)
Location Hierarchy
1 − Global
2 − Super Region
3 − Region
4 − Country
5 − Subnational (Level 1)
Uncertainty Interval
5 − Subnational (Level 1)

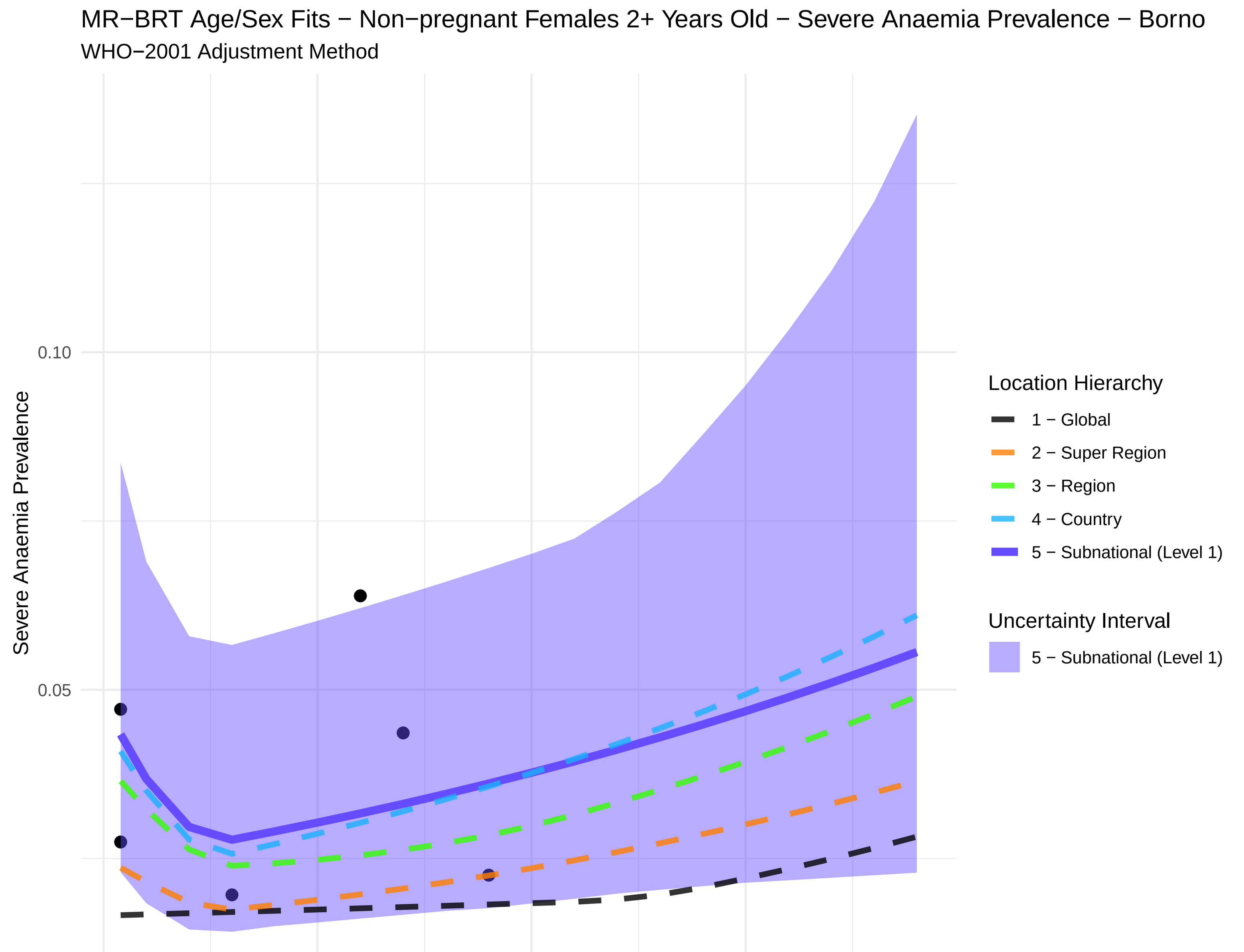
MR−BRT Age/Sex Fits − Non−pregnant Females 2+ Years Old − Severe Anaemia Prevalence − Borno
Severe Anaemia Prevalence
Age (year)
Location Hierarchy
1 − Global
2 − Super Region
3 − Region
4 − Country
5 − Subnational (Level 1)
Uncertainty Interval
5 − Subnational (Level 1)

MR−BRT Age/Sex Fits − Non−pregnant Females 2+ Years Old − Severe Anaemia Prevalence − Cross River
WHO−2001 Adjustment Method

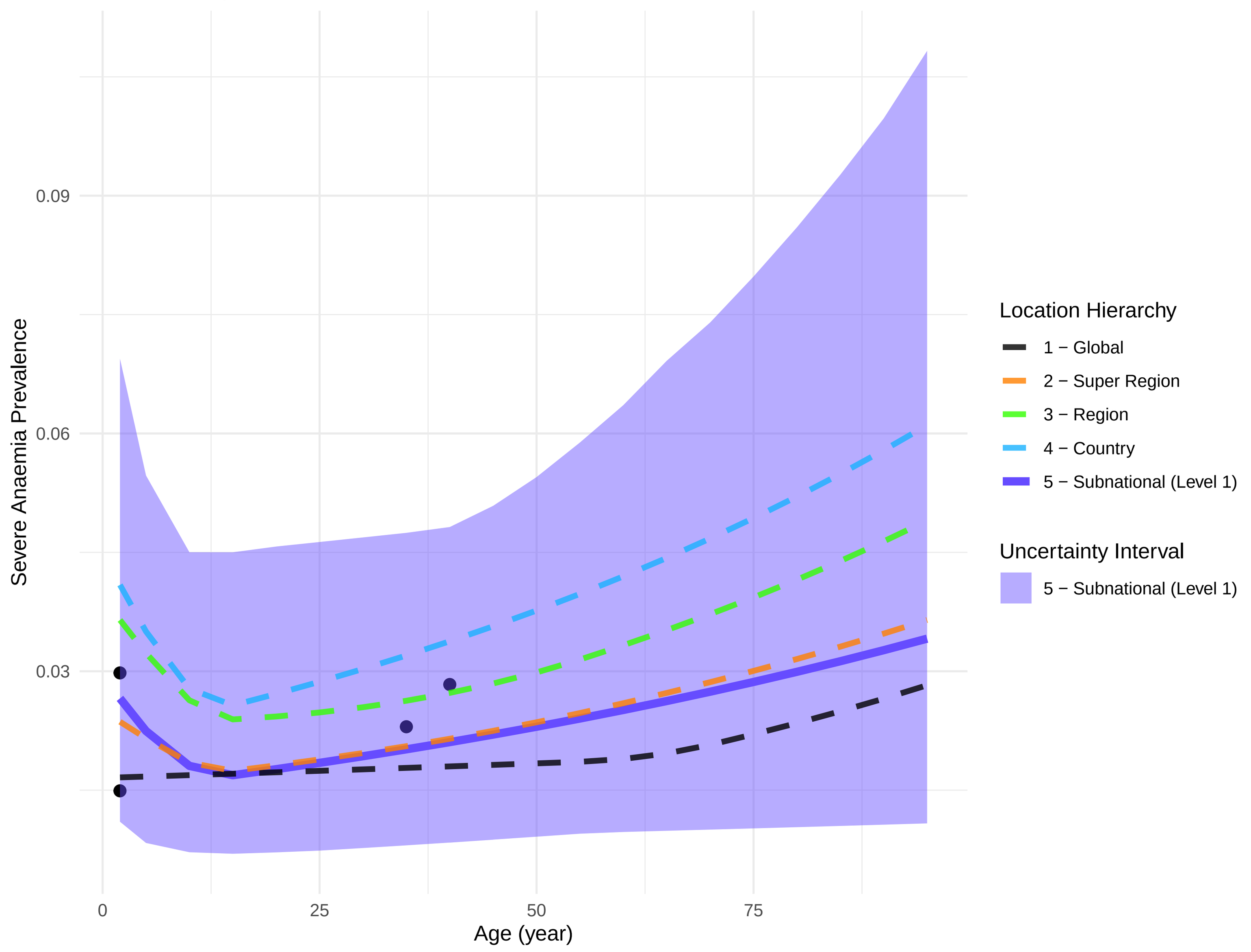

MR−BRT Age/Sex Fits − Non−pregnant Females 2+ Years Old − Severe Anaemia Prevalence − Delta
WHO−2001 Adjustment Method

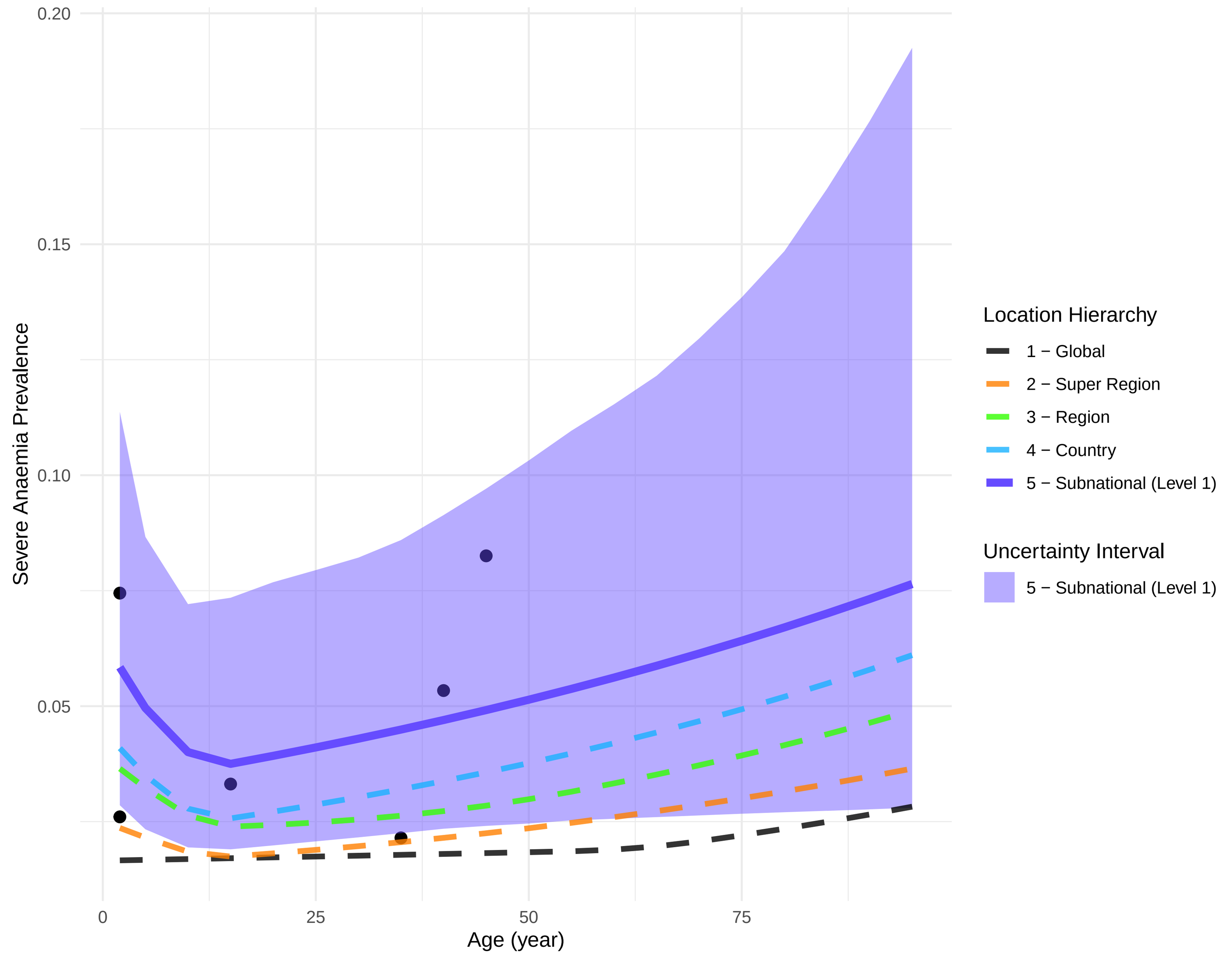

MR−BRT Age/Sex Fits − Non−pregnant Females 2+ Years Old − Severe Anaemia Prevalence − Ebonyi
WHO−2001 Adjustment Method

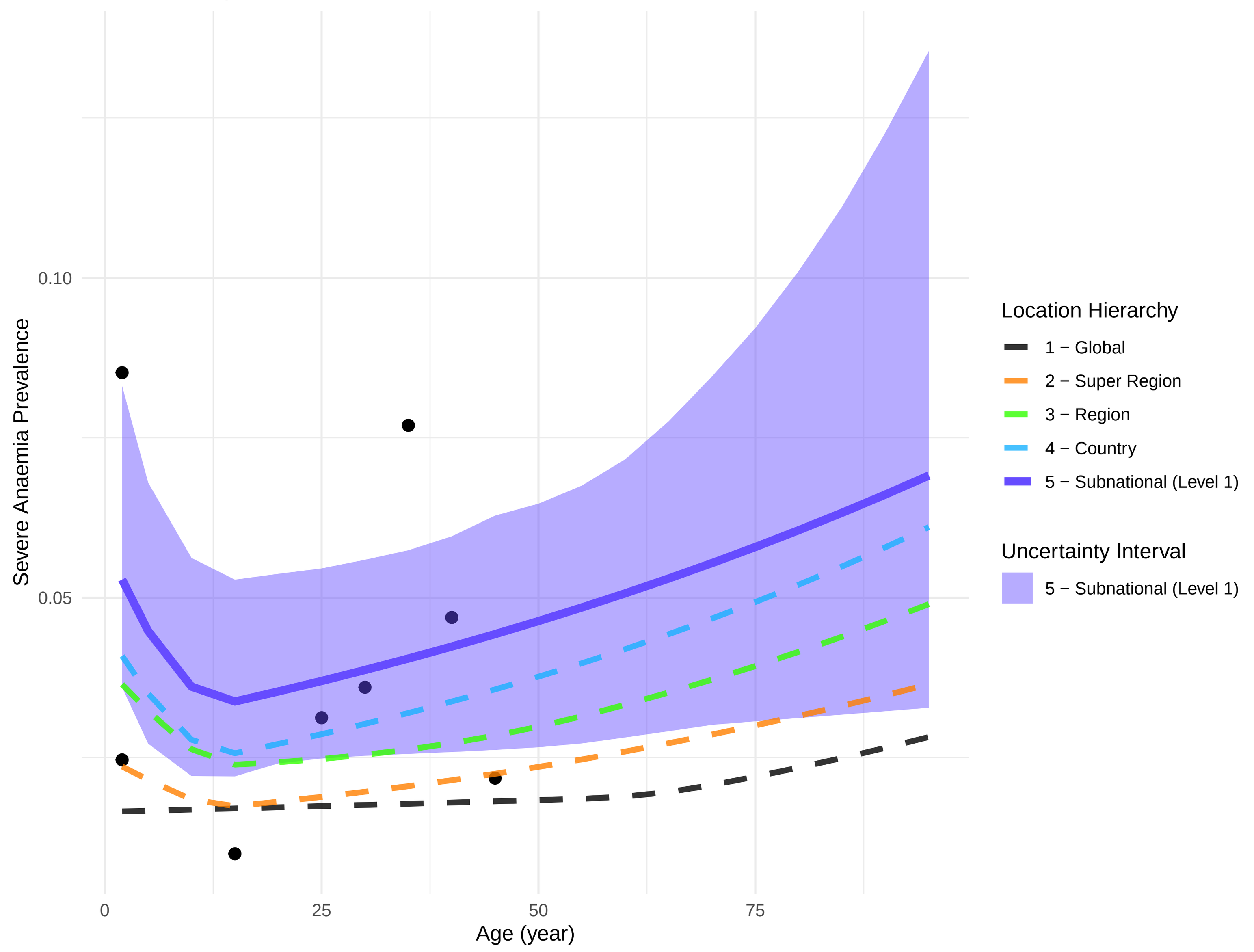

MR−BRT Age/Sex Fits − Non−pregnant Females 2+ Years Old − Severe Anaemia Prevalence − Edo
WHO−2001 Adjustment Method

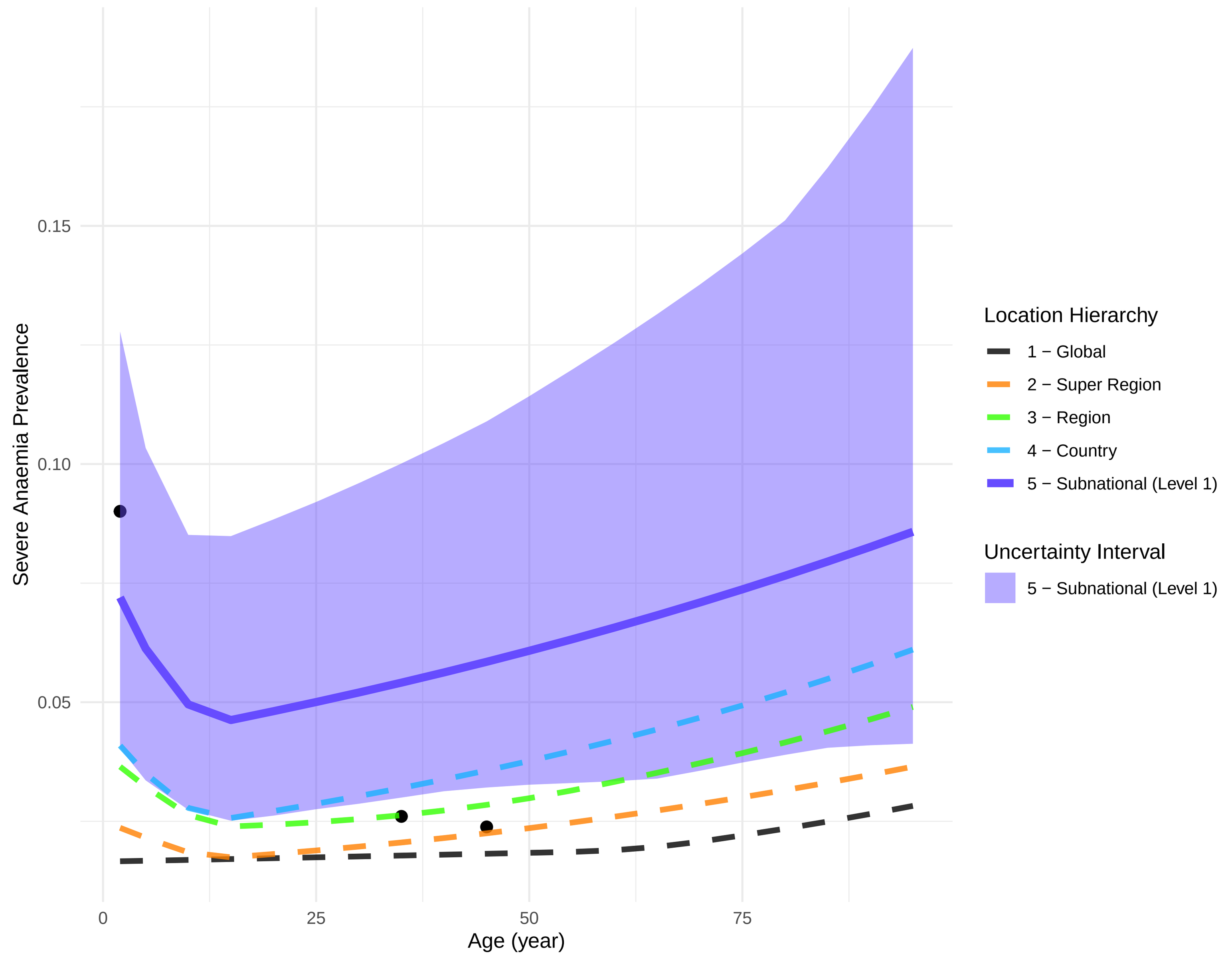

MR−BRT Age/Sex Fits − Non−pregnant Females 2+ Years Old − Severe Anaemia Prevalence − Ekiti
WHO−2001 Adjustment Method

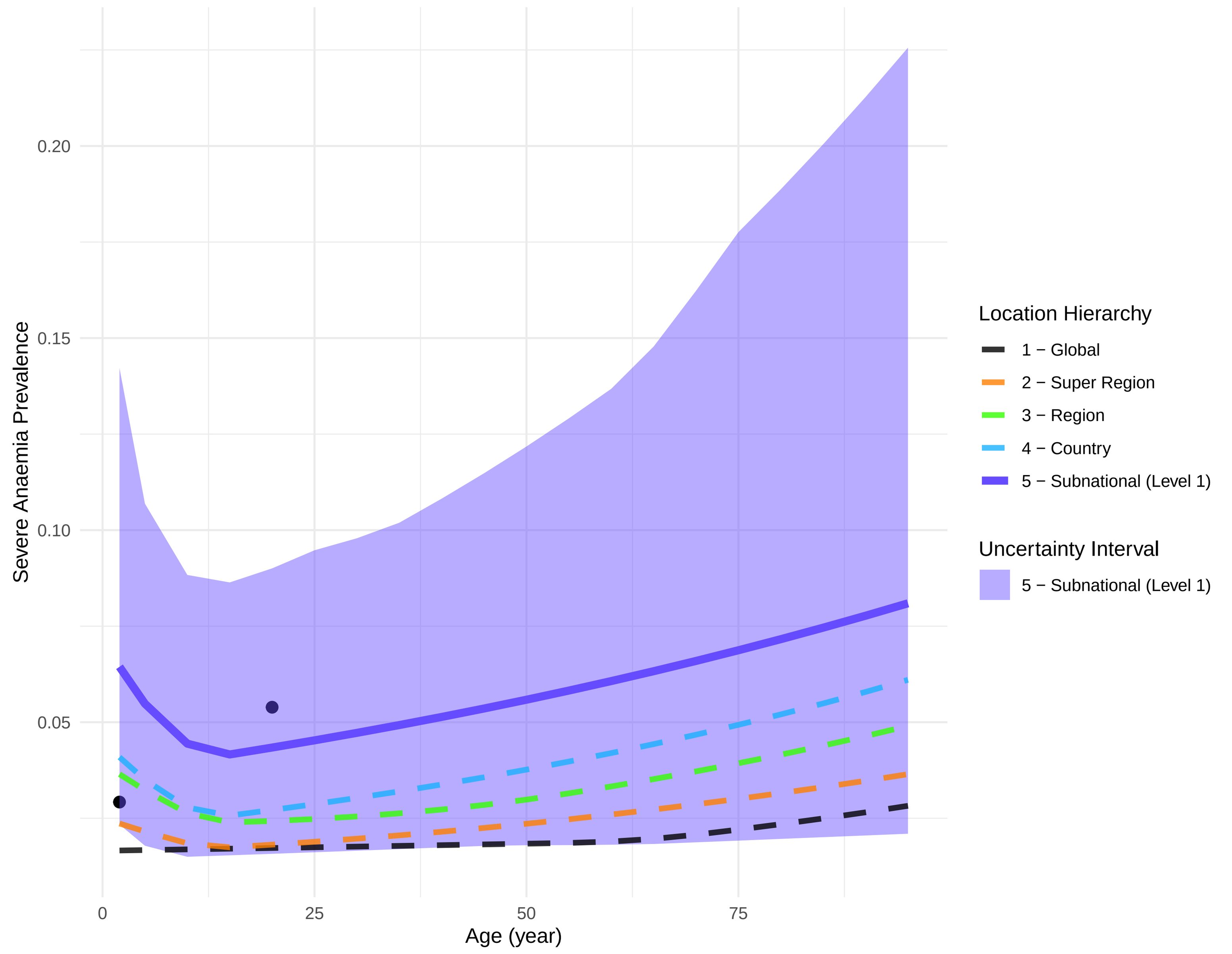

MR−BRT Age/Sex Fits − Non−pregnant Females 2+ Years Old − Severe Anaemia Prevalence − Enugu
WHO−2001 Adjustment Method

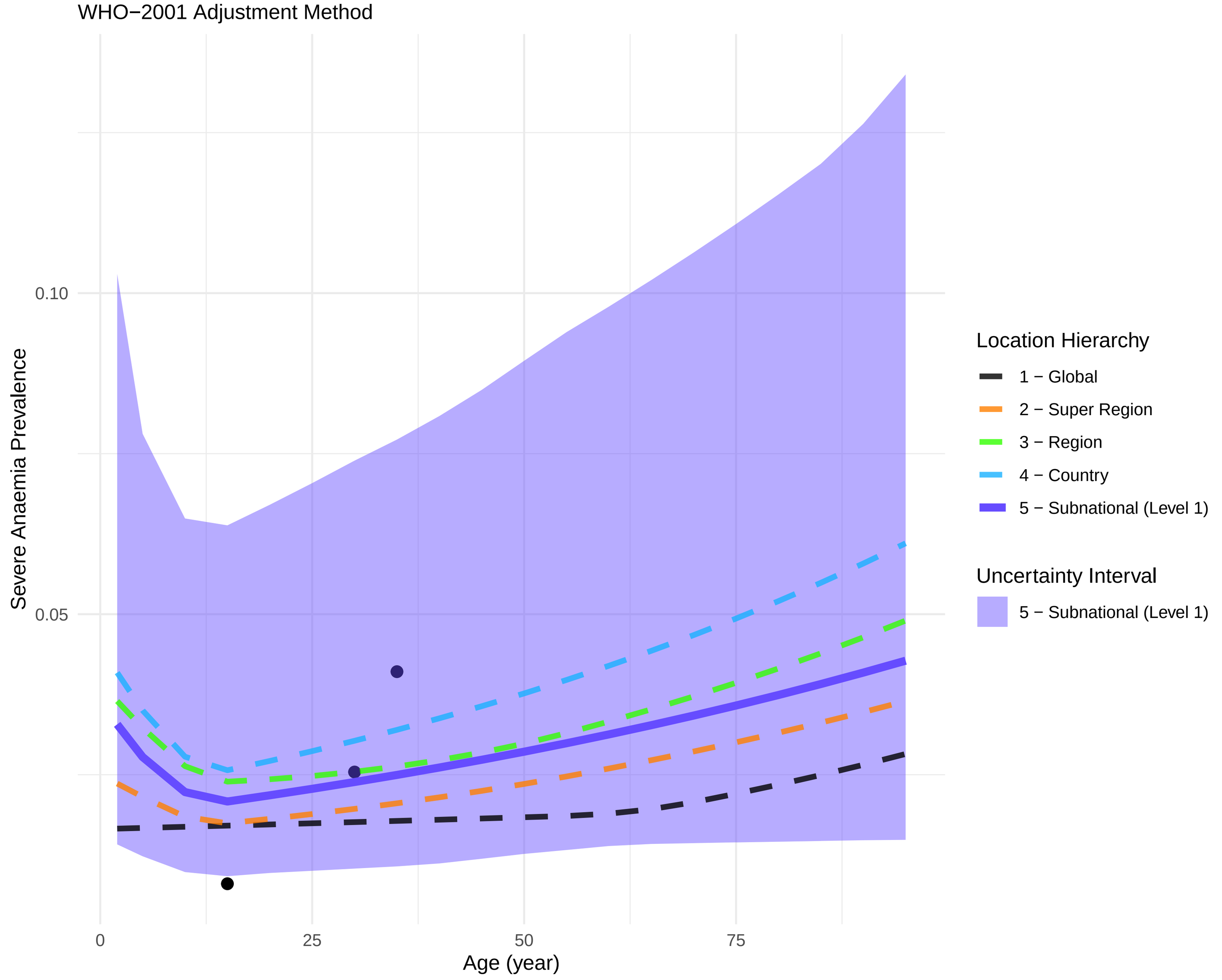

MR−BRT Age/Sex Fits − Non−pregnant Females 2+ Years Old − Severe Anaemia Prevalence − FCT (Abuja)
WHO−2001 Adjustment Method

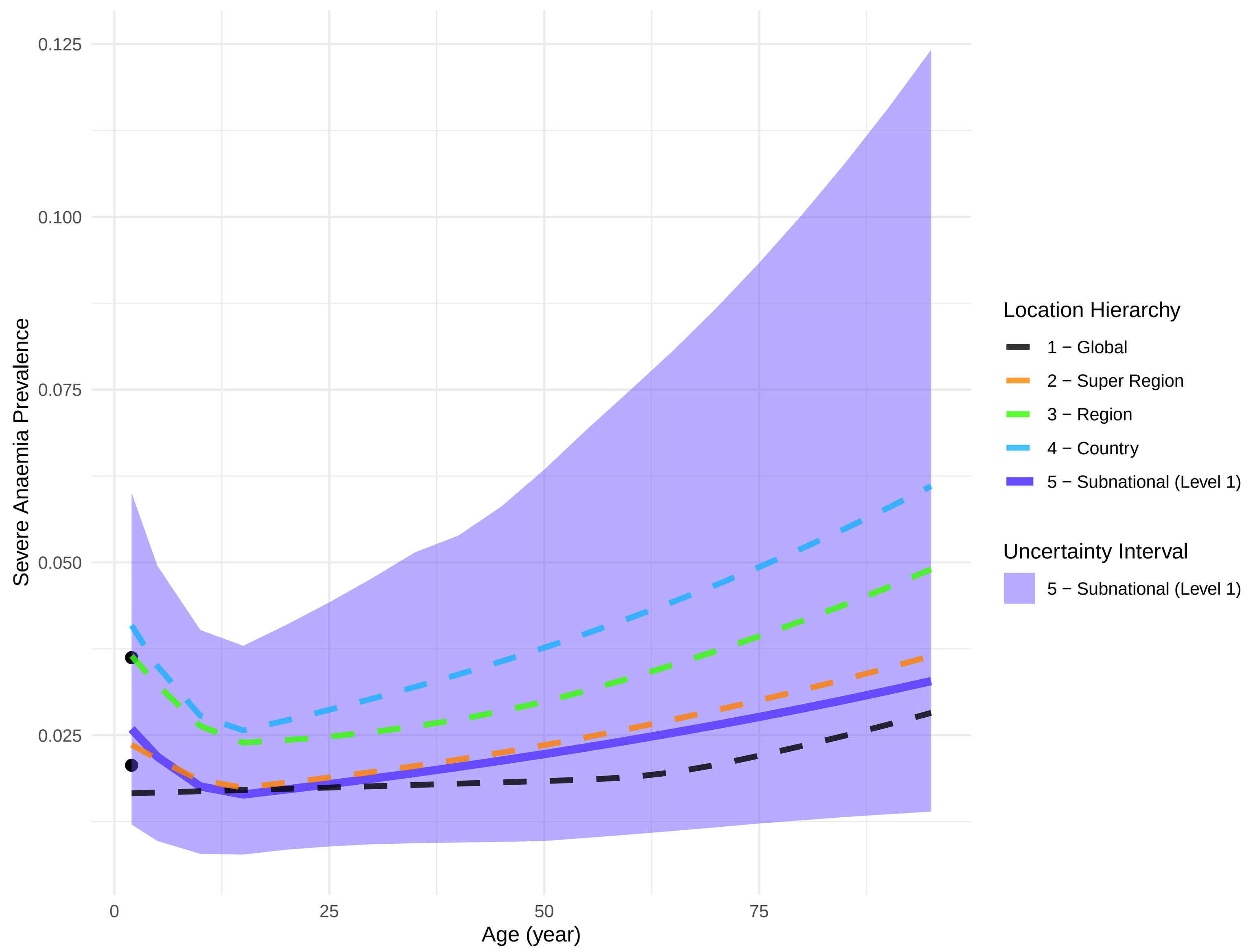

MR−BRT Age/Sex Fits − Non−pregnant Females 2+ Years Old − Severe Anaemia Prevalence − Gombe
WHO−2001 Adjustment Method

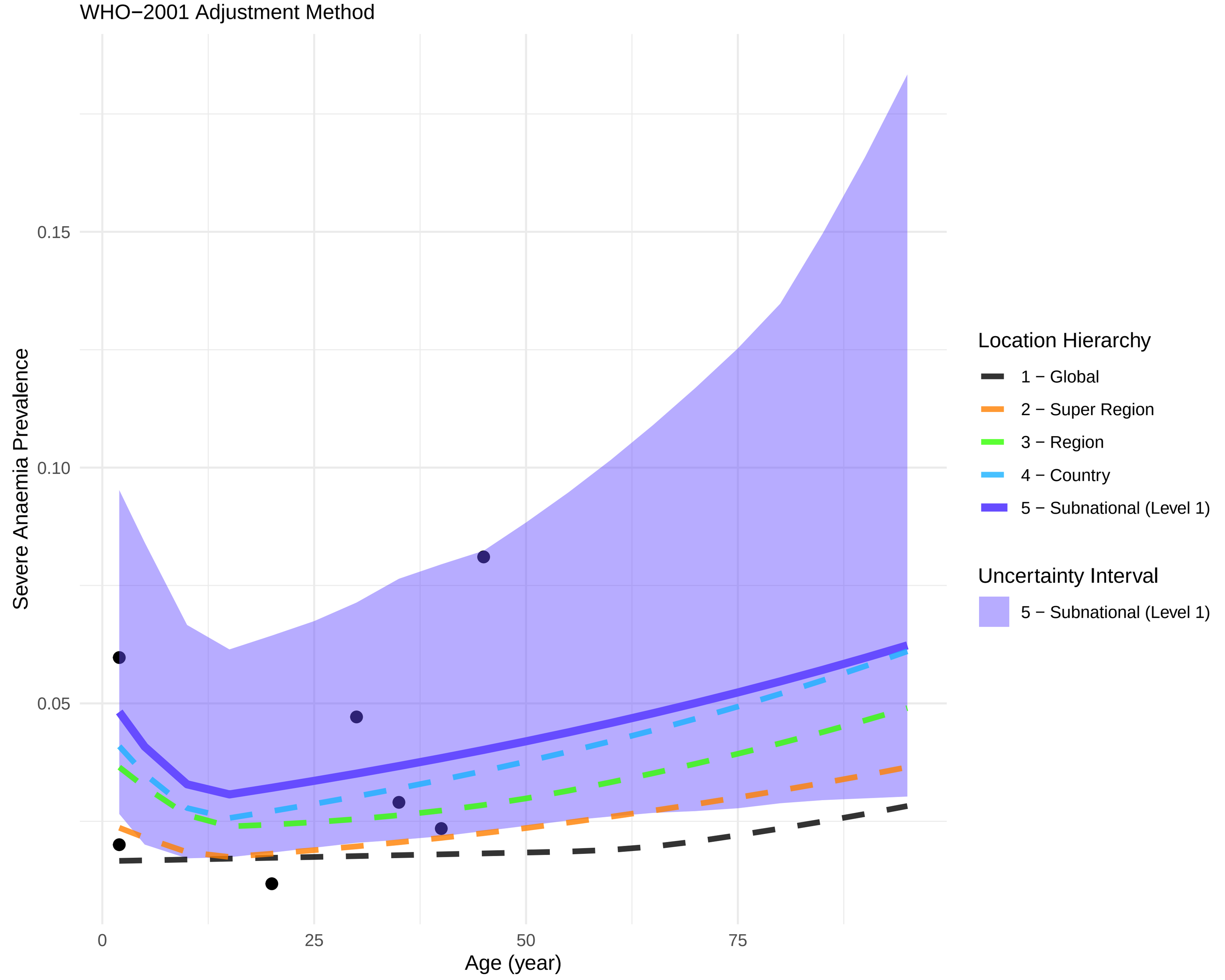

MR−BRT Age/Sex Fits − Non−pregnant Females 2+ Years Old − Severe Anaemia Prevalence − Imo
WHO−2001 Adjustment Method

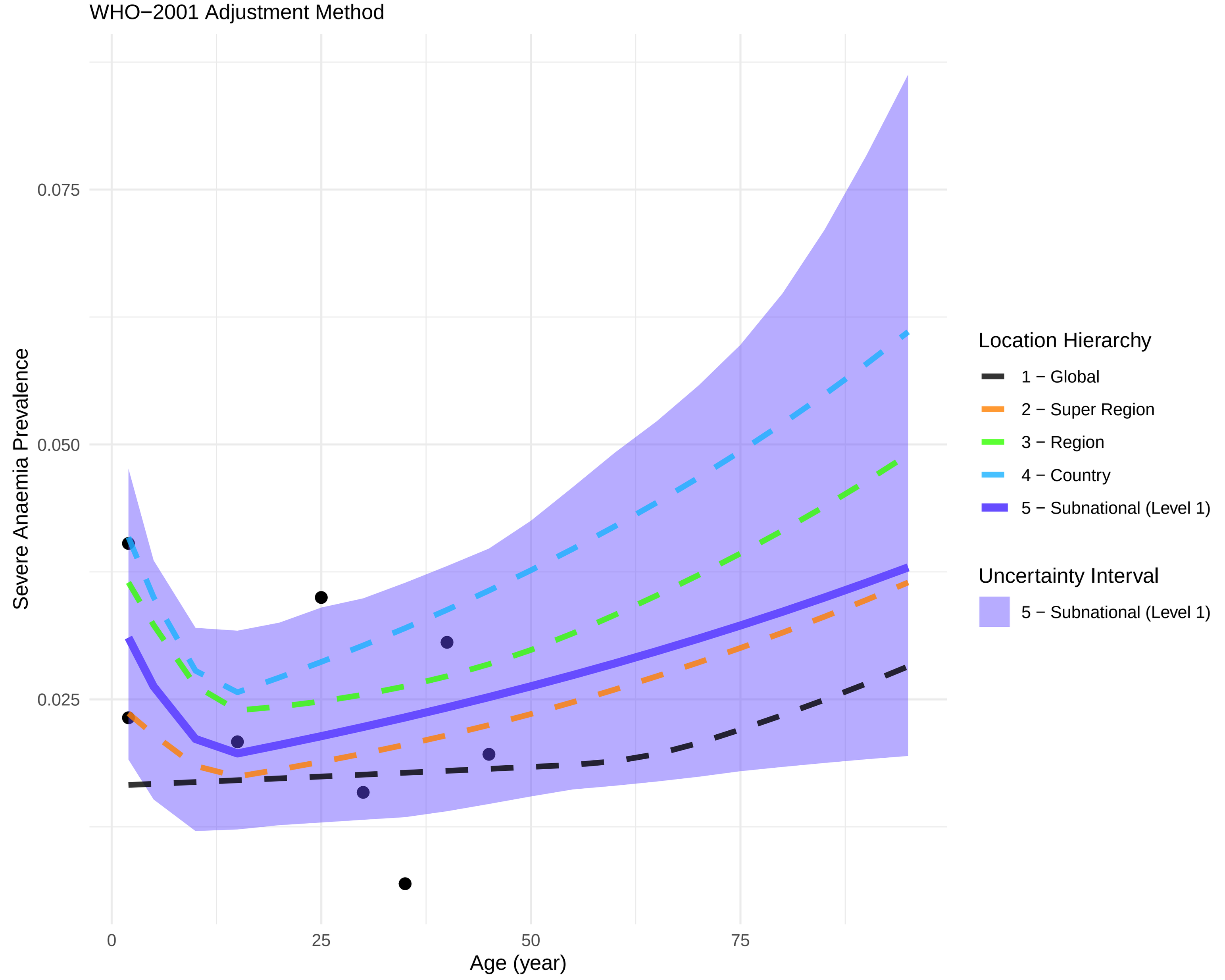

MR−BRT Age/Sex Fits − Non−pregnant Females 2+ Years Old − Severe Anaemia Prevalence − Jigawa
WHO−2001 Adjustment Method

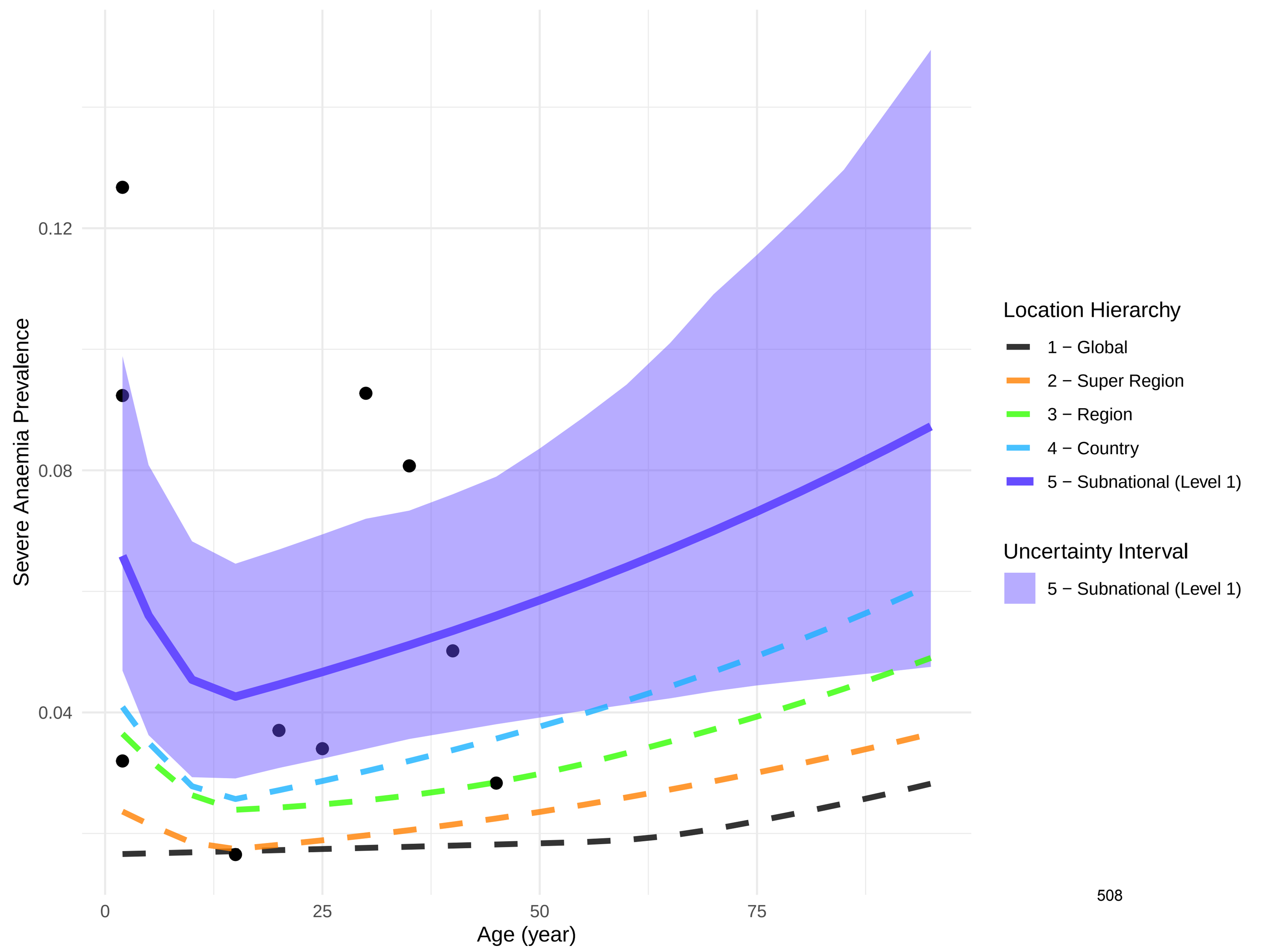

MR−BRT Age/Sex Fits − Non−pregnant Females 2+ Years Old − Severe Anaemia Prevalence − Kaduna
WHO−2001 Adjustment Method

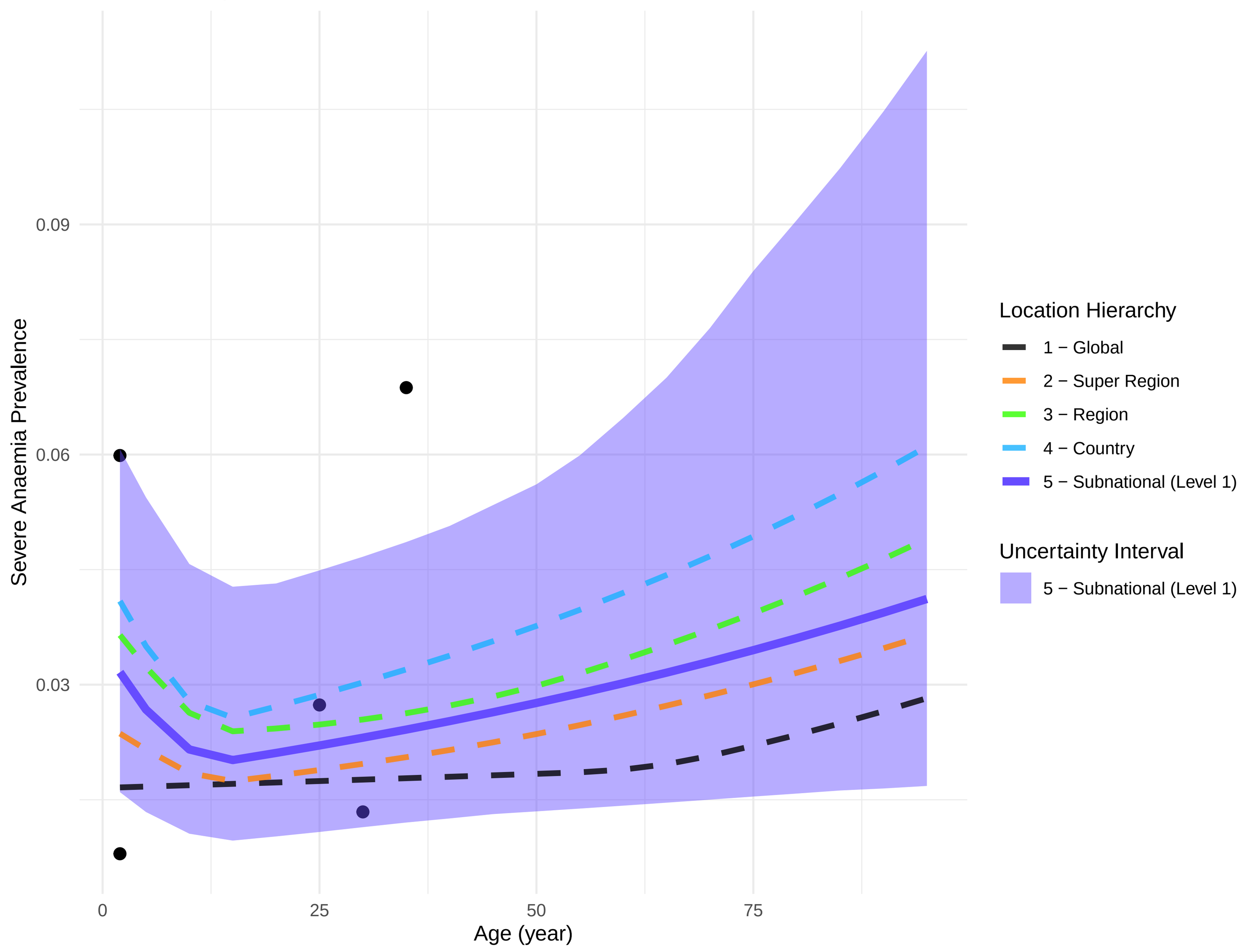

MR−BRT Age/Sex Fits − Non−pregnant Females 2+ Years Old − Severe Anaemia Prevalence − Kano
WHO−2001 Adjustment Method

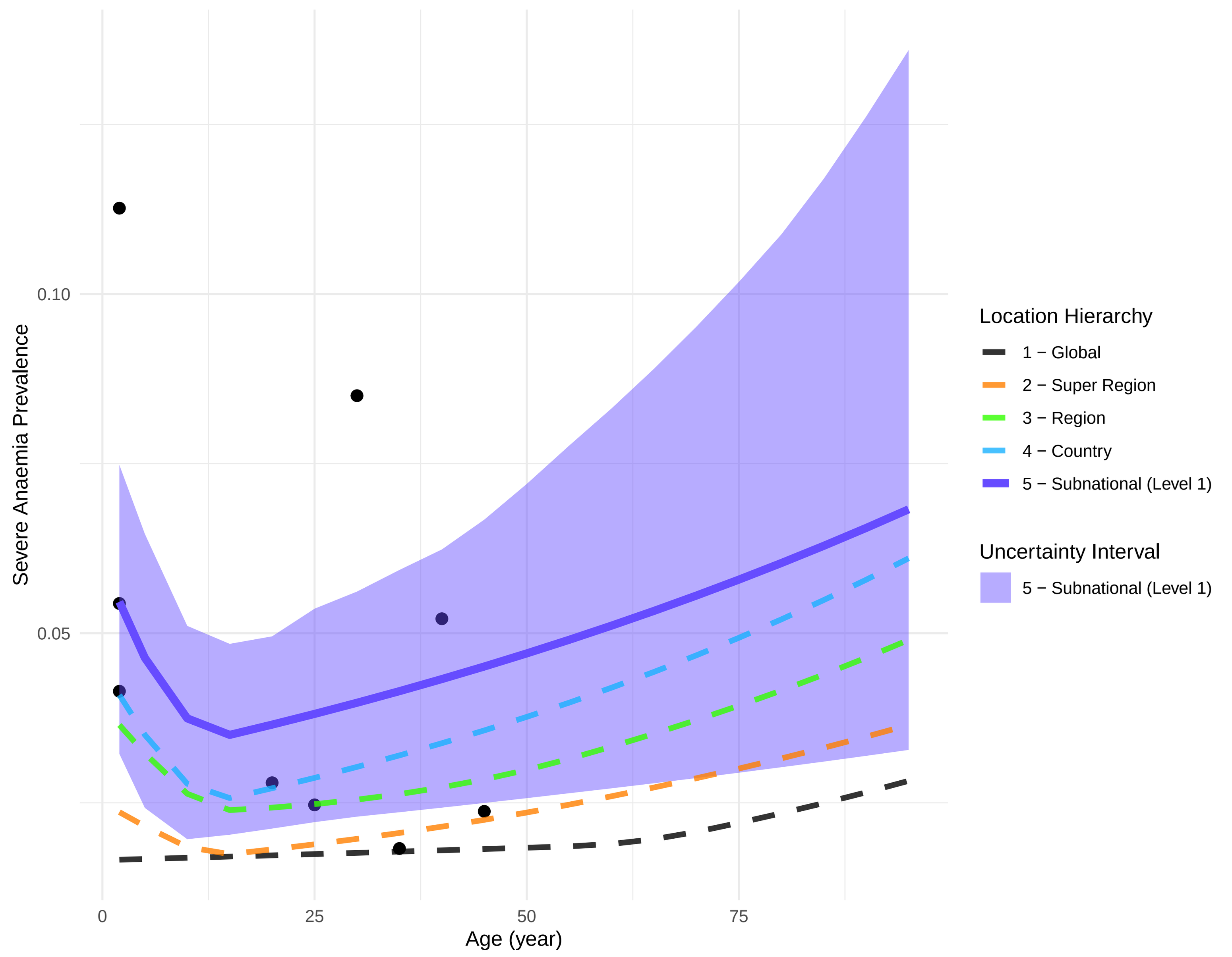

MR−BRT Age/Sex Fits − Non−pregnant Females 2+ Years Old − Severe Anaemia Prevalence − Katsina
WHO−2001 Adjustment Method

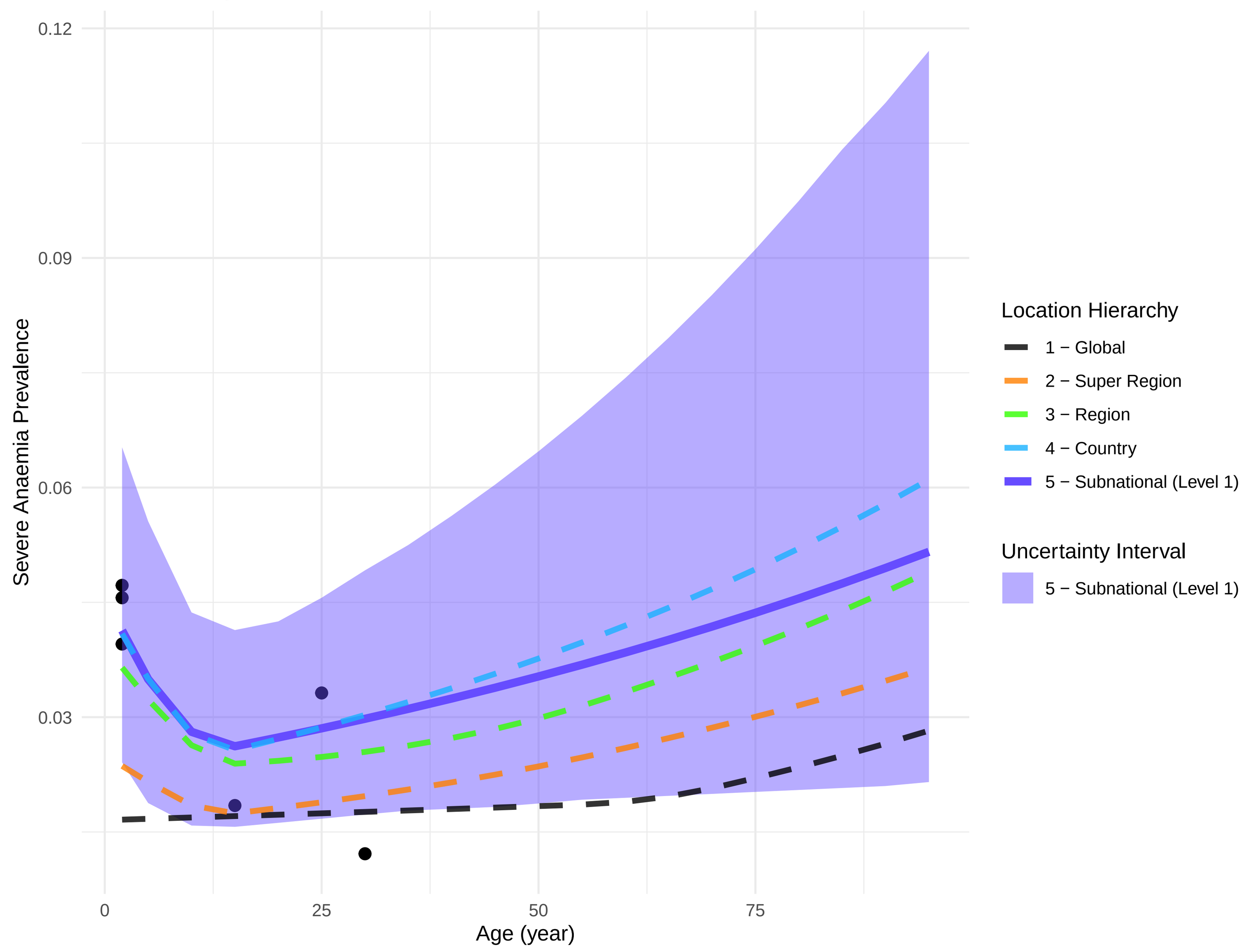

MR−BRT Age/Sex Fits − Non−pregnant Females 2+ Years Old − Severe Anaemia Prevalence − Kebbi
WHO−2001 Adjustment Method

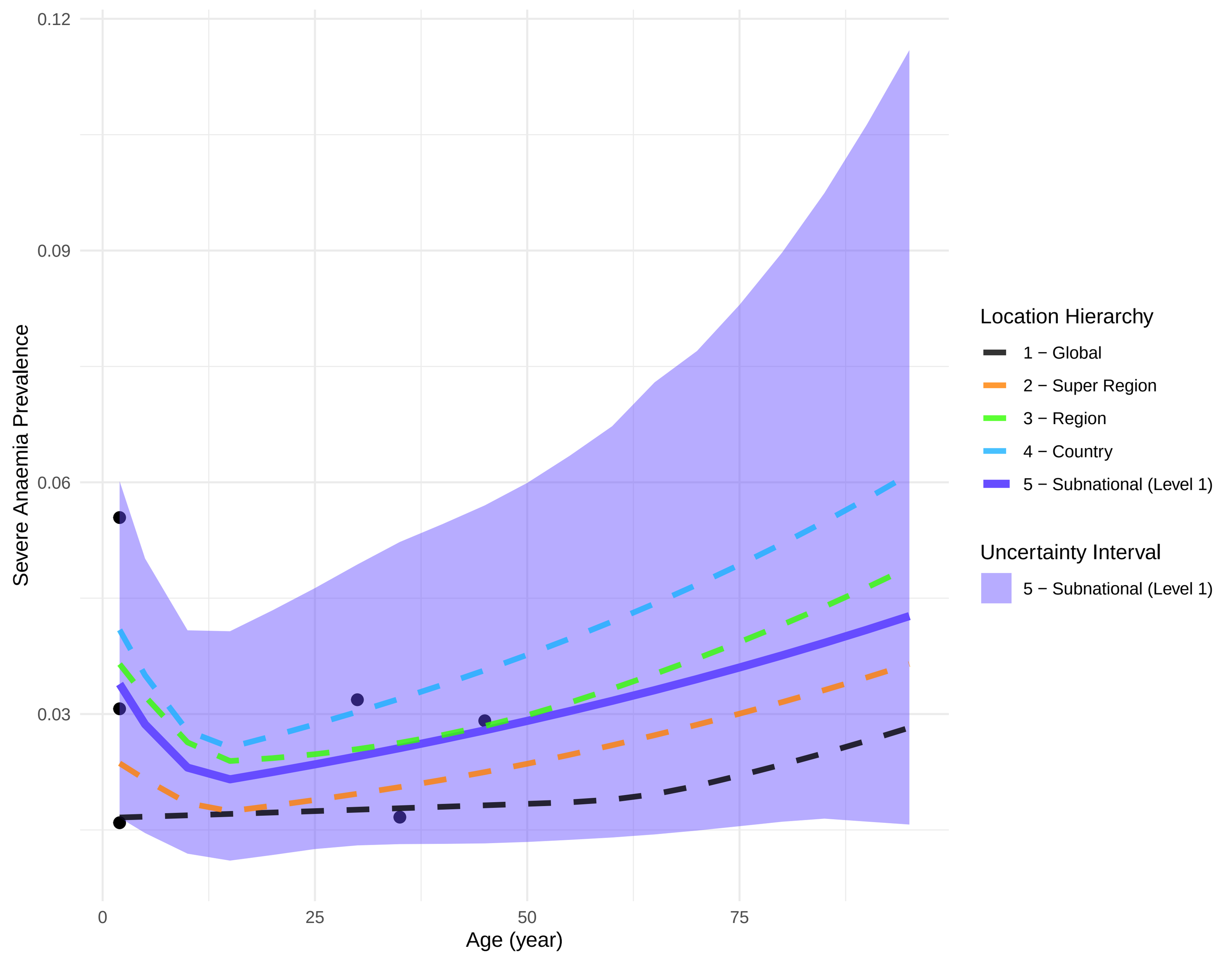

MR−BRT Age/Sex Fits − Non−pregnant Females 2+ Years Old − Severe Anaemia Prevalence − Kogi
WHO−2001 Adjustment Method

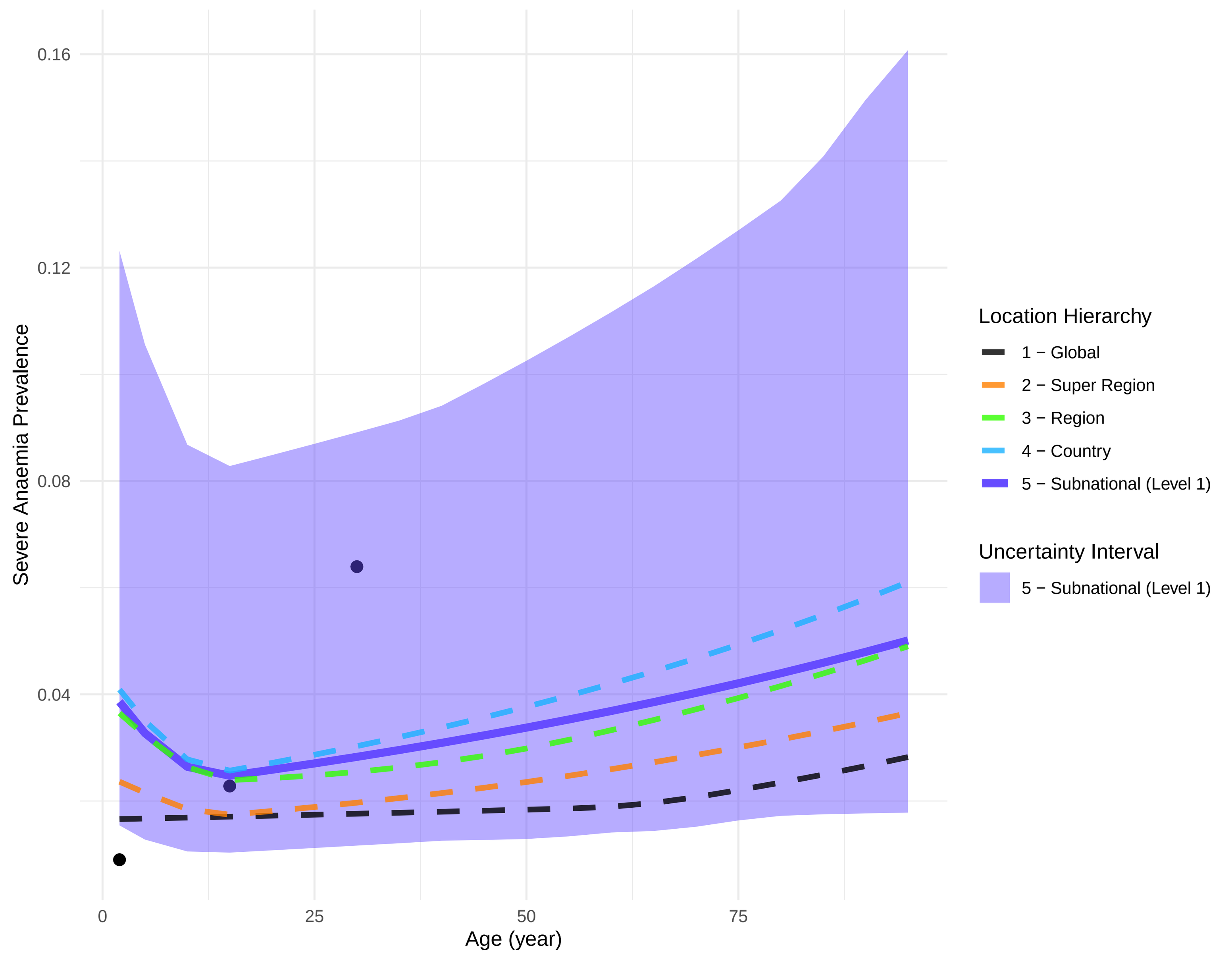

MR−BRT Age/Sex Fits − Non−pregnant Females 2+ Years Old − Severe Anaemia Prevalence − Kwara
WHO−2001 Adjustment Method

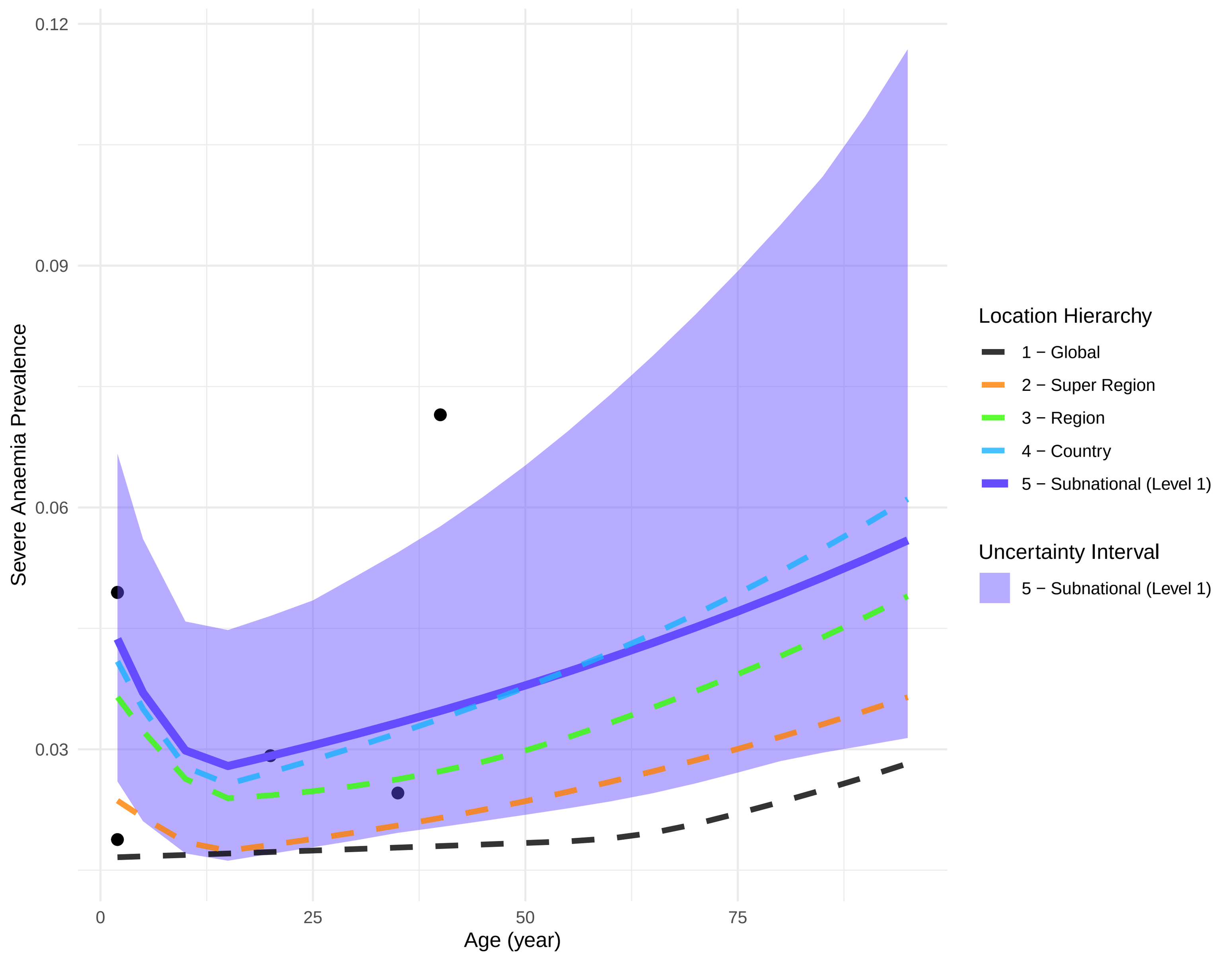

MR−BRT Age/Sex Fits − Non−pregnant Females 2+ Years Old − Severe Anaemia Prevalence − Lagos
WHO−2001 Adjustment Method

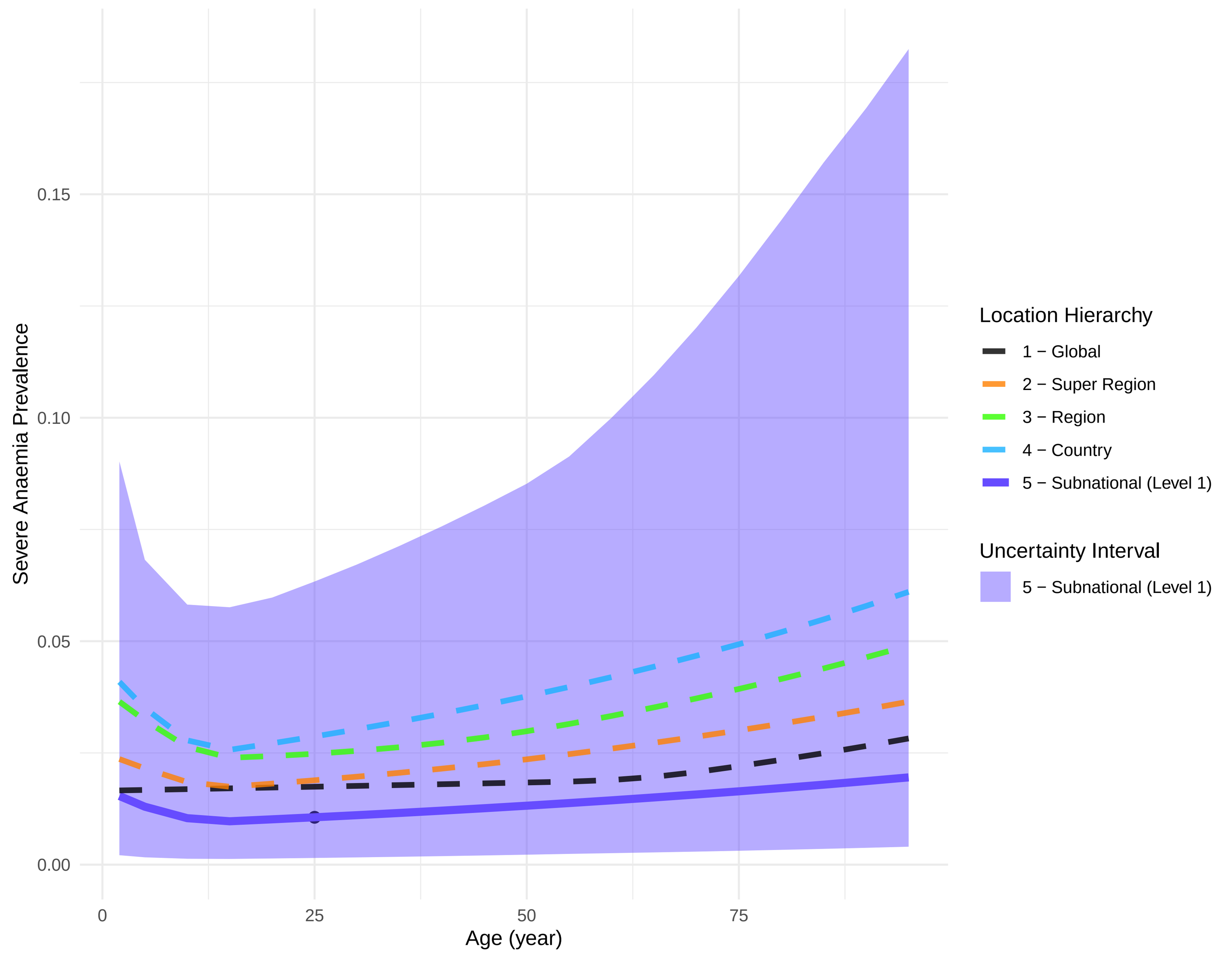

MR−BRT Age/Sex Fits − Non−pregnant Females 2+ Years Old − Severe Anaemia Prevalence − Nasarawa
WHO−2001 Adjustment Method

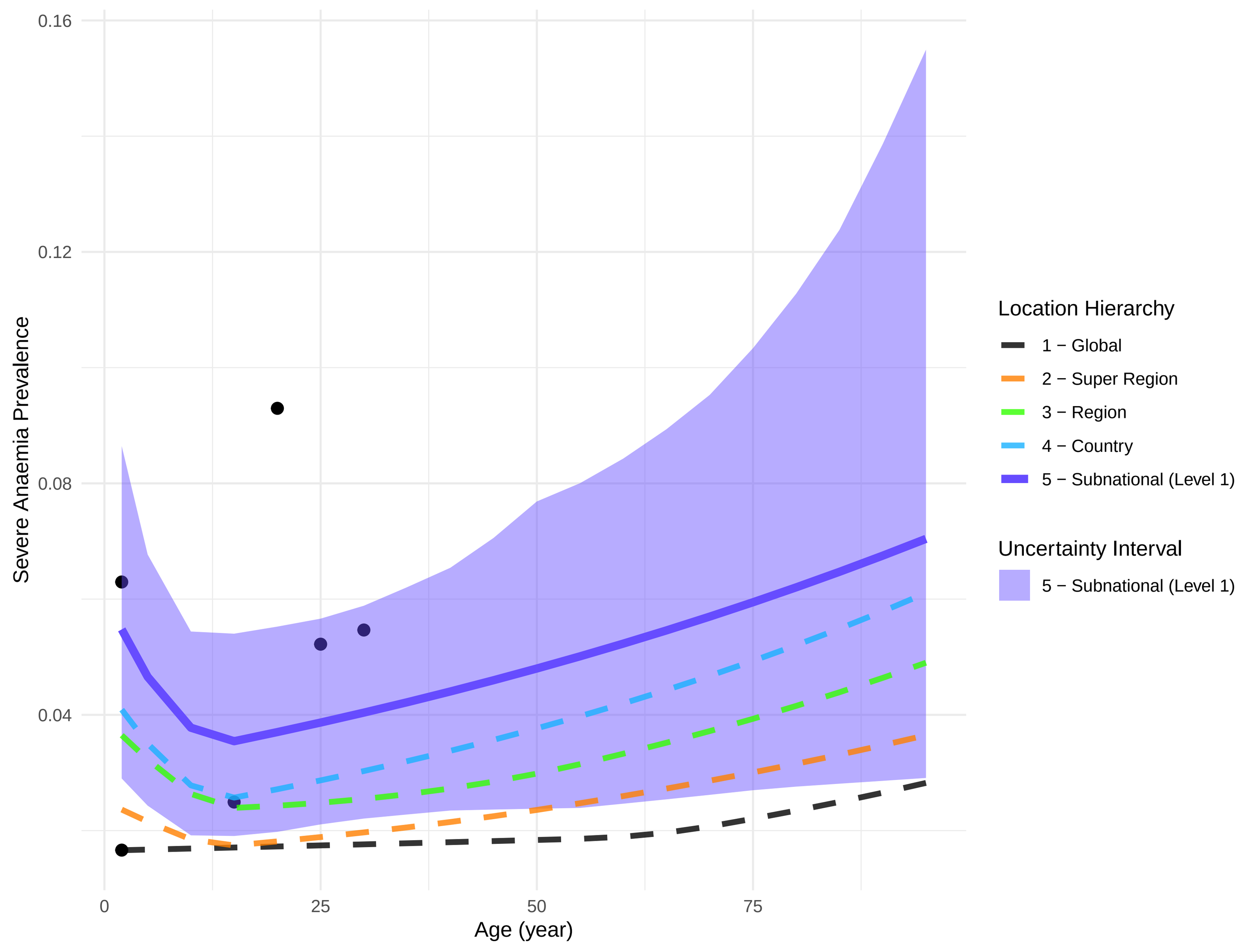

MR−BRT Age/Sex Fits − Non−pregnant Females 2+ Years Old − Severe Anaemia Prevalence − Niger
WHO−2001 Adjustment Method

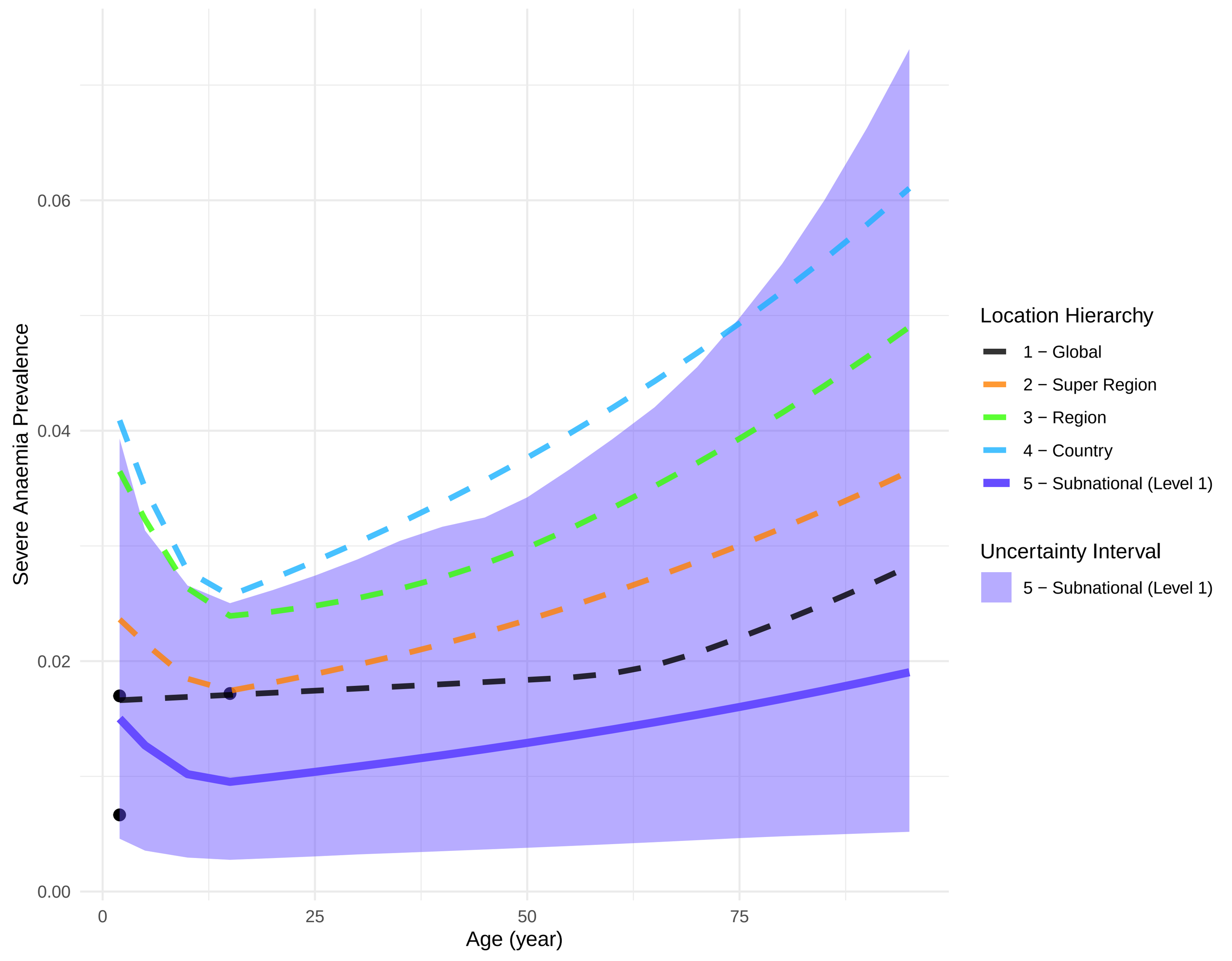

MR−BRT Age/Sex Fits − Non−pregnant Females 2+ Years Old − Severe Anaemia Prevalence − Ogun
WHO−2001 Adjustment Method

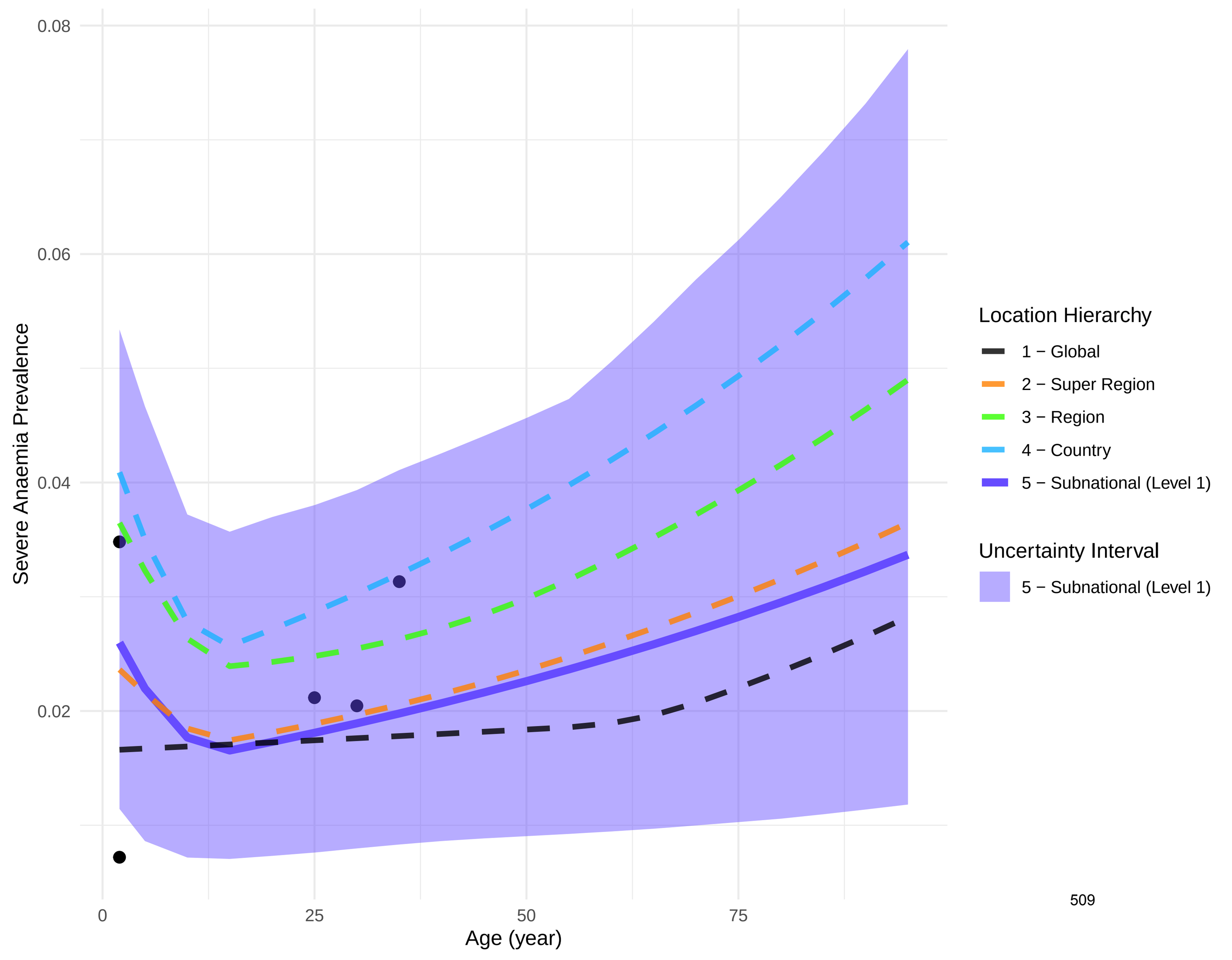

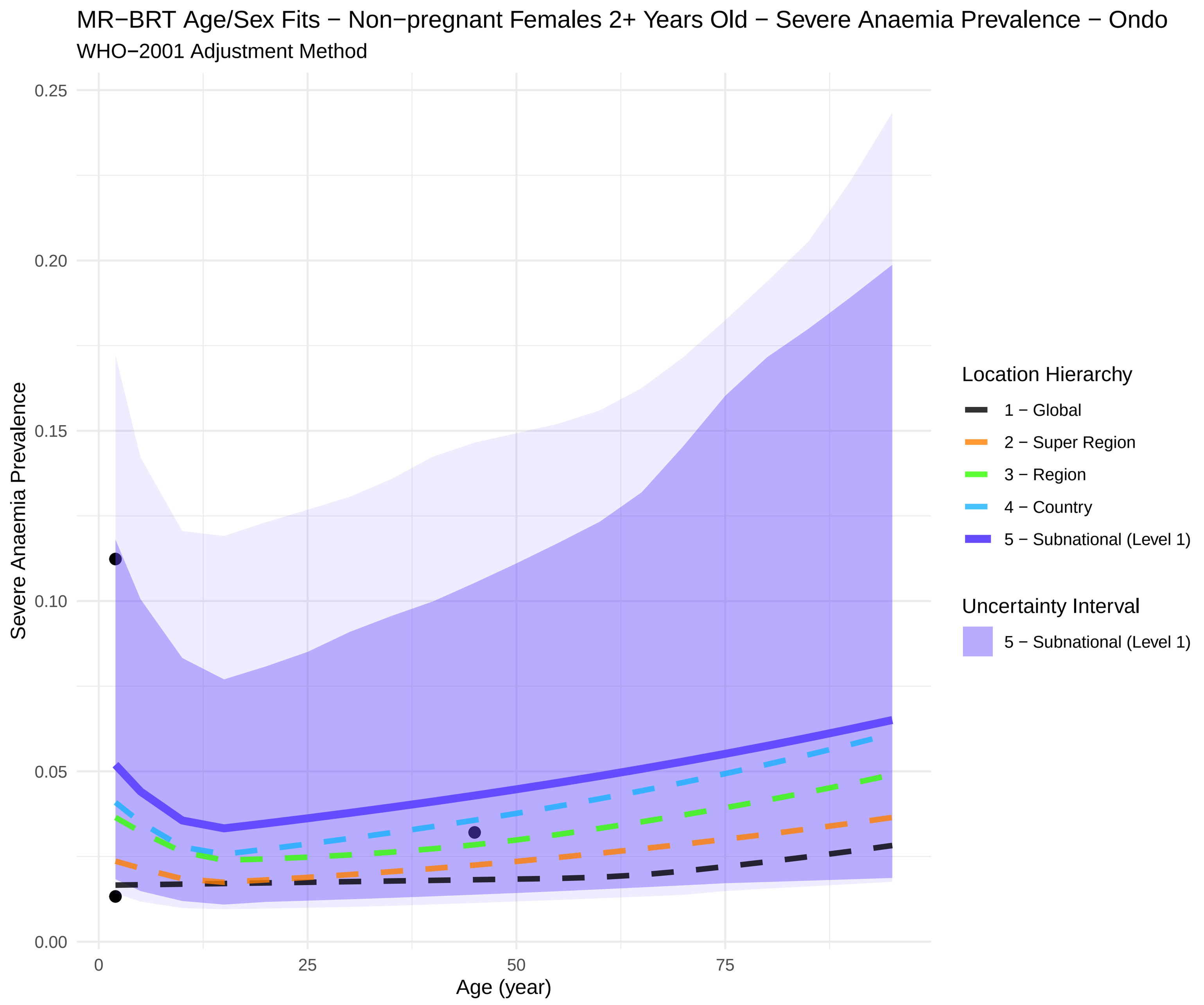
MR−BRT Age/Sex Fits − Non−pregnant Females 2+ Years Old − Severe Anaemia Prevalence − Ondo
WHO−2001 Adjustment Method
Severe Anaemia Prevalence
Age (year)
Location Hierarchy
1 − Global
2 − Super Region
3 − Region
4 − Country
5 − Subnational (Level 1)
Uncertainty Interval
5 − Subnational (Level 1)

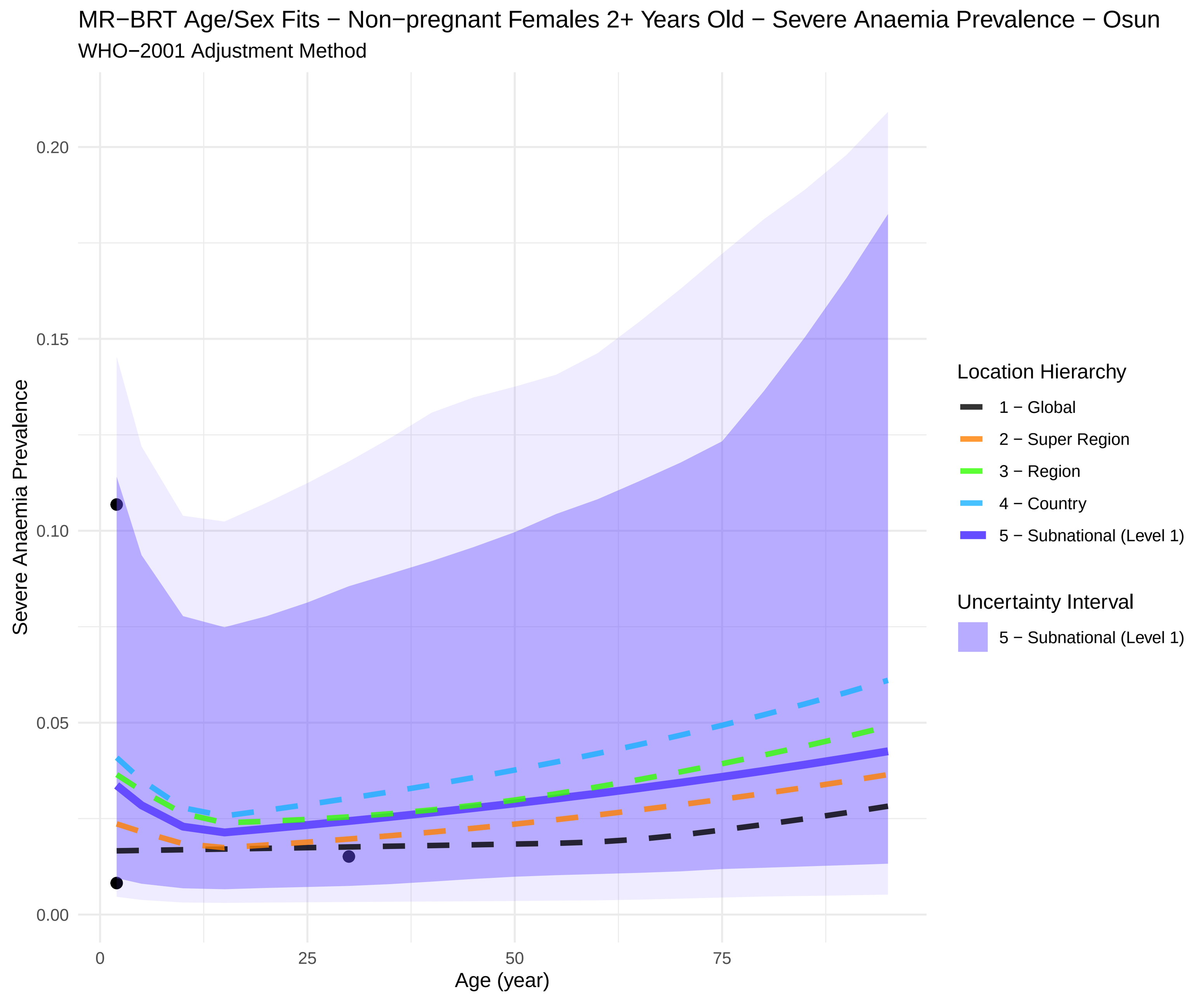
MR−BRT Age/Sex Fits − Non−pregnant Females 2+ Years Old − Severe Anaemia Prevalence − Osun
WHO−2001 Adjustment Method
Severe Anaemia Prevalence
Age (year)
Location Hierarchy
1 − Global
2 − Super Region
3 − Region
4 − Country
5 − Subnational (Level 1)
Uncertainty Interval
5 − Subnational (Level 1)

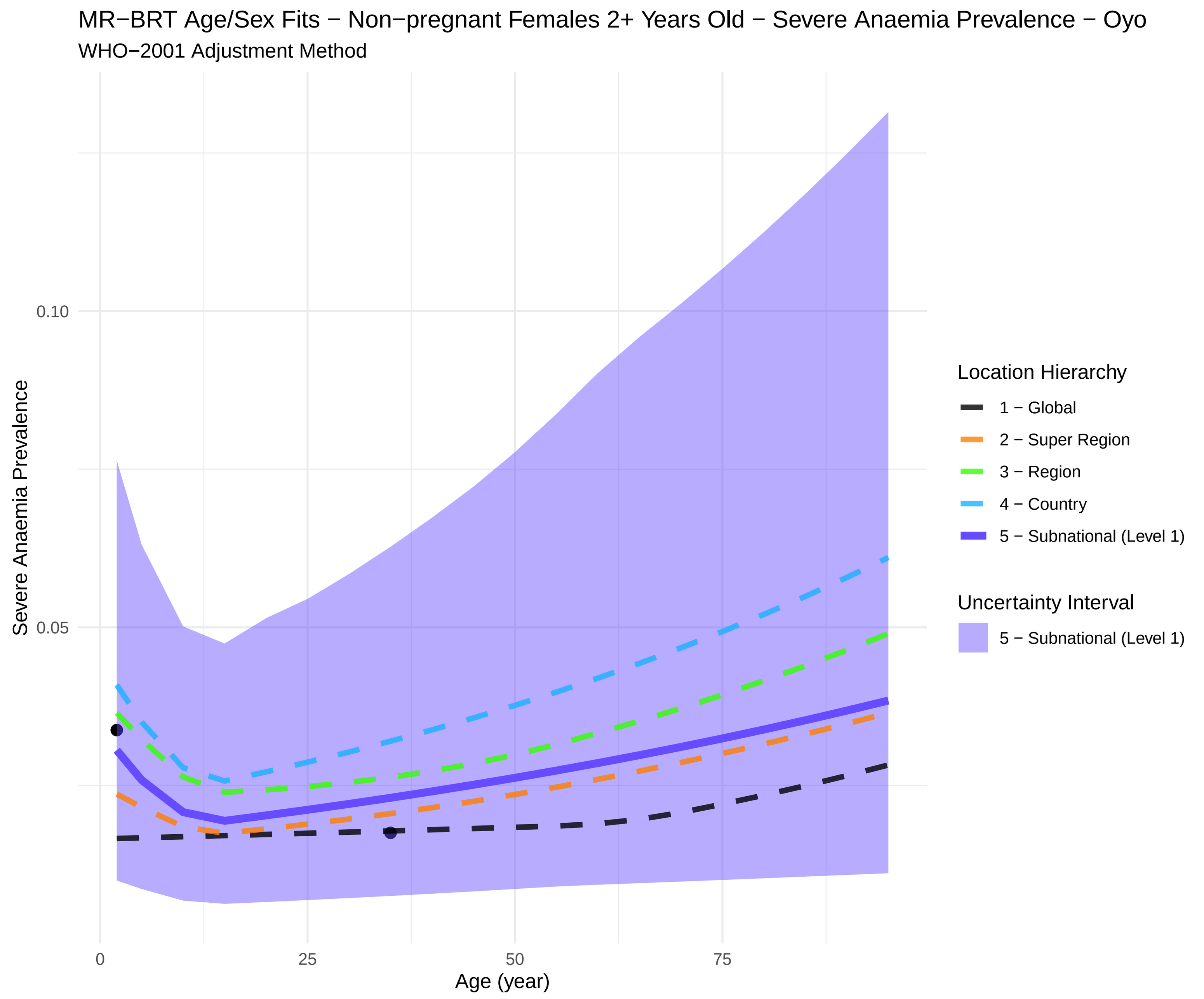
MR−BRT Age/Sex Fits − Non−pregnant Females 2+ Years Old − Severe Anaemia Prevalence − Oyo
WHO−2001 Adjustment Method
Severe Anaemia Prevalence
Age (year)
Location Hierarchy
1 − Global
2 − Super Region
3 − Region
4 − Country
5 − Subnational (Level 1)
Uncertainty Interval
5 − Subnational (Level 1)

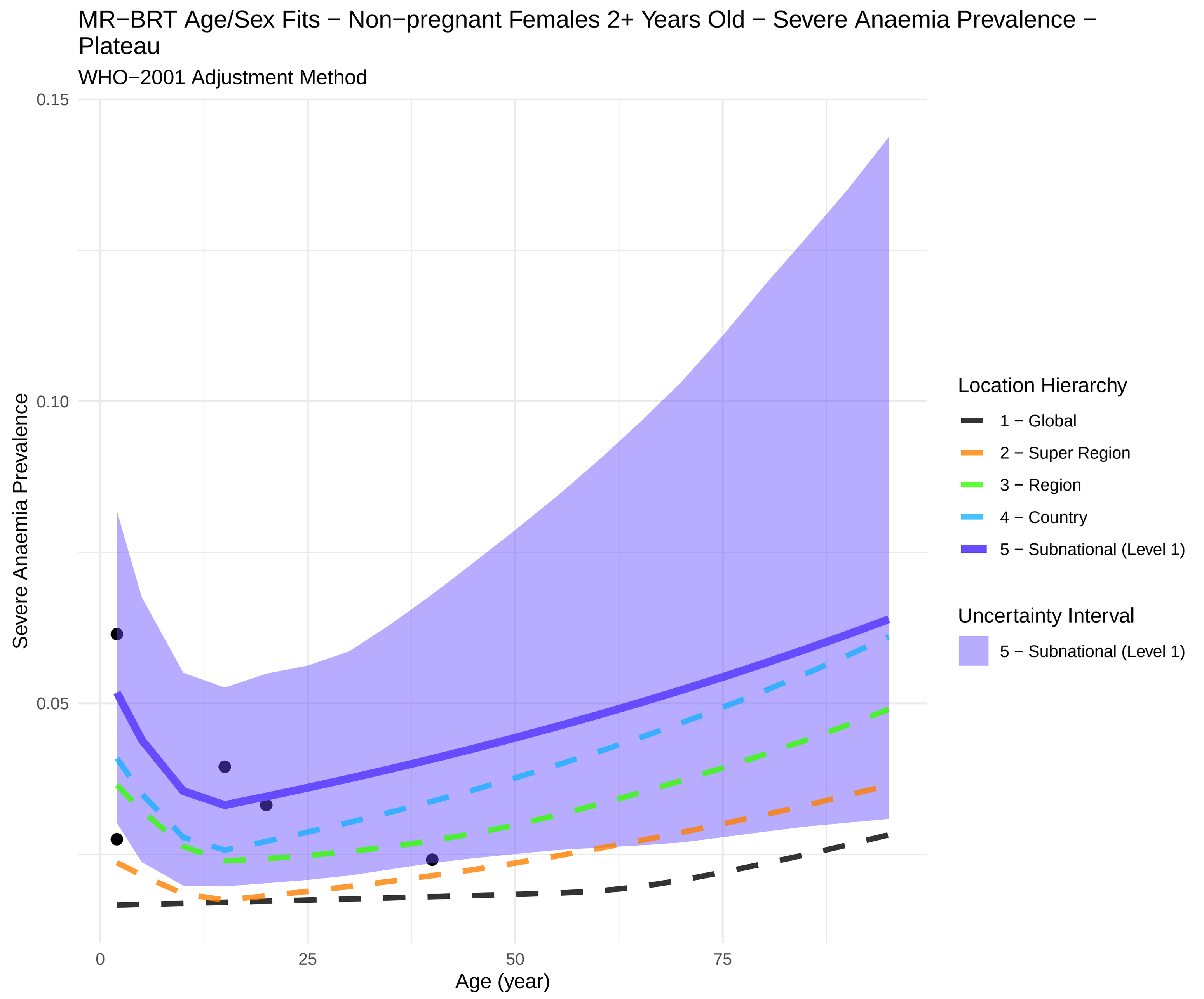
MR−BRT Age/Sex Fits − Non−pregnant Females 2+ Years Old − Severe Anaemia Prevalence − Plateau
WHO−2001 Adjustment Method
Severe Anaemia Prevalence
Age (year)
Location Hierarchy
1 − Global
2 − Super Region
3 − Region
4 − Country
5 − Subnational (Level 1)
Uncertainty Interval
5 − Subnational (Level 1)

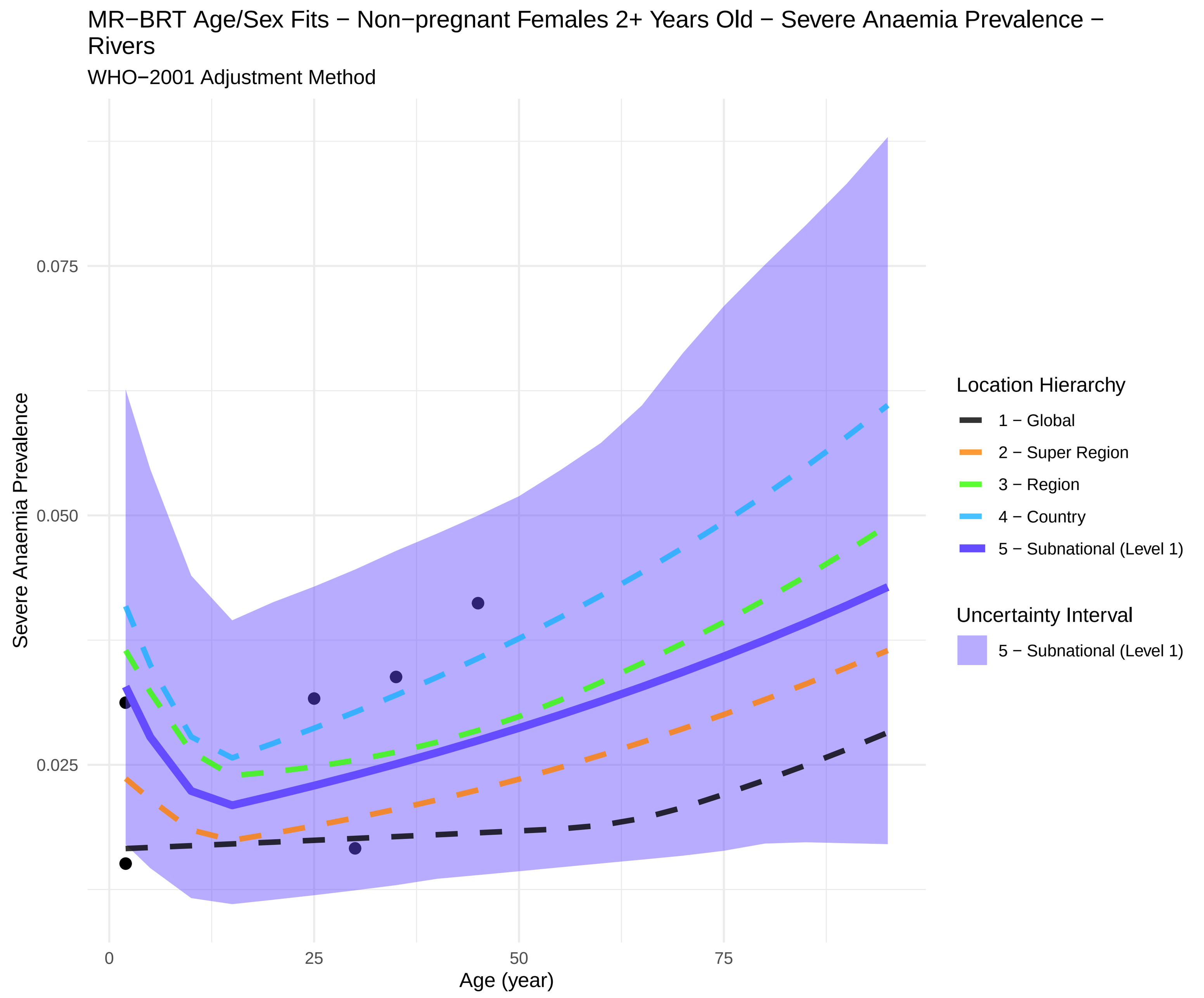
MR−BRT Age/Sex Fits − Non−pregnant Females 2+ Years Old − Severe Anaemia Prevalence − Rivers
WHO−2001 Adjustment Method
Severe Anaemia Prevalence
Age (year)
Location Hierarchy
1 − Global
2 − Super Region
3 − Region
4 − Country
5 − Subnational (Level 1)
Uncertainty Interval
5 − Subnational (Level 1)

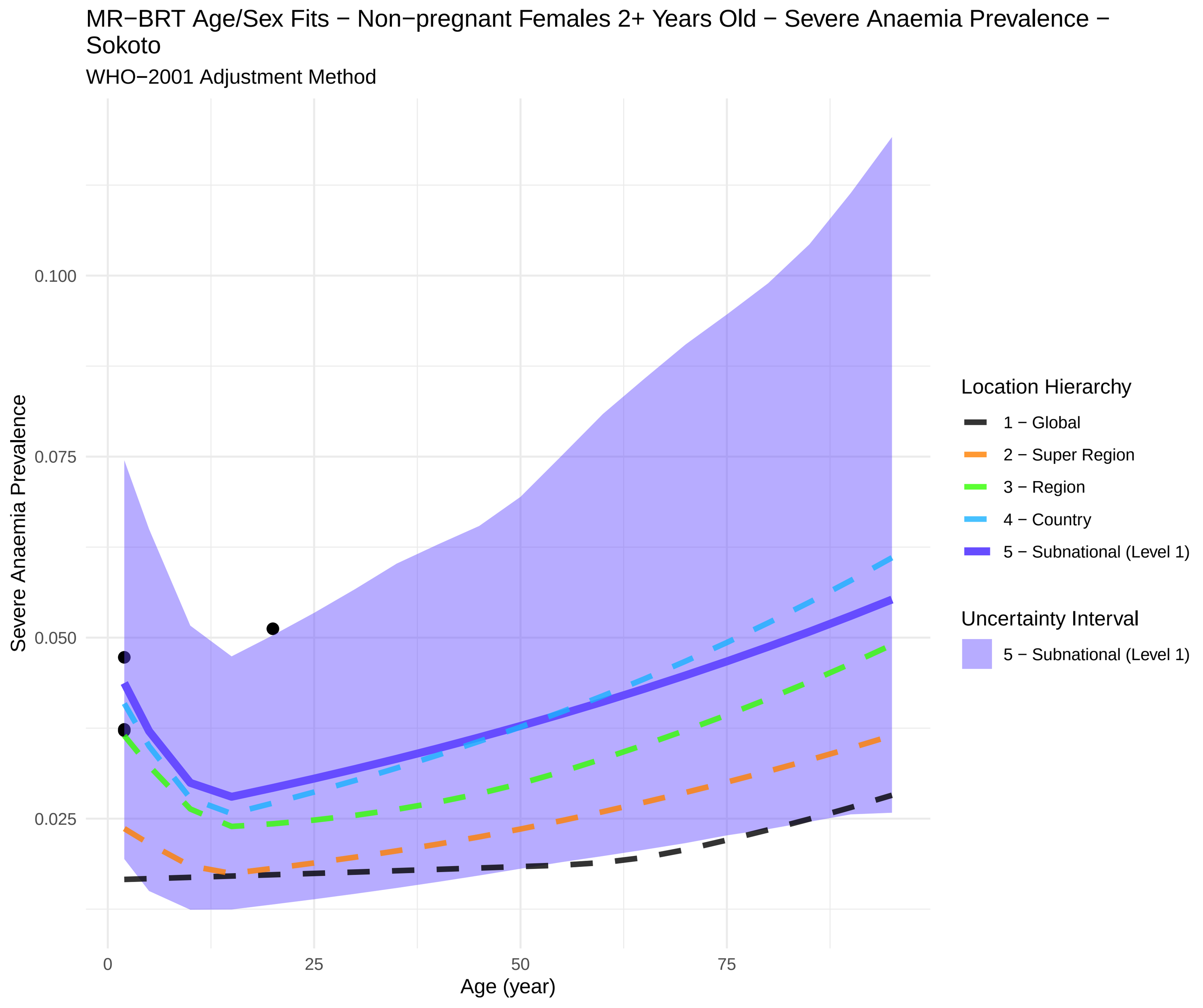
MR−BRT Age/Sex Fits − Non−pregnant Females 2+ Years Old − Severe Anaemia Prevalence − Sokoto
WHO−2001 Adjustment Method
Severe Anaemia Prevalence
Age (year)
Location Hierarchy
1 − Global
2 − Super Region
3 − Region
4 − Country
5 − Subnational (Level 1)
Uncertainty Interval
5 − Subnational (Level 1)

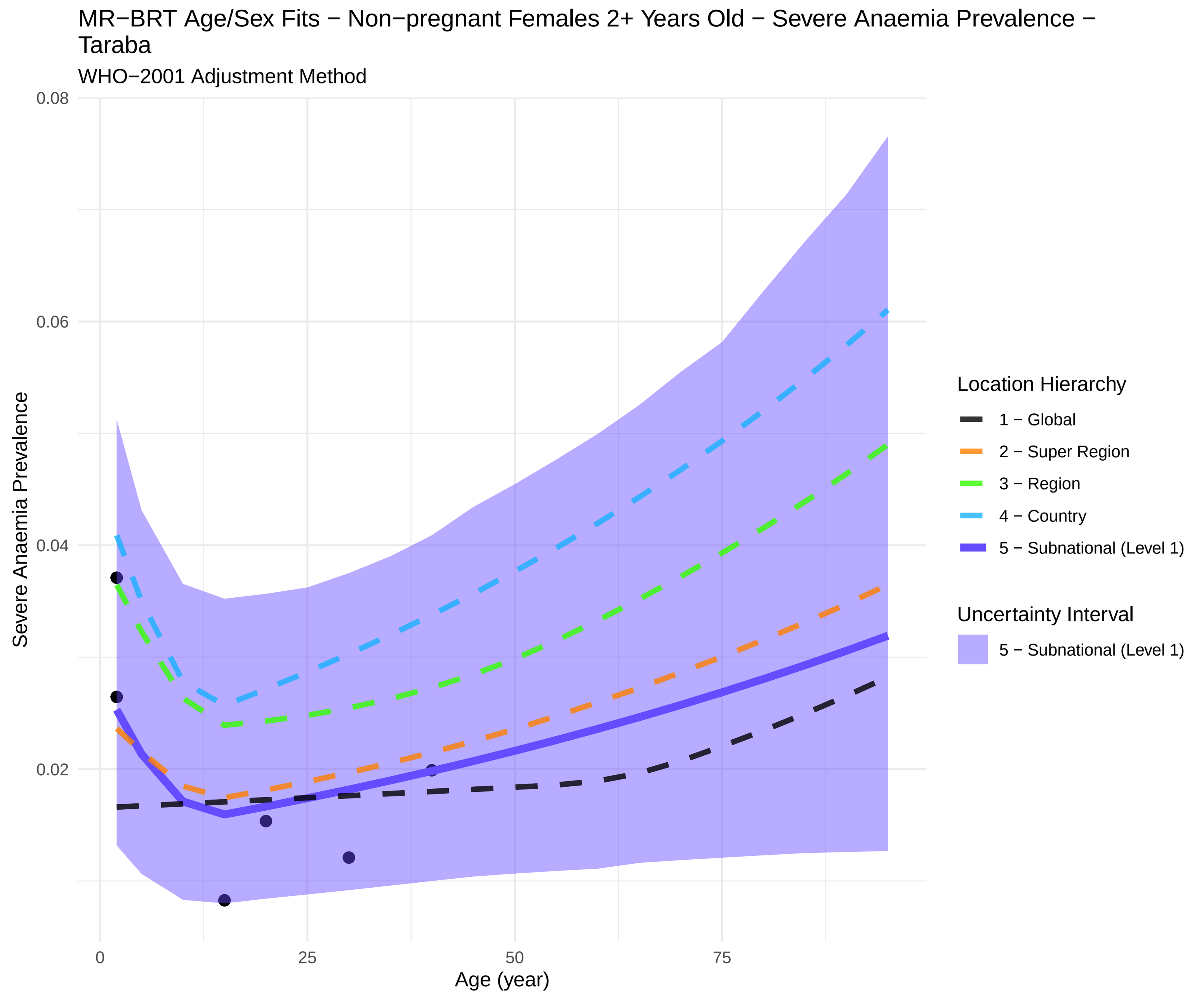
MR−BRT Age/Sex Fits − Non−pregnant Females 2+ Years Old − Severe Anaemia Prevalence − Taraba
WHO−2001 Adjustment Method
Severe Anaemia Prevalence
Age (year)
Location Hierarchy
1 − Global
2 − Super Region
3 − Region
4 − Country
5 − Subnational (Level 1)
Uncertainty Interval
5 − Subnational (Level 1)

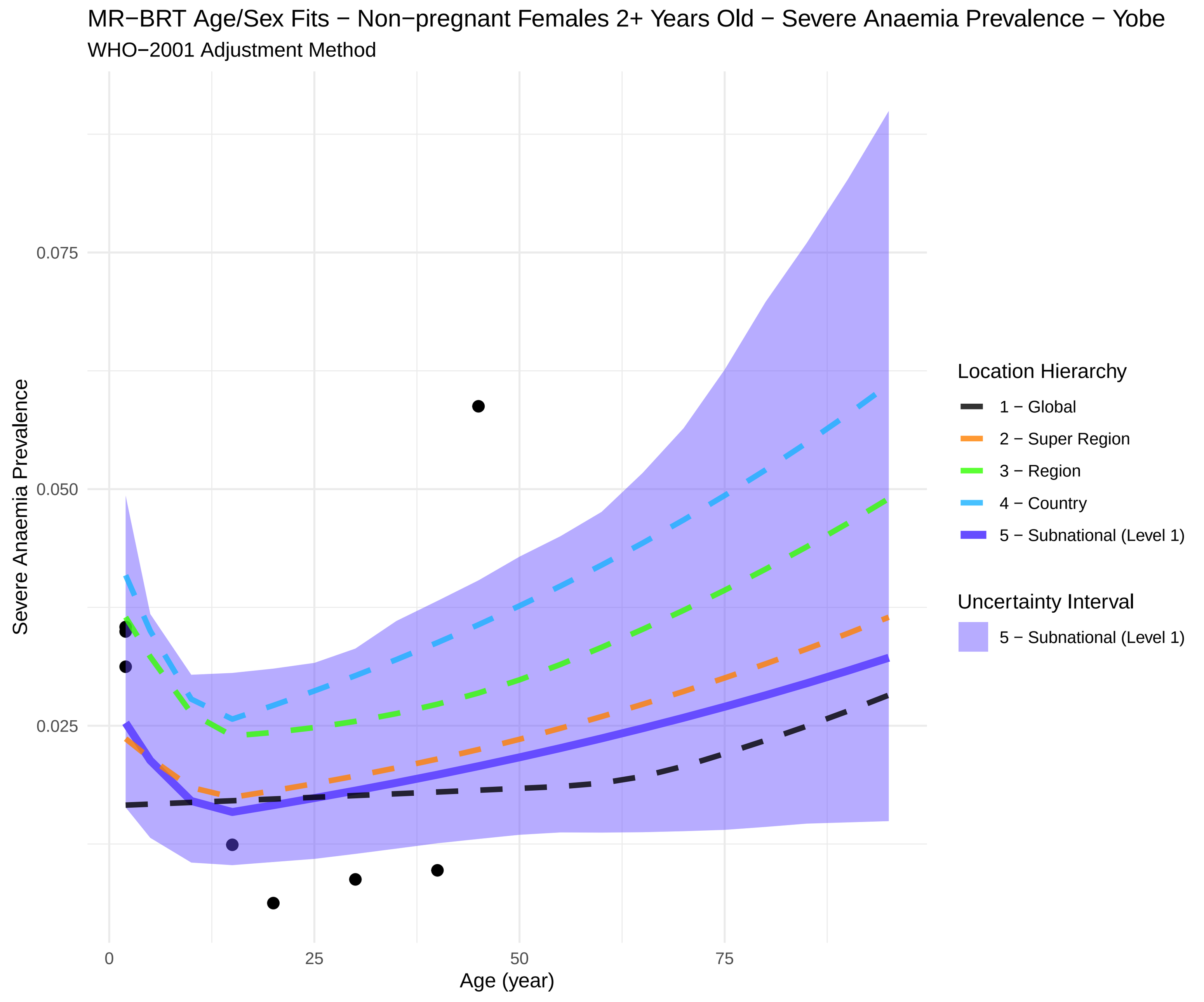
MR−BRT Age/Sex Fits − Non−pregnant Females 2+ Years Old − Severe Anaemia Prevalence − Yobe
WHO−2001 Adjustment Method
Severe Anaemia Prevalence
Age (year)
Location Hierarchy
1 − Global
2 − Super Region
3 − Region
4 − Country
5 − Subnational (Level 1)
Uncertainty Interval
5 − Subnational (Level 1)

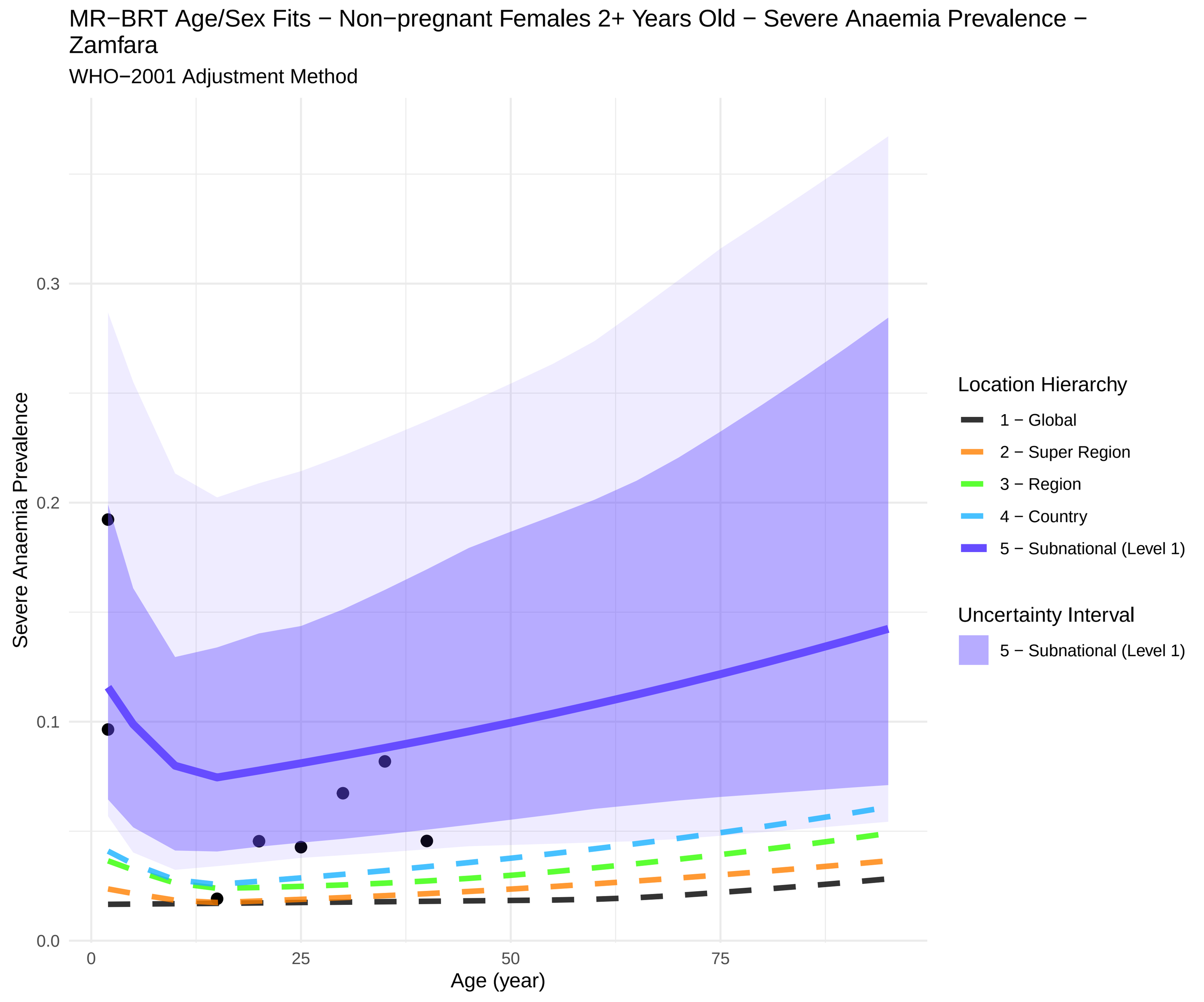
MR−BRT Age/Sex Fits − Non−pregnant Females 2+ Years Old − Severe Anaemia Prevalence − Zamfara
WHO−2001 Adjustment Method
Severe Anaemia Prevalence
Age (year)
Location Hierarchy
1 − Global
2 − Super Region
3 − Region
4 − Country
5 − Subnational (Level 1)
Uncertainty Interval
5 − Subnational (Level 1)

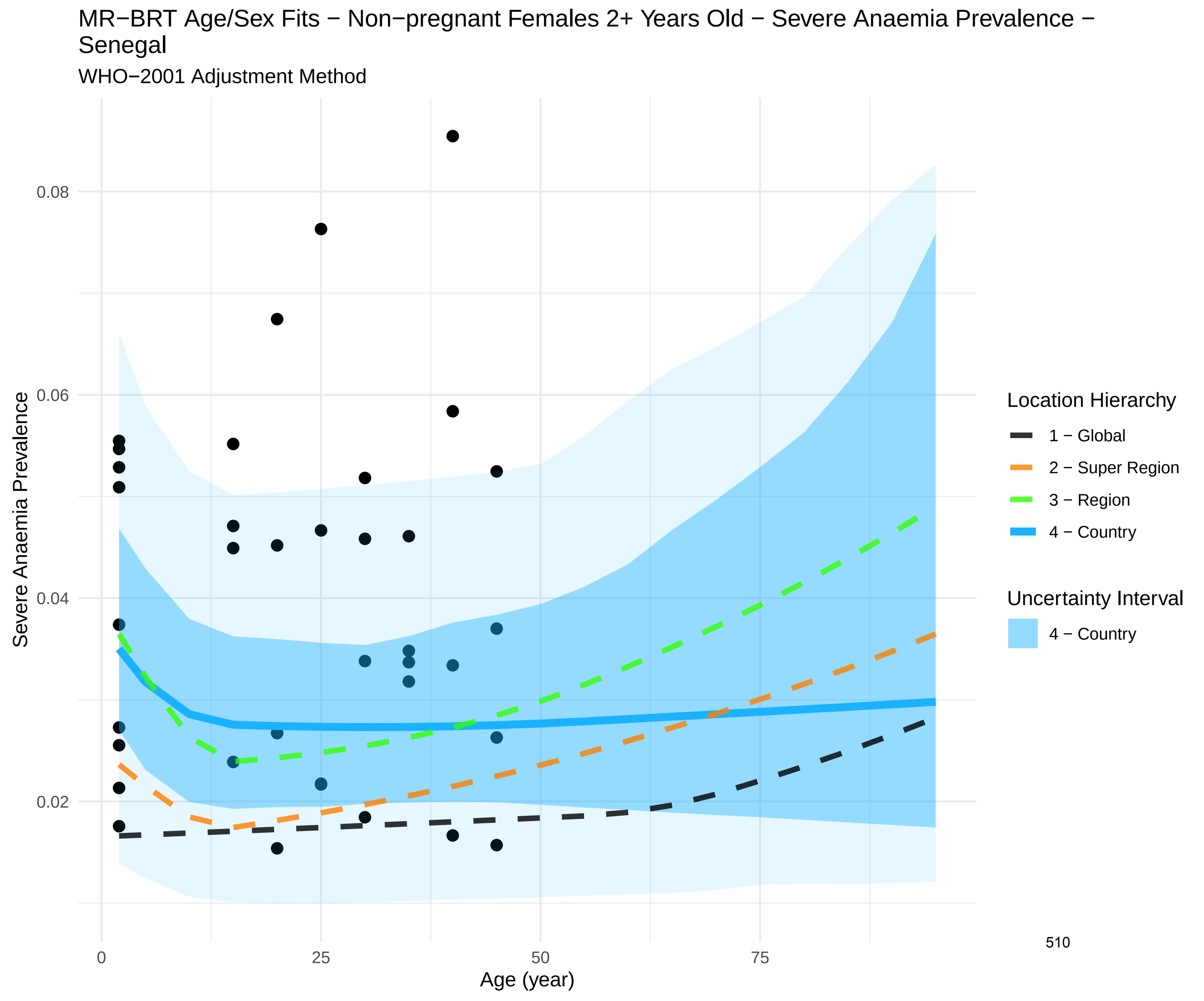
MR−BRT Age/Sex Fits − Non−pregnant Females 2+ Years Old − Severe Anaemia Prevalence − Senegal
WHO−2001 Adjustment Method
Severe Anaemia Prevalence
Age (year)
Location Hierarchy
1 − Global
2 − Super Region
3 − Region
4 − Country
Uncertainty Interval
4 − Country

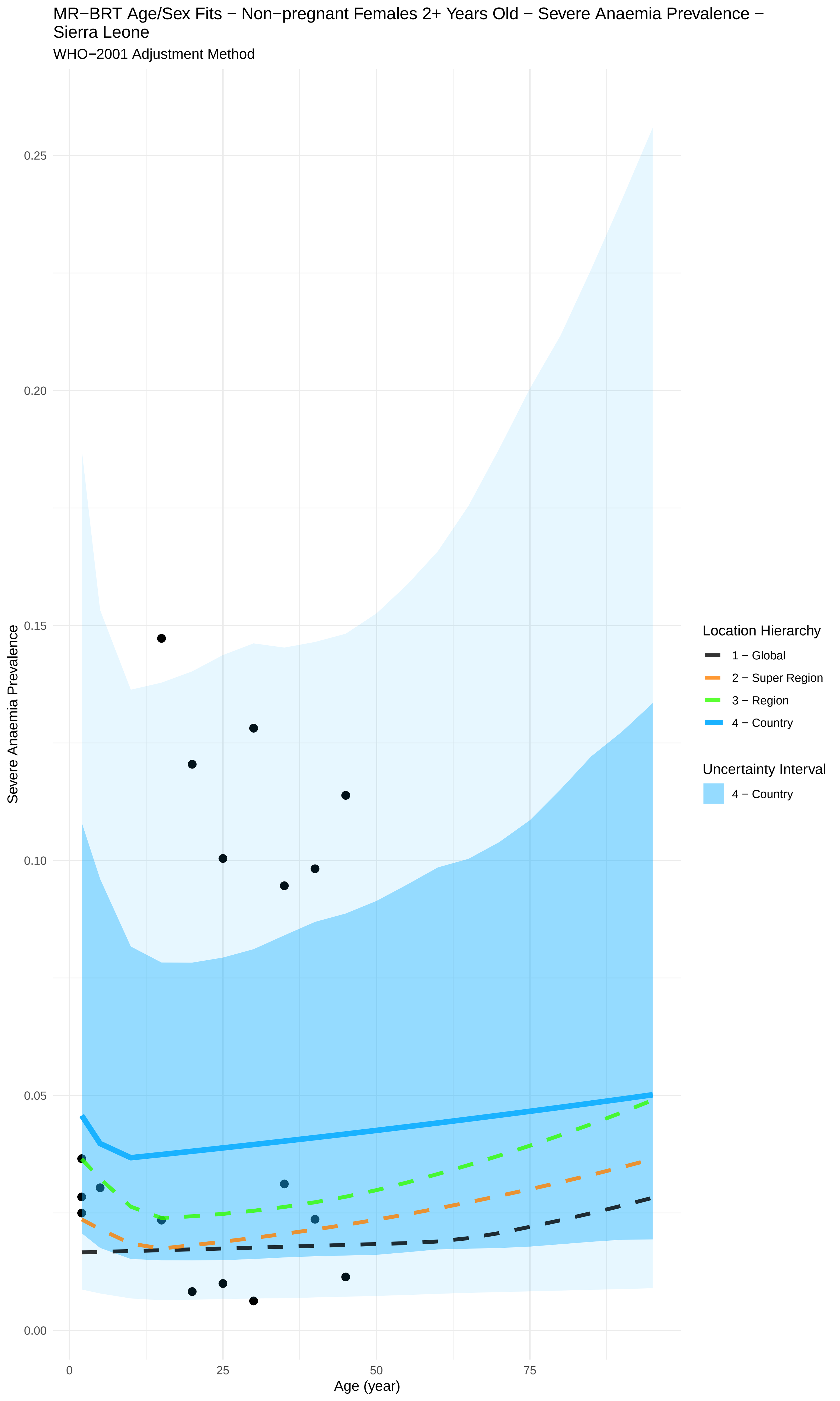
MR−BRT Age/Sex Fits − Non−pregnant Females 2+ Years Old − Severe Anaemia Prevalence − Sierra Leone
WHO−2001 Adjustment Method
Severe Anaemia Prevalence
0.00
0.05
0.10
0.15
0.20
0.25
0
25
50
75
Age (year)
Location Hierarchy
1 − Global
2 − Super Region
3 − Region
4 − Country
Uncertainty Interval
4 − Country

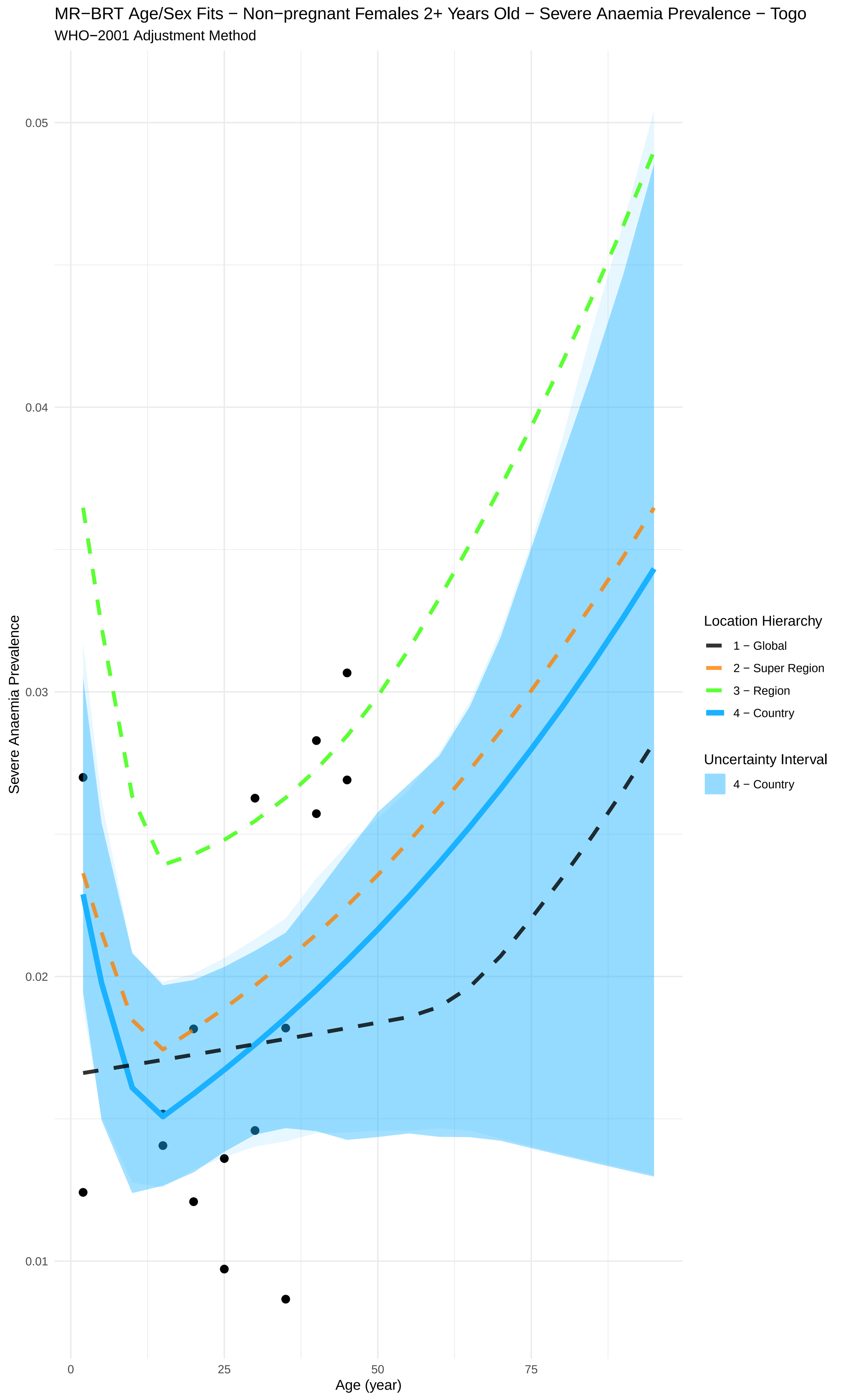
MR−BRT Age/Sex Fits − Non−pregnant Females 2+ Years Old − Severe Anaemia Prevalence − Togo
WHO−2001 Adjustment Method
Severe Anaemia Prevalence
0.01
0.02
0.03
0.04
0.05
0
25
50
75
Age (year)
Location Hierarchy
1 − Global
2 − Super Region
3 − Region
4 − Country
Uncertainty Interval
4 − Country

MR−BRT Age/Sex Fits − Males 2+ Years Old − Mean Haemglobin − Global
WHO−2024 Adjustment Method

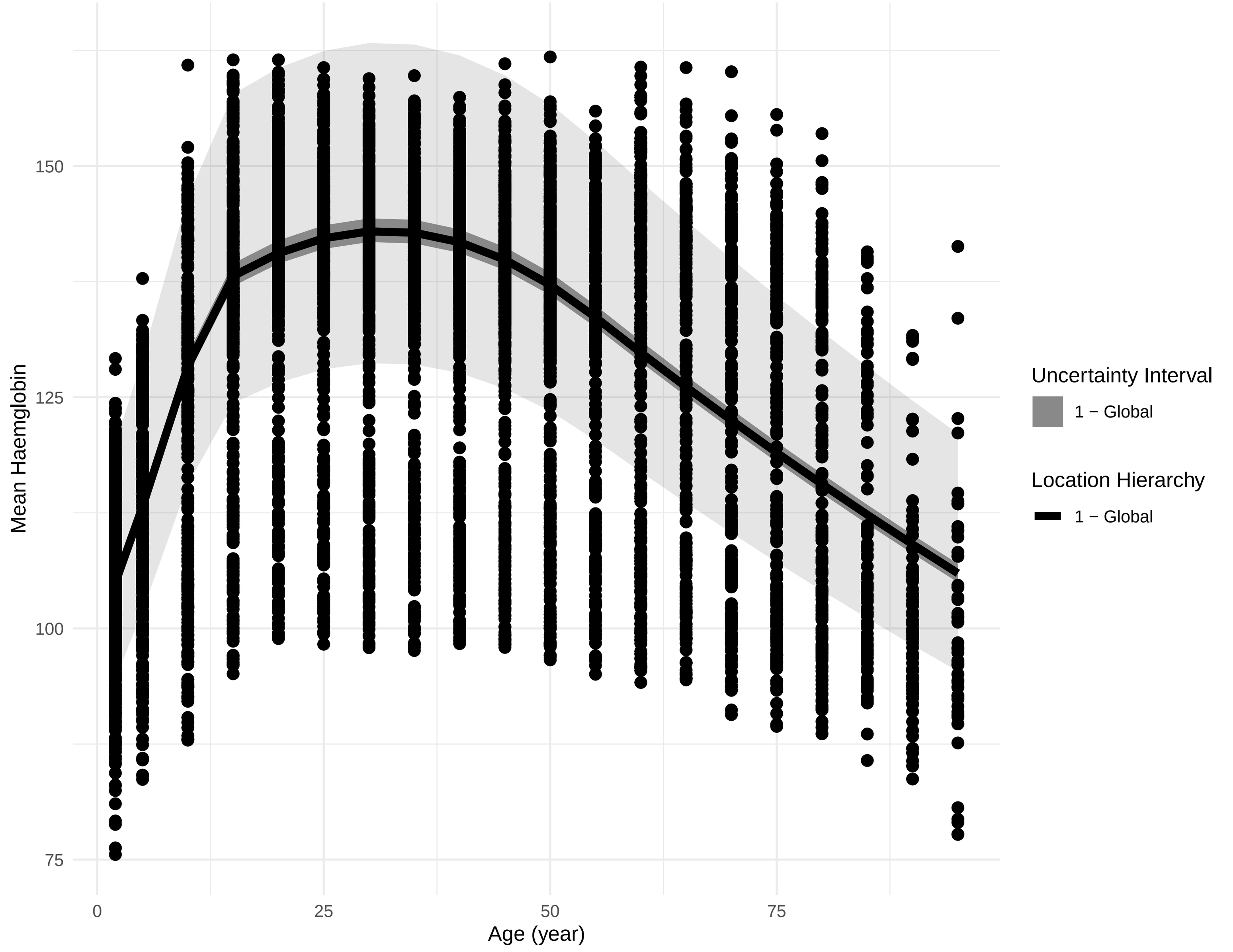

MR−BRT Age/Sex Fits − Males 2+ Years Old − Mean Haemglobin − Central Europe, Eastern Europe, and Central Asia
WHO−2024 Adjustment Method

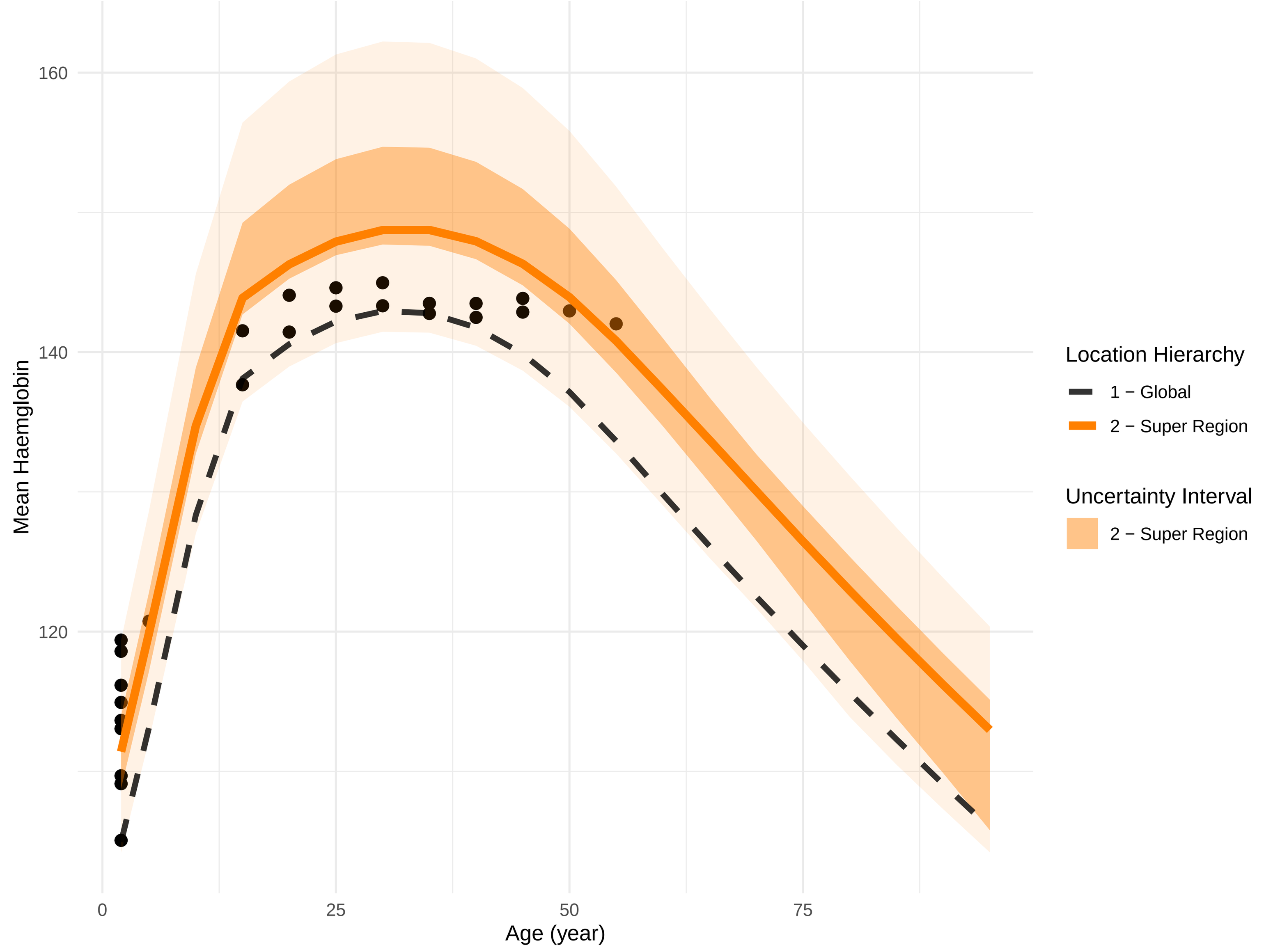

MR−BRT Age/Sex Fits − Males 2+ Years Old − Mean Haemglobin − Central Asia
WHO−2024 Adjustment Method

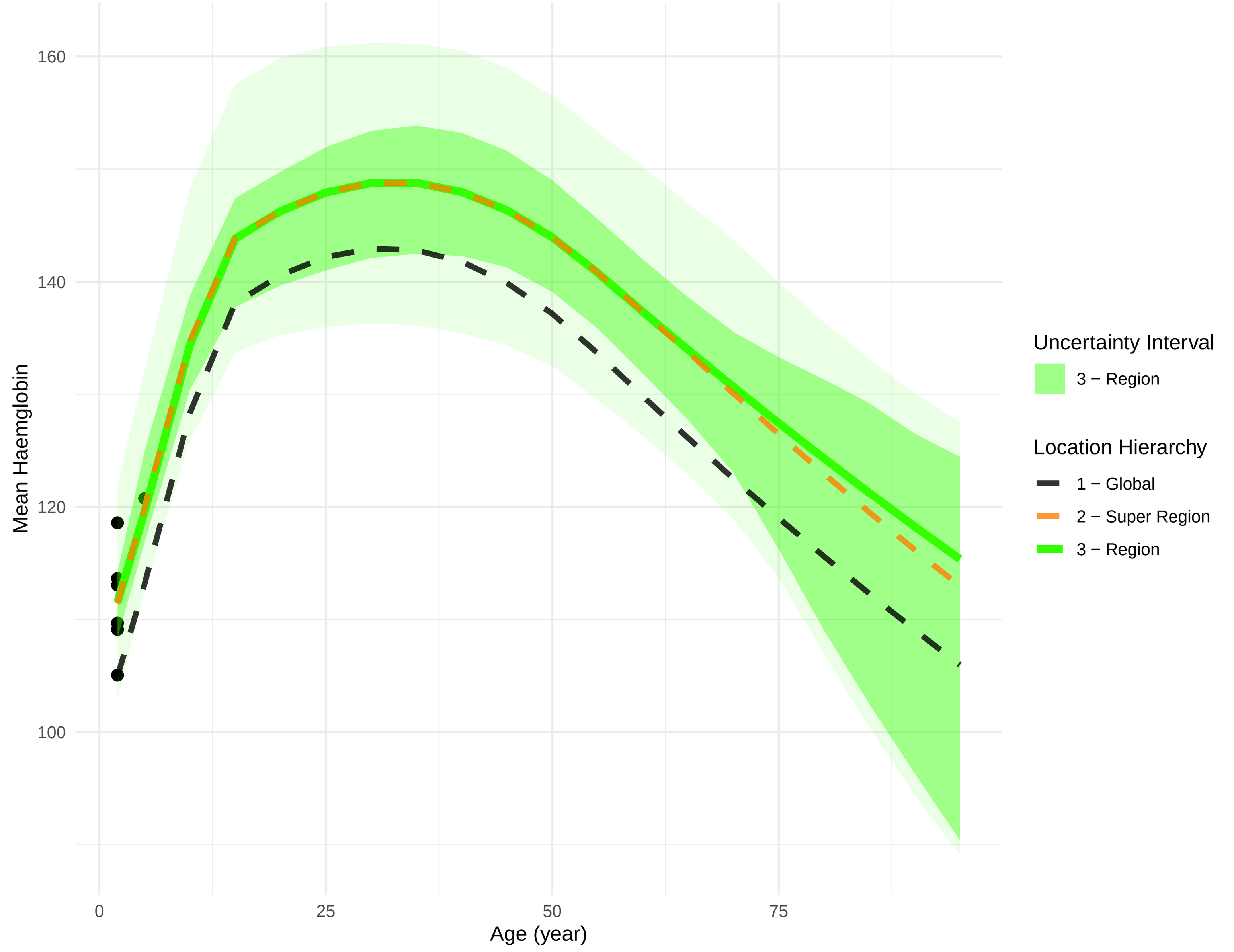

MR−BRT Age/Sex Fits − Males 2+ Years Old − Mean Haemglobin − Armenia
WHO−2024 Adjustment Method

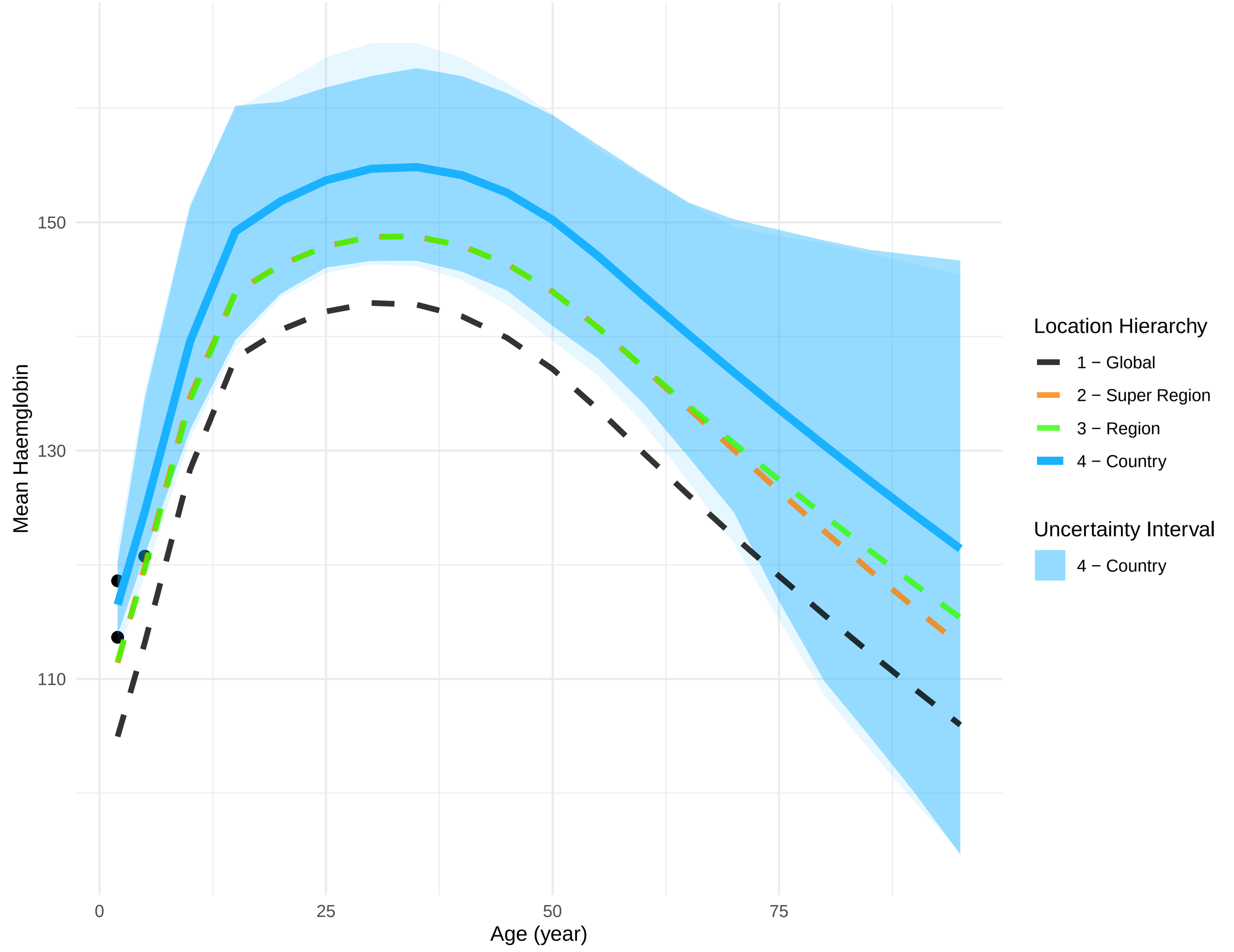

MR−BRT Age/Sex Fits − Males 2+ Years Old − Mean Haemglobin − Azerbaijan
WHO−2024 Adjustment Method

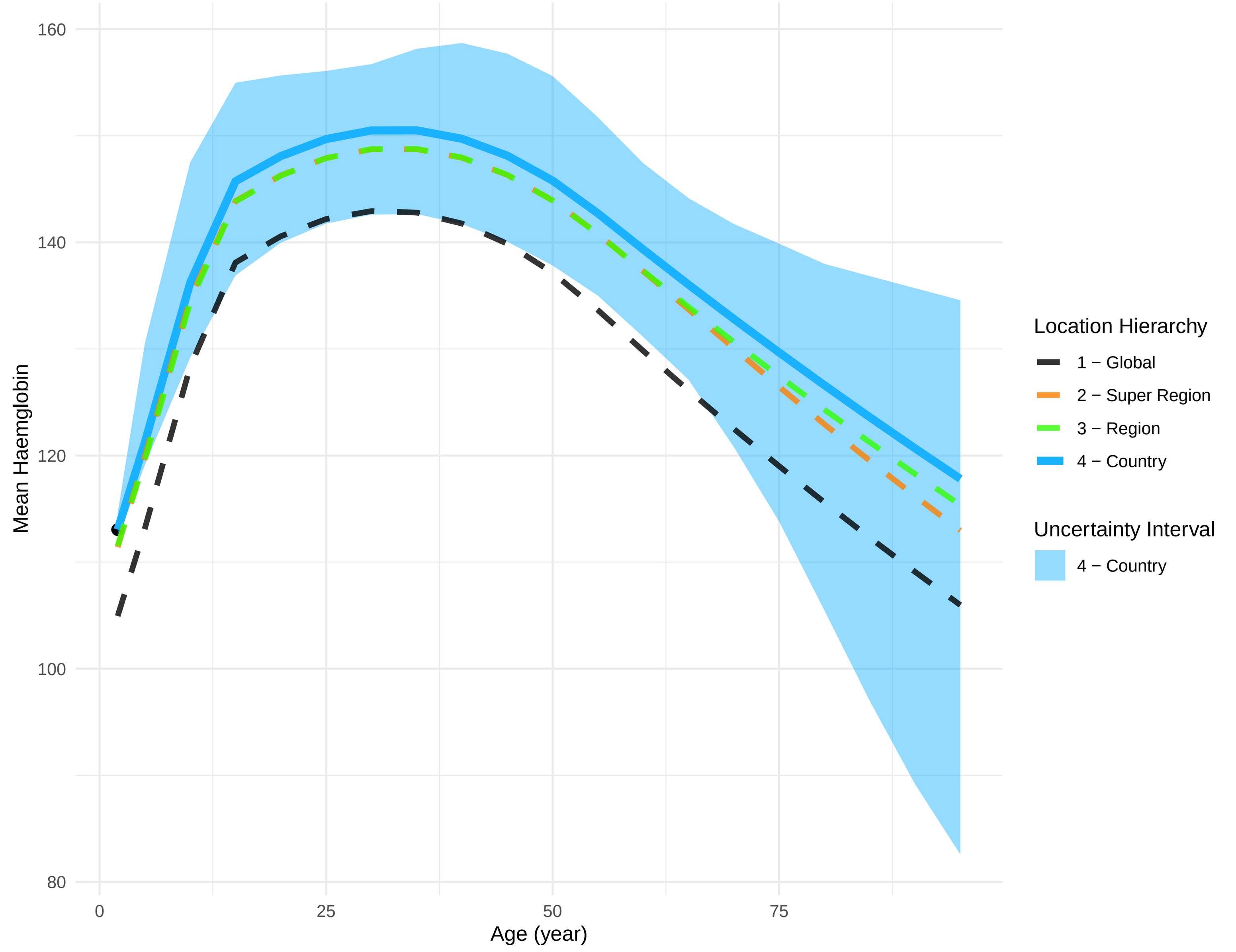

MR−BRT Age/Sex Fits − Males 2+ Years Old − Mean Haemglobin − Kyrgyzstan
WHO−2024 Adjustment Method

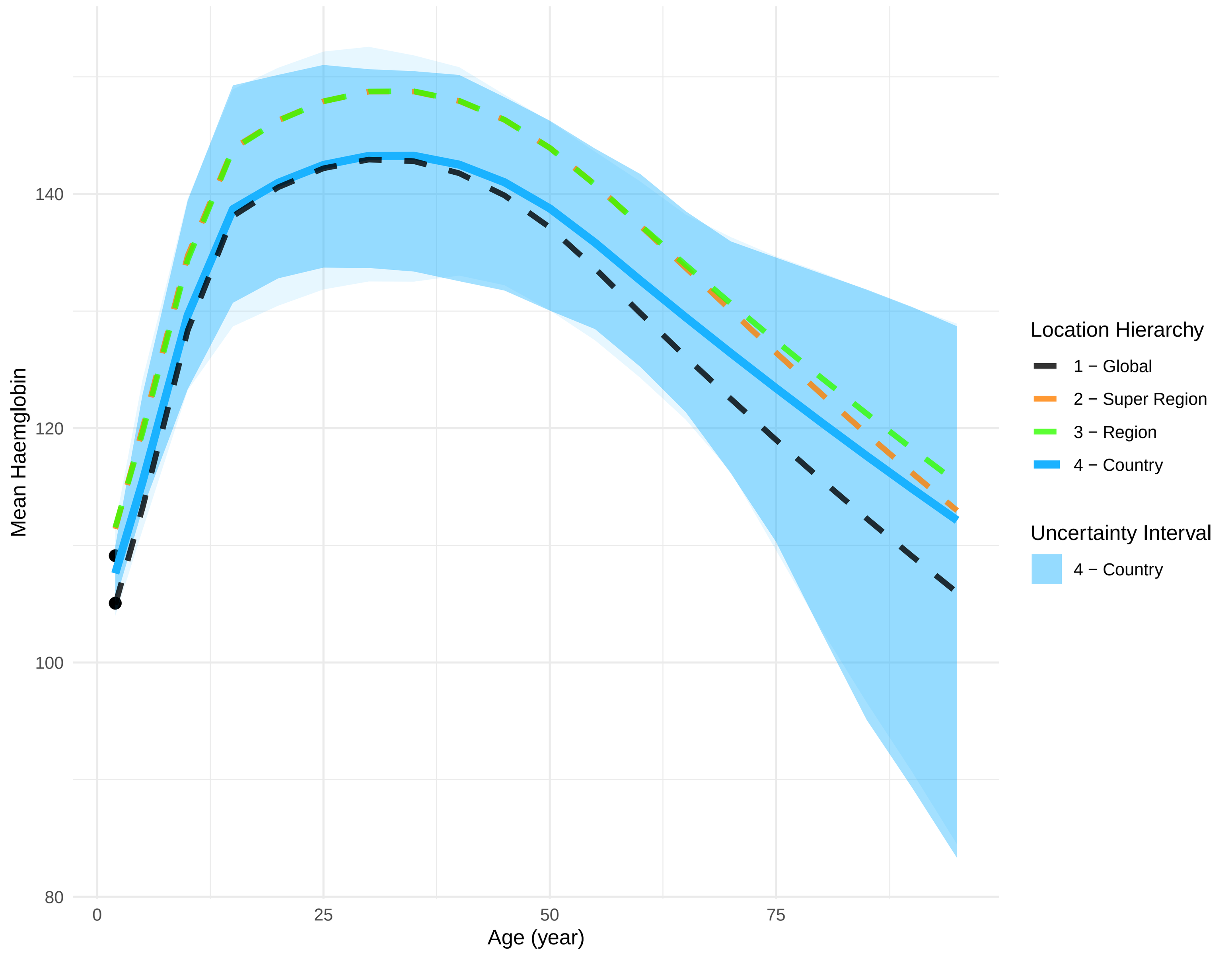

MR−BRT Age/Sex Fits − Males 2+ Years Old − Mean Haemglobin − Uzbekistan
WHO−2024 Adjustment Method

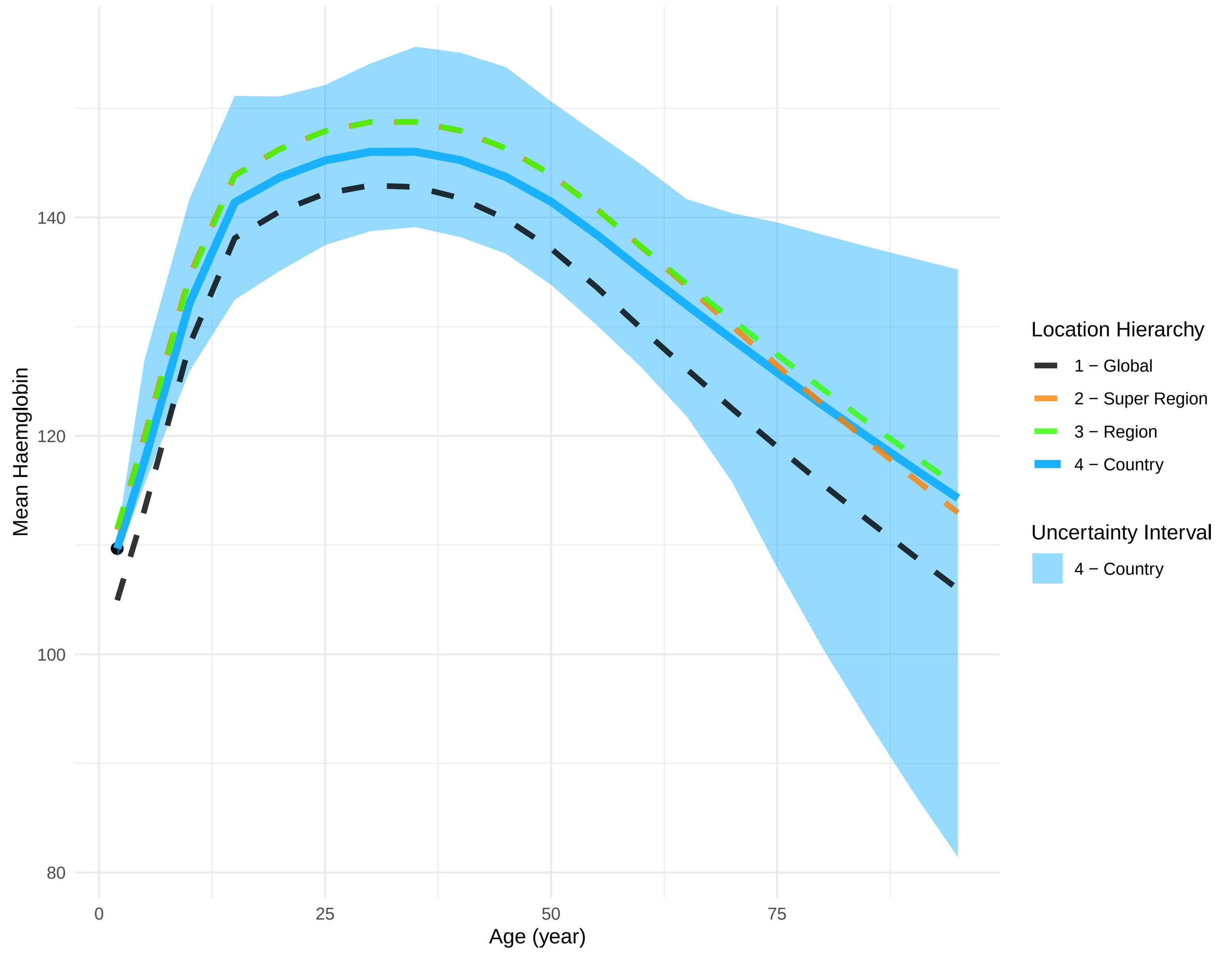

MR−BRT Age/Sex Fits − Males 2+ Years Old − Mean Haemglobin − Central Europe
WHO−2024 Adjustment Method

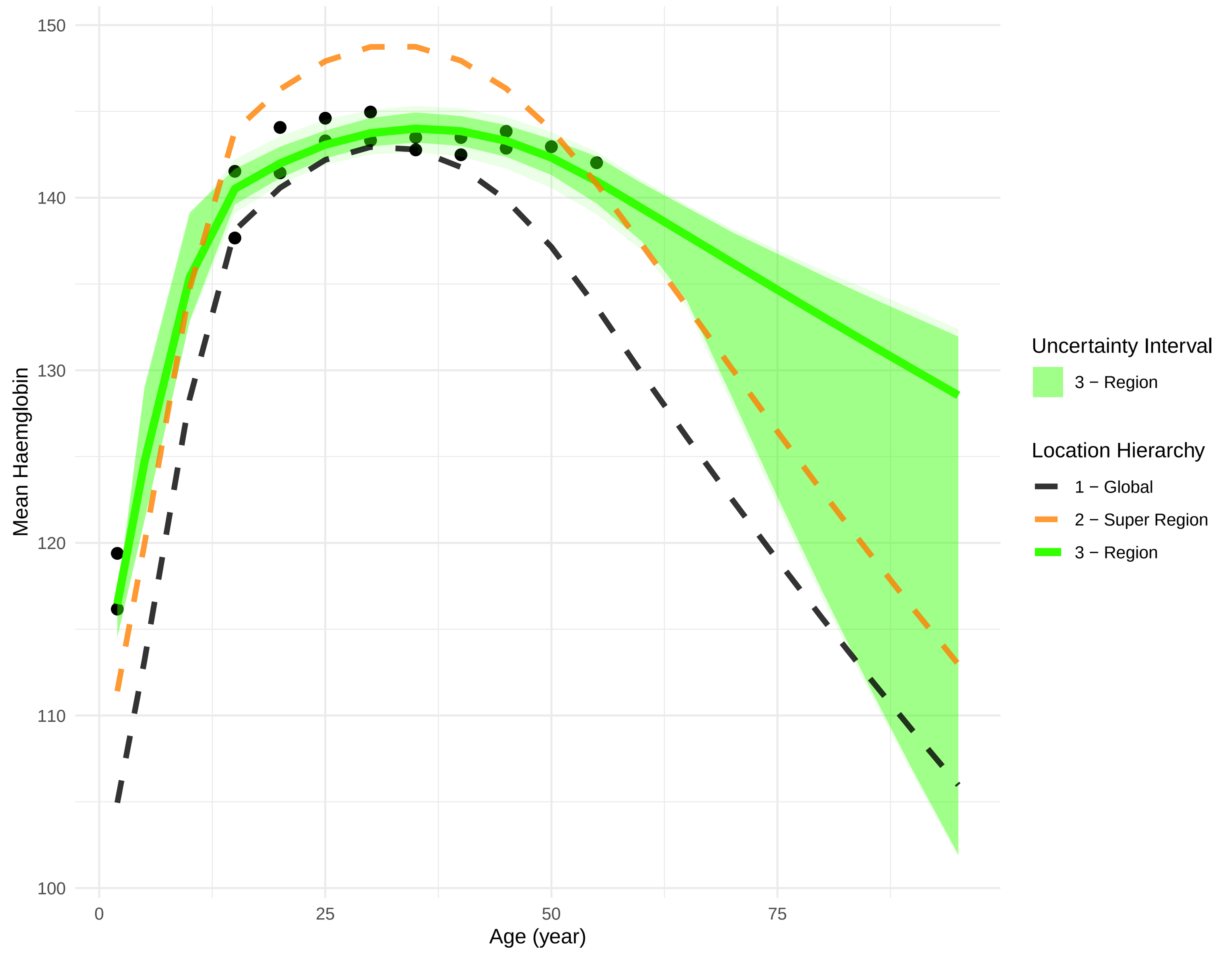

MR−BRT Age/Sex Fits − Males 2+ Years Old − Mean Haemglobin − Albania
WHO−2024 Adjustment Method

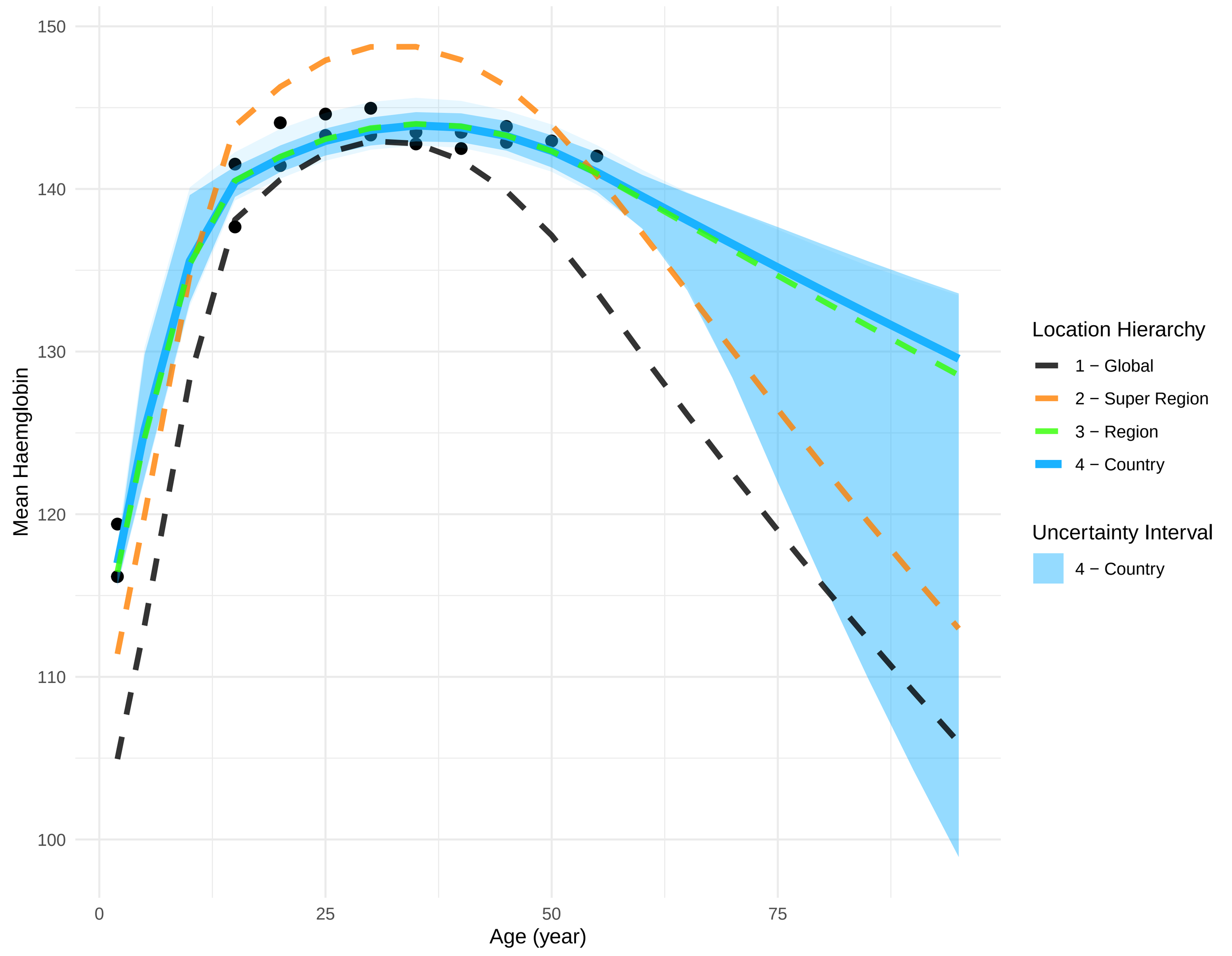

MR−BRT Age/Sex Fits − Males 2+ Years Old − Mean Haemglobin − Eastern Europe
WHO−2024 Adjustment Method

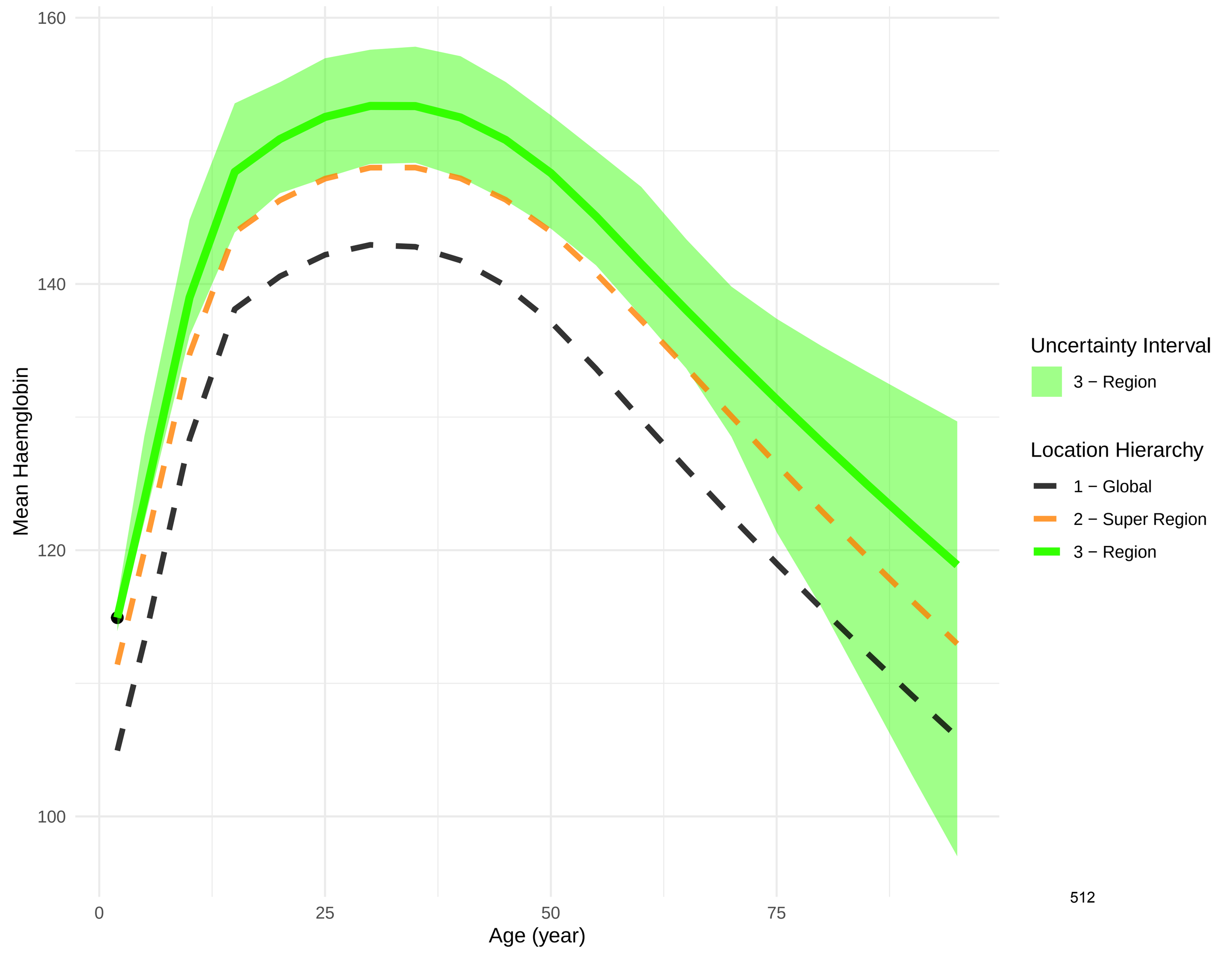

MR−BRT Age/Sex Fits − Males 2+ Years Old − Mean Haemglobin − Republic of Moldova
WHO−2024 Adjustment Method

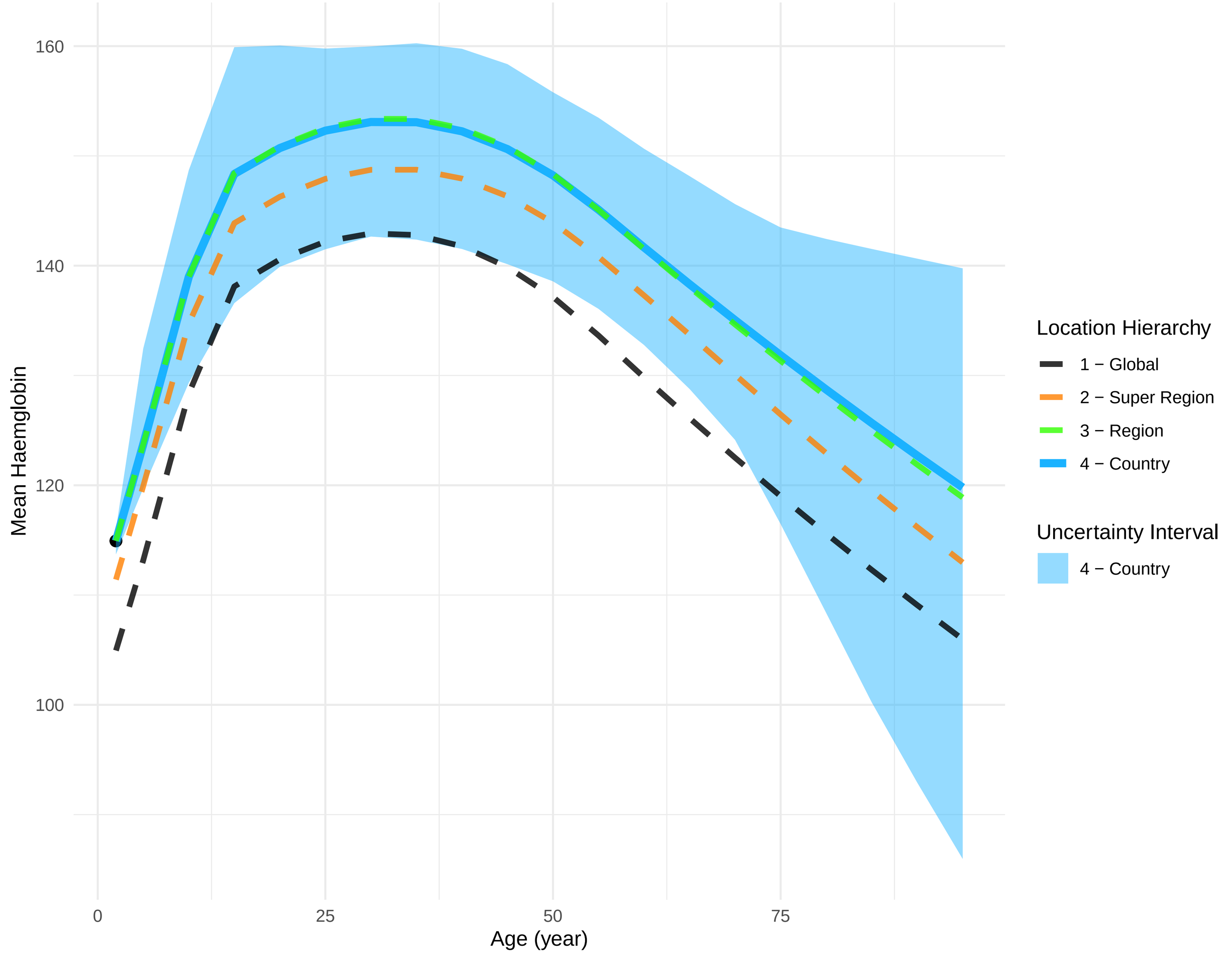

MR−BRT Age/Sex Fits − Males 2+ Years Old − Mean Haemglobin − High−income
WHO−2024 Adjustment Method

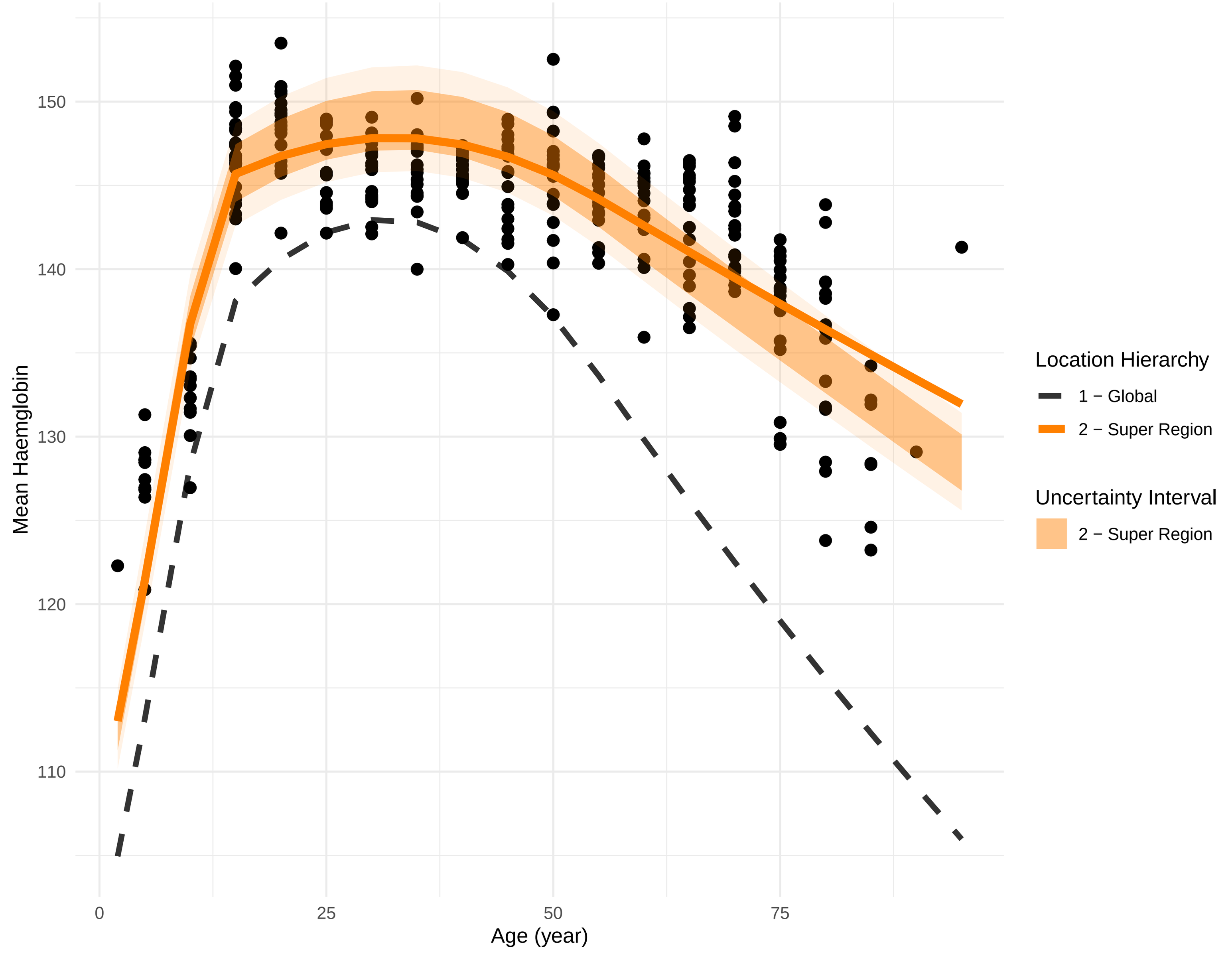

MR−BRT Age/Sex Fits − Males 2+ Years Old − Mean Haemglobin − Western Europe
WHO−2024 Adjustment Method

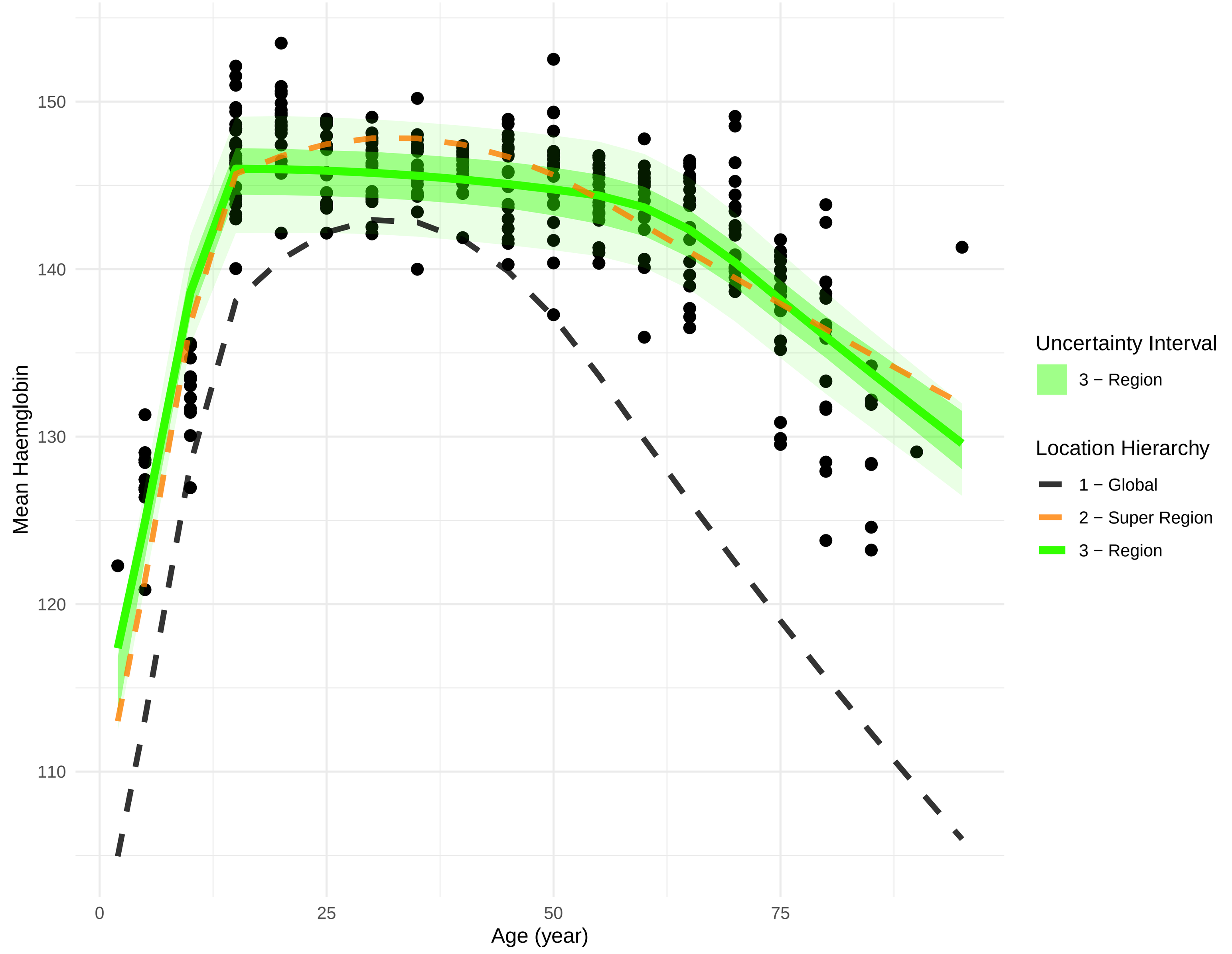

MR−BRT Age/Sex Fits − Males 2+ Years Old − Mean Haemglobin − United Kingdom
WHO−2024 Adjustment Method

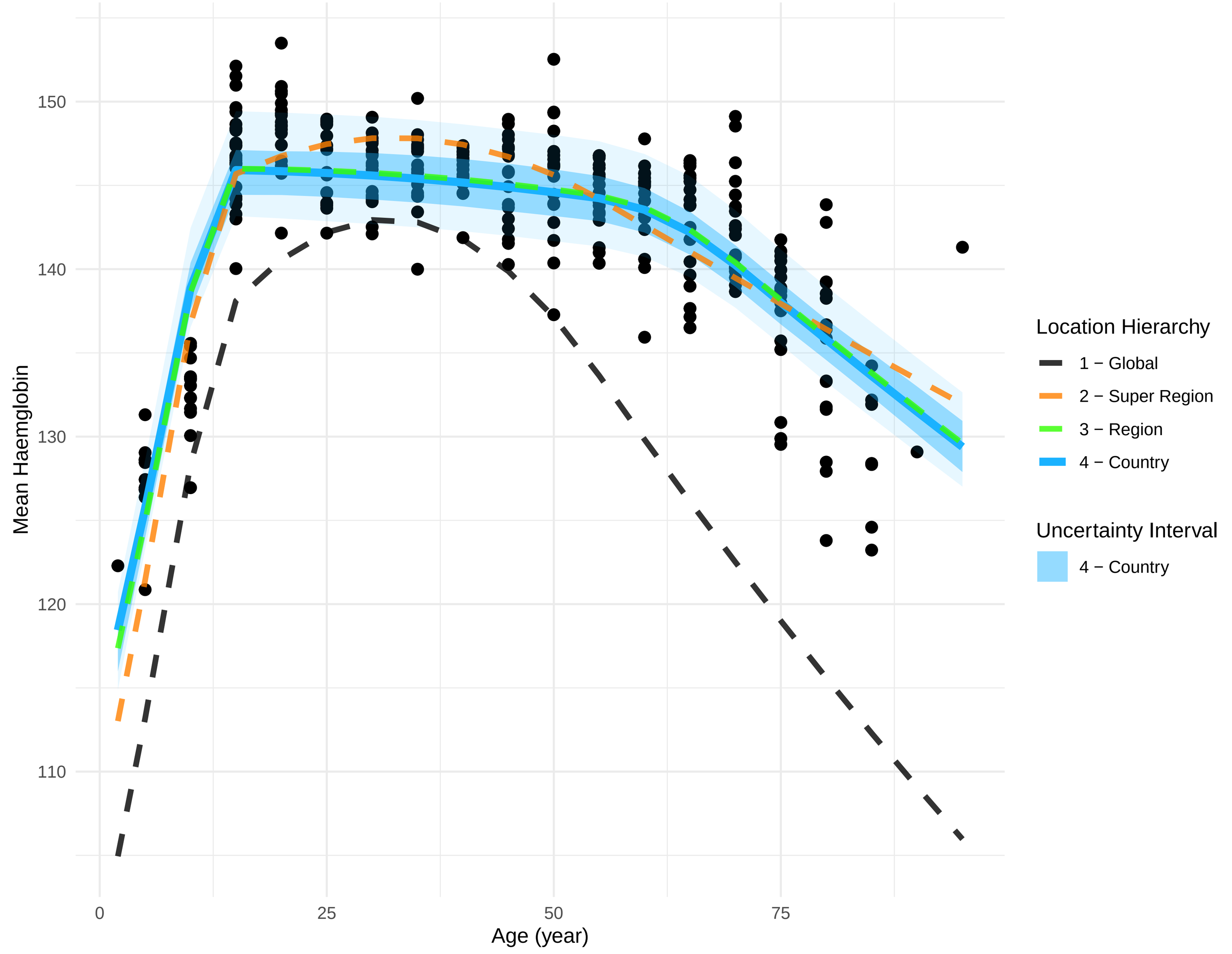

MR−BRT Age/Sex Fits − Males 2+ Years Old − Mean Haemglobin − England
WHO−2024 Adjustment Method

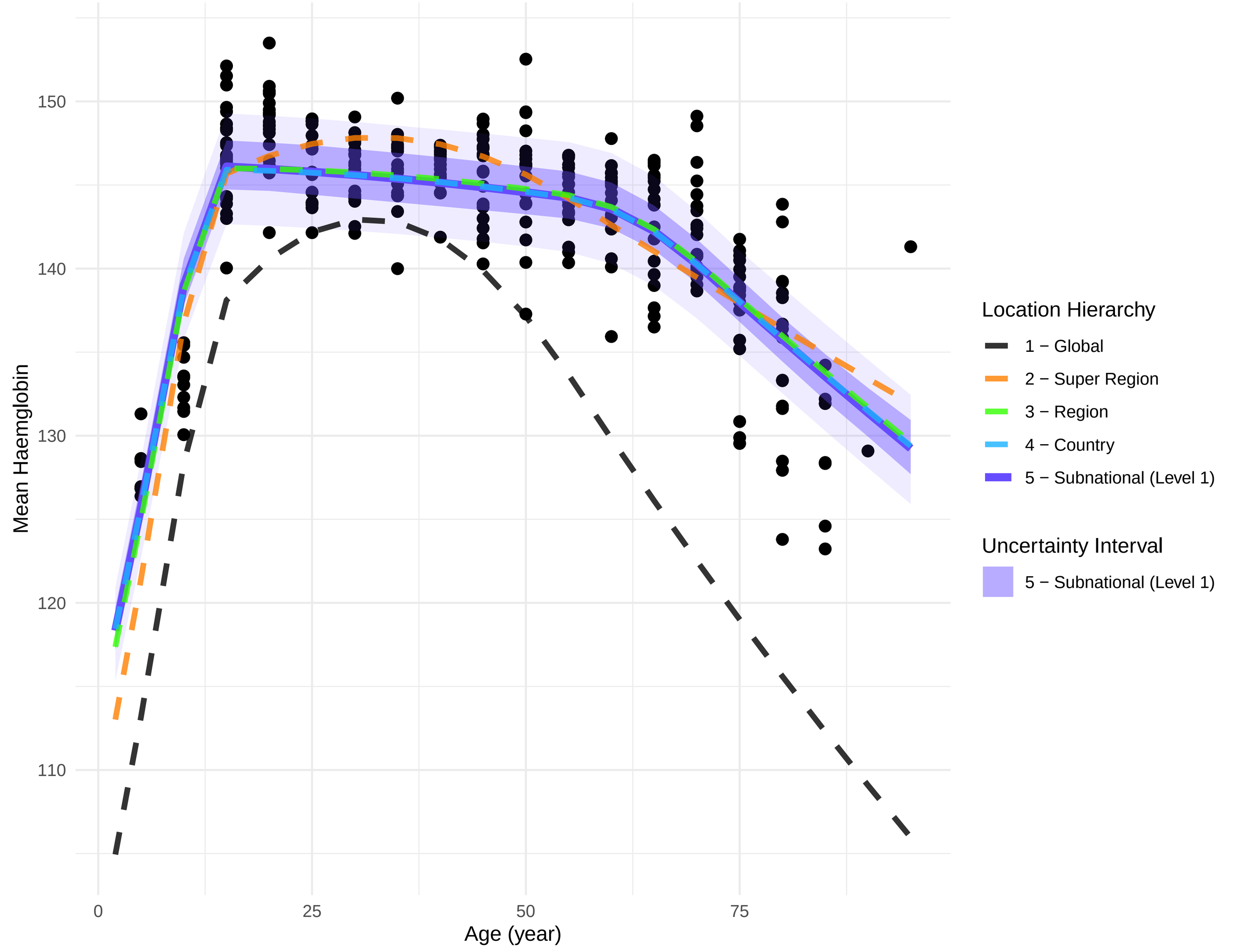

MR−BRT Age/Sex Fits − Males 2+ Years Old − Mean Haemglobin − East Midlands
WHO−2024 Adjustment Method

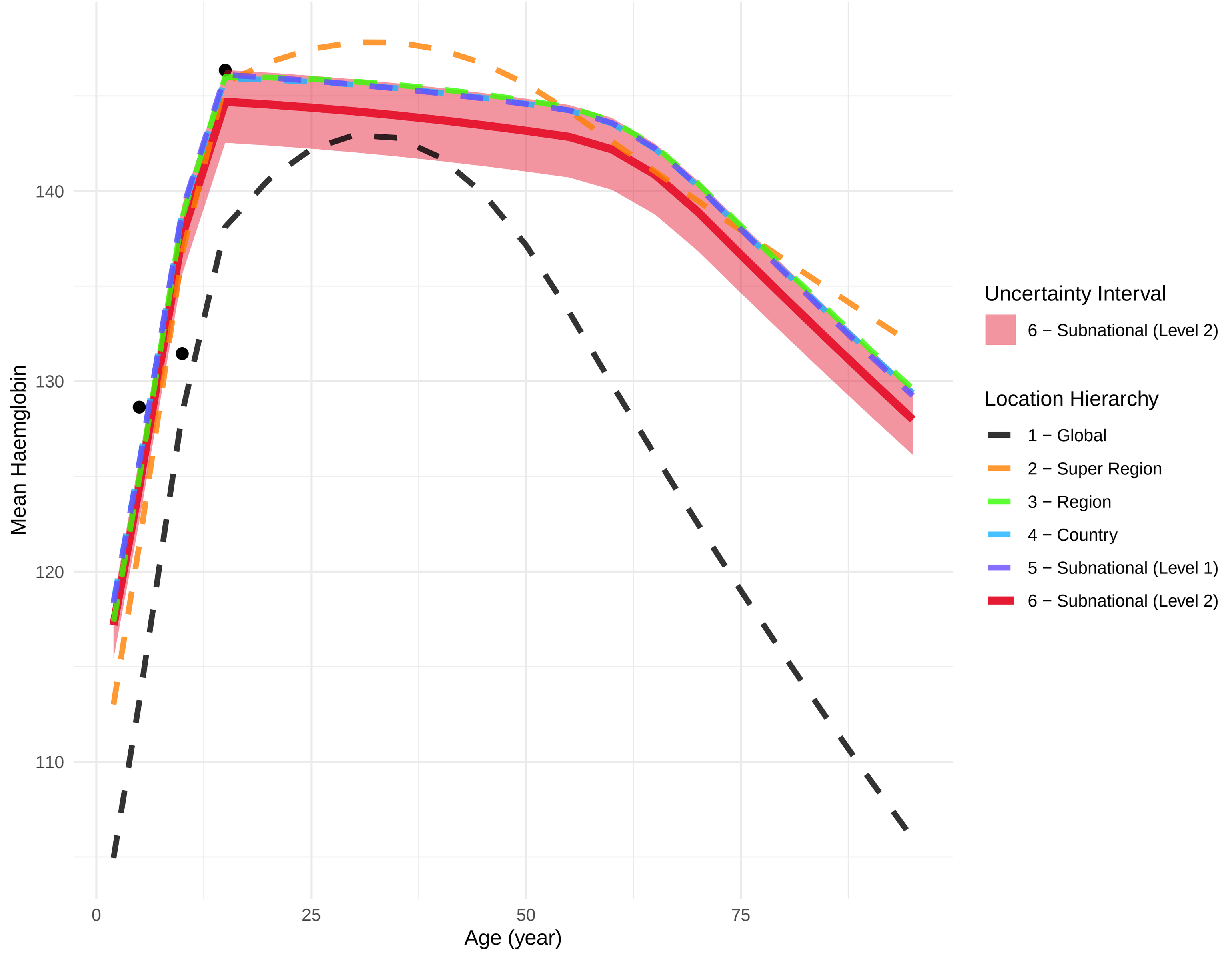

MR−BRT Age/Sex Fits − Males 2+ Years Old − Mean Haemglobin − East of England
WHO−2024 Adjustment Method

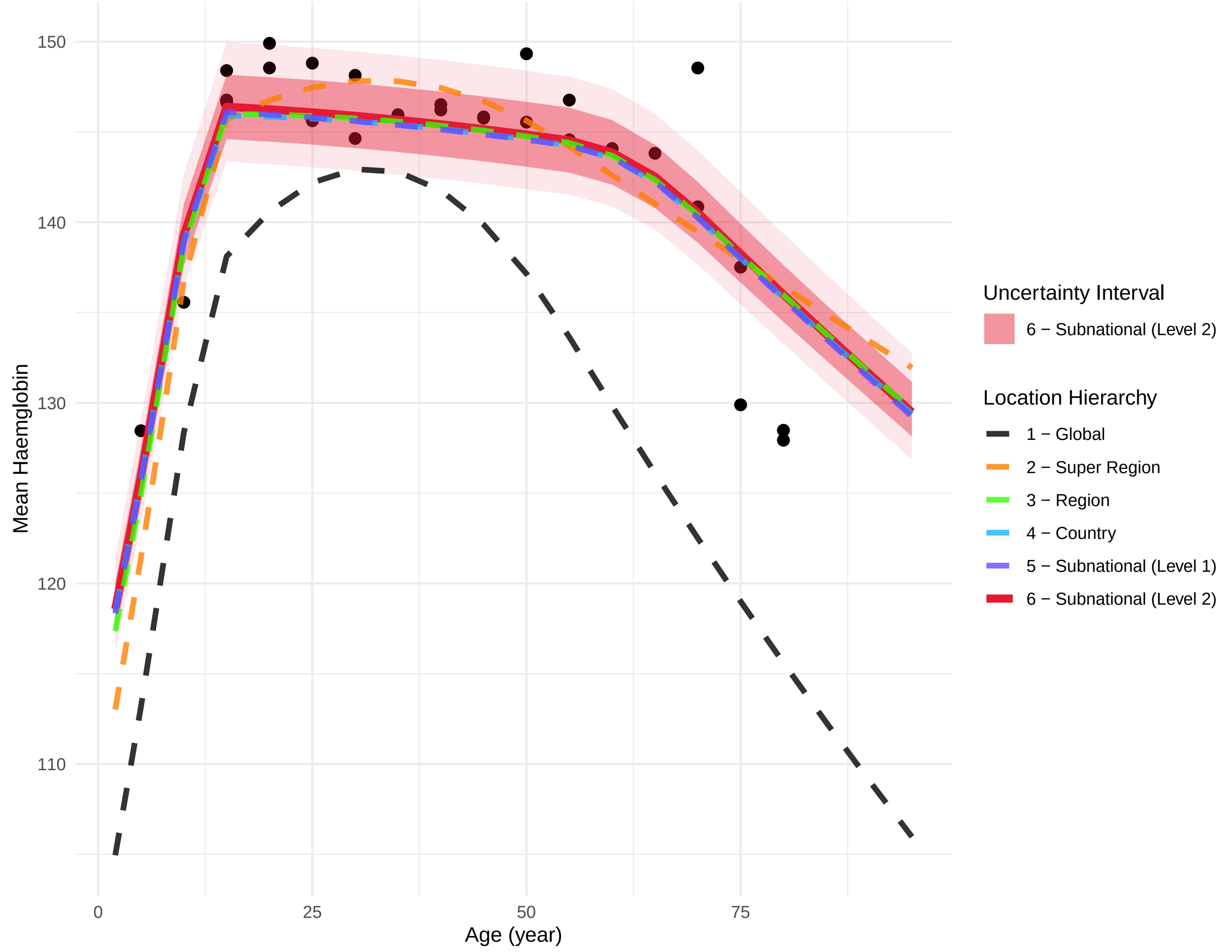

MR−BRT Age/Sex Fits − Males 2+ Years Old − Mean Haemglobin − Greater London
WHO−2024 Adjustment Method

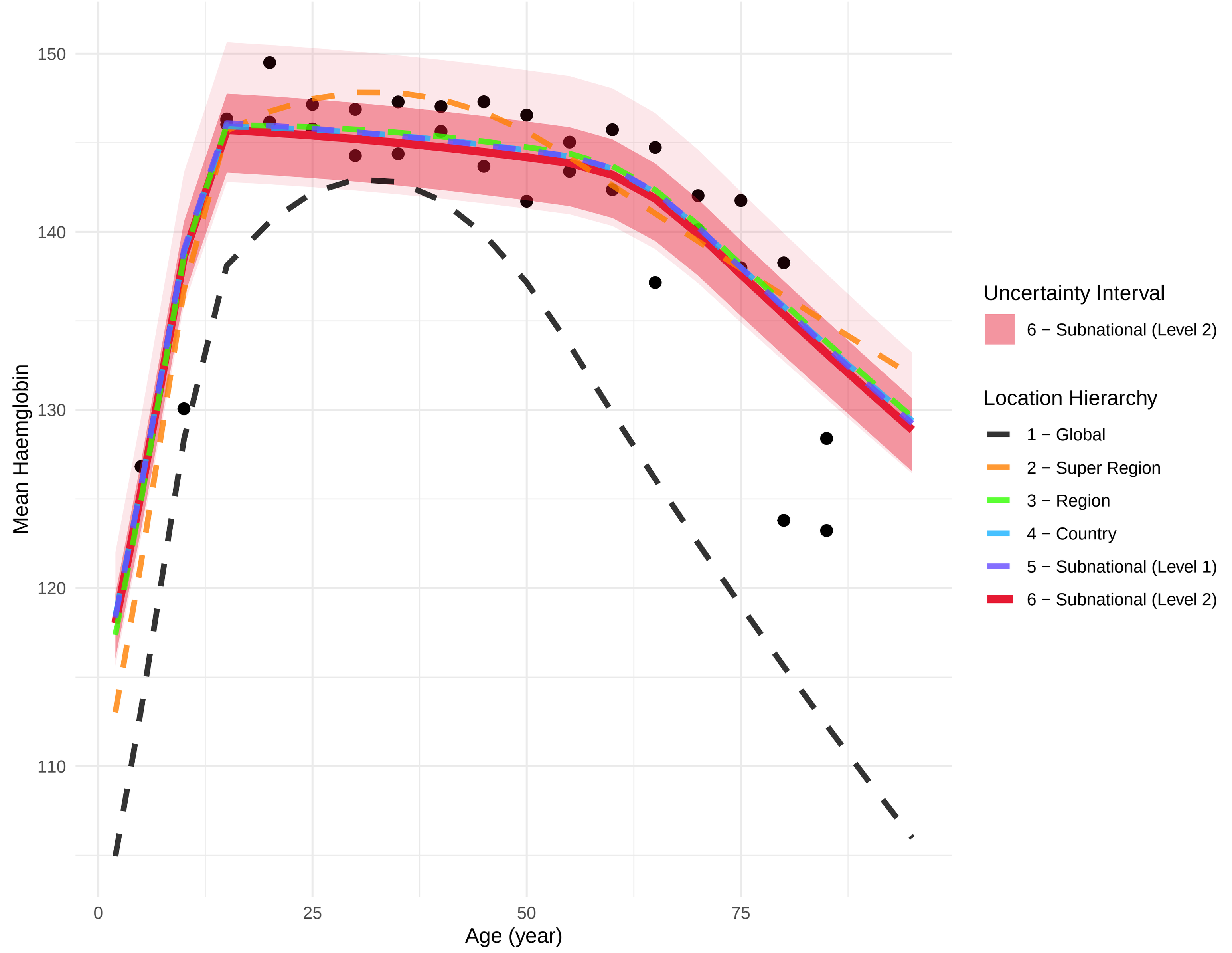

MR−BRT Age/Sex Fits − Males 2+ Years Old − Mean Haemglobin − North East England
WHO−2024 Adjustment Method

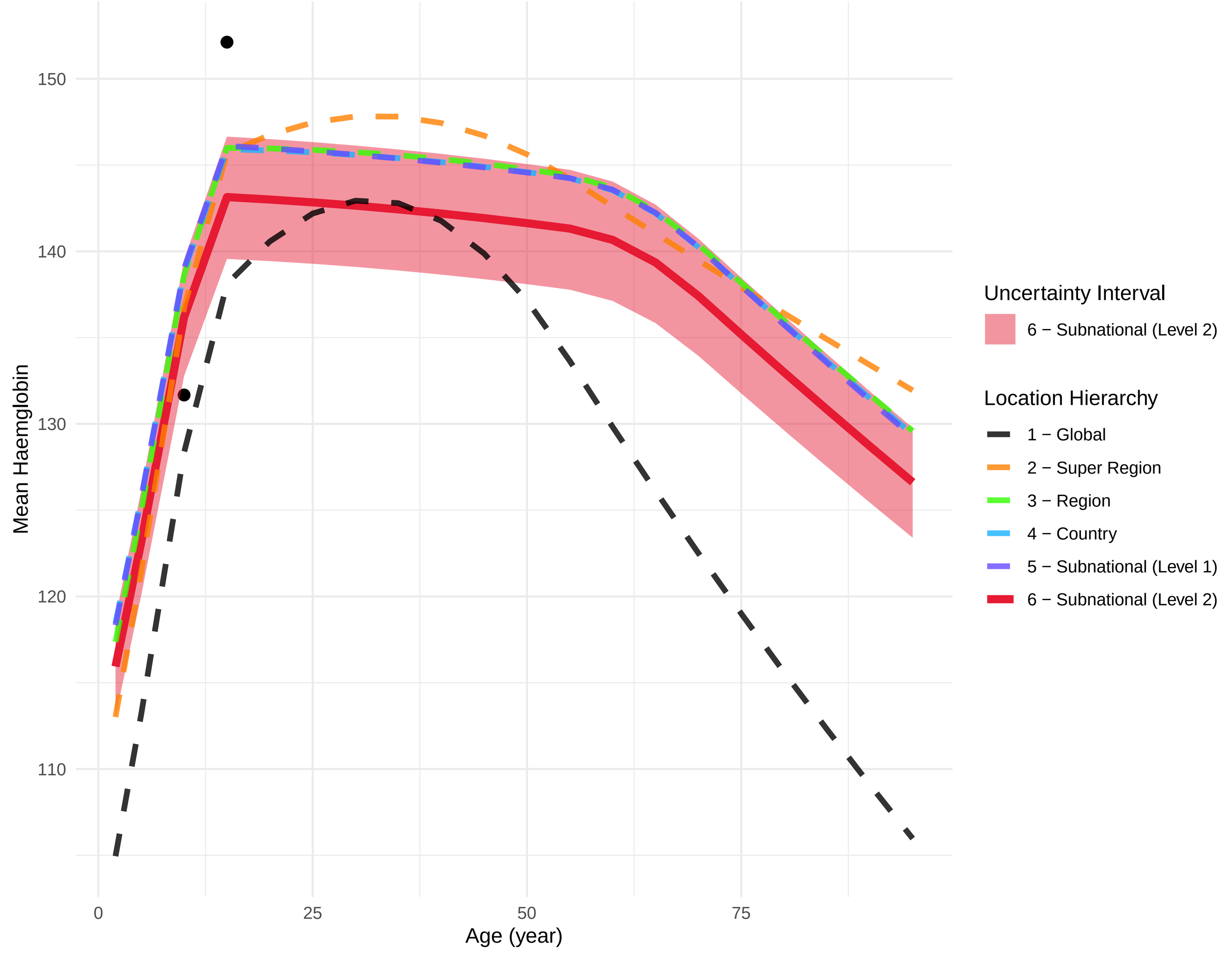

MR−BRT Age/Sex Fits − Males 2+ Years Old − Mean Haemglobin − North West England
WHO−2024 Adjustment Method

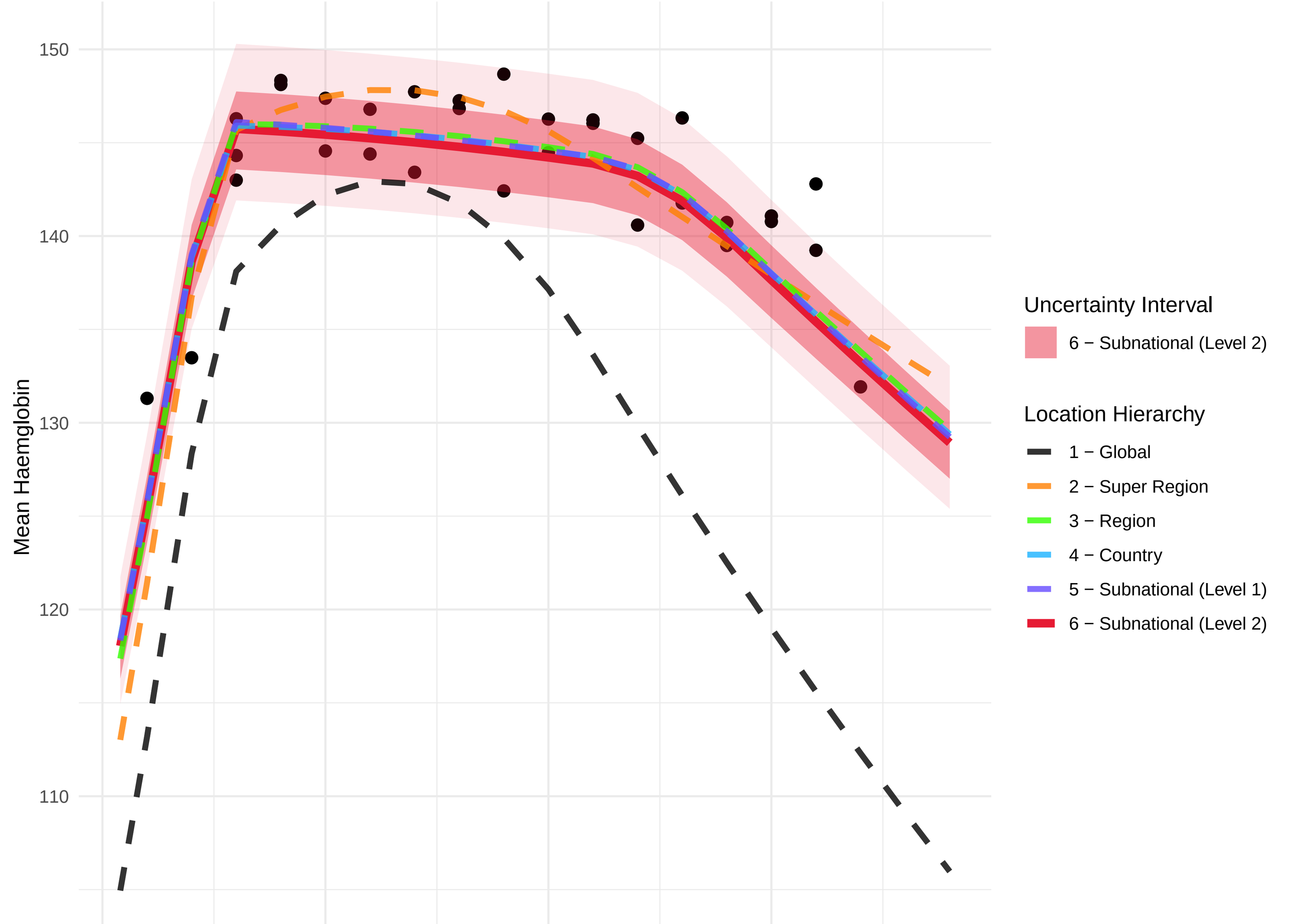

MR−BRT Age/Sex Fits − Males 2+ Years Old − Mean Haemglobin − South East England
WHO−2024 Adjustment Method

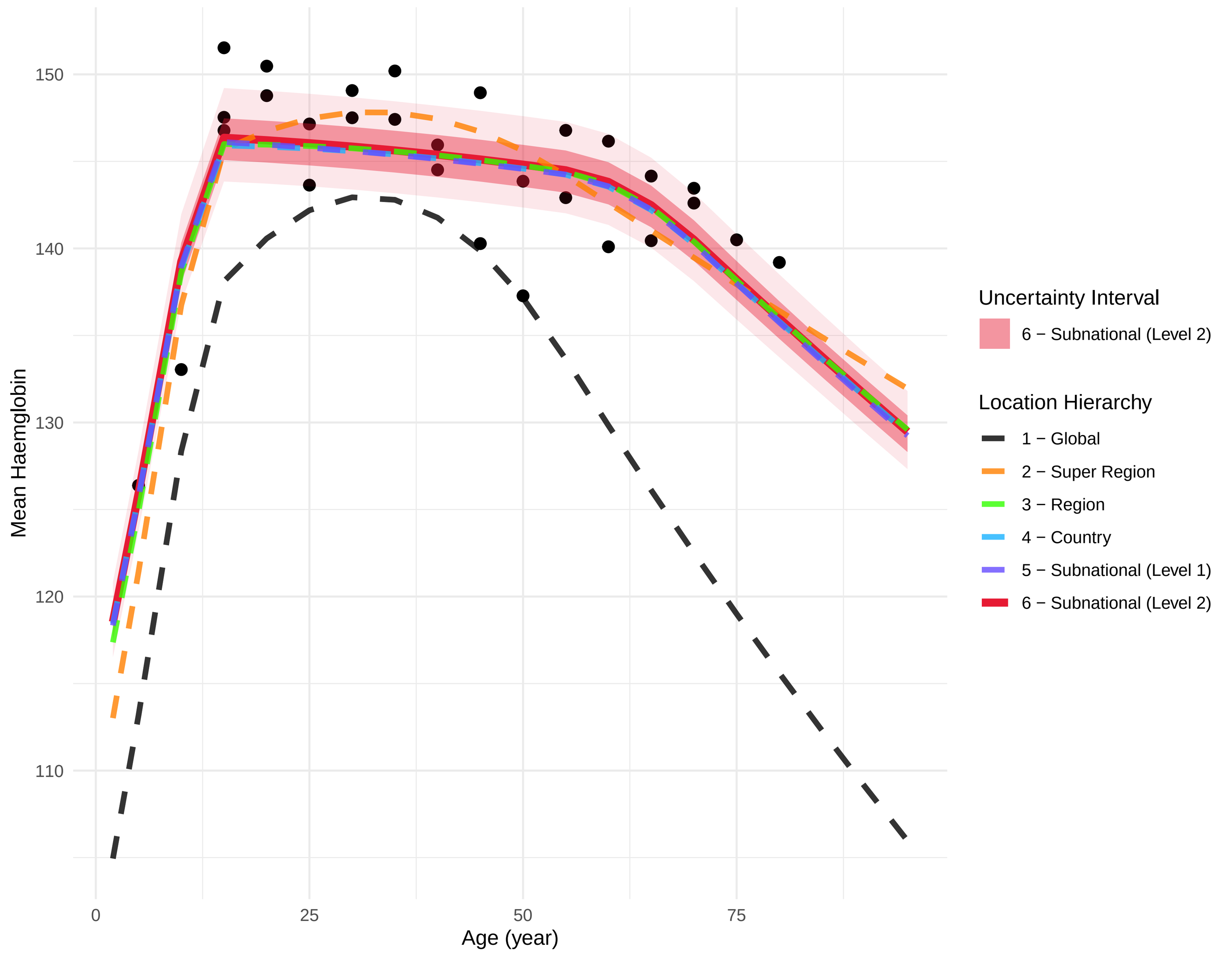

MR−BRT Age/Sex Fits − Males 2+ Years Old − Mean Haemglobin − South West England
WHO−2024 Adjustment Method

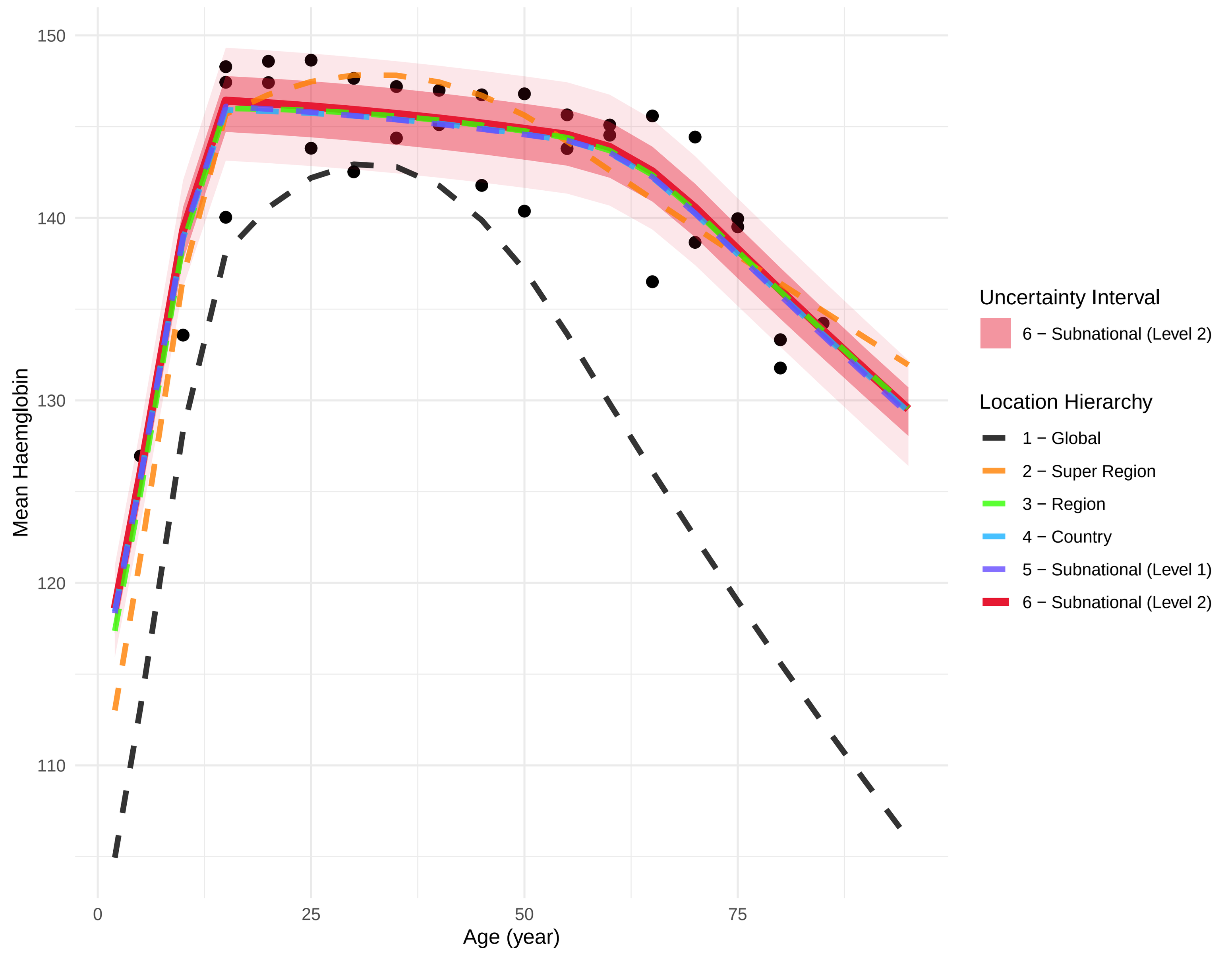

MR−BRT Age/Sex Fits − Males 2+ Years Old − Mean Haemglobin − West Midlands
WHO−2024 Adjustment Method

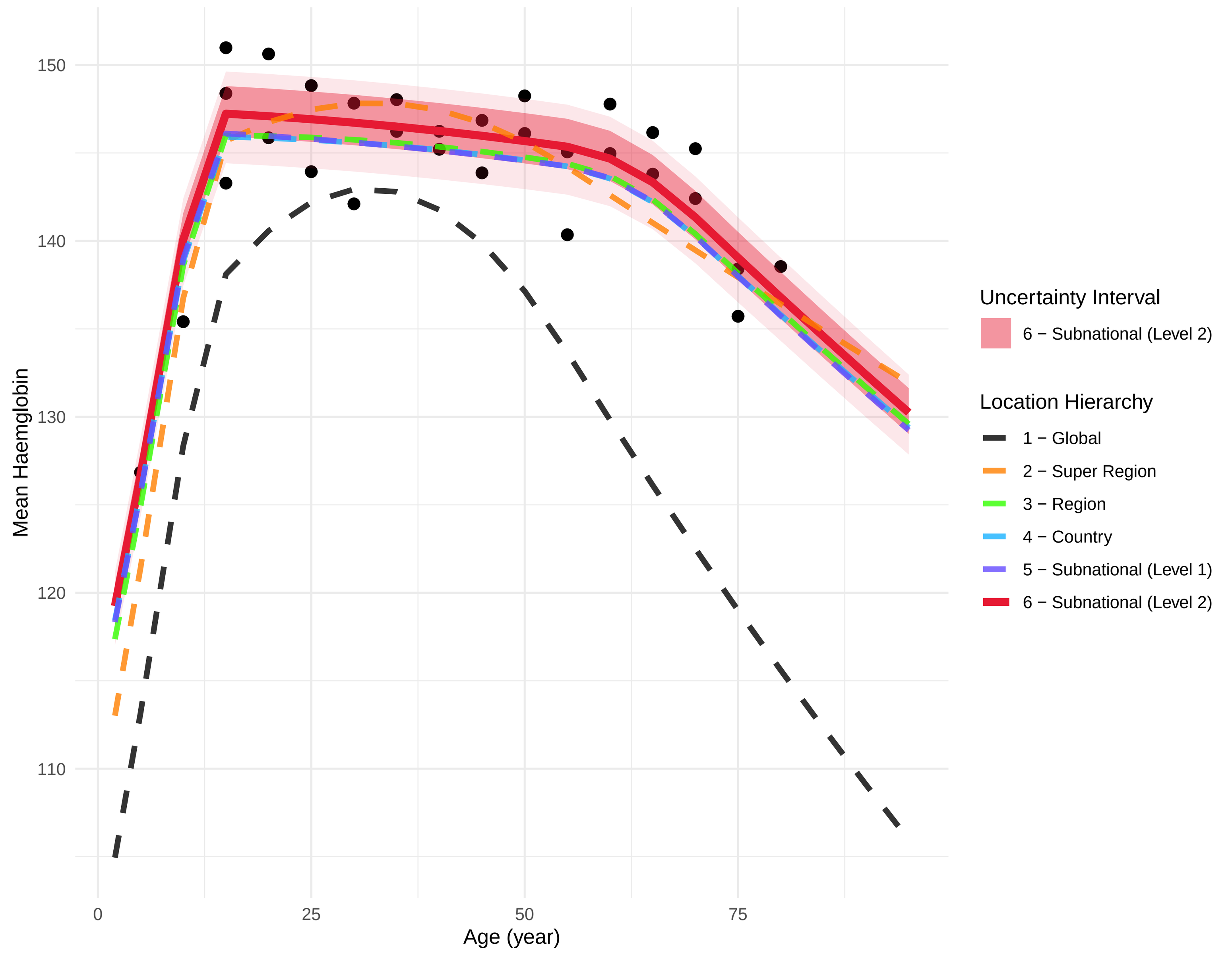

MR−BRT Age/Sex Fits − Males 2+ Years Old − Mean Haemglobin − Yorkshire and the Humber
WHO−2024 Adjustment Method

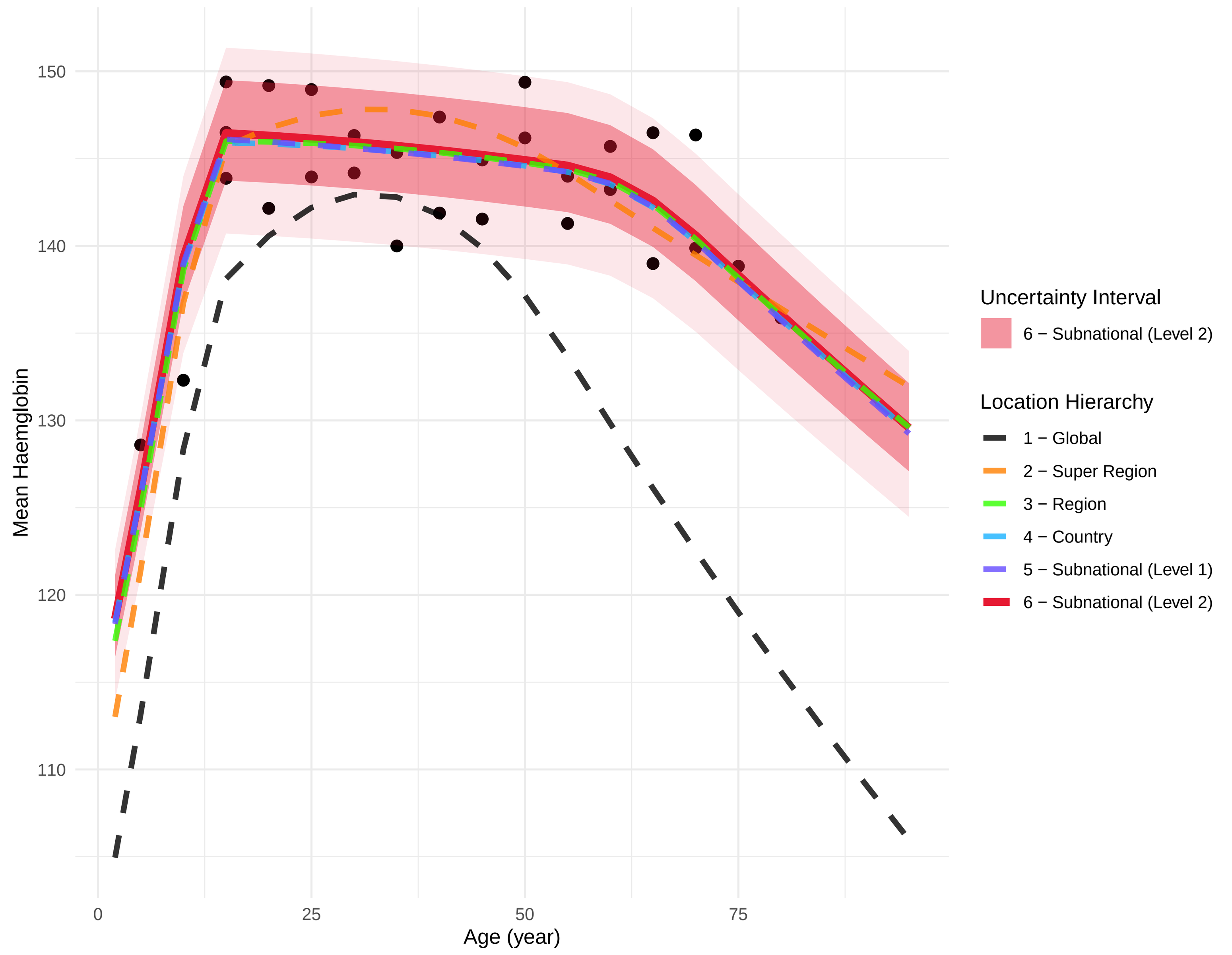

MR−BRT Age/Sex Fits − Males 2+ Years Old − Mean Haemglobin − Scotland
WHO−2024 Adjustment Method

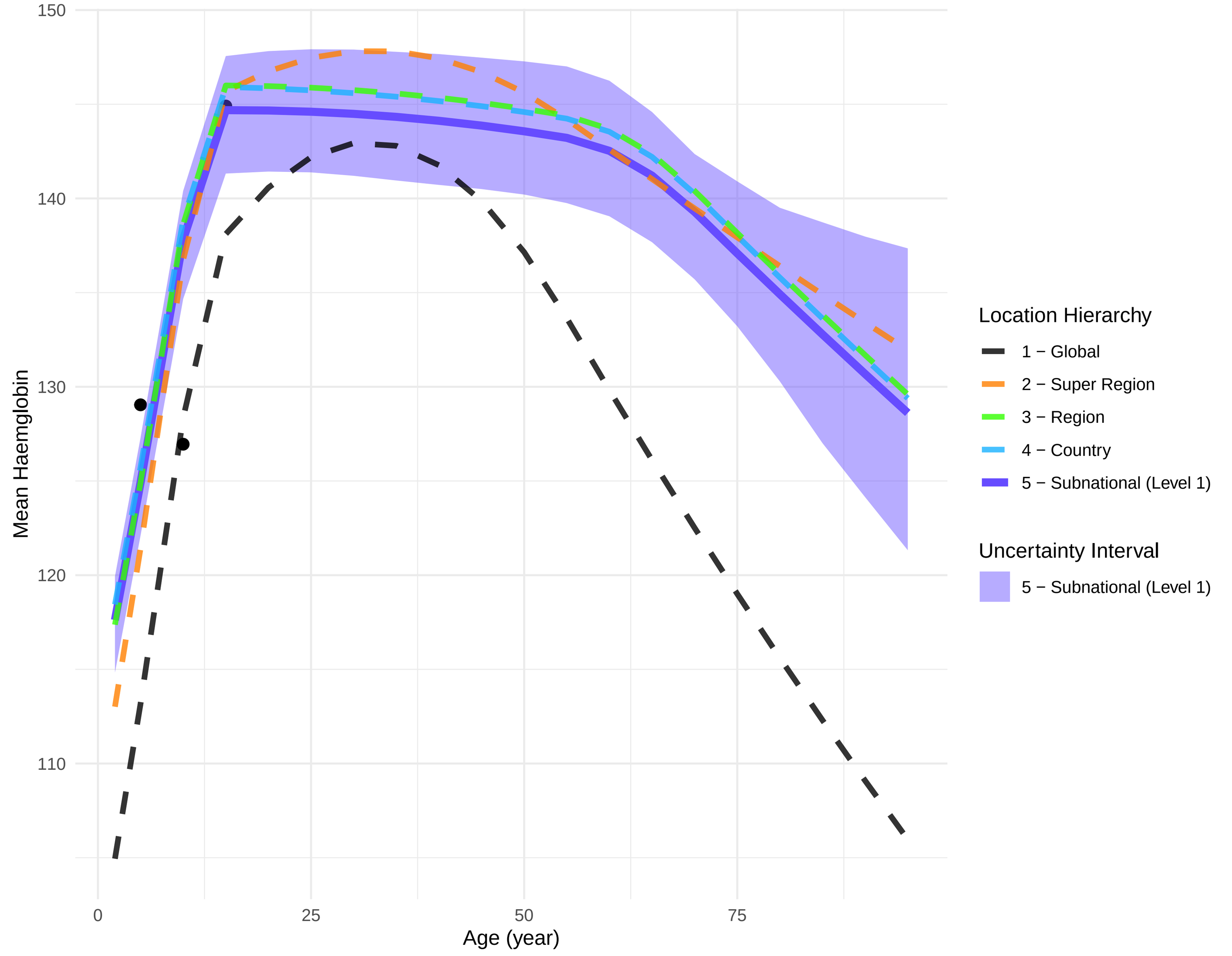

MR−BRT Age/Sex Fits − Males 2+ Years Old − Mean Haemglobin − Wales
WHO−2024 Adjustment Method

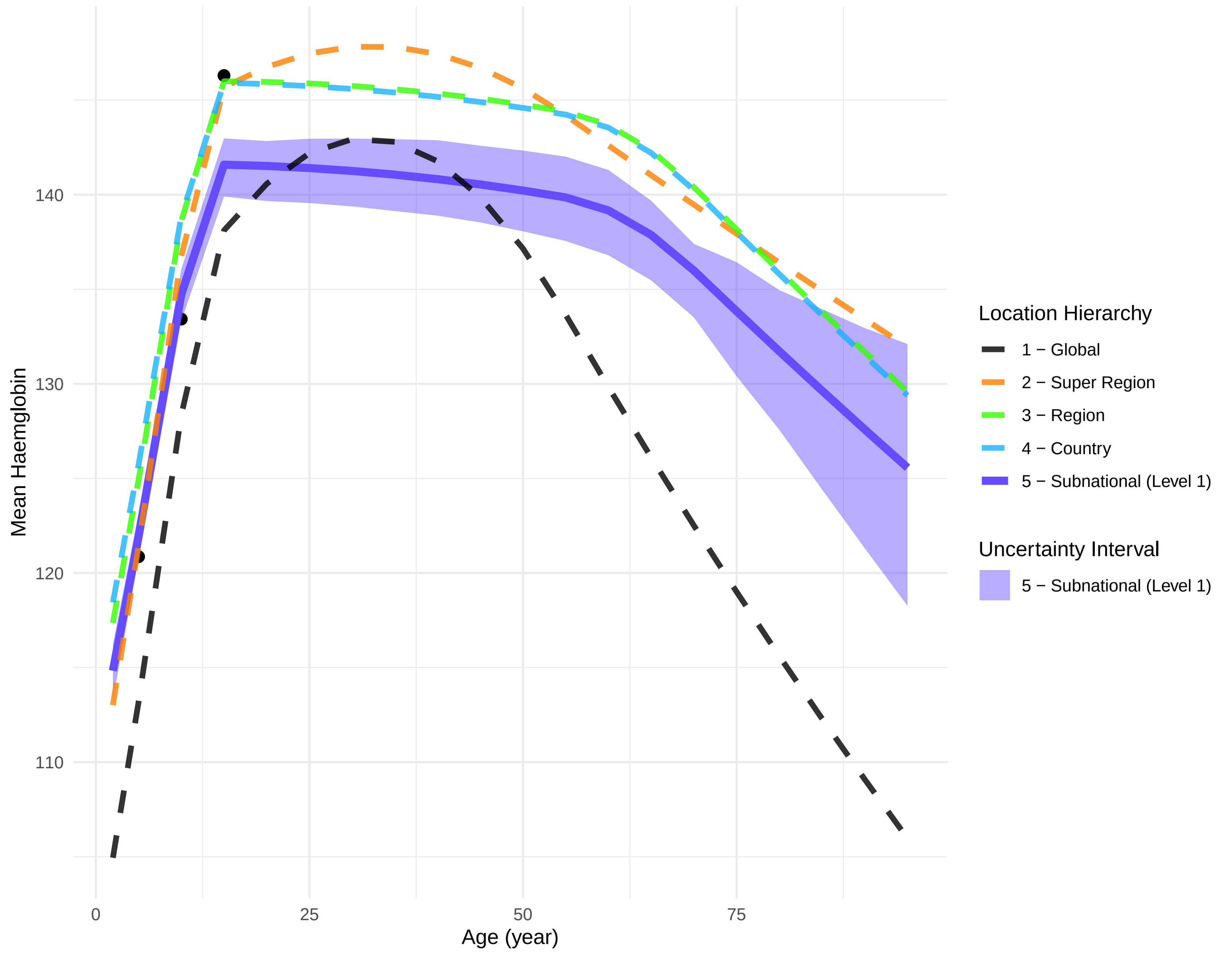

MR−BRT Age/Sex Fits − Males 2+ Years Old − Mean Haemglobin − Latin America and Caribbean
WHO−2024 Adjustment Method

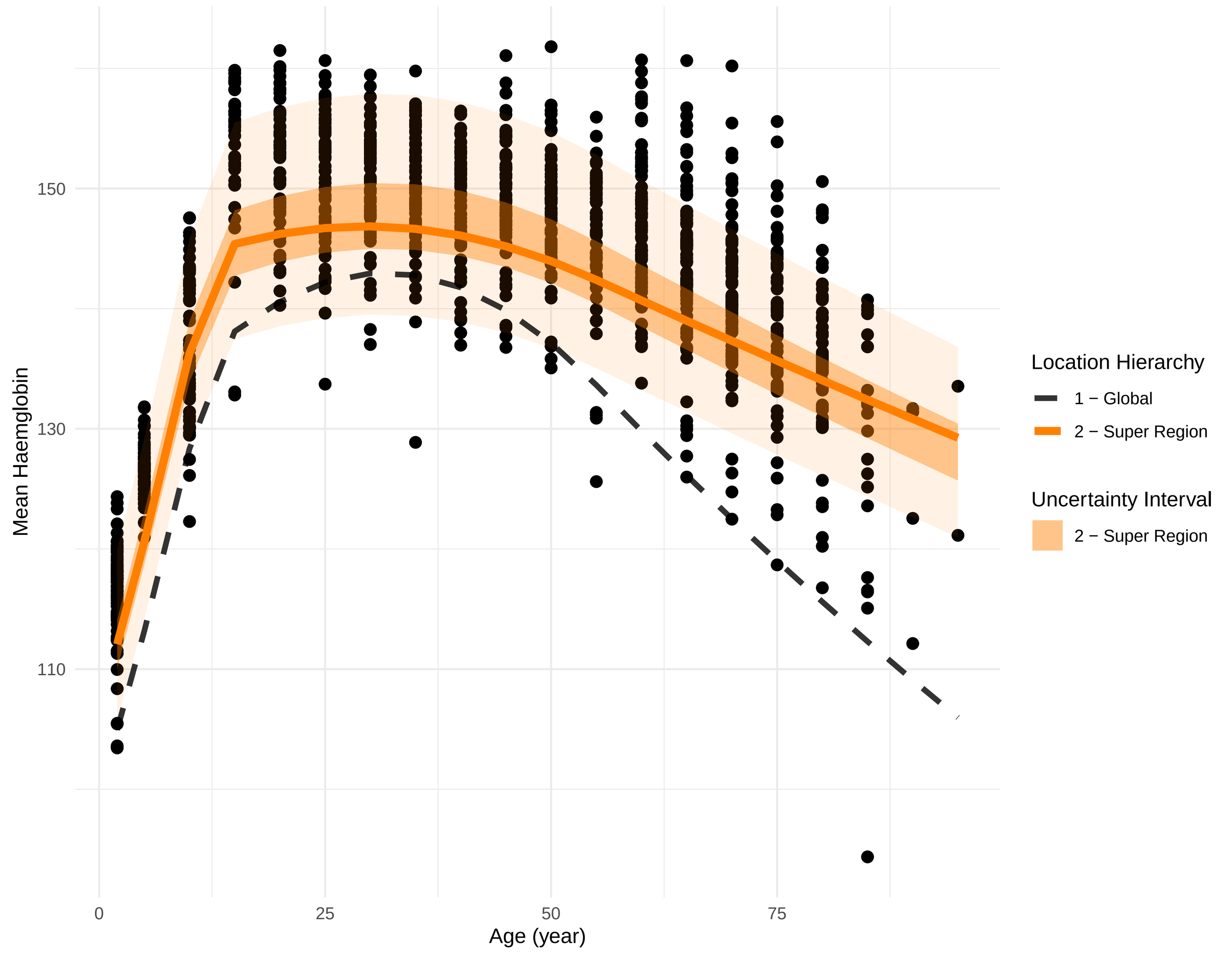

MR−BRT Age/Sex Fits − Males 2+ Years Old − Mean Haemglobin − Andean Latin America
WHO−2024 Adjustment Method

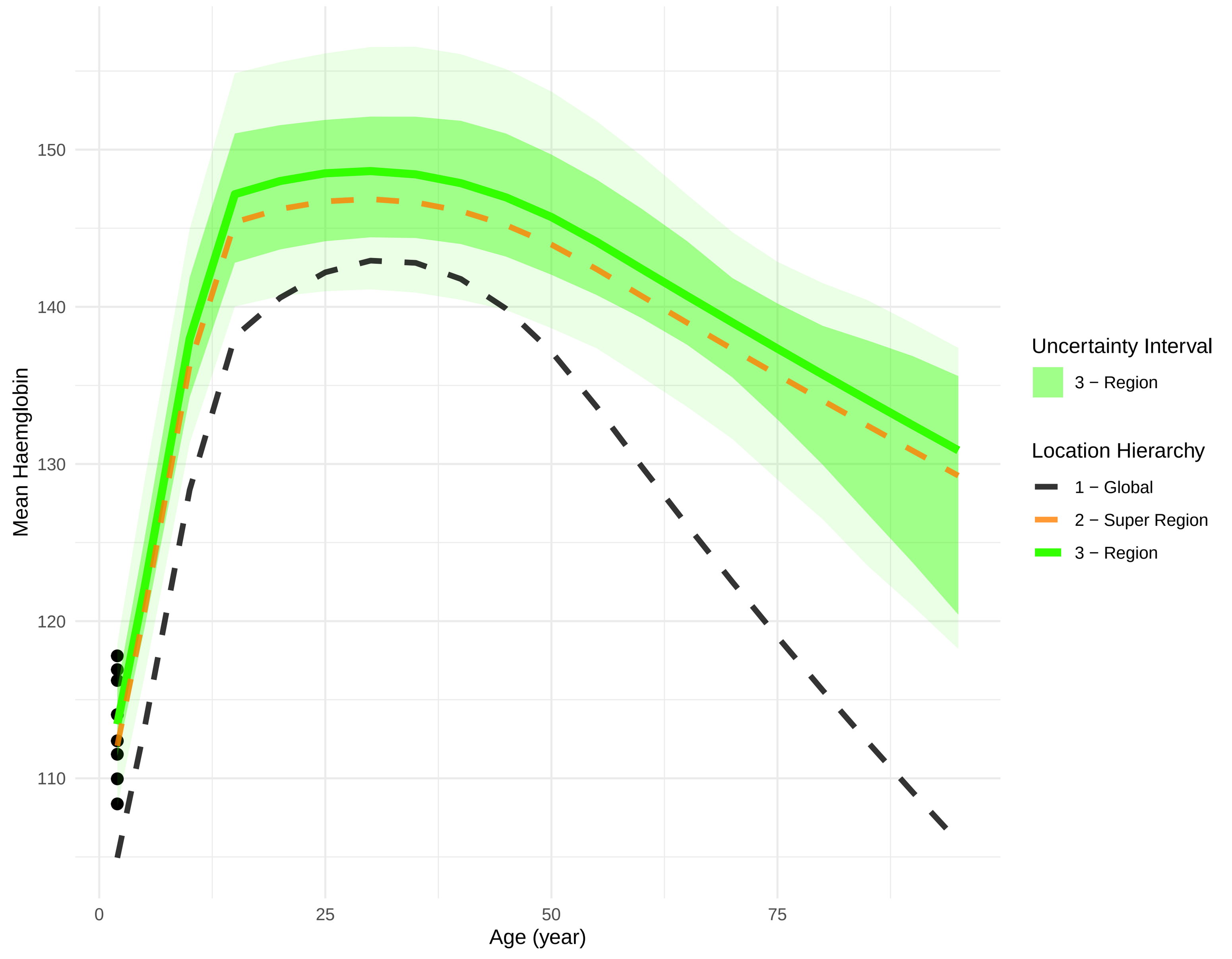

MR−BRT Age/Sex Fits − Males 2+ Years Old − Mean Haemglobin − Bolivia (Plurinational State of)
WHO−2024 Adjustment Method

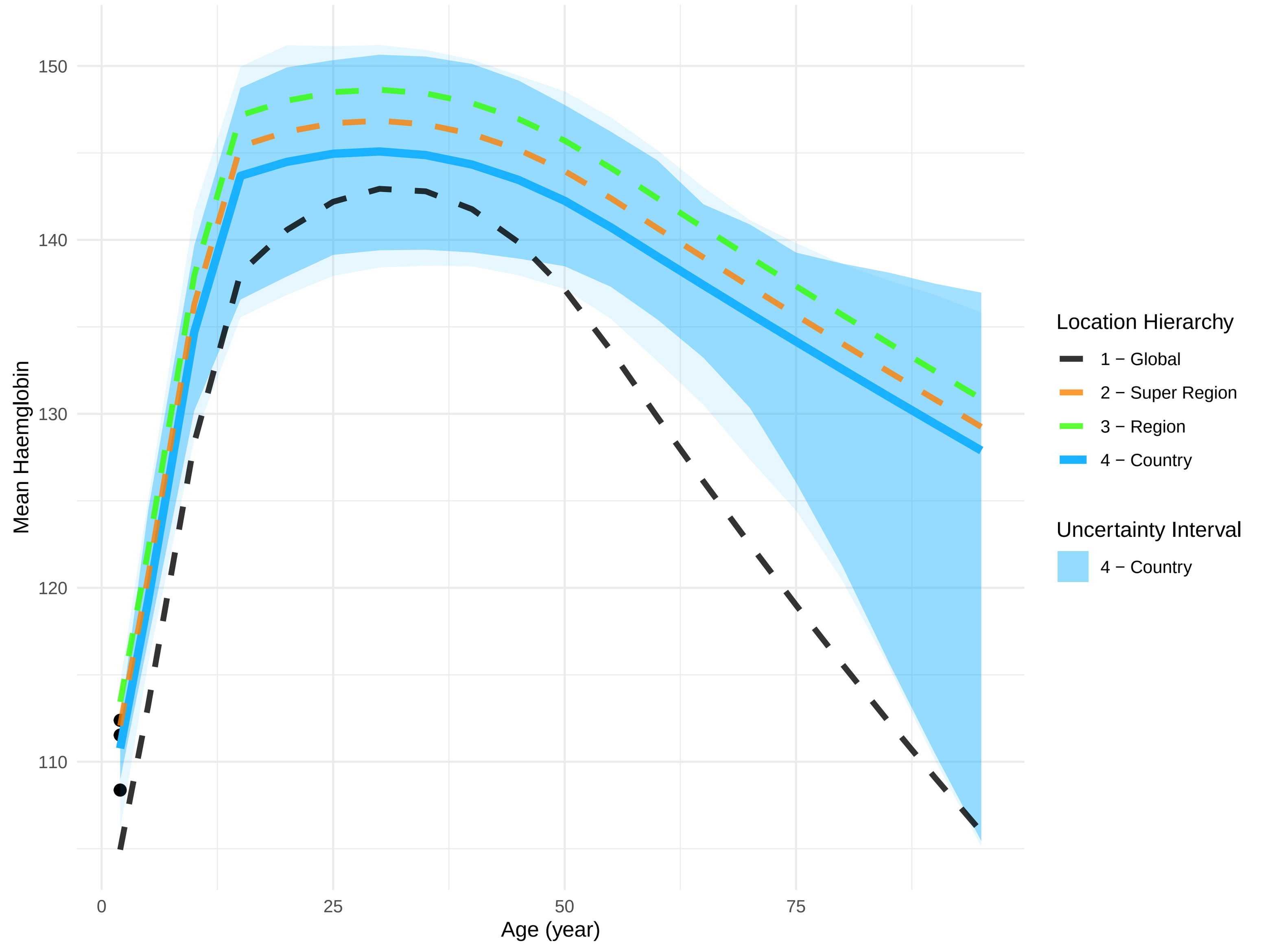

MR−BRT Age/Sex Fits − Males 2+ Years Old − Mean Haemglobin − Peru
WHO−2024 Adjustment Method

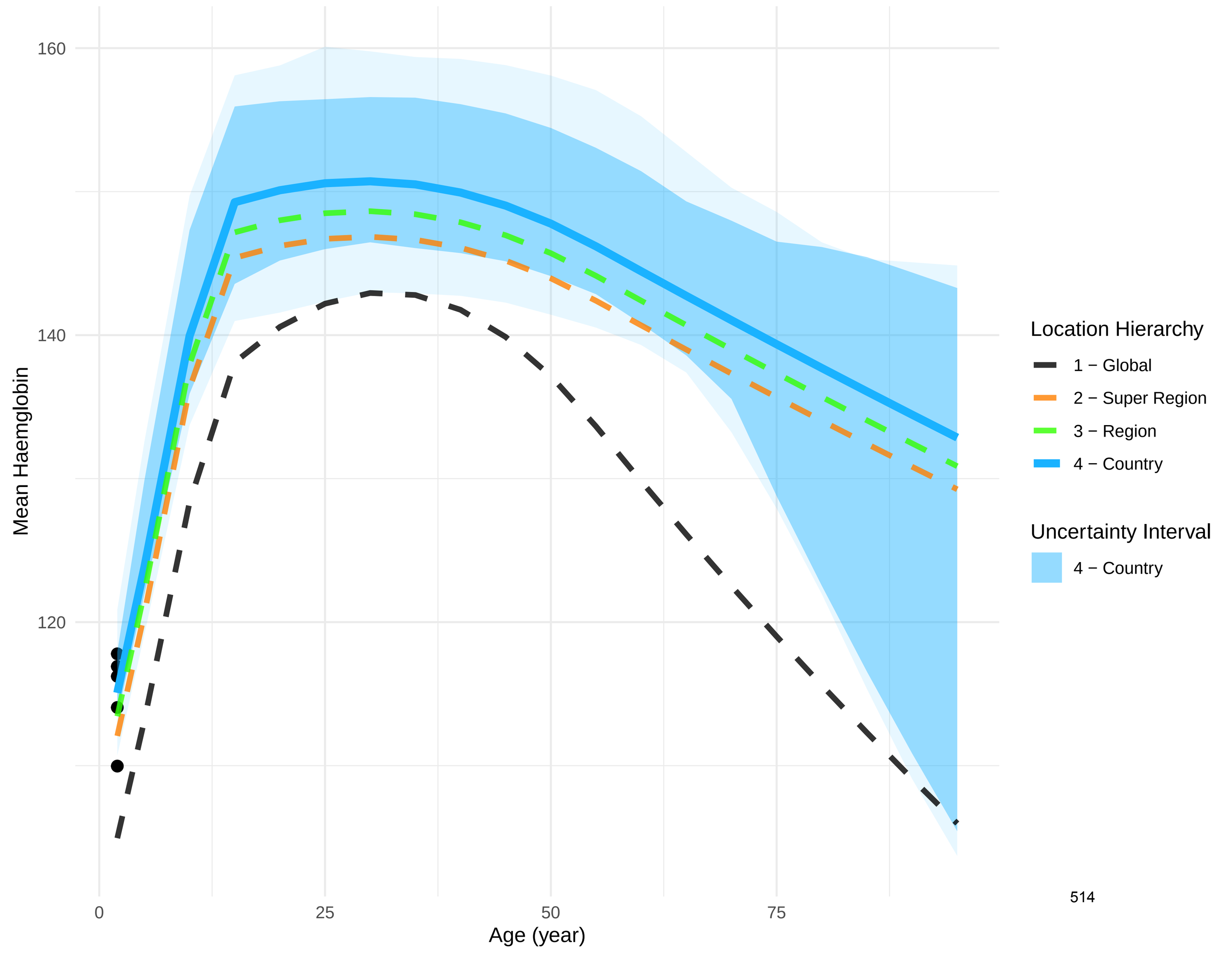

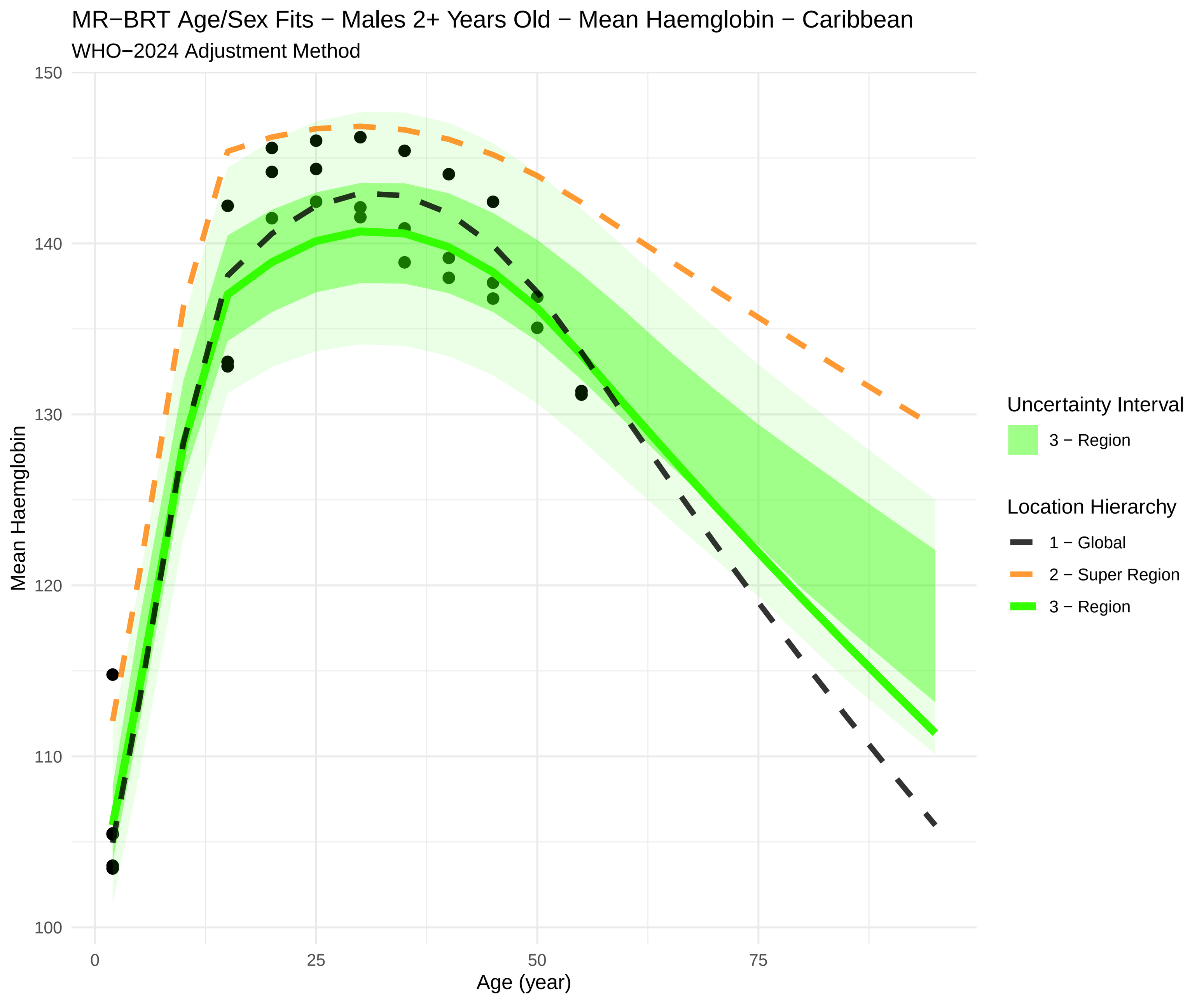
MR−BRT Age/Sex Fits − Males 2+ Years Old − Mean Haemglobin − Caribbean
WHO−2024 Adjustment Method
Mean Haemglobin
Age (year)
Uncertainty Interval
3 − Region
Location Hierarchy
1 − Global
2 − Super Region
3 − Region

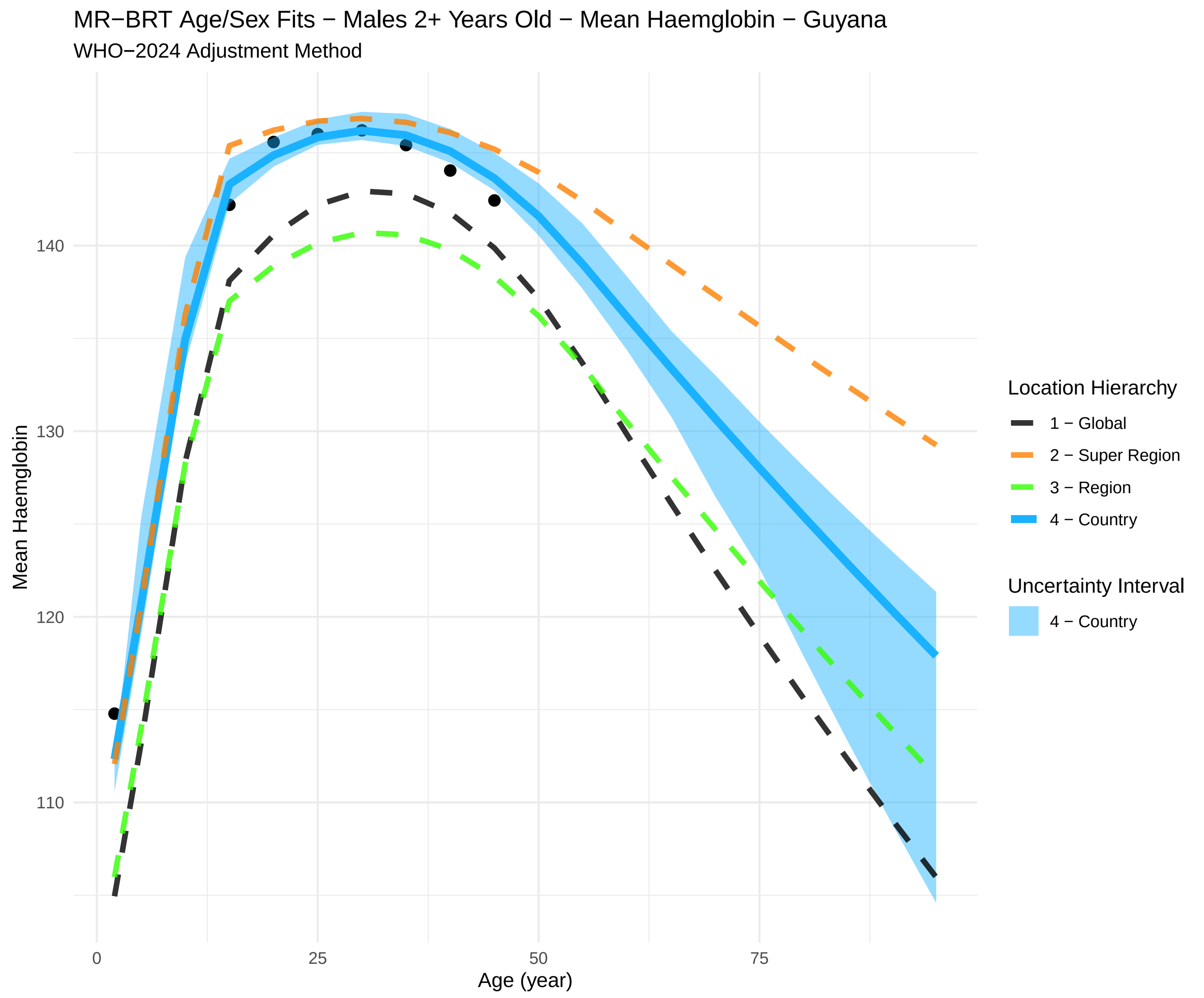
MR−BRT Age/Sex Fits − Males 2+ Years Old − Mean Haemglobin − Guyana
WHO−2024 Adjustment Method
Mean Haemglobin
Age (year)
Location Hierarchy
1 − Global
2 − Super Region
3 − Region
4 − Country
Uncertainty Interval
4 − Country

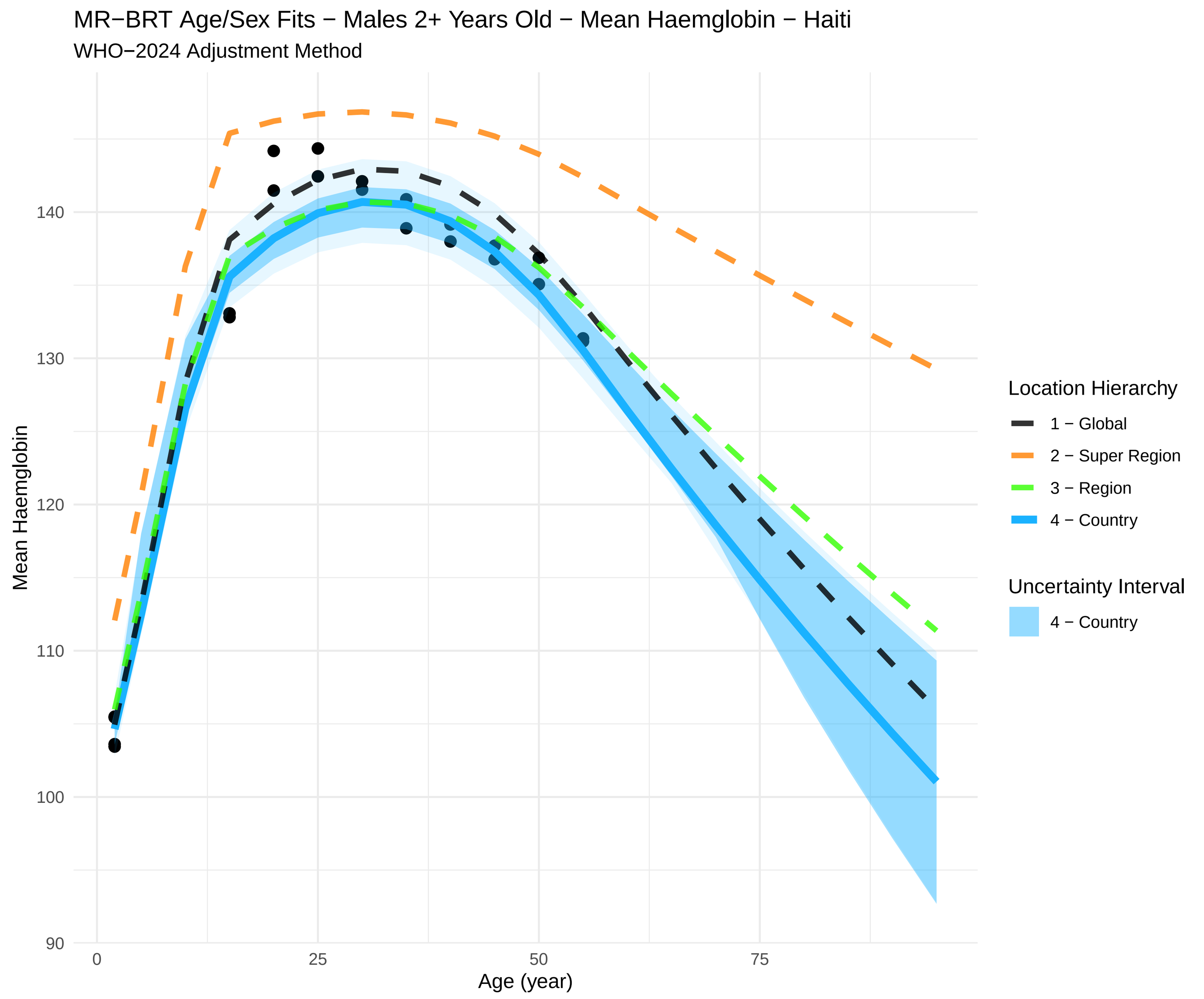
MR−BRT Age/Sex Fits − Males 2+ Years Old − Mean Haemglobin − Haiti
WHO−2024 Adjustment Method
Mean Haemglobin
Age (year)
Location Hierarchy
1 − Global
2 − Super Region
3 − Region
4 − Country
Uncertainty Interval
4 − Country

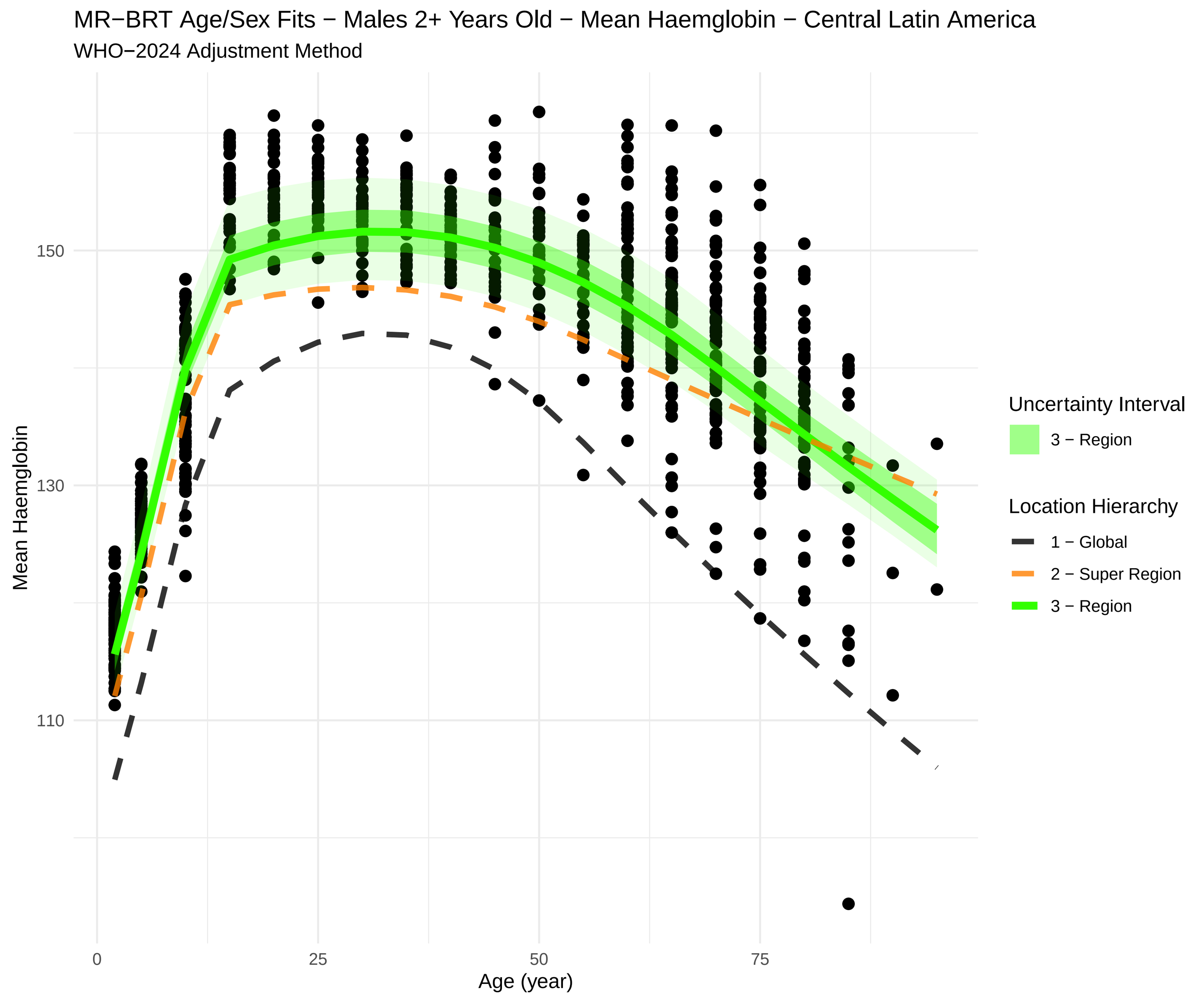
MR−BRT Age/Sex Fits − Males 2+ Years Old − Mean Haemglobin − Central Latin America
WHO−2024 Adjustment Method
Mean Haemglobin
Age (year)
Uncertainty Interval
3 − Region
Location Hierarchy
1 − Global
2 − Super Region
3 − Region

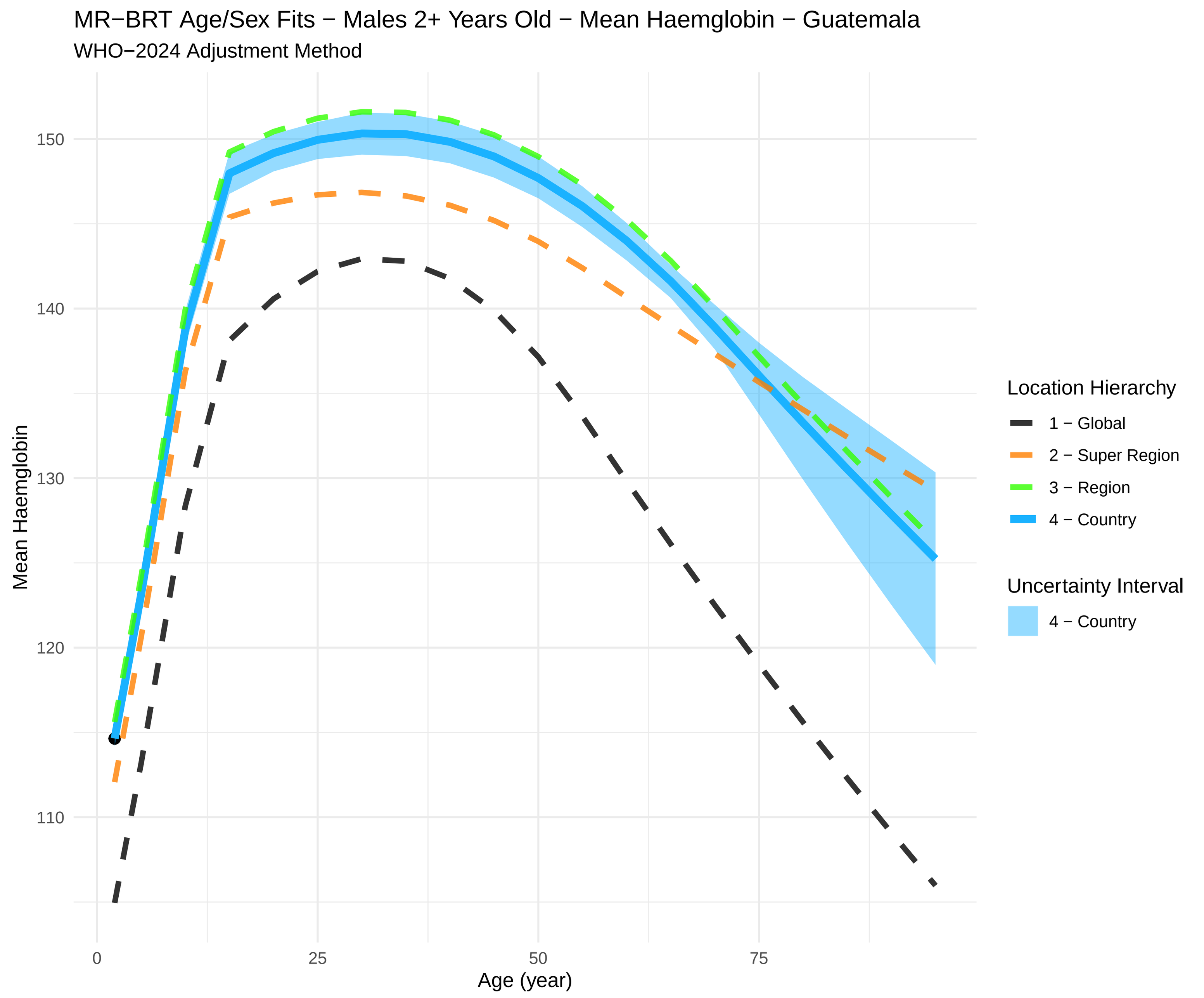
MR−BRT Age/Sex Fits − Males 2+ Years Old − Mean Haemglobin − Guatemala
WHO−2024 Adjustment Method
Mean Haemglobin
Age (year)
Location Hierarchy
1 − Global
2 − Super Region
3 − Region
4 − Country
Uncertainty Interval
4 − Country

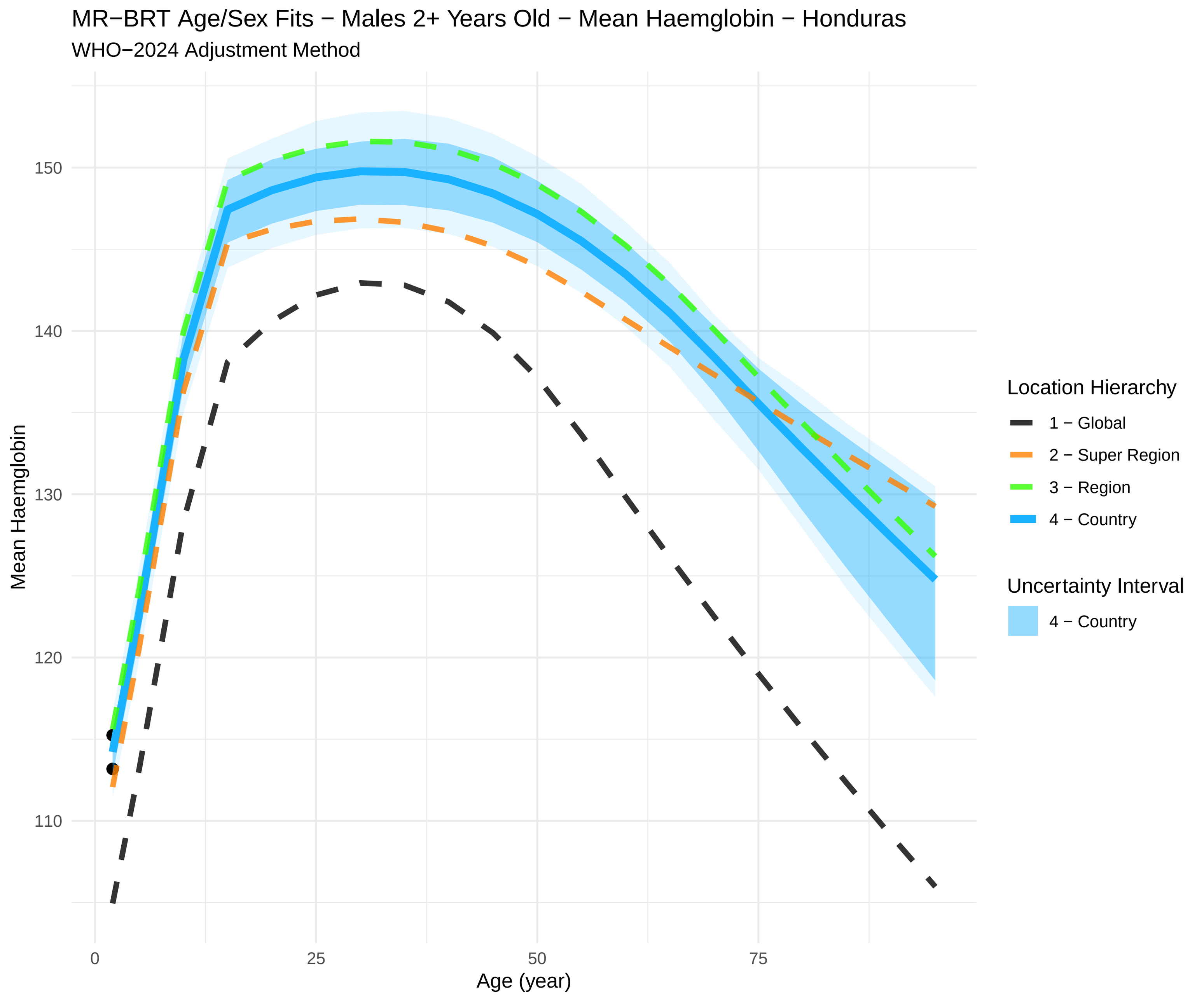
MR−BRT Age/Sex Fits − Males 2+ Years Old − Mean Haemglobin − Honduras
WHO−2024 Adjustment Method
Mean Haemglobin
Age (year)
Location Hierarchy
1 − Global
2 − Super Region
3 − Region
4 − Country
Uncertainty Interval
4 − Country

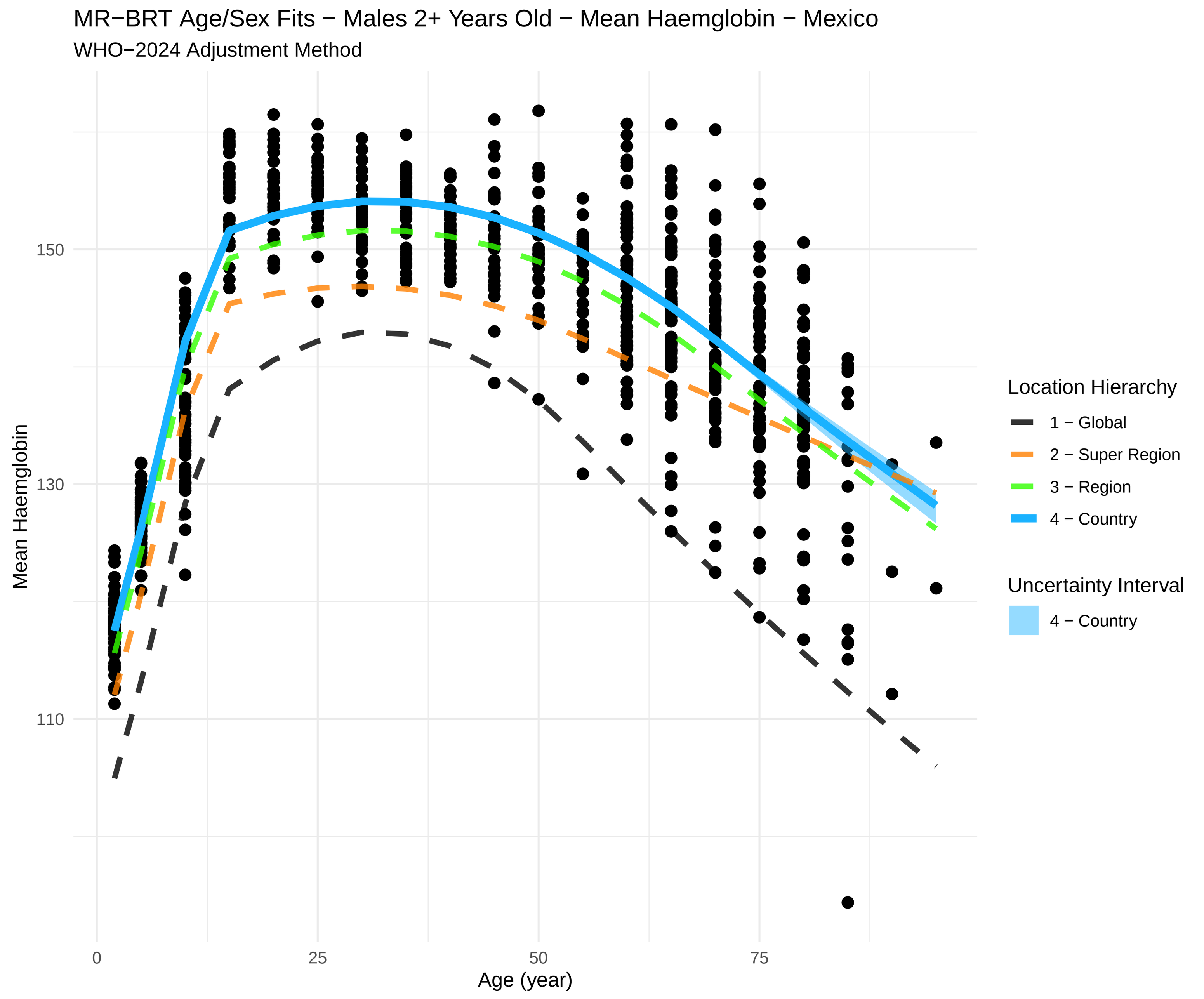
MR−BRT Age/Sex Fits − Males 2+ Years Old − Mean Haemglobin − Mexico
WHO−2024 Adjustment Method
Mean Haemglobin
Age (year)
Location Hierarchy
1 − Global
2 − Super Region
3 − Region
4 − Country
Uncertainty Interval
4 − Country

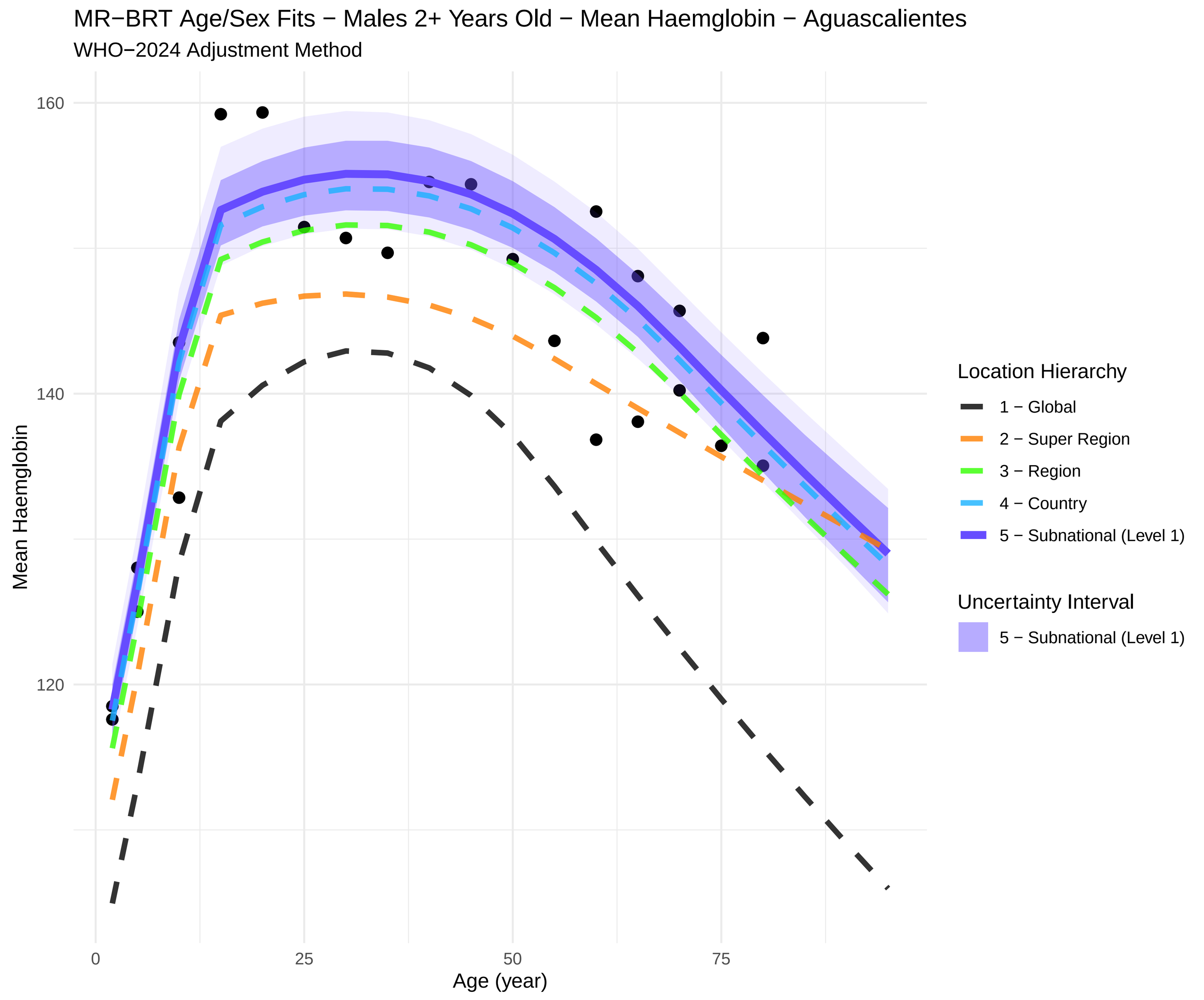
MR−BRT Age/Sex Fits − Males 2+ Years Old − Mean Haemglobin − Aguascalientes
WHO−2024 Adjustment Method
Mean Haemglobin
Age (year)
Location Hierarchy
1 − Global
2 − Super Region
3 − Region
4 − Country
5 − Subnational (Level 1)
Uncertainty Interval
5 − Subnational (Level 1)

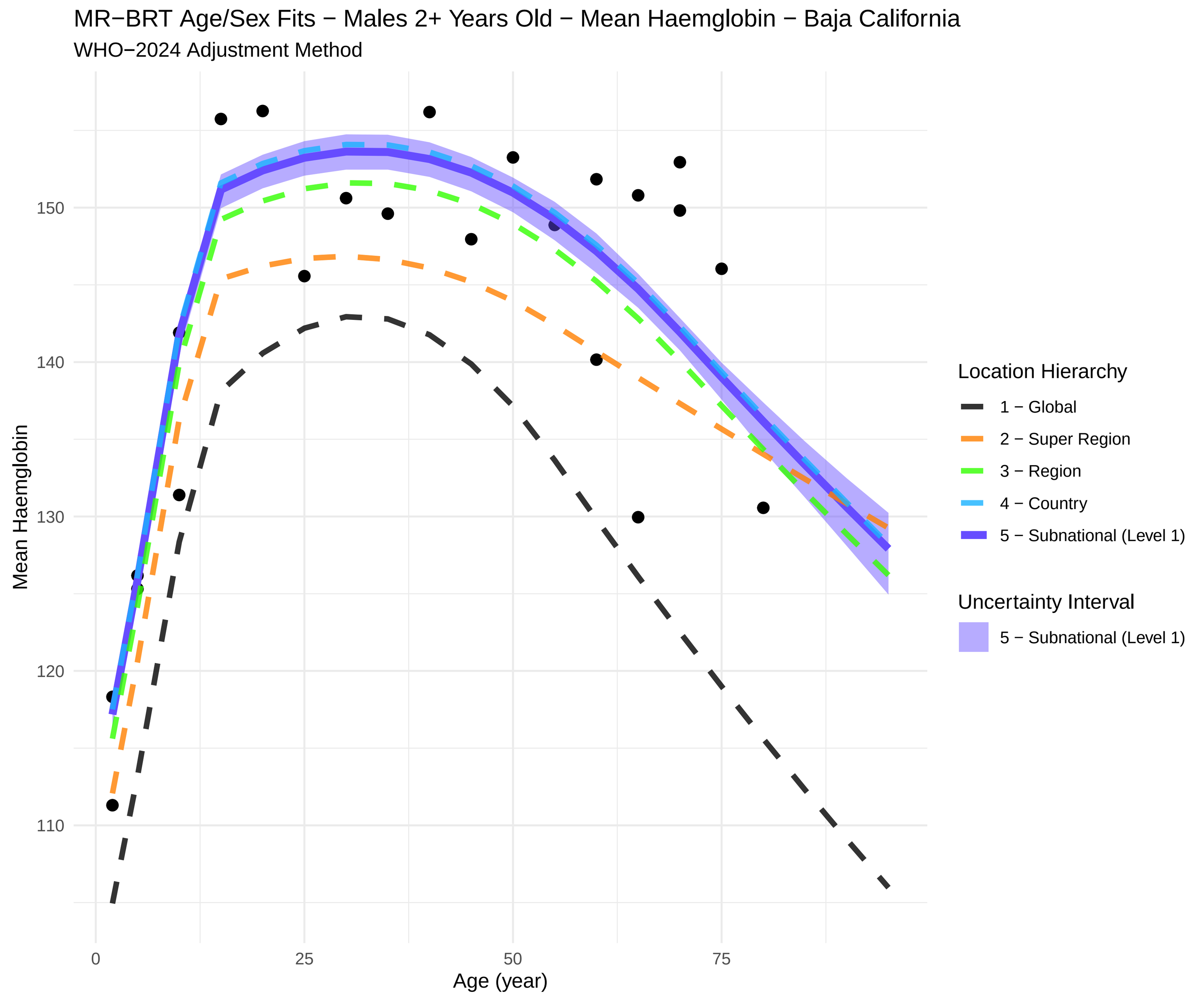
MR−BRT Age/Sex Fits − Males 2+ Years Old − Mean Haemglobin − Baja California
WHO−2024 Adjustment Method
Mean Haemglobin
Age (year)
Location Hierarchy
1 − Global
2 − Super Region
3 − Region
4 − Country
5 − Subnational (Level 1)
Uncertainty Interval
5 − Subnational (Level 1)

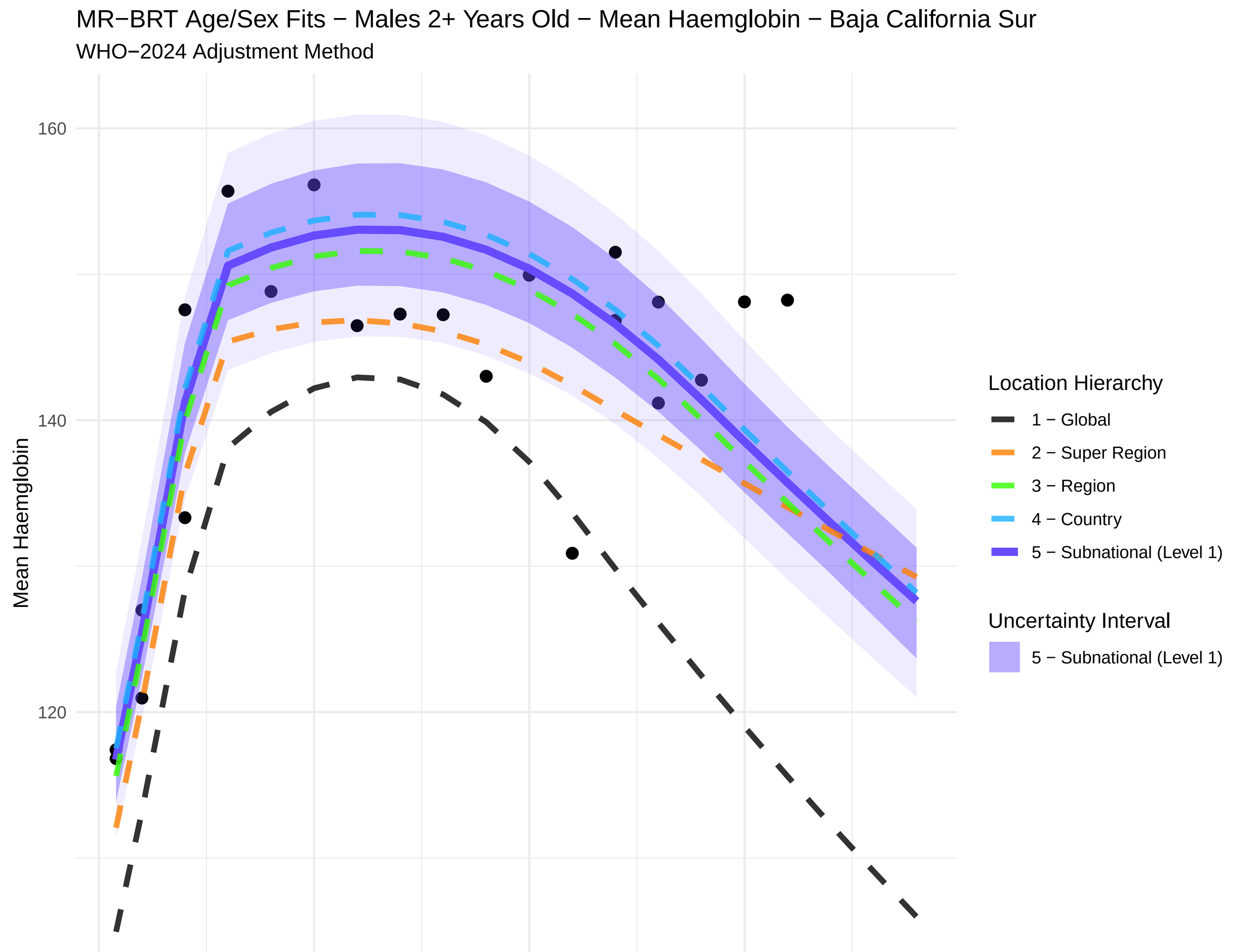
MR−BRT Age/Sex Fits − Males 2+ Years Old − Mean Haemglobin − Baja California Sur
WHO−2024 Adjustment Method
Mean Haemglobin
Age (year)
Location Hierarchy
1 − Global
2 − Super Region
3 − Region
4 − Country
5 − Subnational (Level 1)
Uncertainty Interval
5 − Subnational (Level 1)

MR−BRT Age/Sex Fits − Males 2+ Years Old − Mean Haemoglobin − Campeche
WHO−2024 Adjustment Method

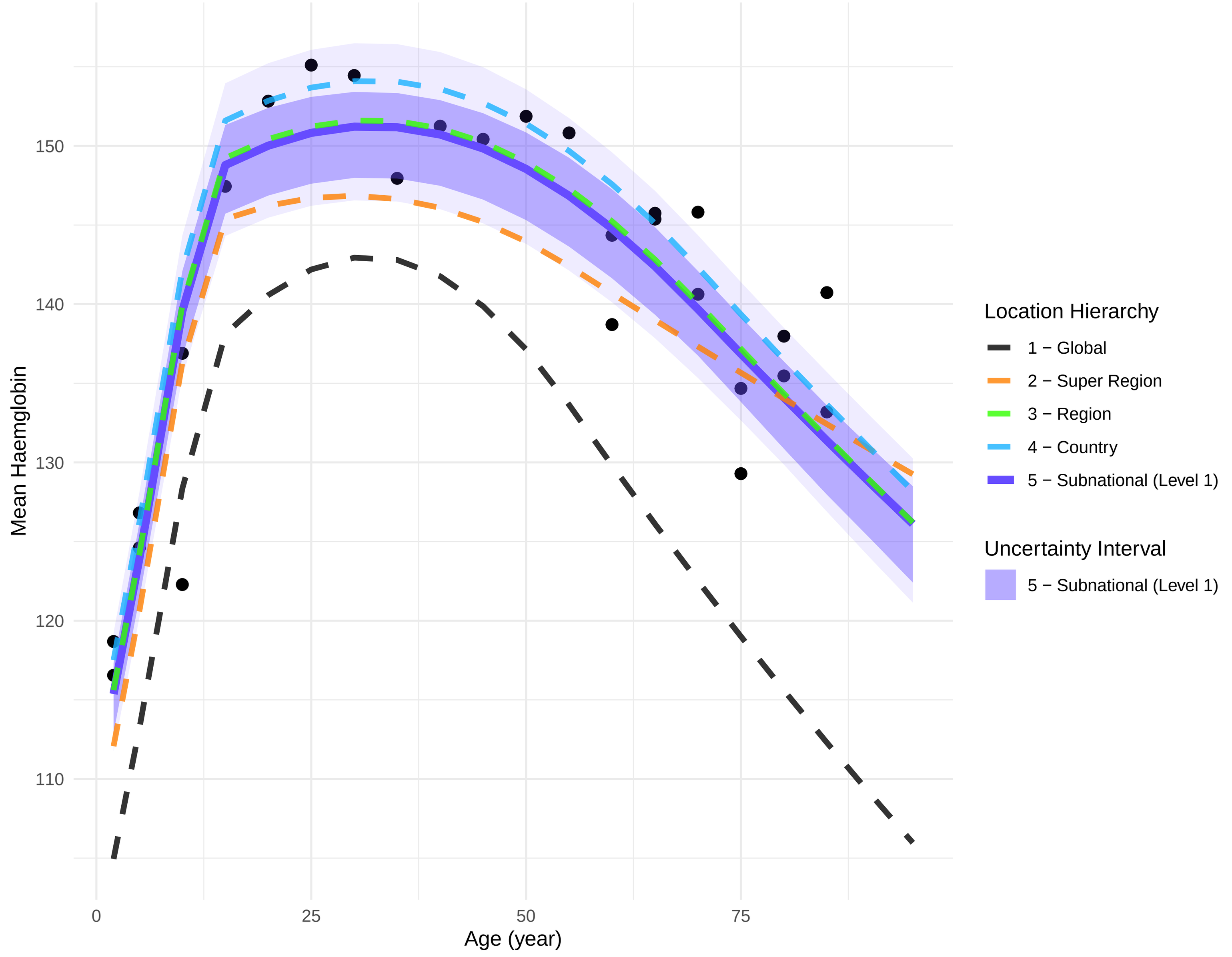

MR−BRT Age/Sex Fits − Males 2+ Years Old − Mean Haemoglobin − Chiapas
WHO−2024 Adjustment Method

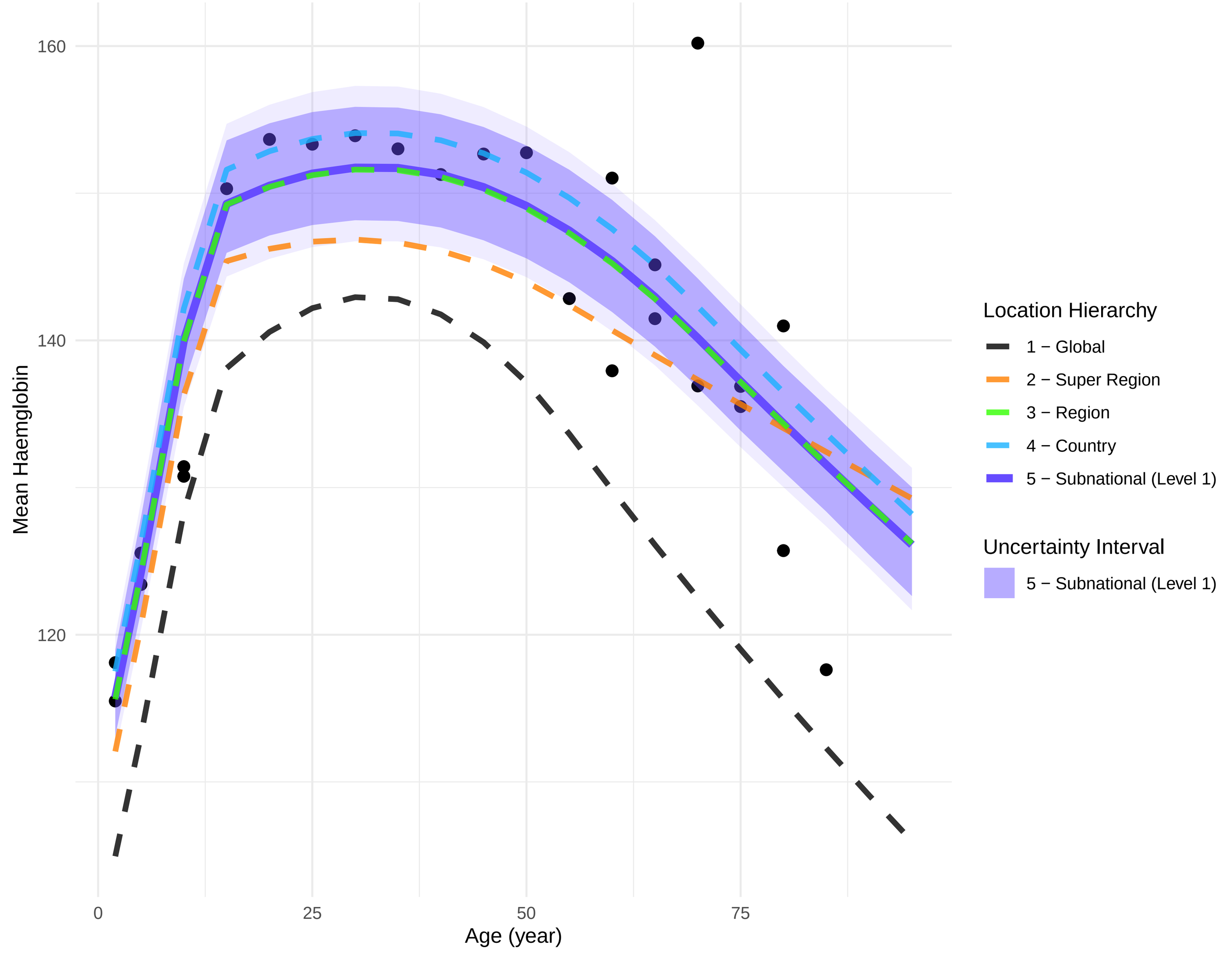

MR−BRT Age/Sex Fits − Males 2+ Years Old − Mean Haemoglobin − Chihuahua
WHO−2024 Adjustment Method

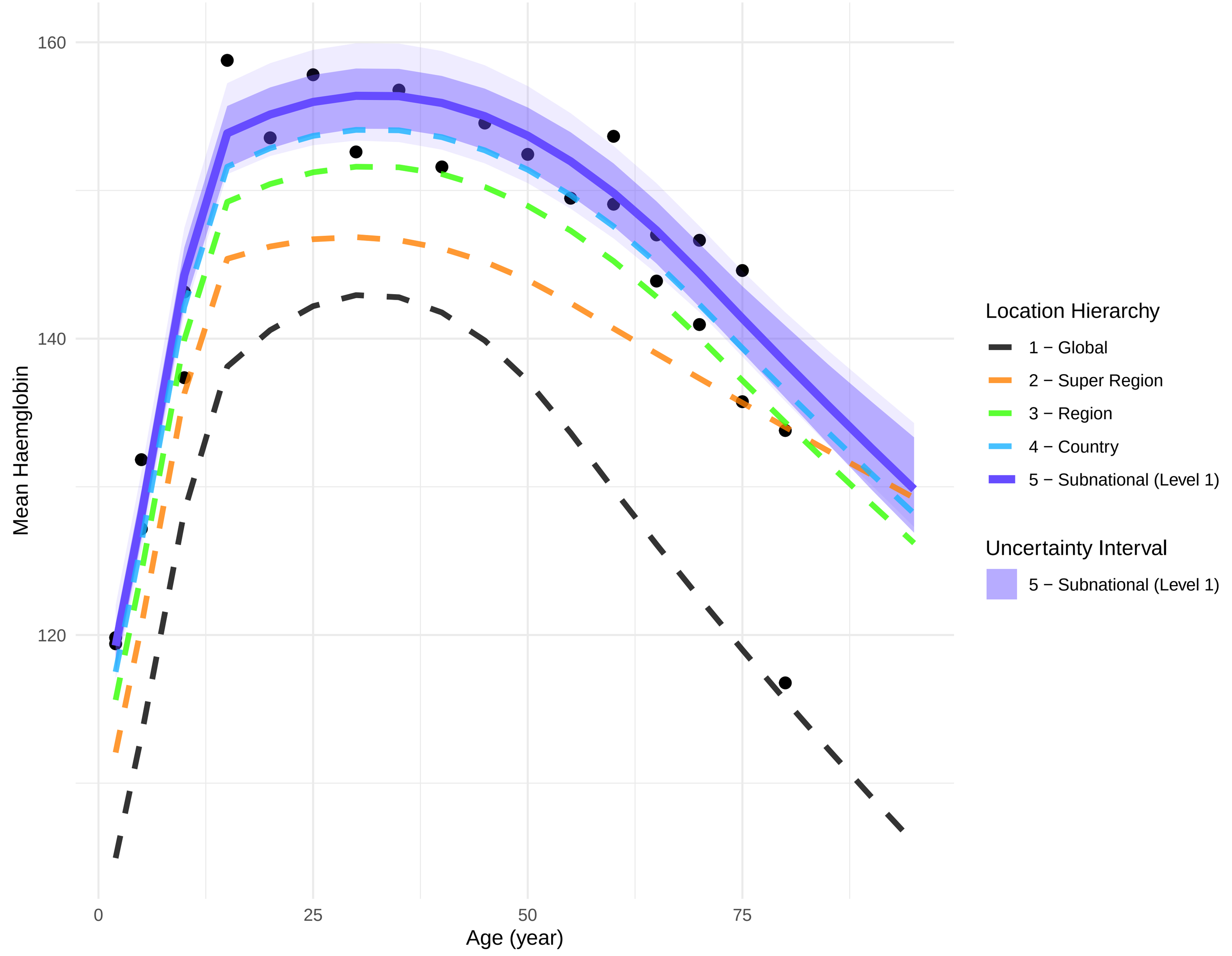

MR−BRT Age/Sex Fits − Males 2+ Years Old − Mean Haemoglobin − Coahuila
WHO−2024 Adjustment Method

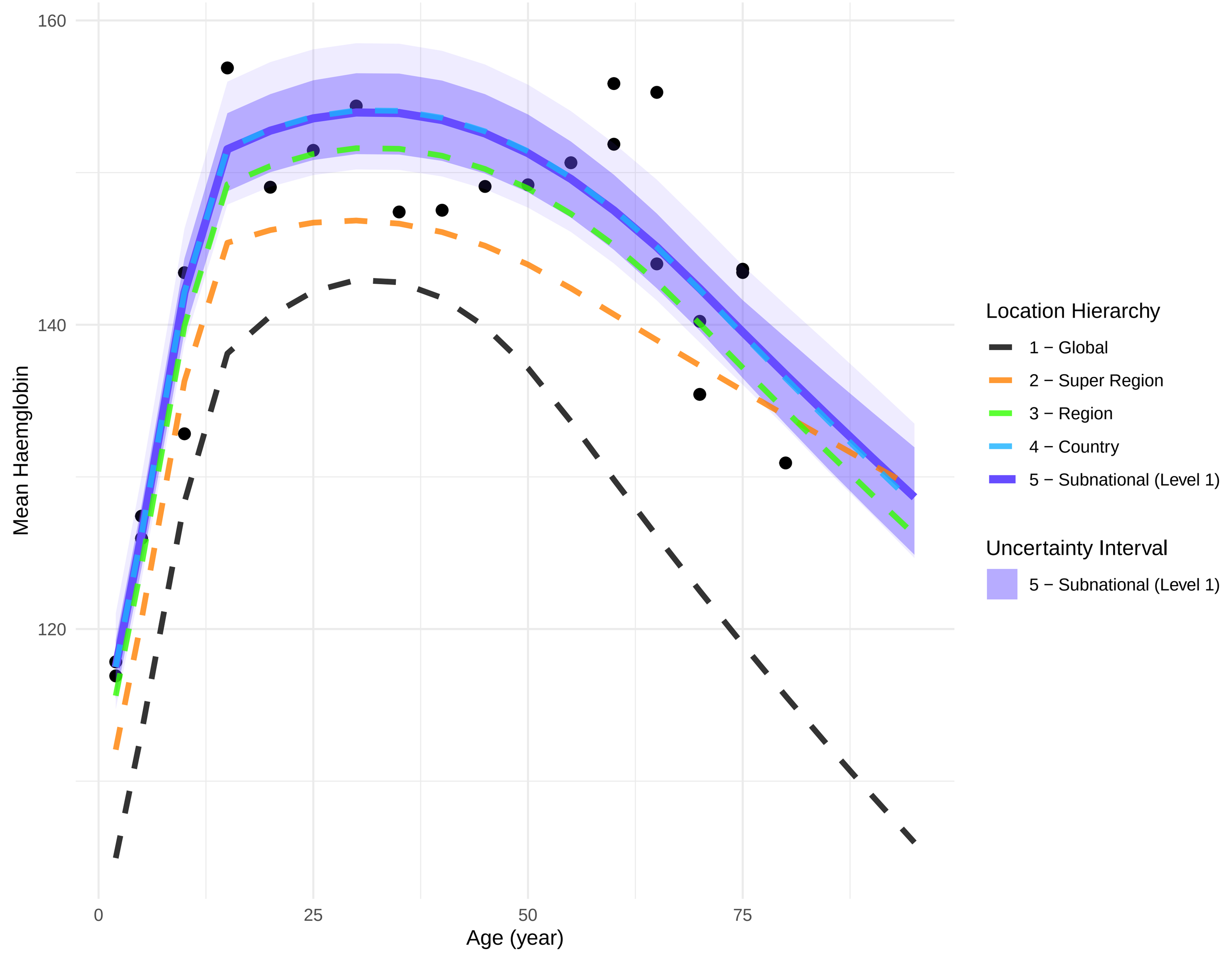

MR−BRT Age/Sex Fits − Males 2+ Years Old − Mean Haemoglobin − Colima
WHO−2024 Adjustment Method

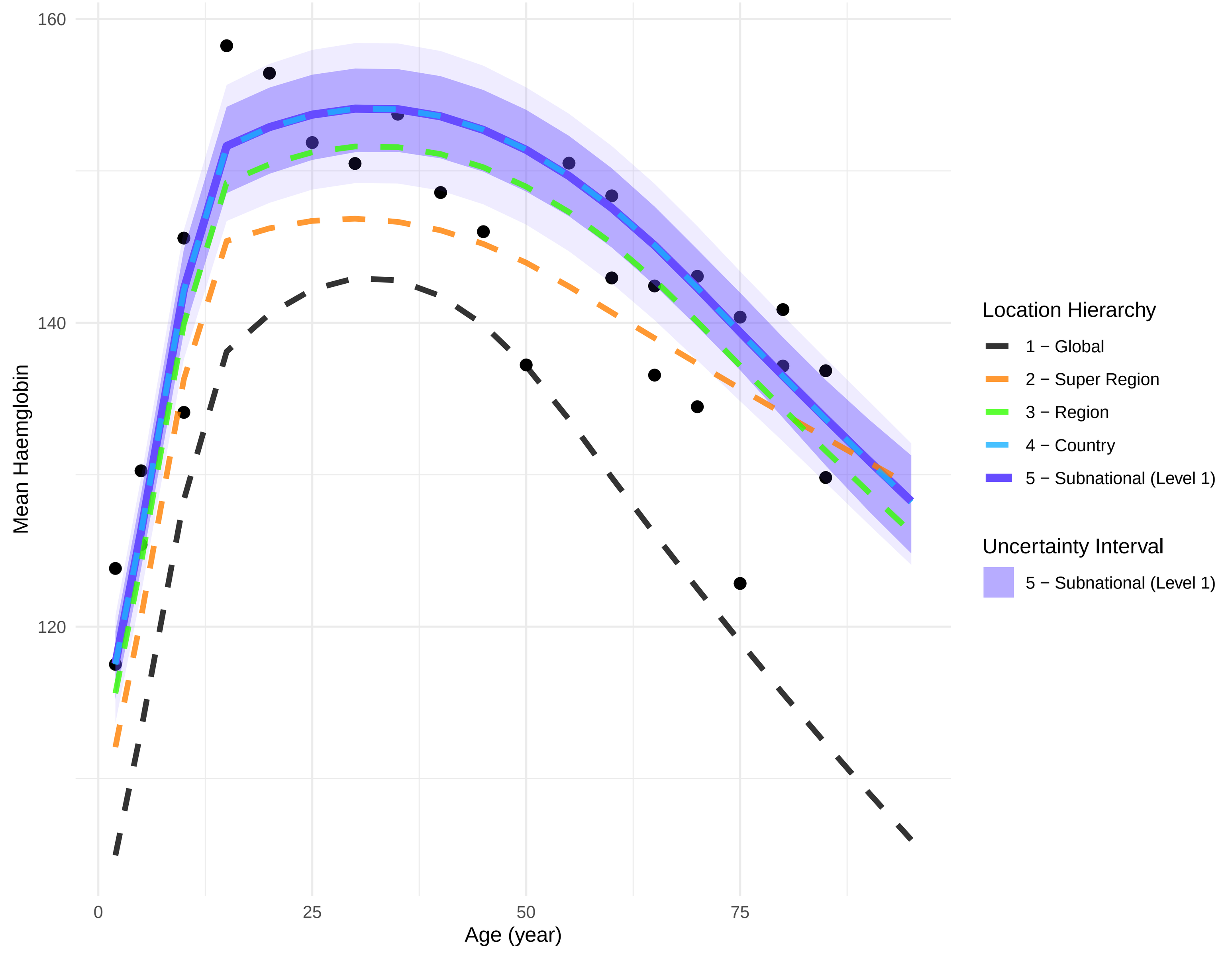

MR−BRT Age/Sex Fits − Males 2+ Years Old − Mean Haemoglobin − Durango
WHO−2024 Adjustment Method

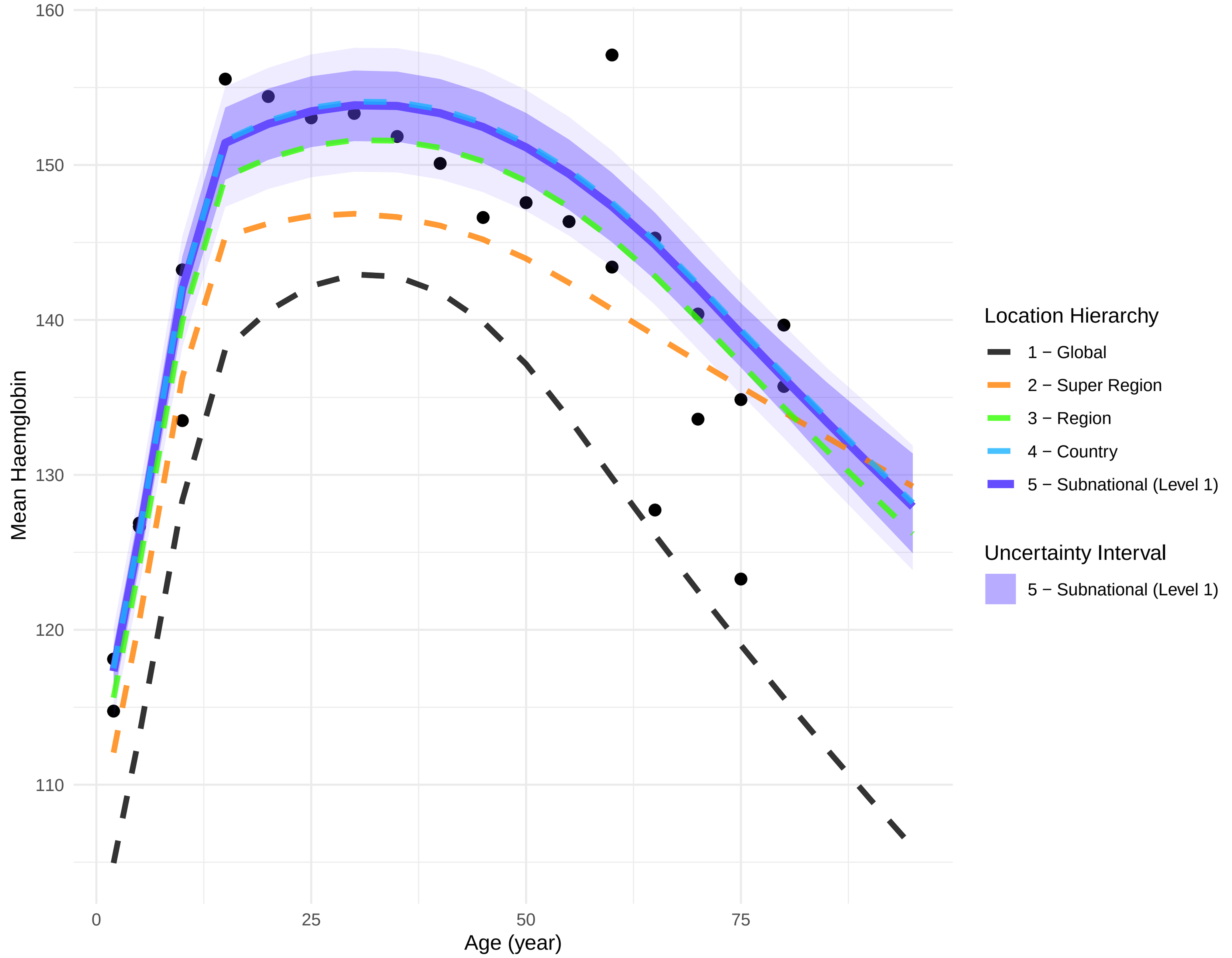

MR−BRT Age/Sex Fits − Males 2+ Years Old − Mean Haemoglobin − Guanajuato
WHO−2024 Adjustment Method

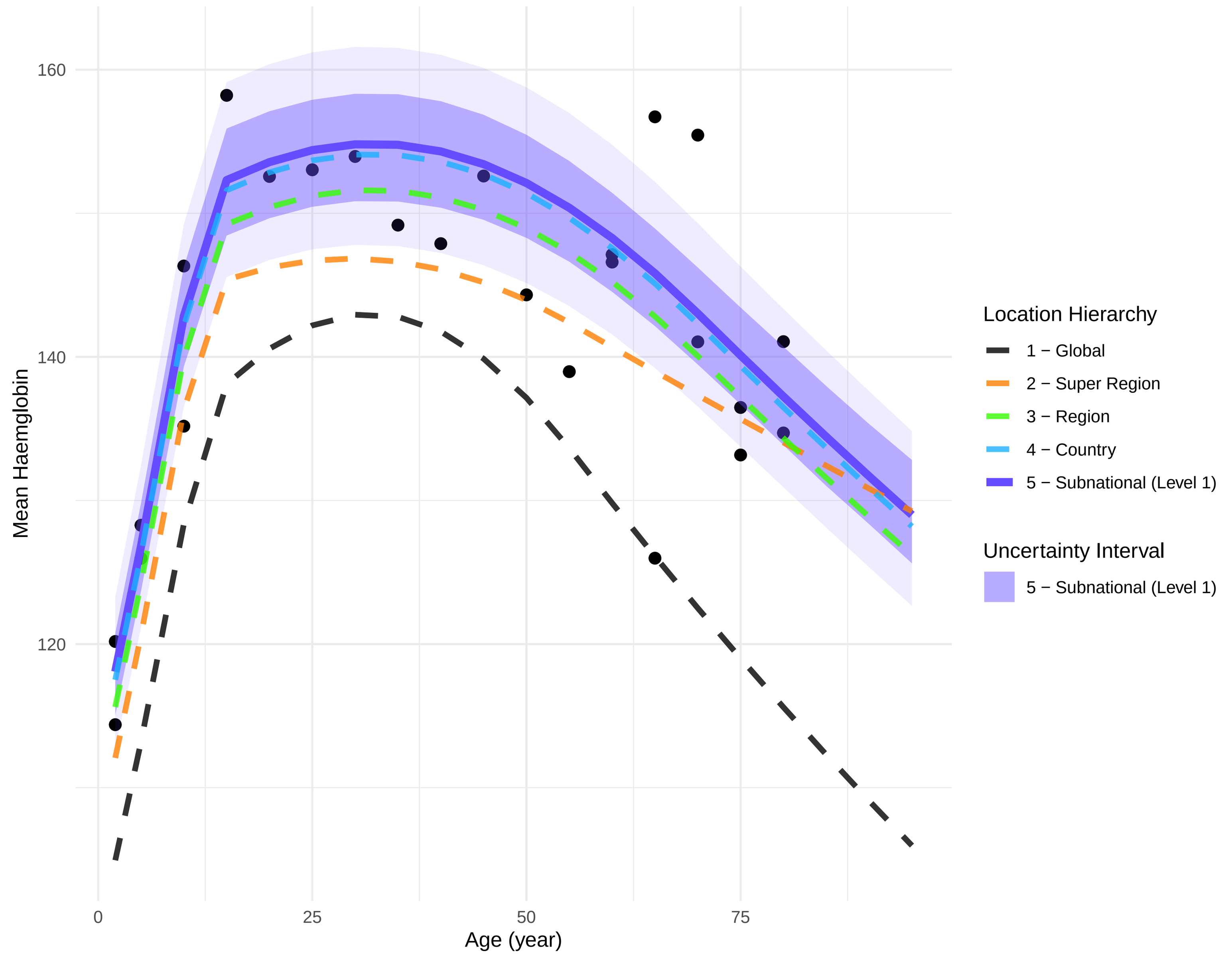

MR−BRT Age/Sex Fits − Males 2+ Years Old − Mean Haemoglobin − Guerrero
WHO−2024 Adjustment Method

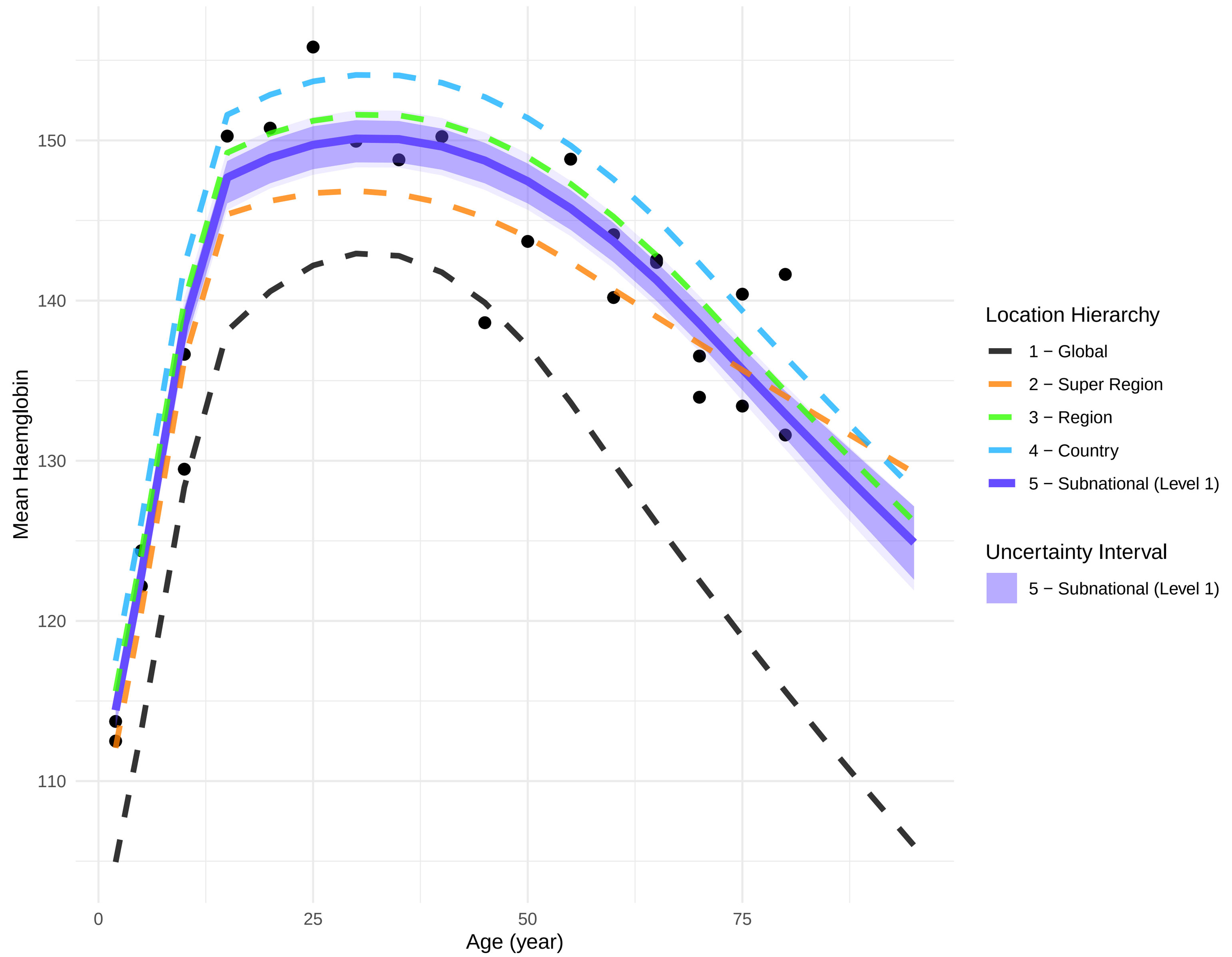

MR−BRT Age/Sex Fits − Males 2+ Years Old − Mean Haemoglobin − Hidalgo
WHO−2024 Adjustment Method

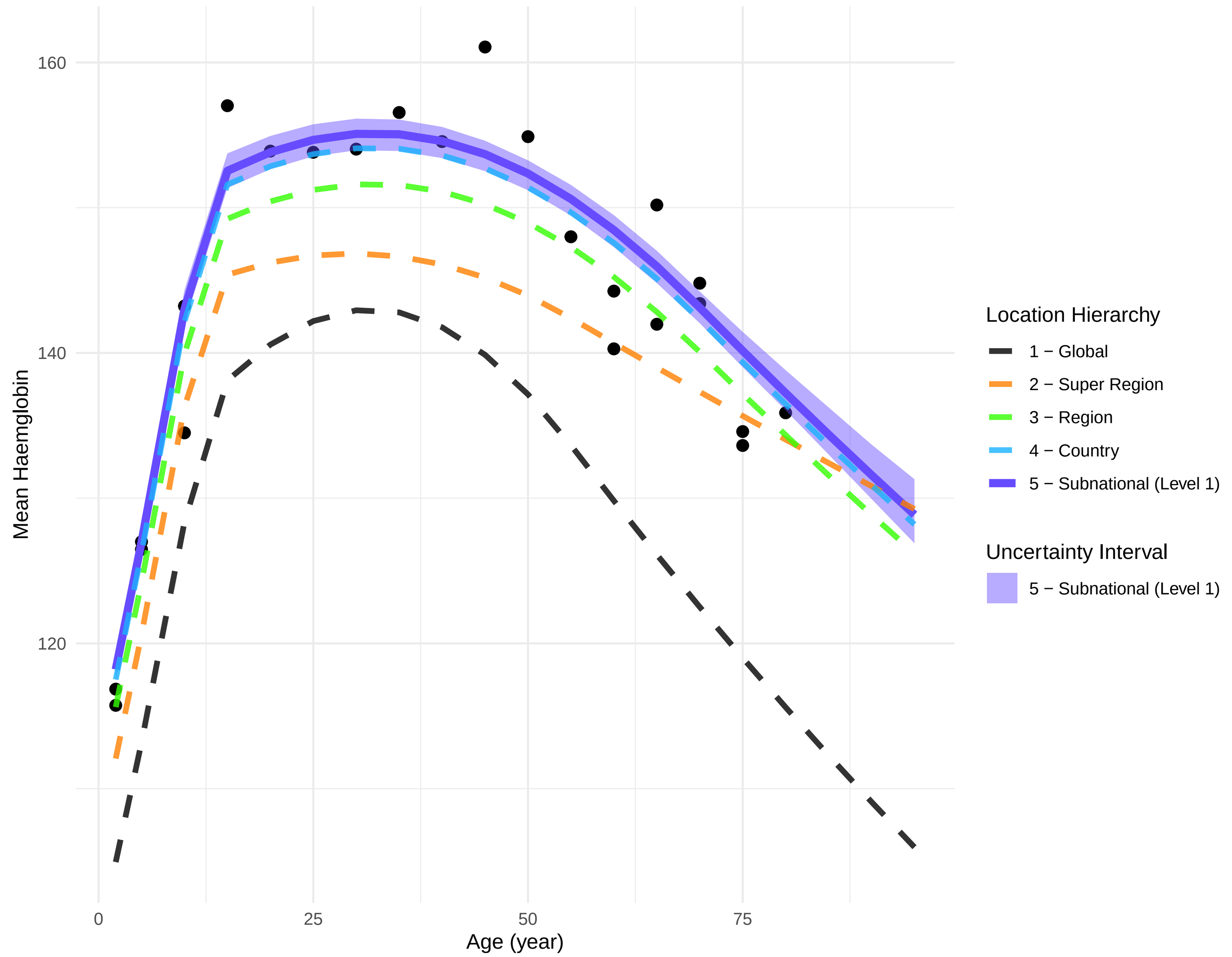

MR−BRT Age/Sex Fits − Males 2+ Years Old − Mean Haemoglobin − Jalisco
WHO−2024 Adjustment Method

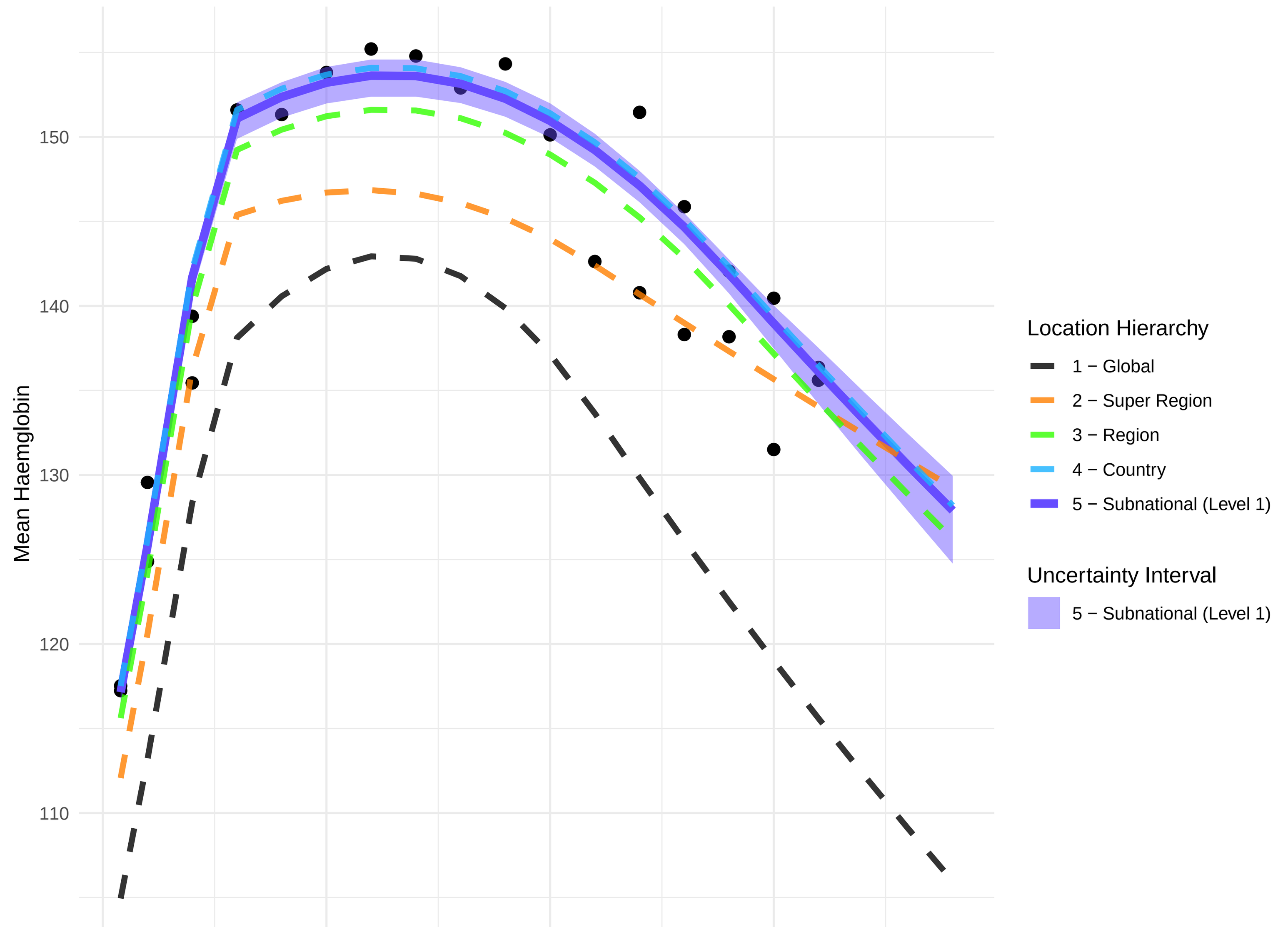

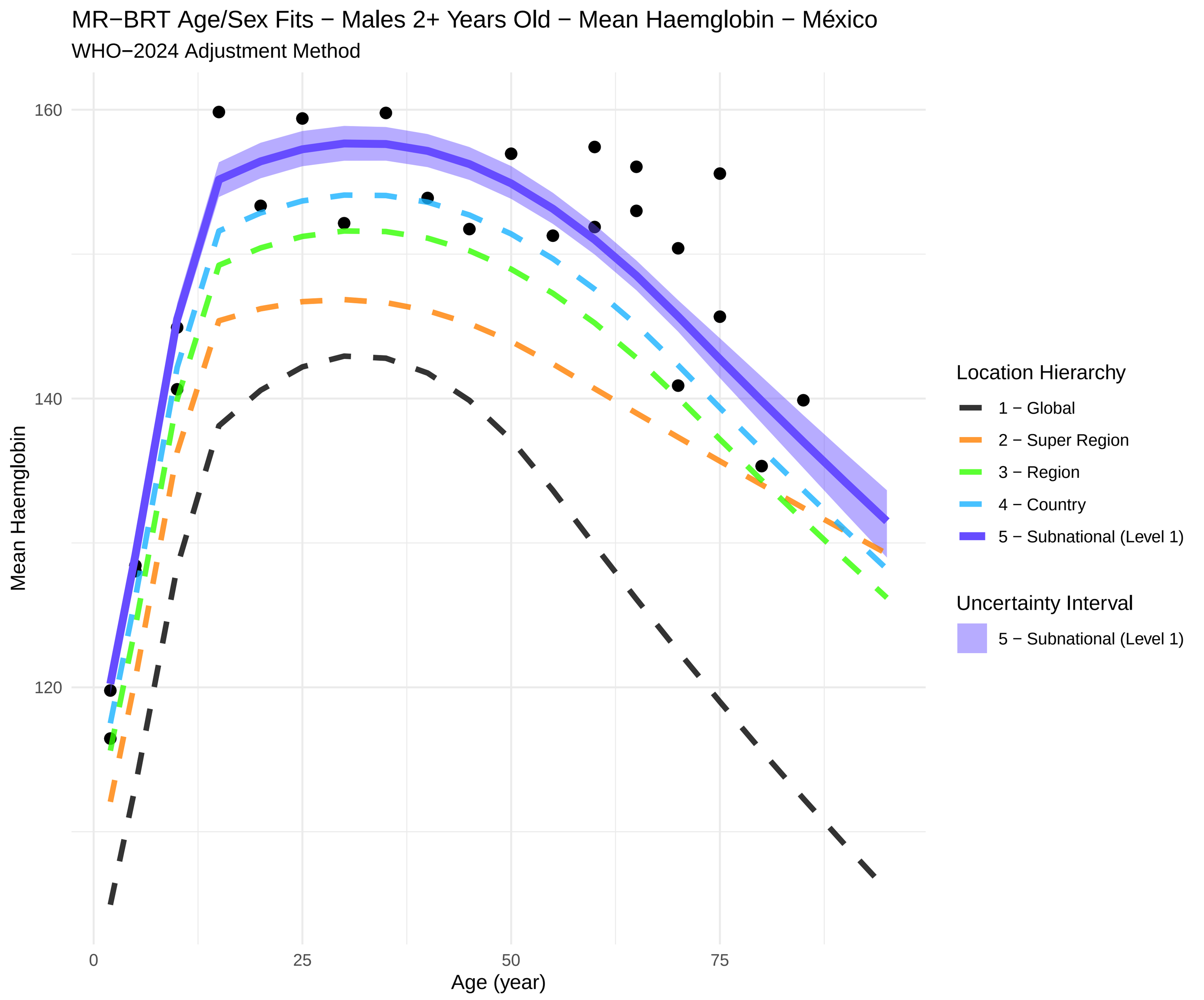
MR−BRT Age/Sex Fits − Males 2+ Years Old − Mean Haemoglobin − México
WHO−2024 Adjustment Method
Mean Haemoglobin
Age (year)
Location Hierarchy
1 − Global
2 − Super Region
3 − Region
4 − Country
5 − Subnational (Level 1)
Uncertainty Interval
5 − Subnational (Level 1)

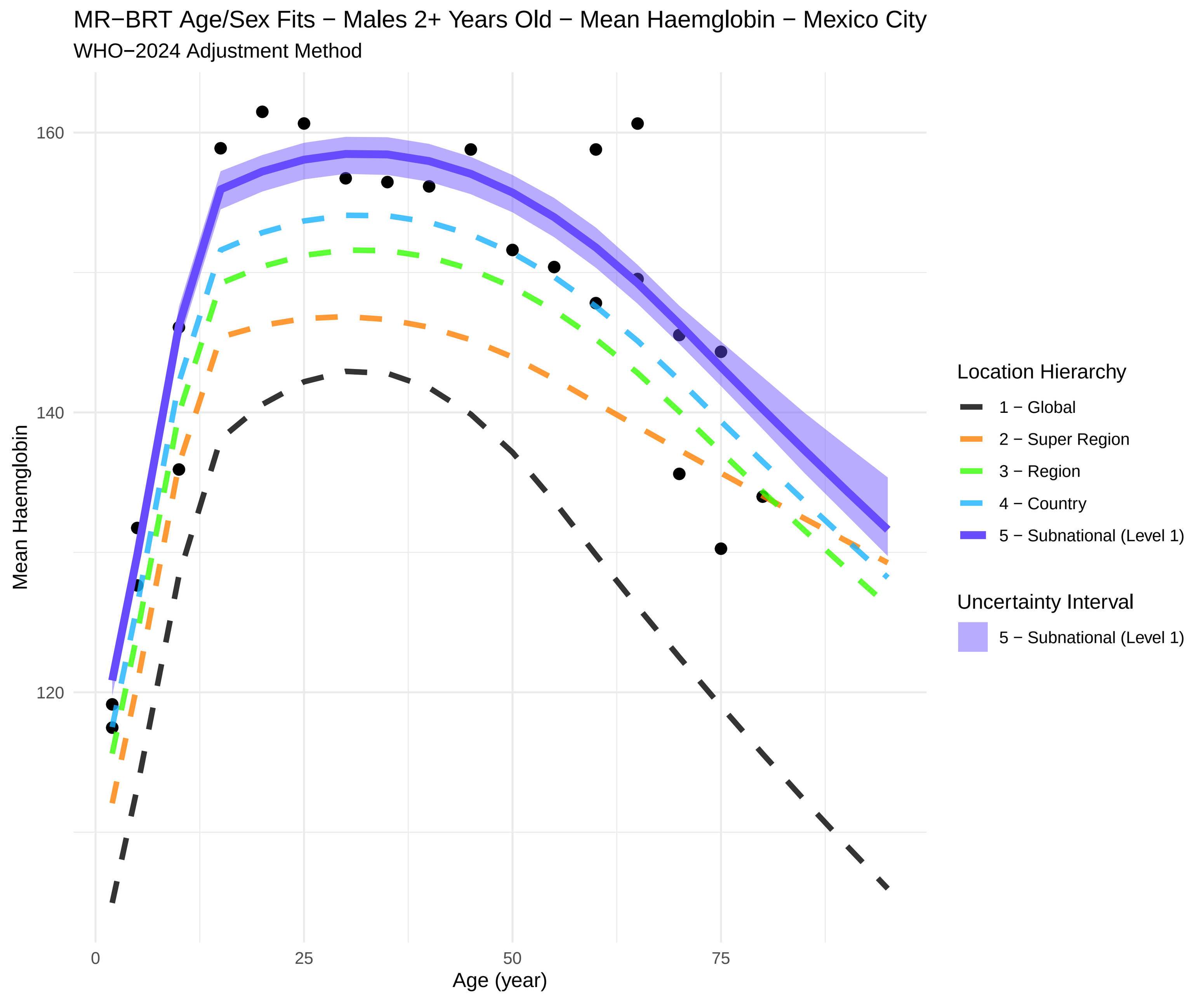
MR−BRT Age/Sex Fits − Males 2+ Years Old − Mean Haemoglobin − Mexico City
WHO−2024 Adjustment Method
Mean Haemoglobin
Age (year)
Location Hierarchy
1 − Global
2 − Super Region
3 − Region
4 − Country
5 − Subnational (Level 1)
Uncertainty Interval
5 − Subnational (Level 1)

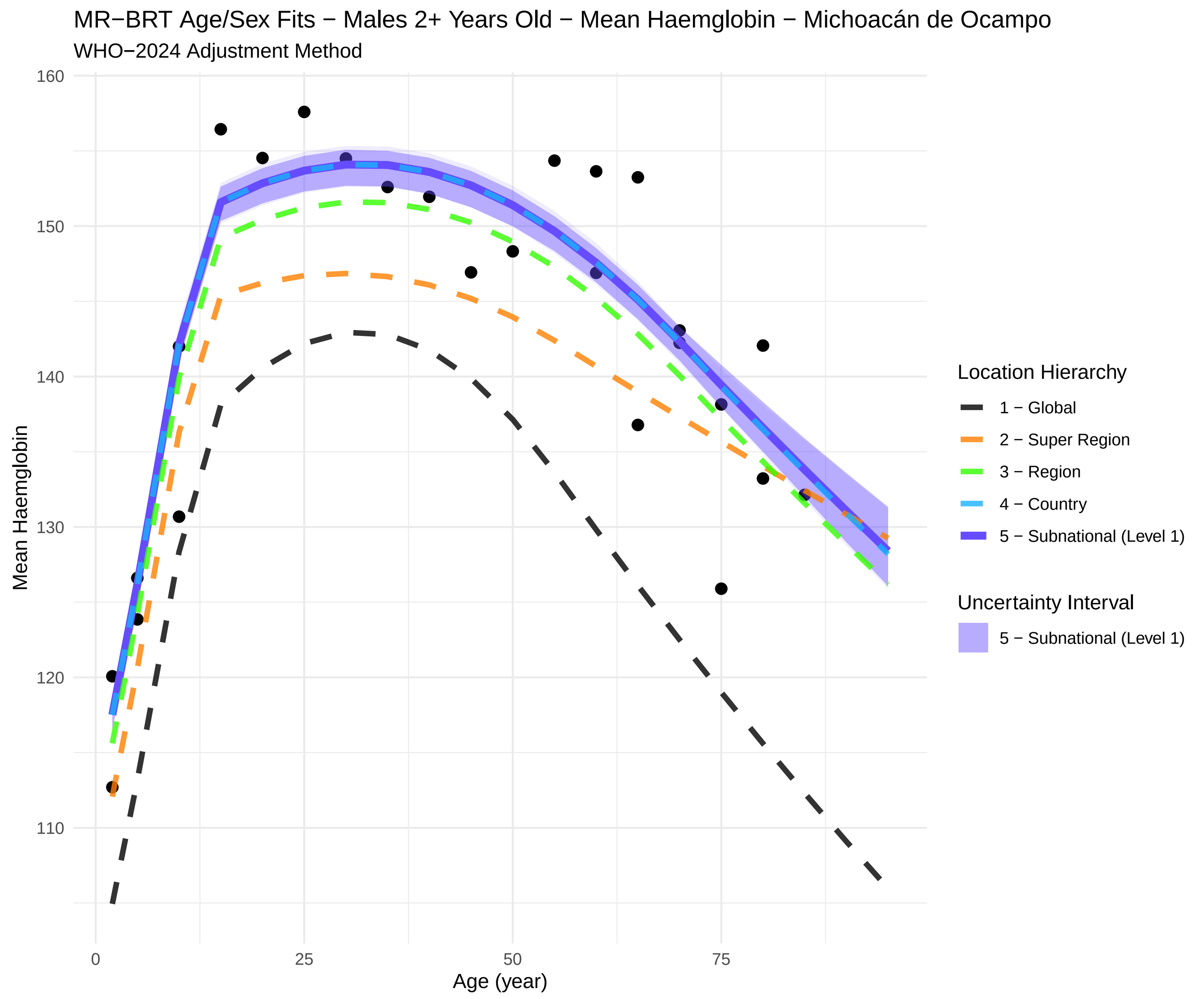
MR−BRT Age/Sex Fits − Males 2+ Years Old − Mean Haemoglobin − Michoacán de Ocampo
WHO−2024 Adjustment Method
Mean Haemoglobin
Age (year)
Location Hierarchy
1 − Global
2 − Super Region
3 − Region
4 − Country
5 − Subnational (Level 1)
Uncertainty Interval
5 − Subnational (Level 1)

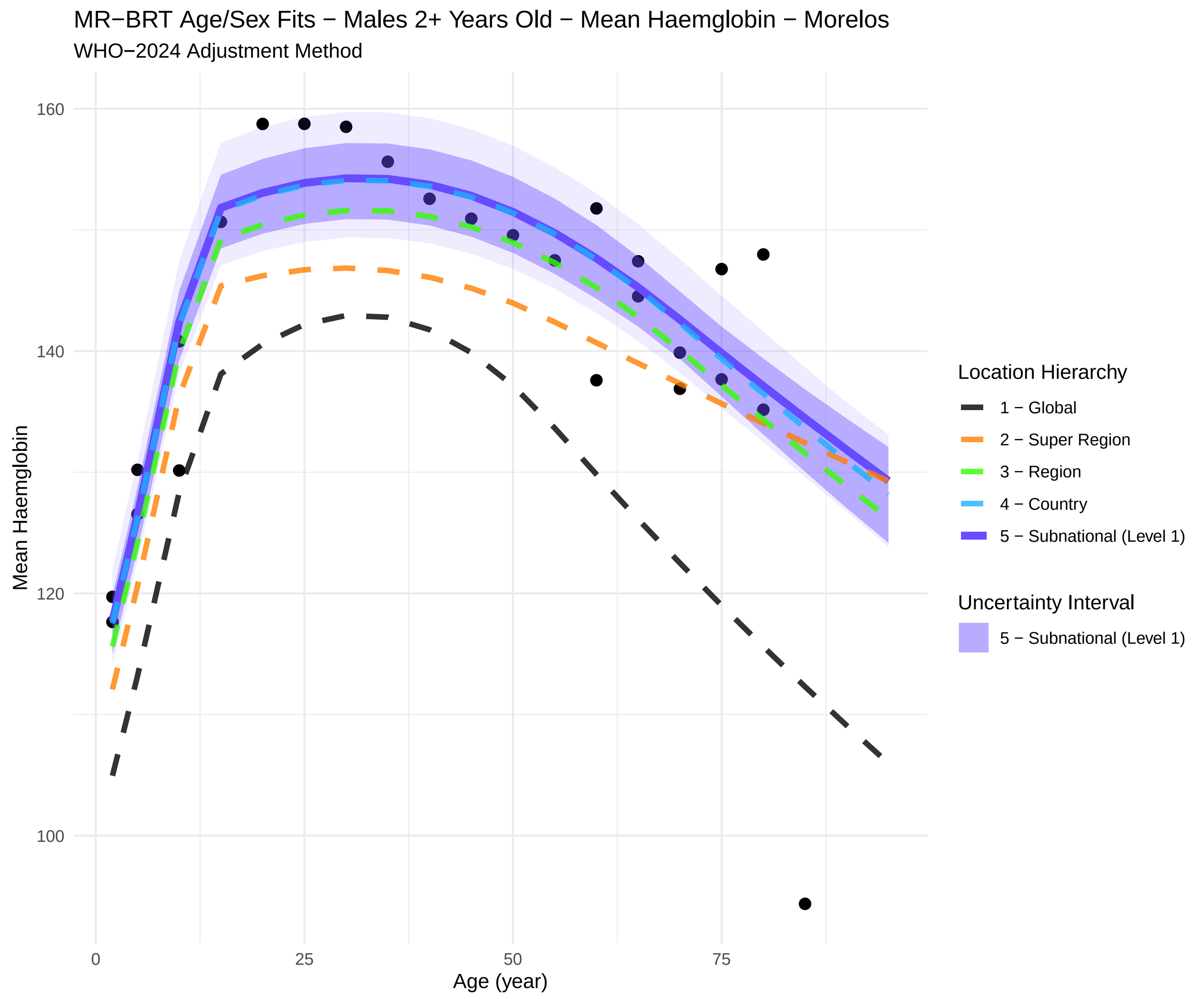
MR−BRT Age/Sex Fits − Males 2+ Years Old − Mean Haemoglobin − Morelos
WHO−2024 Adjustment Method
Mean Haemoglobin
Age (year)
Location Hierarchy
1 − Global
2 − Super Region
3 − Region
4 − Country
5 − Subnational (Level 1)
Uncertainty Interval
5 − Subnational (Level 1)

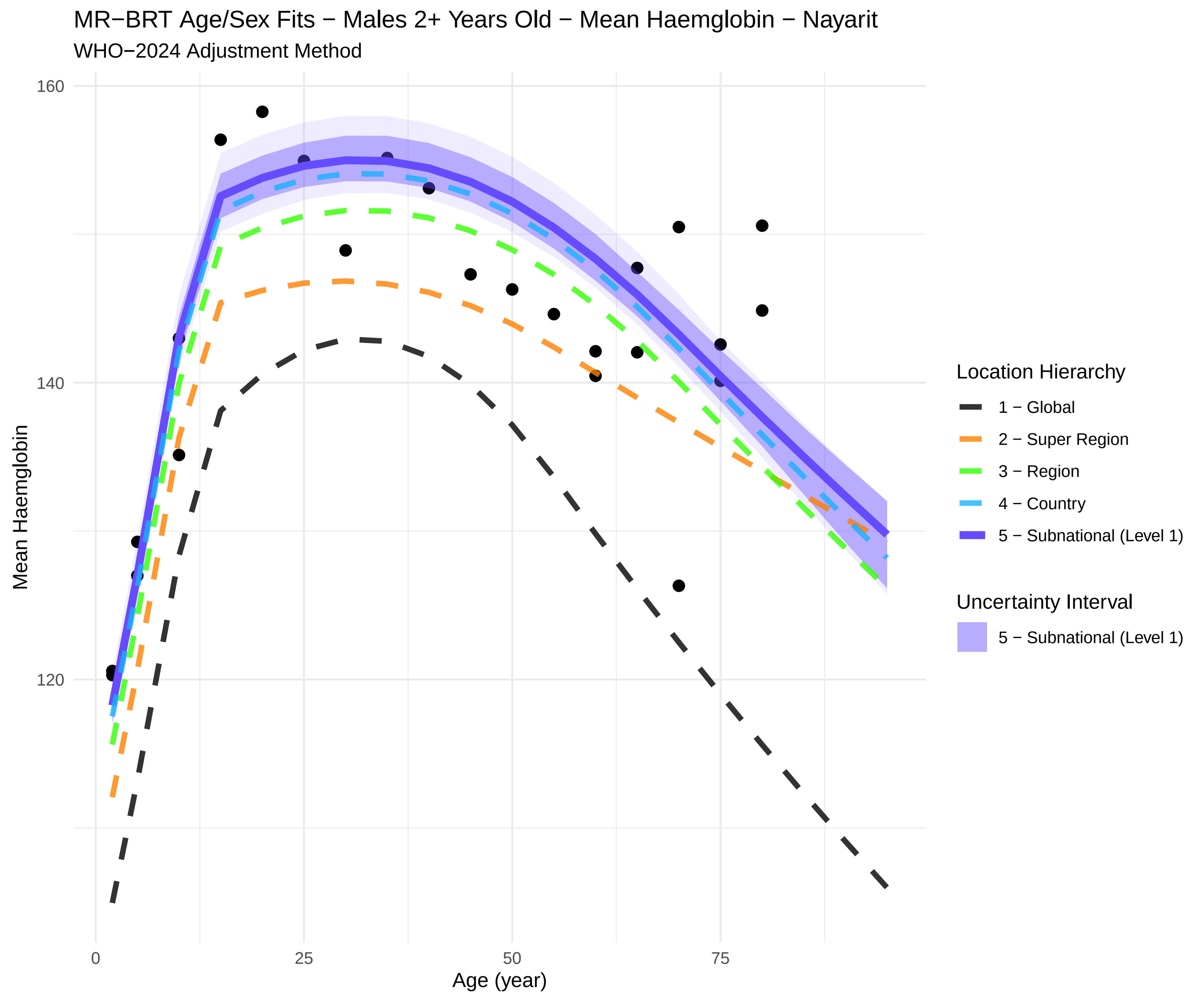
MR−BRT Age/Sex Fits − Males 2+ Years Old − Mean Haemoglobin − Nayarit
WHO−2024 Adjustment Method
Mean Haemoglobin
Age (year)
Location Hierarchy
1 − Global
2 − Super Region
3 − Region
4 − Country
5 − Subnational (Level 1)
Uncertainty Interval
5 − Subnational (Level 1)

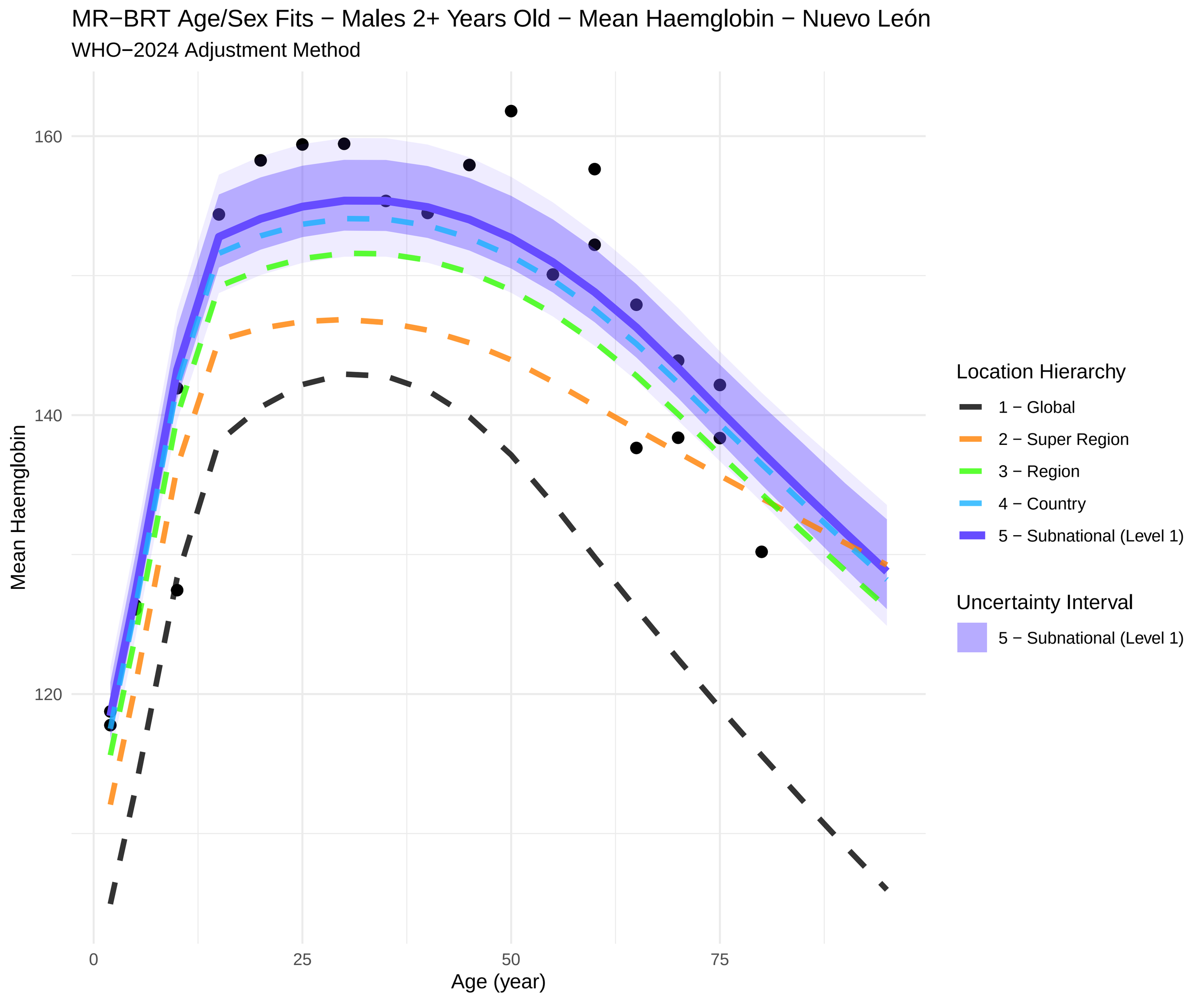
MR−BRT Age/Sex Fits − Males 2+ Years Old − Mean Haemoglobin − Nuevo León
WHO−2024 Adjustment Method
Mean Haemoglobin
Age (year)
Location Hierarchy
1 − Global
2 − Super Region
3 − Region
4 − Country
5 − Subnational (Level 1)
Uncertainty Interval
5 − Subnational (Level 1)

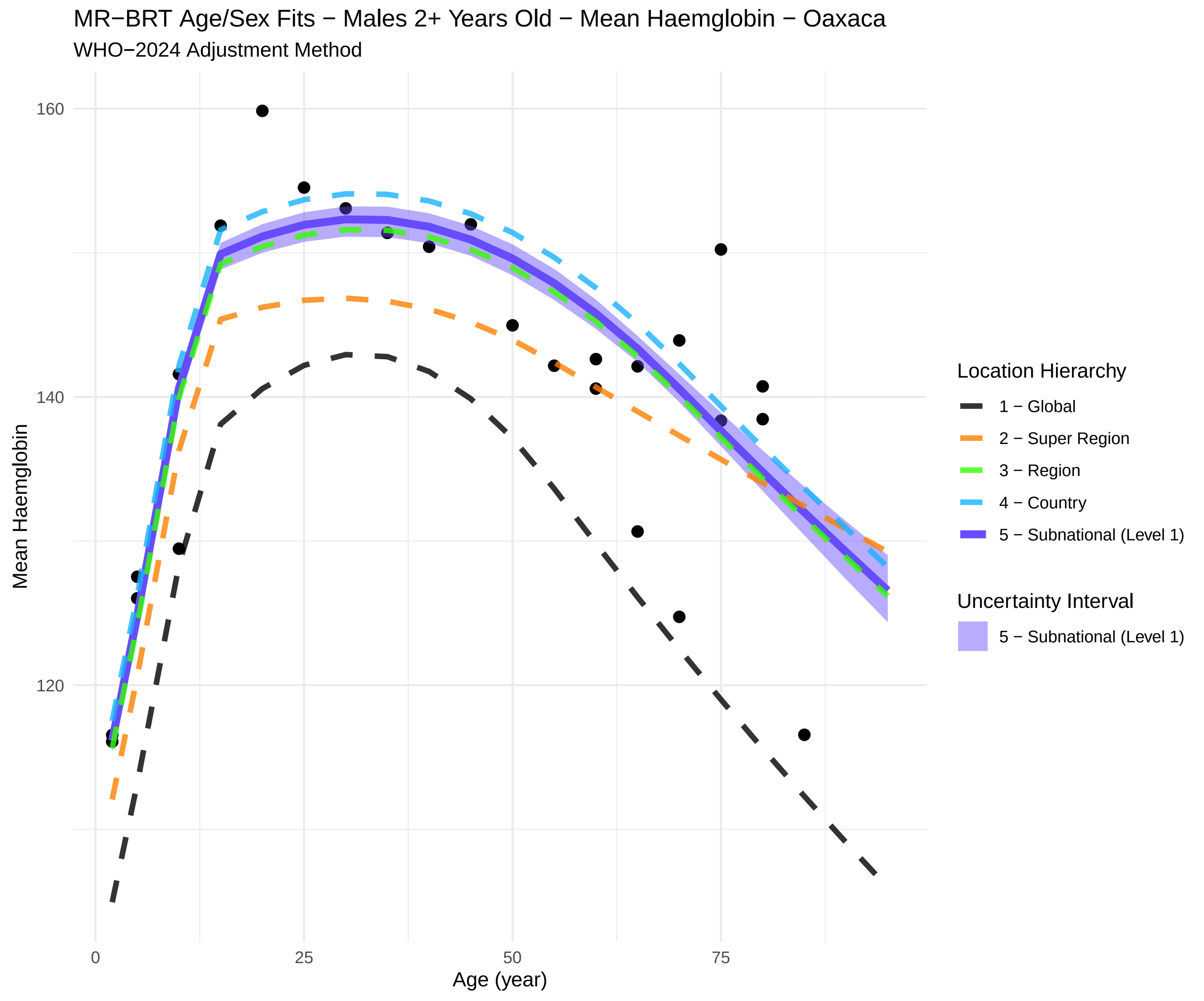
MR−BRT Age/Sex Fits − Males 2+ Years Old − Mean Haemoglobin − Oaxaca
WHO−2024 Adjustment Method
Mean Haemoglobin
Age (year)
Location Hierarchy
1 − Global
2 − Super Region
3 − Region
4 − Country
5 − Subnational (Level 1)
Uncertainty Interval
5 − Subnational (Level 1)

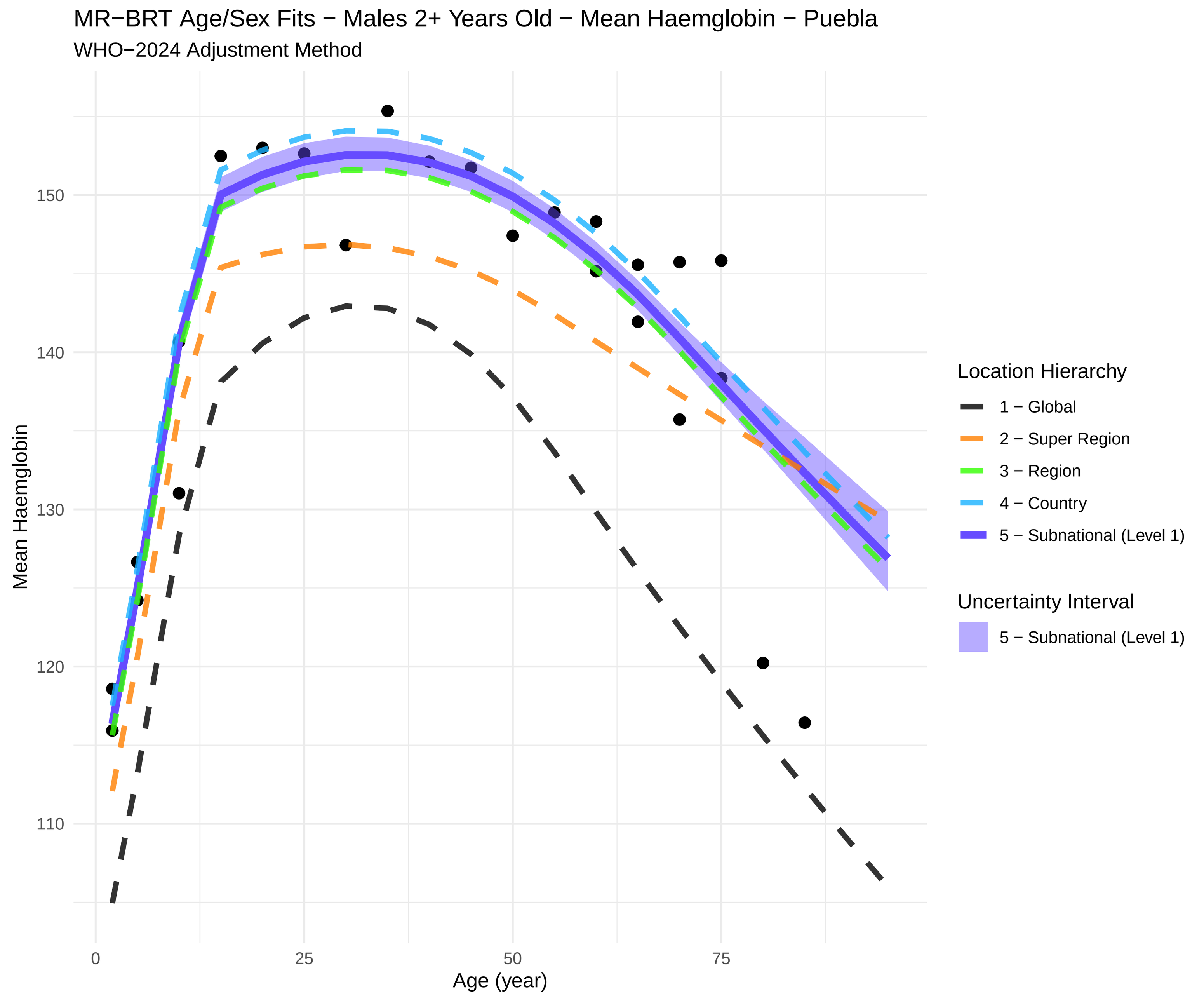
MR−BRT Age/Sex Fits − Males 2+ Years Old − Mean Haemoglobin − Puebla
WHO−2024 Adjustment Method
Mean Haemoglobin
Age (year)
Location Hierarchy
1 − Global
2 − Super Region
3 − Region
4 − Country
5 − Subnational (Level 1)
Uncertainty Interval
5 − Subnational (Level 1)

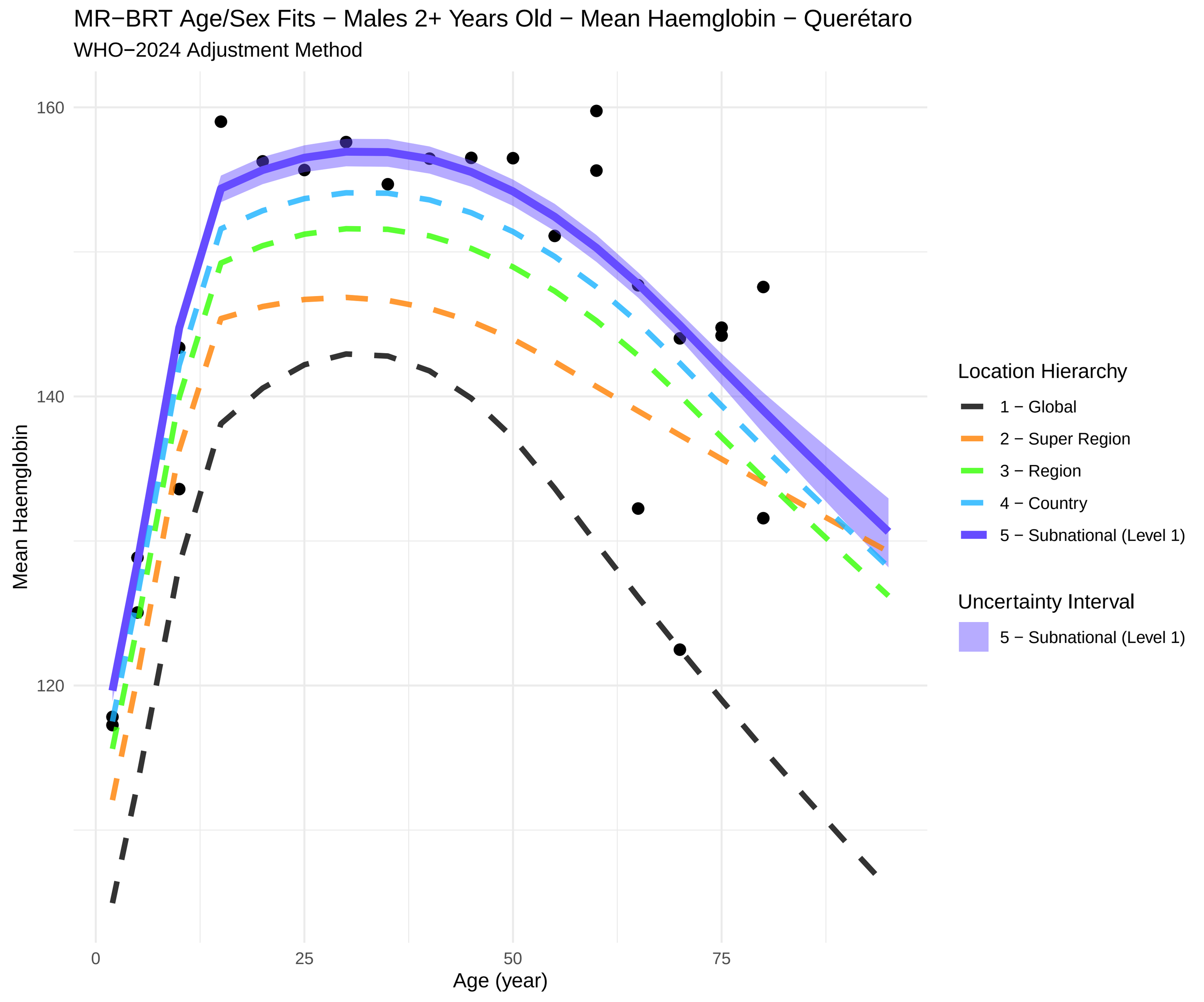
MR−BRT Age/Sex Fits − Males 2+ Years Old − Mean Haemoglobin − Querétaro
WHO−2024 Adjustment Method
Mean Haemoglobin
Age (year)
Location Hierarchy
1 − Global
2 − Super Region
3 − Region
4 − Country
5 − Subnational (Level 1)
Uncertainty Interval
5 − Subnational (Level 1)

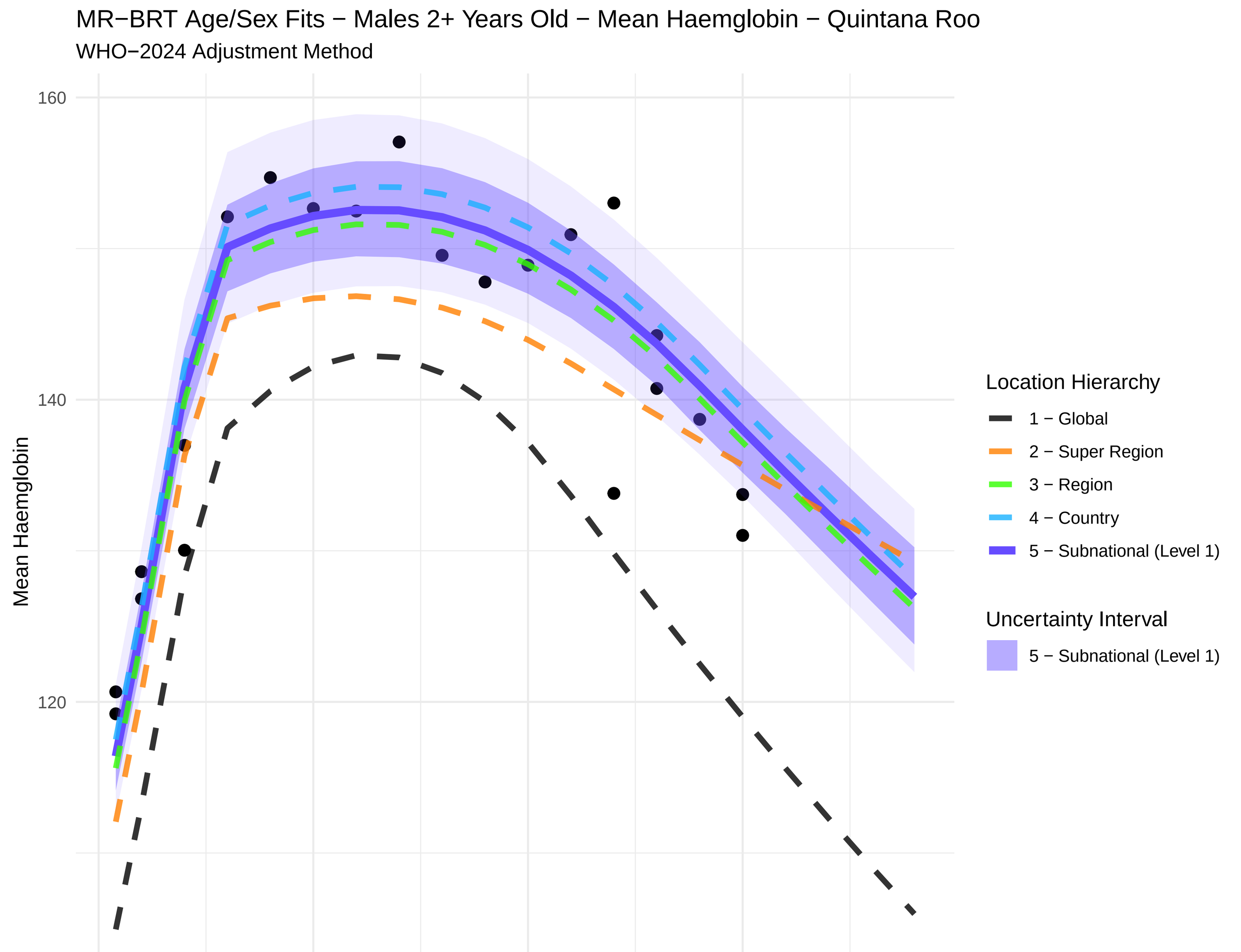
MR−BRT Age/Sex Fits − Males 2+ Years Old − Mean Haemoglobin − Quintana Roo
WHO−2024 Adjustment Method
Mean Haemoglobin
Age (year)
Location Hierarchy
1 − Global
2 − Super Region
3 − Region
4 − Country
5 − Subnational (Level 1)
Uncertainty Interval
5 − Subnational (Level 1)

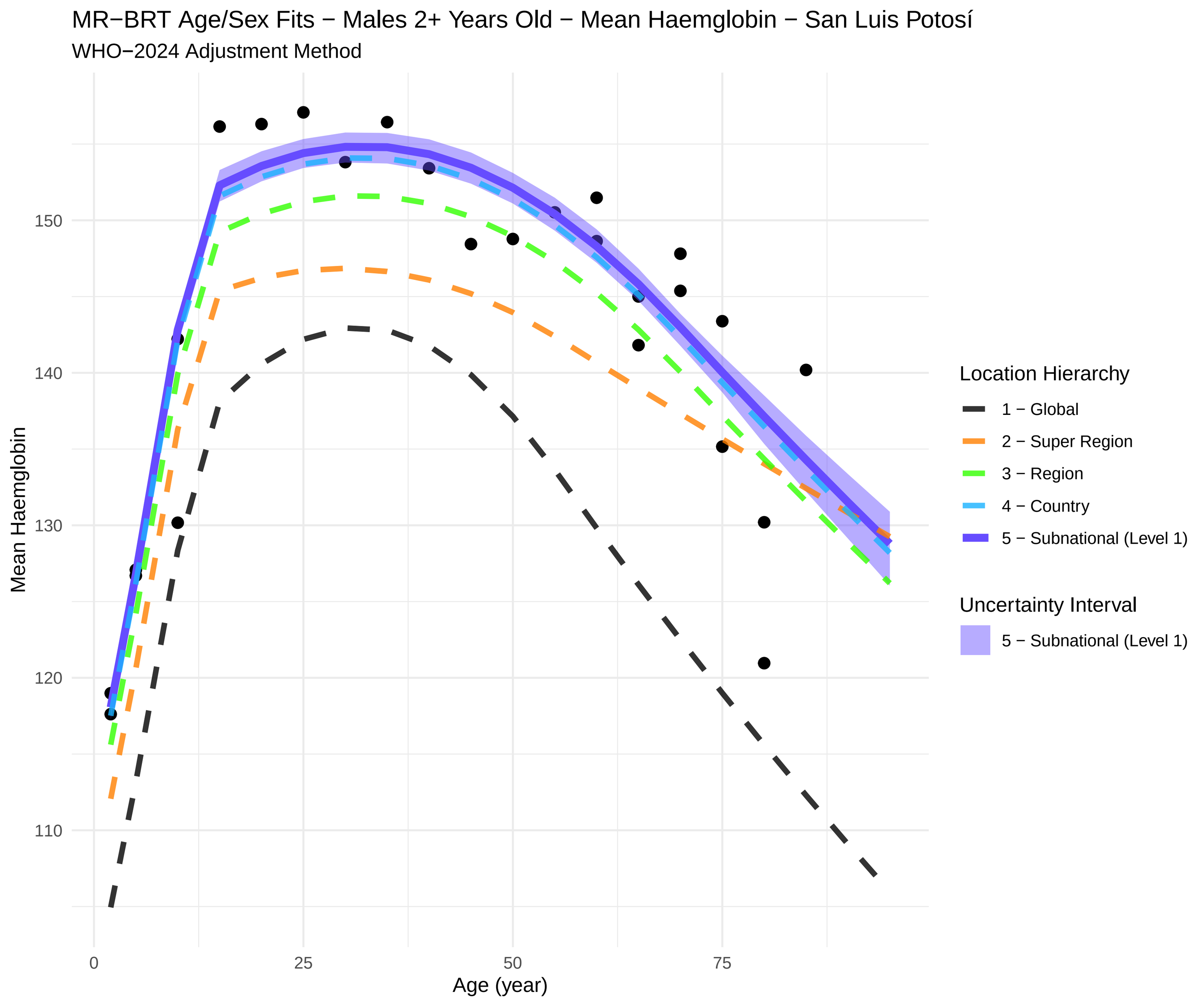
MR−BRT Age/Sex Fits − Males 2+ Years Old − Mean Haemglobin − San Luis Potosí
WHO−2024 Adjustment Method
Mean Haemglobin
Age (year)
Location Hierarchy
1 − Global
2 − Super Region
3 − Region
4 − Country
5 − Subnational (Level 1)
Uncertainty Interval
5 − Subnational (Level 1)

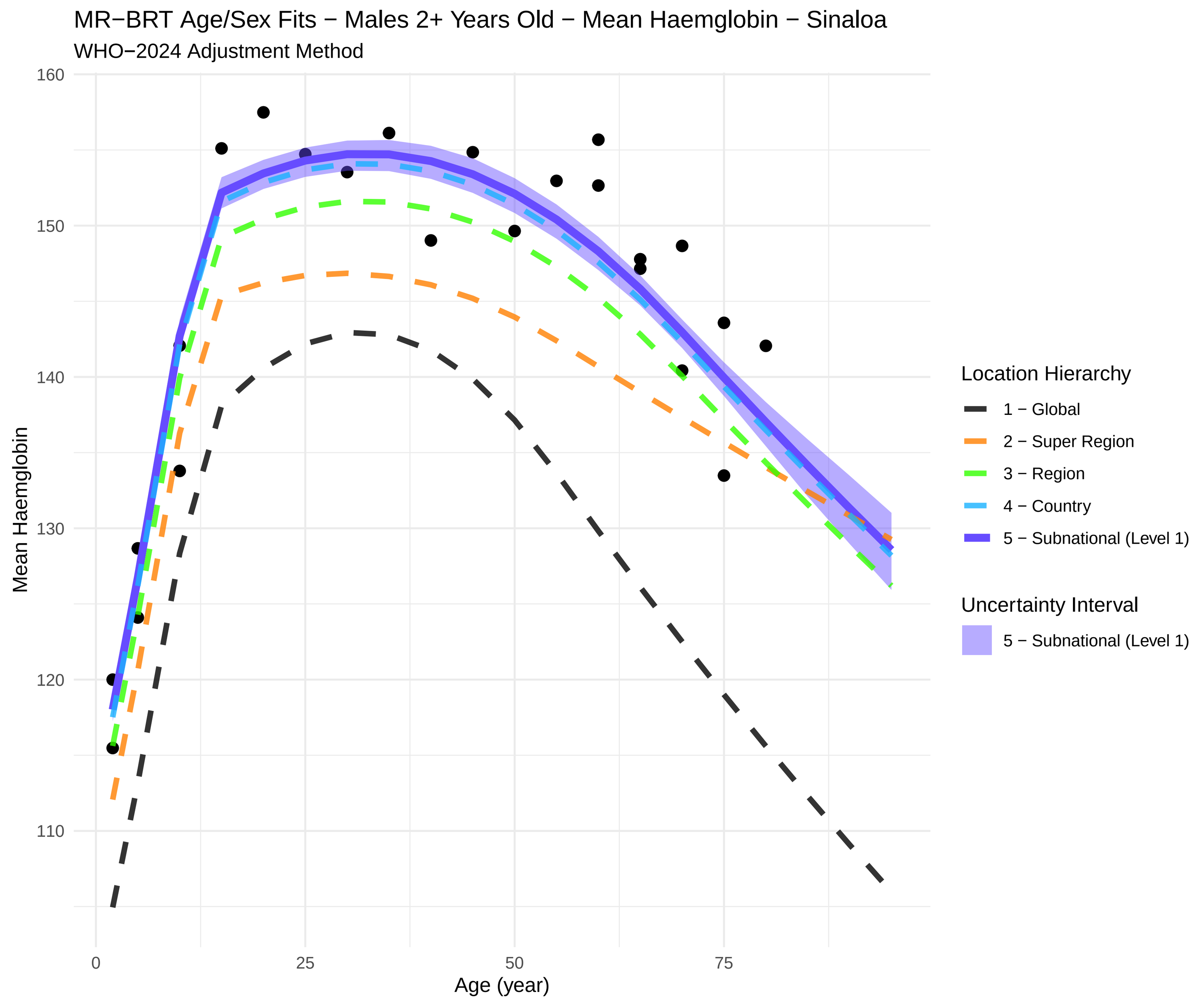
MR−BRT Age/Sex Fits − Males 2+ Years Old − Mean Haemglobin − Sinaloa
WHO−2024 Adjustment Method
Mean Haemglobin
Age (year)
Location Hierarchy
1 − Global
2 − Super Region
3 − Region
4 − Country
5 − Subnational (Level 1)
Uncertainty Interval
5 − Subnational (Level 1)

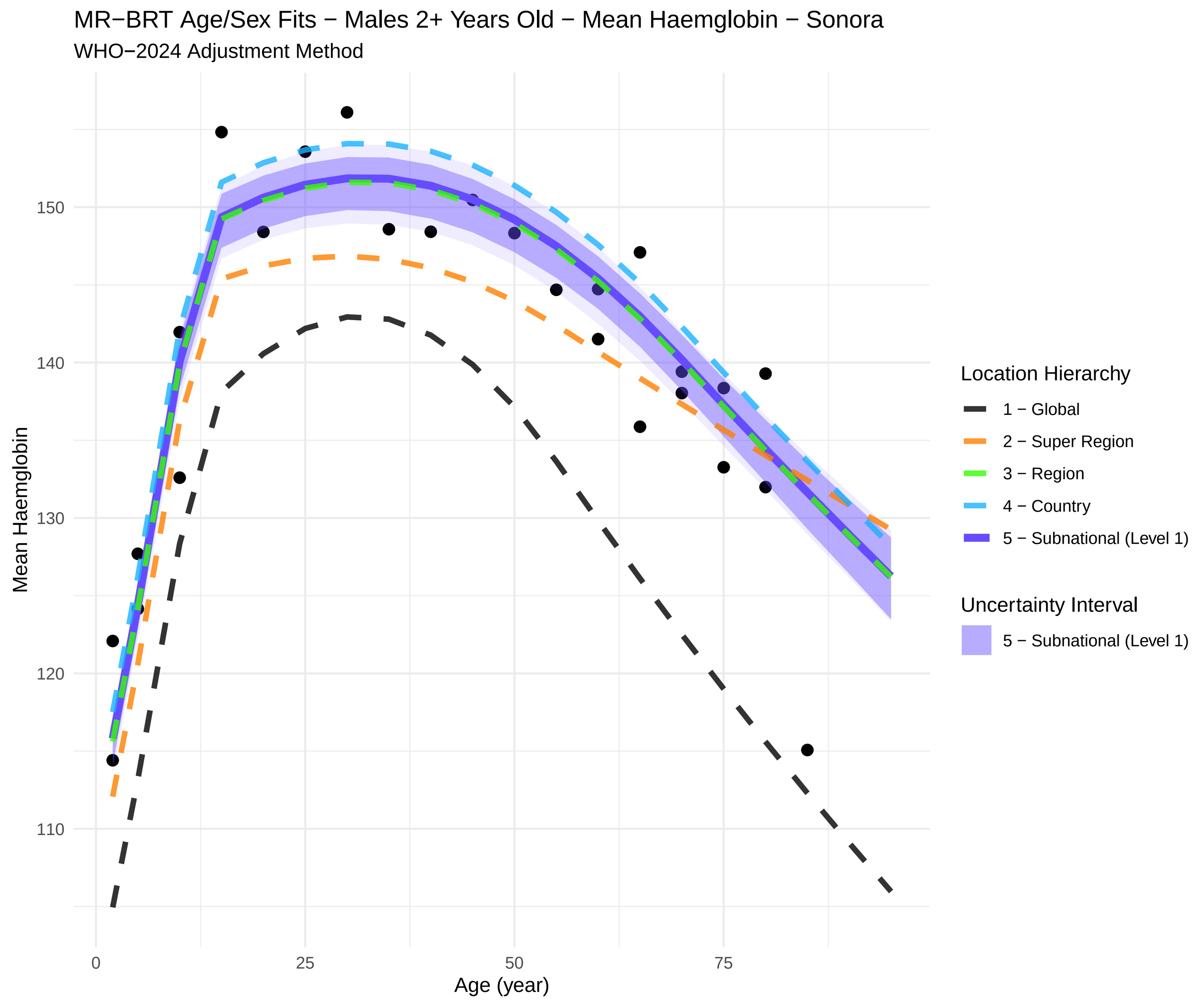
MR−BRT Age/Sex Fits − Males 2+ Years Old − Mean Haemglobin − Sonora
WHO−2024 Adjustment Method
Mean Haemglobin
Age (year)
Location Hierarchy
1 − Global
2 − Super Region
3 − Region
4 − Country
5 − Subnational (Level 1)
Uncertainty Interval
5 − Subnational (Level 1)

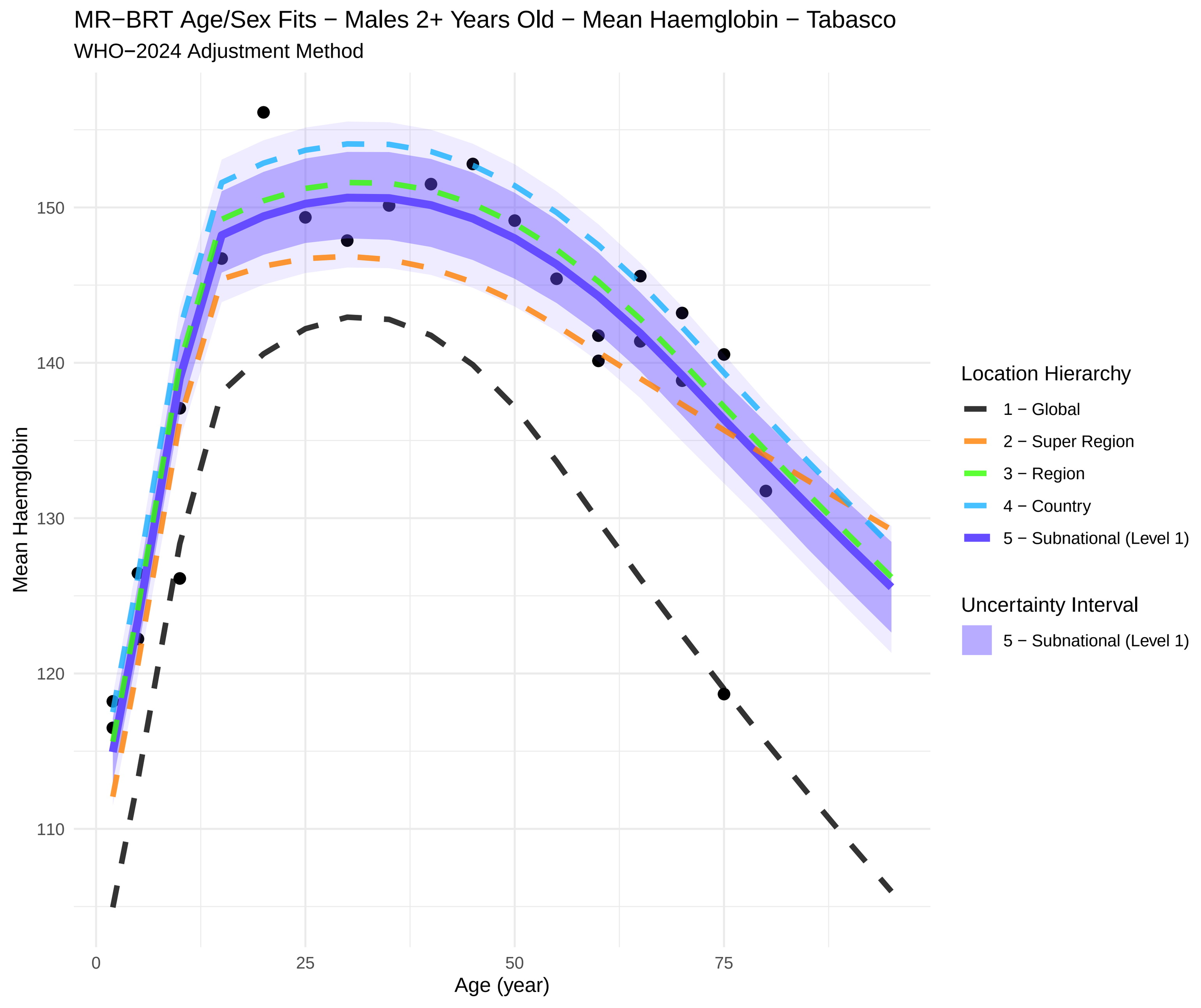
MR−BRT Age/Sex Fits − Males 2+ Years Old − Mean Haemglobin − Tabasco
WHO−2024 Adjustment Method
Mean Haemglobin
Age (year)
Location Hierarchy
1 − Global
2 − Super Region
3 − Region
4 − Country
5 − Subnational (Level 1)
Uncertainty Interval
5 − Subnational (Level 1)

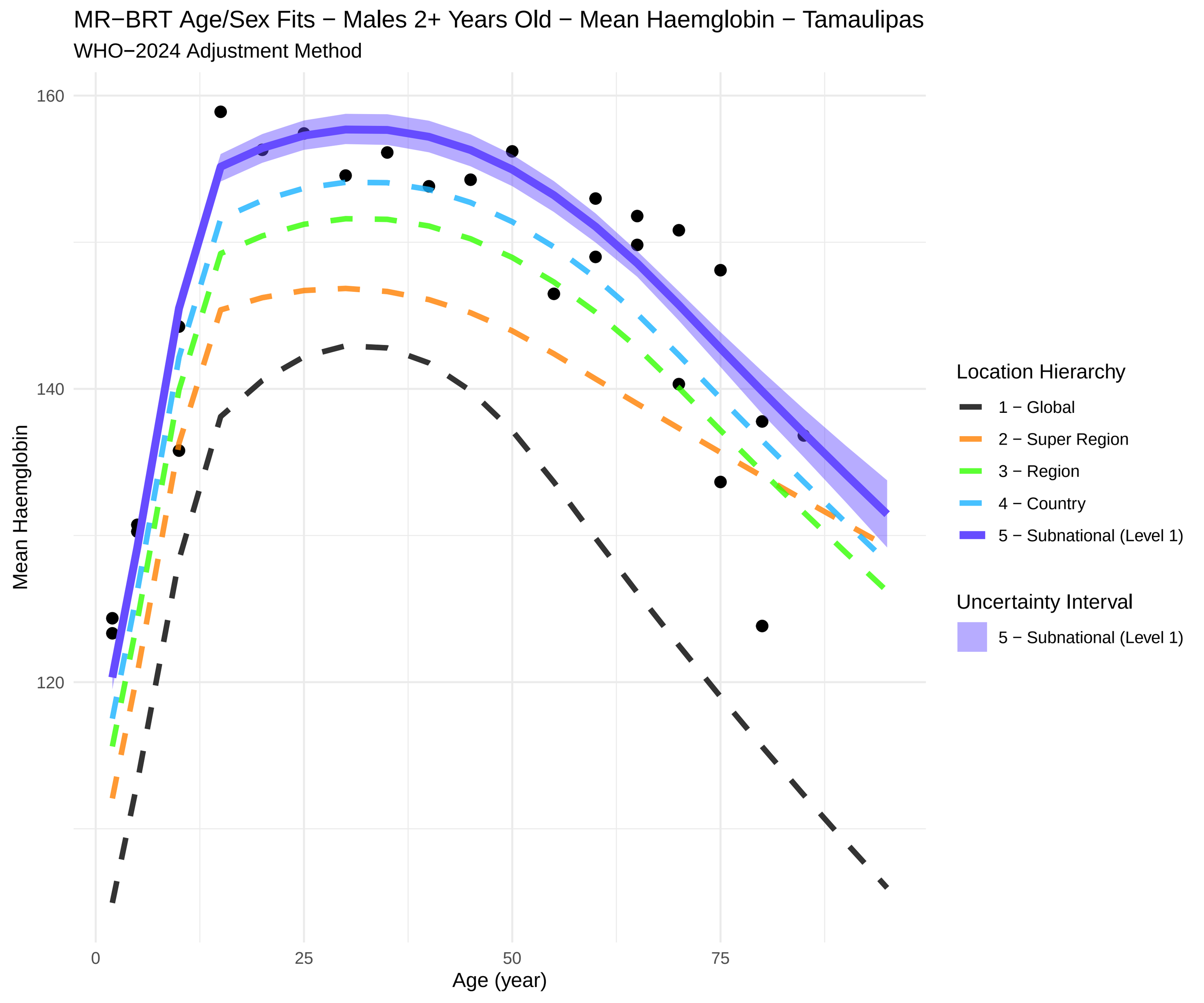
MR−BRT Age/Sex Fits − Males 2+ Years Old − Mean Haemglobin − Tamaulipas
WHO−2024 Adjustment Method
Mean Haemglobin
Age (year)
Location Hierarchy
1 − Global
2 − Super Region
3 − Region
4 − Country
5 − Subnational (Level 1)
Uncertainty Interval
5 − Subnational (Level 1)

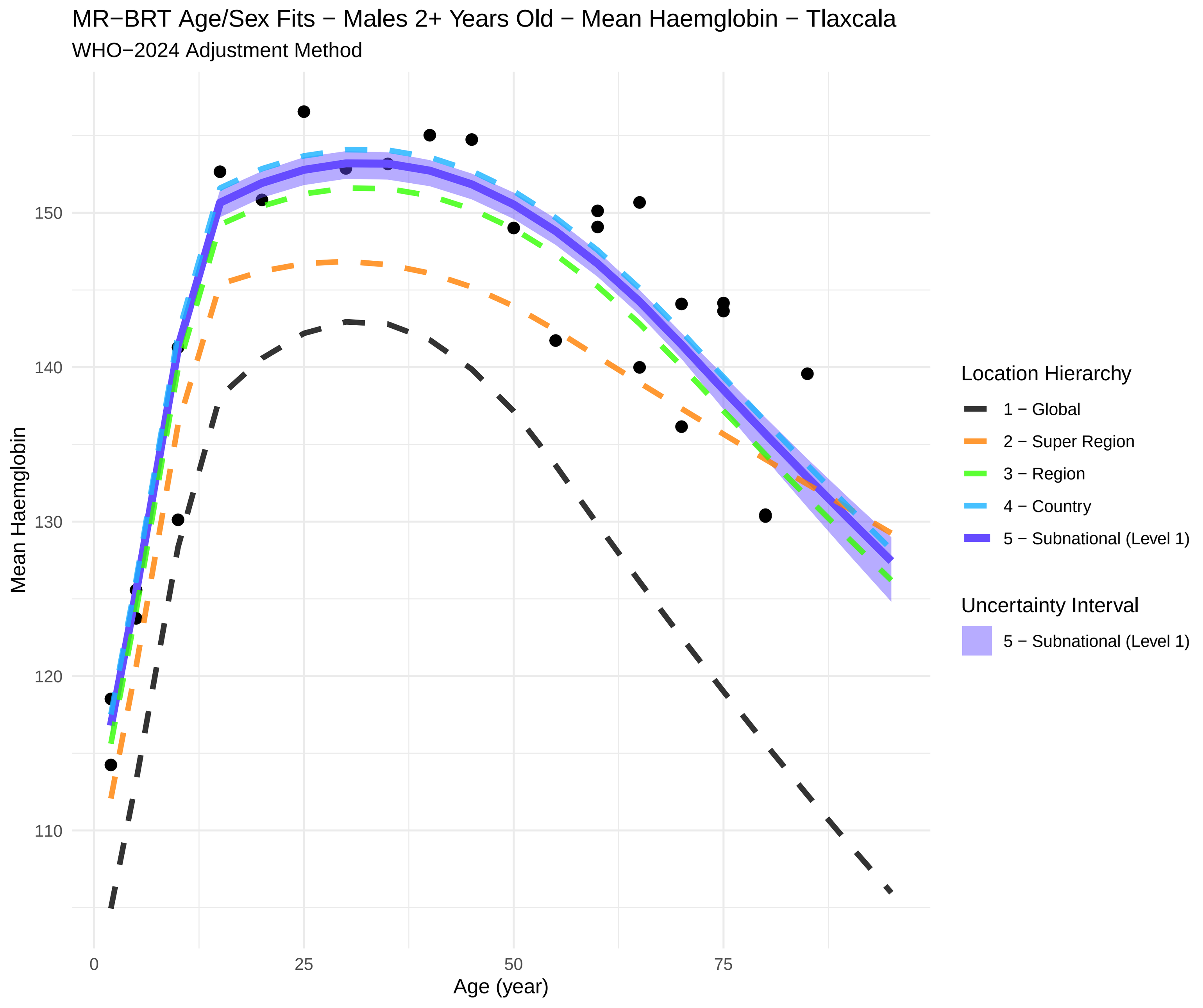
MR−BRT Age/Sex Fits − Males 2+ Years Old − Mean Haemglobin − Tlaxcala
WHO−2024 Adjustment Method
Mean Haemglobin
Age (year)
Location Hierarchy
1 − Global
2 − Super Region
3 − Region
4 − Country
5 − Subnational (Level 1)
Uncertainty Interval
5 − Subnational (Level 1)

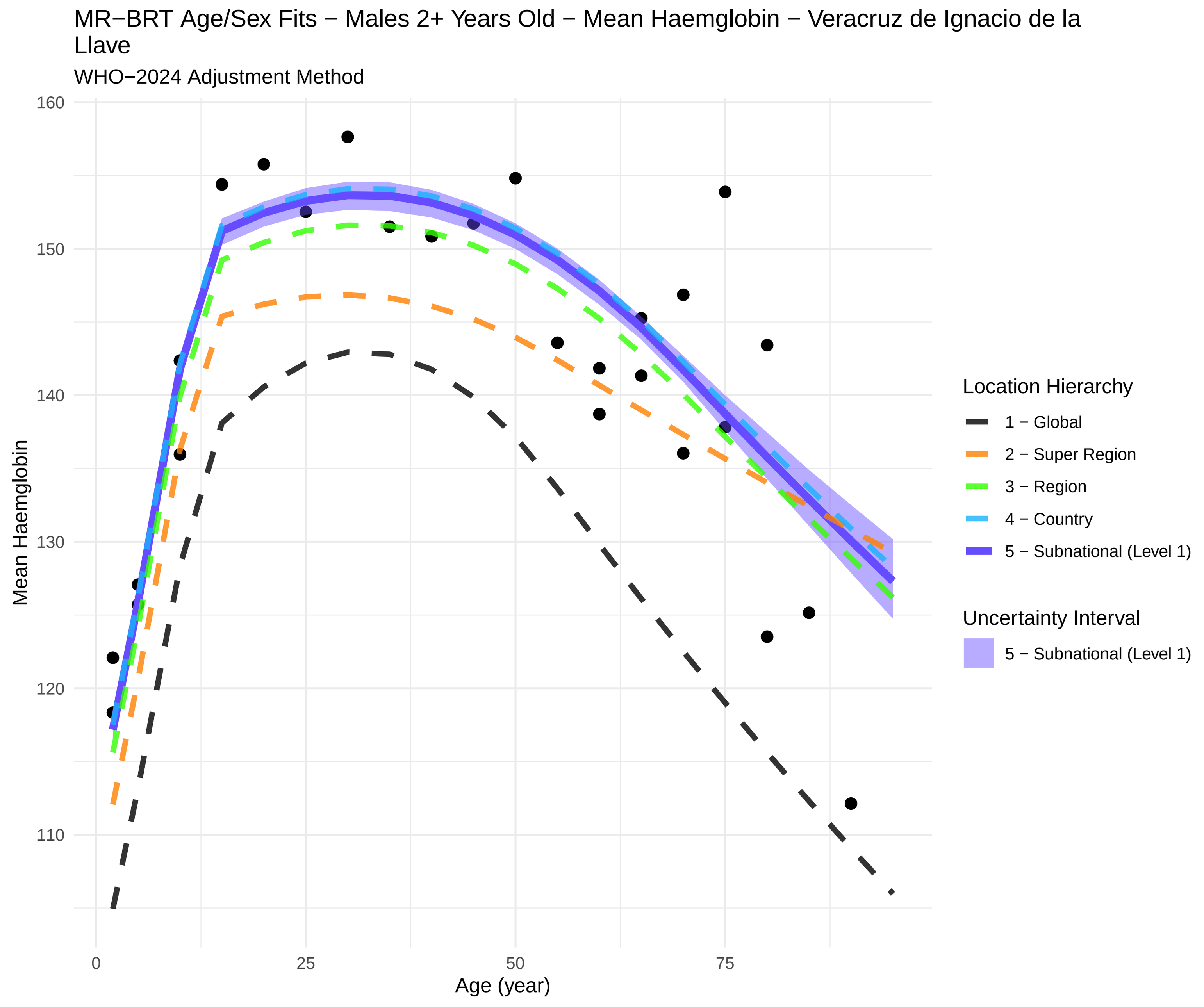
MR−BRT Age/Sex Fits − Males 2+ Years Old − Mean Haemglobin − Veracruz de Ignacio de la Llave
WHO−2024 Adjustment Method
Mean Haemglobin
Age (year)
Location Hierarchy
1 − Global
2 − Super Region
3 − Region
4 − Country
5 − Subnational (Level 1)
Uncertainty Interval
5 − Subnational (Level 1)

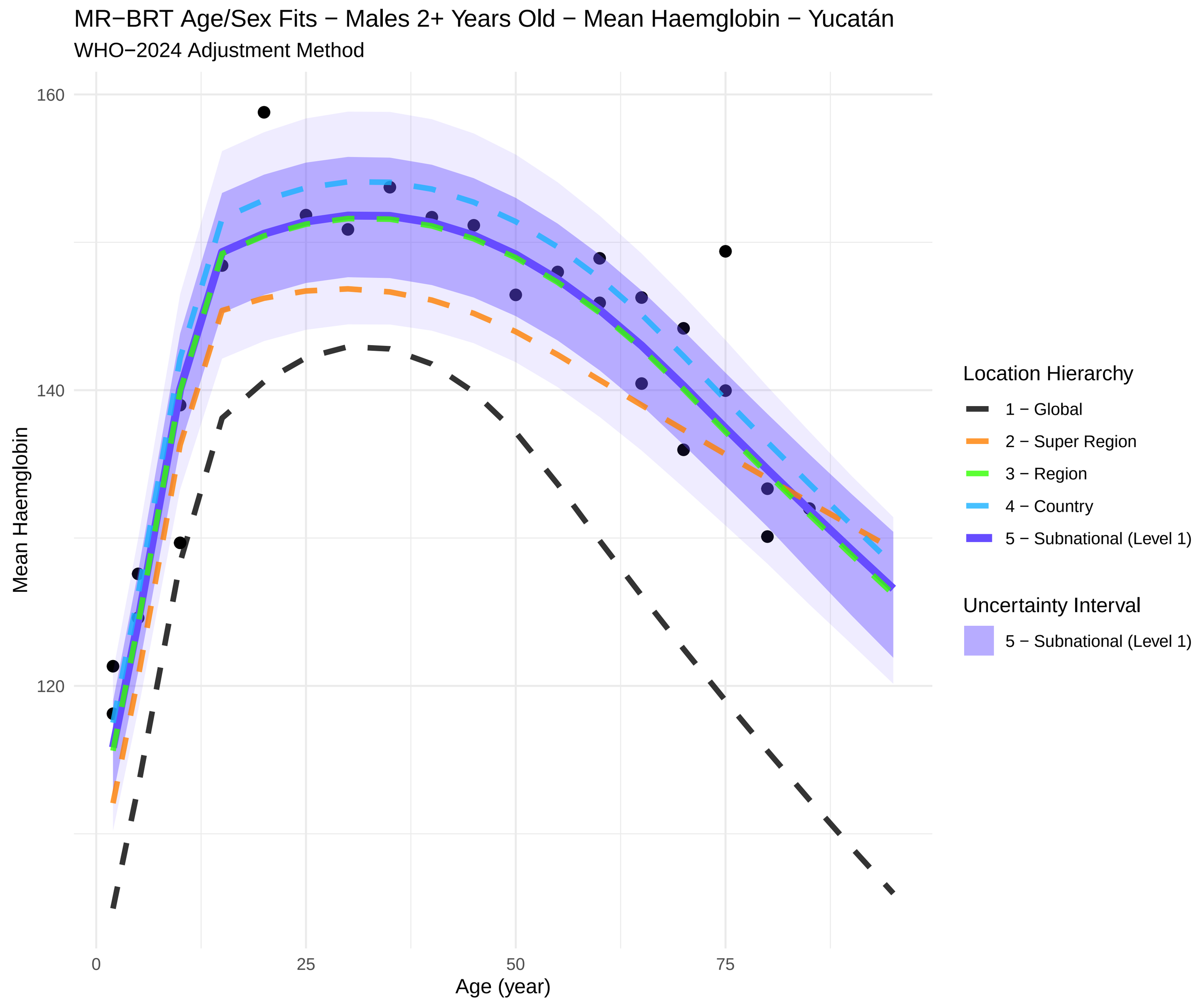
MR−BRT Age/Sex Fits − Males 2+ Years Old − Mean Haemglobin − Yucatán
WHO−2024 Adjustment Method
Mean Haemglobin
Age (year)
Location Hierarchy
1 − Global
2 − Super Region
3 − Region
4 − Country
5 − Subnational (Level 1)
Uncertainty Interval
5 − Subnational (Level 1)

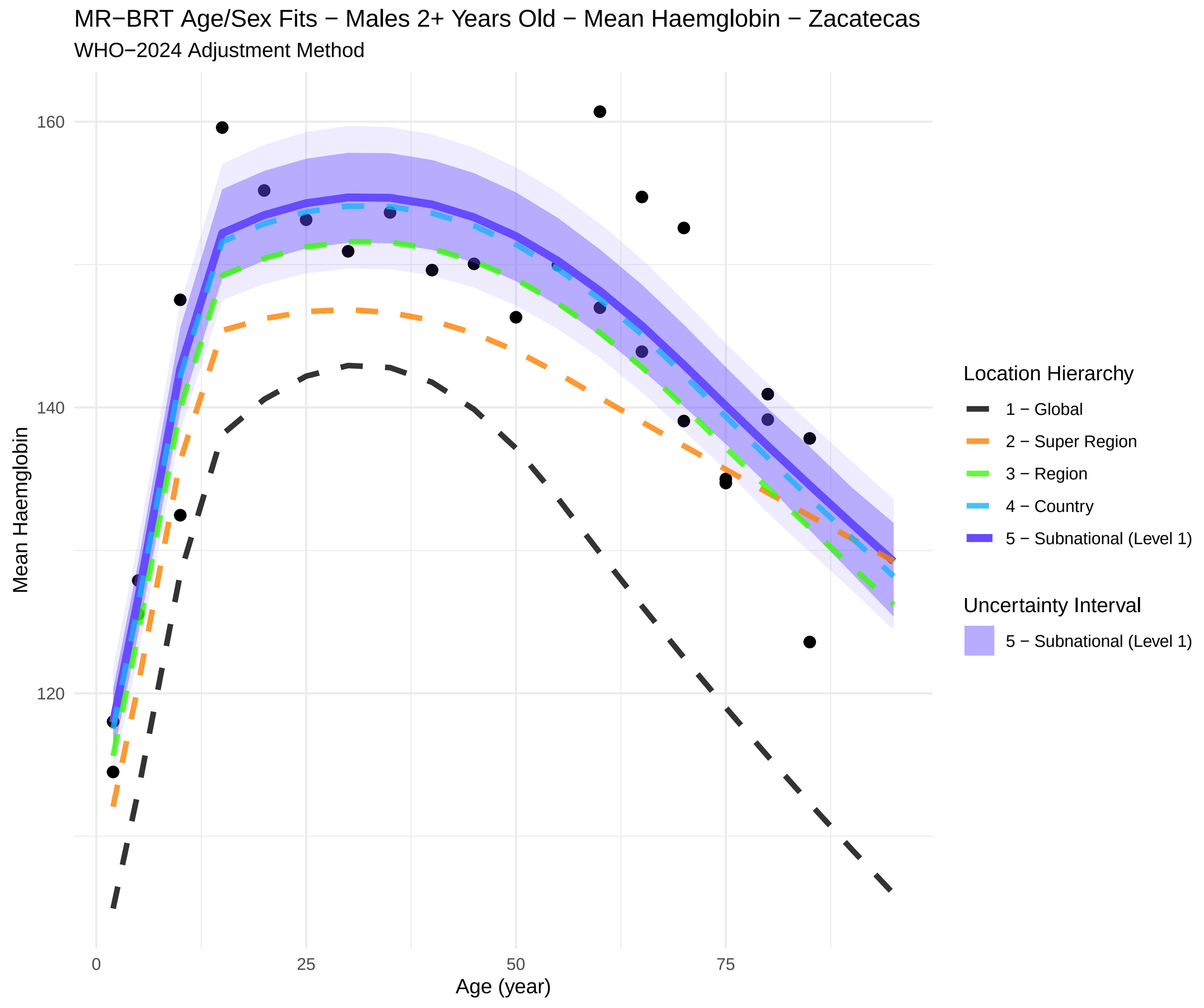
MR−BRT Age/Sex Fits − Males 2+ Years Old − Mean Haemglobin − Zacatecas
WHO−2024 Adjustment Method
Mean Haemglobin
Age (year)
Location Hierarchy
1 − Global
2 − Super Region
3 − Region
4 − Country
5 − Subnational (Level 1)
Uncertainty Interval
5 − Subnational (Level 1)

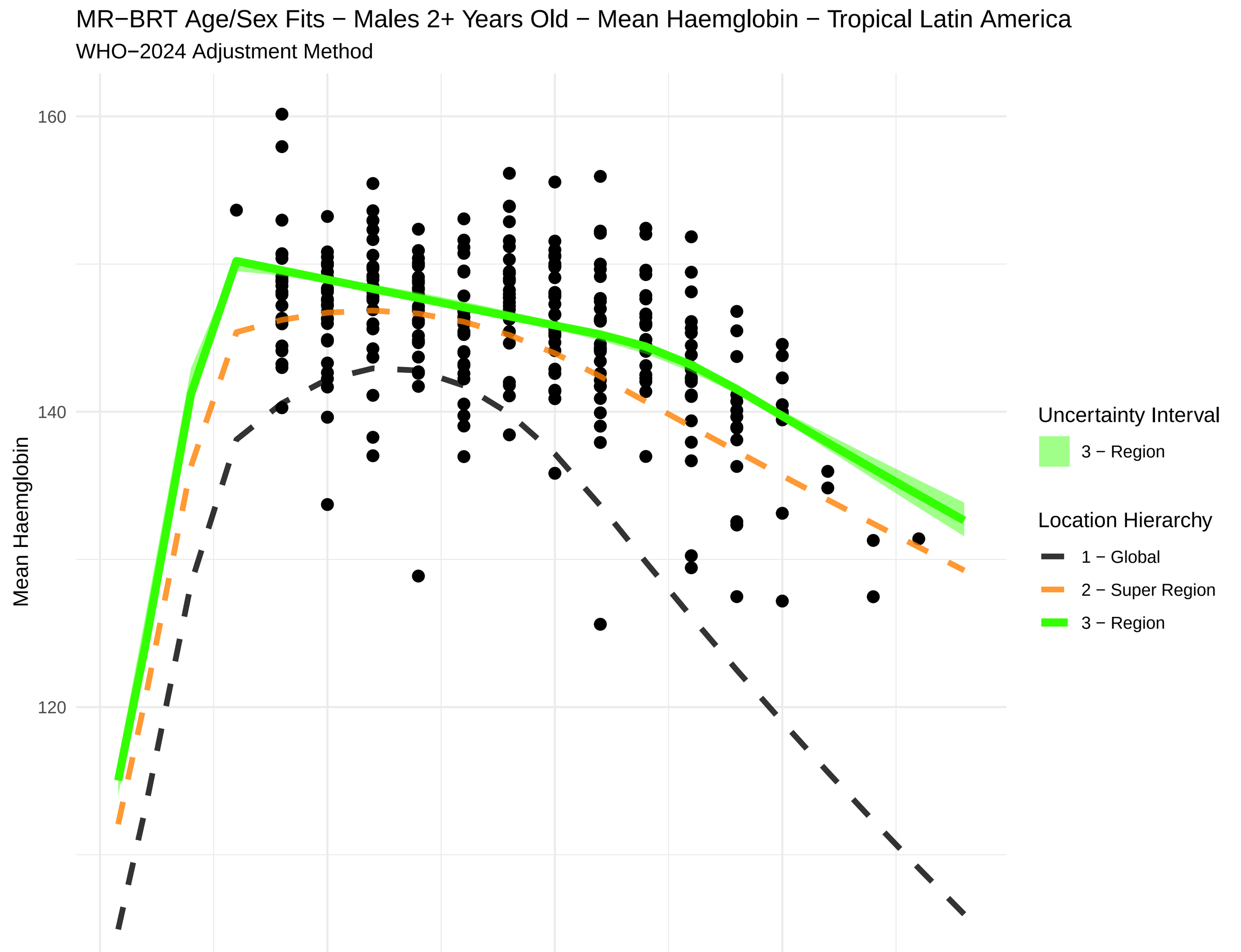
MR−BRT Age/Sex Fits − Males 2+ Years Old − Mean Haemglobin − Tropical Latin America
WHO−2024 Adjustment Method
Mean Haemglobin
Age (year)
Uncertainty Interval
3 − Region
Location Hierarchy
1 − Global
2 − Super Region
3 − Region

MR−BRT Age/Sex Fits − Males 2+ Years Old − Mean Haemglobin − Brazil
WHO−2024 Adjustment Method

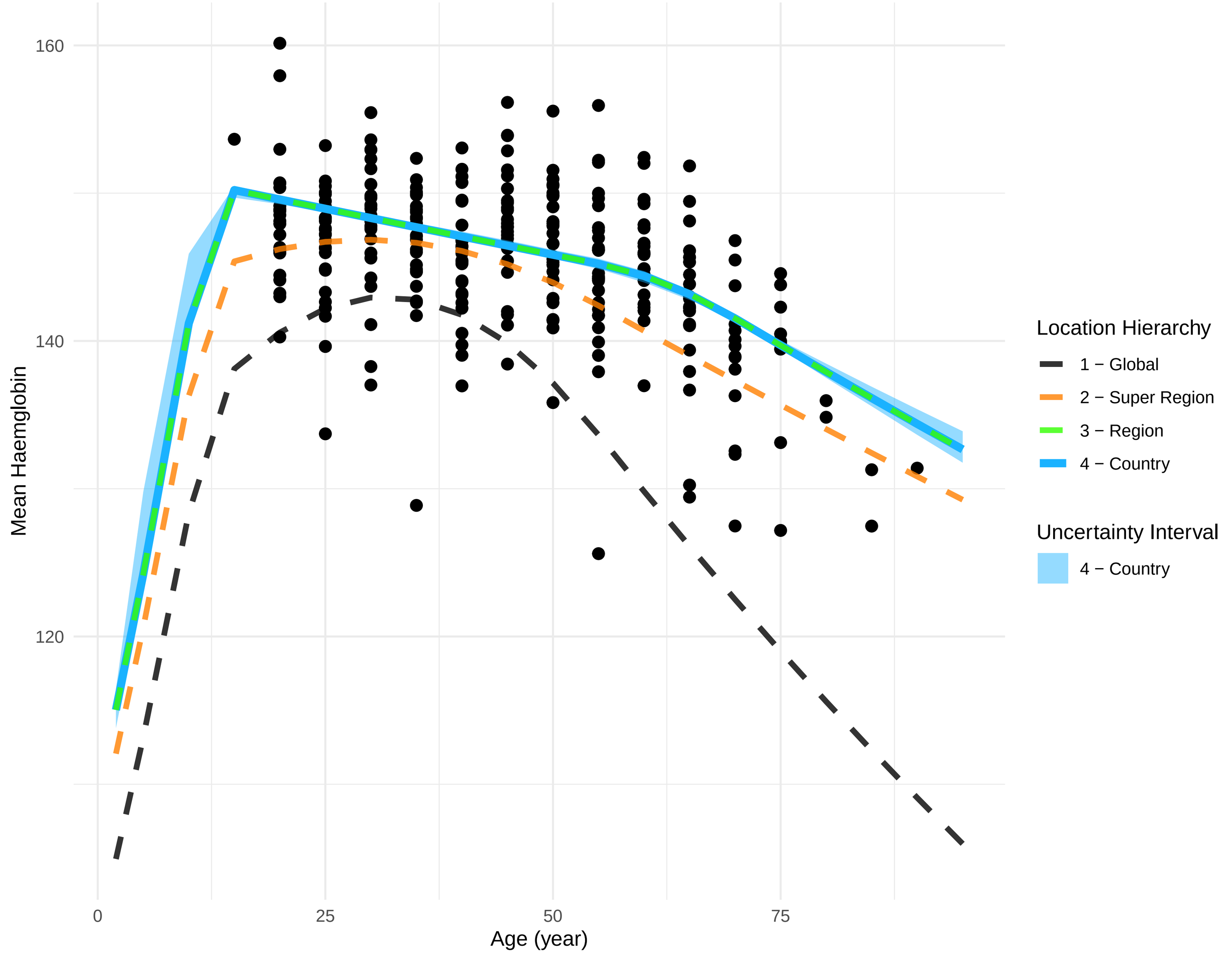

MR−BRT Age/Sex Fits − Males 2+ Years Old − Mean Haemglobin − Acre
WHO−2024 Adjustment Method

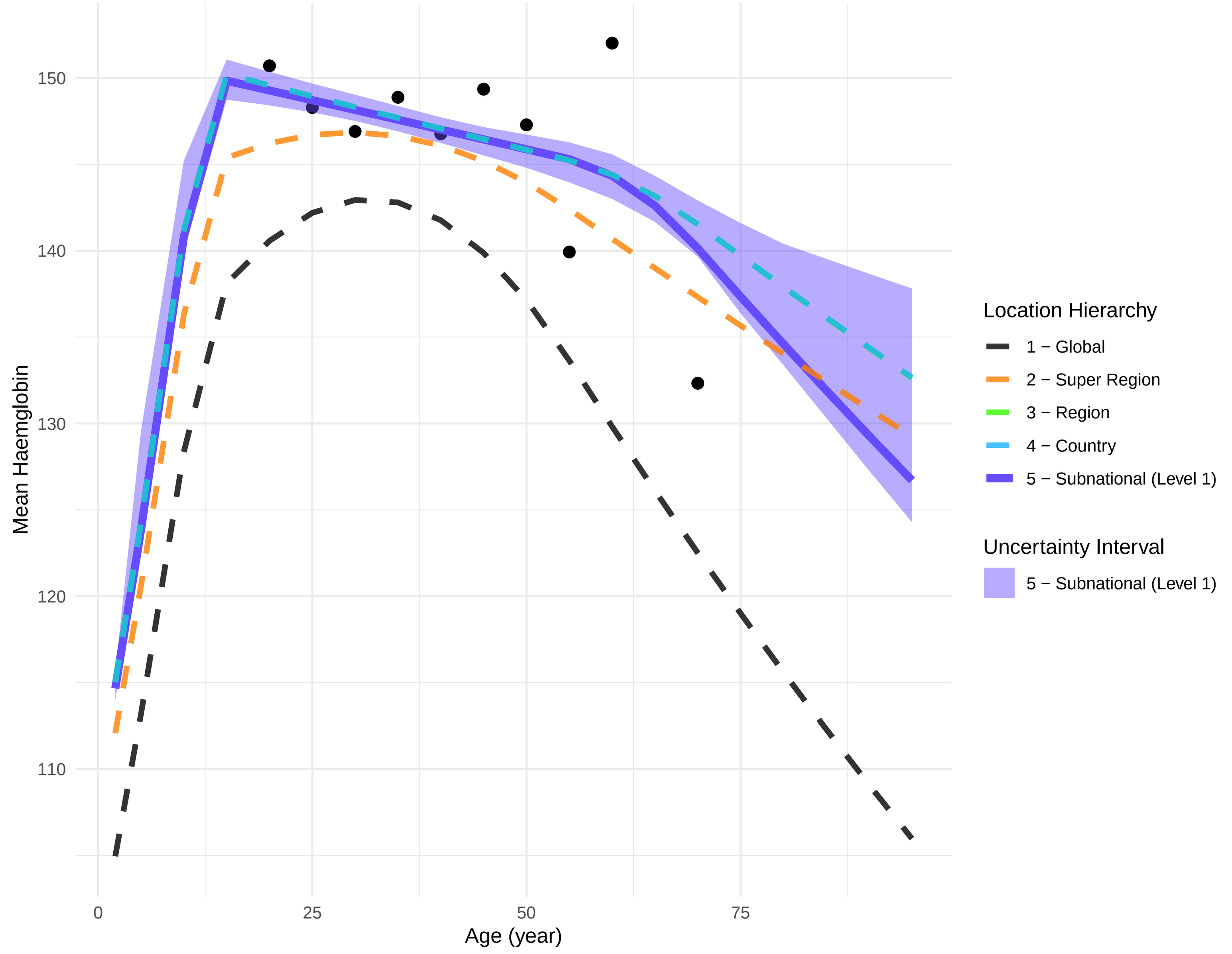

MR−BRT Age/Sex Fits − Males 2+ Years Old − Mean Haemglobin − Alagoas
WHO−2024 Adjustment Method

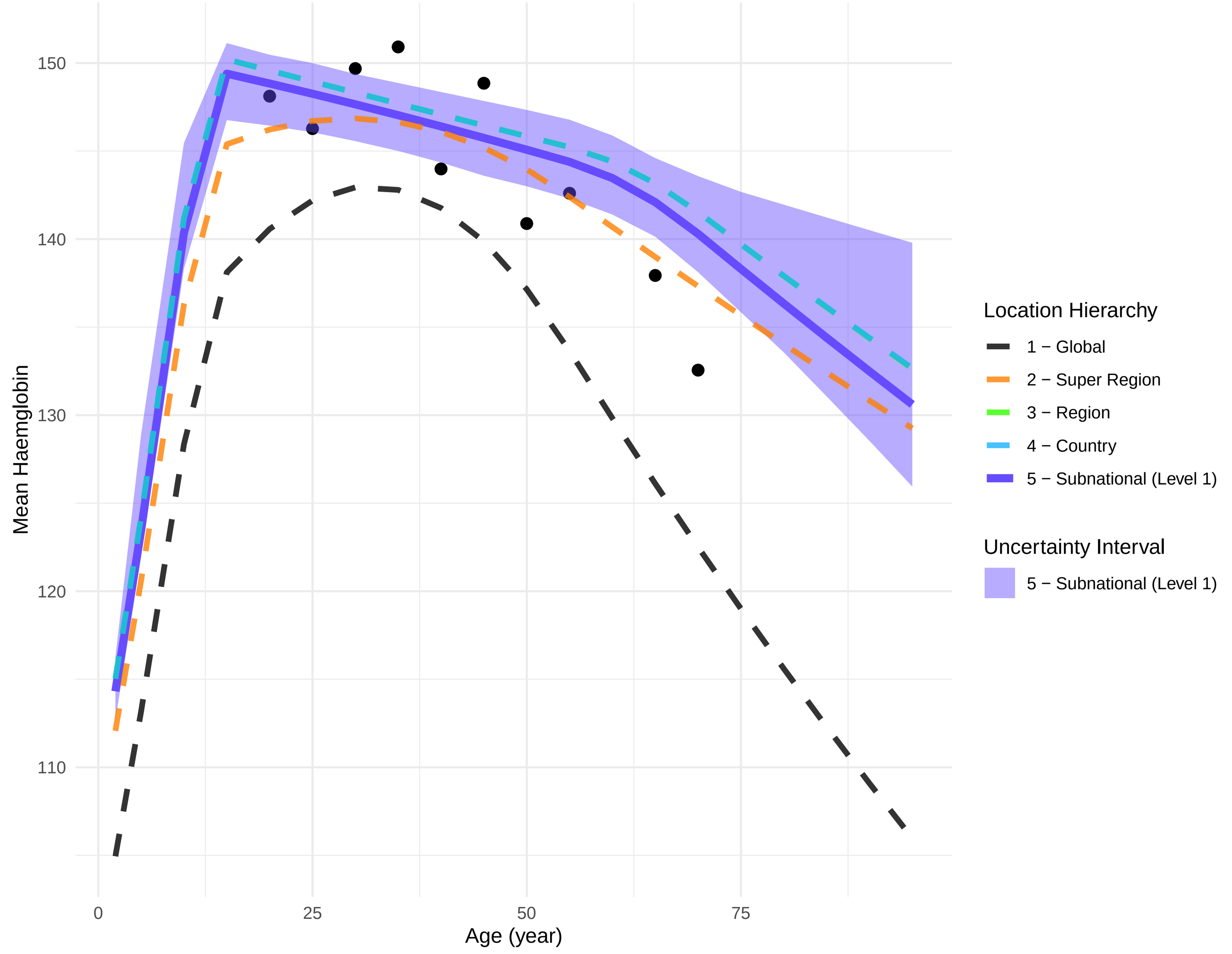

MR−BRT Age/Sex Fits − Males 2+ Years Old − Mean Haemglobin − Amapá
WHO−2024 Adjustment Method

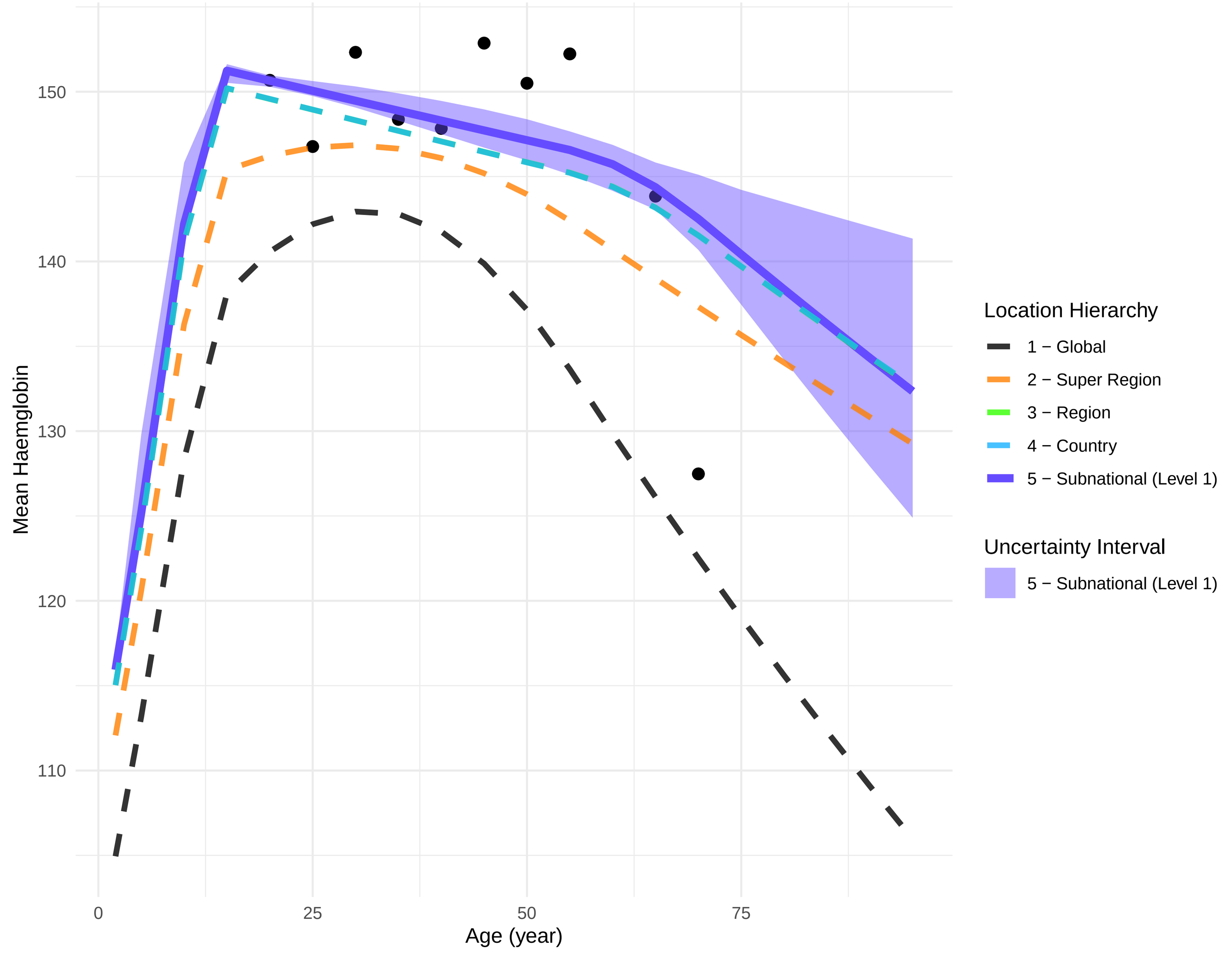

MR−BRT Age/Sex Fits − Males 2+ Years Old − Mean Haemglobin − Amazonas
WHO−2024 Adjustment Method

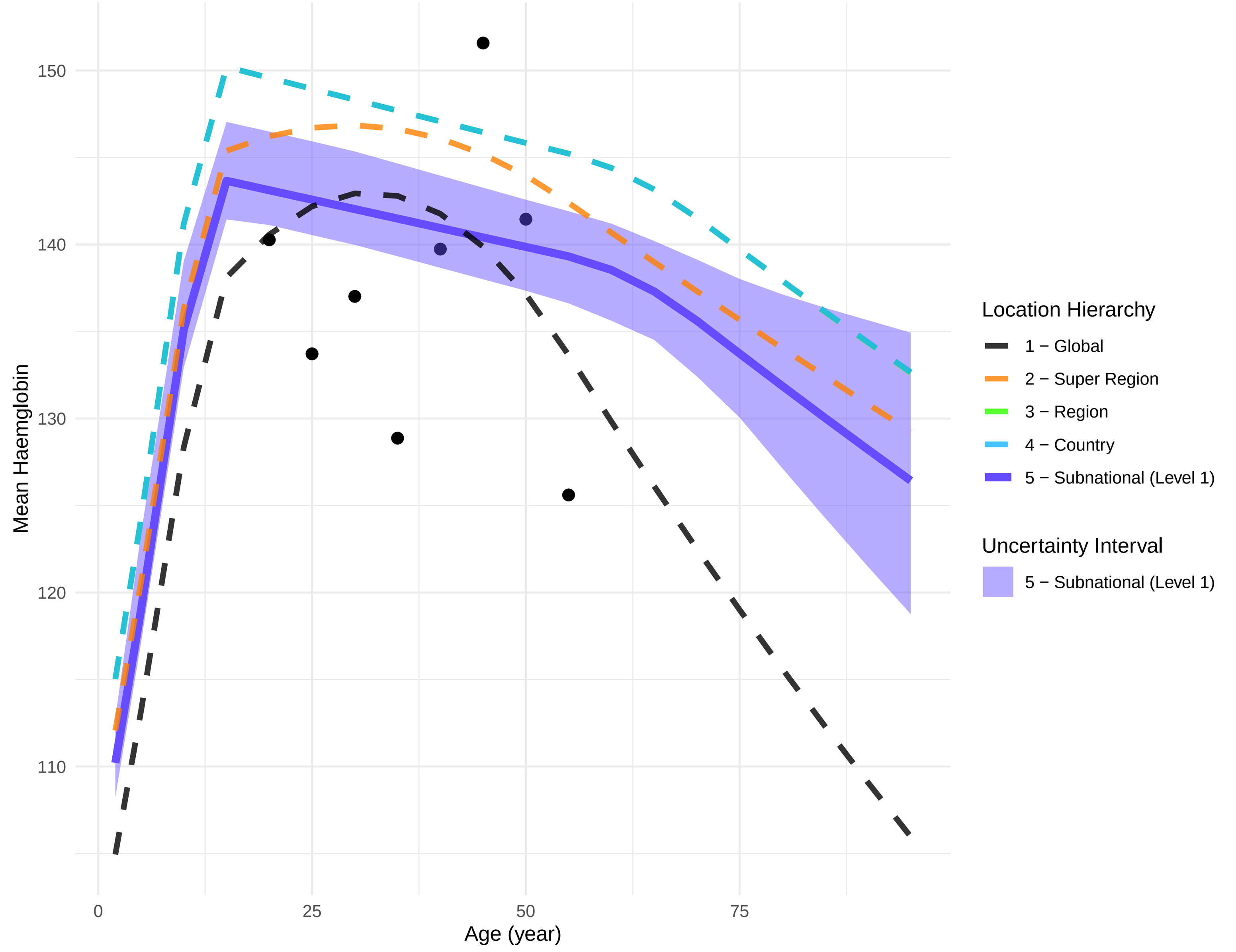

MR−BRT Age/Sex Fits − Males 2+ Years Old − Mean Haemglobin − Bahia
WHO−2024 Adjustment Method

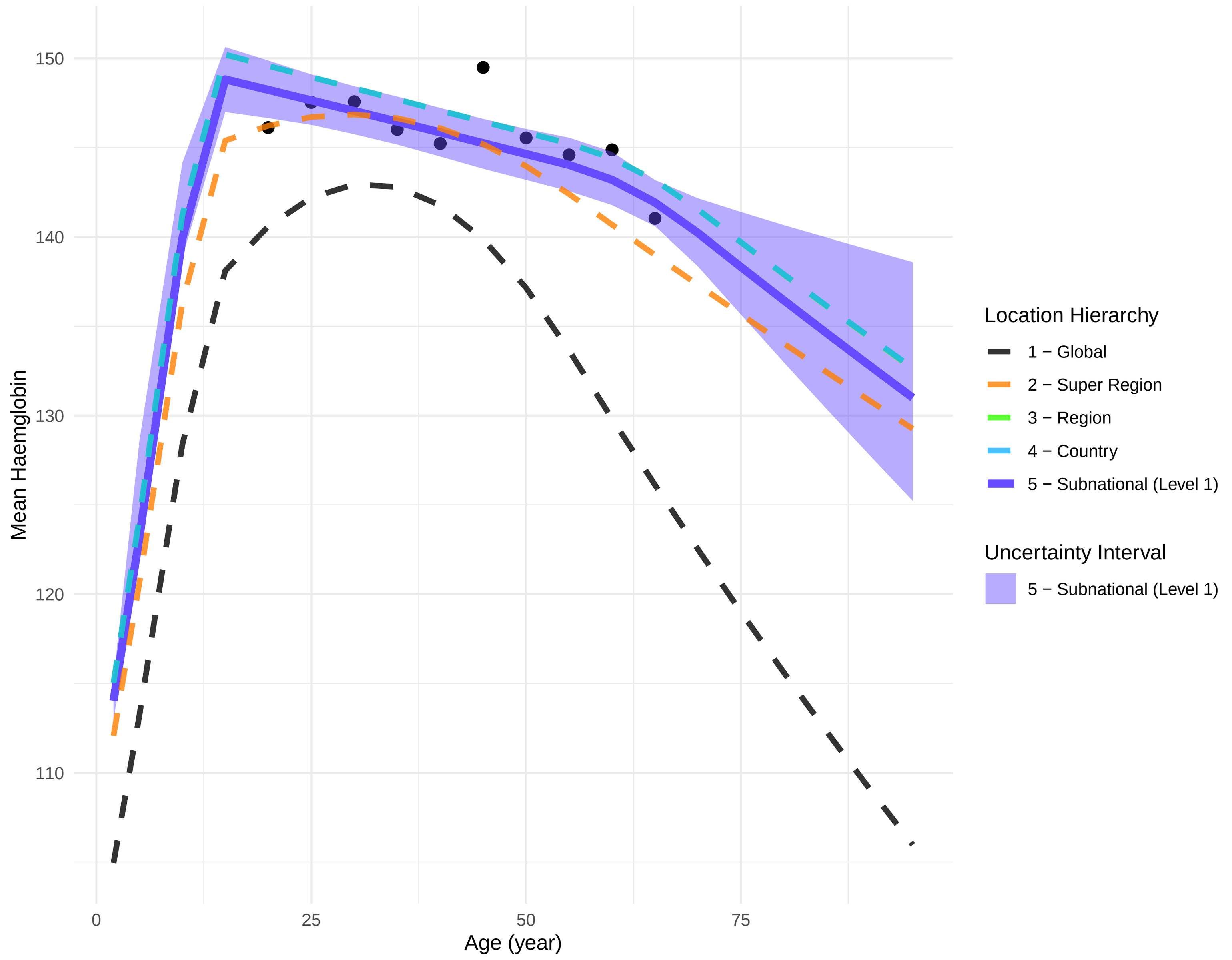

MR−BRT Age/Sex Fits − Males 2+ Years Old − Mean Haemglobin − Ceará
WHO−2024 Adjustment Method

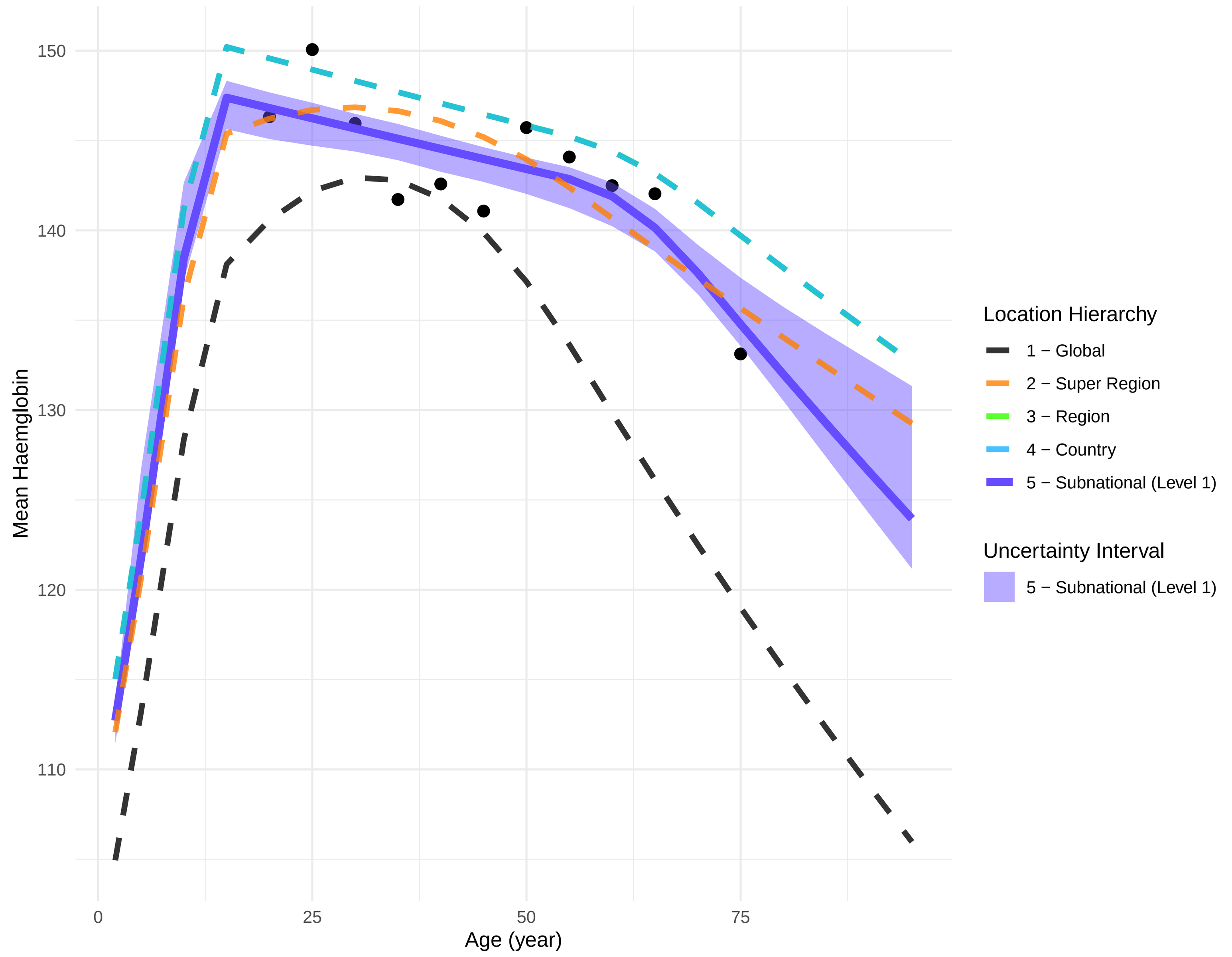

MR−BRT Age/Sex Fits − Males 2+ Years Old − Mean Haemglobin − Distrito Federal
WHO−2024 Adjustment Method

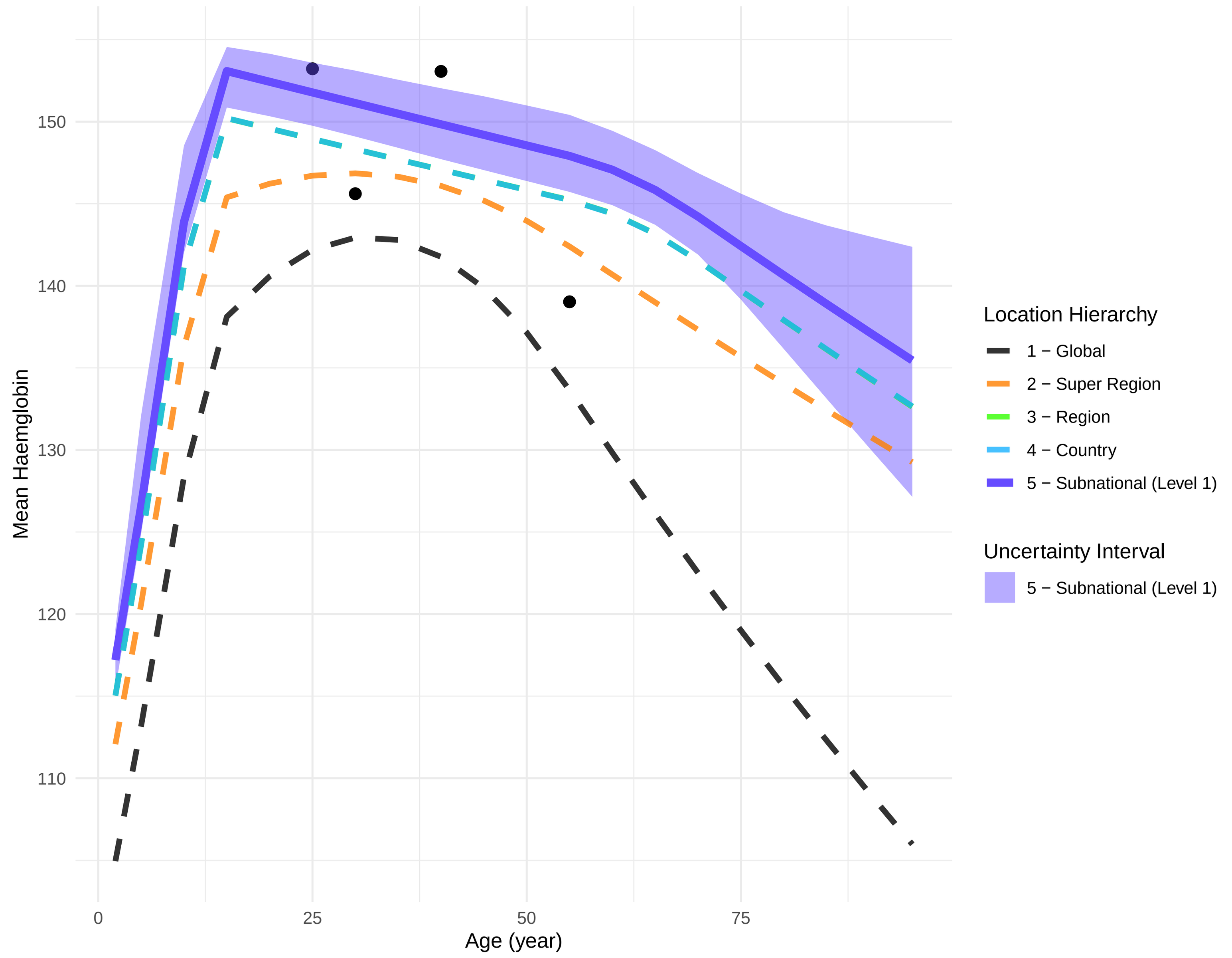

MR−BRT Age/Sex Fits − Males 2+ Years Old − Mean Haemglobin − Espírito Santo
WHO−2024 Adjustment Method

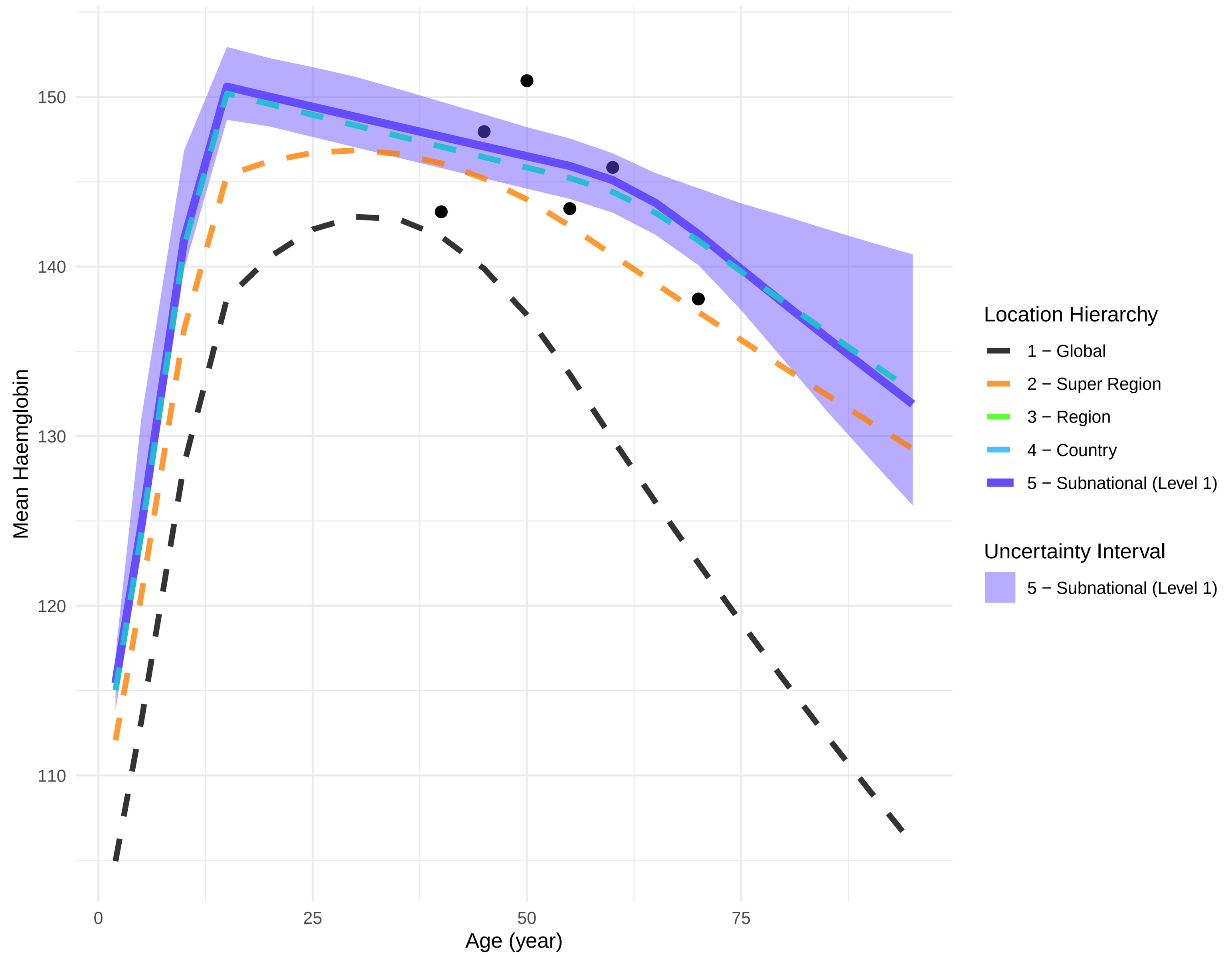

MR−BRT Age/Sex Fits − Males 2+ Years Old − Mean Haemglobin − Goiás
WHO−2024 Adjustment Method

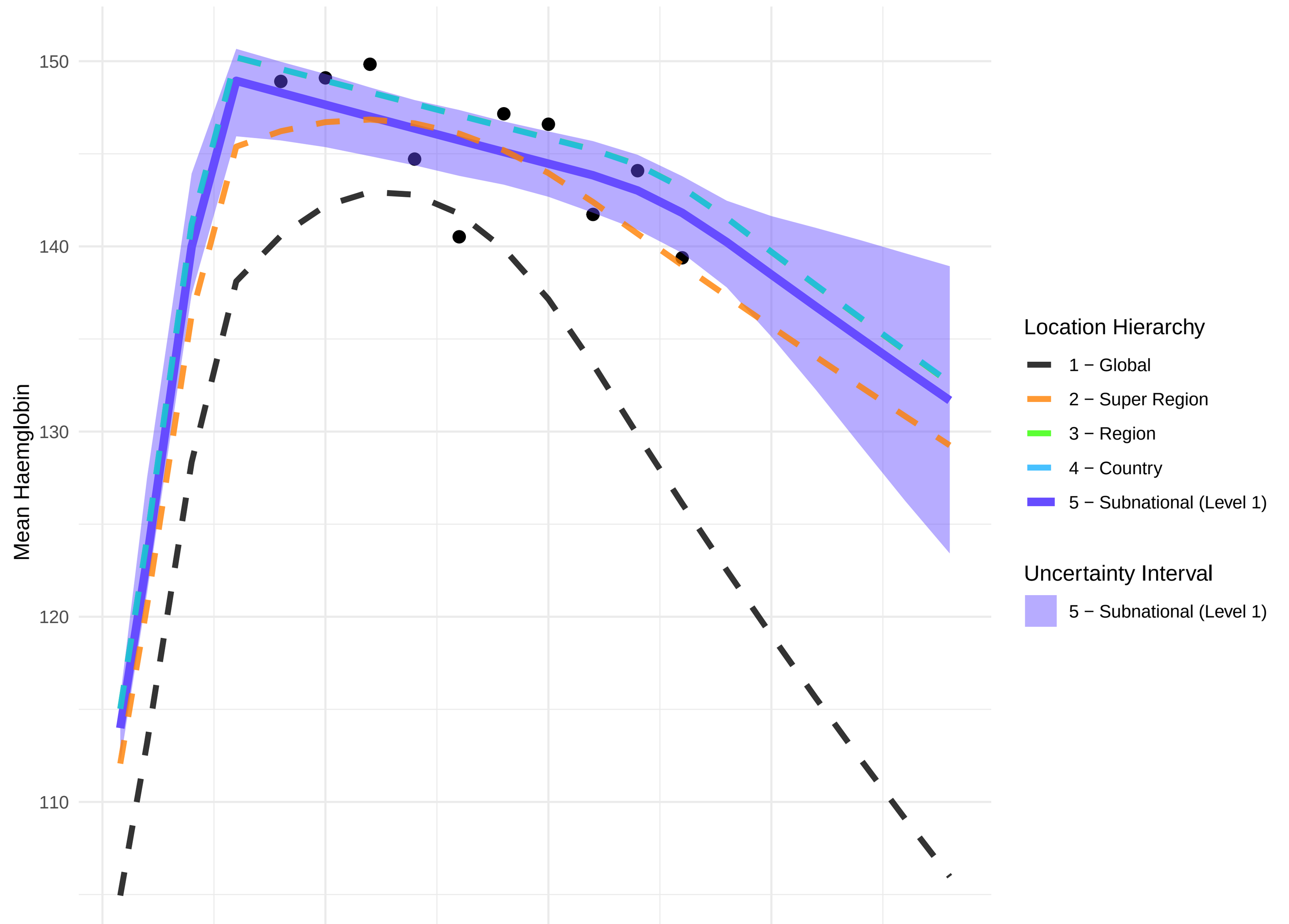

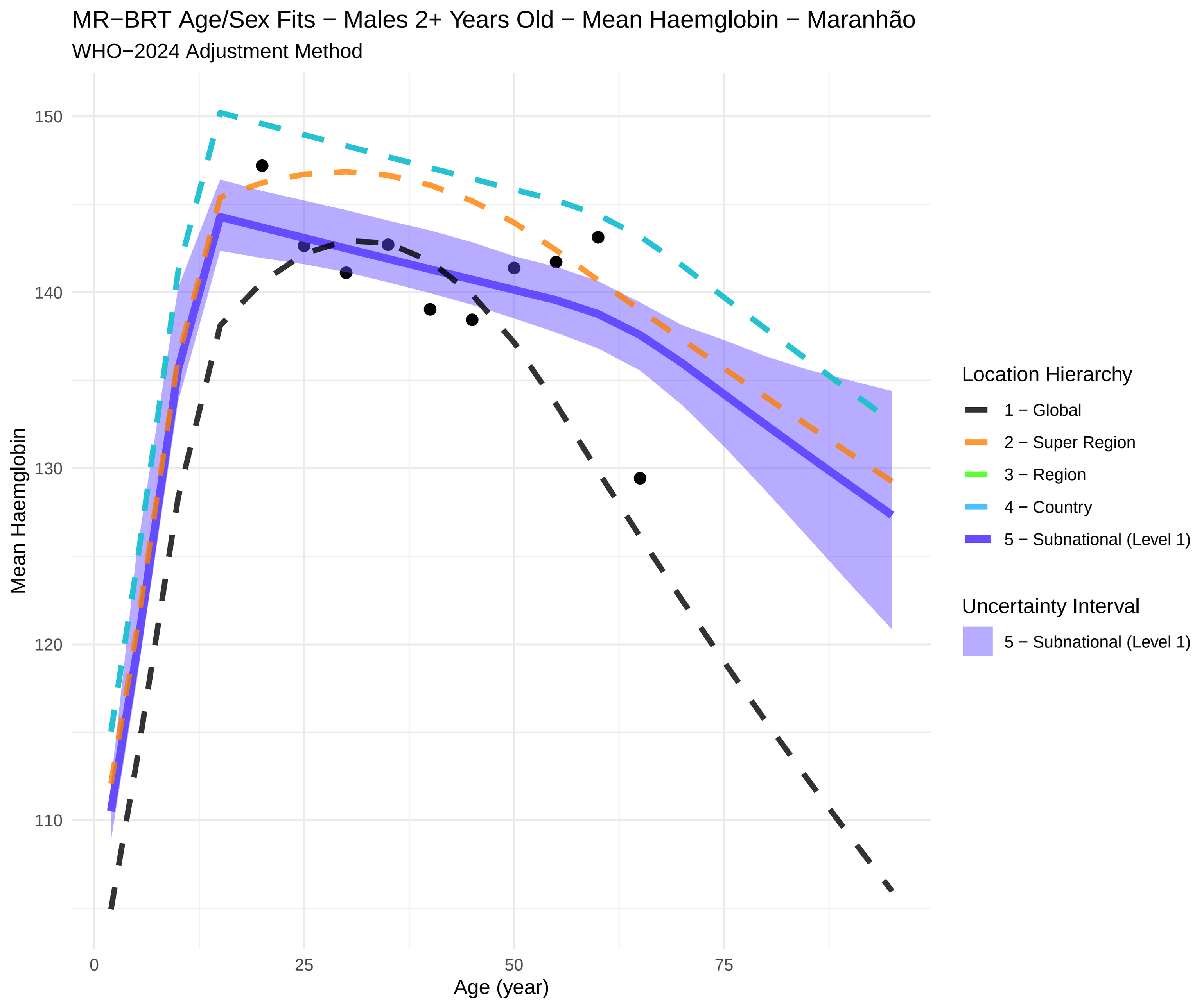
MR−BRT Age/Sex Fits − Males 2+ Years Old − Mean Haemglobin − Maranhão
WHO−2024 Adjustment Method
Mean Haemglobin
Age (year)
Location Hierarchy
1 − Global
2 − Super Region
3 − Region
4 − Country
5 − Subnational (Level 1)
Uncertainty Interval
5 − Subnational (Level 1)

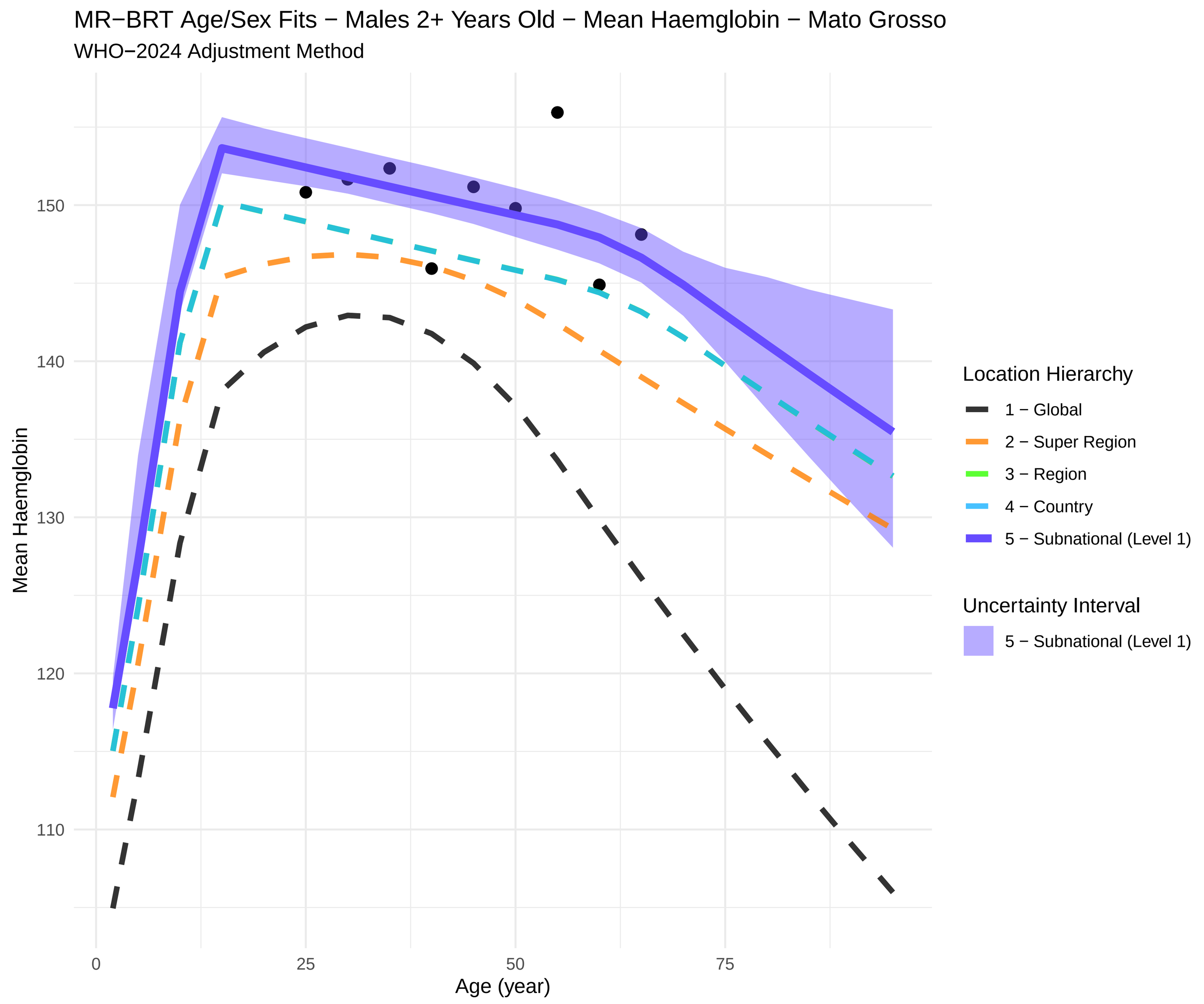
MR−BRT Age/Sex Fits − Males 2+ Years Old − Mean Haemglobin − Mato Grosso
WHO−2024 Adjustment Method
Mean Haemglobin
Age (year)
Location Hierarchy
1 − Global
2 − Super Region
3 − Region
4 − Country
5 − Subnational (Level 1)
Uncertainty Interval
5 − Subnational (Level 1)

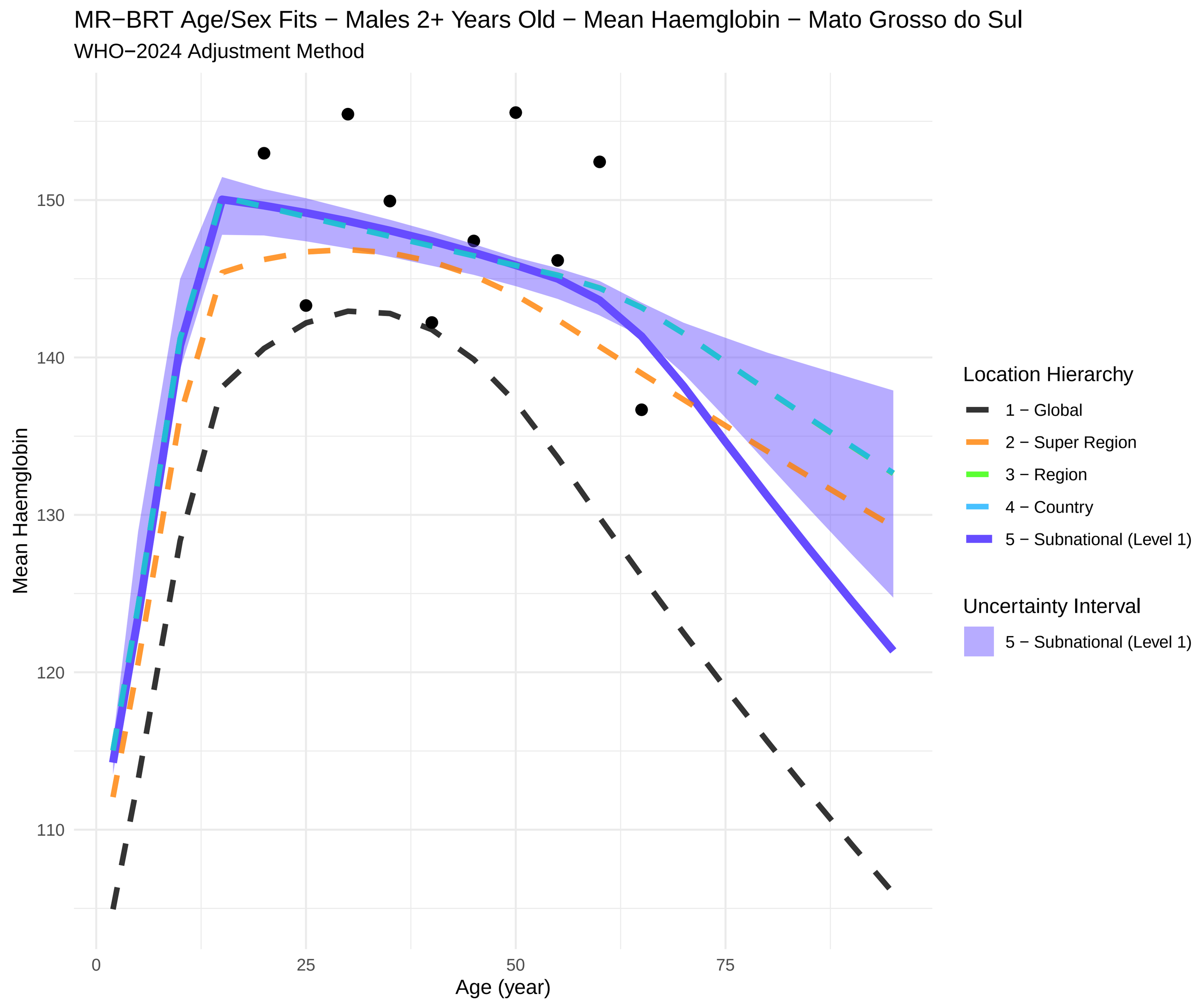
MR−BRT Age/Sex Fits − Males 2+ Years Old − Mean Haemglobin − Mato Grosso do Sul
WHO−2024 Adjustment Method
Mean Haemglobin
Age (year)
Location Hierarchy
1 − Global
2 − Super Region
3 − Region
4 − Country
5 − Subnational (Level 1)
Uncertainty Interval
5 − Subnational (Level 1)

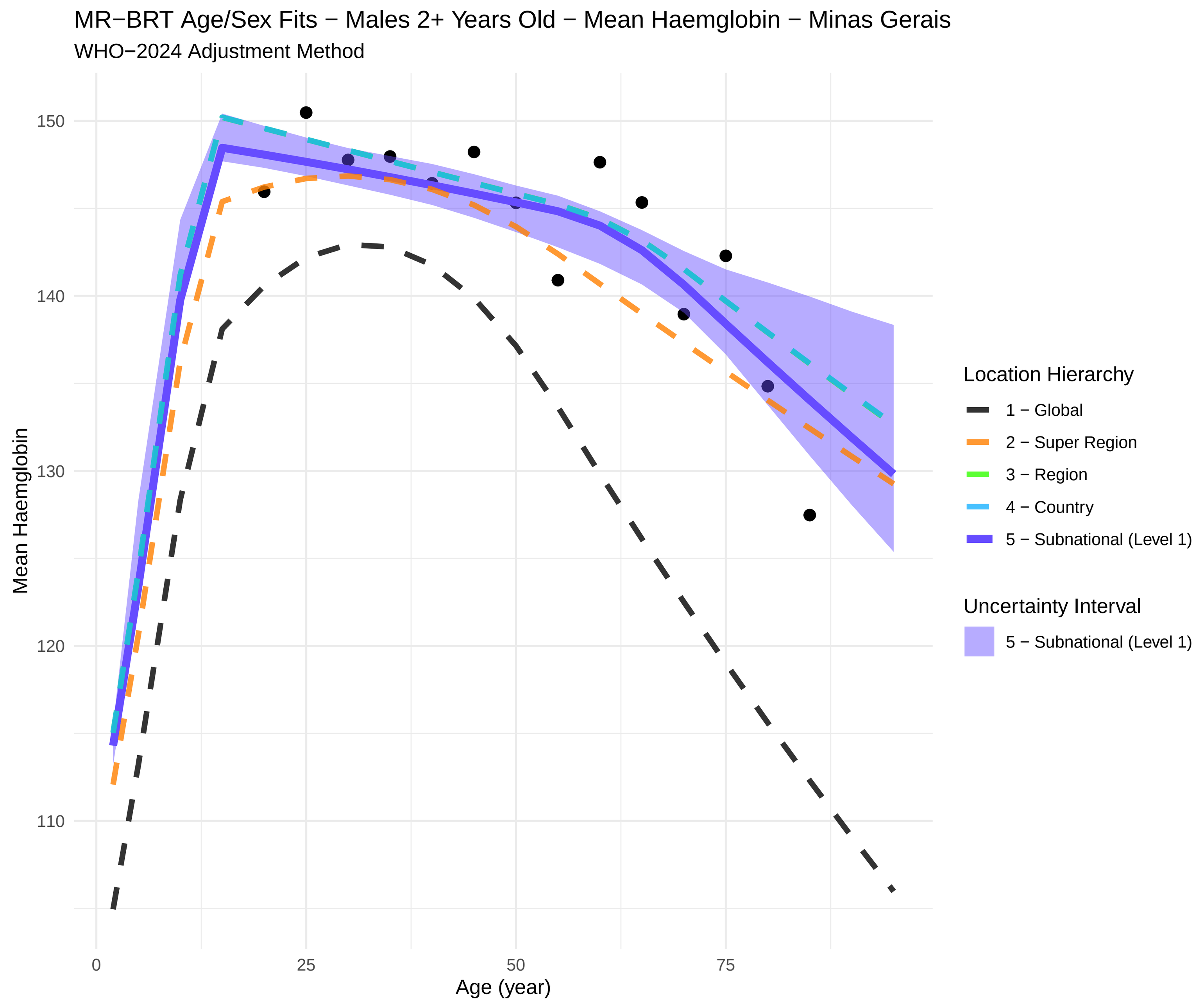
MR−BRT Age/Sex Fits − Males 2+ Years Old − Mean Haemglobin − Minas Gerais
WHO−2024 Adjustment Method
Mean Haemglobin
Age (year)
Location Hierarchy
1 − Global
2 − Super Region
3 − Region
4 − Country
5 − Subnational (Level 1)
Uncertainty Interval
5 − Subnational (Level 1)

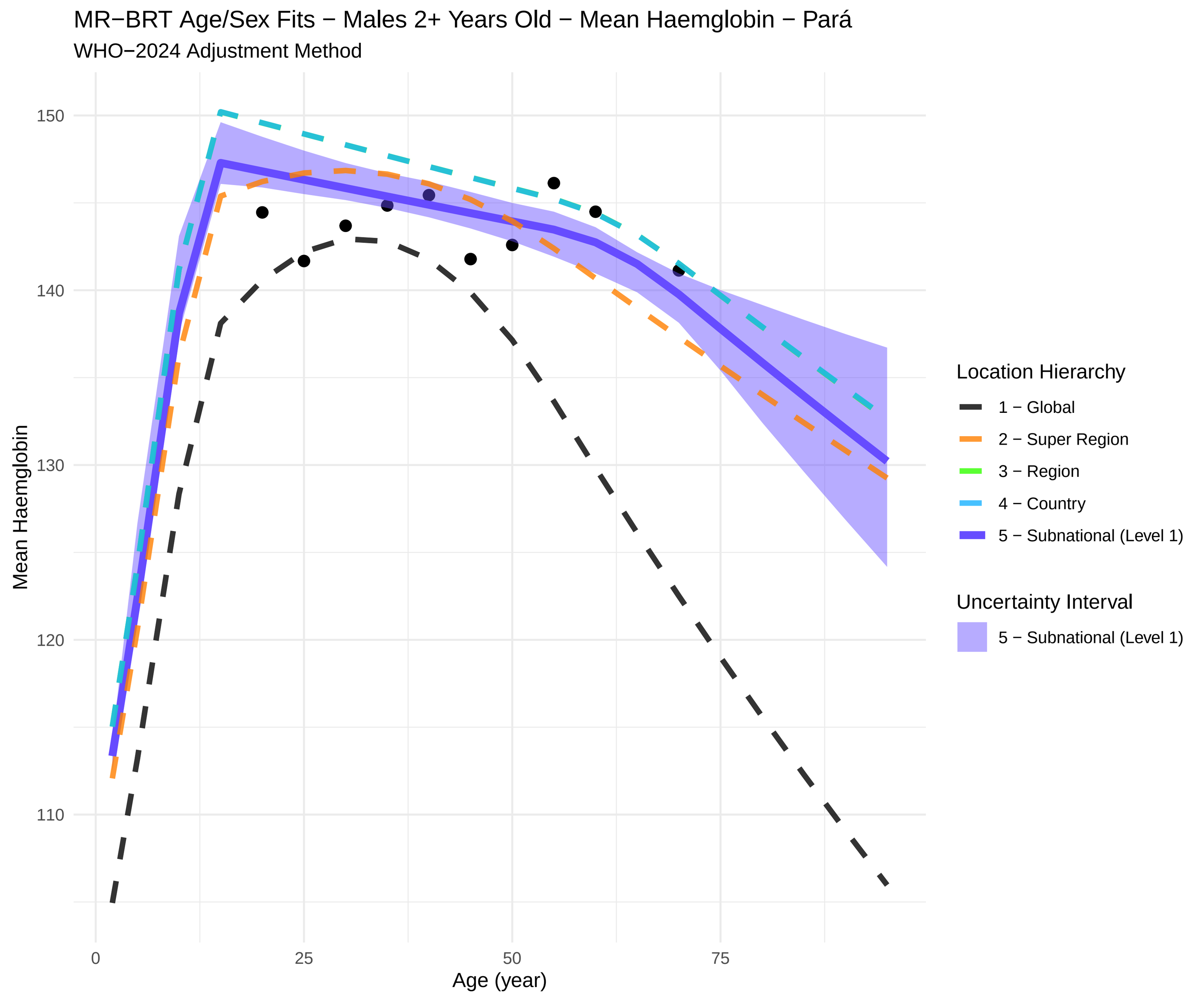
MR−BRT Age/Sex Fits − Males 2+ Years Old − Mean Haemglobin − Pará
WHO−2024 Adjustment Method
Mean Haemglobin
Age (year)
Location Hierarchy
1 − Global
2 − Super Region
3 − Region
4 − Country
5 − Subnational (Level 1)
Uncertainty Interval
5 − Subnational (Level 1)

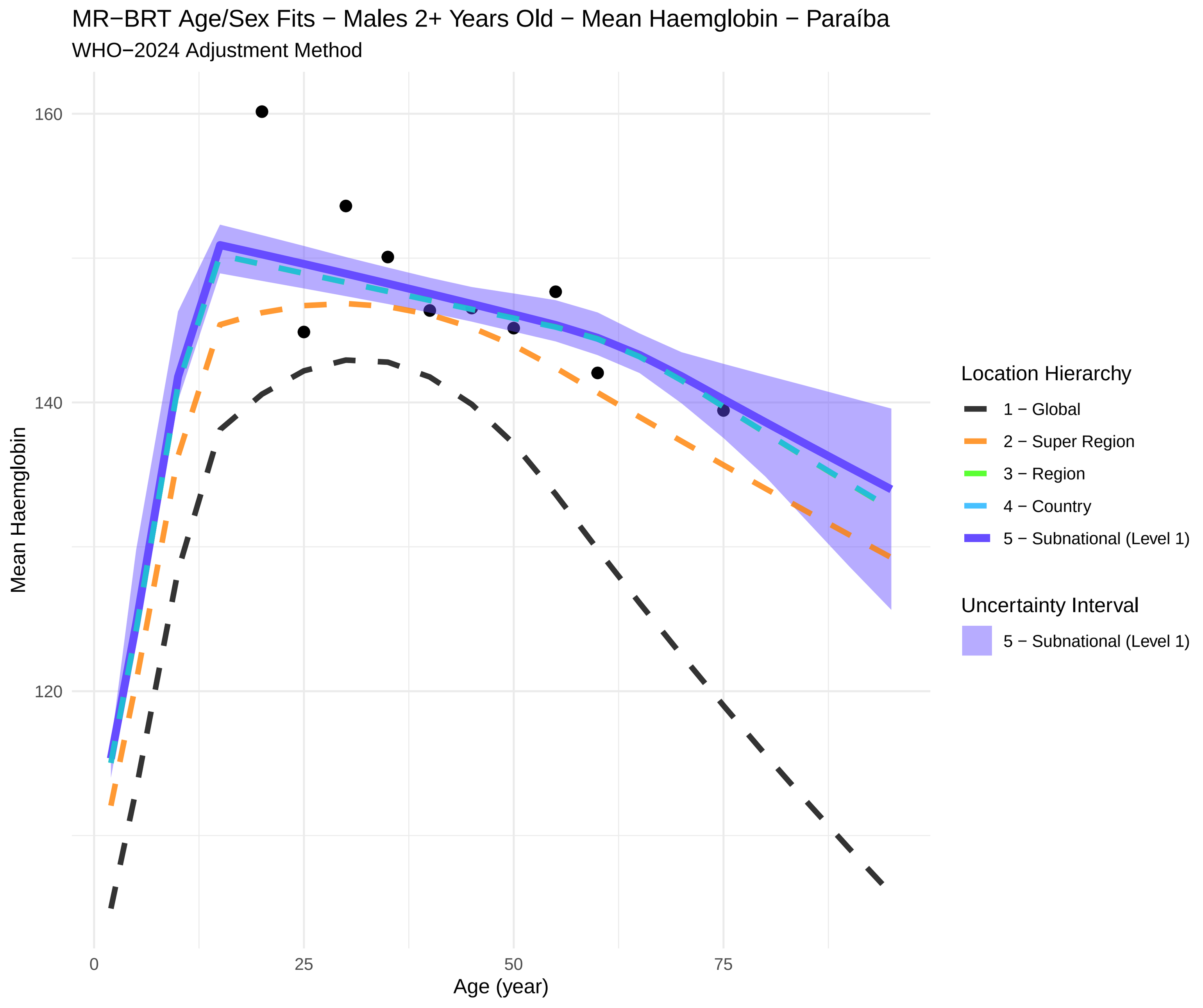
MR−BRT Age/Sex Fits − Males 2+ Years Old − Mean Haemglobin − Paraíba
WHO−2024 Adjustment Method
Mean Haemglobin
Age (year)
Location Hierarchy
1 − Global
2 − Super Region
3 − Region
4 − Country
5 − Subnational (Level 1)
Uncertainty Interval
5 − Subnational (Level 1)

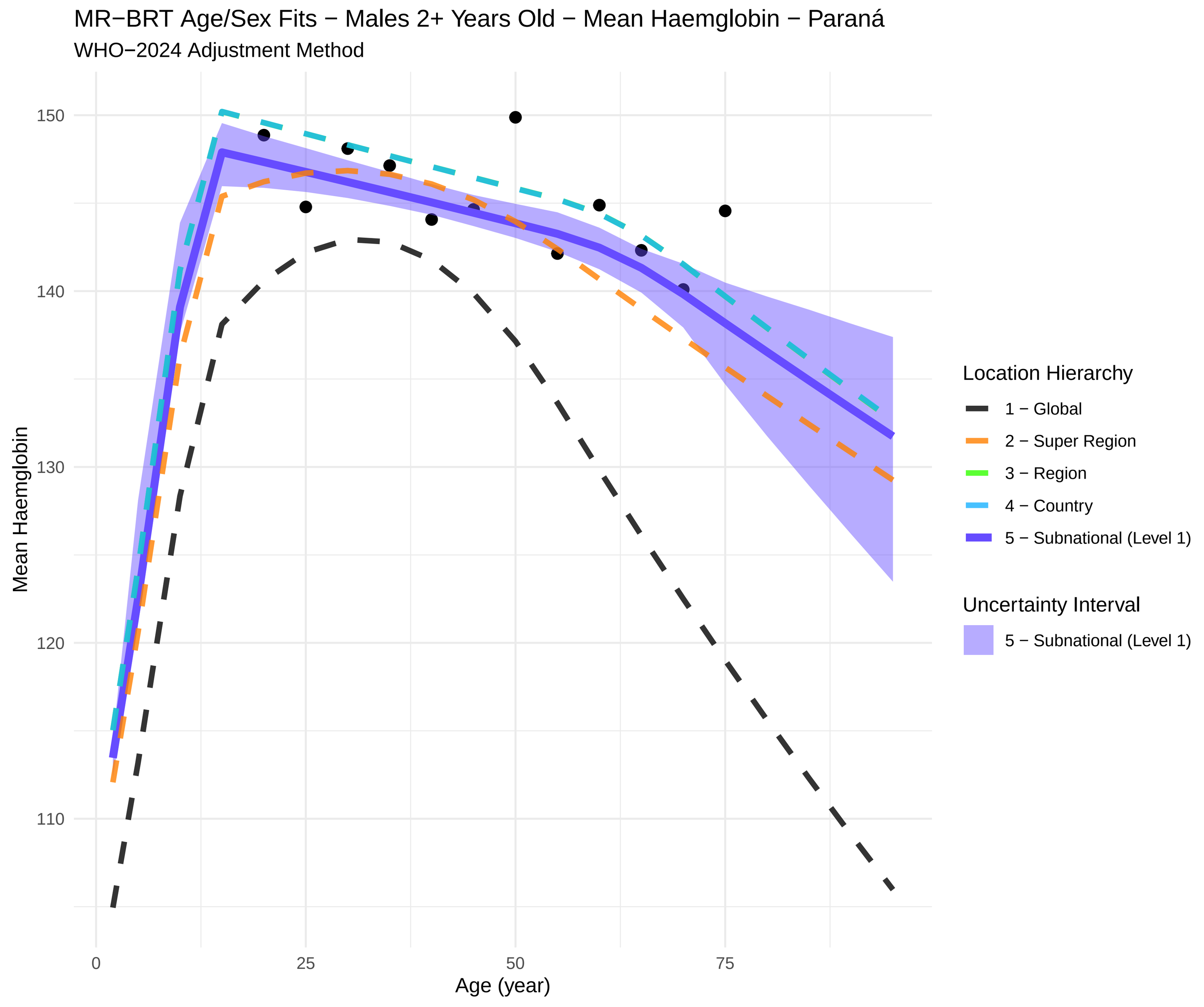
MR−BRT Age/Sex Fits − Males 2+ Years Old − Mean Haemglobin − Paraná
WHO−2024 Adjustment Method
Mean Haemglobin
Age (year)
Location Hierarchy
1 − Global
2 − Super Region
3 − Region
4 − Country
5 − Subnational (Level 1)
Uncertainty Interval
5 − Subnational (Level 1)

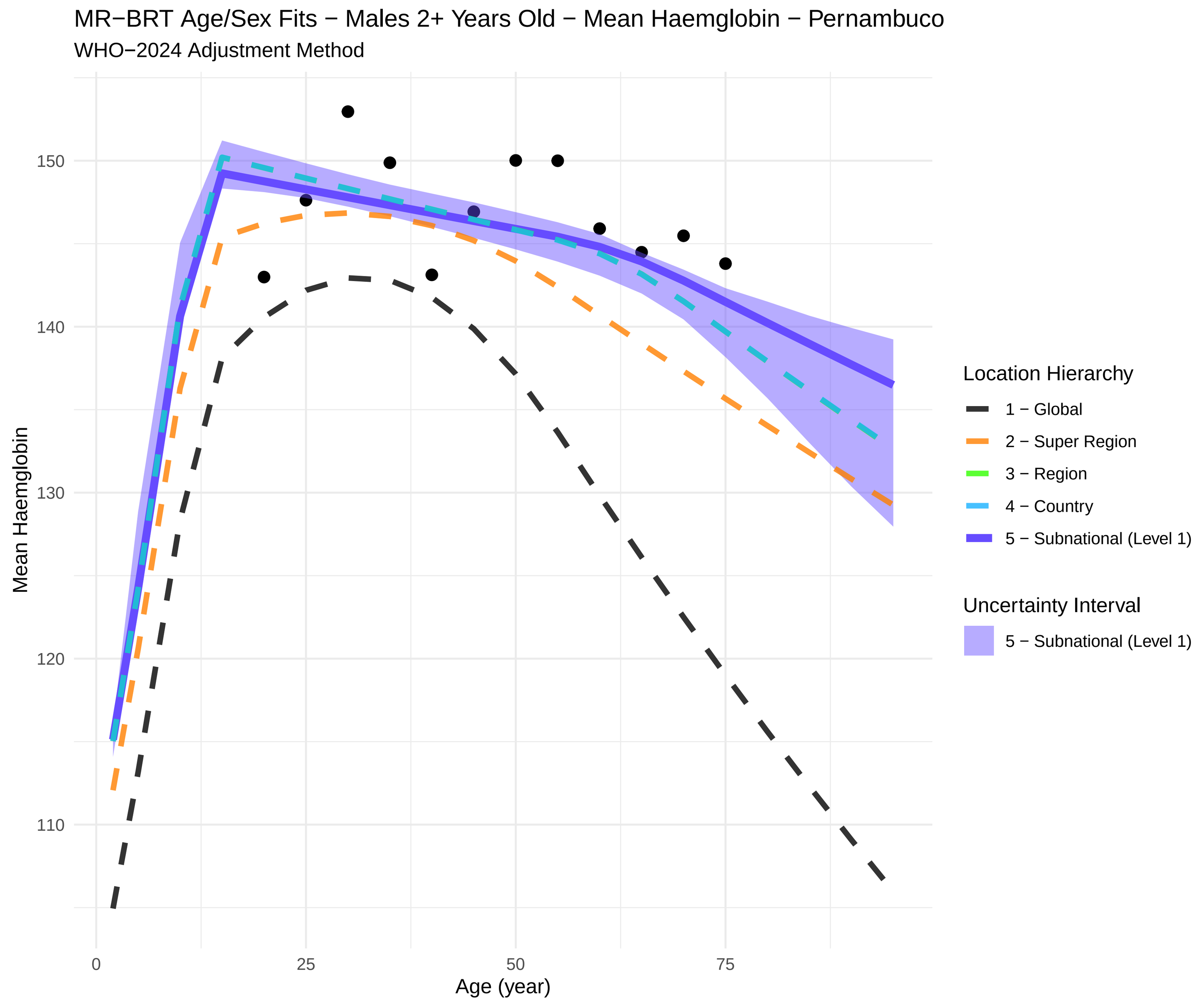
MR−BRT Age/Sex Fits − Males 2+ Years Old − Mean Haemglobin − Pernambuco
WHO−2024 Adjustment Method
Mean Haemglobin
Age (year)
Location Hierarchy
1 − Global
2 − Super Region
3 − Region
4 − Country
5 − Subnational (Level 1)
Uncertainty Interval
5 − Subnational (Level 1)

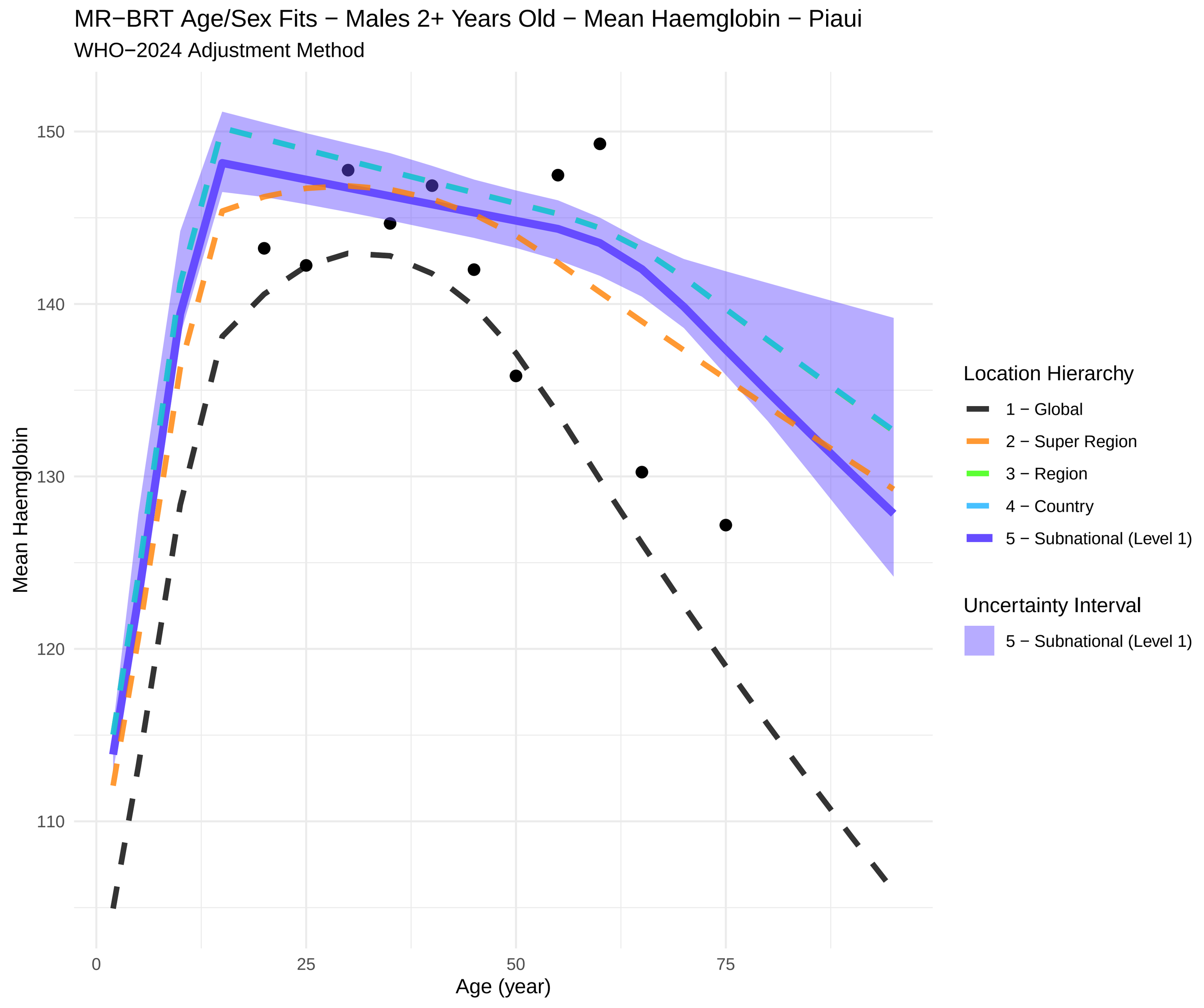
MR−BRT Age/Sex Fits − Males 2+ Years Old − Mean Haemglobin − Piaui
WHO−2024 Adjustment Method
Mean Haemglobin
Age (year)
Location Hierarchy
1 − Global
2 − Super Region
3 − Region
4 − Country
5 − Subnational (Level 1)
Uncertainty Interval
5 − Subnational (Level 1)

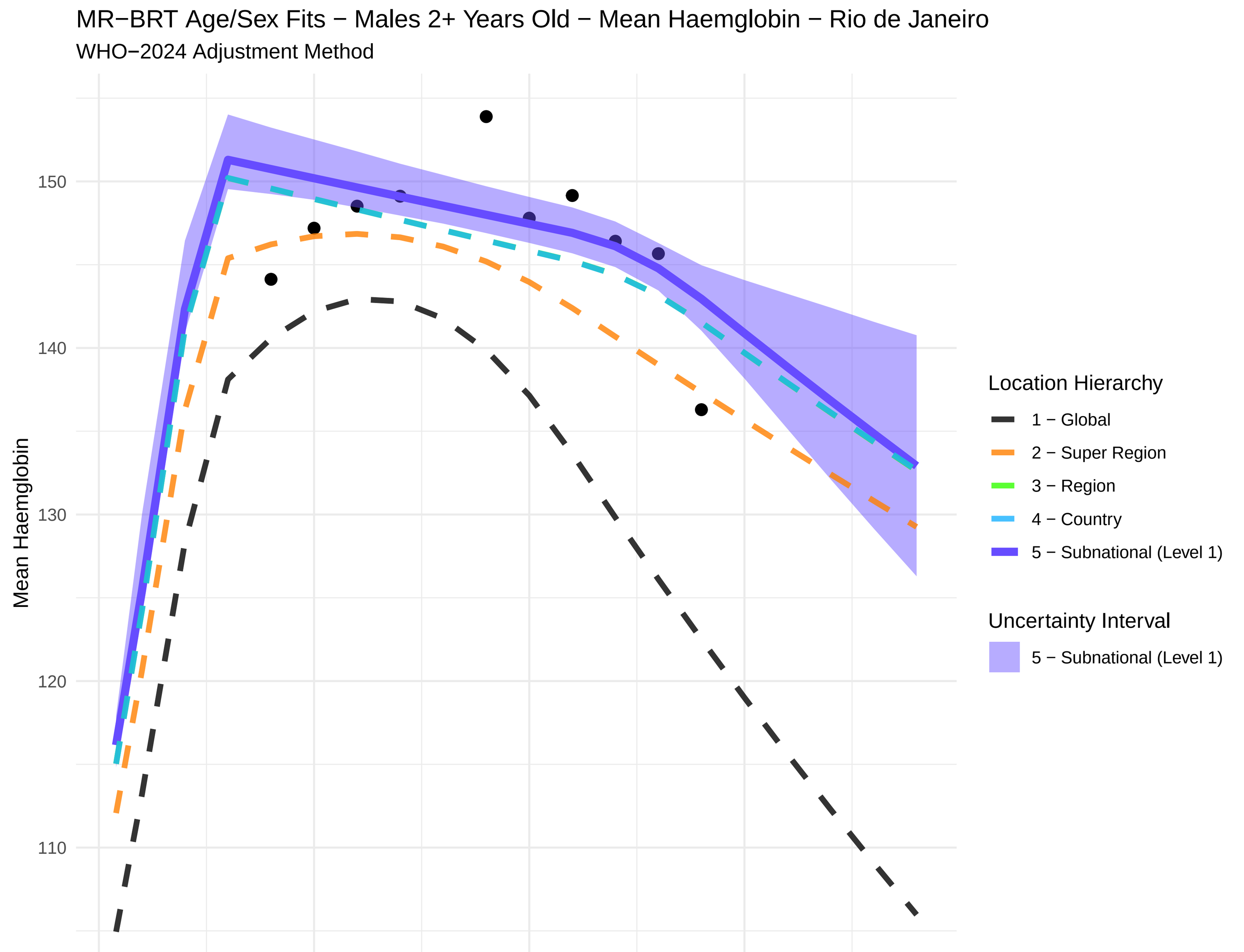
MR−BRT Age/Sex Fits − Males 2+ Years Old − Mean Haemglobin − Rio de Janeiro
WHO−2024 Adjustment Method
Mean Haemglobin
Age (year)
Location Hierarchy
1 − Global
2 − Super Region
3 − Region
4 − Country
5 − Subnational (Level 1)
Uncertainty Interval
5 − Subnational (Level 1)

MR–BRT Age/Sex Fits – Males 2+ Years Old – Mean Haemglobin – Rio Grande do Norte
WHO–2024 Adjustment Method

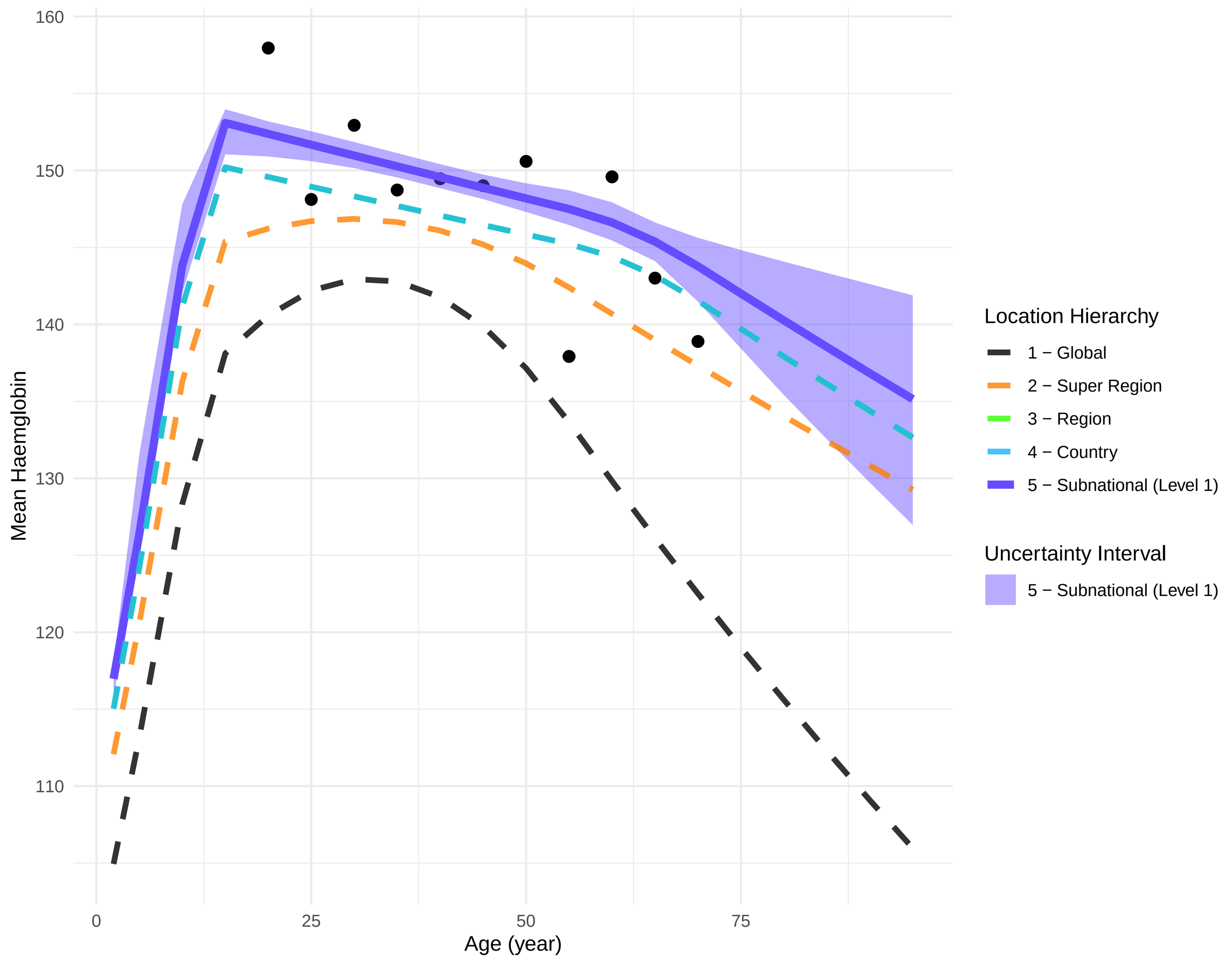

MR–BRT Age/Sex Fits – Males 2+ Years Old – Mean Haemglobin – Rio Grande do Sul
WHO–2024 Adjustment Method

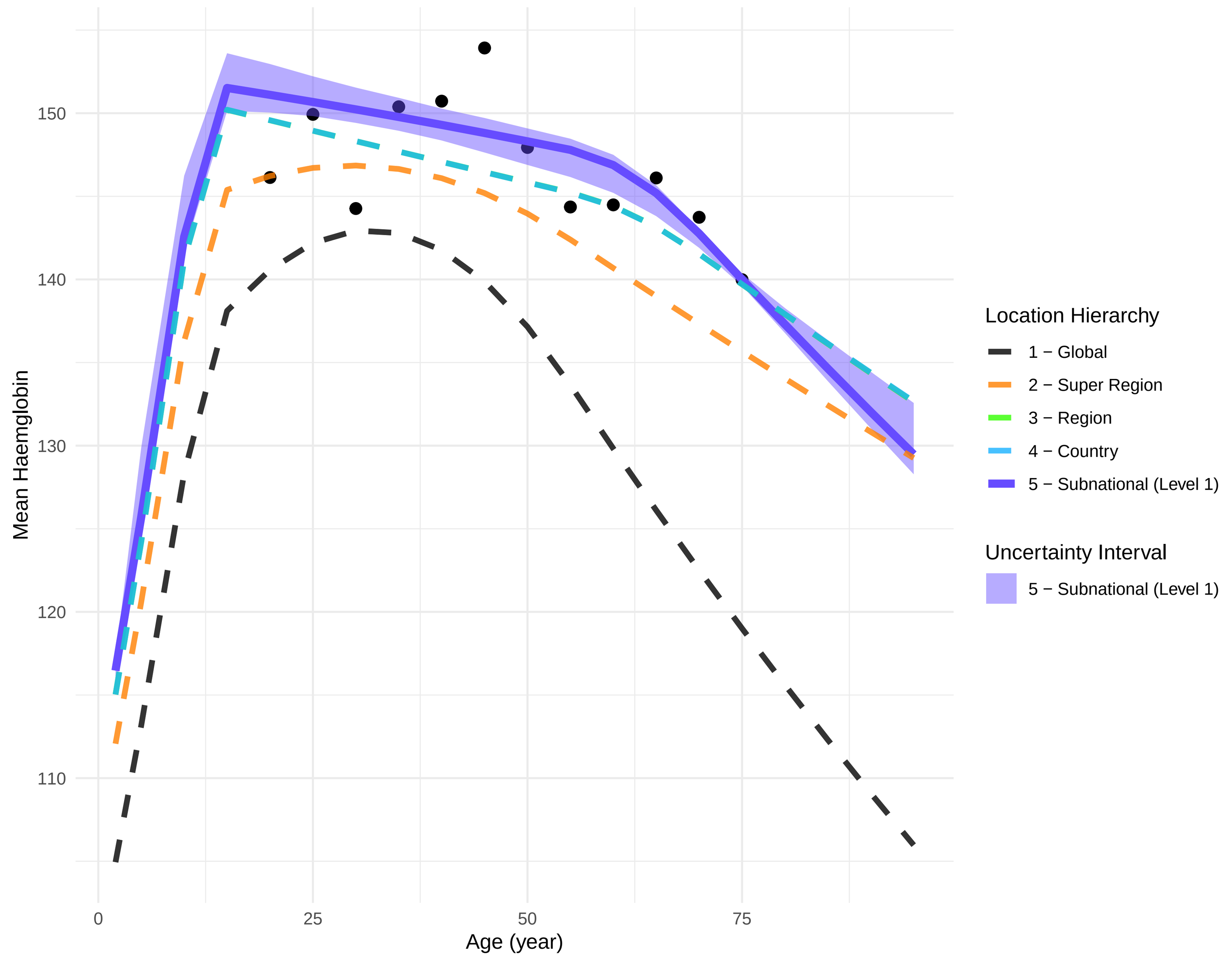

MR–BRT Age/Sex Fits – Males 2+ Years Old – Mean Haemglobin – Rondônia
WHO–2024 Adjustment Method

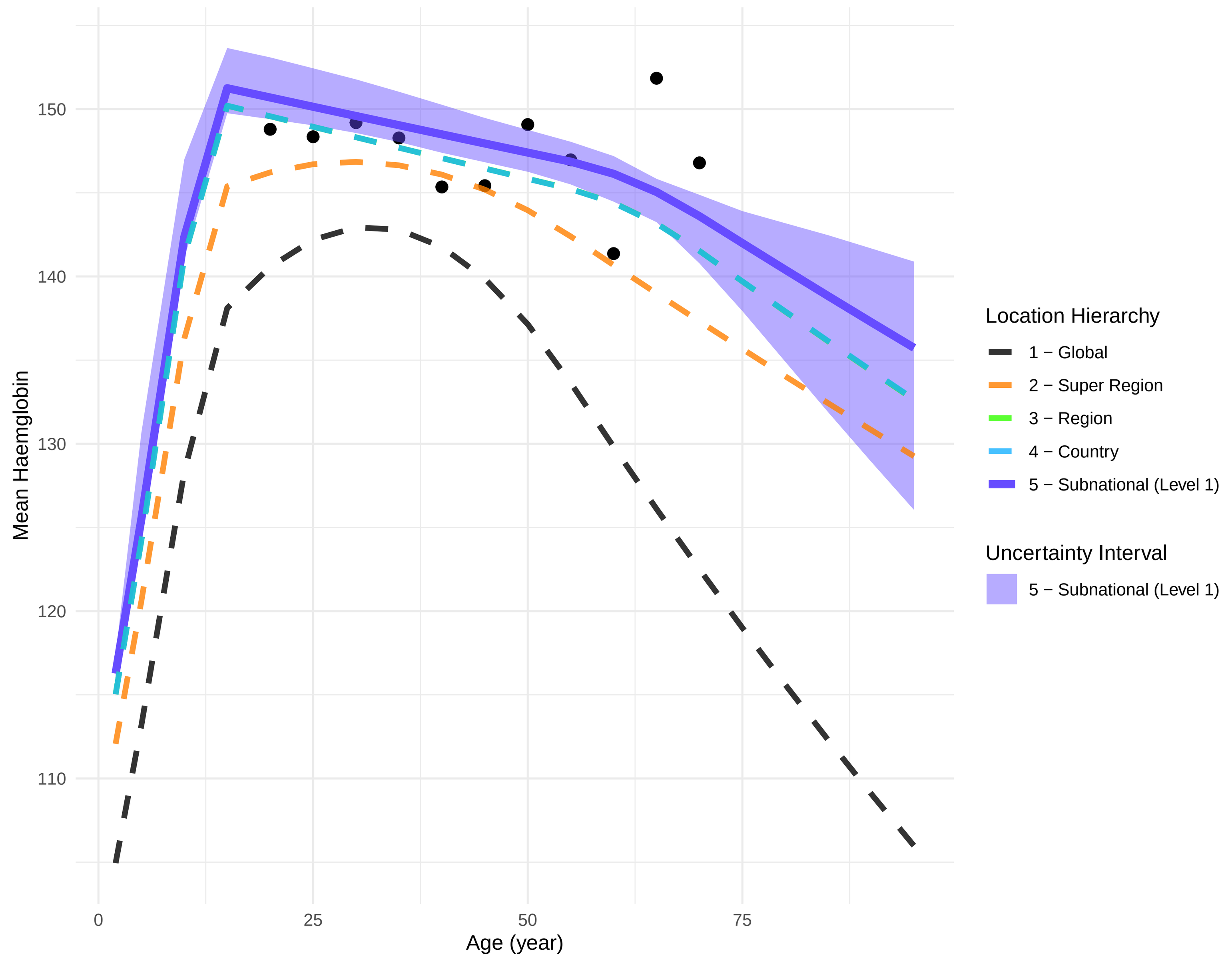

MR–BRT Age/Sex Fits – Males 2+ Years Old – Mean Haemglobin – Roraima
WHO–2024 Adjustment Method

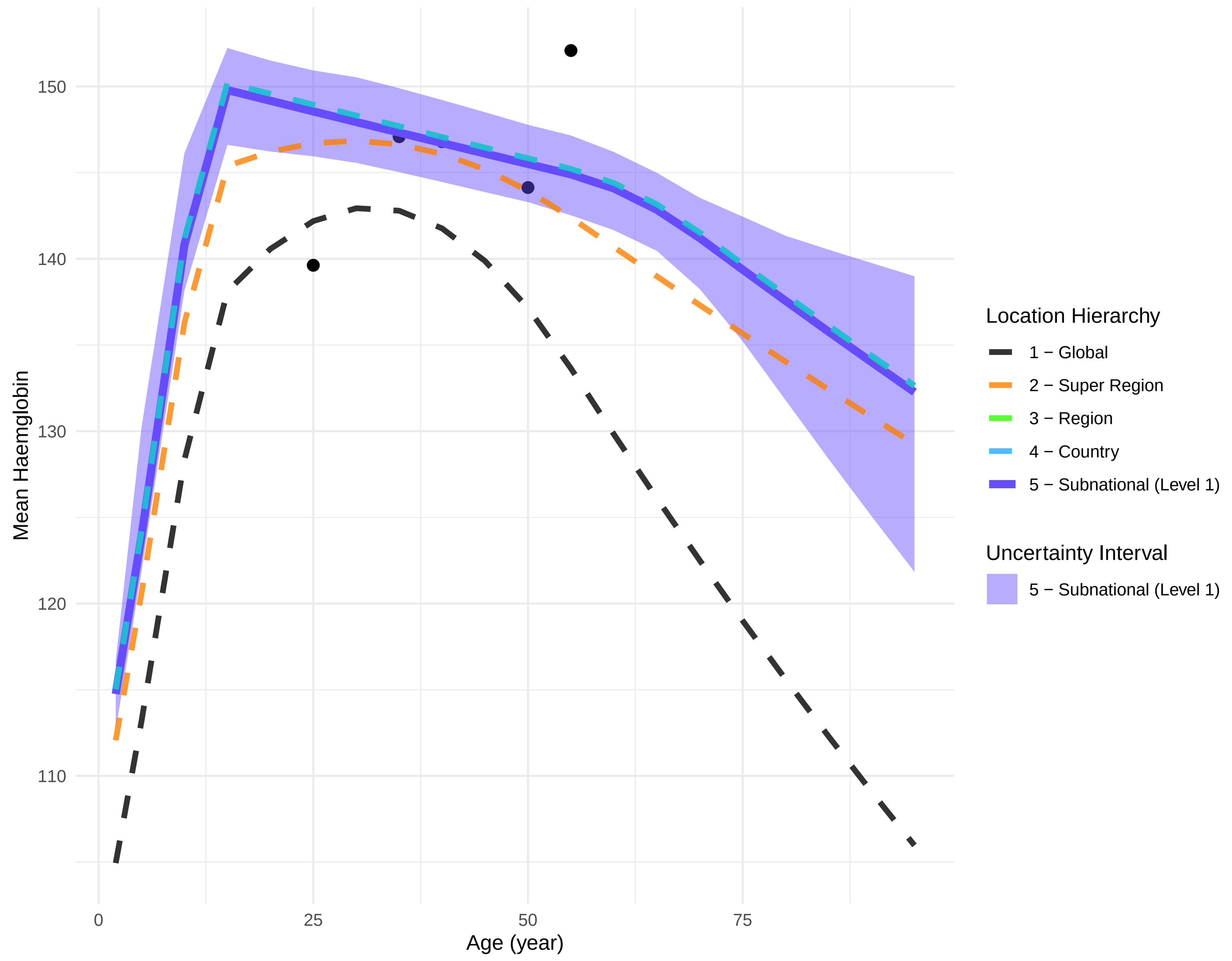

MR–BRT Age/Sex Fits – Males 2+ Years Old – Mean Haemglobin – Santa Catarina
WHO–2024 Adjustment Method

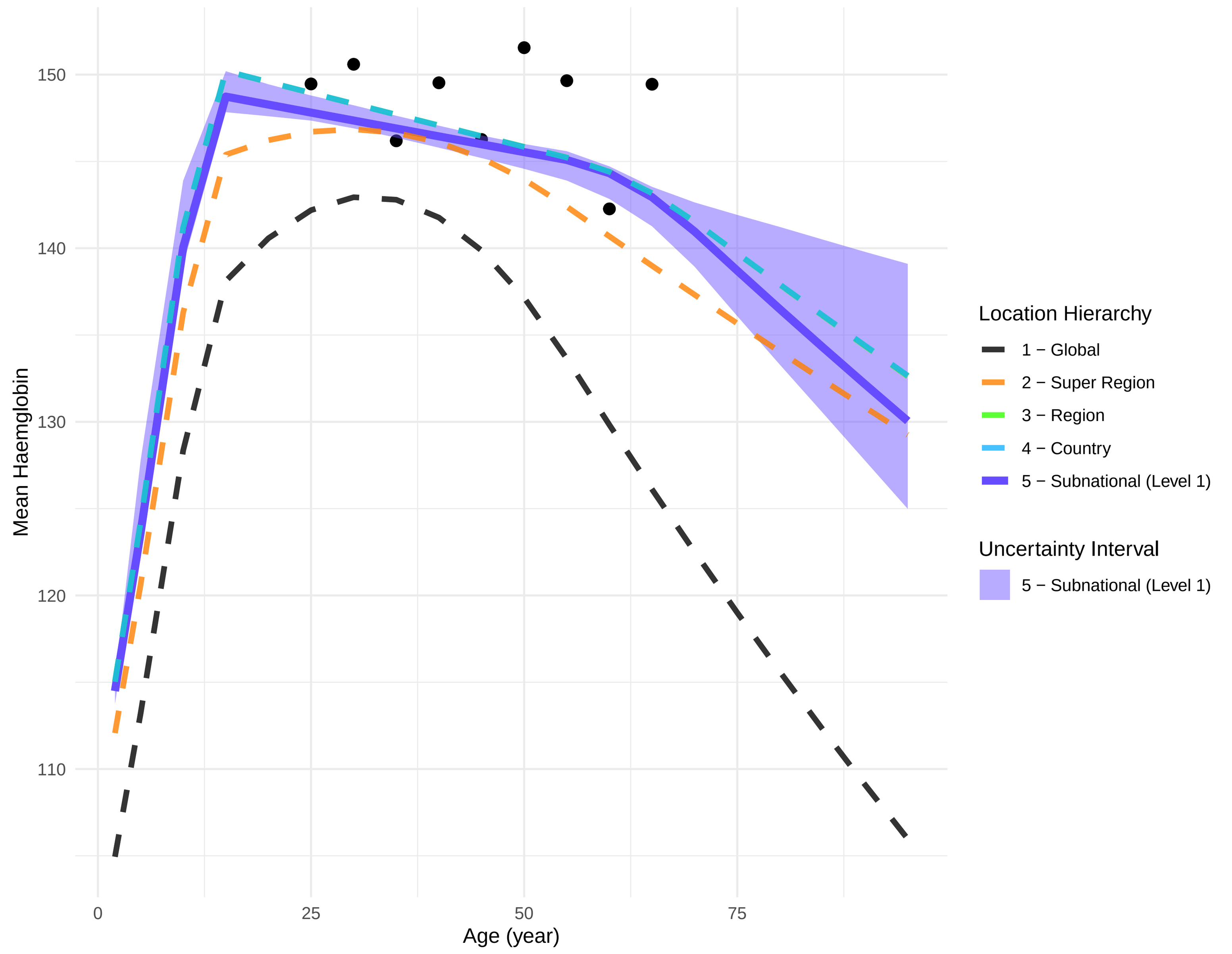

MR–BRT Age/Sex Fits – Males 2+ Years Old – Mean Haemglobin – São Paulo
WHO–2024 Adjustment Method

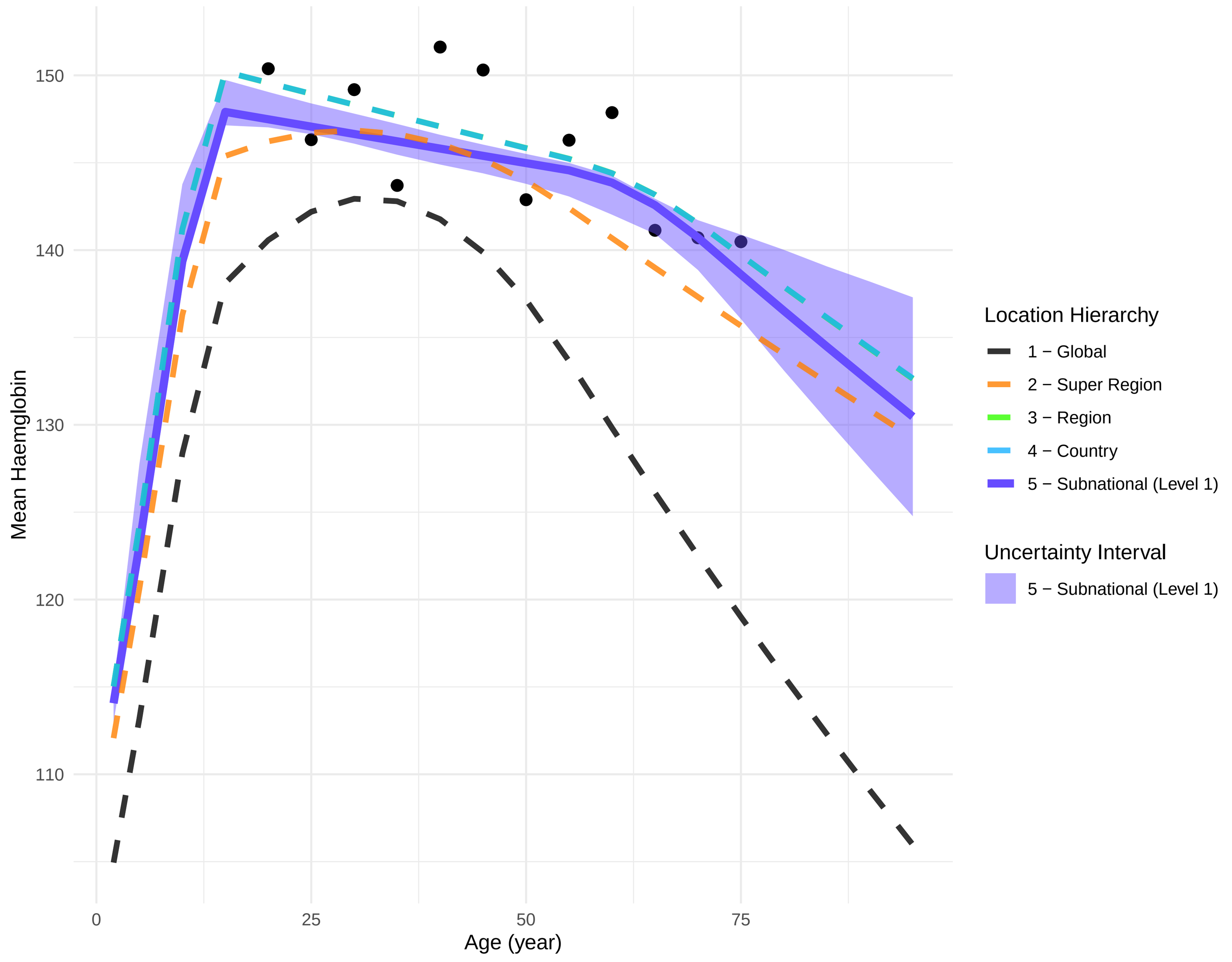

MR–BRT Age/Sex Fits – Males 2+ Years Old – Mean Haemglobin – Sergipe
WHO–2024 Adjustment Method

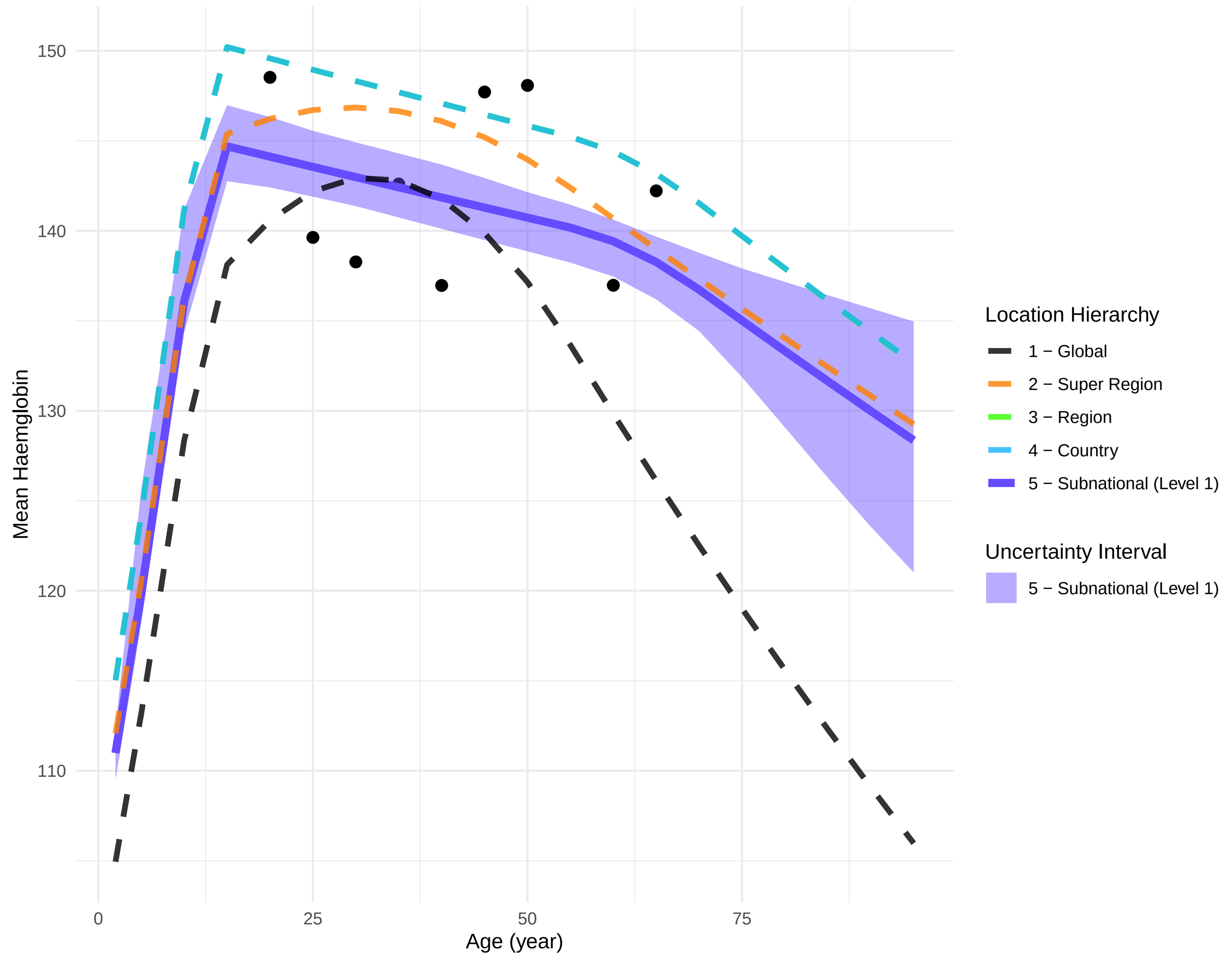

MR–BRT Age/Sex Fits – Males 2+ Years Old – Mean Haemglobin – Tocantins
WHO–2024 Adjustment Method

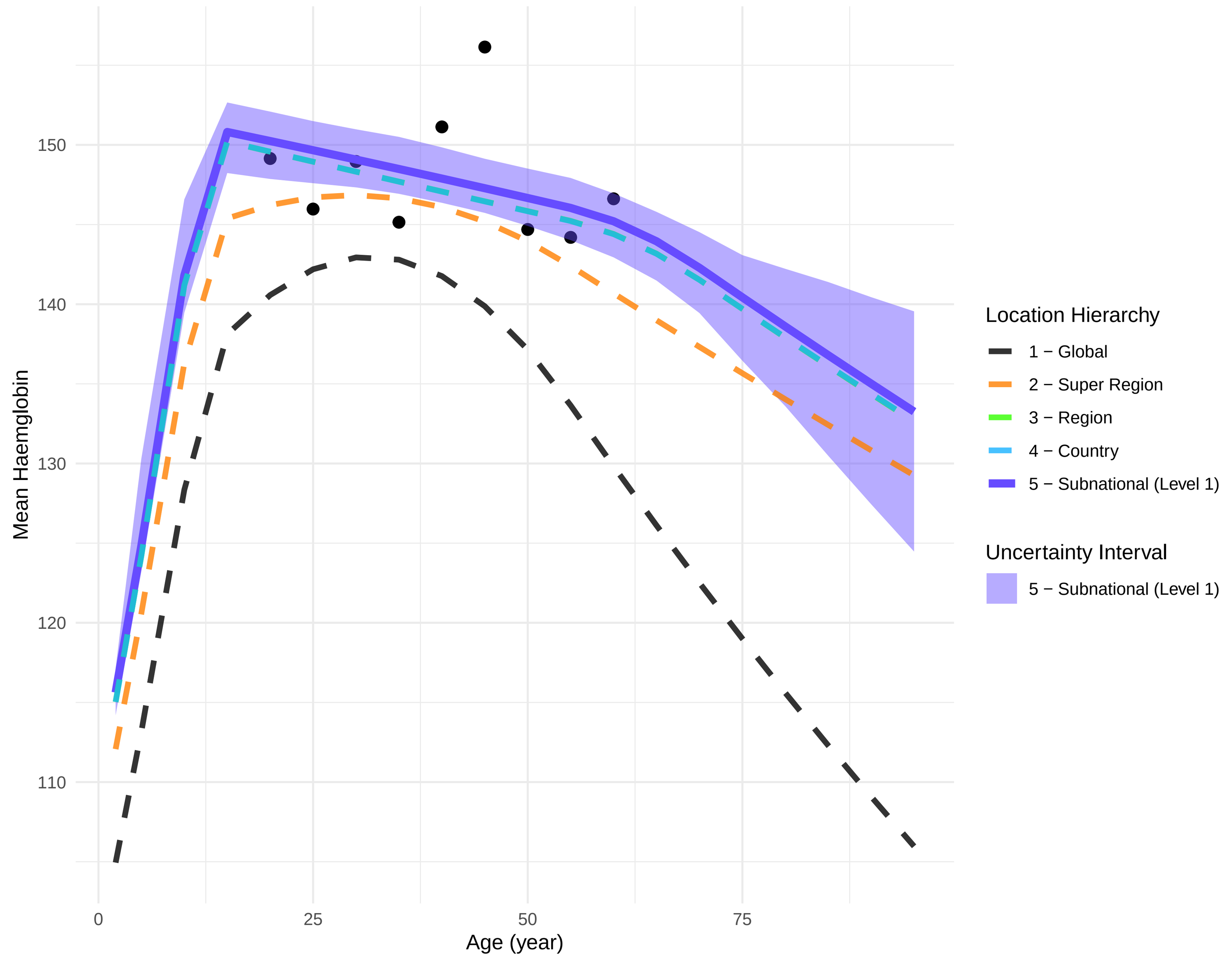

MR–BRT Age/Sex Fits – Males 2+ Years Old – Mean Haemglobin – North Africa and Middle East
WHO–2024 Adjustment Method

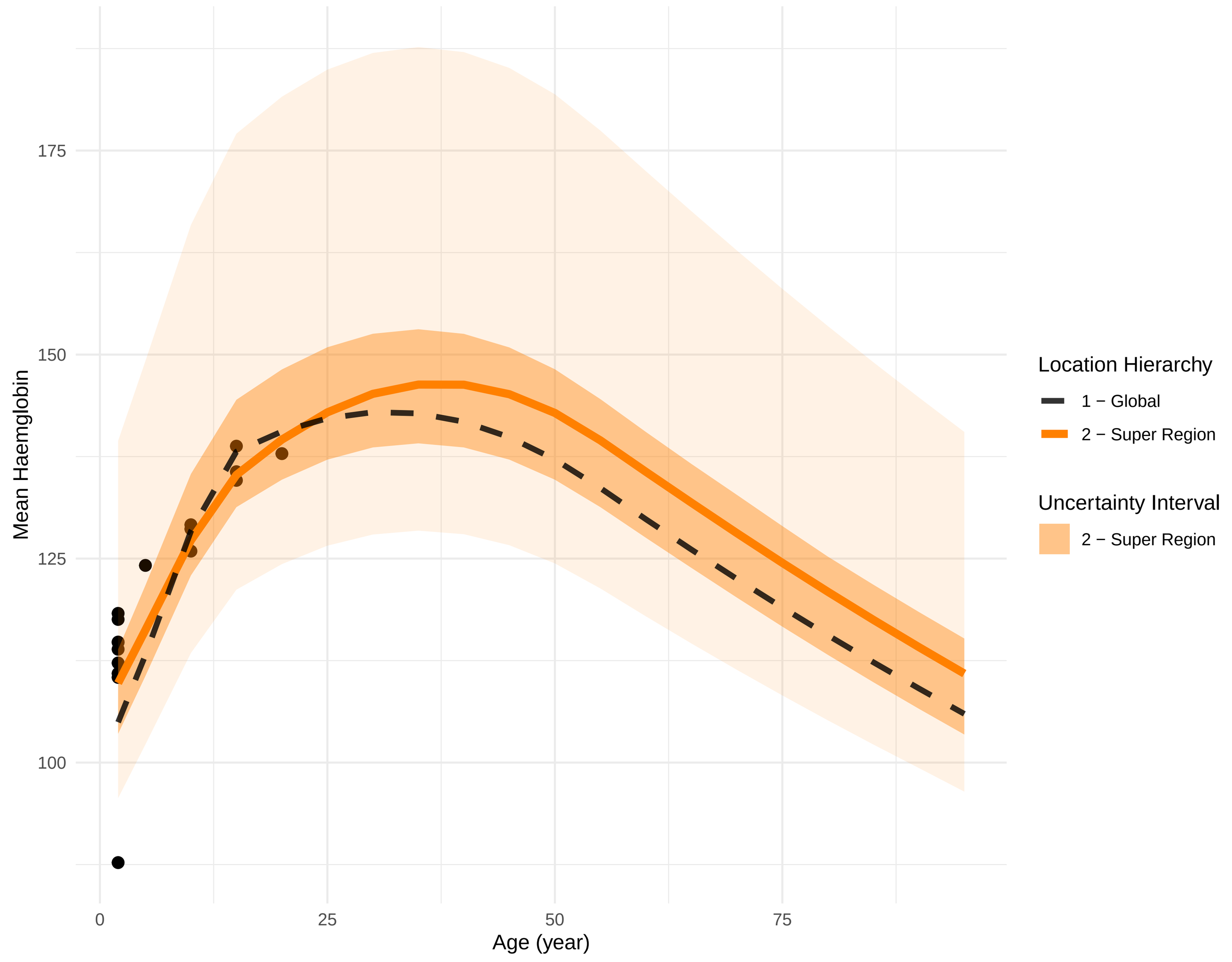

MR–BRT Age/Sex Fits – Males 2+ Years Old – Mean Haemglobin – North Africa and Middle East
WHO–2024 Adjustment Method

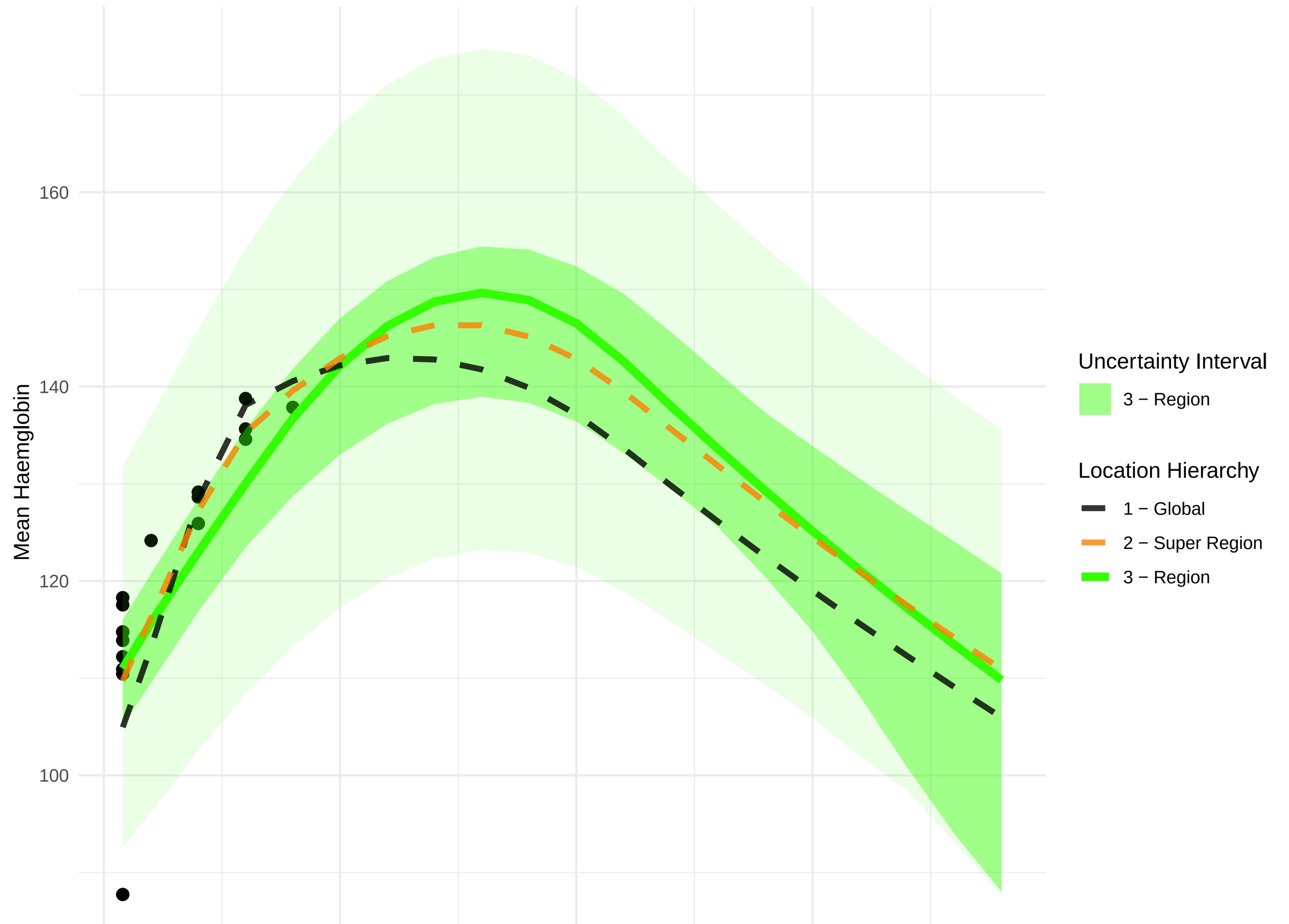

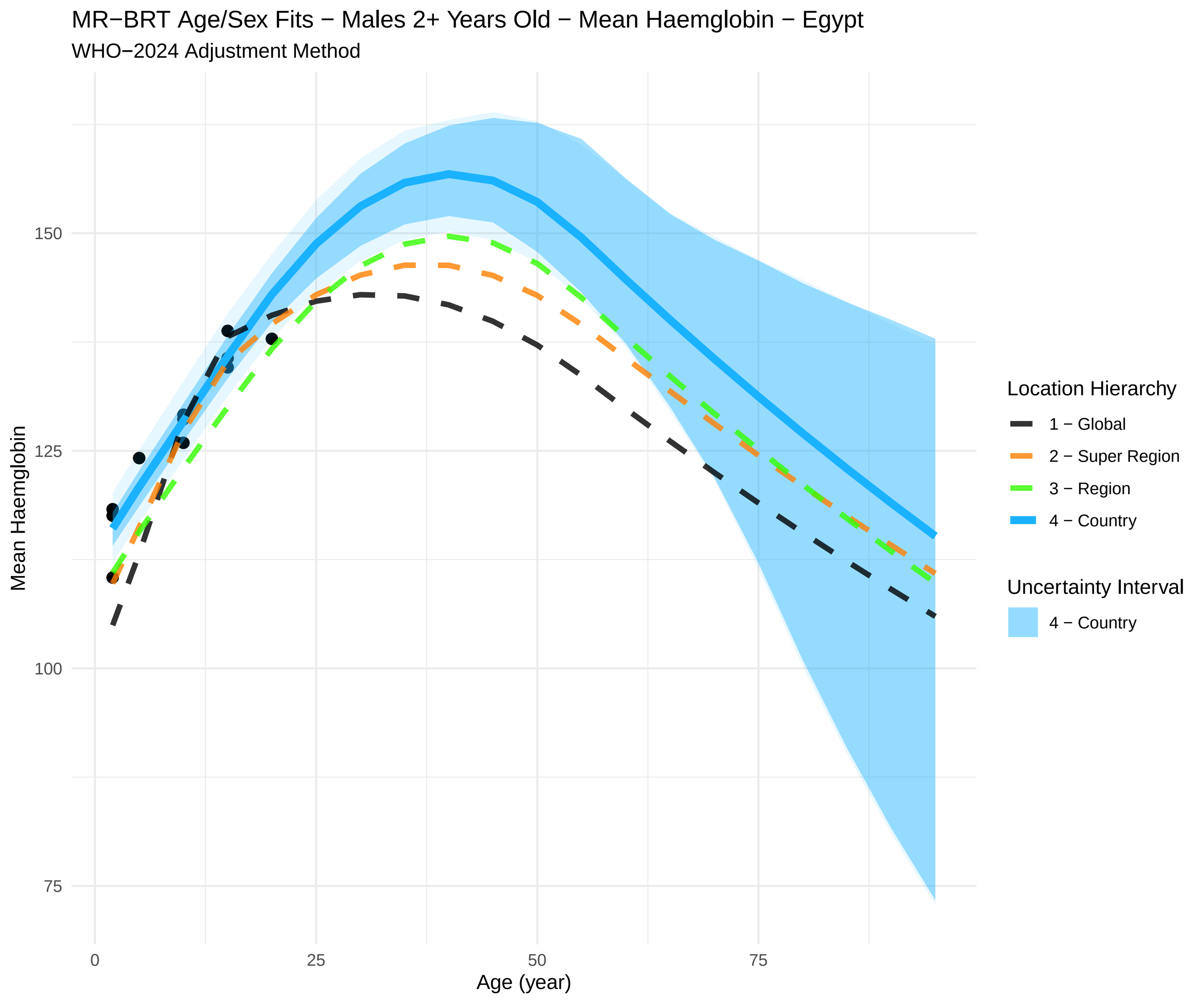
MR−BRT Age/Sex Fits − Males 2+ Years Old − Mean Haemoglobin − Egypt
WHO−2024 Adjustment Method
Mean Haemoglobin
Age (year)
Location Hierarchy
1 − Global
2 − Super Region
3 − Region
4 − Country
Uncertainty Interval
4 − Country

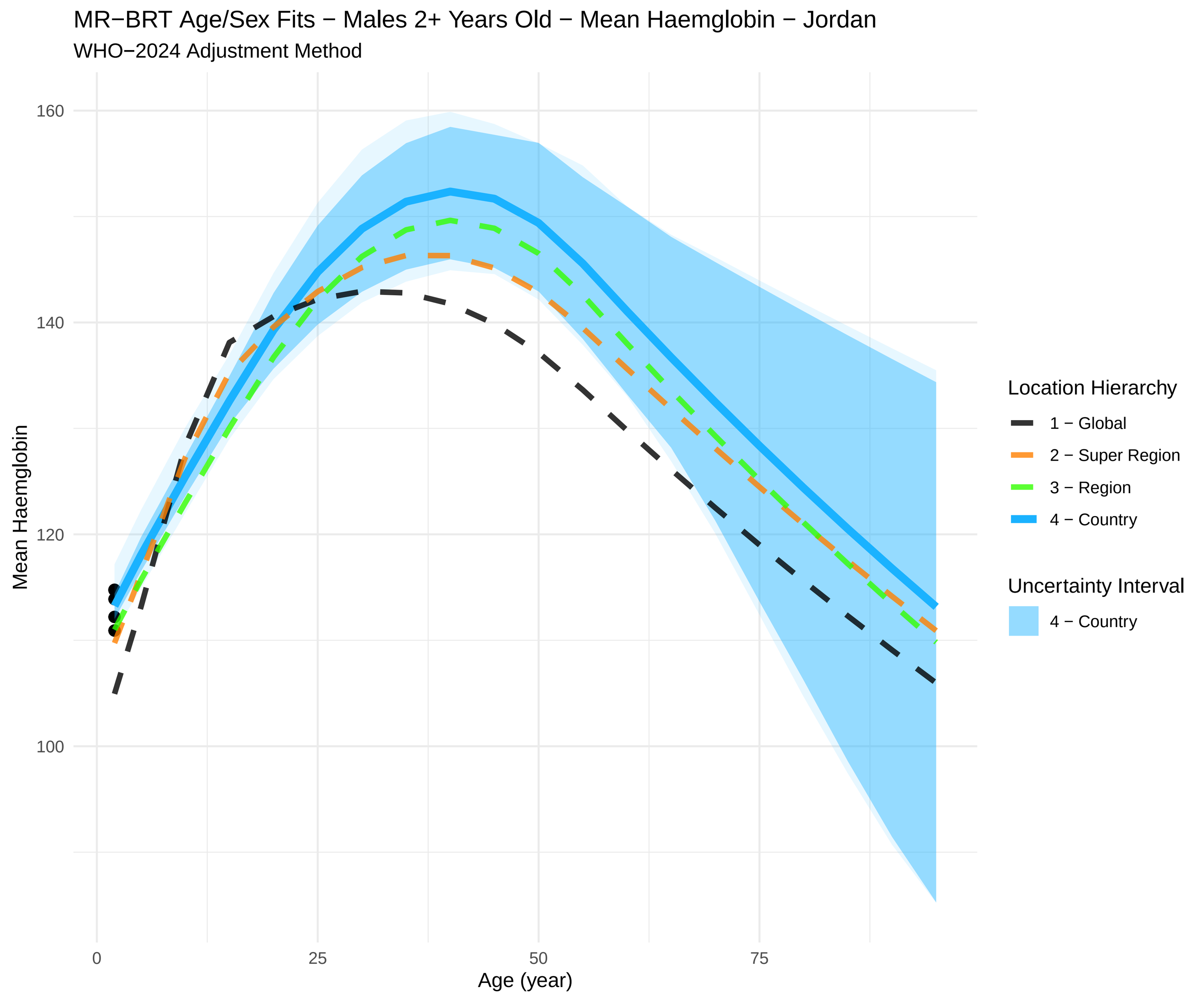
MR−BRT Age/Sex Fits − Males 2+ Years Old − Mean Haemoglobin − Jordan
WHO−2024 Adjustment Method
Mean Haemoglobin
Age (year)
Location Hierarchy
1 − Global
2 − Super Region
3 − Region
4 − Country
Uncertainty Interval
4 − Country

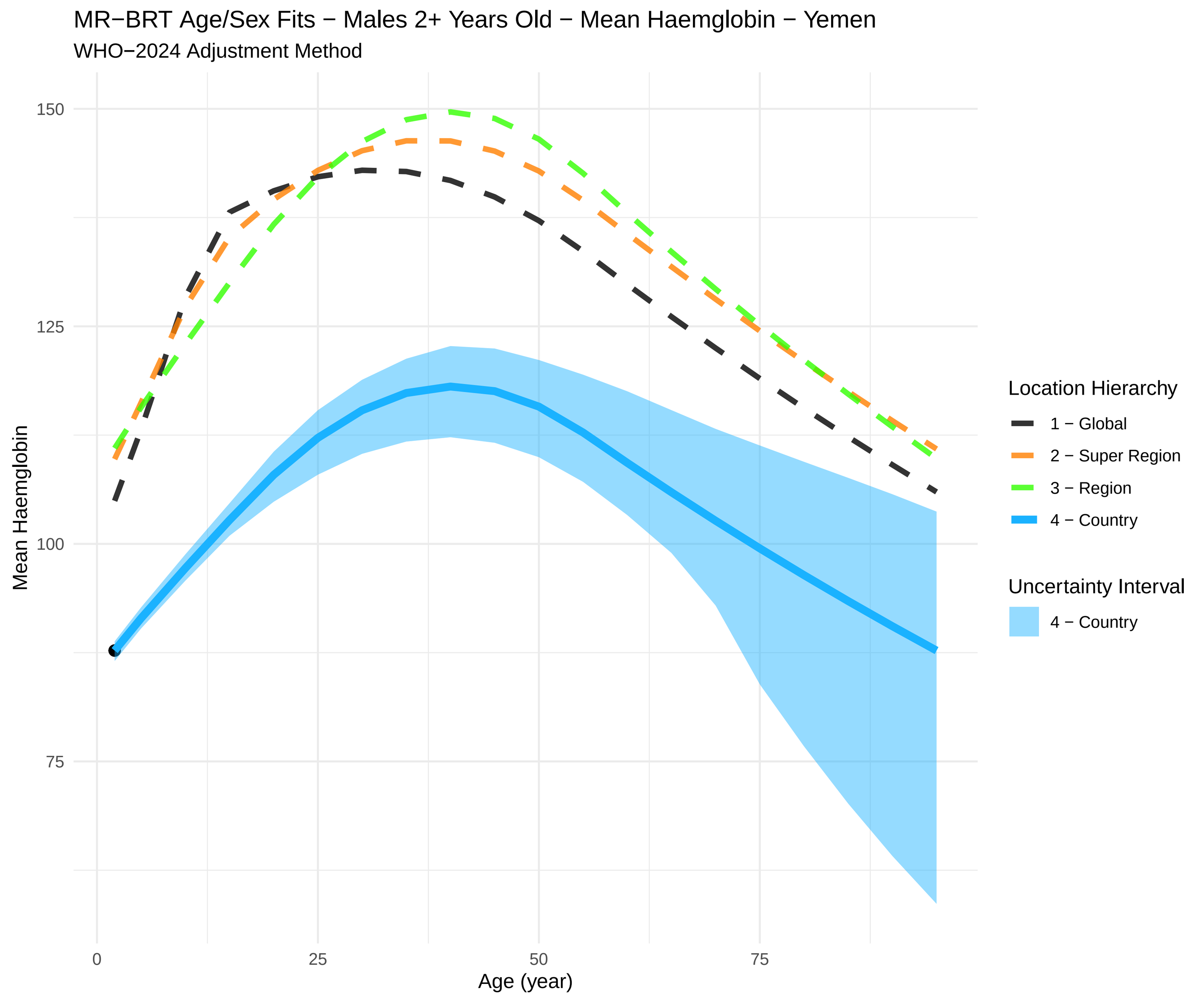
MR−BRT Age/Sex Fits − Males 2+ Years Old − Mean Haemoglobin − Yemen
WHO−2024 Adjustment Method
Mean Haemoglobin
Age (year)
Location Hierarchy
1 − Global
2 − Super Region
3 − Region
4 − Country
Uncertainty Interval
4 − Country

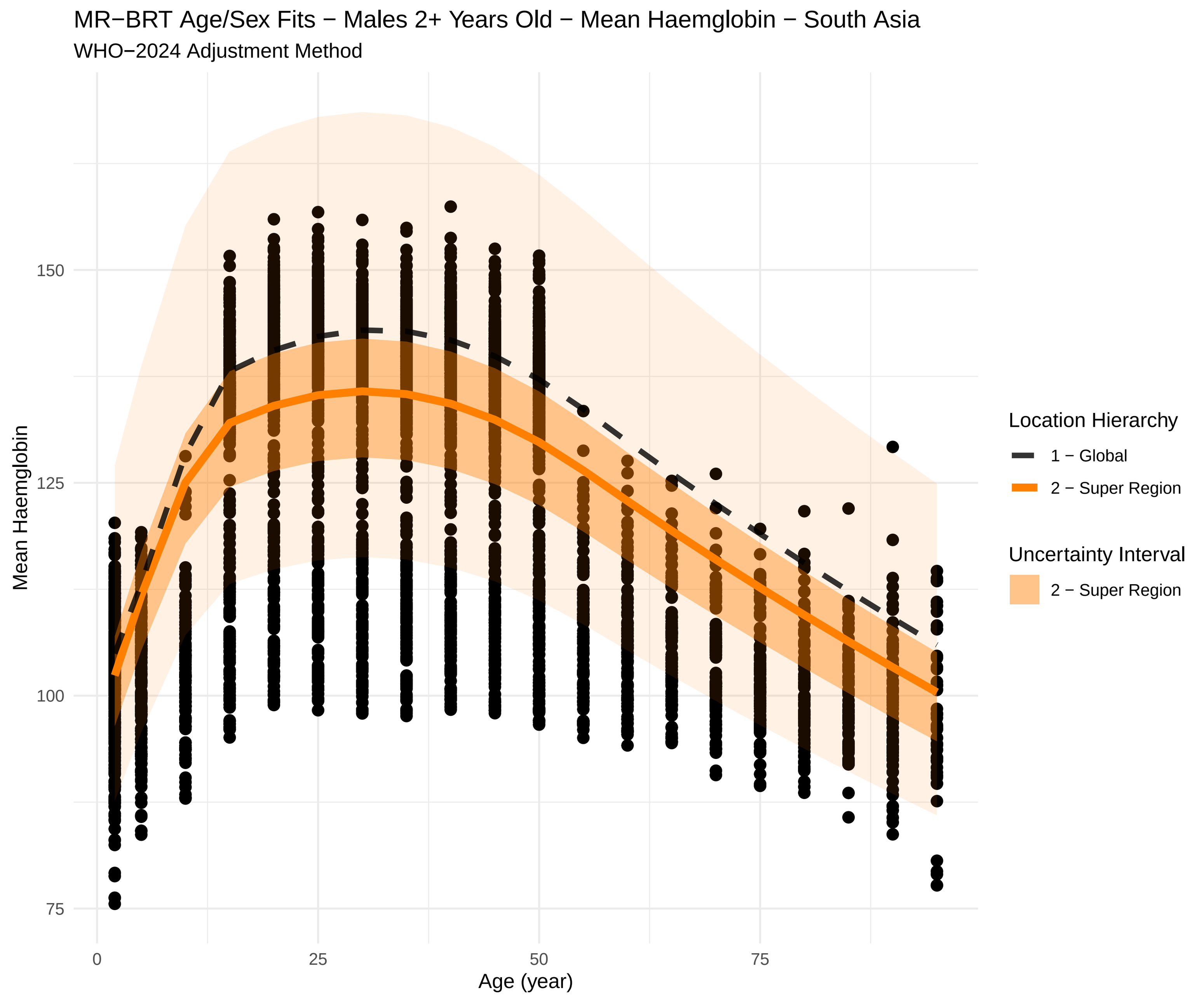
MR−BRT Age/Sex Fits − Males 2+ Years Old − Mean Haemoglobin − South Asia
WHO−2024 Adjustment Method
Mean Haemoglobin
Age (year)
Location Hierarchy
1 − Global
2 − Super Region
Uncertainty Interval
2 − Super Region

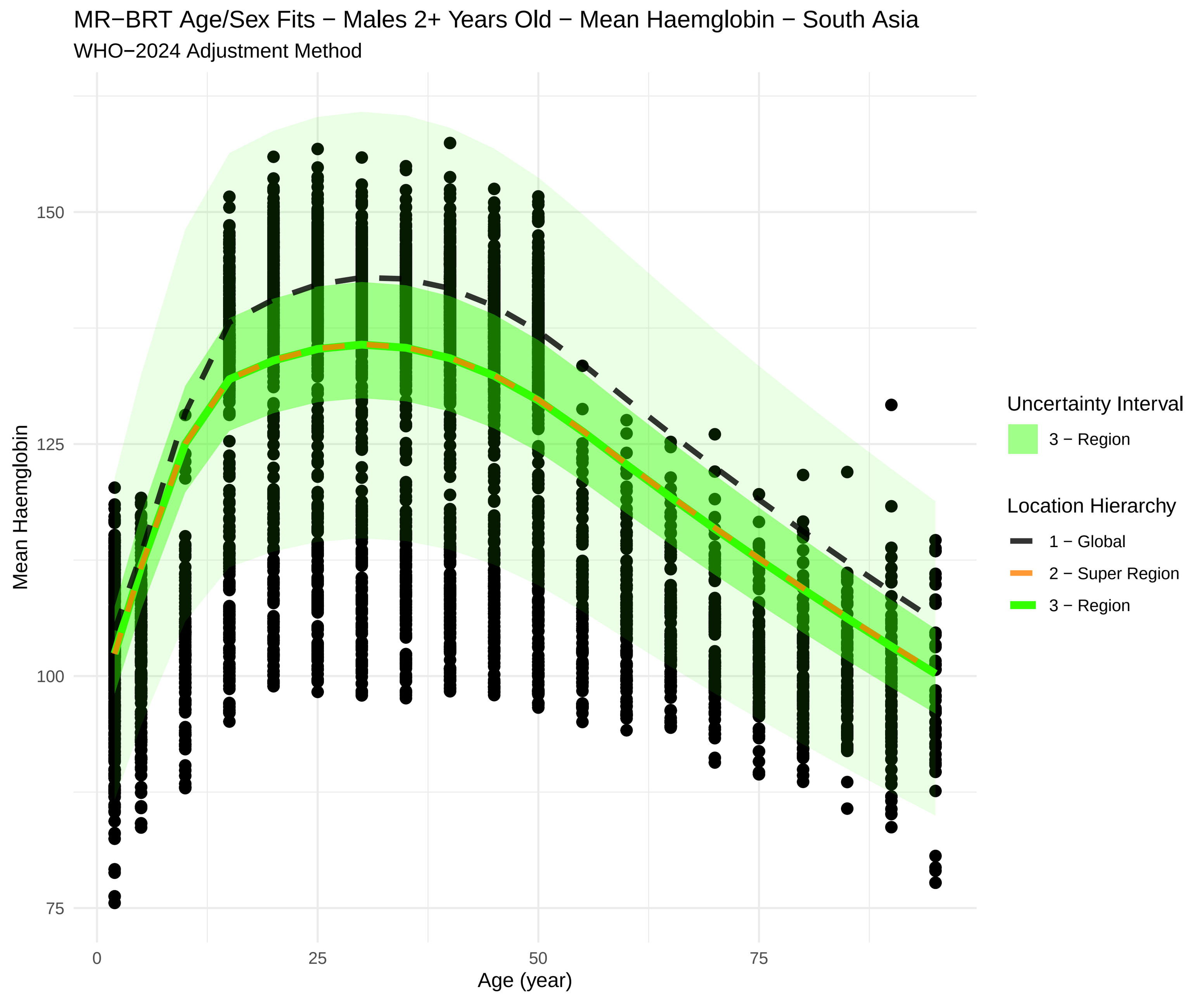
MR−BRT Age/Sex Fits − Males 2+ Years Old − Mean Haemoglobin − South Asia
WHO−2024 Adjustment Method
Mean Haemoglobin
Age (year)
Uncertainty Interval
3 − Region
Location Hierarchy
1 − Global
2 − Super Region
3 − Region

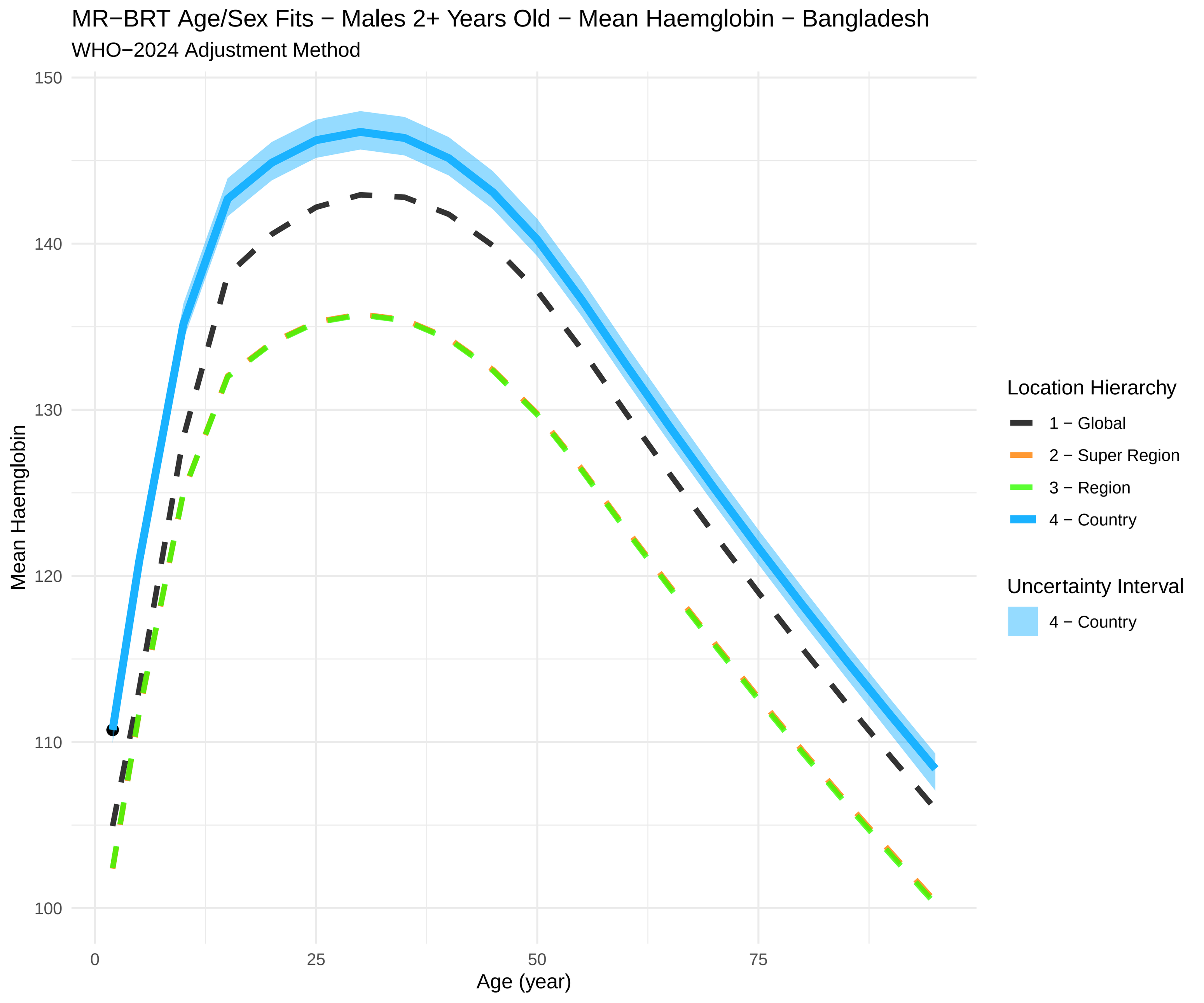
MR−BRT Age/Sex Fits − Males 2+ Years Old − Mean Haemoglobin − Bangladesh
WHO−2024 Adjustment Method
Mean Haemoglobin
Age (year)
Location Hierarchy
1 − Global
2 − Super Region
3 − Region
4 − Country
Uncertainty Interval
4 − Country

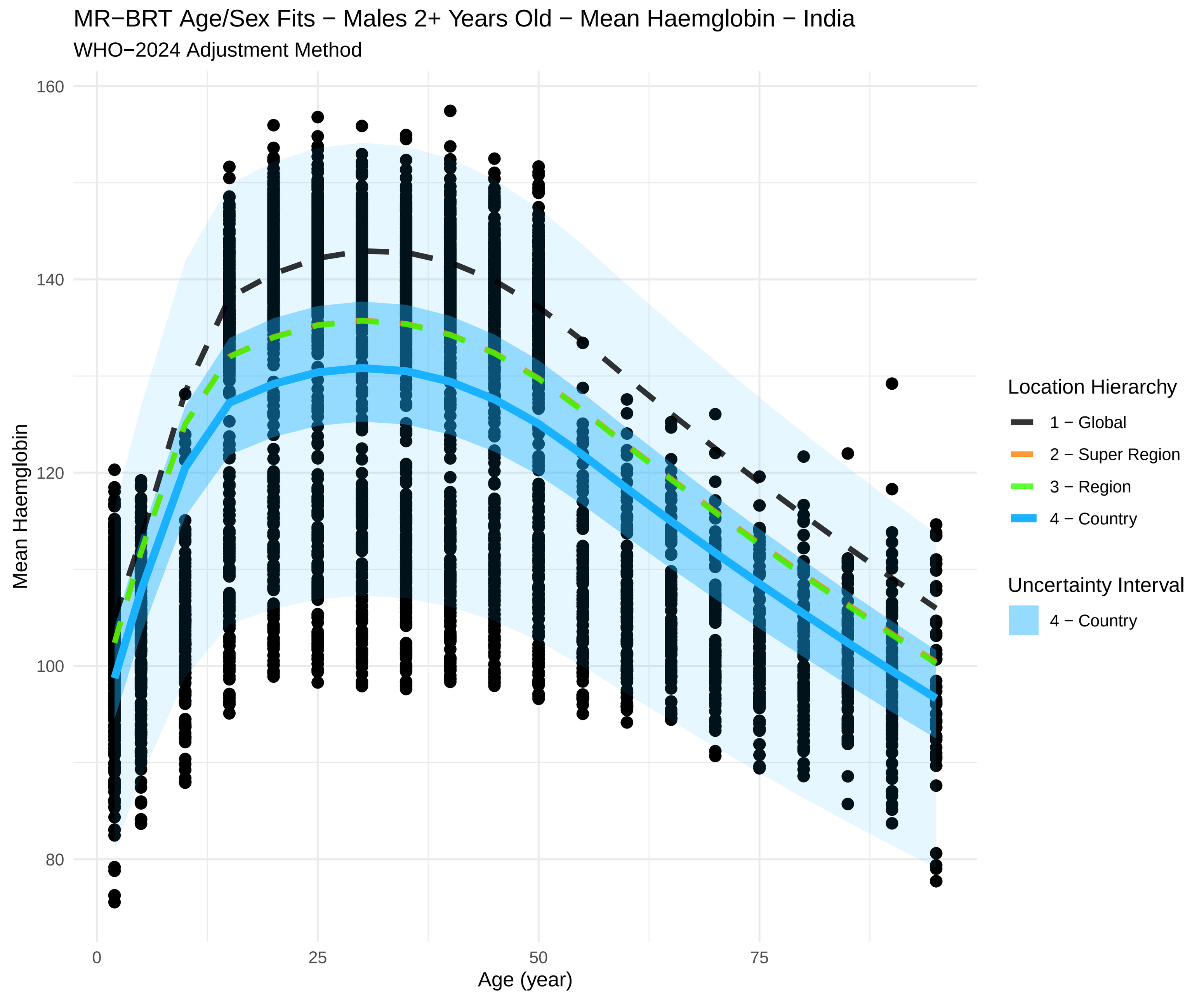
MR−BRT Age/Sex Fits − Males 2+ Years Old − Mean Haemoglobin − India
WHO−2024 Adjustment Method
Mean Haemoglobin
Age (year)
Location Hierarchy
1 − Global
2 − Super Region
3 − Region
4 − Country
Uncertainty Interval
4 − Country

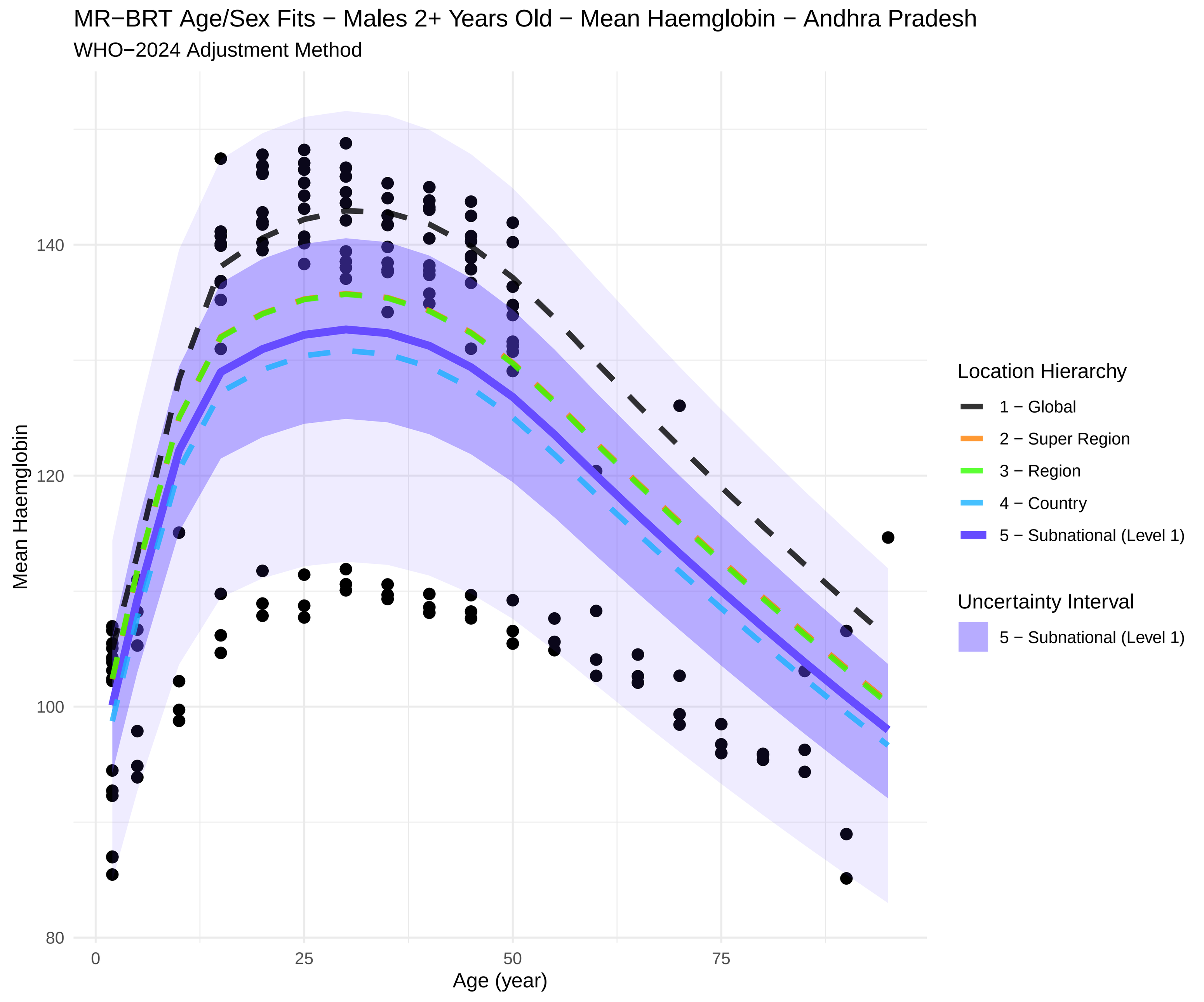
MR−BRT Age/Sex Fits − Males 2+ Years Old − Mean Haemoglobin − Andhra Pradesh
WHO−2024 Adjustment Method
Mean Haemoglobin
Age (year)
Location Hierarchy
1 − Global
2 − Super Region
3 − Region
4 − Country
5 − Subnational (Level 1)
Uncertainty Interval
5 − Subnational (Level 1)

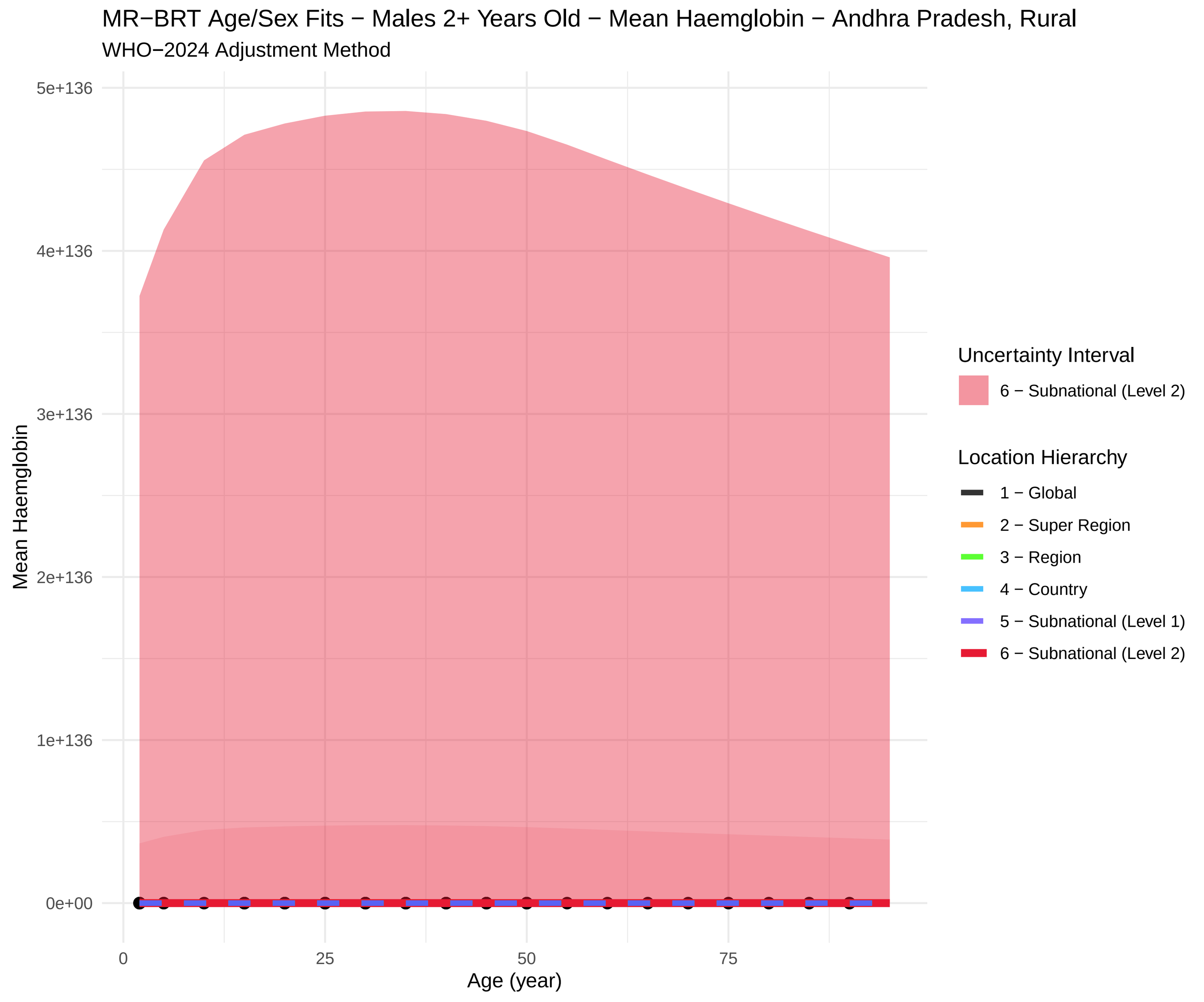
MR−BRT Age/Sex Fits − Males 2+ Years Old − Mean Haemoglobin − Andhra Pradesh, Rural
WHO−2024 Adjustment Method
Mean Haemoglobin
Age (year)
Uncertainty Interval
6 − Subnational (Level 2)
Location Hierarchy
1 − Global
2 − Super Region
3 − Region
4 − Country
5 − Subnational (Level 1)
6 − Subnational (Level 2)

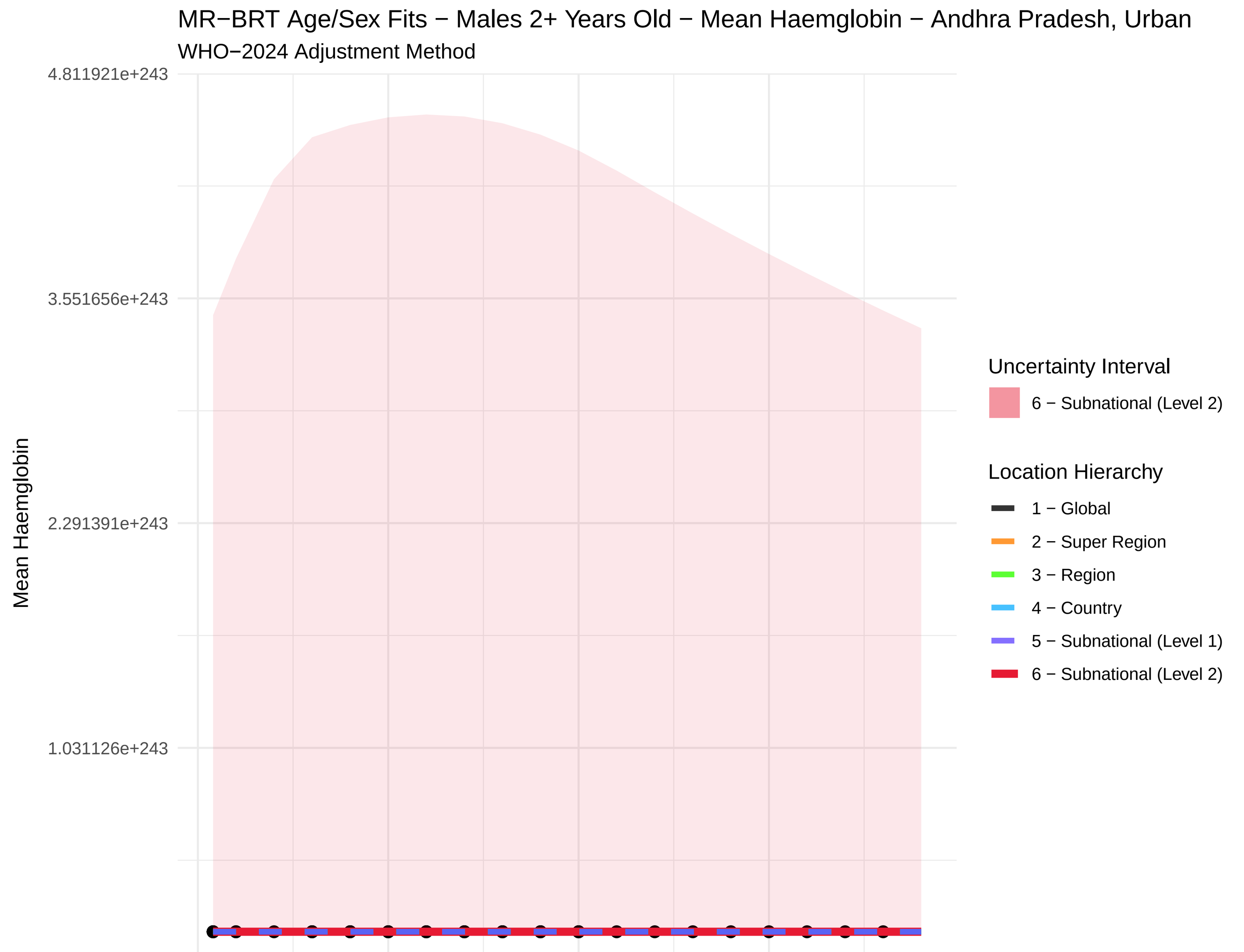
MR−BRT Age/Sex Fits − Males 2+ Years Old − Mean Haemoglobin − Andhra Pradesh, Urban
WHO−2024 Adjustment Method
Mean Haemoglobin
Age (year)
Uncertainty Interval
6 − Subnational (Level 2)
Location Hierarchy
1 − Global
2 − Super Region
3 − Region
4 − Country
5 − Subnational (Level 1)
6 − Subnational (Level 2)

MR−BRT Age/Sex Fits − Males 2+ Years Old − Mean Haemglobin − Arunachal Pradesh
WHO−2024 Adjustment Method

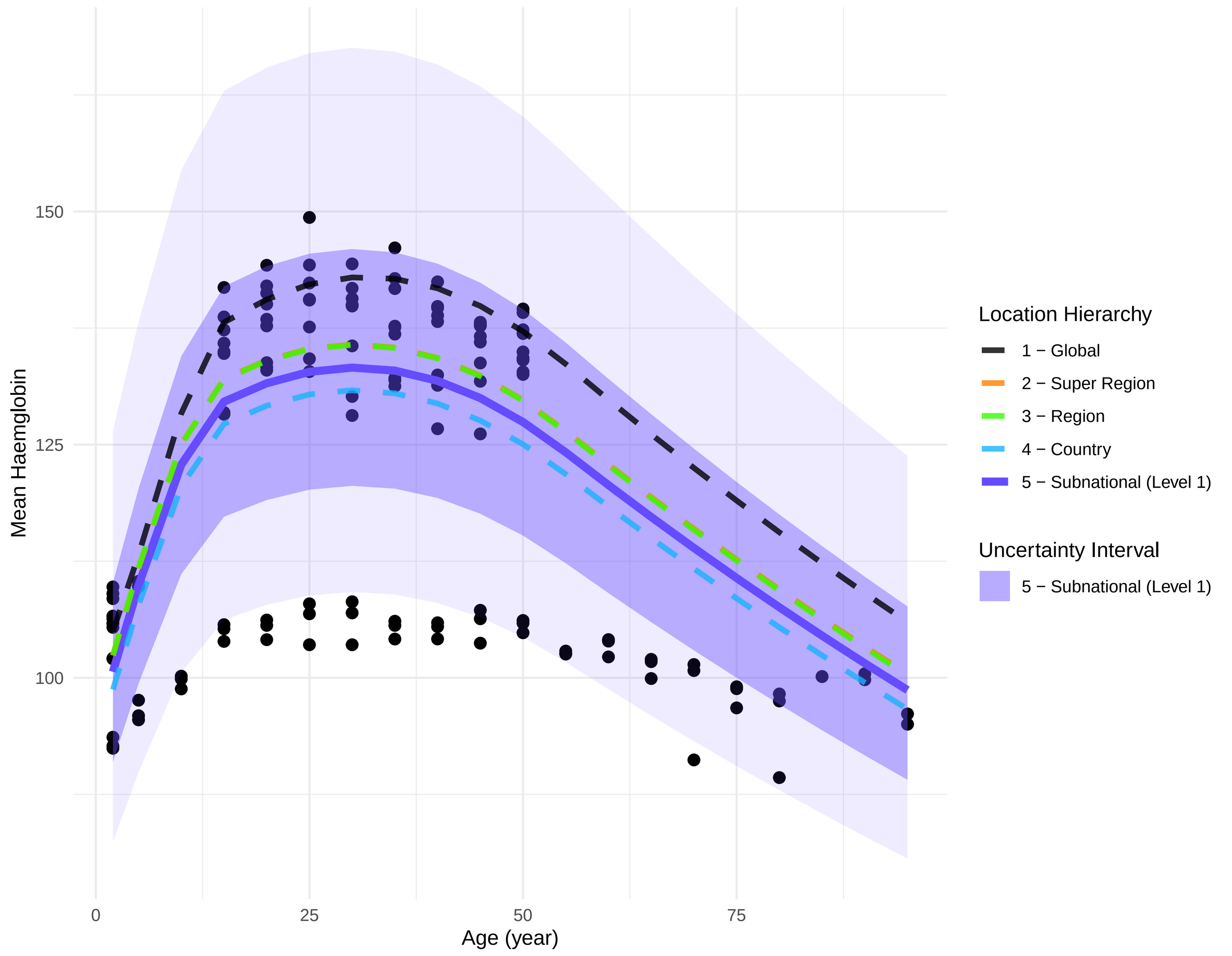

MR−BRT Age/Sex Fits − Males 2+ Years Old − Mean Haemglobin − Arunachal Pradesh, Rural
WHO−2024 Adjustment Method

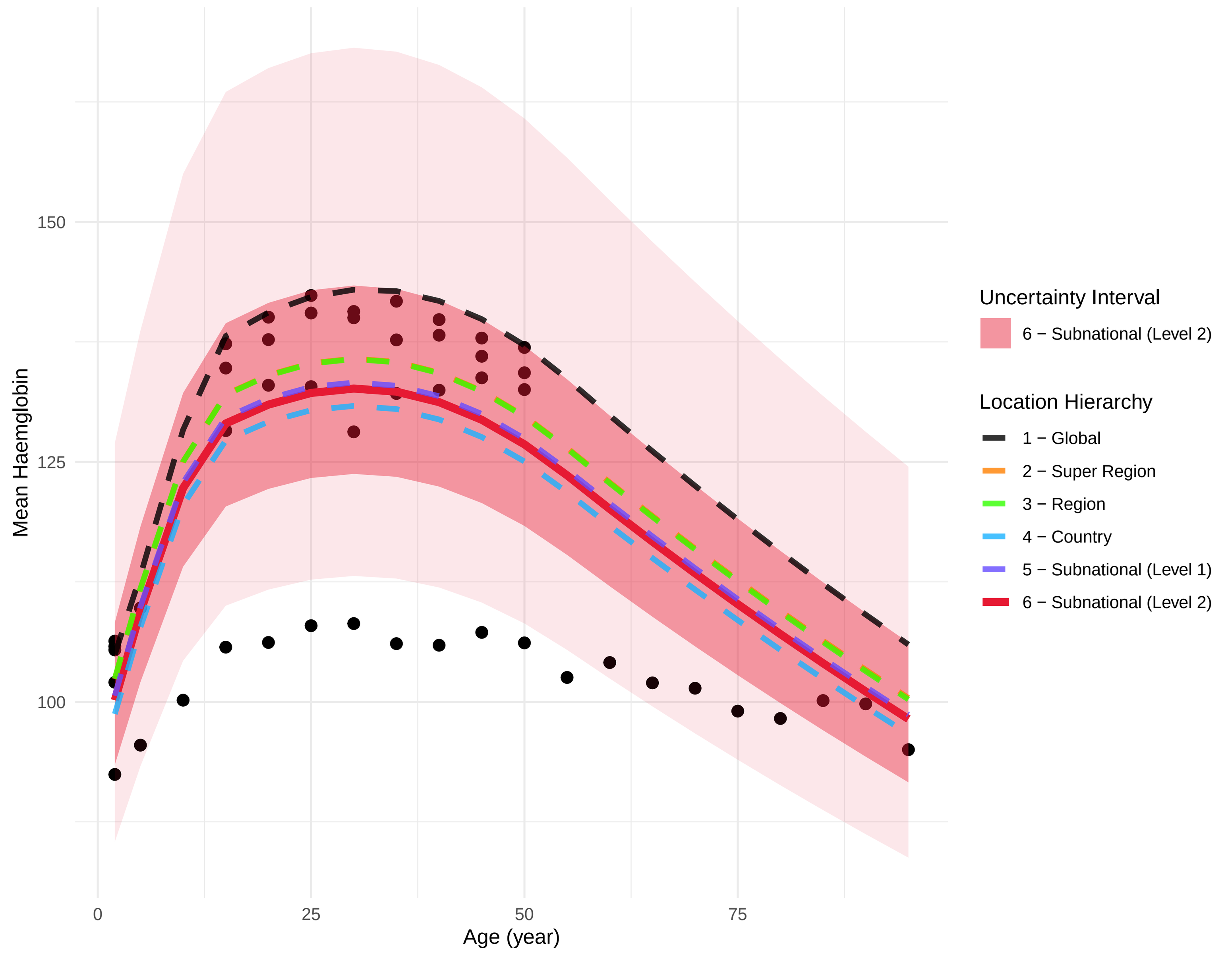

MR−BRT Age/Sex Fits − Males 2+ Years Old − Mean Haemglobin − Arunachal Pradesh, Urban
WHO−2024 Adjustment Method

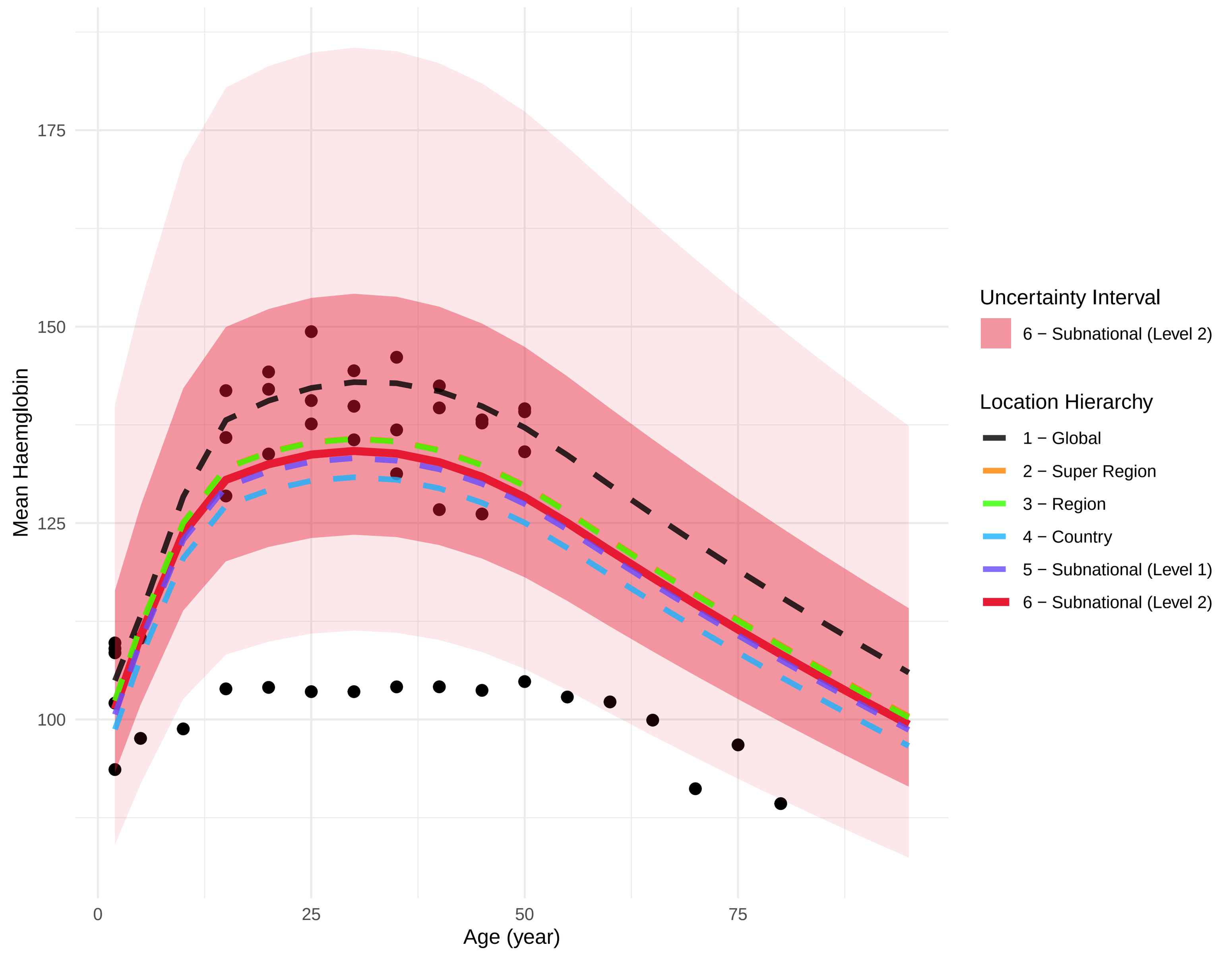

MR−BRT Age/Sex Fits − Males 2+ Years Old − Mean Haemglobin − Assam
WHO−2024 Adjustment Method

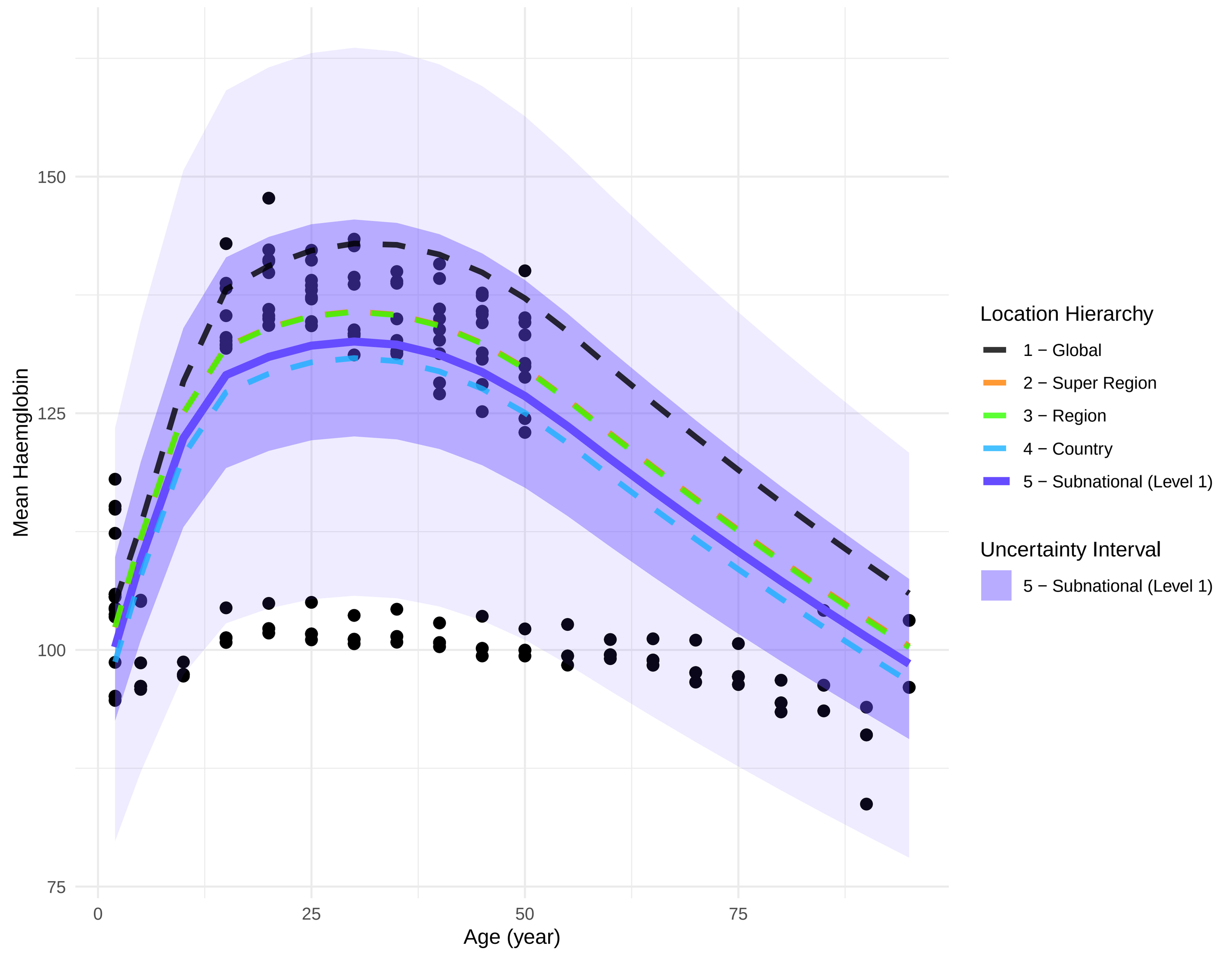

MR−BRT Age/Sex Fits − Males 2+ Years Old − Mean Haemglobin − Assam, Rural
WHO−2024 Adjustment Method

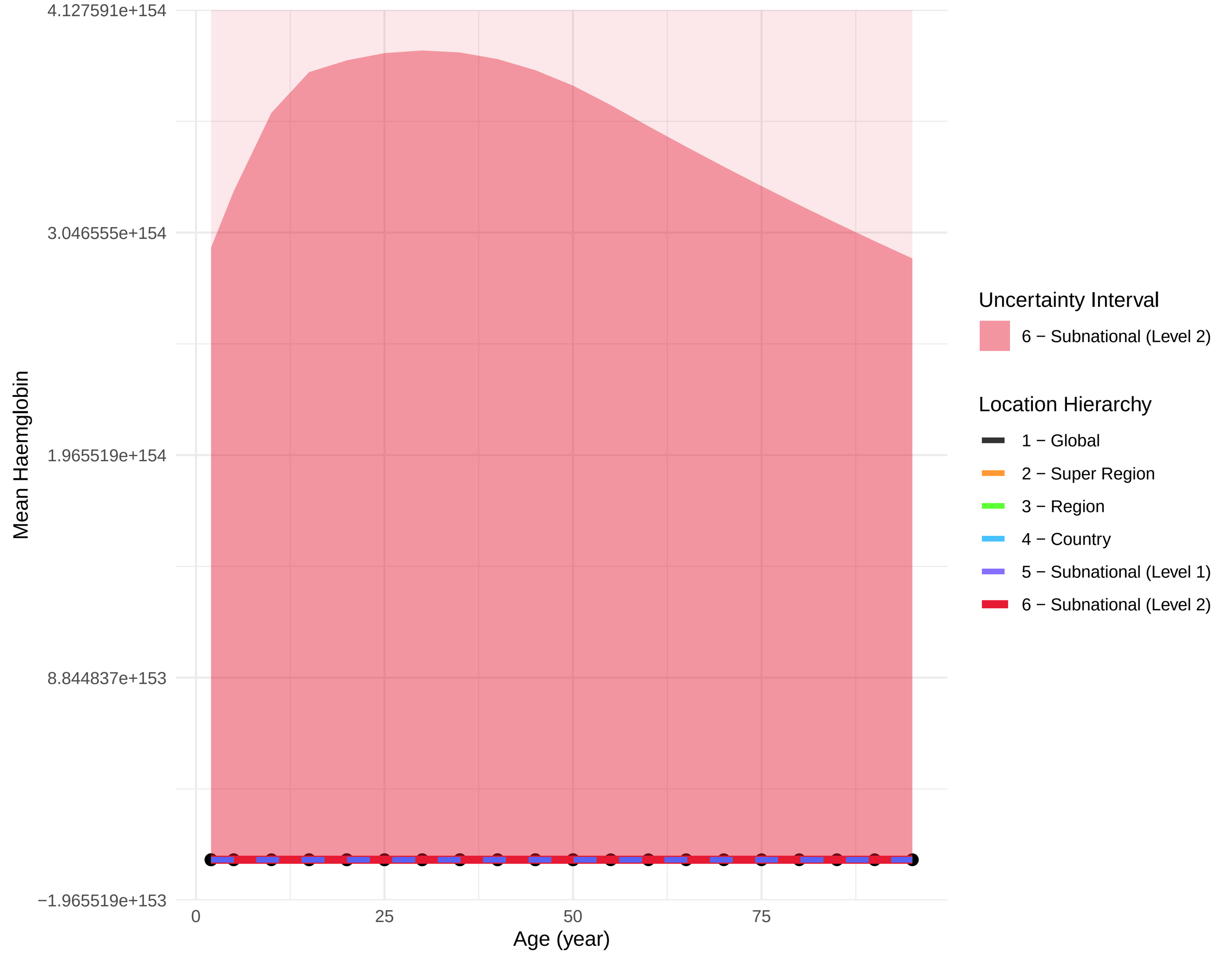

MR−BRT Age/Sex Fits − Males 2+ Years Old − Mean Haemglobin − Assam, Urban
WHO−2024 Adjustment Method

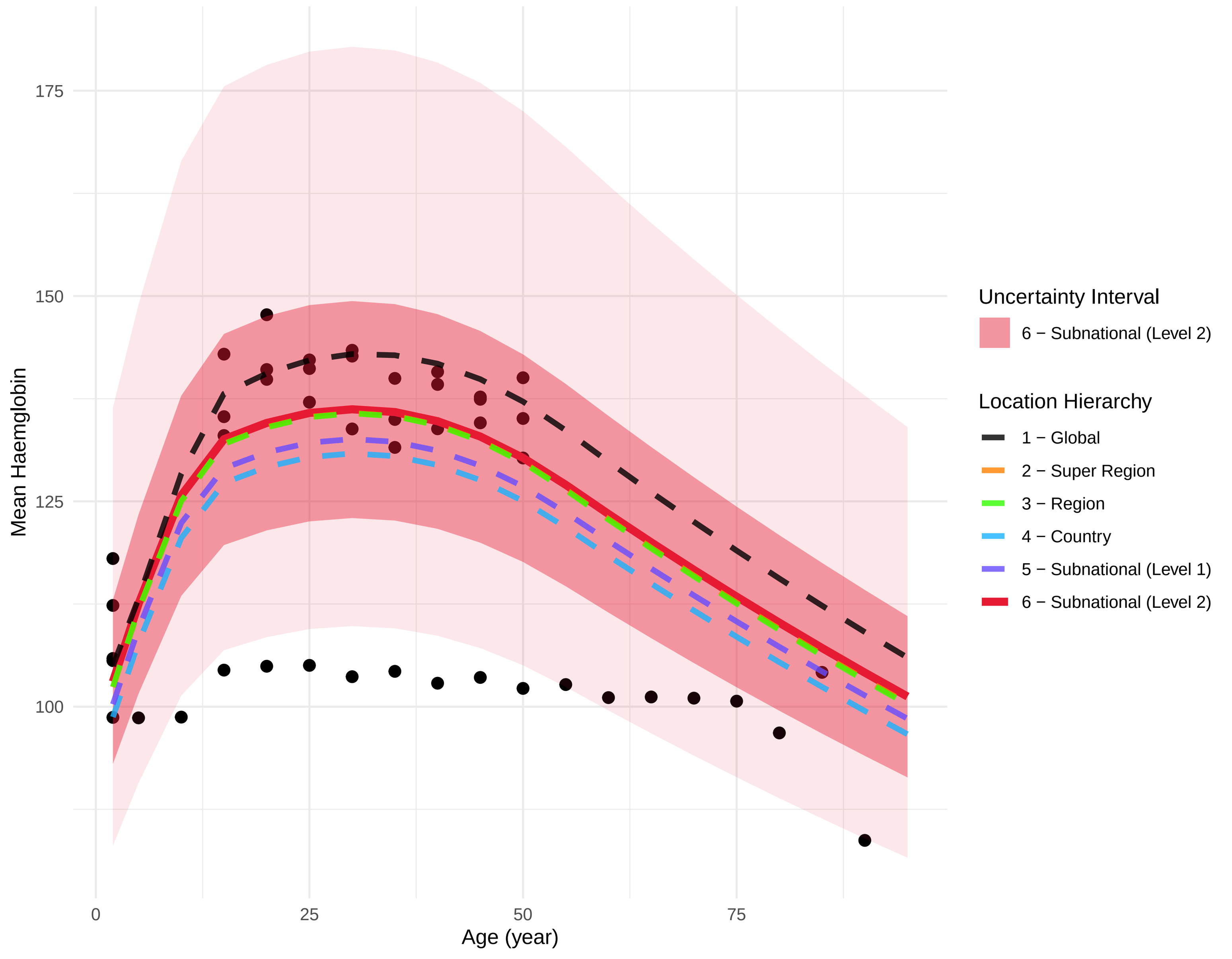

MR−BRT Age/Sex Fits − Males 2+ Years Old − Mean Haemglobin − Bihar
WHO−2024 Adjustment Method

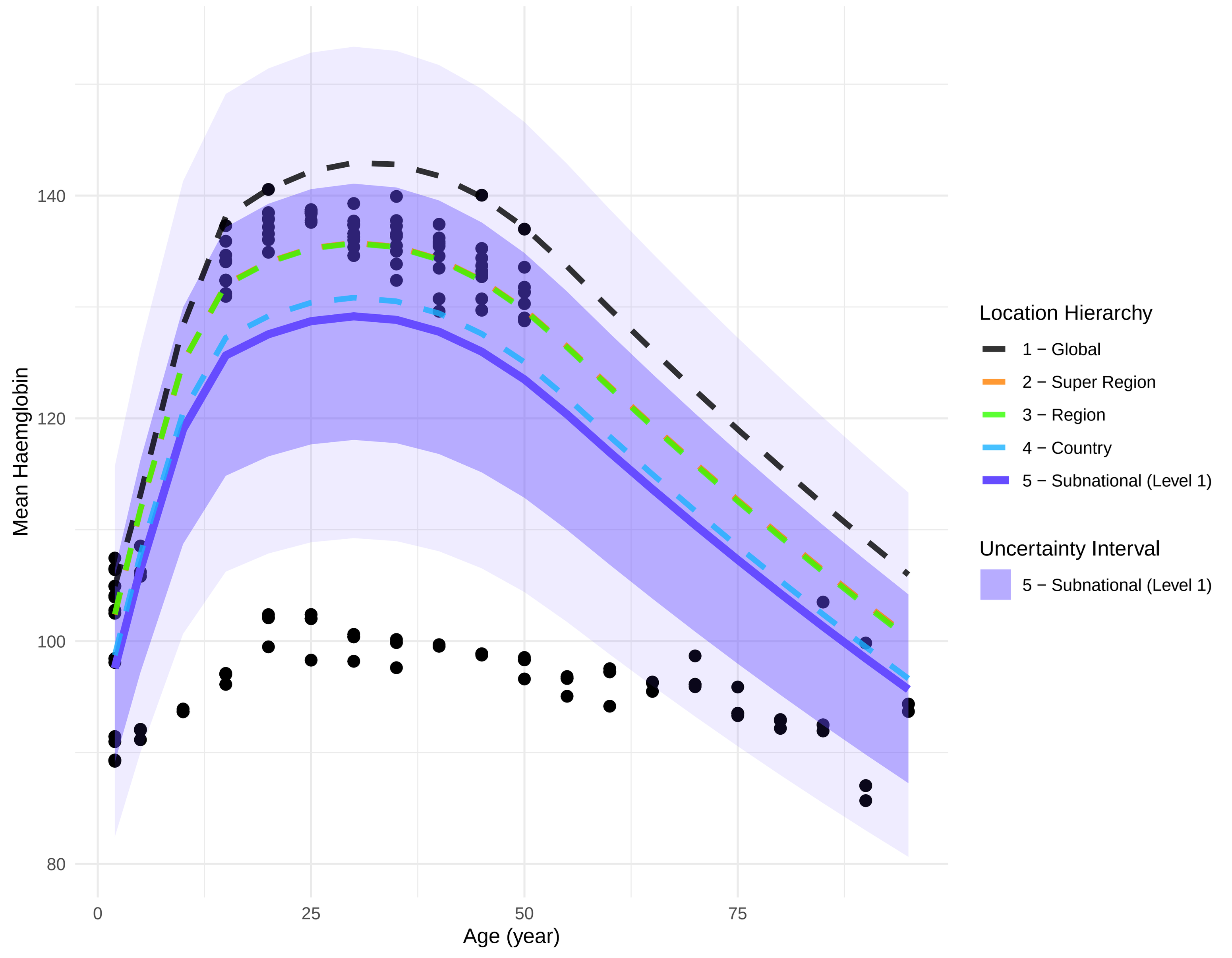

MR−BRT Age/Sex Fits − Males 2+ Years Old − Mean Haemglobin − Bihar, Rural
WHO−2024 Adjustment Method

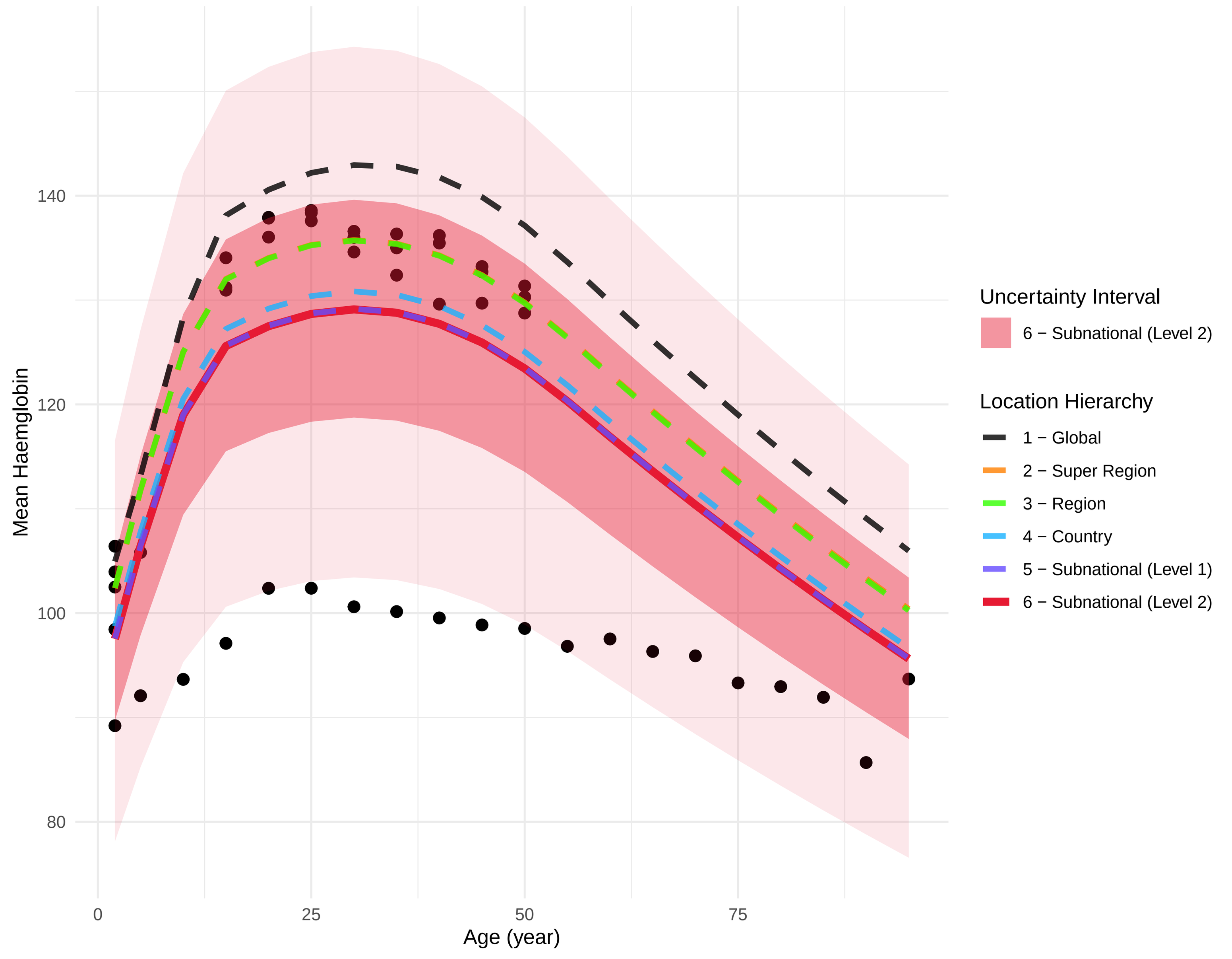

MR−BRT Age/Sex Fits − Males 2+ Years Old − Mean Haemglobin − Bihar, Urban
WHO−2024 Adjustment Method

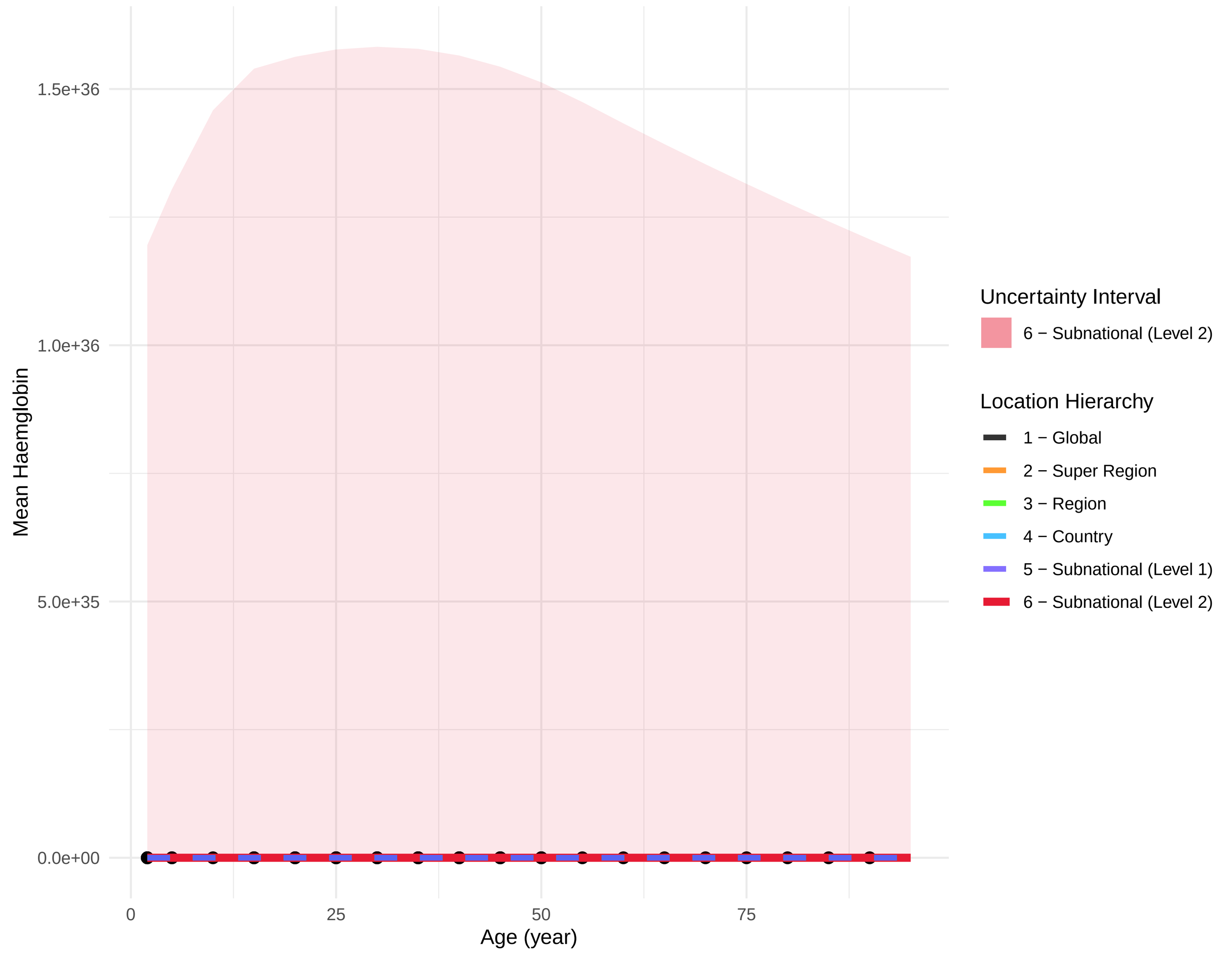

MR−BRT Age/Sex Fits − Males 2+ Years Old − Mean Haemglobin − Chhattisgarh
WHO−2024 Adjustment Method

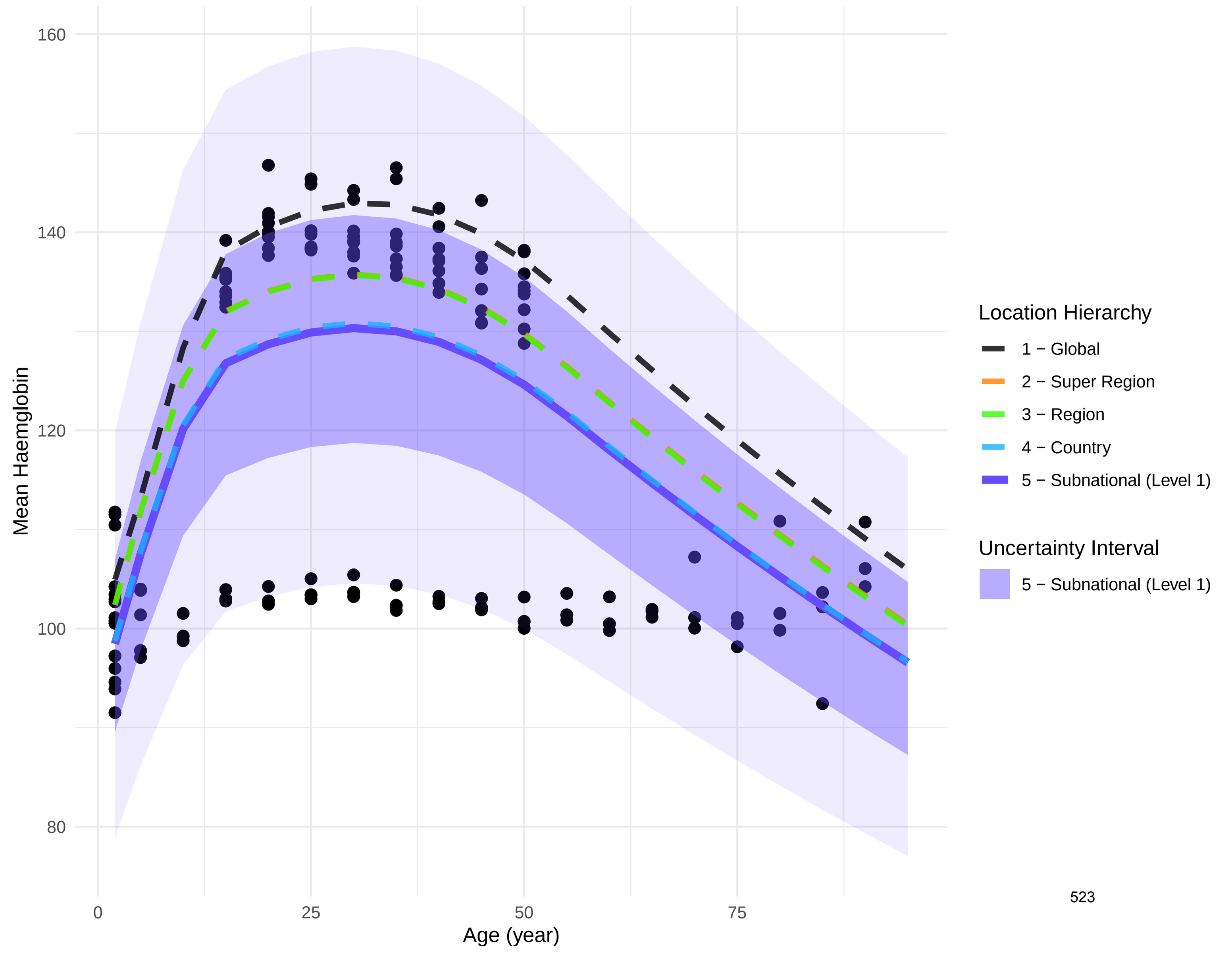

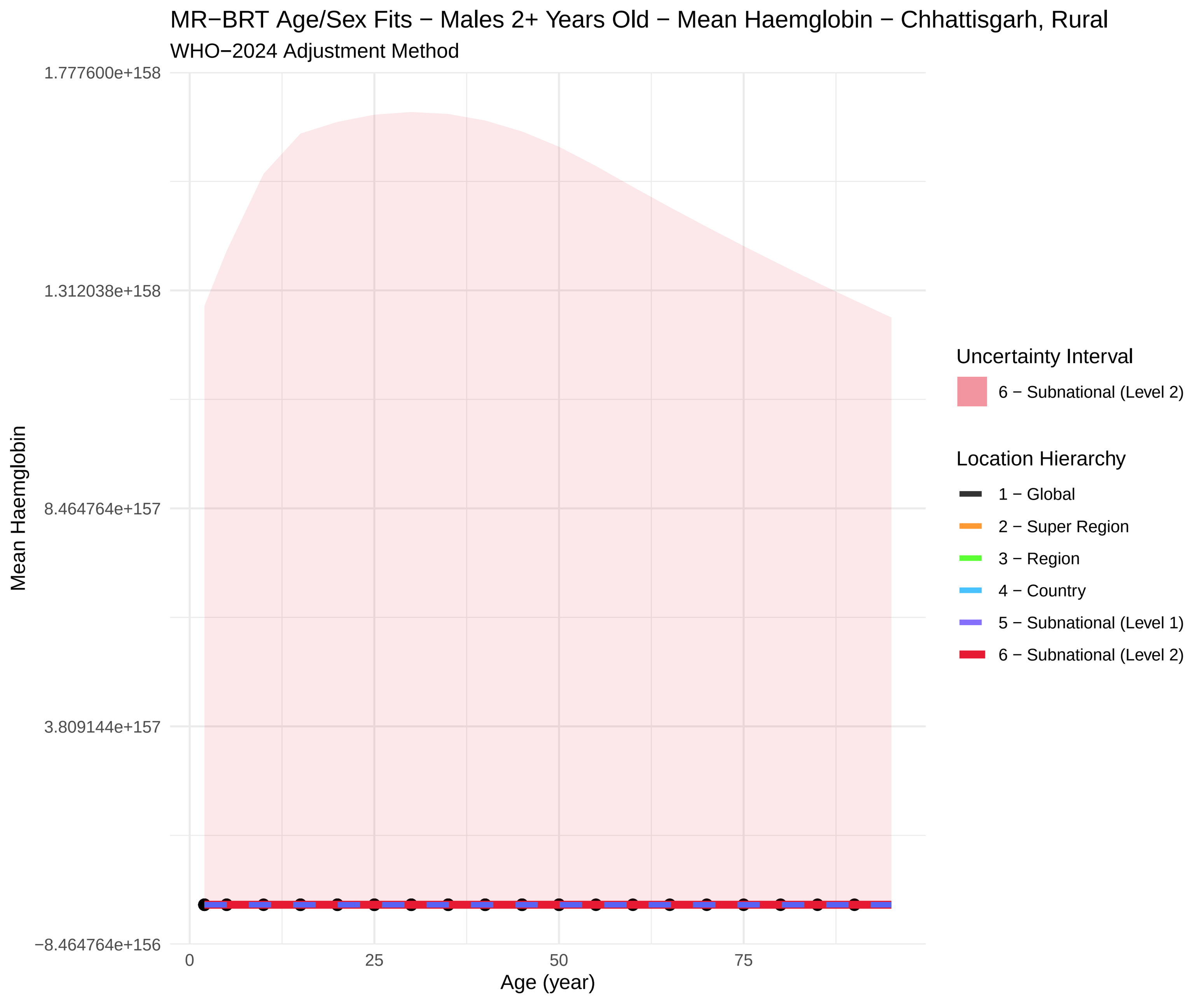
MR−BRT Age/Sex Fits − Males 2+ Years Old − Mean Haemglobin − Chhattisgarh, Rural
WHO−2024 Adjustment Method
Mean Haemglobin
1.777600e+158
1.312038e+158
8.464764e+157
3.809144e+157
−8.464764e+156
0
25
50
75
Age (year)
Uncertainty Interval
6 − Subnational (Level 2)
Location Hierarchy
1 − Global
2 − Super Region
3 − Region
4 − Country
5 − Subnational (Level 1)
6 − Subnational (Level 2)
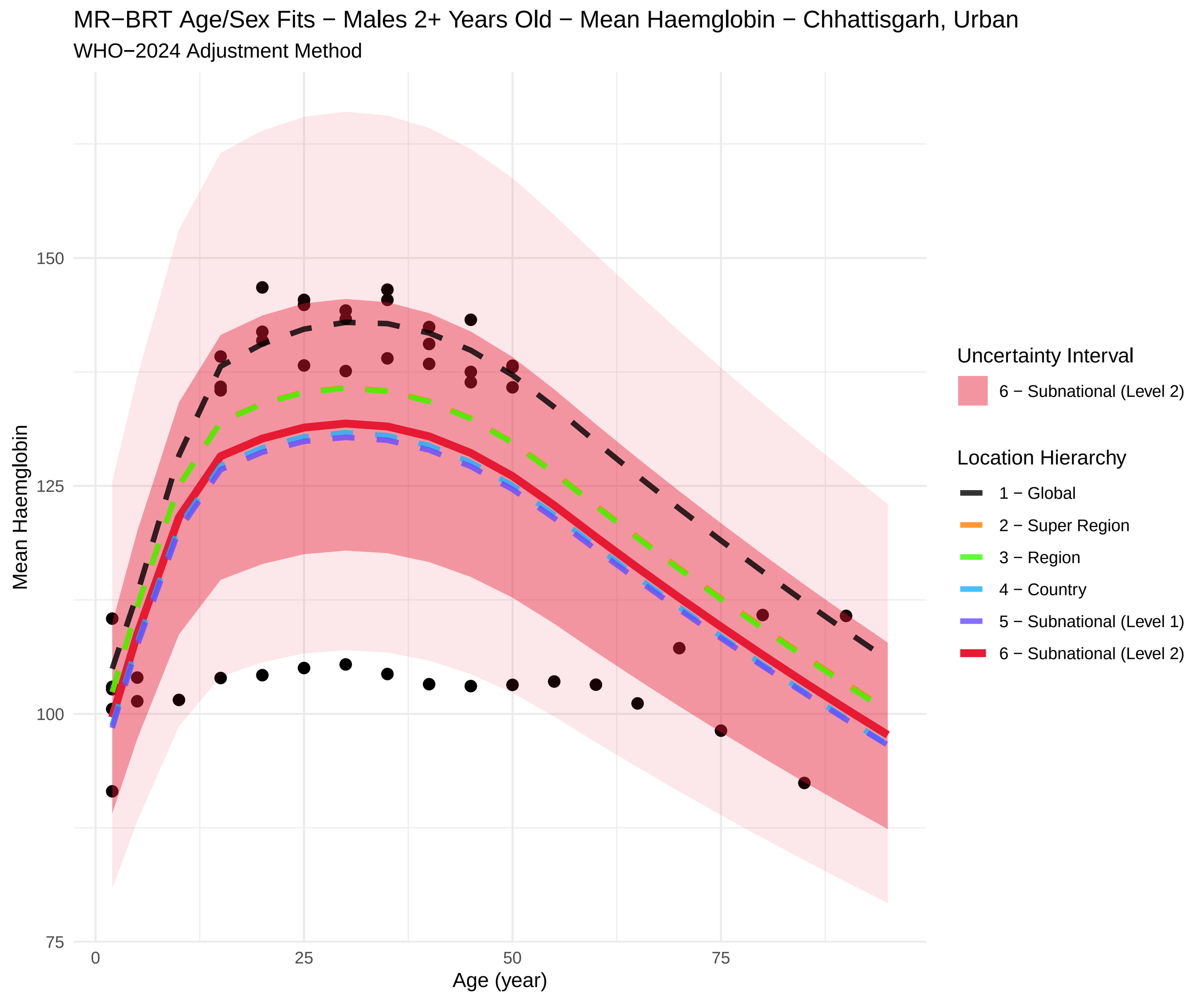
MR−BRT Age/Sex Fits − Males 2+ Years Old − Mean Haemglobin − Chhattisgarh, Urban
WHO−2024 Adjustment Method
Mean Haemglobin
150
125
100
75
0
25
50
75
Age (year)
Uncertainty Interval
6 − Subnational (Level 2)
Location Hierarchy
1 − Global
2 − Super Region
3 − Region
4 − Country
5 − Subnational (Level 1)
6 − Subnational (Level 2)
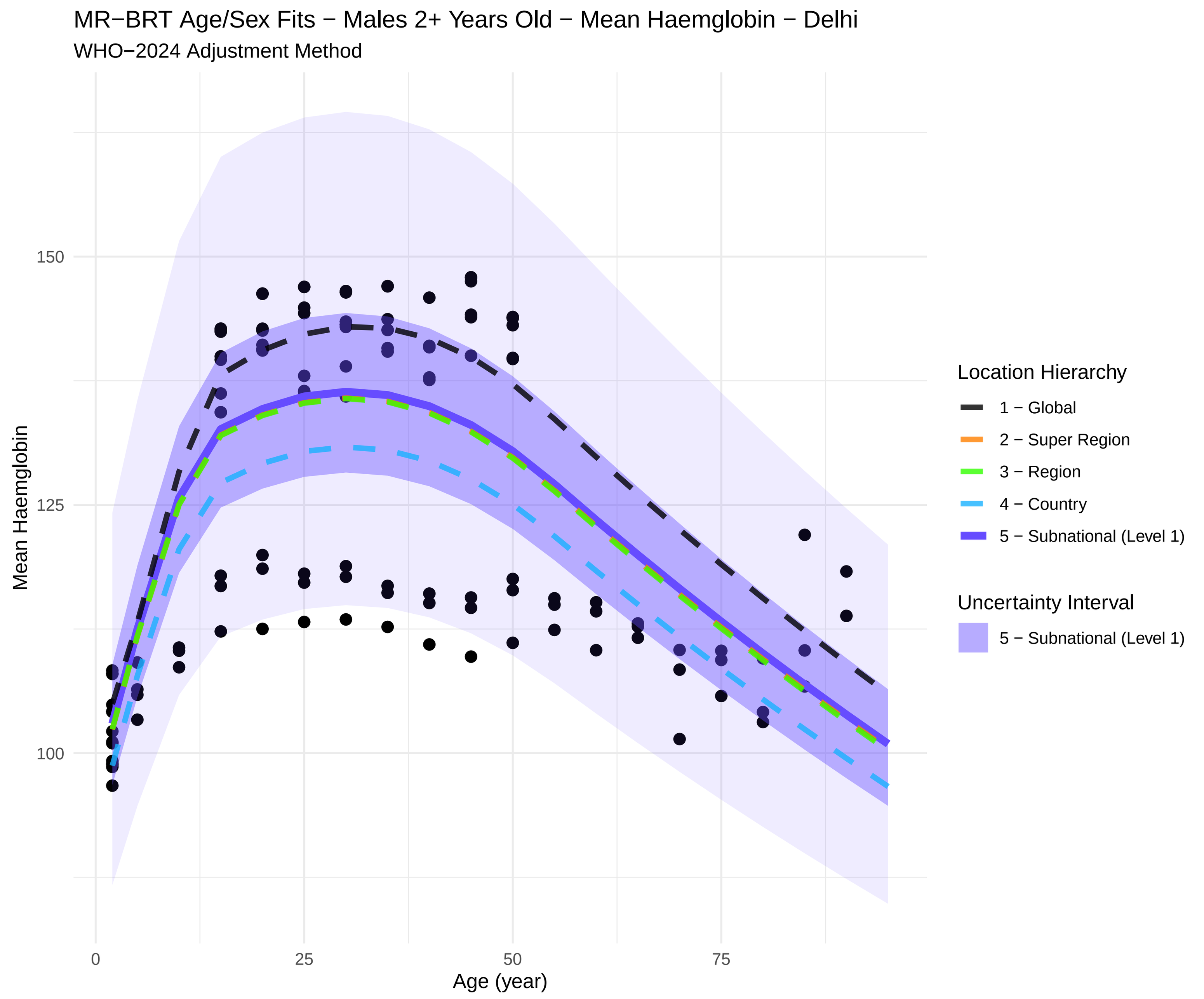
MR−BRT Age/Sex Fits − Males 2+ Years Old − Mean Haemglobin − Delhi
WHO−2024 Adjustment Method
Mean Haemglobin
150
125
100
0
25
50
75
Age (year)
Location Hierarchy
1 − Global
2 − Super Region
3 − Region
4 − Country
5 − Subnational (Level 1)
Uncertainty Interval
5 − Subnational (Level 1)
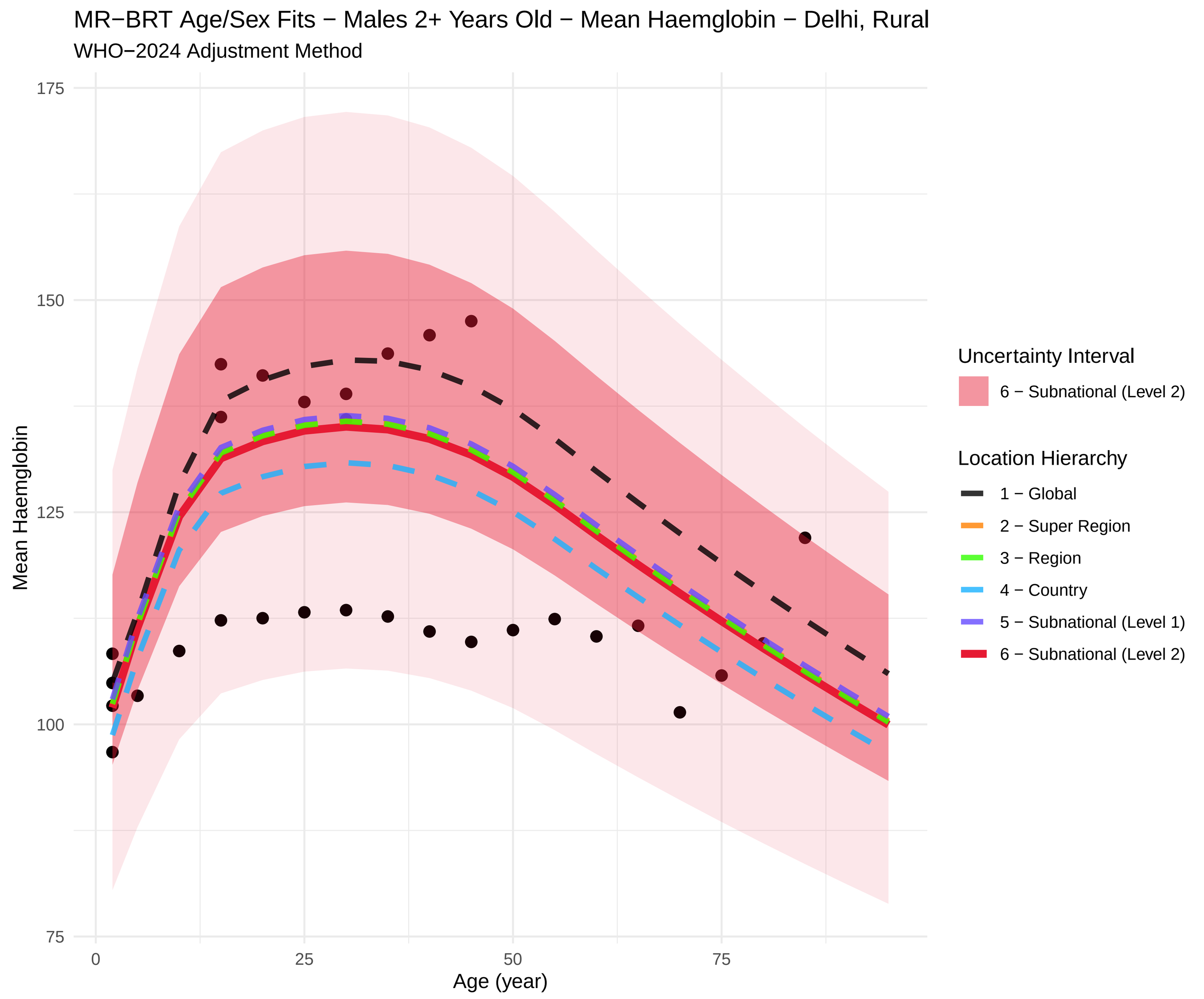
MR−BRT Age/Sex Fits − Males 2+ Years Old − Mean Haemglobin − Delhi, Rural
WHO−2024 Adjustment Method
Mean Haemglobin
175
150
125
100
75
0
25
50
75
Age (year)
Uncertainty Interval
6 − Subnational (Level 2)
Location Hierarchy
1 − Global
2 − Super Region
3 − Region
4 − Country
5 − Subnational (Level 1)
6 − Subnational (Level 2)
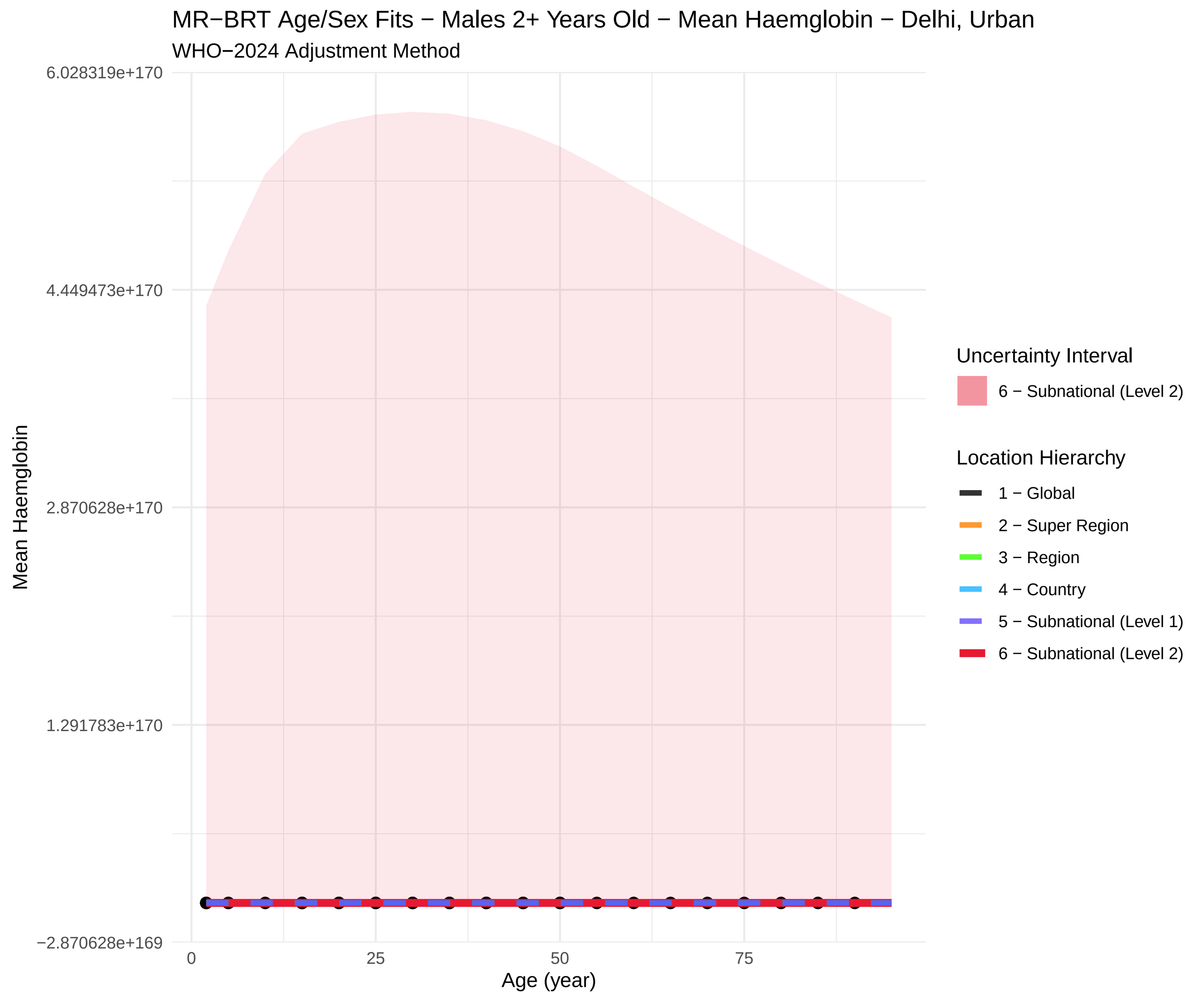
MR−BRT Age/Sex Fits − Males 2+ Years Old − Mean Haemglobin − Delhi, Urban
WHO−2024 Adjustment Method
Mean Haemglobin
6.028319e+170
4.449473e+170
2.870628e+170
1.291783e+170
−2.870628e+169
0
25
50
75
Age (year)
Uncertainty Interval
6 − Subnational (Level 2)
Location Hierarchy
1 − Global
2 − Super Region
3 − Region
4 − Country
5 − Subnational (Level 1)
6 − Subnational (Level 2)
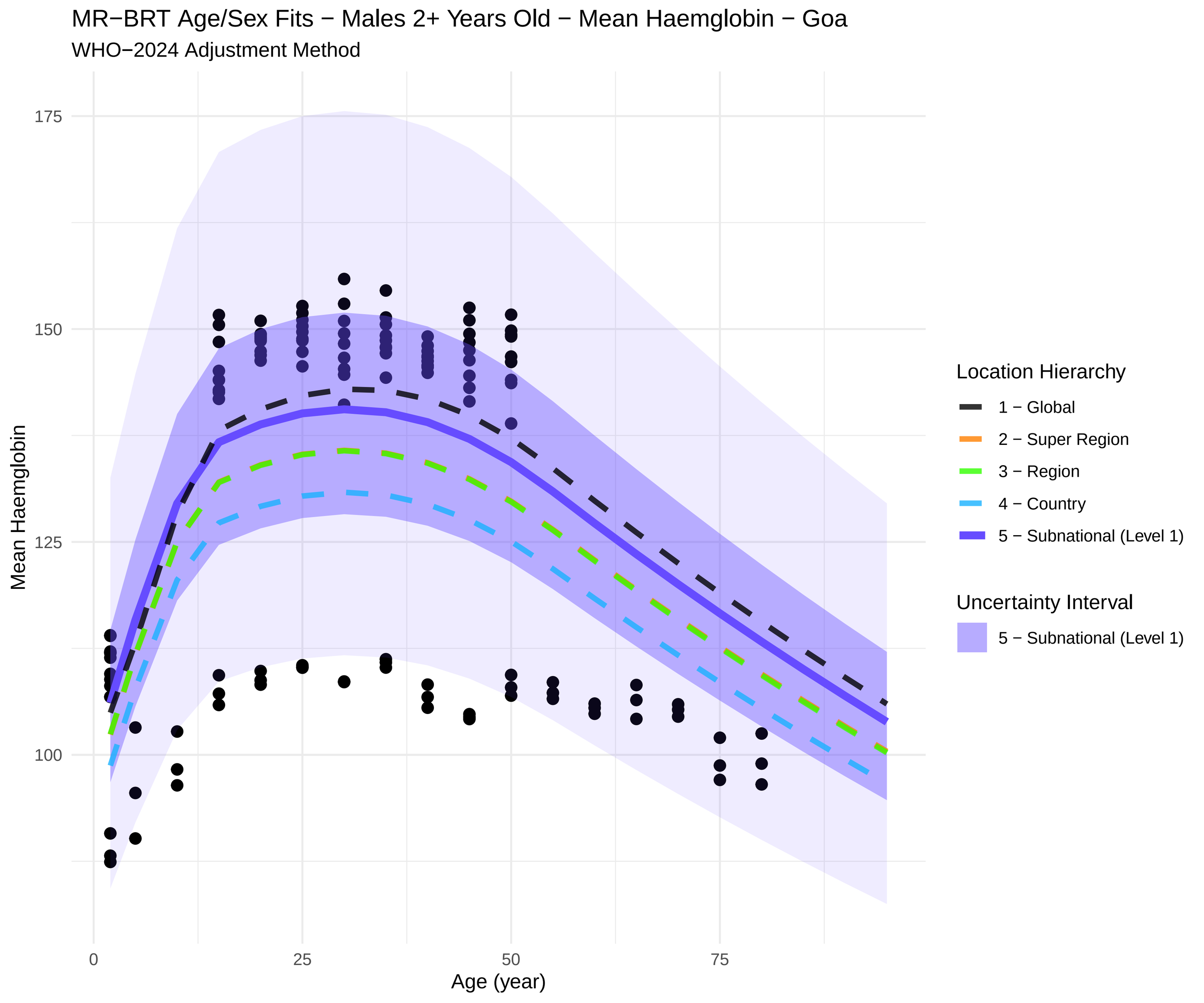
MR−BRT Age/Sex Fits − Males 2+ Years Old − Mean Haemglobin − Goa
WHO−2024 Adjustment Method
Mean Haemglobin
175
150
125
100
0
25
50
75
Age (year)
Location Hierarchy
1 − Global
2 − Super Region
3 − Region
4 − Country
5 − Subnational (Level 1)
Uncertainty Interval
5 − Subnational (Level 1)
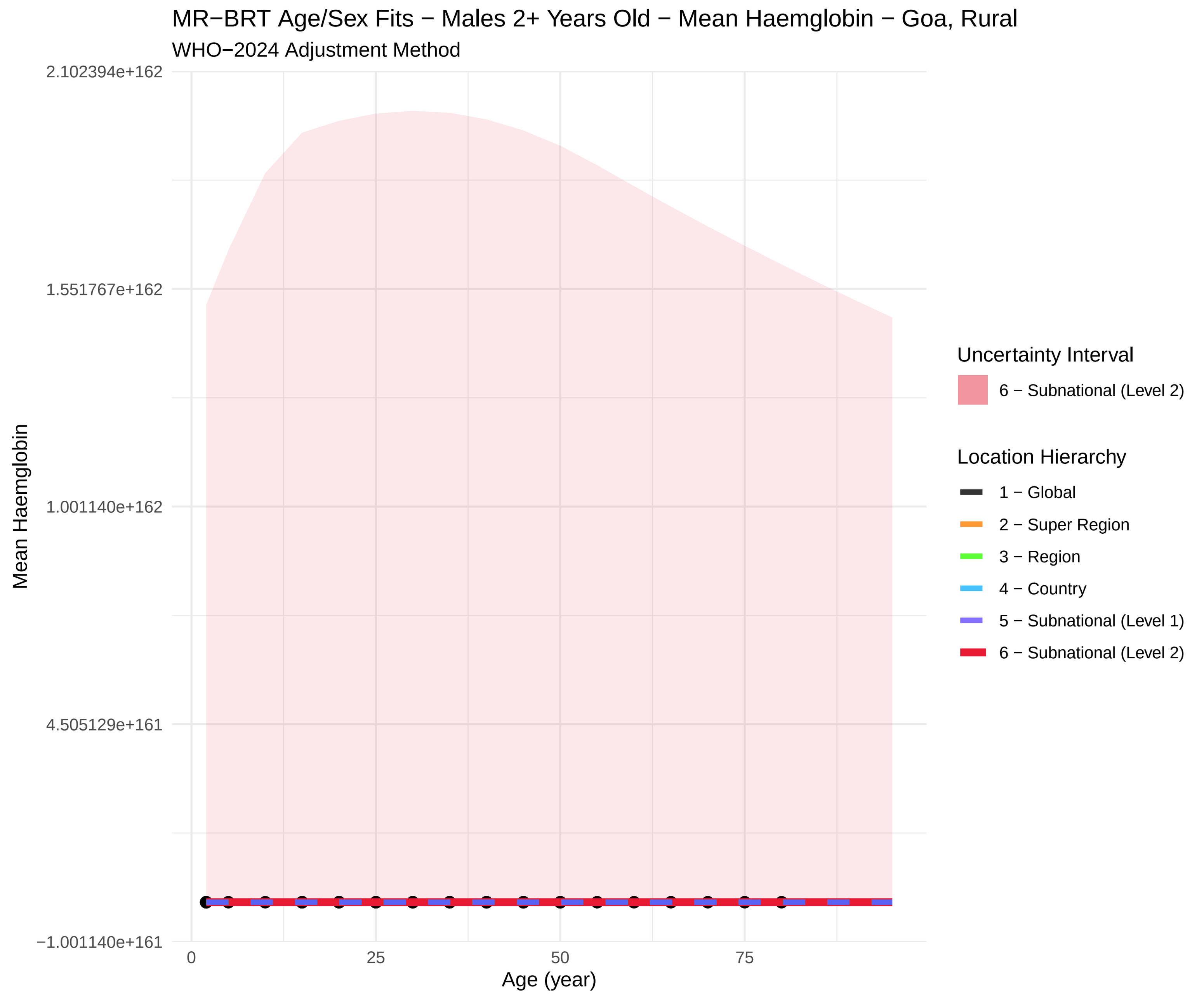
MR−BRT Age/Sex Fits − Males 2+ Years Old − Mean Haemglobin − Goa, Rural
WHO−2024 Adjustment Method
Mean Haemglobin
2.102394e+162
1.551767e+162
1.001140e+162
4.505129e+161
−1.001140e+161
0
25
50
75
Age (year)
Uncertainty Interval
6 − Subnational (Level 2)
Location Hierarchy
1 − Global
2 − Super Region
3 − Region
4 − Country
5 − Subnational (Level 1)
6 − Subnational (Level 2)
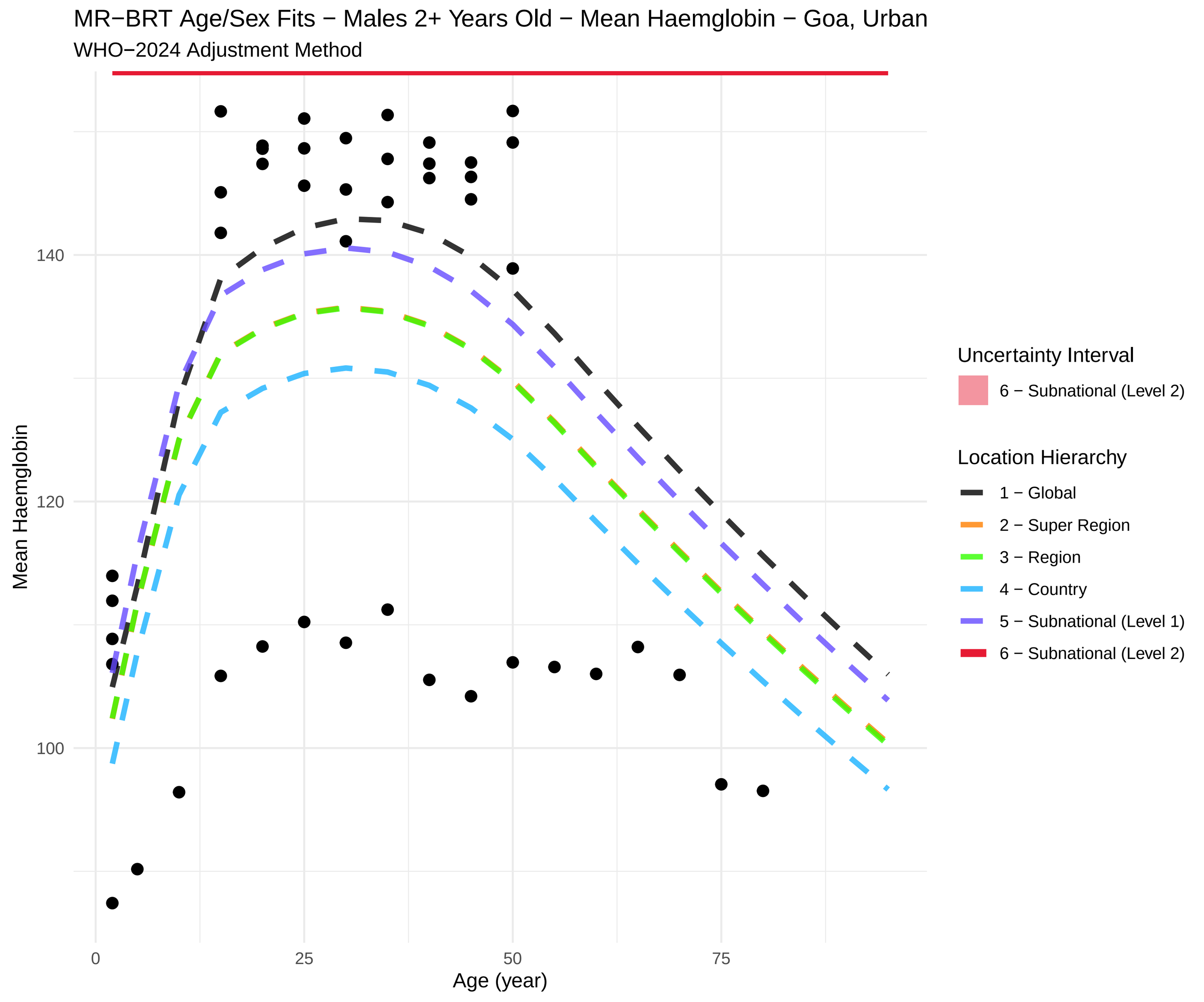
MR−BRT Age/Sex Fits − Males 2+ Years Old − Mean Haemglobin − Goa, Urban
WHO−2024 Adjustment Method
Mean Haemglobin
140
120
100
0
25
50
75
Age (year)
Uncertainty Interval
6 − Subnational (Level 2)
Location Hierarchy
1 − Global
2 − Super Region
3 − Region
4 − Country
5 − Subnational (Level 1)
6 − Subnational (Level 2)
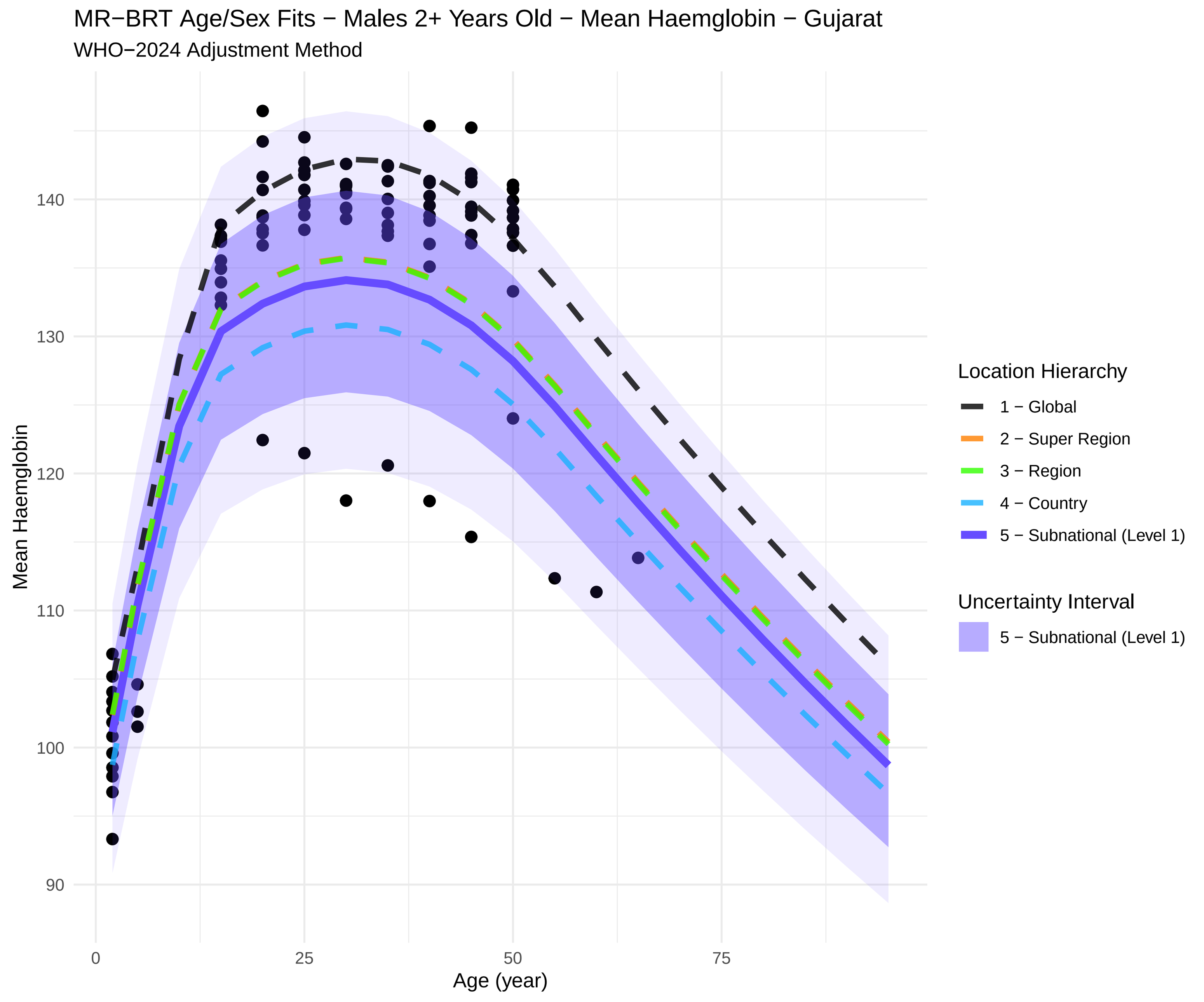
MR−BRT Age/Sex Fits − Males 2+ Years Old − Mean Haemglobin − Gujarat
WHO−2024 Adjustment Method
Mean Haemglobin
140
130
120
110
100
90
0
25
50
75
Age (year)
Location Hierarchy
1 − Global
2 − Super Region
3 − Region
4 − Country
5 − Subnational (Level 1)
Uncertainty Interval
5 − Subnational (Level 1)
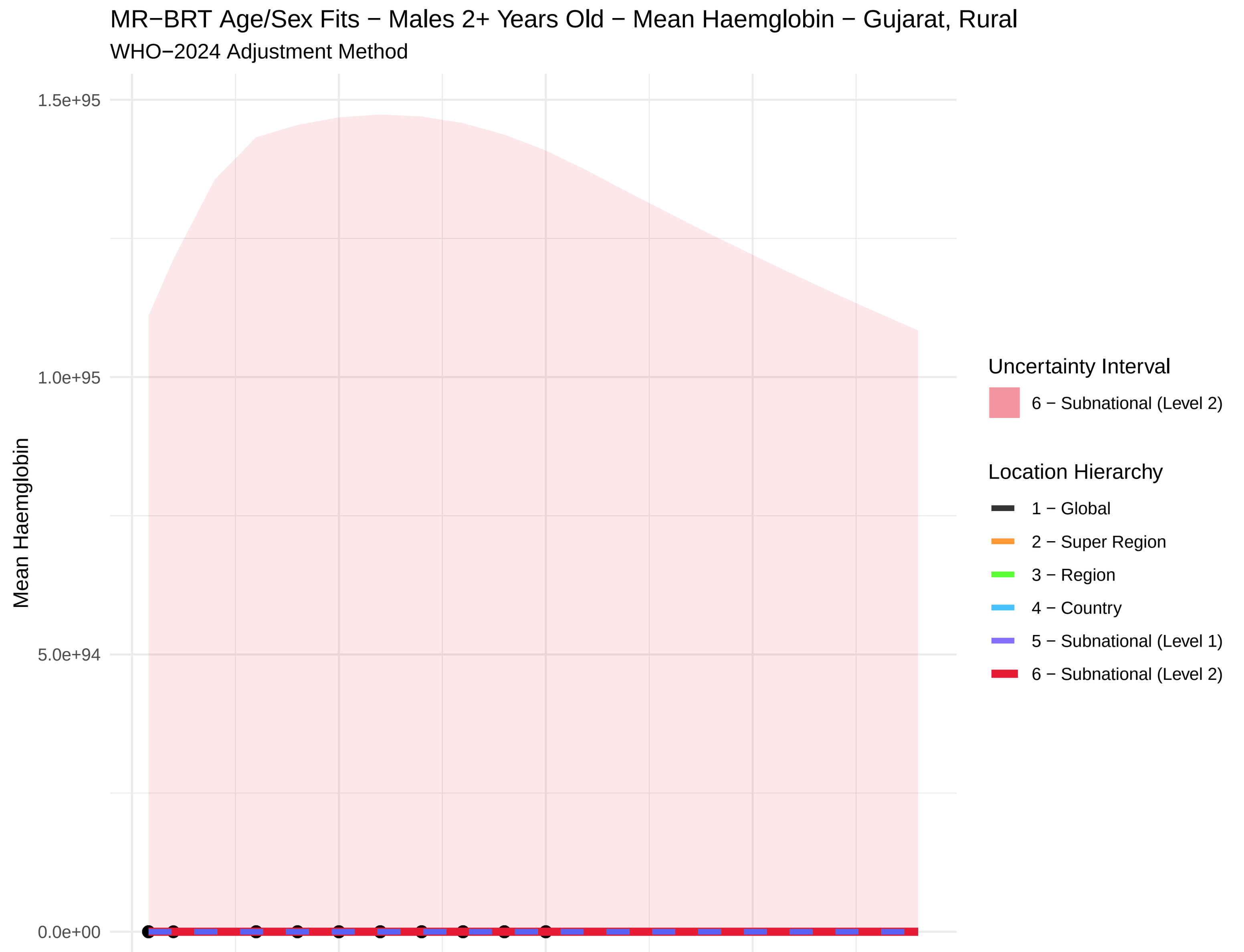
MR−BRT Age/Sex Fits − Males 2+ Years Old − Mean Haemglobin − Gujarat, Rural
WHO−2024 Adjustment Method
Mean Haemglobin
1.5e+95
1.0e+95
5.0e+94
0.0e+00
0
25
50
75
Age (year)
Uncertainty Interval
6 − Subnational (Level 2)
Location Hierarchy
1 − Global
2 − Super Region
3 − Region
4 − Country
5 − Subnational (Level 1)
6 − Subnational (Level 2)

MR−BRT Age/Sex Fits − Males 2+ Years Old − Mean Haemglobin − Gujarat, Urban
WHO−2024 Adjustment Method

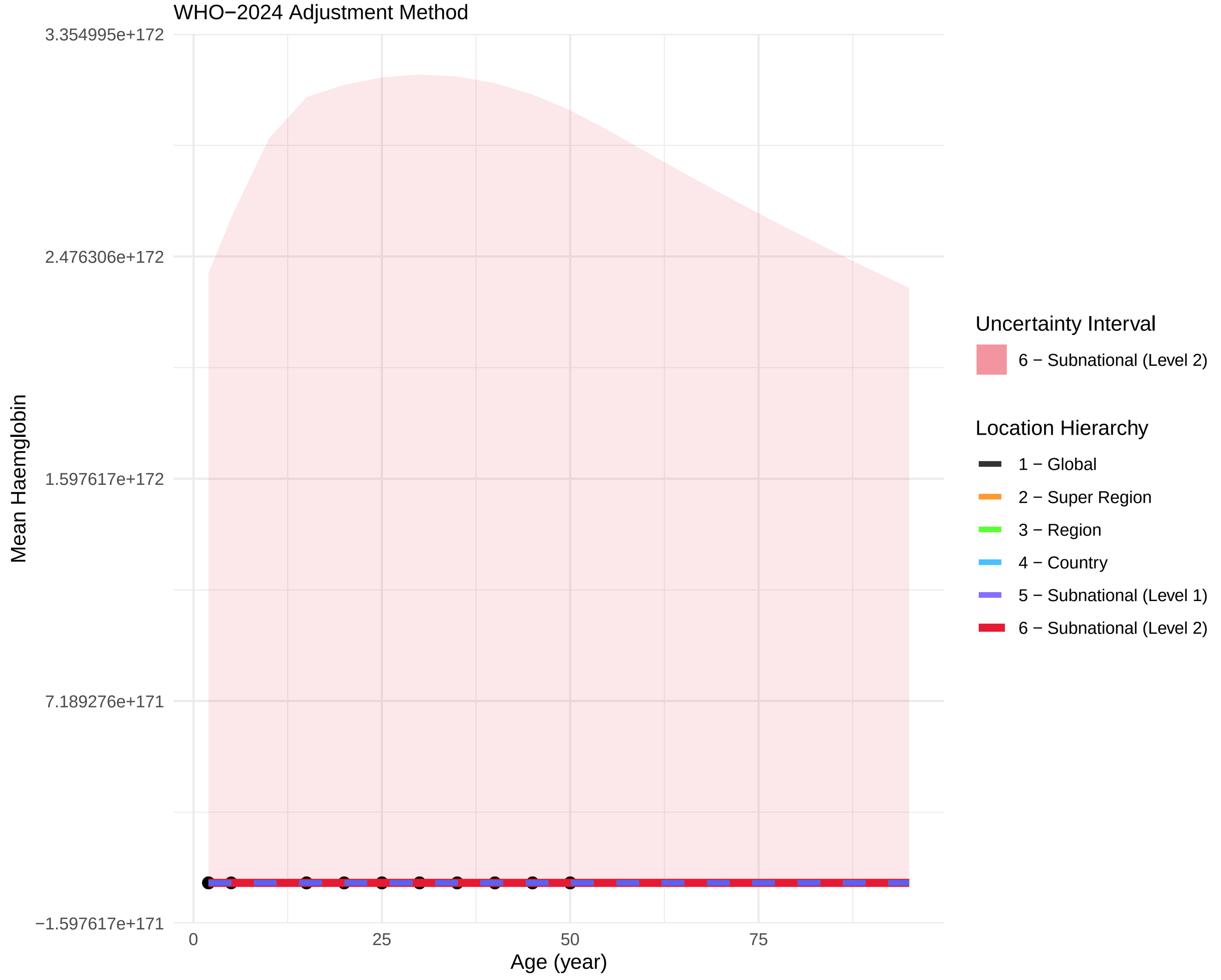

MR−BRT Age/Sex Fits − Males 2+ Years Old − Mean Haemglobin − Haryana
WHO−2024 Adjustment Method

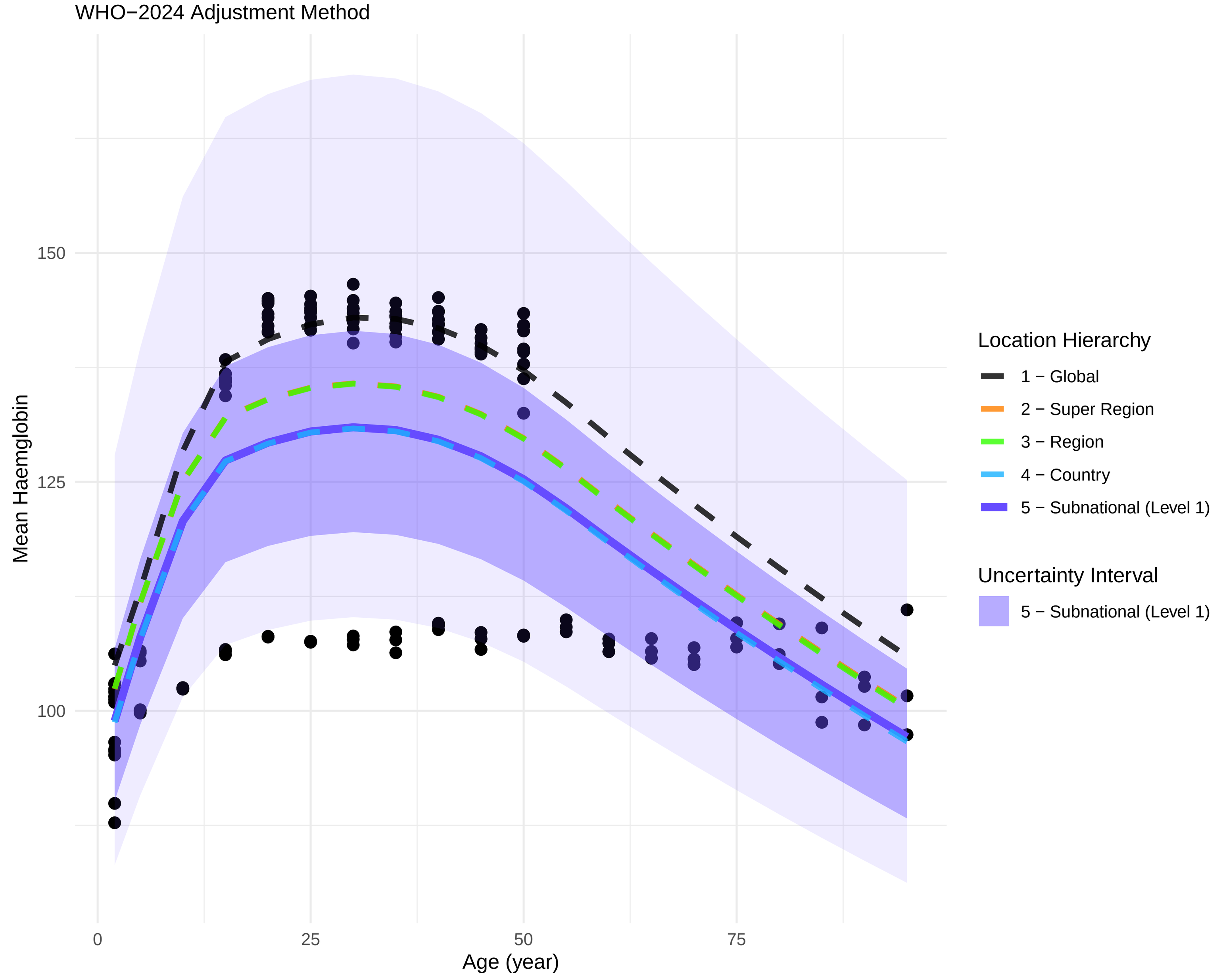

MR−BRT Age/Sex Fits − Males 2+ Years Old − Mean Haemglobin − Haryana, Rural
WHO−2024 Adjustment Method

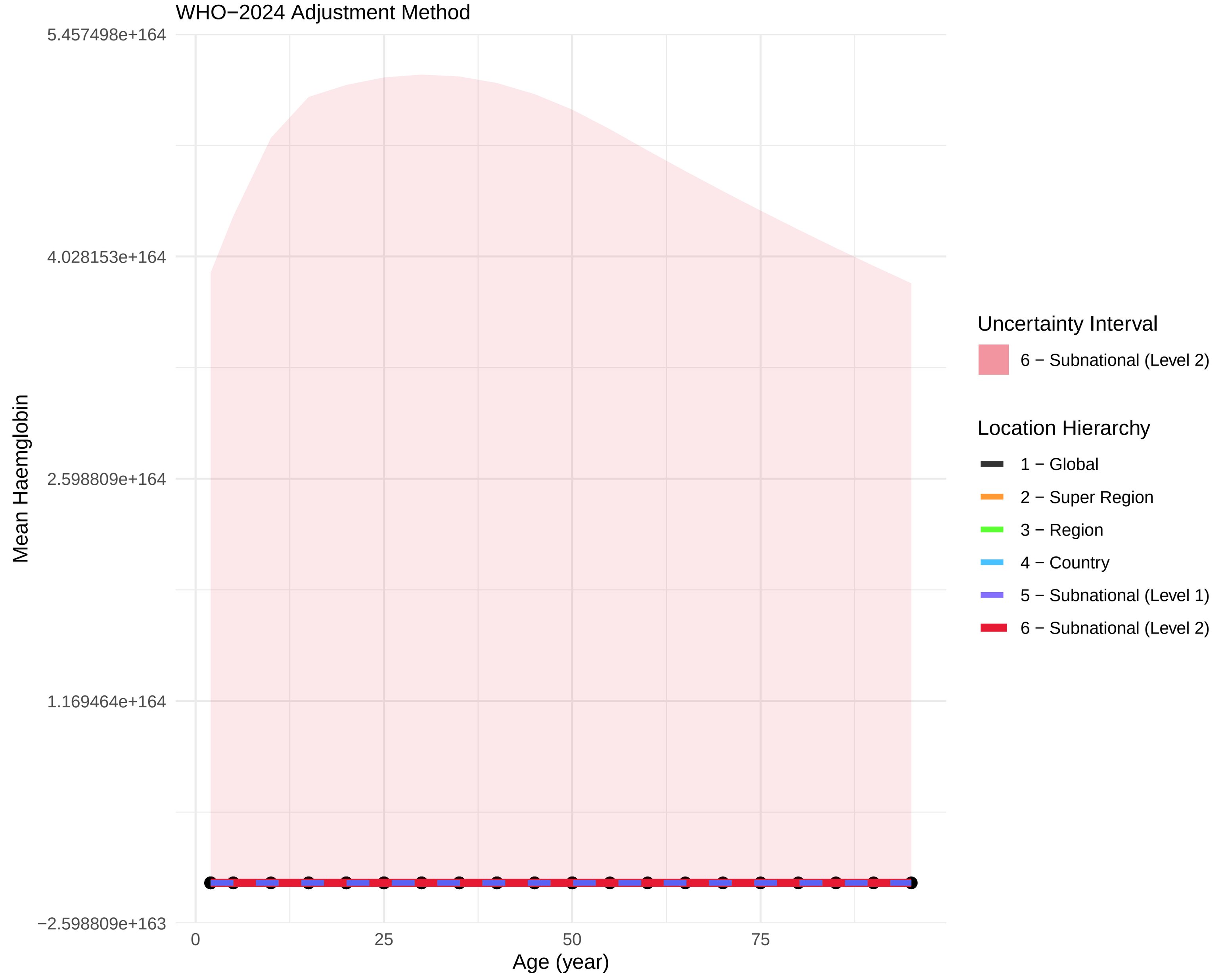

MR−BRT Age/Sex Fits − Males 2+ Years Old − Mean Haemglobin − Haryana, Urban
WHO−2024 Adjustment Method

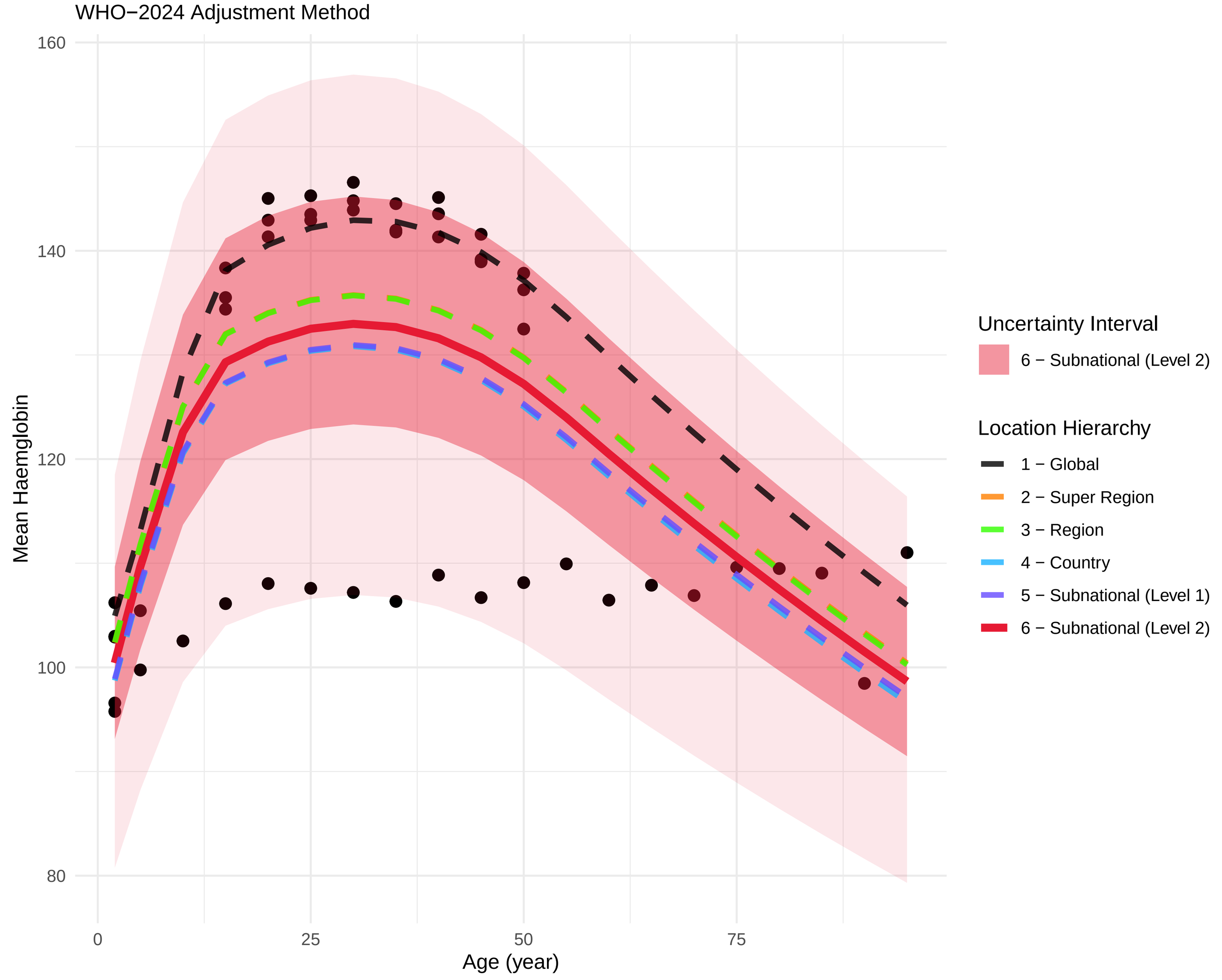

MR−BRT Age/Sex Fits − Males 2+ Years Old − Mean Haemglobin − Himachal Pradesh
WHO−2024 Adjustment Method

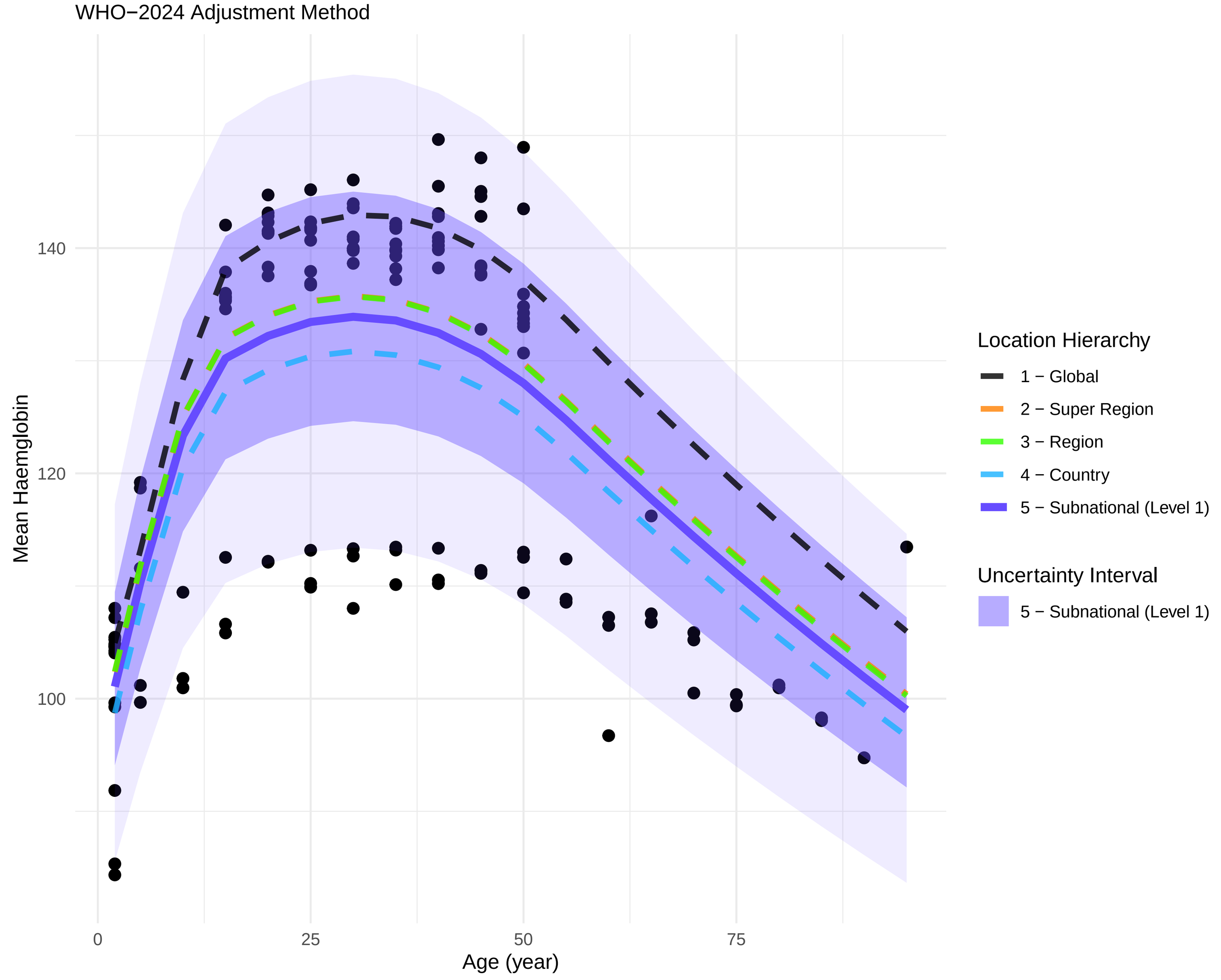

MR−BRT Age/Sex Fits − Males 2+ Years Old − Mean Haemglobin − Himachal Pradesh, Rural
WHO−2024 Adjustment Method

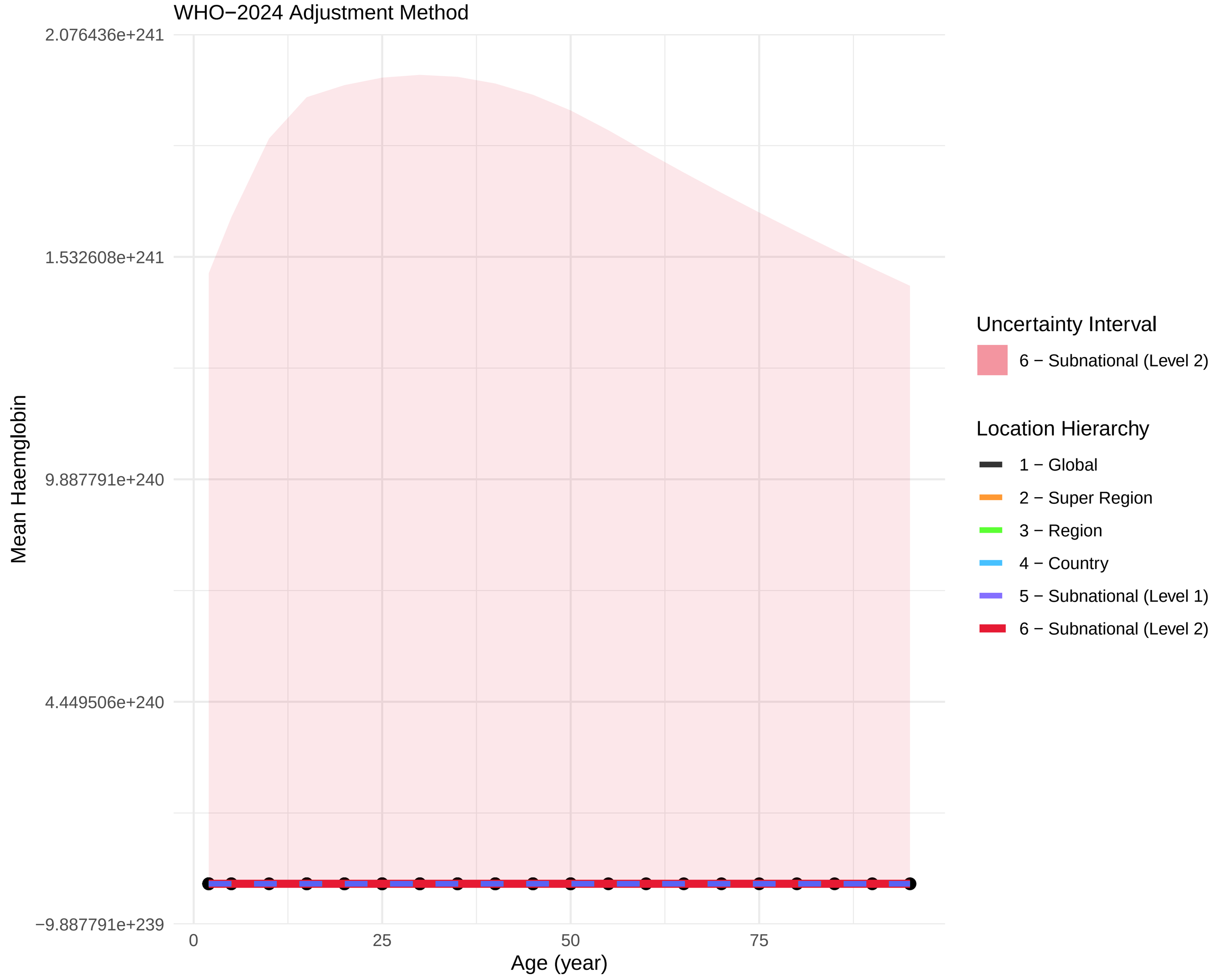

MR−BRT Age/Sex Fits − Males 2+ Years Old − Mean Haemglobin − Himachal Pradesh, Urban
WHO−2024 Adjustment Method

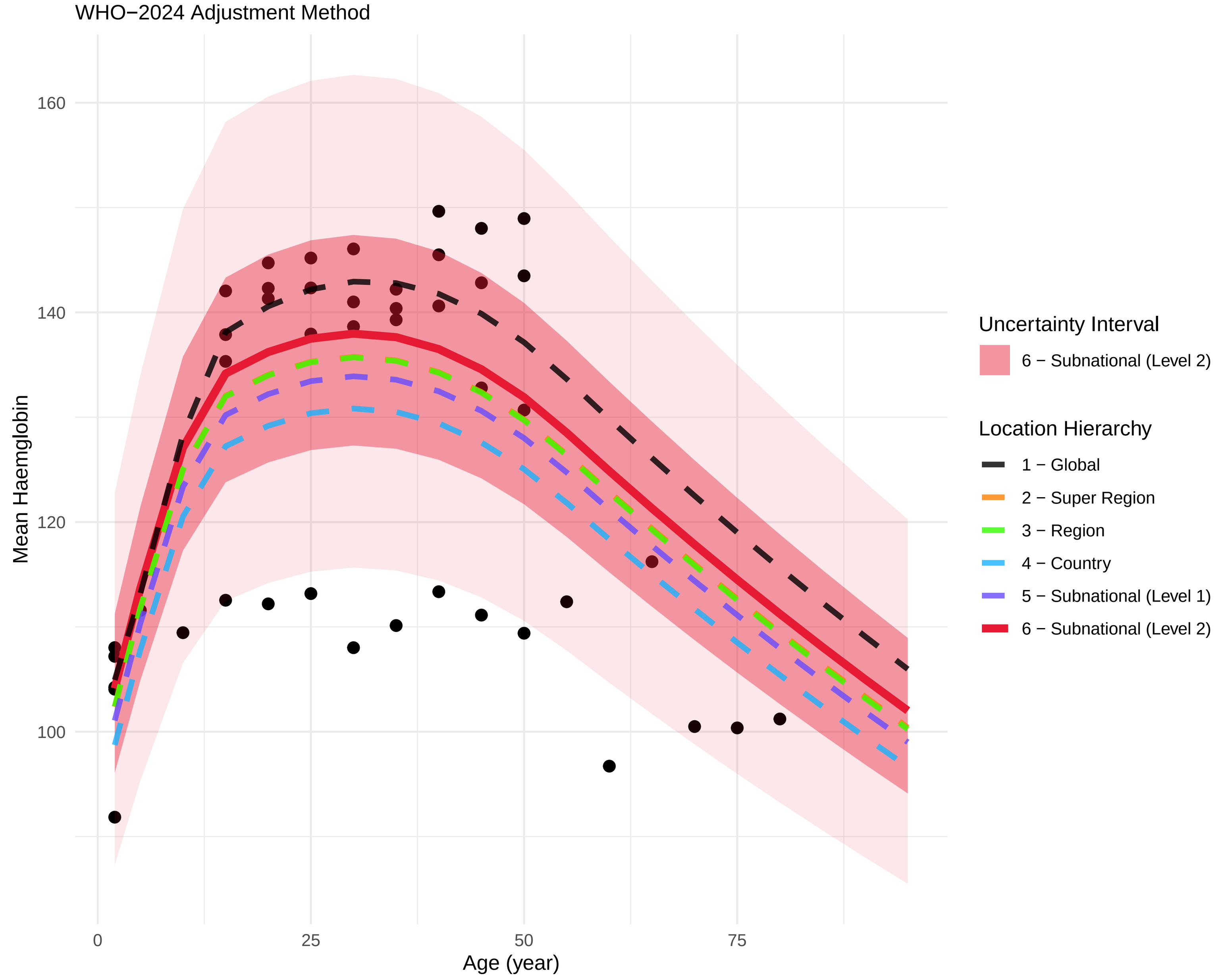

MR−BRT Age/Sex Fits − Males 2+ Years Old − Mean Haemglobin − Jammu & Kashmir and Ladakh
WHO−2024 Adjustment Method

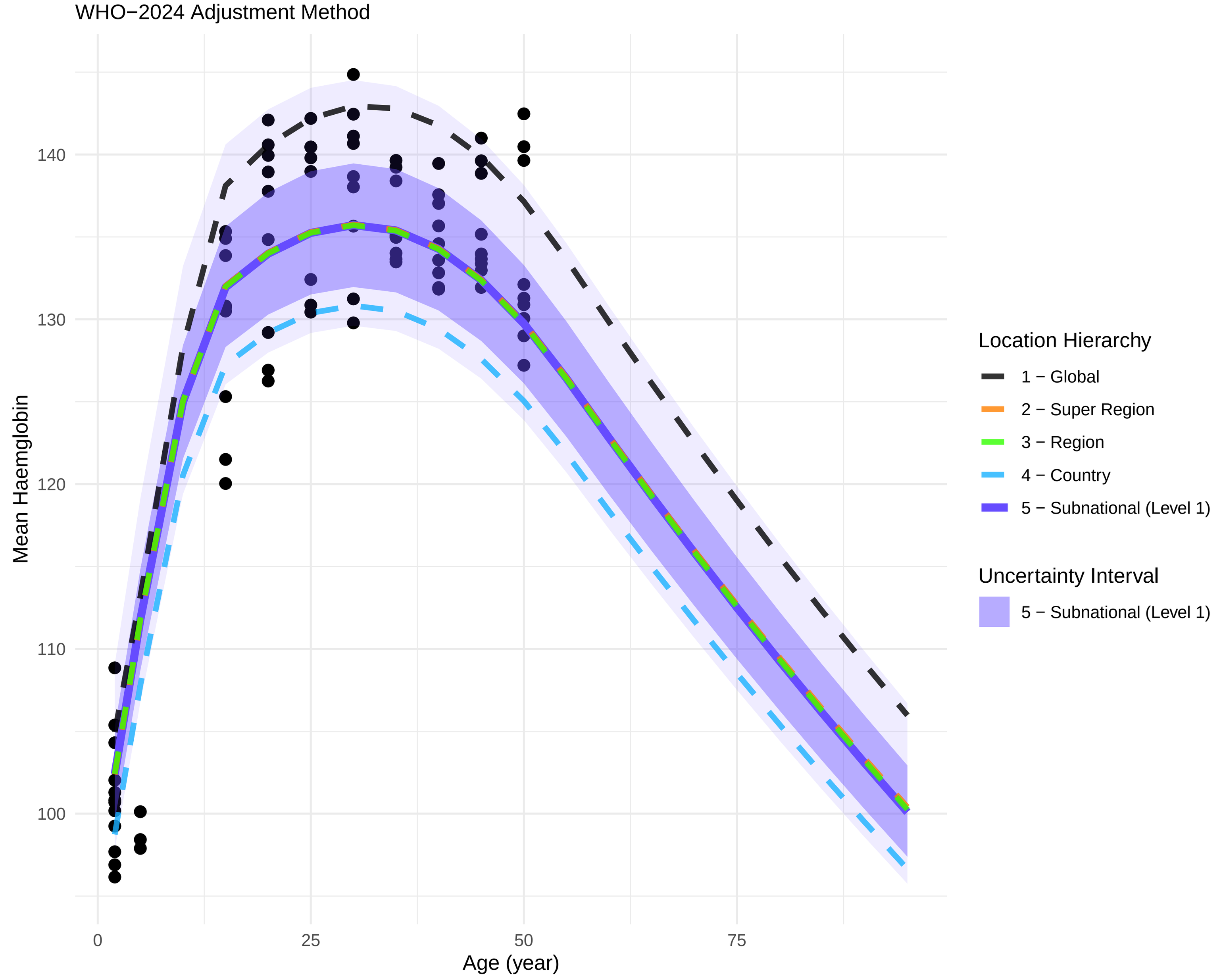

MR−BRT Age/Sex Fits − Males 2+ Years Old − Mean Haemglobin − Jammu & Kashmir and Ladakh, Rural
WHO−2024 Adjustment Method

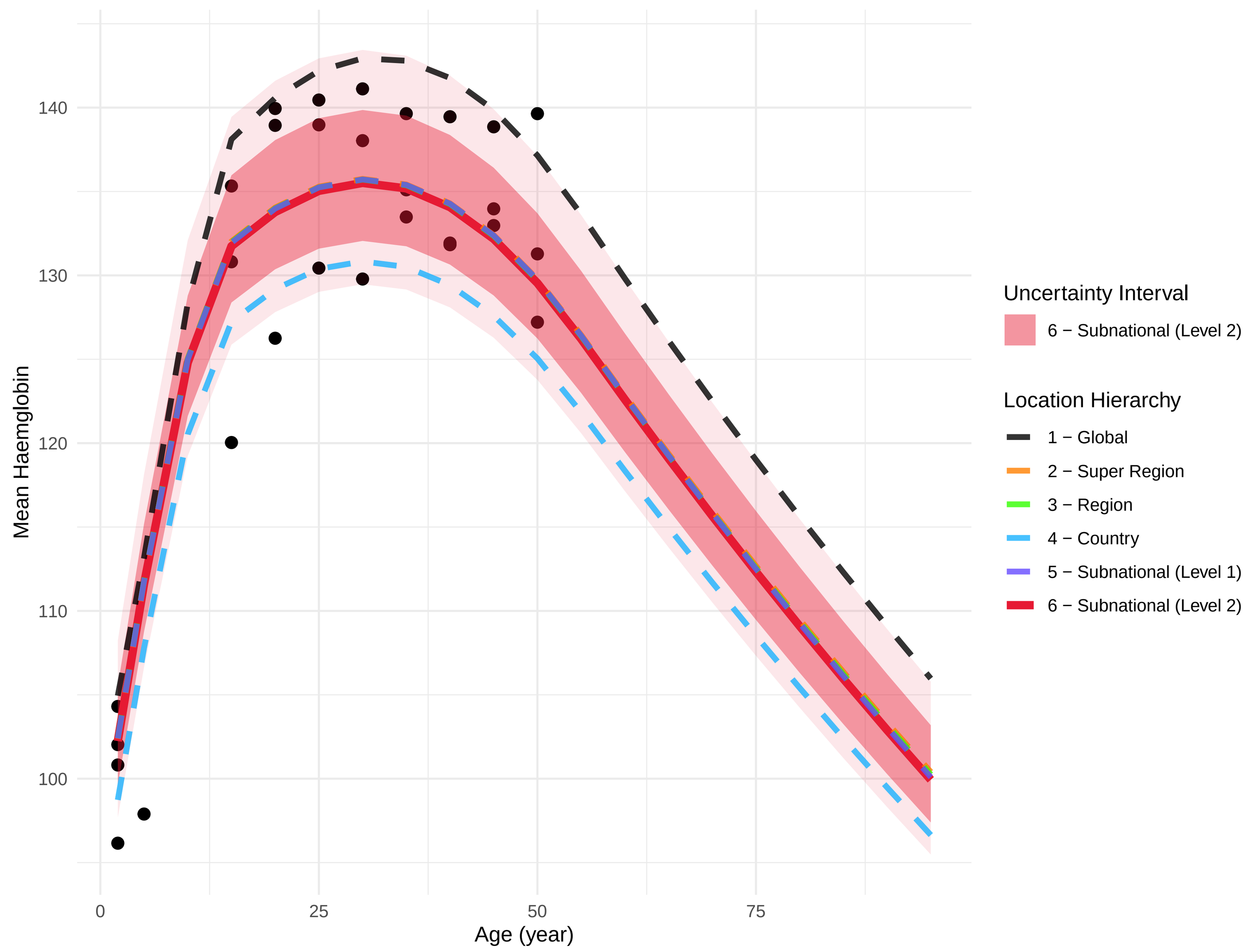

MR−BRT Age/Sex Fits − Males 2+ Years Old − Mean Haemglobin − Jammu & Kashmir and Ladakh, Urban
WHO−2024 Adjustment Method

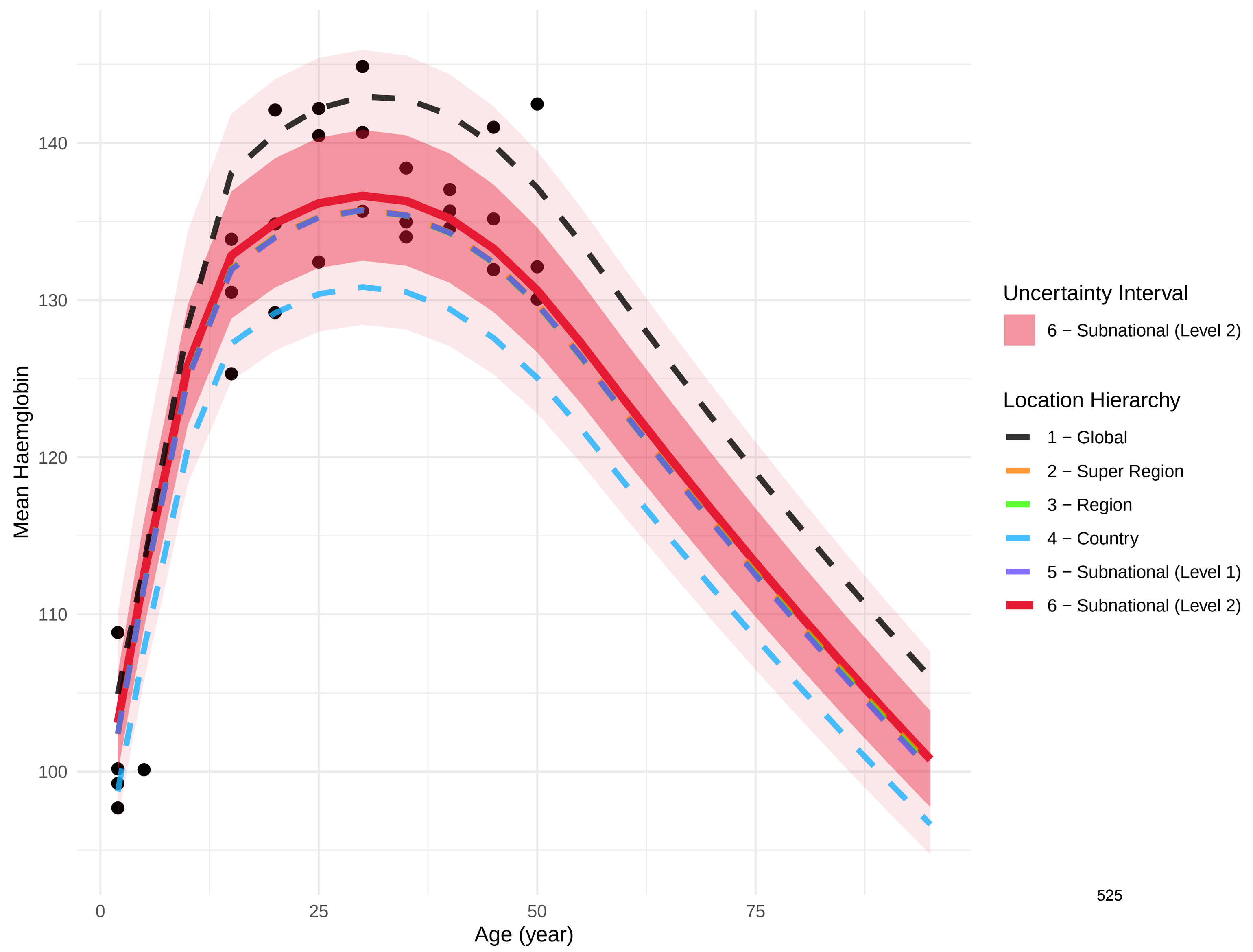

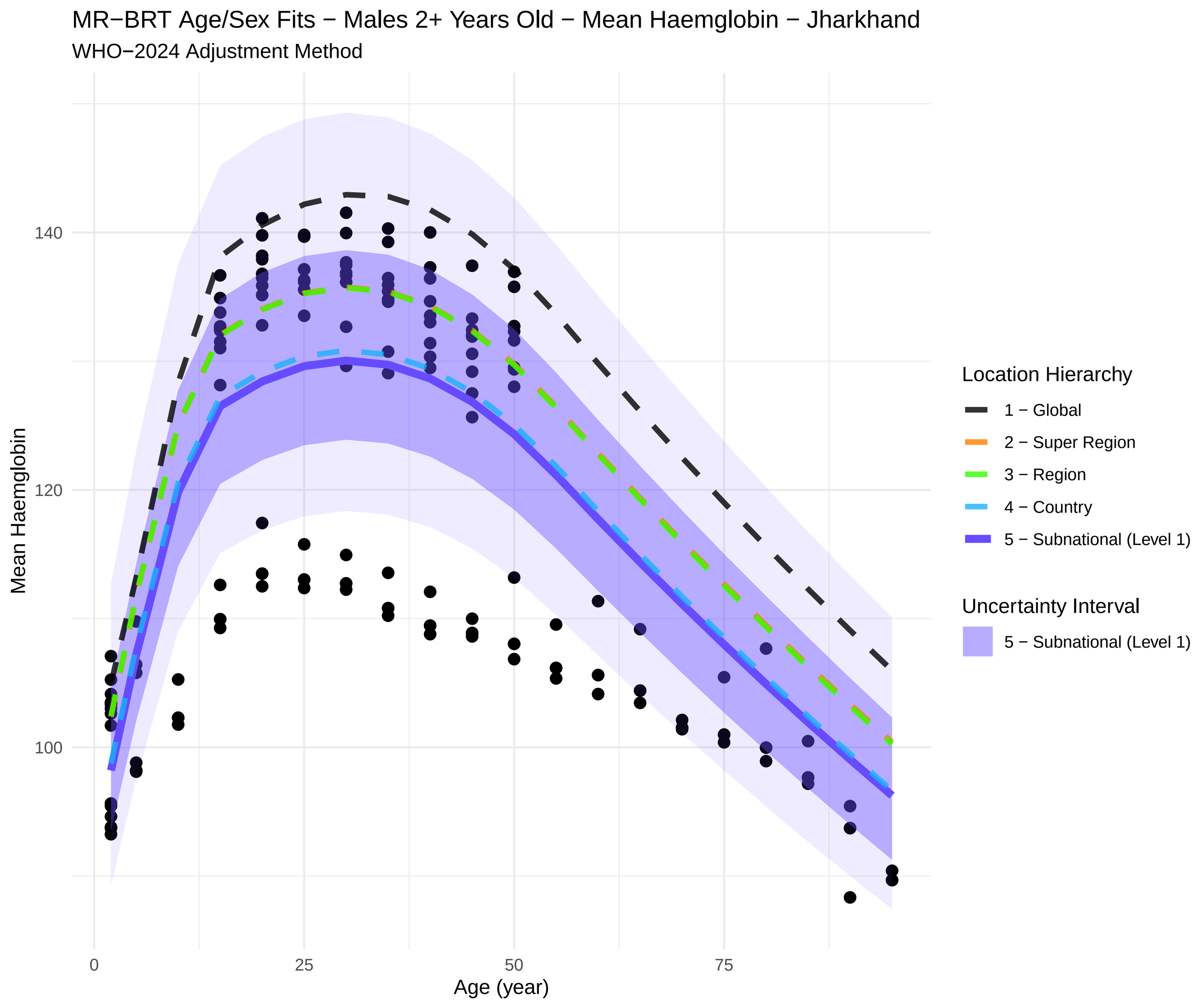
MR−BRT Age/Sex Fits − Males 2+ Years Old − Mean Haemglobin − Jharkhand
WHO−2024 Adjustment Method
Mean Haemglobin
Age (year)
Location Hierarchy
1 − Global
2 − Super Region
3 − Region
4 − Country
5 − Subnational (Level 1)
Uncertainty Interval
5 − Subnational (Level 1)

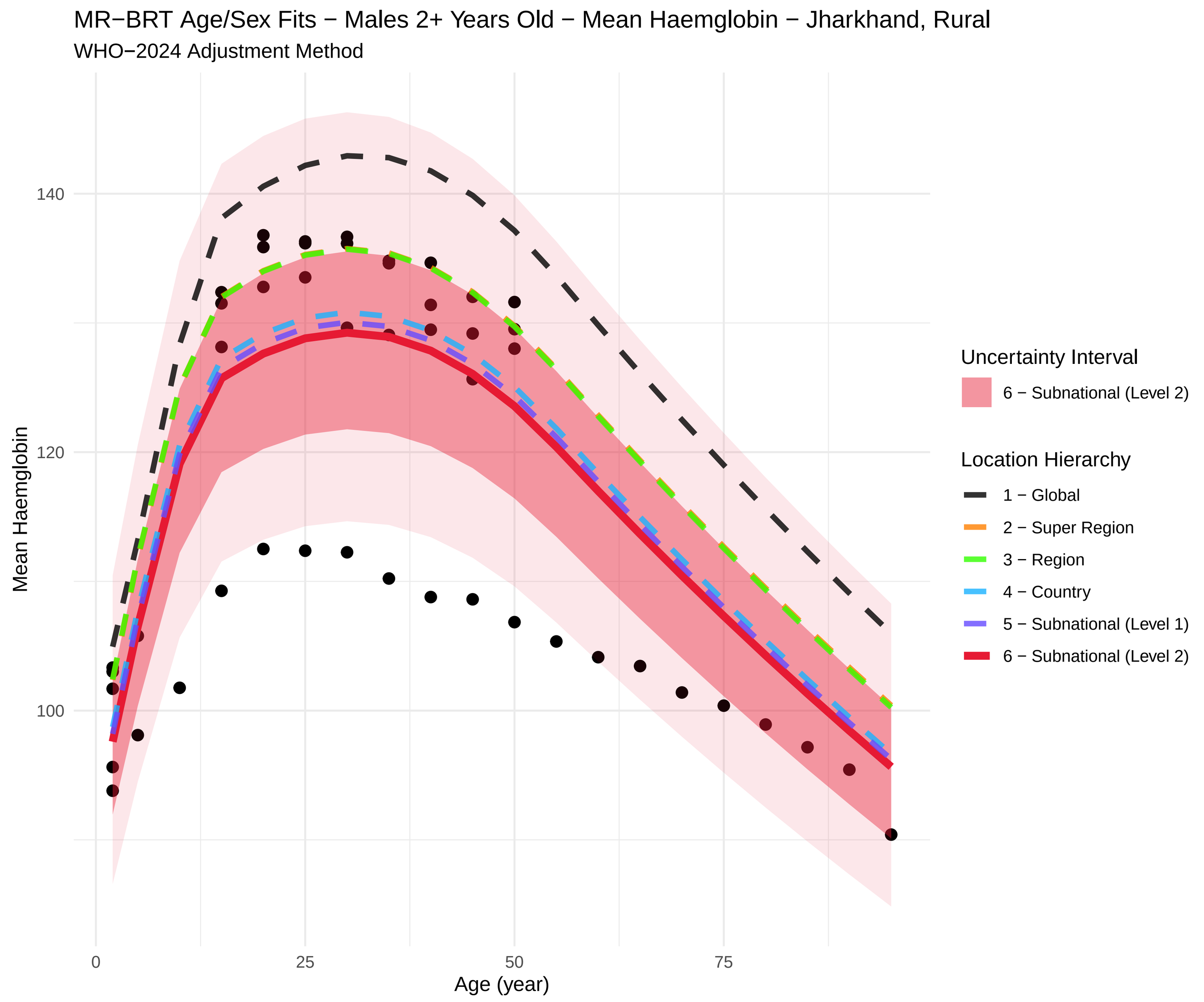
MR−BRT Age/Sex Fits − Males 2+ Years Old − Mean Haemglobin − Jharkhand, Rural
WHO−2024 Adjustment Method
Mean Haemglobin
Age (year)
Uncertainty Interval
6 − Subnational (Level 2)
Location Hierarchy
1 − Global
2 − Super Region
3 − Region
4 − Country
5 − Subnational (Level 1)
6 − Subnational (Level 2)

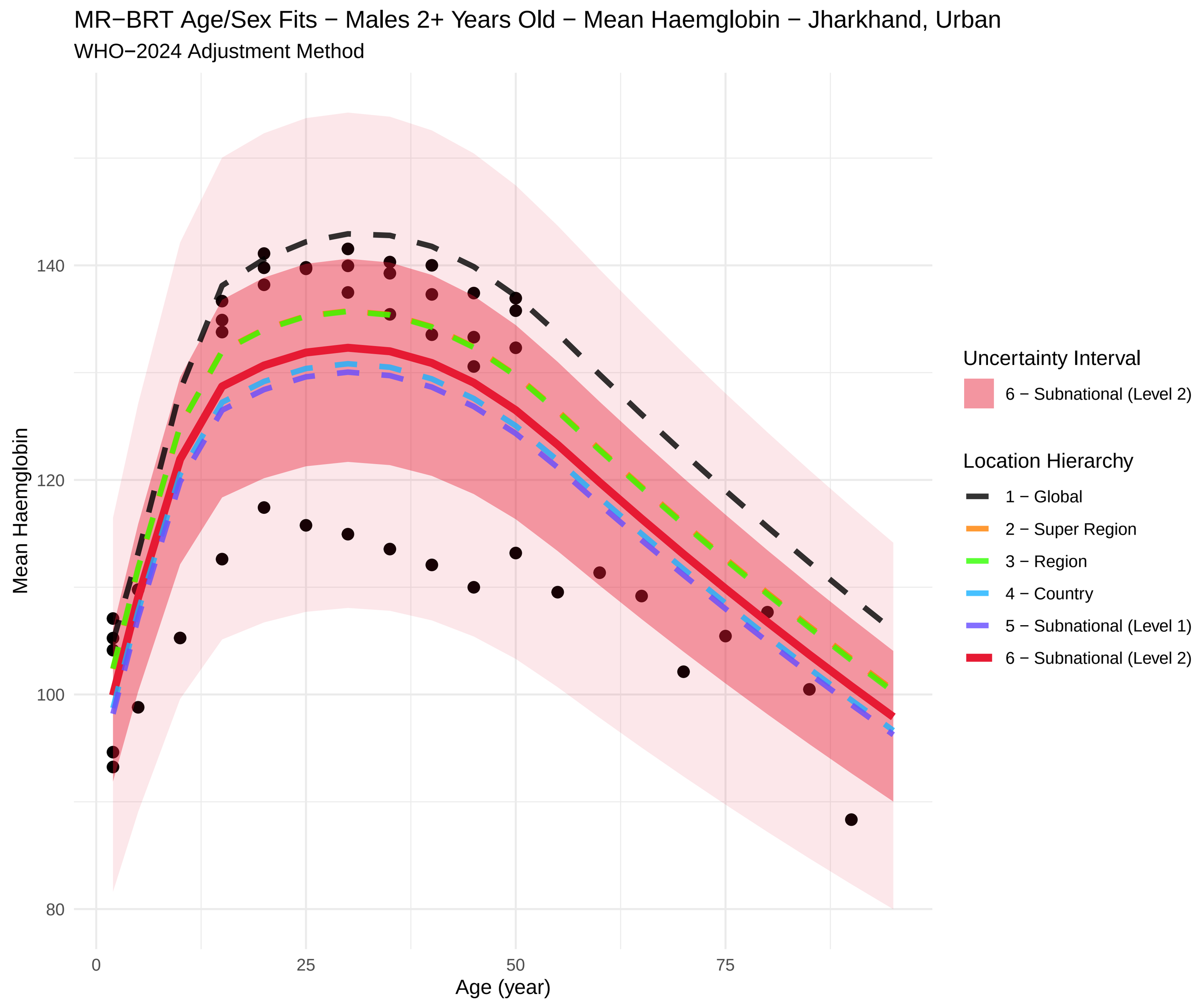
MR−BRT Age/Sex Fits − Males 2+ Years Old − Mean Haemglobin − Jharkhand, Urban
WHO−2024 Adjustment Method
Mean Haemglobin
Age (year)
Uncertainty Interval
6 − Subnational (Level 2)
Location Hierarchy
1 − Global
2 − Super Region
3 − Region
4 − Country
5 − Subnational (Level 1)
6 − Subnational (Level 2)

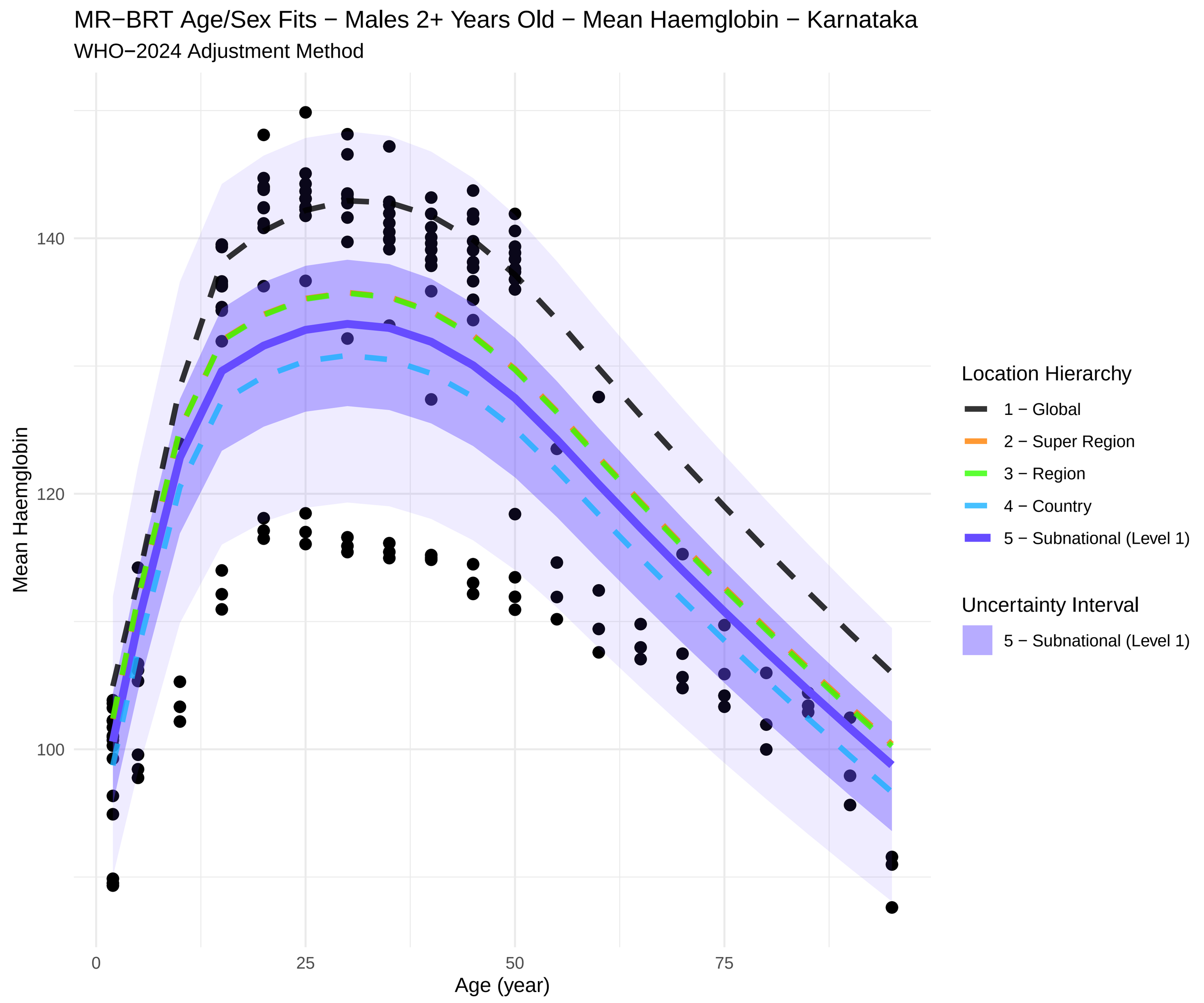
MR−BRT Age/Sex Fits − Males 2+ Years Old − Mean Haemglobin − Karnataka
WHO−2024 Adjustment Method
Mean Haemglobin
Age (year)
Location Hierarchy
1 − Global
2 − Super Region
3 − Region
4 − Country
5 − Subnational (Level 1)
Uncertainty Interval
5 − Subnational (Level 1)

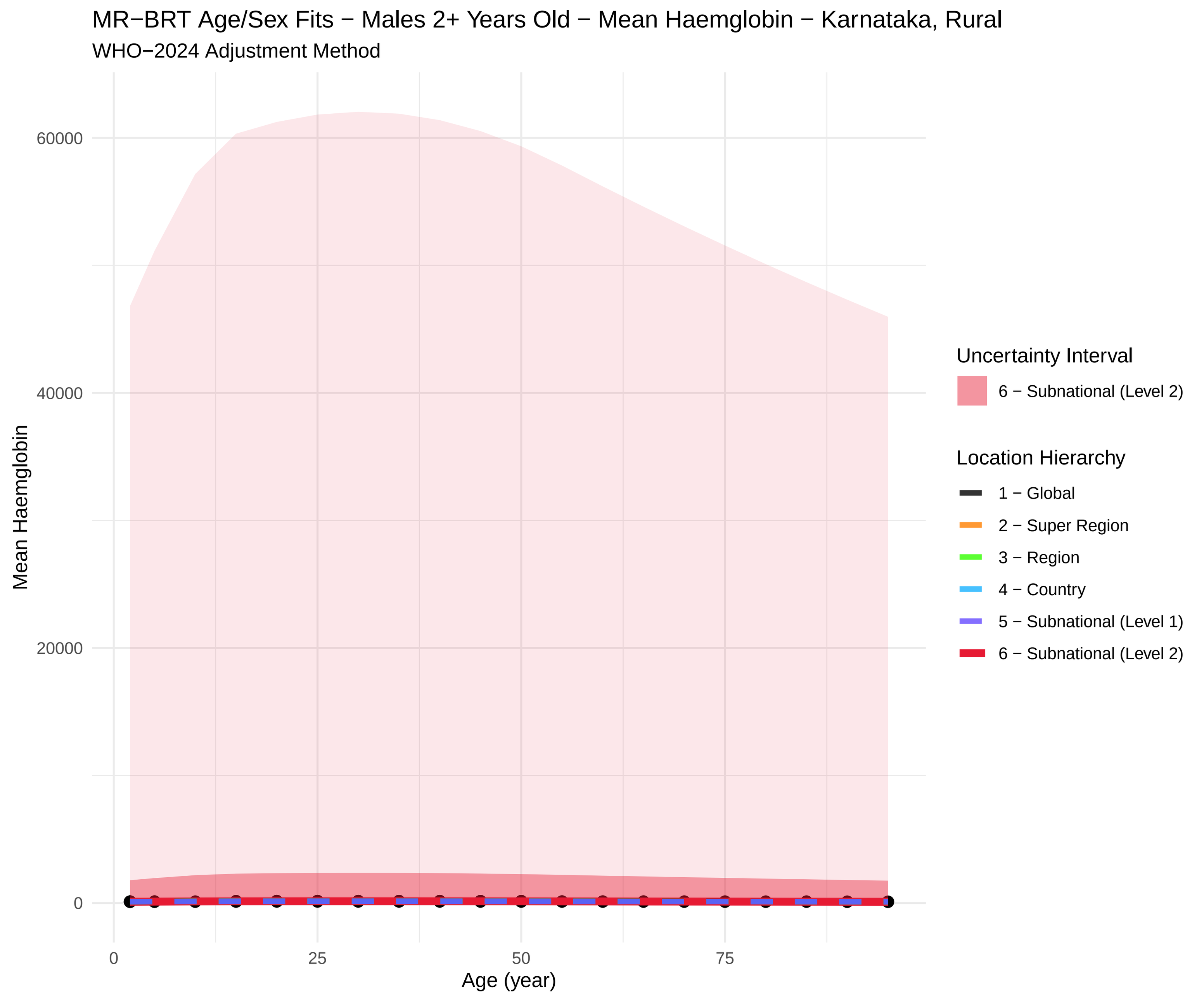
MR−BRT Age/Sex Fits − Males 2+ Years Old − Mean Haemglobin − Karnataka, Rural
WHO−2024 Adjustment Method
Mean Haemglobin
Age (year)
Uncertainty Interval
6 − Subnational (Level 2)
Location Hierarchy
1 − Global
2 − Super Region
3 − Region
4 − Country
5 − Subnational (Level 1)
6 − Subnational (Level 2)

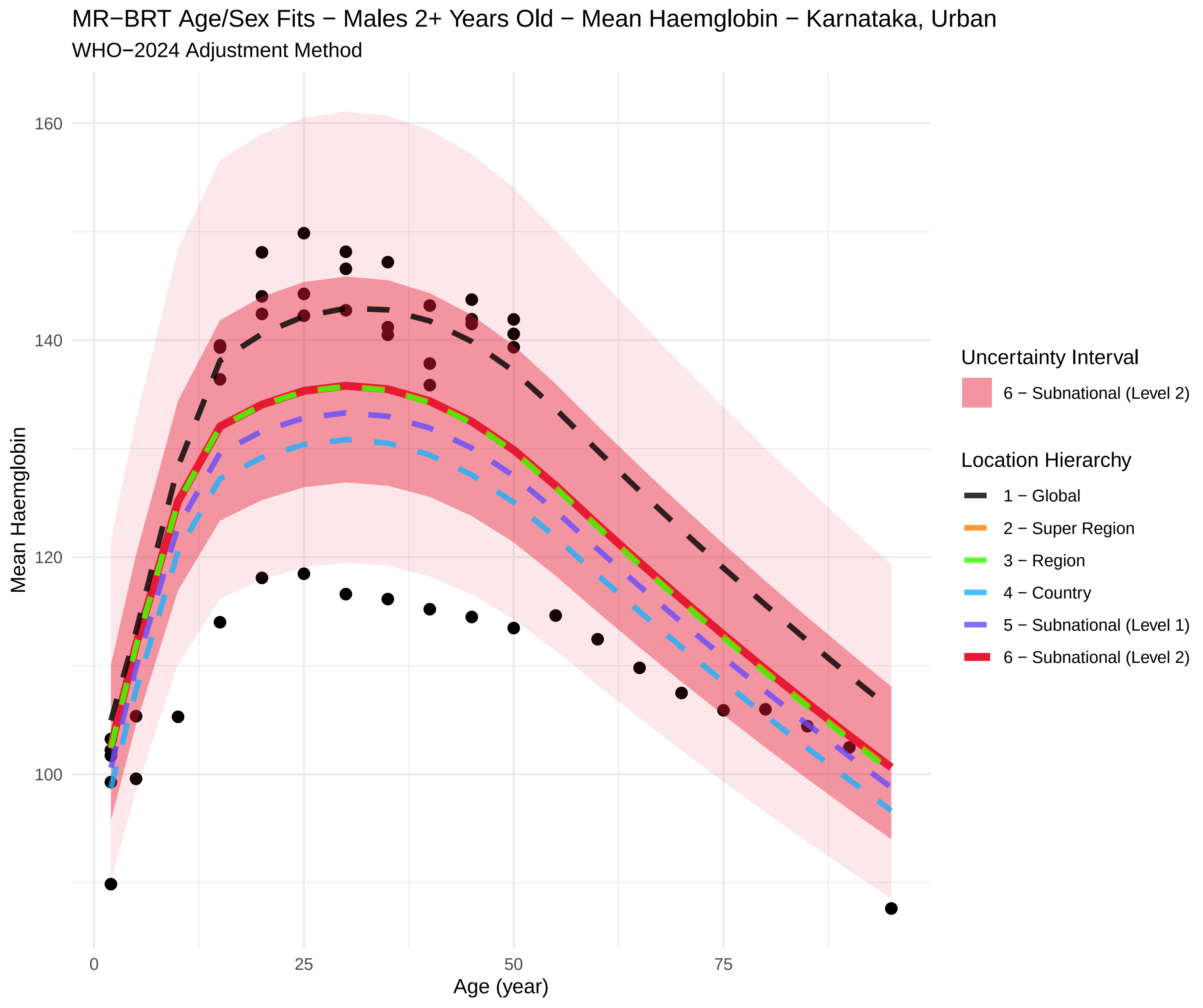
MR−BRT Age/Sex Fits − Males 2+ Years Old − Mean Haemglobin − Karnataka, Urban
WHO−2024 Adjustment Method
Mean Haemglobin
Age (year)
Uncertainty Interval
6 − Subnational (Level 2)
Location Hierarchy
1 − Global
2 − Super Region
3 − Region
4 − Country
5 − Subnational (Level 1)
6 − Subnational (Level 2)

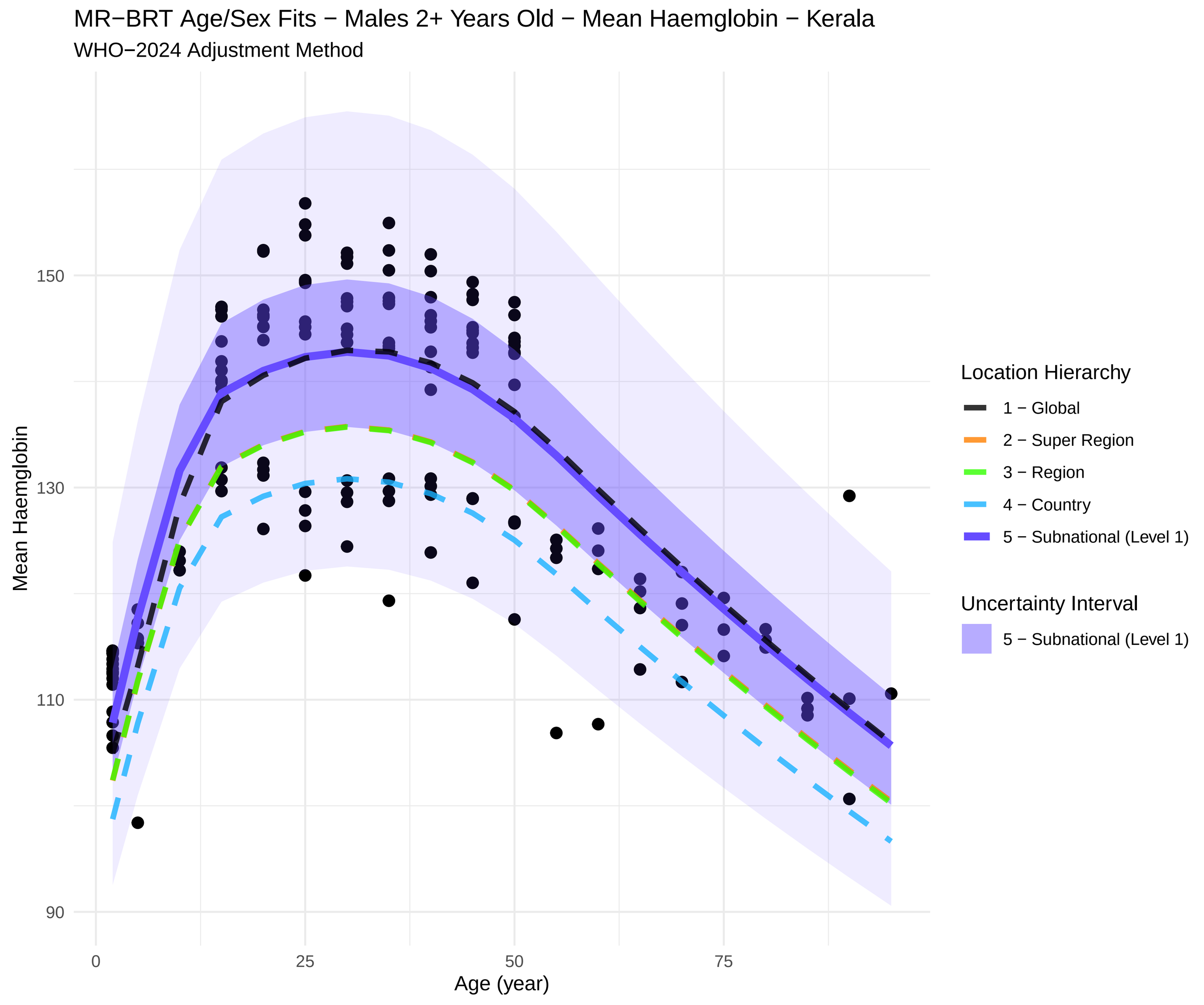
MR−BRT Age/Sex Fits − Males 2+ Years Old − Mean Haemglobin − Kerala
WHO−2024 Adjustment Method
Mean Haemglobin
Age (year)
Location Hierarchy
1 − Global
2 − Super Region
3 − Region
4 − Country
5 − Subnational (Level 1)
Uncertainty Interval
5 − Subnational (Level 1)

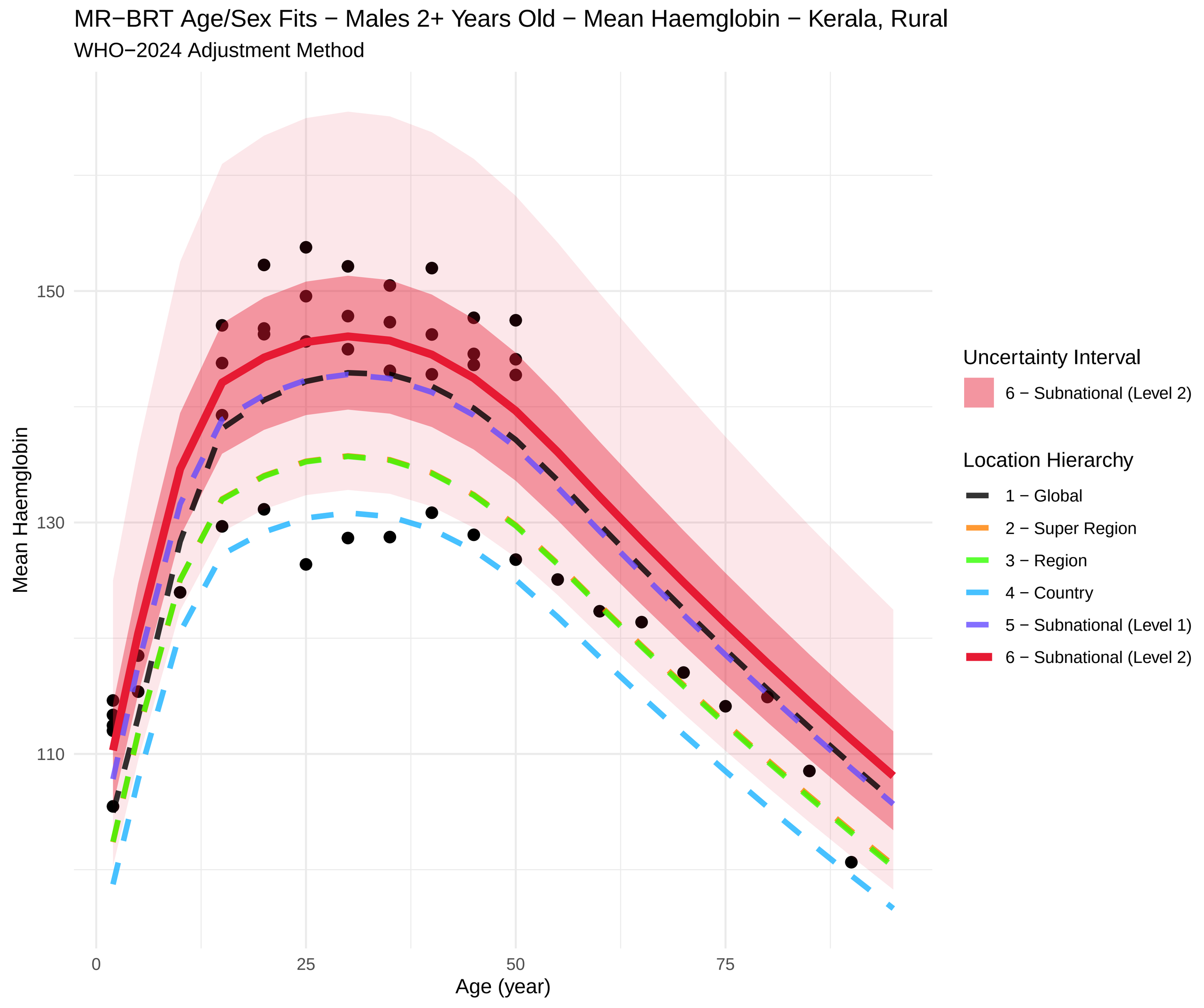
MR−BRT Age/Sex Fits − Males 2+ Years Old − Mean Haemglobin − Kerala, Rural
WHO−2024 Adjustment Method
Mean Haemglobin
Age (year)
Uncertainty Interval
6 − Subnational (Level 2)
Location Hierarchy
1 − Global
2 − Super Region
3 − Region
4 − Country
5 − Subnational (Level 1)
6 − Subnational (Level 2)

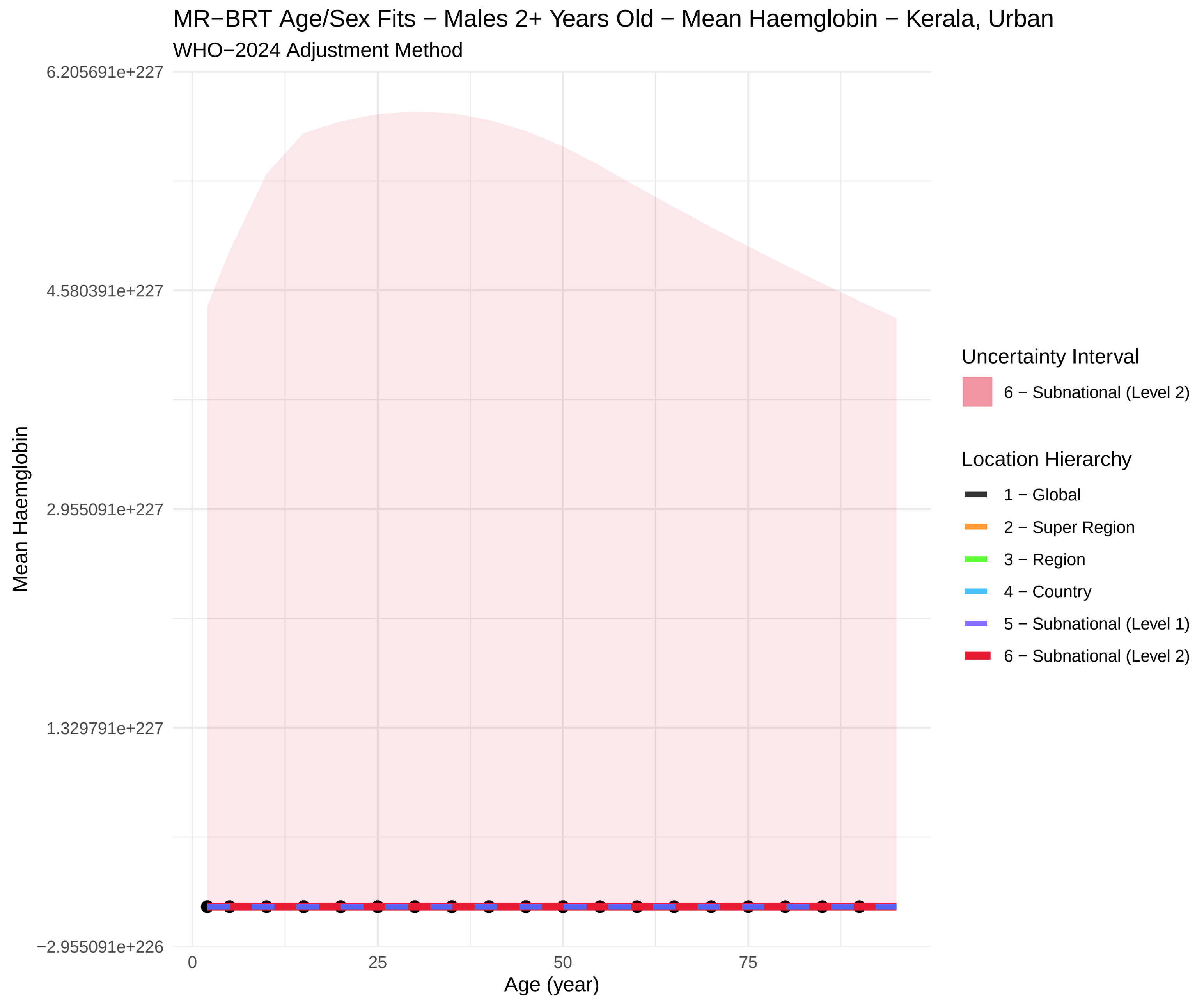
MR−BRT Age/Sex Fits − Males 2+ Years Old − Mean Haemglobin − Kerala, Urban
WHO−2024 Adjustment Method
Mean Haemglobin
Age (year)
Uncertainty Interval
6 − Subnational (Level 2)
Location Hierarchy
1 − Global
2 − Super Region
3 − Region
4 − Country
5 − Subnational (Level 1)
6 − Subnational (Level 2)

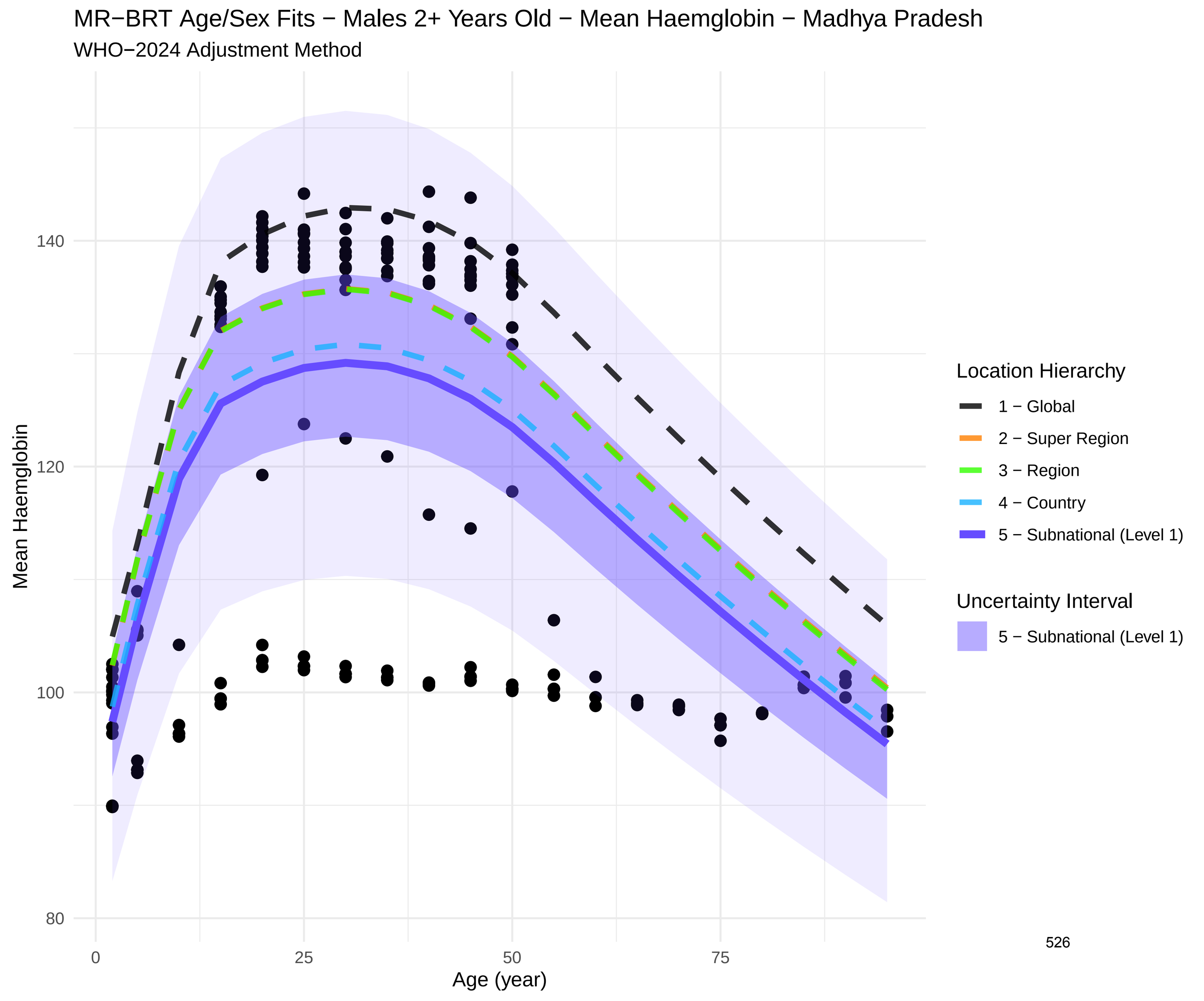
MR−BRT Age/Sex Fits − Males 2+ Years Old − Mean Haemglobin − Madhya Pradesh
WHO−2024 Adjustment Method
Mean Haemglobin
Age (year)
Location Hierarchy
1 − Global
2 − Super Region
3 − Region
4 − Country
5 − Subnational (Level 1)
Uncertainty Interval
5 − Subnational (Level 1)

MR−BRT Age/Sex Fits − Males 2+ Years Old − Mean Haemglobin − Madhya Pradesh, Rural
WHO−2024 Adjustment Method

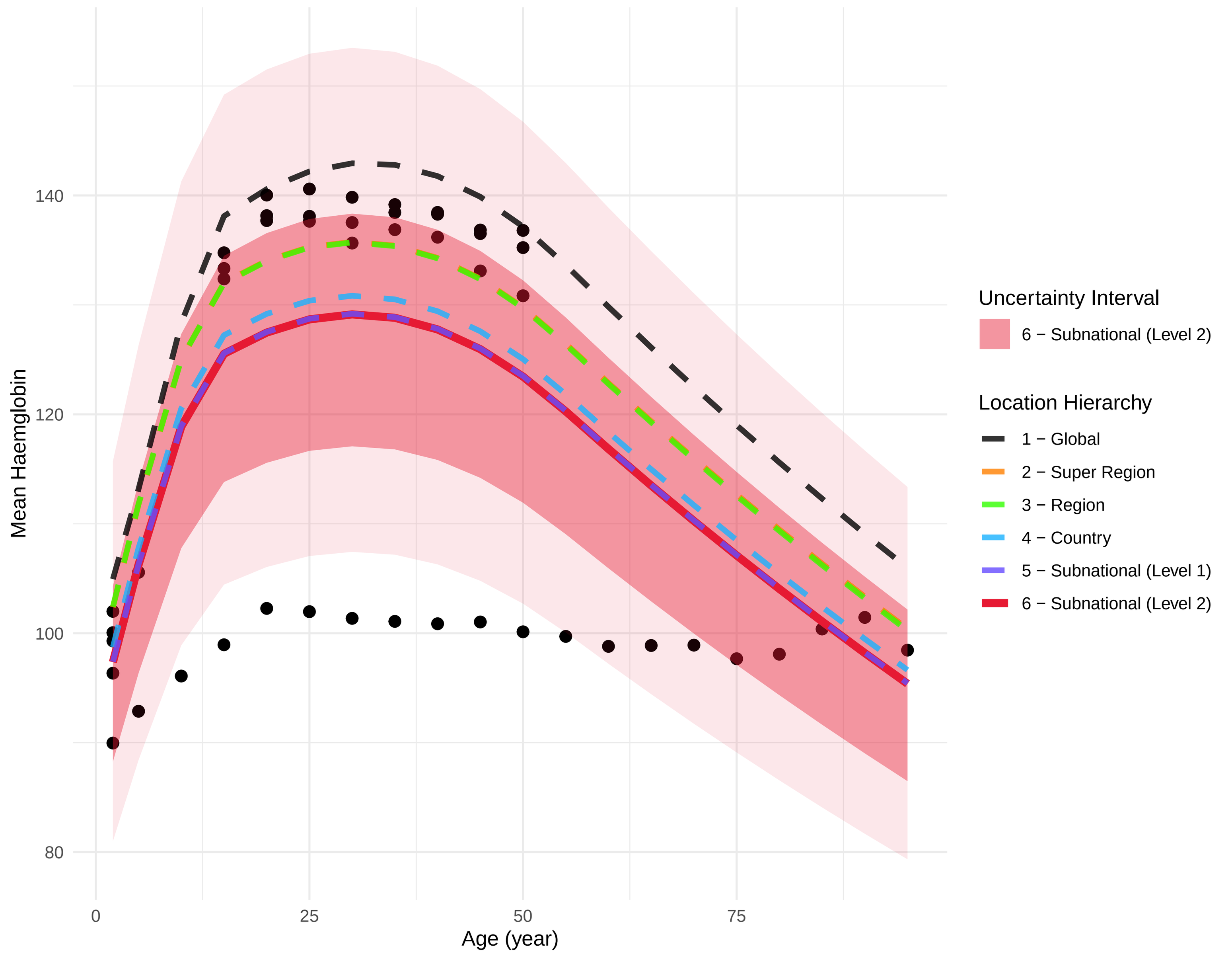

MR−BRT Age/Sex Fits − Males 2+ Years Old − Mean Haemglobin − Madhya Pradesh, Urban
WHO−2024 Adjustment Method

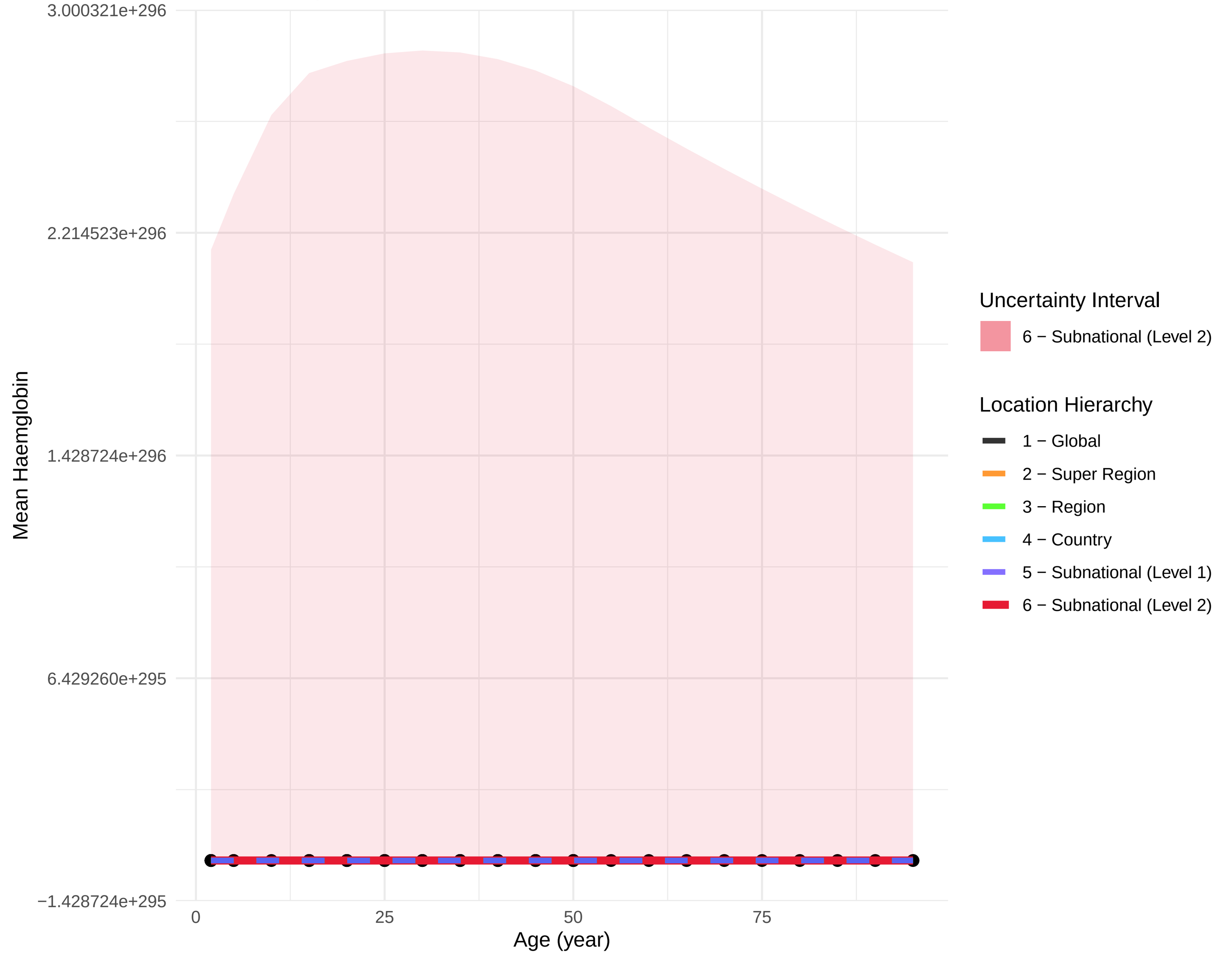

MR−BRT Age/Sex Fits − Males 2+ Years Old − Mean Haemglobin − Maharashtra
WHO−2024 Adjustment Method

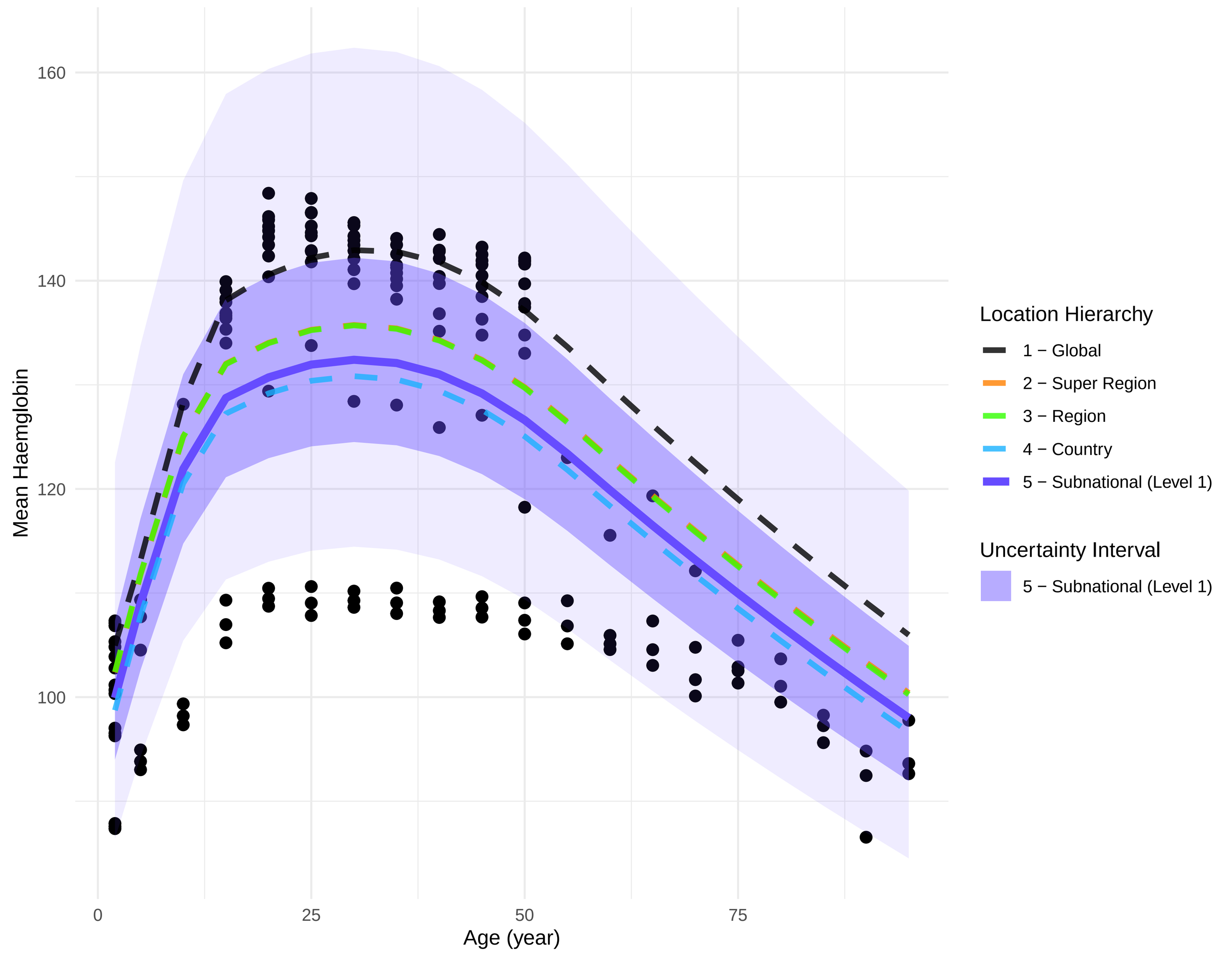

MR−BRT Age/Sex Fits − Males 2+ Years Old − Mean Haemglobin − Maharashtra, Rural
WHO−2024 Adjustment Method

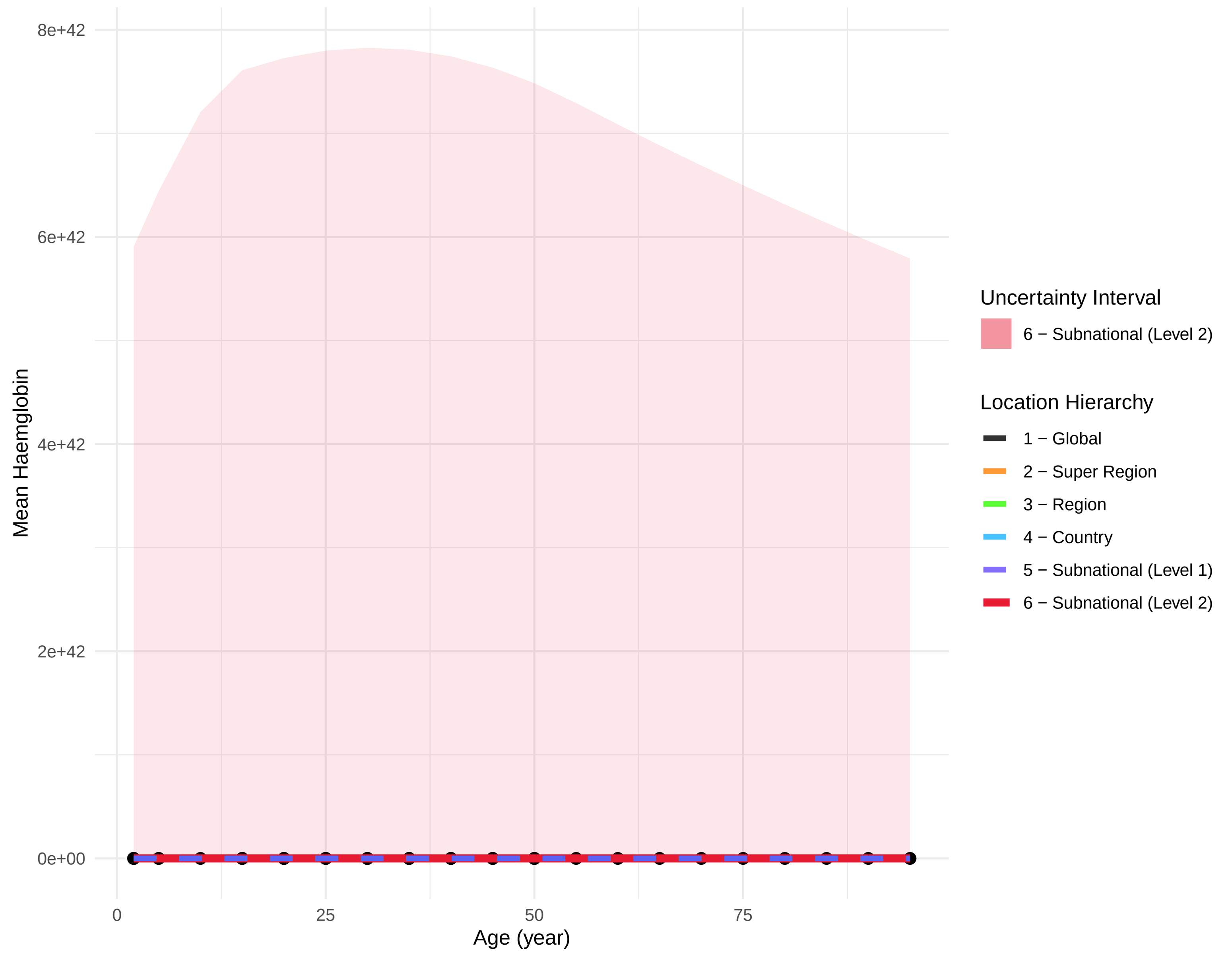

MR−BRT Age/Sex Fits − Males 2+ Years Old − Mean Haemglobin − Maharashtra, Urban
WHO−2024 Adjustment Method

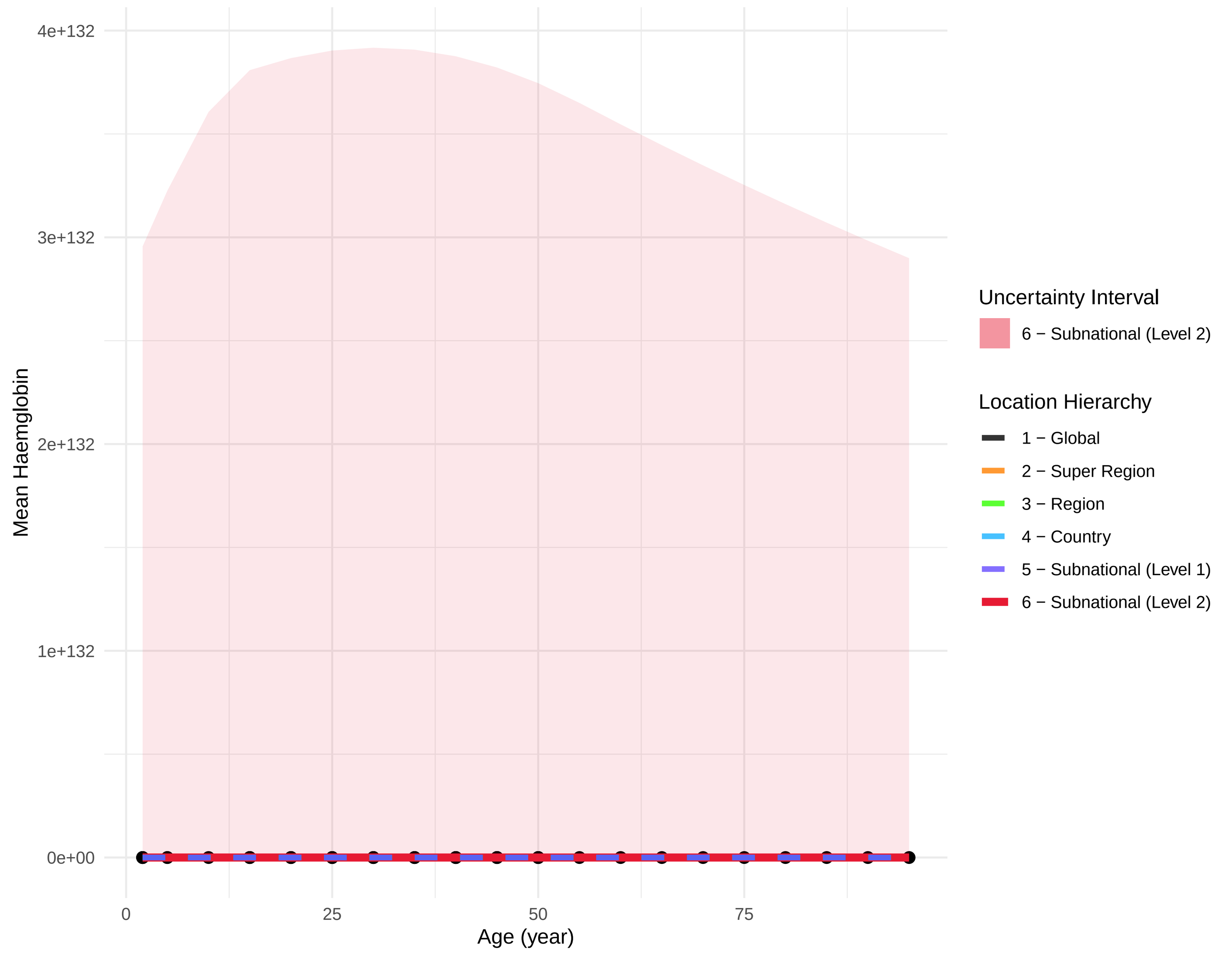

MR−BRT Age/Sex Fits − Males 2+ Years Old − Mean Haemglobin − Manipur
WHO−2024 Adjustment Method

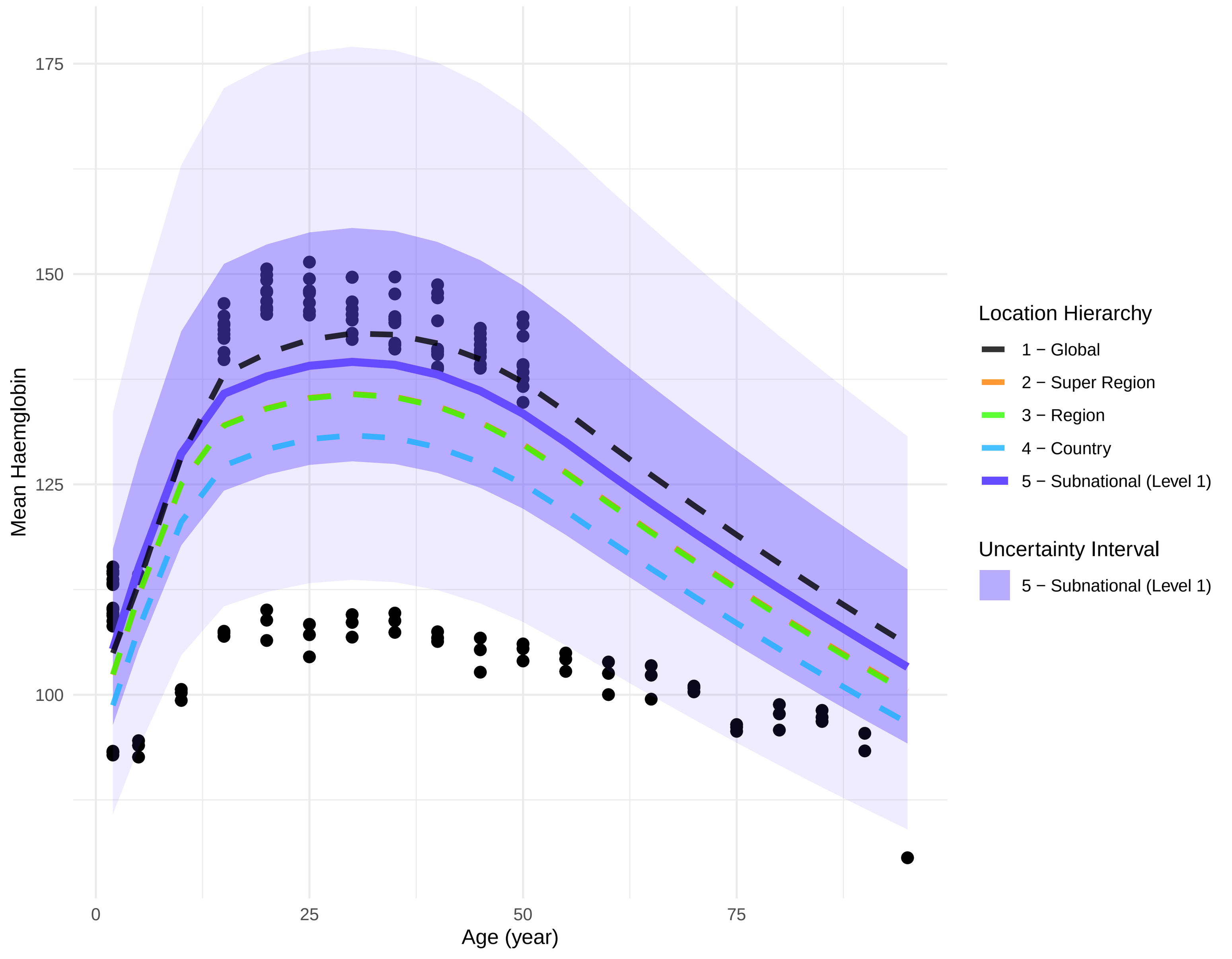

MR−BRT Age/Sex Fits − Males 2+ Years Old − Mean Haemglobin − Manipur, Rural
WHO−2024 Adjustment Method

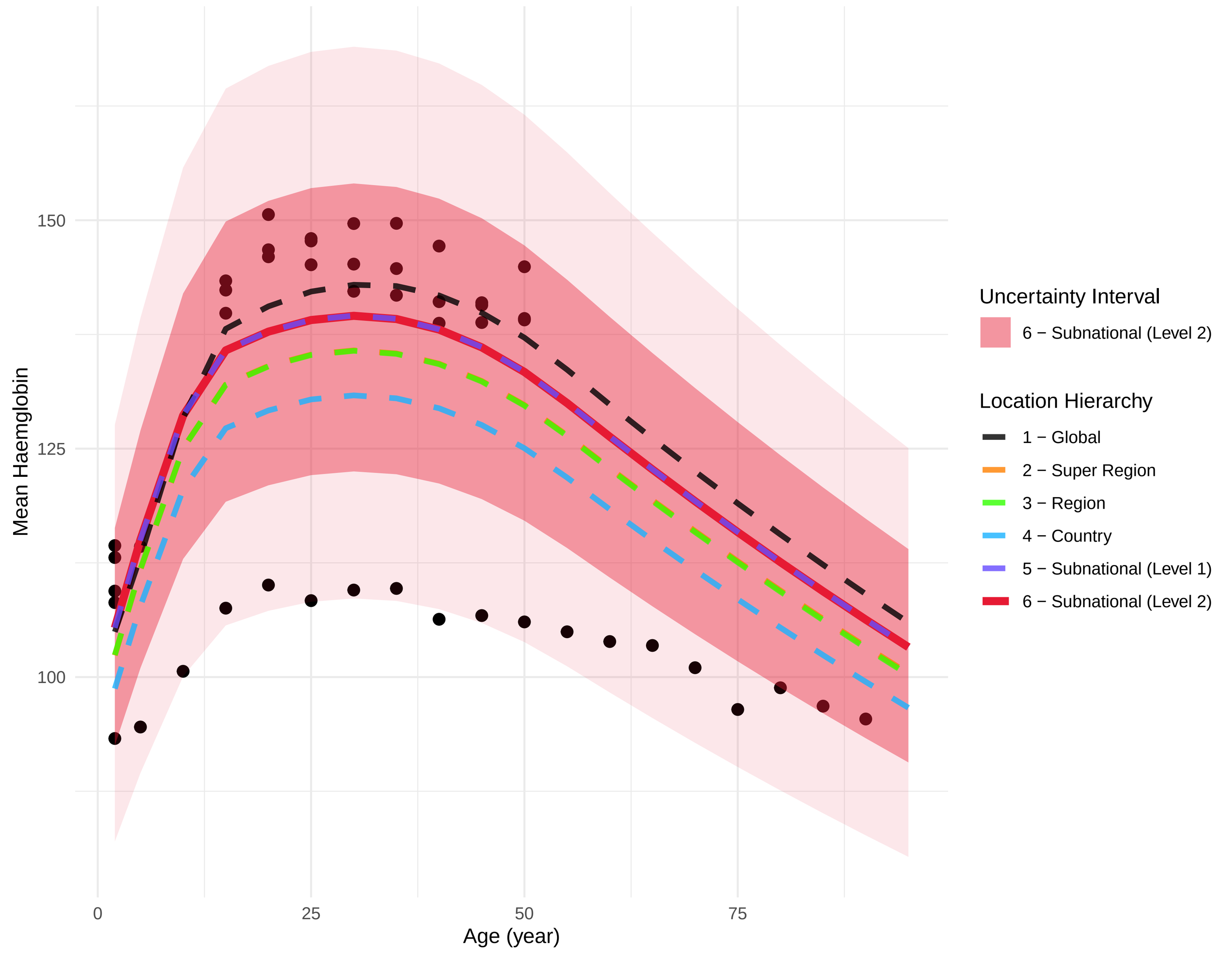

MR−BRT Age/Sex Fits − Males 2+ Years Old − Mean Haemglobin − Manipur, Urban
WHO−2024 Adjustment Method

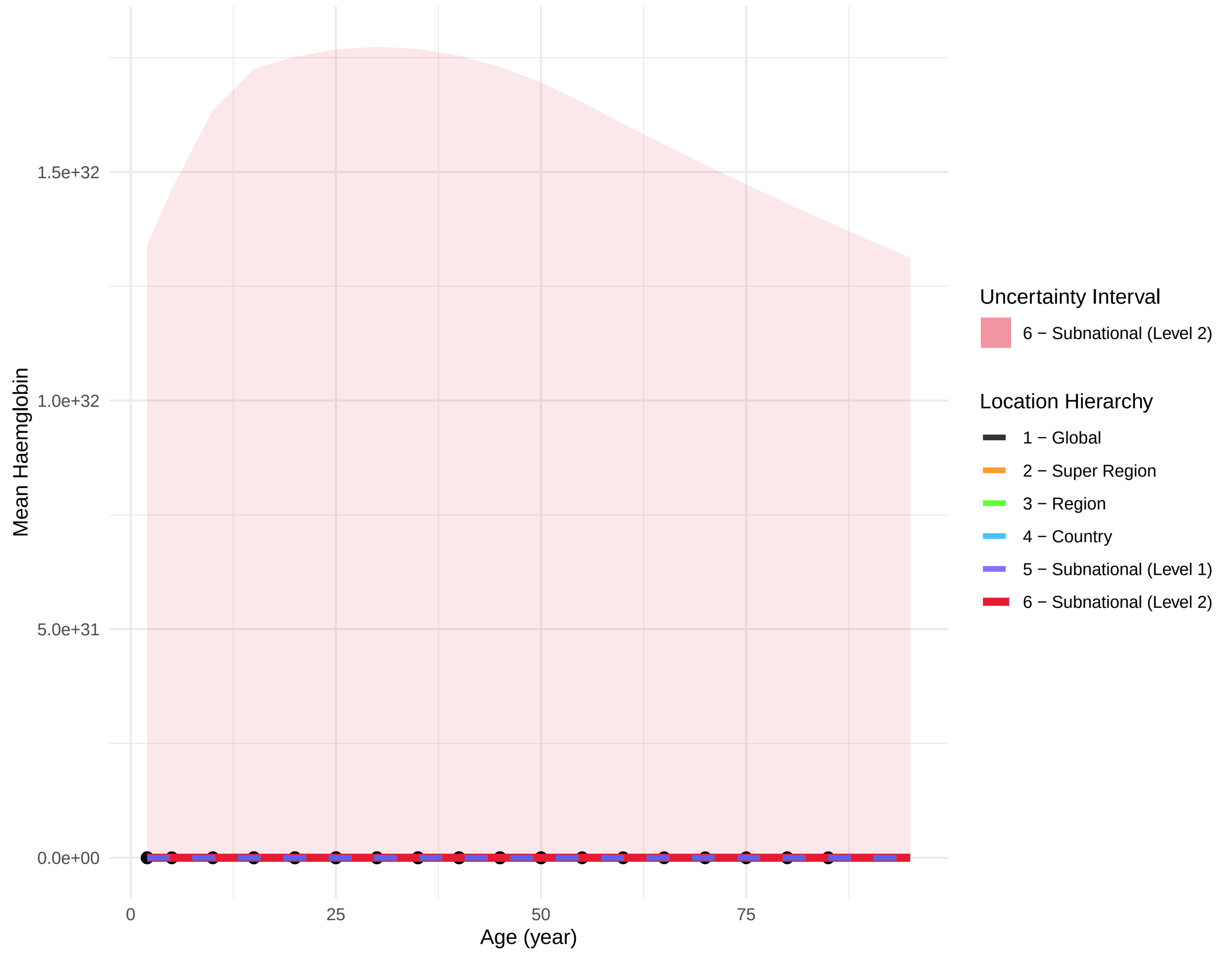

MR−BRT Age/Sex Fits − Males 2+ Years Old − Mean Haemglobin − Meghalaya
WHO−2024 Adjustment Method

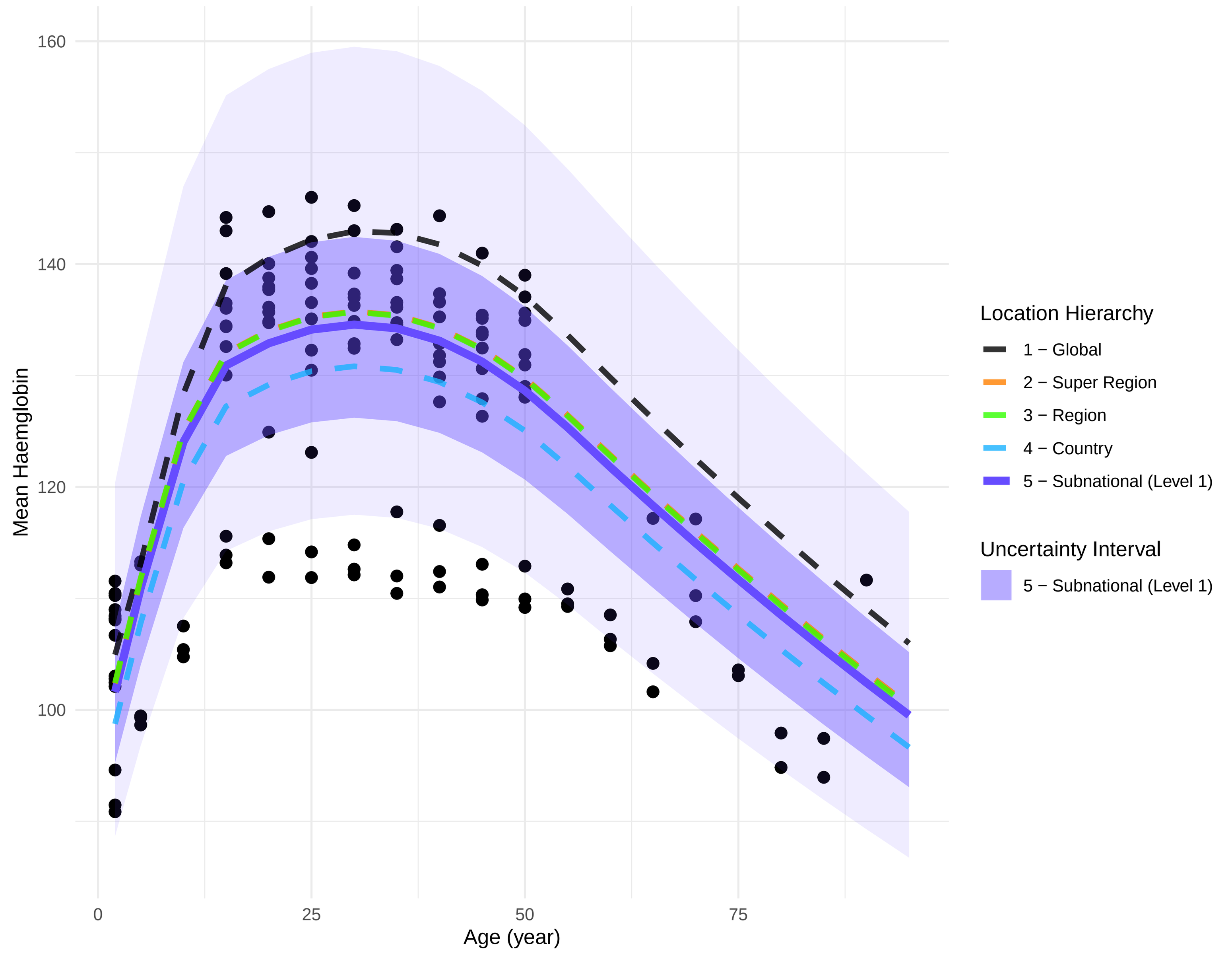

MR−BRT Age/Sex Fits − Males 2+ Years Old − Mean Haemglobin − Meghalaya, Rural
WHO−2024 Adjustment Method

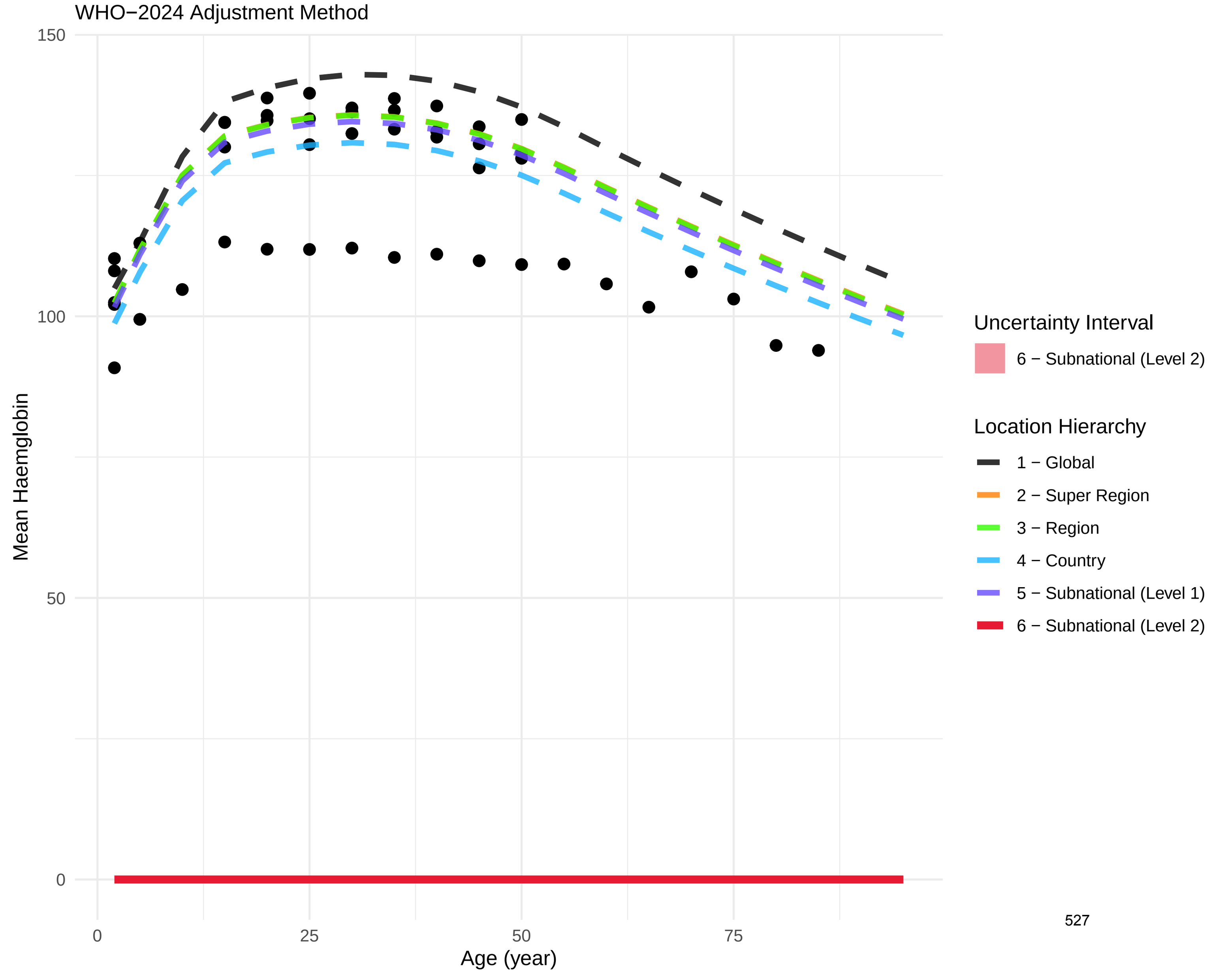

MR−BRT Age/Sex Fits − Males 2+ Years Old − Mean Haemglobin − Meghalaya, Urban
WHO−2024 Adjustment Method

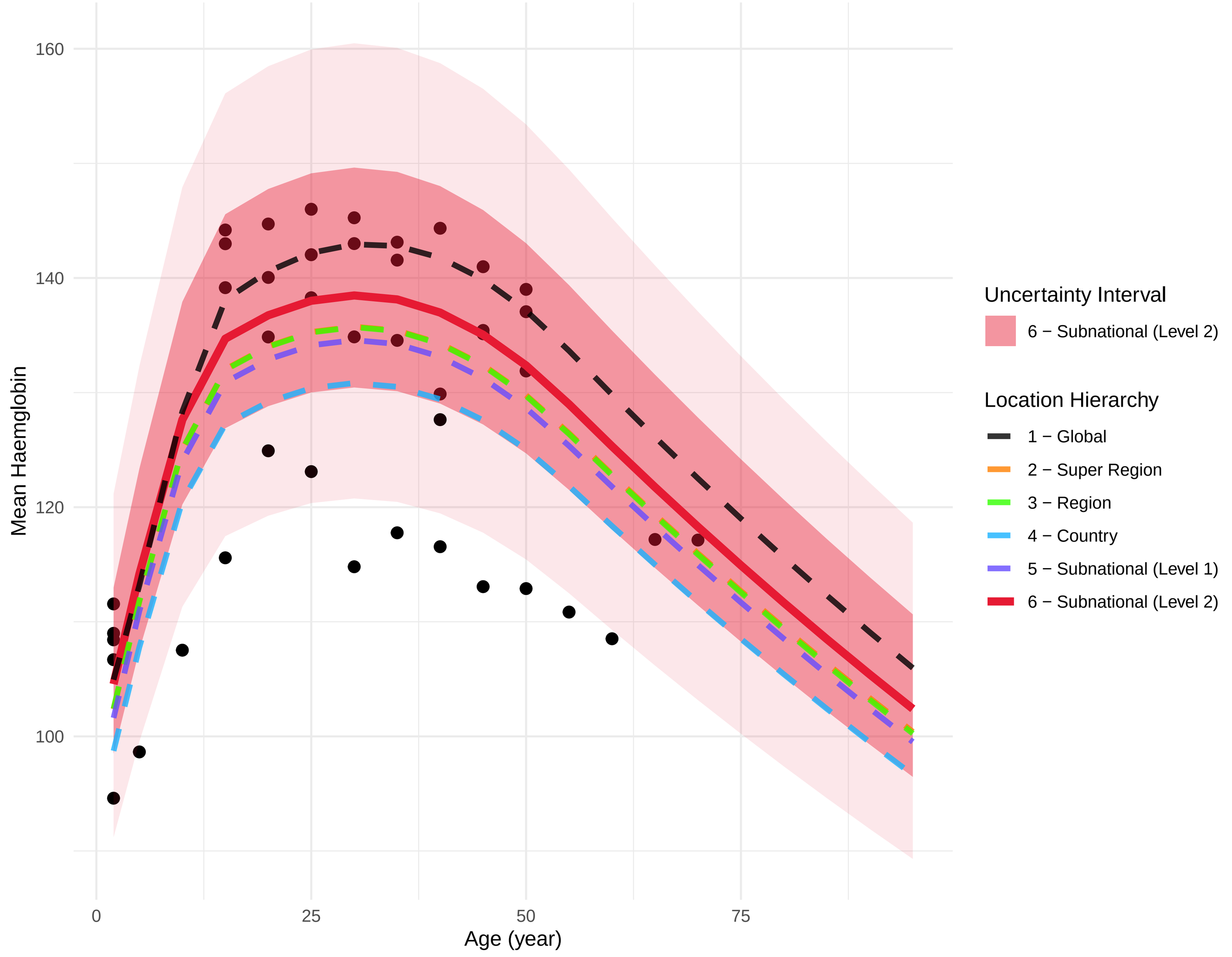

MR−BRT Age/Sex Fits − Males 2+ Years Old − Mean Haemglobin − Mizoram
WHO−2024 Adjustment Method

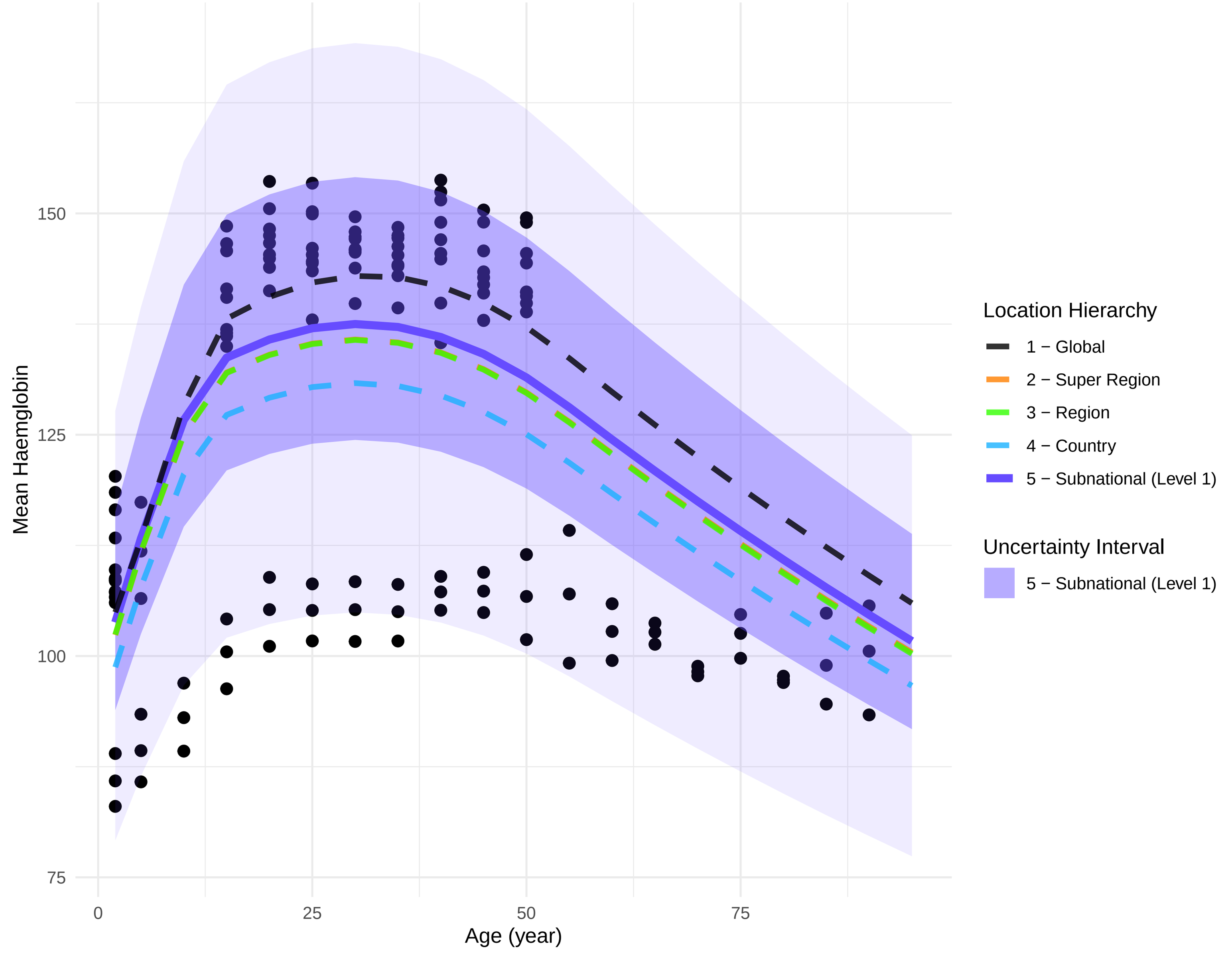

MR−BRT Age/Sex Fits − Males 2+ Years Old − Mean Haemglobin − Mizoram, Rural
WHO−2024 Adjustment Method

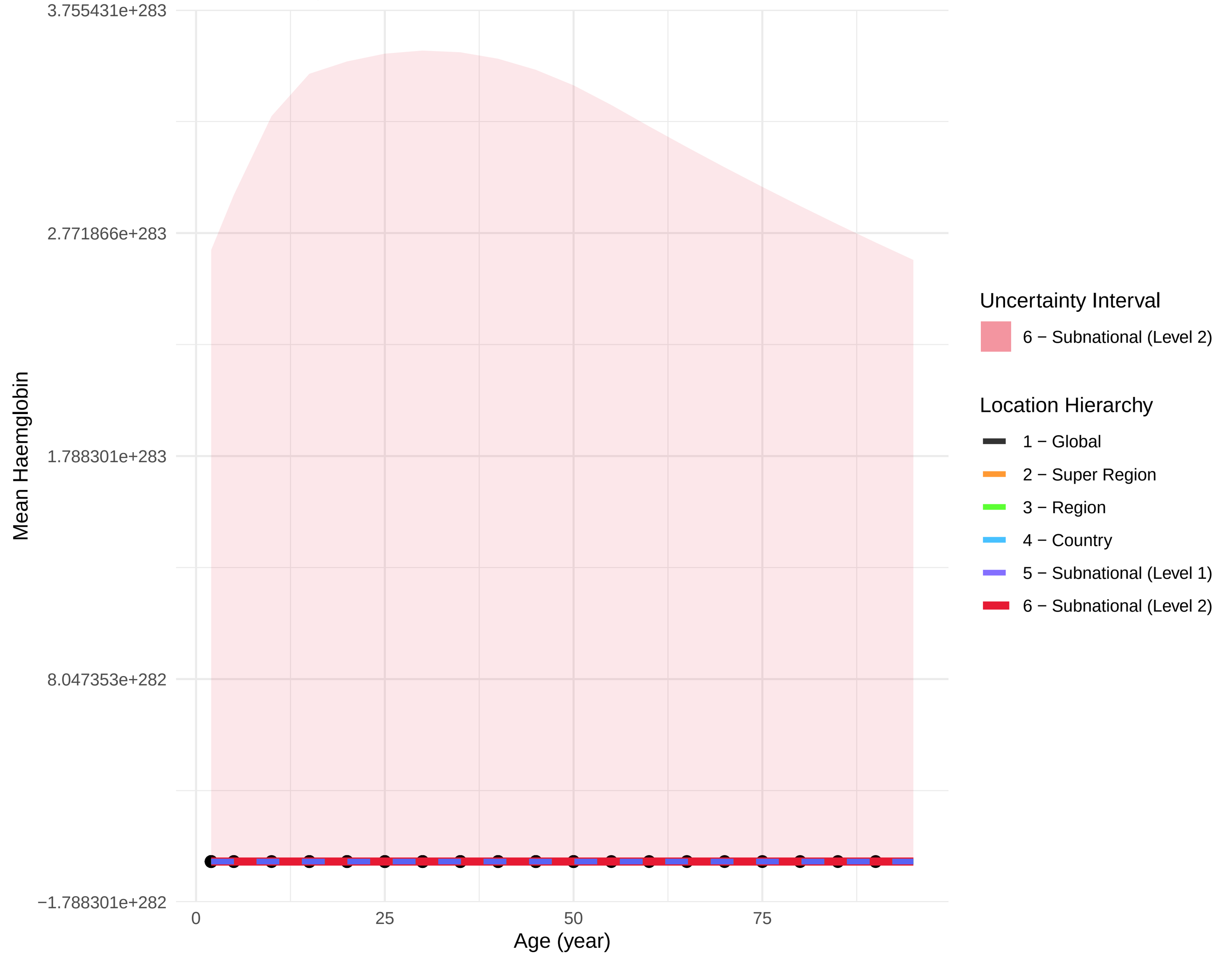

MR−BRT Age/Sex Fits − Males 2+ Years Old − Mean Haemglobin − Mizoram, Urban
WHO−2024 Adjustment Method

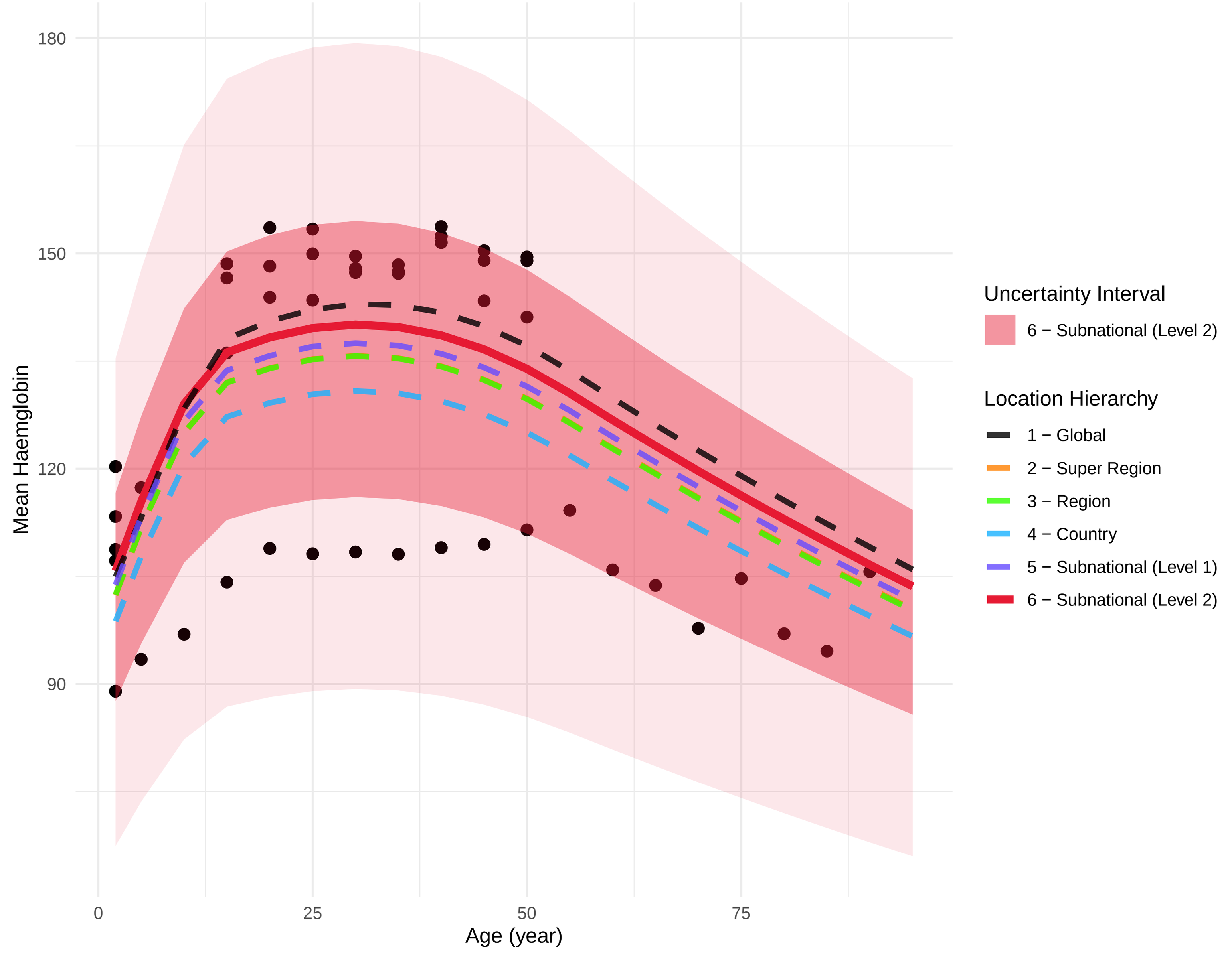

MR−BRT Age/Sex Fits − Males 2+ Years Old − Mean Haemglobin − Nagaland
WHO−2024 Adjustment Method

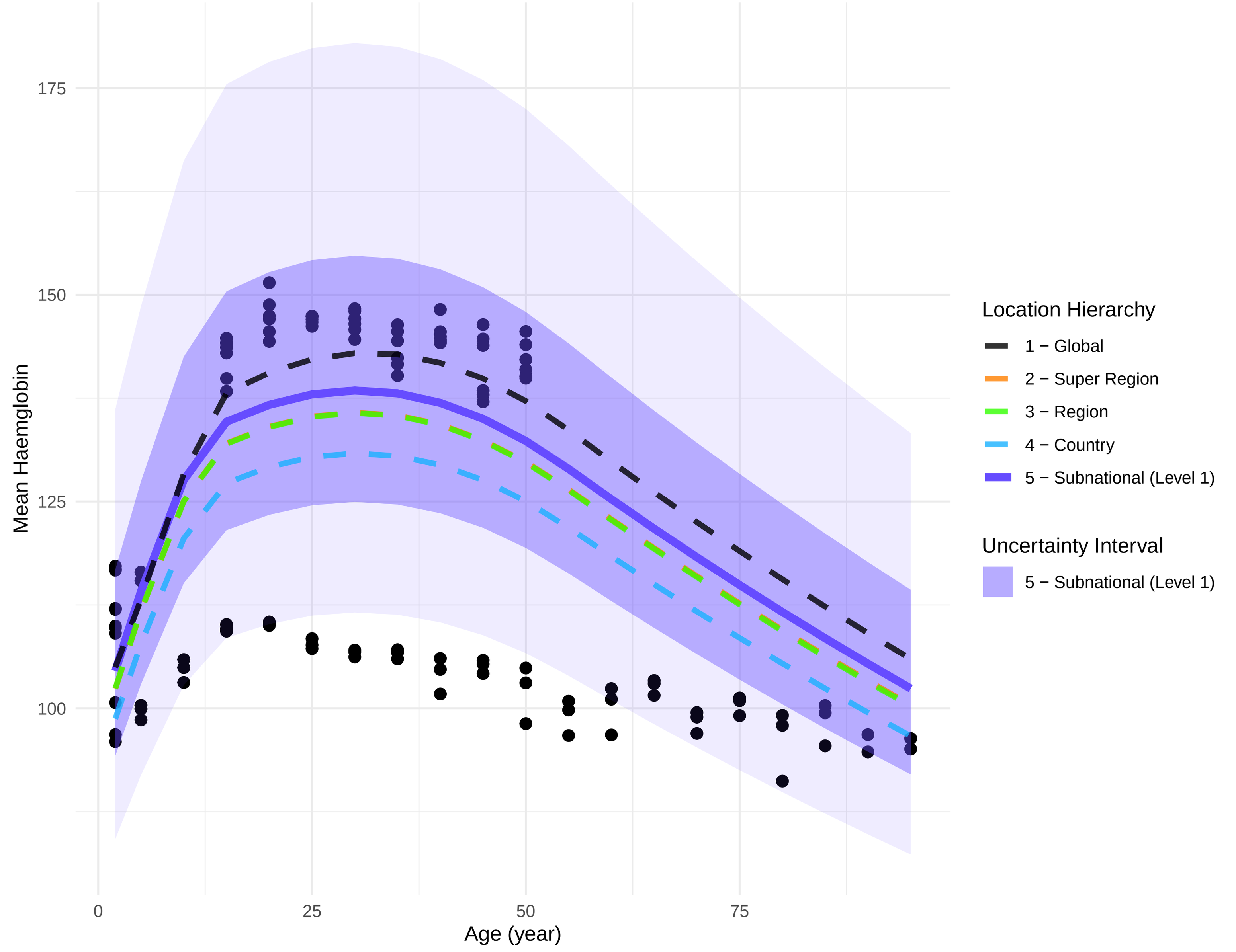

MR−BRT Age/Sex Fits − Males 2+ Years Old − Mean Haemglobin − Nagaland, Rural
WHO−2024 Adjustment Method

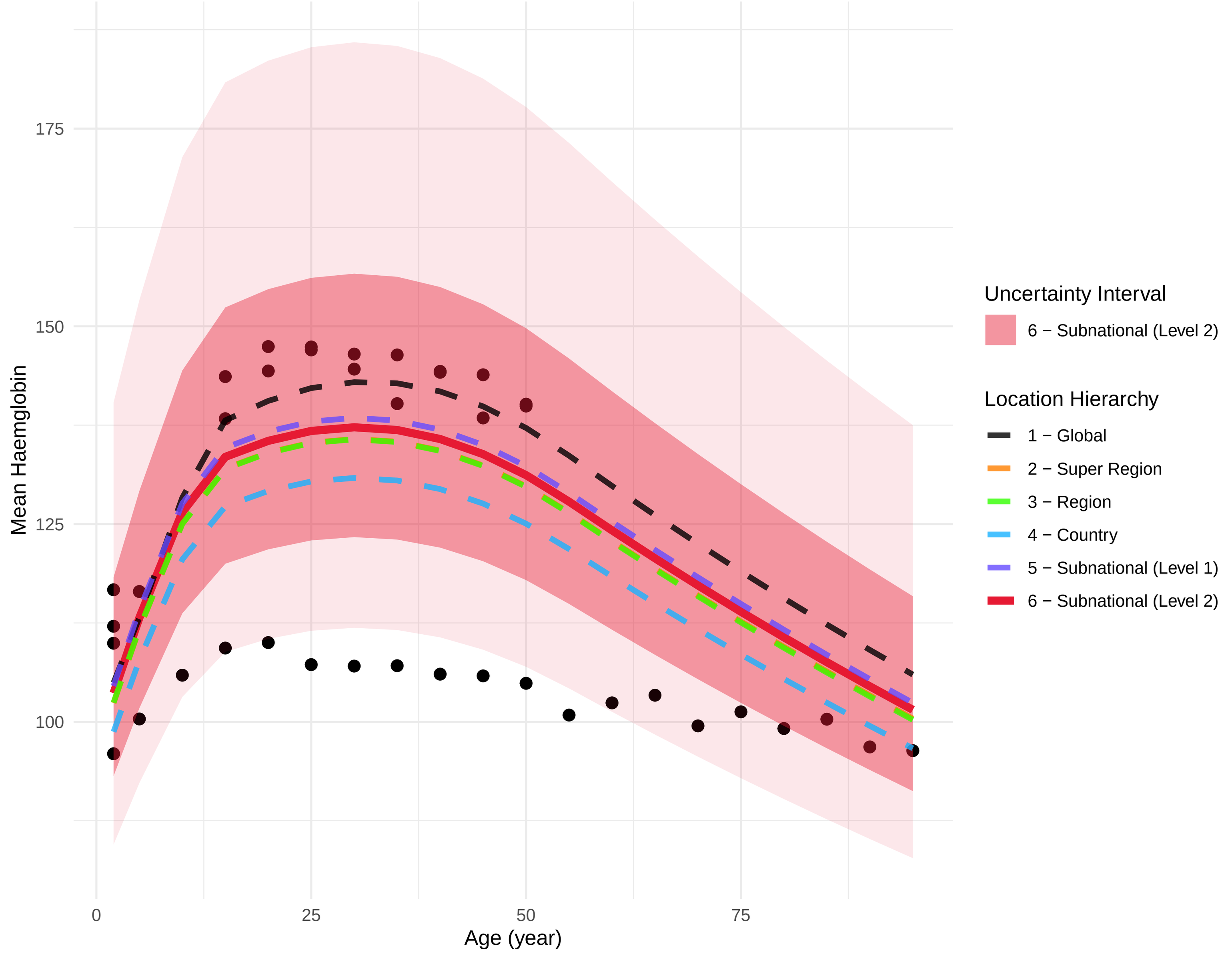

MR−BRT Age/Sex Fits − Males 2+ Years Old − Mean Haemglobin − Nagaland, Urban
WHO−2024 Adjustment Method

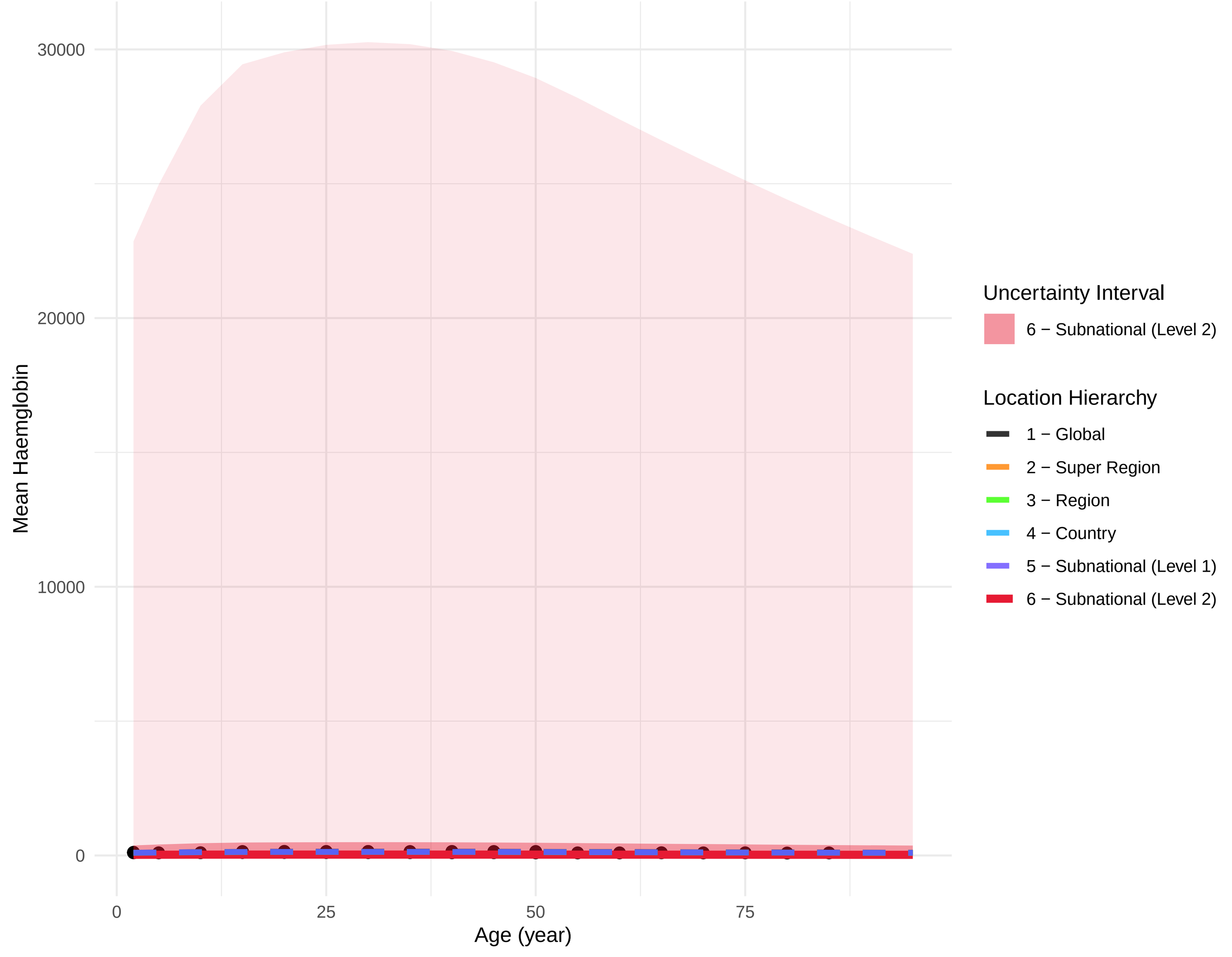

MR−BRT Age/Sex Fits − Males 2+ Years Old − Mean Haemglobin − Odisha
WHO−2024 Adjustment Method

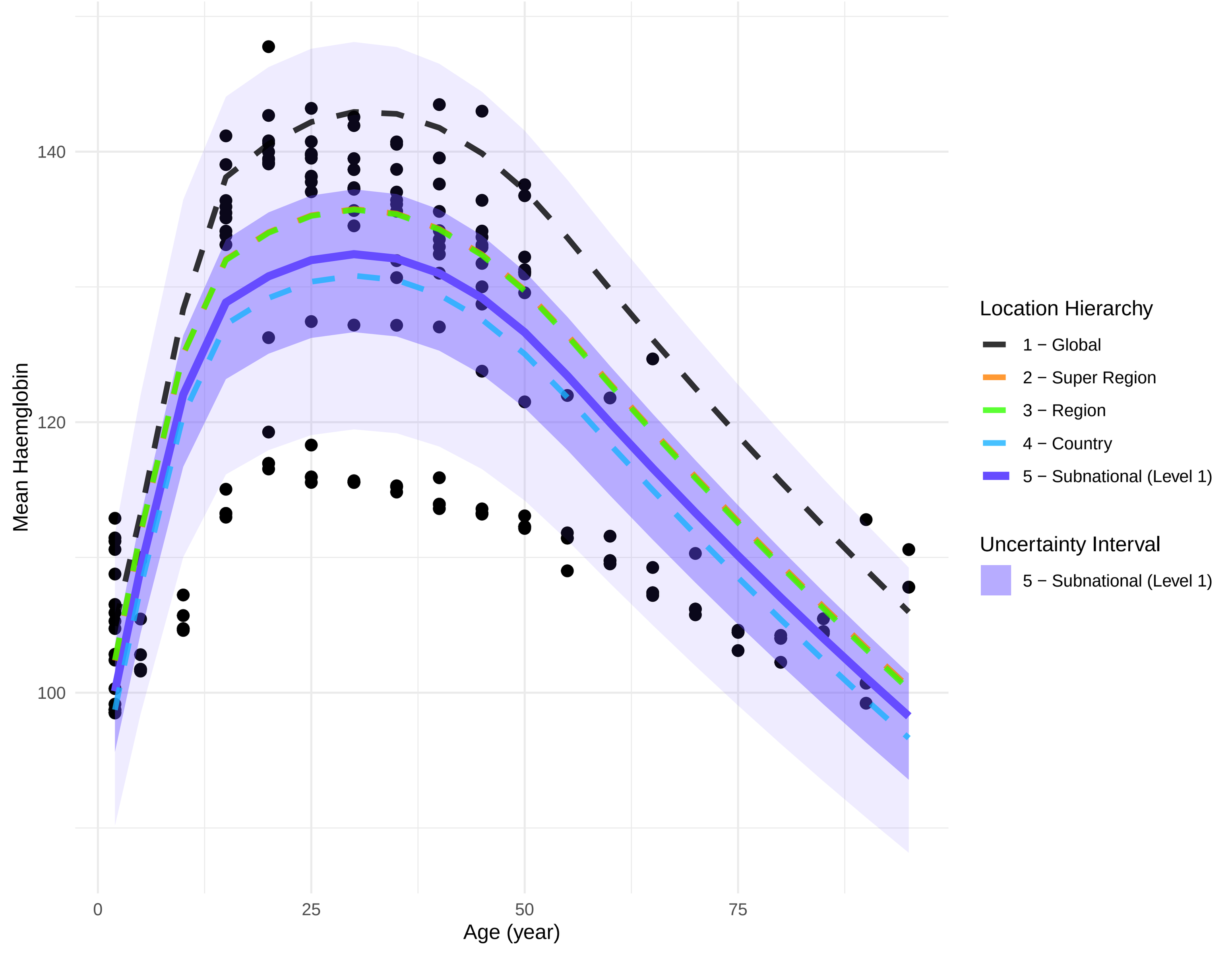

MR−BRT Age/Sex Fits − Males 2+ Years Old − Mean Haemglobin − Odisha, Rural
WHO−2024 Adjustment Method

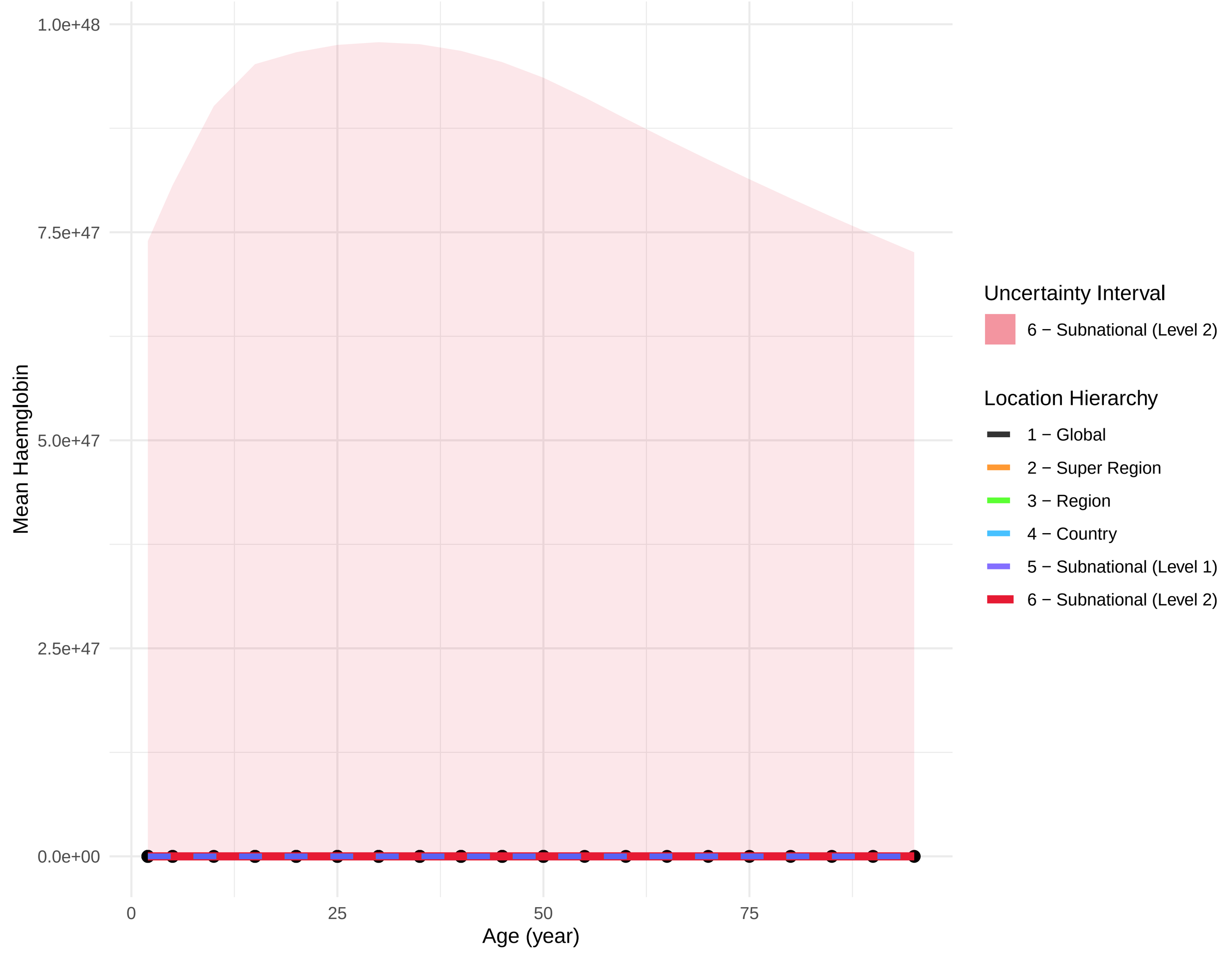

MR−BRT Age/Sex Fits − Males 2+ Years Old − Mean Haemglobin − Odisha, Urban
WHO−2024 Adjustment Method

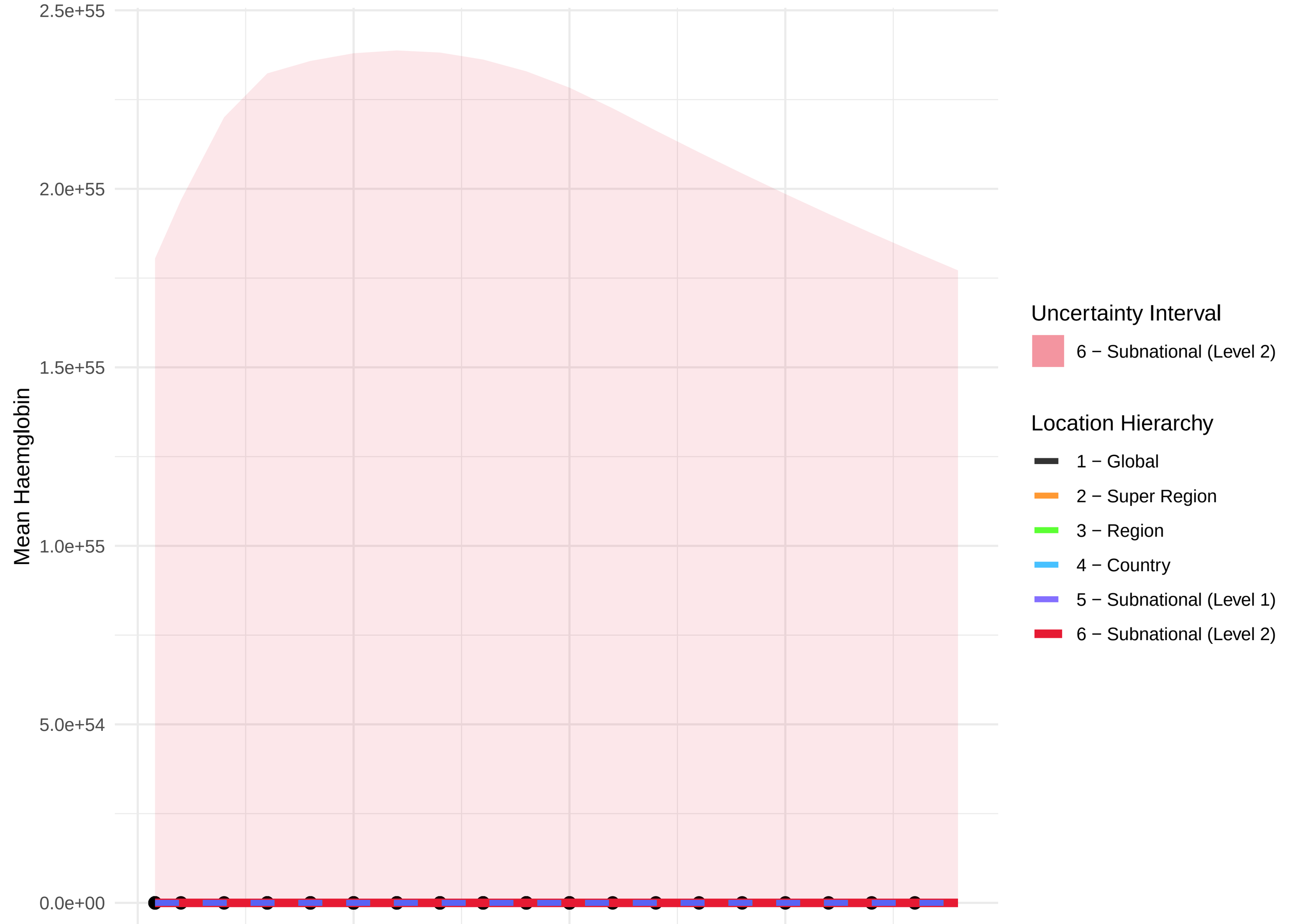

MR−BRT Age/Sex Fits − Males 2+ Years Old − Mean Haemglobin − Other Union Territories
WHO−2024 Adjustment Method

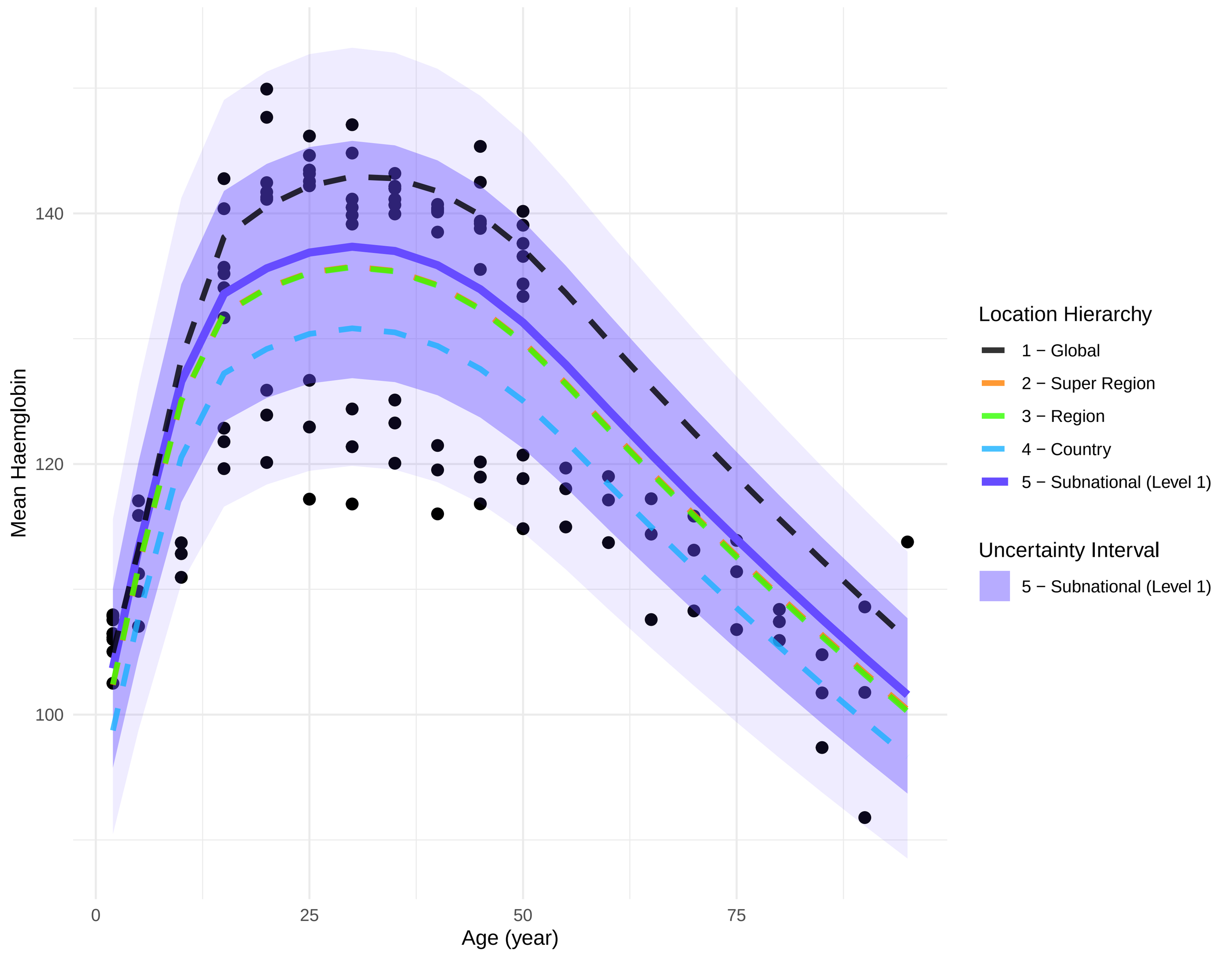

MR−BRT Age/Sex Fits − Males 2+ Years Old − Mean Haemglobin − Other Union Territories, Rural
WHO−2024 Adjustment Method

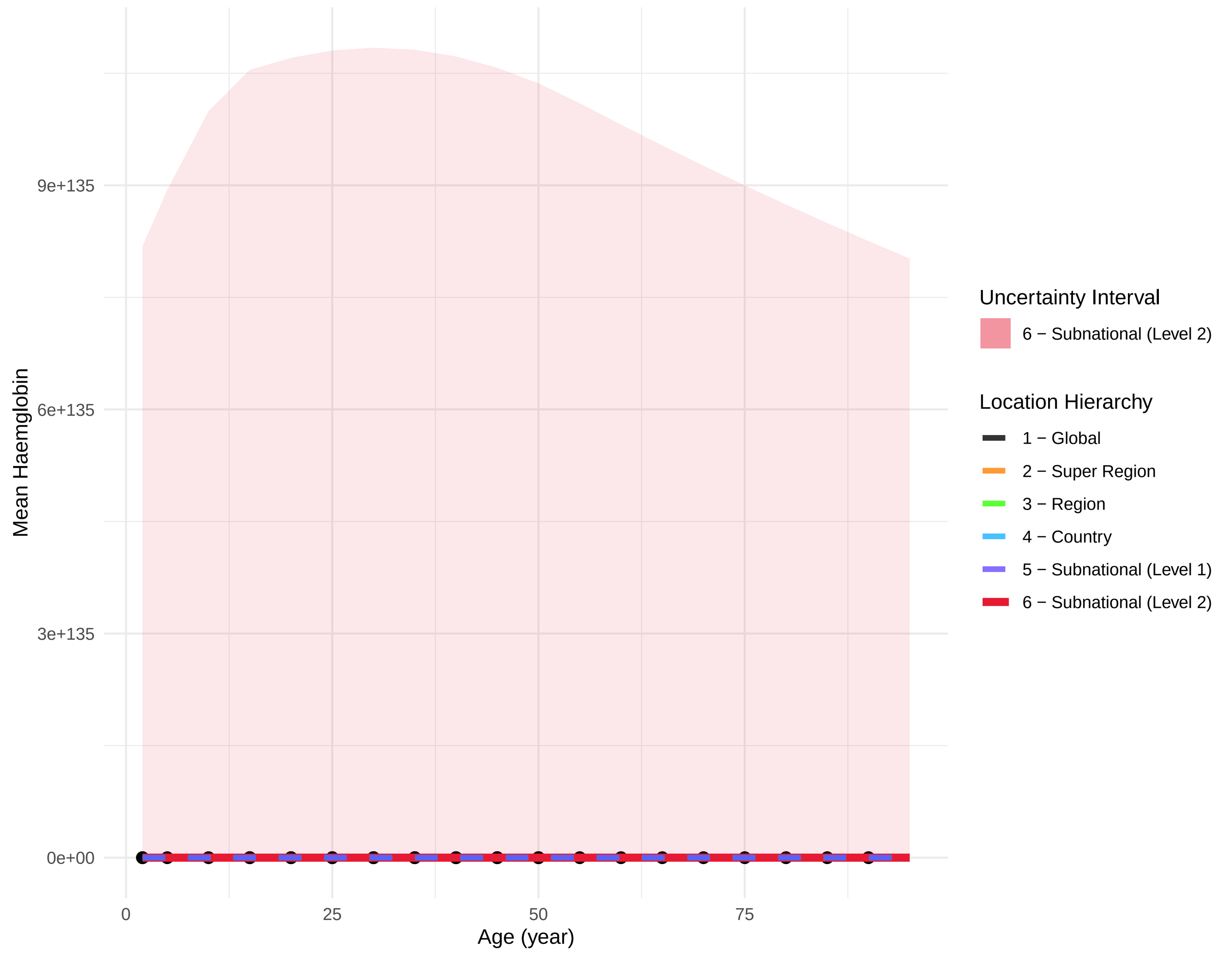

MR−BRT Age/Sex Fits − Males 2+ Years Old − Mean Haemglobin − Other Union Territories, Urban
WHO−2024 Adjustment Method

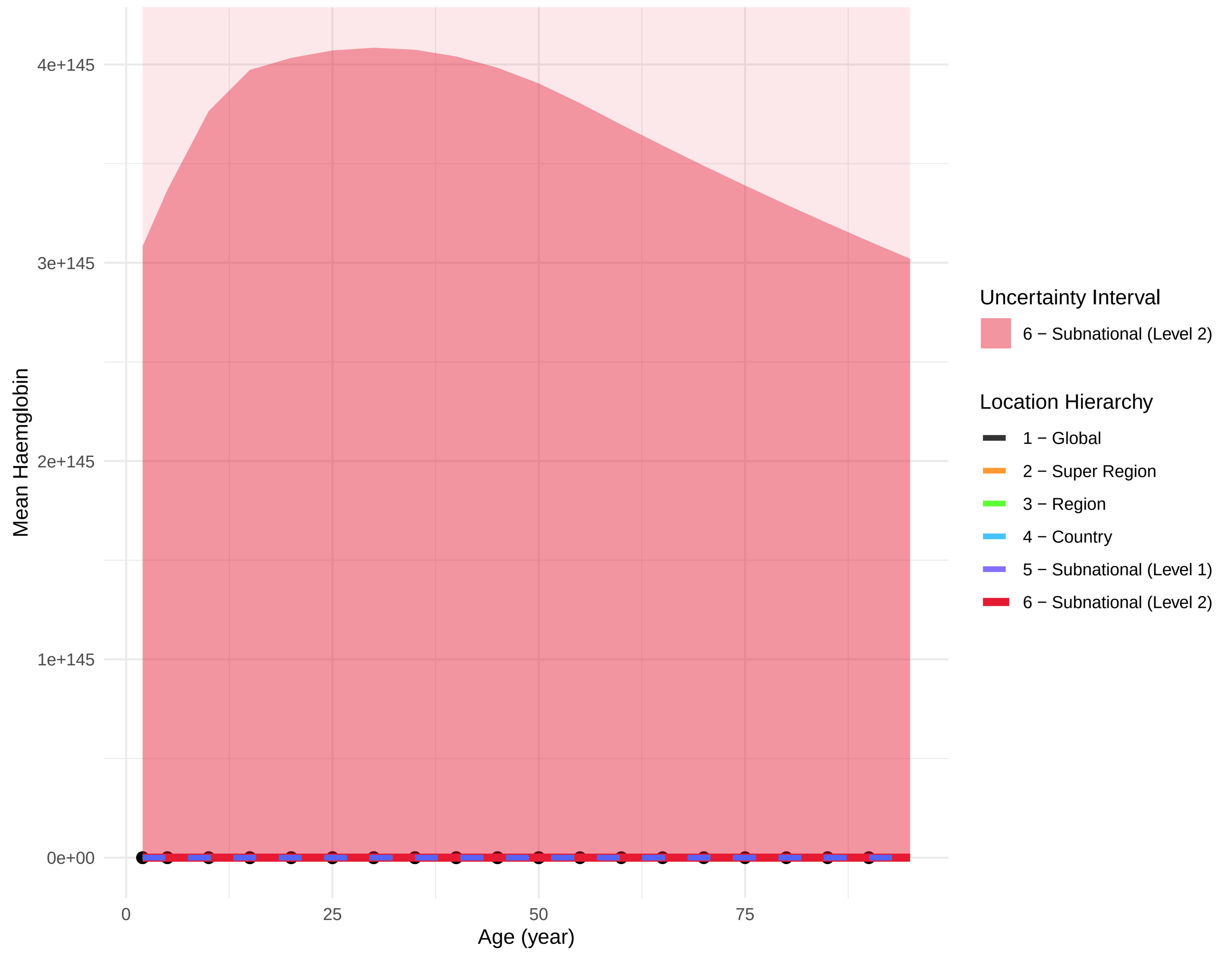

MR−BRT Age/Sex Fits − Males 2+ Years Old − Mean Haemglobin − Punjab
WHO−2024 Adjustment Method

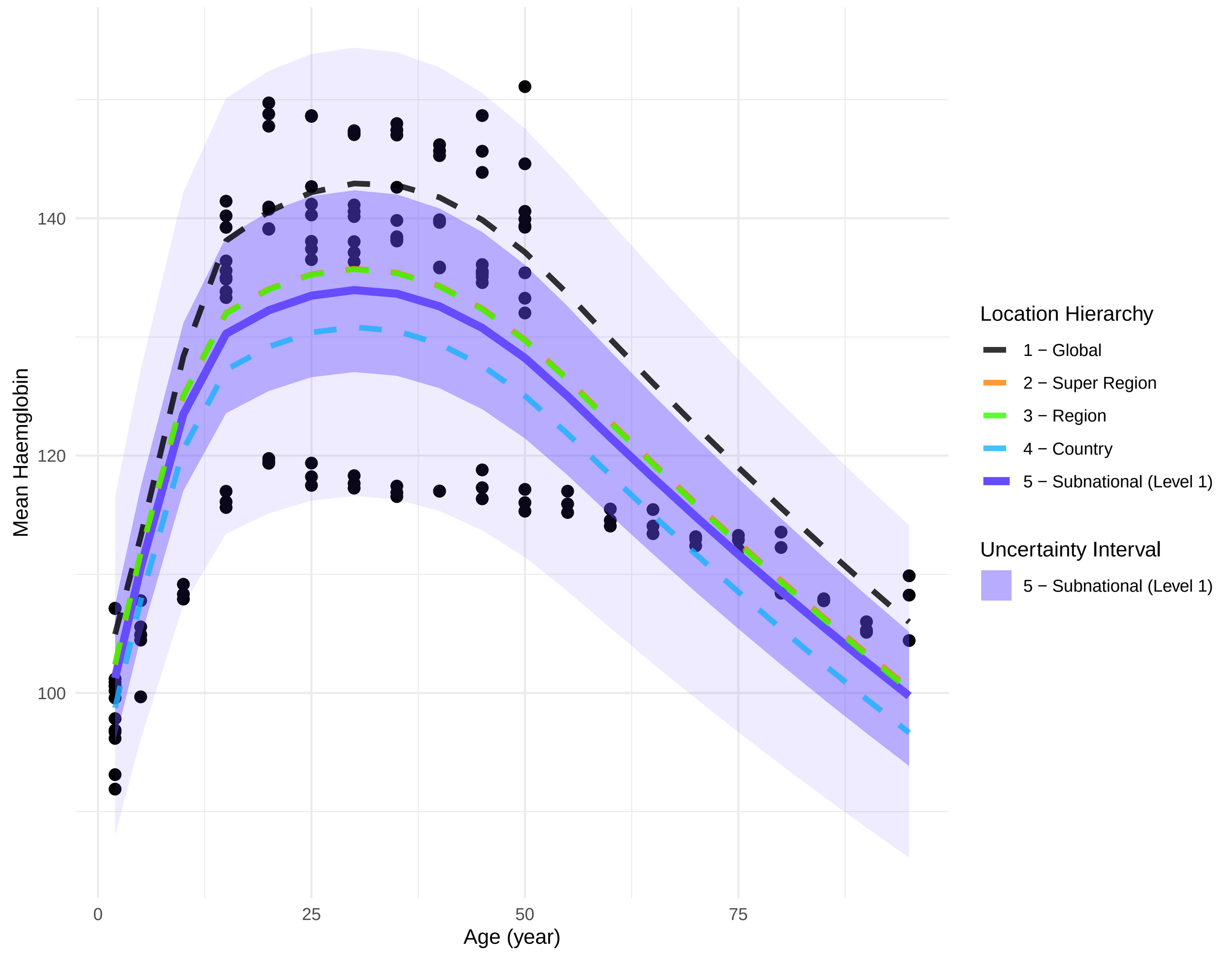

MR−BRT Age/Sex Fits − Males 2+ Years Old − Mean Haemglobin − Punjab, Rural
WHO−2024 Adjustment Method

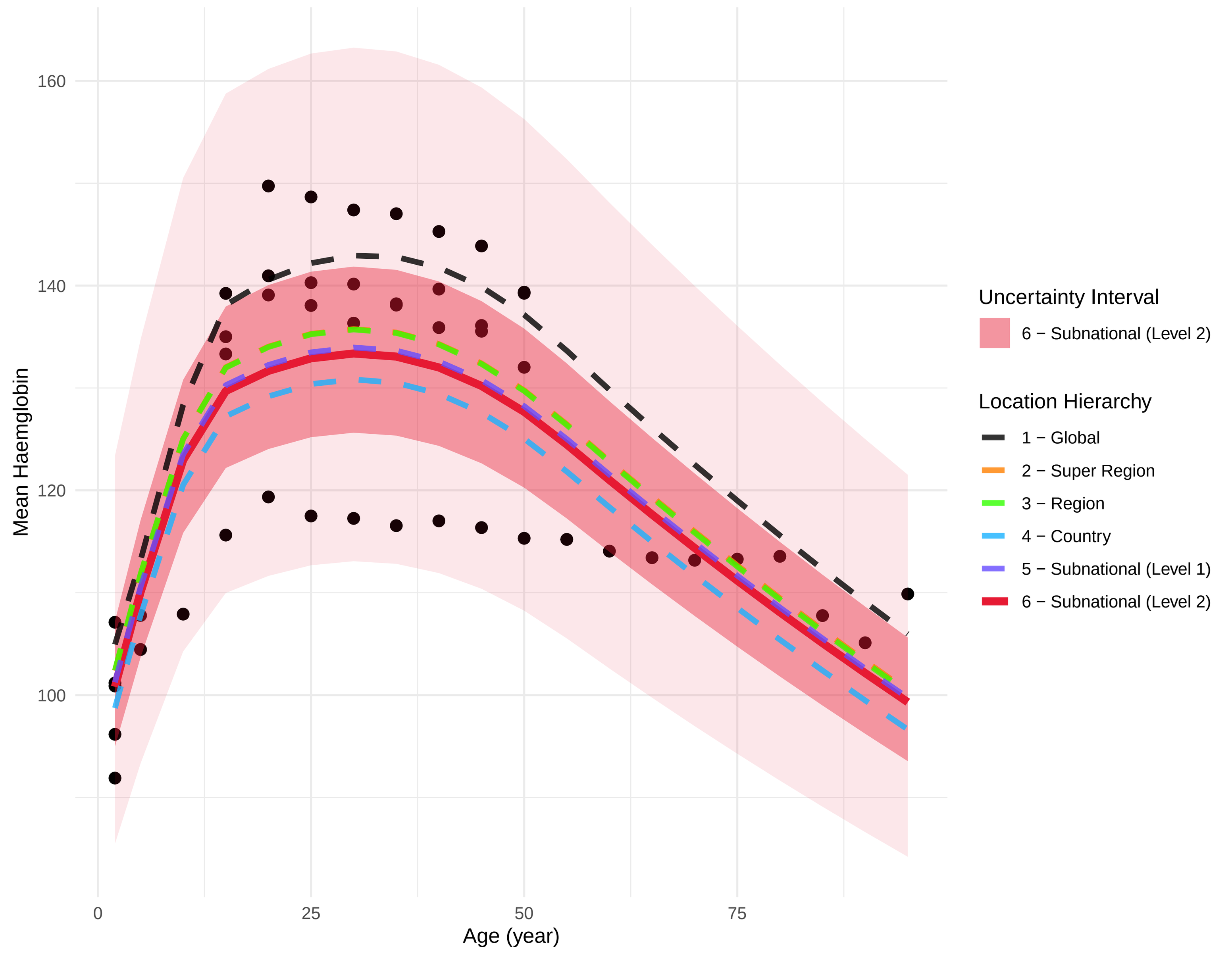

MR−BRT Age/Sex Fits − Males 2+ Years Old − Mean Haemglobin − Punjab, Urban
WHO−2024 Adjustment Method

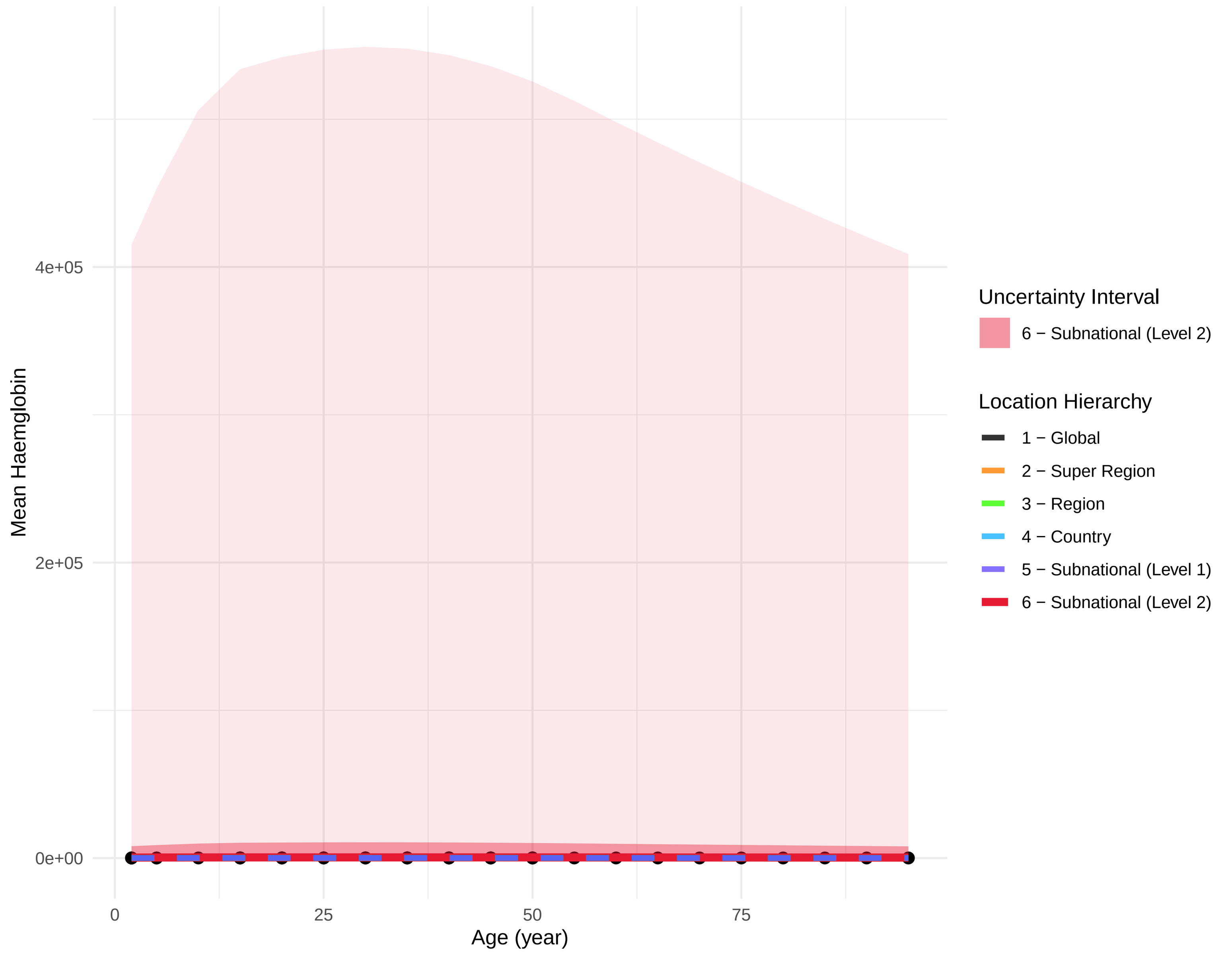

MR−BRT Age/Sex Fits − Males 2+ Years Old − Mean Haemglobin − Rajasthan
WHO−2024 Adjustment Method

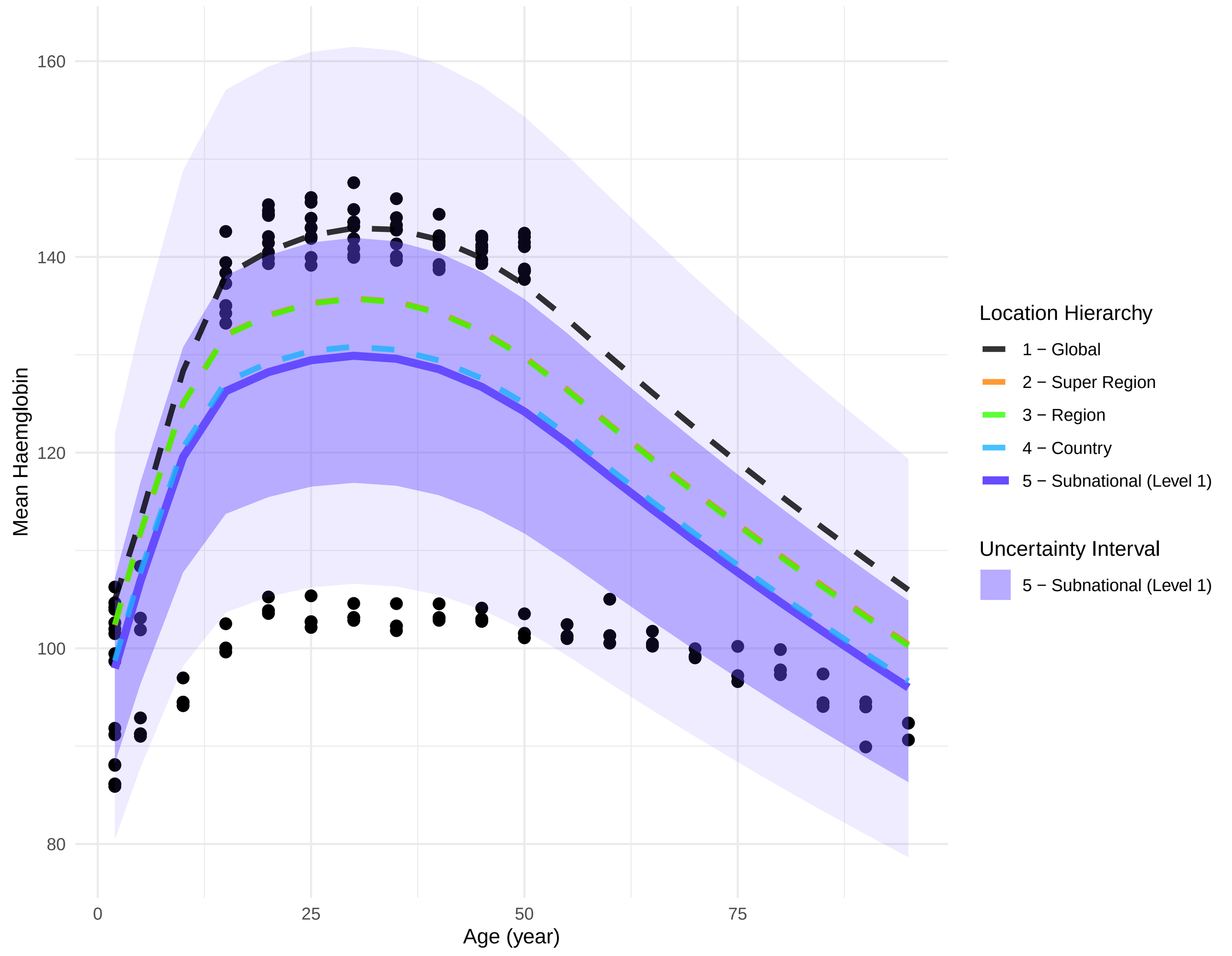

MR−BRT Age/Sex Fits − Males 2+ Years Old − Mean Haemglobin − Rajasthan, Rural
WHO−2024 Adjustment Method

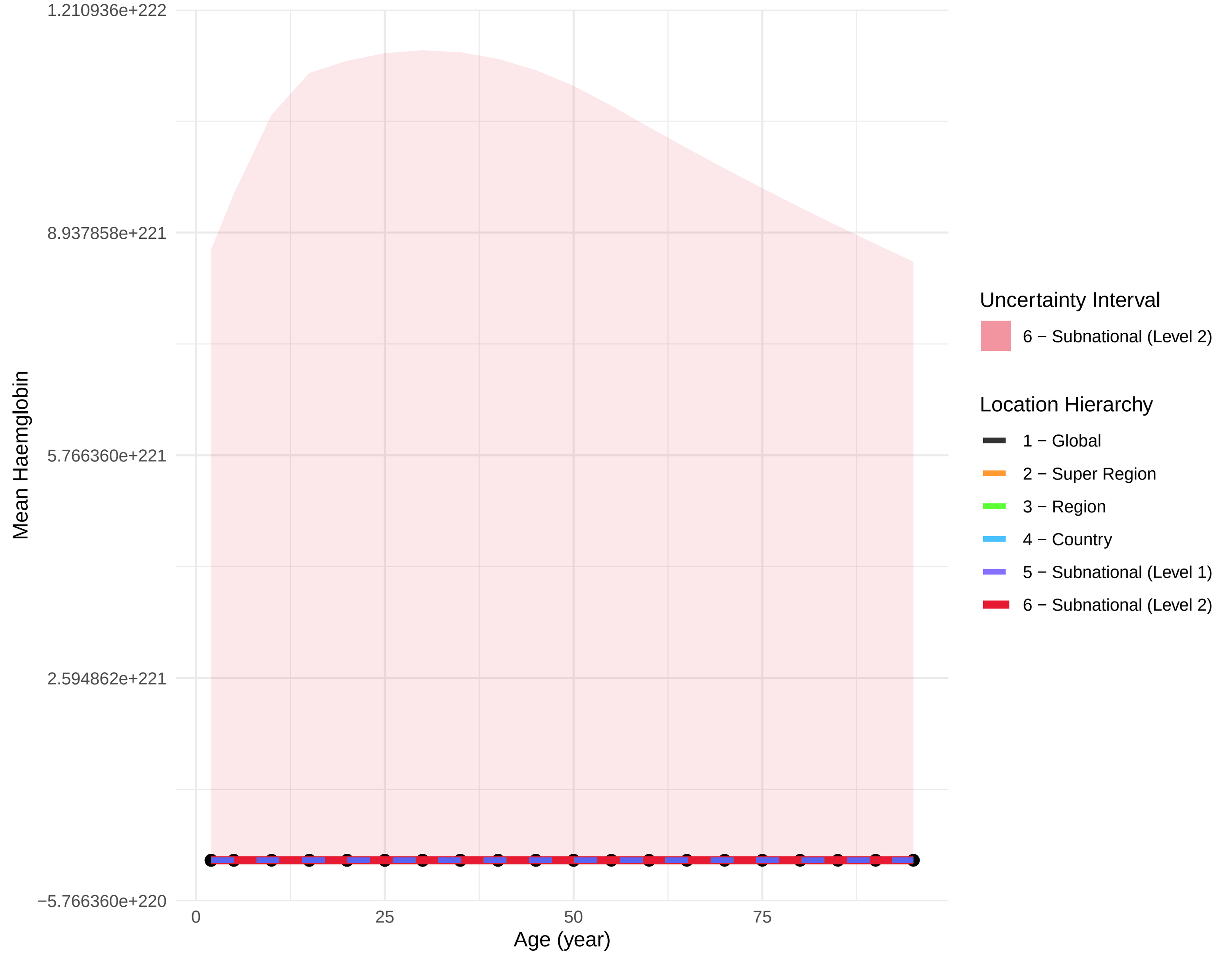

MR−BRT Age/Sex Fits − Males 2+ Years Old − Mean Haemglobin − Rajasthan, Urban
WHO−2024 Adjustment Method

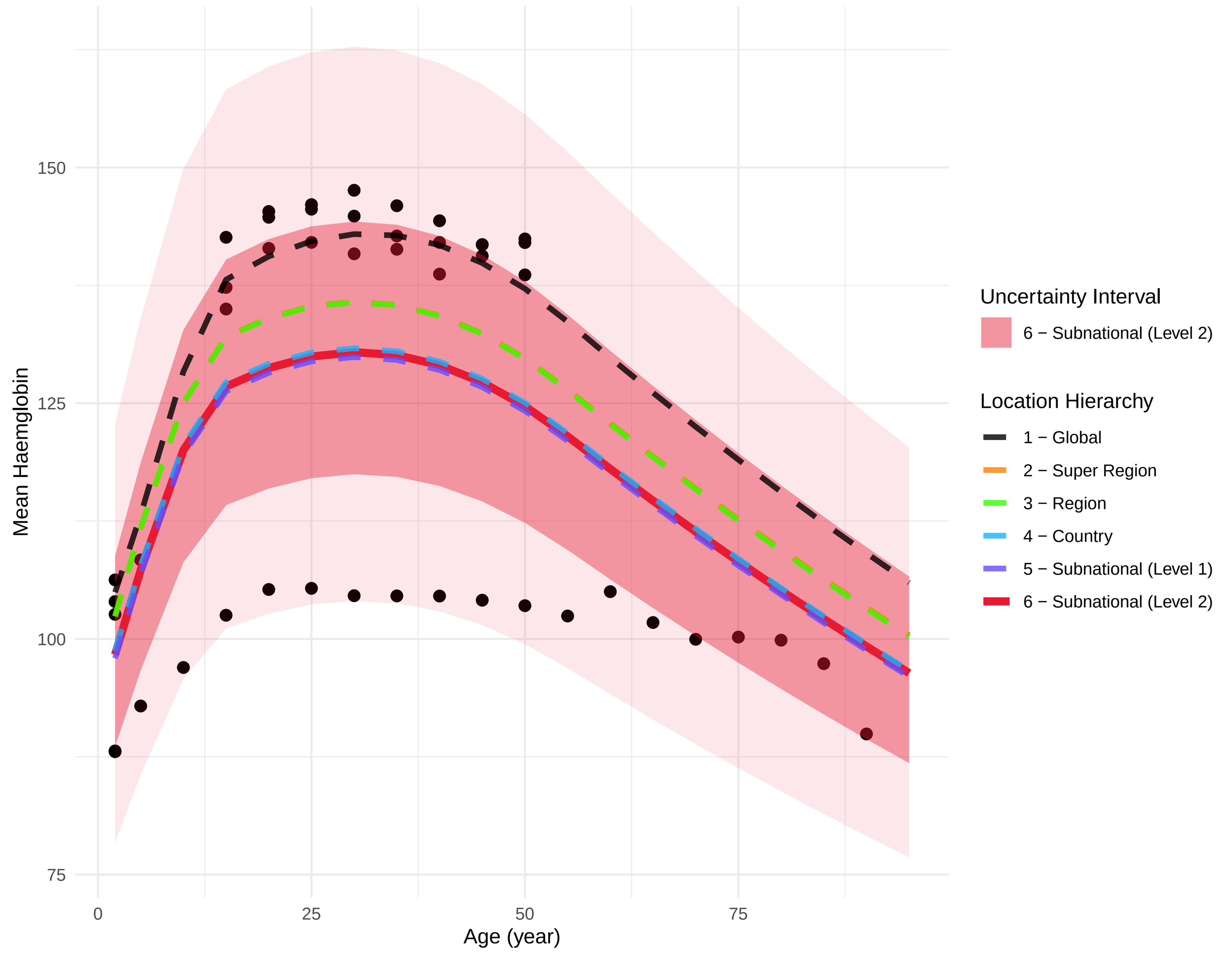

MR−BRT Age/Sex Fits − Males 2+ Years Old − Mean Haemglobin − Sikkim
WHO−2024 Adjustment Method

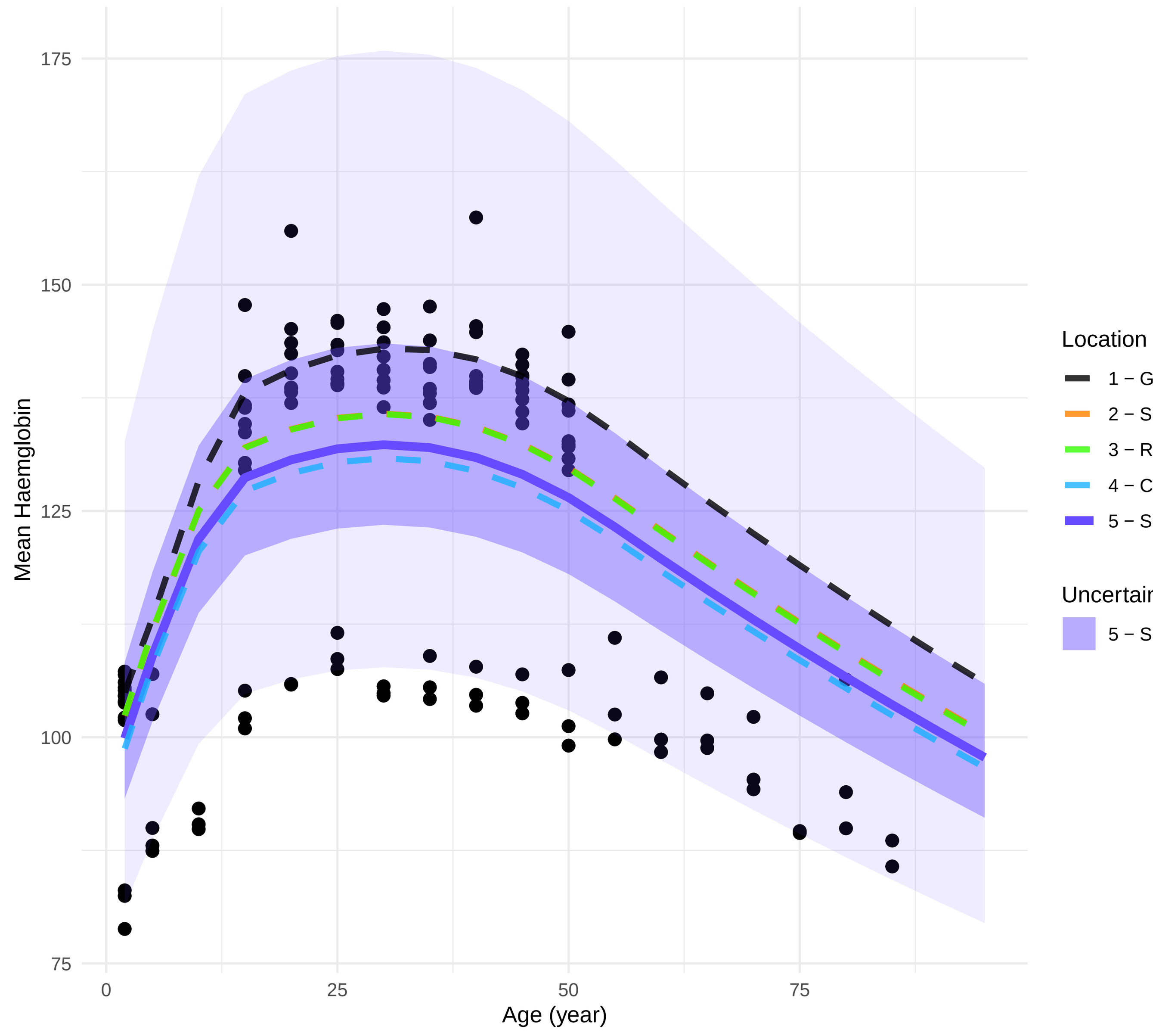

MR−BRT Age/Sex Fits − Males 2+ Years Old − Mean Haemglobin − Sikkim, Rural
WHO−2024 Adjustment Method

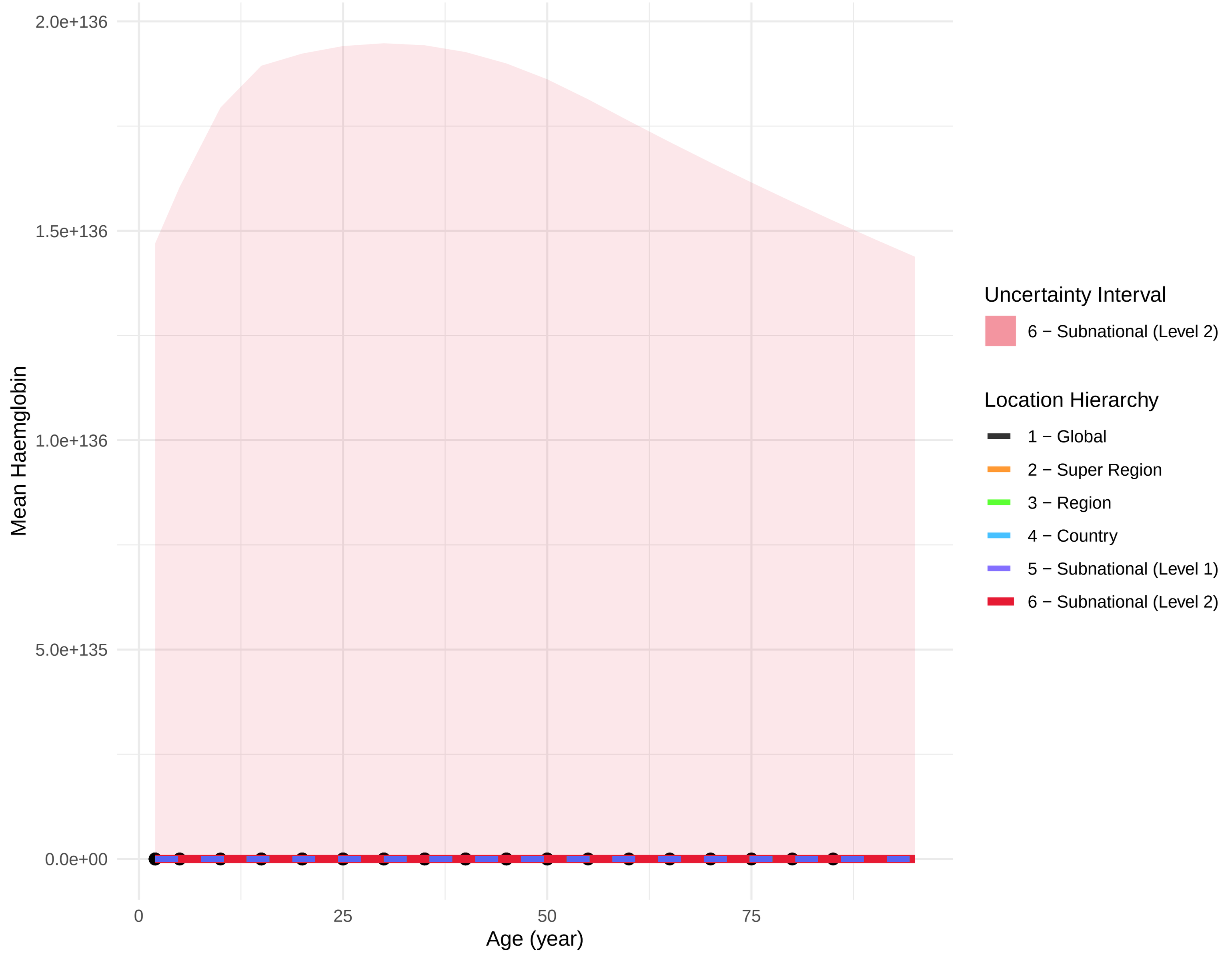

MR−BRT Age/Sex Fits − Males 2+ Years Old − Mean Haemglobin − Sikkim, Urban
WHO−2024 Adjustment Method

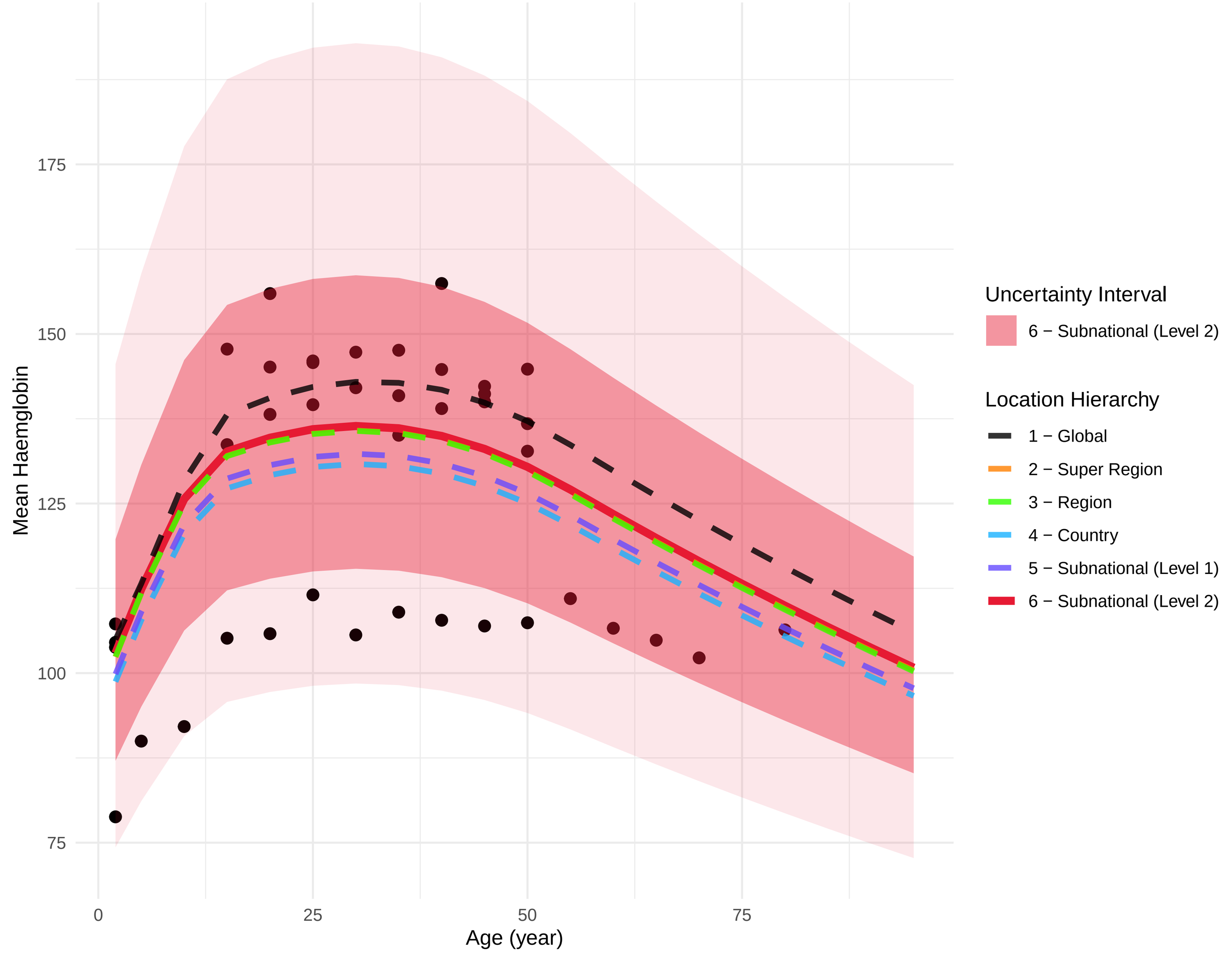

MR−BRT Age/Sex Fits − Males 2+ Years Old − Mean Haemglobin − Tamil Nadu
WHO−2024 Adjustment Method

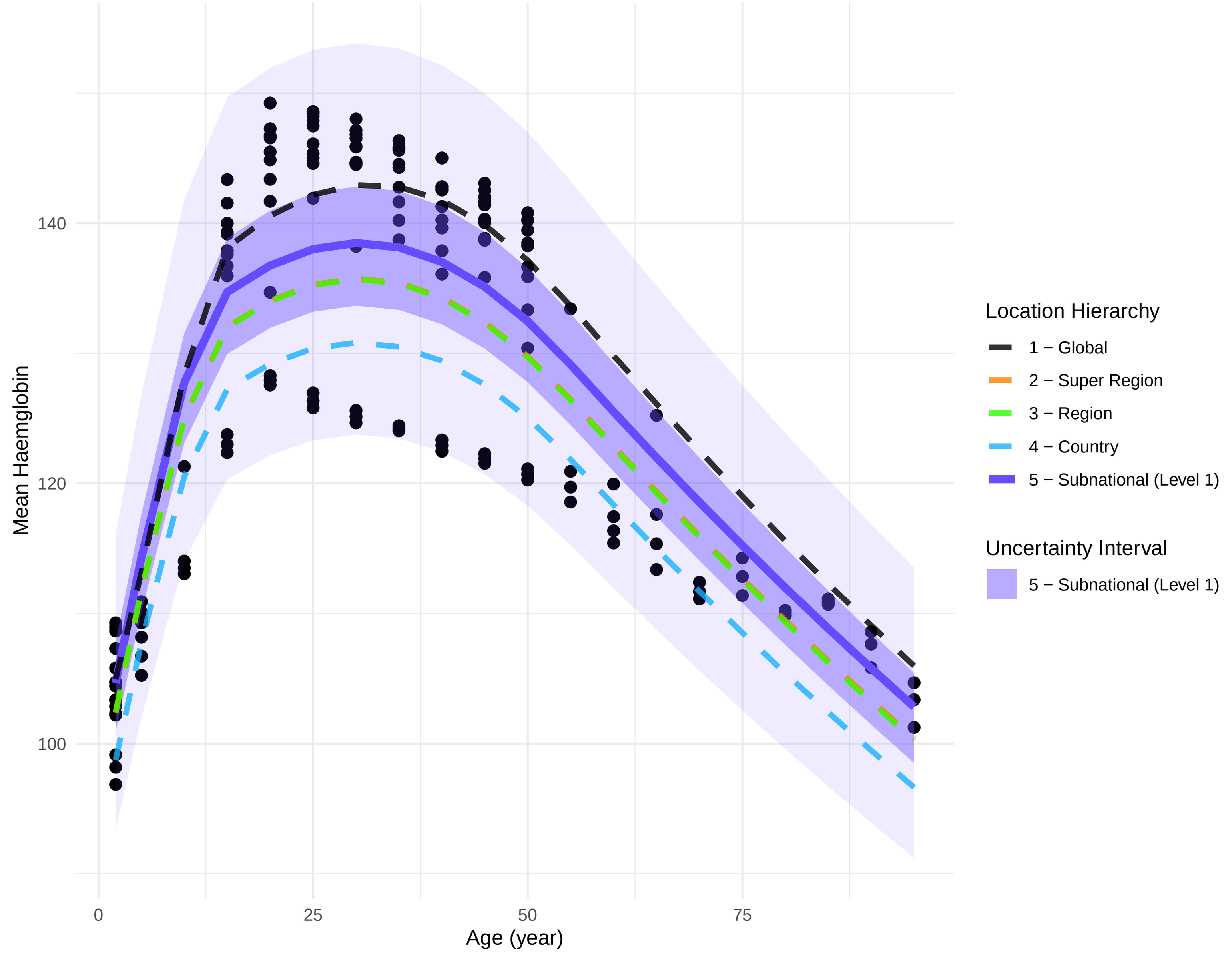

MR−BRT Age/Sex Fits − Males 2+ Years Old − Mean Haemglobin − Tamil Nadu, Rural
WHO−2024 Adjustment Method

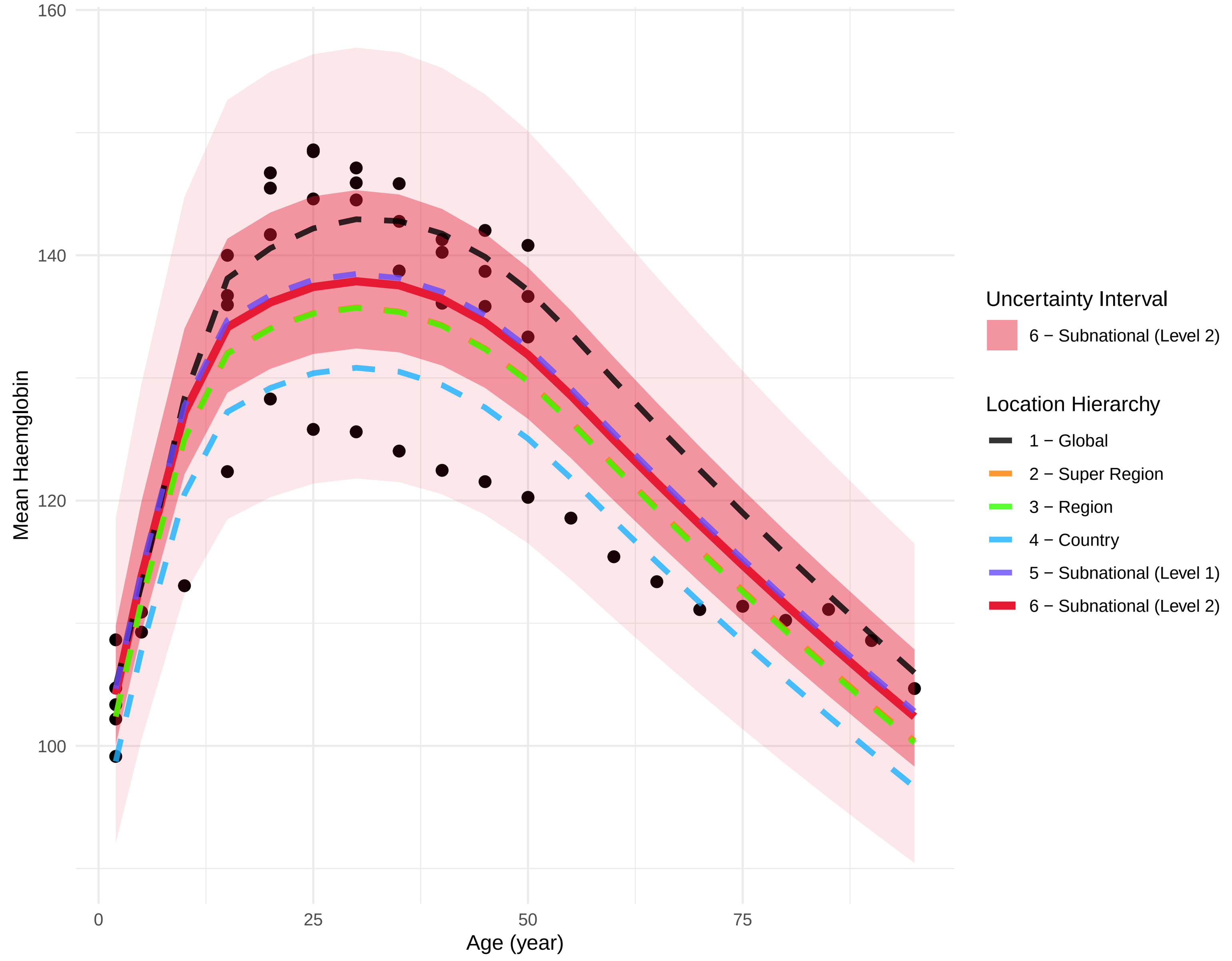

MR−BRT Age/Sex Fits − Males 2+ Years Old − Mean Haemglobin − Tamil Nadu, Urban
WHO−2024 Adjustment Method

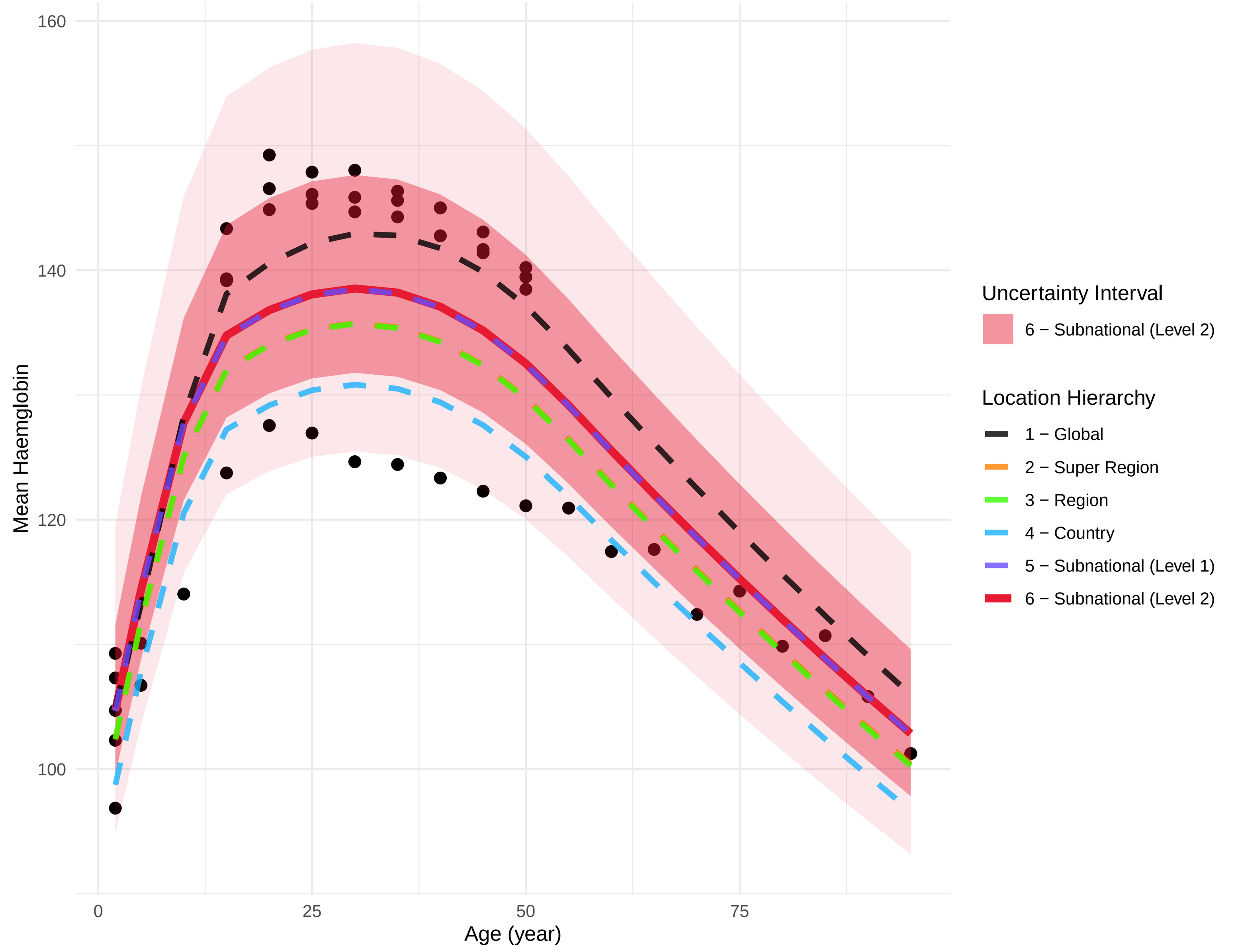

MR−BRT Age/Sex Fits − Males 2+ Years Old − Mean Haemglobin − Telangana
WHO−2024 Adjustment Method

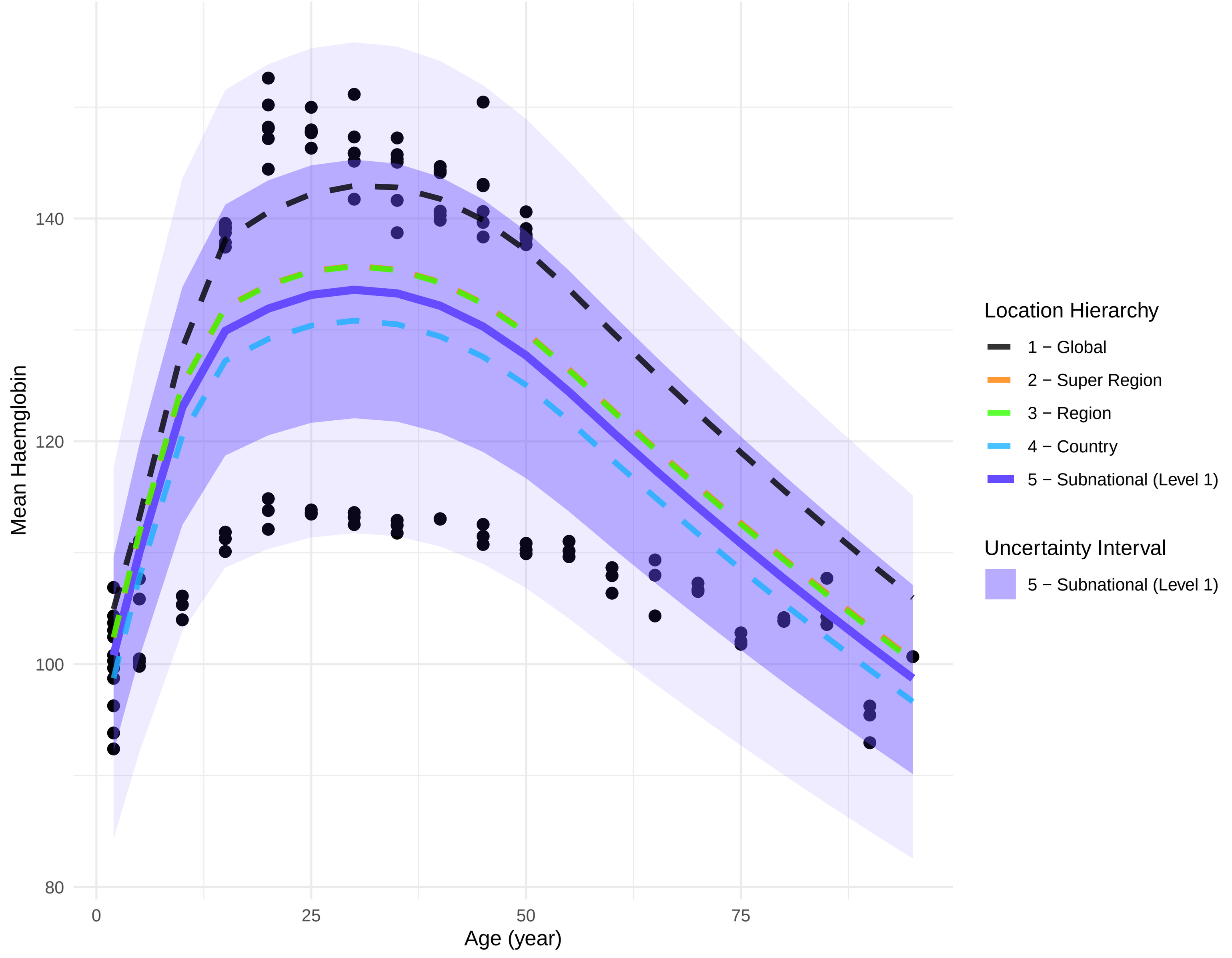

MR−BRT Age/Sex Fits − Males 2+ Years Old − Mean Haemglobin − Telangana, Rural
WHO−2024 Adjustment Method

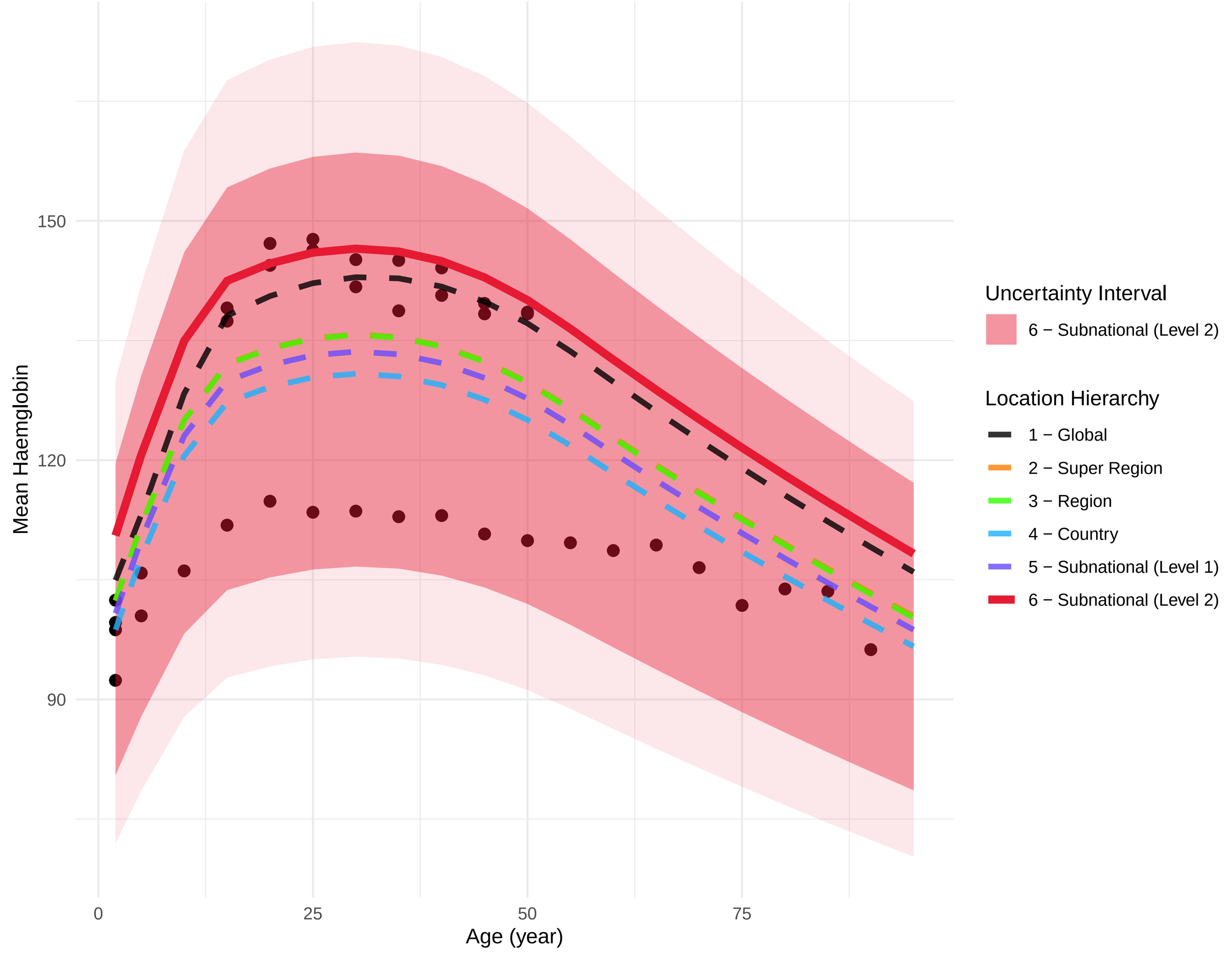

MR−BRT Age/Sex Fits − Males 2+ Years Old − Mean Haemglobin − Telangana, Urban
WHO−2024 Adjustment Method

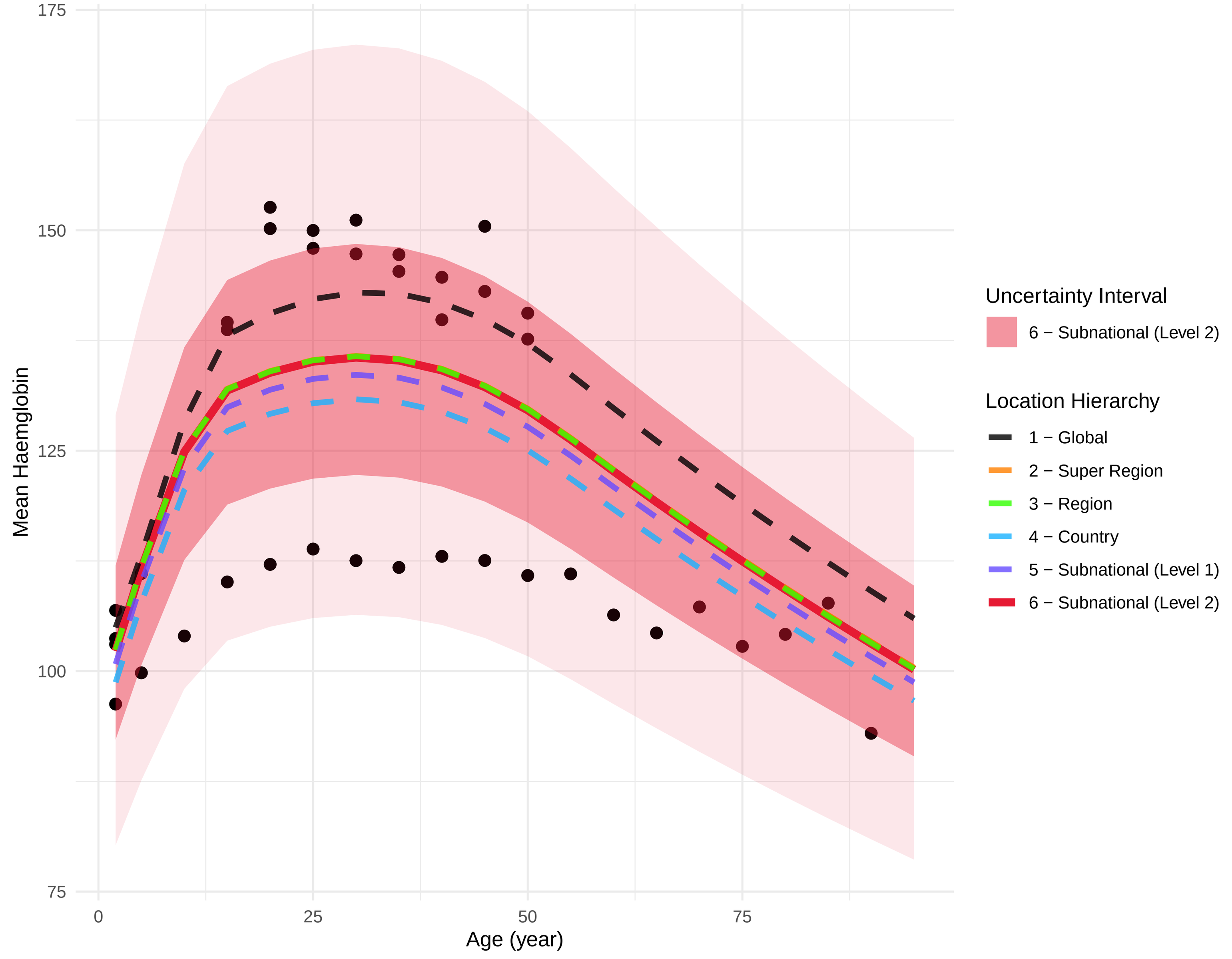

MR−BRT Age/Sex Fits − Males 2+ Years Old − Mean Haemglobin − Tripura
WHO−2024 Adjustment Method

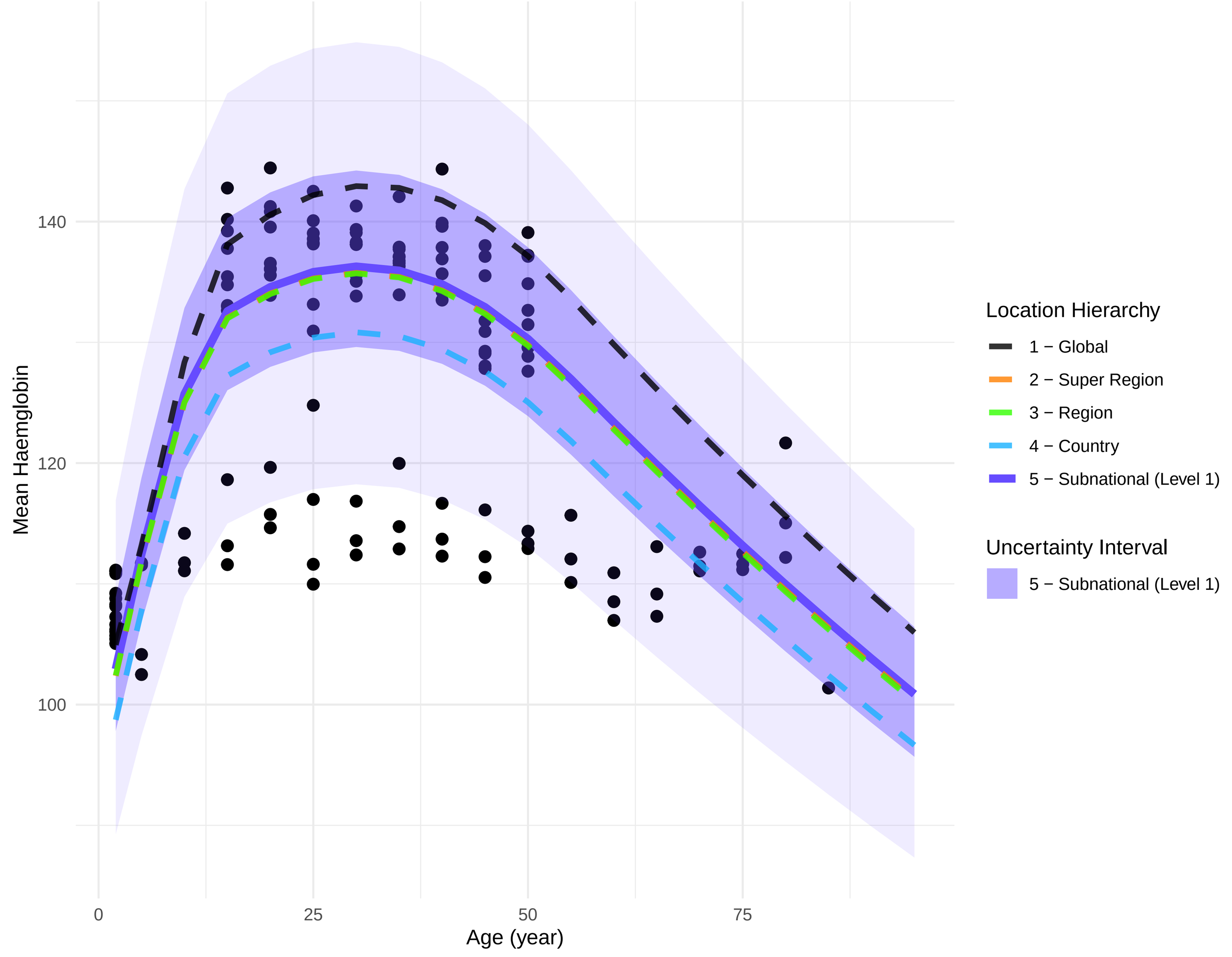

MR−BRT Age/Sex Fits − Males 2+ Years Old − Mean Haemglobin − Tripura, Rural
WHO−2024 Adjustment Method

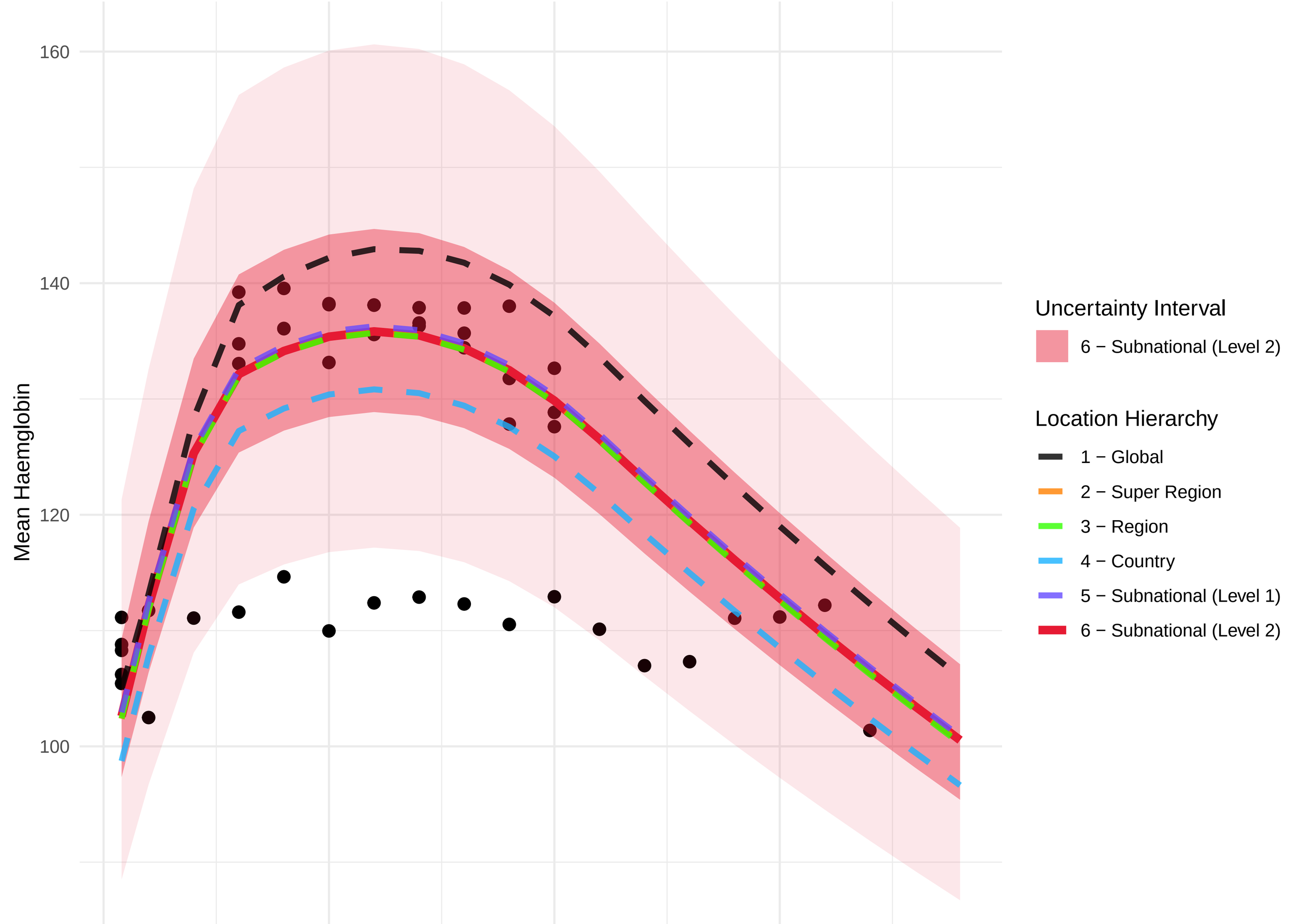

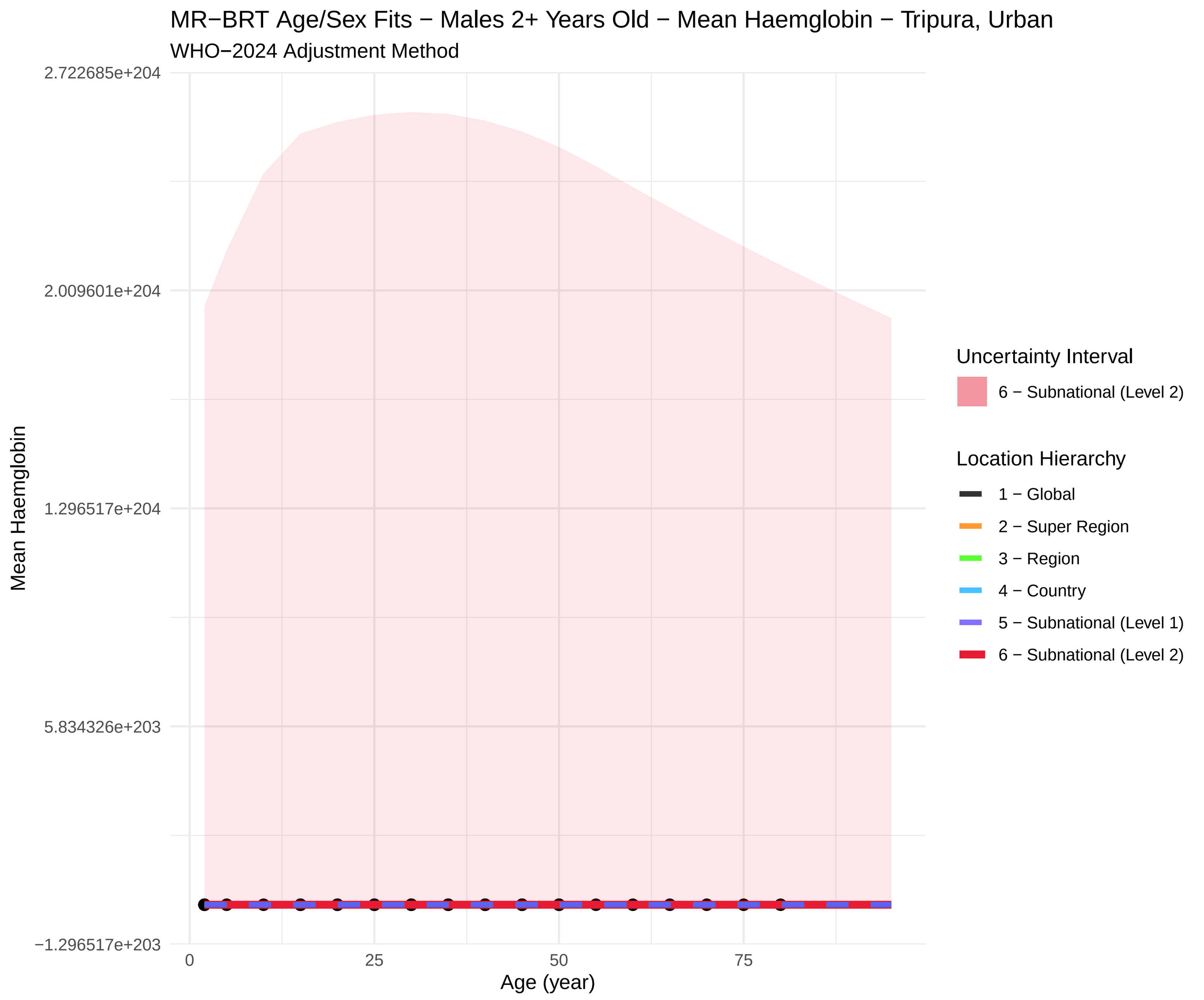
MR−BRT Age/Sex Fits − Males 2+ Years Old − Mean Haemglobin − Tripura, Urban
WHO−2024 Adjustment Method
Mean Haemglobin
Age (year)
Uncertainty Interval
6 − Subnational (Level 2)
Location Hierarchy
1 − Global
2 − Super Region
3 − Region
4 − Country
5 − Subnational (Level 1)
6 − Subnational (Level 2)

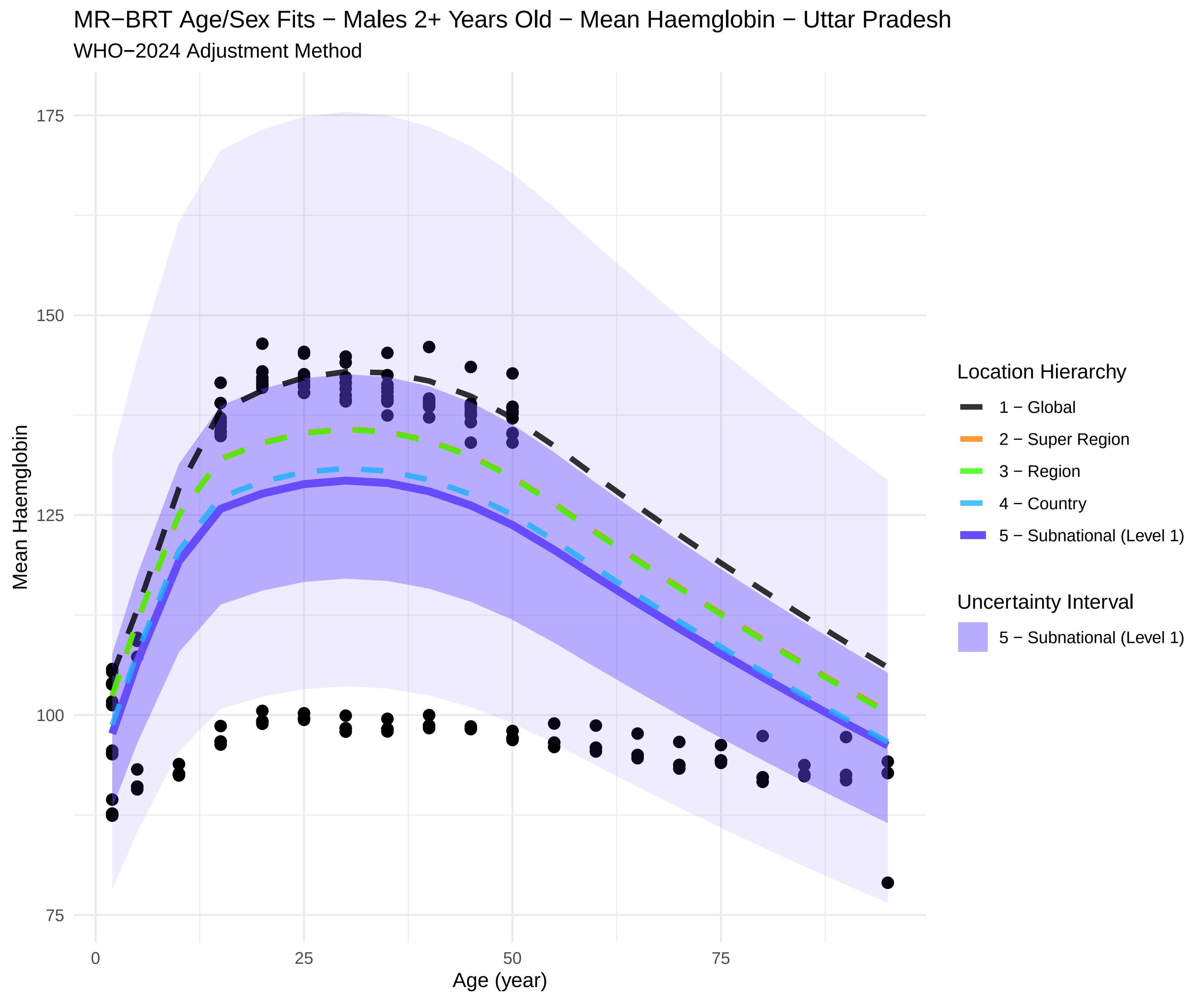
MR−BRT Age/Sex Fits − Males 2+ Years Old − Mean Haemglobin − Uttar Pradesh
WHO−2024 Adjustment Method
Mean Haemglobin
Age (year)
Location Hierarchy
1 − Global
2 − Super Region
3 − Region
4 − Country
5 − Subnational (Level 1)
Uncertainty Interval
5 − Subnational (Level 1)

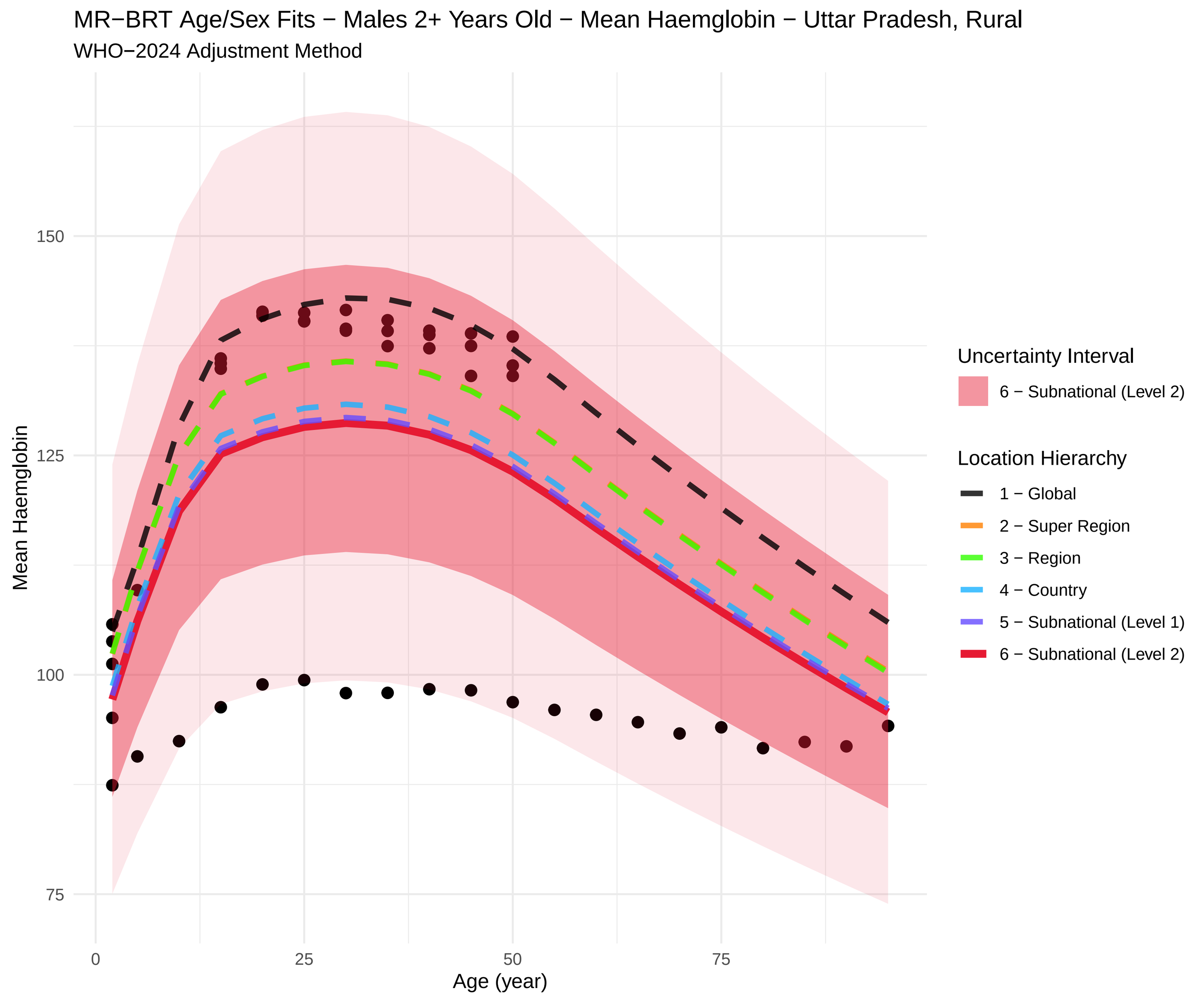
MR−BRT Age/Sex Fits − Males 2+ Years Old − Mean Haemglobin − Uttar Pradesh, Rural
WHO−2024 Adjustment Method
Mean Haemglobin
Age (year)
Uncertainty Interval
6 − Subnational (Level 2)
Location Hierarchy
1 − Global
2 − Super Region
3 − Region
4 − Country
5 − Subnational (Level 1)
6 − Subnational (Level 2)

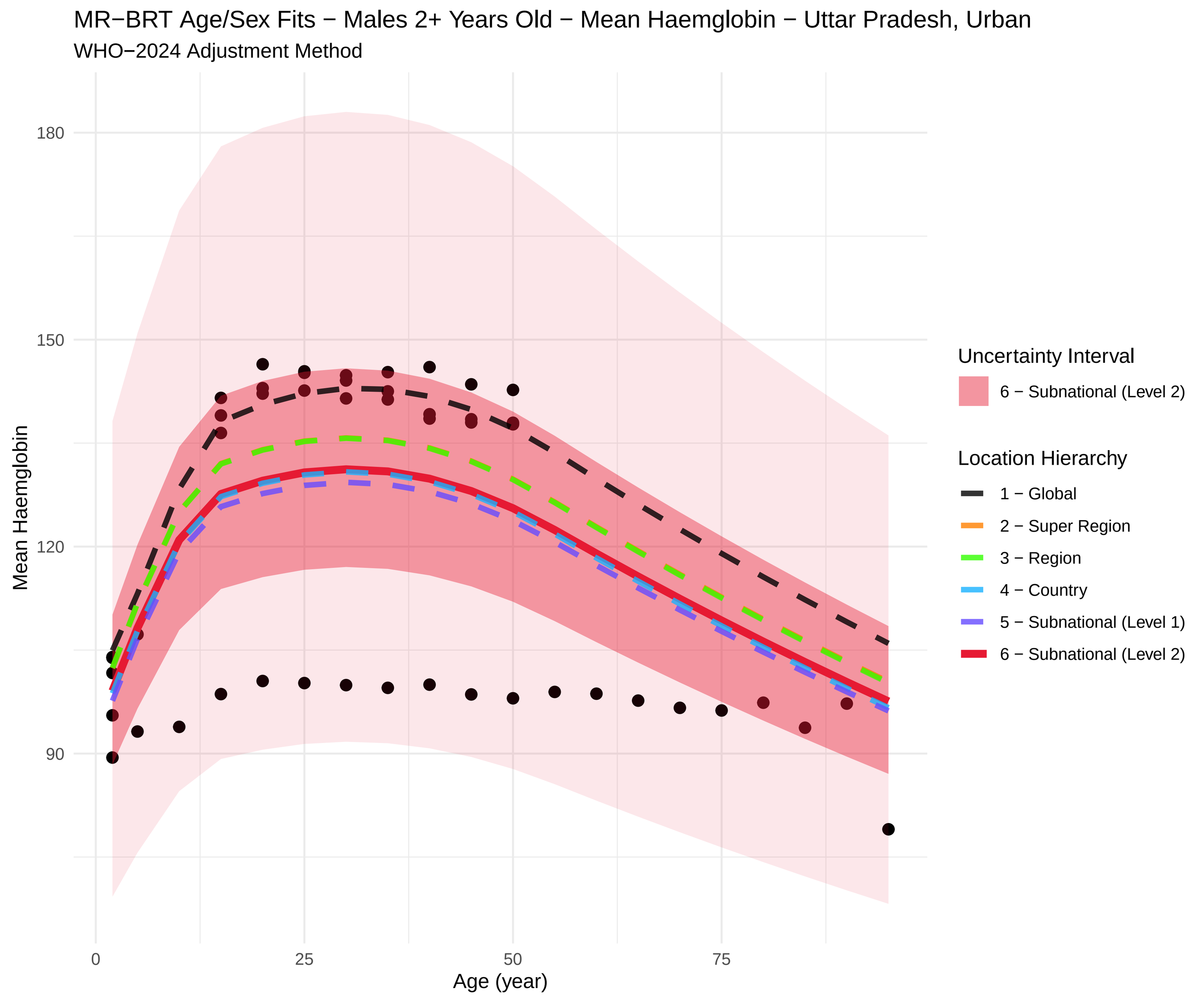
MR−BRT Age/Sex Fits − Males 2+ Years Old − Mean Haemglobin − Uttar Pradesh, Urban
WHO−2024 Adjustment Method
Mean Haemglobin
Age (year)
Uncertainty Interval
6 − Subnational (Level 2)
Location Hierarchy
1 − Global
2 − Super Region
3 − Region
4 − Country
5 − Subnational (Level 1)
6 − Subnational (Level 2)

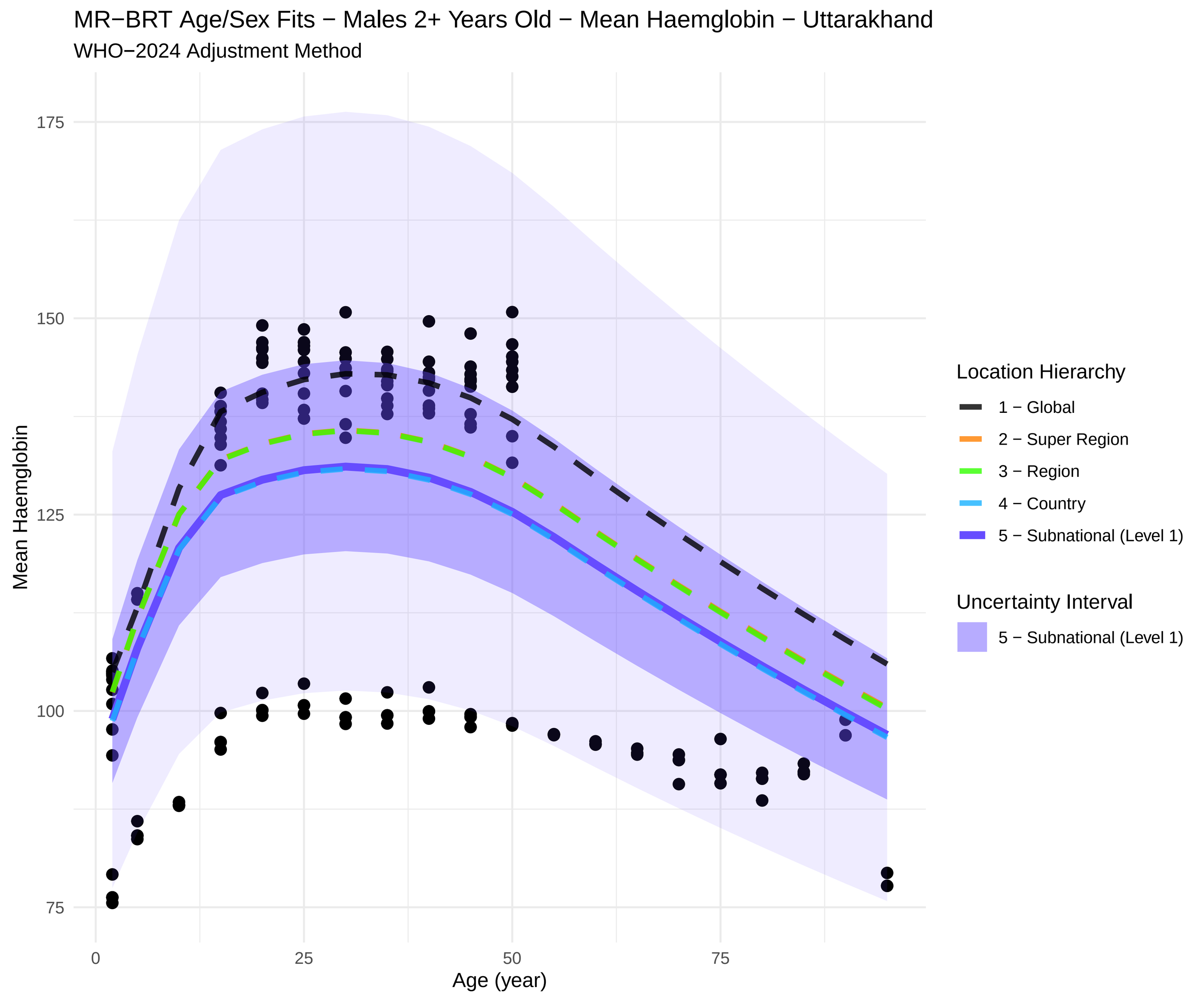
MR−BRT Age/Sex Fits − Males 2+ Years Old − Mean Haemglobin − Uttarakhand
WHO−2024 Adjustment Method
Mean Haemglobin
Age (year)
Location Hierarchy
1 − Global
2 − Super Region
3 − Region
4 − Country
5 − Subnational (Level 1)
Uncertainty Interval
5 − Subnational (Level 1)

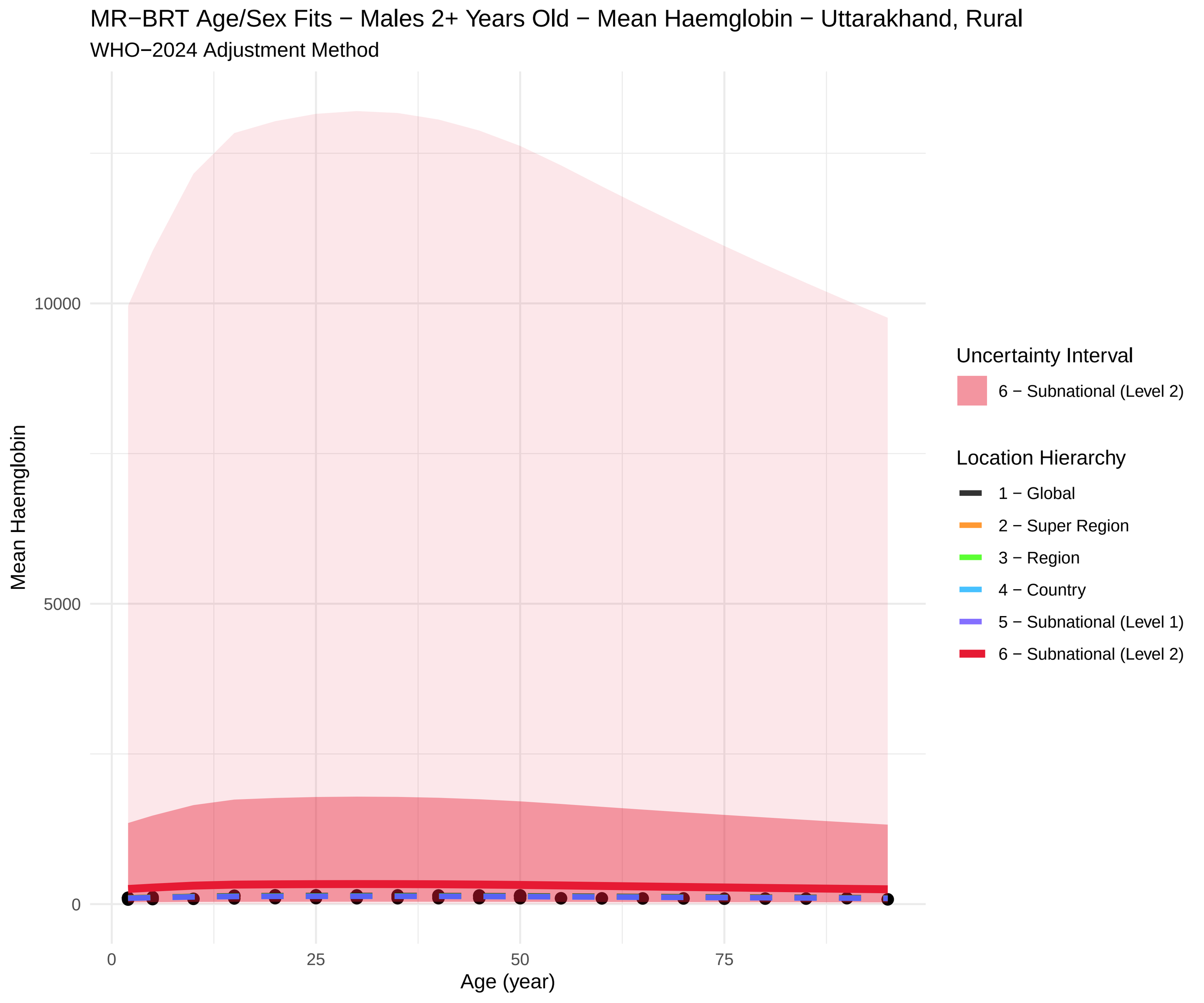
MR−BRT Age/Sex Fits − Males 2+ Years Old − Mean Haemglobin − Uttarakhand, Rural
WHO−2024 Adjustment Method
Mean Haemglobin
Age (year)
Uncertainty Interval
6 − Subnational (Level 2)
Location Hierarchy
1 − Global
2 − Super Region
3 − Region
4 − Country
5 − Subnational (Level 1)
6 − Subnational (Level 2)

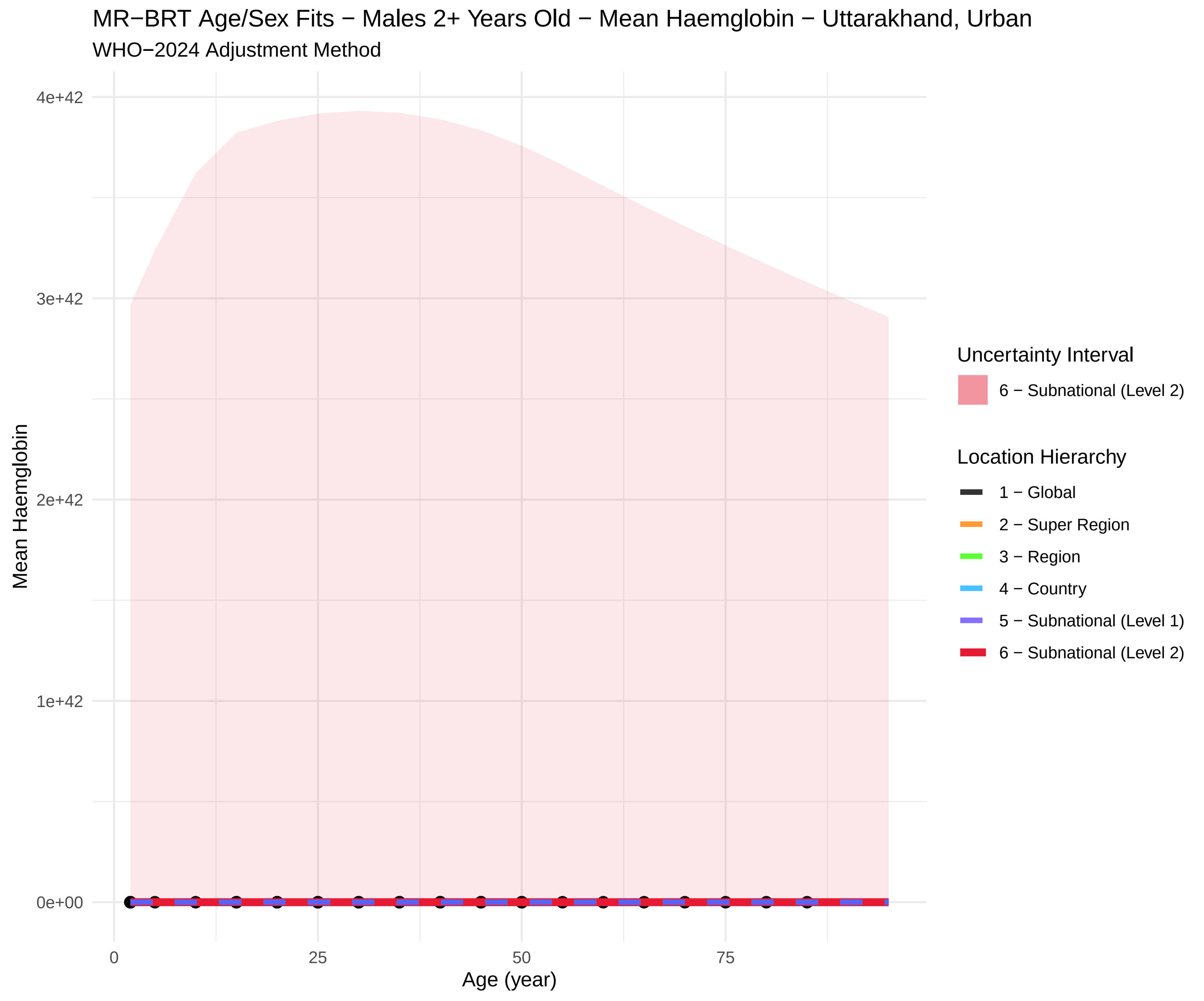
MR−BRT Age/Sex Fits − Males 2+ Years Old − Mean Haemglobin − Uttarakhand, Urban
WHO−2024 Adjustment Method
Mean Haemglobin
Age (year)
Uncertainty Interval
6 − Subnational (Level 2)
Location Hierarchy
1 − Global
2 − Super Region
3 − Region
4 − Country
5 − Subnational (Level 1)
6 − Subnational (Level 2)

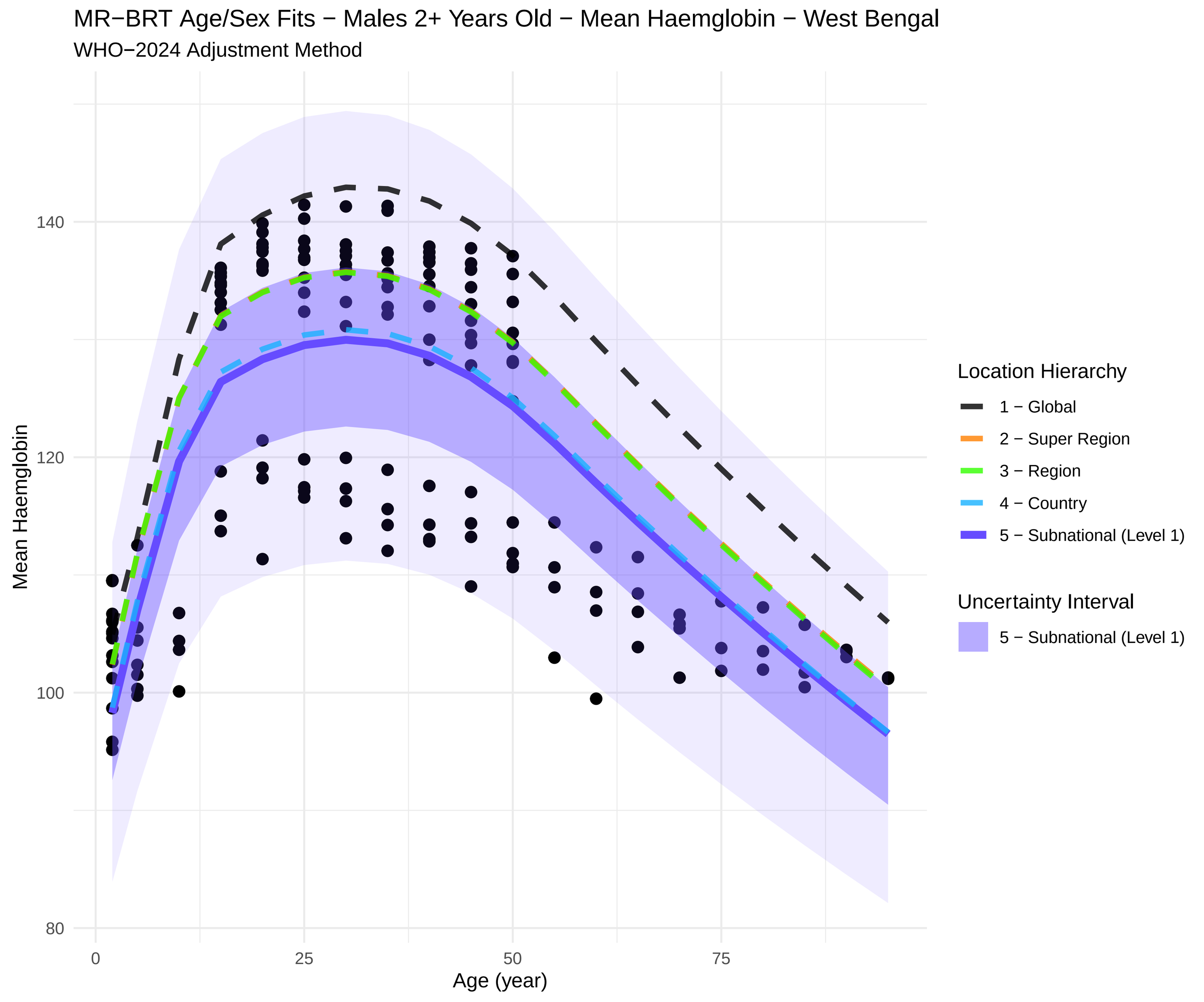
MR−BRT Age/Sex Fits − Males 2+ Years Old − Mean Haemglobin − West Bengal
WHO−2024 Adjustment Method
Mean Haemglobin
Age (year)
Location Hierarchy
1 − Global
2 − Super Region
3 − Region
4 − Country
5 − Subnational (Level 1)
Uncertainty Interval
5 − Subnational (Level 1)

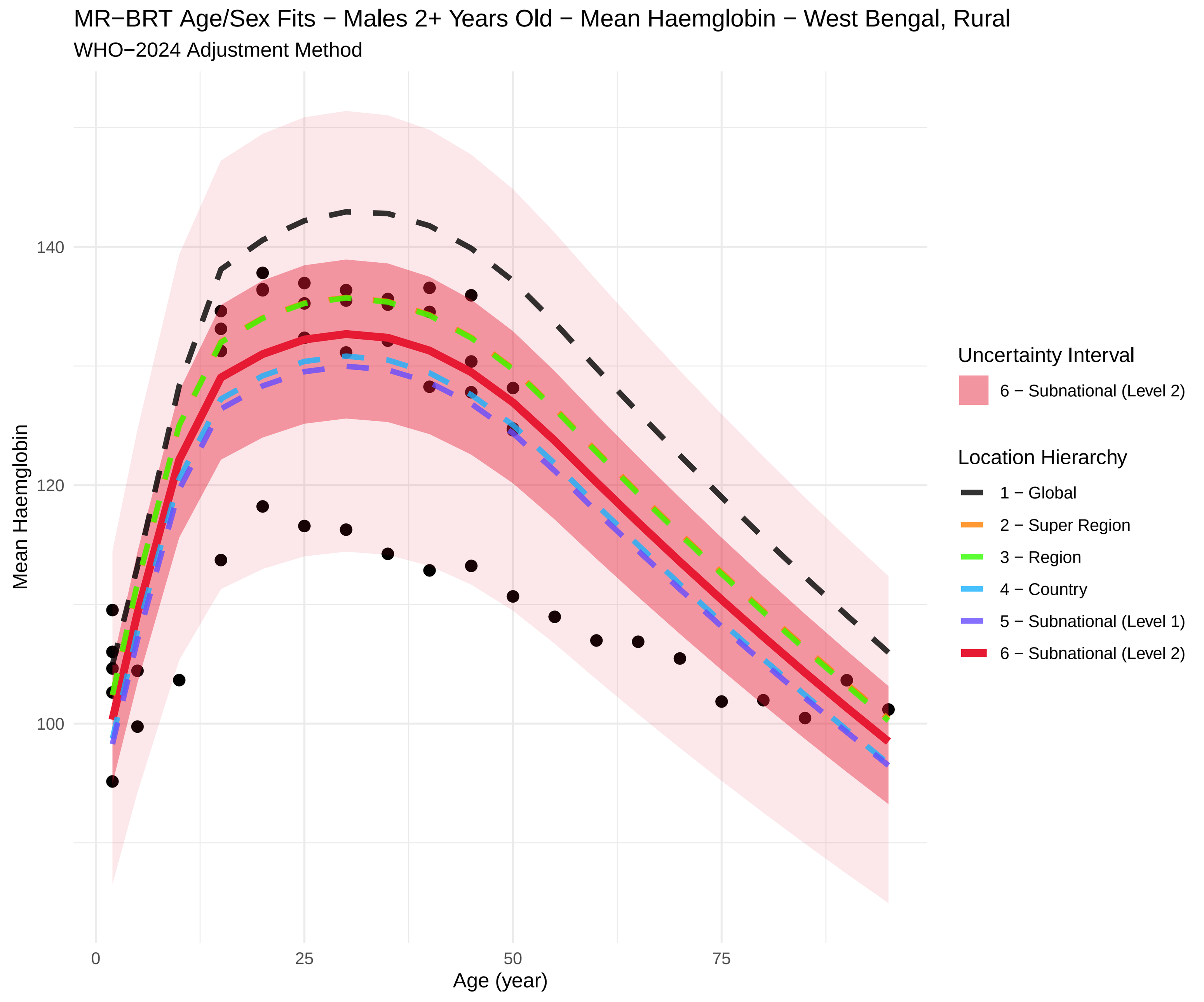
MR−BRT Age/Sex Fits − Males 2+ Years Old − Mean Haemglobin − West Bengal, Rural
WHO−2024 Adjustment Method
Mean Haemglobin
Age (year)
Uncertainty Interval
6 − Subnational (Level 2)
Location Hierarchy
1 − Global
2 − Super Region
3 − Region
4 − Country
5 − Subnational (Level 1)
6 − Subnational (Level 2)

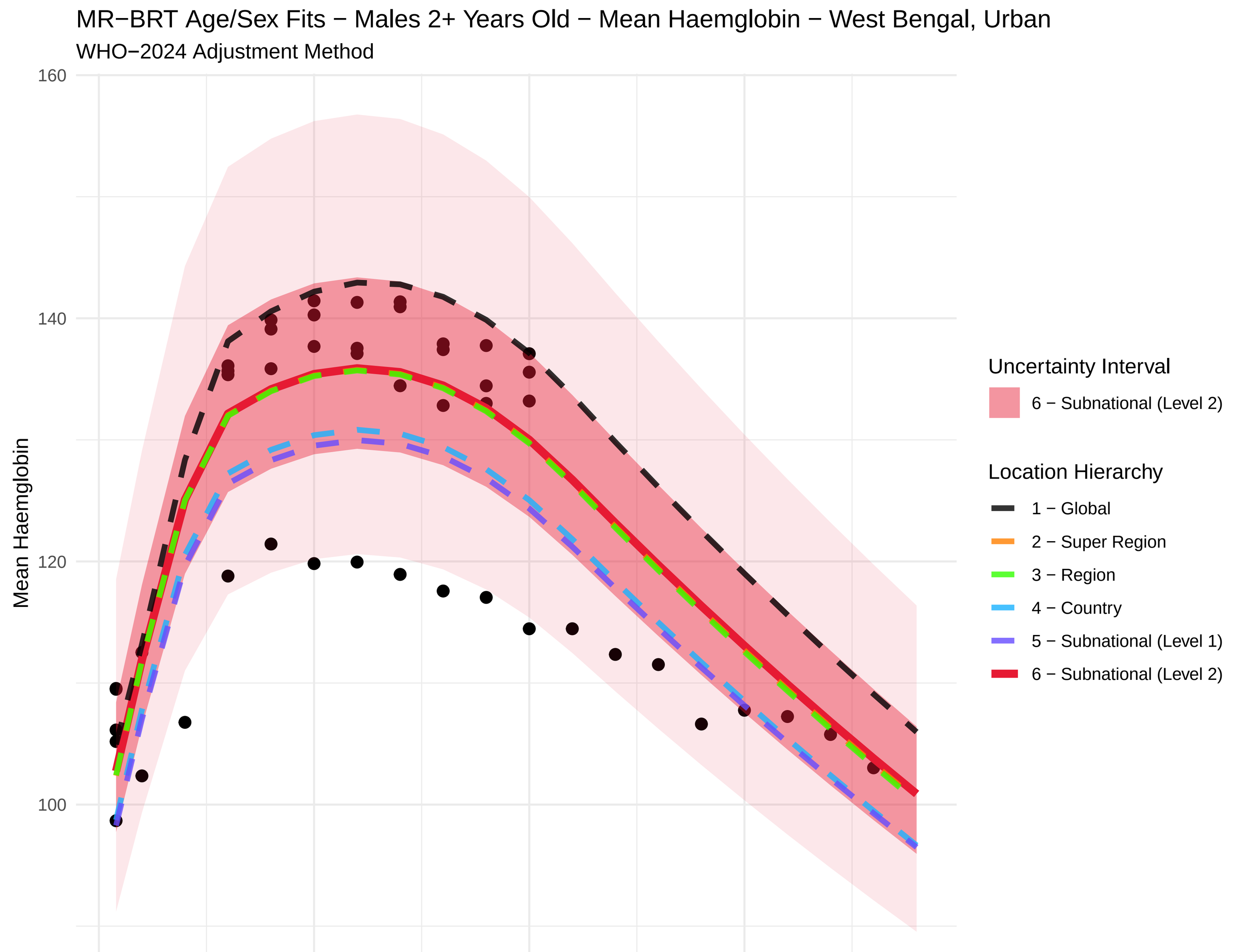
MR−BRT Age/Sex Fits − Males 2+ Years Old − Mean Haemglobin − West Bengal, Urban
WHO−2024 Adjustment Method
Mean Haemglobin
Age (year)
Uncertainty Interval
6 − Subnational (Level 2)
Location Hierarchy
1 − Global
2 − Super Region
3 − Region
4 − Country
5 − Subnational (Level 1)
6 − Subnational (Level 2)

MR−BRT Age/Sex Fits − Males 2+ Years Old − Mean Haemglobin − Nepal
WHO−2024 Adjustment Method

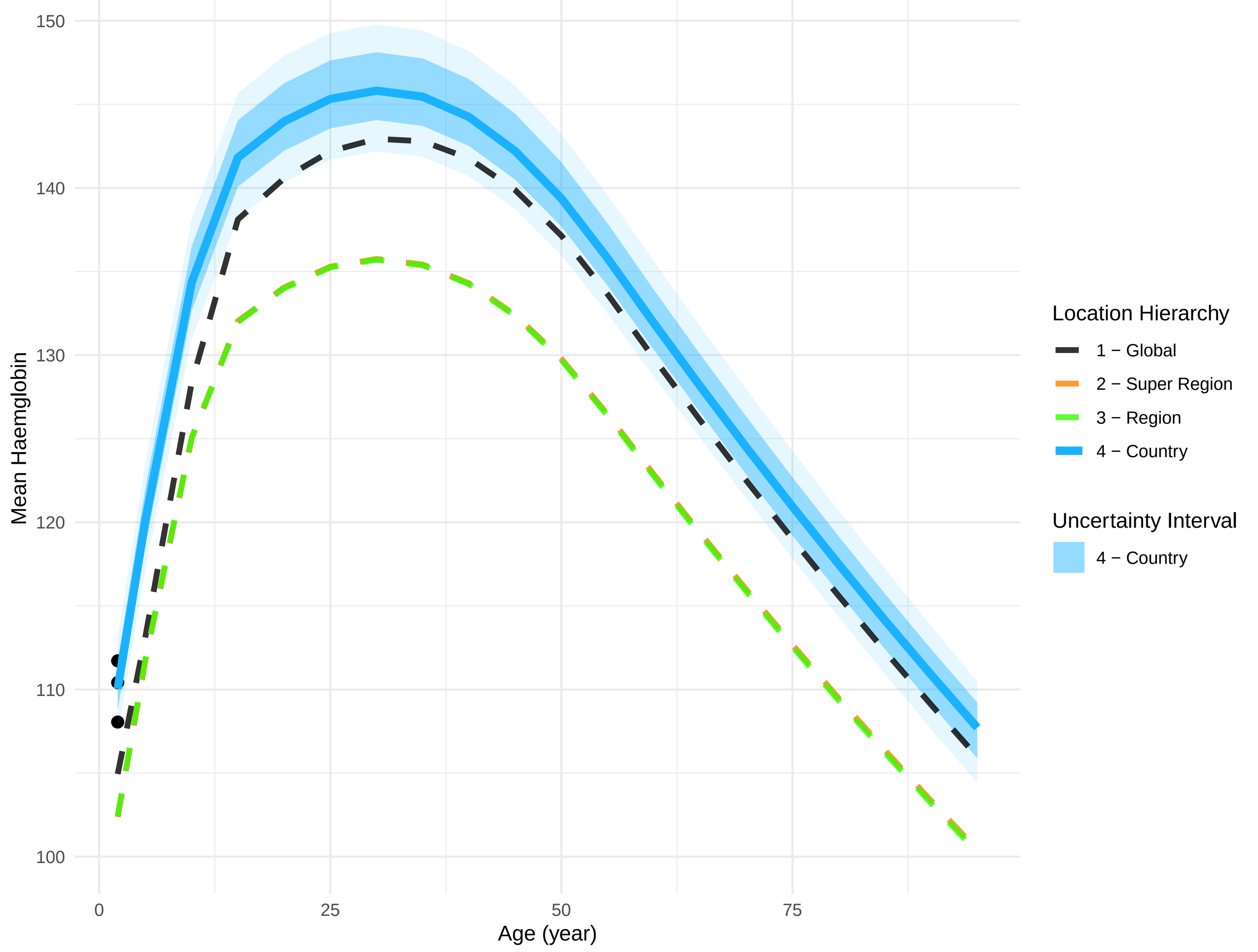

MR−BRT Age/Sex Fits − Males 2+ Years Old − Mean Haemglobin − Southeast Asia, East Asia, and Oceania
WHO−2024 Adjustment Method

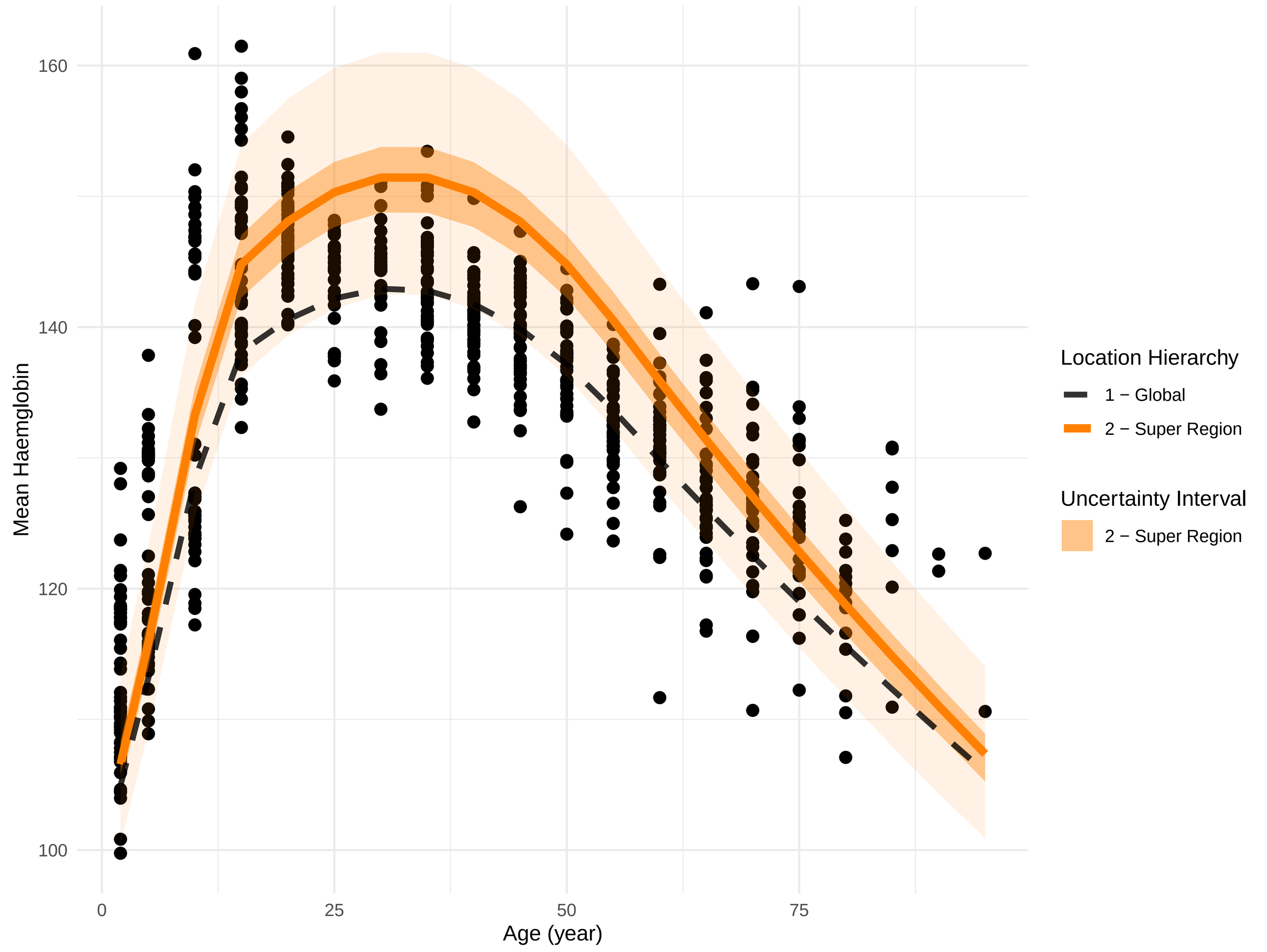

MR−BRT Age/Sex Fits − Males 2+ Years Old − Mean Haemglobin − Southeast Asia
WHO−2024 Adjustment Method

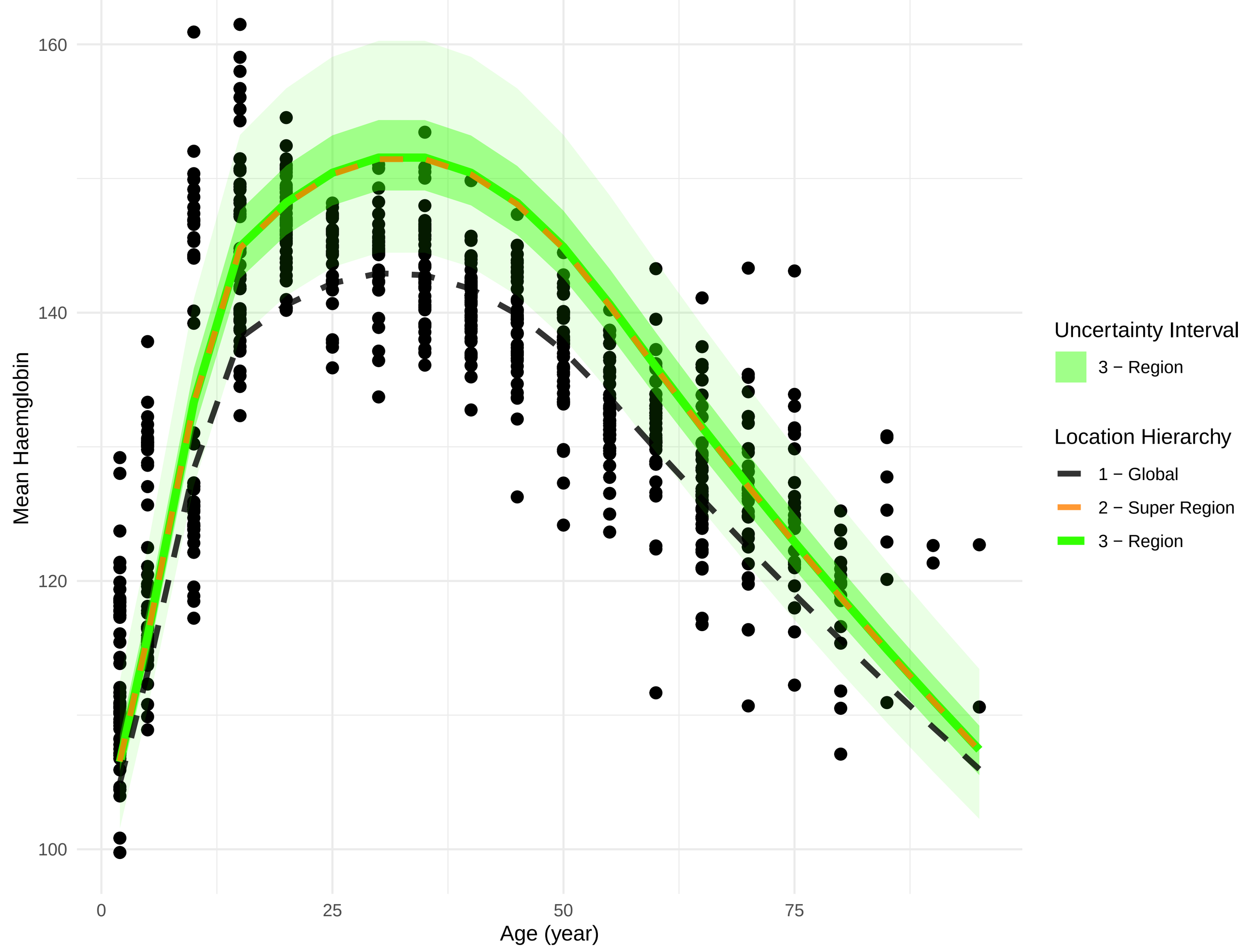

MR−BRT Age/Sex Fits − Males 2+ Years Old − Mean Haemglobin − Cambodia
WHO−2024 Adjustment Method

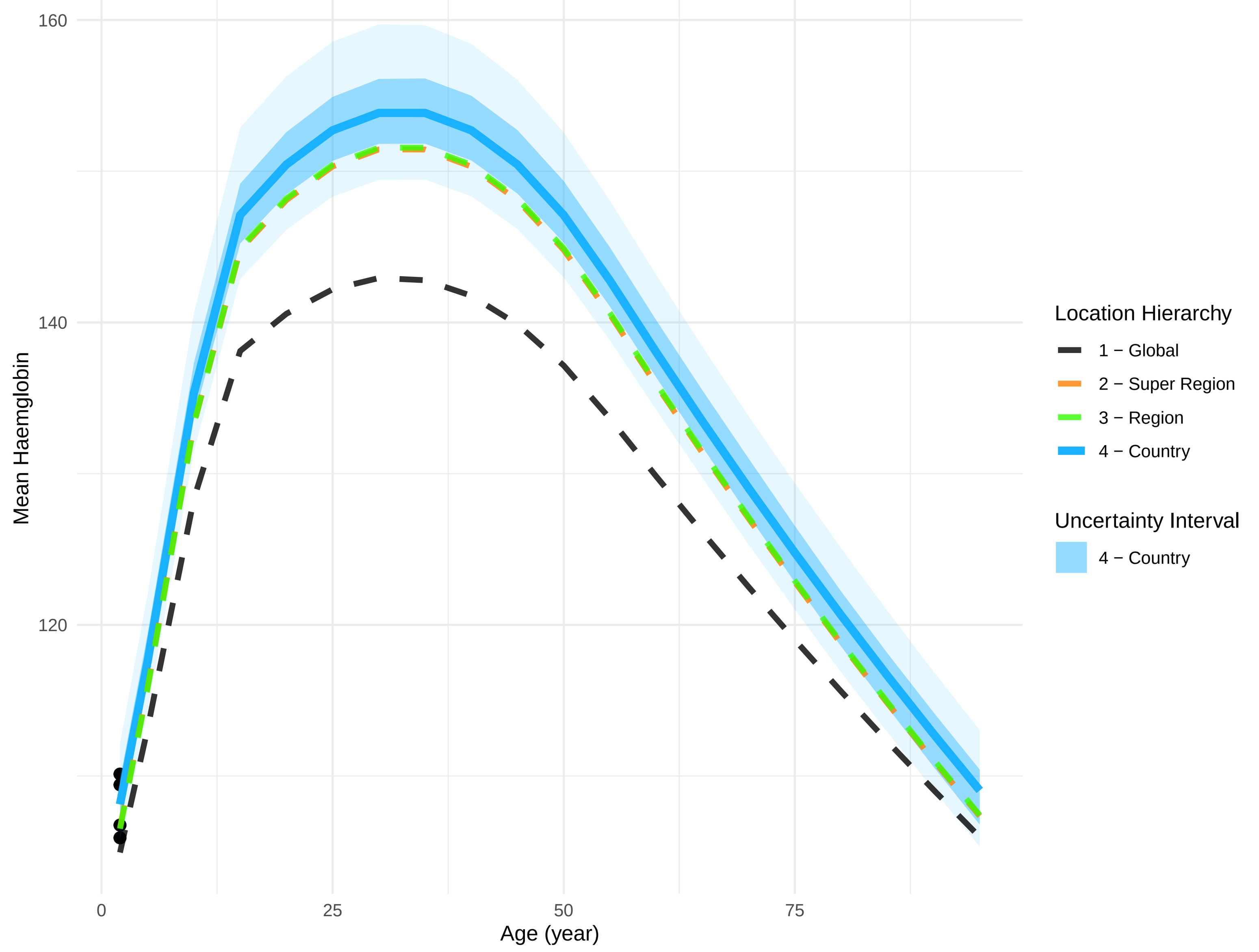

MR−BRT Age/Sex Fits − Males 2+ Years Old − Mean Haemglobin − Indonesia
WHO−2024 Adjustment Method

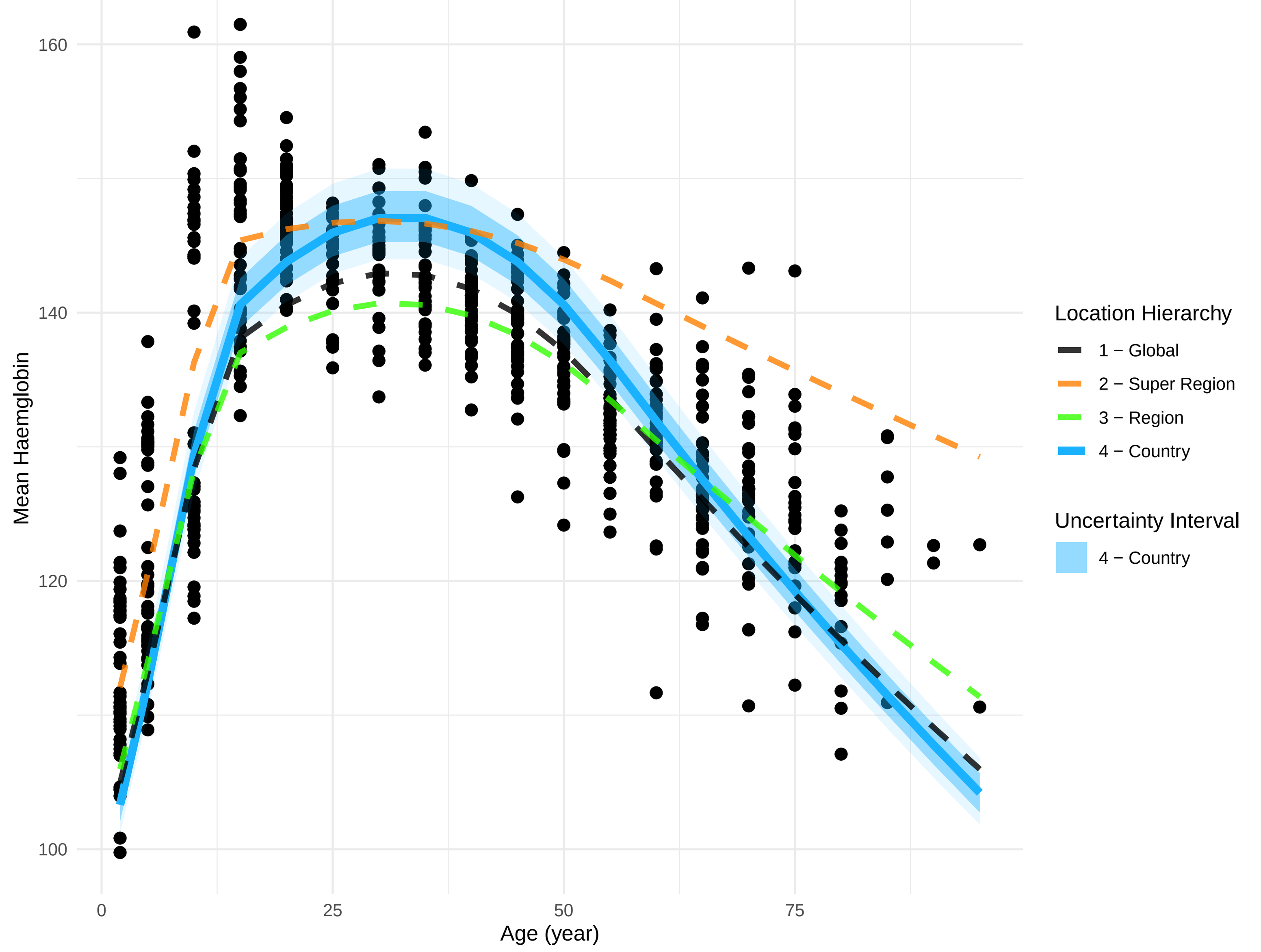

MR−BRT Age/Sex Fits − Males 2+ Years Old − Mean Haemglobin − Bali
WHO−2024 Adjustment Method

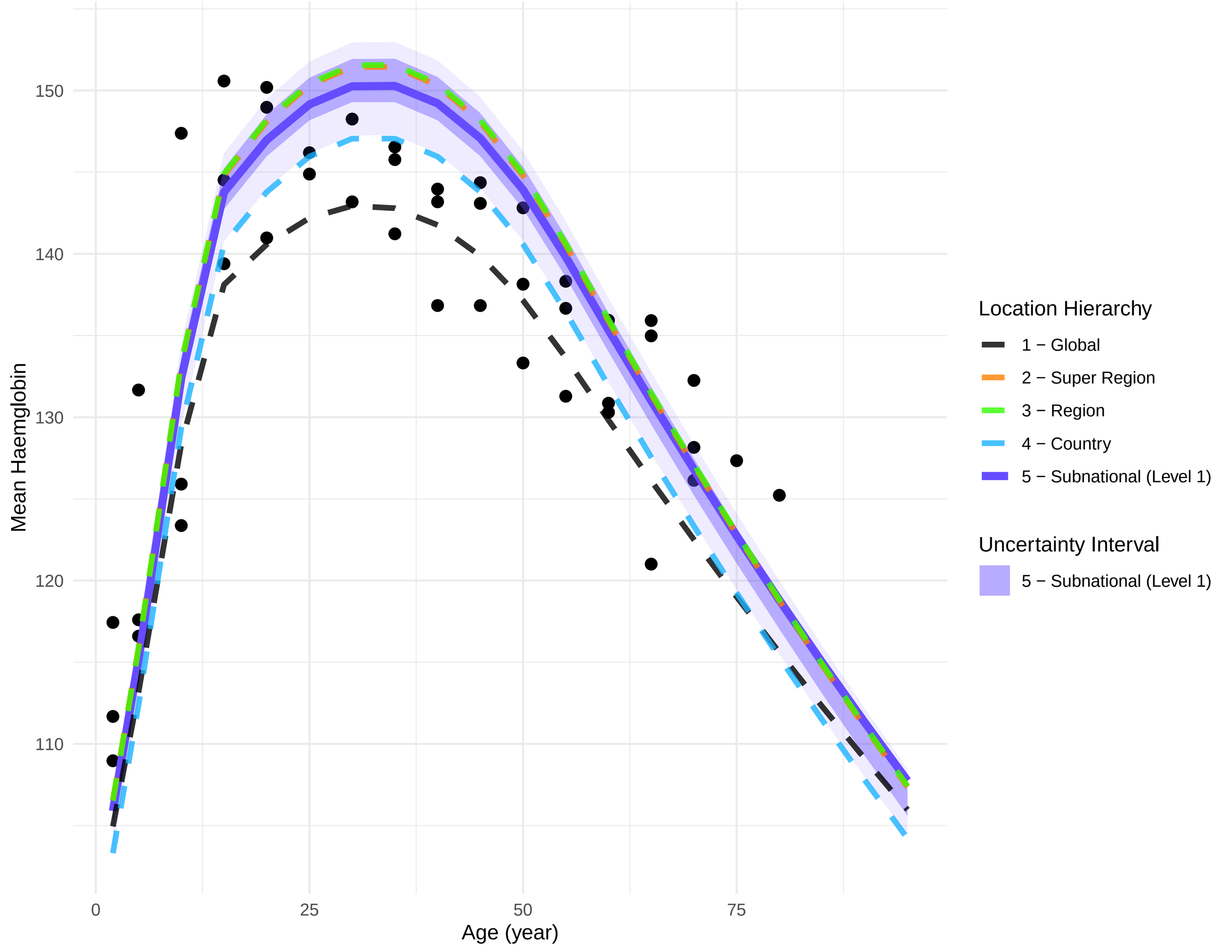

MR−BRT Age/Sex Fits − Males 2+ Years Old − Mean Haemglobin − Bangka−Belitung Islands
WHO−2024 Adjustment Method

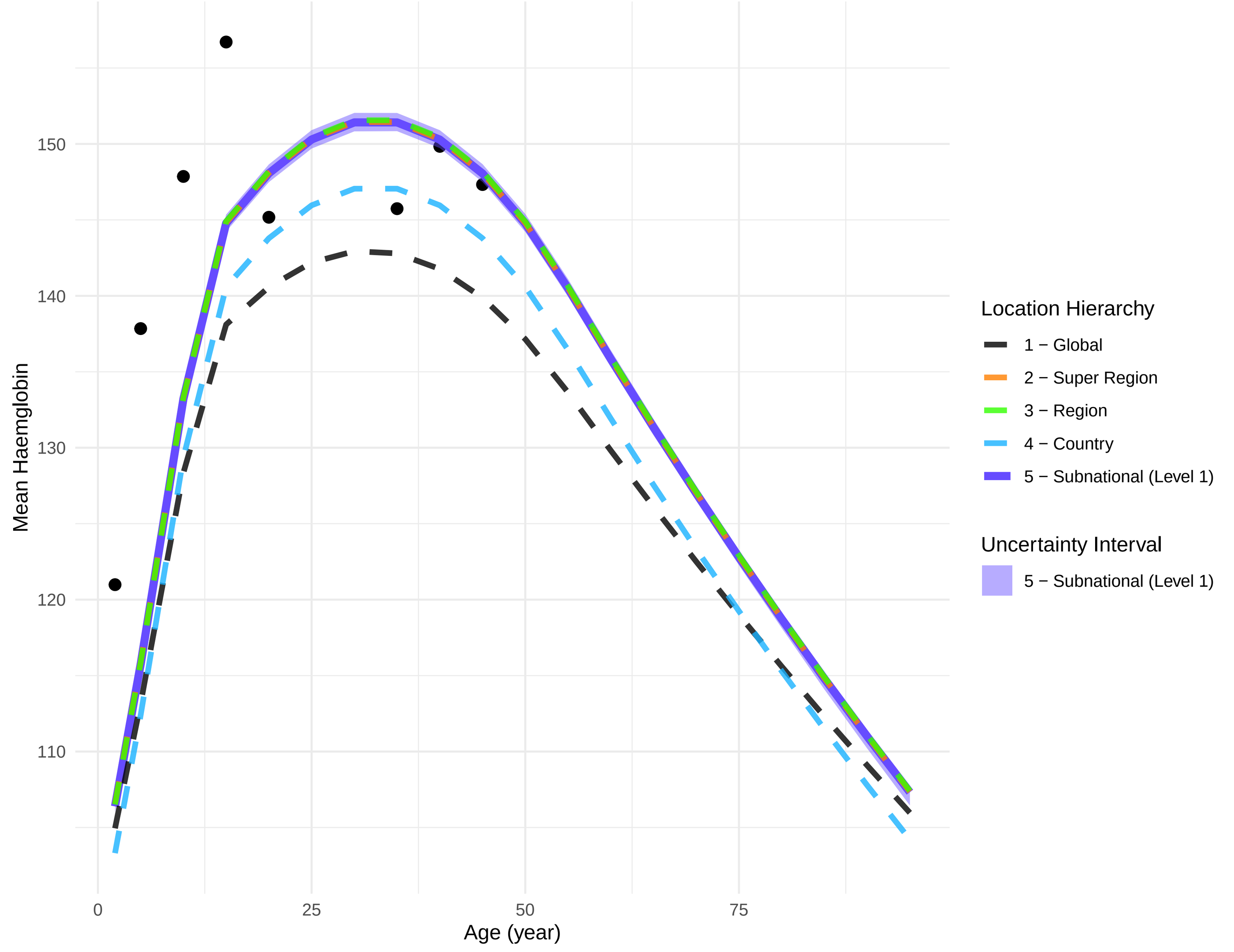

MR−BRT Age/Sex Fits − Males 2+ Years Old − Mean Haemglobin − Banten
WHO−2024 Adjustment Method

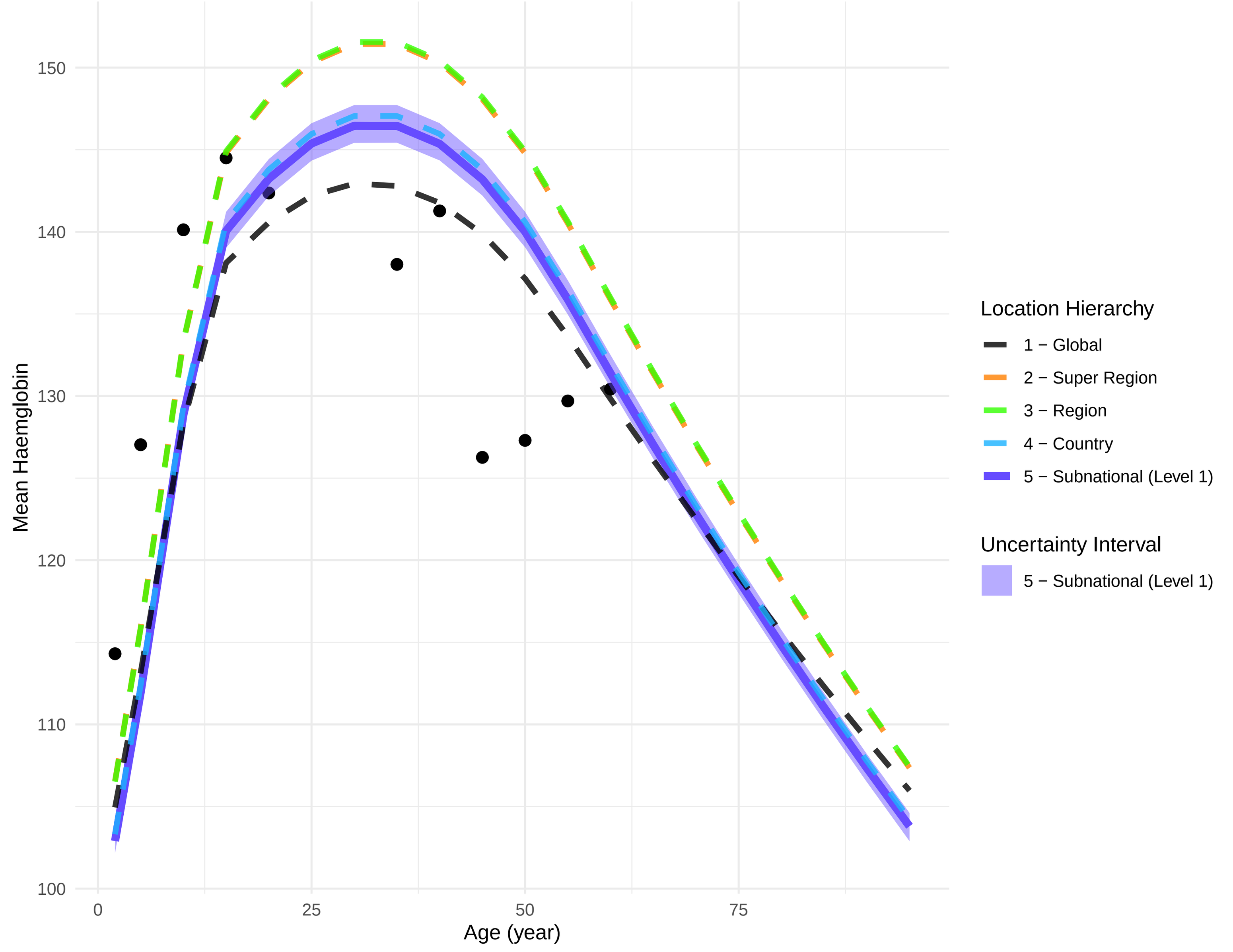

MR−BRT Age/Sex Fits − Males 2+ Years Old − Mean Haemglobin − Jakarta
WHO−2024 Adjustment Method

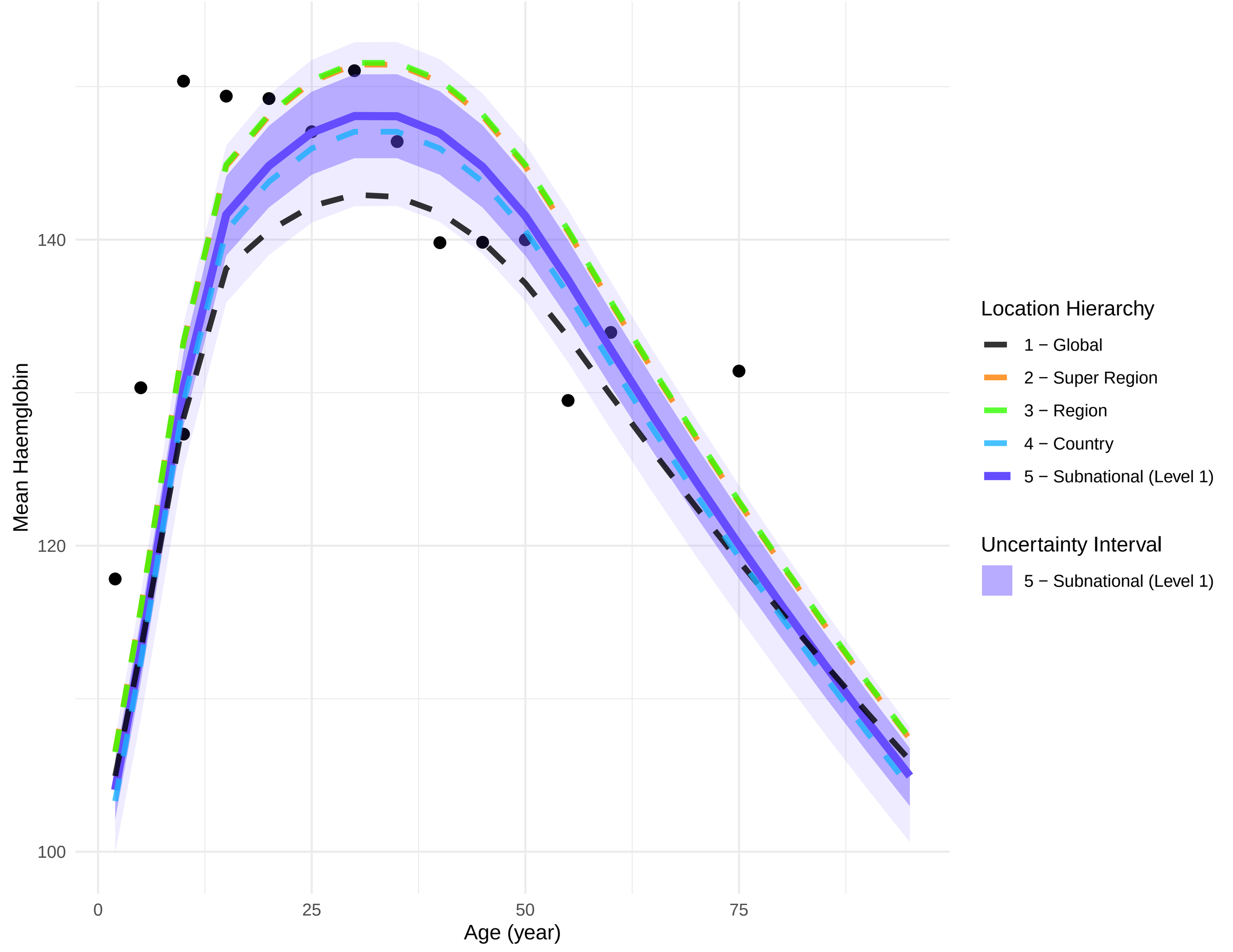

MR−BRT Age/Sex Fits − Males 2+ Years Old − Mean Haemglobin − West Java
WHO−2024 Adjustment Method

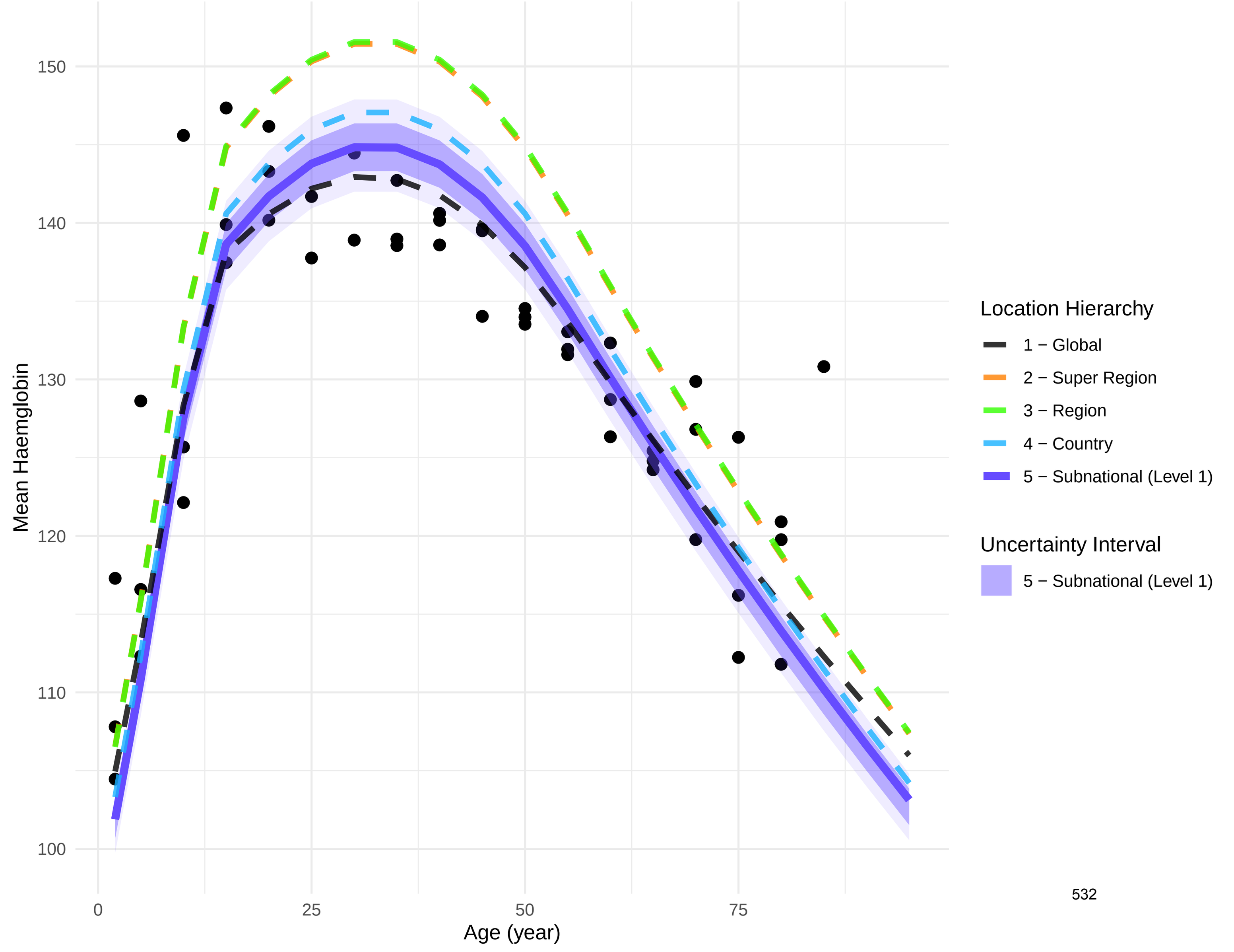

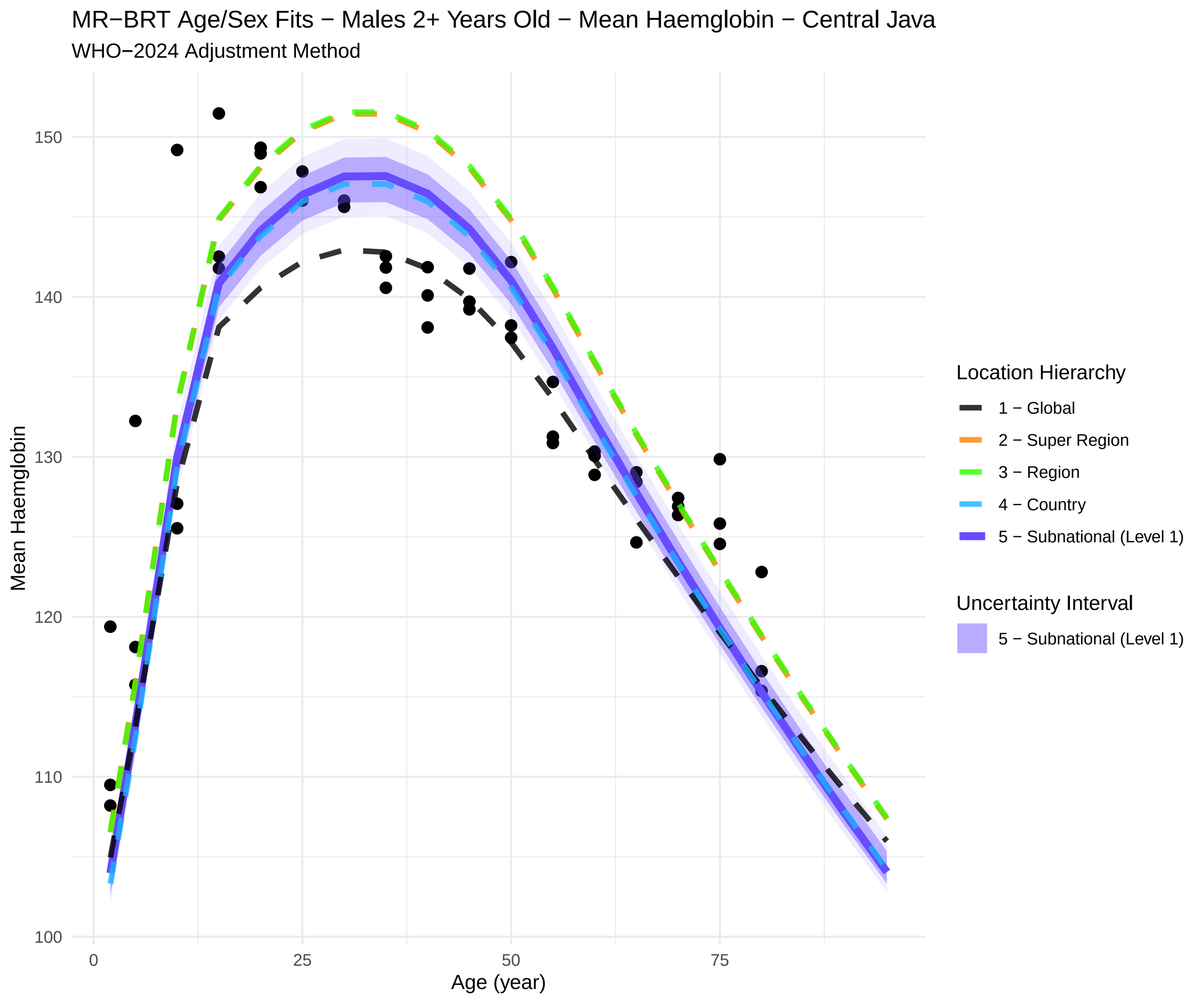
MR−BRT Age/Sex Fits − Males 2+ Years Old − Mean Haemglobin − Central Java
WHO−2024 Adjustment Method
Mean Haemglobin
Age (year)
Location Hierarchy
1 − Global
2 − Super Region
3 − Region
4 − Country
5 − Subnational (Level 1)
Uncertainty Interval
5 − Subnational (Level 1)

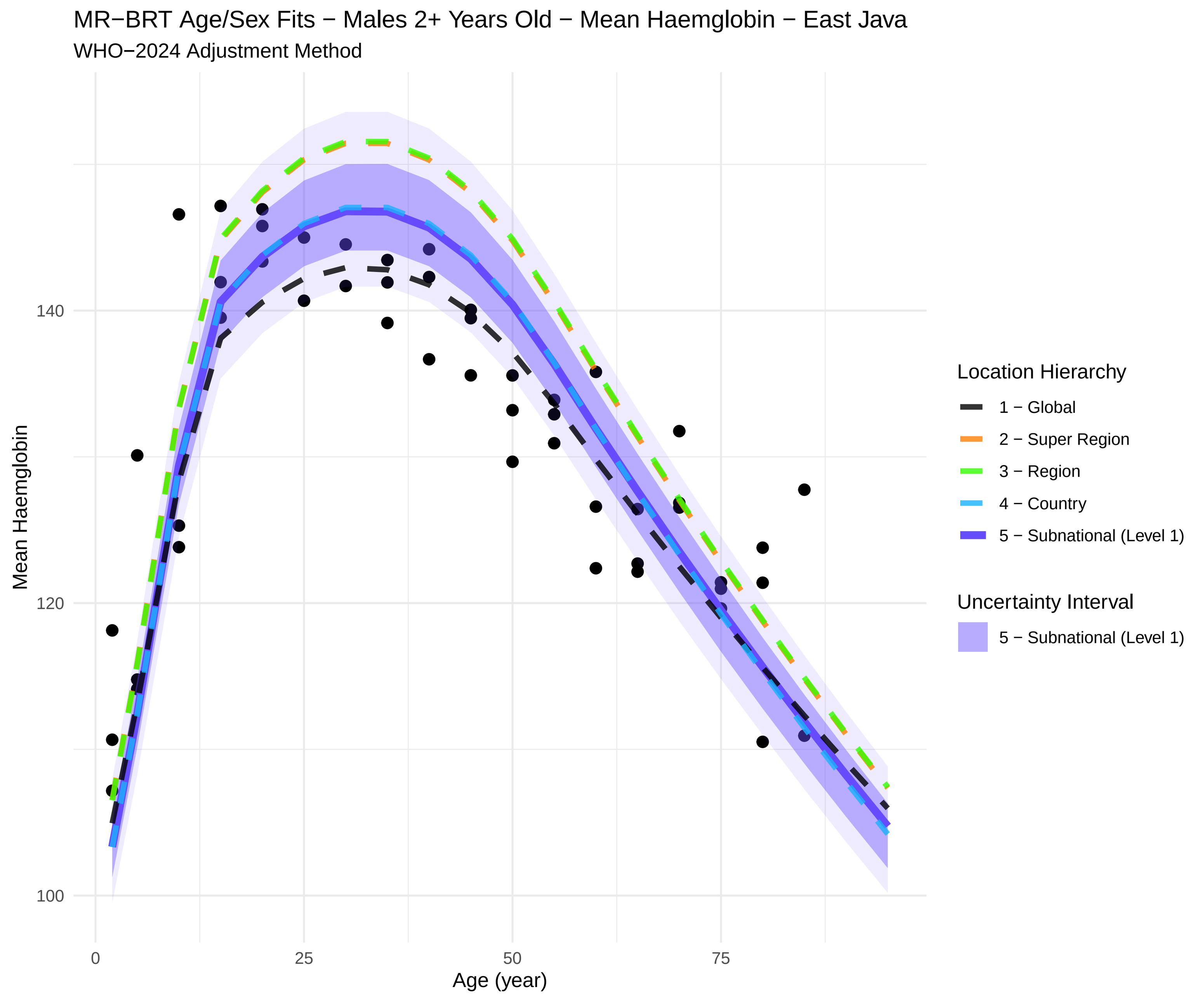
MR−BRT Age/Sex Fits − Males 2+ Years Old − Mean Haemglobin − East Java
WHO−2024 Adjustment Method
Mean Haemglobin
Age (year)
Location Hierarchy
1 − Global
2 − Super Region
3 − Region
4 − Country
5 − Subnational (Level 1)
Uncertainty Interval
5 − Subnational (Level 1)

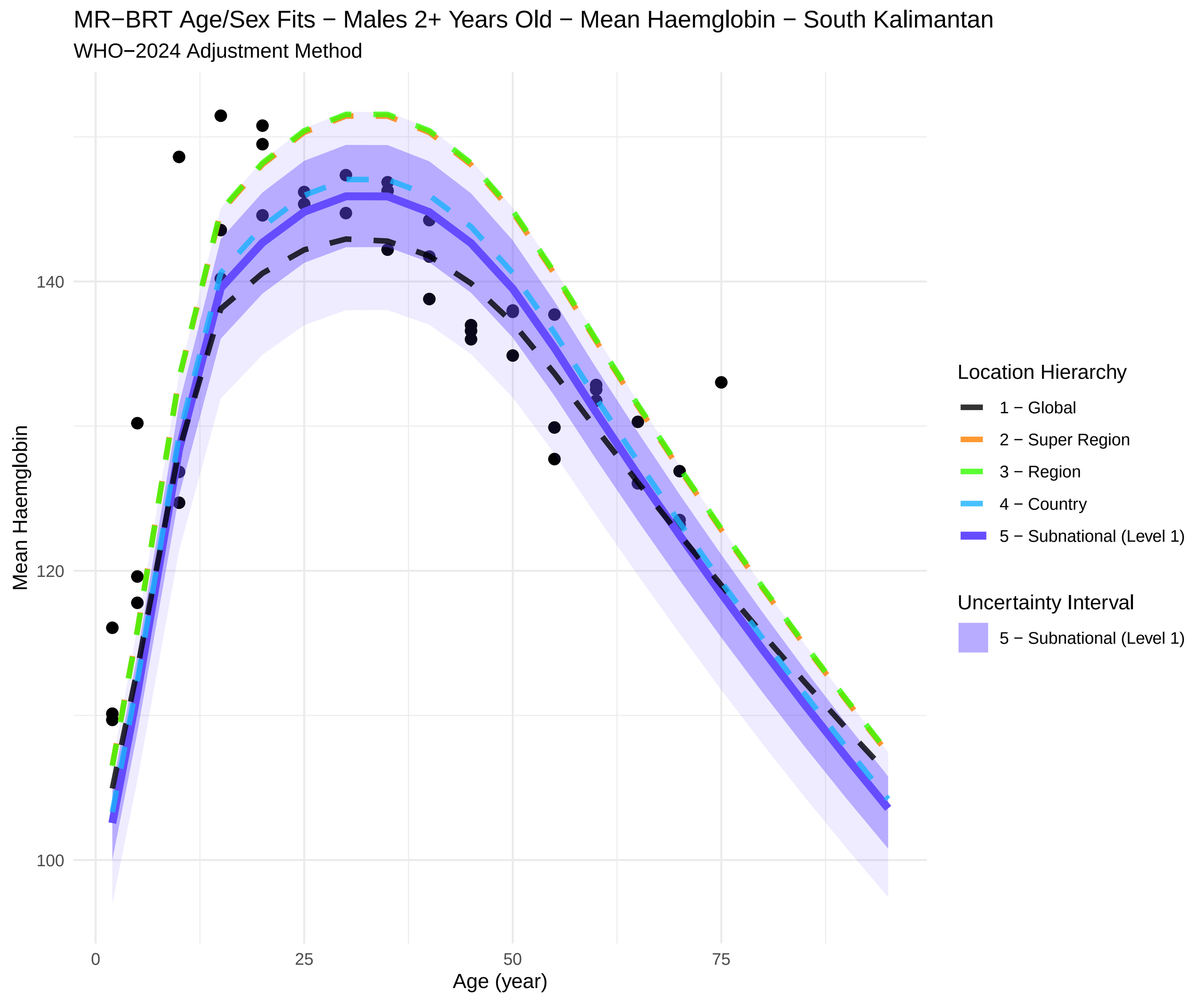
MR−BRT Age/Sex Fits − Males 2+ Years Old − Mean Haemglobin − South Kalimantan
WHO−2024 Adjustment Method
Mean Haemglobin
Age (year)
Location Hierarchy
1 − Global
2 − Super Region
3 − Region
4 − Country
5 − Subnational (Level 1)
Uncertainty Interval
5 − Subnational (Level 1)

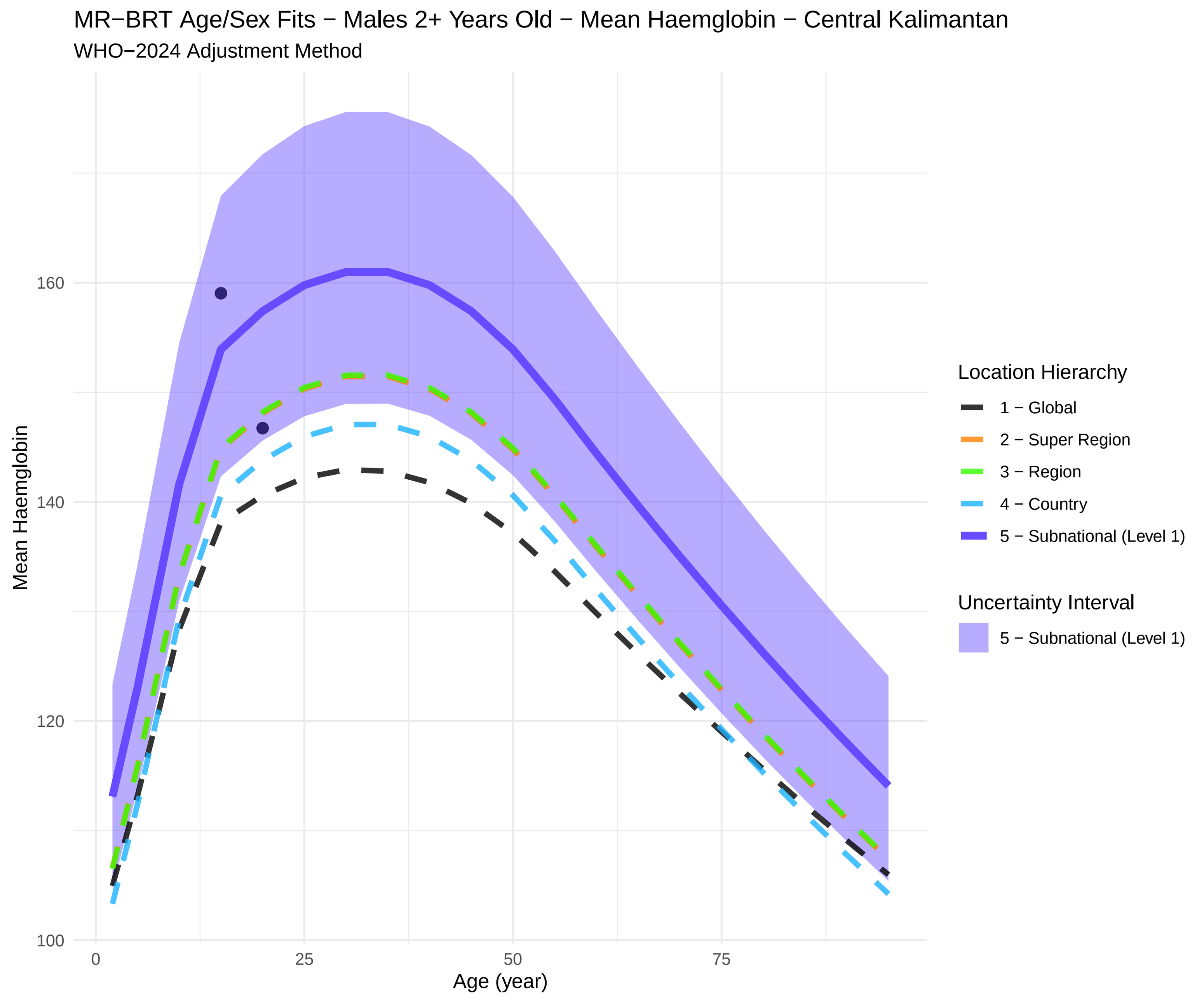
MR−BRT Age/Sex Fits − Males 2+ Years Old − Mean Haemglobin − Central Kalimantan
WHO−2024 Adjustment Method
Mean Haemglobin
Age (year)
Location Hierarchy
1 − Global
2 − Super Region
3 − Region
4 − Country
5 − Subnational (Level 1)
Uncertainty Interval
5 − Subnational (Level 1)

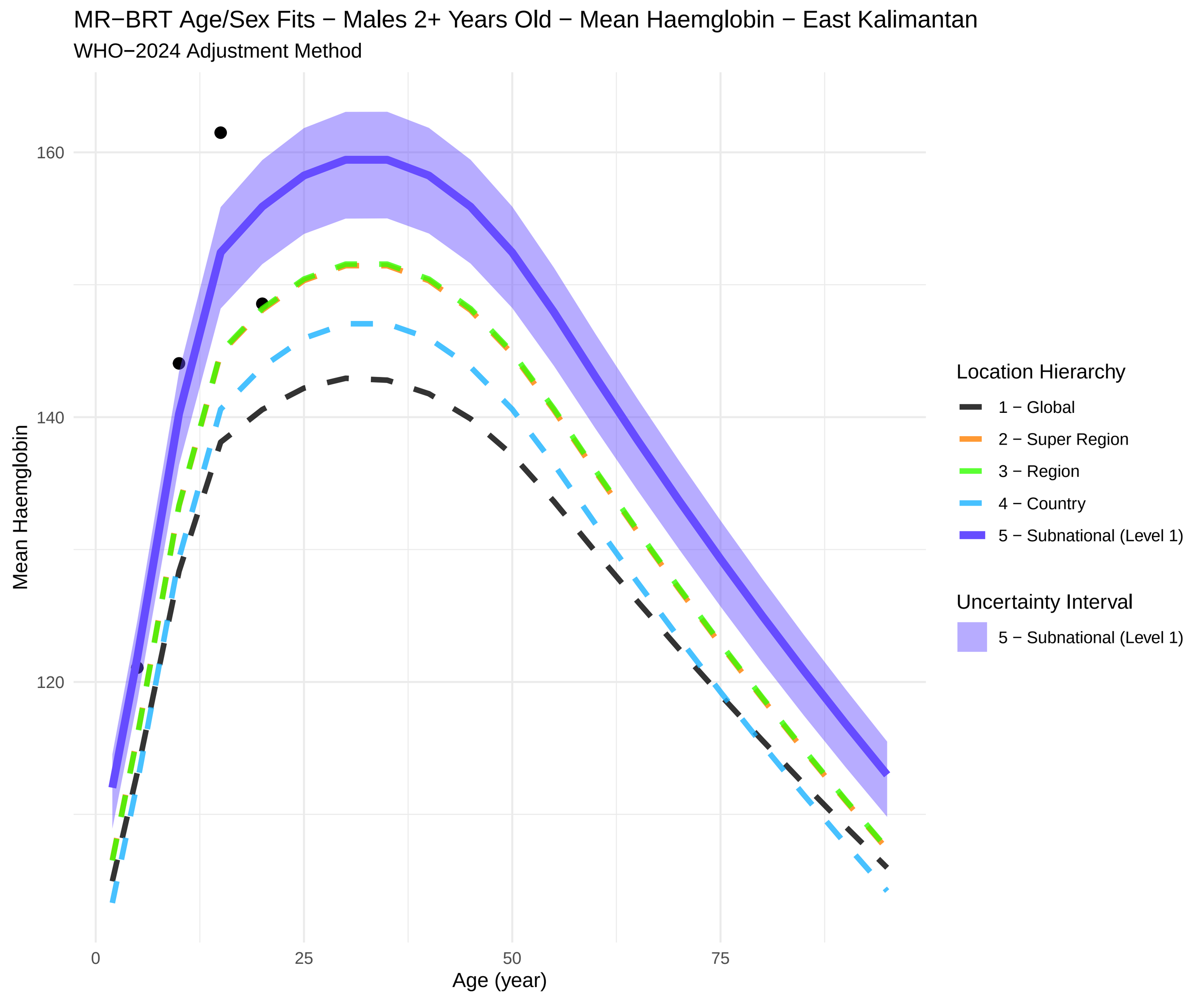
MR−BRT Age/Sex Fits − Males 2+ Years Old − Mean Haemglobin − East Kalimantan
WHO−2024 Adjustment Method
Mean Haemglobin
Age (year)
Location Hierarchy
1 − Global
2 − Super Region
3 − Region
4 − Country
5 − Subnational (Level 1)
Uncertainty Interval
5 − Subnational (Level 1)

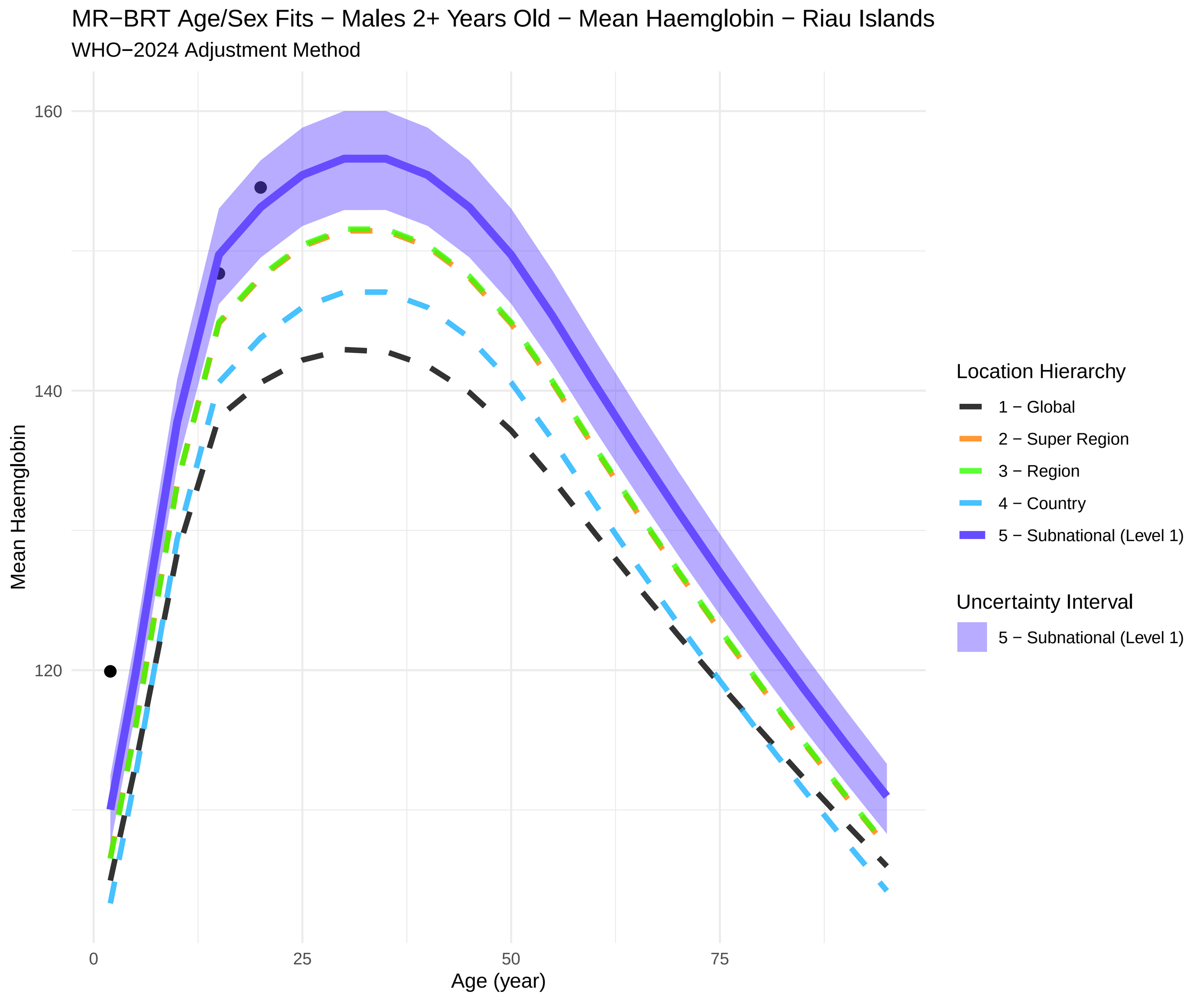
MR−BRT Age/Sex Fits − Males 2+ Years Old − Mean Haemglobin − Riau Islands
WHO−2024 Adjustment Method
Mean Haemglobin
Age (year)
Location Hierarchy
1 − Global
2 − Super Region
3 − Region
4 − Country
5 − Subnational (Level 1)
Uncertainty Interval
5 − Subnational (Level 1)

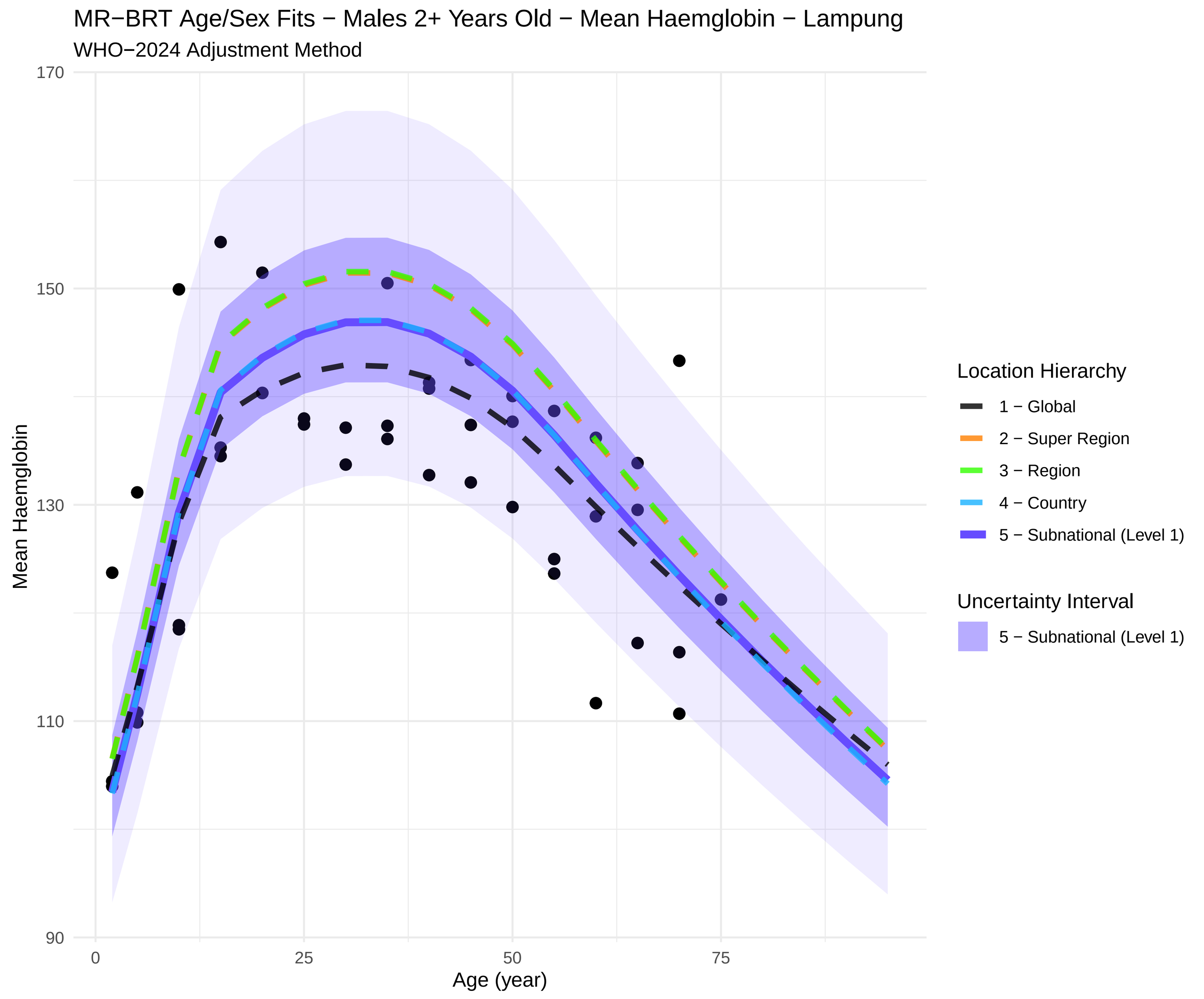
MR−BRT Age/Sex Fits − Males 2+ Years Old − Mean Haemglobin − Lampung
WHO−2024 Adjustment Method
Mean Haemglobin
Age (year)
Location Hierarchy
1 − Global
2 − Super Region
3 − Region
4 − Country
5 − Subnational (Level 1)
Uncertainty Interval
5 − Subnational (Level 1)

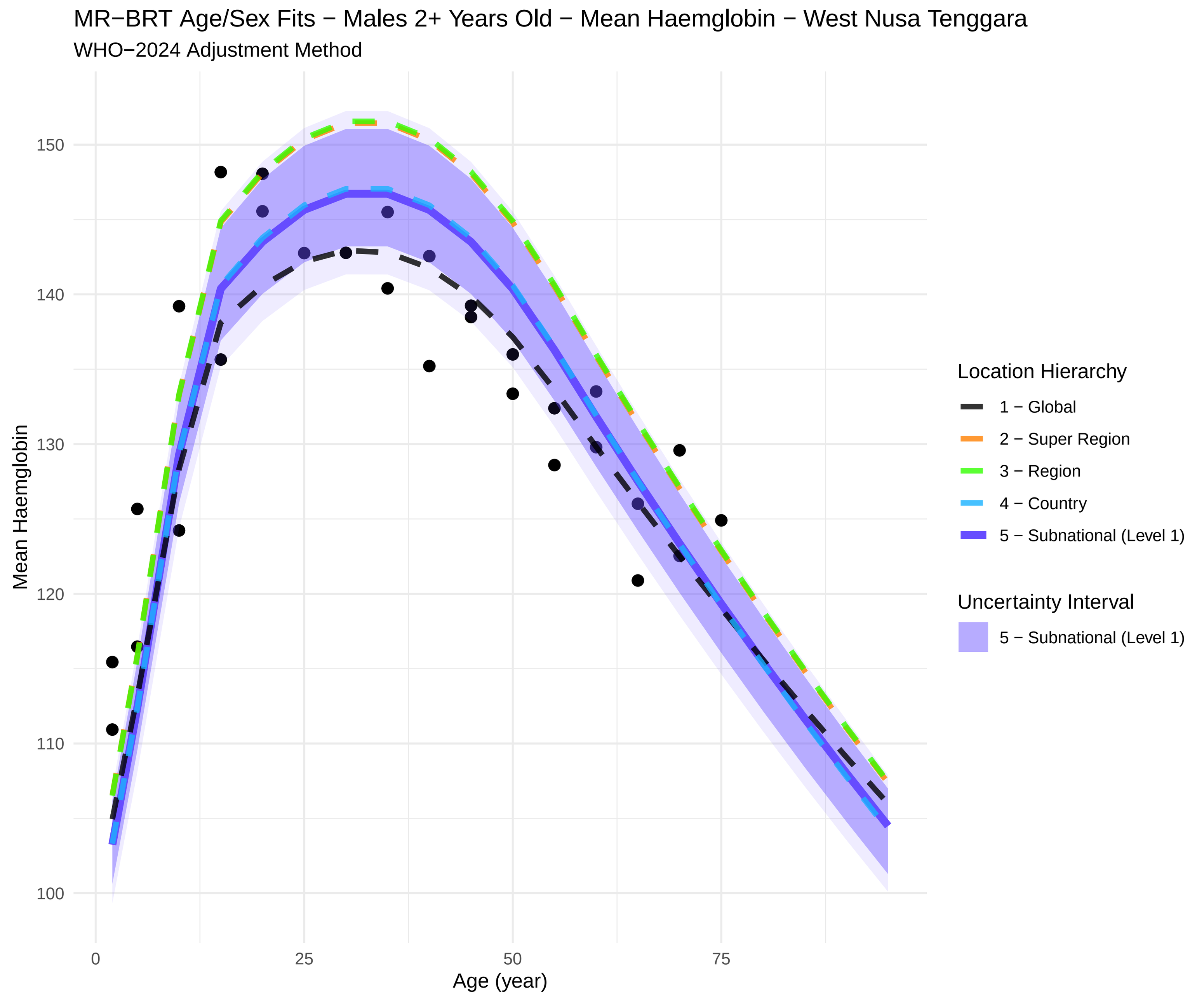
MR−BRT Age/Sex Fits − Males 2+ Years Old − Mean Haemglobin − West Nusa Tenggara
WHO−2024 Adjustment Method
Mean Haemglobin
Age (year)
Location Hierarchy
1 − Global
2 − Super Region
3 − Region
4 − Country
5 − Subnational (Level 1)
Uncertainty Interval
5 − Subnational (Level 1)

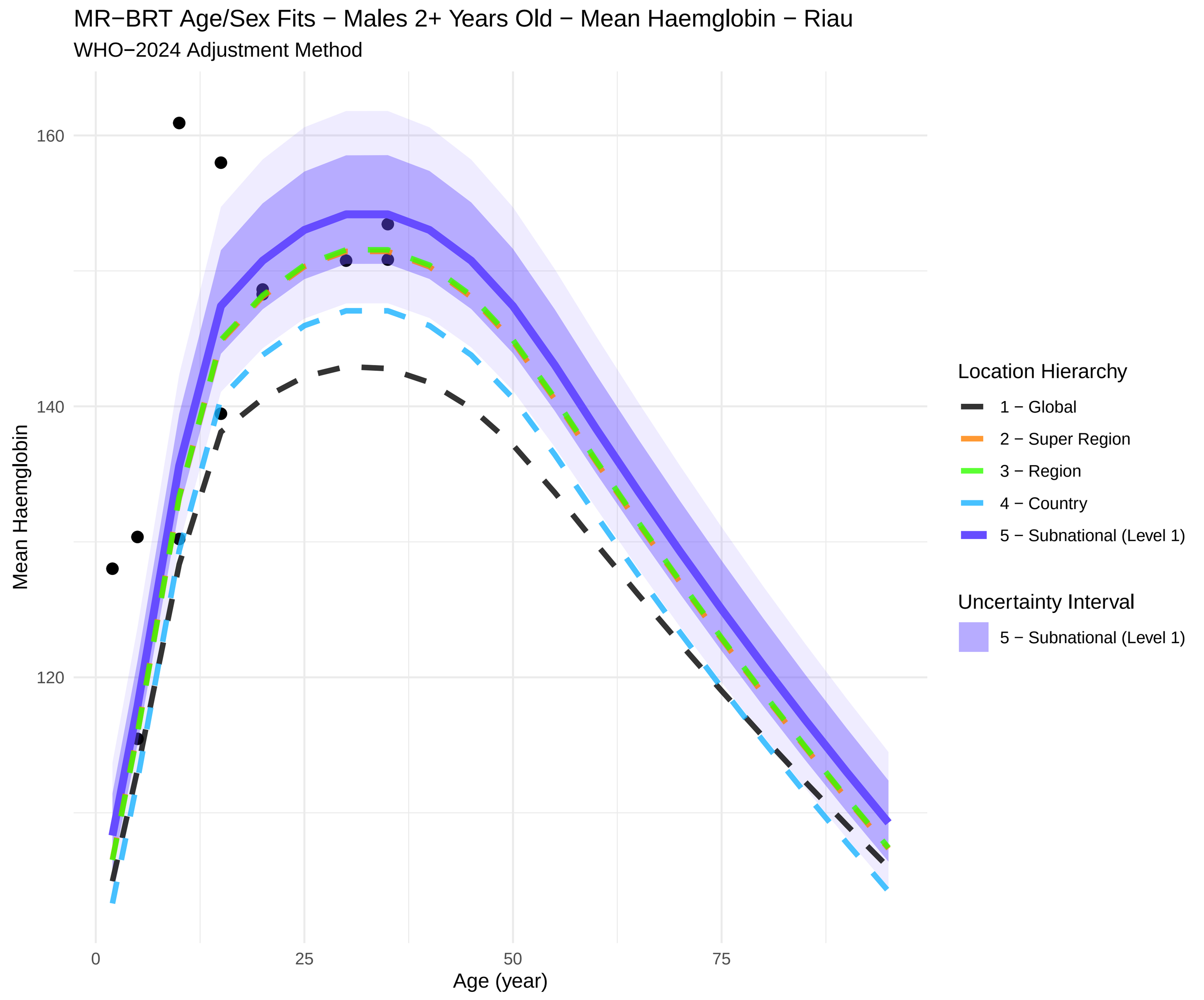
MR−BRT Age/Sex Fits − Males 2+ Years Old − Mean Haemglobin − Riau
WHO−2024 Adjustment Method
Mean Haemglobin
Age (year)
Location Hierarchy
1 − Global
2 − Super Region
3 − Region
4 − Country
5 − Subnational (Level 1)
Uncertainty Interval
5 − Subnational (Level 1)

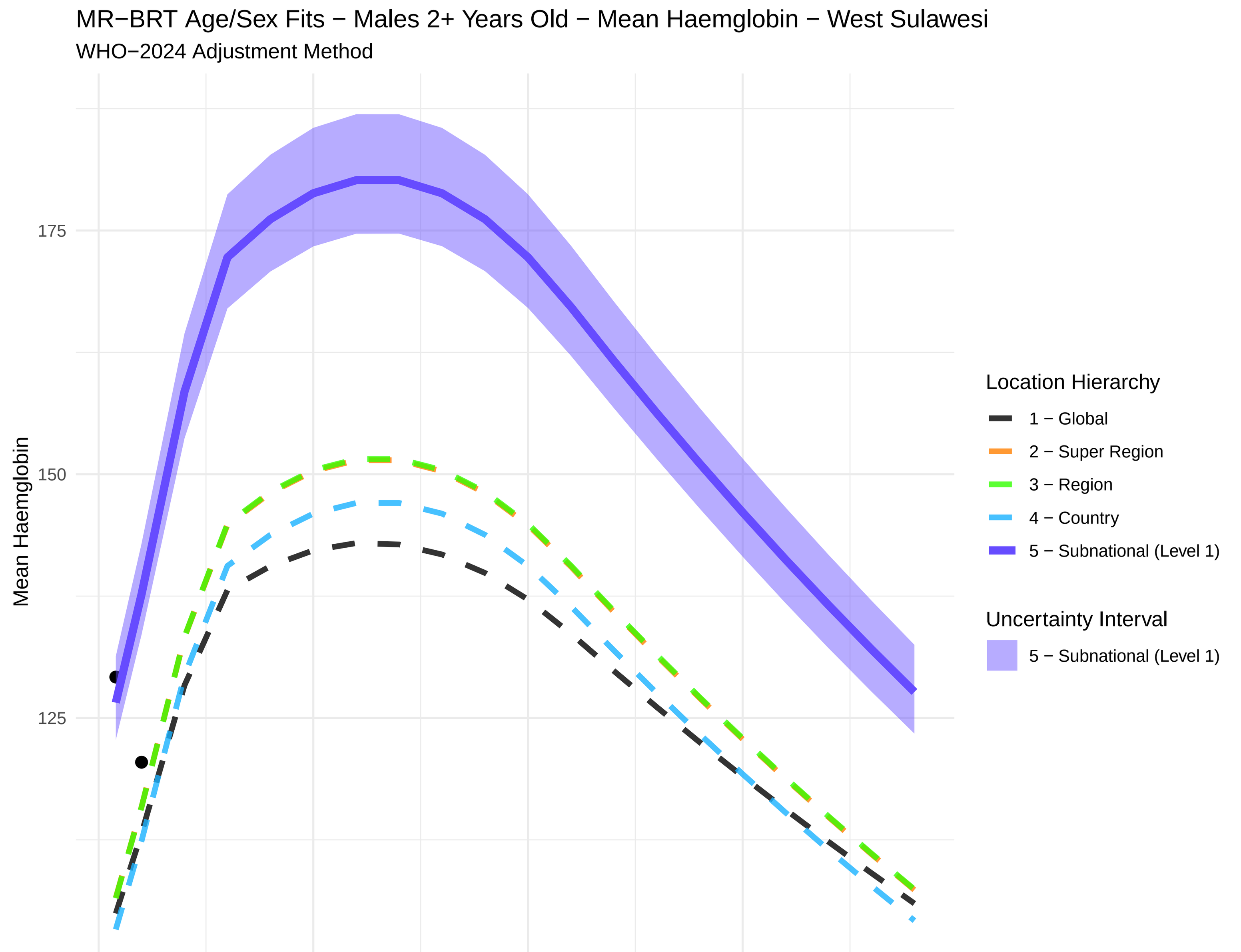
MR−BRT Age/Sex Fits − Males 2+ Years Old − Mean Haemglobin − West Sulawesi
WHO−2024 Adjustment Method
Mean Haemglobin
Age (year)
Location Hierarchy
1 − Global
2 − Super Region
3 − Region
4 − Country
5 − Subnational (Level 1)
Uncertainty Interval
5 − Subnational (Level 1)

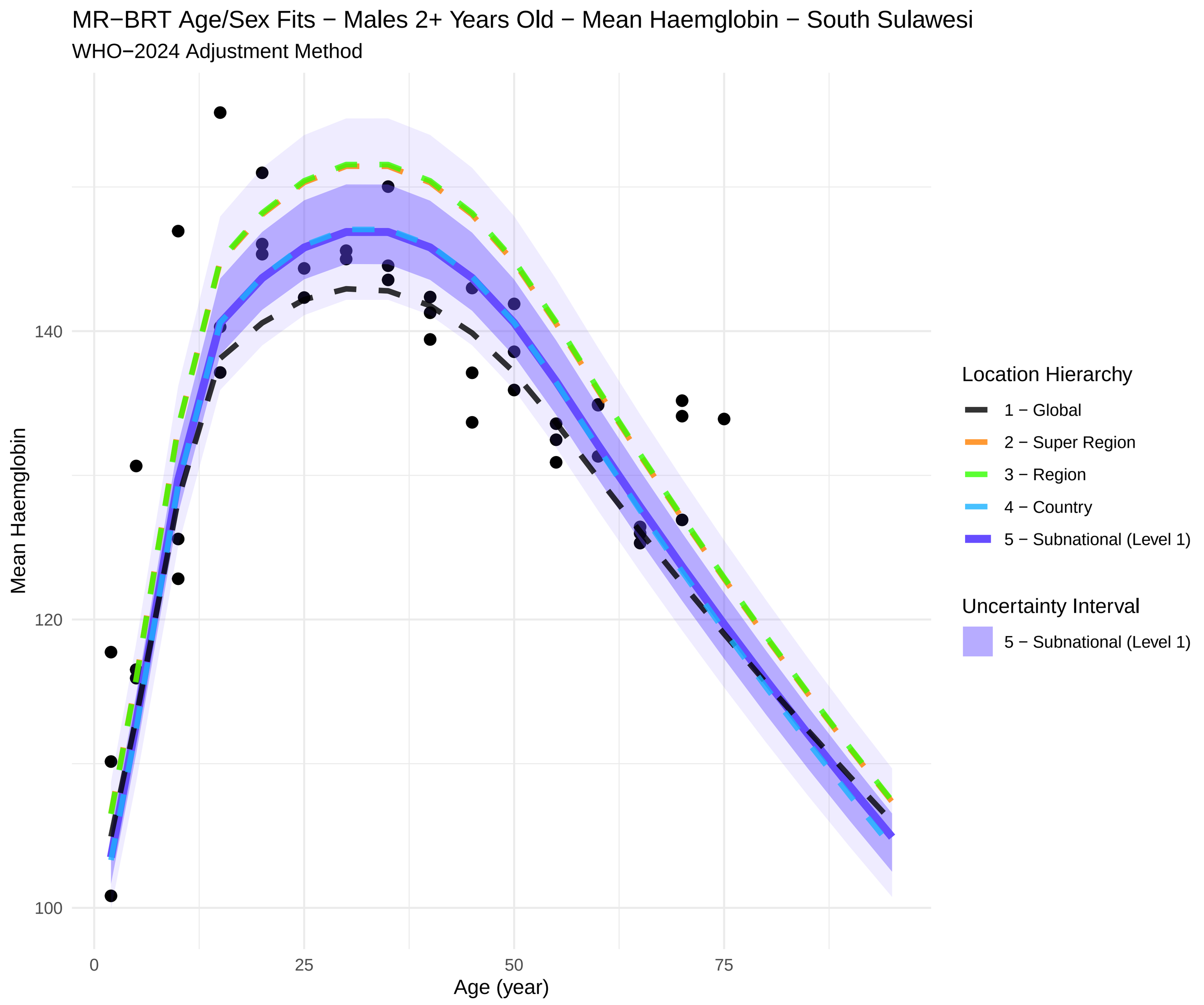
MR−BRT Age/Sex Fits − Males 2+ Years Old − Mean Haemglobin − South Sulawesi
WHO−2024 Adjustment Method
Mean Haemglobin
Age (year)
Location Hierarchy
1 − Global
2 − Super Region
3 − Region
4 − Country
5 − Subnational (Level 1)
Uncertainty Interval
5 − Subnational (Level 1)

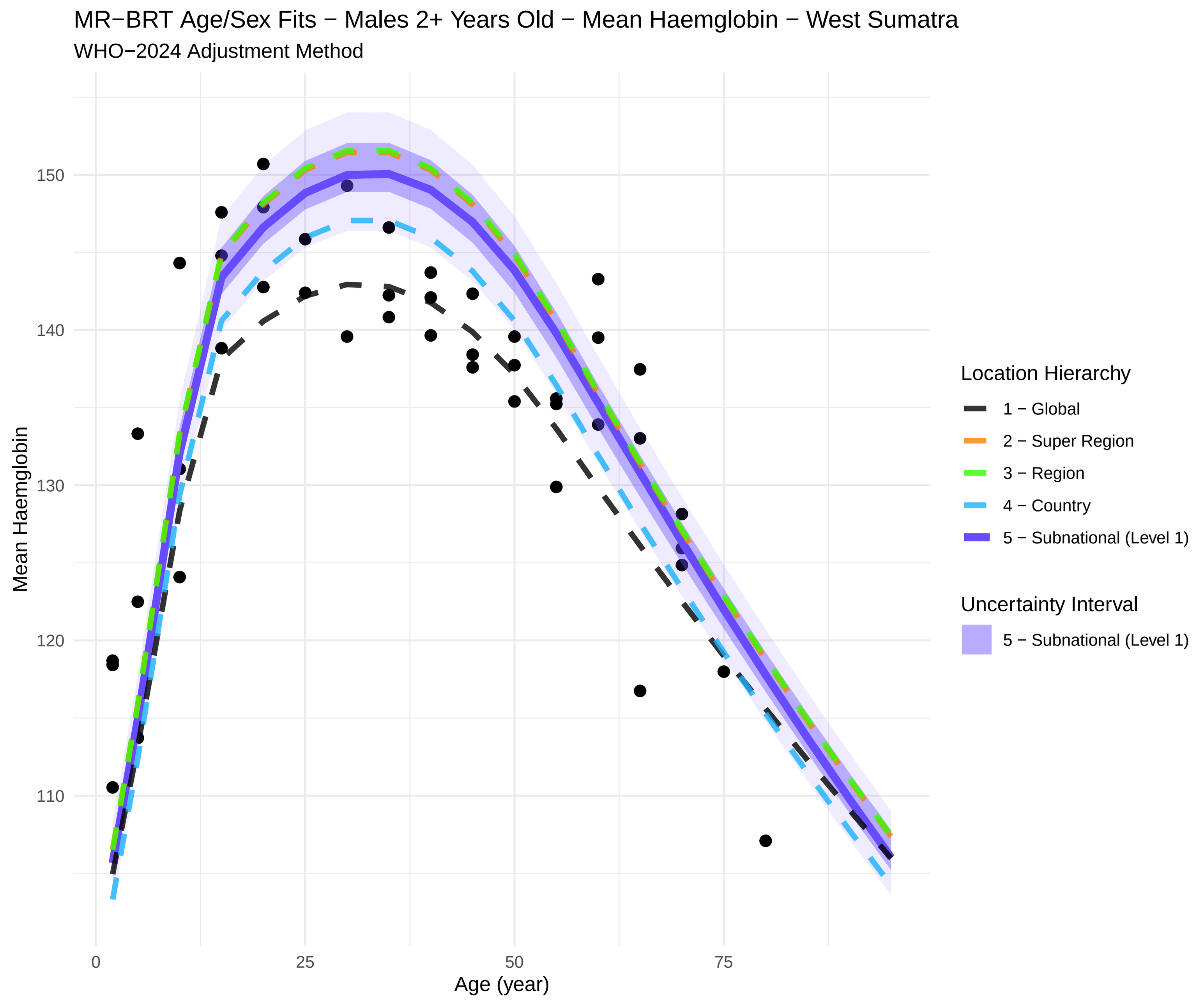
MR−BRT Age/Sex Fits − Males 2+ Years Old − Mean Haemglobin − West Sumatra
WHO−2024 Adjustment Method
Mean Haemglobin
Age (year)
Location Hierarchy
1 − Global
2 − Super Region
3 − Region
4 − Country
5 − Subnational (Level 1)
Uncertainty Interval
5 − Subnational (Level 1)

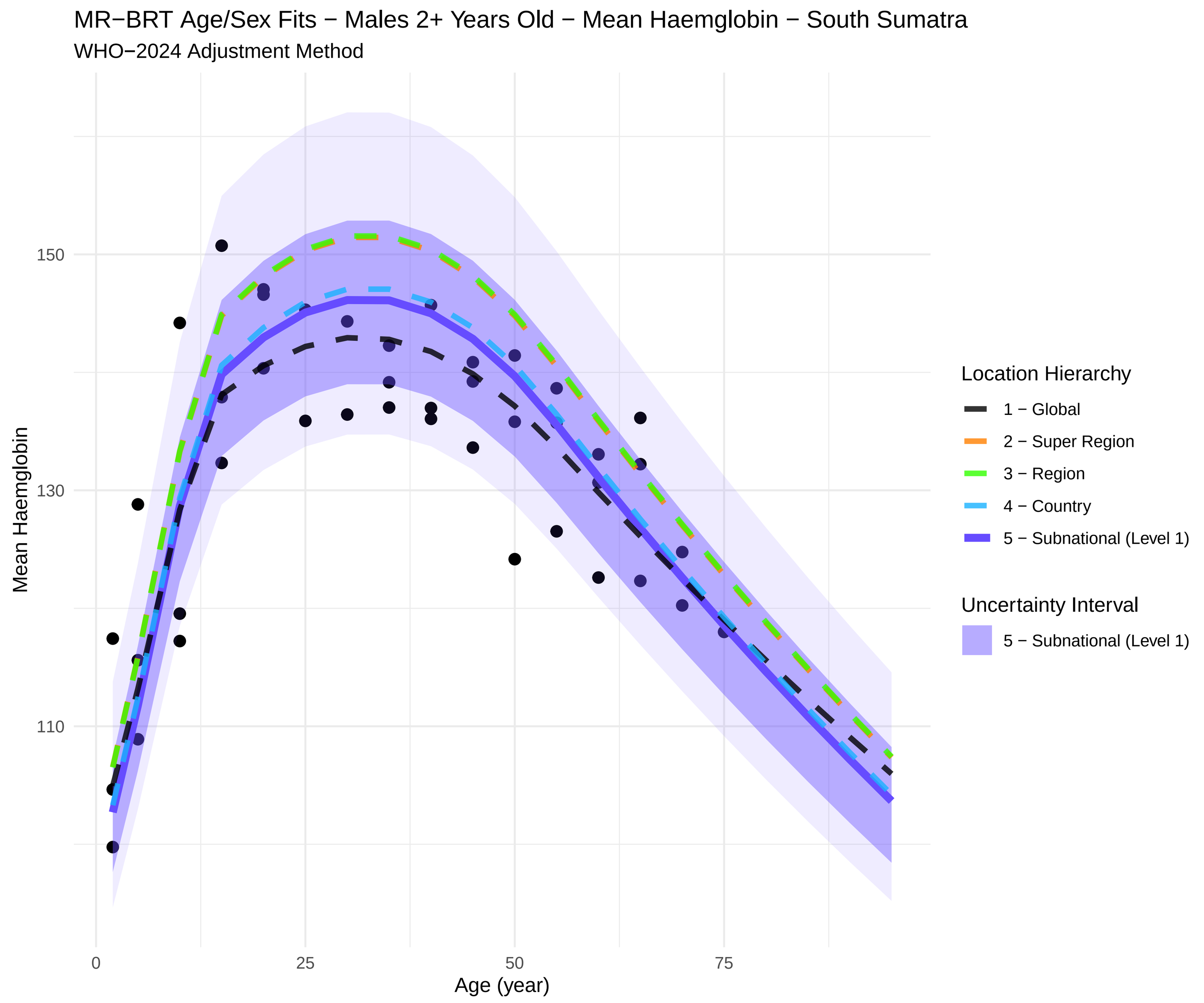
MR−BRT Age/Sex Fits − Males 2+ Years Old − Mean Haemglobin − South Sumatra
WHO−2024 Adjustment Method
Mean Haemglobin
Age (year)
Location Hierarchy
1 − Global
2 − Super Region
3 − Region
4 − Country
5 − Subnational (Level 1)
Uncertainty Interval
5 − Subnational (Level 1)

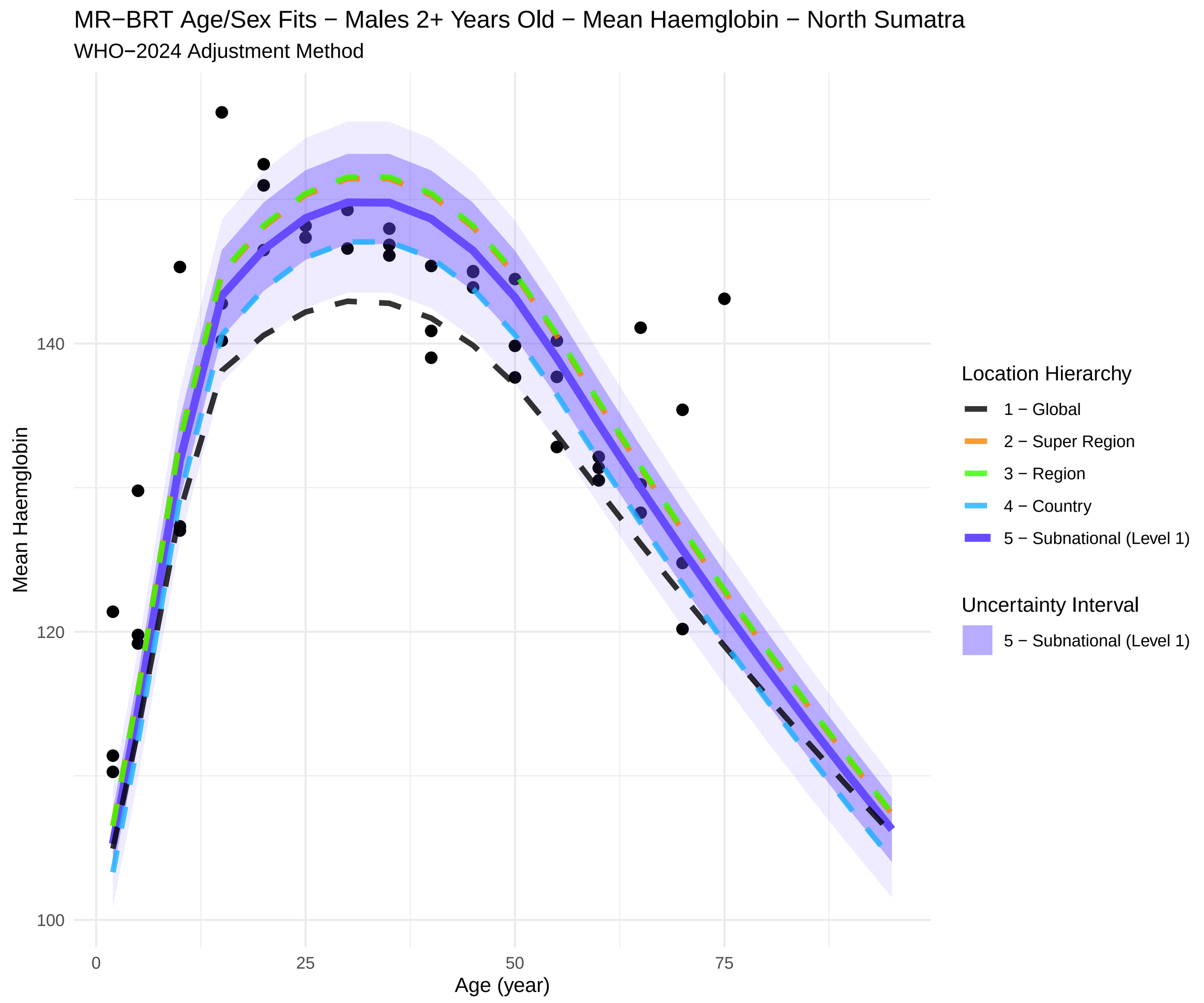
MR−BRT Age/Sex Fits − Males 2+ Years Old − Mean Haemglobin − North Sumatra
WHO−2024 Adjustment Method
Mean Haemglobin
Age (year)
Location Hierarchy
1 − Global
2 − Super Region
3 − Region
4 − Country
5 − Subnational (Level 1)
Uncertainty Interval
5 − Subnational (Level 1)

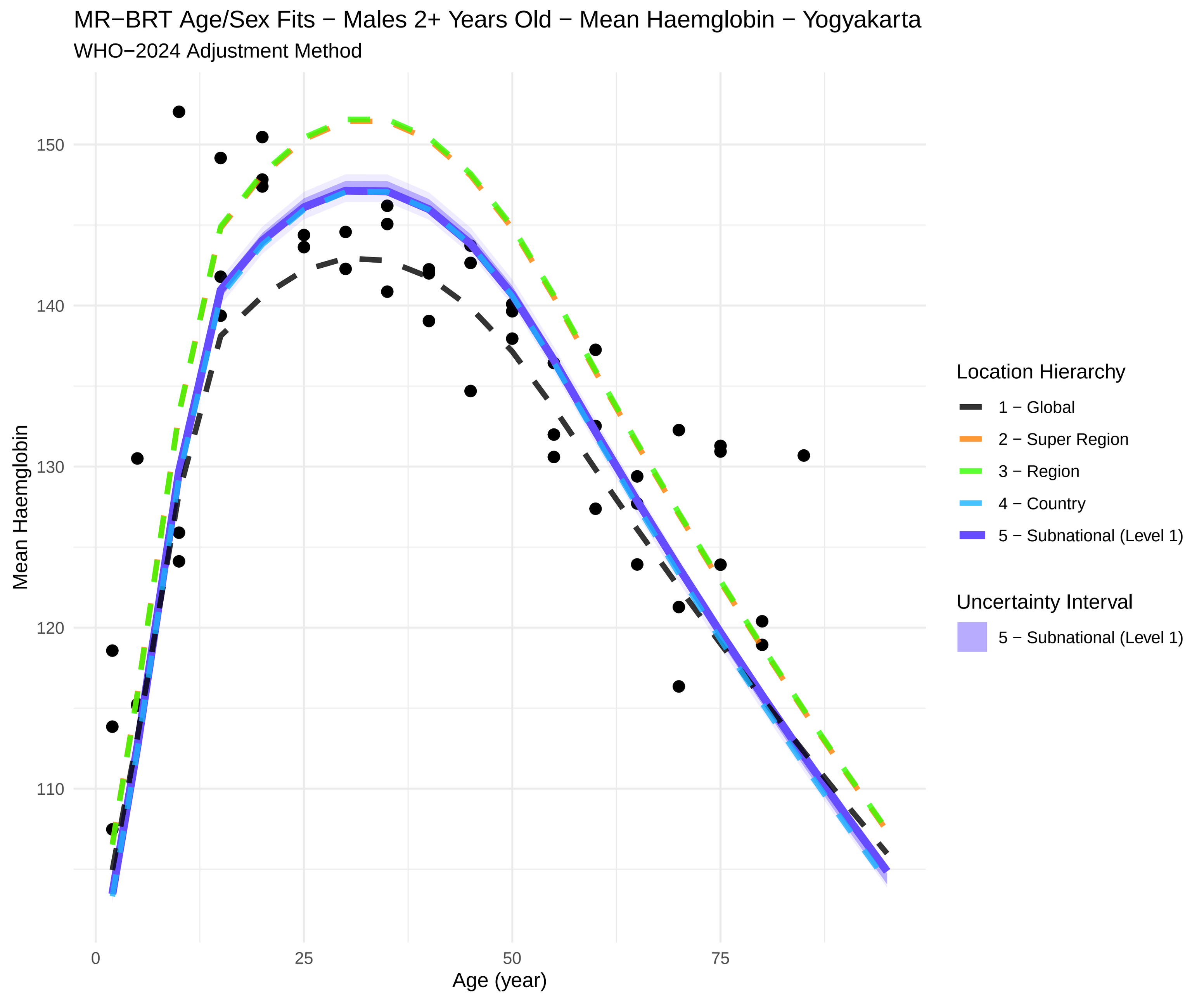
MR−BRT Age/Sex Fits − Males 2+ Years Old − Mean Haemglobin − Yogyakarta
WHO−2024 Adjustment Method
Mean Haemglobin
Age (year)
Location Hierarchy
1 − Global
2 − Super Region
3 − Region
4 − Country
5 − Subnational (Level 1)
Uncertainty Interval
5 − Subnational (Level 1)

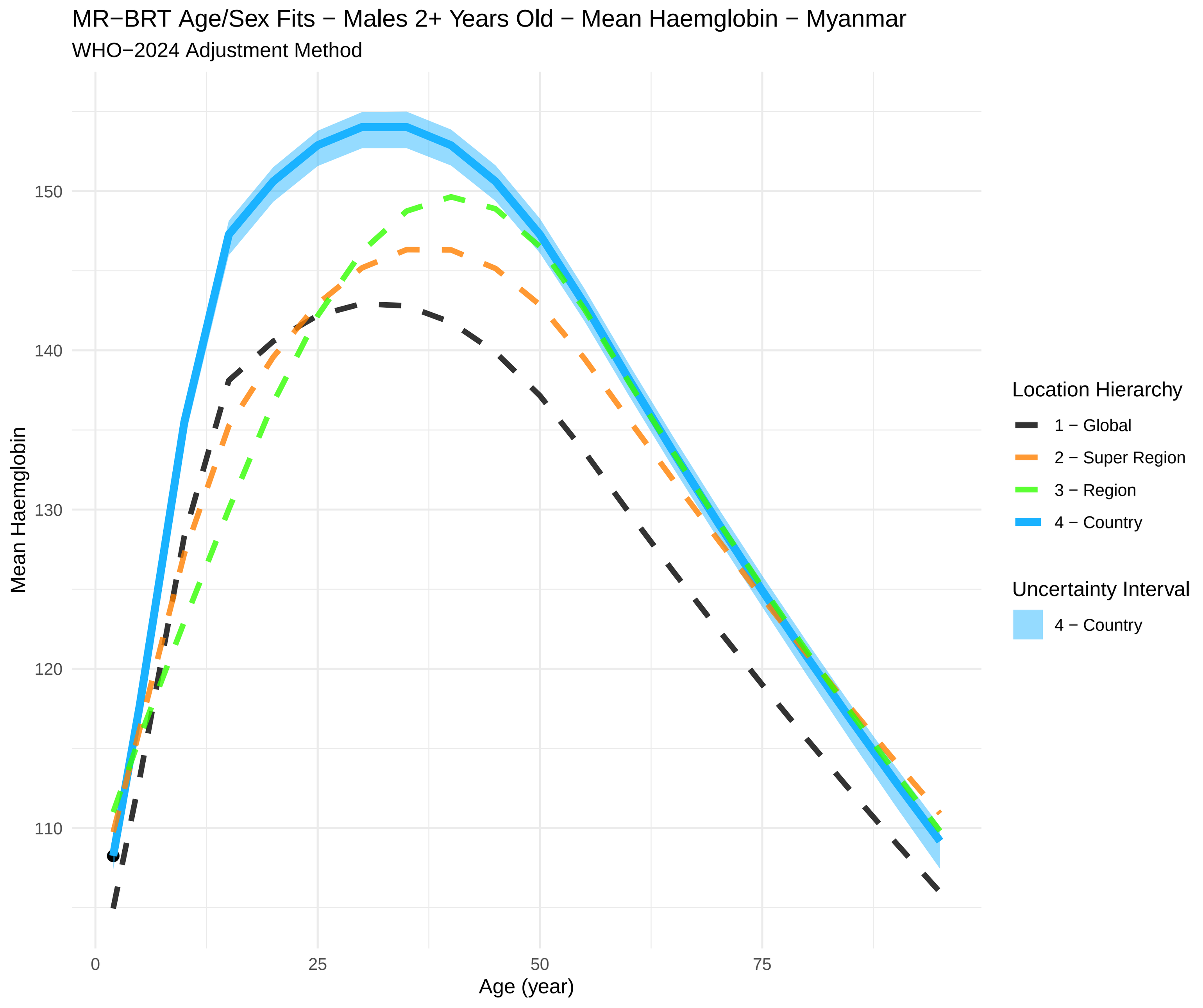
MR−BRT Age/Sex Fits − Males 2+ Years Old − Mean Haemglobin − Myanmar
WHO−2024 Adjustment Method
Mean Haemglobin
Age (year)
Location Hierarchy
1 − Global
2 − Super Region
3 − Region
4 − Country
Uncertainty Interval
4 − Country

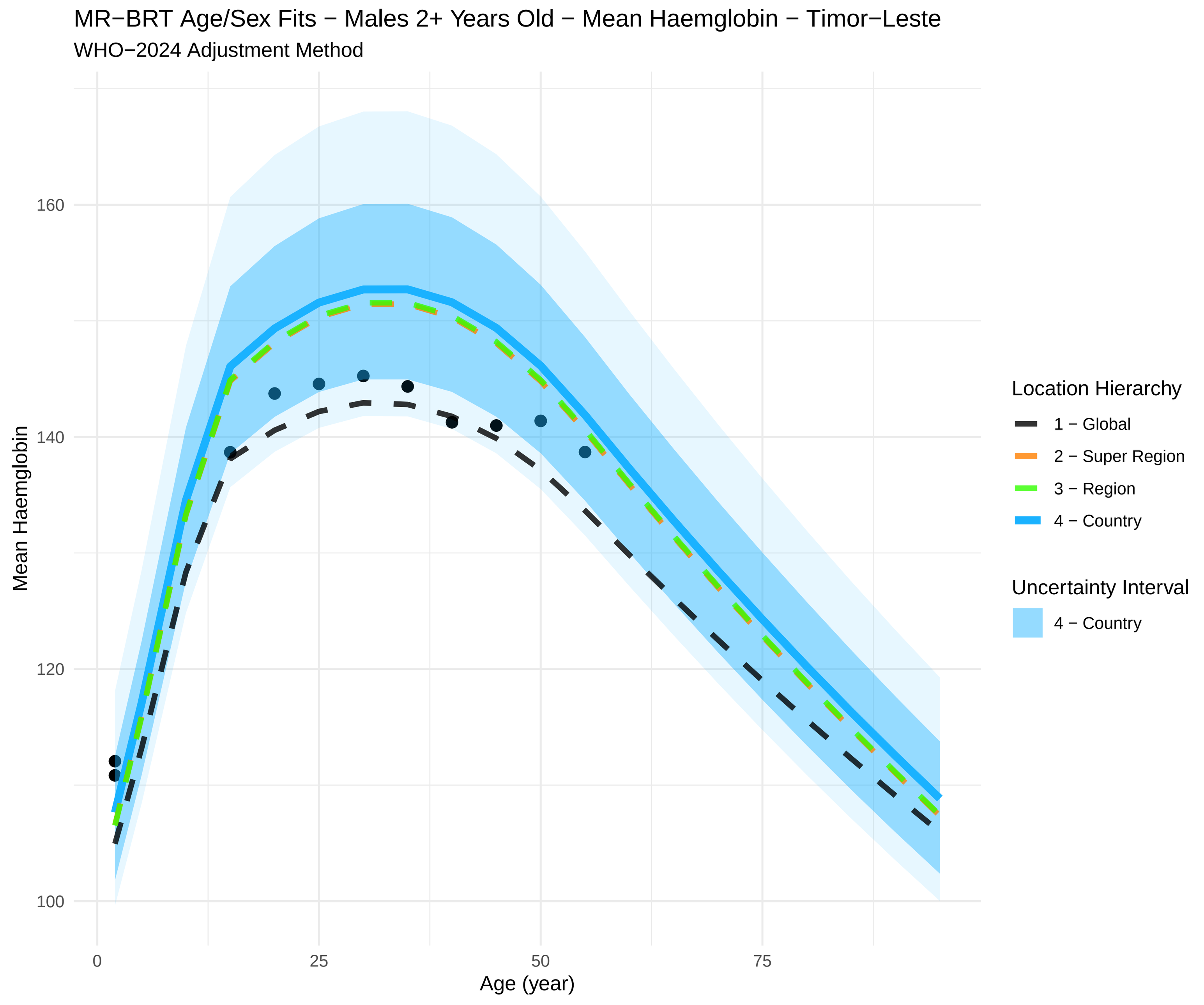
MR−BRT Age/Sex Fits − Males 2+ Years Old − Mean Haemglobin − Timor−Leste
WHO−2024 Adjustment Method
Mean Haemglobin
Age (year)
Location Hierarchy
1 − Global
2 − Super Region
3 − Region
4 − Country
Uncertainty Interval
4 − Country

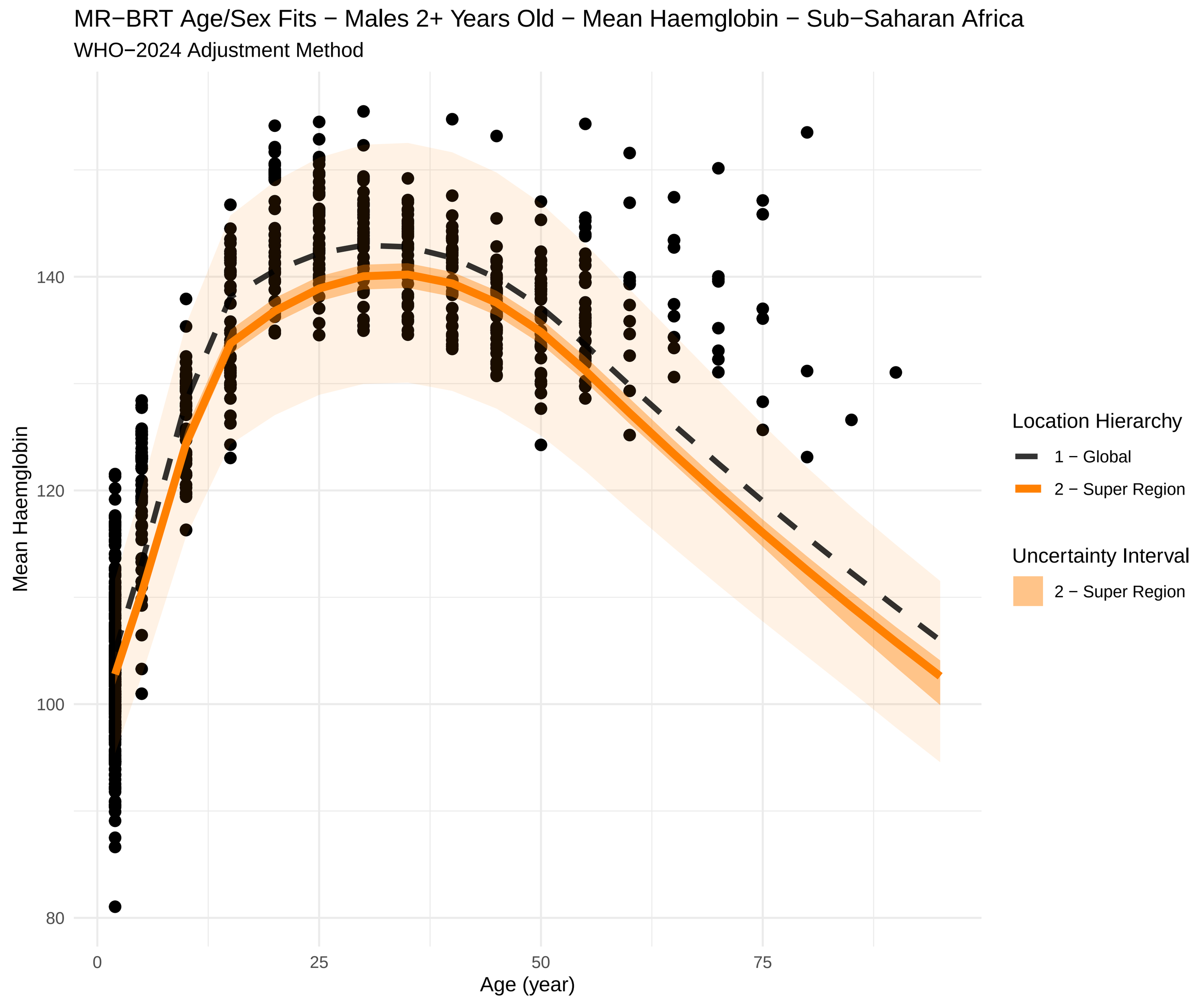
MR−BRT Age/Sex Fits − Males 2+ Years Old − Mean Haemglobin − Sub−Saharan Africa
WHO−2024 Adjustment Method
Mean Haemglobin
Age (year)
Location Hierarchy
1 − Global
2 − Super Region
Uncertainty Interval
2 − Super Region

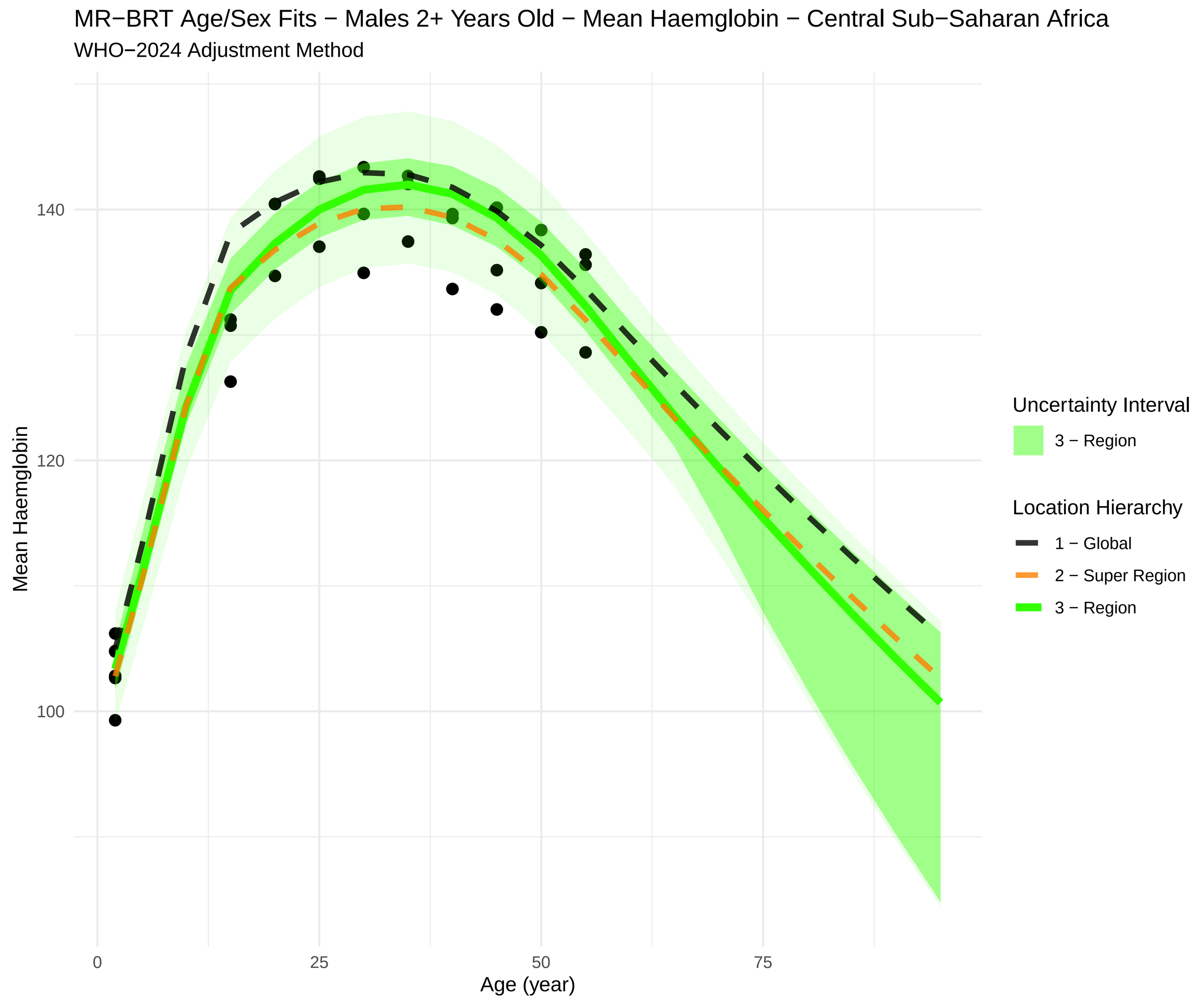
MR−BRT Age/Sex Fits − Males 2+ Years Old − Mean Haemglobin − Central Sub−Saharan Africa
WHO−2024 Adjustment Method
Mean Haemglobin
Age (year)
Uncertainty Interval
3 − Region
Location Hierarchy
1 − Global
2 − Super Region
3 − Region

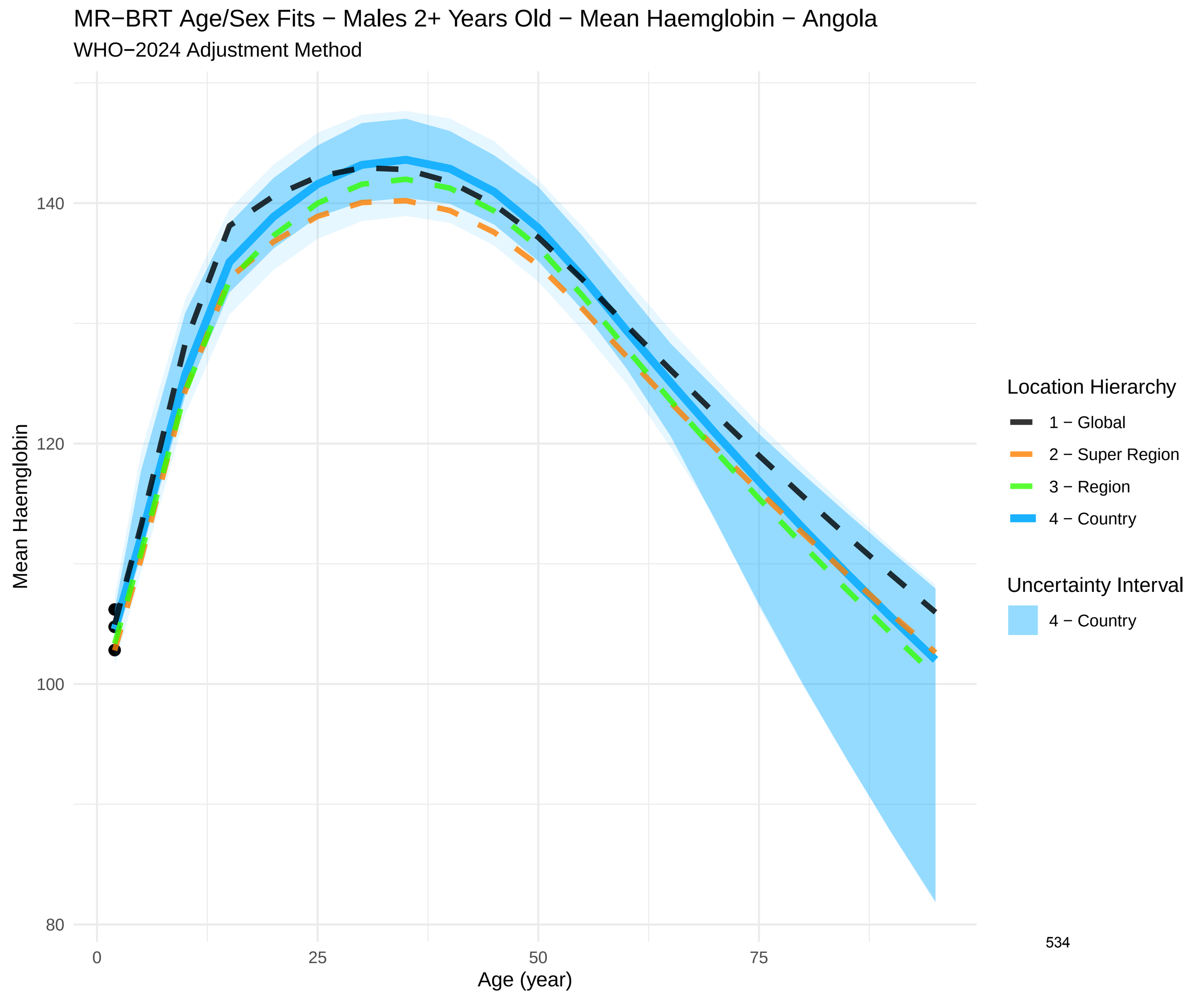
MR−BRT Age/Sex Fits − Males 2+ Years Old − Mean Haemglobin − Angola
WHO−2024 Adjustment Method
Mean Haemglobin
Age (year)
Location Hierarchy
1 − Global
2 − Super Region
3 − Region
4 − Country
Uncertainty Interval
4 − Country

MR−BRT Age/Sex Fits − Males 2+ Years Old − Mean Haemglobin − Democratic Republic of the Congo
WHO−2024 Adjustment Method

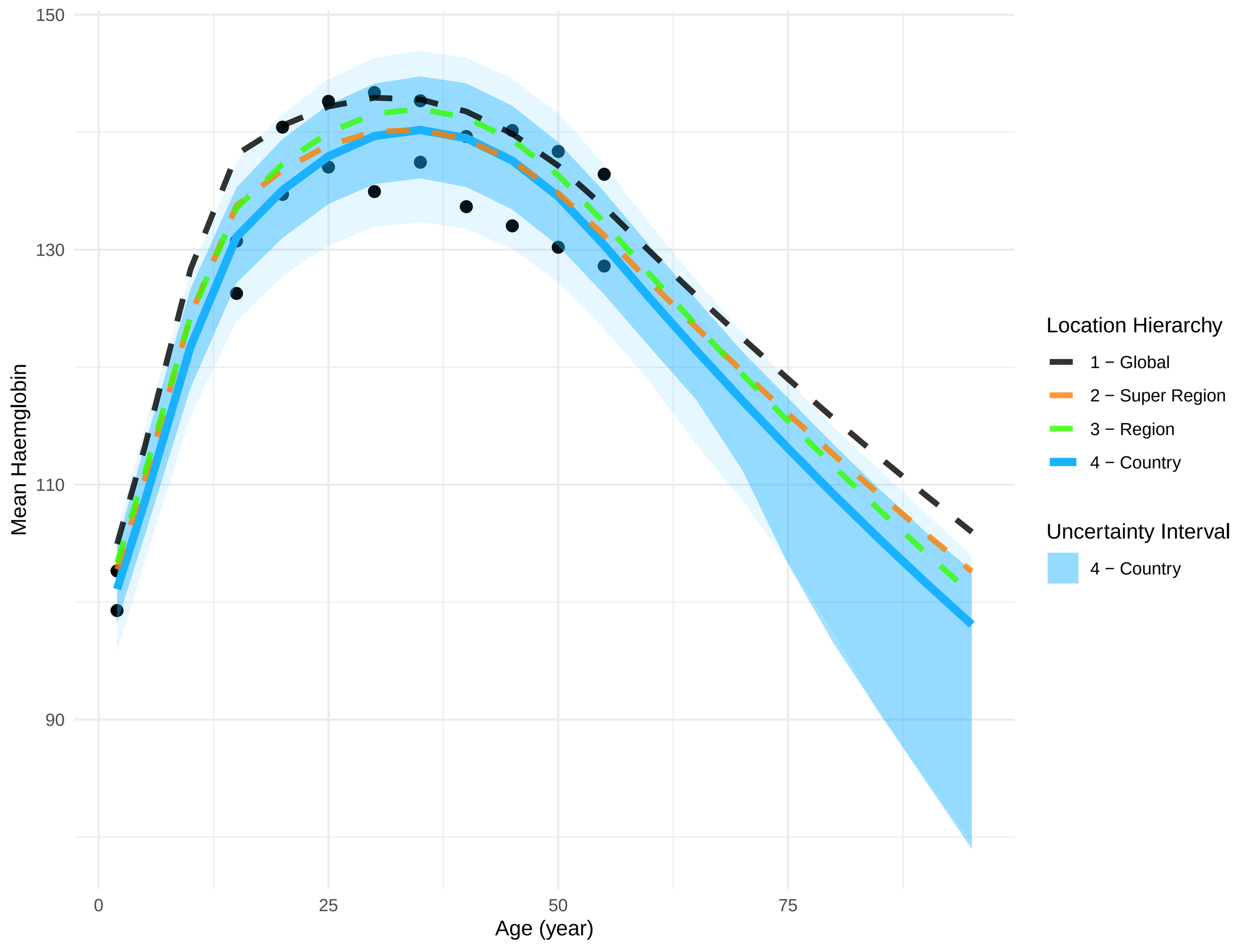

MR−BRT Age/Sex Fits − Males 2+ Years Old − Mean Haemglobin − Gabon
WHO−2024 Adjustment Method

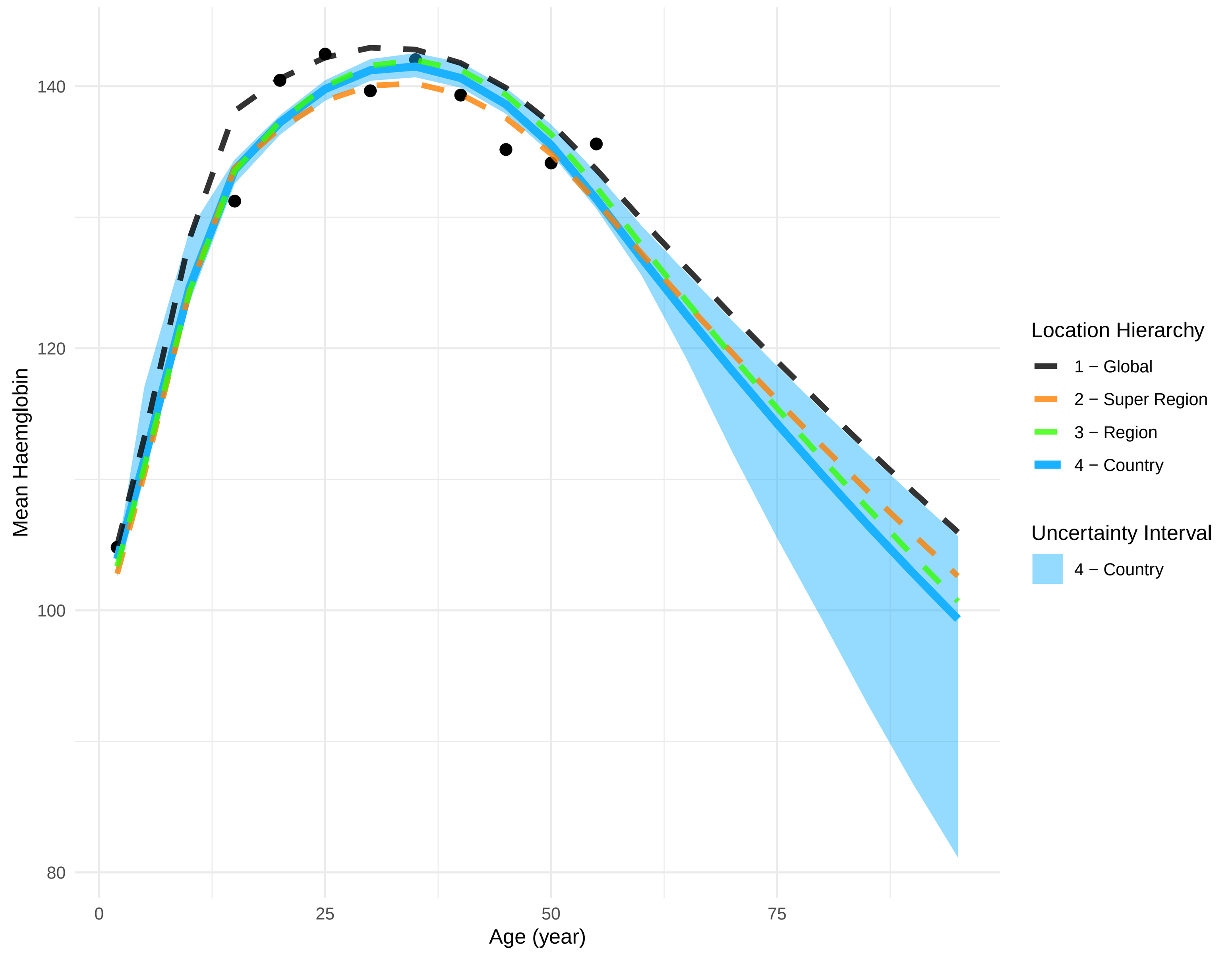

MR−BRT Age/Sex Fits − Males 2+ Years Old − Mean Haemglobin − Eastern Sub−Saharan Africa
WHO−2024 Adjustment Method

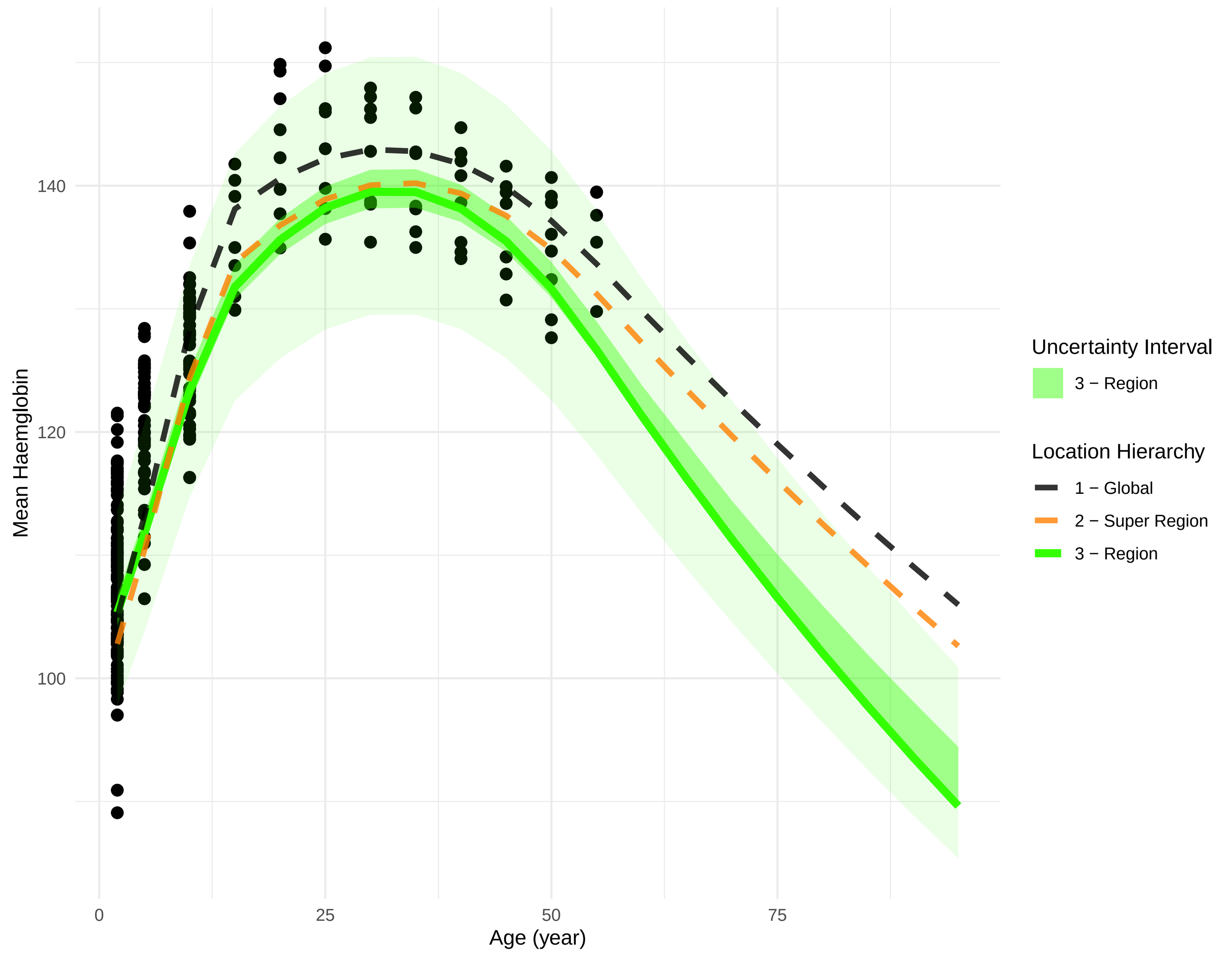

MR−BRT Age/Sex Fits − Males 2+ Years Old − Mean Haemglobin − Burundi
WHO−2024 Adjustment Method

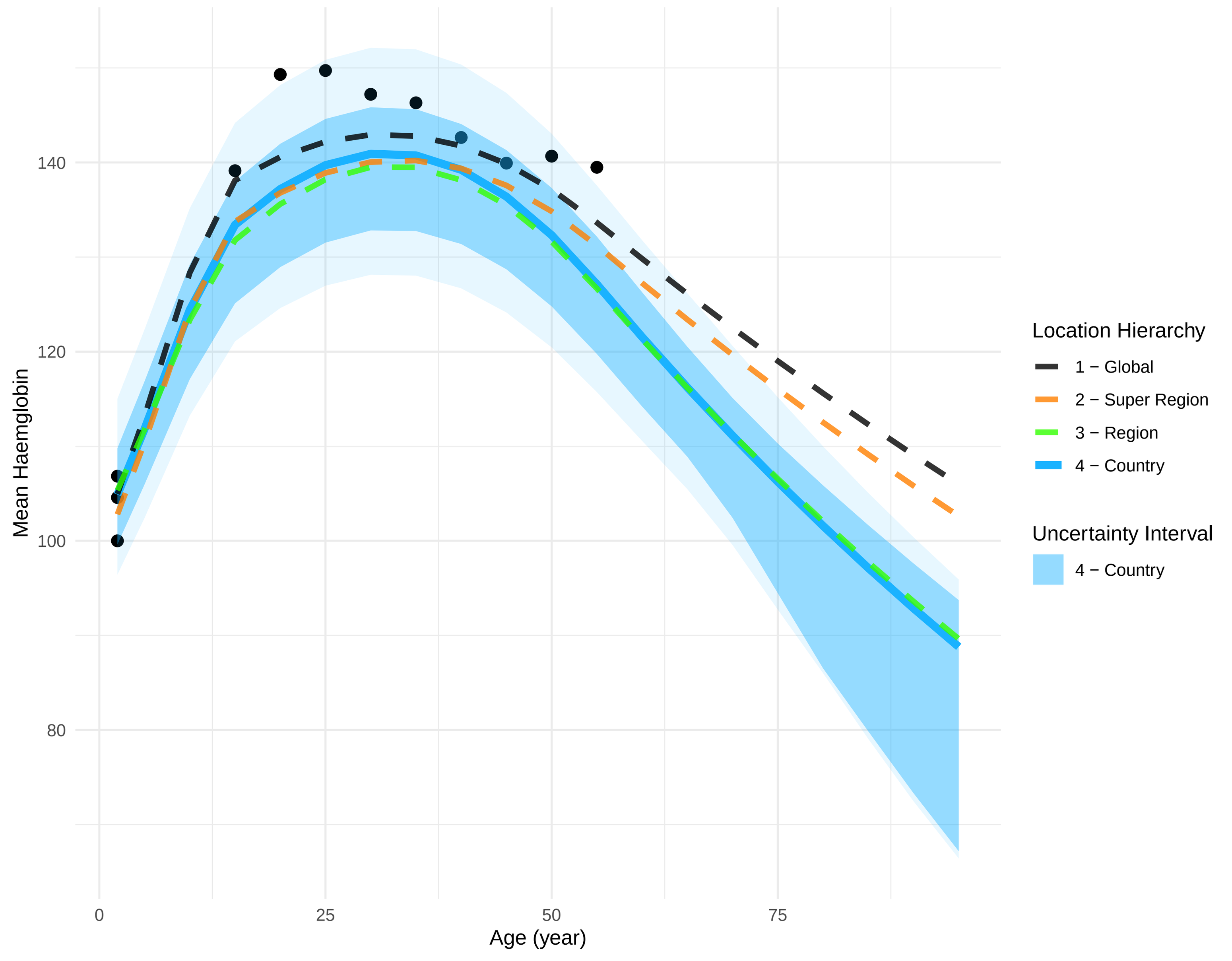

MR−BRT Age/Sex Fits − Males 2+ Years Old − Mean Haemglobin − Ethiopia
WHO−2024 Adjustment Method

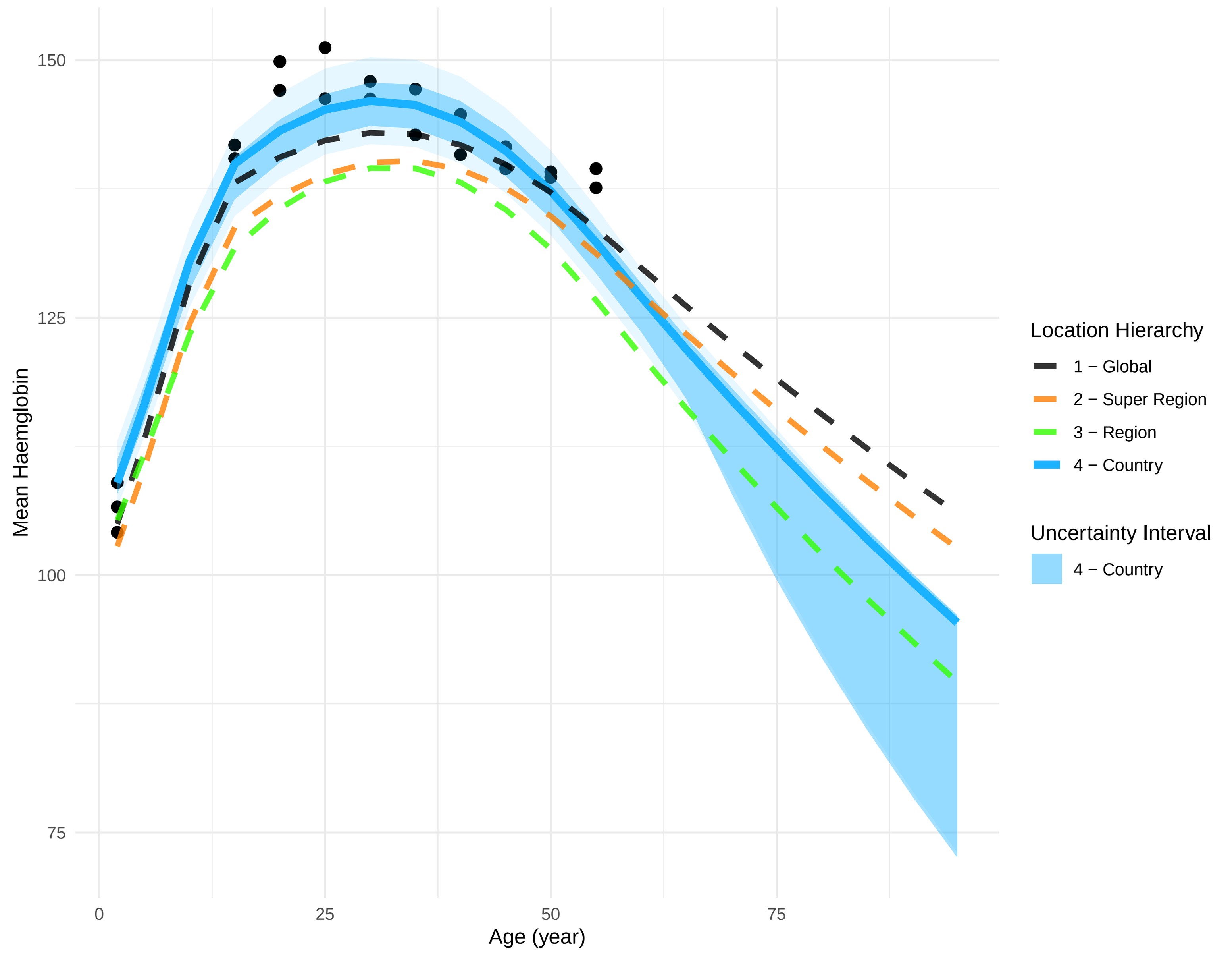

MR−BRT Age/Sex Fits − Males 2+ Years Old − Mean Haemglobin − Kenya
WHO−2024 Adjustment Method

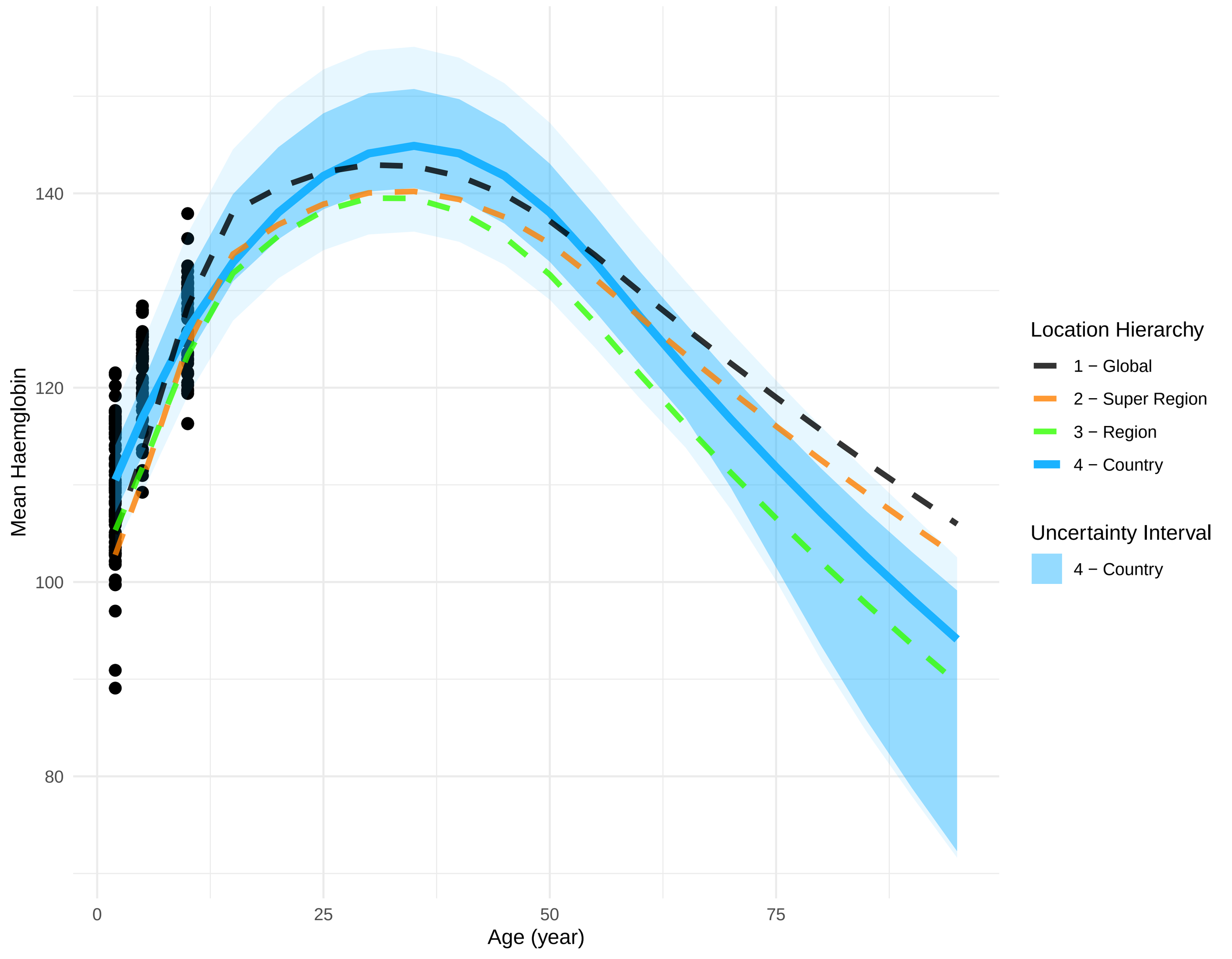

MR−BRT Age/Sex Fits − Males 2+ Years Old − Mean Haemglobin − Baringo
WHO−2024 Adjustment Method

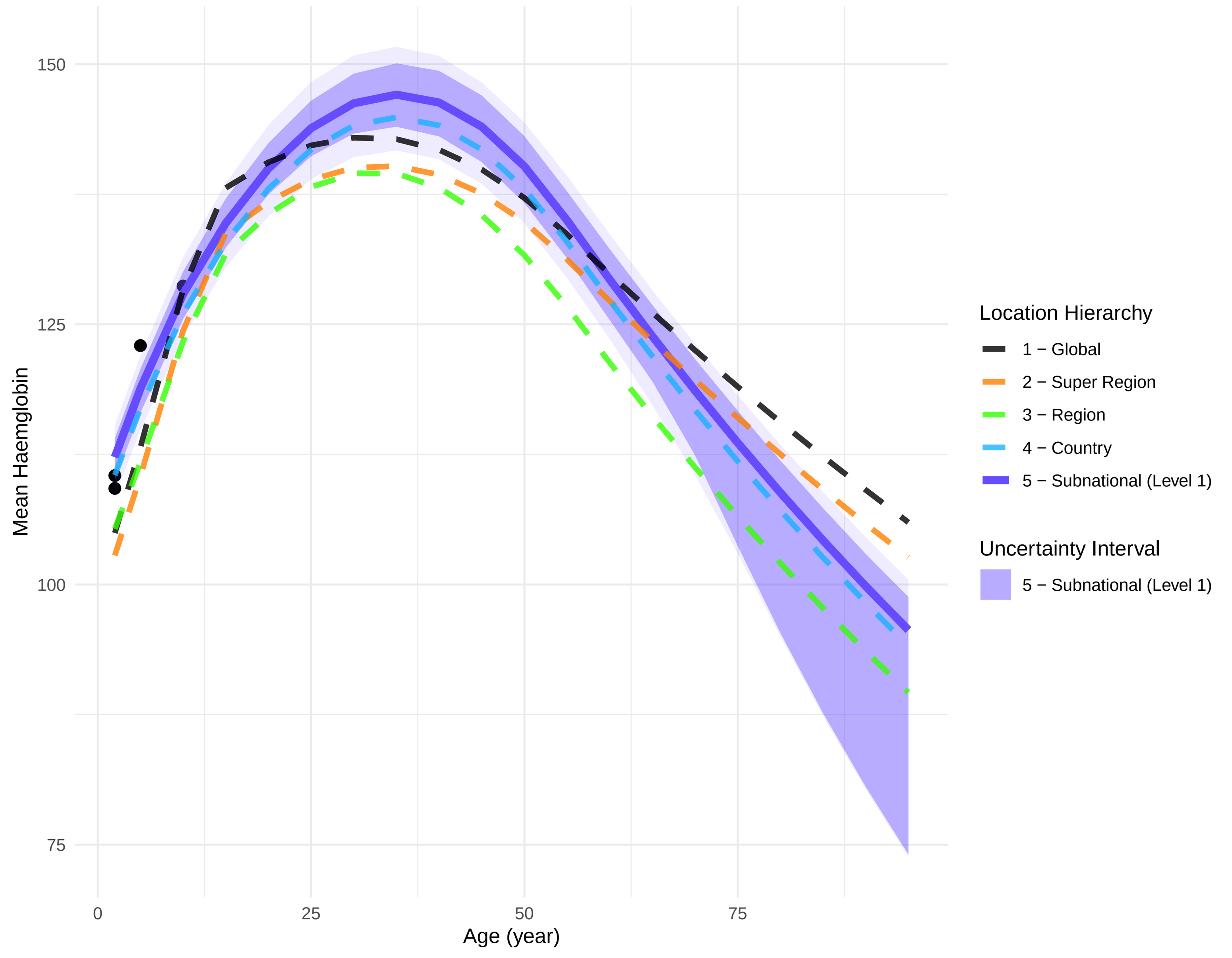

MR−BRT Age/Sex Fits − Males 2+ Years Old − Mean Haemglobin − Bomet
WHO−2024 Adjustment Method

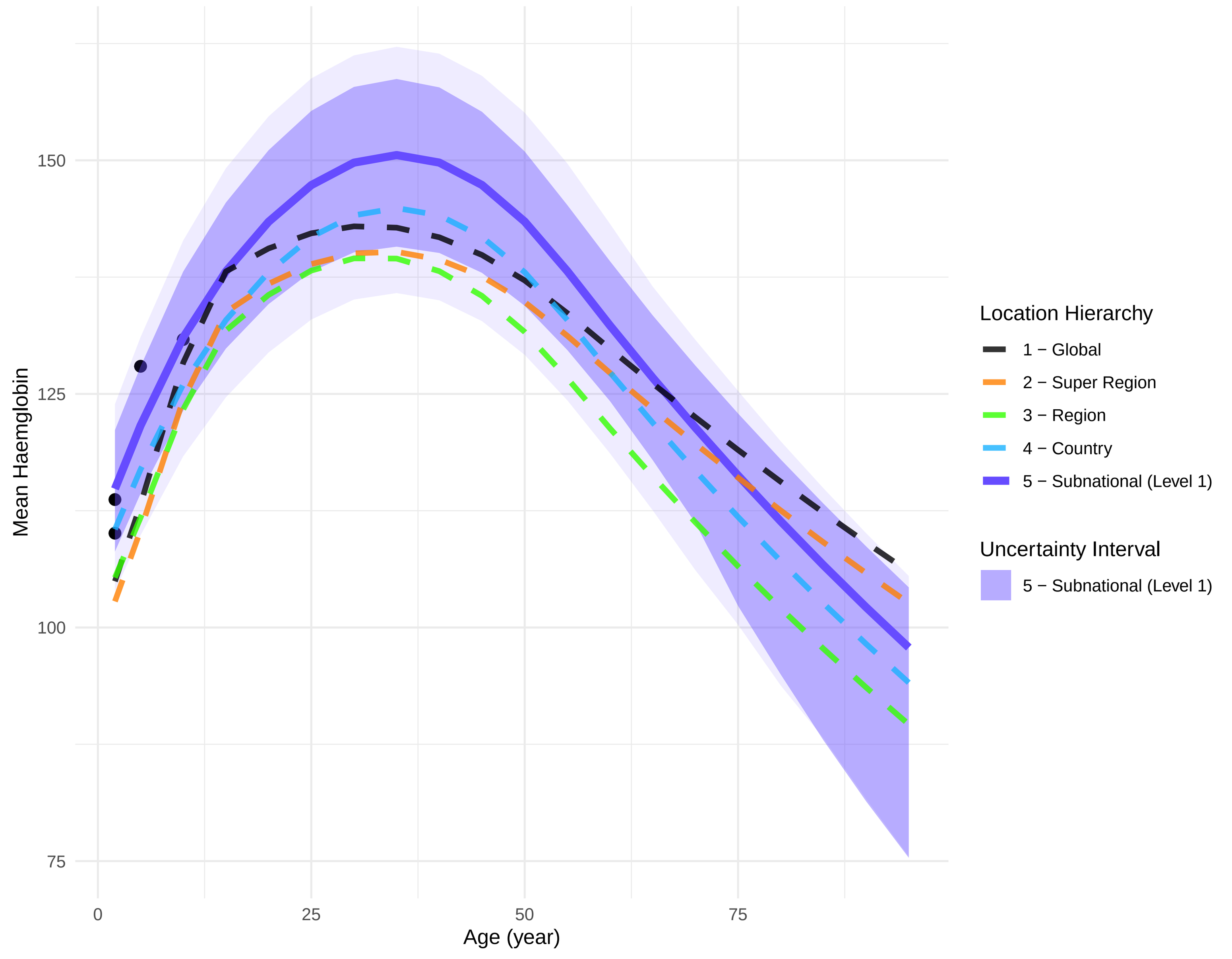

MR−BRT Age/Sex Fits − Males 2+ Years Old − Mean Haemglobin − Bungoma
WHO−2024 Adjustment Method

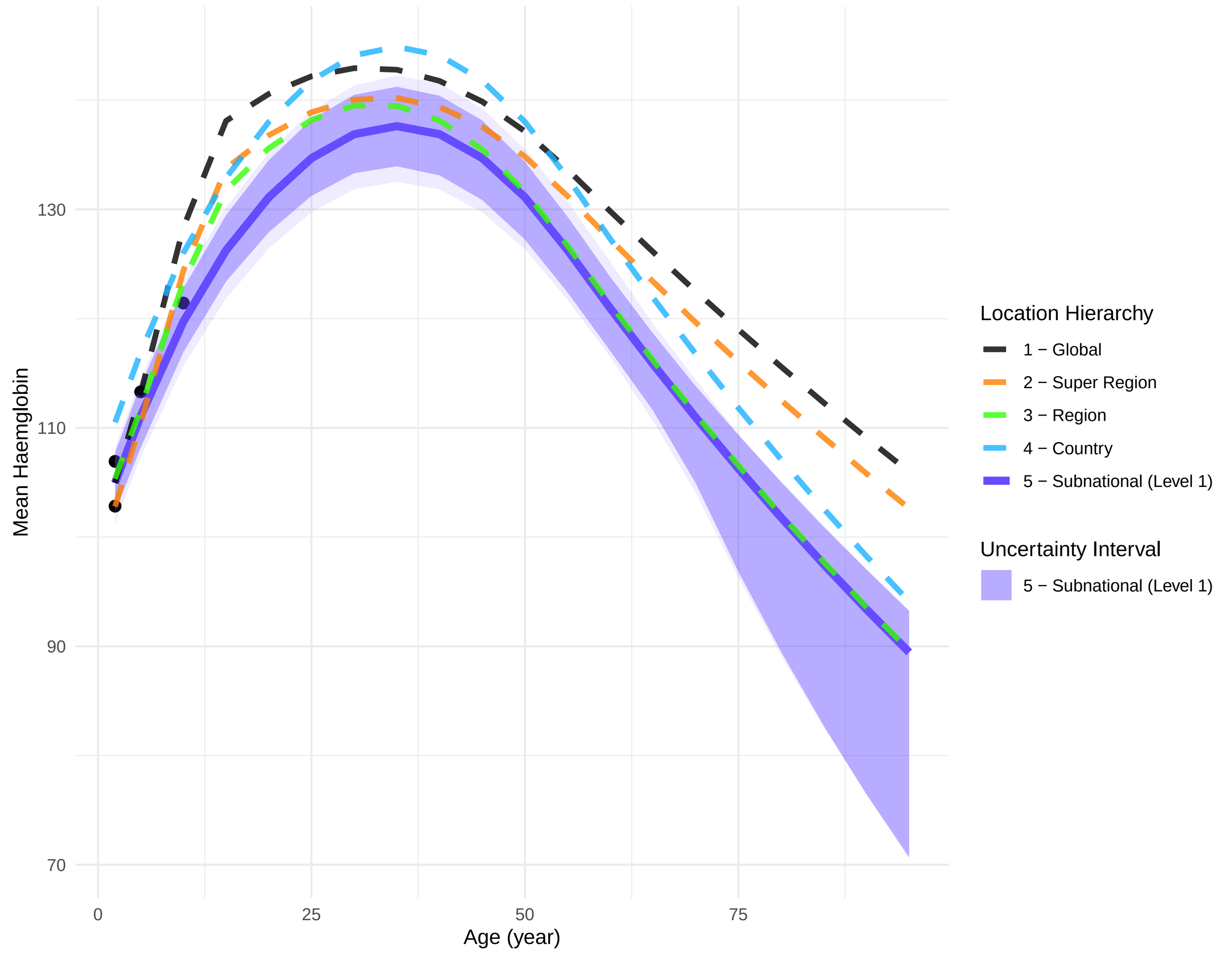

MR−BRT Age/Sex Fits − Males 2+ Years Old − Mean Haemglobin − Busia
WHO−2024 Adjustment Method

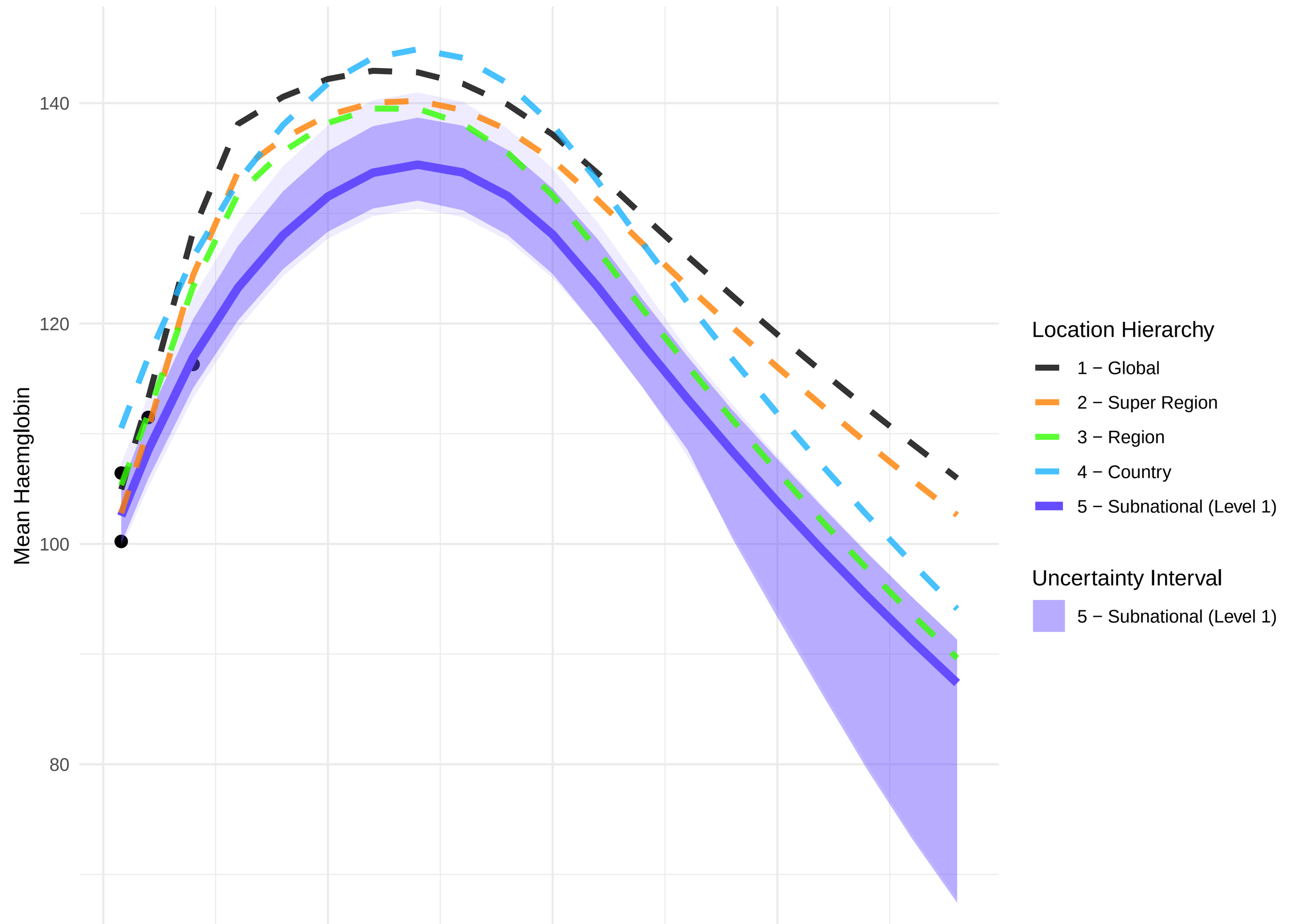

MR−BRT Age/Sex Fits − Males 2+ Years Old − Mean Haemglobin − Elgeyo−Marakwet
WHO−2024 Adjustment Method

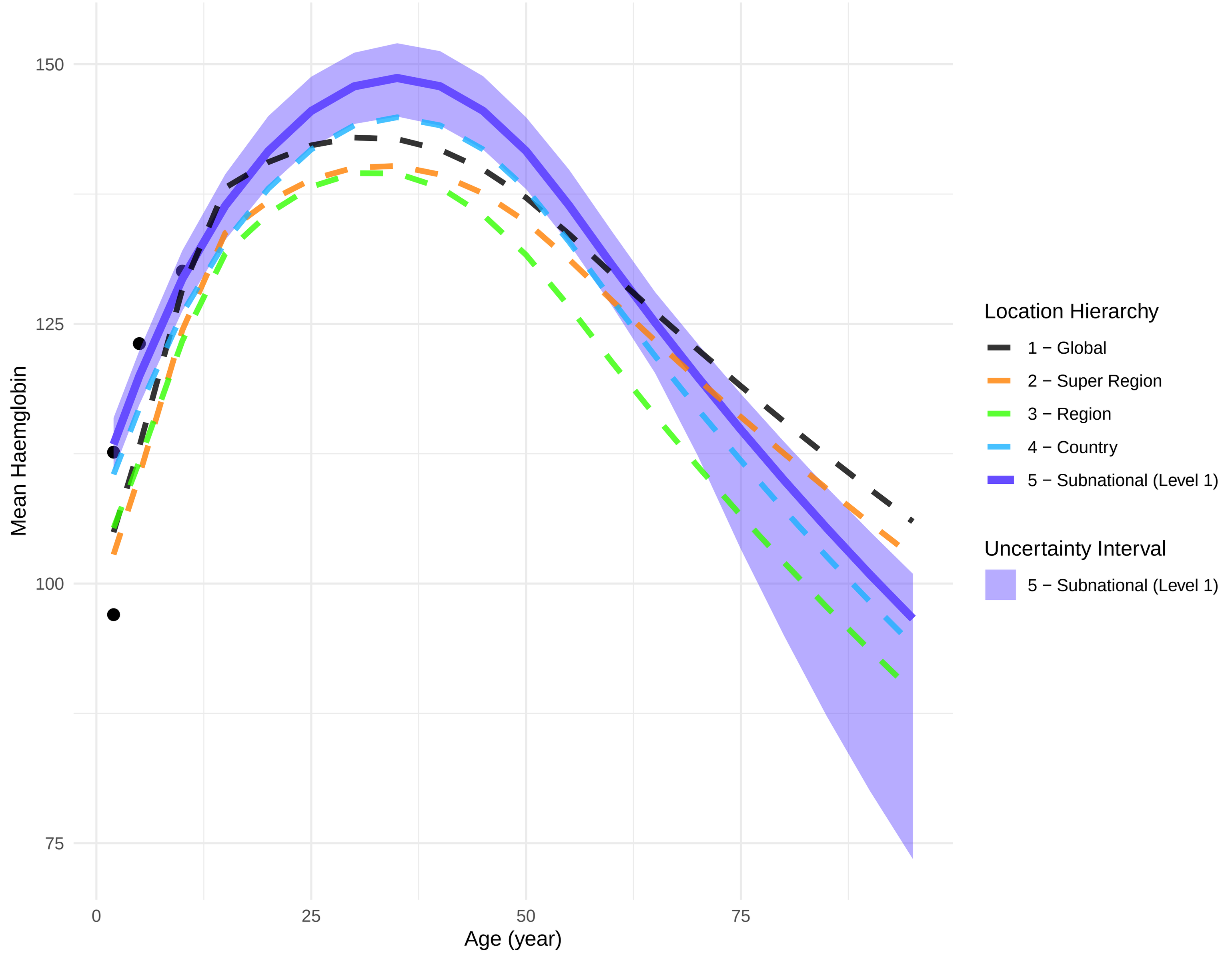

MR−BRT Age/Sex Fits − Males 2+ Years Old − Mean Haemglobin − Embu
WHO−2024 Adjustment Method

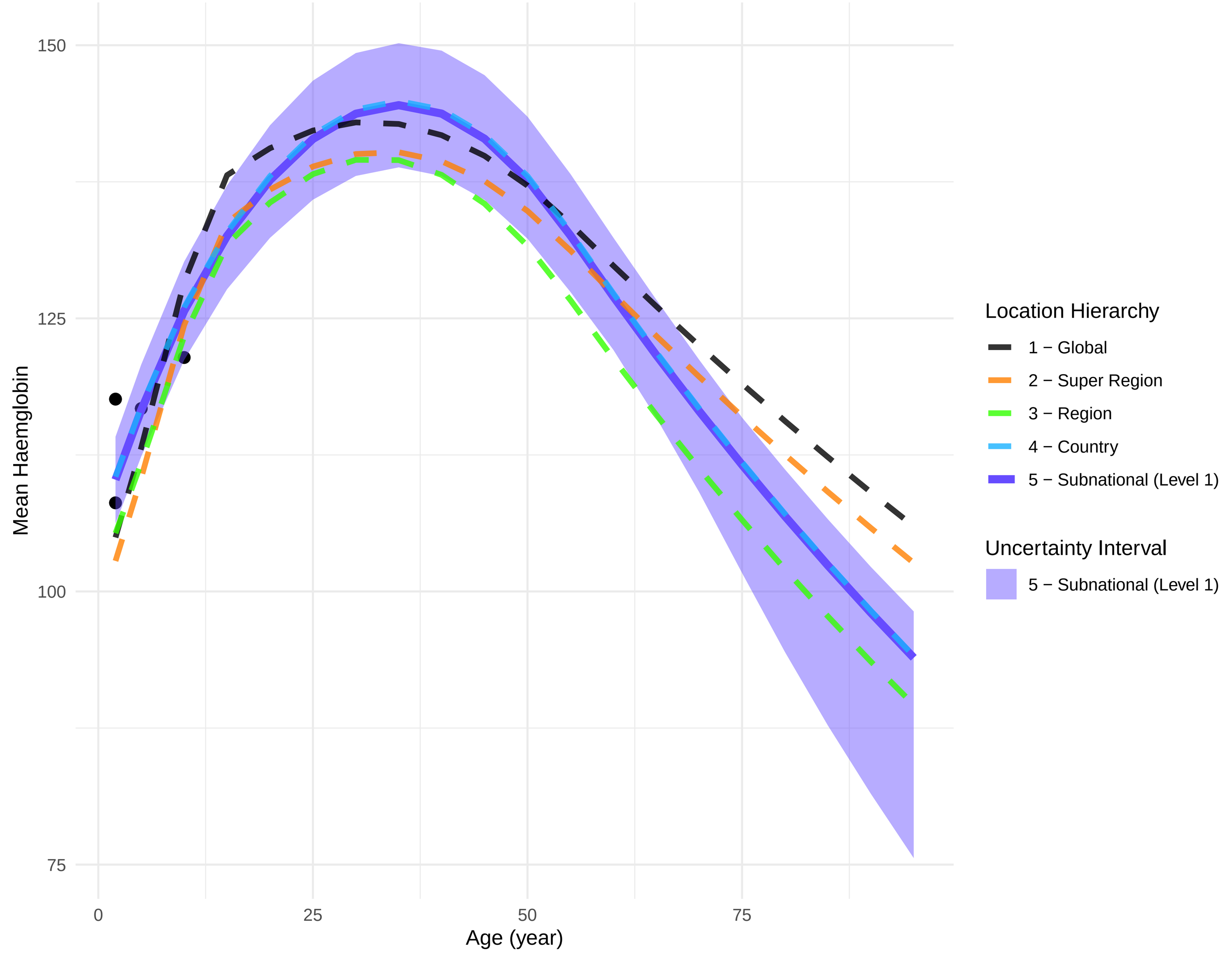

MR−BRT Age/Sex Fits − Males 2+ Years Old − Mean Haemglobin − Garissa
WHO−2024 Adjustment Method

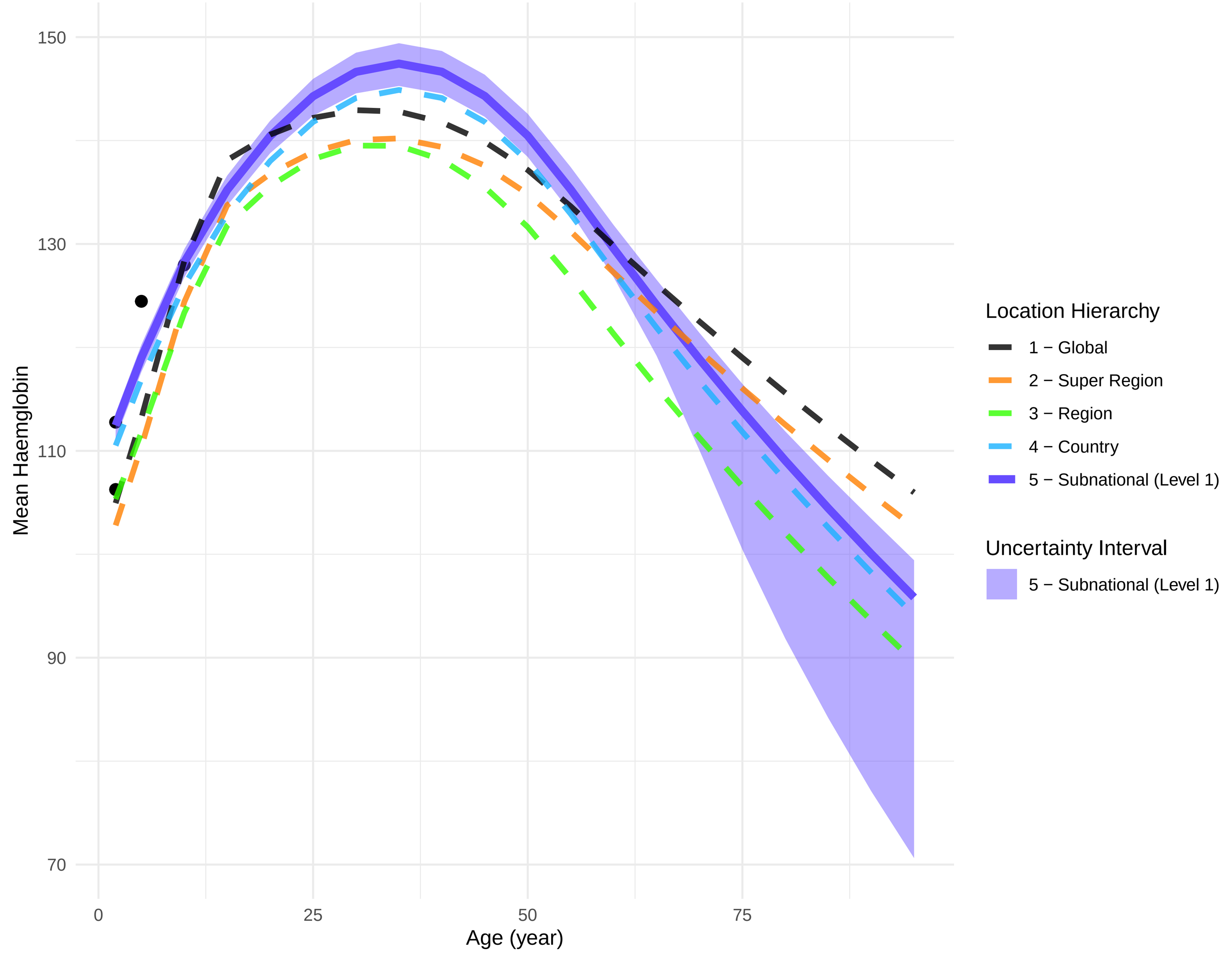

MR−BRT Age/Sex Fits − Males 2+ Years Old − Mean Haemglobin − Homa Bay
WHO−2024 Adjustment Method

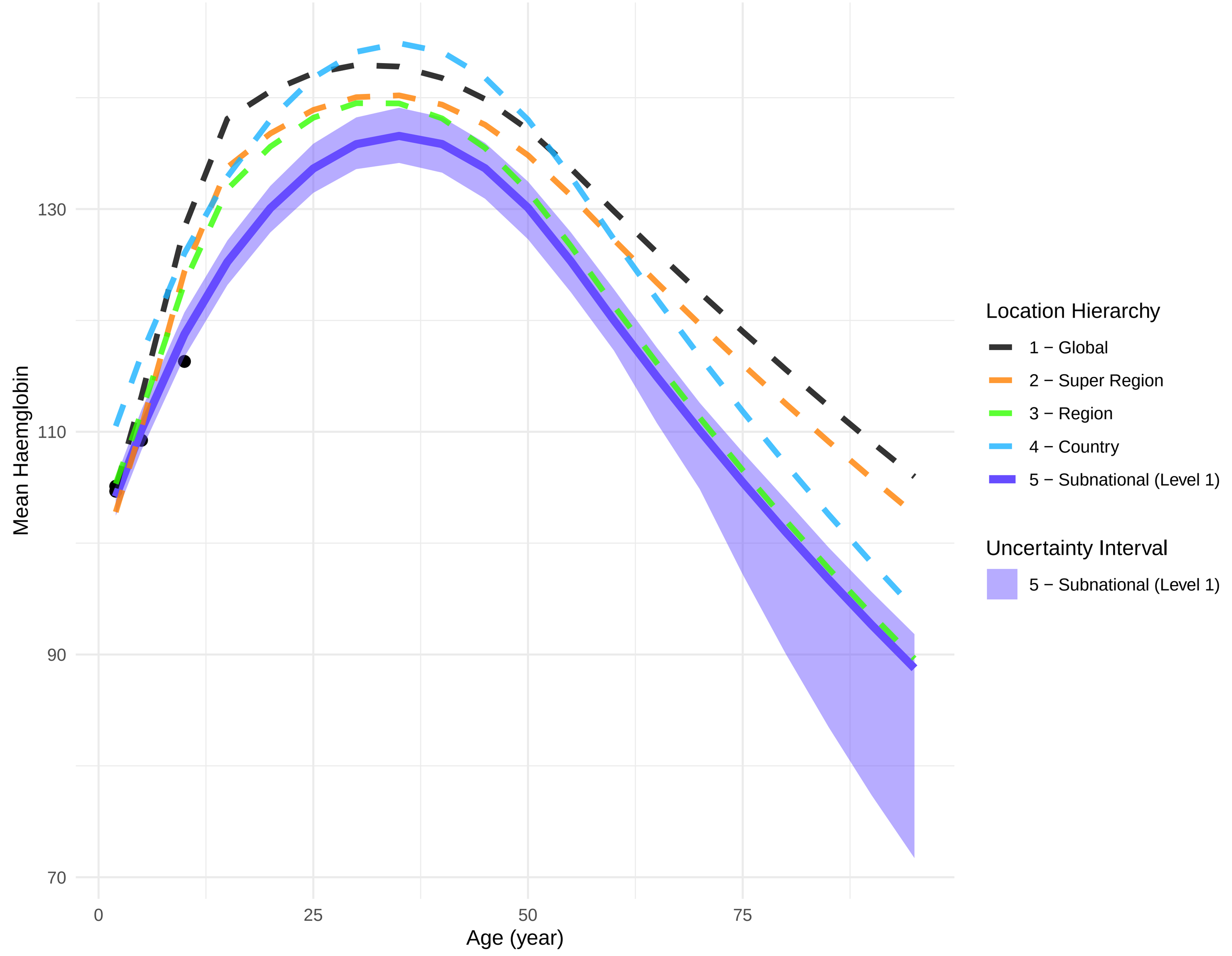

MR−BRT Age/Sex Fits − Males 2+ Years Old − Mean Haemglobin − Isiolo
WHO−2024 Adjustment Method

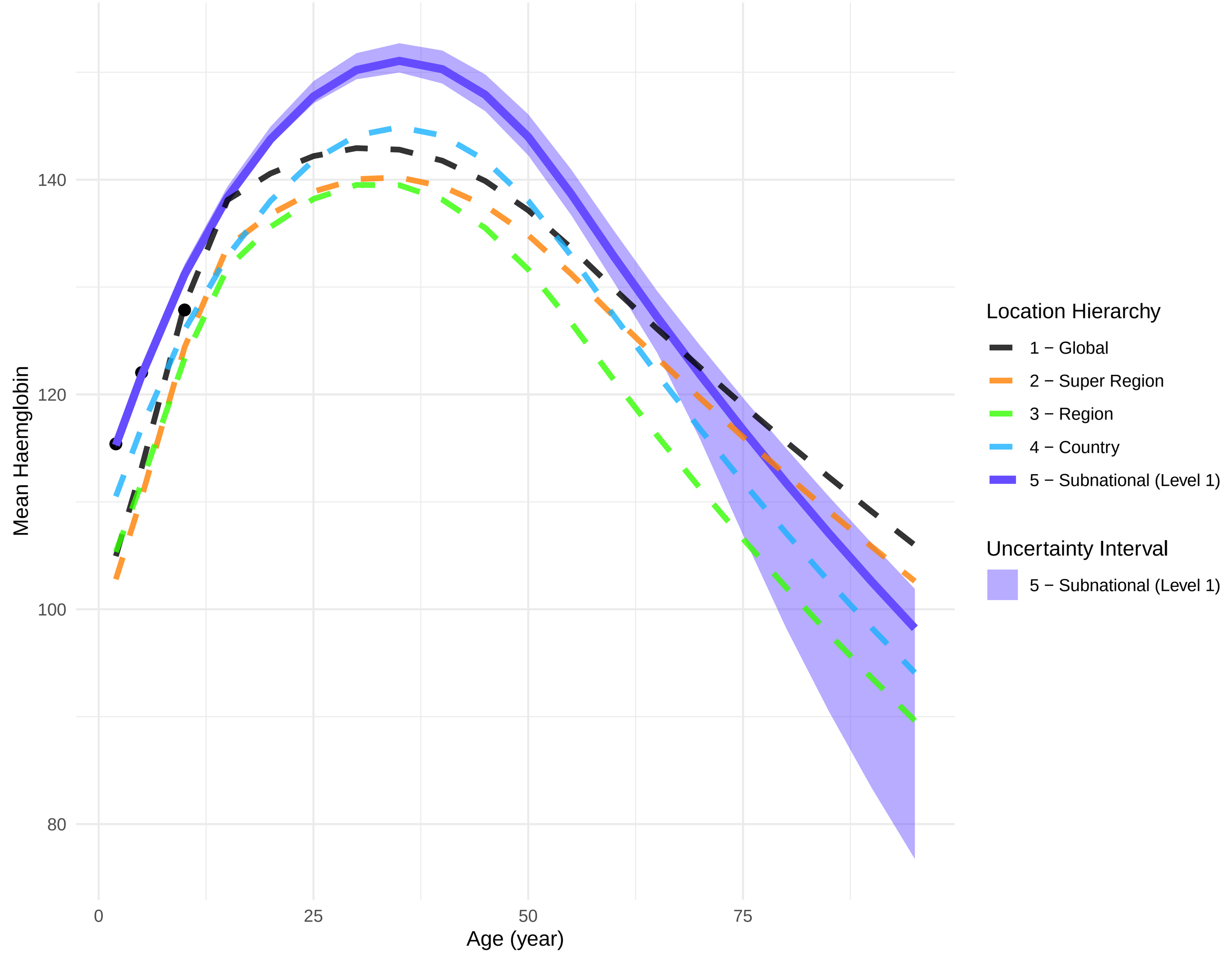

MR−BRT Age/Sex Fits − Males 2+ Years Old − Mean Haemglobin − Kajiado
WHO−2024 Adjustment Method

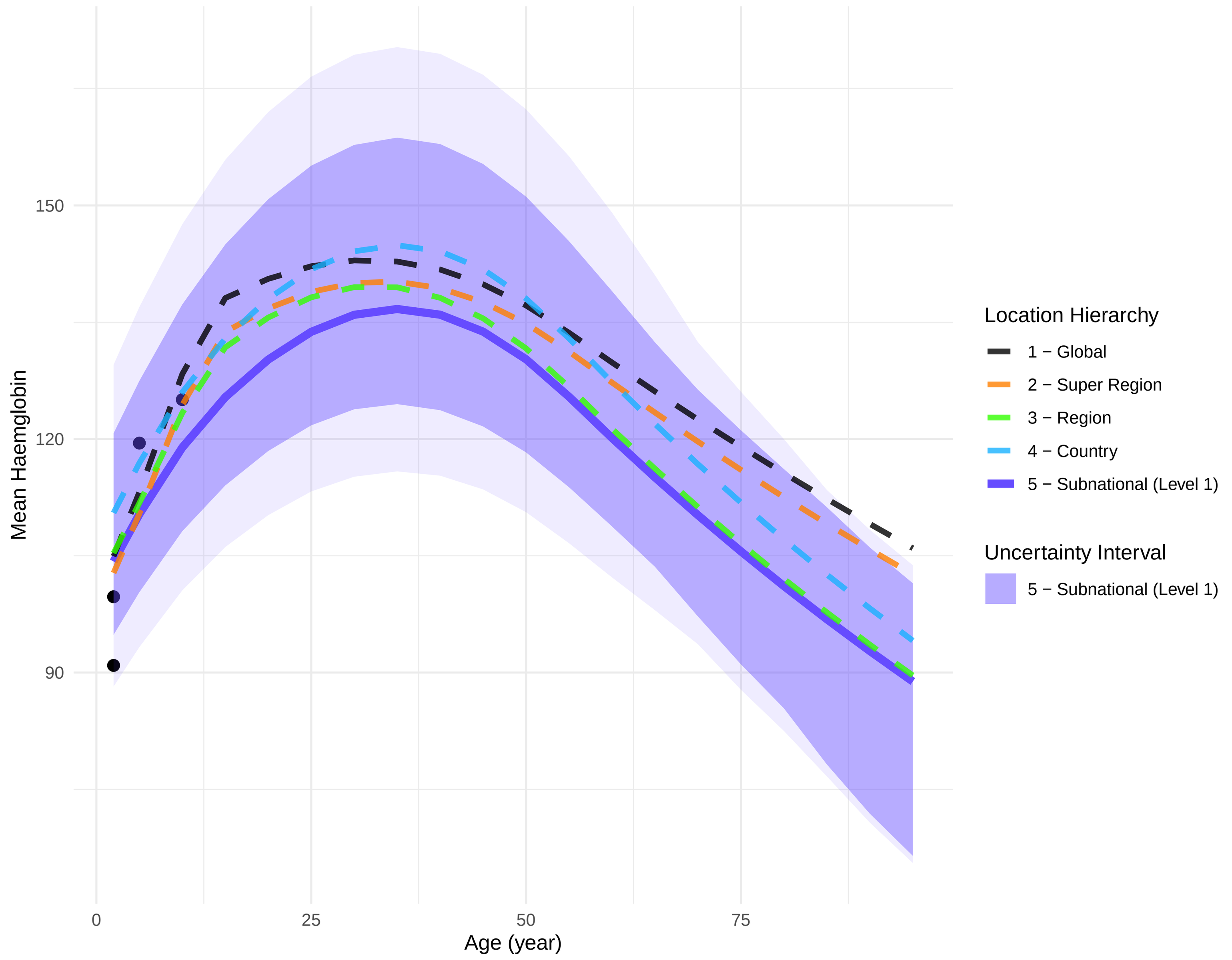

MR−BRT Age/Sex Fits − Males 2+ Years Old − Mean Haemglobin − Kakamega
WHO−2024 Adjustment Method

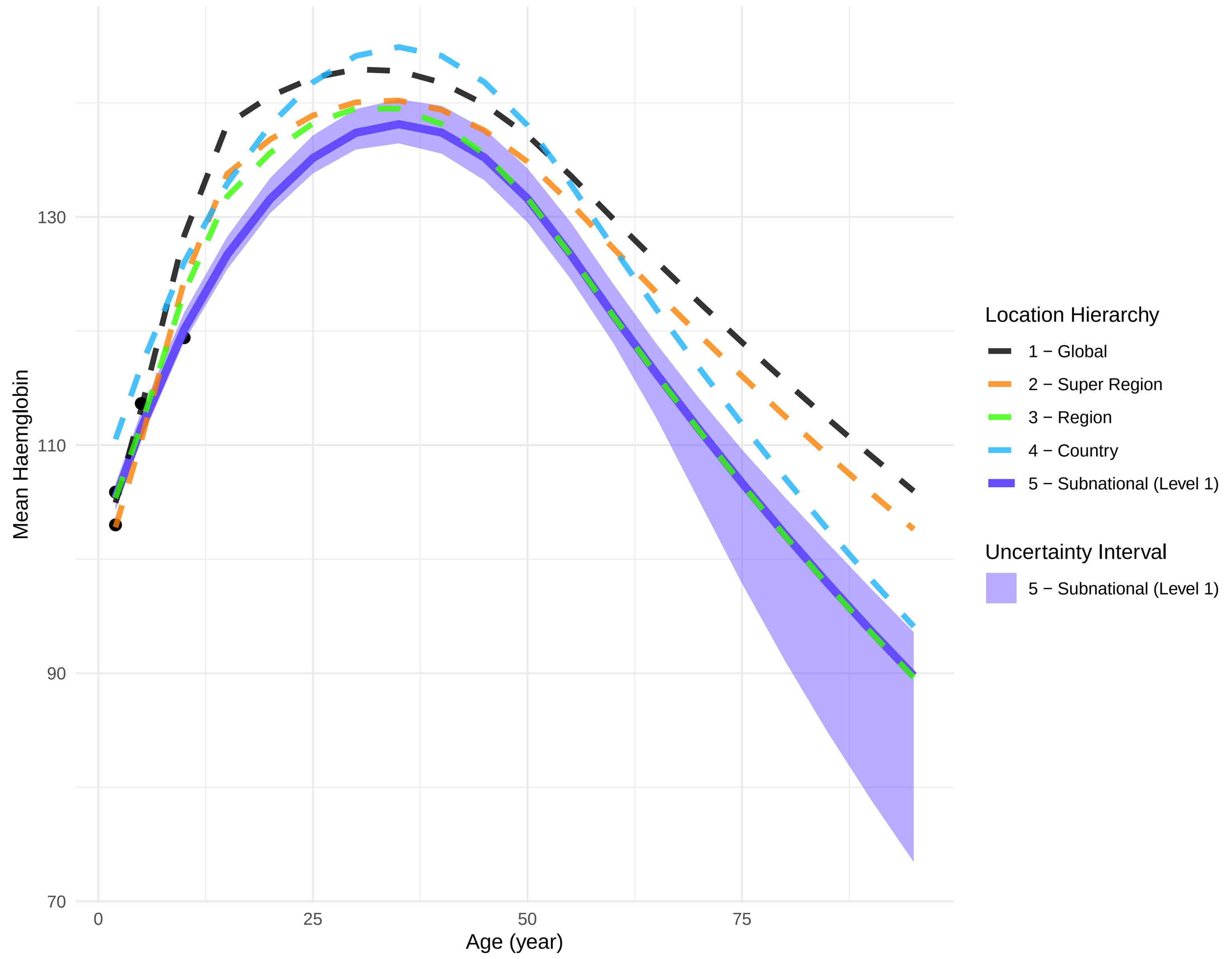

MR−BRT Age/Sex Fits − Males 2+ Years Old − Mean Haemglobin − Kericho
WHO−2024 Adjustment Method

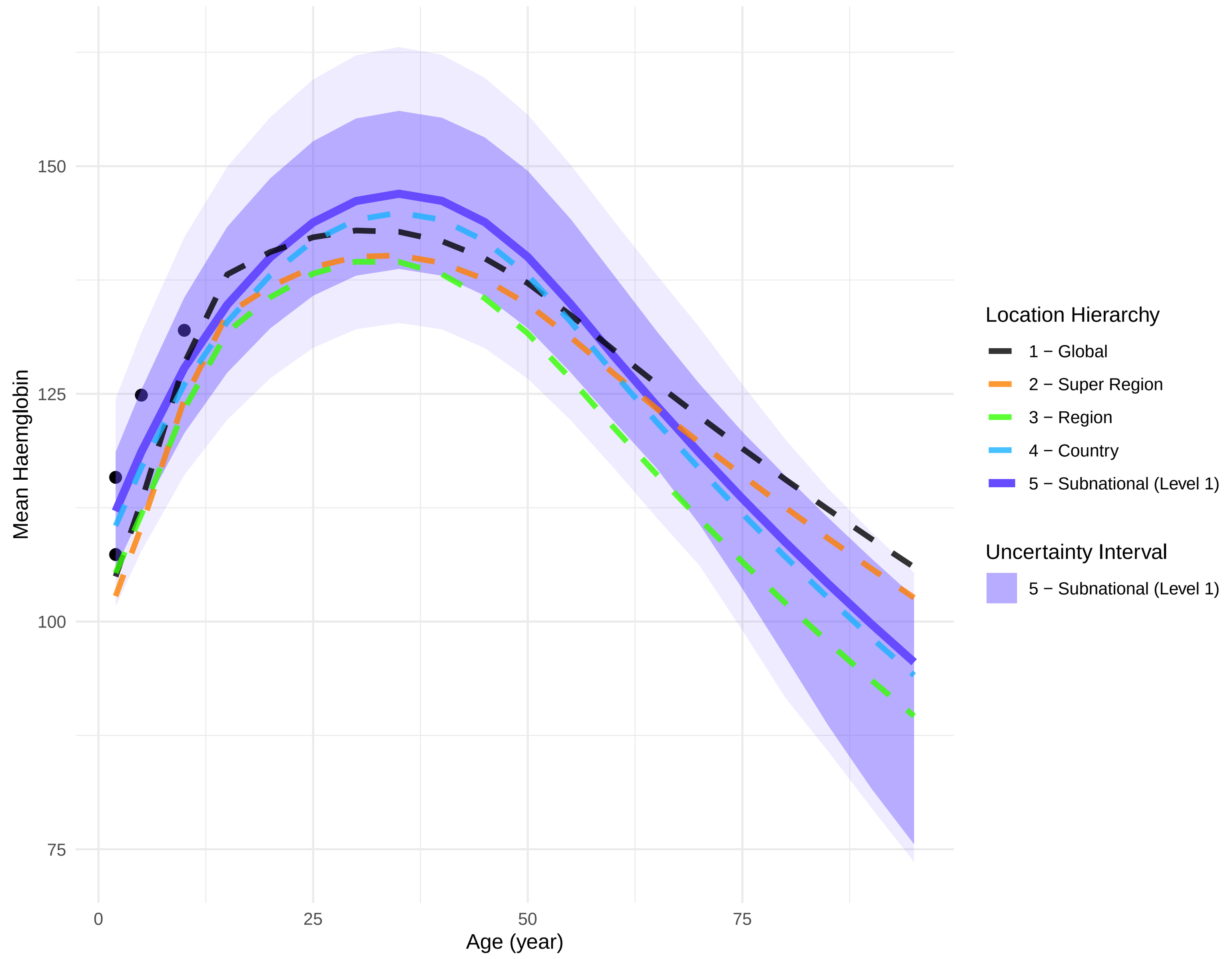

MR−BRT Age/Sex Fits − Males 2+ Years Old − Mean Haemglobin − Kiambu
WHO−2024 Adjustment Method

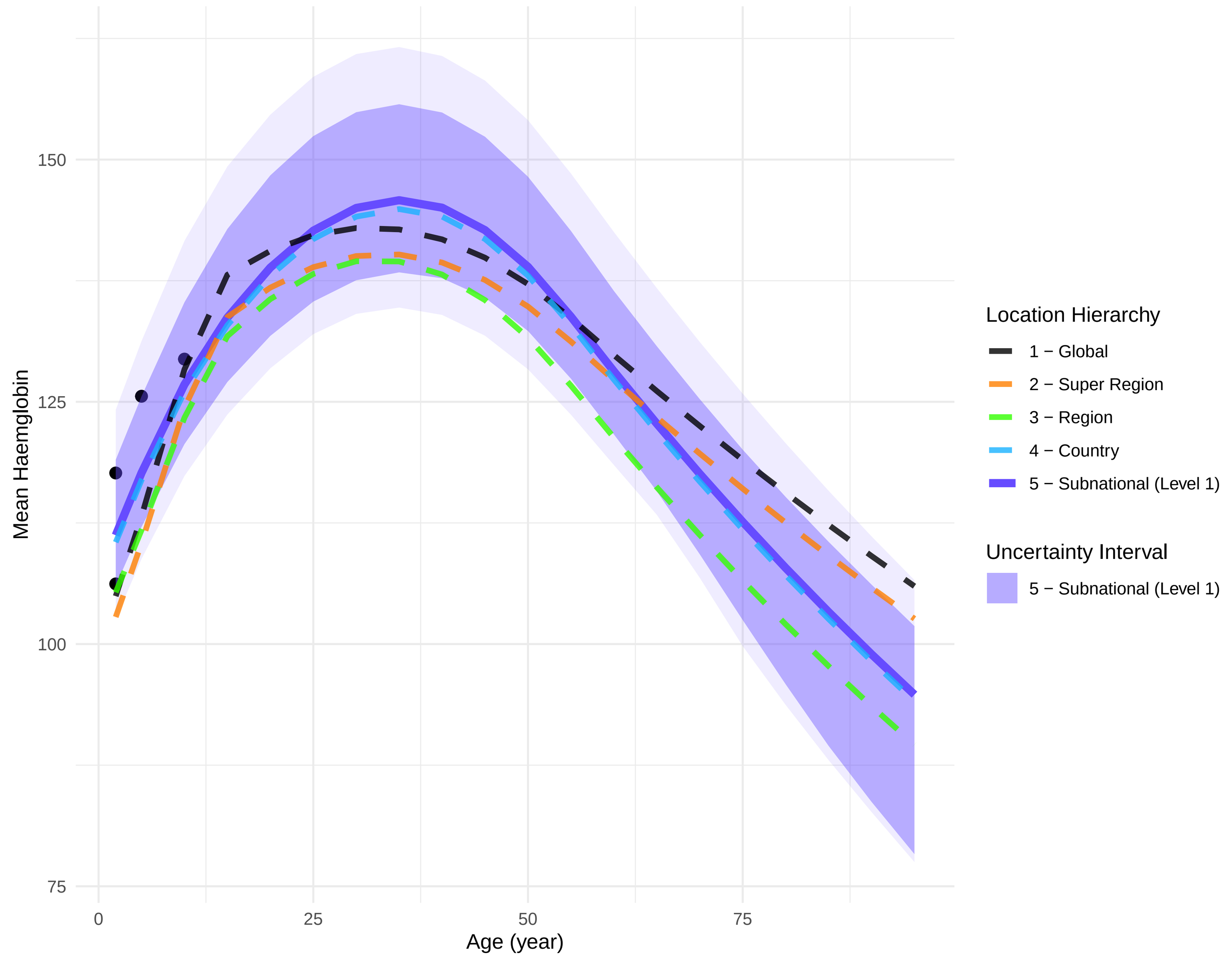

MR−BRT Age/Sex Fits − Males 2+ Years Old − Mean Haemglobin − Kilifi
WHO−2024 Adjustment Method

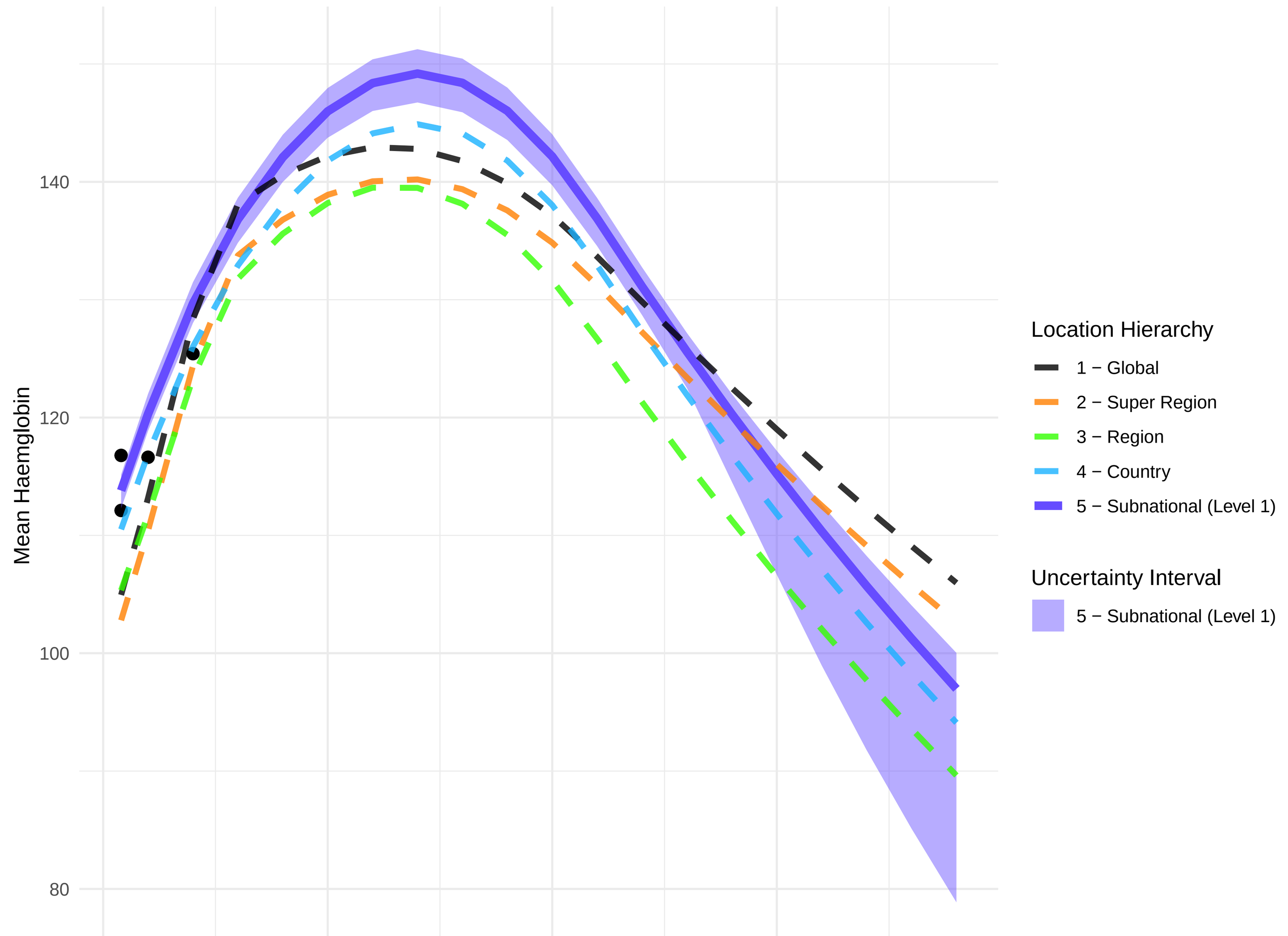

MR−BRT Age/Sex Fits − Males 2+ Years Old − Mean Haemglobin − Kirinyaga
WHO−2024 Adjustment Method

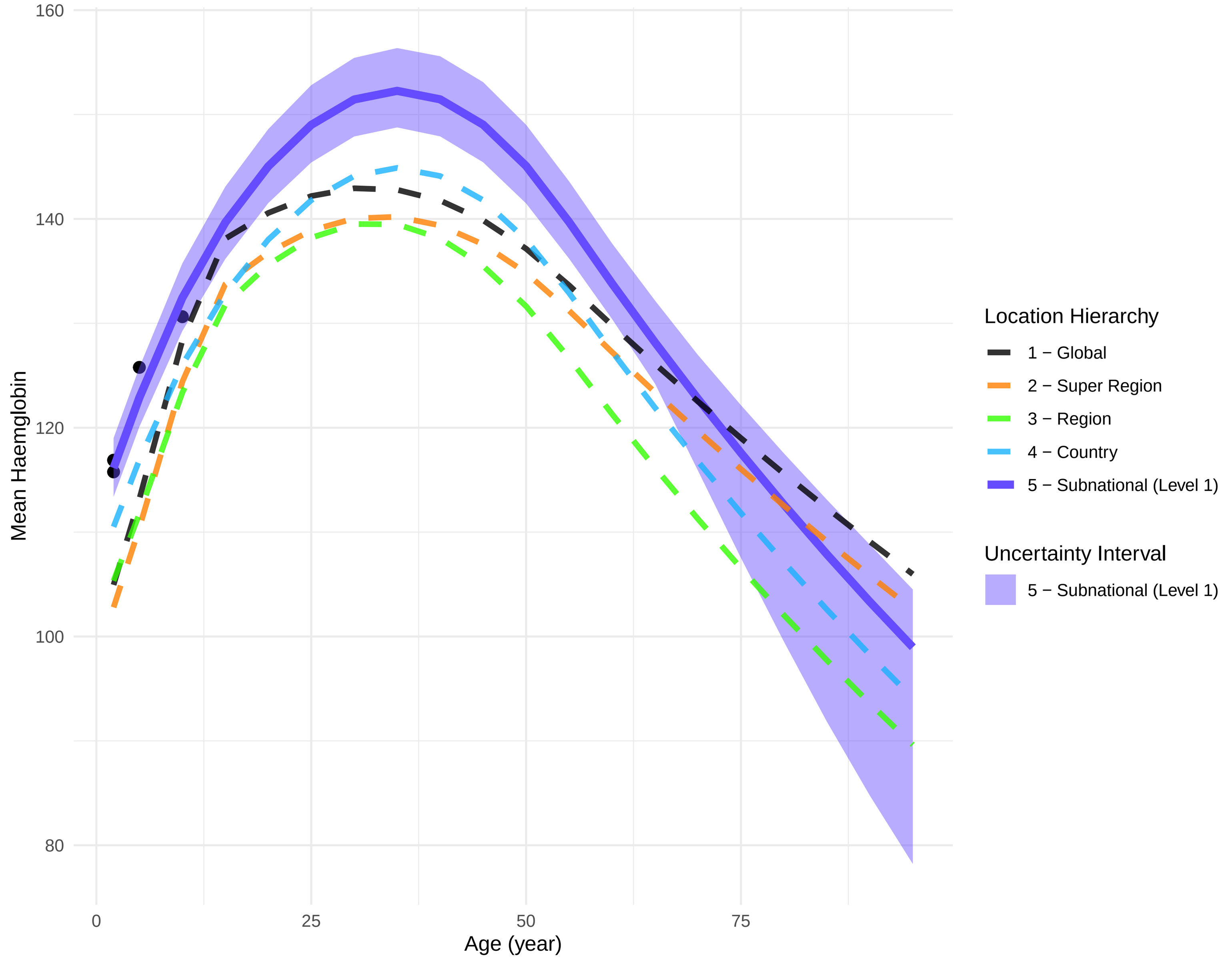

MR−BRT Age/Sex Fits − Males 2+ Years Old − Mean Haemglobin − Kisii
WHO−2024 Adjustment Method

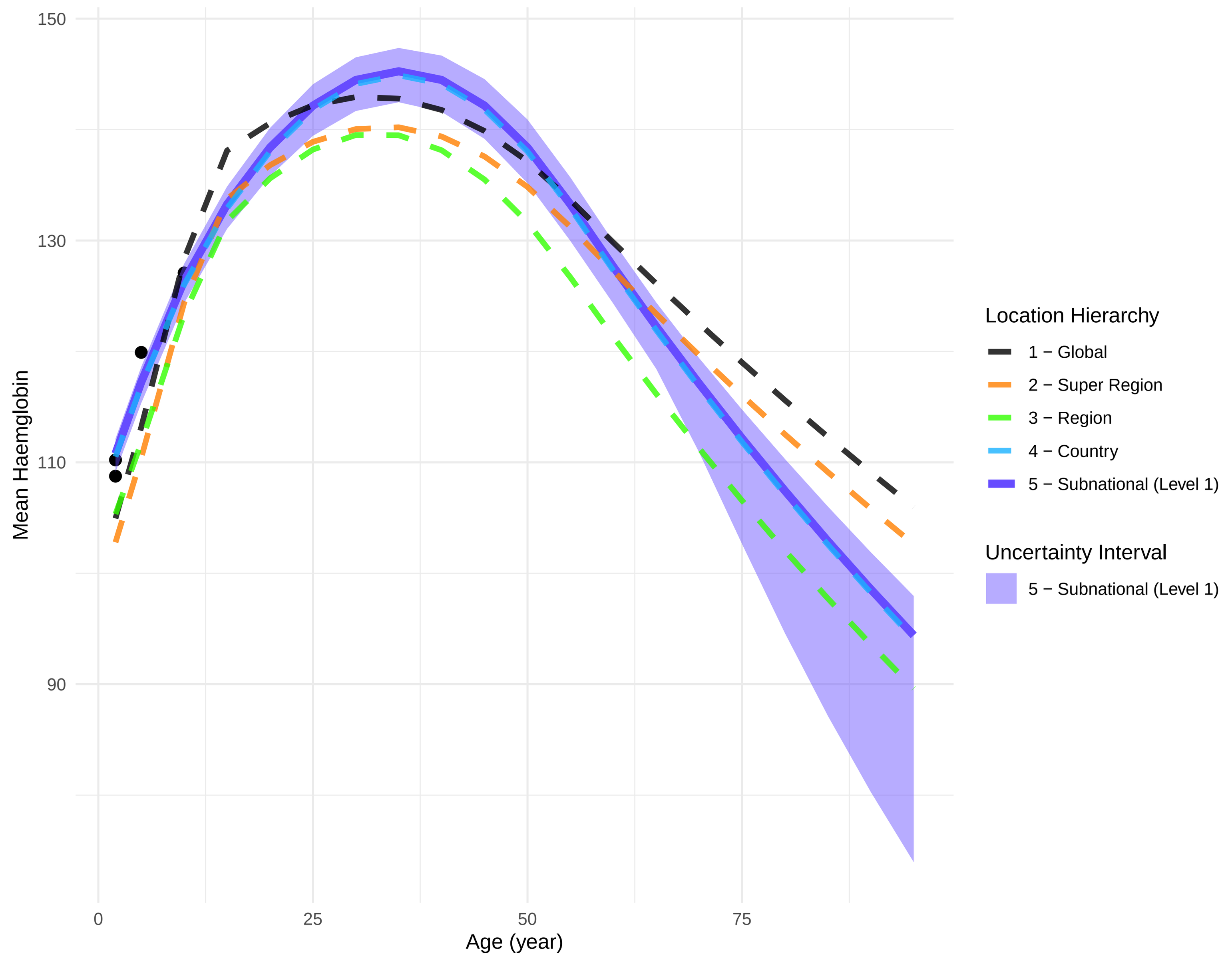

MR−BRT Age/Sex Fits − Males 2+ Years Old − Mean Haemglobin − Kisumu
WHO−2024 Adjustment Method

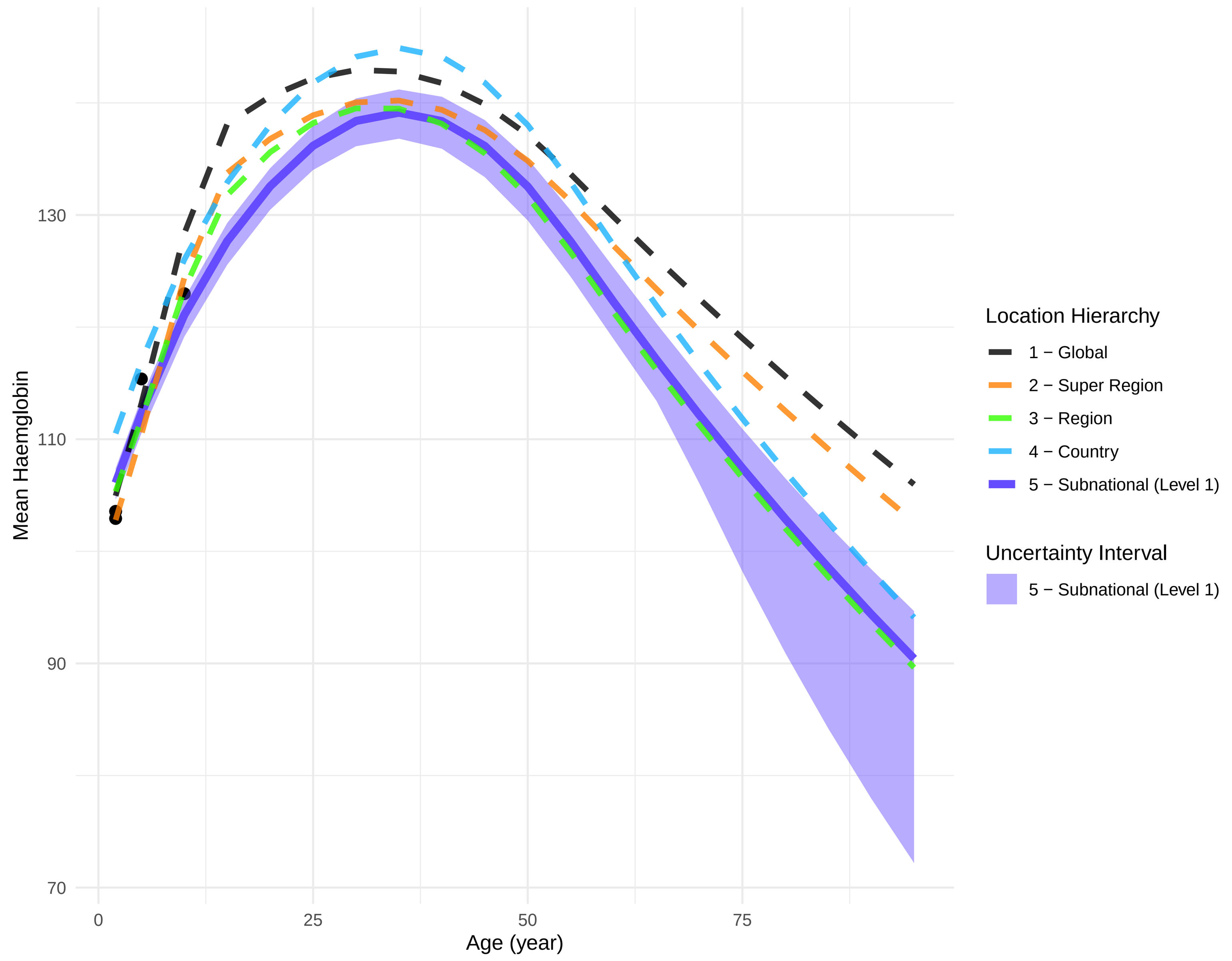

MR−BRT Age/Sex Fits − Males 2+ Years Old − Mean Haemglobin − Kitui
WHO−2024 Adjustment Method

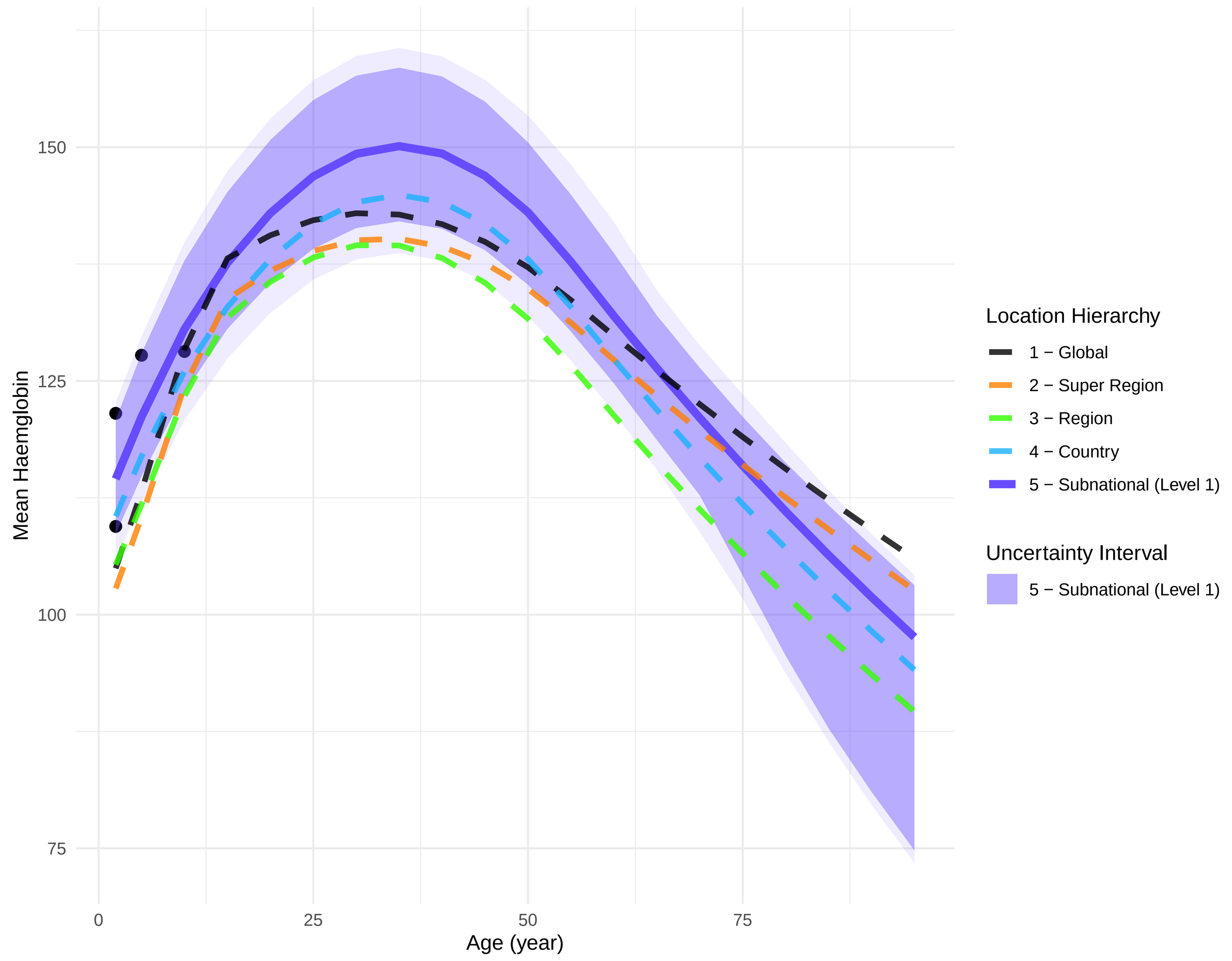

MR−BRT Age/Sex Fits − Males 2+ Years Old − Mean Haemglobin − Kwale
WHO−2024 Adjustment Method

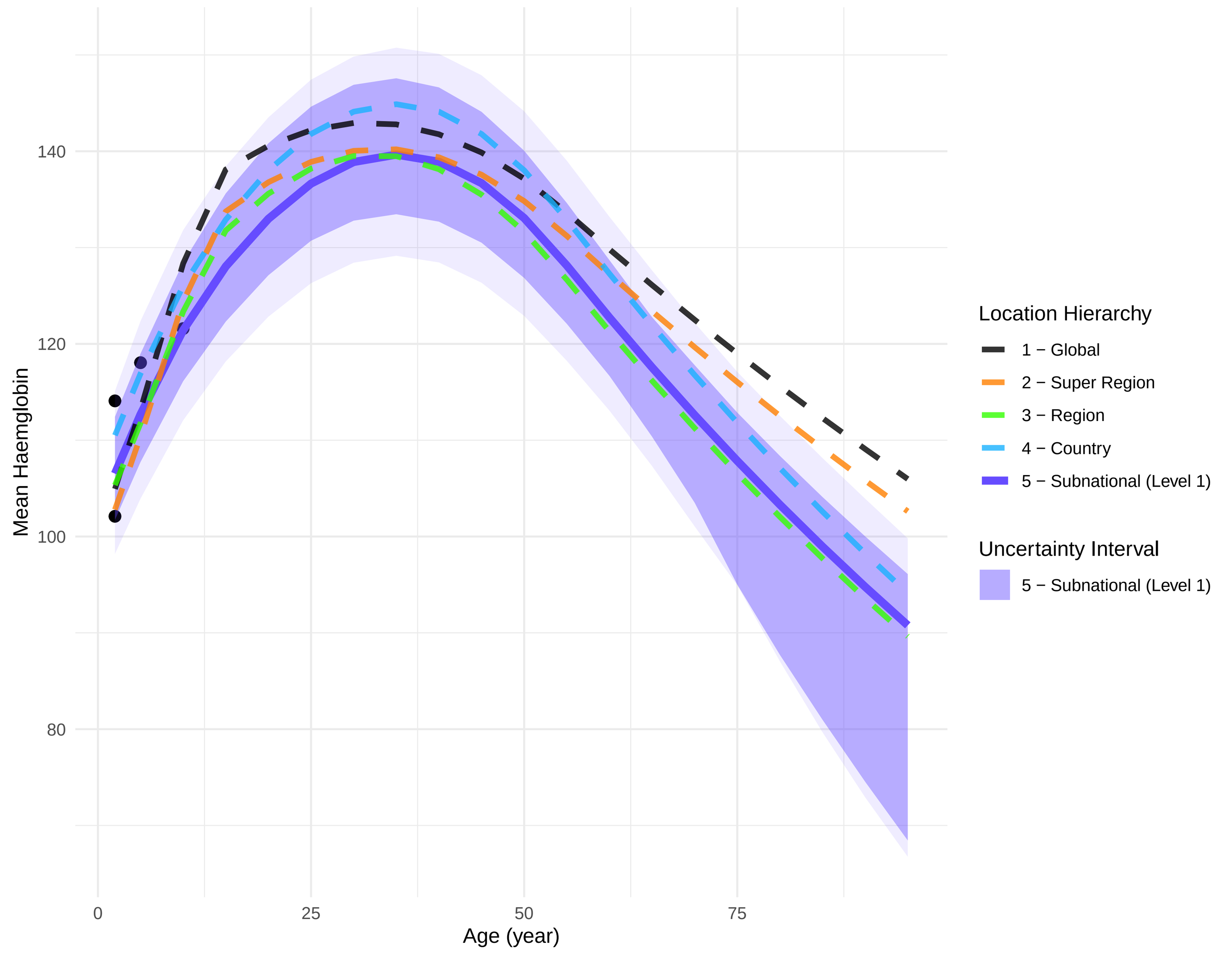

MR−BRT Age/Sex Fits − Males 2+ Years Old − Mean Haemglobin − Laikipia
WHO−2024 Adjustment Method

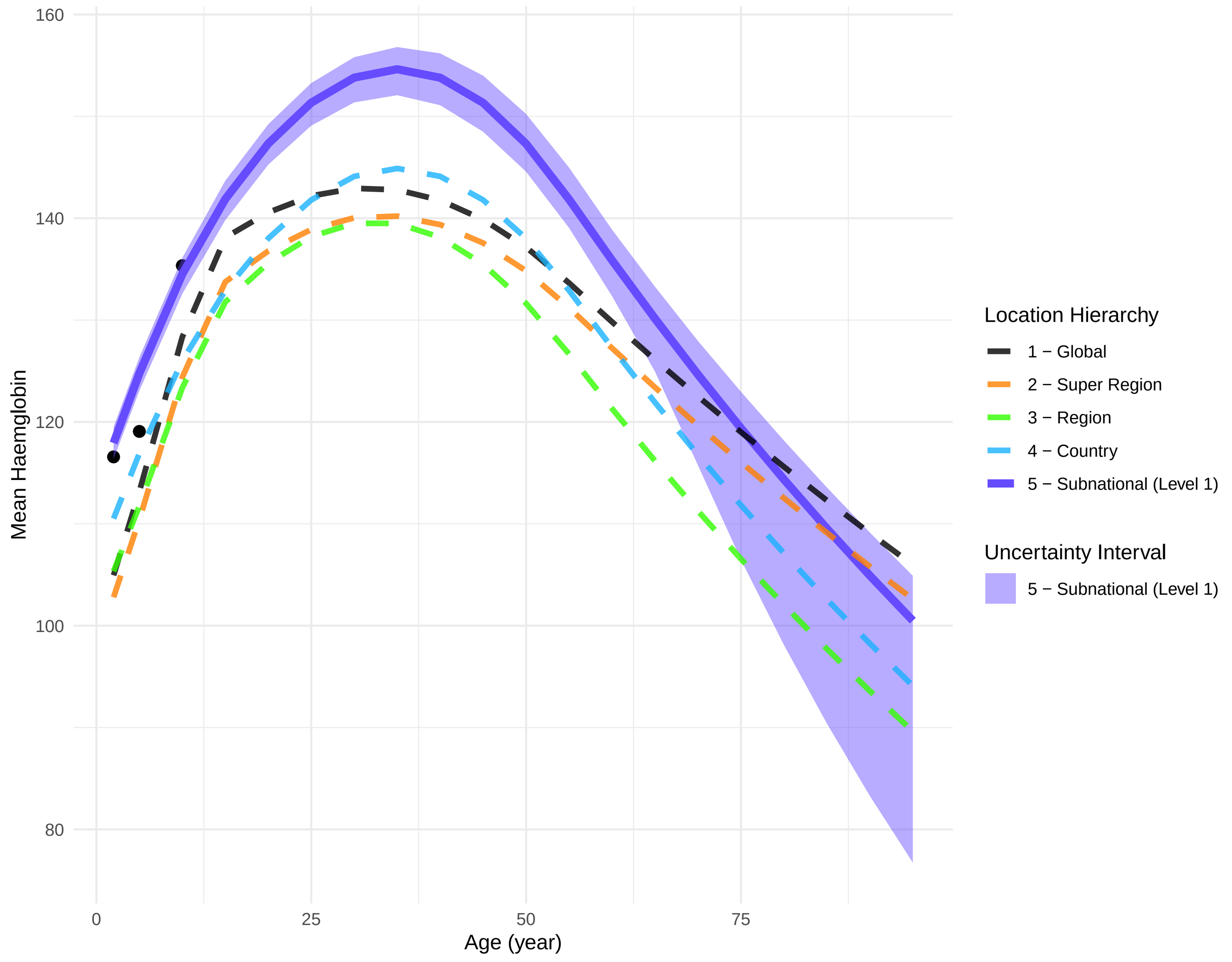

MR−BRT Age/Sex Fits − Males 2+ Years Old − Mean Haemglobin − Lamu
WHO−2024 Adjustment Method

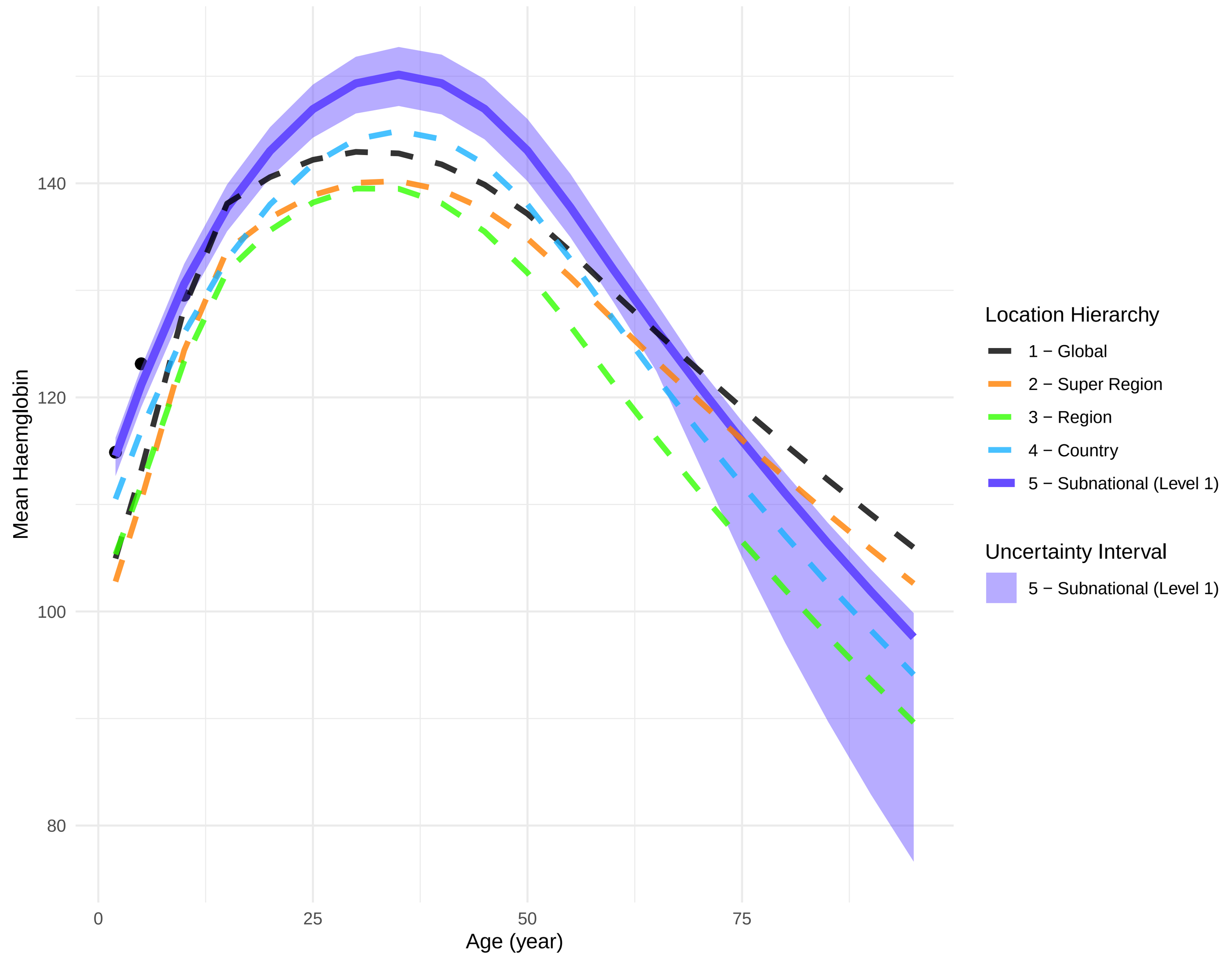

MR−BRT Age/Sex Fits − Males 2+ Years Old − Mean Haemglobin − Machakos
WHO−2024 Adjustment Method

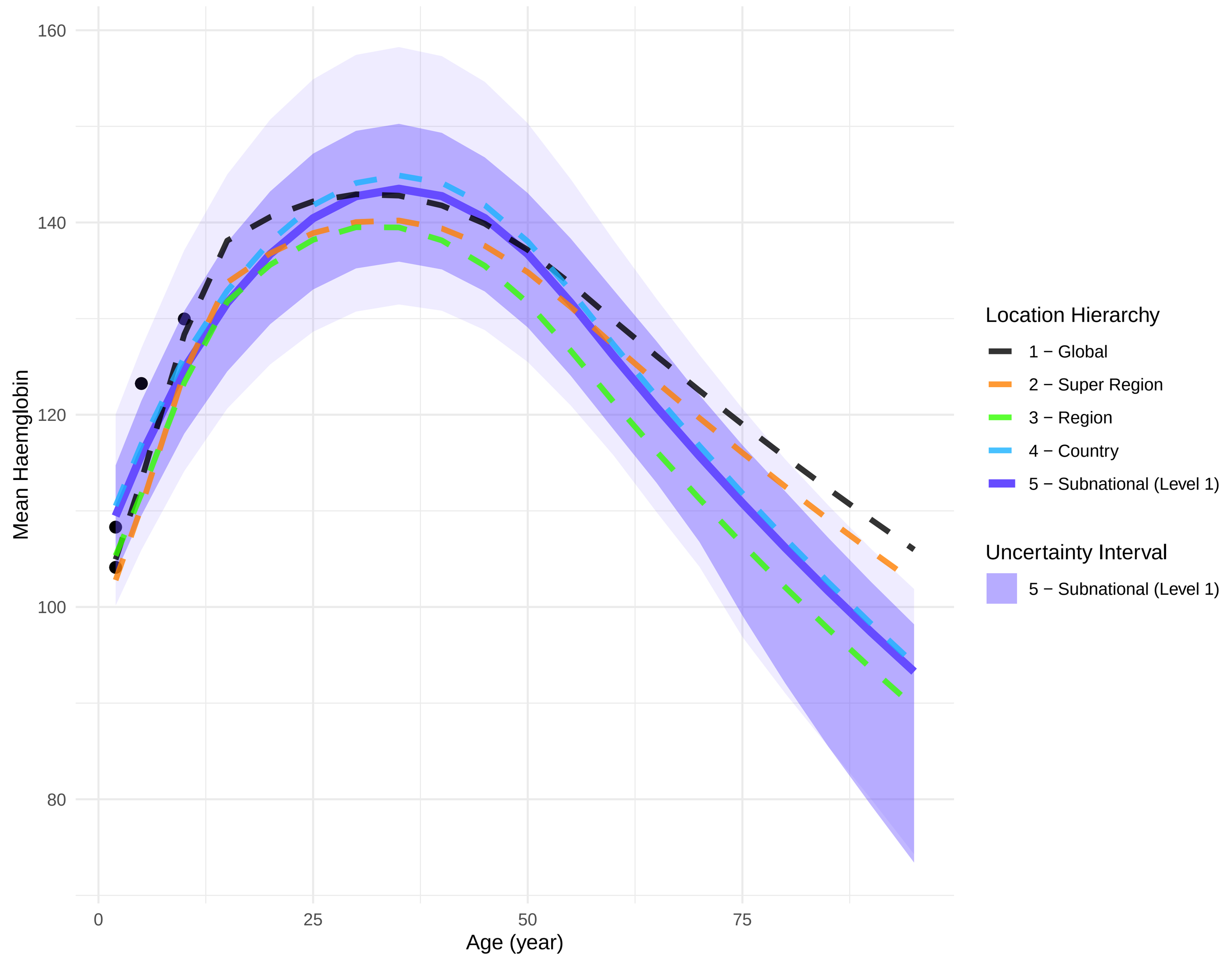

MR−BRT Age/Sex Fits − Males 2+ Years Old − Mean Haemglobin − Makueni
WHO−2024 Adjustment Method

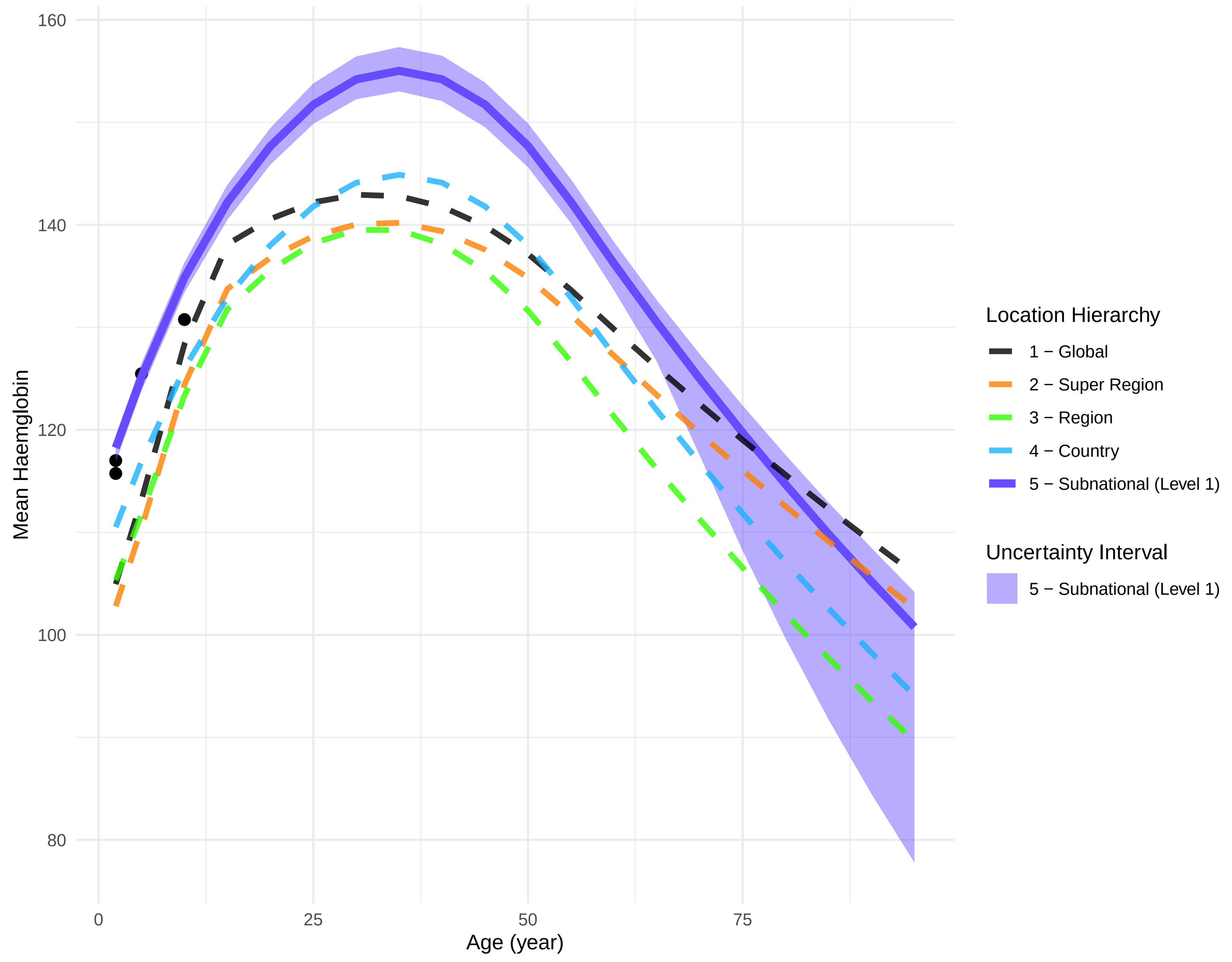

MR−BRT Age/Sex Fits − Males 2+ Years Old − Mean Haemglobin − Mandera
WHO−2024 Adjustment Method

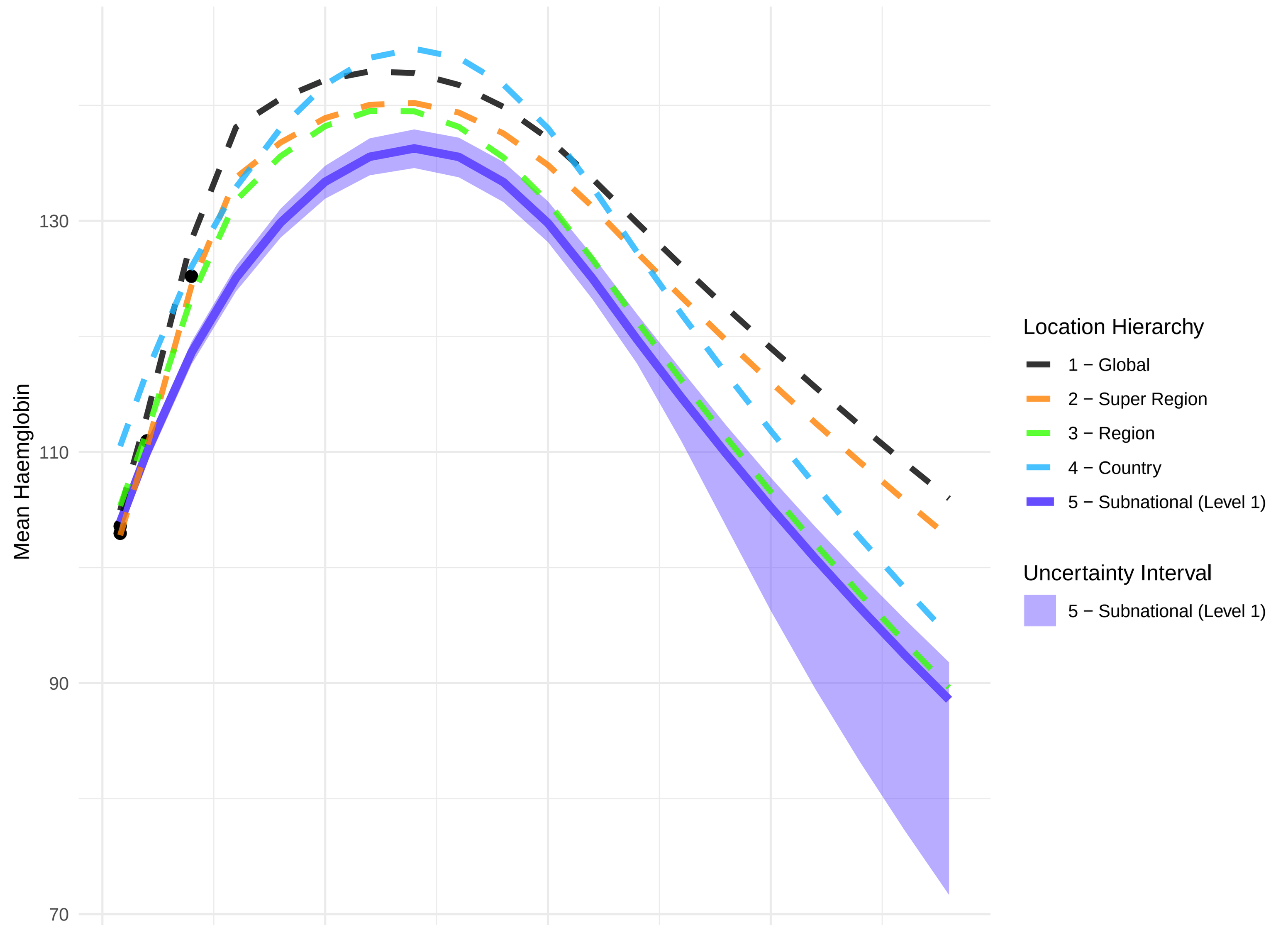

MR−BRT Age/Sex Fits − Males 2+ Years Old − Mean Haemglobin − Marsabit
WHO−2024 Adjustment Method

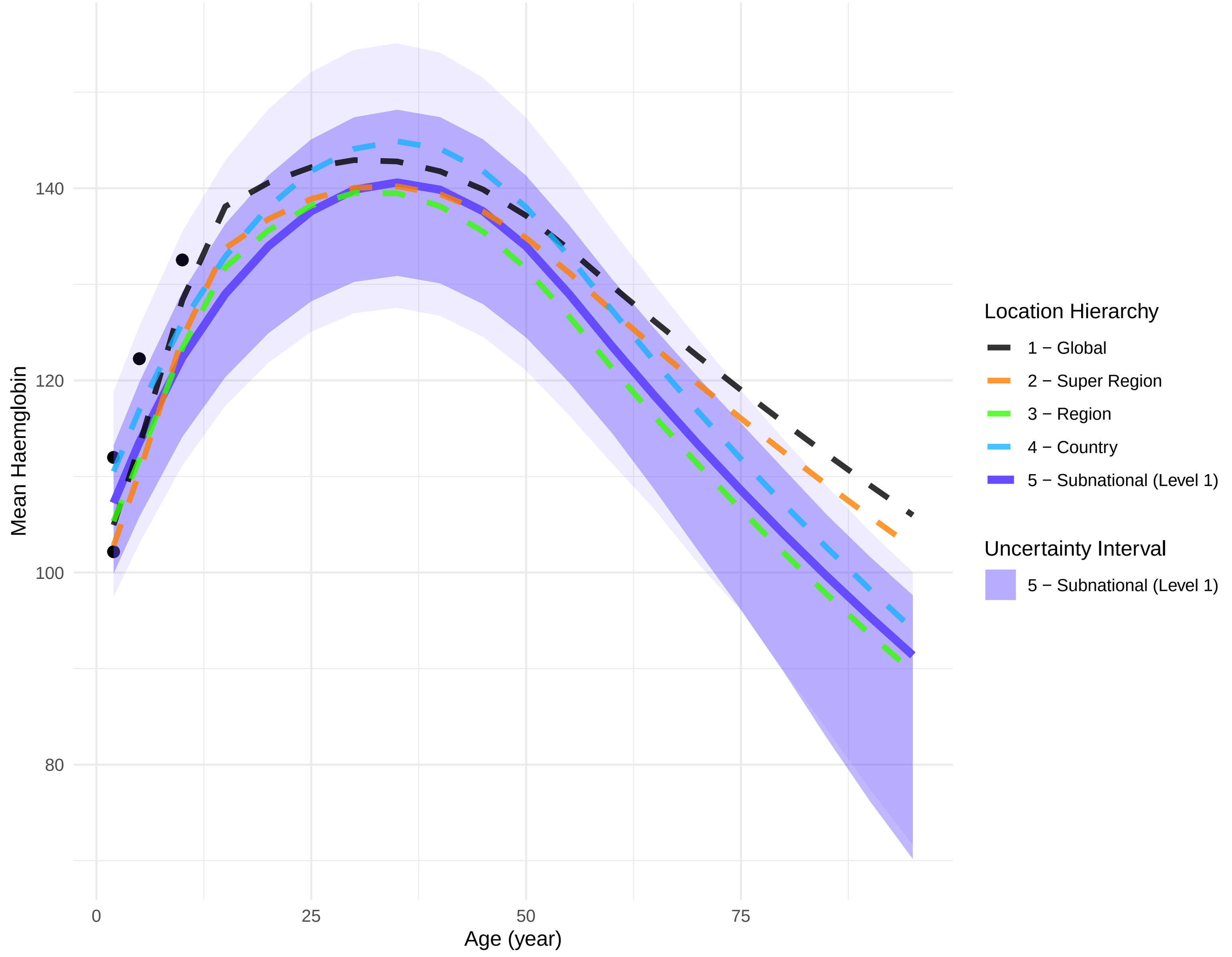

MR−BRT Age/Sex Fits − Males 2+ Years Old − Mean Haemglobin − Meru
WHO−2024 Adjustment Method

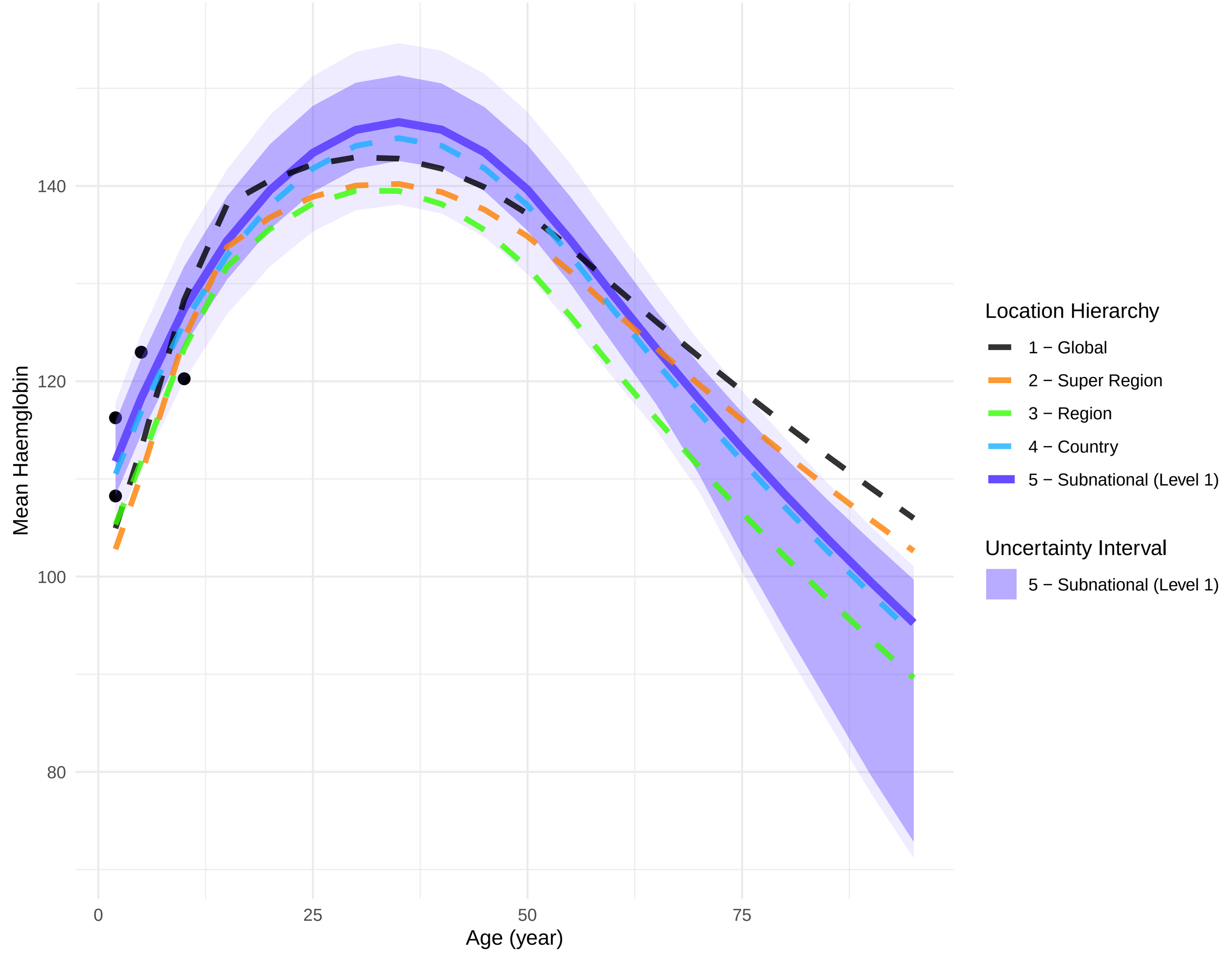

MR−BRT Age/Sex Fits − Males 2+ Years Old − Mean Haemglobin − Migori
WHO−2024 Adjustment Method

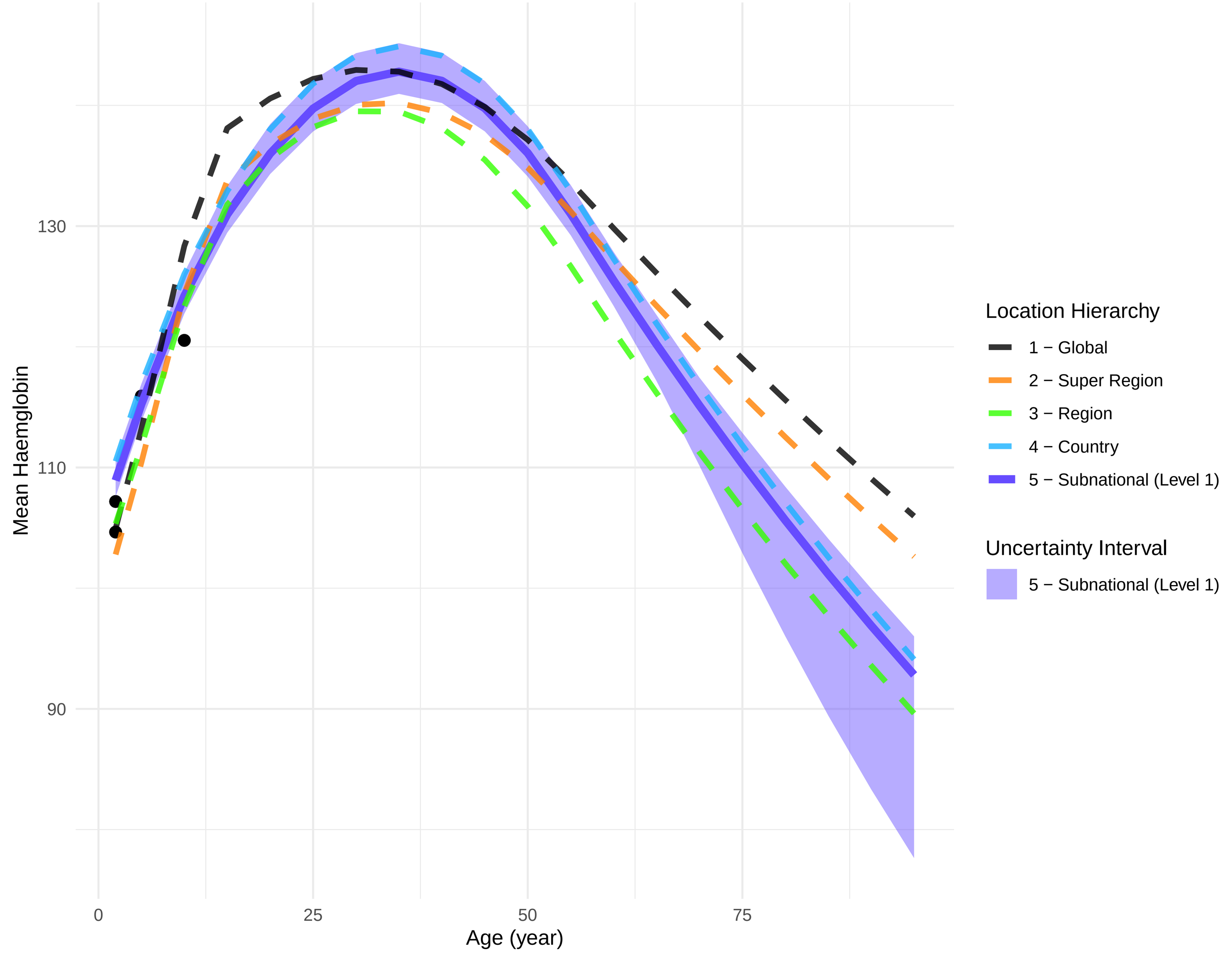

MR−BRT Age/Sex Fits − Males 2+ Years Old − Mean Haemglobin − Mombasa
WHO−2024 Adjustment Method

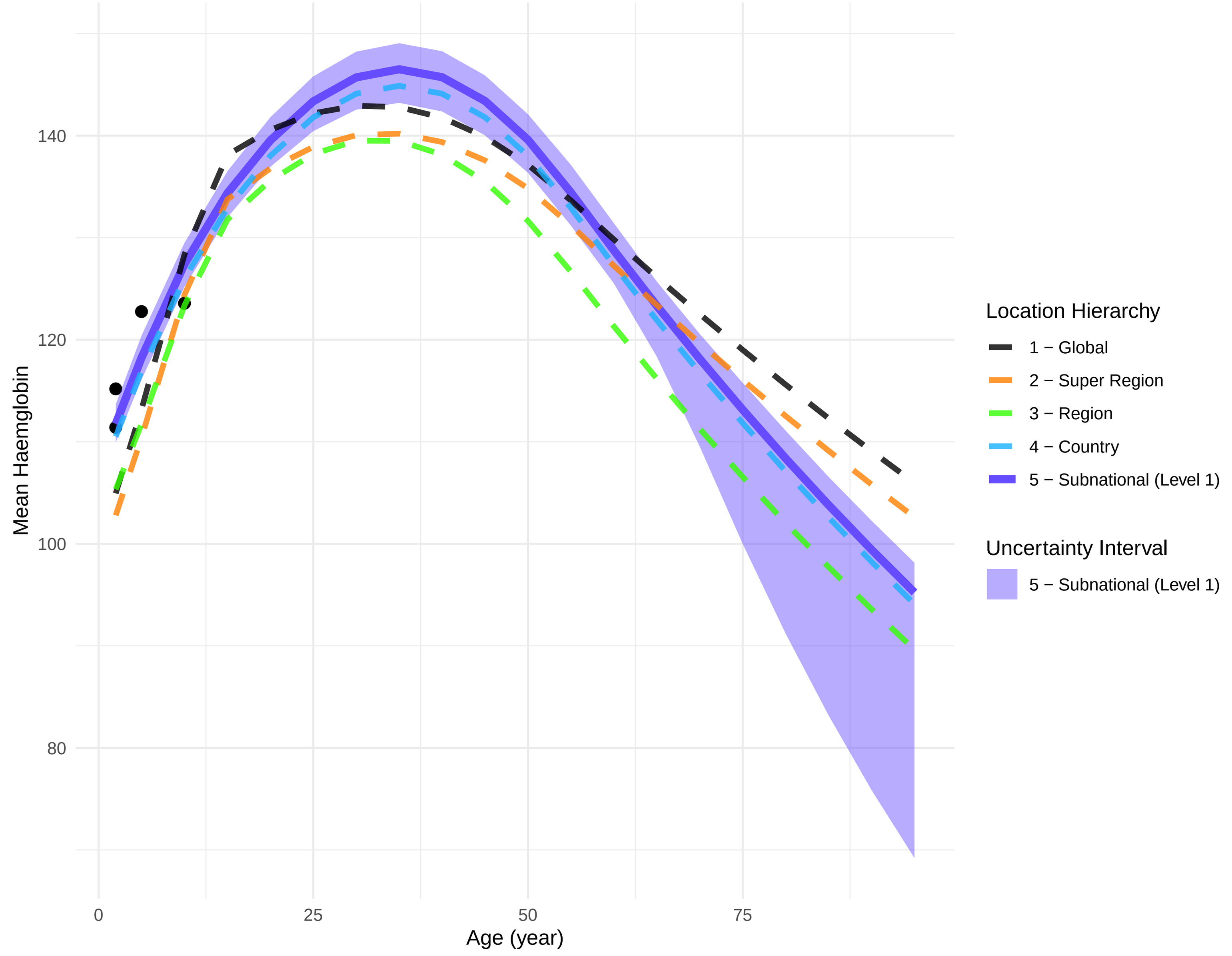

MR−BRT Age/Sex Fits − Males 2+ Years Old − Mean Haemglobin − Murang'a
WHO−2024 Adjustment Method

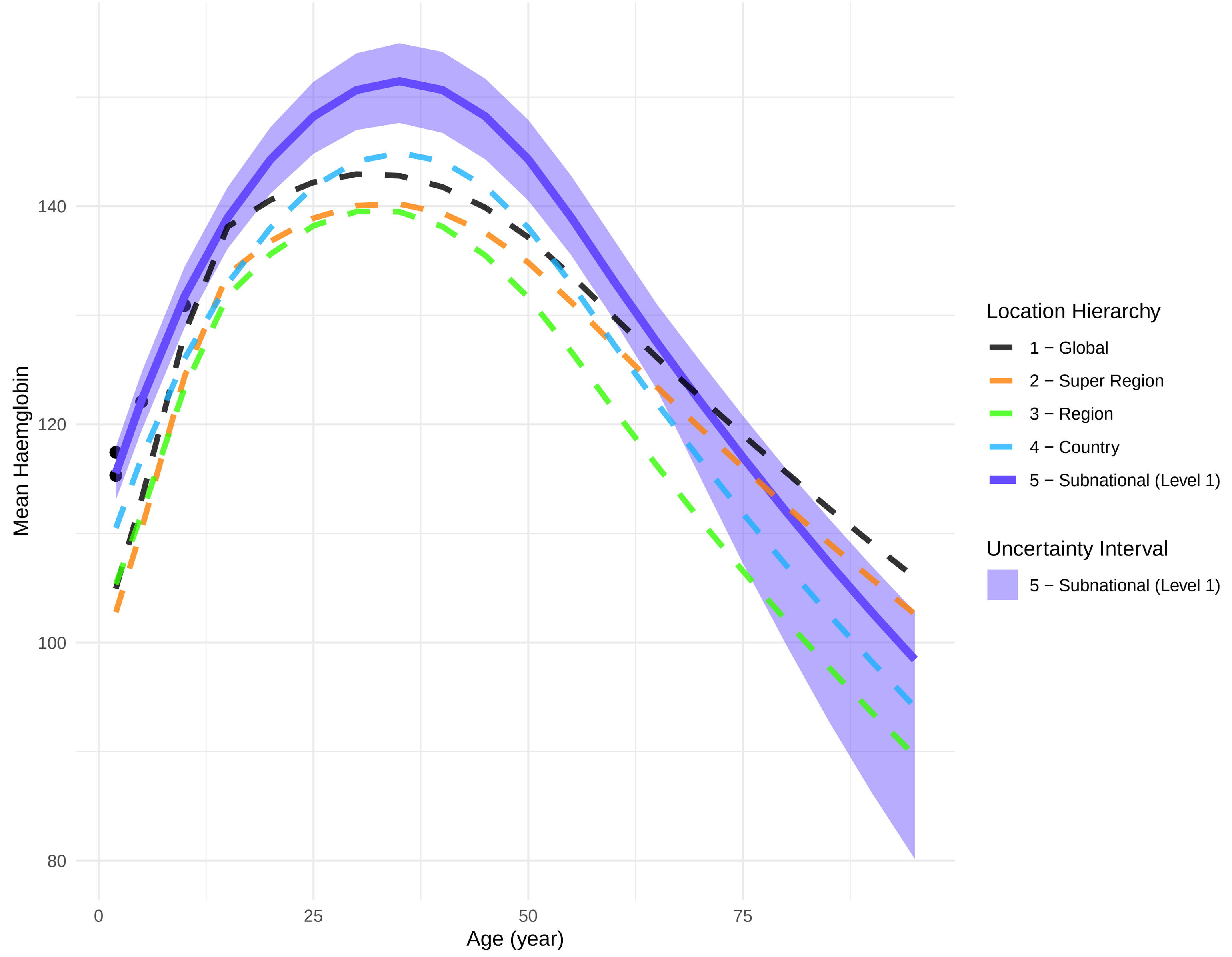

MR−BRT Age/Sex Fits − Males 2+ Years Old − Mean Haemglobin − Nairobi
WHO−2024 Adjustment Method

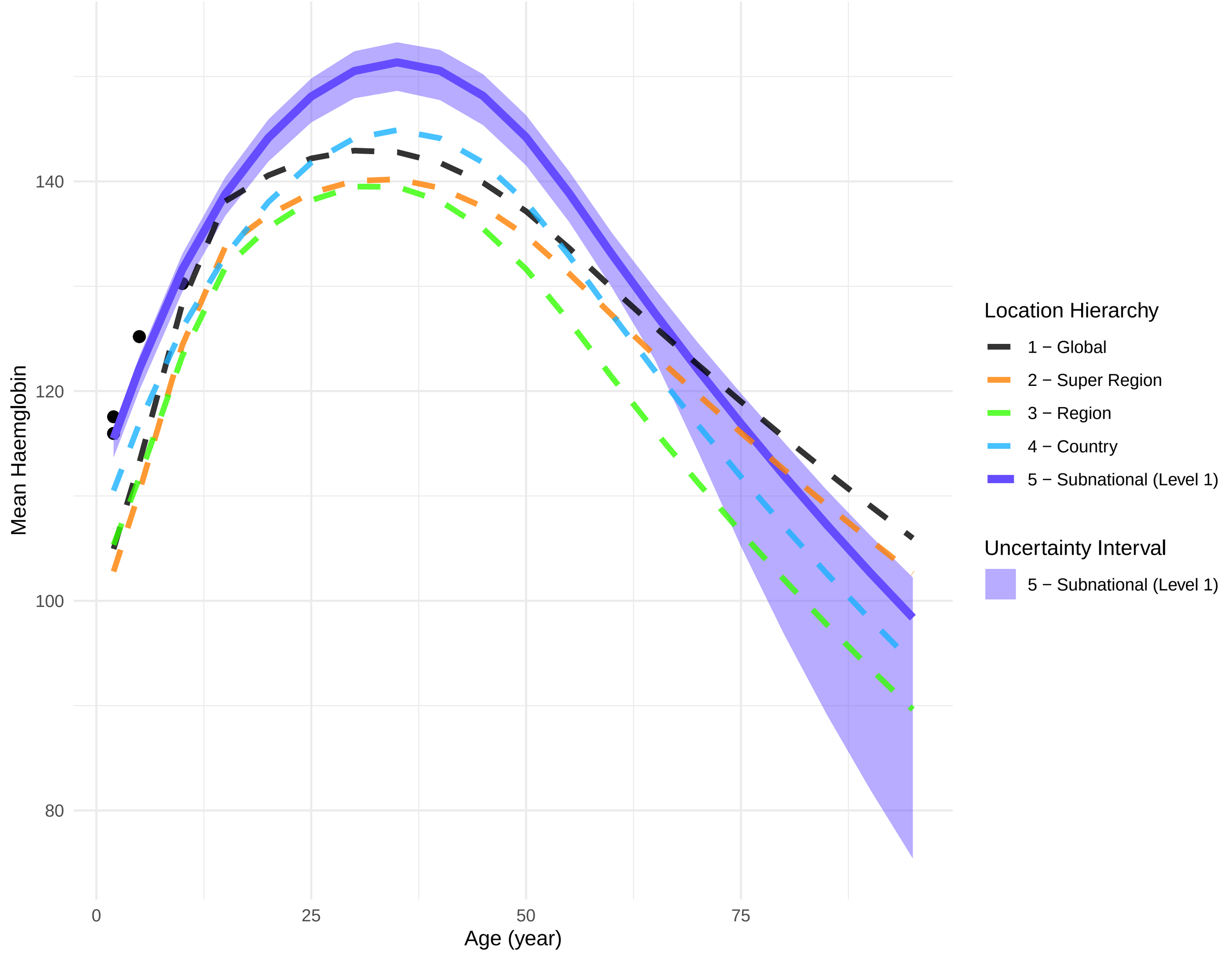

MR−BRT Age/Sex Fits − Males 2+ Years Old − Mean Haemglobin − Nakuru
WHO−2024 Adjustment Method

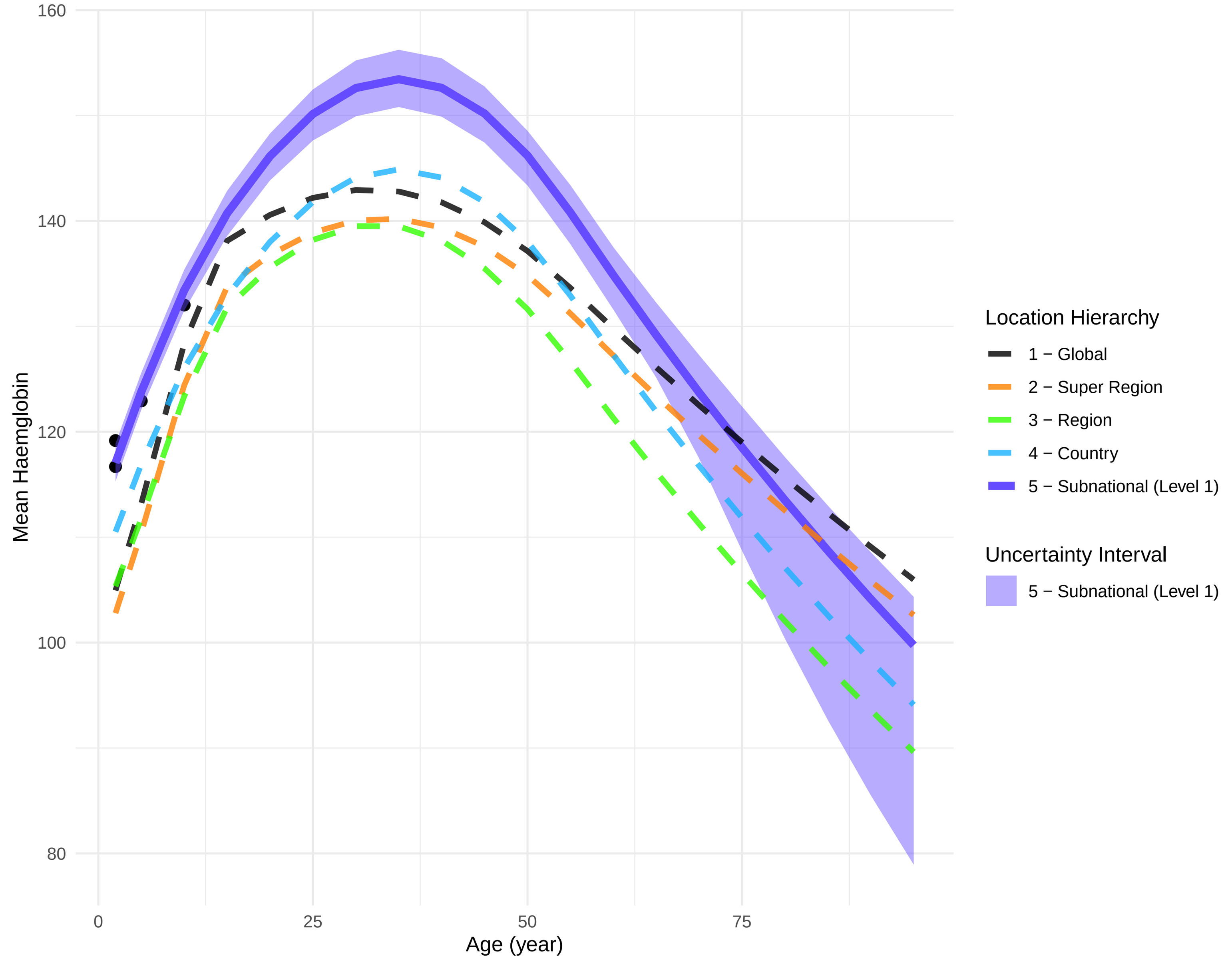

MR−BRT Age/Sex Fits − Males 2+ Years Old − Mean Haemglobin − Nandi
WHO−2024 Adjustment Method

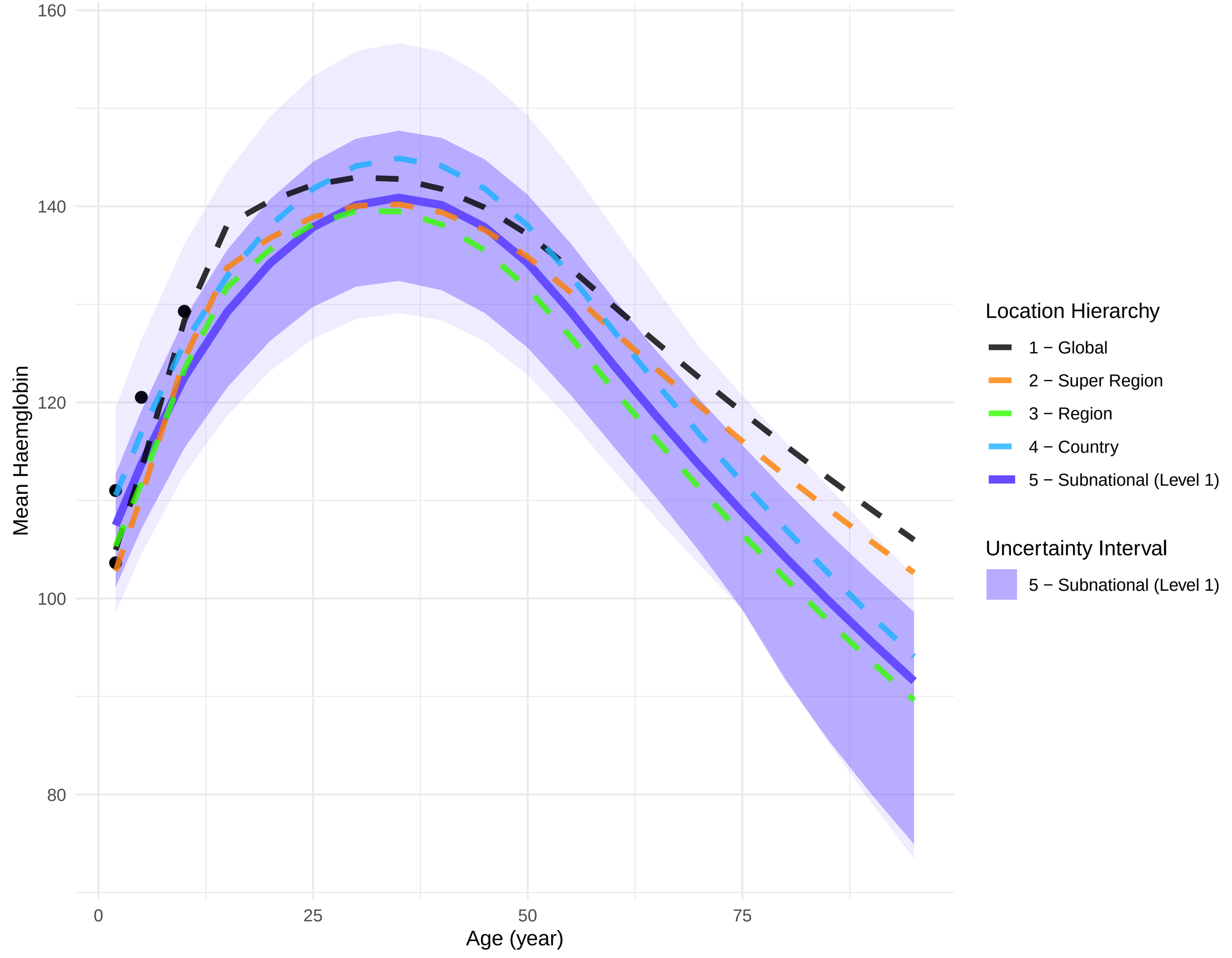

MR−BRT Age/Sex Fits − Males 2+ Years Old − Mean Haemglobin − Narok
WHO−2024 Adjustment Method

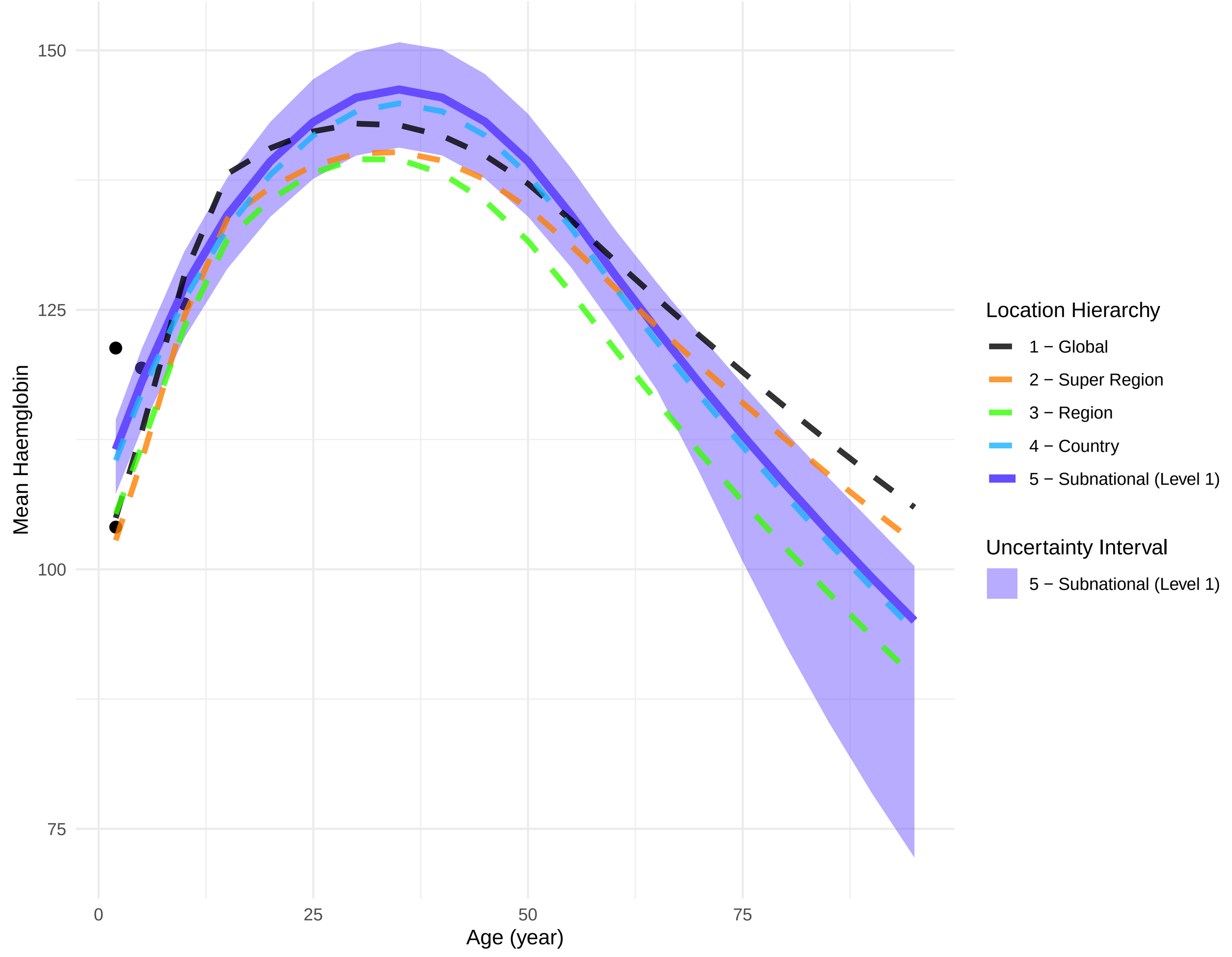

MR−BRT Age/Sex Fits − Males 2+ Years Old − Mean Haemglobin − Nyamira
WHO−2024 Adjustment Method

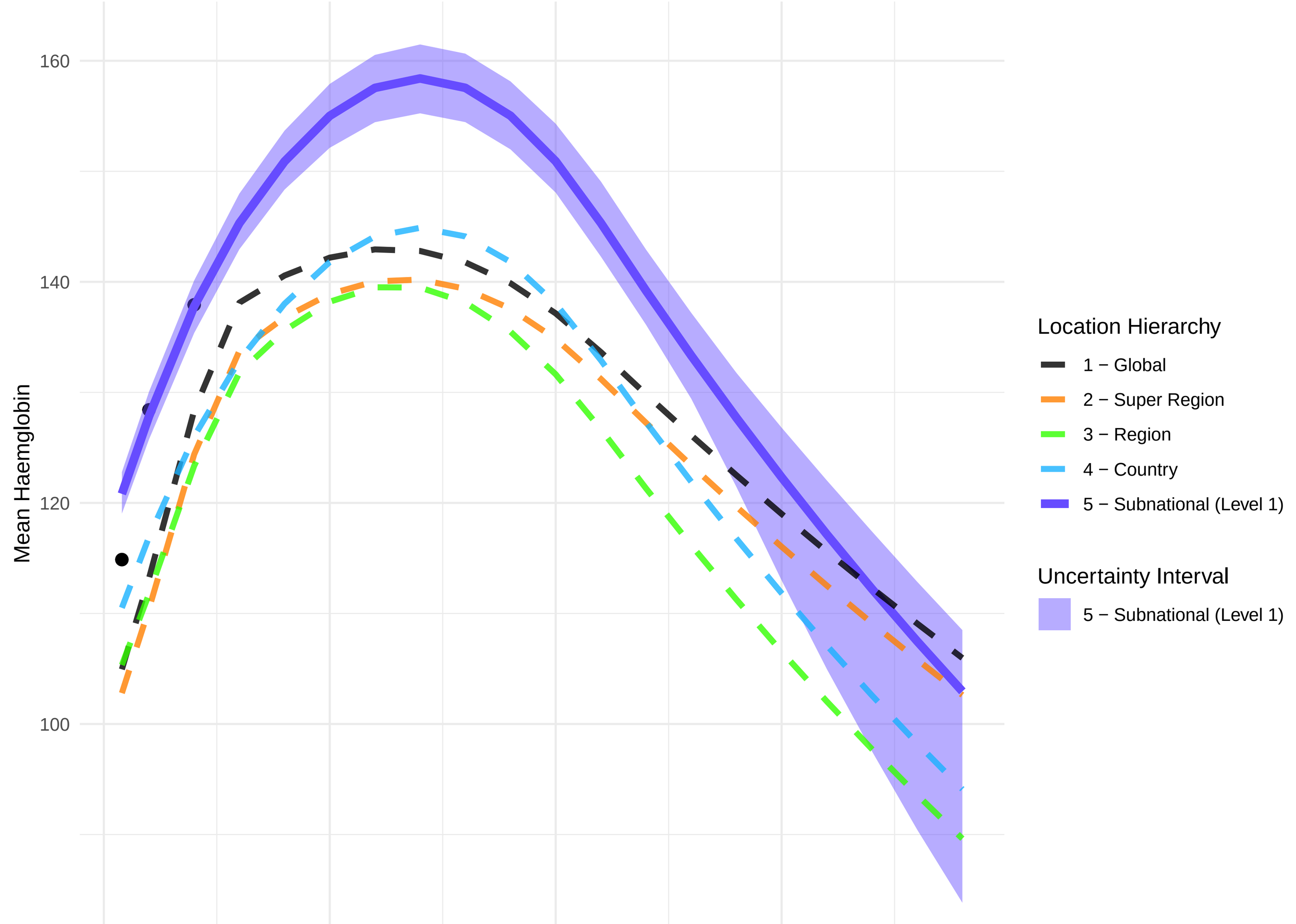

MR−BRT Age/Sex Fits − Males 2+ Years Old − Mean Haemglobin − Nyandarua
WHO−2024 Adjustment Method

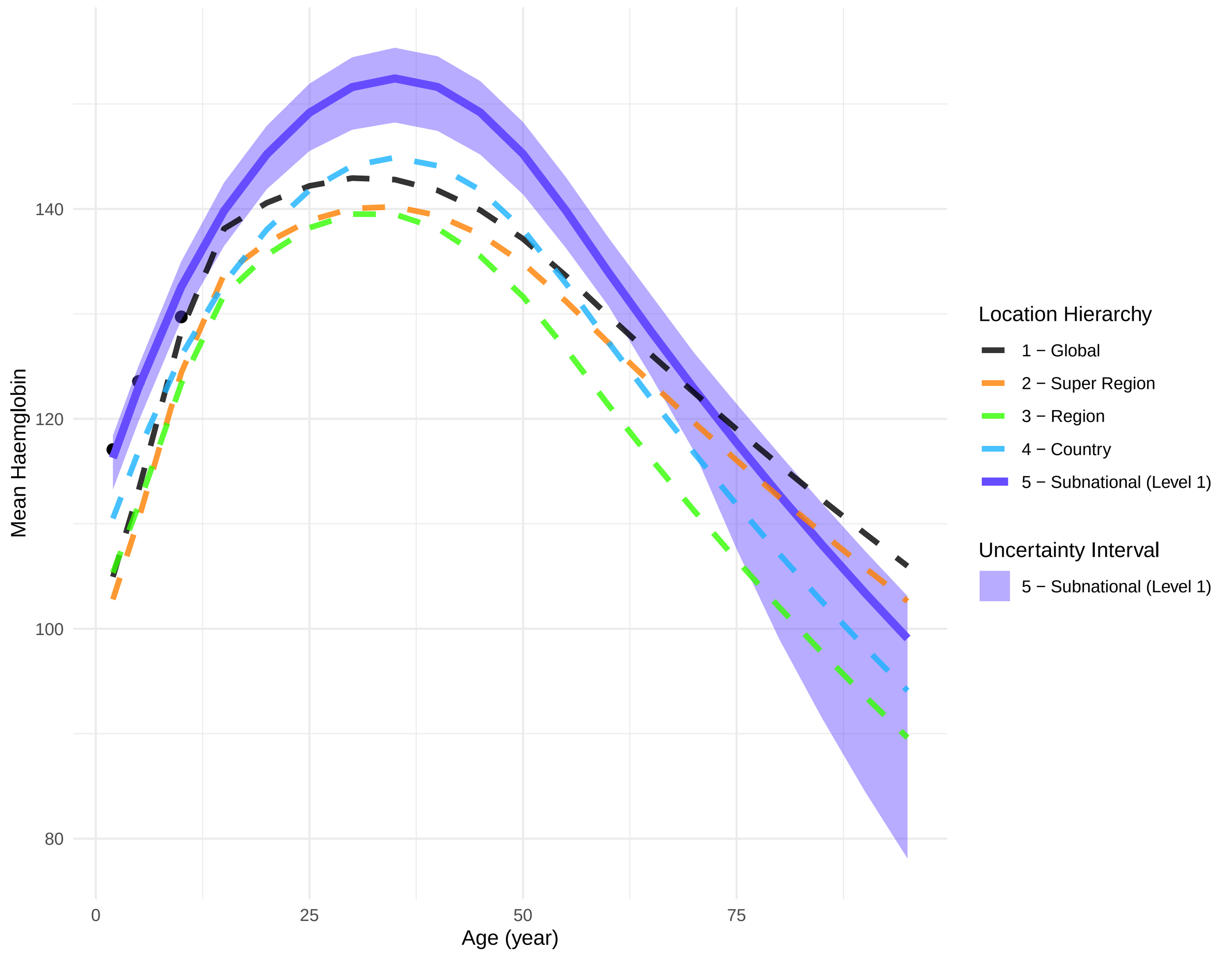

MR−BRT Age/Sex Fits − Males 2+ Years Old − Mean Haemglobin − Nyeri
WHO−2024 Adjustment Method

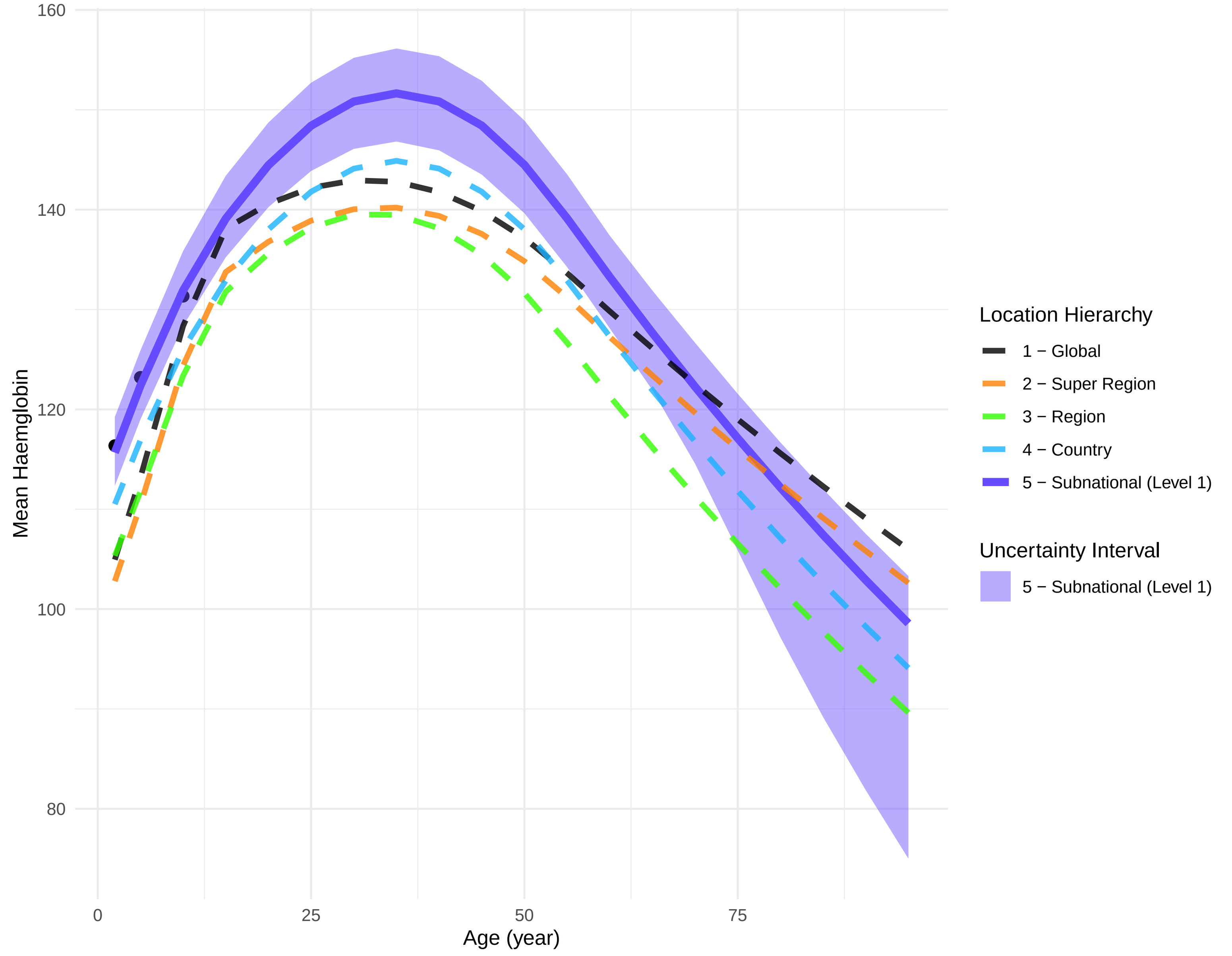

MR−BRT Age/Sex Fits − Males 2+ Years Old − Mean Haemglobin − Samburu
WHO−2024 Adjustment Method

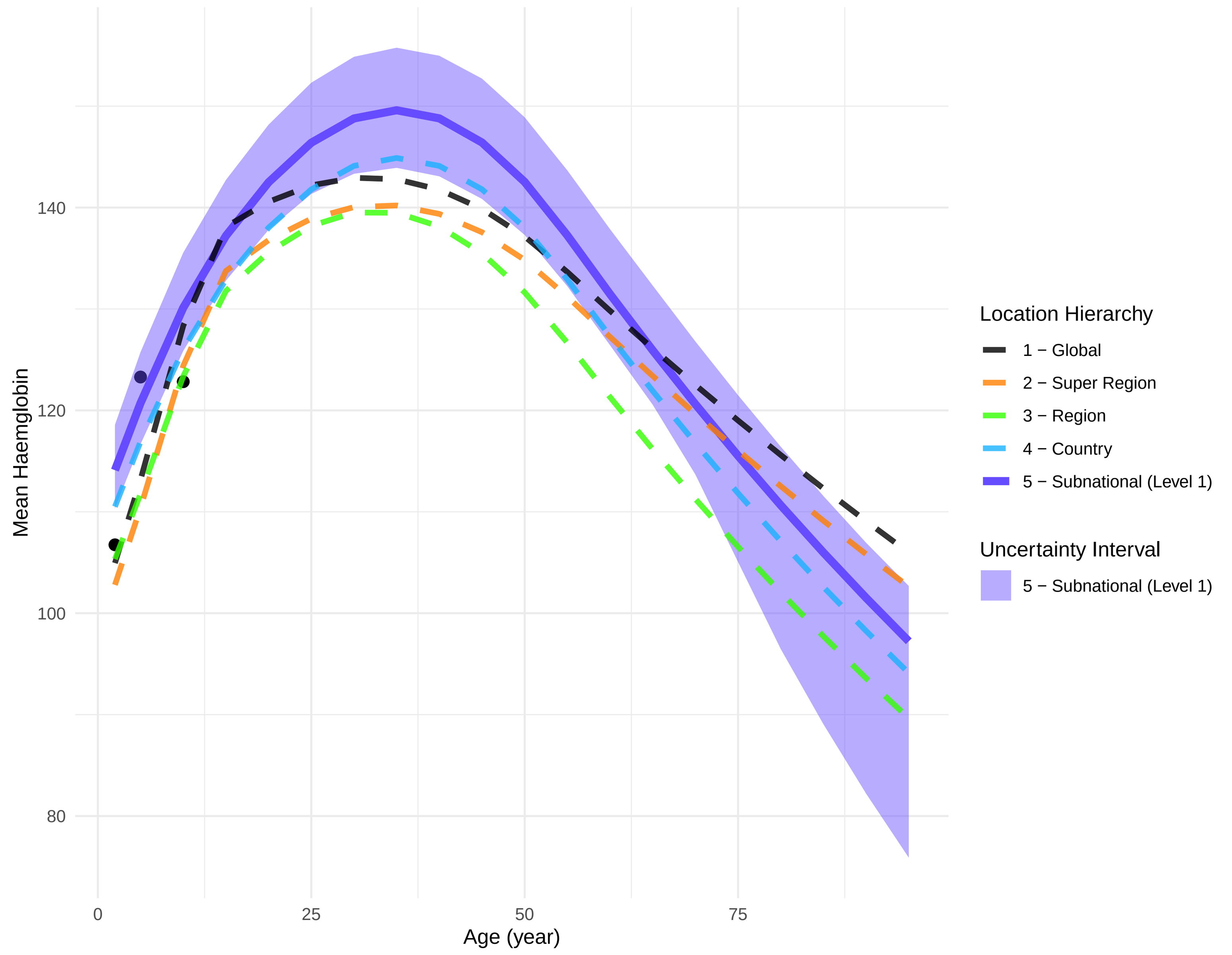

MR−BRT Age/Sex Fits − Males 2+ Years Old − Mean Haemglobin − Siaya
WHO−2024 Adjustment Method

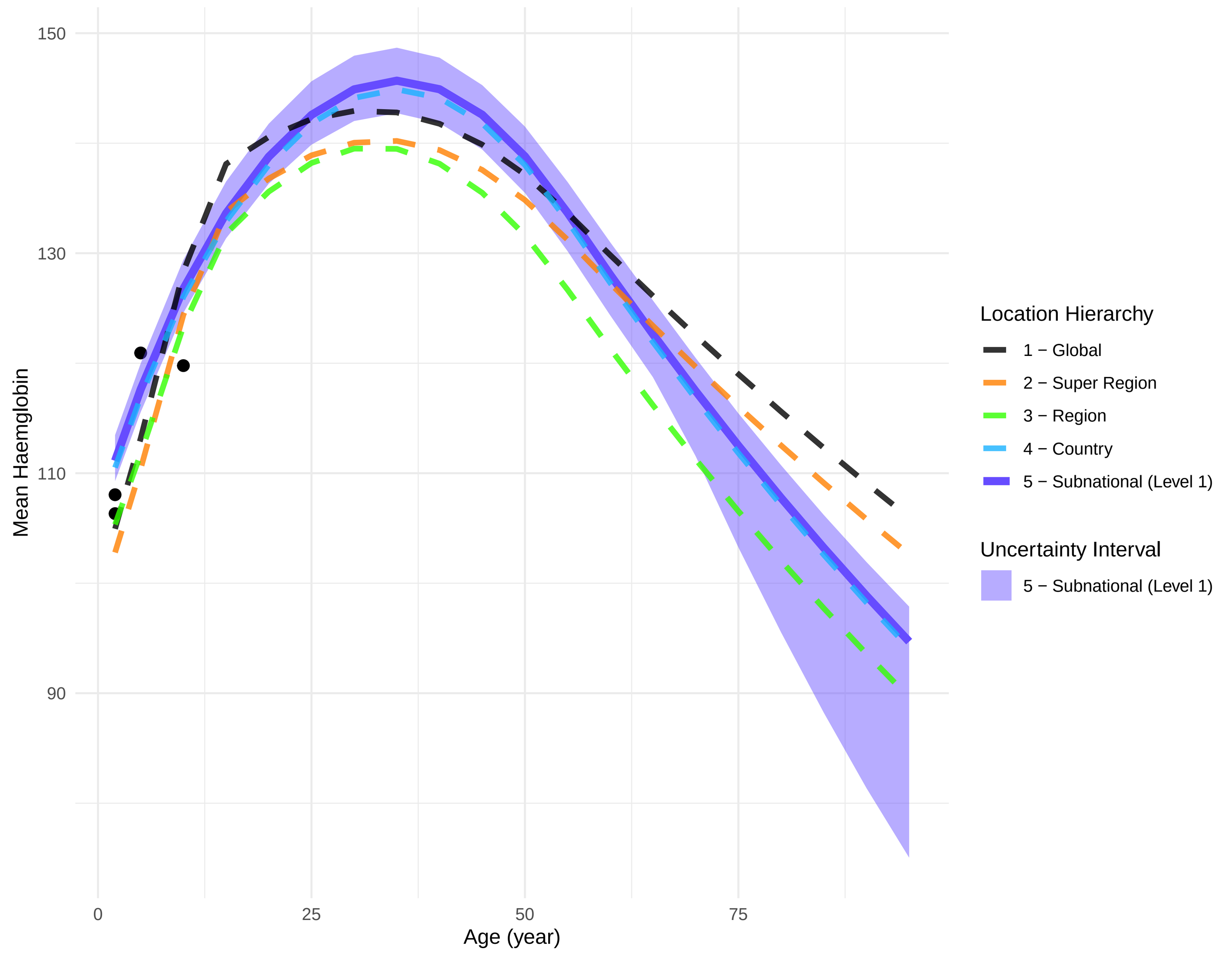

MR−BRT Age/Sex Fits − Males 2+ Years Old − Mean Haemglobin − Taita Taveta
WHO−2024 Adjustment Method

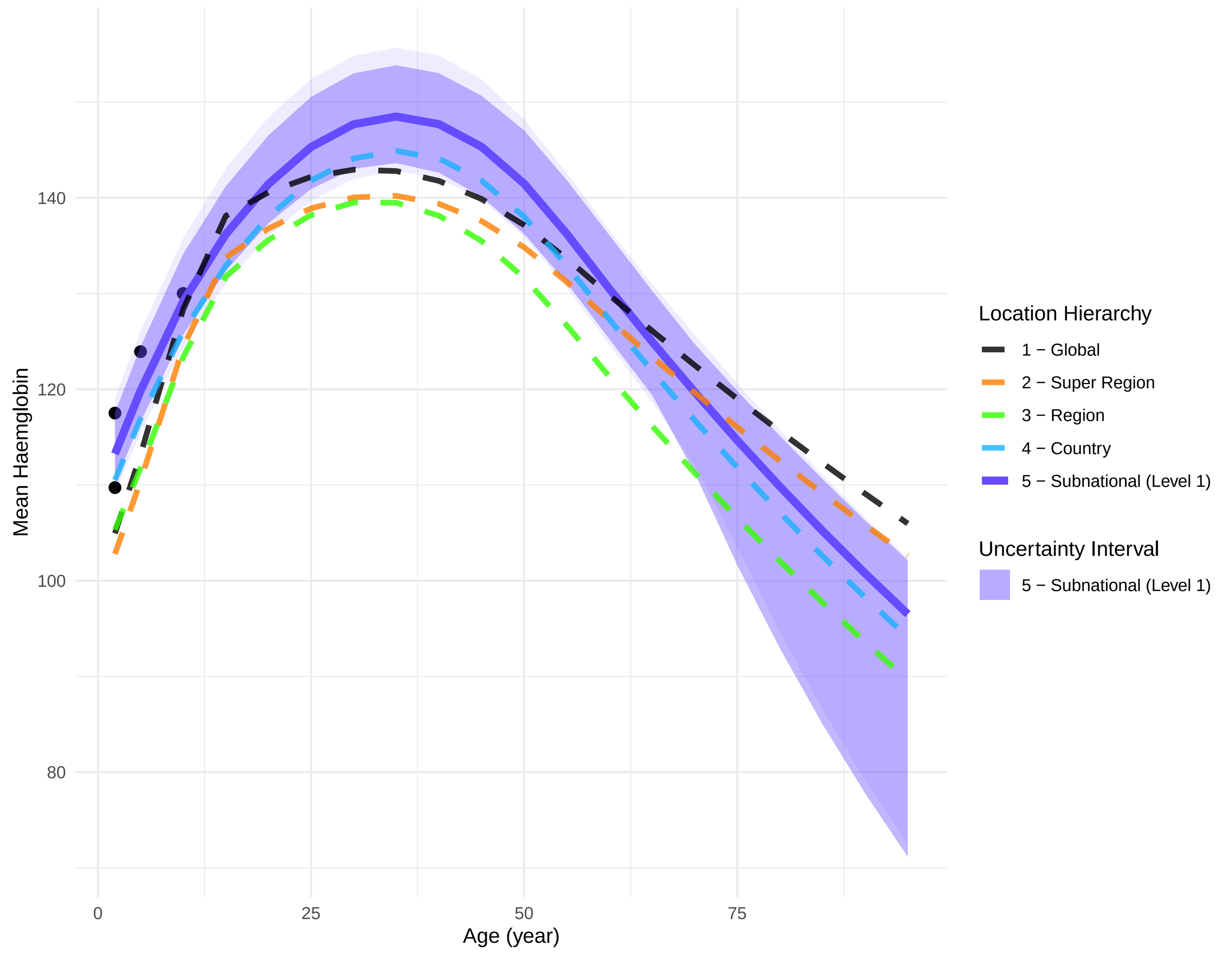

MR−BRT Age/Sex Fits − Males 2+ Years Old − Mean Haemglobin − Tana River
WHO−2024 Adjustment Method

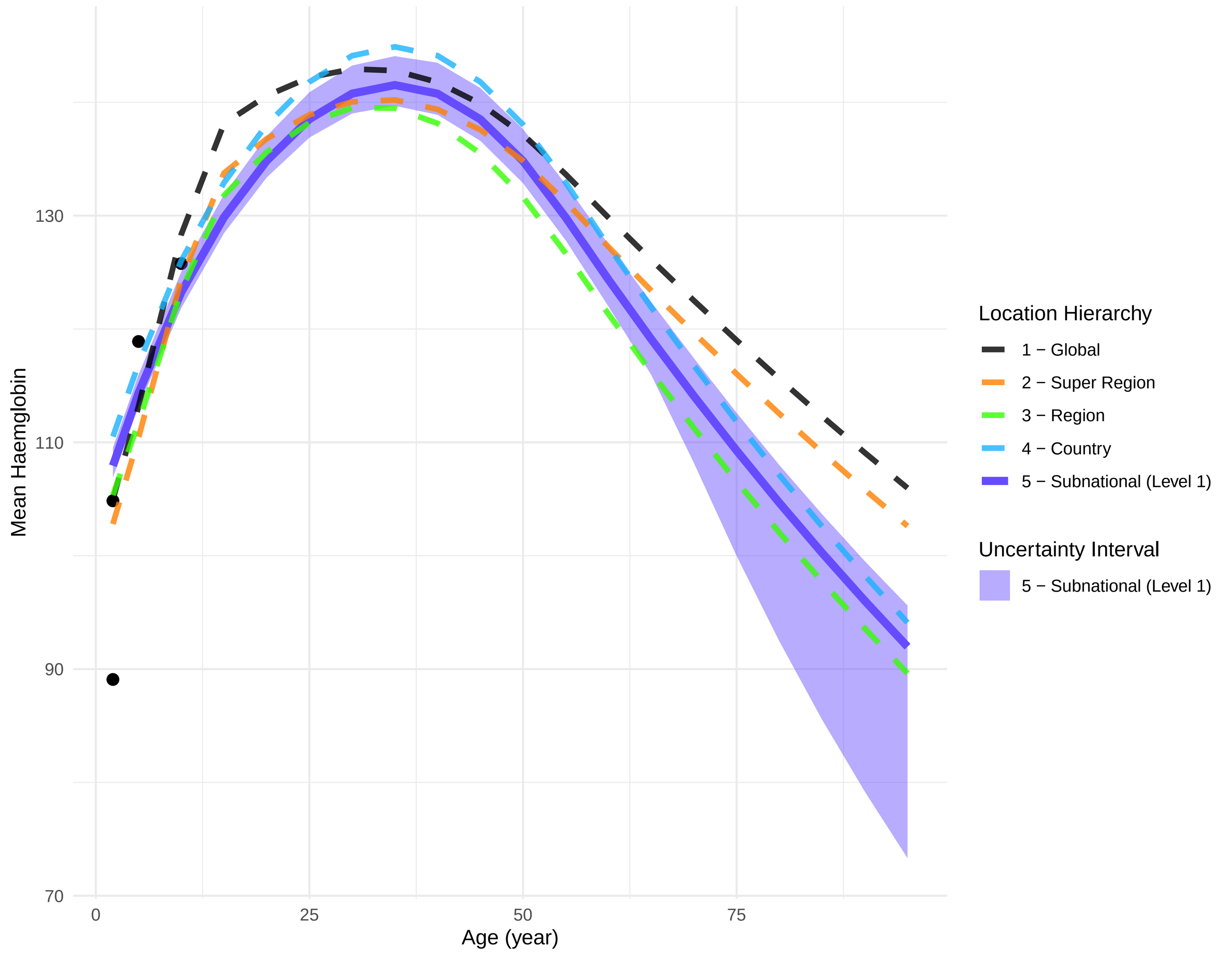

MR−BRT Age/Sex Fits − Males 2+ Years Old − Mean Haemglobin − Tharaka Nithi
WHO−2024 Adjustment Method

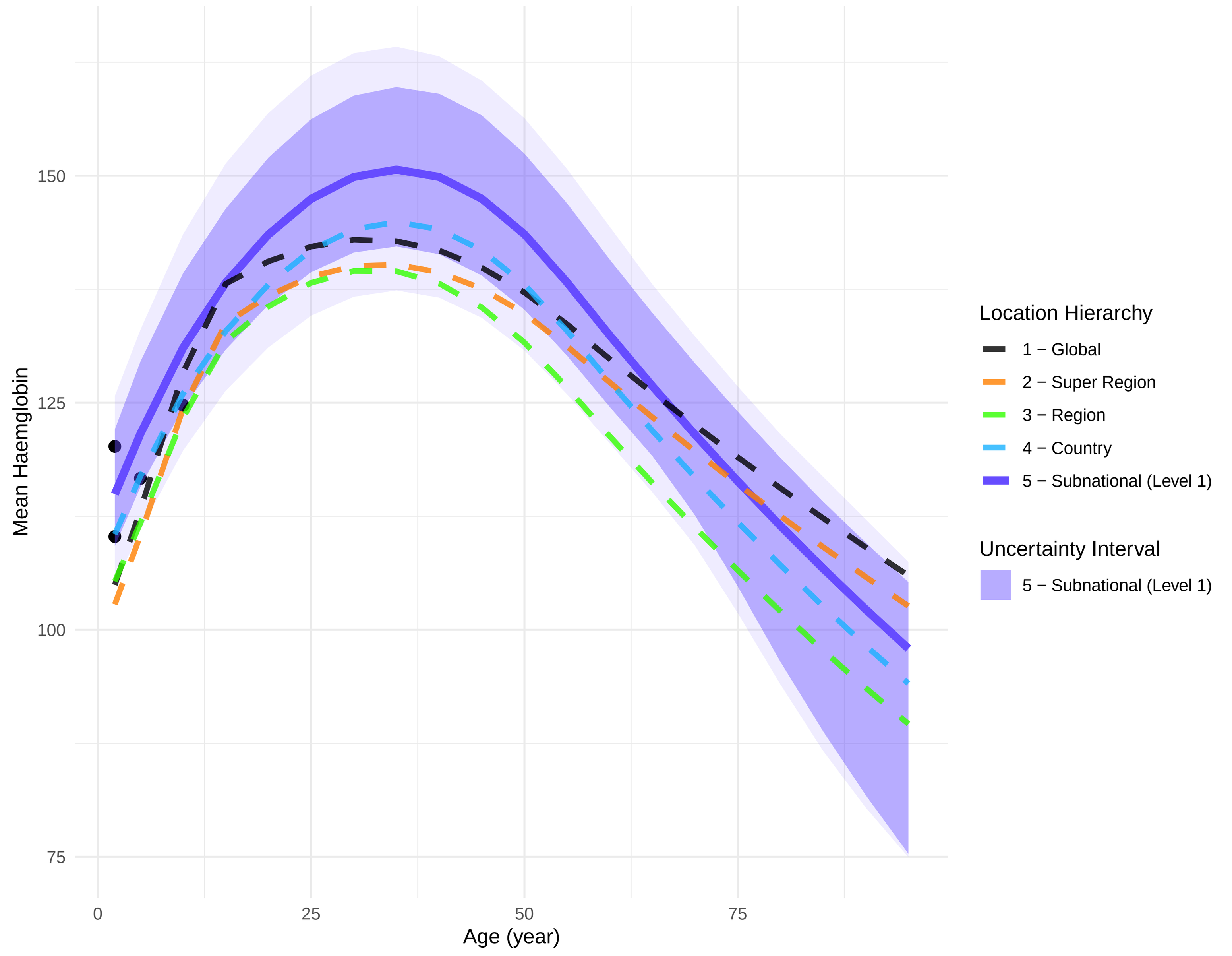

MR−BRT Age/Sex Fits − Males 2+ Years Old − Mean Haemglobin − Trans Nzoia
WHO−2024 Adjustment Method

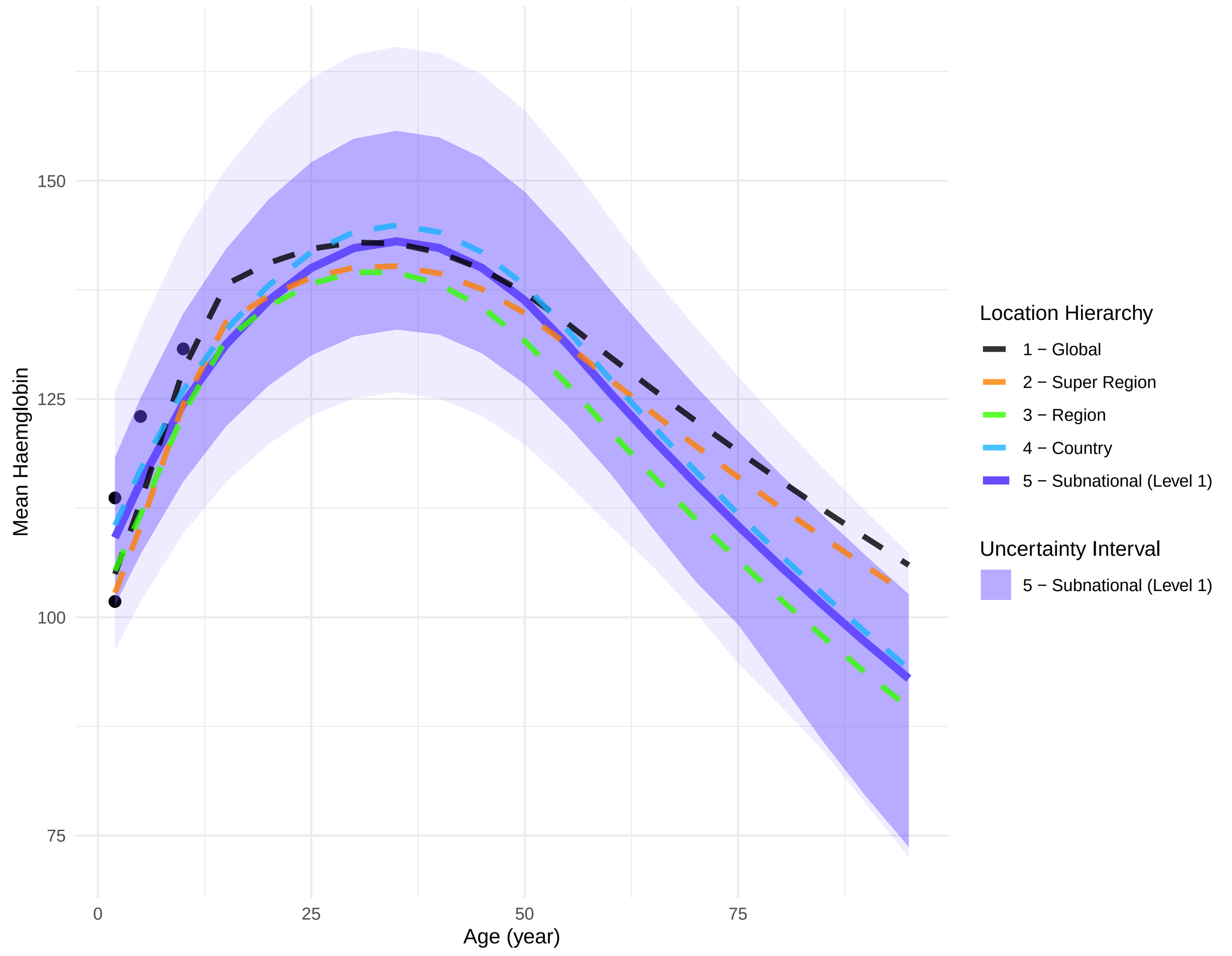

MR−BRT Age/Sex Fits − Males 2+ Years Old − Mean Haemglobin − Turkana
WHO−2024 Adjustment Method

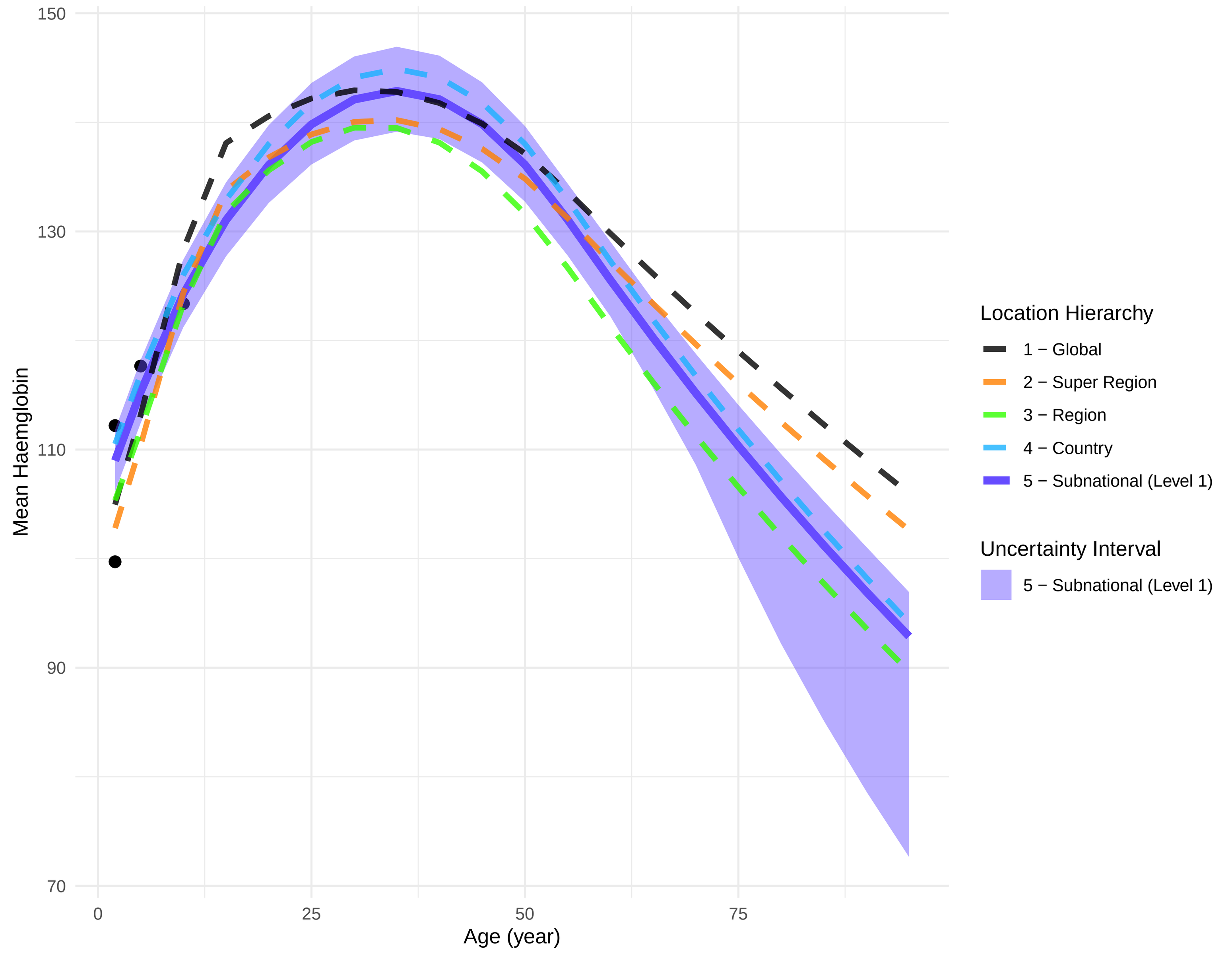

MR−BRT Age/Sex Fits − Males 2+ Years Old − Mean Haemglobin − Uasin Gishu
WHO−2024 Adjustment Method

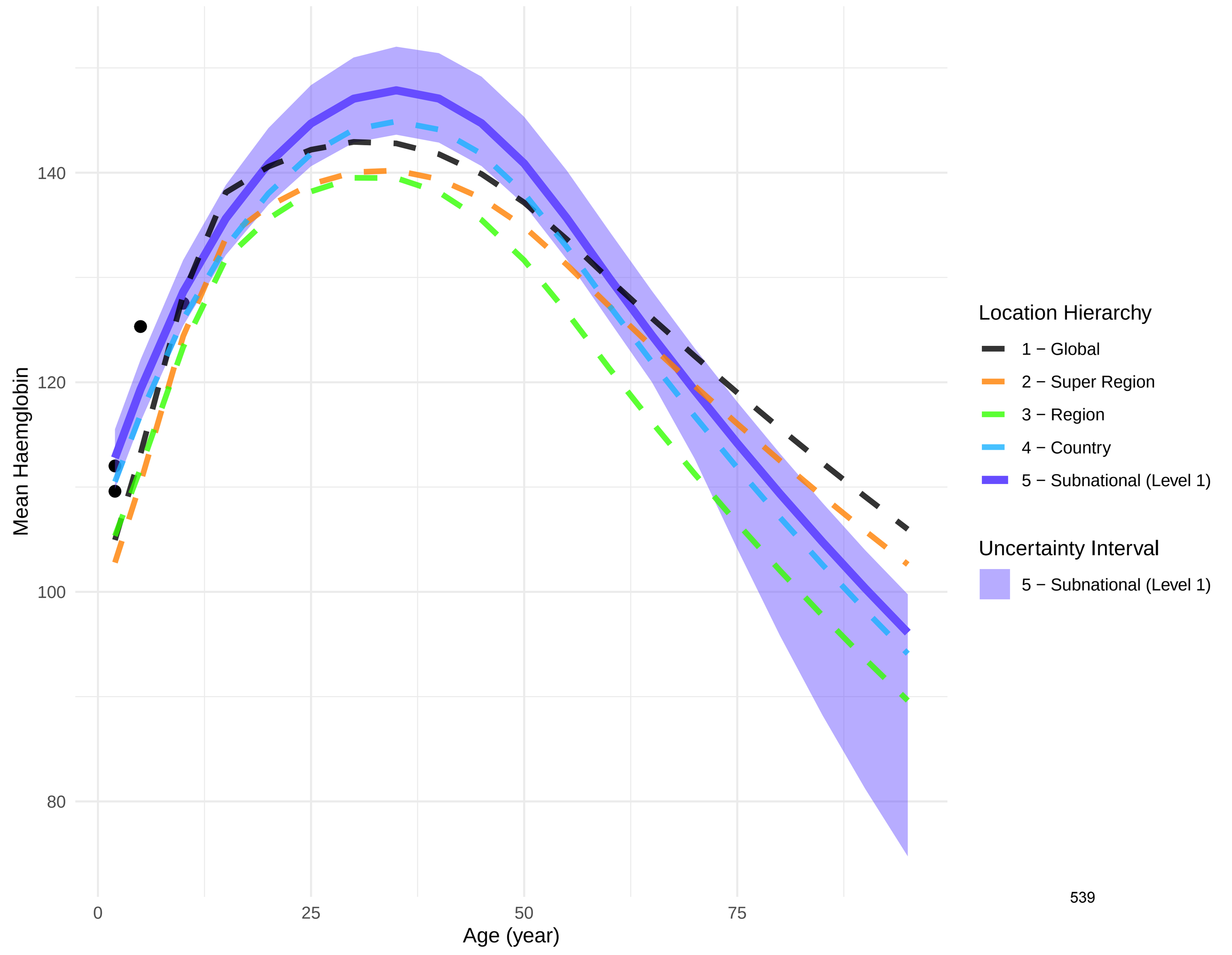

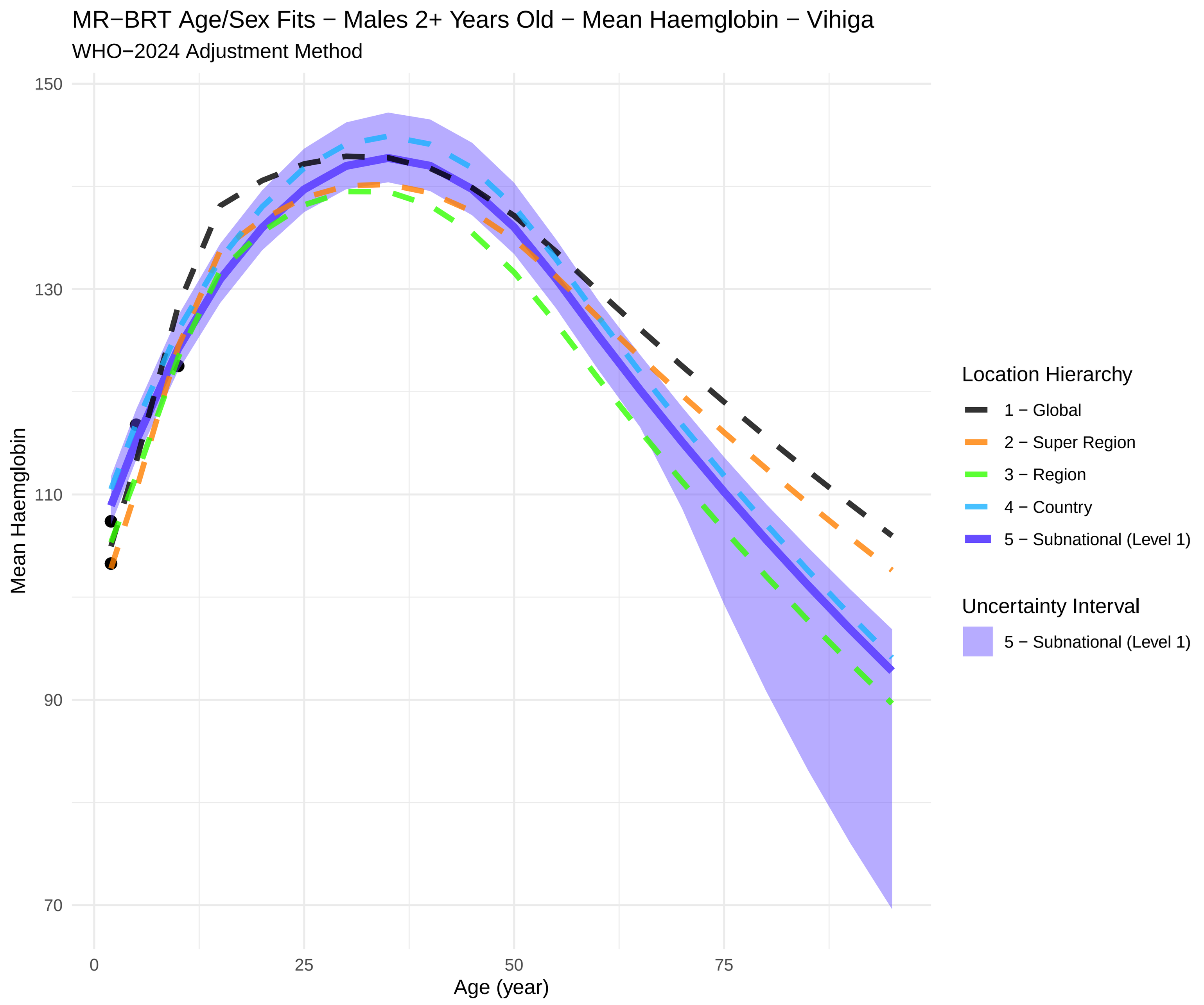
MR−BRT Age/Sex Fits − Males 2+ Years Old − Mean Haemglobin − Vihiga
WHO−2024 Adjustment Method
Mean Haemglobin
Age (year)
Location Hierarchy
1 − Global
2 − Super Region
3 − Region
4 − Country
5 − Subnational (Level 1)
Uncertainty Interval
5 − Subnational (Level 1)

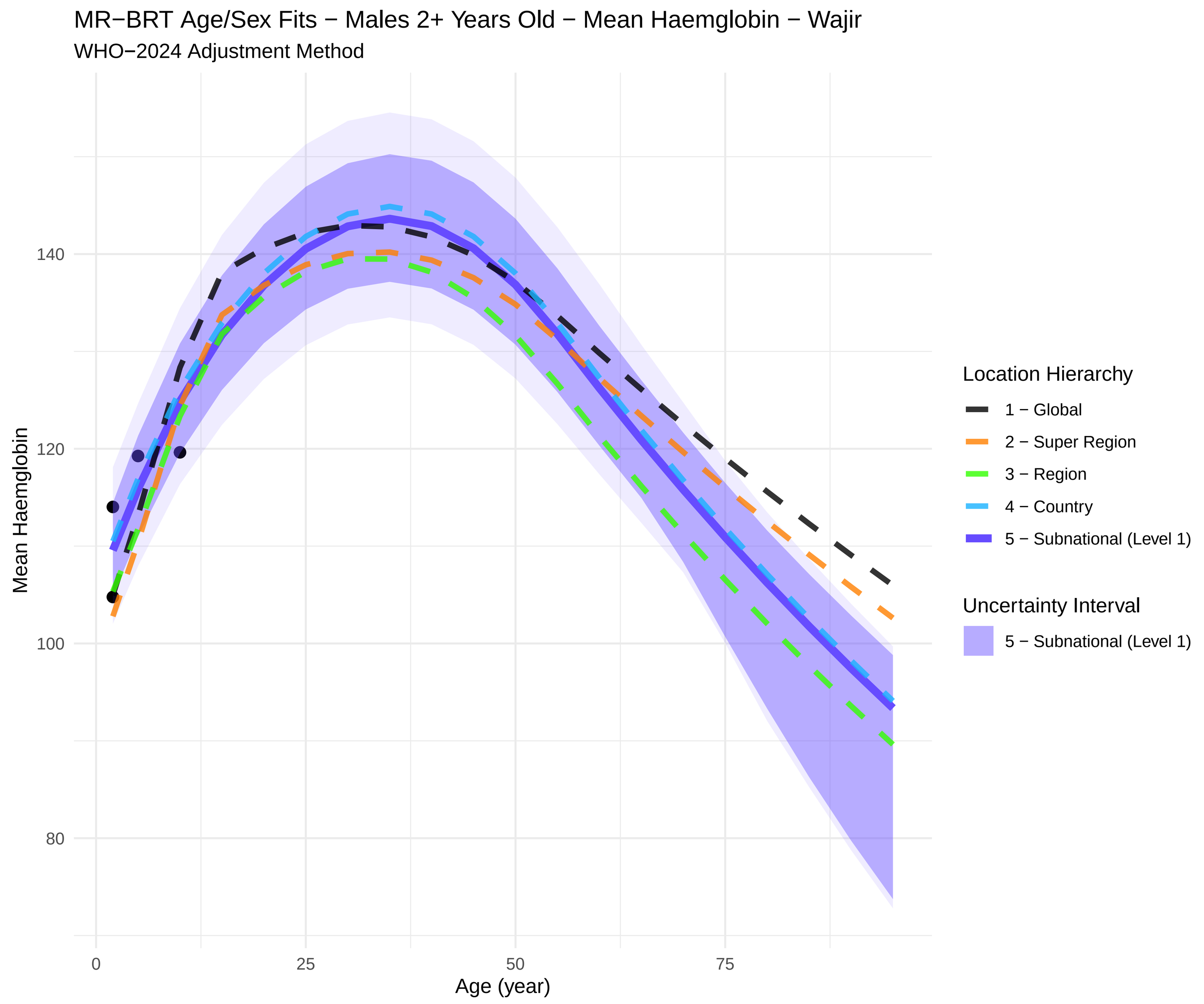
MR−BRT Age/Sex Fits − Males 2+ Years Old − Mean Haemglobin − Wajir
WHO−2024 Adjustment Method
Mean Haemglobin
Age (year)
Location Hierarchy
1 − Global
2 − Super Region
3 − Region
4 − Country
5 − Subnational (Level 1)
Uncertainty Interval
5 − Subnational (Level 1)

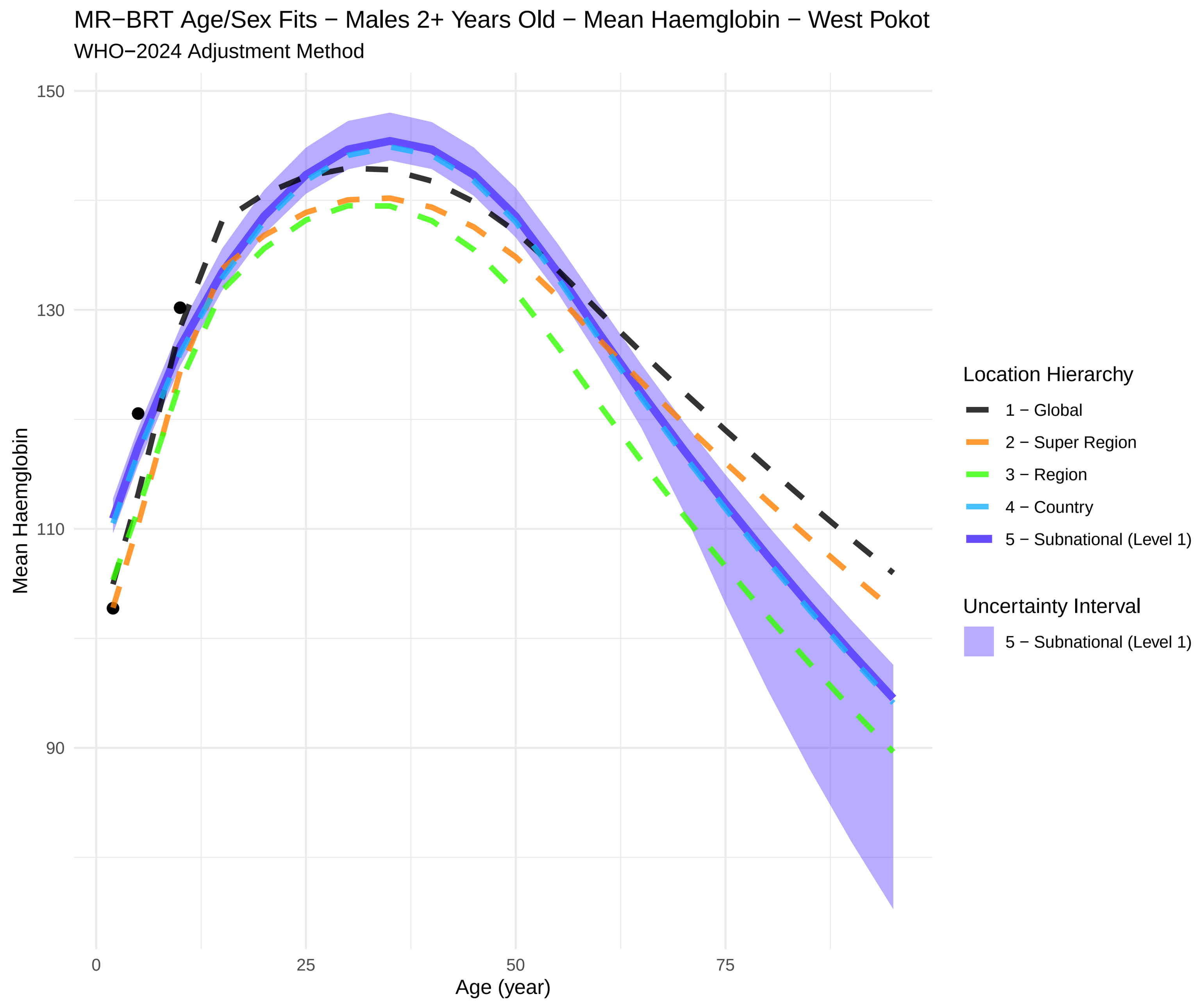
MR−BRT Age/Sex Fits − Males 2+ Years Old − Mean Haemglobin − West Pokot
WHO−2024 Adjustment Method
Mean Haemglobin
Age (year)
Location Hierarchy
1 − Global
2 − Super Region
3 − Region
4 − Country
5 − Subnational (Level 1)
Uncertainty Interval
5 − Subnational (Level 1)

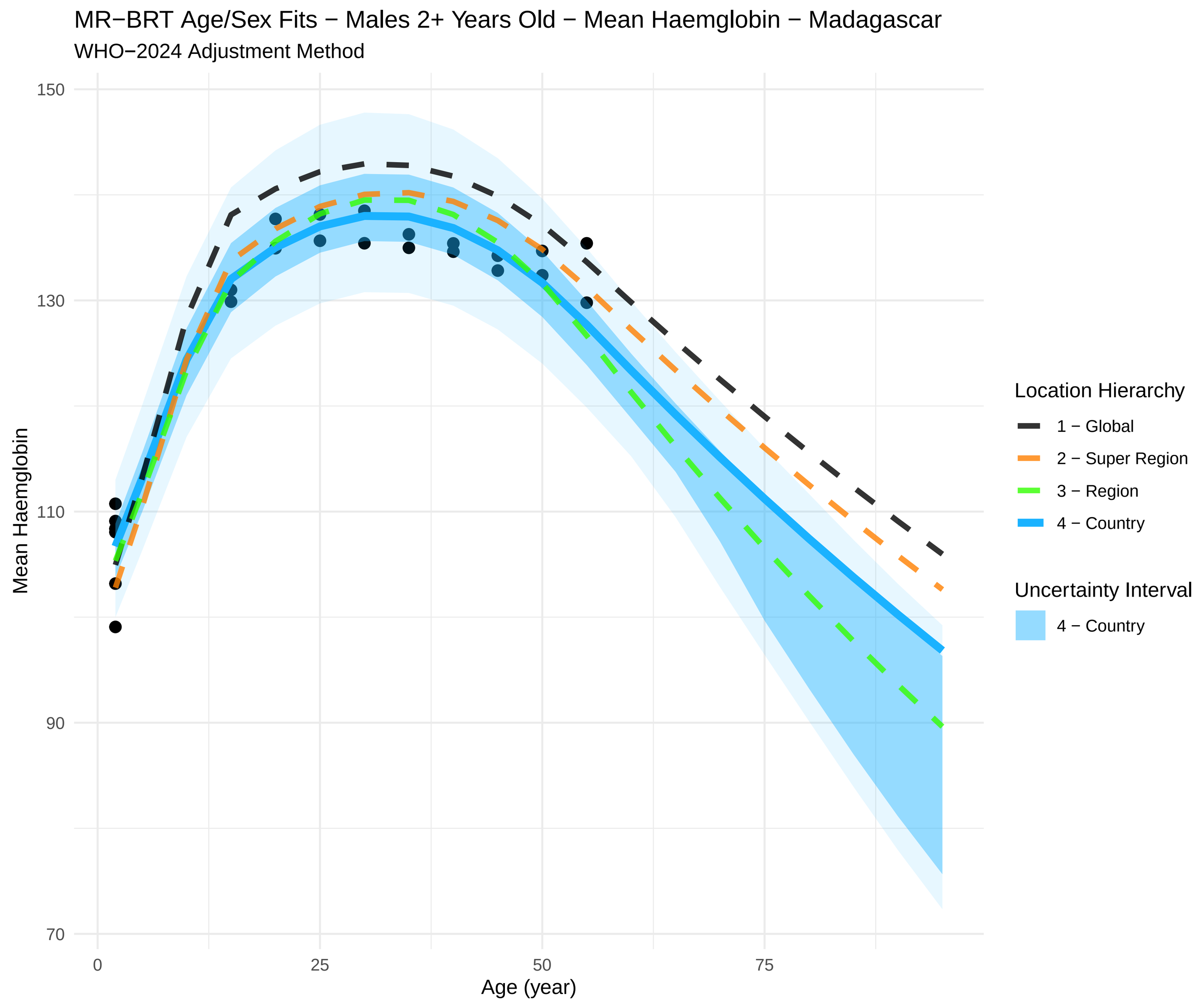
MR−BRT Age/Sex Fits − Males 2+ Years Old − Mean Haemglobin − Madagascar
WHO−2024 Adjustment Method
Mean Haemglobin
Age (year)
Location Hierarchy
1 − Global
2 − Super Region
3 − Region
4 − Country
Uncertainty Interval
4 − Country

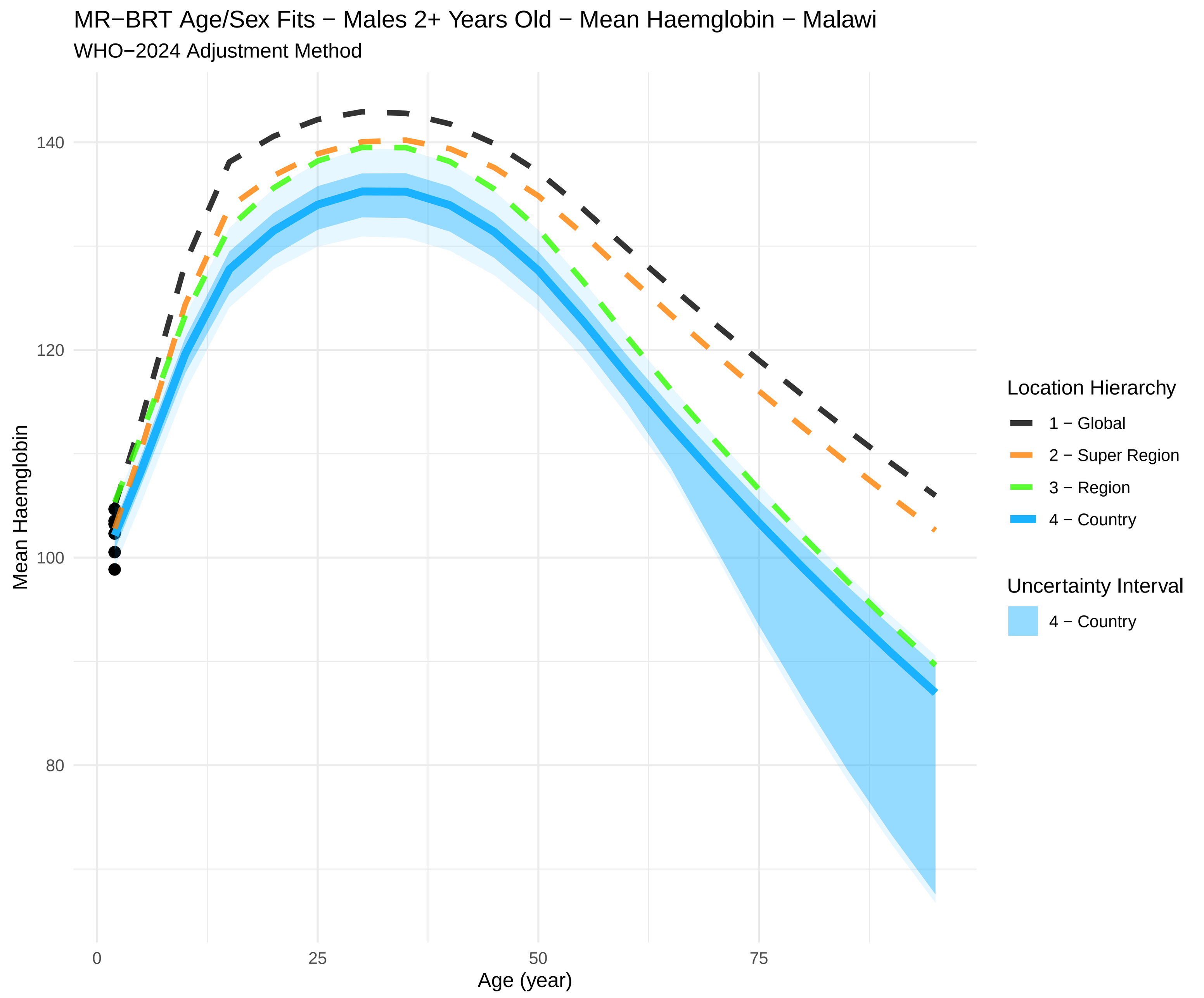
MR−BRT Age/Sex Fits − Males 2+ Years Old − Mean Haemglobin − Malawi
WHO−2024 Adjustment Method
Mean Haemglobin
Age (year)
Location Hierarchy
1 − Global
2 − Super Region
3 − Region
4 − Country
Uncertainty Interval
4 − Country

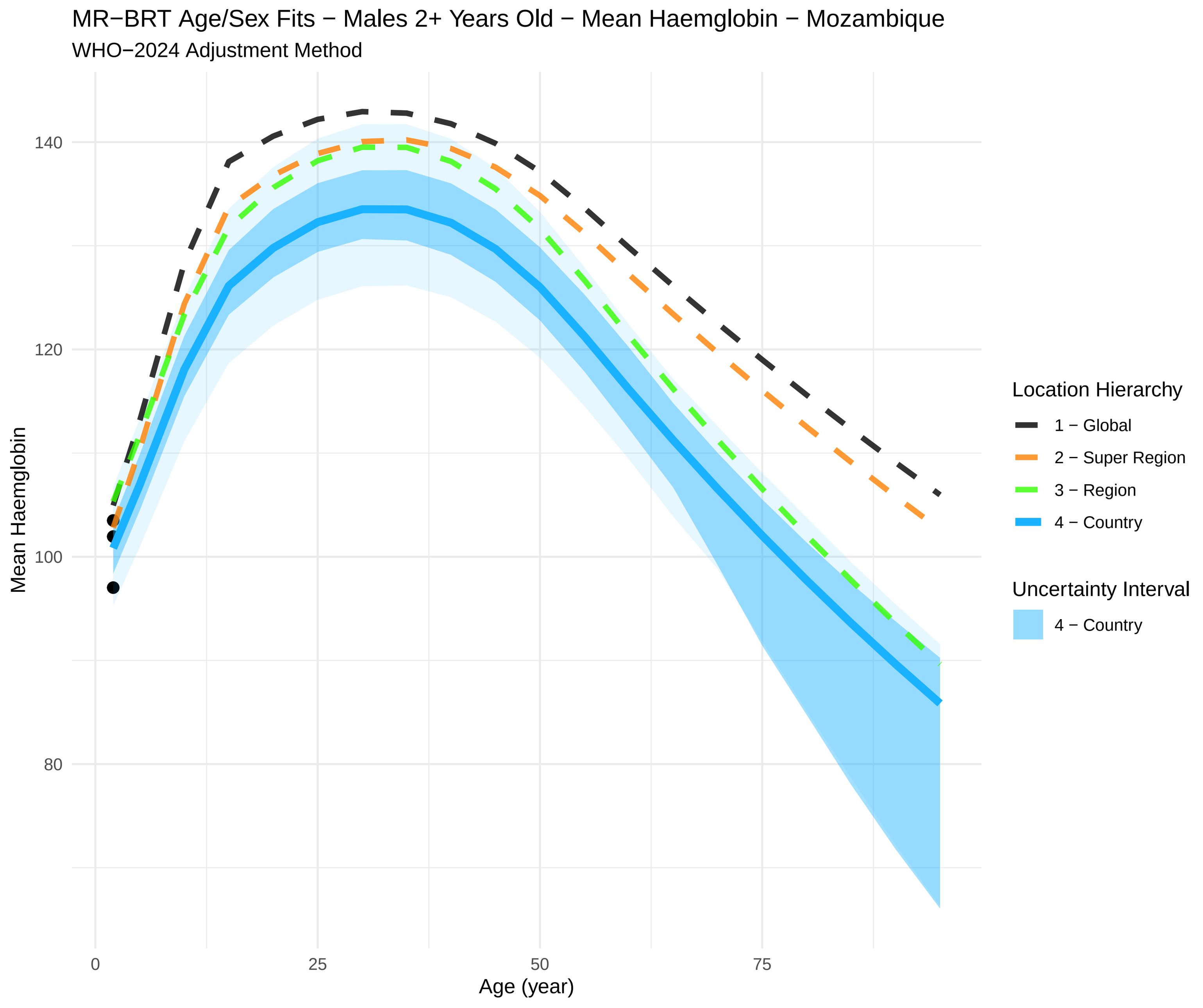
MR−BRT Age/Sex Fits − Males 2+ Years Old − Mean Haemglobin − Mozambique
WHO−2024 Adjustment Method
Mean Haemglobin
Age (year)
Location Hierarchy
1 − Global
2 − Super Region
3 − Region
4 − Country
Uncertainty Interval
4 − Country

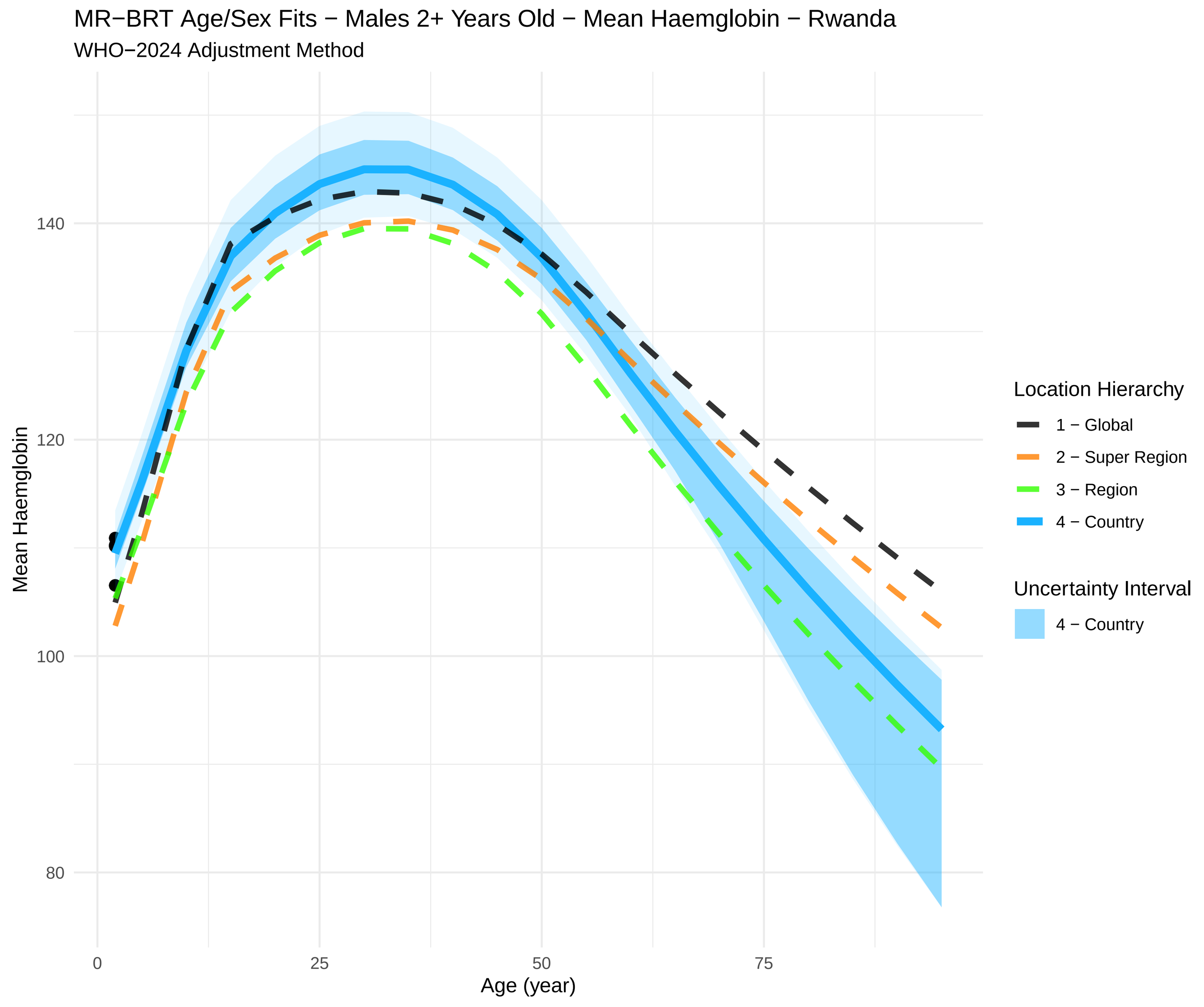
MR−BRT Age/Sex Fits − Males 2+ Years Old − Mean Haemglobin − Rwanda
WHO−2024 Adjustment Method
Mean Haemglobin
Age (year)
Location Hierarchy
1 − Global
2 − Super Region
3 − Region
4 − Country
Uncertainty Interval
4 − Country

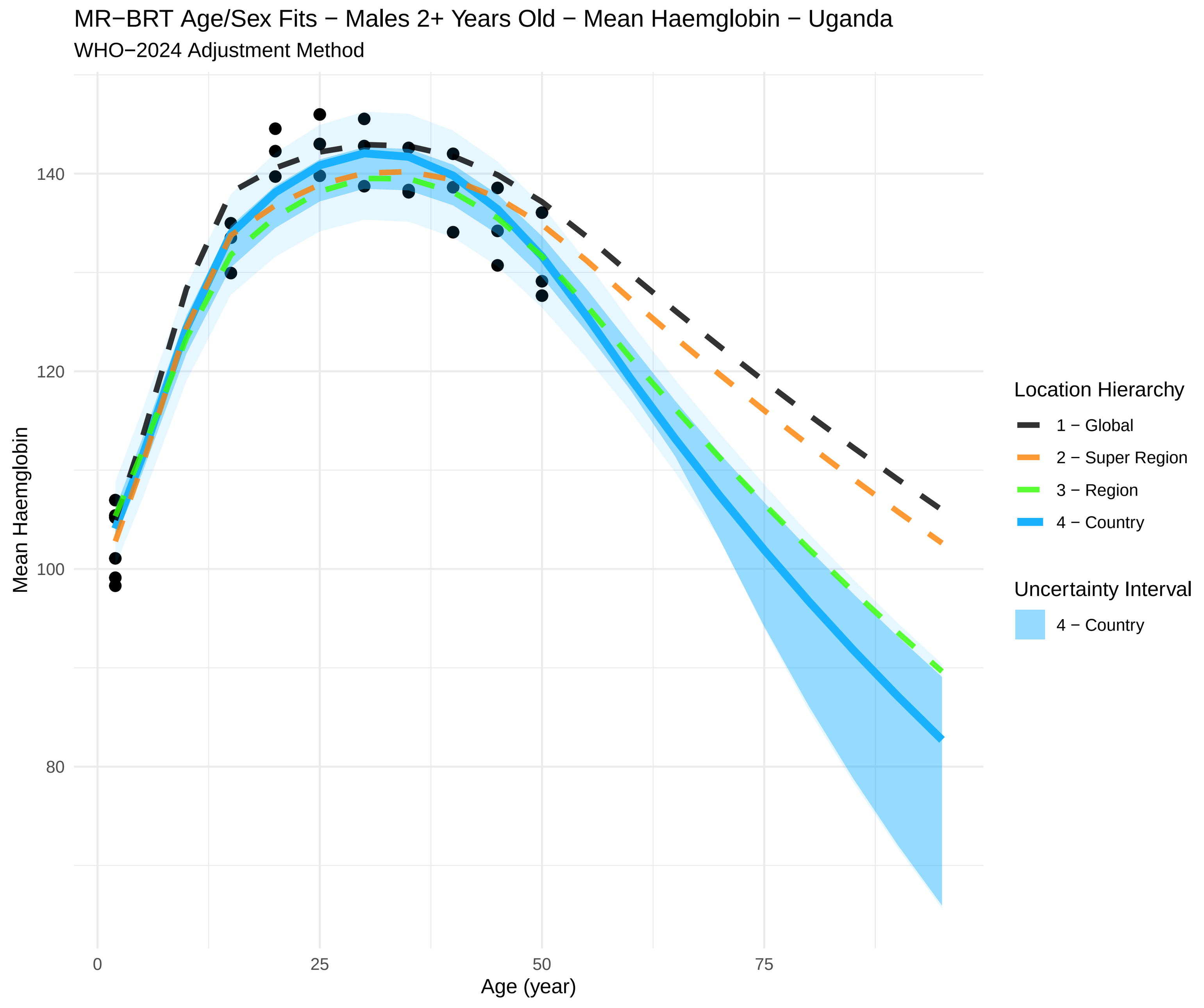
MR−BRT Age/Sex Fits − Males 2+ Years Old − Mean Haemglobin − Uganda
WHO−2024 Adjustment Method
Mean Haemglobin
Age (year)
Location Hierarchy
1 − Global
2 − Super Region
3 − Region
4 − Country
Uncertainty Interval
4 − Country

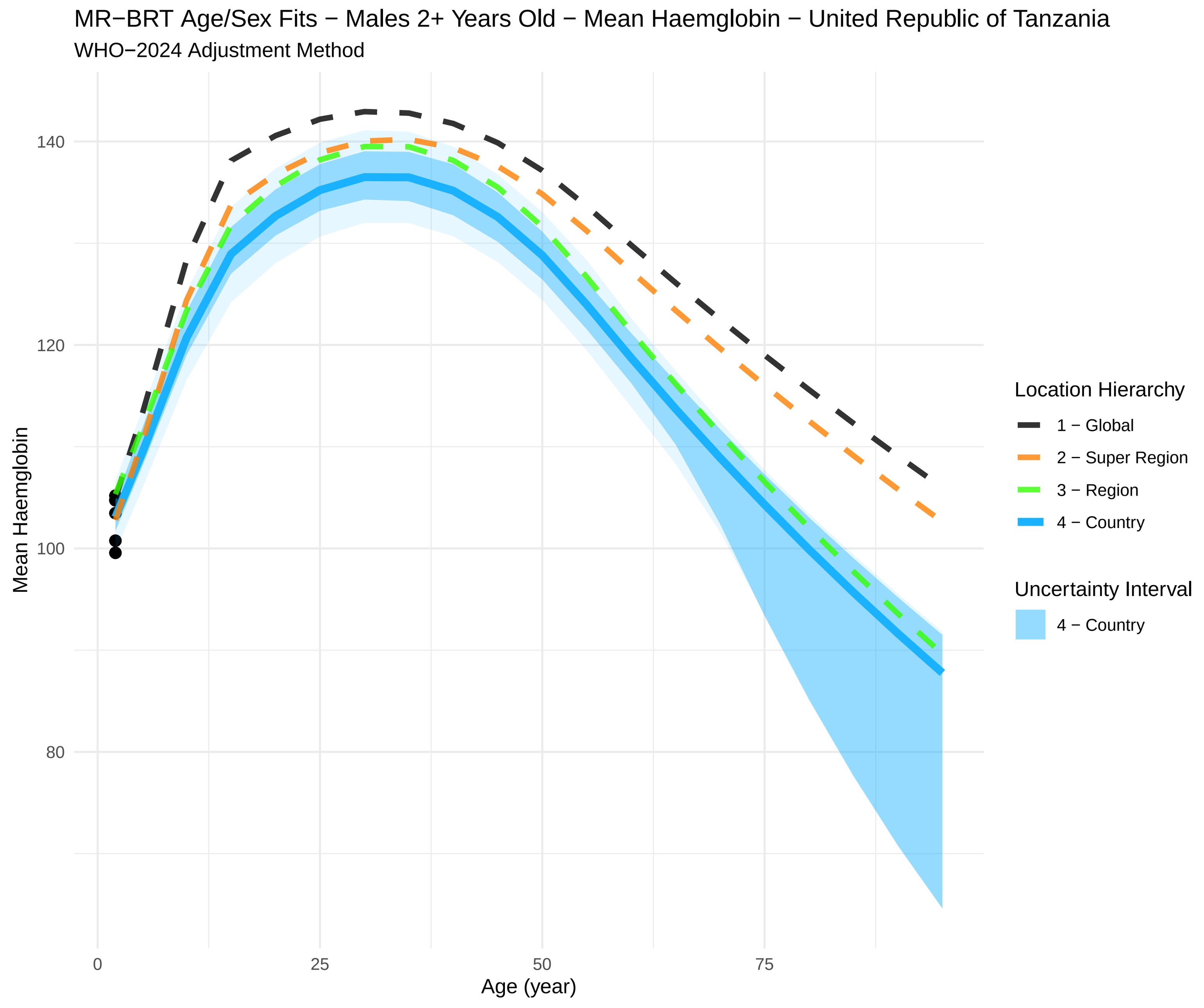
MR−BRT Age/Sex Fits − Males 2+ Years Old − Mean Haemglobin − United Republic of Tanzania
WHO−2024 Adjustment Method
Mean Haemglobin
Age (year)
Location Hierarchy
1 − Global
2 − Super Region
3 − Region
4 − Country
Uncertainty Interval
4 − Country

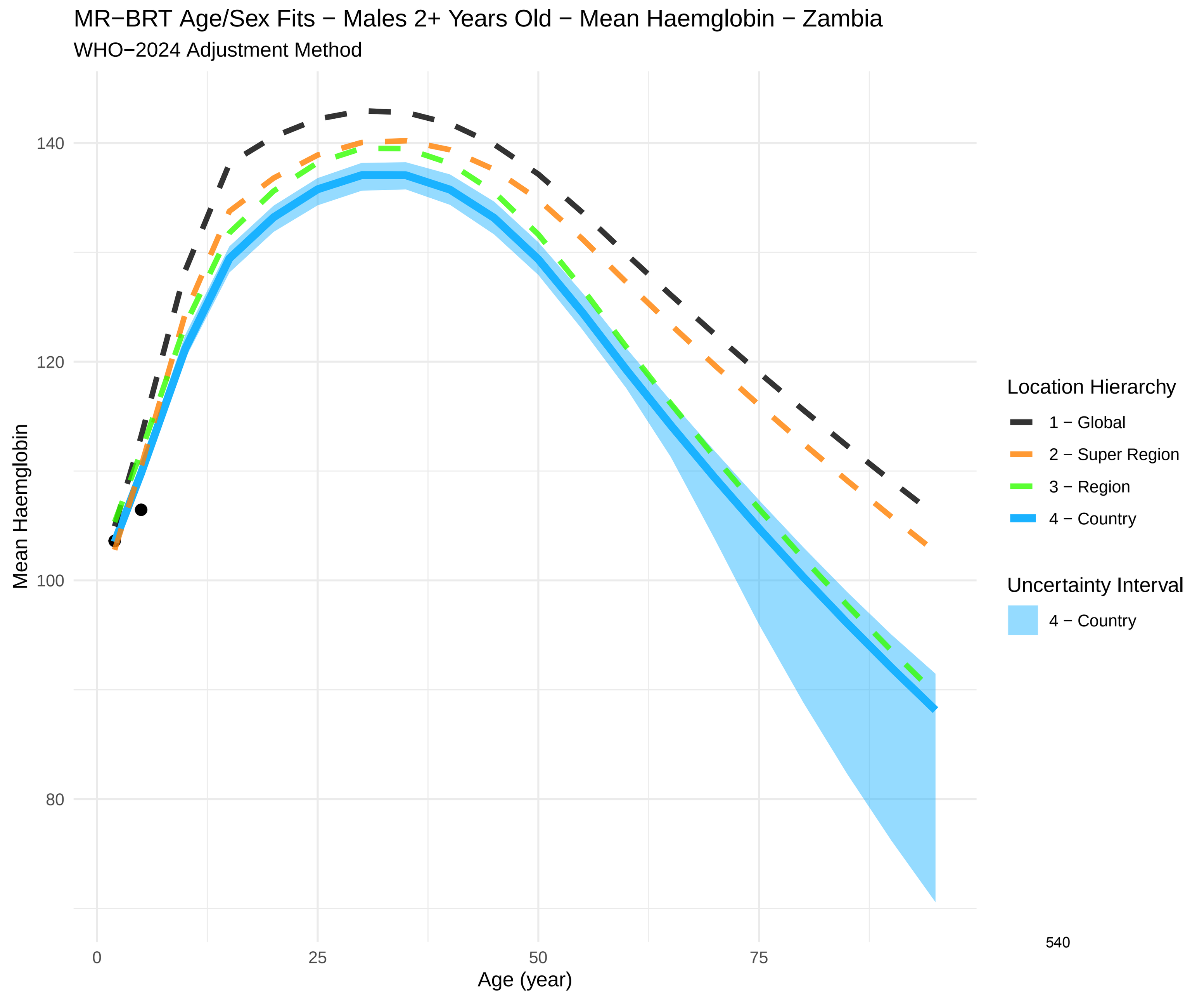
MR−BRT Age/Sex Fits − Males 2+ Years Old − Mean Haemglobin − Zambia
WHO−2024 Adjustment Method
Mean Haemglobin
Age (year)
Location Hierarchy
1 − Global
2 − Super Region
3 − Region
4 − Country
Uncertainty Interval
4 − Country

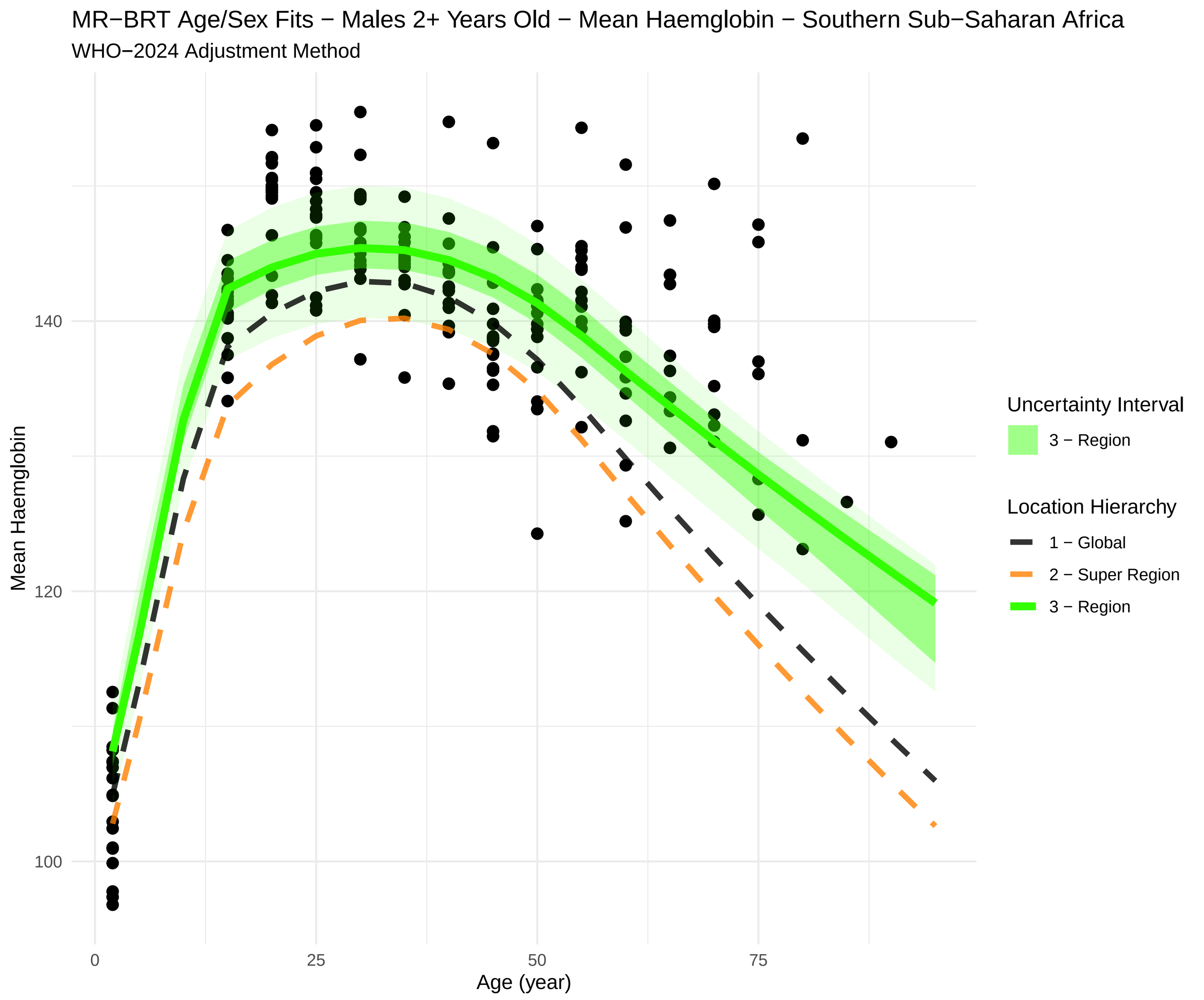
MR−BRT Age/Sex Fits − Males 2+ Years Old − Mean Haemoglobin − Southern Sub−Saharan Africa
WHO−2024 Adjustment Method
Mean Haemoglobin
Age (year)
Uncertainty Interval
3 − Region
Location Hierarchy
1 − Global
2 − Super Region
3 − Region

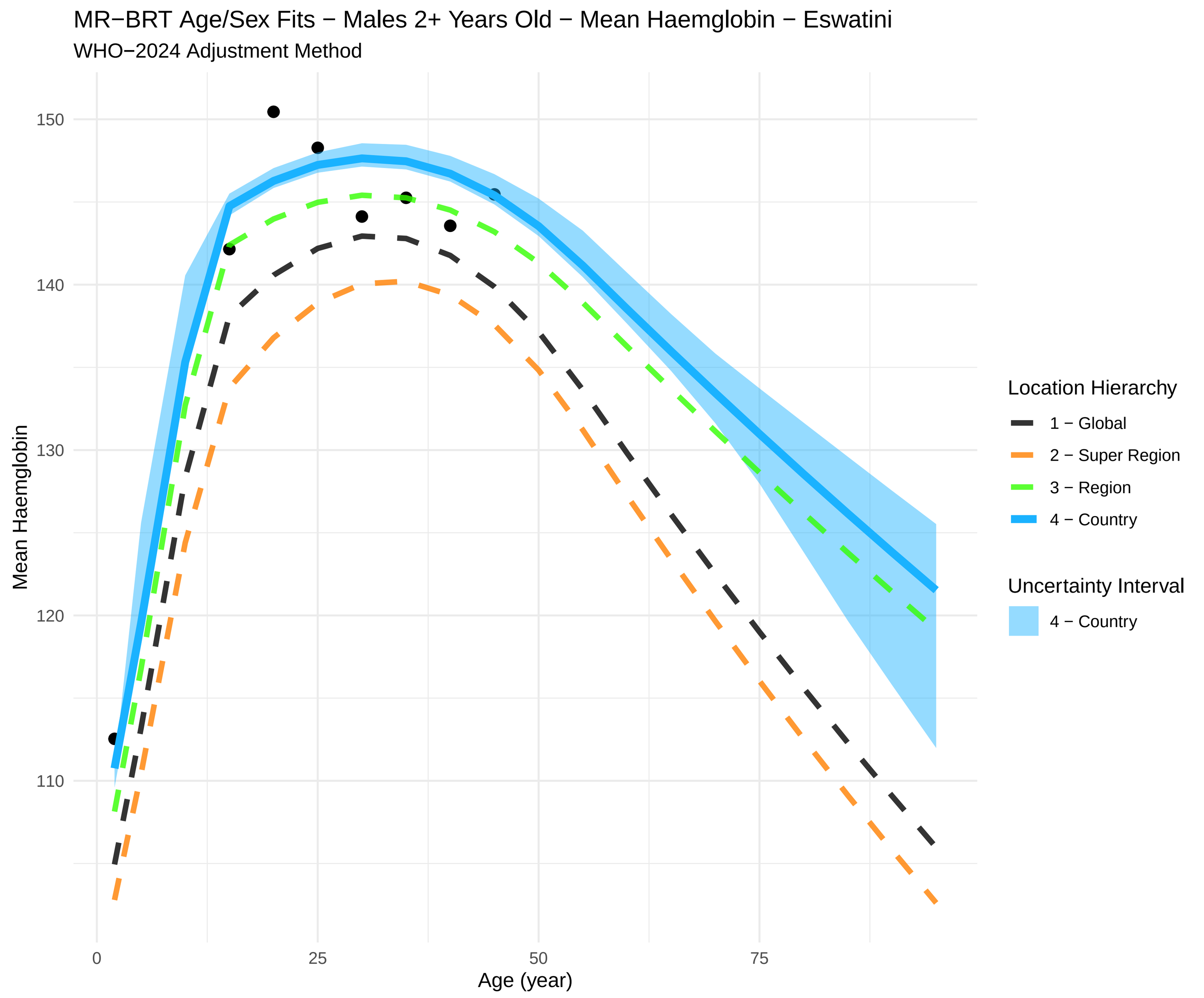
MR−BRT Age/Sex Fits − Males 2+ Years Old − Mean Haemoglobin − Eswatini
WHO−2024 Adjustment Method
Mean Haemoglobin
Age (year)
Location Hierarchy
1 − Global
2 − Super Region
3 − Region
4 − Country
Uncertainty Interval
4 − Country

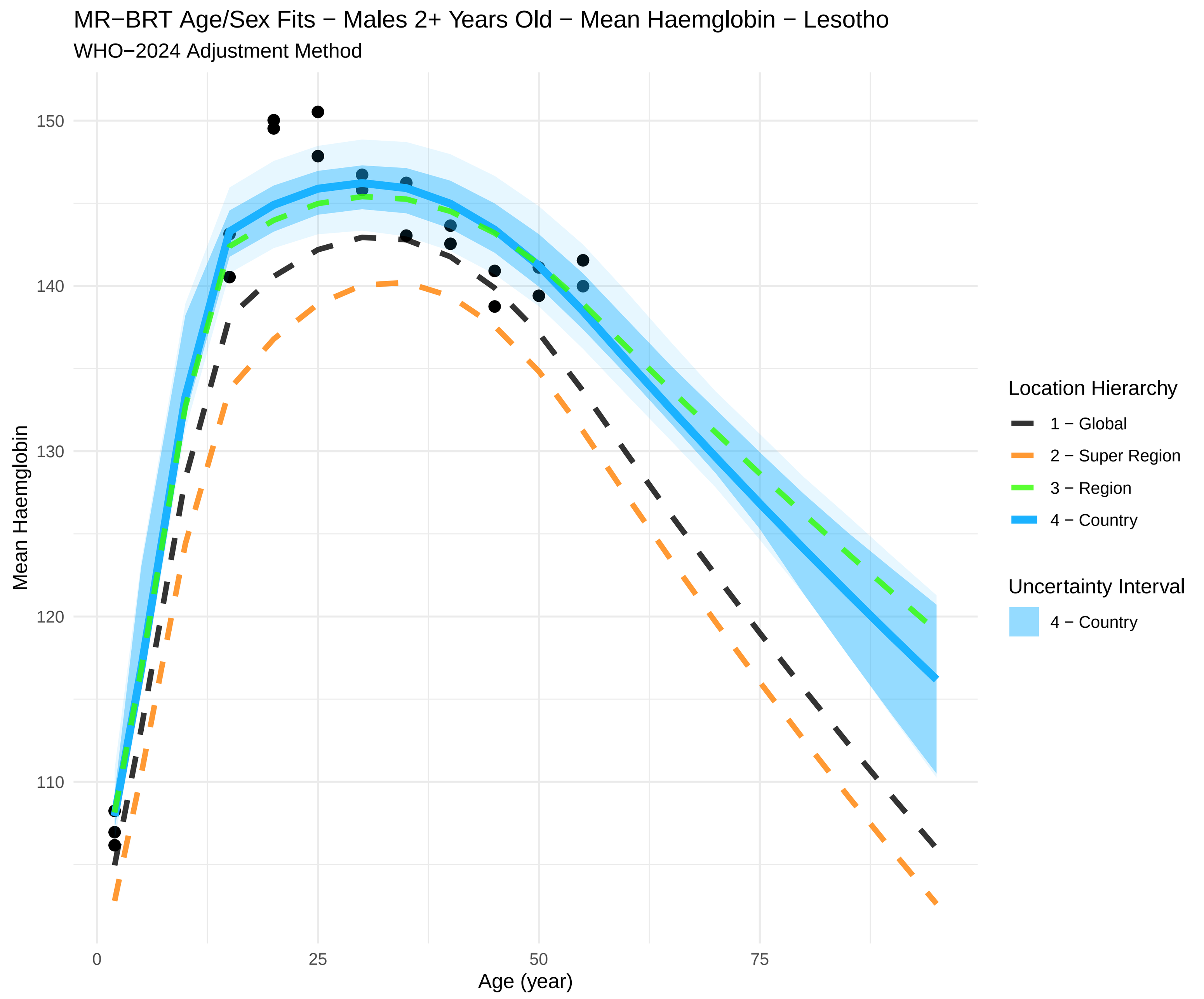
MR−BRT Age/Sex Fits − Males 2+ Years Old − Mean Haemoglobin − Lesotho
WHO−2024 Adjustment Method
Mean Haemoglobin
Age (year)
Location Hierarchy
1 − Global
2 − Super Region
3 − Region
4 − Country
Uncertainty Interval
4 − Country

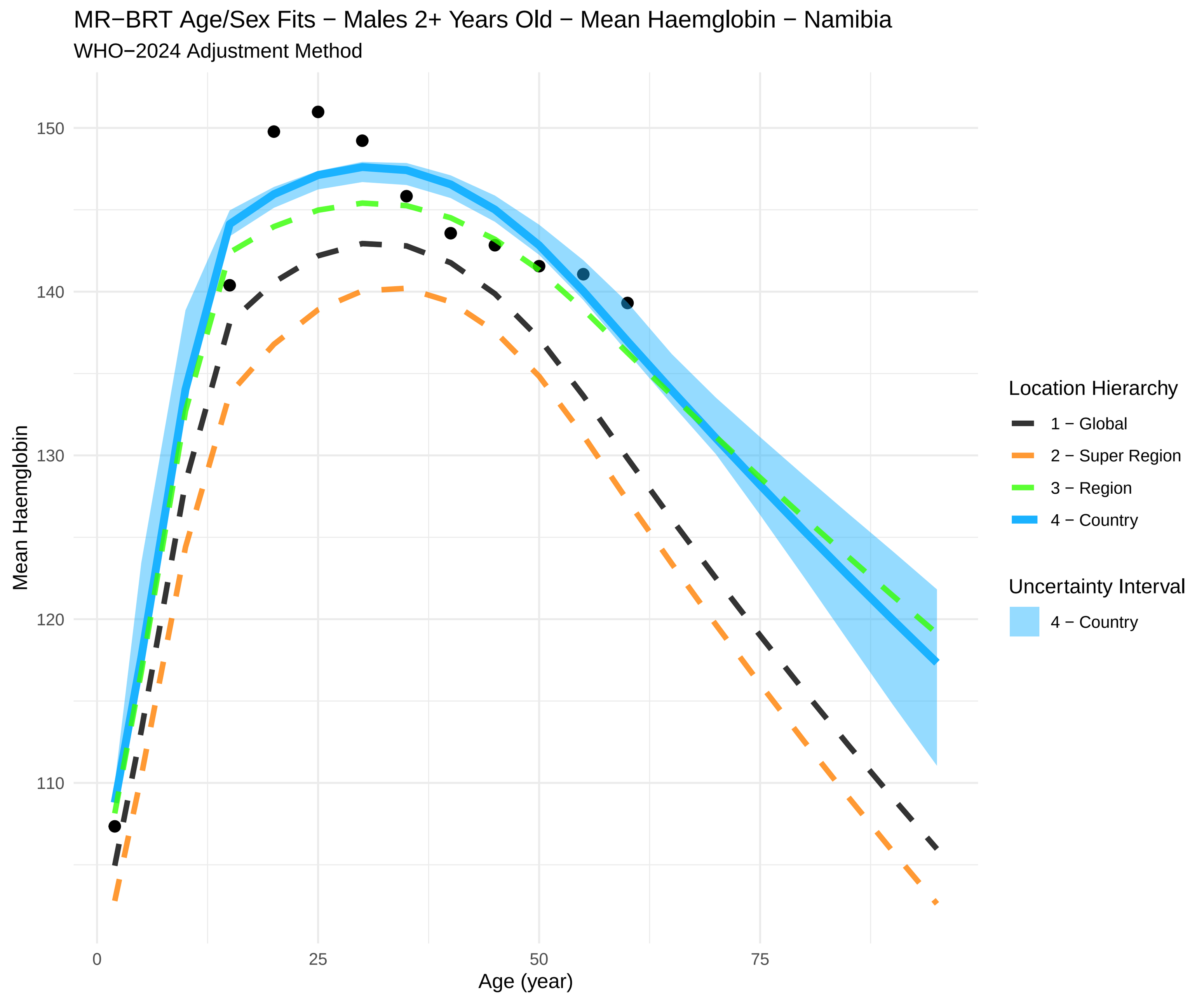
MR−BRT Age/Sex Fits − Males 2+ Years Old − Mean Haemoglobin − Namibia
WHO−2024 Adjustment Method
Mean Haemoglobin
Age (year)
Location Hierarchy
1 − Global
2 − Super Region
3 − Region
4 − Country
Uncertainty Interval
4 − Country

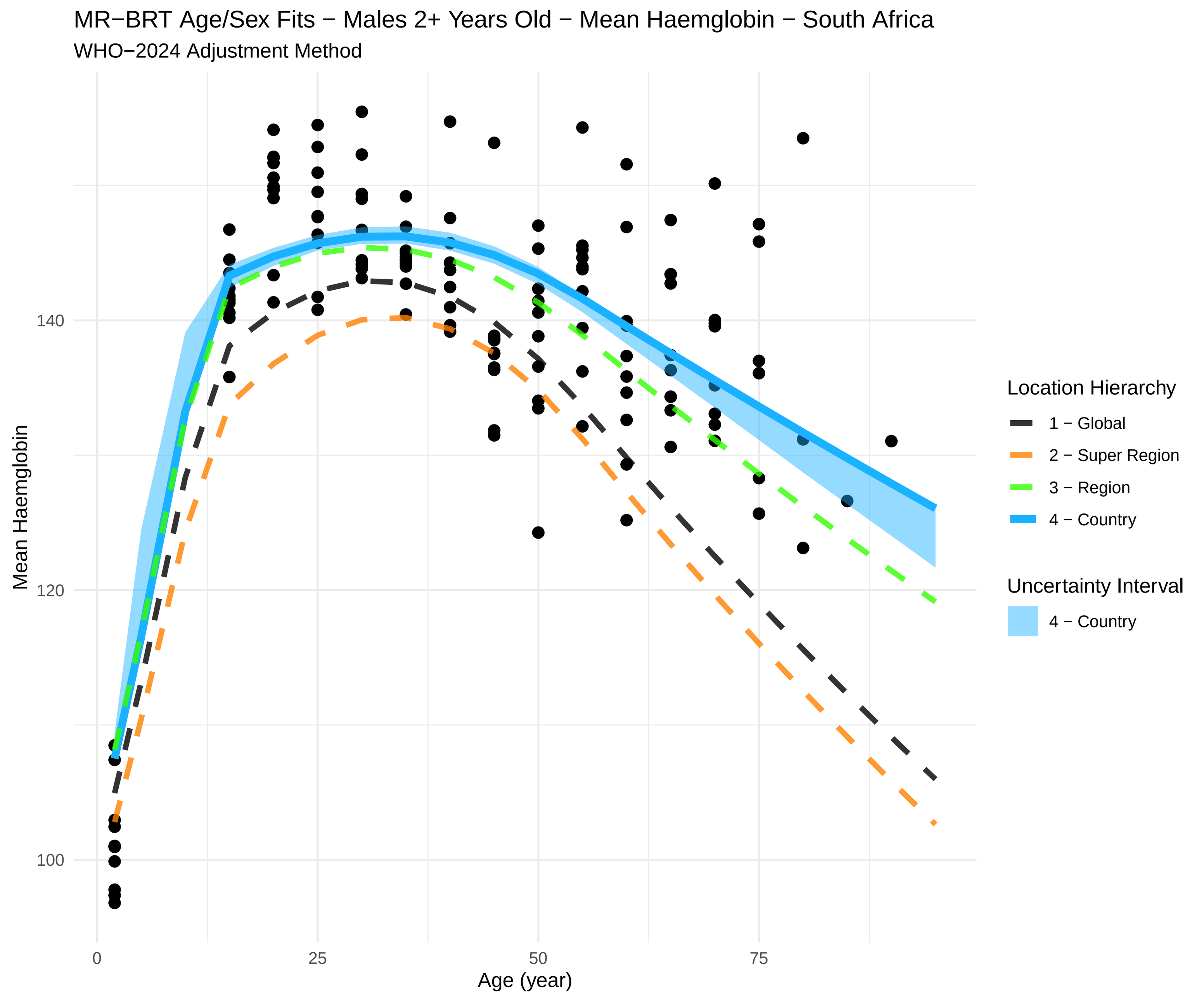
MR−BRT Age/Sex Fits − Males 2+ Years Old − Mean Haemoglobin − South Africa
WHO−2024 Adjustment Method
Mean Haemoglobin
Age (year)
Location Hierarchy
1 − Global
2 − Super Region
3 − Region
4 − Country
Uncertainty Interval
4 − Country

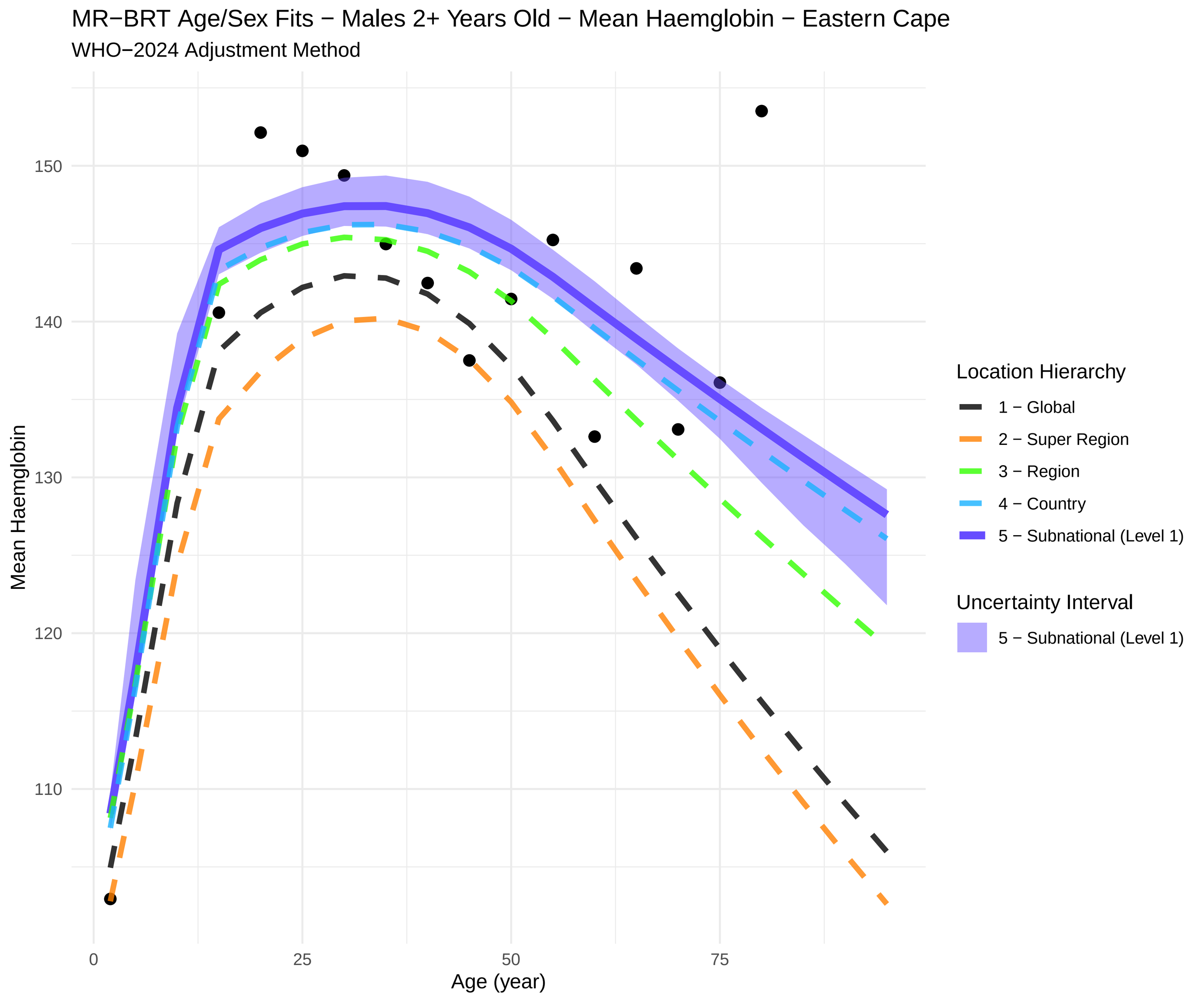
MR−BRT Age/Sex Fits − Males 2+ Years Old − Mean Haemoglobin − Eastern Cape
WHO−2024 Adjustment Method
Mean Haemoglobin
Age (year)
Location Hierarchy
1 − Global
2 − Super Region
3 − Region
4 − Country
5 − Subnational (Level 1)
Uncertainty Interval
5 − Subnational (Level 1)

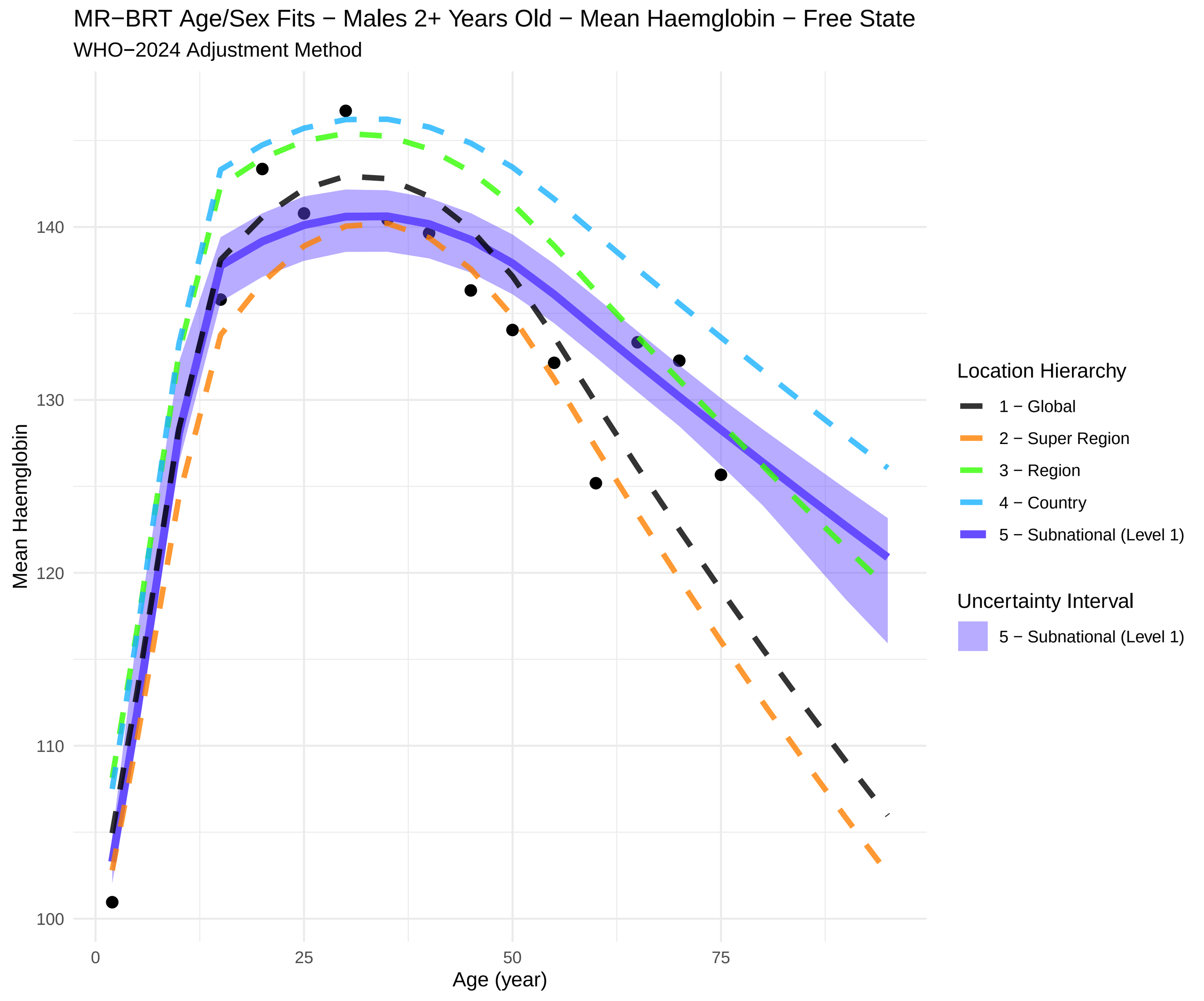
MR−BRT Age/Sex Fits − Males 2+ Years Old − Mean Haemoglobin − Free State
WHO−2024 Adjustment Method
Mean Haemoglobin
Age (year)
Location Hierarchy
1 − Global
2 − Super Region
3 − Region
4 − Country
5 − Subnational (Level 1)
Uncertainty Interval
5 − Subnational (Level 1)

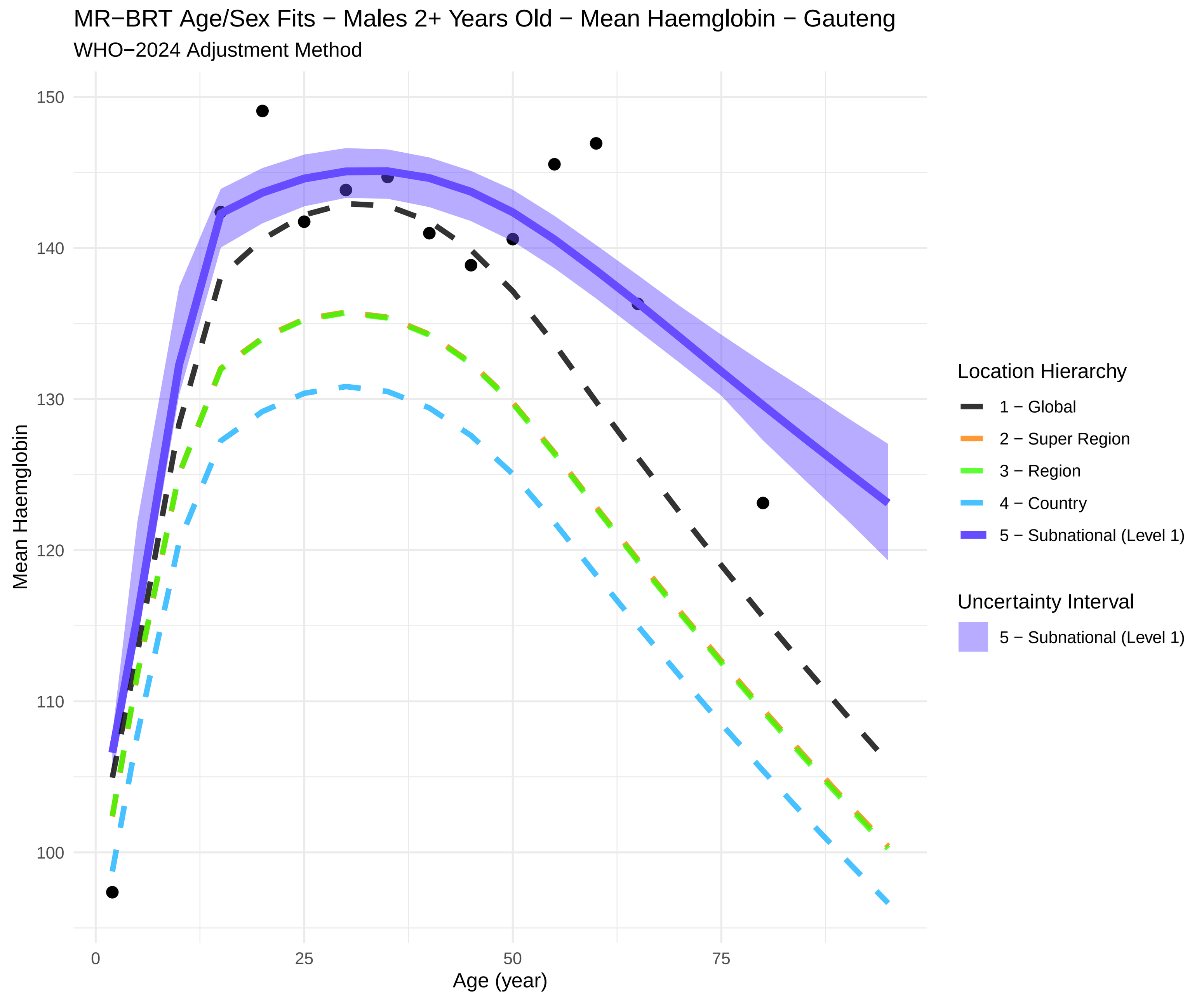
MR−BRT Age/Sex Fits − Males 2+ Years Old − Mean Haemoglobin − Gauteng
WHO−2024 Adjustment Method
Mean Haemoglobin
Age (year)
Location Hierarchy
1 − Global
2 − Super Region
3 − Region
4 − Country
5 − Subnational (Level 1)
Uncertainty Interval
5 − Subnational (Level 1)

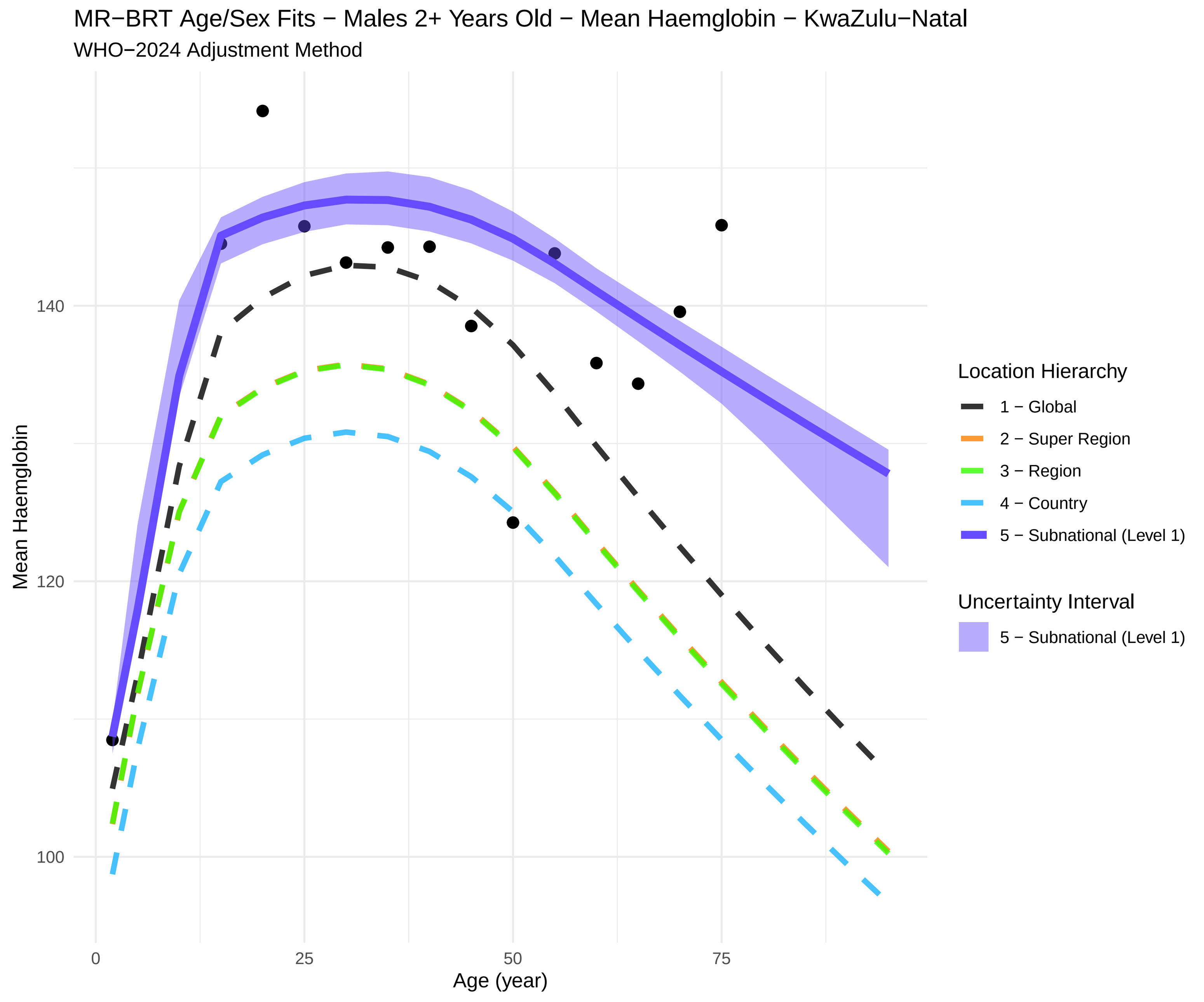
MR−BRT Age/Sex Fits − Males 2+ Years Old − Mean Haemoglobin − KwaZulu−Natal
WHO−2024 Adjustment Method
Mean Haemoglobin
Age (year)
Location Hierarchy
1 − Global
2 − Super Region
3 − Region
4 − Country
5 − Subnational (Level 1)
Uncertainty Interval
5 − Subnational (Level 1)

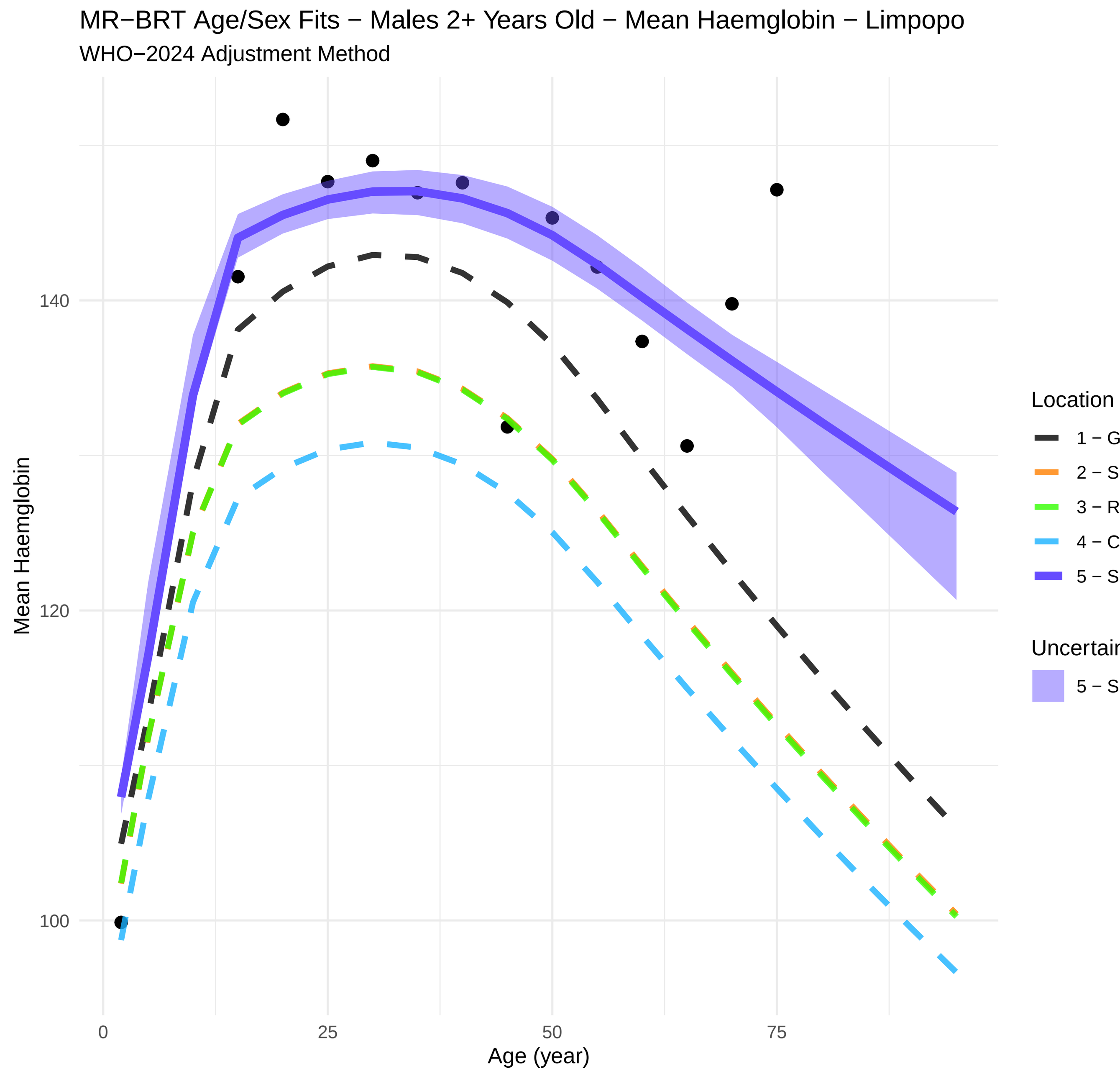
MR−BRT Age/Sex Fits − Males 2+ Years Old − Mean Haemoglobin − Limpopo
WHO−2024 Adjustment Method
Mean Haemoglobin
Age (year)
Location Hierarchy
1 − Global
2 − Super Region
3 − Region
4 − Country
5 − Subnational (Level 1)
Uncertainty Interval
5 − Subnational (Level 1)

MR−BRT Age/Sex Fits − Males 2+ Years Old − Mean Haemglobin − Mpumalanga

WHO−2024 Adjustment Method

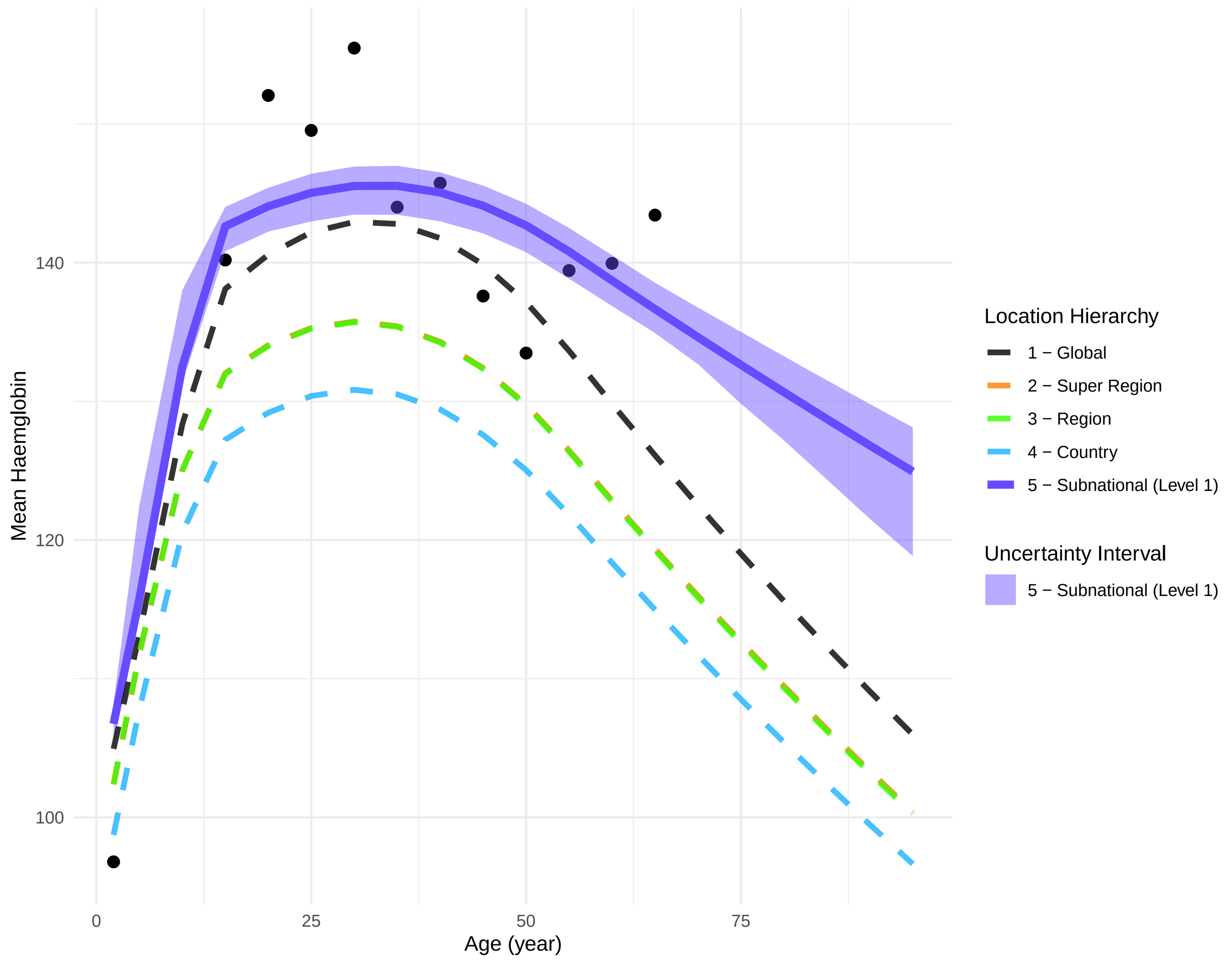

MR−BRT Age/Sex Fits − Males 2+ Years Old − Mean Haemglobin − North−West

WHO−2024 Adjustment Method

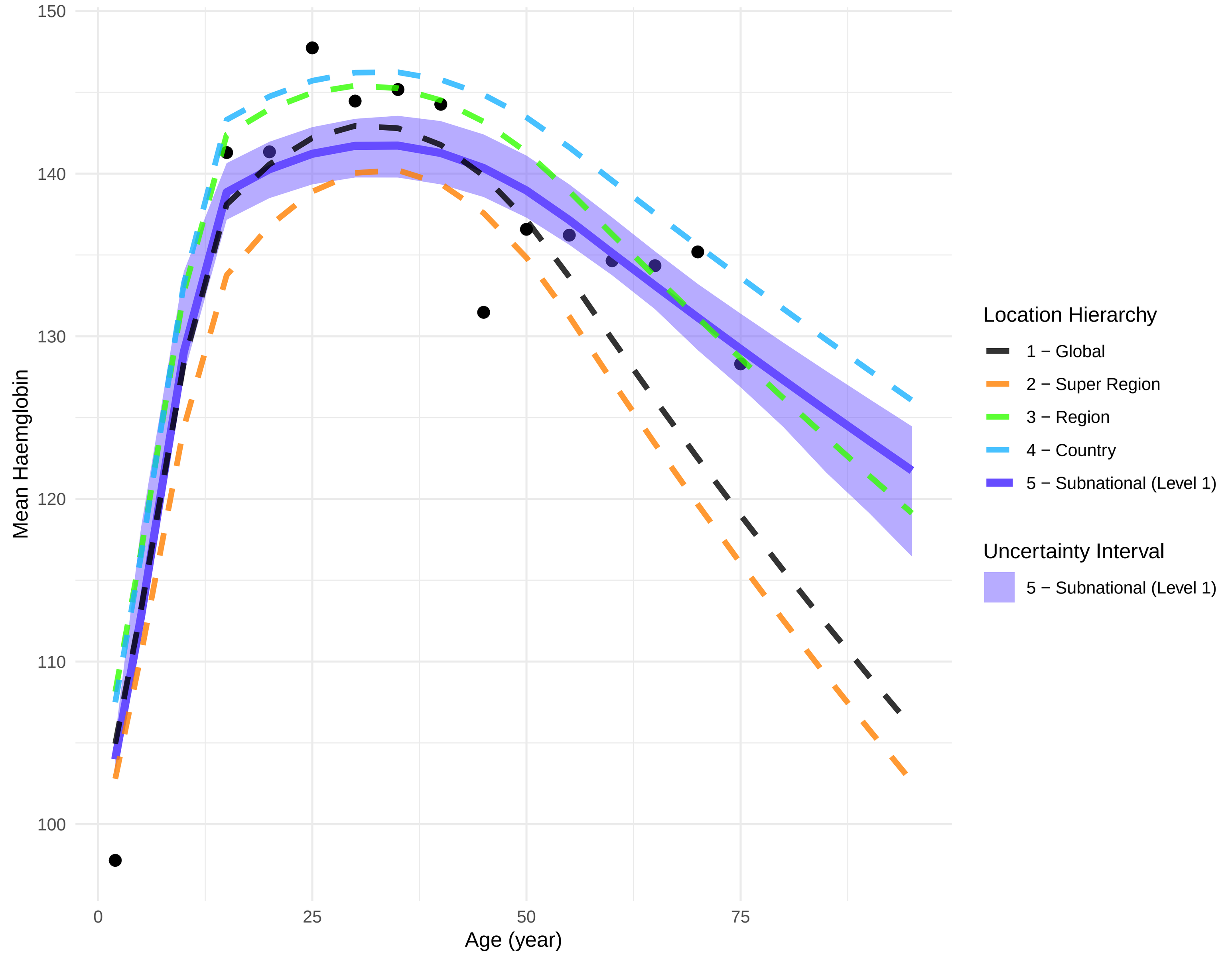

MR−BRT Age/Sex Fits − Males 2+ Years Old − Mean Haemglobin − Northern Cape

WHO−2024 Adjustment Method

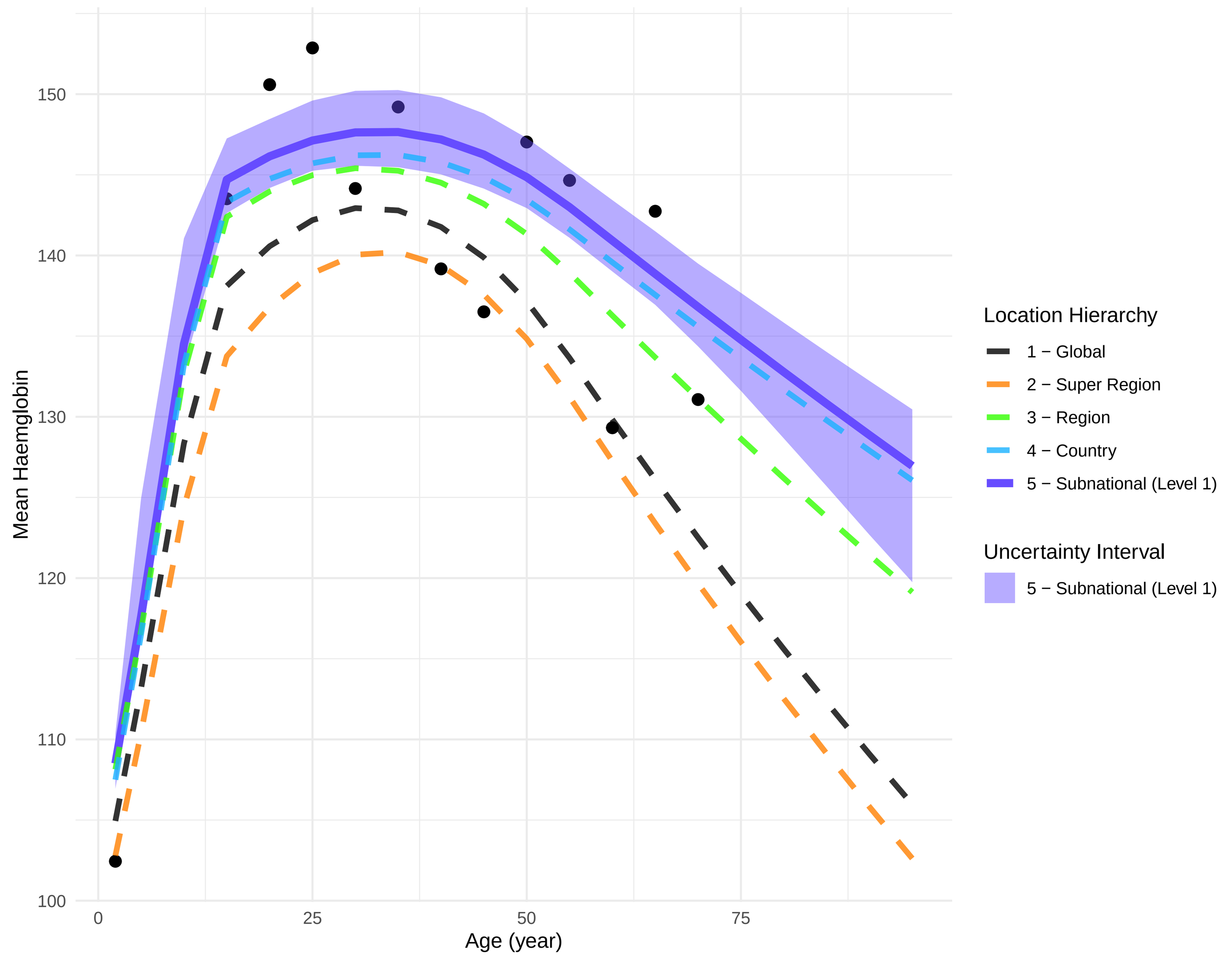

MR−BRT Age/Sex Fits − Males 2+ Years Old − Mean Haemglobin − Western Cape

WHO−2024 Adjustment Method

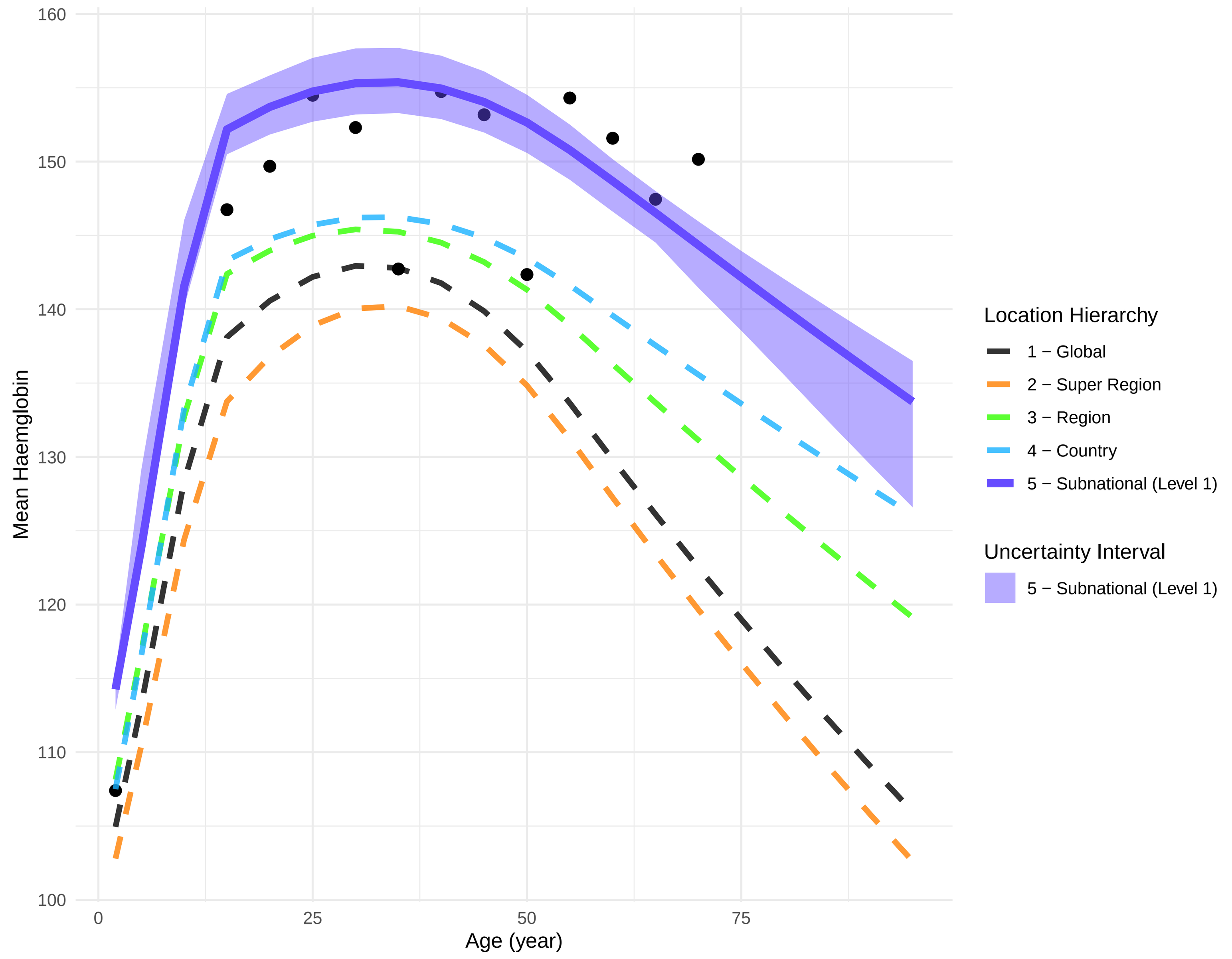

MR−BRT Age/Sex Fits − Males 2+ Years Old − Mean Haemglobin − Zimbabwe

WHO−2024 Adjustment Method

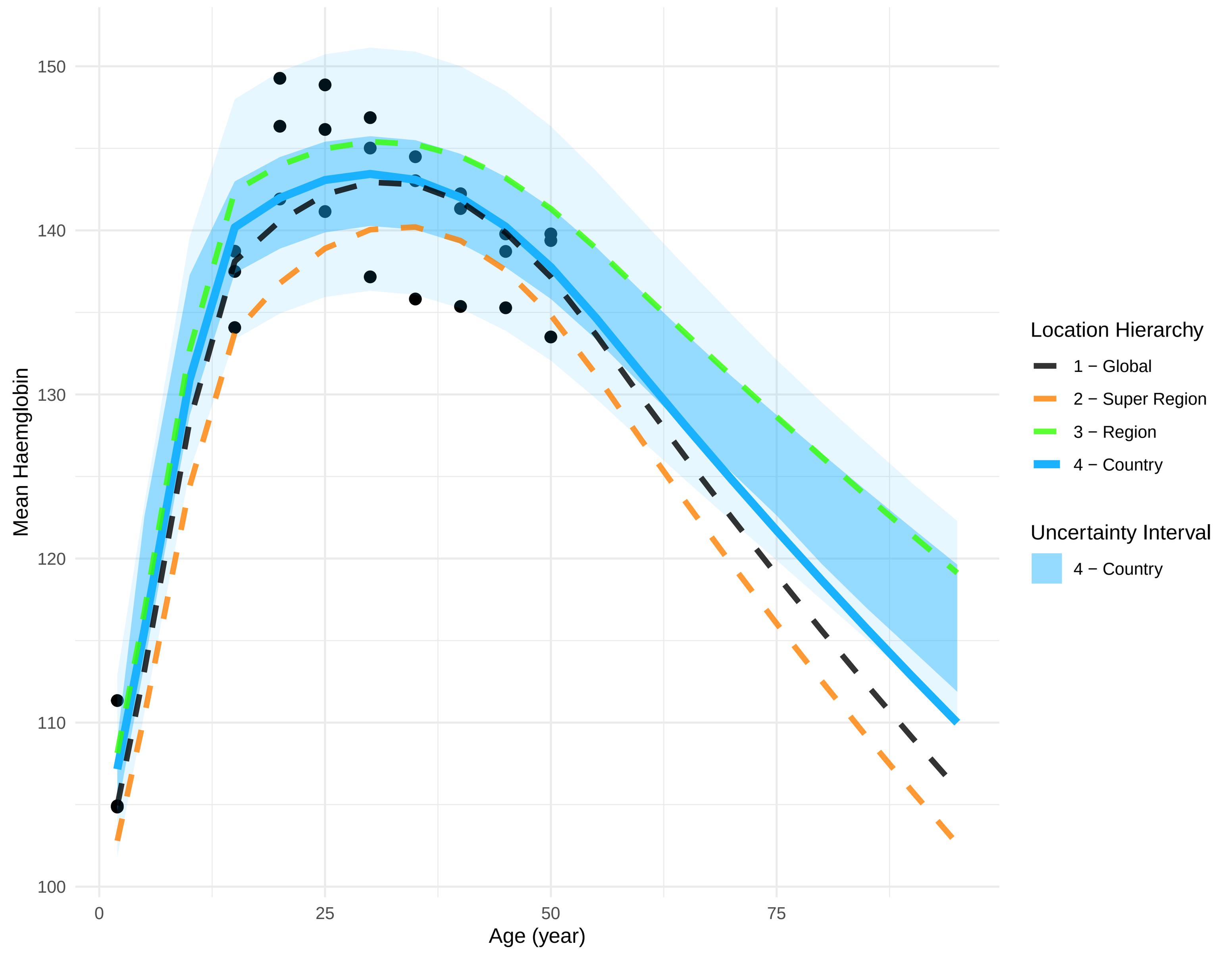

MR−BRT Age/Sex Fits − Males 2+ Years Old − Mean Haemglobin − Western Sub−Saharan Africa

WHO−2024 Adjustment Method

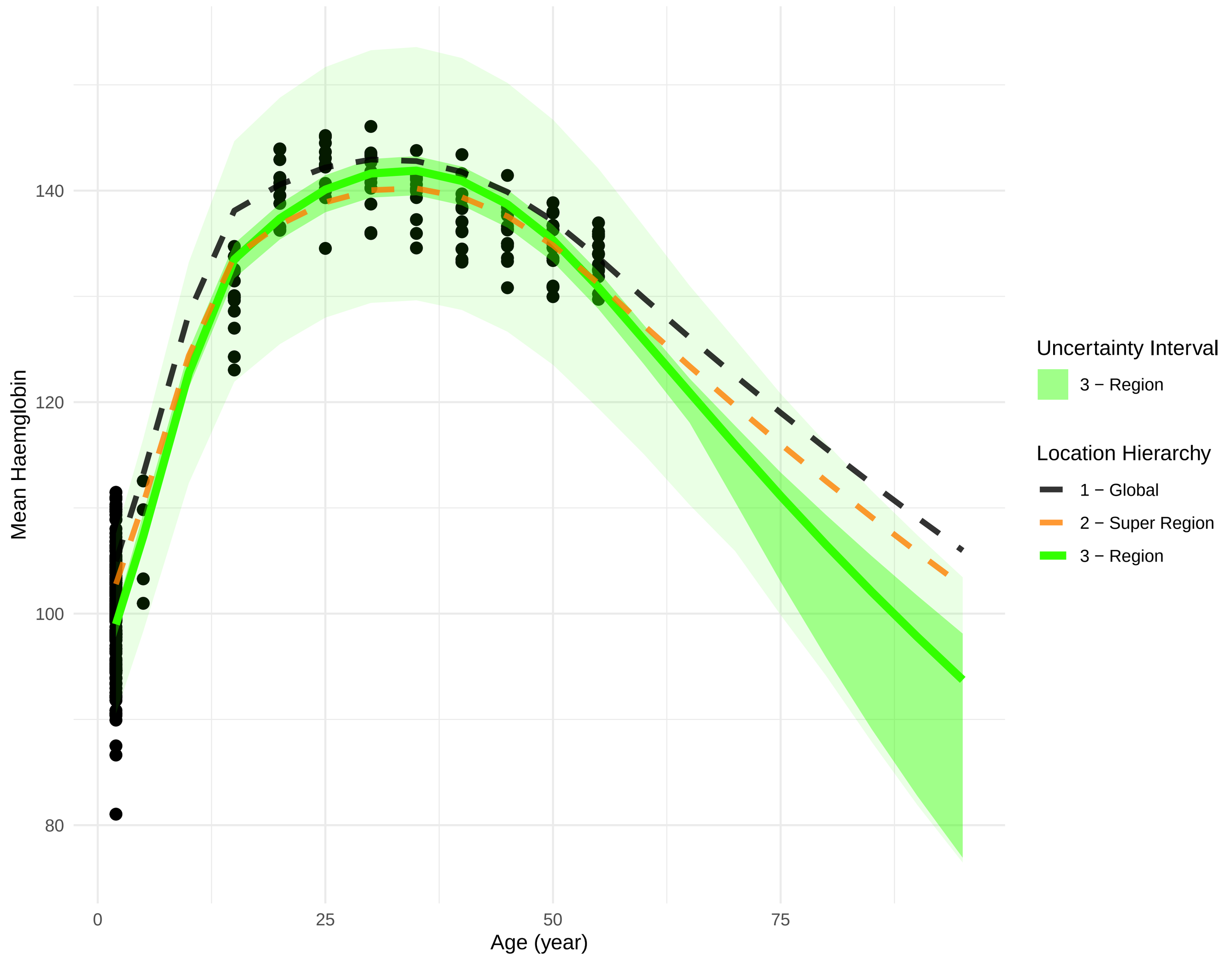

MR−BRT Age/Sex Fits − Males 2+ Years Old − Mean Haemglobin − Benin

WHO−2024 Adjustment Method

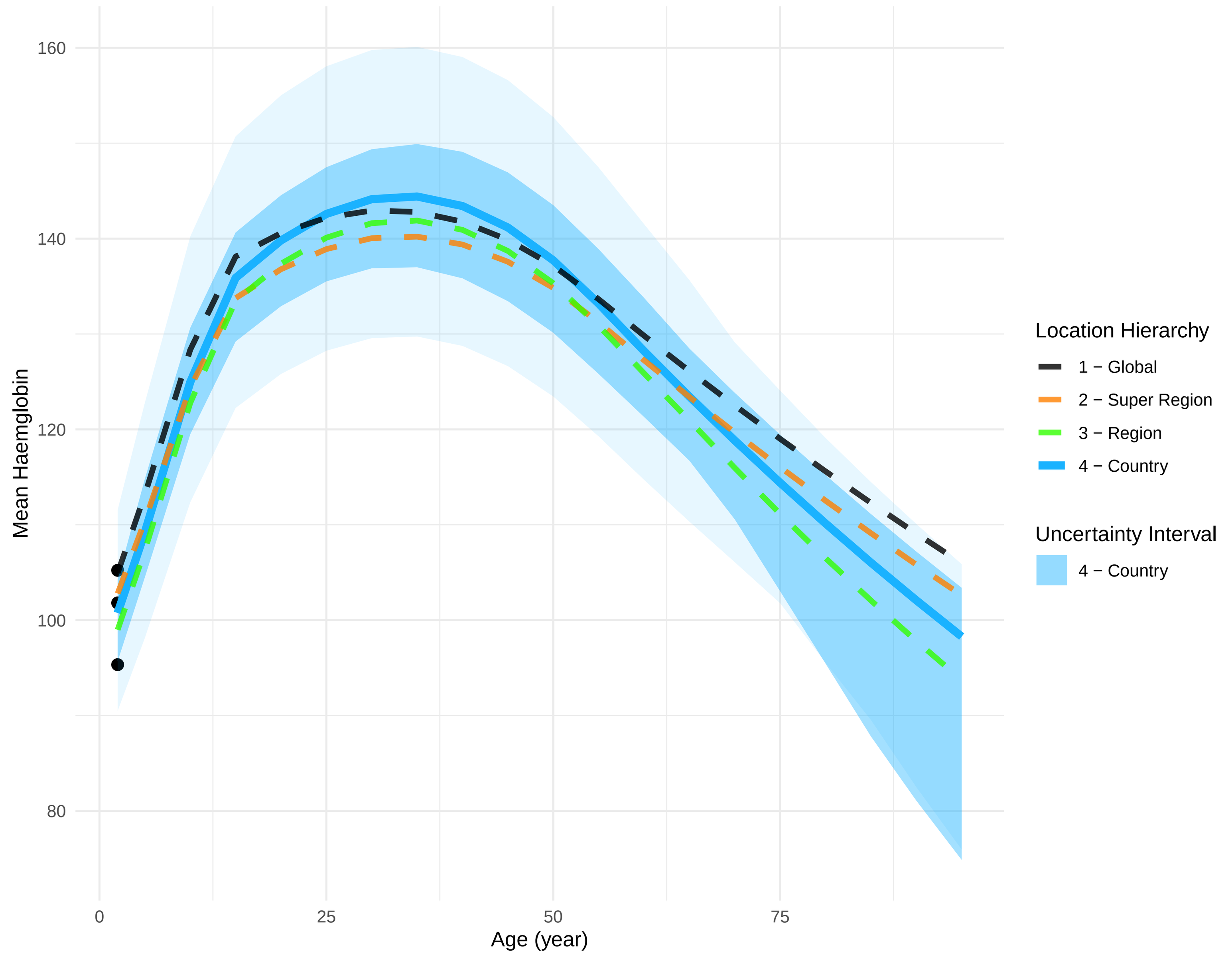

MR−BRT Age/Sex Fits − Males 2+ Years Old − Mean Haemglobin − Burkina Faso

WHO−2024 Adjustment Method

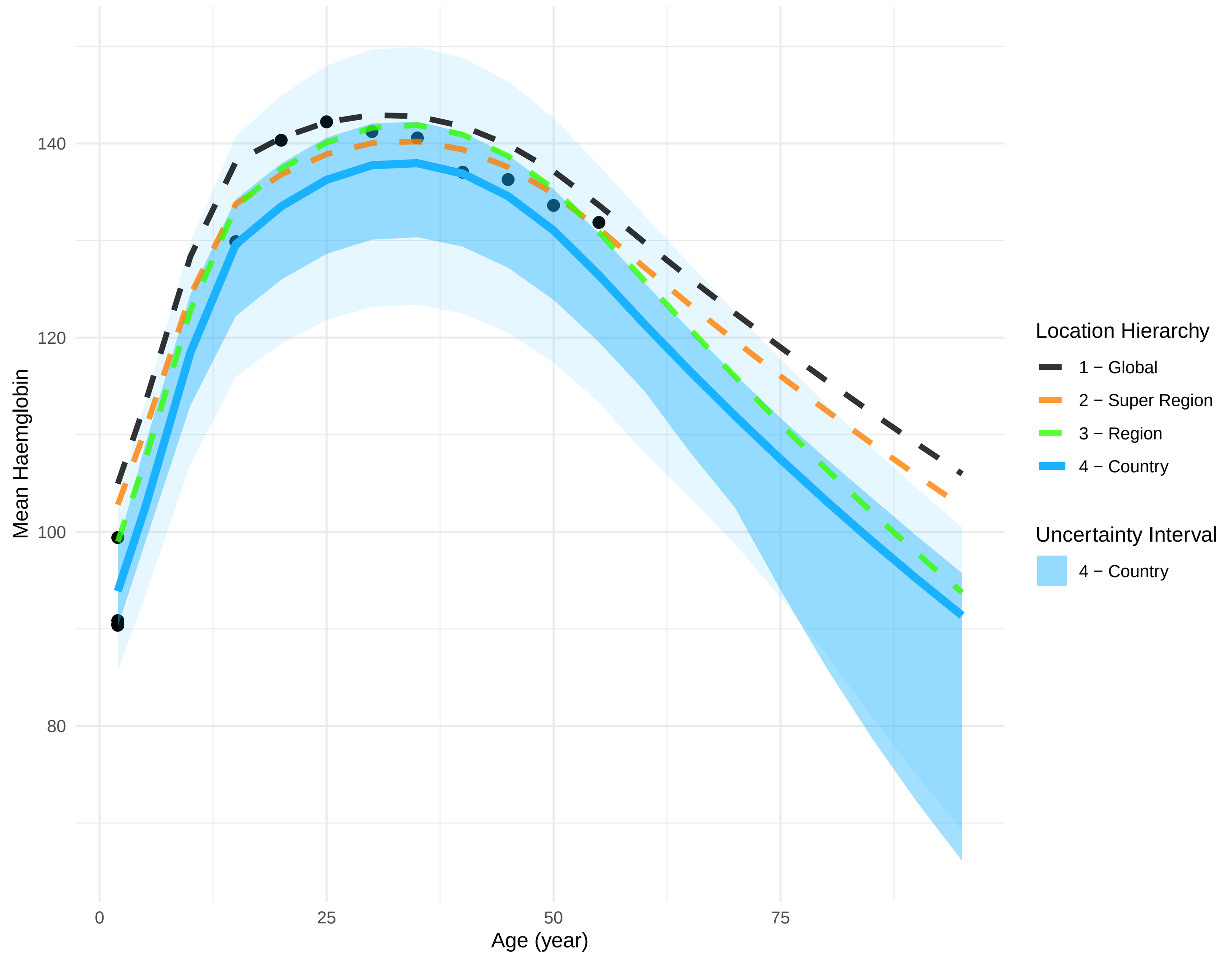

MR−BRT Age/Sex Fits − Males 2+ Years Old − Mean Haemglobin − Cameroon

WHO−2024 Adjustment Method

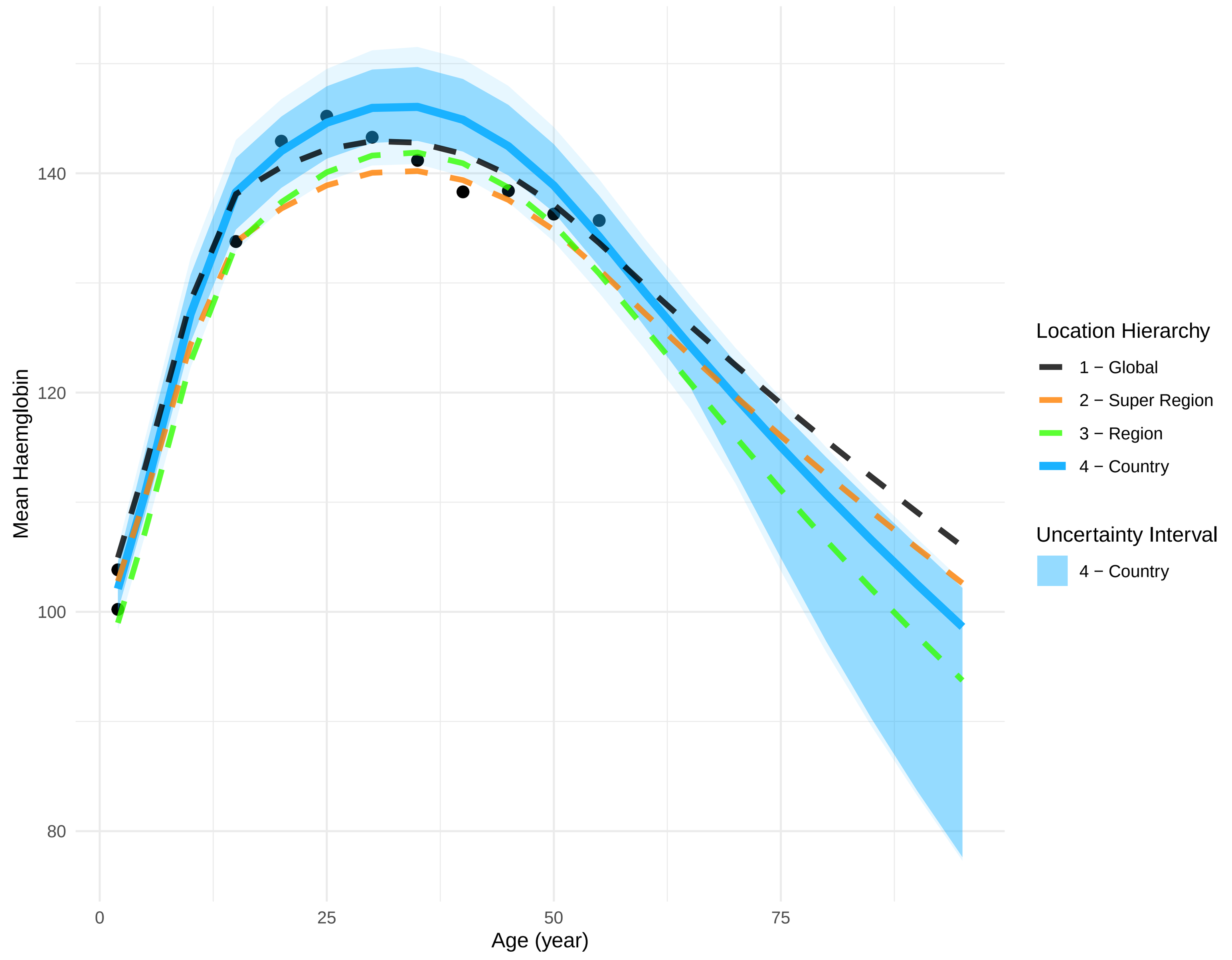

MR−BRT Age/Sex Fits − Males 2+ Years Old − Mean Haemglobin − Côte d'Ivoire

WHO−2024 Adjustment Method

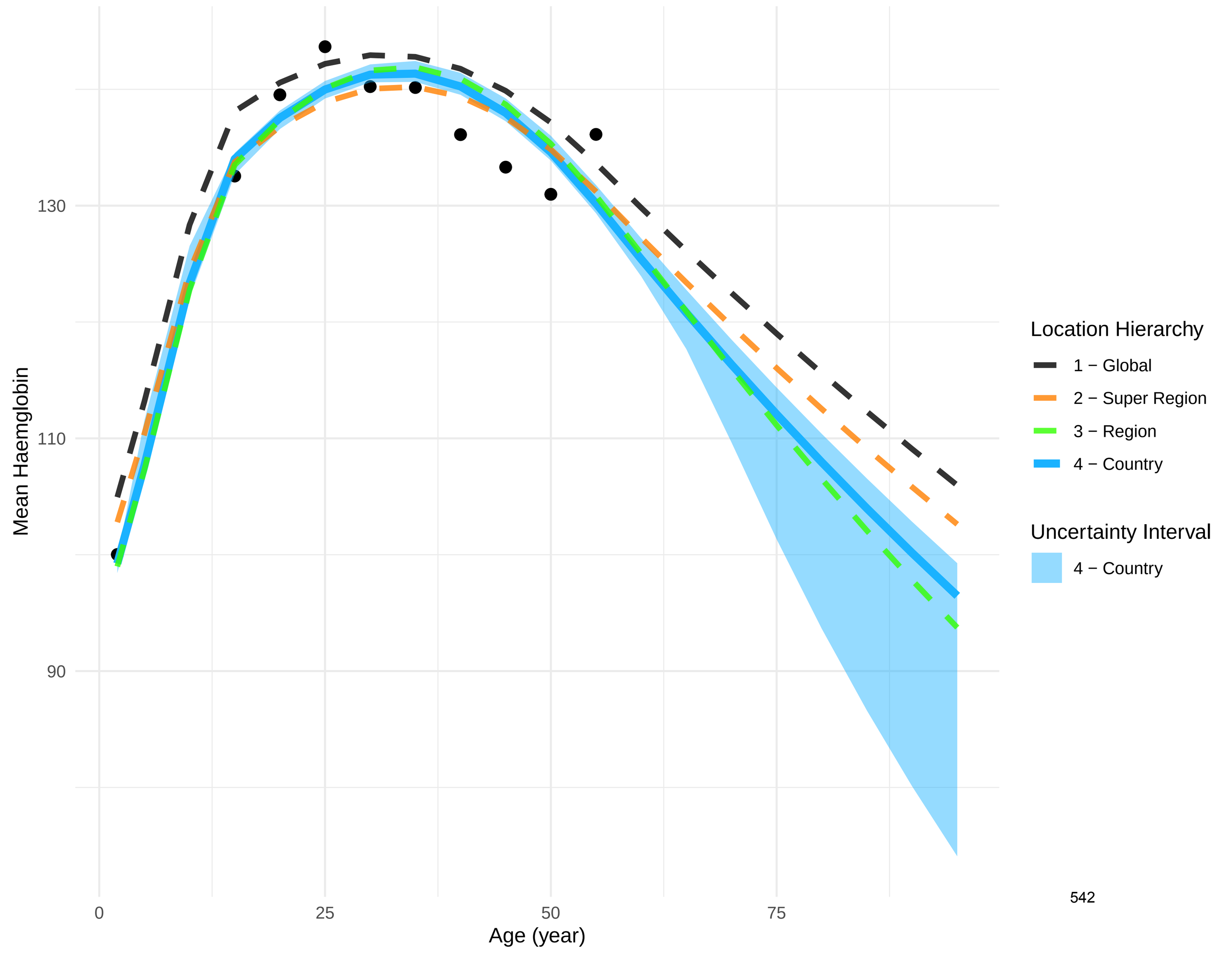

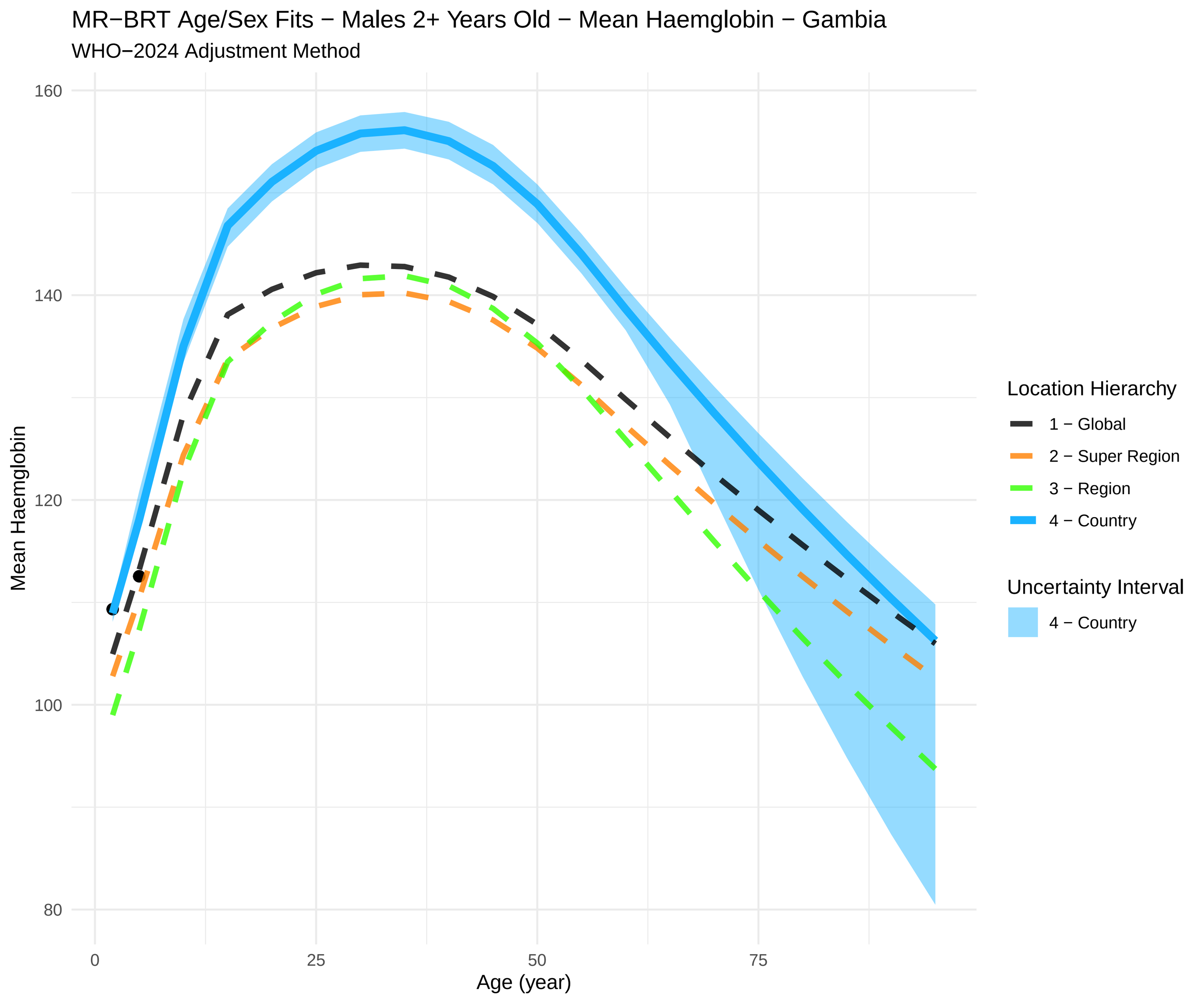
MR−BRT Age/Sex Fits − Males 2+ Years Old − Mean Haemglobin − Gambia
WHO−2024 Adjustment Method
Mean Haemglobin
Age (year)
Location Hierarchy
1 − Global
2 − Super Region
3 − Region
4 − Country
Uncertainty Interval
4 − Country

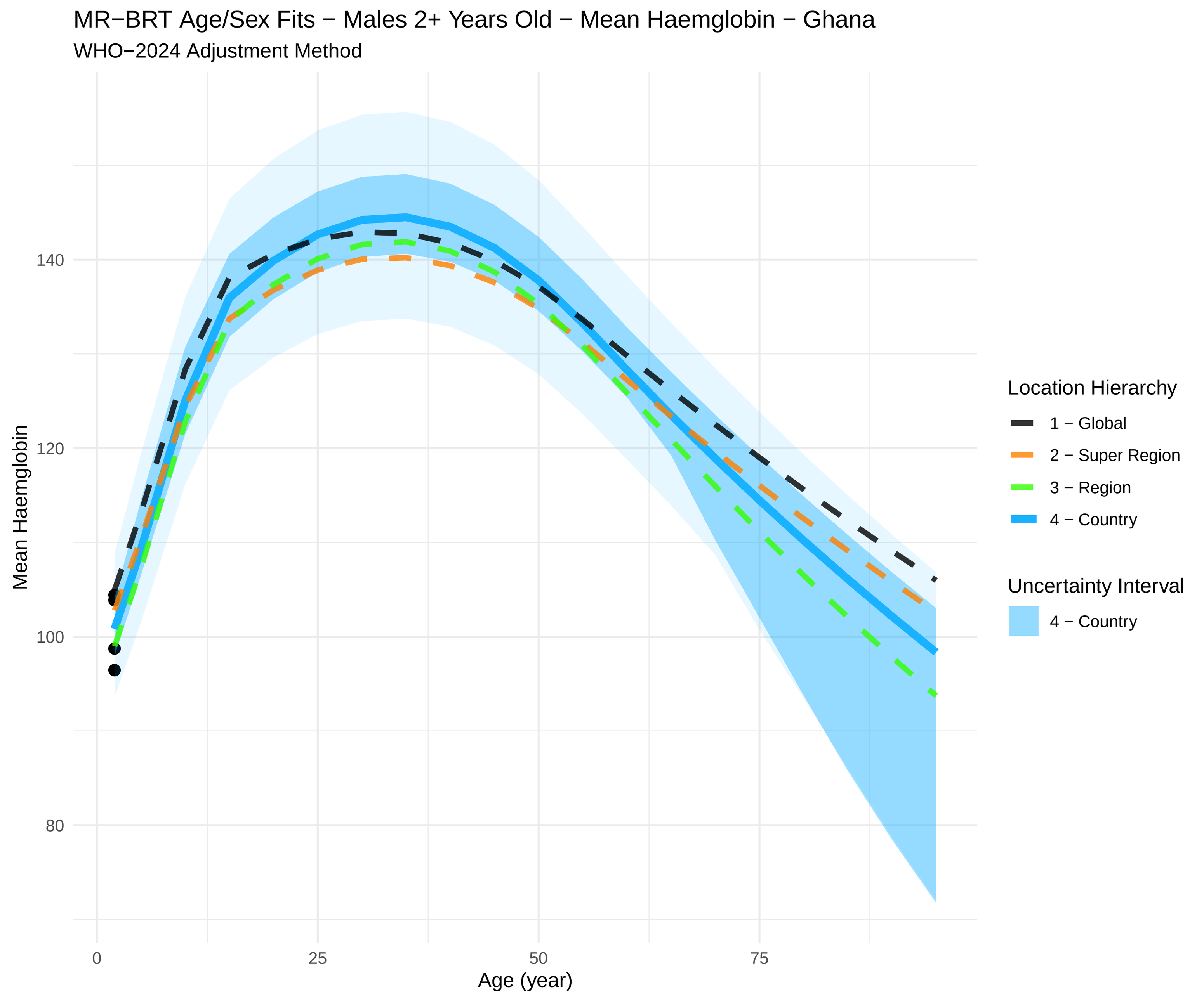
MR−BRT Age/Sex Fits − Males 2+ Years Old − Mean Haemglobin − Ghana
WHO−2024 Adjustment Method
Mean Haemglobin
Age (year)
Location Hierarchy
1 − Global
2 − Super Region
3 − Region
4 − Country
Uncertainty Interval
4 − Country

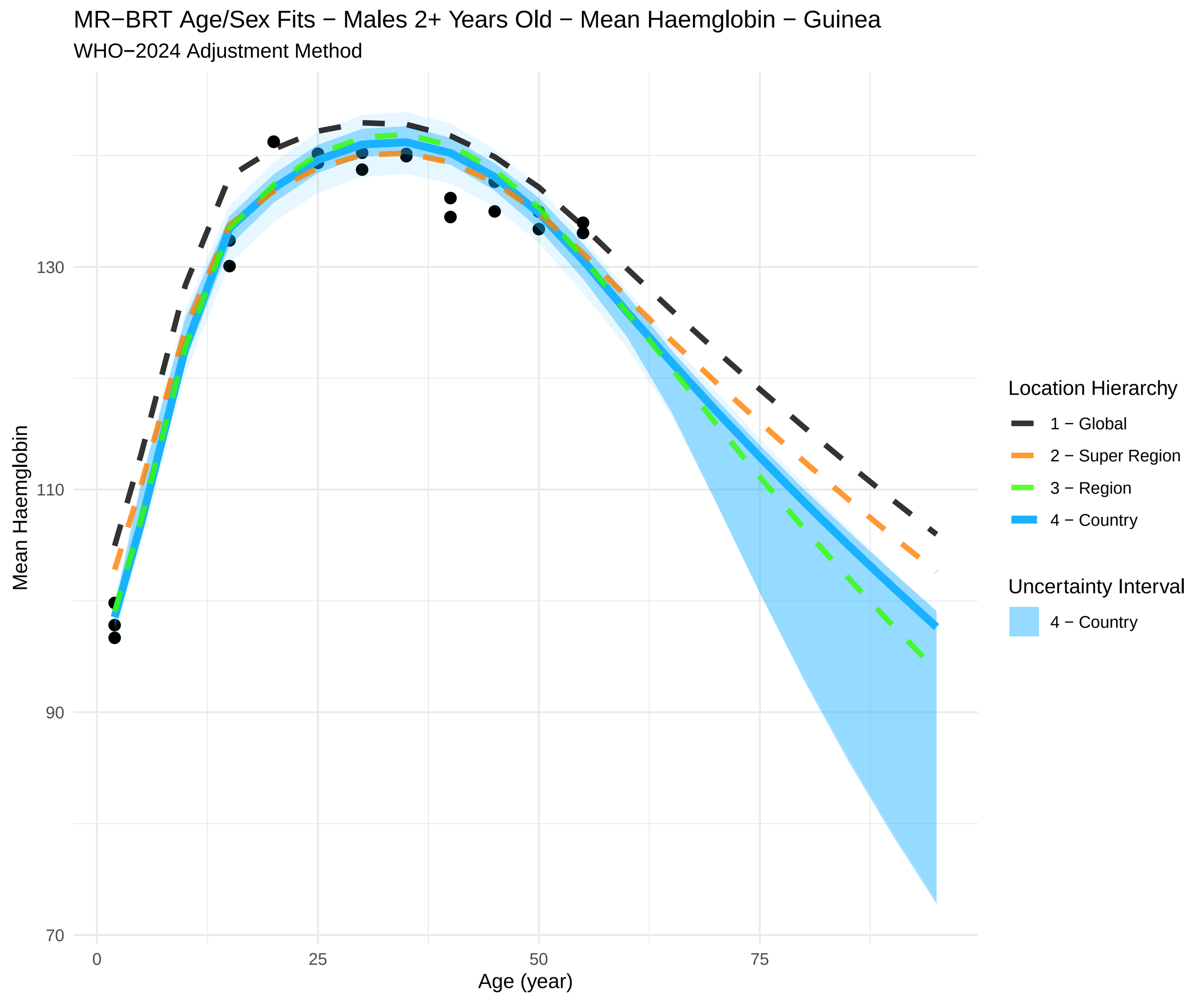
MR−BRT Age/Sex Fits − Males 2+ Years Old − Mean Haemglobin − Guinea
WHO−2024 Adjustment Method
Mean Haemglobin
Age (year)
Location Hierarchy
1 − Global
2 − Super Region
3 − Region
4 − Country
Uncertainty Interval
4 − Country

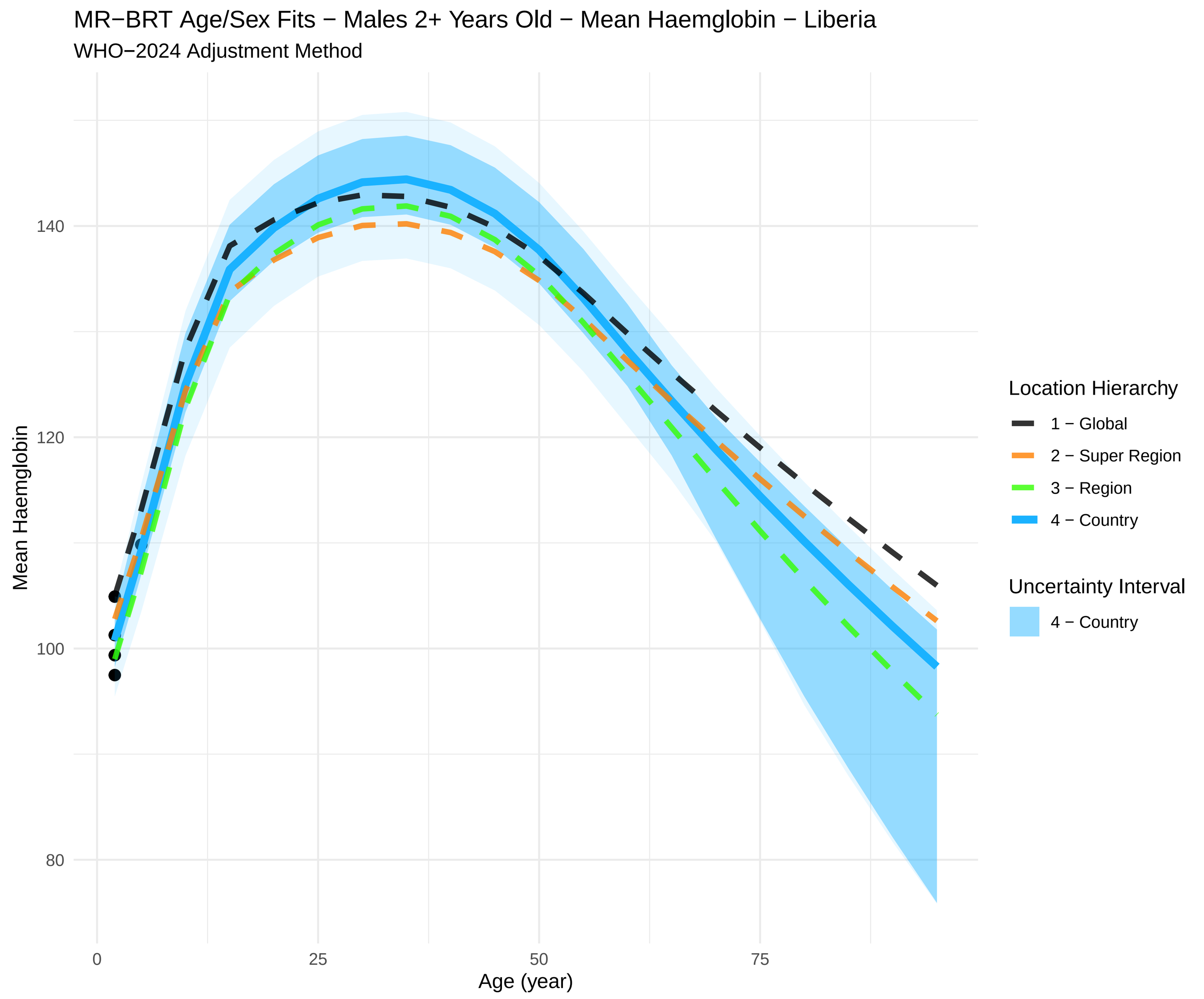
MR−BRT Age/Sex Fits − Males 2+ Years Old − Mean Haemglobin − Liberia
WHO−2024 Adjustment Method
Mean Haemglobin
Age (year)
Location Hierarchy
1 − Global
2 − Super Region
3 − Region
4 − Country
Uncertainty Interval
4 − Country

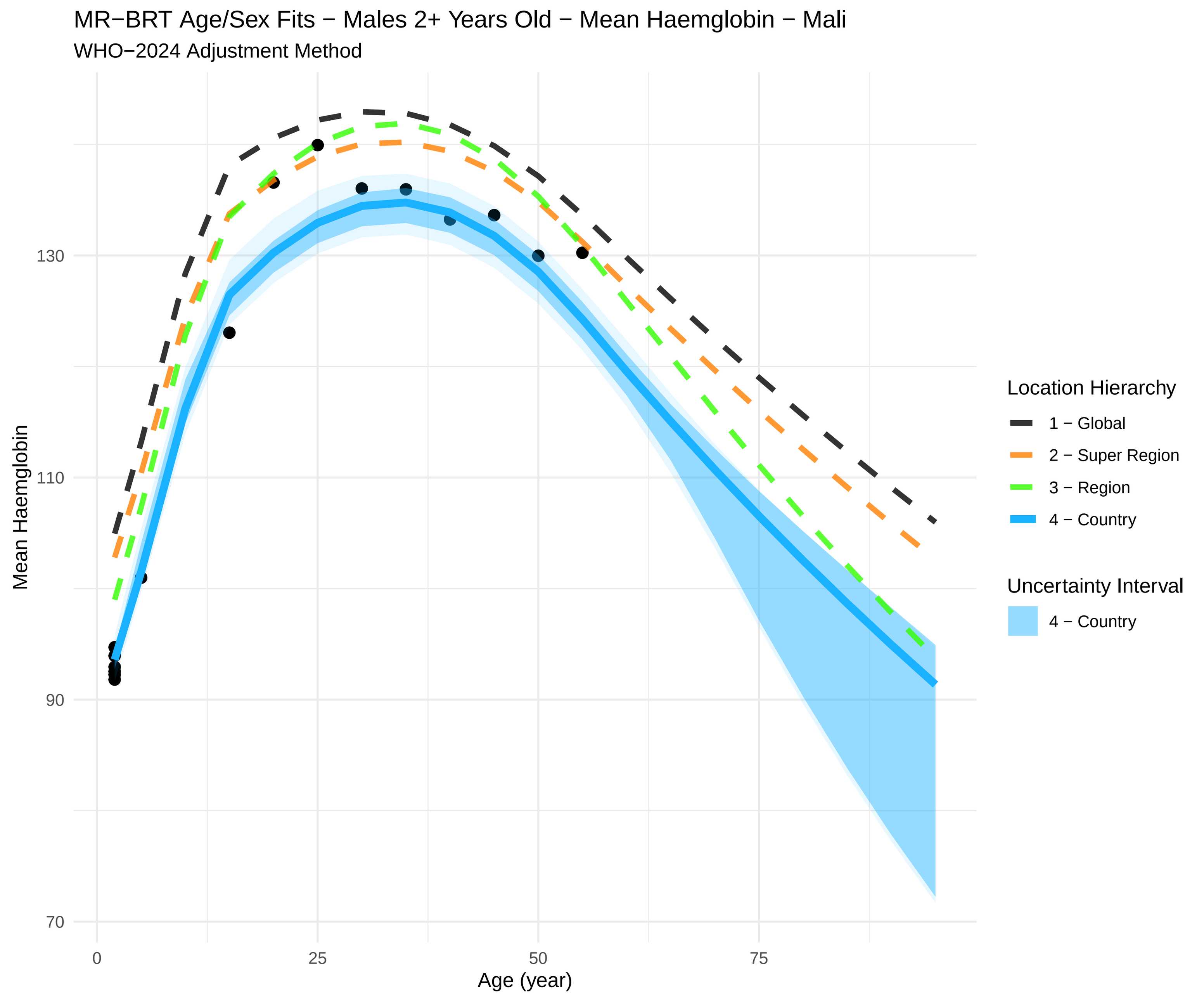
MR−BRT Age/Sex Fits − Males 2+ Years Old − Mean Haemglobin − Mali
WHO−2024 Adjustment Method
Mean Haemglobin
Age (year)
Location Hierarchy
1 − Global
2 − Super Region
3 − Region
4 − Country
Uncertainty Interval
4 − Country

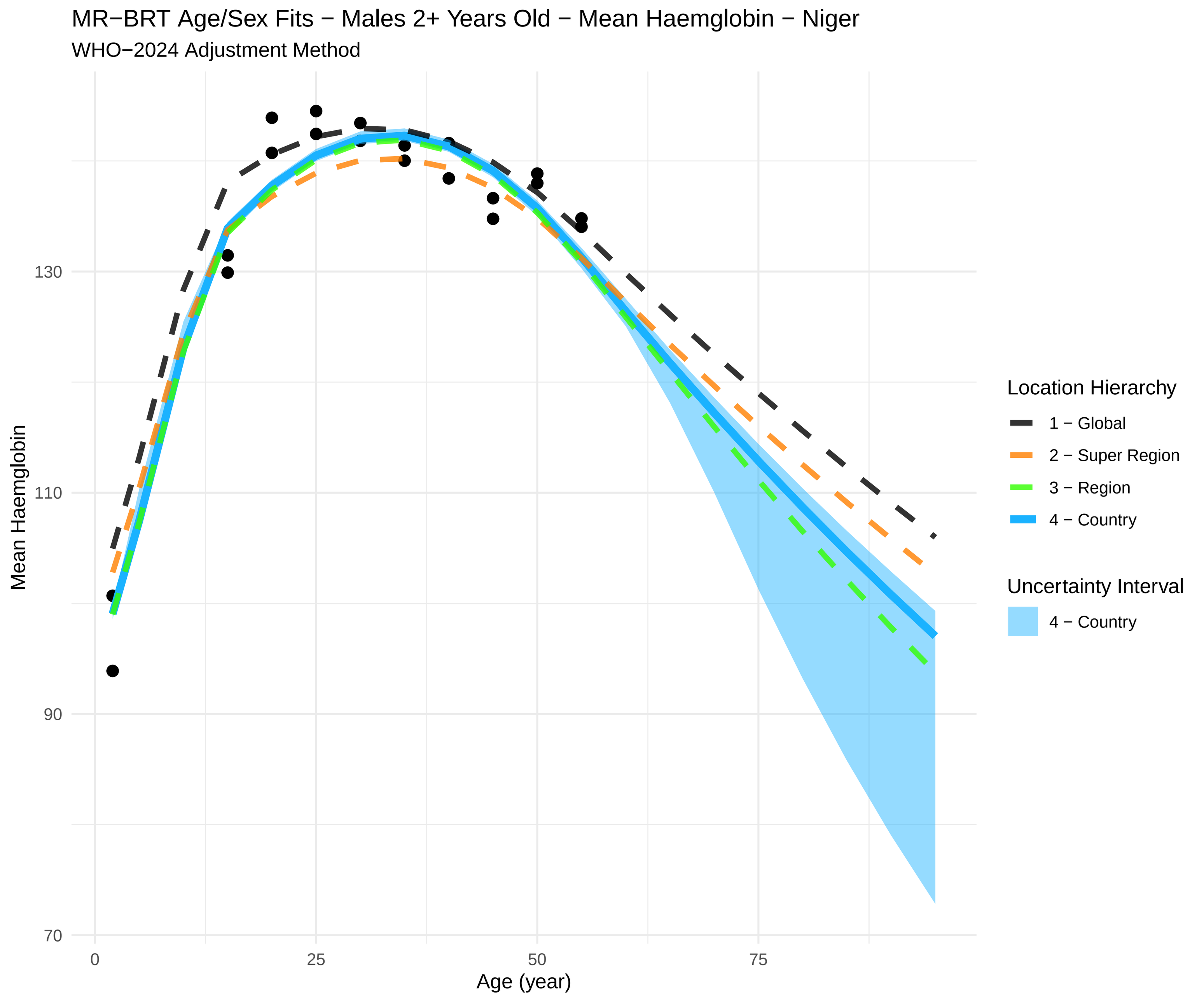
MR−BRT Age/Sex Fits − Males 2+ Years Old − Mean Haemglobin − Niger
WHO−2024 Adjustment Method
Mean Haemglobin
Age (year)
Location Hierarchy
1 − Global
2 − Super Region
3 − Region
4 − Country
Uncertainty Interval
4 − Country

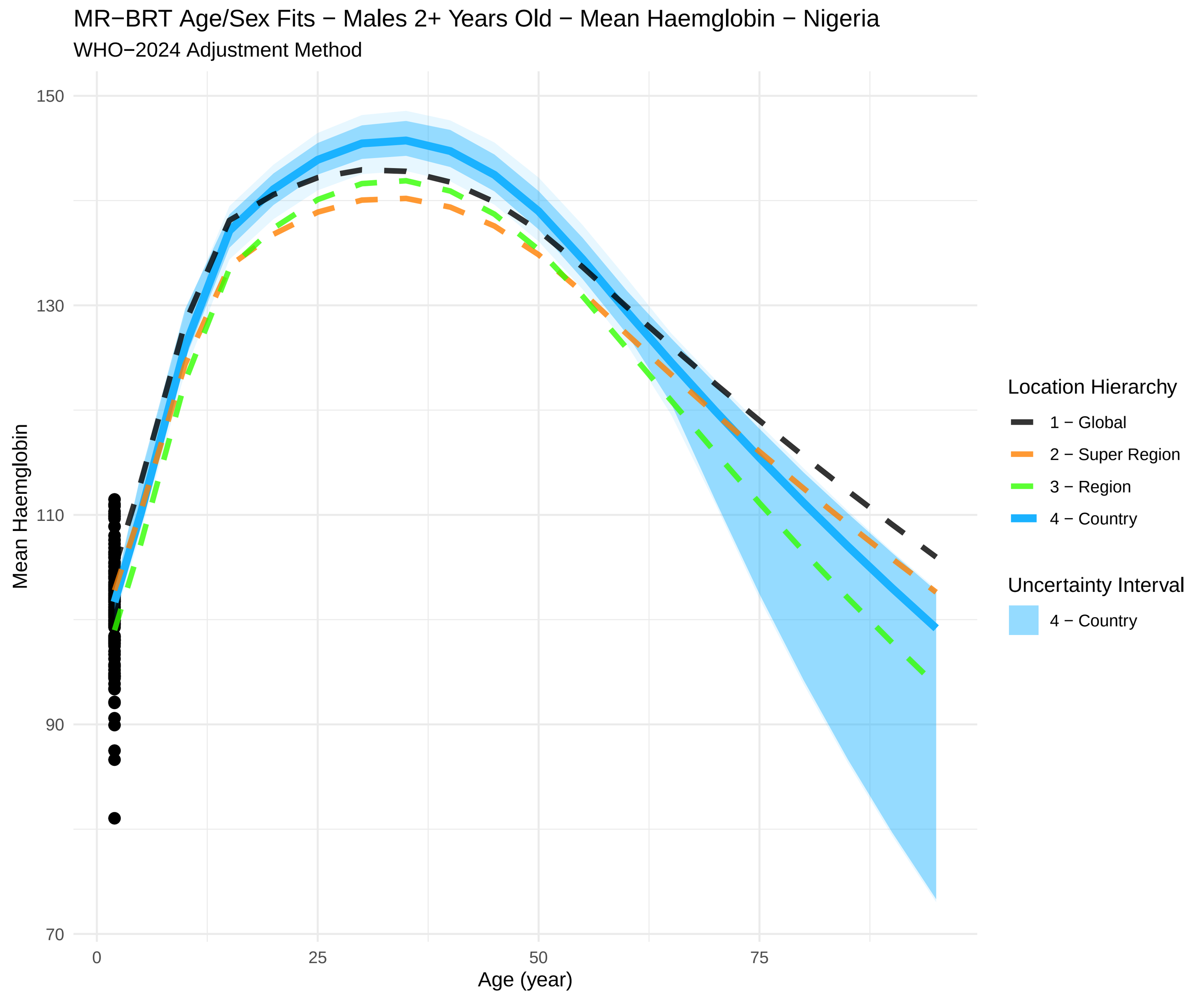
MR−BRT Age/Sex Fits − Males 2+ Years Old − Mean Haemglobin − Nigeria
WHO−2024 Adjustment Method
Mean Haemglobin
Age (year)
Location Hierarchy
1 − Global
2 − Super Region
3 − Region
4 − Country
Uncertainty Interval
4 − Country

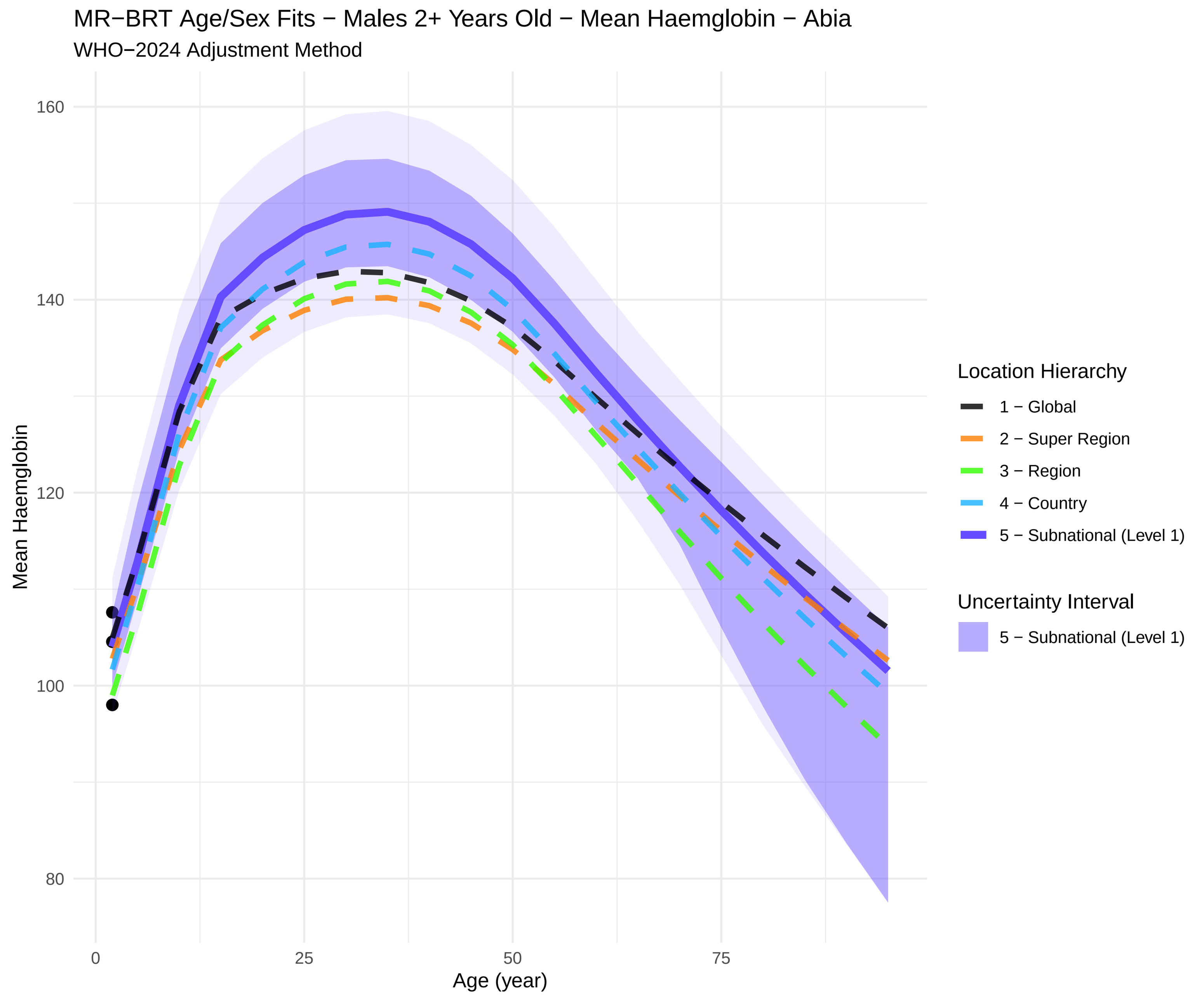
MR−BRT Age/Sex Fits − Males 2+ Years Old − Mean Haemglobin − Abia
WHO−2024 Adjustment Method
Mean Haemglobin
Age (year)
Location Hierarchy
1 − Global
2 − Super Region
3 − Region
4 − Country
5 − Subnational (Level 1)
Uncertainty Interval
5 − Subnational (Level 1)

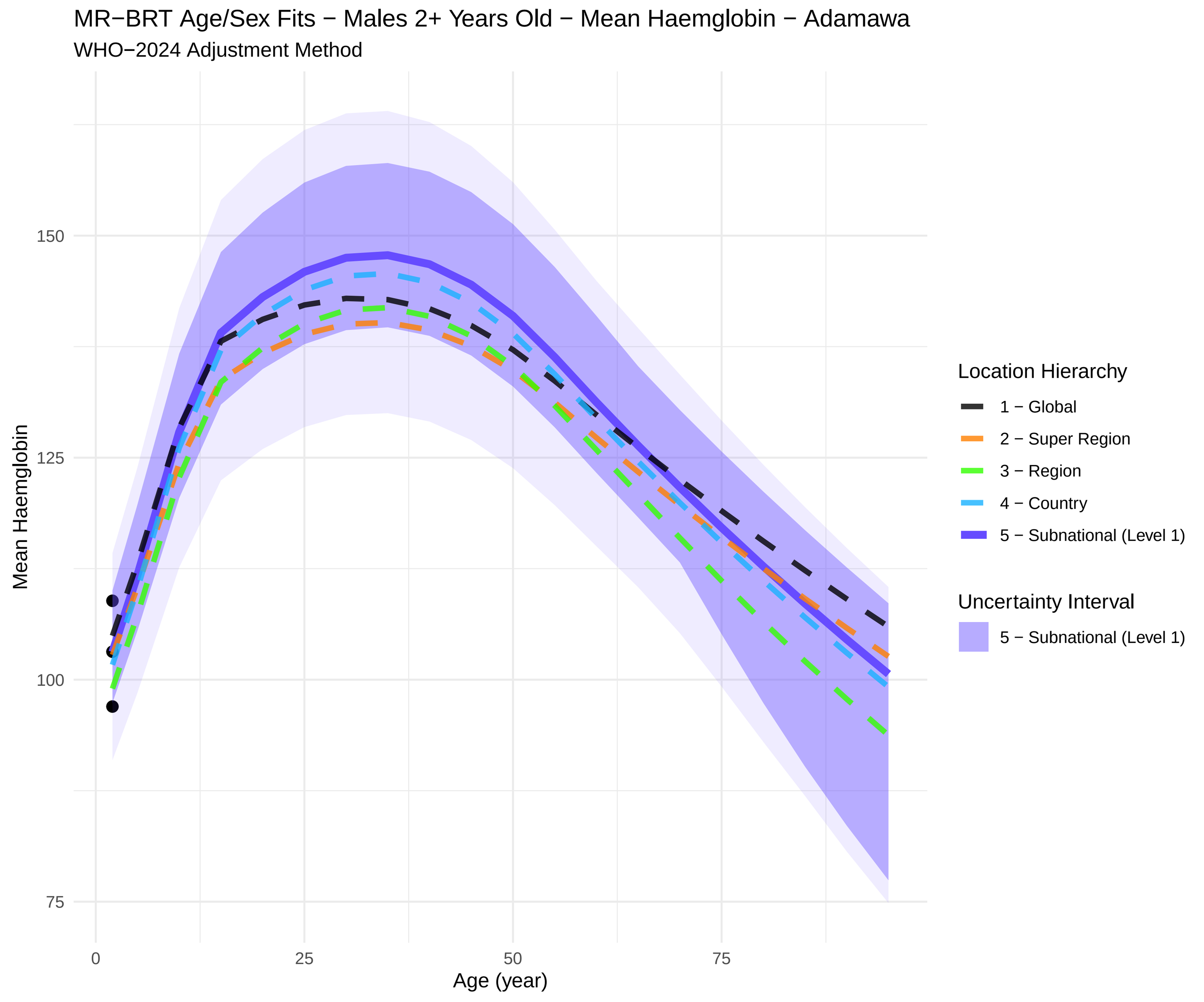
MR−BRT Age/Sex Fits − Males 2+ Years Old − Mean Haemglobin − Adamawa
WHO−2024 Adjustment Method
Mean Haemglobin
Age (year)
Location Hierarchy
1 − Global
2 − Super Region
3 − Region
4 − Country
5 − Subnational (Level 1)
Uncertainty Interval
5 − Subnational (Level 1)

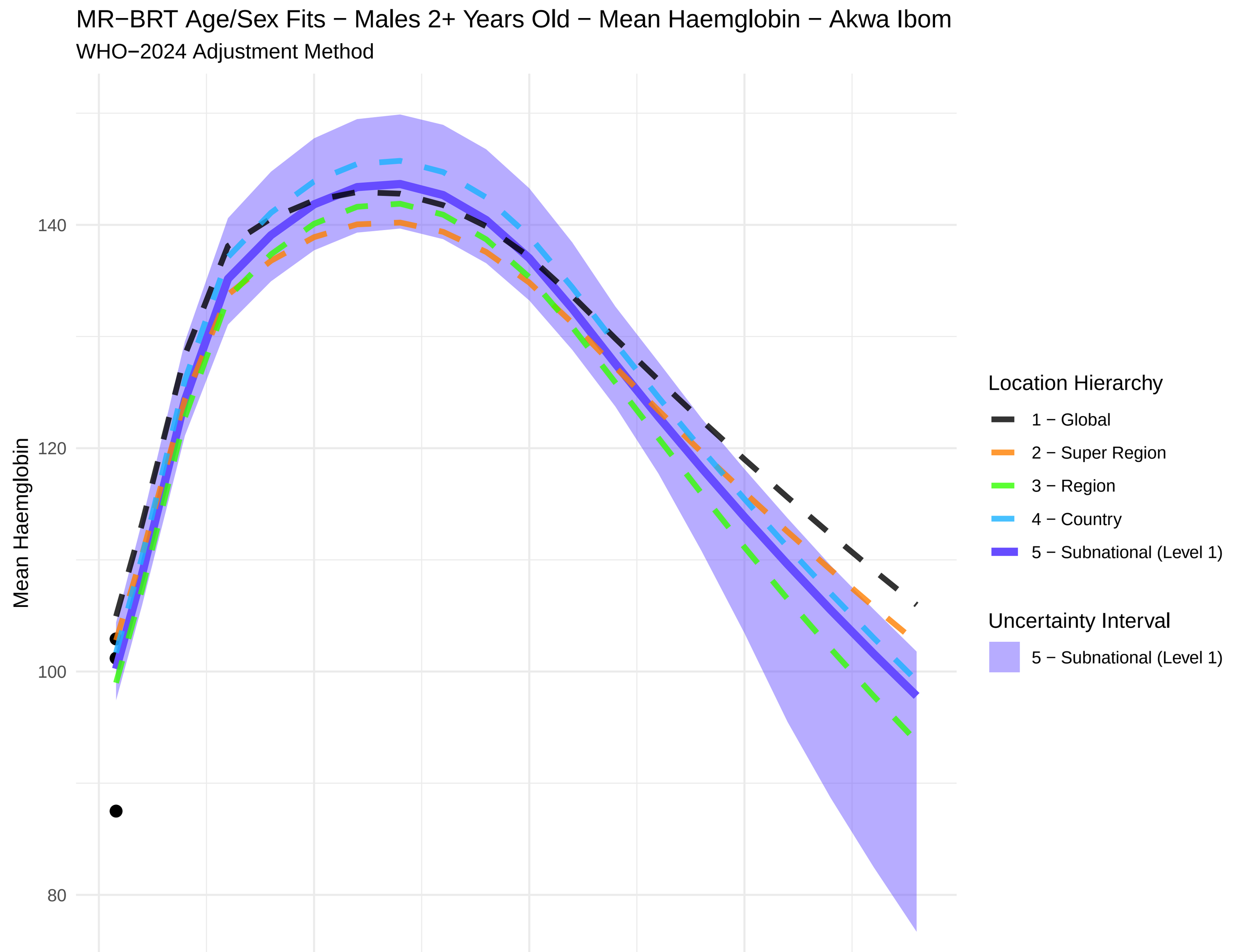
MR−BRT Age/Sex Fits − Males 2+ Years Old − Mean Haemglobin − Akwa Ibom
WHO−2024 Adjustment Method
Mean Haemglobin
Age (year)
Location Hierarchy
1 − Global
2 − Super Region
3 − Region
4 − Country
5 − Subnational (Level 1)
Uncertainty Interval
5 − Subnational (Level 1)

MR−BRT Age/Sex Fits − Males 2+ Years Old − Mean Haemglobin − Anambra
WHO−2024 Adjustment Method

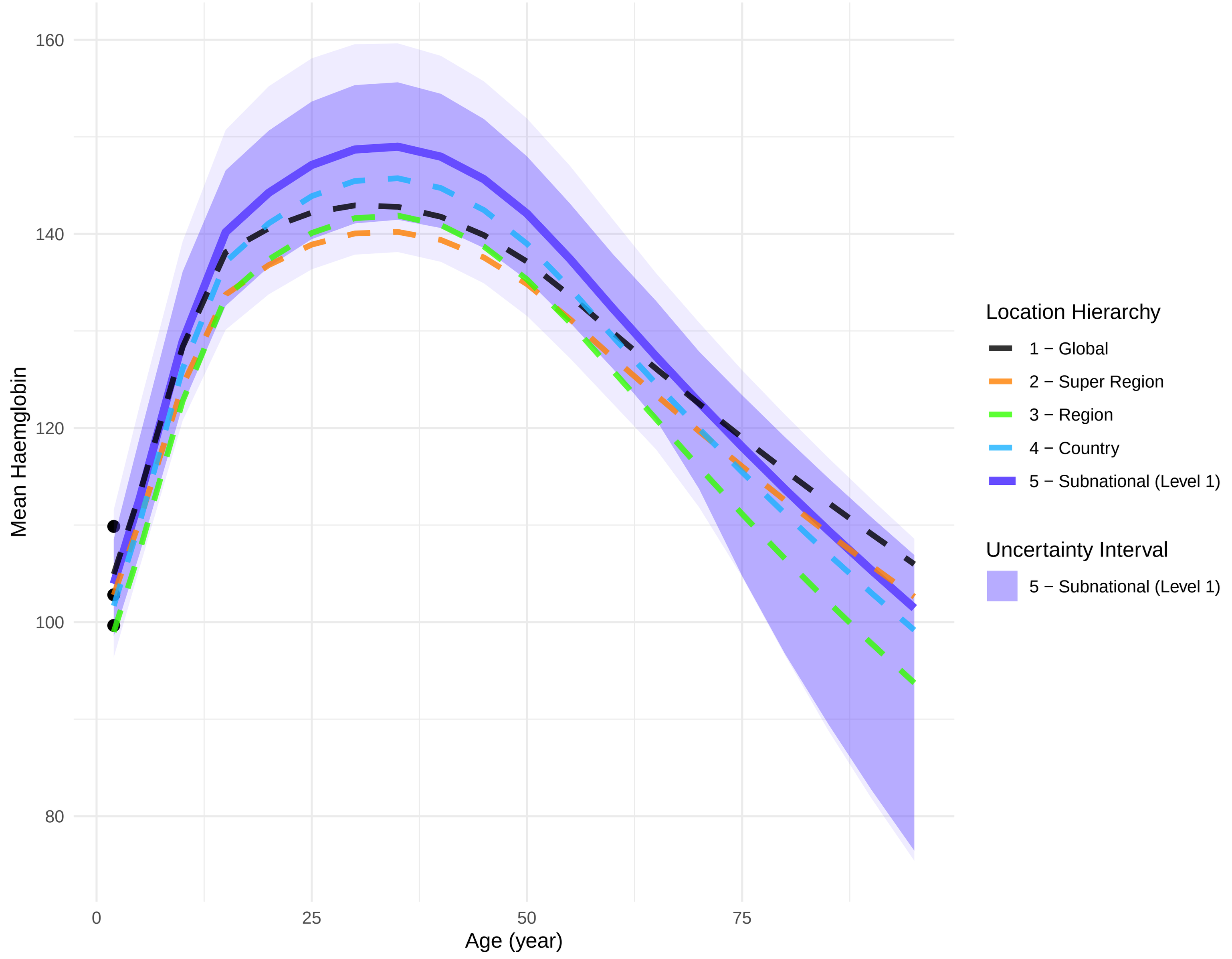

MR−BRT Age/Sex Fits − Males 2+ Years Old − Mean Haemglobin − Bauchi
WHO−2024 Adjustment Method

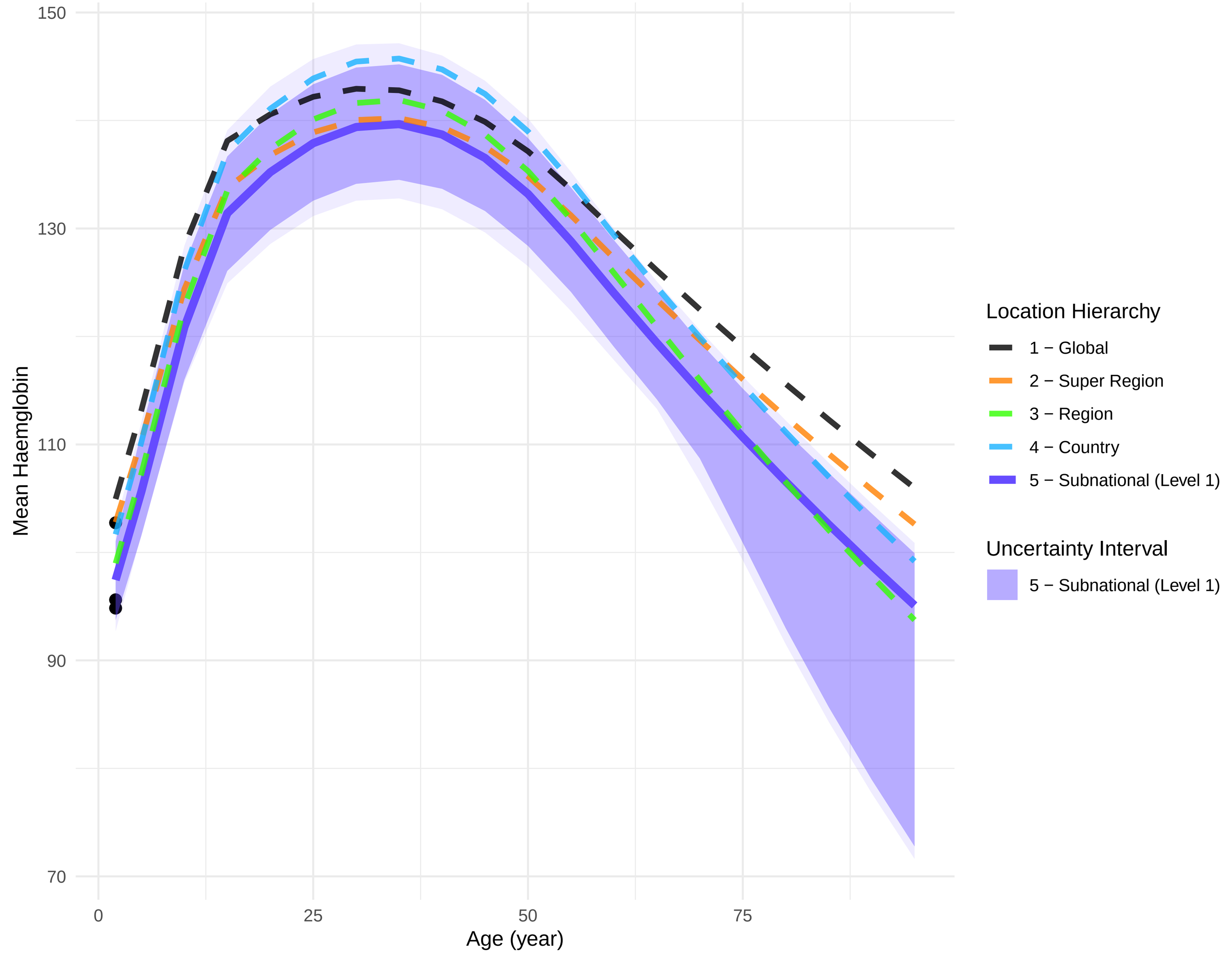

MR−BRT Age/Sex Fits − Males 2+ Years Old − Mean Haemglobin − Bayelsa
WHO−2024 Adjustment Method

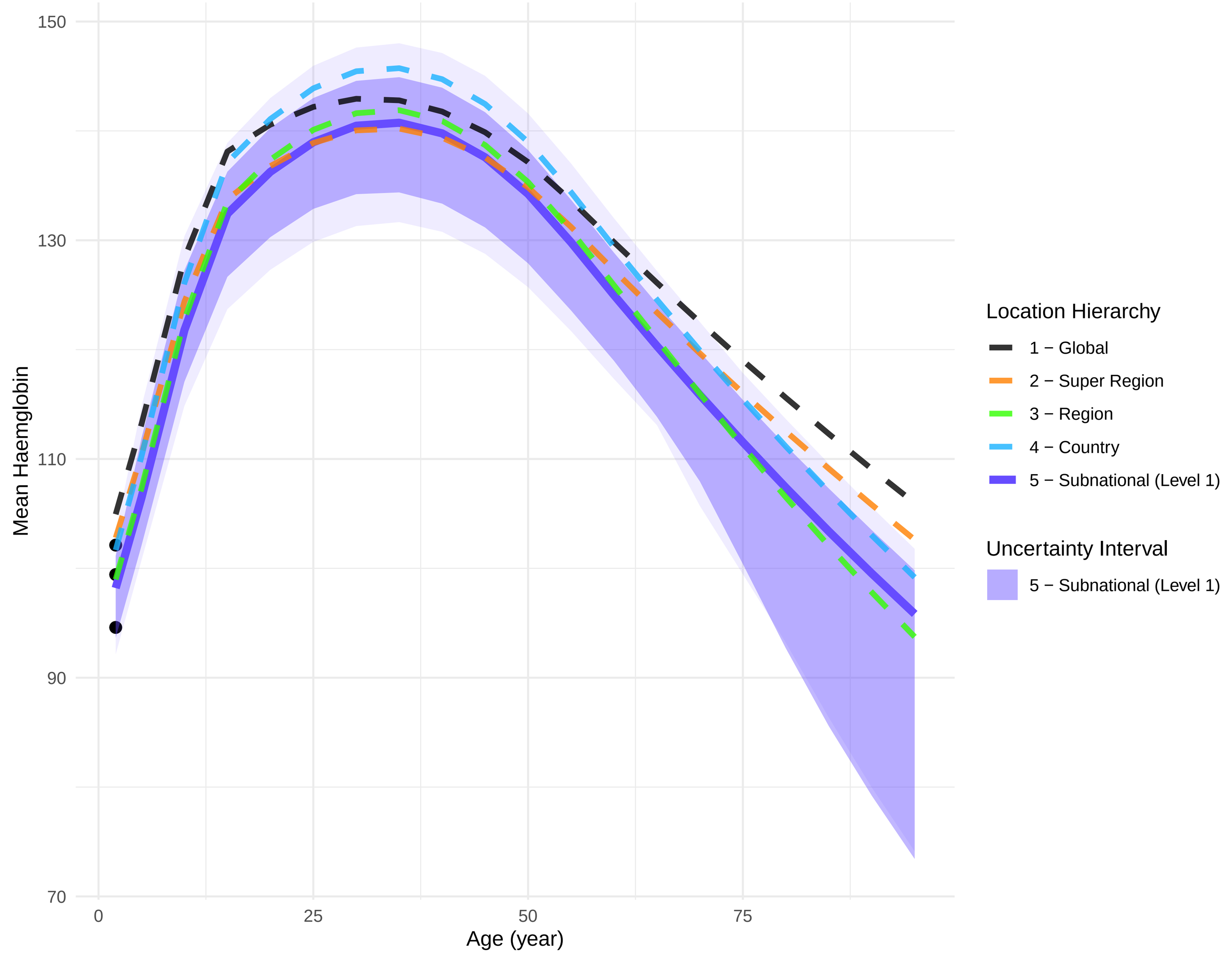

MR−BRT Age/Sex Fits − Males 2+ Years Old − Mean Haemglobin − Benue
WHO−2024 Adjustment Method

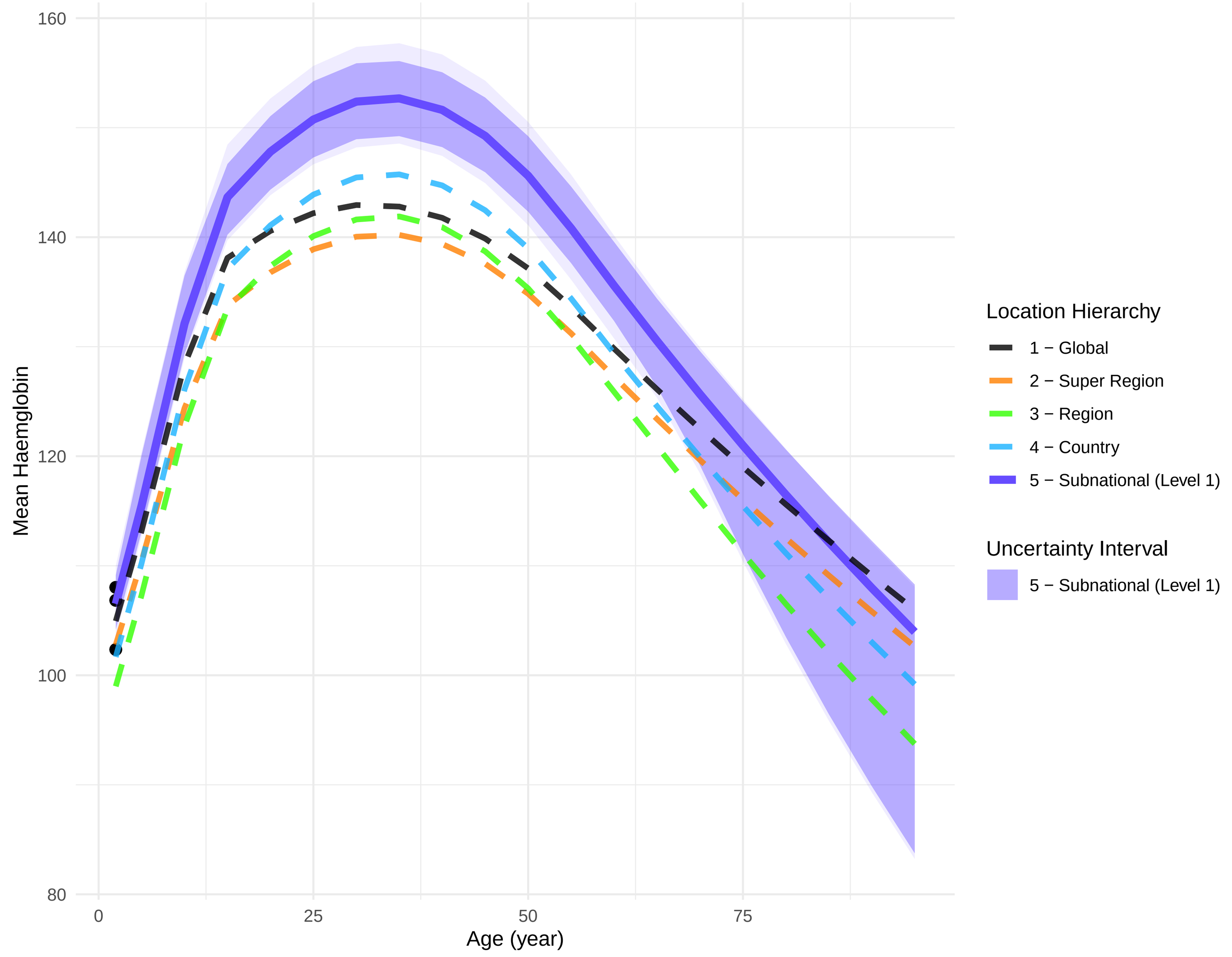

MR−BRT Age/Sex Fits − Males 2+ Years Old − Mean Haemglobin − Borno
WHO−2024 Adjustment Method

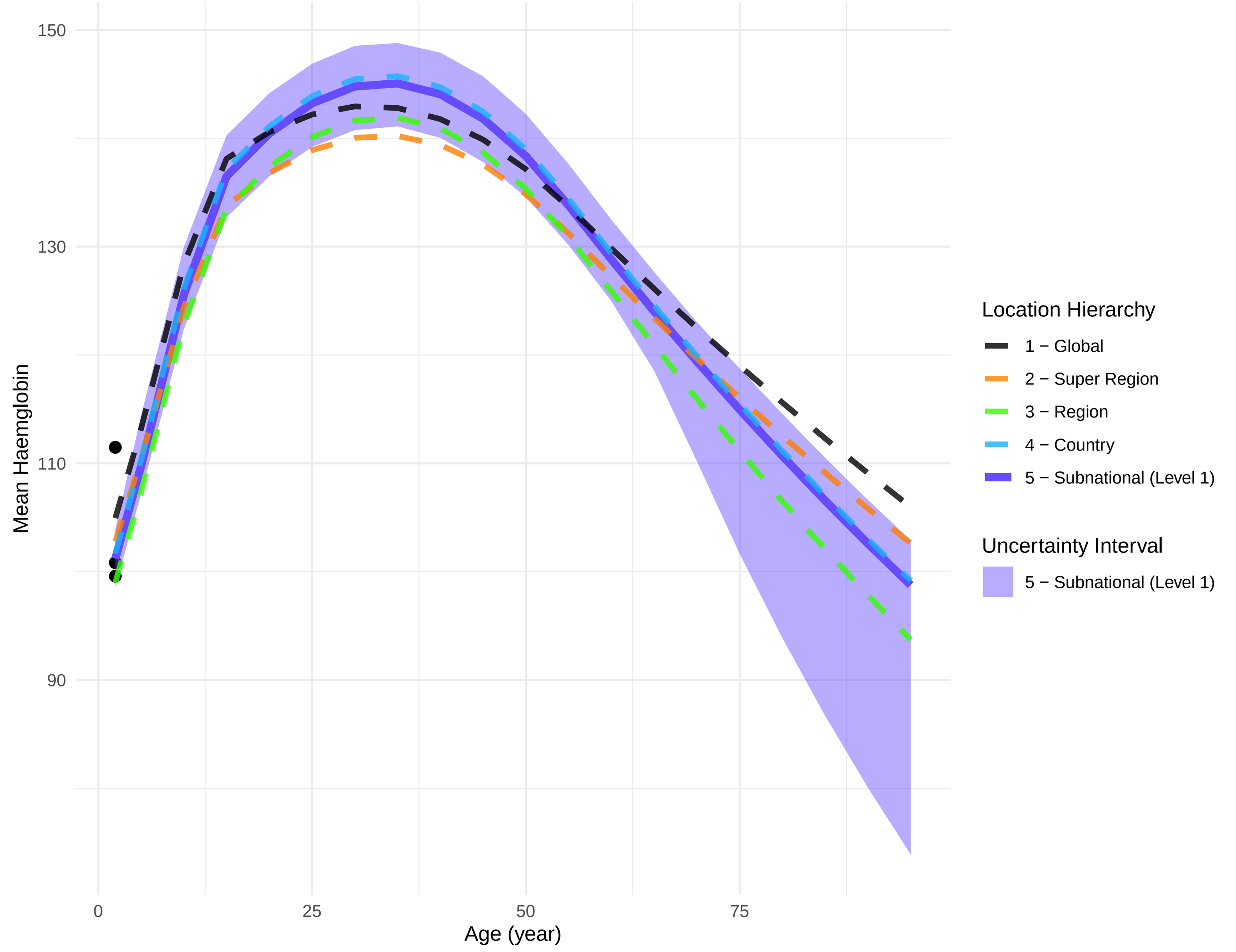

MR−BRT Age/Sex Fits − Males 2+ Years Old − Mean Haemglobin − Cross River
WHO−2024 Adjustment Method

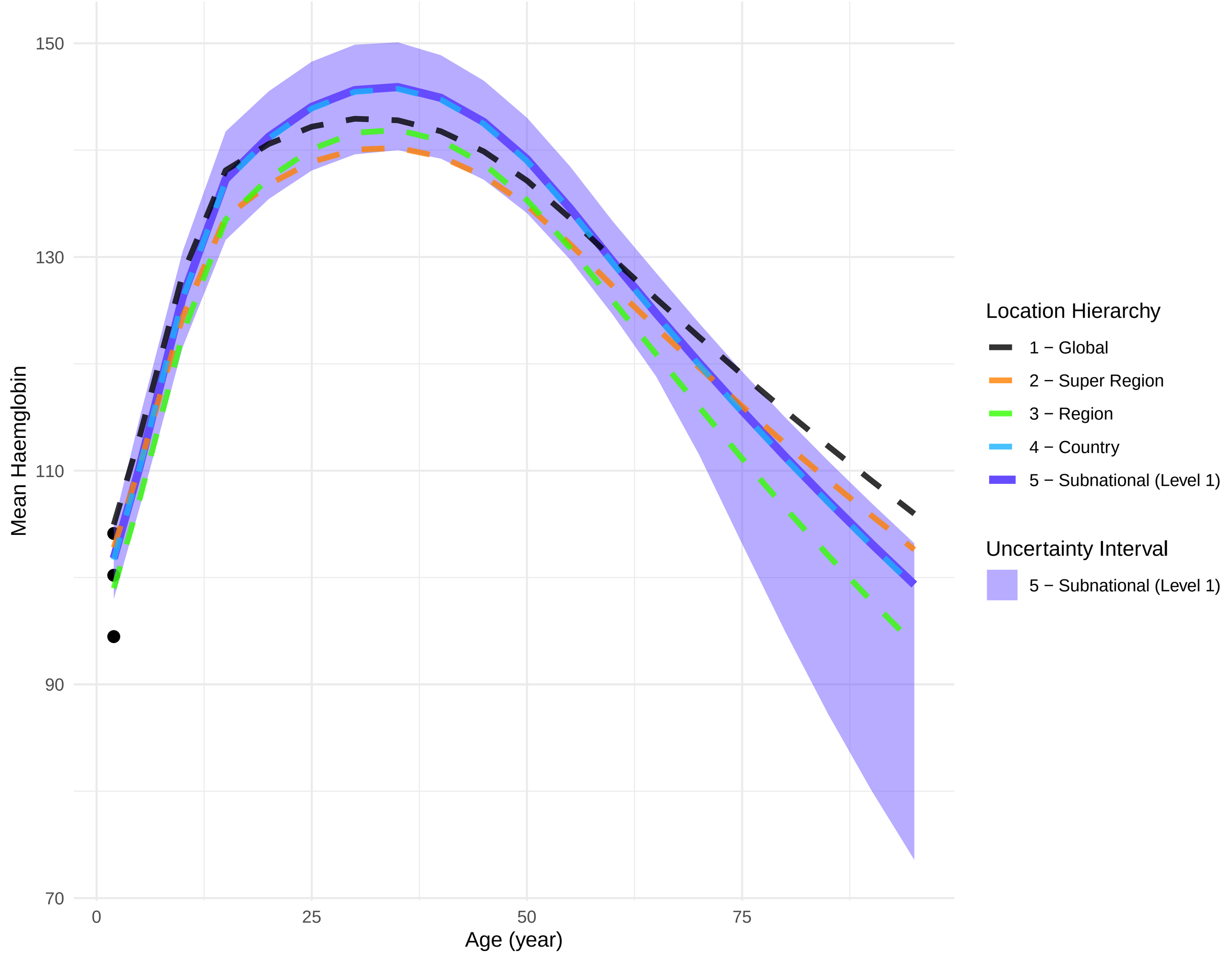

MR−BRT Age/Sex Fits − Males 2+ Years Old − Mean Haemglobin − Delta
WHO−2024 Adjustment Method

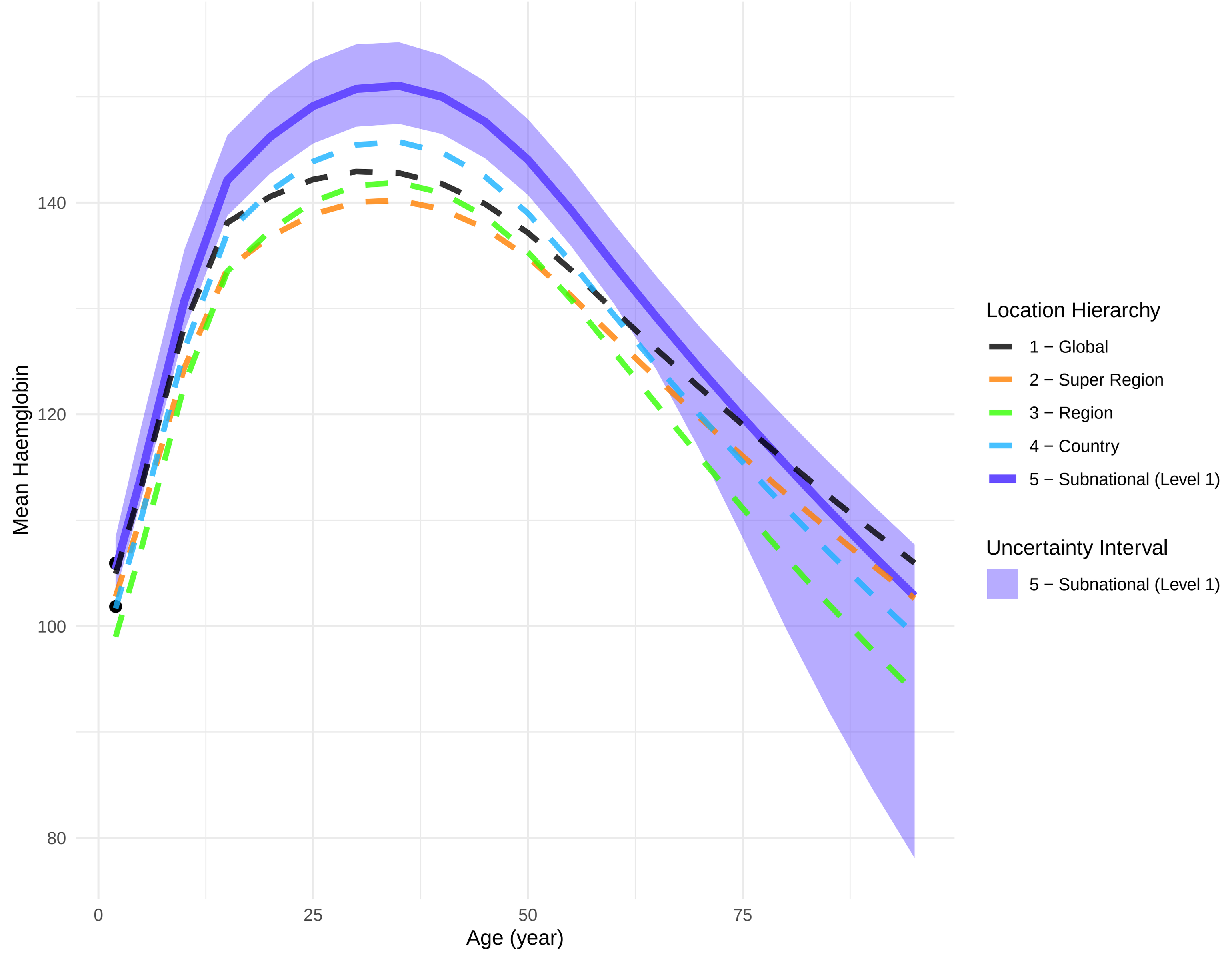

MR−BRT Age/Sex Fits − Males 2+ Years Old − Mean Haemglobin − Ebonyi
WHO−2024 Adjustment Method

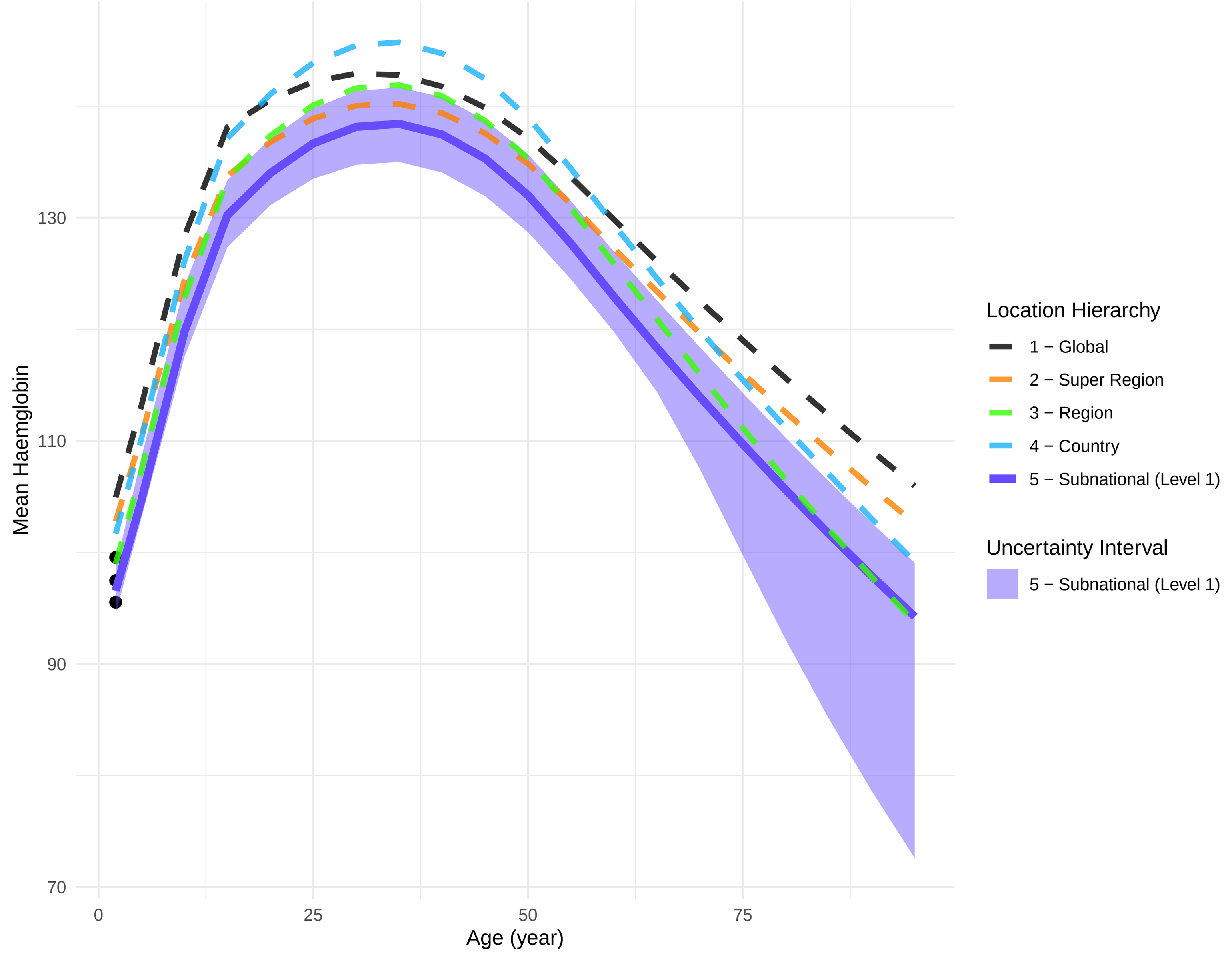

MR−BRT Age/Sex Fits − Males 2+ Years Old − Mean Haemglobin − Edo
WHO−2024 Adjustment Method

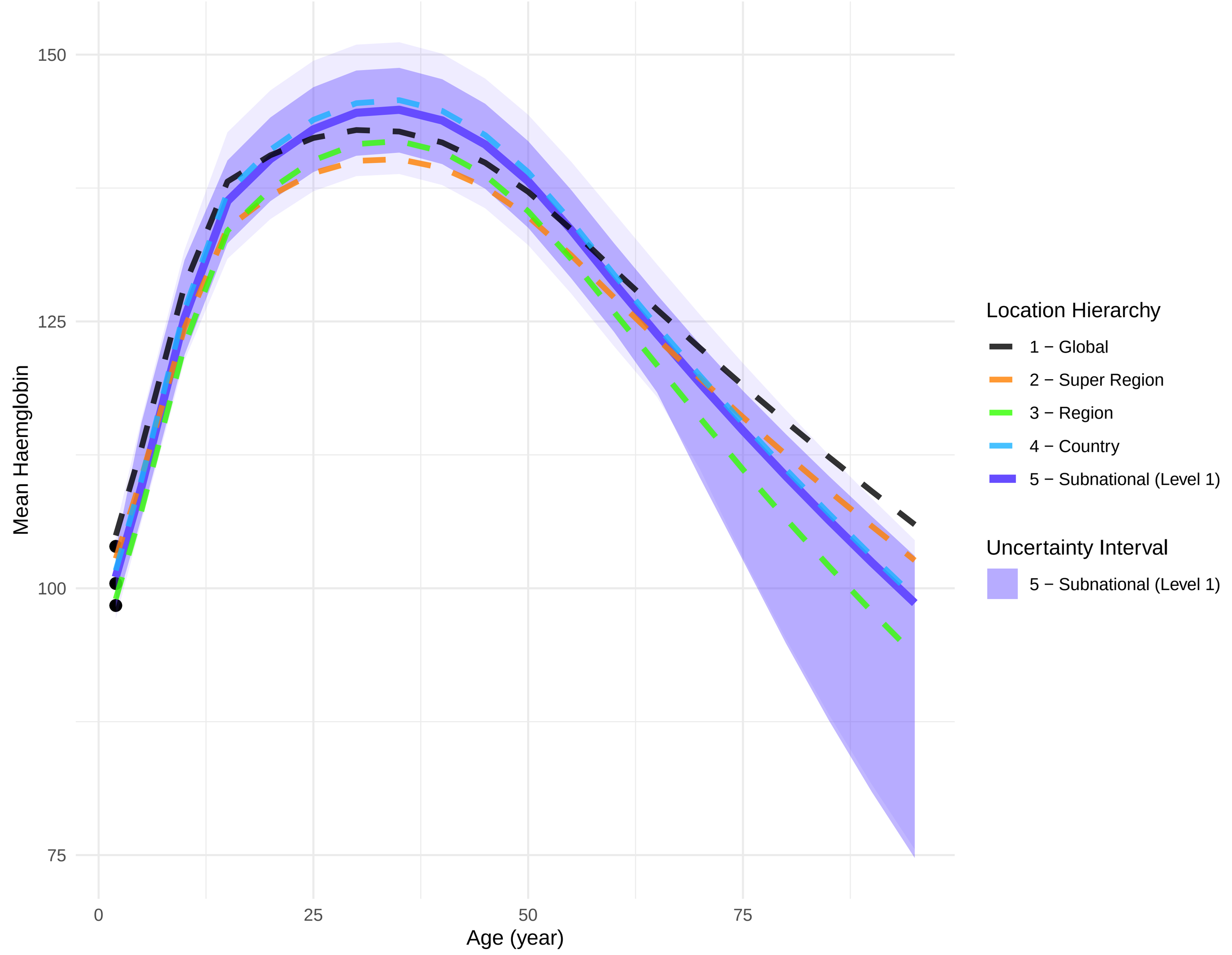

MR−BRT Age/Sex Fits − Males 2+ Years Old − Mean Haemglobin − Ekiti
WHO−2024 Adjustment Method

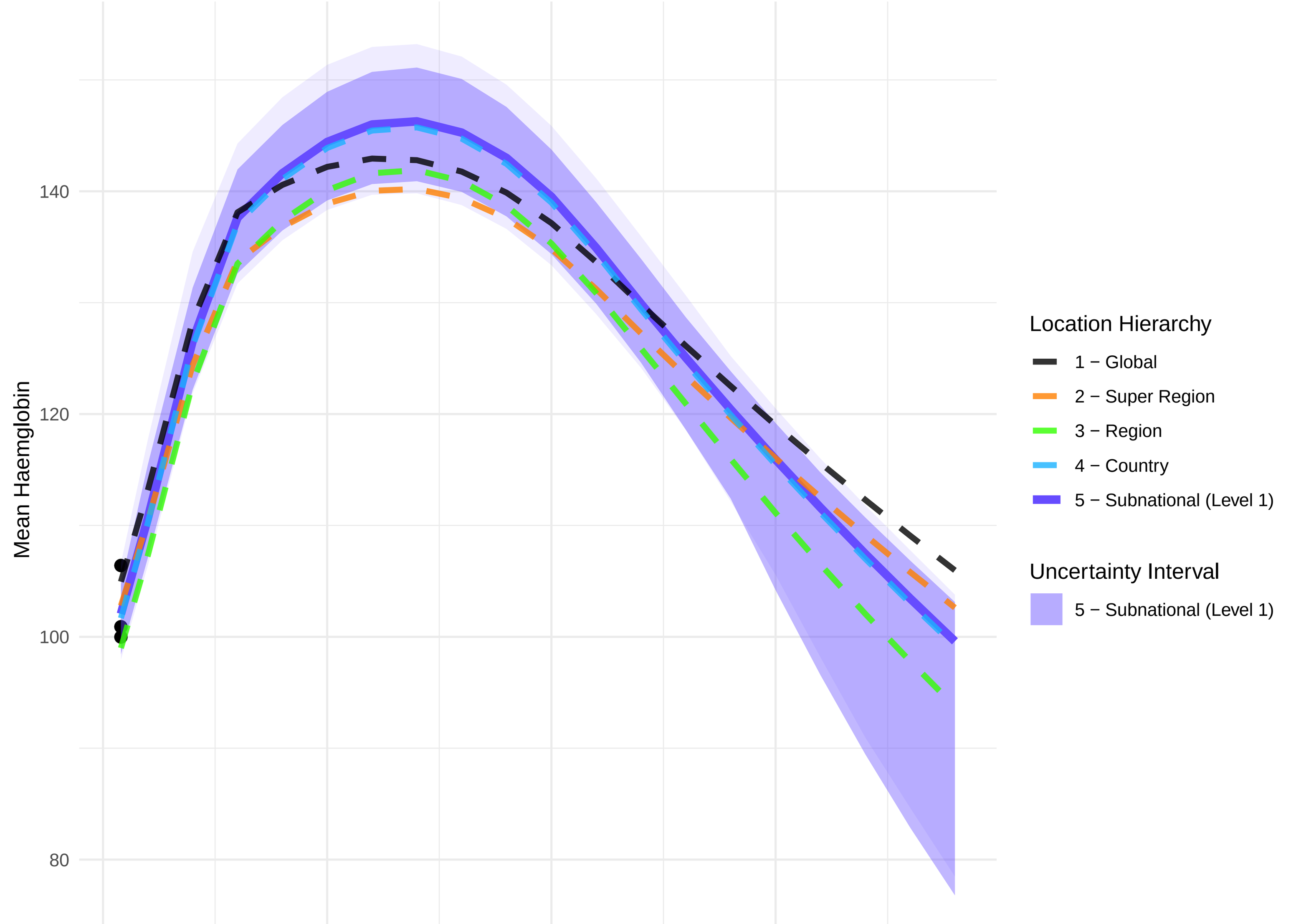

MR−BRT Age/Sex Fits − Males 2+ Years Old − Mean Haemglobin − Enugu
WHO−2024 Adjustment Method

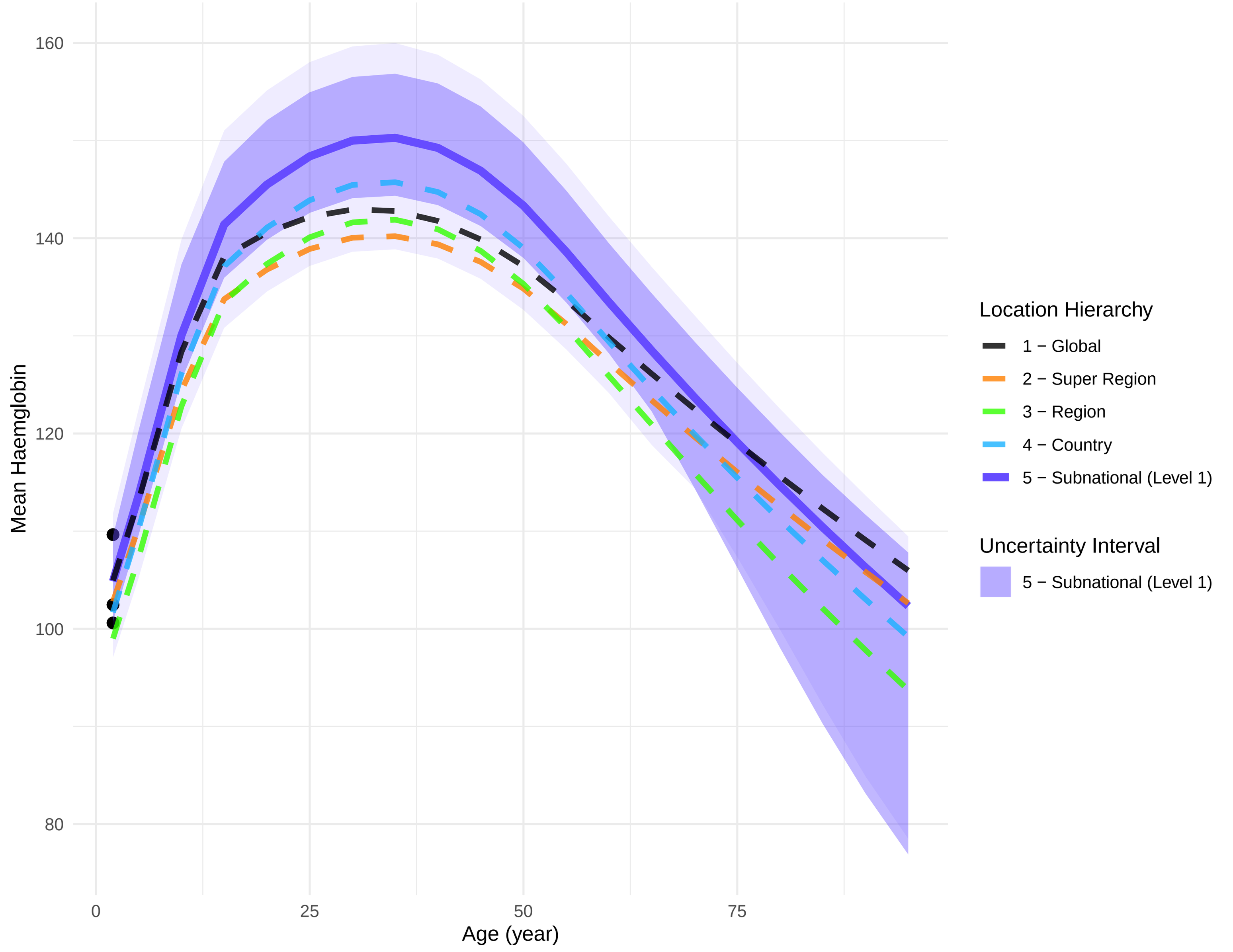

MR−BRT Age/Sex Fits − Males 2+ Years Old − Mean Haemglobin − FCT (Abuja)
WHO−2024 Adjustment Method

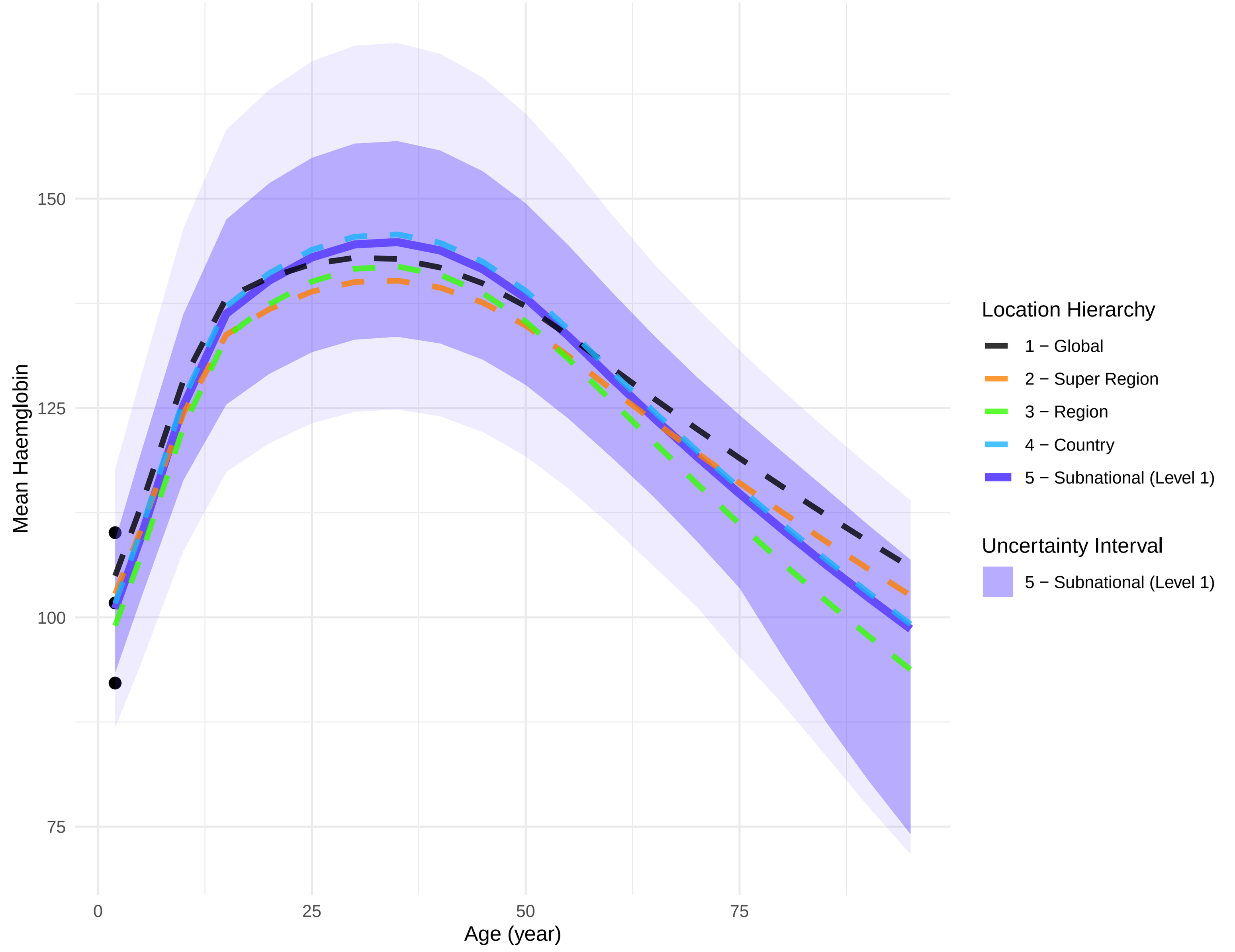

MR−BRT Age/Sex Fits − Males 2+ Years Old − Mean Haemglobin − Gombe
WHO−2024 Adjustment Method

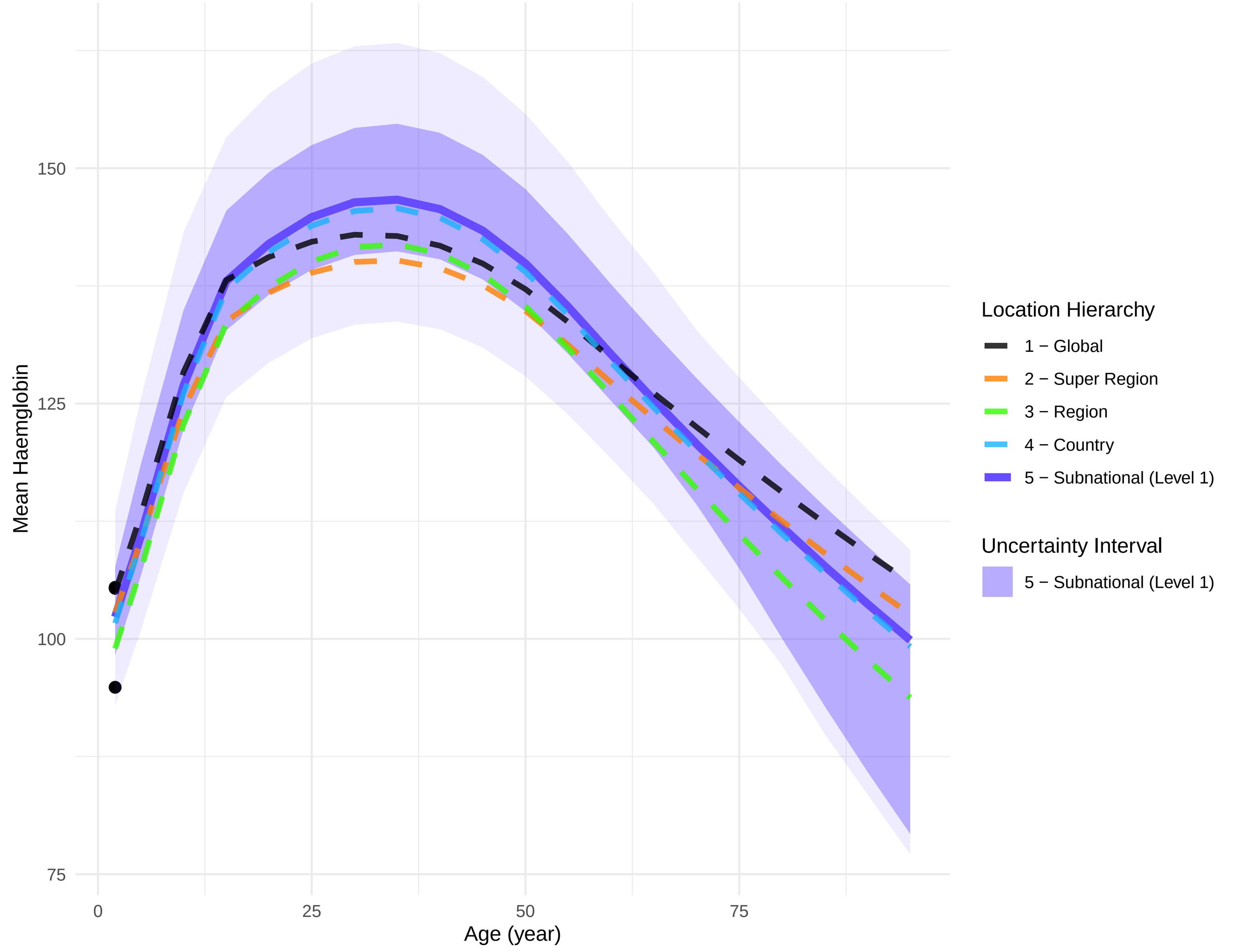

MR−BRT Age/Sex Fits − Males 2+ Years Old − Mean Haemglobin − Imo
WHO−2024 Adjustment Method

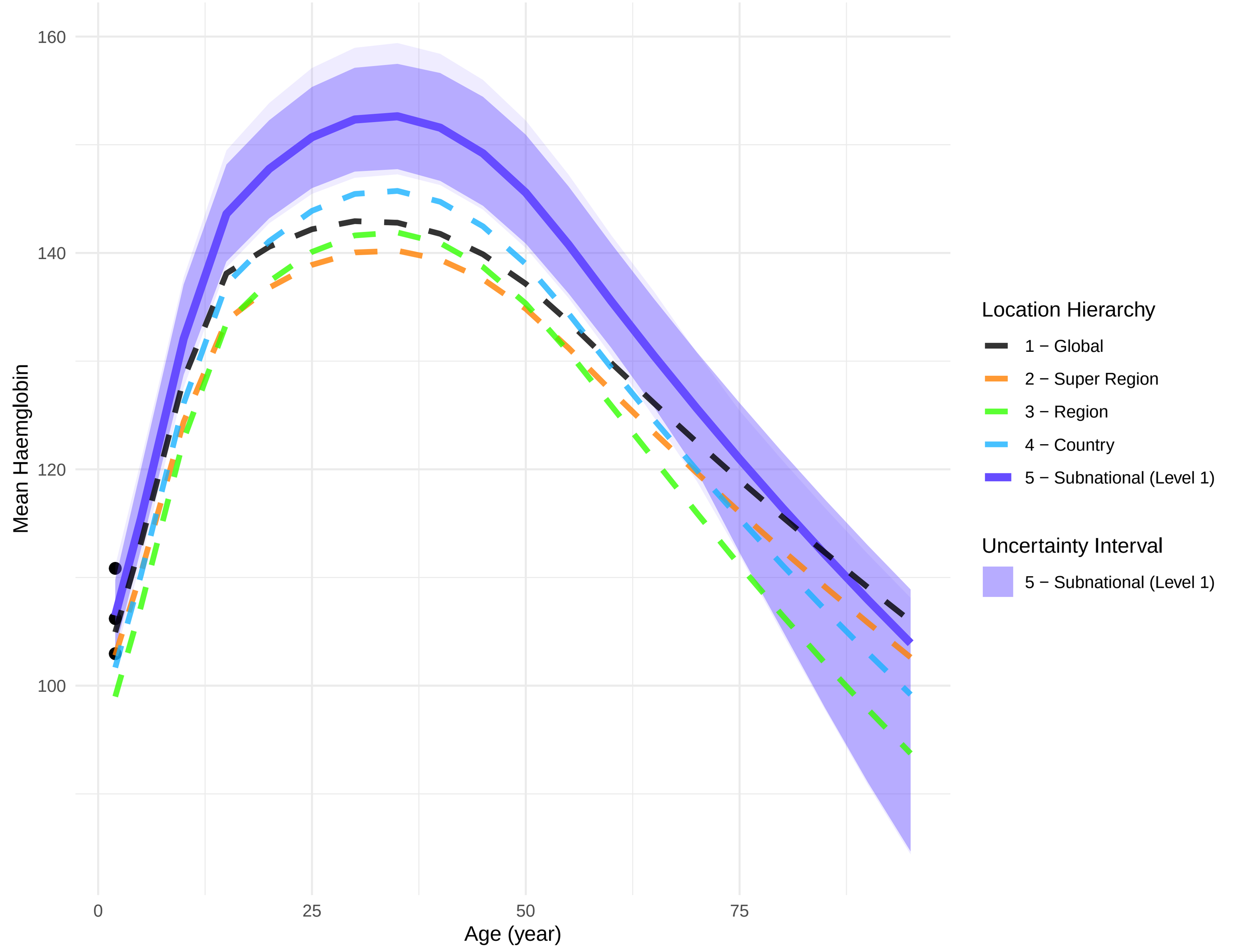

MR−BRT Age/Sex Fits − Males 2+ Years Old − Mean Haemglobin − Jigawa
WHO−2024 Adjustment Method

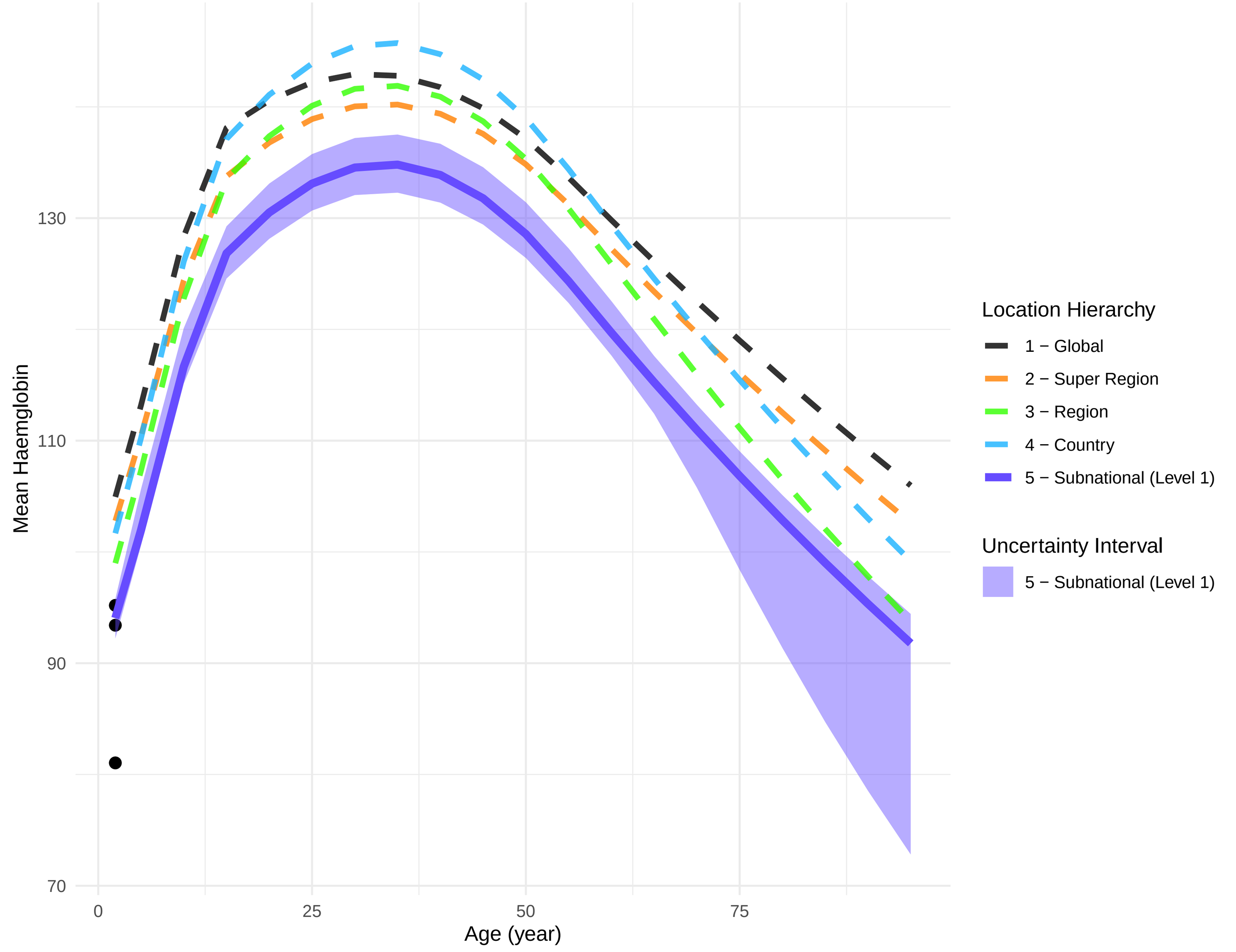

MR−BRT Age/Sex Fits − Males 2+ Years Old − Mean Haemglobin − Kaduna
WHO−2024 Adjustment Method

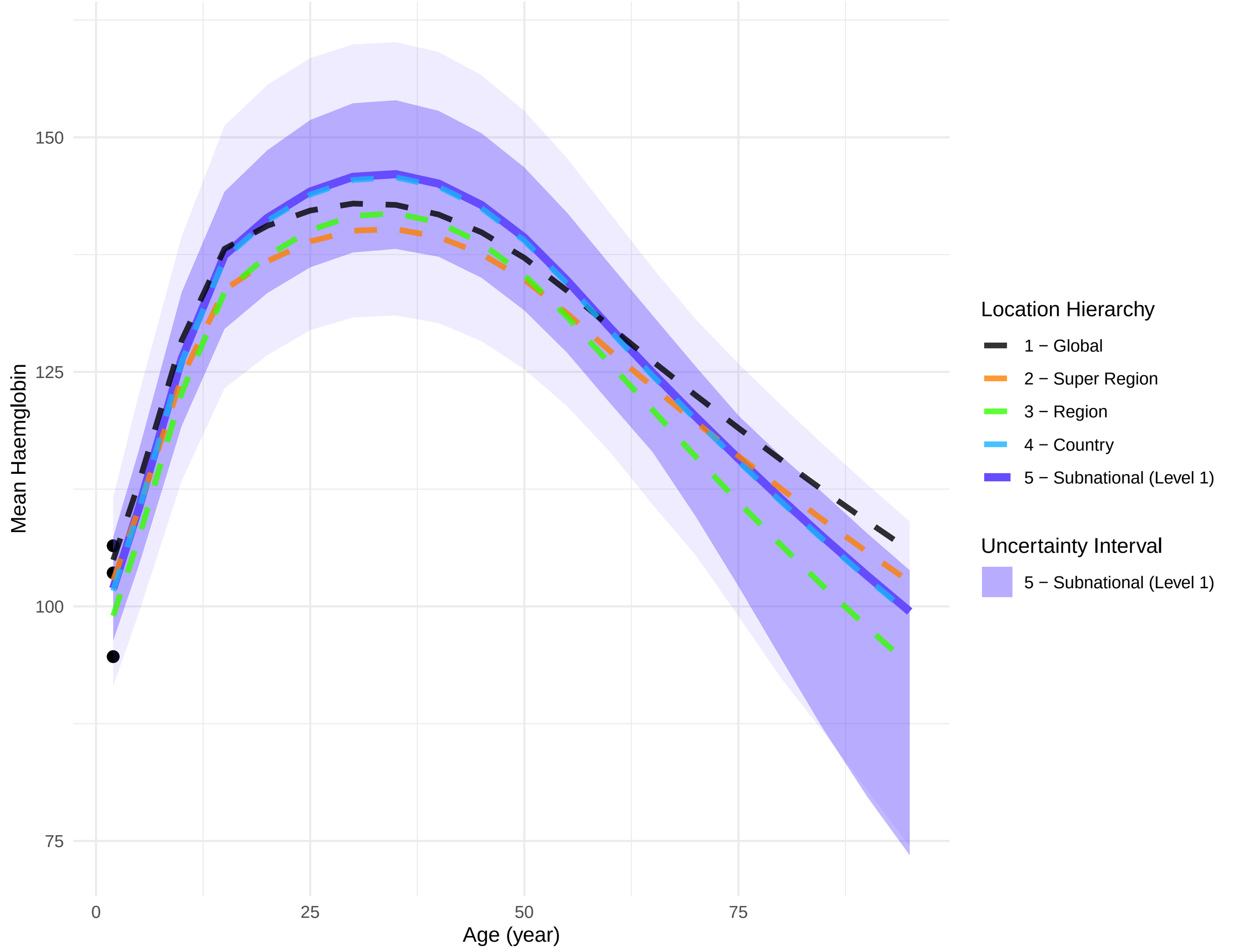

MR−BRT Age/Sex Fits − Males 2+ Years Old − Mean Haemglobin − Kano
WHO−2024 Adjustment Method

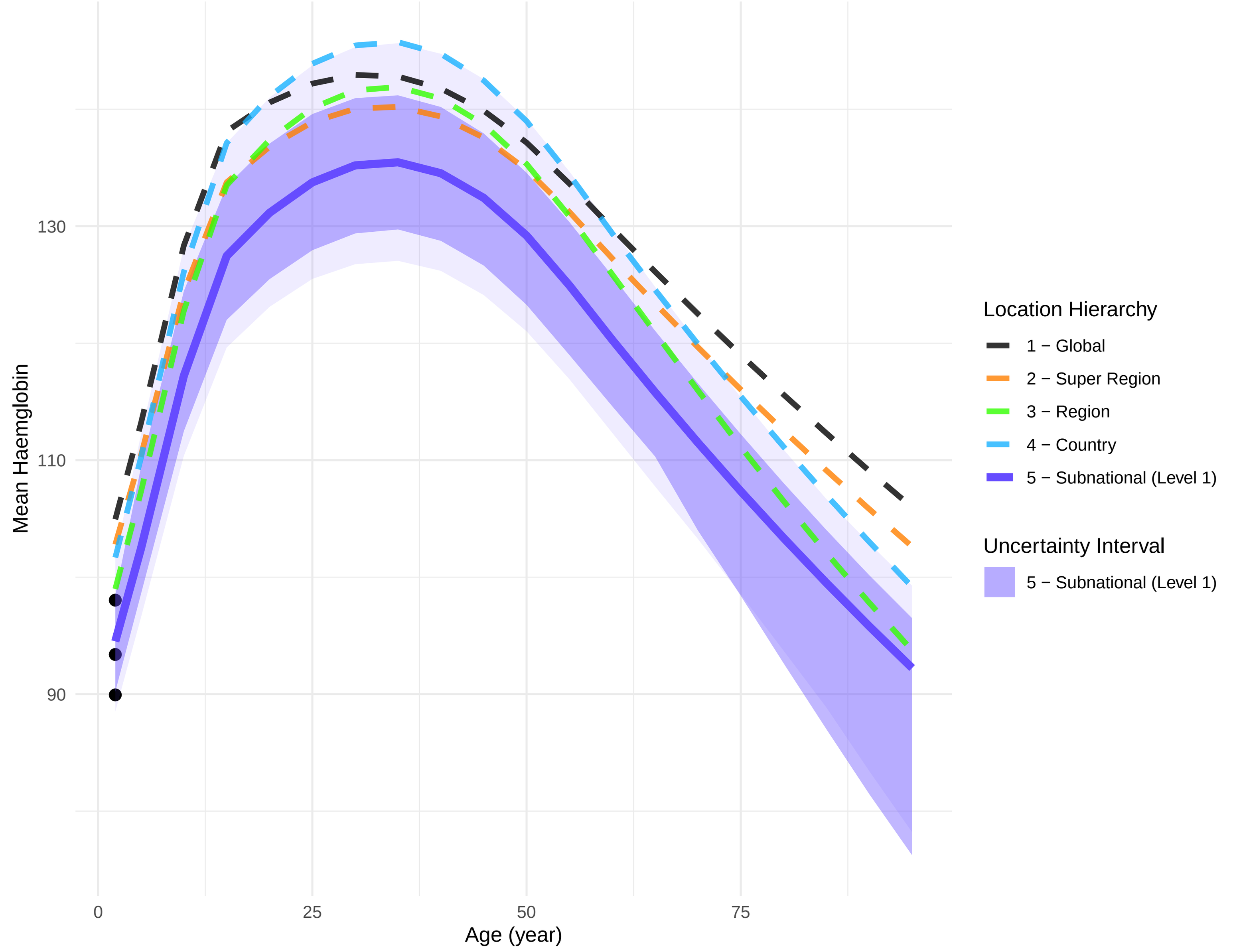

MR−BRT Age/Sex Fits − Males 2+ Years Old − Mean Haemglobin − Katsina
WHO−2024 Adjustment Method

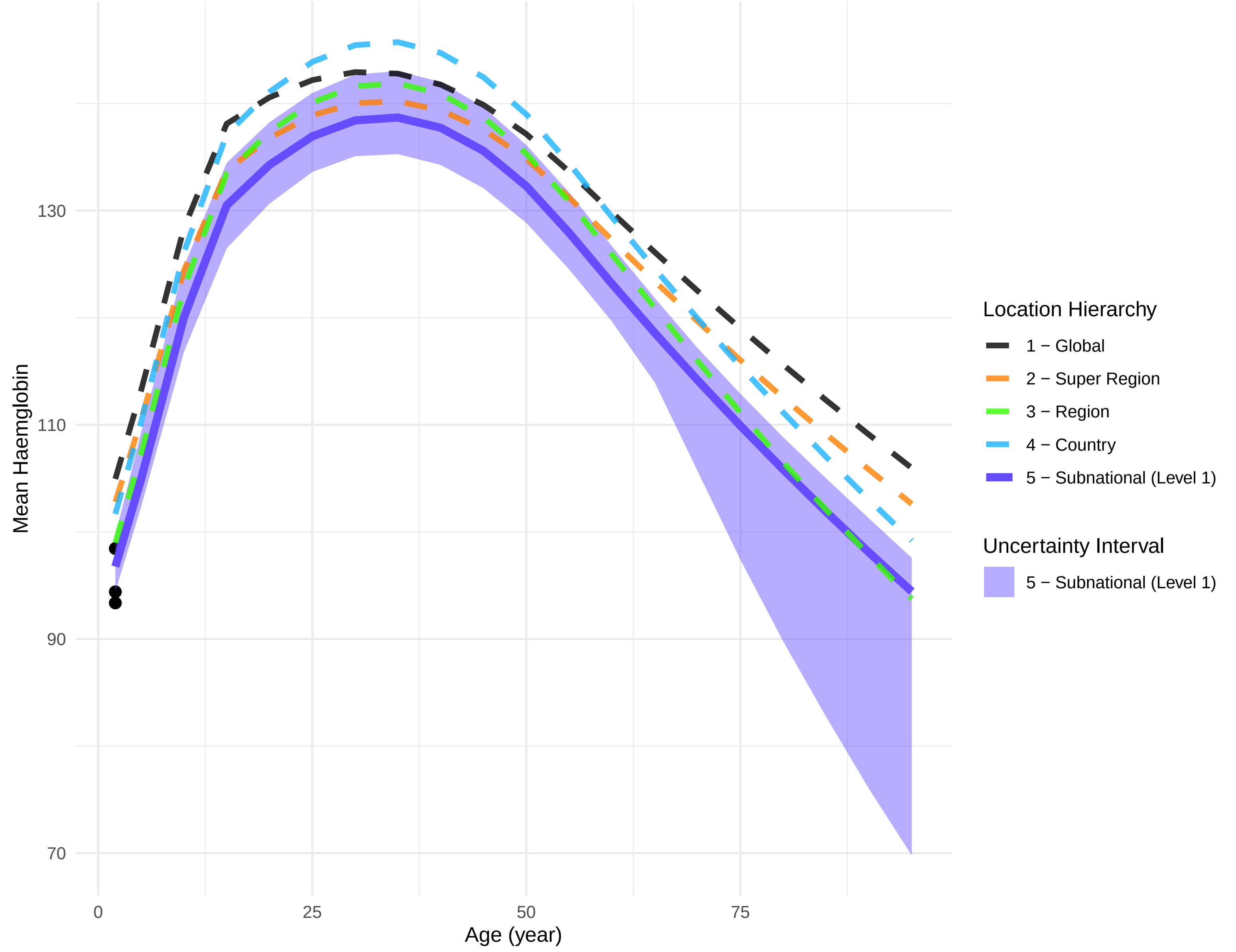

MR−BRT Age/Sex Fits − Males 2+ Years Old − Mean Haemglobin − Kebbi
WHO−2024 Adjustment Method

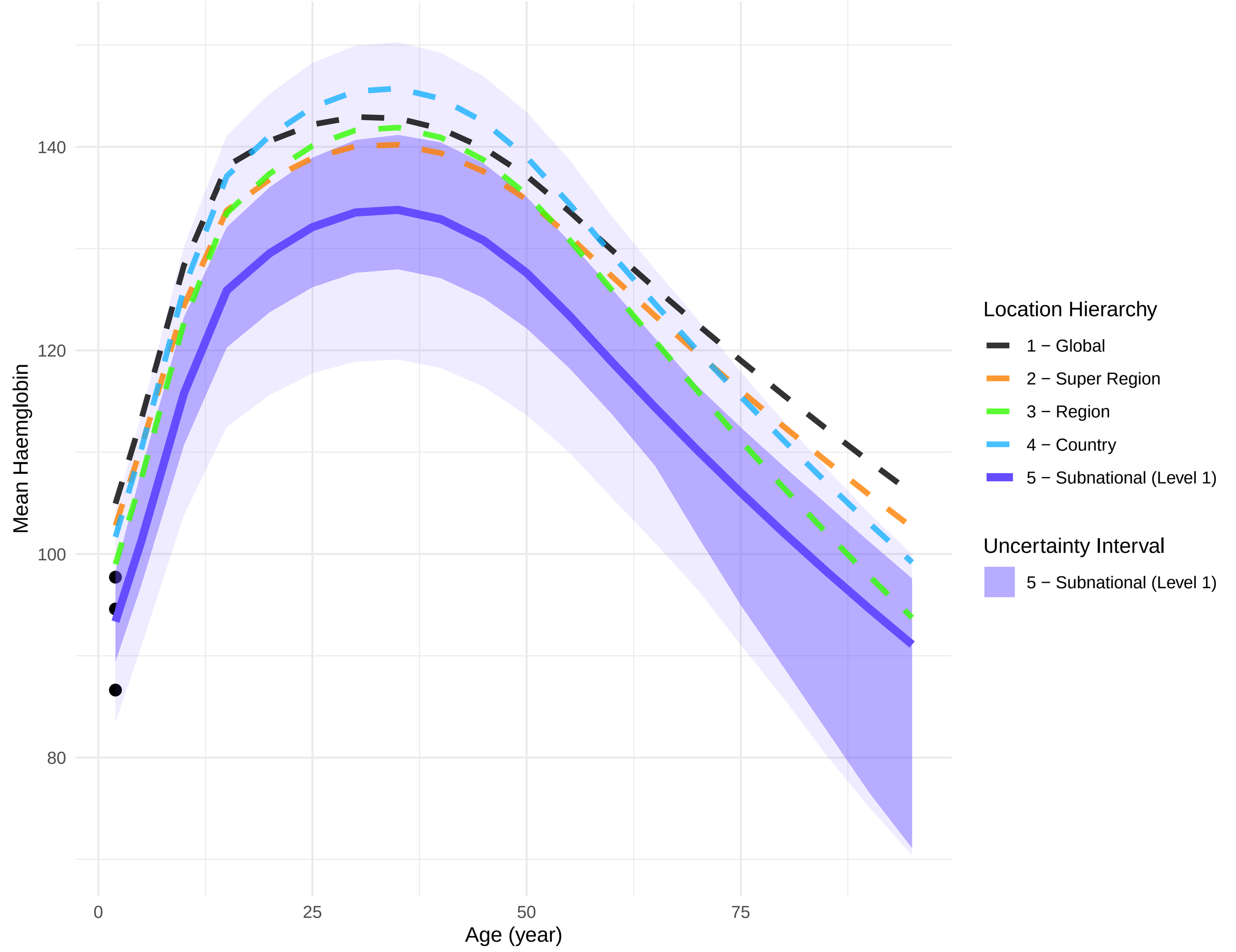

MR−BRT Age/Sex Fits − Males 2+ Years Old − Mean Haemglobin − Kogi
WHO−2024 Adjustment Method

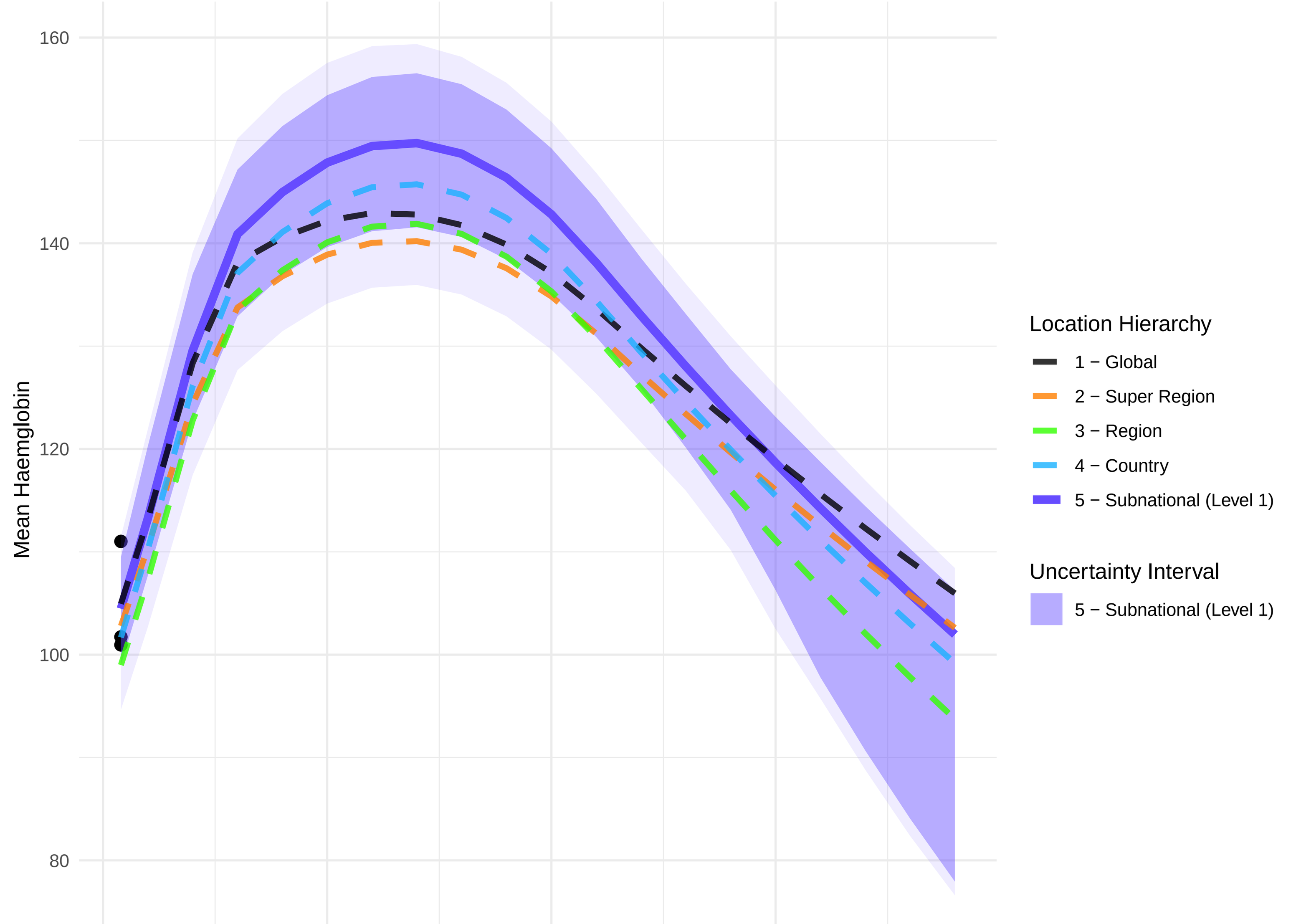

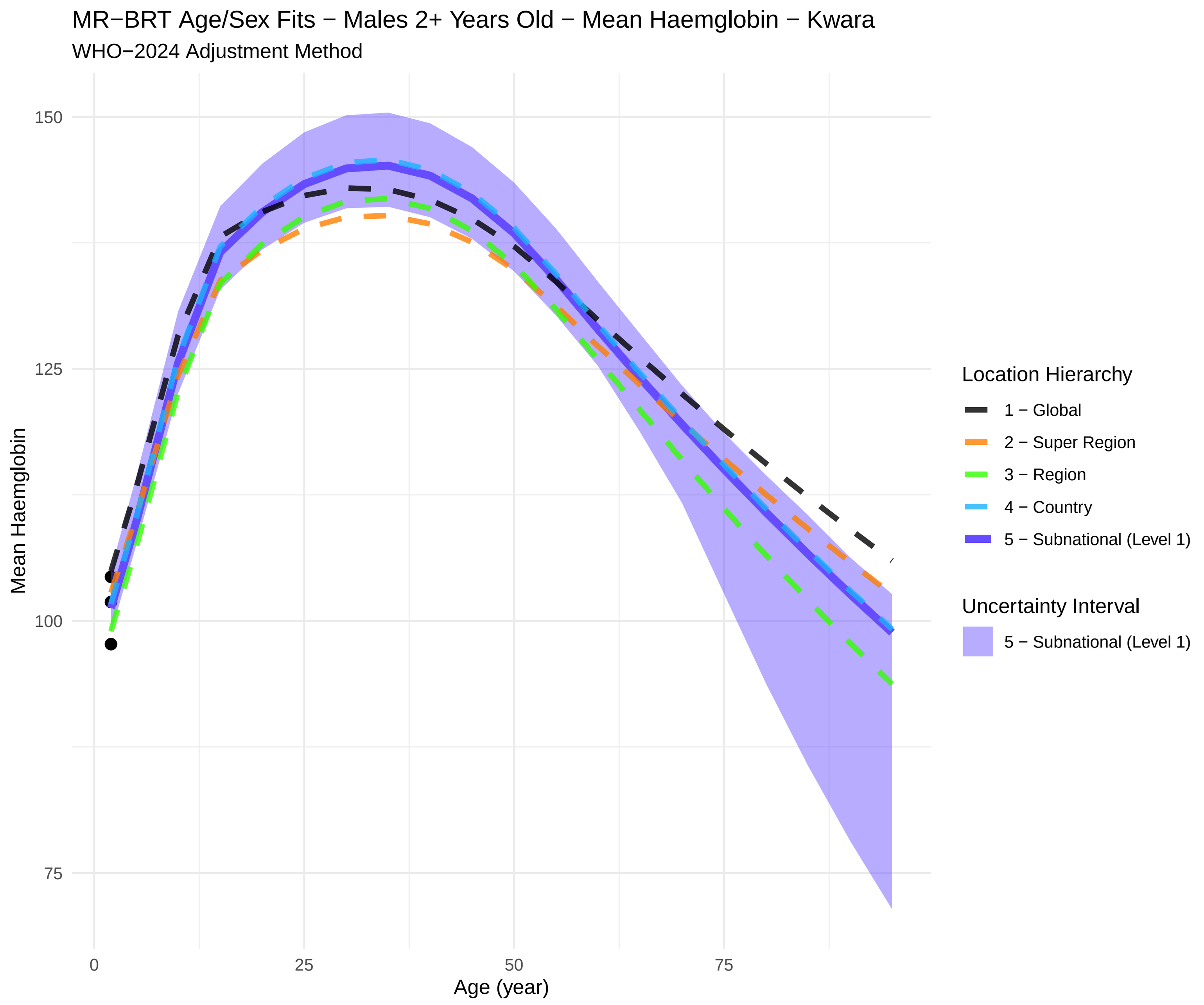
MR−BRT Age/Sex Fits − Males 2+ Years Old − Mean Haemglobin − Kwara
WHO−2024 Adjustment Method
Mean Haemglobin
150
125
100
75
0
25
50
75
Age (year)
Location Hierarchy
1 − Global
2 − Super Region
3 − Region
4 − Country
5 − Subnational (Level 1)
Uncertainty Interval
5 − Subnational (Level 1)

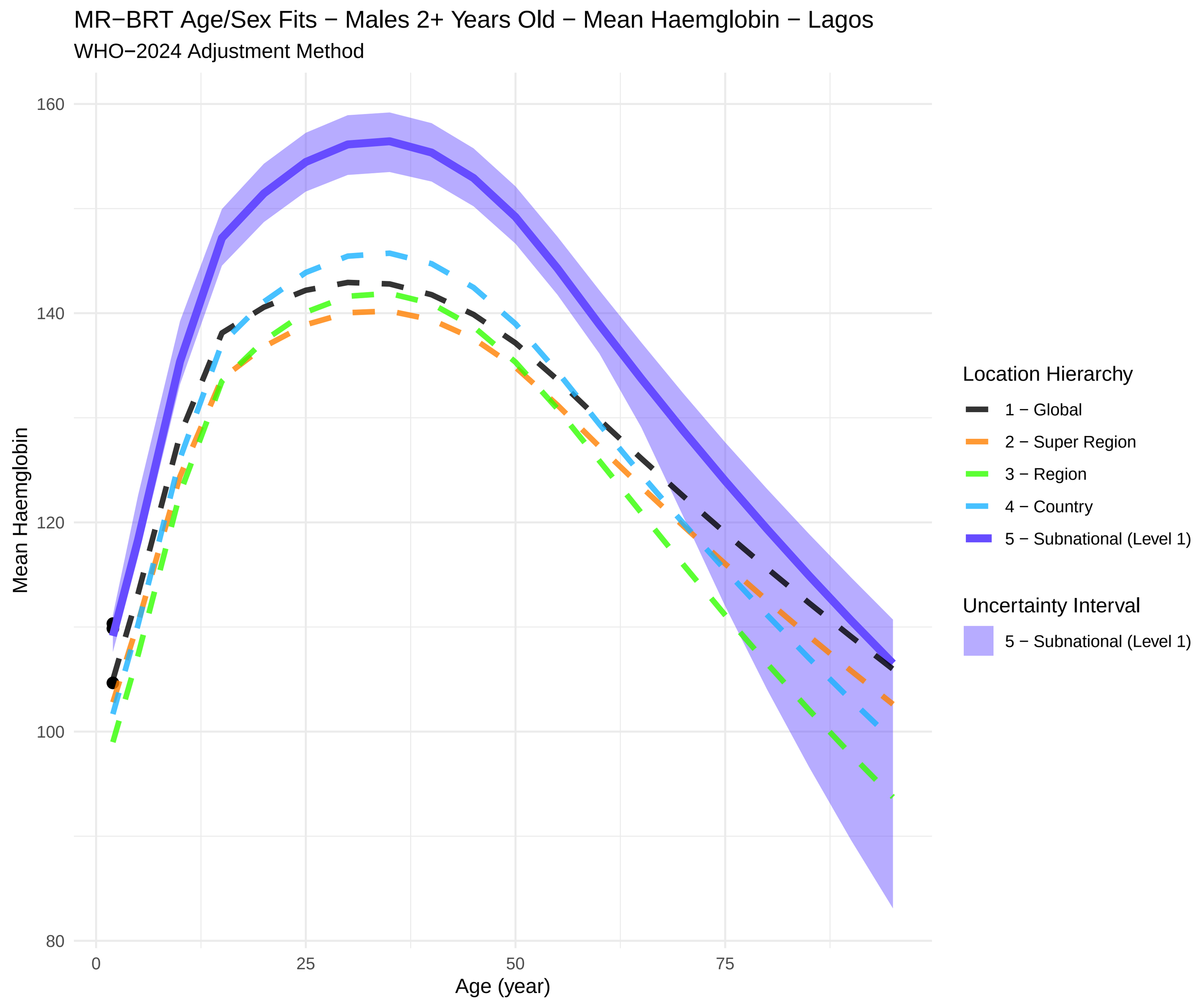
MR−BRT Age/Sex Fits − Males 2+ Years Old − Mean Haemglobin − Lagos
WHO−2024 Adjustment Method
Mean Haemglobin
160
140
120
100
80
0
25
50
75
Age (year)
Location Hierarchy
1 − Global
2 − Super Region
3 − Region
4 − Country
5 − Subnational (Level 1)
Uncertainty Interval
5 − Subnational (Level 1)

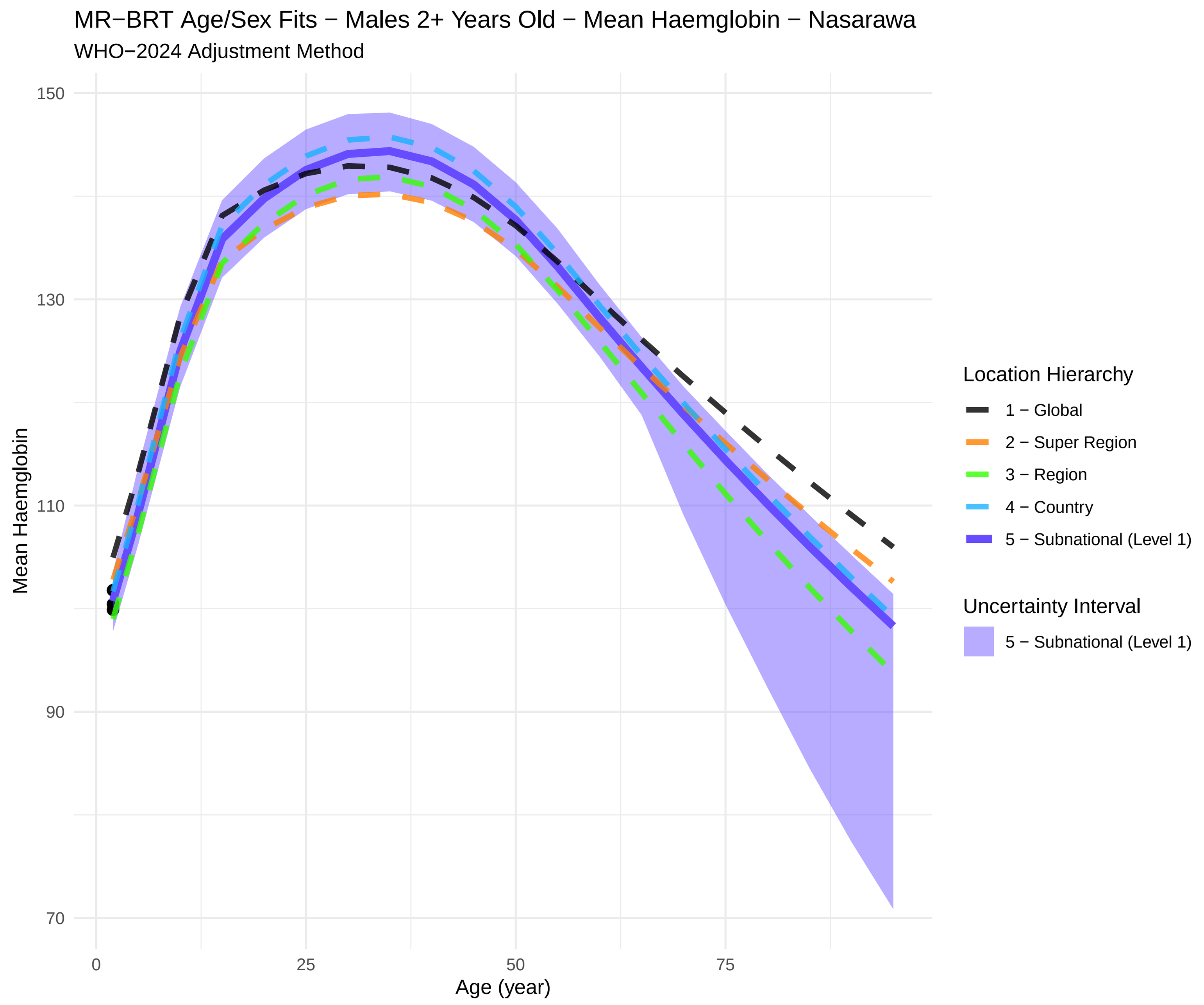
MR−BRT Age/Sex Fits − Males 2+ Years Old − Mean Haemglobin − Nasarawa
WHO−2024 Adjustment Method
Mean Haemglobin
150
130
110
90
70
0
25
50
75
Age (year)
Location Hierarchy
1 − Global
2 − Super Region
3 − Region
4 − Country
5 − Subnational (Level 1)
Uncertainty Interval
5 − Subnational (Level 1)

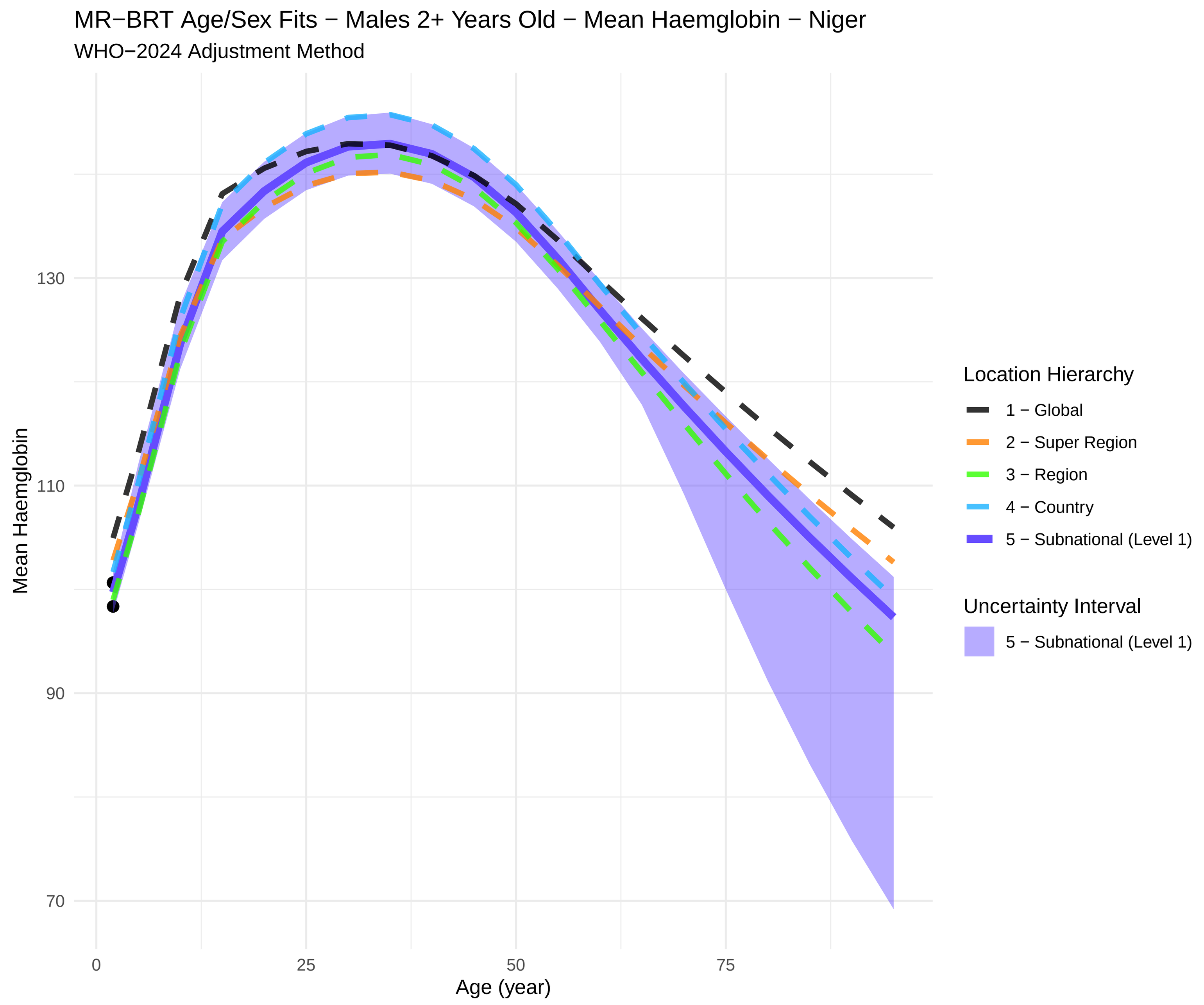
MR−BRT Age/Sex Fits − Males 2+ Years Old − Mean Haemglobin − Niger
WHO−2024 Adjustment Method
Mean Haemglobin
130
110
90
70
0
25
50
75
Age (year)
Location Hierarchy
1 − Global
2 − Super Region
3 − Region
4 − Country
5 − Subnational (Level 1)
Uncertainty Interval
5 − Subnational (Level 1)

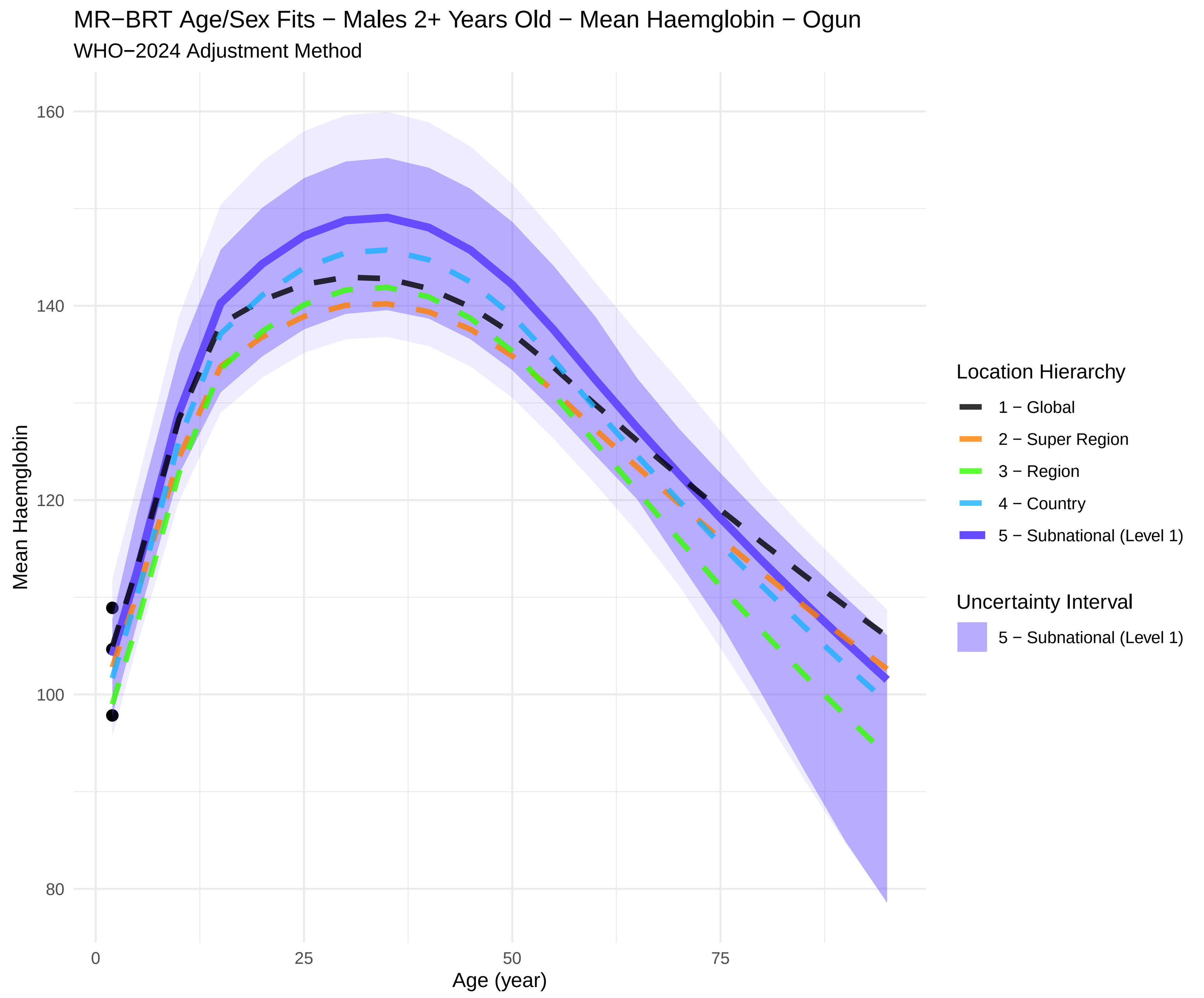
MR−BRT Age/Sex Fits − Males 2+ Years Old − Mean Haemglobin − Ogun
WHO−2024 Adjustment Method
Mean Haemglobin
160
140
120
100
80
0
25
50
75
Age (year)
Location Hierarchy
1 − Global
2 − Super Region
3 − Region
4 − Country
5 − Subnational (Level 1)
Uncertainty Interval
5 − Subnational (Level 1)

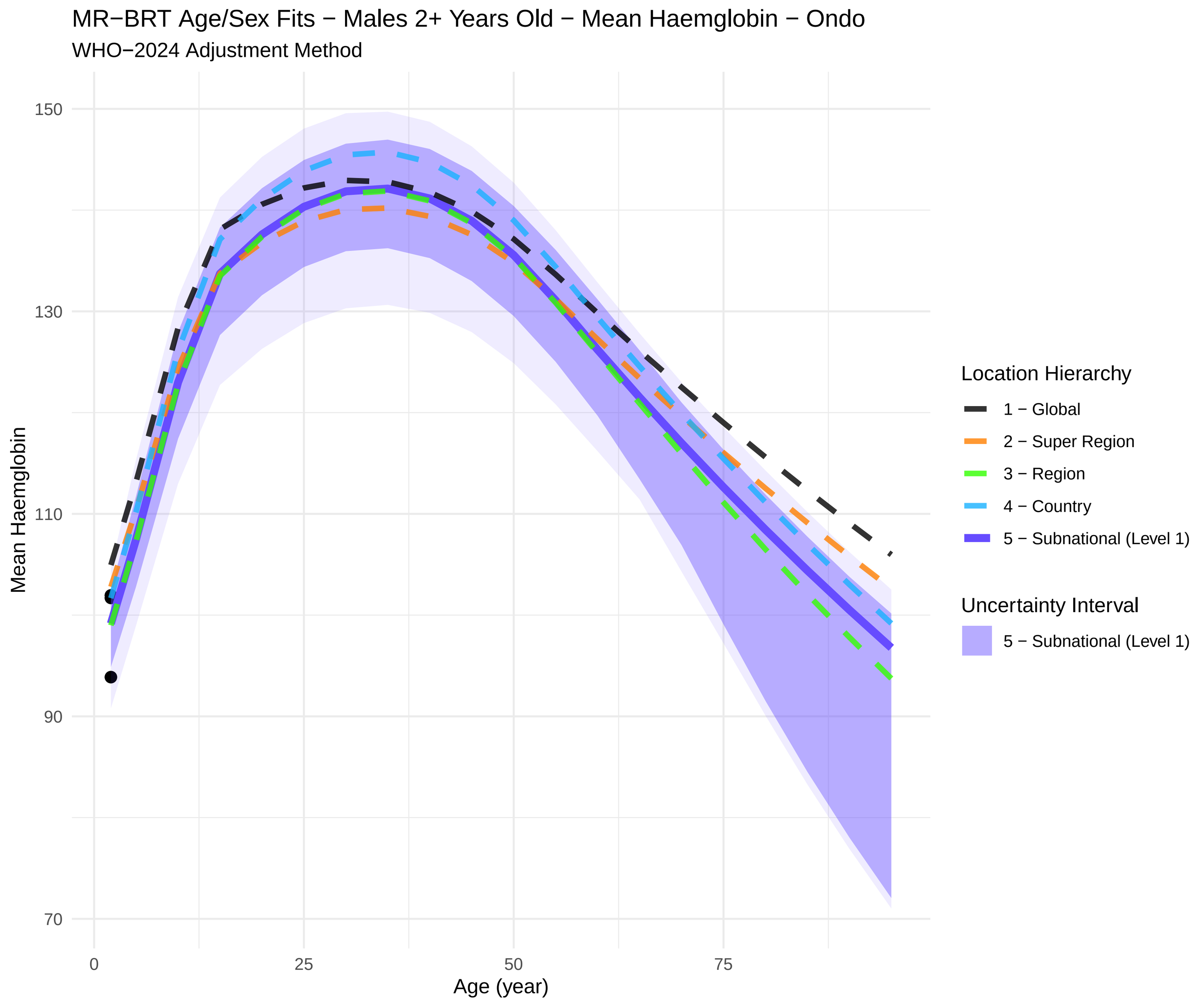
MR−BRT Age/Sex Fits − Males 2+ Years Old − Mean Haemglobin − Ondo
WHO−2024 Adjustment Method
Mean Haemglobin
150
130
110
90
70
0
25
50
75
Age (year)
Location Hierarchy
1 − Global
2 − Super Region
3 − Region
4 − Country
5 − Subnational (Level 1)
Uncertainty Interval
5 − Subnational (Level 1)

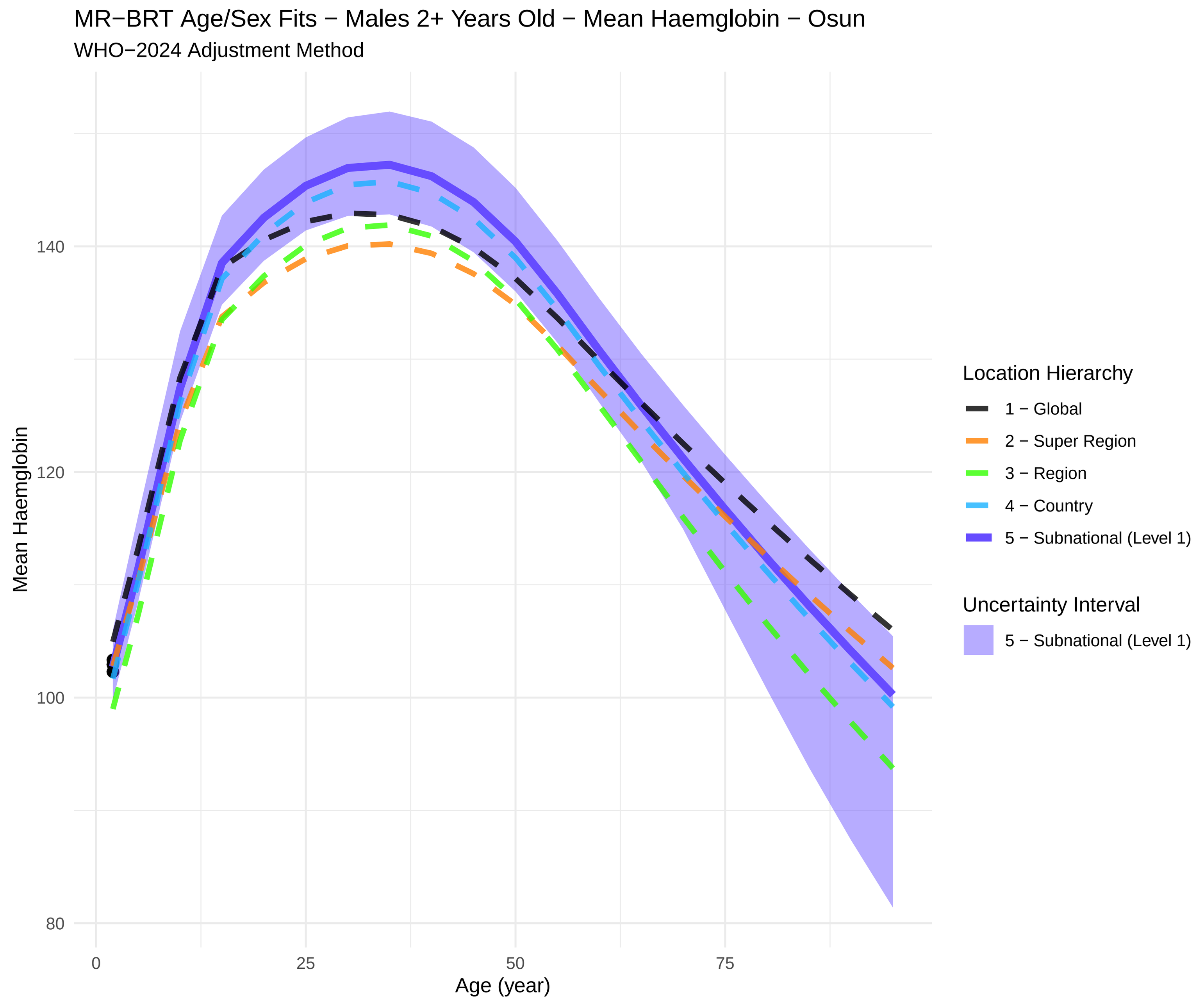
MR−BRT Age/Sex Fits − Males 2+ Years Old − Mean Haemglobin − Osun
WHO−2024 Adjustment Method
Mean Haemglobin
140
120
100
80
0
25
50
75
Age (year)
Location Hierarchy
1 − Global
2 − Super Region
3 − Region
4 − Country
5 − Subnational (Level 1)
Uncertainty Interval
5 − Subnational (Level 1)

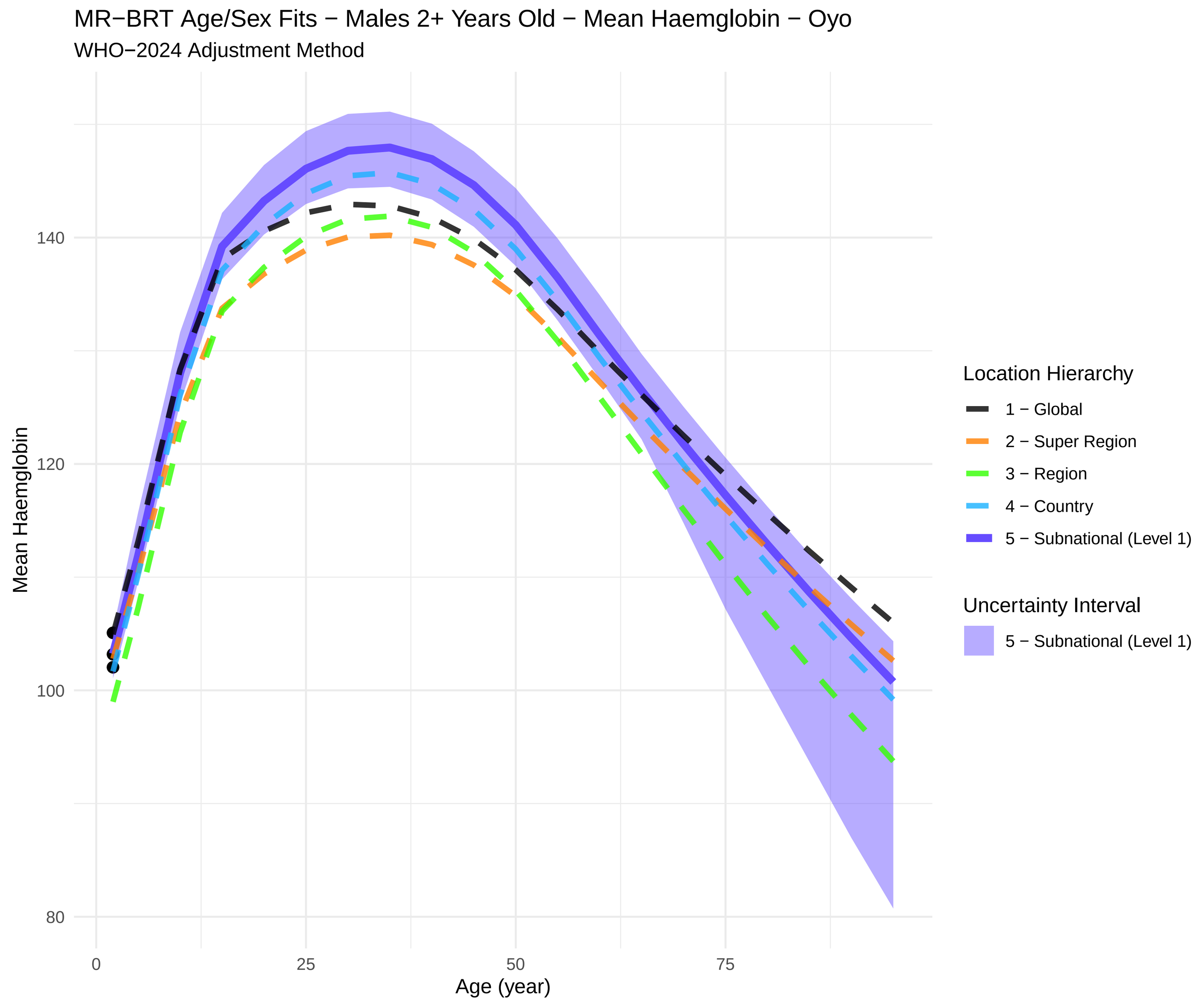
MR−BRT Age/Sex Fits − Males 2+ Years Old − Mean Haemglobin − Oyo
WHO−2024 Adjustment Method
Mean Haemglobin
140
120
100
80
0
25
50
75
Age (year)
Location Hierarchy
1 − Global
2 − Super Region
3 − Region
4 − Country
5 − Subnational (Level 1)
Uncertainty Interval
5 − Subnational (Level 1)

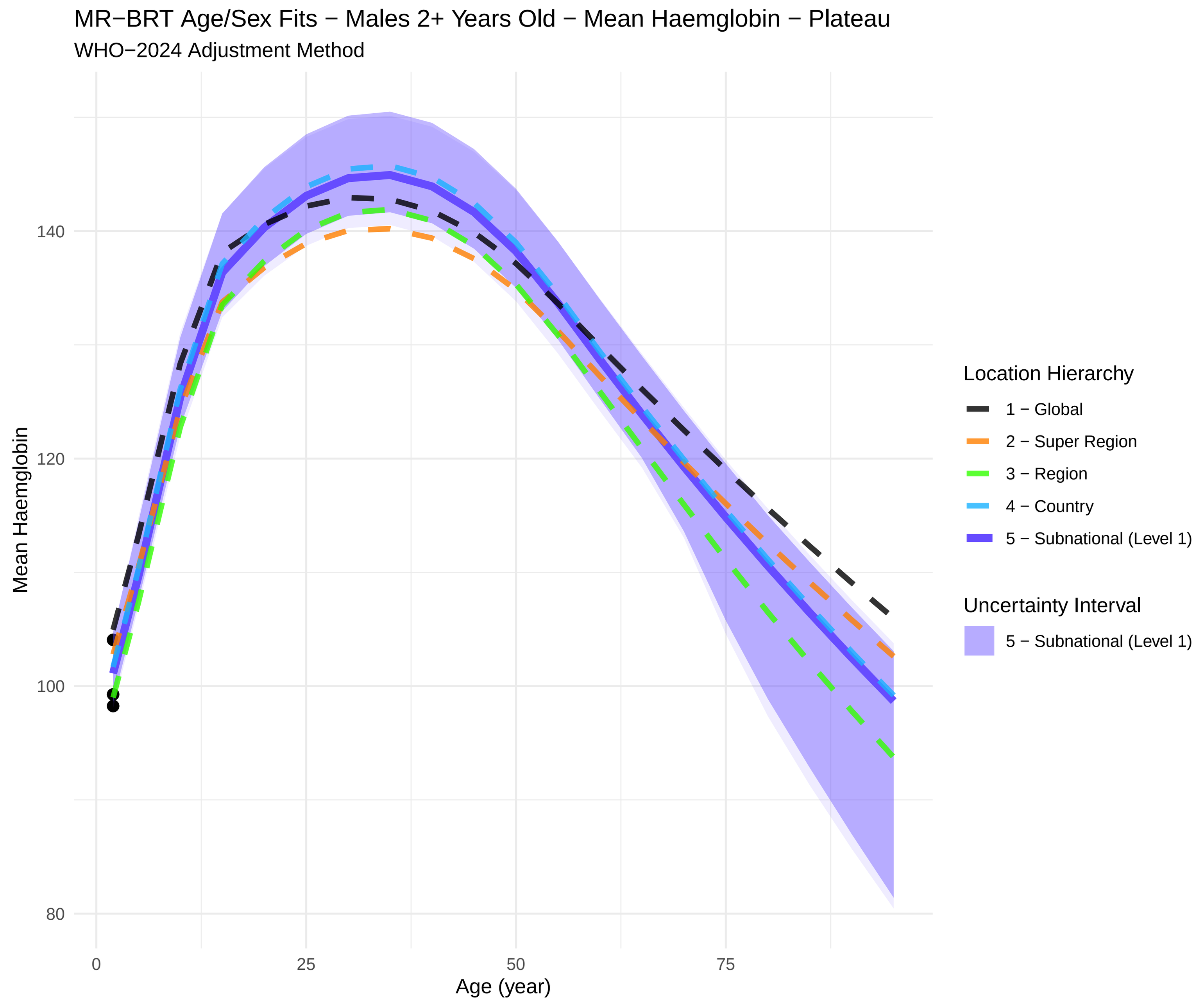
MR−BRT Age/Sex Fits − Males 2+ Years Old − Mean Haemglobin − Plateau
WHO−2024 Adjustment Method
Mean Haemglobin
140
120
100
80
0
25
50
75
Age (year)
Location Hierarchy
1 − Global
2 − Super Region
3 − Region
4 − Country
5 − Subnational (Level 1)
Uncertainty Interval
5 − Subnational (Level 1)

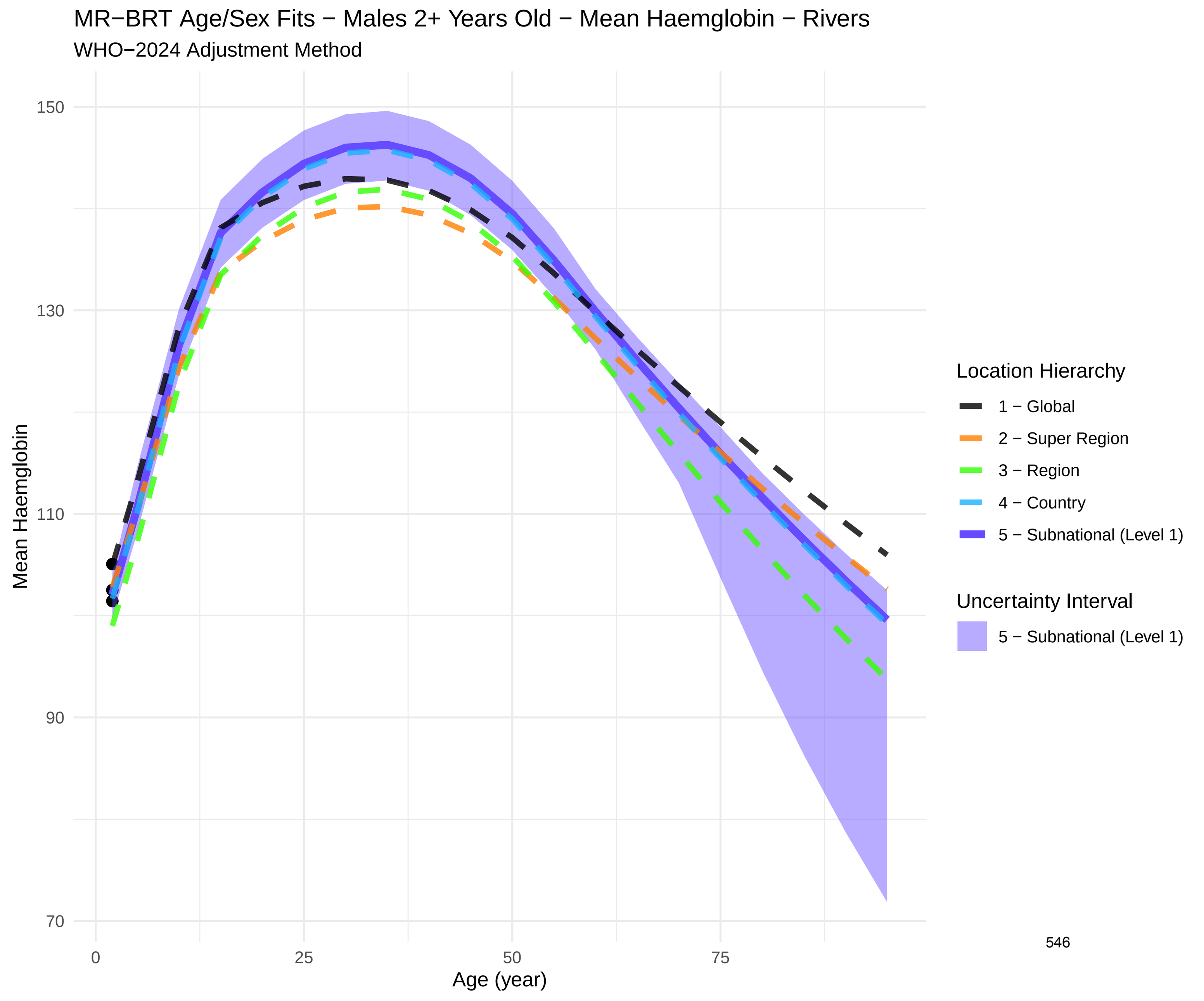
MR−BRT Age/Sex Fits − Males 2+ Years Old − Mean Haemglobin − Rivers
WHO−2024 Adjustment Method
Mean Haemglobin
150
130
110
90
70
0
25
50
75
Age (year)
Location Hierarchy
1 − Global
2 − Super Region
3 − Region
4 − Country
5 − Subnational (Level 1)
Uncertainty Interval
5 − Subnational (Level 1)

MR−BRT Age/Sex Fits − Males 2+ Years Old − Mean Haemglobin − Sokoto
WHO−2024 Adjustment Method

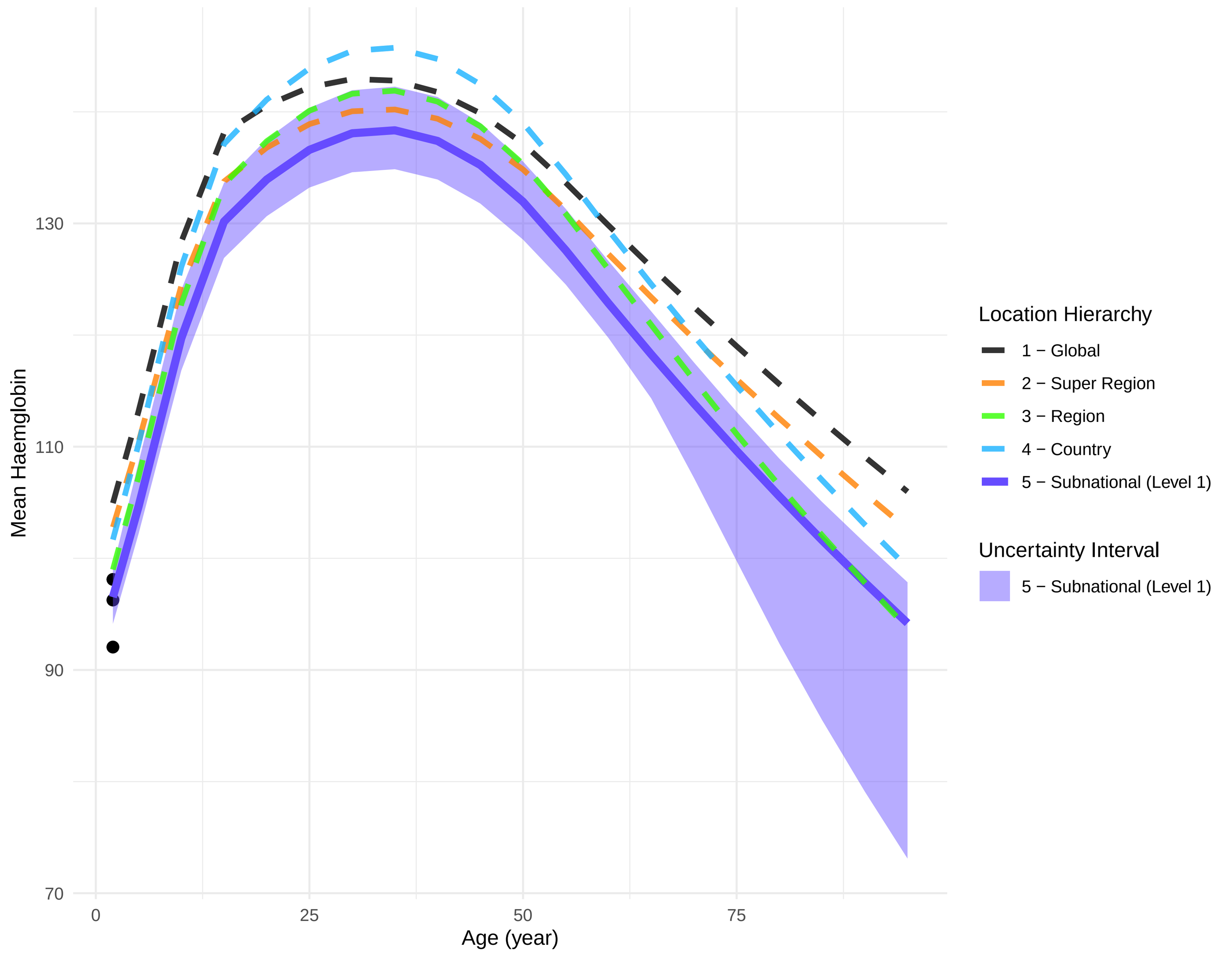

MR−BRT Age/Sex Fits − Males 2+ Years Old − Mean Haemglobin − Taraba
WHO−2024 Adjustment Method

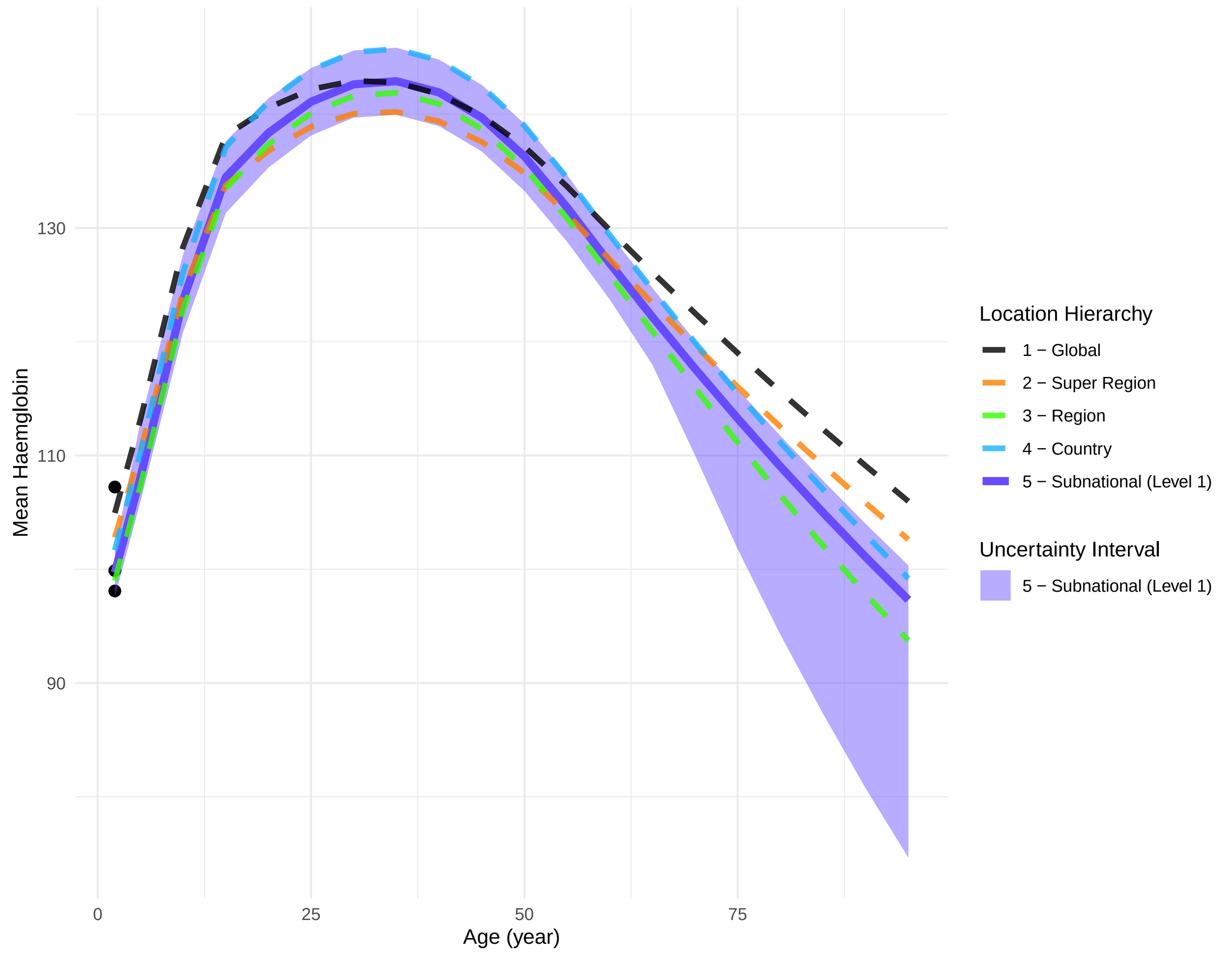

MR−BRT Age/Sex Fits − Males 2+ Years Old − Mean Haemglobin − Yobe
WHO−2024 Adjustment Method

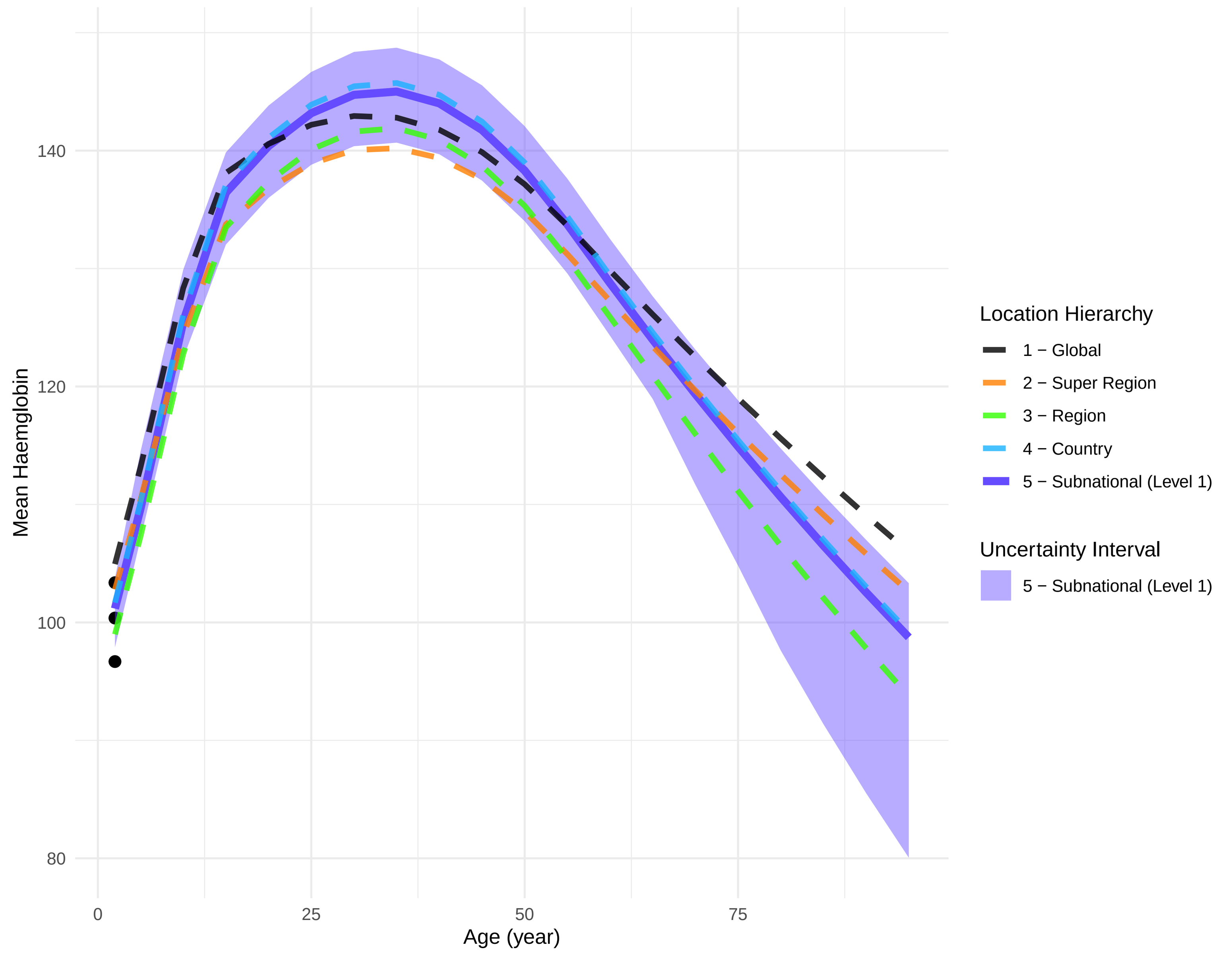

MR−BRT Age/Sex Fits − Males 2+ Years Old − Mean Haemglobin − Zamfara
WHO−2024 Adjustment Method

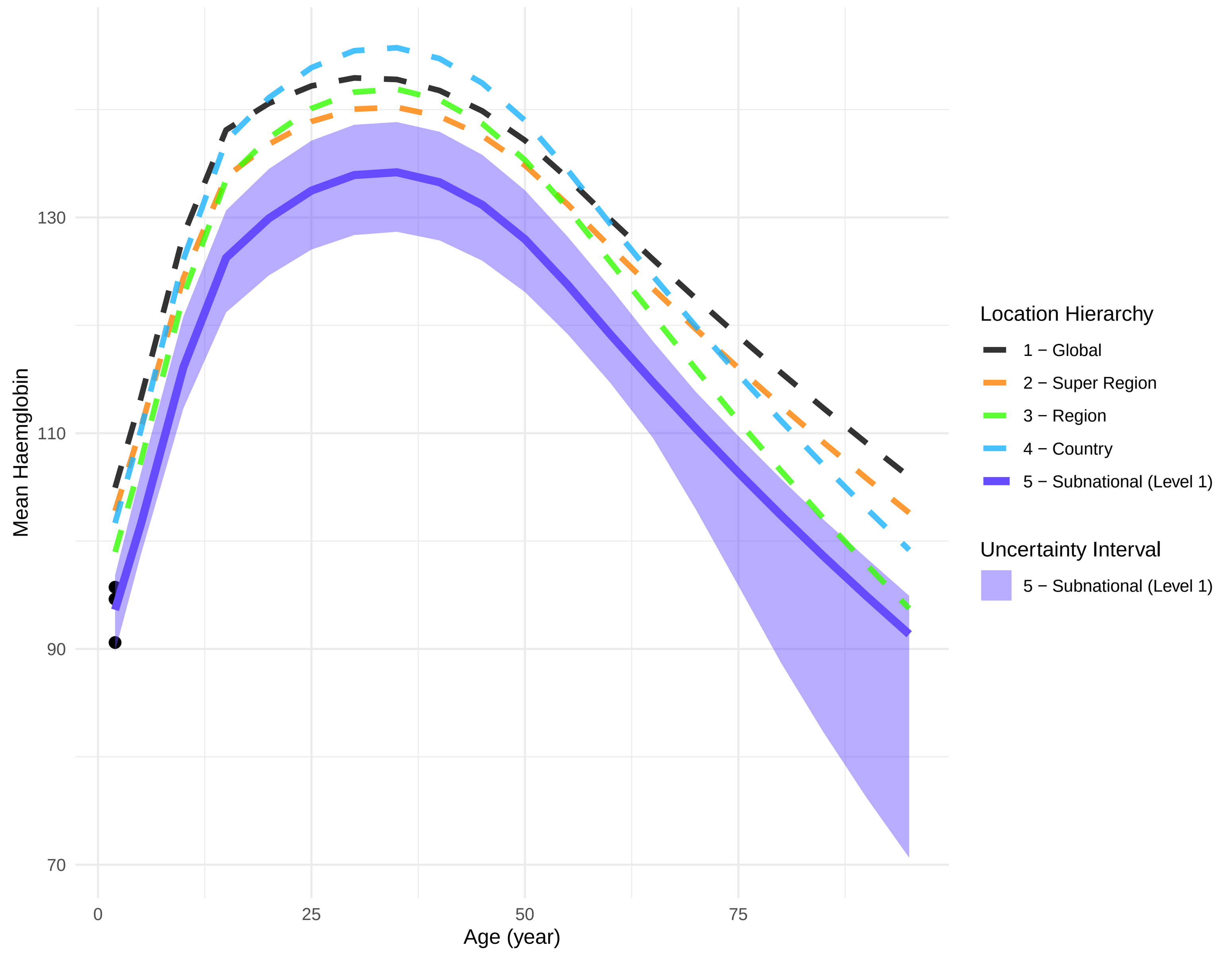

MR−BRT Age/Sex Fits − Males 2+ Years Old − Mean Haemglobin − Senegal
WHO−2024 Adjustment Method

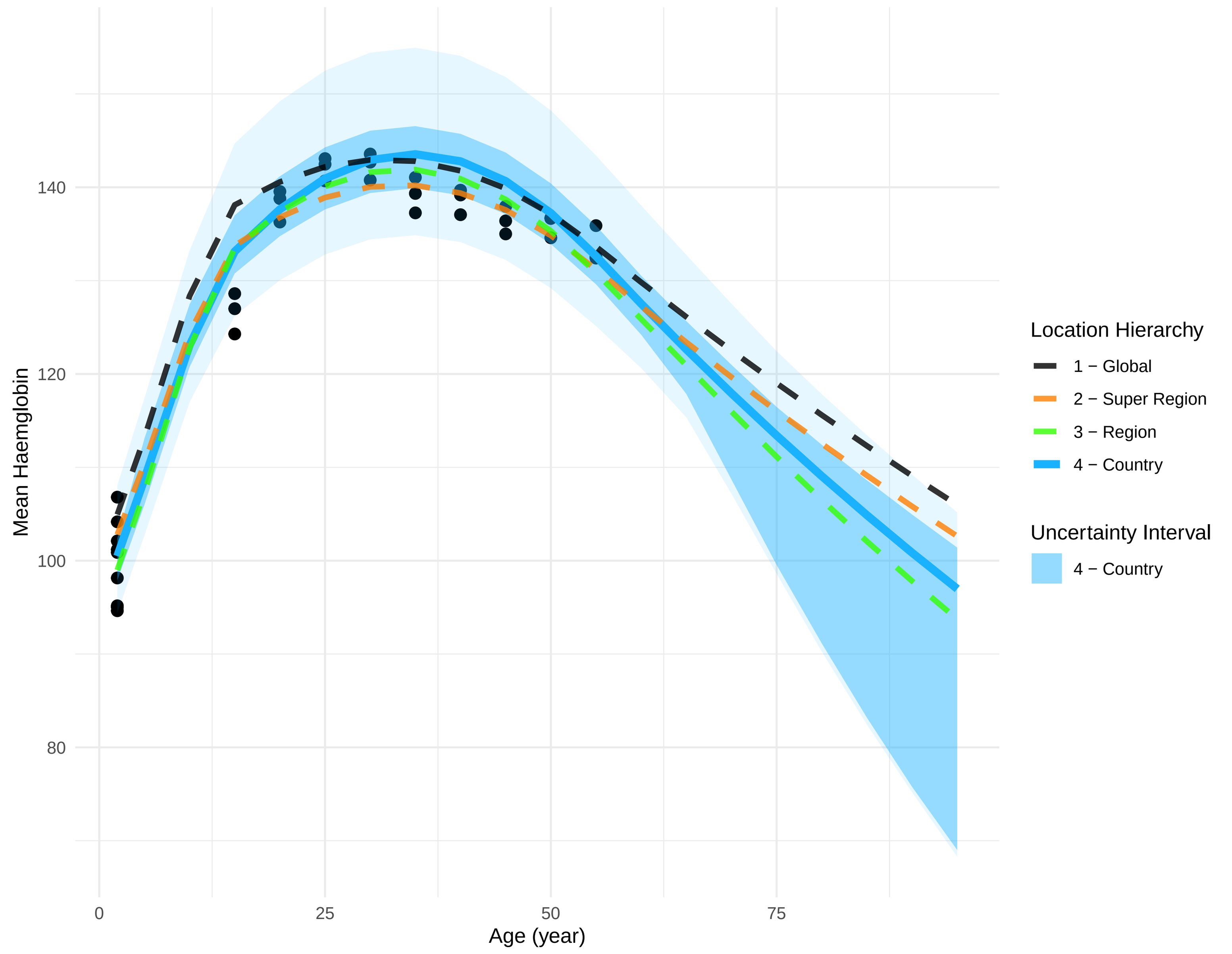

MR−BRT Age/Sex Fits − Males 2+ Years Old − Mean Haemglobin − Sierra Leone
WHO−2024 Adjustment Method

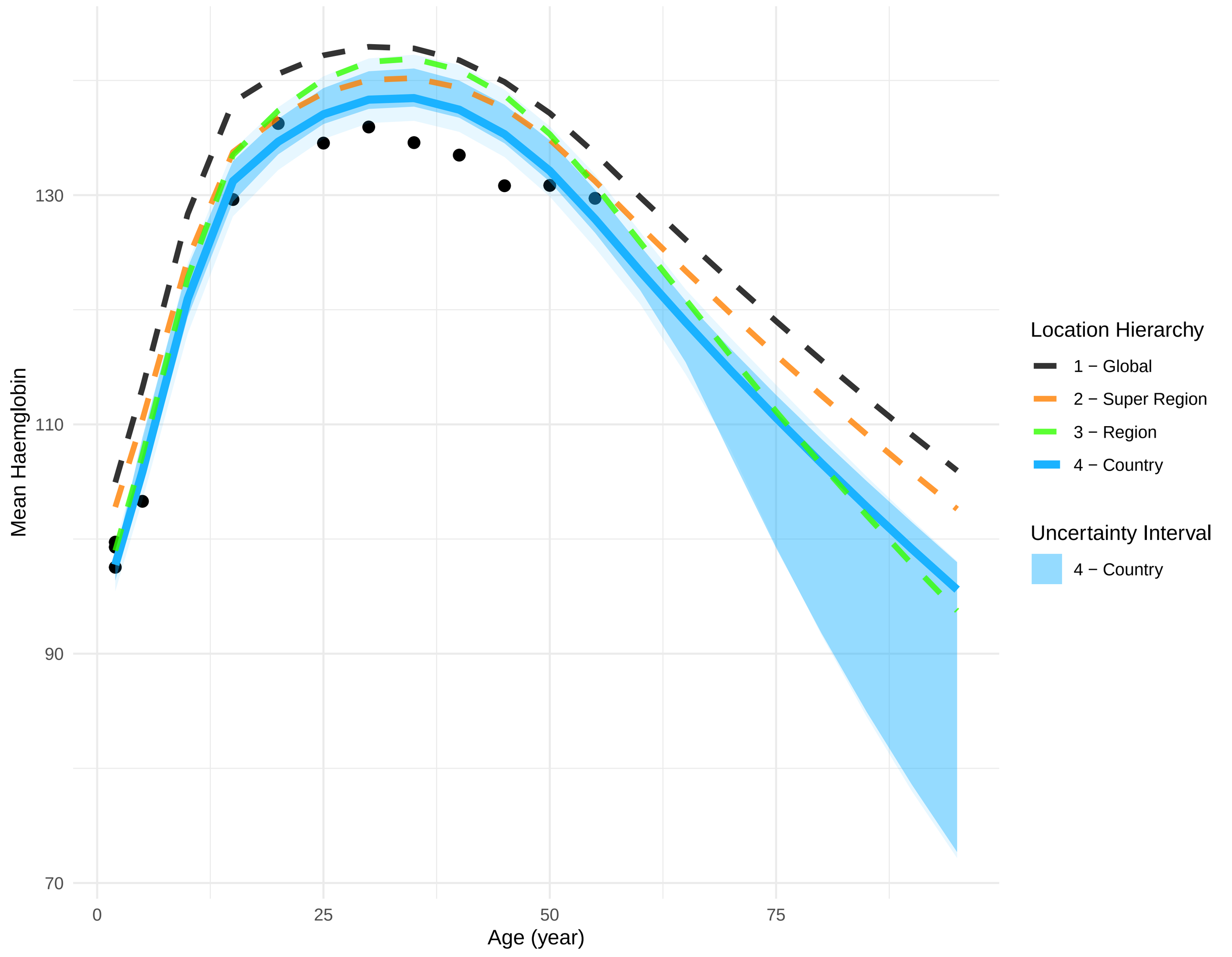

MR−BRT Age/Sex Fits − Males 2+ Years Old − Mean Haemglobin − Togo
WHO−2024 Adjustment Method

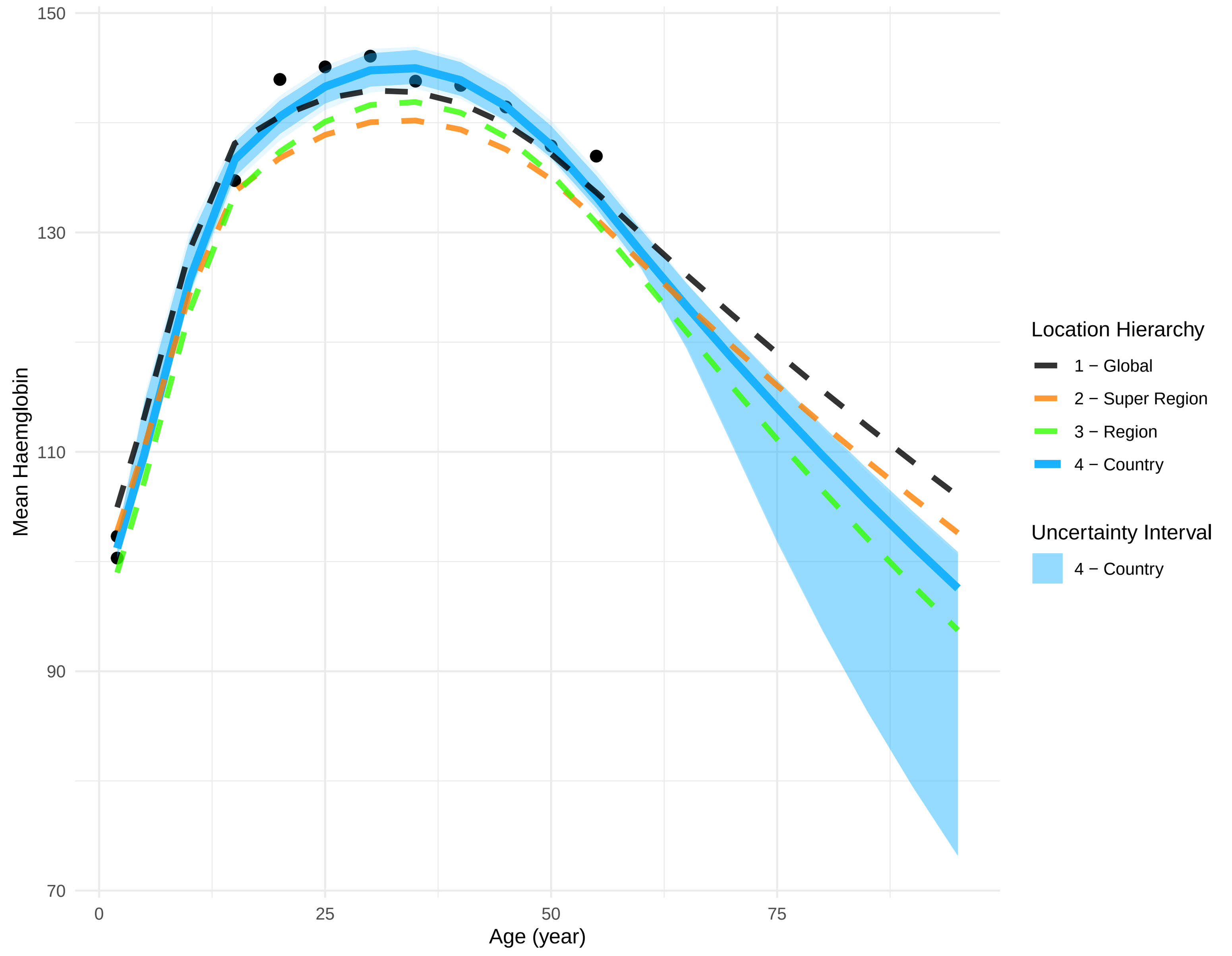

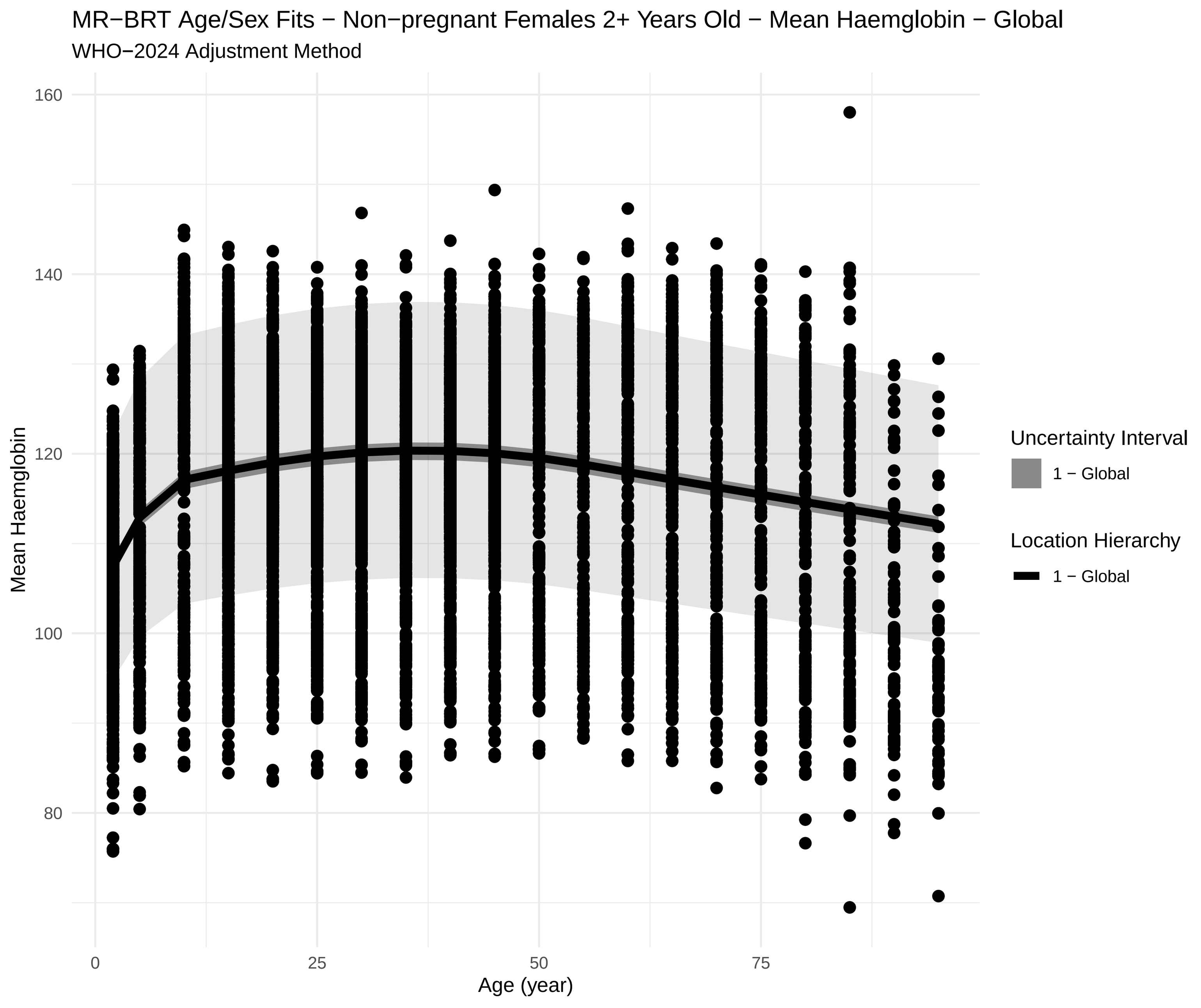
MR−BRT Age/Sex Fits − Non−pregnant Females 2+ Years Old − Mean Haemglobin − Global
WHO−2024 Adjustment Method
Mean Haemglobin
Age (year)
Uncertainty Interval
1 − Global
Location Hierarchy
1 − Global

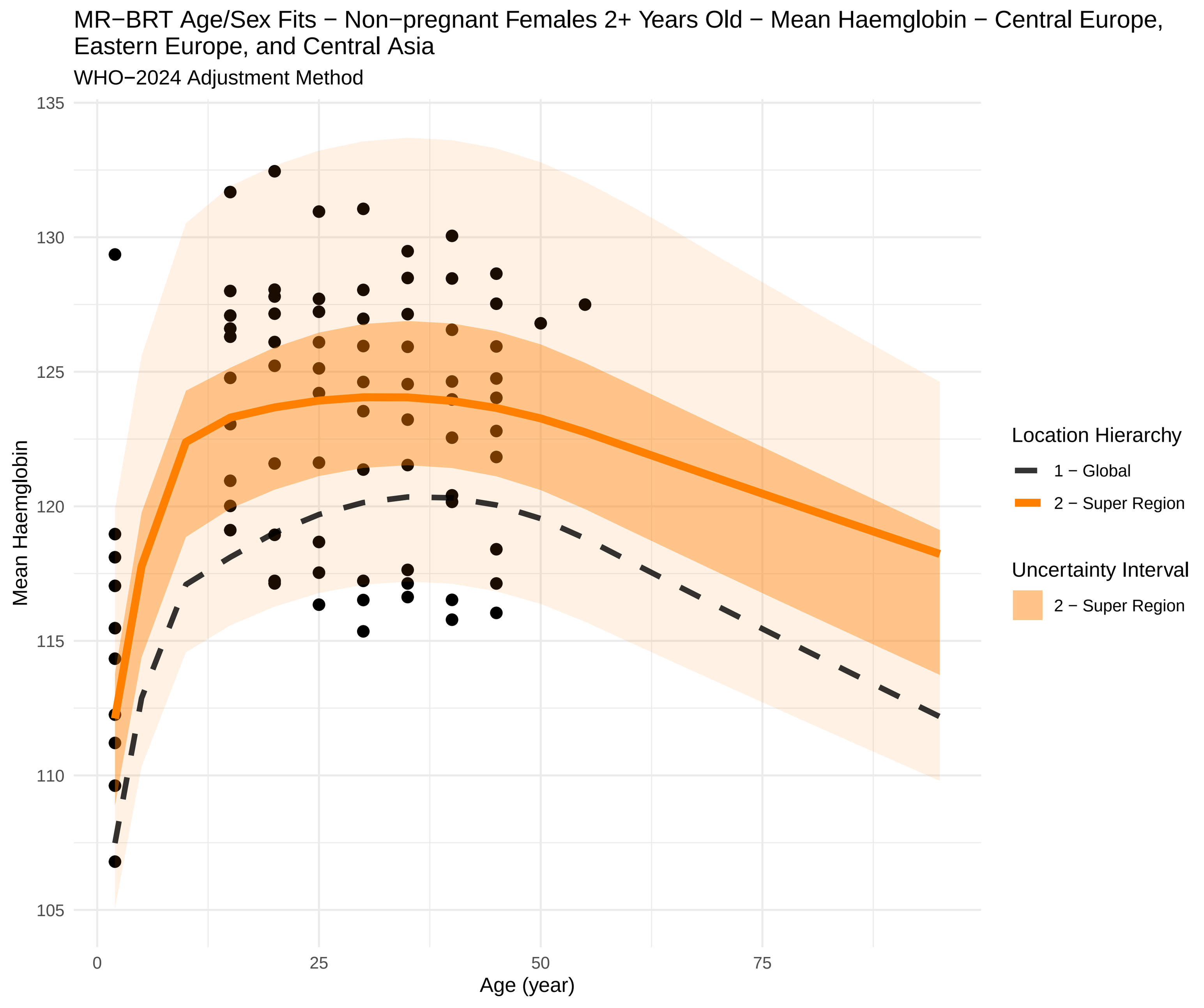
MR−BRT Age/Sex Fits − Non−pregnant Females 2+ Years Old − Mean Haemglobin − Central Europe, Eastern Europe, and Central Asia
WHO−2024 Adjustment Method
Mean Haemglobin
Age (year)
Location Hierarchy
1 − Global
2 − Super Region
Uncertainty Interval
2 − Super Region

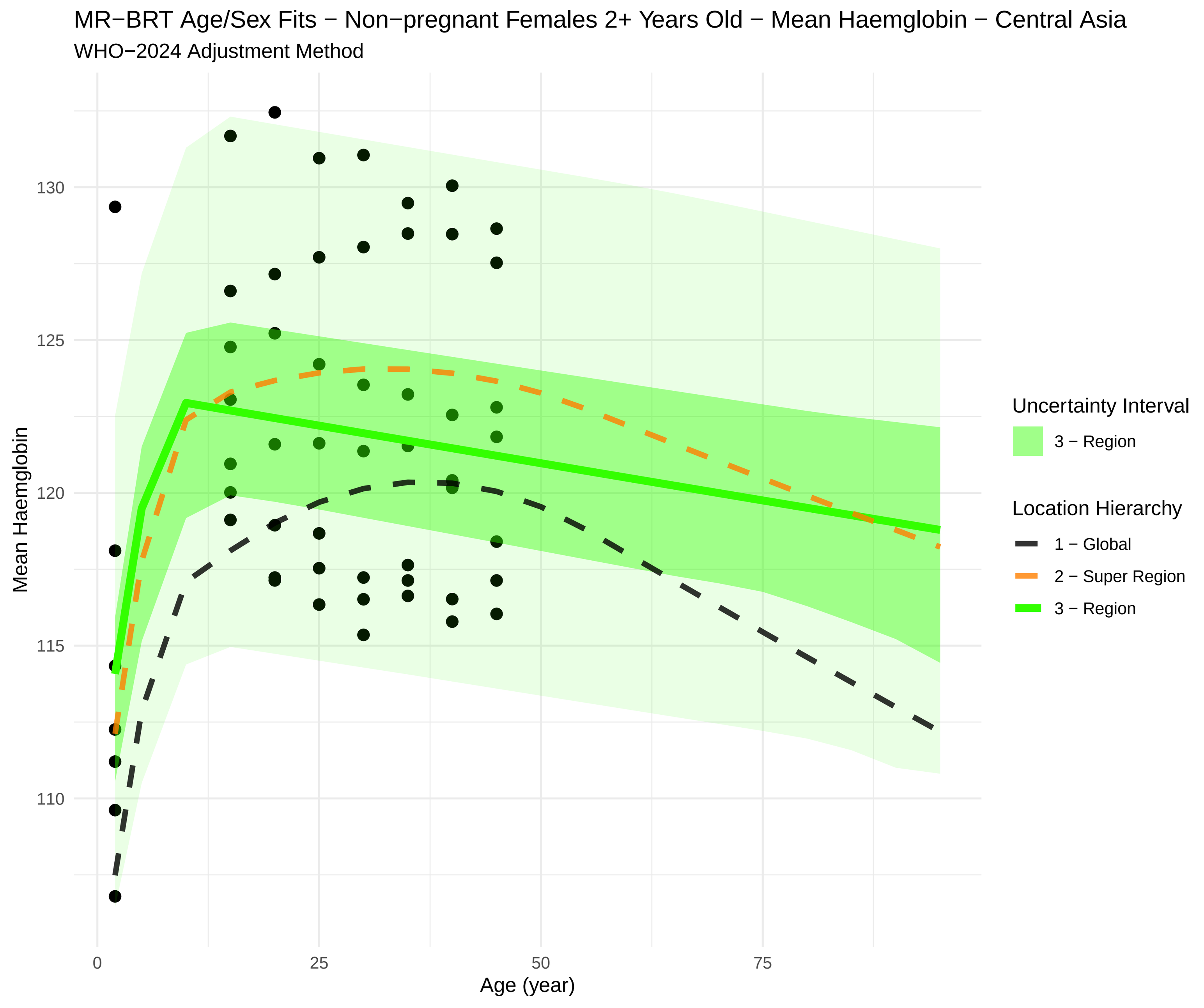
MR−BRT Age/Sex Fits − Non−pregnant Females 2+ Years Old − Mean Haemglobin − Central Asia
WHO−2024 Adjustment Method
Mean Haemglobin
Age (year)
Uncertainty Interval
3 − Region
Location Hierarchy
1 − Global
2 − Super Region
3 − Region

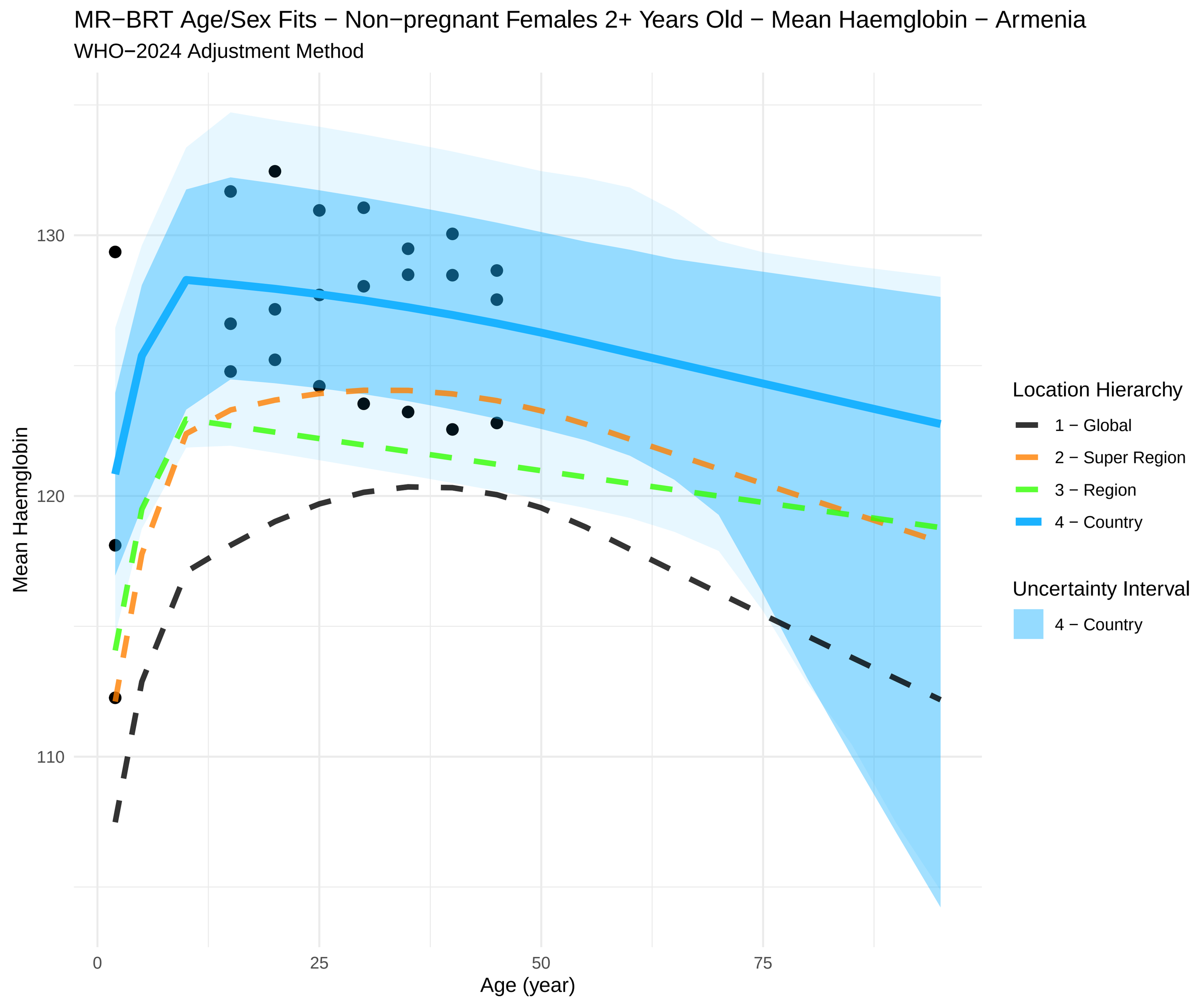
MR−BRT Age/Sex Fits − Non−pregnant Females 2+ Years Old − Mean Haemglobin − Armenia
WHO−2024 Adjustment Method
Mean Haemglobin
Age (year)
Location Hierarchy
1 − Global
2 − Super Region
3 − Region
4 − Country
Uncertainty Interval
4 − Country

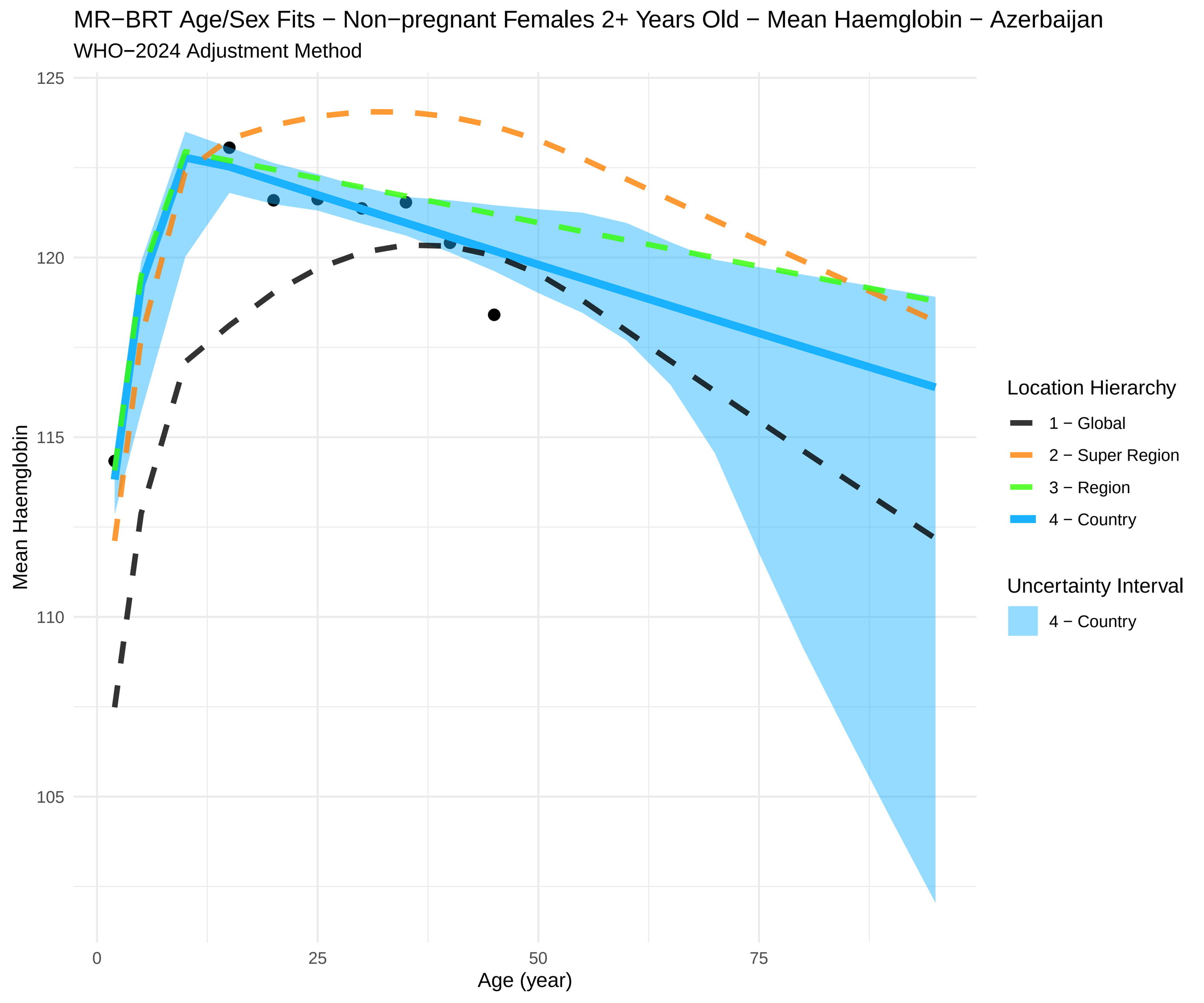
MR−BRT Age/Sex Fits − Non−pregnant Females 2+ Years Old − Mean Haemglobin − Azerbaijan
WHO−2024 Adjustment Method
Mean Haemglobin
Age (year)
Location Hierarchy
1 − Global
2 − Super Region
3 − Region
4 − Country
Uncertainty Interval
4 − Country

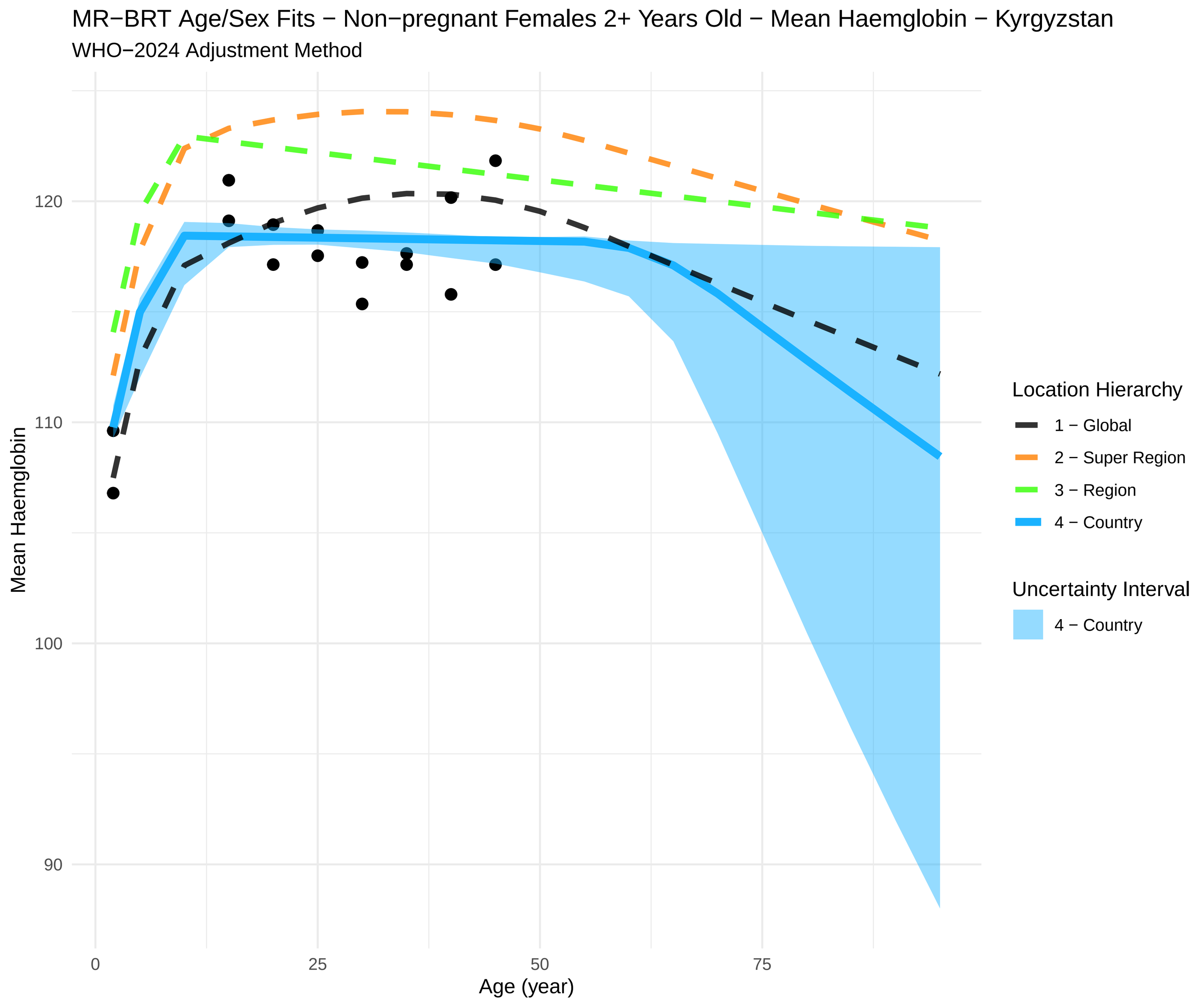
MR−BRT Age/Sex Fits − Non−pregnant Females 2+ Years Old − Mean Haemglobin − Kyrgyzstan
WHO−2024 Adjustment Method
Mean Haemglobin
Age (year)
Location Hierarchy
1 − Global
2 − Super Region
3 − Region
4 − Country
Uncertainty Interval
4 − Country

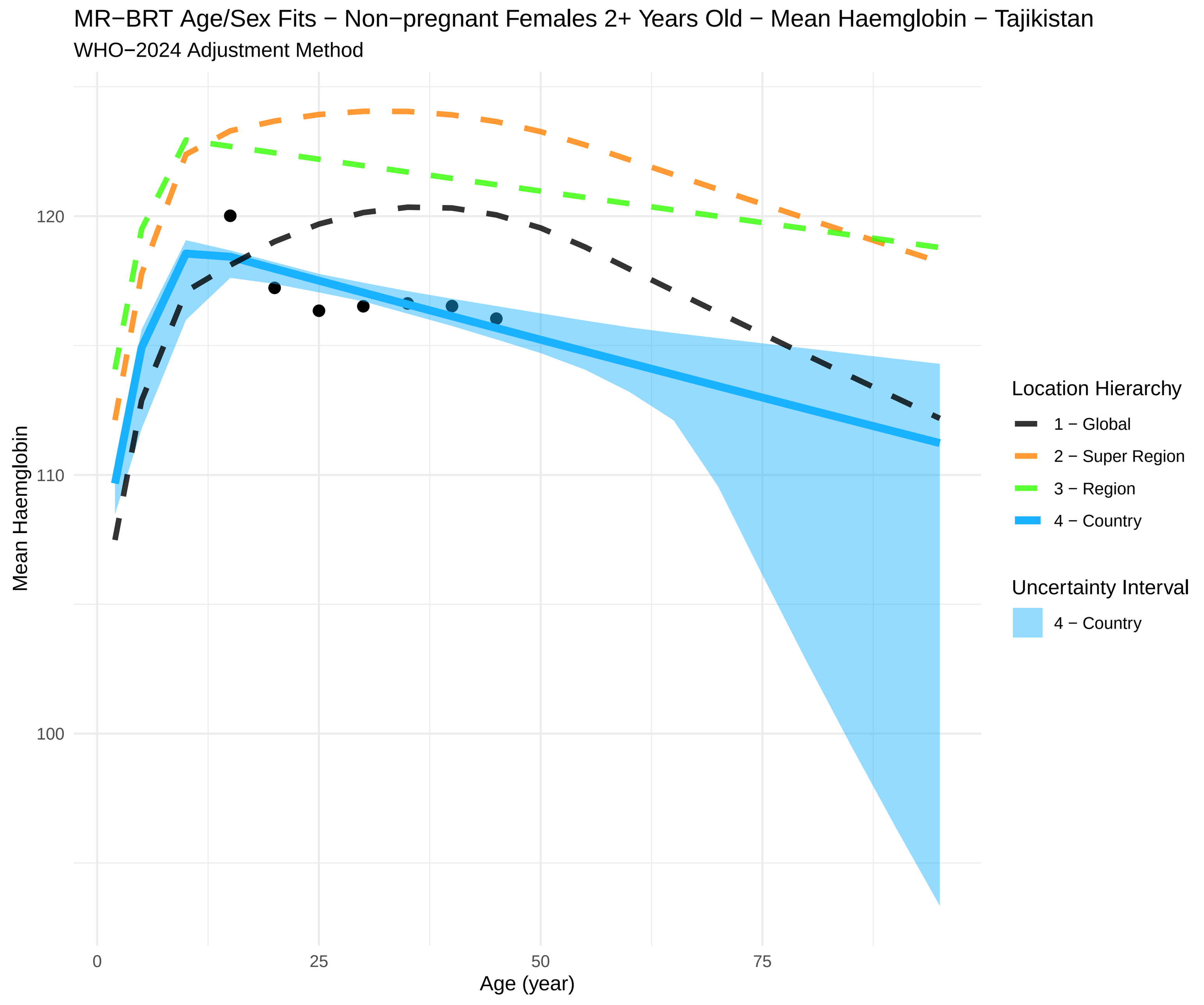
MR−BRT Age/Sex Fits − Non−pregnant Females 2+ Years Old − Mean Haemglobin − Tajikistan
WHO−2024 Adjustment Method
Mean Haemglobin
Age (year)
Location Hierarchy
1 − Global
2 − Super Region
3 − Region
4 − Country
Uncertainty Interval
4 − Country

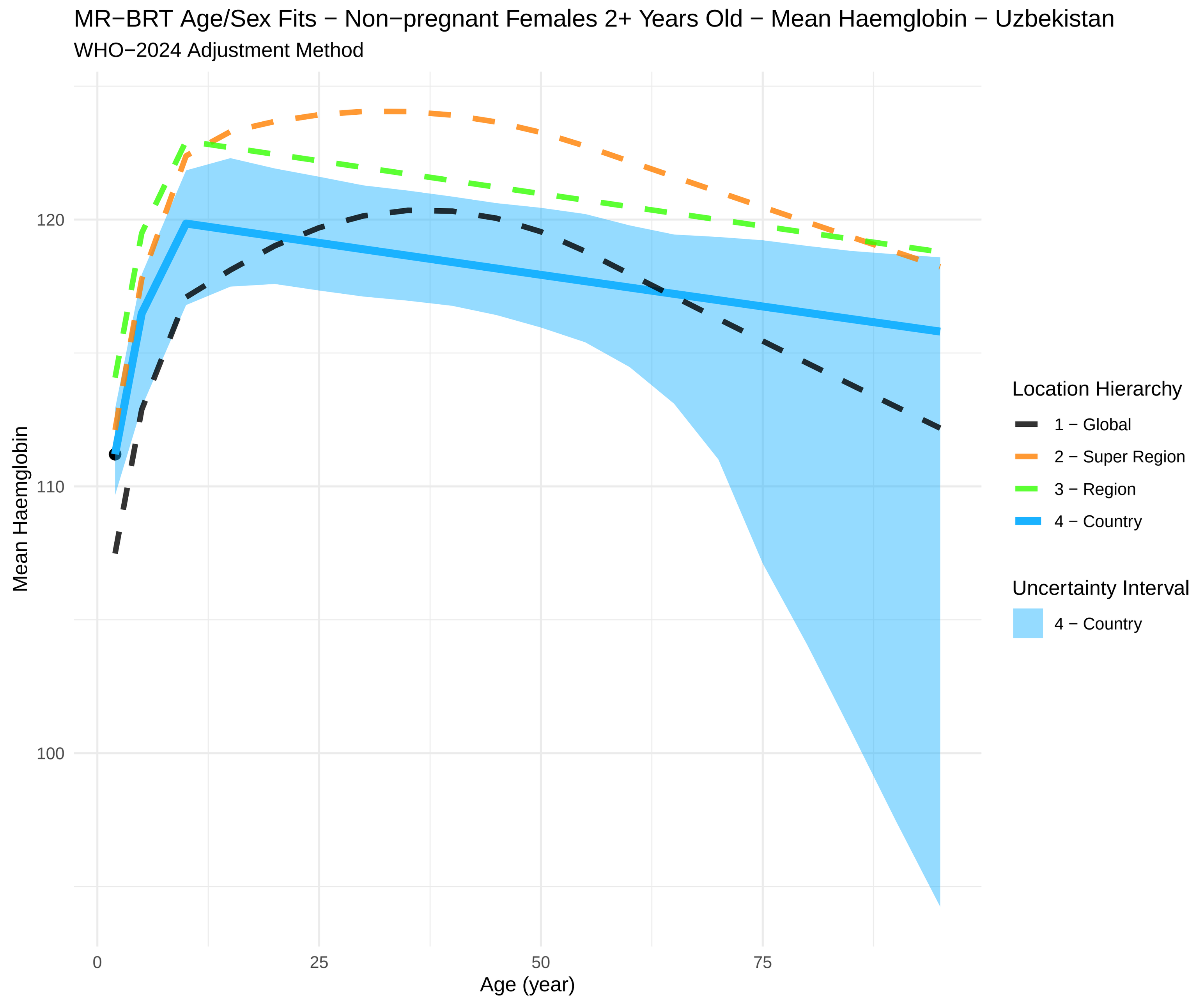
MR−BRT Age/Sex Fits − Non−pregnant Females 2+ Years Old − Mean Haemglobin − Uzbekistan
WHO−2024 Adjustment Method
Mean Haemglobin
Age (year)
Location Hierarchy
1 − Global
2 − Super Region
3 − Region
4 − Country
Uncertainty Interval
4 − Country

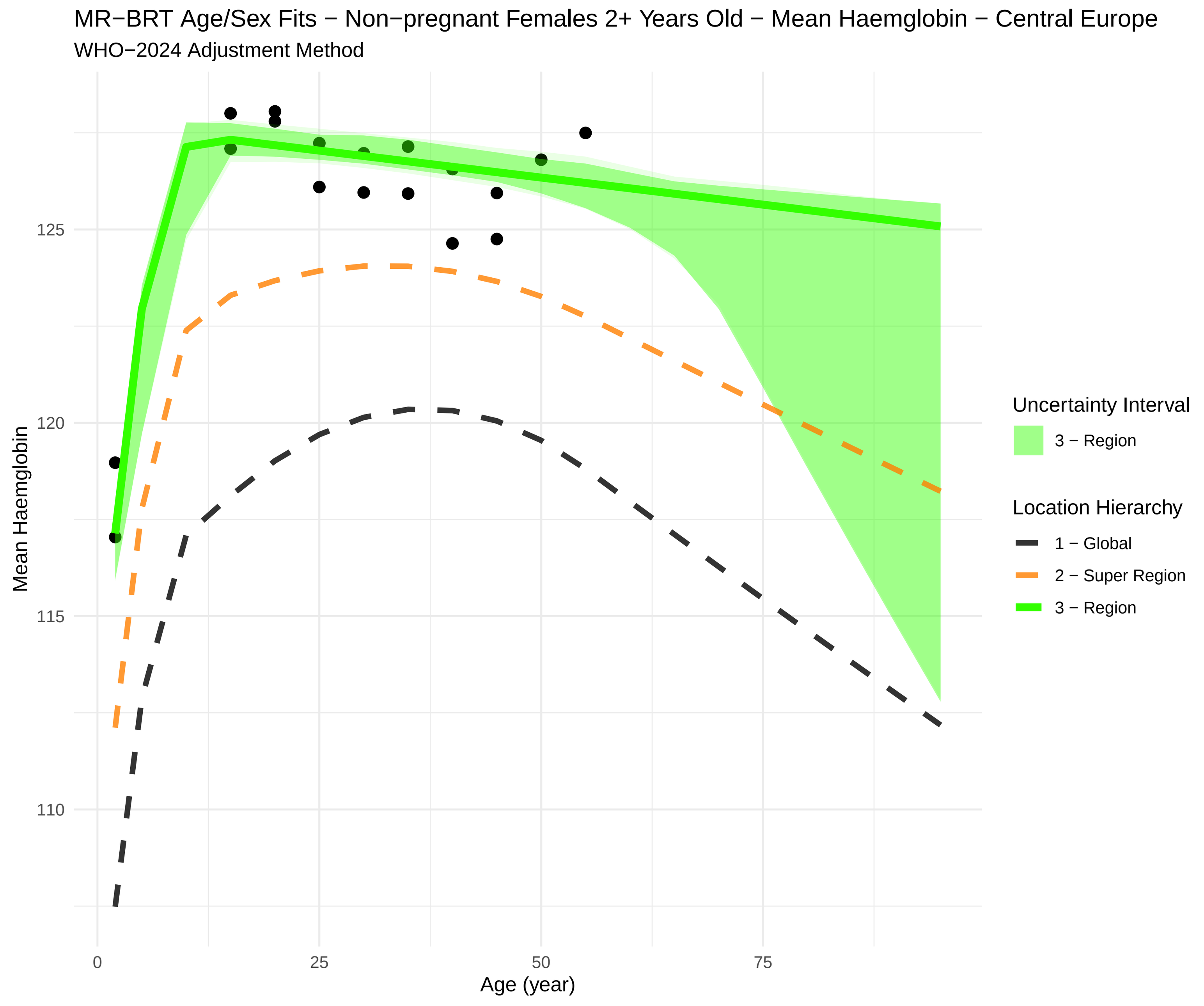
MR−BRT Age/Sex Fits − Non−pregnant Females 2+ Years Old − Mean Haemglobin − Central Europe
WHO−2024 Adjustment Method
Mean Haemglobin
Age (year)
Uncertainty Interval
3 − Region
Location Hierarchy
1 − Global
2 − Super Region
3 − Region

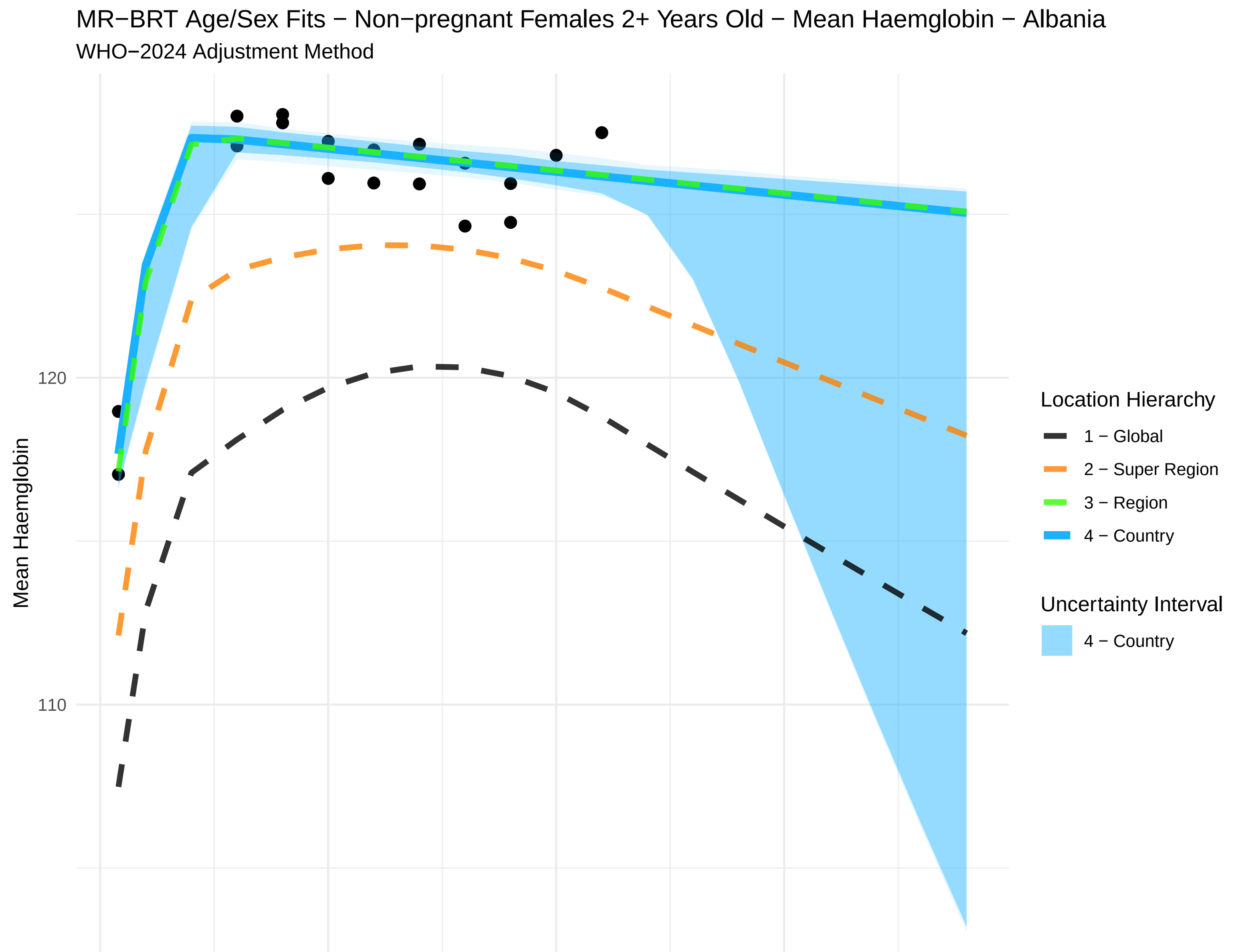
MR−BRT Age/Sex Fits − Non−pregnant Females 2+ Years Old − Mean Haemglobin − Albania
WHO−2024 Adjustment Method
Mean Haemglobin
Age (year)
Location Hierarchy
1 − Global
2 − Super Region
3 − Region
4 − Country
Uncertainty Interval
4 − Country

MR−BRT Age/Sex Fits − Non−pregnant Females 2+ Years Old − Mean Haemglobin − Eastern Europe
WHO−2024 Adjustment Method

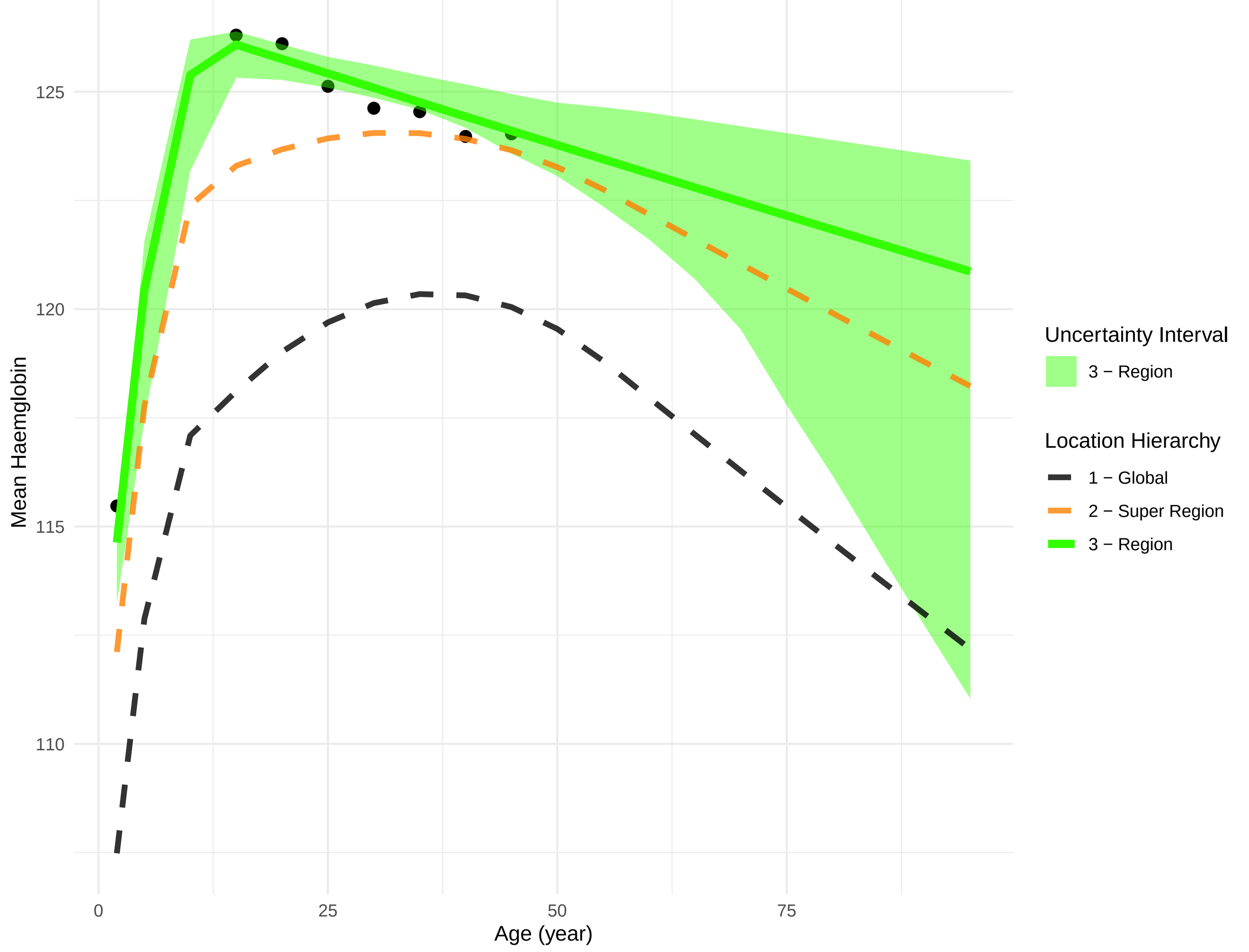

MR−BRT Age/Sex Fits − Non−pregnant Females 2+ Years Old − Mean Haemglobin − Republic of Moldova
WHO−2024 Adjustment Method

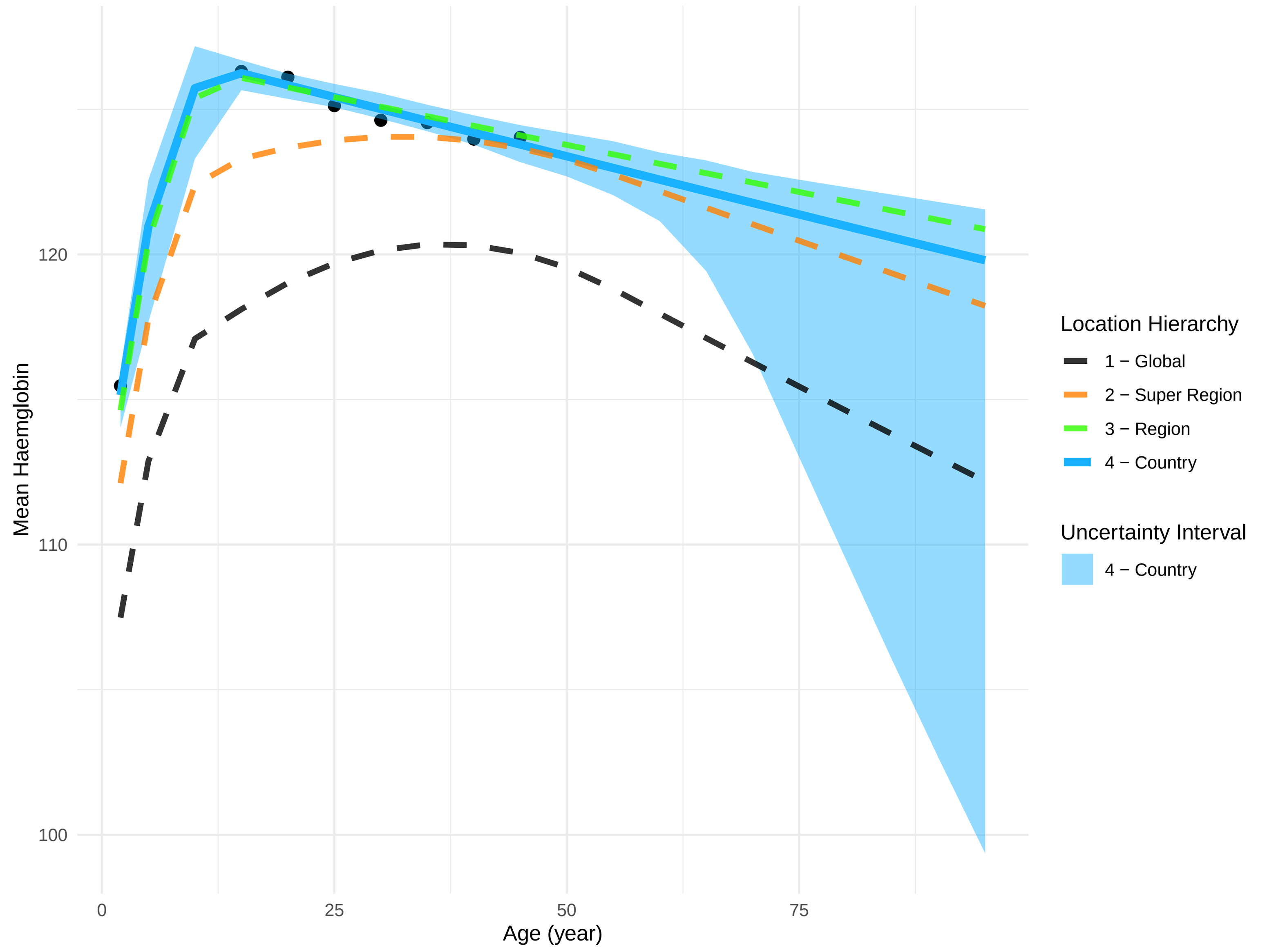

MR−BRT Age/Sex Fits − Non−pregnant Females 2+ Years Old − Mean Haemglobin − High−income
WHO−2024 Adjustment Method

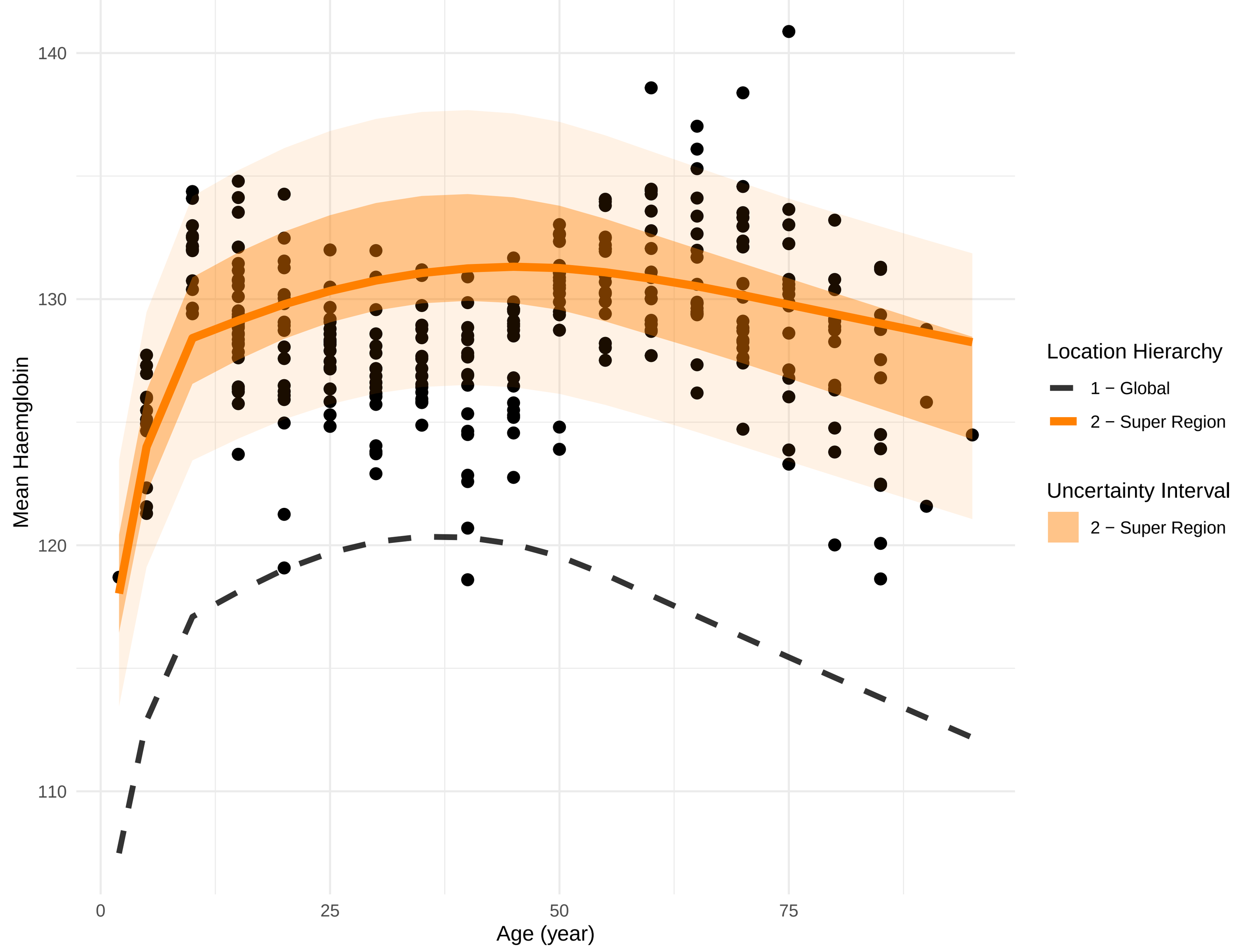

MR−BRT Age/Sex Fits − Non−pregnant Females 2+ Years Old − Mean Haemglobin − Western Europe
WHO−2024 Adjustment Method

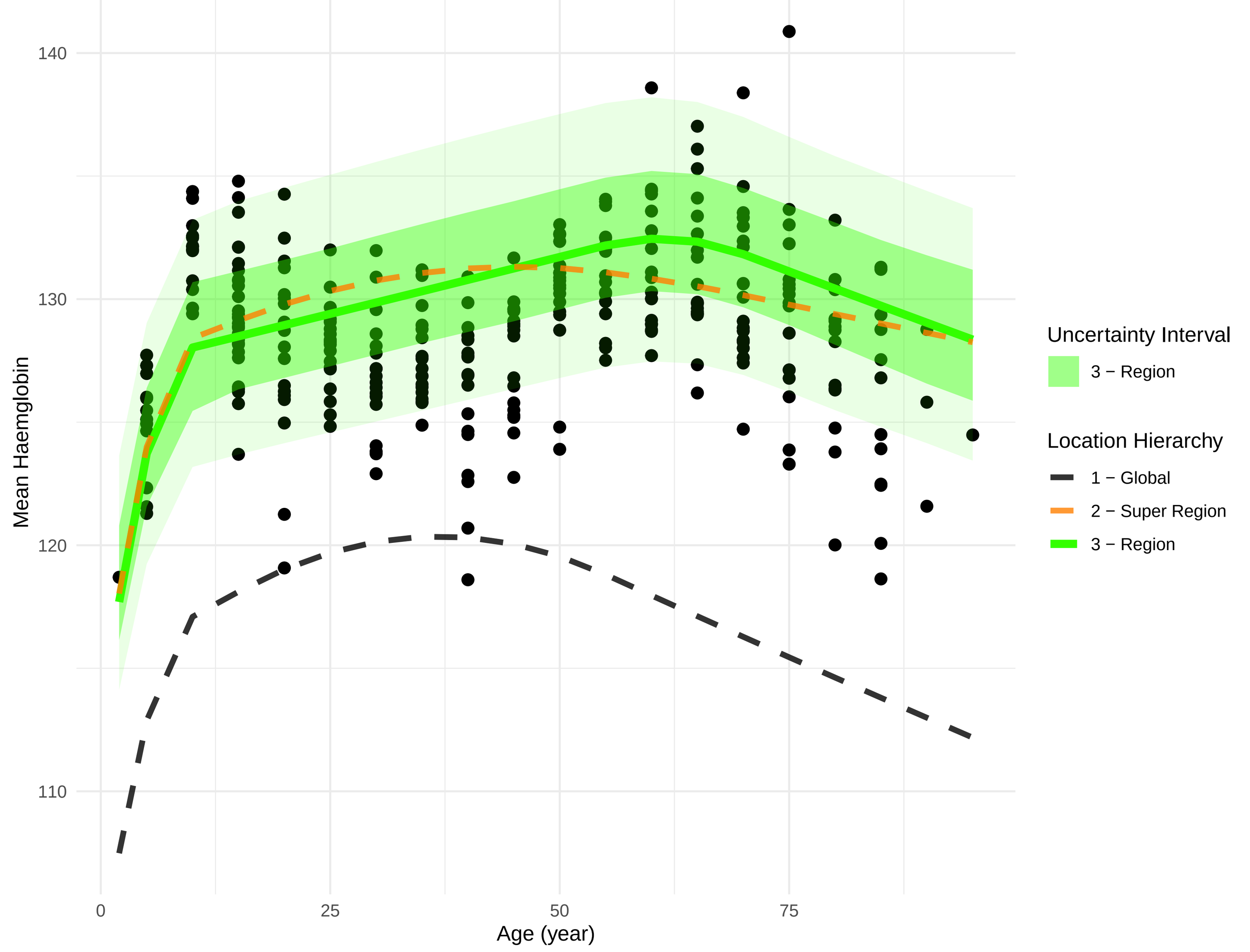

MR−BRT Age/Sex Fits − Non−pregnant Females 2+ Years Old − Mean Haemglobin − United Kingdom
WHO−2024 Adjustment Method

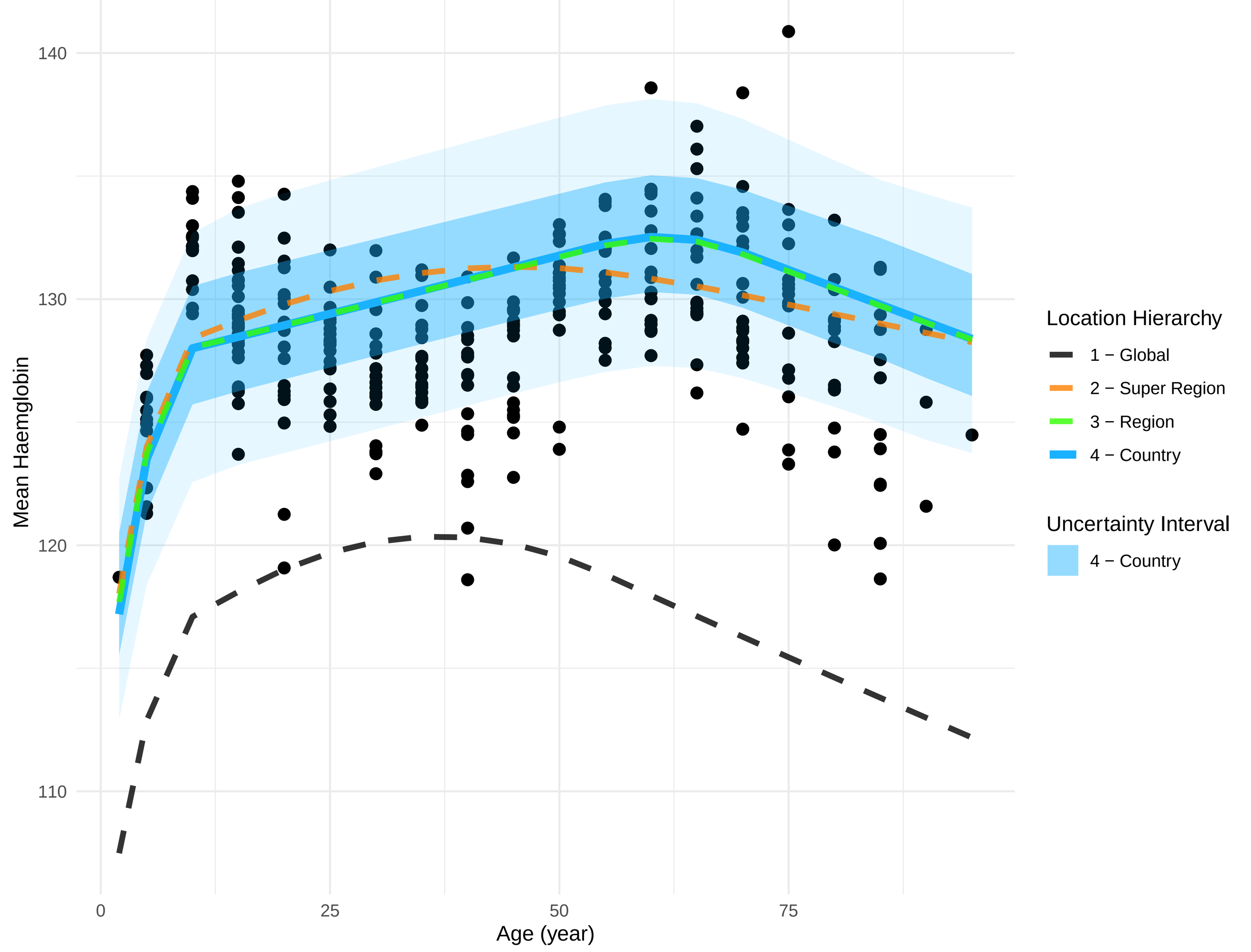

MR−BRT Age/Sex Fits − Non−pregnant Females 2+ Years Old − Mean Haemglobin − England
WHO−2024 Adjustment Method

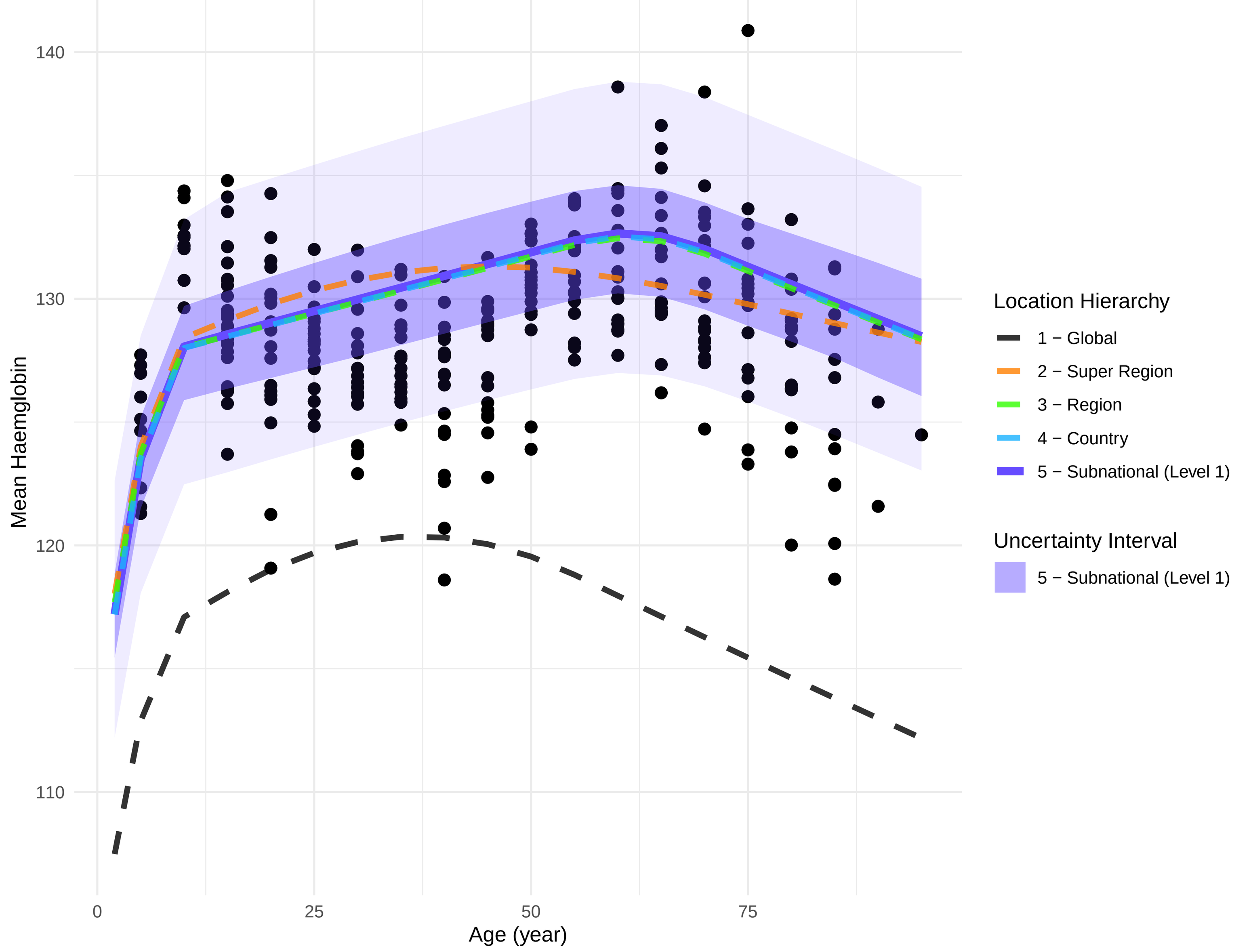

MR−BRT Age/Sex Fits − Non−pregnant Females 2+ Years Old − Mean Haemglobin − East Midlands
WHO−2024 Adjustment Method

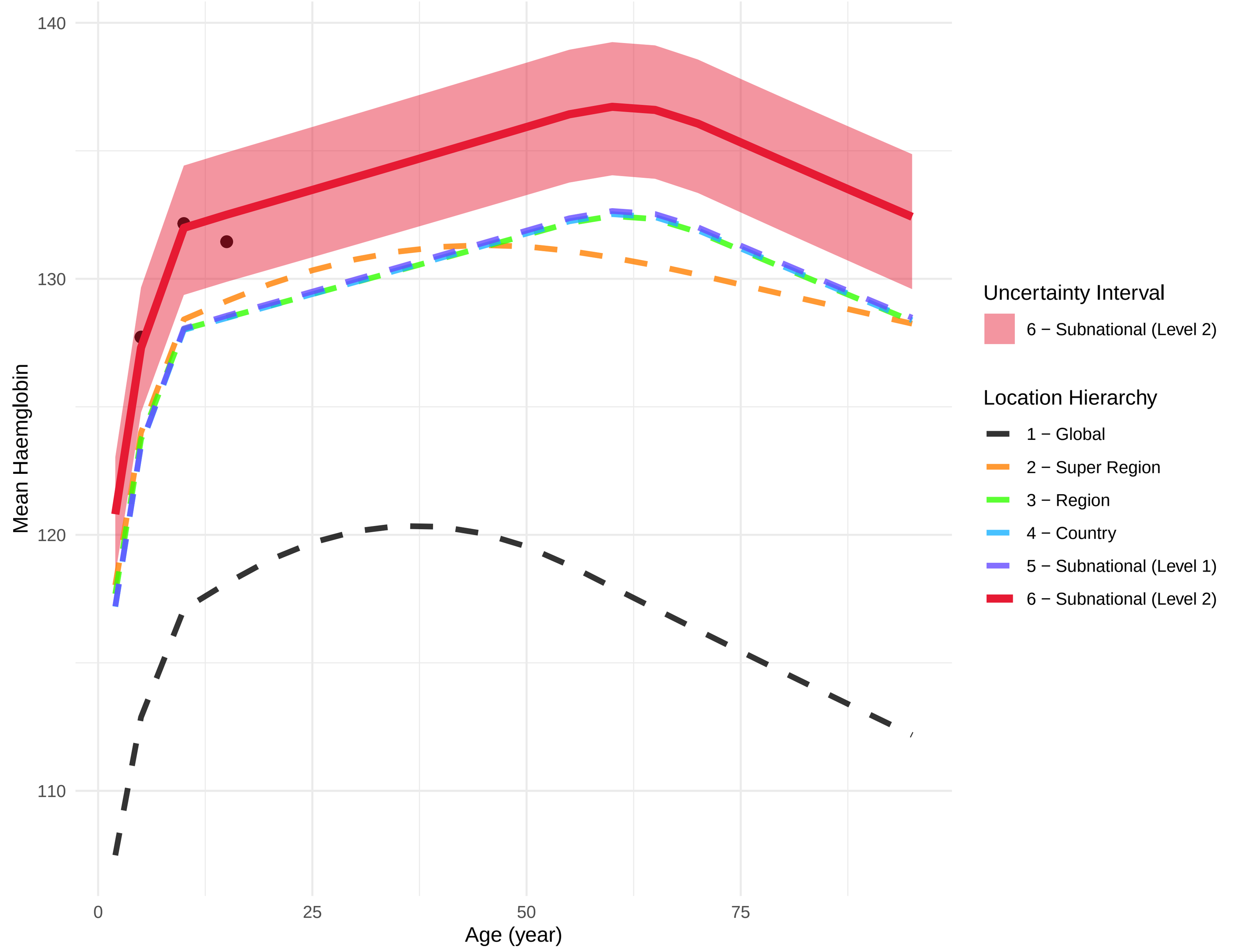

MR−BRT Age/Sex Fits − Non−pregnant Females 2+ Years Old − Mean Haemglobin − East of England
WHO−2024 Adjustment Method

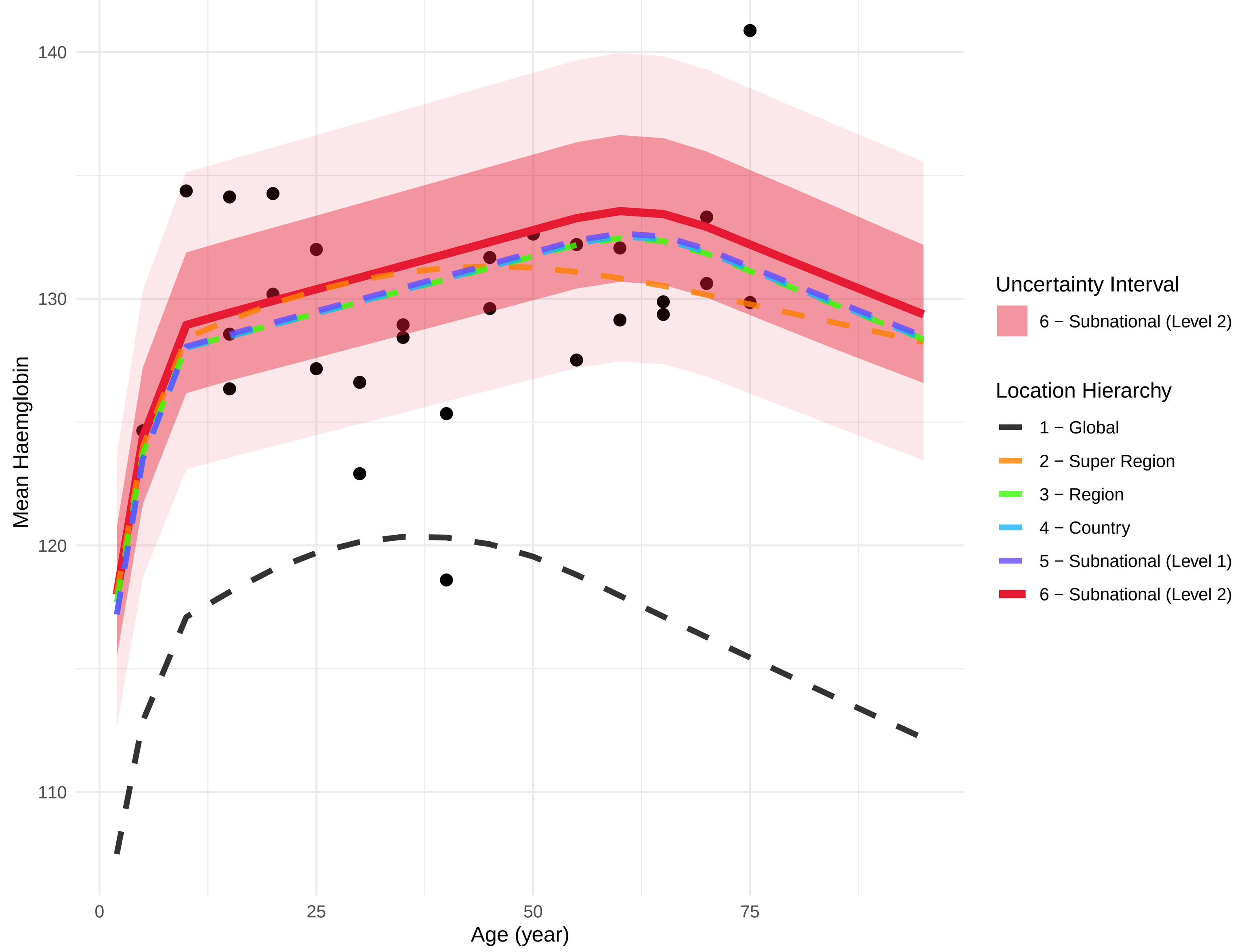

MR−BRT Age/Sex Fits − Non−pregnant Females 2+ Years Old − Mean Haemglobin − Greater London
WHO−2024 Adjustment Method

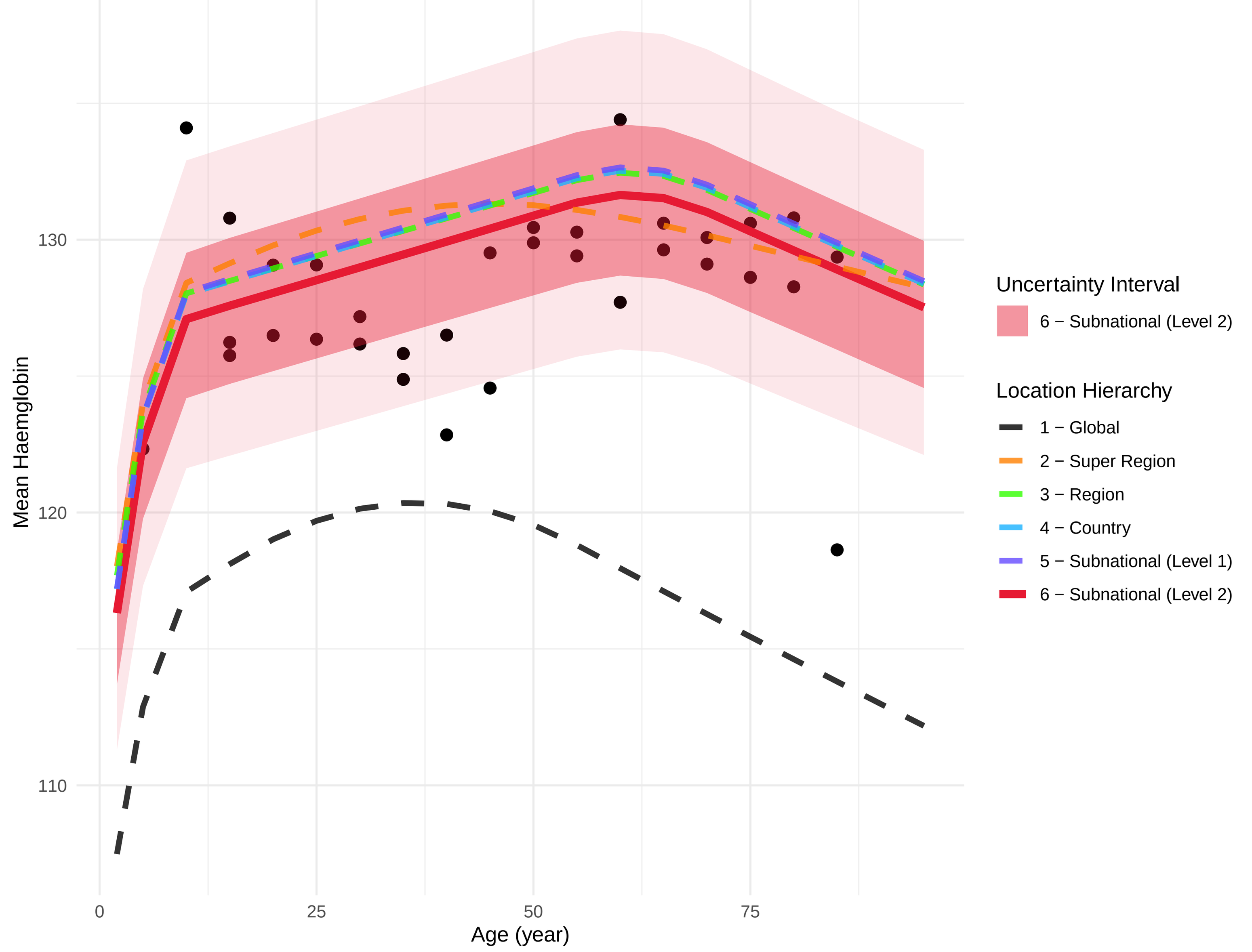

MR−BRT Age/Sex Fits − Non−pregnant Females 2+ Years Old − Mean Haemglobin − North East England
WHO−2024 Adjustment Method

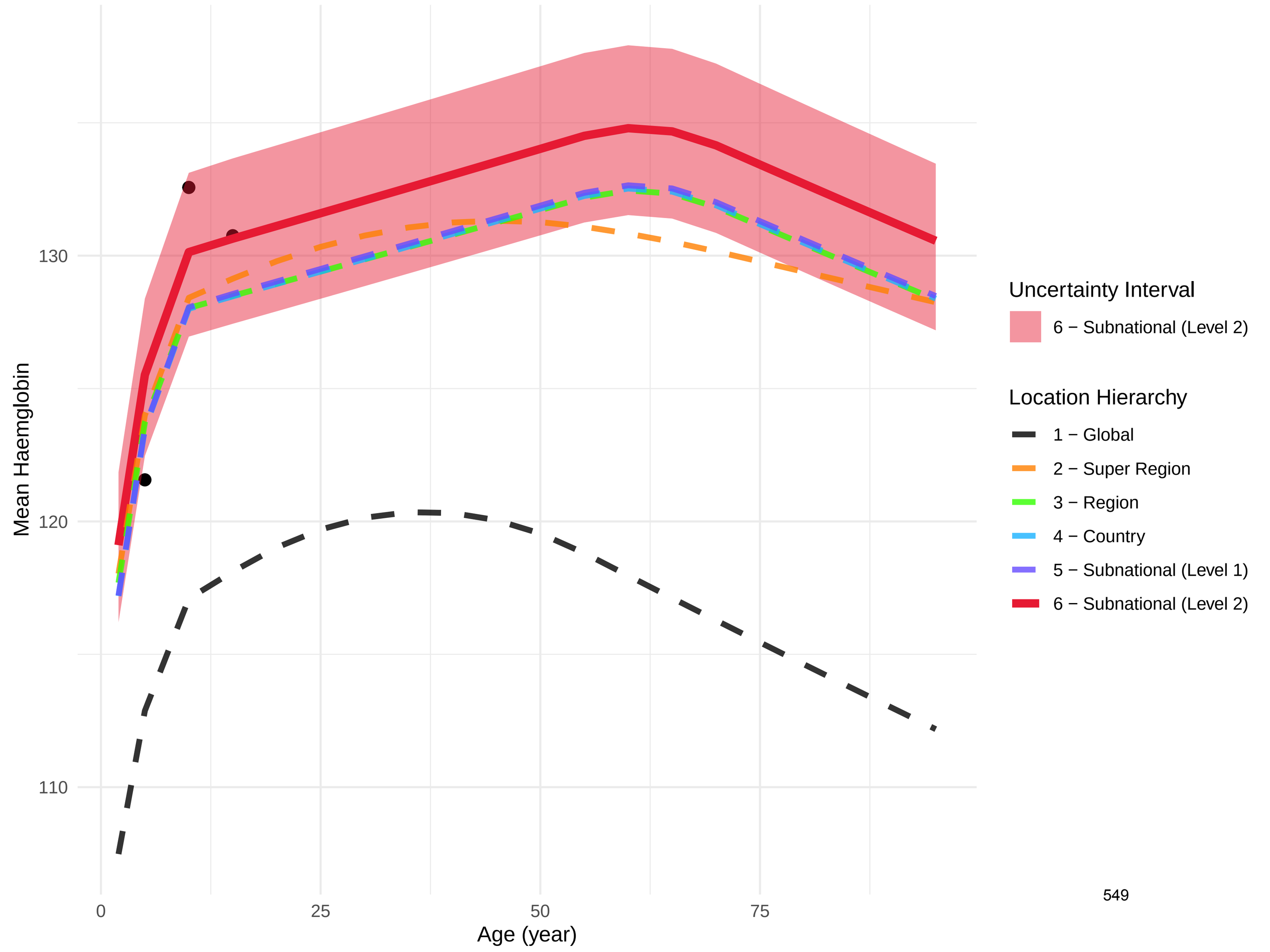

MR−BRT Age/Sex Fits − Non−pregnant Females 2+ Years Old − Mean Haemglobin − North West England
WHO−2024 Adjustment Method

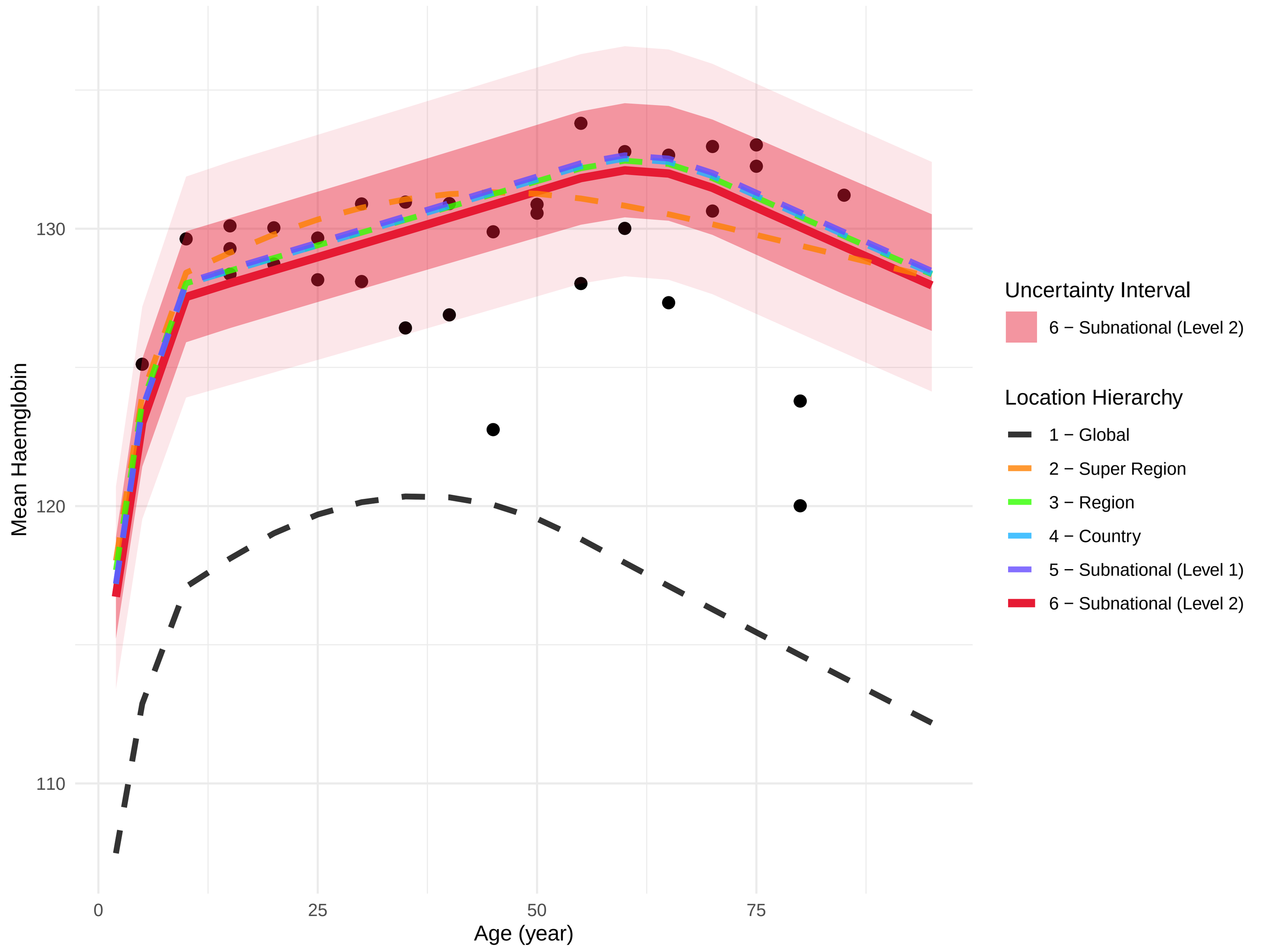

MR−BRT Age/Sex Fits − Non−pregnant Females 2+ Years Old − Mean Haemglobin − South East England
WHO−2024 Adjustment Method

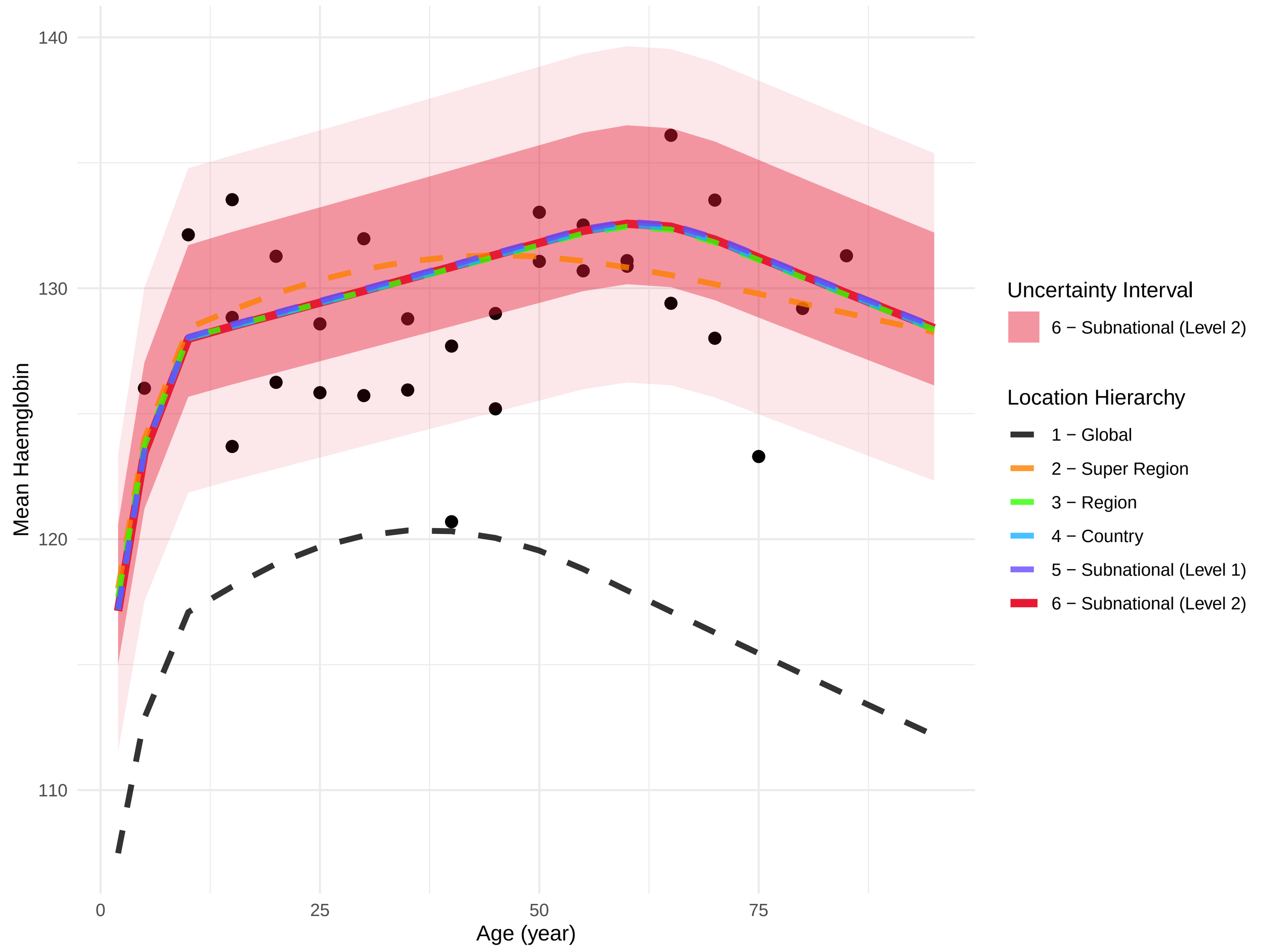

MR−BRT Age/Sex Fits − Non−pregnant Females 2+ Years Old − Mean Haemglobin − South West England
WHO−2024 Adjustment Method

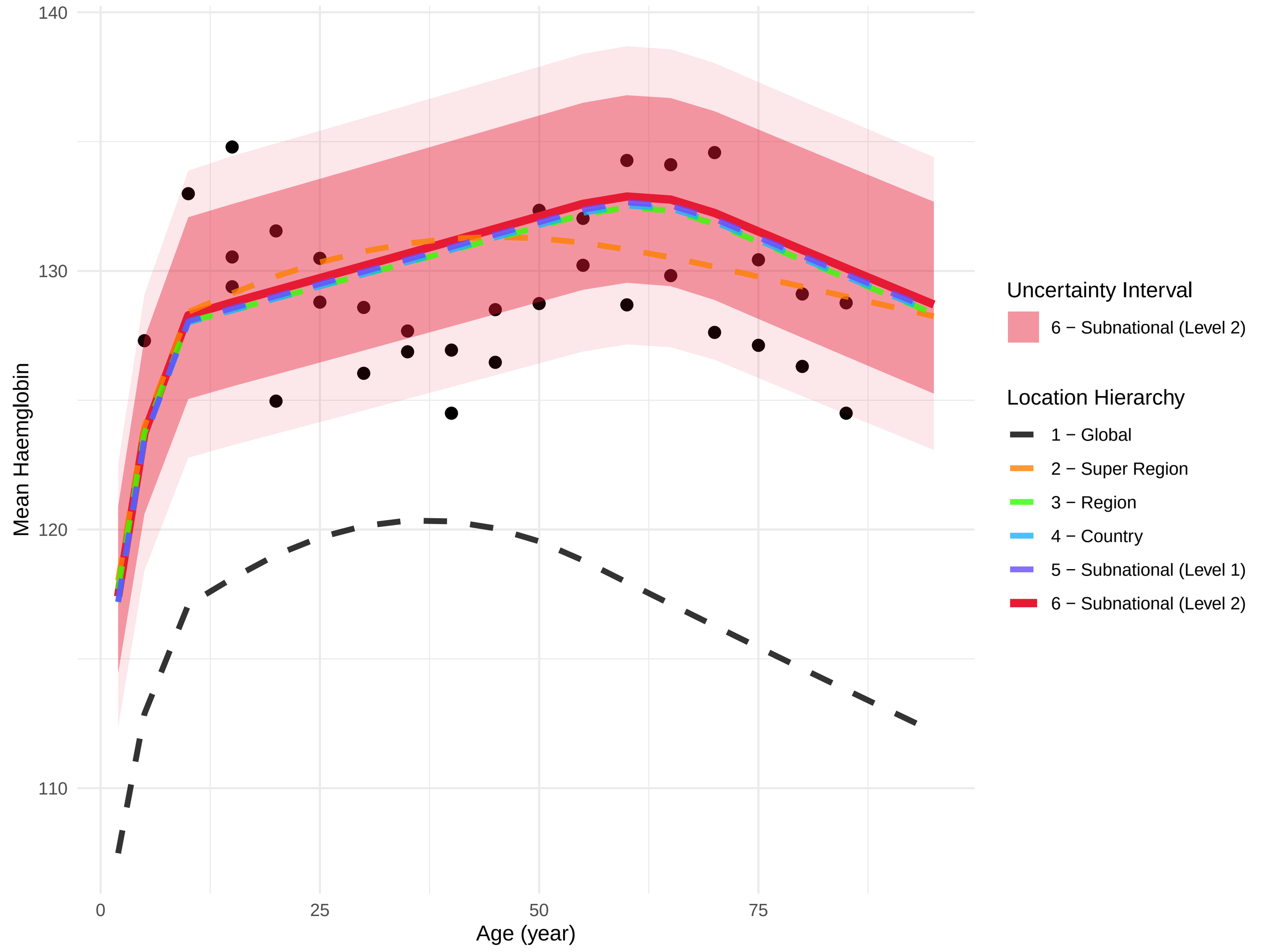

MR−BRT Age/Sex Fits − Non−pregnant Females 2+ Years Old − Mean Haemglobin − West Midlands
WHO−2024 Adjustment Method

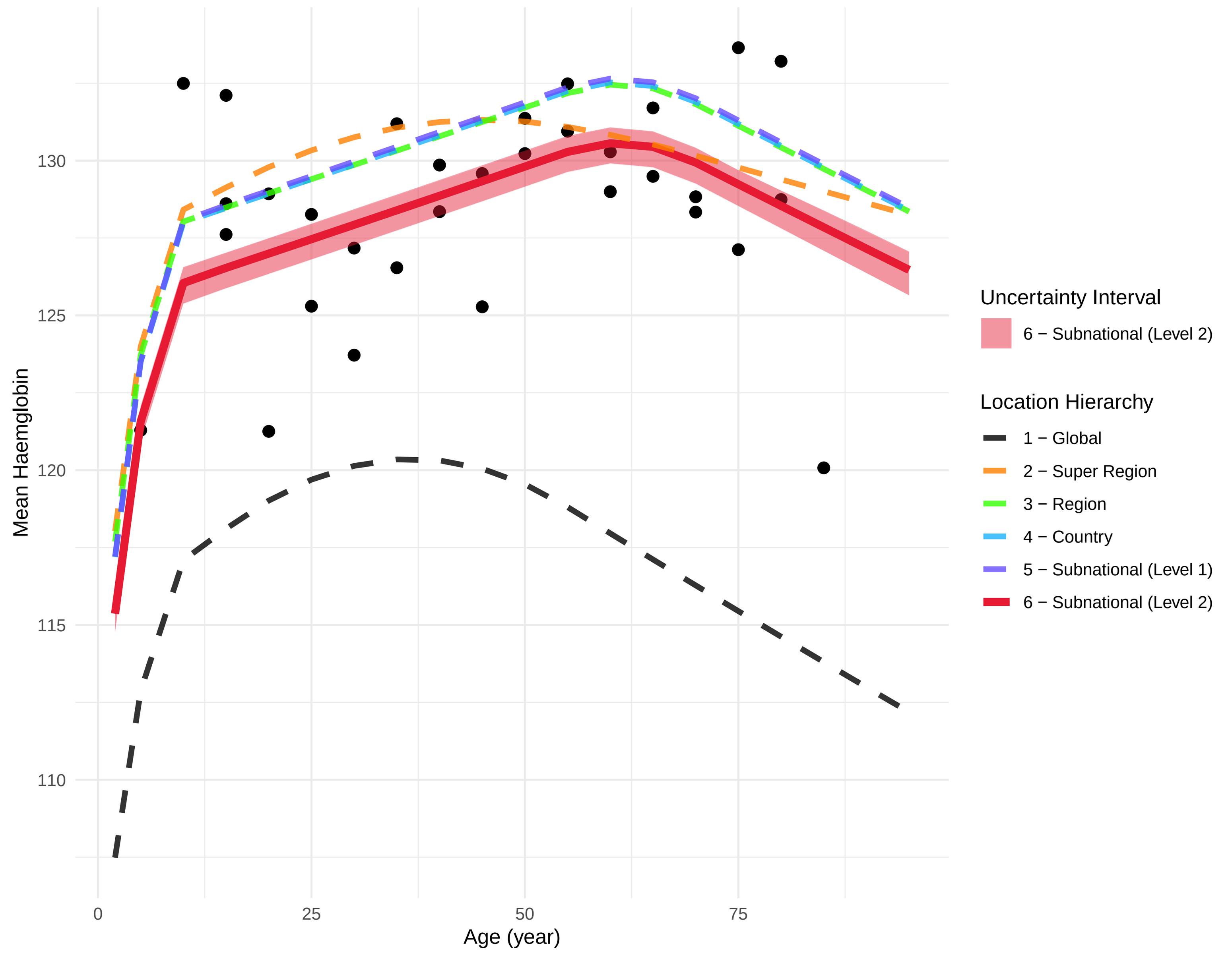

MR−BRT Age/Sex Fits − Non−pregnant Females 2+ Years Old − Mean Haemglobin − Yorkshire and the Humber
WHO−2024 Adjustment Method

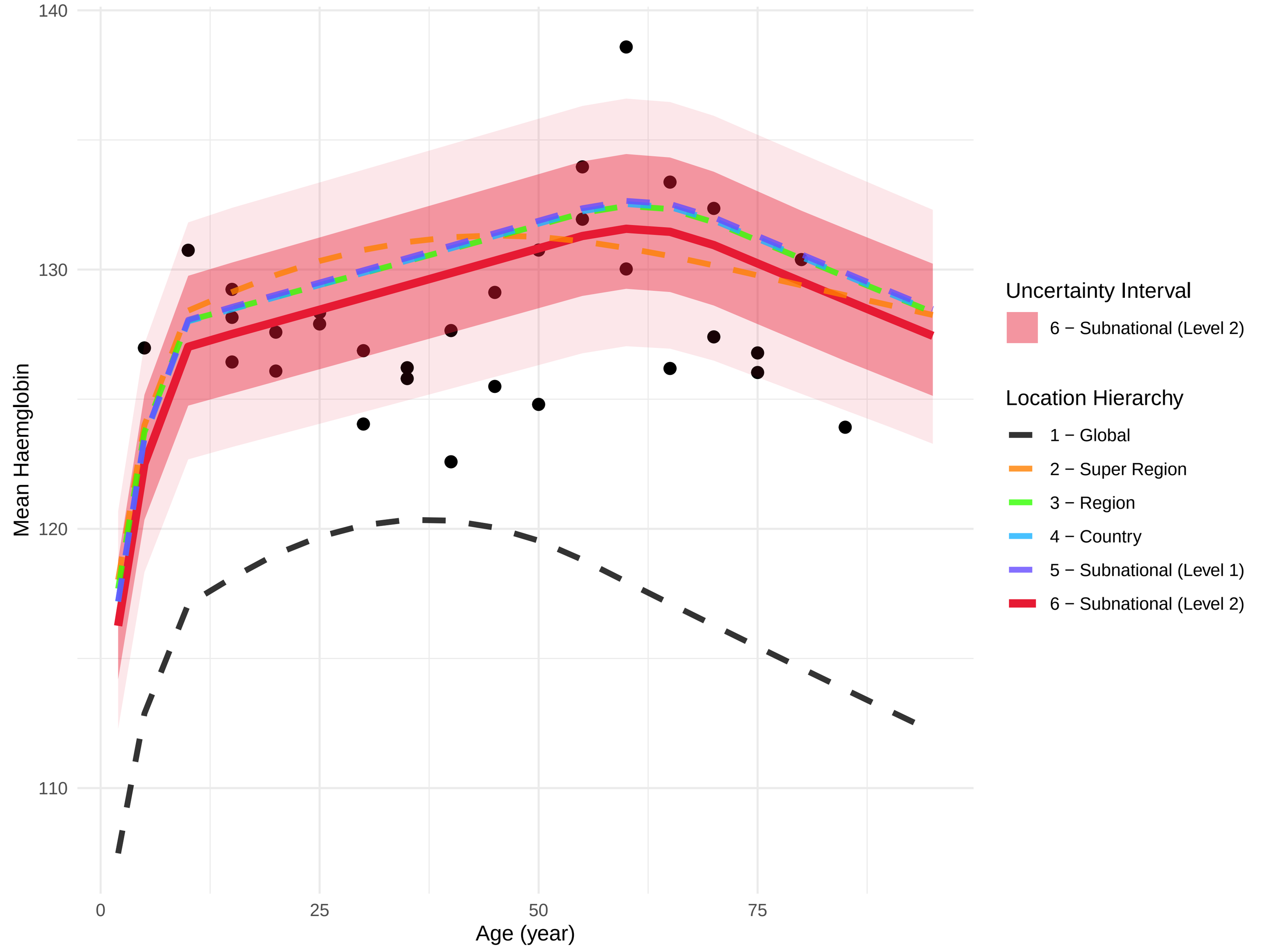

MR−BRT Age/Sex Fits − Non−pregnant Females 2+ Years Old − Mean Haemglobin − Scotland
WHO−2024 Adjustment Method

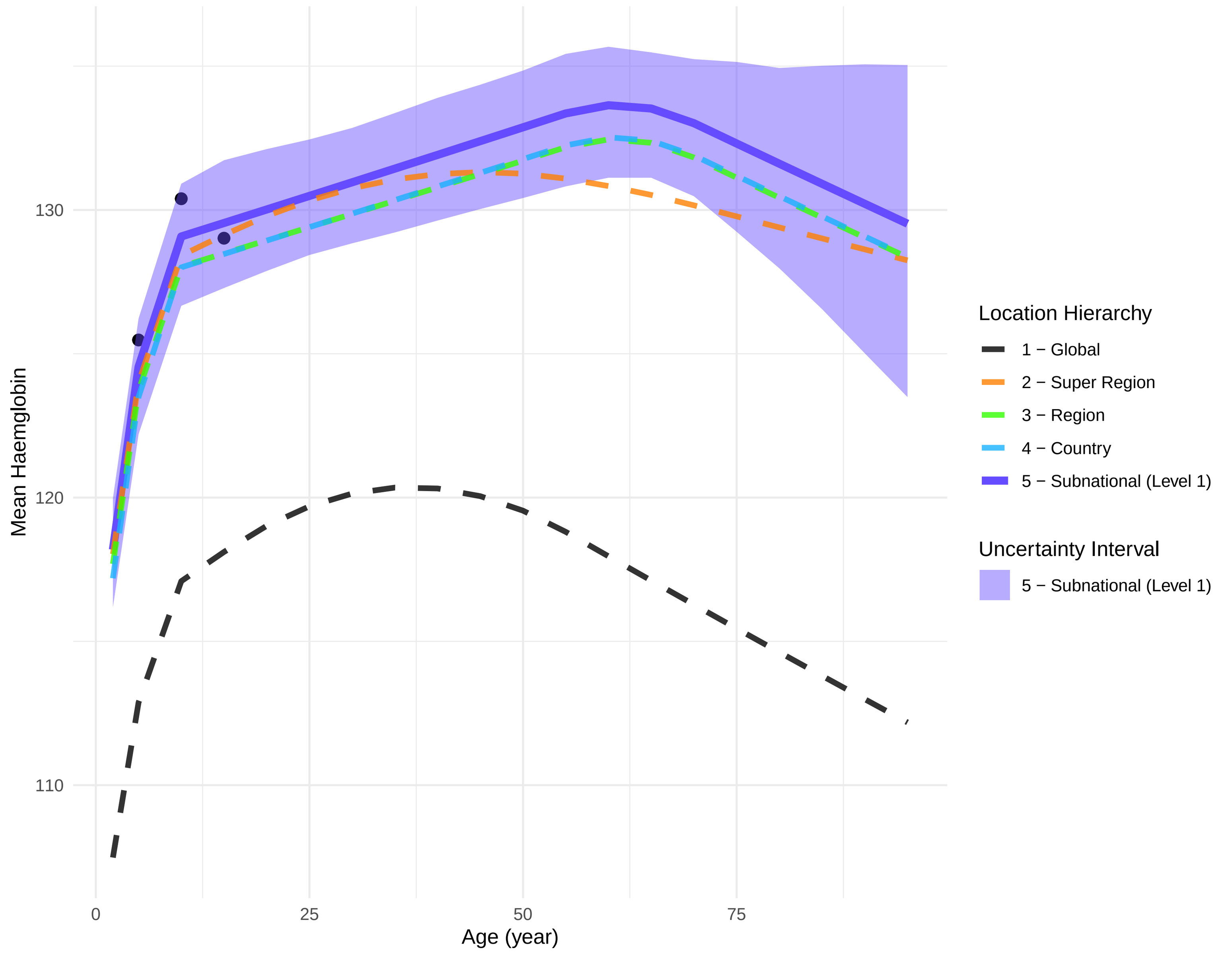

MR−BRT Age/Sex Fits − Non−pregnant Females 2+ Years Old − Mean Haemglobin − Wales
WHO−2024 Adjustment Method

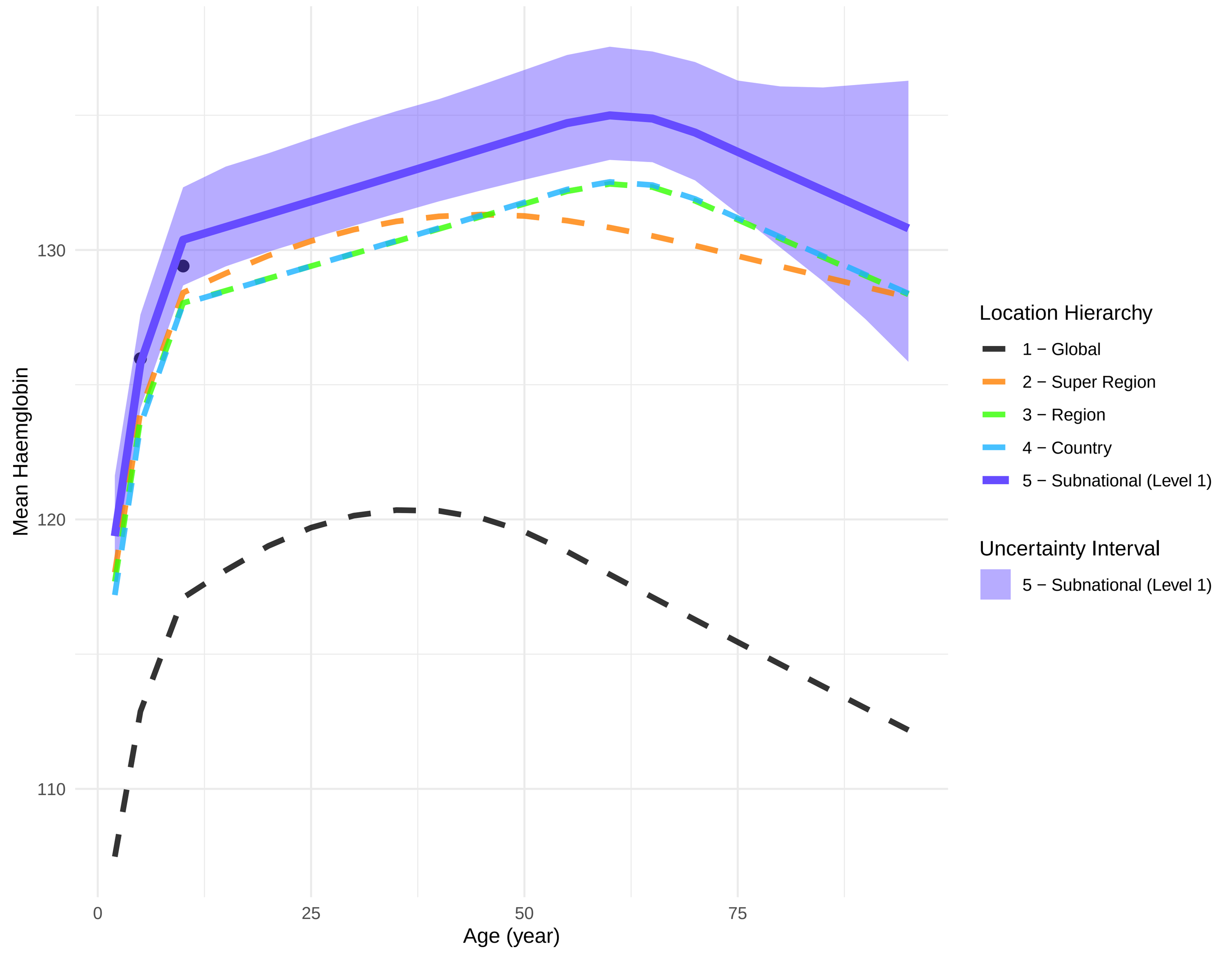

MR−BRT Age/Sex Fits − Non−pregnant Females 2+ Years Old − Mean Haemglobin − Latin America and Caribbean
WHO−2024 Adjustment Method

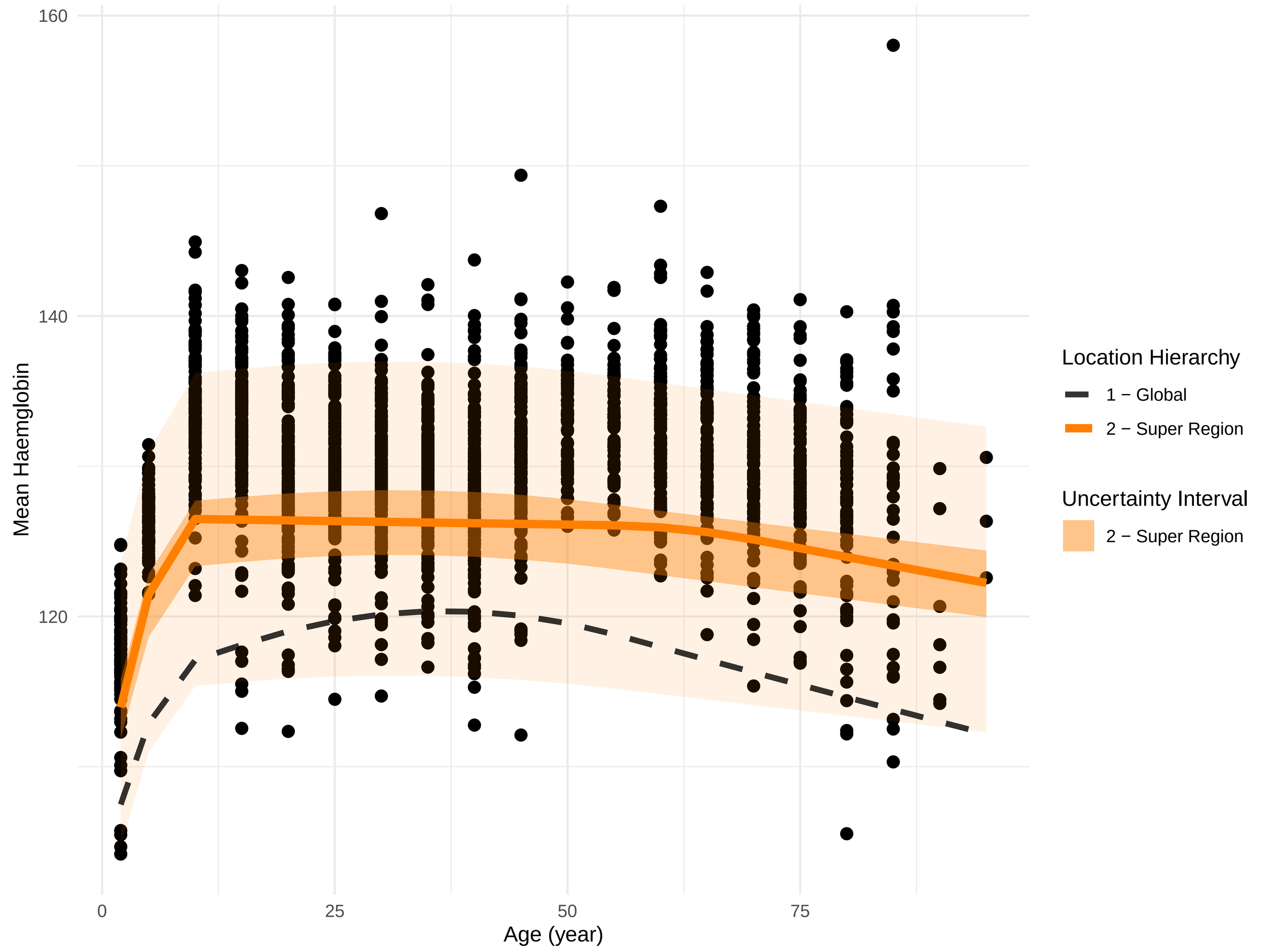

MR−BRT Age/Sex Fits − Non−pregnant Females 2+ Years Old − Mean Haemglobin − Andean Latin America
WHO−2024 Adjustment Method

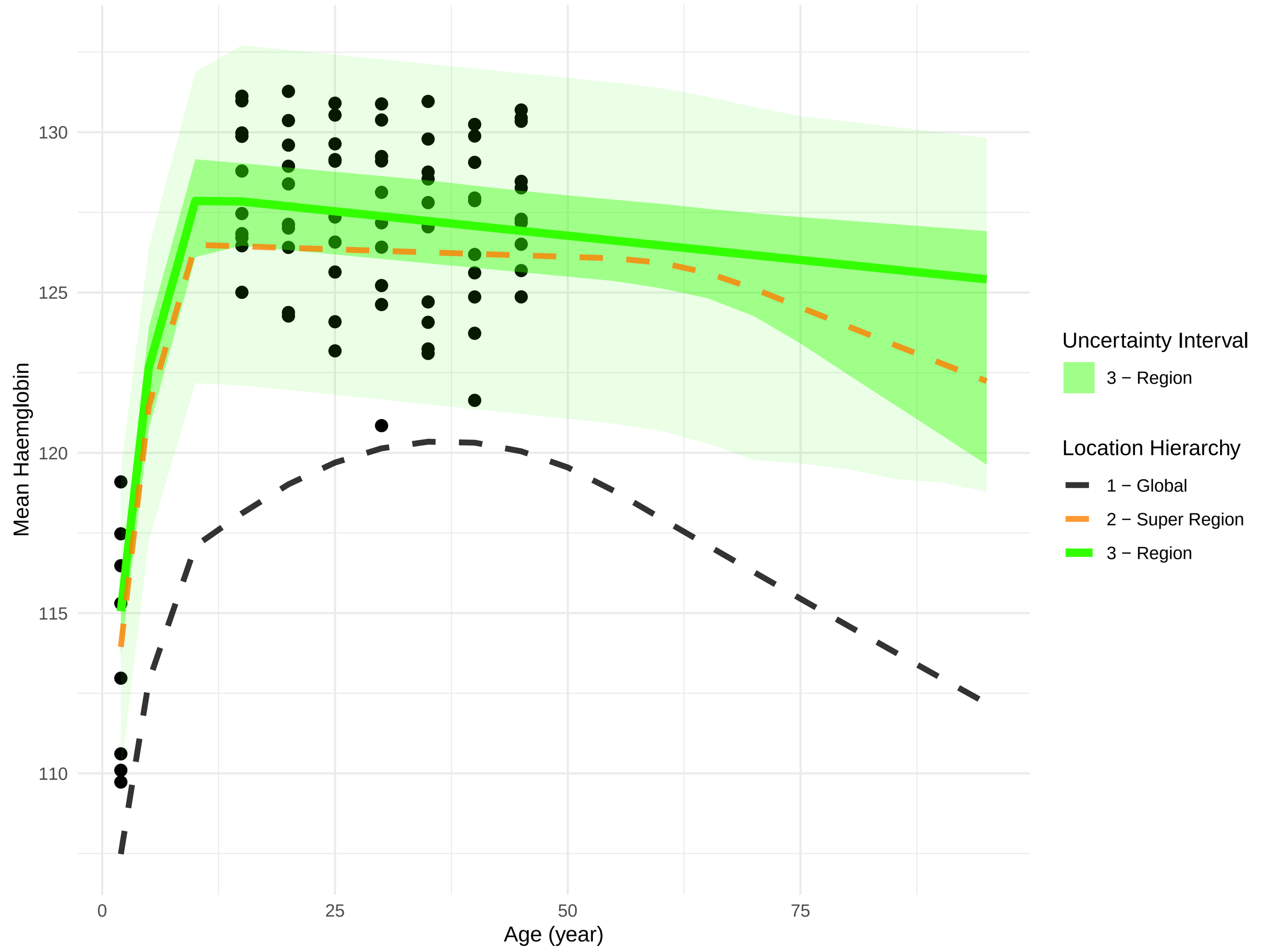

MR−BRT Age/Sex Fits − Non−pregnant Females 2+ Years Old − Mean Haemglobin − Bolivia (Plurinational State of)
WHO−2024 Adjustment Method

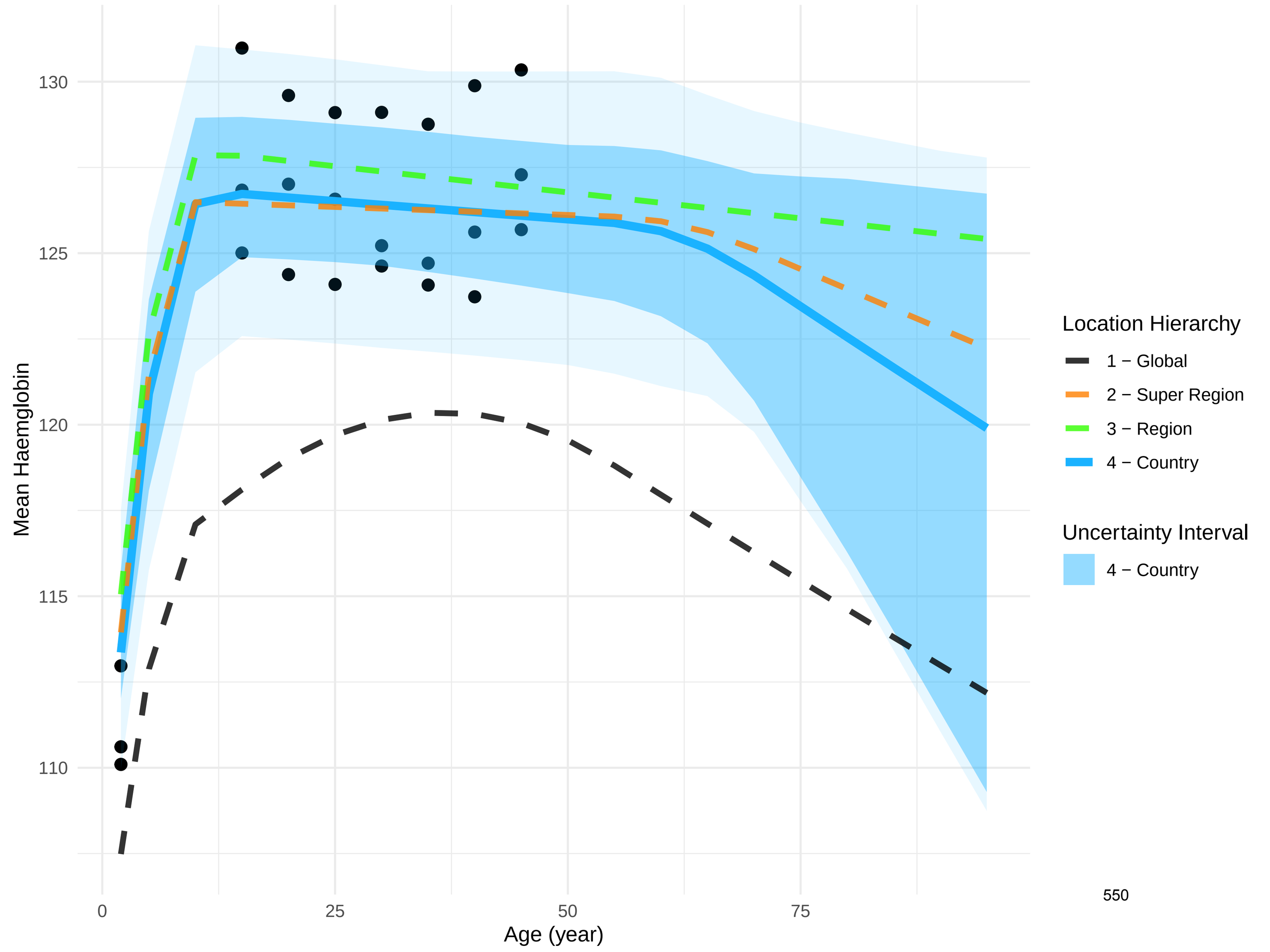

MR−BRT Age/Sex Fits − Non−pregnant Females 2+ Years Old − Mean Haemglobin − Peru
WHO−2024 Adjustment Method

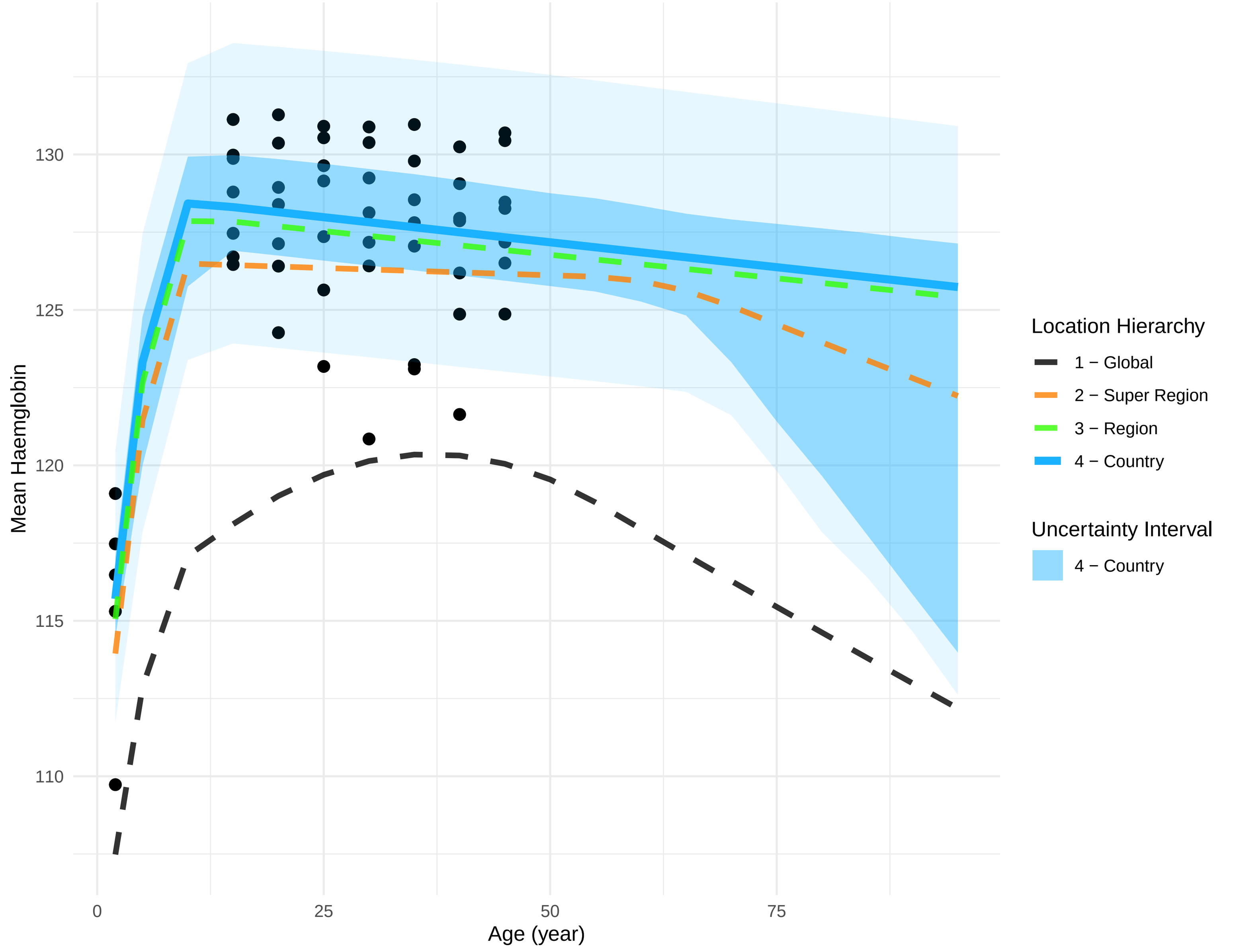

MR−BRT Age/Sex Fits − Non−pregnant Females 2+ Years Old − Mean Haemglobin − Caribbean
WHO−2024 Adjustment Method

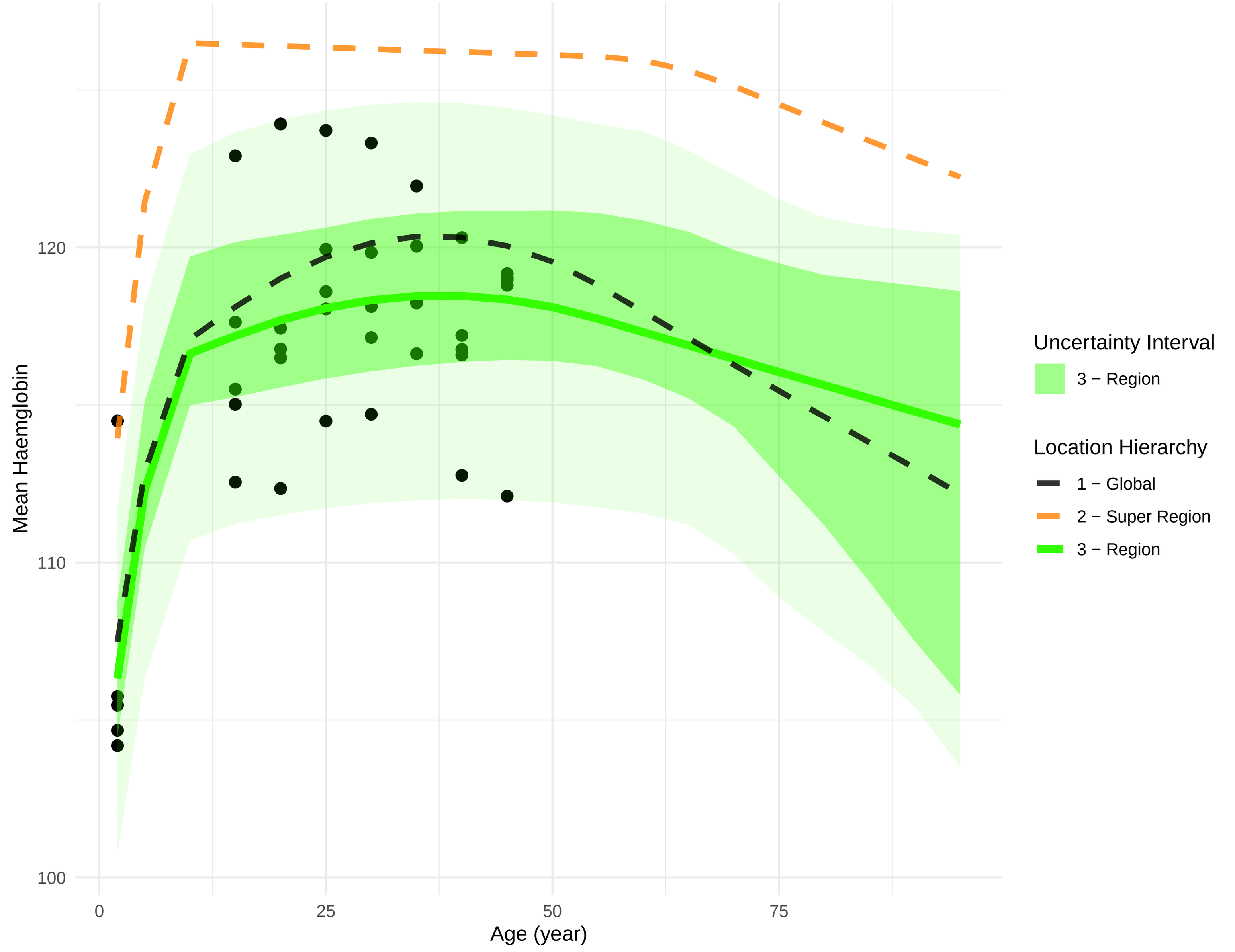

MR−BRT Age/Sex Fits − Non−pregnant Females 2+ Years Old − Mean Haemglobin − Guyana
WHO−2024 Adjustment Method

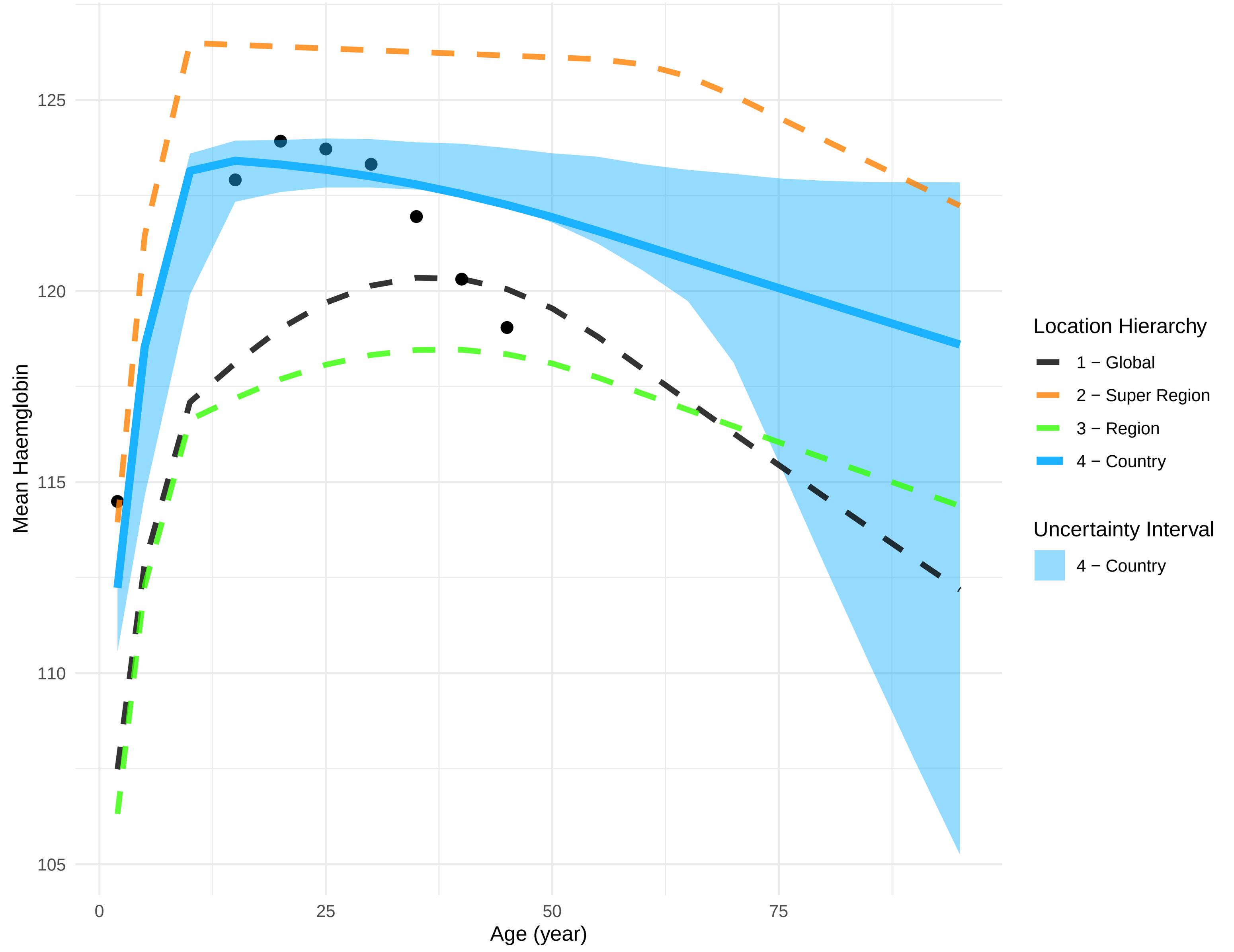

MR−BRT Age/Sex Fits − Non−pregnant Females 2+ Years Old − Mean Haemglobin − Haiti
WHO−2024 Adjustment Method

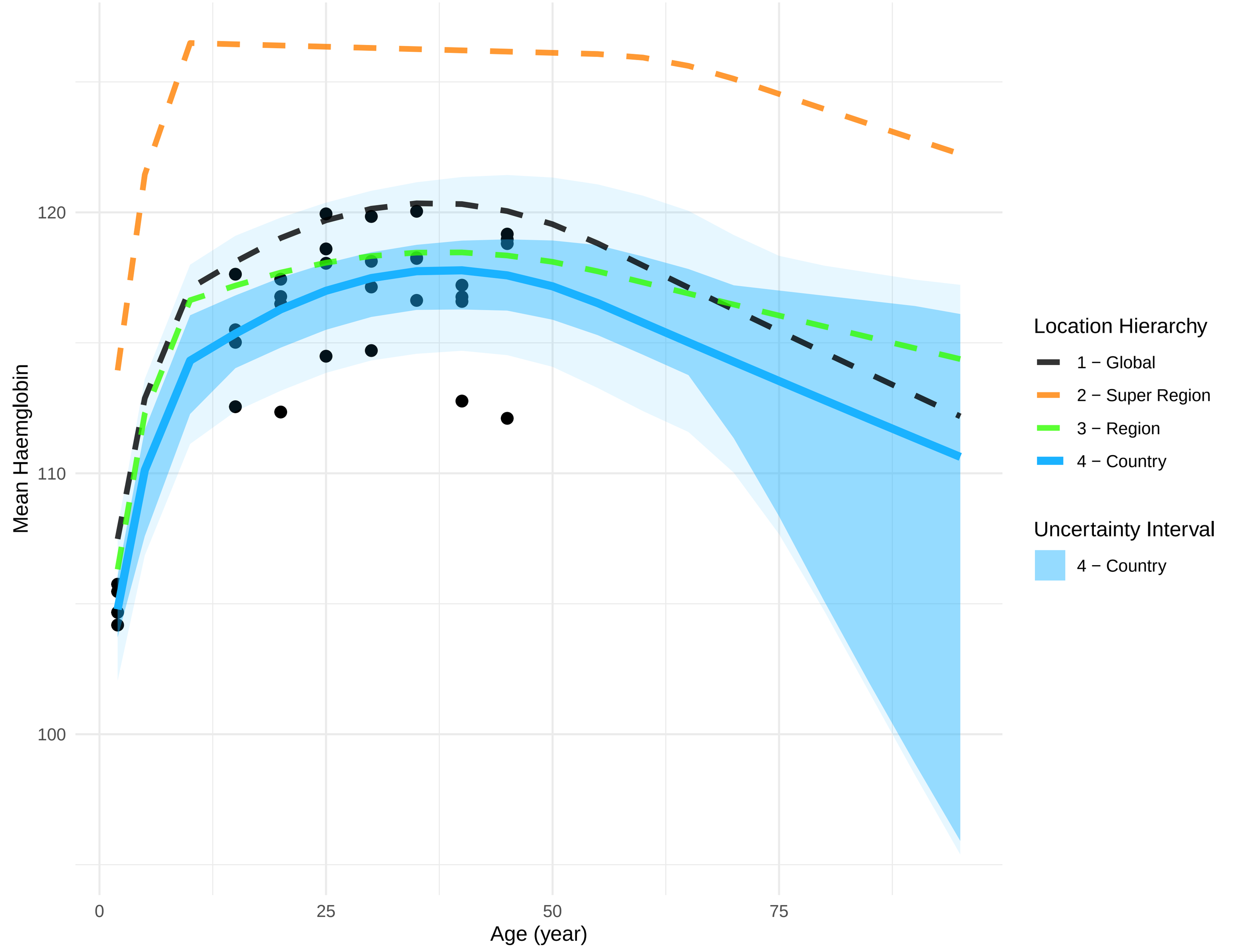

MR−BRT Age/Sex Fits − Non−pregnant Females 2+ Years Old − Mean Haemglobin − Central Latin America
WHO−2024 Adjustment Method

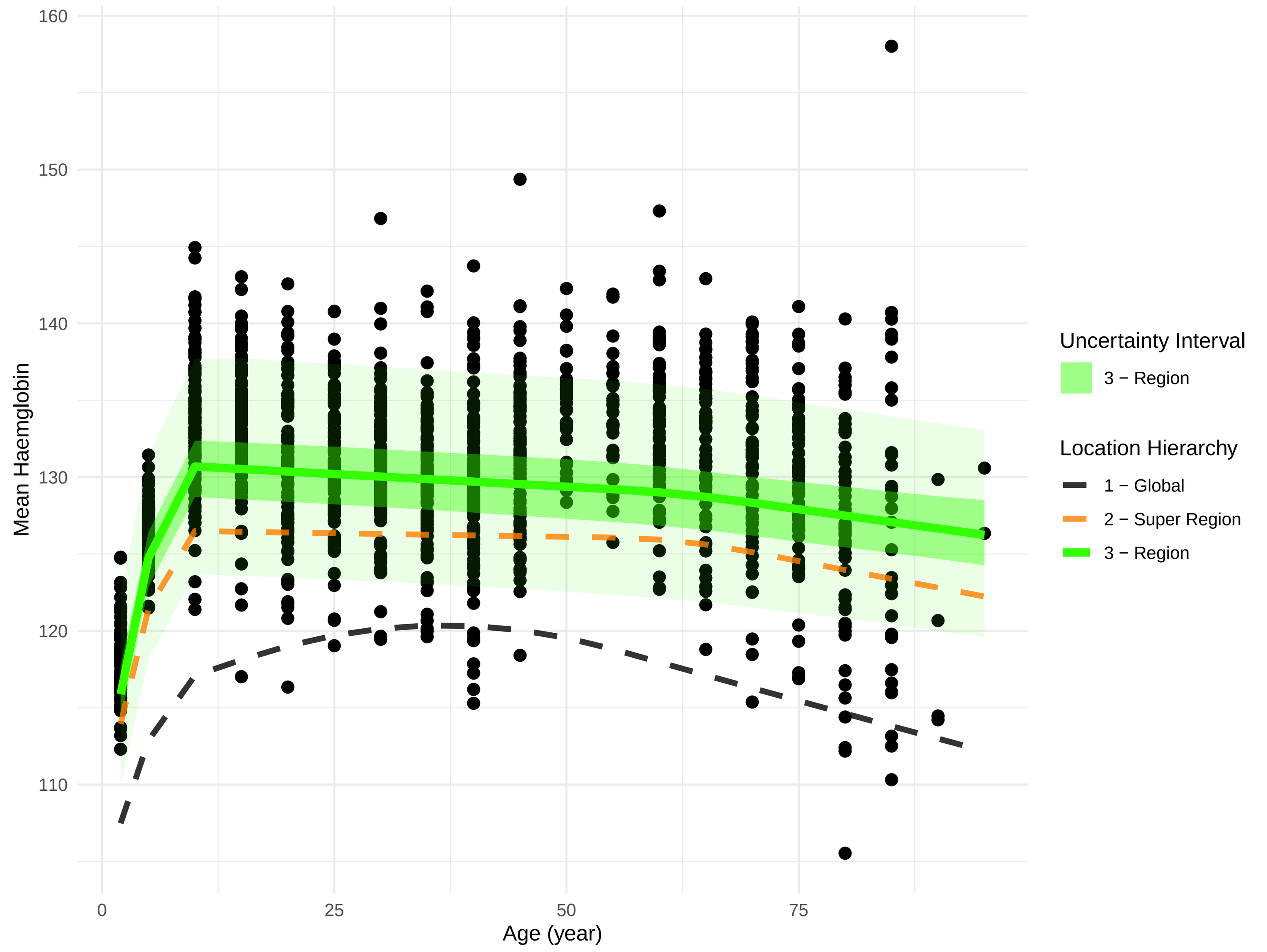

MR−BRT Age/Sex Fits − Non−pregnant Females 2+ Years Old − Mean Haemglobin − El Salvador
WHO−2024 Adjustment Method

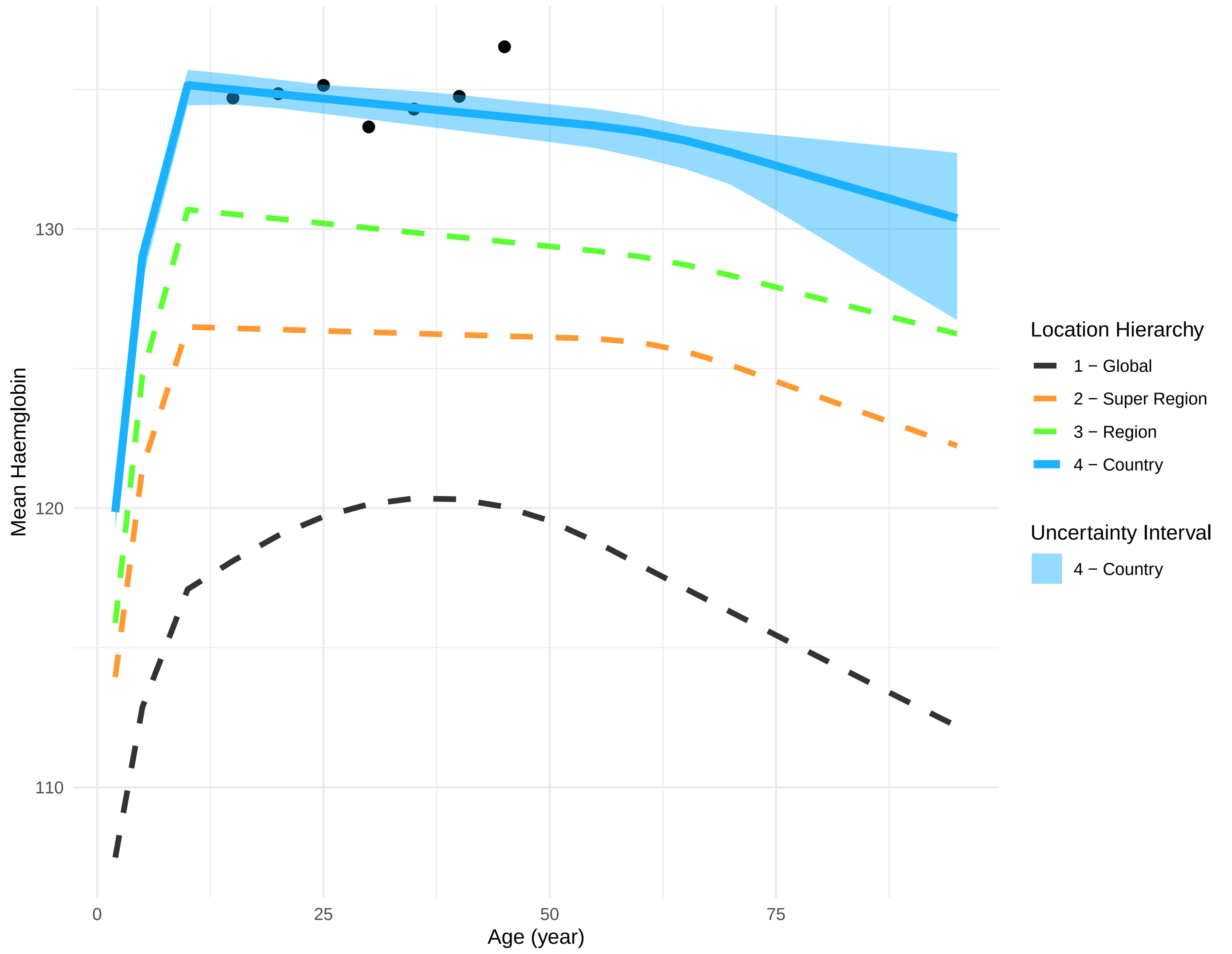

MR−BRT Age/Sex Fits − Non−pregnant Females 2+ Years Old − Mean Haemglobin − Guatemala
WHO−2024 Adjustment Method

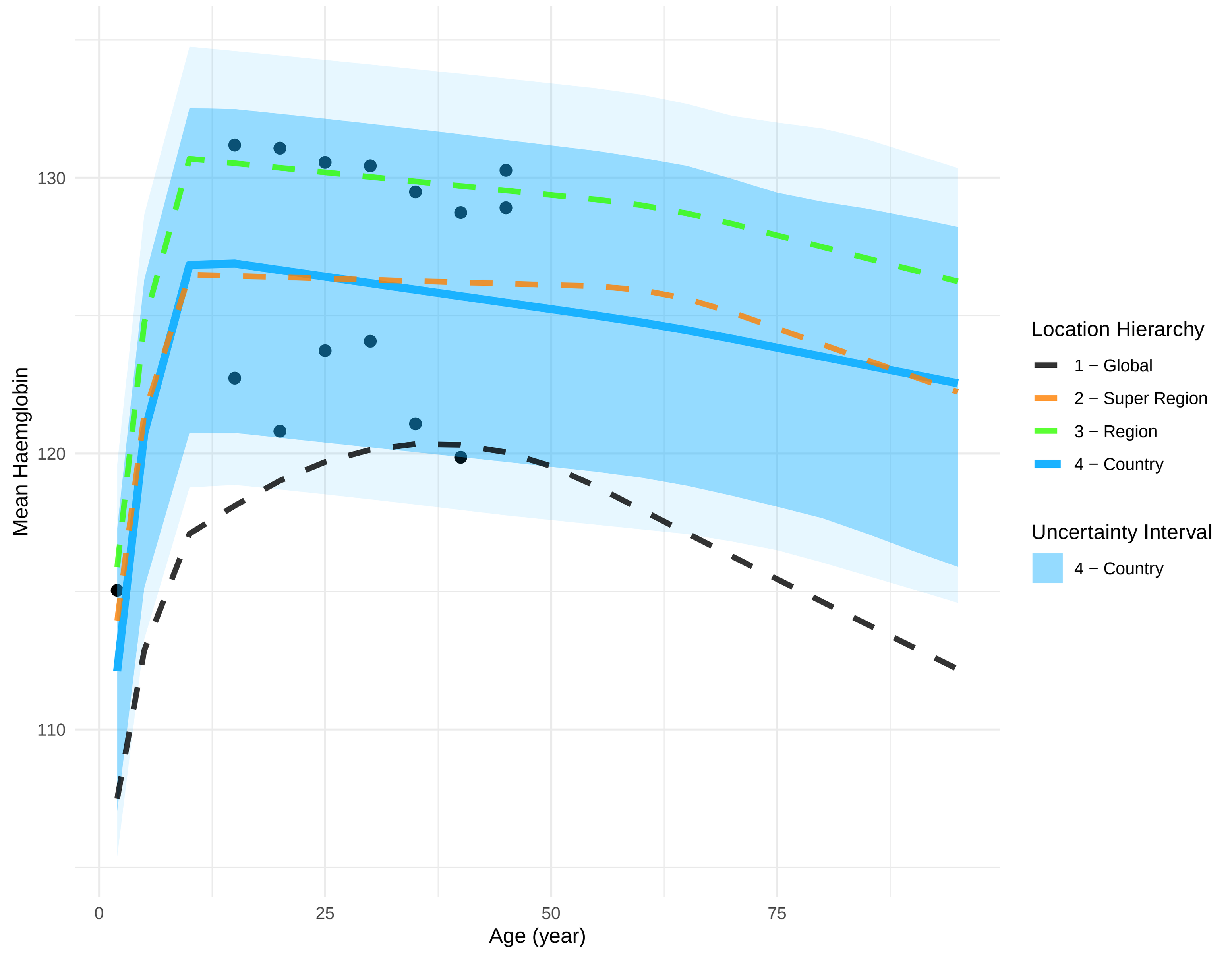

MR−BRT Age/Sex Fits − Non−pregnant Females 2+ Years Old − Mean Haemglobin − Honduras
WHO−2024 Adjustment Method

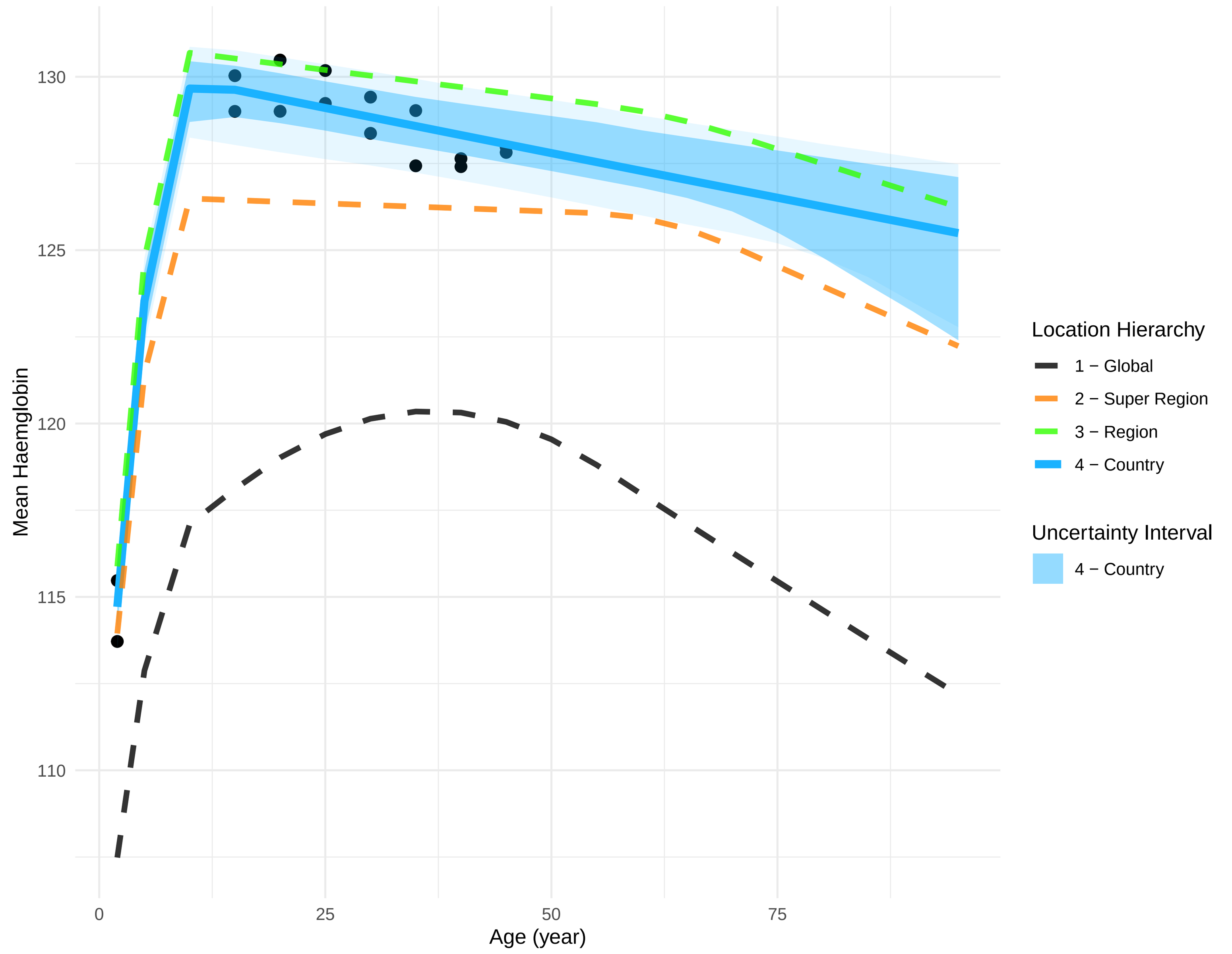

MR−BRT Age/Sex Fits − Non−pregnant Females 2+ Years Old − Mean Haemglobin − Mexico
WHO−2024 Adjustment Method

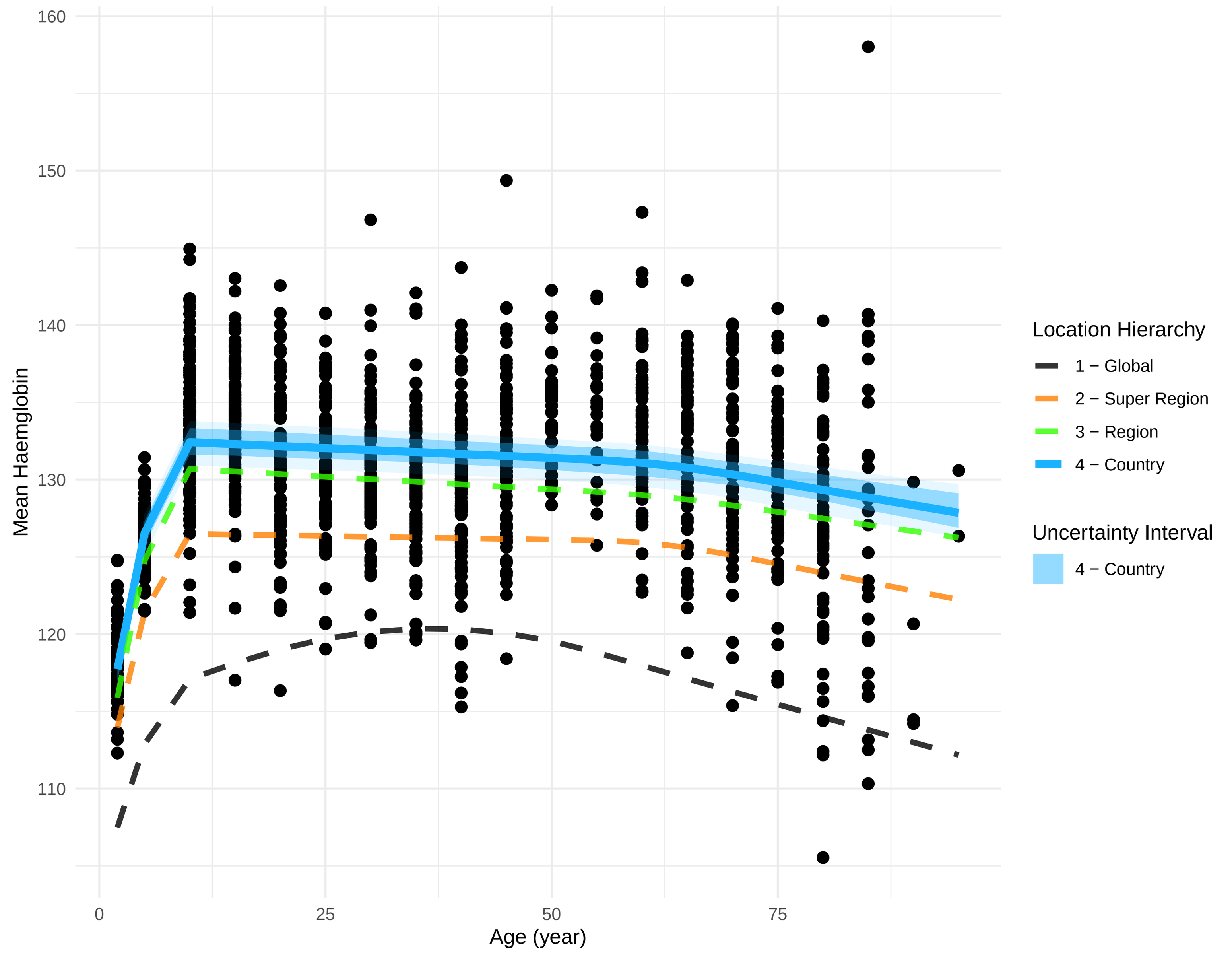

MR−BRT Age/Sex Fits − Non−pregnant Females 2+ Years Old − Mean Haemglobin − Aguascalientes
WHO−2024 Adjustment Method

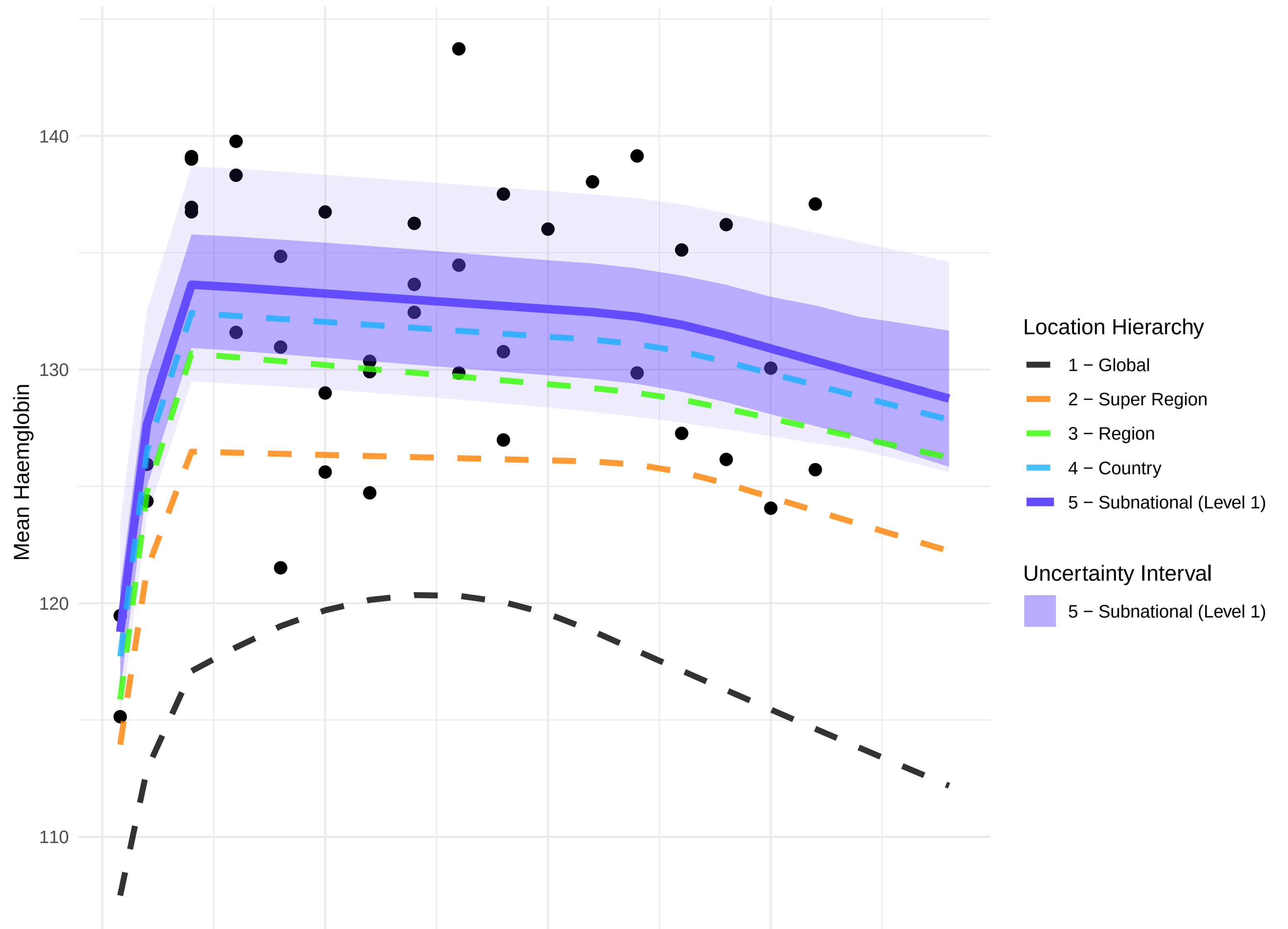

MR−BRT Age/Sex Fits − Non−pregnant Females 2+ Years Old − Mean Haemglobin − Baja California
WHO−2024 Adjustment Method

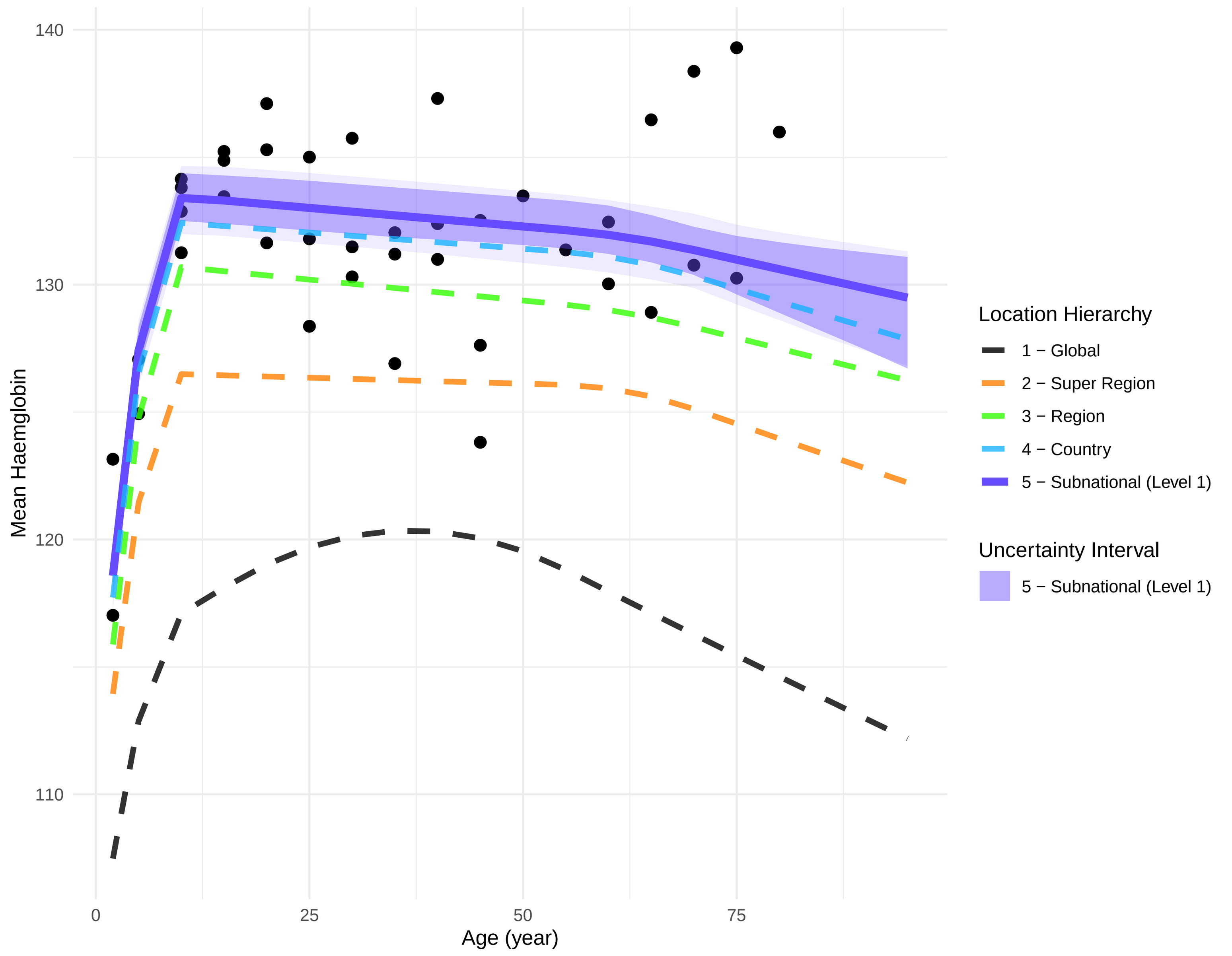

MR−BRT Age/Sex Fits − Non−pregnant Females 2+ Years Old − Mean Haemglobin − Baja California Sur
WHO−2024 Adjustment Method

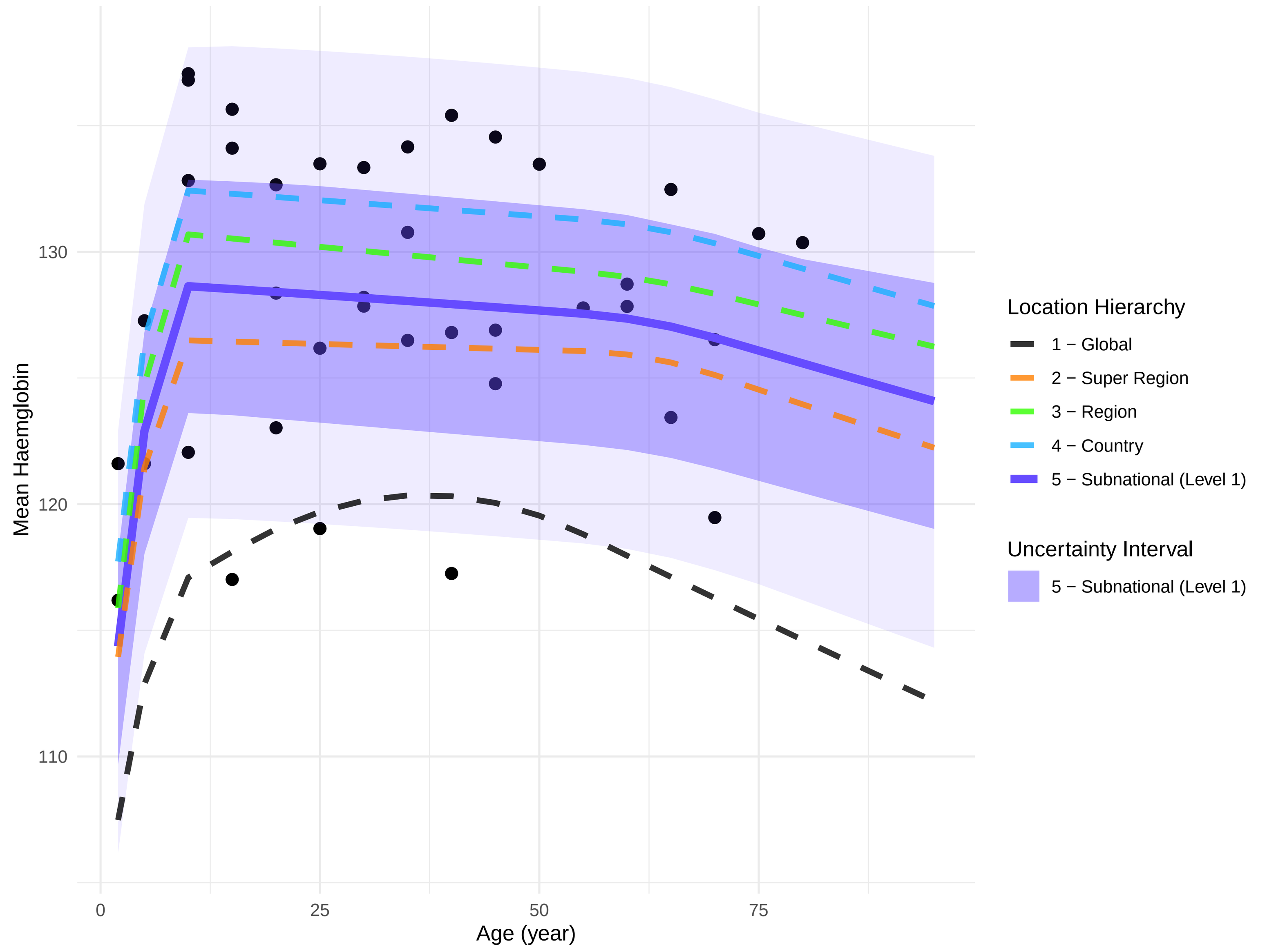

MR−BRT Age/Sex Fits − Non−pregnant Females 2+ Years Old − Mean Haemglobin − Campeche
WHO−2024 Adjustment Method

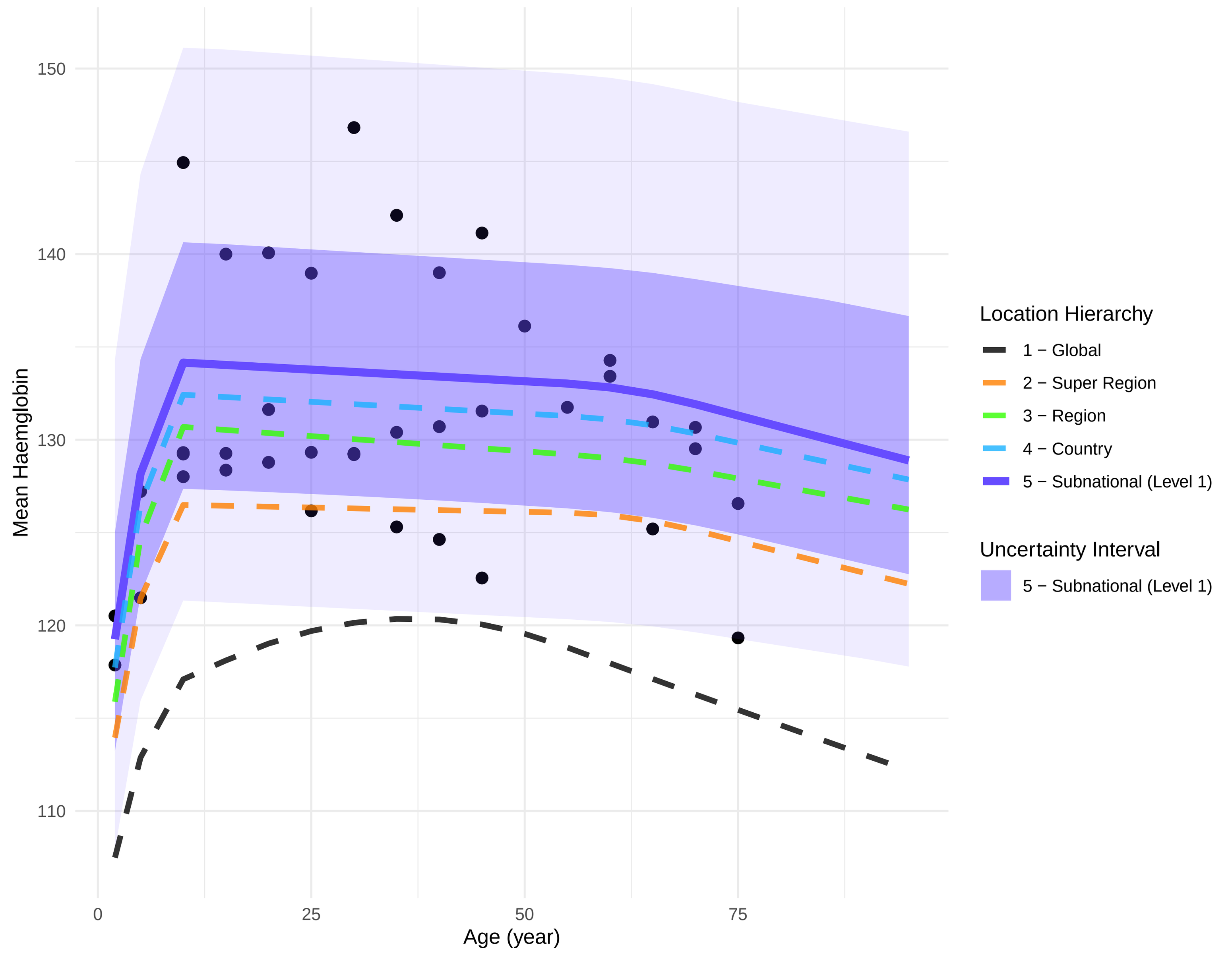

MR−BRT Age/Sex Fits − Non−pregnant Females 2+ Years Old − Mean Haemglobin − Chiapas
WHO−2024 Adjustment Method

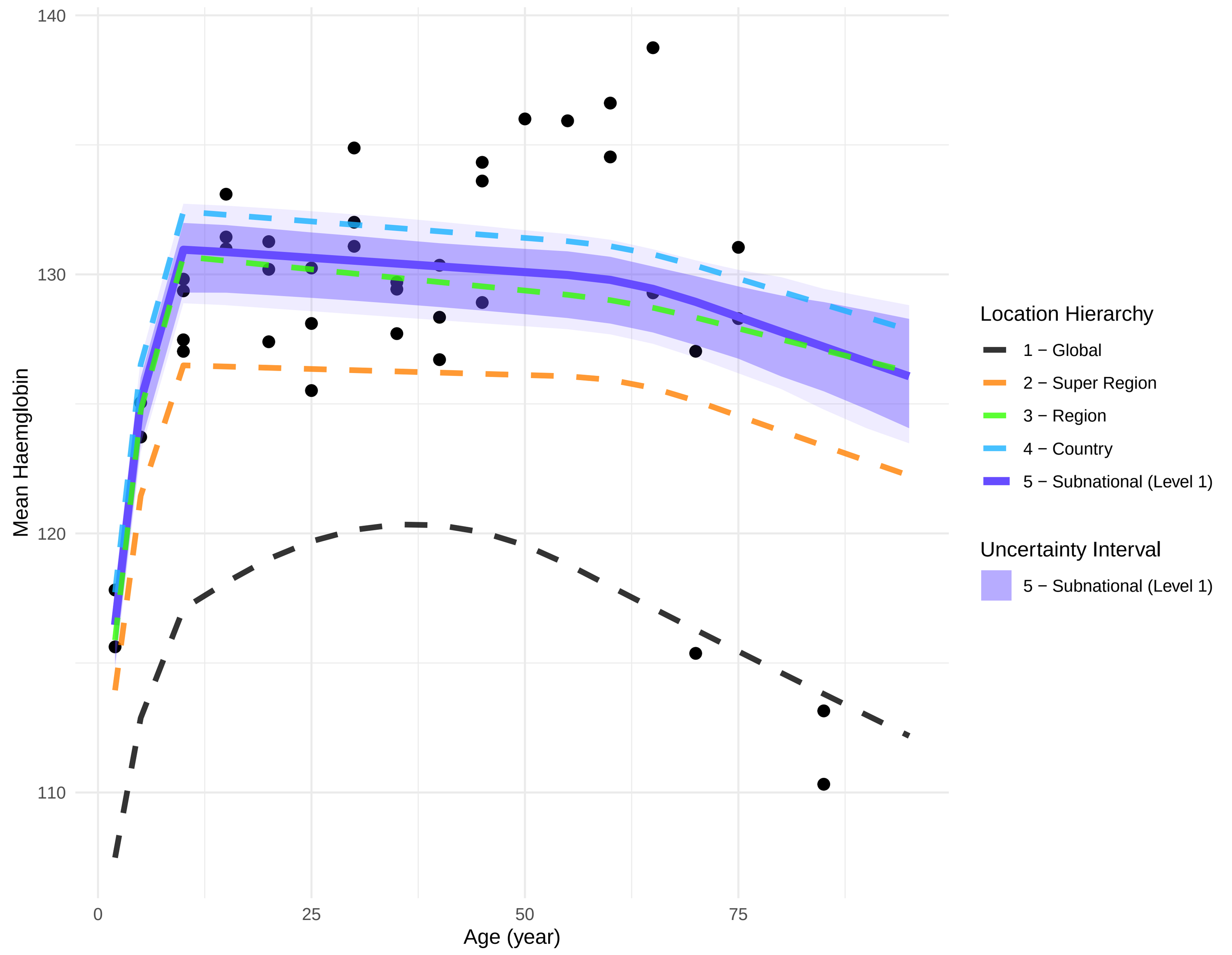

MR−BRT Age/Sex Fits − Non−pregnant Females 2+ Years Old − Mean Haemglobin − Chihuahua
WHO−2024 Adjustment Method

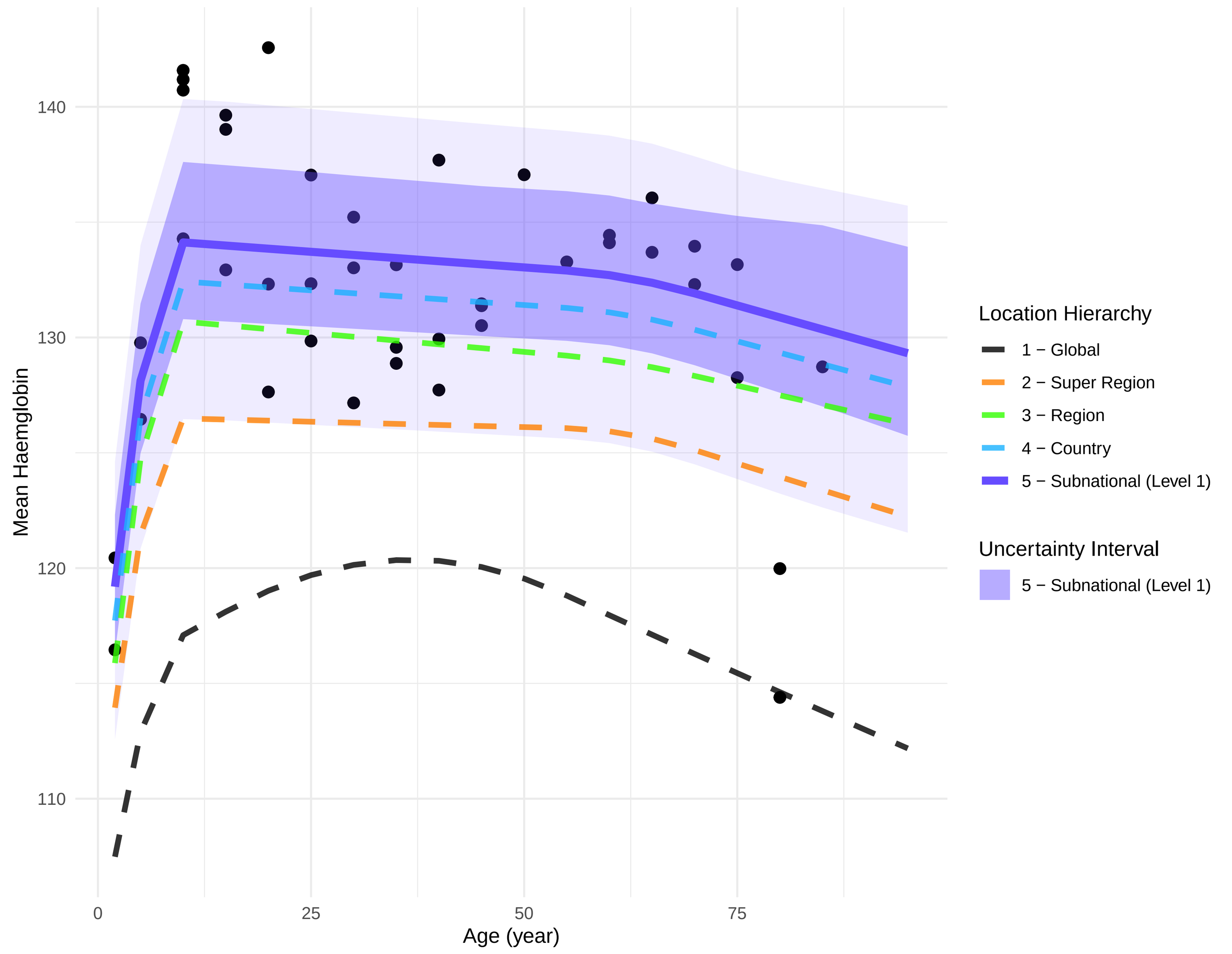

MR−BRT Age/Sex Fits − Non−pregnant Females 2+ Years Old − Mean Haemglobin − Coahuila
WHO−2024 Adjustment Method

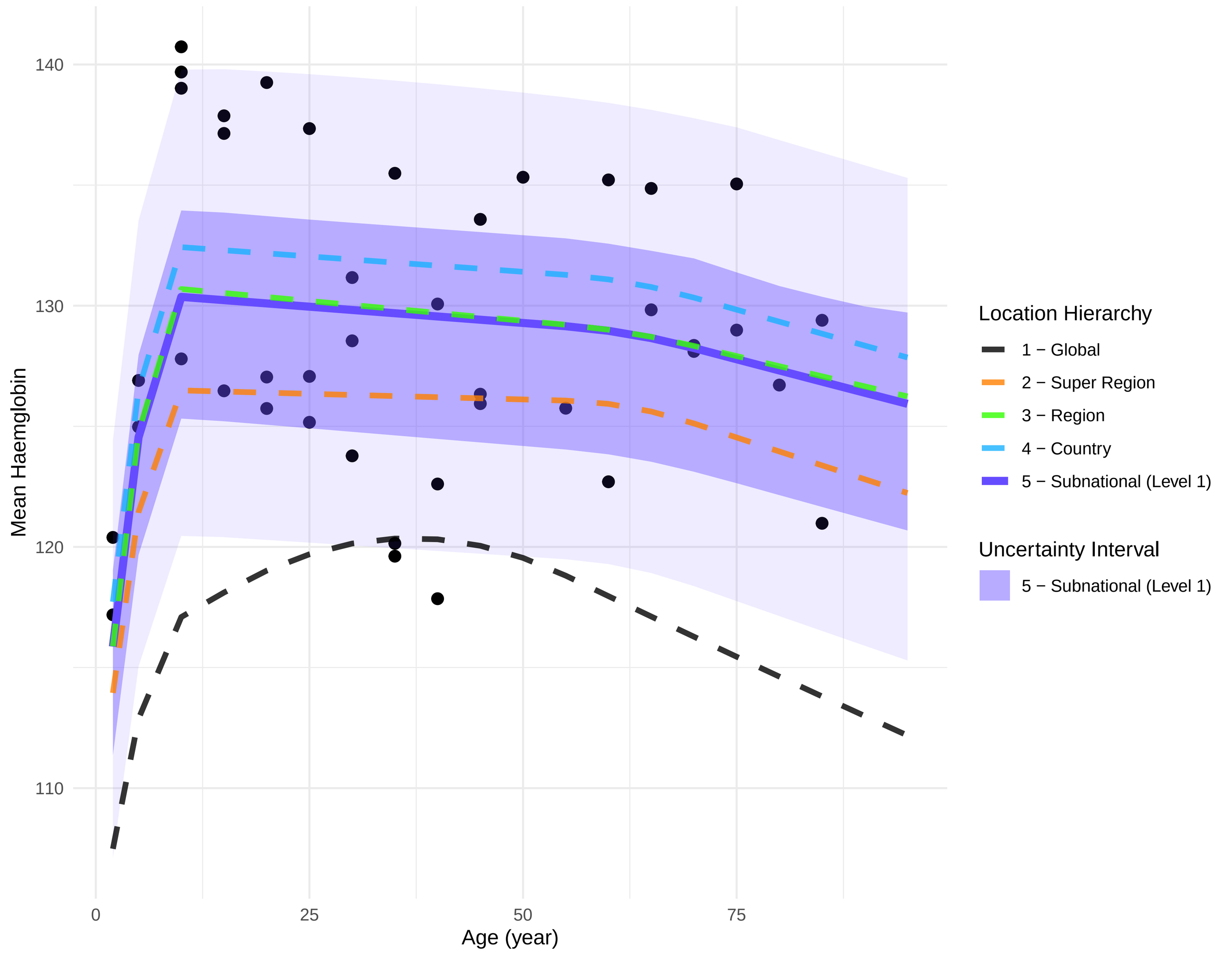

MR−BRT Age/Sex Fits − Non−pregnant Females 2+ Years Old − Mean Haemglobin − Colima
WHO−2024 Adjustment Method

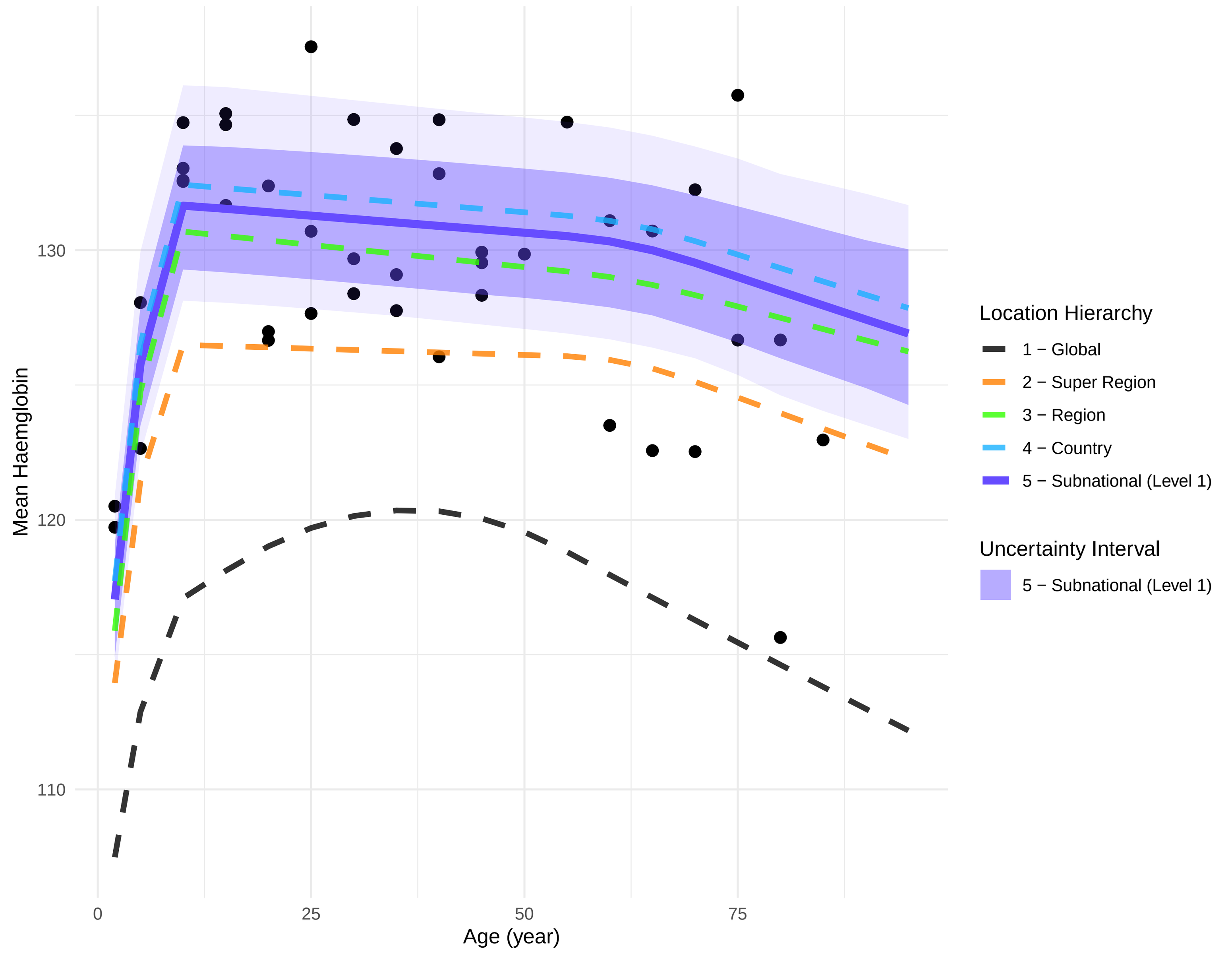

MR−BRT Age/Sex Fits − Non−pregnant Females 2+ Years Old − Mean Haemglobin − Durango
WHO−2024 Adjustment Method

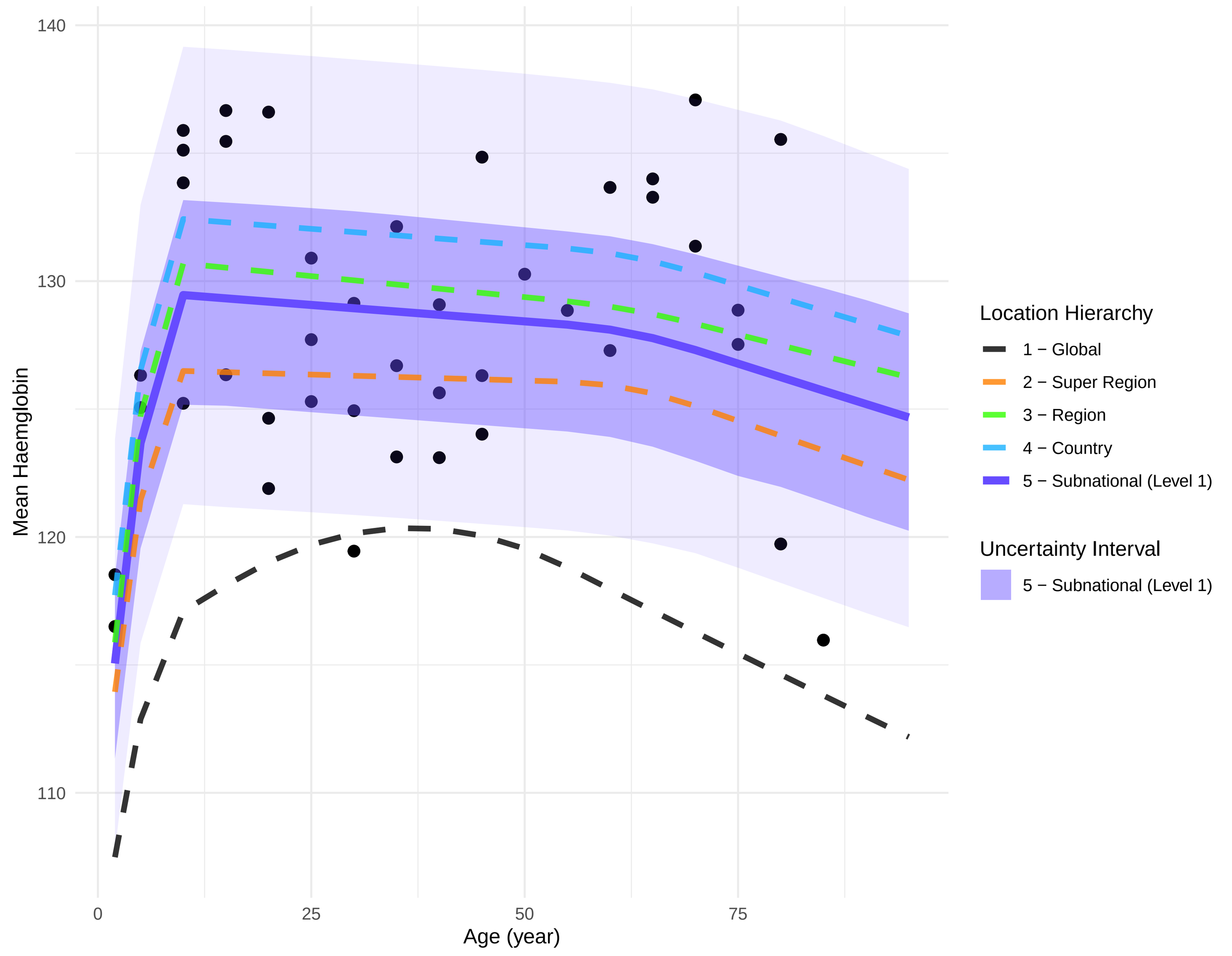

MR−BRT Age/Sex Fits − Non−pregnant Females 2+ Years Old − Mean Haemglobin − Guanajuato
WHO−2024 Adjustment Method

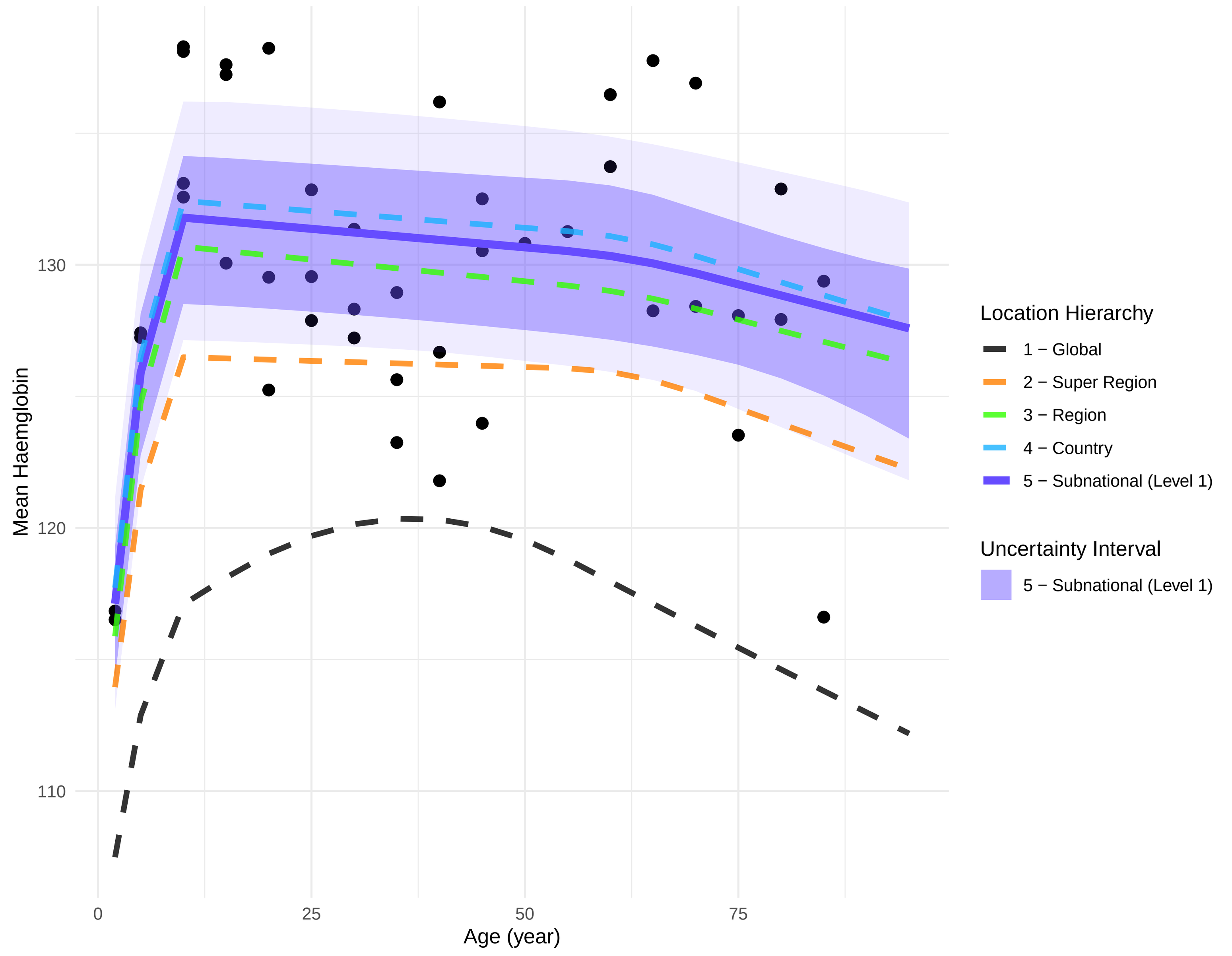

MR−BRT Age/Sex Fits − Non−pregnant Females 2+ Years Old − Mean Haemglobin − Guerrero
WHO−2024 Adjustment Method

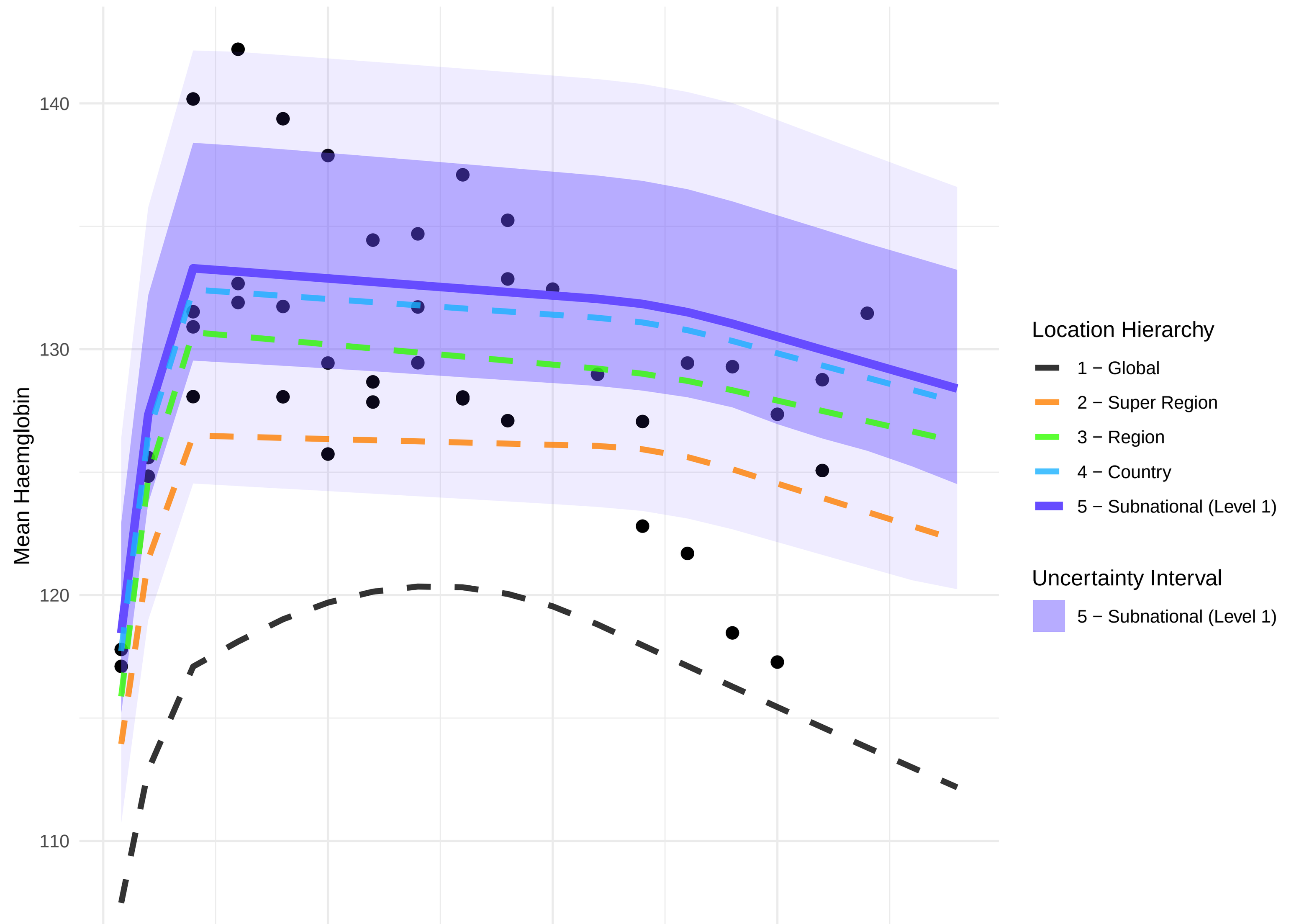

MR−BRT Age/Sex Fits − Non−pregnant Females 2+ Years Old − Mean Haemglobin − Hidalgo
WHO−2024 Adjustment Method

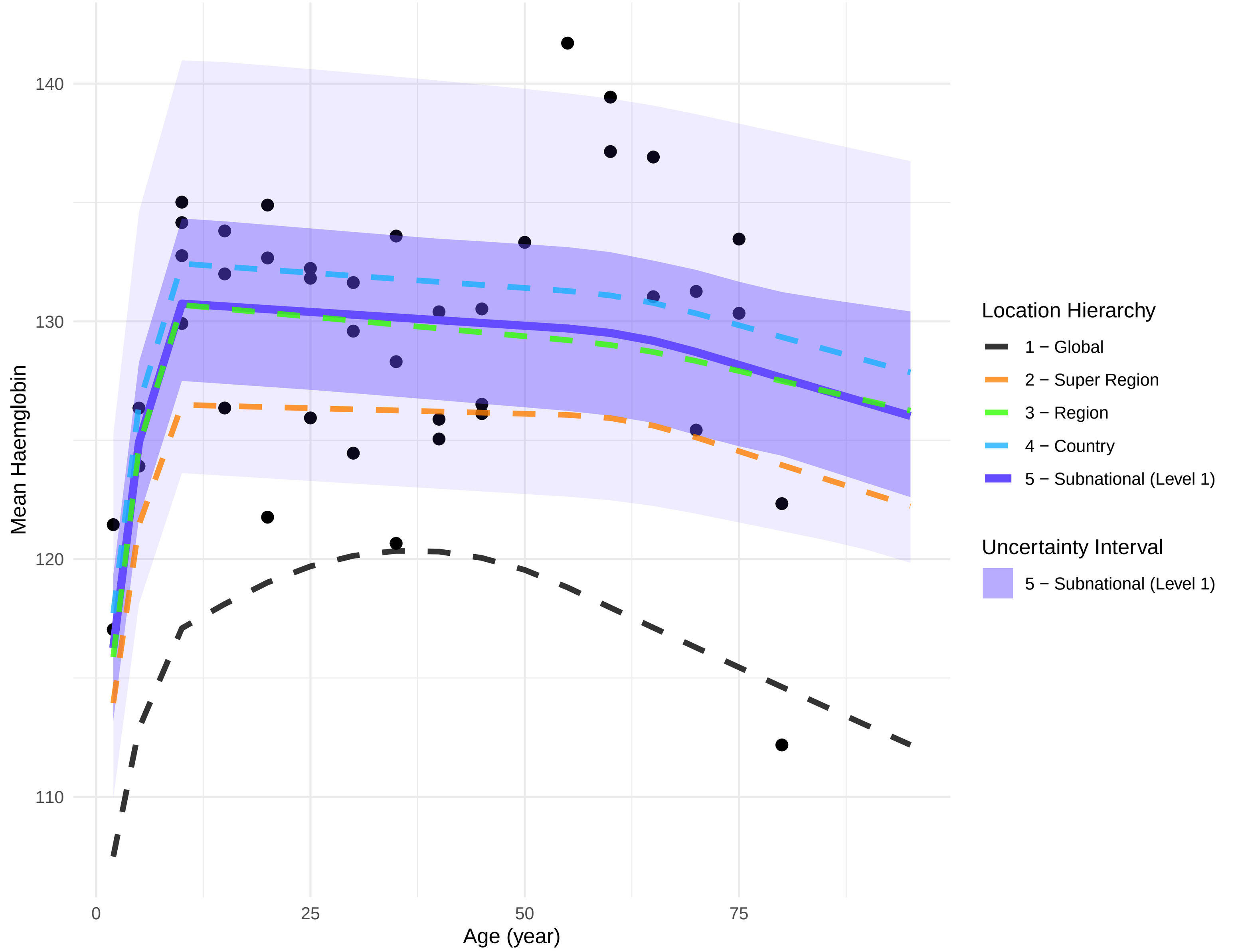

MR−BRT Age/Sex Fits − Non−pregnant Females 2+ Years Old − Mean Haemglobin − Jalisco
WHO−2024 Adjustment Method

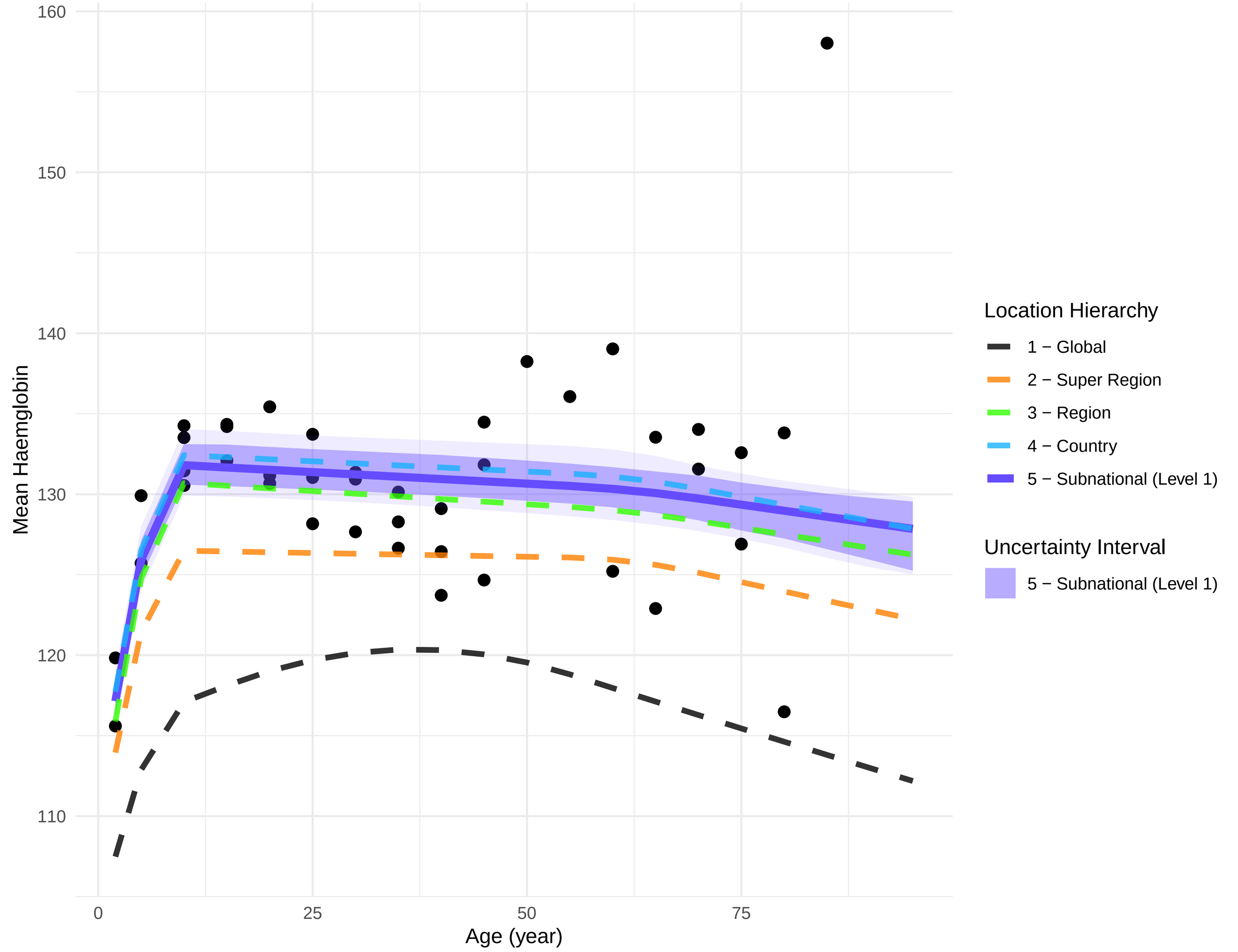

MR−BRT Age/Sex Fits − Non−pregnant Females 2+ Years Old − Mean Haemglobin − México
WHO−2024 Adjustment Method

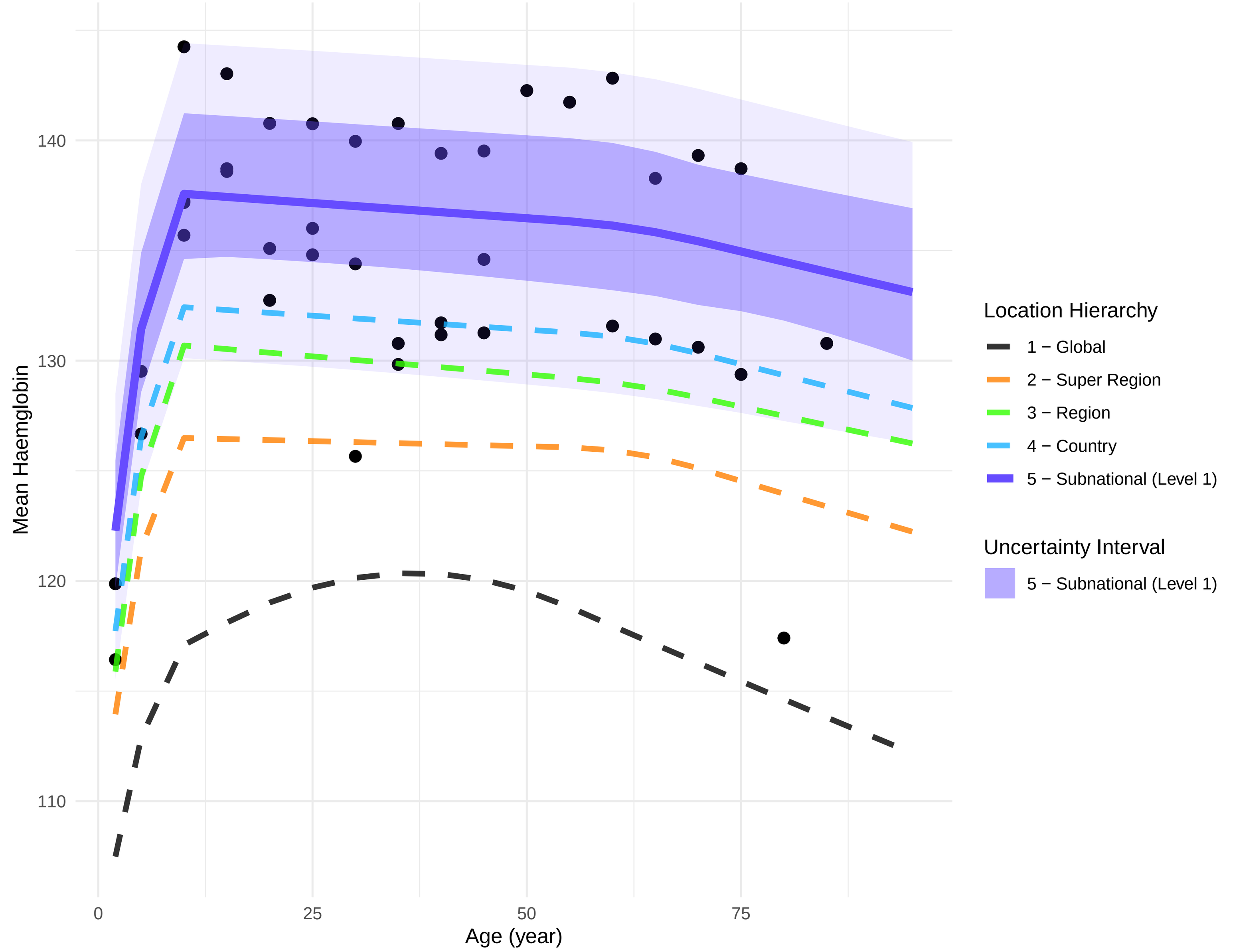

MR−BRT Age/Sex Fits − Non−pregnant Females 2+ Years Old − Mean Haemglobin − Mexico City
WHO−2024 Adjustment Method

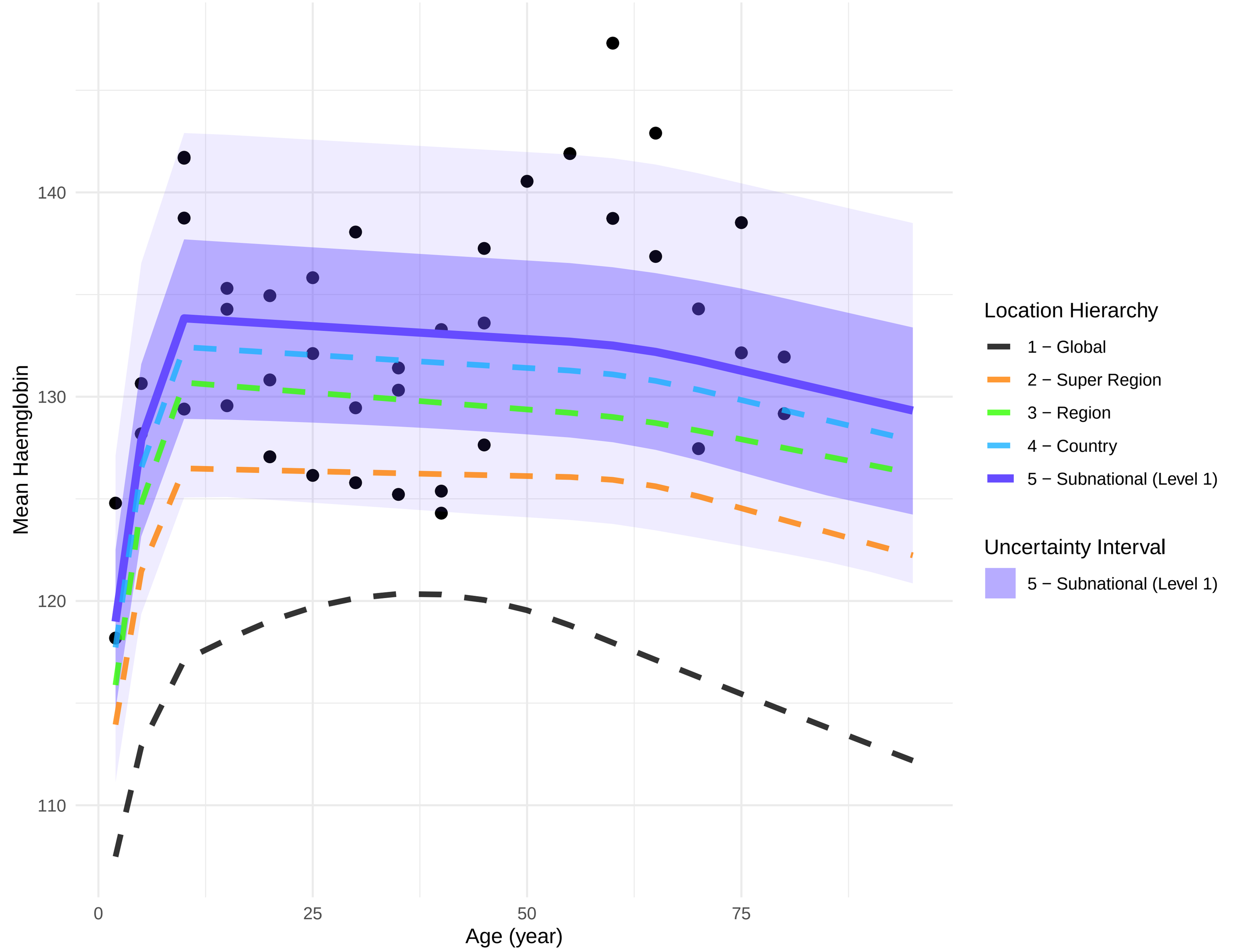

MR−BRT Age/Sex Fits − Non−pregnant Females 2+ Years Old − Mean Haemglobin − Michoacán de Ocampo
WHO−2024 Adjustment Method

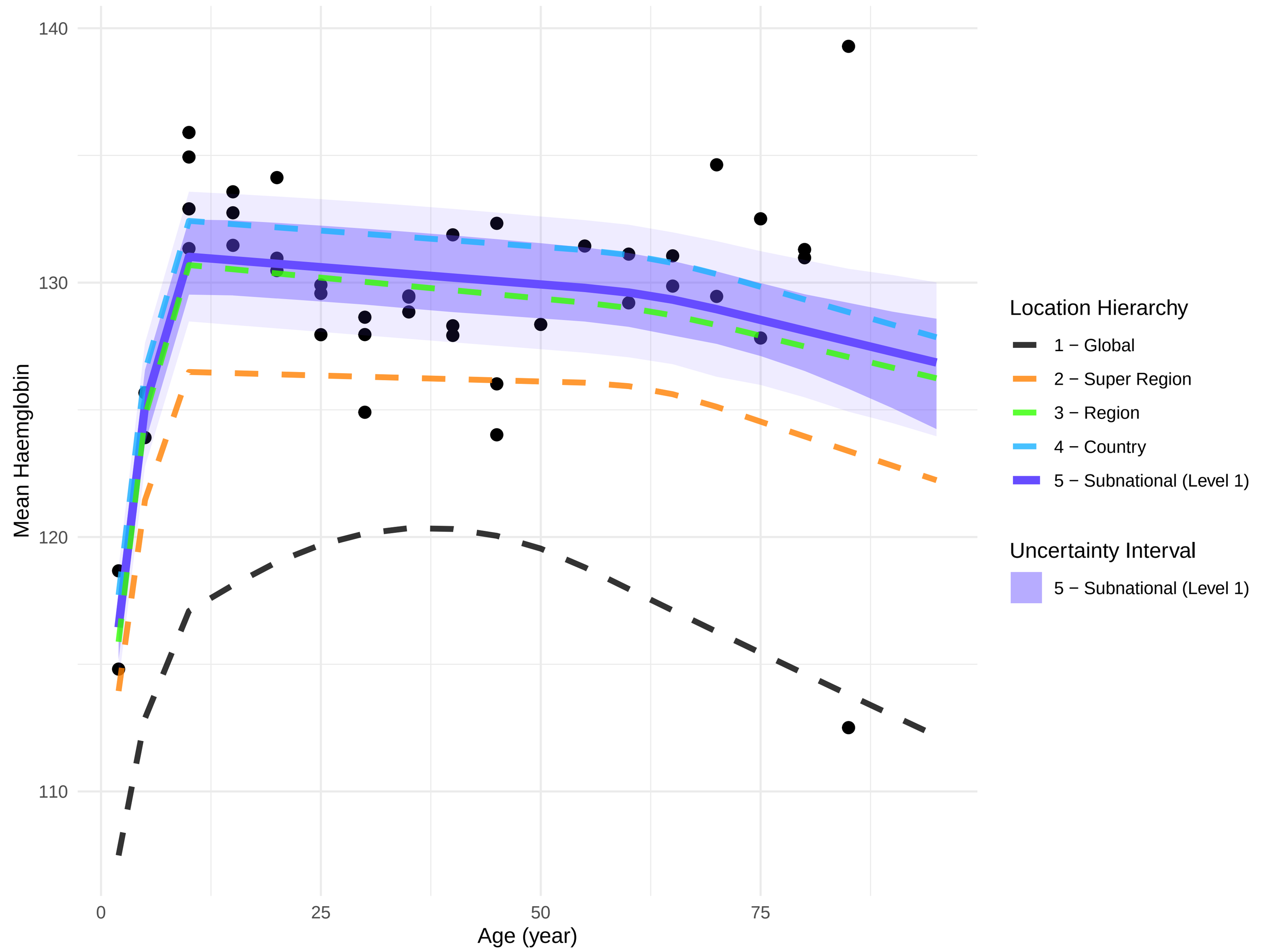

MR−BRT Age/Sex Fits − Non−pregnant Females 2+ Years Old − Mean Haemglobin − Morelos
WHO−2024 Adjustment Method

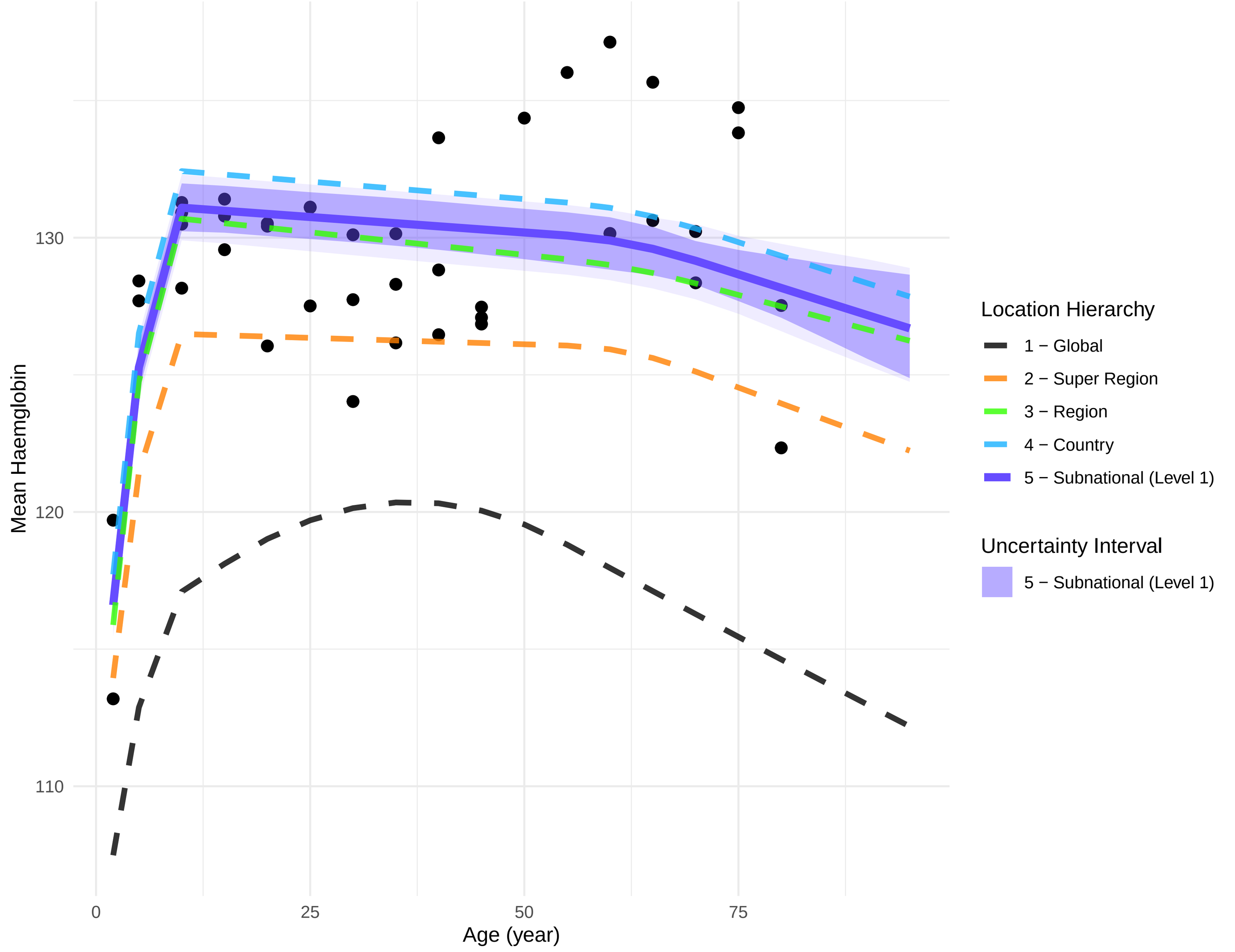

MR−BRT Age/Sex Fits − Non−pregnant Females 2+ Years Old − Mean Haemglobin − Nayarit
WHO−2024 Adjustment Method

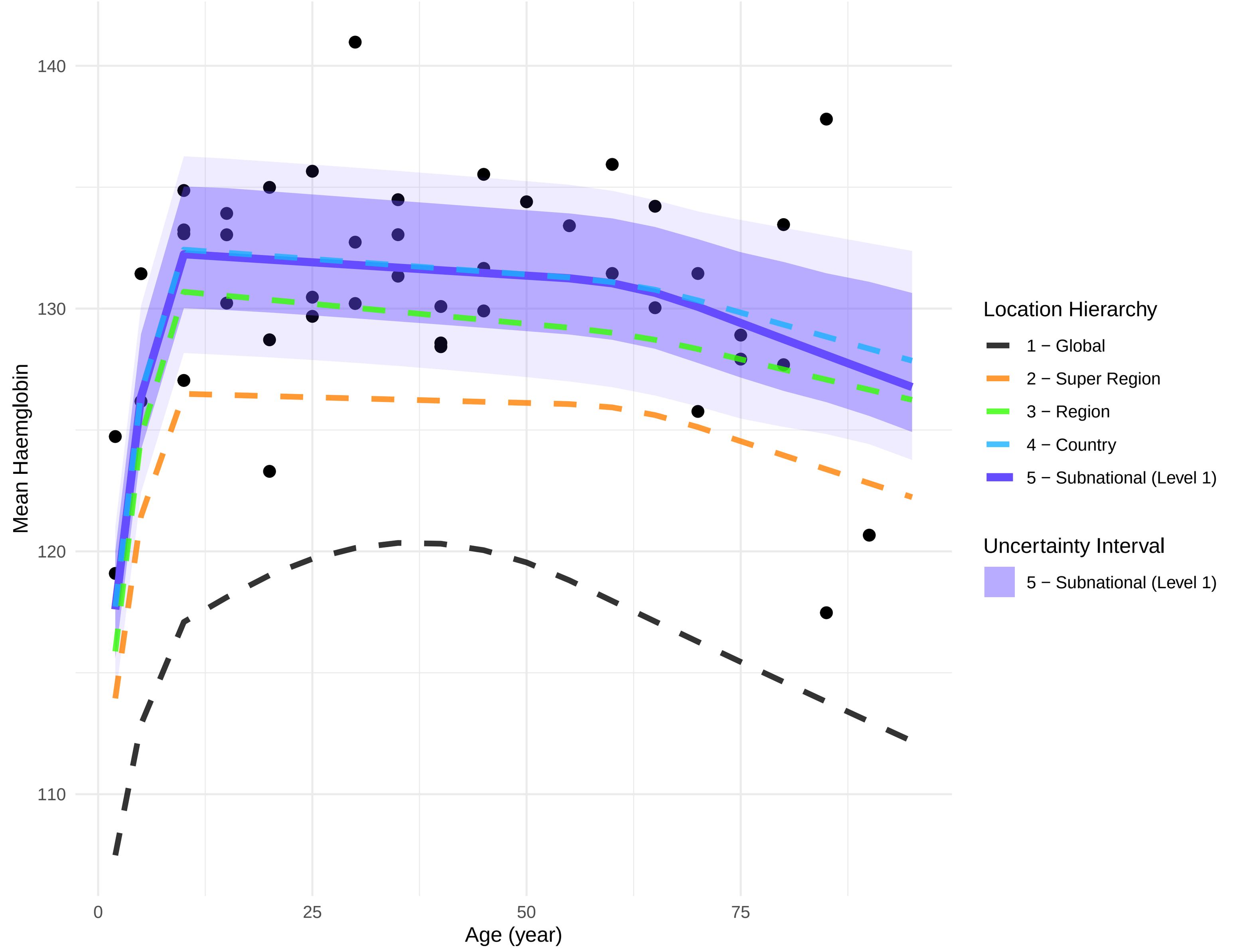

MR−BRT Age/Sex Fits − Non−pregnant Females 2+ Years Old − Mean Haemglobin − Nuevo León
WHO−2024 Adjustment Method

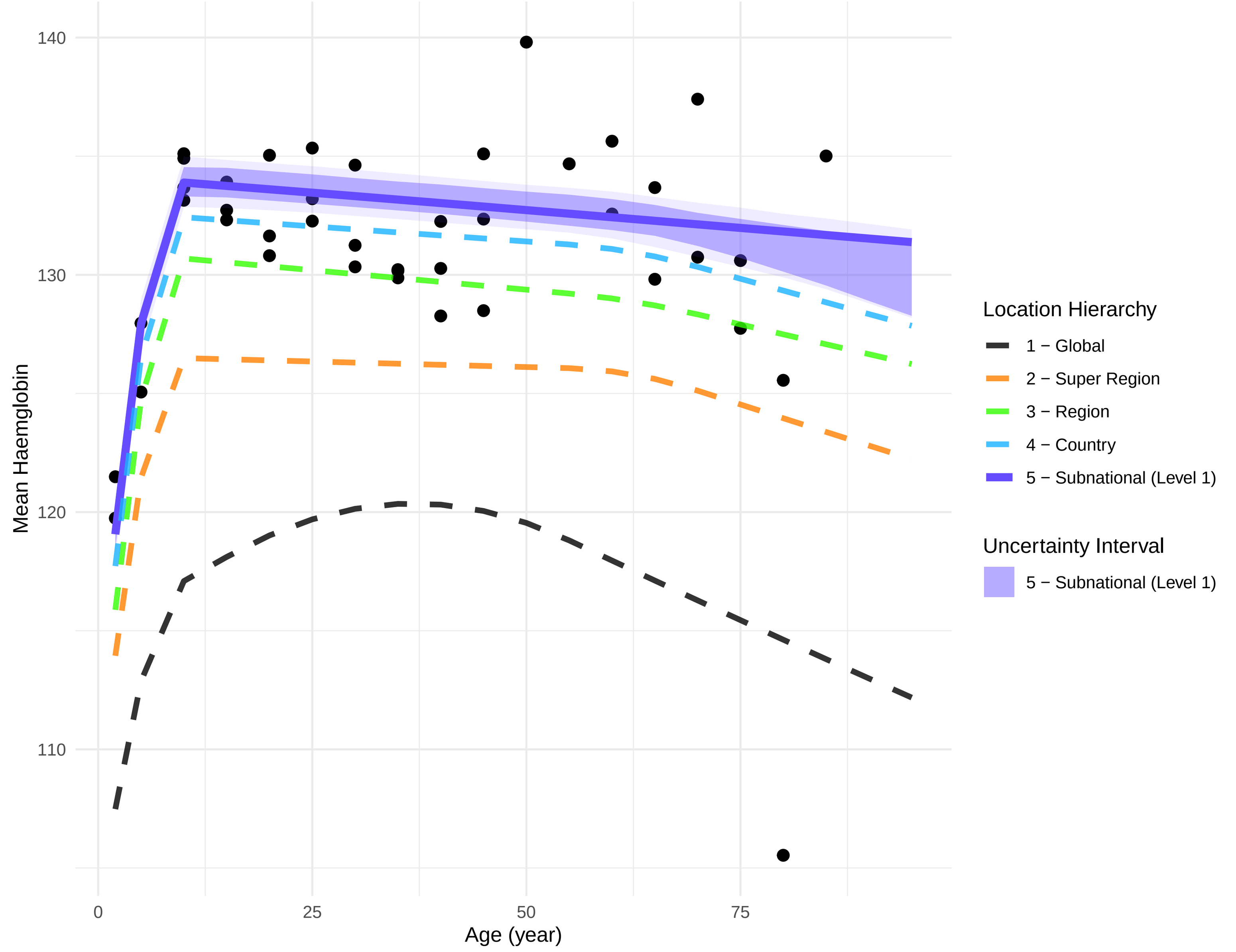

MR−BRT Age/Sex Fits − Non−pregnant Females 2+ Years Old − Mean Haemglobin − Oaxaca
WHO−2024 Adjustment Method

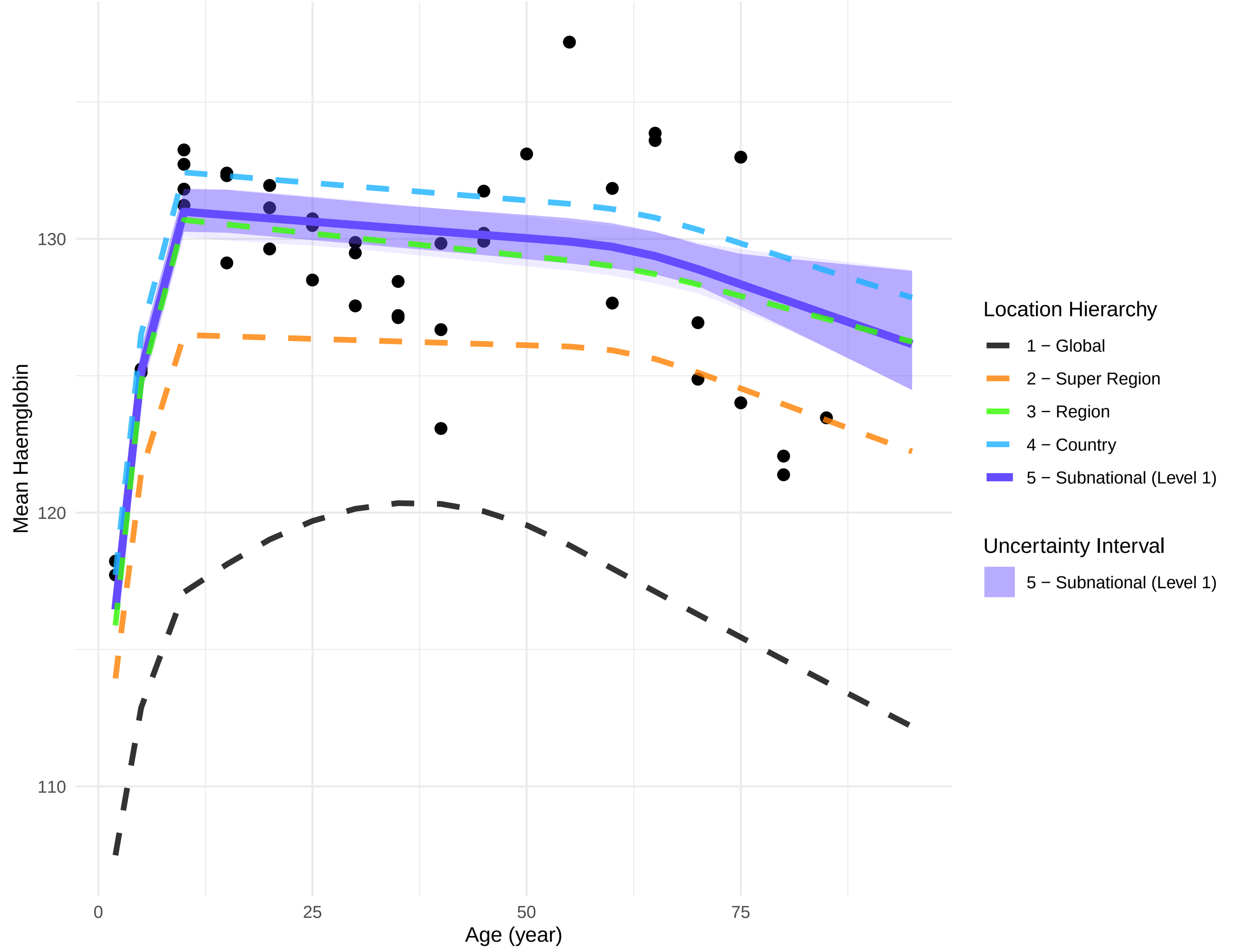

MR−BRT Age/Sex Fits − Non−pregnant Females 2+ Years Old − Mean Haemglobin − Puebla
WHO−2024 Adjustment Method

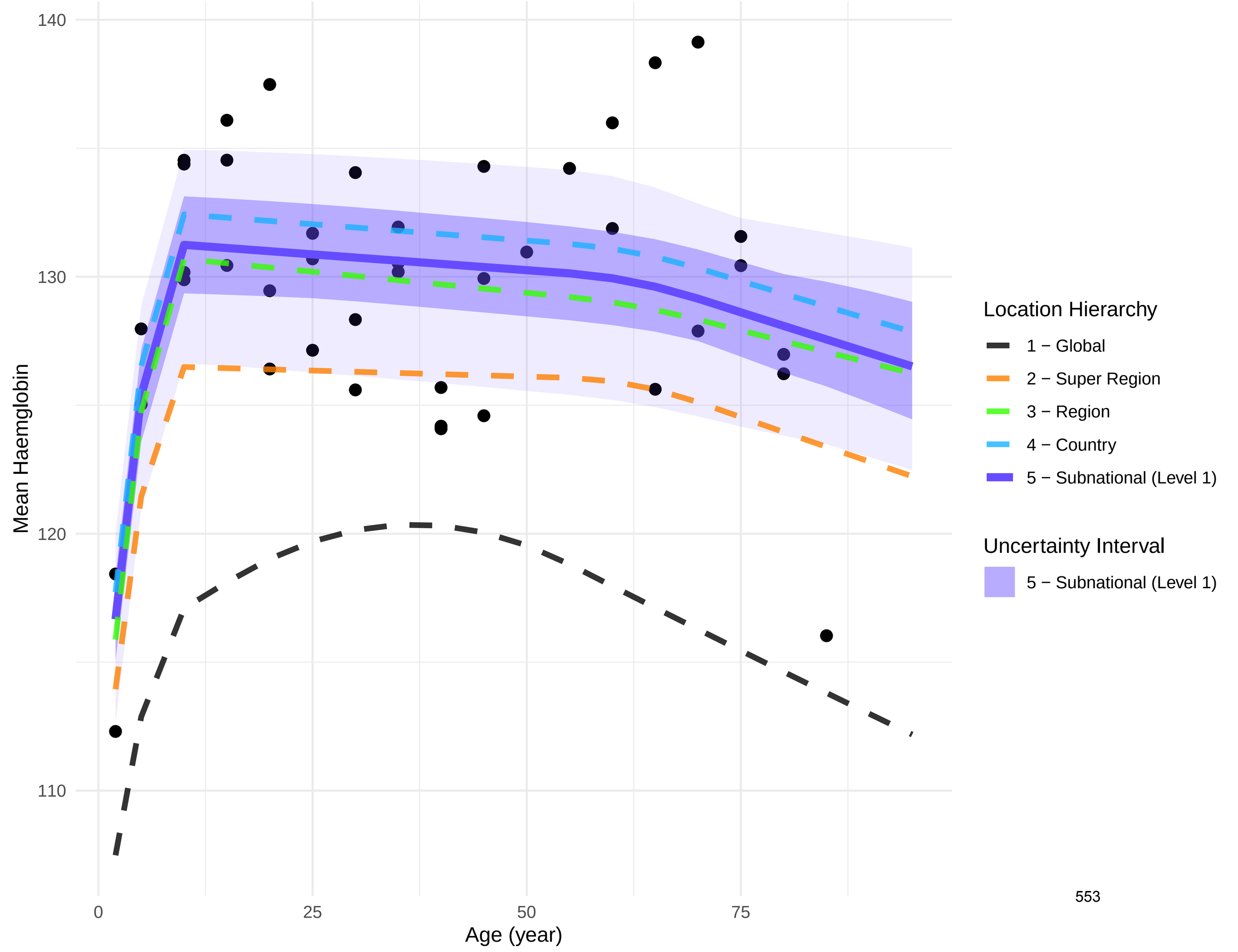

MR−BRT Age/Sex Fits − Non−pregnant Females 2+ Years Old − Mean Haemglobin − Querétaro
WHO−2024 Adjustment Method

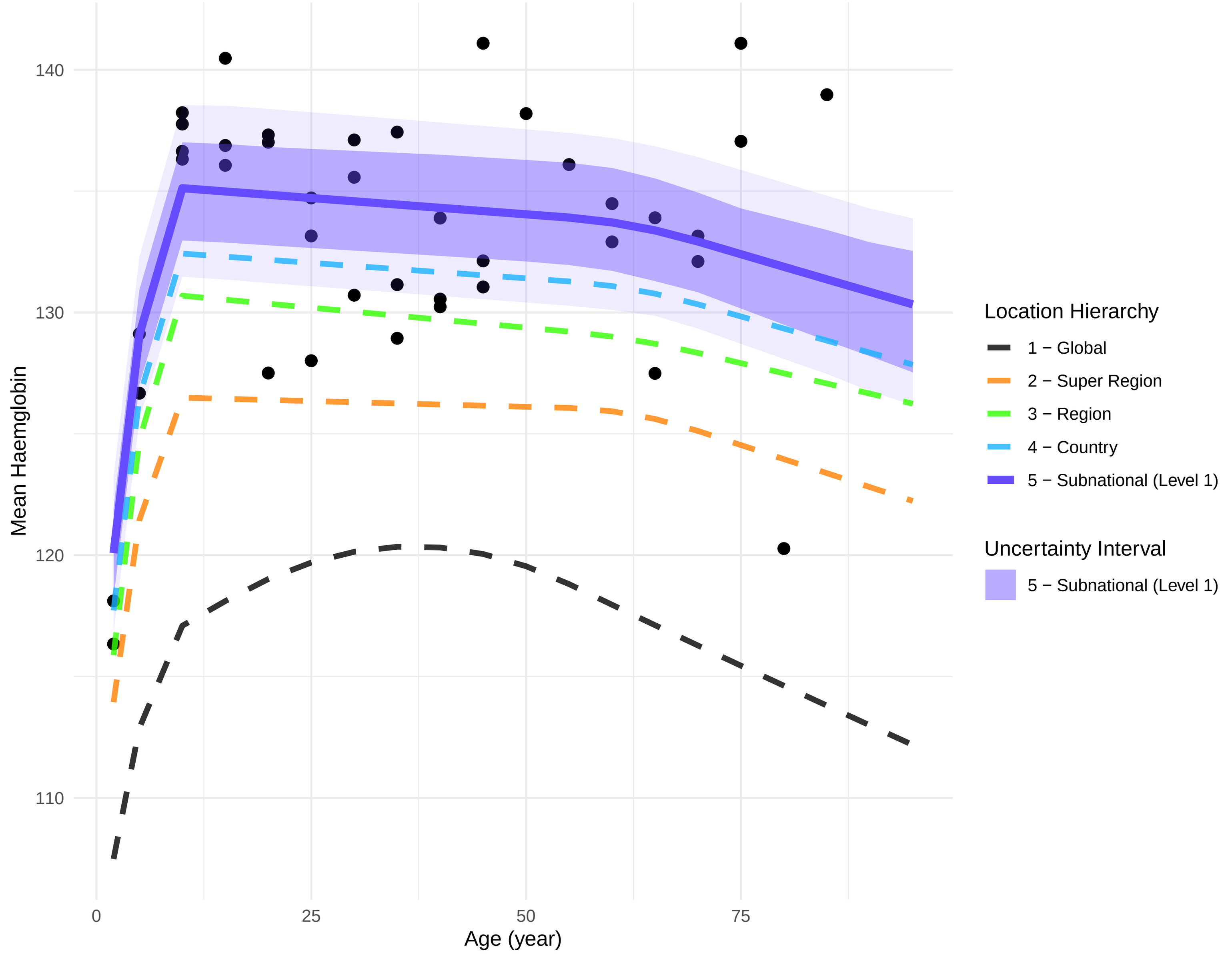

MR−BRT Age/Sex Fits − Non−pregnant Females 2+ Years Old − Mean Haemglobin − Quintana Roo
WHO−2024 Adjustment Method

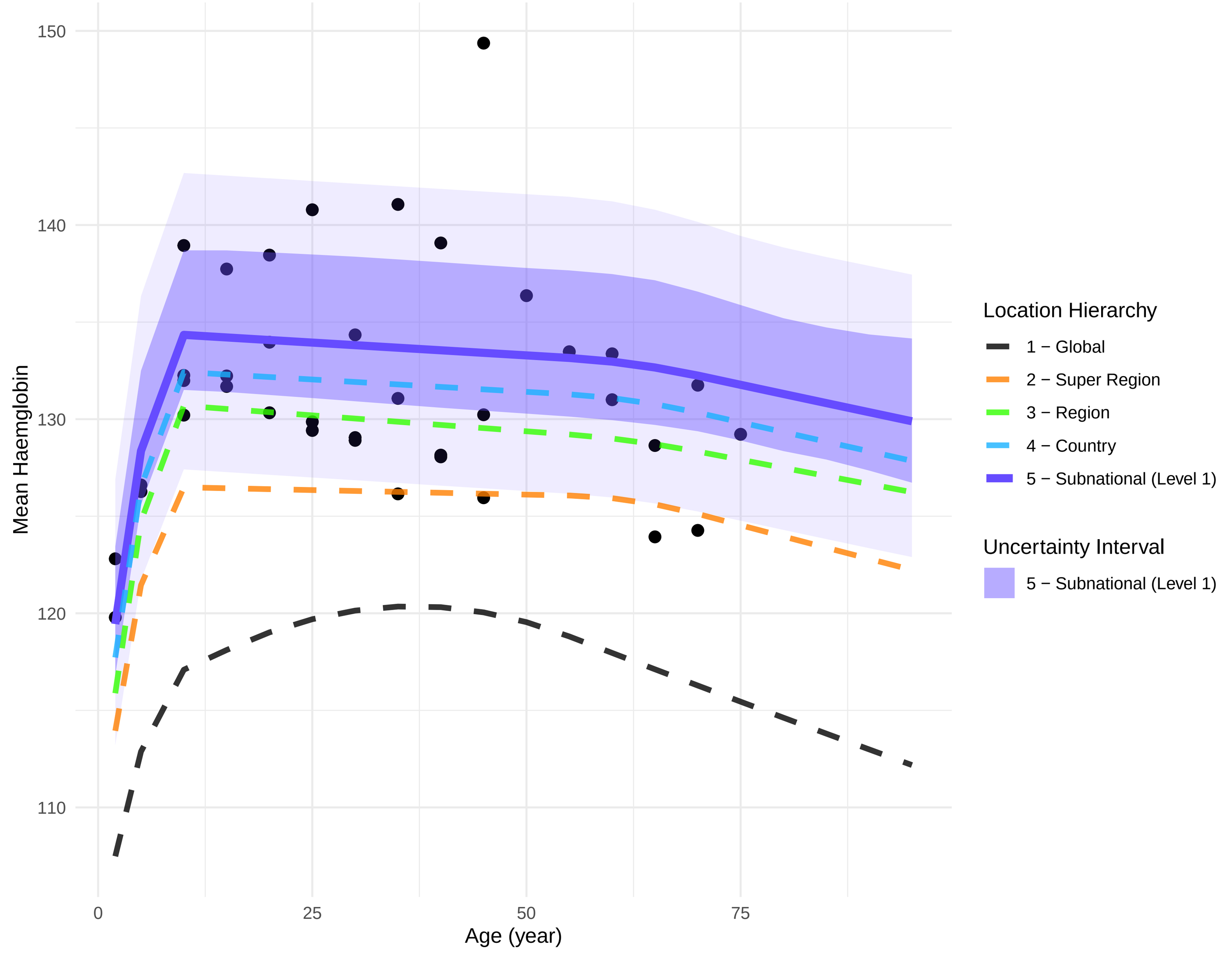

MR−BRT Age/Sex Fits − Non−pregnant Females 2+ Years Old − Mean Haemglobin − San Luis Potosí
WHO−2024 Adjustment Method

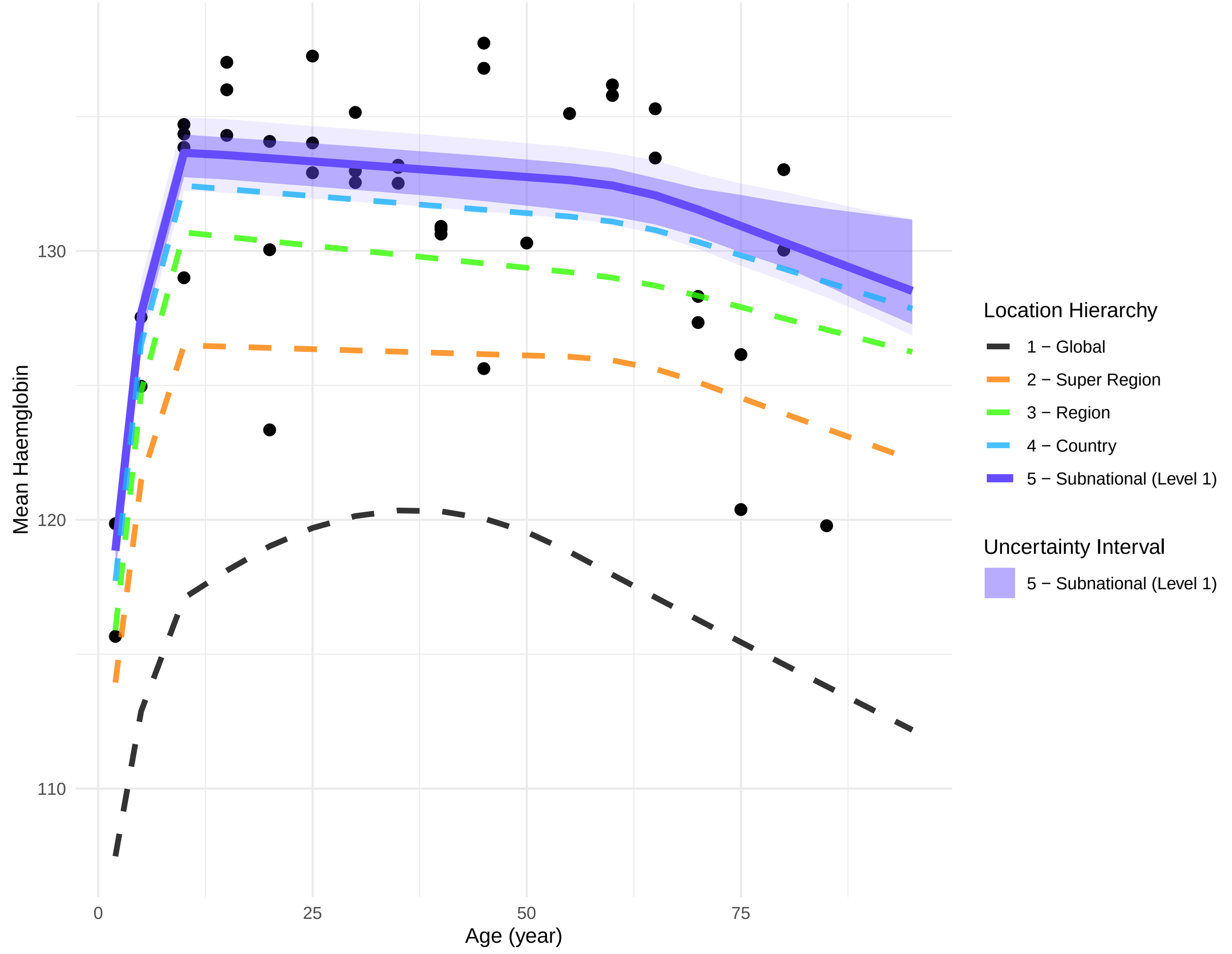

MR−BRT Age/Sex Fits − Non−pregnant Females 2+ Years Old − Mean Haemglobin − Sinaloa
WHO−2024 Adjustment Method

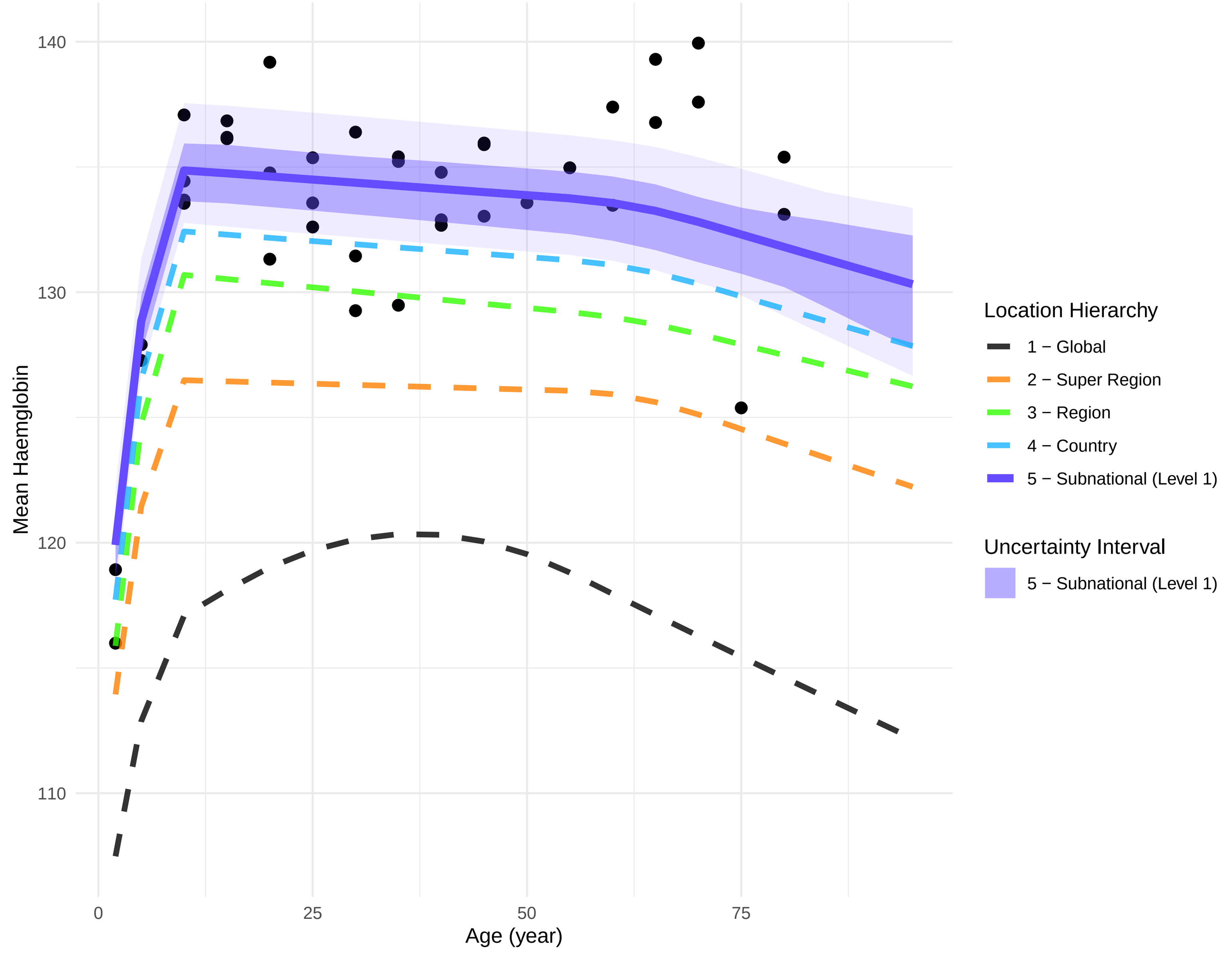

MR−BRT Age/Sex Fits − Non−pregnant Females 2+ Years Old − Mean Haemglobin − Sonora
WHO−2024 Adjustment Method

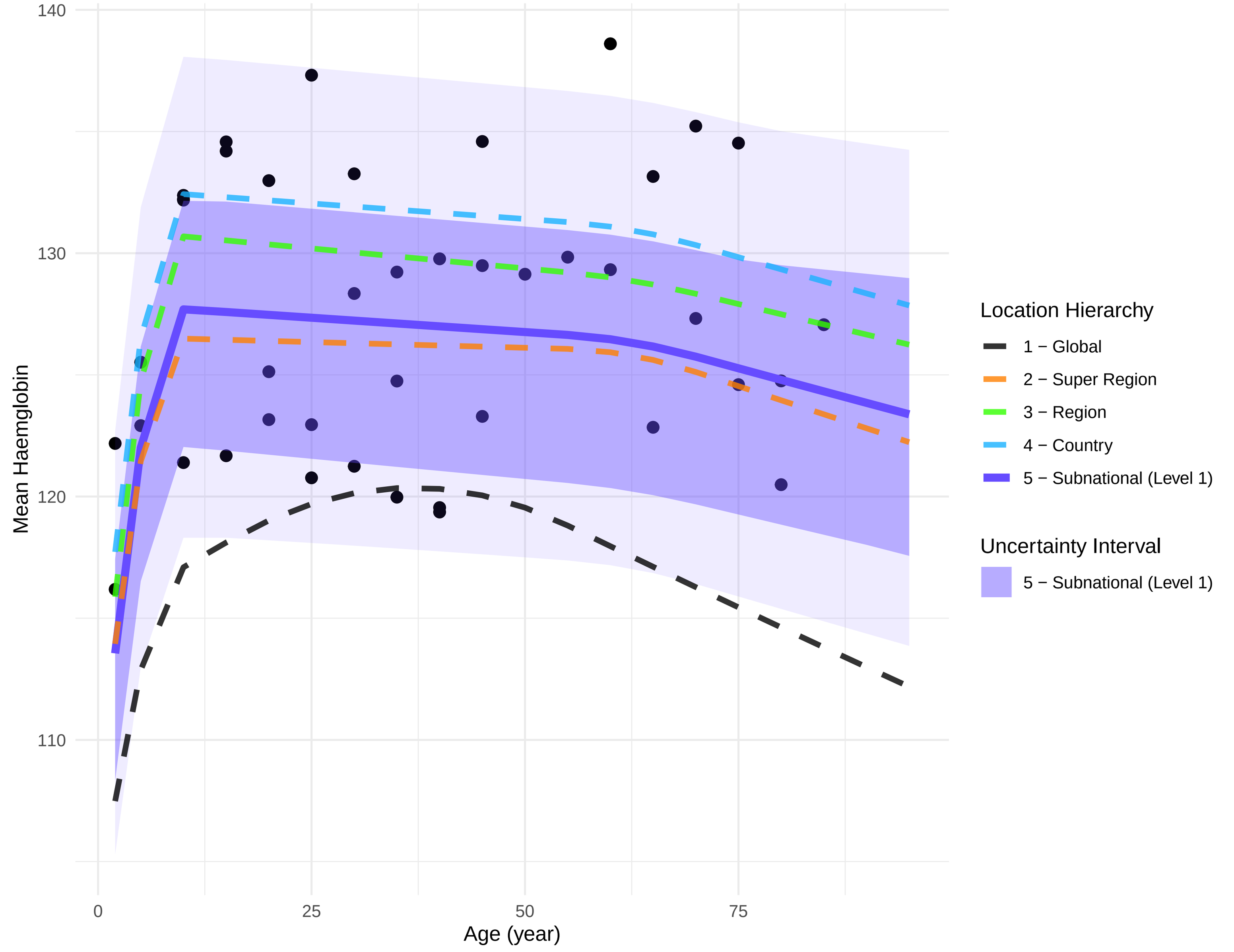

MR−BRT Age/Sex Fits − Non−pregnant Females 2+ Years Old − Mean Haemglobin − Tabasco
WHO−2024 Adjustment Method

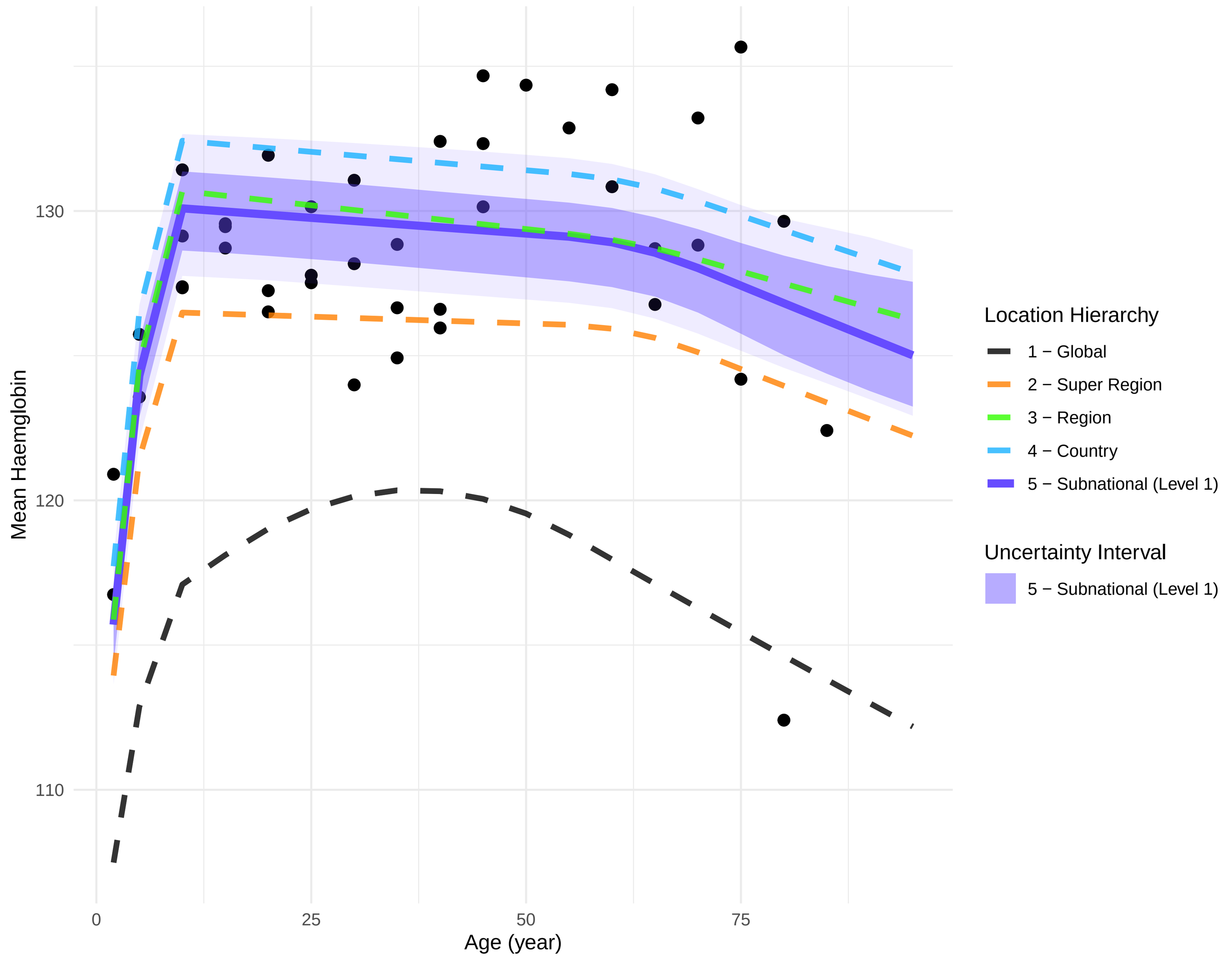

MR−BRT Age/Sex Fits − Non−pregnant Females 2+ Years Old − Mean Haemglobin − Tamaulipas
WHO−2024 Adjustment Method

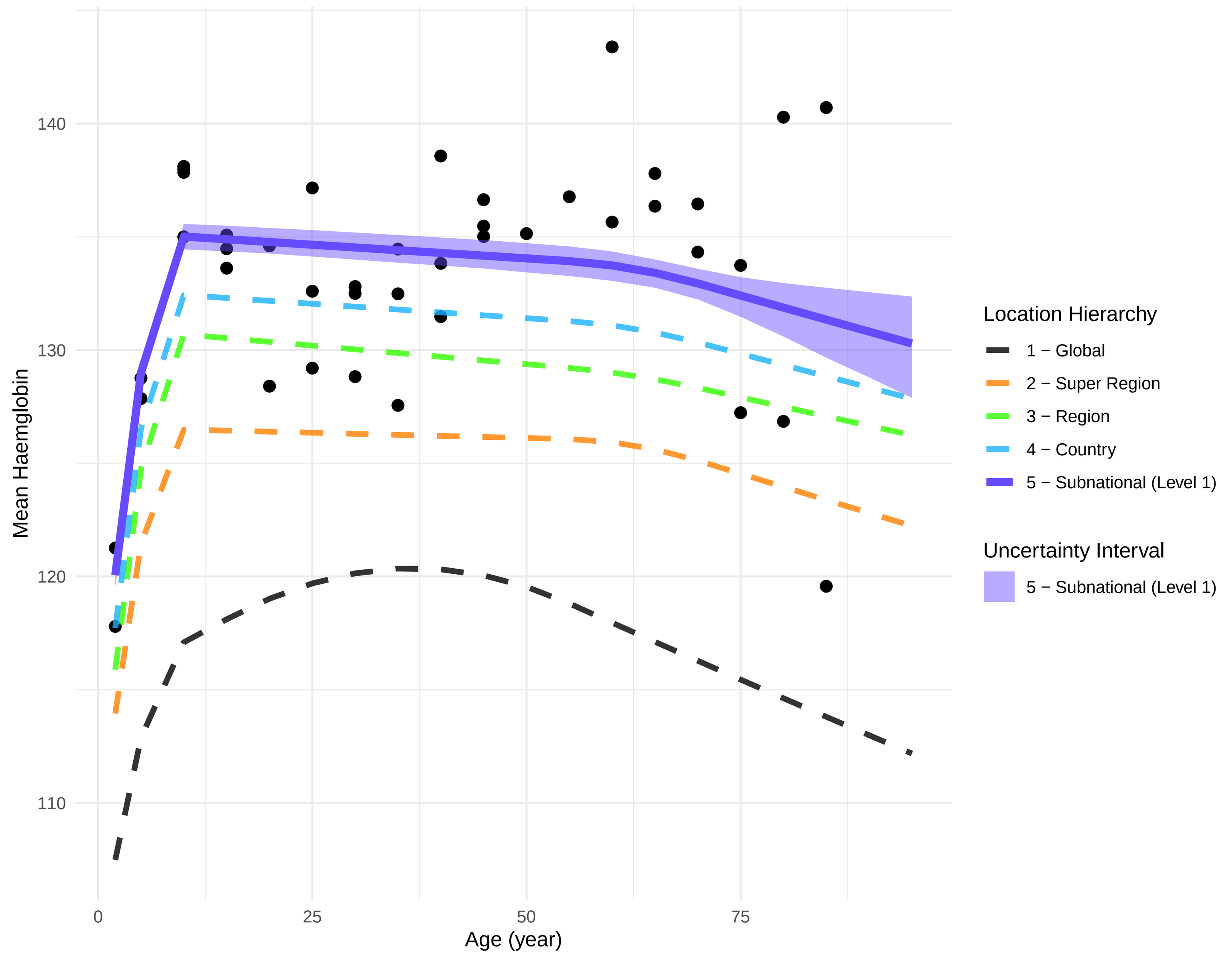

MR−BRT Age/Sex Fits − Non−pregnant Females 2+ Years Old − Mean Haemglobin − Tlaxcala
WHO−2024 Adjustment Method

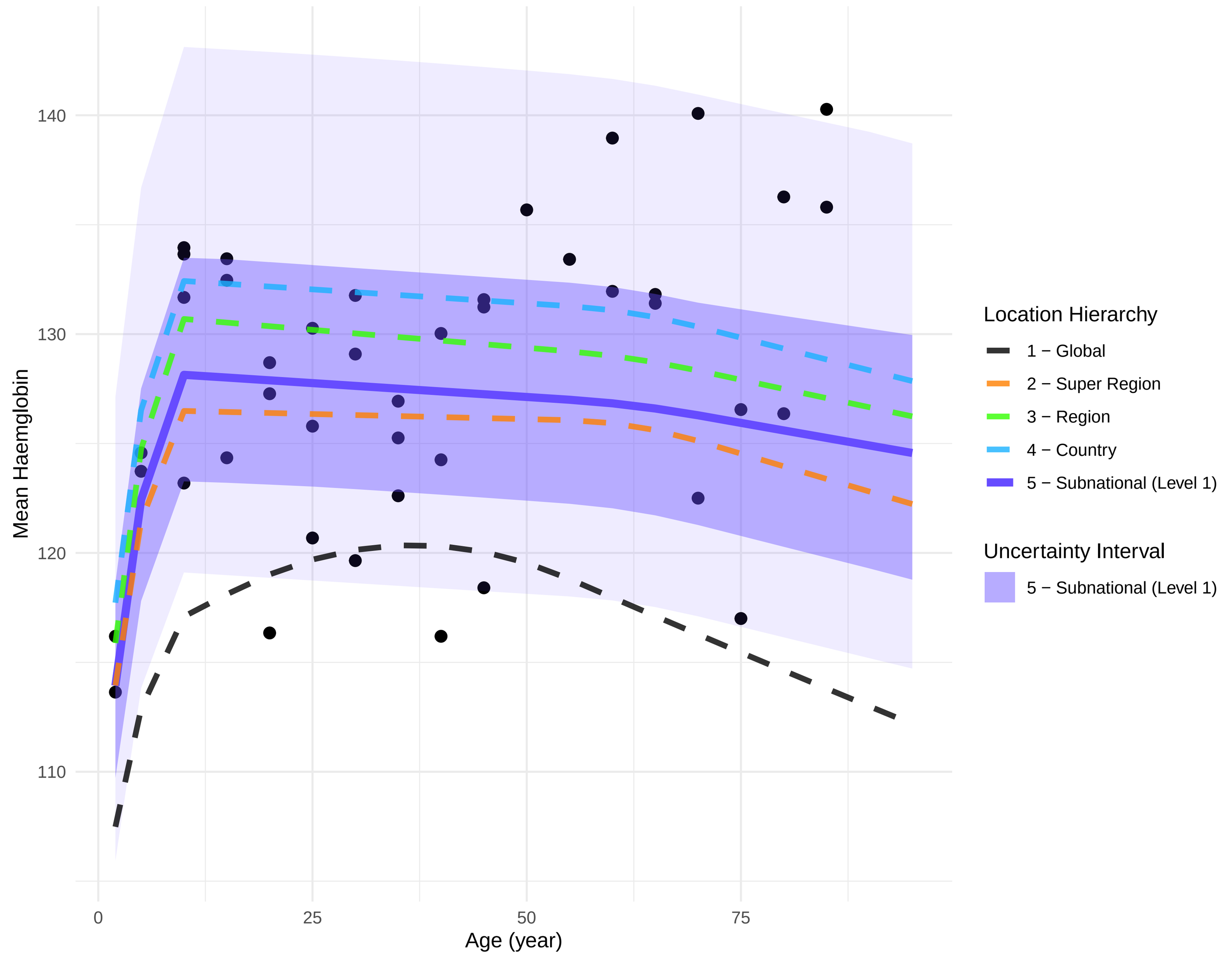

MR−BRT Age/Sex Fits − Non−pregnant Females 2+ Years Old − Mean Haemglobin − Veracruz de Ignacio de la Llave
WHO−2024 Adjustment Method

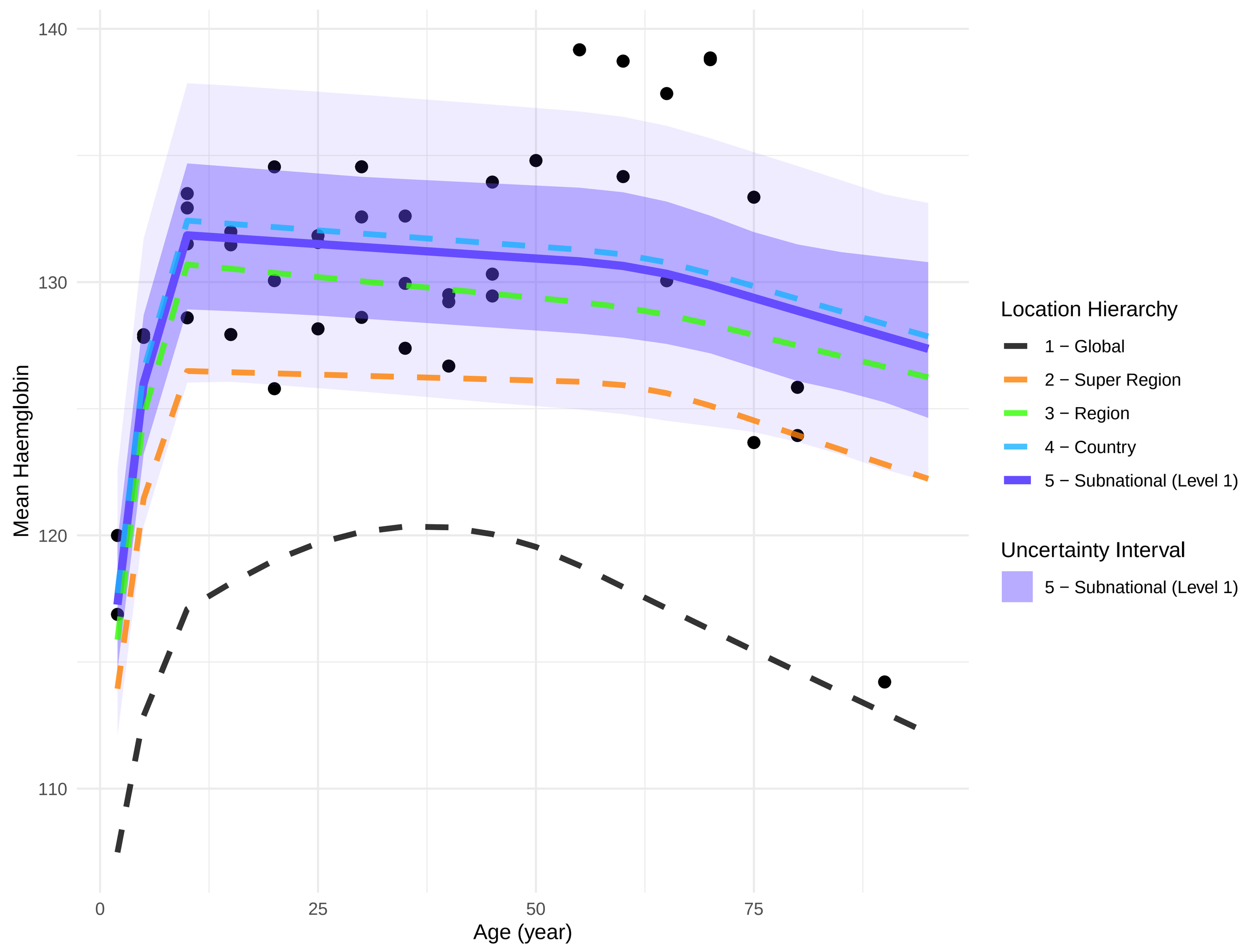

MR−BRT Age/Sex Fits − Non−pregnant Females 2+ Years Old − Mean Haemglobin − Yucatán
WHO−2024 Adjustment Method

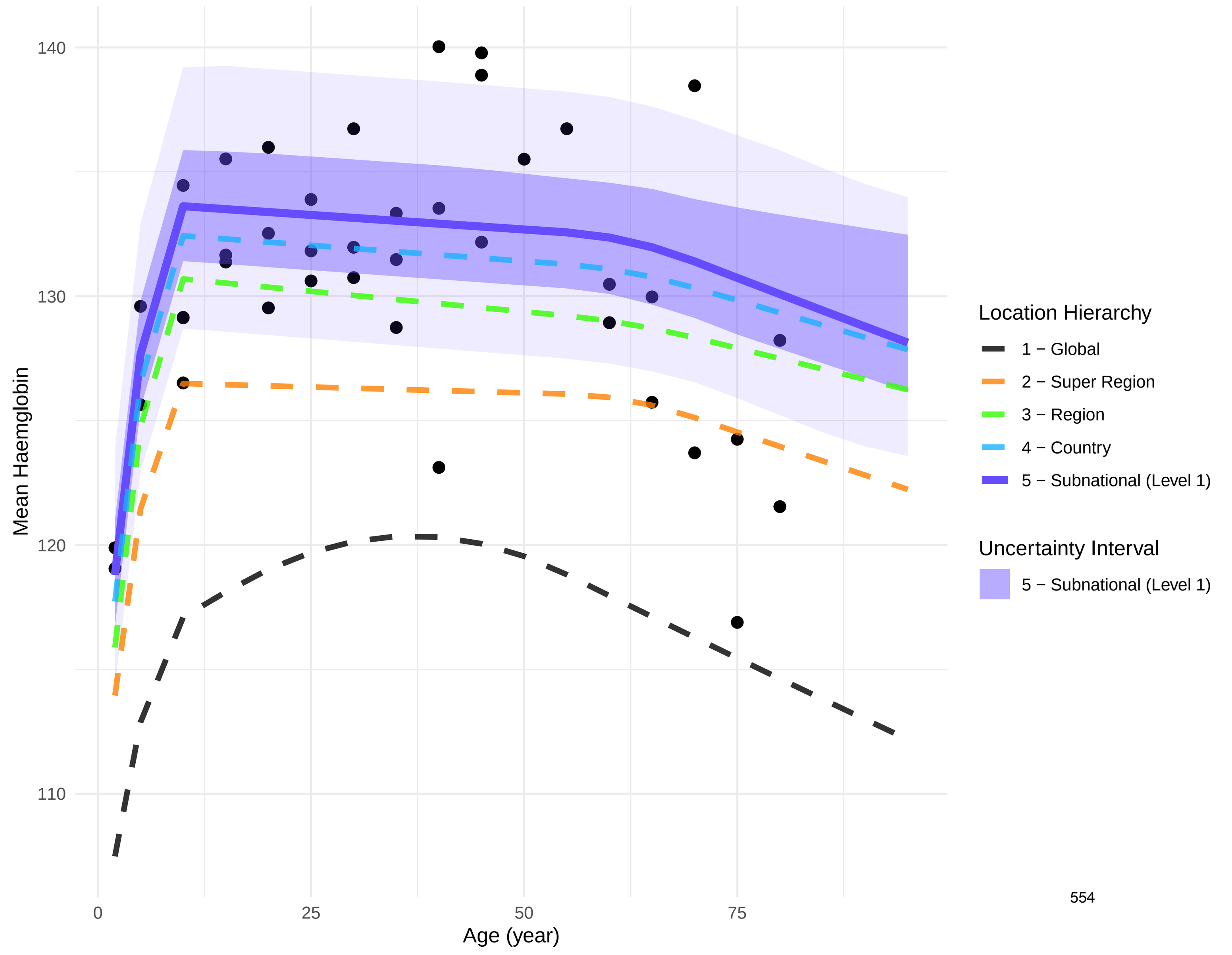

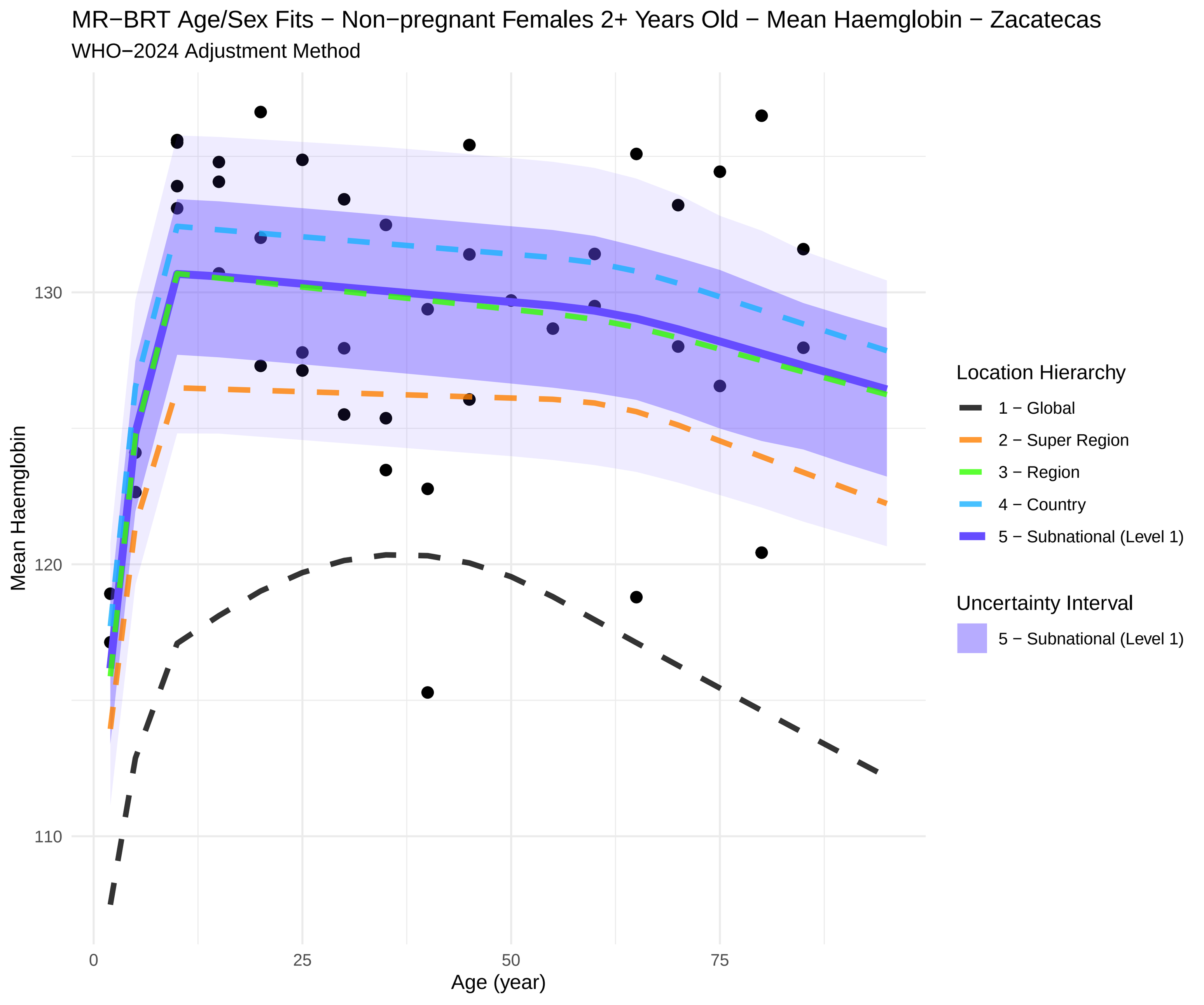
MR−BRT Age/Sex Fits − Non−pregnant Females 2+ Years Old − Mean Haemglobin − Zacatecas
WHO−2024 Adjustment Method
Mean Haemglobin
Age (year)
Location Hierarchy
1 − Global
2 − Super Region
3 − Region
4 − Country
5 − Subnational (Level 1)
Uncertainty Interval
5 − Subnational (Level 1)

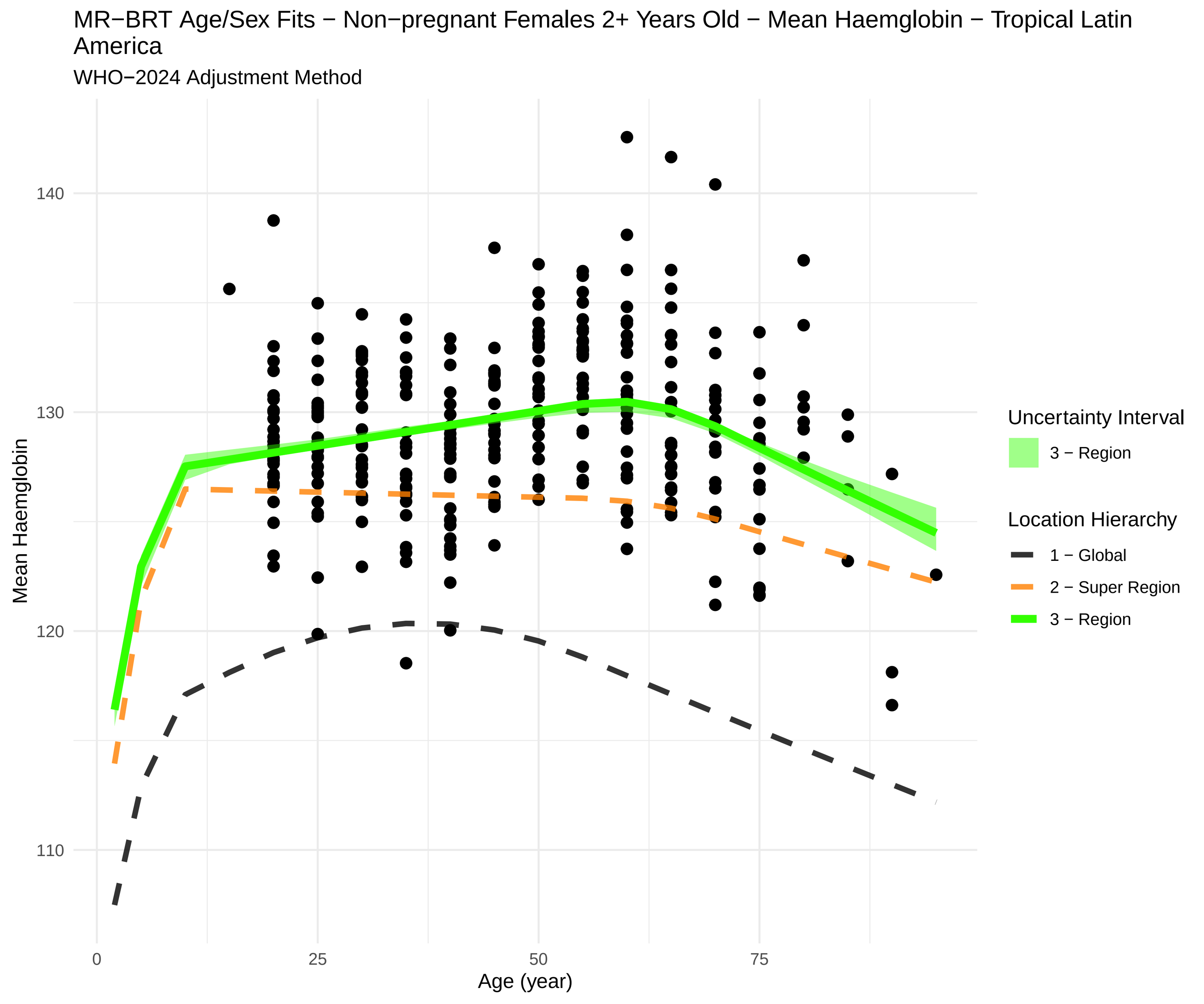
MR−BRT Age/Sex Fits − Non−pregnant Females 2+ Years Old − Mean Haemglobin − Tropical Latin America
WHO−2024 Adjustment Method
Mean Haemglobin
Age (year)
Uncertainty Interval
3 − Region
Location Hierarchy
1 − Global
2 − Super Region
3 − Region

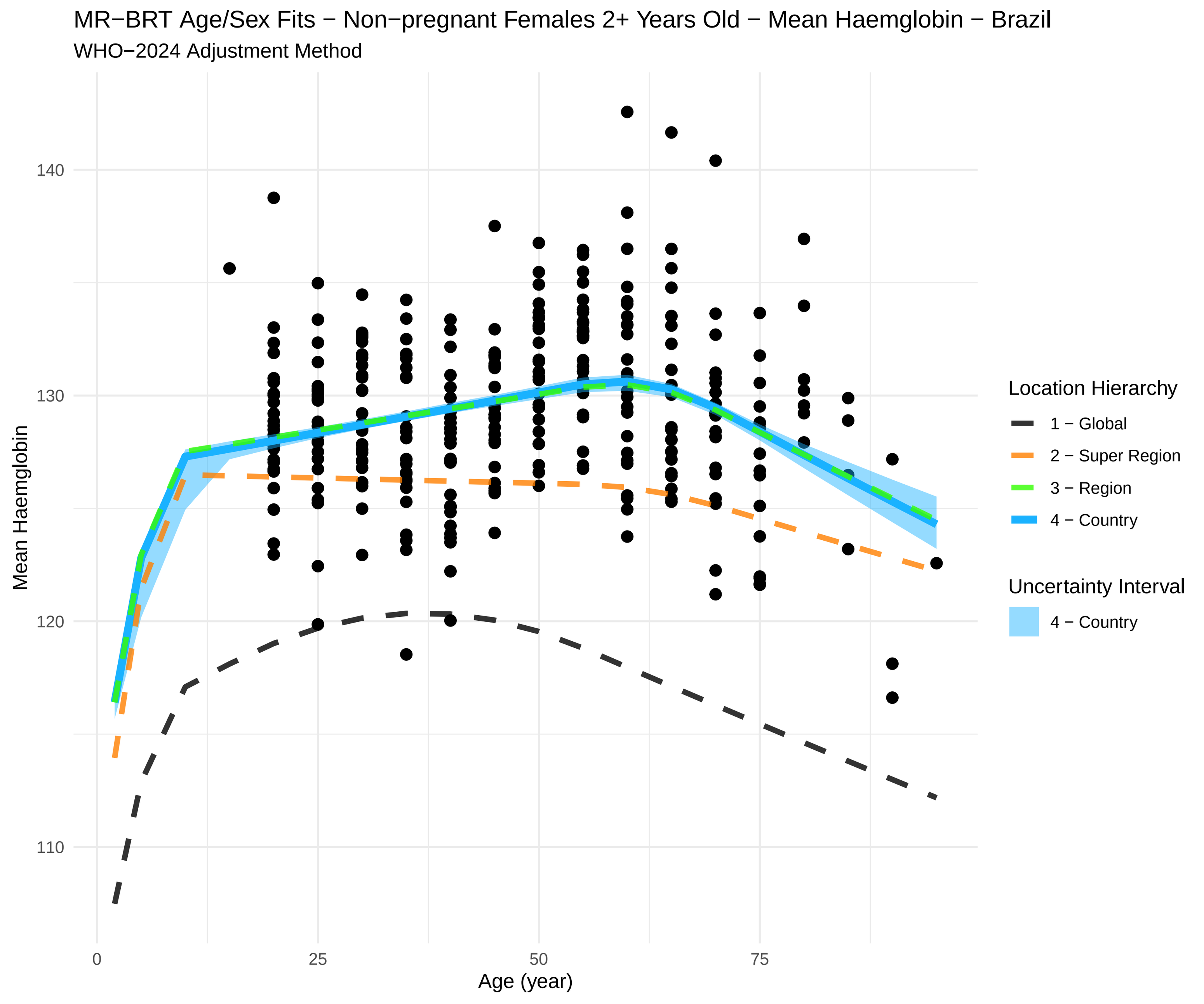
MR−BRT Age/Sex Fits − Non−pregnant Females 2+ Years Old − Mean Haemglobin − Brazil
WHO−2024 Adjustment Method
Mean Haemglobin
Age (year)
Location Hierarchy
1 − Global
2 − Super Region
3 − Region
4 − Country
Uncertainty Interval
4 − Country

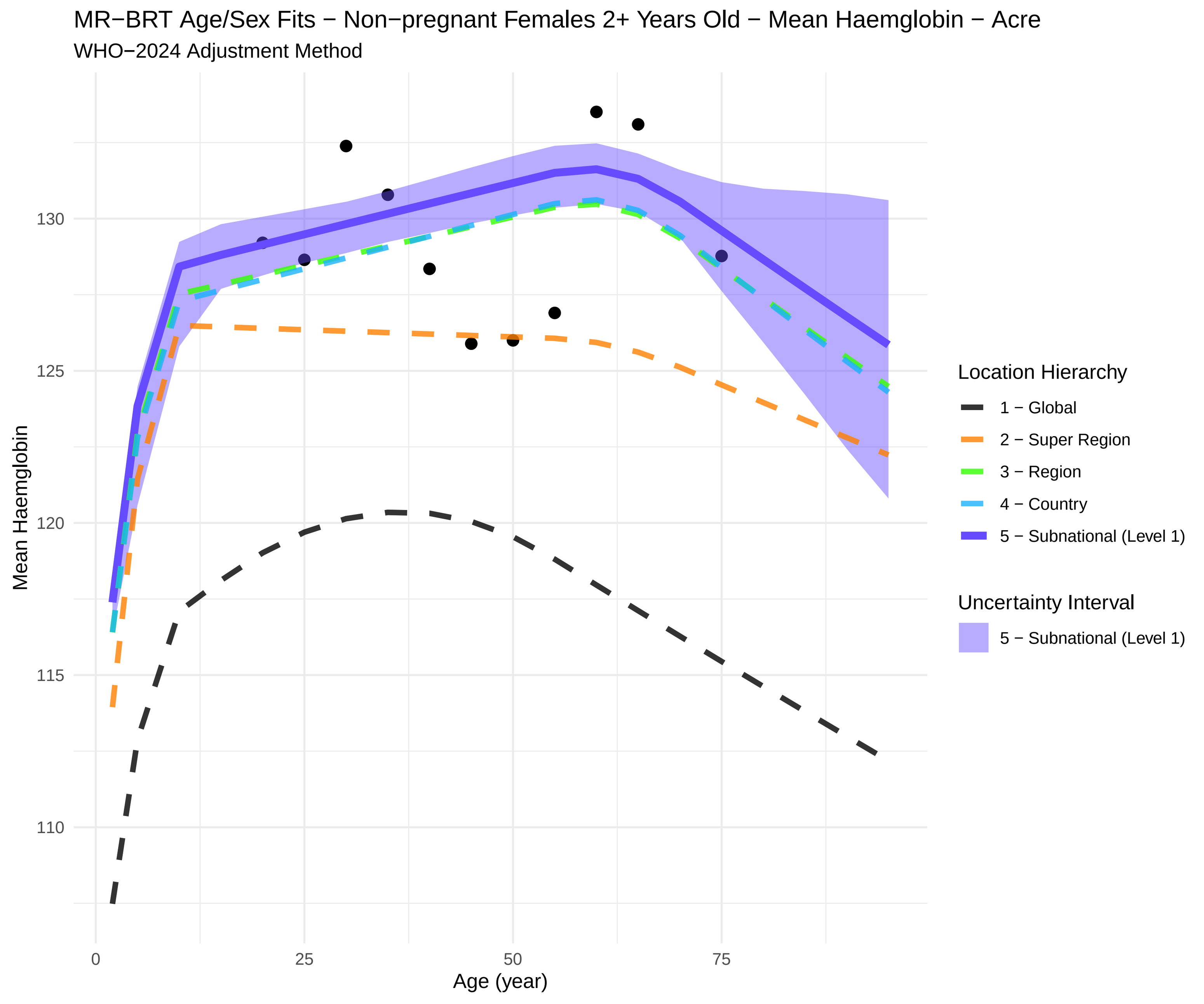
MR−BRT Age/Sex Fits − Non−pregnant Females 2+ Years Old − Mean Haemglobin − Acre
WHO−2024 Adjustment Method
Mean Haemglobin
Age (year)
Location Hierarchy
1 − Global
2 − Super Region
3 − Region
4 − Country
5 − Subnational (Level 1)
Uncertainty Interval
5 − Subnational (Level 1)

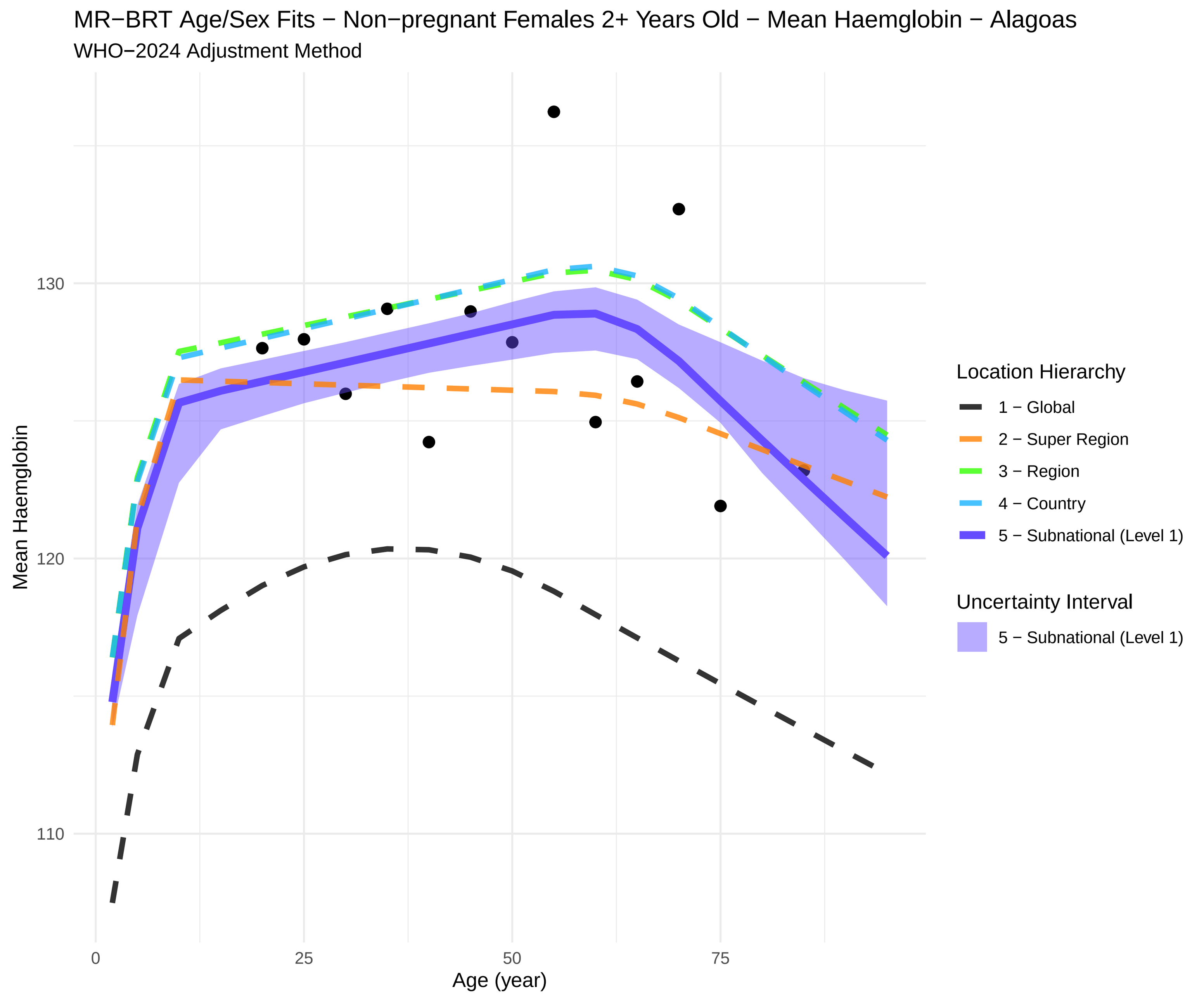
MR−BRT Age/Sex Fits − Non−pregnant Females 2+ Years Old − Mean Haemglobin − Alagoas
WHO−2024 Adjustment Method
Mean Haemglobin
Age (year)
Location Hierarchy
1 − Global
2 − Super Region
3 − Region
4 − Country
5 − Subnational (Level 1)
Uncertainty Interval
5 − Subnational (Level 1)

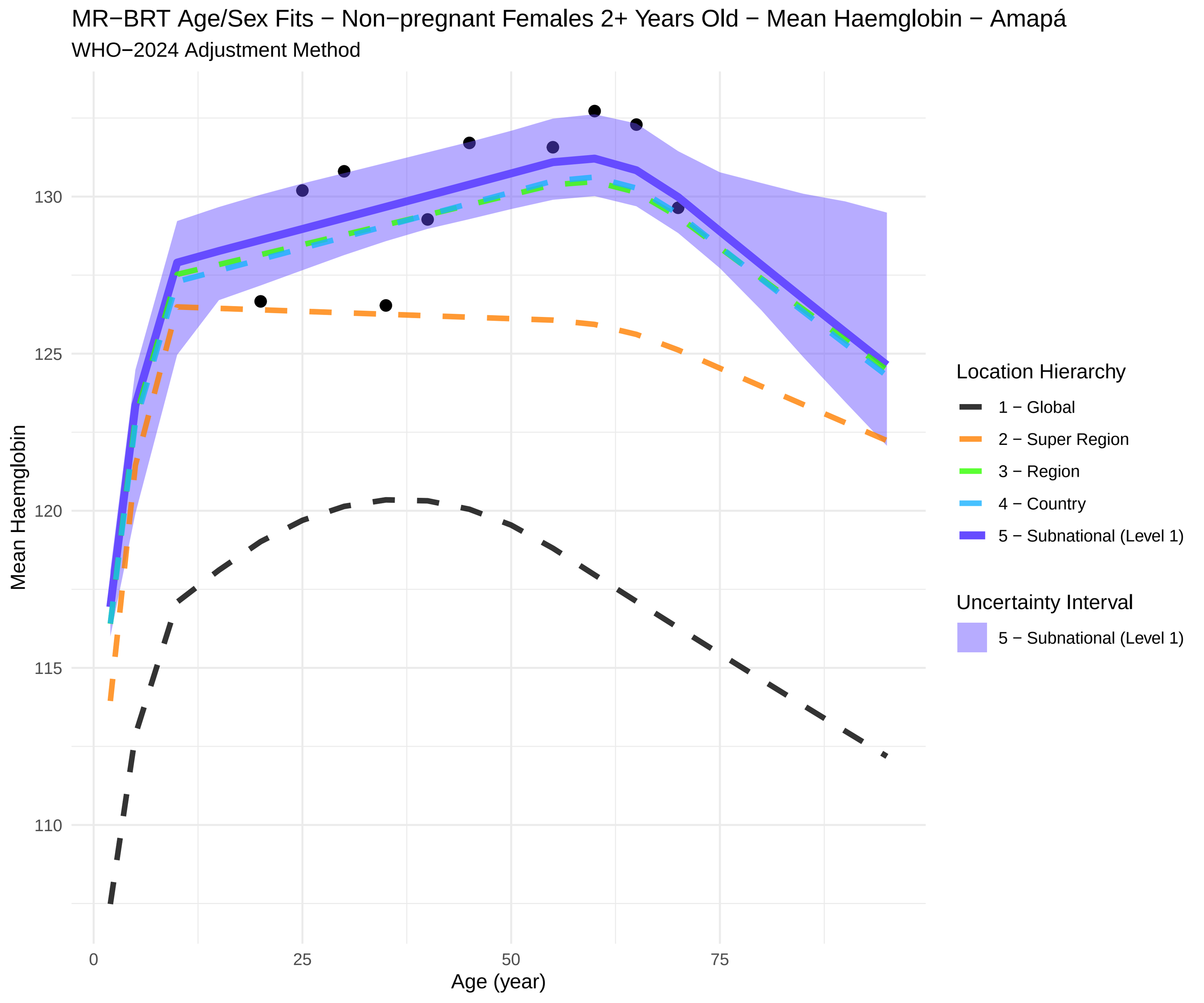
MR−BRT Age/Sex Fits − Non−pregnant Females 2+ Years Old − Mean Haemglobin − Amapá
WHO−2024 Adjustment Method
Mean Haemglobin
Age (year)
Location Hierarchy
1 − Global
2 − Super Region
3 − Region
4 − Country
5 − Subnational (Level 1)
Uncertainty Interval
5 − Subnational (Level 1)

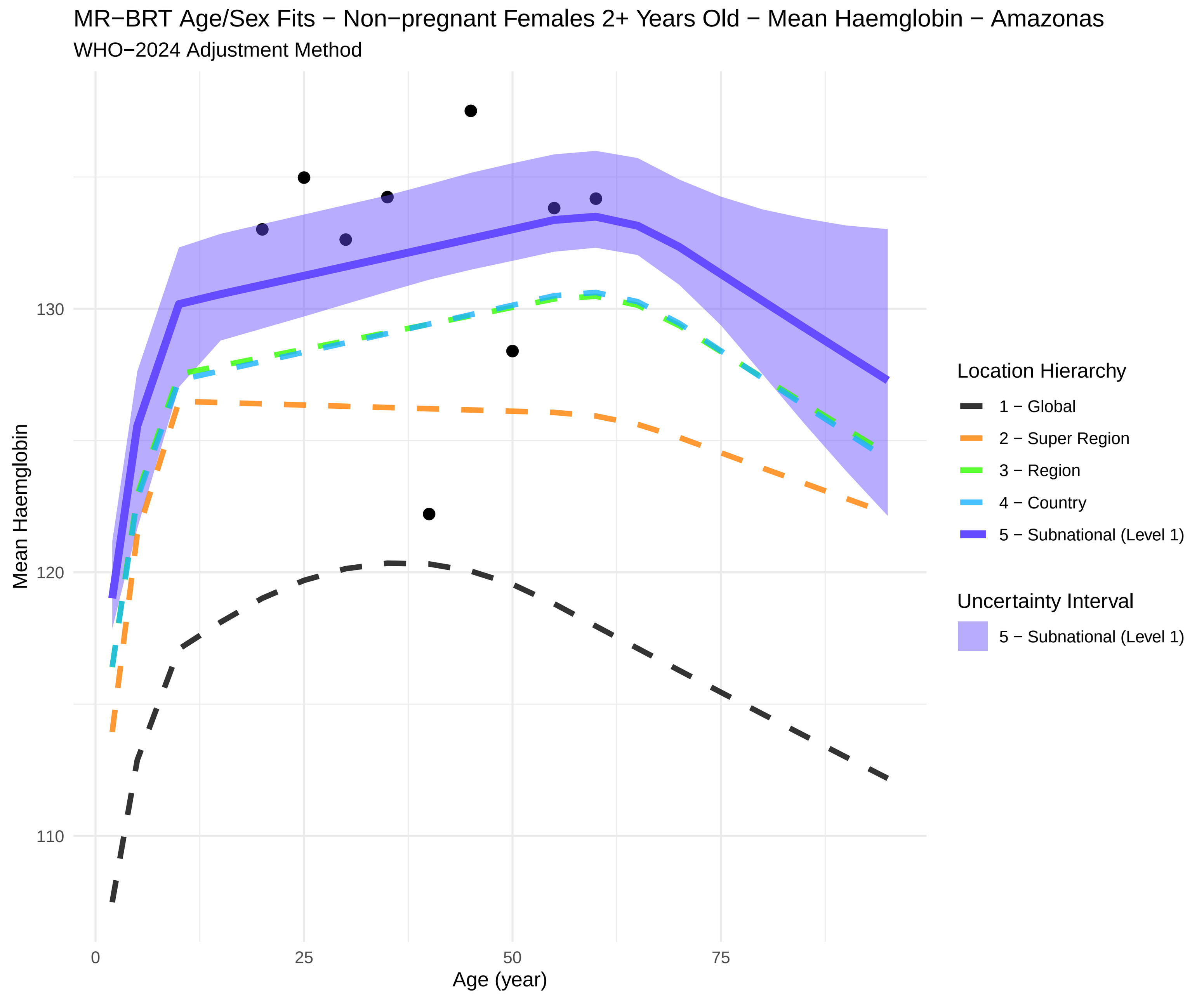
MR−BRT Age/Sex Fits − Non−pregnant Females 2+ Years Old − Mean Haemglobin − Amazonas
WHO−2024 Adjustment Method
Mean Haemglobin
Age (year)
Location Hierarchy
1 − Global
2 − Super Region
3 − Region
4 − Country
5 − Subnational (Level 1)
Uncertainty Interval
5 − Subnational (Level 1)

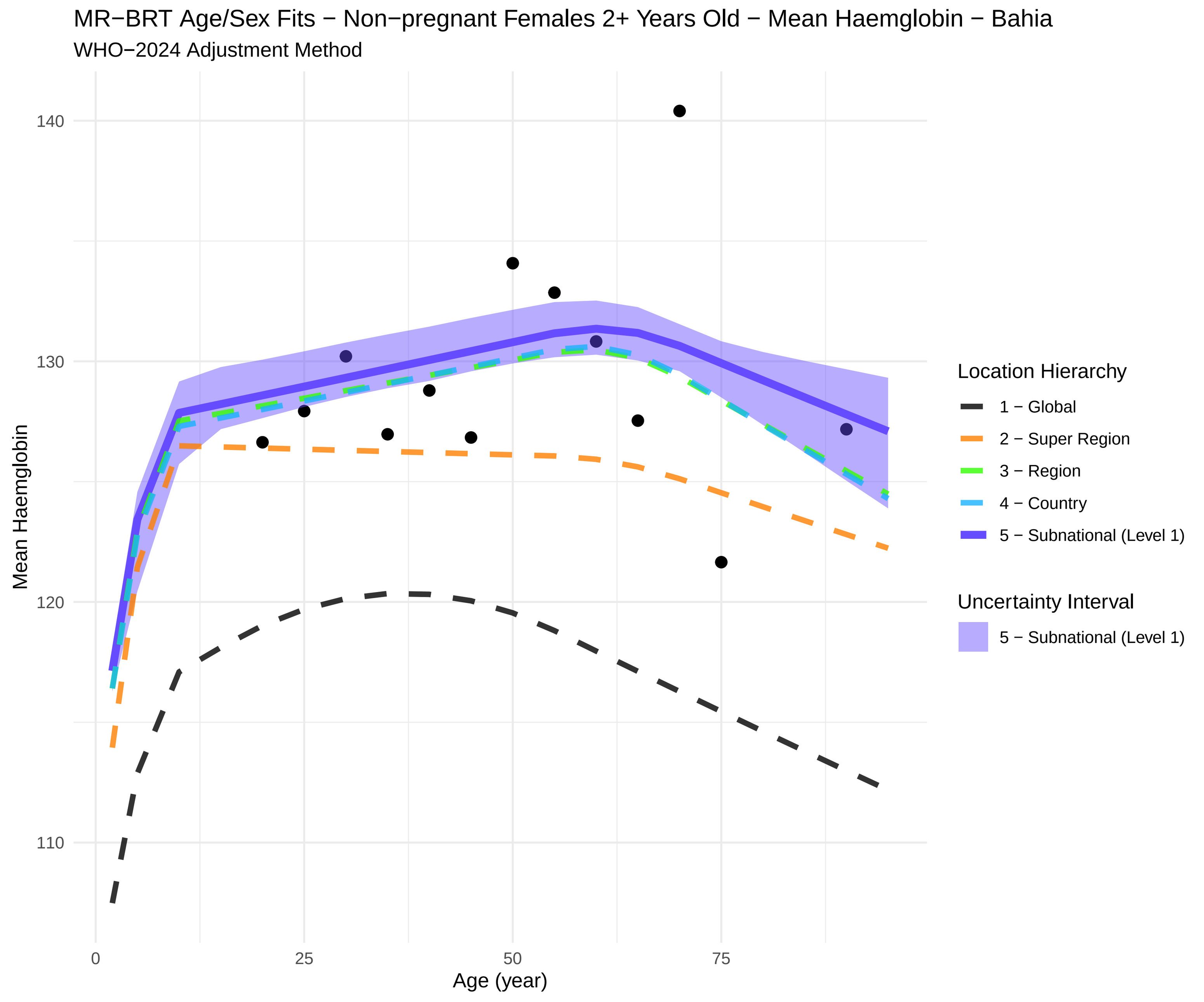
MR−BRT Age/Sex Fits − Non−pregnant Females 2+ Years Old − Mean Haemglobin − Bahia
WHO−2024 Adjustment Method
Mean Haemglobin
Age (year)
Location Hierarchy
1 − Global
2 − Super Region
3 − Region
4 − Country
5 − Subnational (Level 1)
Uncertainty Interval
5 − Subnational (Level 1)

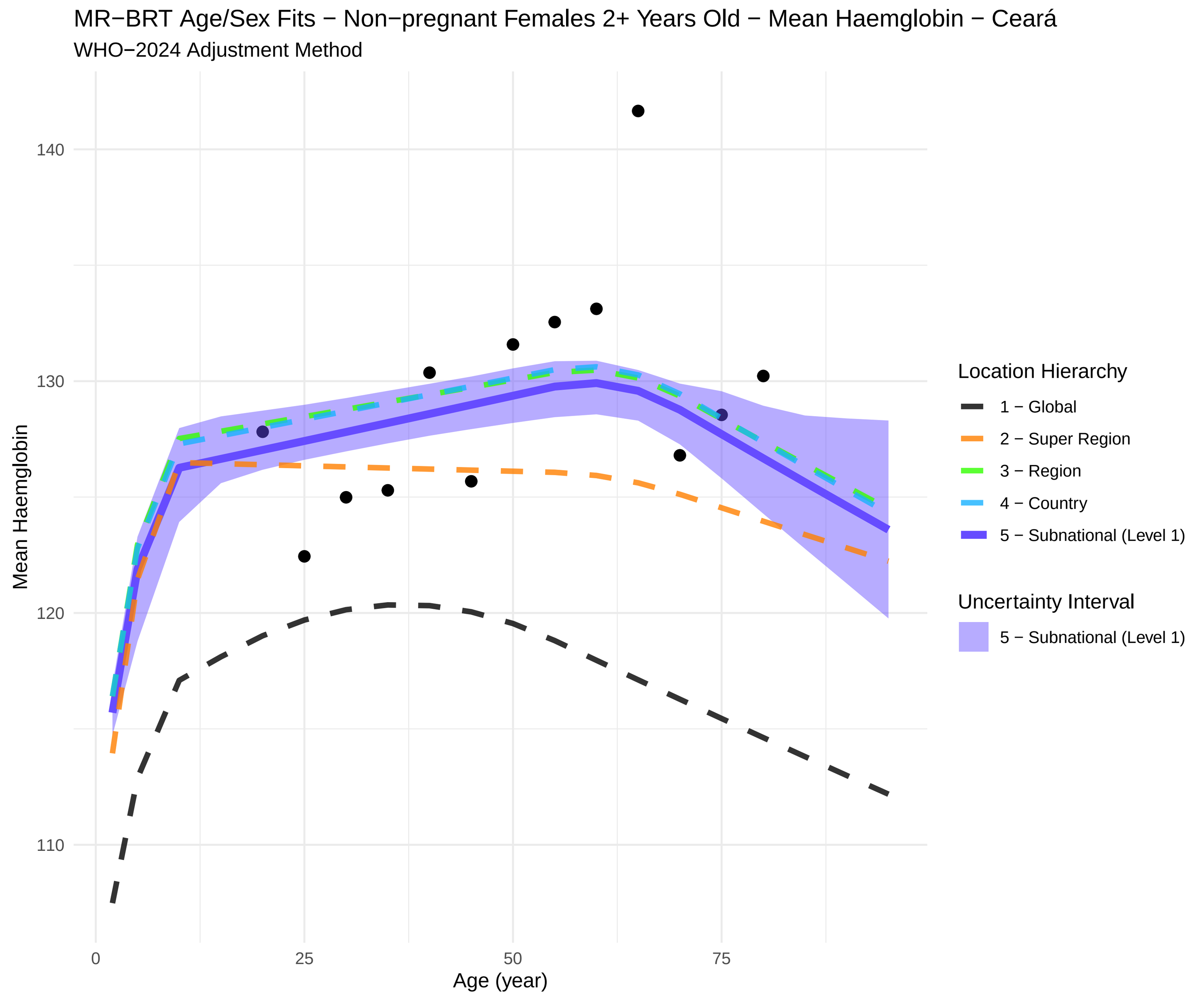
MR−BRT Age/Sex Fits − Non−pregnant Females 2+ Years Old − Mean Haemglobin − Ceará
WHO−2024 Adjustment Method
Mean Haemglobin
Age (year)
Location Hierarchy
1 − Global
2 − Super Region
3 − Region
4 − Country
5 − Subnational (Level 1)
Uncertainty Interval
5 − Subnational (Level 1)

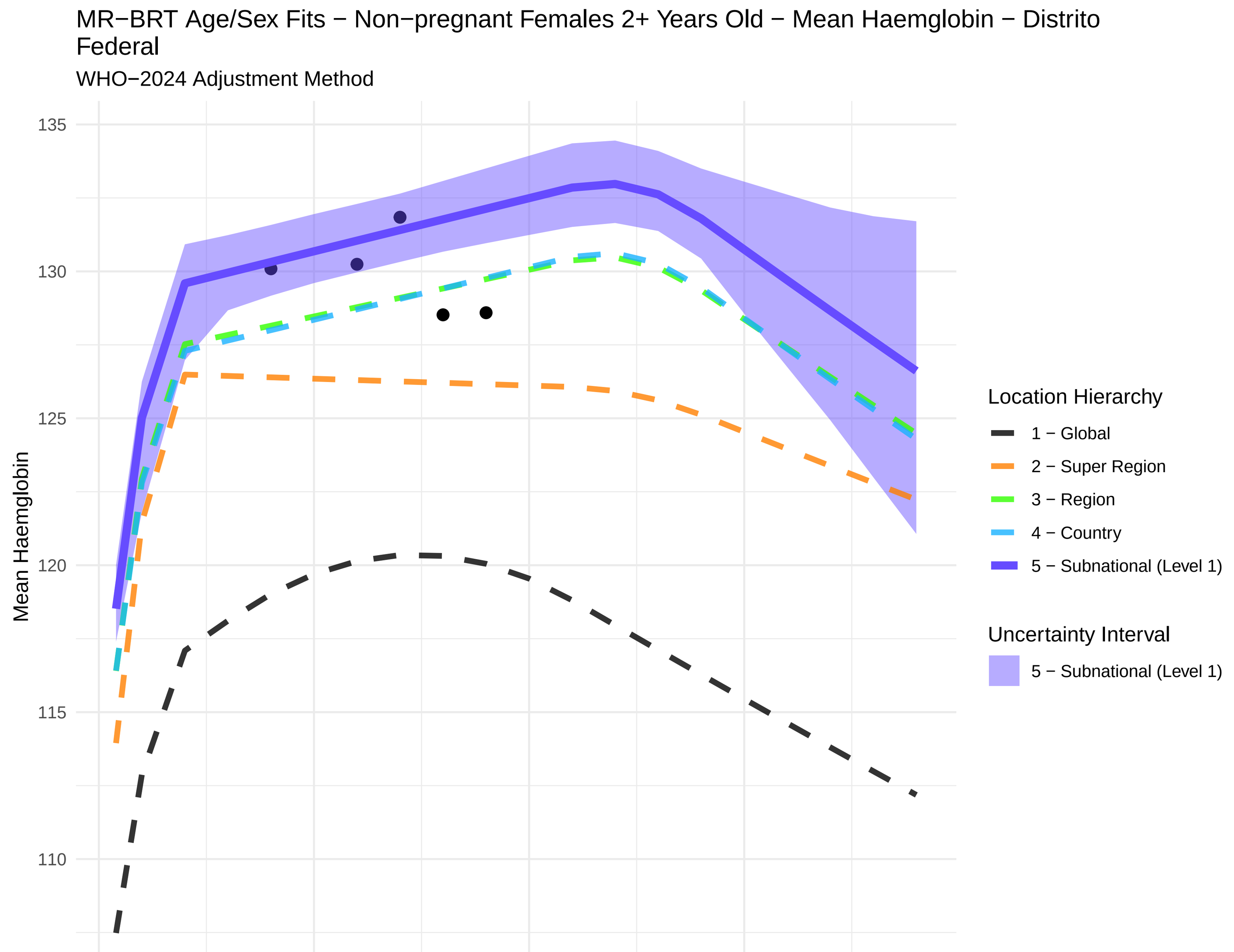
MR−BRT Age/Sex Fits − Non−pregnant Females 2+ Years Old − Mean Haemglobin − Distrito Federal
WHO−2024 Adjustment Method
Mean Haemglobin
Age (year)
Location Hierarchy
1 − Global
2 − Super Region
3 − Region
4 − Country
5 − Subnational (Level 1)
Uncertainty Interval
5 − Subnational (Level 1)

MR−BRT Age/Sex Fits − Non−pregnant Females 2+ Years Old − Mean Haemglobin − Espírito Santo
WHO−2024 Adjustment Method

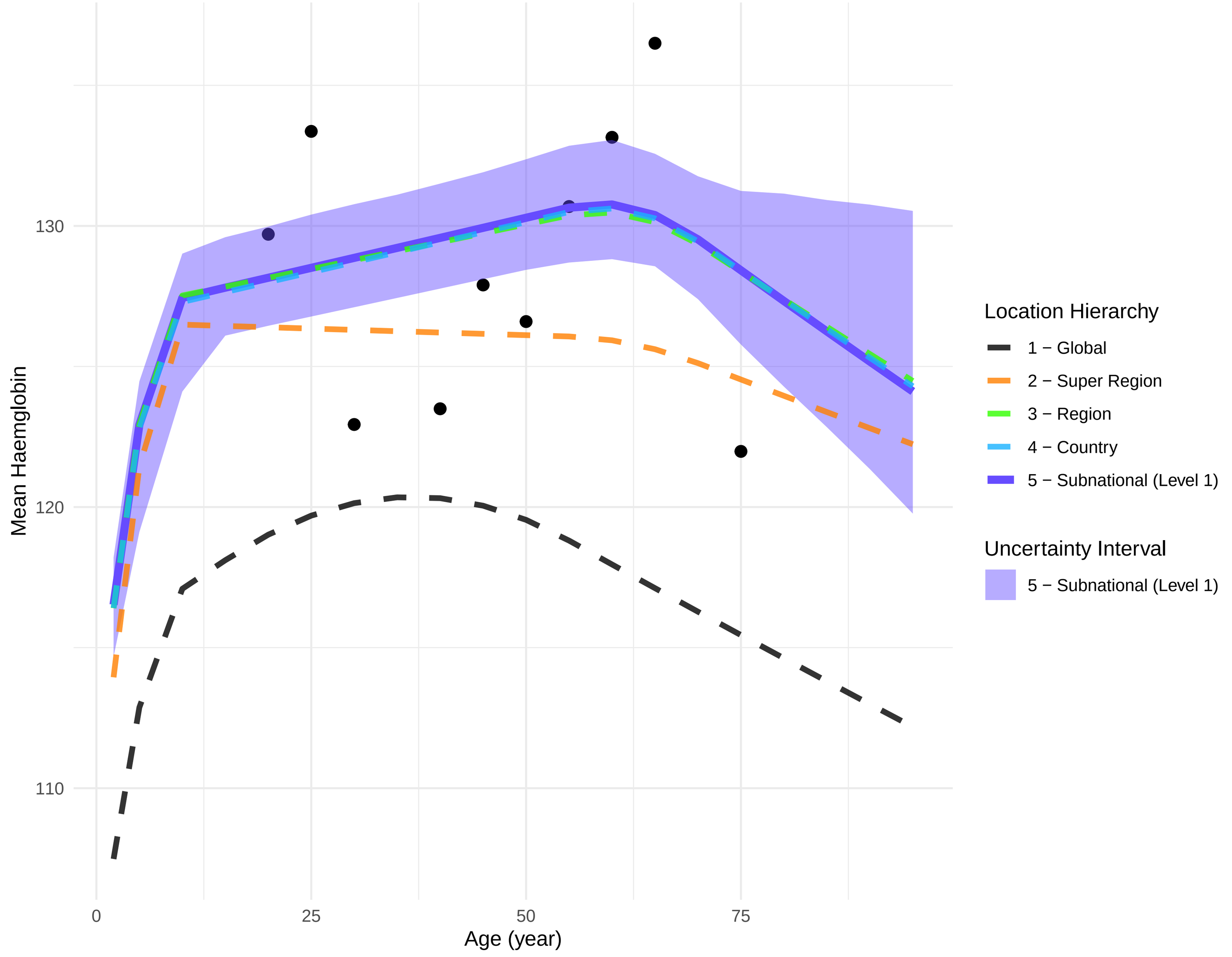

MR−BRT Age/Sex Fits − Non−pregnant Females 2+ Years Old − Mean Haemglobin − Goiás
WHO−2024 Adjustment Method

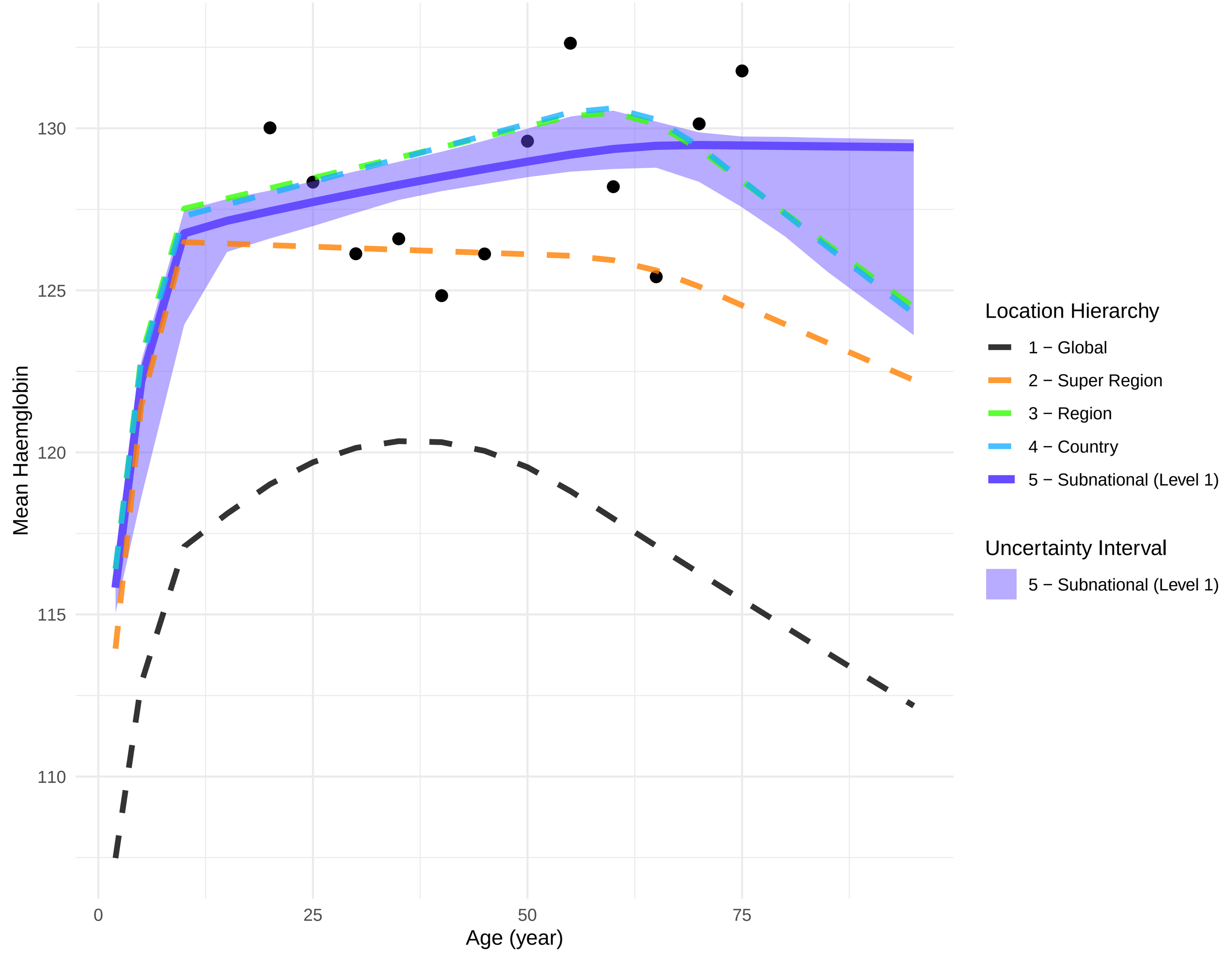

MR−BRT Age/Sex Fits − Non−pregnant Females 2+ Years Old − Mean Haemglobin − Maranhão
WHO−2024 Adjustment Method

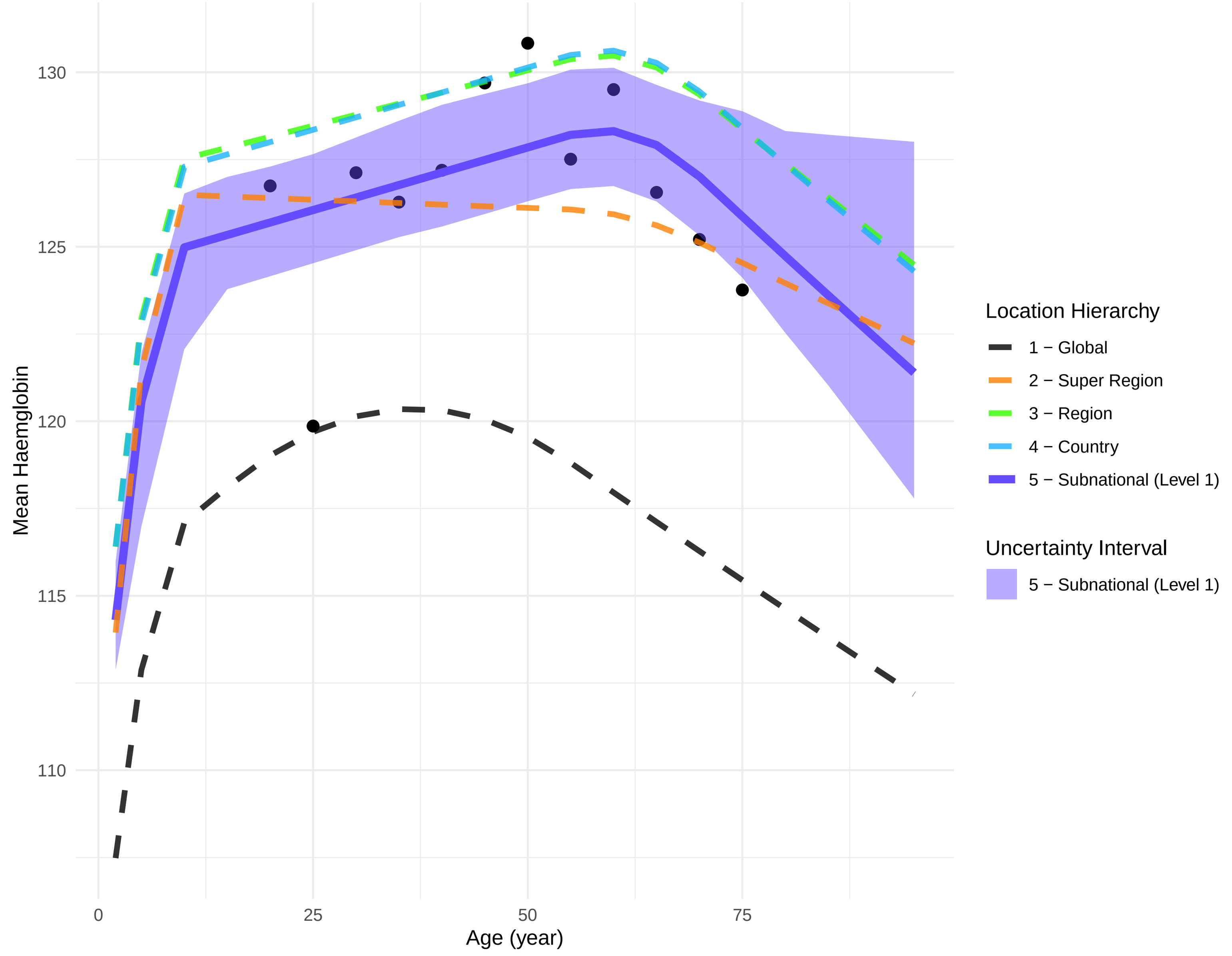

MR−BRT Age/Sex Fits − Non−pregnant Females 2+ Years Old − Mean Haemglobin − Mato Grosso
WHO−2024 Adjustment Method

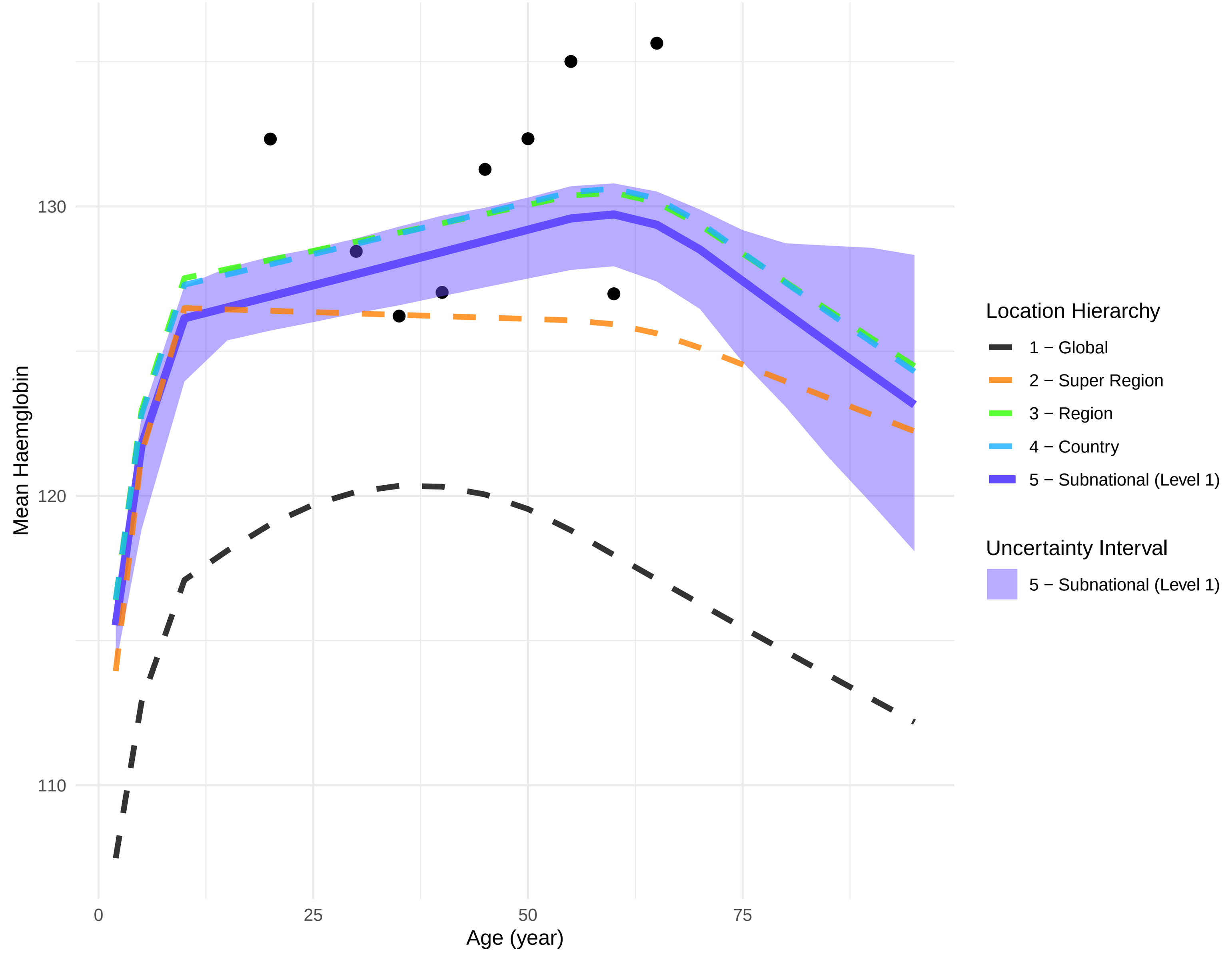

MR−BRT Age/Sex Fits − Non−pregnant Females 2+ Years Old − Mean Haemglobin − Mato Grosso do Sul
WHO−2024 Adjustment Method

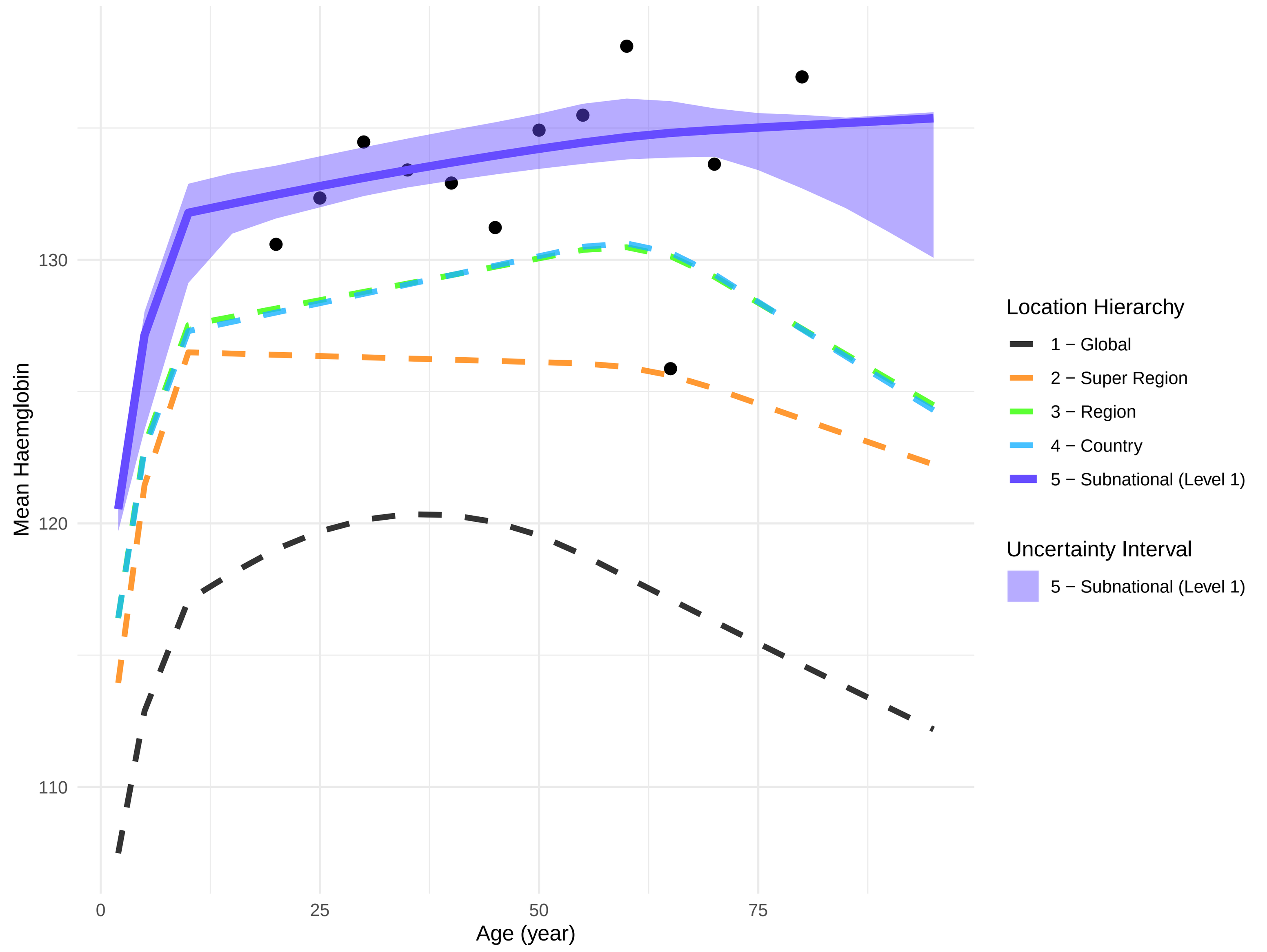

MR−BRT Age/Sex Fits − Non−pregnant Females 2+ Years Old − Mean Haemglobin − Minas Gerais
WHO−2024 Adjustment Method

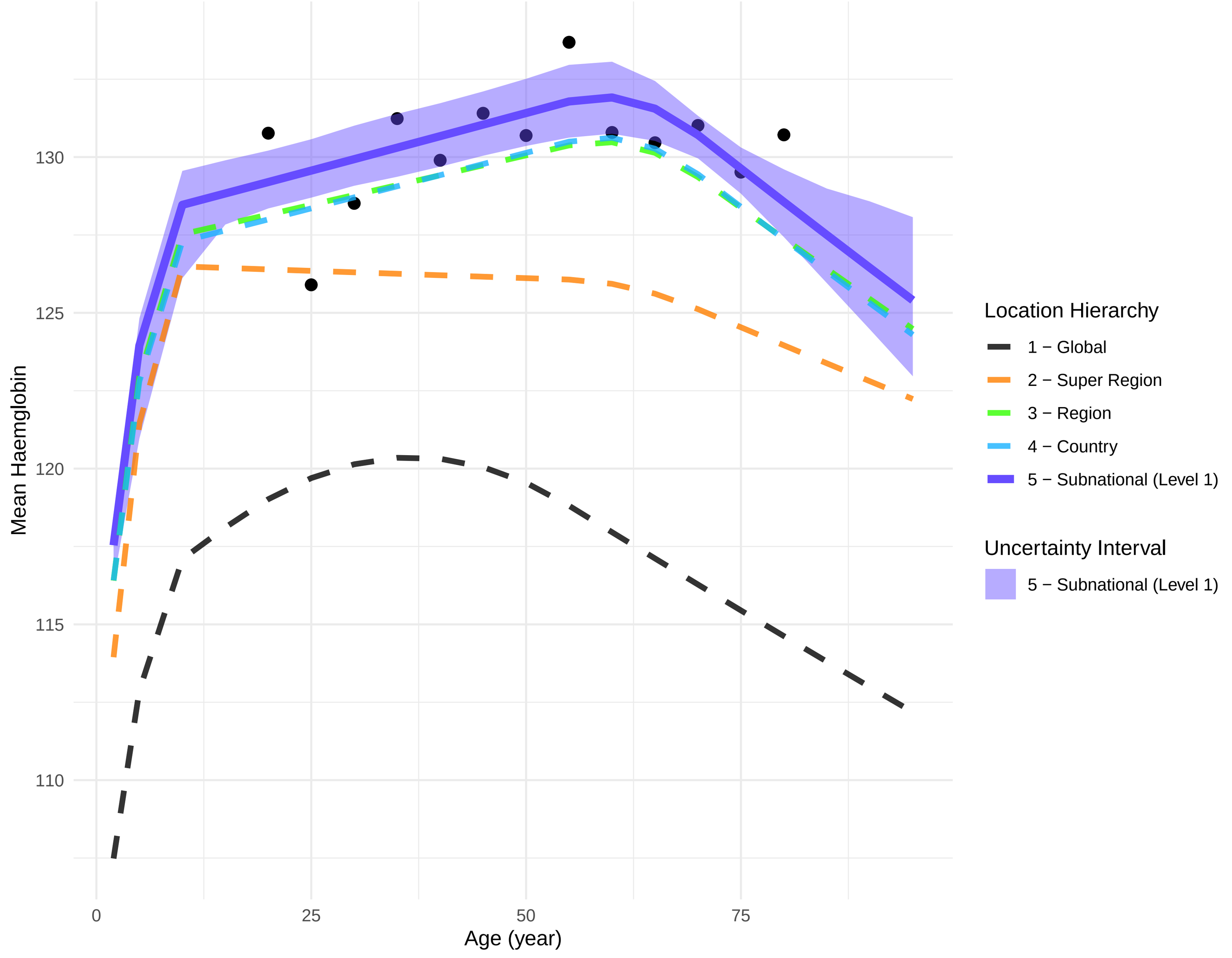

MR−BRT Age/Sex Fits − Non−pregnant Females 2+ Years Old − Mean Haemglobin − Pará
WHO−2024 Adjustment Method

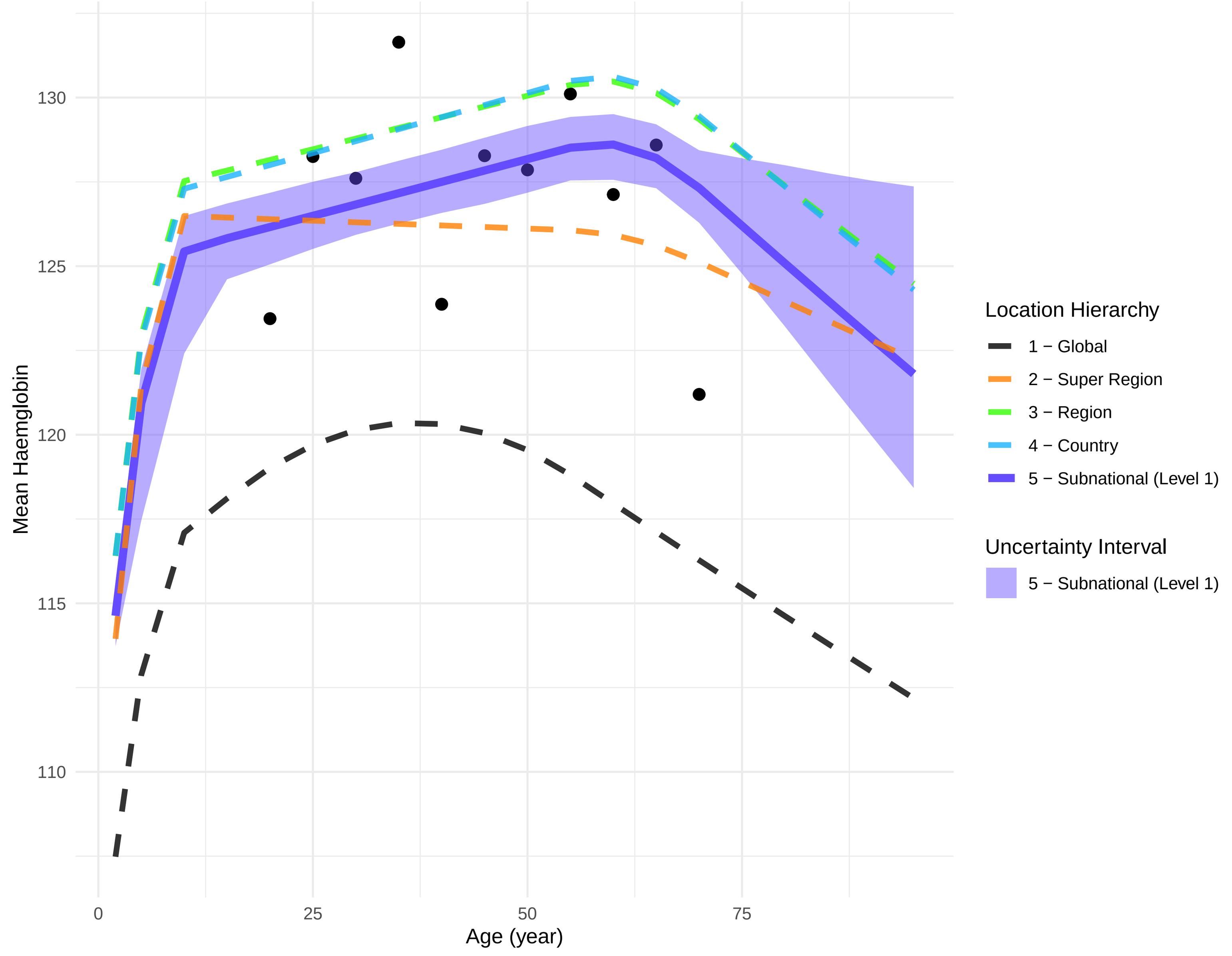

MR−BRT Age/Sex Fits − Non−pregnant Females 2+ Years Old − Mean Haemglobin − Paraíba
WHO−2024 Adjustment Method

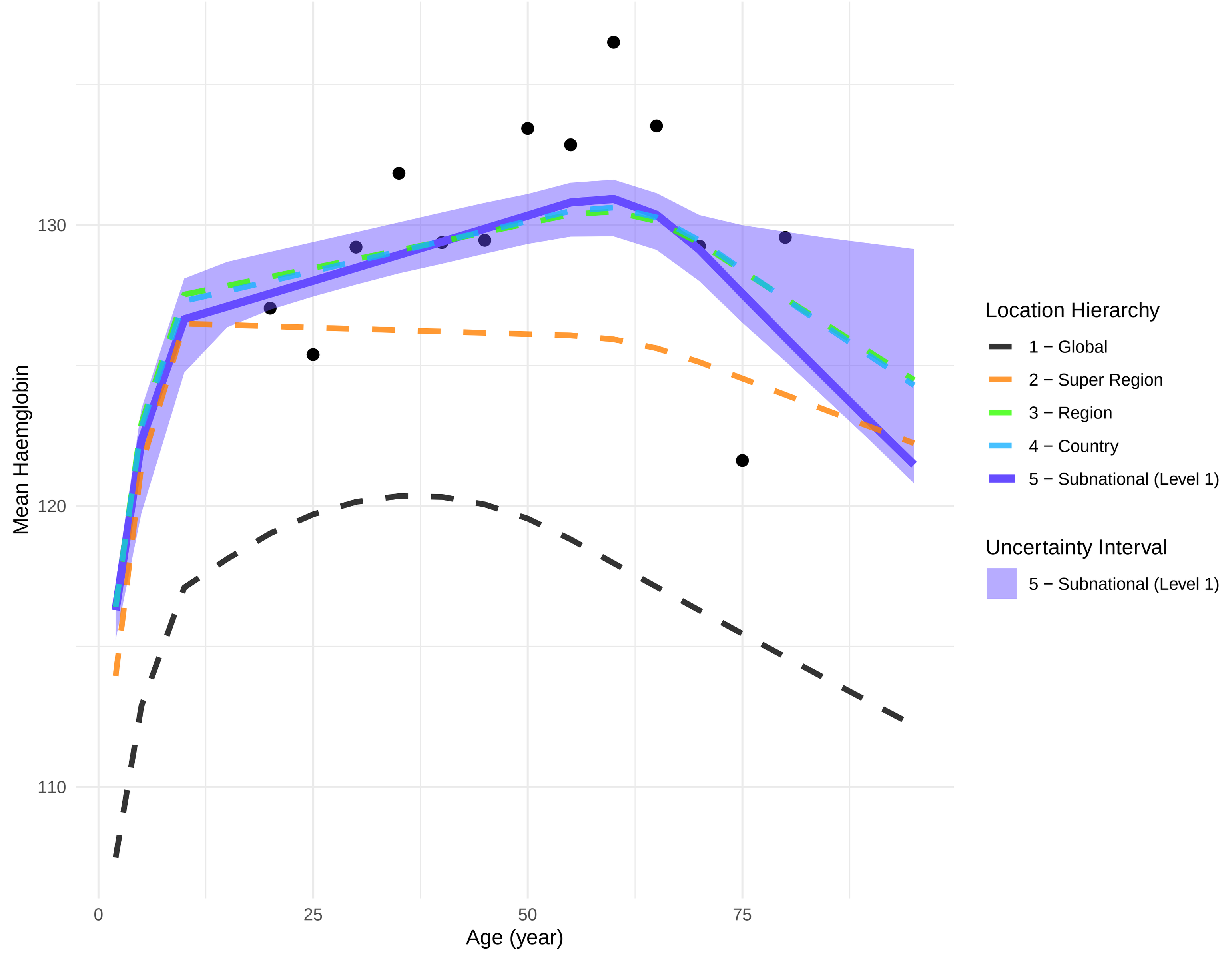

MR−BRT Age/Sex Fits − Non−pregnant Females 2+ Years Old − Mean Haemglobin − Paraná
WHO−2024 Adjustment Method

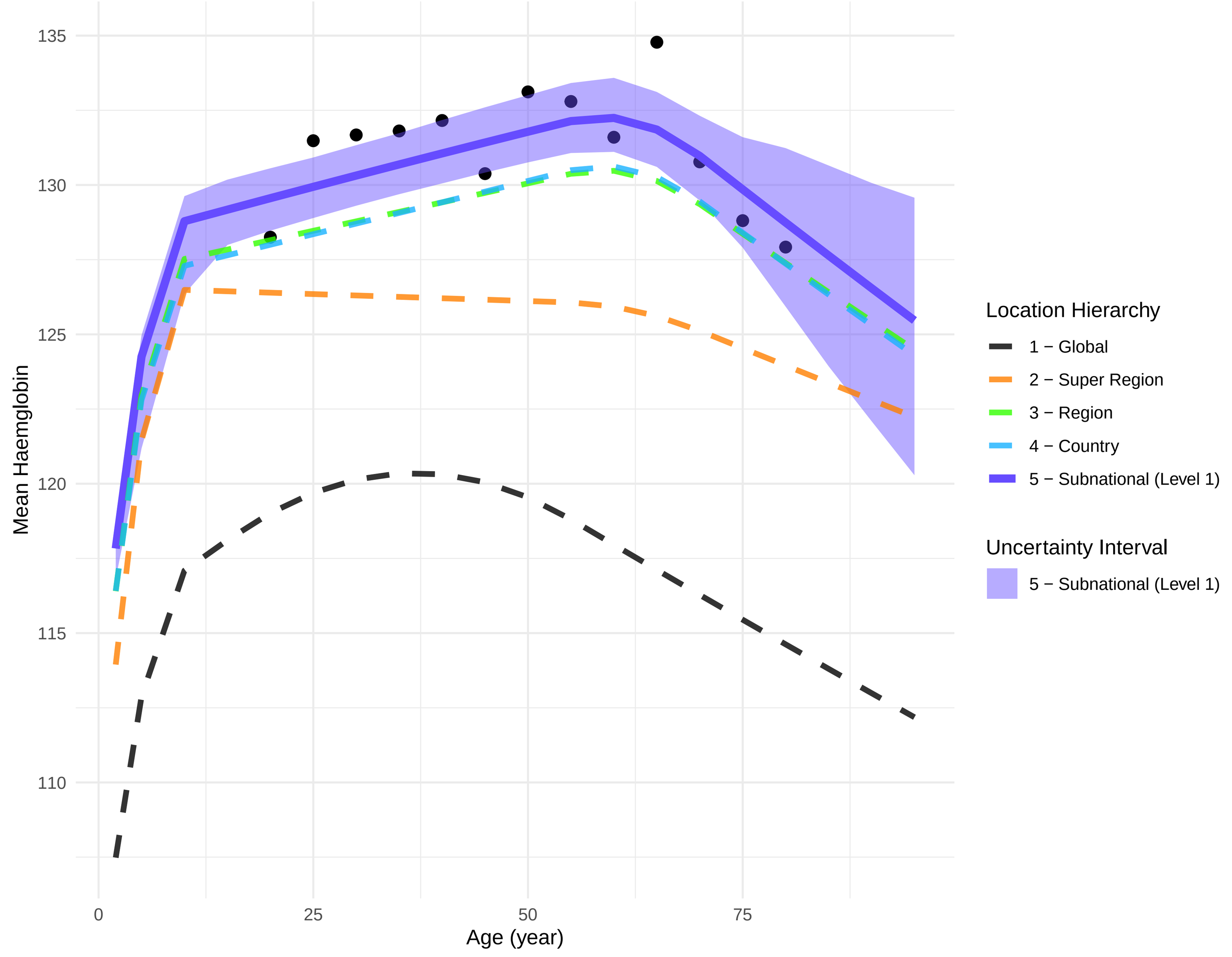

MR−BRT Age/Sex Fits − Non−pregnant Females 2+ Years Old − Mean Haemglobin − Pernambuco
WHO−2024 Adjustment Method

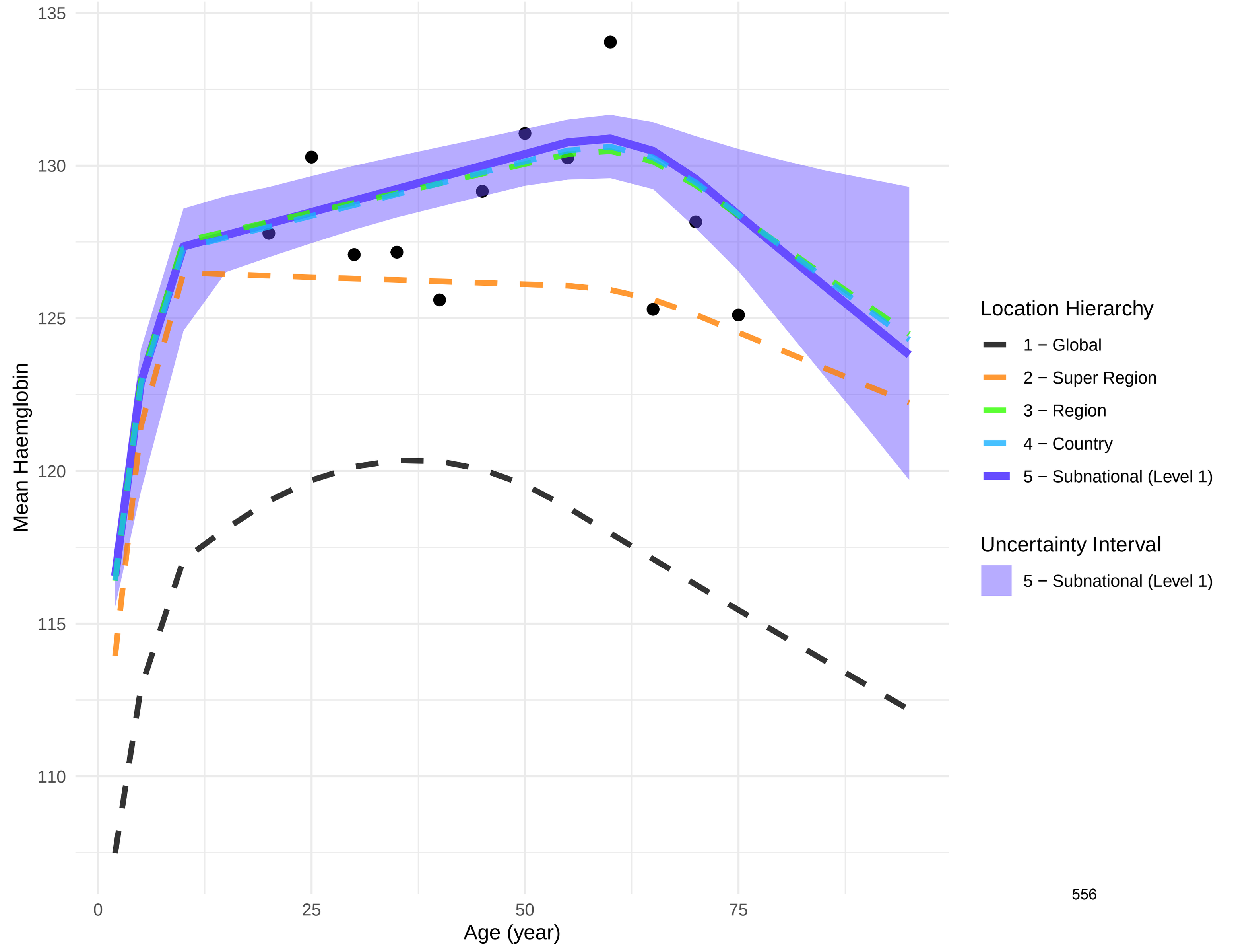

MR−BRT Age/Sex Fits − Non−pregnant Females 2+ Years Old − Mean Haemglobin − Piaui
WHO−2024 Adjustment Method

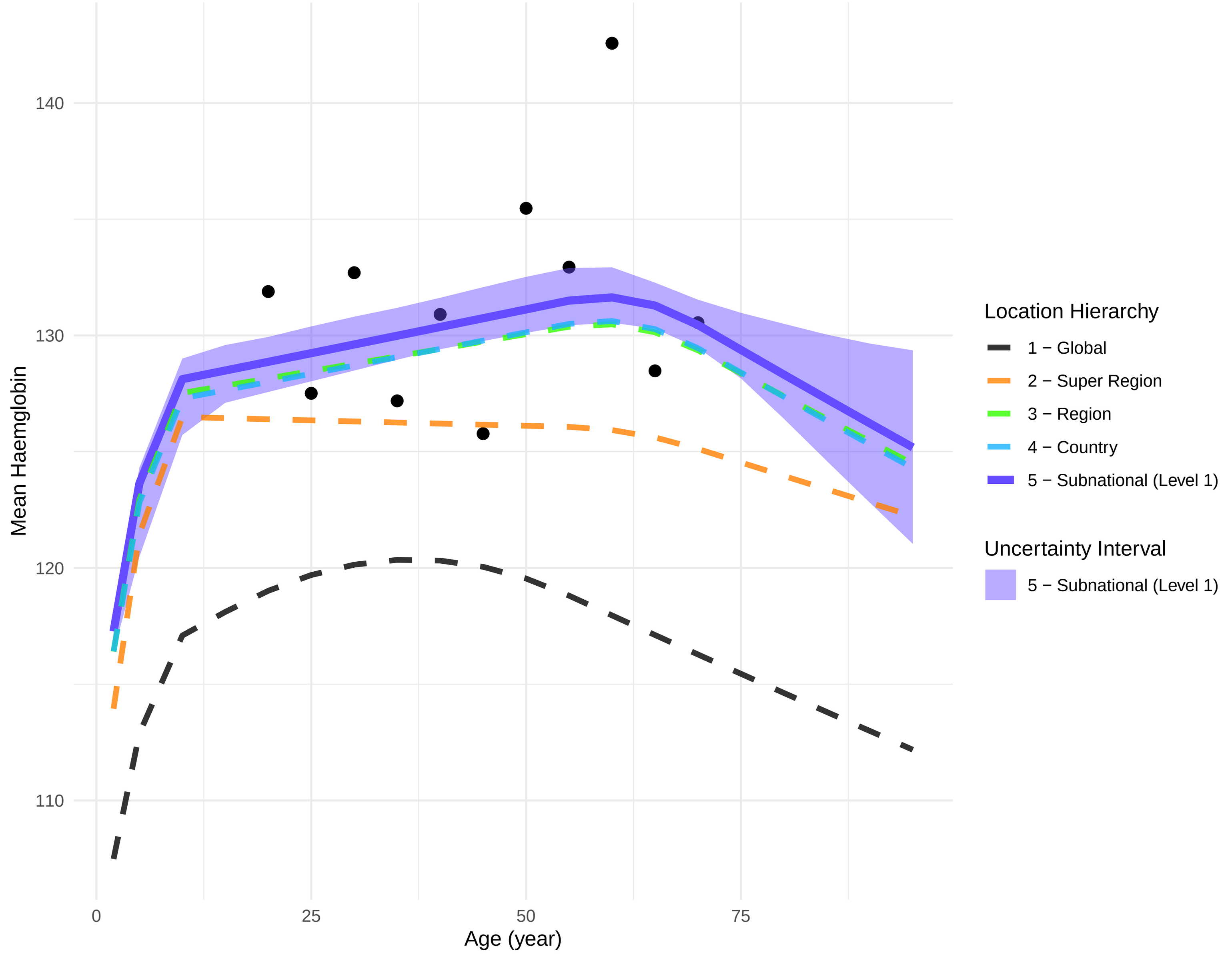

MR−BRT Age/Sex Fits − Non−pregnant Females 2+ Years Old − Mean Haemglobin − Rio de Janeiro
WHO−2024 Adjustment Method

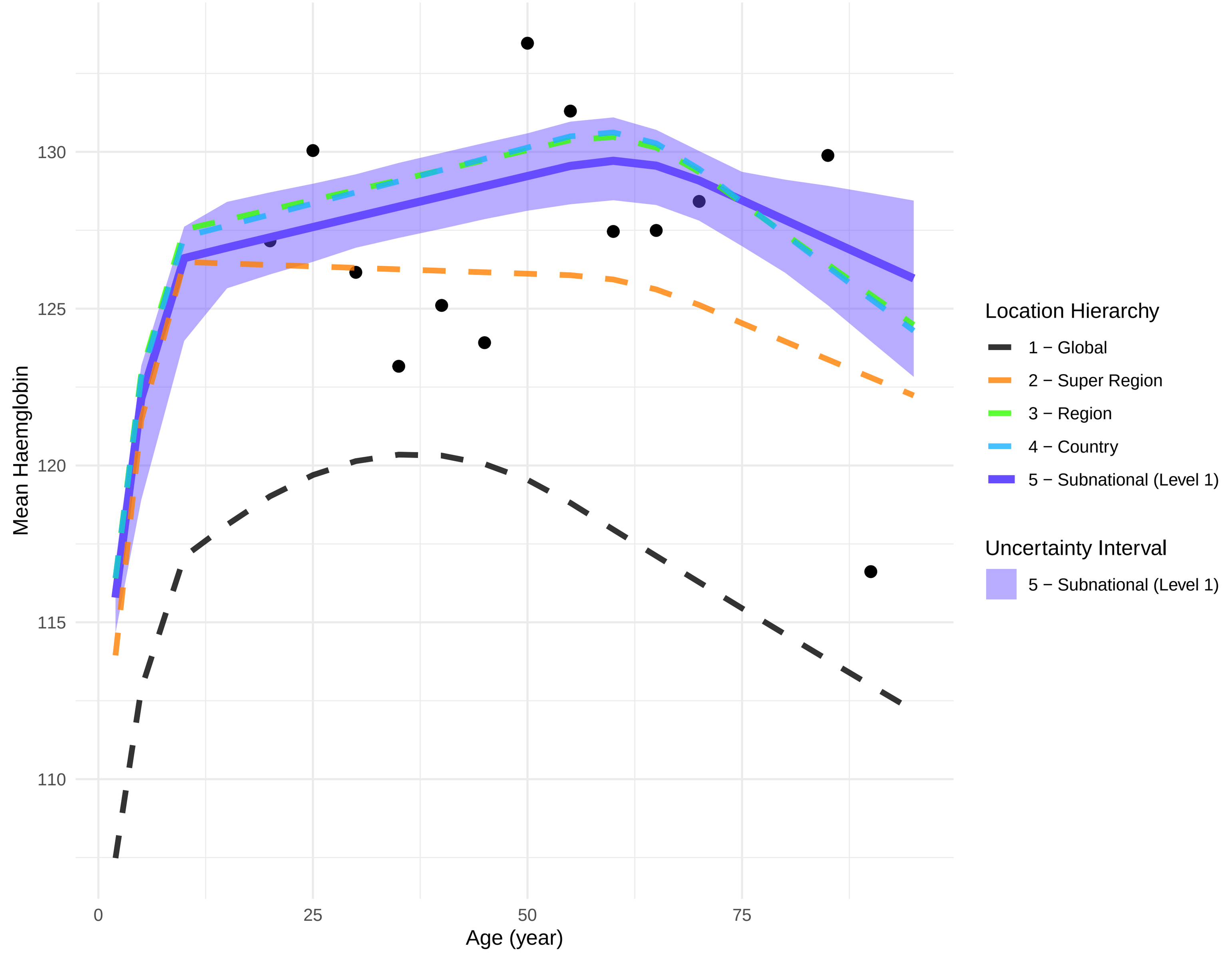

MR−BRT Age/Sex Fits − Non−pregnant Females 2+ Years Old − Mean Haemglobin − Rio Grande do Norte
WHO−2024 Adjustment Method

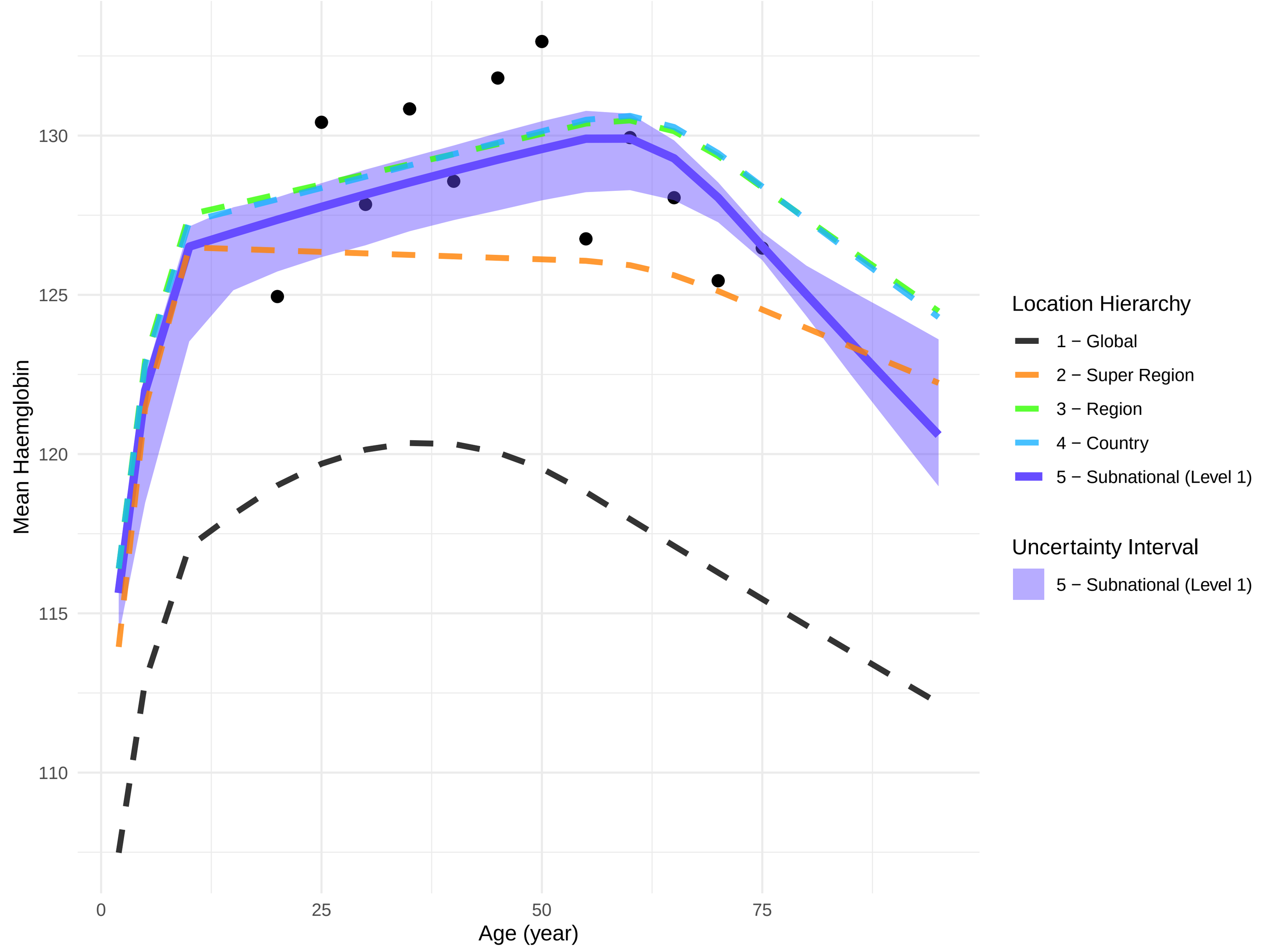

MR−BRT Age/Sex Fits − Non−pregnant Females 2+ Years Old − Mean Haemglobin − Rio Grande do Sul
WHO−2024 Adjustment Method

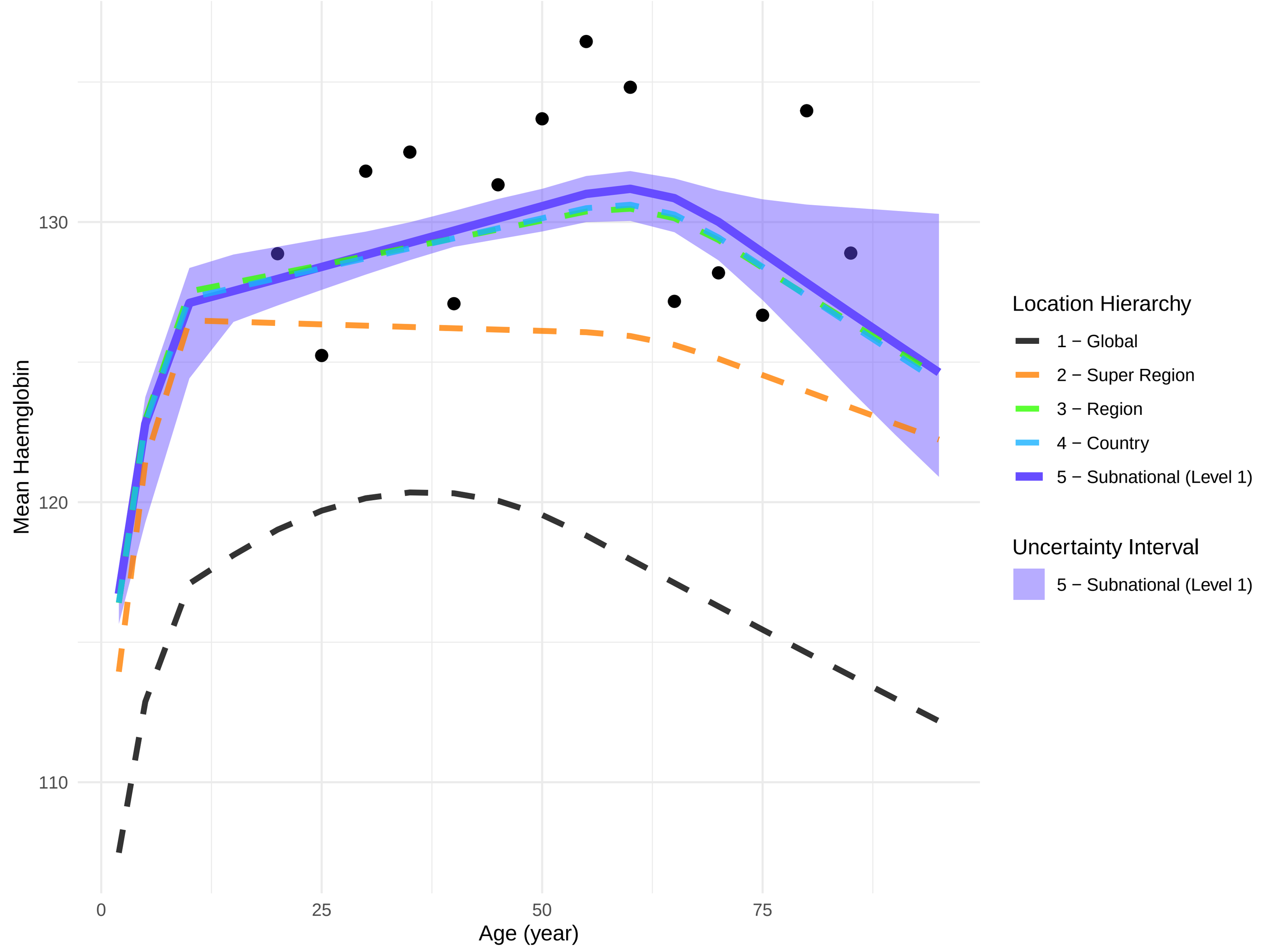

MR−BRT Age/Sex Fits − Non−pregnant Females 2+ Years Old − Mean Haemglobin − Rondônia
WHO−2024 Adjustment Method

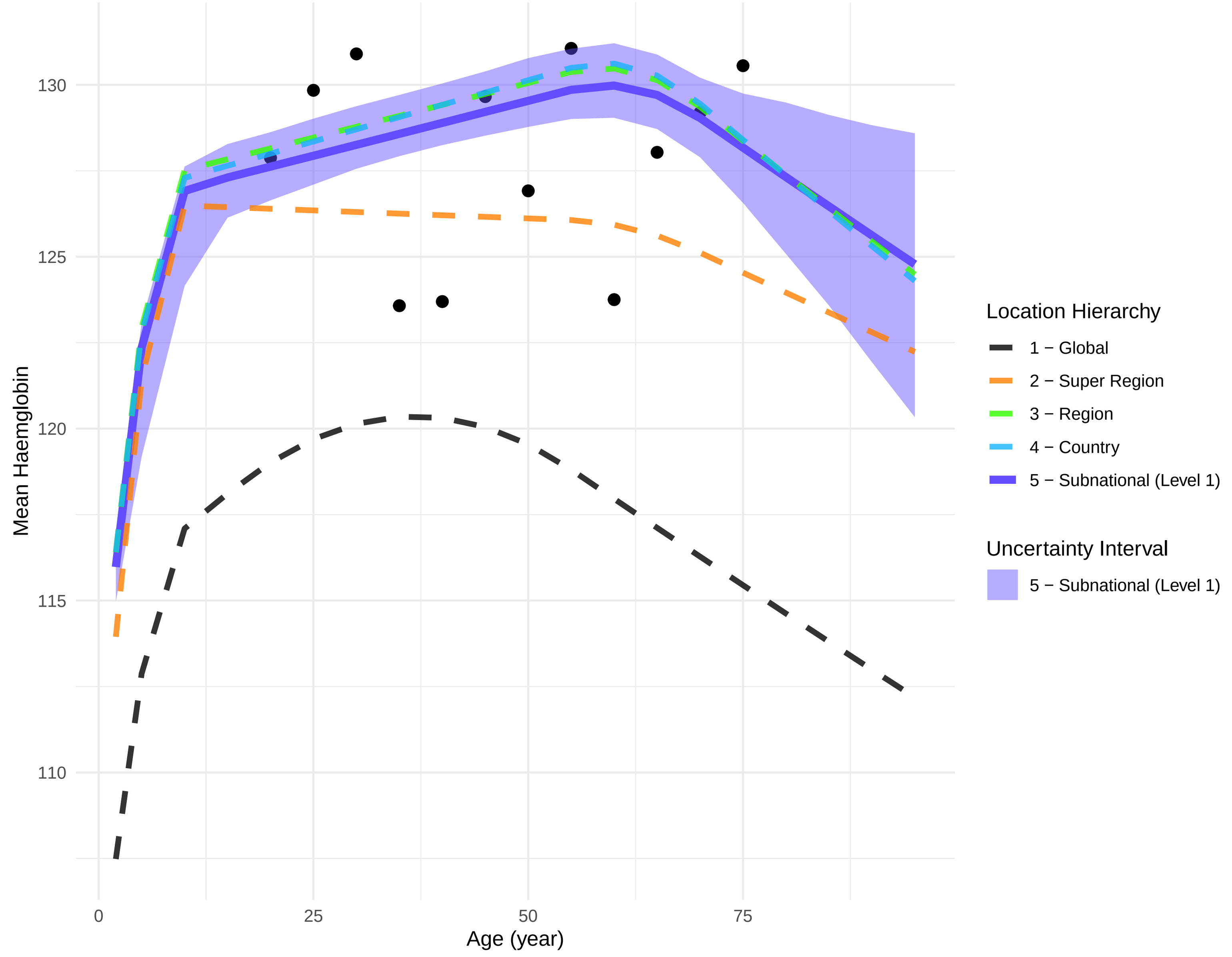

MR−BRT Age/Sex Fits − Non−pregnant Females 2+ Years Old − Mean Haemglobin − Roraima
WHO−2024 Adjustment Method

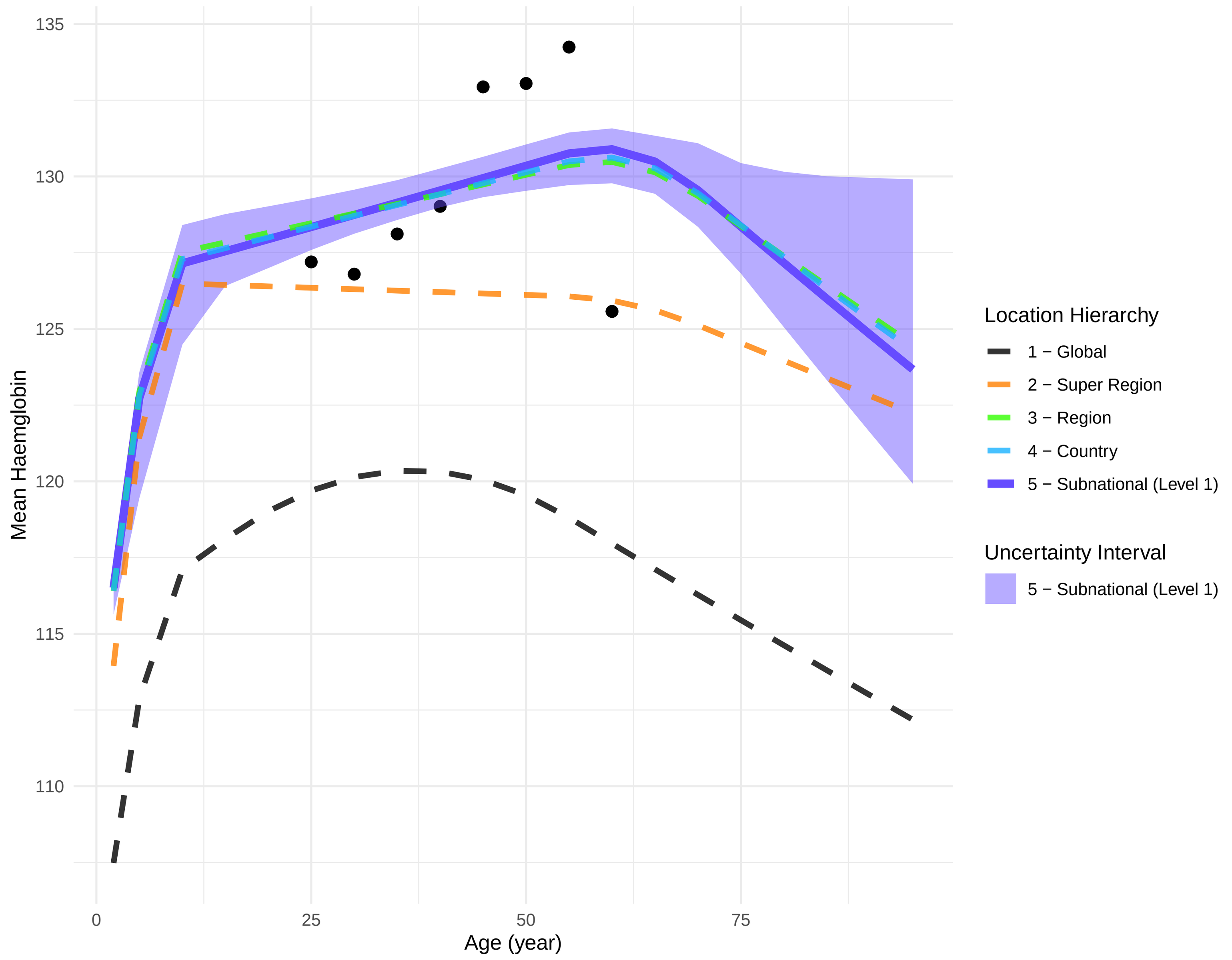

MR−BRT Age/Sex Fits − Non−pregnant Females 2+ Years Old − Mean Haemglobin − Santa Catarina
WHO−2024 Adjustment Method

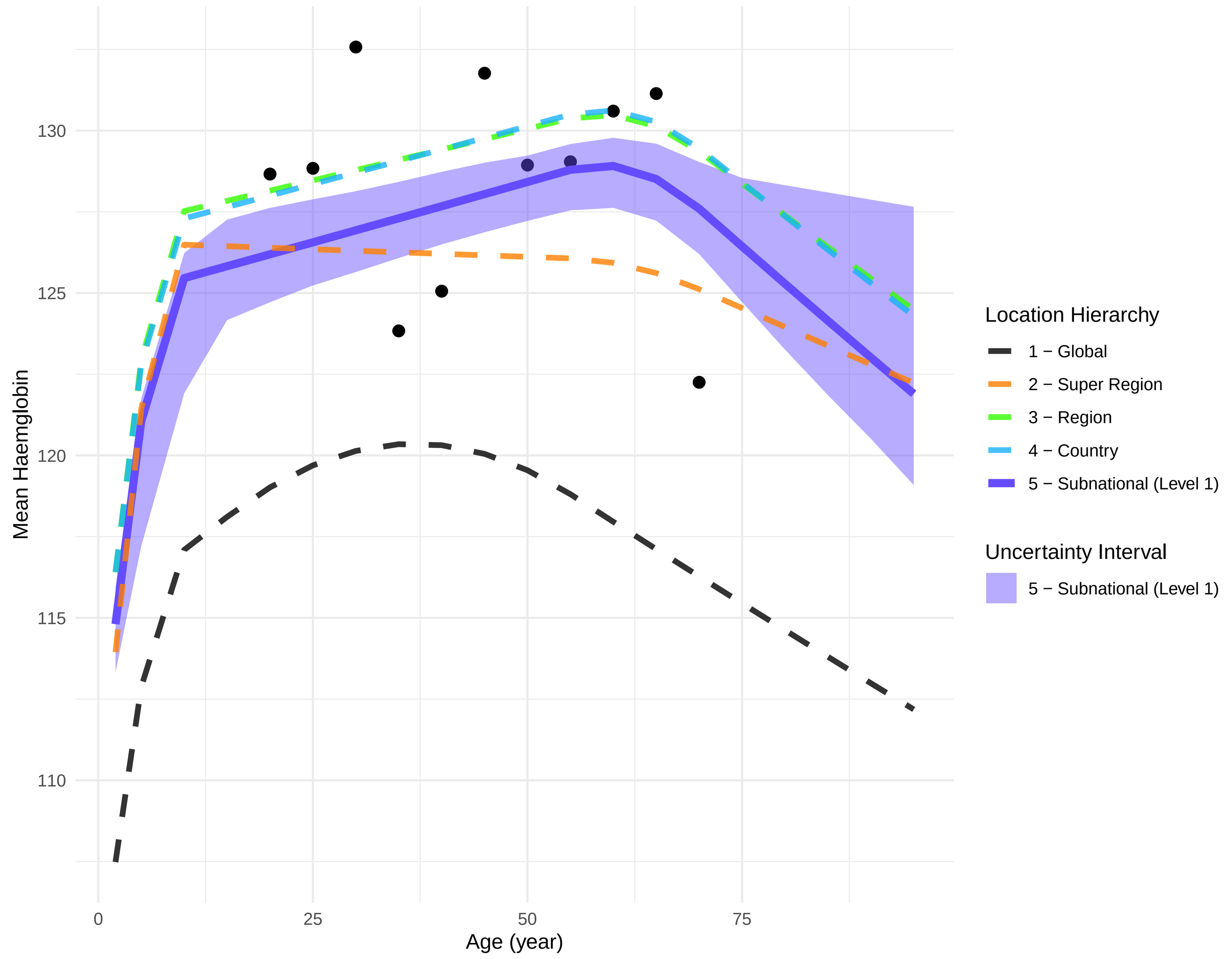

MR−BRT Age/Sex Fits − Non−pregnant Females 2+ Years Old − Mean Haemglobin − São Paulo
WHO−2024 Adjustment Method

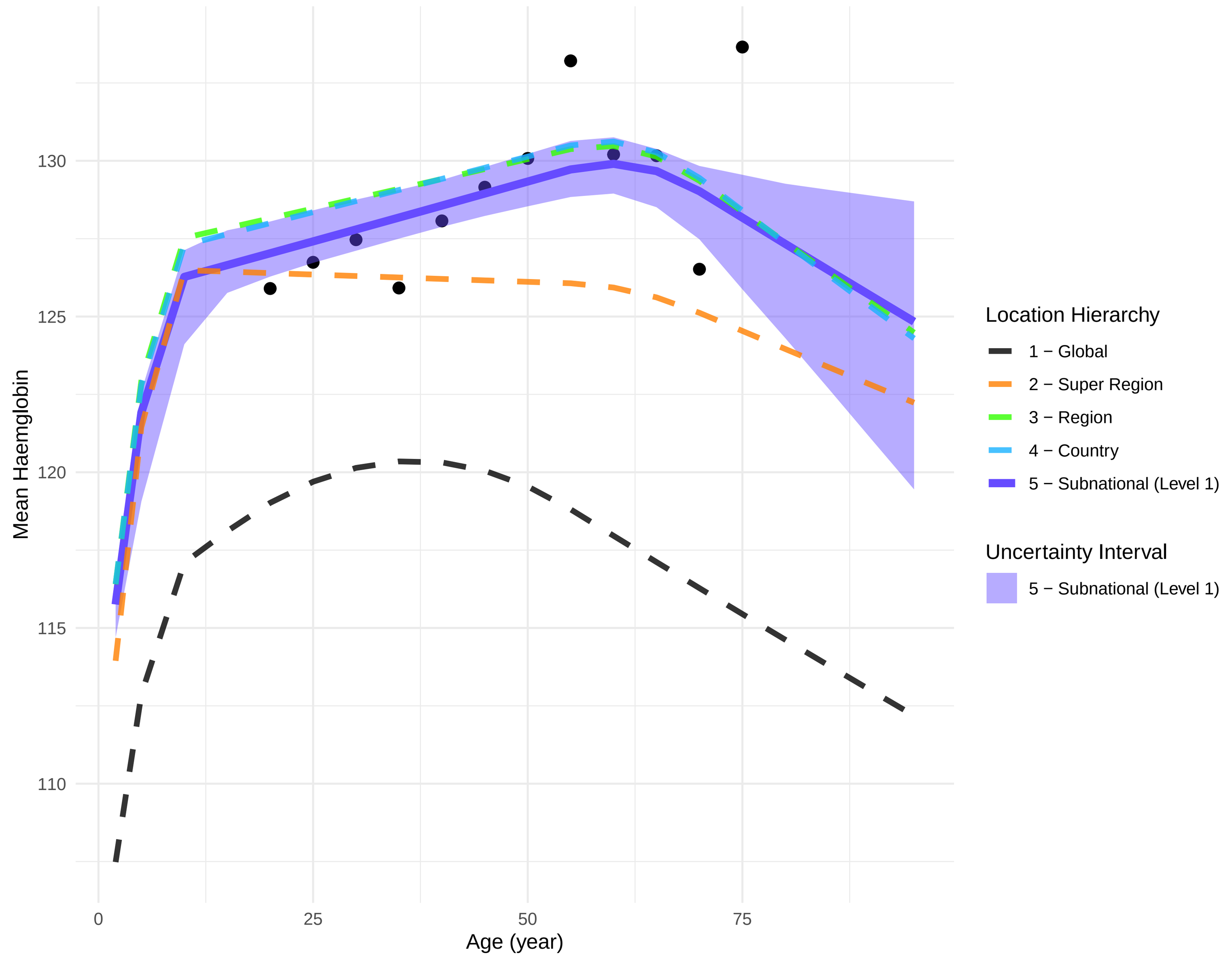

MR−BRT Age/Sex Fits − Non−pregnant Females 2+ Years Old − Mean Haemglobin − Sergipe
WHO−2024 Adjustment Method

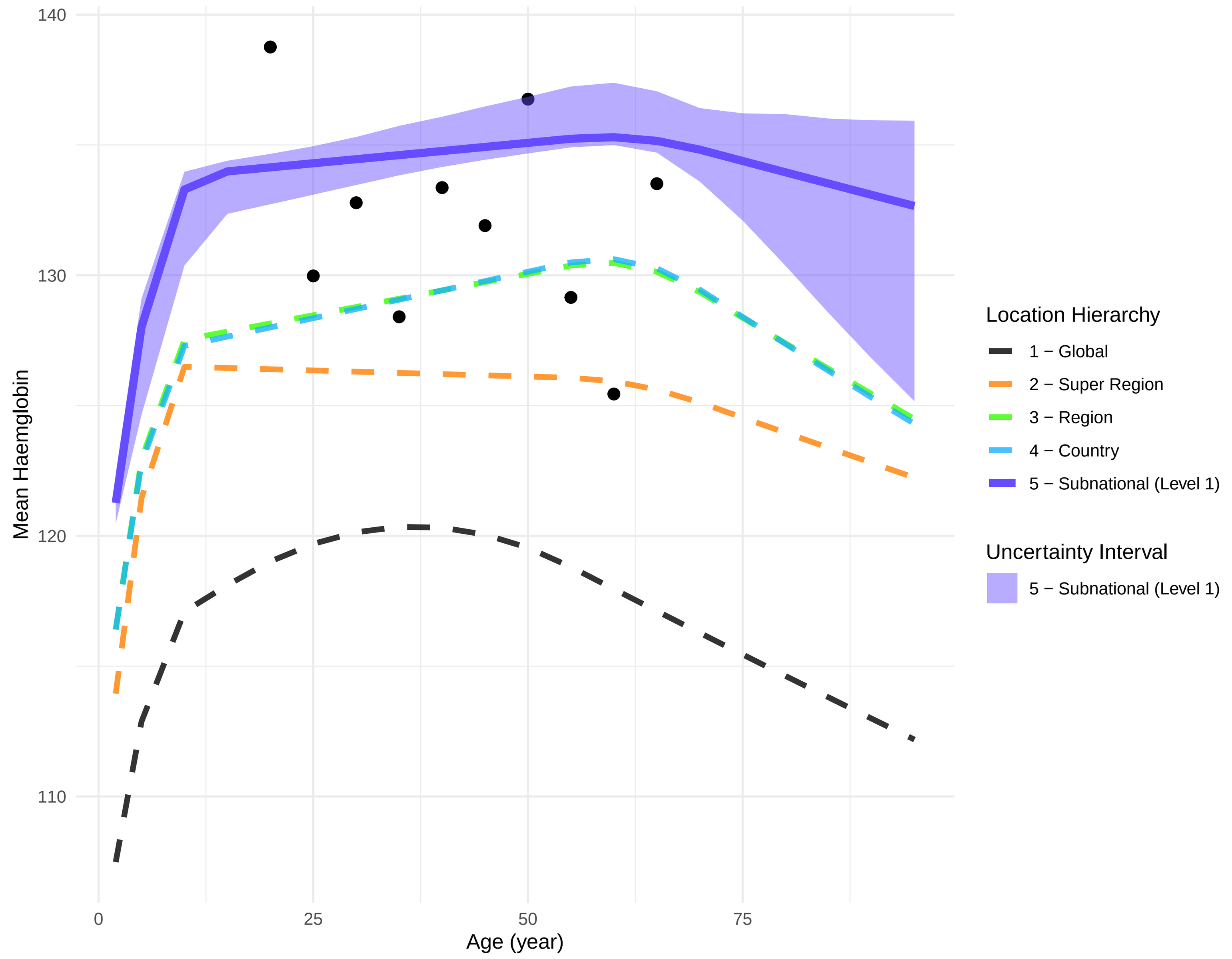

MR−BRT Age/Sex Fits − Non−pregnant Females 2+ Years Old − Mean Haemglobin − Tocantins
WHO−2024 Adjustment Method

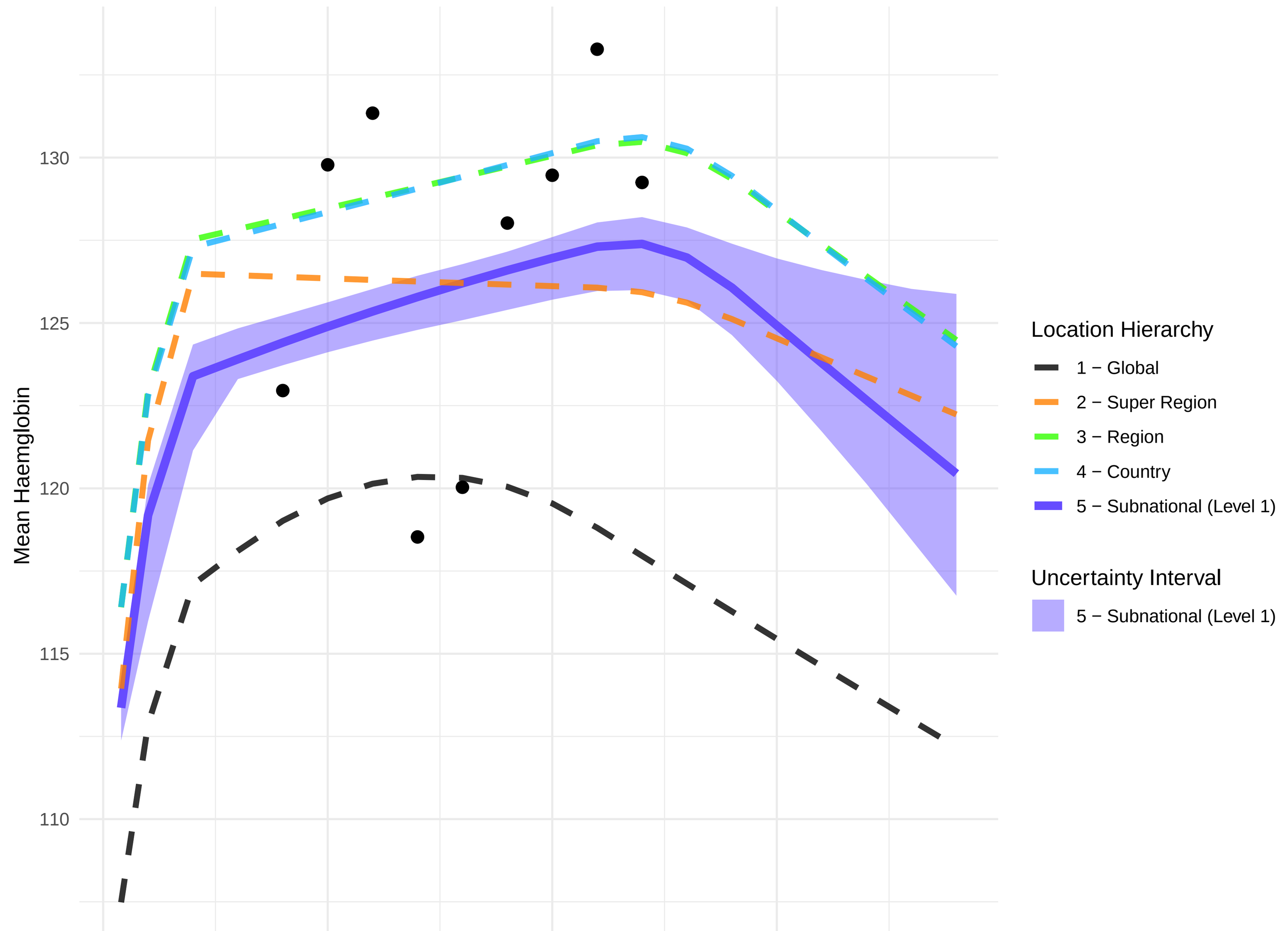

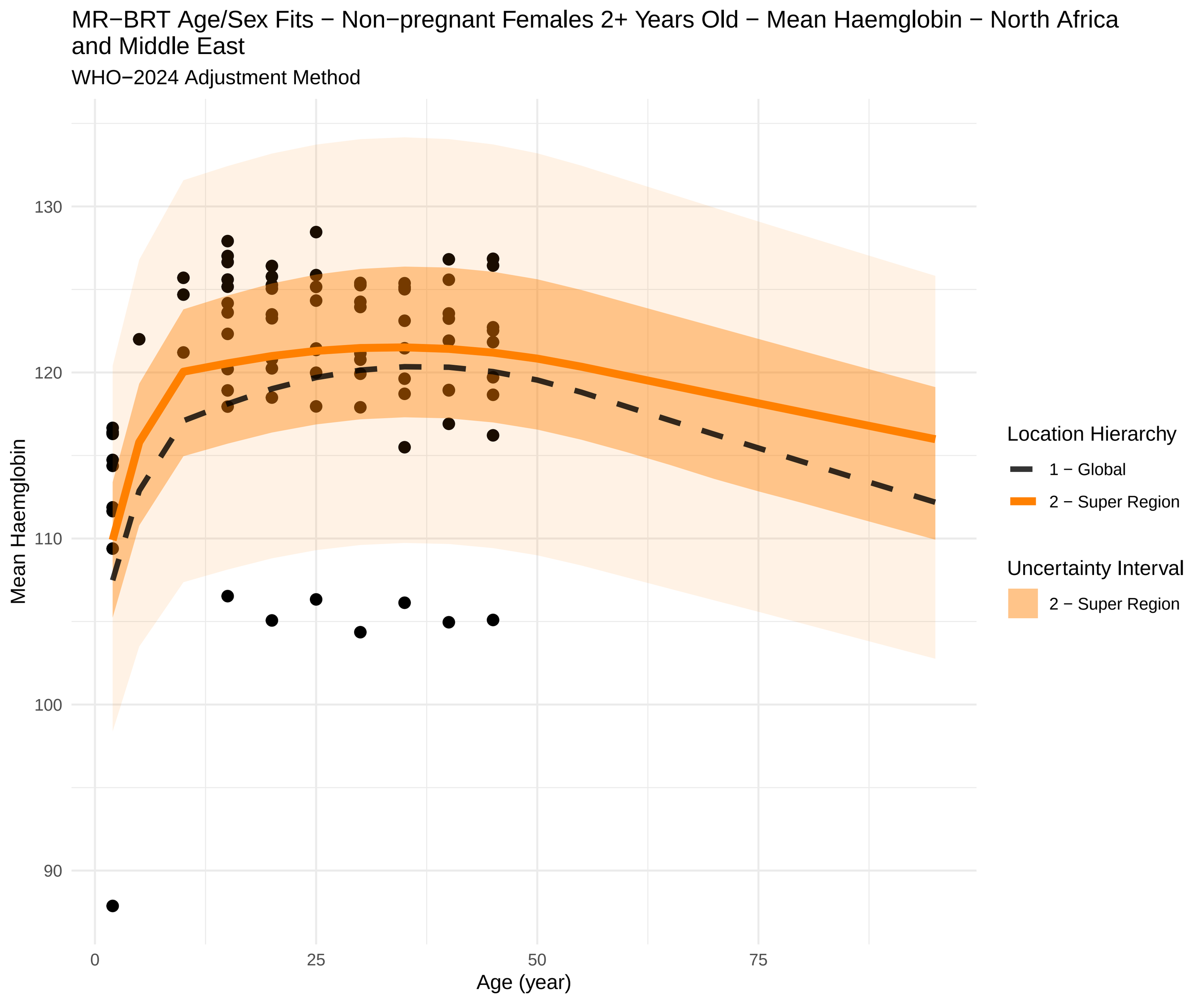
MR−BRT Age/Sex Fits − Non−pregnant Females 2+ Years Old − Mean Haemglobin − North Africa and Middle East
WHO−2024 Adjustment Method
Mean Haemglobin
Age (year)
Location Hierarchy
1 − Global
2 − Super Region
Uncertainty Interval
2 − Super Region

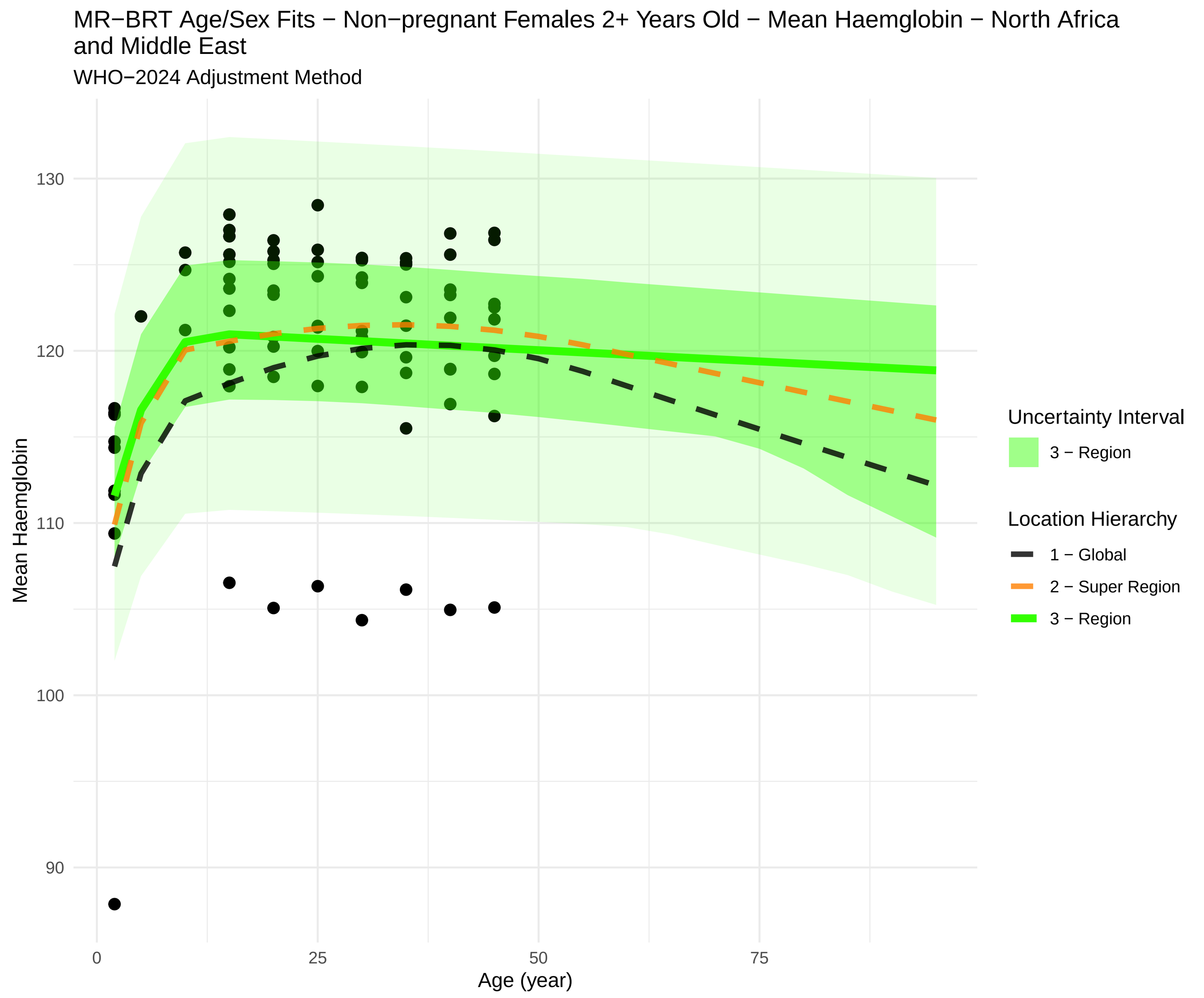
MR−BRT Age/Sex Fits − Non−pregnant Females 2+ Years Old − Mean Haemglobin − North Africa and Middle East
WHO−2024 Adjustment Method
Mean Haemglobin
Age (year)
Uncertainty Interval
3 − Region
Location Hierarchy
1 − Global
2 − Super Region
3 − Region

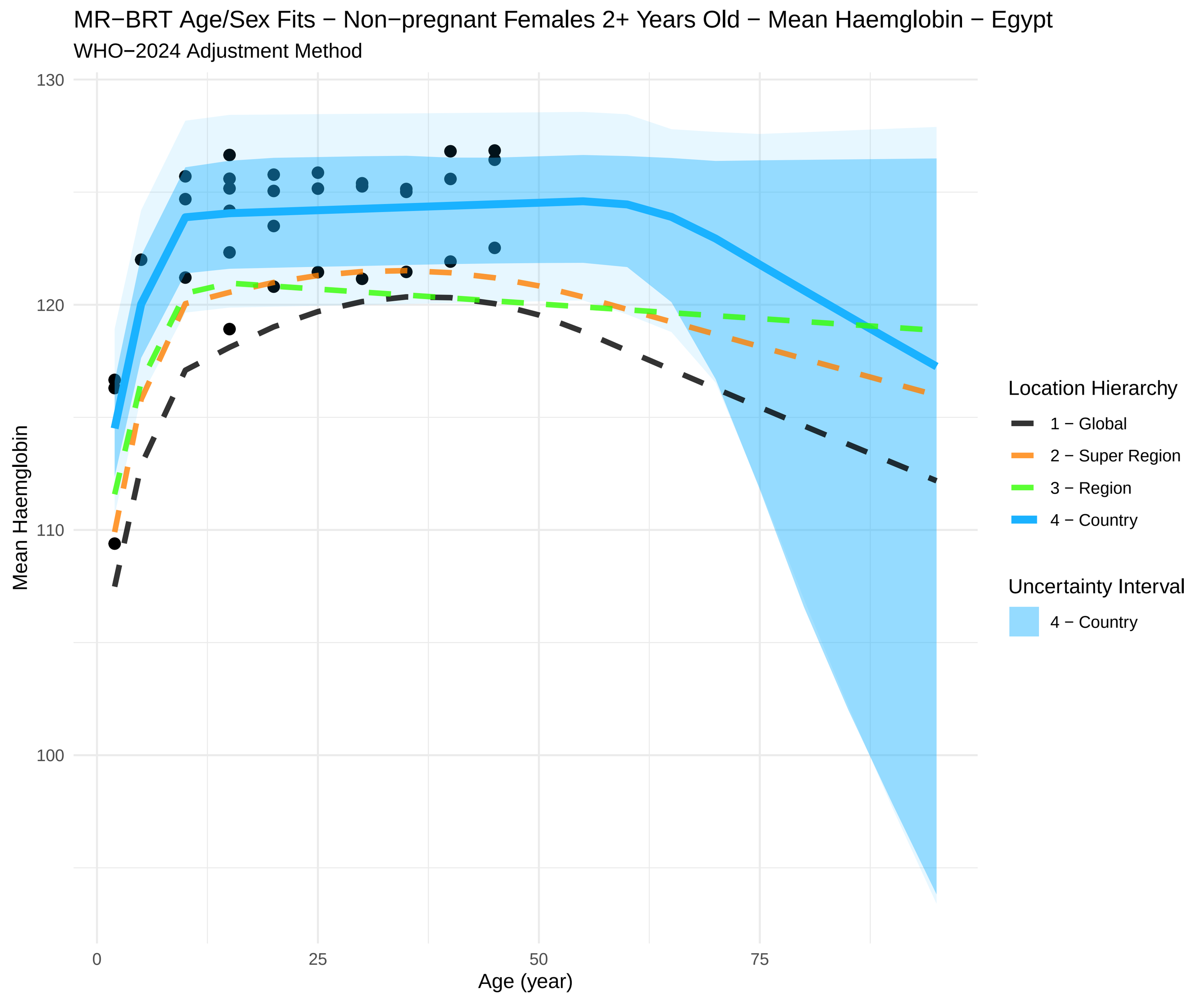
MR−BRT Age/Sex Fits − Non−pregnant Females 2+ Years Old − Mean Haemglobin − Egypt
WHO−2024 Adjustment Method
Mean Haemglobin
Age (year)
Location Hierarchy
1 − Global
2 − Super Region
3 − Region
4 − Country
Uncertainty Interval
4 − Country

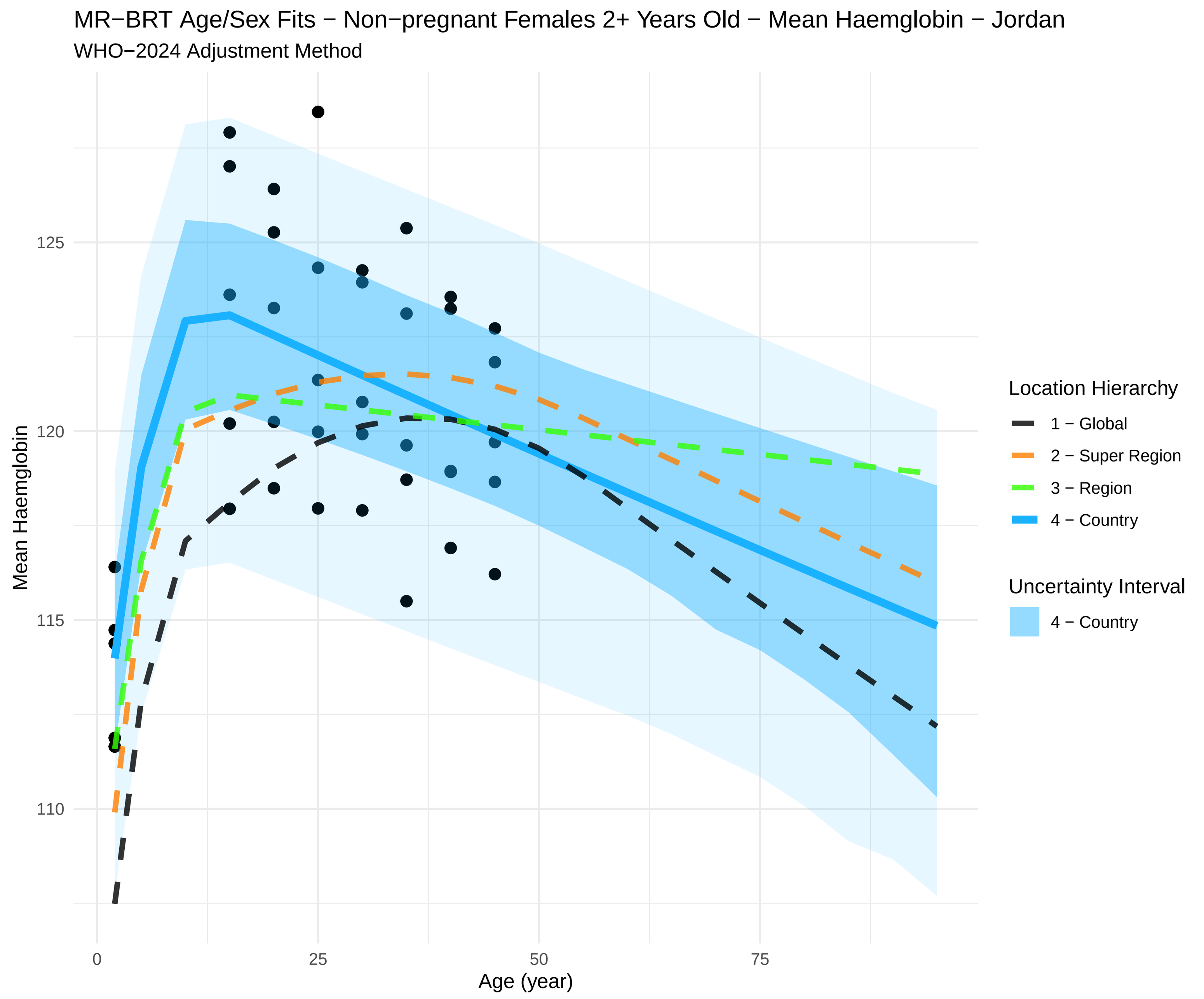
MR−BRT Age/Sex Fits − Non−pregnant Females 2+ Years Old − Mean Haemglobin − Jordan
WHO−2024 Adjustment Method
Mean Haemglobin
Age (year)
Location Hierarchy
1 − Global
2 − Super Region
3 − Region
4 − Country
Uncertainty Interval
4 − Country

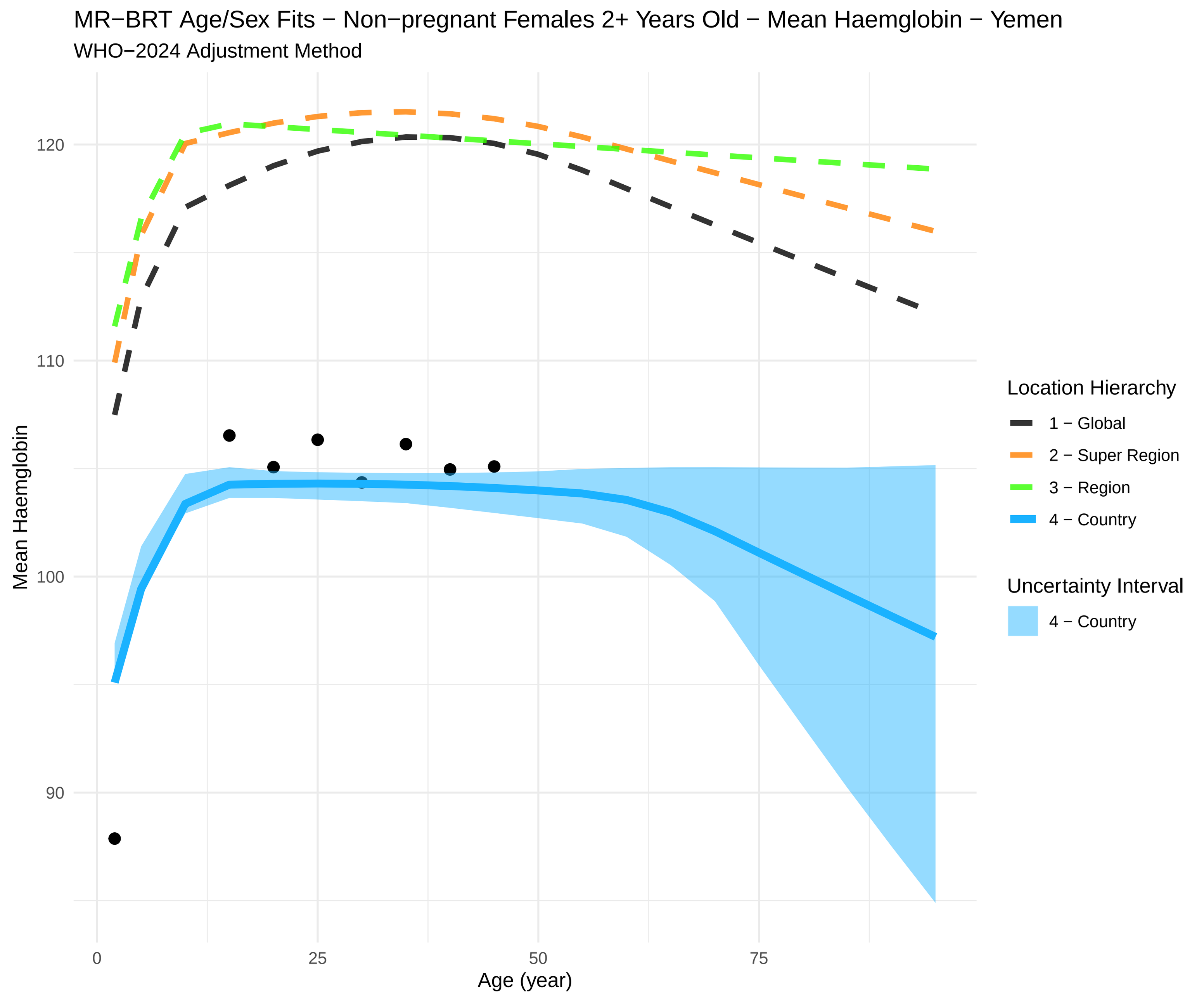
MR−BRT Age/Sex Fits − Non−pregnant Females 2+ Years Old − Mean Haemglobin − Yemen
WHO−2024 Adjustment Method
Mean Haemglobin
Age (year)
Location Hierarchy
1 − Global
2 − Super Region
3 − Region
4 − Country
Uncertainty Interval
4 − Country

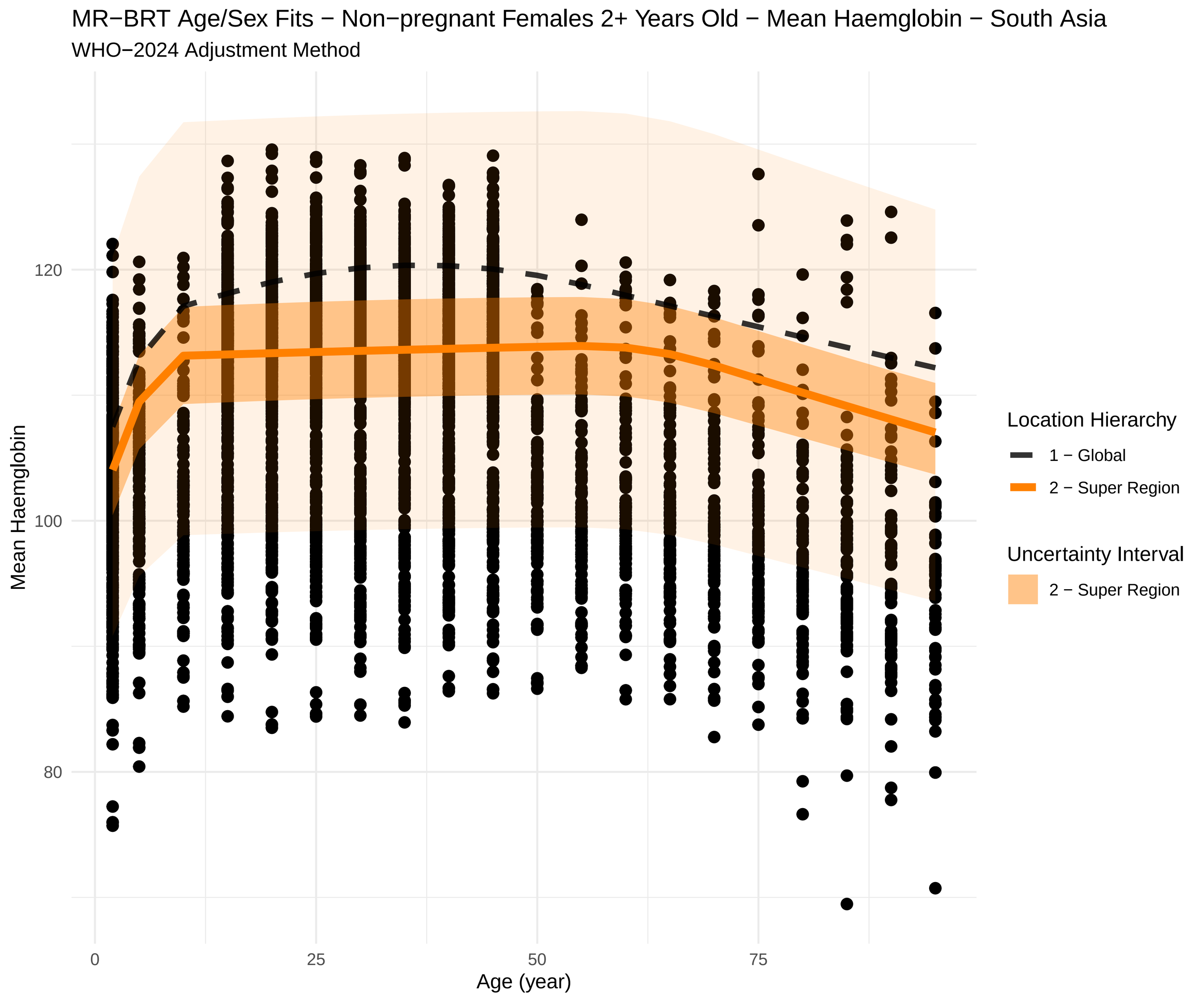
MR−BRT Age/Sex Fits − Non−pregnant Females 2+ Years Old − Mean Haemglobin − South Asia
WHO−2024 Adjustment Method
Mean Haemglobin
Age (year)
Location Hierarchy
1 − Global
2 − Super Region
Uncertainty Interval
2 − Super Region

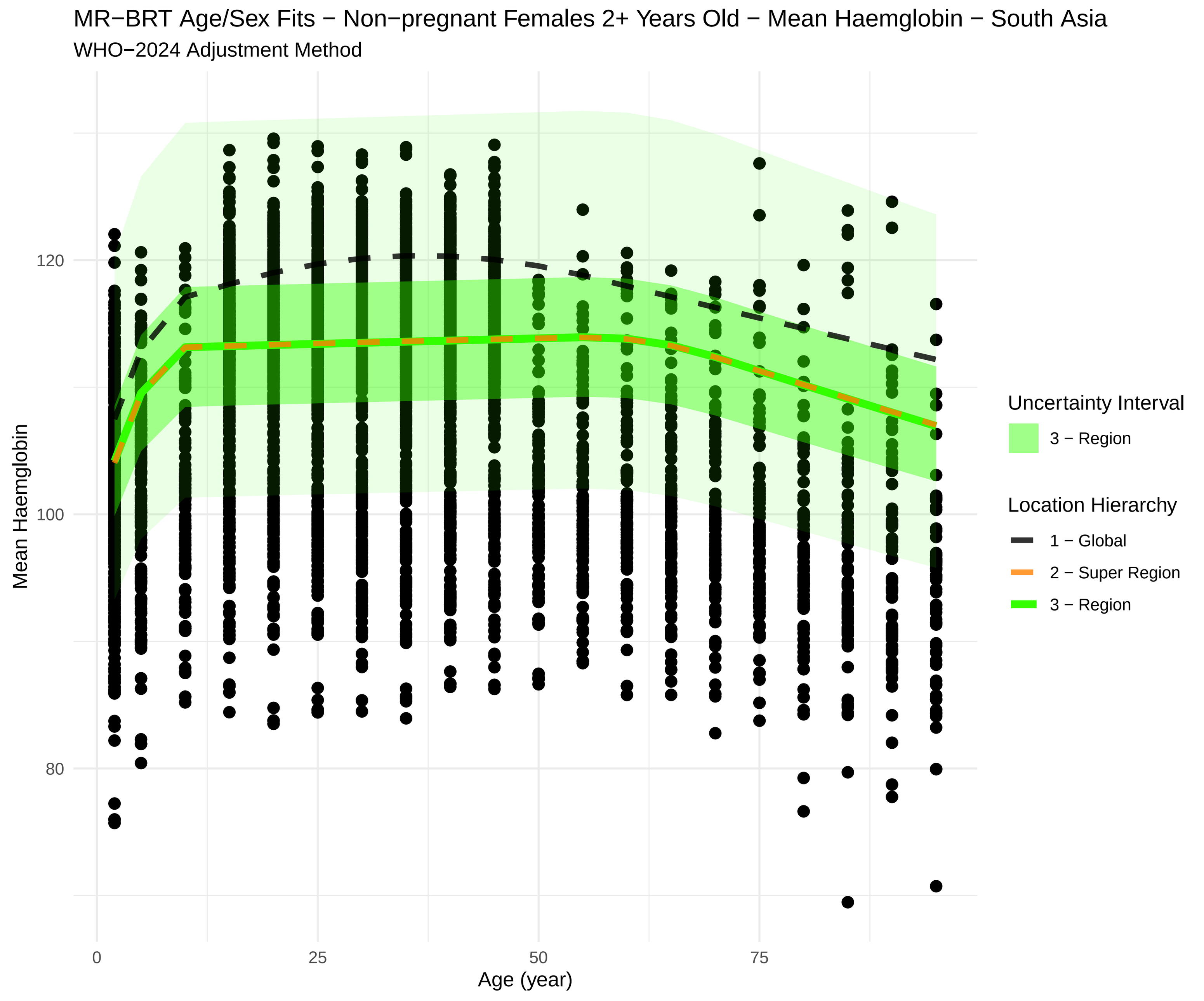
MR−BRT Age/Sex Fits − Non−pregnant Females 2+ Years Old − Mean Haemglobin − South Asia
WHO−2024 Adjustment Method
Mean Haemglobin
Age (year)
Uncertainty Interval
3 − Region
Location Hierarchy
1 − Global
2 − Super Region
3 − Region

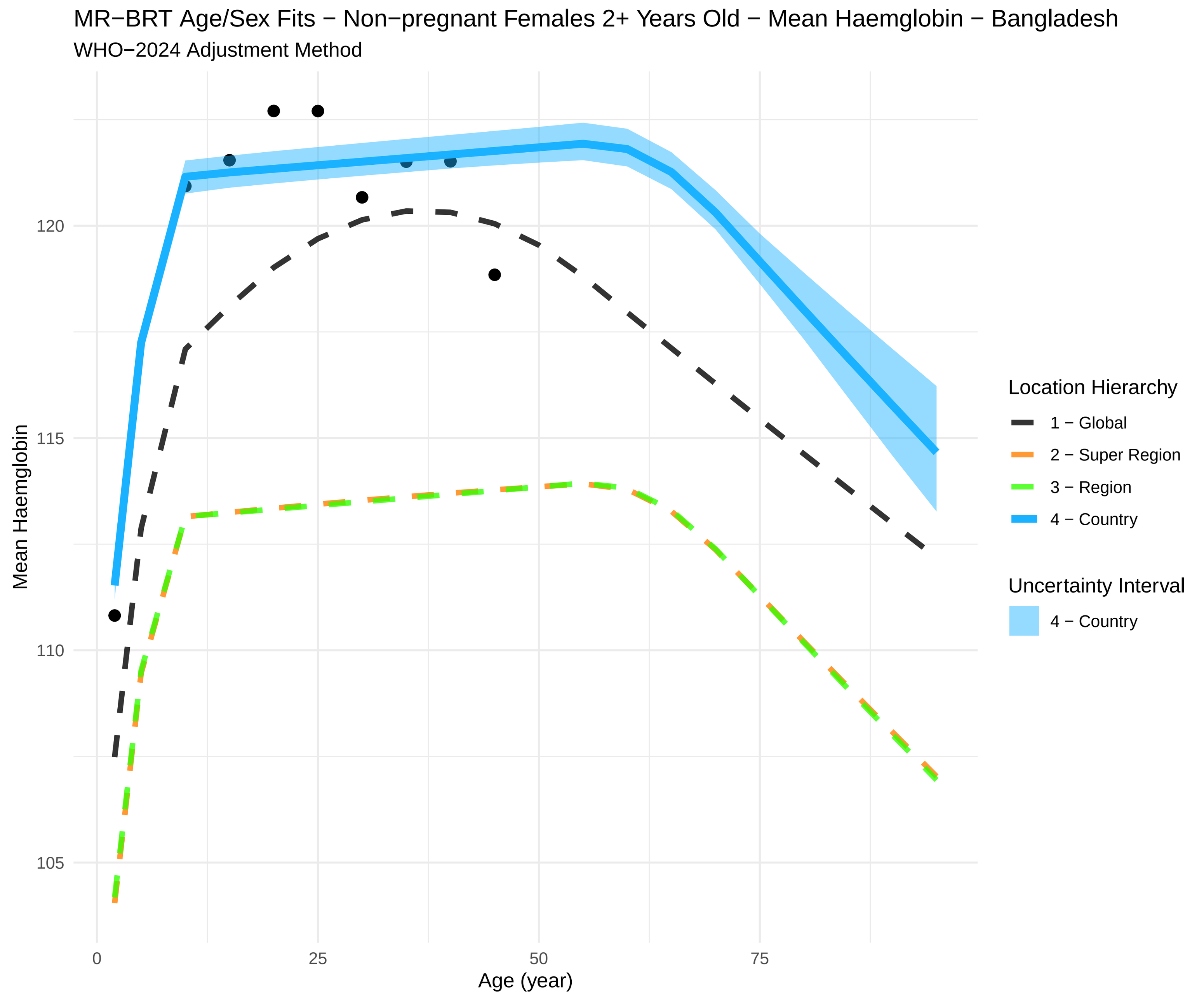
MR−BRT Age/Sex Fits − Non−pregnant Females 2+ Years Old − Mean Haemglobin − Bangladesh
WHO−2024 Adjustment Method
Mean Haemglobin
Age (year)
Location Hierarchy
1 − Global
2 − Super Region
3 − Region
4 − Country
Uncertainty Interval
4 − Country

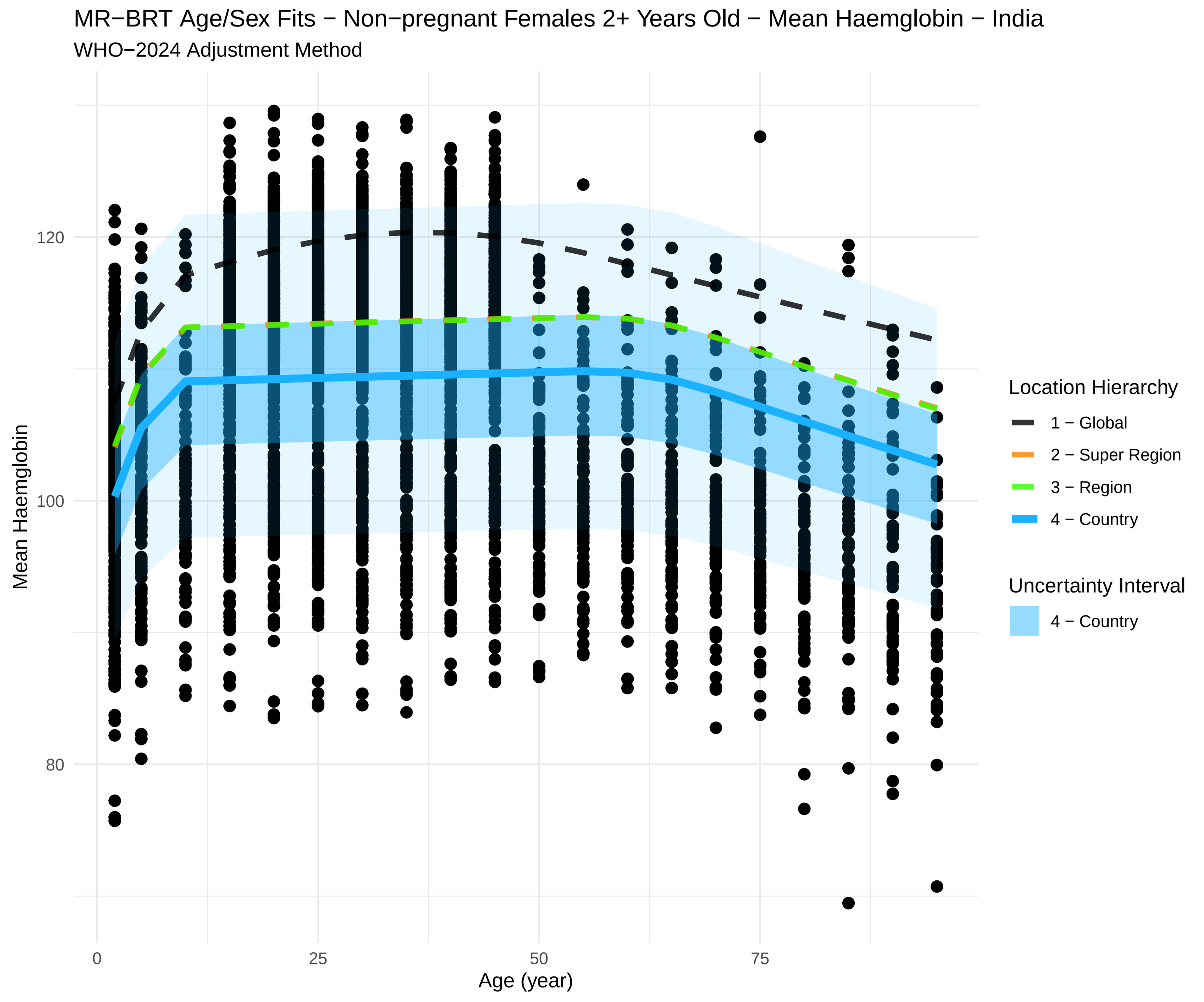
MR−BRT Age/Sex Fits − Non−pregnant Females 2+ Years Old − Mean Haemglobin − India
WHO−2024 Adjustment Method
Mean Haemglobin
Age (year)
Location Hierarchy
1 − Global
2 − Super Region
3 − Region
4 − Country
Uncertainty Interval
4 − Country

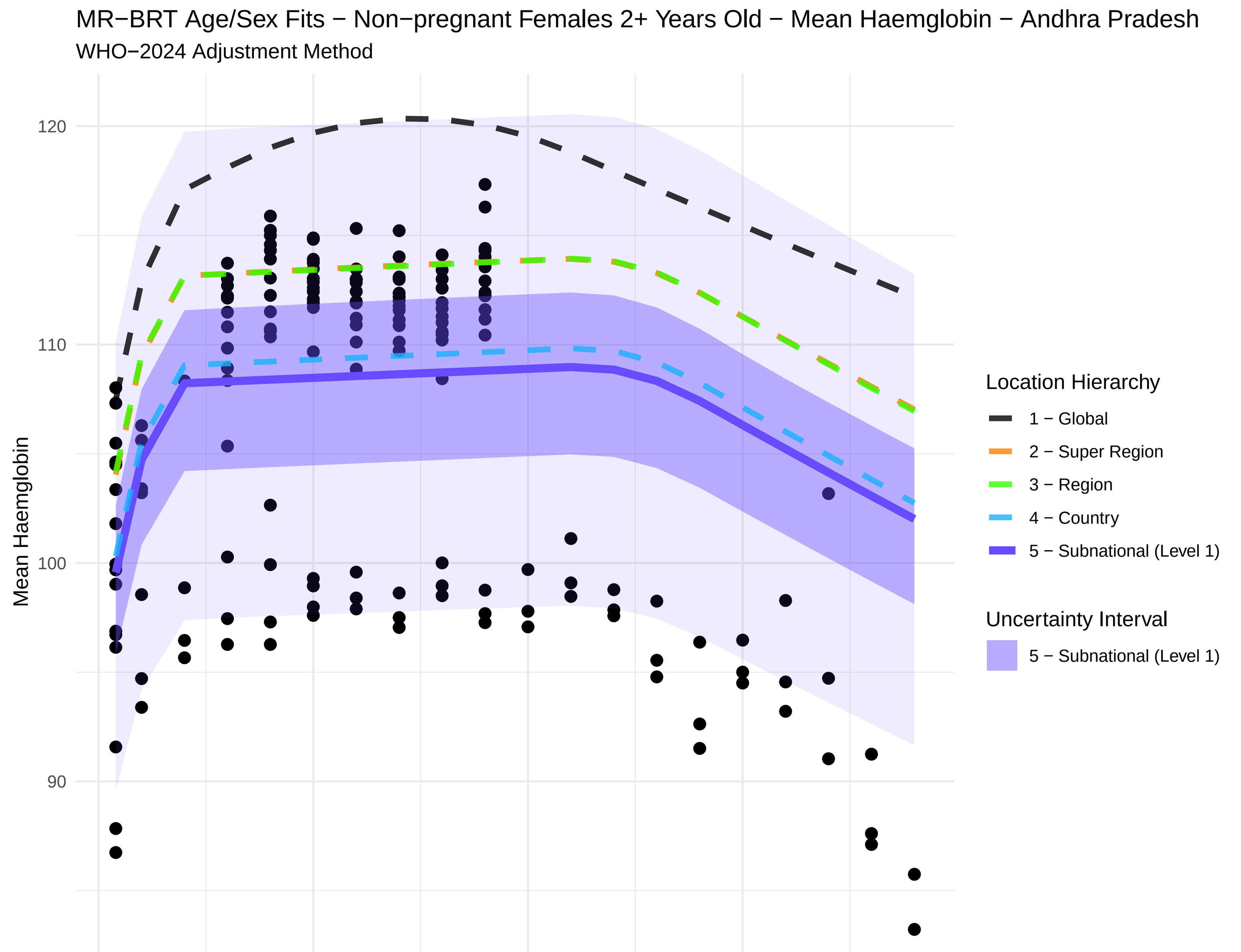
MR−BRT Age/Sex Fits − Non−pregnant Females 2+ Years Old − Mean Haemglobin − Andhra Pradesh
WHO−2024 Adjustment Method
Mean Haemglobin
Age (year)
Location Hierarchy
1 − Global
2 − Super Region
3 − Region
4 − Country
5 − Subnational (Level 1)
Uncertainty Interval
5 − Subnational (Level 1)

MR−BRT Age/Sex Fits − Non−pregnant Females 2+ Years Old − Mean Haemglobin − Andhra Pradesh, Rural
WHO−2024 Adjustment Method

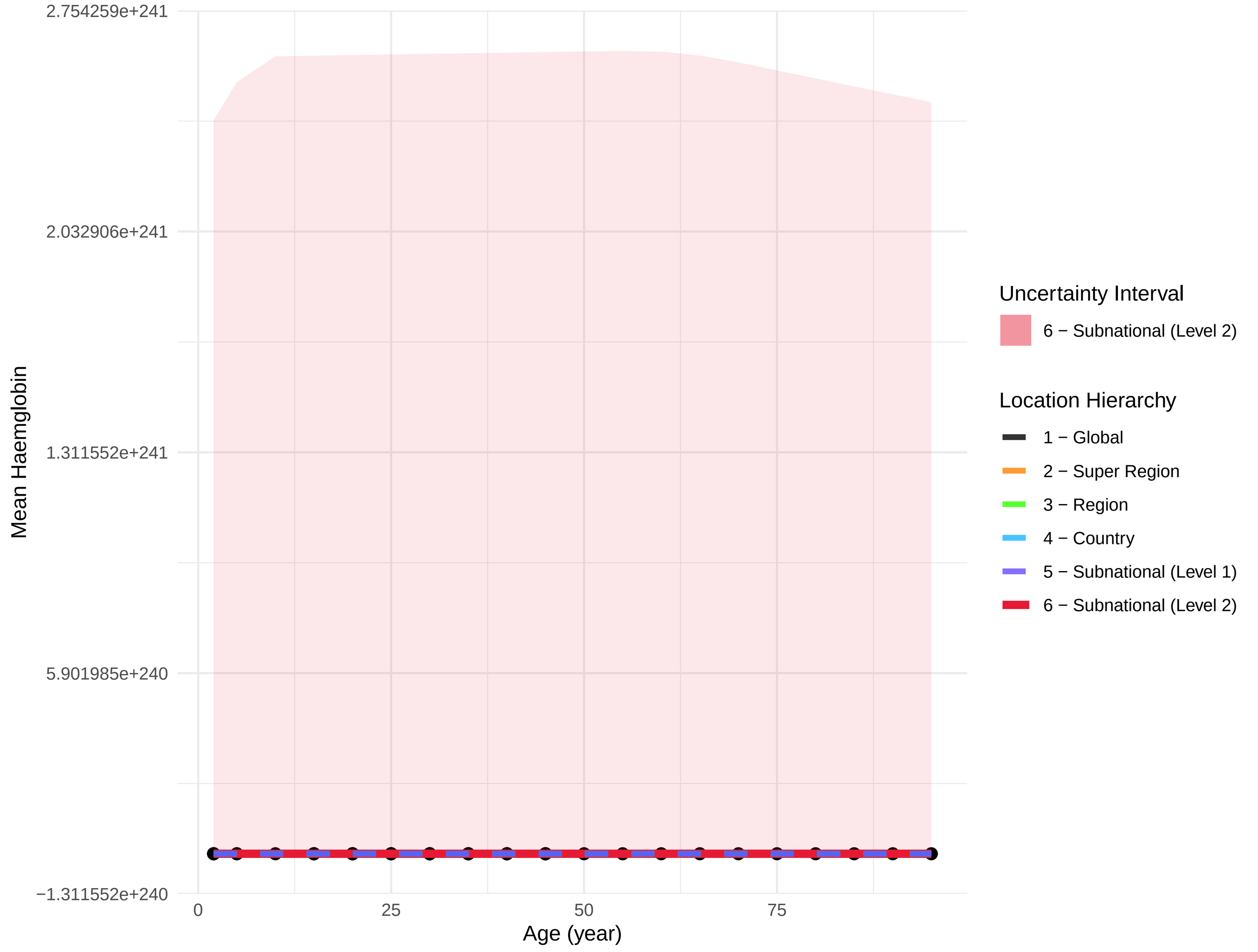

MR−BRT Age/Sex Fits − Non−pregnant Females 2+ Years Old − Mean Haemglobin − Andhra Pradesh, Urban
WHO−2024 Adjustment Method

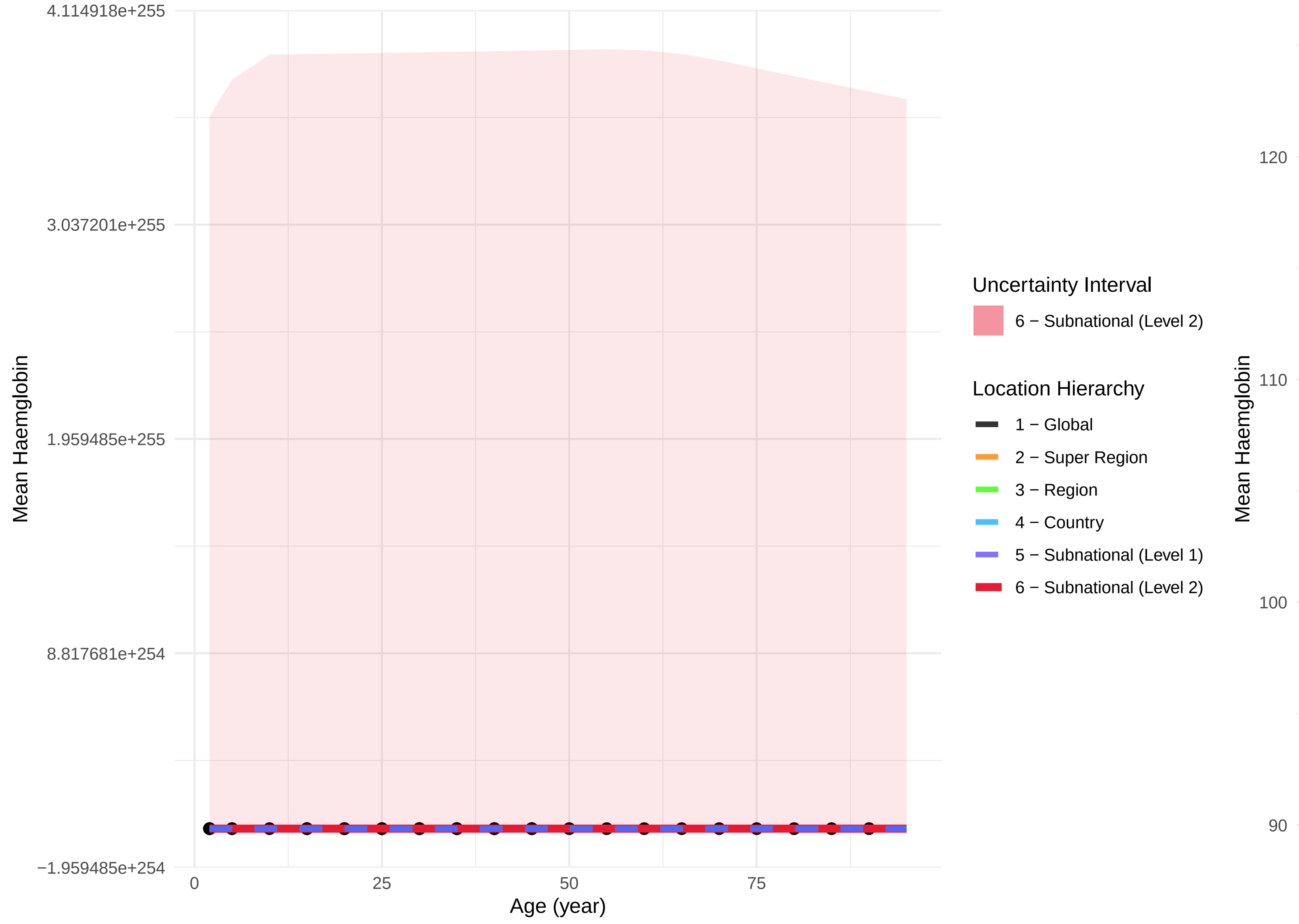

MR−BRT Age/Sex Fits − Non−pregnant Females 2+ Years Old − Mean Haemglobin − Arunachal Pradesh
WHO−2024 Adjustment Method

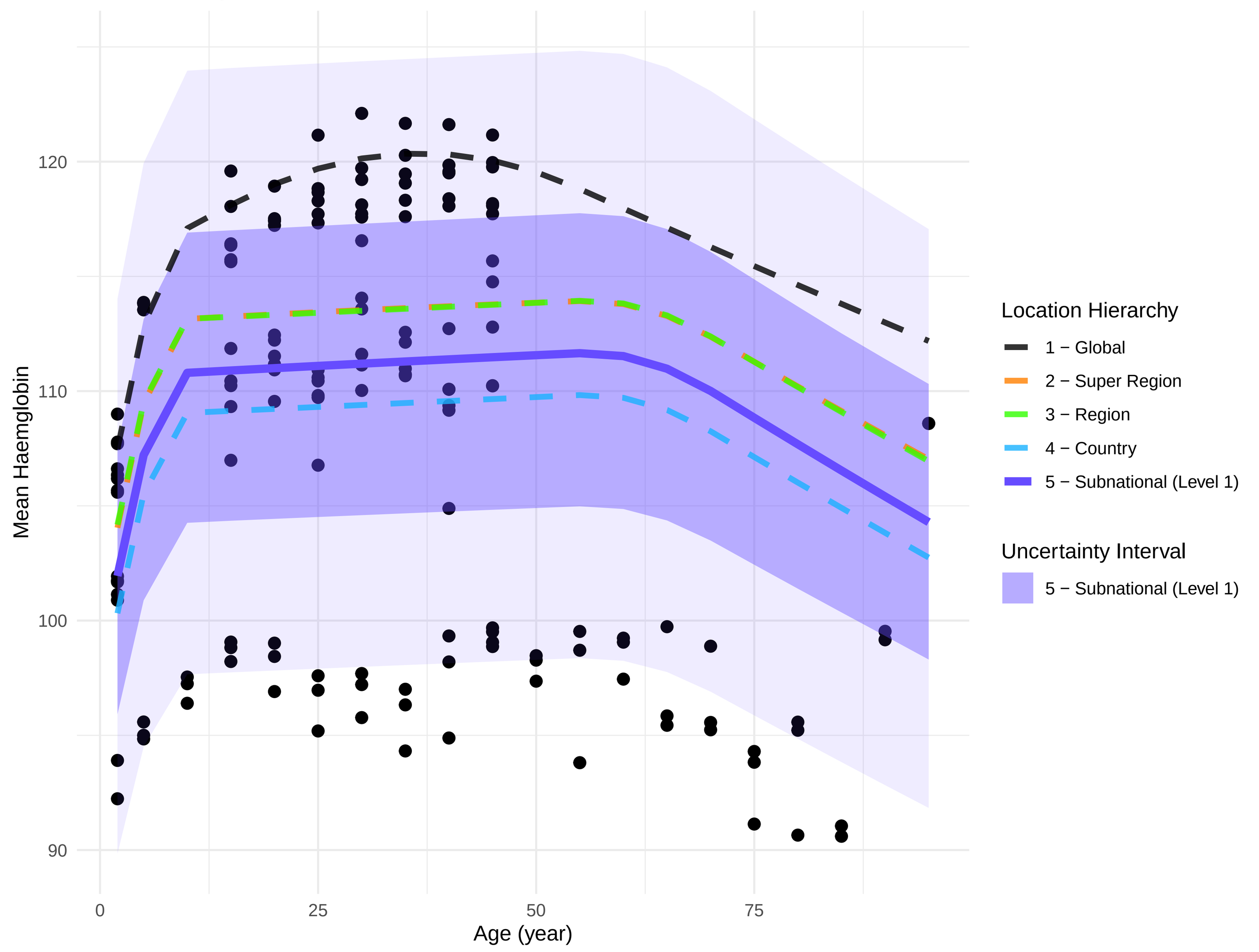

MR−BRT Age/Sex Fits − Non−pregnant Females 2+ Years Old − Mean Haemglobin − Arunachal Pradesh, Rural
WHO−2024 Adjustment Method

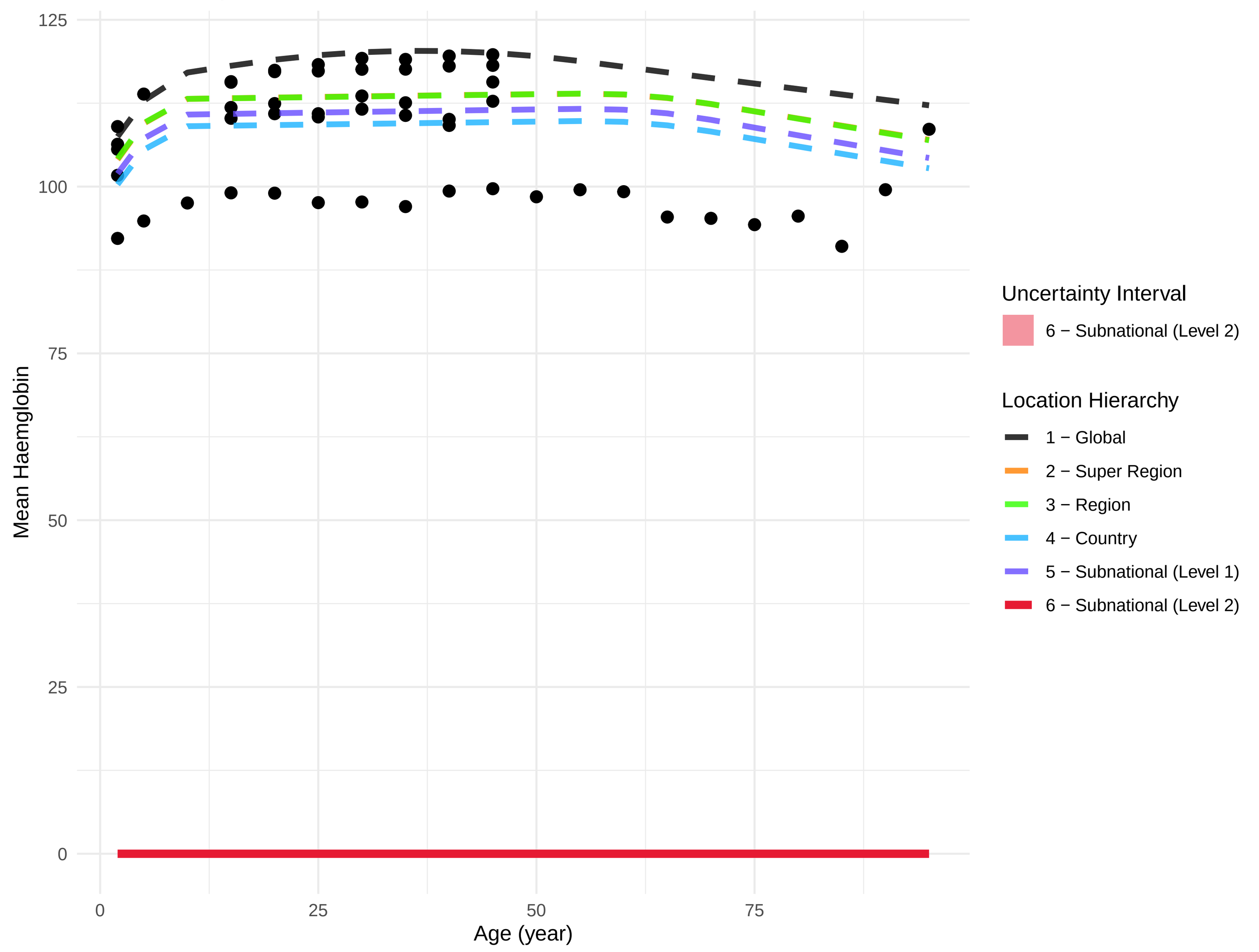

MR−BRT Age/Sex Fits − Non−pregnant Females 2+ Years Old − Mean Haemglobin − Arunachal Pradesh, Urban
WHO−2024 Adjustment Method

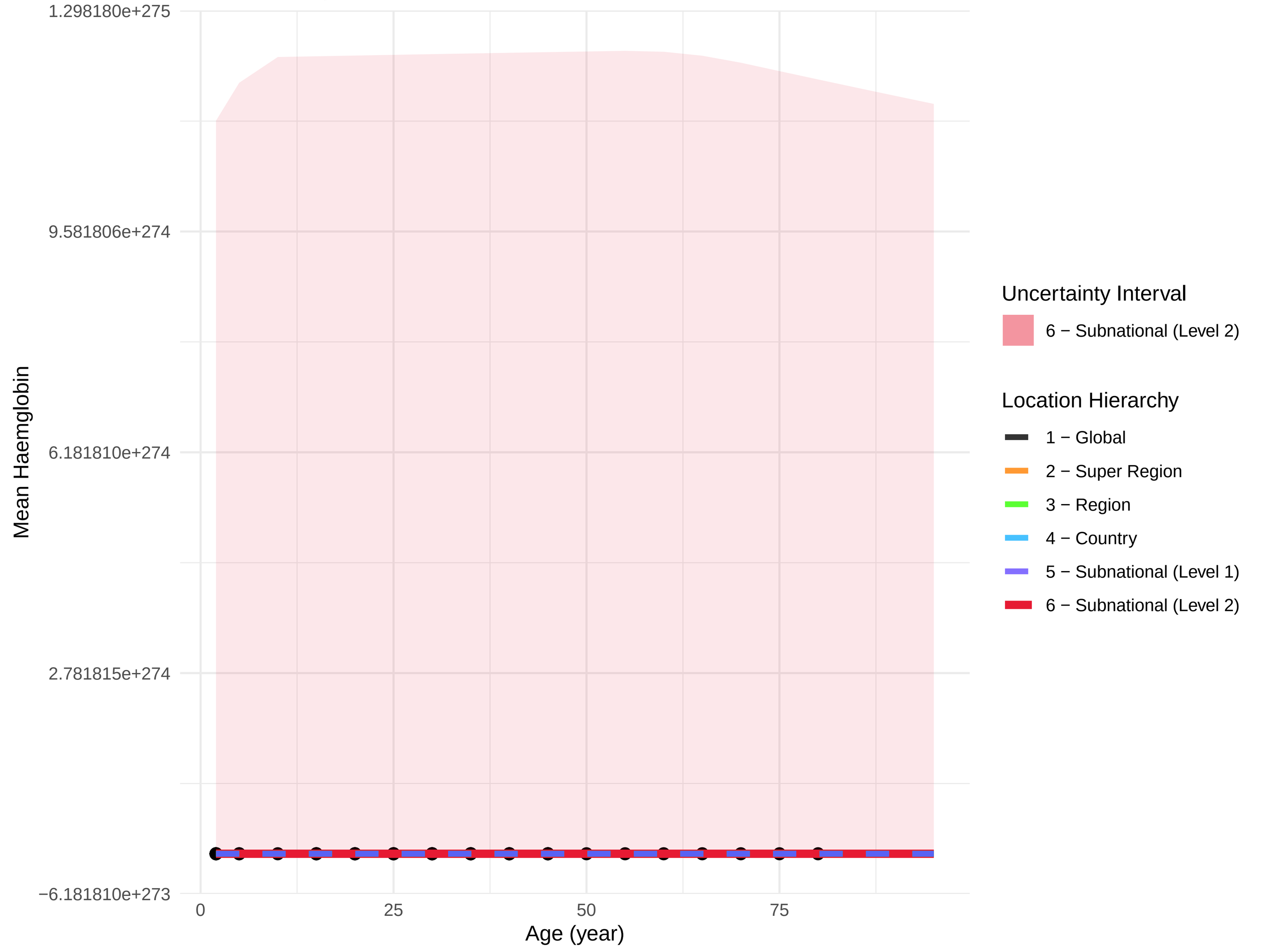

MR−BRT Age/Sex Fits − Non−pregnant Females 2+ Years Old − Mean Haemglobin − Assam
WHO−2024 Adjustment Method

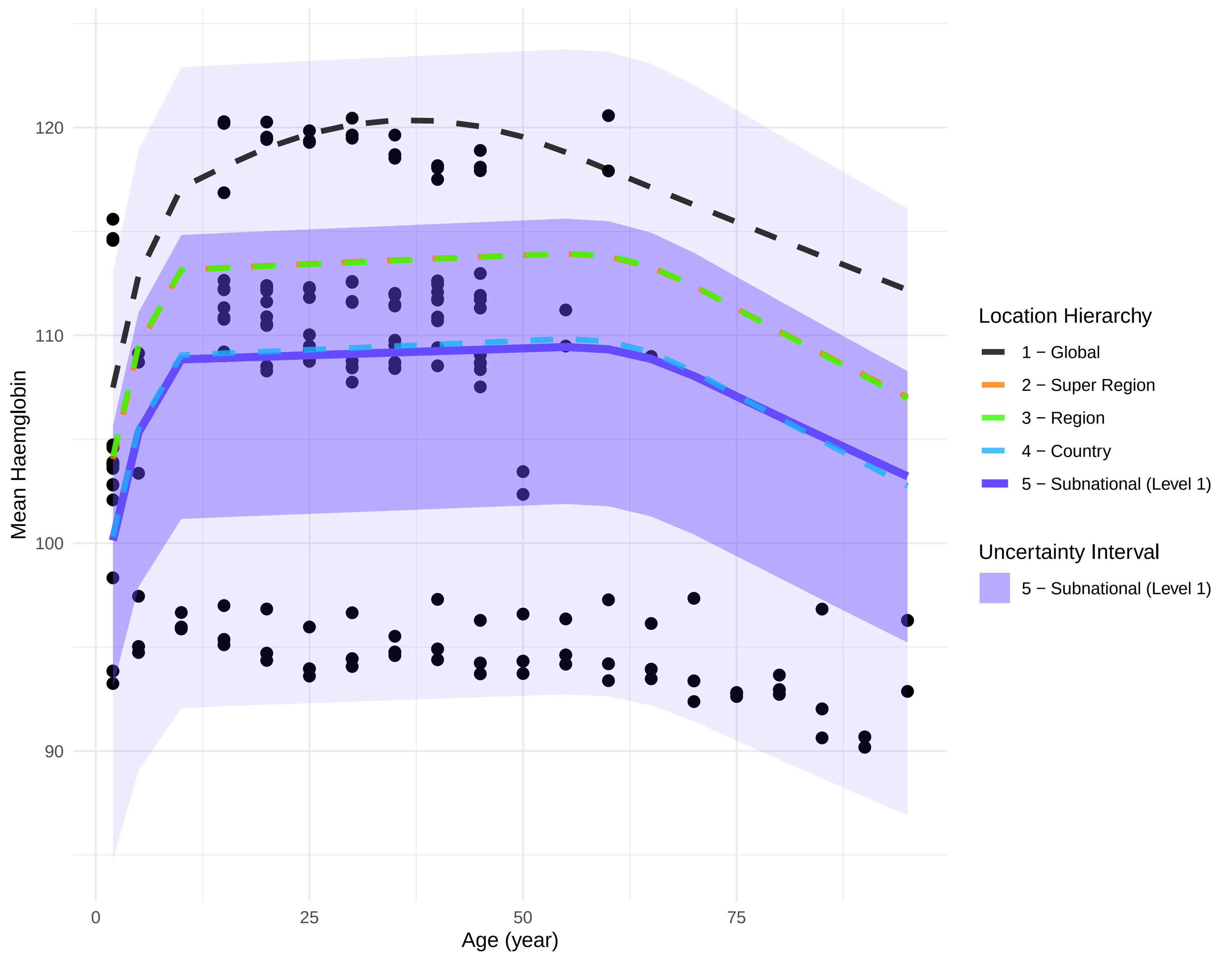

MR−BRT Age/Sex Fits − Non−pregnant Females 2+ Years Old − Mean Haemglobin − Assam, Rural
WHO−2024 Adjustment Method

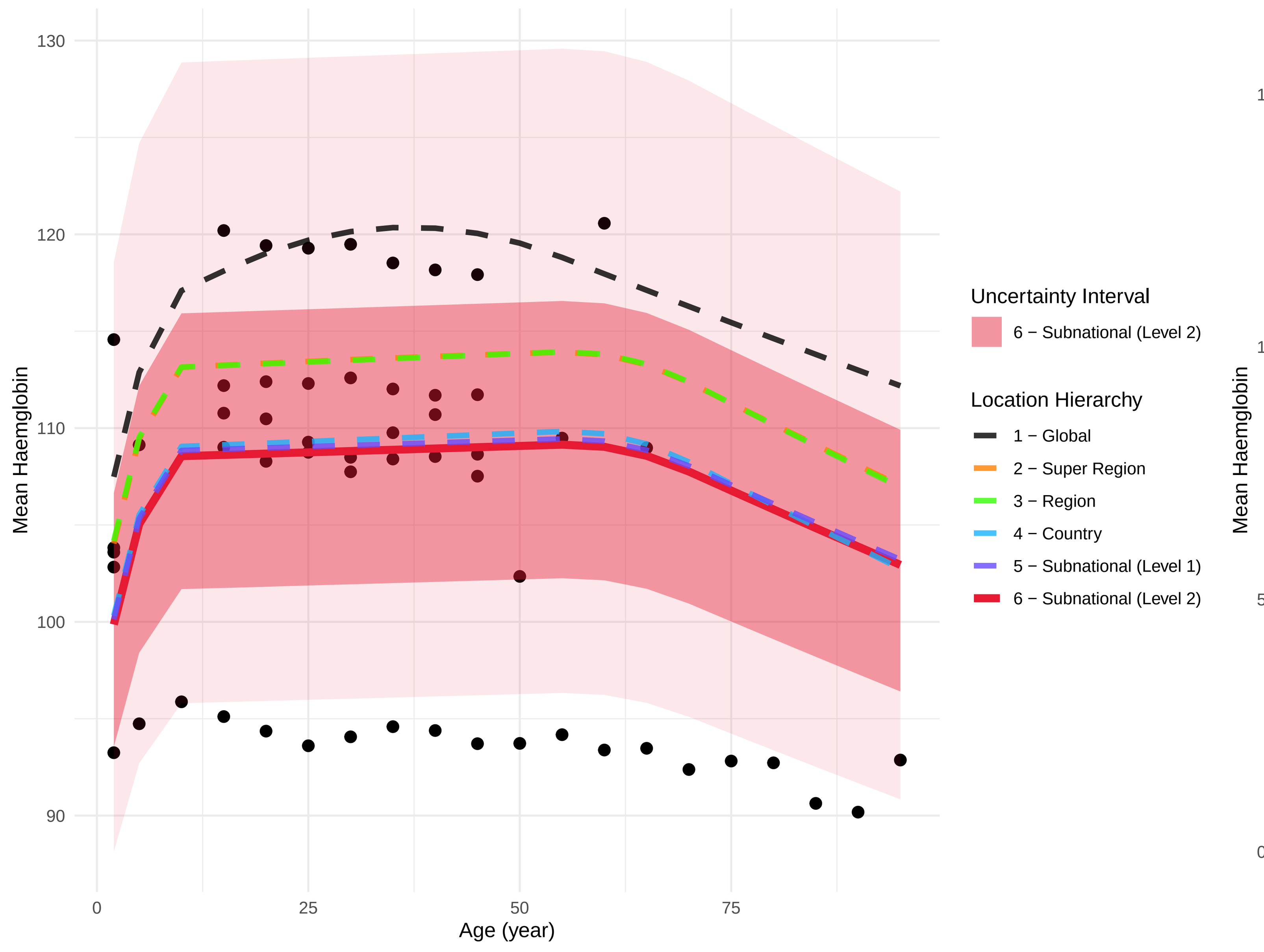

MR−BRT Age/Sex Fits − Non−pregnant Females 2+ Years Old − Mean Haemglobin − Assam, Urban
WHO−2024 Adjustment Method

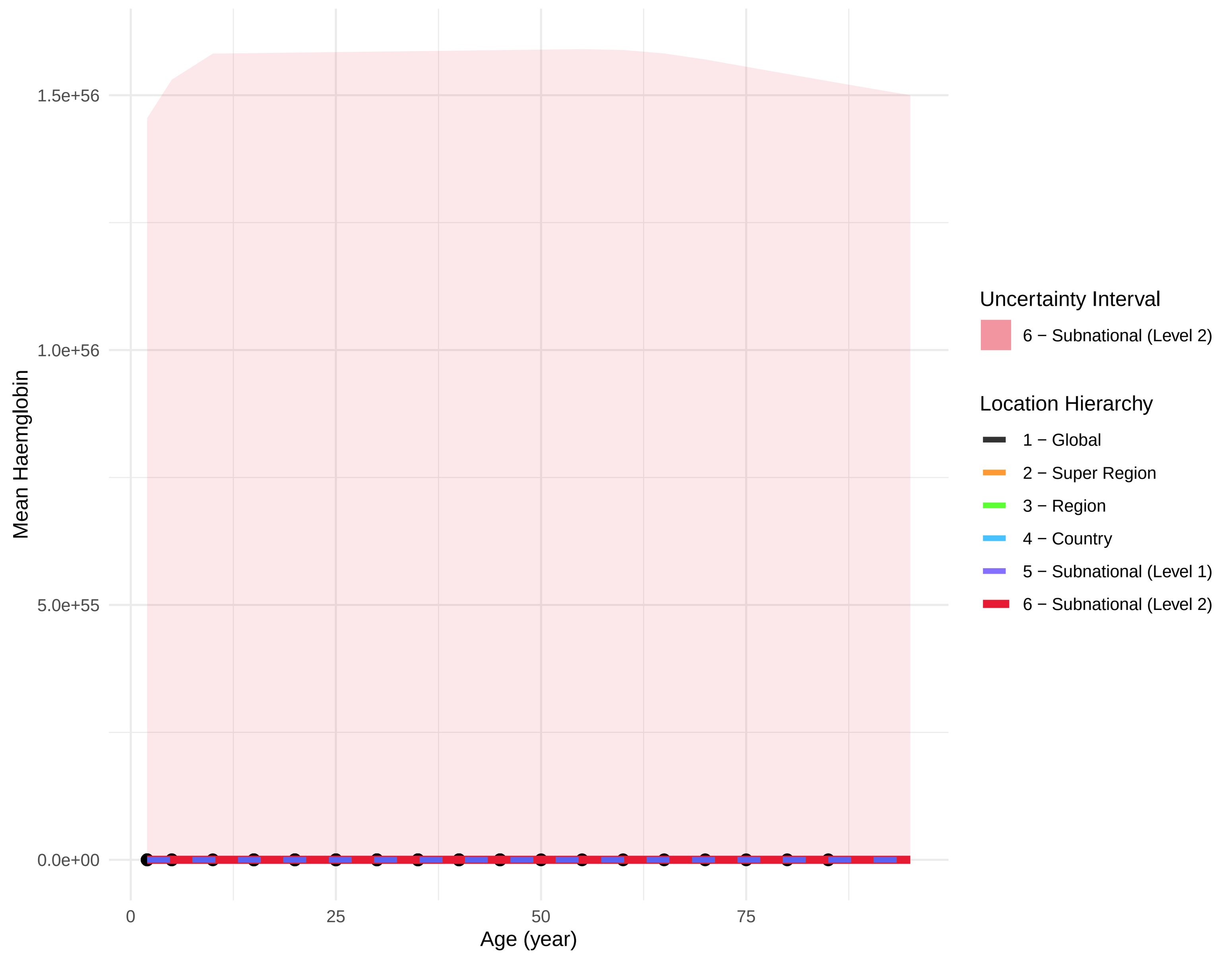

MR−BRT Age/Sex Fits − Non−pregnant Females 2+ Years Old − Mean Haemglobin − Bihar
WHO−2024 Adjustment Method

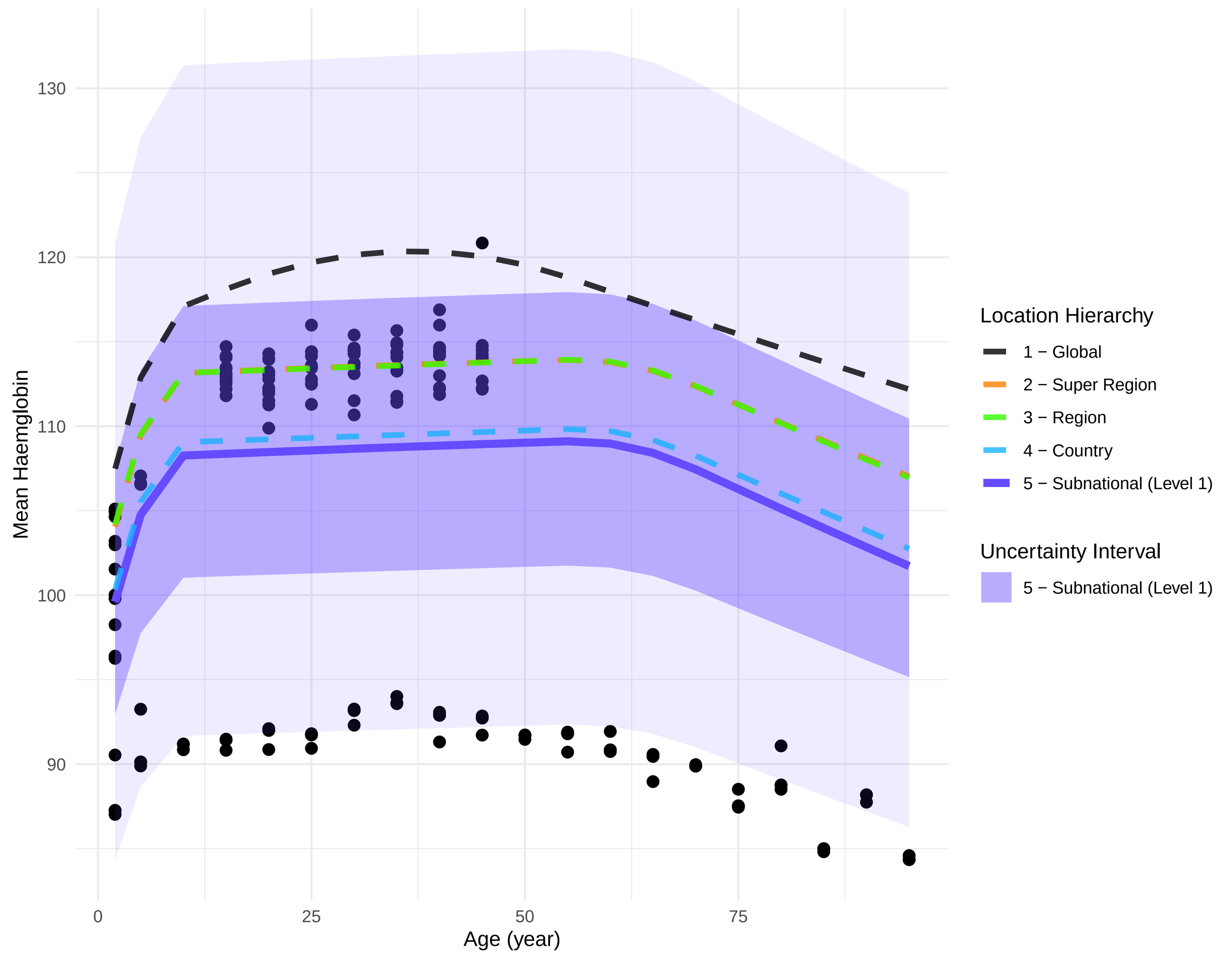

MR−BRT Age/Sex Fits − Non−pregnant Females 2+ Years Old − Mean Haemglobin − Bihar, Rural
WHO−2024 Adjustment Method

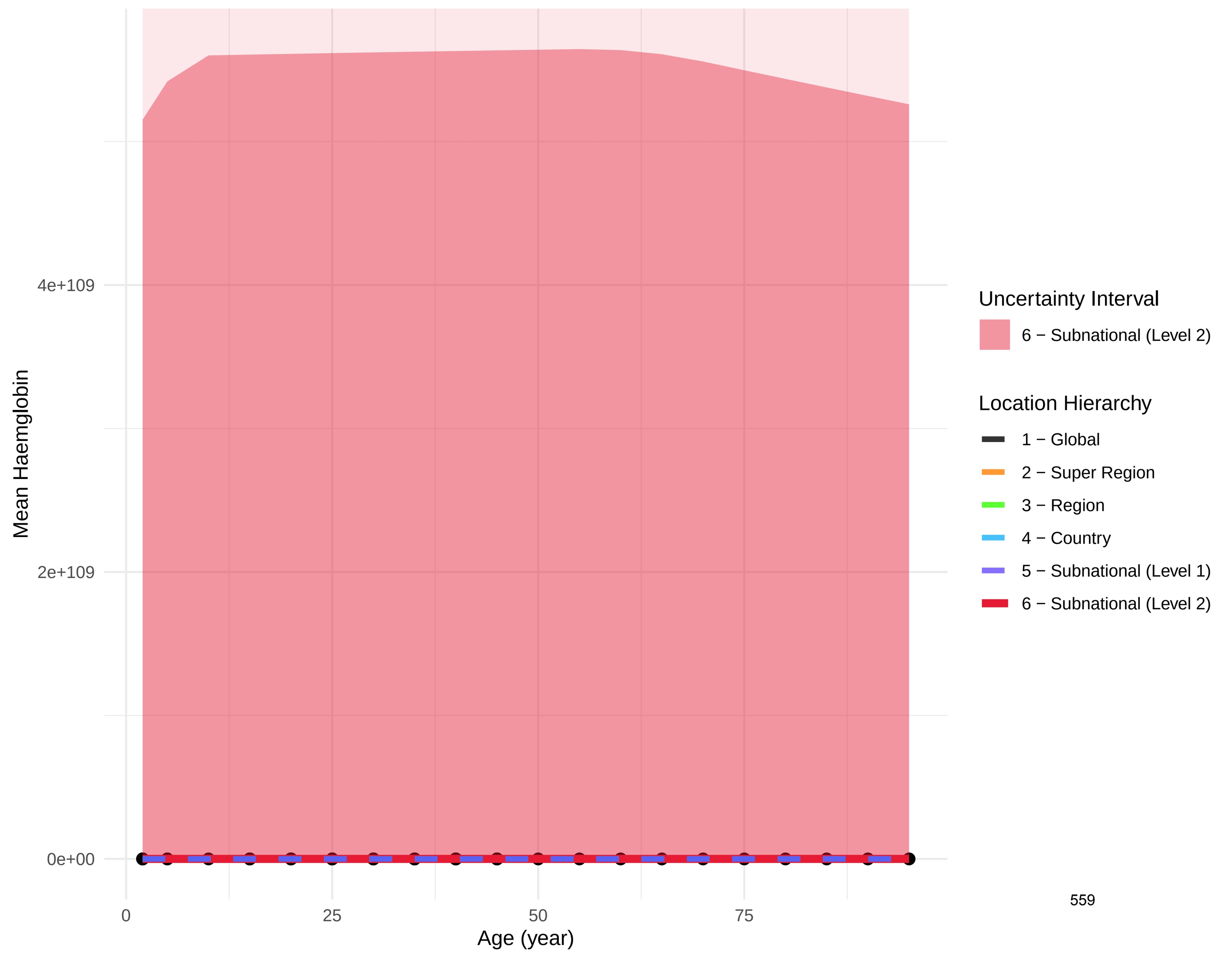

MR−BRT Age/Sex Fits − Non−pregnant Females 2+ Years Old − Mean Haemoglobin − Bihar, Urban
WHO−2024 Adjustment Method

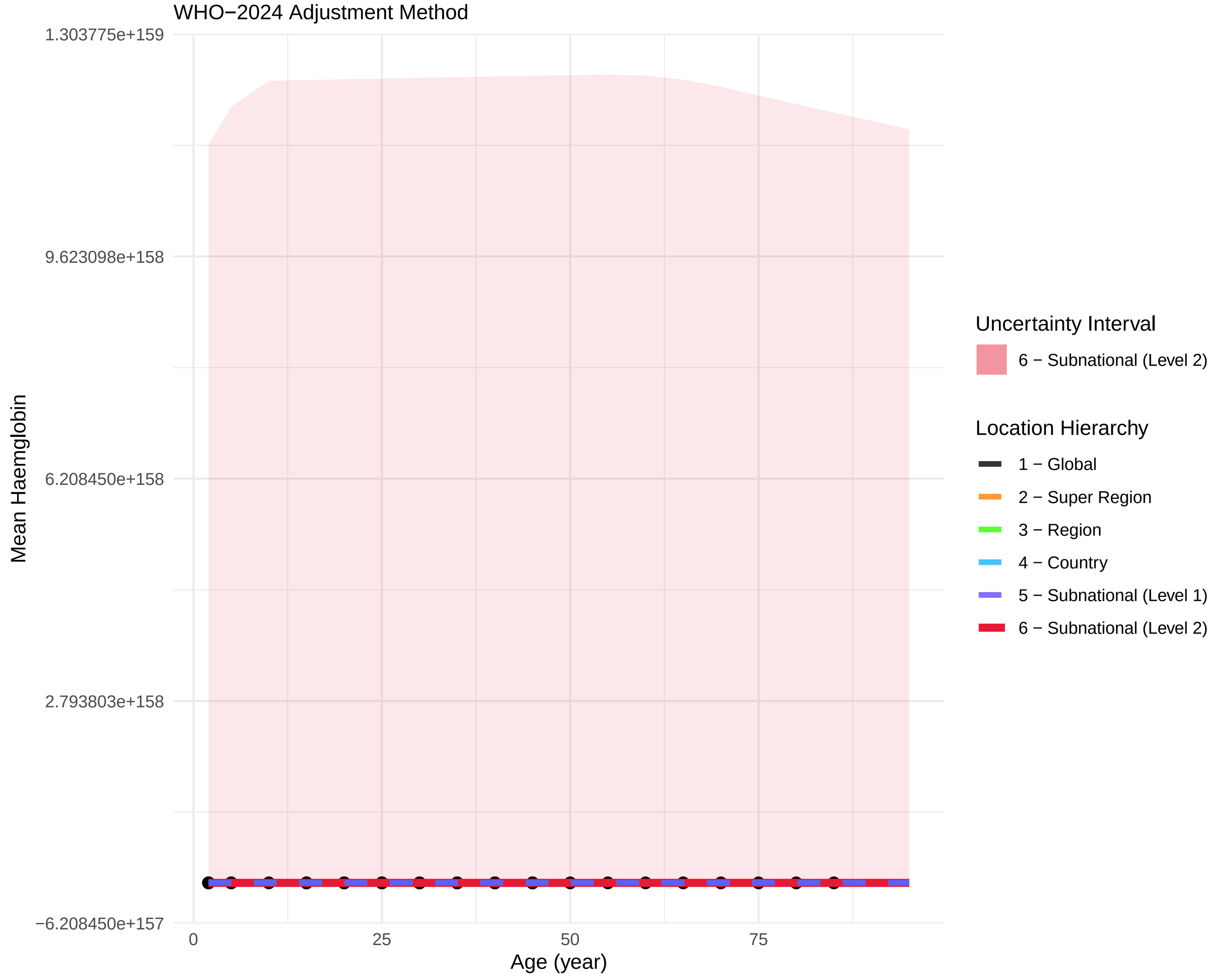

MR−BRT Age/Sex Fits − Non−pregnant Females 2+ Years Old − Mean Haemoglobin − Chhattisgarh
WHO−2024 Adjustment Method

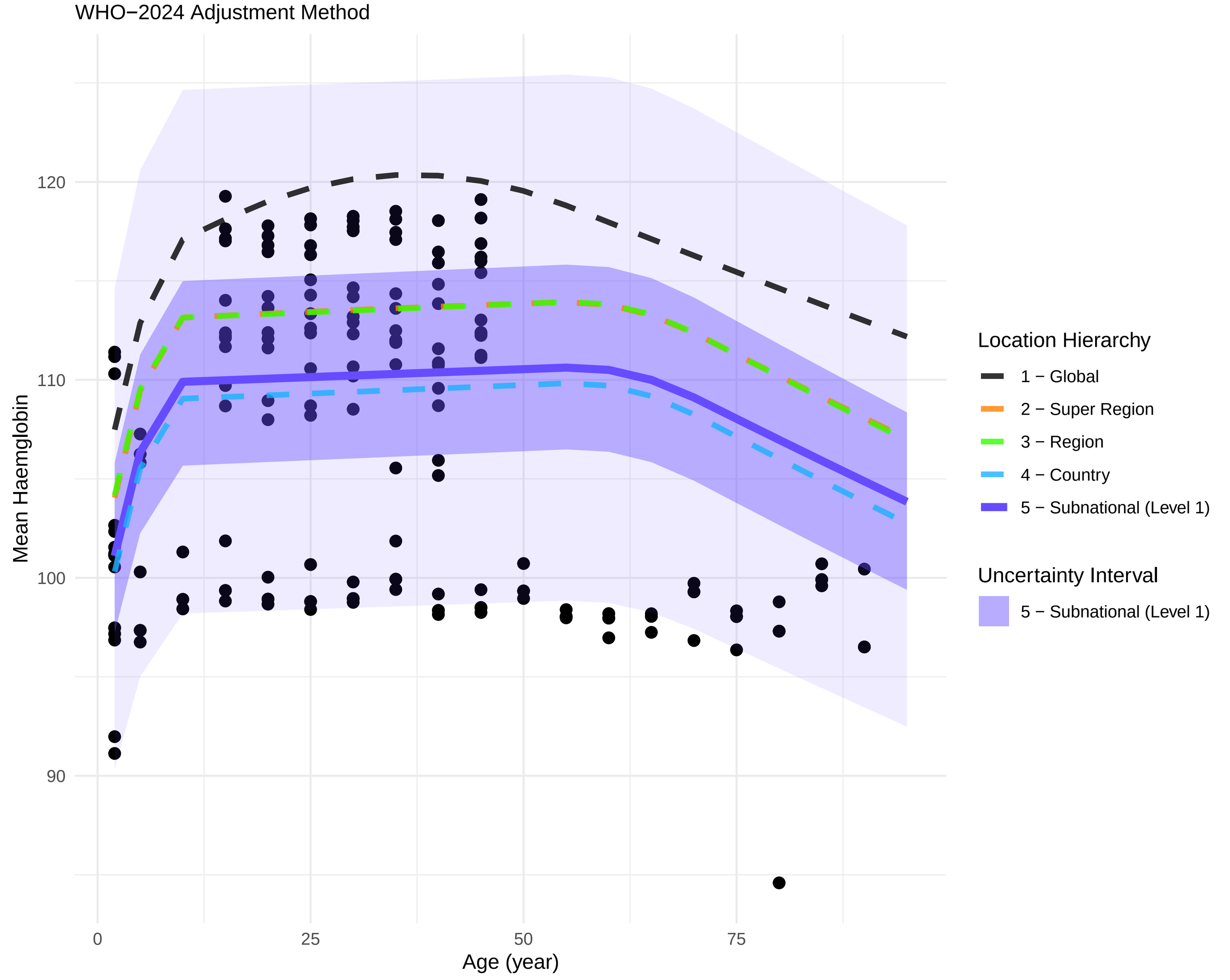

MR−BRT Age/Sex Fits − Non−pregnant Females 2+ Years Old − Mean Haemoglobin − Chhattisgarh, Rural
WHO−2024 Adjustment Method

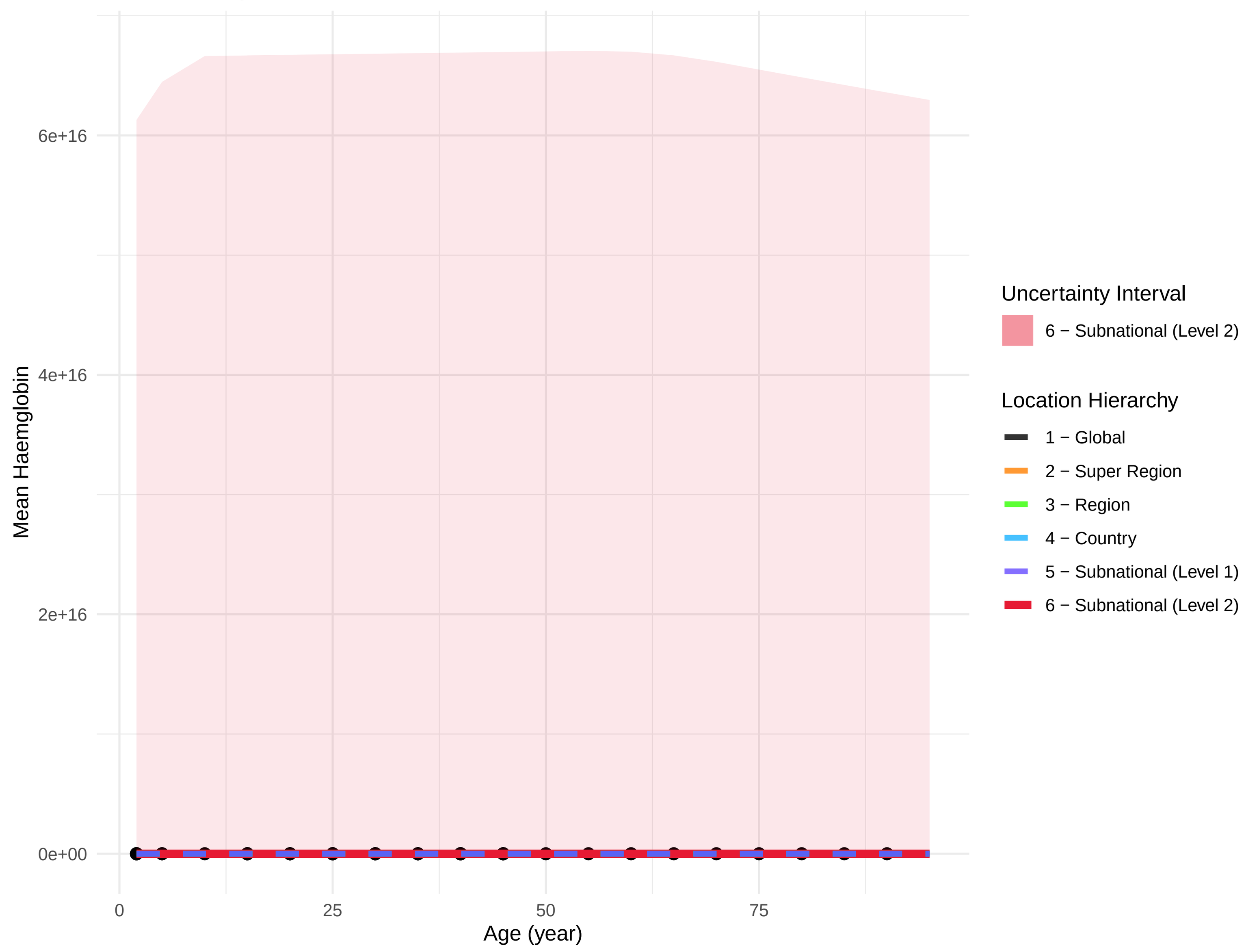

MR−BRT Age/Sex Fits − Non−pregnant Females 2+ Years Old − Mean Haemoglobin − Chhattisgarh, Urban
WHO−2024 Adjustment Method

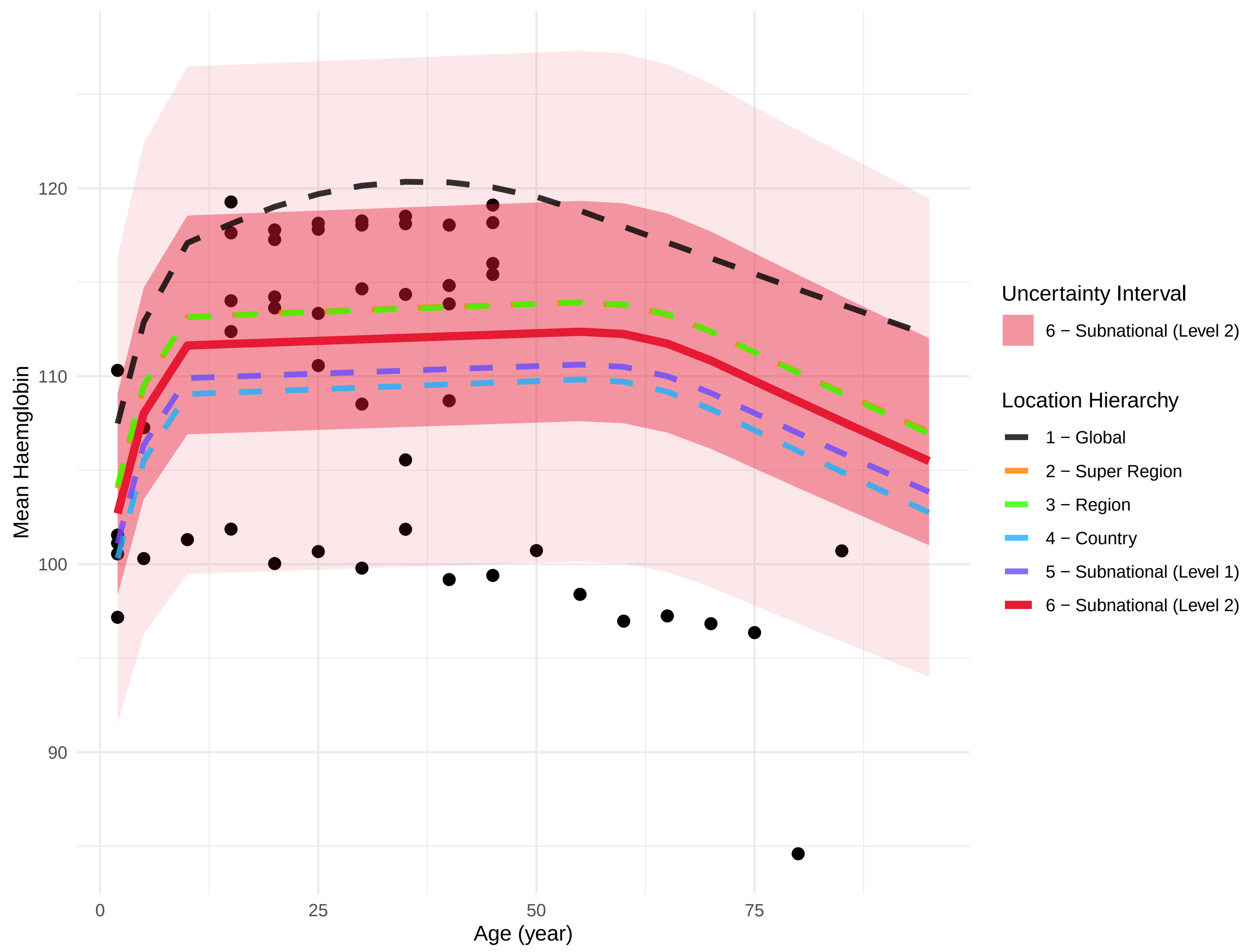

MR−BRT Age/Sex Fits − Non−pregnant Females 2+ Years Old − Mean Haemoglobin − Delhi
WHO−2024 Adjustment Method

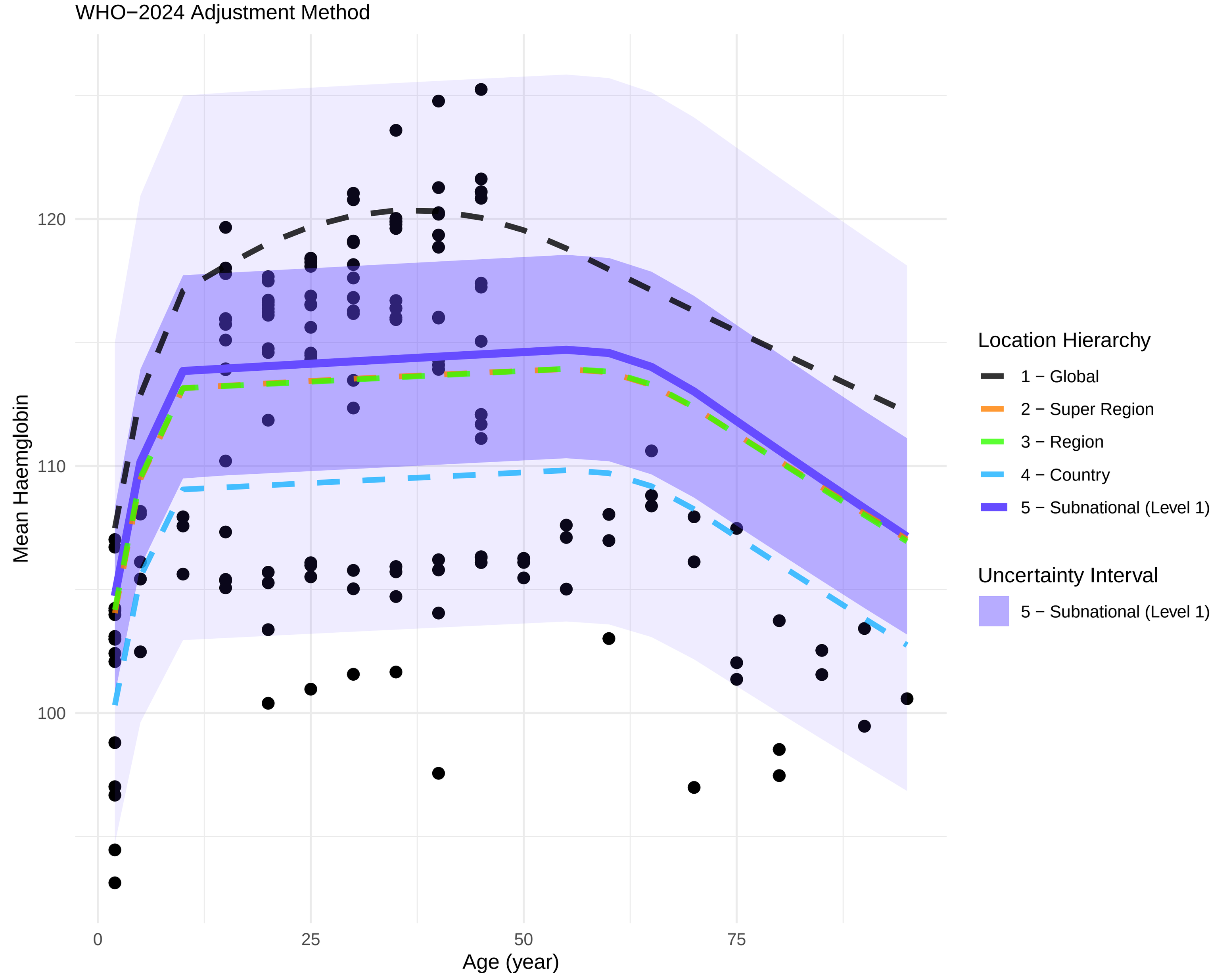

MR−BRT Age/Sex Fits − Non−pregnant Females 2+ Years Old − Mean Haemoglobin − Delhi, Rural
WHO−2024 Adjustment Method

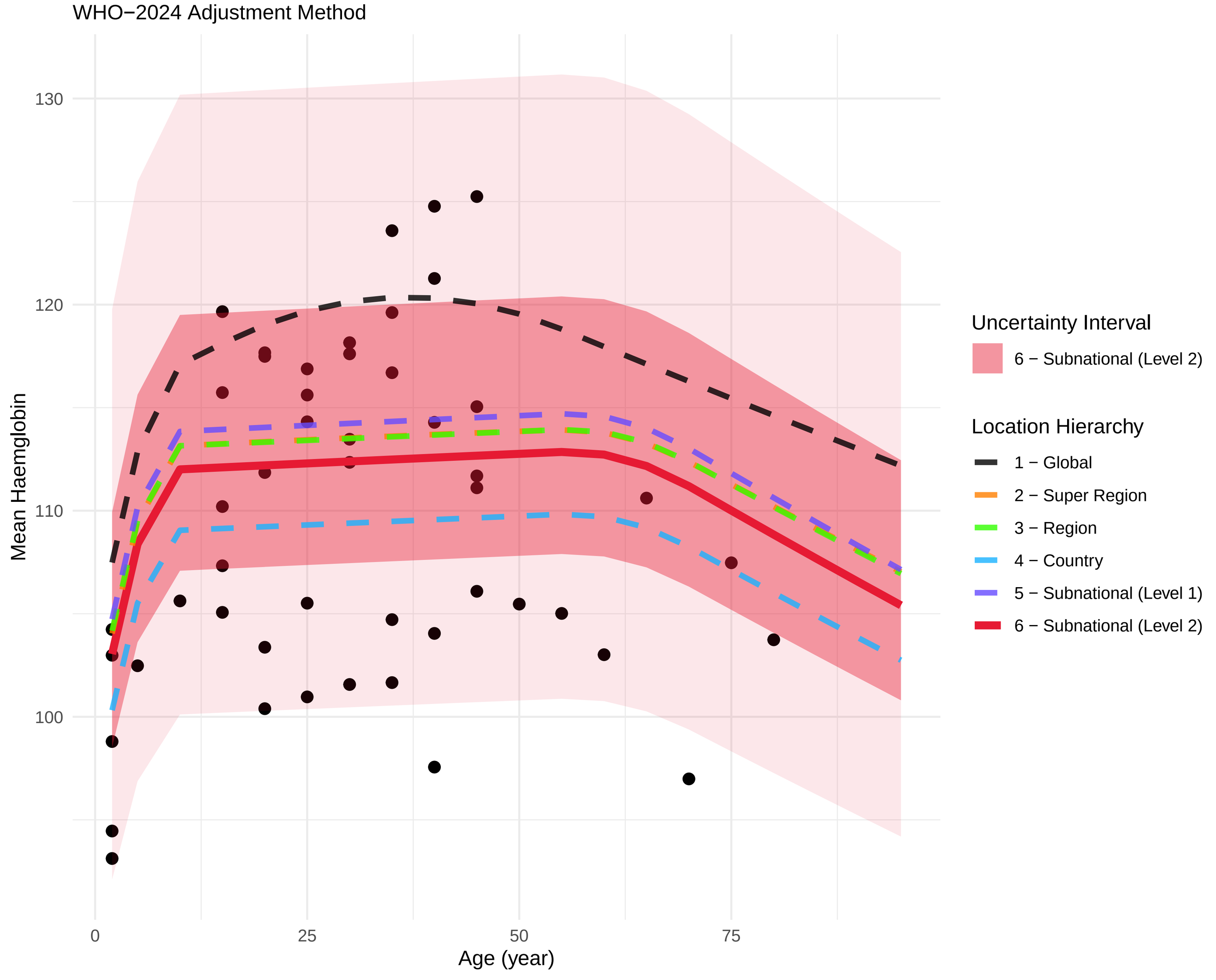

MR−BRT Age/Sex Fits − Non−pregnant Females 2+ Years Old − Mean Haemoglobin − Delhi, Urban
WHO−2024 Adjustment Method

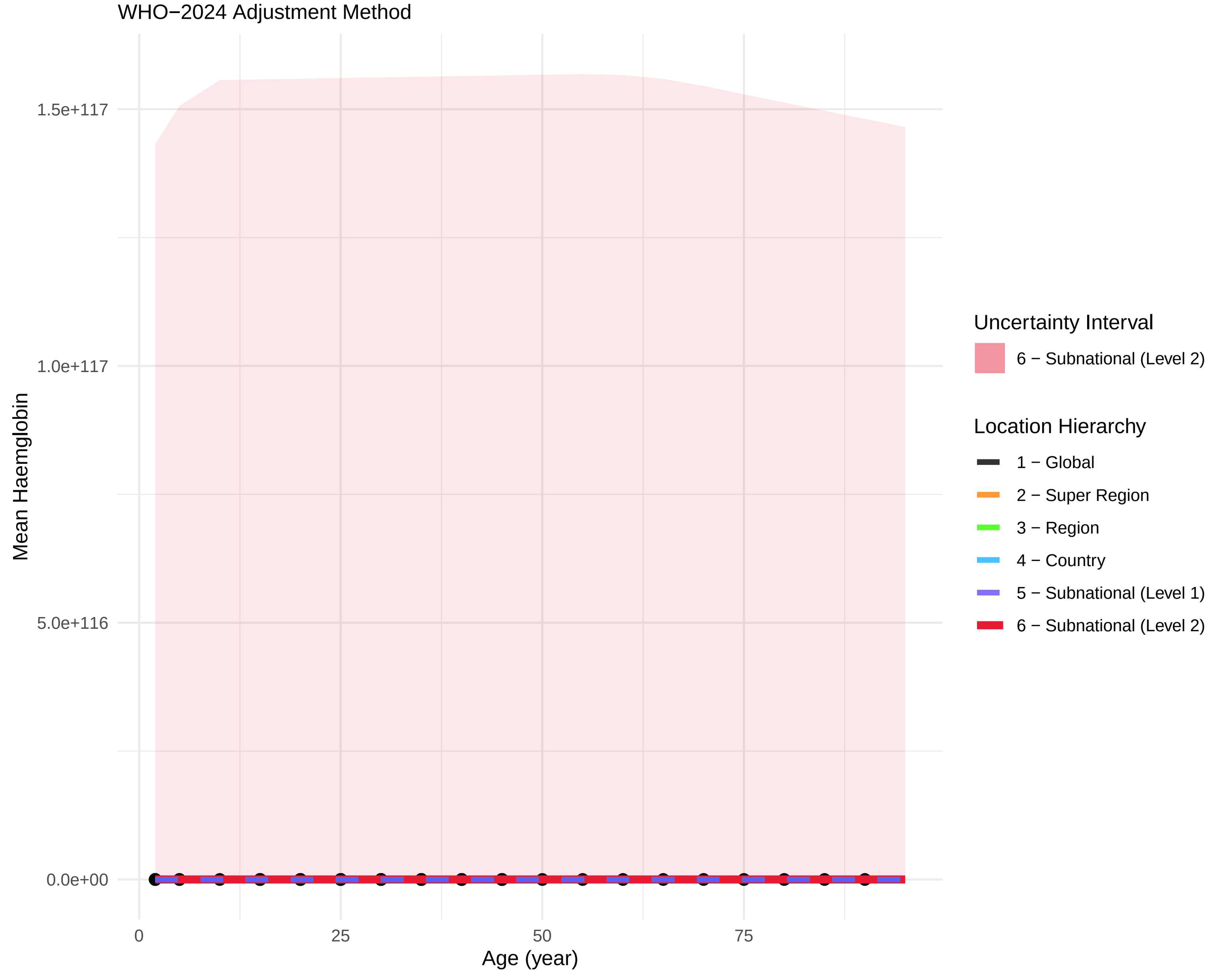

MR−BRT Age/Sex Fits − Non−pregnant Females 2+ Years Old − Mean Haemoglobin − Goa
WHO−2024 Adjustment Method

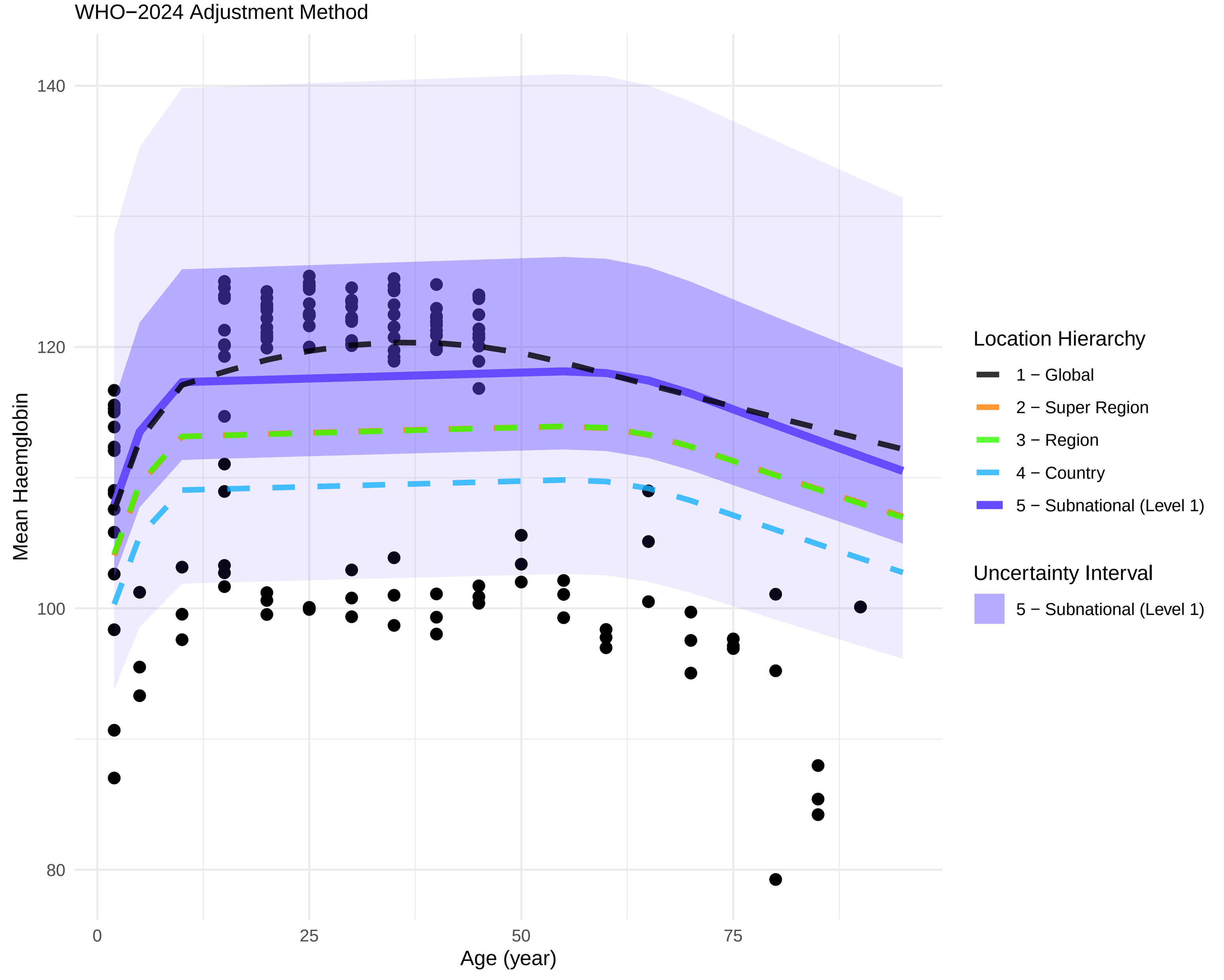

MR−BRT Age/Sex Fits − Non−pregnant Females 2+ Years Old − Mean Haemoglobin − Goa, Rural
WHO−2024 Adjustment Method

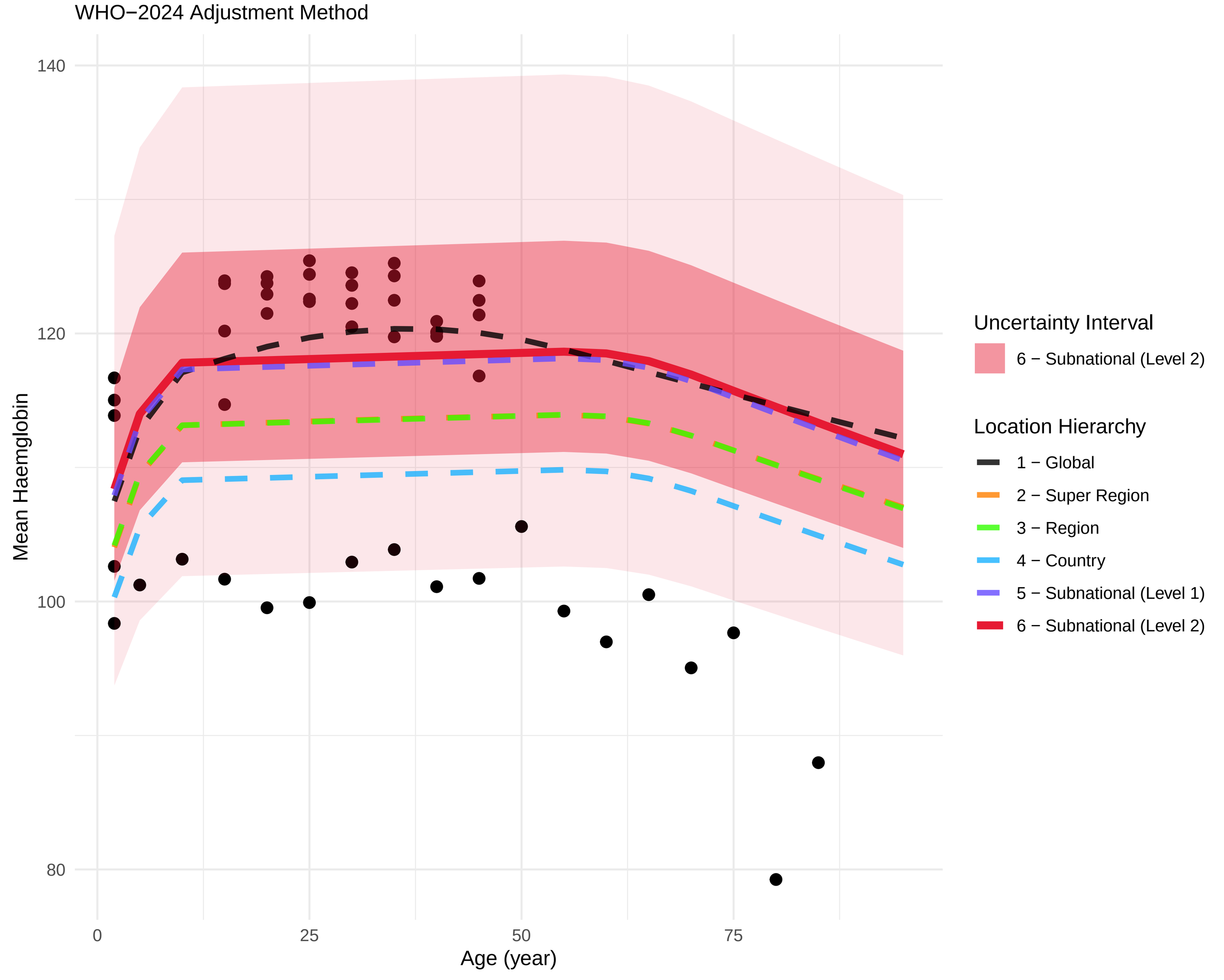

MR−BRT Age/Sex Fits − Non−pregnant Females 2+ Years Old − Mean Haemoglobin − Goa, Urban
WHO−2024 Adjustment Method

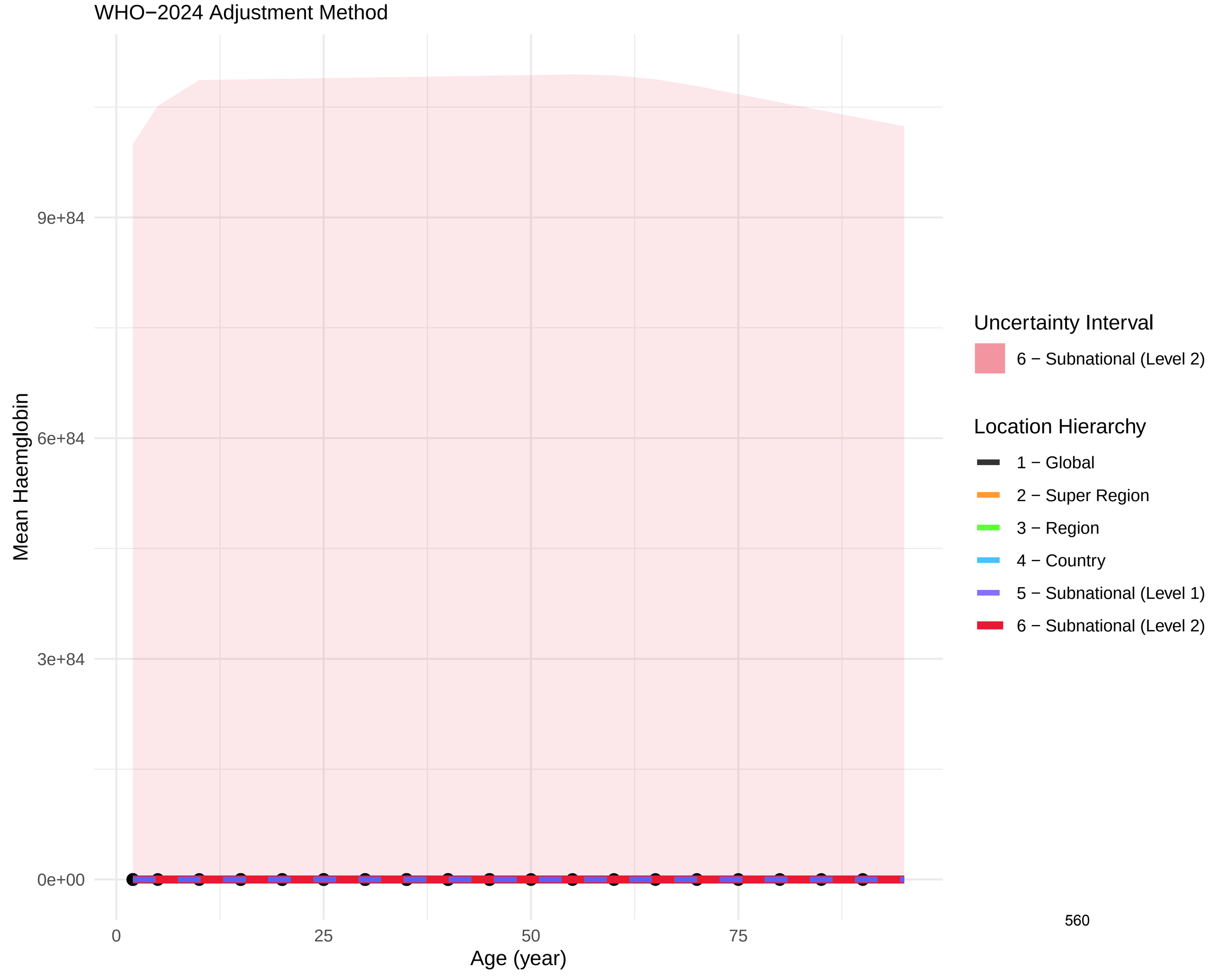

MR−BRT Age/Sex Fits − Non−pregnant Females 2+ Years Old − Mean Haemglobin − Gujarat
WHO−2024 Adjustment Method

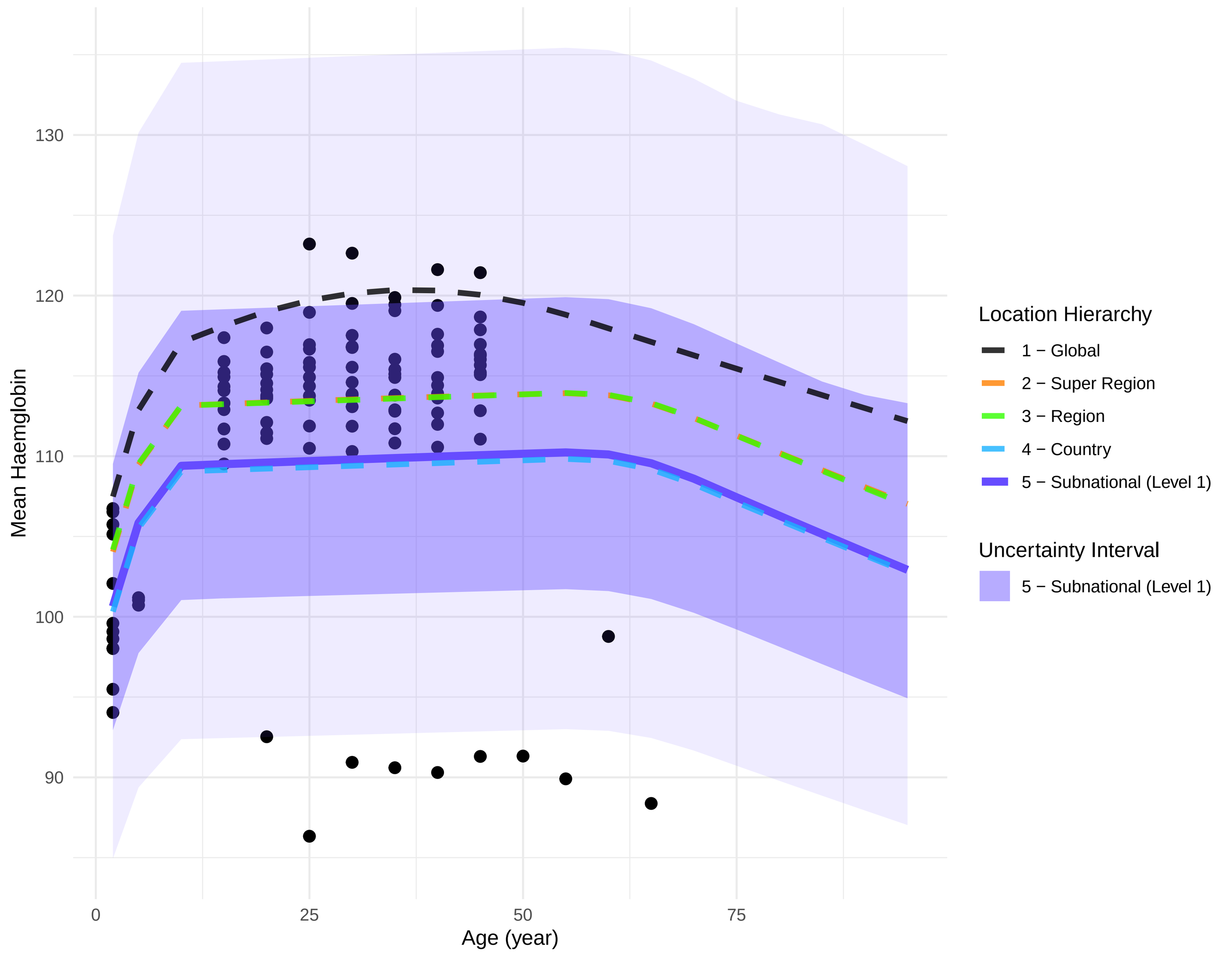

MR−BRT Age/Sex Fits − Non−pregnant Females 2+ Years Old − Mean Haemglobin − Gujarat, Rural
WHO−2024 Adjustment Method

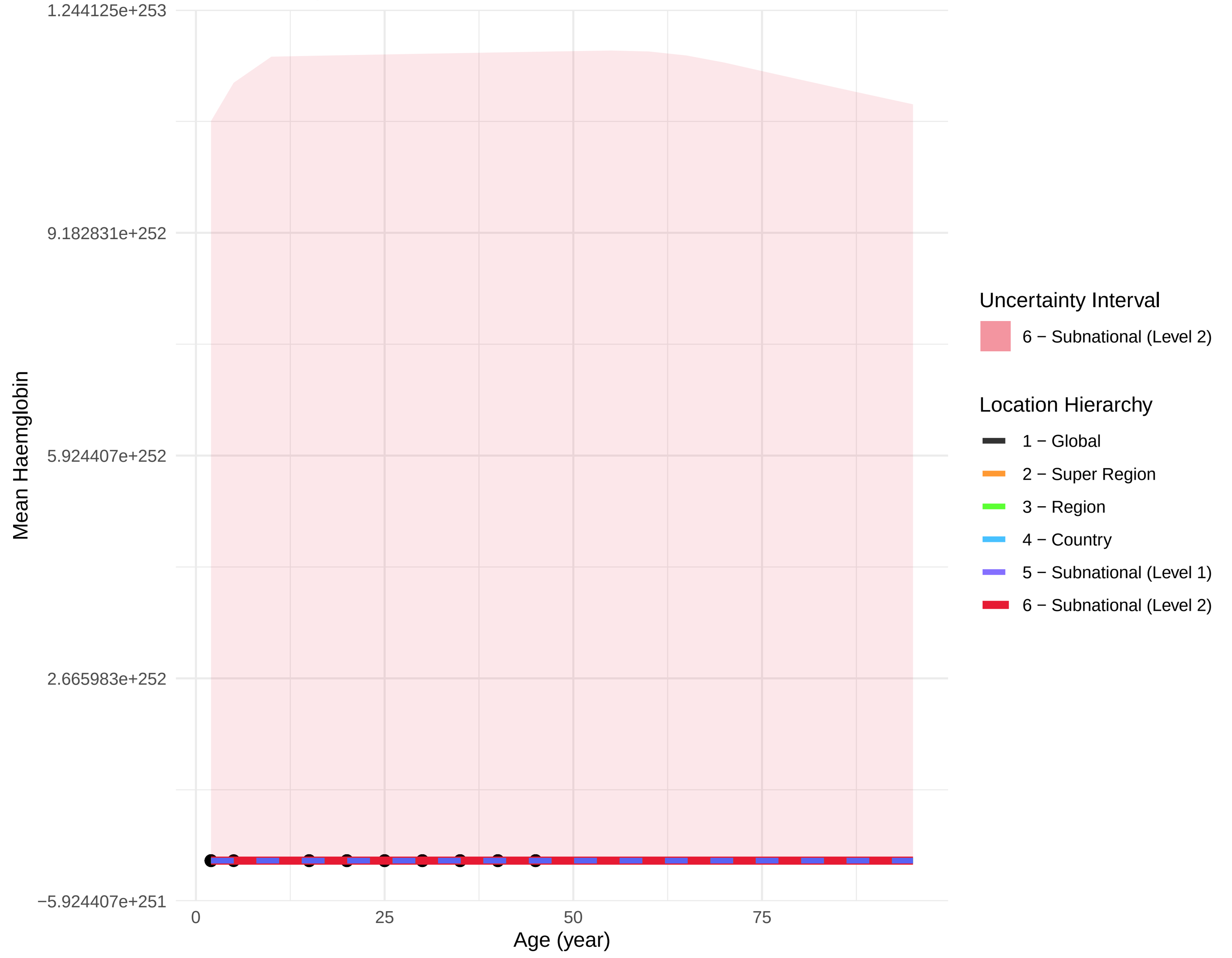

MR−BRT Age/Sex Fits − Non−pregnant Females 2+ Years Old − Mean Haemglobin − Gujarat, Urban
WHO−2024 Adjustment Method

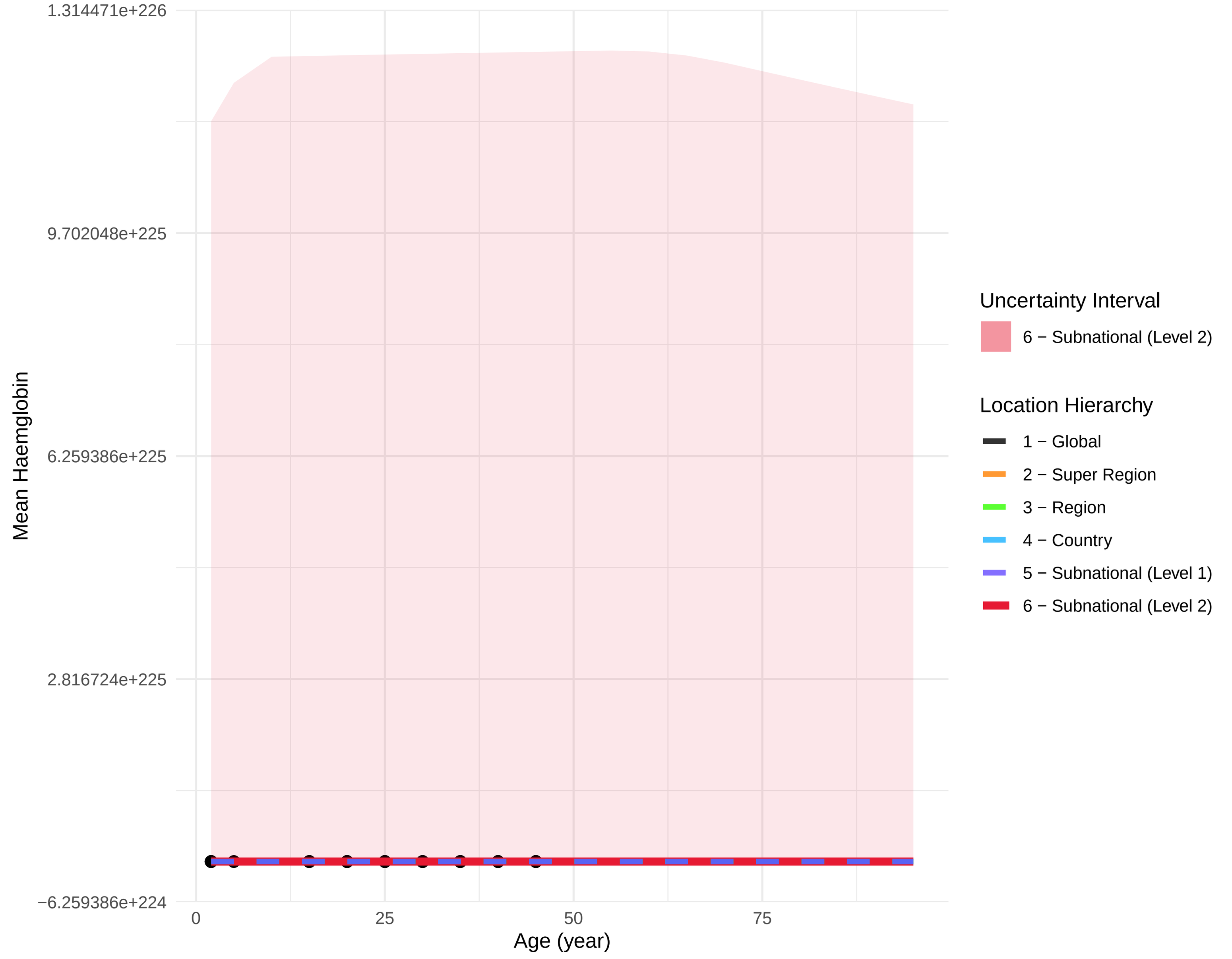

MR−BRT Age/Sex Fits − Non−pregnant Females 2+ Years Old − Mean Haemglobin − Haryana
WHO−2024 Adjustment Method

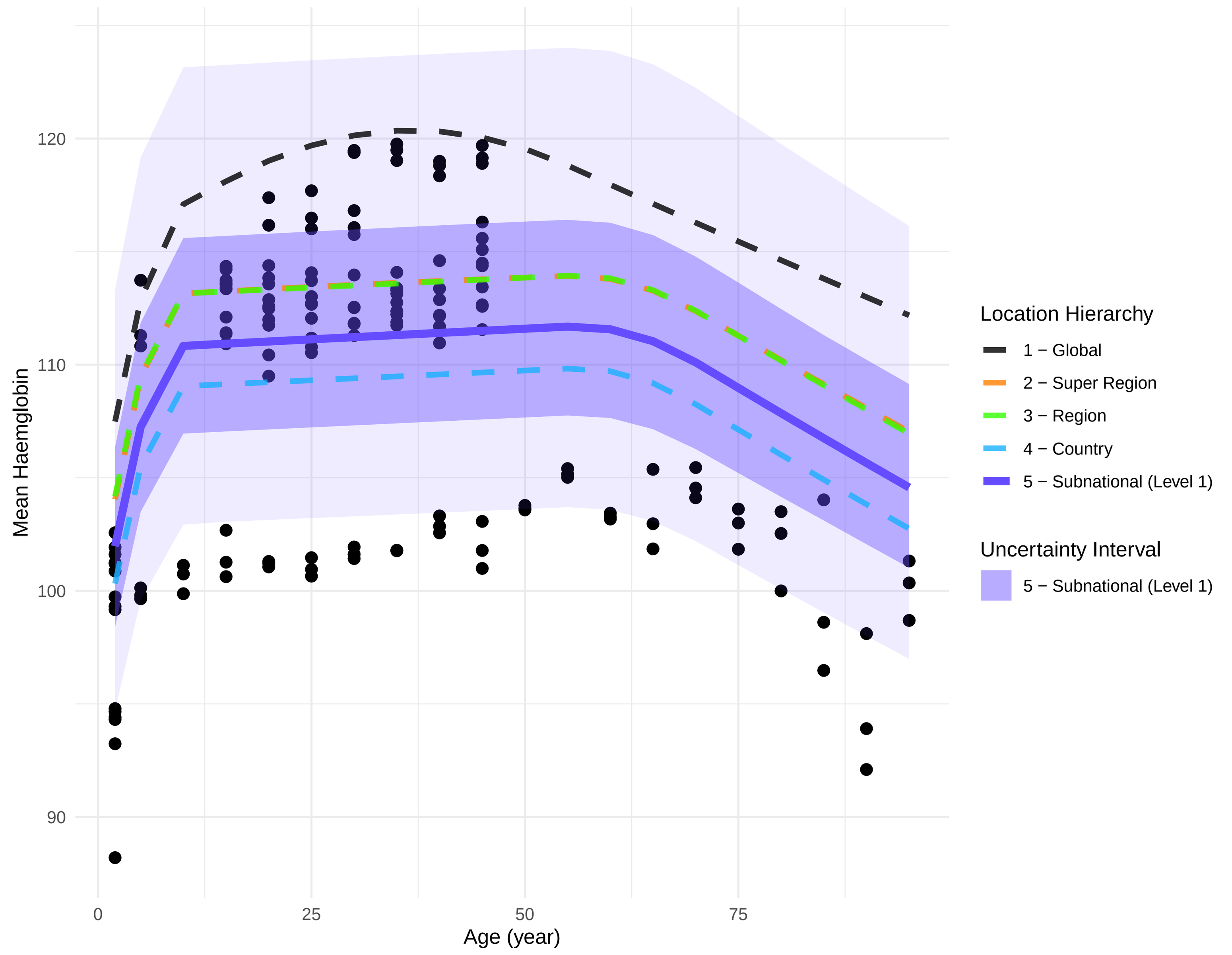

MR−BRT Age/Sex Fits − Non−pregnant Females 2+ Years Old − Mean Haemglobin − Haryana,
WHO−2024 Adjustment Method

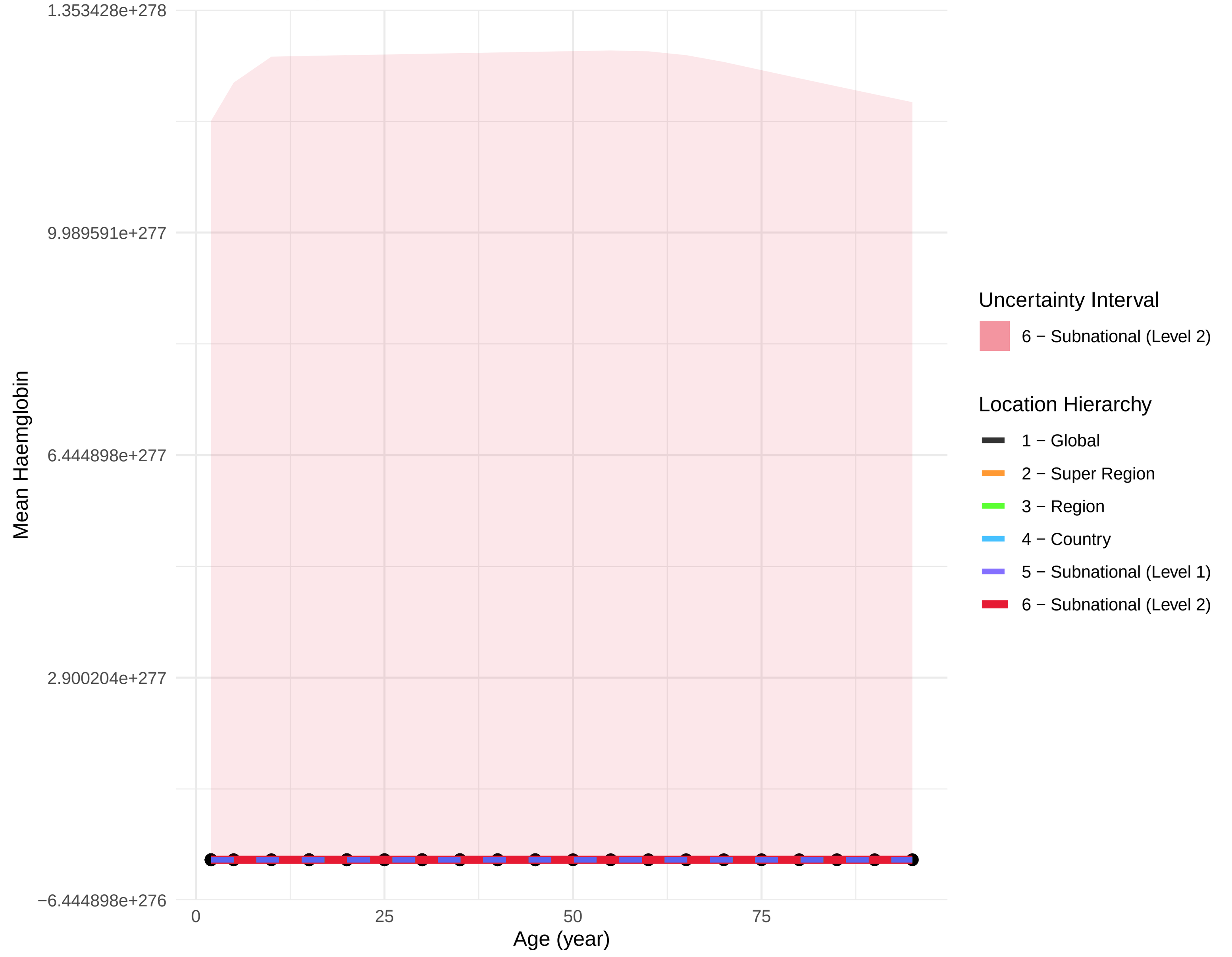

MR−BRT Age/Sex Fits − Non−pregnant Females 2+ Years Old − Mean Haemglobin − Haryana, Urban
WHO−2024 Adjustment Method

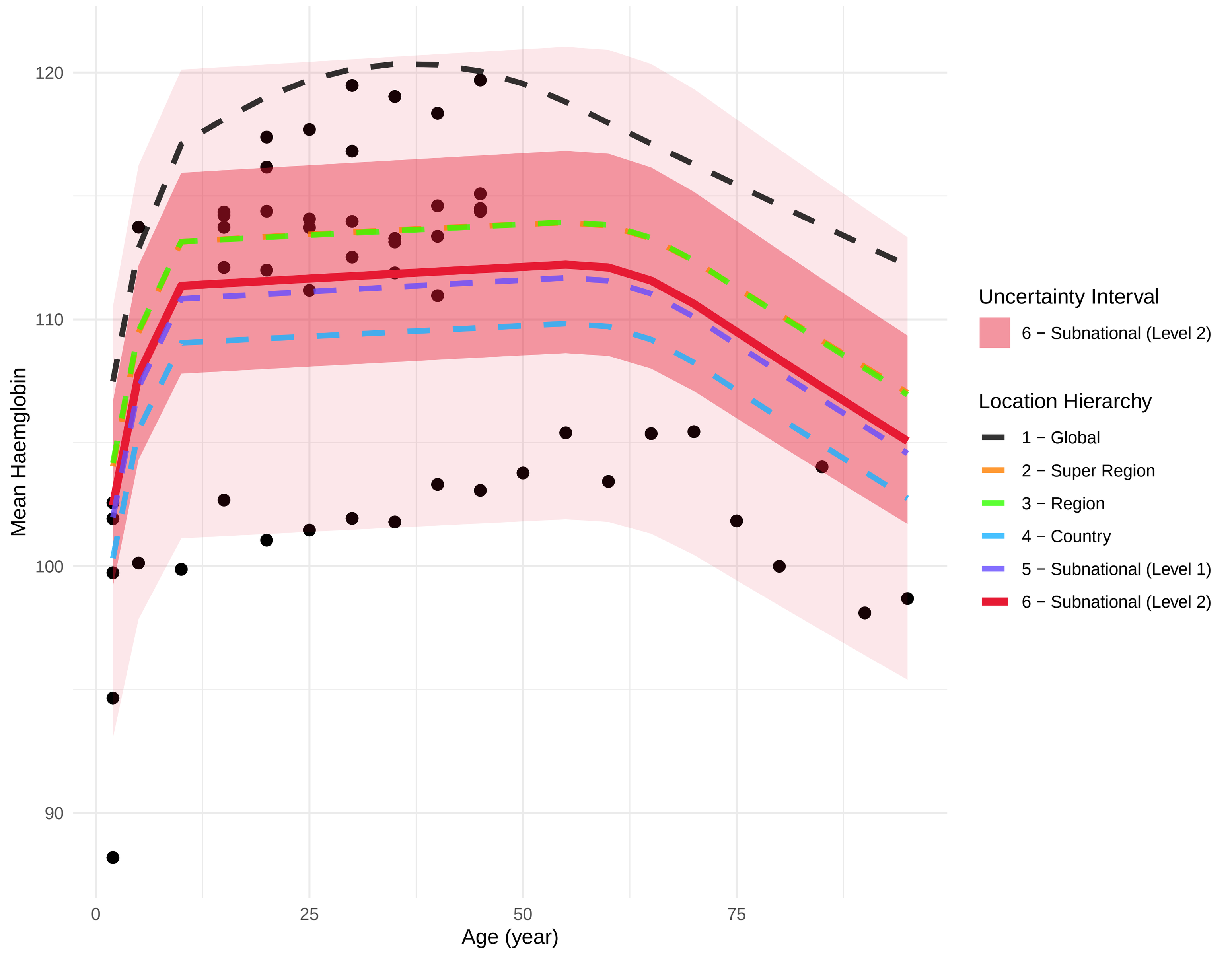

MR−BRT Age/Sex Fits − Non−pregnant Females 2+ Years Old − Mean Haemglobin − Himachal Pradesh
WHO−2024 Adjustment Method

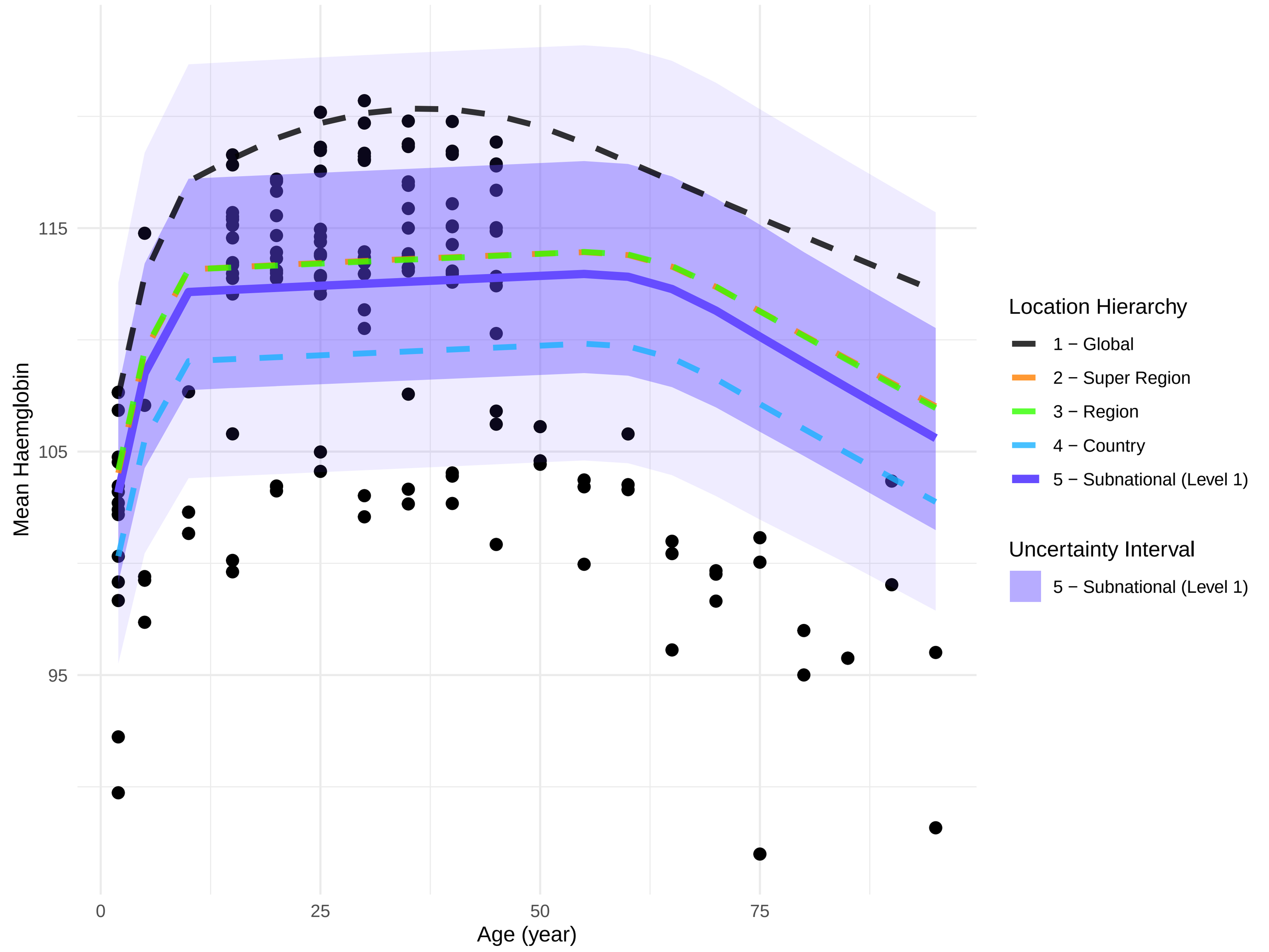

MR−BRT Age/Sex Fits − Non−pregnant Females 2+ Years Old − Mean Haemglobin − Himachal Pradesh, Rural
WHO−2024 Adjustment Method

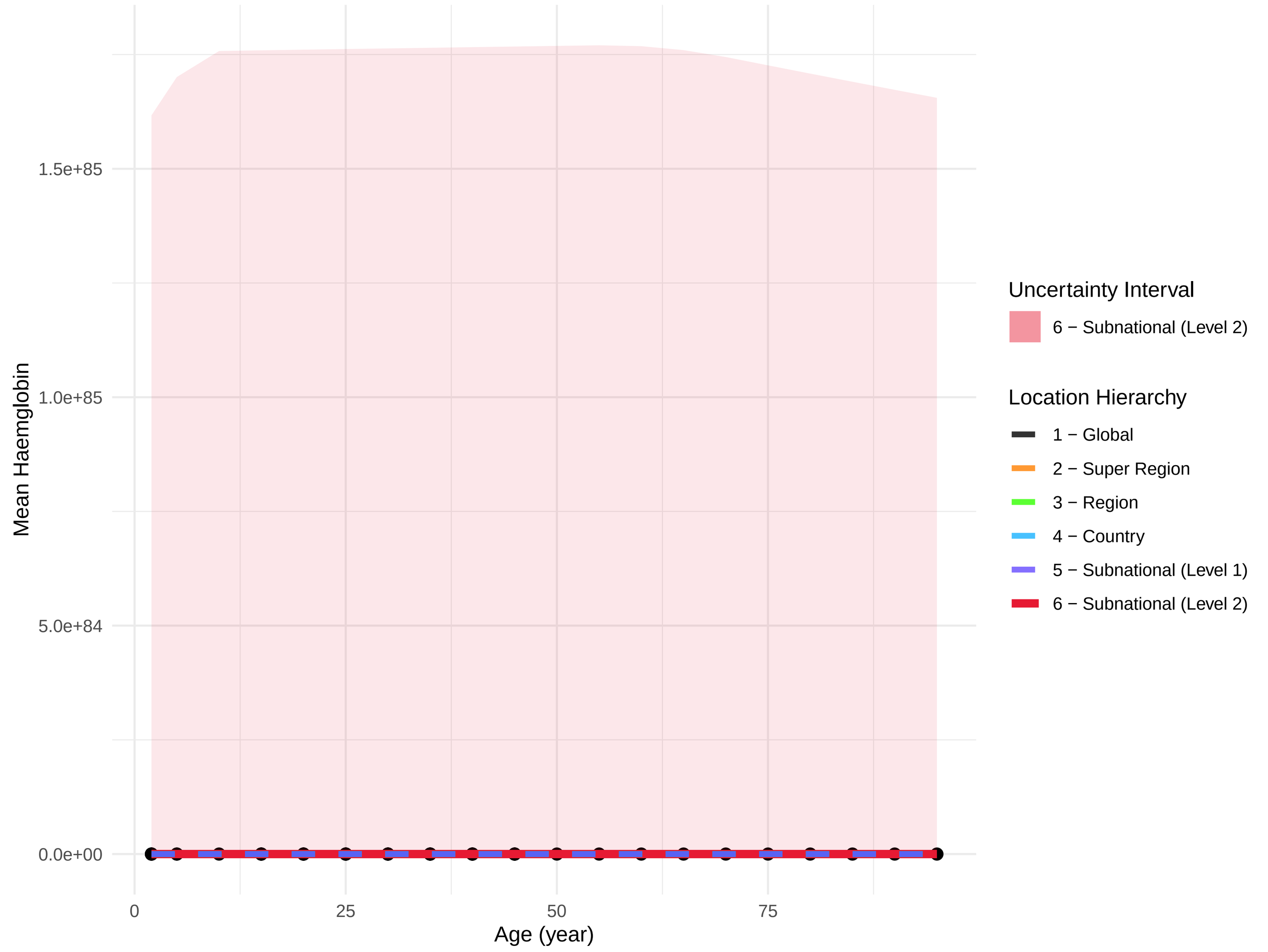

MR−BRT Age/Sex Fits − Non−pregnant Females 2+ Years Old − Mean Haemglobin − Himachal Pradesh, Urban
WHO−2024 Adjustment Method

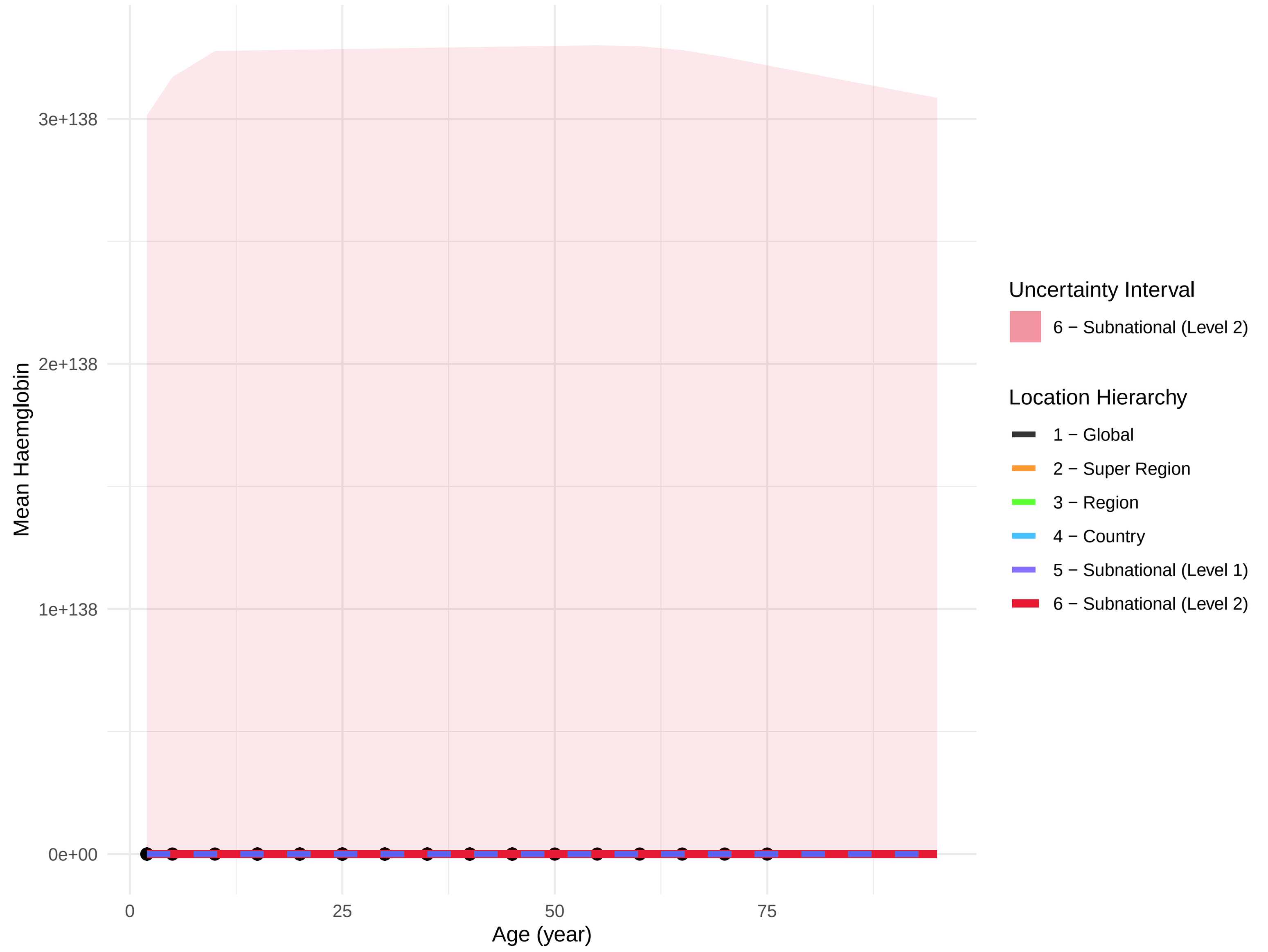

MR−BRT Age/Sex Fits − Non−pregnant Females 2+ Years Old − Mean Haemglobin − Jammu & Kashmir and Ladakh
WHO−2024 Adjustment Method

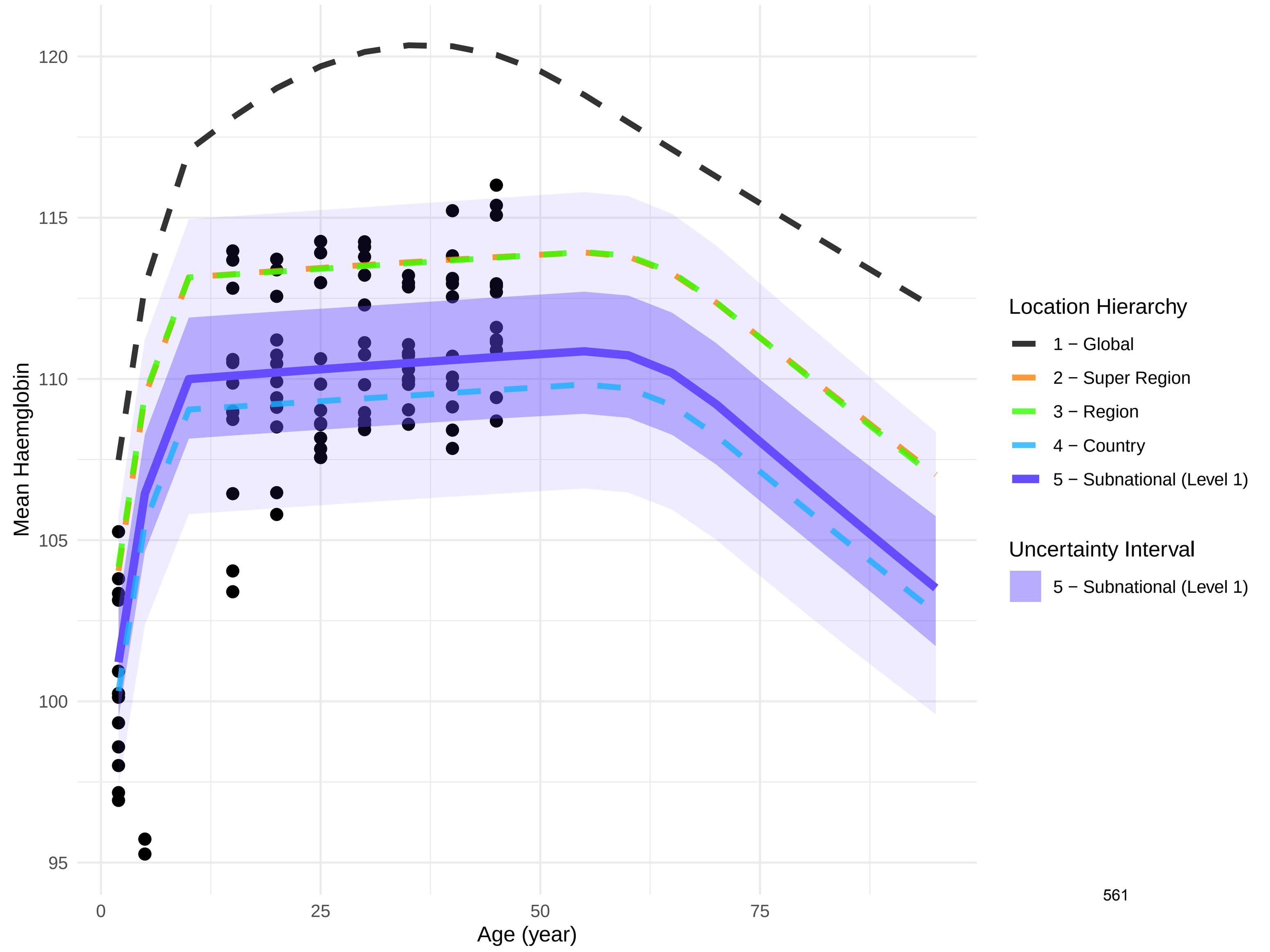

MR−BRT Age/Sex Fits − Non−pregnant Females 2+ Years Old − Mean Haemglobin − Jammu & Kashmir and Ladakh, Rural
WHO−2024 Adjustment Method

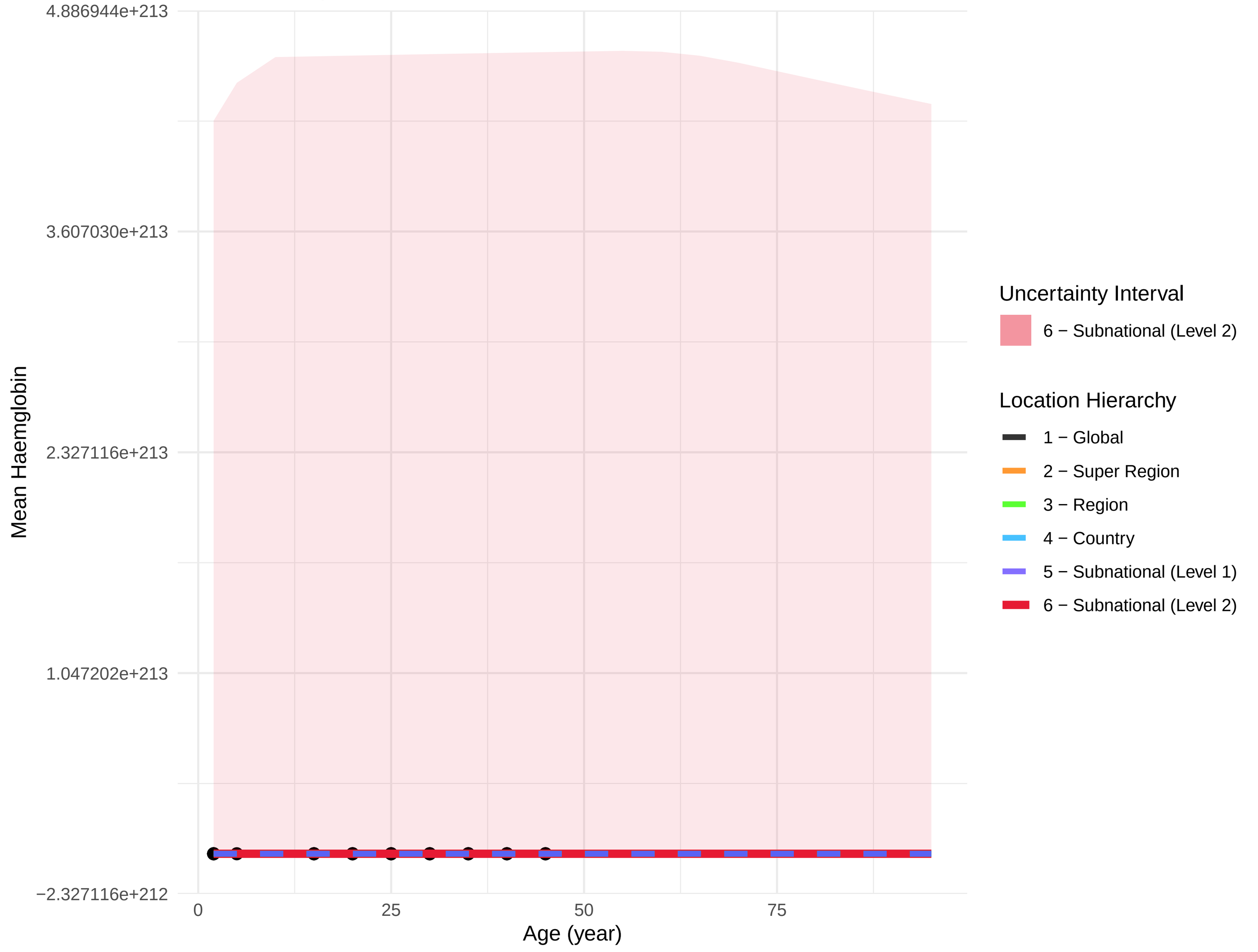

MR−BRT Age/Sex Fits − Non−pregnant Females 2+ Years Old − Mean Haemglobin − Jammu & Kashmir and Ladakh, Urban
WHO−2024 Adjustment Method

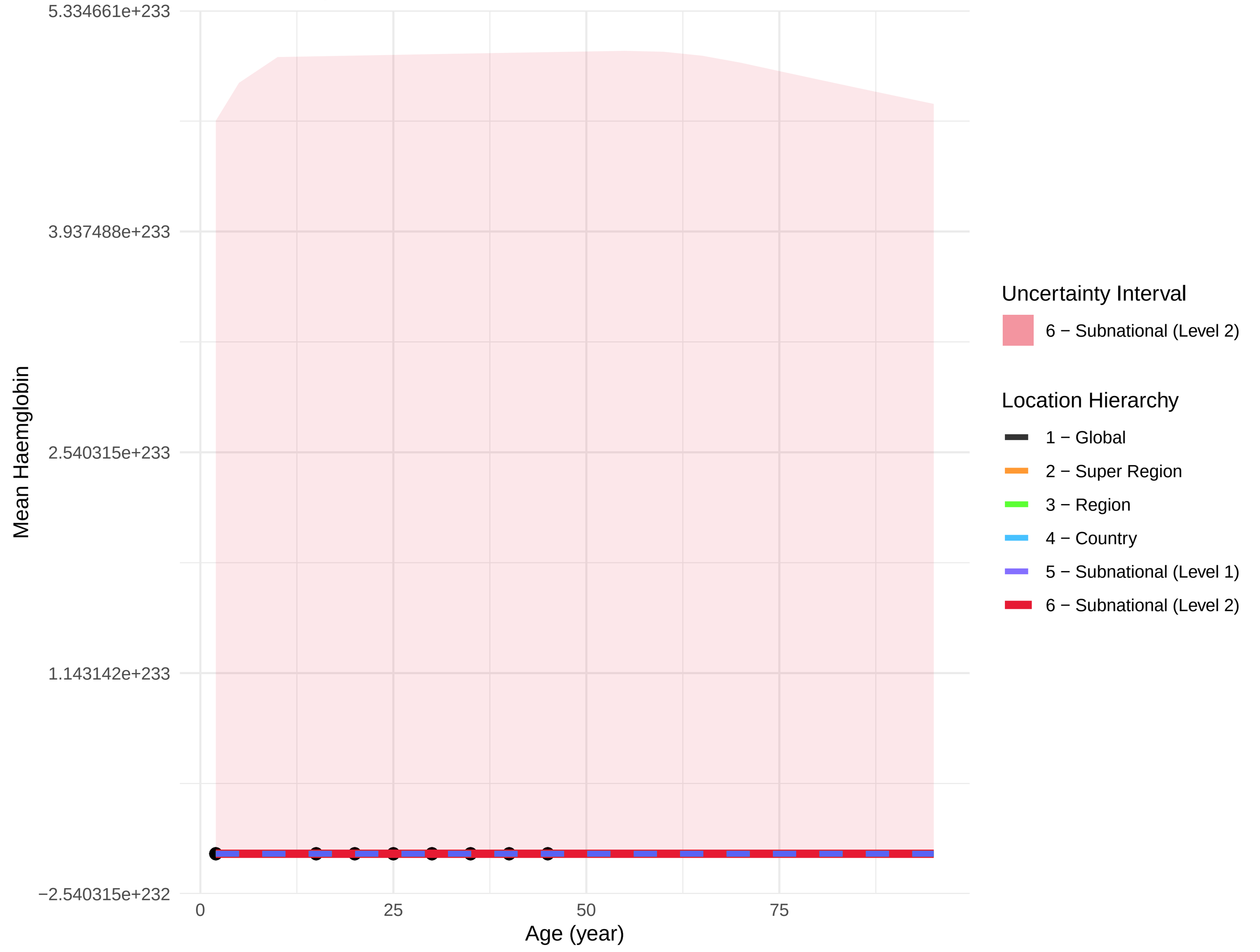

MR−BRT Age/Sex Fits − Non−pregnant Females 2+ Years Old − Mean Haemglobin − Jharkhand
WHO−2024 Adjustment Method

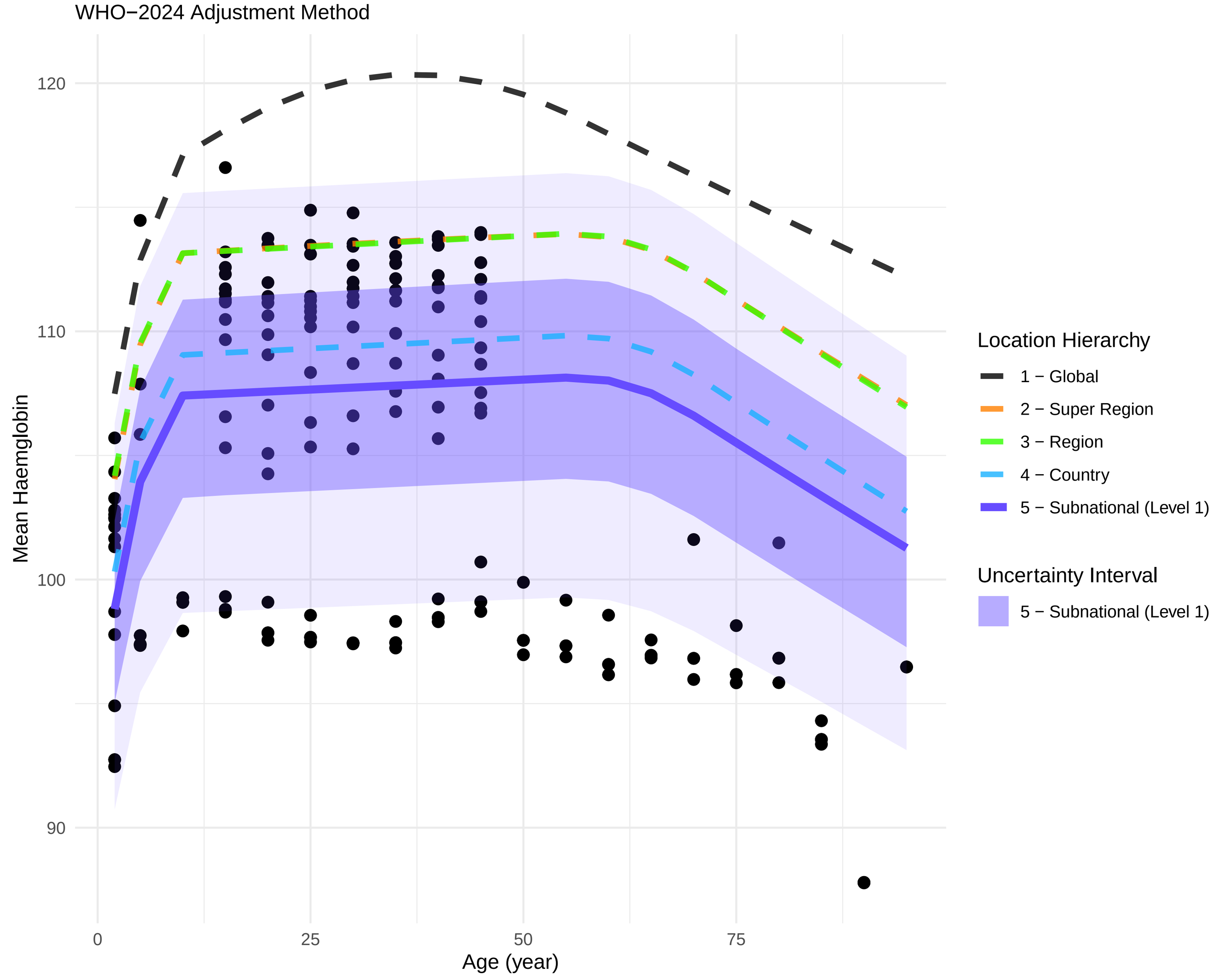

MR−BRT Age/Sex Fits − Non−pregnant Females 2+ Years Old − Mean Haemglobin − Jharkhand, Rural
WHO−2024 Adjustment Method

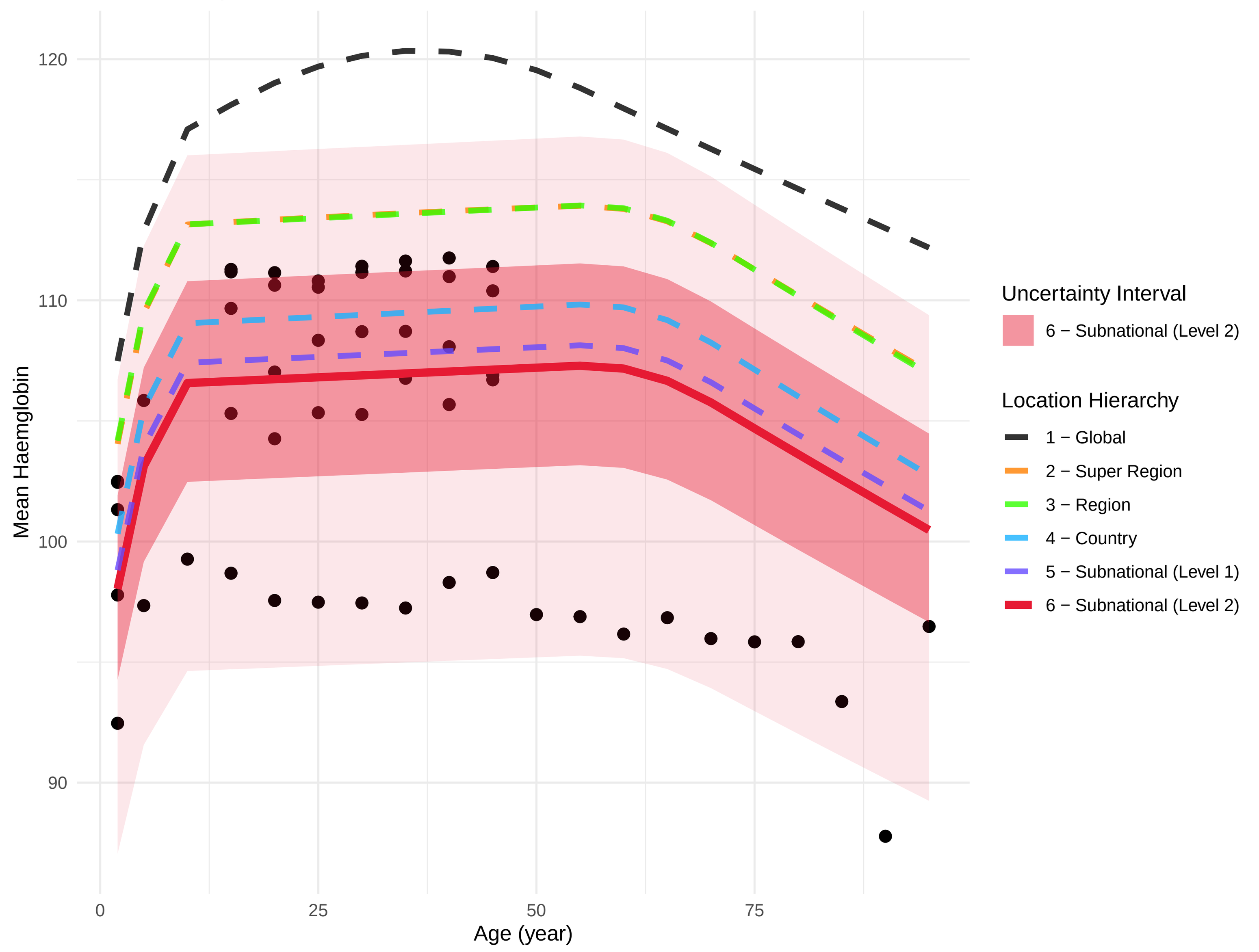

MR−BRT Age/Sex Fits − Non−pregnant Females 2+ Years Old − Mean Haemglobin − Jharkhand, Urban
WHO−2024 Adjustment Method

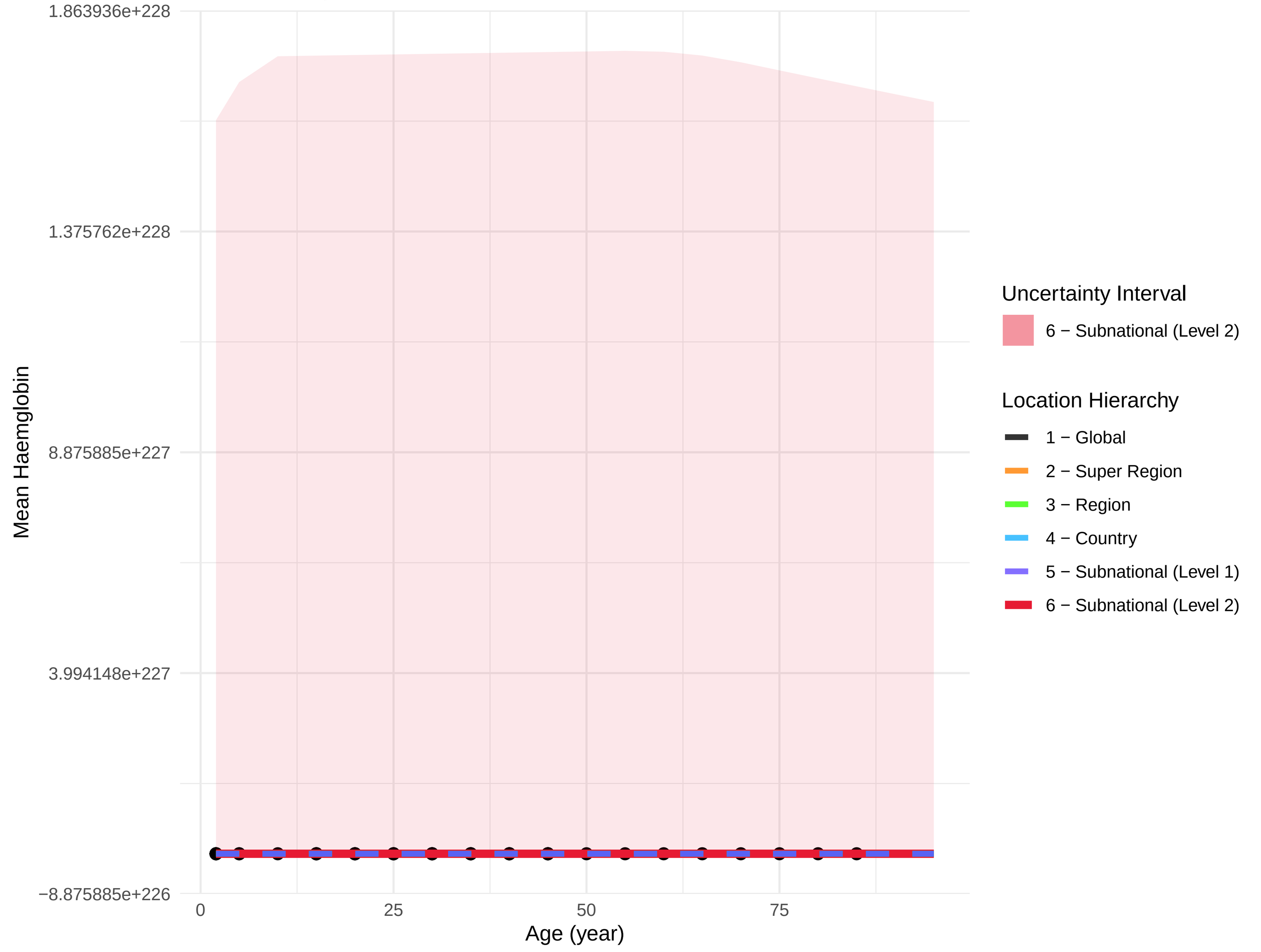

MR−BRT Age/Sex Fits − Non−pregnant Females 2+ Years Old − Mean Haemglobin − Karnataka
WHO−2024 Adjustment Method

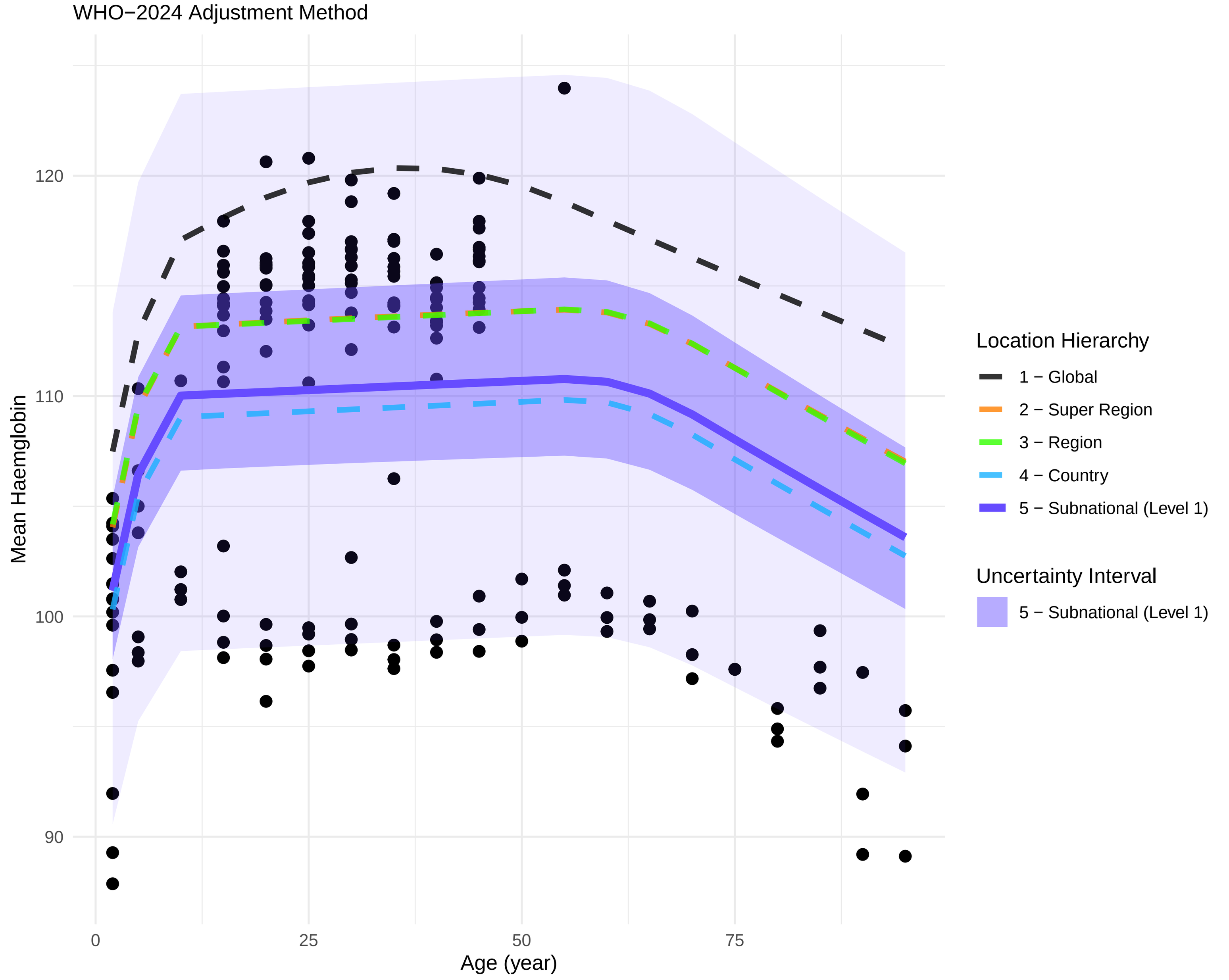

MR−BRT Age/Sex Fits − Non−pregnant Females 2+ Years Old − Mean Haemglobin − Karnataka, Rural
WHO−2024 Adjustment Method

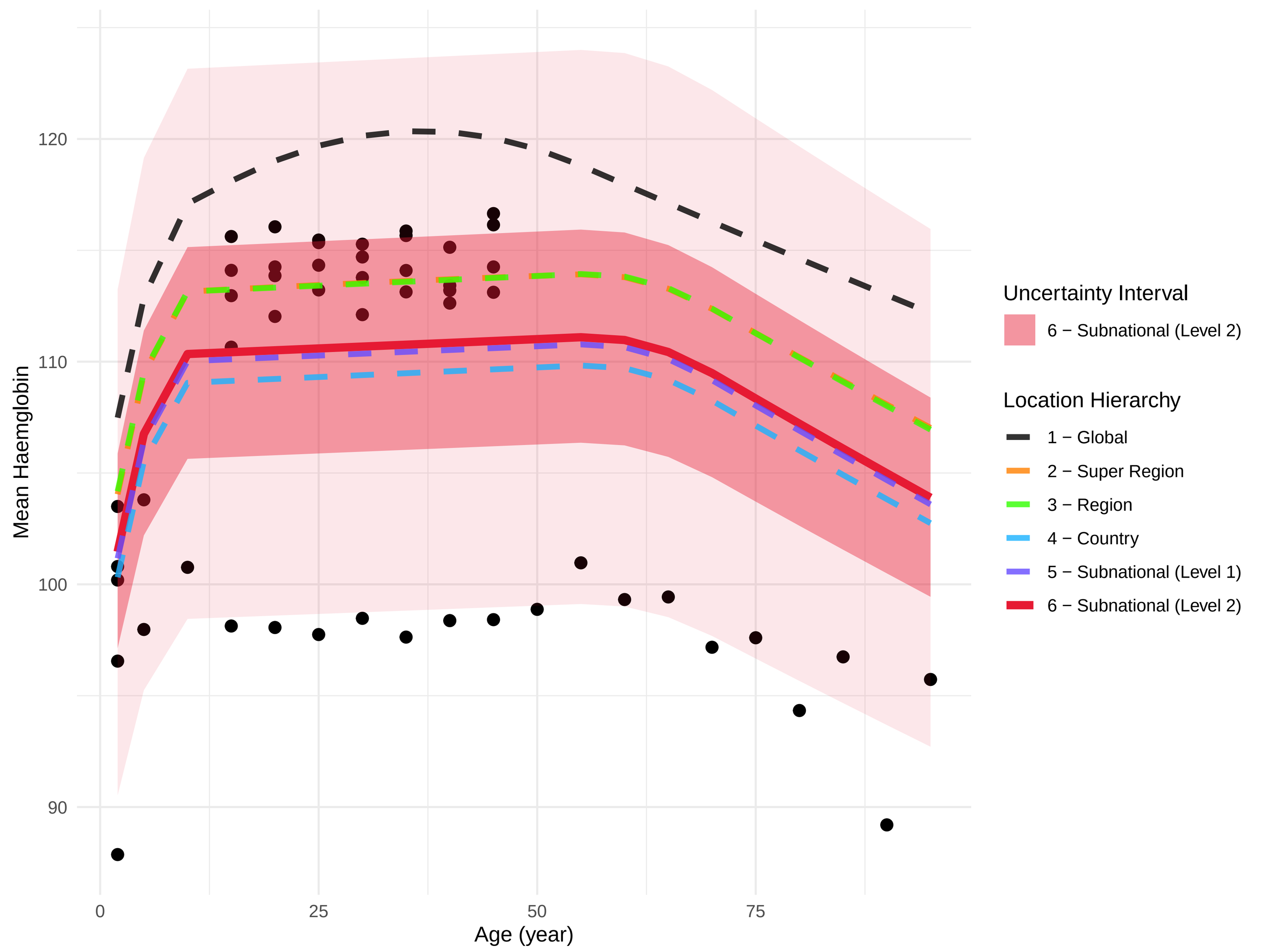

MR−BRT Age/Sex Fits − Non−pregnant Females 2+ Years Old − Mean Haemglobin − Karnataka, Urban
WHO−2024 Adjustment Method

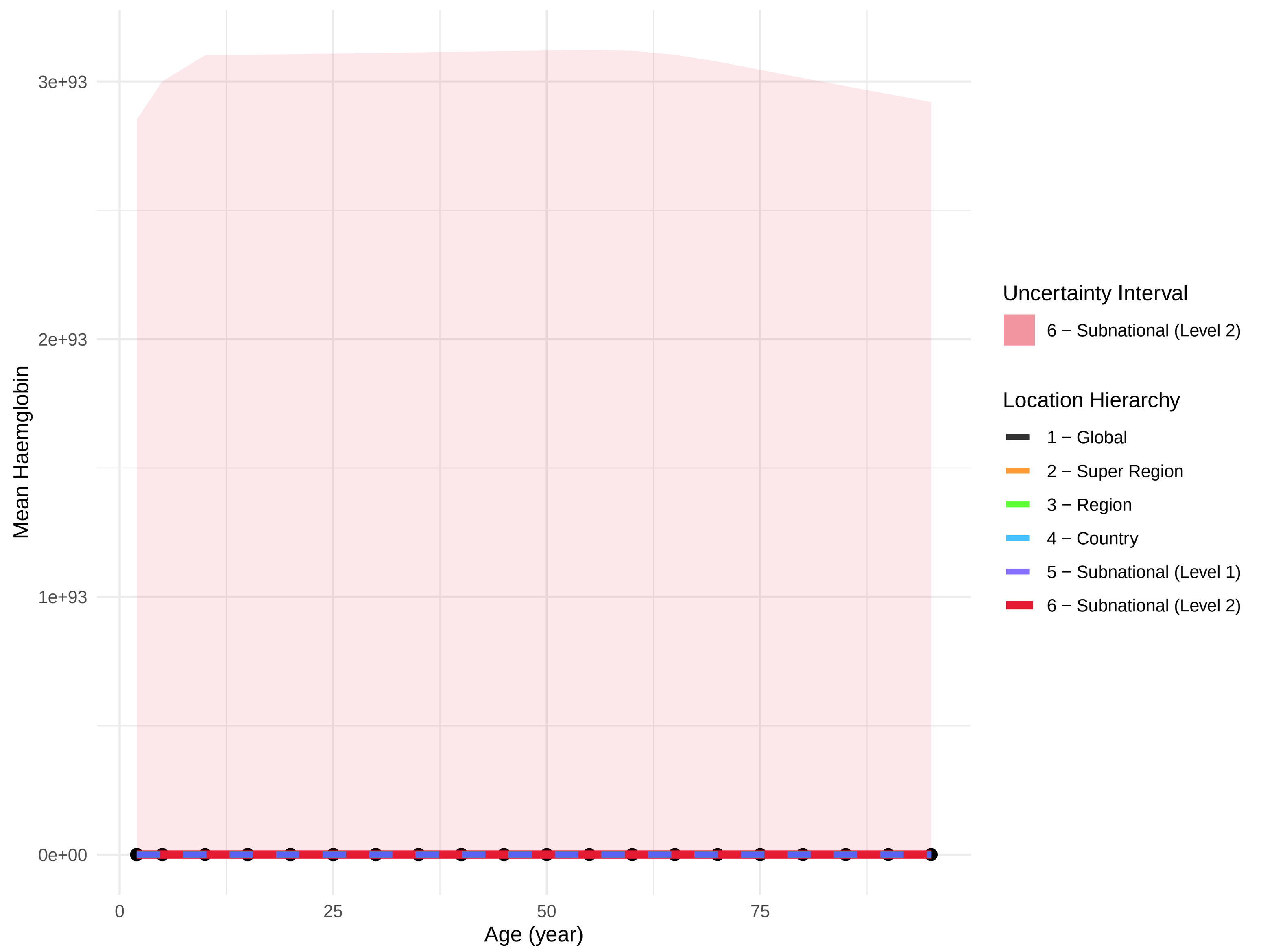

MR−BRT Age/Sex Fits − Non−pregnant Females 2+ Years Old − Mean Haemglobin − Kerala
WHO−2024 Adjustment Method

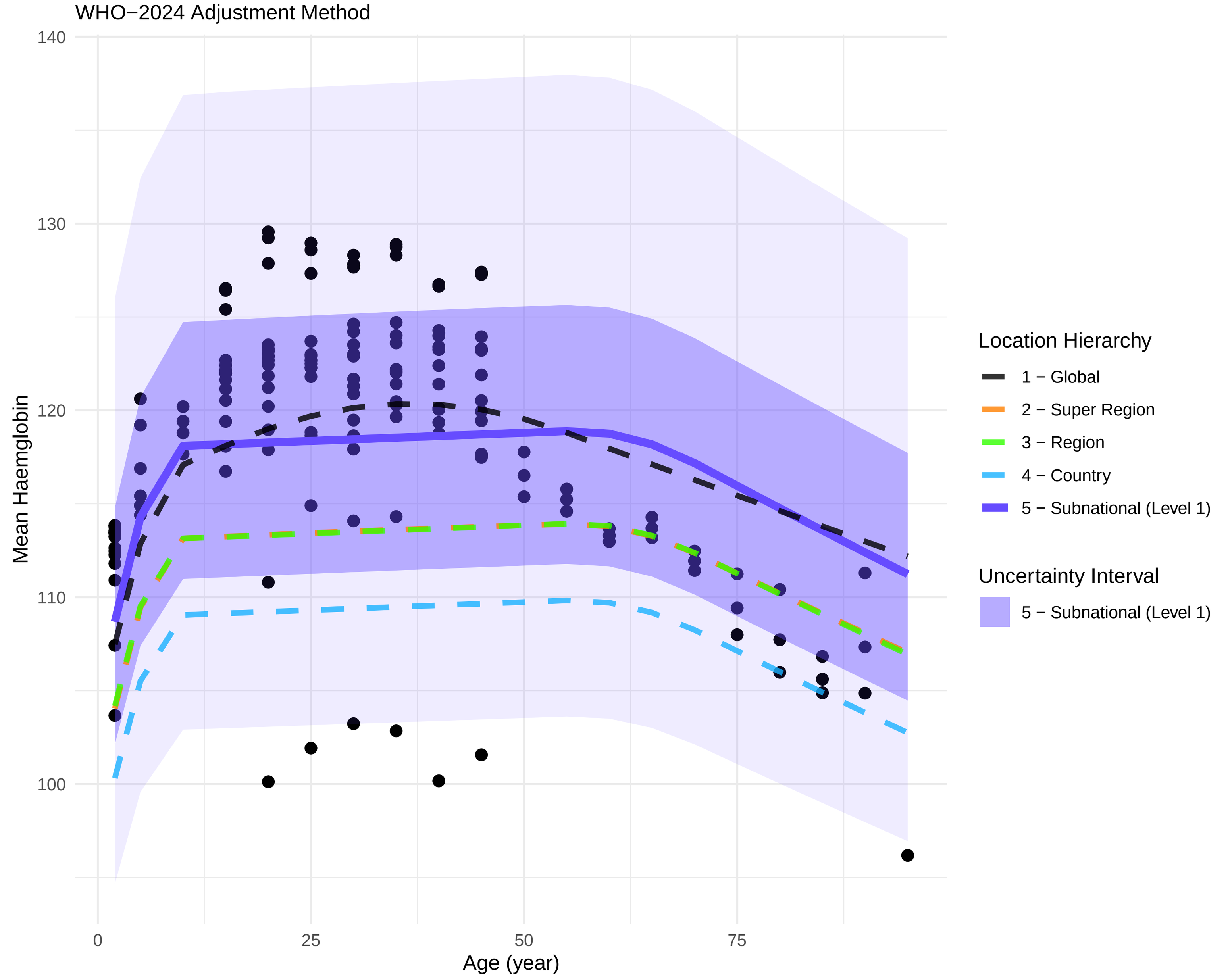

MR−BRT Age/Sex Fits − Non−pregnant Females 2+ Years Old − Mean Haemglobin − Kerala, Rural
WHO−2024 Adjustment Method

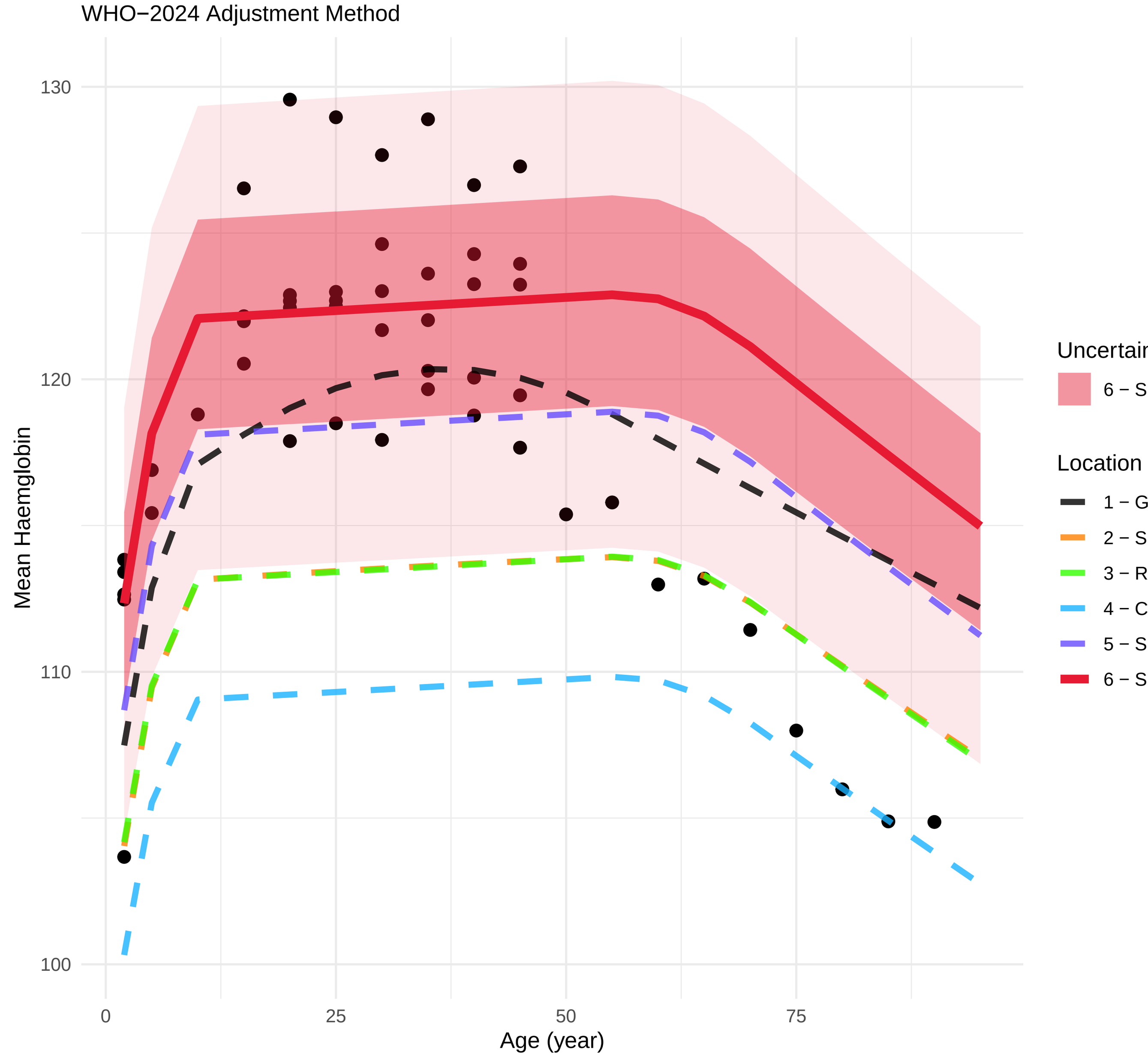

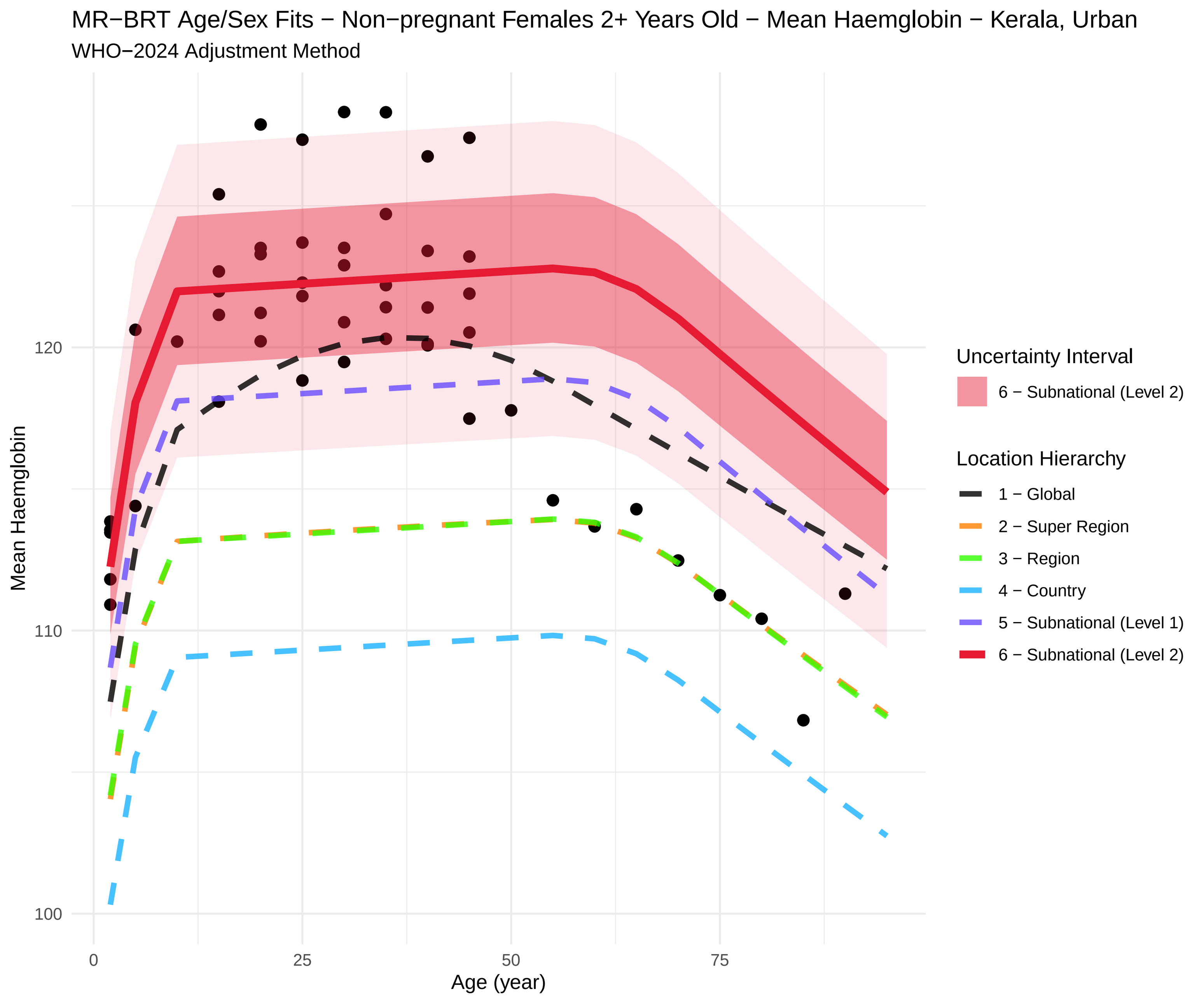
MR−BRT Age/Sex Fits − Non−pregnant Females 2+ Years Old − Mean Haemglobin − Kerala, Urban
WHO−2024 Adjustment Method
Mean Haemglobin
Age (year)
100
110
120
0
25
50
75
Uncertainty Interval
6 − Subnational (Level 2)
Location Hierarchy
1 − Global
2 − Super Region
3 − Region
4 − Country
5 − Subnational (Level 1)
6 − Subnational (Level 2)
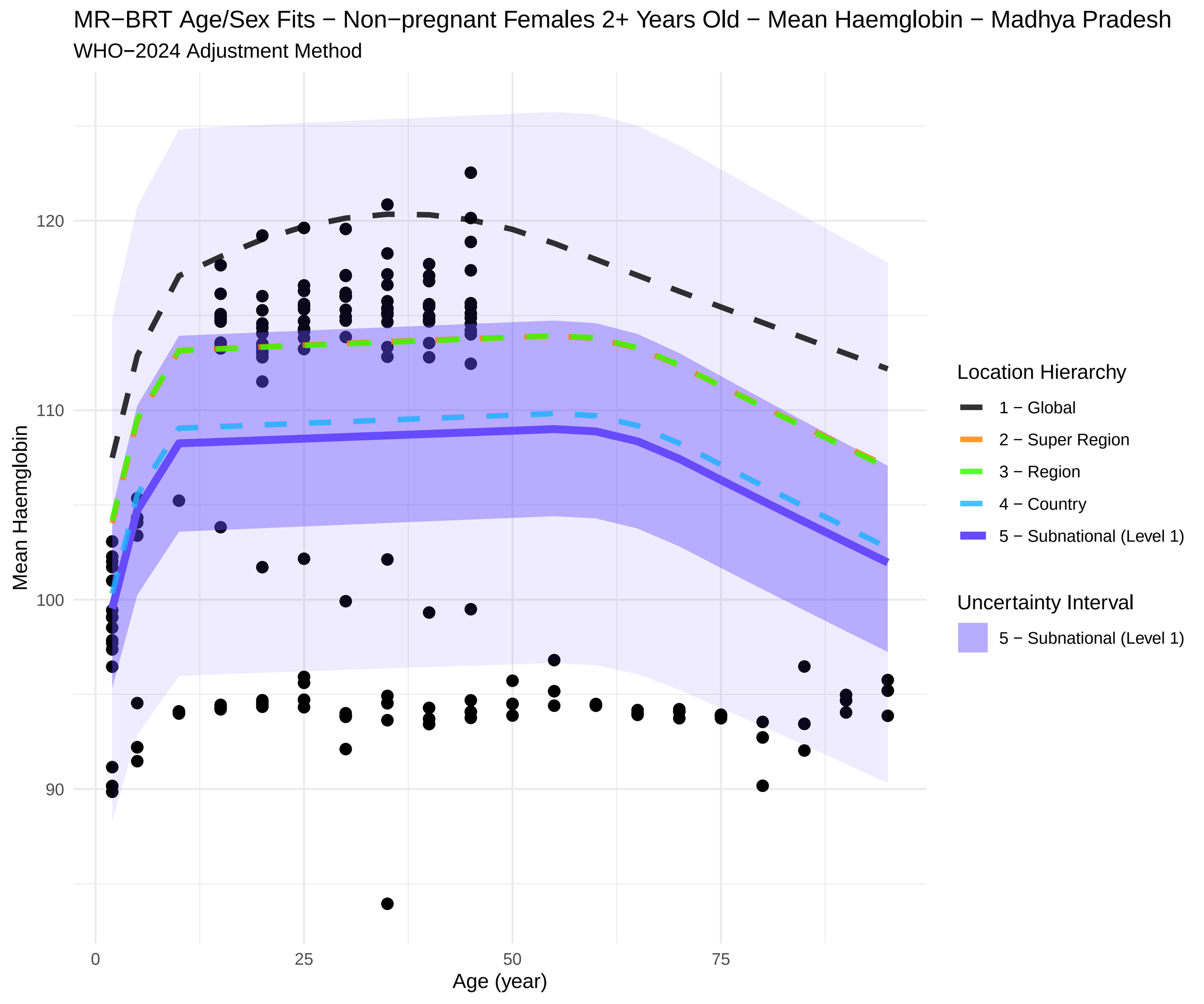
MR−BRT Age/Sex Fits − Non−pregnant Females 2+ Years Old − Mean Haemglobin − Madhya Pradesh
WHO−2024 Adjustment Method
Mean Haemglobin
Age (year)
90
100
110
120
0
25
50
75
Location Hierarchy
1 − Global
2 − Super Region
3 − Region
4 − Country
5 − Subnational (Level 1)
Uncertainty Interval
5 − Subnational (Level 1)
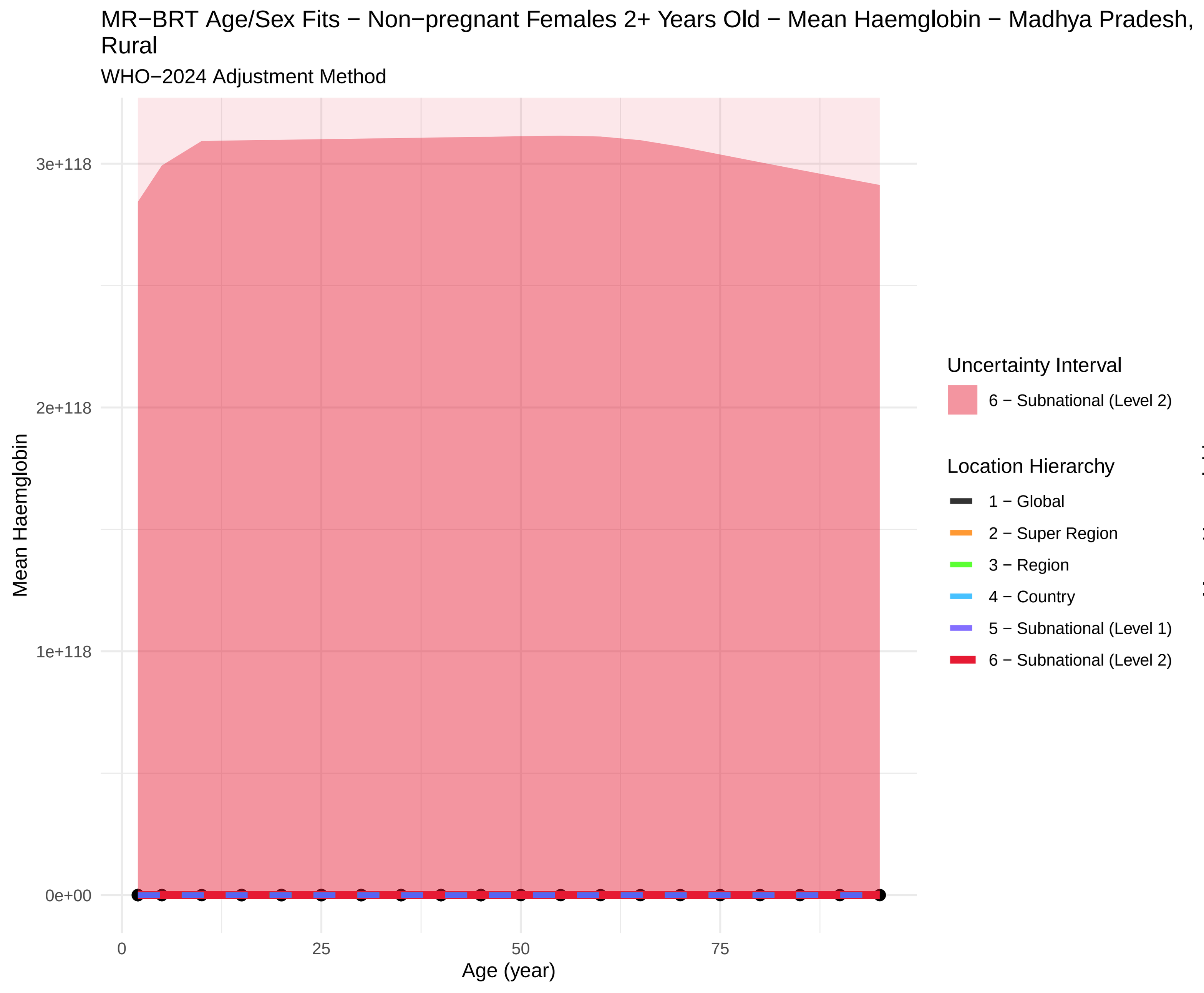
MR−BRT Age/Sex Fits − Non−pregnant Females 2+ Years Old − Mean Haemglobin − Madhya Pradesh, Rural
WHO−2024 Adjustment Method
Mean Haemglobin
Age (year)
0e+00
1e+118
2e+118
3e+118
0
25
50
75
Uncertainty Interval
6 − Subnational (Level 2)
Location Hierarchy
1 − Global
2 − Super Region
3 − Region
4 − Country
5 − Subnational (Level 1)
6 − Subnational (Level 2)
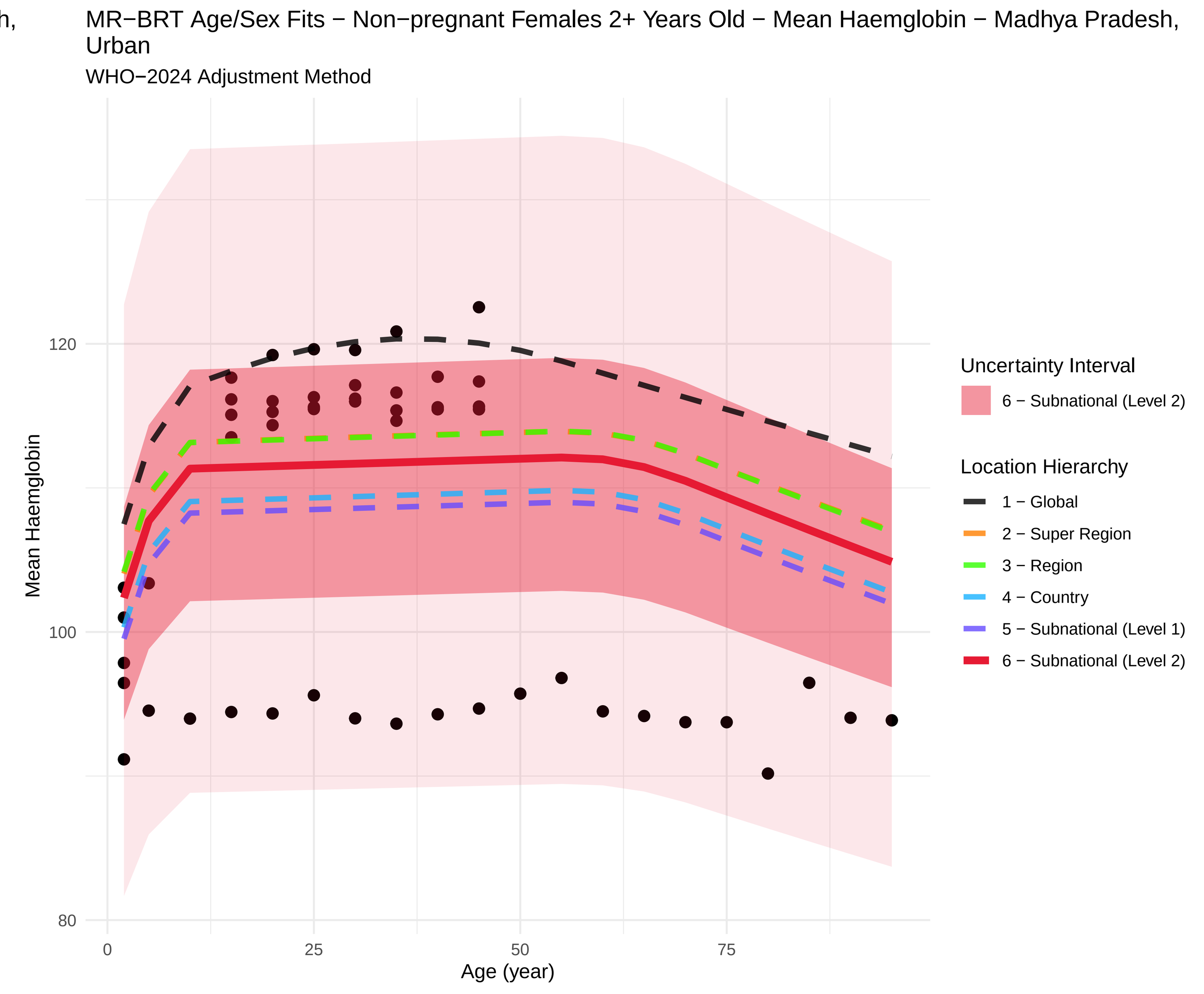
MR−BRT Age/Sex Fits − Non−pregnant Females 2+ Years Old − Mean Haemglobin − Madhya Pradesh, Urban
WHO−2024 Adjustment Method
Mean Haemglobin
Age (year)
80
100
120
0
25
50
75
Uncertainty Interval
6 − Subnational (Level 2)
Location Hierarchy
1 − Global
2 − Super Region
3 − Region
4 − Country
5 − Subnational (Level 1)
6 − Subnational (Level 2)
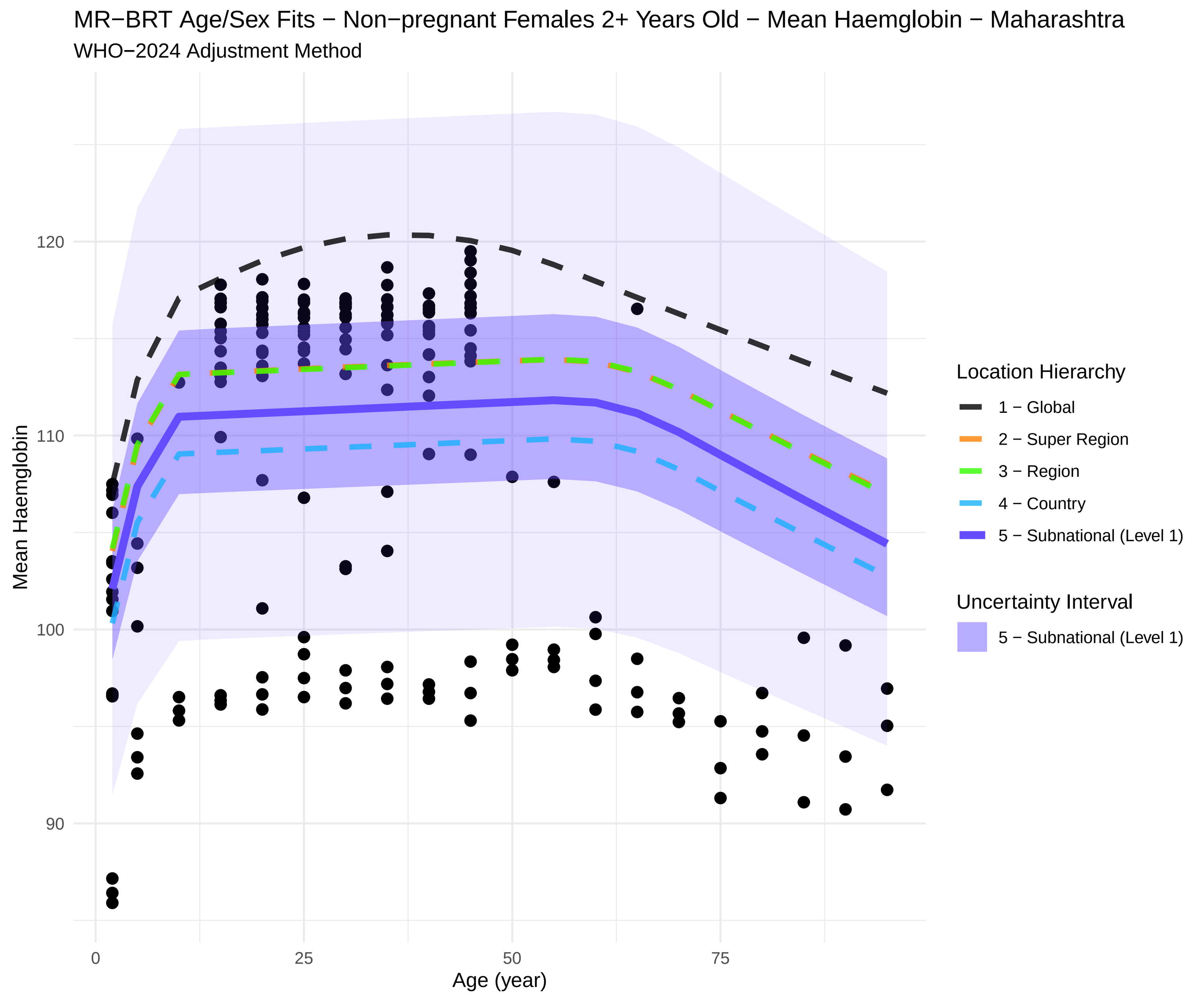
MR−BRT Age/Sex Fits − Non−pregnant Females 2+ Years Old − Mean Haemglobin − Maharashtra
WHO−2024 Adjustment Method
Mean Haemglobin
Age (year)
90
100
110
120
0
25
50
75
Location Hierarchy
1 − Global
2 − Super Region
3 − Region
4 − Country
5 − Subnational (Level 1)
Uncertainty Interval
5 − Subnational (Level 1)
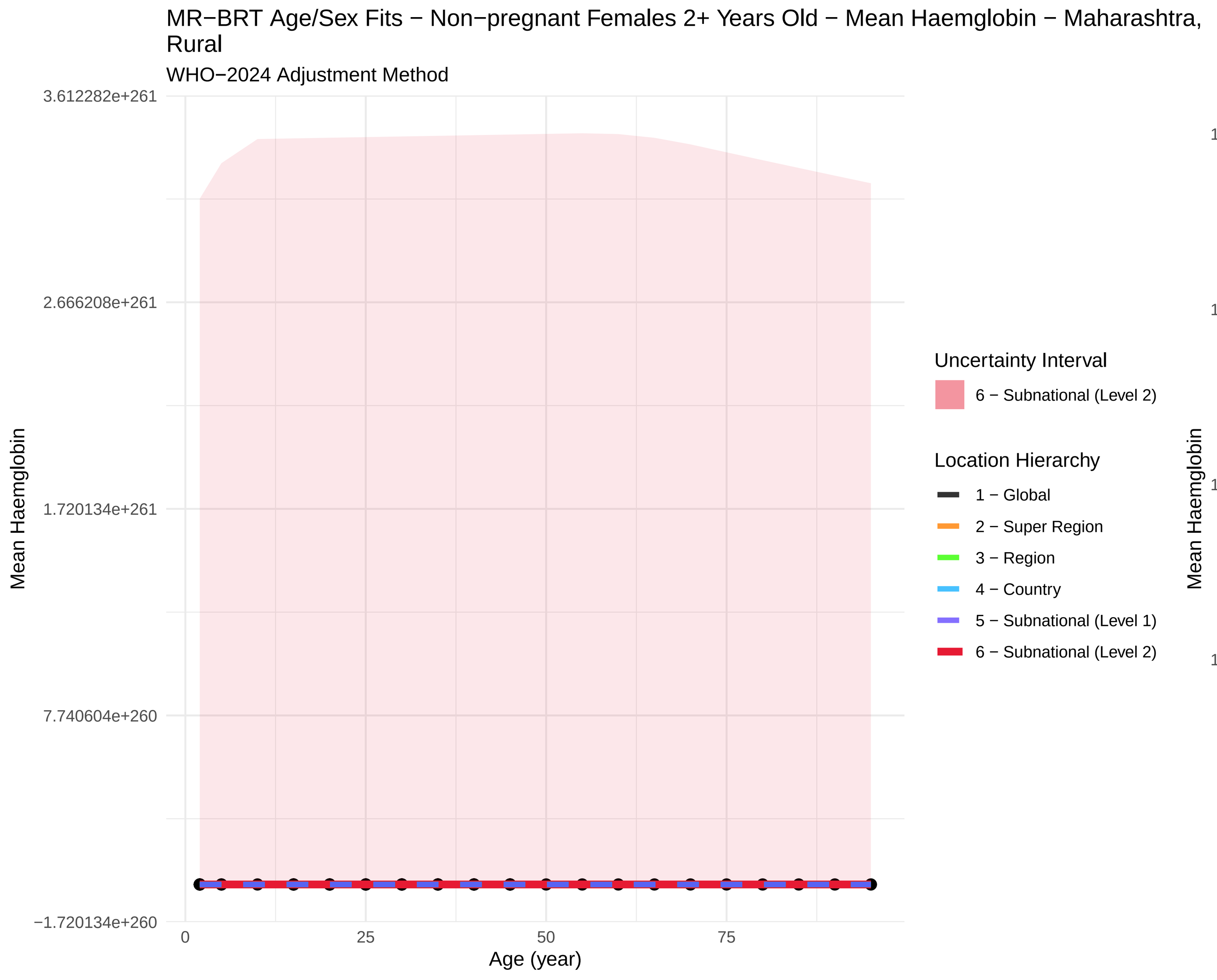
MR−BRT Age/Sex Fits − Non−pregnant Females 2+ Years Old − Mean Haemglobin − Maharashtra, Rural
WHO−2024 Adjustment Method
Mean Haemglobin
Age (year)
−1.720134e+260
7.740604e+260
1.720134e+261
2.666208e+261
3.612282e+261
0
25
50
75
Uncertainty Interval
6 − Subnational (Level 2)
Location Hierarchy
1 − Global
2 − Super Region
3 − Region
4 − Country
5 − Subnational (Level 1)
6 − Subnational (Level 2)
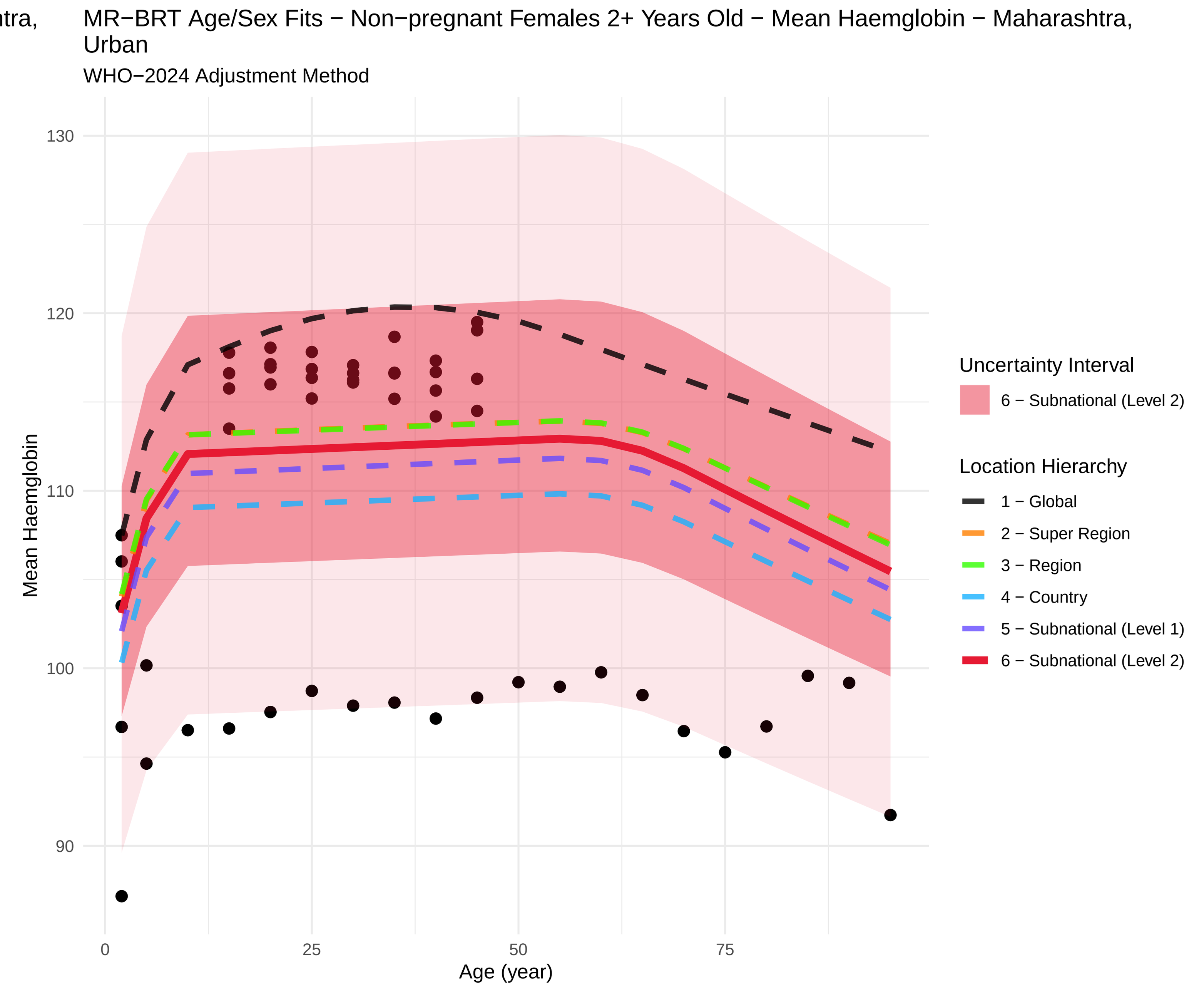
MR−BRT Age/Sex Fits − Non−pregnant Females 2+ Years Old − Mean Haemglobin − Maharashtra, Urban
WHO−2024 Adjustment Method
Mean Haemglobin
Age (year)
90
100
110
120
130
0
25
50
75
Uncertainty Interval
6 − Subnational (Level 2)
Location Hierarchy
1 − Global
2 − Super Region
3 − Region
4 − Country
5 − Subnational (Level 1)
6 − Subnational (Level 2)
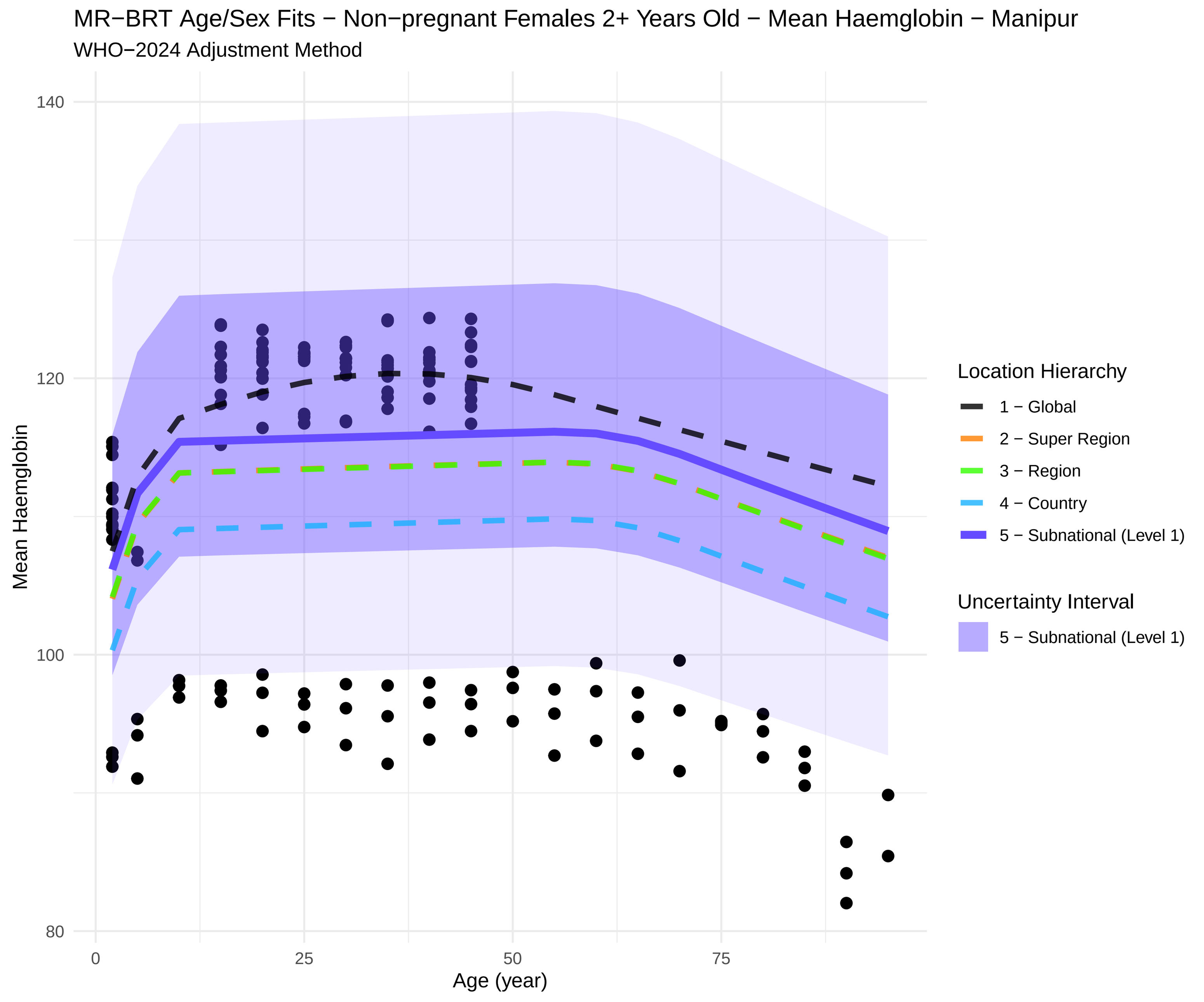
MR−BRT Age/Sex Fits − Non−pregnant Females 2+ Years Old − Mean Haemglobin − Manipur
WHO−2024 Adjustment Method
Mean Haemglobin
Age (year)
80
100
120
140
0
25
50
75
Location Hierarchy
1 − Global
2 − Super Region
3 − Region
4 − Country
5 − Subnational (Level 1)
Uncertainty Interval
5 − Subnational (Level 1)
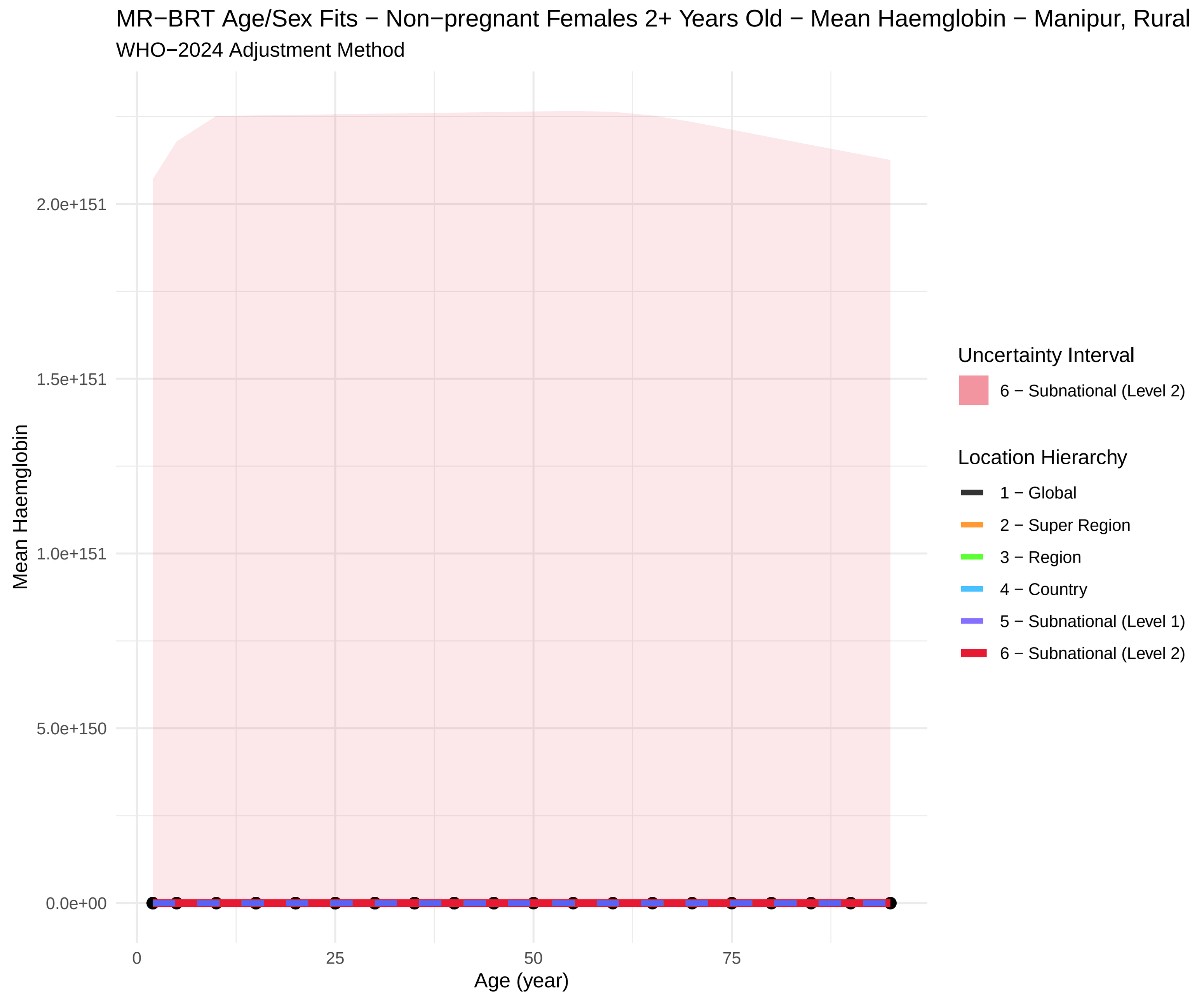
MR−BRT Age/Sex Fits − Non−pregnant Females 2+ Years Old − Mean Haemglobin − Manipur, Rural
WHO−2024 Adjustment Method
Mean Haemglobin
Age (year)
0.0e+00
5.0e+150
1.0e+151
1.5e+151
2.0e+151
0
25
50
75
Uncertainty Interval
6 − Subnational (Level 2)
Location Hierarchy
1 − Global
2 − Super Region
3 − Region
4 − Country
5 − Subnational (Level 1)
6 − Subnational (Level 2)
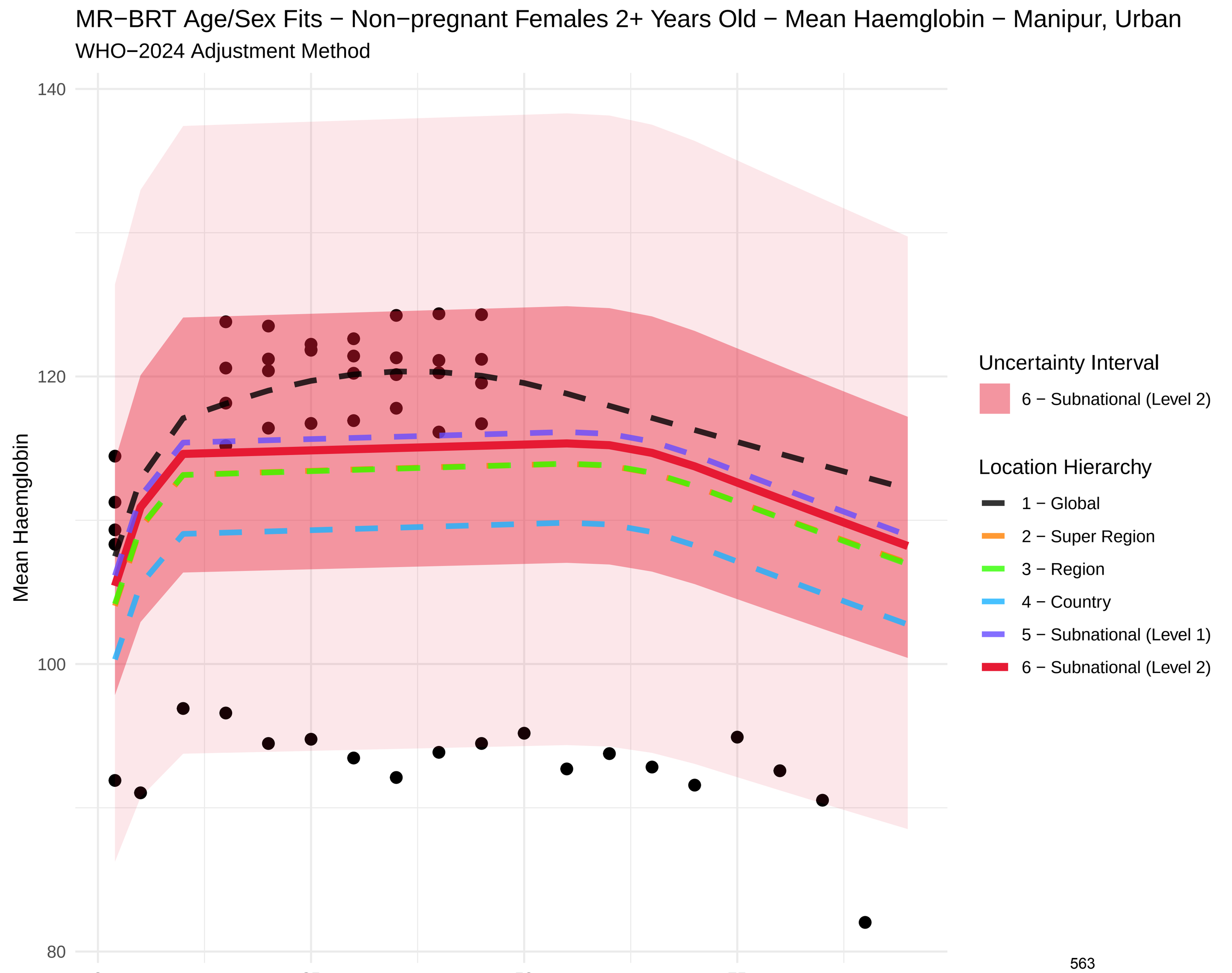
MR−BRT Age/Sex Fits − Non−pregnant Females 2+ Years Old − Mean Haemglobin − Manipur, Urban
WHO−2024 Adjustment Method
Mean Haemglobin
Age (year)
80
100
120
140
0
25
50
75
Uncertainty Interval
6 − Subnational (Level 2)
Location Hierarchy
1 − Global
2 − Super Region
3 − Region
4 − Country
5 − Subnational (Level 1)
6 − Subnational (Level 2)

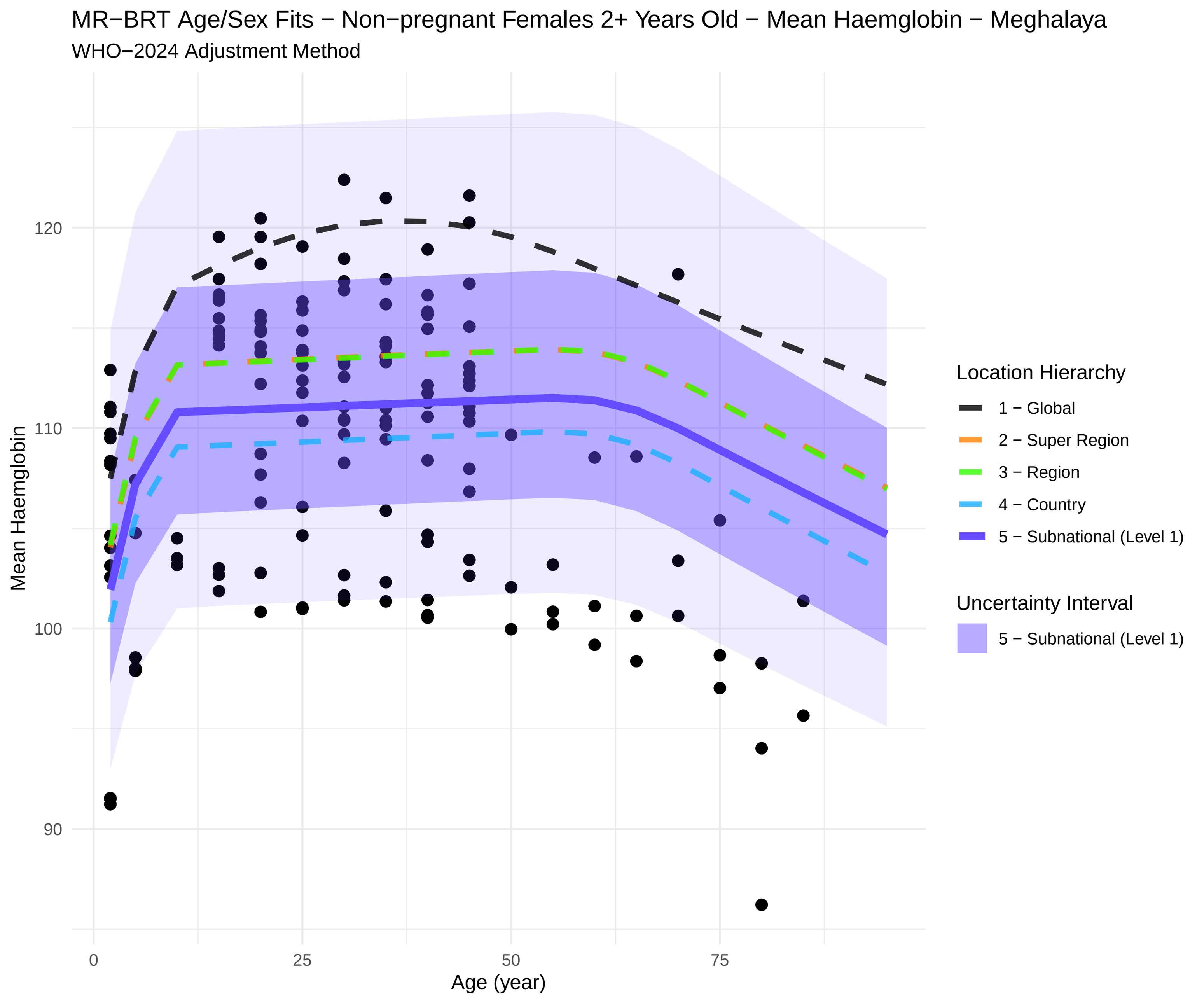
MR−BRT Age/Sex Fits − Non−pregnant Females 2+ Years Old − Mean Haemglobin − Meghalaya
WHO−2024 Adjustment Method
Mean Haemglobin
Age (year)
Location Hierarchy
1 − Global
2 − Super Region
3 − Region
4 − Country
5 − Subnational (Level 1)
Uncertainty Interval
5 − Subnational (Level 1)

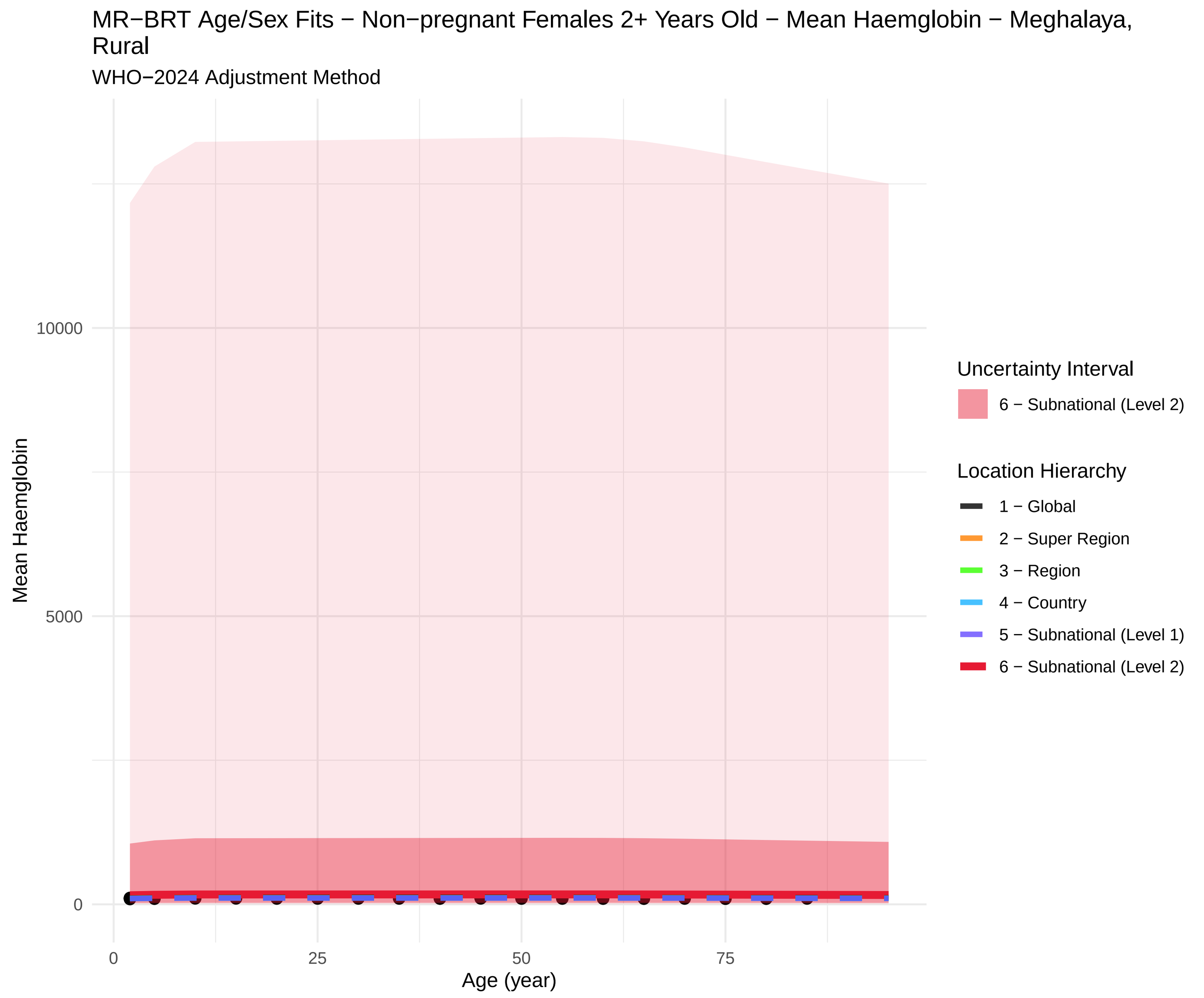
MR−BRT Age/Sex Fits − Non−pregnant Females 2+ Years Old − Mean Haemglobin − Meghalaya, Rural
WHO−2024 Adjustment Method
Mean Haemglobin
Age (year)
Uncertainty Interval
6 − Subnational (Level 2)
Location Hierarchy
1 − Global
2 − Super Region
3 − Region
4 − Country
5 − Subnational (Level 1)
6 − Subnational (Level 2)

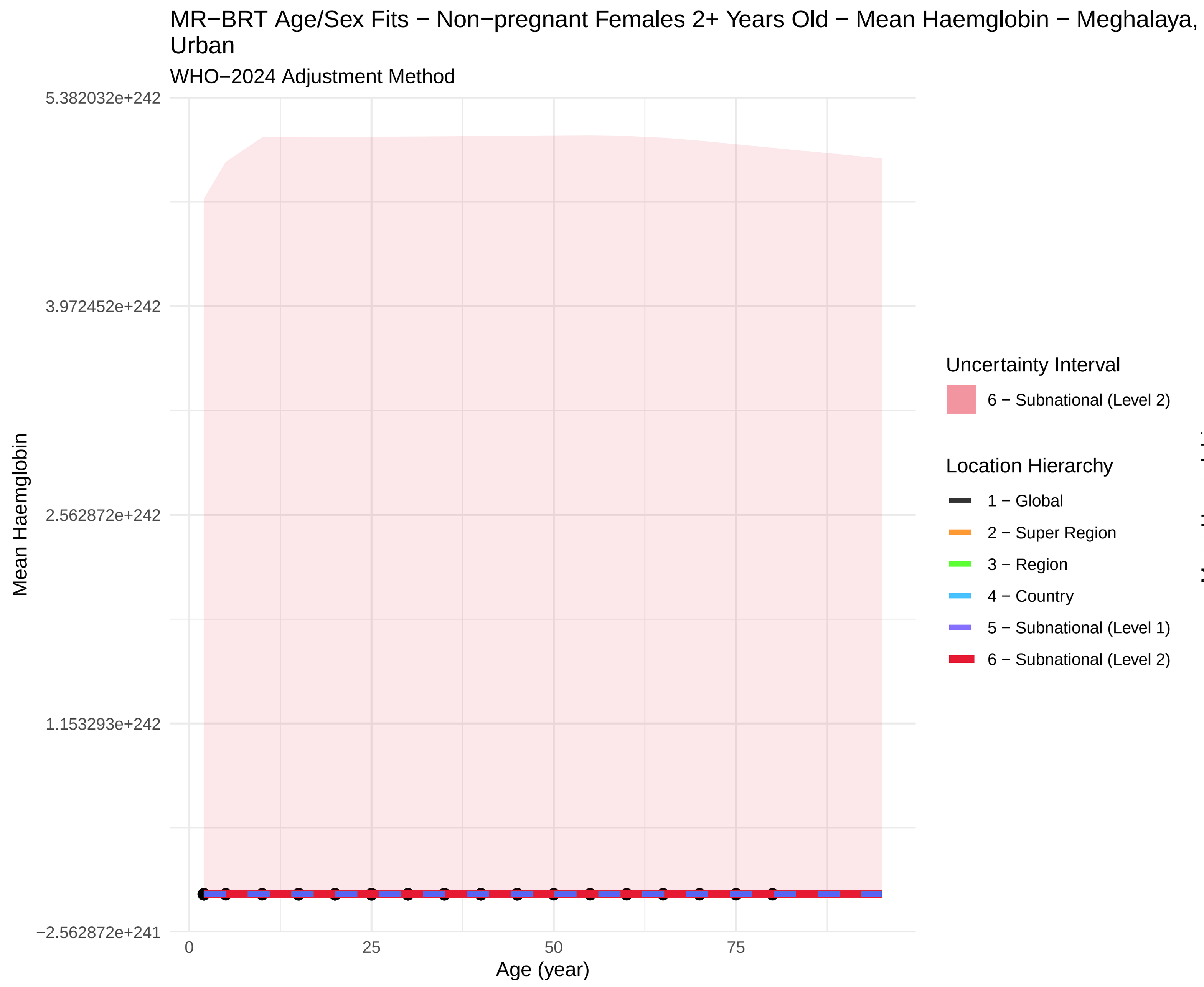
MR−BRT Age/Sex Fits − Non−pregnant Females 2+ Years Old − Mean Haemglobin − Meghalaya, Urban
WHO−2024 Adjustment Method
5.382032e+242
3.972452e+242
2.562872e+242
1.153293e+242
−2.562872e+241
Mean Haemglobin
Age (year)
Uncertainty Interval
6 − Subnational (Level 2)
Location Hierarchy
1 − Global
2 − Super Region
3 − Region
4 − Country
5 − Subnational (Level 1)
6 − Subnational (Level 2)

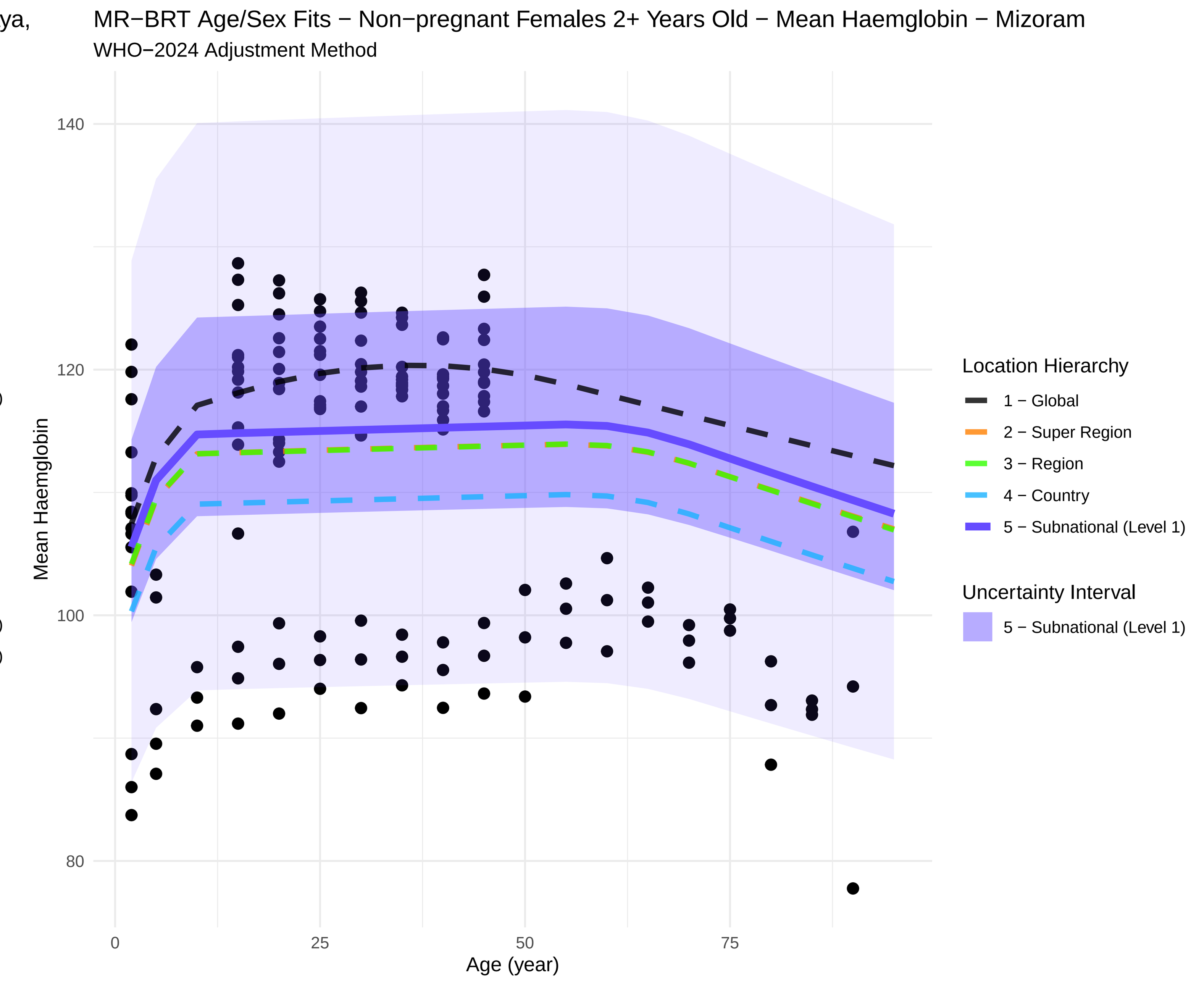
MR−BRT Age/Sex Fits − Non−pregnant Females 2+ Years Old − Mean Haemglobin − Mizoram
WHO−2024 Adjustment Method
Mean Haemglobin
Age (year)
Location Hierarchy
1 − Global
2 − Super Region
3 − Region
4 − Country
5 − Subnational (Level 1)
Uncertainty Interval
5 − Subnational (Level 1)

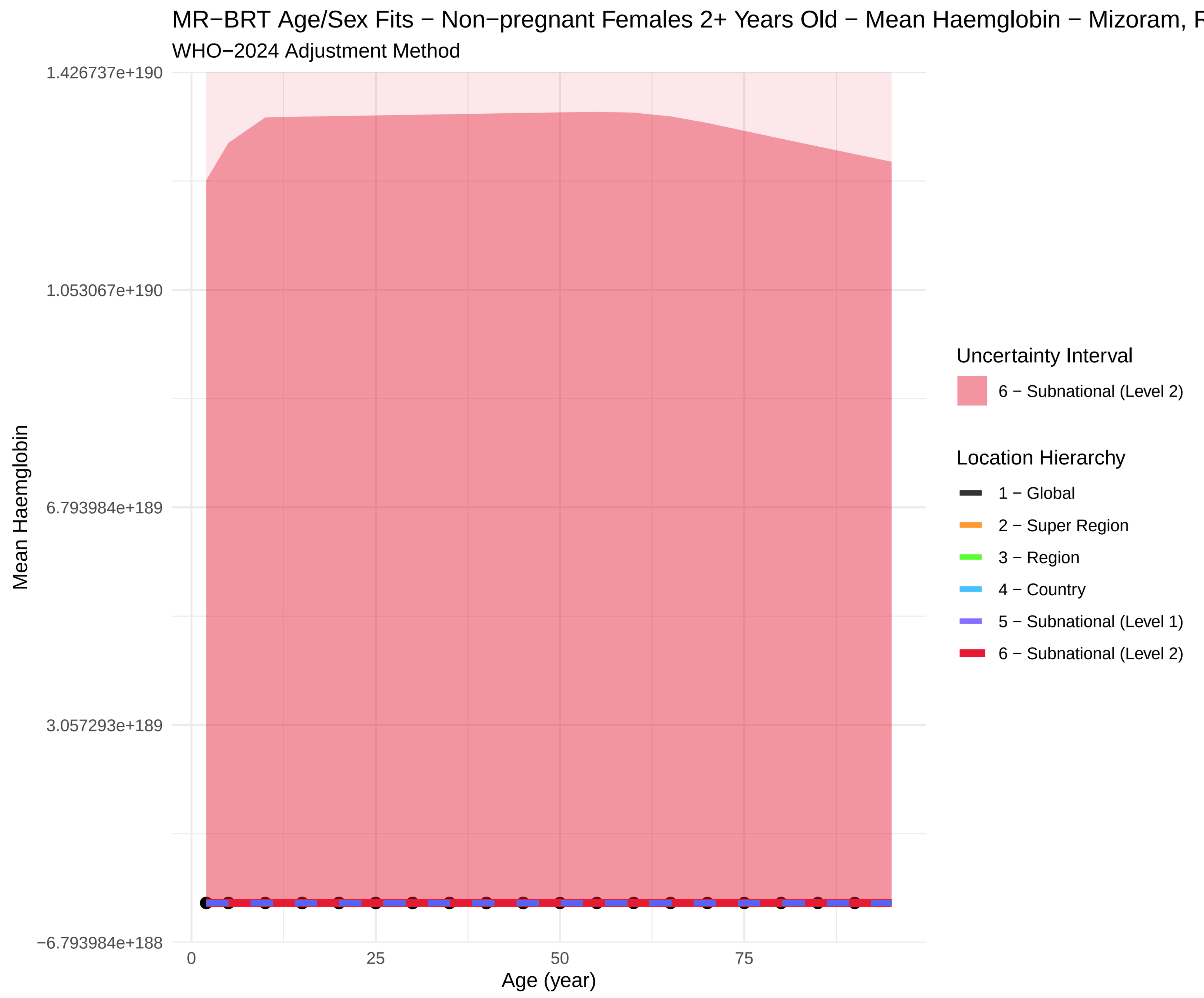
MR−BRT Age/Sex Fits − Non−pregnant Females 2+ Years Old − Mean Haemglobin − Mizoram, Rural
WHO−2024 Adjustment Method
1.426737e+190
1.053067e+190
6.793984e+189
3.057293e+189
−6.793984e+188
Mean Haemglobin
Age (year)
Uncertainty Interval
6 − Subnational (Level 2)
Location Hierarchy
1 − Global
2 − Super Region
3 − Region
4 − Country
5 − Subnational (Level 1)
6 − Subnational (Level 2)

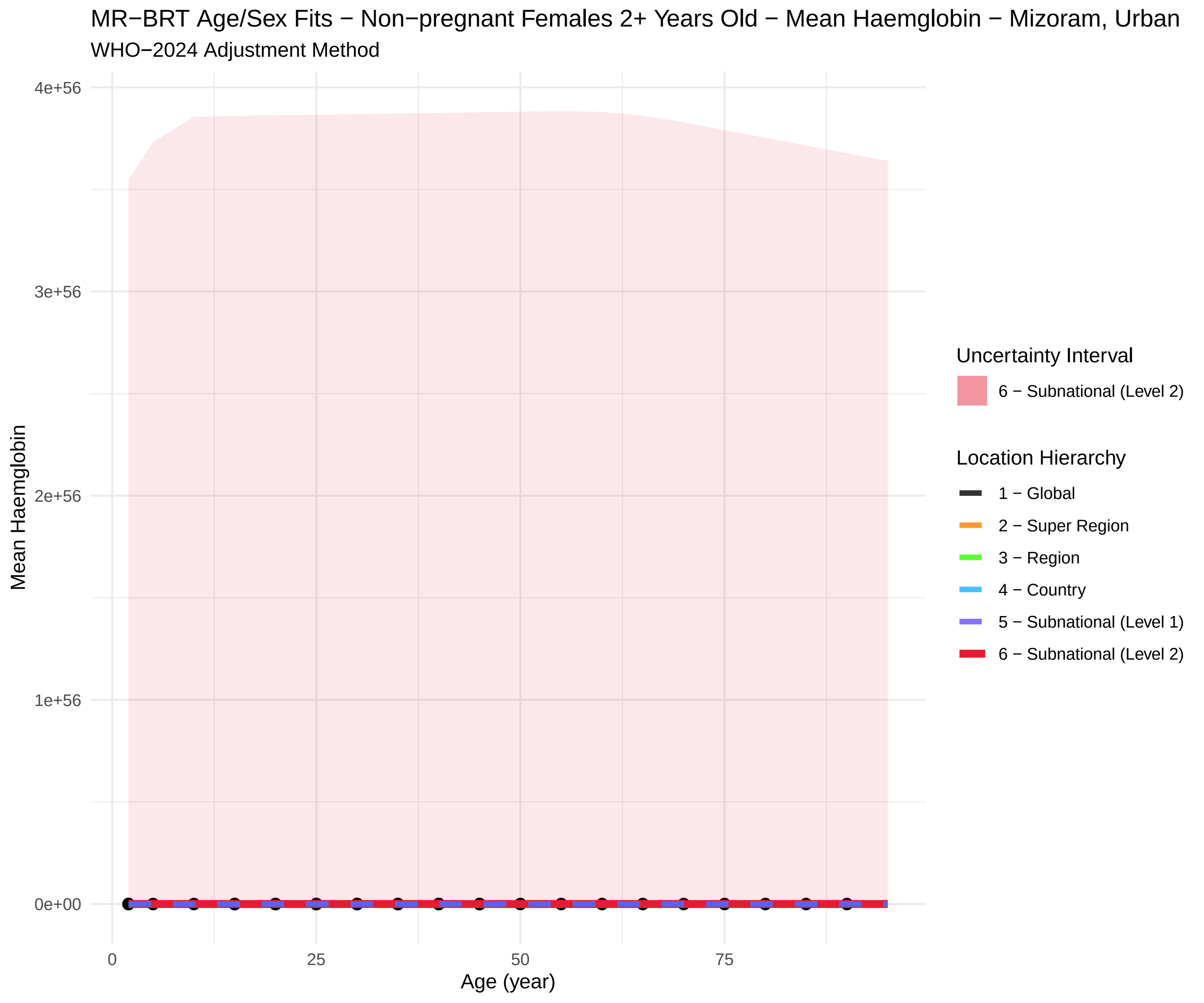
MR−BRT Age/Sex Fits − Non−pregnant Females 2+ Years Old − Mean Haemglobin − Mizoram, Urban
WHO−2024 Adjustment Method
4e+56
3e+56
2e+56
1e+56
0e+00
Mean Haemglobin
Age (year)
Uncertainty Interval
6 − Subnational (Level 2)
Location Hierarchy
1 − Global
2 − Super Region
3 − Region
4 − Country
5 − Subnational (Level 1)
6 − Subnational (Level 2)

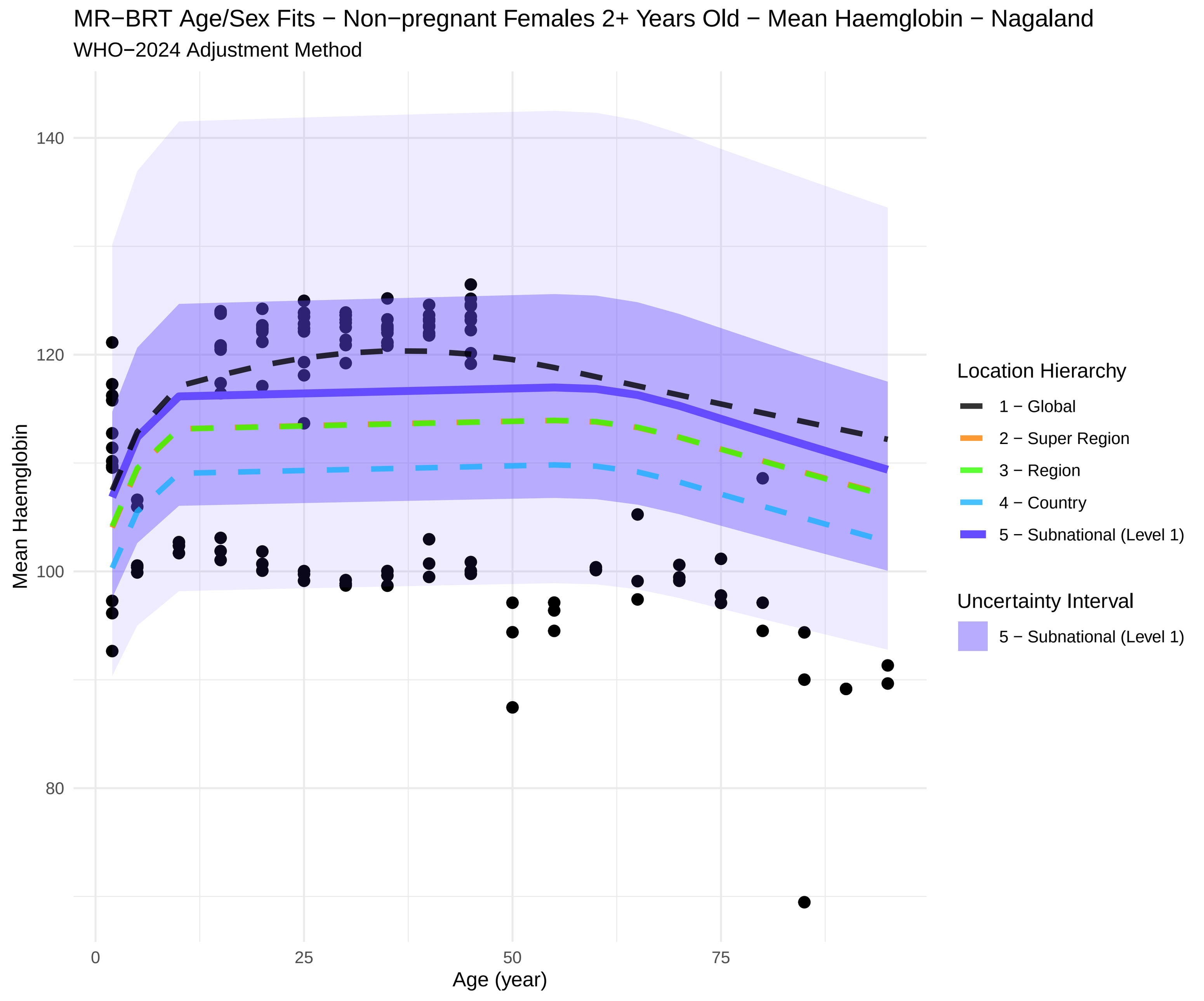
MR−BRT Age/Sex Fits − Non−pregnant Females 2+ Years Old − Mean Haemglobin − Nagaland
WHO−2024 Adjustment Method
Mean Haemglobin
Age (year)
Location Hierarchy
1 − Global
2 − Super Region
3 − Region
4 − Country
5 − Subnational (Level 1)
Uncertainty Interval
5 − Subnational (Level 1)

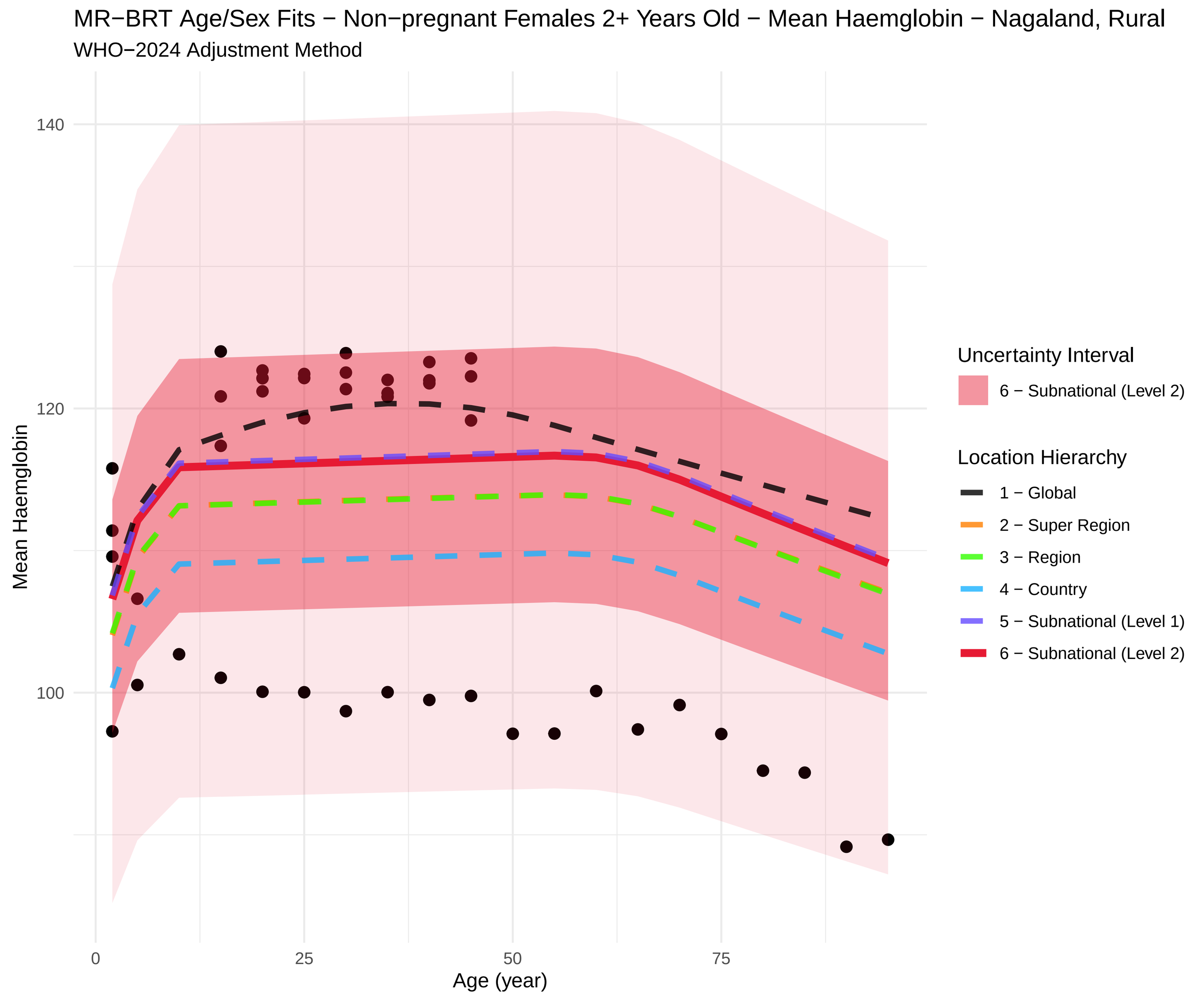
MR−BRT Age/Sex Fits − Non−pregnant Females 2+ Years Old − Mean Haemglobin − Nagaland, Rural
WHO−2024 Adjustment Method
Mean Haemglobin
Age (year)
Uncertainty Interval
6 − Subnational (Level 2)
Location Hierarchy
1 − Global
2 − Super Region
3 − Region
4 − Country
5 − Subnational (Level 1)
6 − Subnational (Level 2)

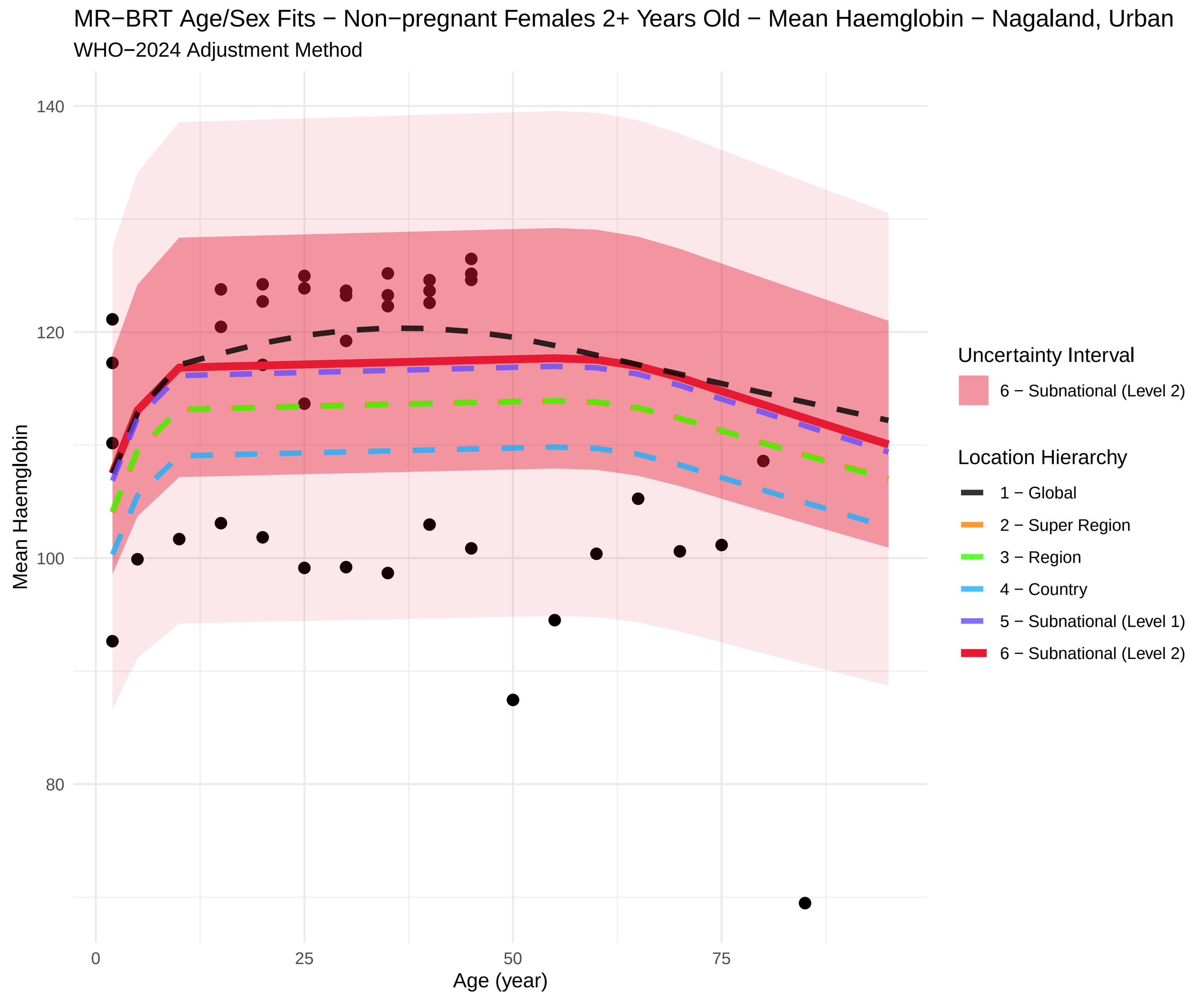
MR−BRT Age/Sex Fits − Non−pregnant Females 2+ Years Old − Mean Haemglobin − Nagaland, Urban
WHO−2024 Adjustment Method
Mean Haemglobin
Age (year)
Uncertainty Interval
6 − Subnational (Level 2)
Location Hierarchy
1 − Global
2 − Super Region
3 − Region
4 − Country
5 − Subnational (Level 1)
6 − Subnational (Level 2)

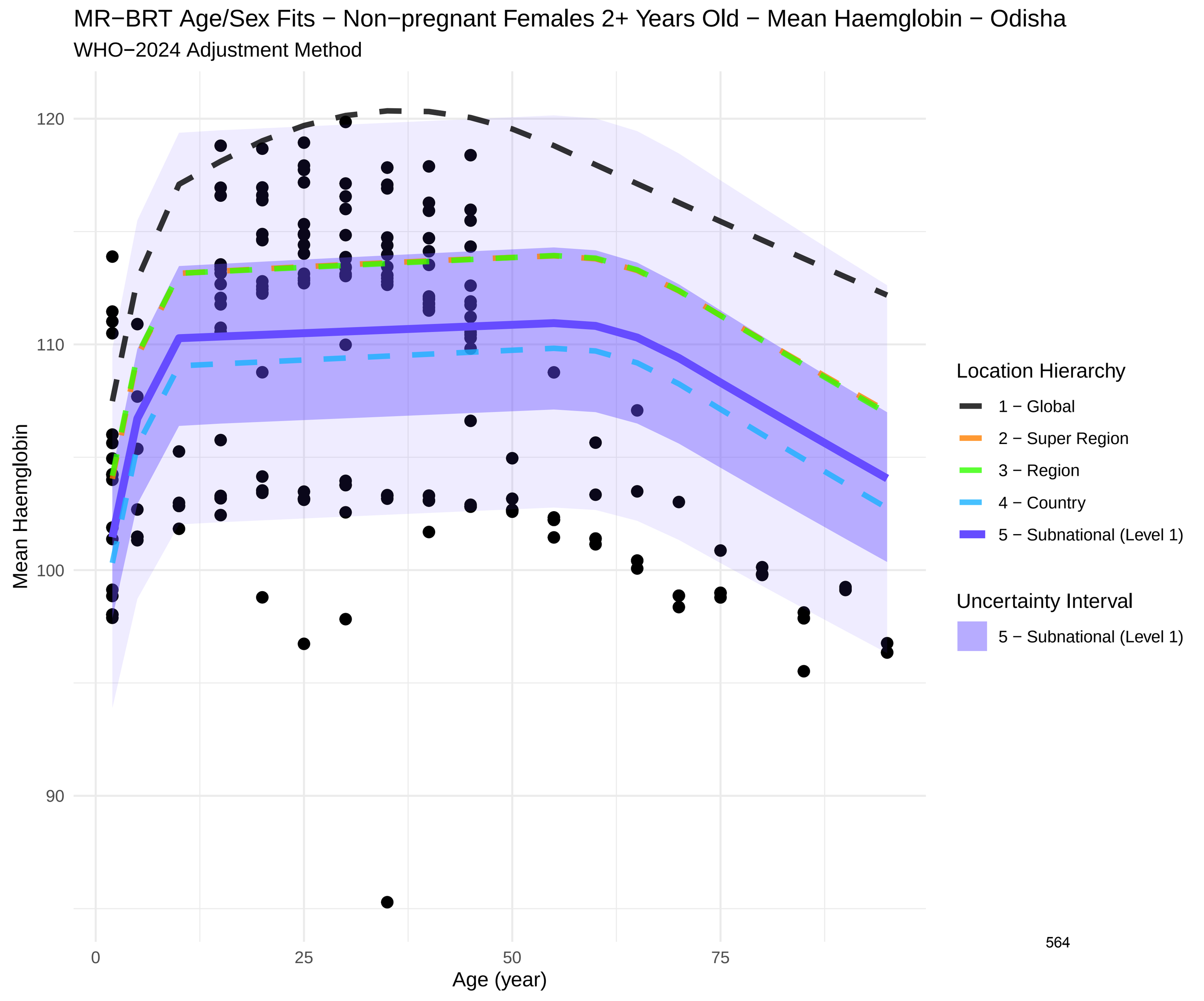
MR−BRT Age/Sex Fits − Non−pregnant Females 2+ Years Old − Mean Haemglobin − Odisha
WHO−2024 Adjustment Method
Mean Haemglobin
Age (year)
Location Hierarchy
1 − Global
2 − Super Region
3 − Region
4 − Country
5 − Subnational (Level 1)
Uncertainty Interval
5 − Subnational (Level 1)

MR−BRT Age/Sex Fits − Non−pregnant Females 2+ Years Old − Mean Haemglobin − Odisha, Rural
WHO−2024 Adjustment Method

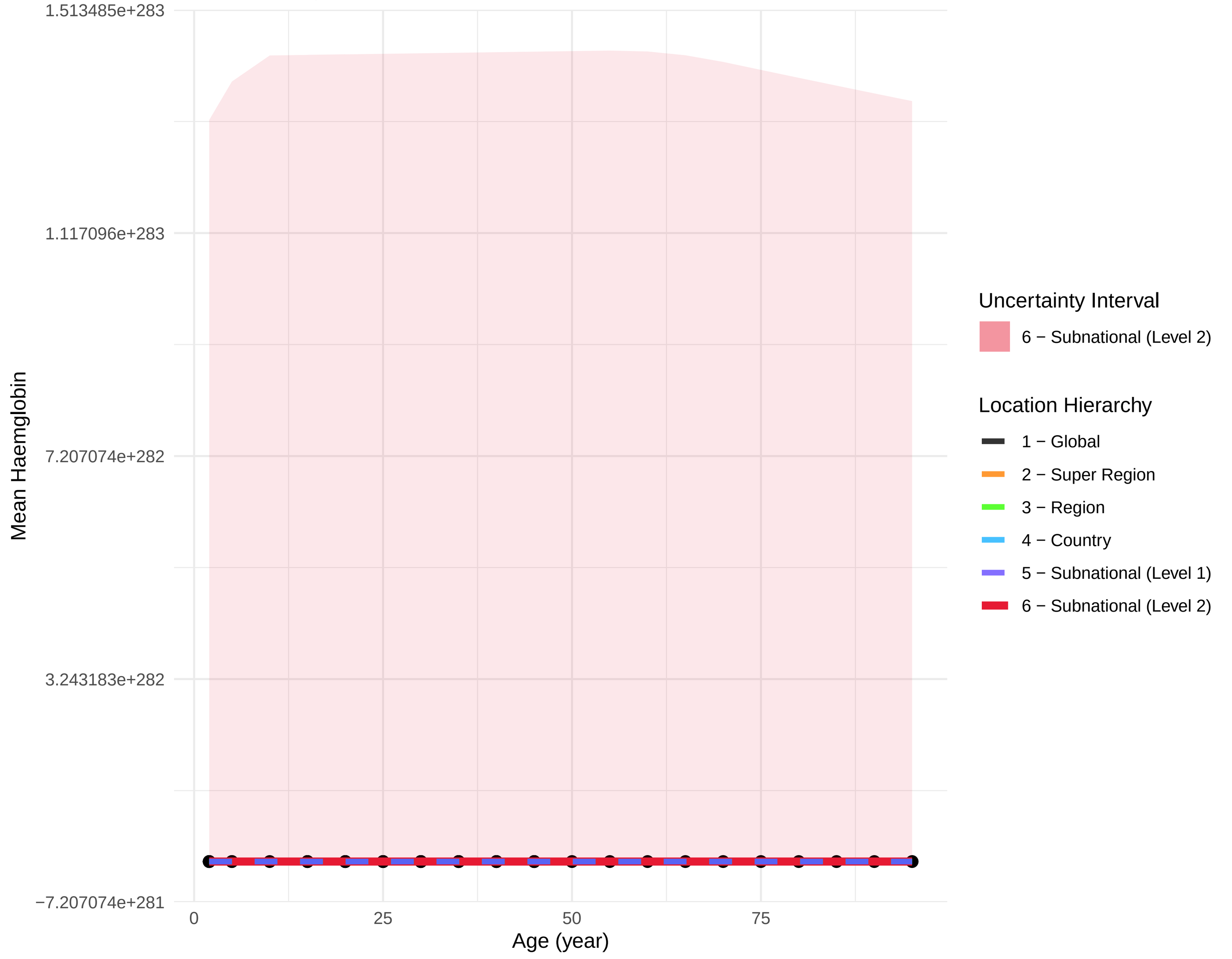

MR−BRT Age/Sex Fits − Non−pregnant Females 2+ Years Old − Mean Haemglobin − Odisha, Urban
WHO−2024 Adjustment Method

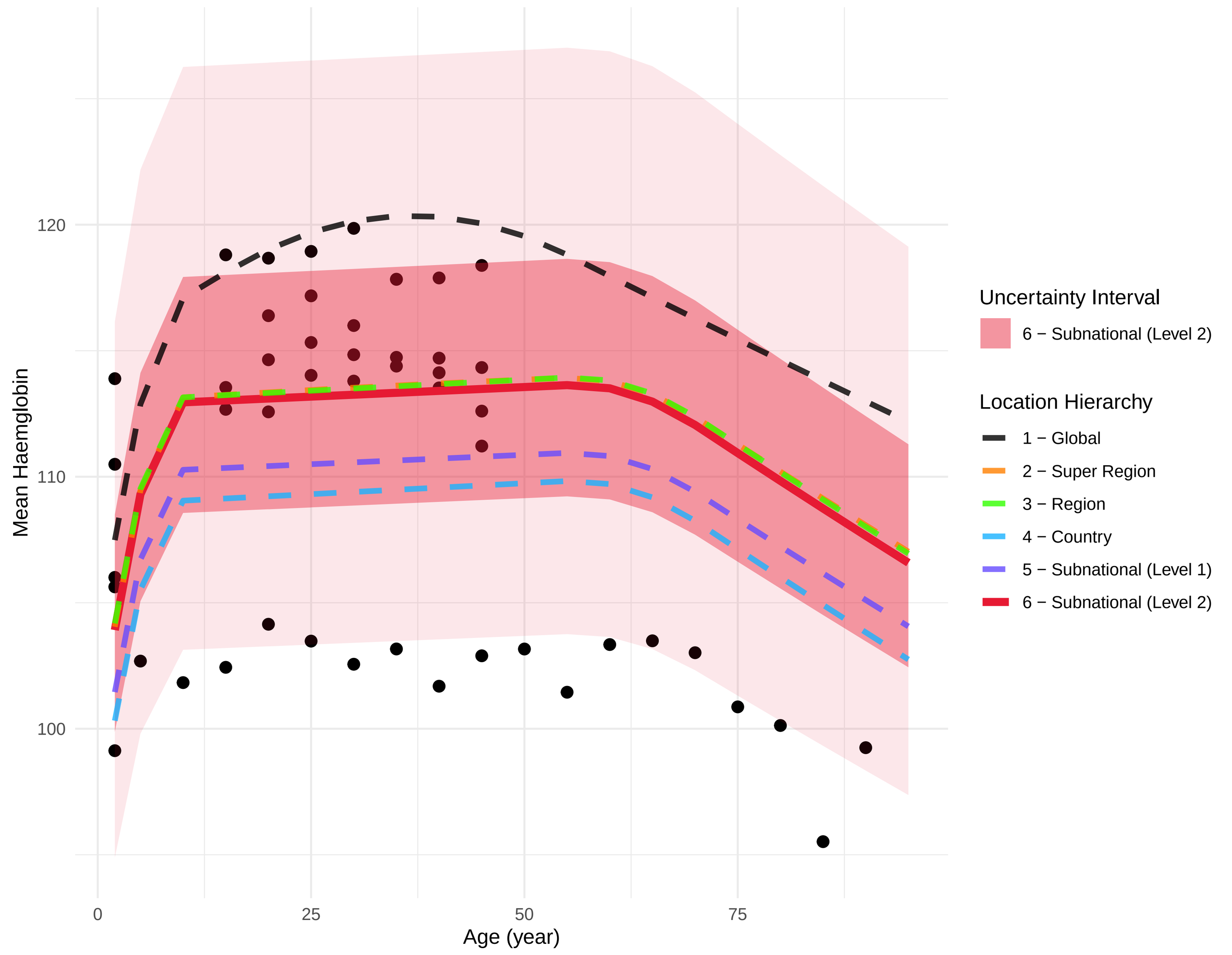

MR−BRT Age/Sex Fits − Non−pregnant Females 2+ Years Old − Mean Haemglobin − Other Union Territories
WHO−2024 Adjustment Method

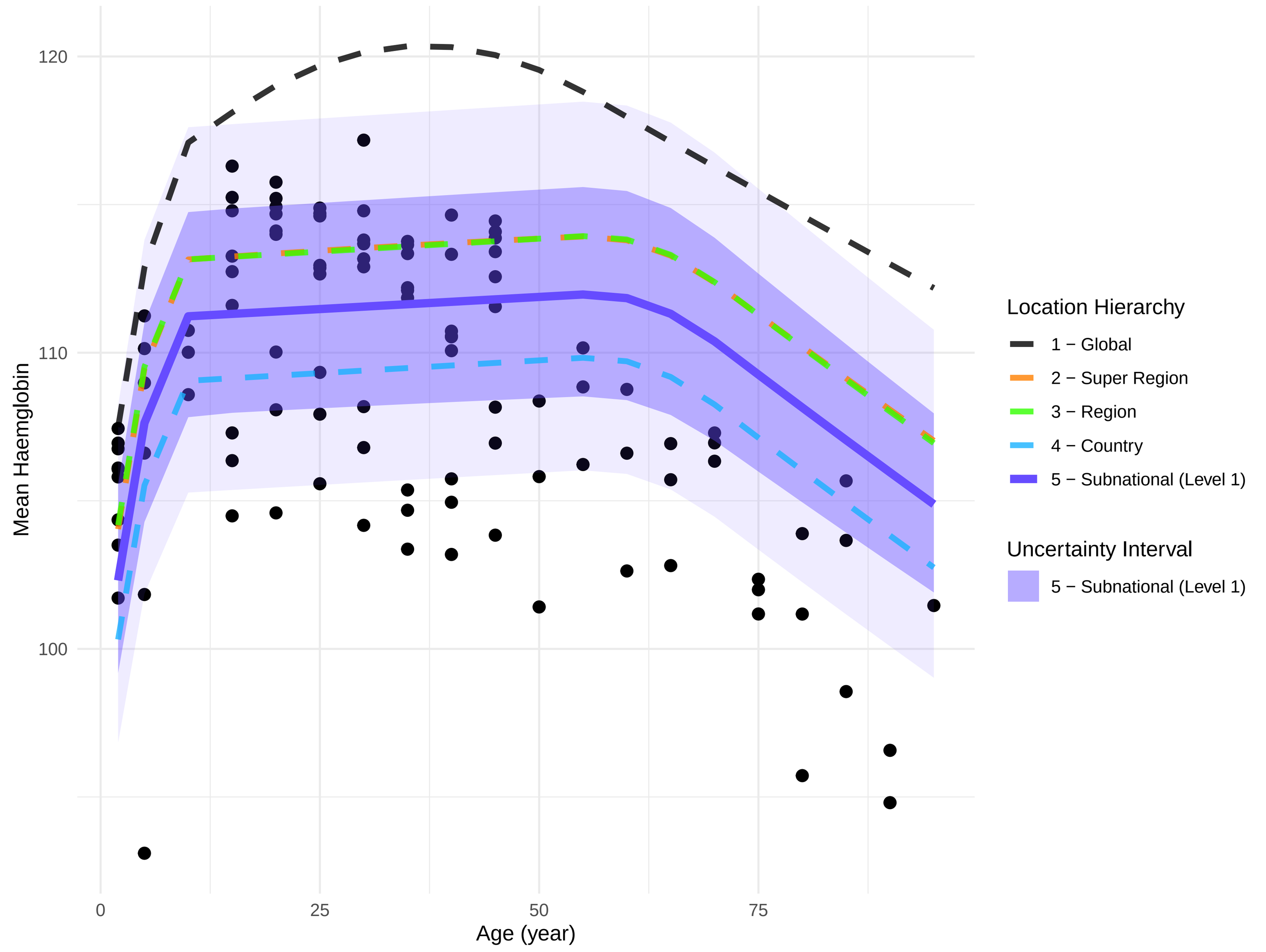

MR−BRT Age/Sex Fits − Non−pregnant Females 2+ Years Old − Mean Haemglobin − Other Union Territories, Rural
WHO−2024 Adjustment Method

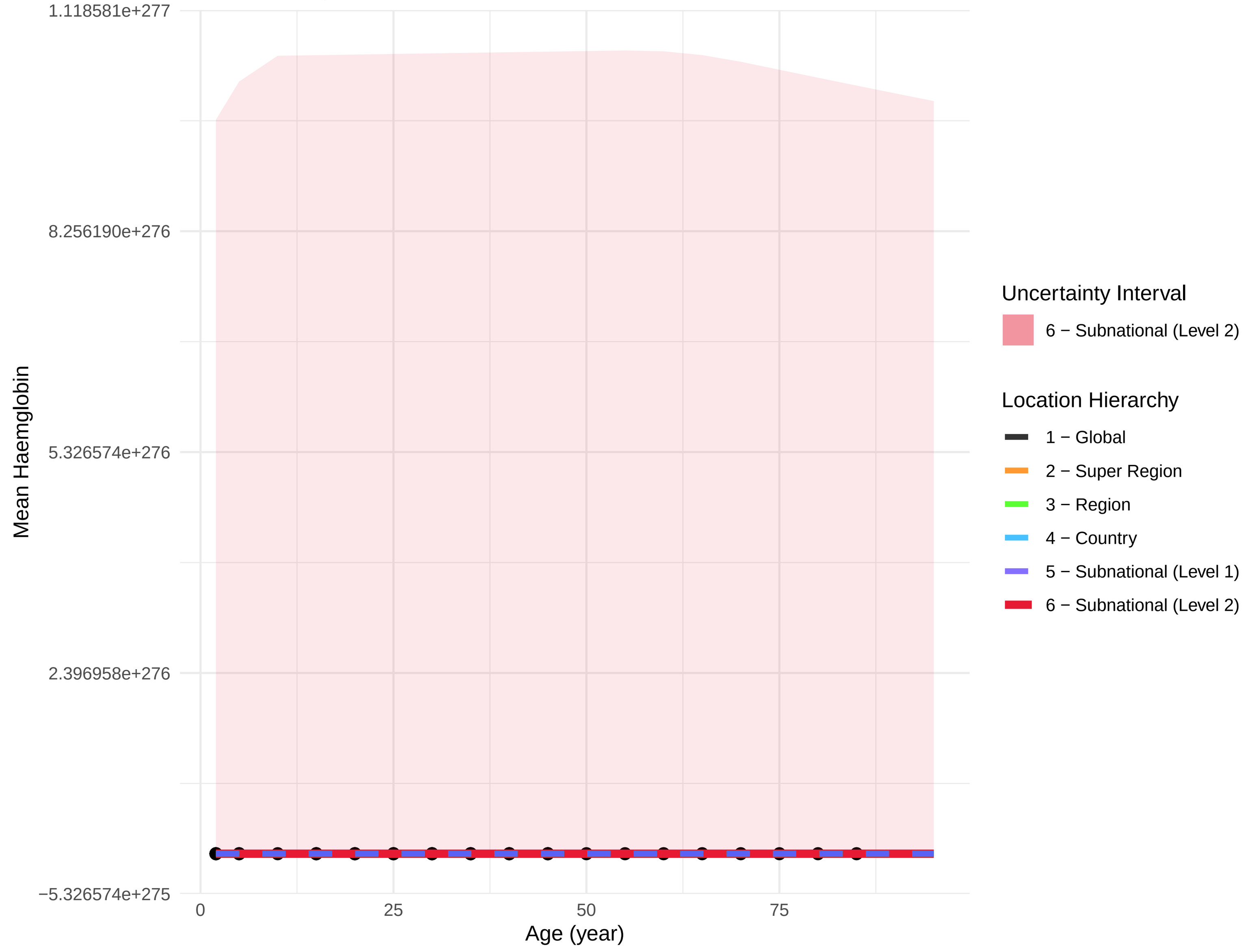

MR−BRT Age/Sex Fits − Non−pregnant Females 2+ Years Old − Mean Haemglobin − Other Union Territories, Urban
WHO−2024 Adjustment Method

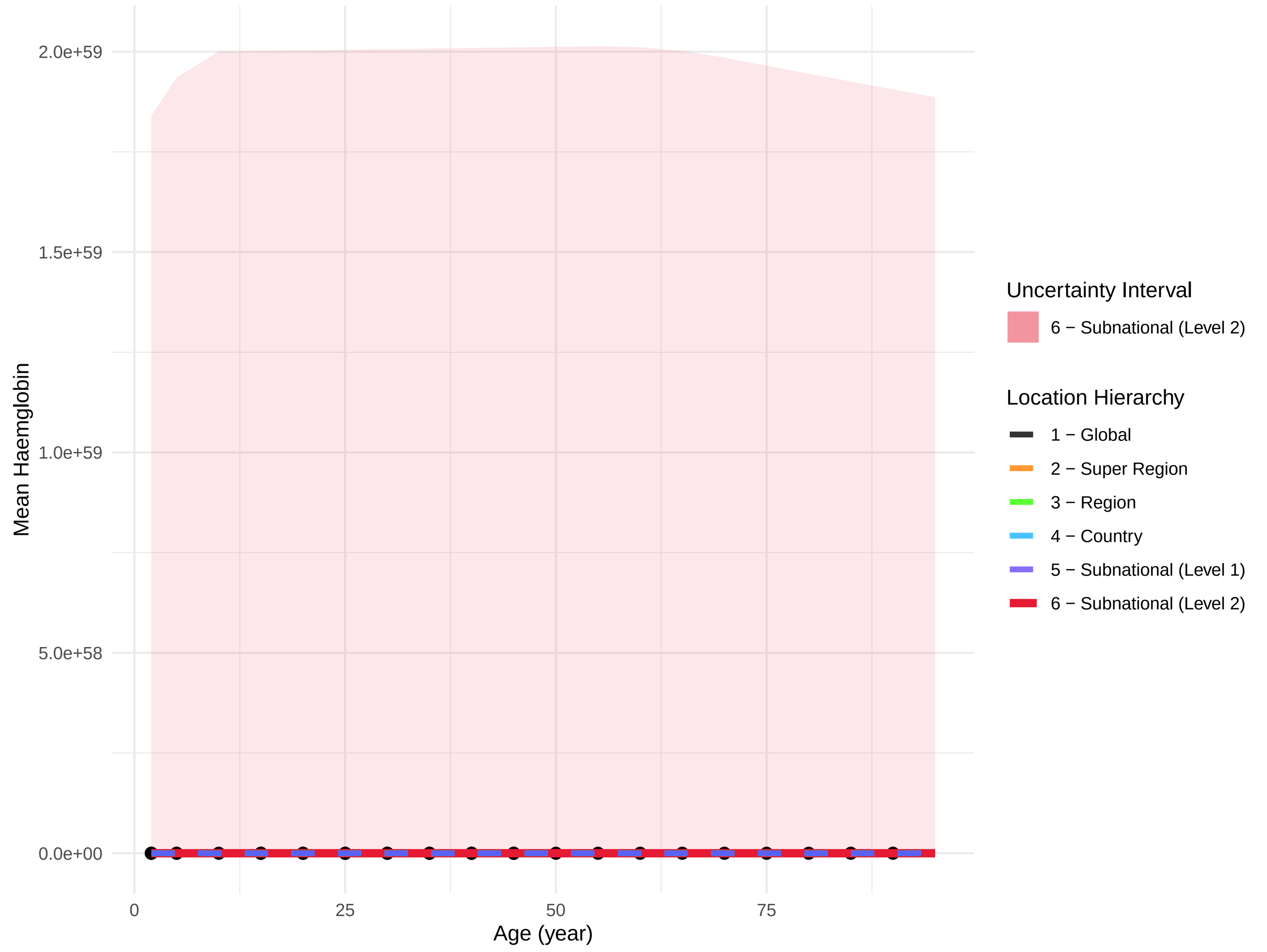

MR−BRT Age/Sex Fits − Non−pregnant Females 2+ Years Old − Mean Haemglobin − Punjab
WHO−2024 Adjustment Method

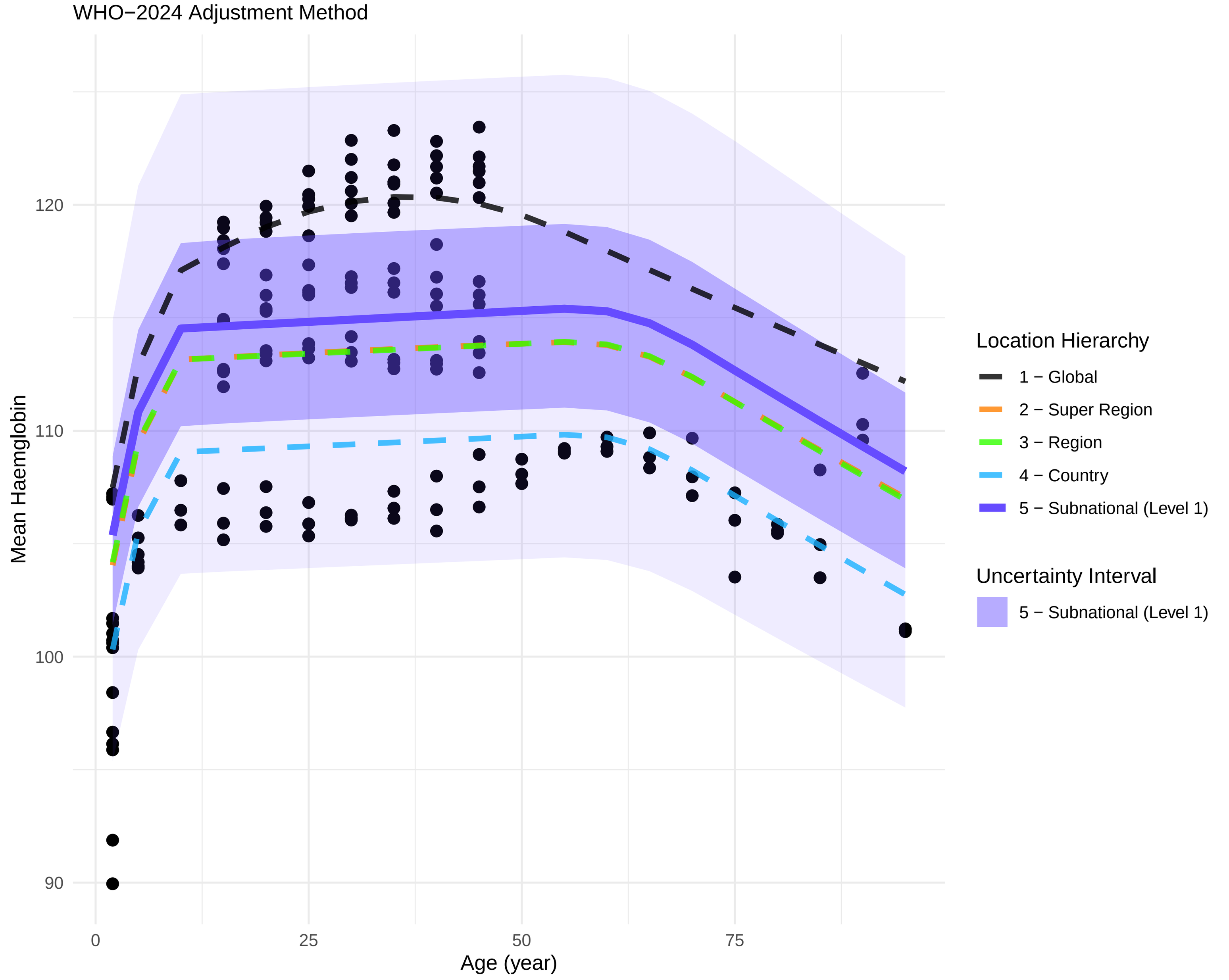

MR−BRT Age/Sex Fits − Non−pregnant Females 2+ Years Old − Mean Haemglobin − Punjab, Rural
WHO−2024 Adjustment Method

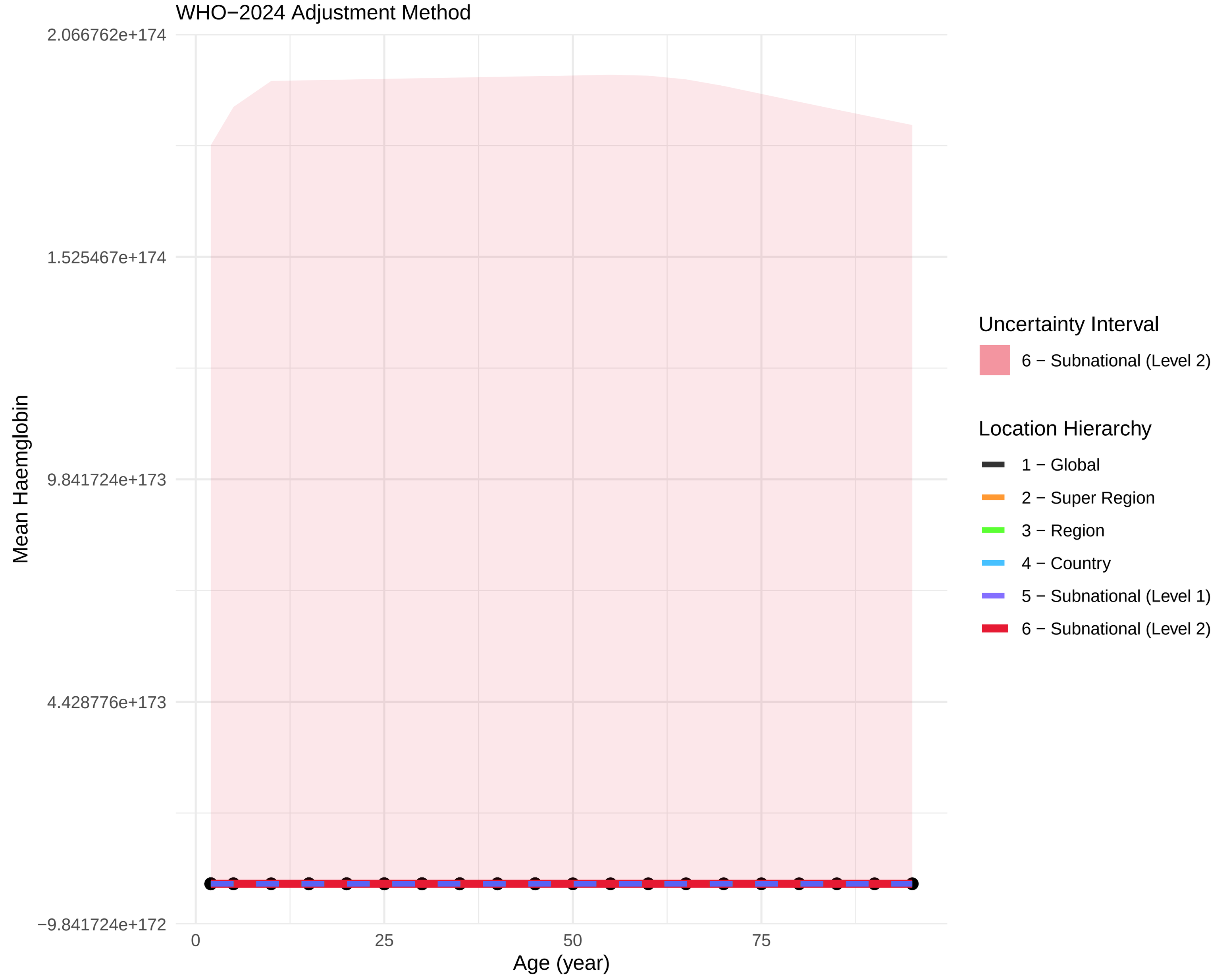

MR−BRT Age/Sex Fits − Non−pregnant Females 2+ Years Old − Mean Haemglobin − Punjab, Urban
WHO−2024 Adjustment Method

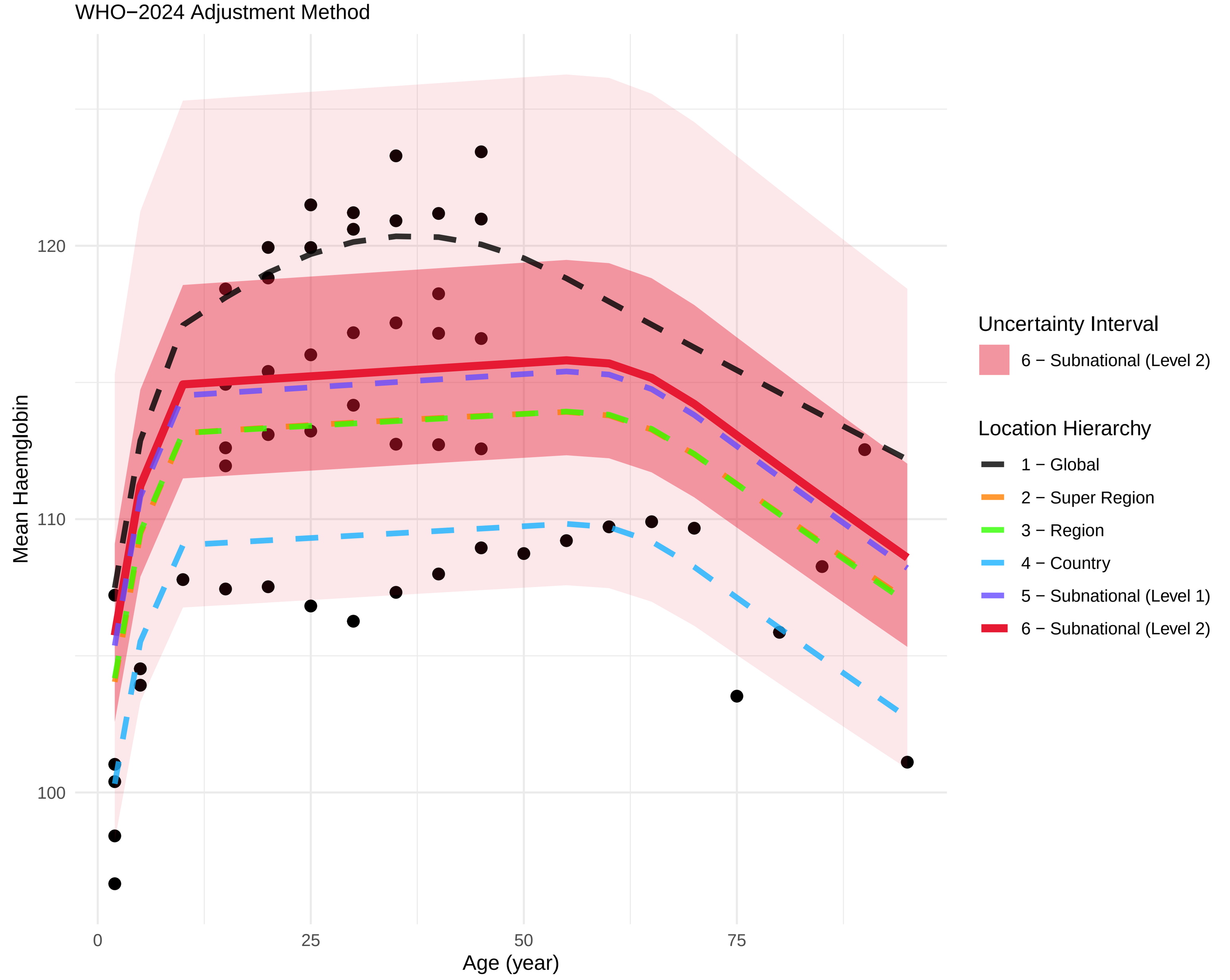

MR−BRT Age/Sex Fits − Non−pregnant Females 2+ Years Old − Mean Haemglobin − Rajasthan
WHO−2024 Adjustment Method

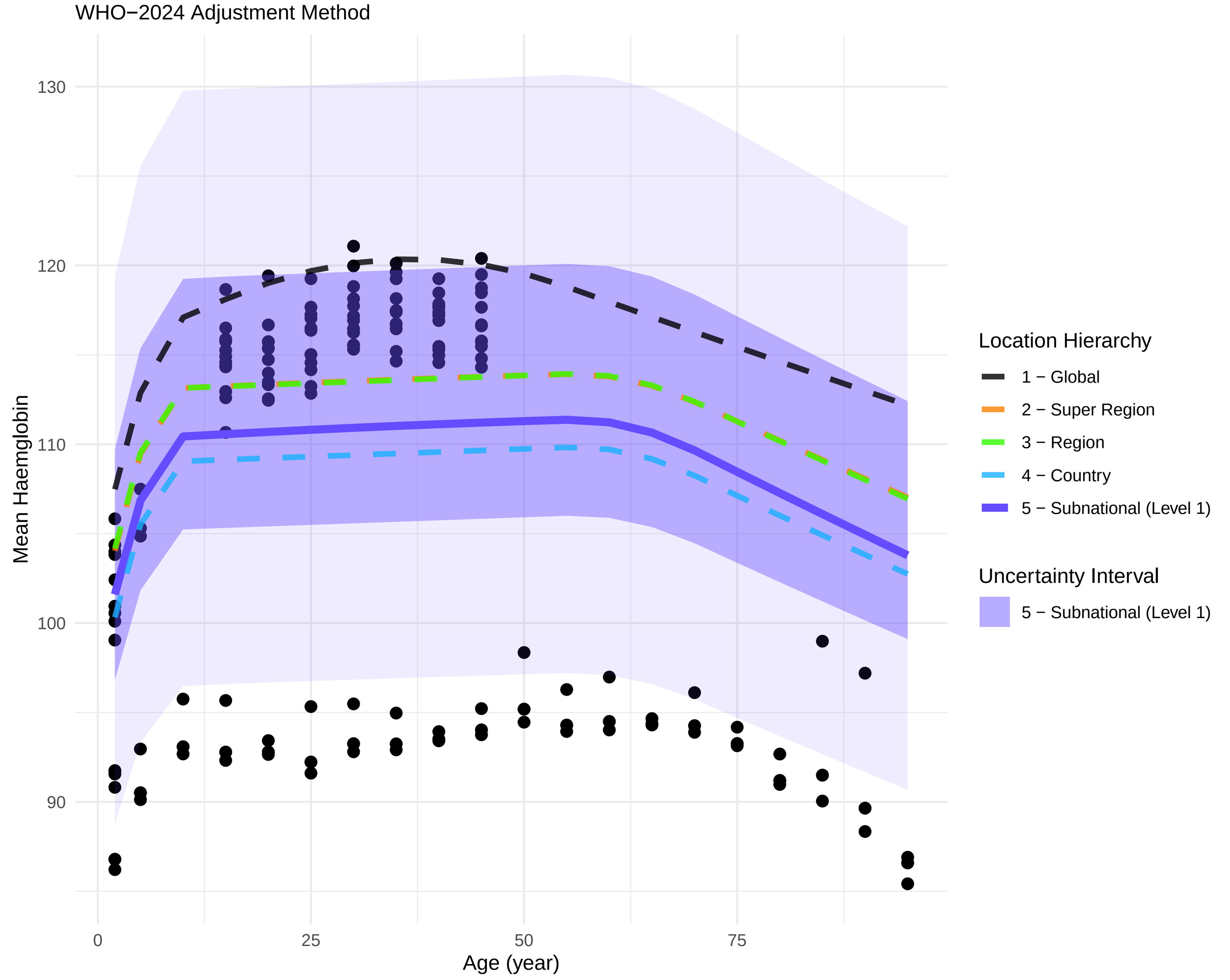

MR−BRT Age/Sex Fits − Non−pregnant Females 2+ Years Old − Mean Haemglobin − Rajasthan, Rural
WHO−2024 Adjustment Method

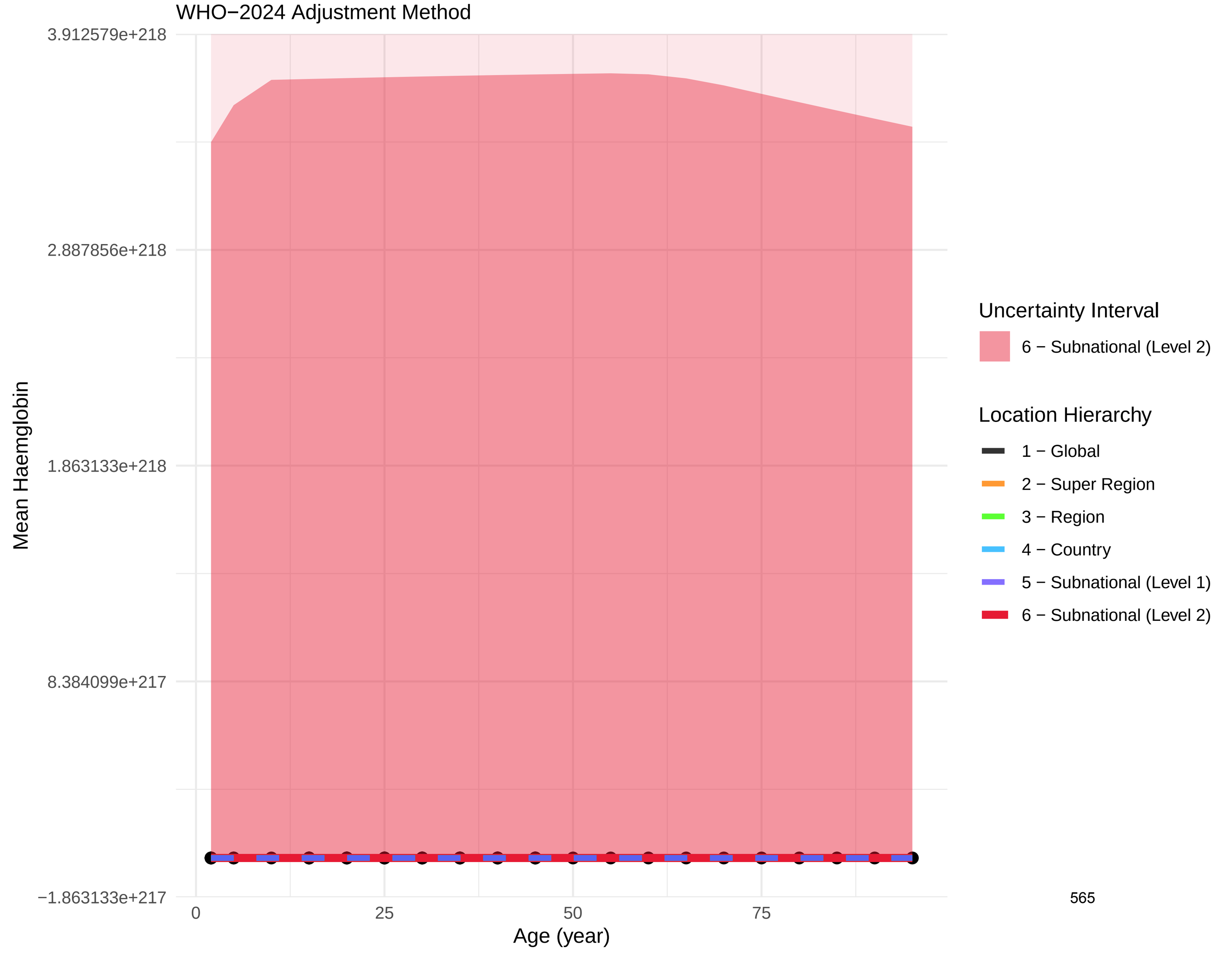

MR−BRT Age/Sex Fits − Non−pregnant Females 2+ Years Old − Mean Haemglobin − Rajasthan, Urban

WHO−2024 Adjustment Method

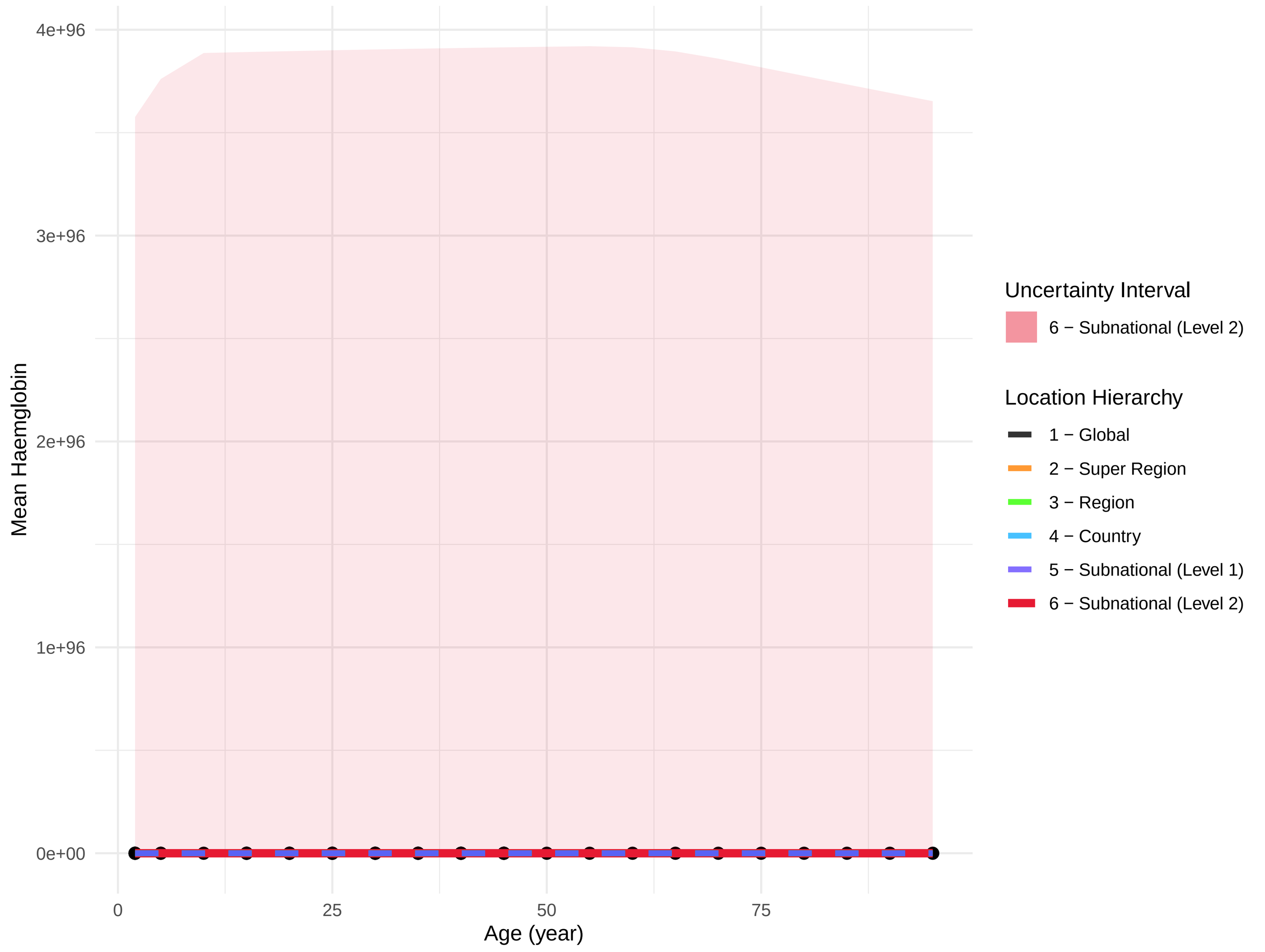

MR−BRT Age/Sex Fits − Non−pregnant Females 2+ Years Old − Mean Haemglobin − Sikkim

WHO−2024 Adjustment Method

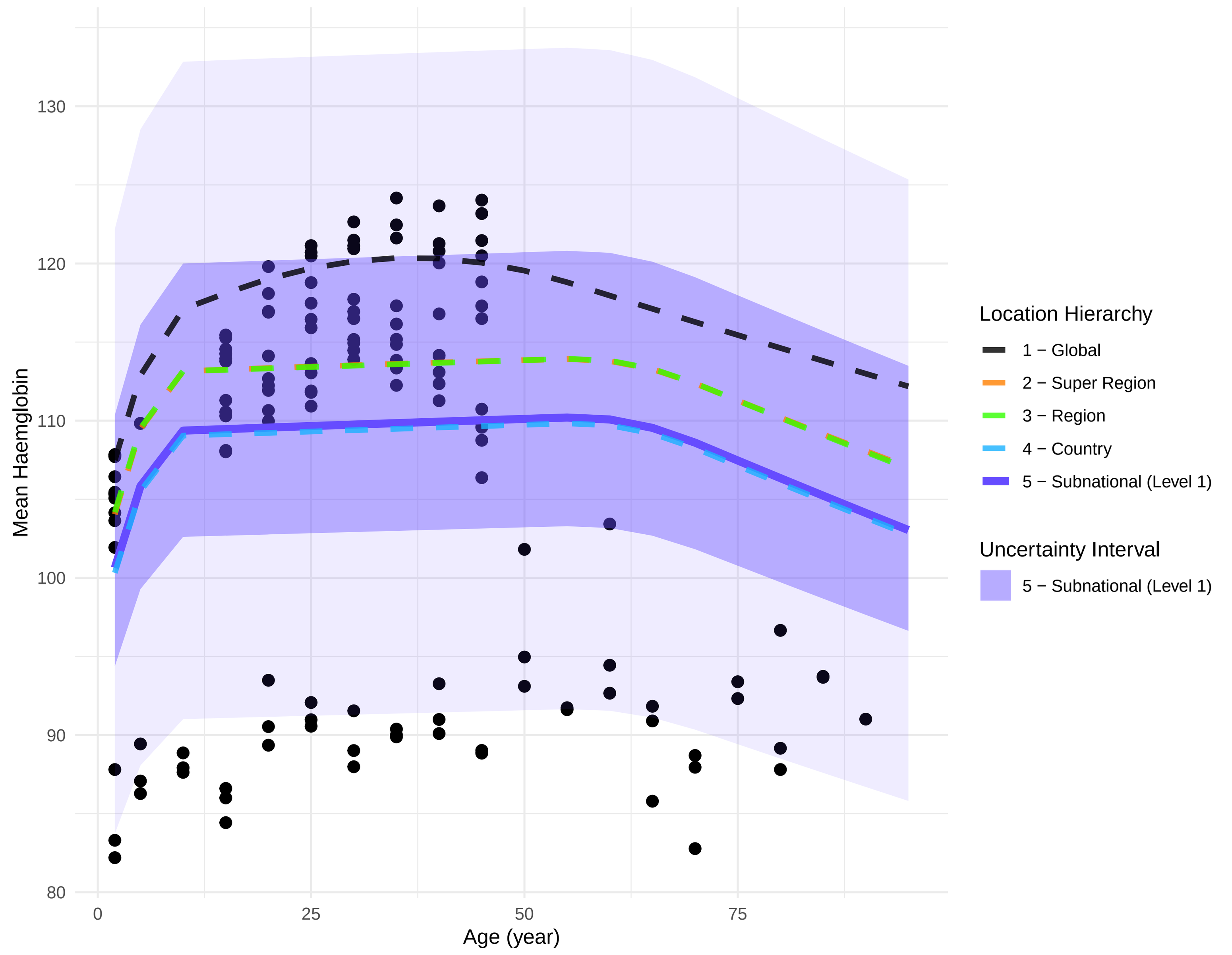

MR−BRT Age/Sex Fits − Non−pregnant Females 2+ Years Old − Mean Haemglobin − Sikkim, Rural

WHO−2024 Adjustment Method

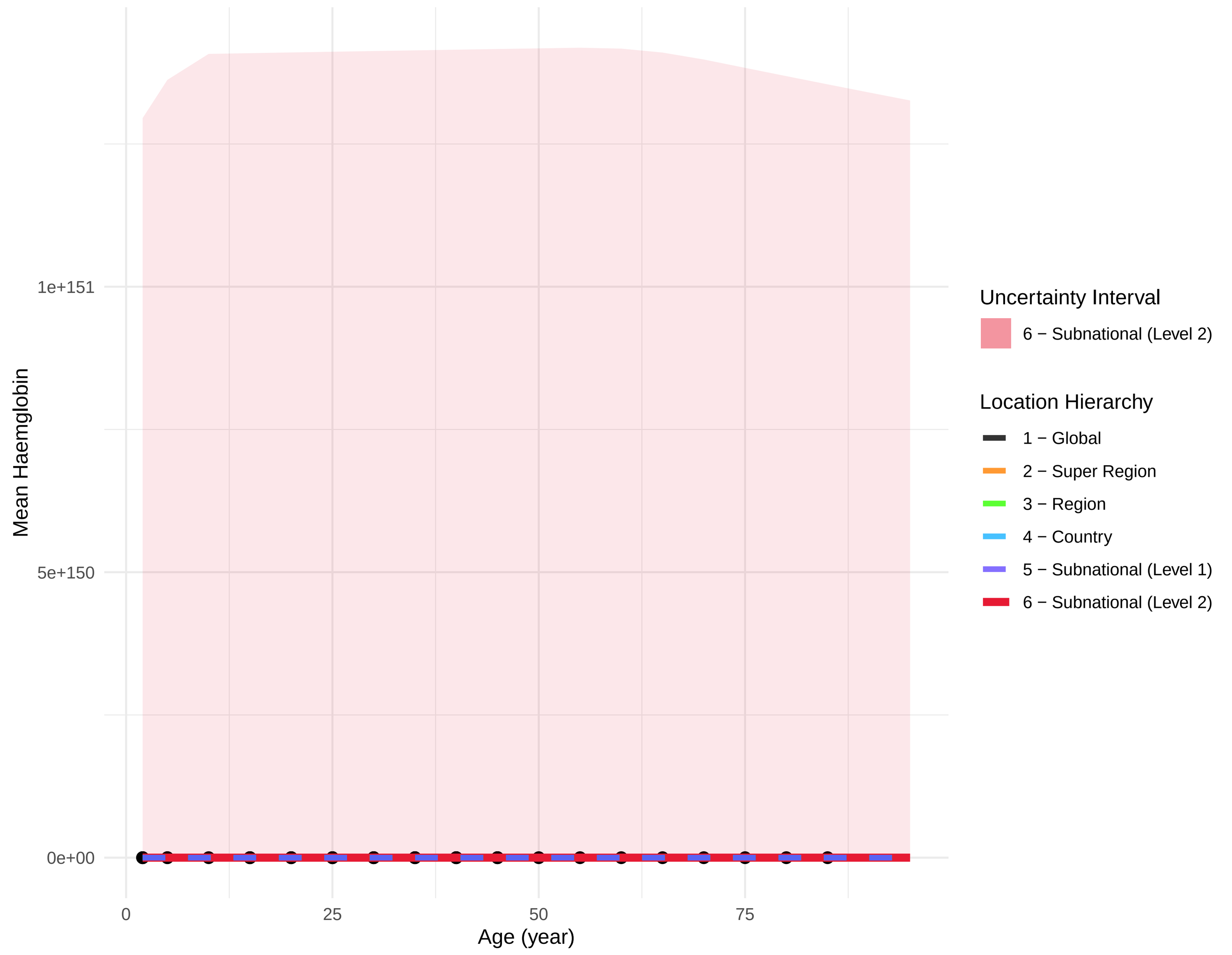

MR−BRT Age/Sex Fits − Non−pregnant Females 2+ Years Old − Mean Haemglobin − Sikkim, Urban

WHO−2024 Adjustment Method

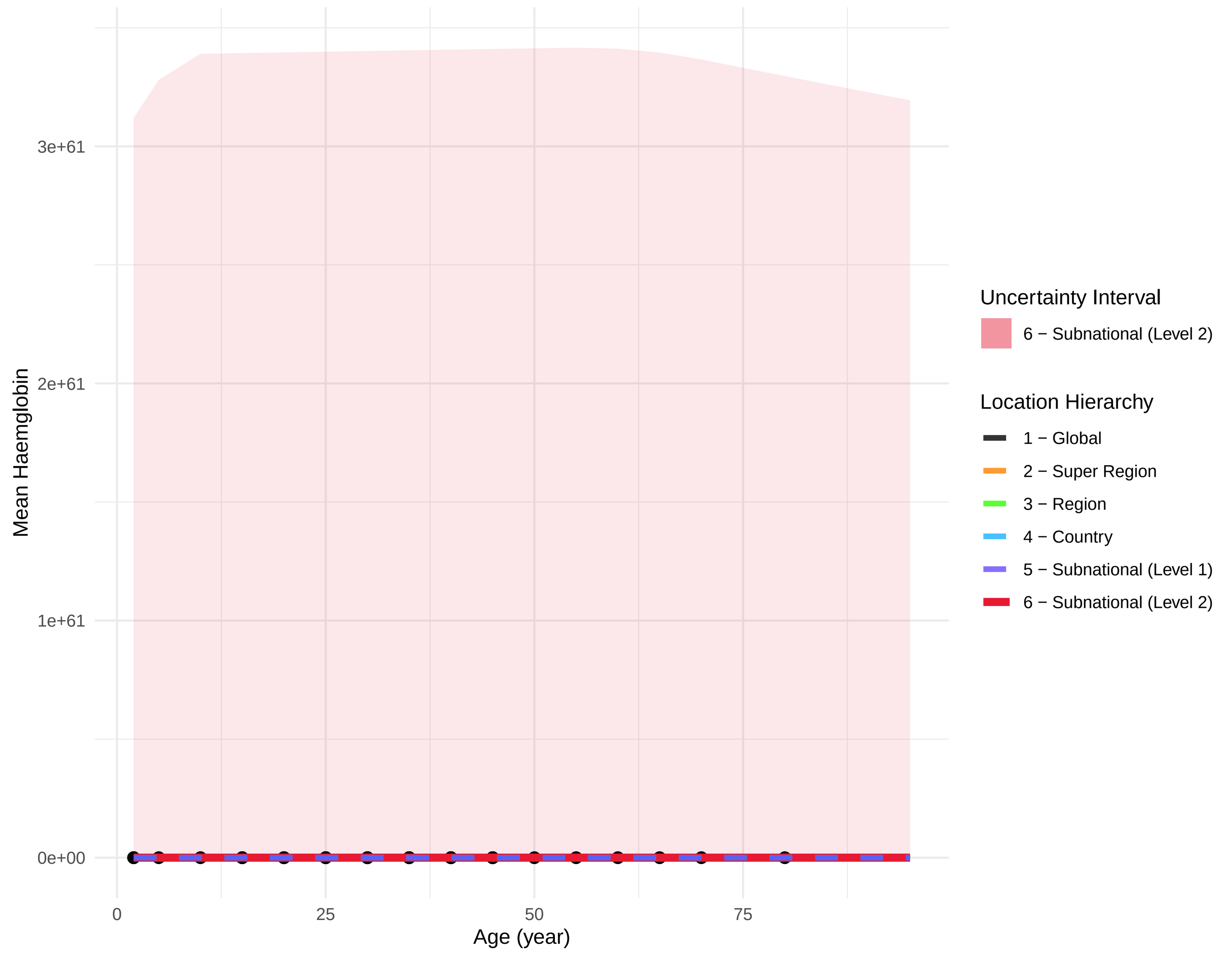

MR−BRT Age/Sex Fits − Non−pregnant Females 2+ Years Old − Mean Haemglobin − Tamil Nadu

WHO−2024 Adjustment Method

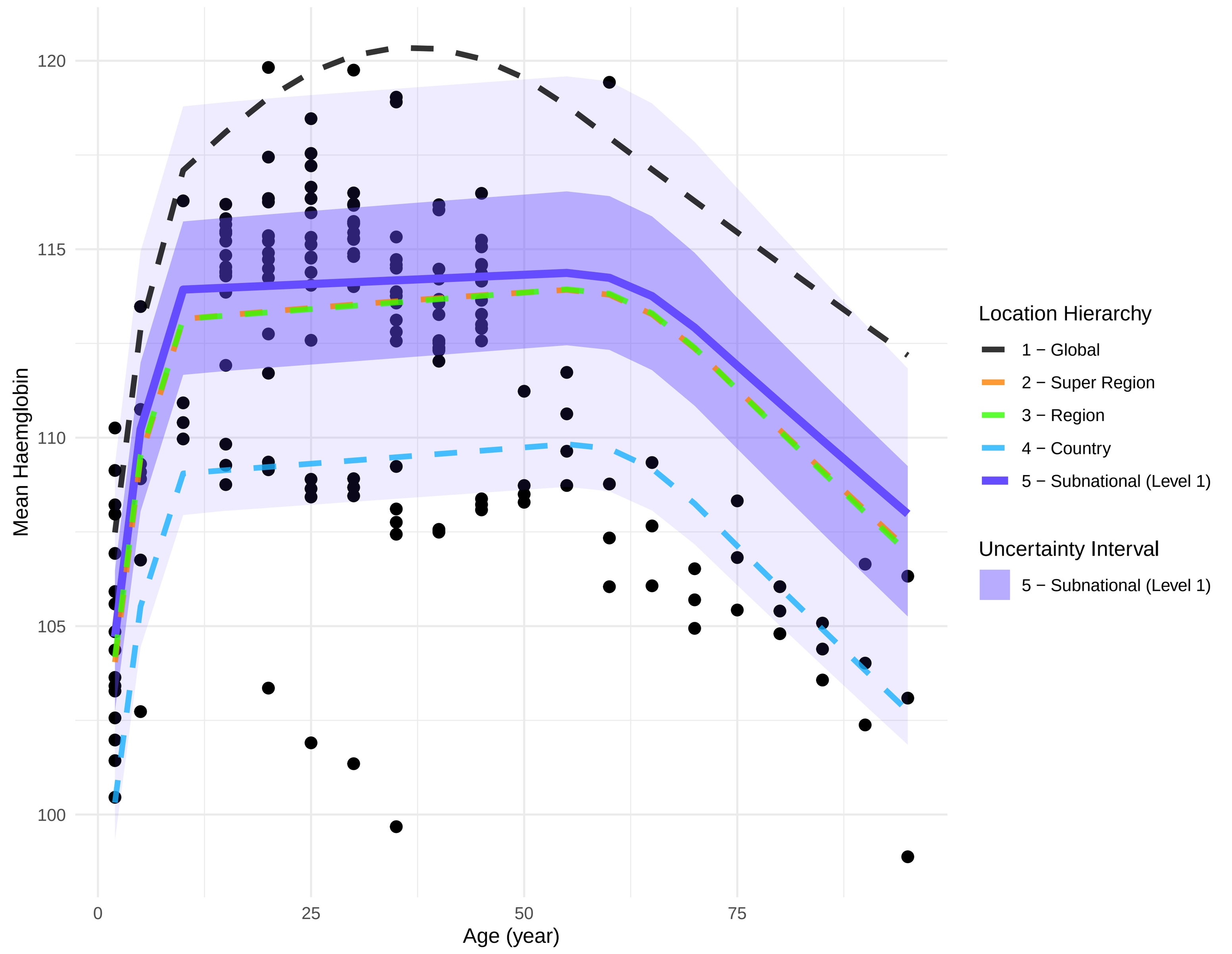

MR−BRT Age/Sex Fits − Non−pregnant Females 2+ Years Old − Mean Haemglobin − Tamil Nadu, Rural

WHO−2024 Adjustment Method

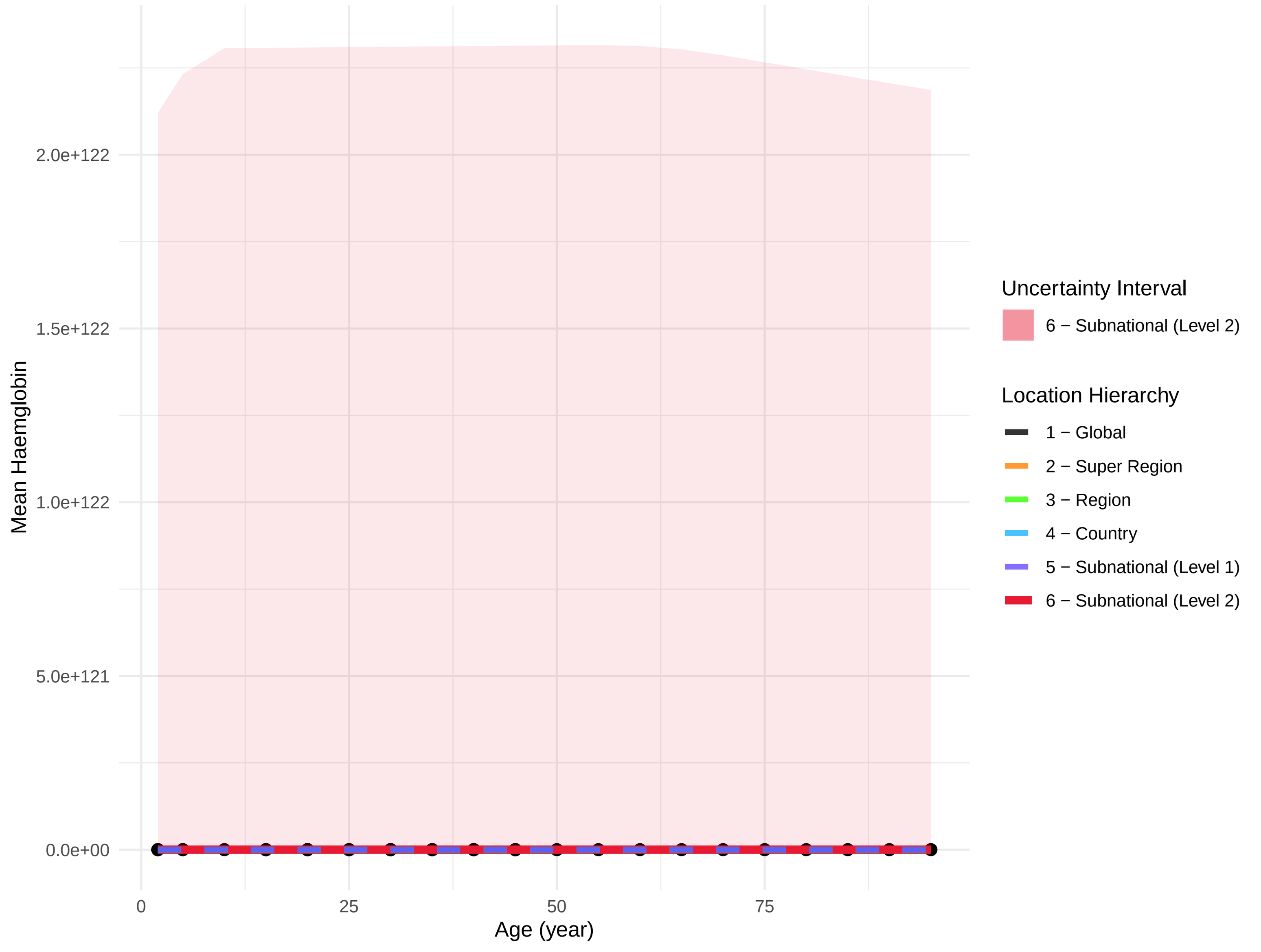

MR−BRT Age/Sex Fits − Non−pregnant Females 2+ Years Old − Mean Haemglobin − Tamil Nadu, Urban

WHO−2024 Adjustment Method

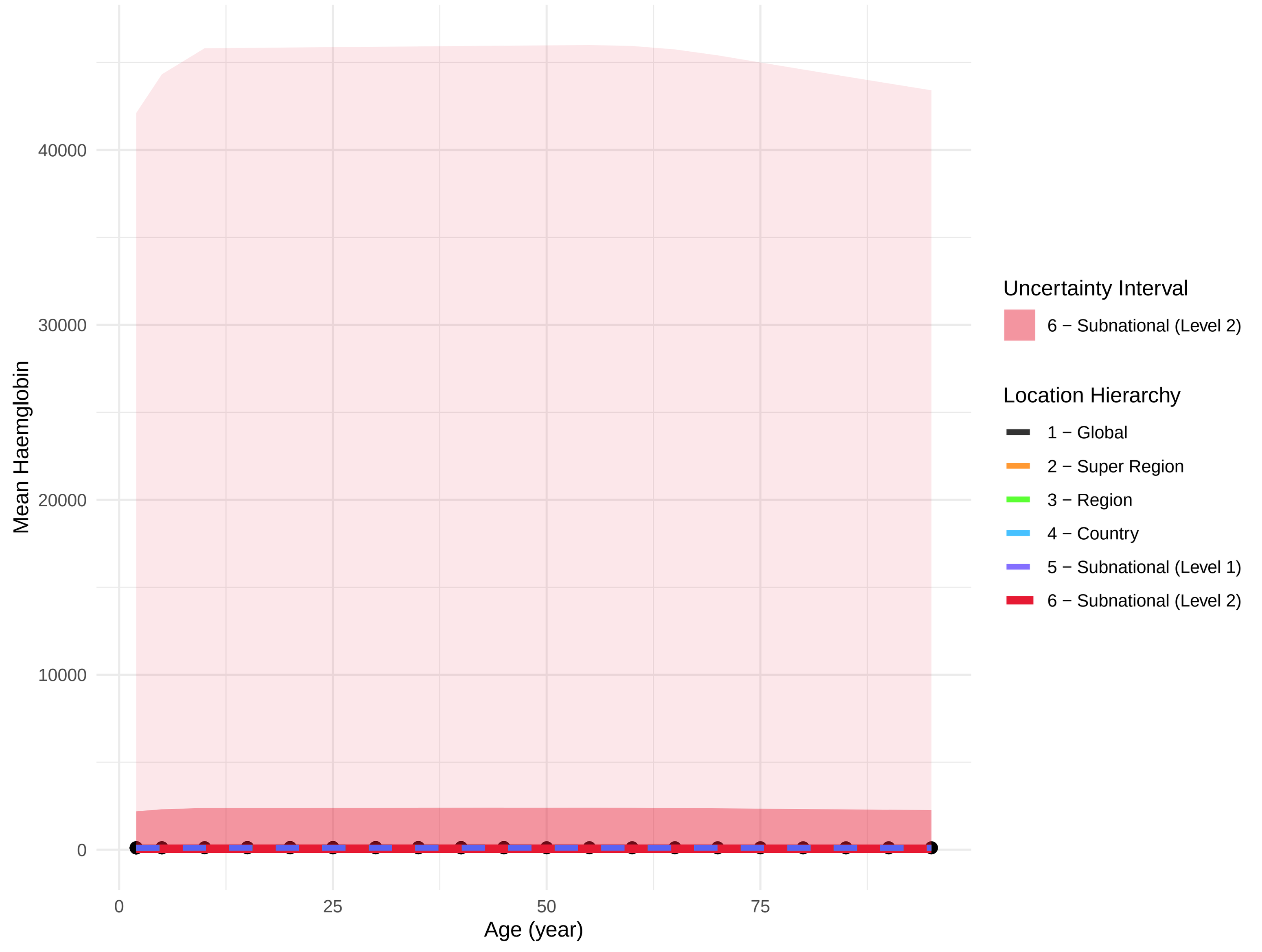

MR−BRT Age/Sex Fits − Non−pregnant Females 2+ Years Old − Mean Haemglobin − Telangana

WHO−2024 Adjustment Method

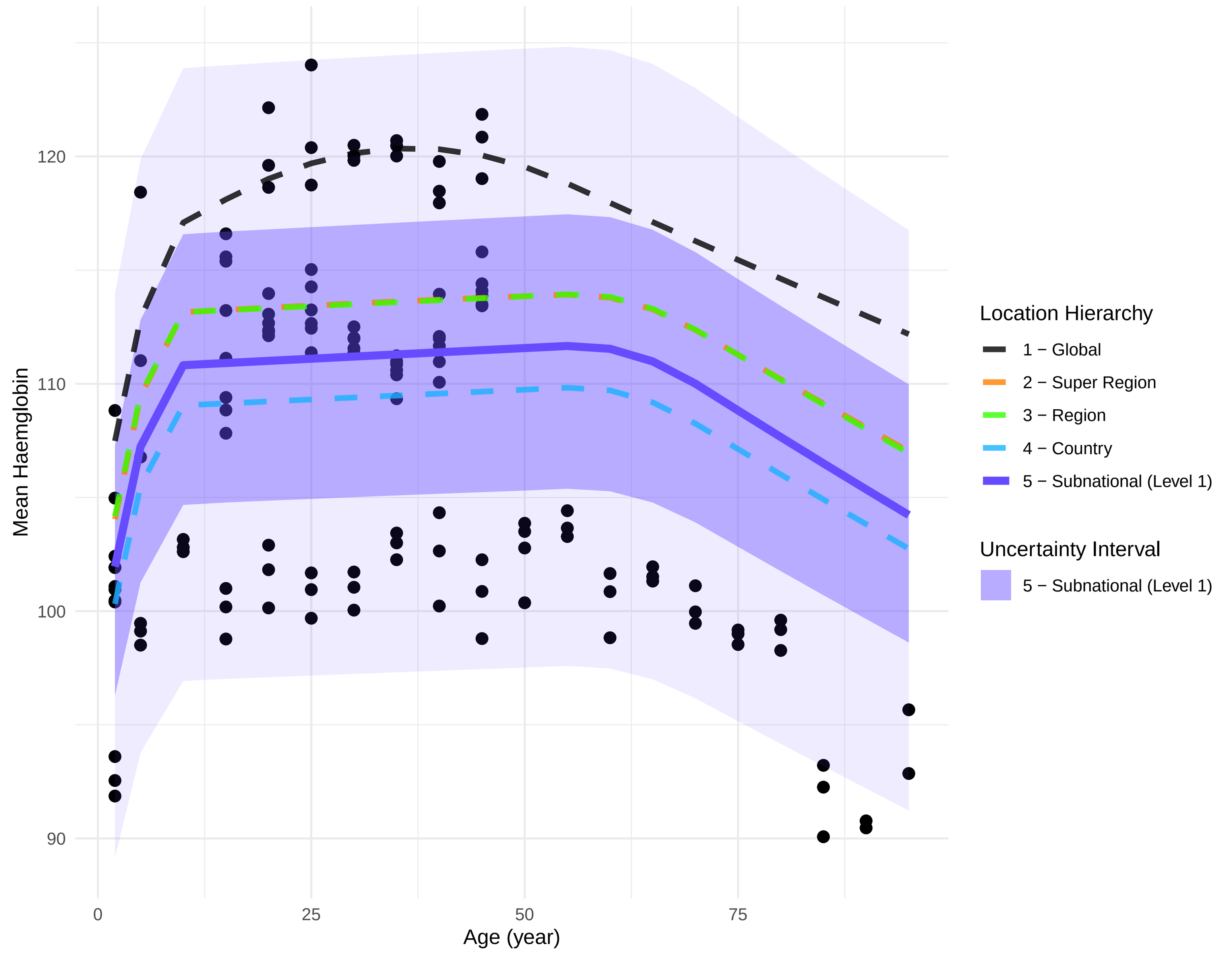

MR−BRT Age/Sex Fits − Non−pregnant Females 2+ Years Old − Mean Haemglobin − Telangana, Rural

WHO−2024 Adjustment Method

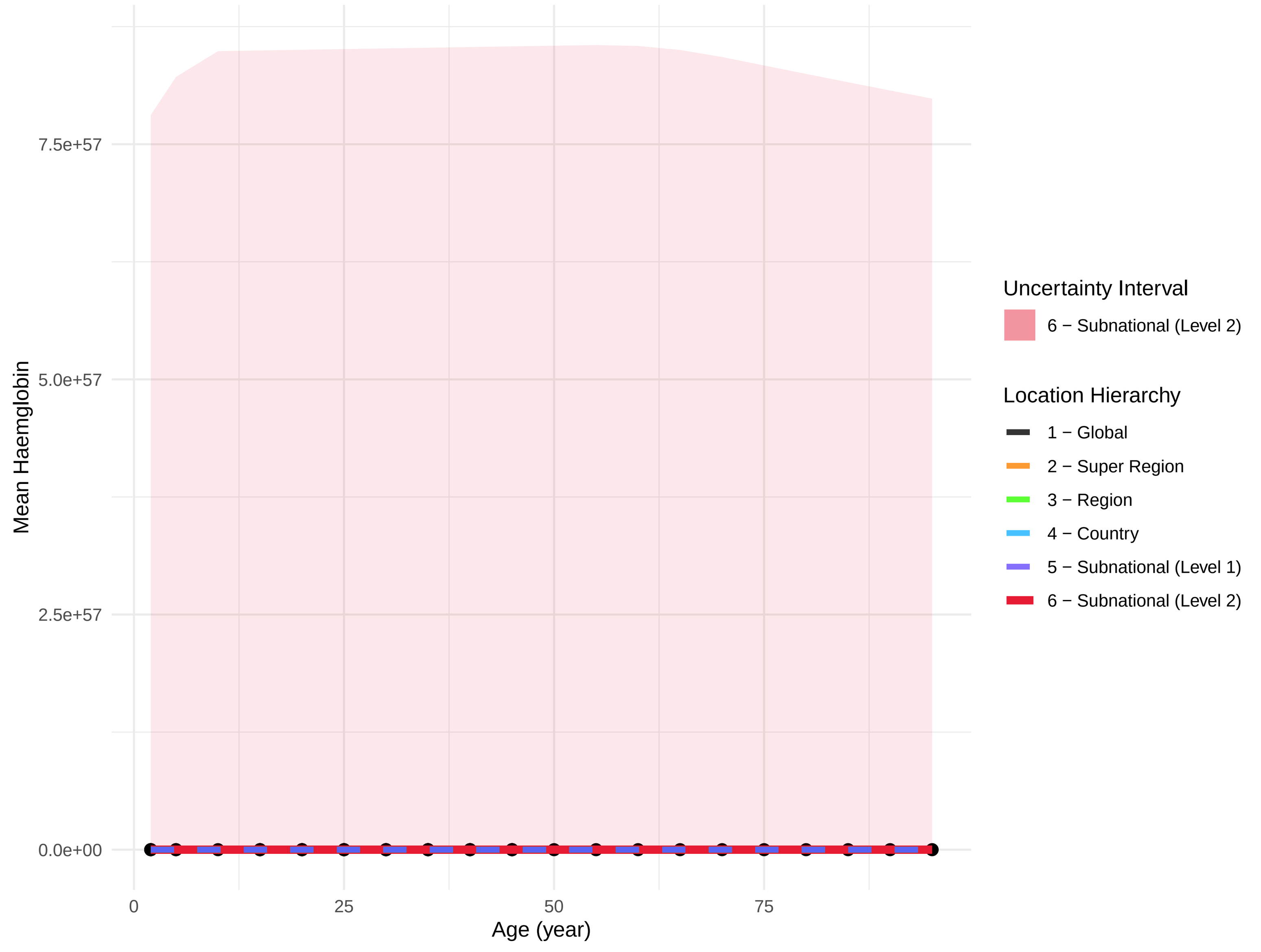

MR−BRT Age/Sex Fits − Non−pregnant Females 2+ Years Old − Mean Haemglobin − Telangana, Urban

WHO−2024 Adjustment Method

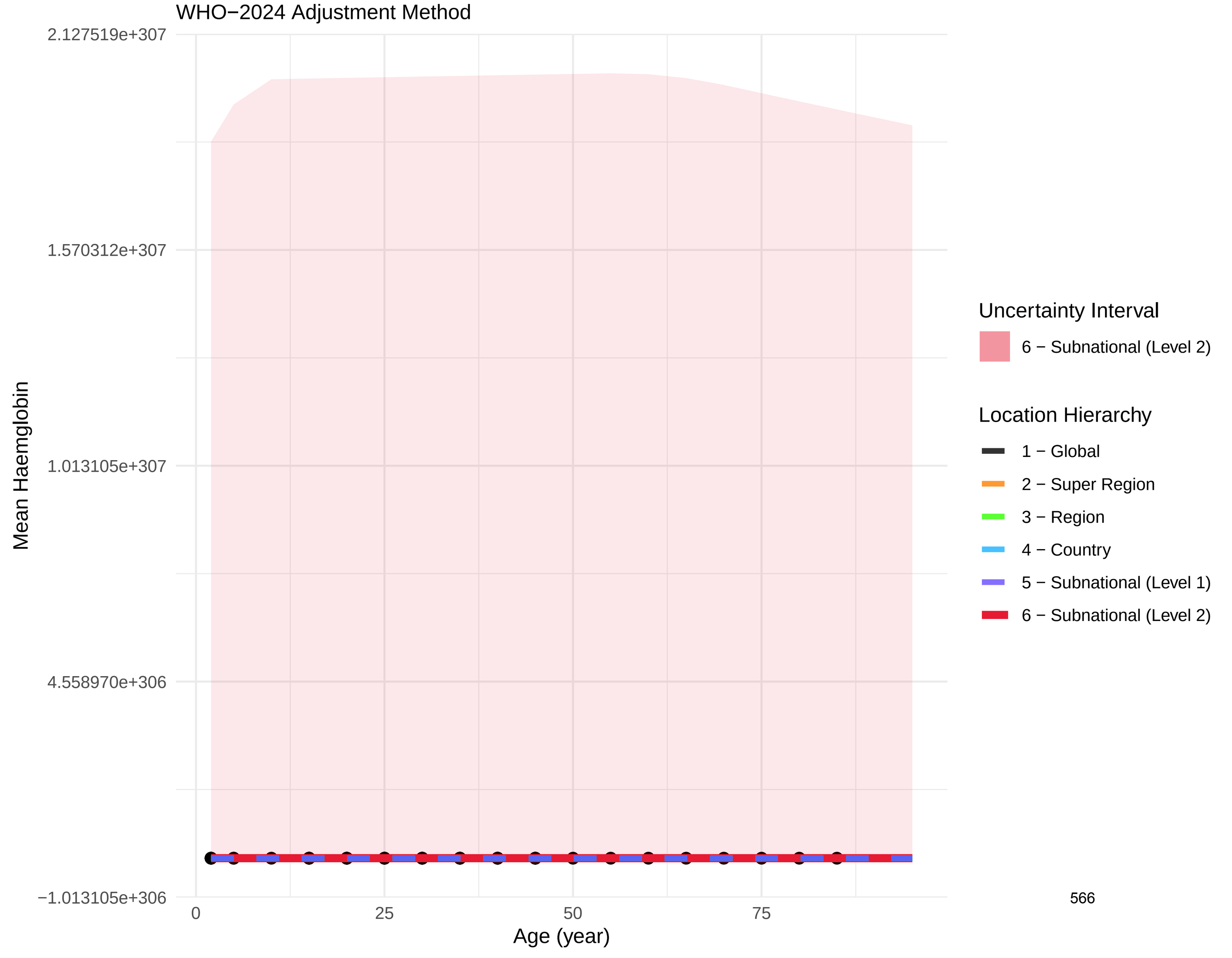

MR−BRT Age/Sex Fits − Non−pregnant Females 2+ Years Old − Mean Haemglobin − Tripura
WHO−2024 Adjustment Method

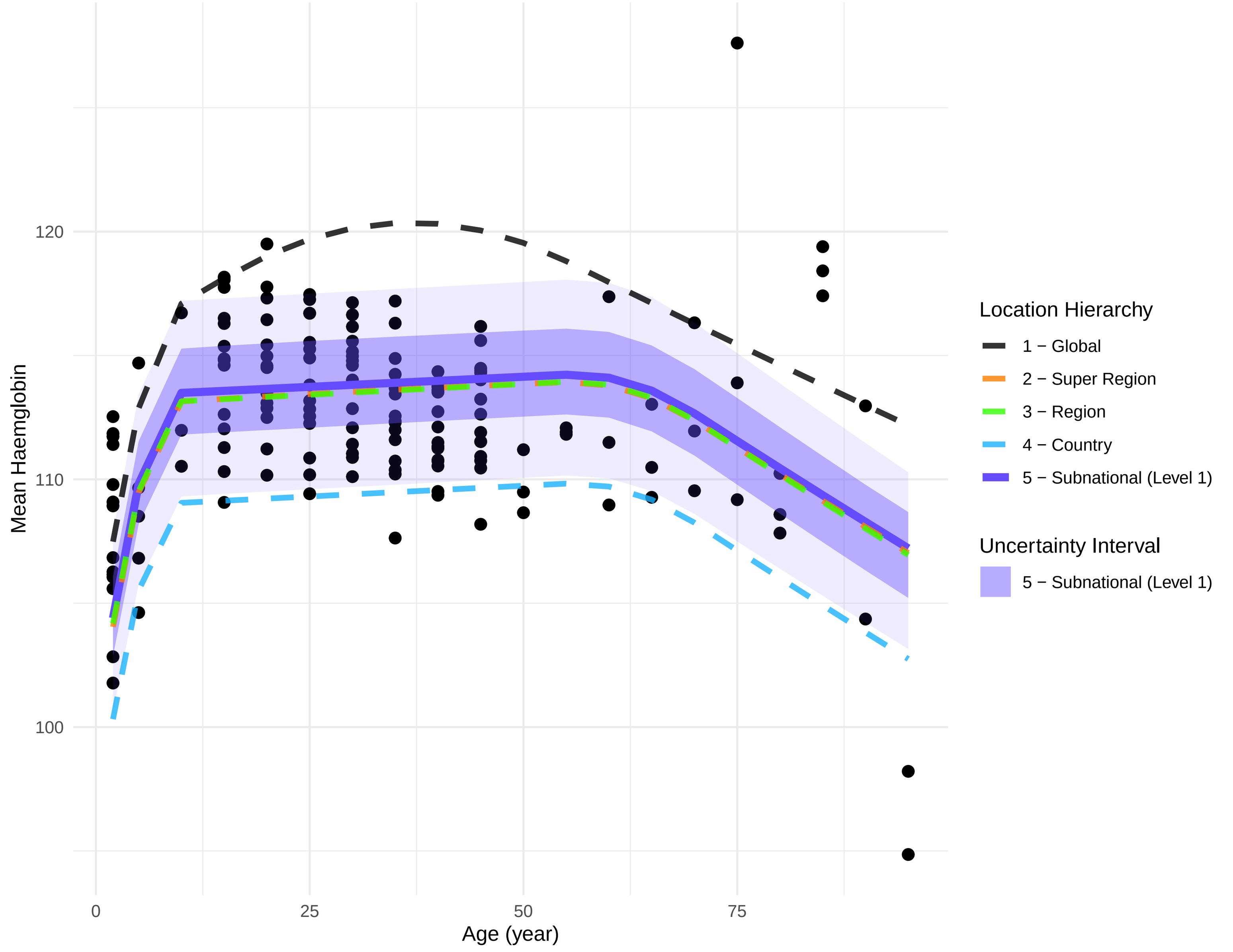

MR−BRT Age/Sex Fits − Non−pregnant Females 2+ Years Old − Mean Haemglobin − Tripura, Rural
WHO−2024 Adjustment Method

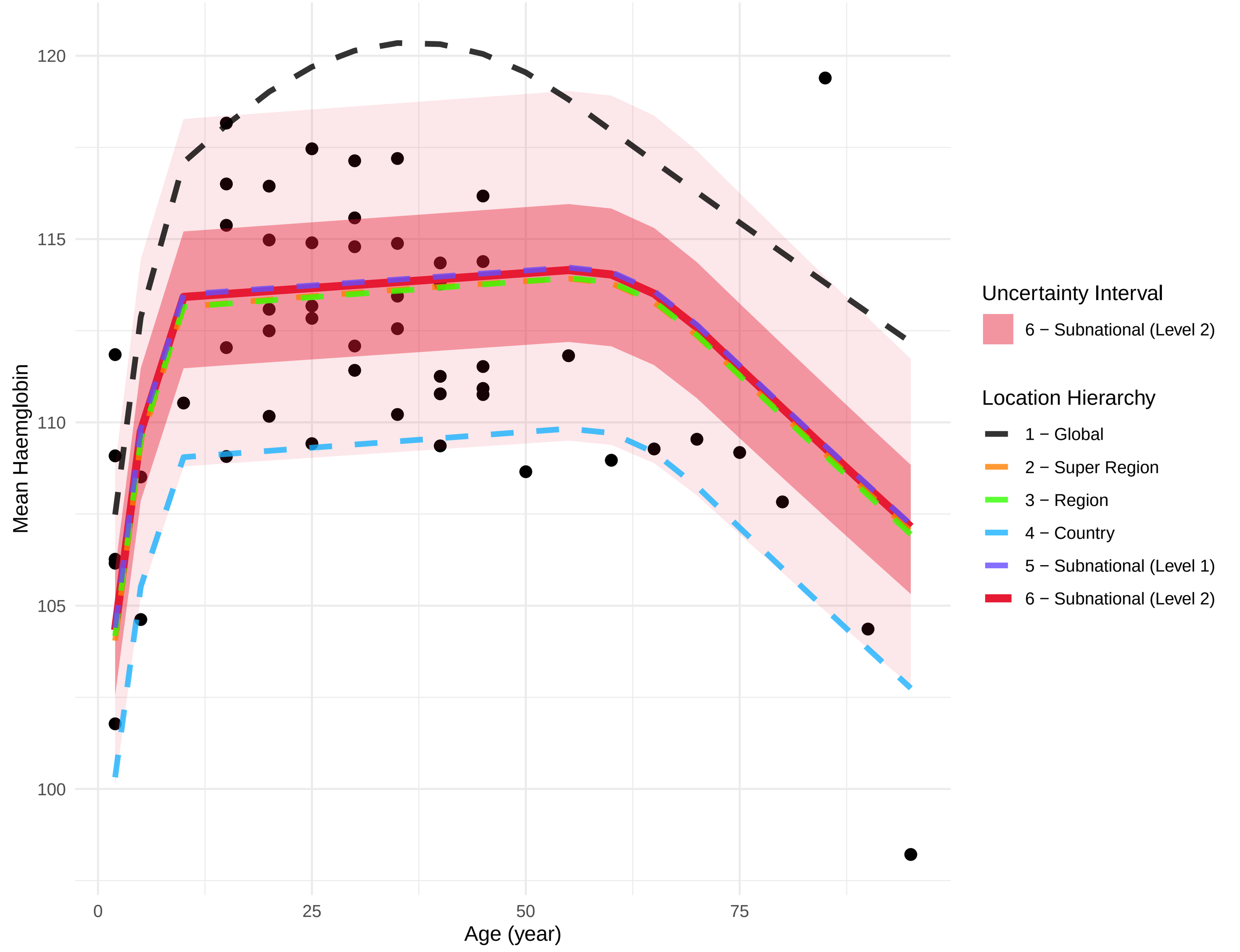

MR−BRT Age/Sex Fits − Non−pregnant Females 2+ Years Old − Mean Haemglobin − Tripura, Urban
WHO−2024 Adjustment Method

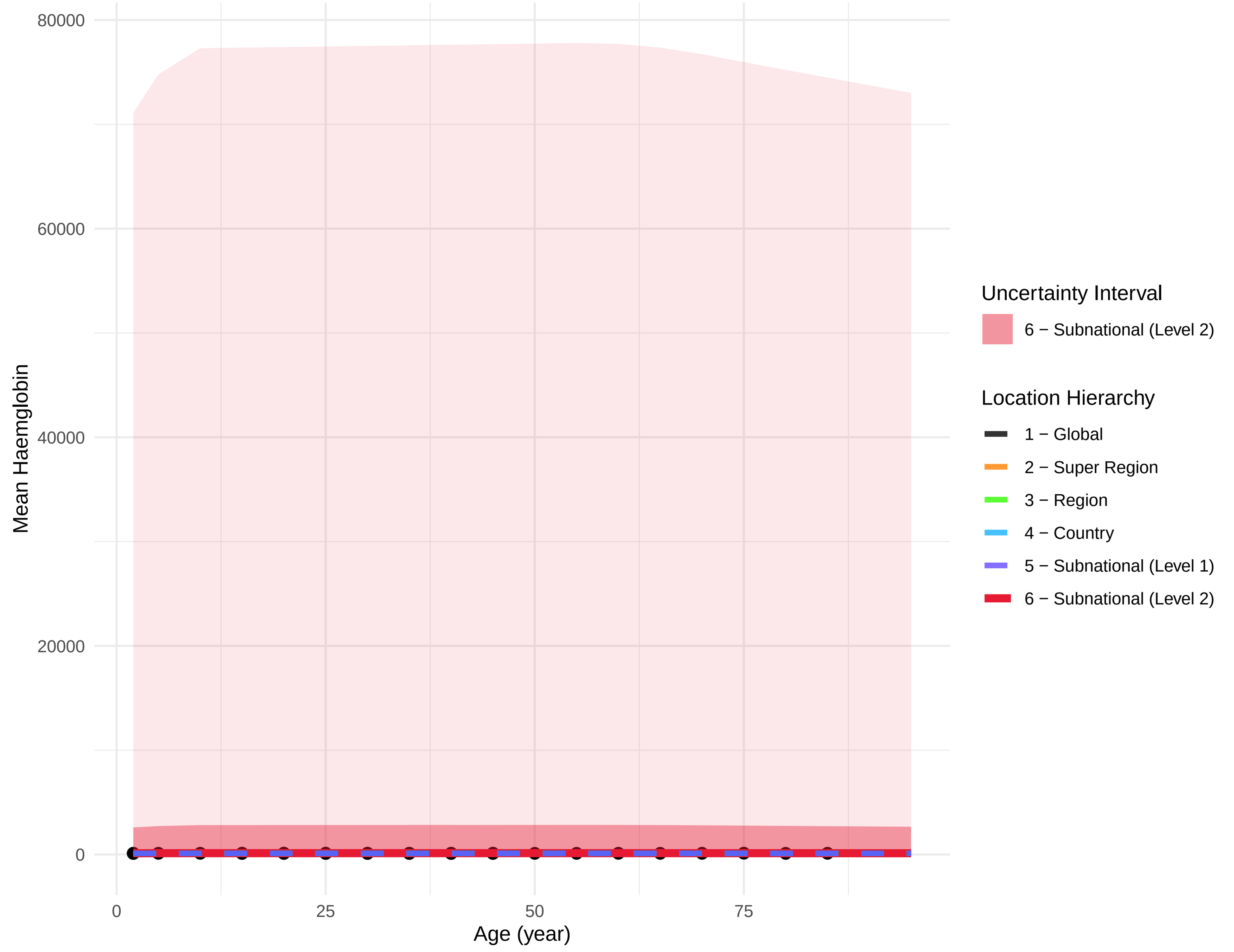

MR−BRT Age/Sex Fits − Non−pregnant Females 2+ Years Old − Mean Haemglobin − Uttar Pradesh
WHO−2024 Adjustment Method

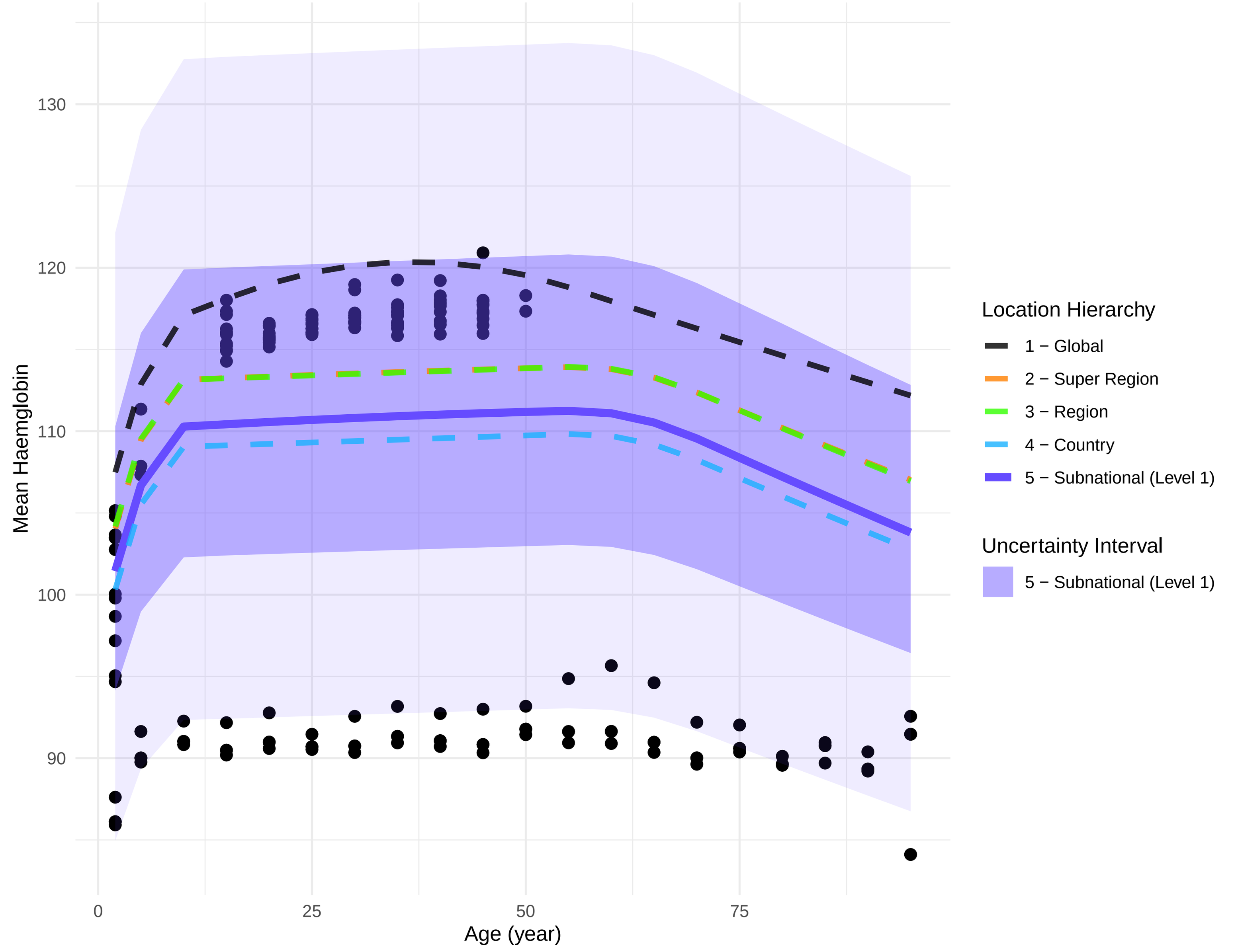

MR−BRT Age/Sex Fits − Non−pregnant Females 2+ Years Old − Mean Haemglobin − Uttar Pradesh, Rural
WHO−2024 Adjustment Method

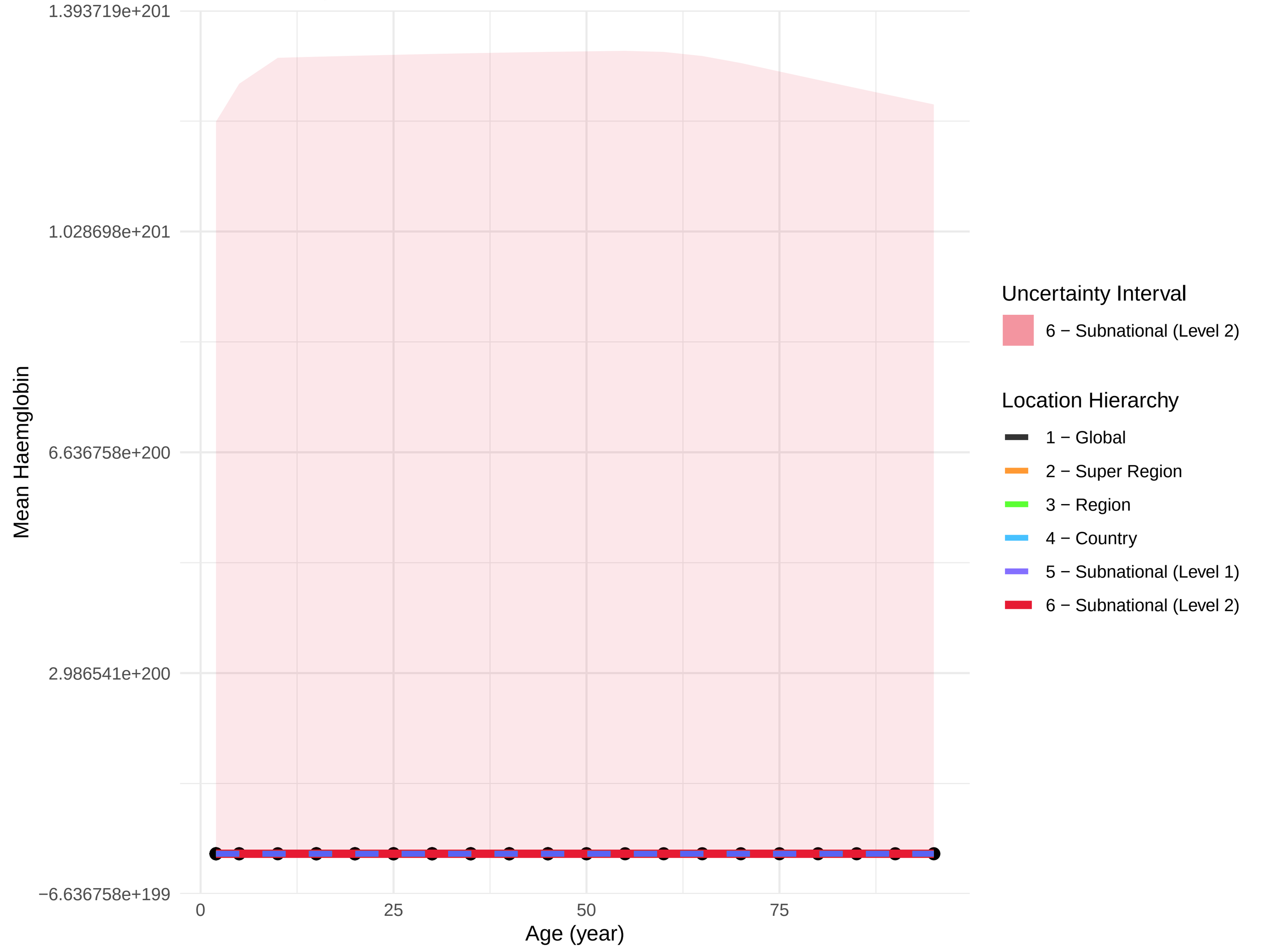

MR−BRT Age/Sex Fits − Non−pregnant Females 2+ Years Old − Mean Haemglobin − Uttar Pradesh, Urban
WHO−2024 Adjustment Method

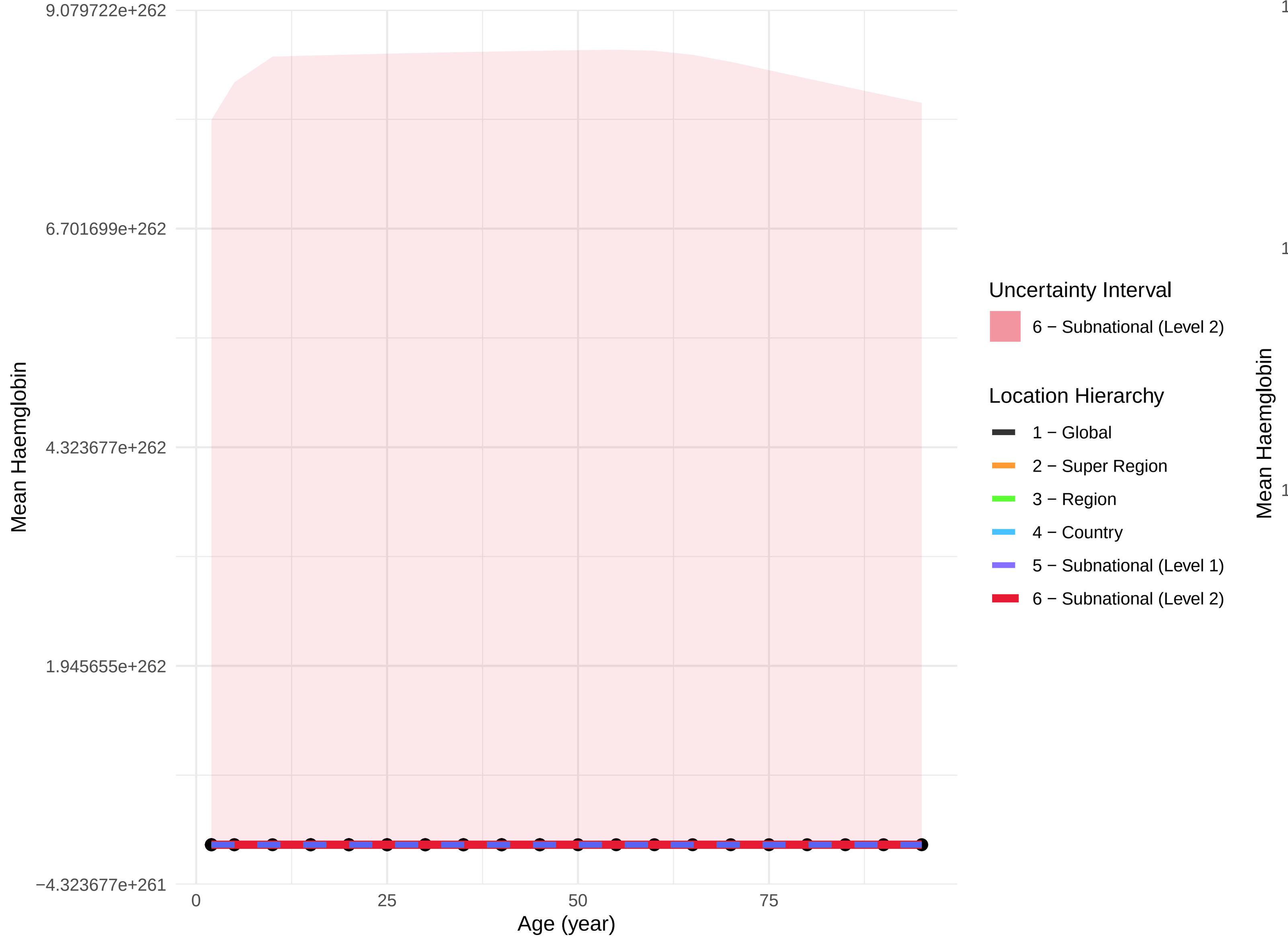

MR−BRT Age/Sex Fits − Non−pregnant Females 2+ Years Old − Mean Haemglobin − Uttarakhand
WHO−2024 Adjustment Method

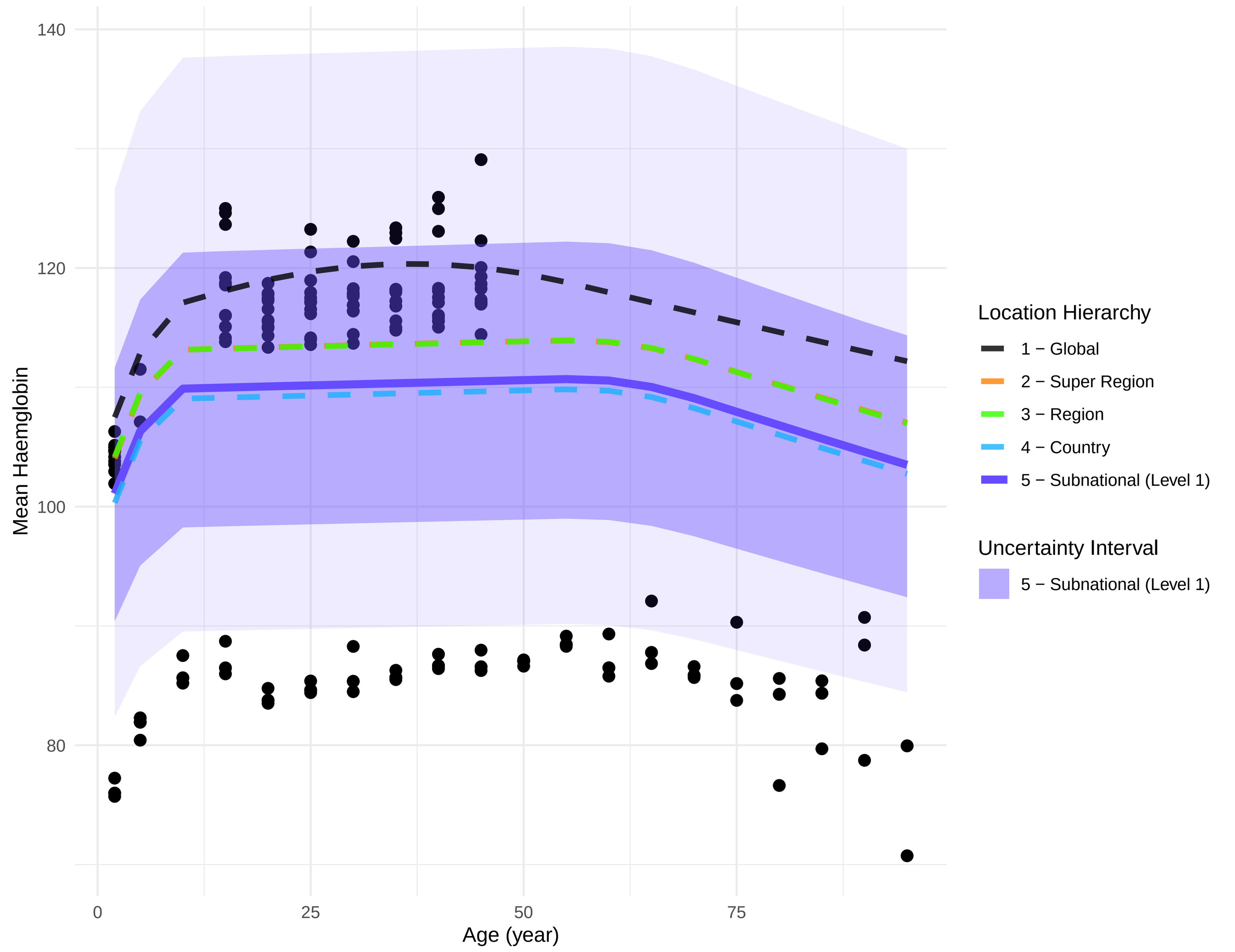

MR−BRT Age/Sex Fits − Non−pregnant Females 2+ Years Old − Mean Haemglobin − Uttarakhand, Rural
WHO−2024 Adjustment Method

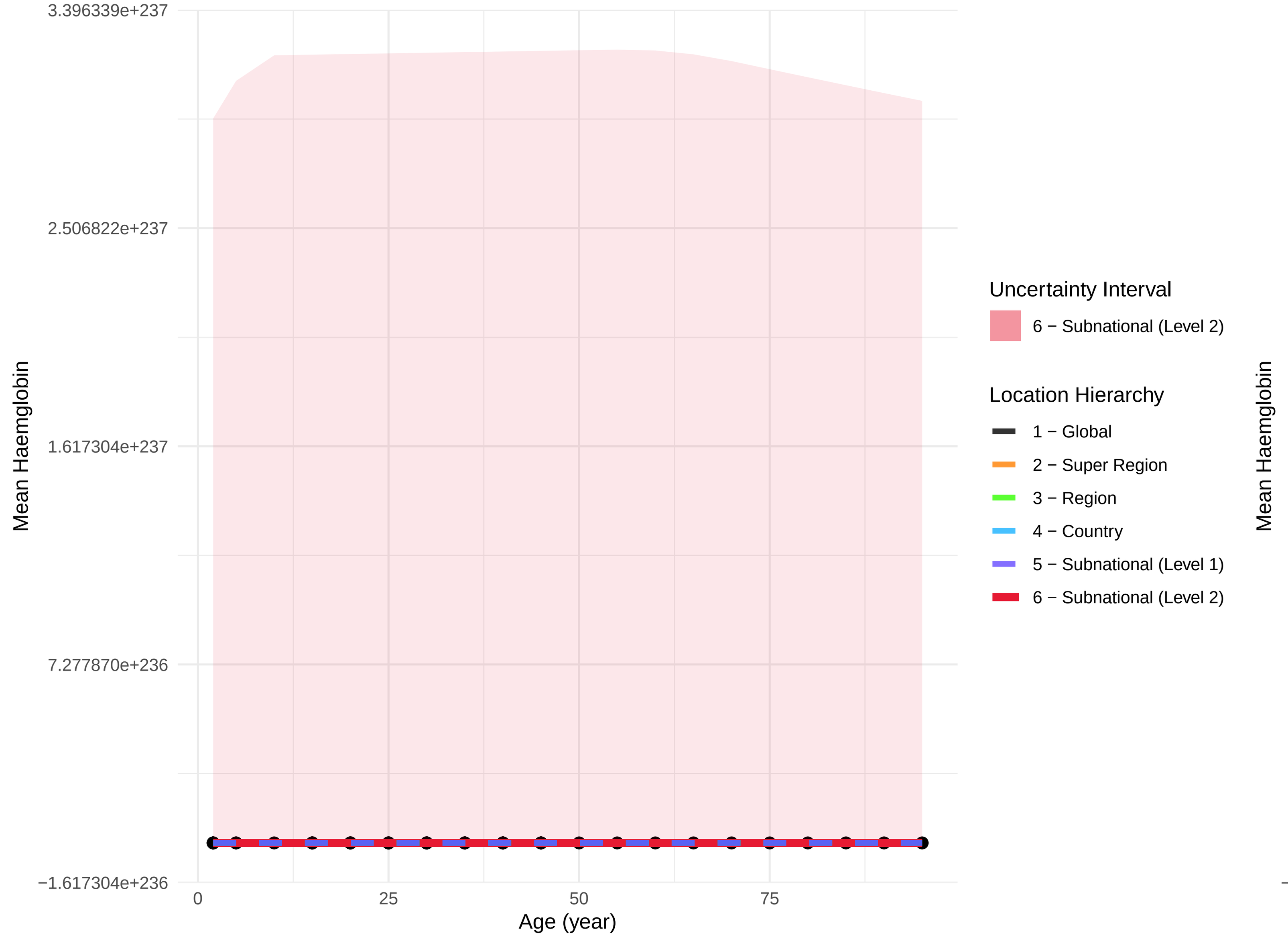

MR−BRT Age/Sex Fits − Non−pregnant Females 2+ Years Old − Mean Haemglobin − Uttarakhand, Urban
WHO−2024 Adjustment Method

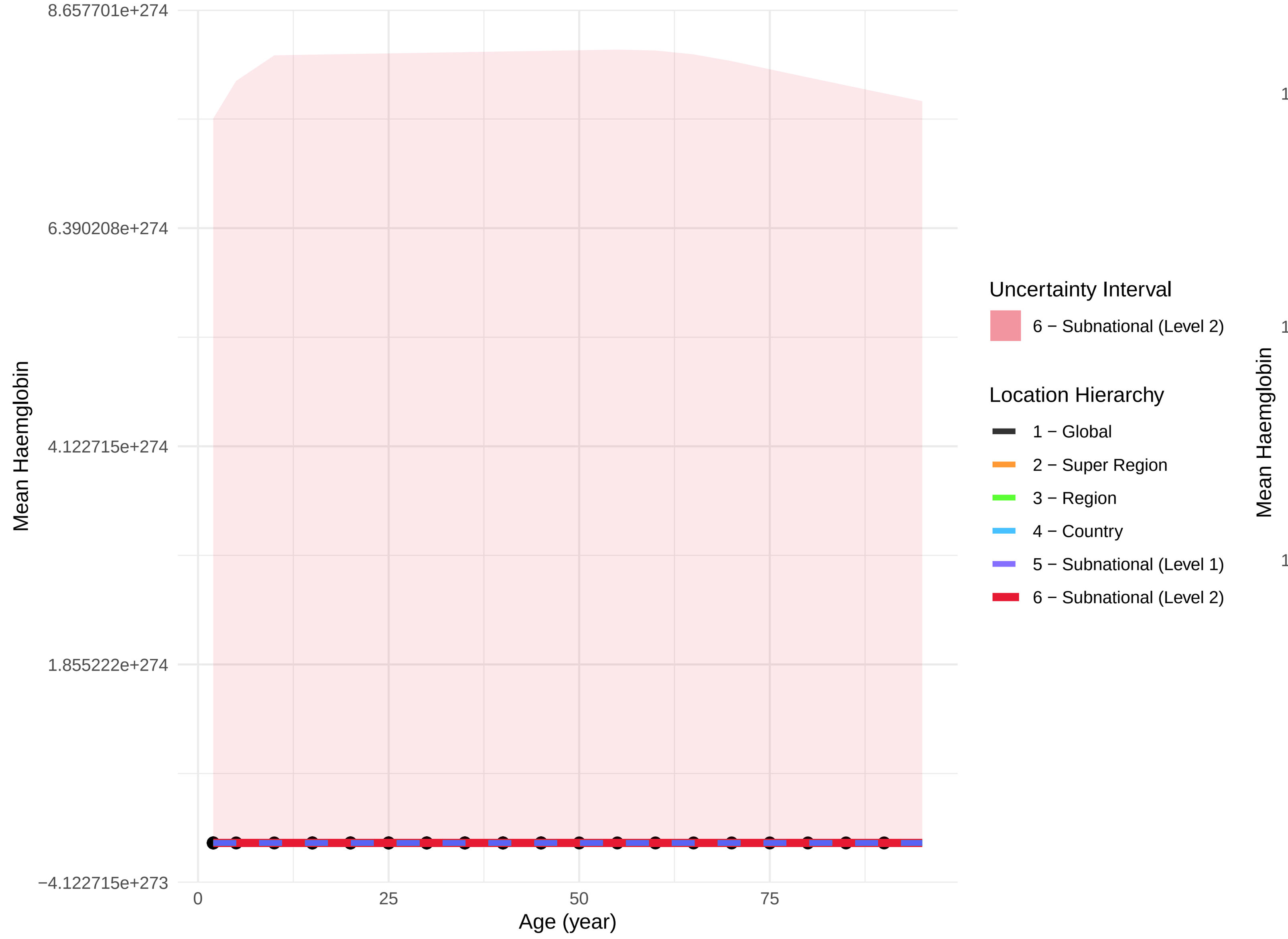

MR−BRT Age/Sex Fits − Non−pregnant Females 2+ Years Old − Mean Haemglobin − West Bengal
WHO−2024 Adjustment Method

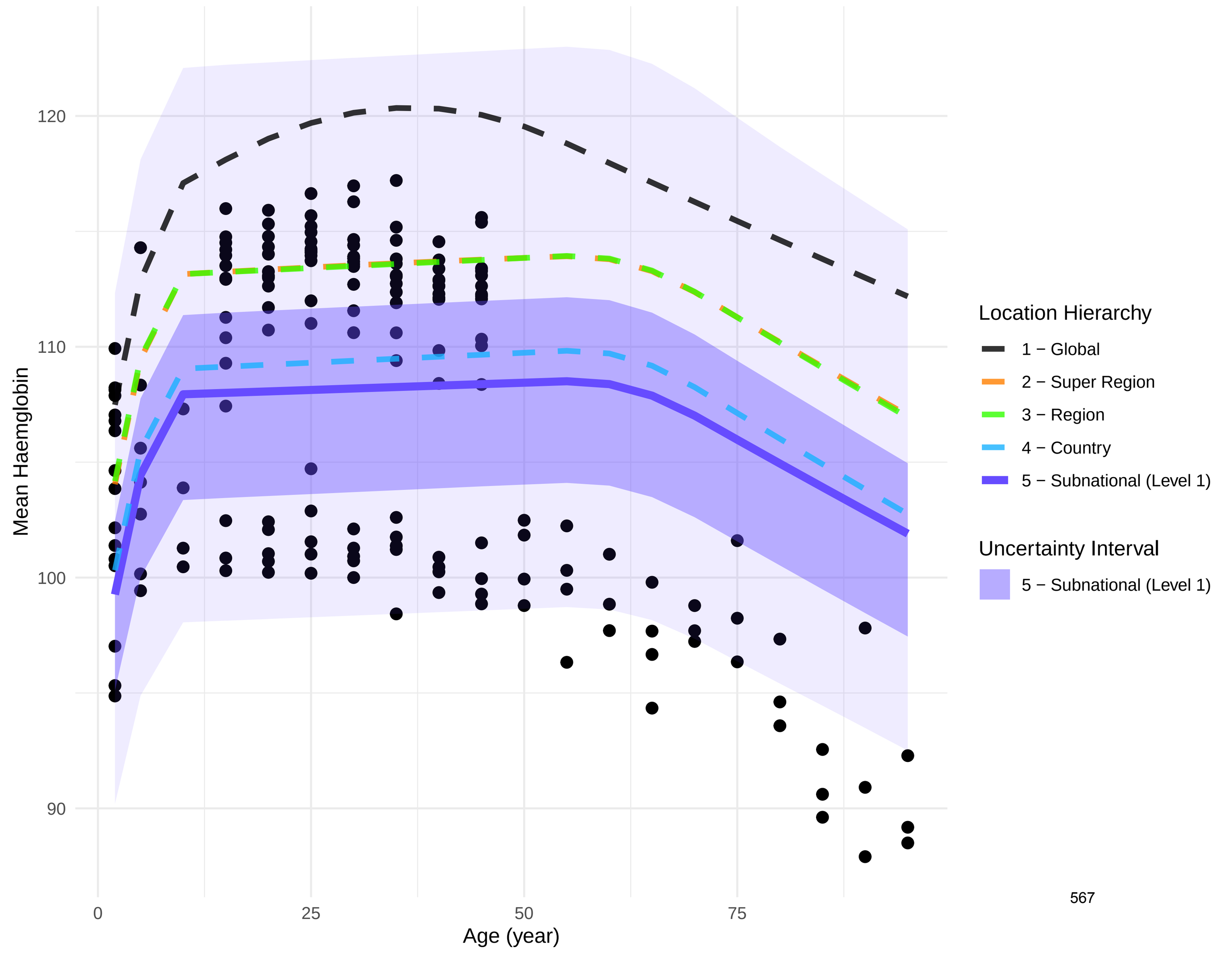

MR−BRT Age/Sex Fits − Non−pregnant Females 2+ Years Old − Mean Haemglobin − West Bengal, Rural
WHO−2024 Adjustment Method

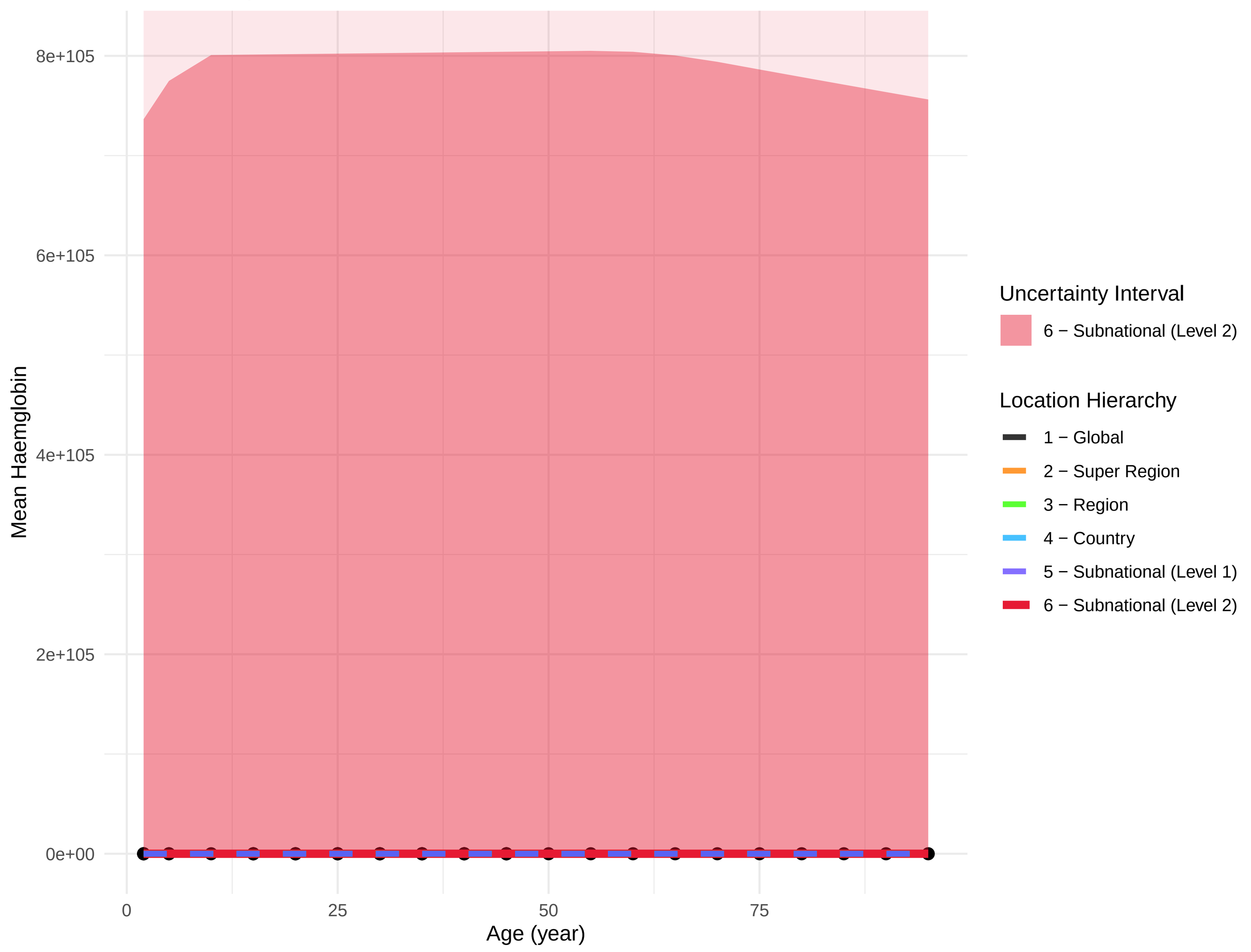

MR−BRT Age/Sex Fits − Non−pregnant Females 2+ Years Old − Mean Haemglobin − West Bengal, Urban
WHO−2024 Adjustment Method

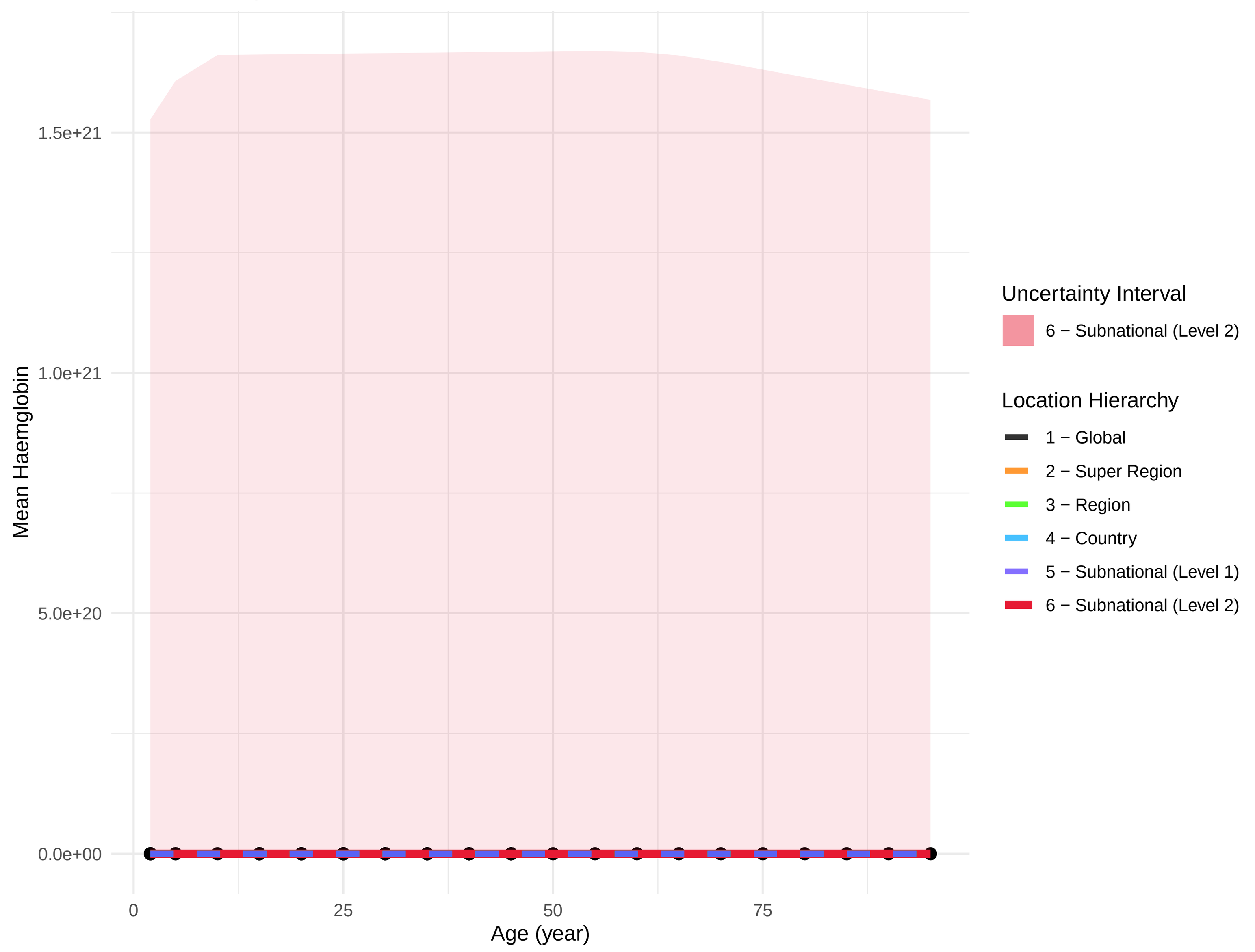

MR−BRT Age/Sex Fits − Non−pregnant Females 2+ Years Old − Mean Haemglobin − Nepal
WHO−2024 Adjustment Method

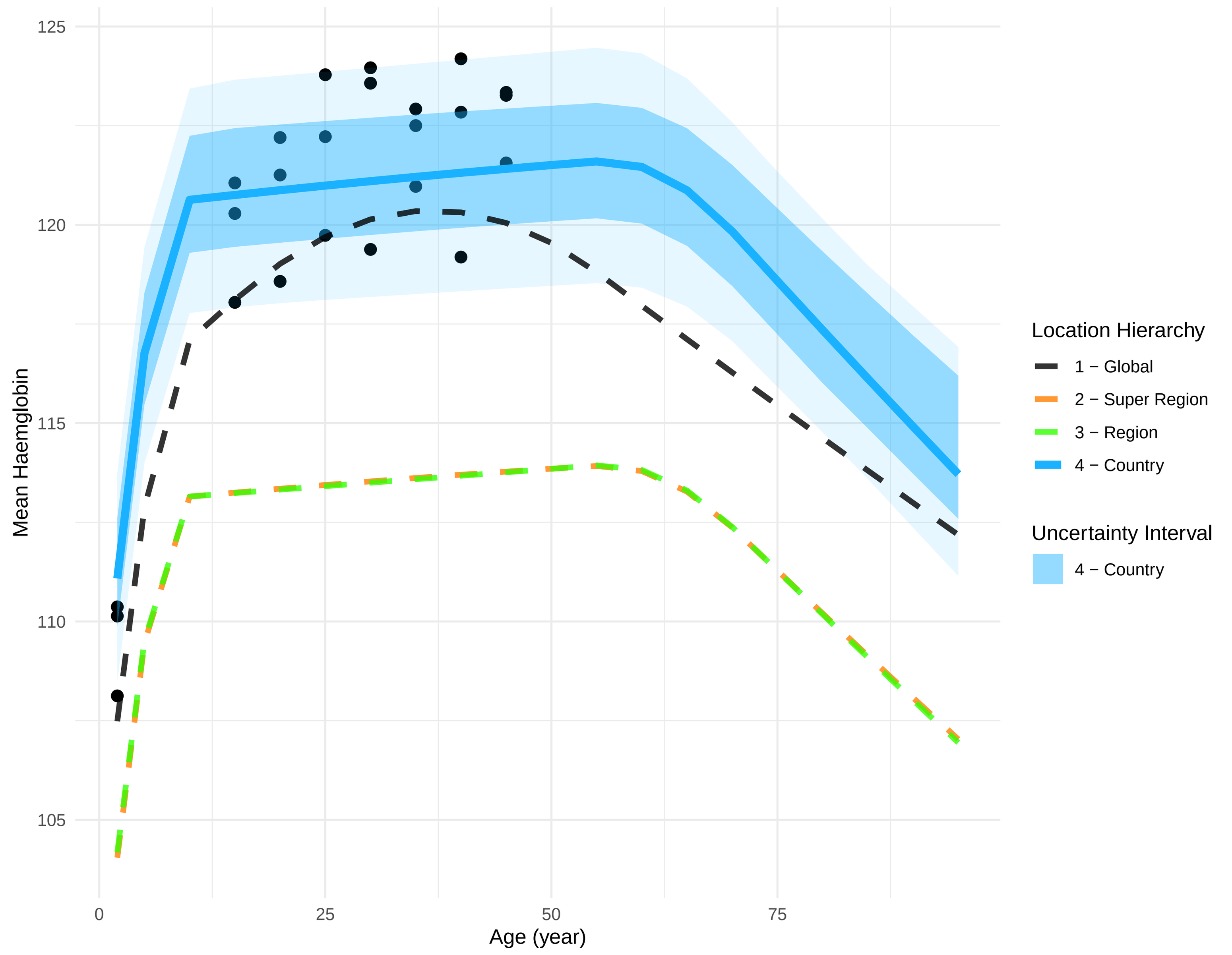

MR−BRT Age/Sex Fits − Non−pregnant Females 2+ Years Old − Mean Haemglobin − Pakistan
WHO−2024 Adjustment Method

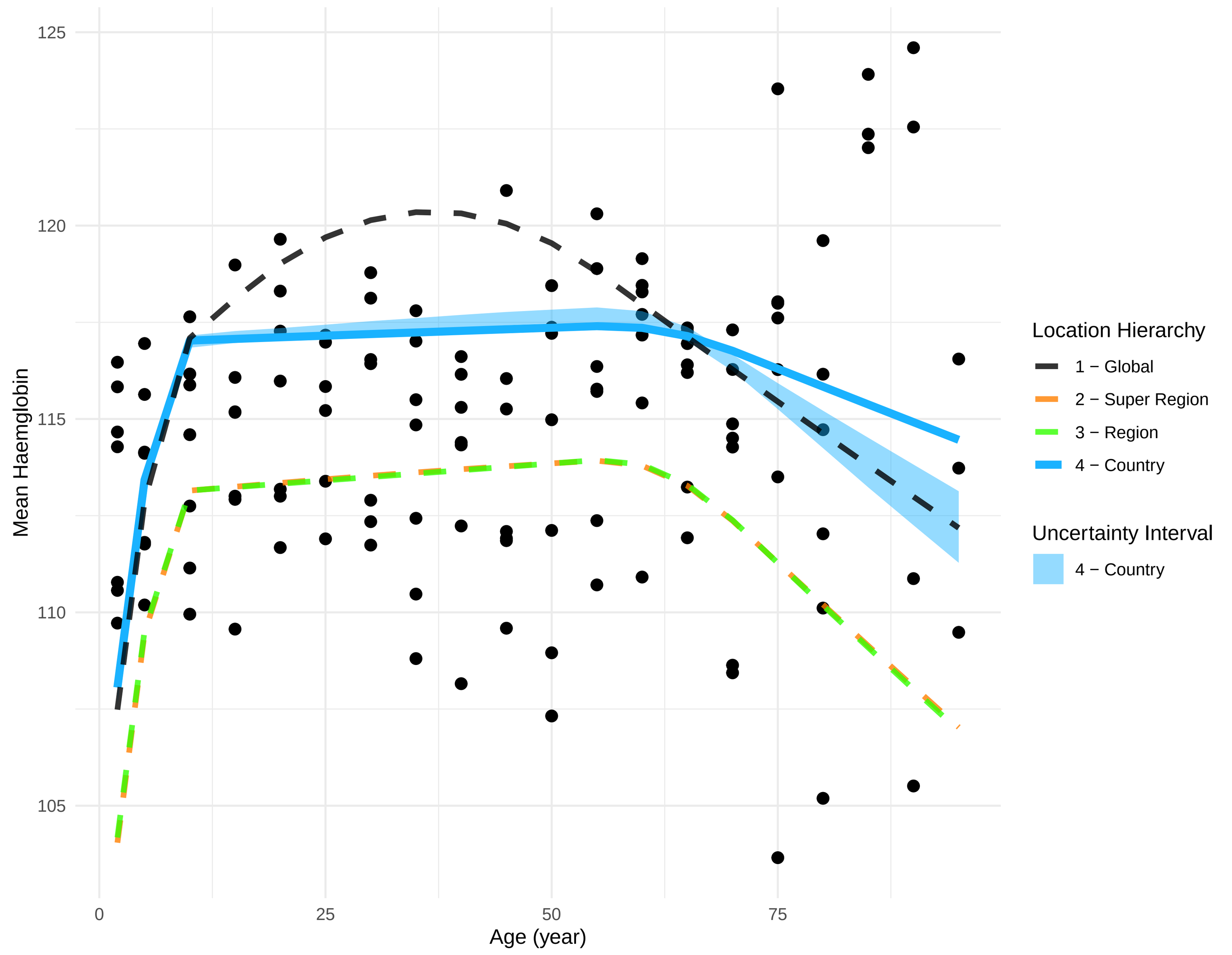

MR−BRT Age/Sex Fits − Non−pregnant Females 2+ Years Old − Mean Haemglobin − Azad Jammu & Kashmir
WHO−2024 Adjustment Method

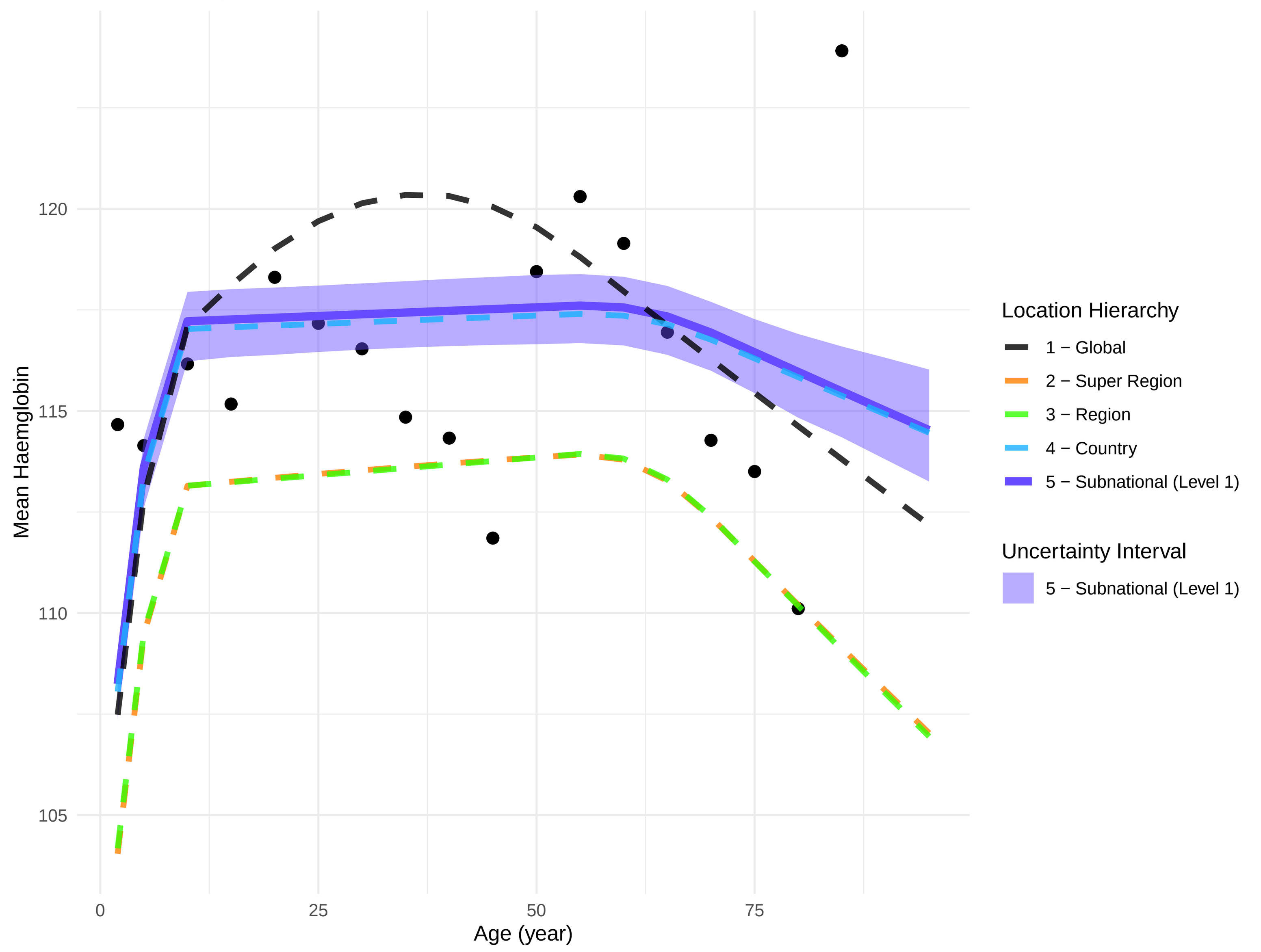

MR−BRT Age/Sex Fits − Non−pregnant Females 2+ Years Old − Mean Haemglobin − Balochistan
WHO−2024 Adjustment Method

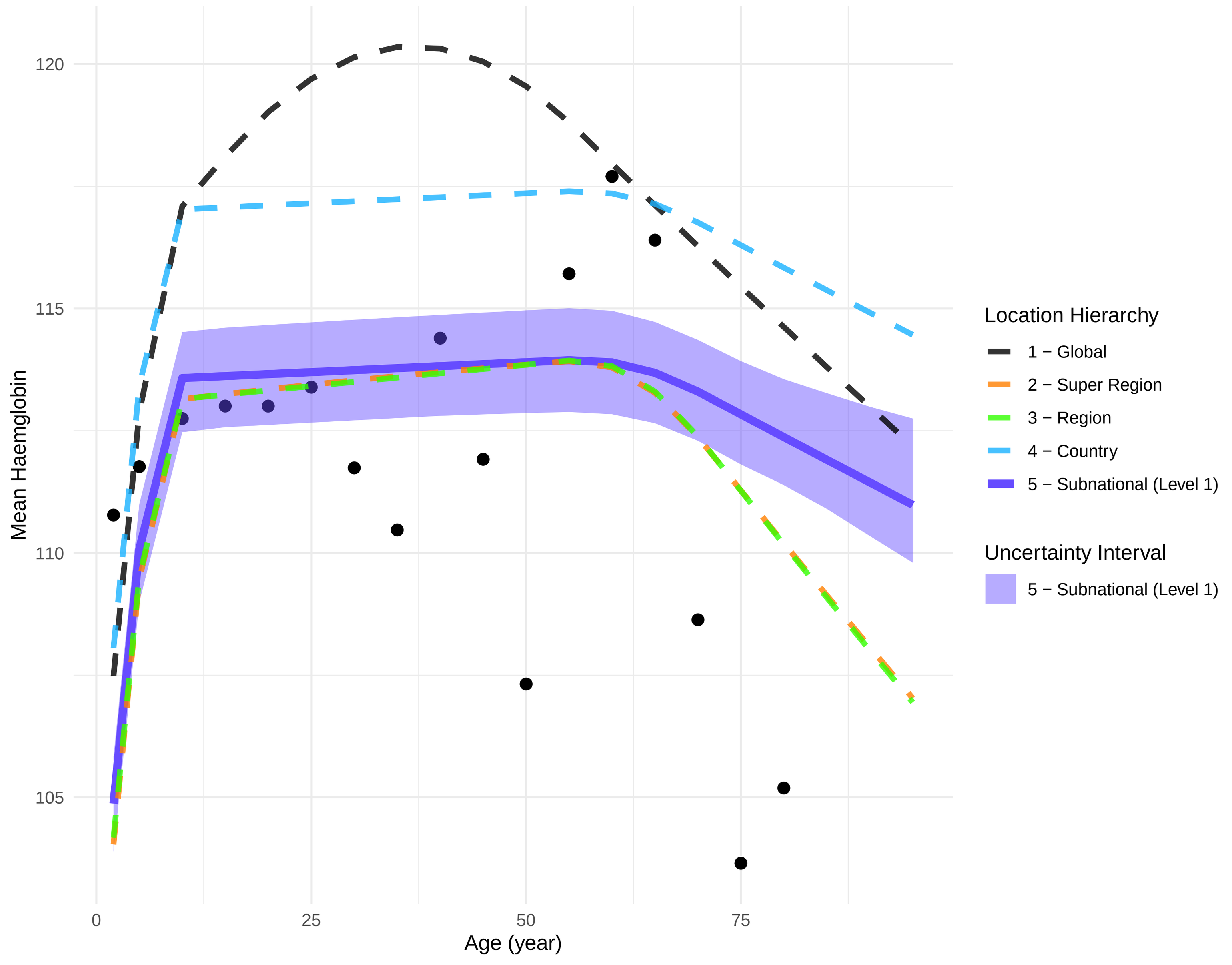

MR−BRT Age/Sex Fits − Non−pregnant Females 2+ Years Old − Mean Haemglobin − Gilgit−Baltistan
WHO−2024 Adjustment Method

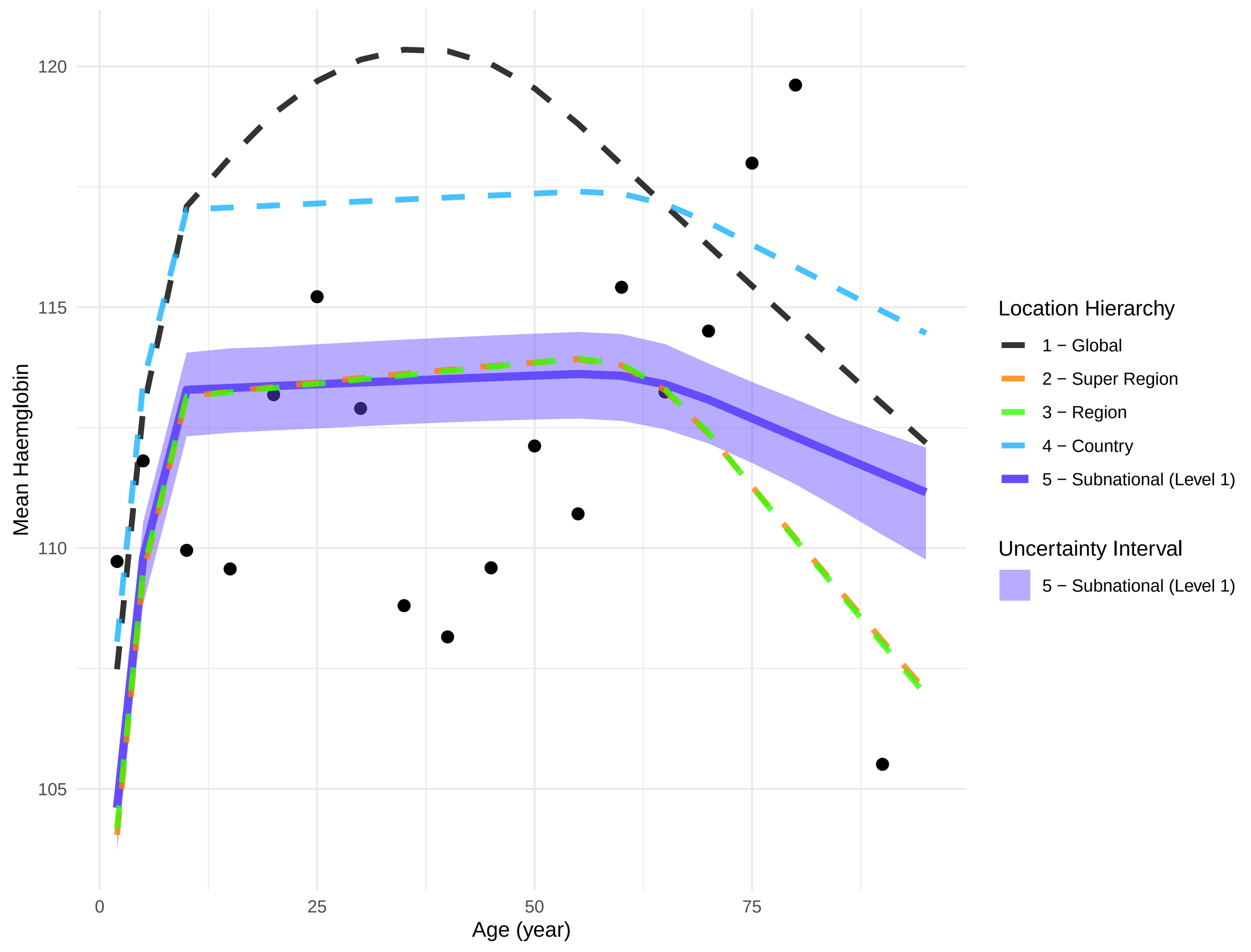

MR−BRT Age/Sex Fits − Non−pregnant Females 2+ Years Old − Mean Haemglobin − Khyber Pakhtunkhwa
WHO−2024 Adjustment Method

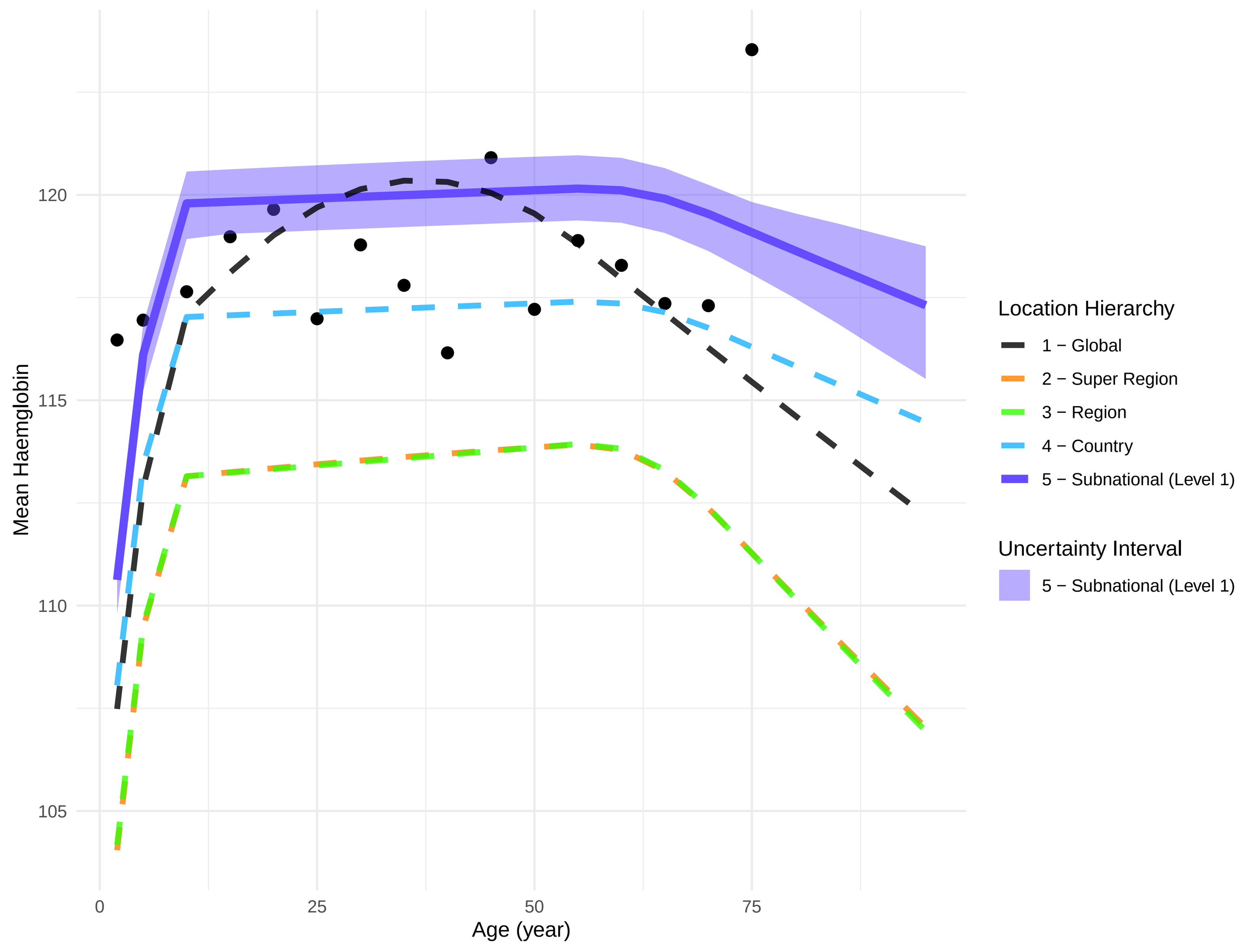

MR−BRT Age/Sex Fits − Non−pregnant Females 2+ Years Old − Mean Haemglobin − Punjab
WHO−2024 Adjustment Method

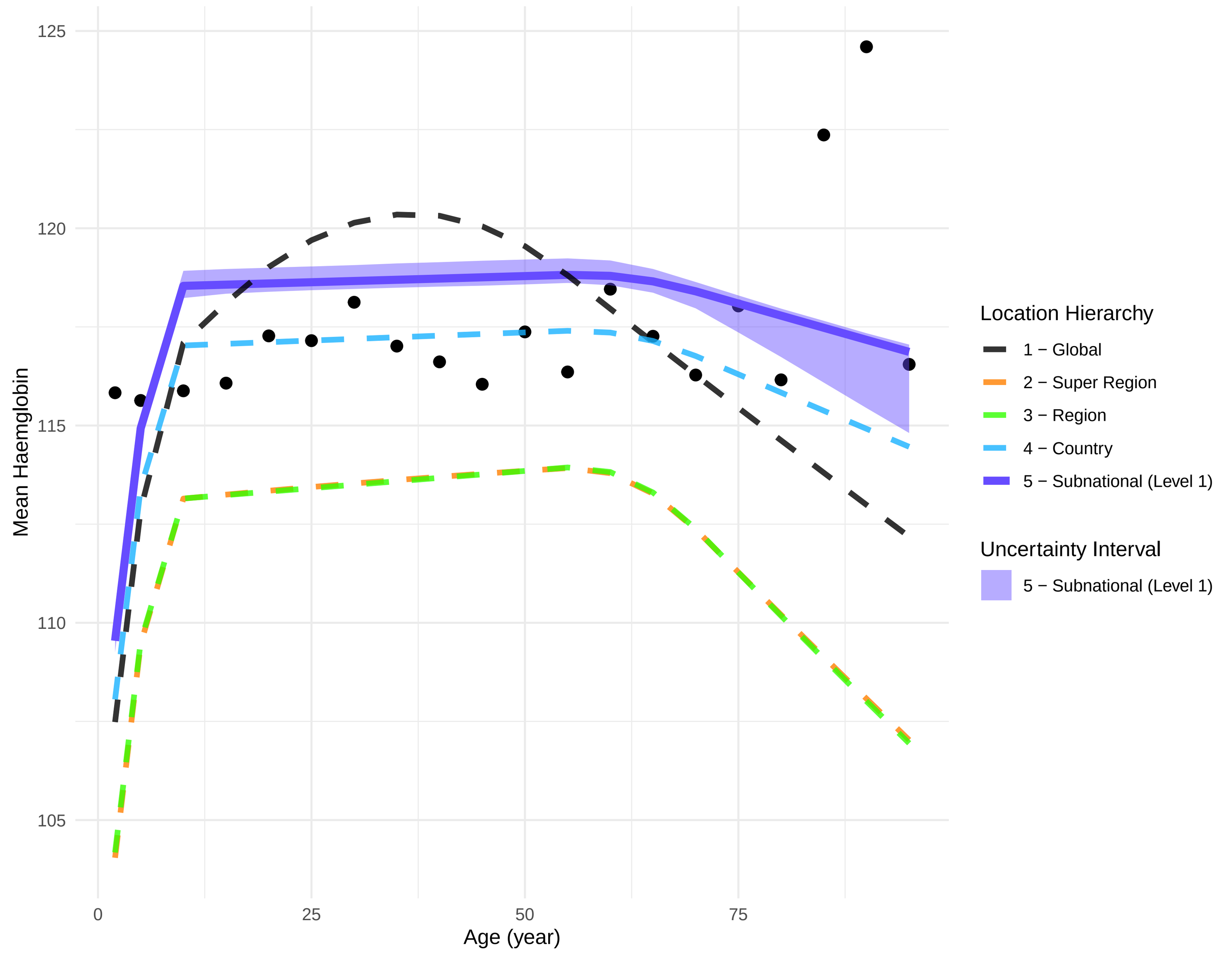

MR−BRT Age/Sex Fits − Non−pregnant Females 2+ Years Old − Mean Haemglobin − Sindh
WHO−2024 Adjustment Method

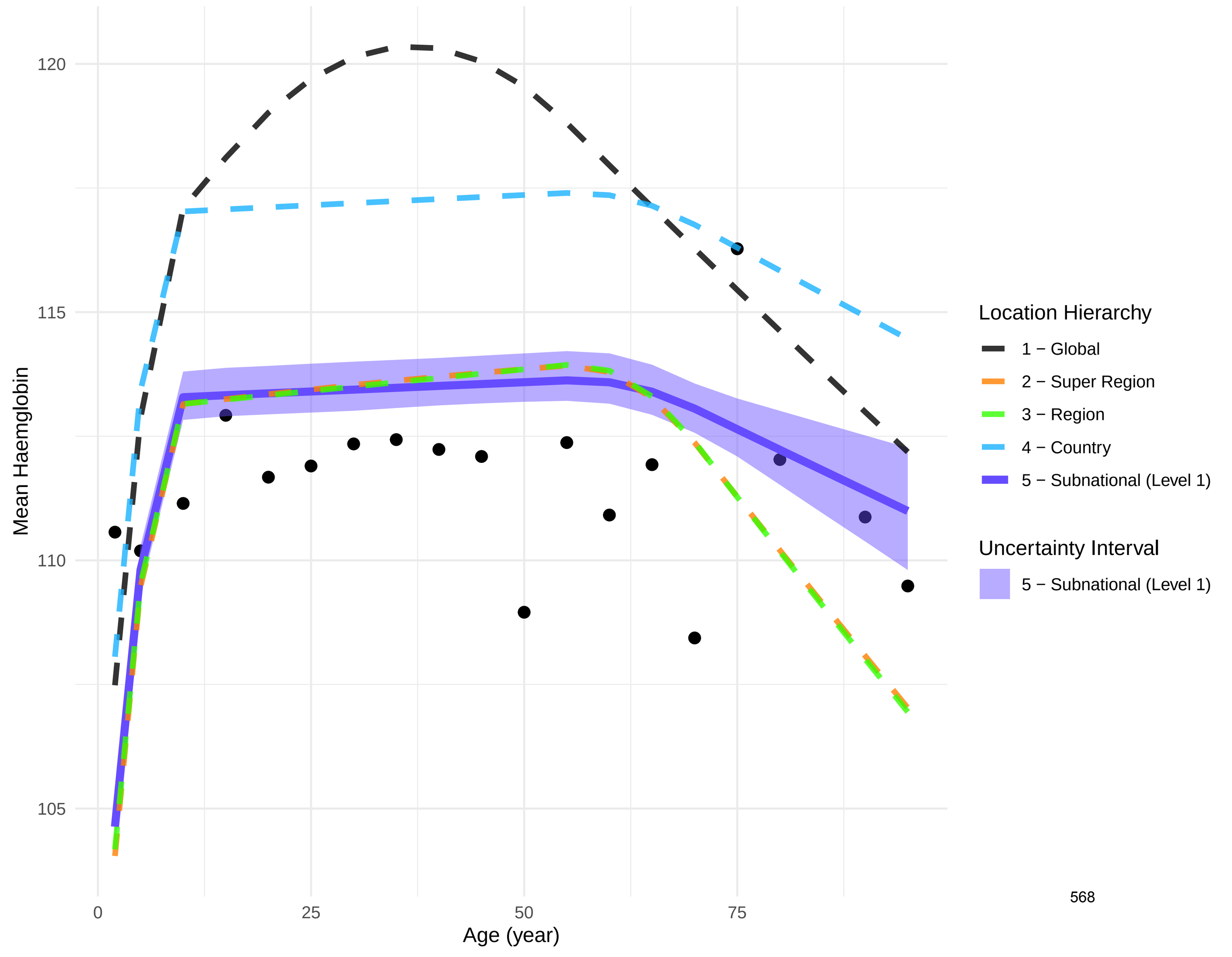

MR−BRT Age/Sex Fits − Non-pregnant Females 2+ Years Old − Mean Haemglobin − Southeast Asia, East Asia, and Oceania
WHO−2024 Adjustment Method

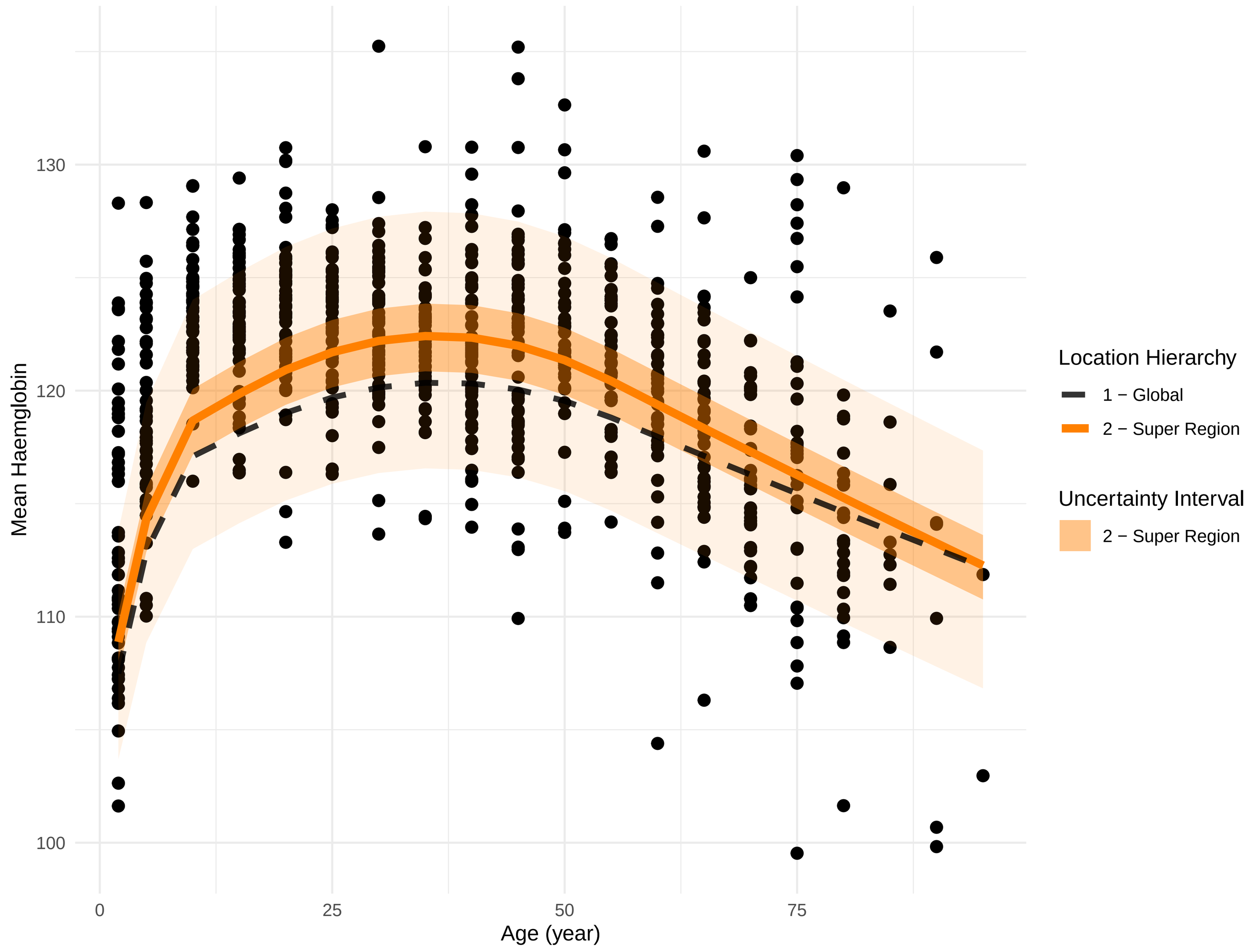

MR−BRT Age/Sex Fits − Non-pregnant Females 2+ Years Old − Mean Haemglobin − Oceania
WHO−2024 Adjustment Method

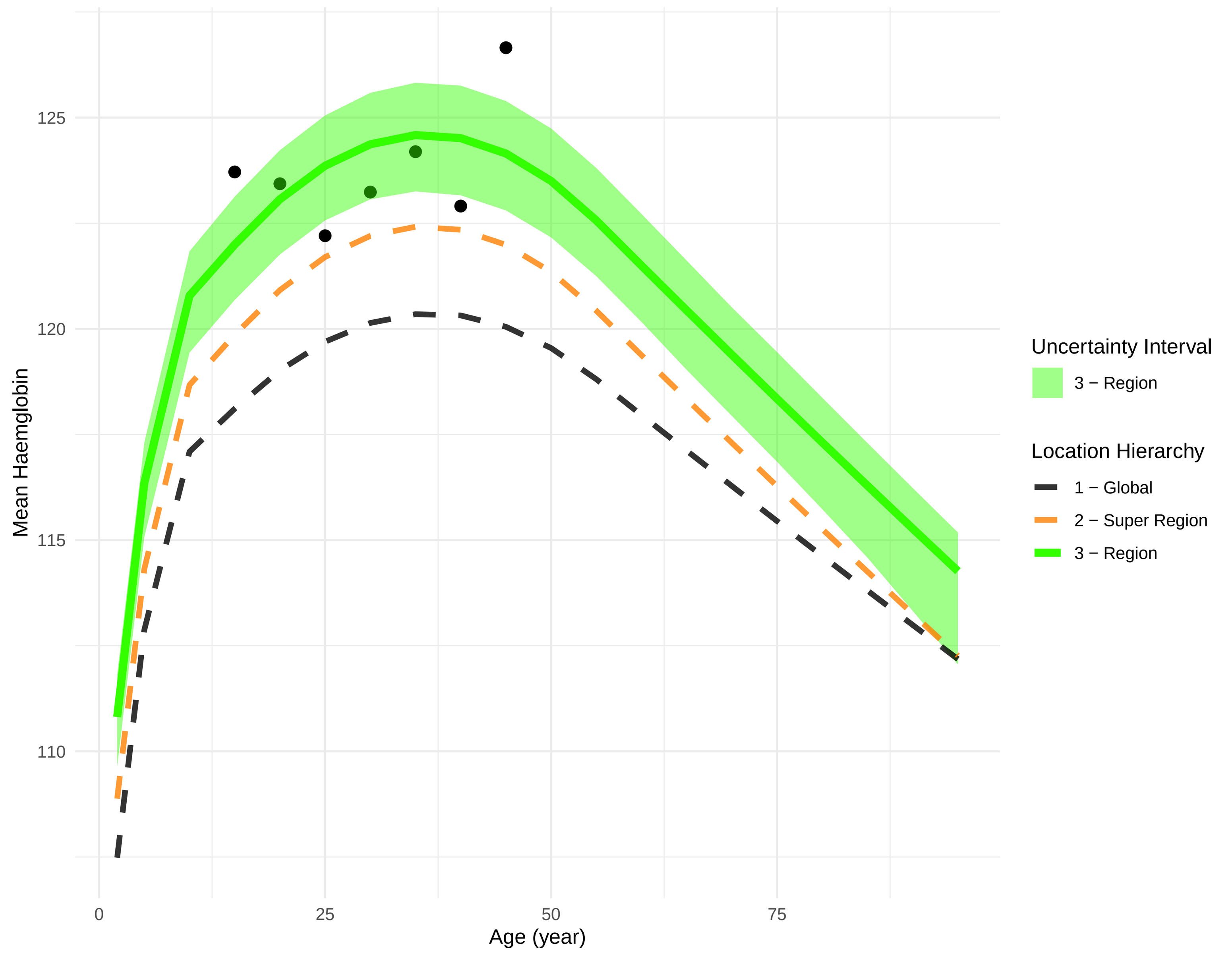

MR−BRT Age/Sex Fits − Non-pregnant Females 2+ Years Old − Mean Haemglobin − Papua New Guinea
WHO−2024 Adjustment Method

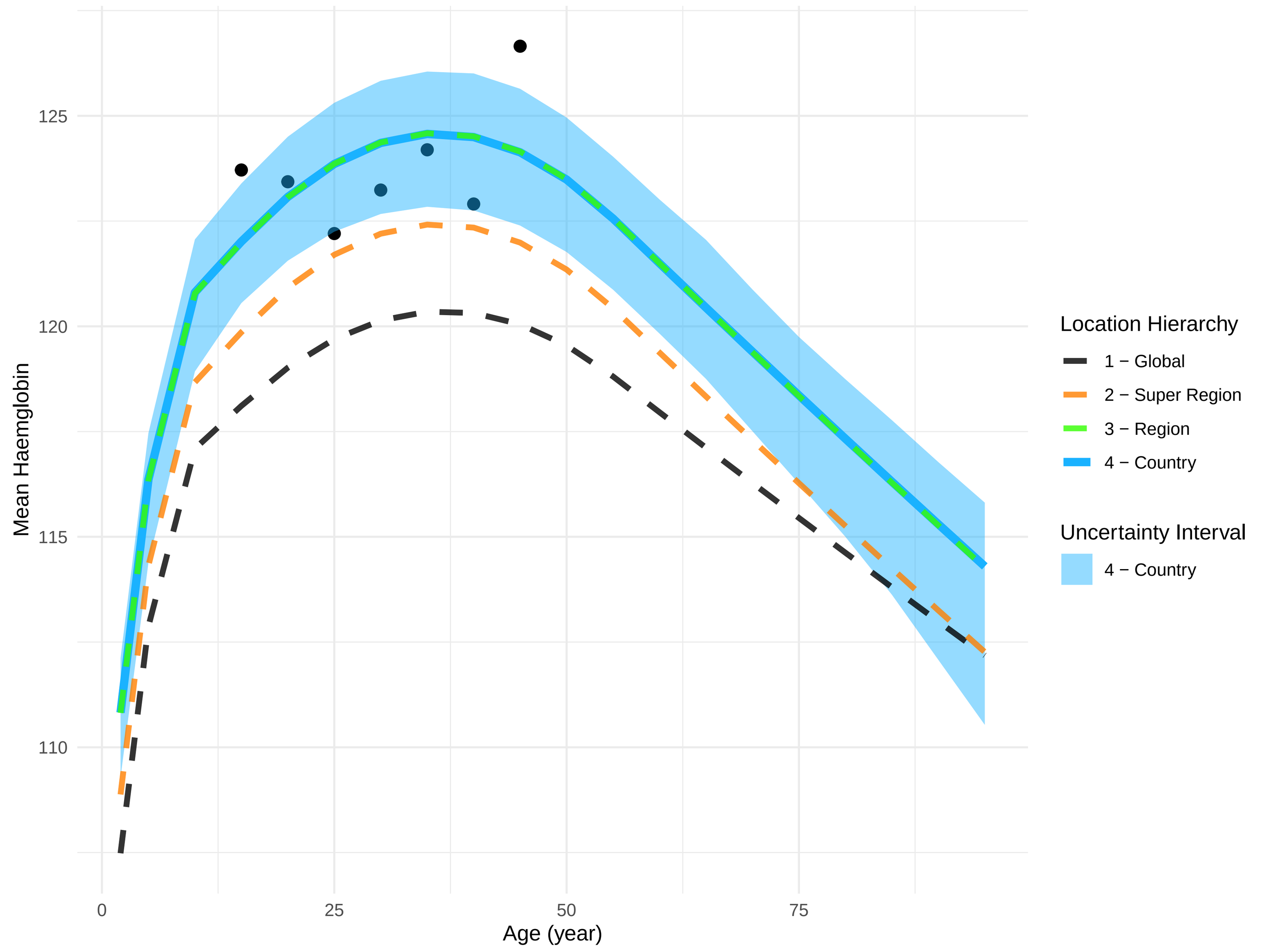

MR−BRT Age/Sex Fits − Non-pregnant Females 2+ Years Old − Mean Haemglobin − Southeast Asia
WHO−2024 Adjustment Method

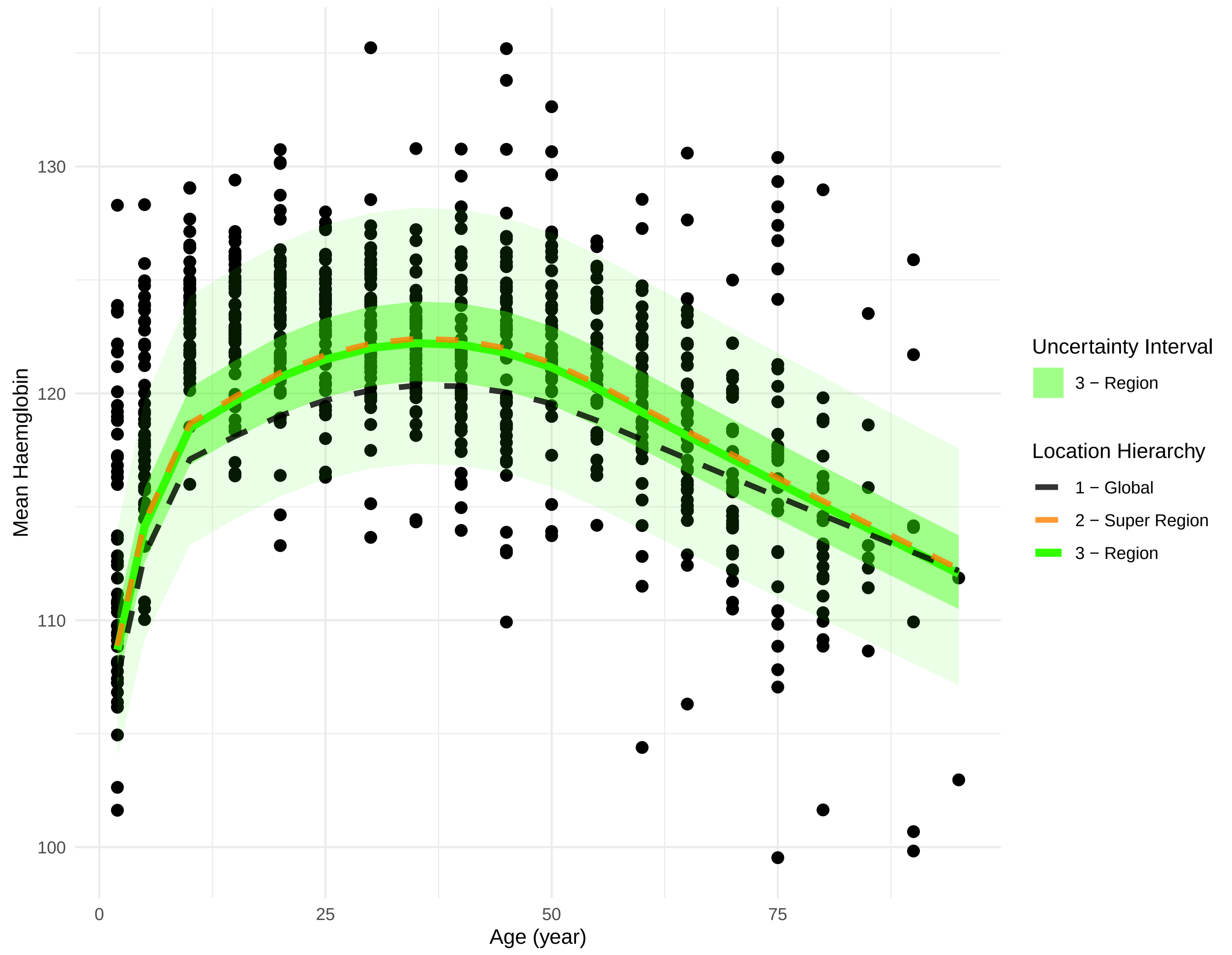

MR−BRT Age/Sex Fits − Non-pregnant Females 2+ Years Old − Mean Haemglobin − Cambodia
WHO−2024 Adjustment Method

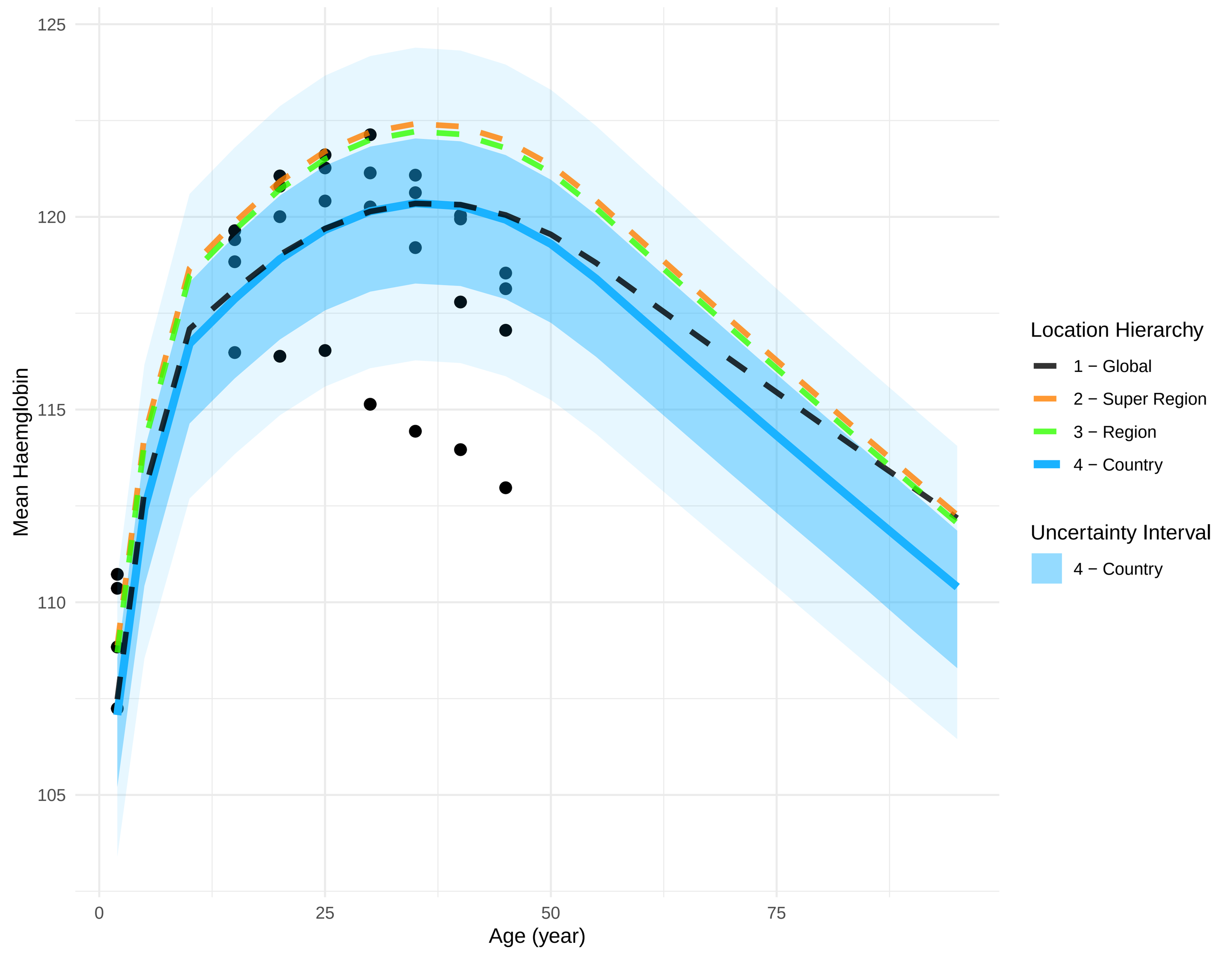

MR−BRT Age/Sex Fits − Non-pregnant Females 2+ Years Old − Mean Haemglobin − Indonesia
WHO−2024 Adjustment Method

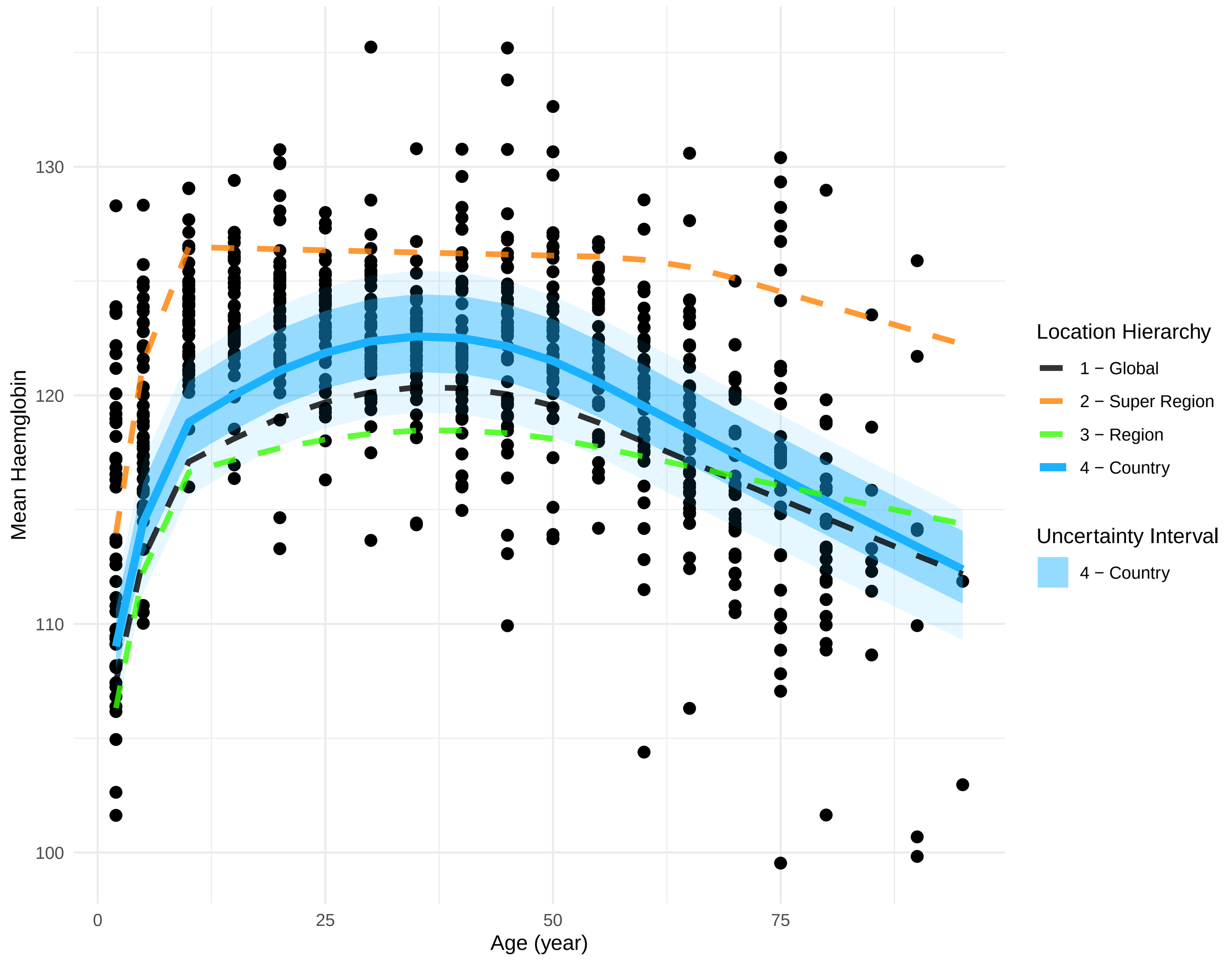

MR−BRT Age/Sex Fits − Non-pregnant Females 2+ Years Old − Mean Haemglobin − Bali
WHO−2024 Adjustment Method

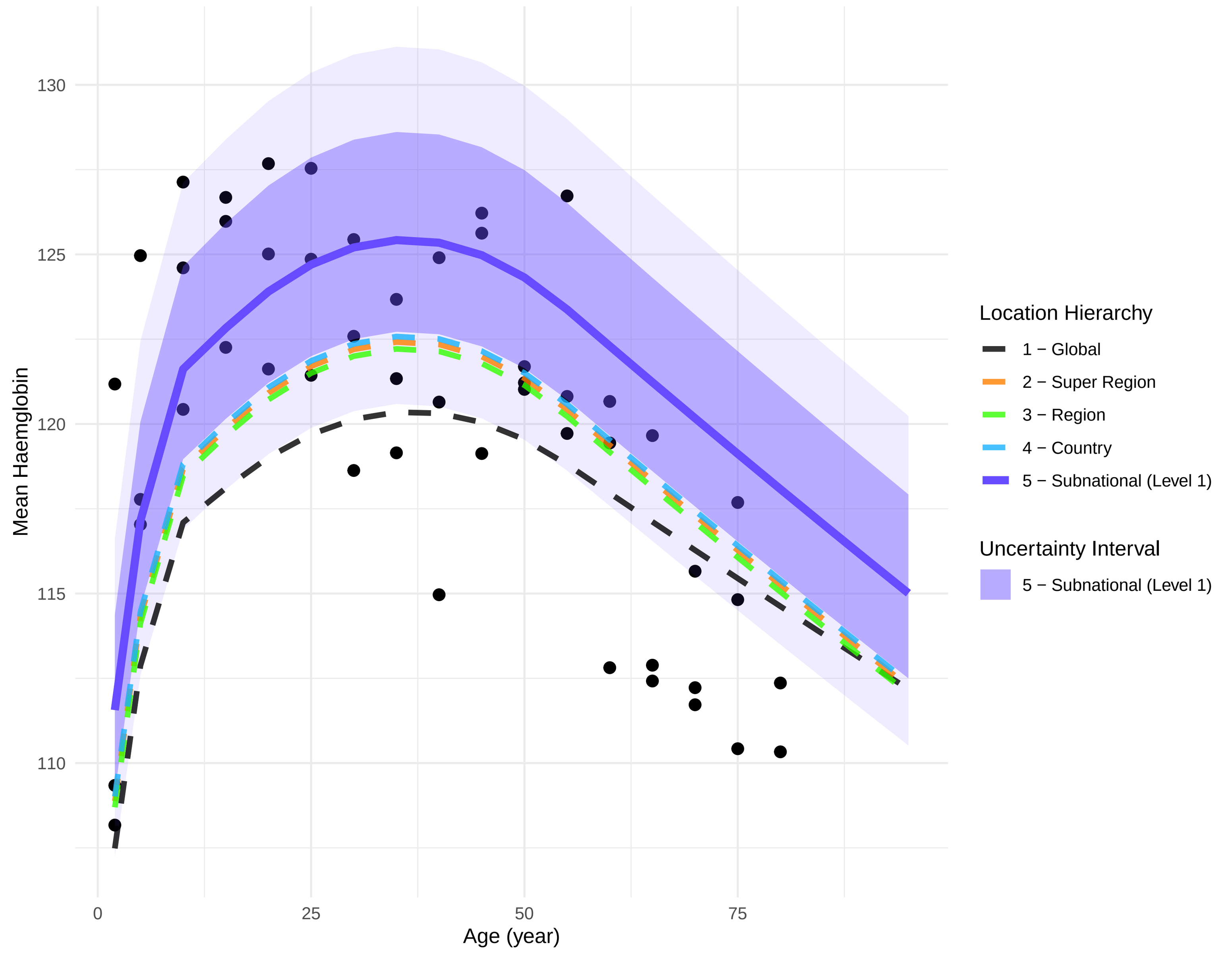

MR−BRT Age/Sex Fits − Non-pregnant Females 2+ Years Old − Mean Haemglobin − Bangka−Belitung Islands
WHO−2024 Adjustment Method

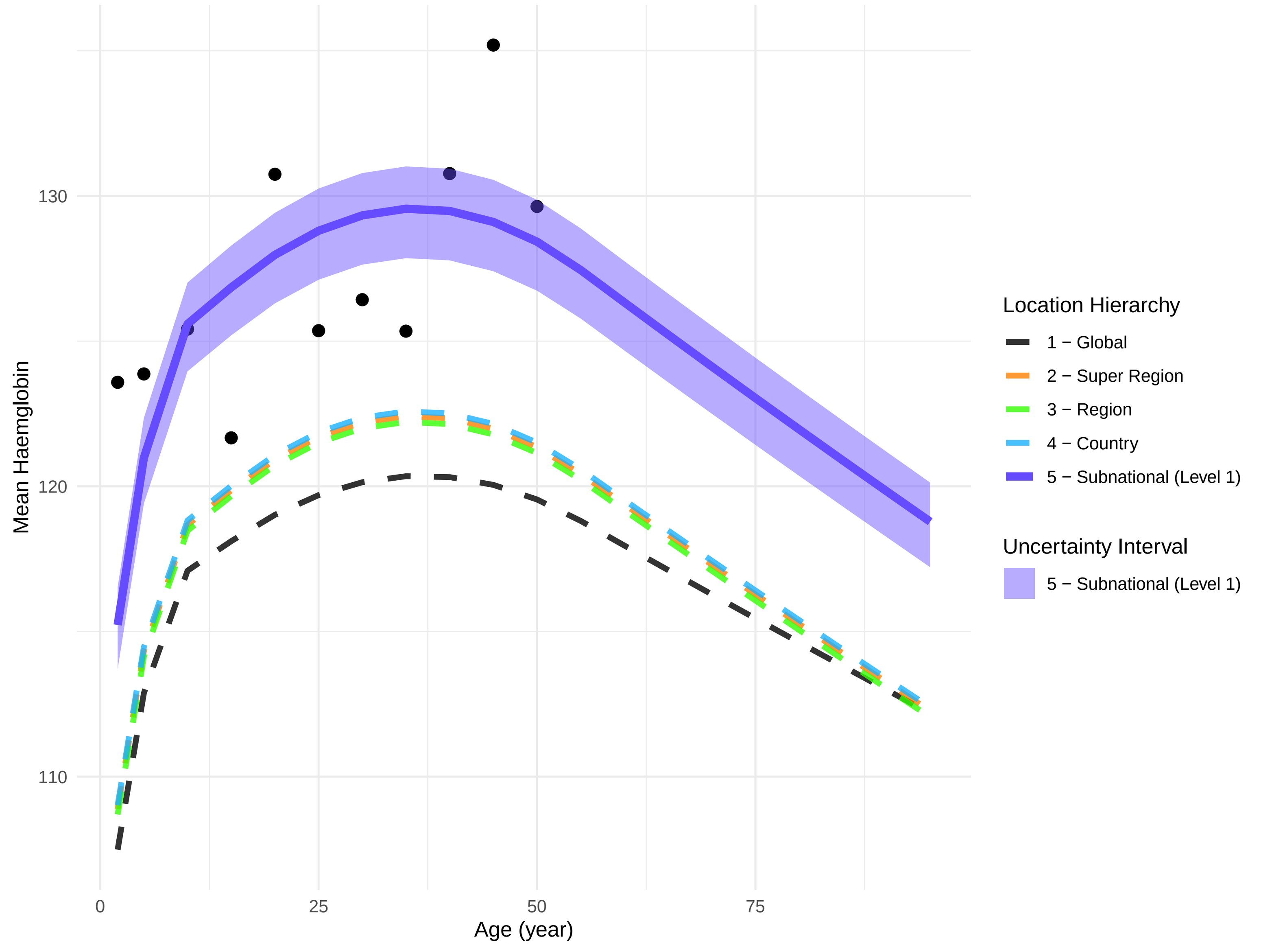

MR−BRT Age/Sex Fits − Non-pregnant Females 2+ Years Old − Mean Haemglobin − Banten
WHO−2024 Adjustment Method

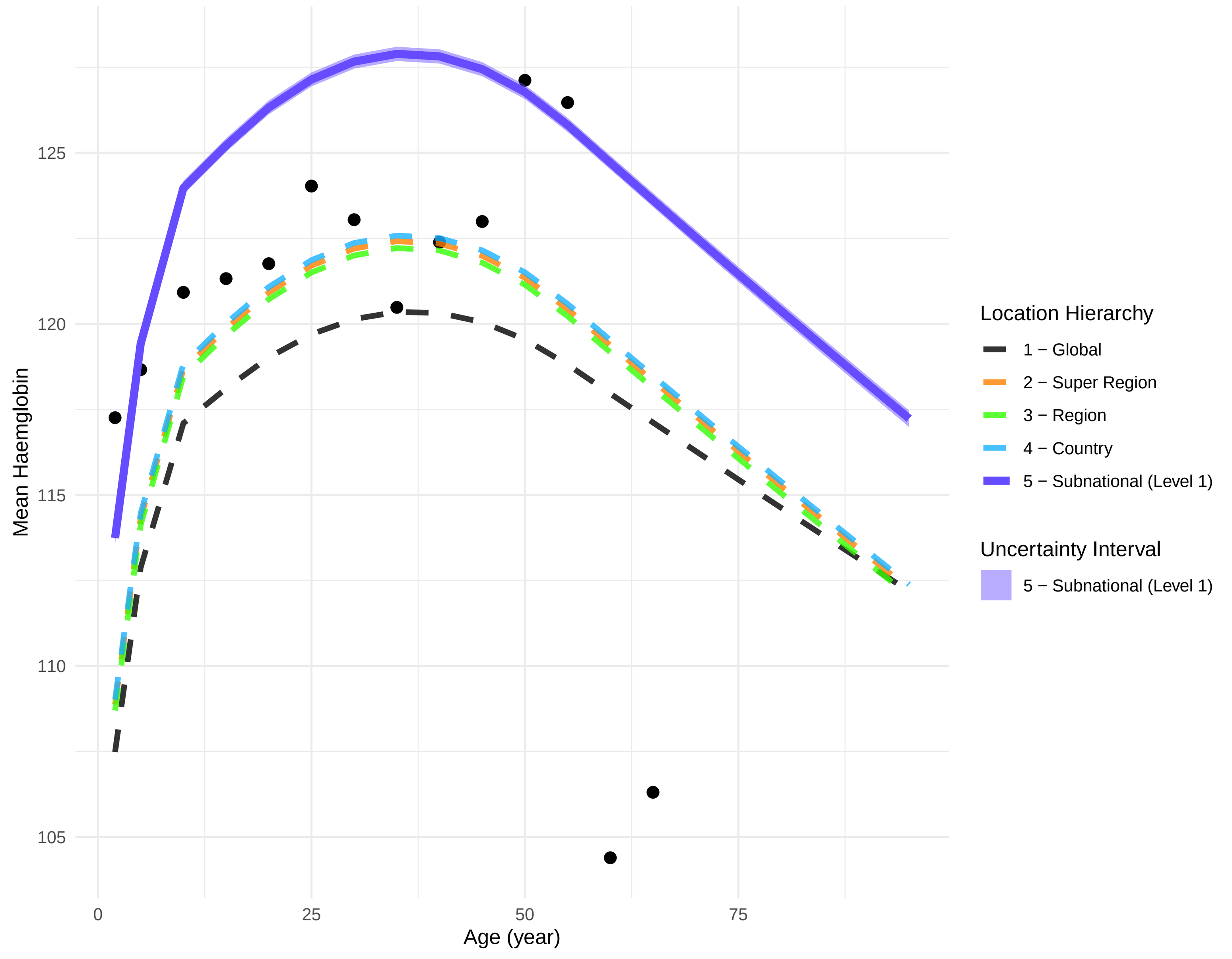

MR−BRT Age/Sex Fits − Non-pregnant Females 2+ Years Old − Mean Haemglobin − Jakarta
WHO−2024 Adjustment Method

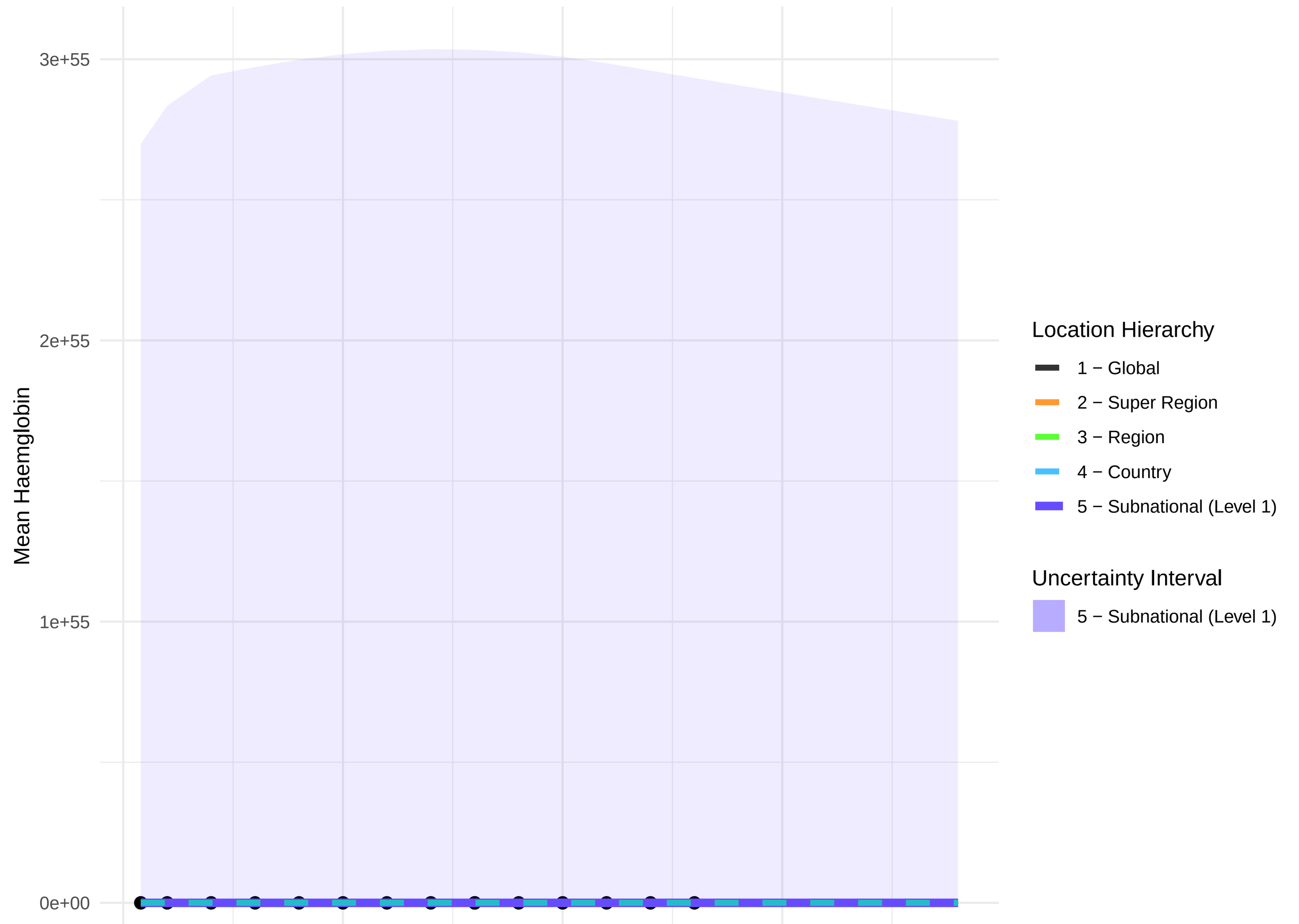

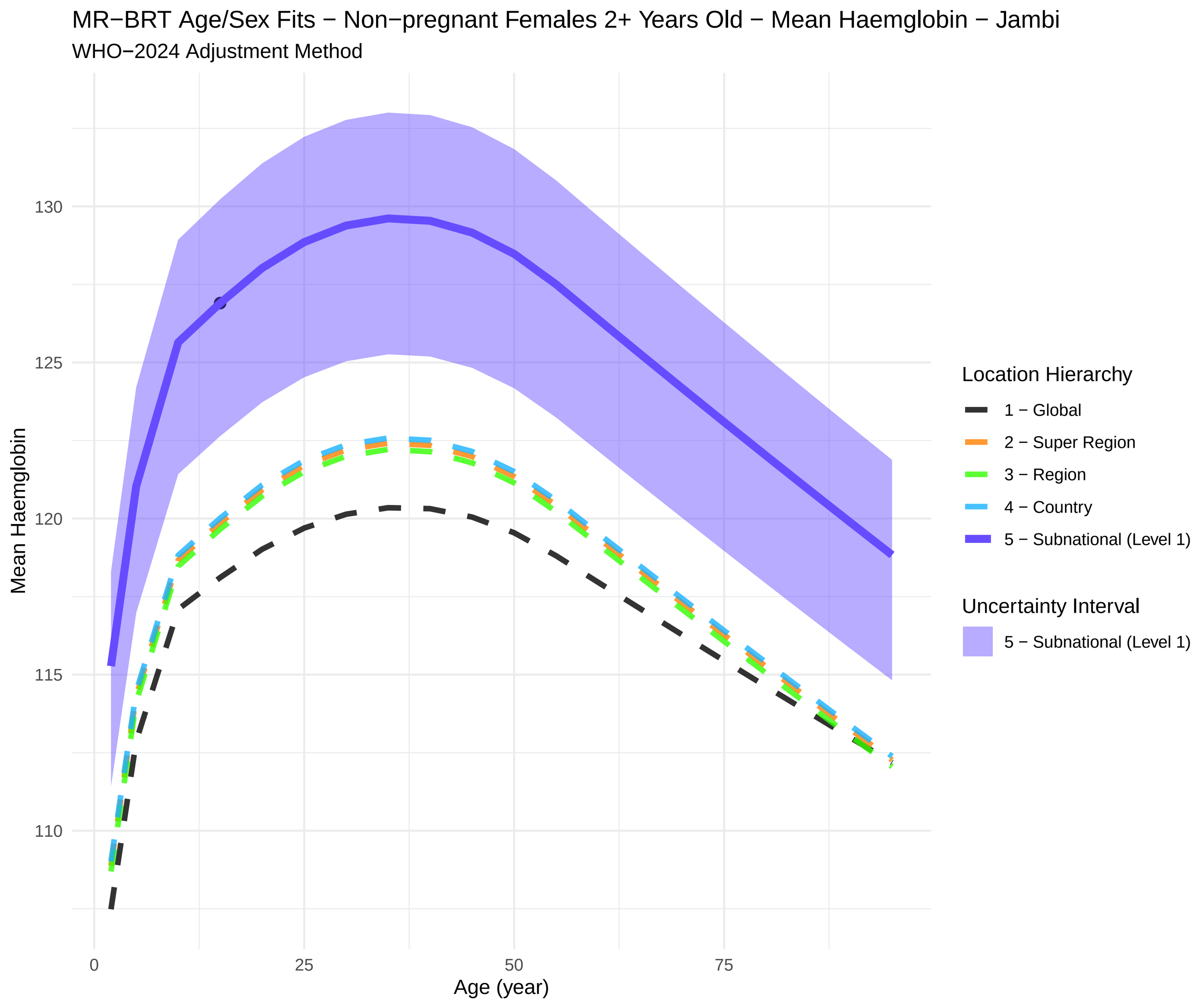
MR−BRT Age/Sex Fits − Non−pregnant Females 2+ Years Old − Mean Haemglobin − Jambi
WHO−2024 Adjustment Method
Mean Haemglobin
Age (year)
Location Hierarchy
1 − Global
2 − Super Region
3 − Region
4 − Country
5 − Subnational (Level 1)
Uncertainty Interval
5 − Subnational (Level 1)

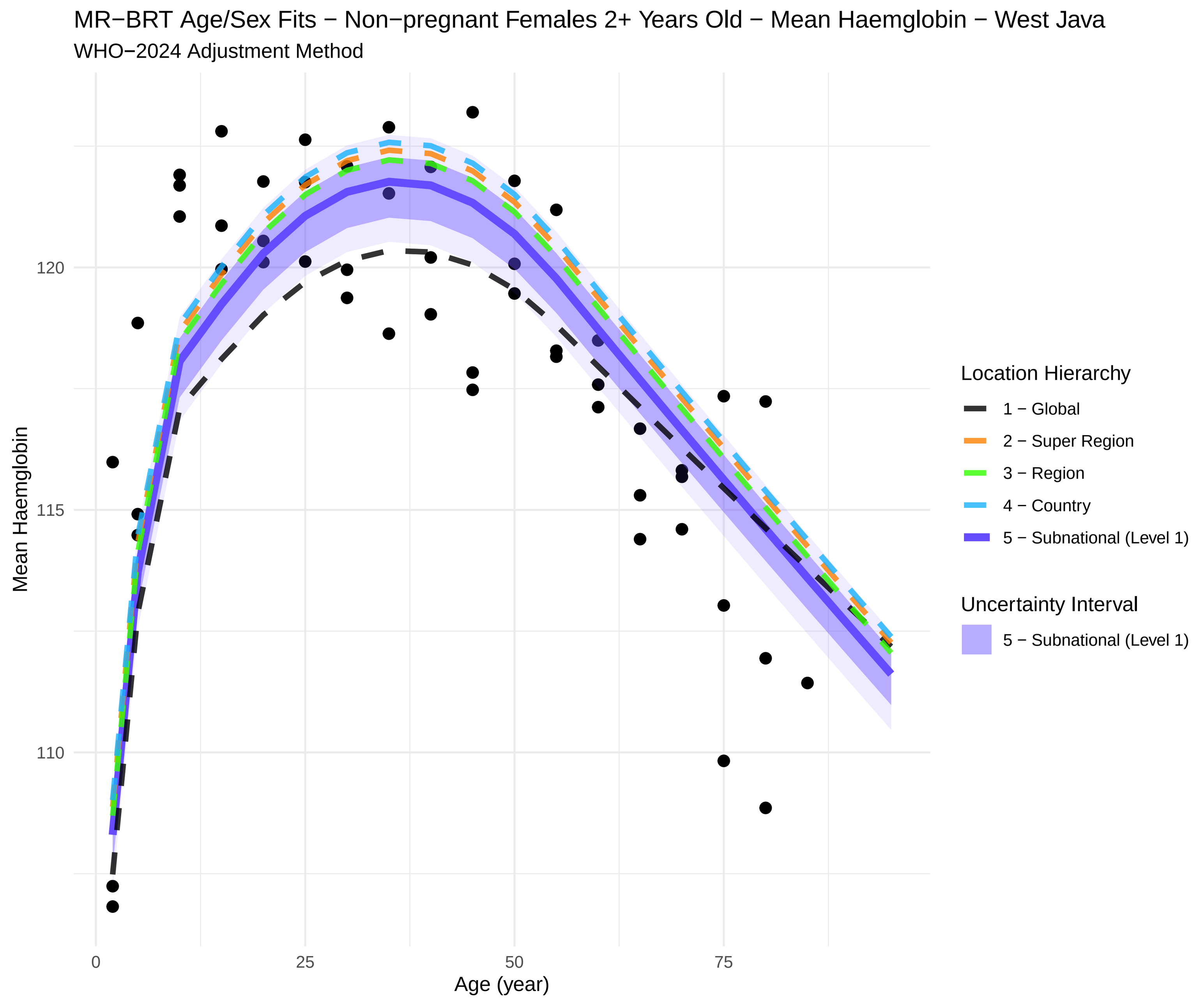
MR−BRT Age/Sex Fits − Non−pregnant Females 2+ Years Old − Mean Haemglobin − West Java
WHO−2024 Adjustment Method
Mean Haemglobin
Age (year)
Location Hierarchy
1 − Global
2 − Super Region
3 − Region
4 − Country
5 − Subnational (Level 1)
Uncertainty Interval
5 − Subnational (Level 1)

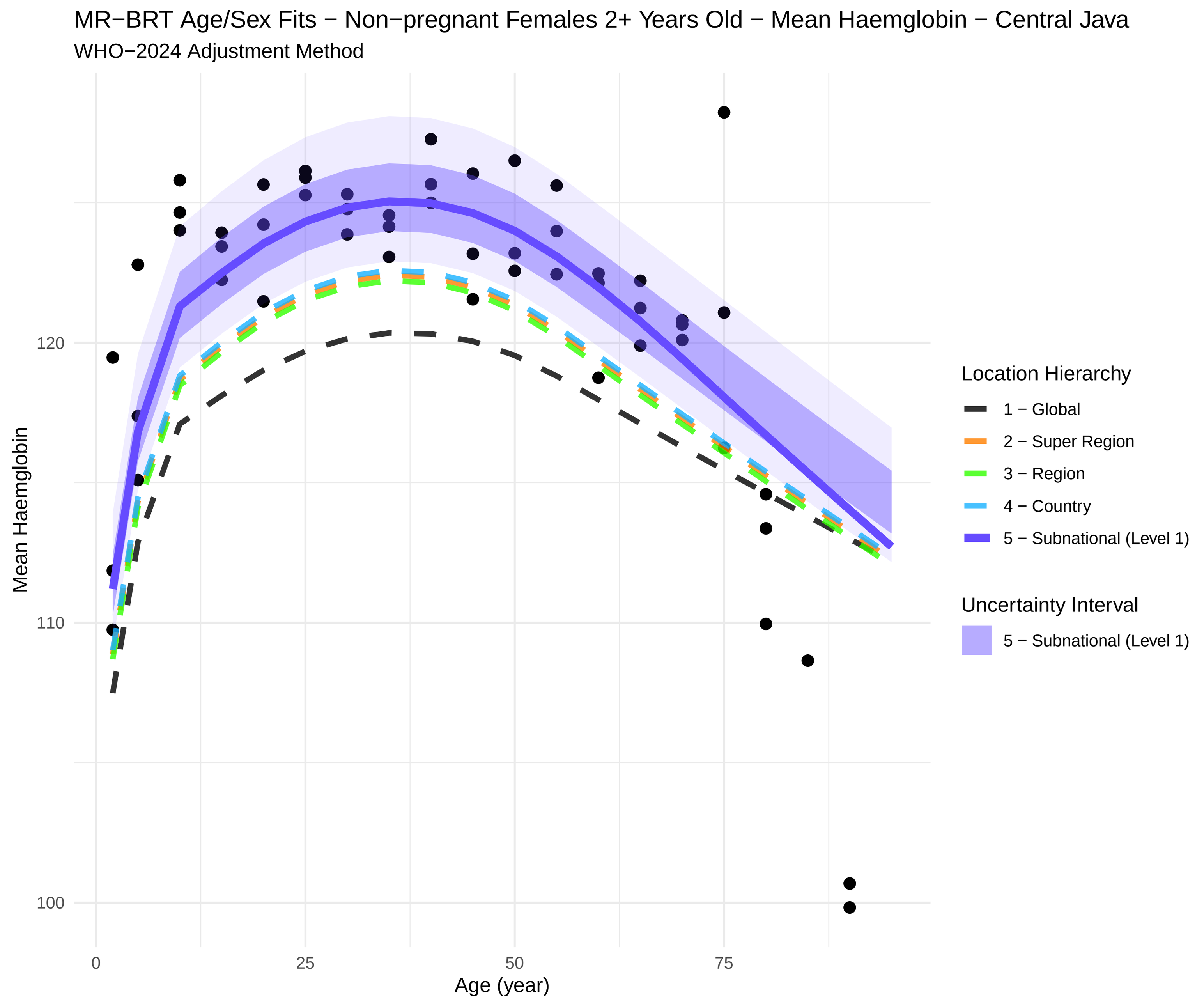
MR−BRT Age/Sex Fits − Non−pregnant Females 2+ Years Old − Mean Haemglobin − Central Java
WHO−2024 Adjustment Method
Mean Haemglobin
Age (year)
Location Hierarchy
1 − Global
2 − Super Region
3 − Region
4 − Country
5 − Subnational (Level 1)
Uncertainty Interval
5 − Subnational (Level 1)

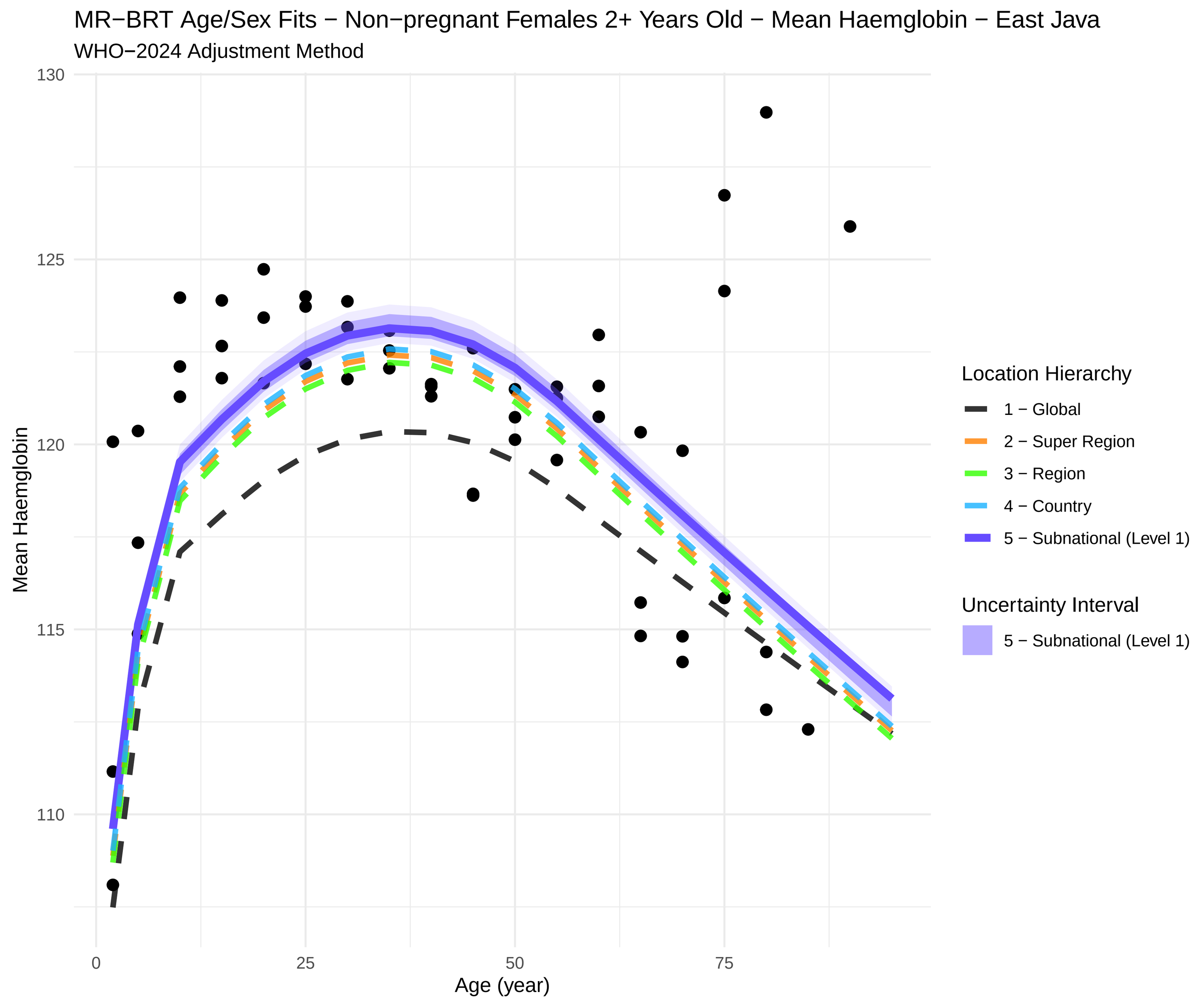
MR−BRT Age/Sex Fits − Non−pregnant Females 2+ Years Old − Mean Haemglobin − East Java
WHO−2024 Adjustment Method
Mean Haemglobin
Age (year)
Location Hierarchy
1 − Global
2 − Super Region
3 − Region
4 − Country
5 − Subnational (Level 1)
Uncertainty Interval
5 − Subnational (Level 1)

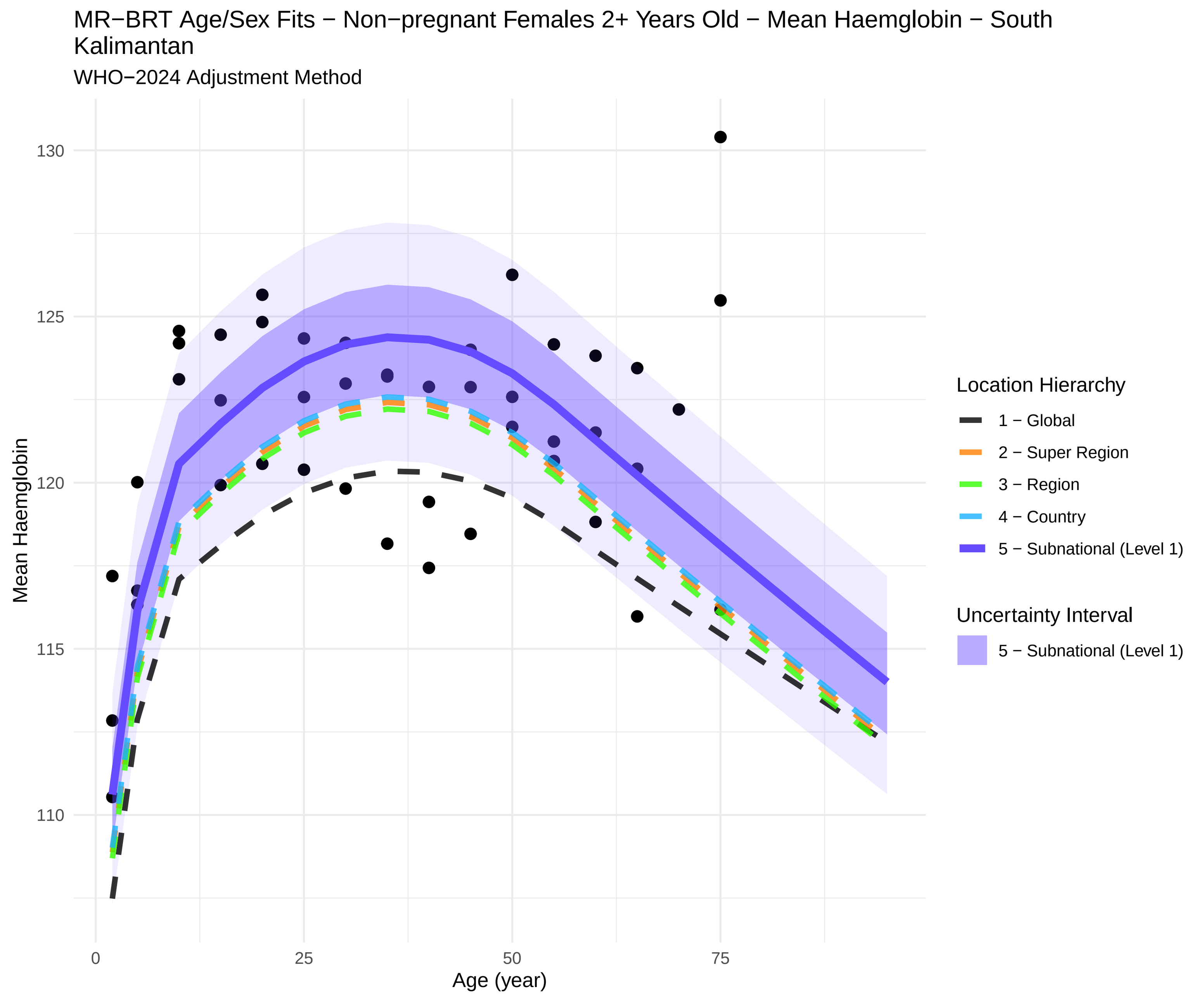
MR−BRT Age/Sex Fits − Non−pregnant Females 2+ Years Old − Mean Haemglobin − South Kalimantan
WHO−2024 Adjustment Method
Mean Haemglobin
Age (year)
Location Hierarchy
1 − Global
2 − Super Region
3 − Region
4 − Country
5 − Subnational (Level 1)
Uncertainty Interval
5 − Subnational (Level 1)

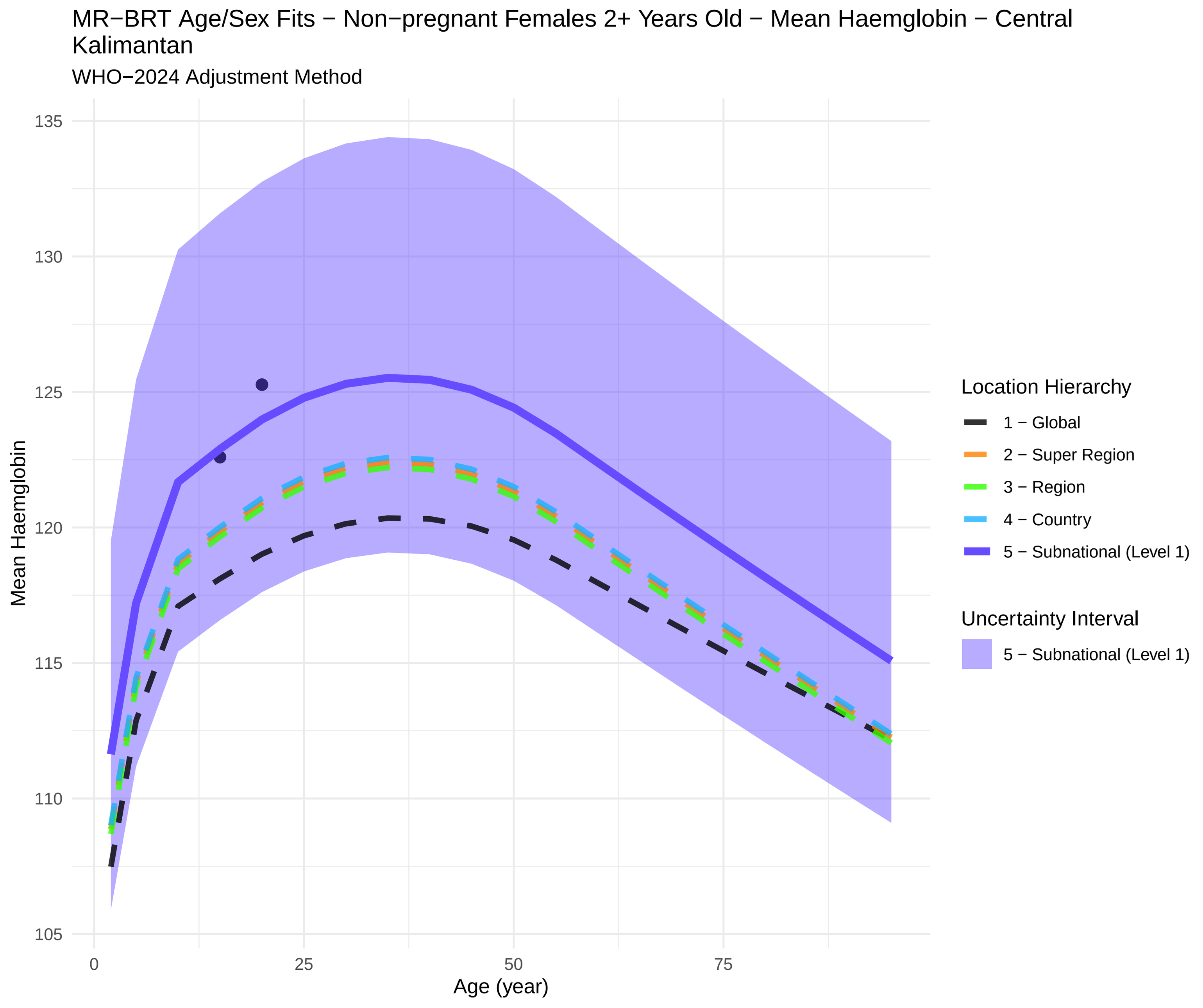
MR−BRT Age/Sex Fits − Non−pregnant Females 2+ Years Old − Mean Haemglobin − Central Kalimantan
WHO−2024 Adjustment Method
Mean Haemglobin
Age (year)
Location Hierarchy
1 − Global
2 − Super Region
3 − Region
4 − Country
5 − Subnational (Level 1)
Uncertainty Interval
5 − Subnational (Level 1)

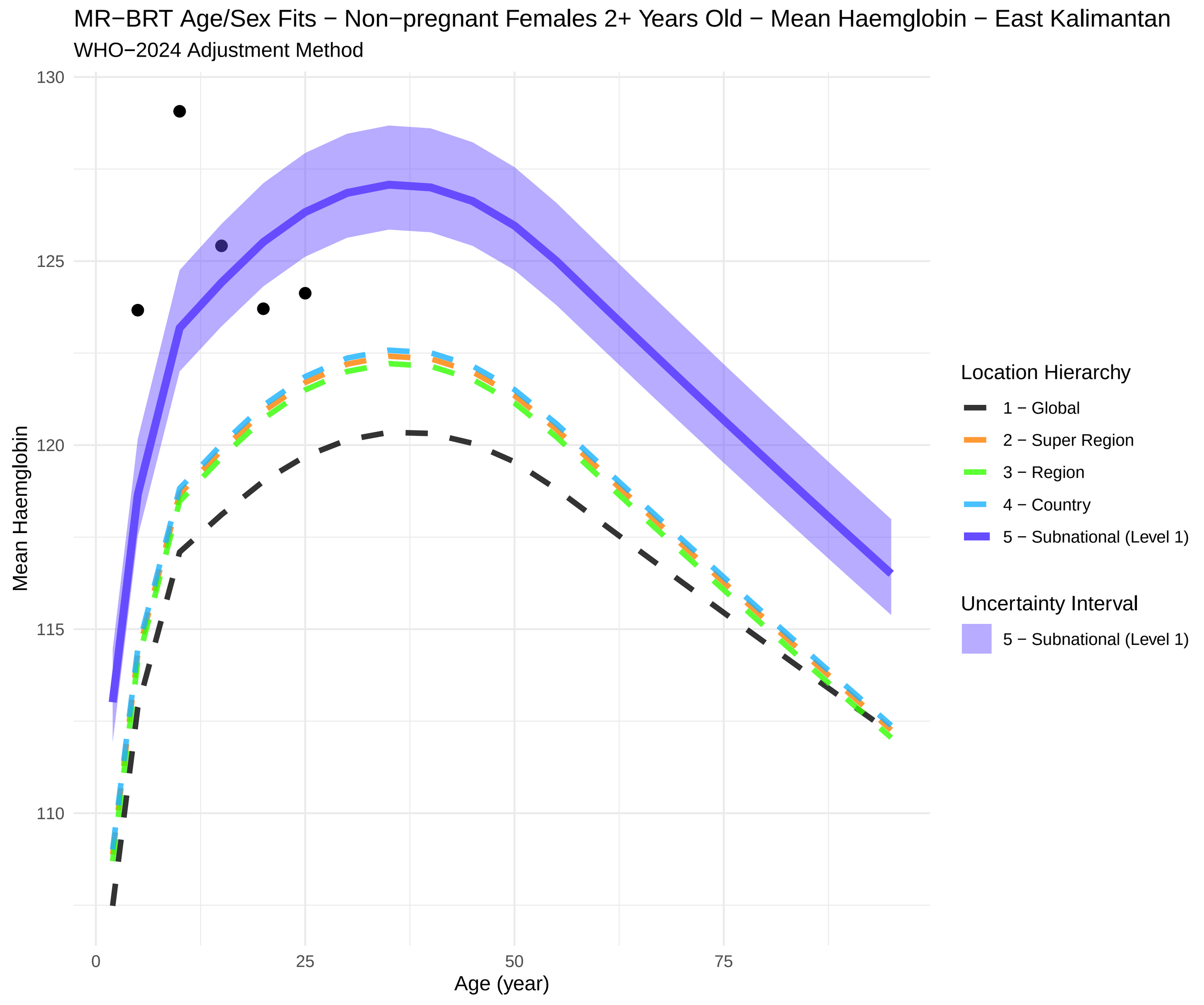
MR−BRT Age/Sex Fits − Non−pregnant Females 2+ Years Old − Mean Haemglobin − East Kalimantan
WHO−2024 Adjustment Method
Mean Haemglobin
Age (year)
Location Hierarchy
1 − Global
2 − Super Region
3 − Region
4 − Country
5 − Subnational (Level 1)
Uncertainty Interval
5 − Subnational (Level 1)

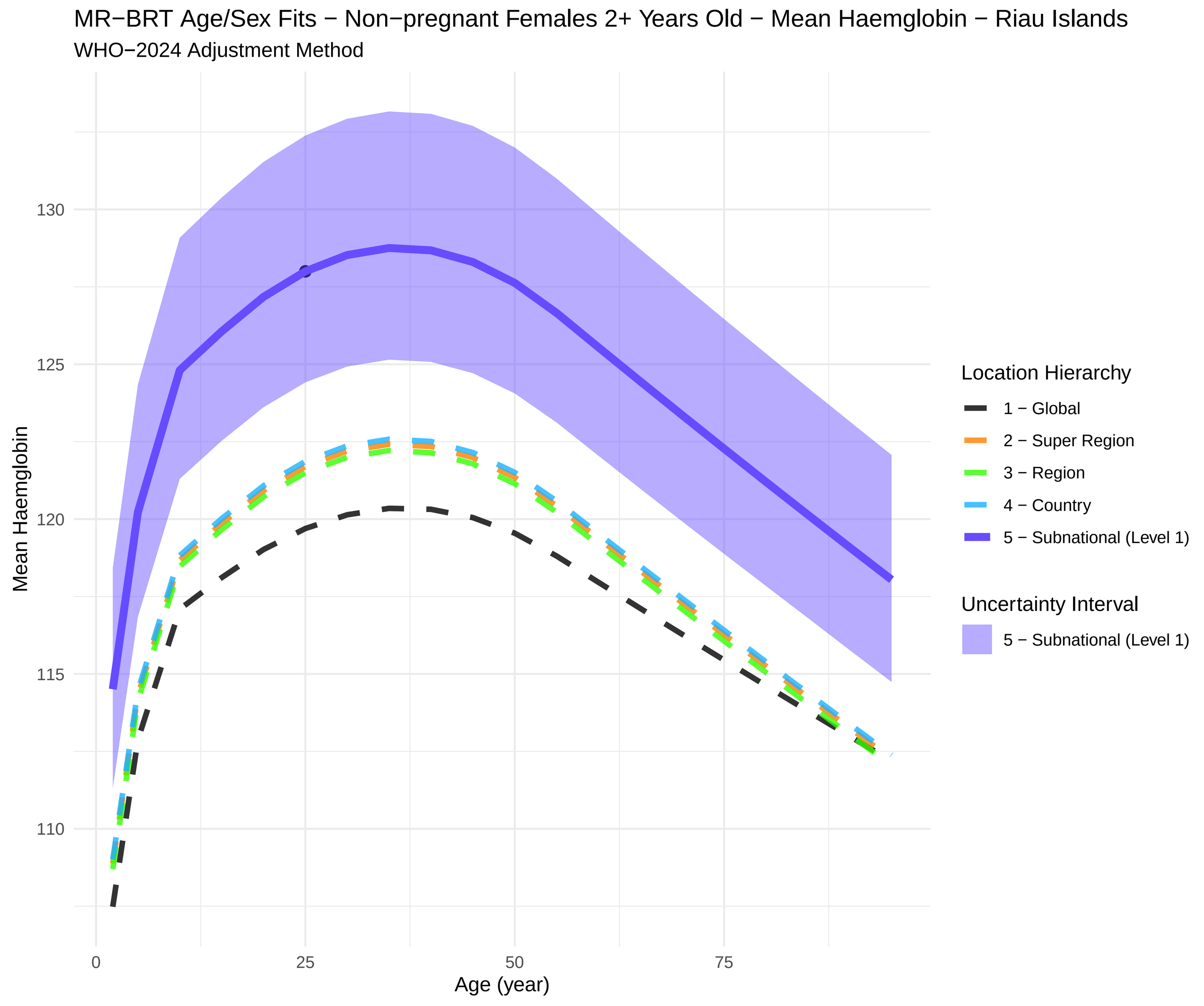
MR−BRT Age/Sex Fits − Non−pregnant Females 2+ Years Old − Mean Haemglobin − Riau Islands
WHO−2024 Adjustment Method
Mean Haemglobin
Age (year)
Location Hierarchy
1 − Global
2 − Super Region
3 − Region
4 − Country
5 − Subnational (Level 1)
Uncertainty Interval
5 − Subnational (Level 1)

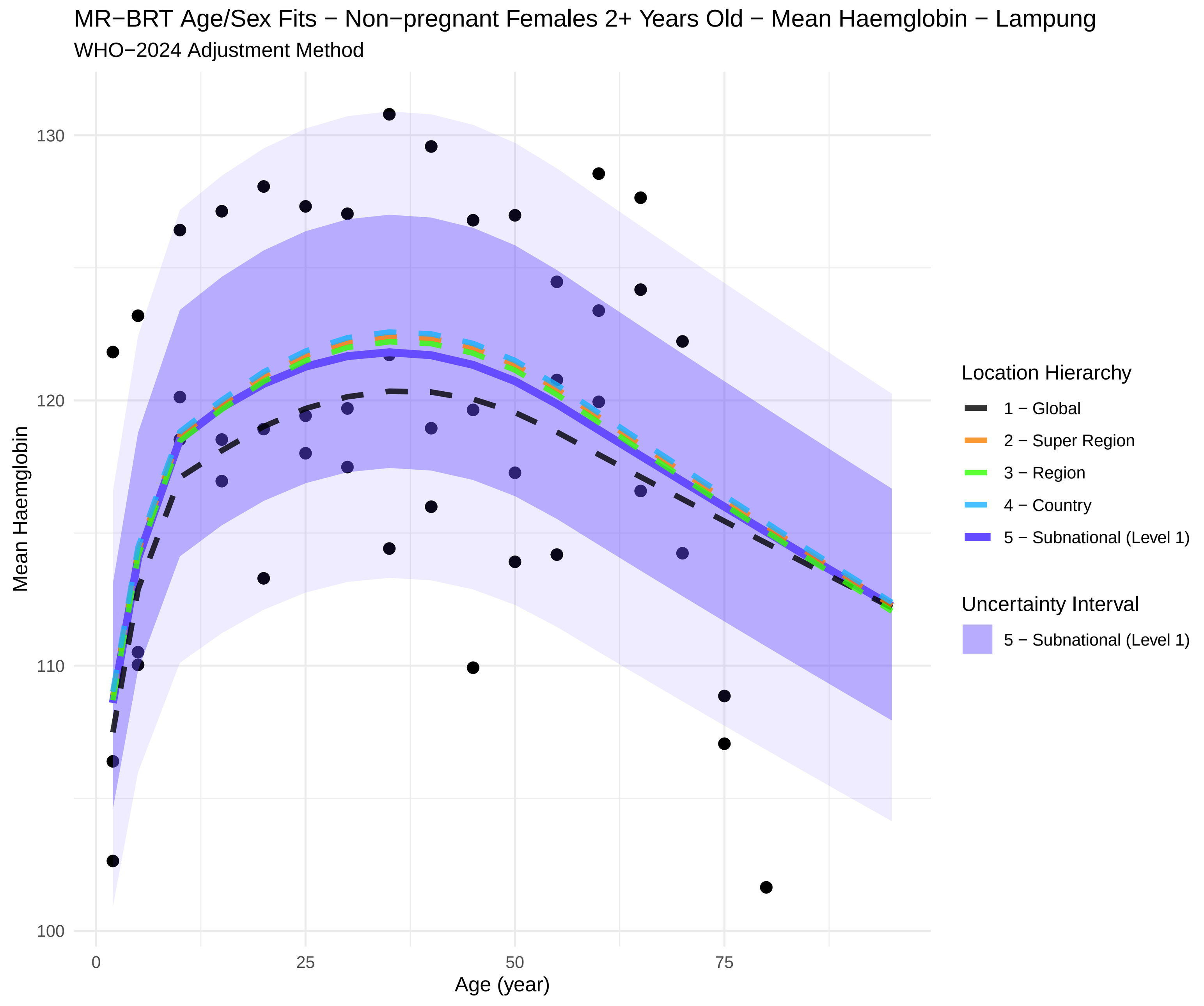
MR−BRT Age/Sex Fits − Non−pregnant Females 2+ Years Old − Mean Haemglobin − Lampung
WHO−2024 Adjustment Method
Mean Haemglobin
Age (year)
Location Hierarchy
1 − Global
2 − Super Region
3 − Region
4 − Country
5 − Subnational (Level 1)
Uncertainty Interval
5 − Subnational (Level 1)

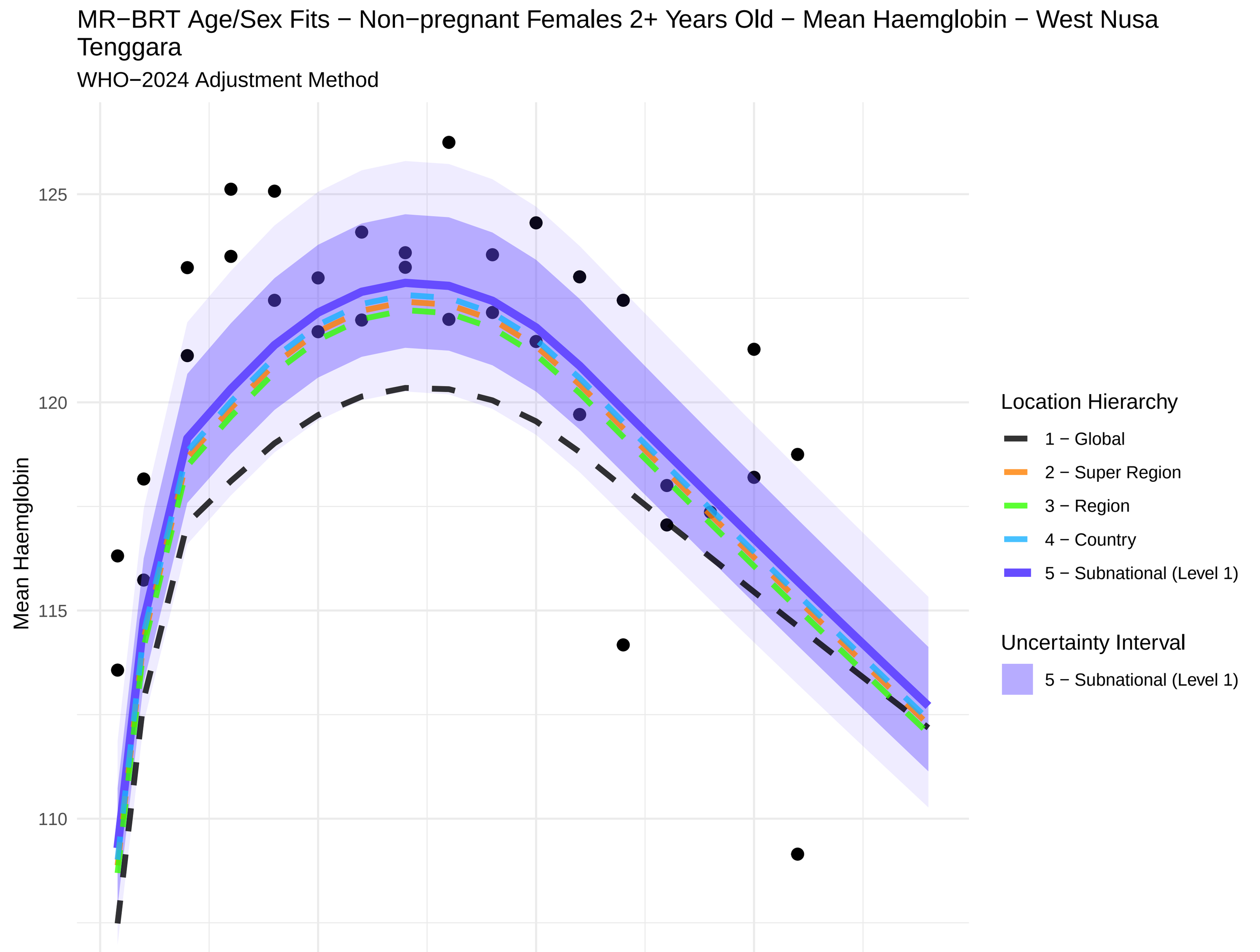
MR−BRT Age/Sex Fits − Non−pregnant Females 2+ Years Old − Mean Haemglobin − West Nusa Tenggara
WHO−2024 Adjustment Method
Mean Haemglobin
Age (year)
Location Hierarchy
1 − Global
2 − Super Region
3 − Region
4 − Country
5 − Subnational (Level 1)
Uncertainty Interval
5 − Subnational (Level 1)

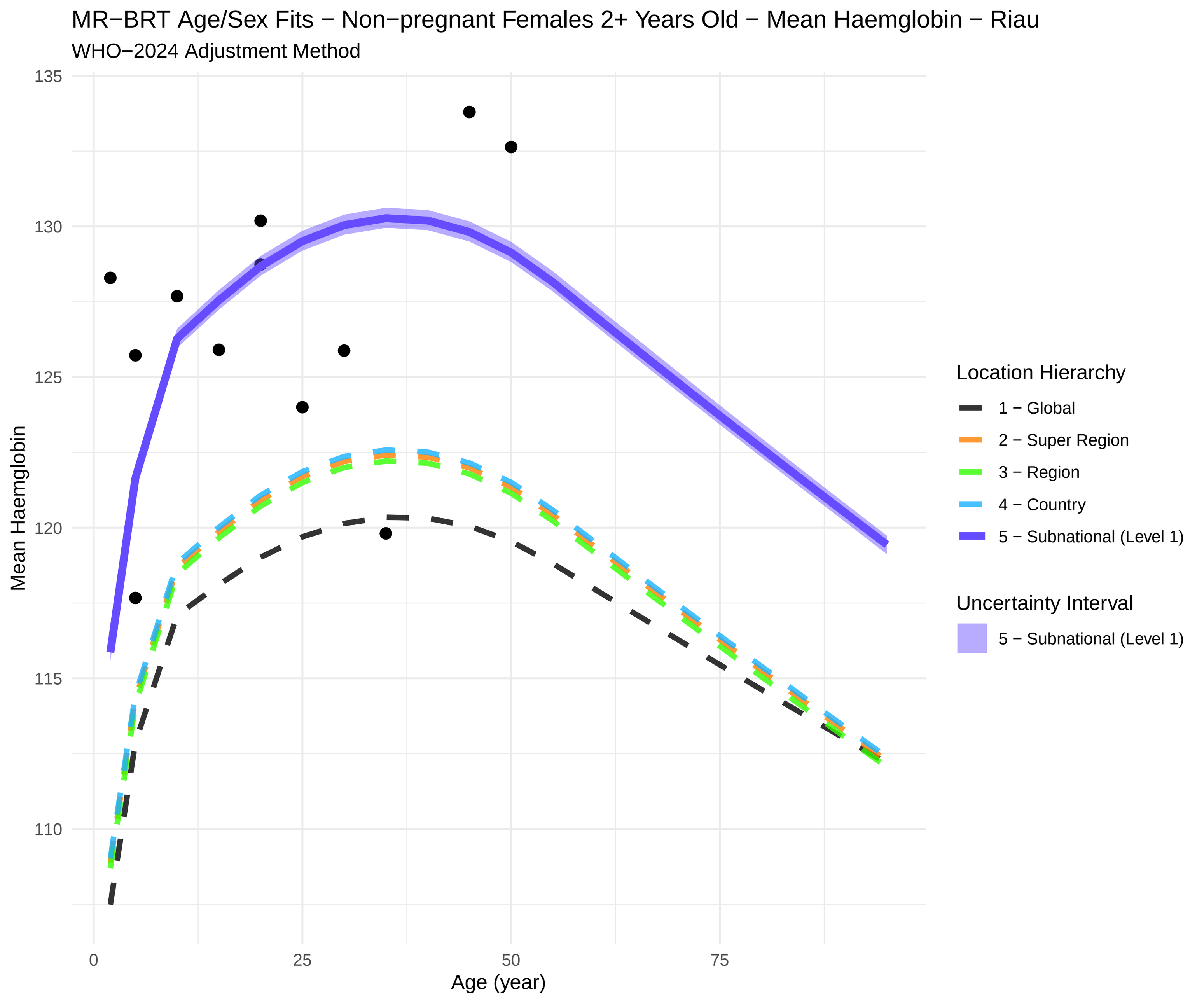
MR−BRT Age/Sex Fits − Non−pregnant Females 2+ Years Old − Mean Haemoglobin − Riau
WHO−2024 Adjustment Method
Mean Haemoglobin
Age (year)
Location Hierarchy
1 − Global
2 − Super Region
3 − Region
4 − Country
5 − Subnational (Level 1)
Uncertainty Interval
5 − Subnational (Level 1)

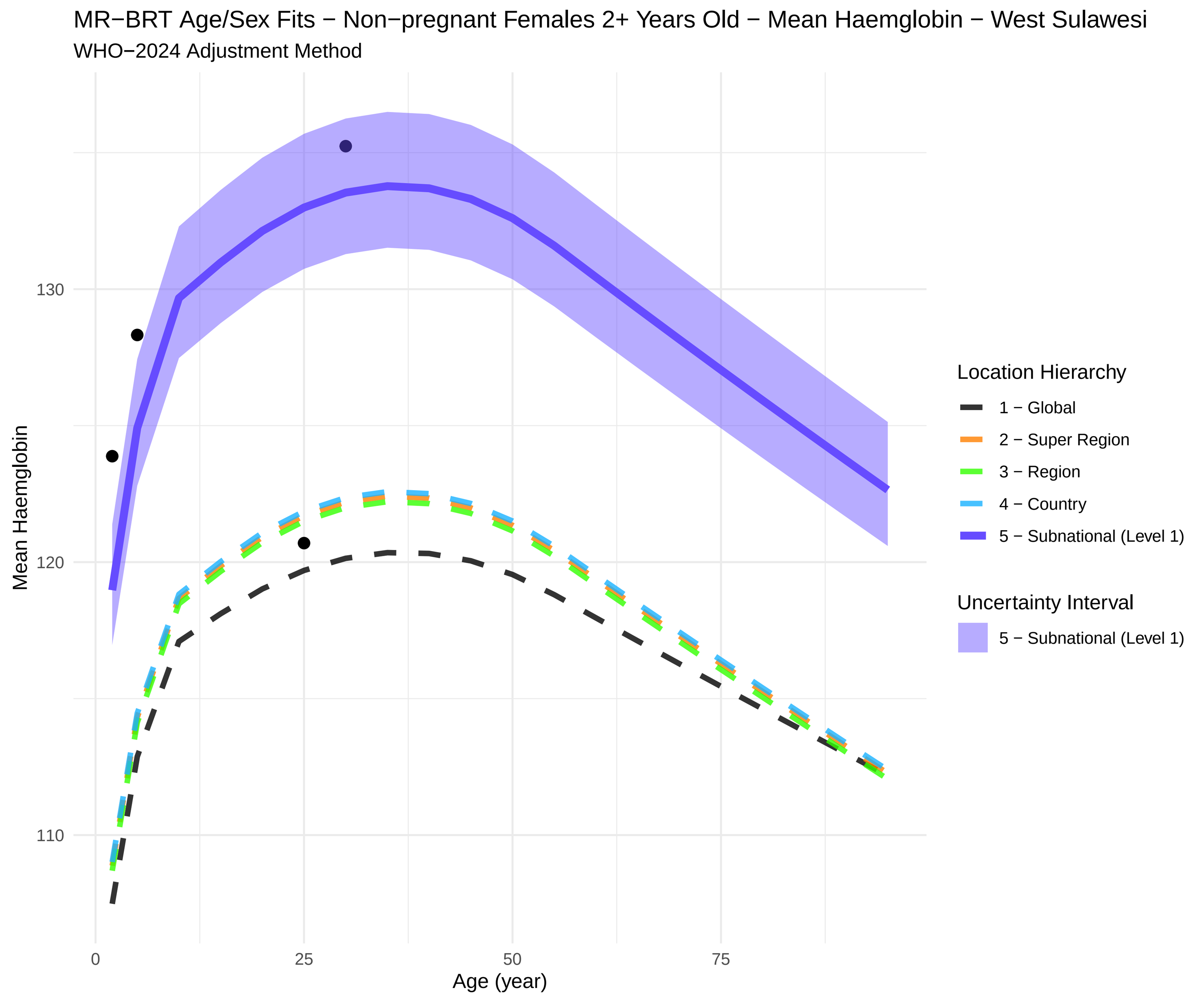
MR−BRT Age/Sex Fits − Non−pregnant Females 2+ Years Old − Mean Haemoglobin − West Sulawesi
WHO−2024 Adjustment Method
Mean Haemoglobin
Age (year)
Location Hierarchy
1 − Global
2 − Super Region
3 − Region
4 − Country
5 − Subnational (Level 1)
Uncertainty Interval
5 − Subnational (Level 1)

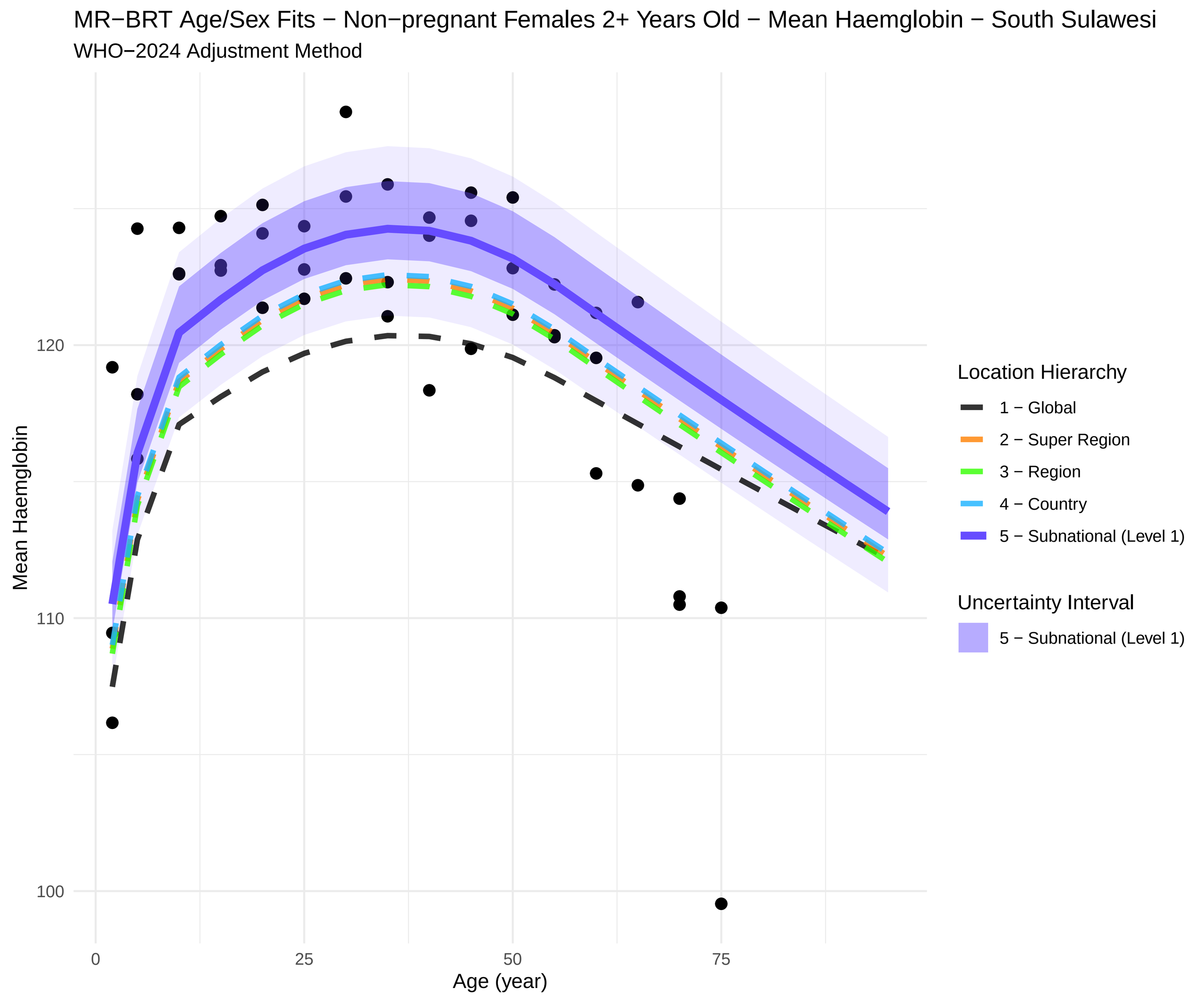
MR−BRT Age/Sex Fits − Non−pregnant Females 2+ Years Old − Mean Haemoglobin − South Sulawesi
WHO−2024 Adjustment Method
Mean Haemoglobin
Age (year)
Location Hierarchy
1 − Global
2 − Super Region
3 − Region
4 − Country
5 − Subnational (Level 1)
Uncertainty Interval
5 − Subnational (Level 1)

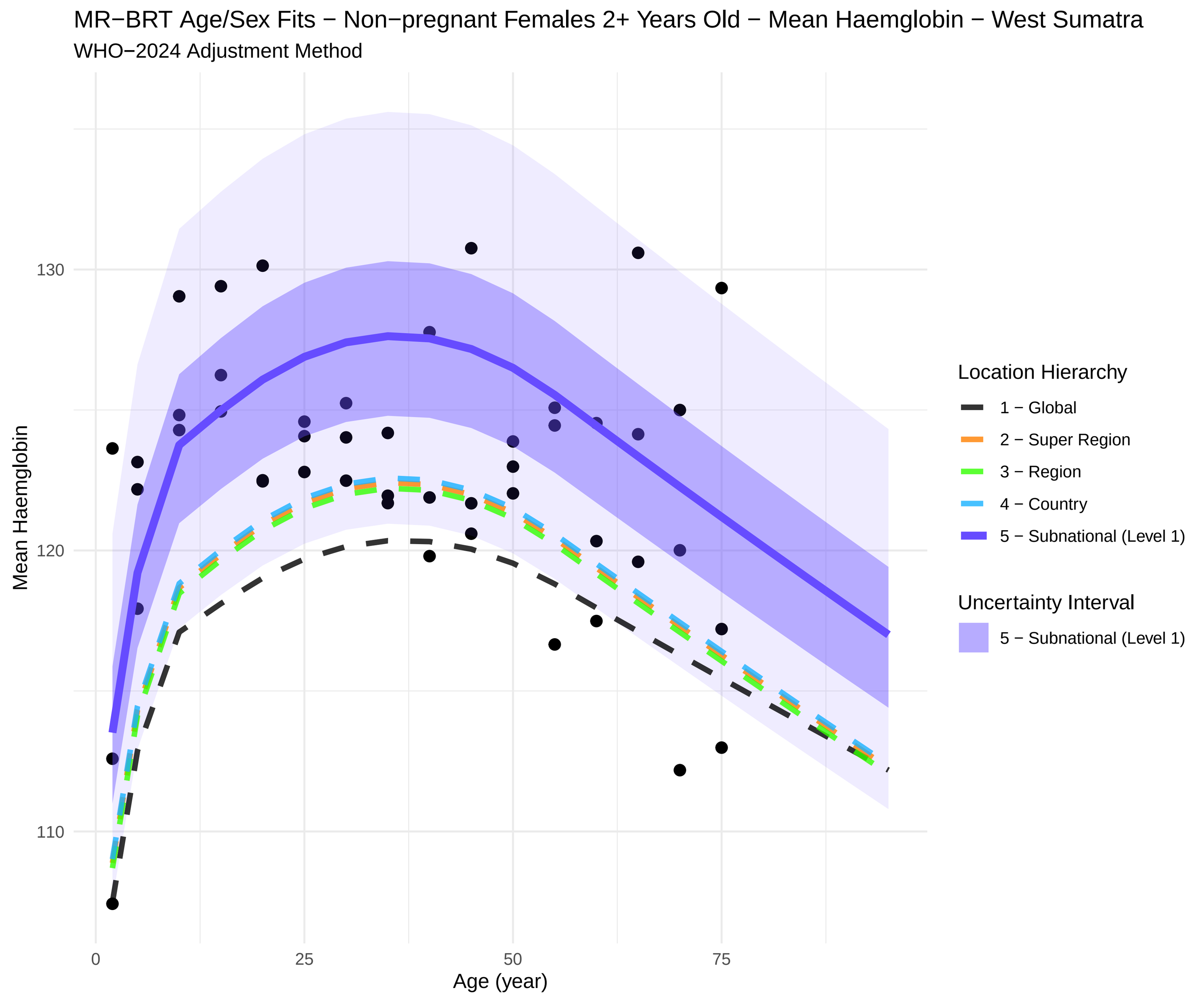
MR−BRT Age/Sex Fits − Non−pregnant Females 2+ Years Old − Mean Haemoglobin − West Sumatra
WHO−2024 Adjustment Method
Mean Haemoglobin
Age (year)
Location Hierarchy
1 − Global
2 − Super Region
3 − Region
4 − Country
5 − Subnational (Level 1)
Uncertainty Interval
5 − Subnational (Level 1)

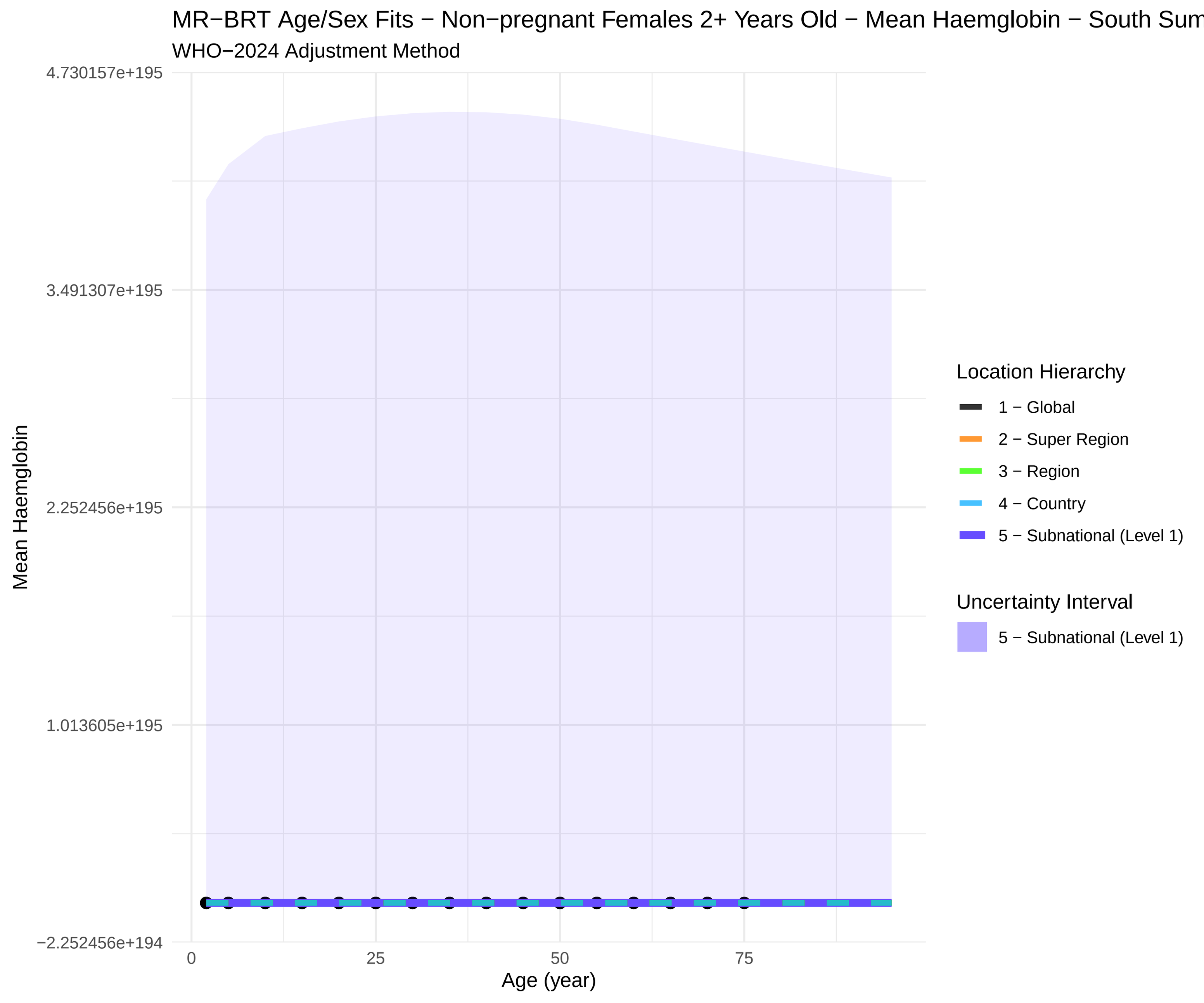
MR−BRT Age/Sex Fits − Non−pregnant Females 2+ Years Old − Mean Haemoglobin − South Su
WHO−2024 Adjustment Method
Mean Haemoglobin
Age (year)
Location Hierarchy
1 − Global
2 − Super Region
3 − Region
4 − Country
5 − Subnational (Level 1)
Uncertainty Interval
5 − Subnational (Level 1)

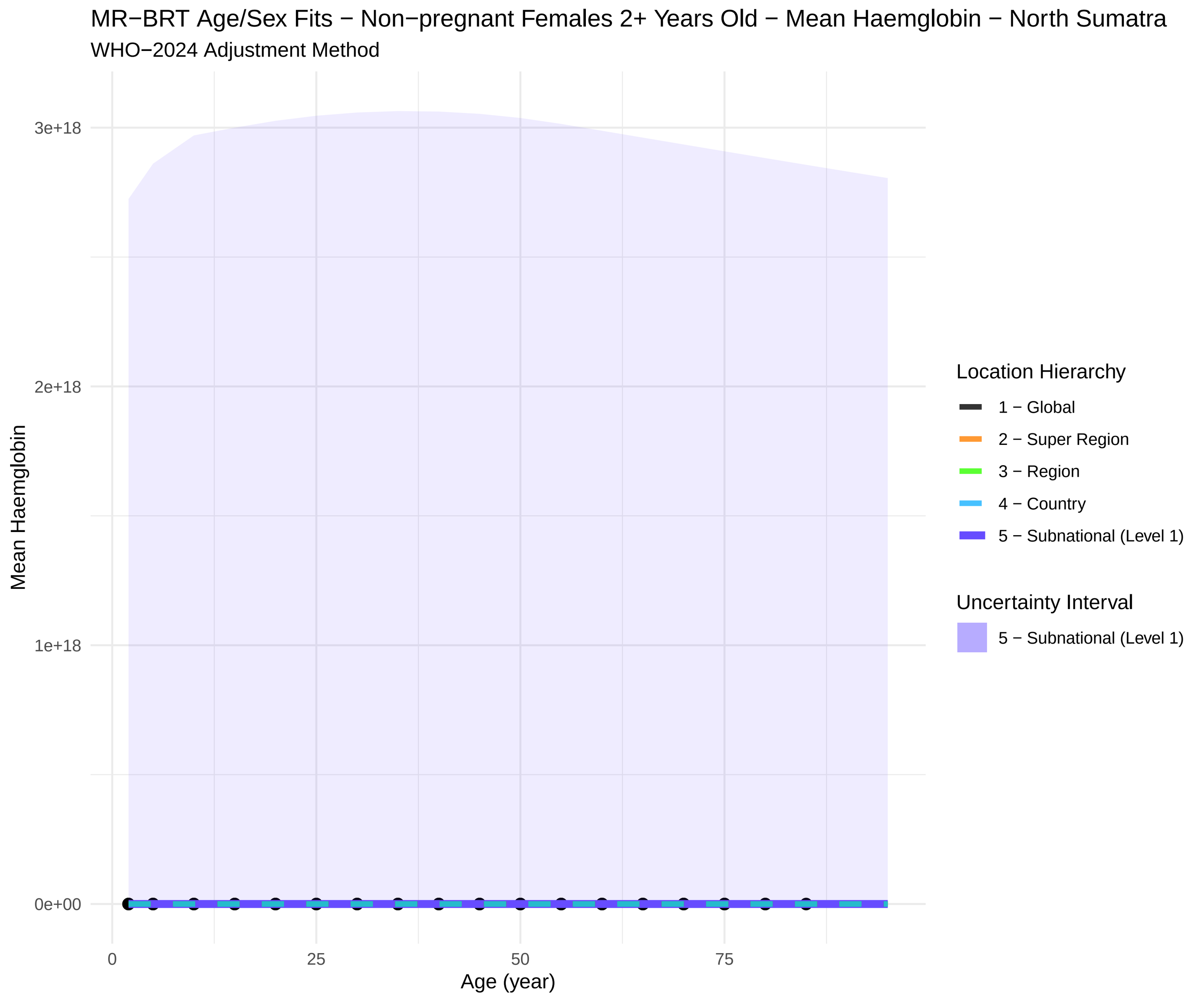
MR−BRT Age/Sex Fits − Non−pregnant Females 2+ Years Old − Mean Haemoglobin − North Sumatra
WHO−2024 Adjustment Method
Mean Haemoglobin
Age (year)
Location Hierarchy
1 − Global
2 − Super Region
3 − Region
4 − Country
5 − Subnational (Level 1)
Uncertainty Interval
5 − Subnational (Level 1)

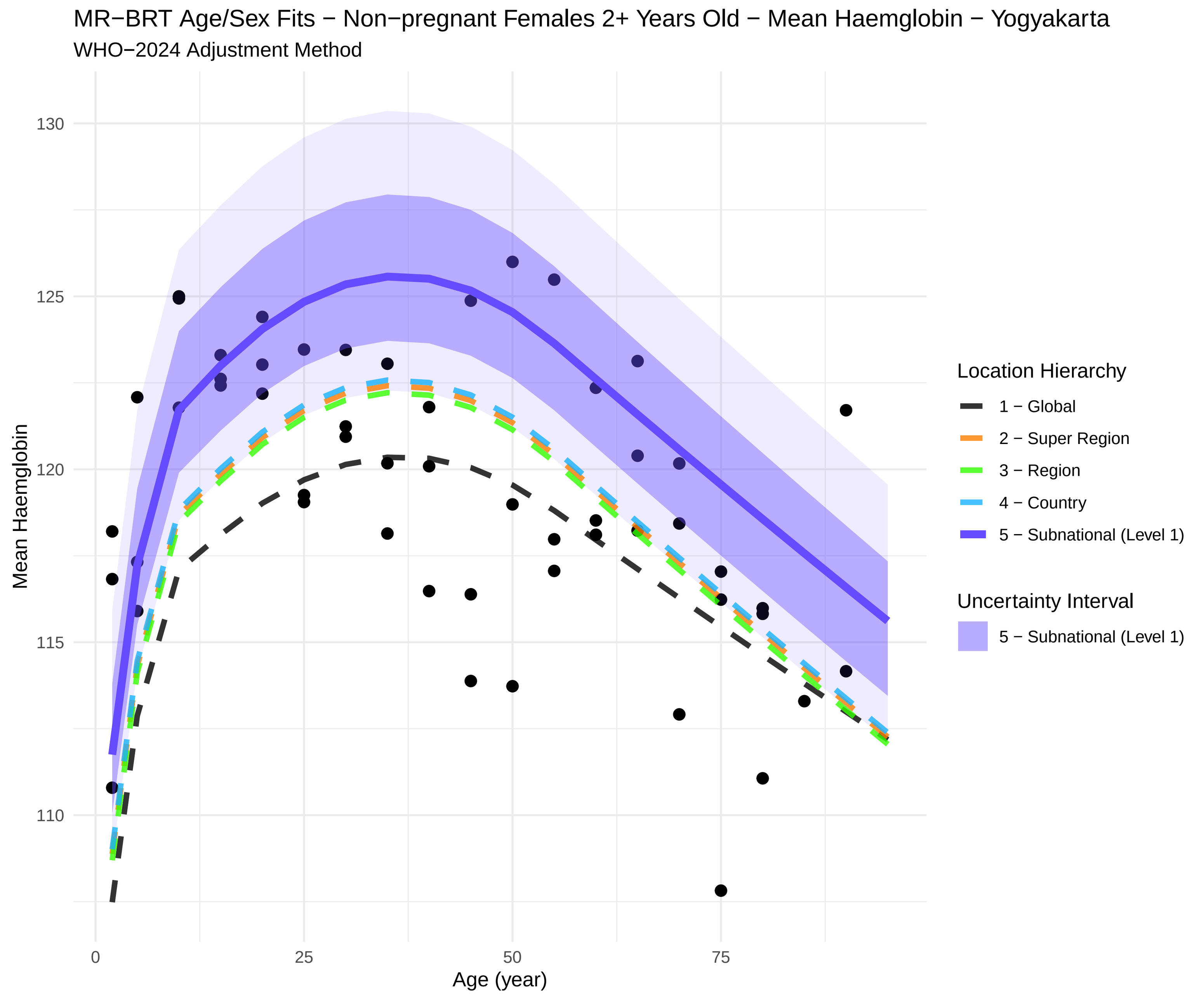
MR−BRT Age/Sex Fits − Non−pregnant Females 2+ Years Old − Mean Haemoglobin − Yogyakarta
WHO−2024 Adjustment Method
Mean Haemoglobin
Age (year)
Location Hierarchy
1 − Global
2 − Super Region
3 − Region
4 − Country
5 − Subnational (Level 1)
Uncertainty Interval
5 − Subnational (Level 1)

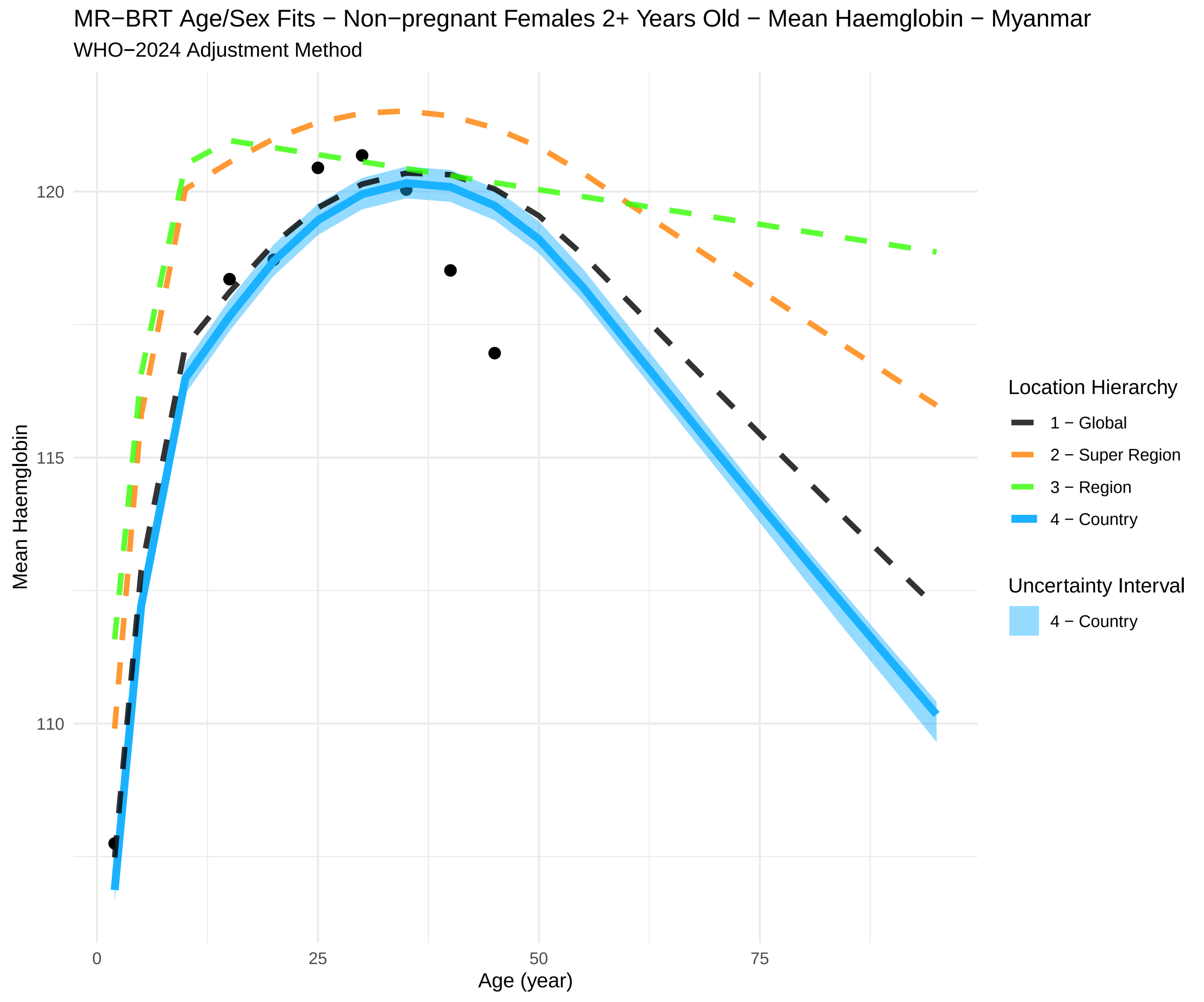
MR−BRT Age/Sex Fits − Non−pregnant Females 2+ Years Old − Mean Haemoglobin − Myanmar
WHO−2024 Adjustment Method
Mean Haemoglobin
Age (year)
Location Hierarchy
1 − Global
2 − Super Region
3 − Region
4 − Country
Uncertainty Interval
4 − Country

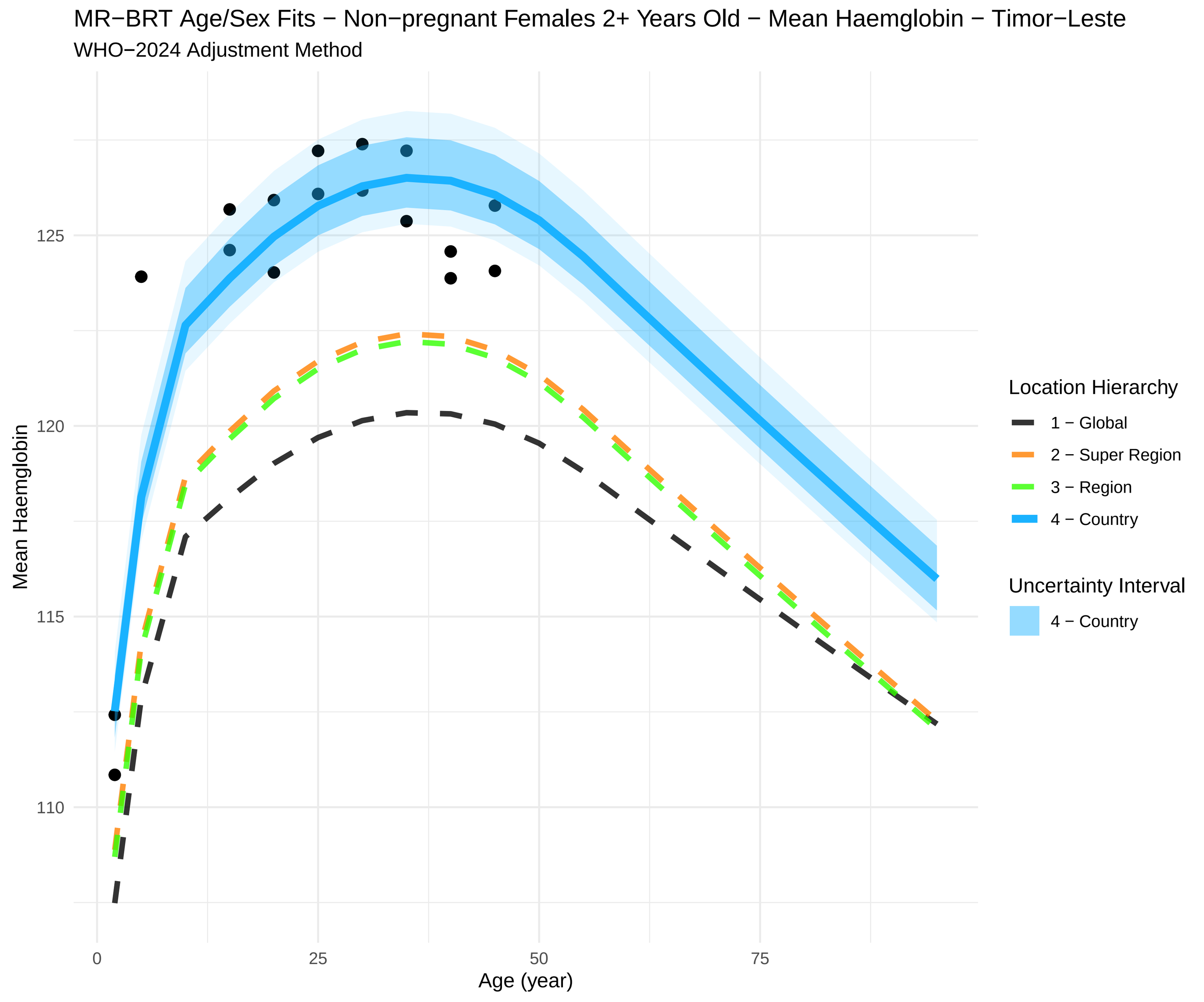
MR−BRT Age/Sex Fits − Non−pregnant Females 2+ Years Old − Mean Haemoglobin − Timor−Leste
WHO−2024 Adjustment Method
Mean Haemoglobin
Age (year)
Location Hierarchy
1 − Global
2 − Super Region
3 − Region
4 − Country
Uncertainty Interval
4 − Country

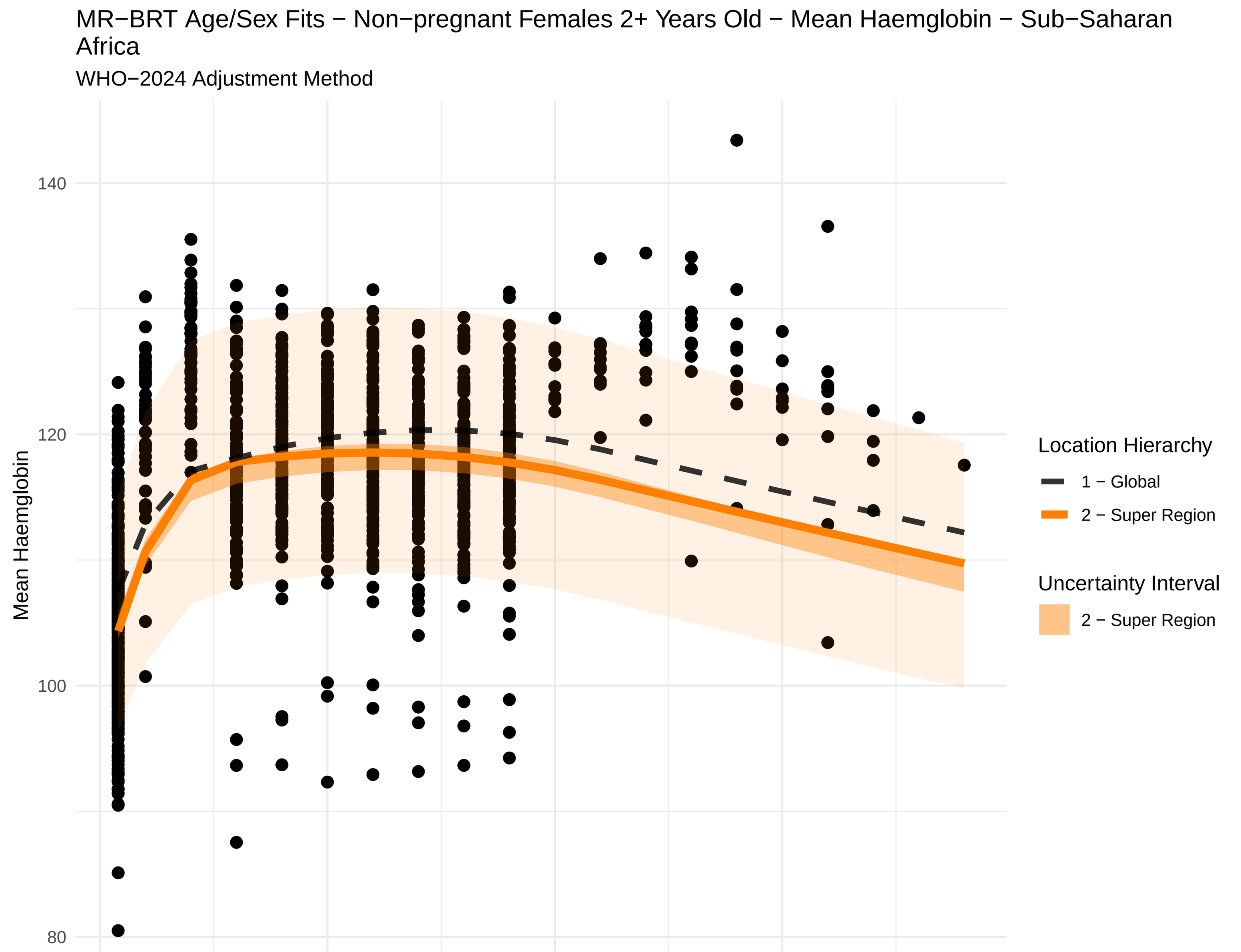
MR−BRT Age/Sex Fits − Non−pregnant Females 2+ Years Old − Mean Haemoglobin − Sub−Saharan Africa
WHO−2024 Adjustment Method
Mean Haemoglobin
Age (year)
Location Hierarchy
1 − Global
2 − Super Region
Uncertainty Interval
2 − Super Region

MR−BRT Age/Sex Fits − Non−pregnant Females 2+ Years Old − Mean Haemglobin − Central Sub−Saharan Africa
WHO−2024 Adjustment Method

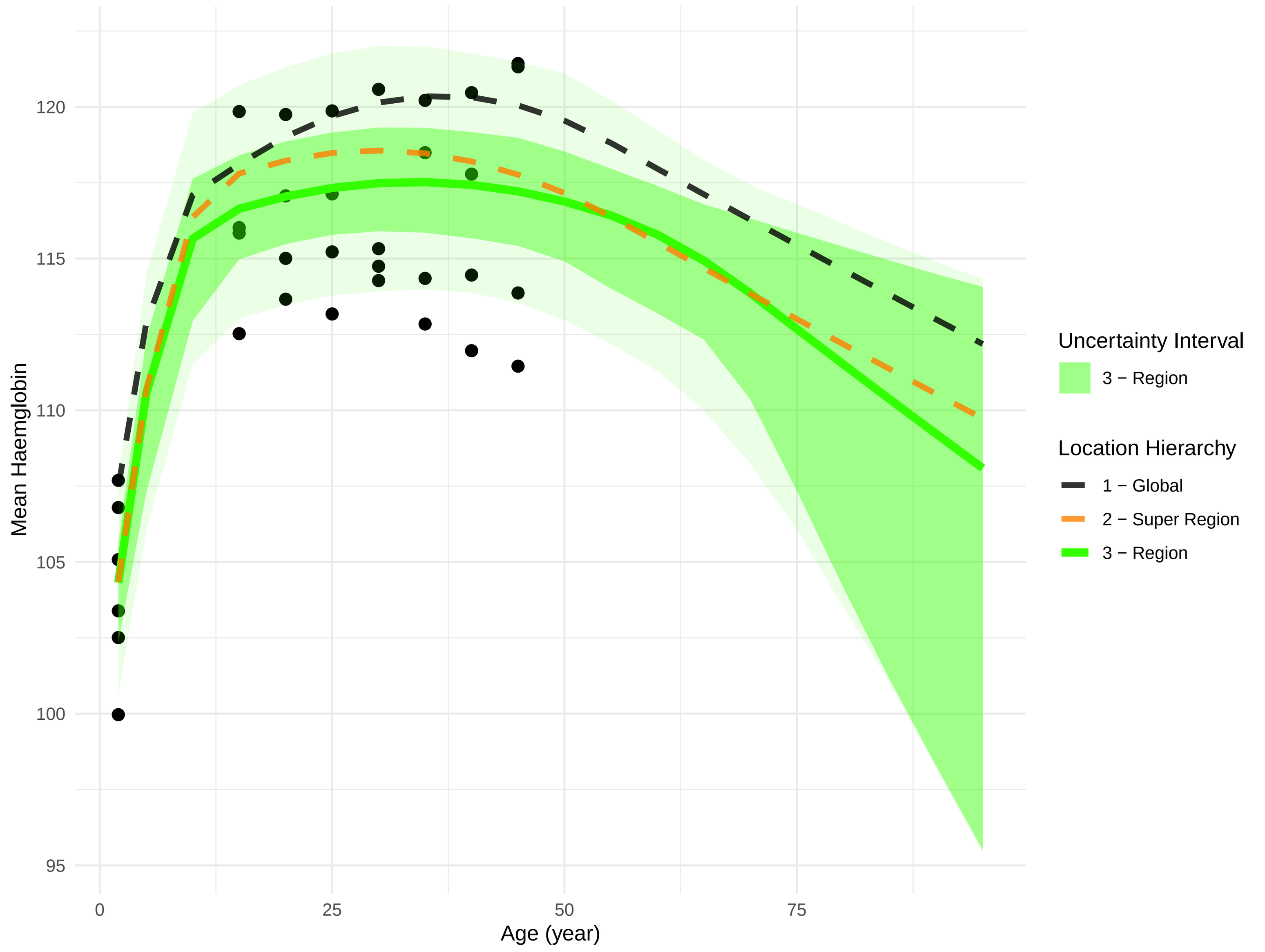

MR−BRT Age/Sex Fits − Non−pregnant Females 2+ Years Old − Mean Haemglobin − Angola
WHO−2024 Adjustment Method

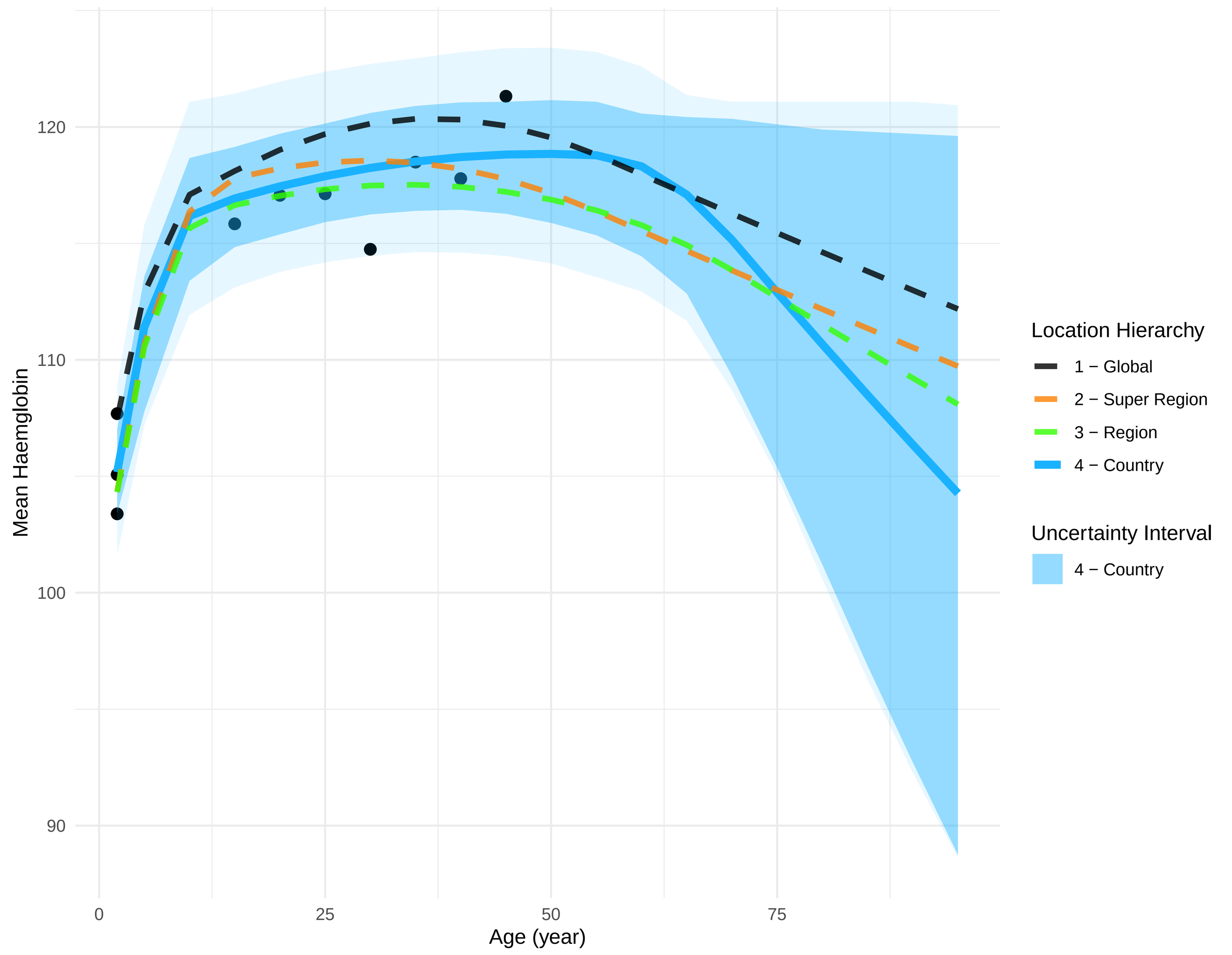

MR−BRT Age/Sex Fits − Non−pregnant Females 2+ Years Old − Mean Haemglobin − Democratic Republic of the Congo
WHO−2024 Adjustment Method

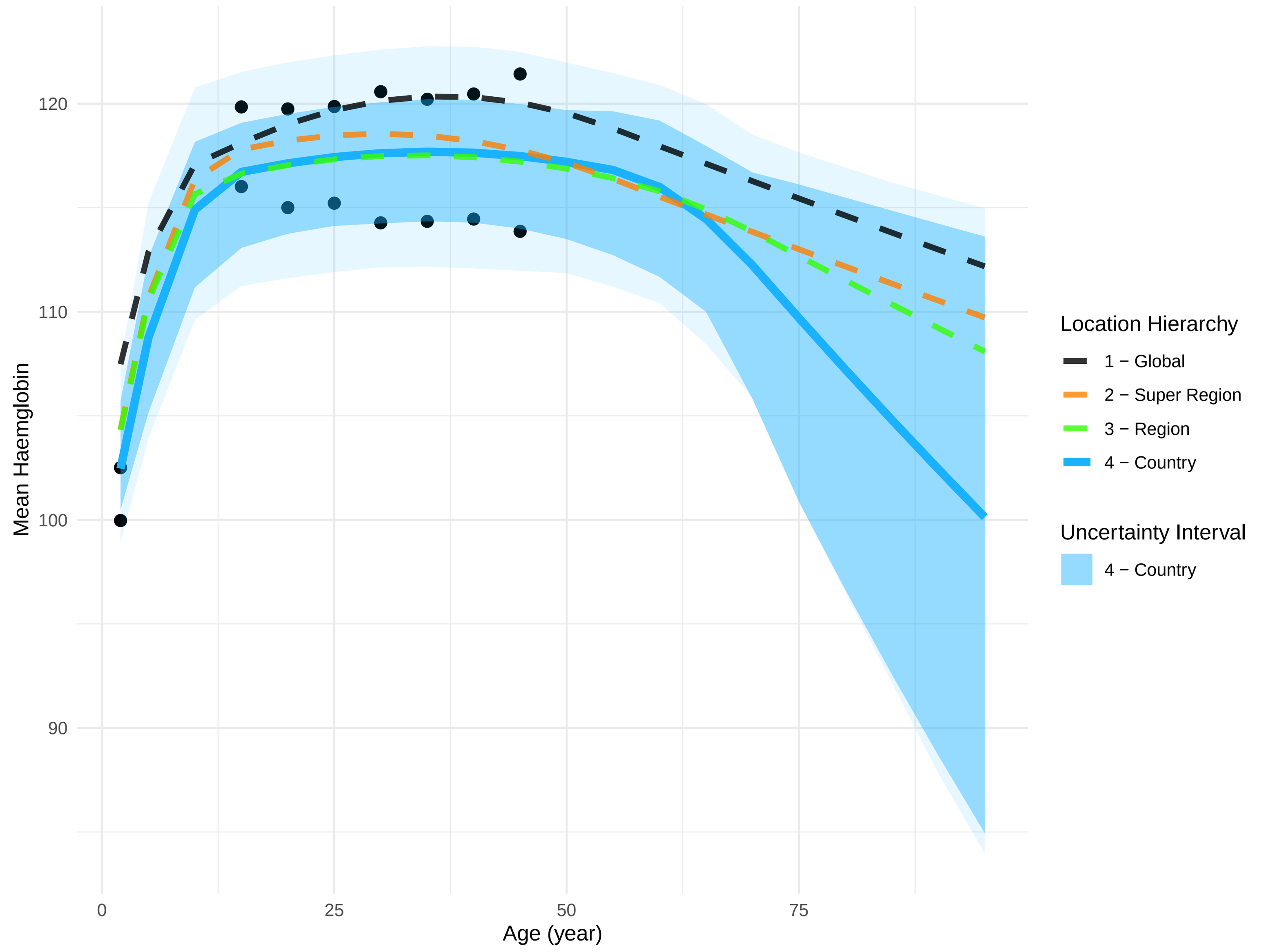

MR−BRT Age/Sex Fits − Non−pregnant Females 2+ Years Old − Mean Haemglobin − Gabon
WHO−2024 Adjustment Method

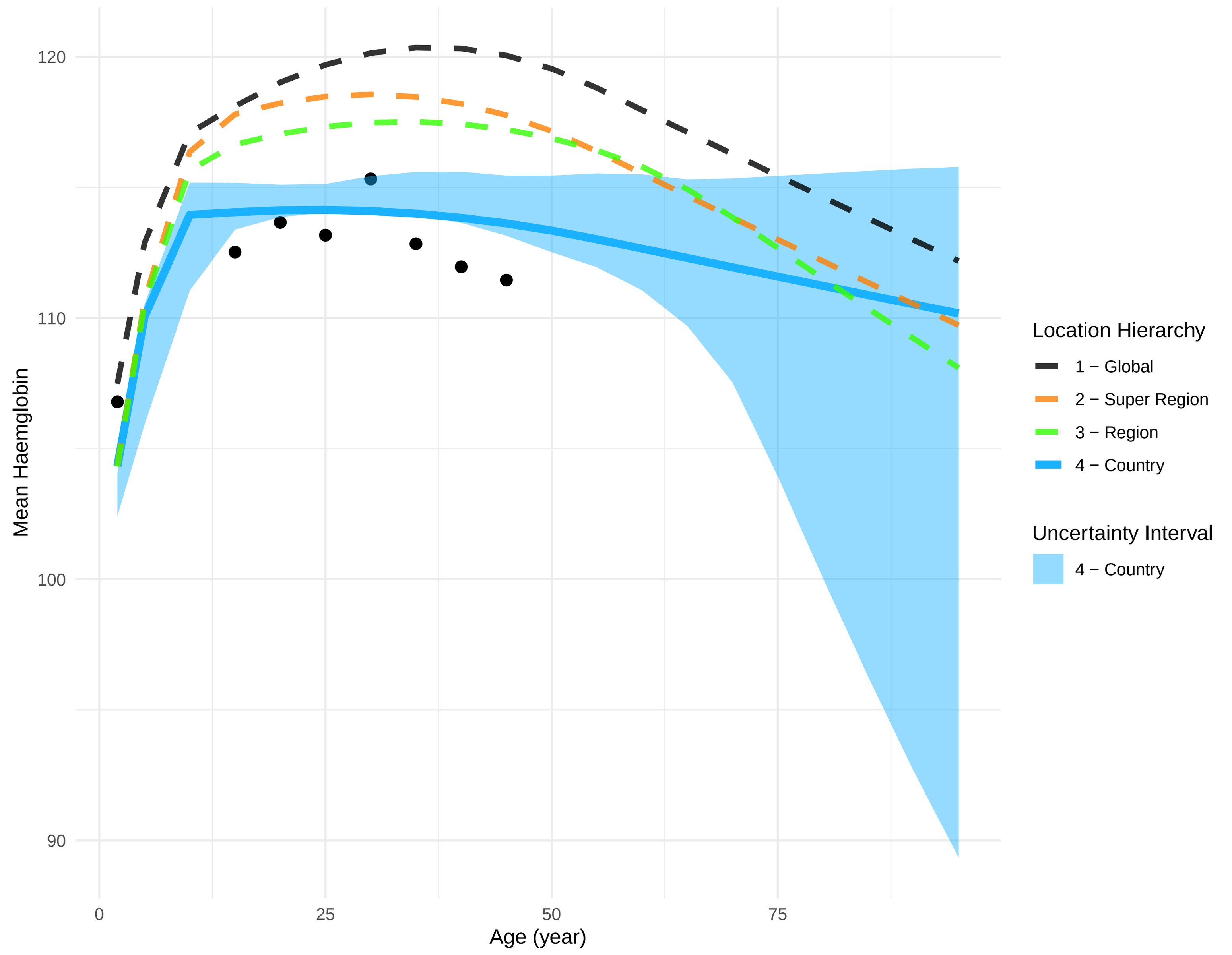

MR−BRT Age/Sex Fits − Non−pregnant Females 2+ Years Old − Mean Haemglobin − Eastern Sub−Saharan Africa
WHO−2024 Adjustment Method

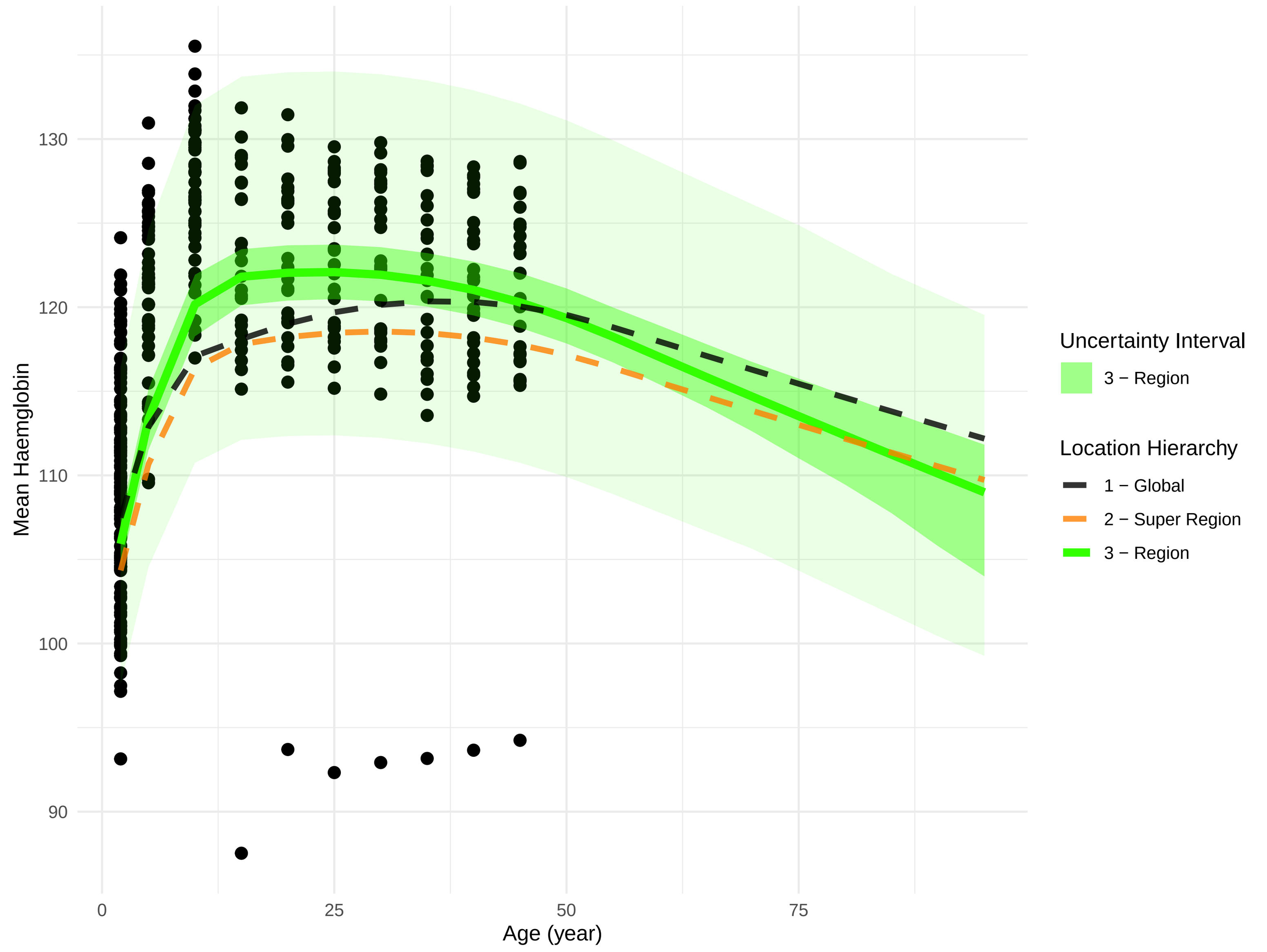

MR−BRT Age/Sex Fits − Non−pregnant Females 2+ Years Old − Mean Haemglobin − Burundi
WHO−2024 Adjustment Method

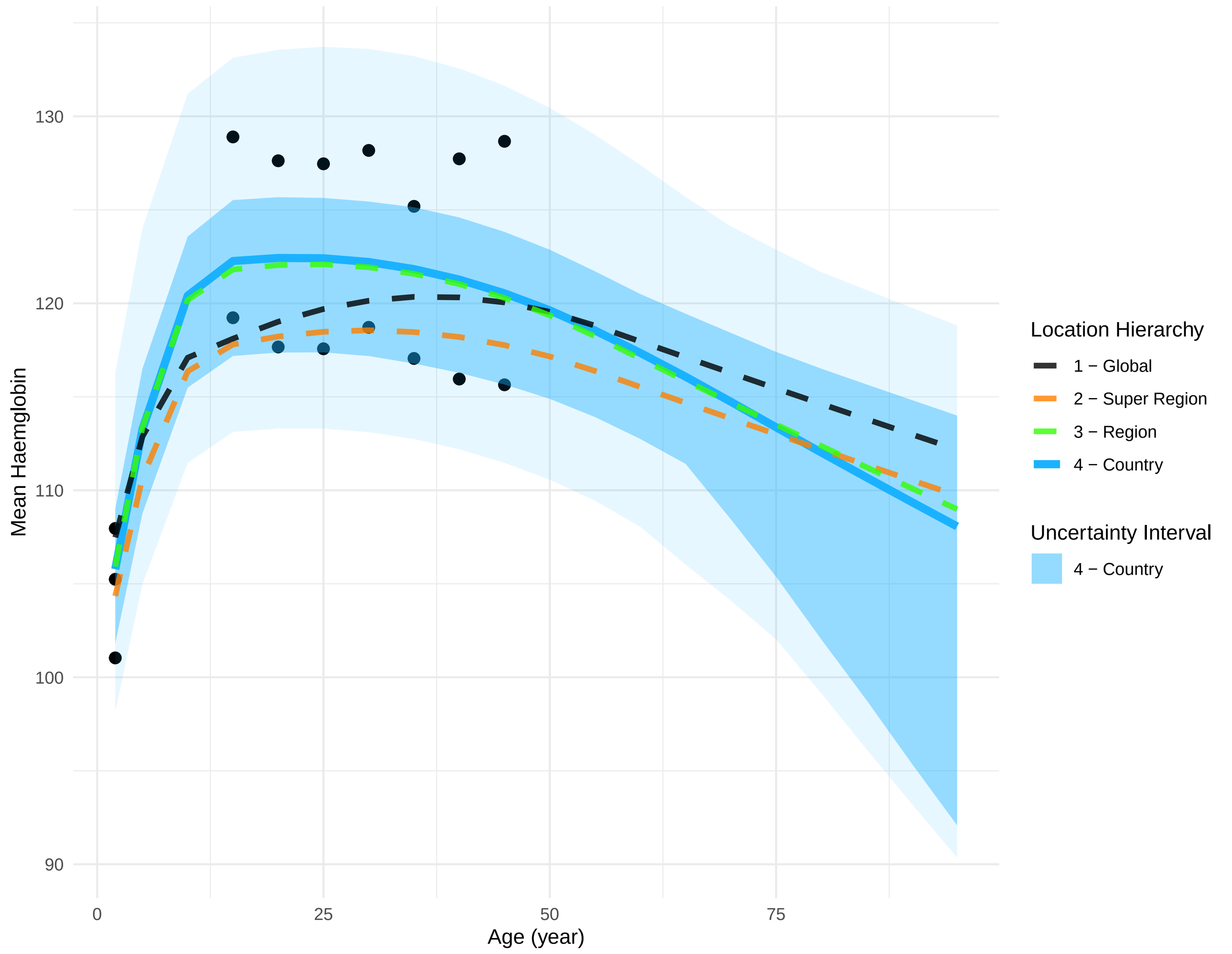

MR−BRT Age/Sex Fits − Non−pregnant Females 2+ Years Old − Mean Haemglobin − Ethiopia
WHO−2024 Adjustment Method

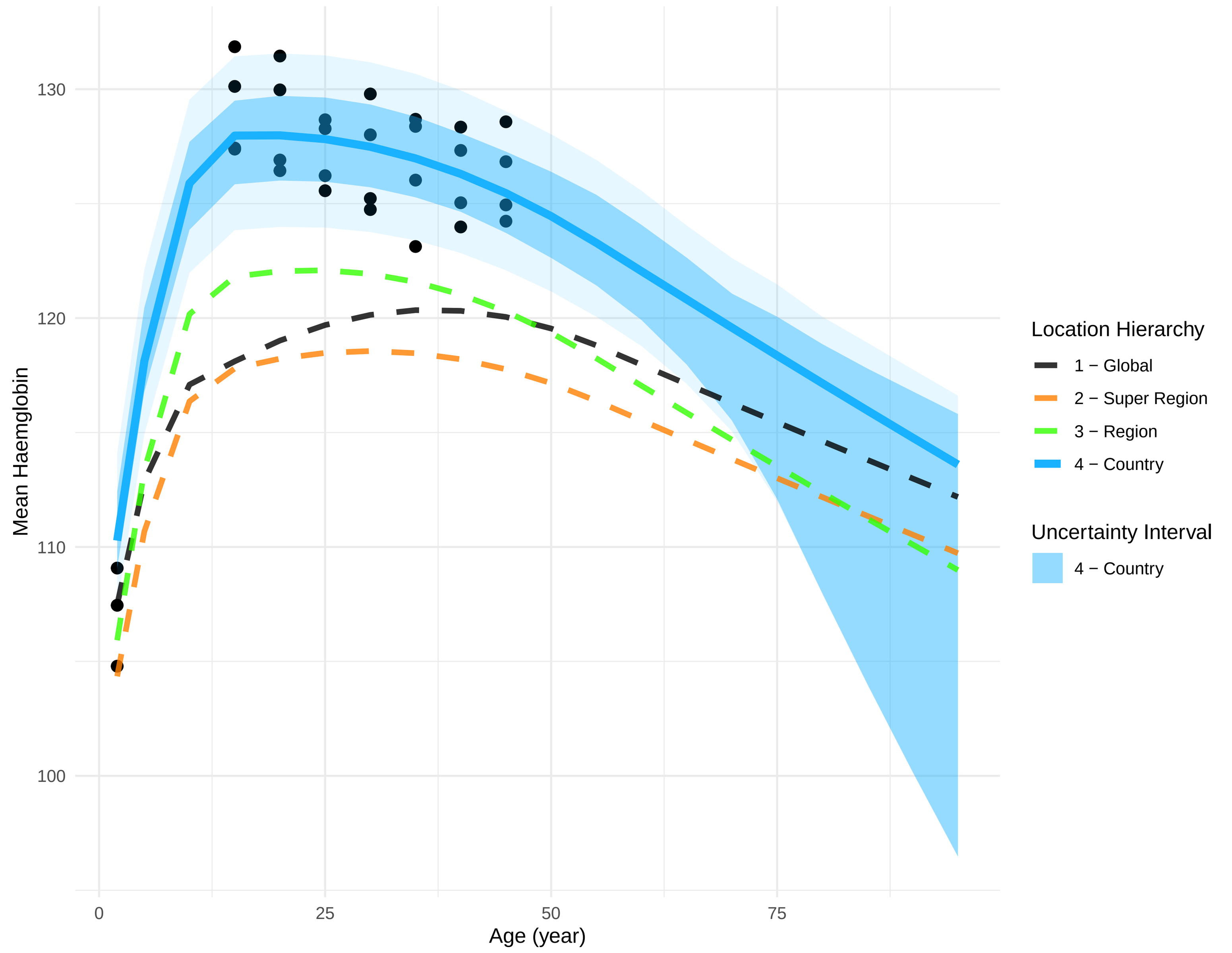

MR−BRT Age/Sex Fits − Non−pregnant Females 2+ Years Old − Mean Haemglobin − Kenya
WHO−2024 Adjustment Method

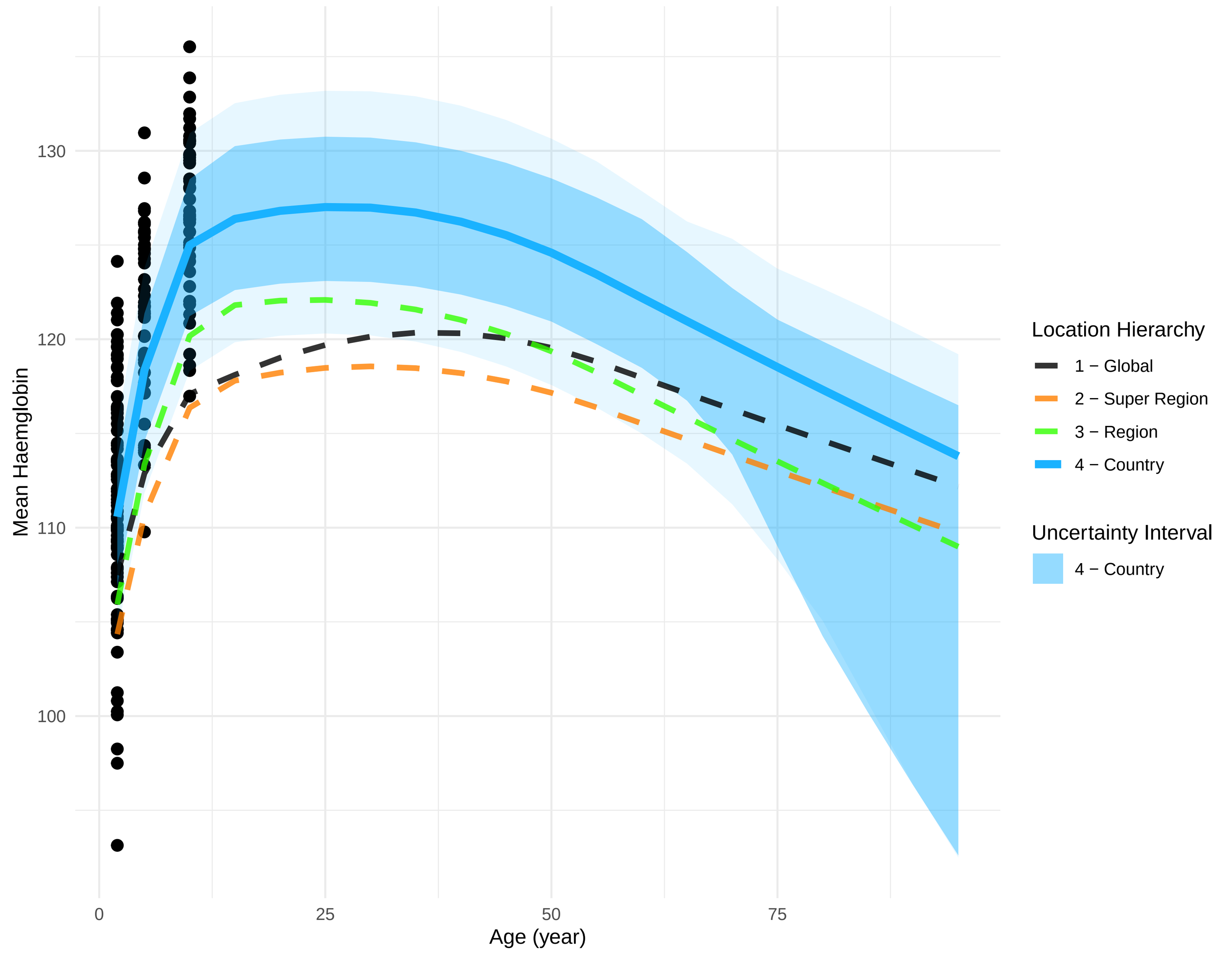

MR−BRT Age/Sex Fits − Non−pregnant Females 2+ Years Old − Mean Haemglobin − Baringo
WHO−2024 Adjustment Method

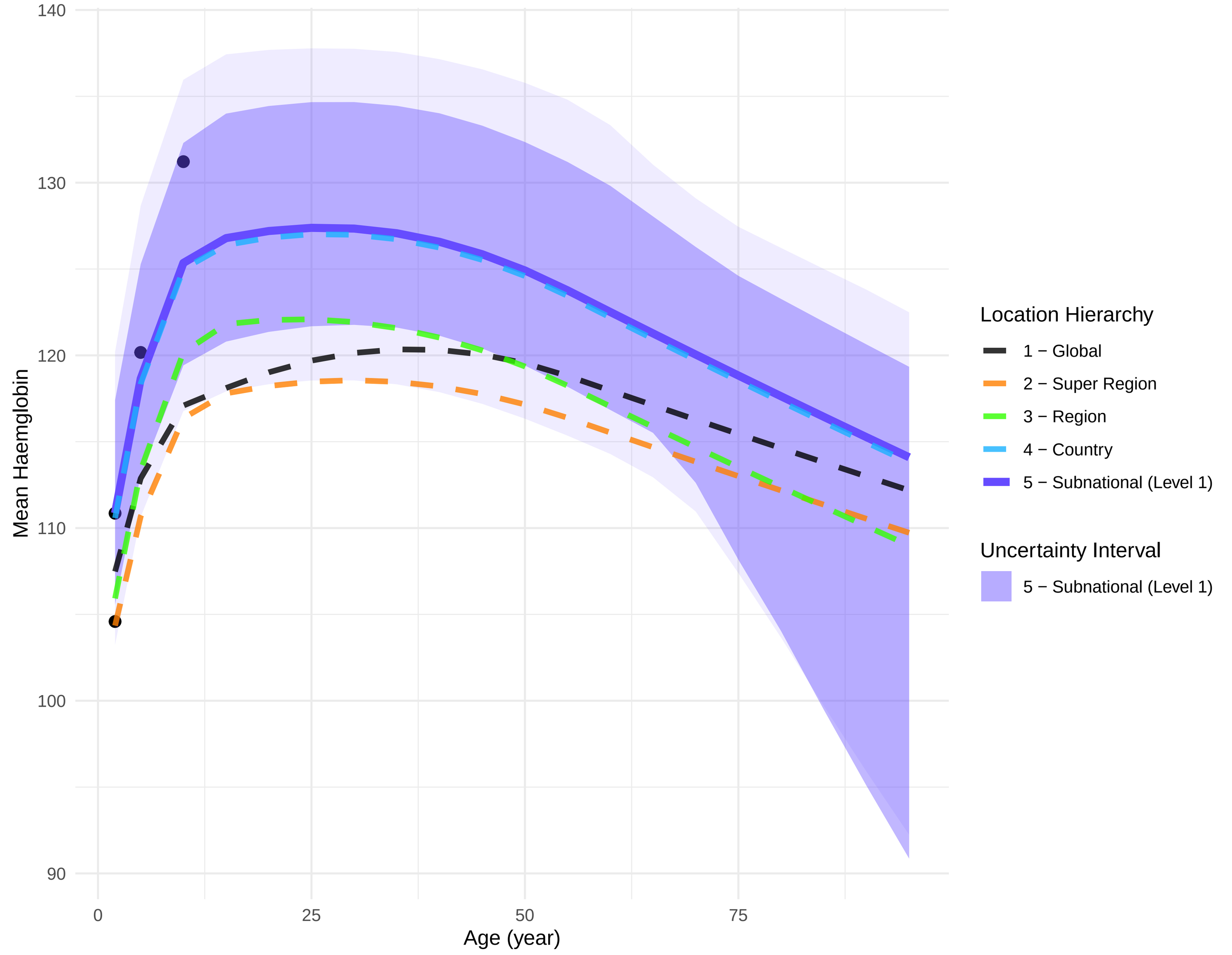

MR−BRT Age/Sex Fits − Non−pregnant Females 2+ Years Old − Mean Haemglobin − Bomet
WHO−2024 Adjustment Method

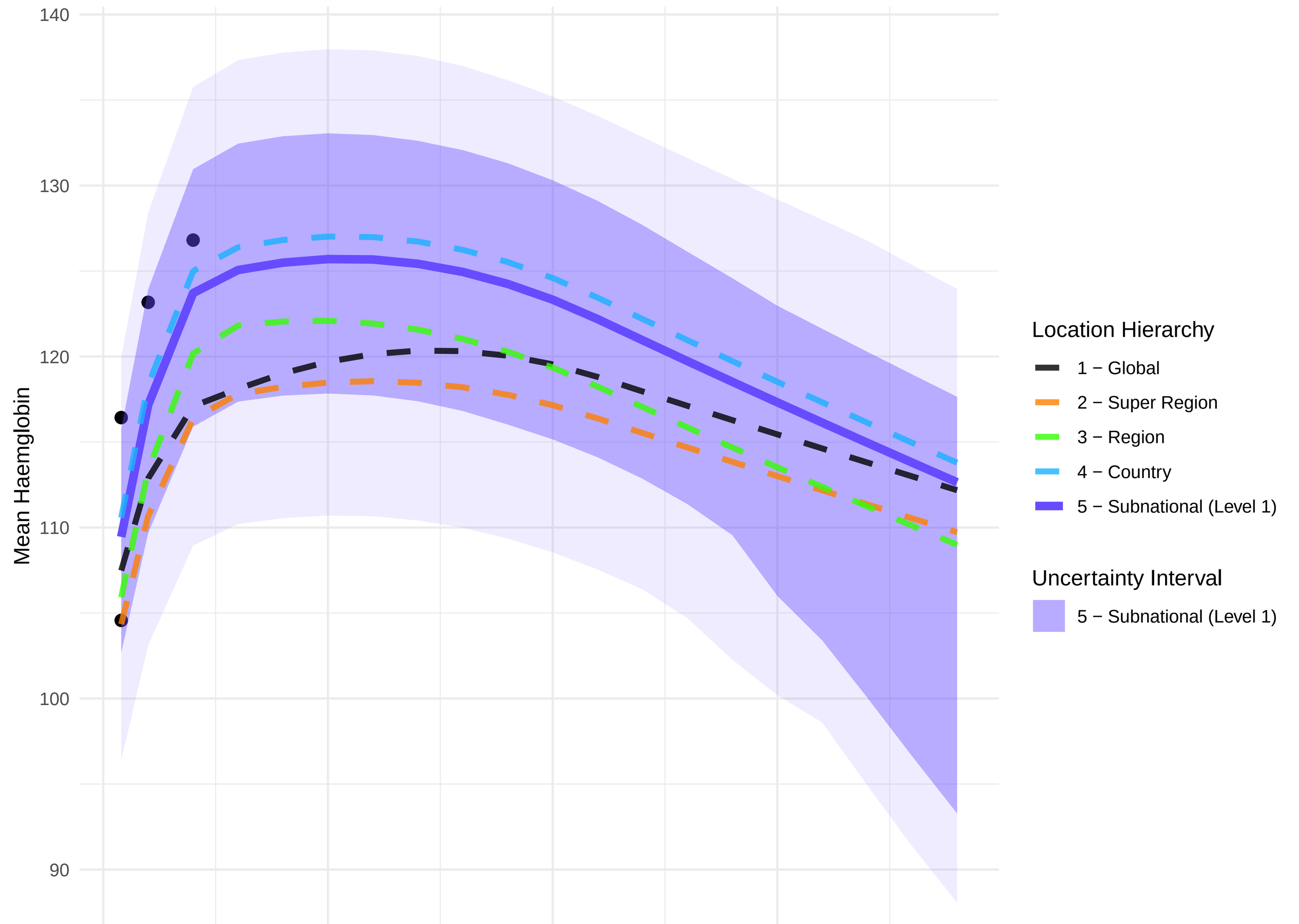

MR−BRT Age/Sex Fits − Non−pregnant Females 2+ Years Old − Mean Haemglobin − Bungoma
WHO−2024 Adjustment Method

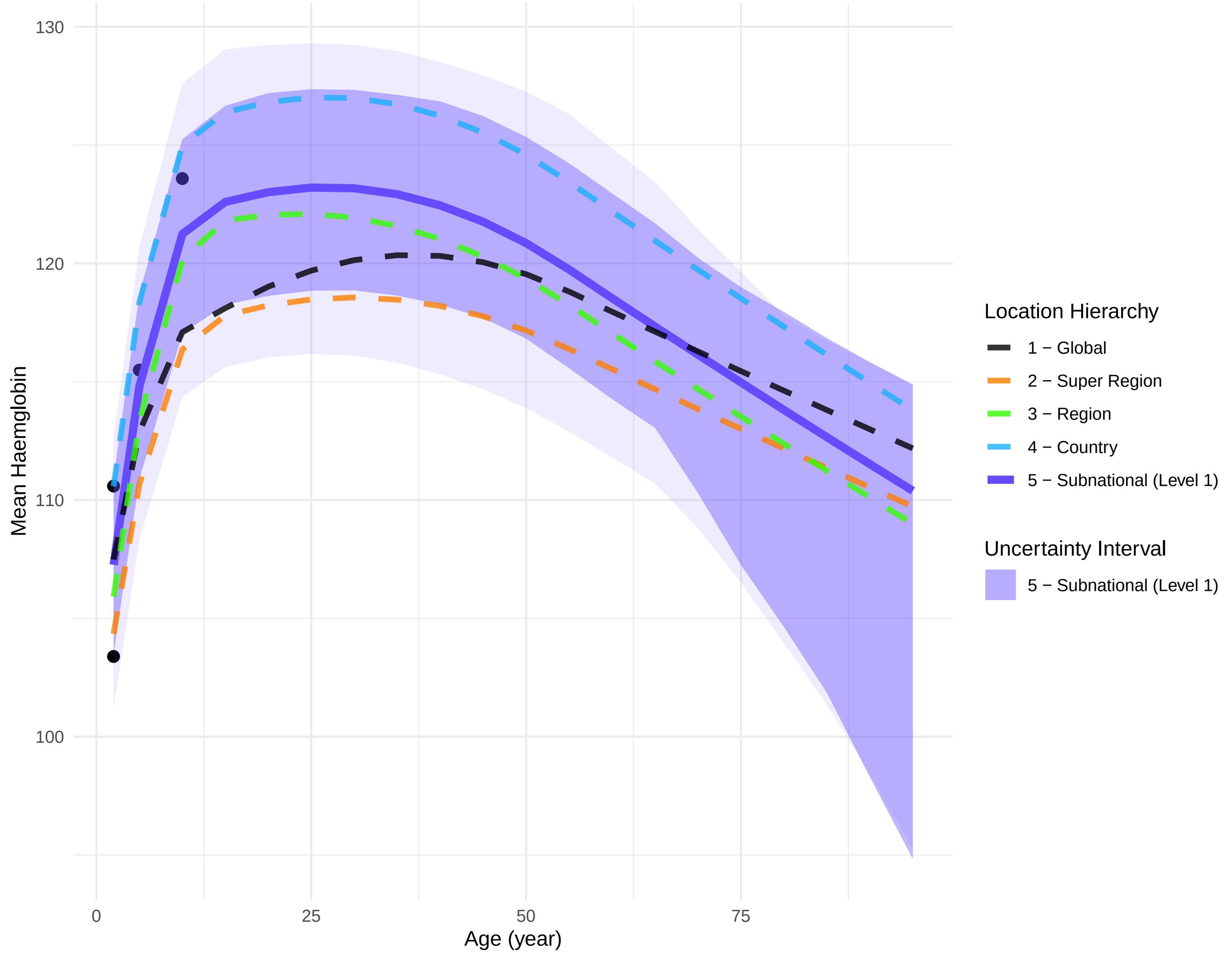

MR−BRT Age/Sex Fits − Non−pregnant Females 2+ Years Old − Mean Haemglobin − Busia
WHO−2024 Adjustment Method

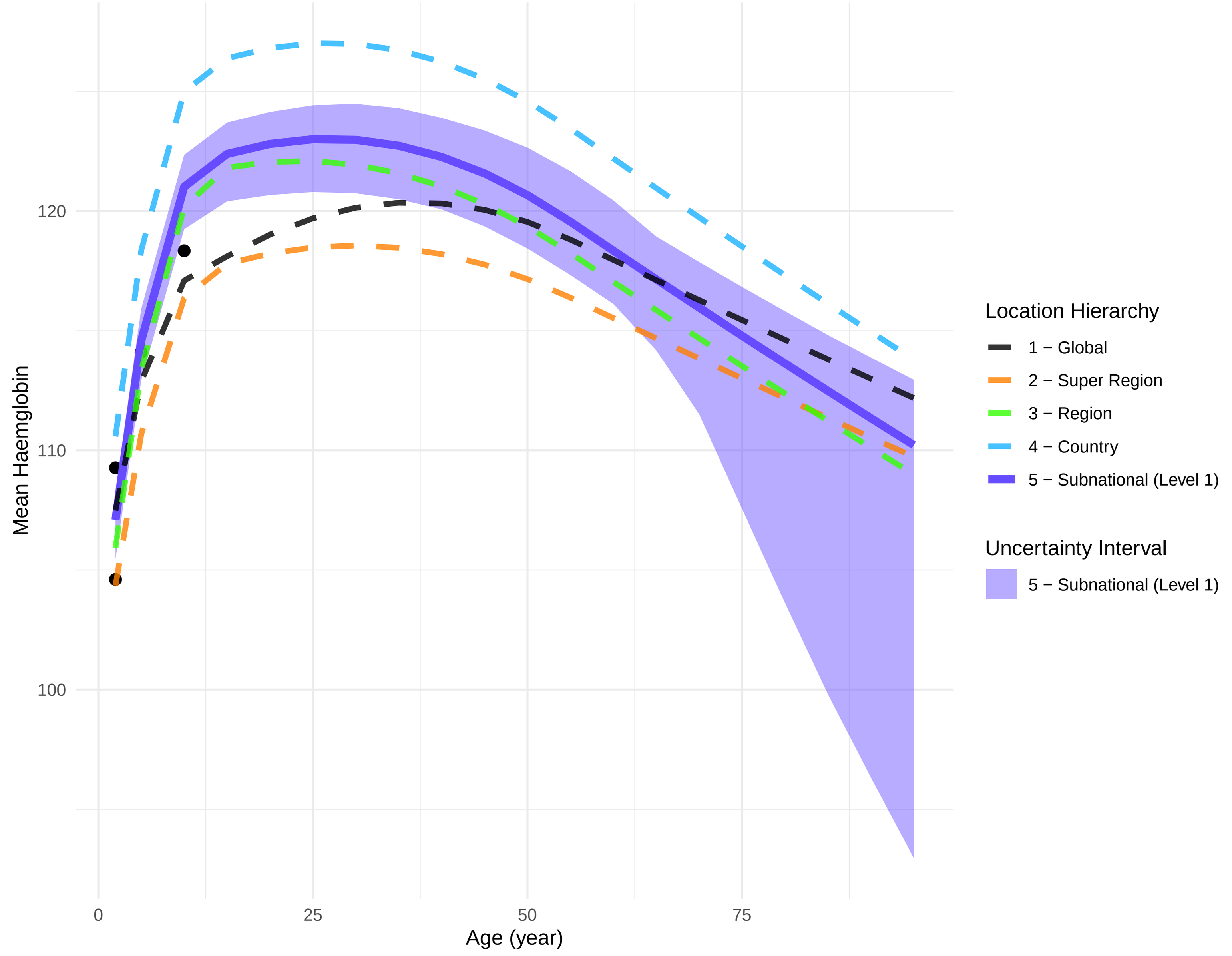

MR−BRT Age/Sex Fits − Non−pregnant Females 2+ Years Old − Mean Haemglobin − Elgeyo−Marakwet
WHO−2024 Adjustment Method

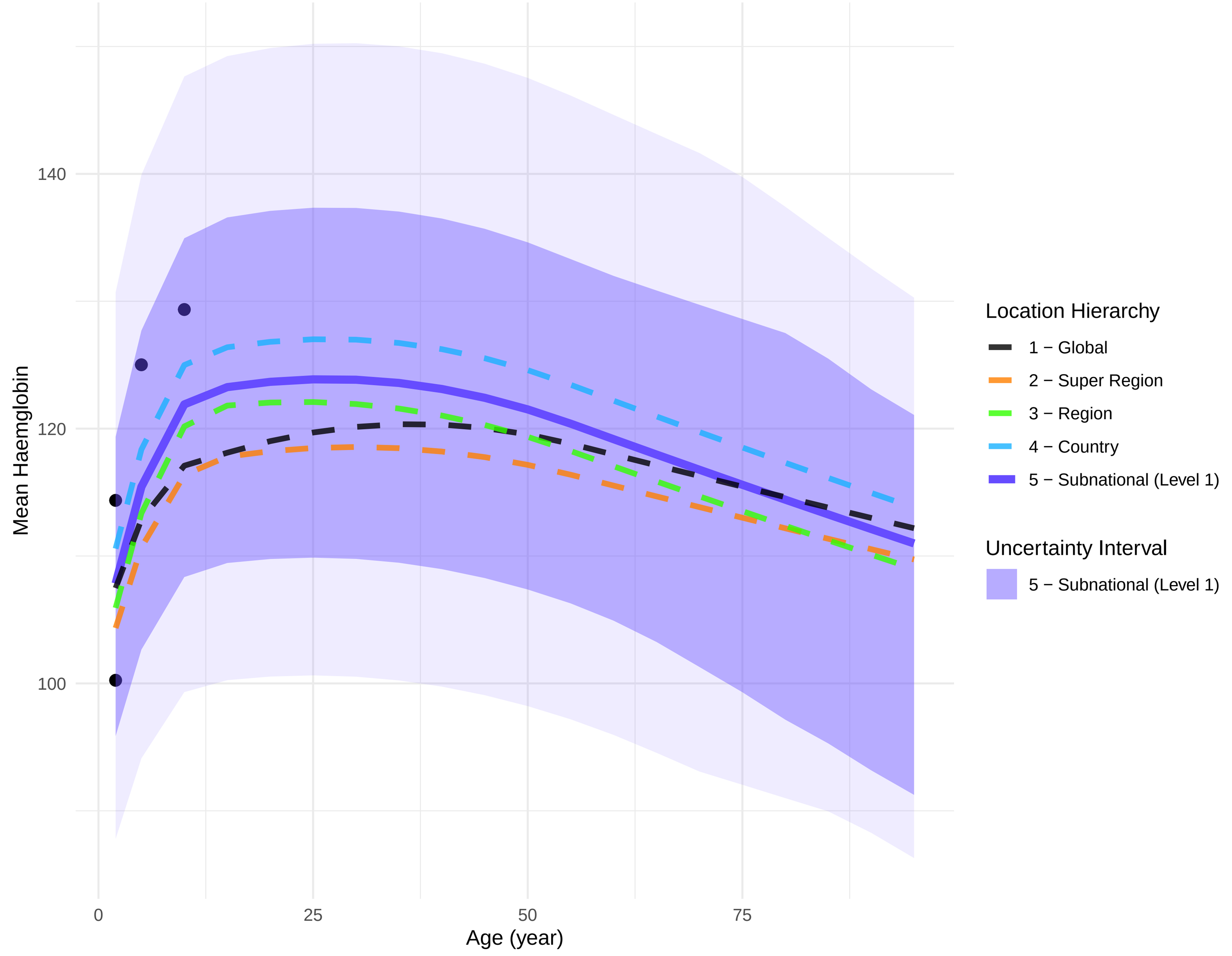

MR−BRT Age/Sex Fits − Non−pregnant Females 2+ Years Old − Mean Haemglobin − Embu
WHO−2024 Adjustment Method

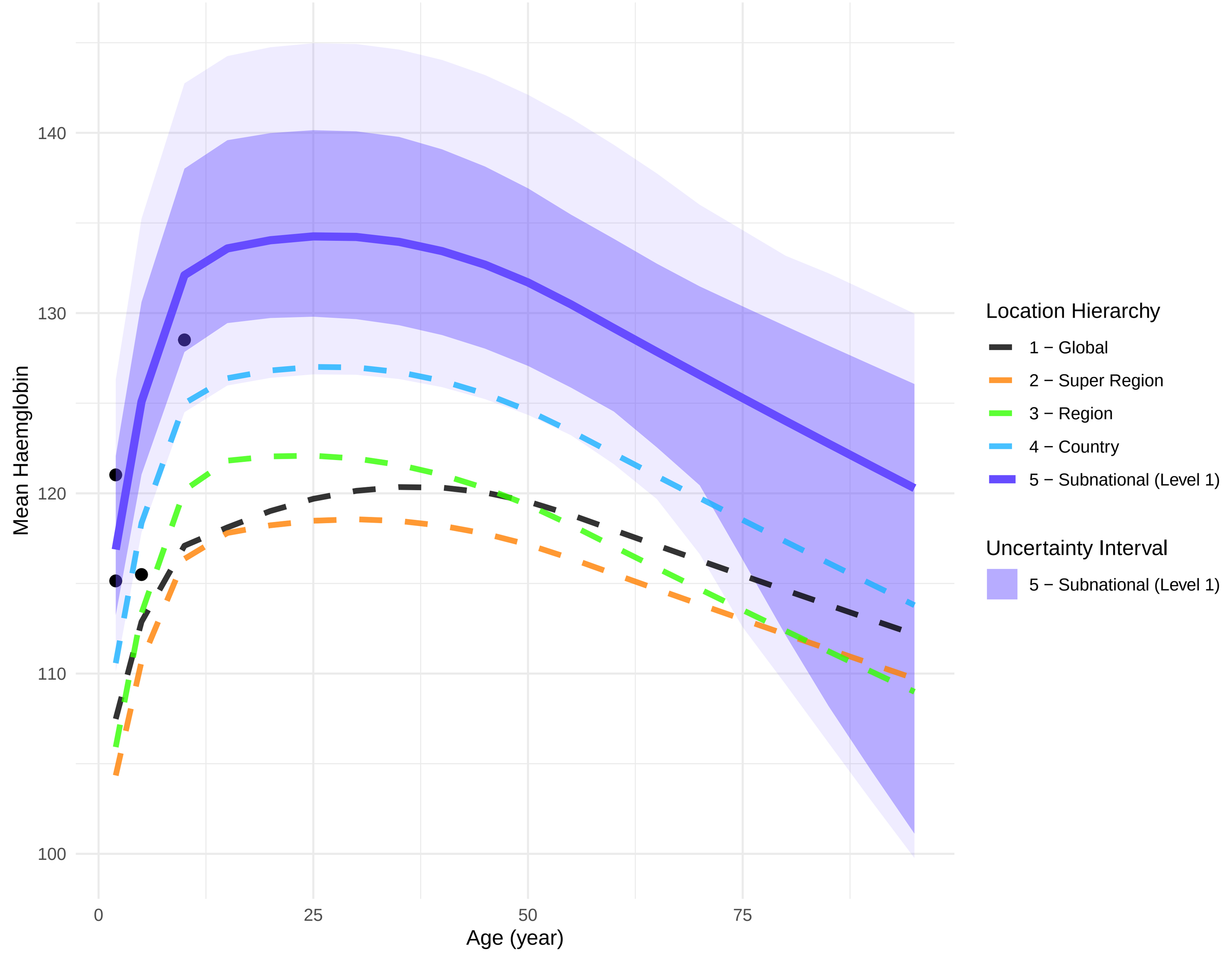

MR−BRT Age/Sex Fits − Non−pregnant Females 2+ Years Old − Mean Haemglobin − Garissa
WHO−2024 Adjustment Method

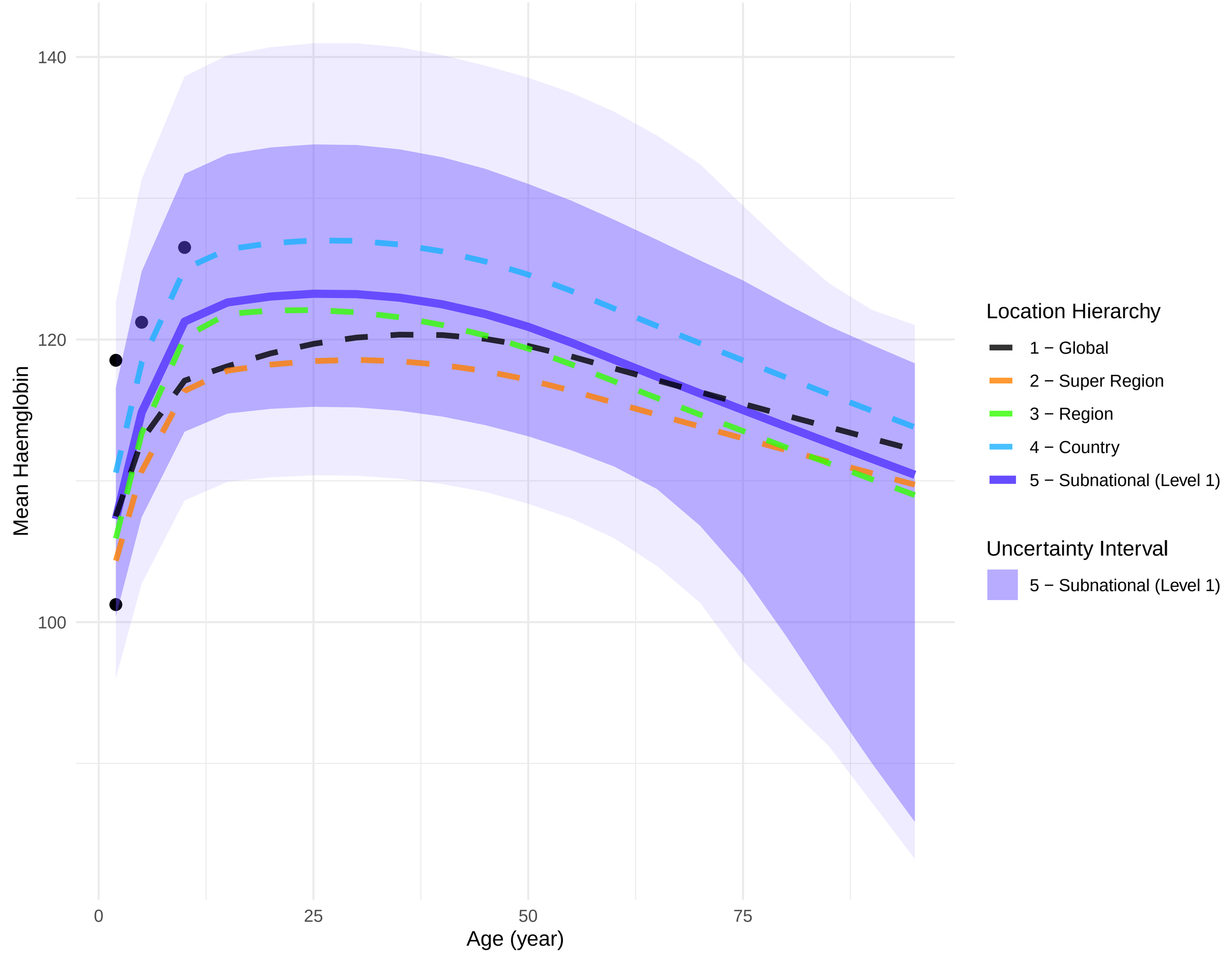

MR−BRT Age/Sex Fits − Non−pregnant Females 2+ Years Old − Mean Haemglobin − Homa Bay
WHO−2024 Adjustment Method

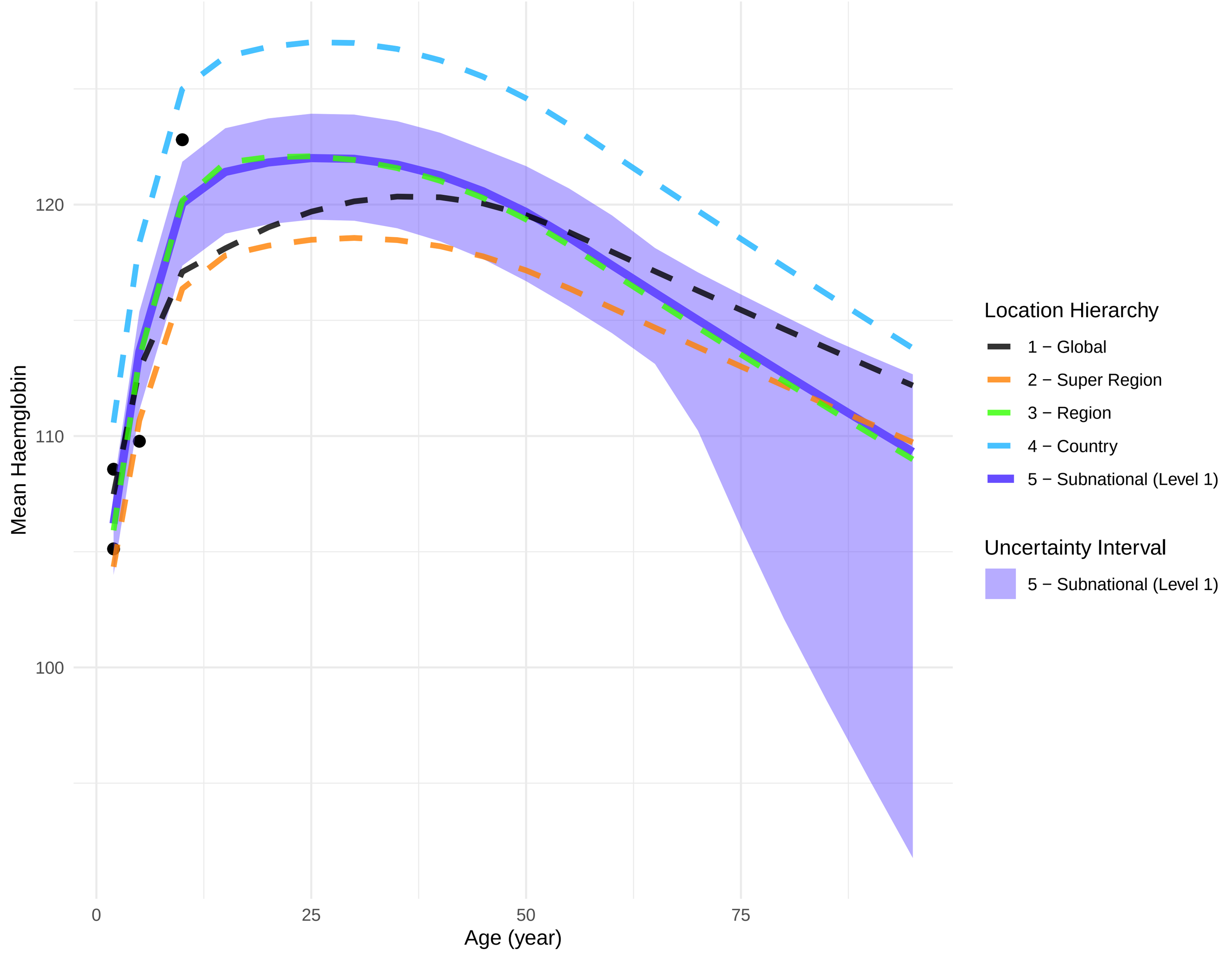

MR−BRT Age/Sex Fits − Non−pregnant Females 2+ Years Old − Mean Haemglobin − Isiolo
WHO−2024 Adjustment Method

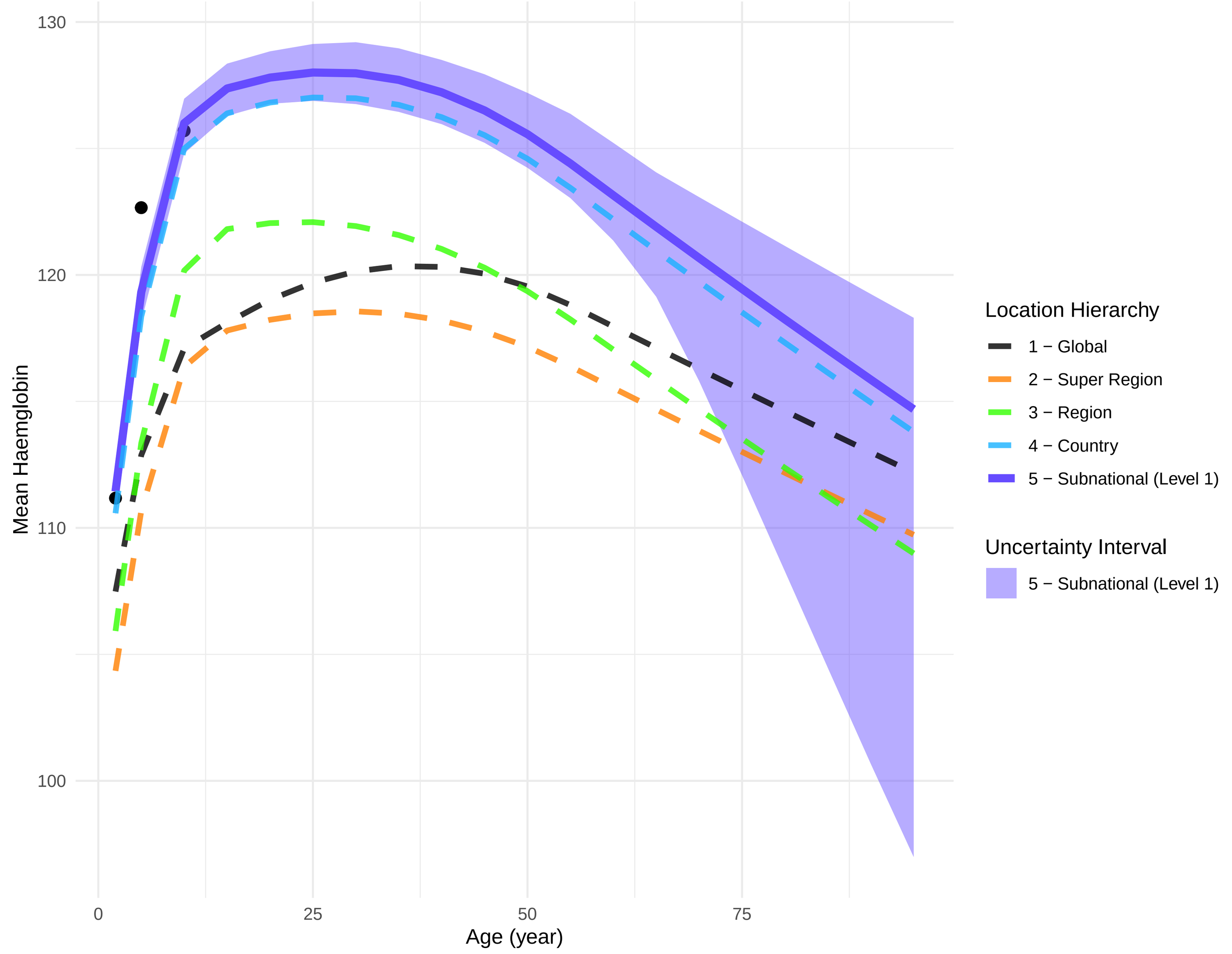

MR−BRT Age/Sex Fits − Non−pregnant Females 2+ Years Old − Mean Haemglobin − Kajiado
WHO−2024 Adjustment Method

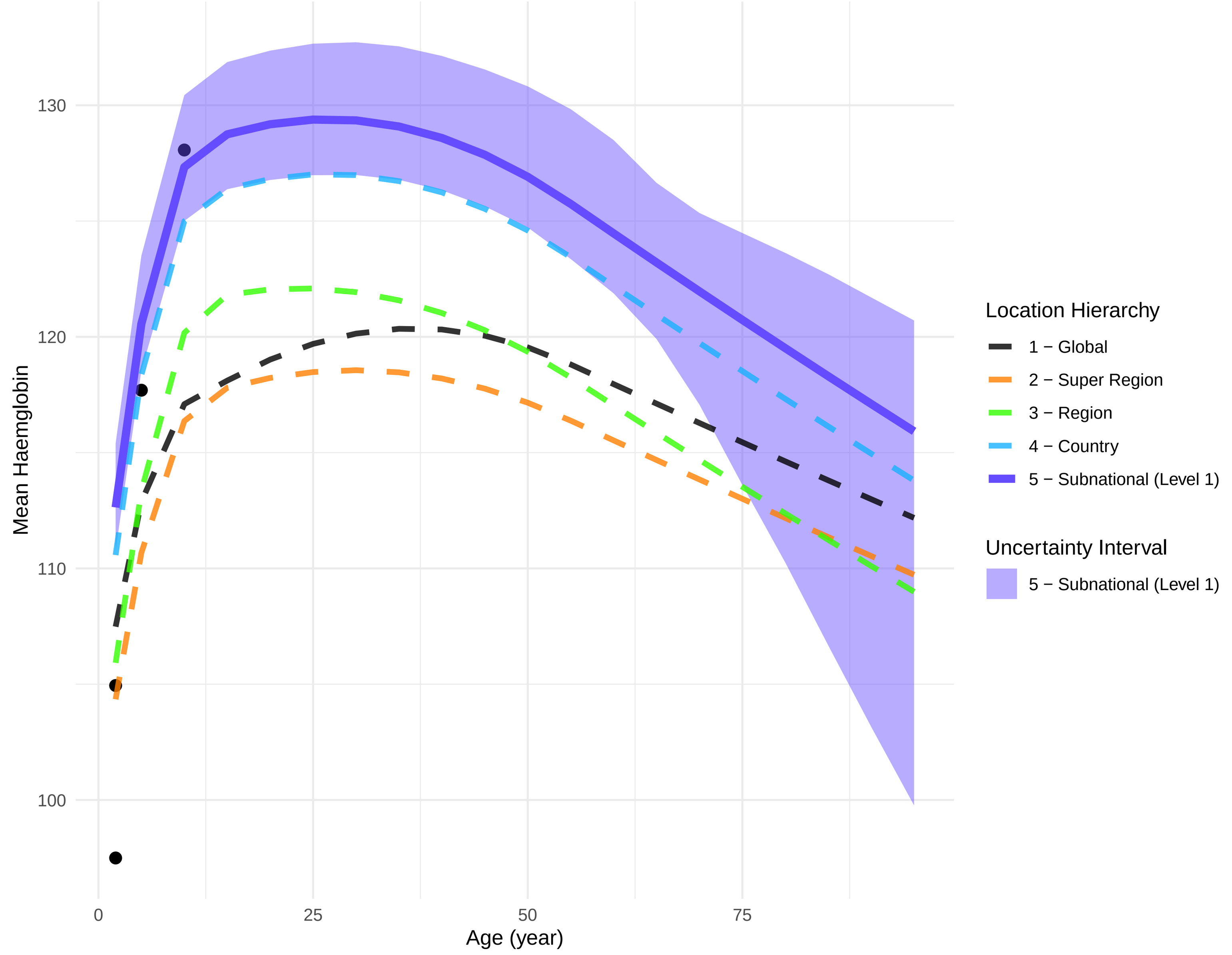

MR−BRT Age/Sex Fits − Non−pregnant Females 2+ Years Old − Mean Haemglobin − Kakamega
WHO−2024 Adjustment Method

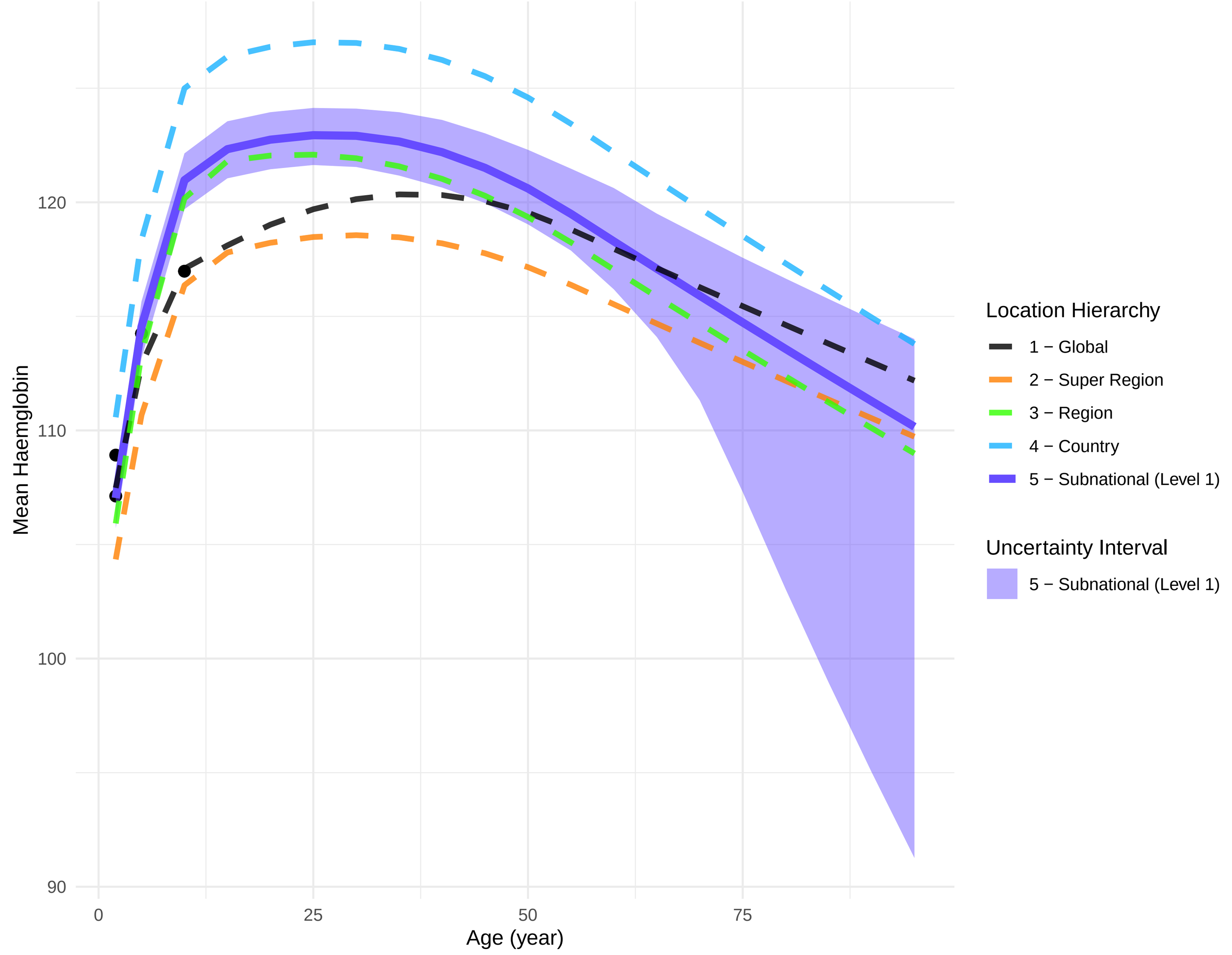

MR−BRT Age/Sex Fits − Non−pregnant Females 2+ Years Old − Mean Haemglobin − Kericho
WHO−2024 Adjustment Method

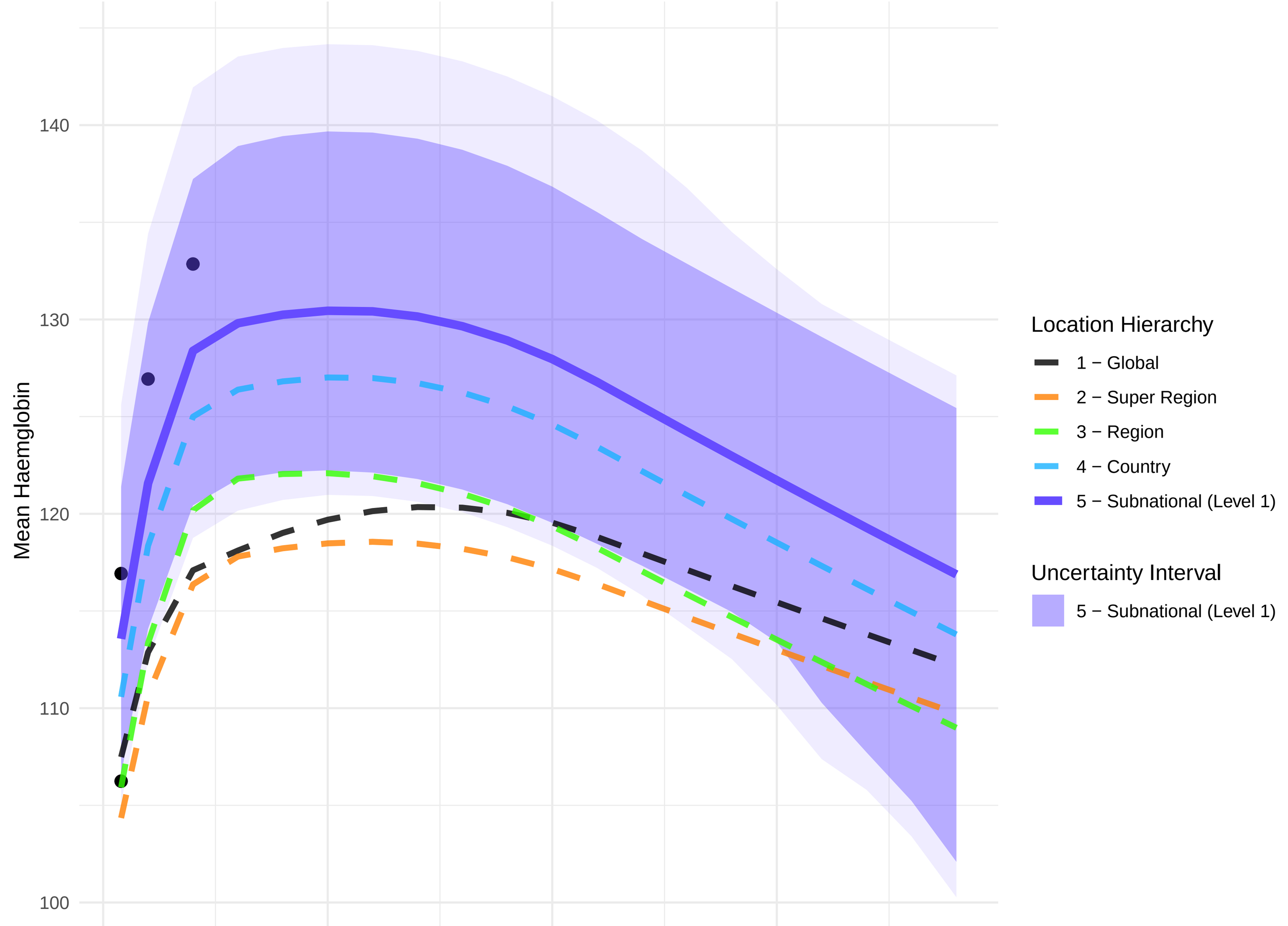

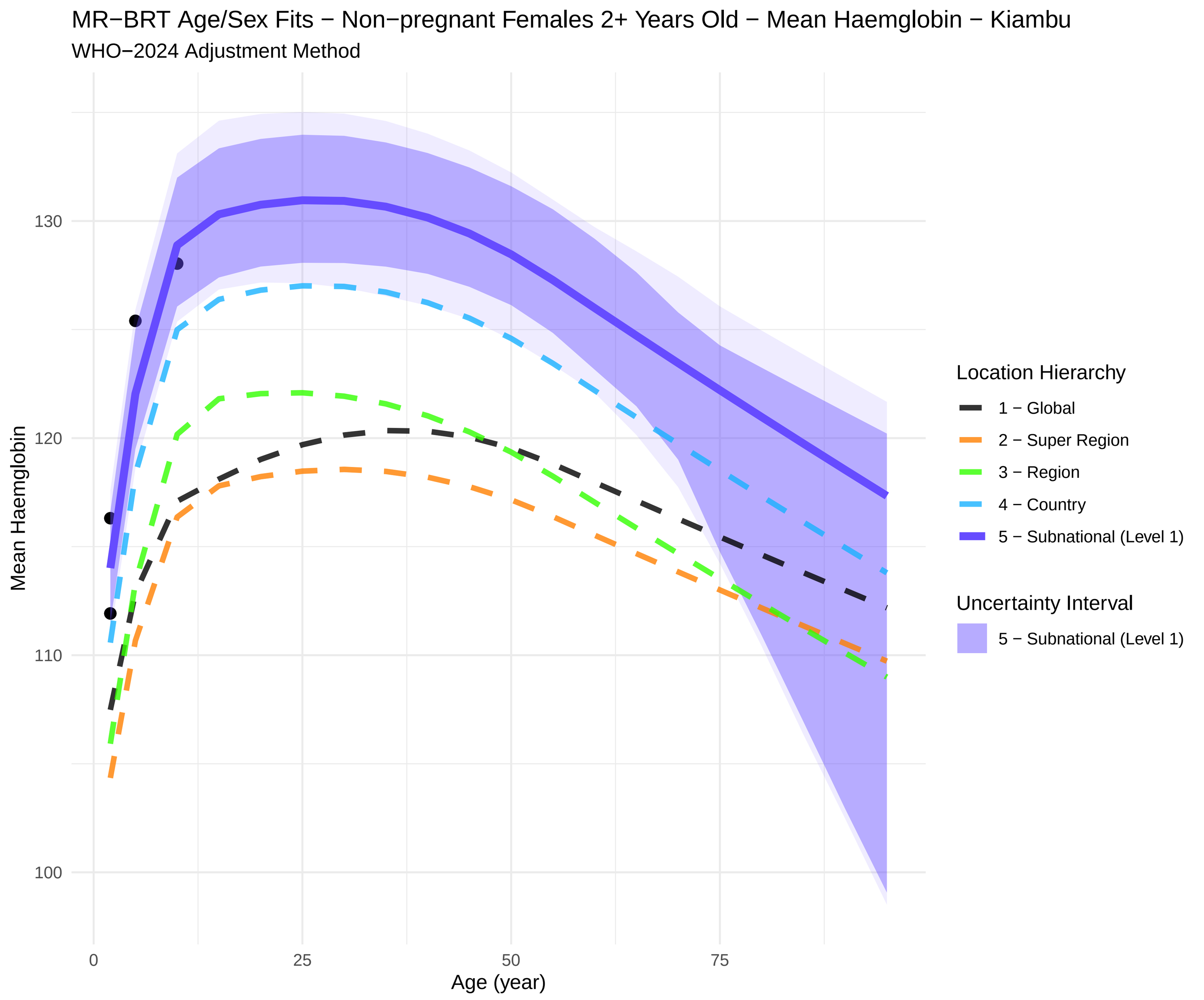
MR−BRT Age/Sex Fits − Non−pregnant Females 2+ Years Old − Mean Haemglobin − Kiambu
WHO−2024 Adjustment Method
Location Hierarchy
1 − Global
2 − Super Region
3 − Region
4 − Country
5 − Subnational (Level 1)
Uncertainty Interval
5 − Subnational (Level 1)
Mean Haemglobin
Age (year)

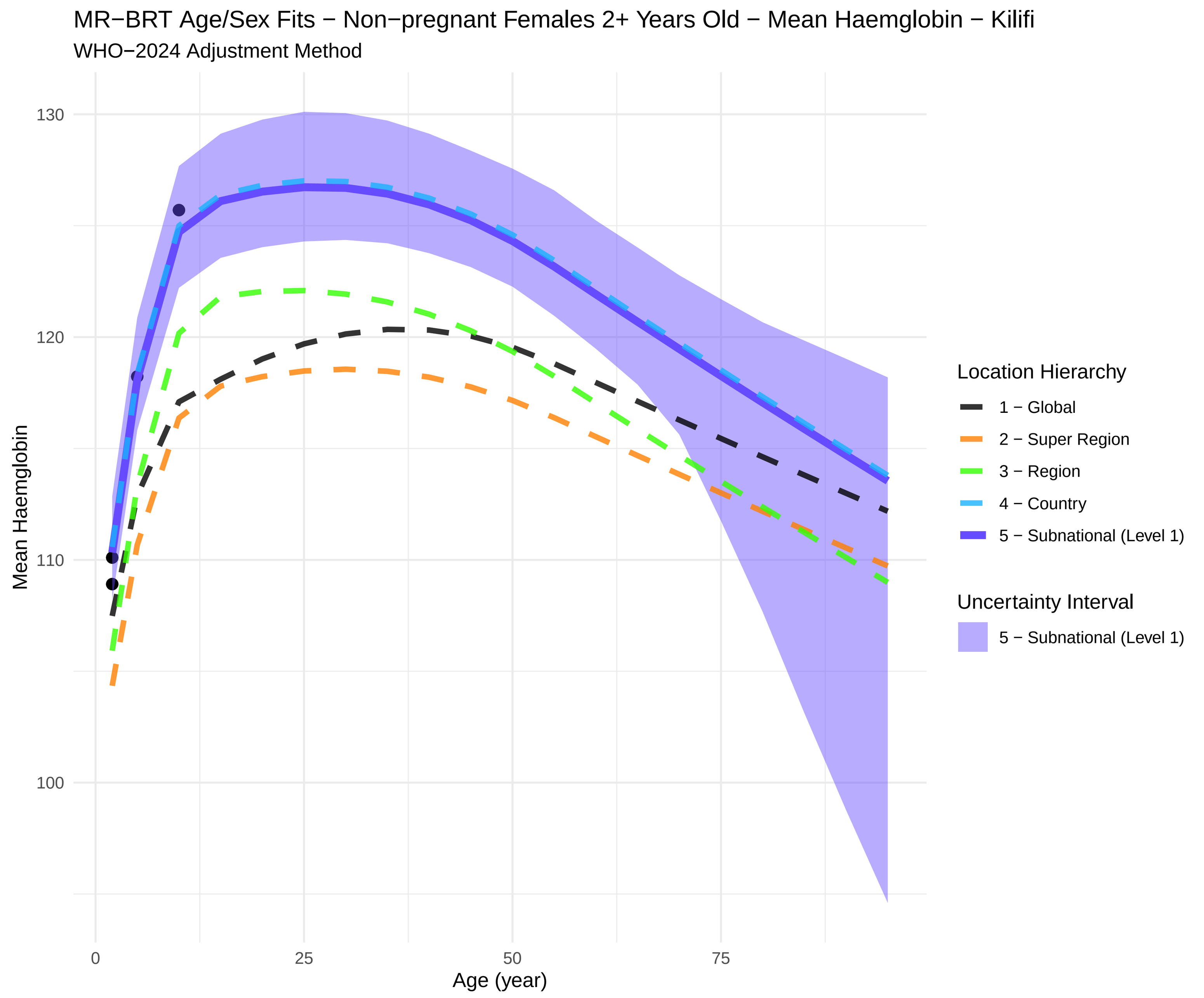
MR−BRT Age/Sex Fits − Non−pregnant Females 2+ Years Old − Mean Haemglobin − Kilifi
WHO−2024 Adjustment Method
Location Hierarchy
1 − Global
2 − Super Region
3 − Region
4 − Country
5 − Subnational (Level 1)
Uncertainty Interval
5 − Subnational (Level 1)
Mean Haemglobin
Age (year)

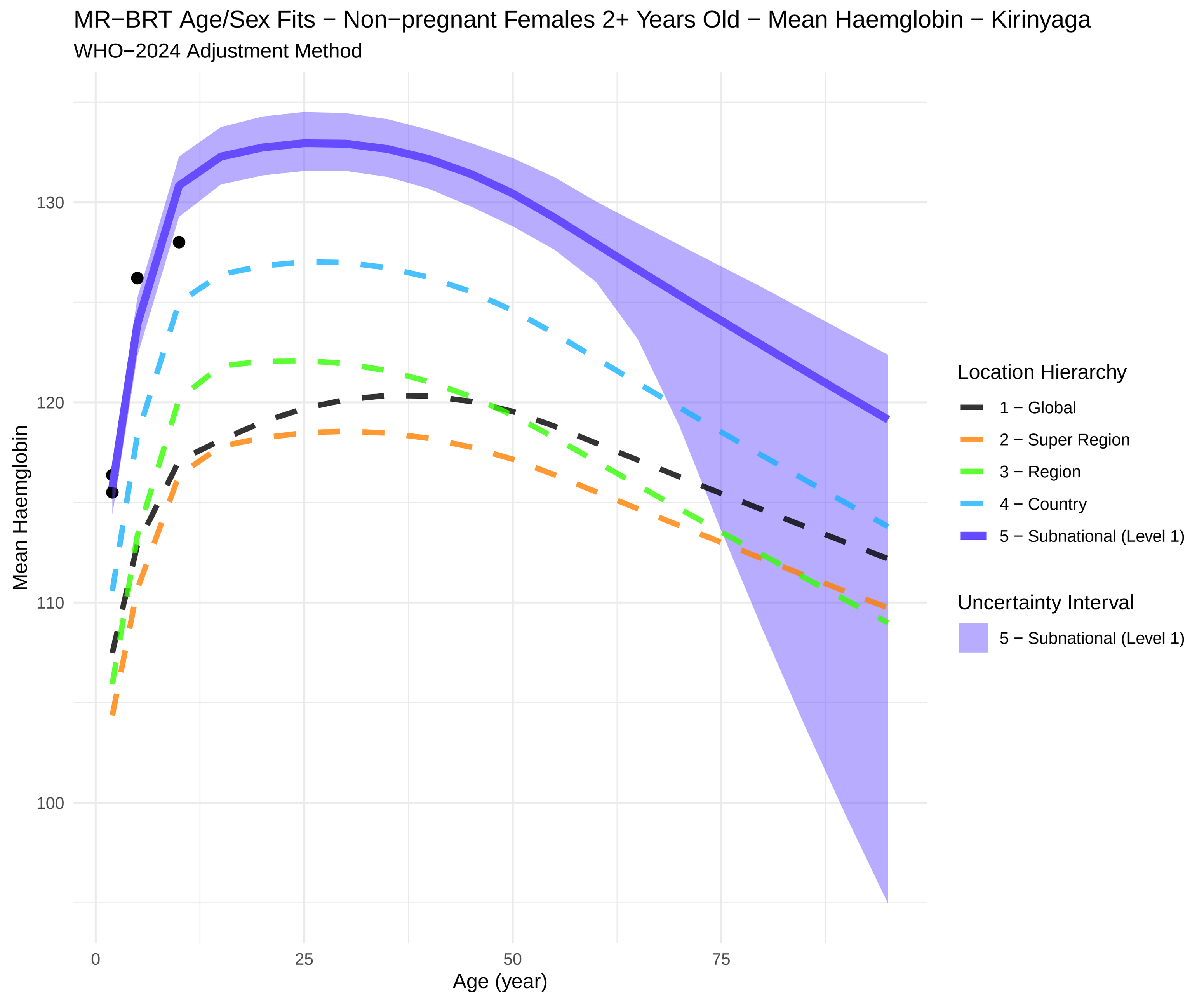
MR−BRT Age/Sex Fits − Non−pregnant Females 2+ Years Old − Mean Haemglobin − Kirinyaga
WHO−2024 Adjustment Method
Location Hierarchy
1 − Global
2 − Super Region
3 − Region
4 − Country
5 − Subnational (Level 1)
Uncertainty Interval
5 − Subnational (Level 1)
Mean Haemglobin
Age (year)

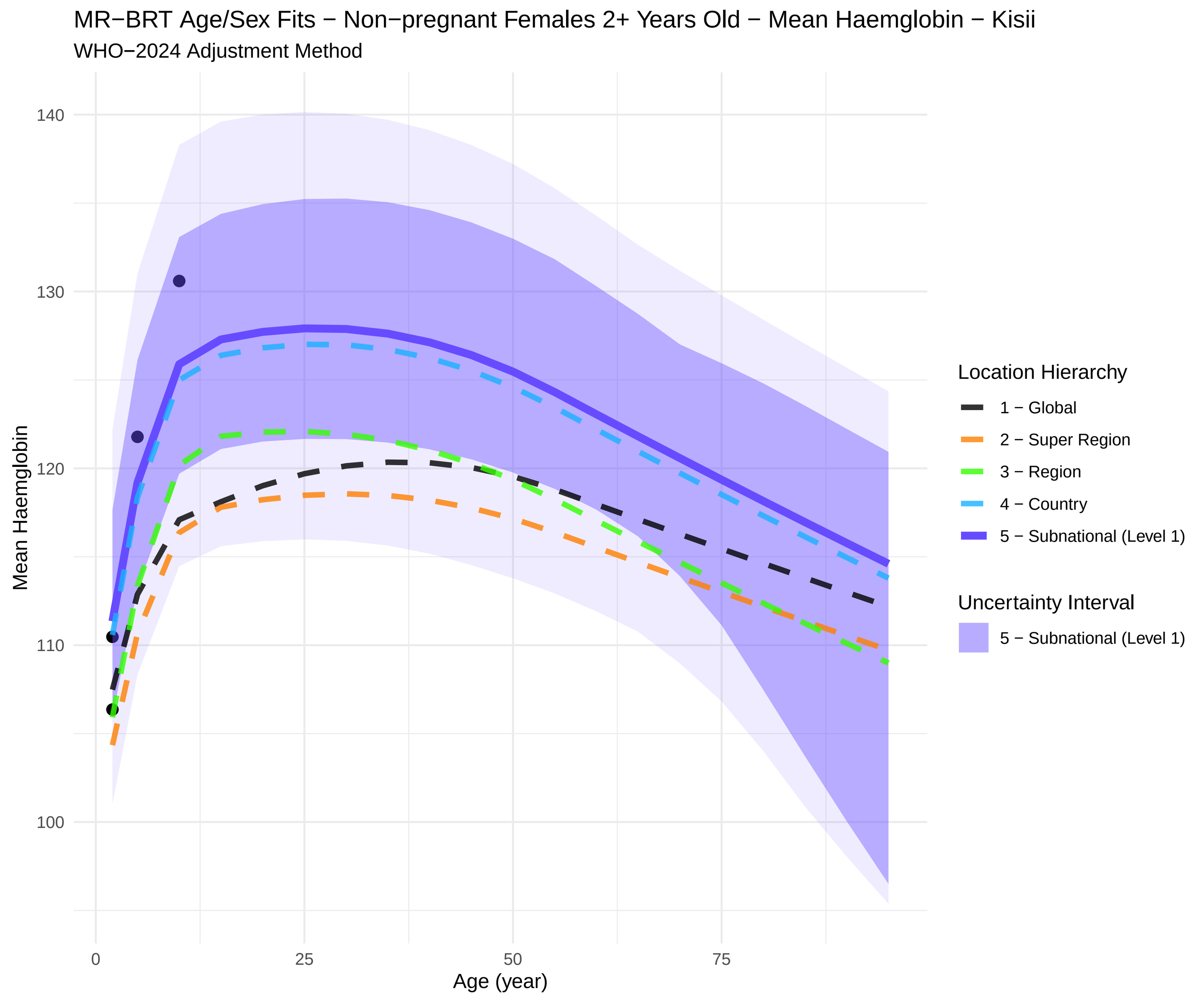
MR−BRT Age/Sex Fits − Non−pregnant Females 2+ Years Old − Mean Haemglobin − Kisii
WHO−2024 Adjustment Method
Location Hierarchy
1 − Global
2 − Super Region
3 − Region
4 − Country
5 − Subnational (Level 1)
Uncertainty Interval
5 − Subnational (Level 1)
Mean Haemglobin
Age (year)

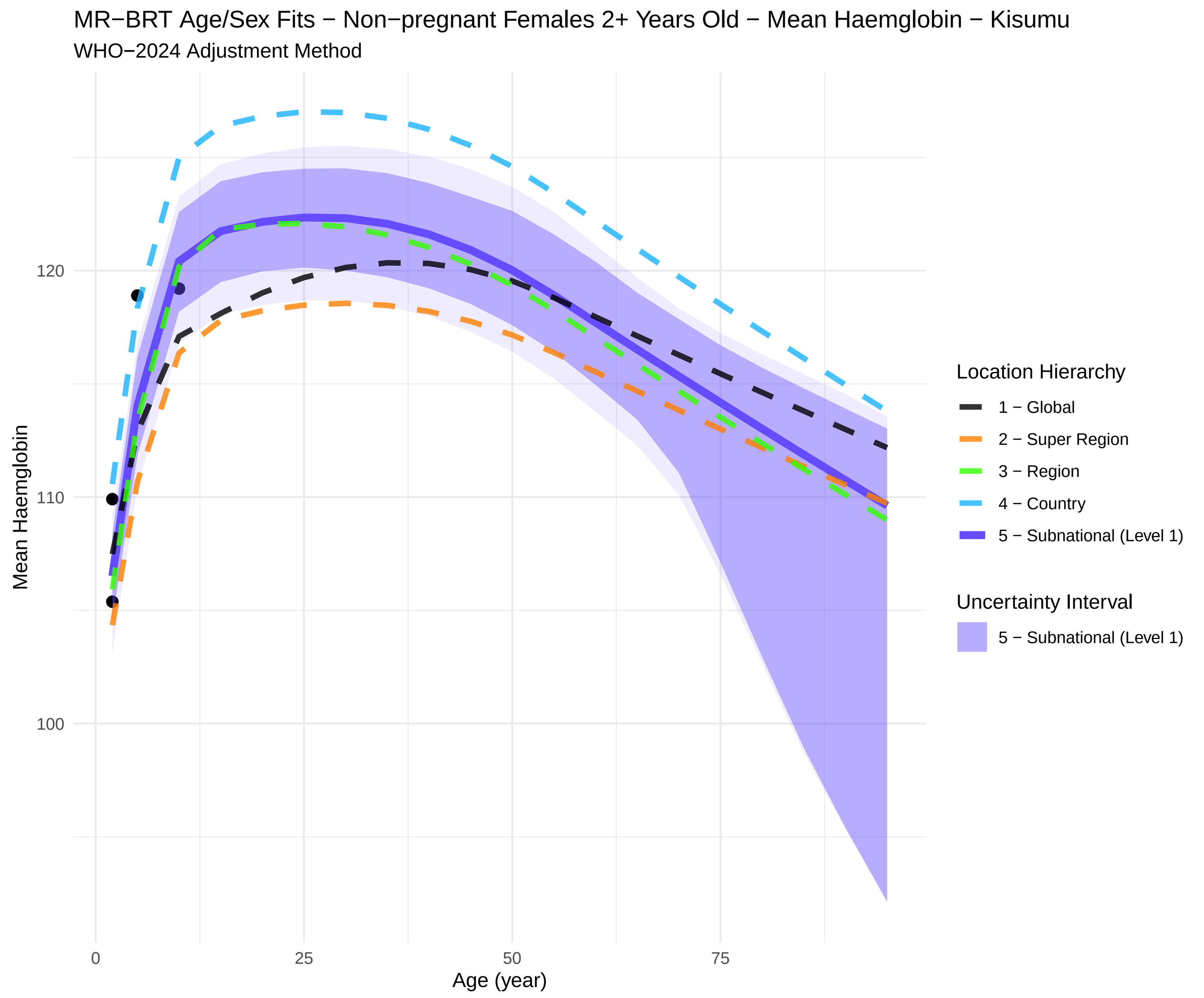
MR−BRT Age/Sex Fits − Non−pregnant Females 2+ Years Old − Mean Haemglobin − Kisumu
WHO−2024 Adjustment Method
Location Hierarchy
1 − Global
2 − Super Region
3 − Region
4 − Country
5 − Subnational (Level 1)
Uncertainty Interval
5 − Subnational (Level 1)
Mean Haemglobin
Age (year)

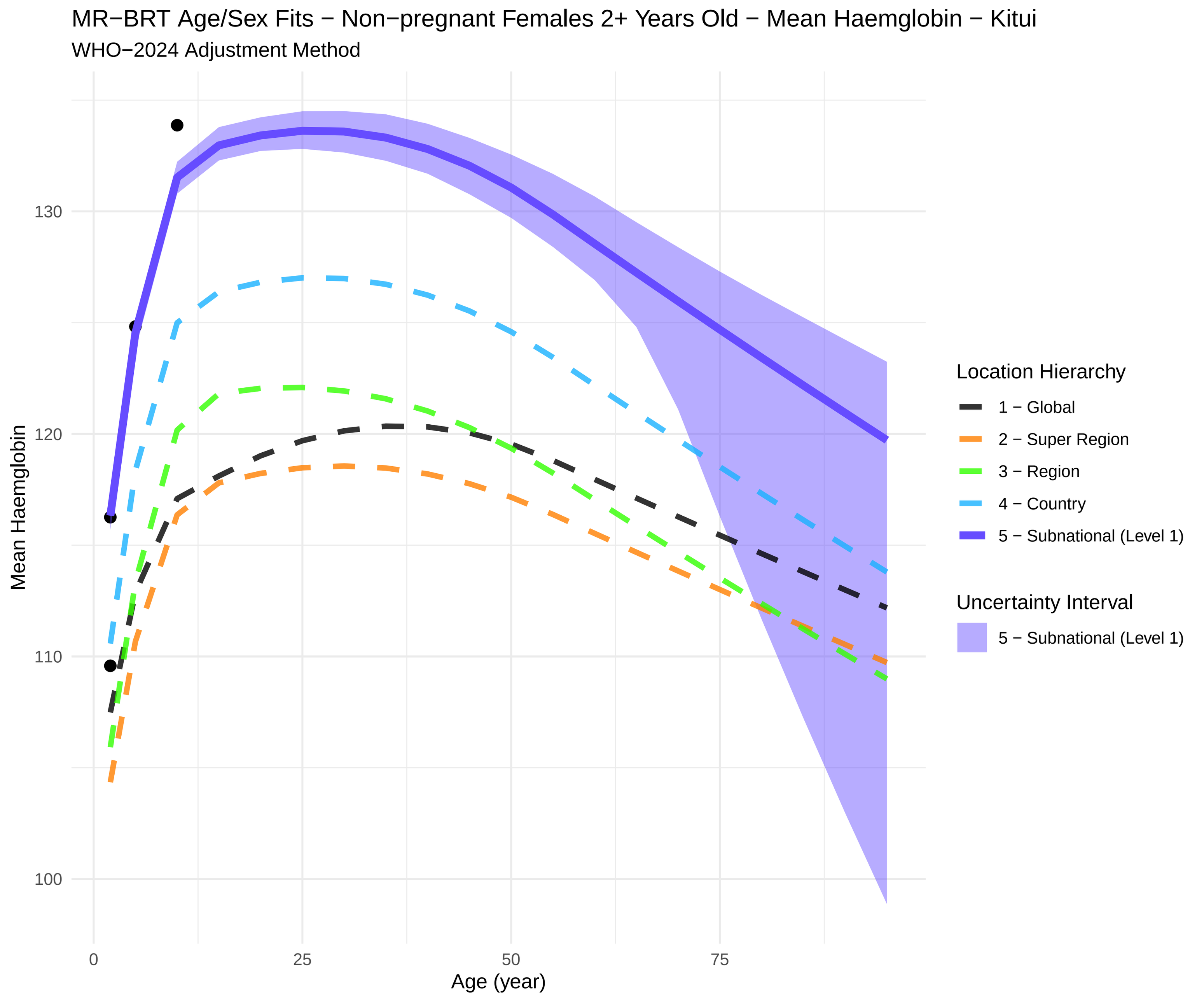
MR−BRT Age/Sex Fits − Non−pregnant Females 2+ Years Old − Mean Haemglobin − Kitui
WHO−2024 Adjustment Method
Location Hierarchy
1 − Global
2 − Super Region
3 − Region
4 − Country
5 − Subnational (Level 1)
Uncertainty Interval
5 − Subnational (Level 1)
Mean Haemglobin
Age (year)

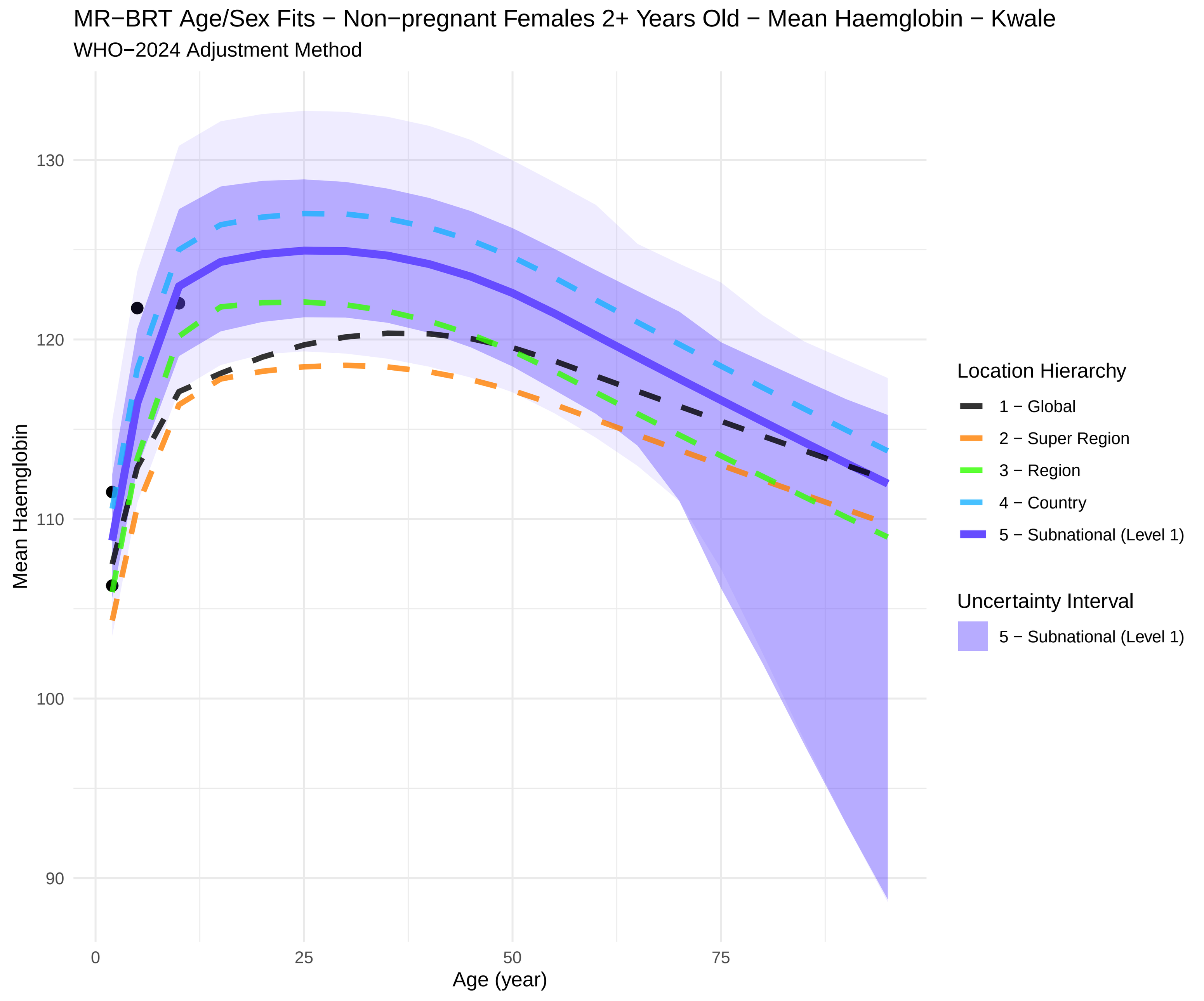
MR−BRT Age/Sex Fits − Non−pregnant Females 2+ Years Old − Mean Haemglobin − Kwale
WHO−2024 Adjustment Method
Location Hierarchy
1 − Global
2 − Super Region
3 − Region
4 − Country
5 − Subnational (Level 1)
Uncertainty Interval
5 − Subnational (Level 1)
Mean Haemglobin
Age (year)

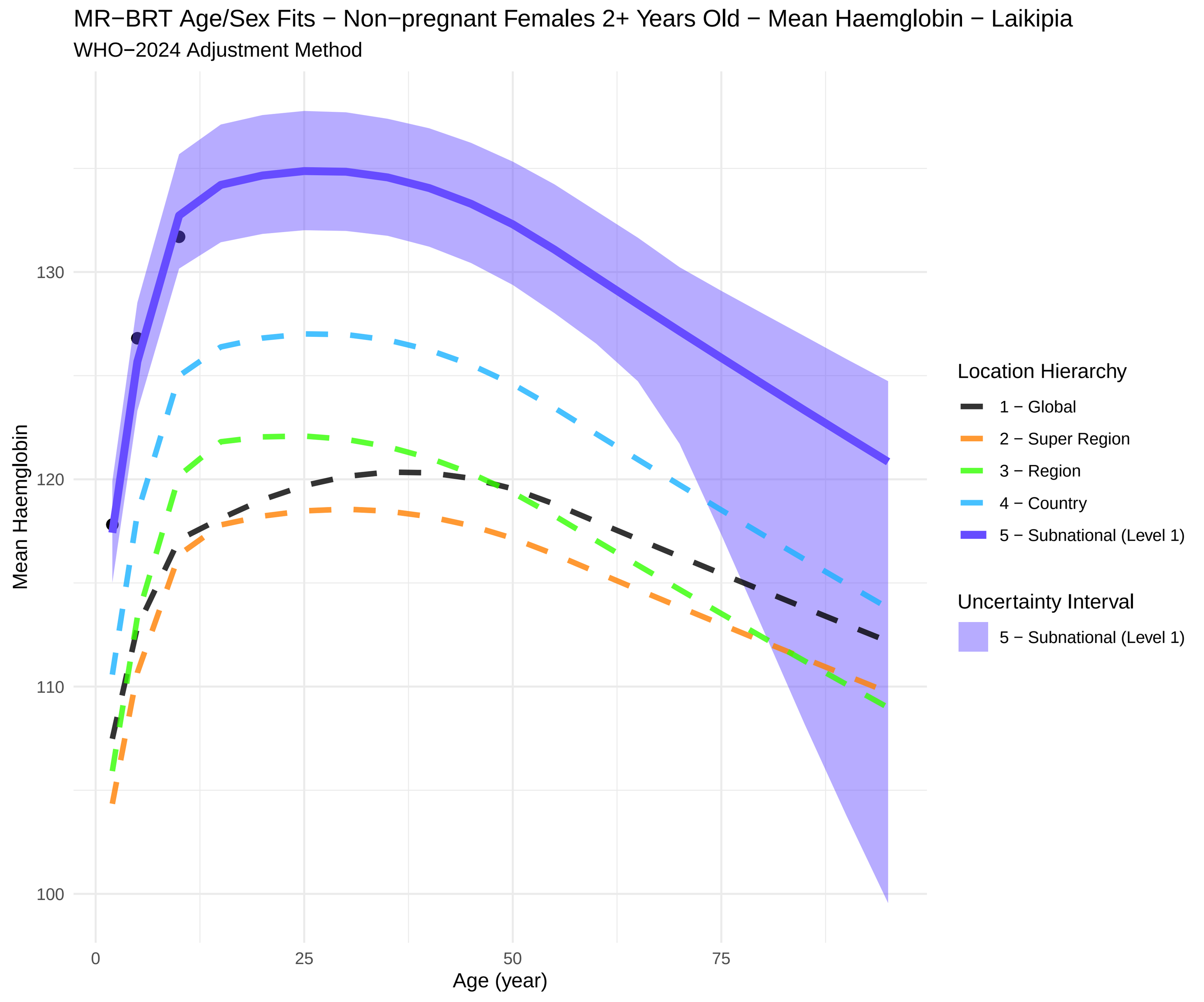
MR−BRT Age/Sex Fits − Non−pregnant Females 2+ Years Old − Mean Haemglobin − Laikipia
WHO−2024 Adjustment Method
Location Hierarchy
1 − Global
2 − Super Region
3 − Region
4 − Country
5 − Subnational (Level 1)
Uncertainty Interval
5 − Subnational (Level 1)
Mean Haemglobin
Age (year)

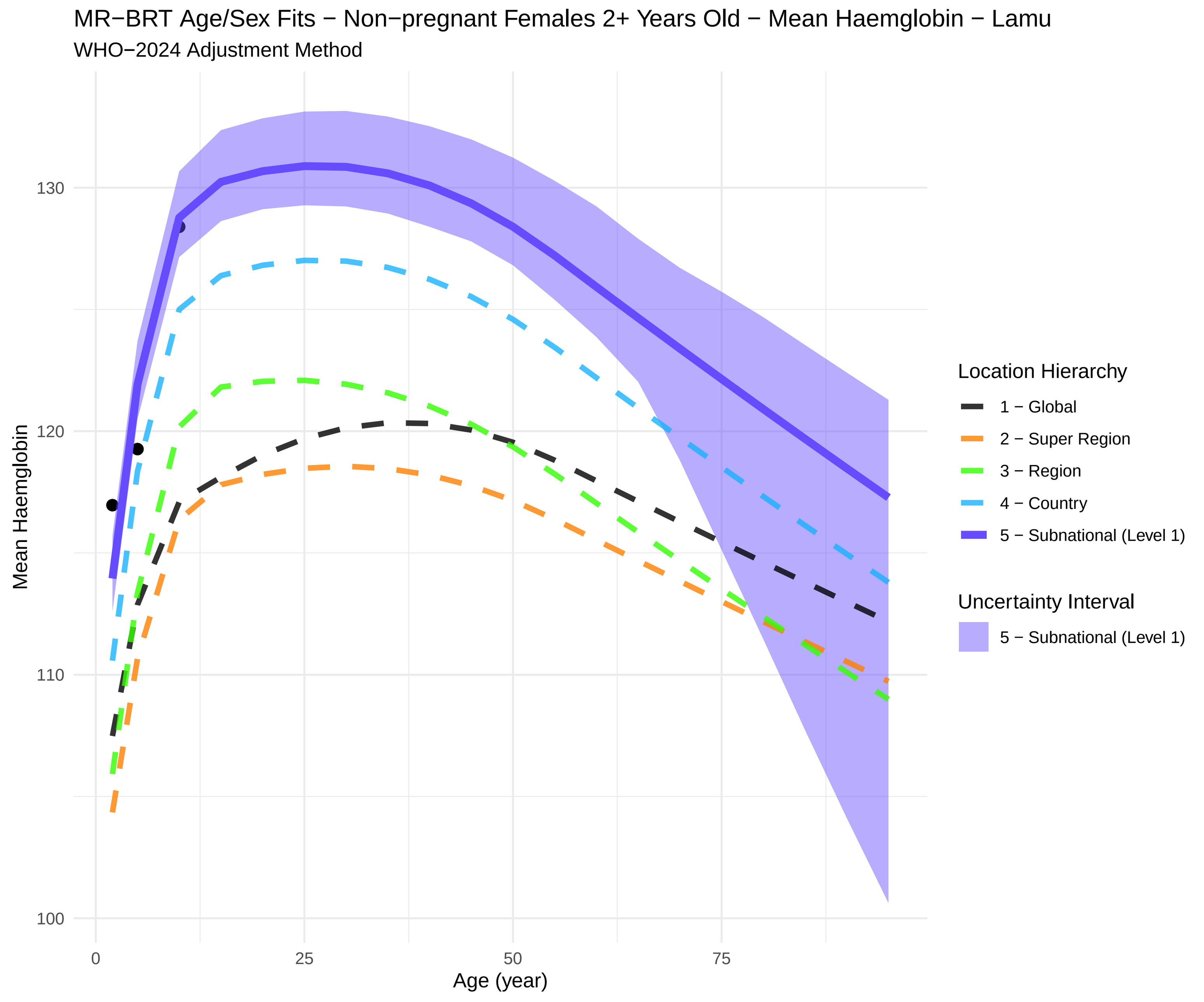
MR−BRT Age/Sex Fits − Non−pregnant Females 2+ Years Old − Mean Haemglobin − Lamu
WHO−2024 Adjustment Method
Location Hierarchy
1 − Global
2 − Super Region
3 − Region
4 − Country
5 − Subnational (Level 1)
Uncertainty Interval
5 − Subnational (Level 1)
Mean Haemglobin
Age (year)

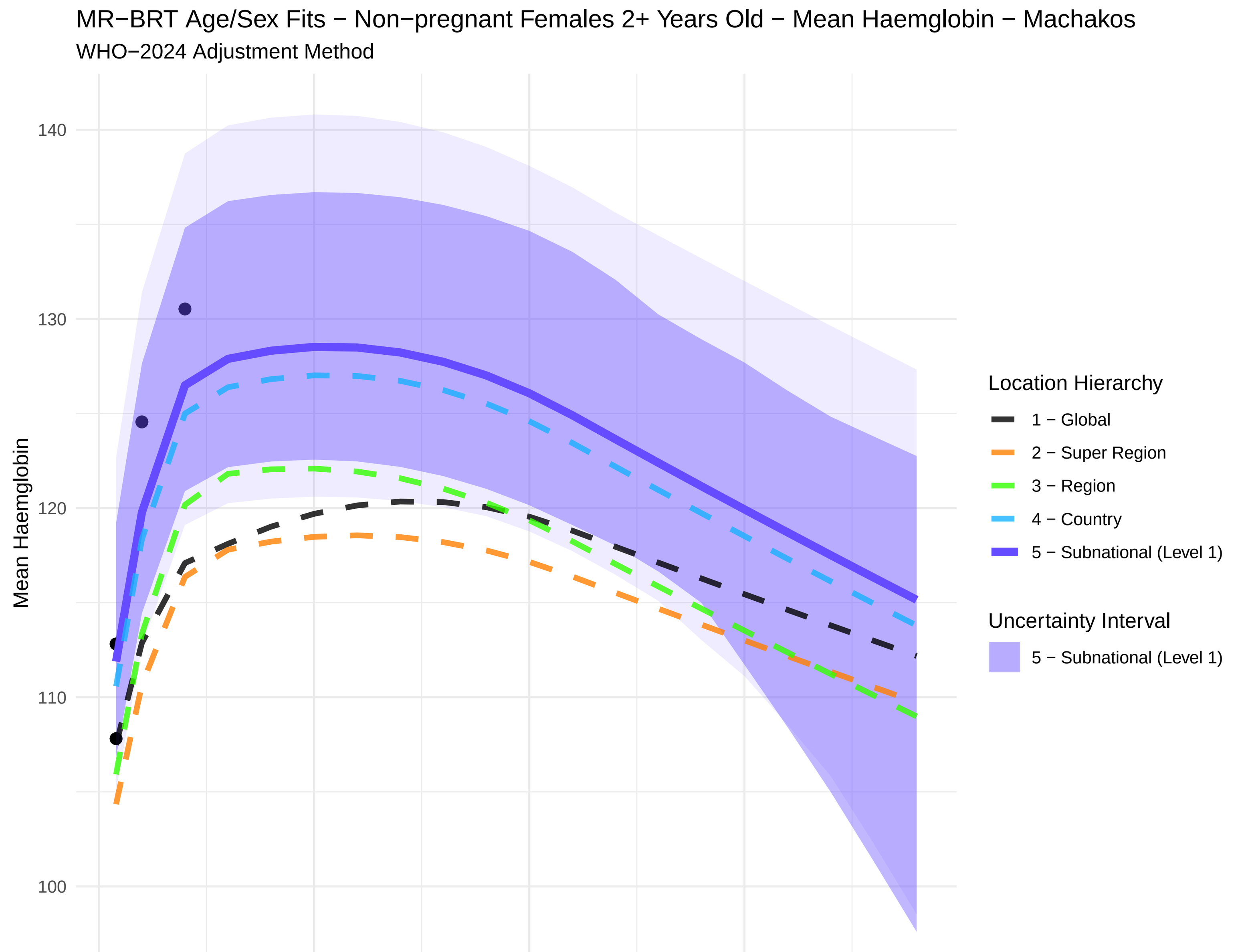
MR−BRT Age/Sex Fits − Non−pregnant Females 2+ Years Old − Mean Haemglobin − Machakos
WHO−2024 Adjustment Method
Location Hierarchy
1 − Global
2 − Super Region
3 − Region
4 − Country
5 − Subnational (Level 1)
Uncertainty Interval
5 − Subnational (Level 1)
Mean Haemglobin
Age (year)

MR−BRT Age/Sex Fits − Non−pregnant Females 2+ Years Old − Mean Haemglobin − Makueni
WHO−2024 Adjustment Method

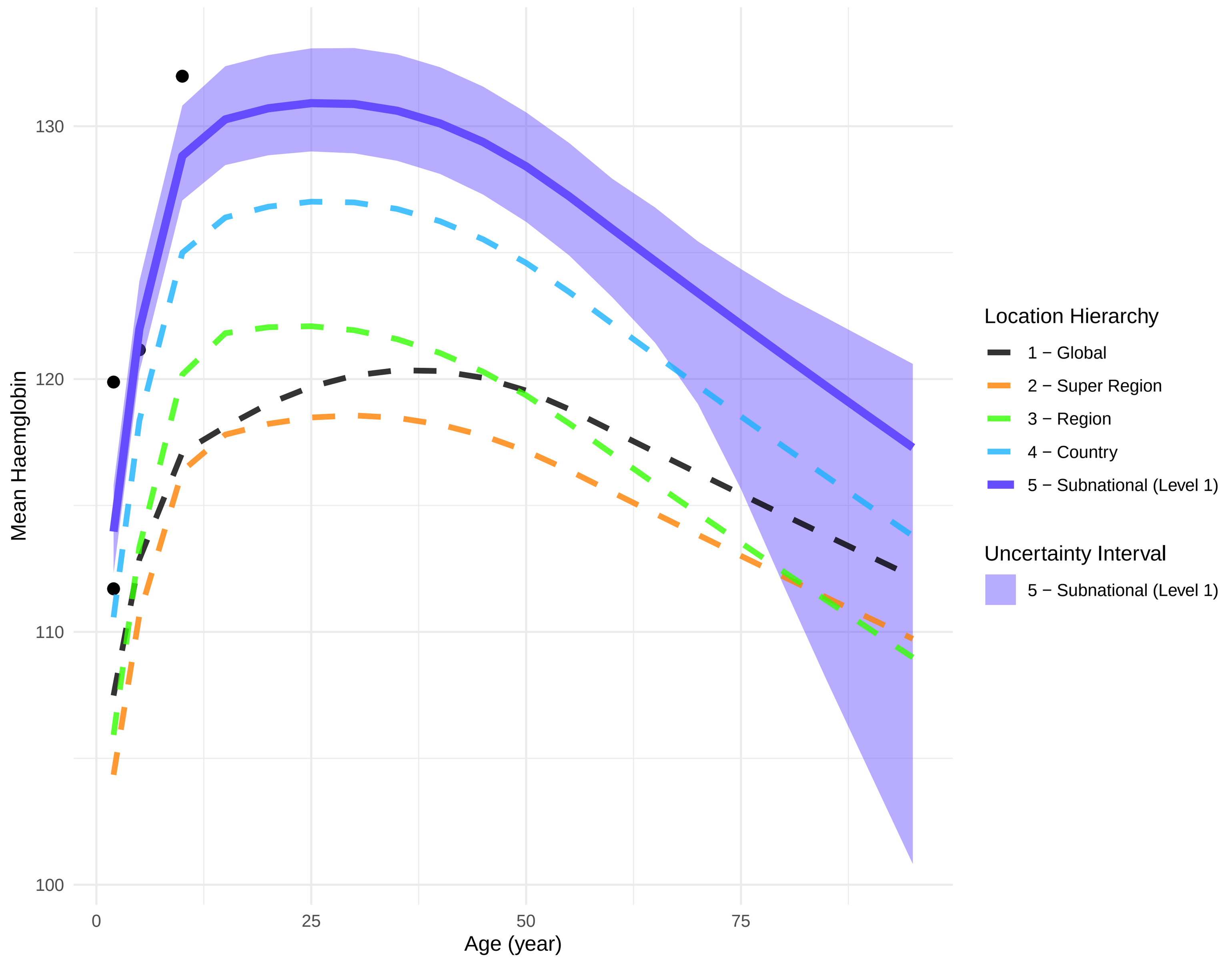

MR−BRT Age/Sex Fits − Non−pregnant Females 2+ Years Old − Mean Haemglobin − Mandera
WHO−2024 Adjustment Method

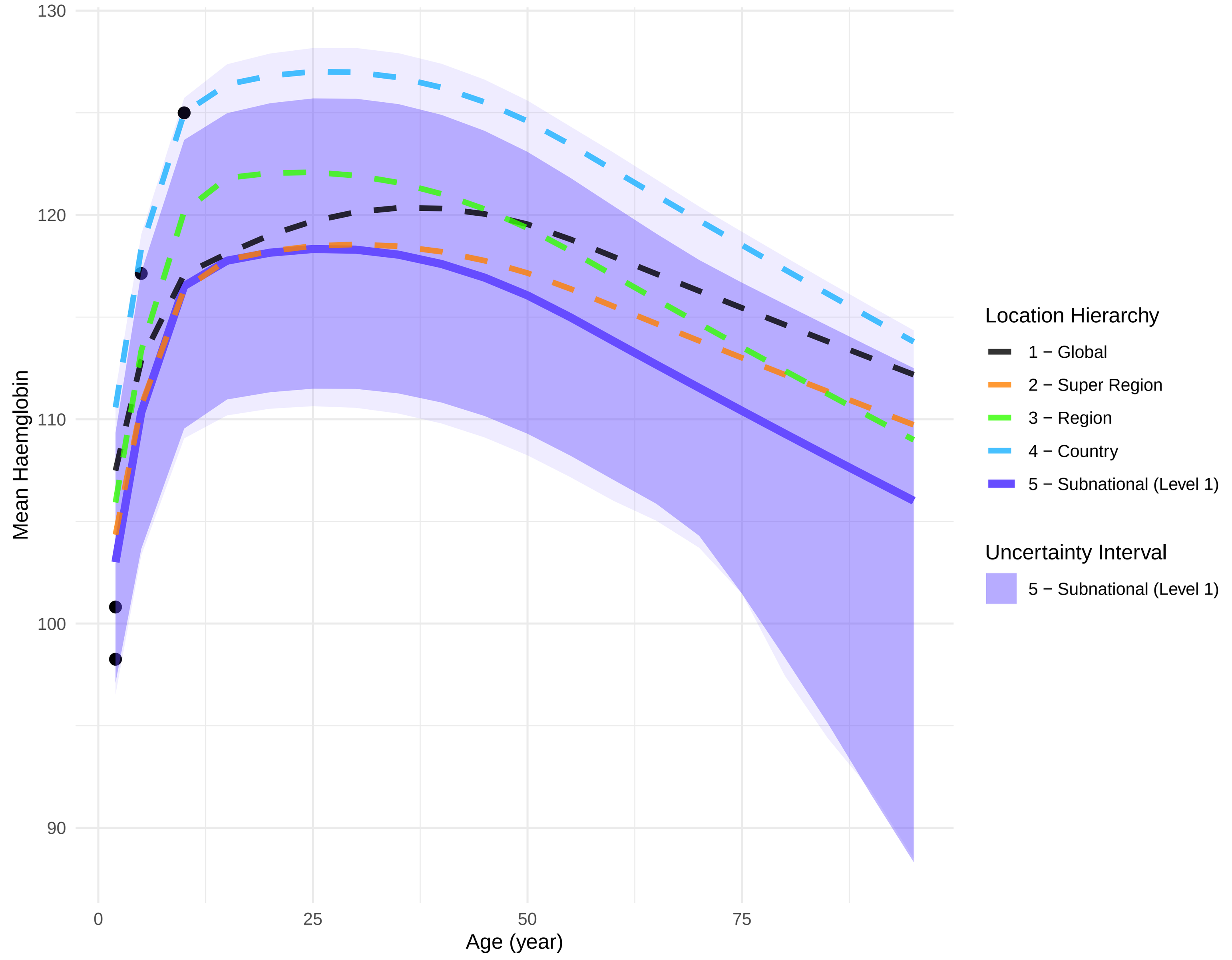

MR−BRT Age/Sex Fits − Non−pregnant Females 2+ Years Old − Mean Haemglobin − Marsabit
WHO−2024 Adjustment Method

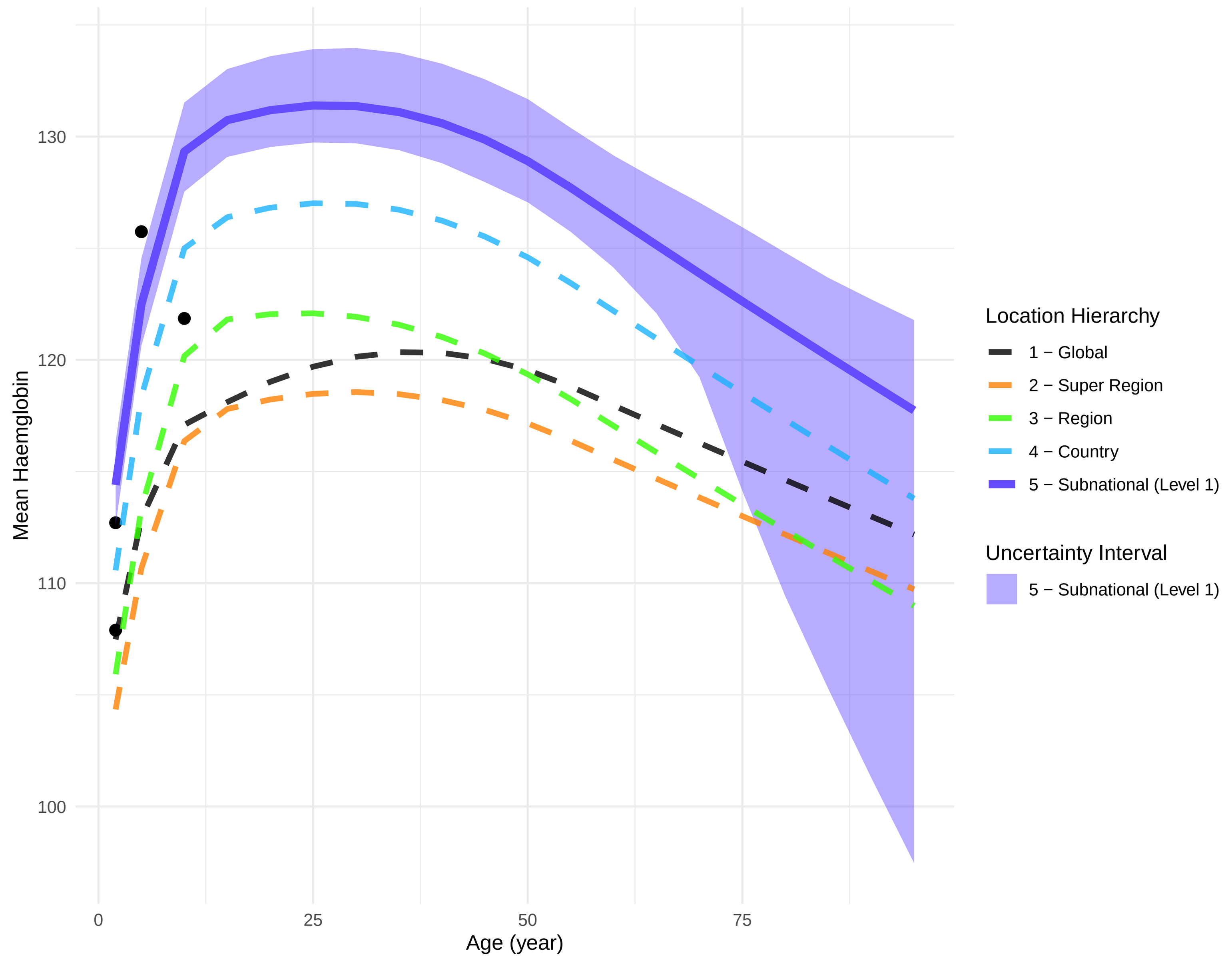

MR−BRT Age/Sex Fits − Non−pregnant Females 2+ Years Old − Mean Haemglobin − Meru
WHO−2024 Adjustment Method

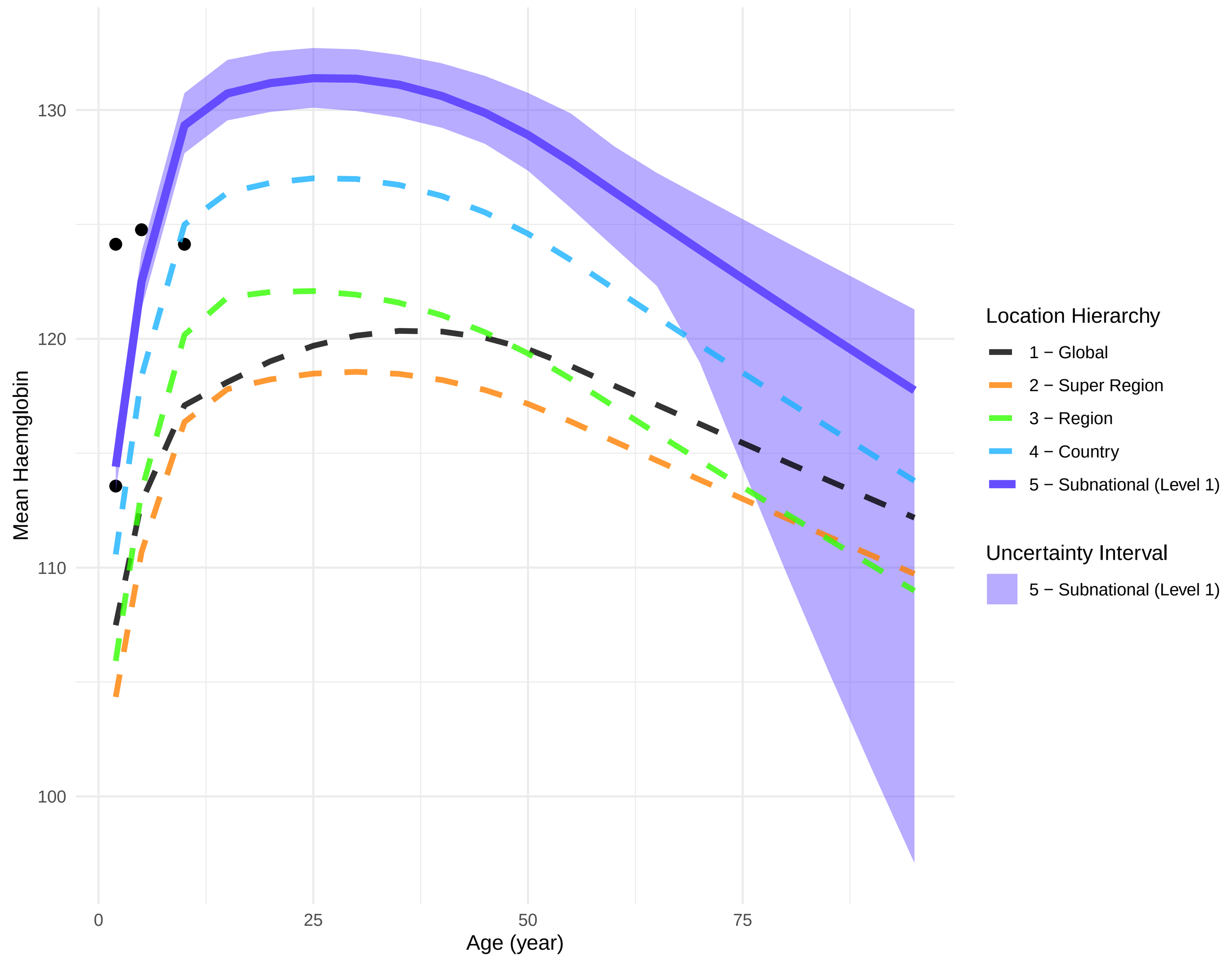

MR−BRT Age/Sex Fits − Non−pregnant Females 2+ Years Old − Mean Haemglobin − Migori
WHO−2024 Adjustment Method

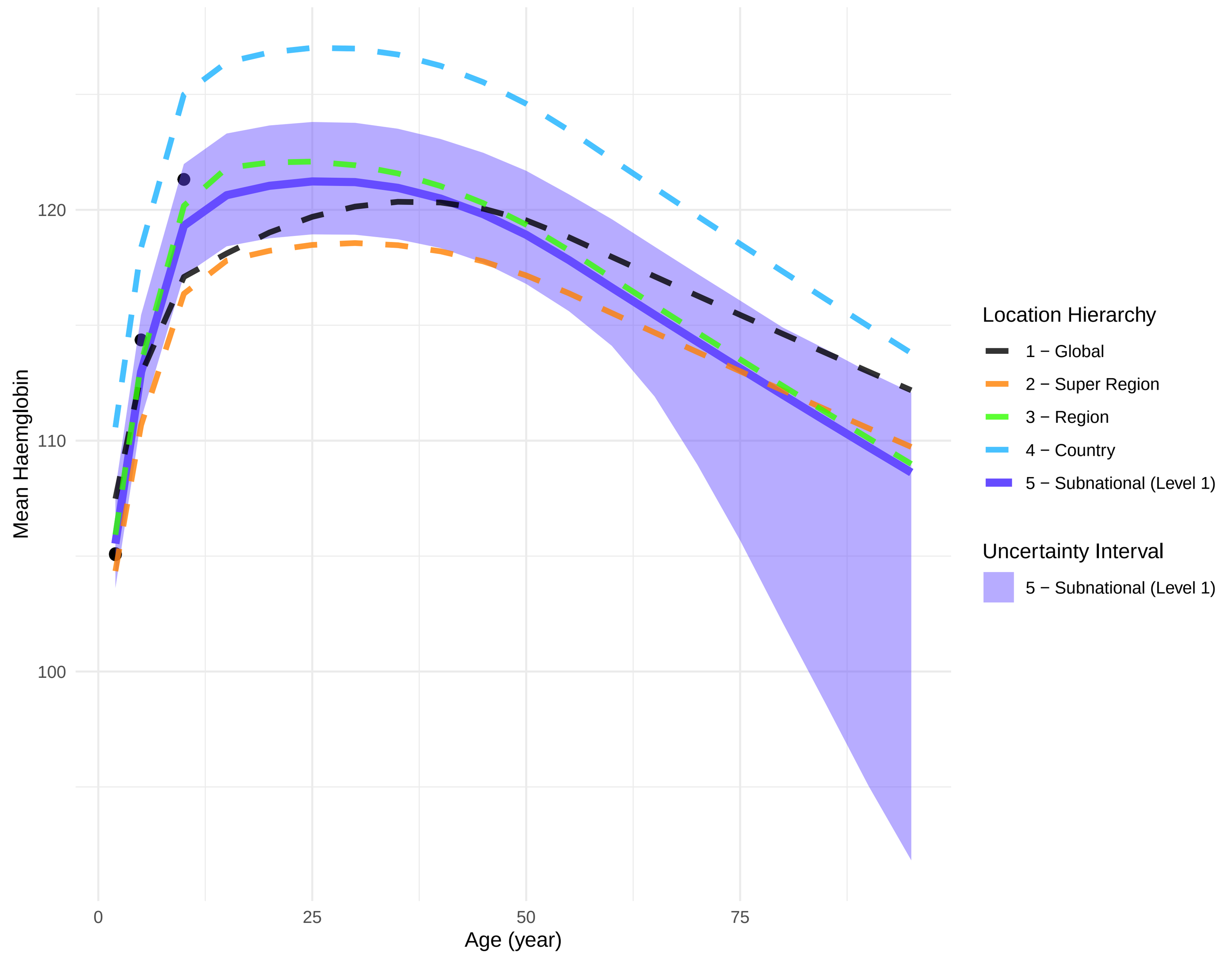

MR−BRT Age/Sex Fits − Non−pregnant Females 2+ Years Old − Mean Haemglobin − Mombasa
WHO−2024 Adjustment Method

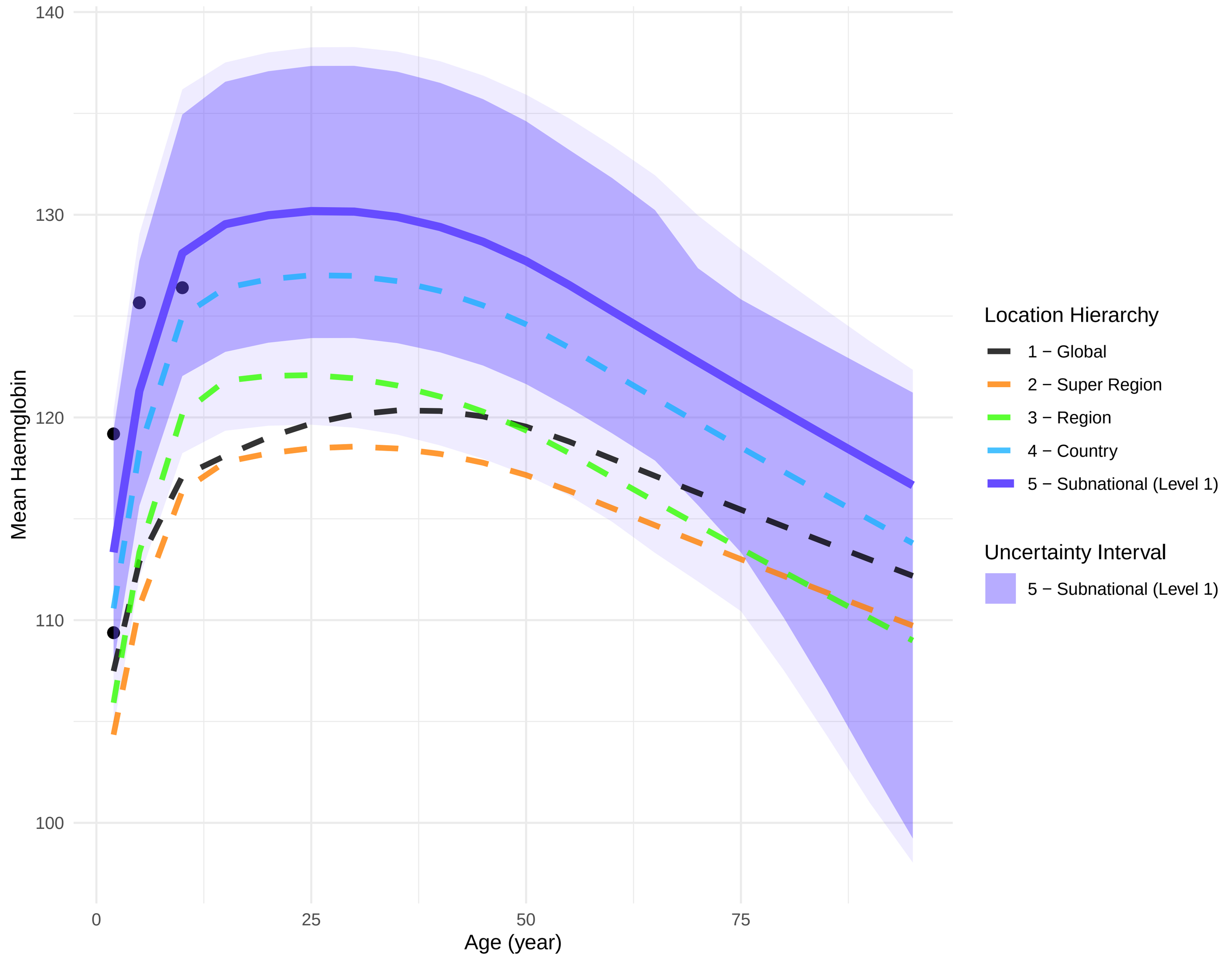

MR−BRT Age/Sex Fits − Non−pregnant Females 2+ Years Old − Mean Haemglobin − Murang'a
WHO−2024 Adjustment Method

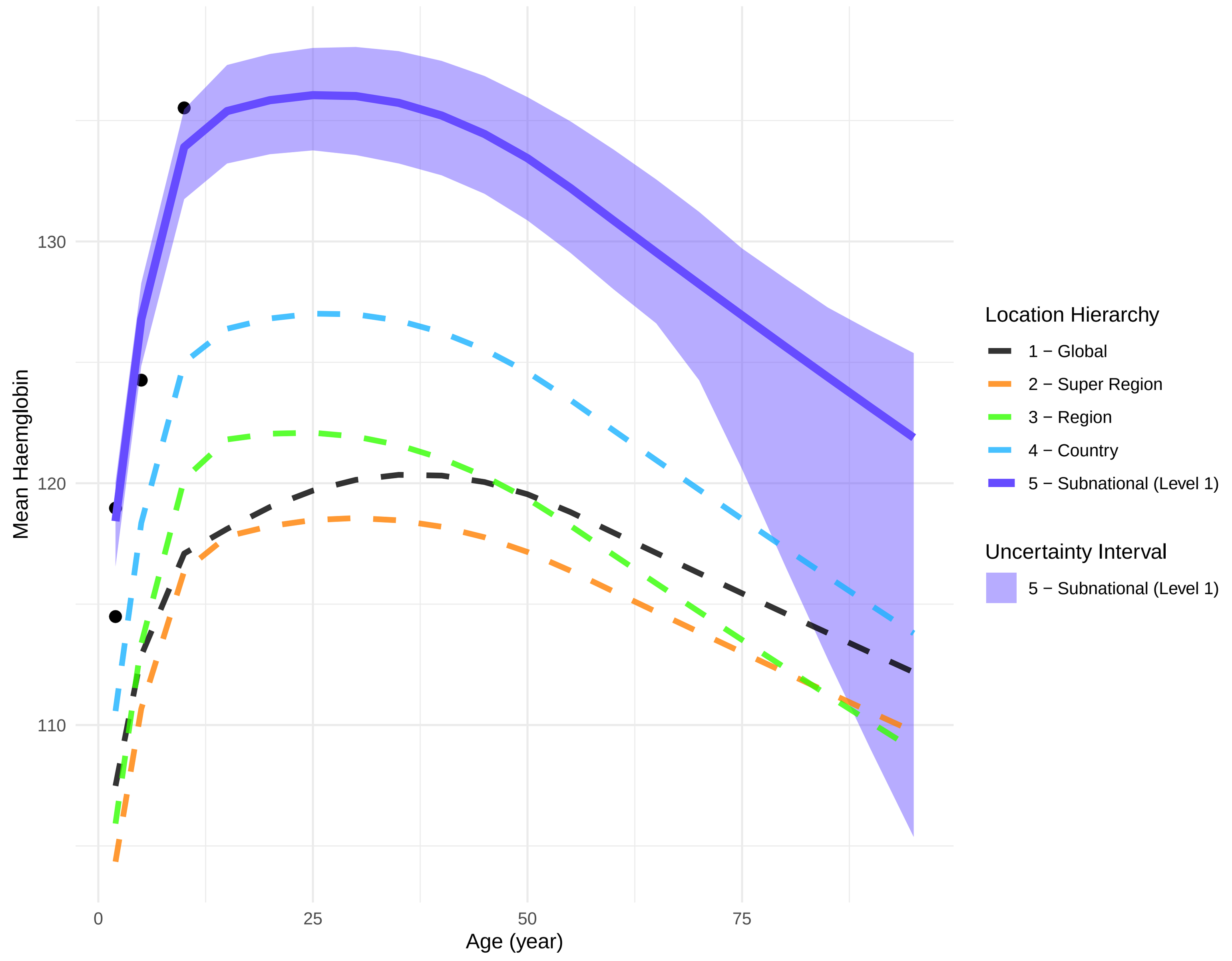

MR−BRT Age/Sex Fits − Non−pregnant Females 2+ Years Old − Mean Haemglobin − Nairobi
WHO−2024 Adjustment Method

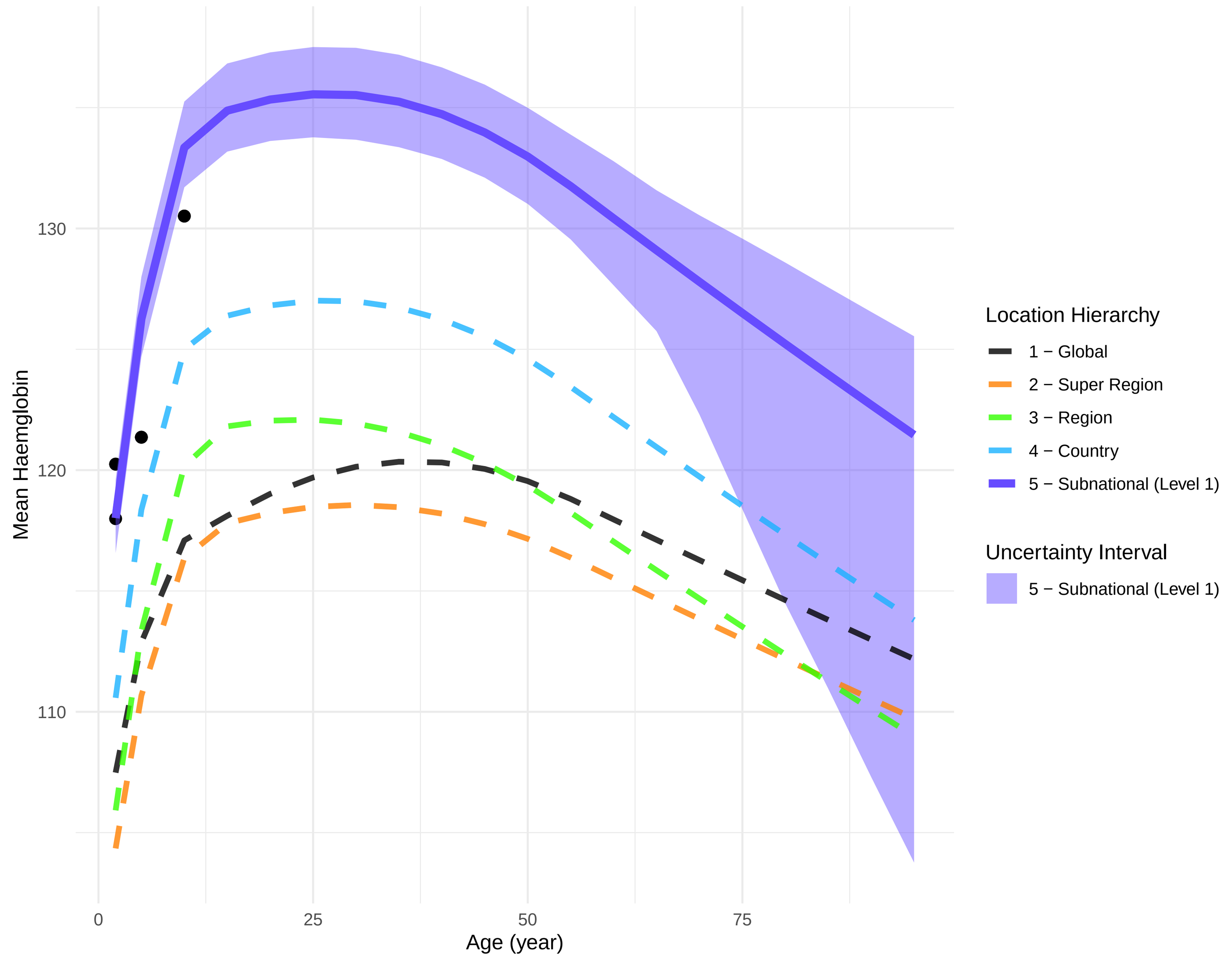

MR−BRT Age/Sex Fits − Non−pregnant Females 2+ Years Old − Mean Haemglobin − Nakuru
WHO−2024 Adjustment Method

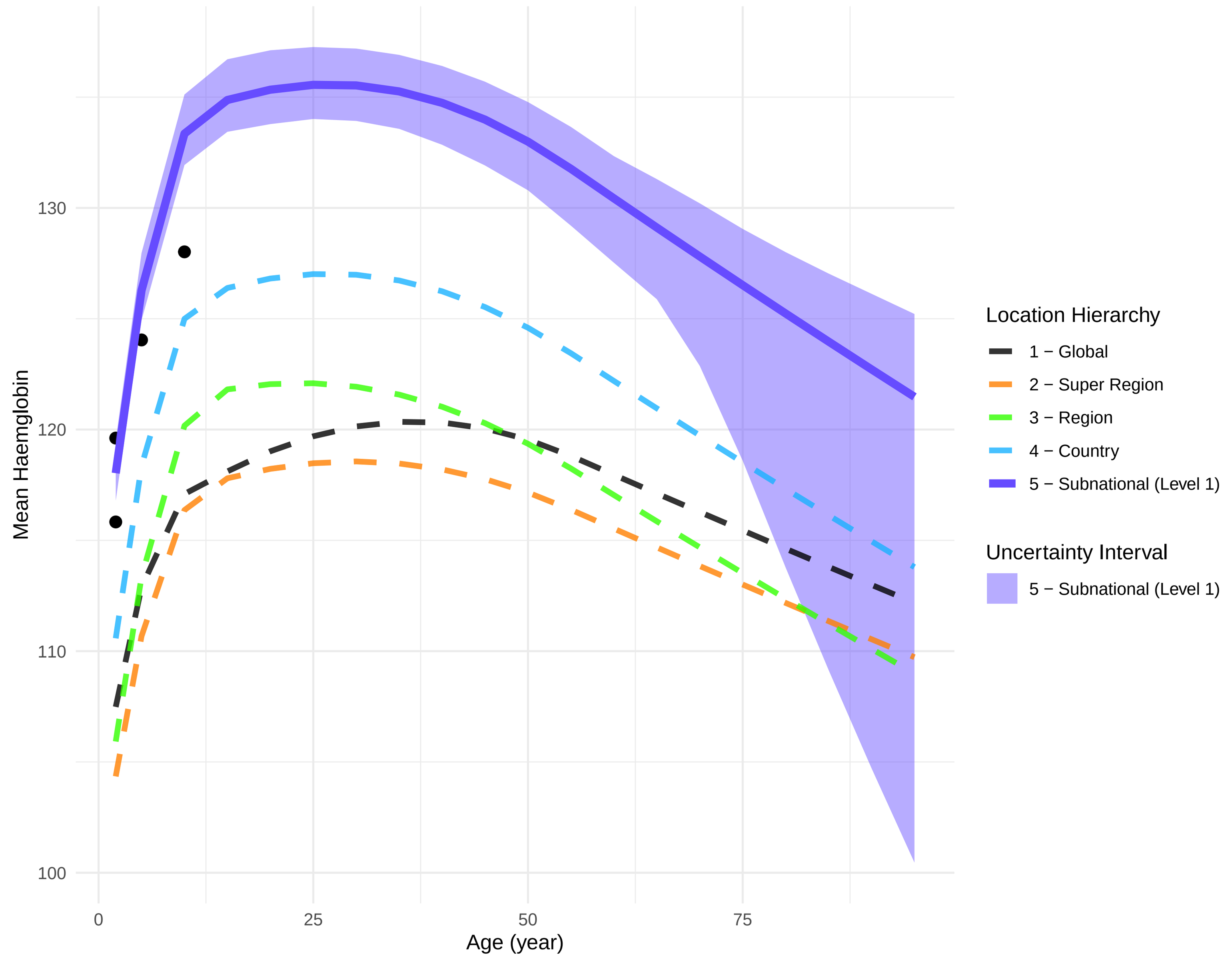

MR−BRT Age/Sex Fits − Non−pregnant Females 2+ Years Old − Mean Haemglobin − Nandi
WHO−2024 Adjustment Method

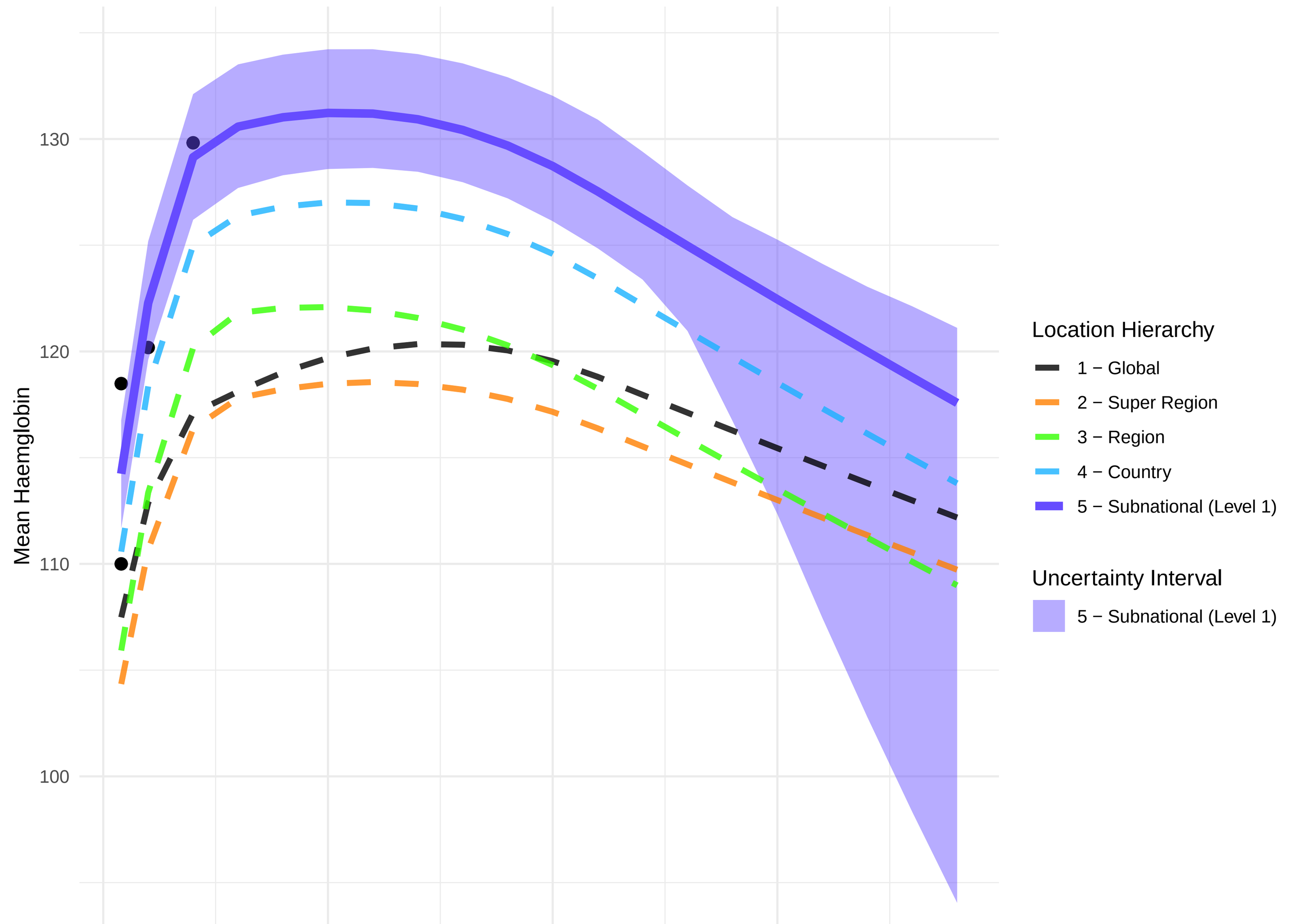

MR−BRT Age/Sex Fits − Non−pregnant Females 2+ Years Old − Mean Haemglobin − Narok
WHO−2024 Adjustment Method

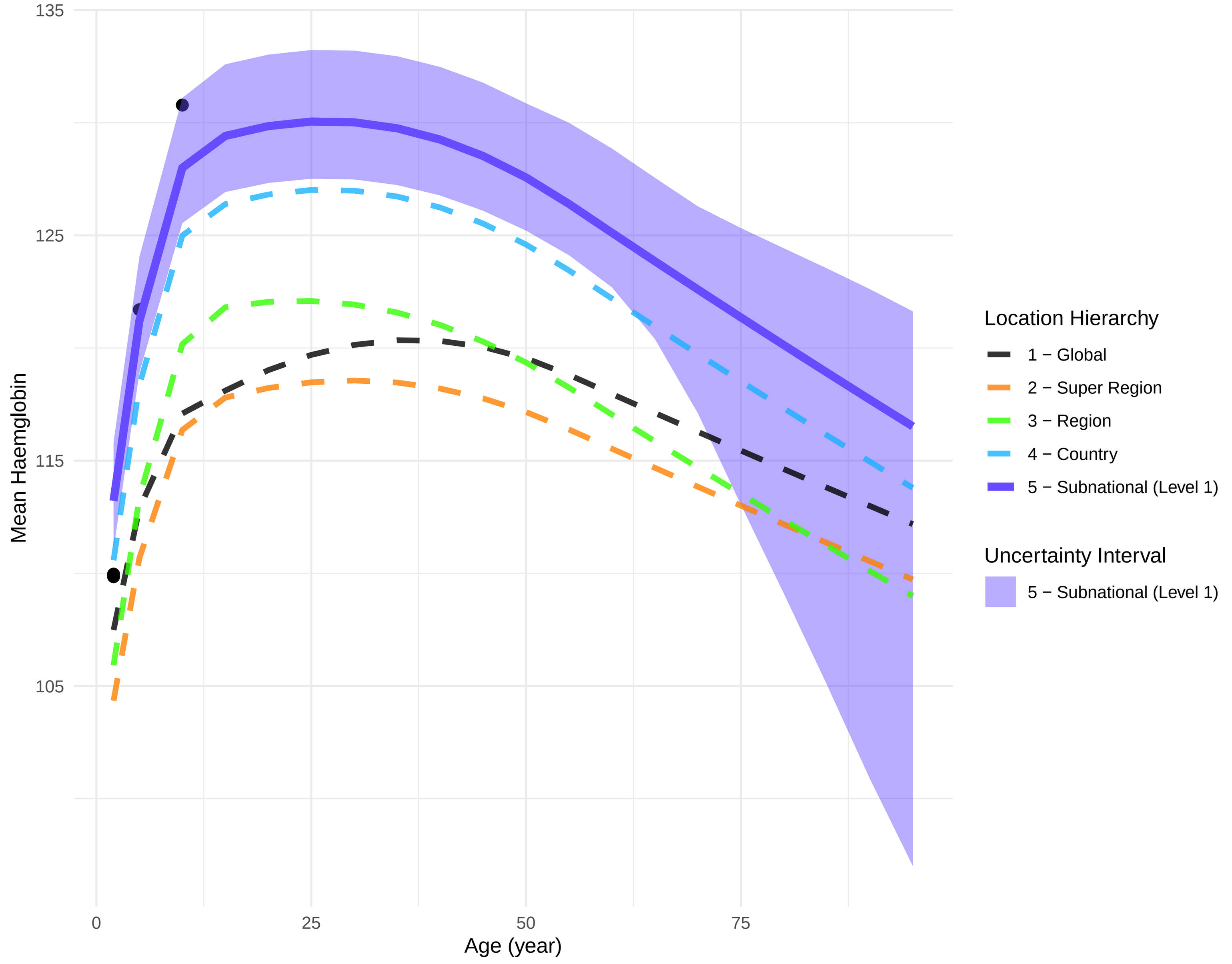

MR−BRT Age/Sex Fits − Non−pregnant Females 2+ Years Old − Mean Haemglobin − Nyamira
WHO−2024 Adjustment Method

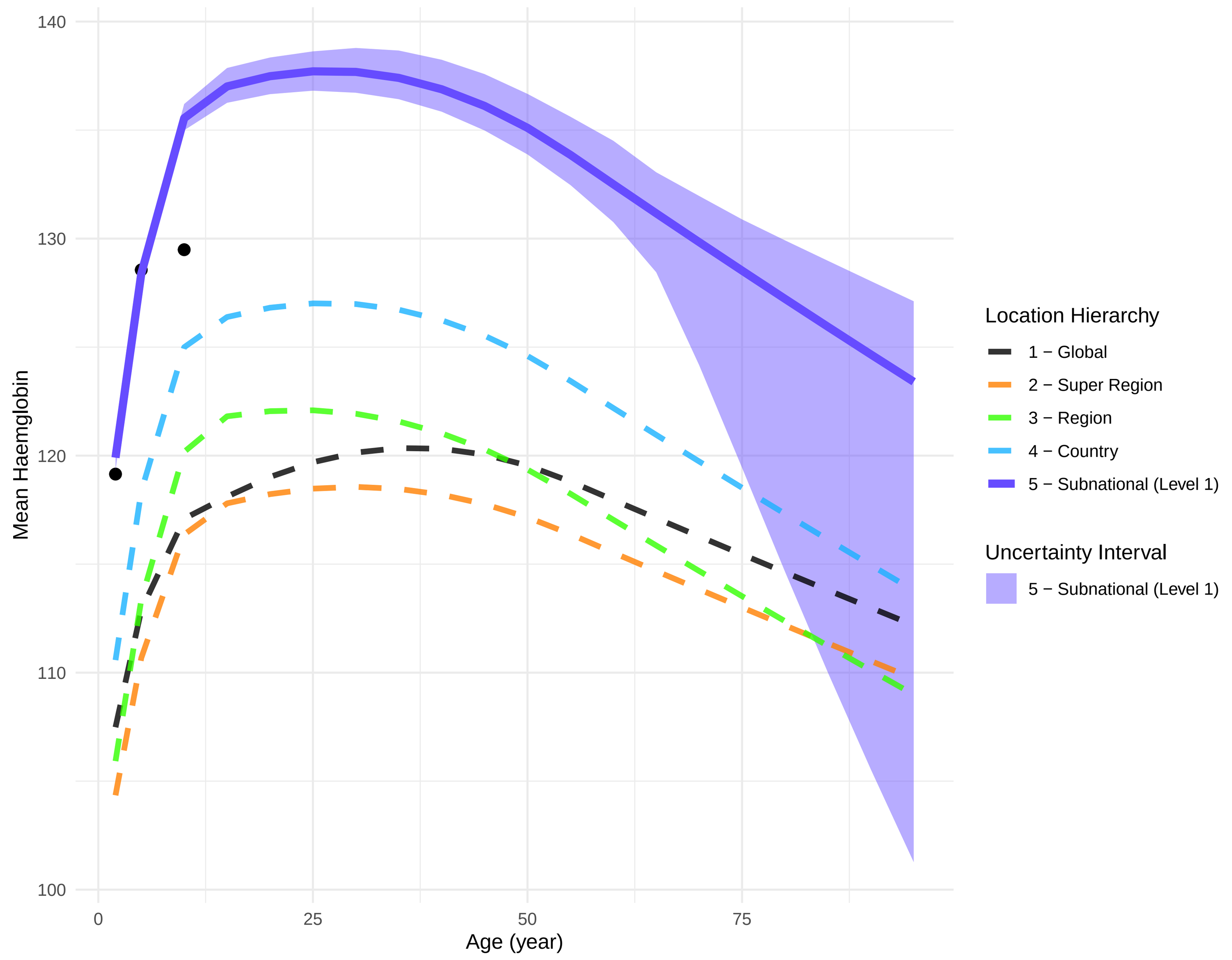

MR−BRT Age/Sex Fits − Non−pregnant Females 2+ Years Old − Mean Haemglobin − Nyandarua
WHO−2024 Adjustment Method

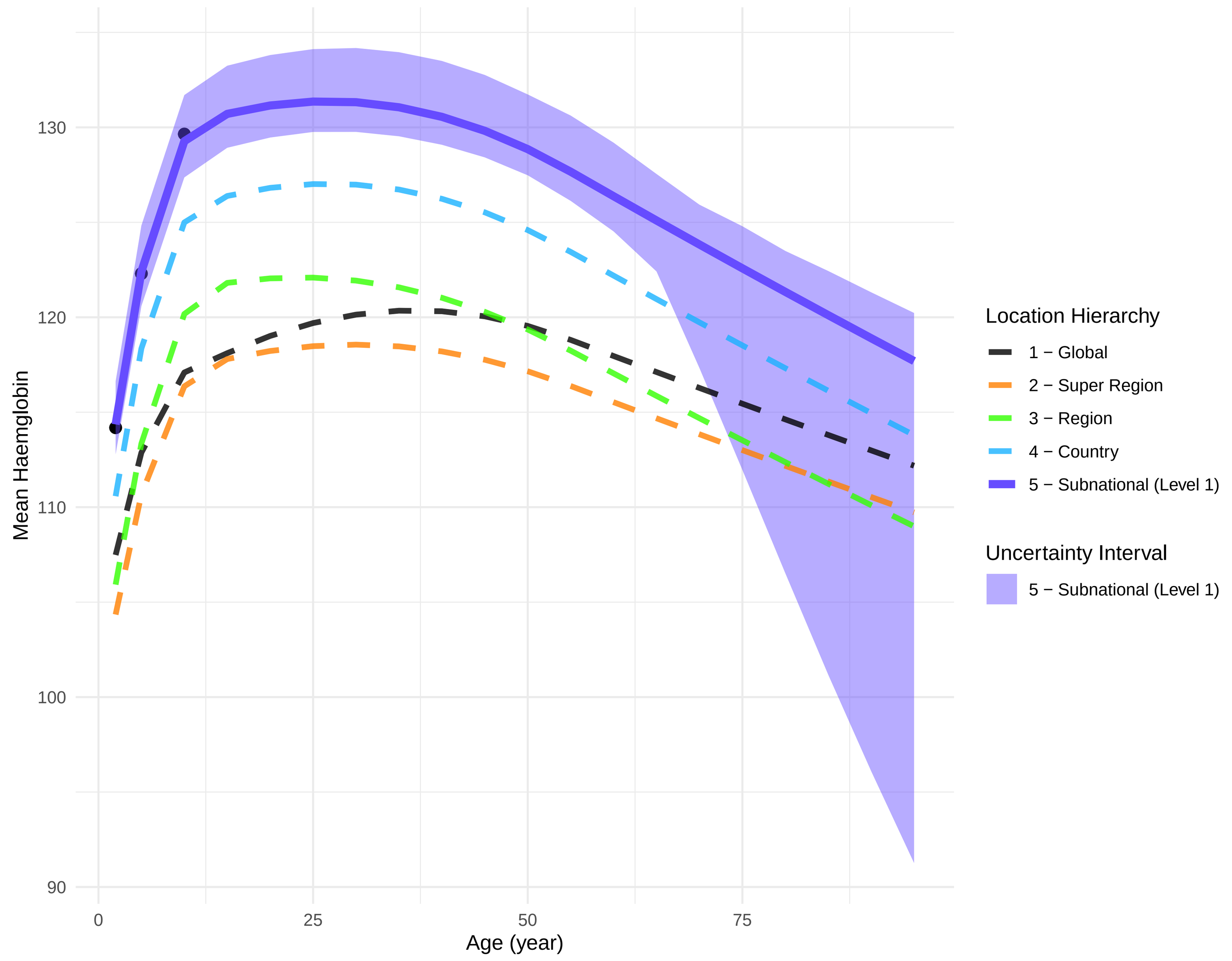

MR−BRT Age/Sex Fits − Non−pregnant Females 2+ Years Old − Mean Haemglobin − Nyeri
WHO−2024 Adjustment Method

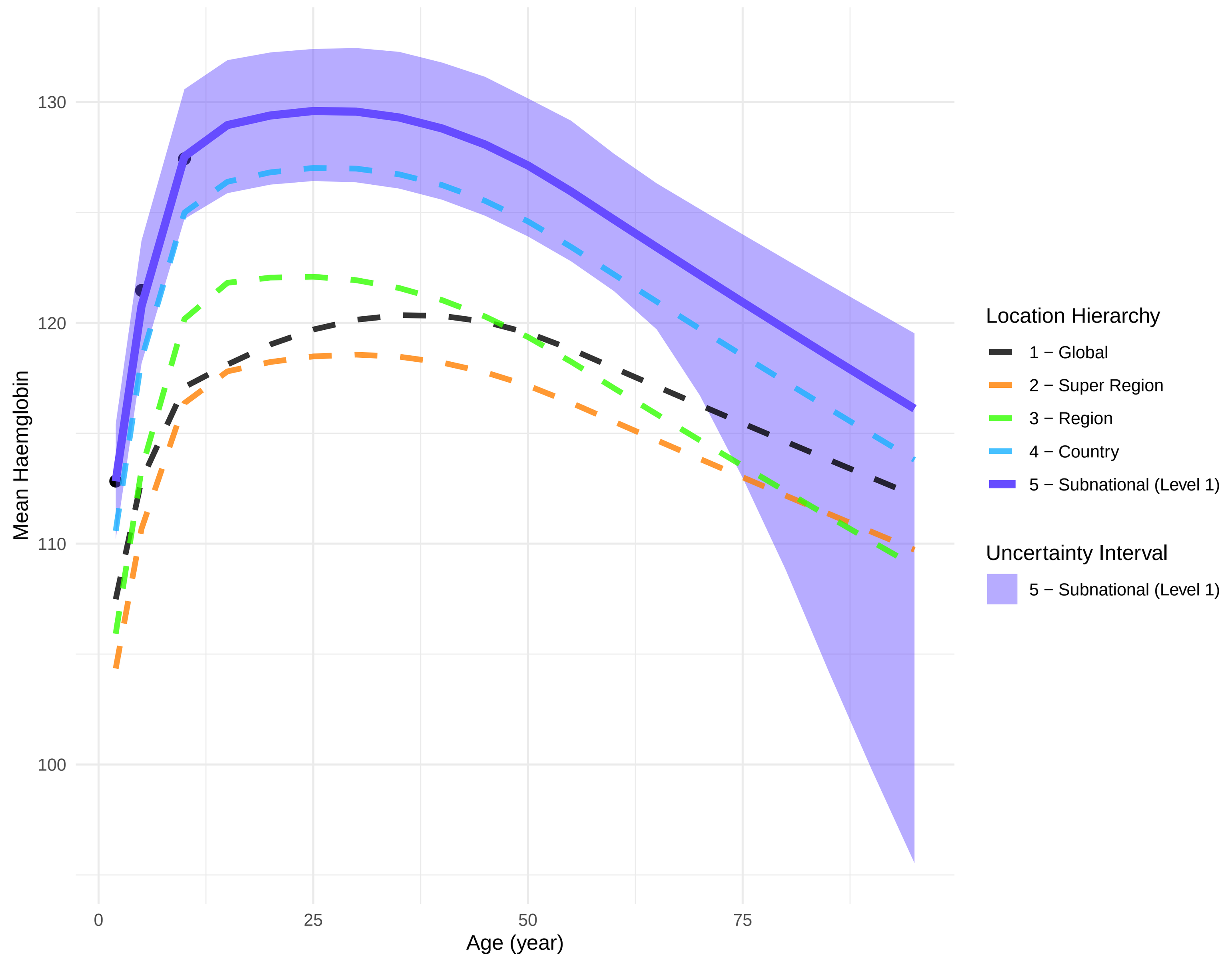

MR−BRT Age/Sex Fits − Non−pregnant Females 2+ Years Old − Mean Haemglobin − Samburu
WHO−2024 Adjustment Method

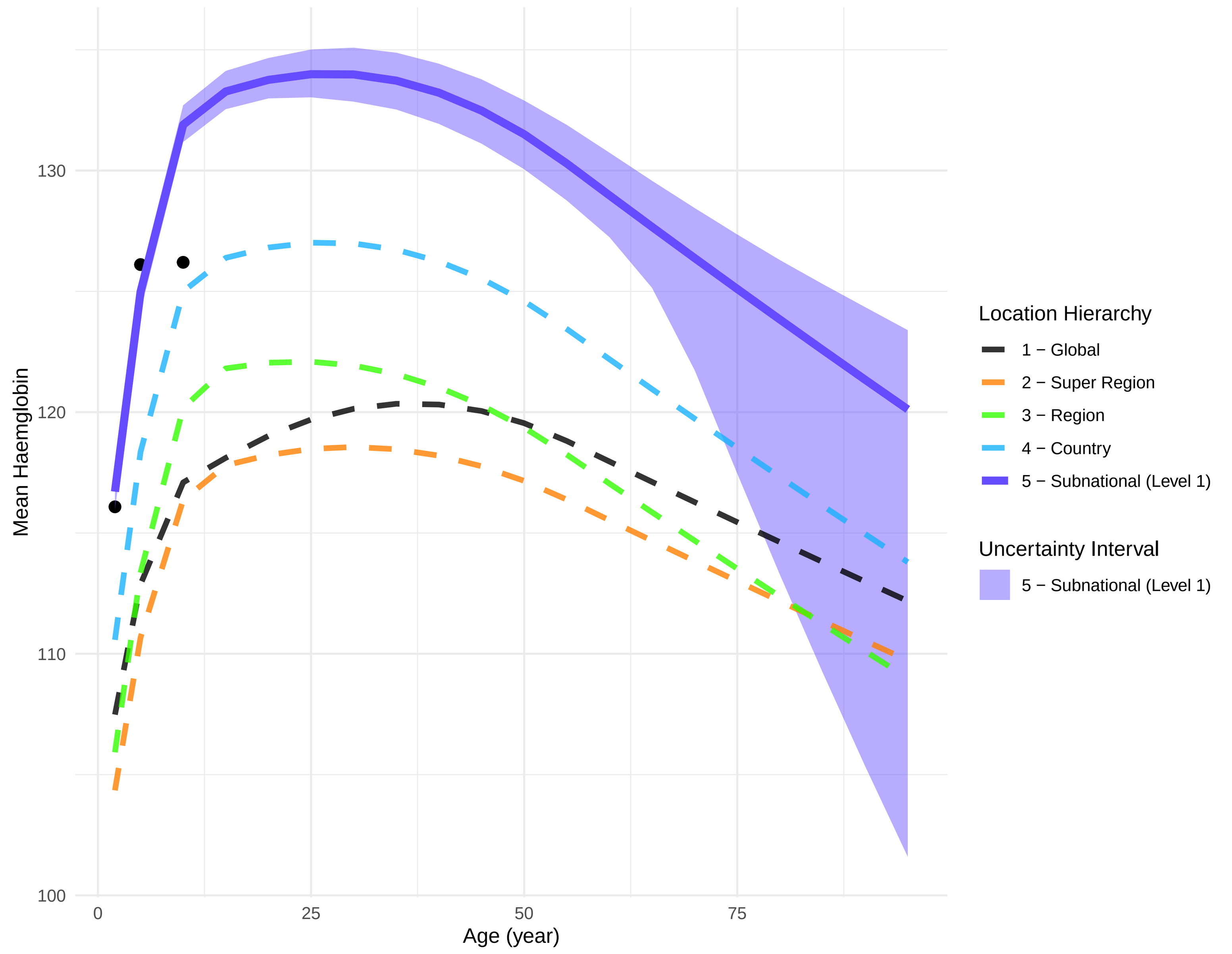

MR−BRT Age/Sex Fits − Non−pregnant Females 2+ Years Old − Mean Haemglobin − Siaya
WHO−2024 Adjustment Method

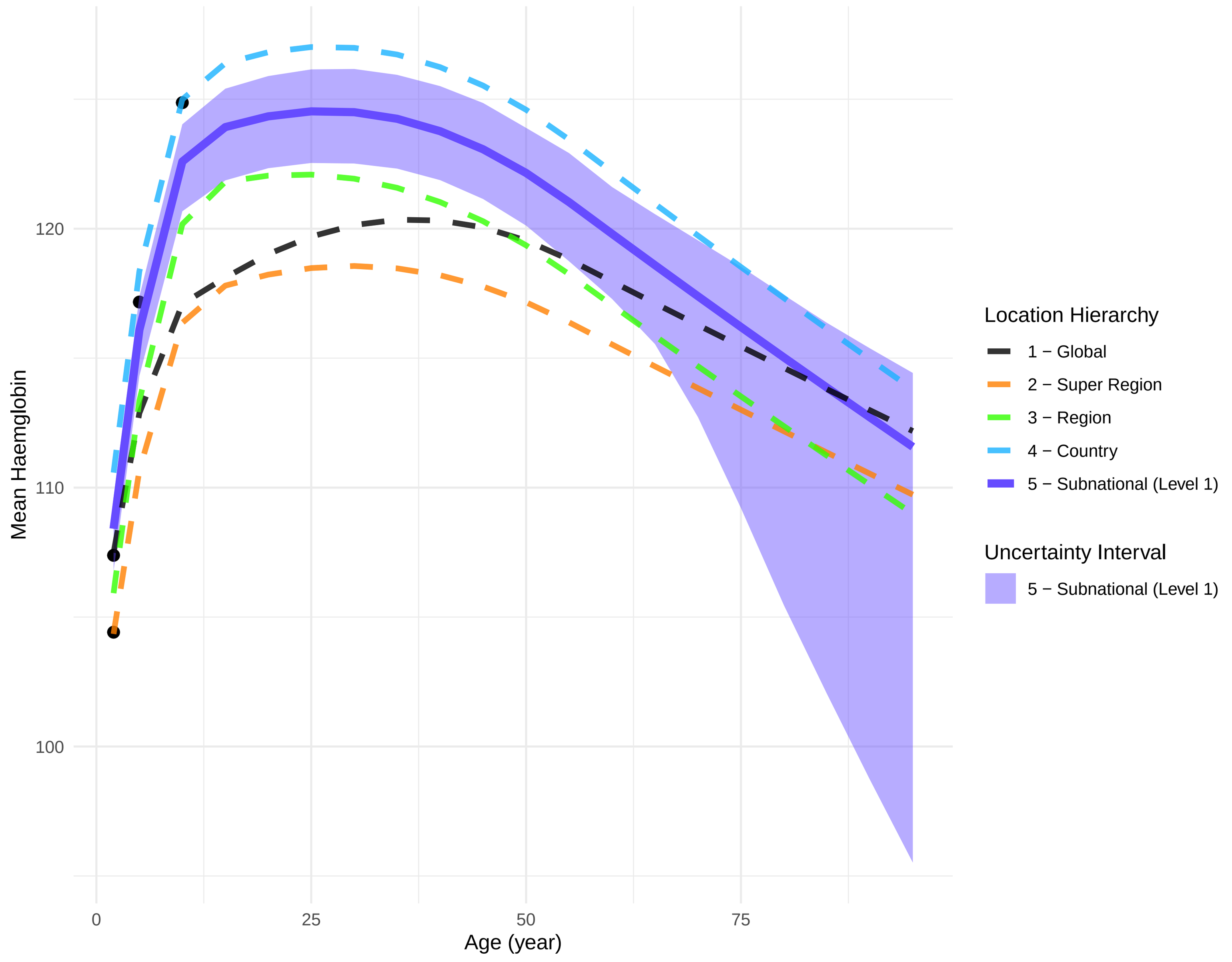

MR−BRT Age/Sex Fits − Non−pregnant Females 2+ Years Old − Mean Haemglobin − Taita Taveta
WHO−2024 Adjustment Method

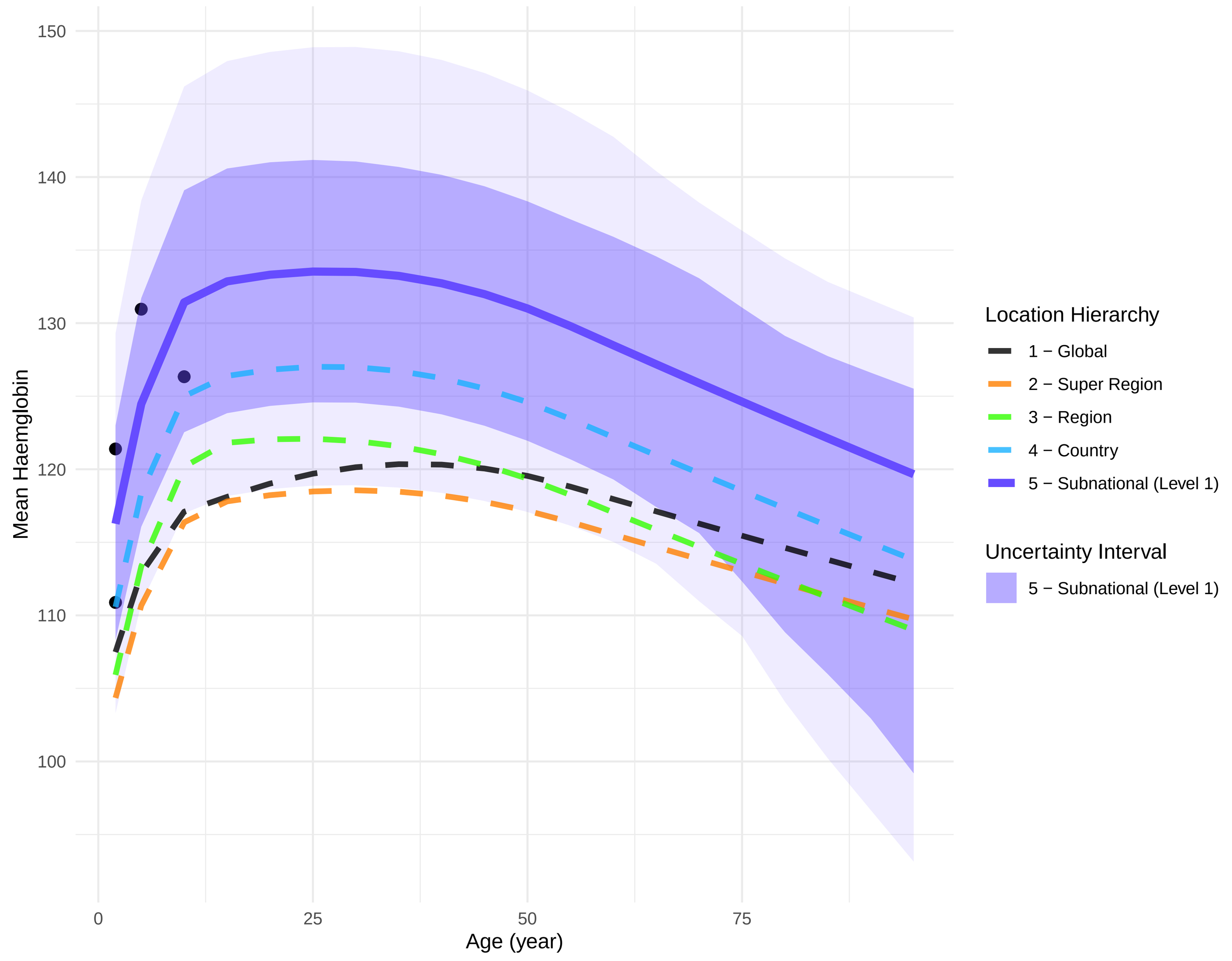

MR−BRT Age/Sex Fits − Non−pregnant Females 2+ Years Old − Mean Haemglobin − Tana River
WHO−2024 Adjustment Method

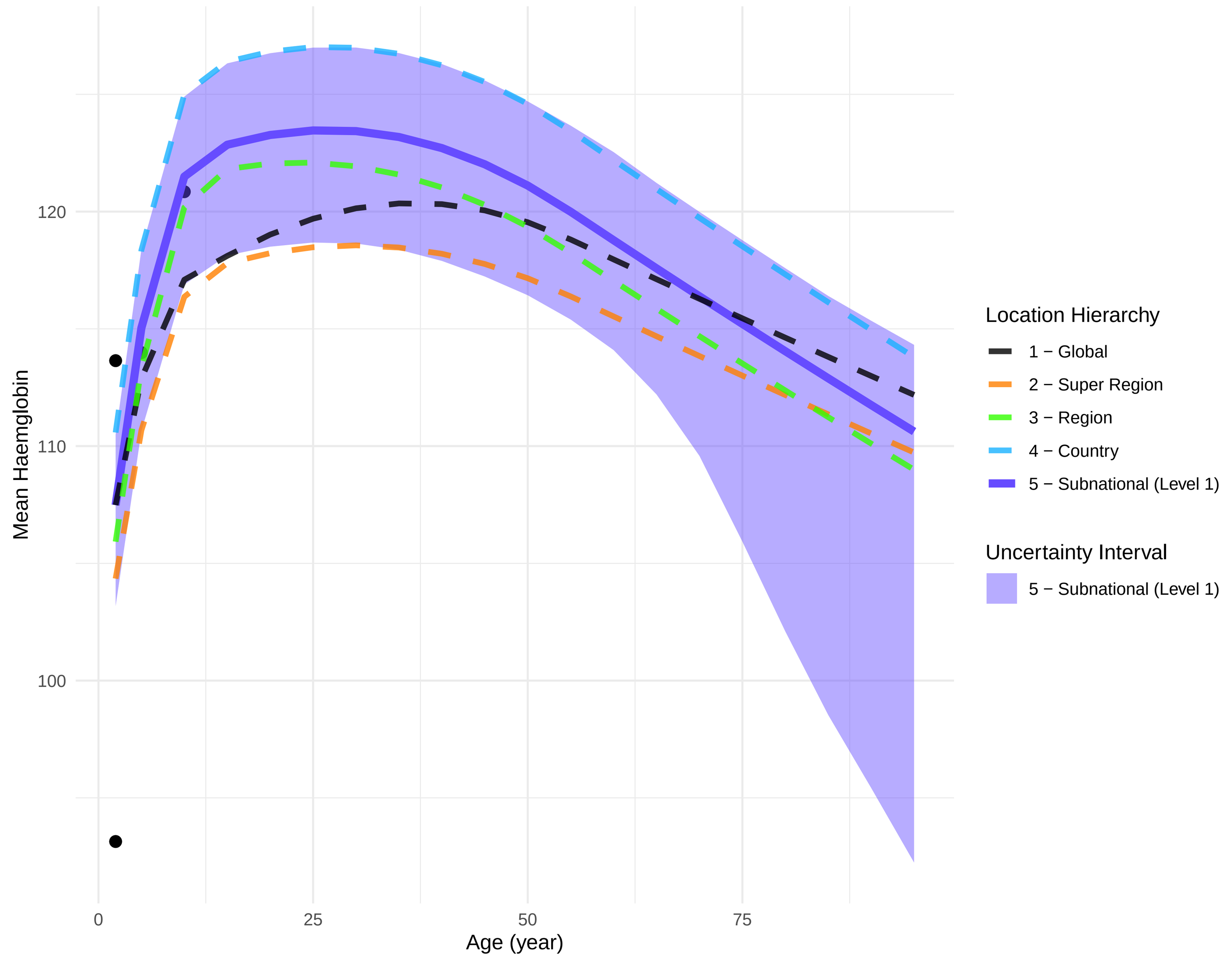

MR−BRT Age/Sex Fits − Non−pregnant Females 2+ Years Old − Mean Haemglobin − Tharaka Nithi
WHO−2024 Adjustment Method

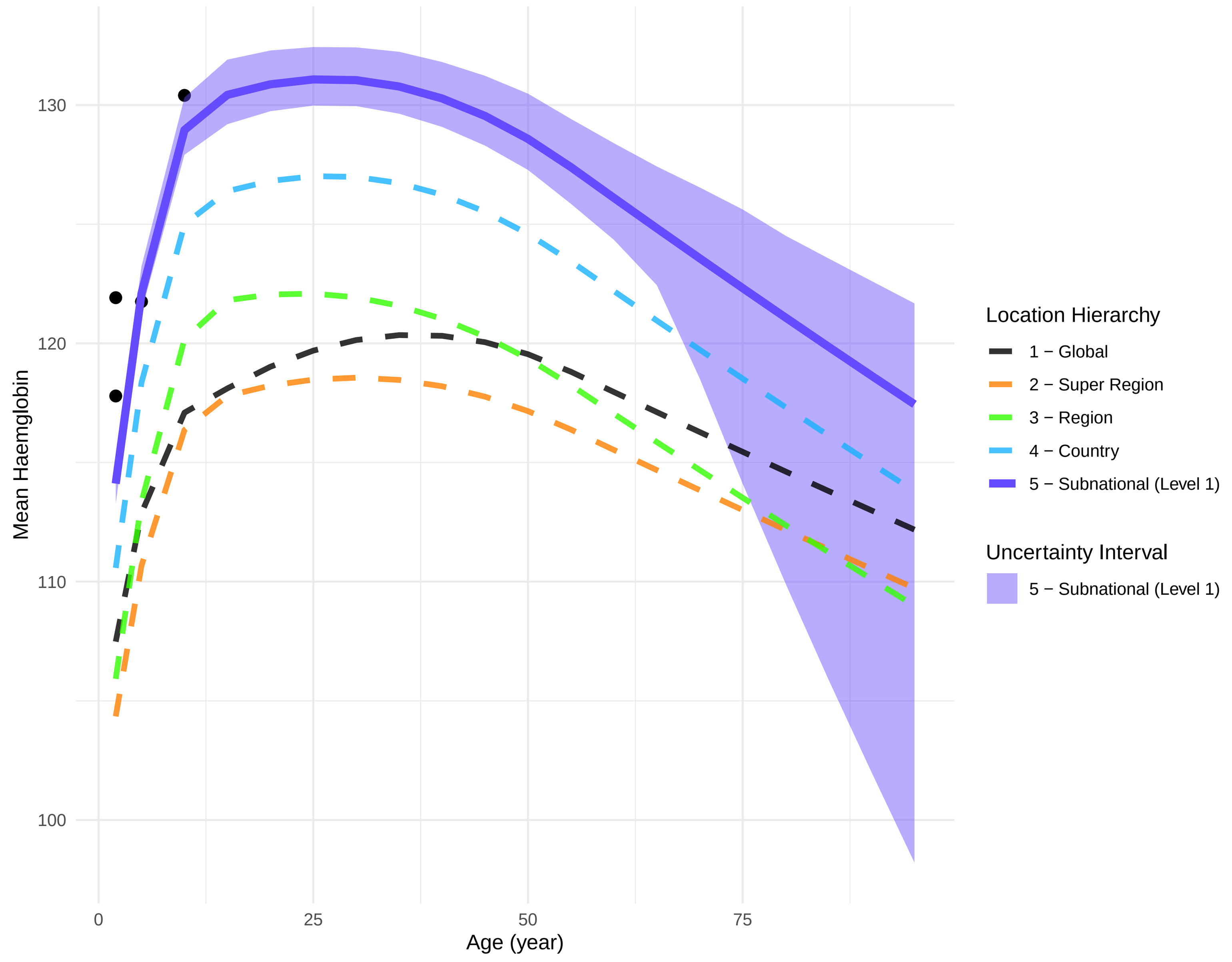

MR−BRT Age/Sex Fits − Non−pregnant Females 2+ Years Old − Mean Haemglobin − Trans Nzoia
WHO−2024 Adjustment Method

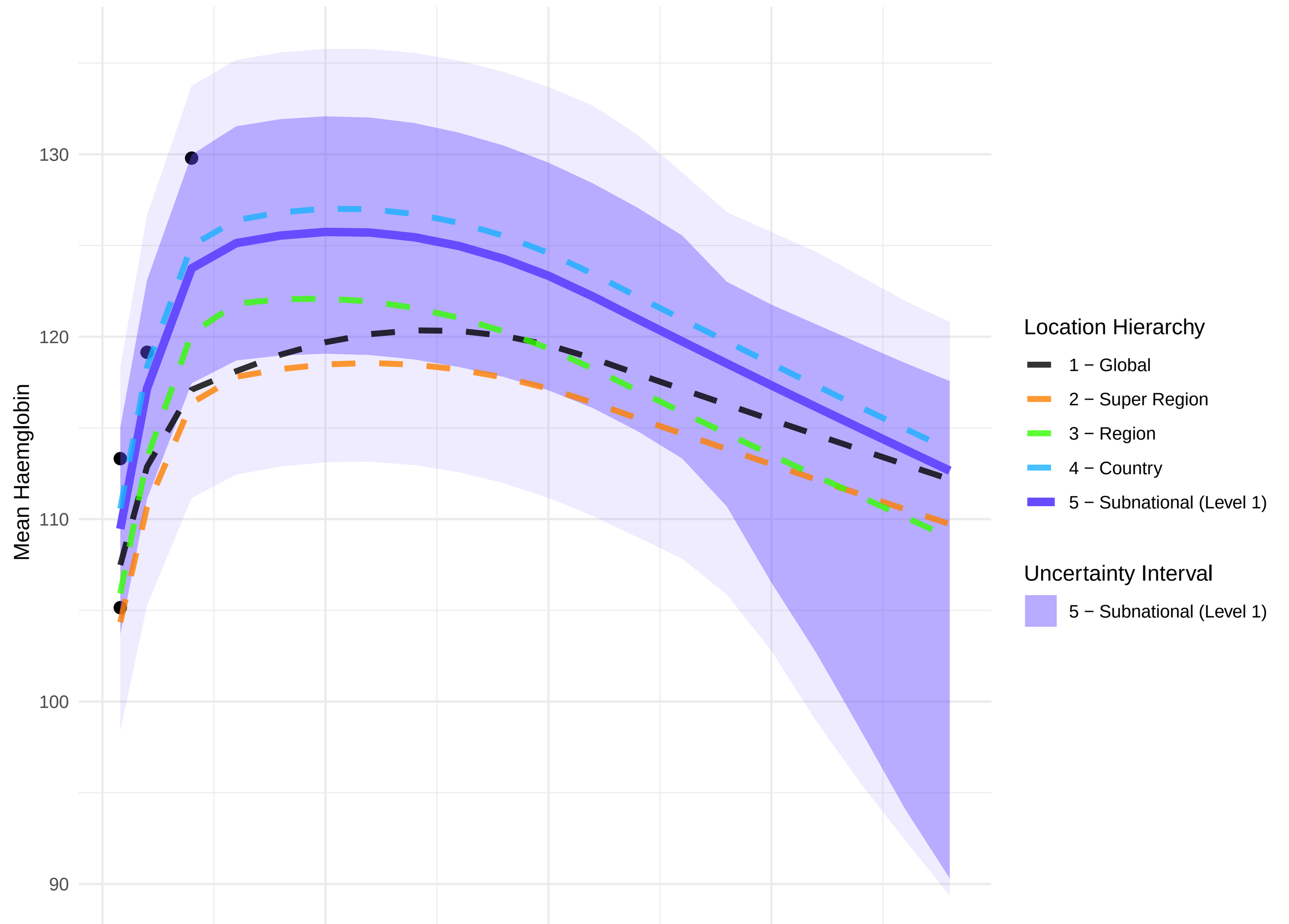

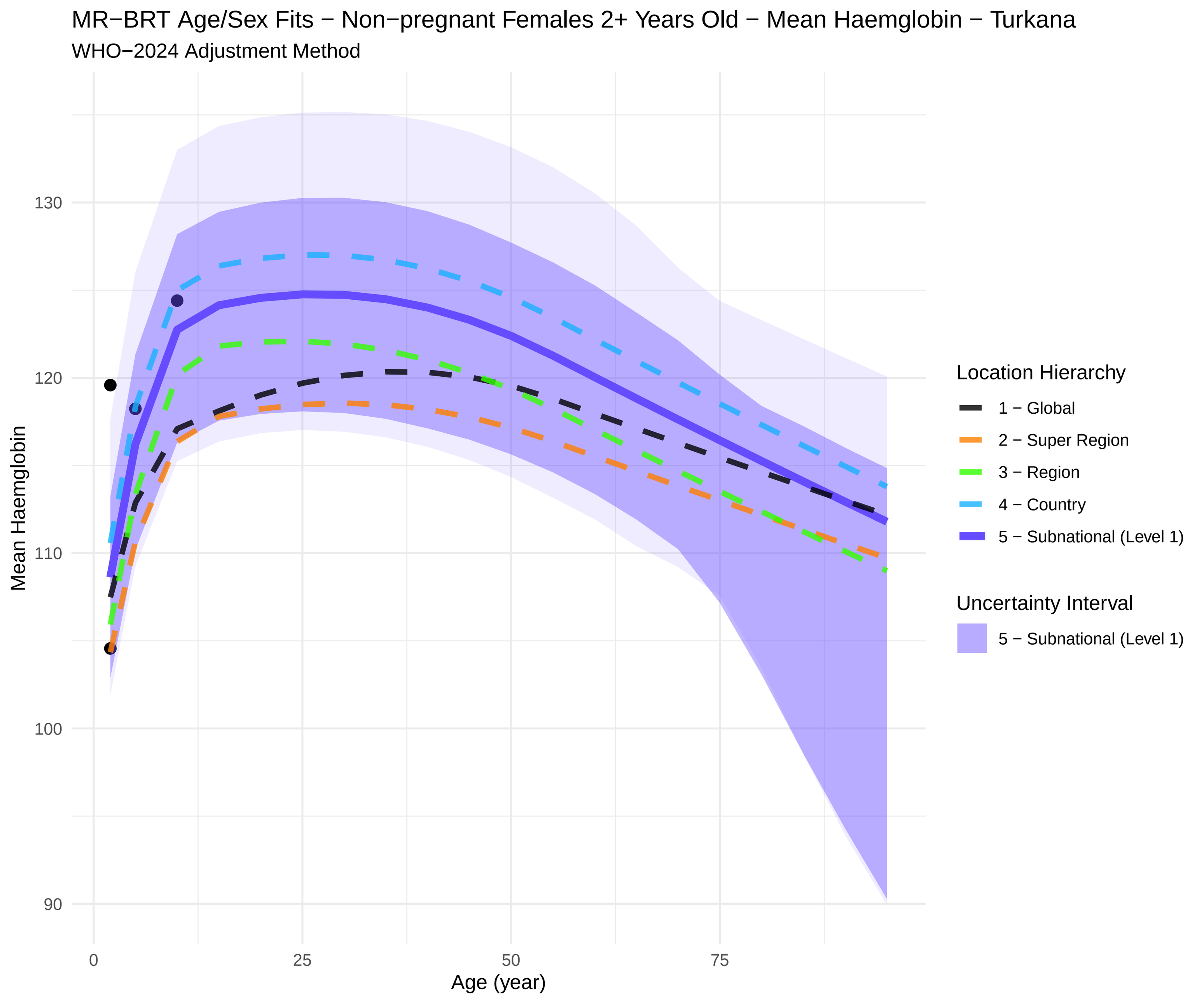
MR−BRT Age/Sex Fits − Non−pregnant Females 2+ Years Old − Mean Haemoglobin − Turkana
WHO−2024 Adjustment Method
Mean Haemoglobin
Age (year)
Location Hierarchy
1 − Global
2 − Super Region
3 − Region
4 − Country
5 − Subnational (Level 1)
Uncertainty Interval
5 − Subnational (Level 1)

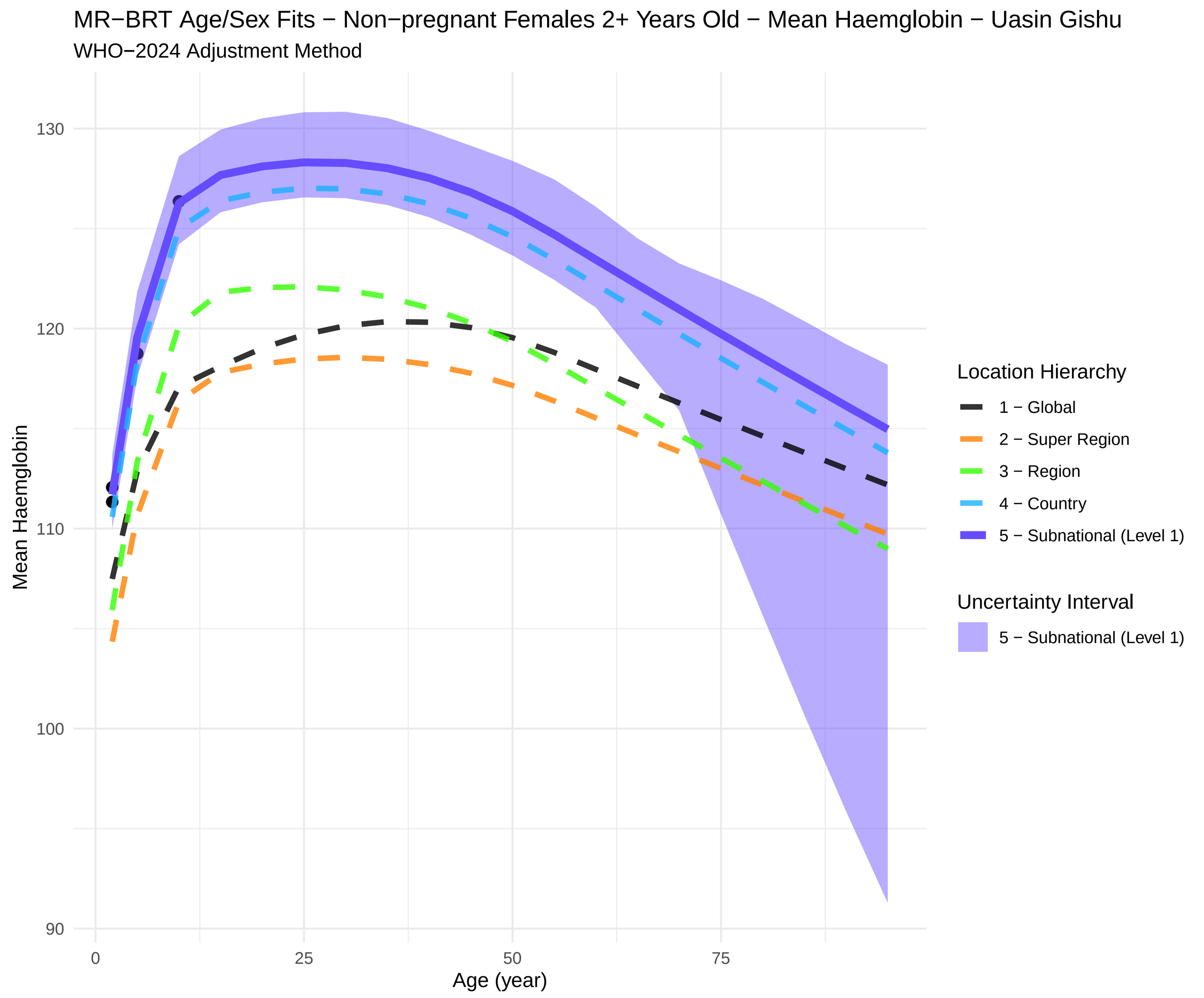
MR−BRT Age/Sex Fits − Non−pregnant Females 2+ Years Old − Mean Haemoglobin − Uasin Gishu
WHO−2024 Adjustment Method
Mean Haemoglobin
Age (year)
Location Hierarchy
1 − Global
2 − Super Region
3 − Region
4 − Country
5 − Subnational (Level 1)
Uncertainty Interval
5 − Subnational (Level 1)

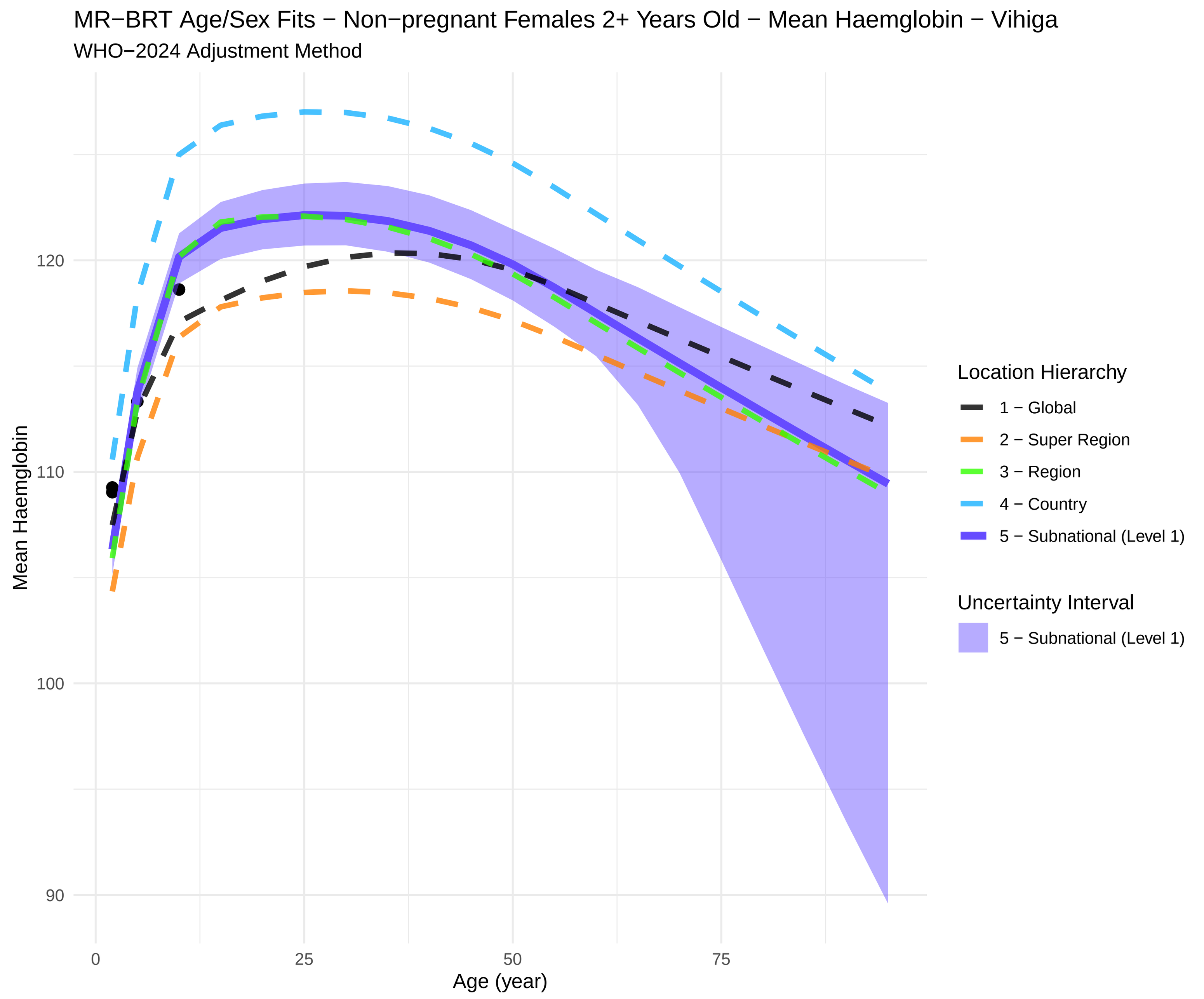
MR−BRT Age/Sex Fits − Non−pregnant Females 2+ Years Old − Mean Haemoglobin − Vihiga
WHO−2024 Adjustment Method
Mean Haemoglobin
Age (year)
Location Hierarchy
1 − Global
2 − Super Region
3 − Region
4 − Country
5 − Subnational (Level 1)
Uncertainty Interval
5 − Subnational (Level 1)

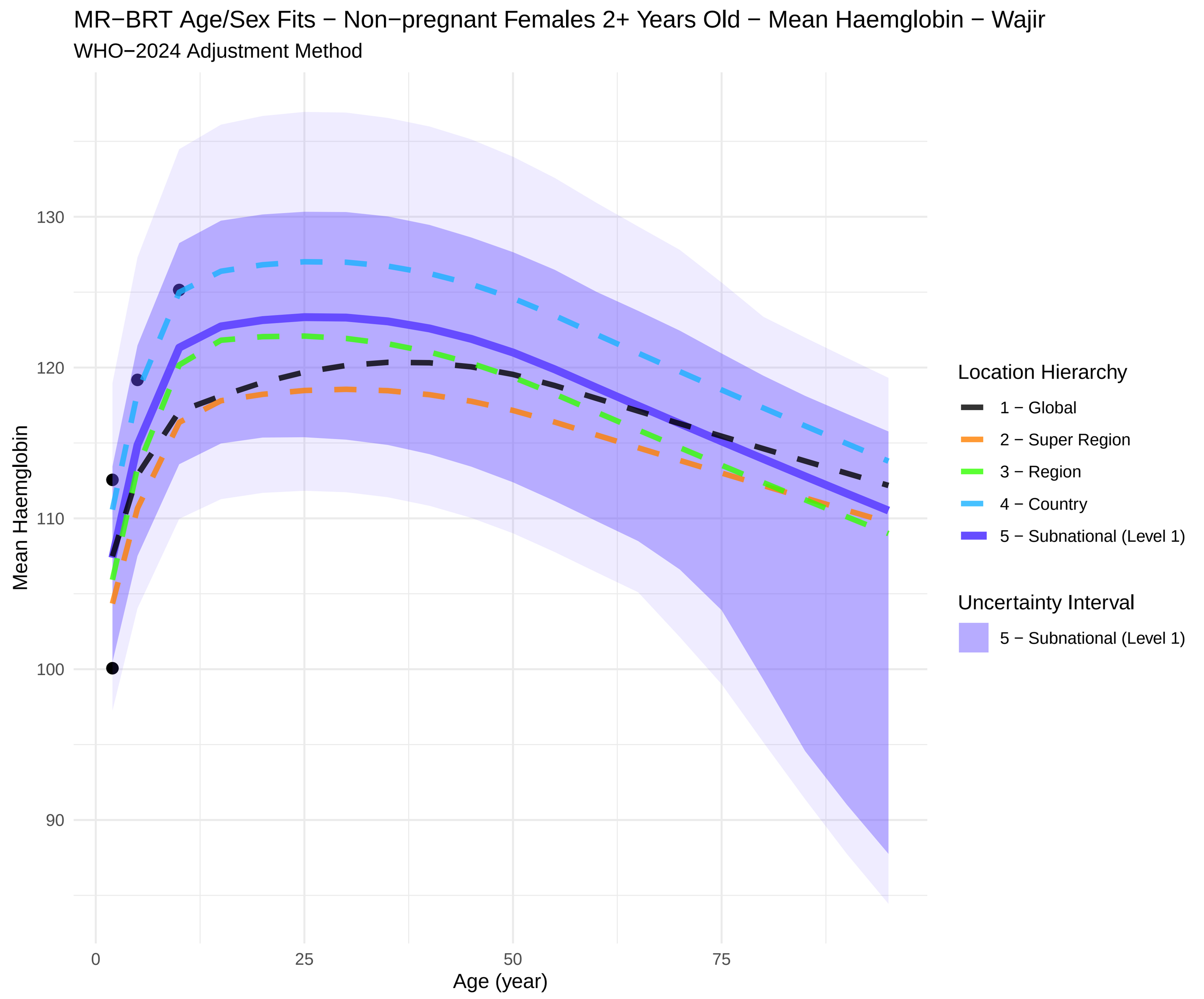
MR−BRT Age/Sex Fits − Non−pregnant Females 2+ Years Old − Mean Haemoglobin − Wajir
WHO−2024 Adjustment Method
Mean Haemoglobin
Age (year)
Location Hierarchy
1 − Global
2 − Super Region
3 − Region
4 − Country
5 − Subnational (Level 1)
Uncertainty Interval
5 − Subnational (Level 1)

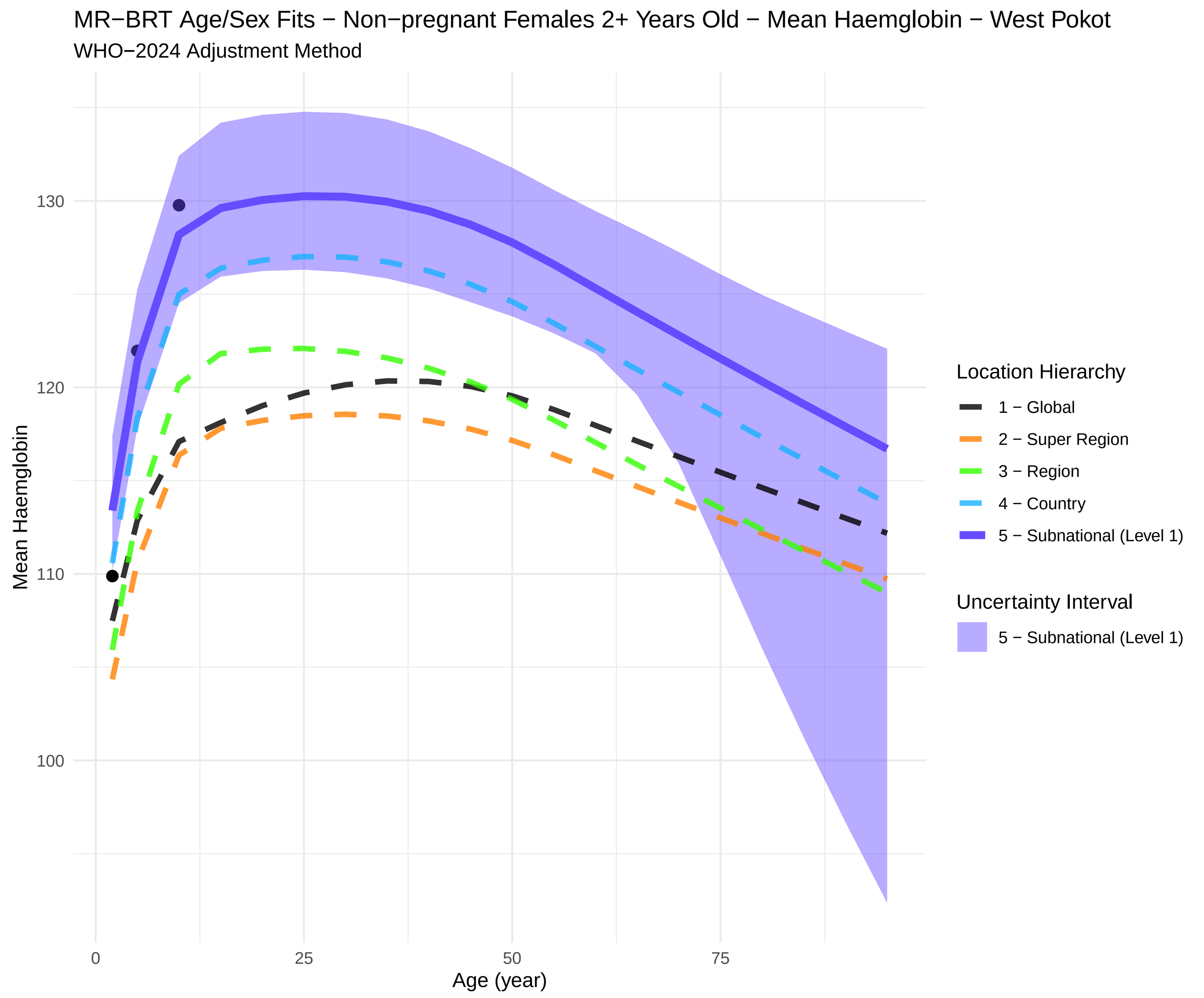
MR−BRT Age/Sex Fits − Non−pregnant Females 2+ Years Old − Mean Haemoglobin − West Pokot
WHO−2024 Adjustment Method
Mean Haemoglobin
Age (year)
Location Hierarchy
1 − Global
2 − Super Region
3 − Region
4 − Country
5 − Subnational (Level 1)
Uncertainty Interval
5 − Subnational (Level 1)

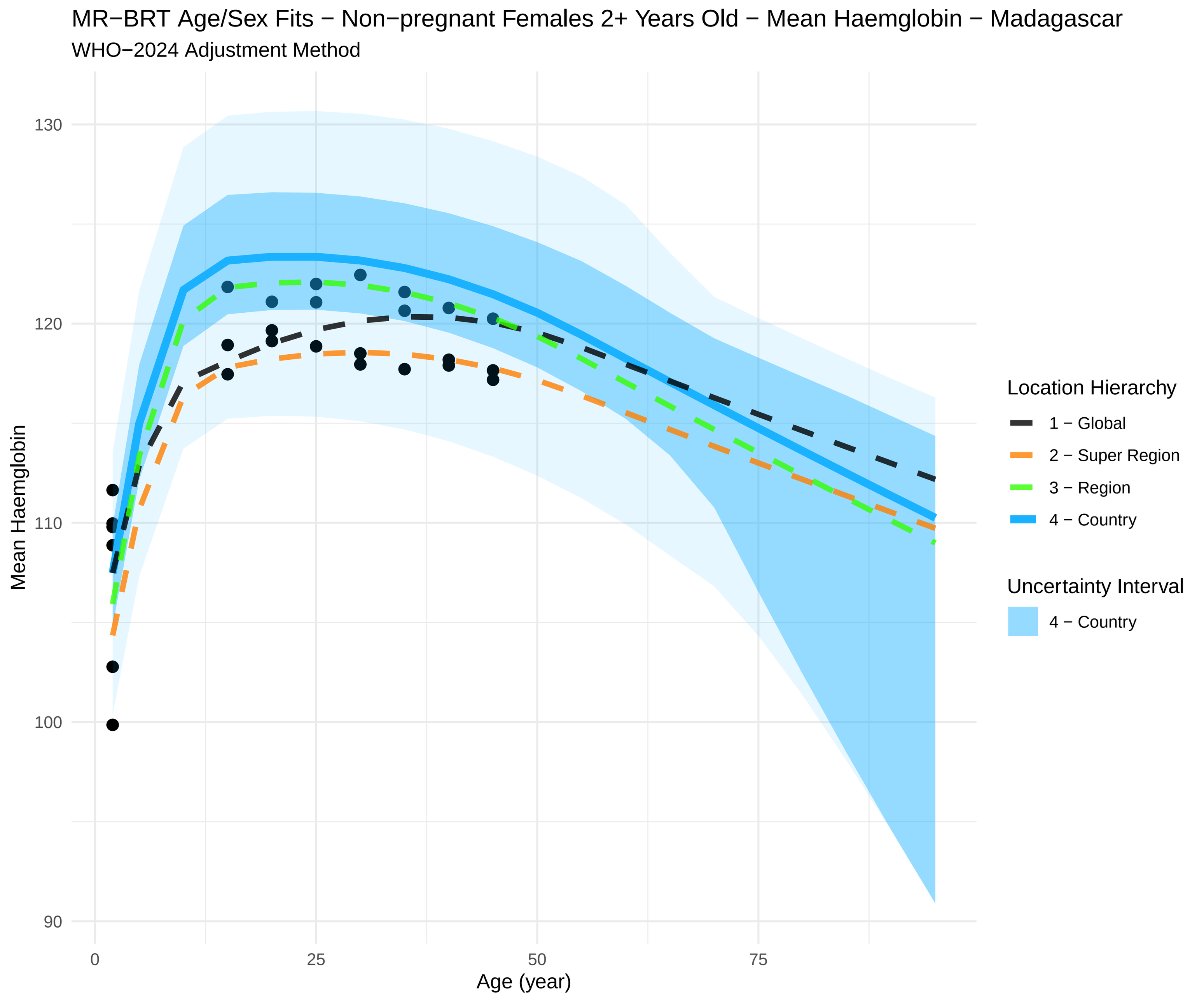
MR−BRT Age/Sex Fits − Non−pregnant Females 2+ Years Old − Mean Haemoglobin − Madagascar
WHO−2024 Adjustment Method
Mean Haemoglobin
Age (year)
Location Hierarchy
1 − Global
2 − Super Region
3 − Region
4 − Country
Uncertainty Interval
4 − Country

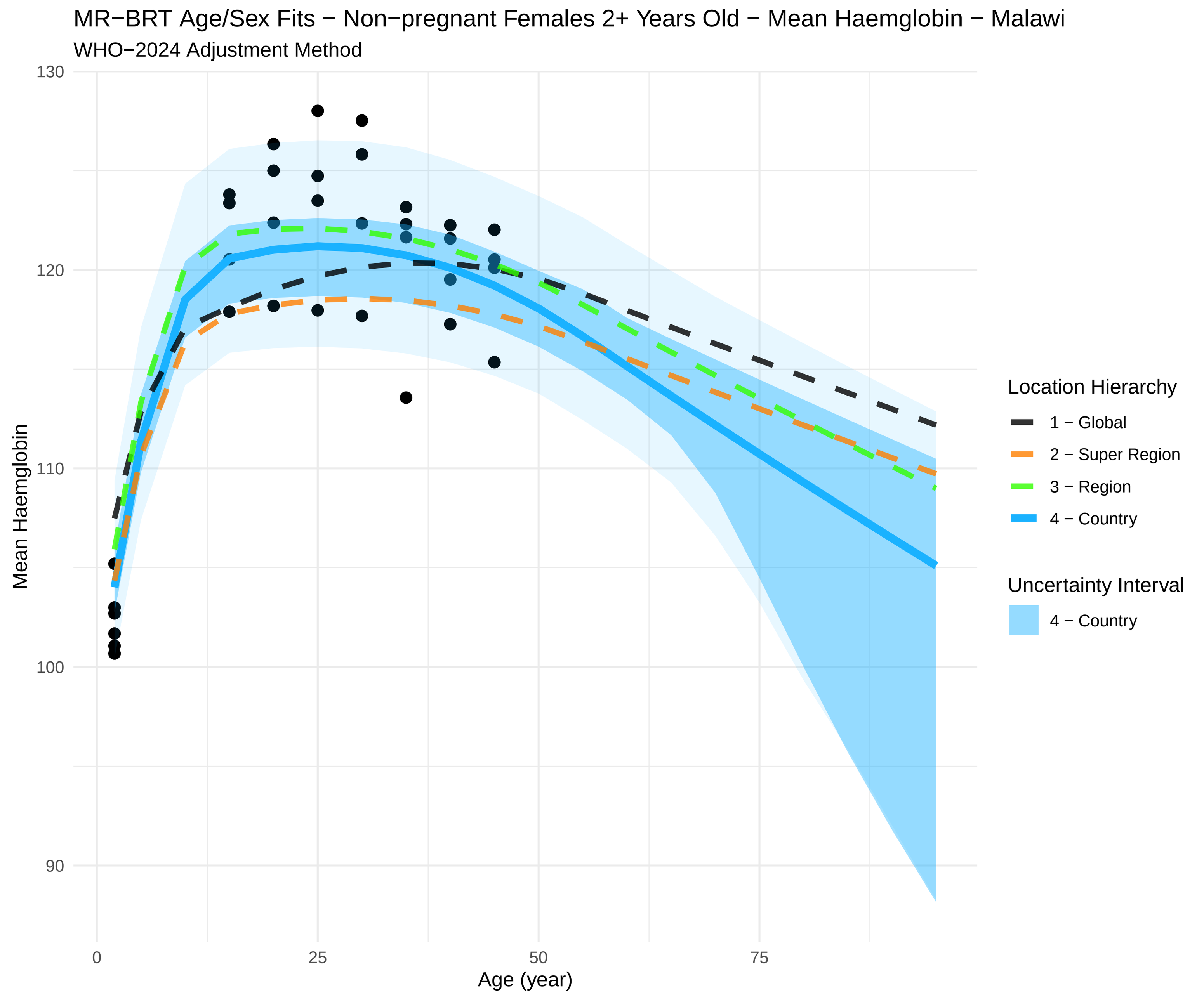
MR−BRT Age/Sex Fits − Non−pregnant Females 2+ Years Old − Mean Haemoglobin − Malawi
WHO−2024 Adjustment Method
Mean Haemoglobin
Age (year)
Location Hierarchy
1 − Global
2 − Super Region
3 − Region
4 − Country
Uncertainty Interval
4 − Country

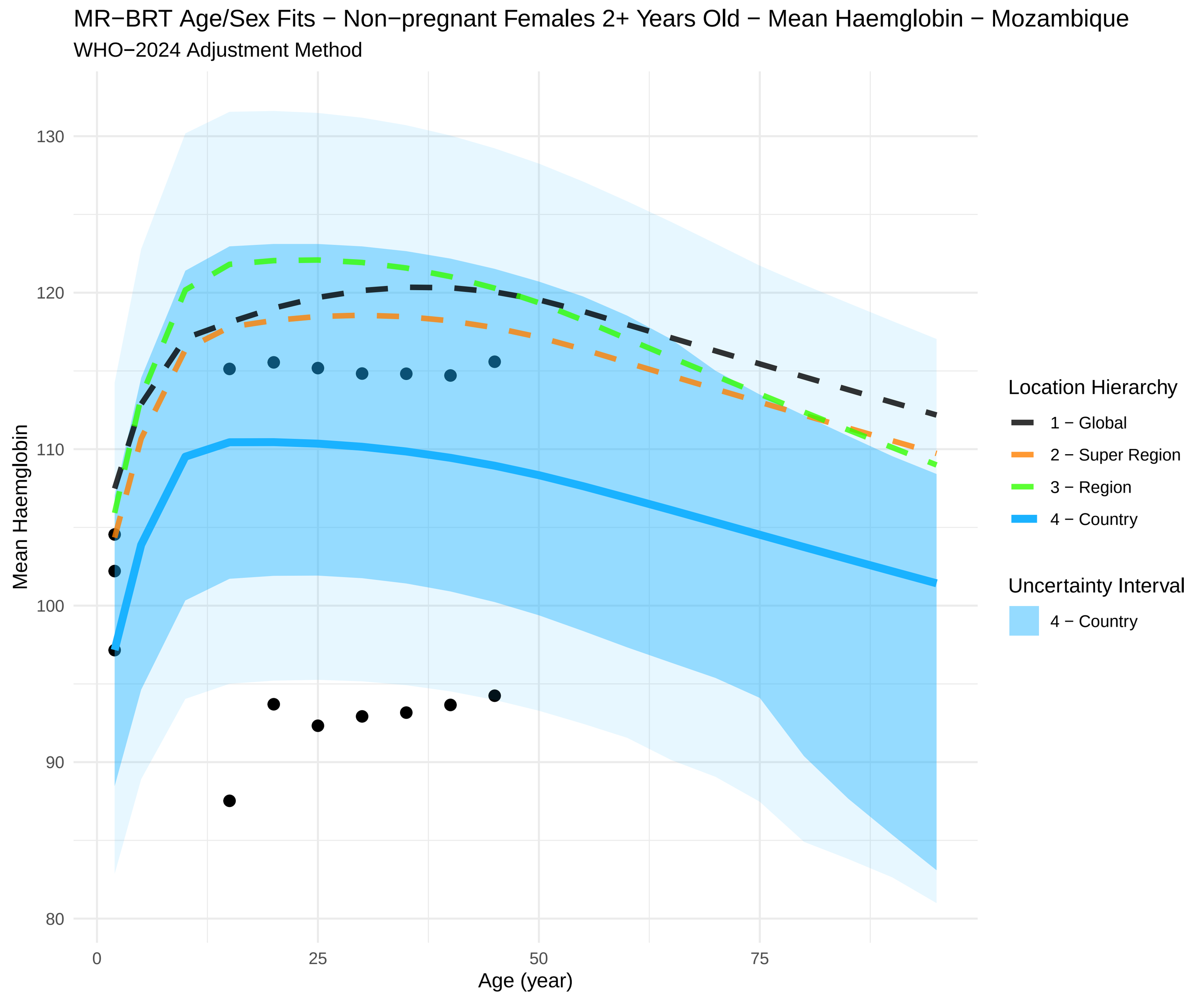
MR−BRT Age/Sex Fits − Non−pregnant Females 2+ Years Old − Mean Haemoglobin − Mozambique
WHO−2024 Adjustment Method
Mean Haemoglobin
Age (year)
Location Hierarchy
1 − Global
2 − Super Region
3 − Region
4 − Country
Uncertainty Interval
4 − Country

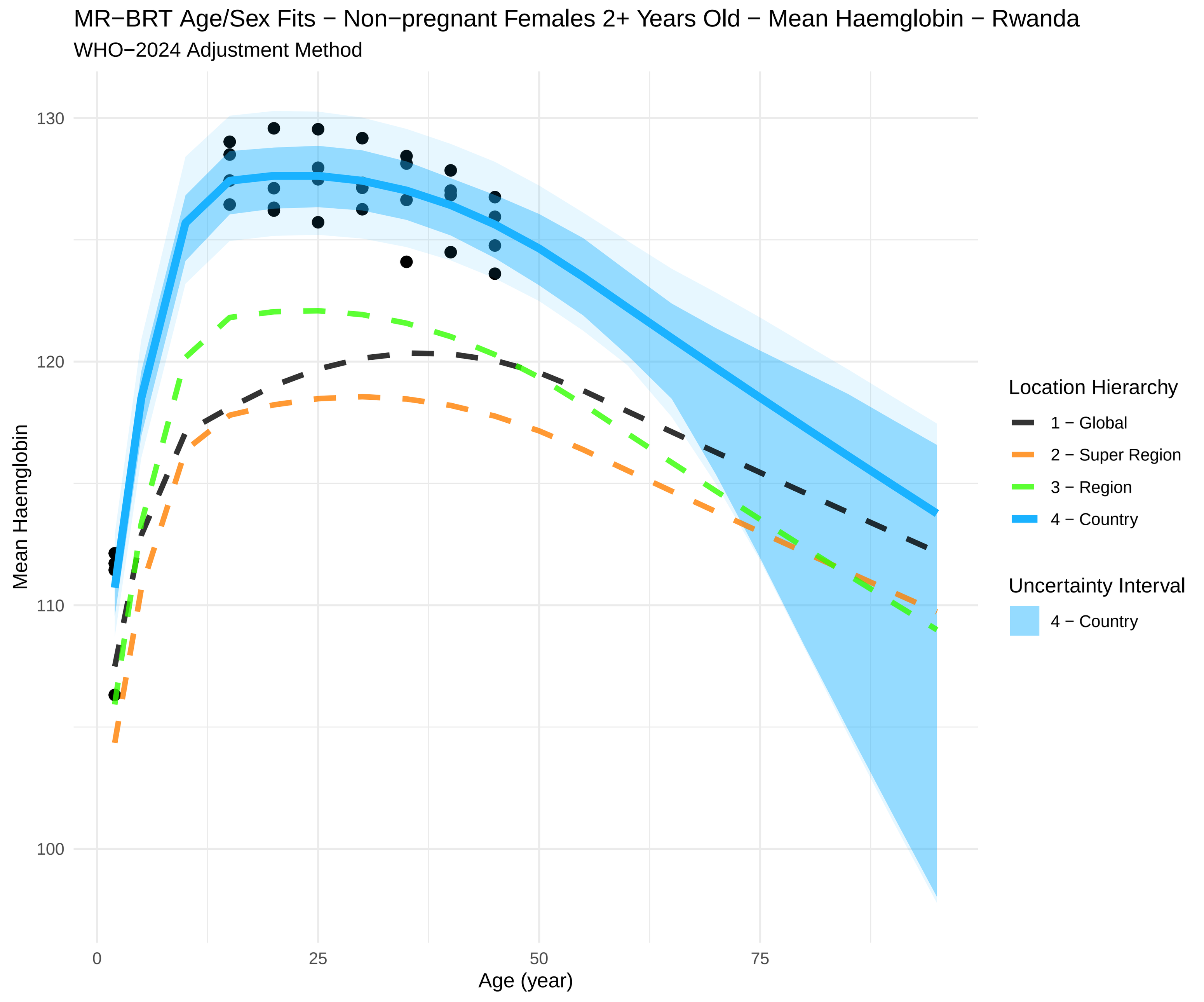
MR−BRT Age/Sex Fits − Non−pregnant Females 2+ Years Old − Mean Haemoglobin − Rwanda
WHO−2024 Adjustment Method
Mean Haemoglobin
Age (year)
Location Hierarchy
1 − Global
2 − Super Region
3 − Region
4 − Country
Uncertainty Interval
4 − Country

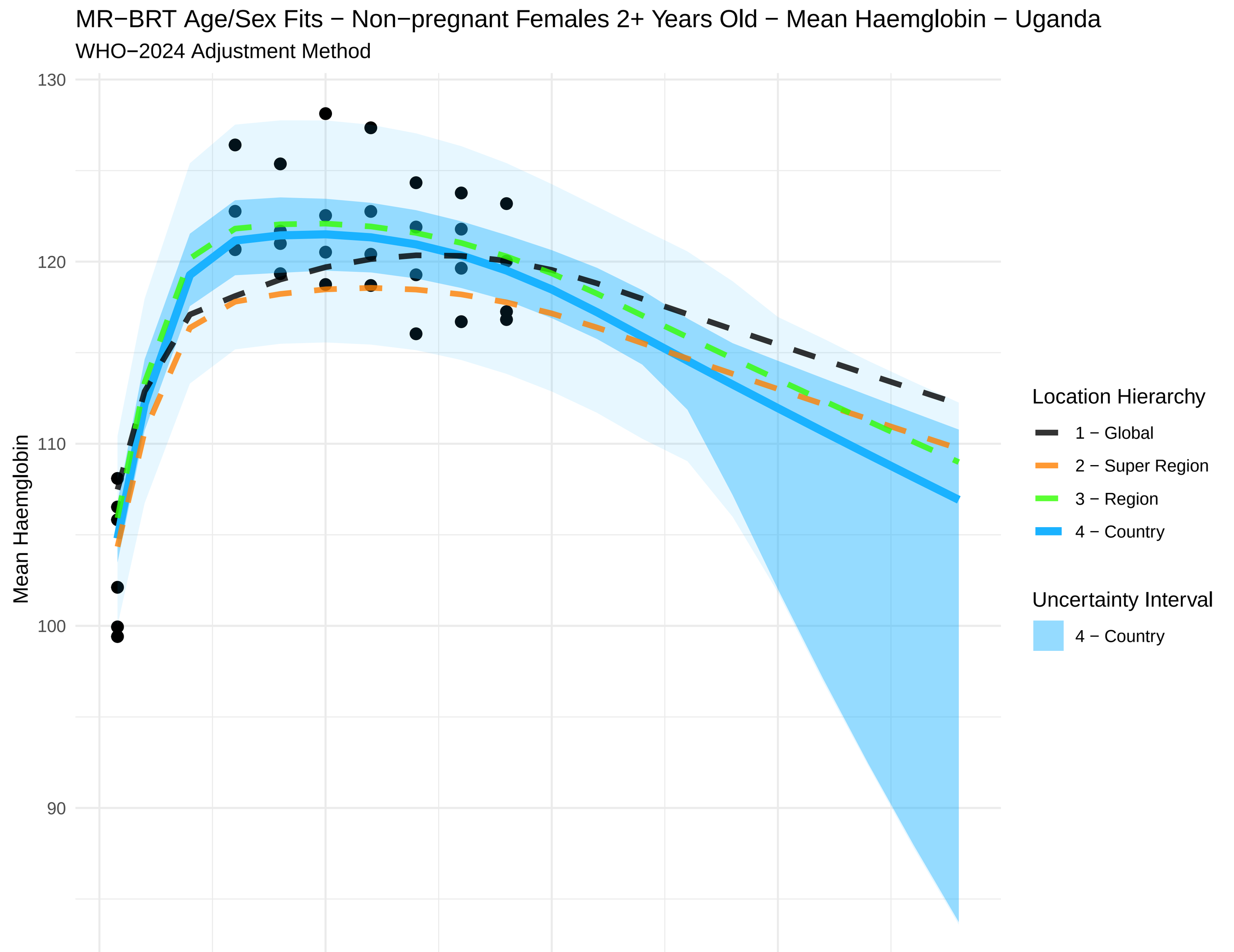
MR−BRT Age/Sex Fits − Non−pregnant Females 2+ Years Old − Mean Haemoglobin − Uganda
WHO−2024 Adjustment Method
Mean Haemoglobin
Age (year)
Location Hierarchy
1 − Global
2 − Super Region
3 − Region
4 − Country
Uncertainty Interval
4 − Country

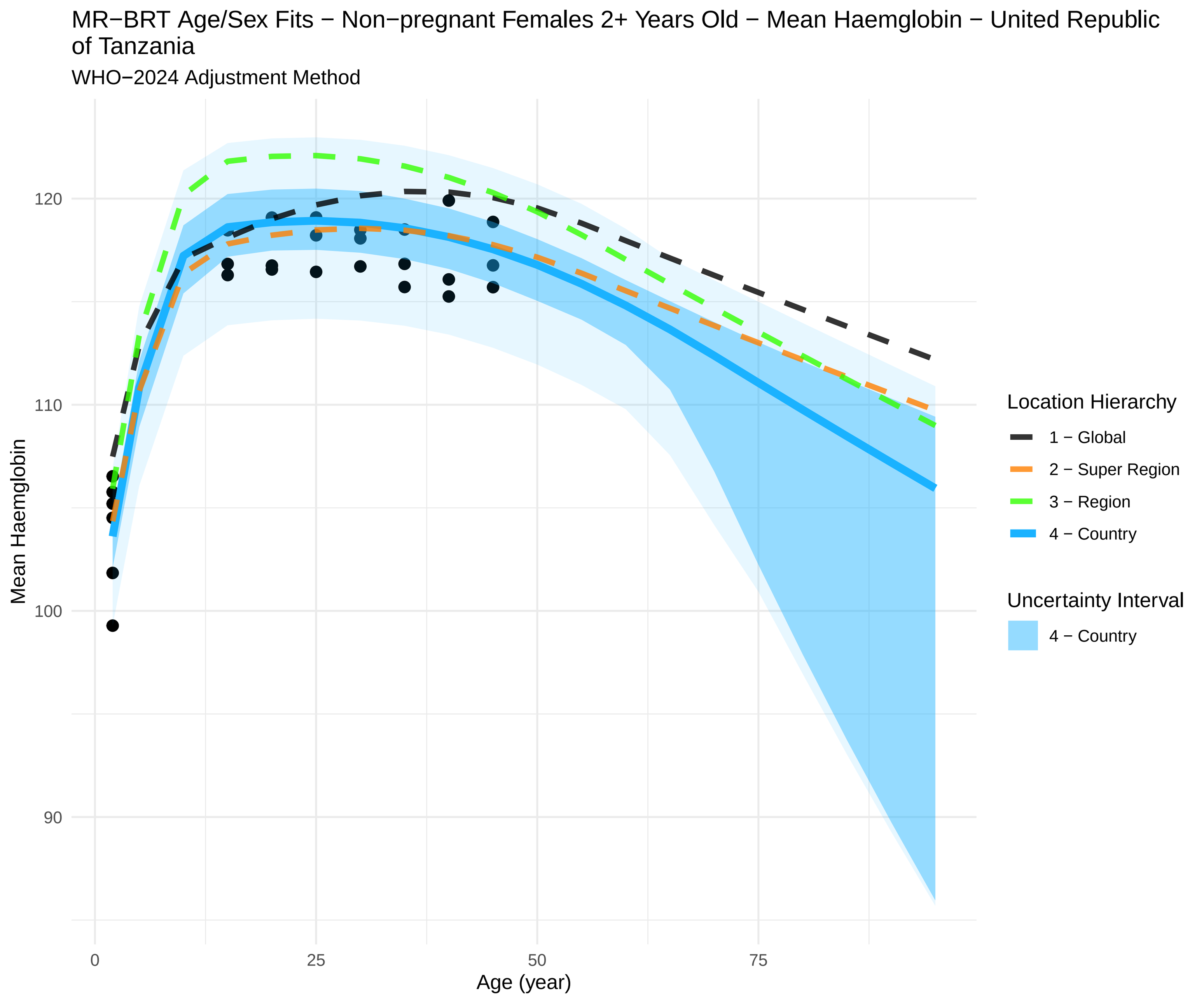
MR−BRT Age/Sex Fits − Non−pregnant Females 2+ Years Old − Mean Haemglobin − United Republic of Tanzania
WHO−2024 Adjustment Method
Mean Haemglobin
Age (year)
Location Hierarchy
1 − Global
2 − Super Region
3 − Region
4 − Country
Uncertainty Interval
4 − Country

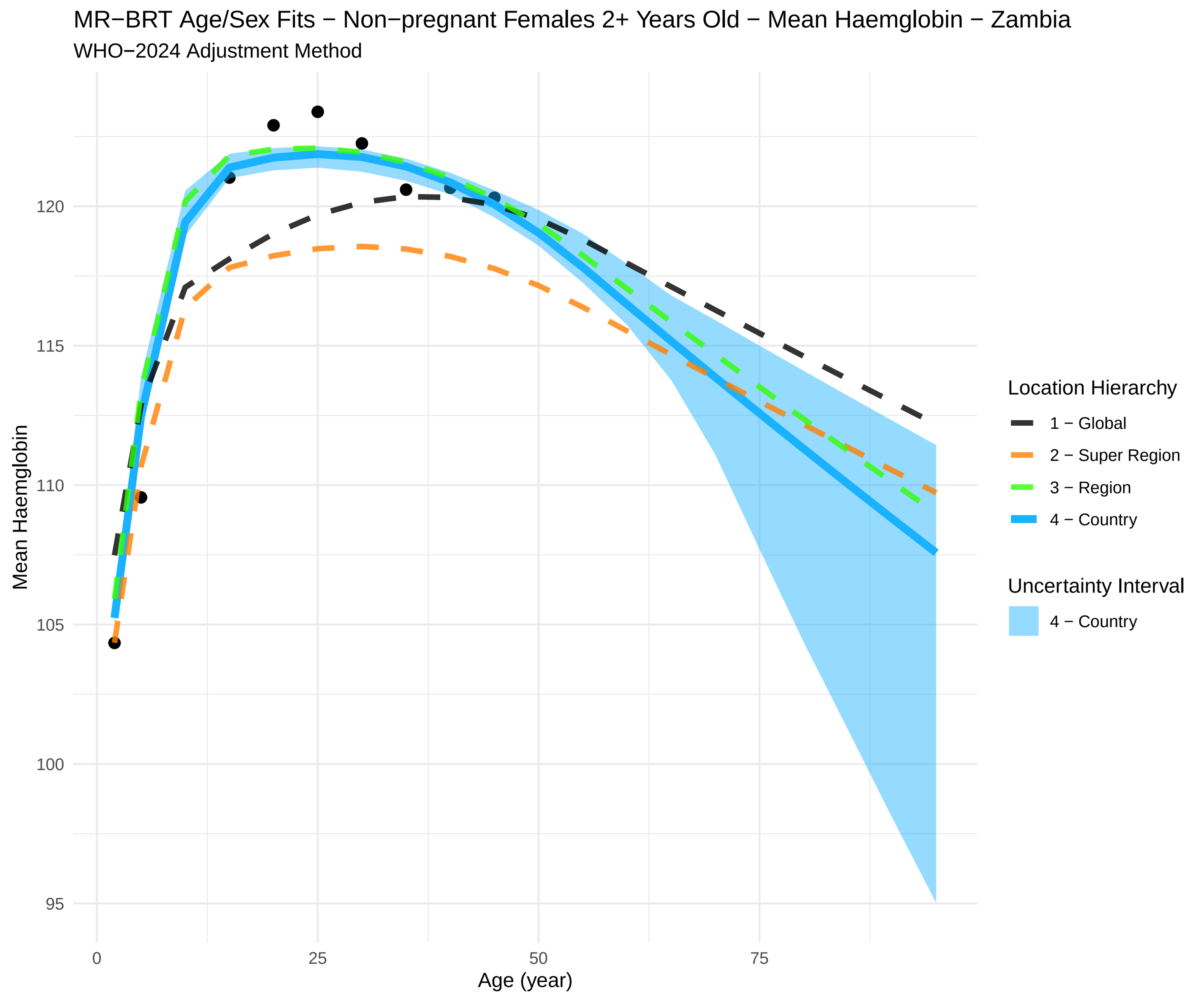
MR−BRT Age/Sex Fits − Non−pregnant Females 2+ Years Old − Mean Haemglobin − Zambia
WHO−2024 Adjustment Method
Mean Haemglobin
Age (year)
Location Hierarchy
1 − Global
2 − Super Region
3 − Region
4 − Country
Uncertainty Interval
4 − Country

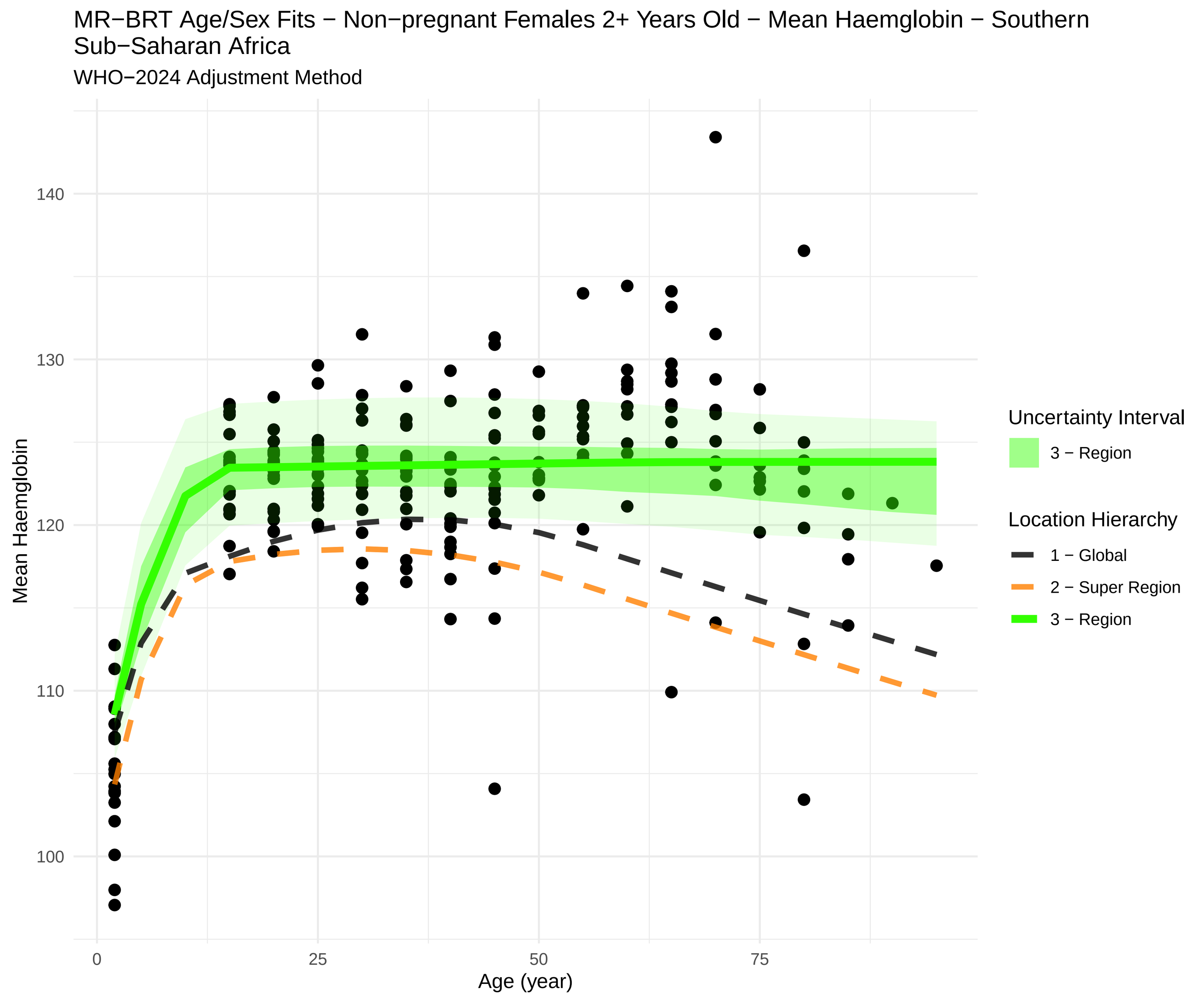
MR−BRT Age/Sex Fits − Non−pregnant Females 2+ Years Old − Mean Haemglobin − Southern Sub−Saharan Africa
WHO−2024 Adjustment Method
Mean Haemglobin
Age (year)
Uncertainty Interval
3 − Region
Location Hierarchy
1 − Global
2 − Super Region
3 − Region

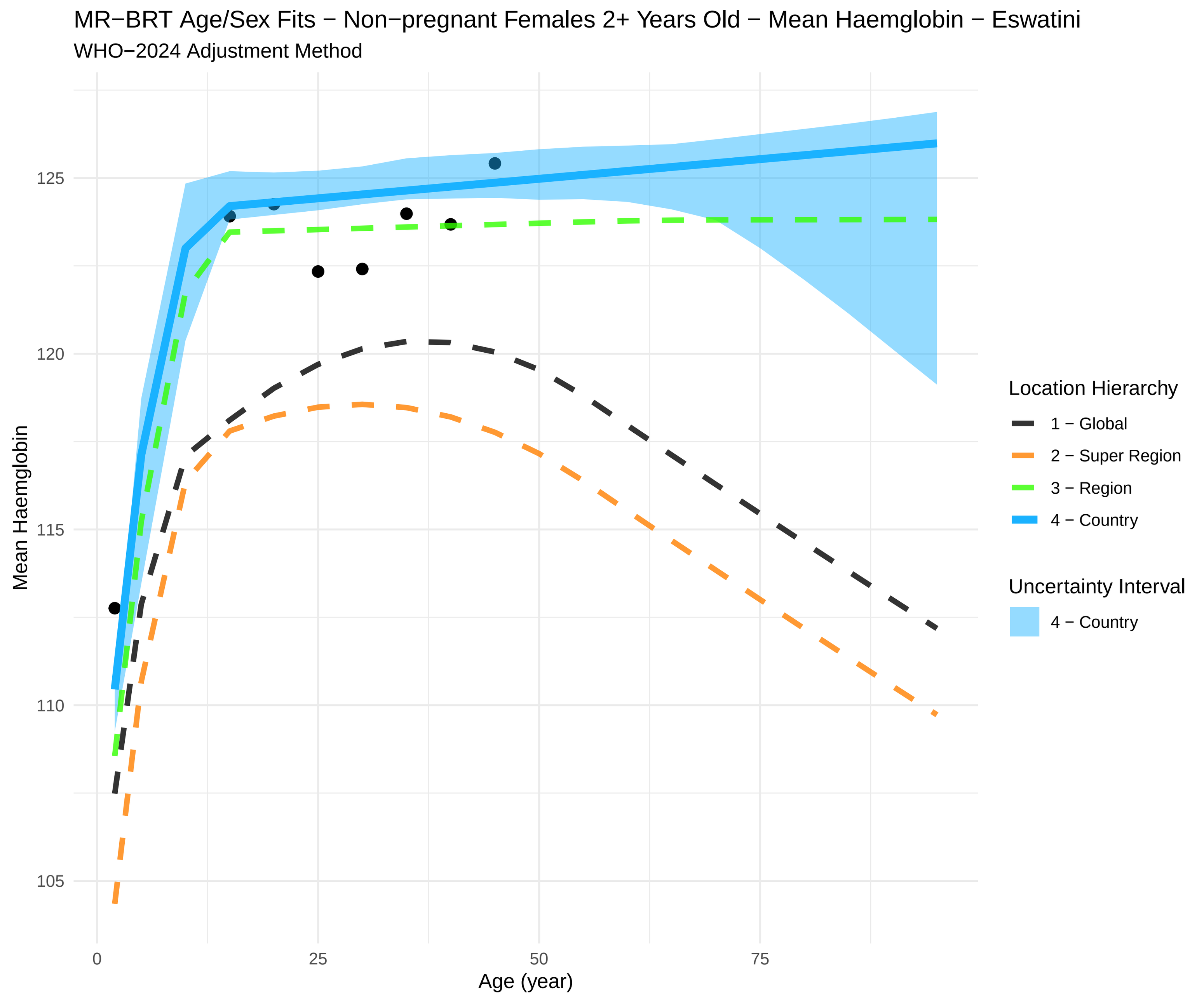
MR−BRT Age/Sex Fits − Non−pregnant Females 2+ Years Old − Mean Haemglobin − Eswatini
WHO−2024 Adjustment Method
Mean Haemglobin
Age (year)
Location Hierarchy
1 − Global
2 − Super Region
3 − Region
4 − Country
Uncertainty Interval
4 − Country

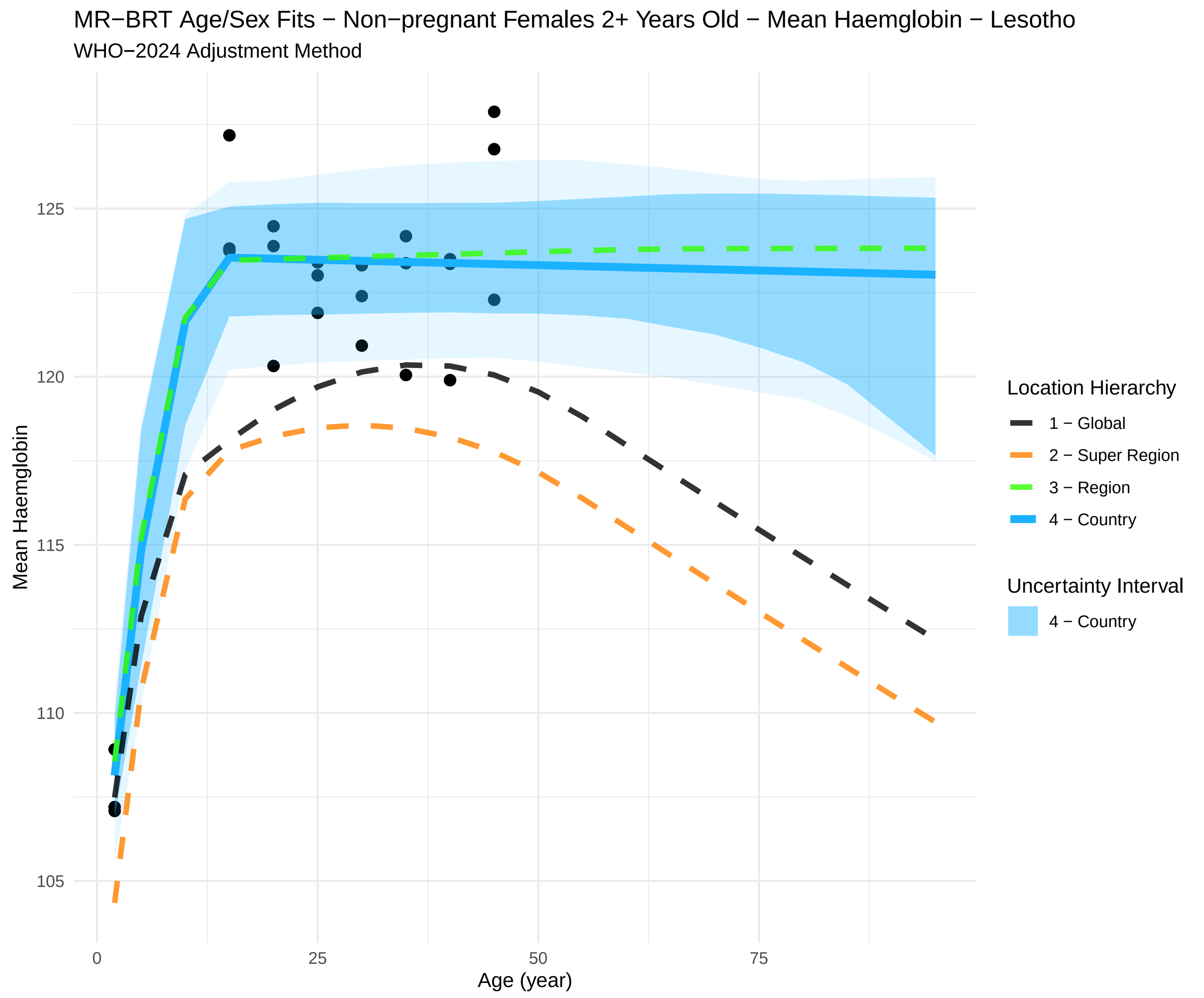
MR−BRT Age/Sex Fits − Non−pregnant Females 2+ Years Old − Mean Haemglobin − Lesotho
WHO−2024 Adjustment Method
Mean Haemglobin
Age (year)
Location Hierarchy
1 − Global
2 − Super Region
3 − Region
4 − Country
Uncertainty Interval
4 − Country

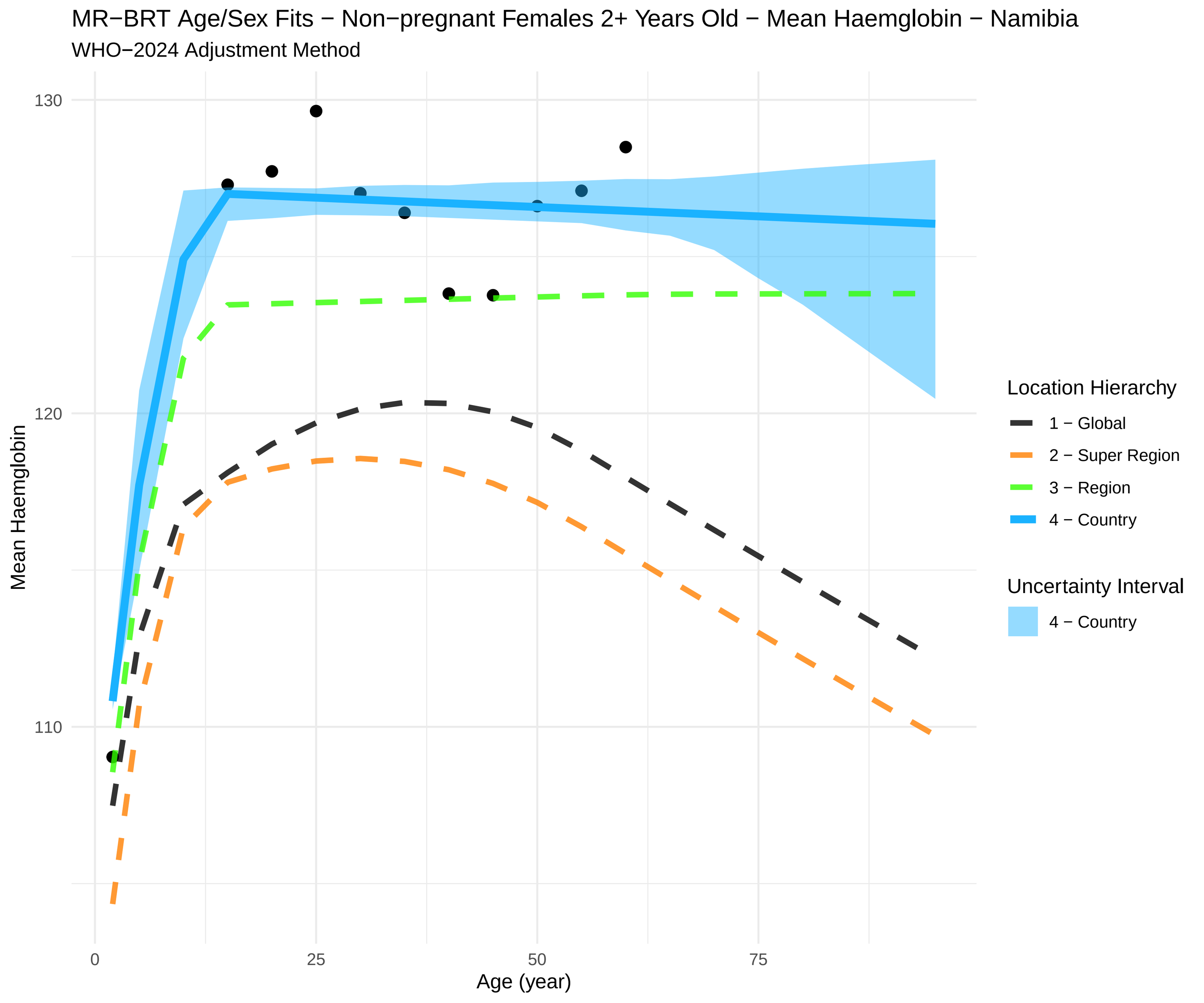
MR−BRT Age/Sex Fits − Non−pregnant Females 2+ Years Old − Mean Haemglobin − Namibia
WHO−2024 Adjustment Method
Mean Haemglobin
Age (year)
Location Hierarchy
1 − Global
2 − Super Region
3 − Region
4 − Country
Uncertainty Interval
4 − Country

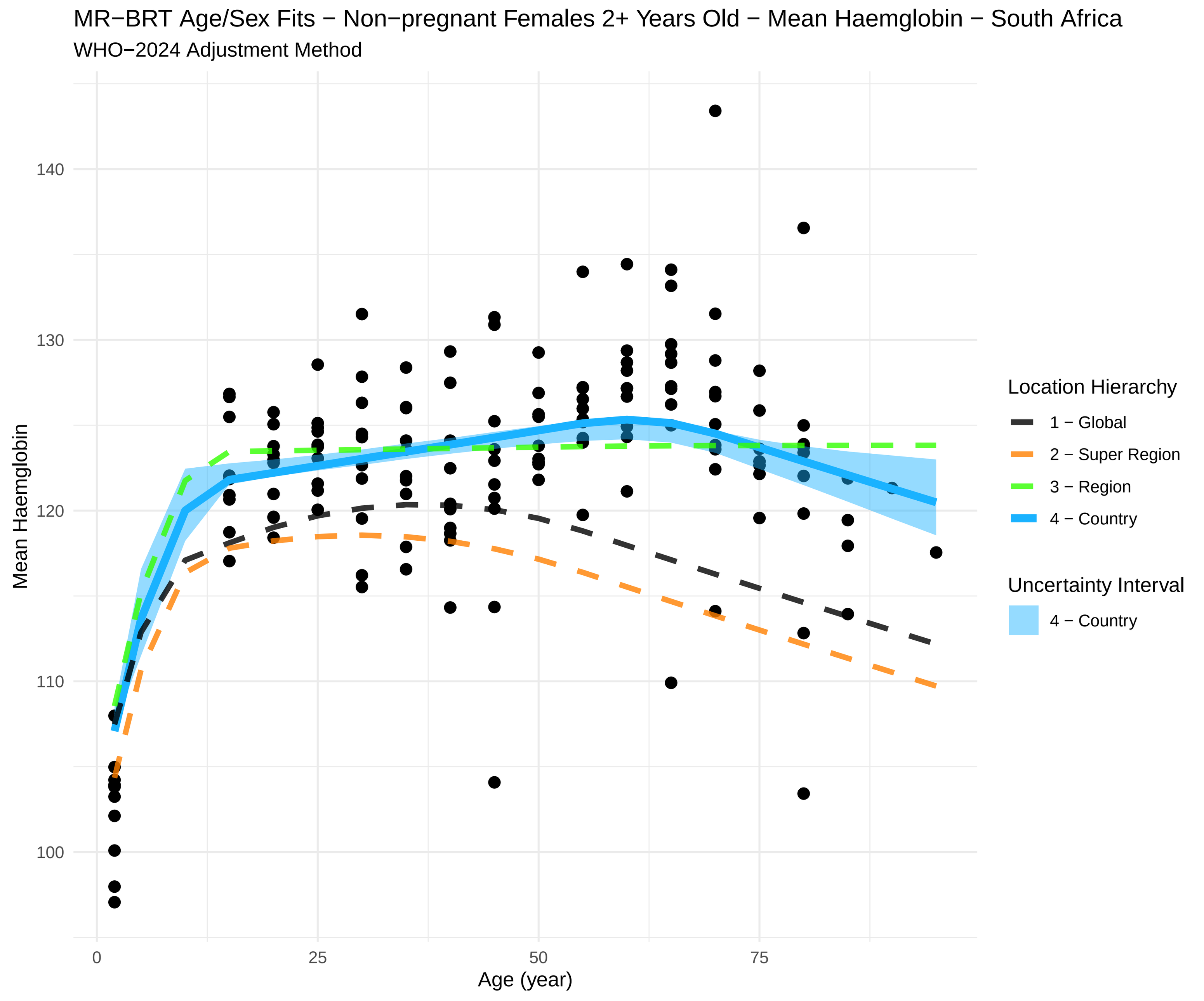
MR−BRT Age/Sex Fits − Non−pregnant Females 2+ Years Old − Mean Haemglobin − South Africa
WHO−2024 Adjustment Method
Mean Haemglobin
Age (year)
Location Hierarchy
1 − Global
2 − Super Region
3 − Region
4 − Country
Uncertainty Interval
4 − Country

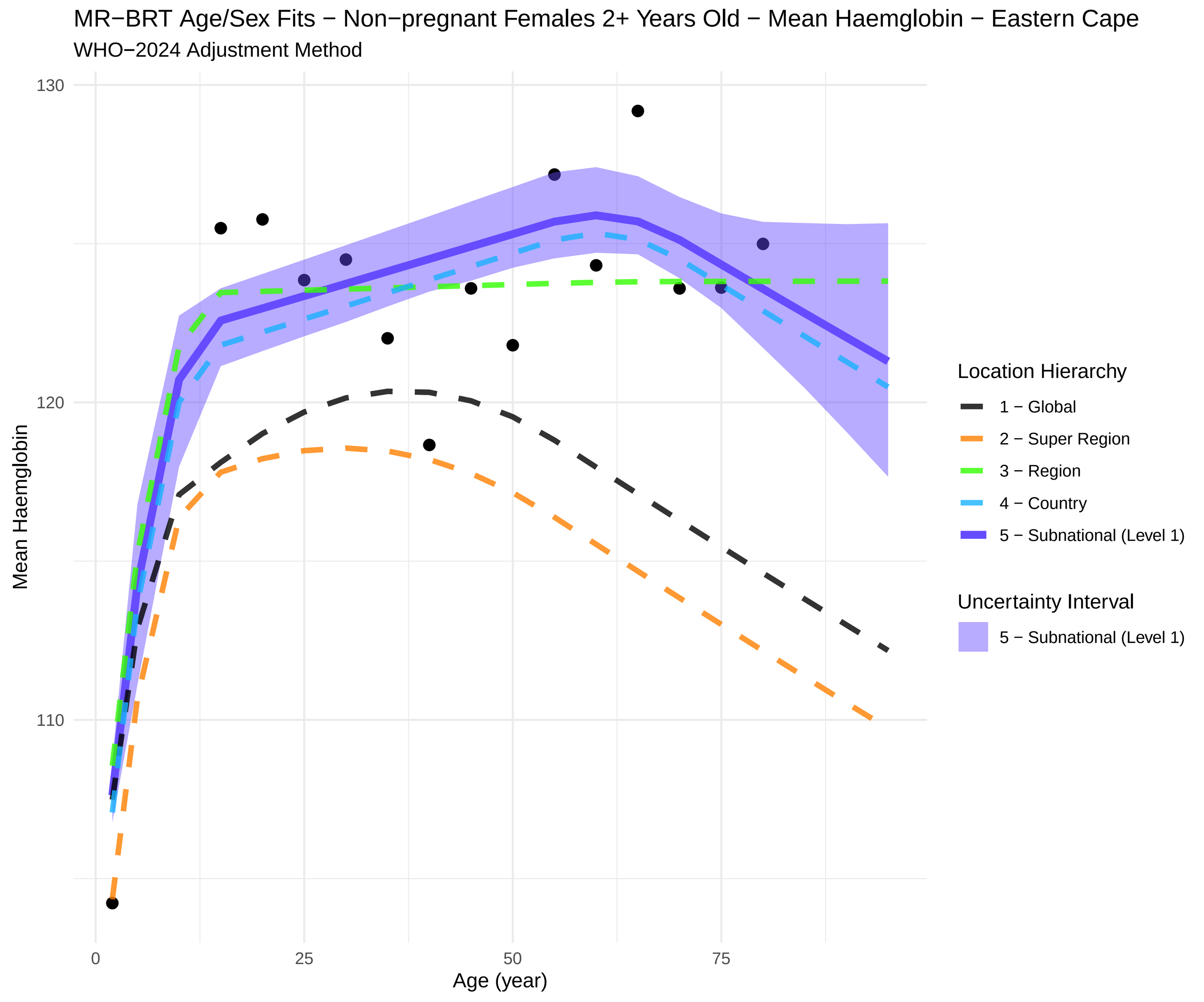
MR−BRT Age/Sex Fits − Non−pregnant Females 2+ Years Old − Mean Haemglobin − Eastern Cape
WHO−2024 Adjustment Method
Mean Haemglobin
Age (year)
Location Hierarchy
1 − Global
2 − Super Region
3 − Region
4 − Country
5 − Subnational (Level 1)
Uncertainty Interval
5 − Subnational (Level 1)

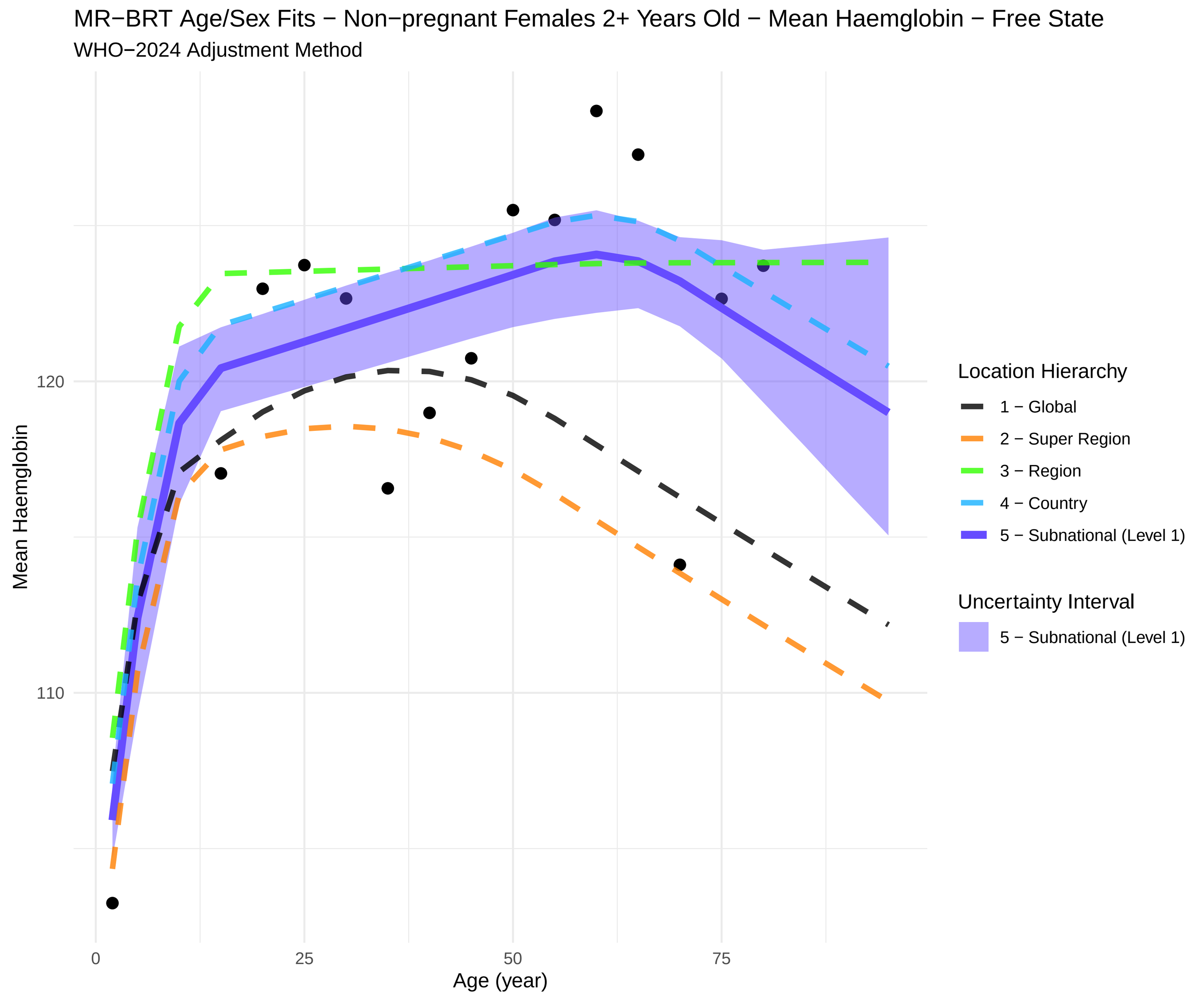
MR−BRT Age/Sex Fits − Non−pregnant Females 2+ Years Old − Mean Haemglobin − Free State
WHO−2024 Adjustment Method
Mean Haemglobin
Age (year)
Location Hierarchy
1 − Global
2 − Super Region
3 − Region
4 − Country
5 − Subnational (Level 1)
Uncertainty Interval
5 − Subnational (Level 1)

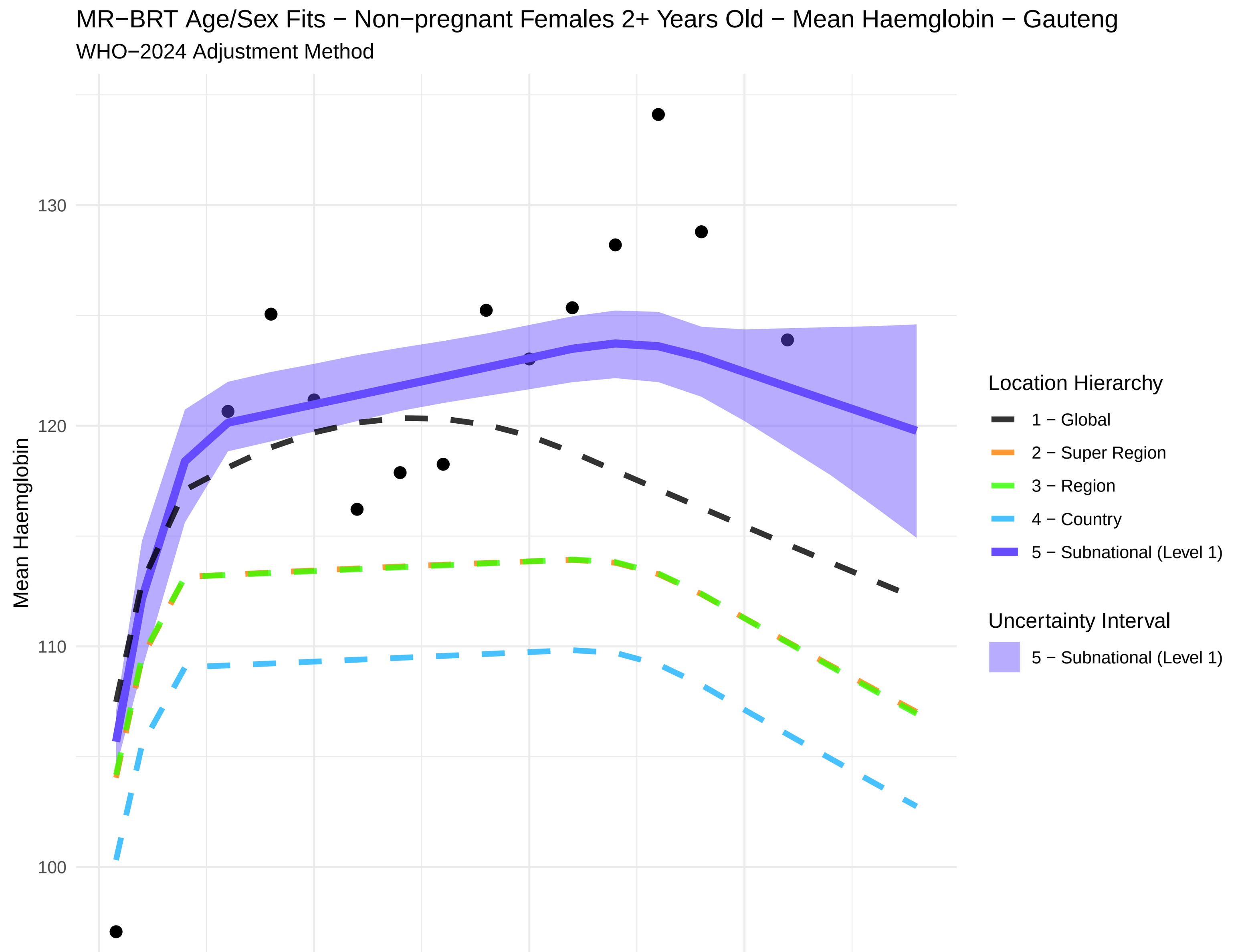
MR−BRT Age/Sex Fits − Non−pregnant Females 2+ Years Old − Mean Haemglobin − Gauteng
WHO−2024 Adjustment Method
Mean Haemglobin
Age (year)
Location Hierarchy
1 − Global
2 − Super Region
3 − Region
4 − Country
5 − Subnational (Level 1)
Uncertainty Interval
5 − Subnational (Level 1)

MR−BRT Age/Sex Fits − Non-pregnant Females 2+ Years Old − Mean Haemglobin − KwaZulu−Natal
WHO−2024 Adjustment Method

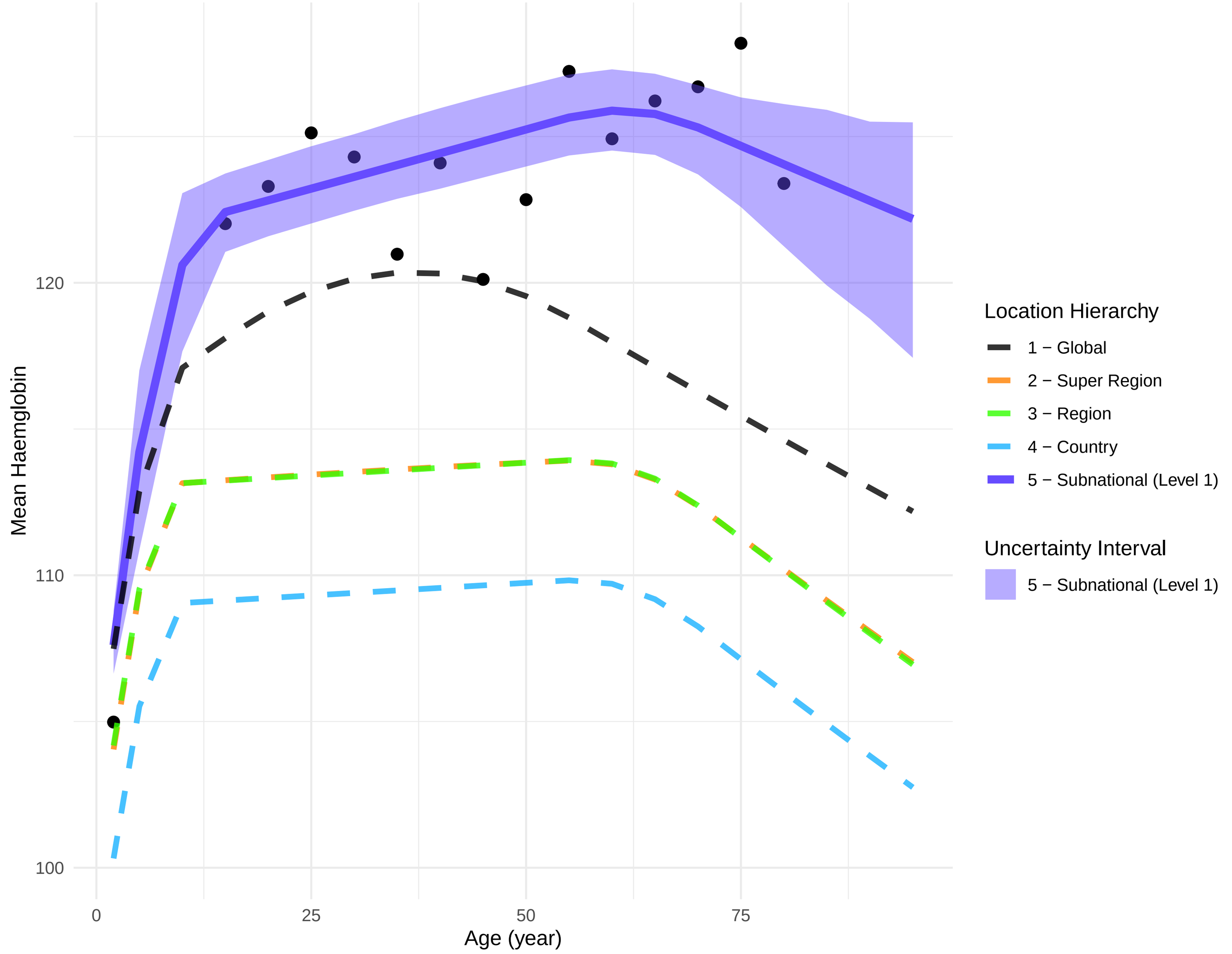

MR−BRT Age/Sex Fits − Non-pregnant Females 2+ Years Old − Mean Haemglobin − Limpopo
WHO−2024 Adjustment Method

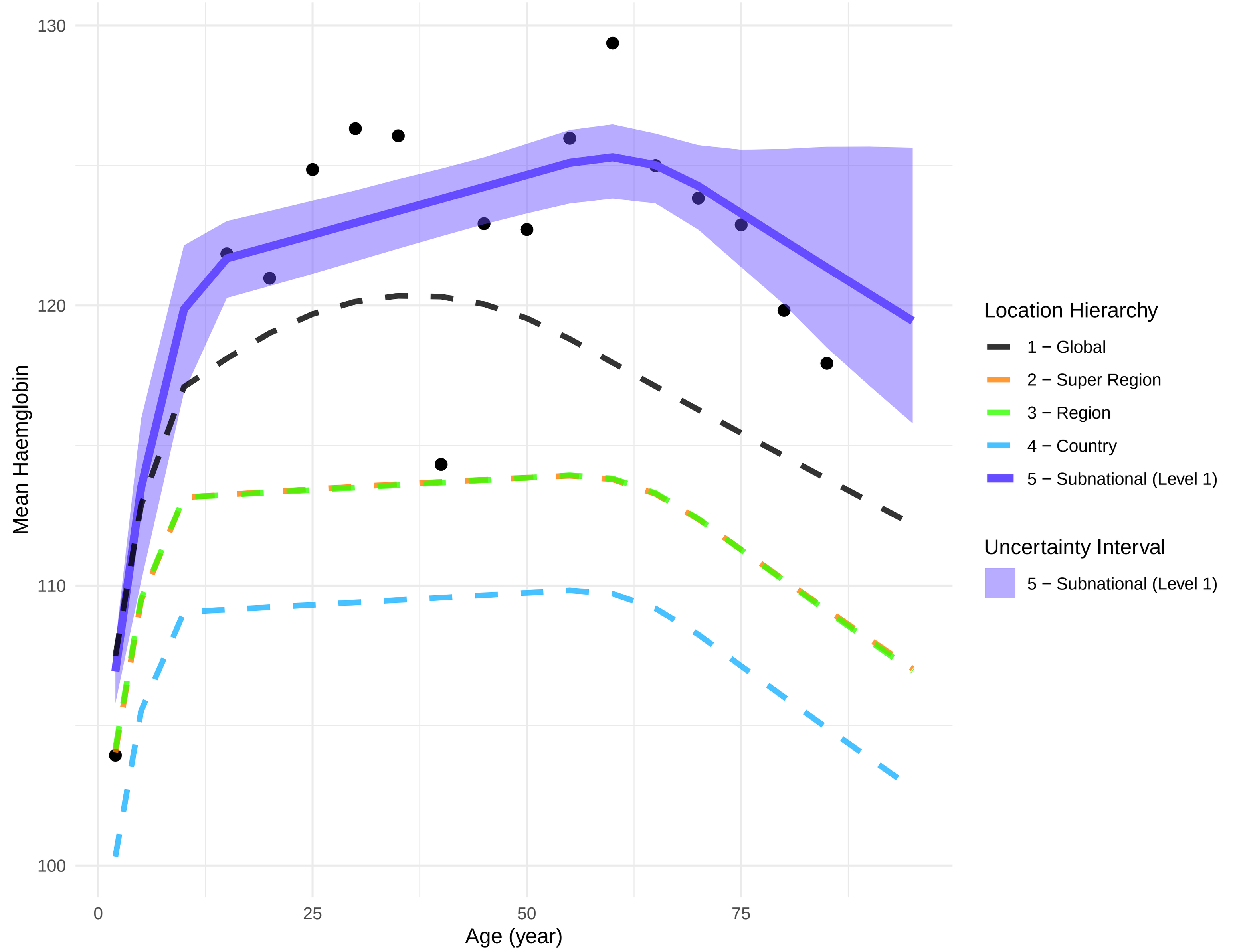

MR−BRT Age/Sex Fits − Non-pregnant Females 2+ Years Old − Mean Haemglobin − Mpumalanga
WHO−2024 Adjustment Method

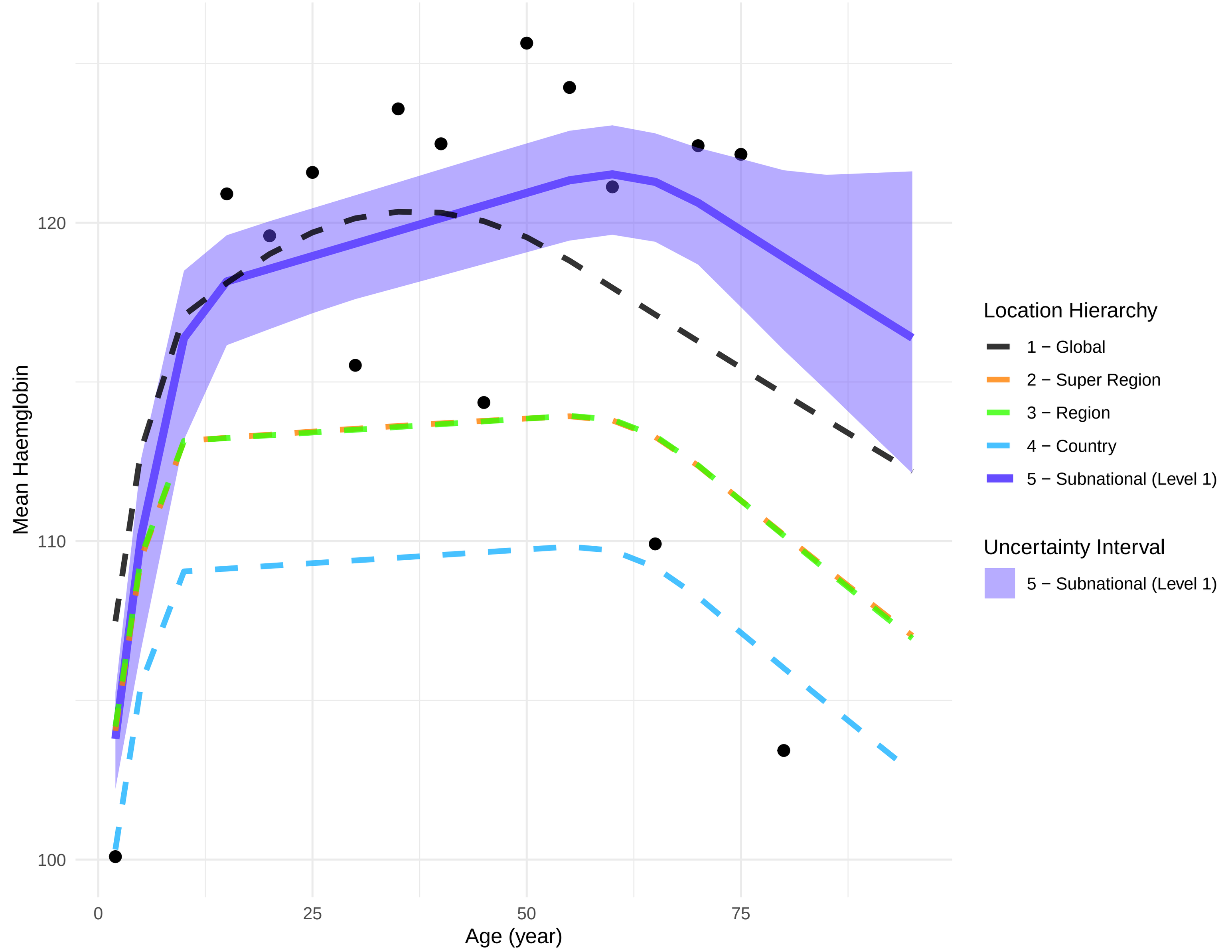

MR−BRT Age/Sex Fits − Non-pregnant Females 2+ Years Old − Mean Haemglobin − North−West
WHO−2024 Adjustment Method

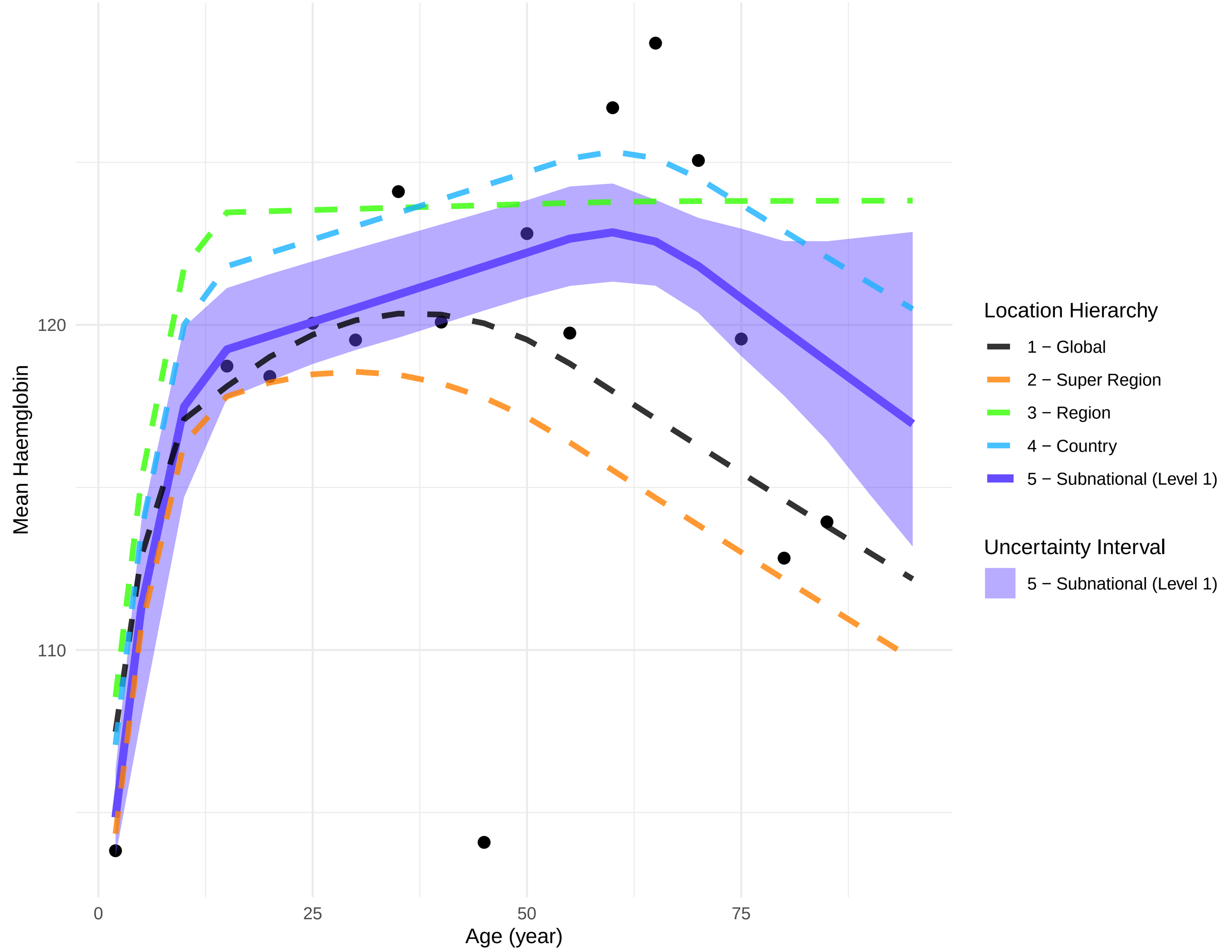

MR−BRT Age/Sex Fits − Non-pregnant Females 2+ Years Old − Mean Haemglobin − Northern Cape
WHO−2024 Adjustment Method

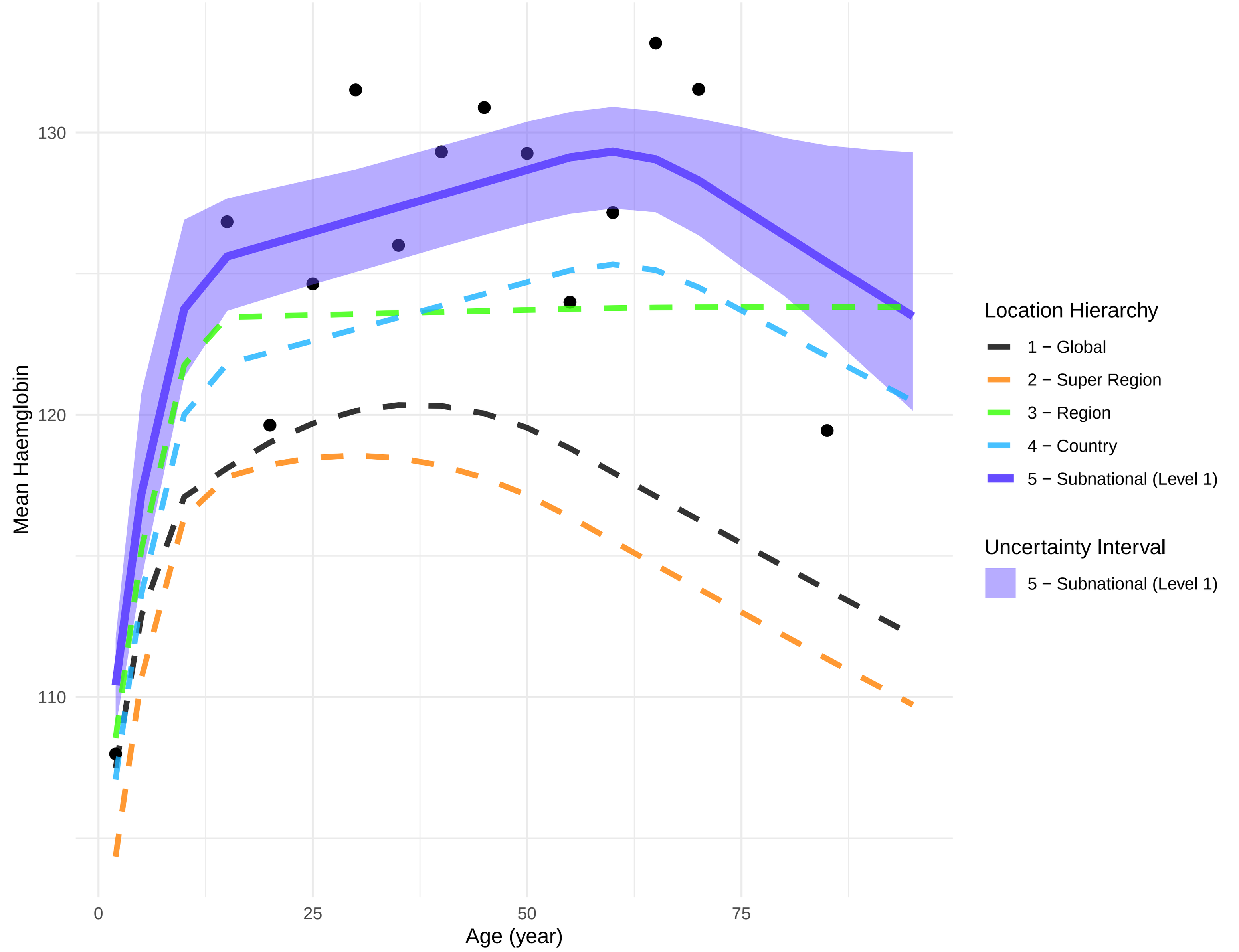

MR−BRT Age/Sex Fits − Non-pregnant Females 2+ Years Old − Mean Haemglobin − Western Cape
WHO−2024 Adjustment Method

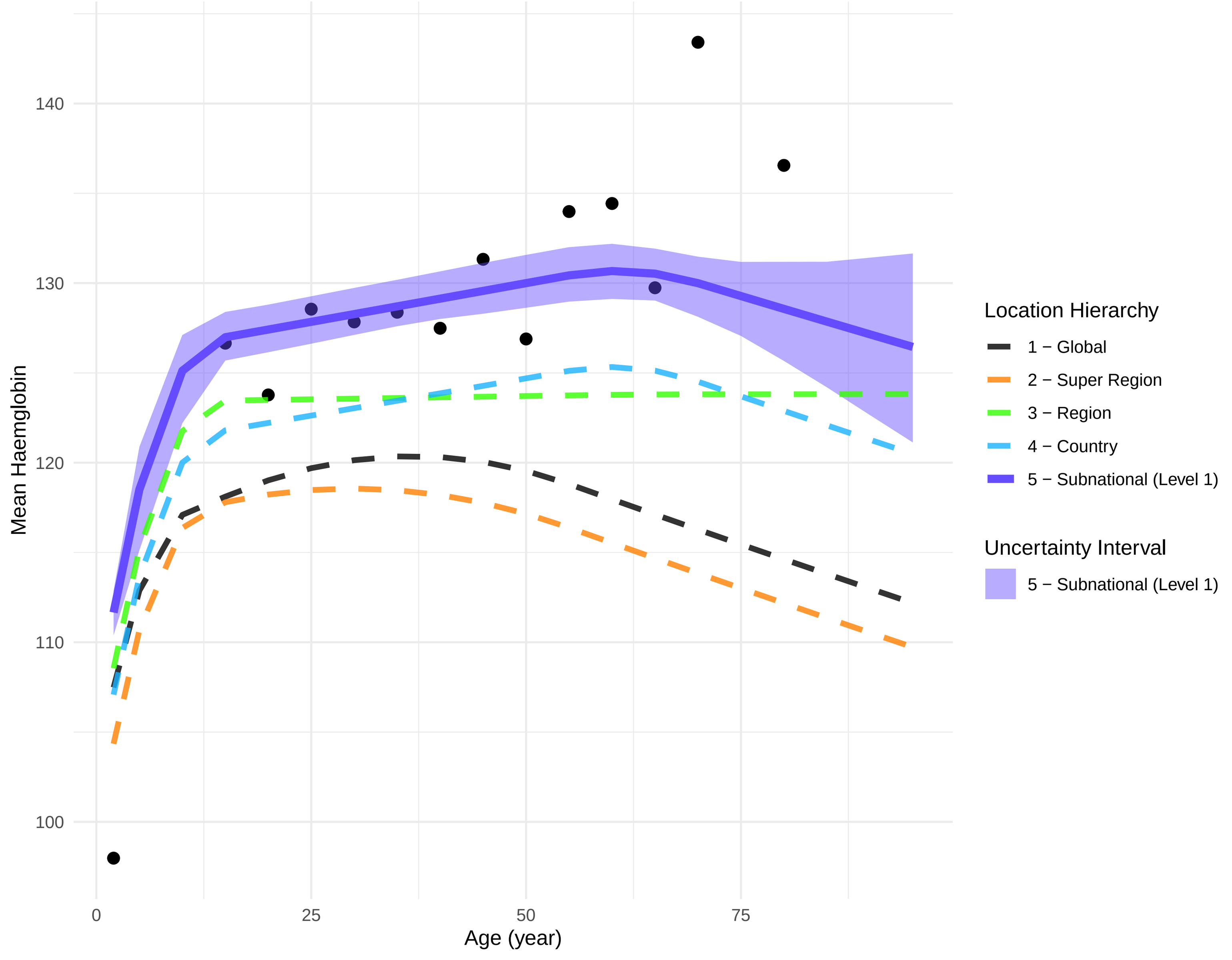

MR−BRT Age/Sex Fits − Non-pregnant Females 2+ Years Old − Mean Haemglobin − Zimbabwe
WHO−2024 Adjustment Method

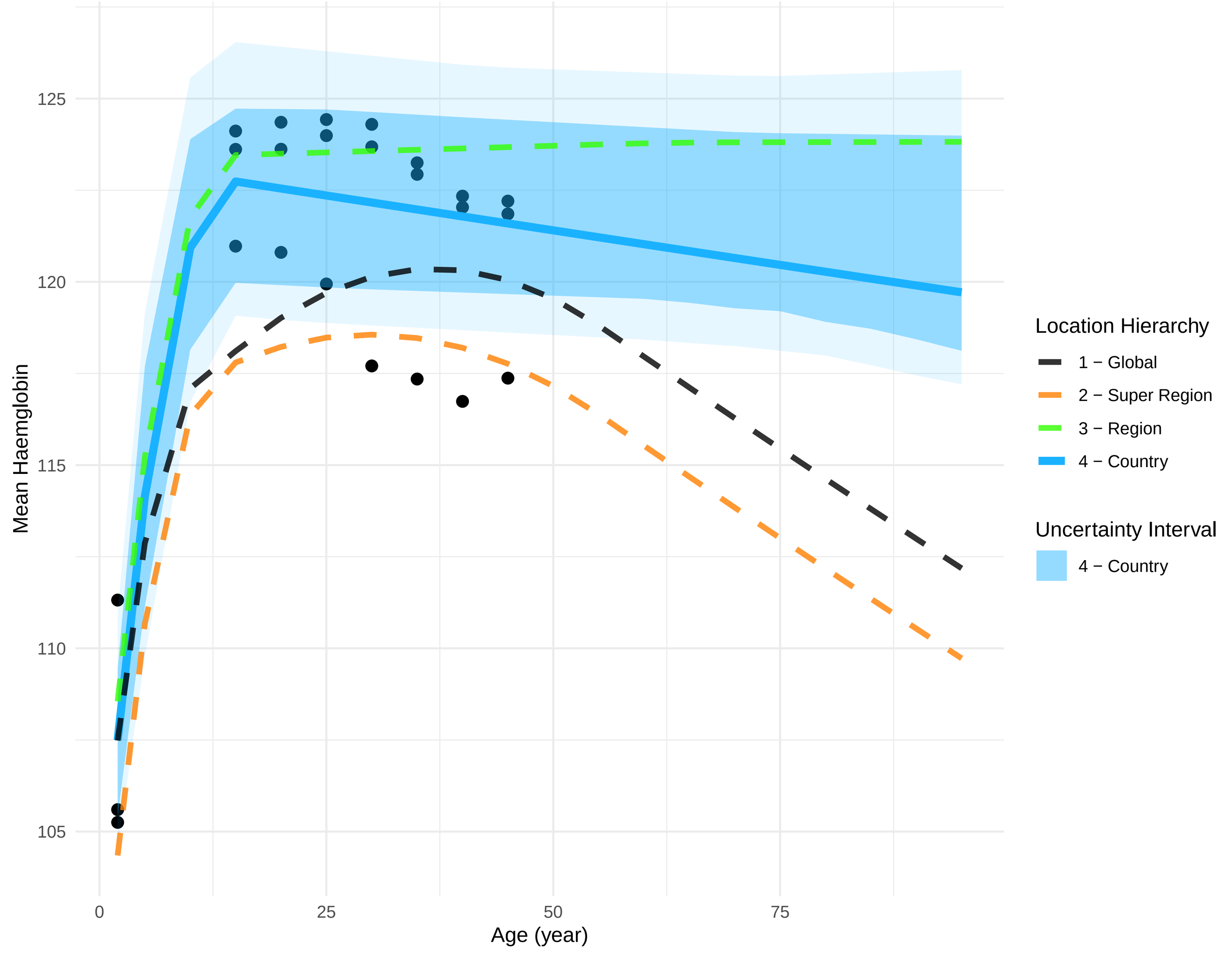

MR−BRT Age/Sex Fits − Non-pregnant Females 2+ Years Old − Mean Haemglobin − Western Sub−Saharan Africa
WHO−2024 Adjustment Method

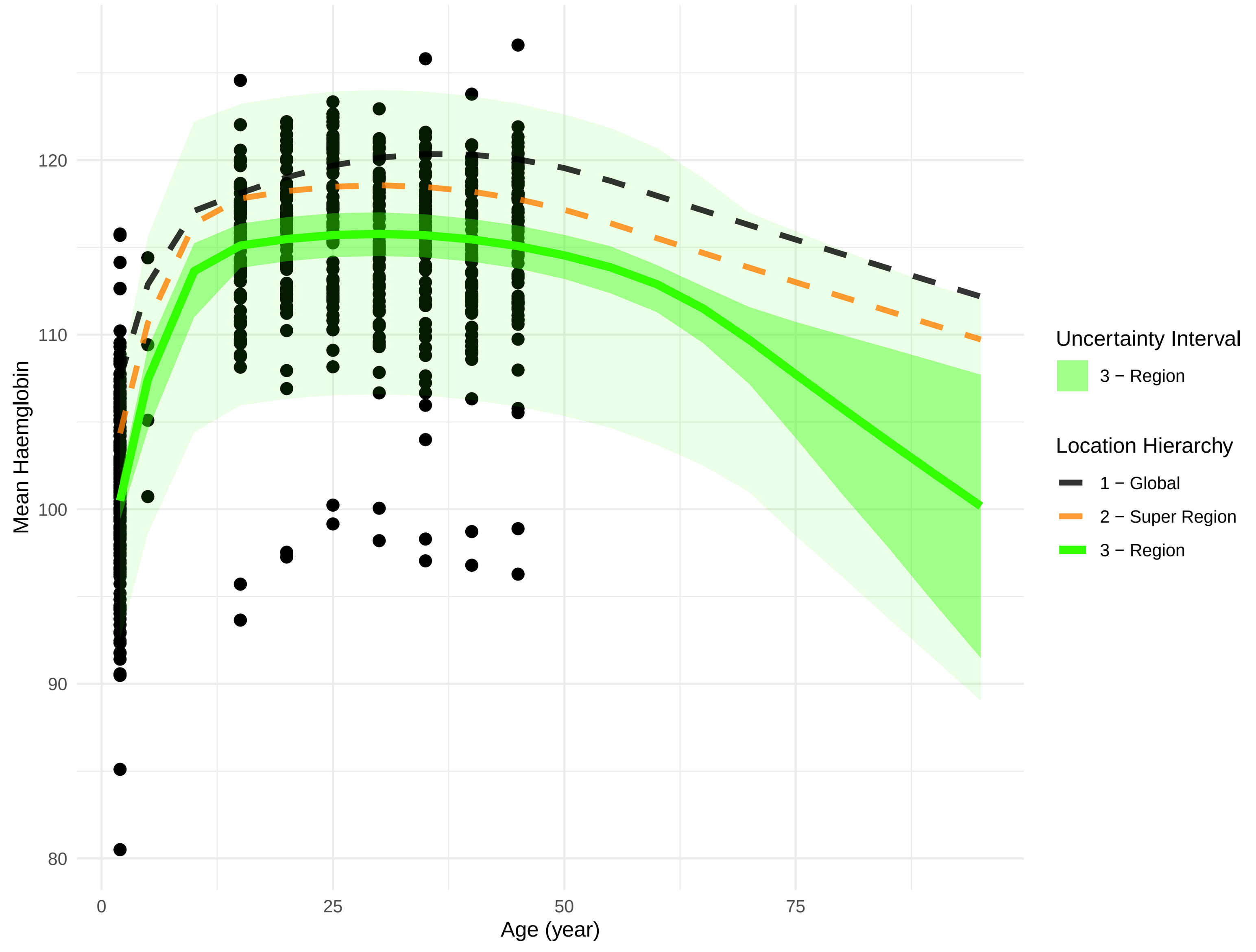

MR−BRT Age/Sex Fits − Non-pregnant Females 2+ Years Old − Mean Haemglobin − Benin
WHO−2024 Adjustment Method

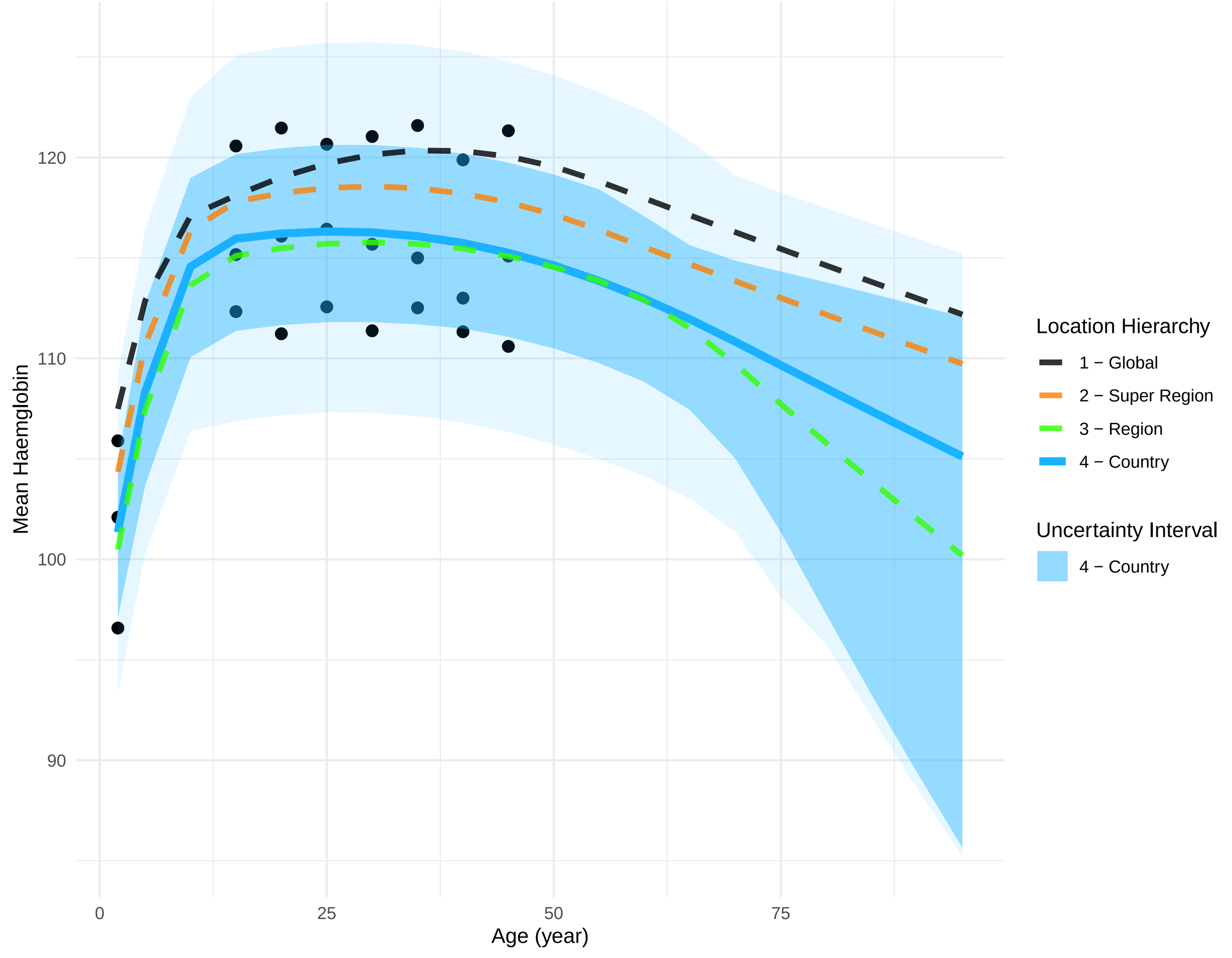

MR−BRT Age/Sex Fits − Non-pregnant Females 2+ Years Old − Mean Haemglobin − Burkina Faso
WHO−2024 Adjustment Method

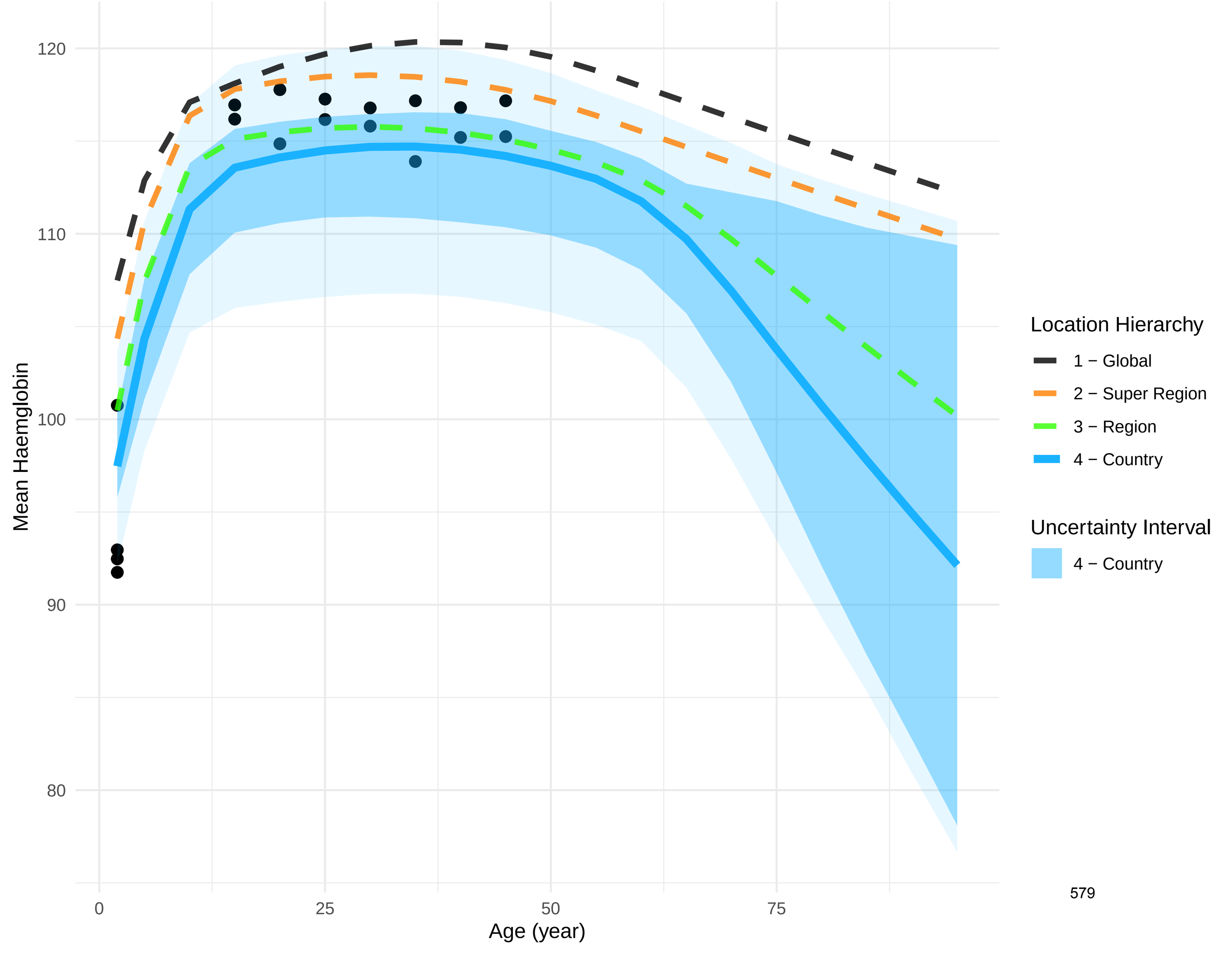

MR−BRT Age/Sex Fits − Non−pregnant Females 2+ Years Old − Mean Haemglobin − Cameroon
WHO−2024 Adjustment Method

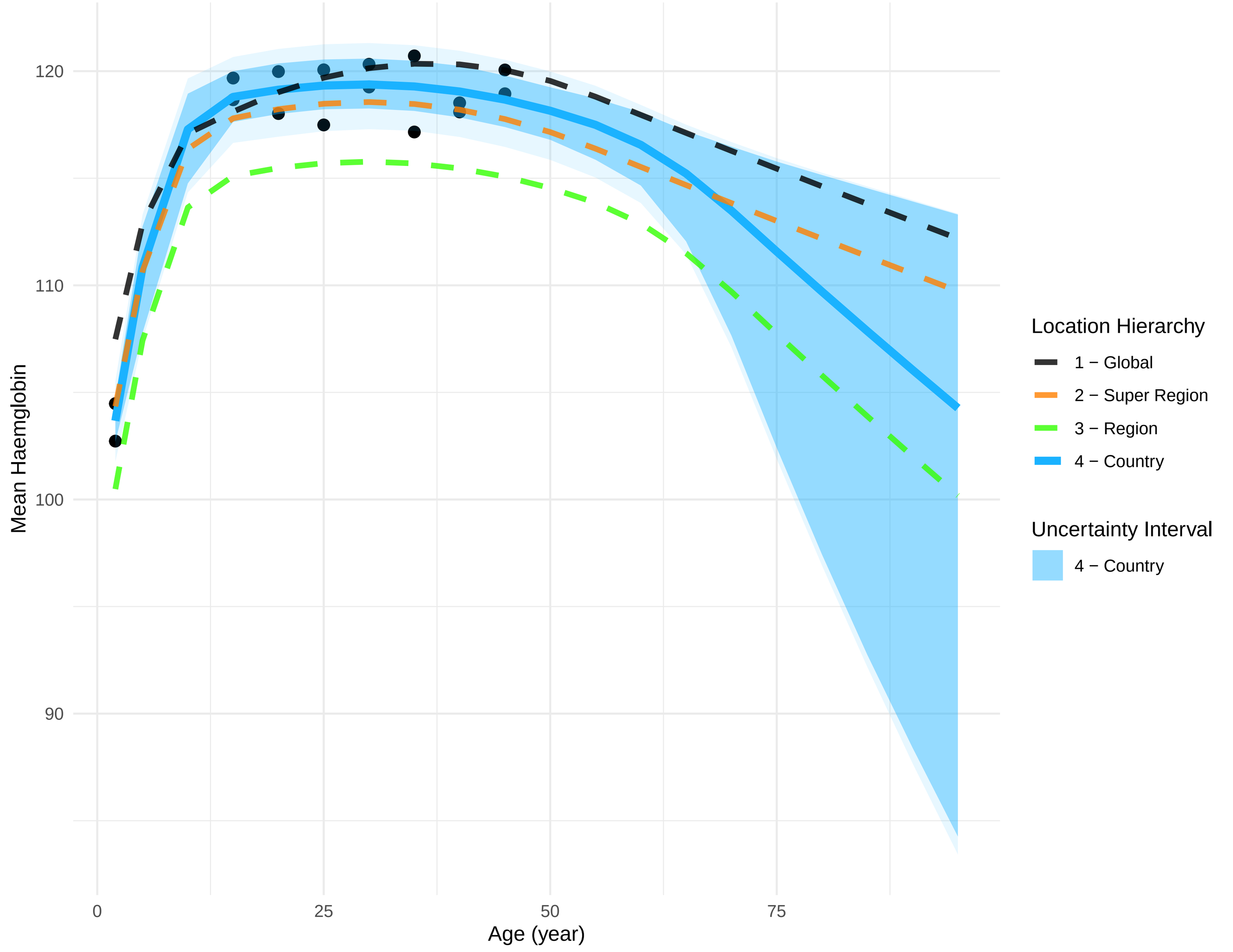

MR−BRT Age/Sex Fits − Non−pregnant Females 2+ Years Old − Mean Haemglobin − Côte d'Ivoire
WHO−2024 Adjustment Method

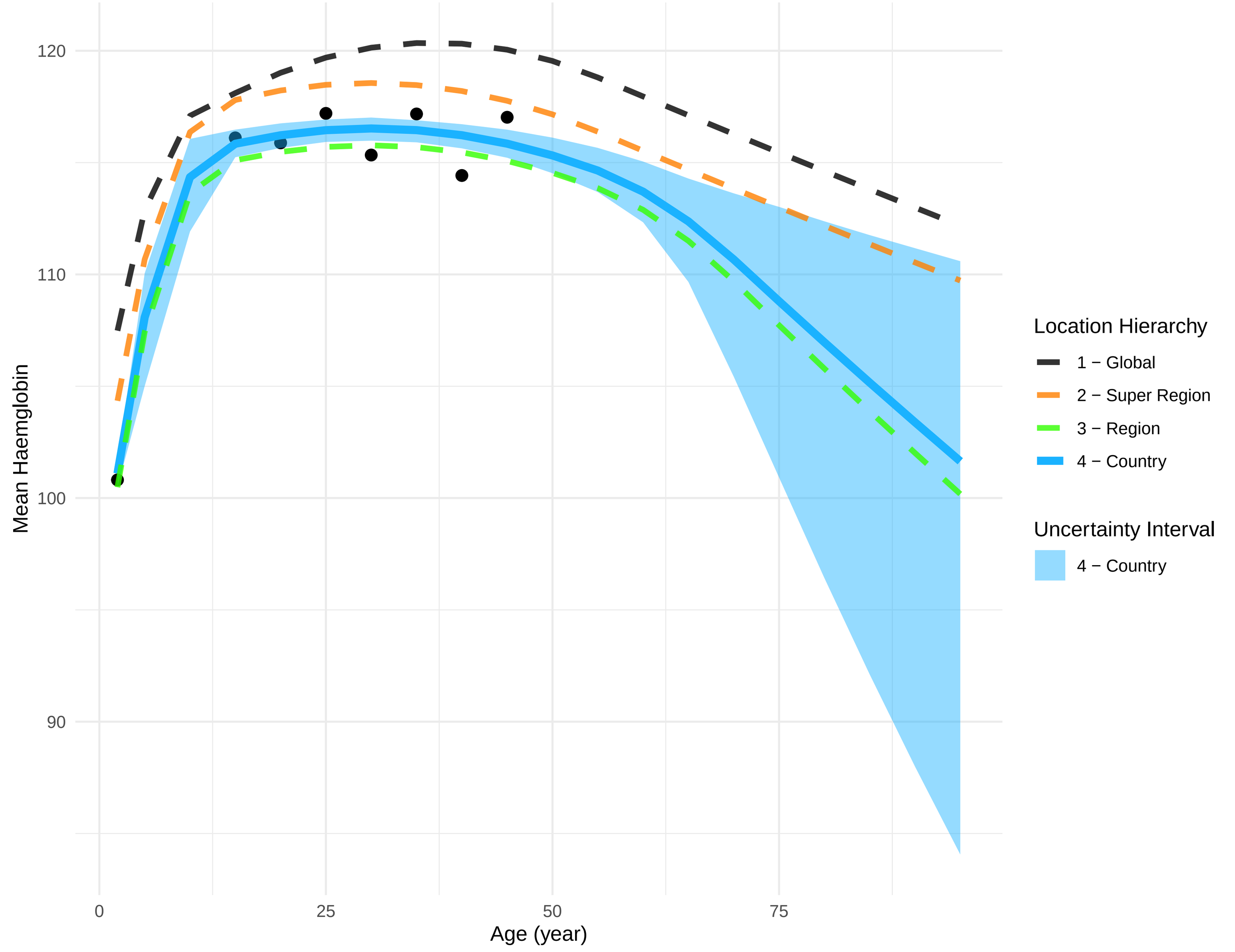

MR−BRT Age/Sex Fits − Non−pregnant Females 2+ Years Old − Mean Haemglobin − Gambia
WHO−2024 Adjustment Method

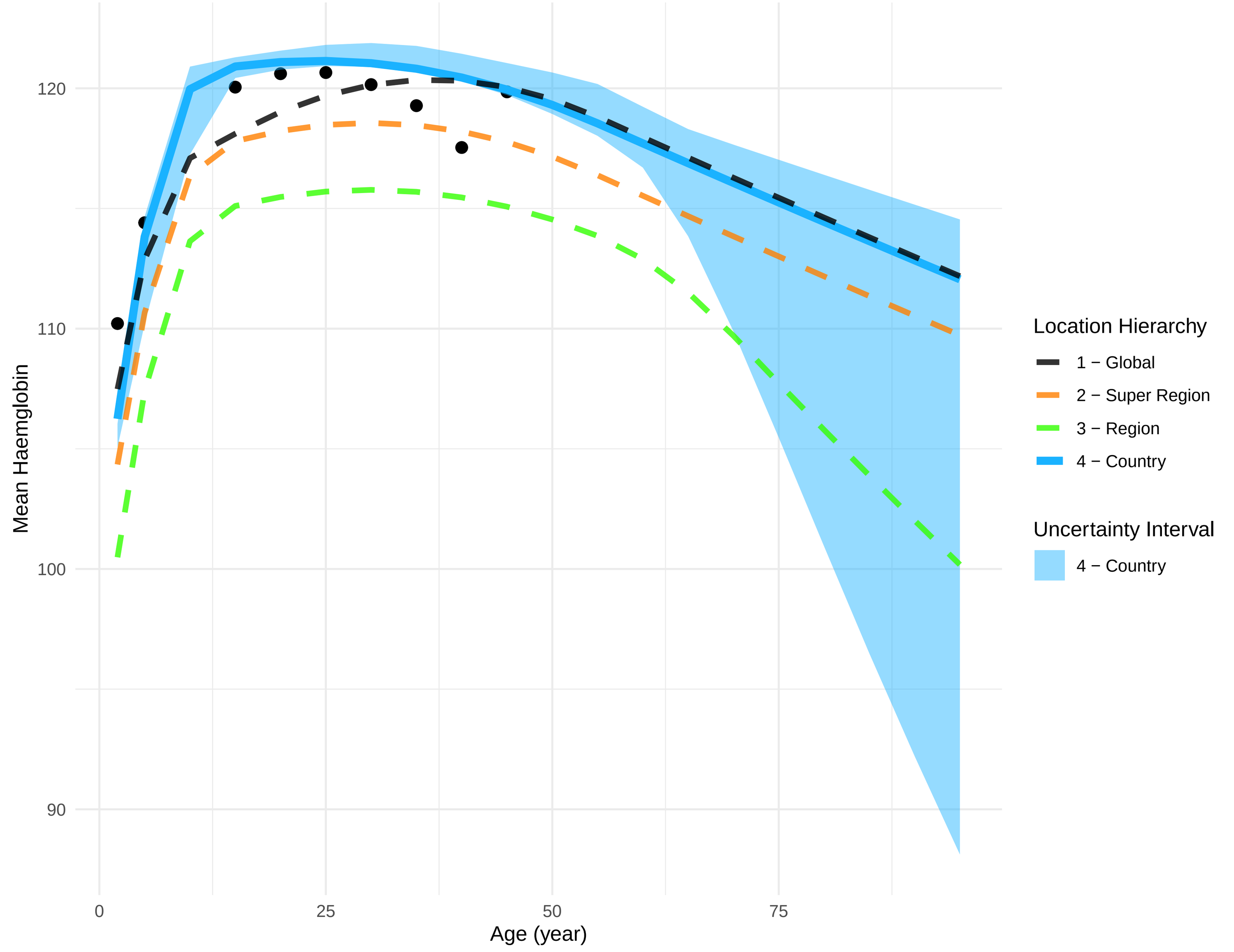

MR−BRT Age/Sex Fits − Non−pregnant Females 2+ Years Old − Mean Haemglobin − Ghana
WHO−2024 Adjustment Method

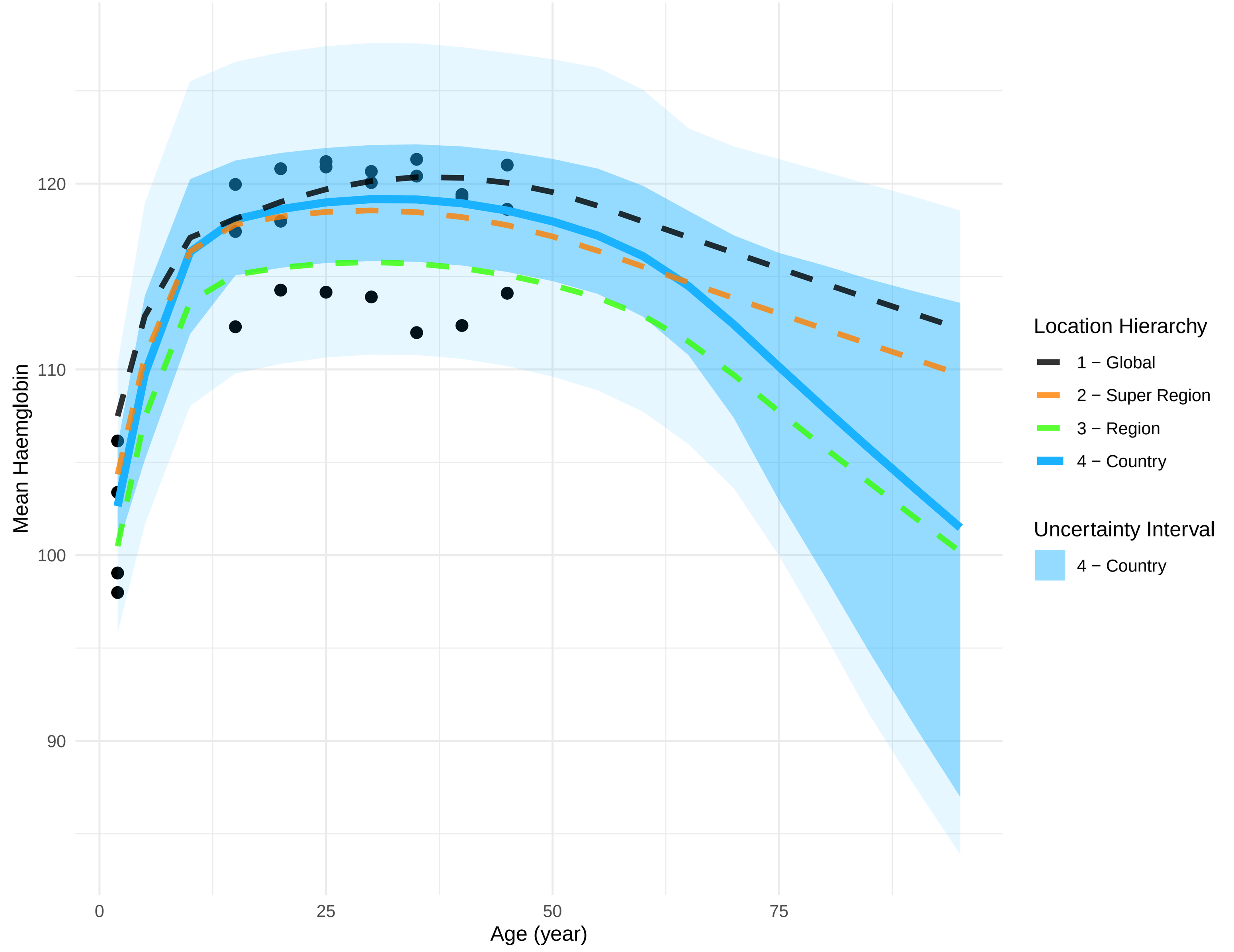

MR−BRT Age/Sex Fits − Non−pregnant Females 2+ Years Old − Mean Haemglobin − Guinea
WHO−2024 Adjustment Method

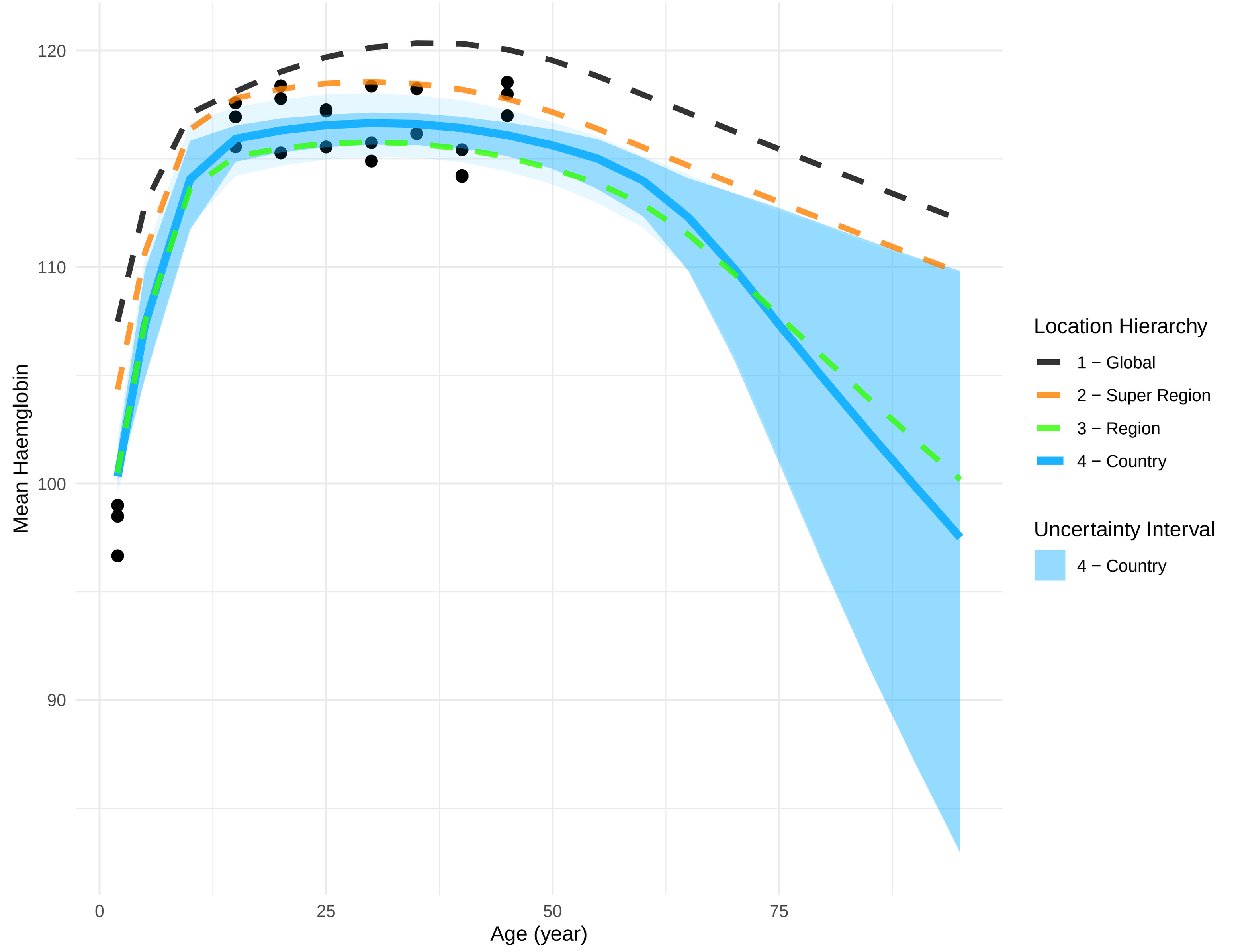

MR−BRT Age/Sex Fits − Non−pregnant Females 2+ Years Old − Mean Haemglobin − Liberia
WHO−2024 Adjustment Method

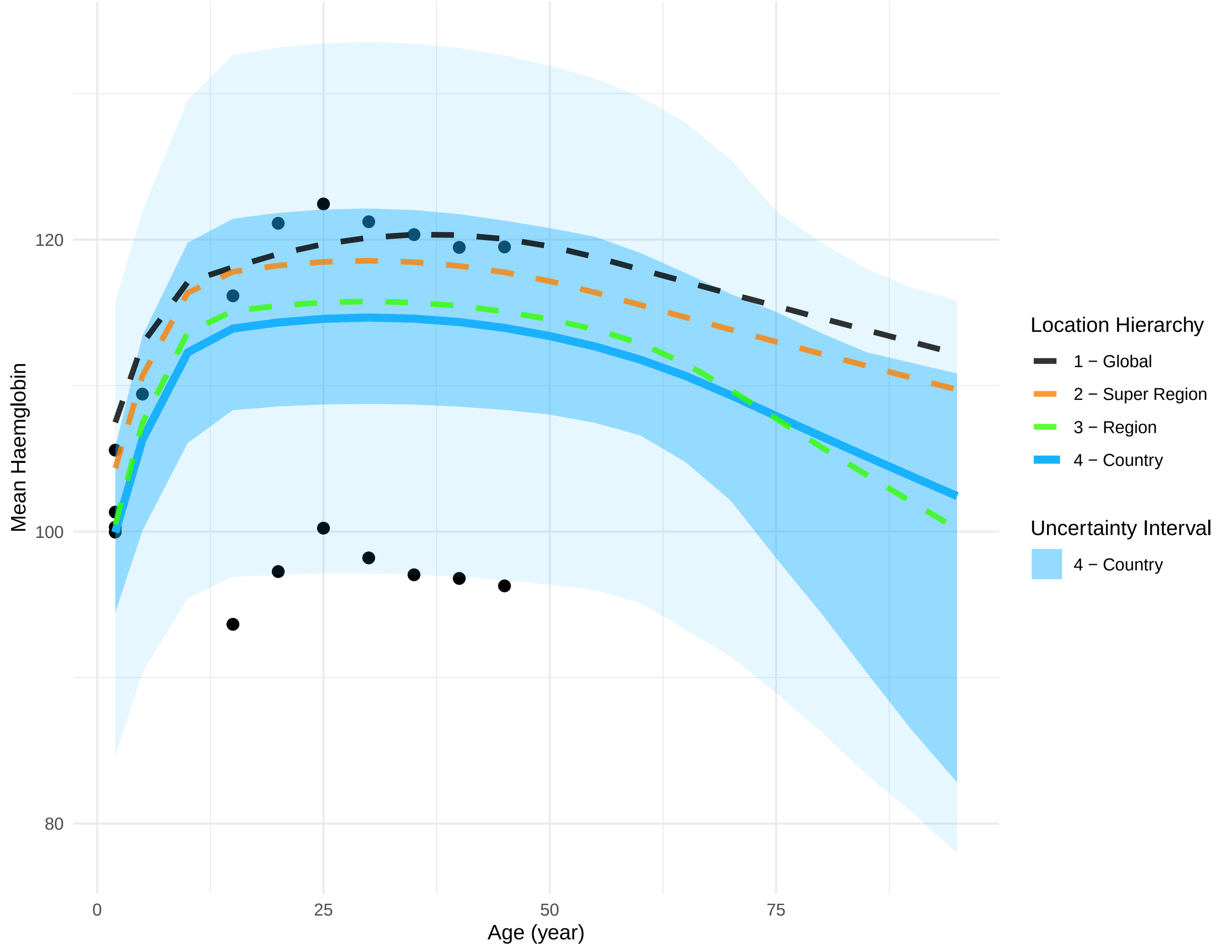

MR−BRT Age/Sex Fits − Non−pregnant Females 2+ Years Old − Mean Haemglobin − Mali
WHO−2024 Adjustment Method

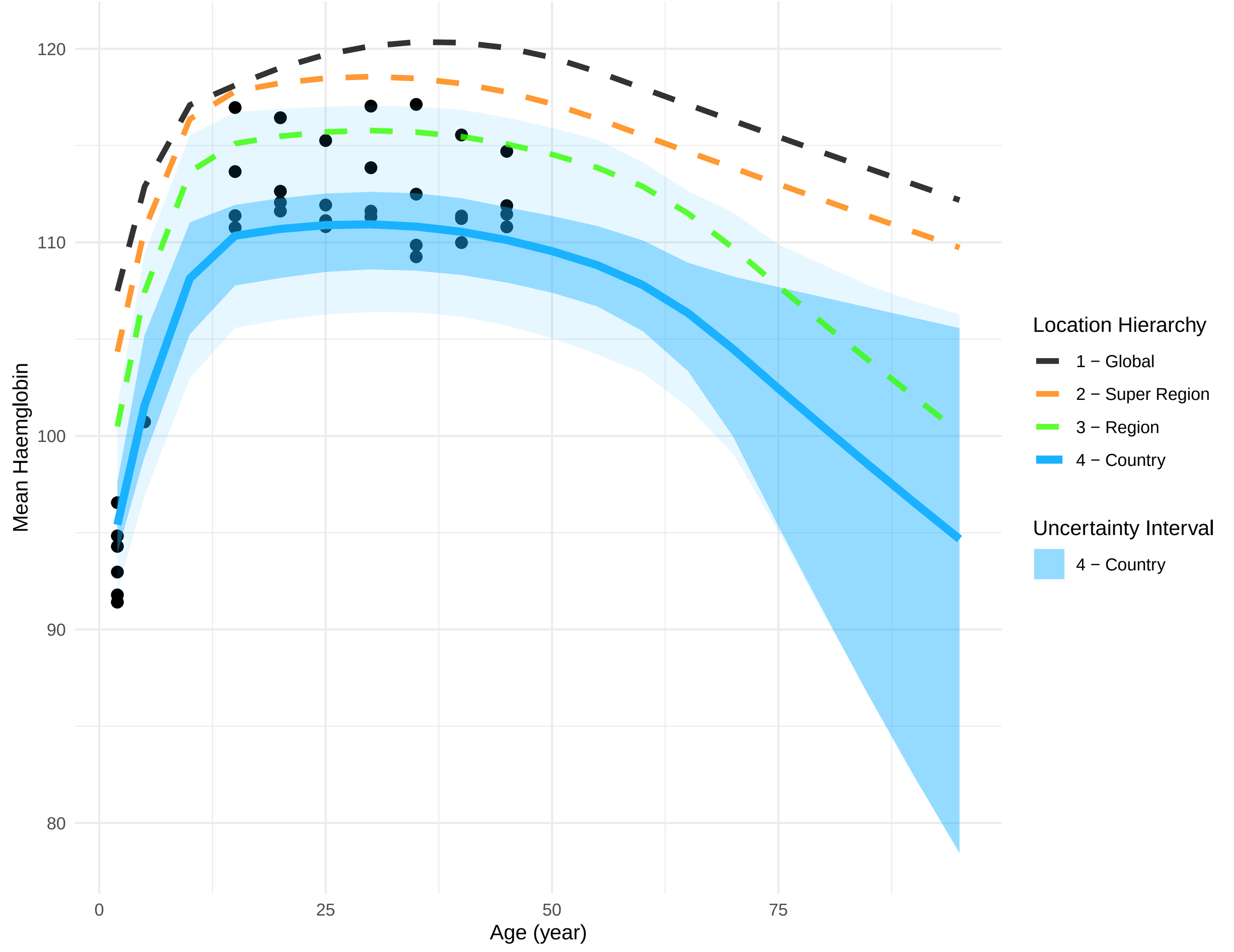

MR−BRT Age/Sex Fits − Non−pregnant Females 2+ Years Old − Mean Haemglobin − Niger
WHO−2024 Adjustment Method

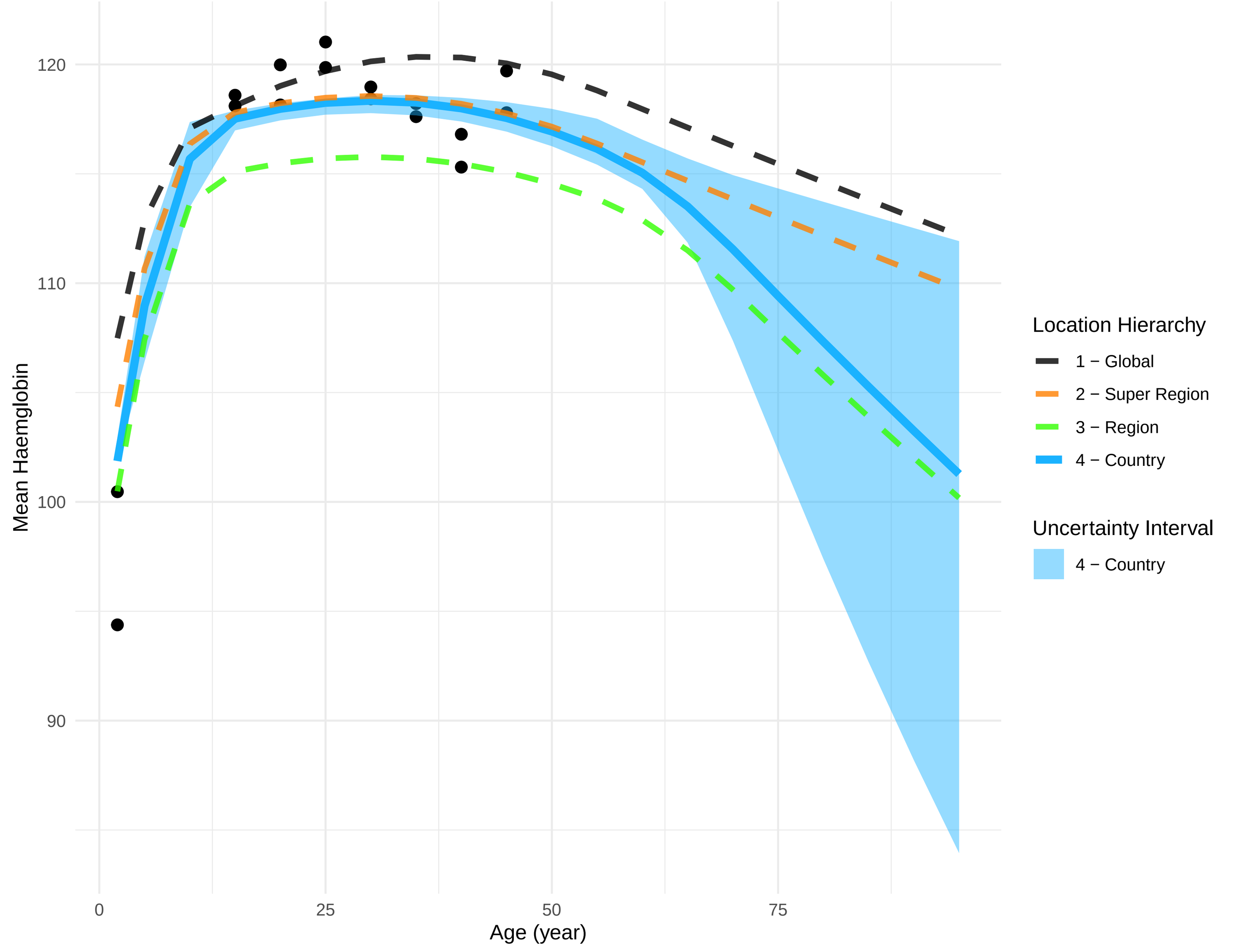

MR−BRT Age/Sex Fits − Non−pregnant Females 2+ Years Old − Mean Haemglobin − Nigeria
WHO−2024 Adjustment Method

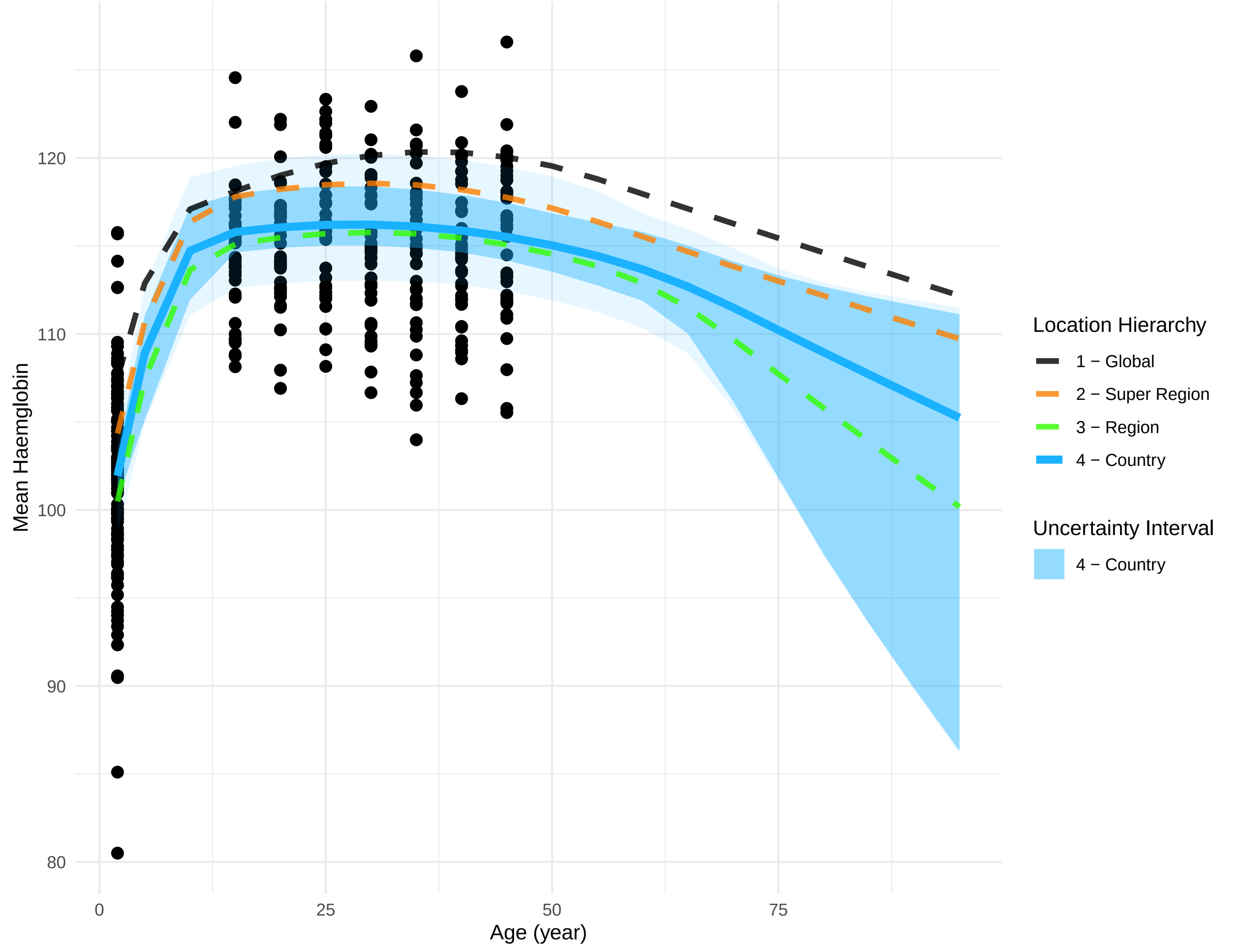

MR−BRT Age/Sex Fits − Non−pregnant Females 2+ Years Old − Mean Haemglobin − Abia
WHO−2024 Adjustment Method

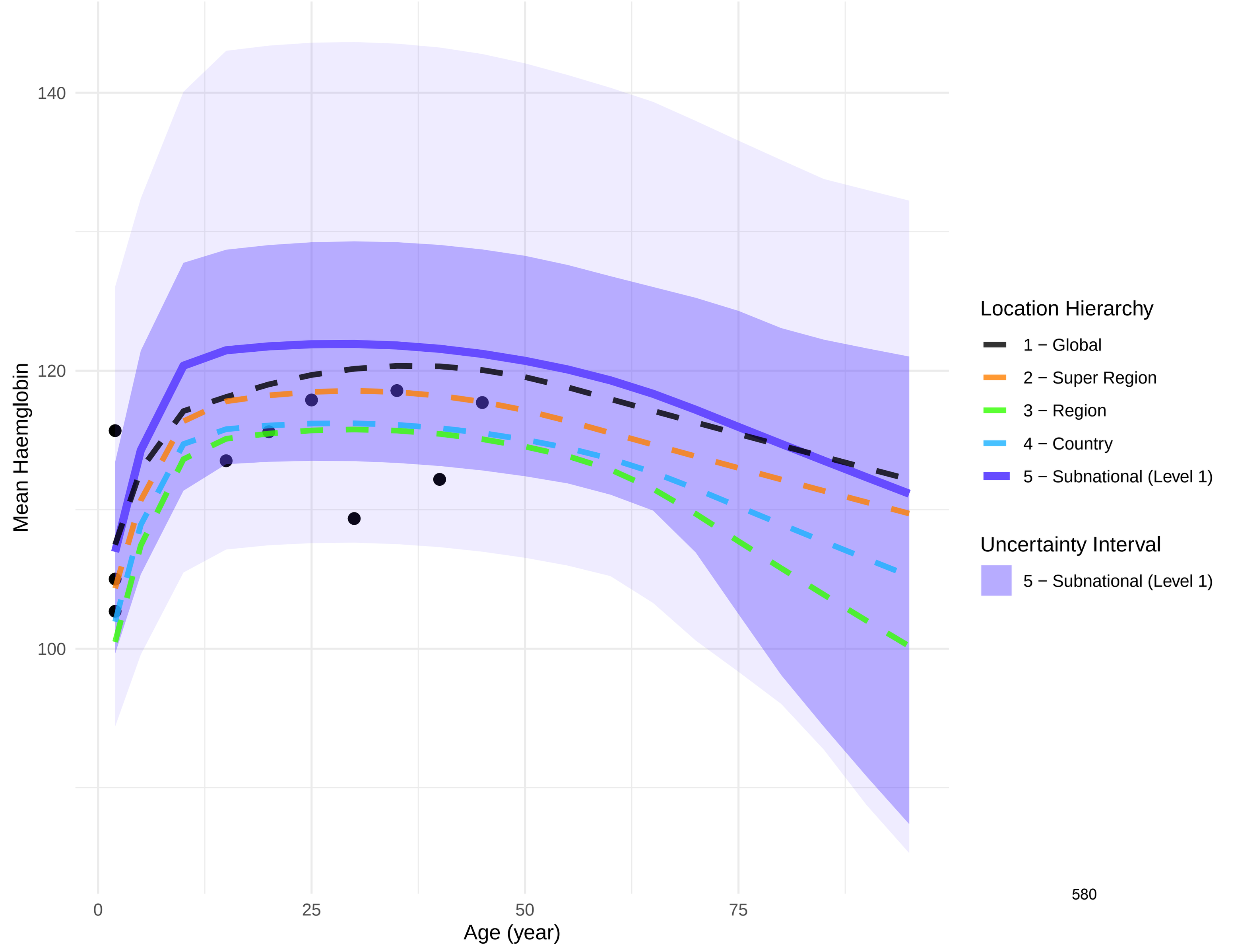

MR−BRT Age/Sex Fits − Non−pregnant Females 2+ Years Old − Mean Haemglobin − Adamawa
WHO−2024 Adjustment Method

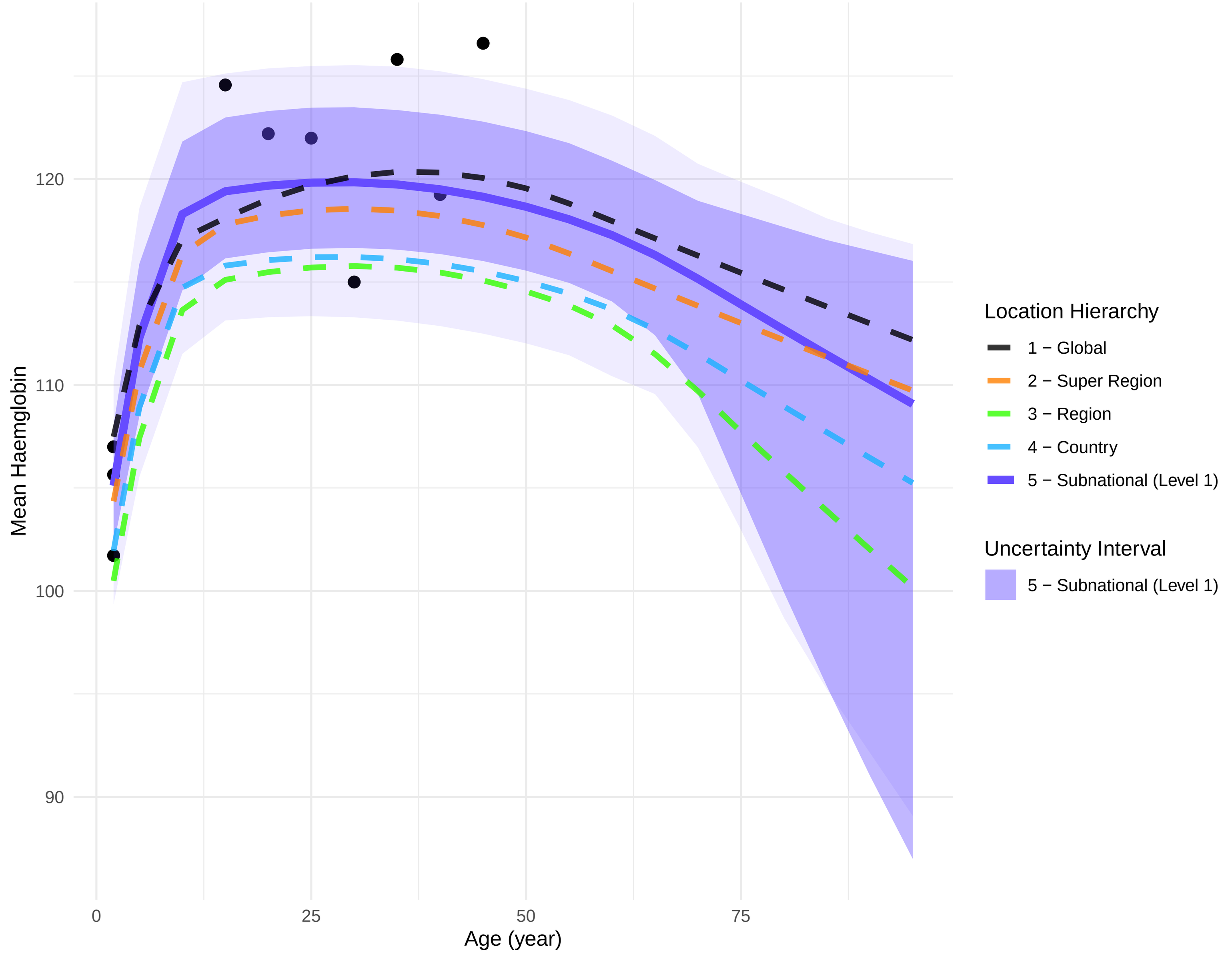

MR−BRT Age/Sex Fits − Non−pregnant Females 2+ Years Old − Mean Haemglobin − Akwa Ibom
WHO−2024 Adjustment Method

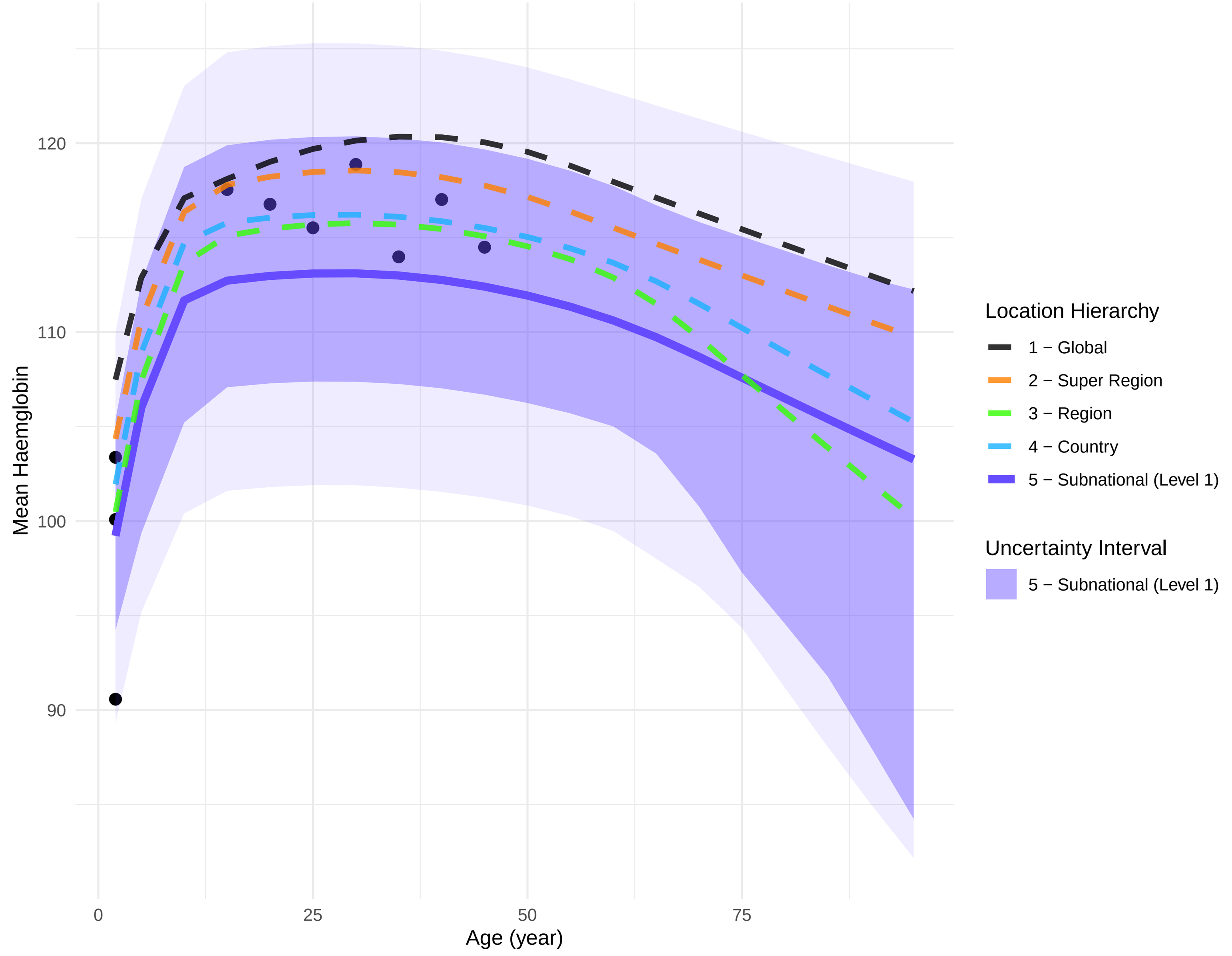

MR−BRT Age/Sex Fits − Non−pregnant Females 2+ Years Old − Mean Haemglobin − Anambra
WHO−2024 Adjustment Method

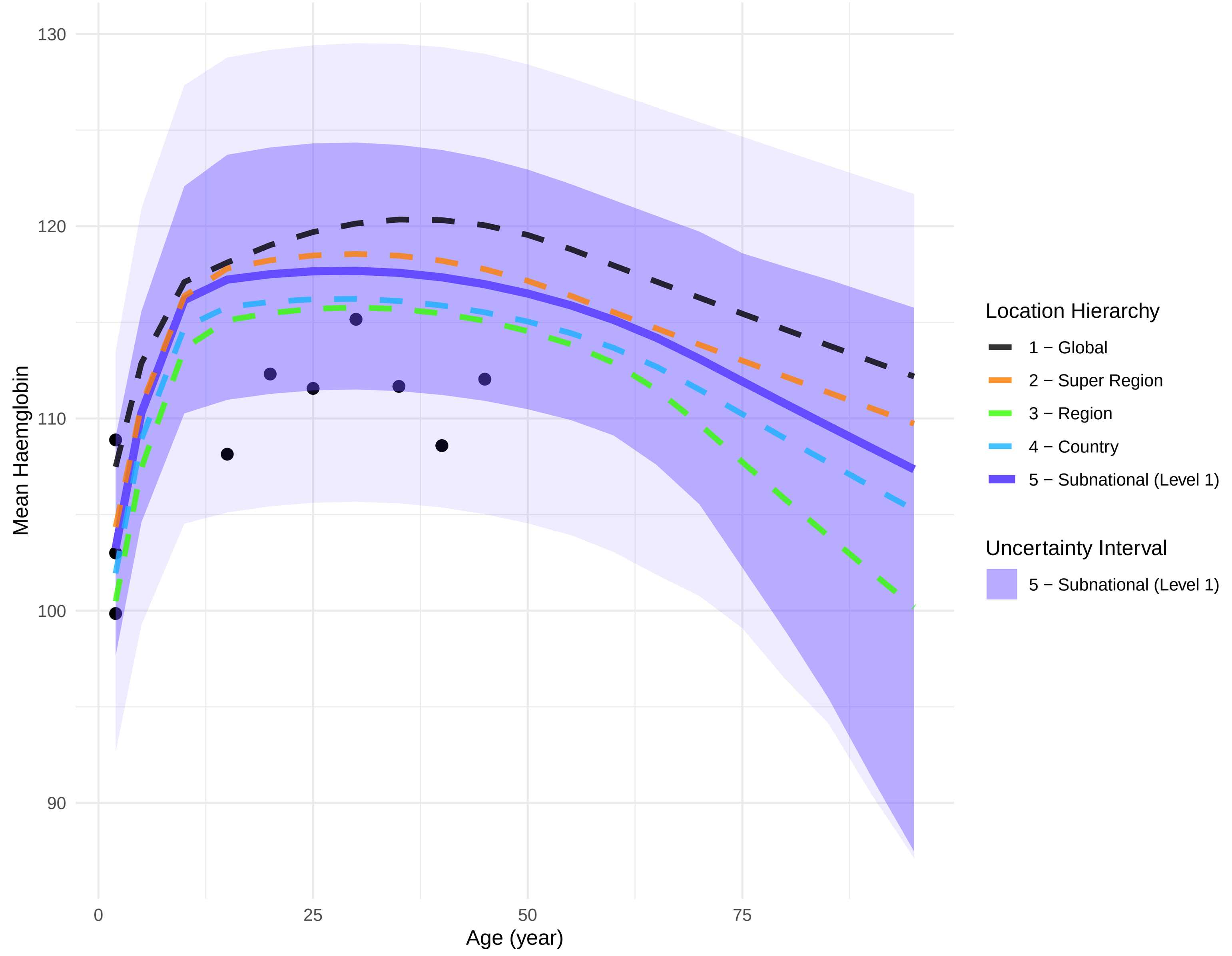

MR−BRT Age/Sex Fits − Non−pregnant Females 2+ Years Old − Mean Haemglobin − Bauchi
WHO−2024 Adjustment Method

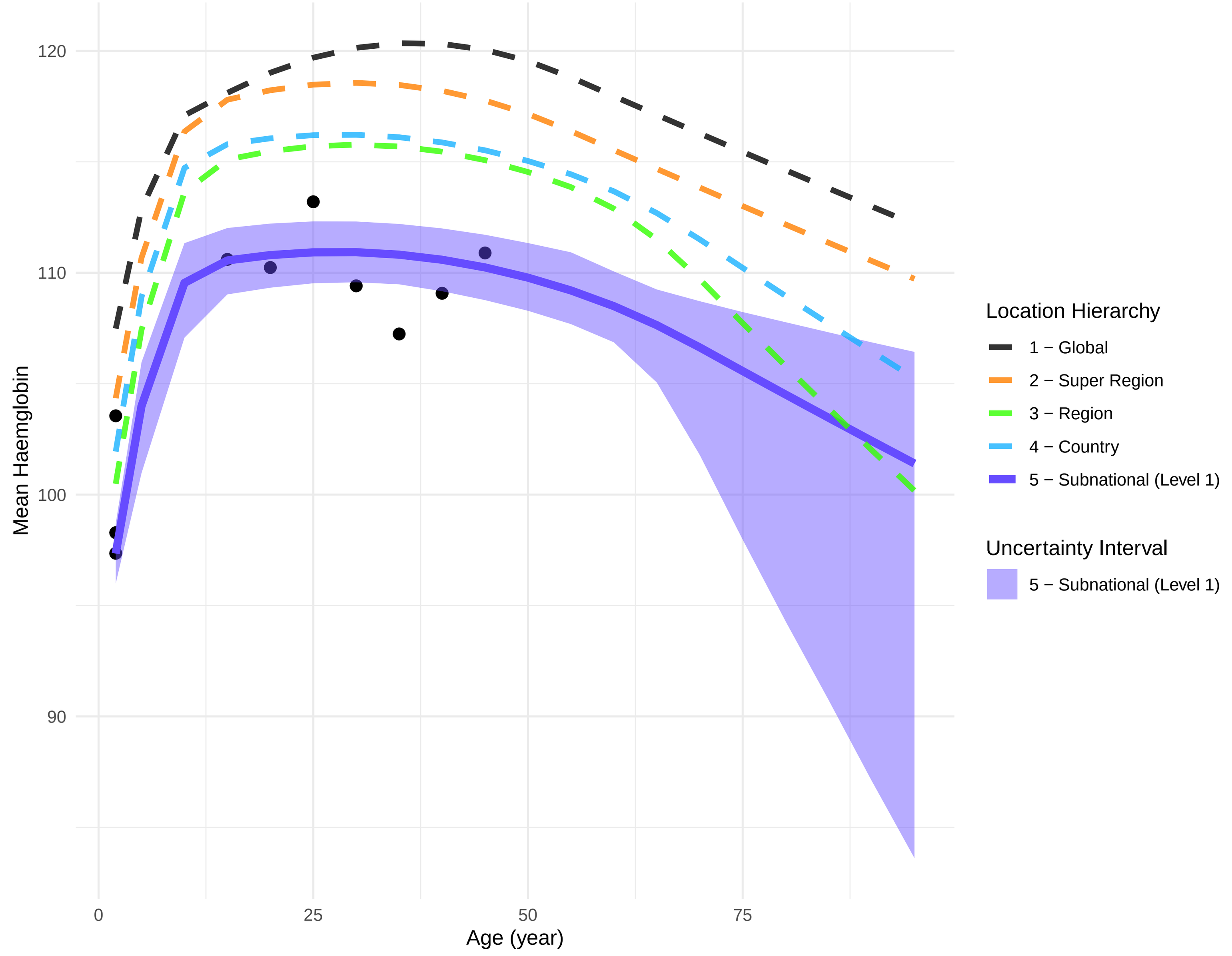

MR−BRT Age/Sex Fits − Non−pregnant Females 2+ Years Old − Mean Haemglobin − Bayelsa
WHO−2024 Adjustment Method

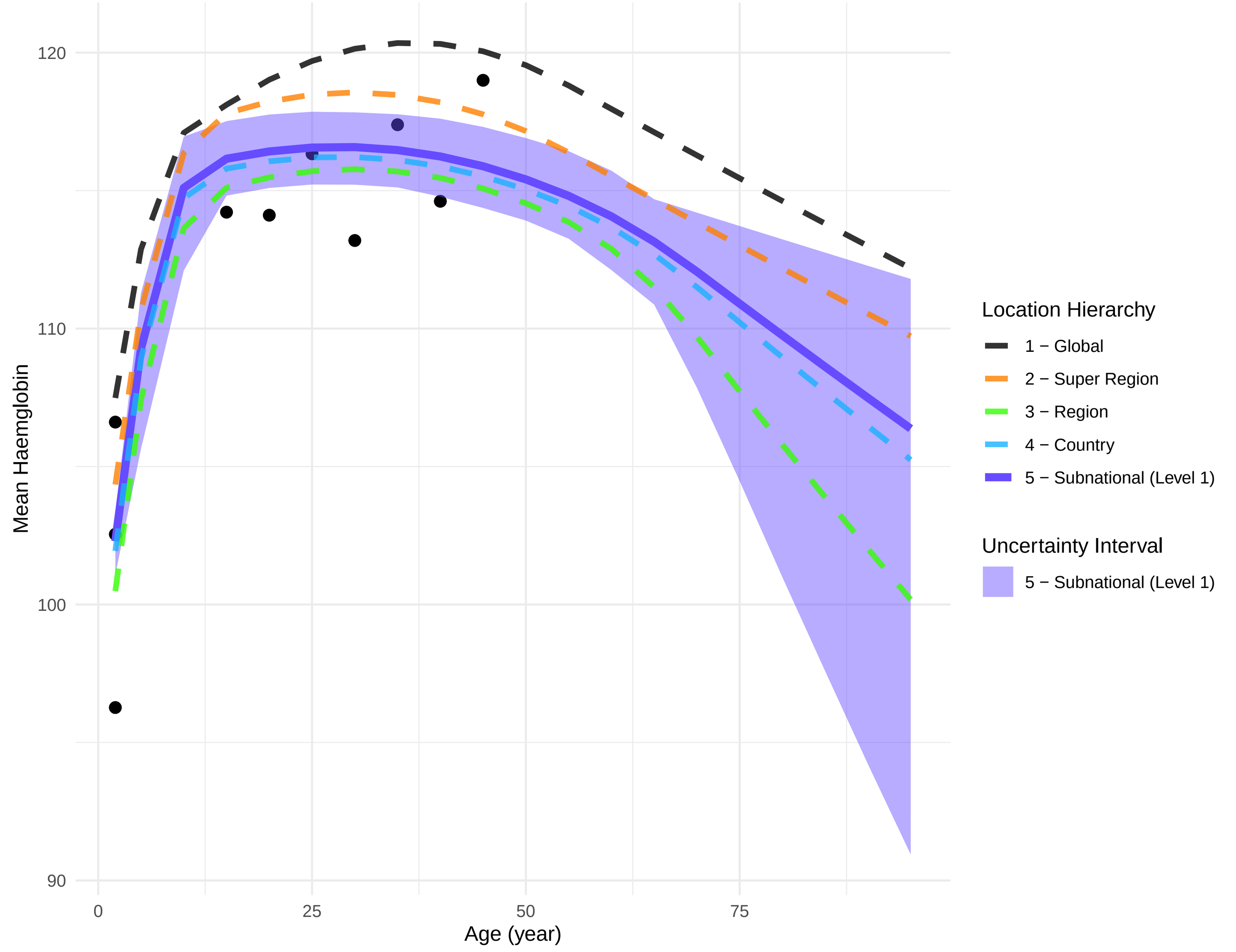

MR−BRT Age/Sex Fits − Non−pregnant Females 2+ Years Old − Mean Haemglobin − Benue
WHO−2024 Adjustment Method

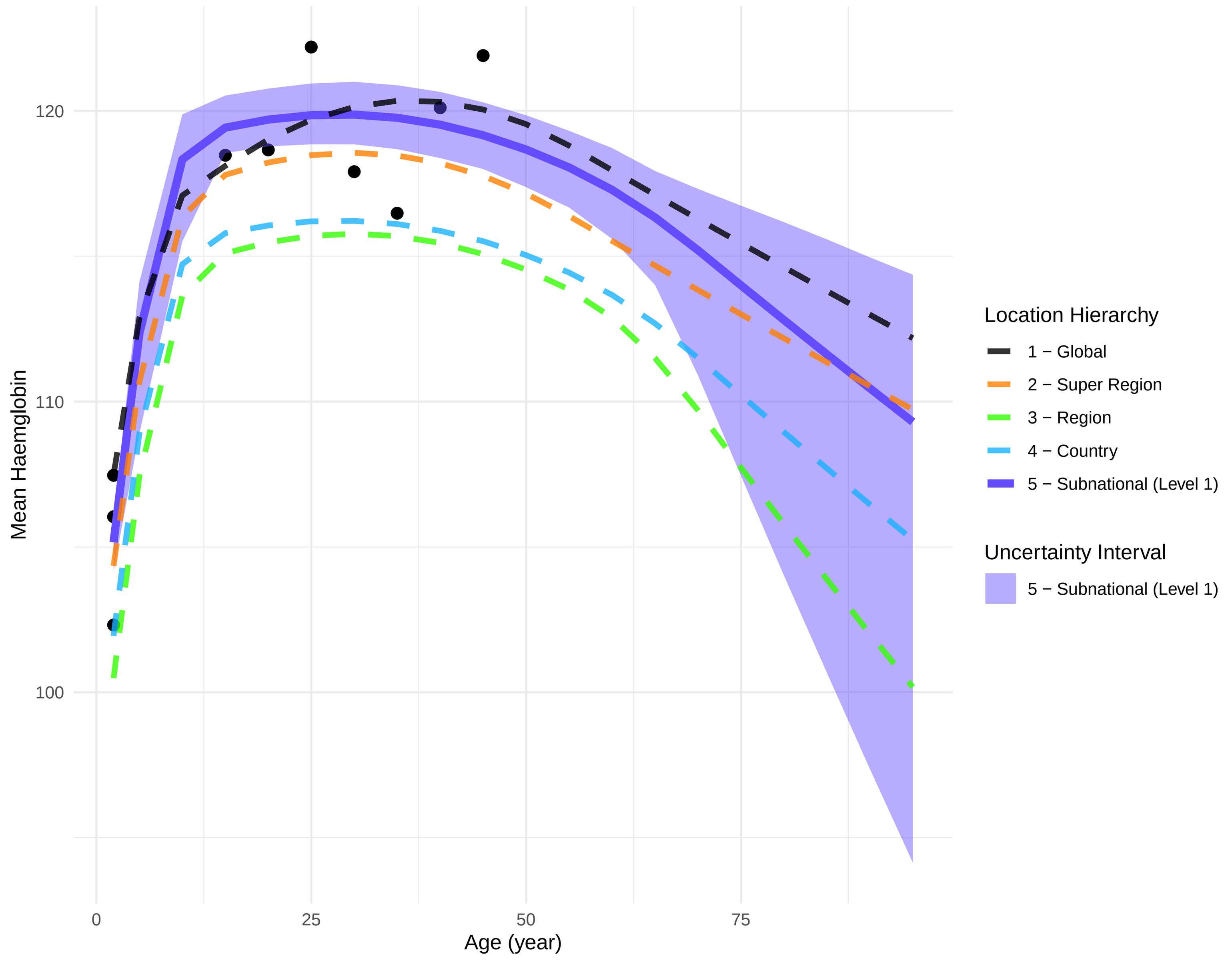

MR−BRT Age/Sex Fits − Non−pregnant Females 2+ Years Old − Mean Haemglobin − Borno
WHO−2024 Adjustment Method

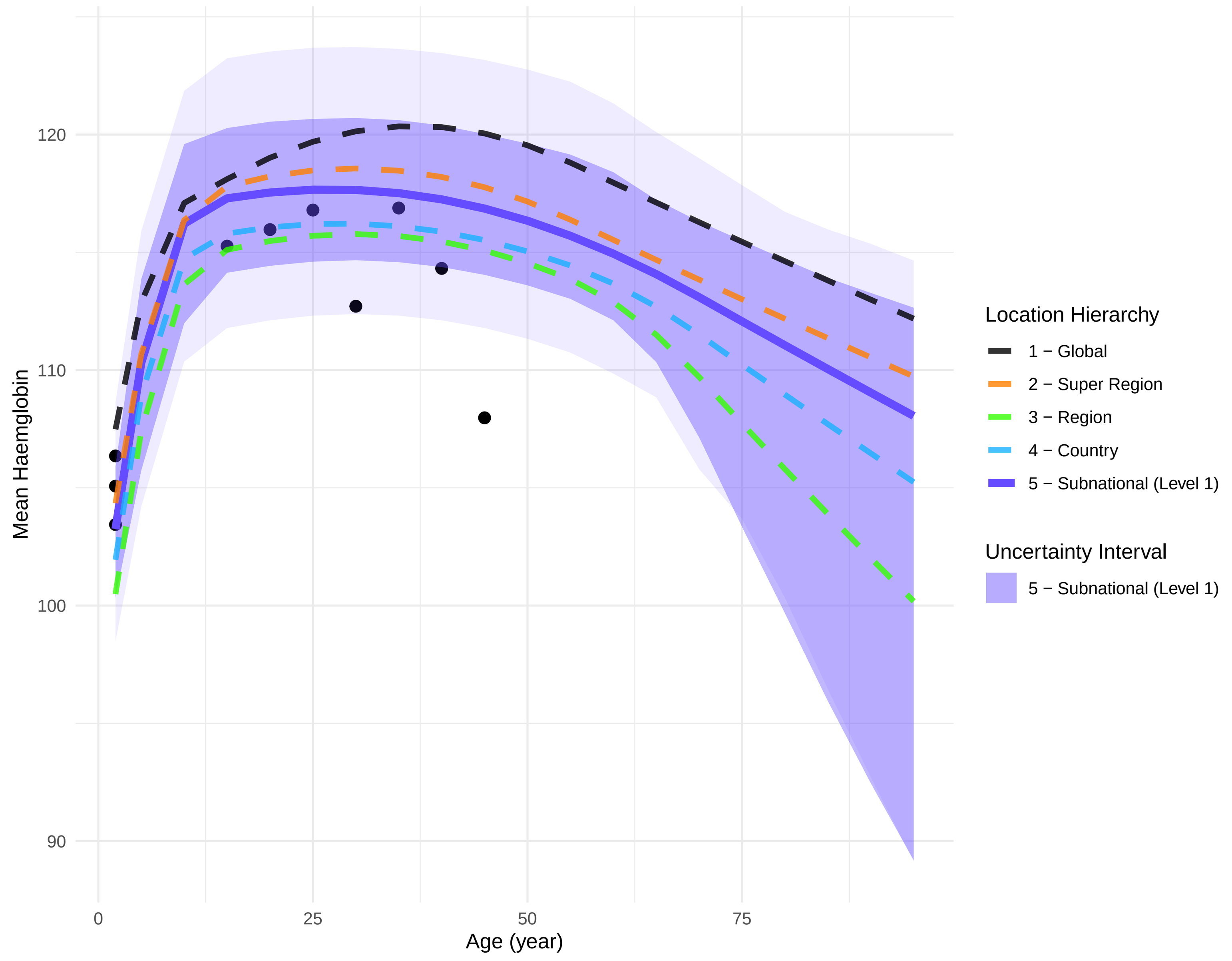

MR−BRT Age/Sex Fits − Non−pregnant Females 2+ Years Old − Mean Haemglobin − Cross River
WHO−2024 Adjustment Method

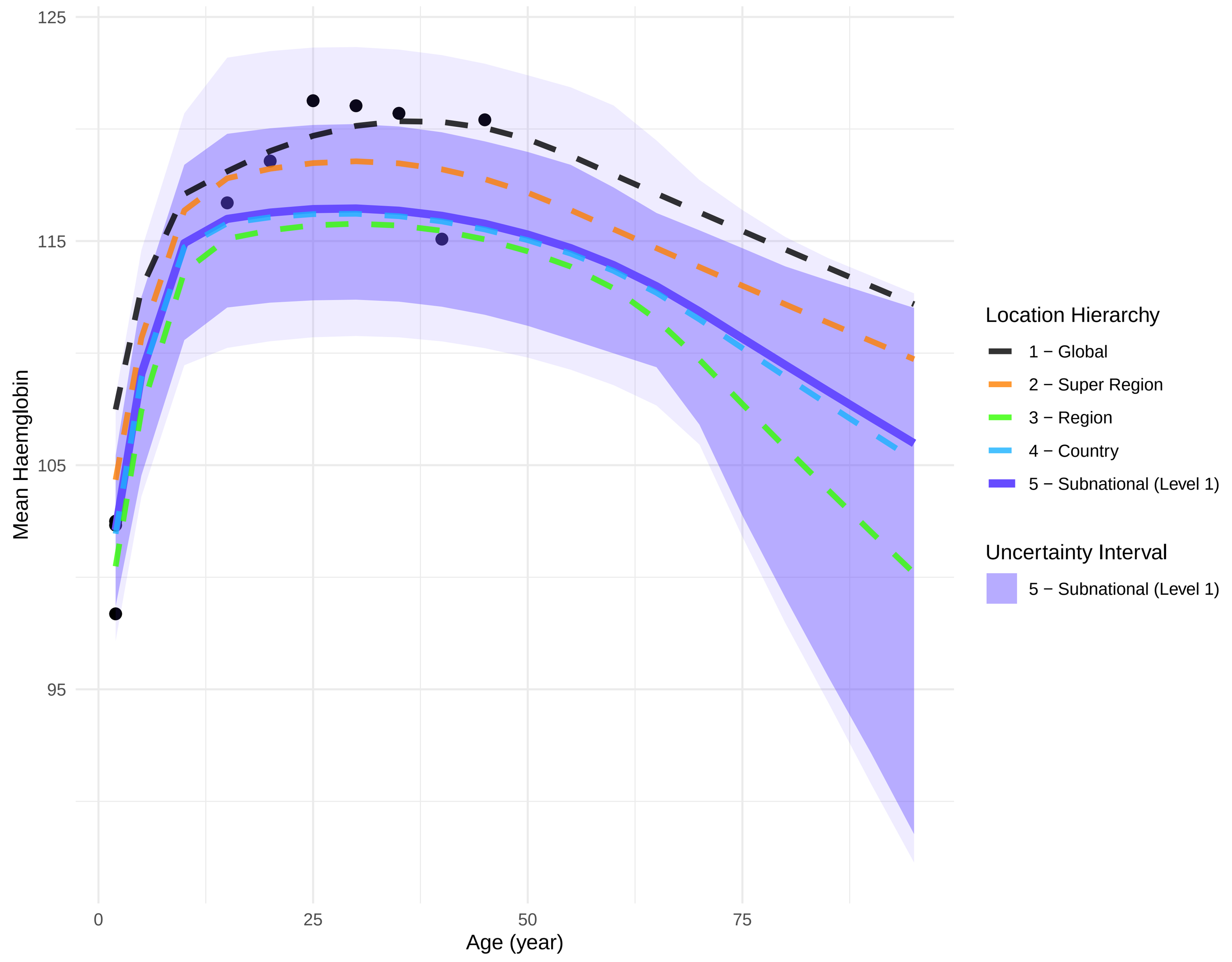

MR−BRT Age/Sex Fits − Non−pregnant Females 2+ Years Old − Mean Haemglobin − Delta
WHO−2024 Adjustment Method

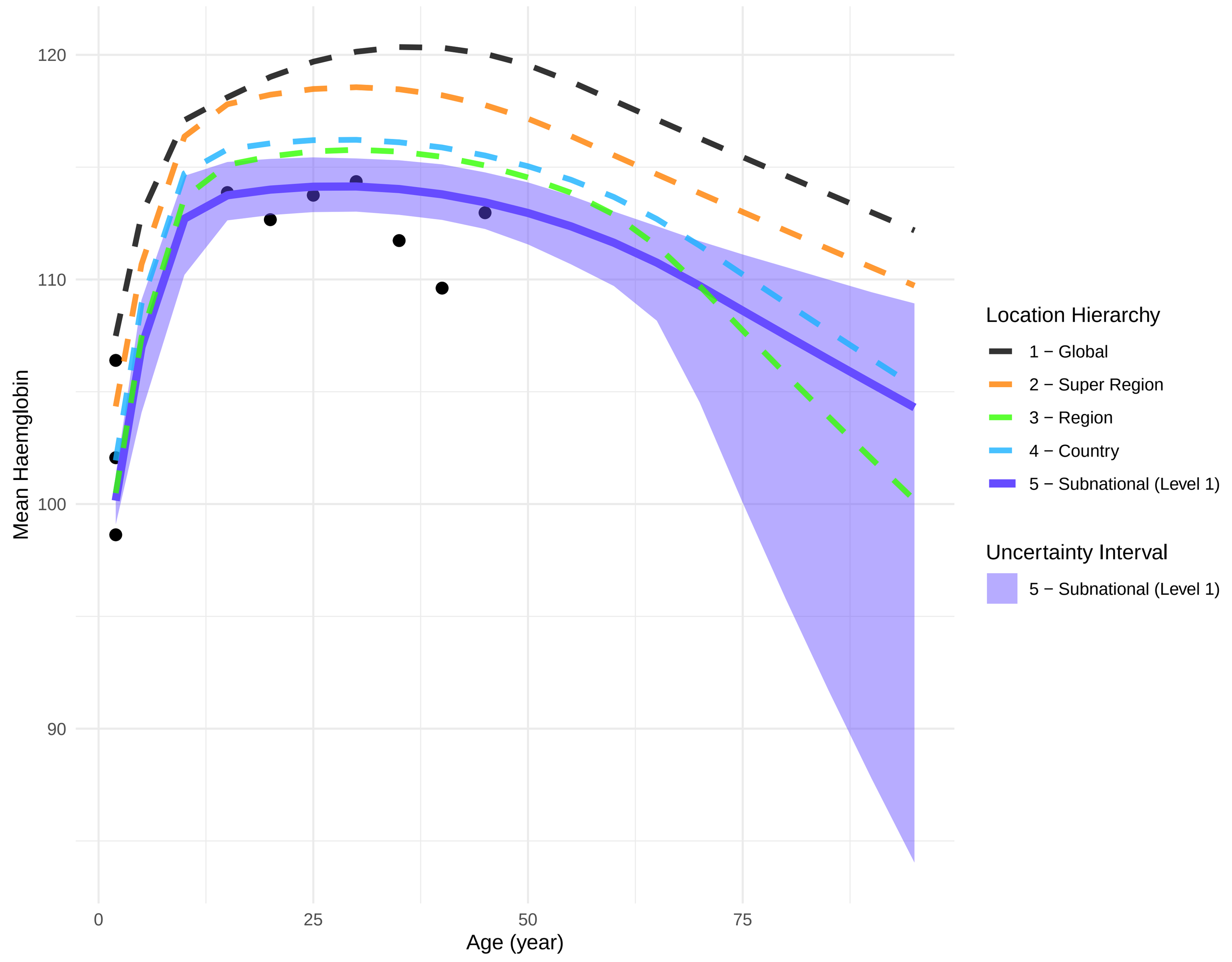

MR−BRT Age/Sex Fits − Non−pregnant Females 2+ Years Old − Mean Haemglobin − Ebonyi
WHO−2024 Adjustment Method

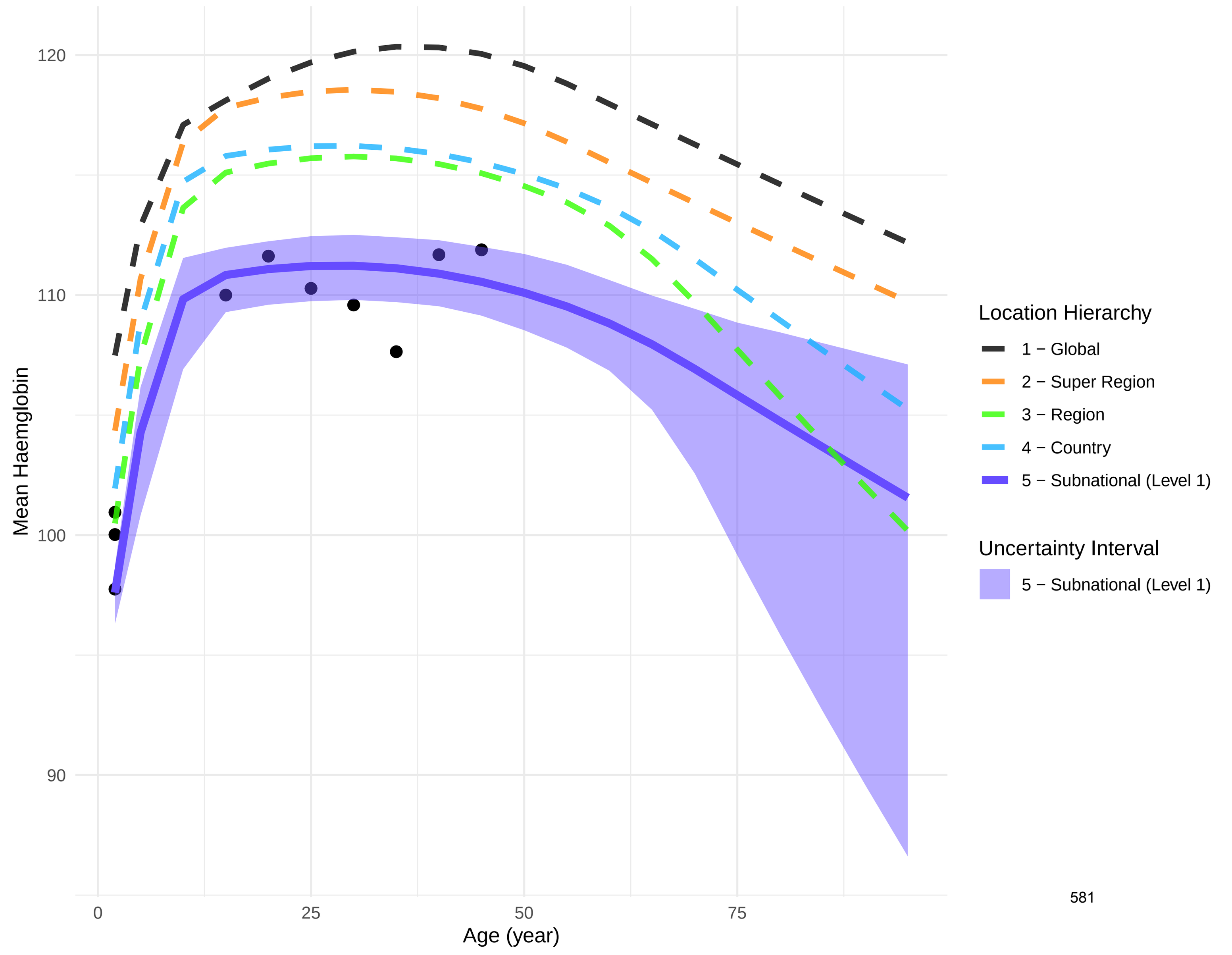

MR−BRT Age/Sex Fits − Non−pregnant Females 2+ Years Old − Mean Haemglobin − Edo
WHO−2024 Adjustment Method

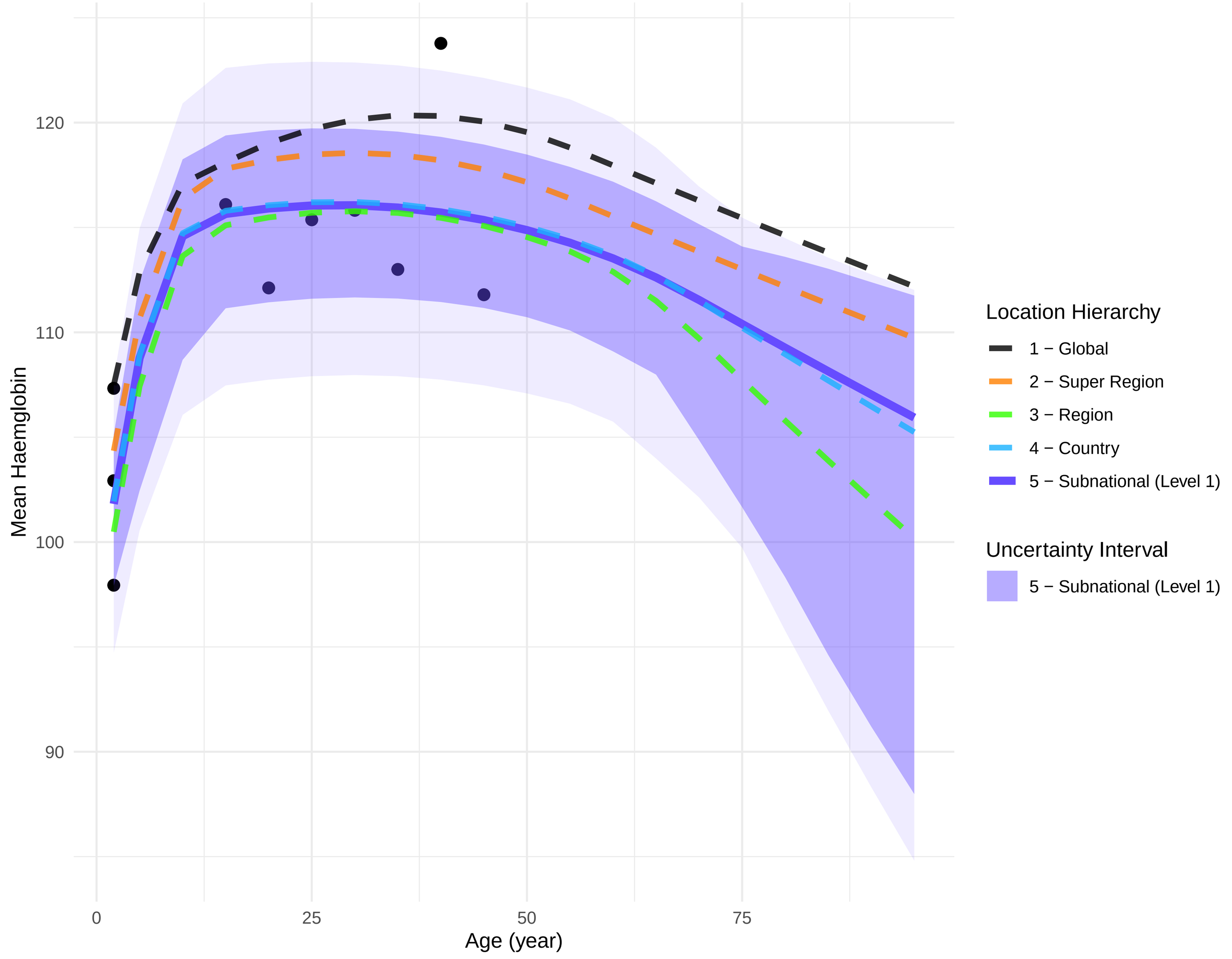

MR−BRT Age/Sex Fits − Non−pregnant Females 2+ Years Old − Mean Haemglobin − Ekiti
WHO−2024 Adjustment Method

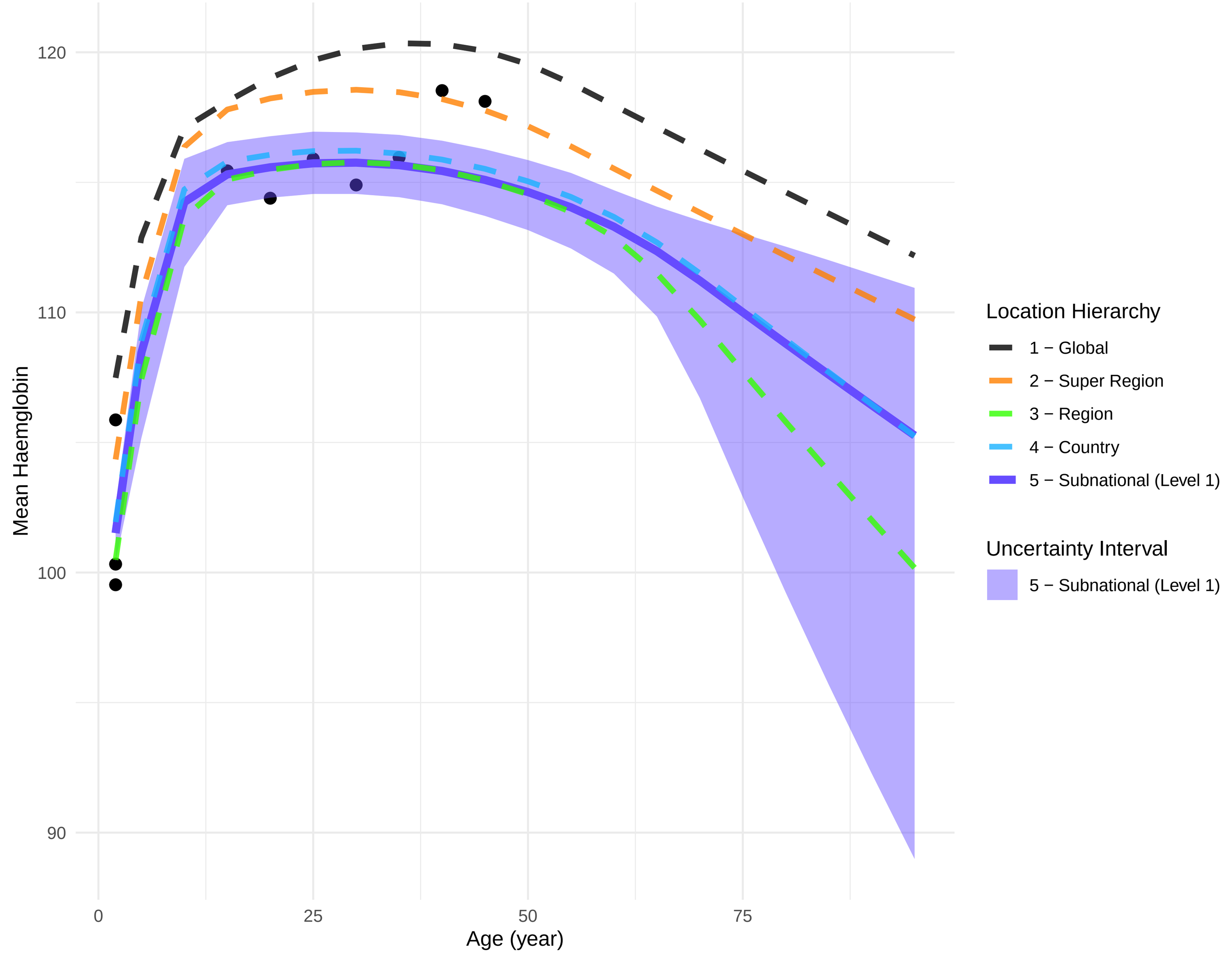

MR−BRT Age/Sex Fits − Non−pregnant Females 2+ Years Old − Mean Haemglobin − Enugu
WHO−2024 Adjustment Method

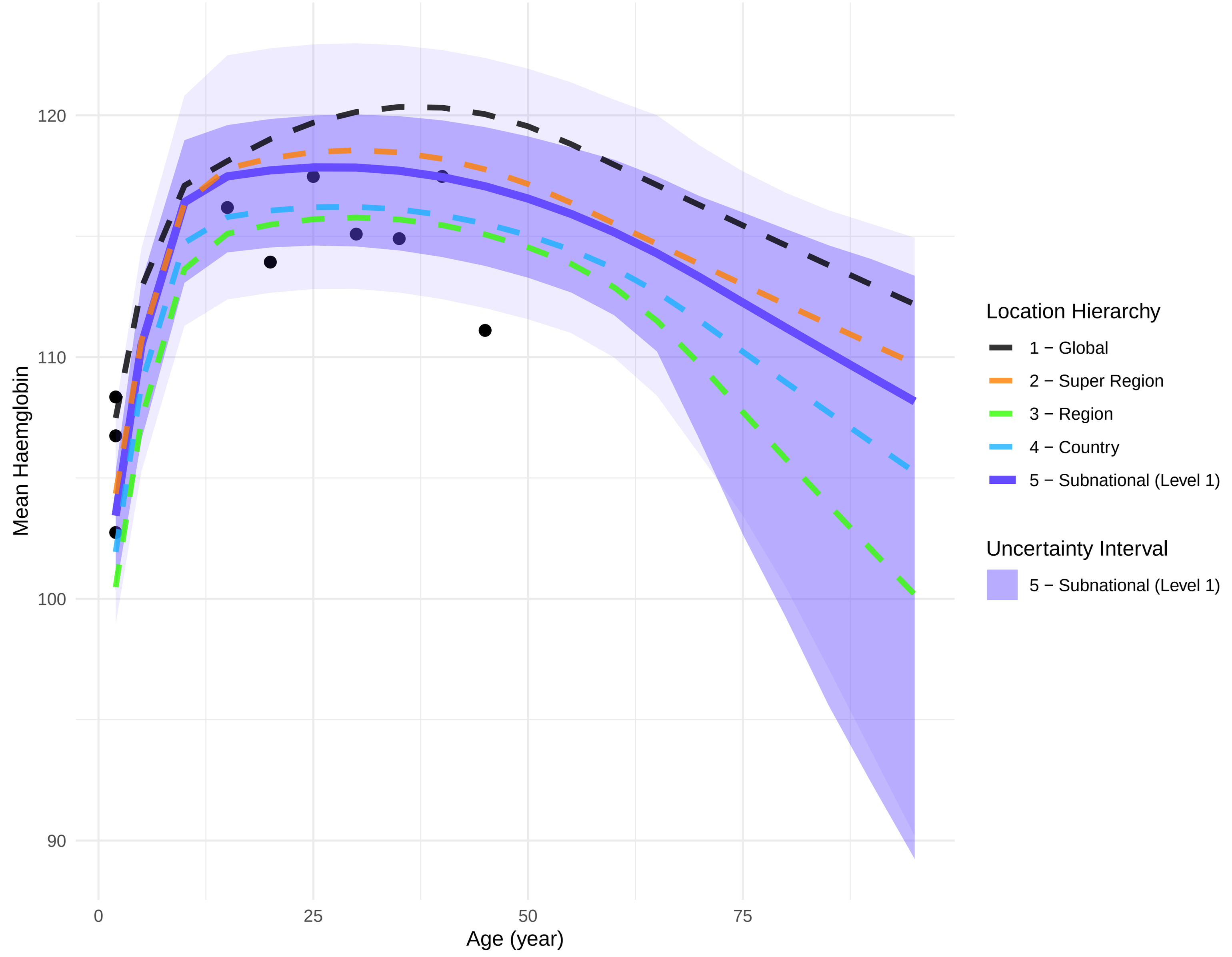

MR−BRT Age/Sex Fits − Non−pregnant Females 2+ Years Old − Mean Haemglobin − FCT (Abuja)
WHO−2024 Adjustment Method

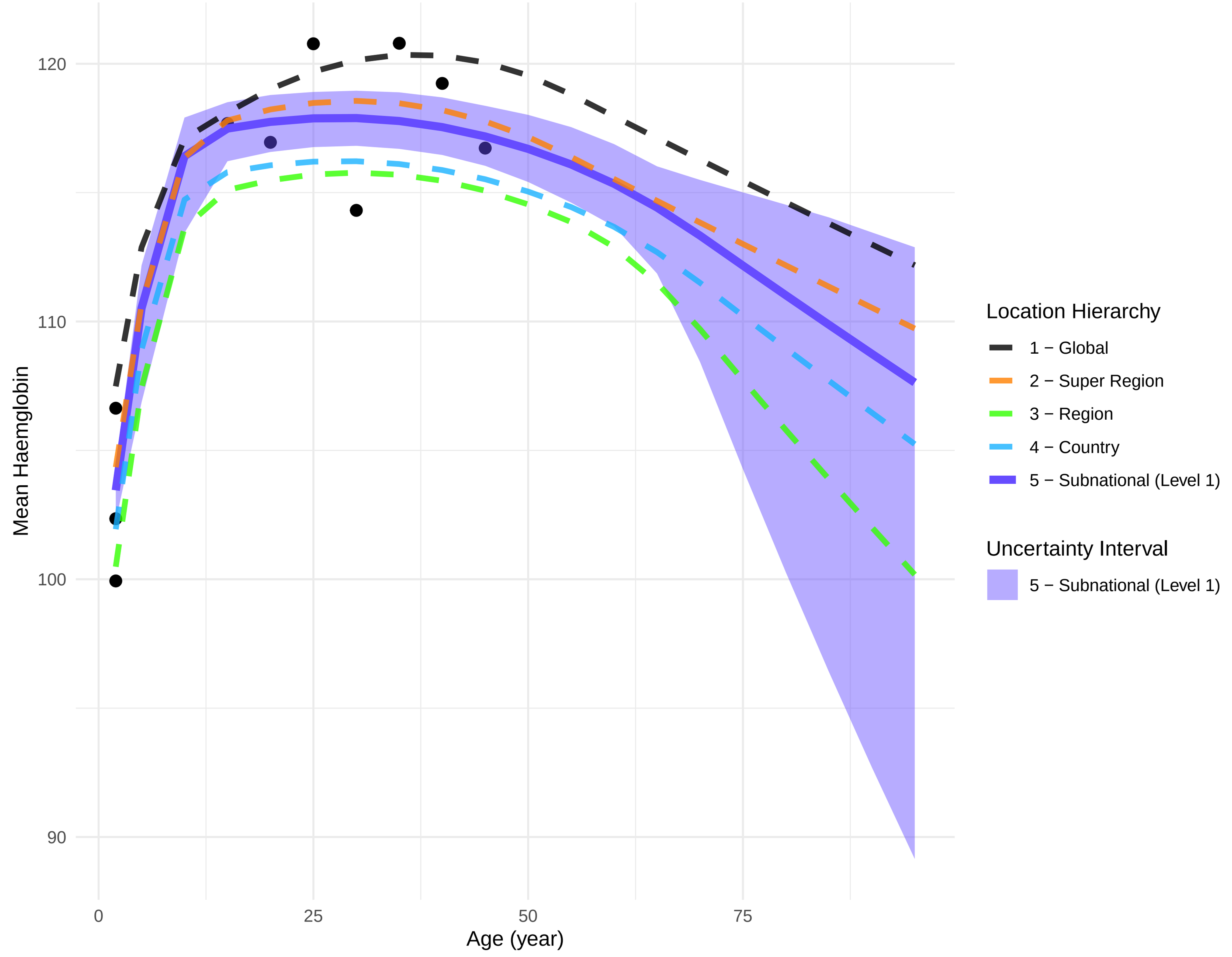

MR−BRT Age/Sex Fits − Non−pregnant Females 2+ Years Old − Mean Haemglobin − Gombe
WHO−2024 Adjustment Method

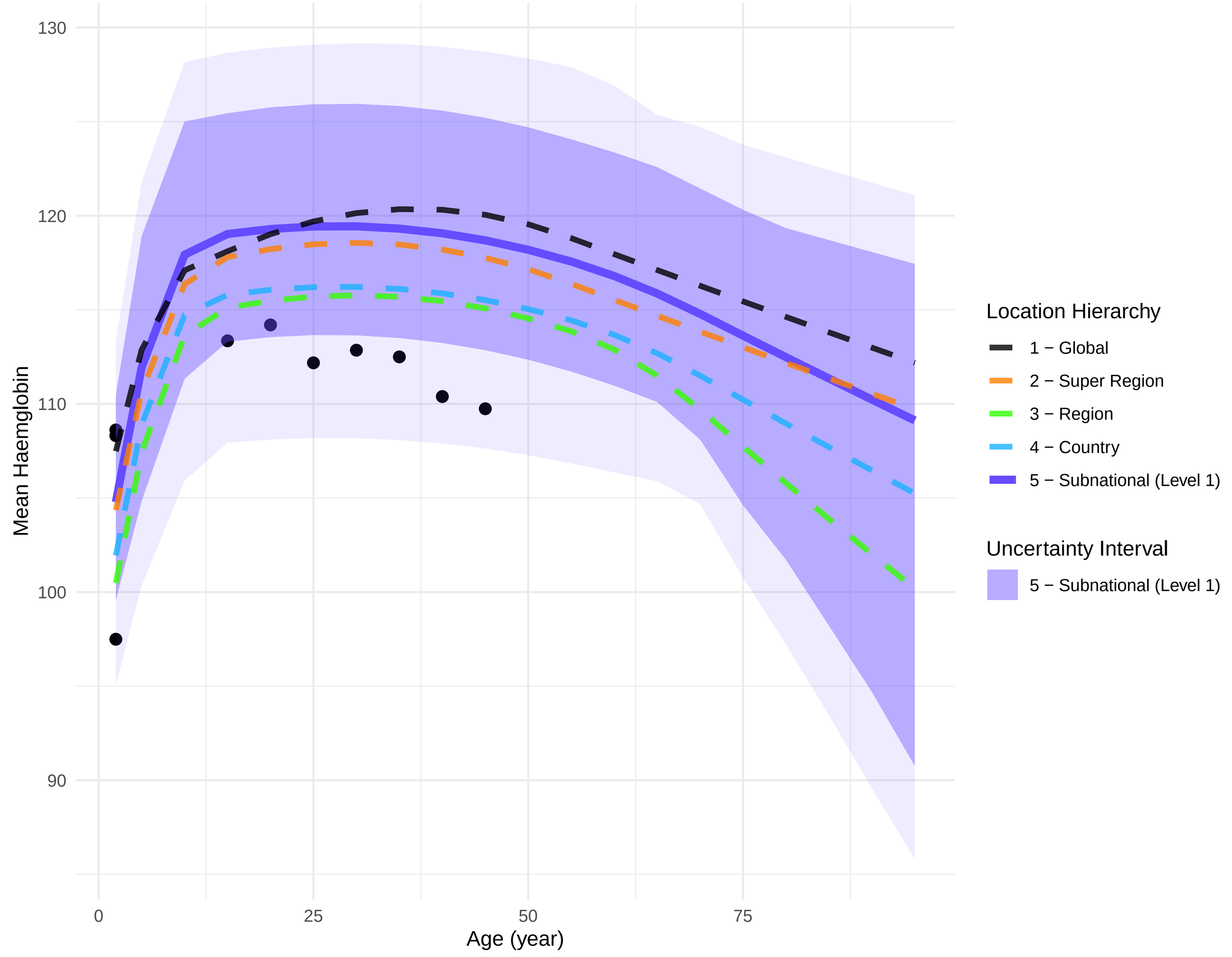

MR−BRT Age/Sex Fits − Non−pregnant Females 2+ Years Old − Mean Haemglobin − Imo
WHO−2024 Adjustment Method

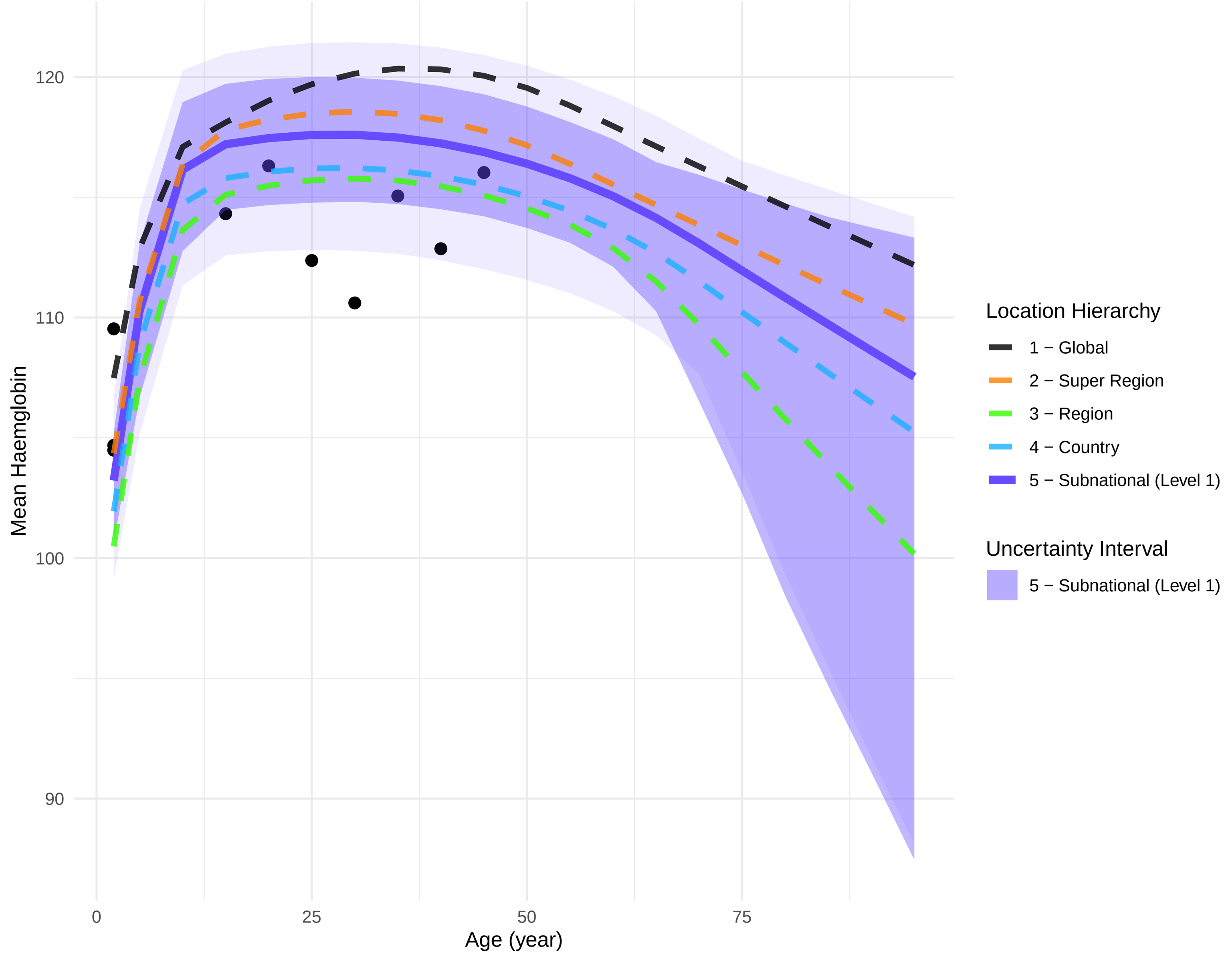

MR−BRT Age/Sex Fits − Non−pregnant Females 2+ Years Old − Mean Haemglobin − Jigawa
WHO−2024 Adjustment Method

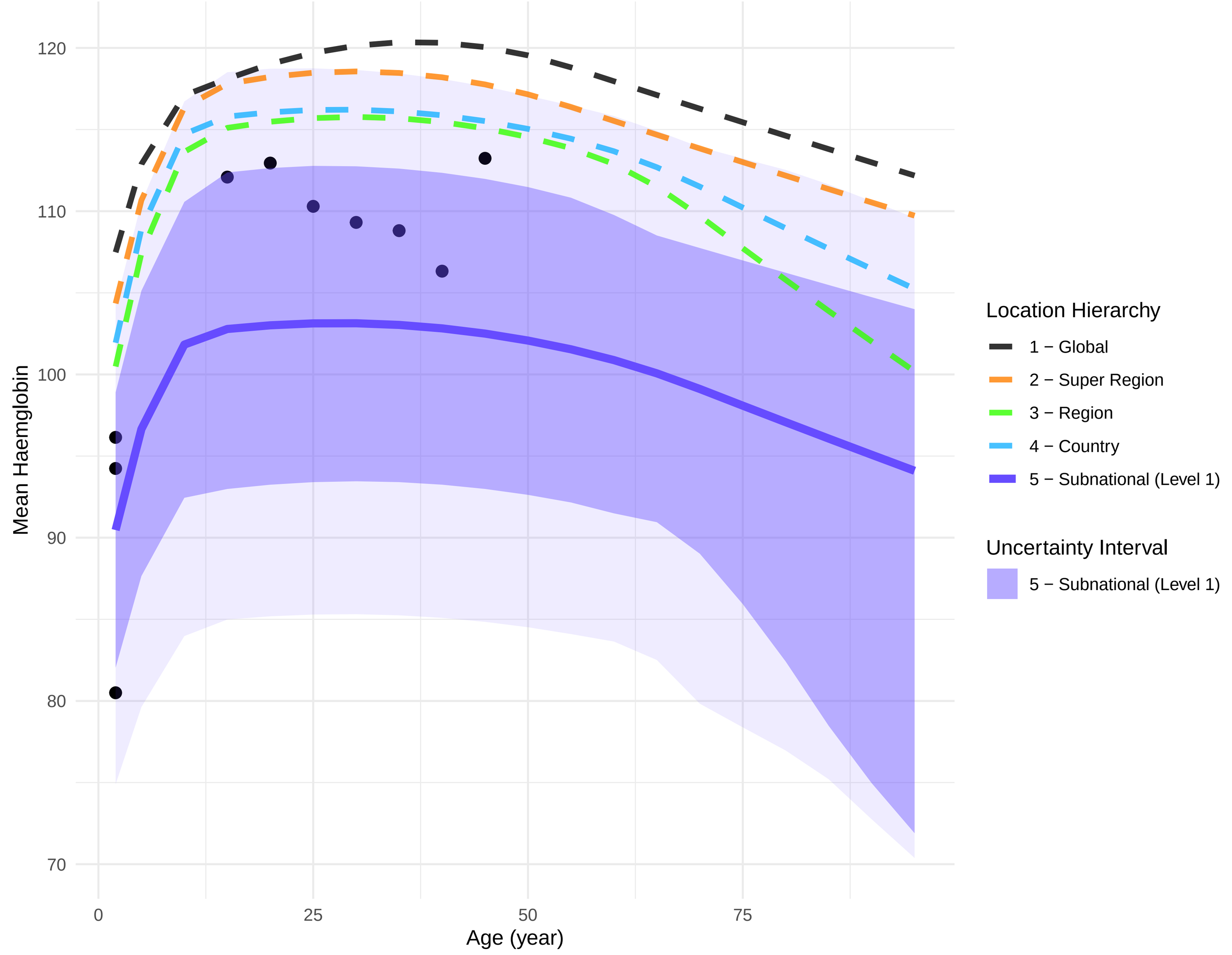

MR−BRT Age/Sex Fits − Non−pregnant Females 2+ Years Old − Mean Haemglobin − Kaduna
WHO−2024 Adjustment Method

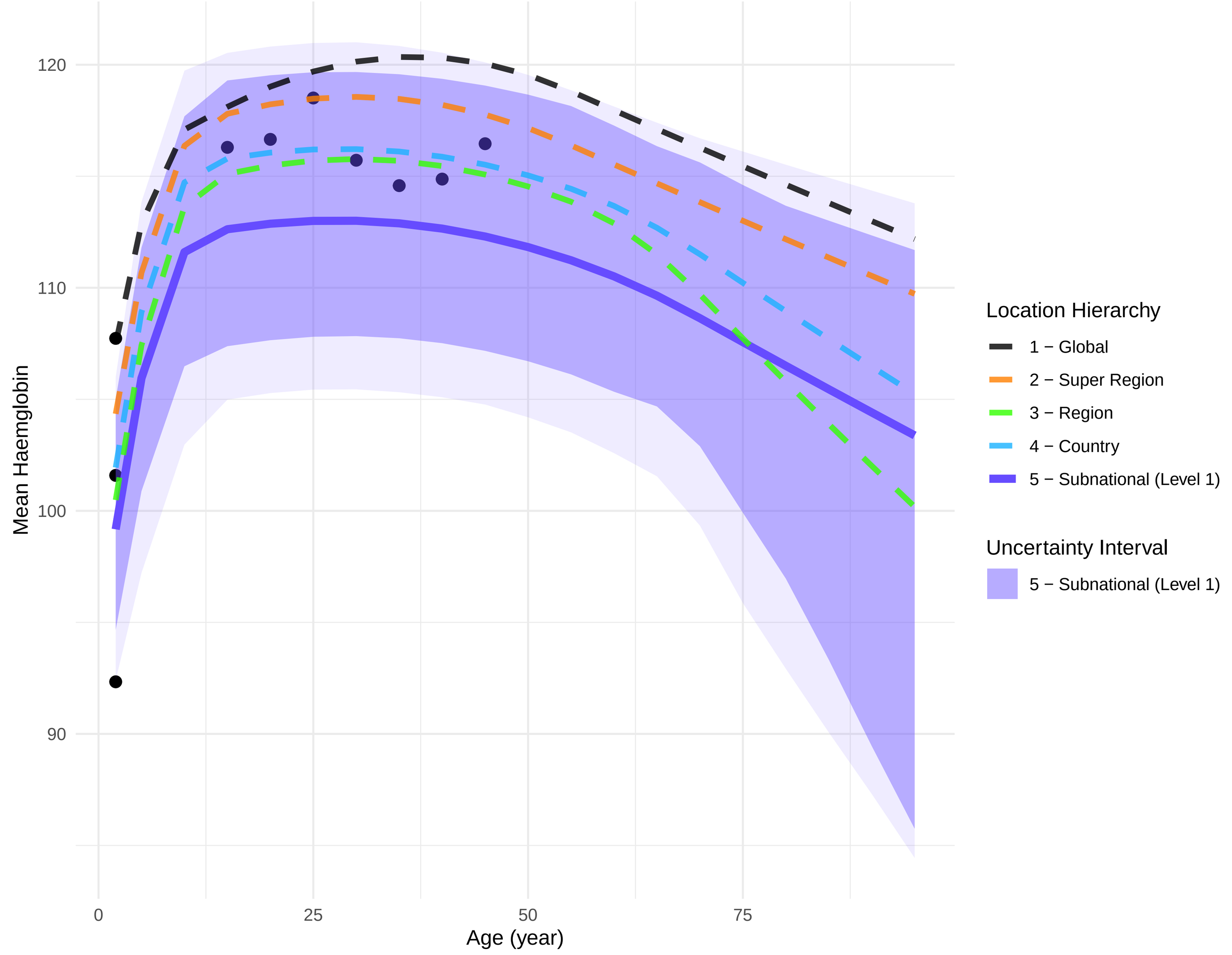

MR−BRT Age/Sex Fits − Non−pregnant Females 2+ Years Old − Mean Haemglobin − Kano
WHO−2024 Adjustment Method

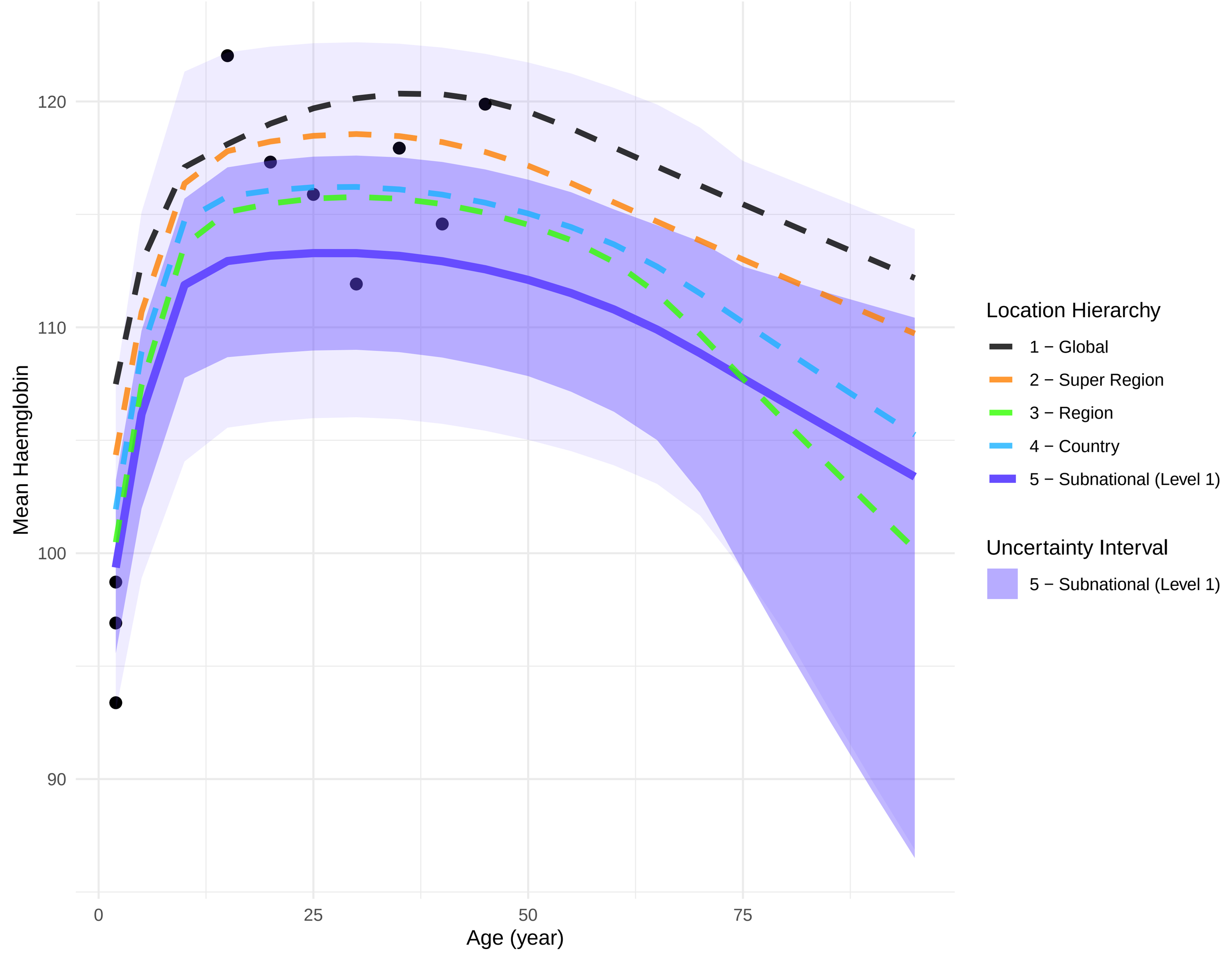

MR−BRT Age/Sex Fits − Non−pregnant Females 2+ Years Old − Mean Haemglobin − Katsina
WHO−2024 Adjustment Method

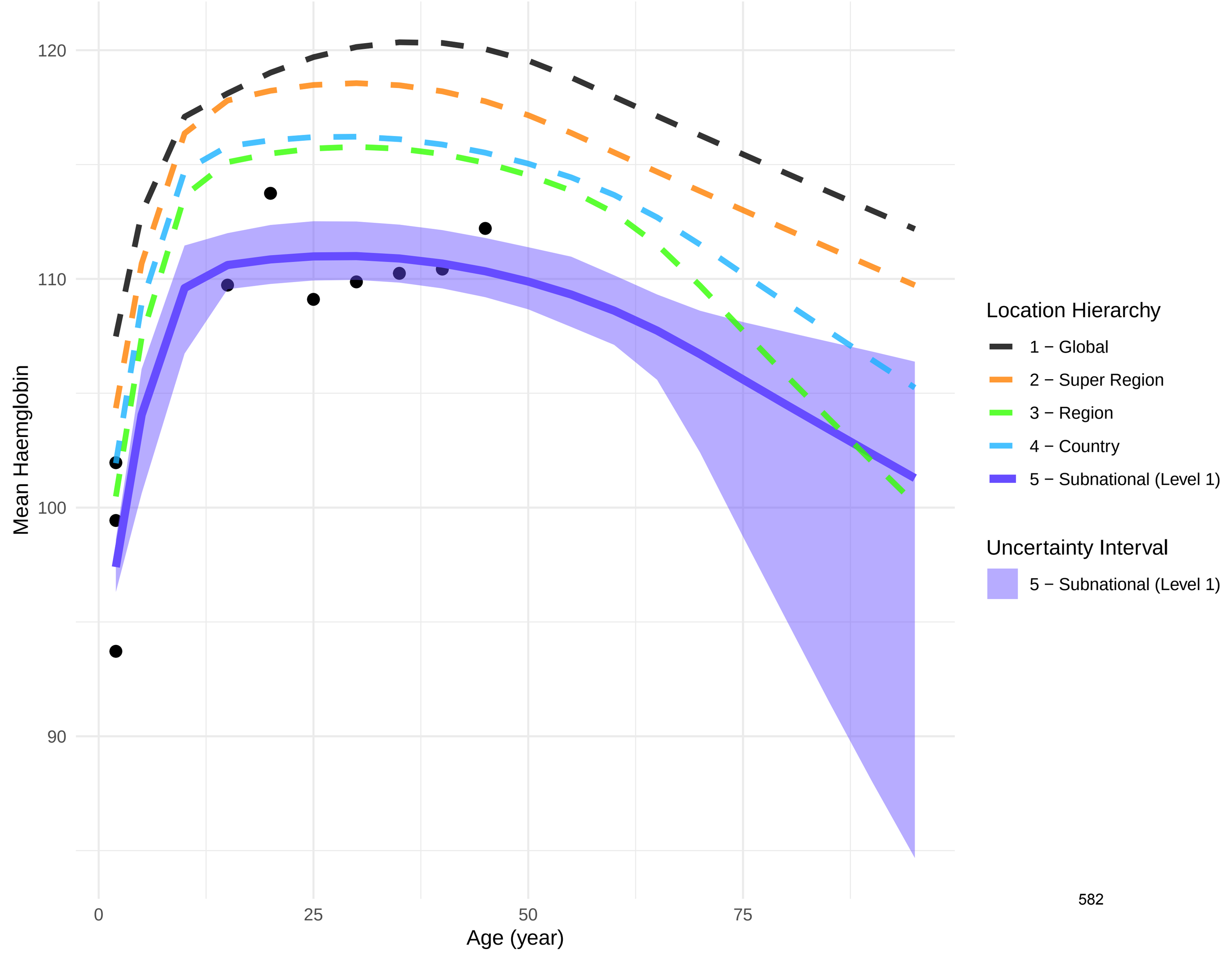

MR−BRT Age/Sex Fits − Non−pregnant Females 2+ Years Old − Mean Haemglobin − Kebbi
WHO−2024 Adjustment Method

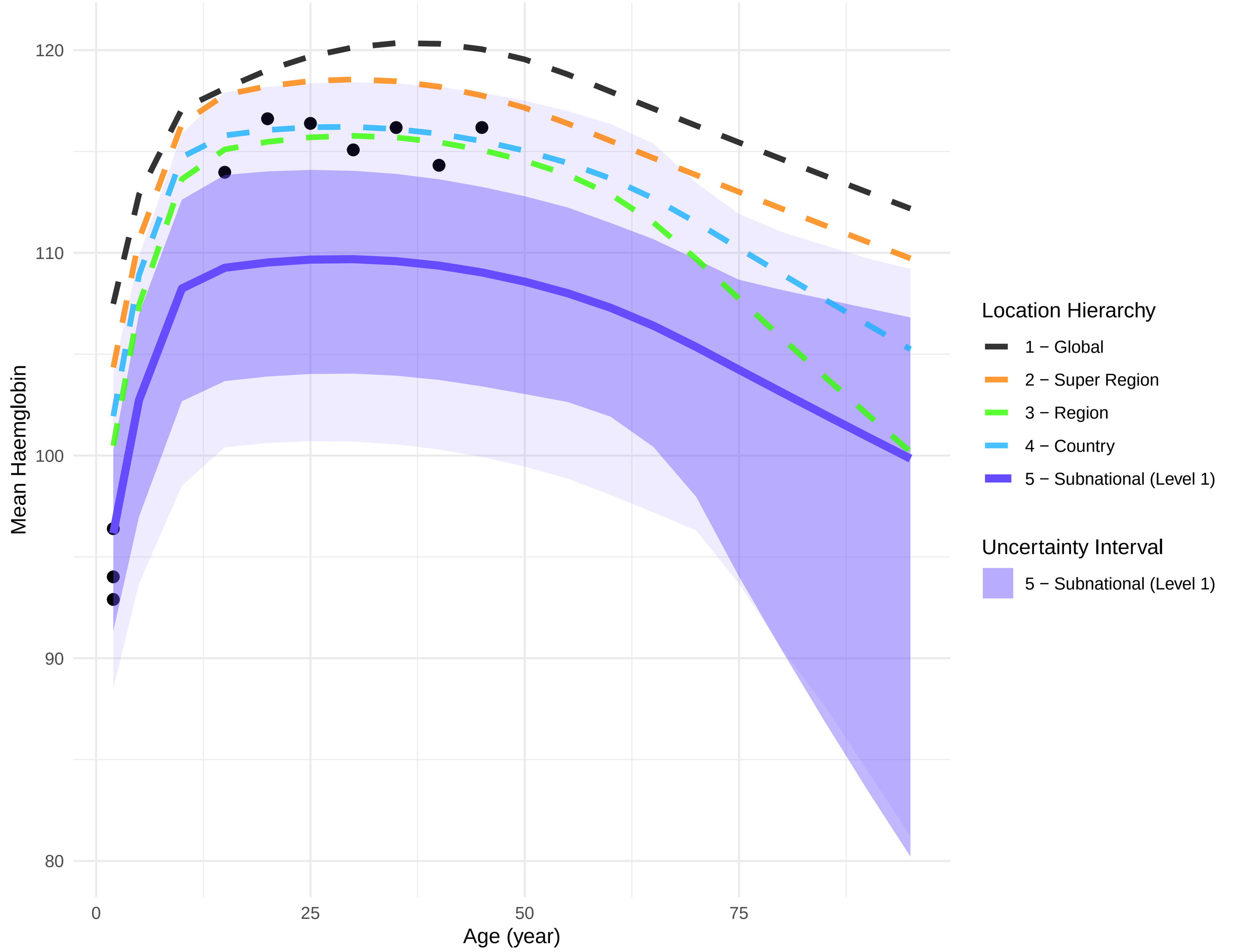

MR−BRT Age/Sex Fits − Non−pregnant Females 2+ Years Old − Mean Haemglobin − Kogi
WHO−2024 Adjustment Method

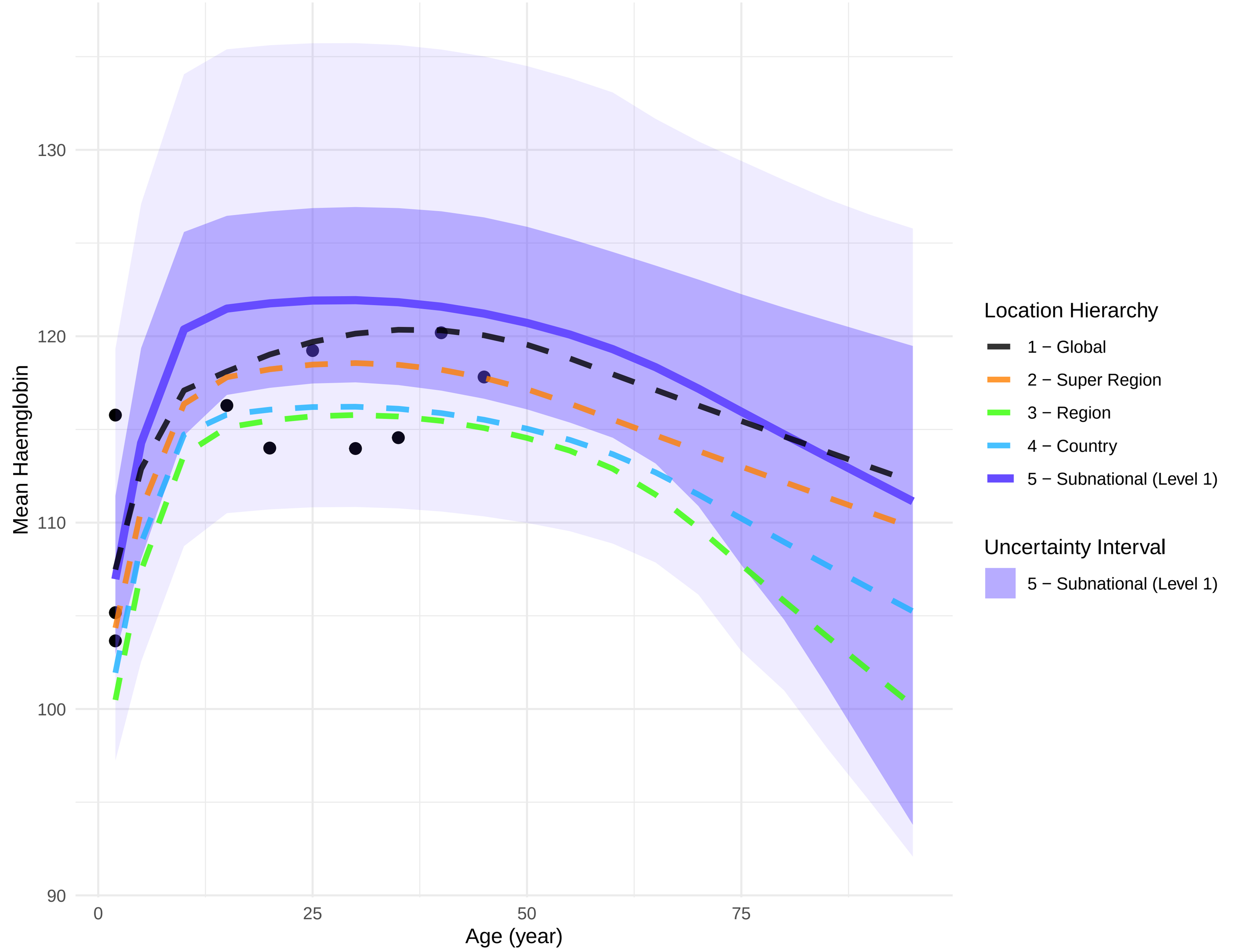

MR−BRT Age/Sex Fits − Non−pregnant Females 2+ Years Old − Mean Haemglobin − Kwara
WHO−2024 Adjustment Method

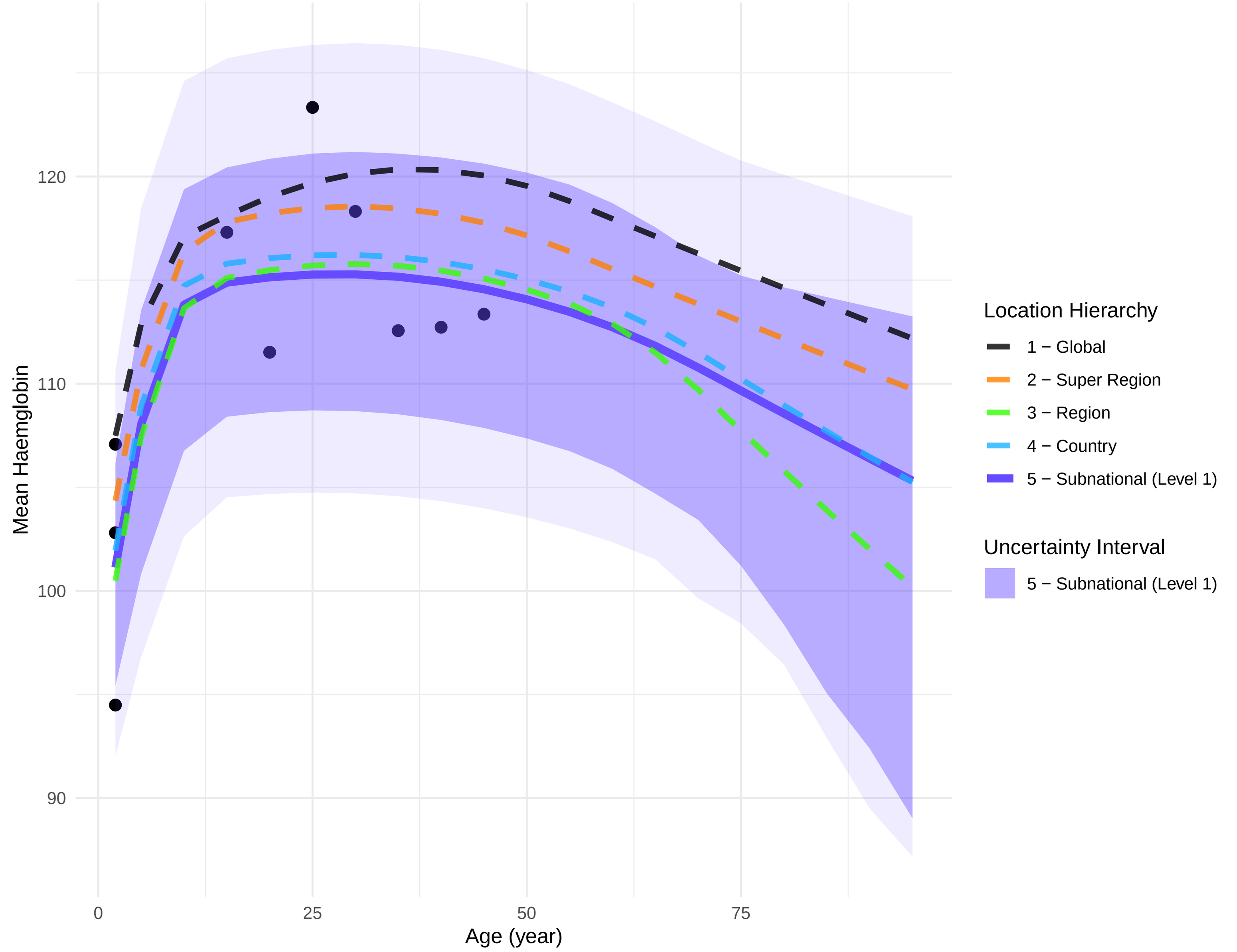

MR−BRT Age/Sex Fits − Non−pregnant Females 2+ Years Old − Mean Haemglobin − Lagos
WHO−2024 Adjustment Method

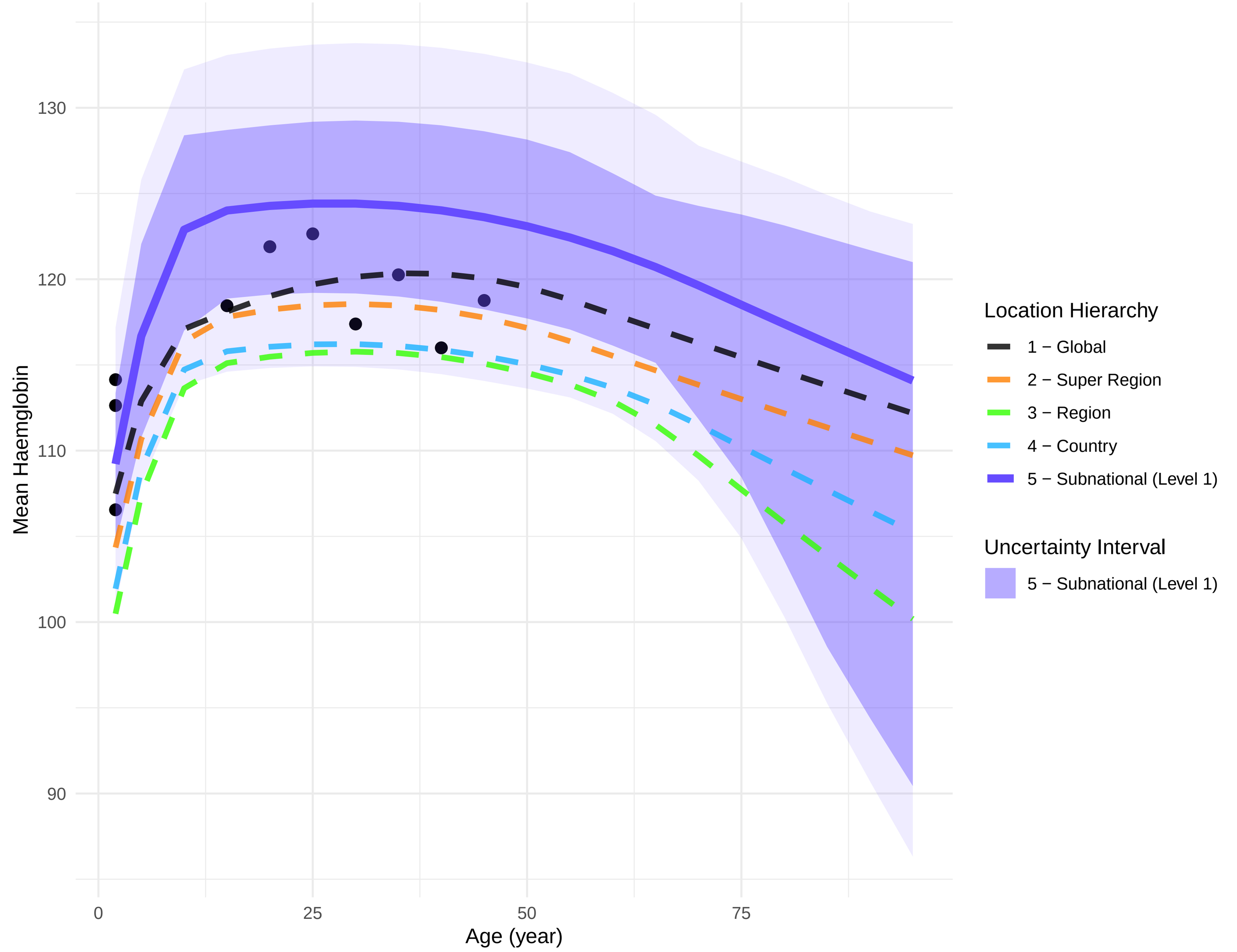

MR−BRT Age/Sex Fits − Non−pregnant Females 2+ Years Old − Mean Haemglobin − Nasarawa
WHO−2024 Adjustment Method

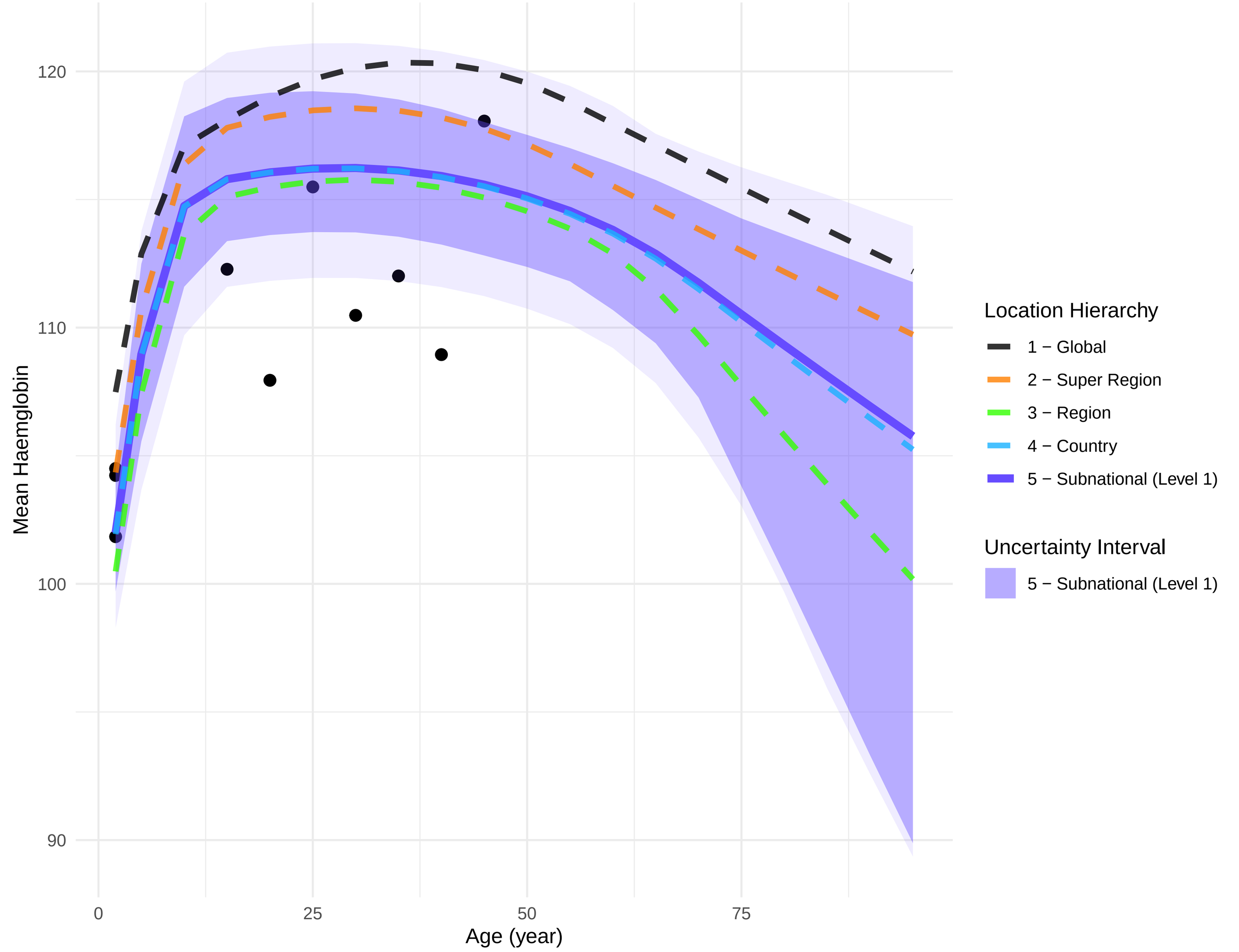

MR−BRT Age/Sex Fits − Non−pregnant Females 2+ Years Old − Mean Haemglobin − Niger
WHO−2024 Adjustment Method

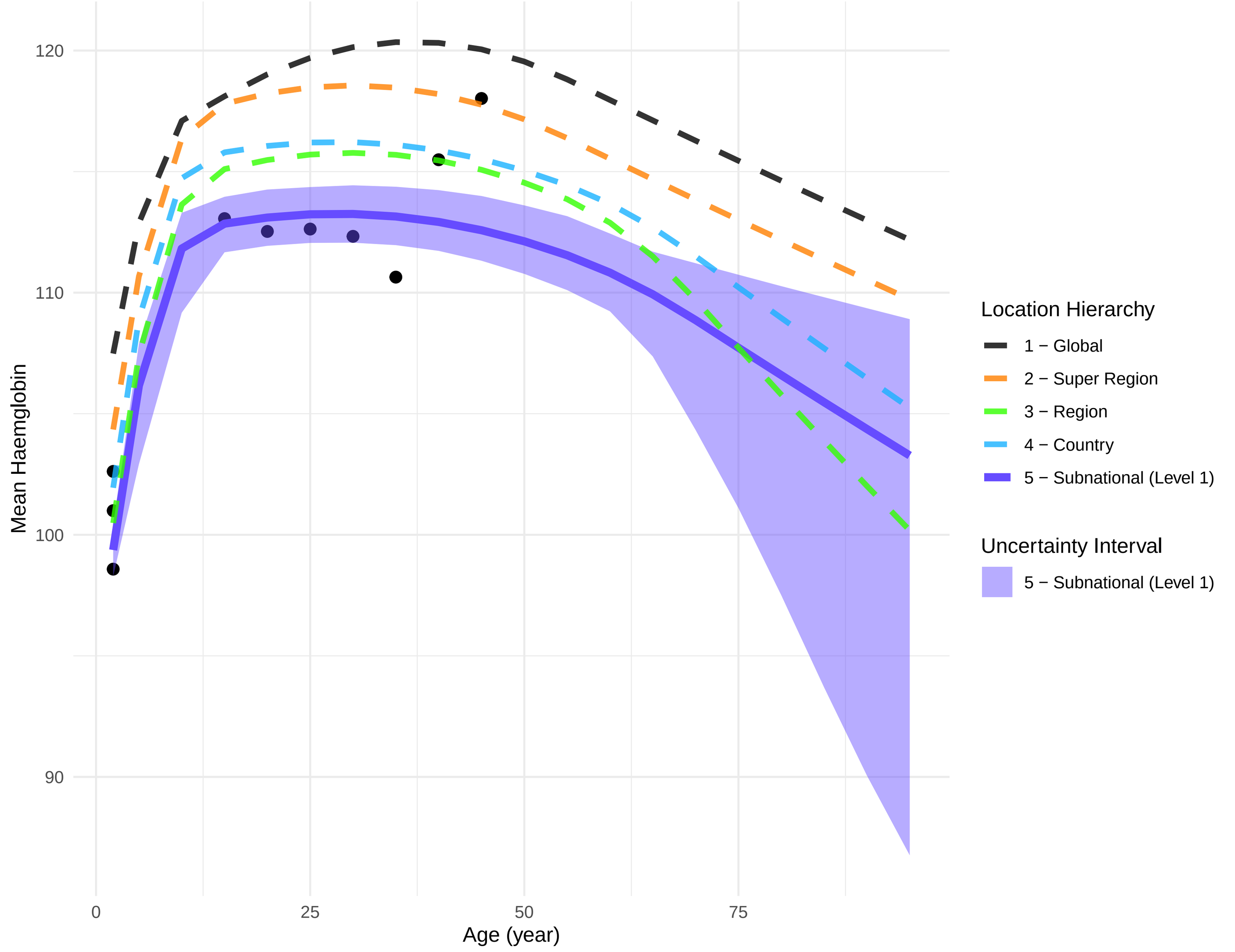

MR−BRT Age/Sex Fits − Non−pregnant Females 2+ Years Old − Mean Haemglobin − Ogun
WHO−2024 Adjustment Method

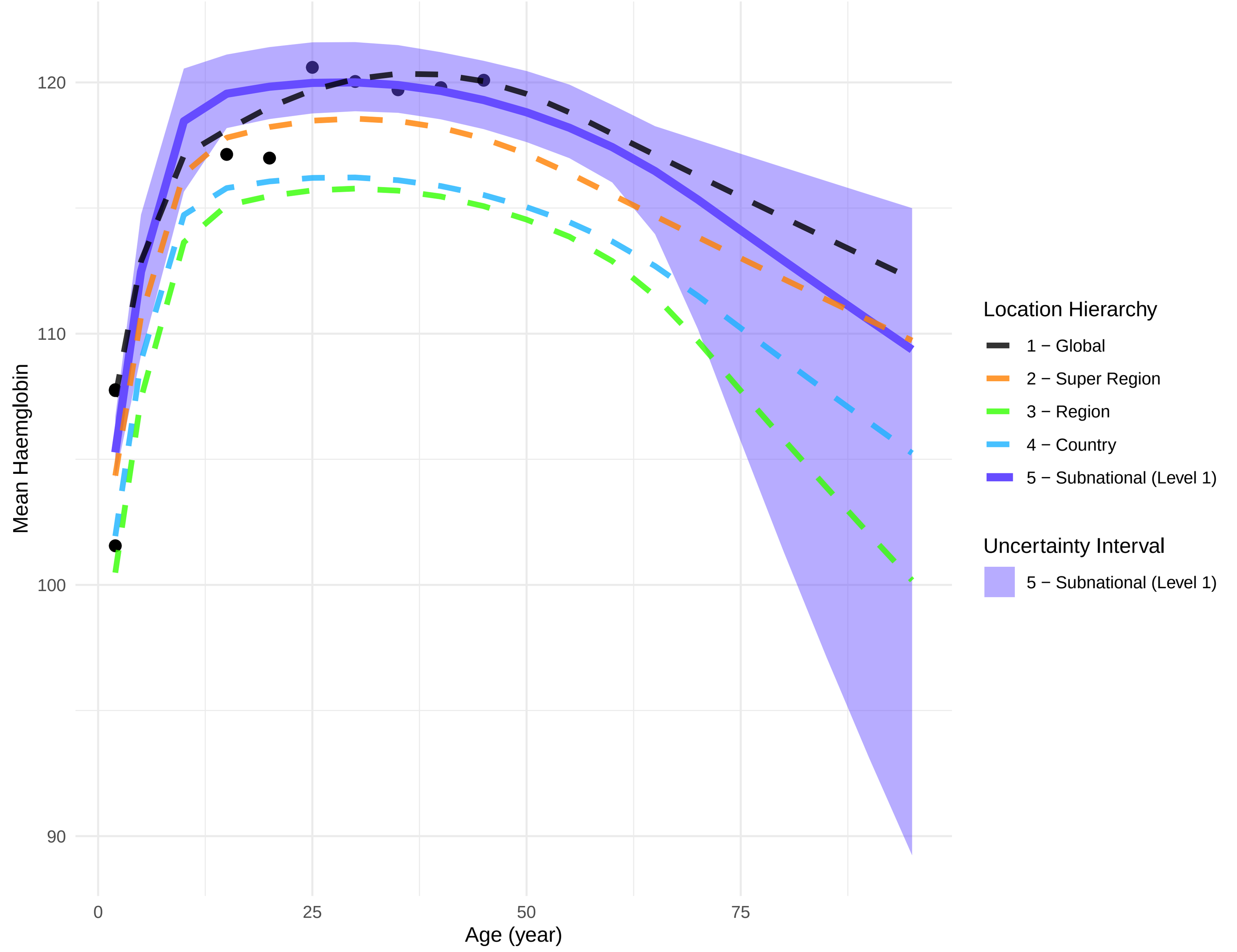

MR−BRT Age/Sex Fits − Non−pregnant Females 2+ Years Old − Mean Haemglobin − Ondo
WHO−2024 Adjustment Method

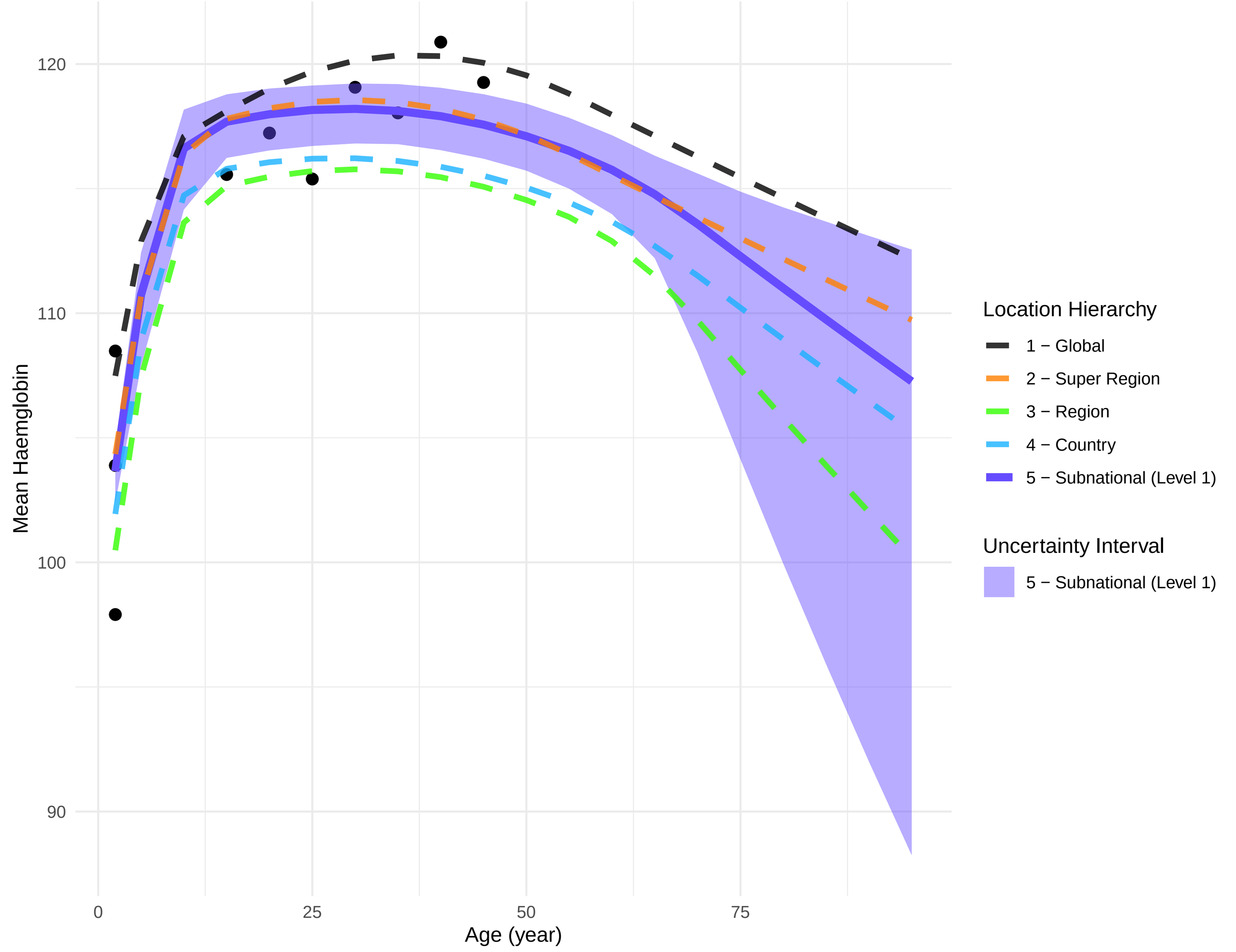

MR−BRT Age/Sex Fits − Non−pregnant Females 2+ Years Old − Mean Haemglobin − Osun
WHO−2024 Adjustment Method

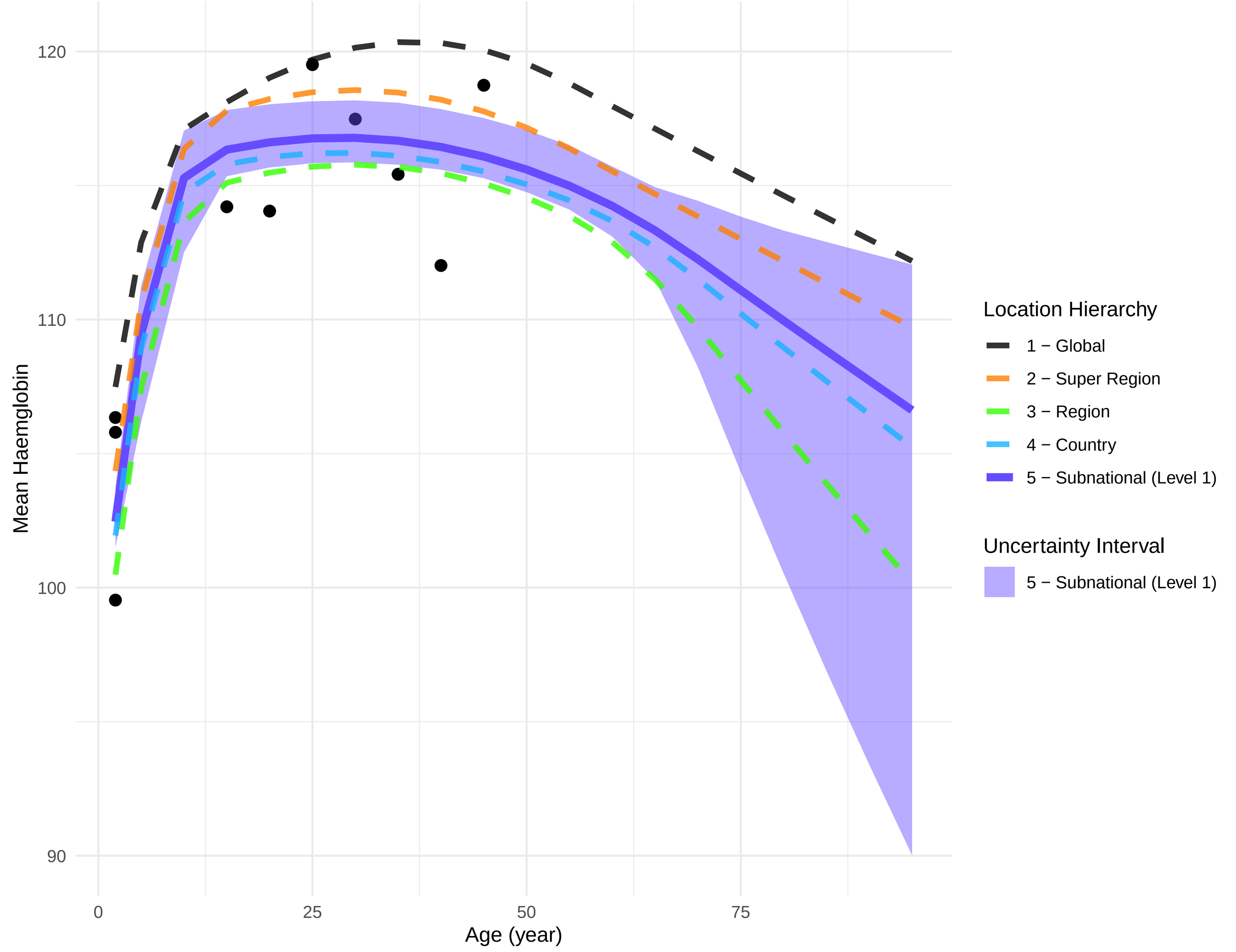

MR−BRT Age/Sex Fits − Non−pregnant Females 2+ Years Old − Mean Haemglobin − Oyo
WHO−2024 Adjustment Method

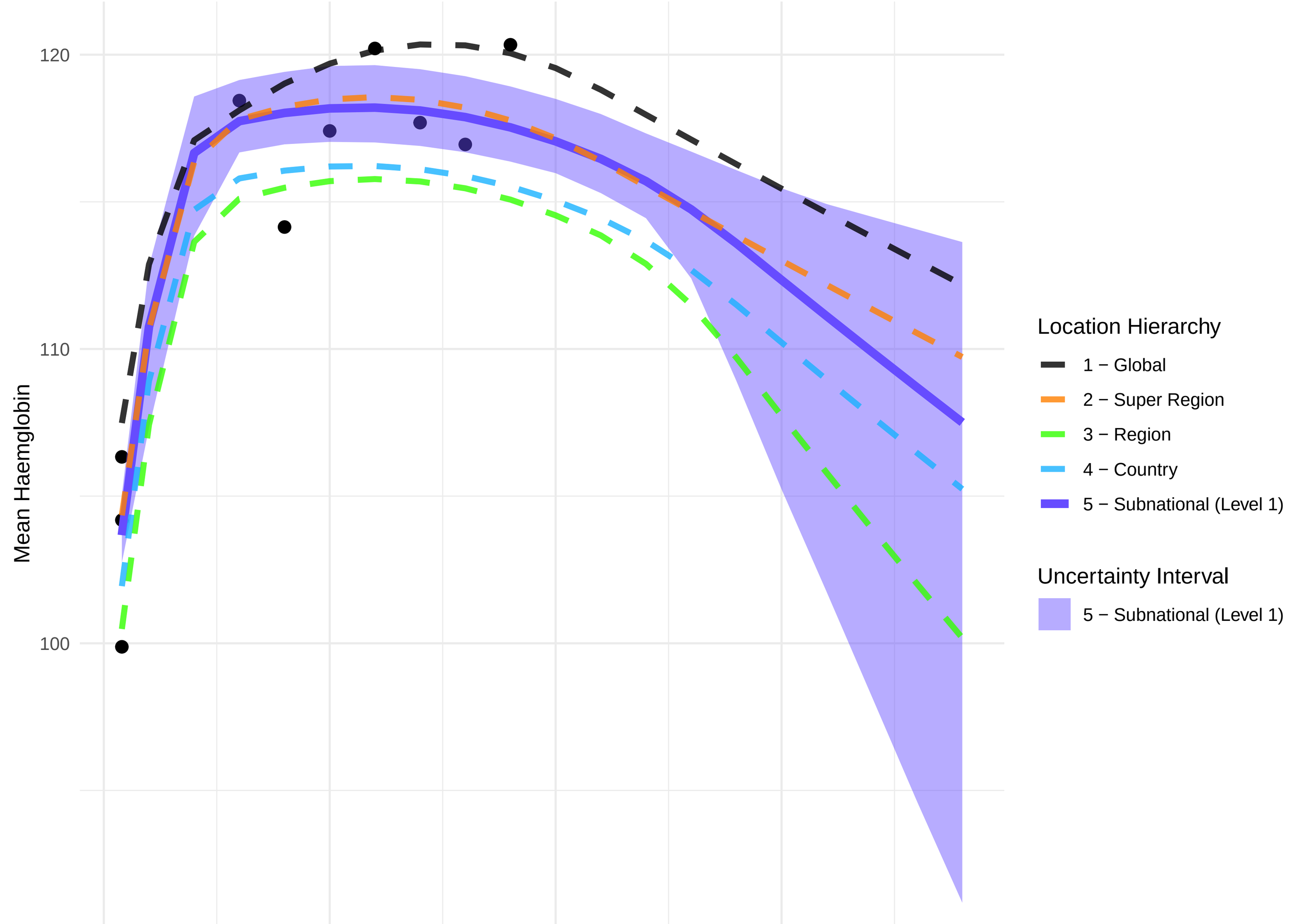

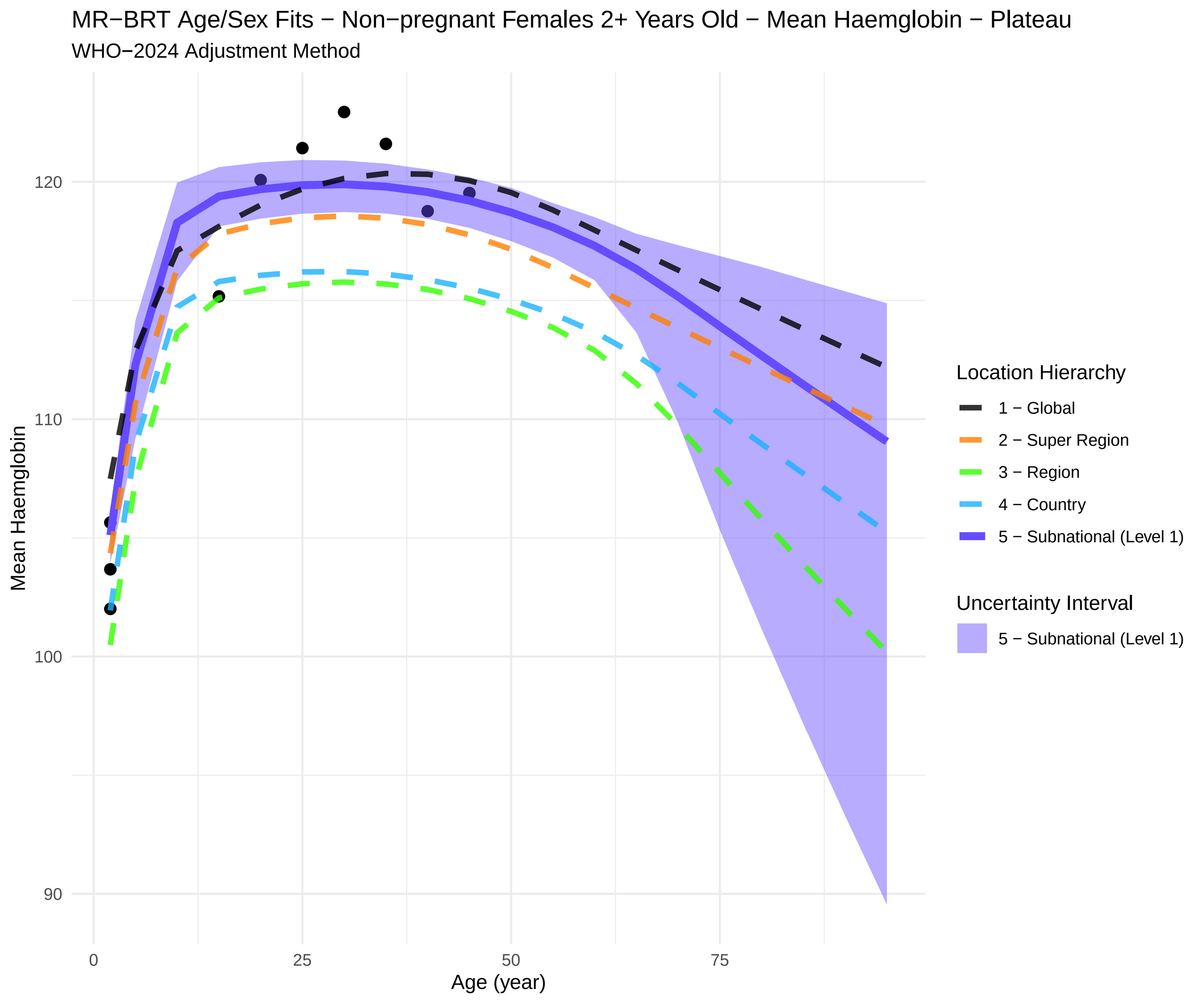
MR−BRT Age/Sex Fits − Non−pregnant Females 2+ Years Old − Mean Haemglobin − Plateau
WHO−2024 Adjustment Method
Mean Haemglobin
Age (year)
Location Hierarchy
1 − Global
2 − Super Region
3 − Region
4 − Country
5 − Subnational (Level 1)
Uncertainty Interval
5 − Subnational (Level 1)

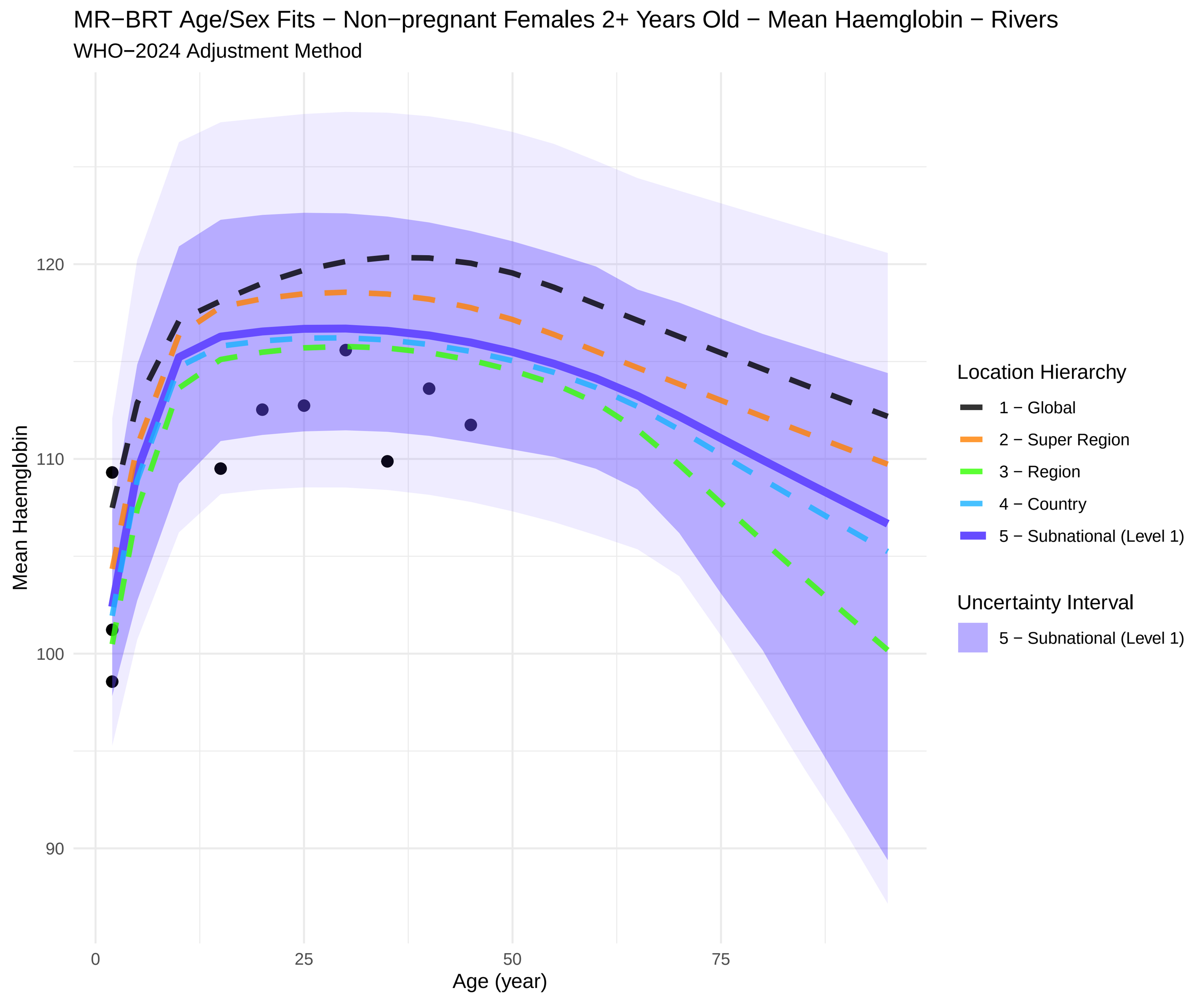
MR−BRT Age/Sex Fits − Non−pregnant Females 2+ Years Old − Mean Haemglobin − Rivers
WHO−2024 Adjustment Method
Mean Haemglobin
Age (year)
Location Hierarchy
1 − Global
2 − Super Region
3 − Region
4 − Country
5 − Subnational (Level 1)
Uncertainty Interval
5 − Subnational (Level 1)

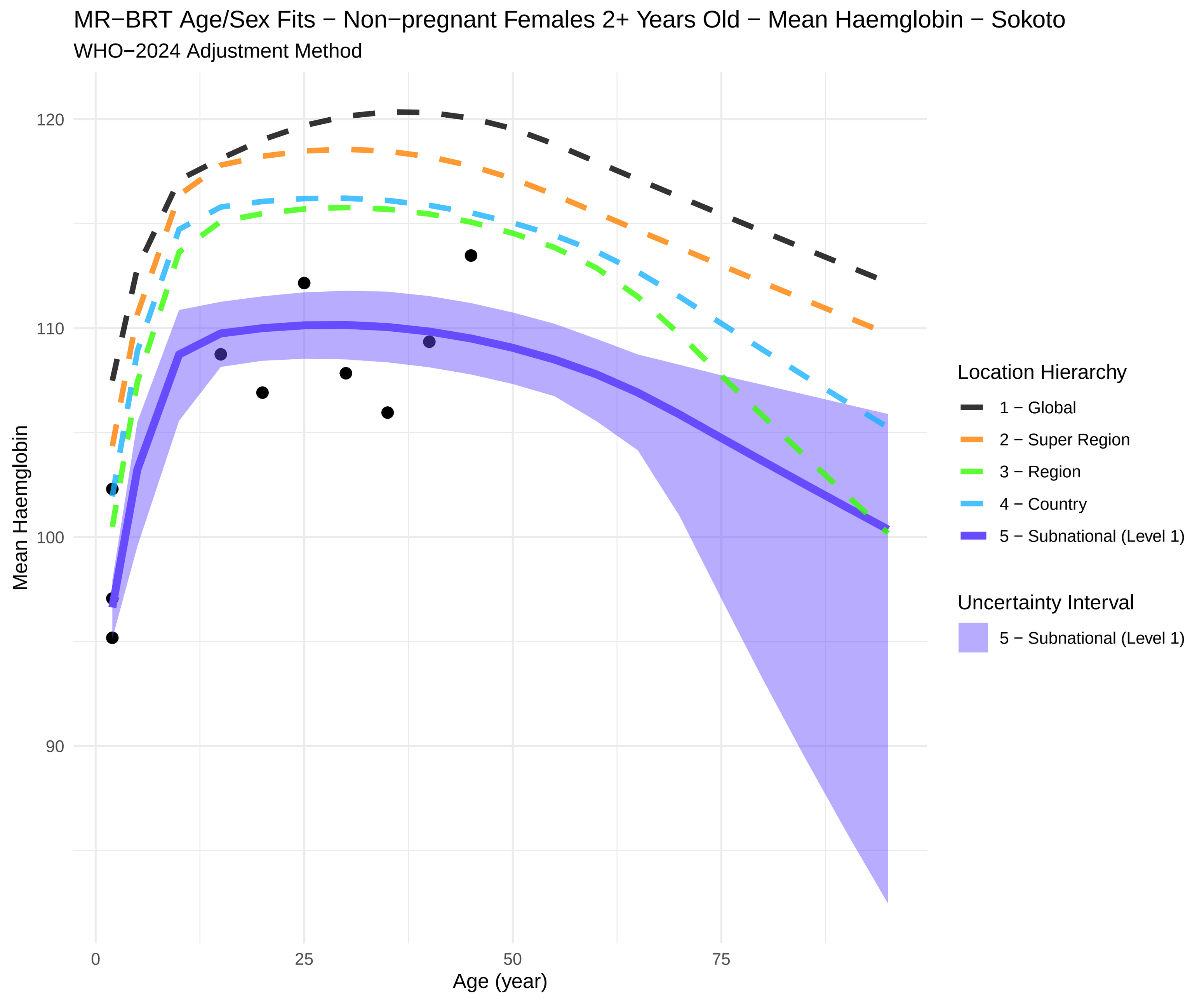
MR−BRT Age/Sex Fits − Non−pregnant Females 2+ Years Old − Mean Haemglobin − Sokoto
WHO−2024 Adjustment Method
Mean Haemglobin
Age (year)
Location Hierarchy
1 − Global
2 − Super Region
3 − Region
4 − Country
5 − Subnational (Level 1)
Uncertainty Interval
5 − Subnational (Level 1)

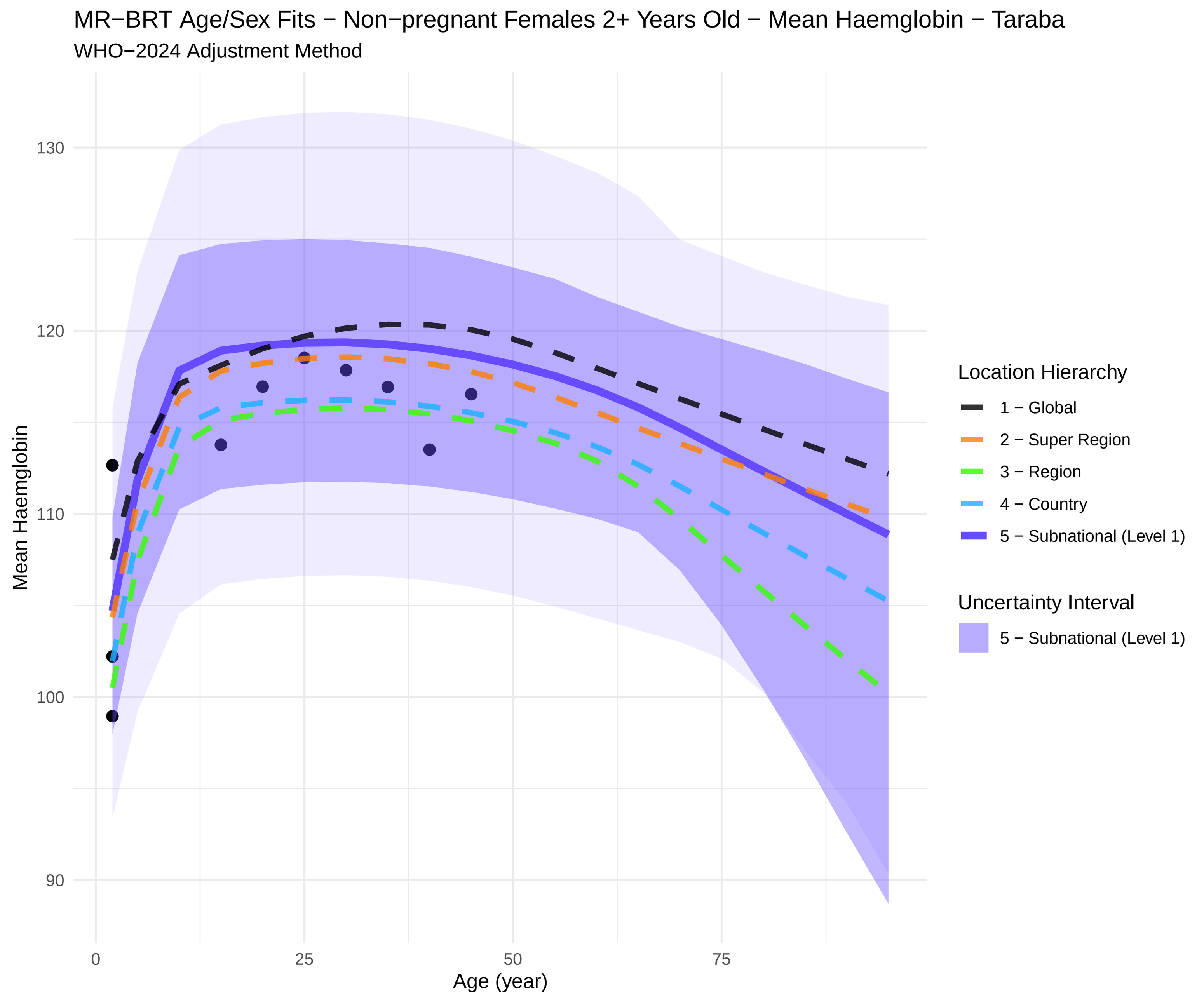
MR−BRT Age/Sex Fits − Non−pregnant Females 2+ Years Old − Mean Haemglobin − Taraba
WHO−2024 Adjustment Method
Mean Haemglobin
Age (year)
Location Hierarchy
1 − Global
2 − Super Region
3 − Region
4 − Country
5 − Subnational (Level 1)
Uncertainty Interval
5 − Subnational (Level 1)

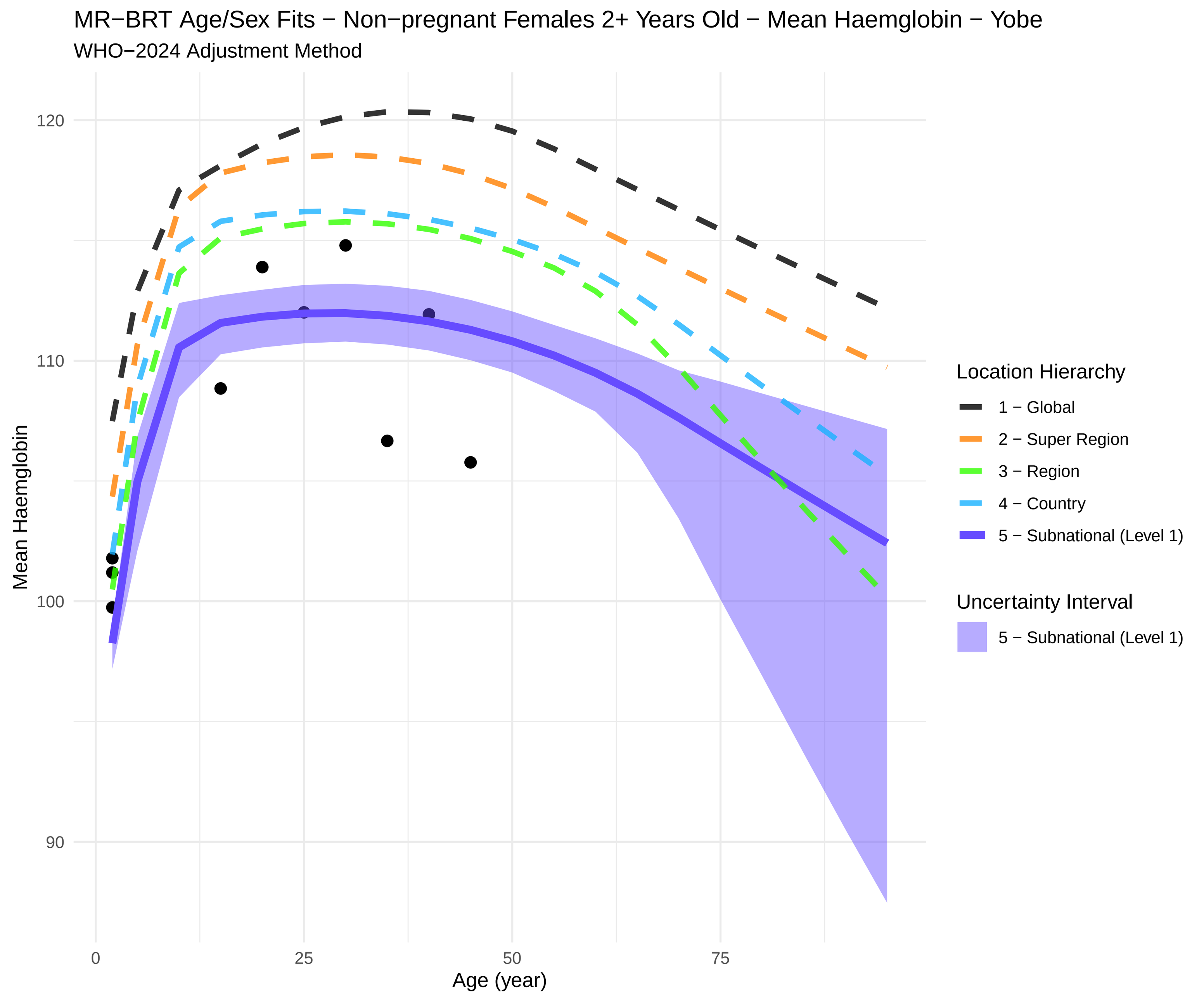
MR−BRT Age/Sex Fits − Non−pregnant Females 2+ Years Old − Mean Haemglobin − Yobe
WHO−2024 Adjustment Method
Mean Haemglobin
Age (year)
Location Hierarchy
1 − Global
2 − Super Region
3 − Region
4 − Country
5 − Subnational (Level 1)
Uncertainty Interval
5 − Subnational (Level 1)

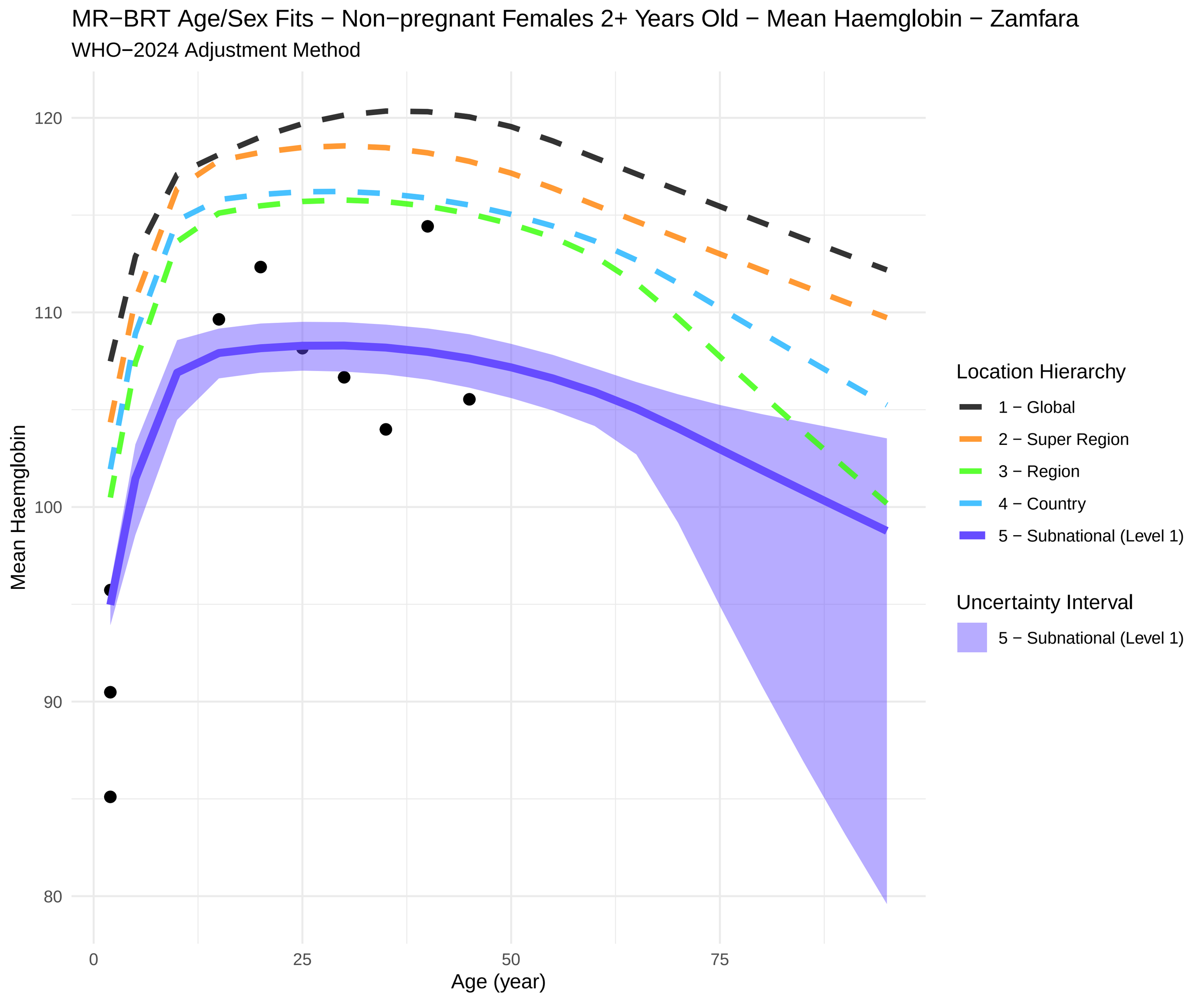
MR−BRT Age/Sex Fits − Non−pregnant Females 2+ Years Old − Mean Haemglobin − Zamfara
WHO−2024 Adjustment Method
Mean Haemglobin
Age (year)
Location Hierarchy
1 − Global
2 − Super Region
3 − Region
4 − Country
5 − Subnational (Level 1)
Uncertainty Interval
5 − Subnational (Level 1)

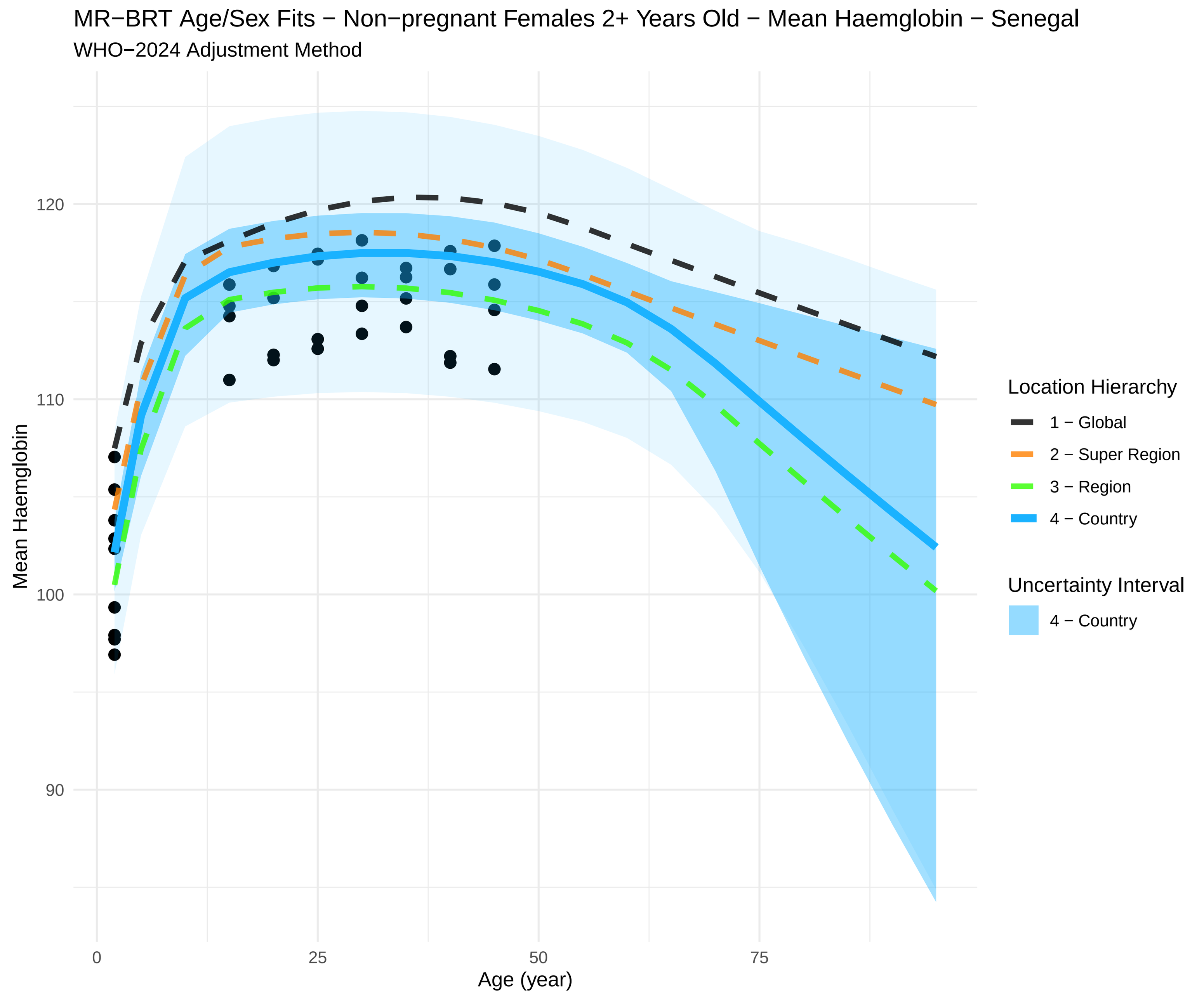
MR−BRT Age/Sex Fits − Non−pregnant Females 2+ Years Old − Mean Haemglobin − Senegal
WHO−2024 Adjustment Method
Mean Haemglobin
Age (year)
Location Hierarchy
1 − Global
2 − Super Region
3 − Region
4 − Country
Uncertainty Interval
4 − Country

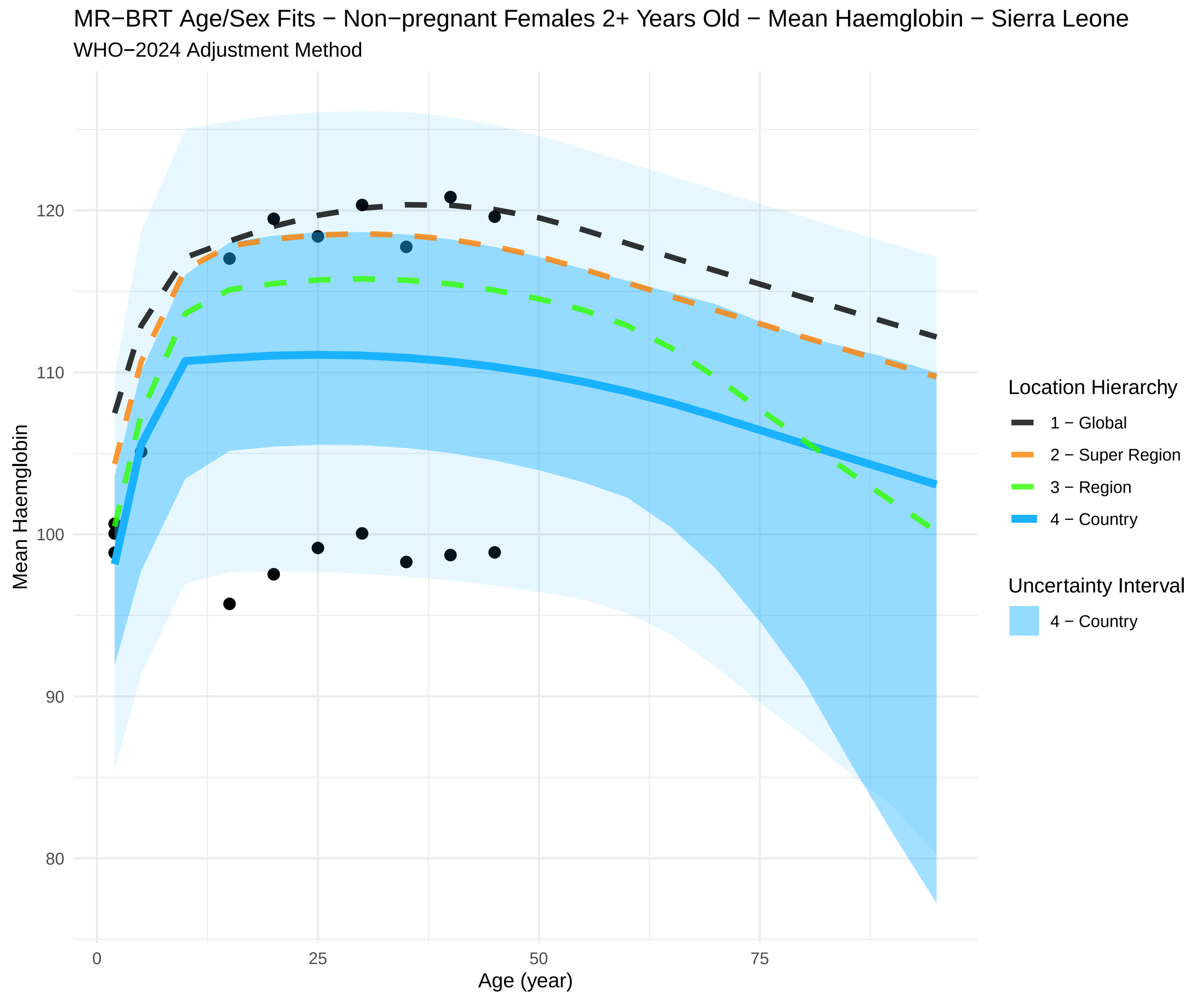
MR−BRT Age/Sex Fits − Non−pregnant Females 2+ Years Old − Mean Haemglobin − Sierra Leone
WHO−2024 Adjustment Method
Mean Haemglobin
Age (year)
Location Hierarchy
1 − Global
2 − Super Region
3 − Region
4 − Country
Uncertainty Interval
4 − Country

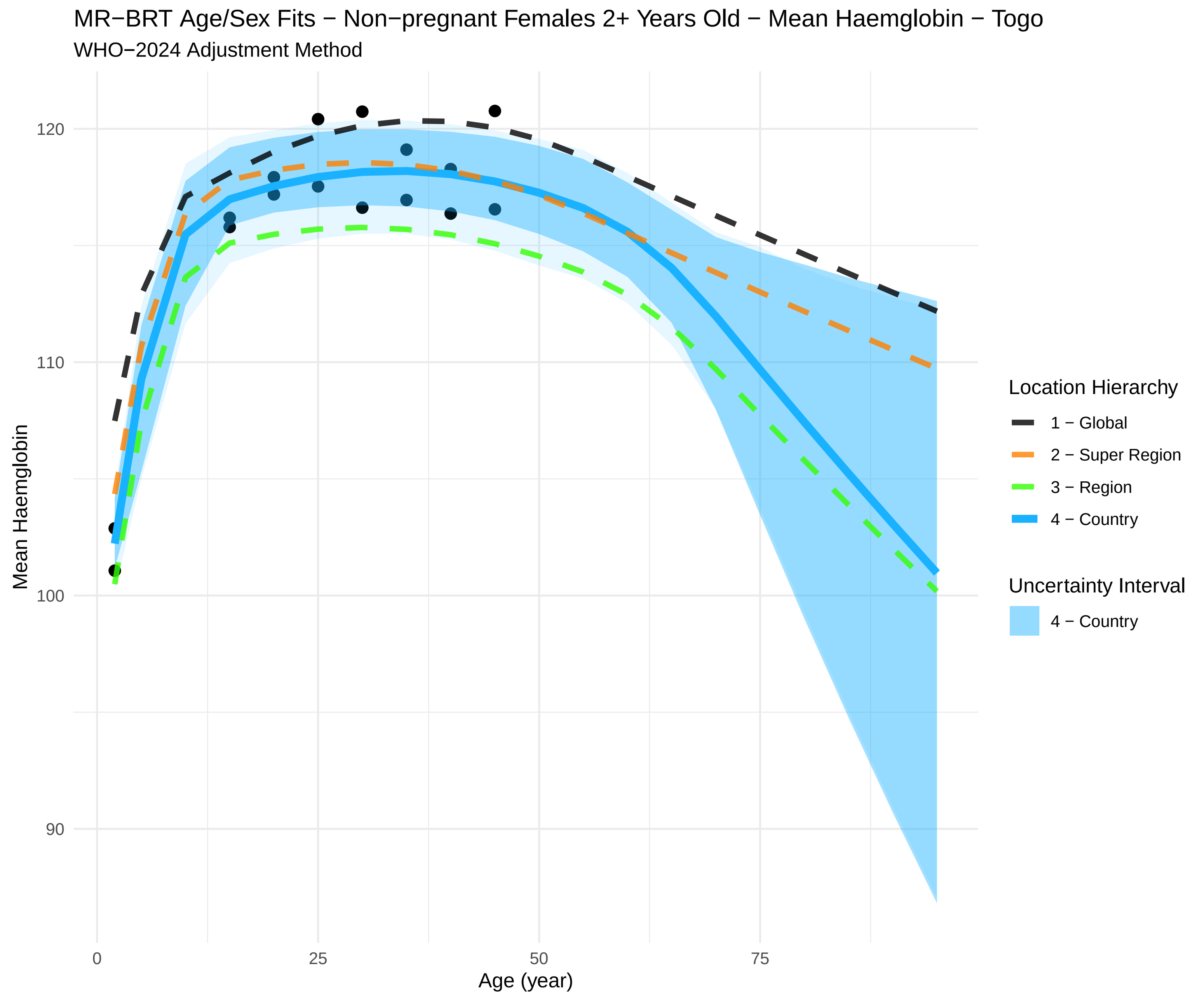
MR−BRT Age/Sex Fits − Non−pregnant Females 2+ Years Old − Mean Haemglobin − Togo
WHO−2024 Adjustment Method
Mean Haemglobin
Age (year)
Location Hierarchy
1 − Global
2 − Super Region
3 − Region
4 − Country
Uncertainty Interval
4 − Country

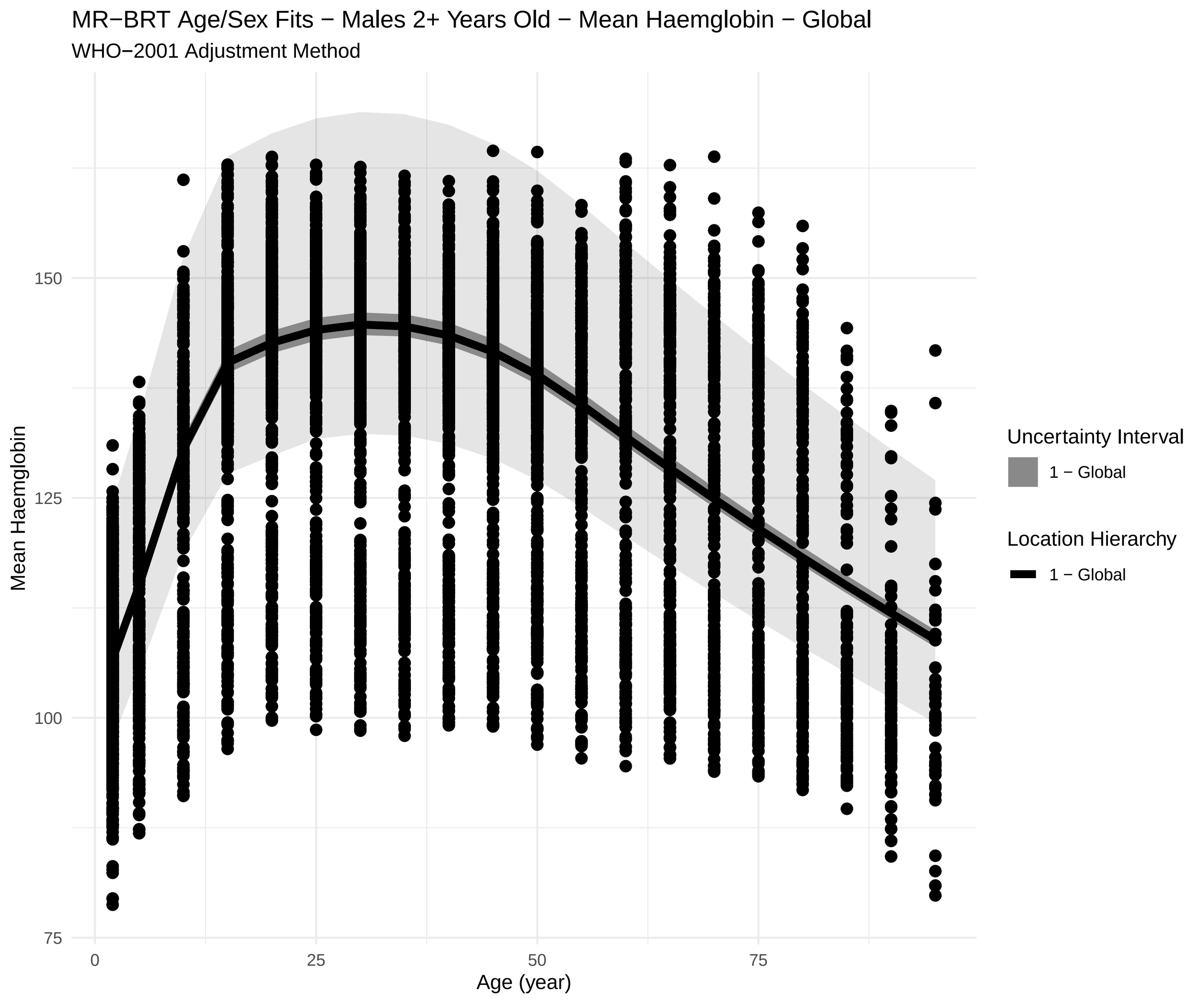
MR−BRT Age/Sex Fits − Males 2+ Years Old − Mean Haemglobin − Global
WHO−2001 Adjustment Method
Mean Haemglobin
Age (year)
Uncertainty Interval
1 − Global
Location Hierarchy
1 − Global

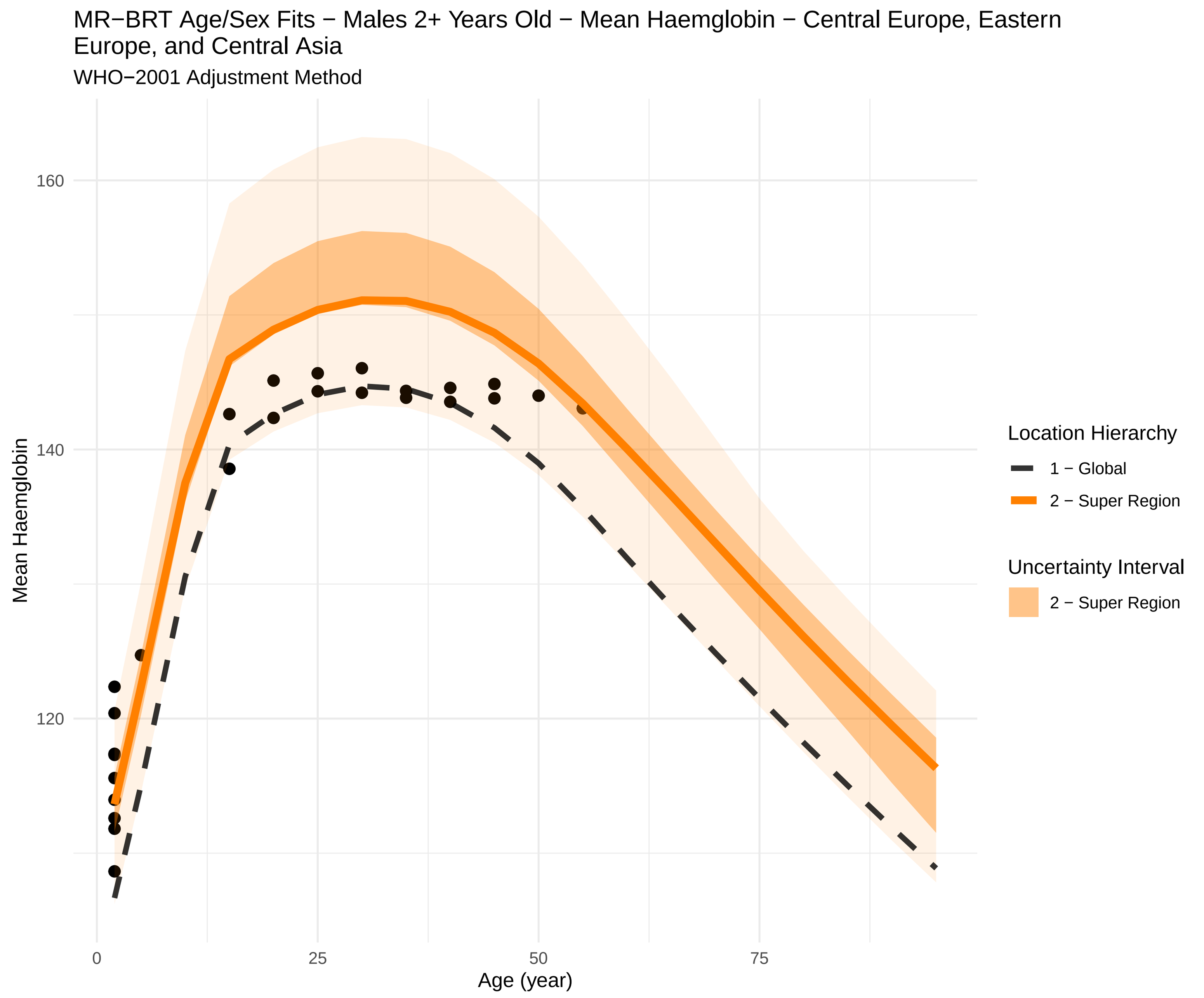
MR−BRT Age/Sex Fits − Males 2+ Years Old − Mean Haemglobin − Central Europe, Eastern Europe, and Central Asia
WHO−2001 Adjustment Method
Mean Haemglobin
Age (year)
Location Hierarchy
1 − Global
2 − Super Region
Uncertainty Interval
2 − Super Region

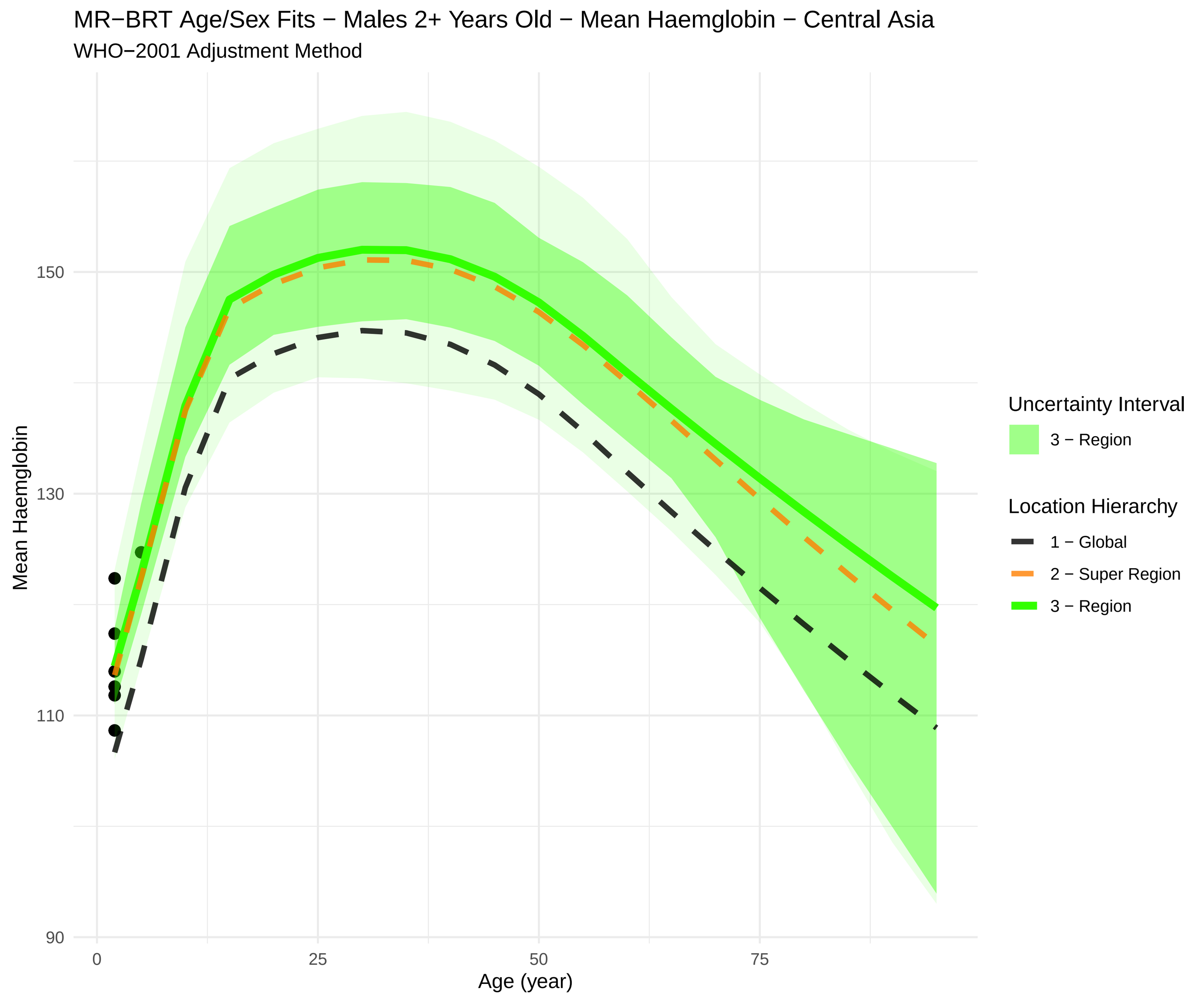
MR−BRT Age/Sex Fits − Males 2+ Years Old − Mean Haemglobin − Central Asia
WHO−2001 Adjustment Method
Mean Haemglobin
Age (year)
Uncertainty Interval
3 − Region
Location Hierarchy
1 − Global
2 − Super Region
3 − Region

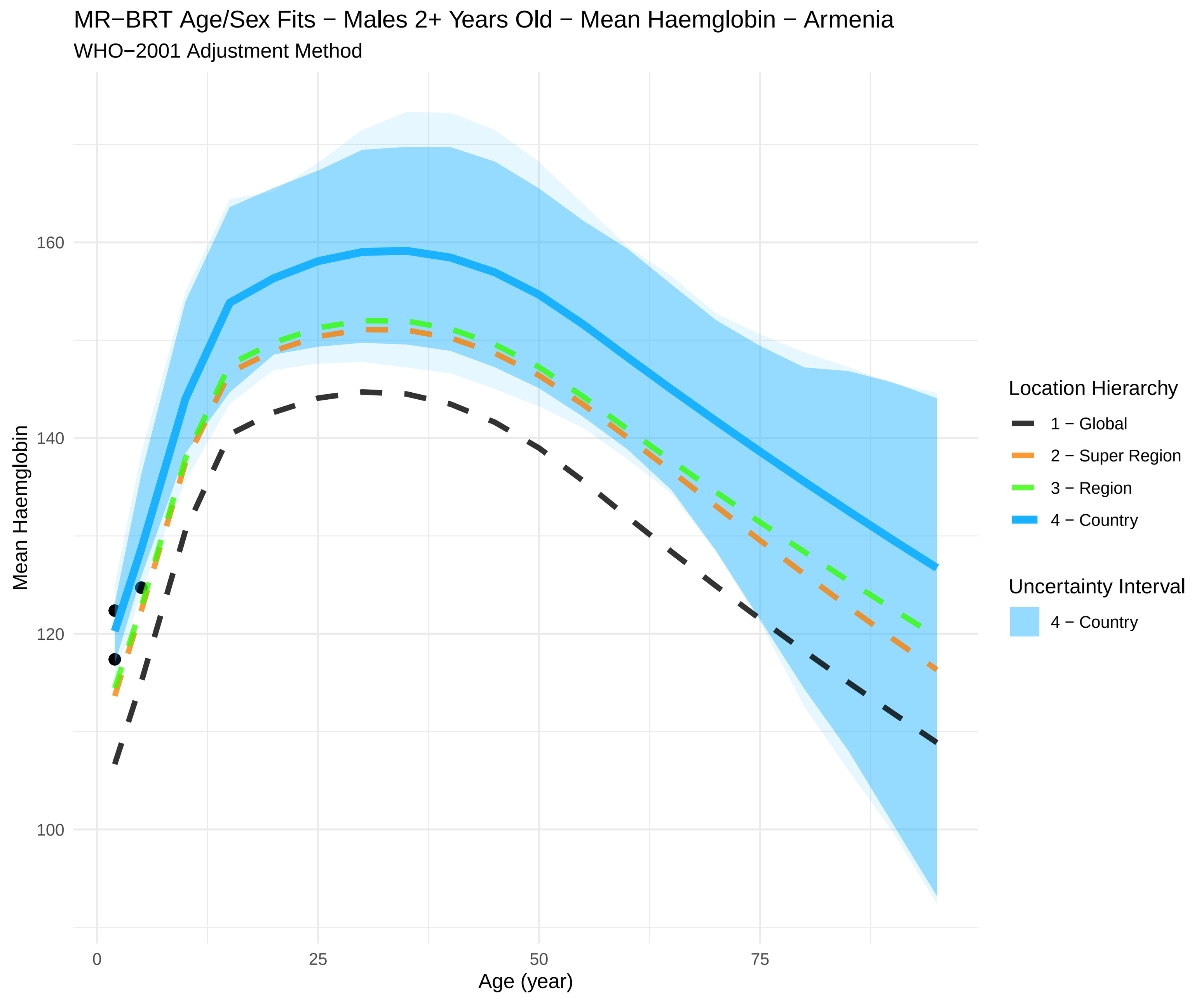
MR−BRT Age/Sex Fits − Males 2+ Years Old − Mean Haemglobin − Armenia
WHO−2001 Adjustment Method
Mean Haemglobin
Age (year)
Location Hierarchy
1 − Global
2 − Super Region
3 − Region
4 − Country
Uncertainty Interval
4 − Country

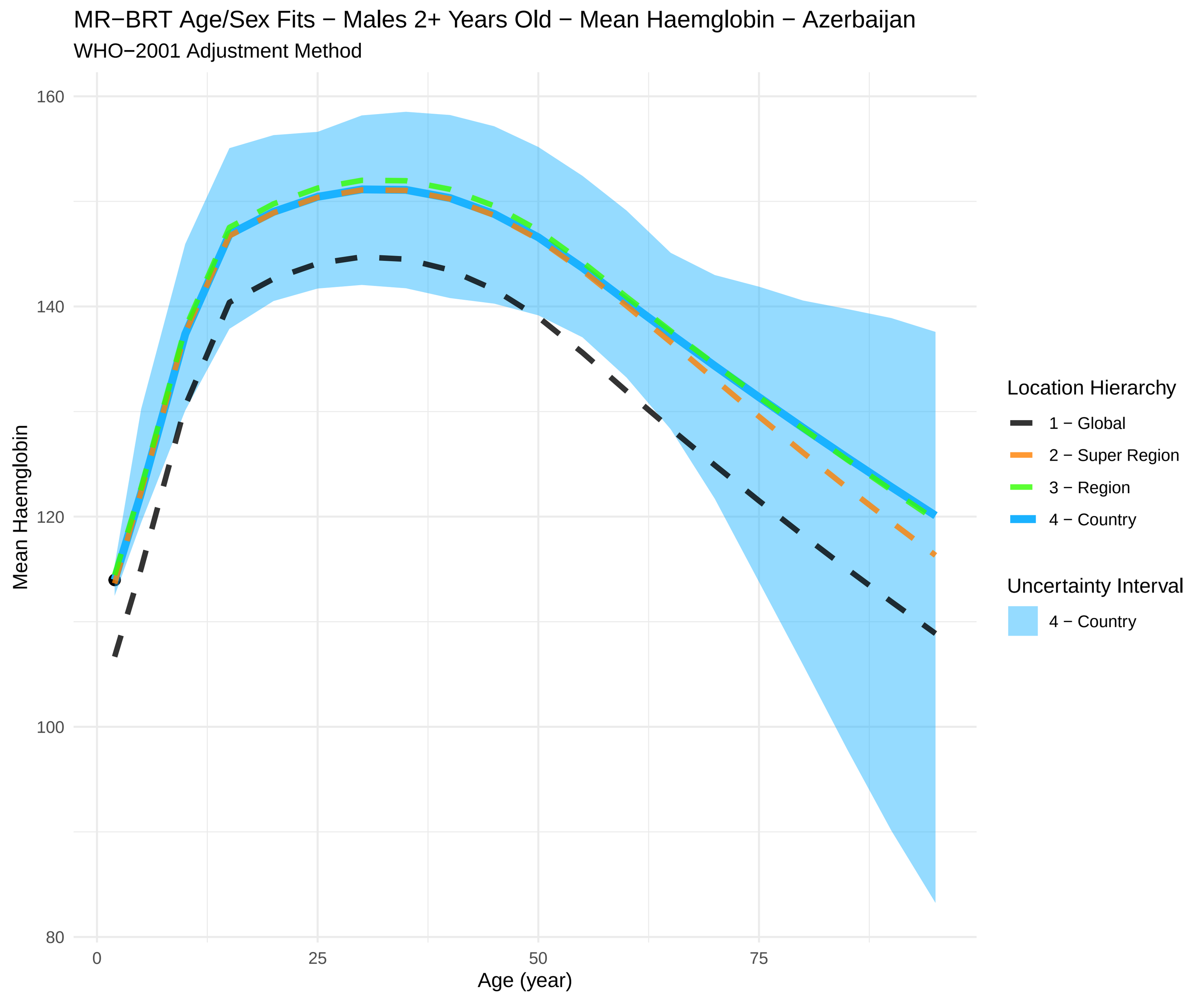
MR−BRT Age/Sex Fits − Males 2+ Years Old − Mean Haemglobin − Azerbaijan
WHO−2001 Adjustment Method
Mean Haemglobin
Age (year)
Location Hierarchy
1 − Global
2 − Super Region
3 − Region
4 − Country
Uncertainty Interval
4 − Country

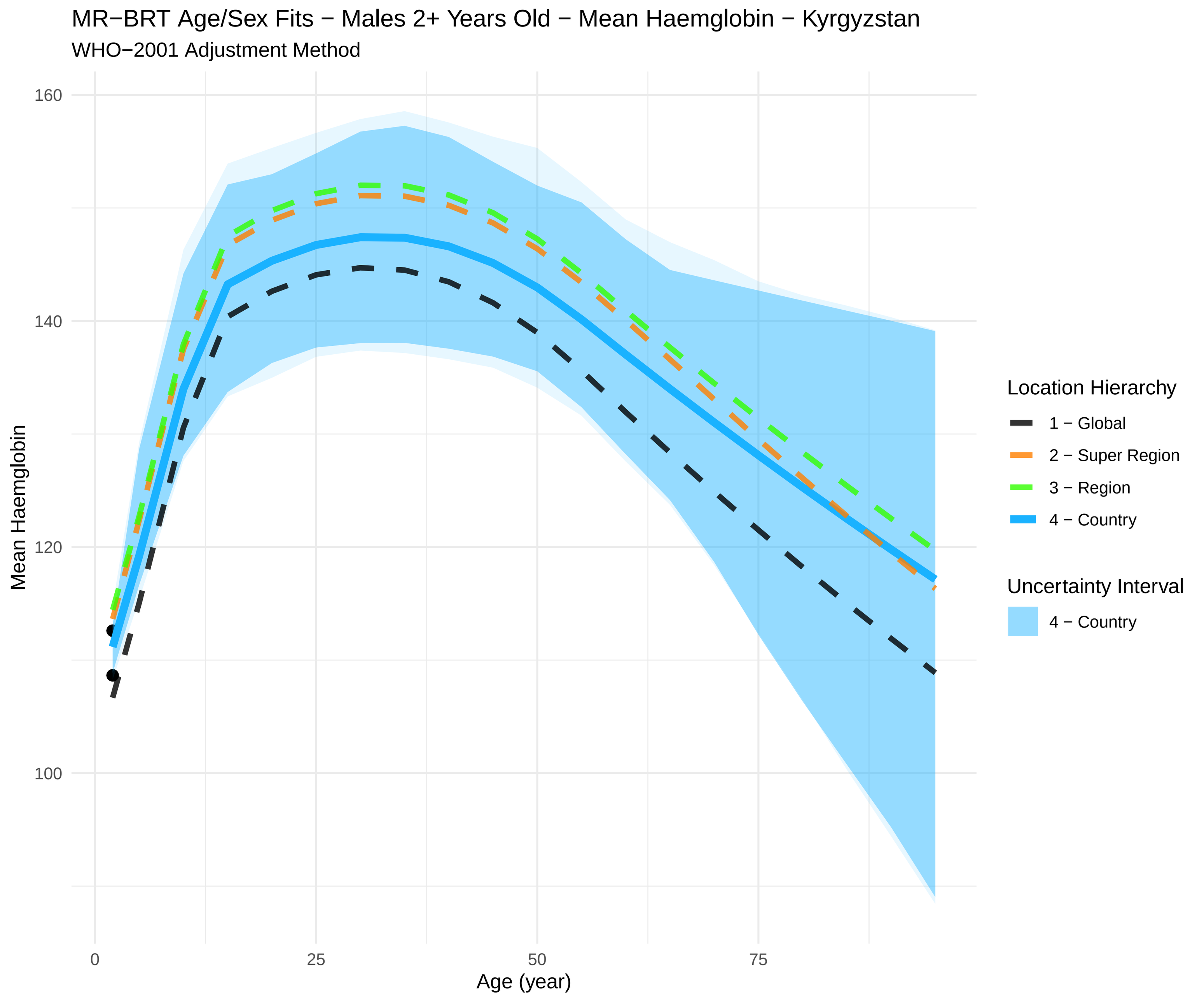
MR−BRT Age/Sex Fits − Males 2+ Years Old − Mean Haemglobin − Kyrgyzstan
WHO−2001 Adjustment Method
Mean Haemglobin
Age (year)
Location Hierarchy
1 − Global
2 − Super Region
3 − Region
4 − Country
Uncertainty Interval
4 − Country

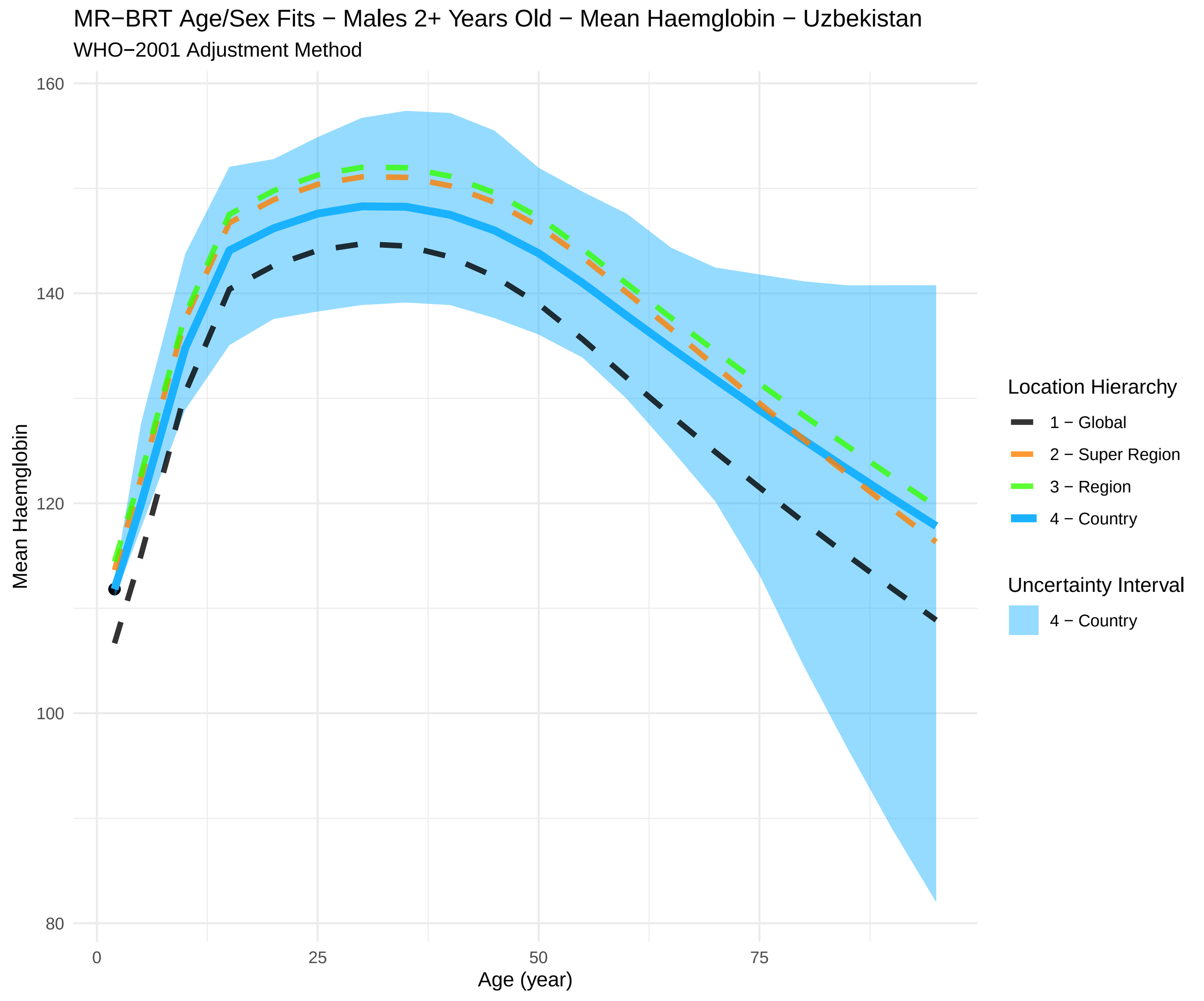
MR−BRT Age/Sex Fits − Males 2+ Years Old − Mean Haemglobin − Uzbekistan
WHO−2001 Adjustment Method
Mean Haemglobin
Age (year)
Location Hierarchy
1 − Global
2 − Super Region
3 − Region
4 − Country
Uncertainty Interval
4 − Country

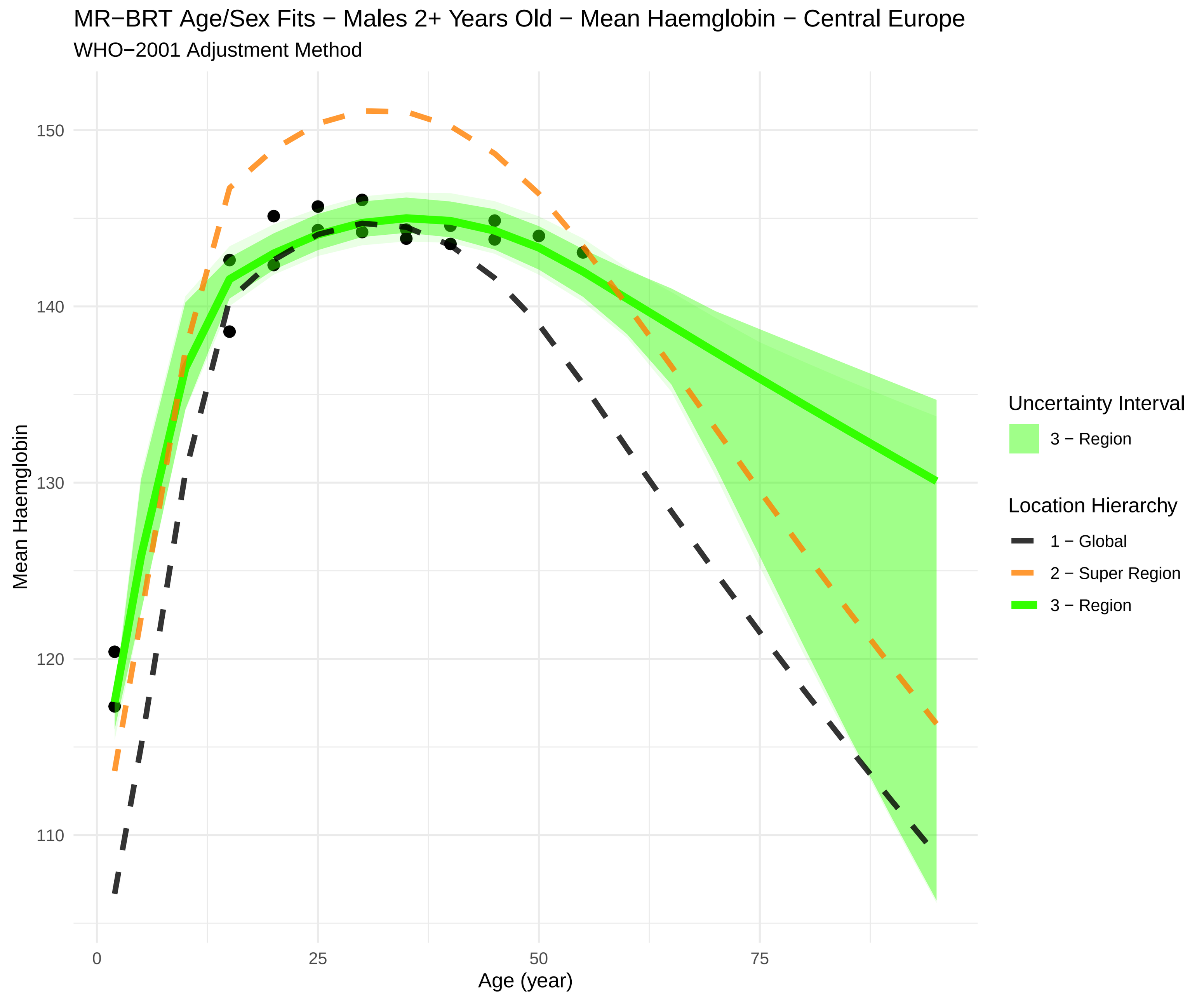
MR−BRT Age/Sex Fits − Males 2+ Years Old − Mean Haemglobin − Central Europe
WHO−2001 Adjustment Method
Mean Haemglobin
Age (year)
Uncertainty Interval
3 − Region
Location Hierarchy
1 − Global
2 − Super Region
3 − Region

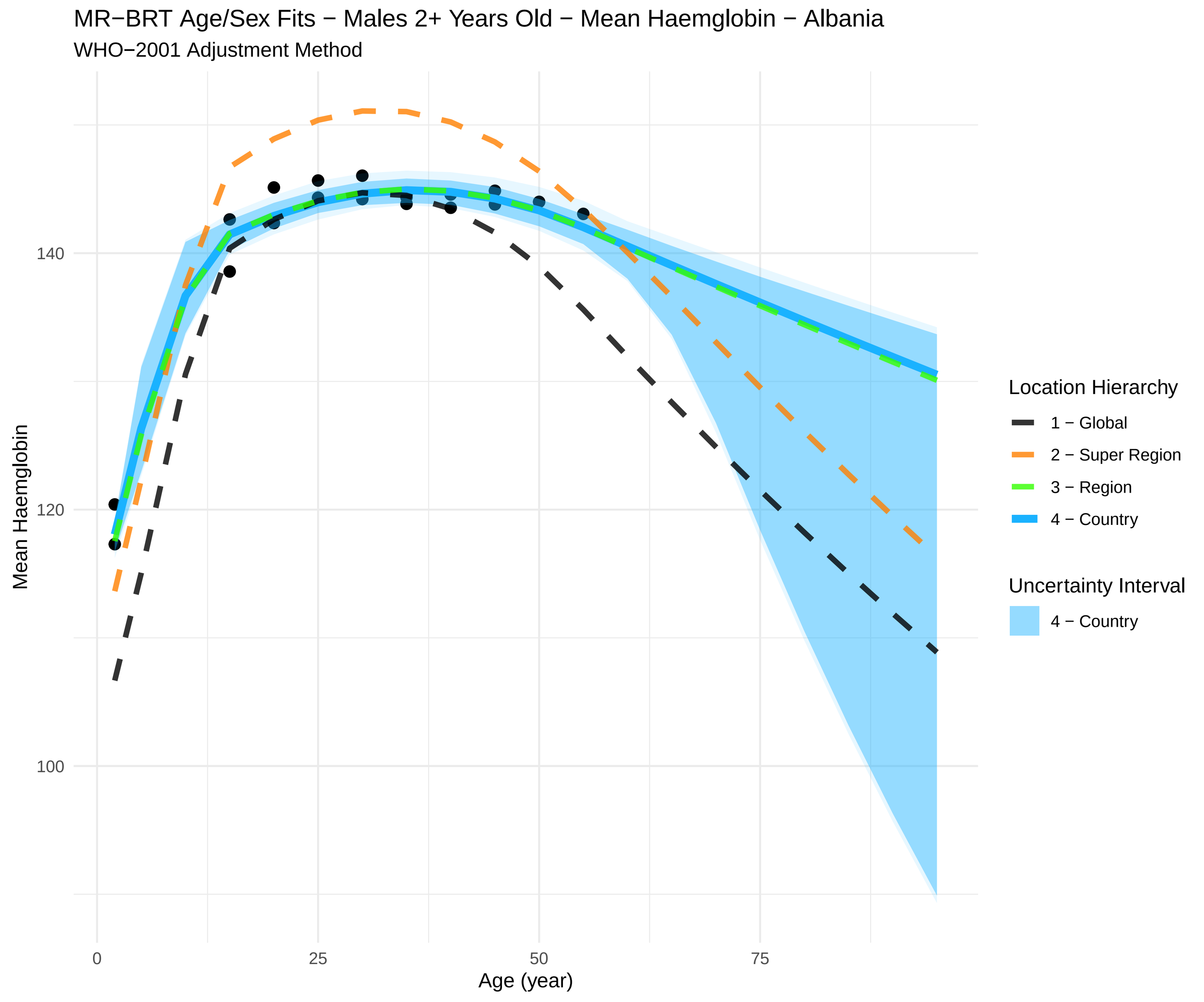
MR−BRT Age/Sex Fits − Males 2+ Years Old − Mean Haemglobin − Albania
WHO−2001 Adjustment Method
Mean Haemglobin
Age (year)
Location Hierarchy
1 − Global
2 − Super Region
3 − Region
4 − Country
Uncertainty Interval
4 − Country

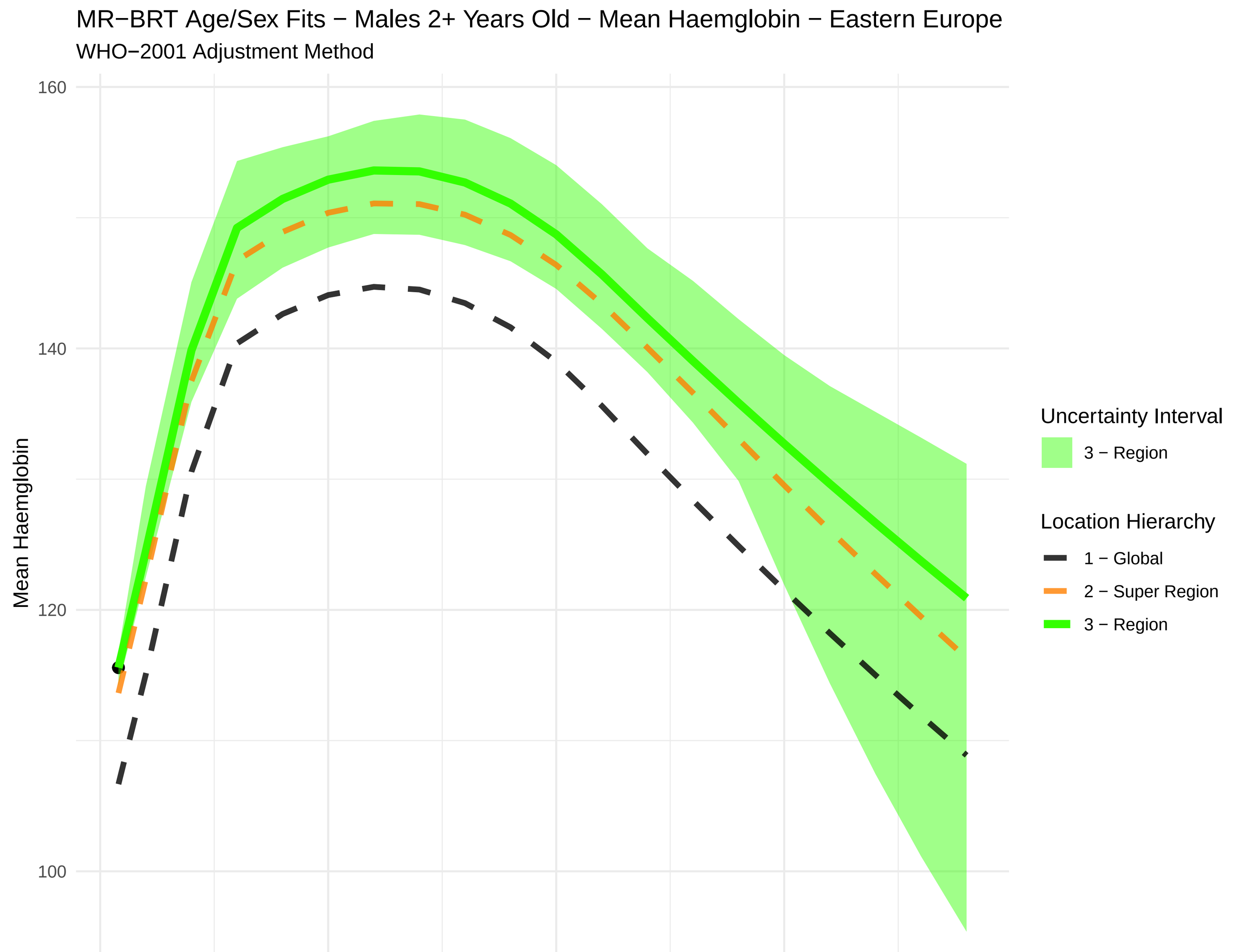
MR−BRT Age/Sex Fits − Males 2+ Years Old − Mean Haemglobin − Eastern Europe
WHO−2001 Adjustment Method
Mean Haemglobin
Age (year)
Uncertainty Interval
3 − Region
Location Hierarchy
1 − Global
2 − Super Region
3 − Region

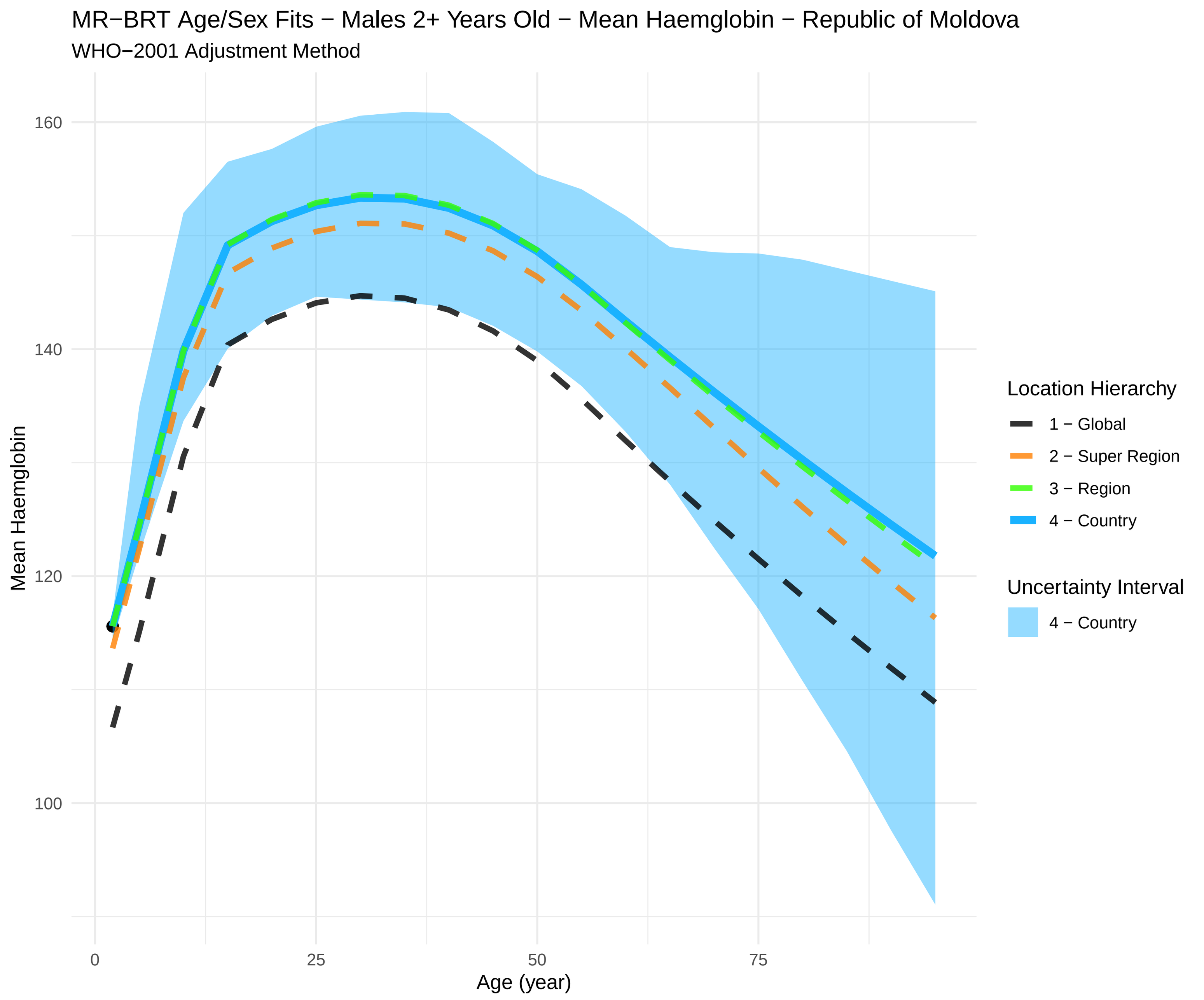
MR−BRT Age/Sex Fits − Males 2+ Years Old − Mean Haemglobin − Republic of Moldova
WHO−2001 Adjustment Method
Mean Haemglobin
Age (year)
Location Hierarchy
1 − Global
2 − Super Region
3 − Region
4 − Country
Uncertainty Interval
4 − Country

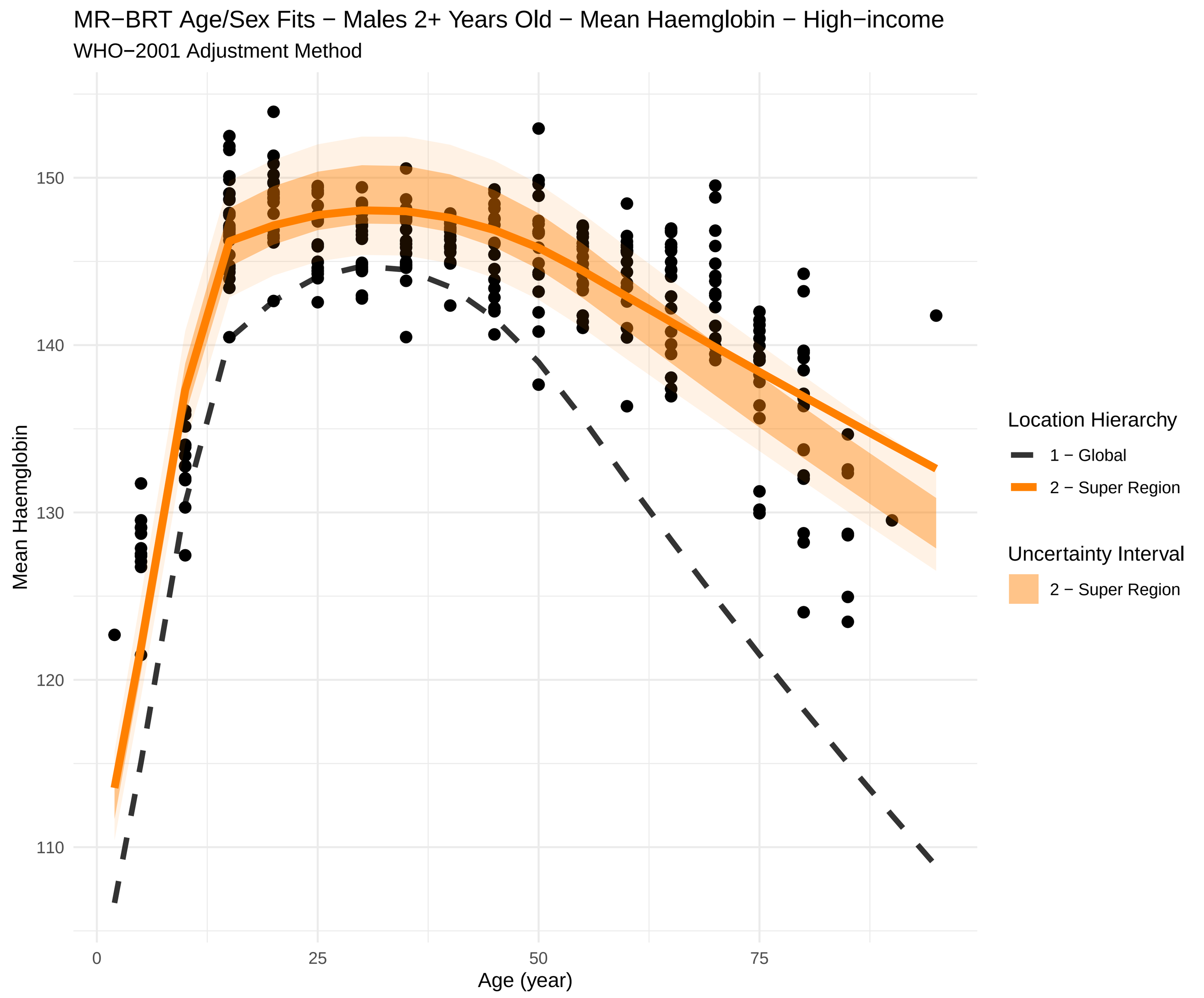
MR−BRT Age/Sex Fits − Males 2+ Years Old − Mean Haemglobin − High−income
WHO−2001 Adjustment Method
Mean Haemglobin
Age (year)
Location Hierarchy
1 − Global
2 − Super Region
Uncertainty Interval
2 − Super Region

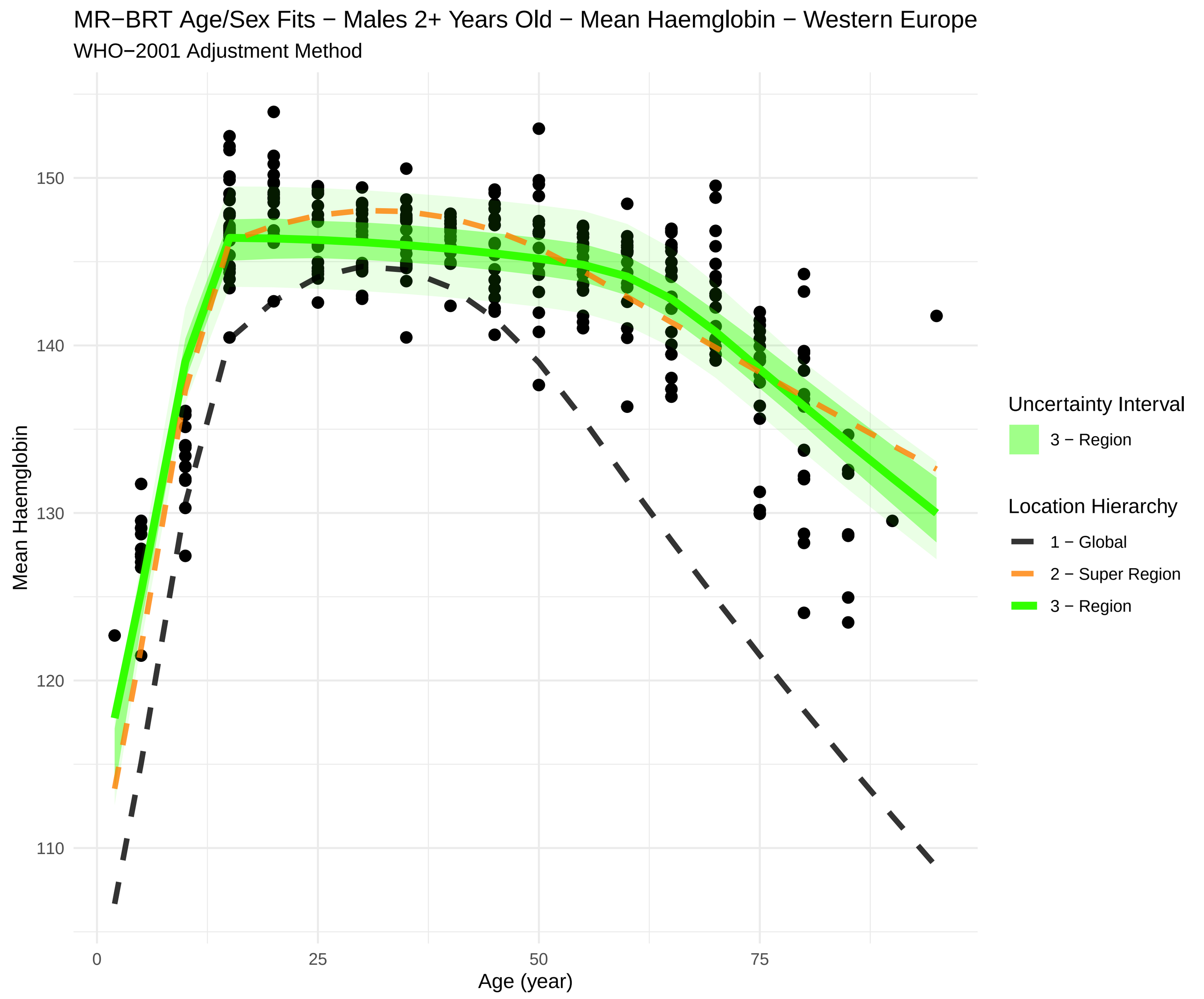
MR−BRT Age/Sex Fits − Males 2+ Years Old − Mean Haemglobin − Western Europe
WHO−2001 Adjustment Method
Mean Haemglobin
Age (year)
Uncertainty Interval
3 − Region
Location Hierarchy
1 − Global
2 − Super Region
3 − Region

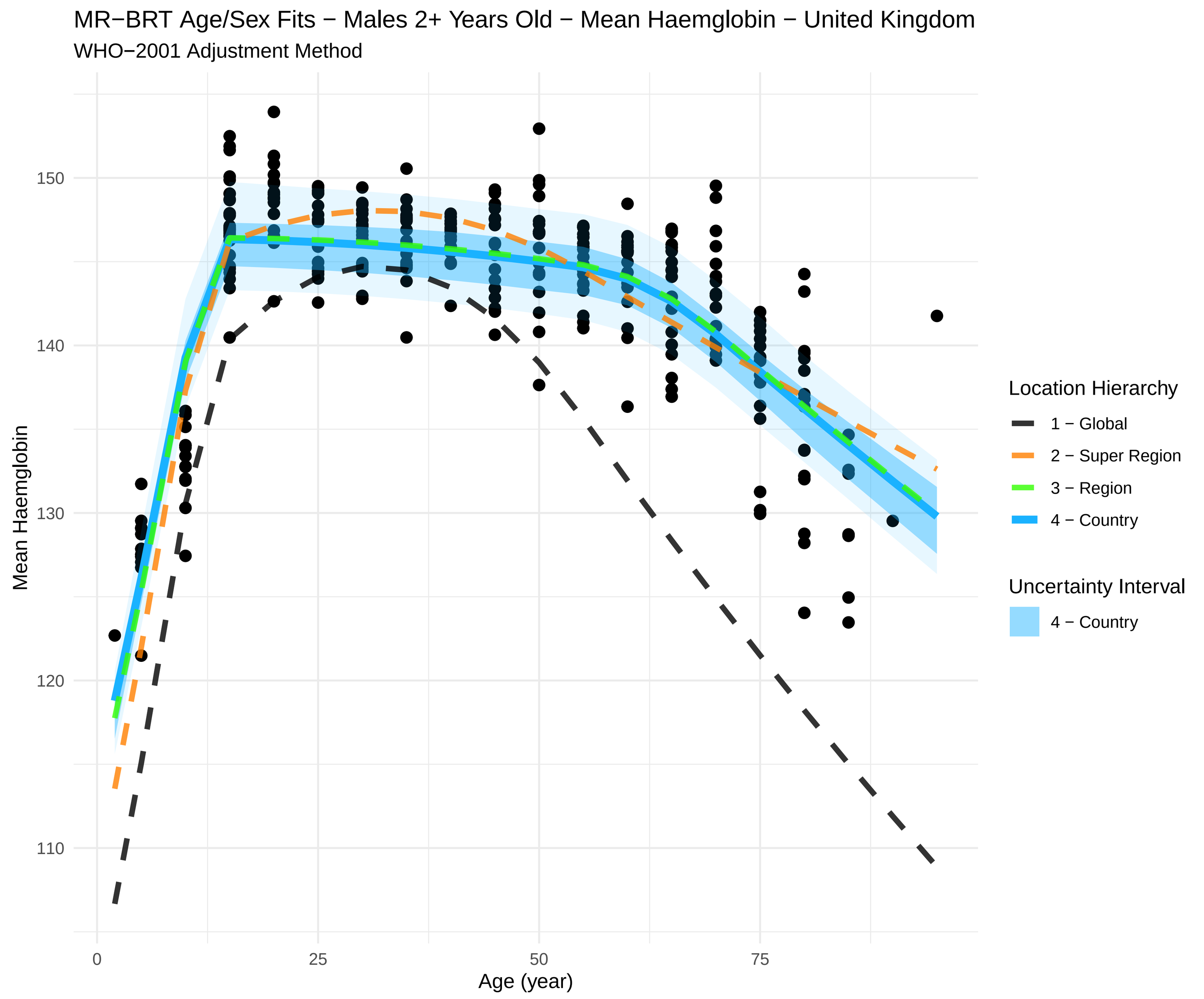
MR−BRT Age/Sex Fits − Males 2+ Years Old − Mean Haemglobin − United Kingdom
WHO−2001 Adjustment Method
Mean Haemglobin
Age (year)
Location Hierarchy
1 − Global
2 − Super Region
3 − Region
4 − Country
Uncertainty Interval
4 − Country

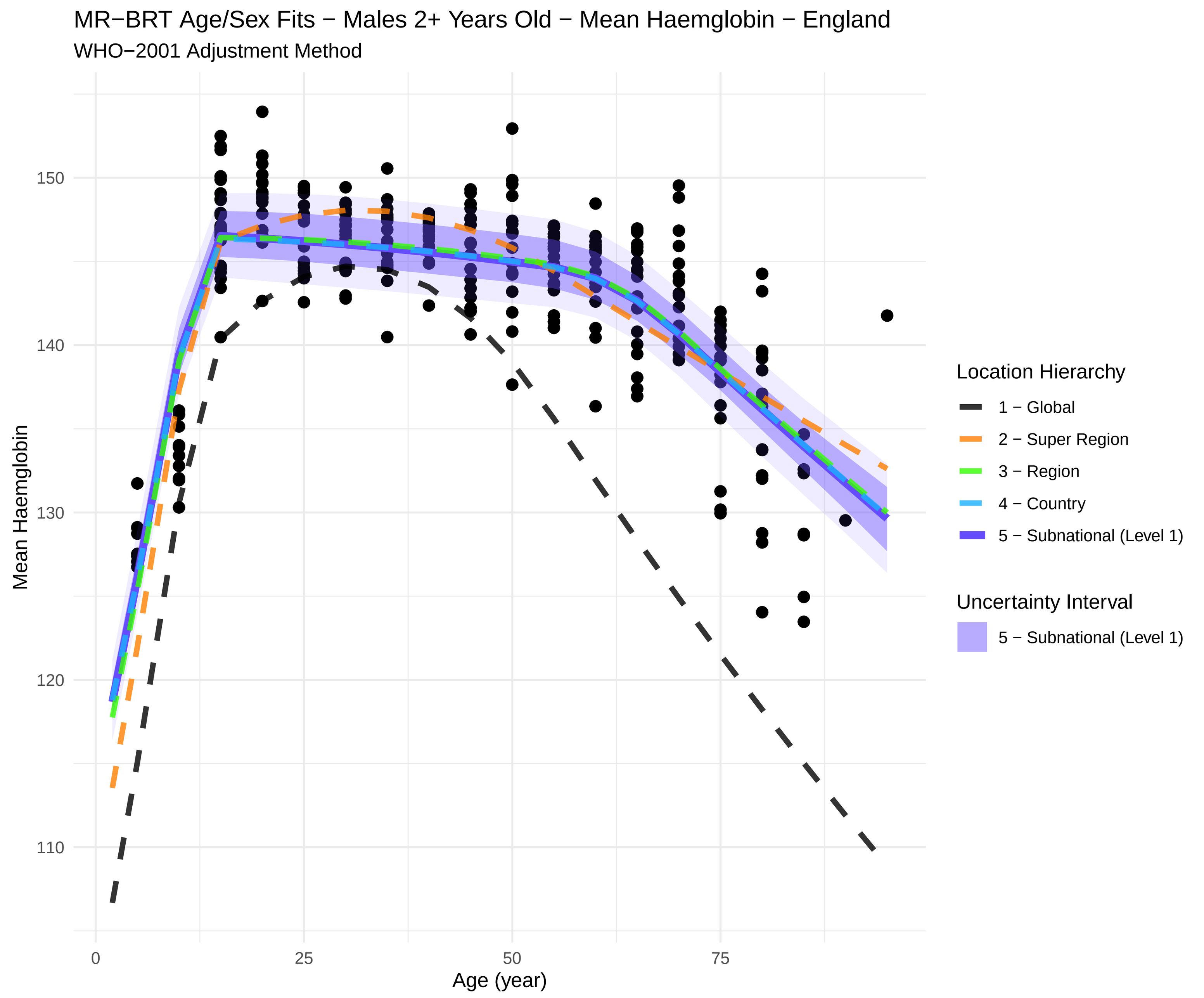
MR−BRT Age/Sex Fits − Males 2+ Years Old − Mean Haemglobin − England
WHO−2001 Adjustment Method
Mean Haemglobin
Age (year)
Location Hierarchy
1 − Global
2 − Super Region
3 − Region
4 − Country
5 − Subnational (Level 1)
Uncertainty Interval
5 − Subnational (Level 1)

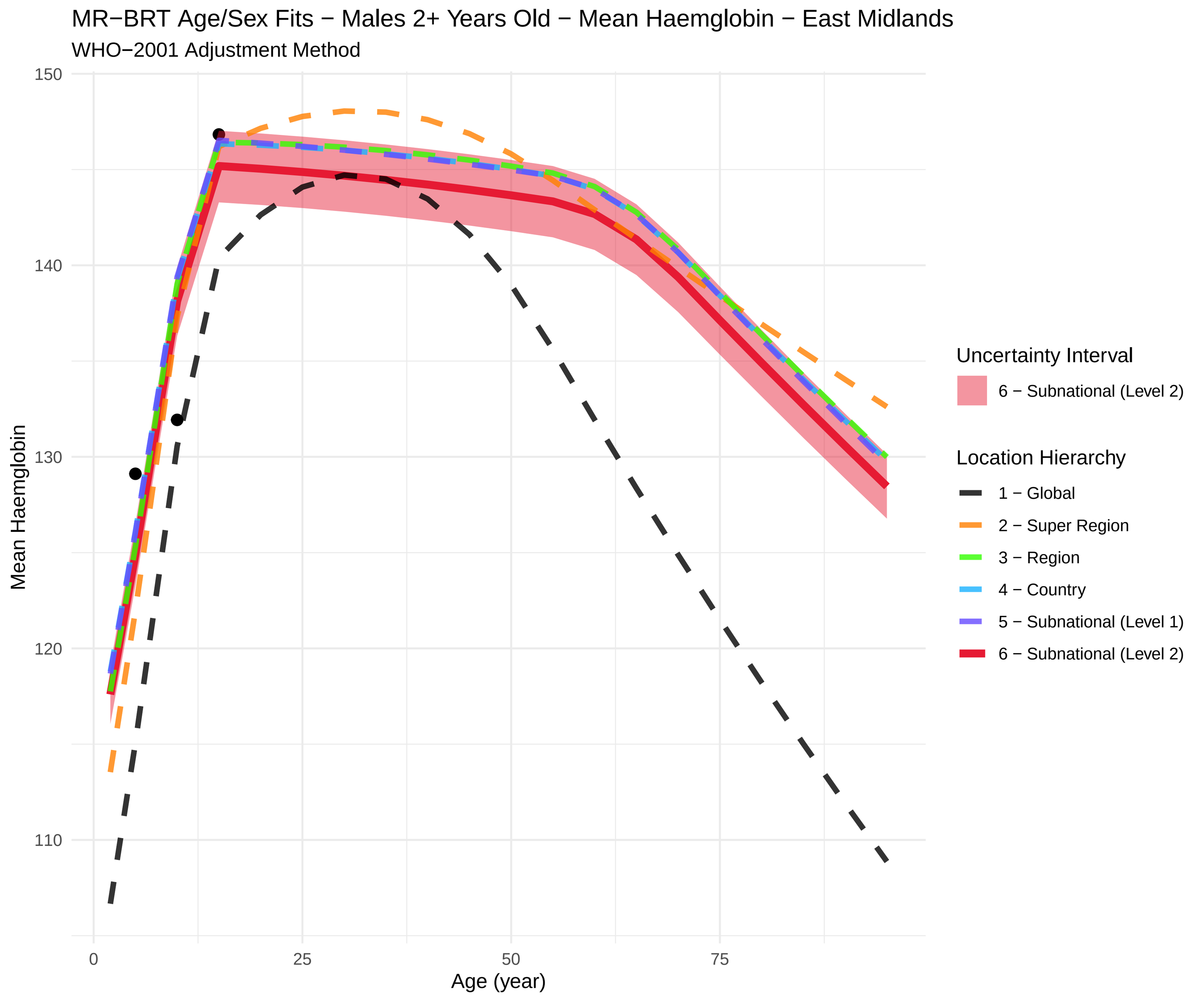
MR−BRT Age/Sex Fits − Males 2+ Years Old − Mean Haemglobin − East Midlands
WHO−2001 Adjustment Method
Mean Haemglobin
Age (year)
Uncertainty Interval
6 − Subnational (Level 2)
Location Hierarchy
1 − Global
2 − Super Region
3 − Region
4 − Country
5 − Subnational (Level 1)
6 − Subnational (Level 2)

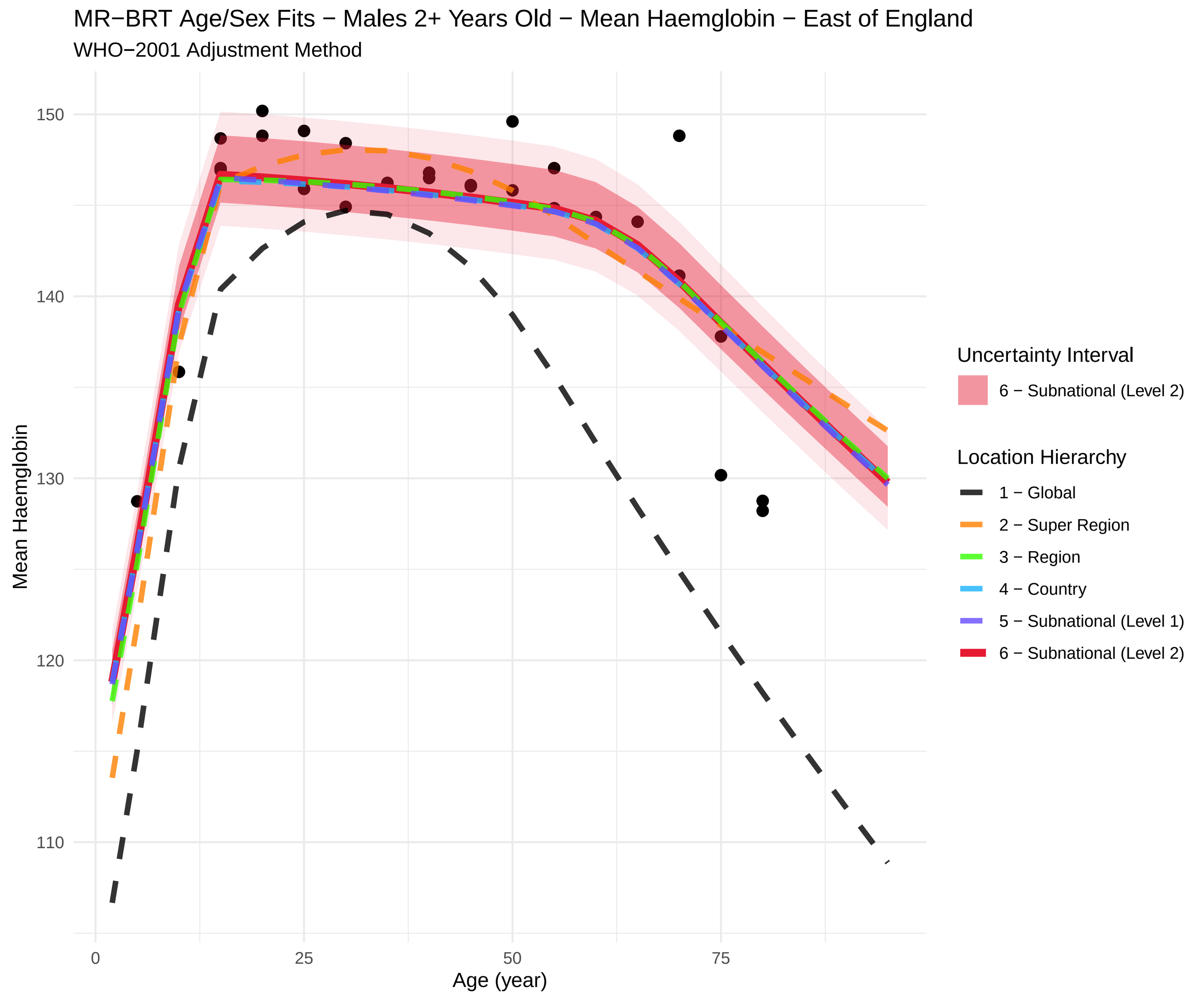
MR−BRT Age/Sex Fits − Males 2+ Years Old − Mean Haemglobin − East of England
WHO−2001 Adjustment Method
Mean Haemglobin
Age (year)
Uncertainty Interval
6 − Subnational (Level 2)
Location Hierarchy
1 − Global
2 − Super Region
3 − Region
4 − Country
5 − Subnational (Level 1)
6 − Subnational (Level 2)

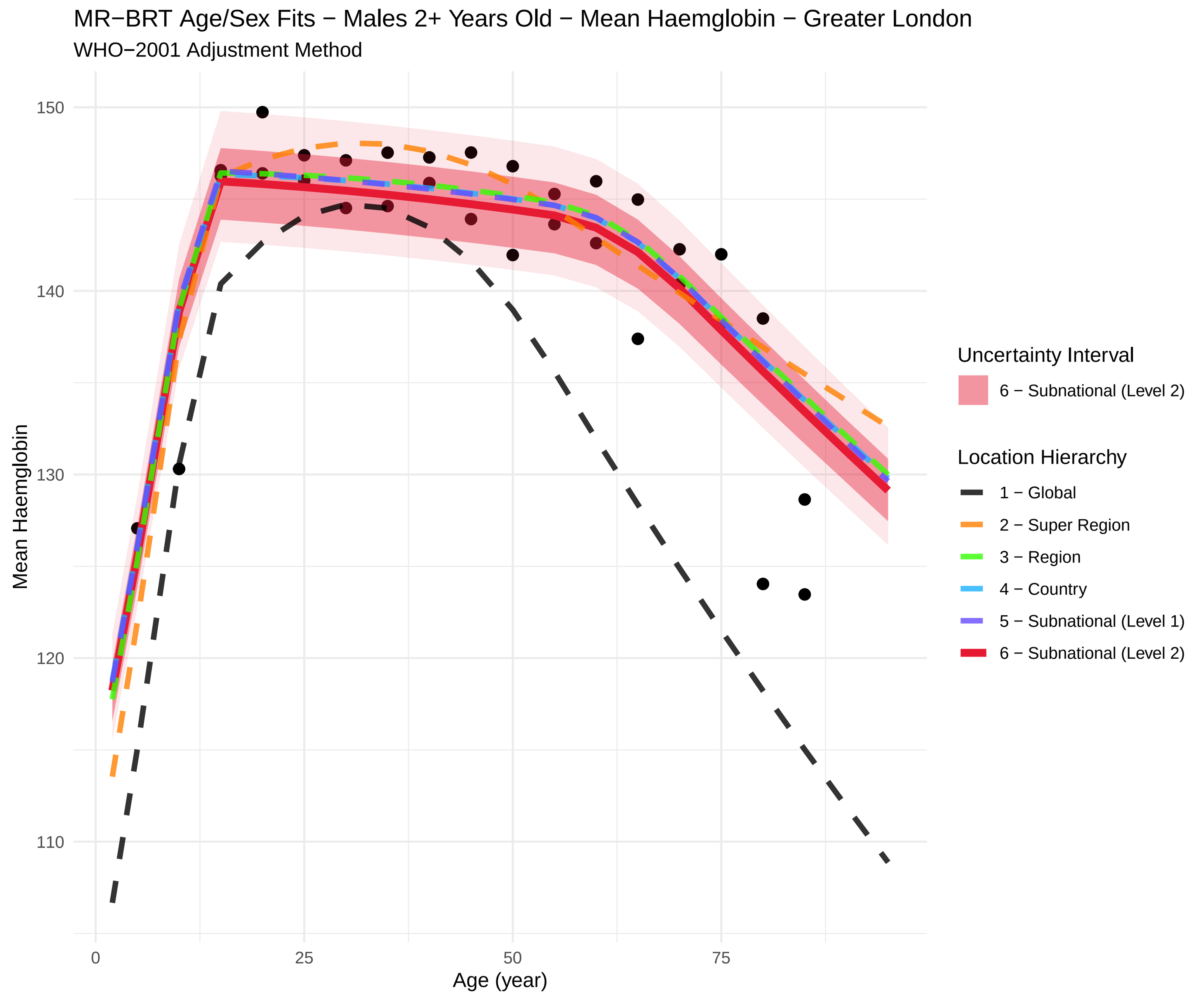
MR−BRT Age/Sex Fits − Males 2+ Years Old − Mean Haemglobin − Greater London
WHO−2001 Adjustment Method
Mean Haemglobin
Age (year)
Uncertainty Interval
6 − Subnational (Level 2)
Location Hierarchy
1 − Global
2 − Super Region
3 − Region
4 − Country
5 − Subnational (Level 1)
6 − Subnational (Level 2)

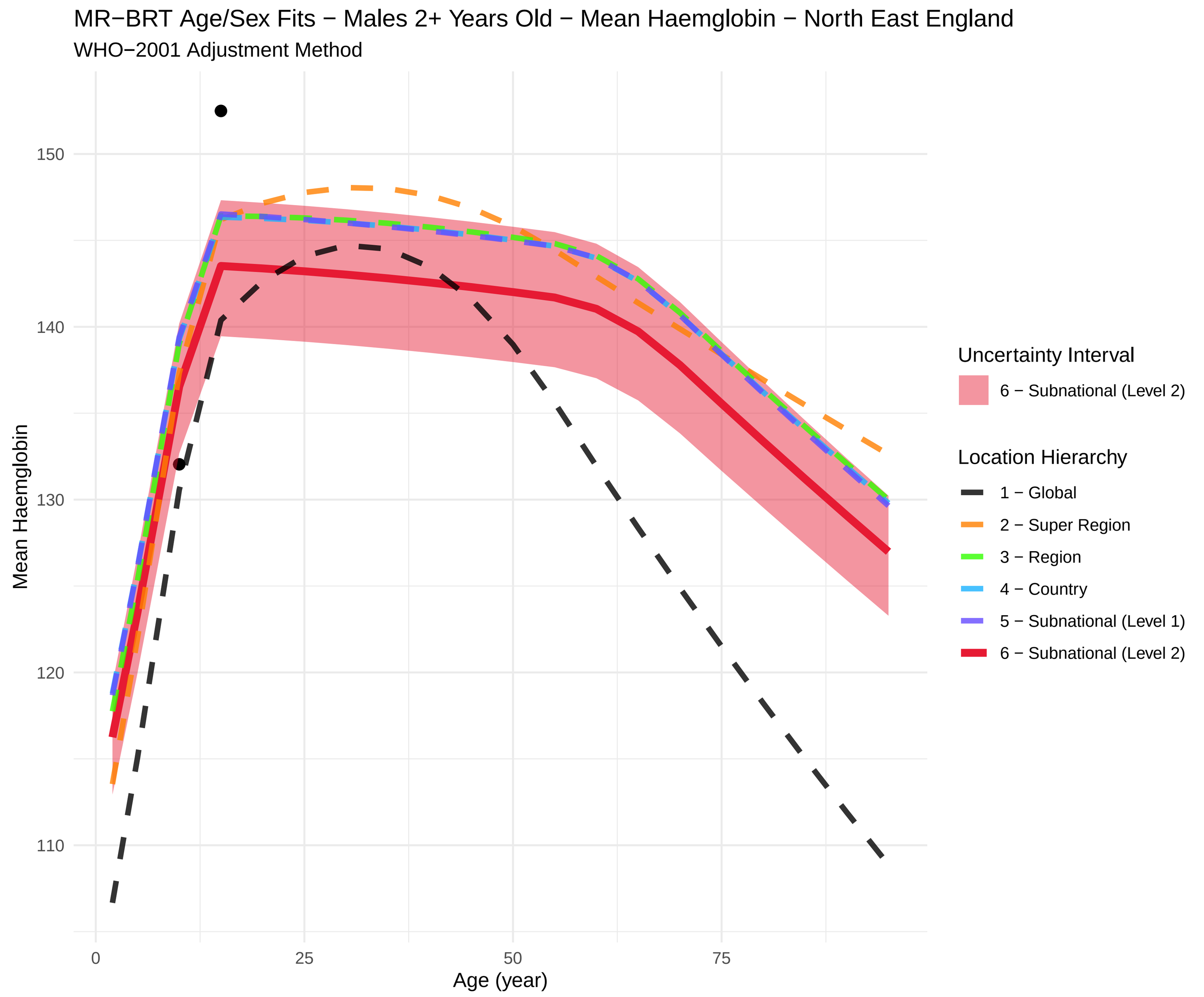
MR−BRT Age/Sex Fits − Males 2+ Years Old − Mean Haemglobin − North East England
WHO−2001 Adjustment Method
Mean Haemglobin
Age (year)
Uncertainty Interval
6 − Subnational (Level 2)
Location Hierarchy
1 − Global
2 − Super Region
3 − Region
4 − Country
5 − Subnational (Level 1)
6 − Subnational (Level 2)

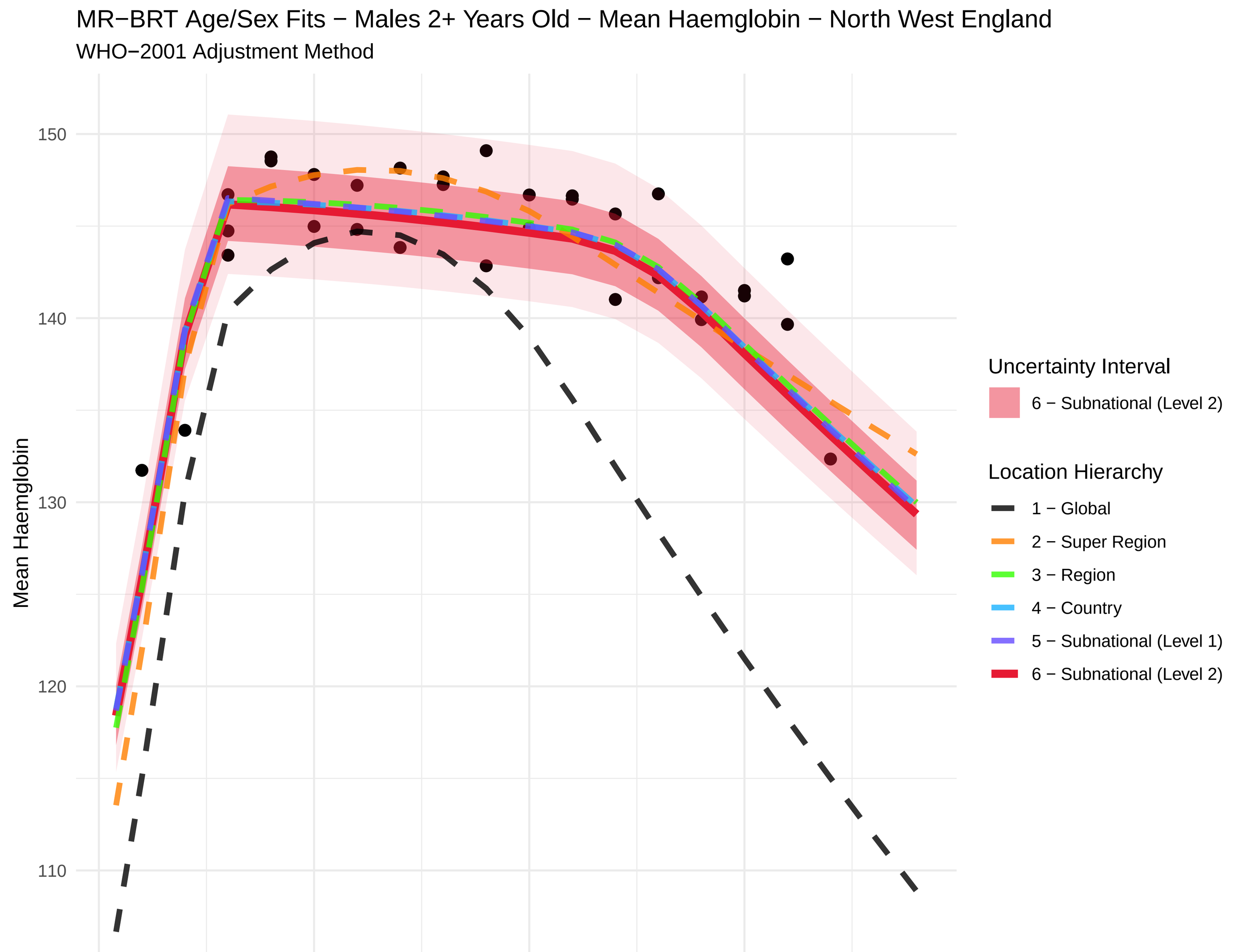
MR−BRT Age/Sex Fits − Males 2+ Years Old − Mean Haemglobin − North West England
WHO−2001 Adjustment Method
Mean Haemglobin
Age (year)
Uncertainty Interval
6 − Subnational (Level 2)
Location Hierarchy
1 − Global
2 − Super Region
3 − Region
4 − Country
5 − Subnational (Level 1)
6 − Subnational (Level 2)

MR−BRT Age/Sex Fits − Males 2+ Years Old − Mean Haemglobin − South East England
WHO−2001 Adjustment Method

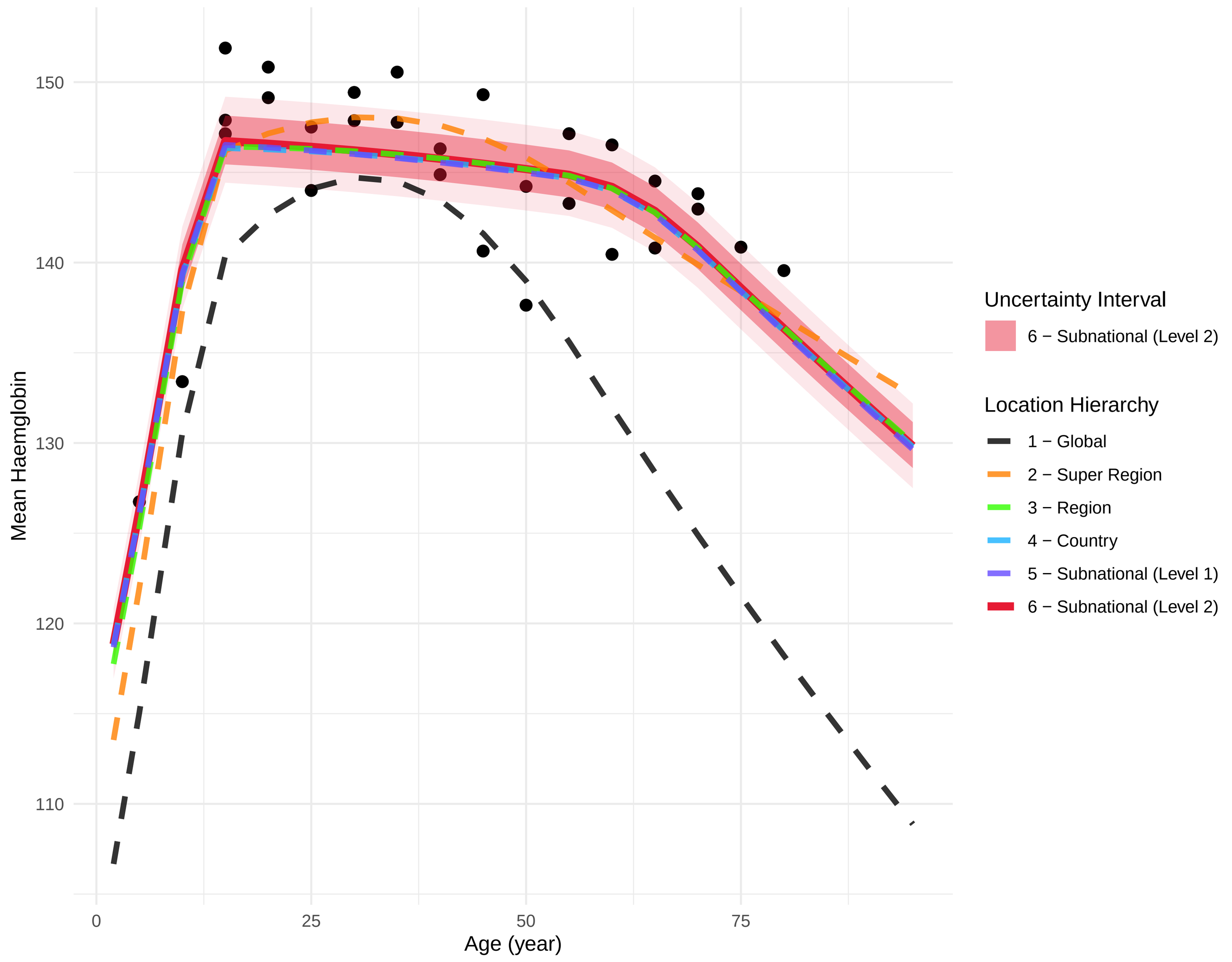

MR−BRT Age/Sex Fits − Males 2+ Years Old − Mean Haemglobin − South West England
WHO−2001 Adjustment Method

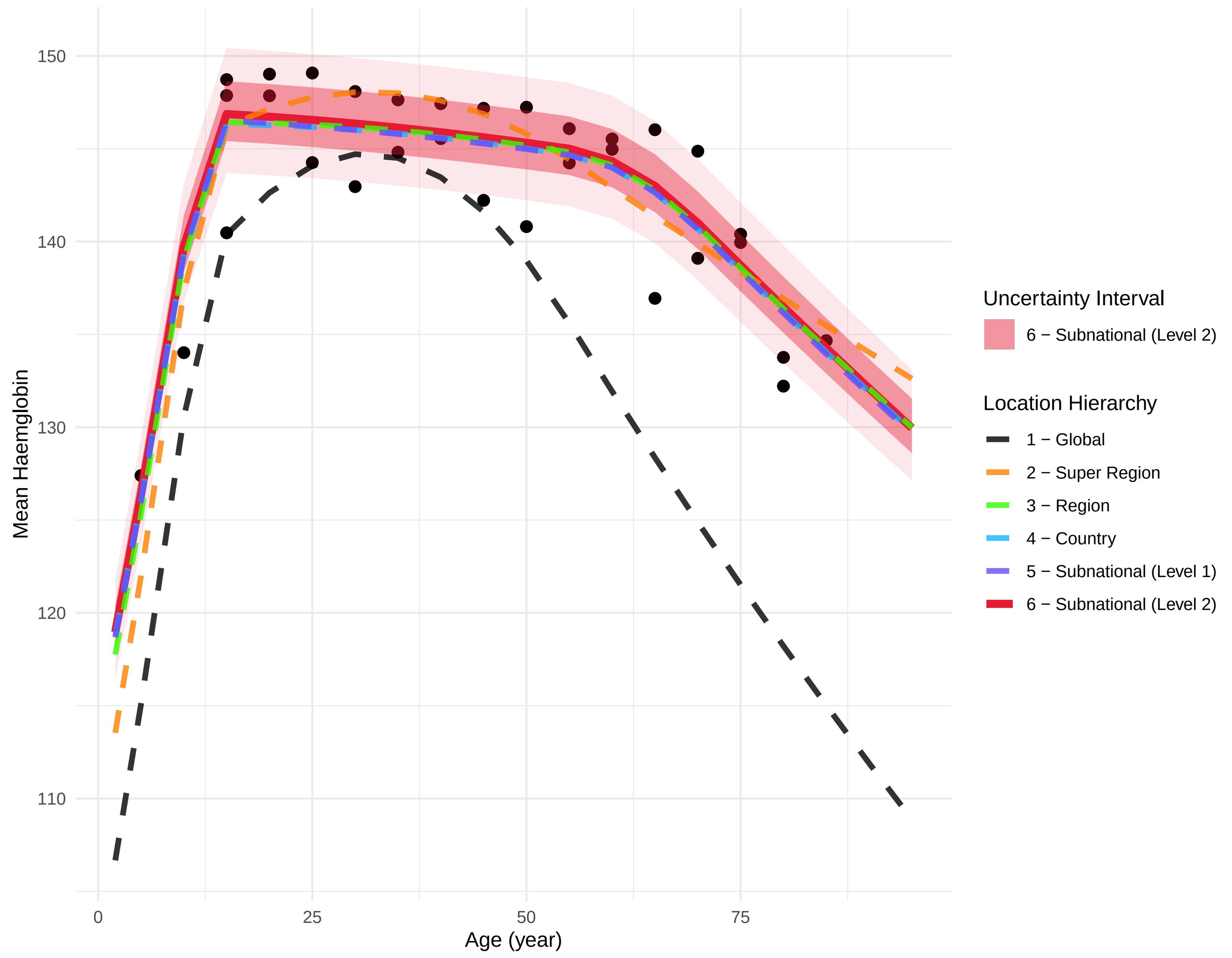

MR−BRT Age/Sex Fits − Males 2+ Years Old − Mean Haemglobin − West Midlands
WHO−2001 Adjustment Method

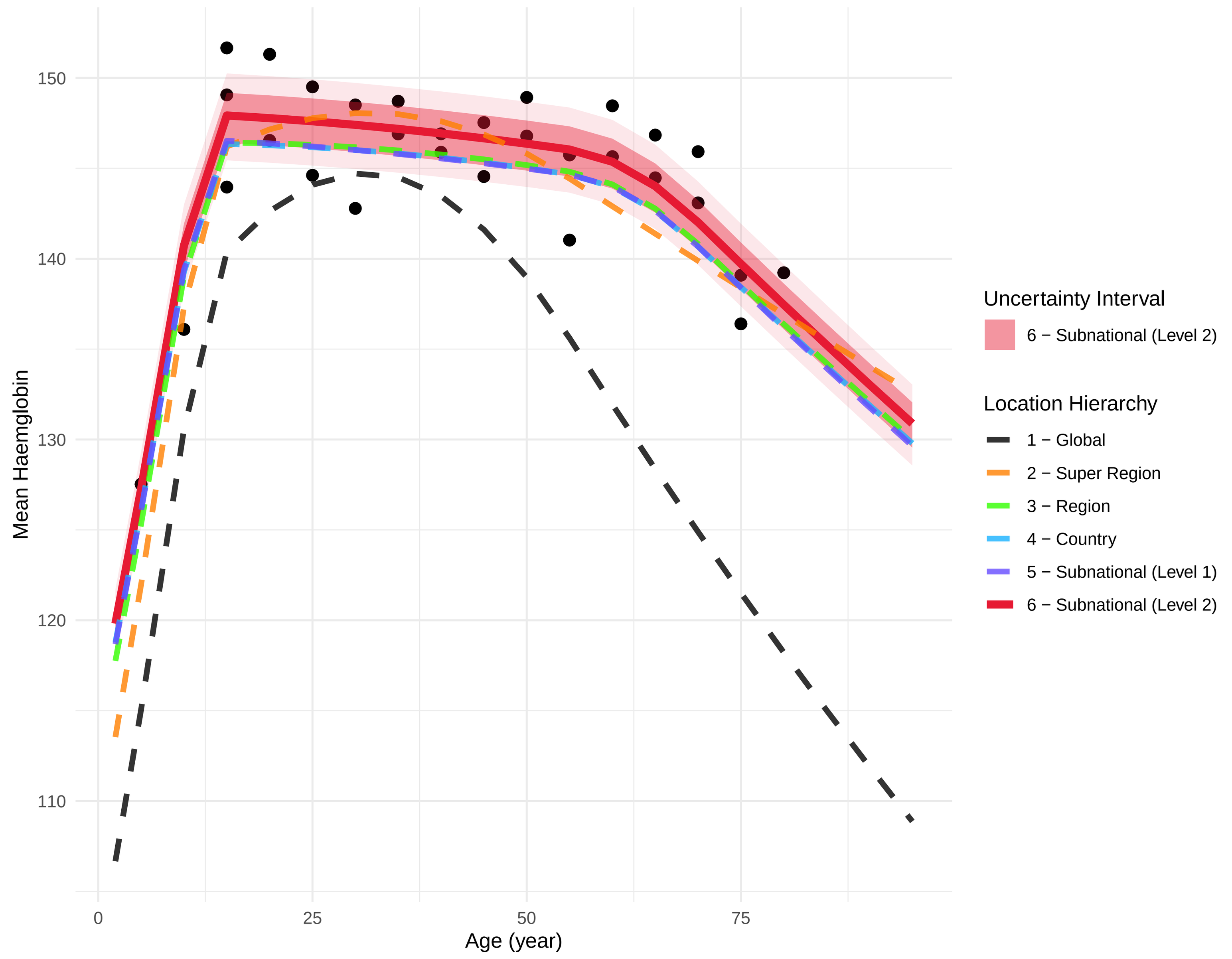

MR−BRT Age/Sex Fits − Males 2+ Years Old − Mean Haemglobin − Yorkshire and the Humber
WHO−2001 Adjustment Method

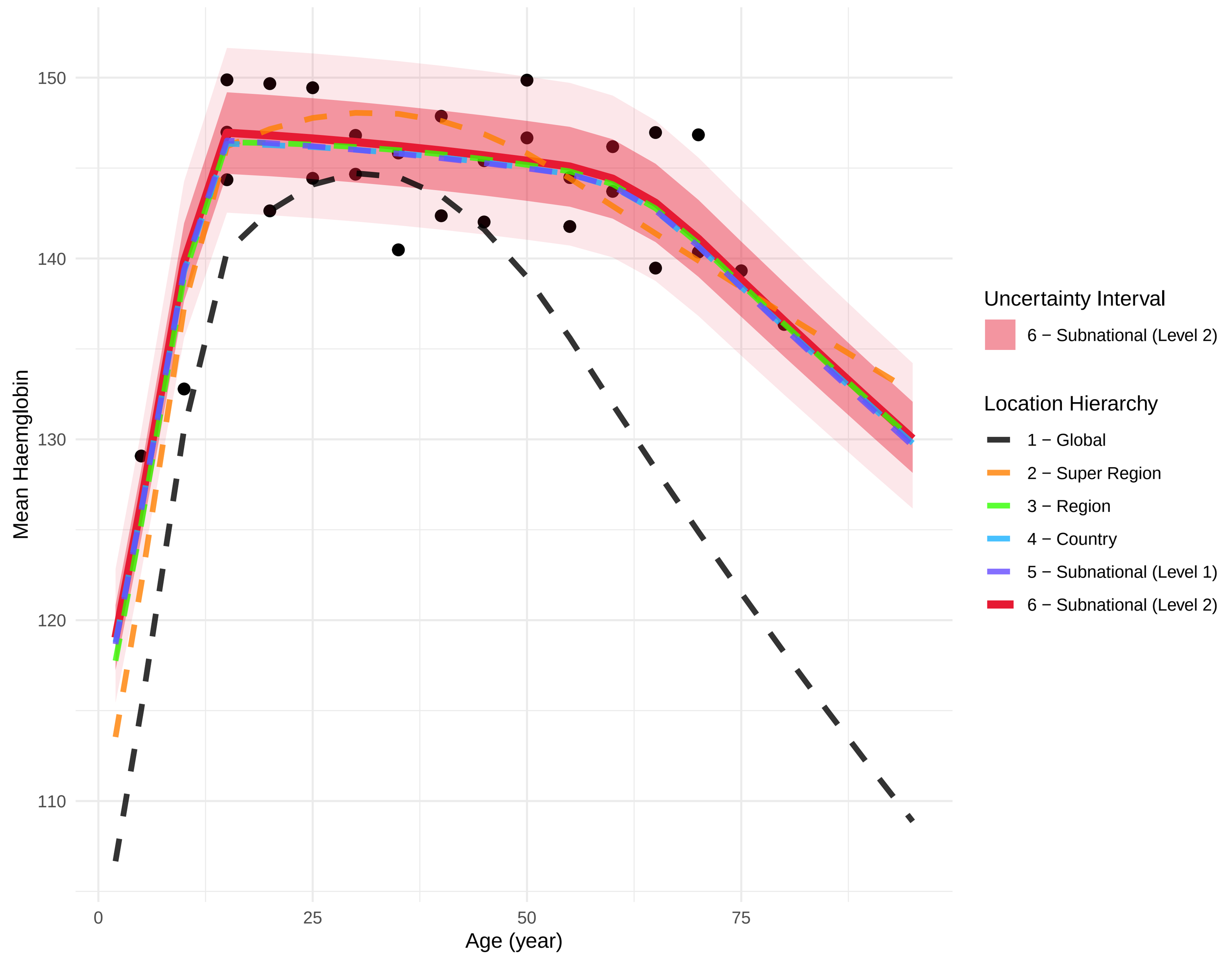

MR−BRT Age/Sex Fits − Males 2+ Years Old − Mean Haemglobin − Scotland
WHO−2001 Adjustment Method

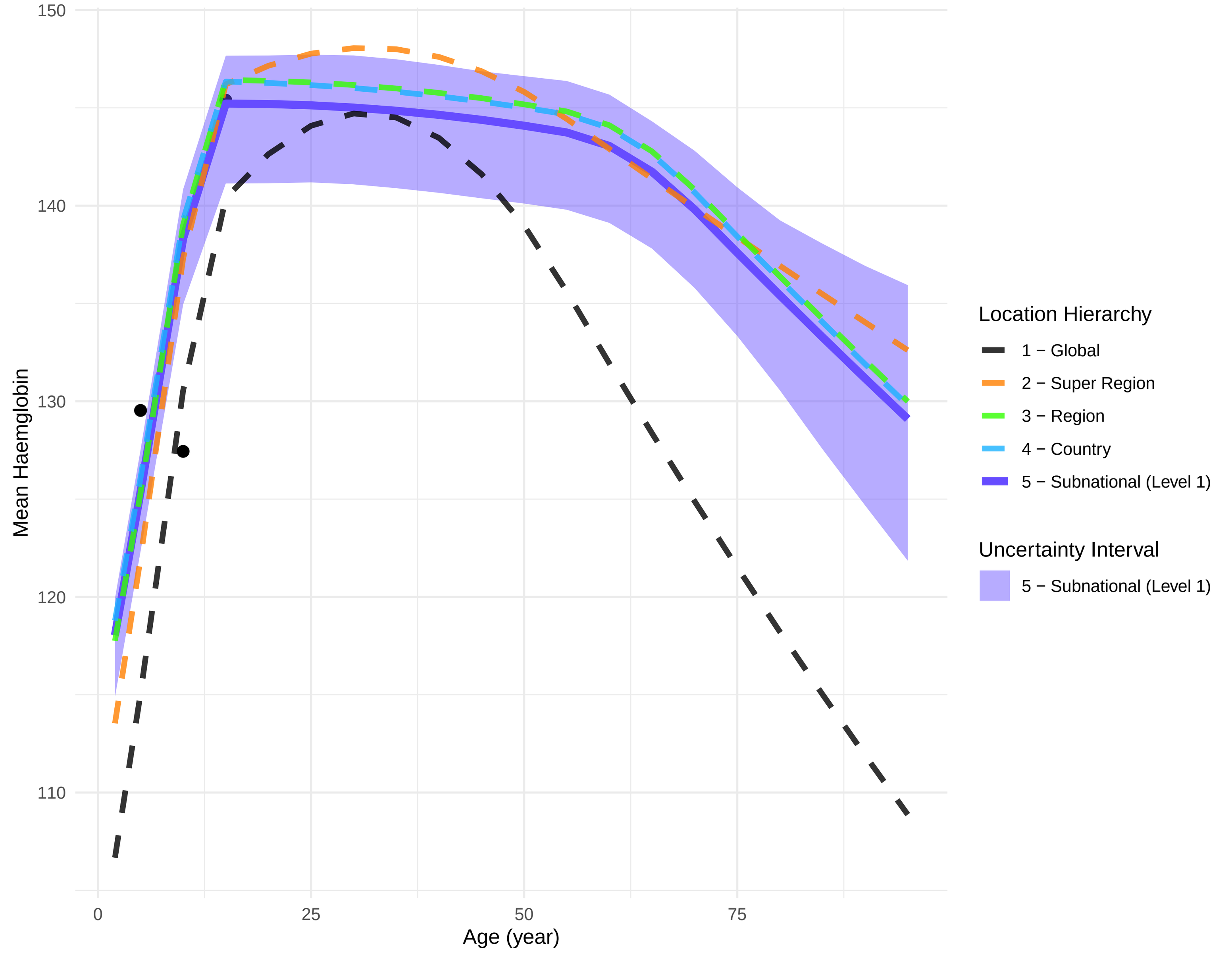

MR−BRT Age/Sex Fits − Males 2+ Years Old − Mean Haemglobin − Wales
WHO−2001 Adjustment Method

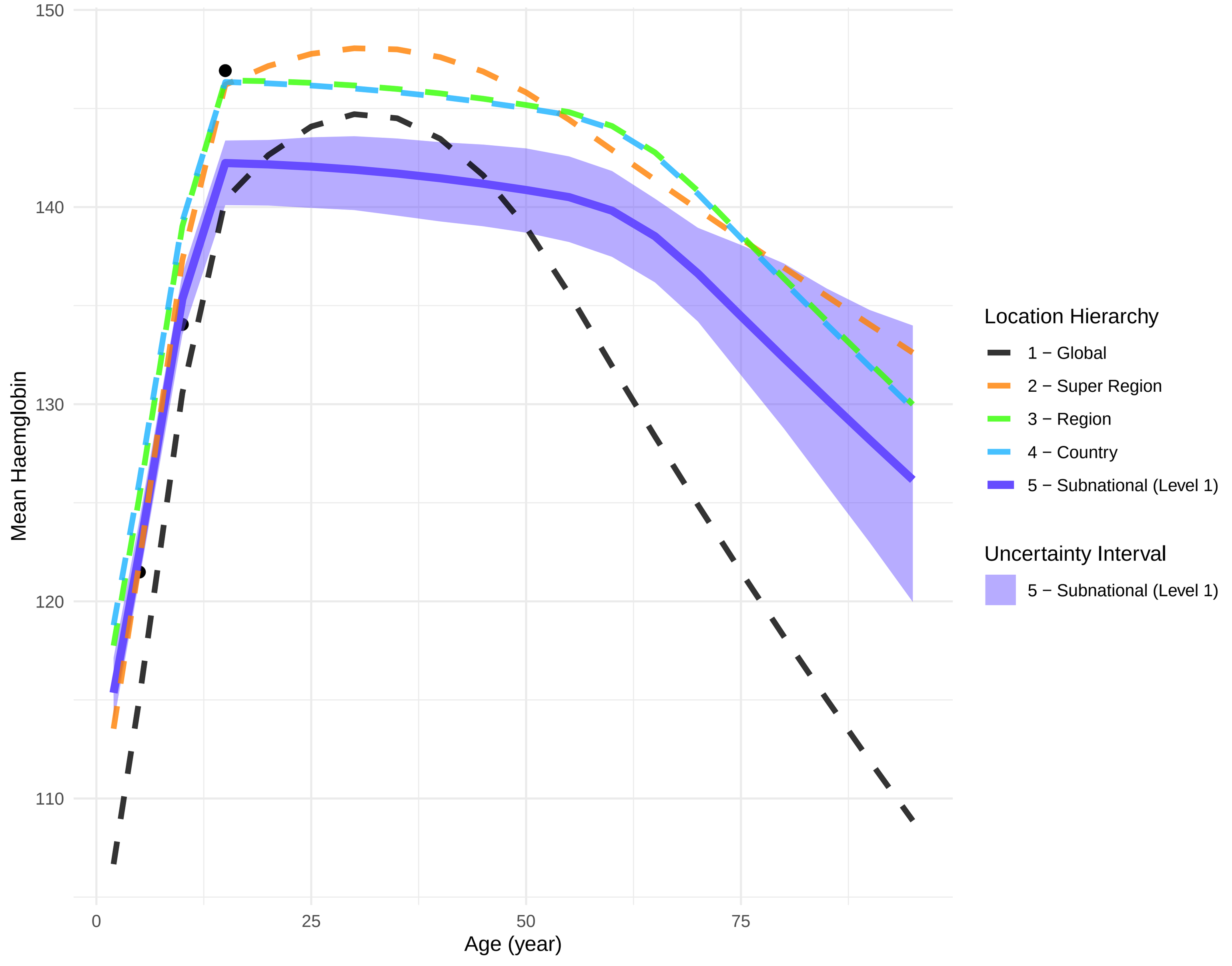

MR−BRT Age/Sex Fits − Males 2+ Years Old − Mean Haemglobin − Latin America and Caribbean
WHO−2001 Adjustment Method

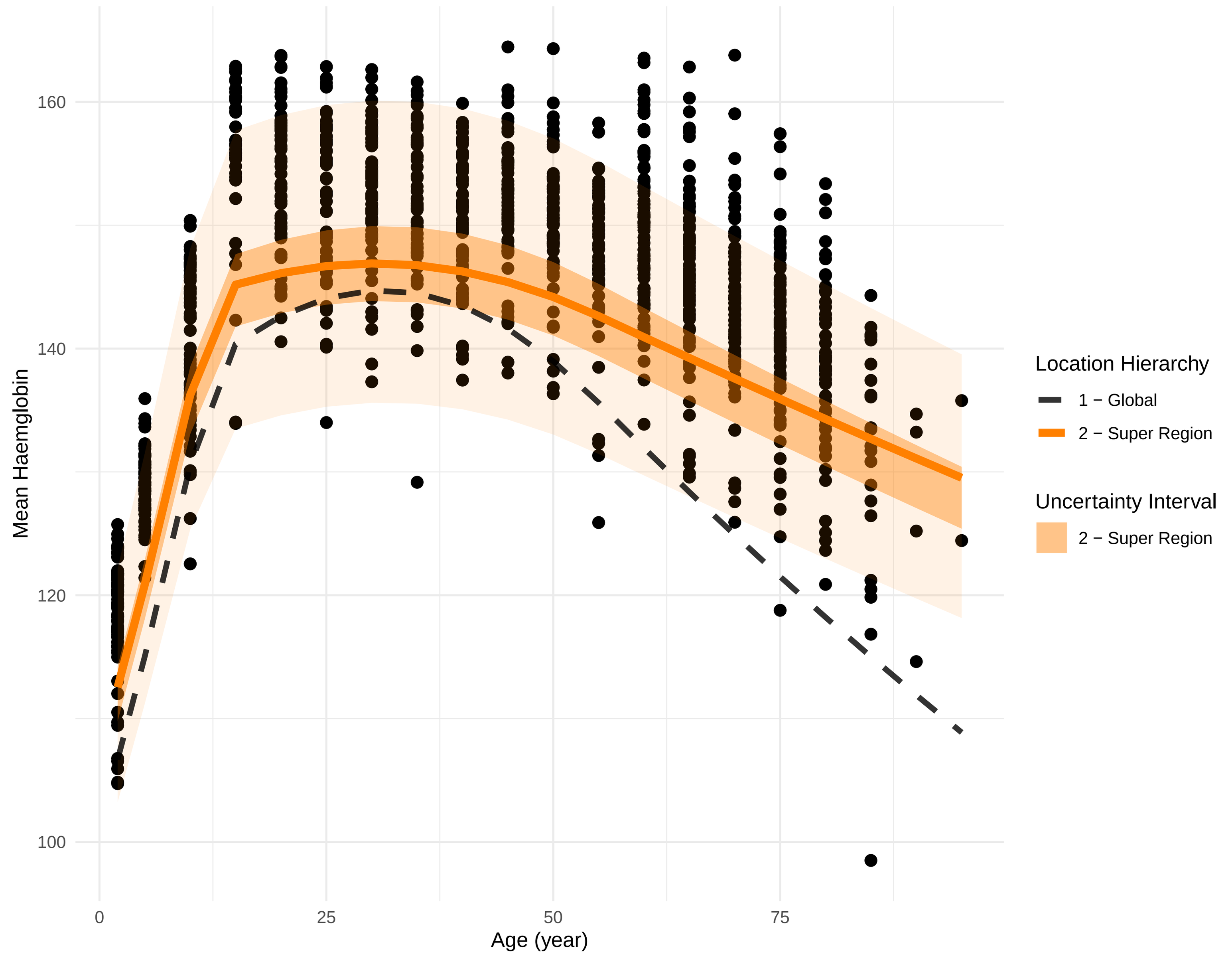

MR−BRT Age/Sex Fits − Males 2+ Years Old − Mean Haemglobin − Andean Latin America
WHO−2001 Adjustment Method

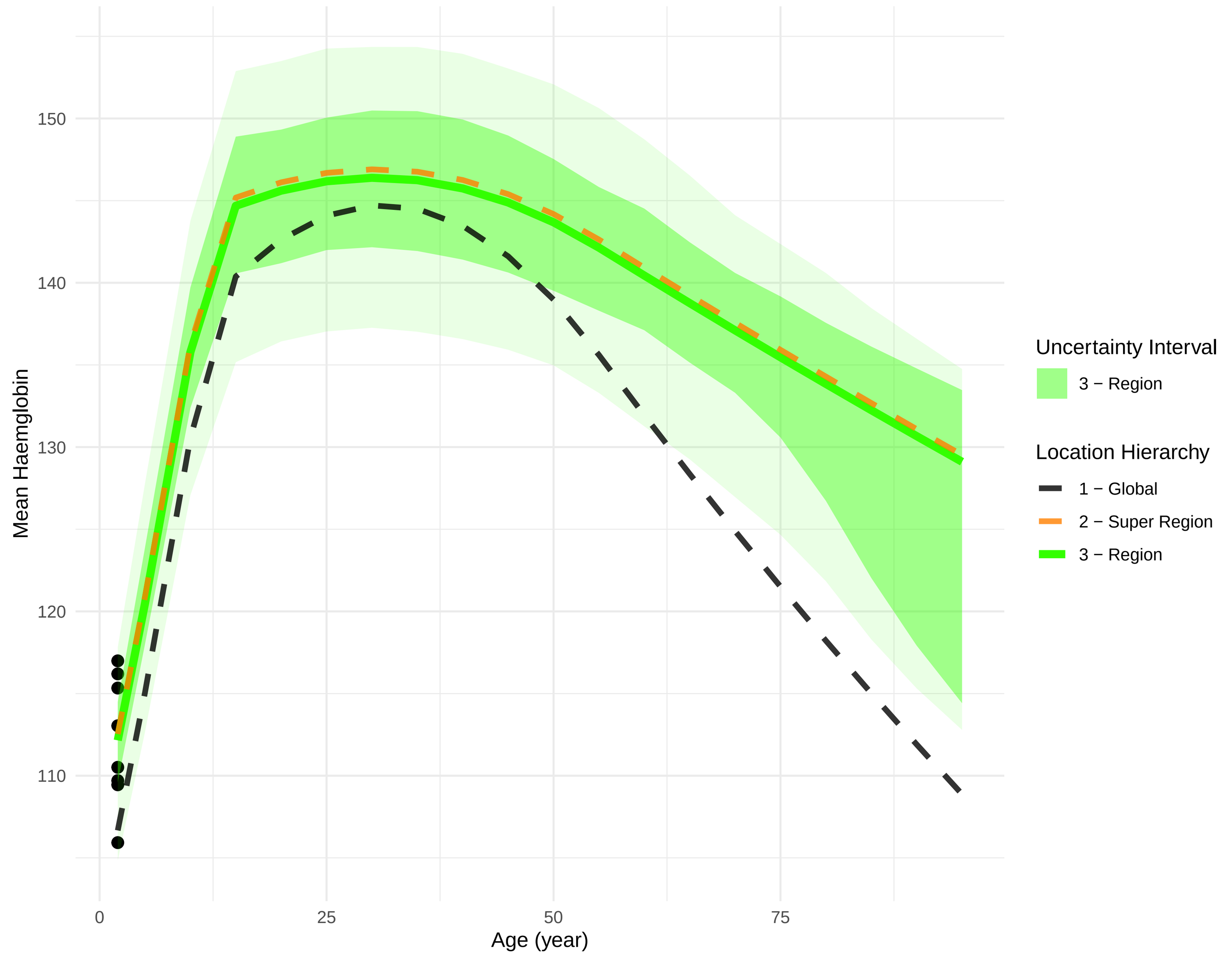

MR−BRT Age/Sex Fits − Males 2+ Years Old − Mean Haemglobin − Bolivia (Plurinational State of)
WHO−2001 Adjustment Method

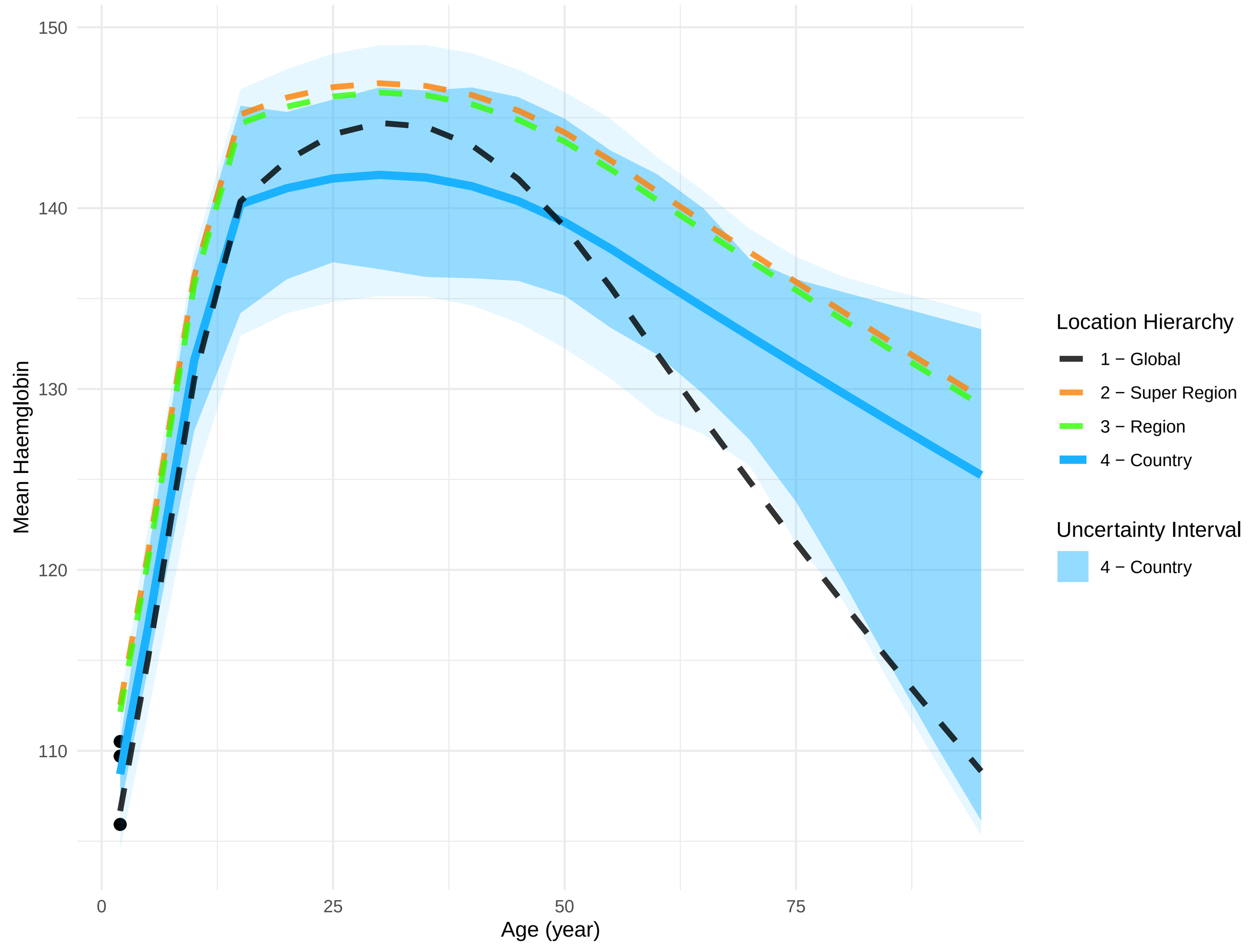

MR−BRT Age/Sex Fits − Males 2+ Years Old − Mean Haemglobin − Peru
WHO−2001 Adjustment Method

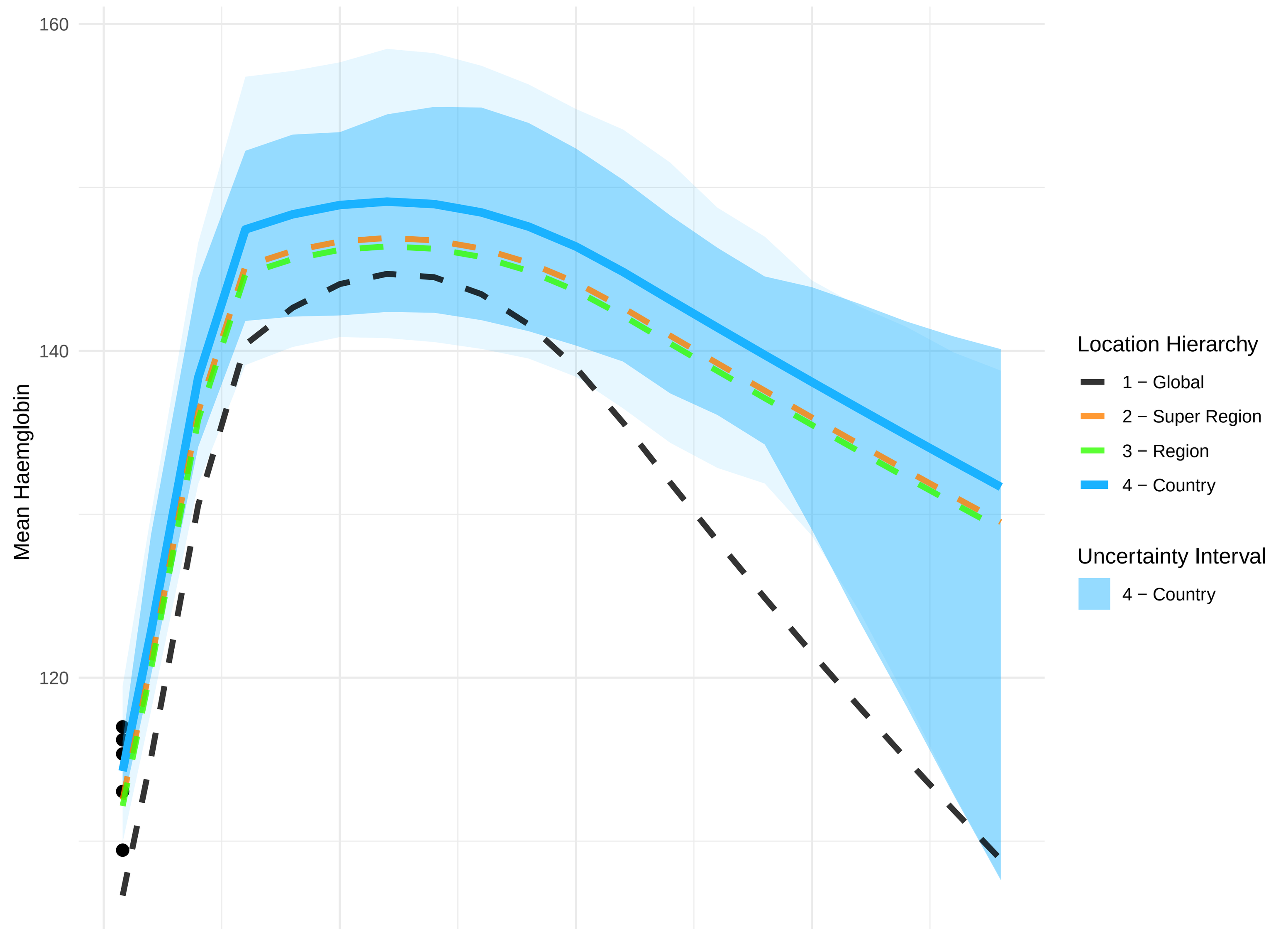

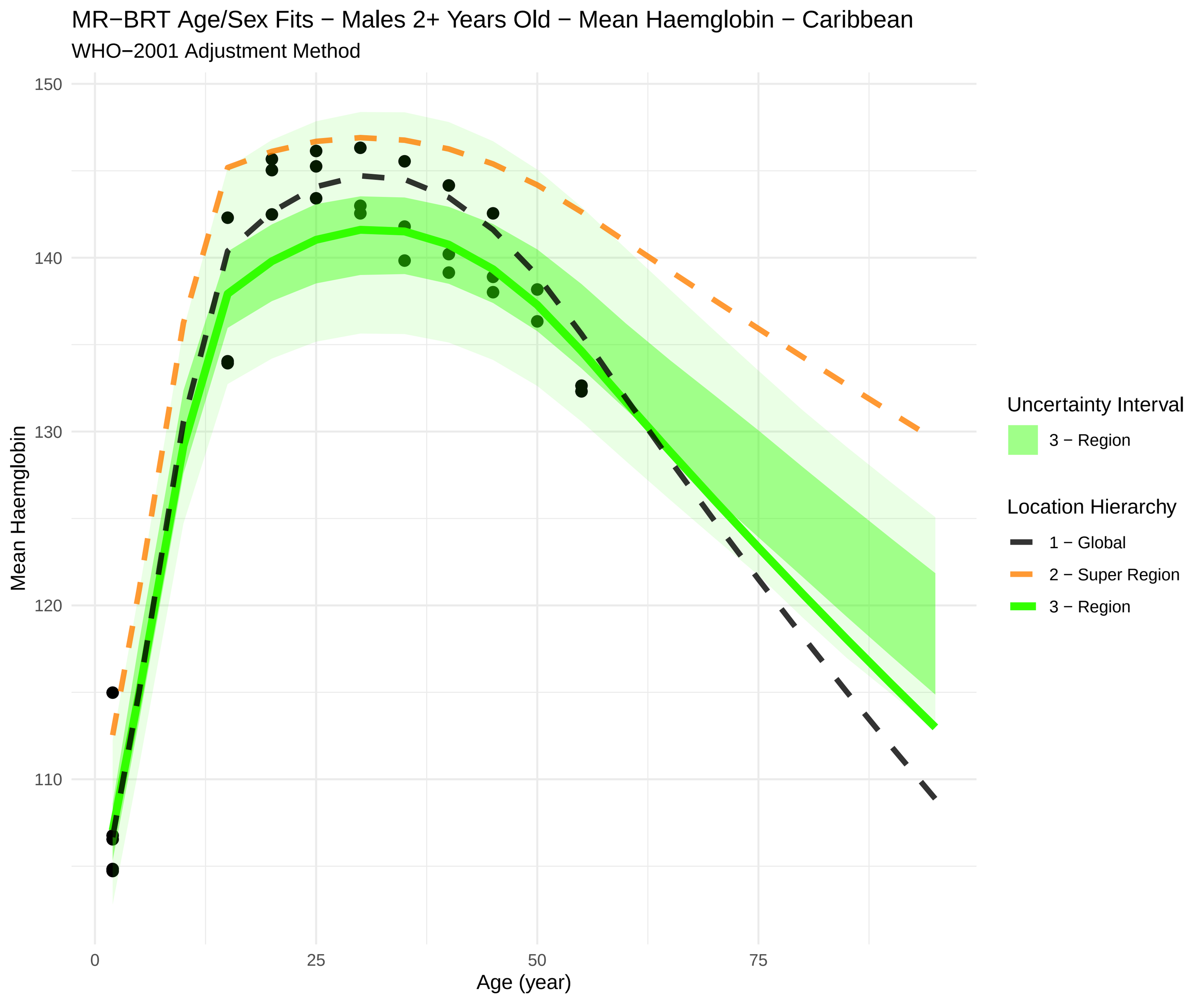
MR−BRT Age/Sex Fits − Males 2+ Years Old − Mean Haemoglobin − Caribbean
WHO−2001 Adjustment Method
Mean Haemoglobin
Age (year)
Uncertainty Interval
3 − Region
Location Hierarchy
1 − Global
2 − Super Region
3 − Region

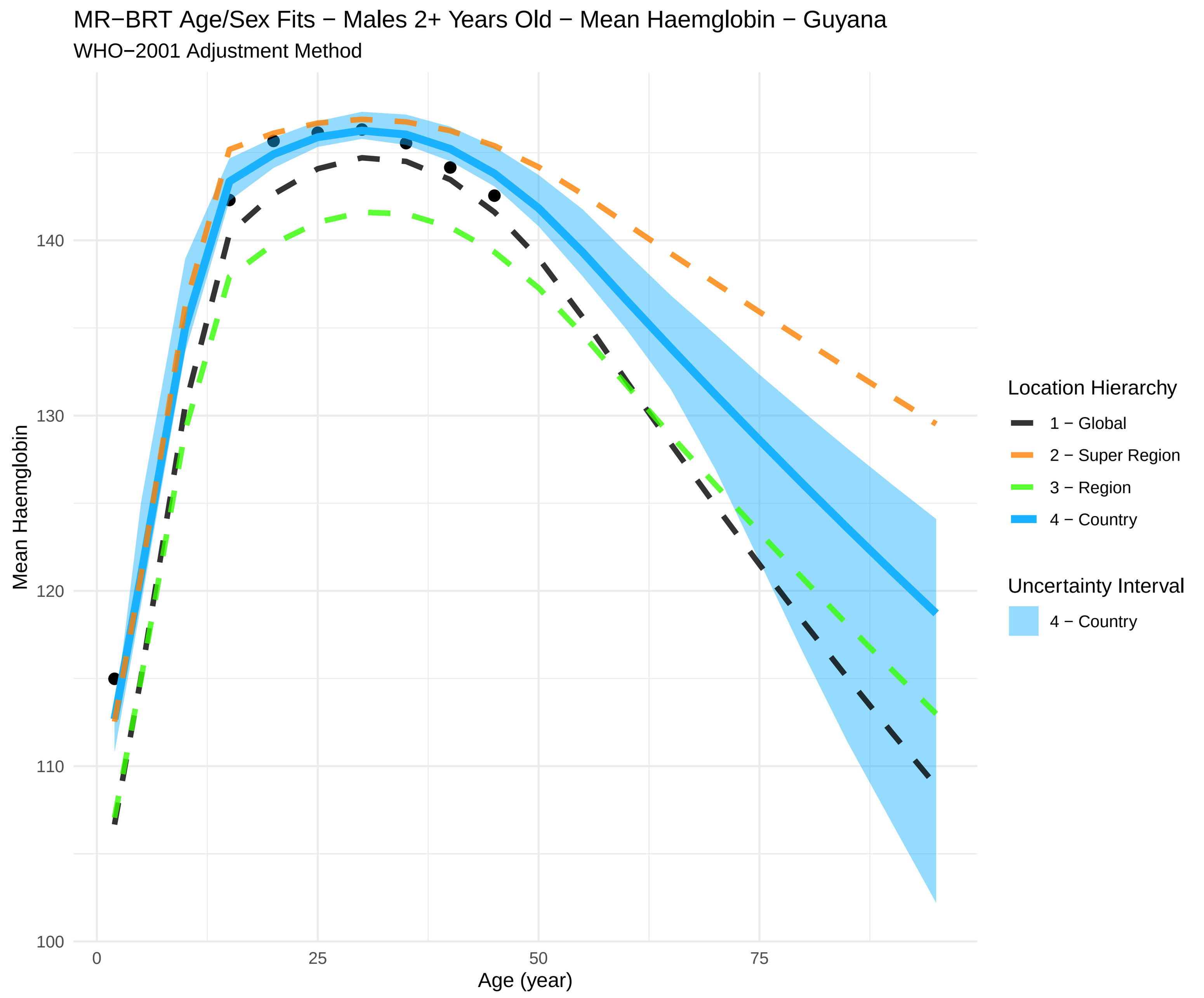
MR−BRT Age/Sex Fits − Males 2+ Years Old − Mean Haemoglobin − Guyana
WHO−2001 Adjustment Method
Mean Haemoglobin
Age (year)
Location Hierarchy
1 − Global
2 − Super Region
3 − Region
4 − Country
Uncertainty Interval
4 − Country

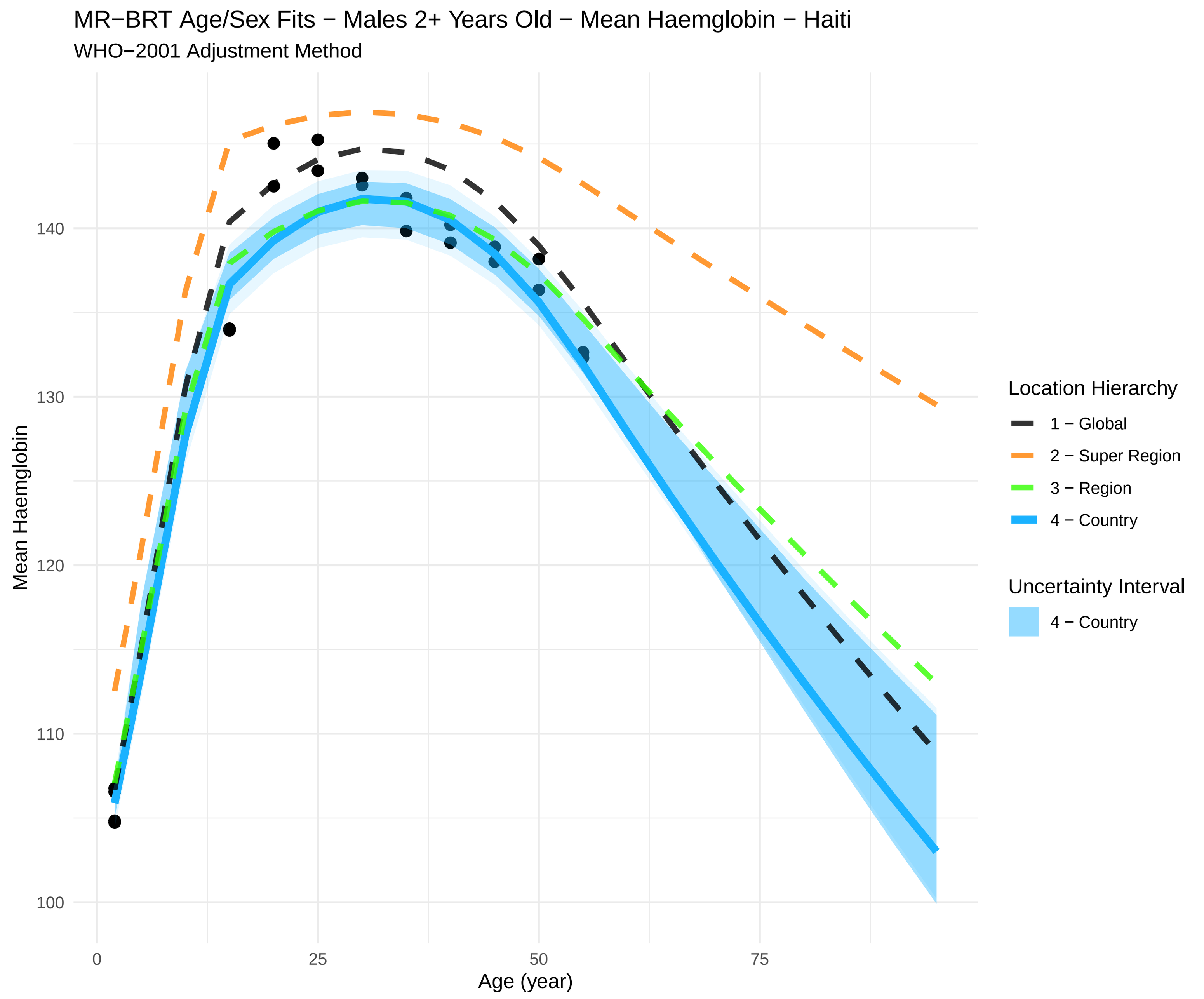
MR−BRT Age/Sex Fits − Males 2+ Years Old − Mean Haemoglobin − Haiti
WHO−2001 Adjustment Method
Mean Haemoglobin
Age (year)
Location Hierarchy
1 − Global
2 − Super Region
3 − Region
4 − Country
Uncertainty Interval
4 − Country

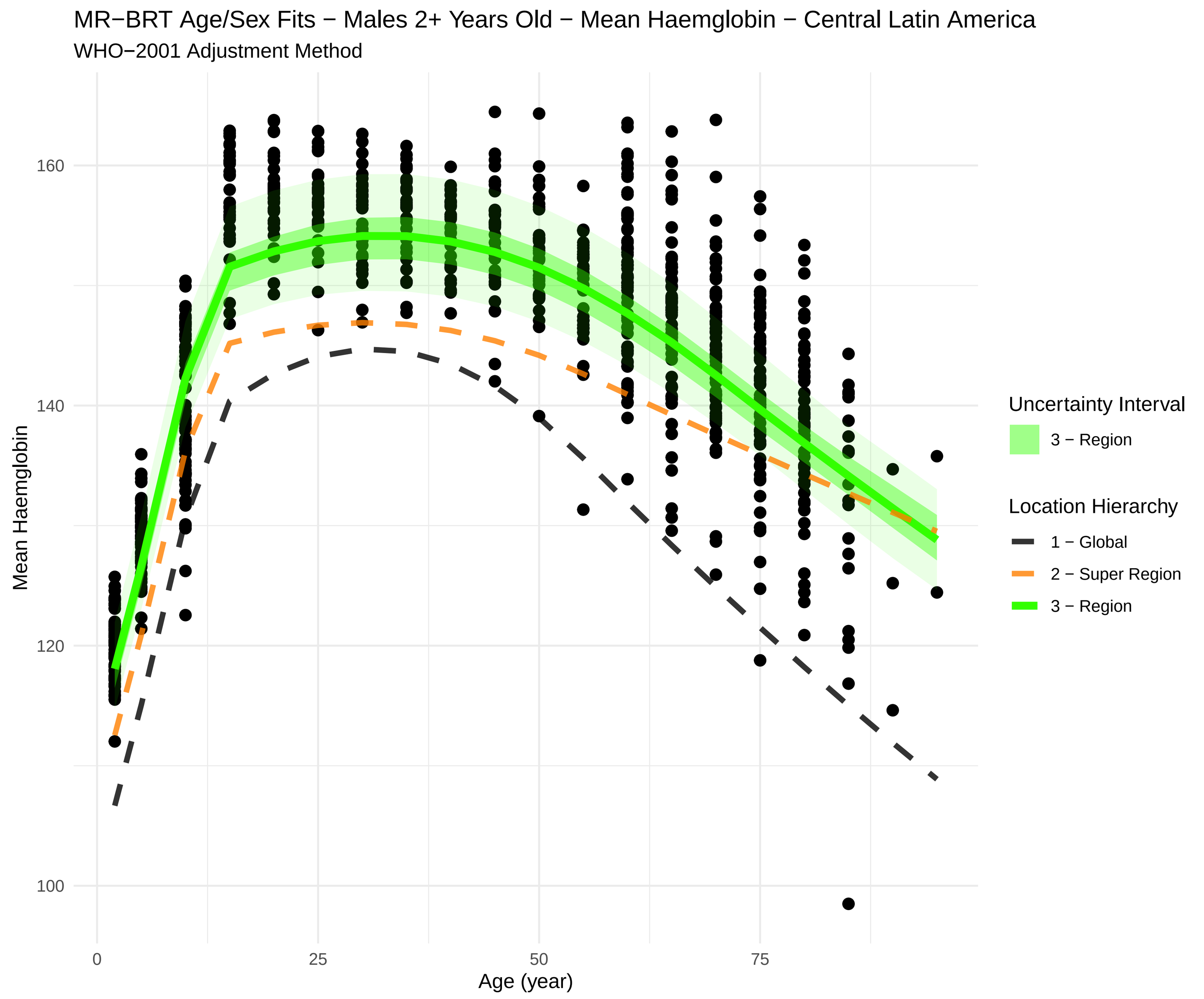
MR−BRT Age/Sex Fits − Males 2+ Years Old − Mean Haemoglobin − Central Latin America
WHO−2001 Adjustment Method
Mean Haemoglobin
Age (year)
Uncertainty Interval
3 − Region
Location Hierarchy
1 − Global
2 − Super Region
3 − Region

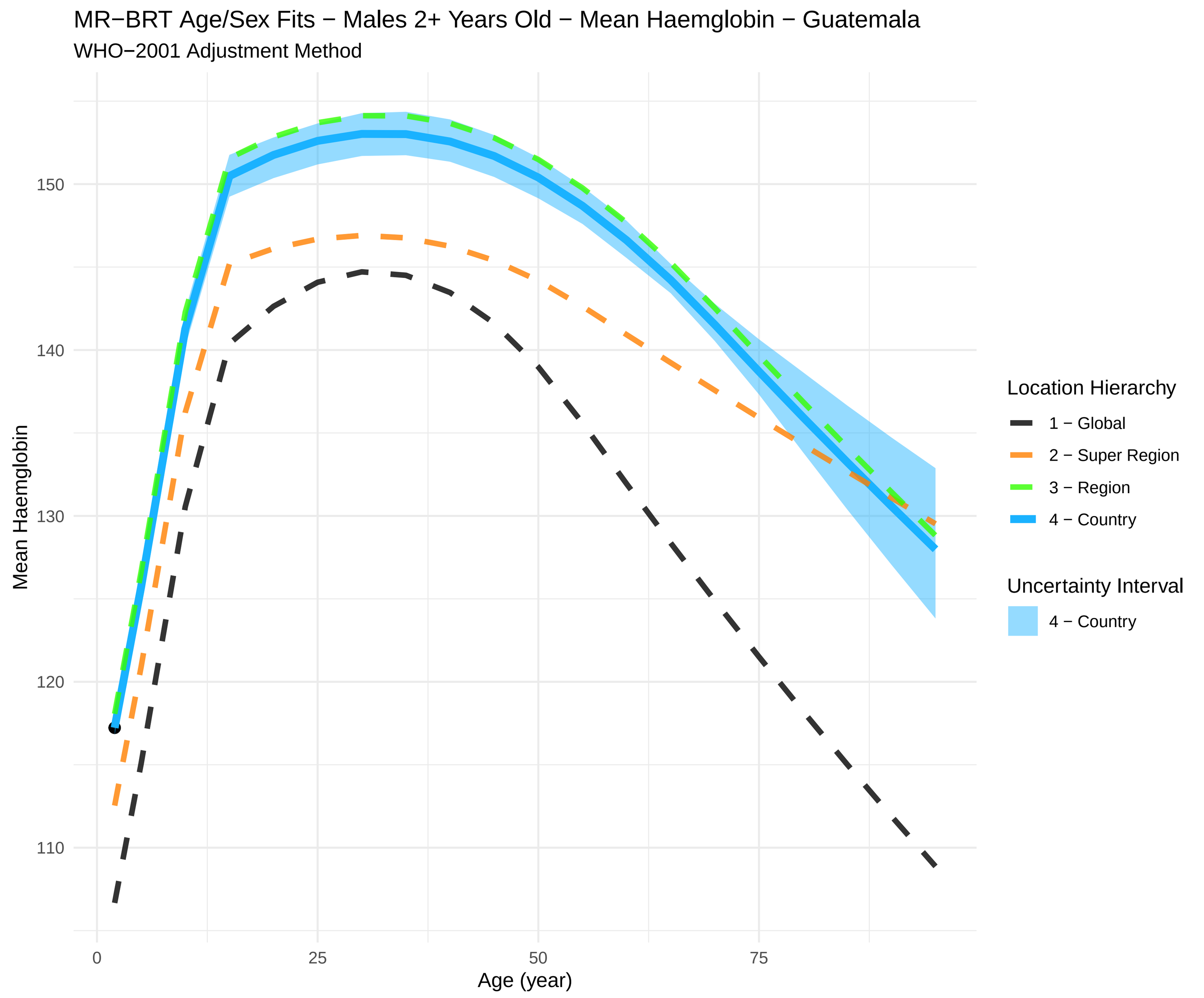
MR−BRT Age/Sex Fits − Males 2+ Years Old − Mean Haemoglobin − Guatemala
WHO−2001 Adjustment Method
Mean Haemoglobin
Age (year)
Location Hierarchy
1 − Global
2 − Super Region
3 − Region
4 − Country
Uncertainty Interval
4 − Country

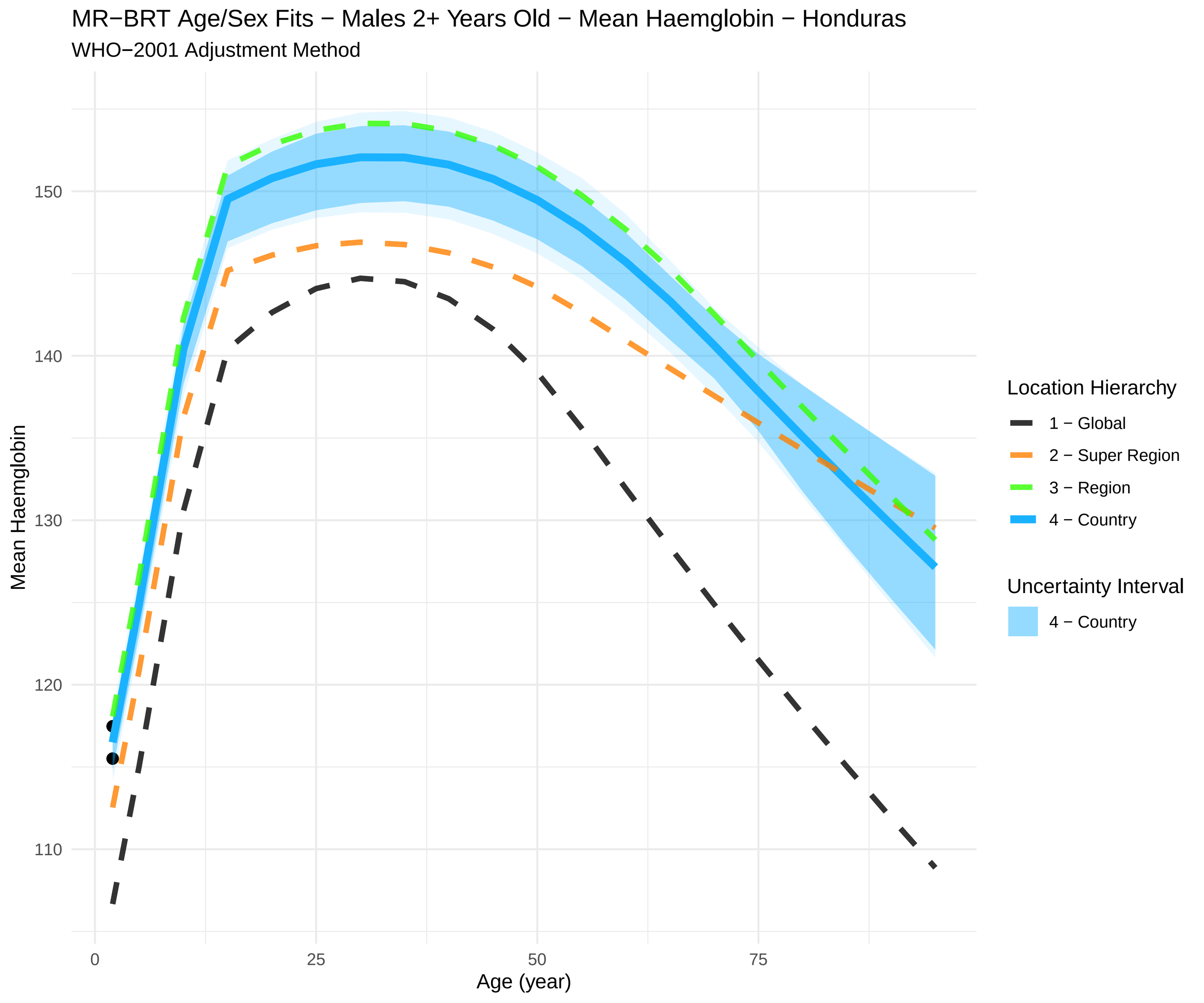
MR−BRT Age/Sex Fits − Males 2+ Years Old − Mean Haemoglobin − Honduras
WHO−2001 Adjustment Method
Mean Haemoglobin
Age (year)
Location Hierarchy
1 − Global
2 − Super Region
3 − Region
4 − Country
Uncertainty Interval
4 − Country

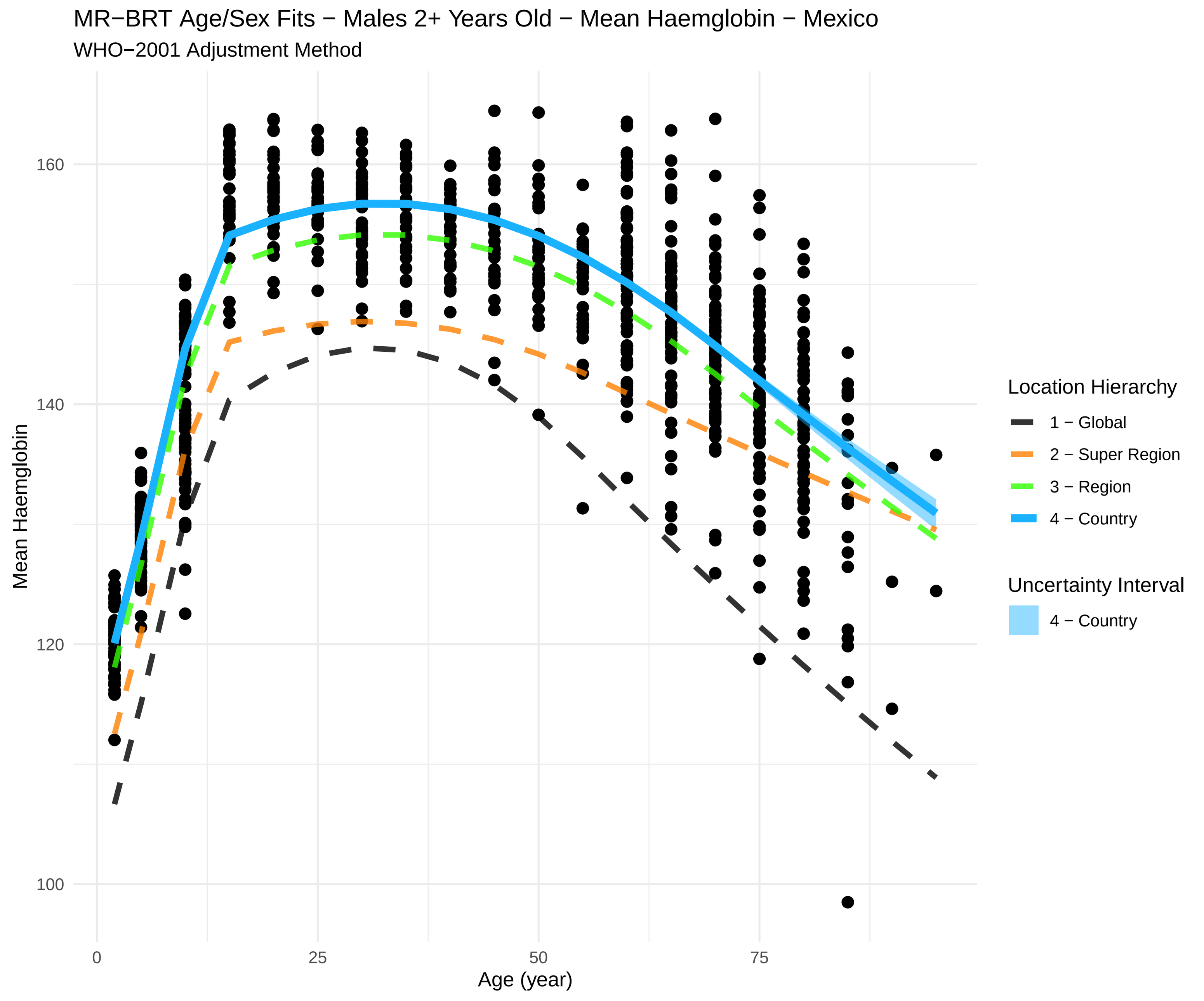
MR−BRT Age/Sex Fits − Males 2+ Years Old − Mean Haemoglobin − Mexico
WHO−2001 Adjustment Method
Mean Haemoglobin
Age (year)
Location Hierarchy
1 − Global
2 − Super Region
3 − Region
4 − Country
Uncertainty Interval
4 − Country

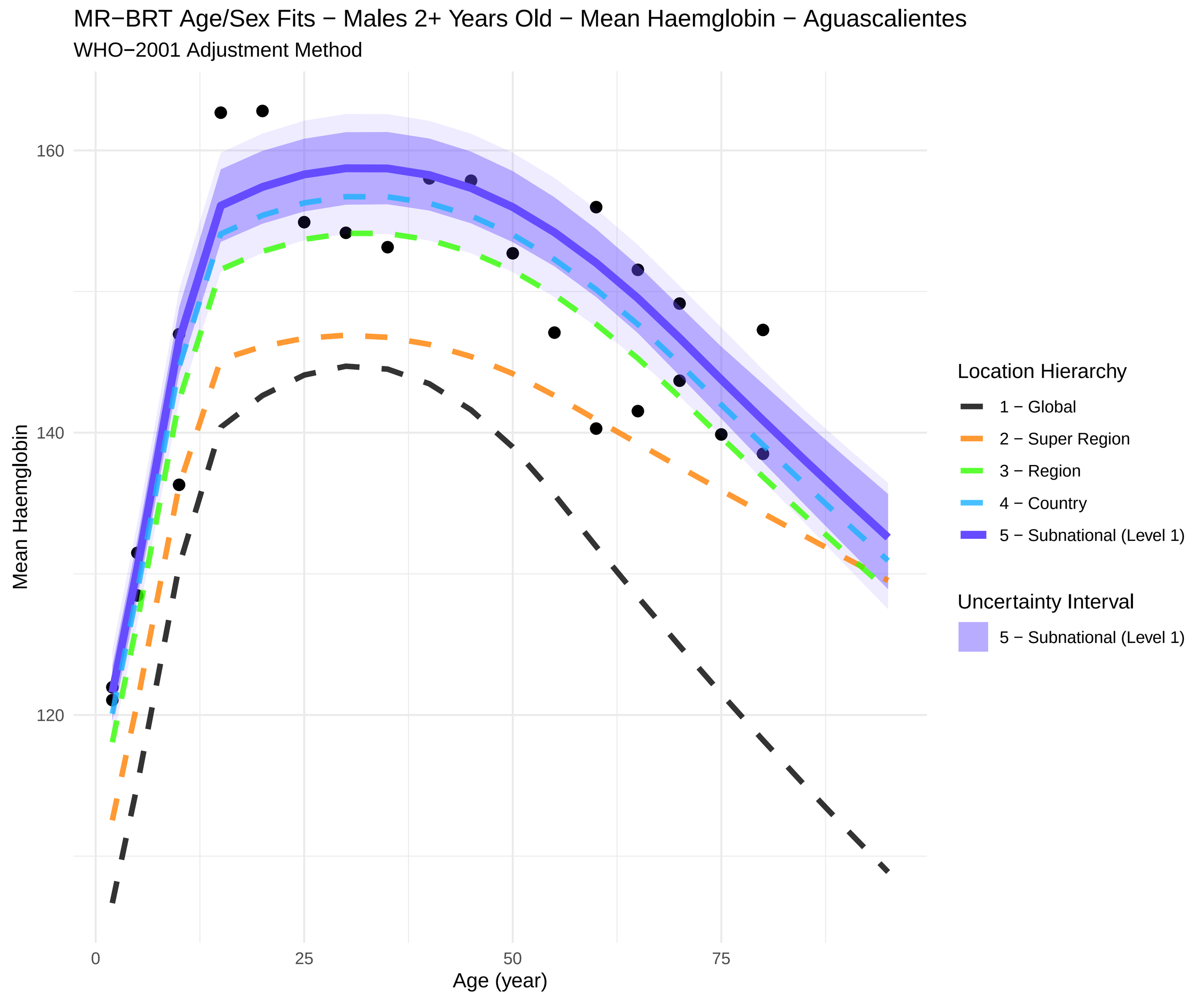
MR−BRT Age/Sex Fits − Males 2+ Years Old − Mean Haemoglobin − Aguascalientes
WHO−2001 Adjustment Method
Mean Haemoglobin
Age (year)
Location Hierarchy
1 − Global
2 − Super Region
3 − Region
4 − Country
5 − Subnational (Level 1)
Uncertainty Interval
5 − Subnational (Level 1)

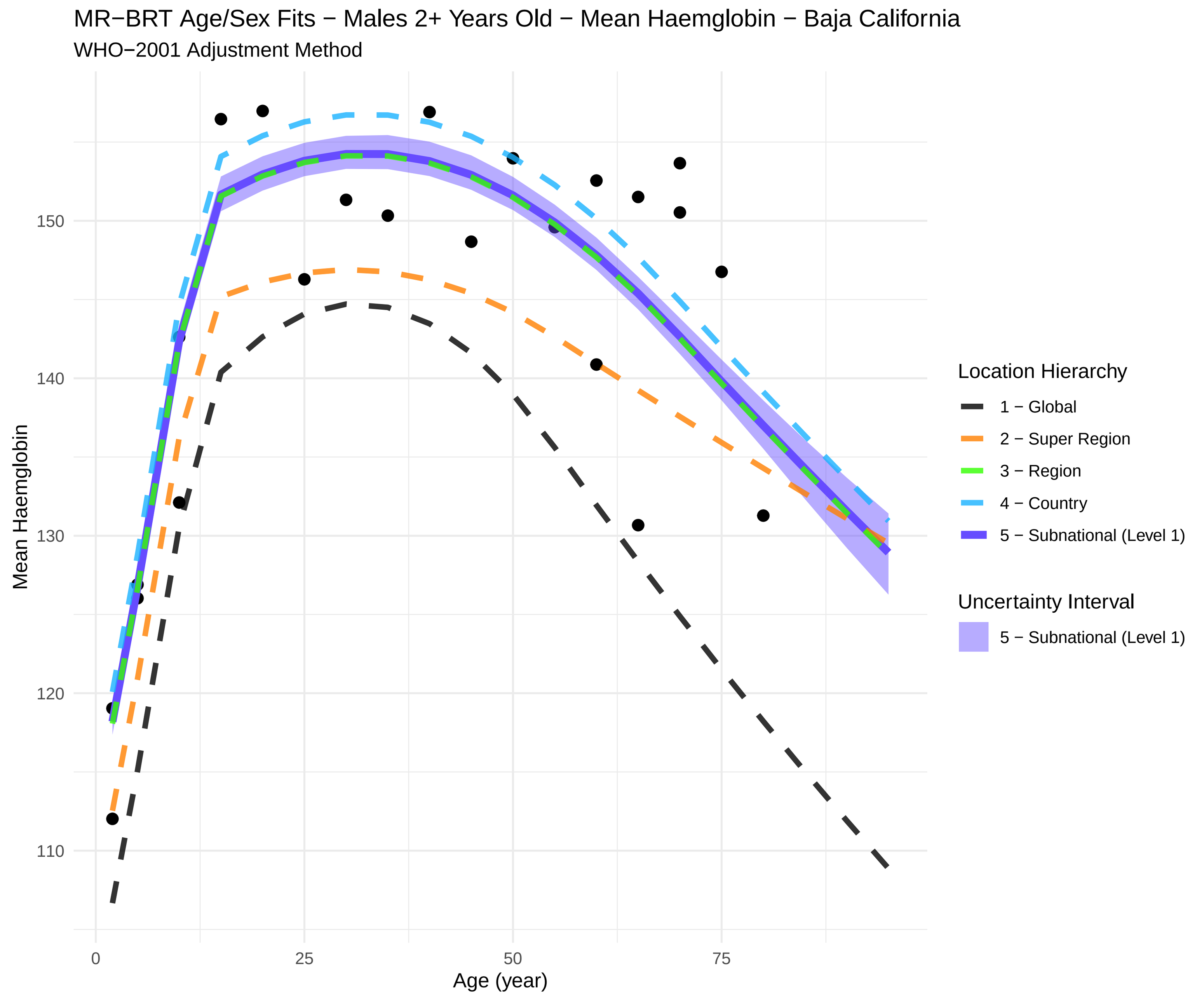
MR−BRT Age/Sex Fits − Males 2+ Years Old − Mean Haemoglobin − Baja California
WHO−2001 Adjustment Method
Mean Haemoglobin
Age (year)
Location Hierarchy
1 − Global
2 − Super Region
3 − Region
4 − Country
5 − Subnational (Level 1)
Uncertainty Interval
5 − Subnational (Level 1)

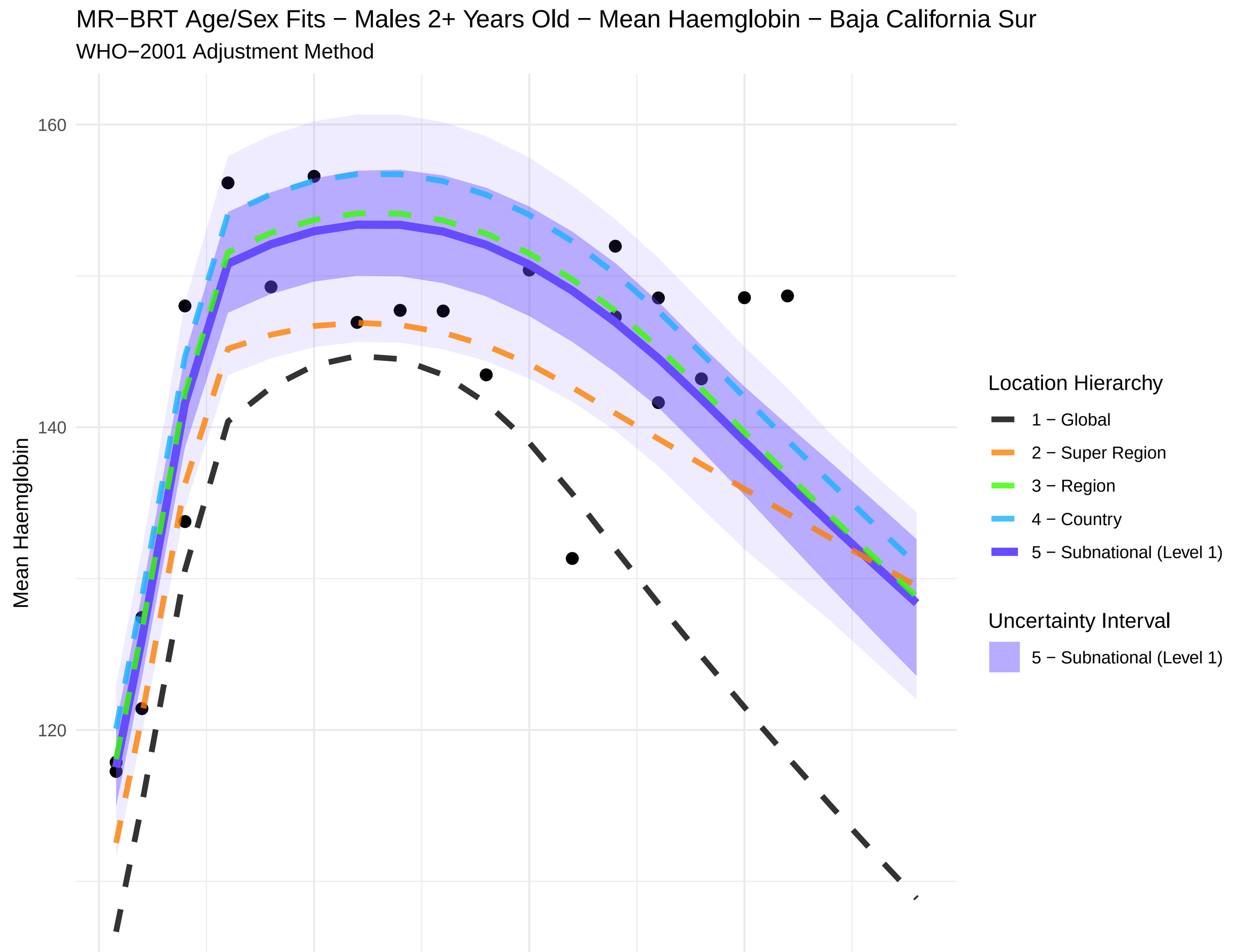
MR−BRT Age/Sex Fits − Males 2+ Years Old − Mean Haemoglobin − Baja California Sur
WHO−2001 Adjustment Method
Mean Haemoglobin
Age (year)
Location Hierarchy
1 − Global
2 − Super Region
3 − Region
4 − Country
5 − Subnational (Level 1)
Uncertainty Interval
5 − Subnational (Level 1)

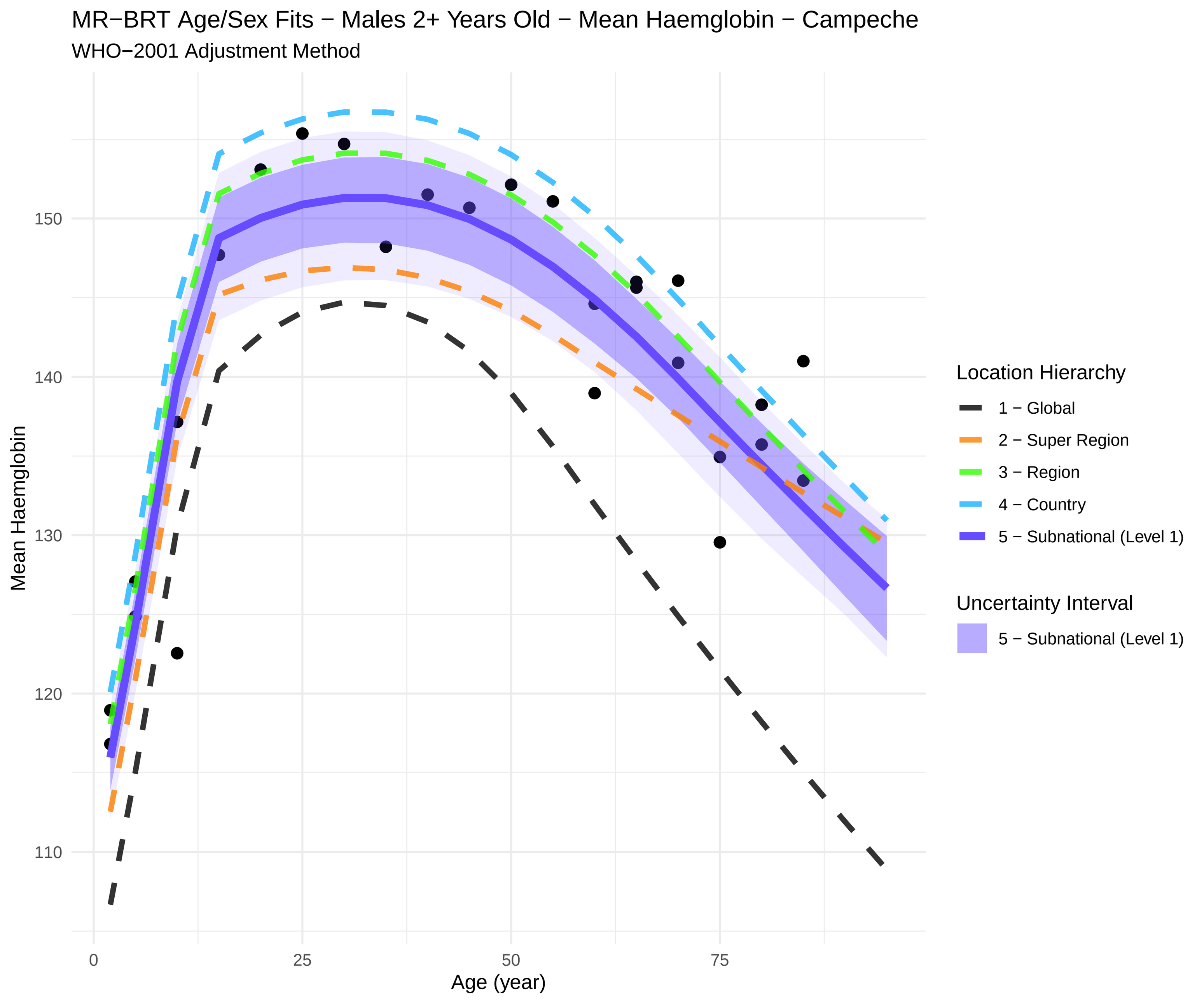
MR−BRT Age/Sex Fits − Males 2+ Years Old − Mean Haemglobin − Campeche
WHO−2001 Adjustment Method
Mean Haemglobin
Age (year)
Location Hierarchy
1 − Global
2 − Super Region
3 − Region
4 − Country
5 − Subnational (Level 1)
Uncertainty Interval
5 − Subnational (Level 1)

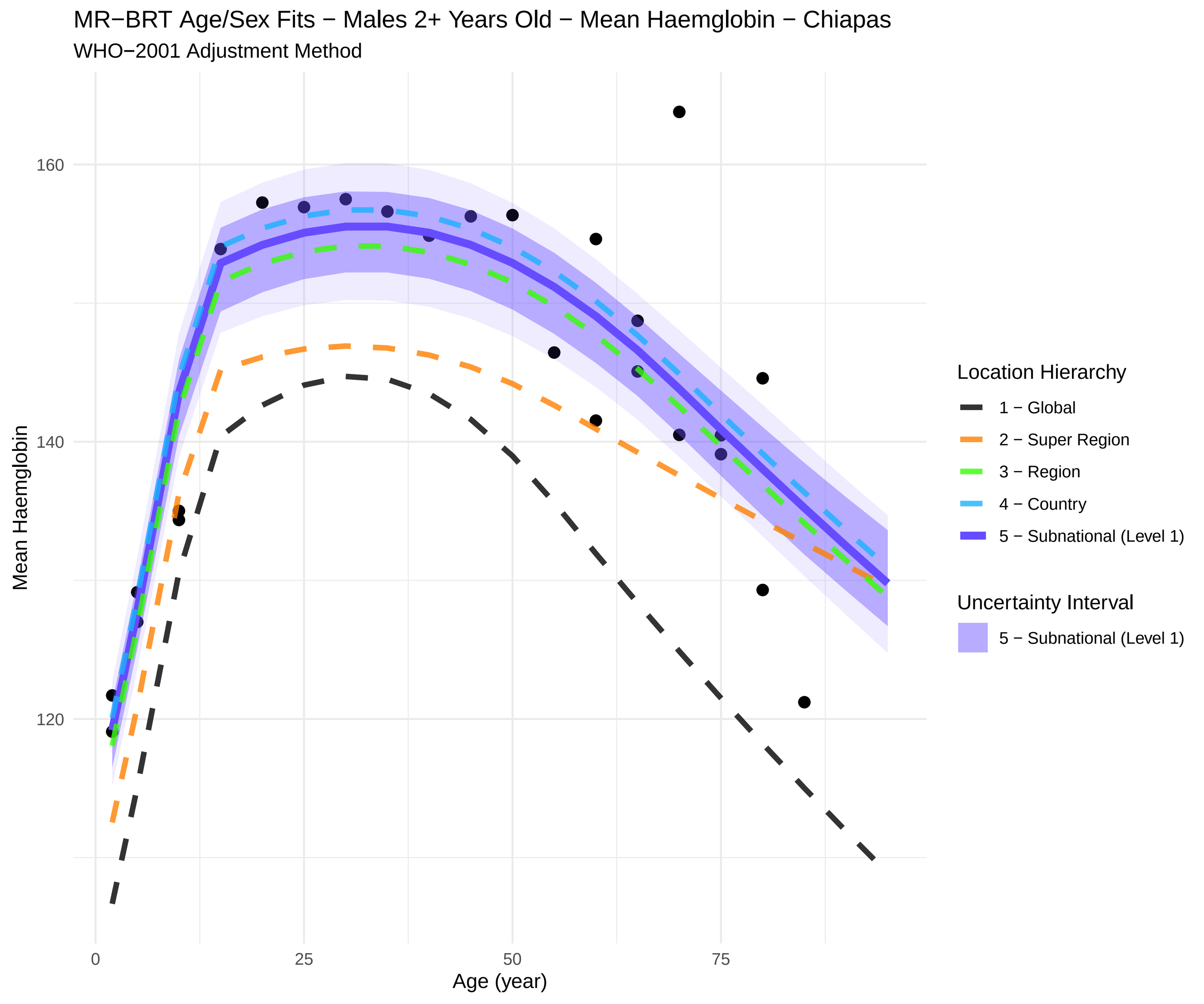
MR−BRT Age/Sex Fits − Males 2+ Years Old − Mean Haemglobin − Chiapas
WHO−2001 Adjustment Method
Mean Haemglobin
Age (year)
Location Hierarchy
1 − Global
2 − Super Region
3 − Region
4 − Country
5 − Subnational (Level 1)
Uncertainty Interval
5 − Subnational (Level 1)

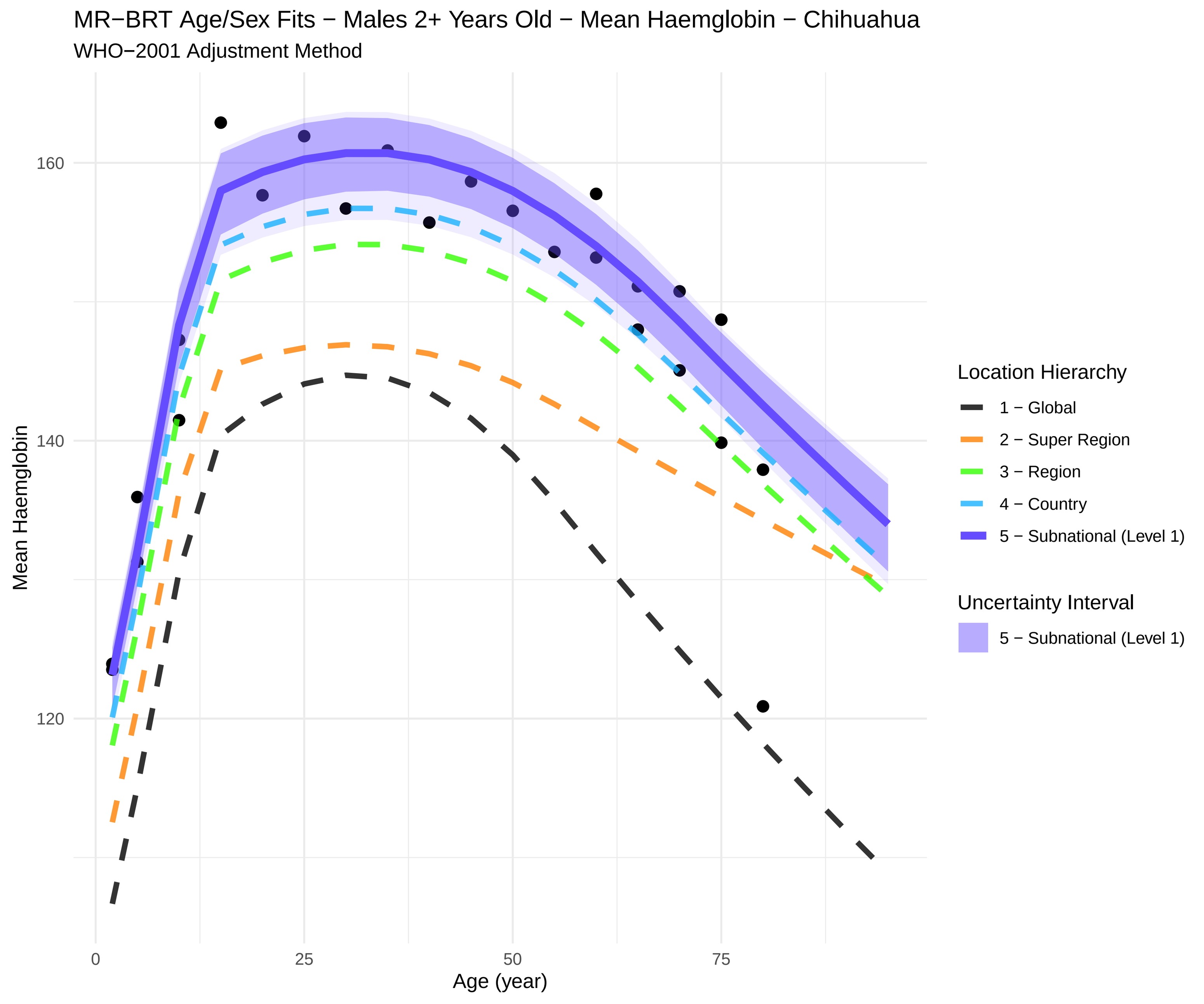
MR−BRT Age/Sex Fits − Males 2+ Years Old − Mean Haemglobin − Chihuahua
WHO−2001 Adjustment Method
Mean Haemglobin
Age (year)
Location Hierarchy
1 − Global
2 − Super Region
3 − Region
4 − Country
5 − Subnational (Level 1)
Uncertainty Interval
5 − Subnational (Level 1)

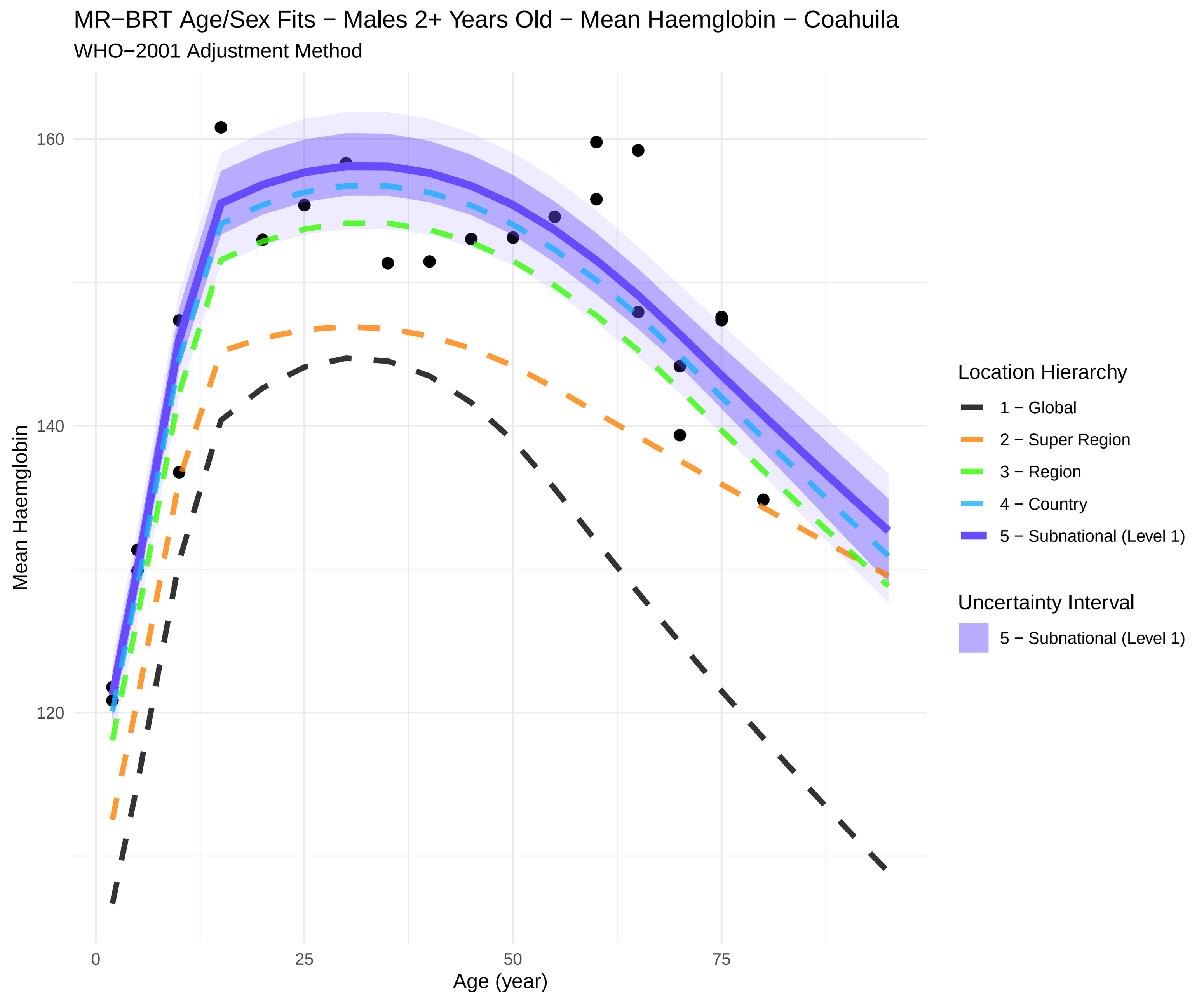
MR−BRT Age/Sex Fits − Males 2+ Years Old − Mean Haemglobin − Coahuila
WHO−2001 Adjustment Method
Mean Haemglobin
Age (year)
Location Hierarchy
1 − Global
2 − Super Region
3 − Region
4 − Country
5 − Subnational (Level 1)
Uncertainty Interval
5 − Subnational (Level 1)

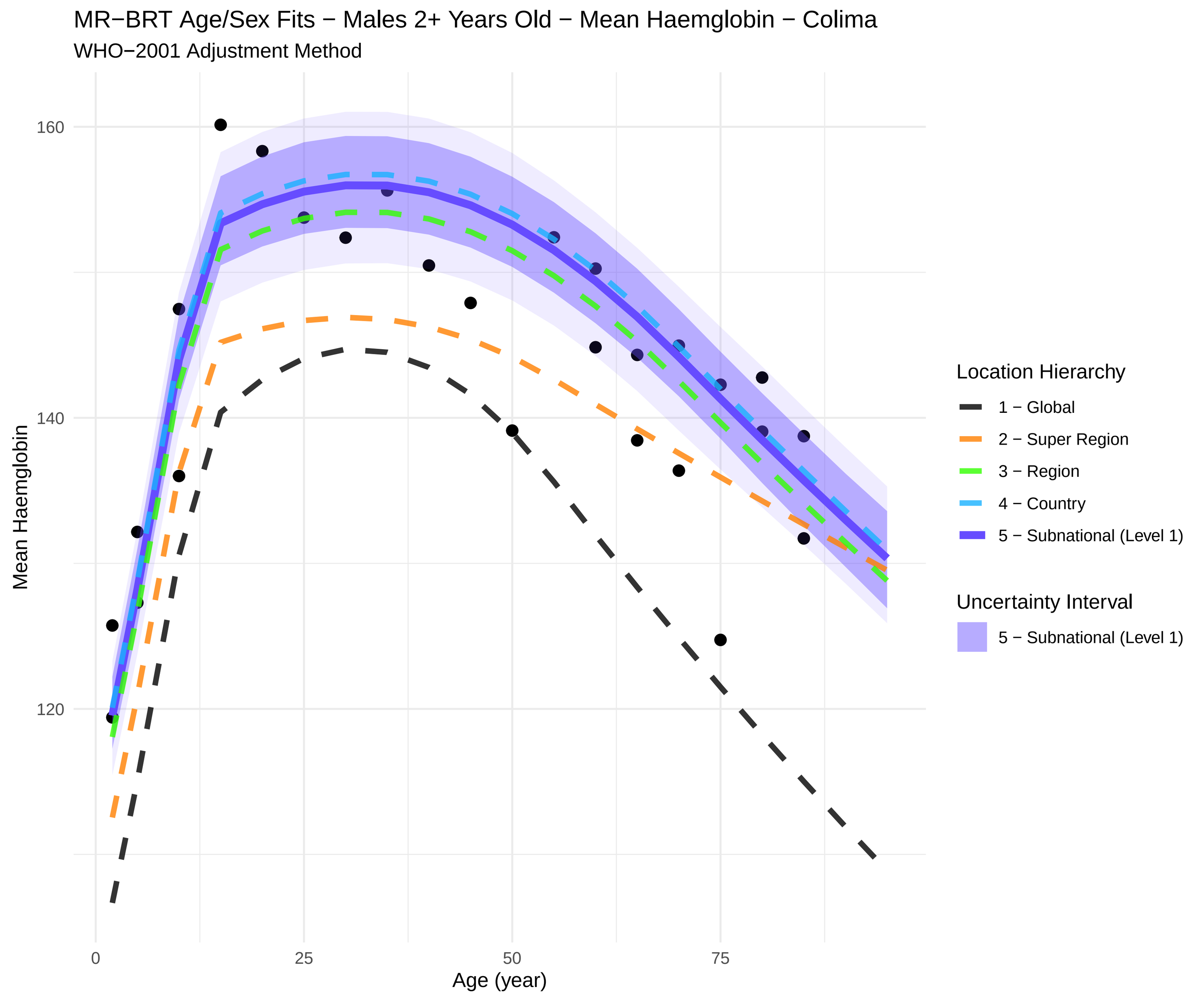
MR−BRT Age/Sex Fits − Males 2+ Years Old − Mean Haemglobin − Colima
WHO−2001 Adjustment Method
Mean Haemglobin
Age (year)
Location Hierarchy
1 − Global
2 − Super Region
3 − Region
4 − Country
5 − Subnational (Level 1)
Uncertainty Interval
5 − Subnational (Level 1)

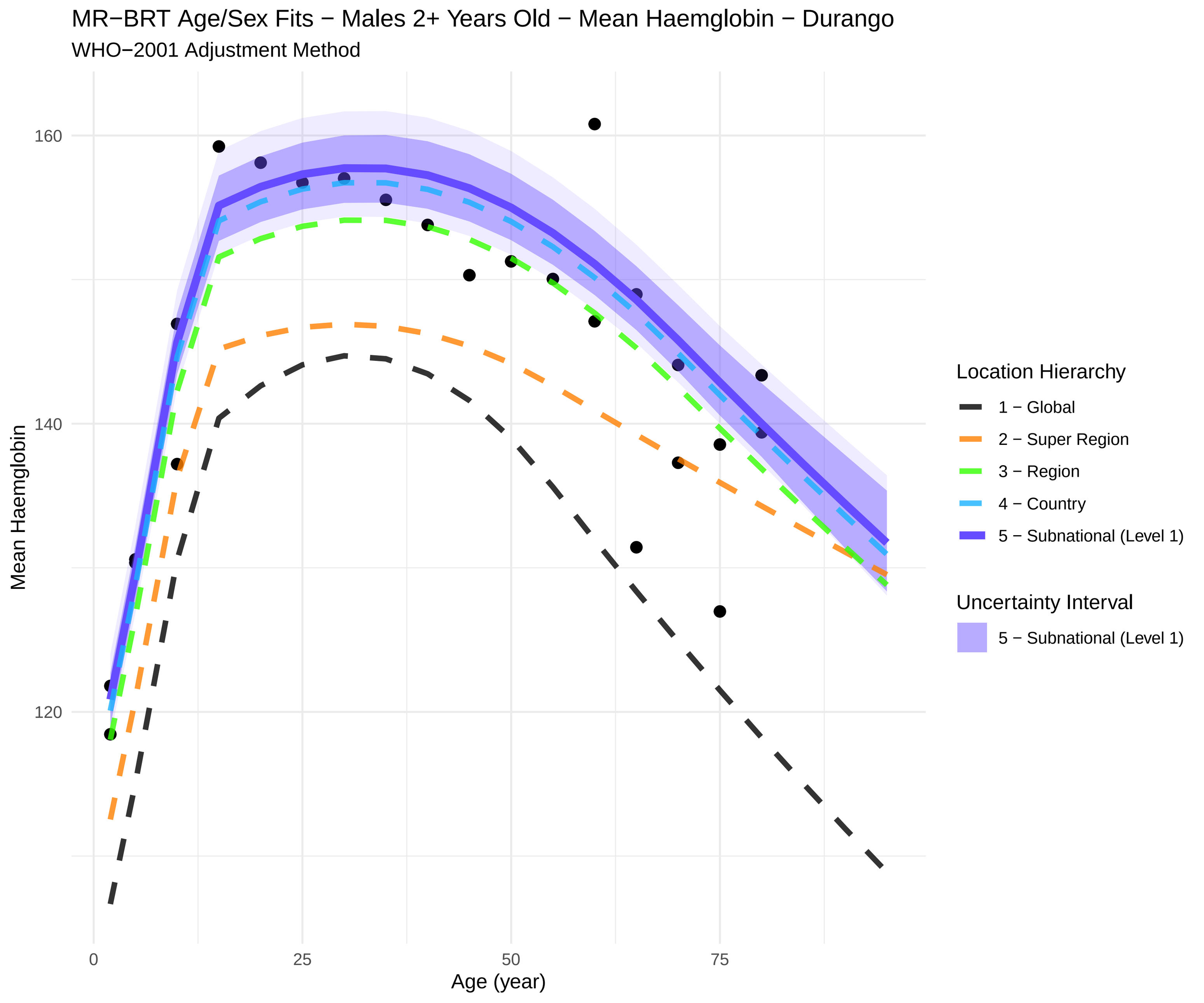
MR−BRT Age/Sex Fits − Males 2+ Years Old − Mean Haemglobin − Durango
WHO−2001 Adjustment Method
Mean Haemglobin
Age (year)
Location Hierarchy
1 − Global
2 − Super Region
3 − Region
4 − Country
5 − Subnational (Level 1)
Uncertainty Interval
5 − Subnational (Level 1)

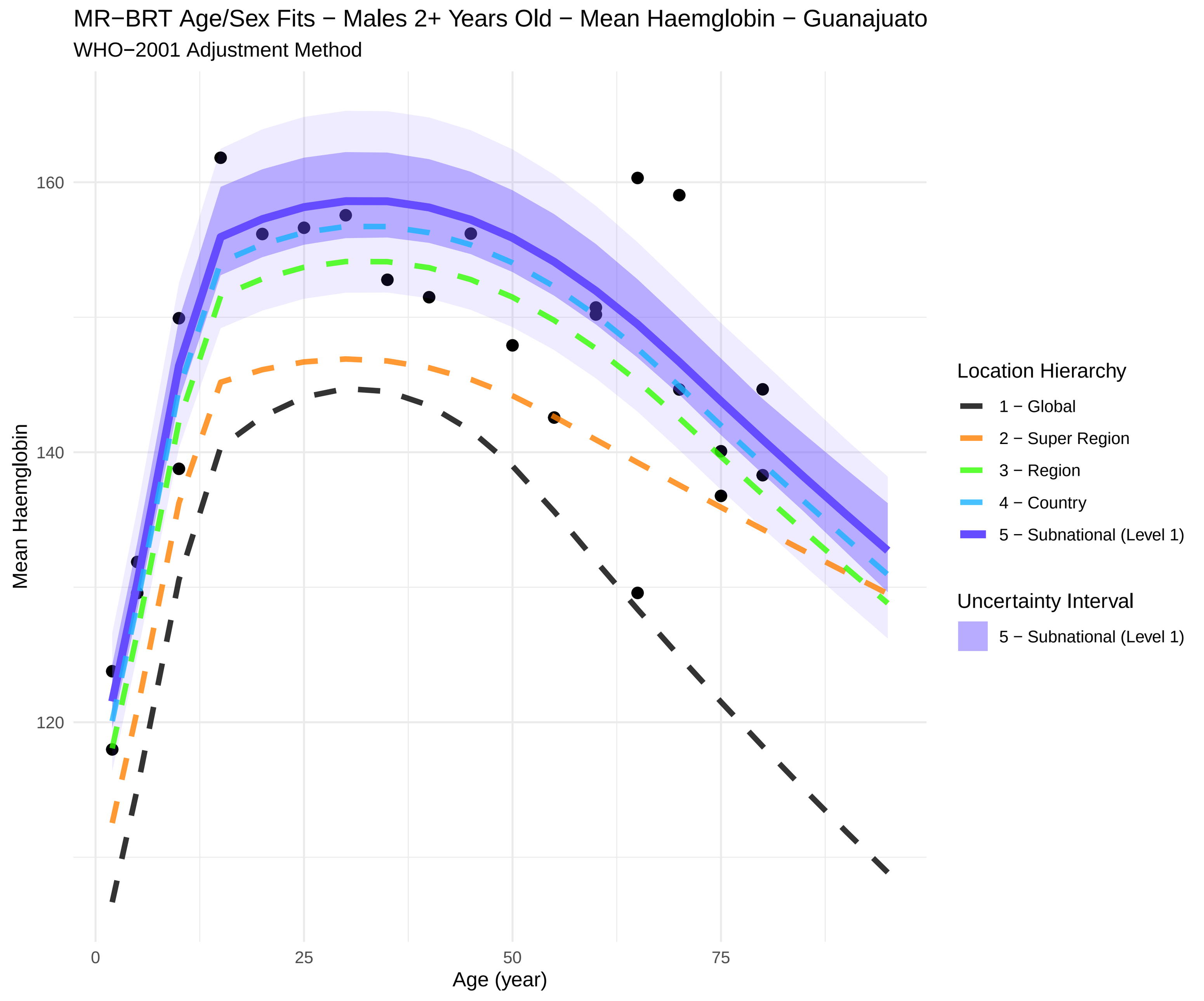
MR−BRT Age/Sex Fits − Males 2+ Years Old − Mean Haemglobin − Guanajuato
WHO−2001 Adjustment Method
Mean Haemglobin
Age (year)
Location Hierarchy
1 − Global
2 − Super Region
3 − Region
4 − Country
5 − Subnational (Level 1)
Uncertainty Interval
5 − Subnational (Level 1)

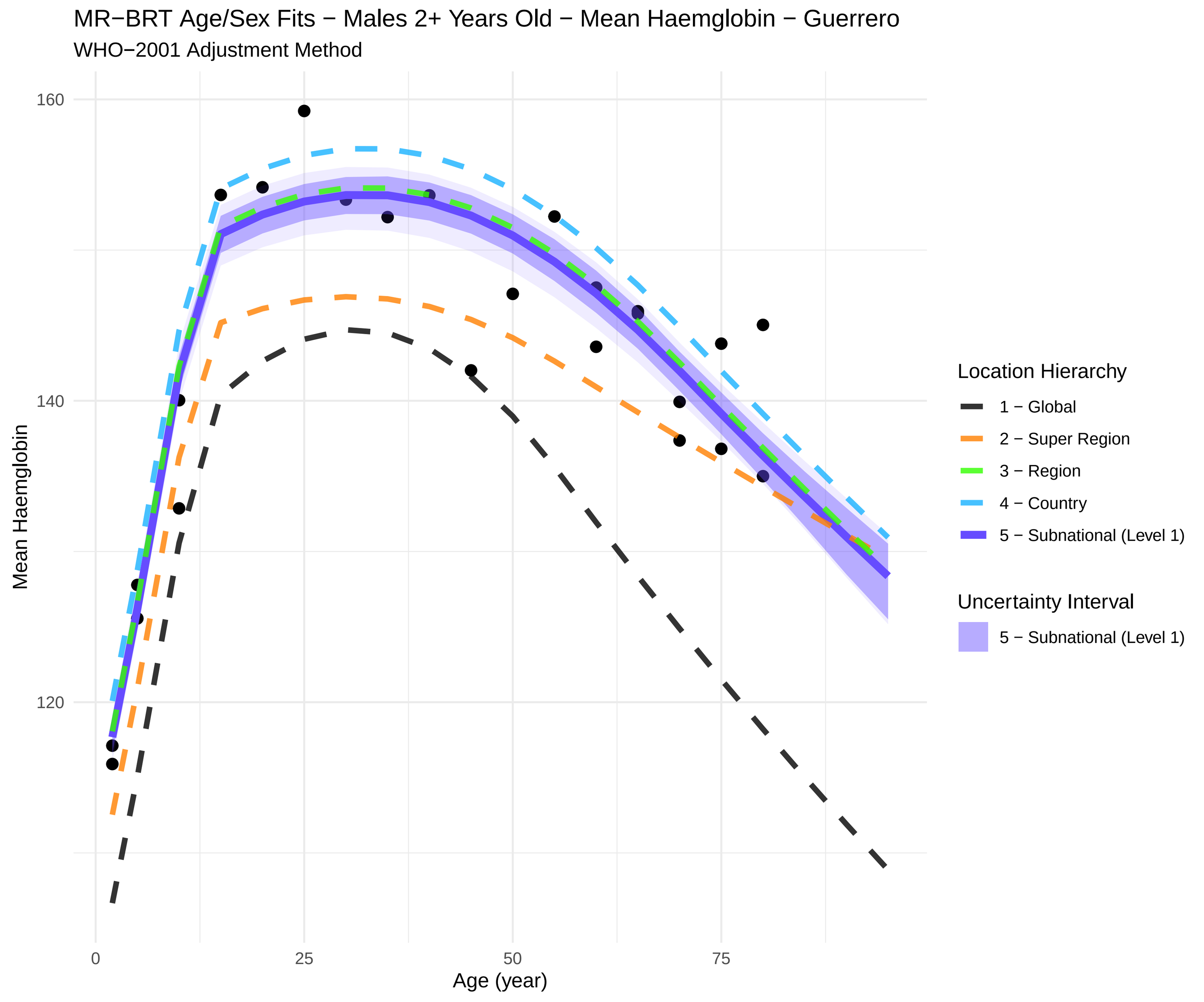
MR−BRT Age/Sex Fits − Males 2+ Years Old − Mean Haemglobin − Guerrero
WHO−2001 Adjustment Method
Mean Haemglobin
Age (year)
Location Hierarchy
1 − Global
2 − Super Region
3 − Region
4 − Country
5 − Subnational (Level 1)
Uncertainty Interval
5 − Subnational (Level 1)

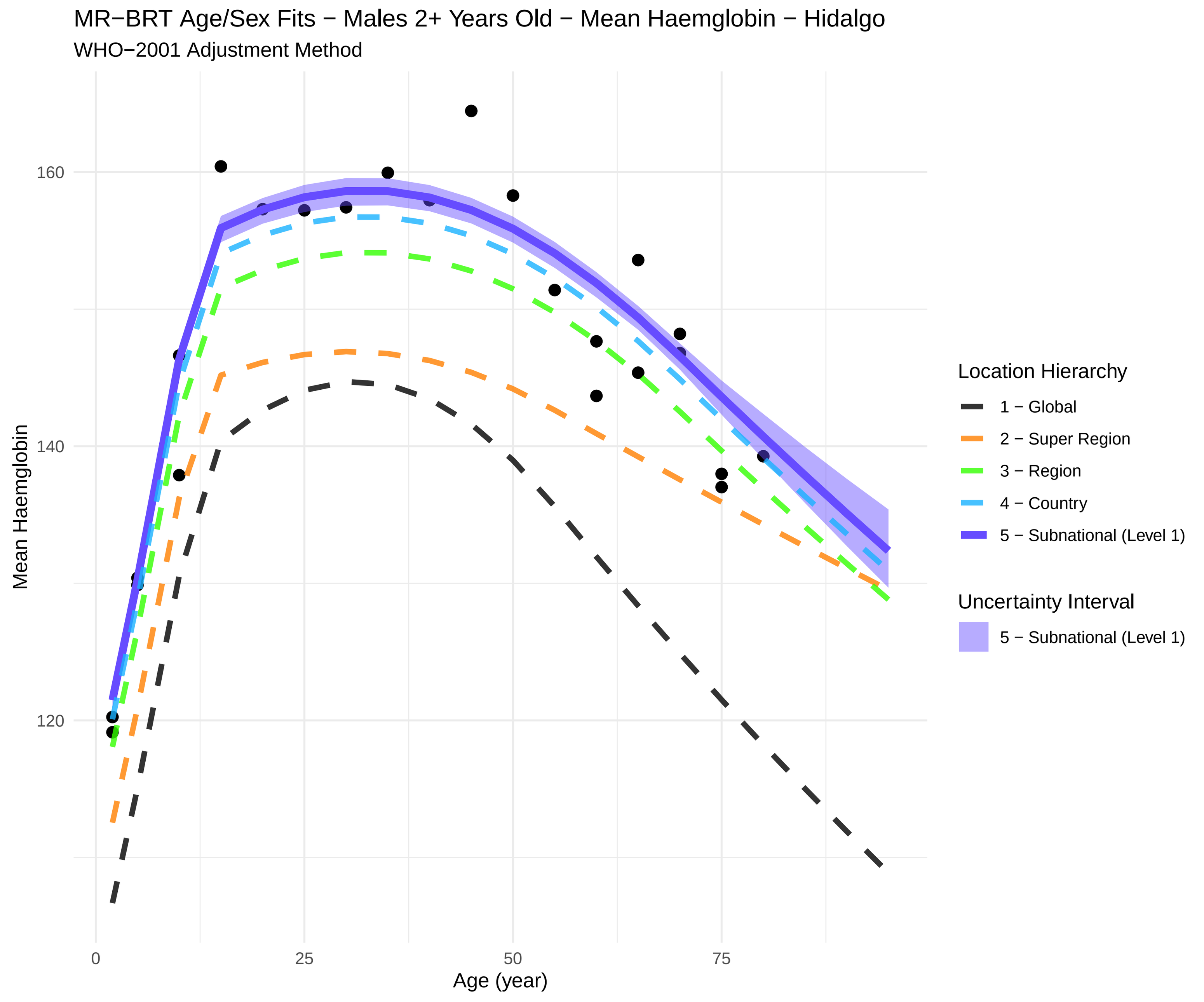
MR−BRT Age/Sex Fits − Males 2+ Years Old − Mean Haemglobin − Hidalgo
WHO−2001 Adjustment Method
Mean Haemglobin
Age (year)
Location Hierarchy
1 − Global
2 − Super Region
3 − Region
4 − Country
5 − Subnational (Level 1)
Uncertainty Interval
5 − Subnational (Level 1)

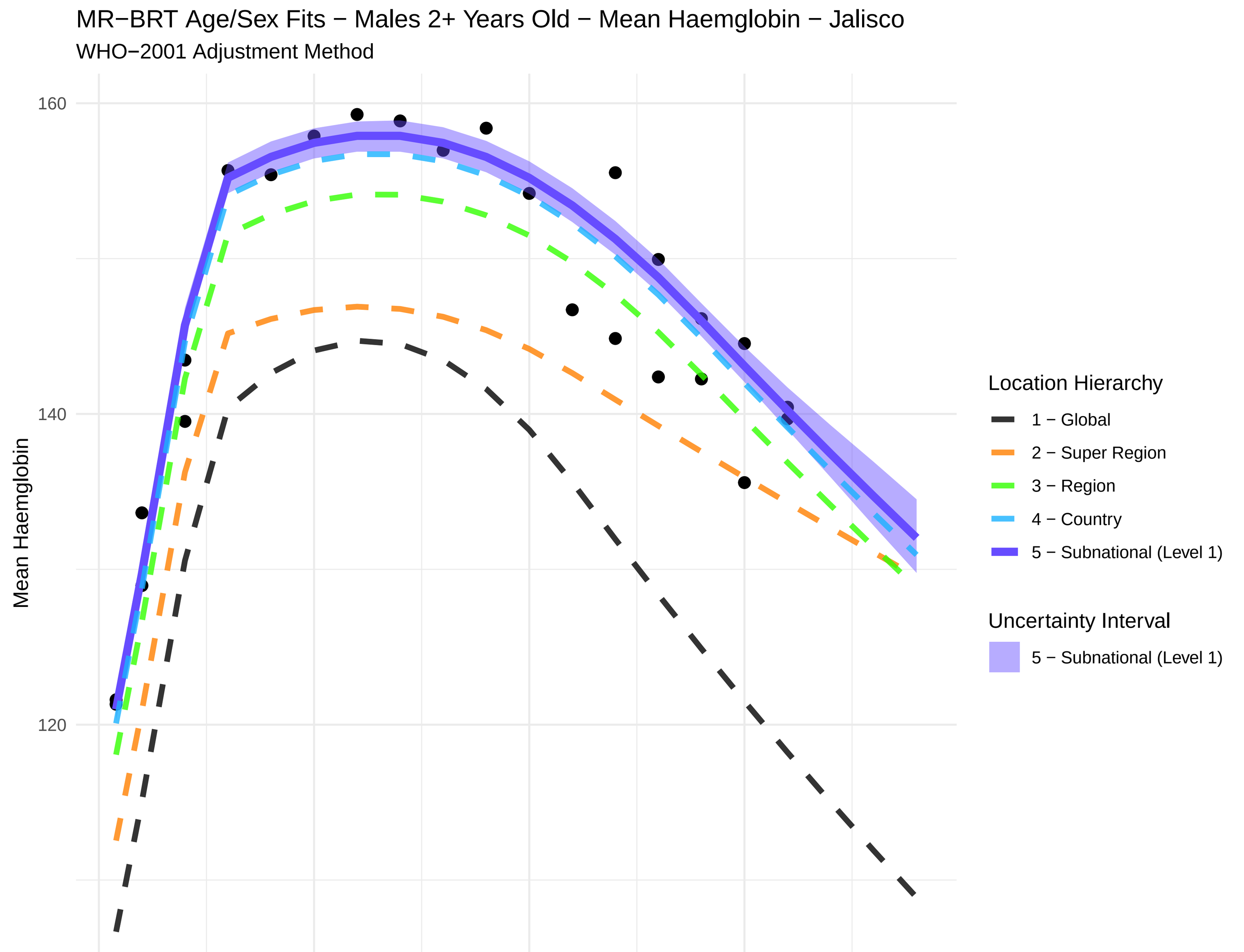
MR−BRT Age/Sex Fits − Males 2+ Years Old − Mean Haemglobin − Jalisco
WHO−2001 Adjustment Method
Mean Haemglobin
Age (year)
Location Hierarchy
1 − Global
2 − Super Region
3 − Region
4 − Country
5 − Subnational (Level 1)
Uncertainty Interval
5 − Subnational (Level 1)

MR−BRT Age/Sex Fits − Males 2+ Years Old − Mean Haemglobin − México
WHO−2001 Adjustment Method

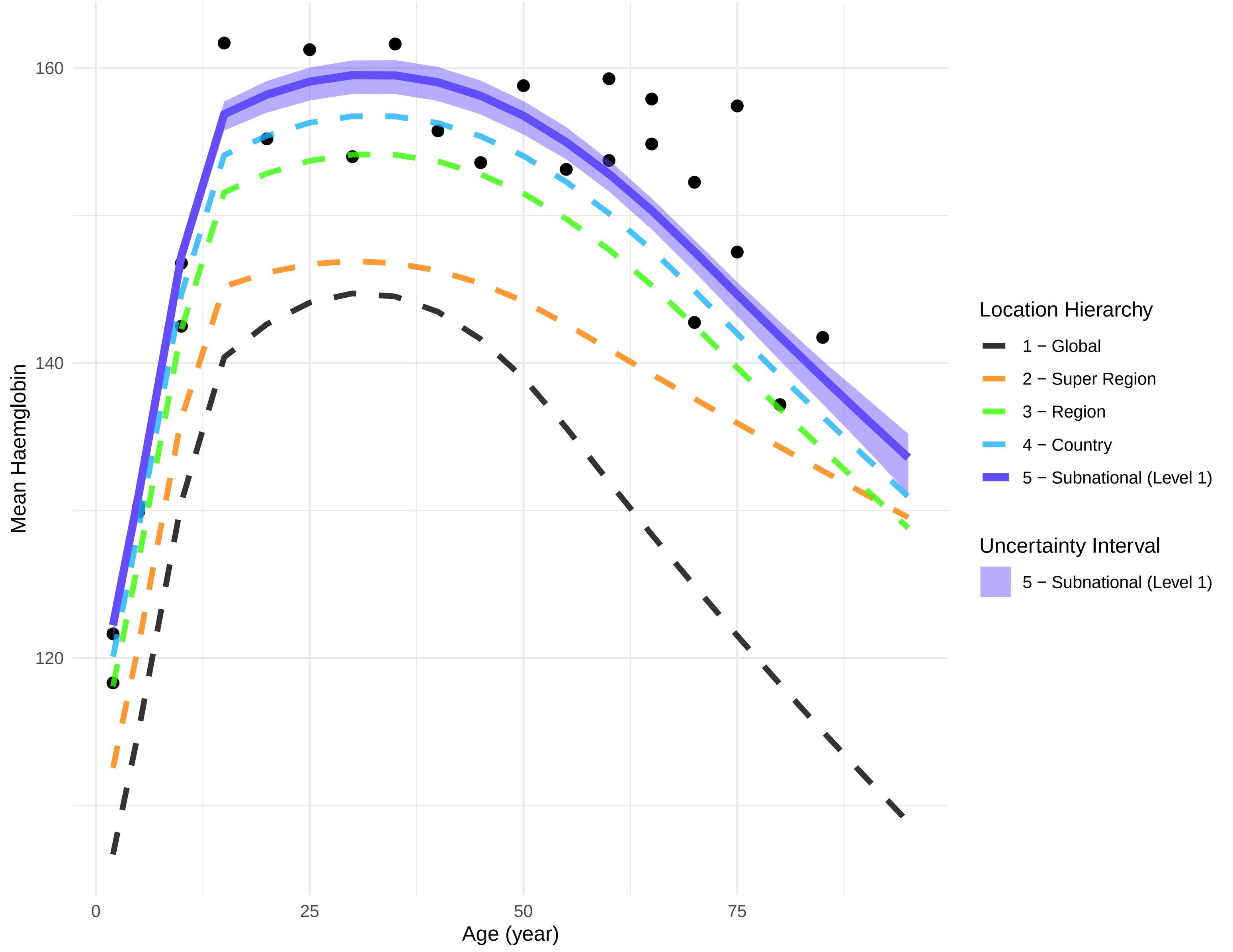

MR−BRT Age/Sex Fits − Males 2+ Years Old − Mean Haemglobin − Mexico City
WHO−2001 Adjustment Method

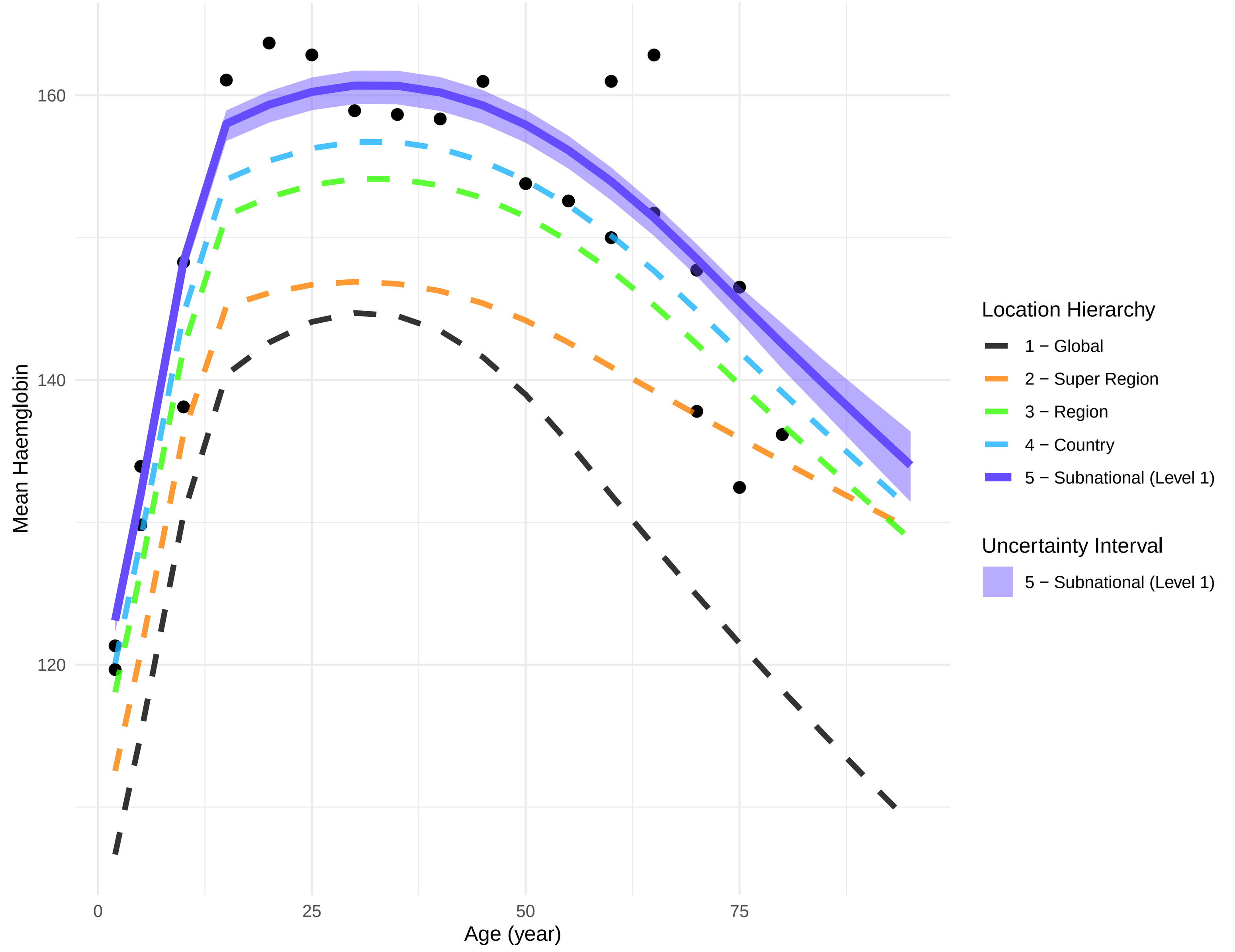

MR−BRT Age/Sex Fits − Males 2+ Years Old − Mean Haemglobin − Michoacán de Ocampo
WHO−2001 Adjustment Method

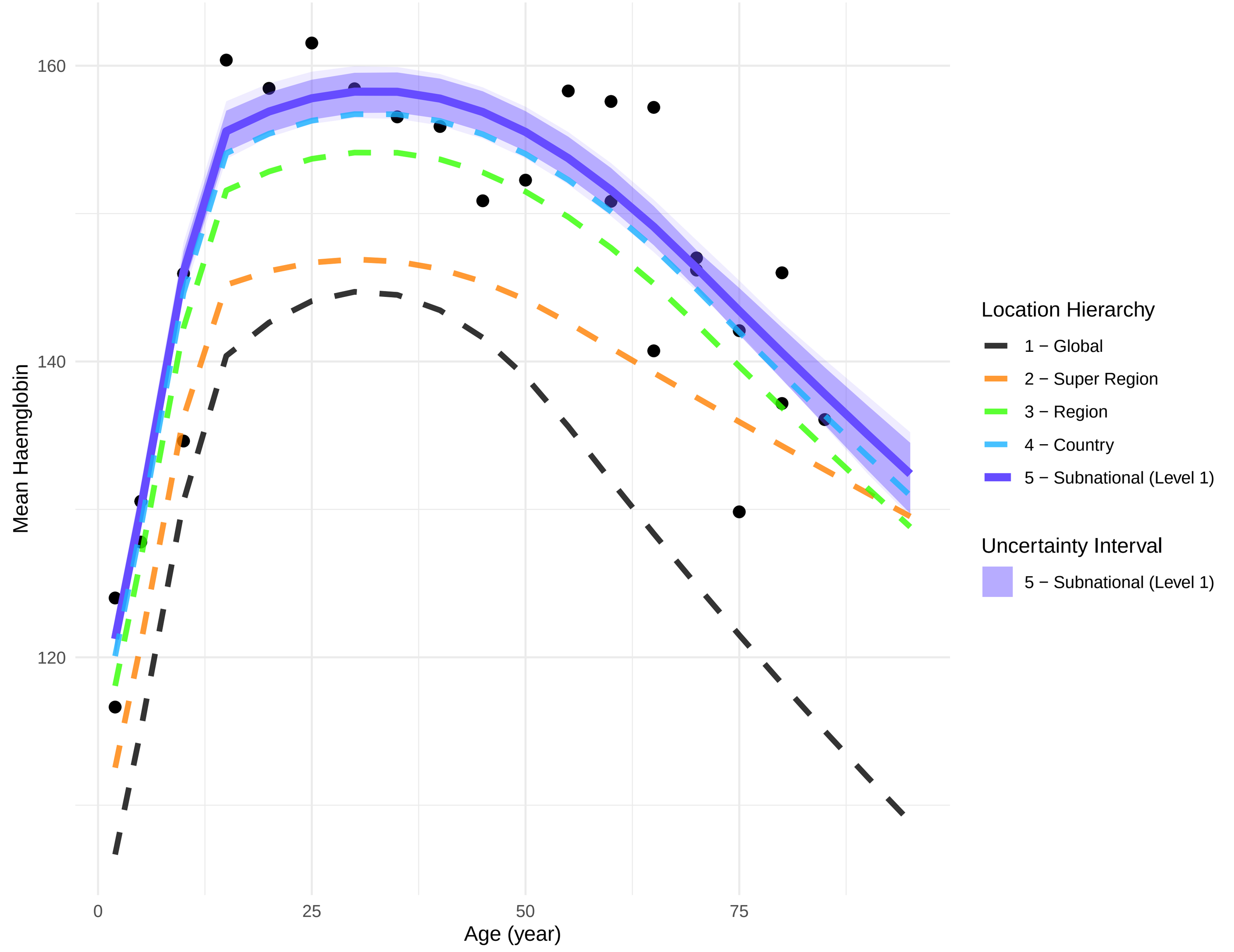

MR−BRT Age/Sex Fits − Males 2+ Years Old − Mean Haemglobin − Morelos
WHO−2001 Adjustment Method

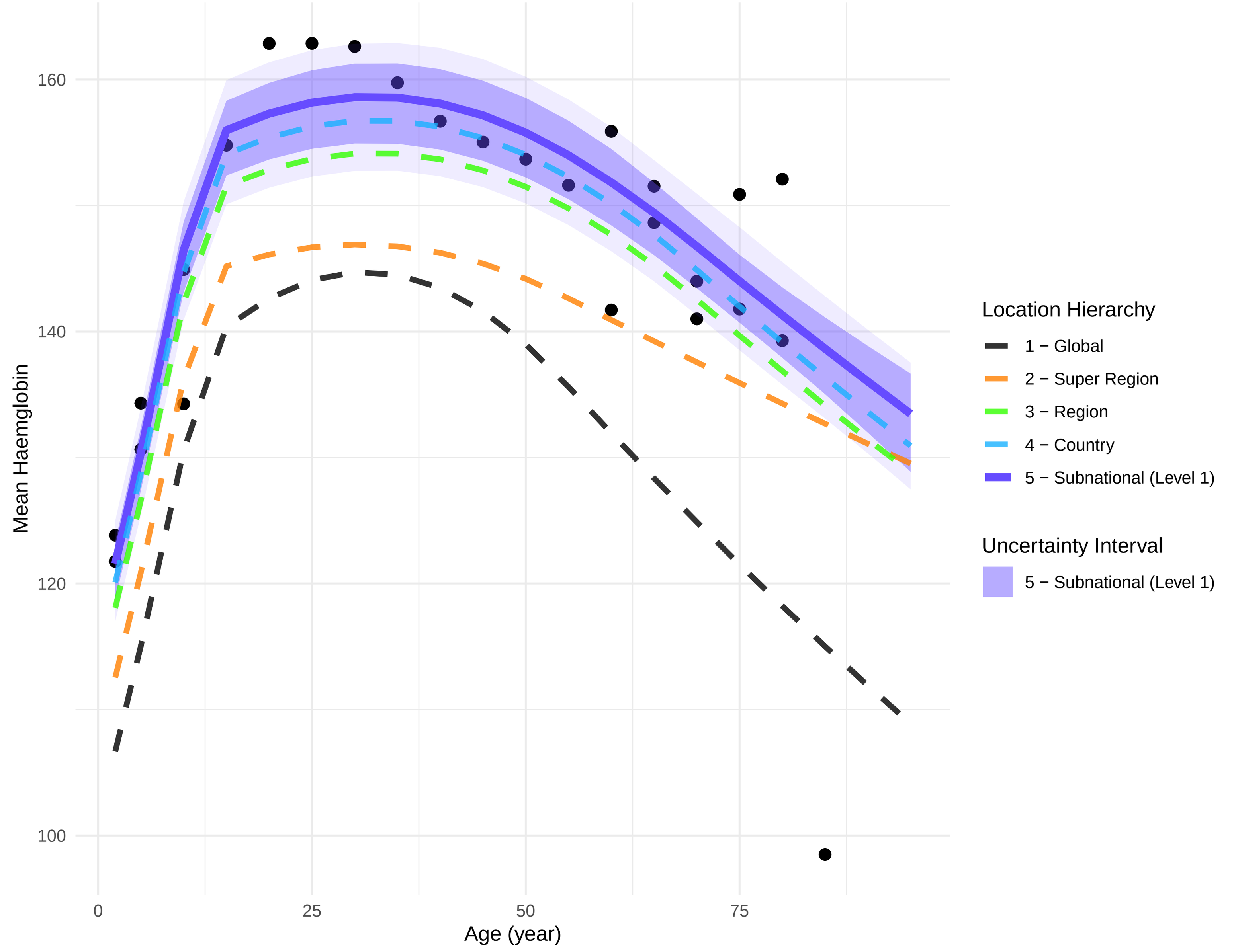

MR−BRT Age/Sex Fits − Males 2+ Years Old − Mean Haemglobin − Nayarit
WHO−2001 Adjustment Method

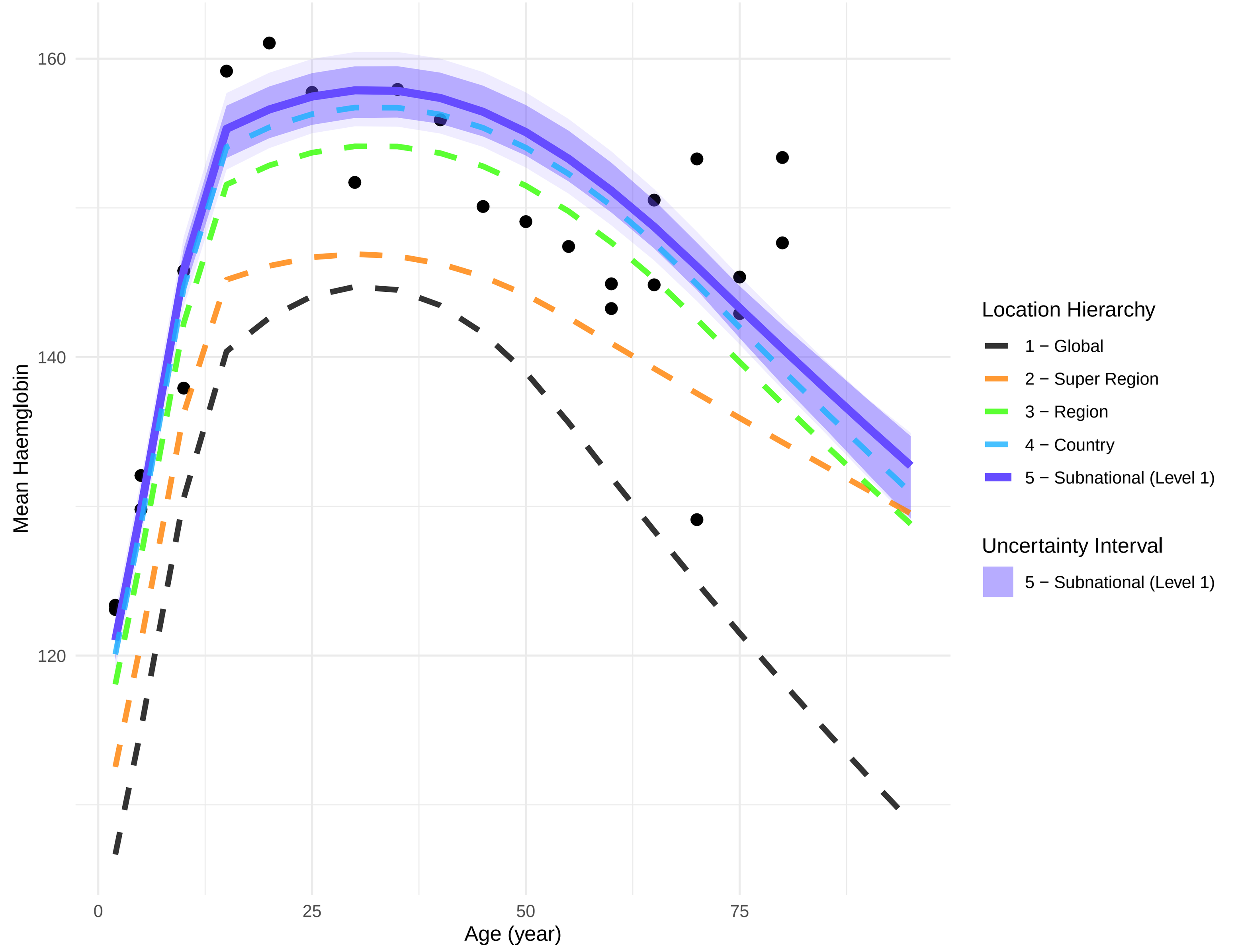

MR−BRT Age/Sex Fits − Males 2+ Years Old − Mean Haemglobin − Nuevo León
WHO−2001 Adjustment Method

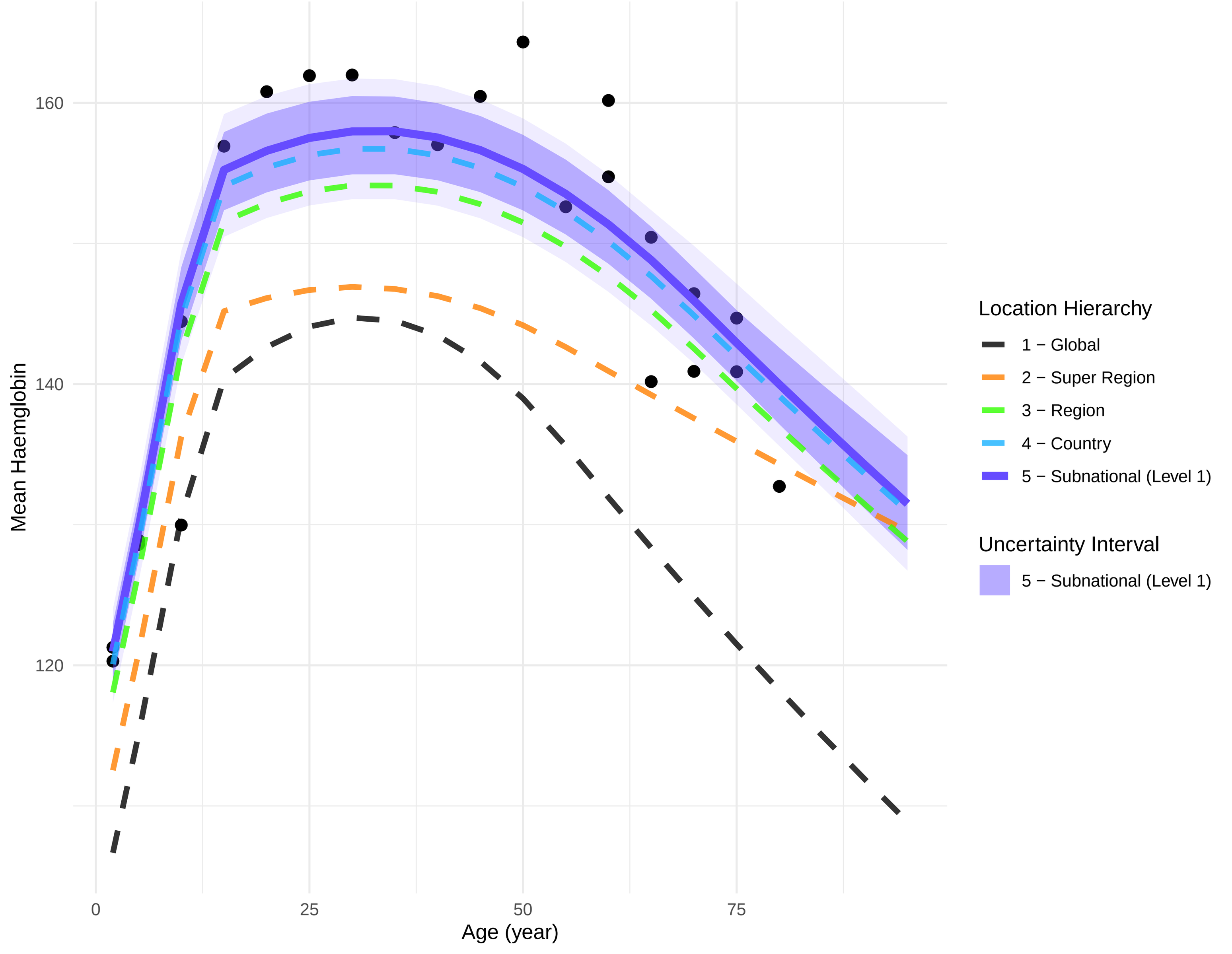

MR−BRT Age/Sex Fits − Males 2+ Years Old − Mean Haemglobin − Oaxaca
WHO−2001 Adjustment Method

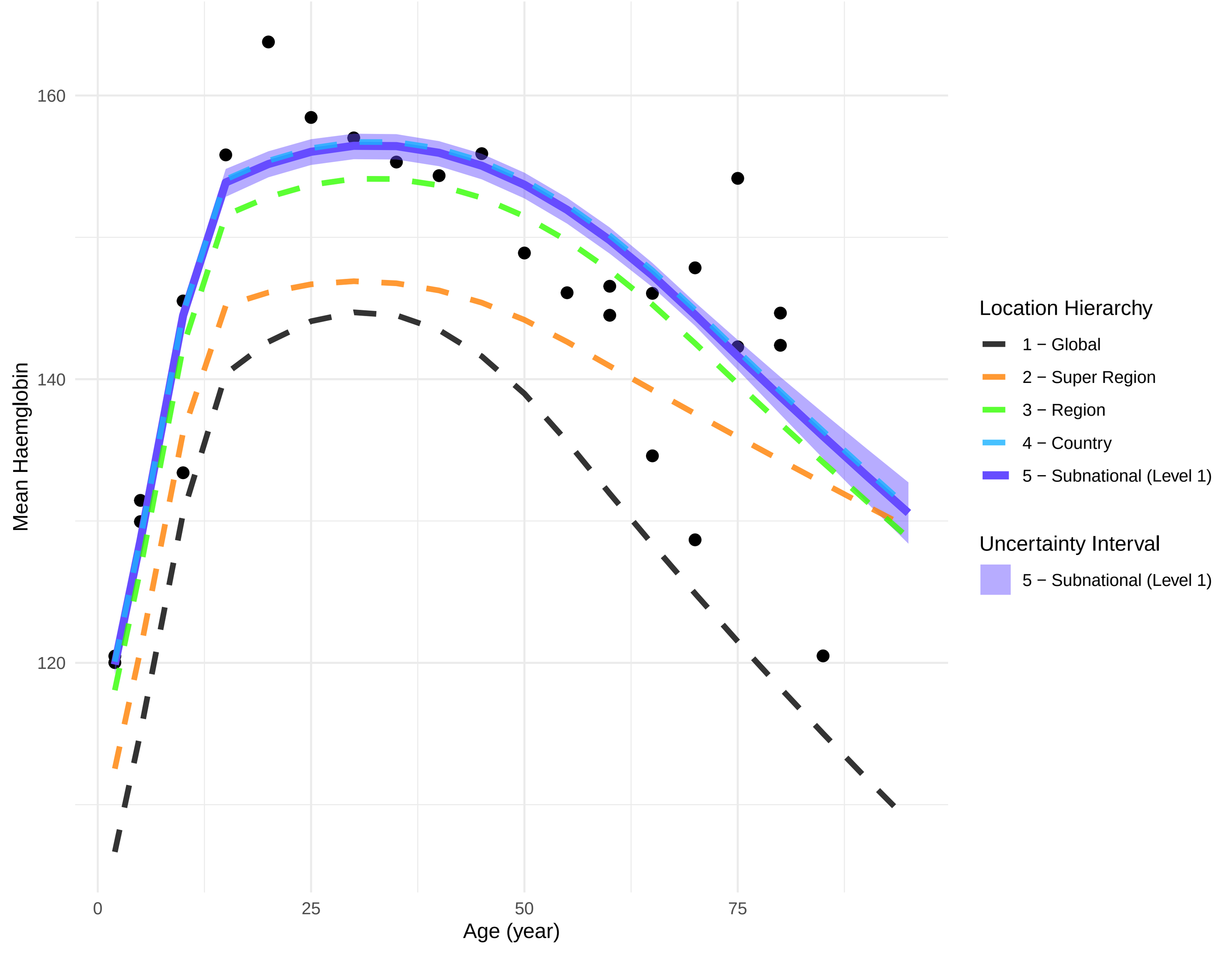

MR−BRT Age/Sex Fits − Males 2+ Years Old − Mean Haemglobin − Puebla
WHO−2001 Adjustment Method

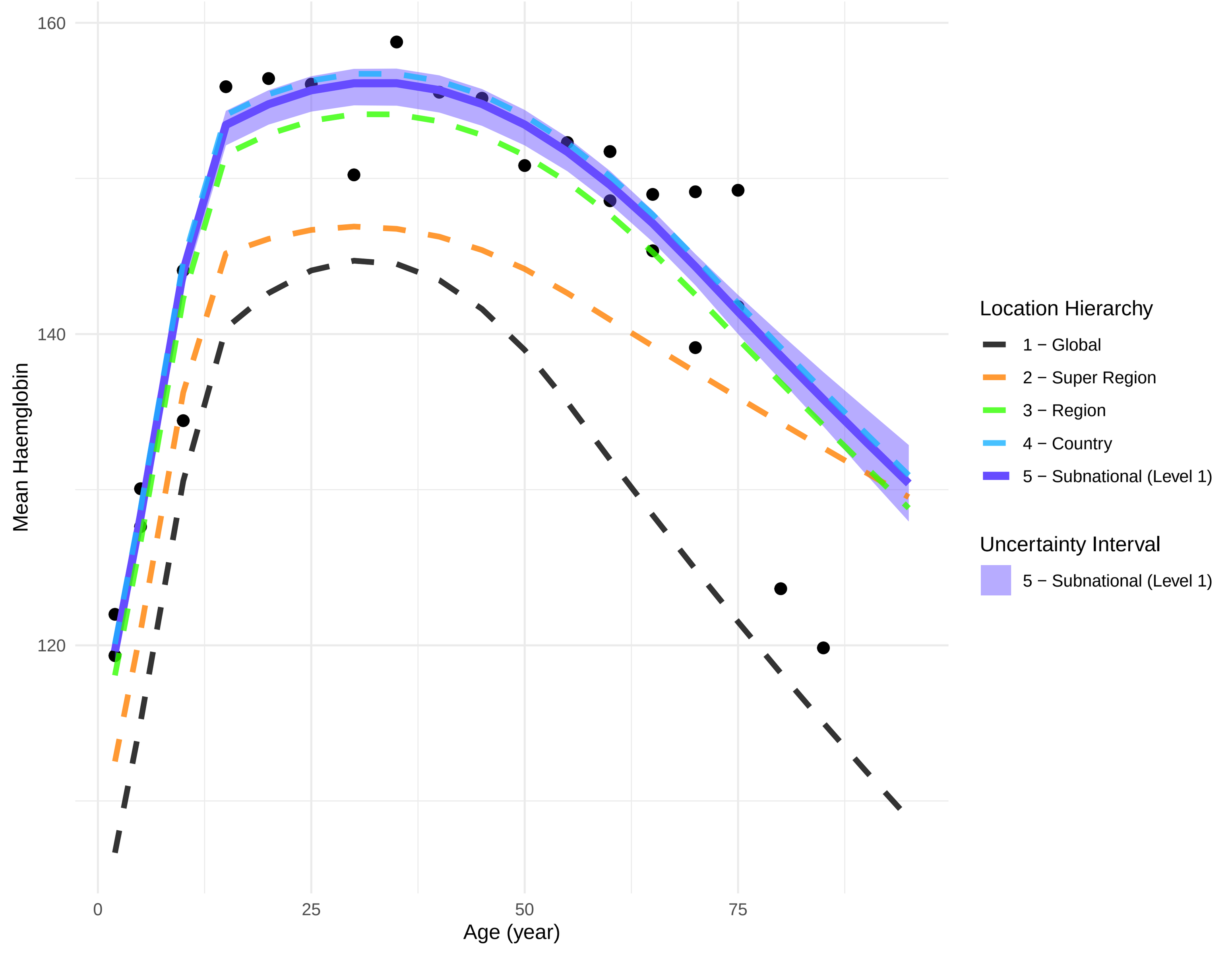

MR−BRT Age/Sex Fits − Males 2+ Years Old − Mean Haemglobin − Querétaro
WHO−2001 Adjustment Method

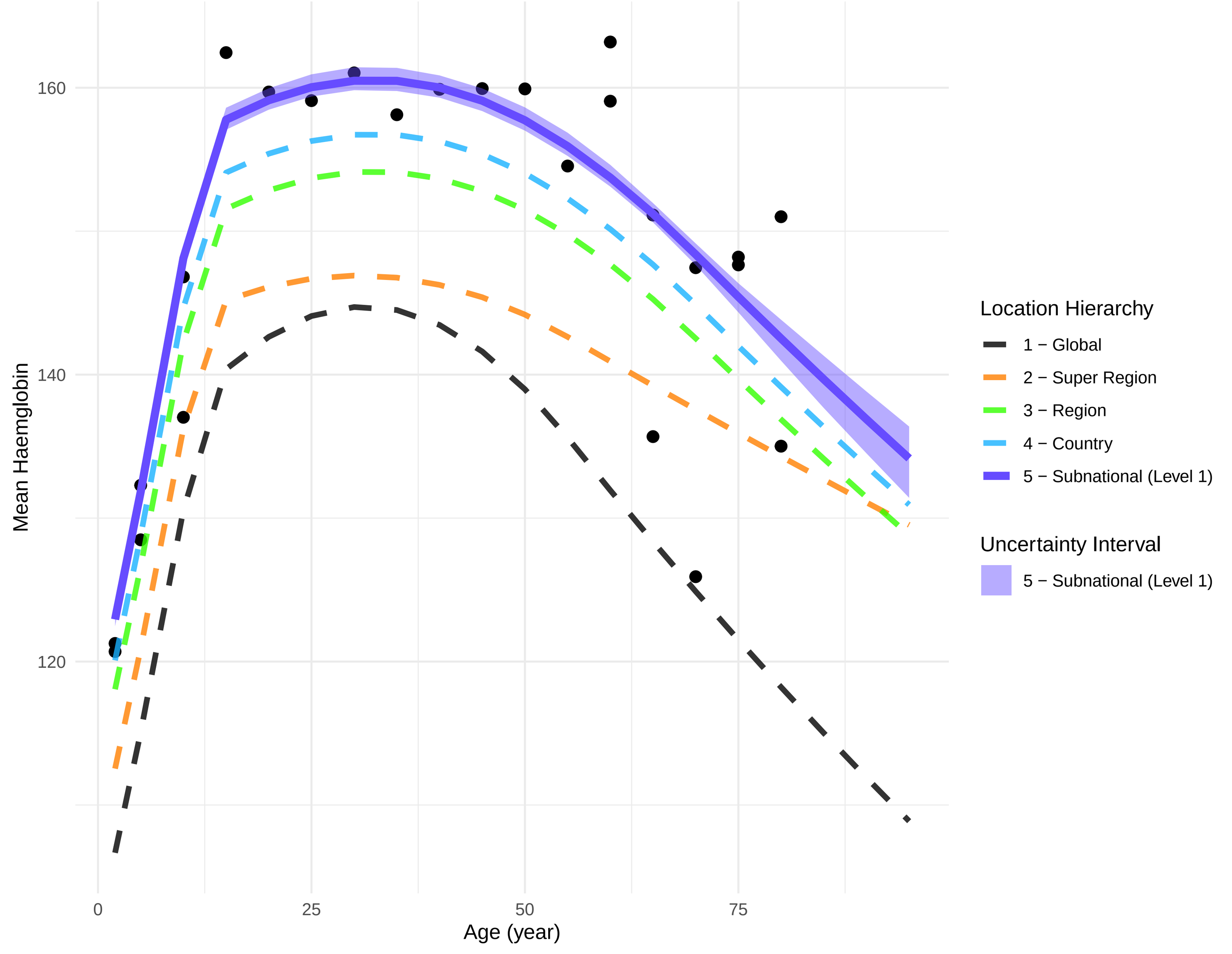

MR−BRT Age/Sex Fits − Males 2+ Years Old − Mean Haemglobin − Quintana Roo
WHO−2001 Adjustment Method

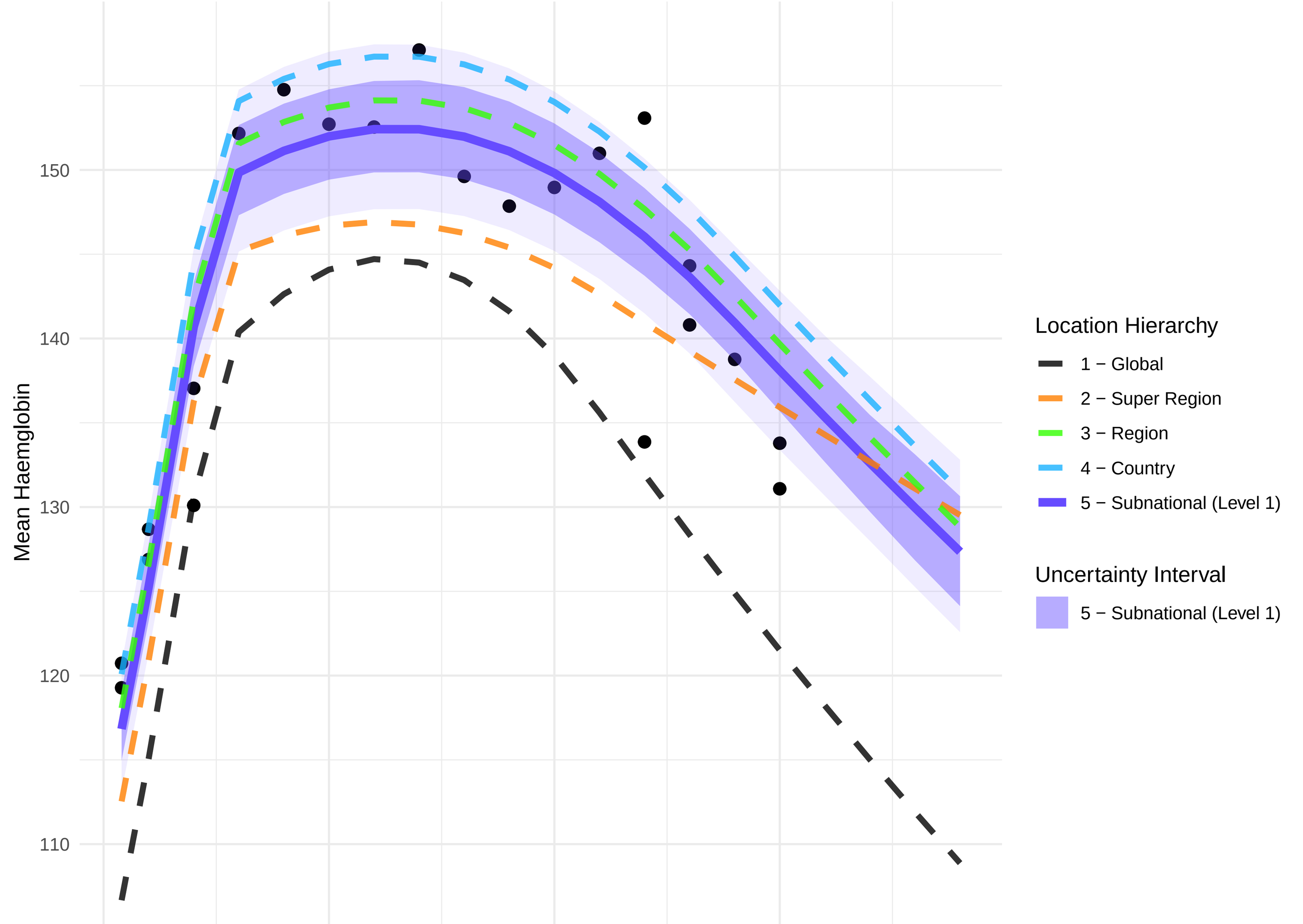

MR−BRT Age/Sex Fits − Males 2+ Years Old − Mean Haemglobin − San Luis Potosí
WHO−2001 Adjustment Method

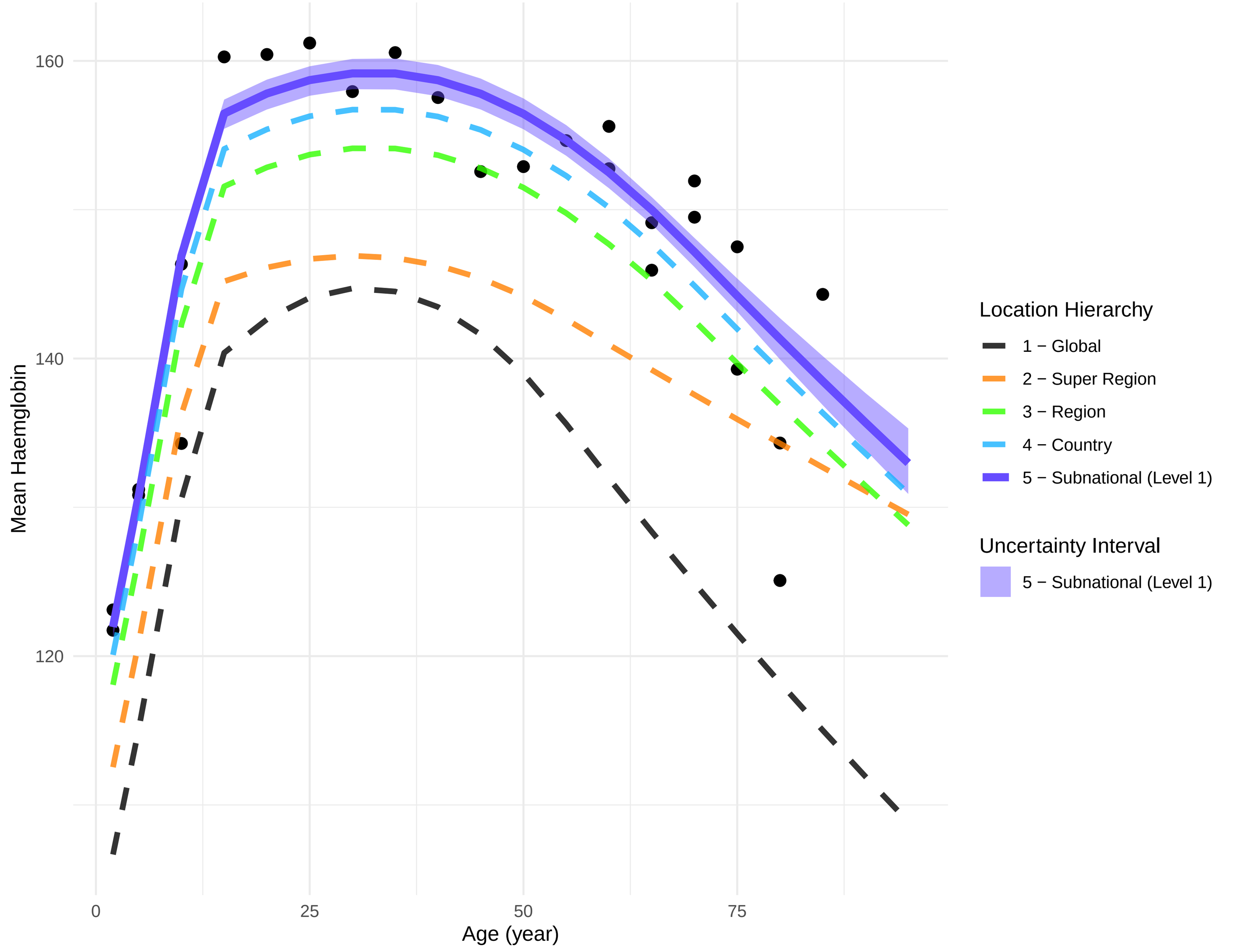

MR−BRT Age/Sex Fits − Males 2+ Years Old − Mean Haemglobin − Sinaloa
WHO−2001 Adjustment Method

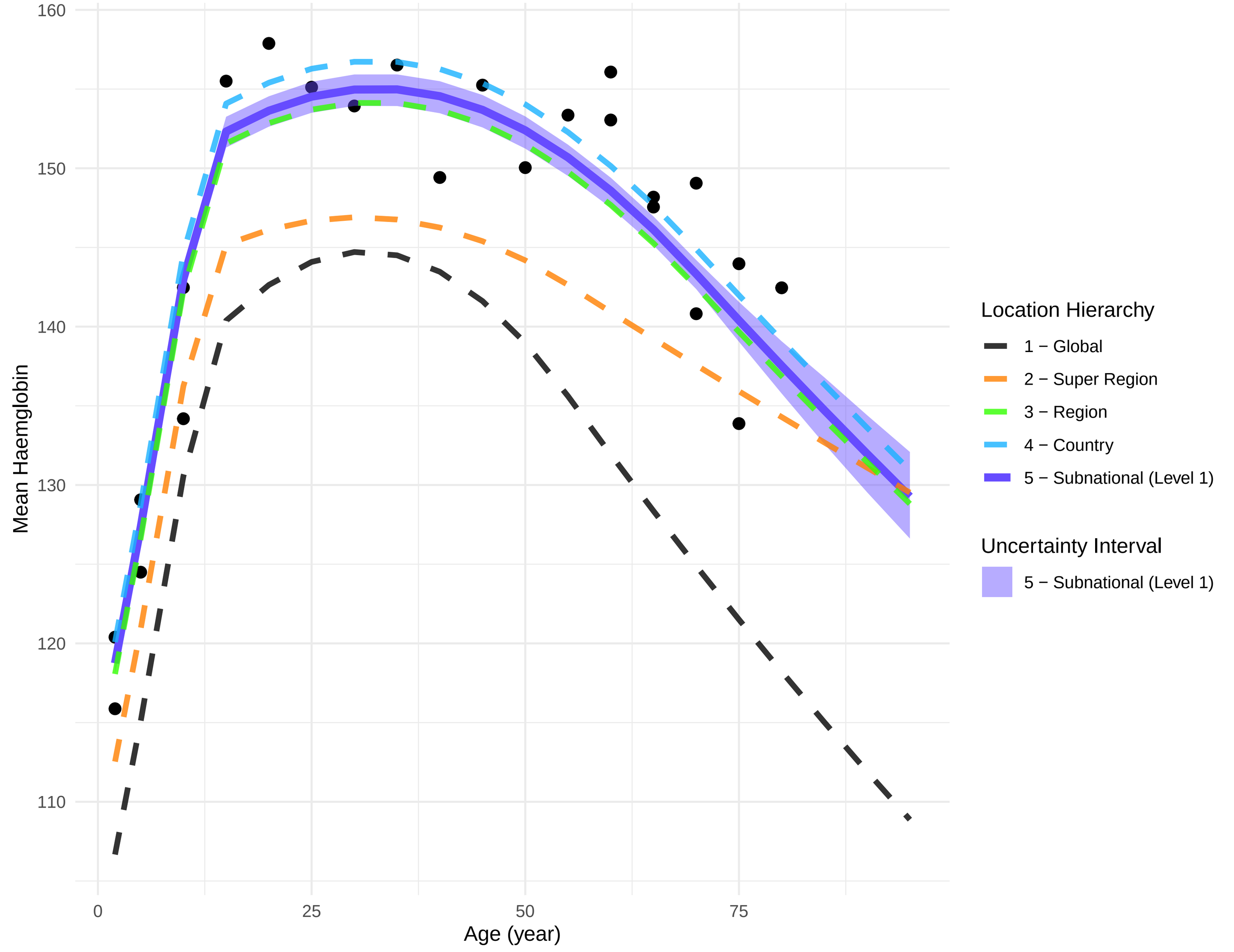

MR−BRT Age/Sex Fits − Males 2+ Years Old − Mean Haemglobin − Sonora
WHO−2001 Adjustment Method

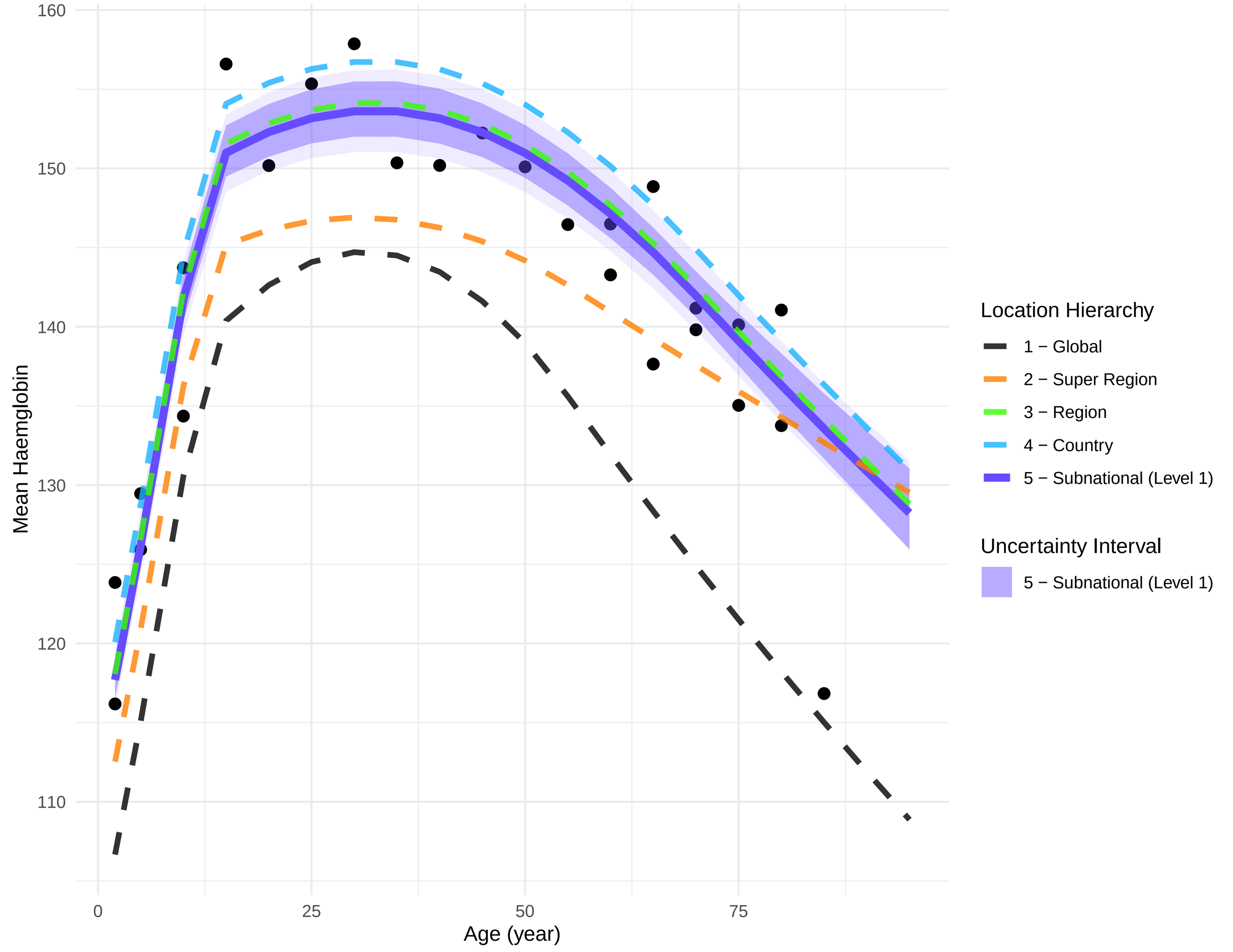

MR−BRT Age/Sex Fits − Males 2+ Years Old − Mean Haemglobin − Tabasco
WHO−2001 Adjustment Method

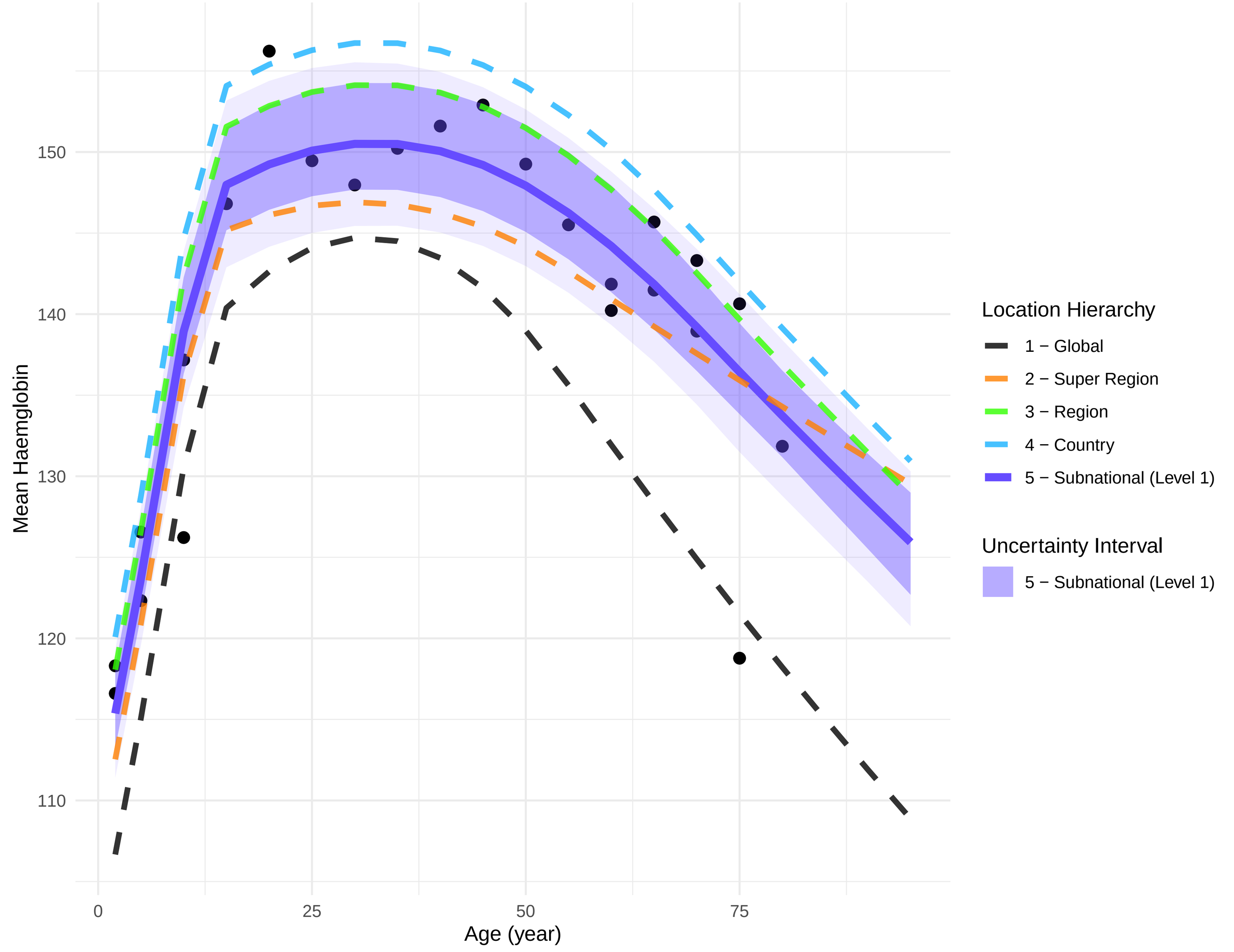

MR−BRT Age/Sex Fits − Males 2+ Years Old − Mean Haemglobin − Tamaulipas
WHO−2001 Adjustment Method

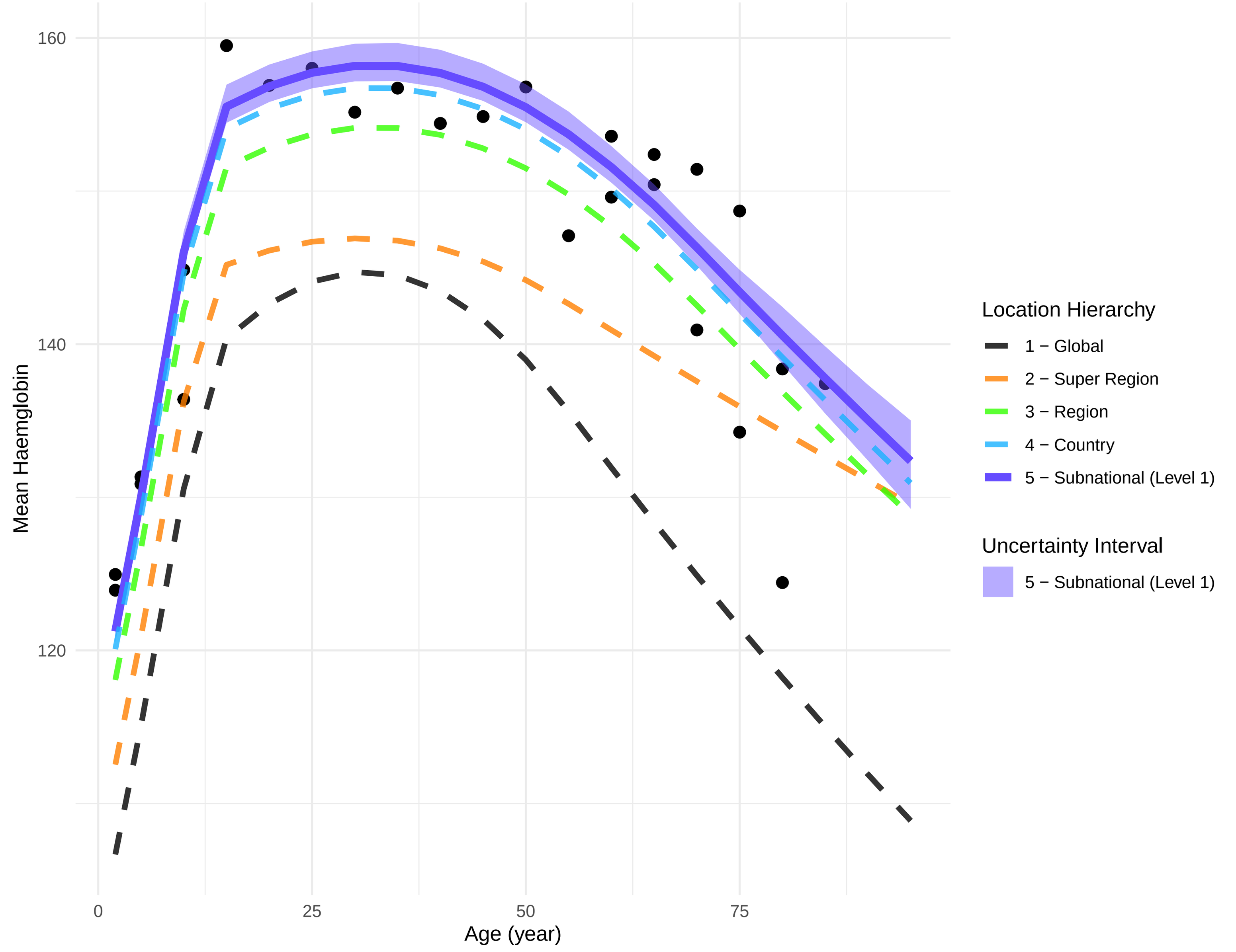

MR−BRT Age/Sex Fits − Males 2+ Years Old − Mean Haemglobin − Tlaxcala
WHO−2001 Adjustment Method

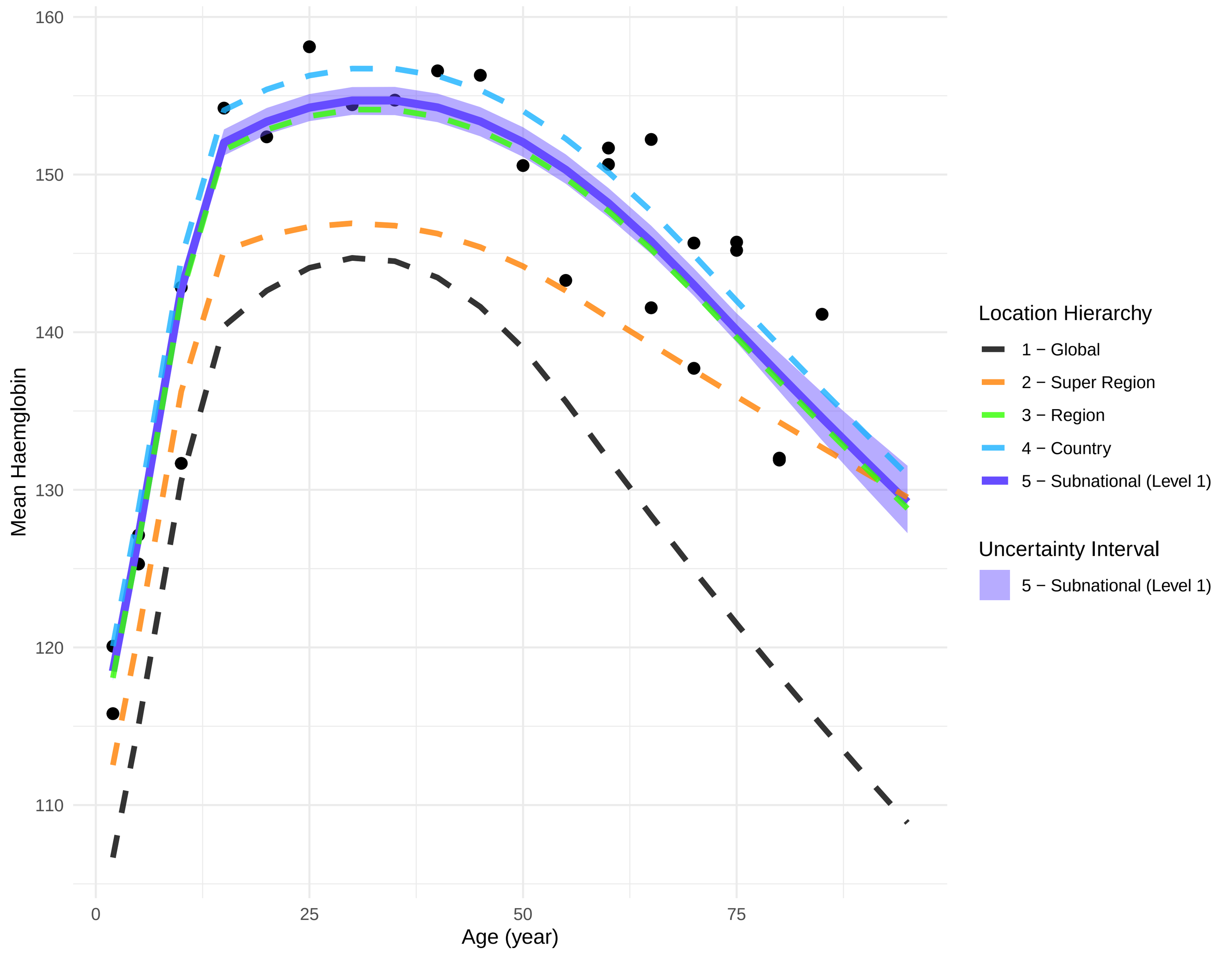

MR−BRT Age/Sex Fits − Males 2+ Years Old − Mean Haemglobin − Veracruz de Ignacio de la Llave
WHO−2001 Adjustment Method

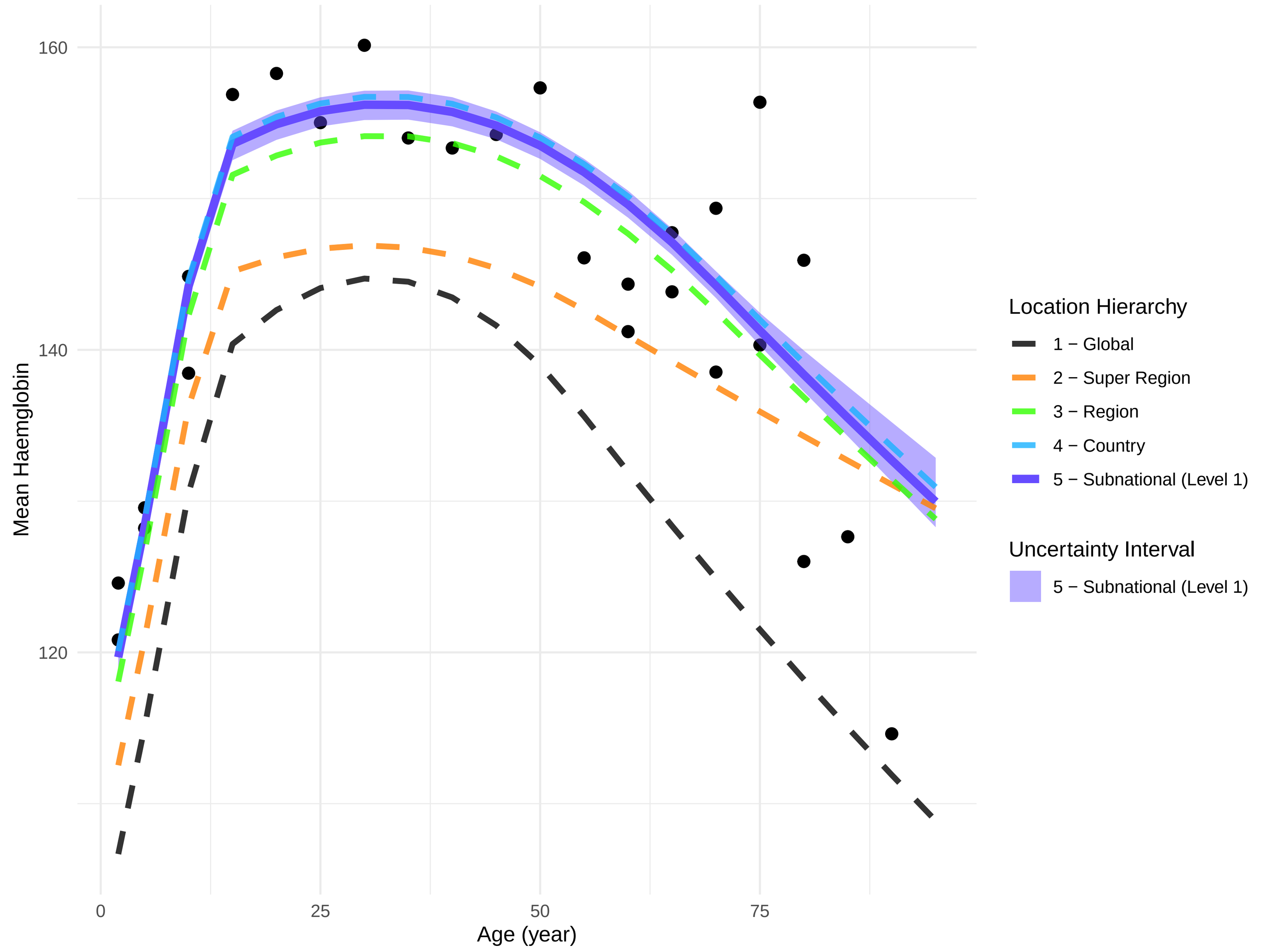

MR−BRT Age/Sex Fits − Males 2+ Years Old − Mean Haemglobin − Yucatán
WHO−2001 Adjustment Method

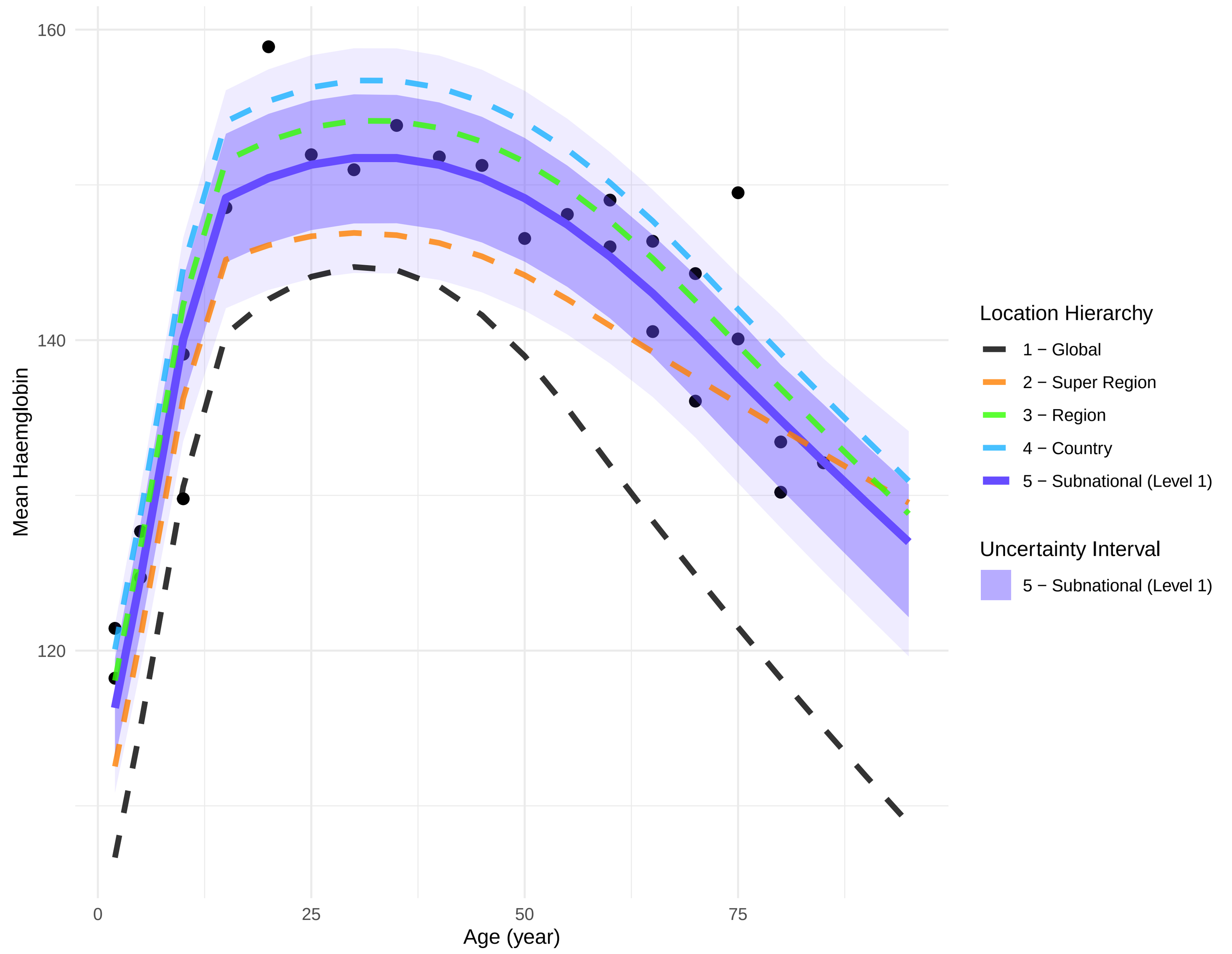

MR−BRT Age/Sex Fits − Males 2+ Years Old − Mean Haemglobin − Zacatecas
WHO−2001 Adjustment Method

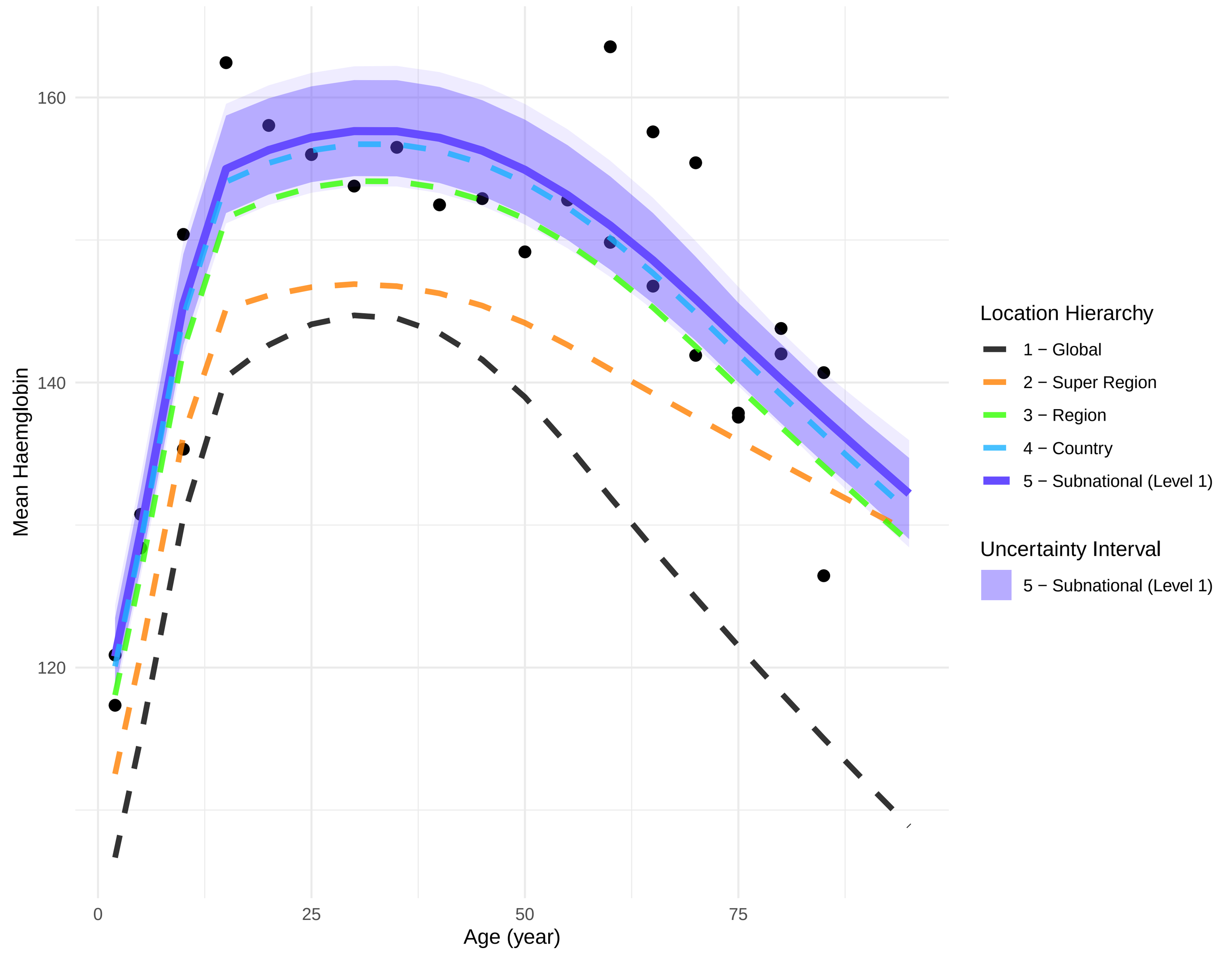

MR−BRT Age/Sex Fits − Males 2+ Years Old − Mean Haemglobin − Tropical Latin America
WHO−2001 Adjustment Method

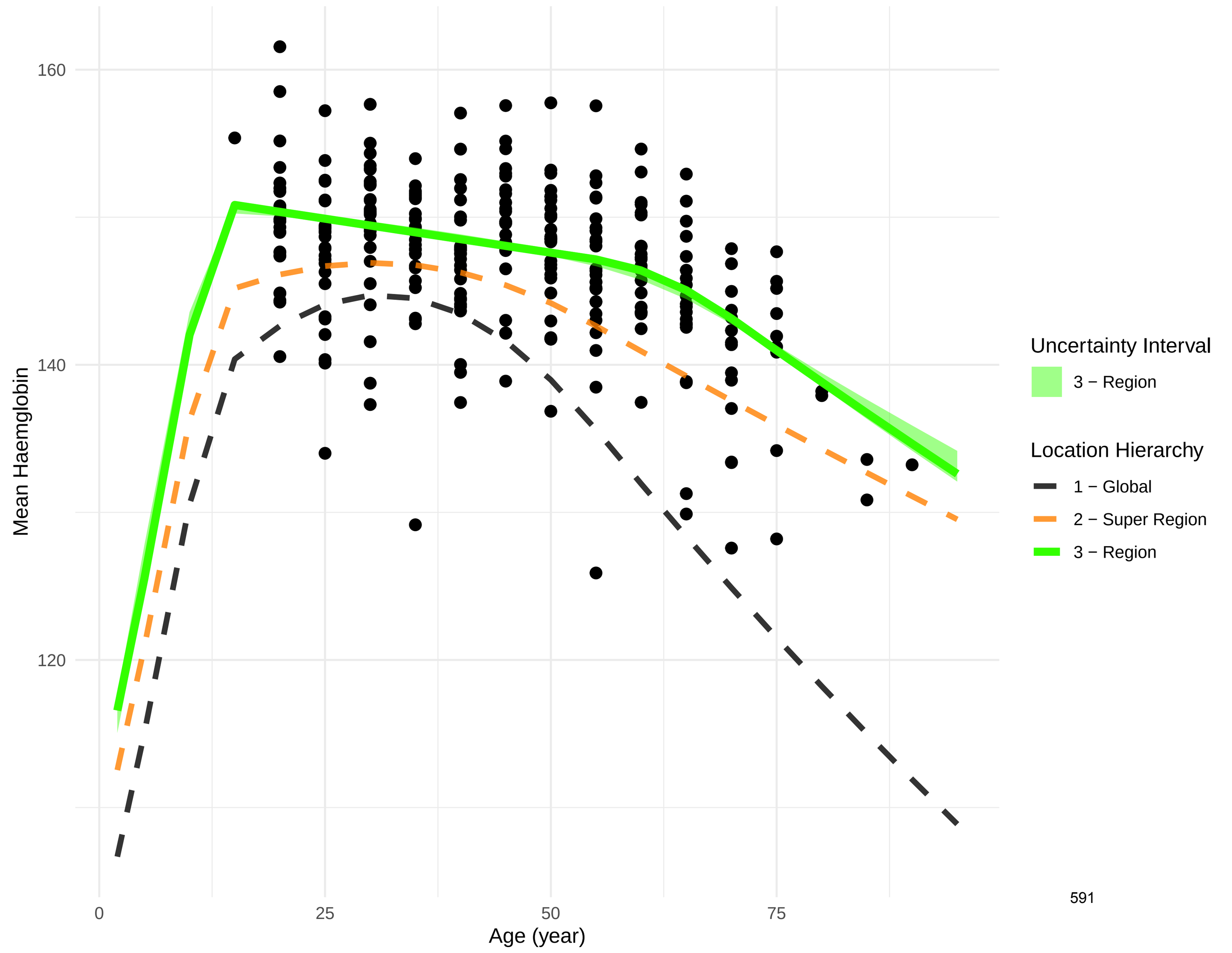

MR−BRT Age/Sex Fits − Males 2+ Years Old − Mean Haemglobin − Brazil
WHO−2001 Adjustment Method

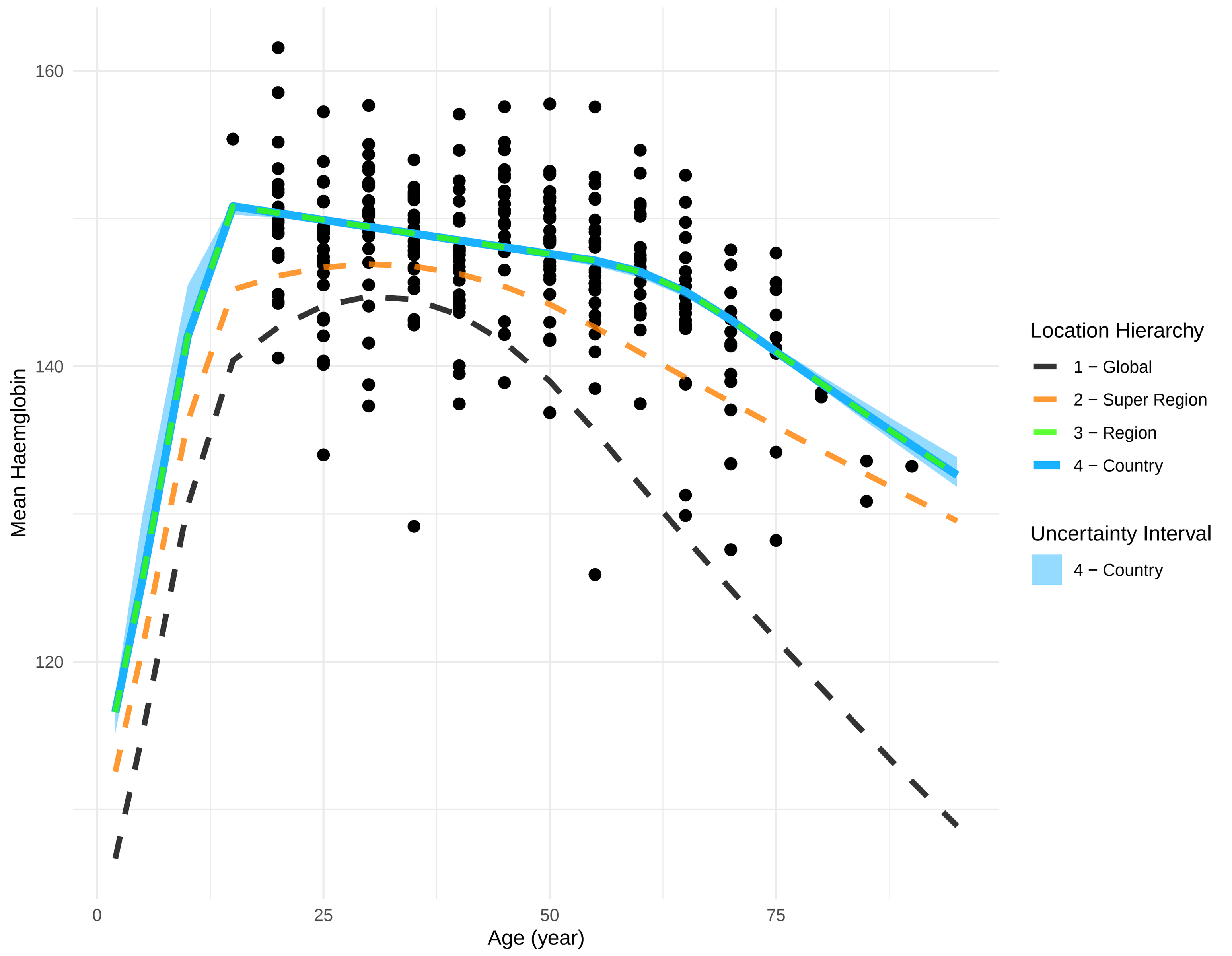

MR−BRT Age/Sex Fits − Males 2+ Years Old − Mean Haemglobin − Acre
WHO−2001 Adjustment Method

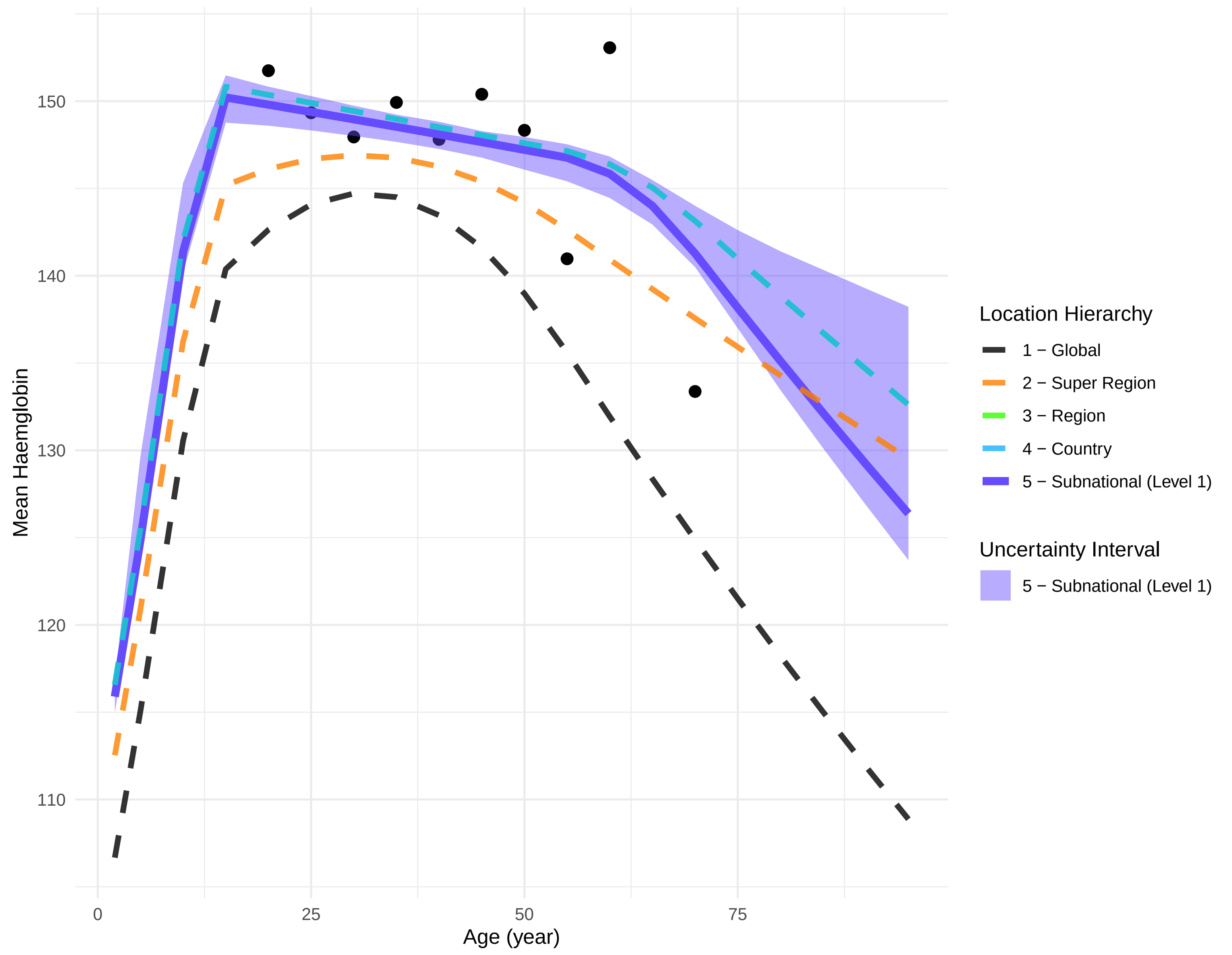

MR−BRT Age/Sex Fits − Males 2+ Years Old − Mean Haemglobin − Alagoas
WHO−2001 Adjustment Method

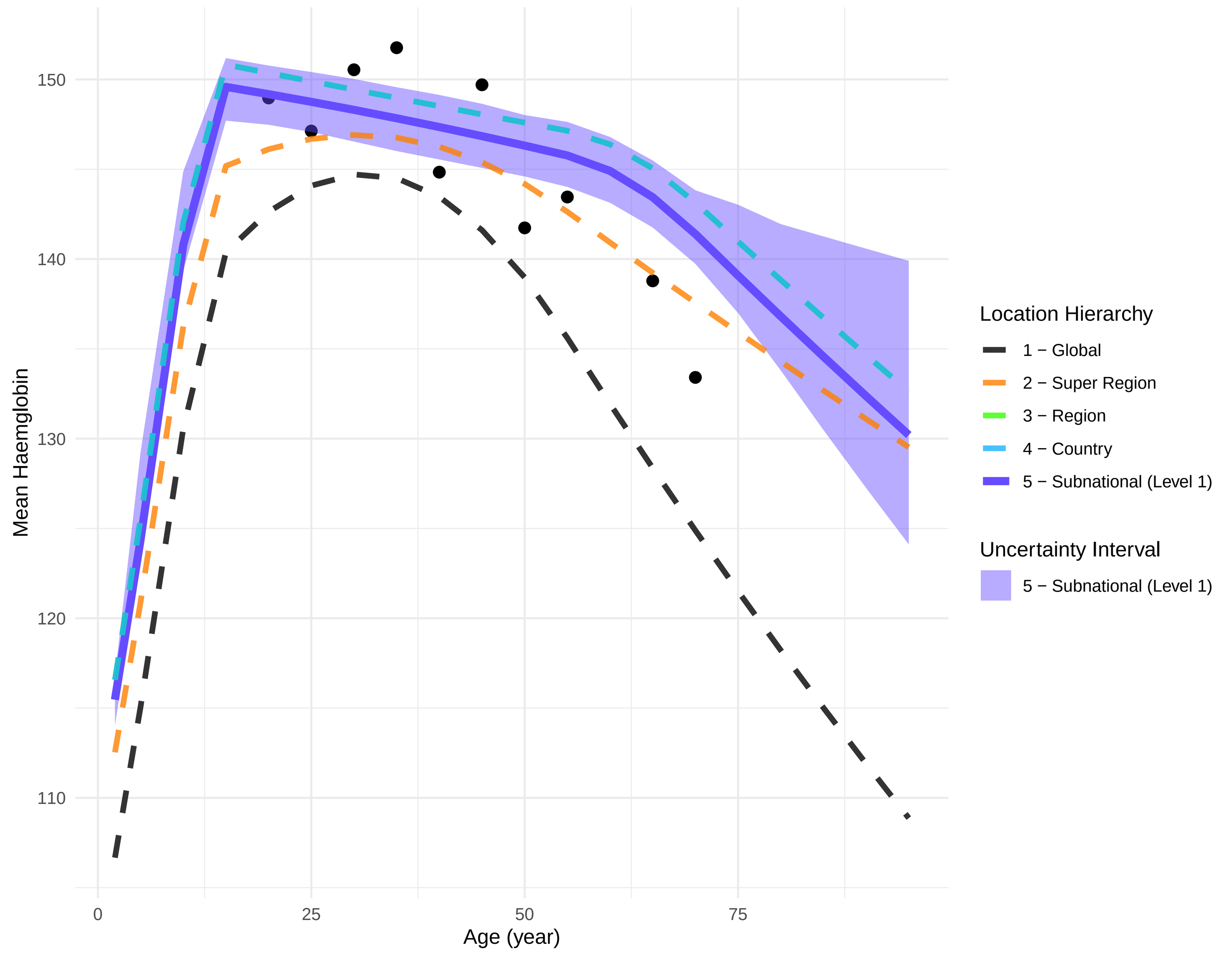

MR−BRT Age/Sex Fits − Males 2+ Years Old − Mean Haemglobin − Amapá
WHO−2001 Adjustment Method

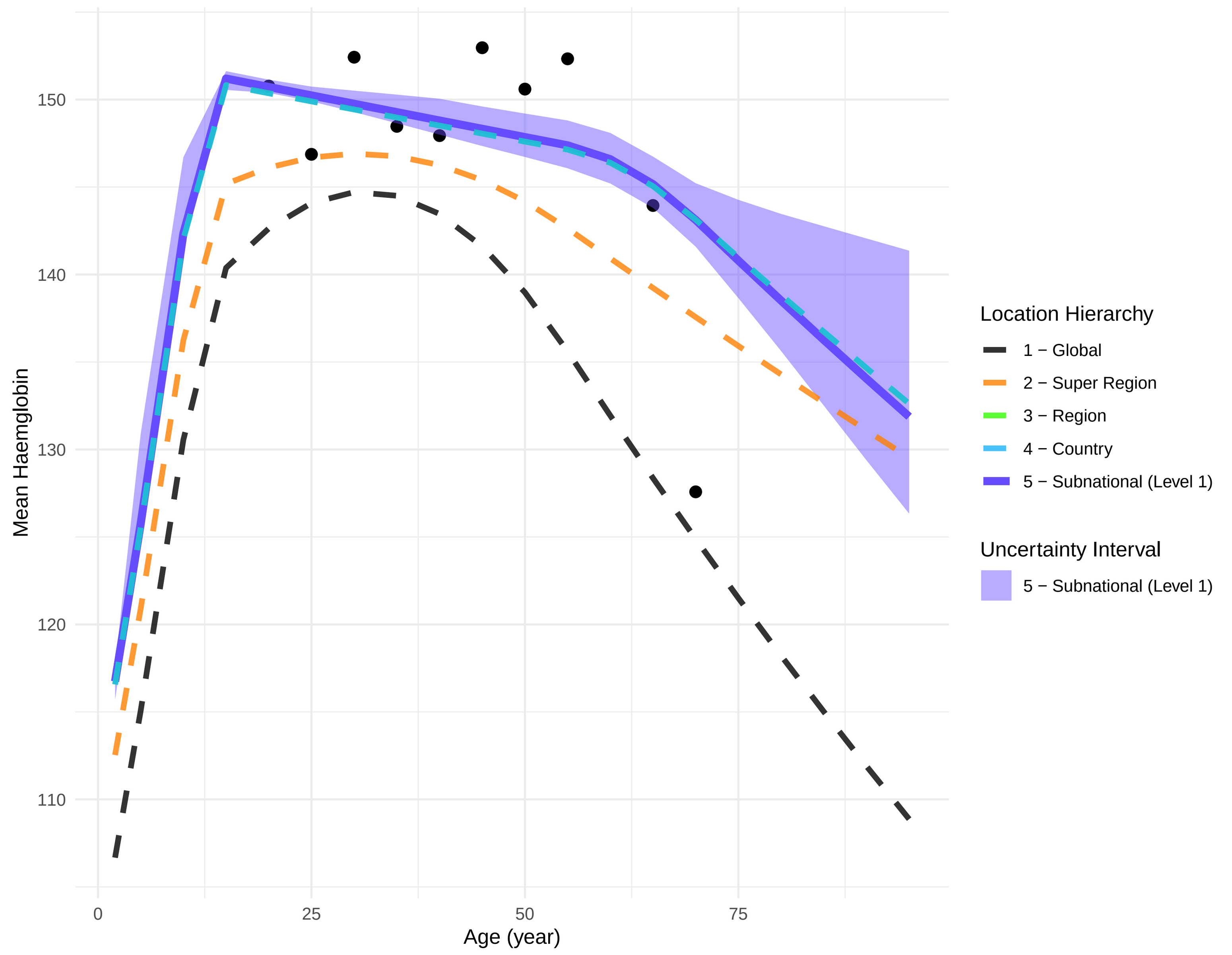

MR−BRT Age/Sex Fits − Males 2+ Years Old − Mean Haemglobin − Amazonas
WHO−2001 Adjustment Method

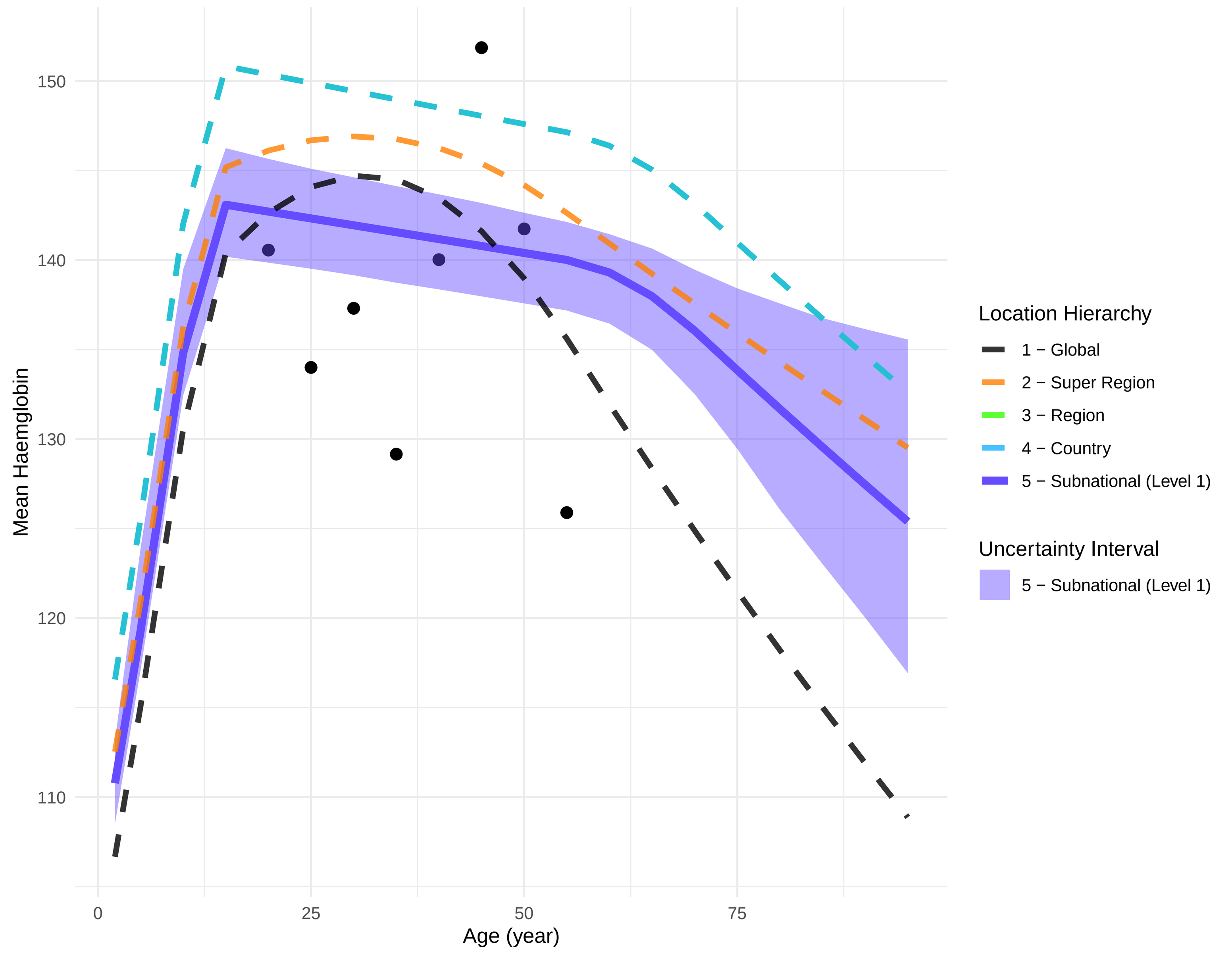

MR−BRT Age/Sex Fits − Males 2+ Years Old − Mean Haemglobin − Bahia
WHO−2001 Adjustment Method

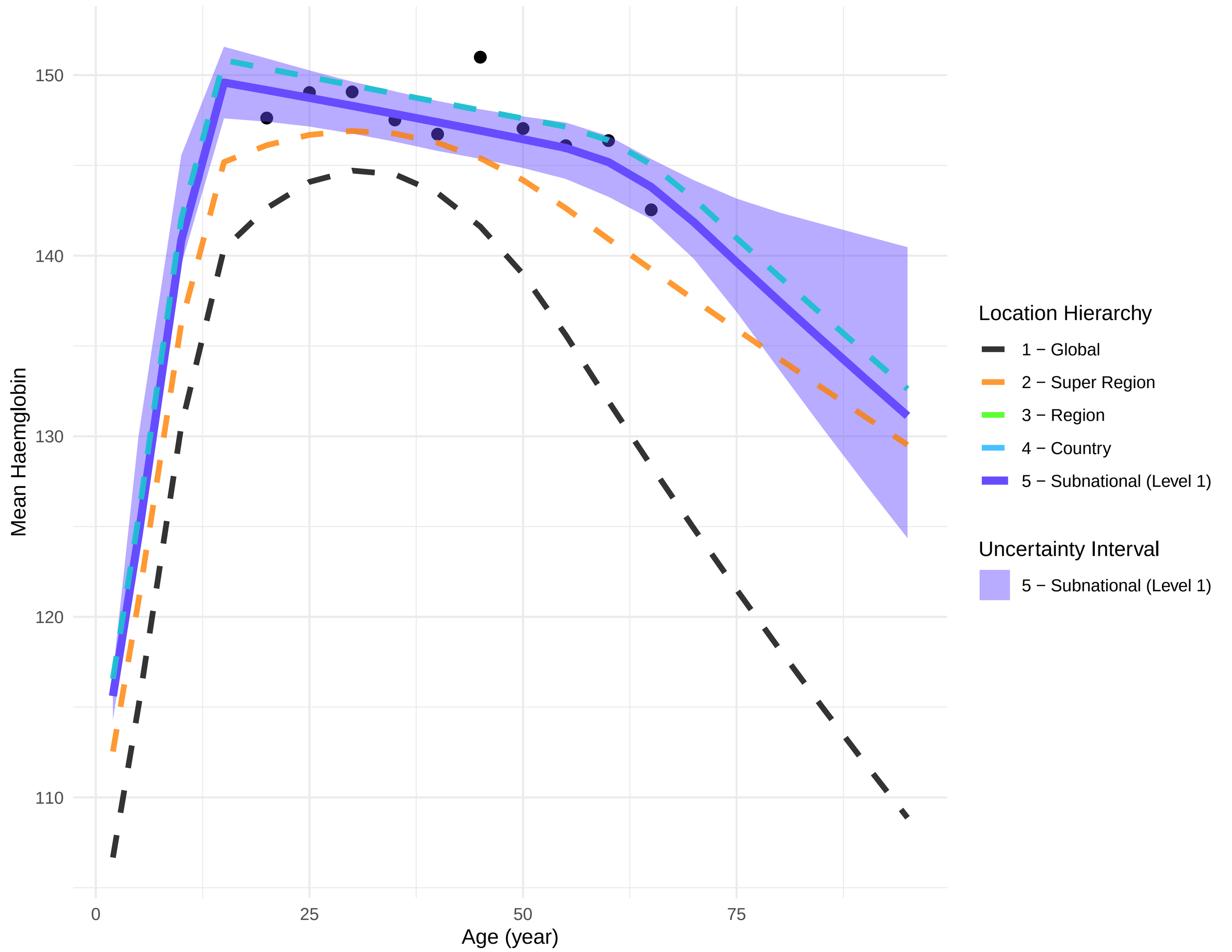

MR−BRT Age/Sex Fits − Males 2+ Years Old − Mean Haemglobin − Ceará
WHO−2001 Adjustment Method

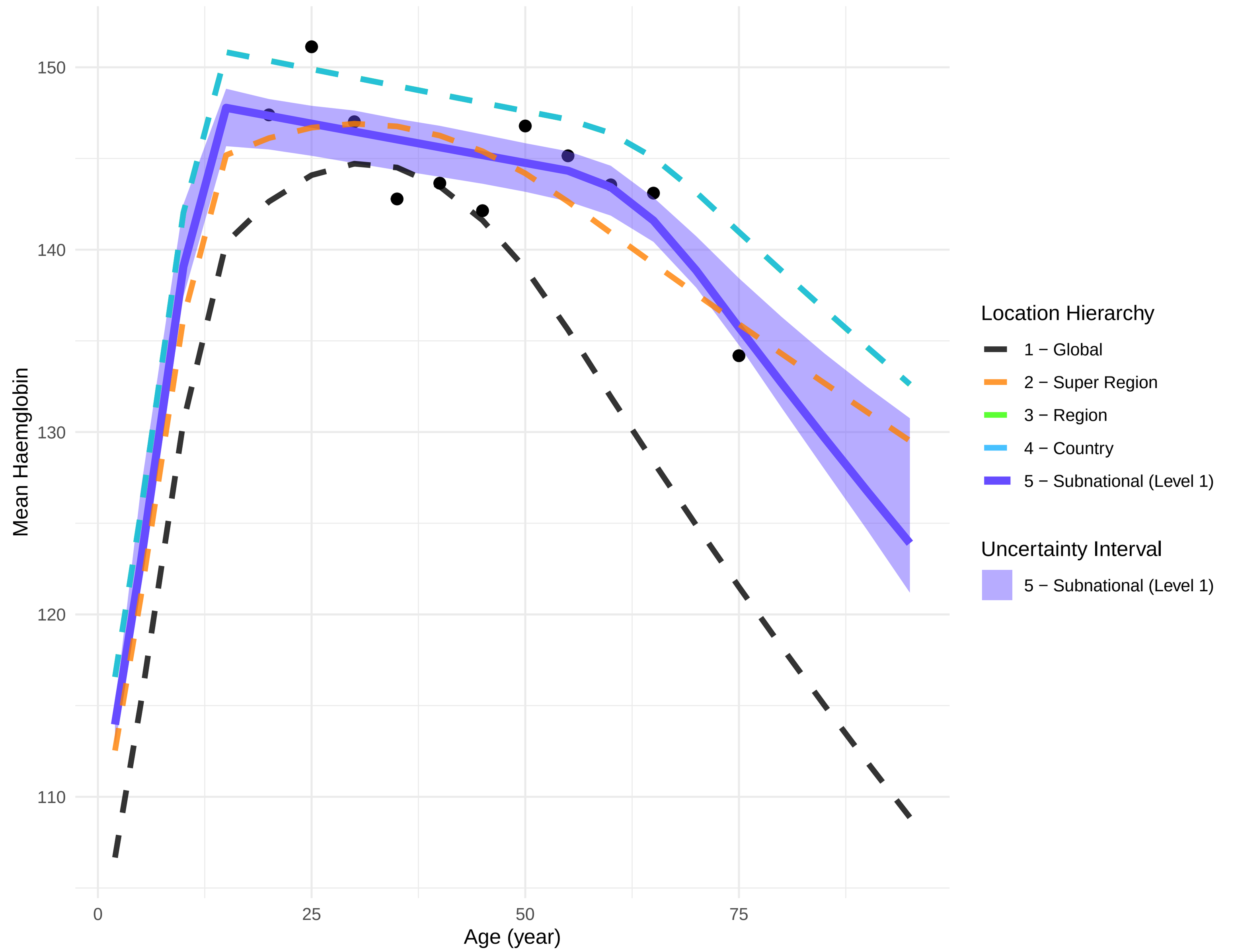

MR−BRT Age/Sex Fits − Males 2+ Years Old − Mean Haemglobin − Distrito Federal
WHO−2001 Adjustment Method

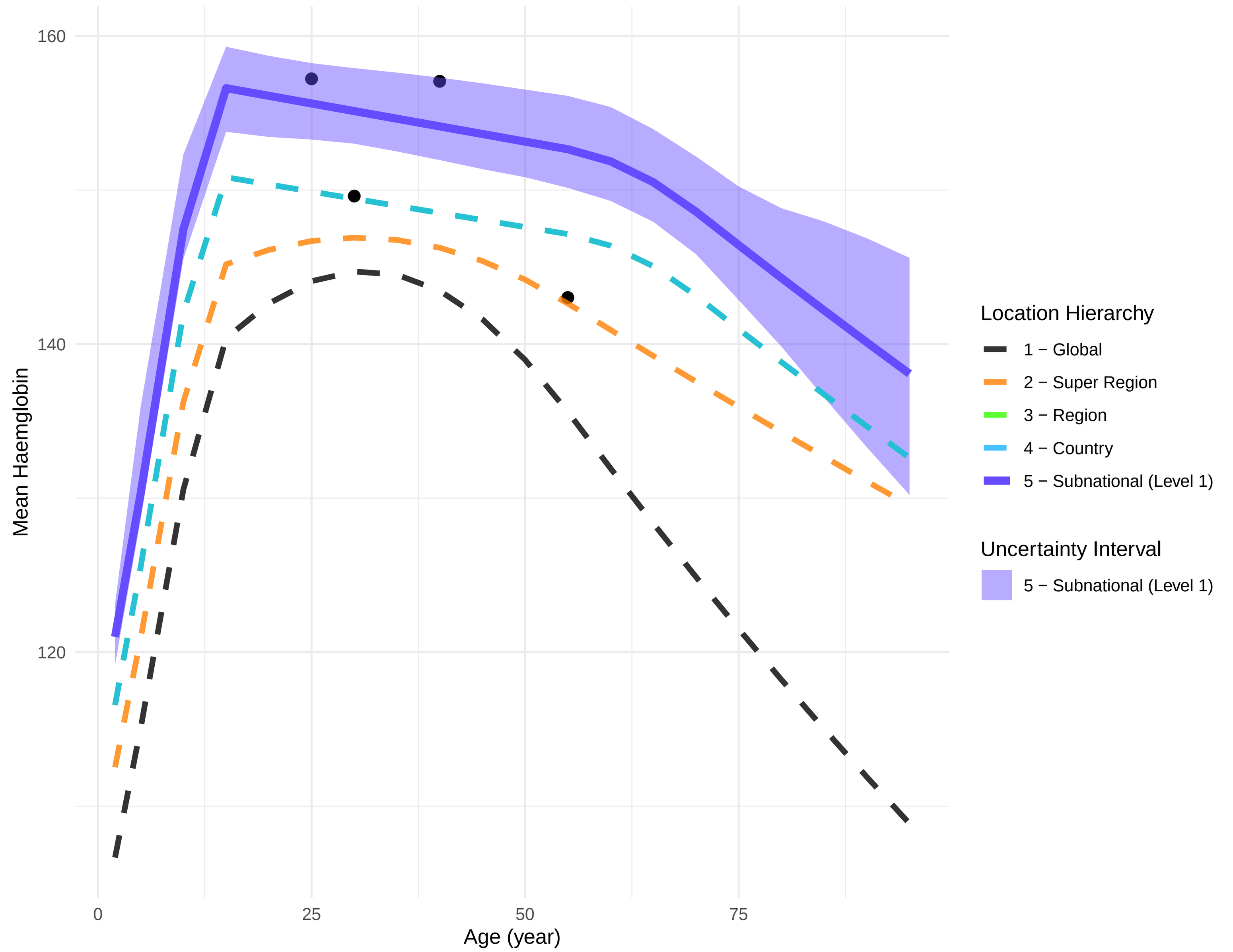

MR−BRT Age/Sex Fits − Males 2+ Years Old − Mean Haemglobin − Espírito Santo
WHO−2001 Adjustment Method

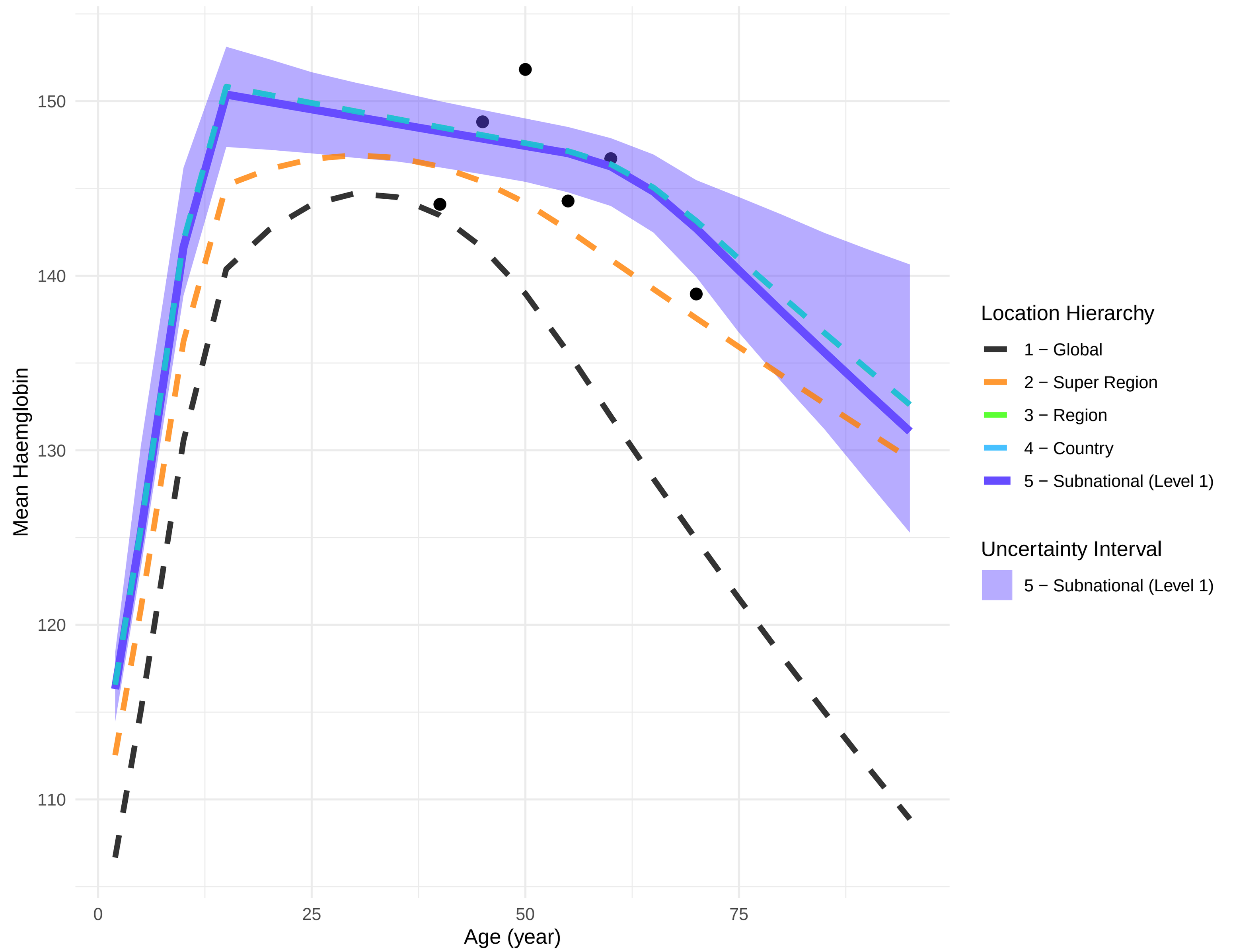

MR−BRT Age/Sex Fits − Males 2+ Years Old − Mean Haemglobin − Goiás
WHO−2001 Adjustment Method

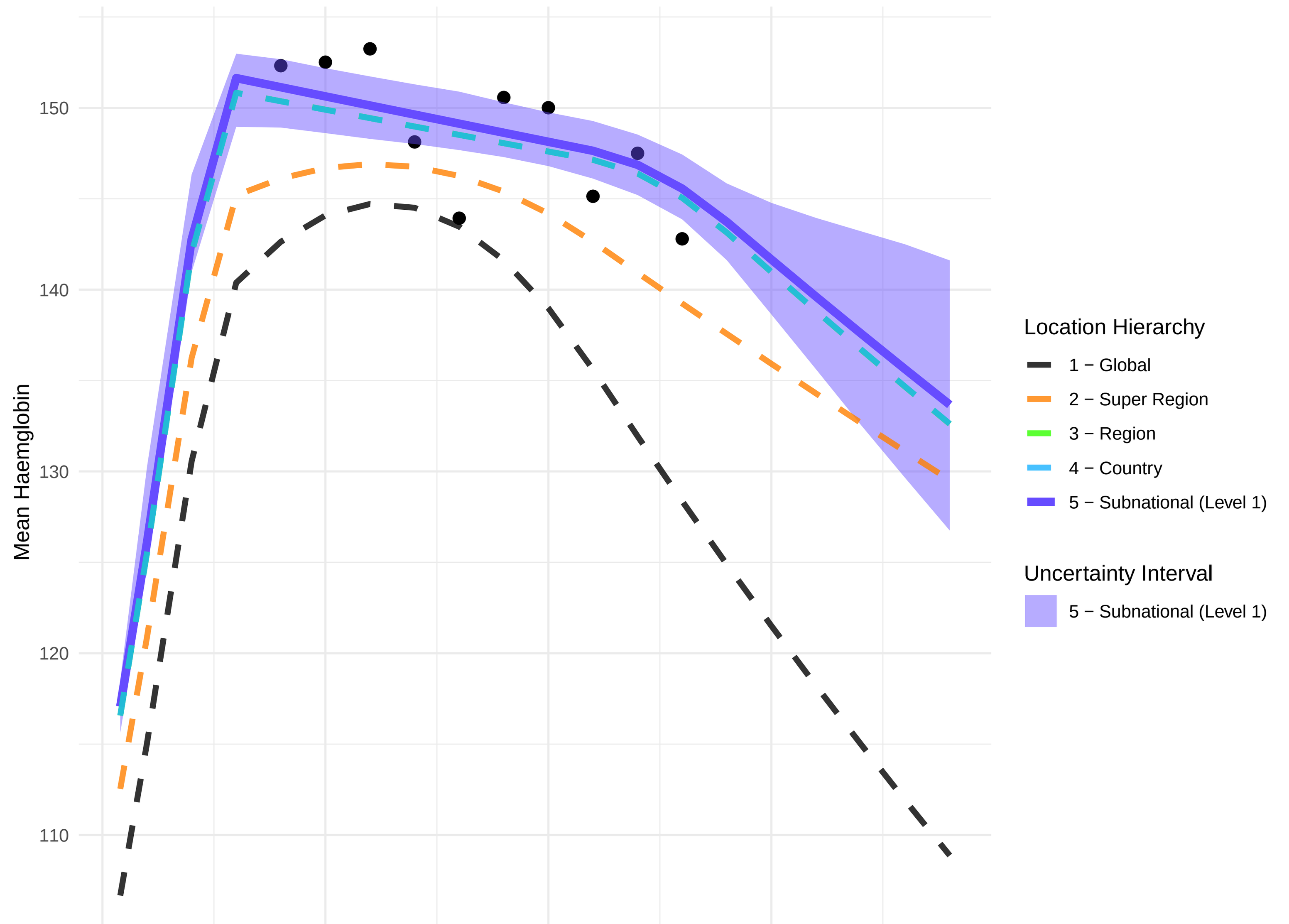

MR−BRT Age/Sex Fits − Males 2+ Years Old − Mean Haemglobin − Maranhão
WHO−2001 Adjustment Method

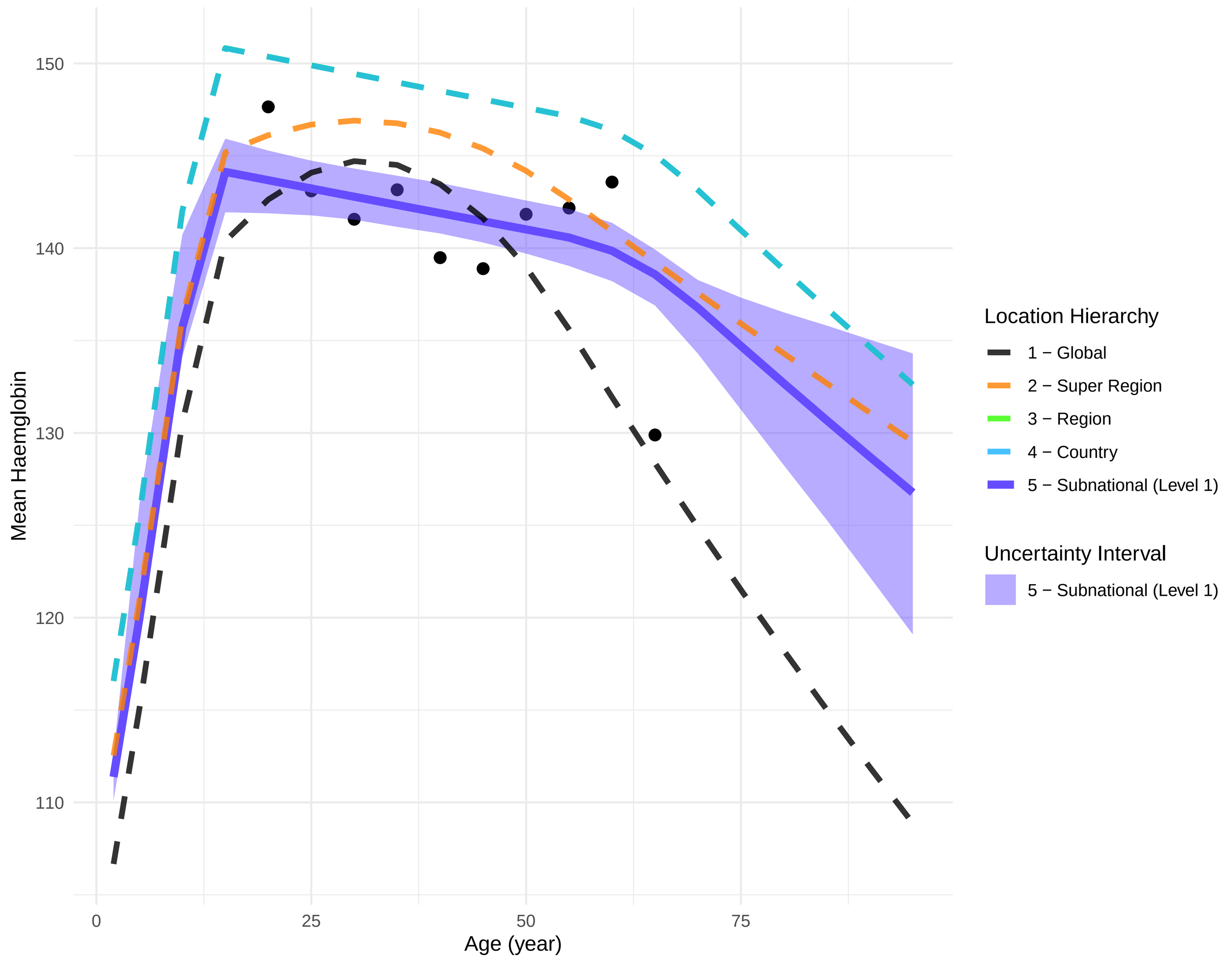

MR−BRT Age/Sex Fits − Males 2+ Years Old − Mean Haemglobin − Mato Grosso
WHO−2001 Adjustment Method

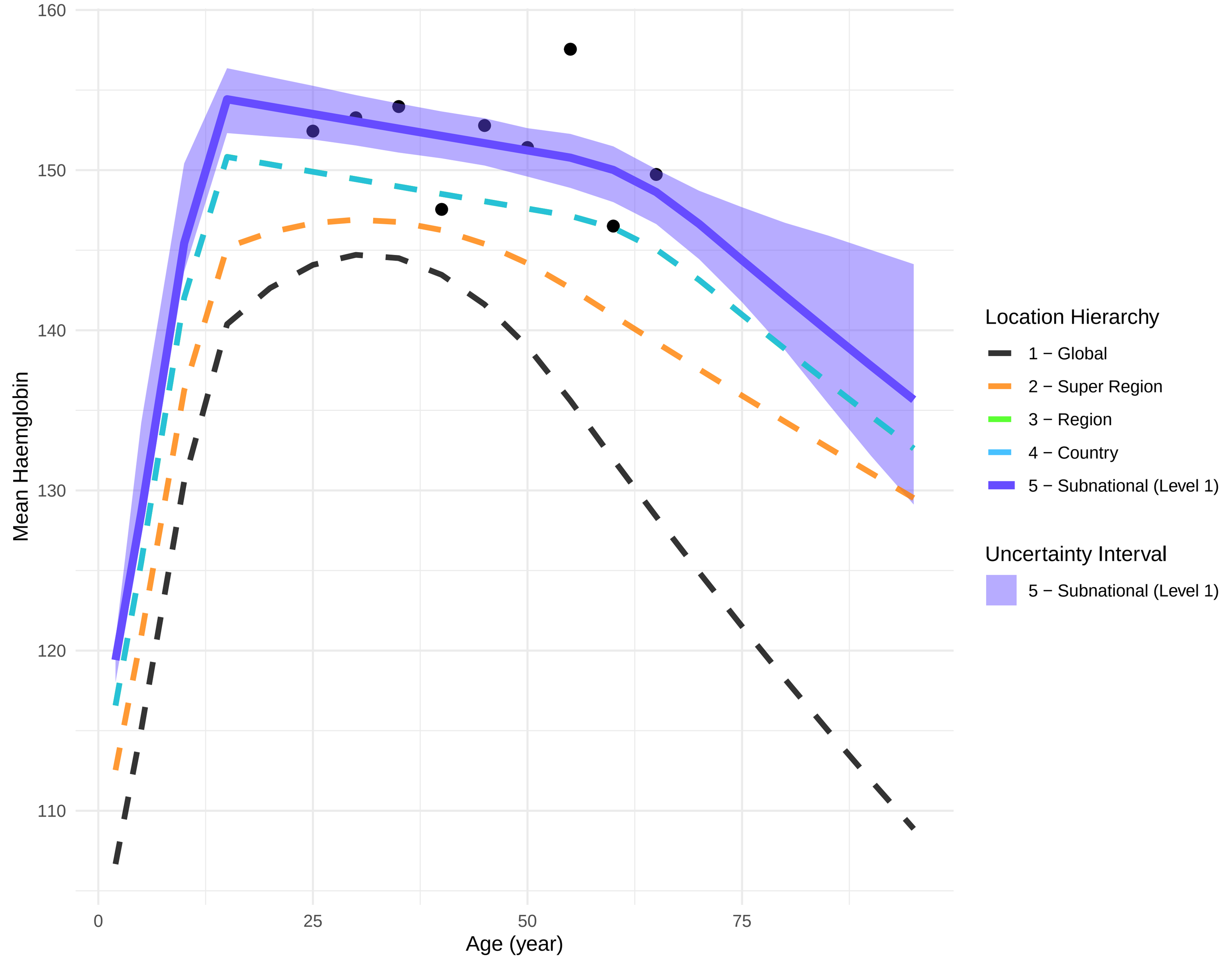

MR−BRT Age/Sex Fits − Males 2+ Years Old − Mean Haemglobin − Mato Grosso do Sul
WHO−2001 Adjustment Method

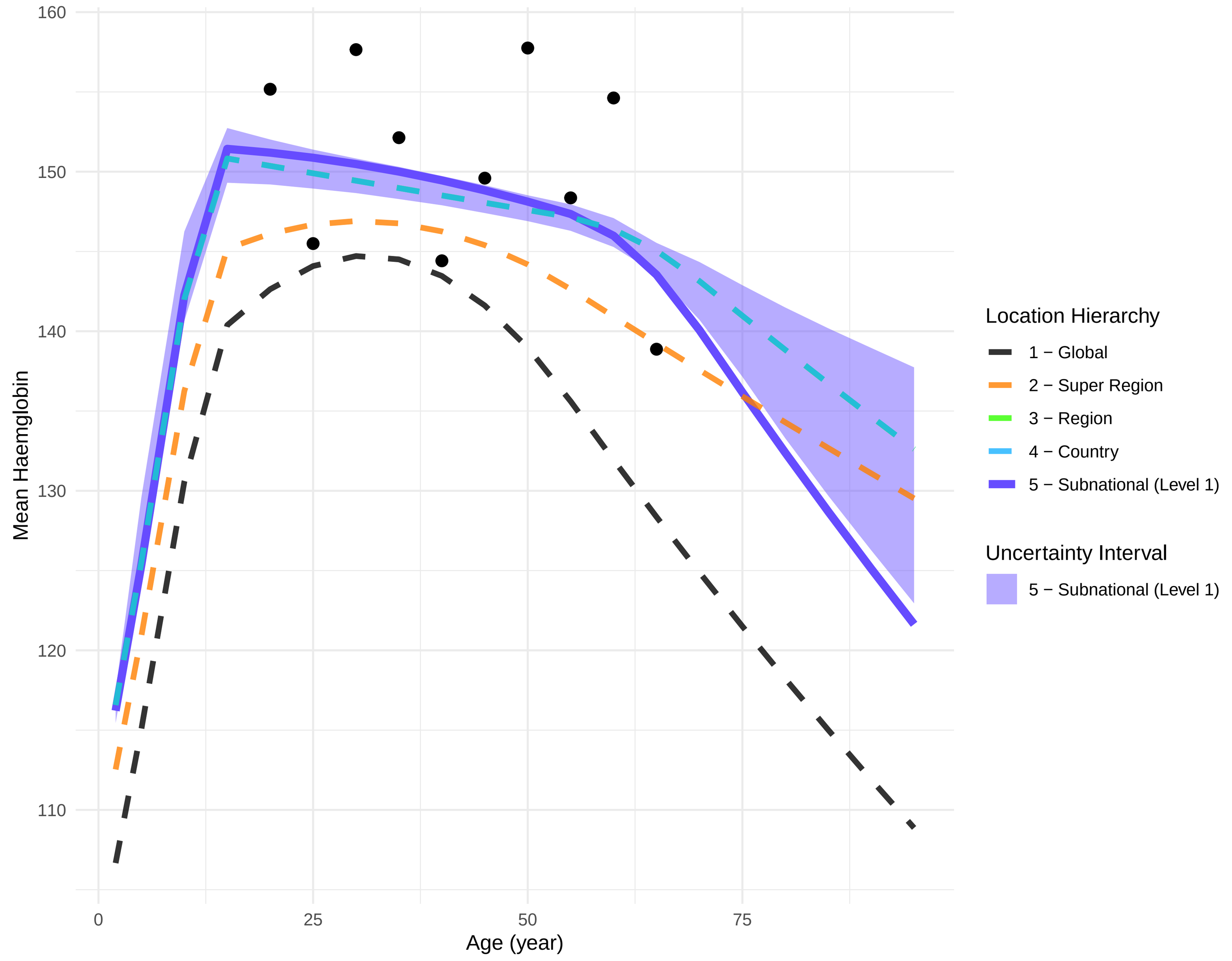

MR−BRT Age/Sex Fits − Males 2+ Years Old − Mean Haemglobin − Minas Gerais
WHO−2001 Adjustment Method

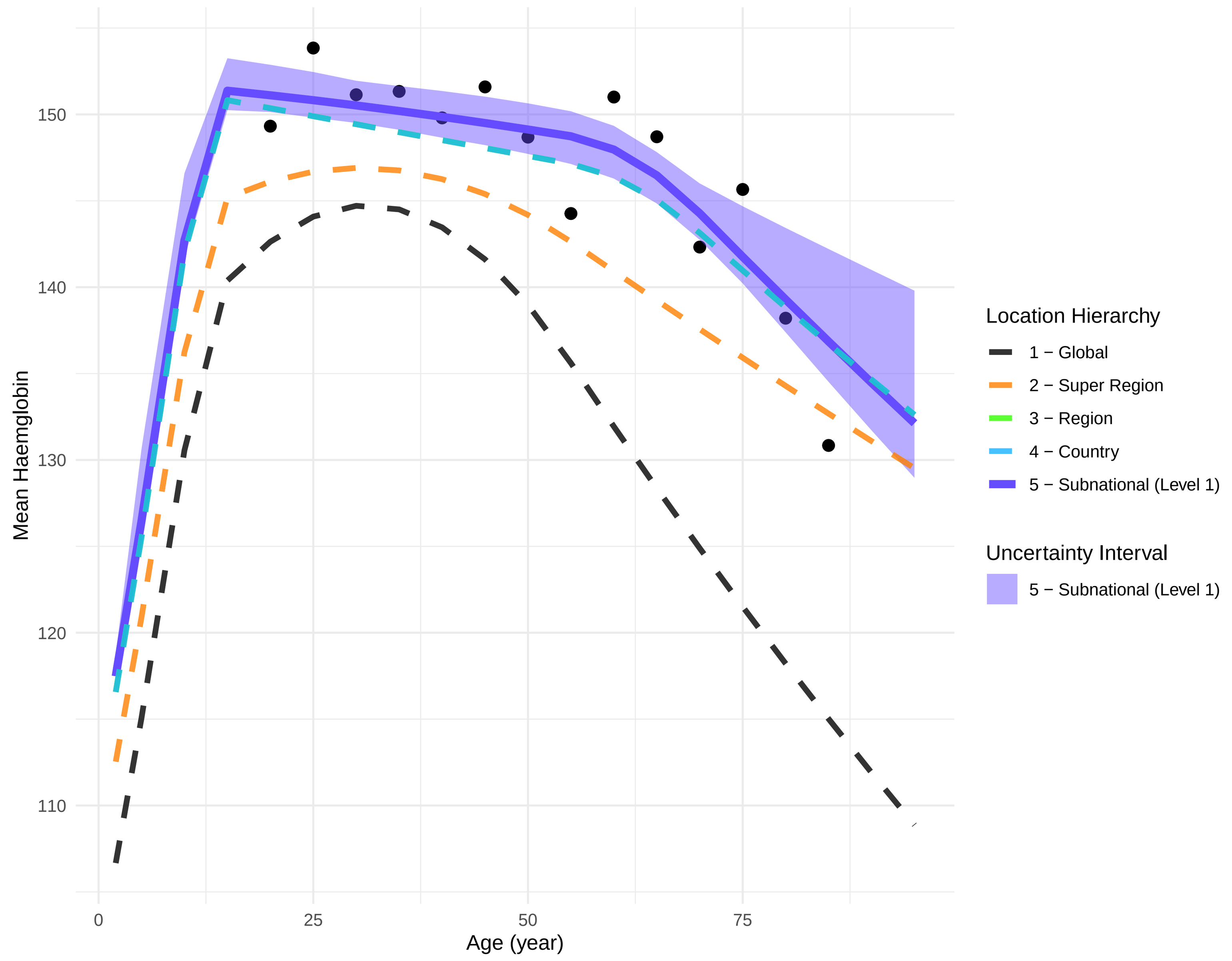

MR−BRT Age/Sex Fits − Males 2+ Years Old − Mean Haemglobin − Pará
WHO−2001 Adjustment Method

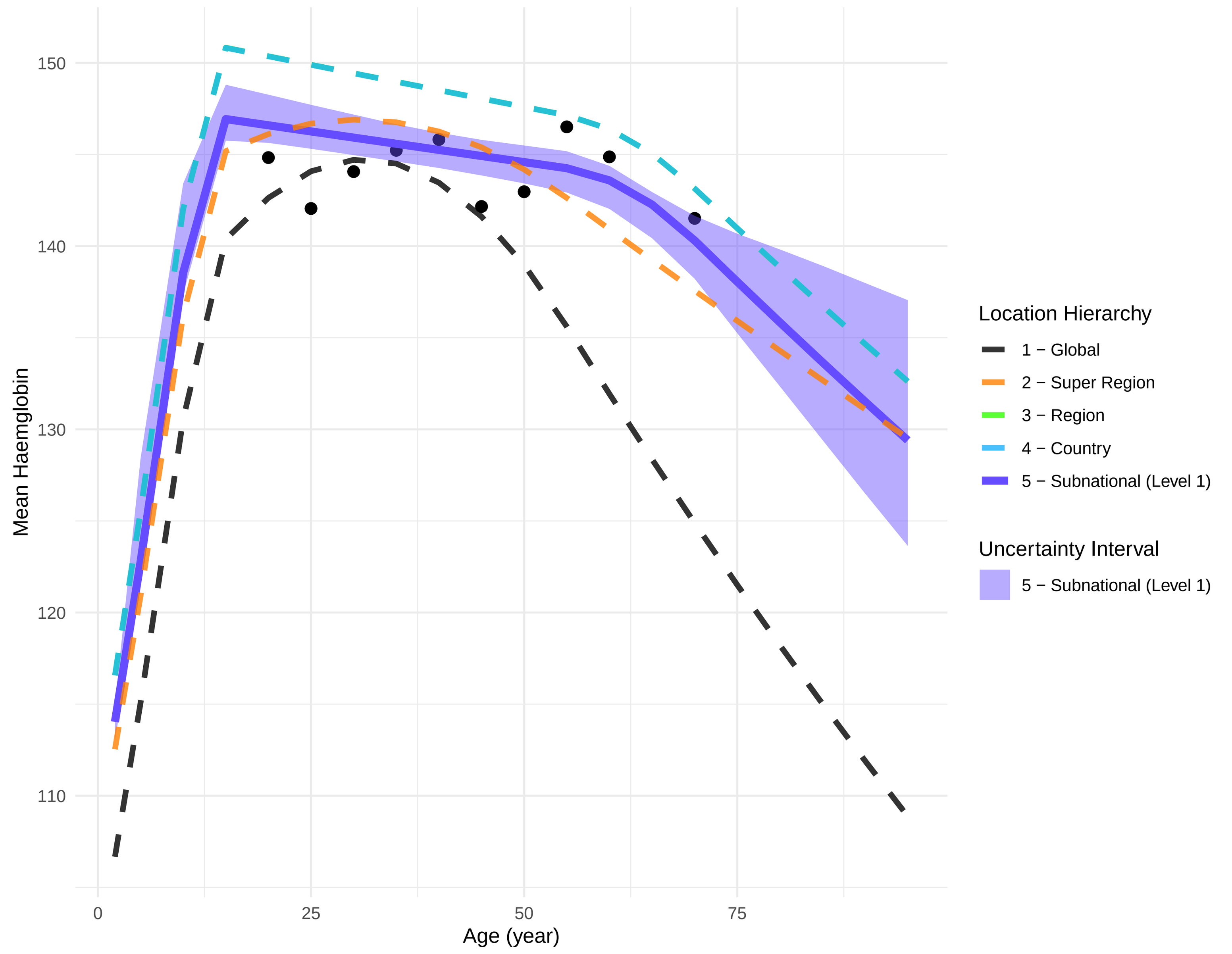

MR−BRT Age/Sex Fits − Males 2+ Years Old − Mean Haemglobin − Paraíba
WHO−2001 Adjustment Method

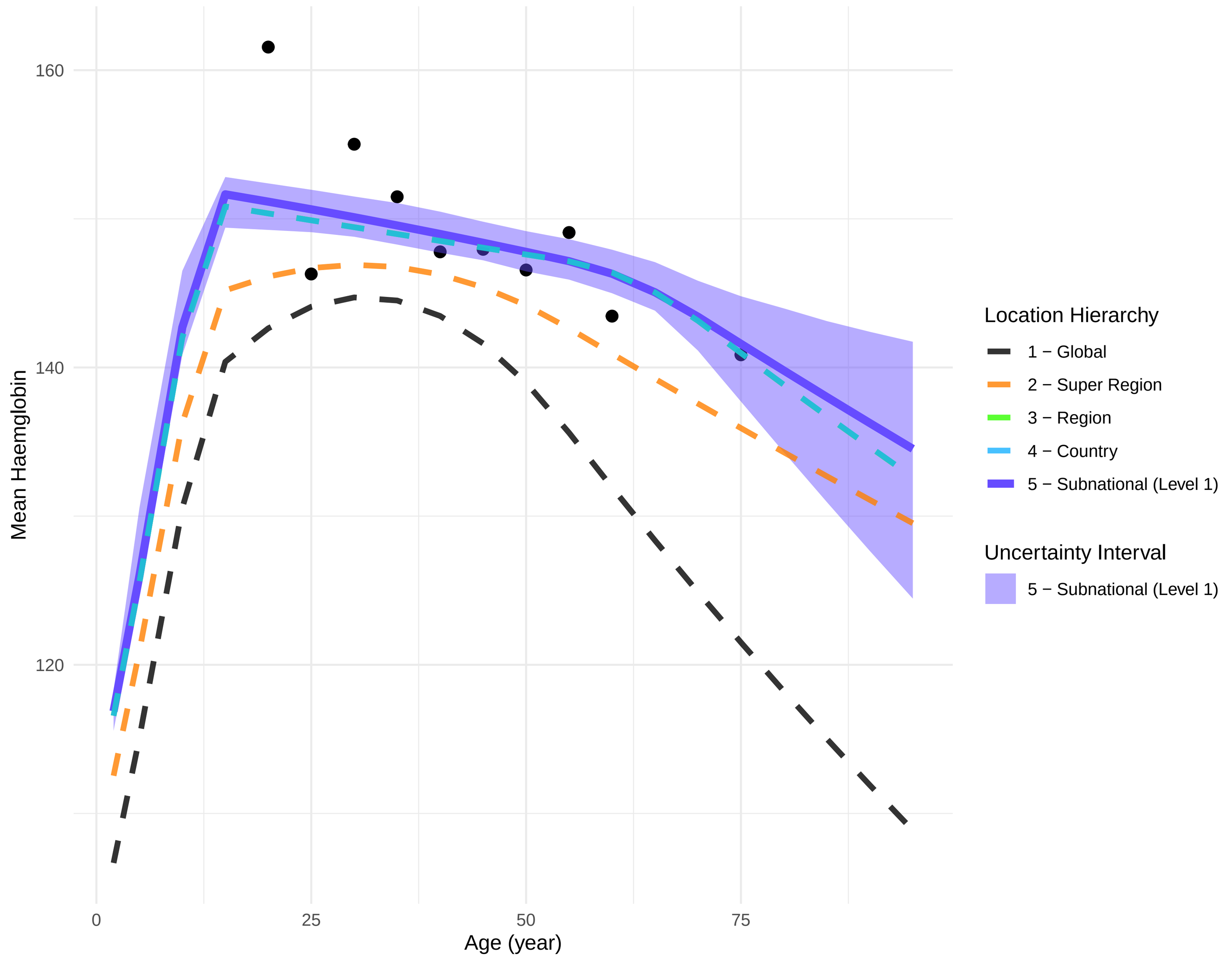

MR−BRT Age/Sex Fits − Males 2+ Years Old − Mean Haemglobin − Paraná
WHO−2001 Adjustment Method

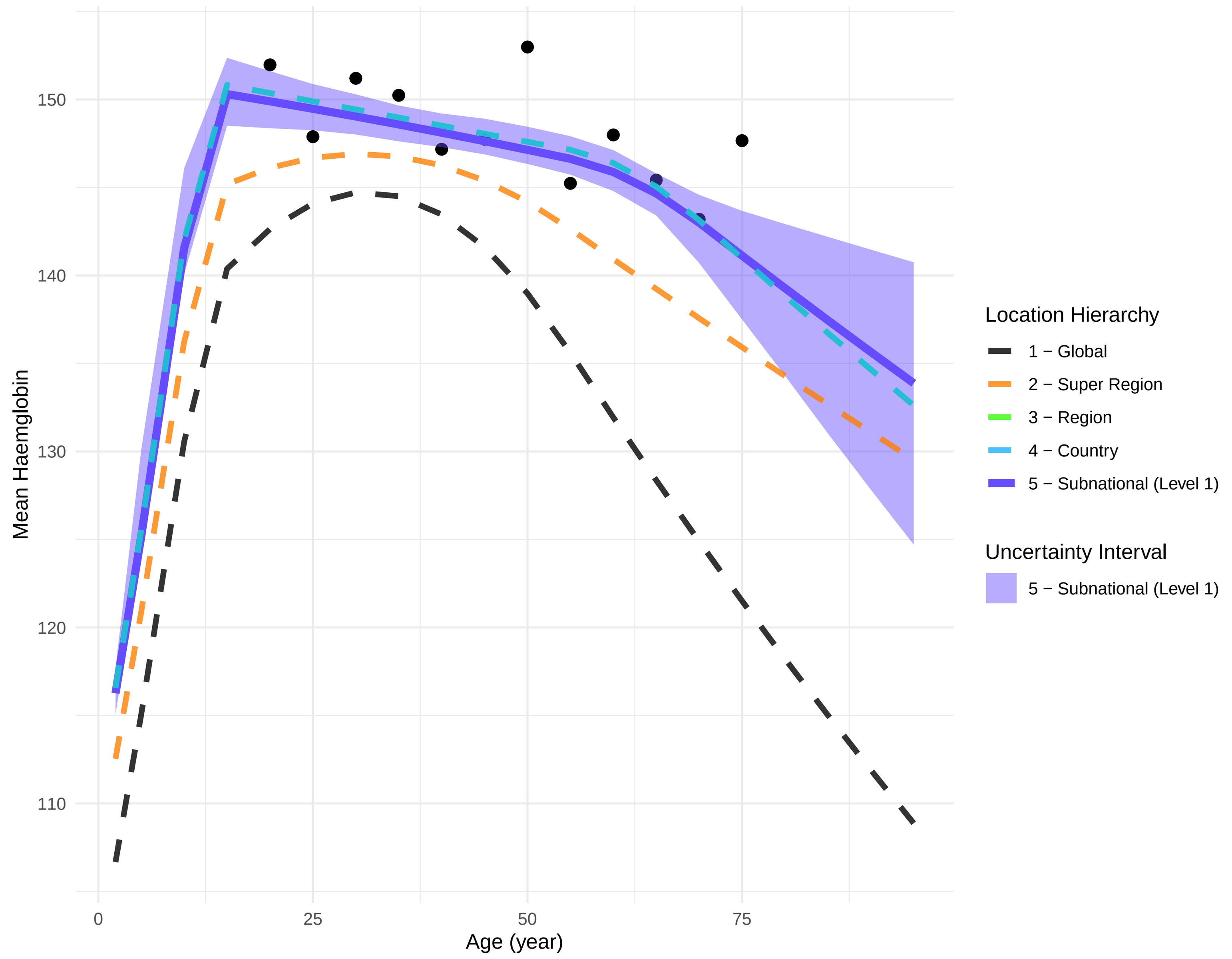

MR−BRT Age/Sex Fits − Males 2+ Years Old − Mean Haemglobin − Pernambuco
WHO−2001 Adjustment Method

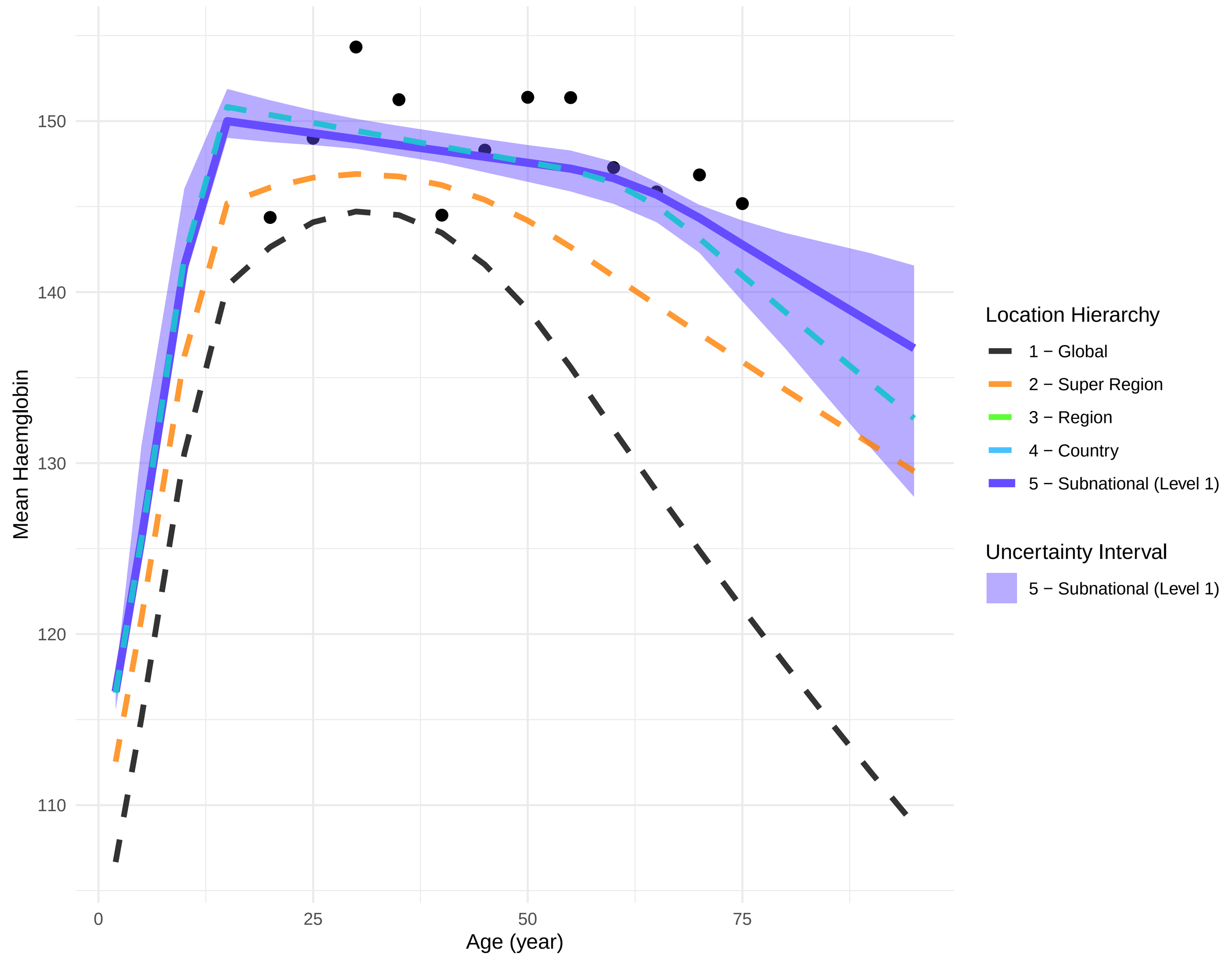

MR−BRT Age/Sex Fits − Males 2+ Years Old − Mean Haemglobin − Piaui
WHO−2001 Adjustment Method

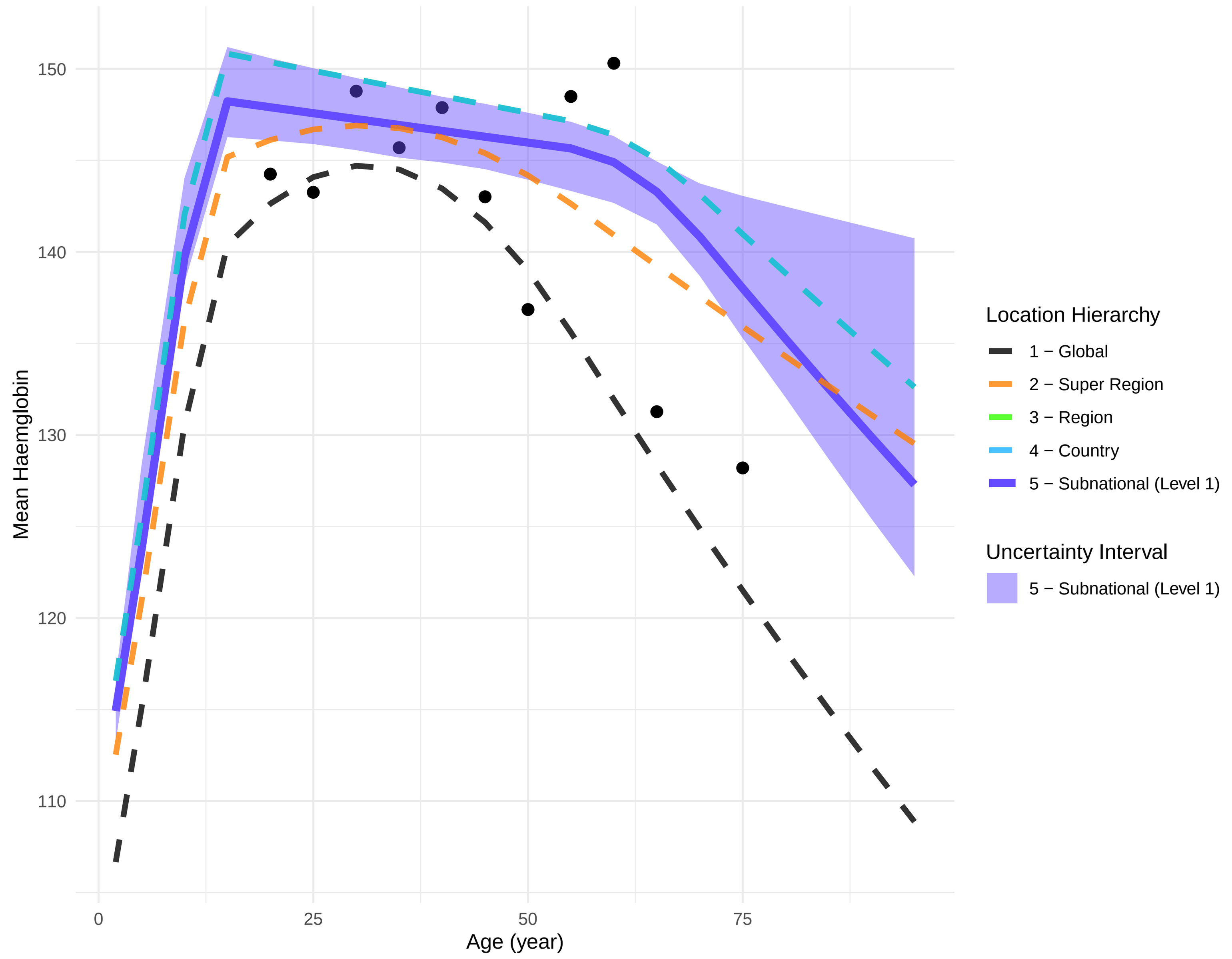

MR−BRT Age/Sex Fits − Males 2+ Years Old − Mean Haemglobin − Rio de Janeiro
WHO−2001 Adjustment Method

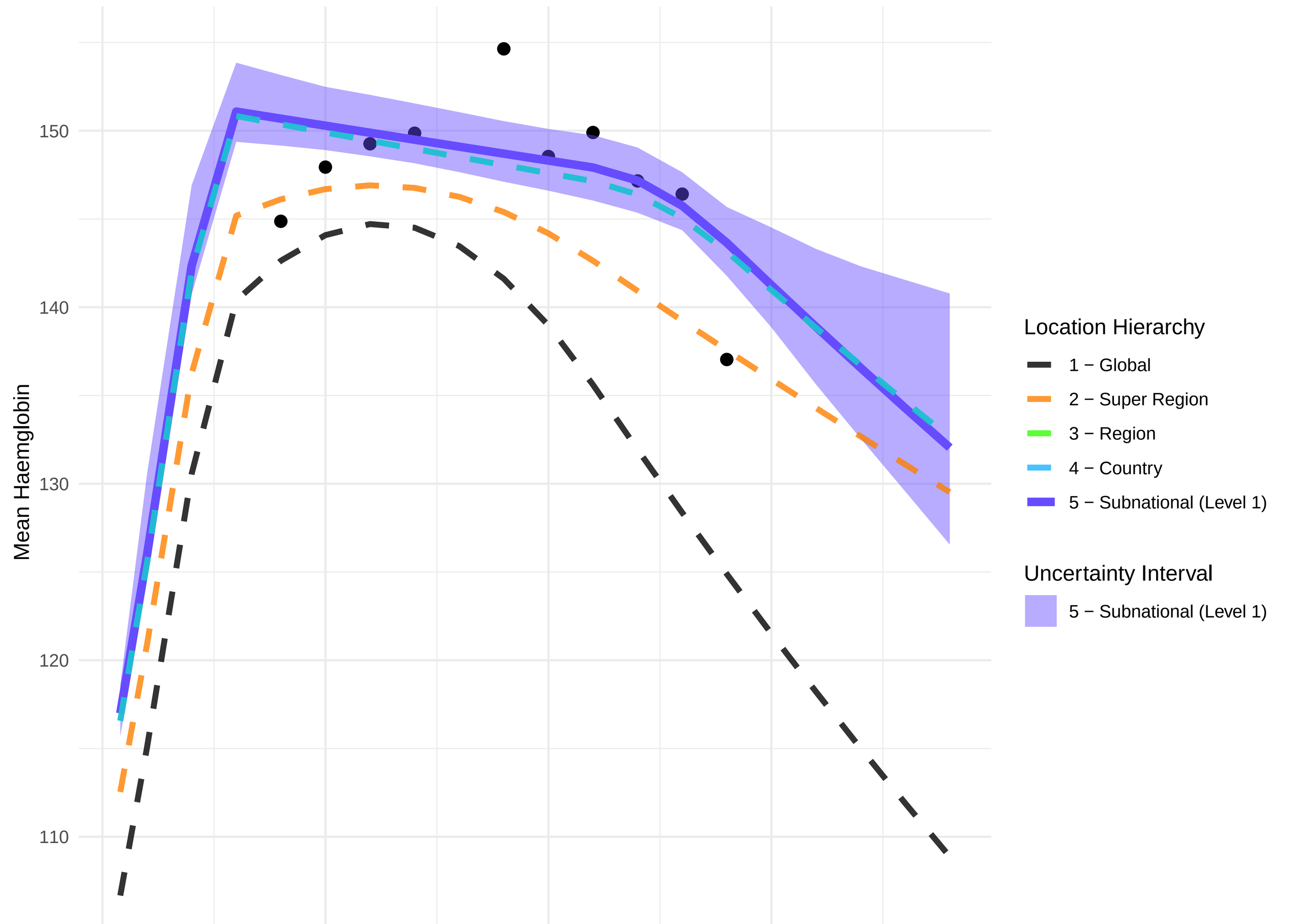

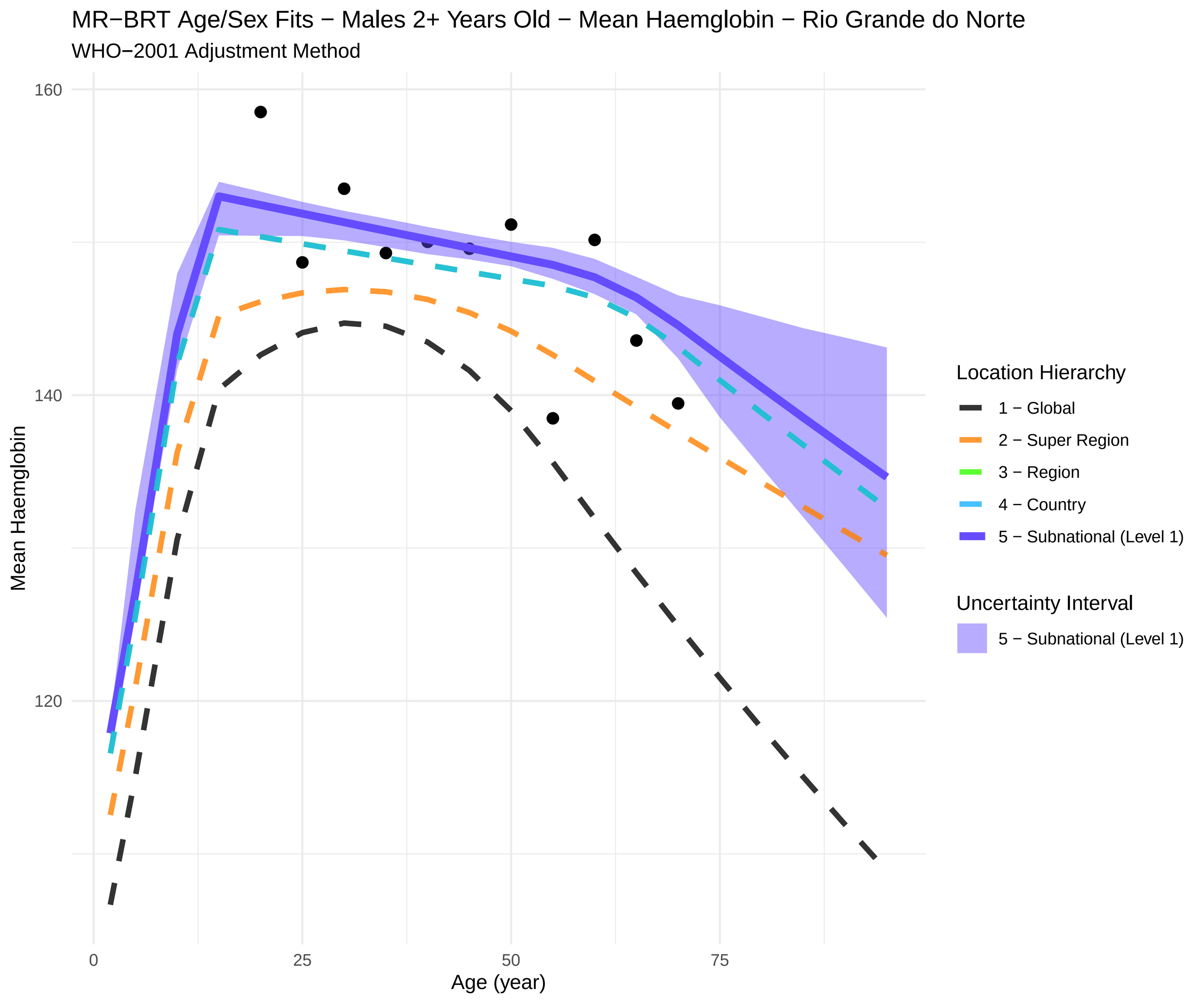
MR−BRT Age/Sex Fits − Males 2+ Years Old − Mean Haemglobin − Rio Grande do Norte
WHO−2001 Adjustment Method
Mean Haemglobin
Age (year)
Location Hierarchy
1 − Global
2 − Super Region
3 − Region
4 − Country
5 − Subnational (Level 1)
Uncertainty Interval
5 − Subnational (Level 1)

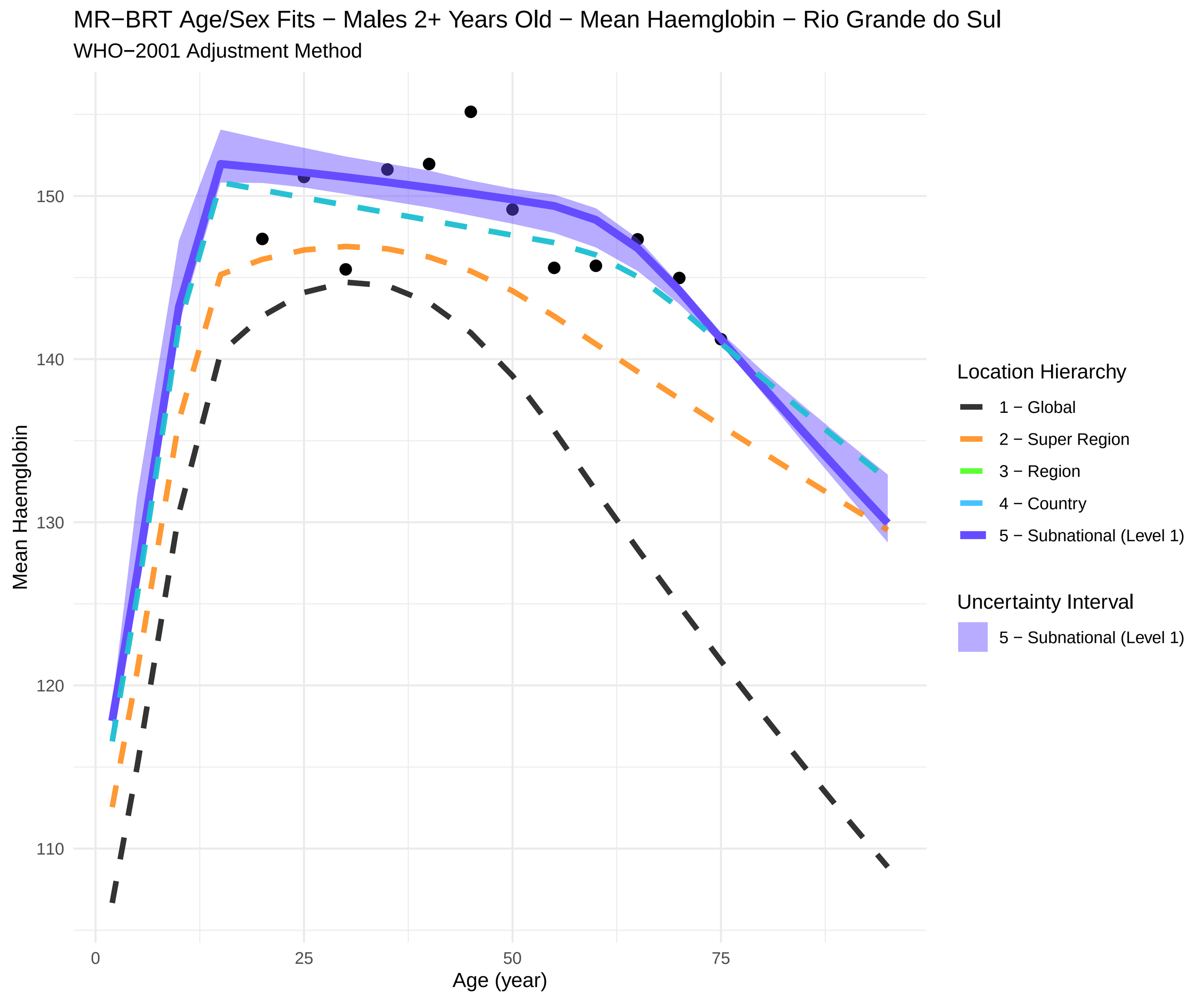
MR−BRT Age/Sex Fits − Males 2+ Years Old − Mean Haemglobin − Rio Grande do Sul
WHO−2001 Adjustment Method
Mean Haemglobin
Age (year)
Location Hierarchy
1 − Global
2 − Super Region
3 − Region
4 − Country
5 − Subnational (Level 1)
Uncertainty Interval
5 − Subnational (Level 1)

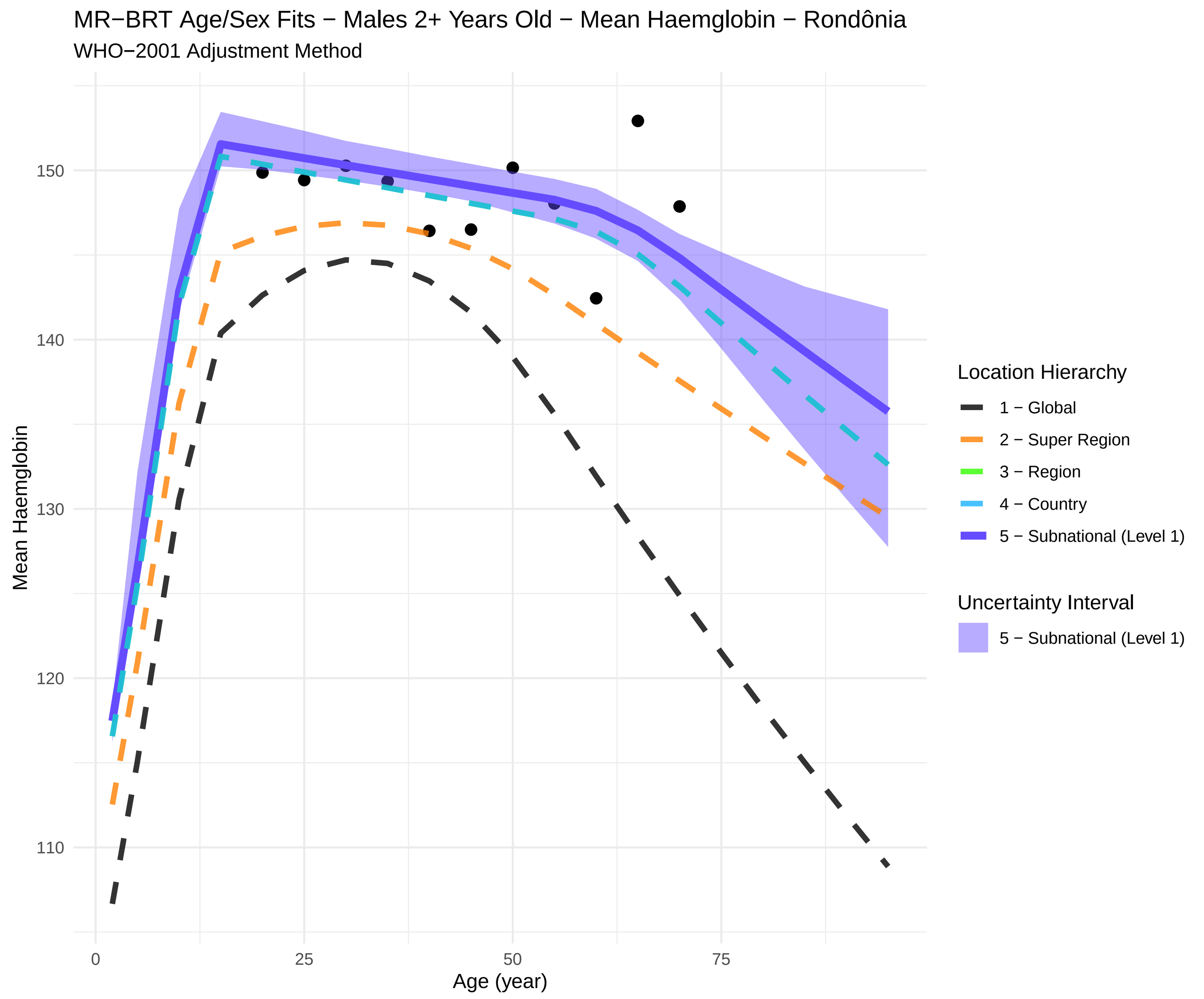
MR−BRT Age/Sex Fits − Males 2+ Years Old − Mean Haemglobin − Rondônia
WHO−2001 Adjustment Method
Mean Haemglobin
Age (year)
Location Hierarchy
1 − Global
2 − Super Region
3 − Region
4 − Country
5 − Subnational (Level 1)
Uncertainty Interval
5 − Subnational (Level 1)

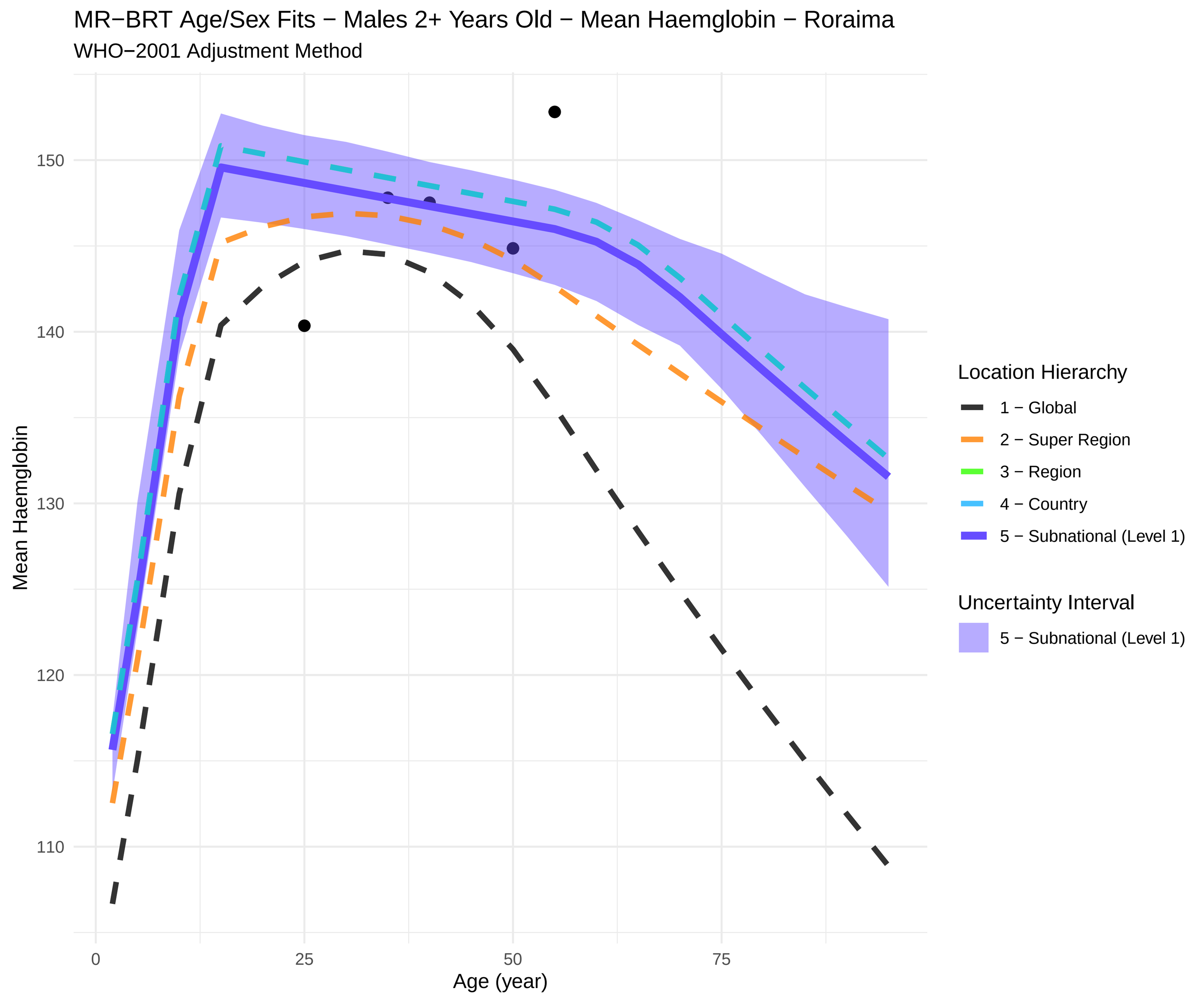
MR−BRT Age/Sex Fits − Males 2+ Years Old − Mean Haemglobin − Roraima
WHO−2001 Adjustment Method
Mean Haemglobin
Age (year)
Location Hierarchy
1 − Global
2 − Super Region
3 − Region
4 − Country
5 − Subnational (Level 1)
Uncertainty Interval
5 − Subnational (Level 1)

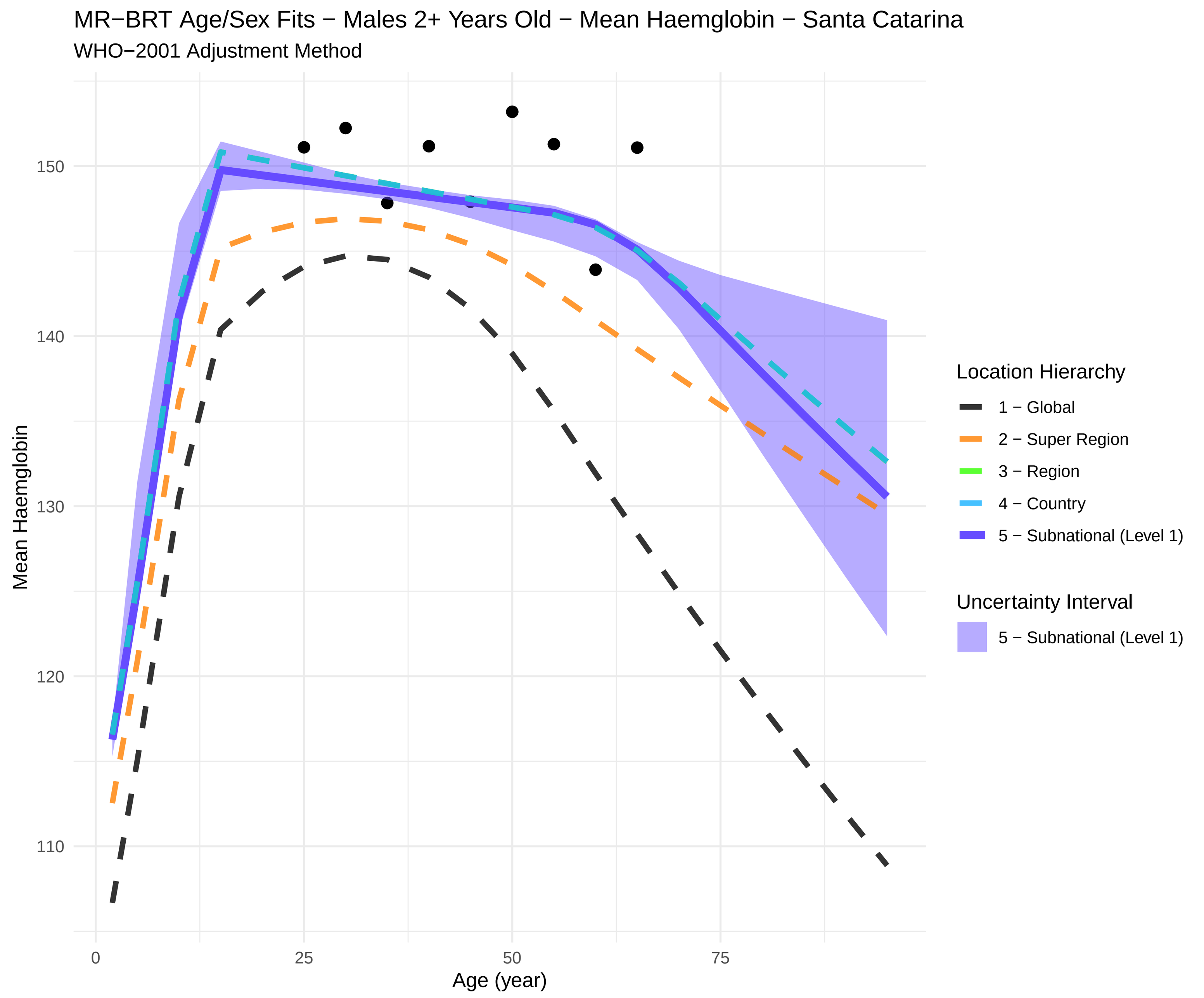
MR−BRT Age/Sex Fits − Males 2+ Years Old − Mean Haemglobin − Santa Catarina
WHO−2001 Adjustment Method
Mean Haemglobin
Age (year)
Location Hierarchy
1 − Global
2 − Super Region
3 − Region
4 − Country
5 − Subnational (Level 1)
Uncertainty Interval
5 − Subnational (Level 1)

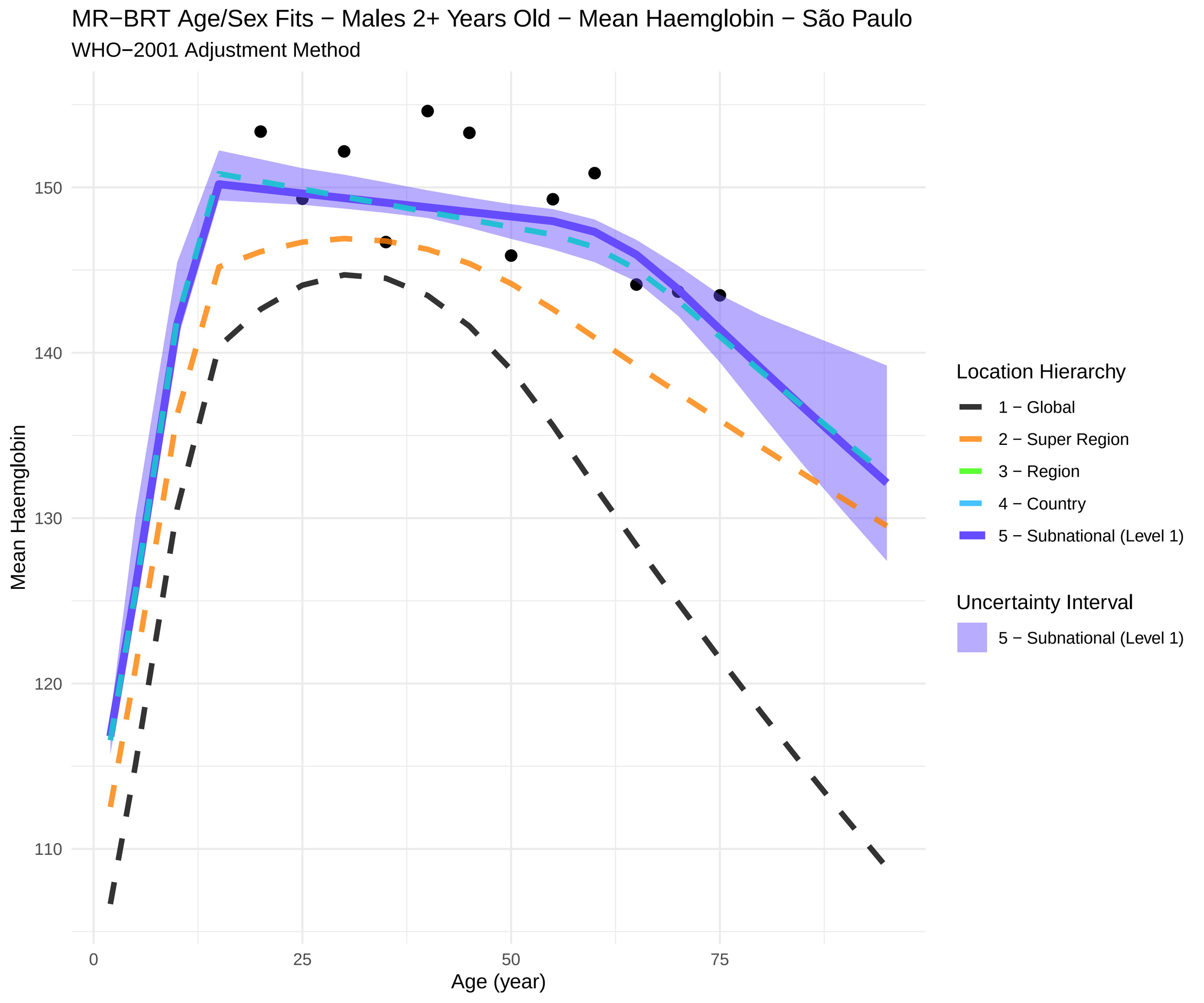
MR−BRT Age/Sex Fits − Males 2+ Years Old − Mean Haemglobin − São Paulo
WHO−2001 Adjustment Method
Mean Haemglobin
Age (year)
Location Hierarchy
1 − Global
2 − Super Region
3 − Region
4 − Country
5 − Subnational (Level 1)
Uncertainty Interval
5 − Subnational (Level 1)

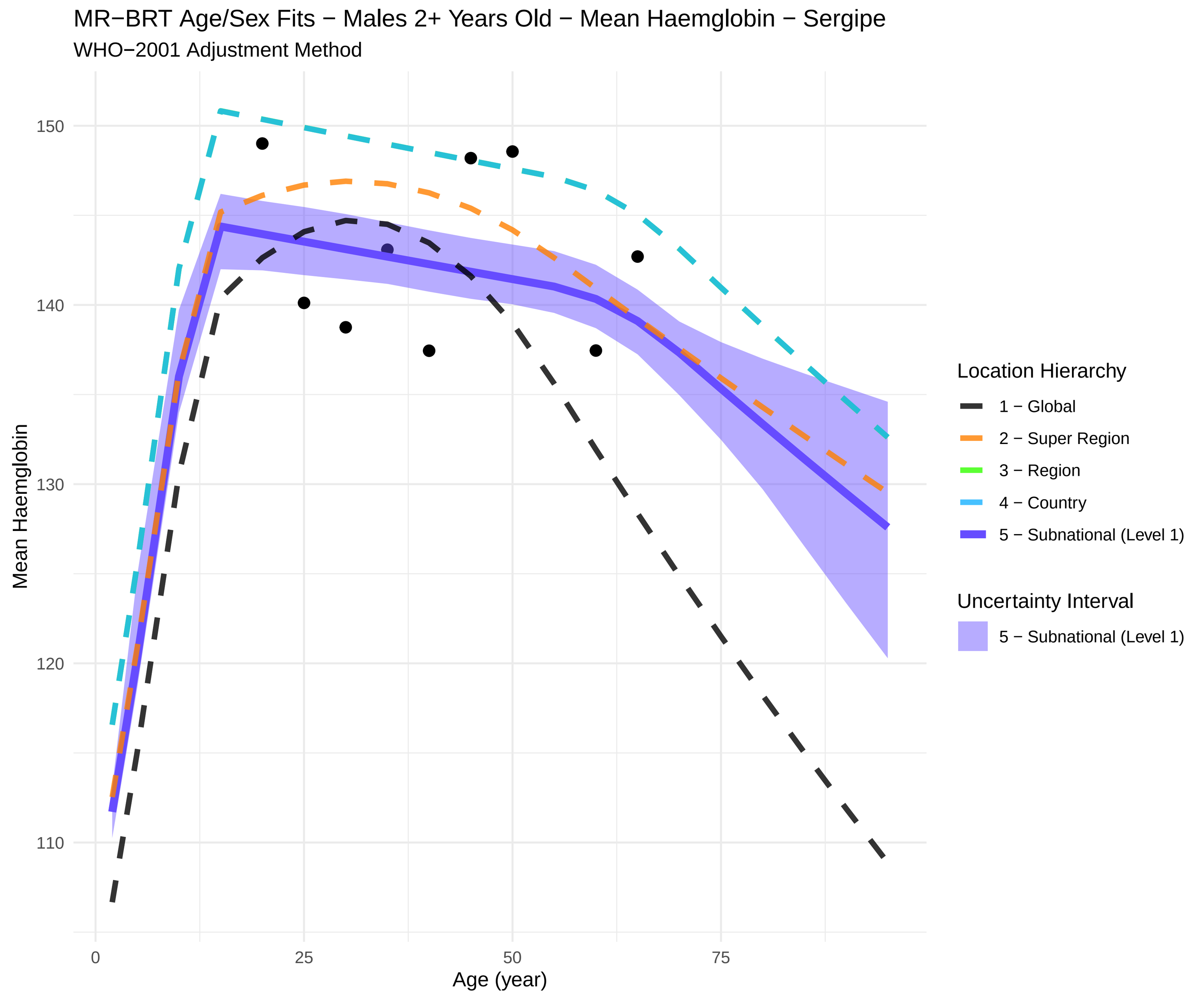
MR−BRT Age/Sex Fits − Males 2+ Years Old − Mean Haemglobin − Sergipe
WHO−2001 Adjustment Method
Mean Haemglobin
Age (year)
Location Hierarchy
1 − Global
2 − Super Region
3 − Region
4 − Country
5 − Subnational (Level 1)
Uncertainty Interval
5 − Subnational (Level 1)

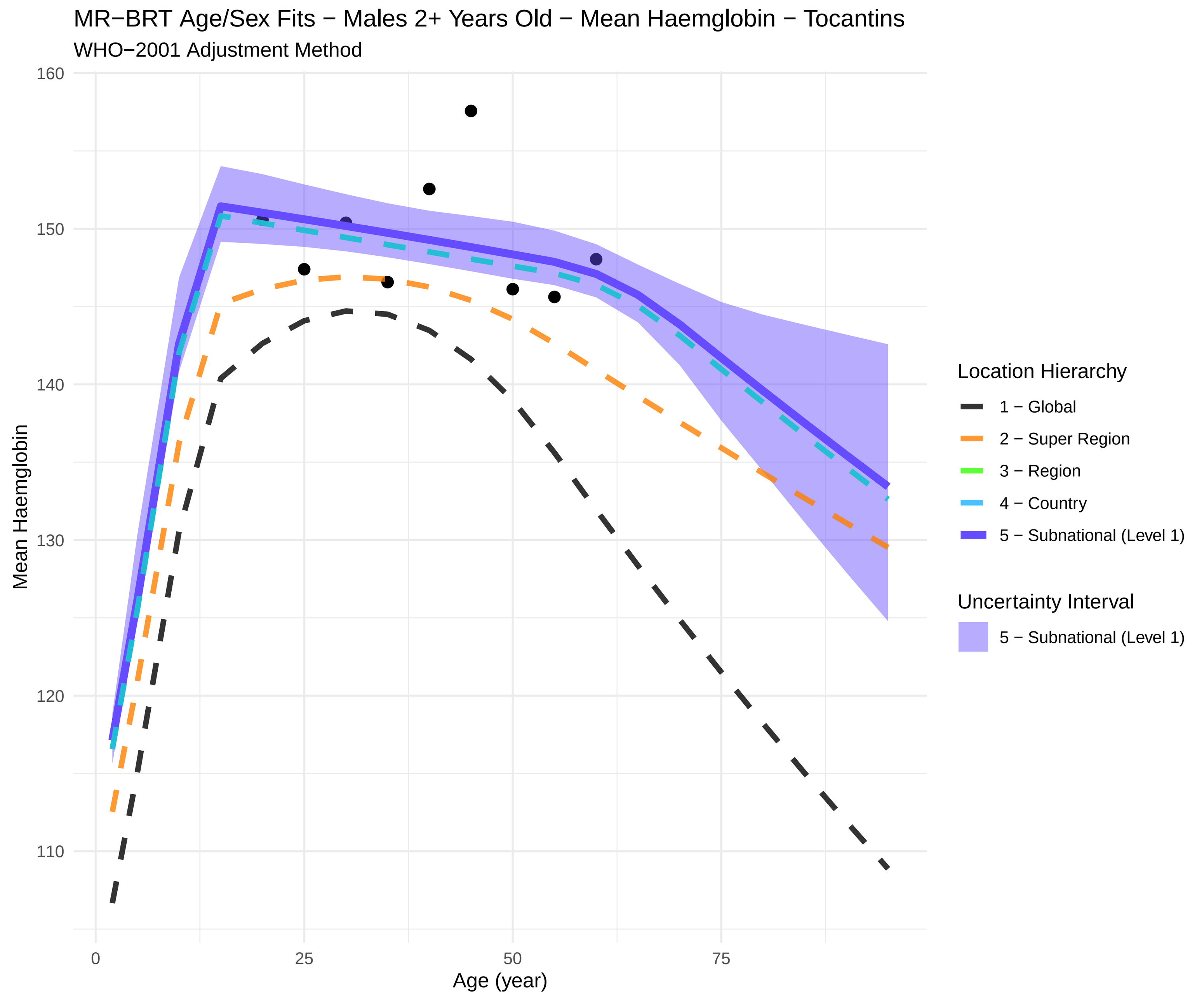
MR−BRT Age/Sex Fits − Males 2+ Years Old − Mean Haemglobin − Tocantins
WHO−2001 Adjustment Method
Mean Haemglobin
Age (year)
Location Hierarchy
1 − Global
2 − Super Region
3 − Region
4 − Country
5 − Subnational (Level 1)
Uncertainty Interval
5 − Subnational (Level 1)

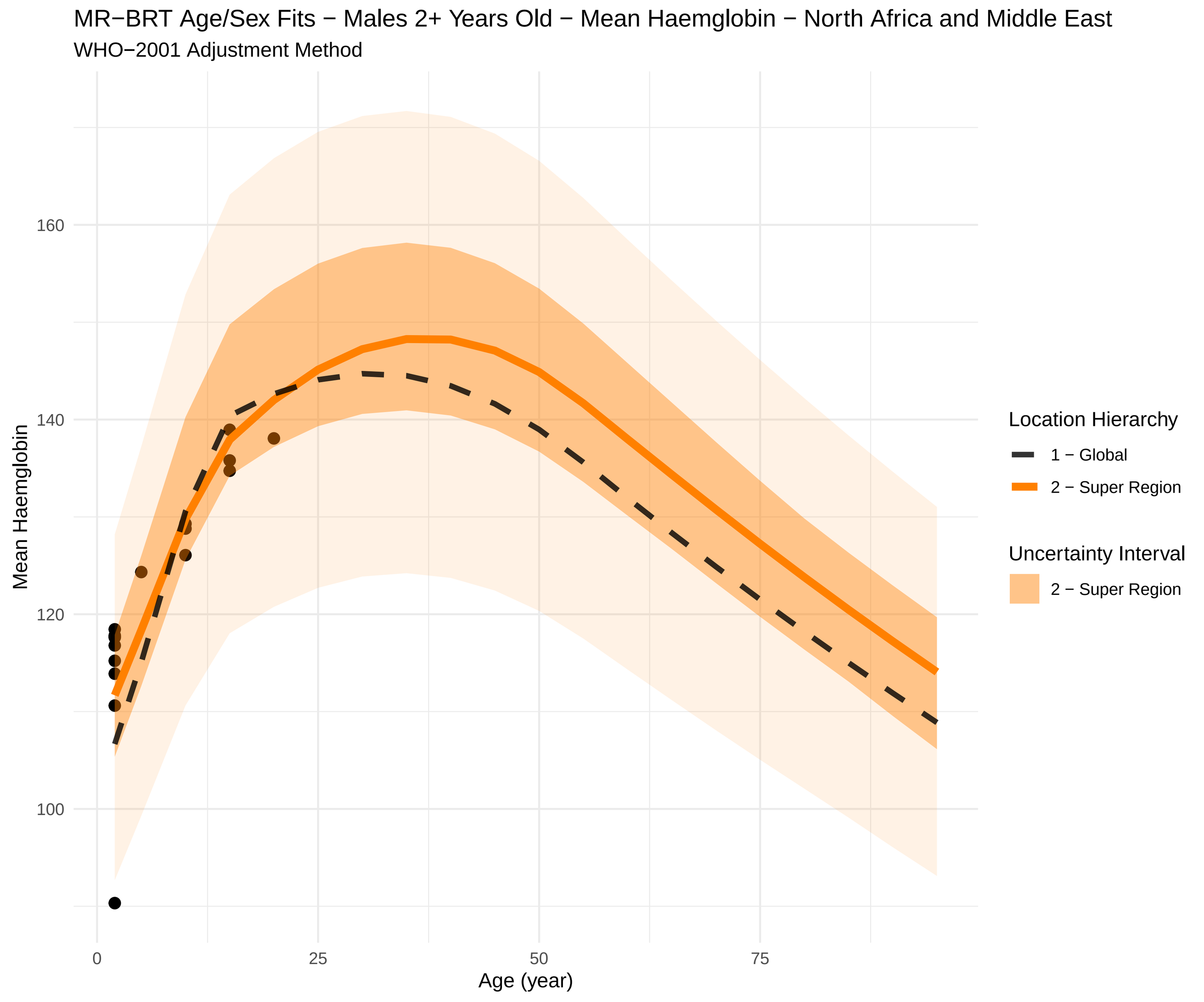
MR−BRT Age/Sex Fits − Males 2+ Years Old − Mean Haemglobin − North Africa and Middle East
WHO−2001 Adjustment Method
Mean Haemglobin
Age (year)
Location Hierarchy
1 − Global
2 − Super Region
Uncertainty Interval
2 − Super Region

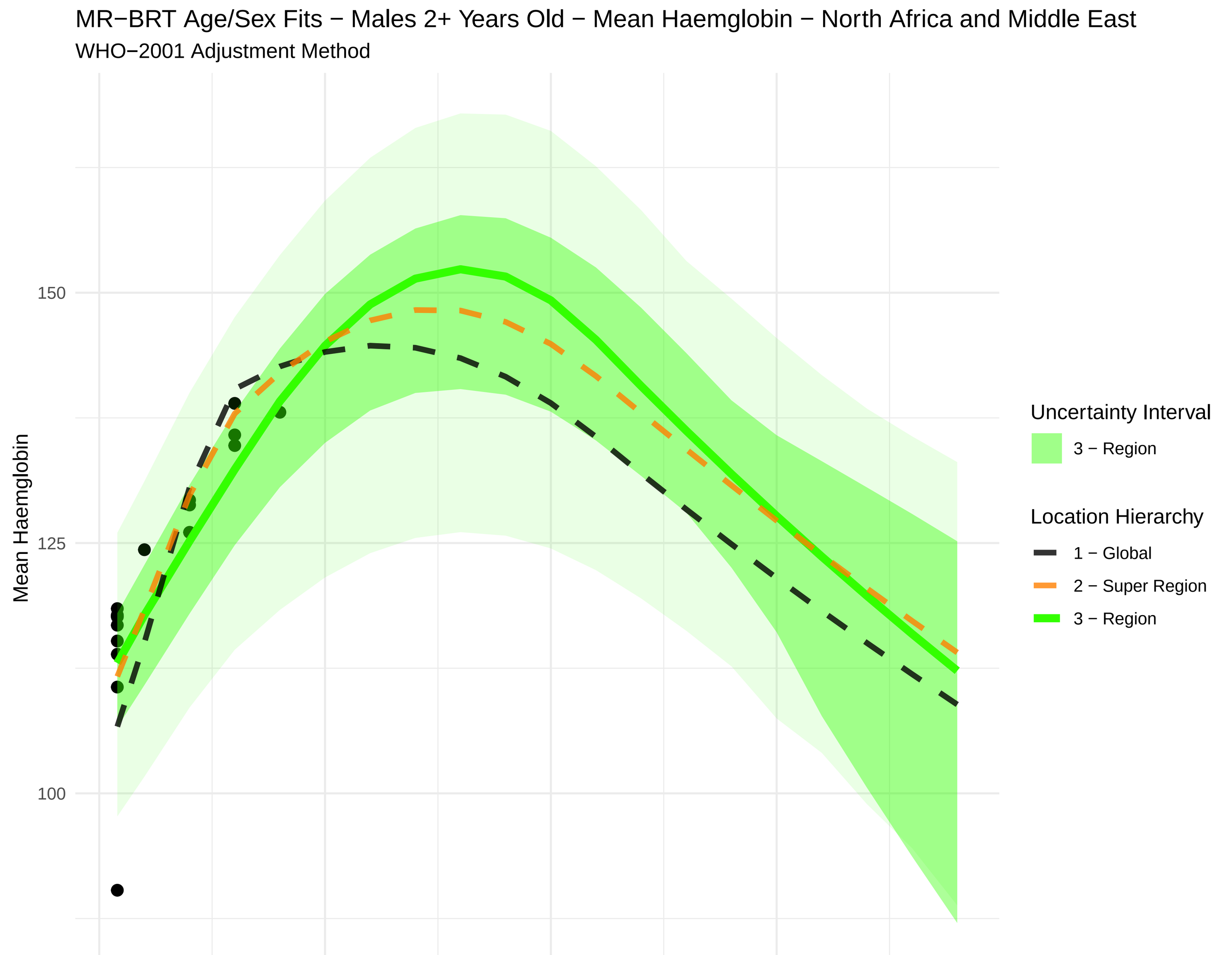
MR−BRT Age/Sex Fits − Males 2+ Years Old − Mean Haemglobin − North Africa and Middle East
WHO−2001 Adjustment Method
Mean Haemglobin
Age (year)
Uncertainty Interval
3 − Region
Location Hierarchy
1 − Global
2 − Super Region
3 − Region

MR−BRT Age/Sex Fits − Males 2+ Years Old − Mean Haemglobin − Egypt
WHO−2001 Adjustment Method

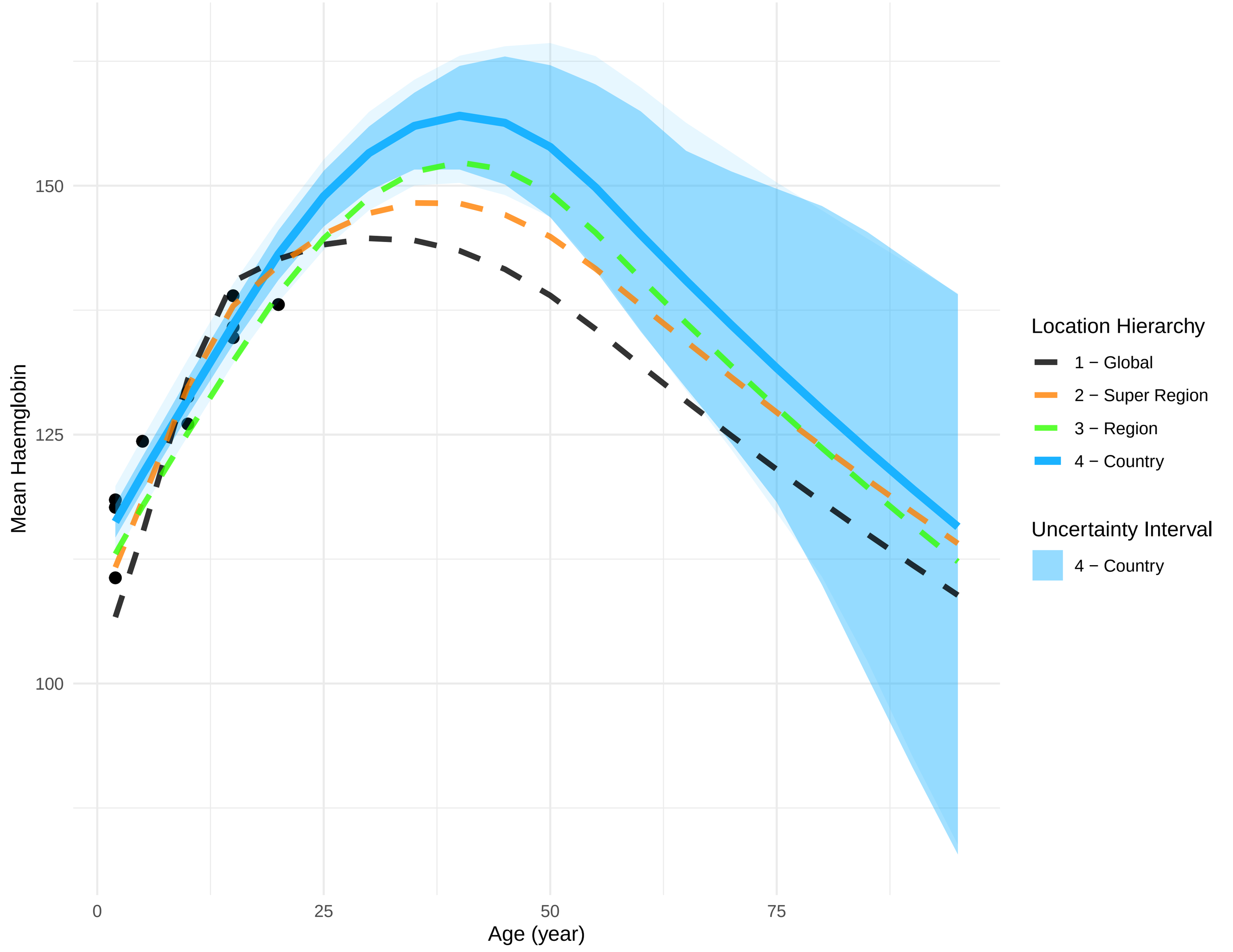

MR−BRT Age/Sex Fits − Males 2+ Years Old − Mean Haemglobin − Jordan
WHO−2001 Adjustment Method

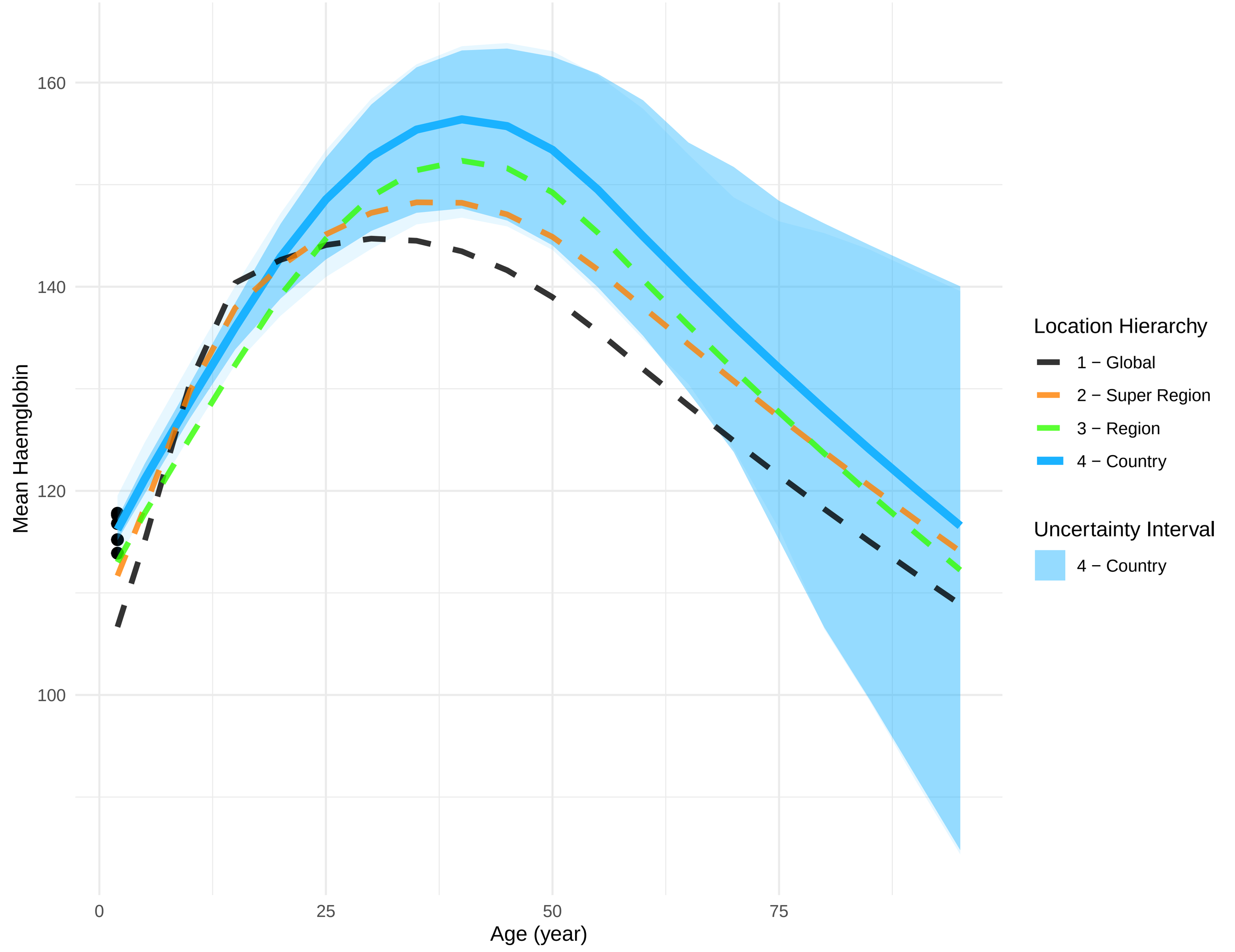

MR−BRT Age/Sex Fits − Males 2+ Years Old − Mean Haemglobin − Yemen
WHO−2001 Adjustment Method

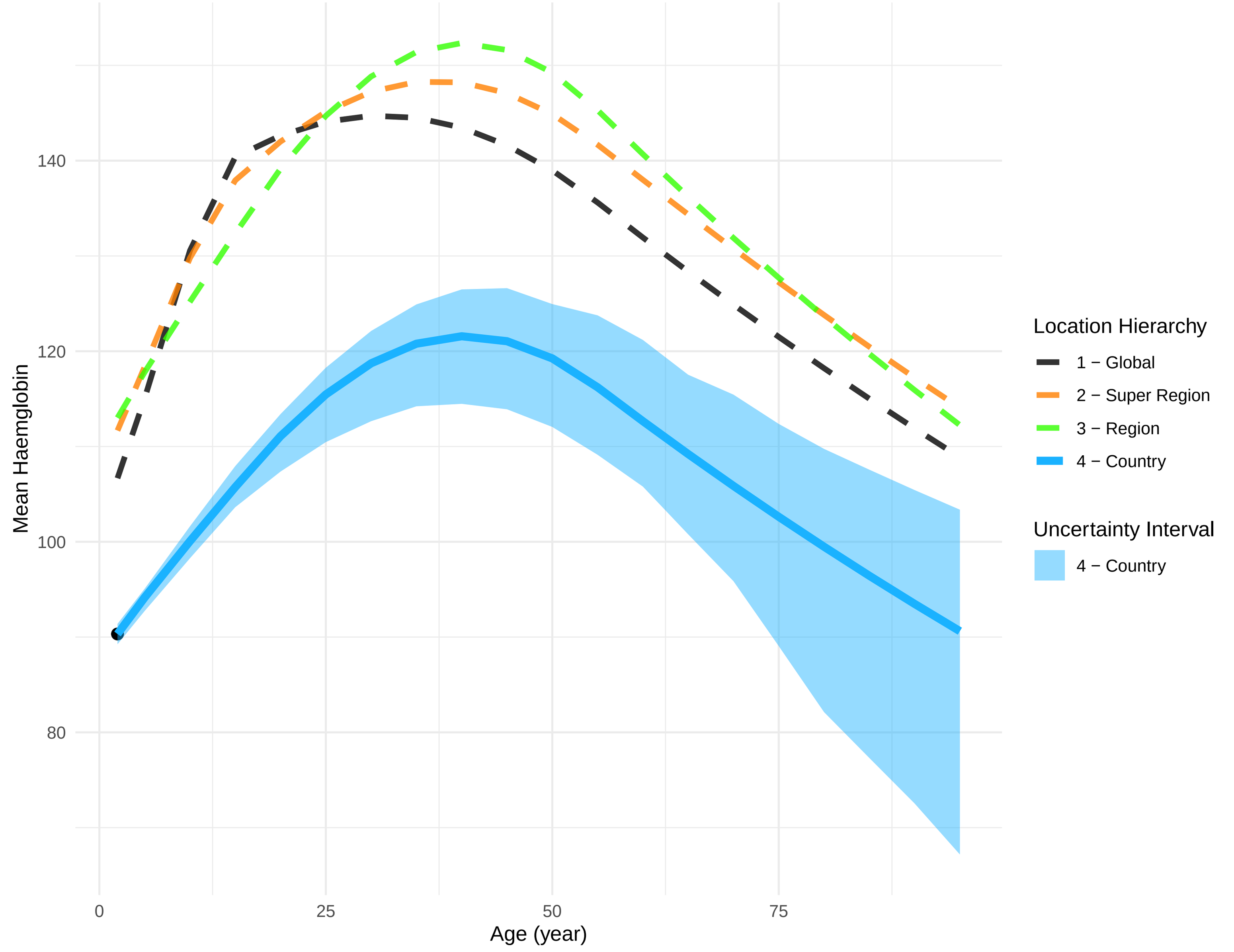

MR−BRT Age/Sex Fits − Males 2+ Years Old − Mean Haemglobin − South Asia
WHO−2001 Adjustment Method

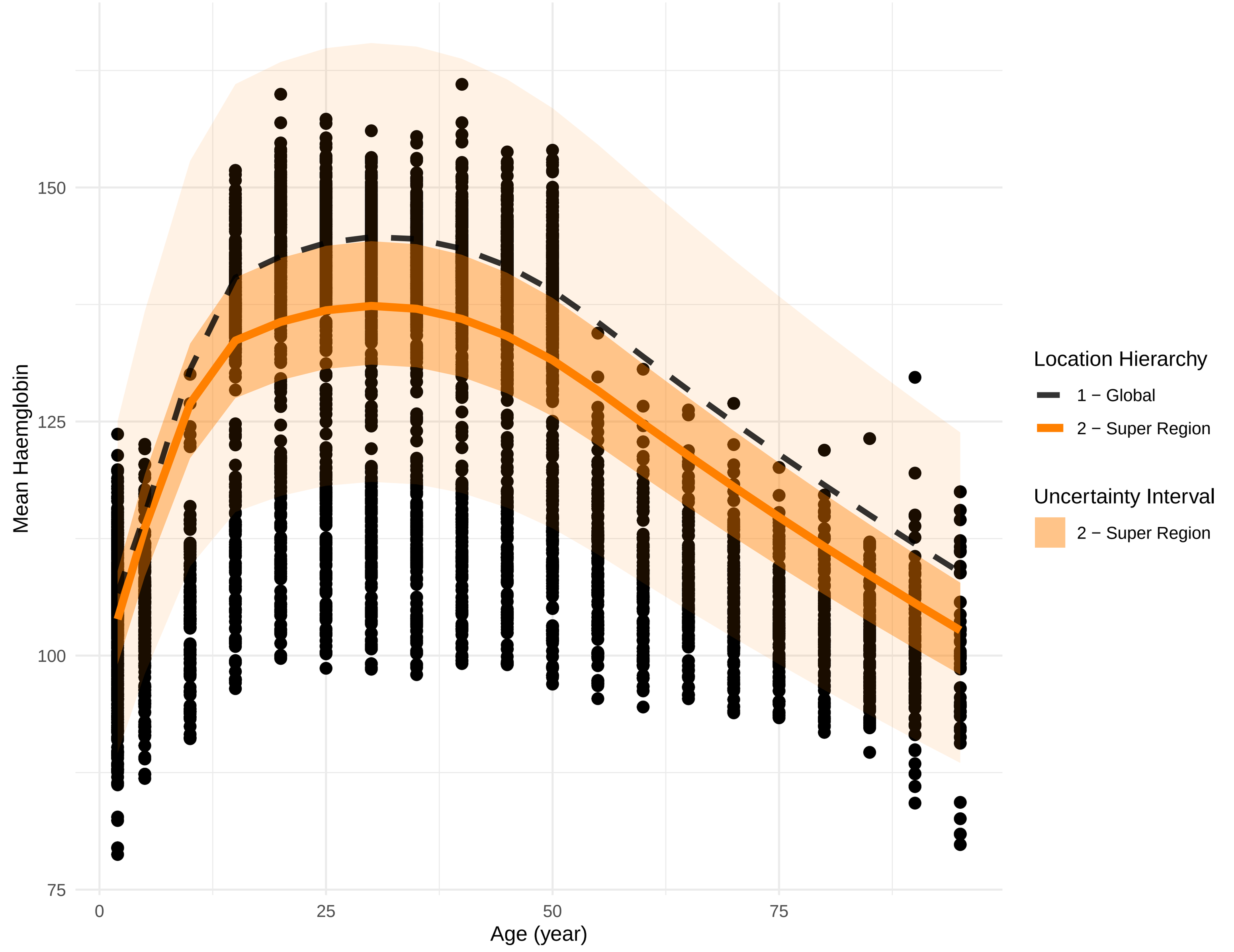

MR−BRT Age/Sex Fits − Males 2+ Years Old − Mean Haemglobin − South Asia
WHO−2001 Adjustment Method

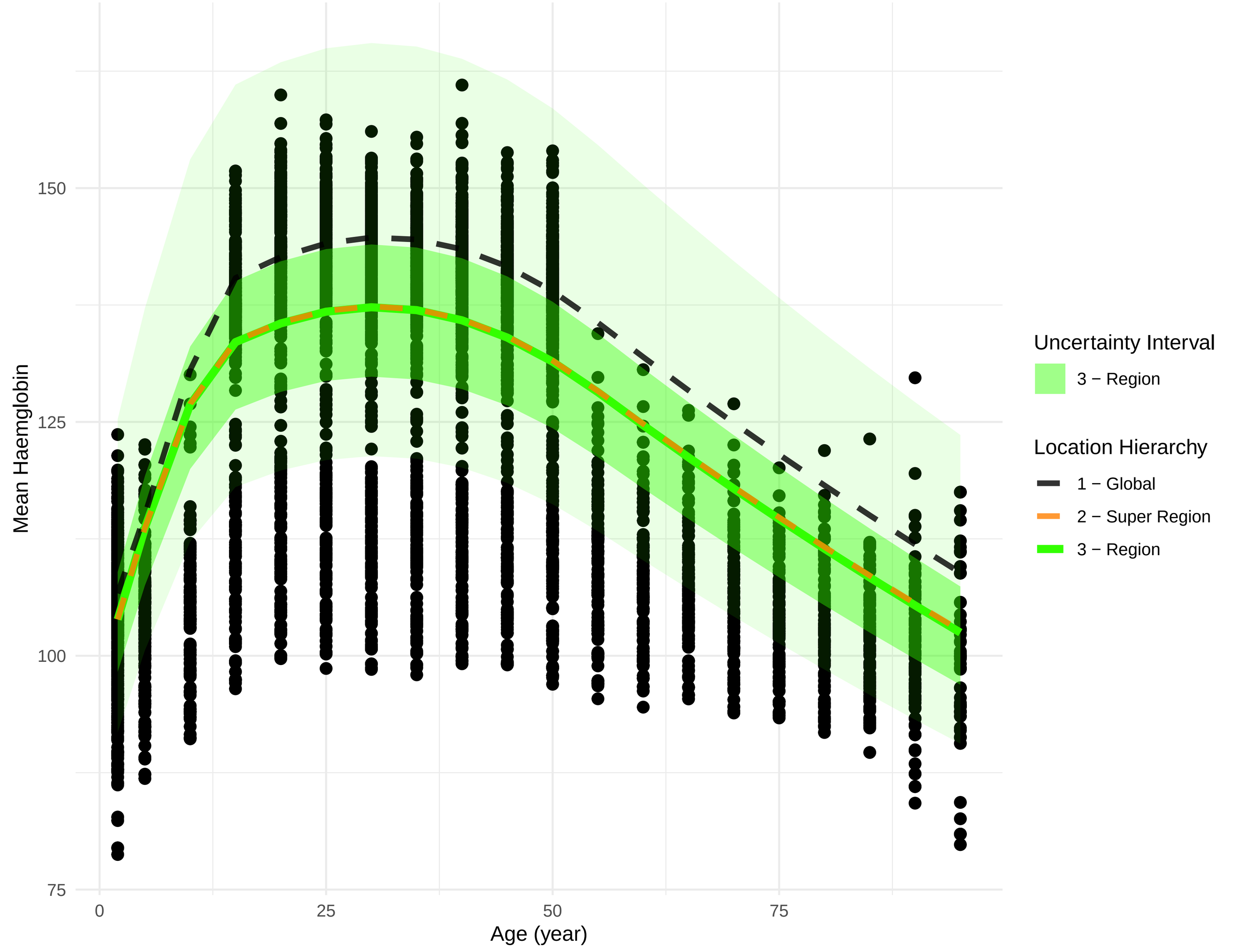

MR−BRT Age/Sex Fits − Males 2+ Years Old − Mean Haemglobin − Bangladesh
WHO−2001 Adjustment Method

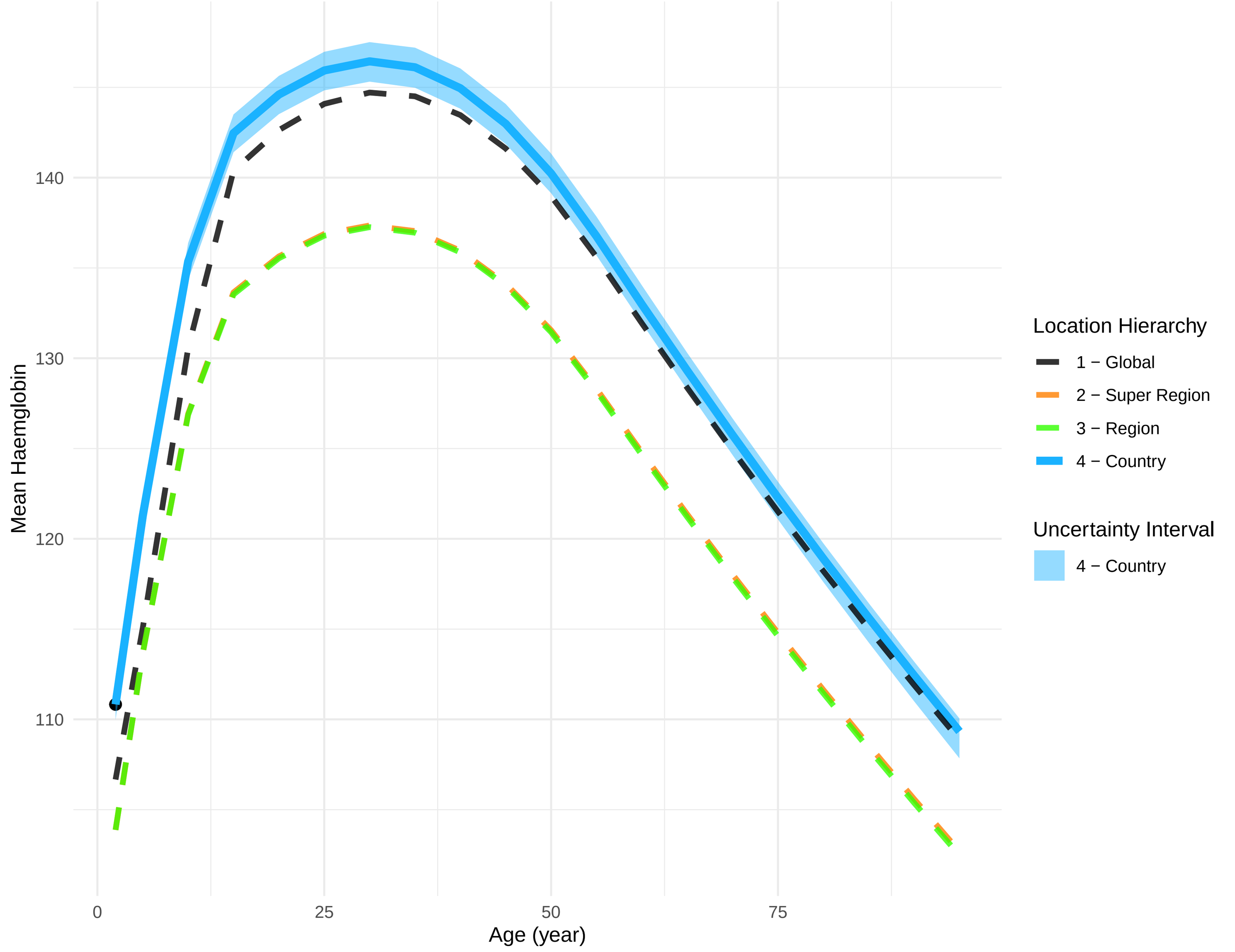

MR−BRT Age/Sex Fits − Males 2+ Years Old − Mean Haemglobin − India
WHO−2001 Adjustment Method

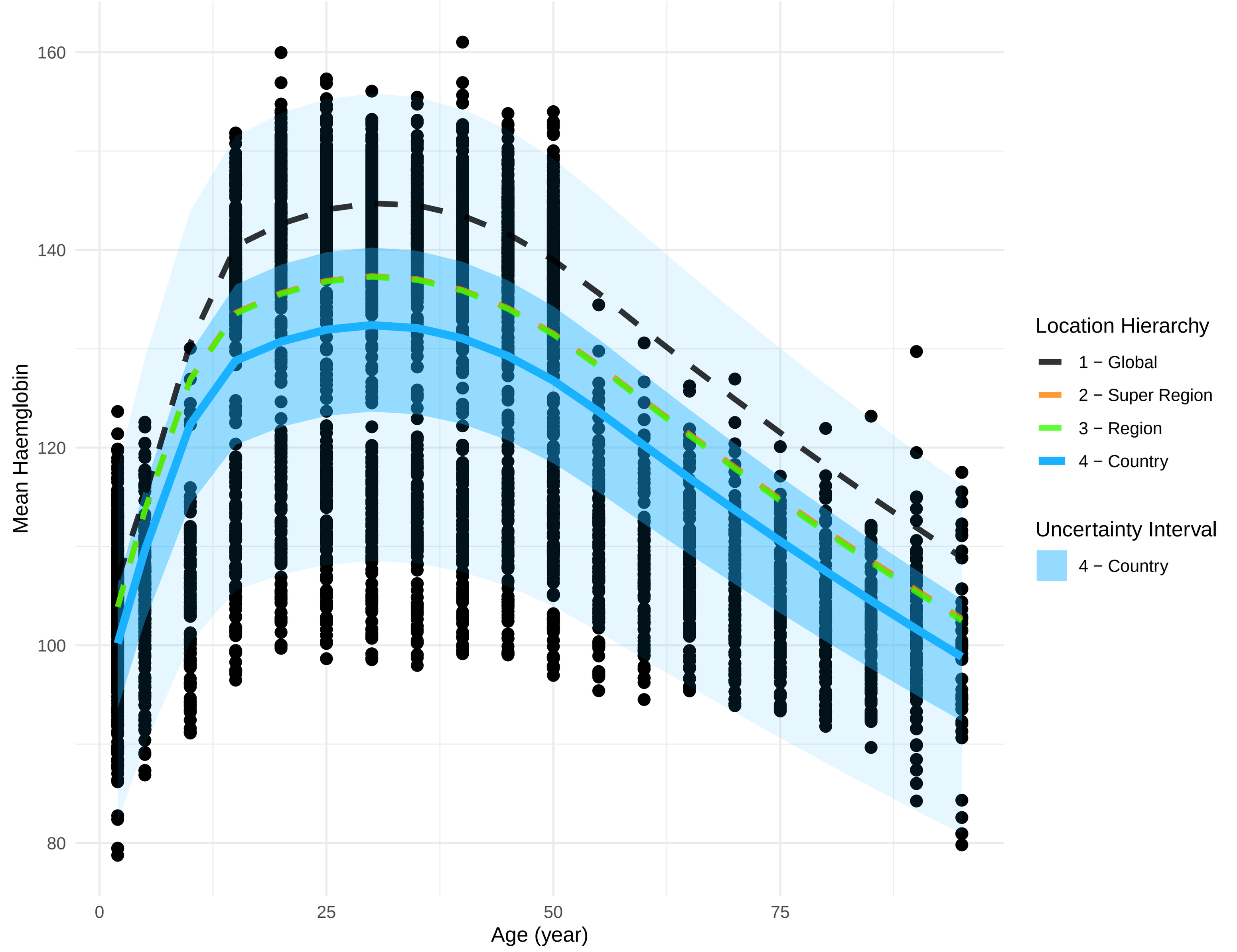

MR−BRT Age/Sex Fits − Males 2+ Years Old − Mean Haemglobin − Andhra Pradesh
WHO−2001 Adjustment Method

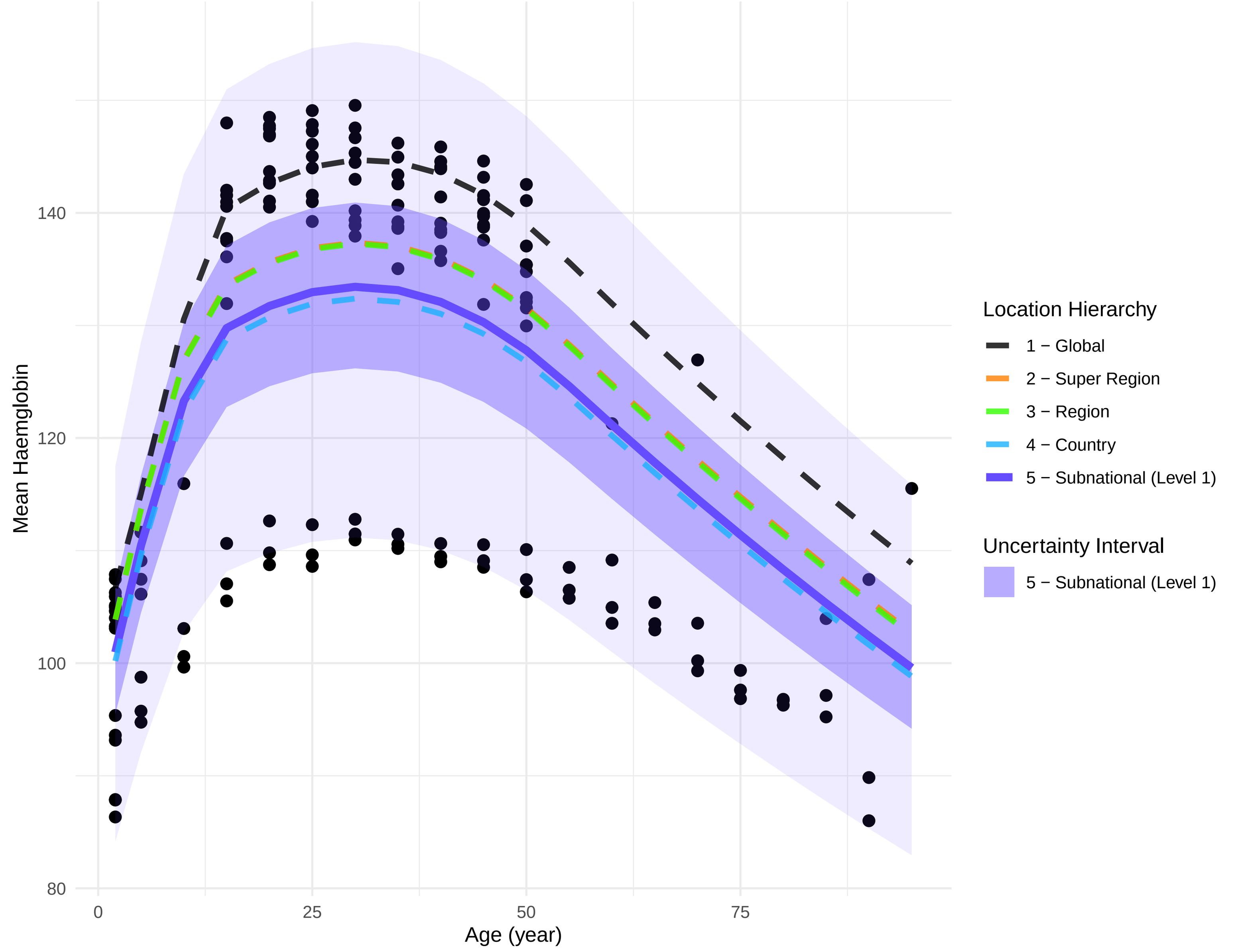

MR−BRT Age/Sex Fits − Males 2+ Years Old − Mean Haemglobin − Andhra Pradesh, Rural
WHO−2001 Adjustment Method

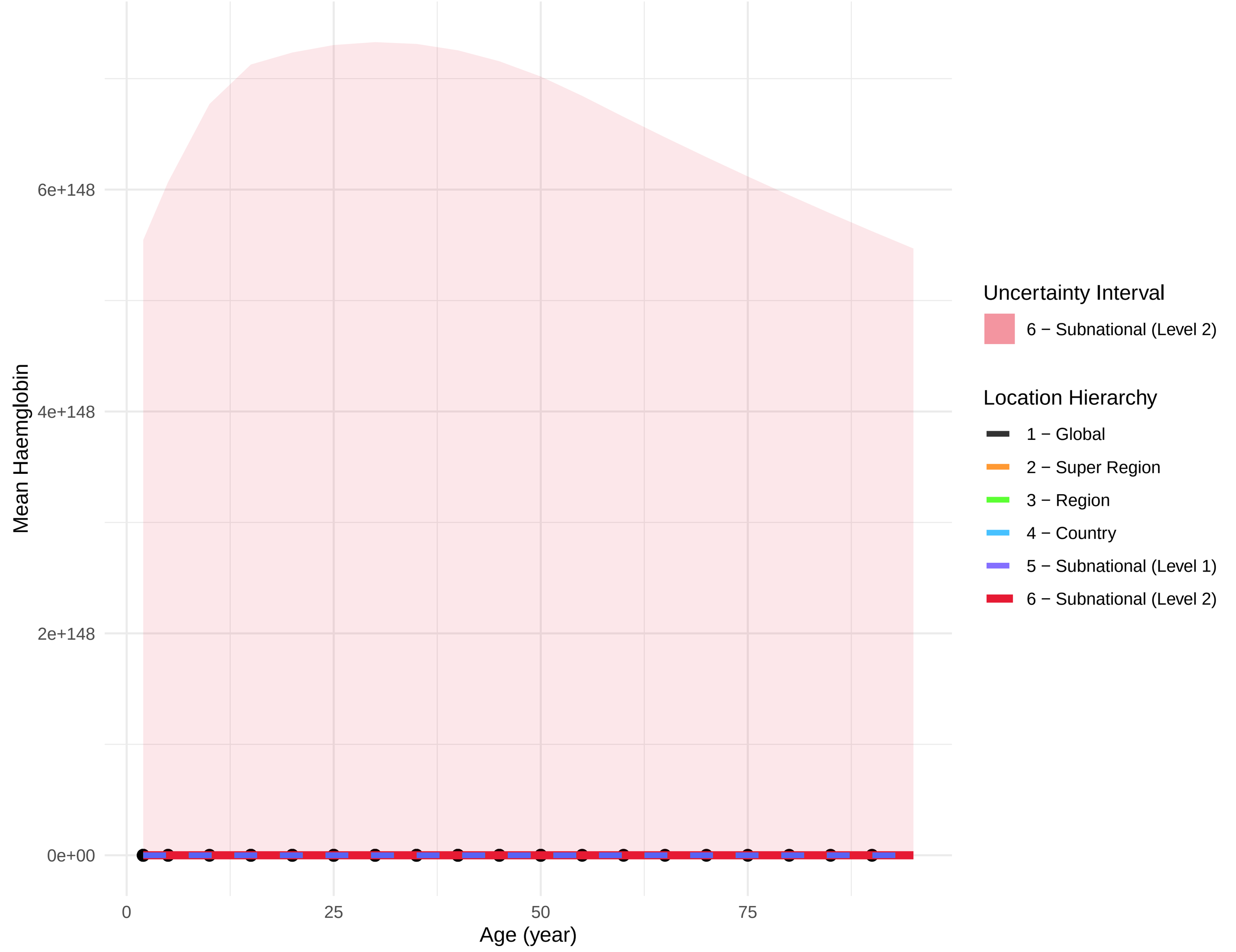

MR−BRT Age/Sex Fits − Males 2+ Years Old − Mean Haemglobin − Andhra Pradesh, Urban
WHO−2001 Adjustment Method

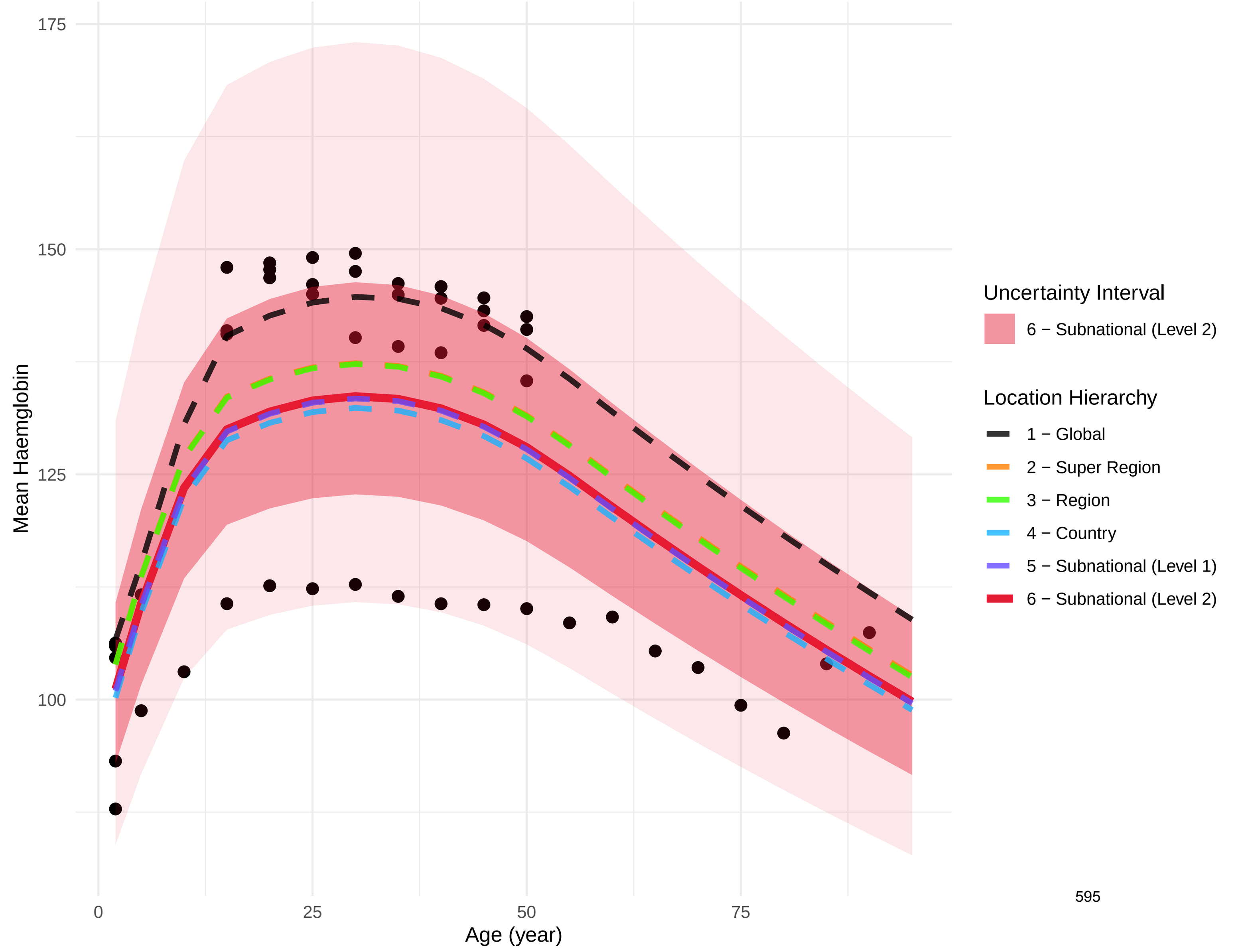

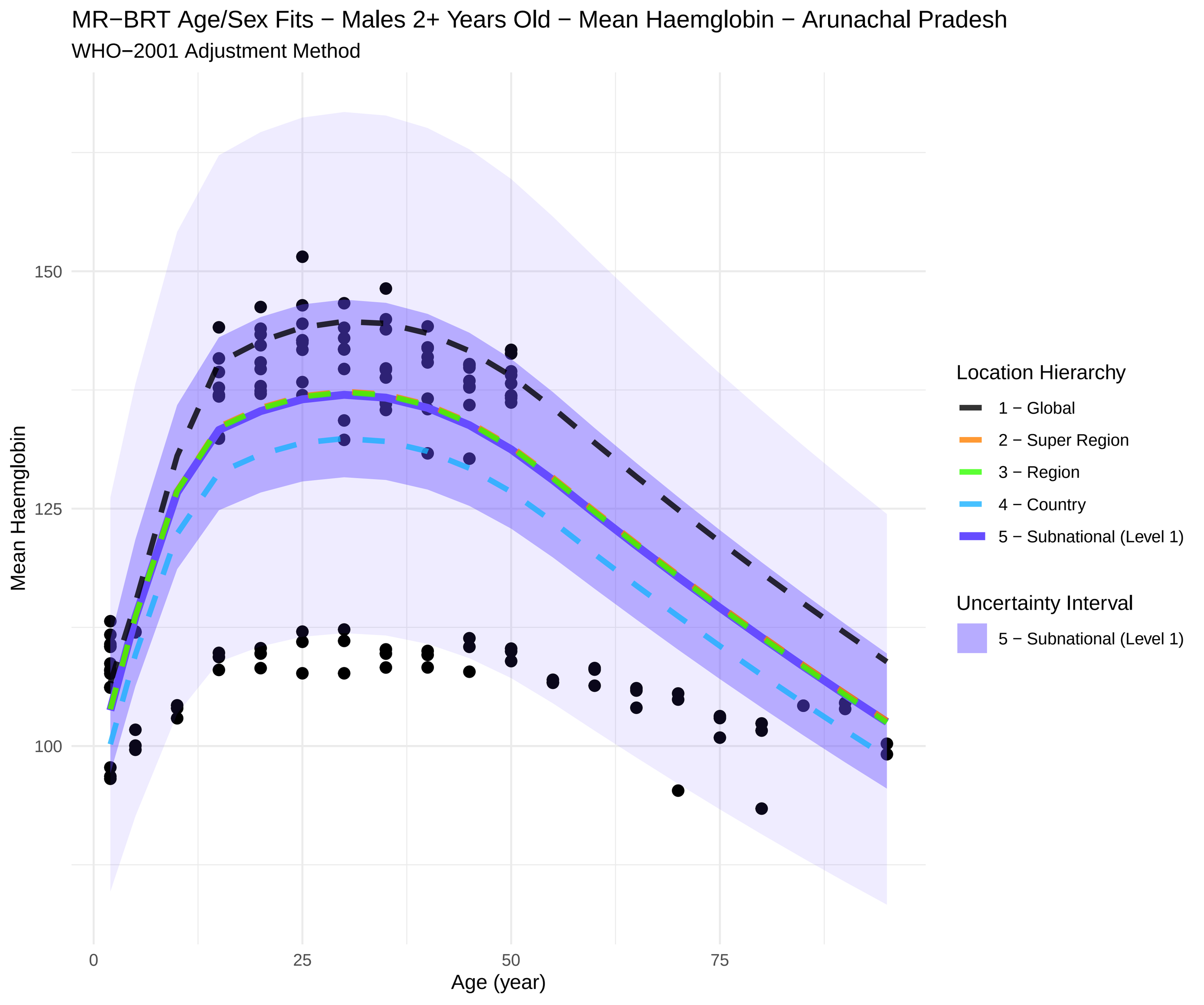
MR−BRT Age/Sex Fits − Males 2+ Years Old − Mean Haemoglobin − Arunachal Pradesh
WHO−2001 Adjustment Method
Mean Haemoglobin
100
125
150
Age (year)
0
25
50
75
Location Hierarchy
1 − Global
2 − Super Region
3 − Region
4 − Country
5 − Subnational (Level 1)
Uncertainty Interval
5 − Subnational (Level 1)

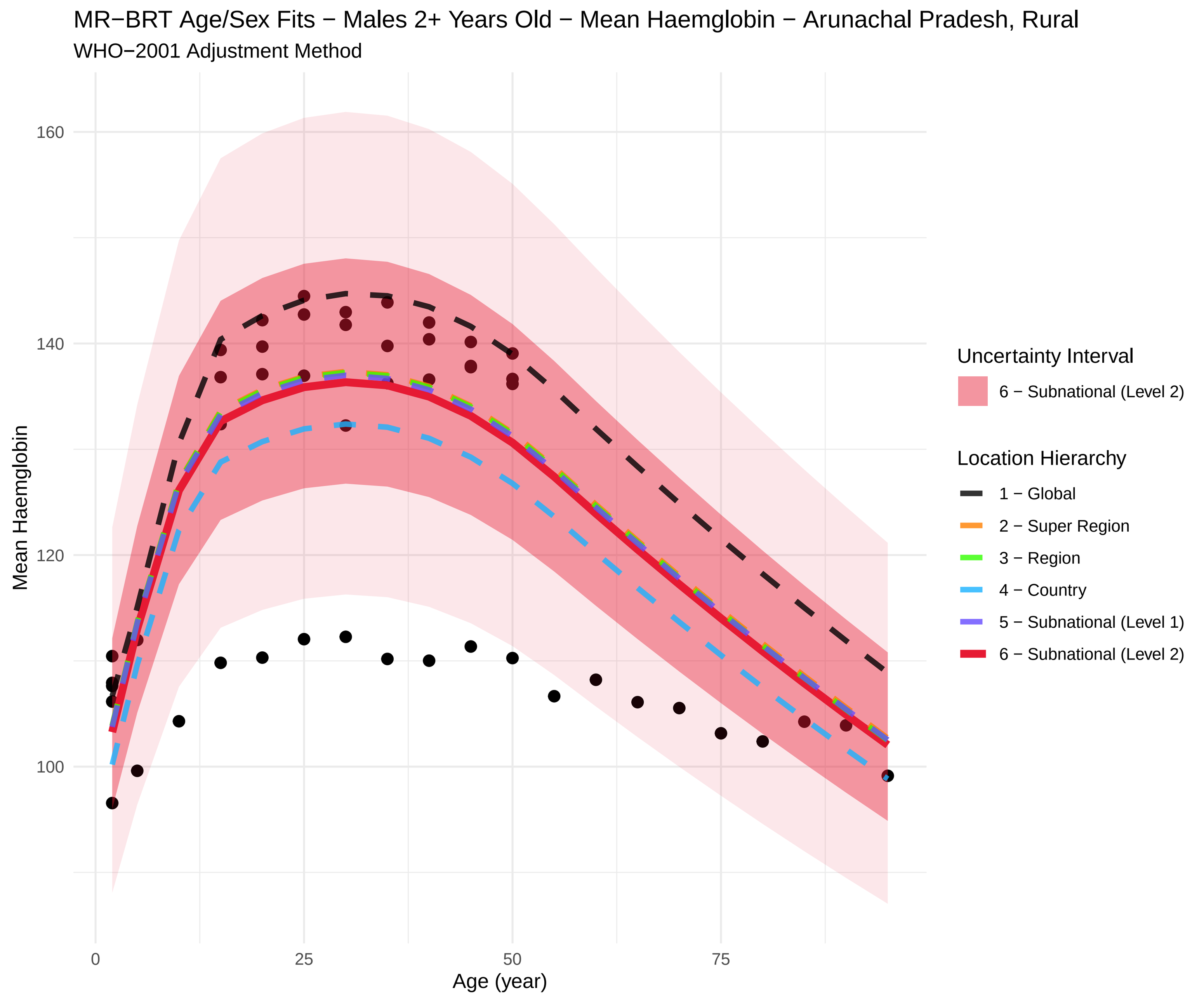
MR−BRT Age/Sex Fits − Males 2+ Years Old − Mean Haemoglobin − Arunachal Pradesh, Rural
WHO−2001 Adjustment Method
Mean Haemoglobin
100
120
140
160
Age (year)
0
25
50
75
Uncertainty Interval
6 − Subnational (Level 2)
Location Hierarchy
1 − Global
2 − Super Region
3 − Region
4 − Country
5 − Subnational (Level 1)
6 − Subnational (Level 2)

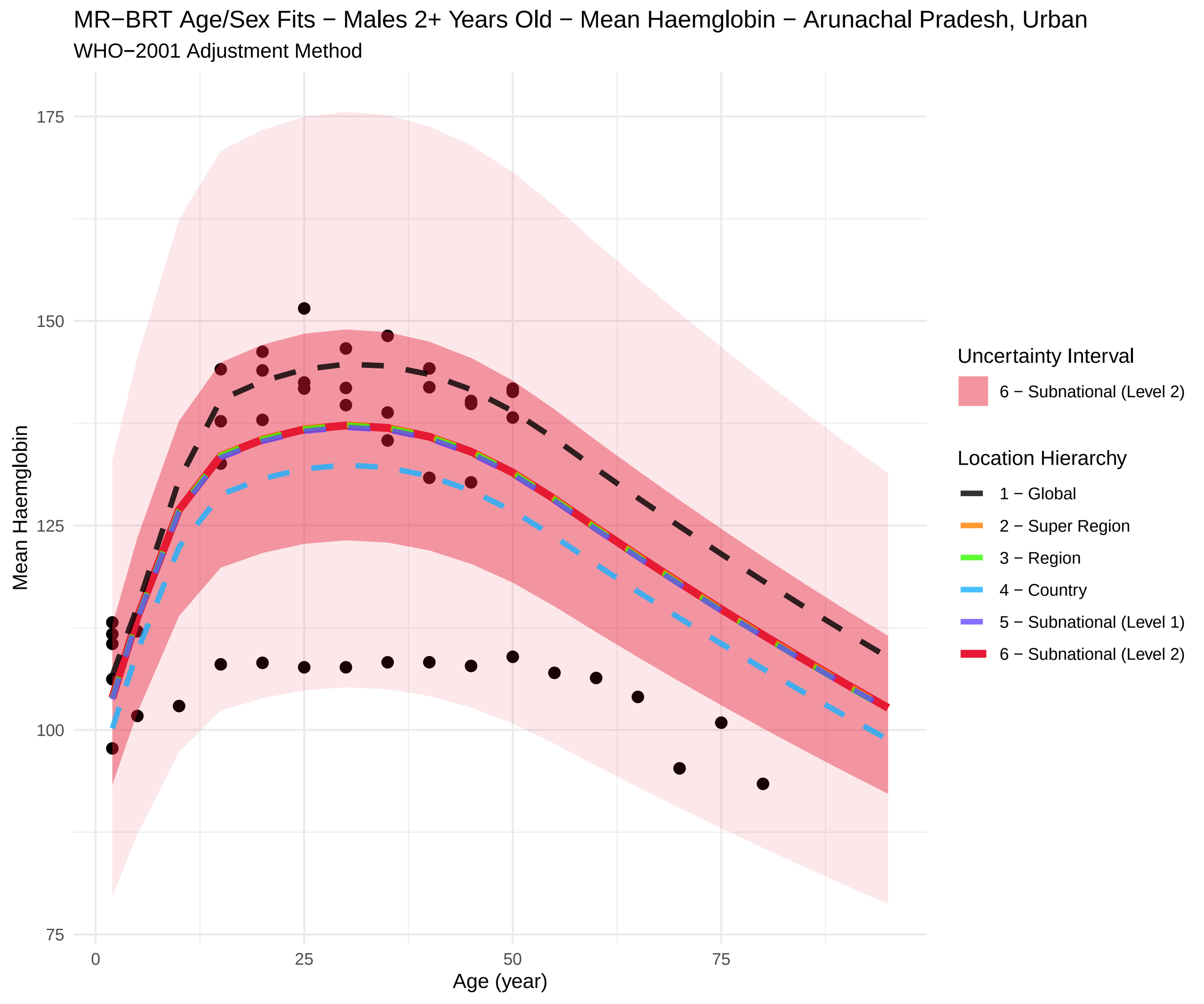
MR−BRT Age/Sex Fits − Males 2+ Years Old − Mean Haemoglobin − Arunachal Pradesh, Urban
WHO−2001 Adjustment Method
Mean Haemoglobin
75
100
125
150
175
Age (year)
0
25
50
75
Uncertainty Interval
6 − Subnational (Level 2)
Location Hierarchy
1 − Global
2 − Super Region
3 − Region
4 − Country
5 − Subnational (Level 1)
6 − Subnational (Level 2)

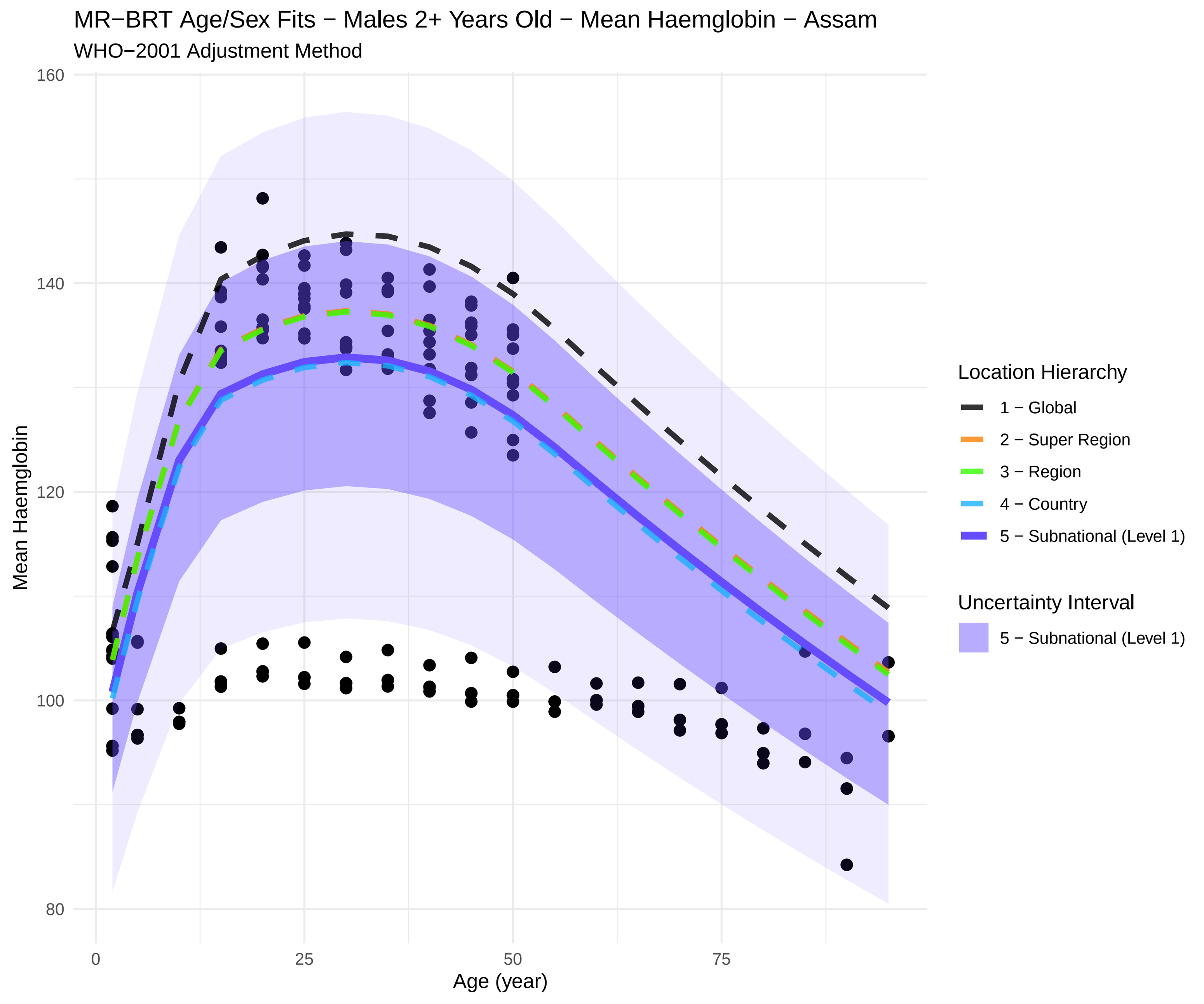
MR−BRT Age/Sex Fits − Males 2+ Years Old − Mean Haemoglobin − Assam
WHO−2001 Adjustment Method
Mean Haemoglobin
80
100
120
140
160
Age (year)
0
25
50
75
Location Hierarchy
1 − Global
2 − Super Region
3 − Region
4 − Country
5 − Subnational (Level 1)
Uncertainty Interval
5 − Subnational (Level 1)

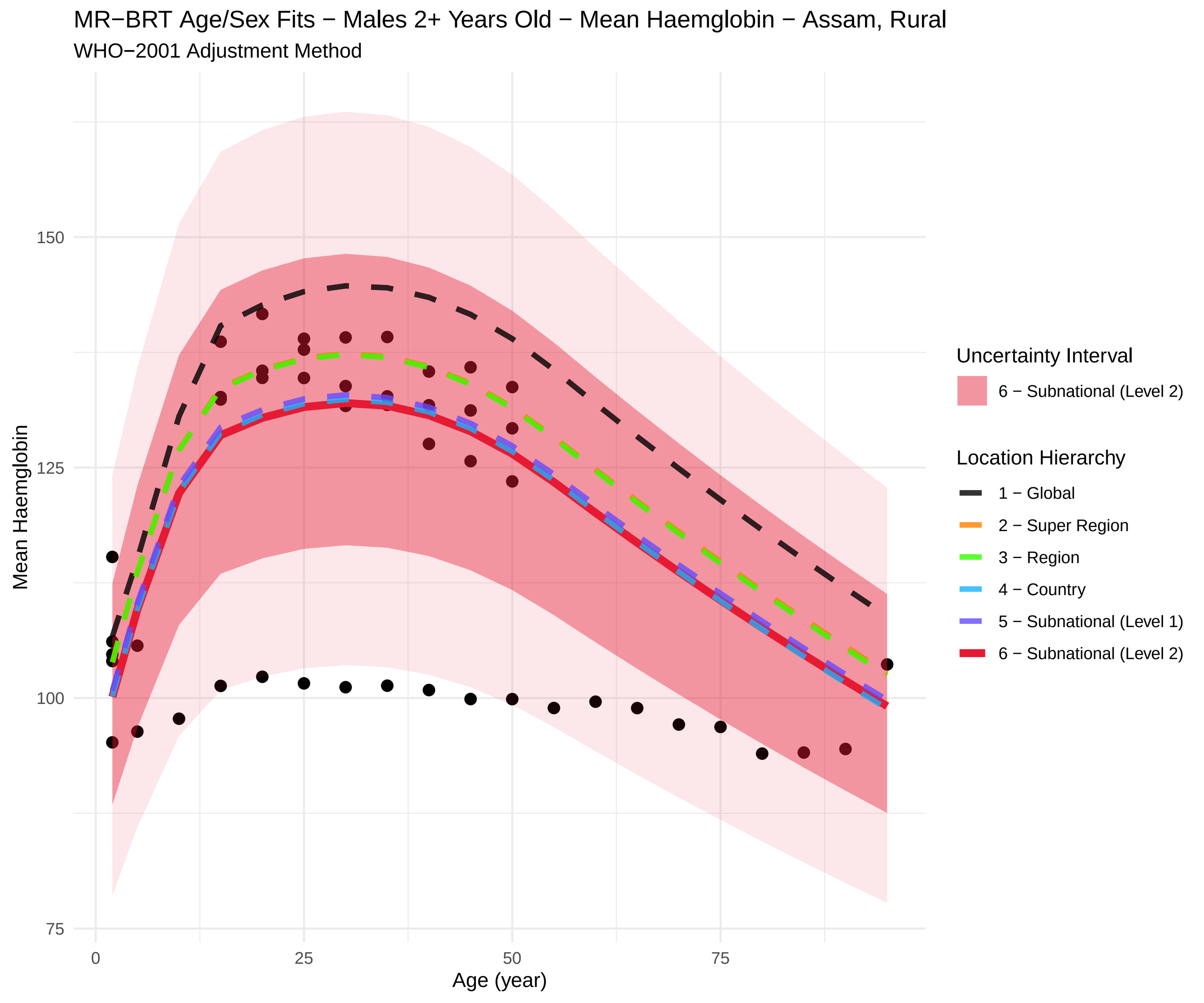
MR−BRT Age/Sex Fits − Males 2+ Years Old − Mean Haemoglobin − Assam, Rural
WHO−2001 Adjustment Method
Mean Haemoglobin
75
100
125
150
Age (year)
0
25
50
75
Uncertainty Interval
6 − Subnational (Level 2)
Location Hierarchy
1 − Global
2 − Super Region
3 − Region
4 − Country
5 − Subnational (Level 1)
6 − Subnational (Level 2)

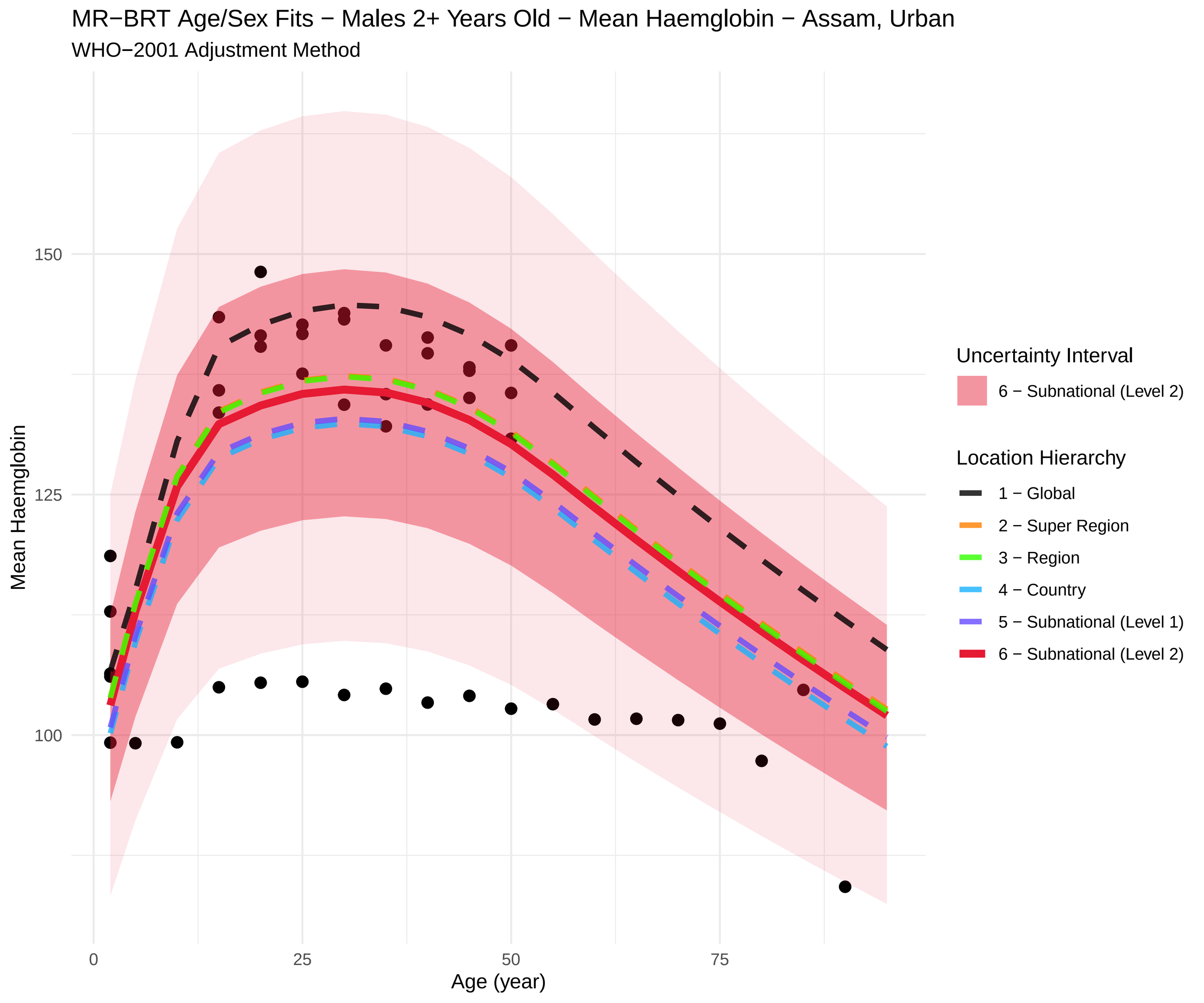
MR−BRT Age/Sex Fits − Males 2+ Years Old − Mean Haemoglobin − Assam, Urban
WHO−2001 Adjustment Method
Mean Haemoglobin
100
125
150
Age (year)
0
25
50
75
Uncertainty Interval
6 − Subnational (Level 2)
Location Hierarchy
1 − Global
2 − Super Region
3 − Region
4 − Country
5 − Subnational (Level 1)
6 − Subnational (Level 2)

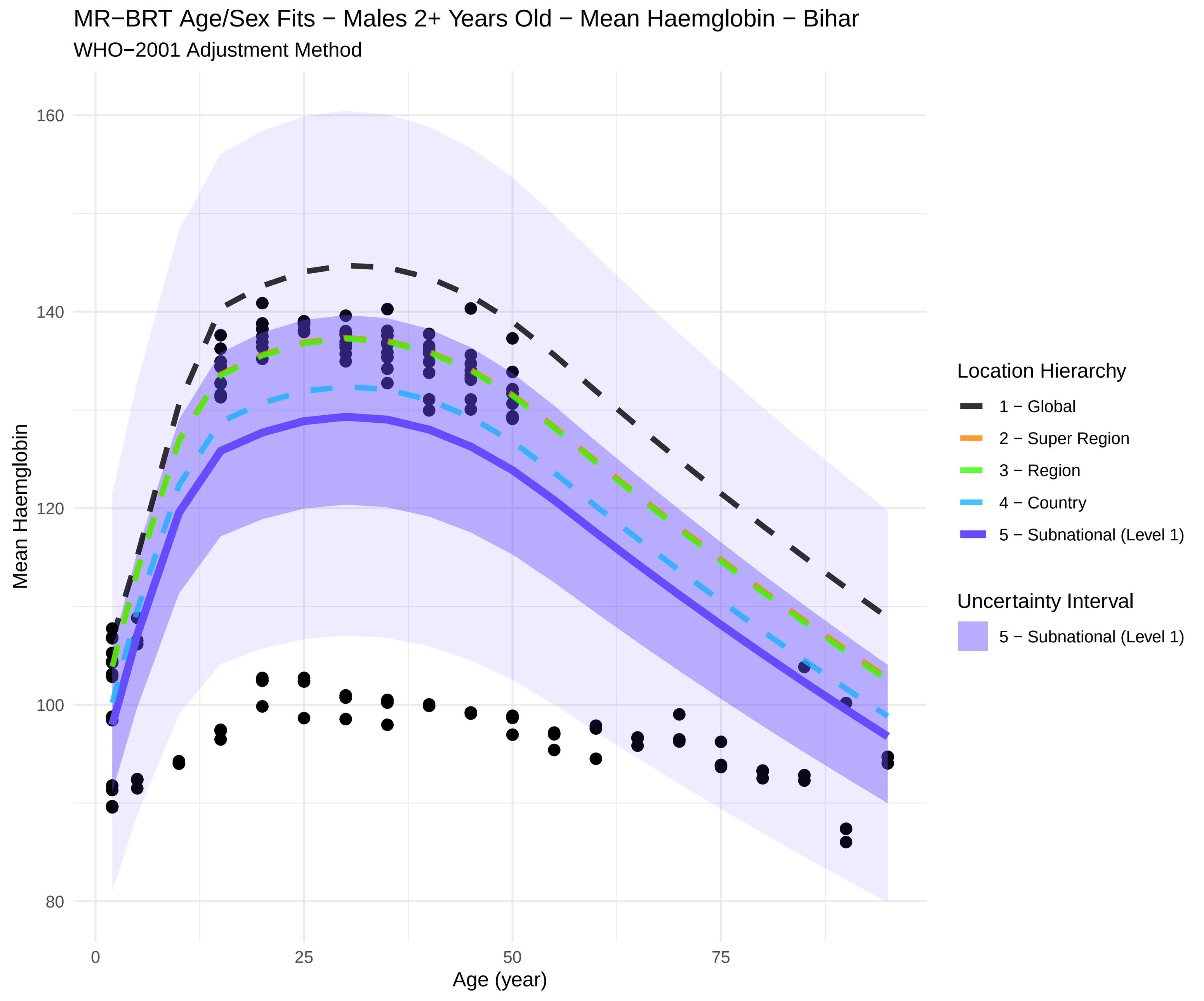
MR−BRT Age/Sex Fits − Males 2+ Years Old − Mean Haemoglobin − Bihar
WHO−2001 Adjustment Method
Mean Haemoglobin
80
100
120
140
160
Age (year)
0
25
50
75
Location Hierarchy
1 − Global
2 − Super Region
3 − Region
4 − Country
5 − Subnational (Level 1)
Uncertainty Interval
5 − Subnational (Level 1)

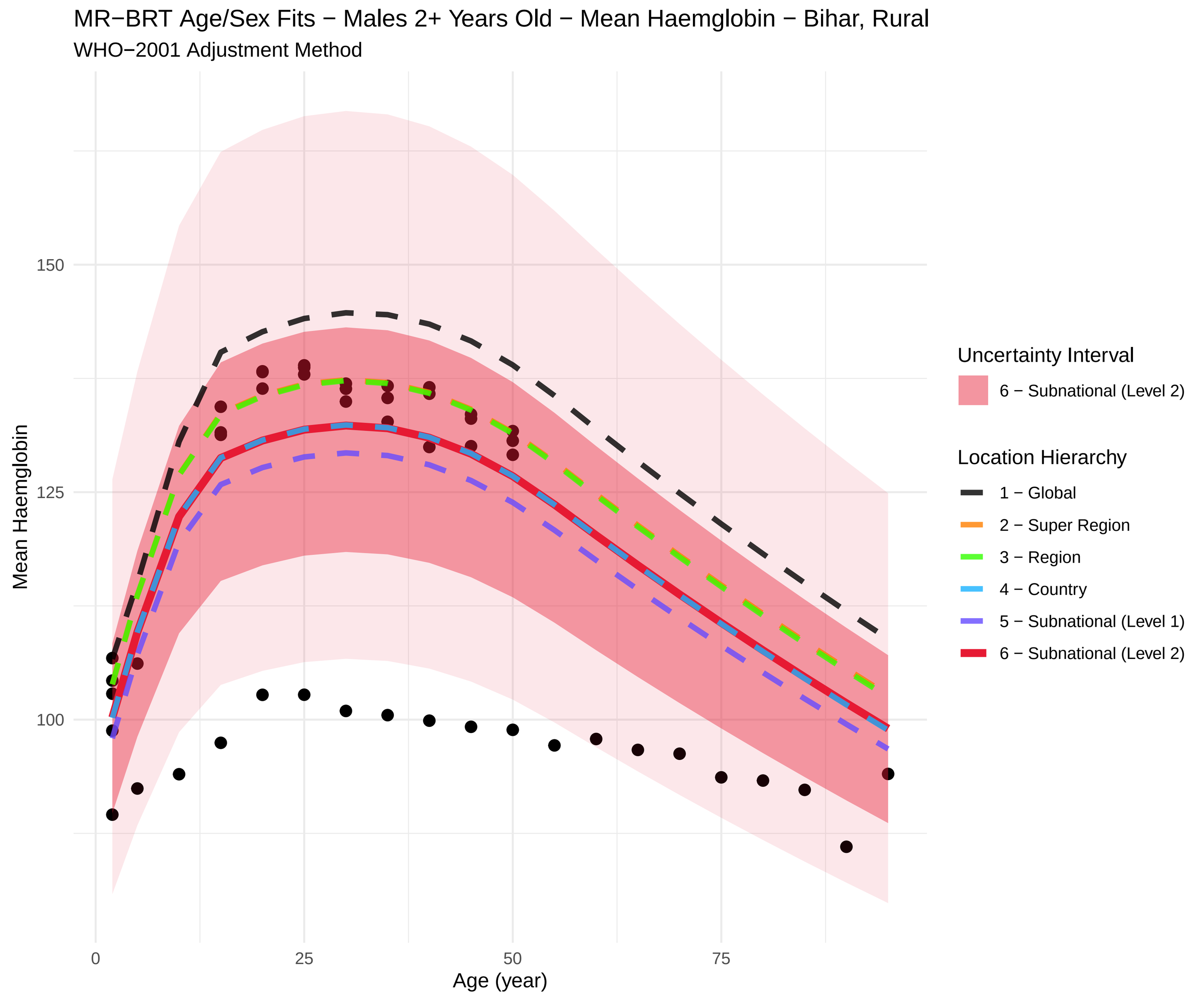
MR−BRT Age/Sex Fits − Males 2+ Years Old − Mean Haemoglobin − Bihar, Rural
WHO−2001 Adjustment Method
Mean Haemoglobin
100
125
150
Age (year)
0
25
50
75
Uncertainty Interval
6 − Subnational (Level 2)
Location Hierarchy
1 − Global
2 − Super Region
3 − Region
4 − Country
5 − Subnational (Level 1)
6 − Subnational (Level 2)

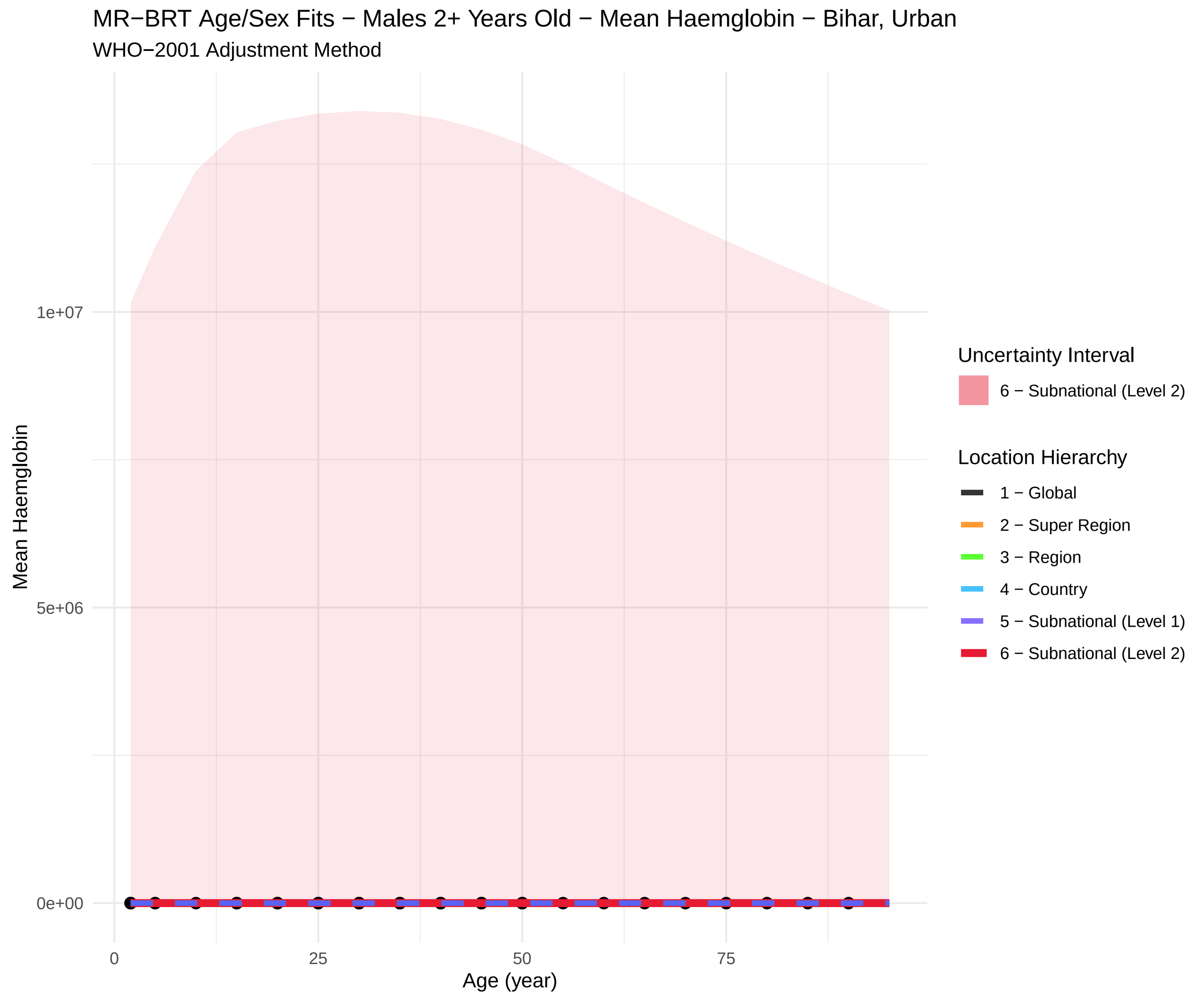
MR−BRT Age/Sex Fits − Males 2+ Years Old − Mean Haemoglobin − Bihar, Urban
WHO−2001 Adjustment Method
Mean Haemoglobin
0e+00
5e+06
1e+07
Age (year)
0
25
50
75
Uncertainty Interval
6 − Subnational (Level 2)
Location Hierarchy
1 − Global
2 − Super Region
3 − Region
4 − Country
5 − Subnational (Level 1)
6 − Subnational (Level 2)

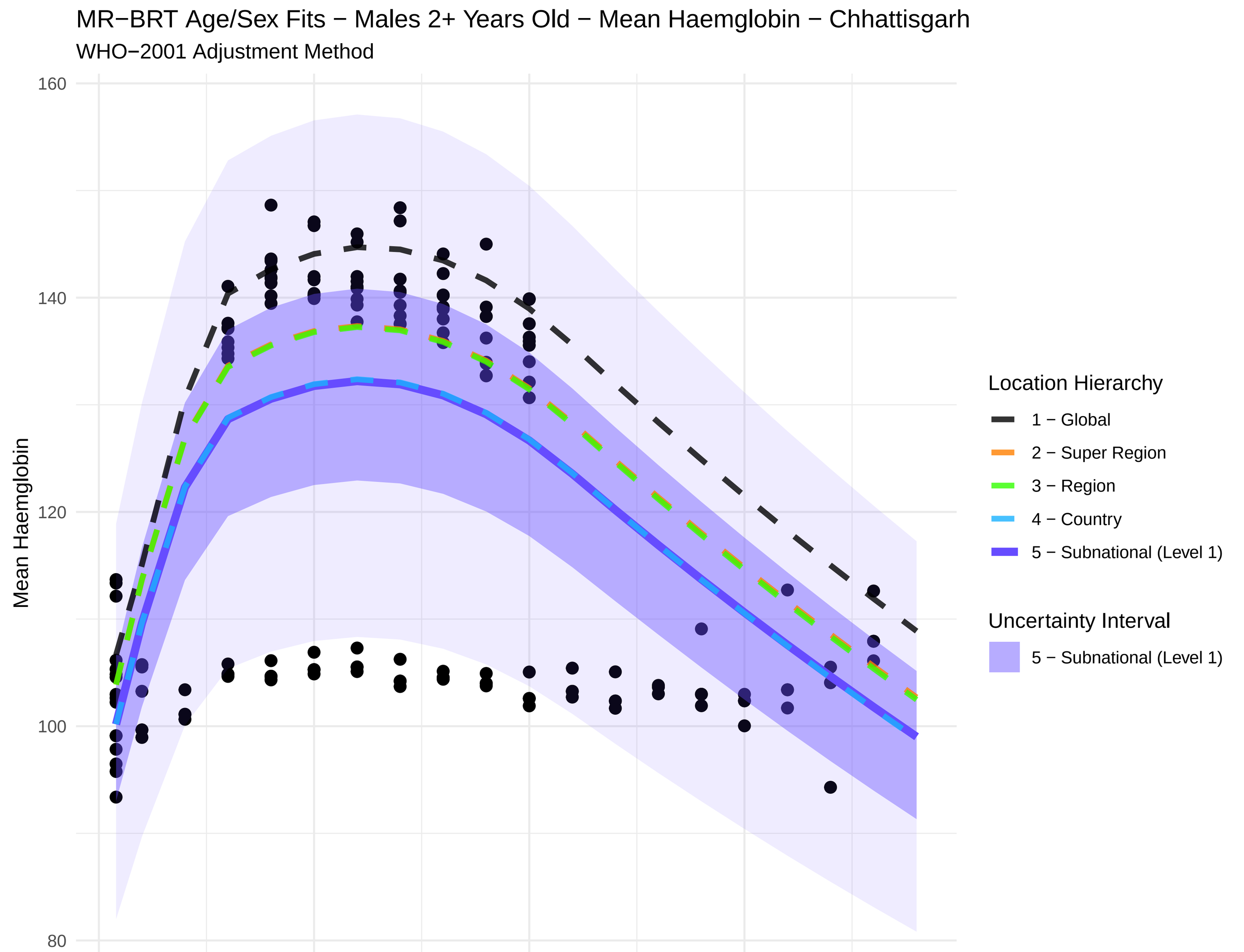
MR−BRT Age/Sex Fits − Males 2+ Years Old − Mean Haemoglobin − Chhattisgarh
WHO−2001 Adjustment Method
Mean Haemoglobin
80
100
120
140
160
Age (year)
0
25
50
75
Location Hierarchy
1 − Global
2 − Super Region
3 − Region
4 − Country
5 − Subnational (Level 1)
Uncertainty Interval
5 − Subnational (Level 1)

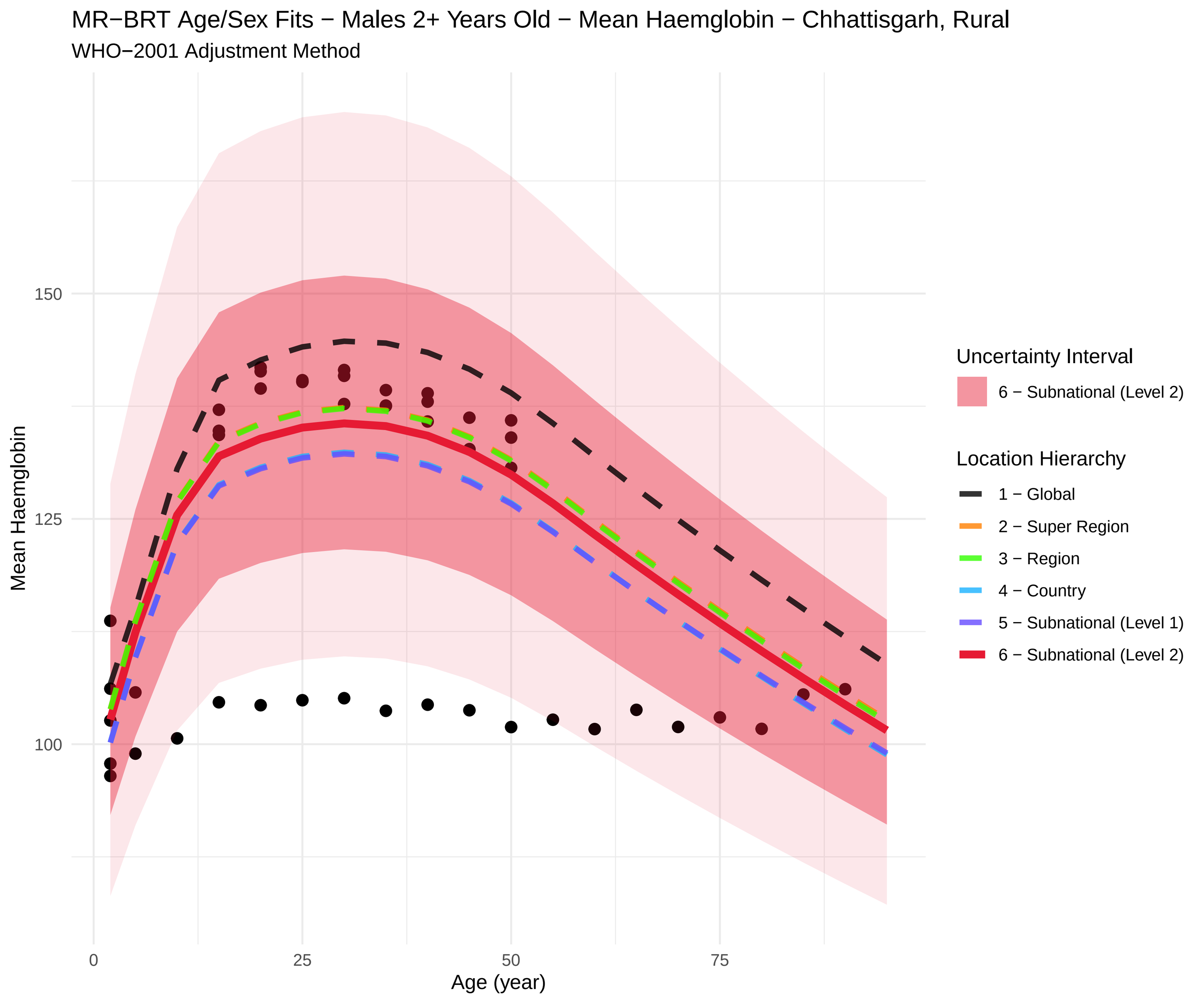
MR−BRT Age/Sex Fits − Males 2+ Years Old − Mean Haemglobin − Chhattisgarh, Rural
WHO−2001 Adjustment Method
Mean Haemglobin
Age (year)
Uncertainty Interval
6 − Subnational (Level 2)
Location Hierarchy
1 − Global
2 − Super Region
3 − Region
4 − Country
5 − Subnational (Level 1)
6 − Subnational (Level 2)

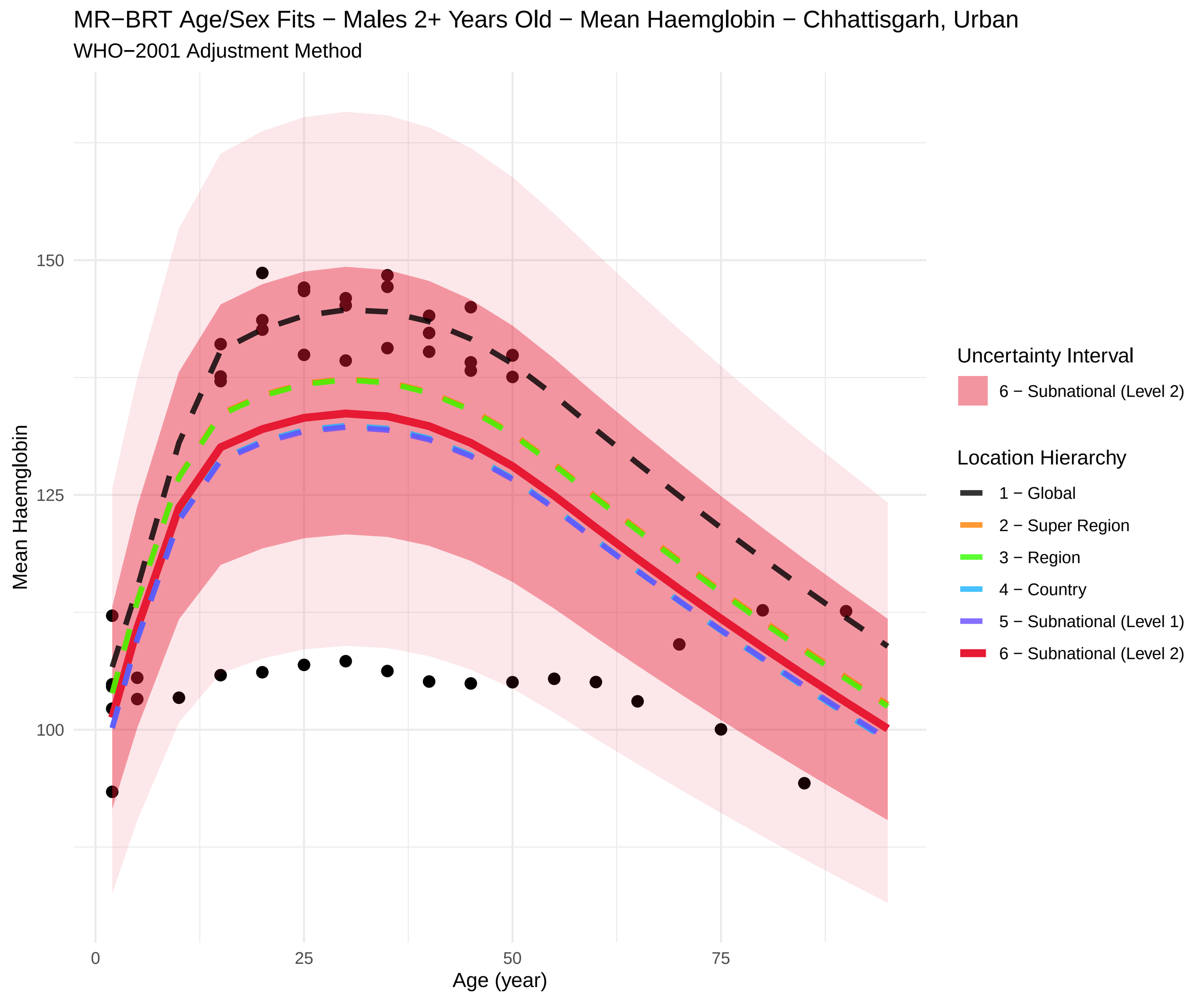
MR−BRT Age/Sex Fits − Males 2+ Years Old − Mean Haemglobin − Chhattisgarh, Urban
WHO−2001 Adjustment Method
Mean Haemglobin
Age (year)
Uncertainty Interval
6 − Subnational (Level 2)
Location Hierarchy
1 − Global
2 − Super Region
3 − Region
4 − Country
5 − Subnational (Level 1)
6 − Subnational (Level 2)

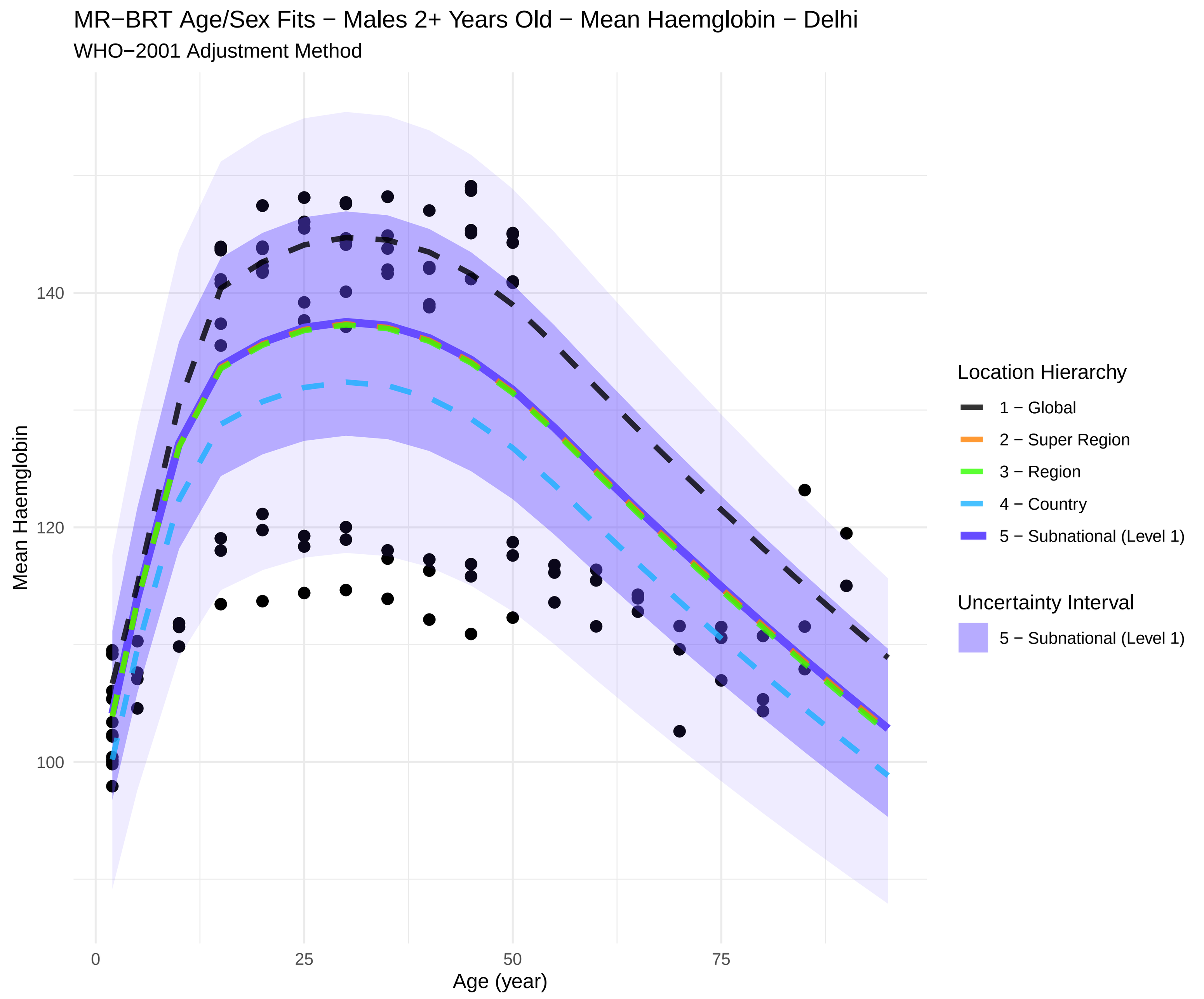
MR−BRT Age/Sex Fits − Males 2+ Years Old − Mean Haemglobin − Delhi
WHO−2001 Adjustment Method
Mean Haemglobin
Age (year)
Location Hierarchy
1 − Global
2 − Super Region
3 − Region
4 − Country
5 − Subnational (Level 1)
Uncertainty Interval
5 − Subnational (Level 1)

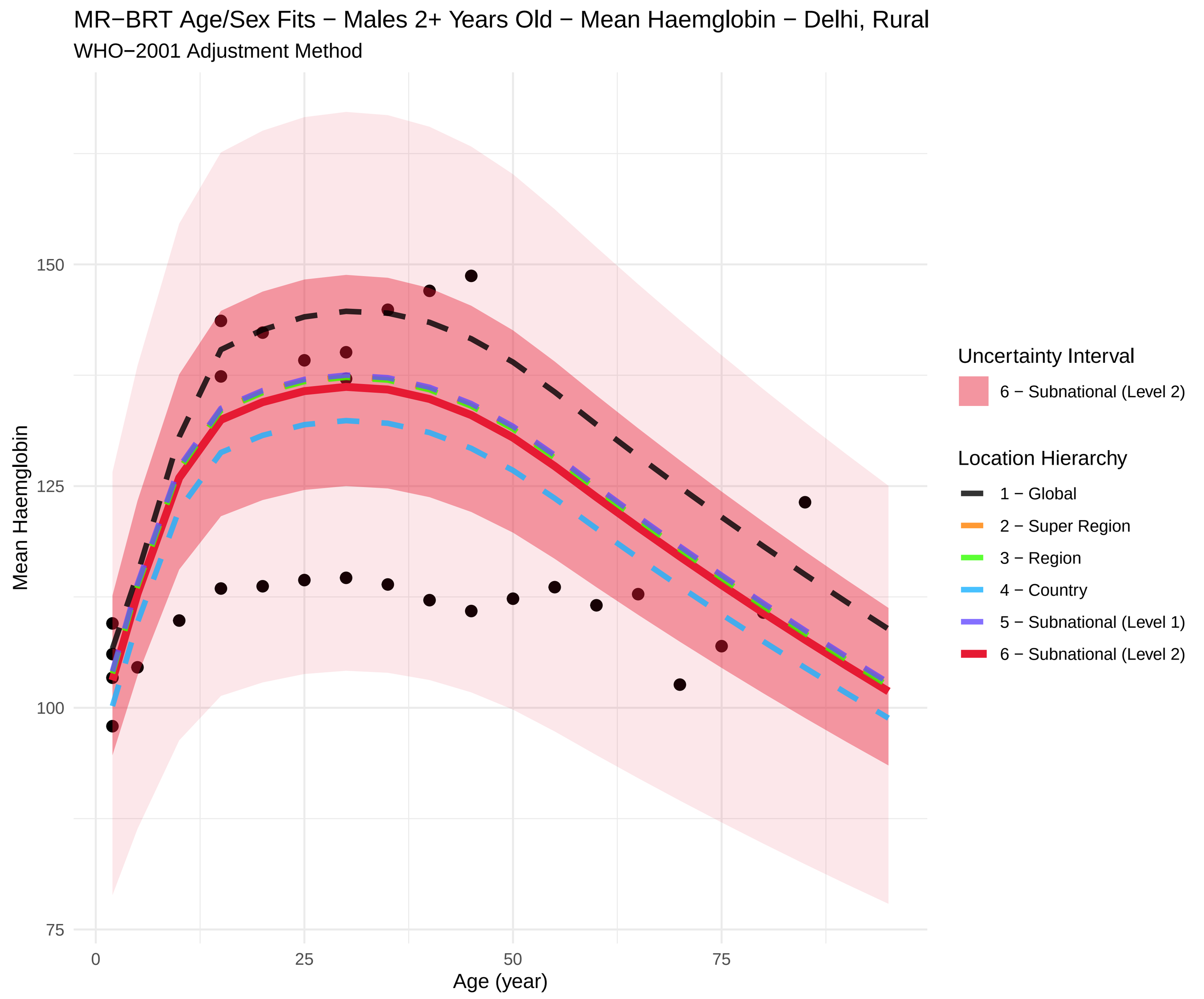
MR−BRT Age/Sex Fits − Males 2+ Years Old − Mean Haemglobin − Delhi, Rural
WHO−2001 Adjustment Method
Mean Haemglobin
Age (year)
Uncertainty Interval
6 − Subnational (Level 2)
Location Hierarchy
1 − Global
2 − Super Region
3 − Region
4 − Country
5 − Subnational (Level 1)
6 − Subnational (Level 2)

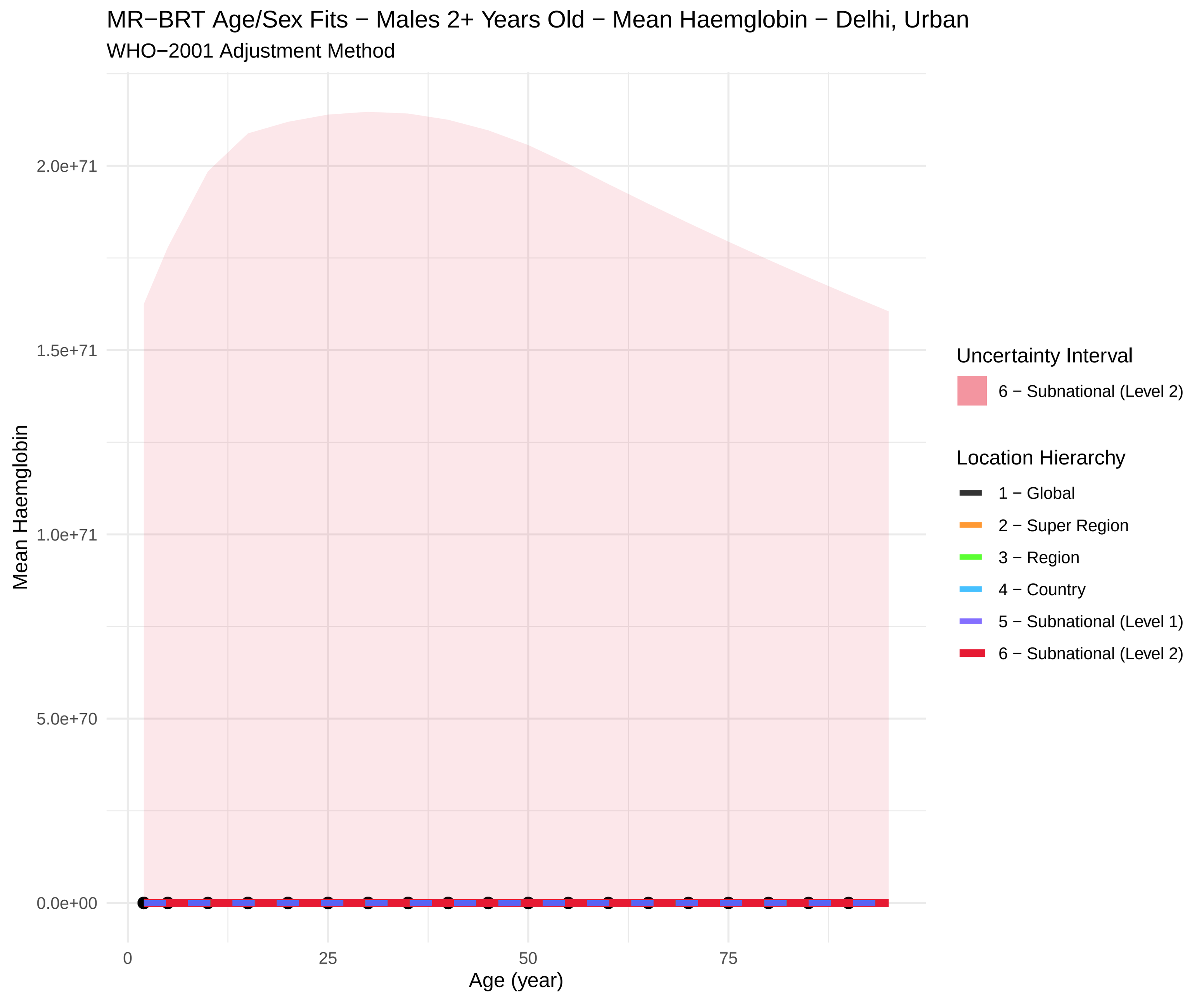
MR−BRT Age/Sex Fits − Males 2+ Years Old − Mean Haemglobin − Delhi, Urban
WHO−2001 Adjustment Method
Mean Haemglobin
Age (year)
Uncertainty Interval
6 − Subnational (Level 2)
Location Hierarchy
1 − Global
2 − Super Region
3 − Region
4 − Country
5 − Subnational (Level 1)
6 − Subnational (Level 2)

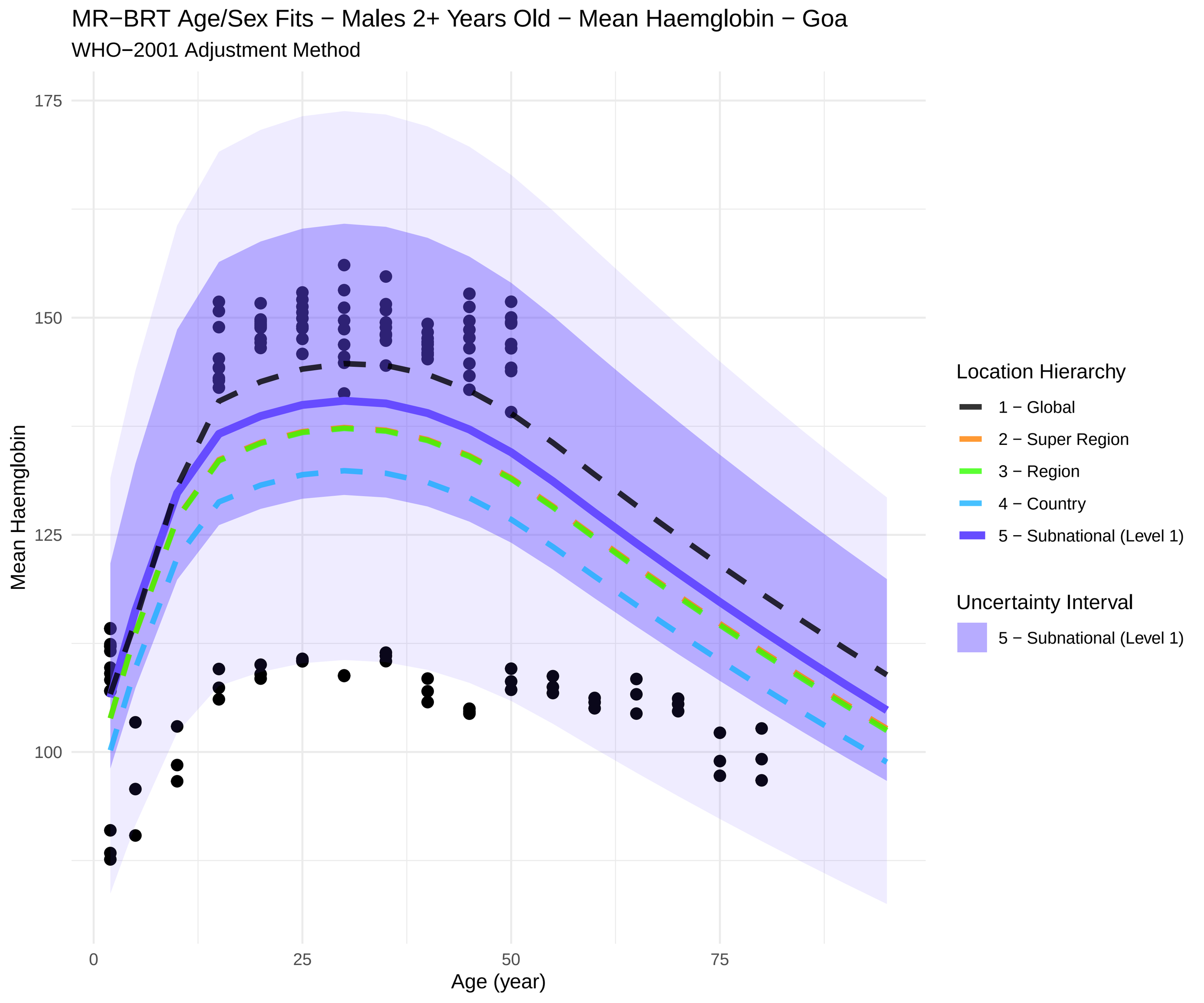
MR−BRT Age/Sex Fits − Males 2+ Years Old − Mean Haemglobin − Goa
WHO−2001 Adjustment Method
Mean Haemglobin
Age (year)
Location Hierarchy
1 − Global
2 − Super Region
3 − Region
4 − Country
5 − Subnational (Level 1)
Uncertainty Interval
5 − Subnational (Level 1)

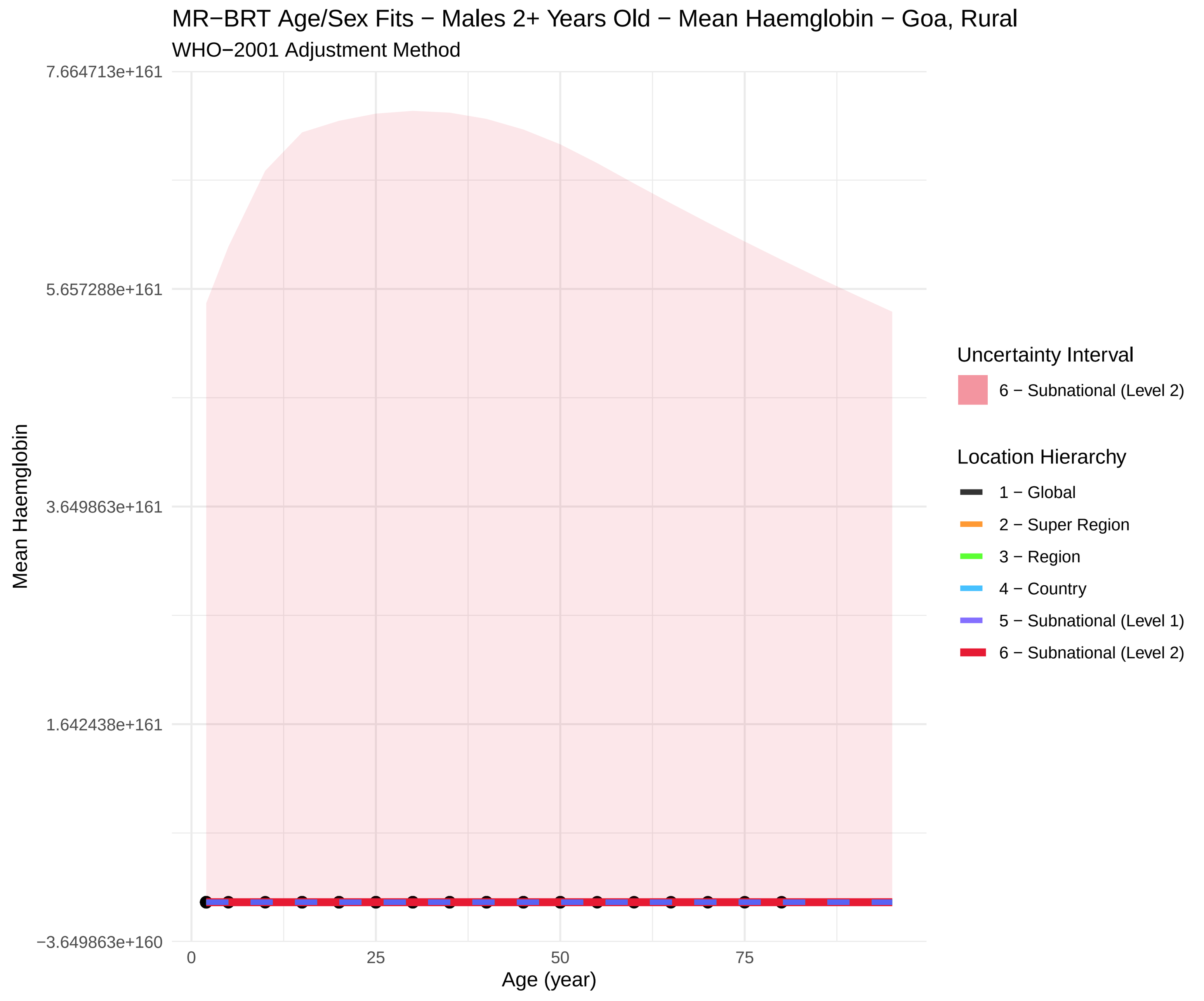
MR−BRT Age/Sex Fits − Males 2+ Years Old − Mean Haemglobin − Goa, Rural
WHO−2001 Adjustment Method
Mean Haemglobin
Age (year)
Uncertainty Interval
6 − Subnational (Level 2)
Location Hierarchy
1 − Global
2 − Super Region
3 − Region
4 − Country
5 − Subnational (Level 1)
6 − Subnational (Level 2)

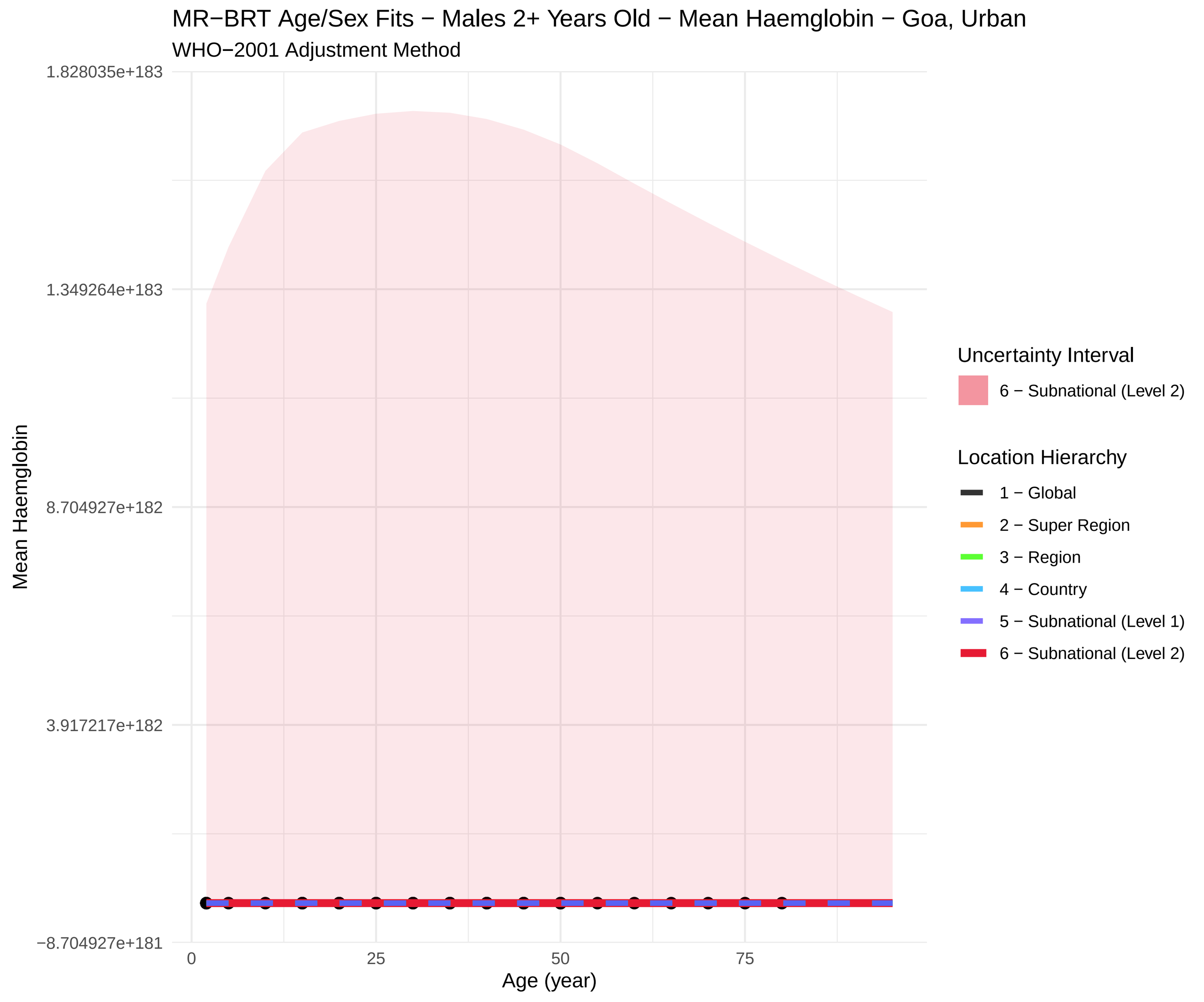
MR−BRT Age/Sex Fits − Males 2+ Years Old − Mean Haemglobin − Goa, Urban
WHO−2001 Adjustment Method
Mean Haemglobin
Age (year)
Uncertainty Interval
6 − Subnational (Level 2)
Location Hierarchy
1 − Global
2 − Super Region
3 − Region
4 − Country
5 − Subnational (Level 1)
6 − Subnational (Level 2)

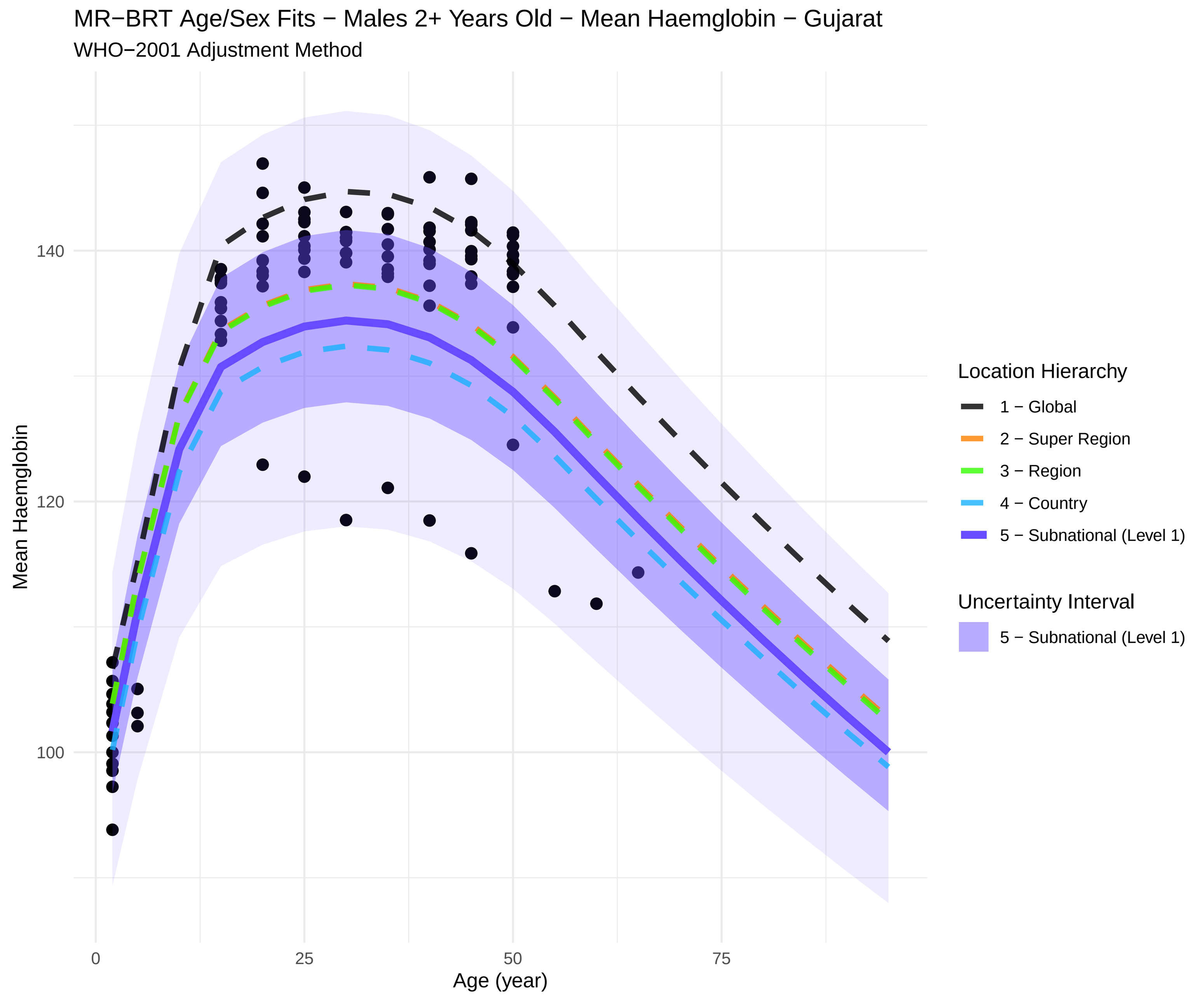
MR−BRT Age/Sex Fits − Males 2+ Years Old − Mean Haemglobin − Gujarat
WHO−2001 Adjustment Method
Mean Haemglobin
Age (year)
Location Hierarchy
1 − Global
2 − Super Region
3 − Region
4 − Country
5 − Subnational (Level 1)
Uncertainty Interval
5 − Subnational (Level 1)

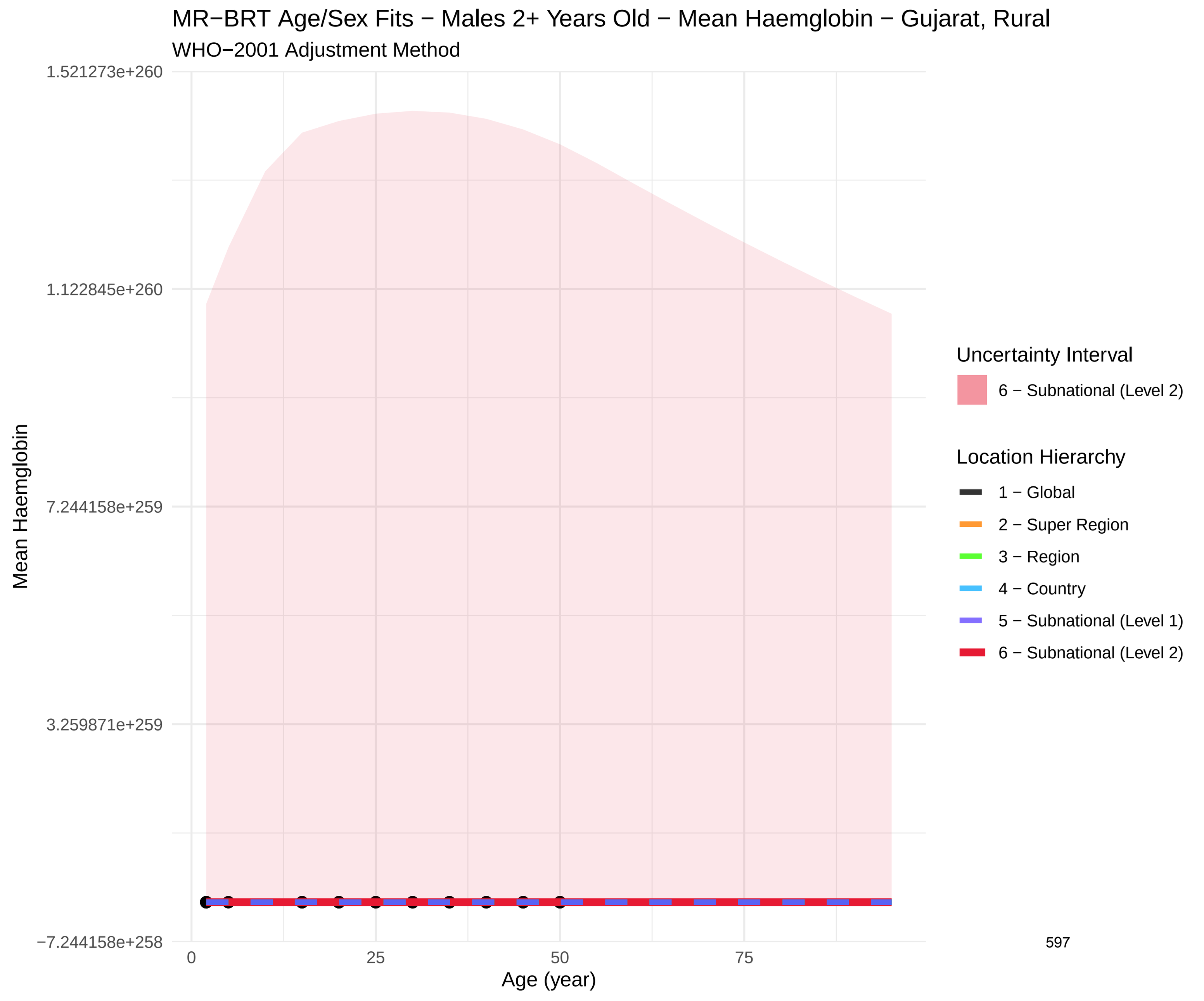
MR−BRT Age/Sex Fits − Males 2+ Years Old − Mean Haemglobin − Gujarat, Rural
WHO−2001 Adjustment Method
Mean Haemglobin
Age (year)
Uncertainty Interval
6 − Subnational (Level 2)
Location Hierarchy
1 − Global
2 − Super Region
3 − Region
4 − Country
5 − Subnational (Level 1)
6 − Subnational (Level 2)

MR−BRT Age/Sex Fits − Males 2+ Years Old − Mean Haemglobin − Gujarat, Urban
WHO−2001 Adjustment Method

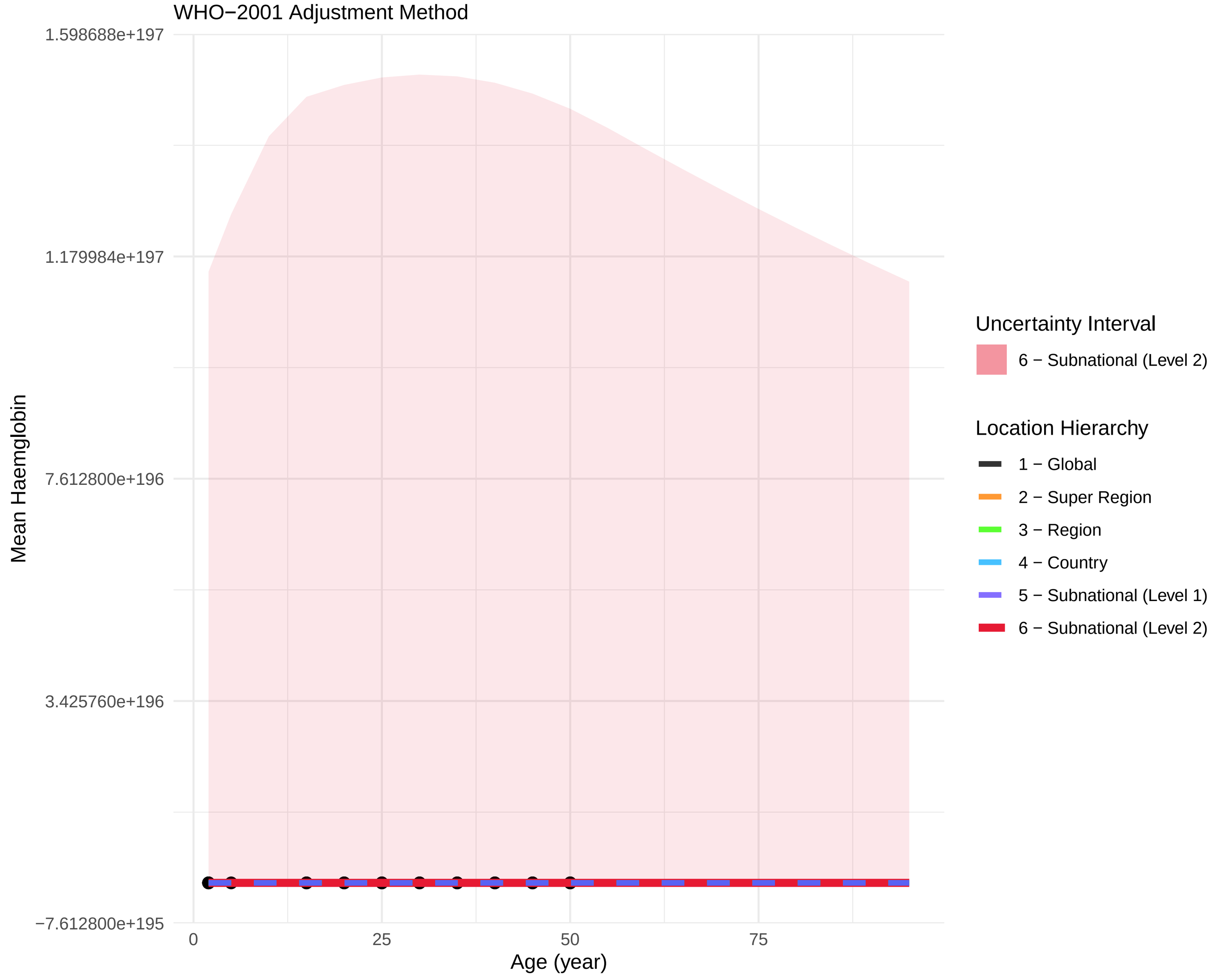

MR−BRT Age/Sex Fits − Males 2+ Years Old − Mean Haemglobin − Haryana
WHO−2001 Adjustment Method

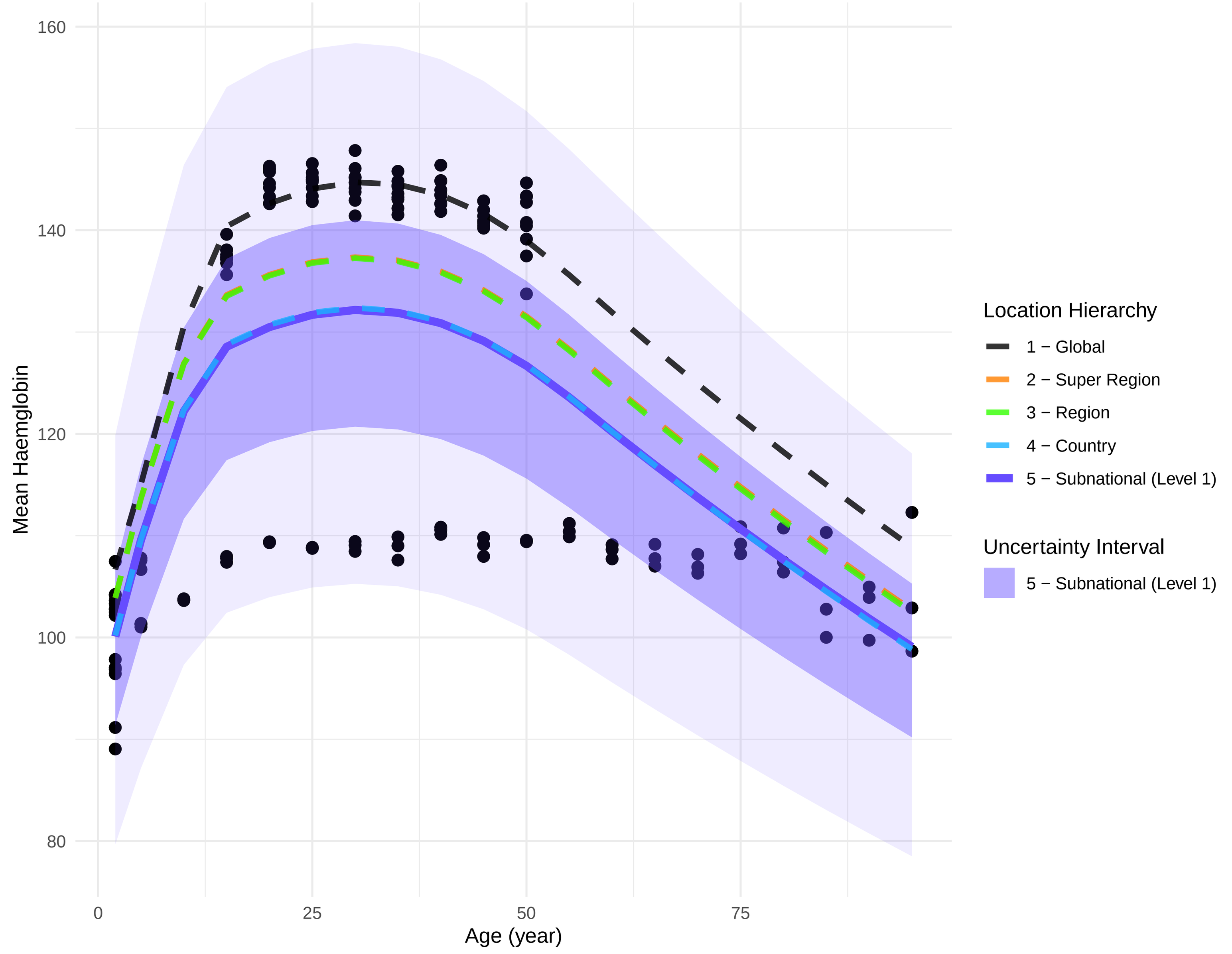

MR−BRT Age/Sex Fits − Males 2+ Years Old − Mean Haemglobin − Haryana, Rural
WHO−2001 Adjustment Method

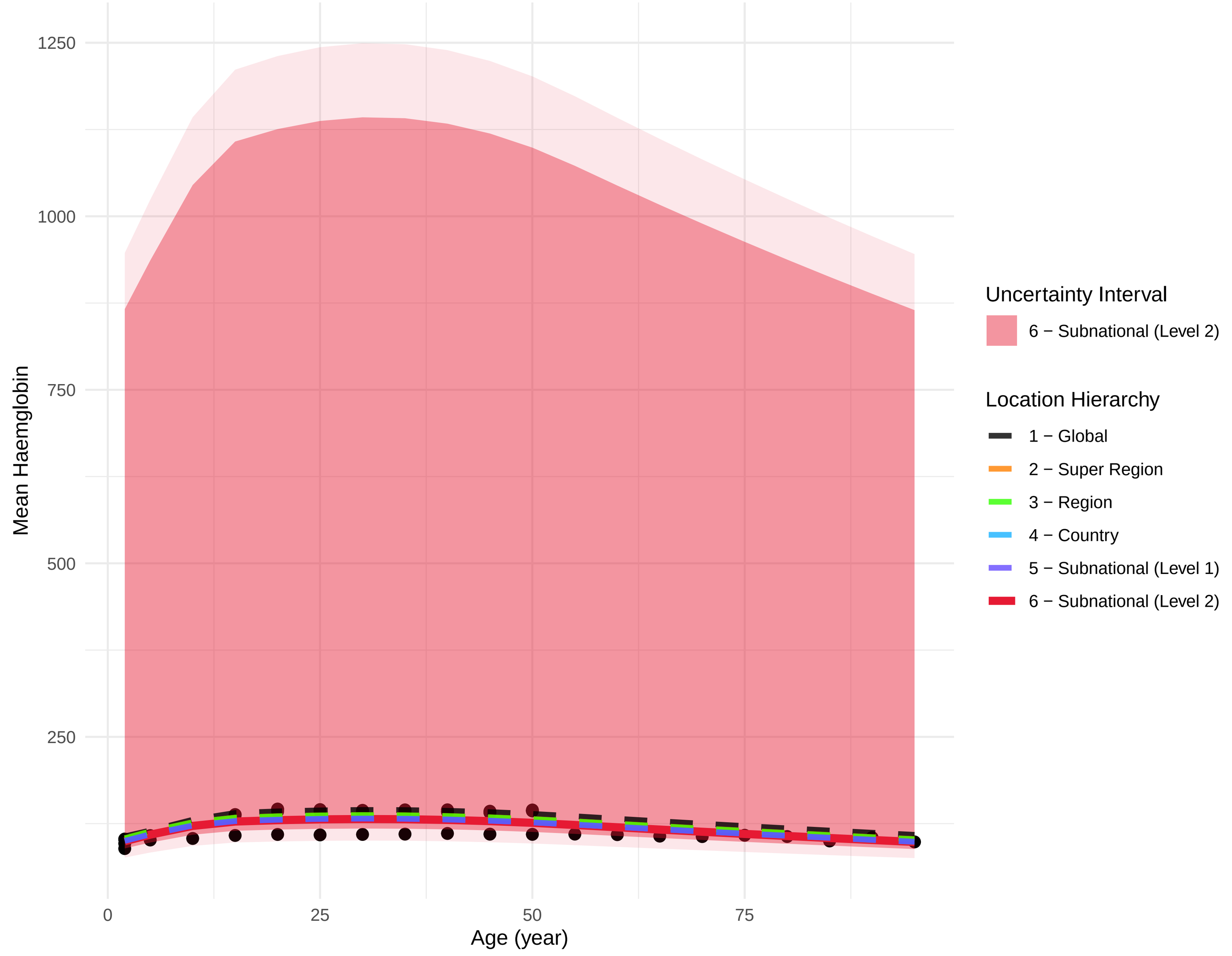

MR−BRT Age/Sex Fits − Males 2+ Years Old − Mean Haemglobin − Haryana, Urban
WHO−2001 Adjustment Method

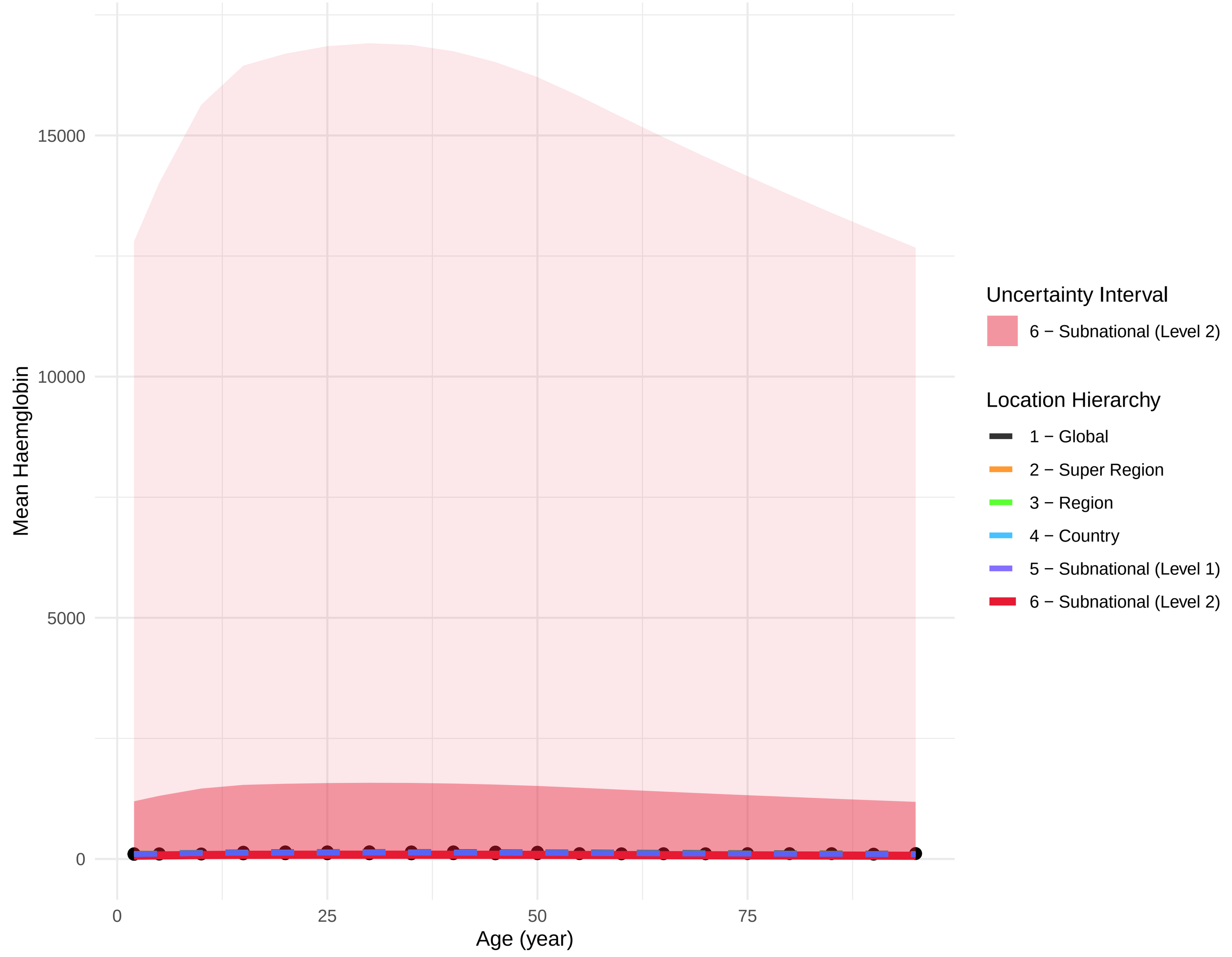

MR−BRT Age/Sex Fits − Males 2+ Years Old − Mean Haemglobin − Himachal Pradesh
WHO−2001 Adjustment Method

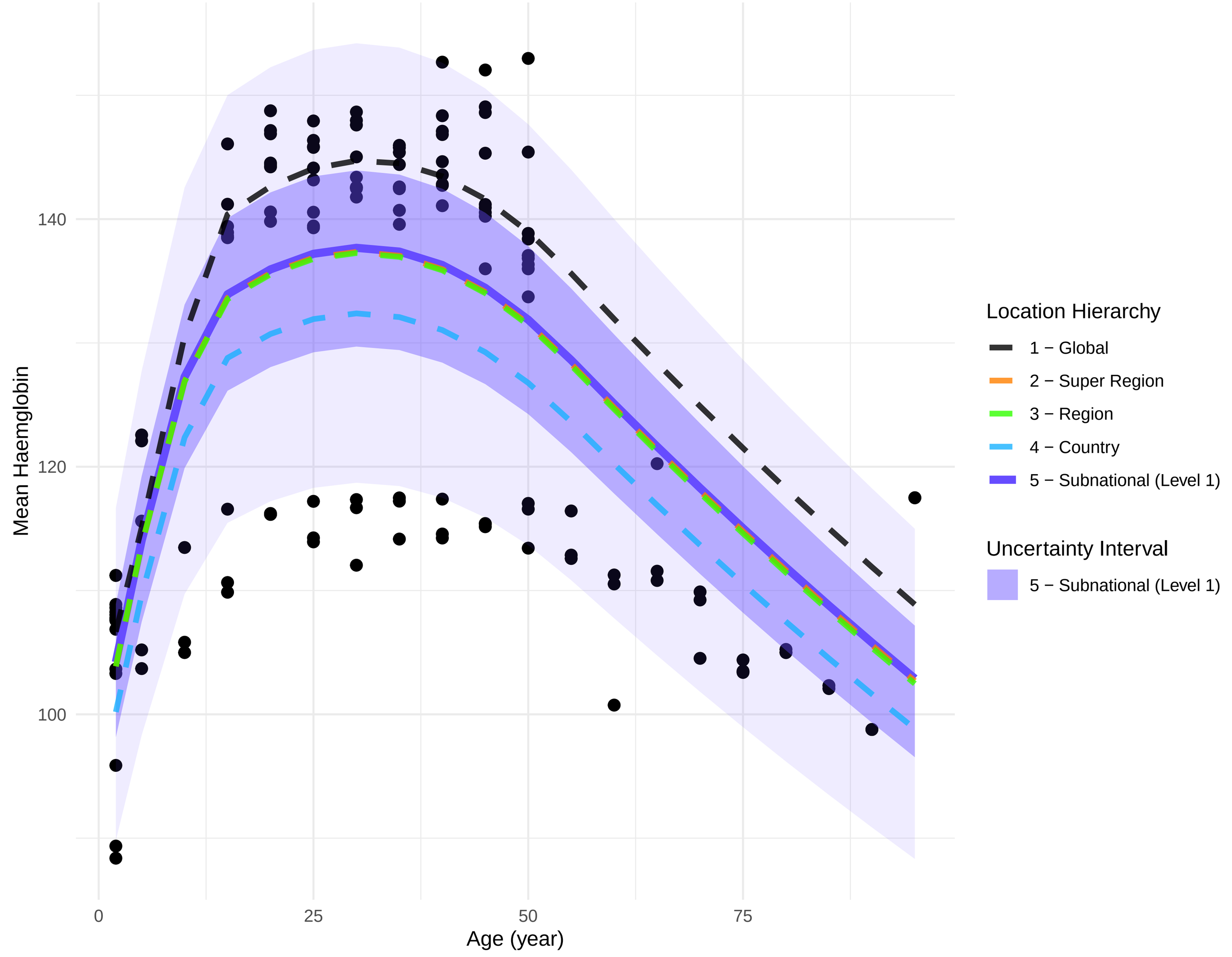

MR−BRT Age/Sex Fits − Males 2+ Years Old − Mean Haemglobin − Himachal Pradesh, Rural
WHO−2001 Adjustment Method

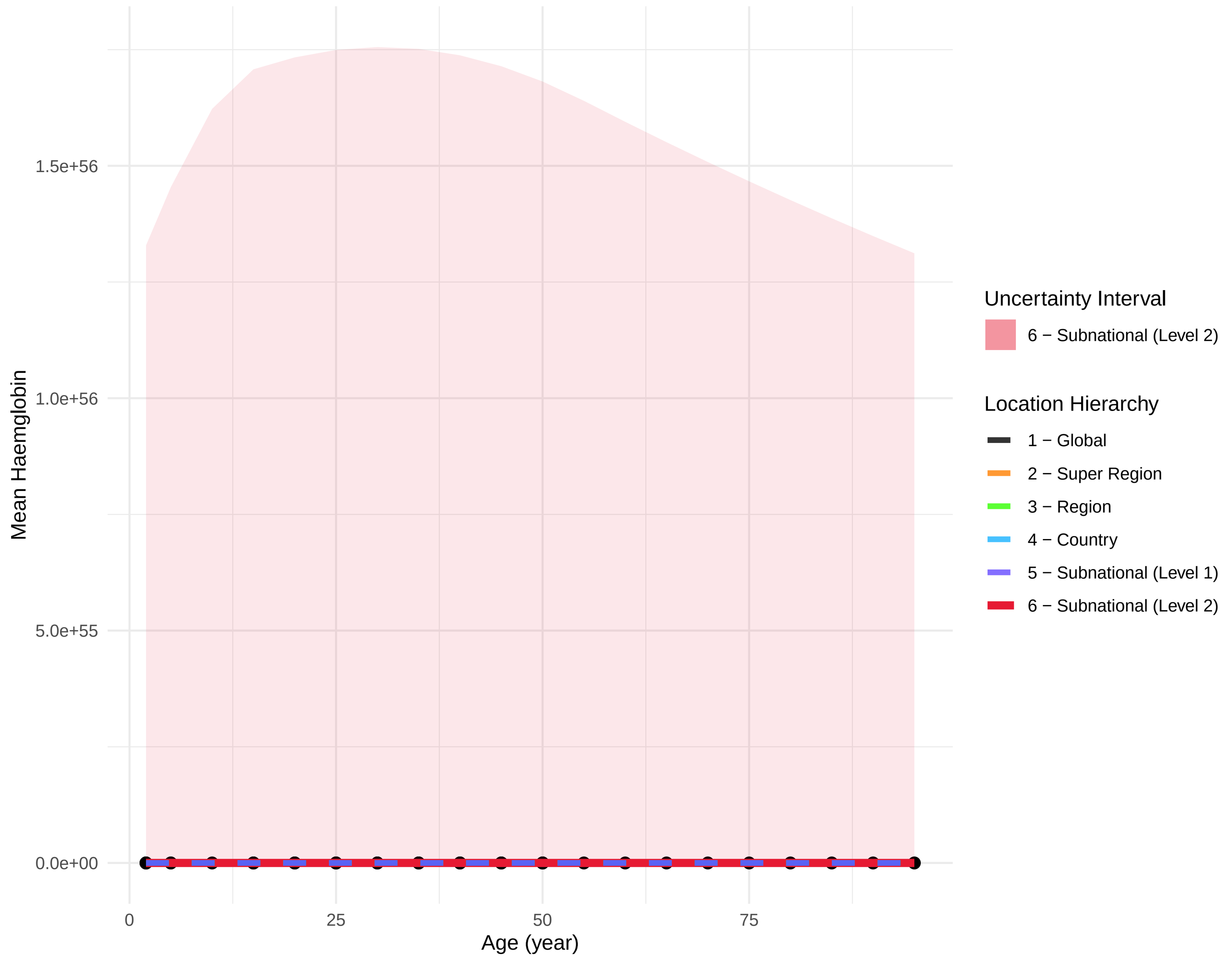

MR−BRT Age/Sex Fits − Males 2+ Years Old − Mean Haemglobin − Himachal Pradesh, Urban
WHO−2001 Adjustment Method

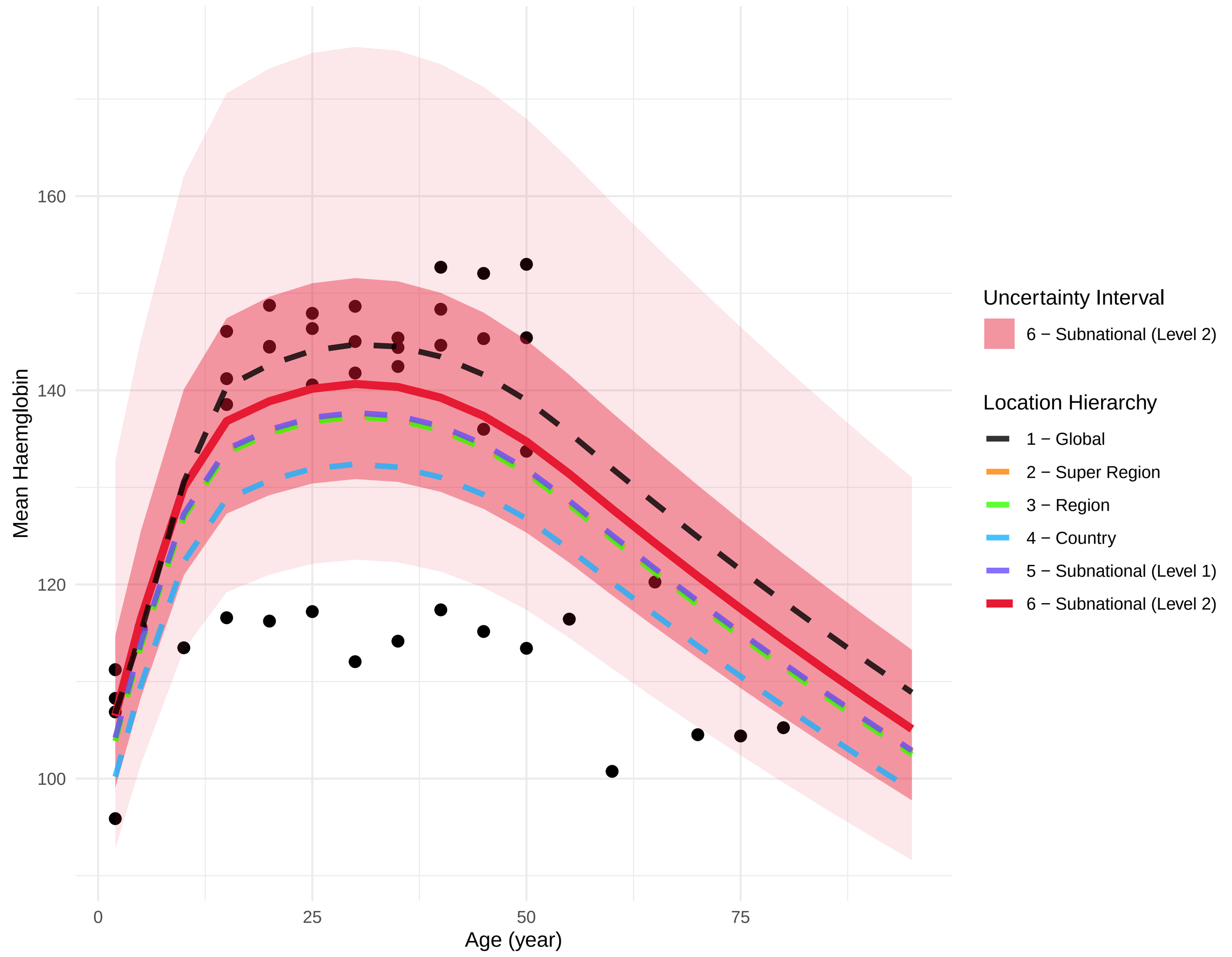

MR−BRT Age/Sex Fits − Males 2+ Years Old − Mean Haemglobin − Jammu & Kashmir and Ladakh
WHO−2001 Adjustment Method

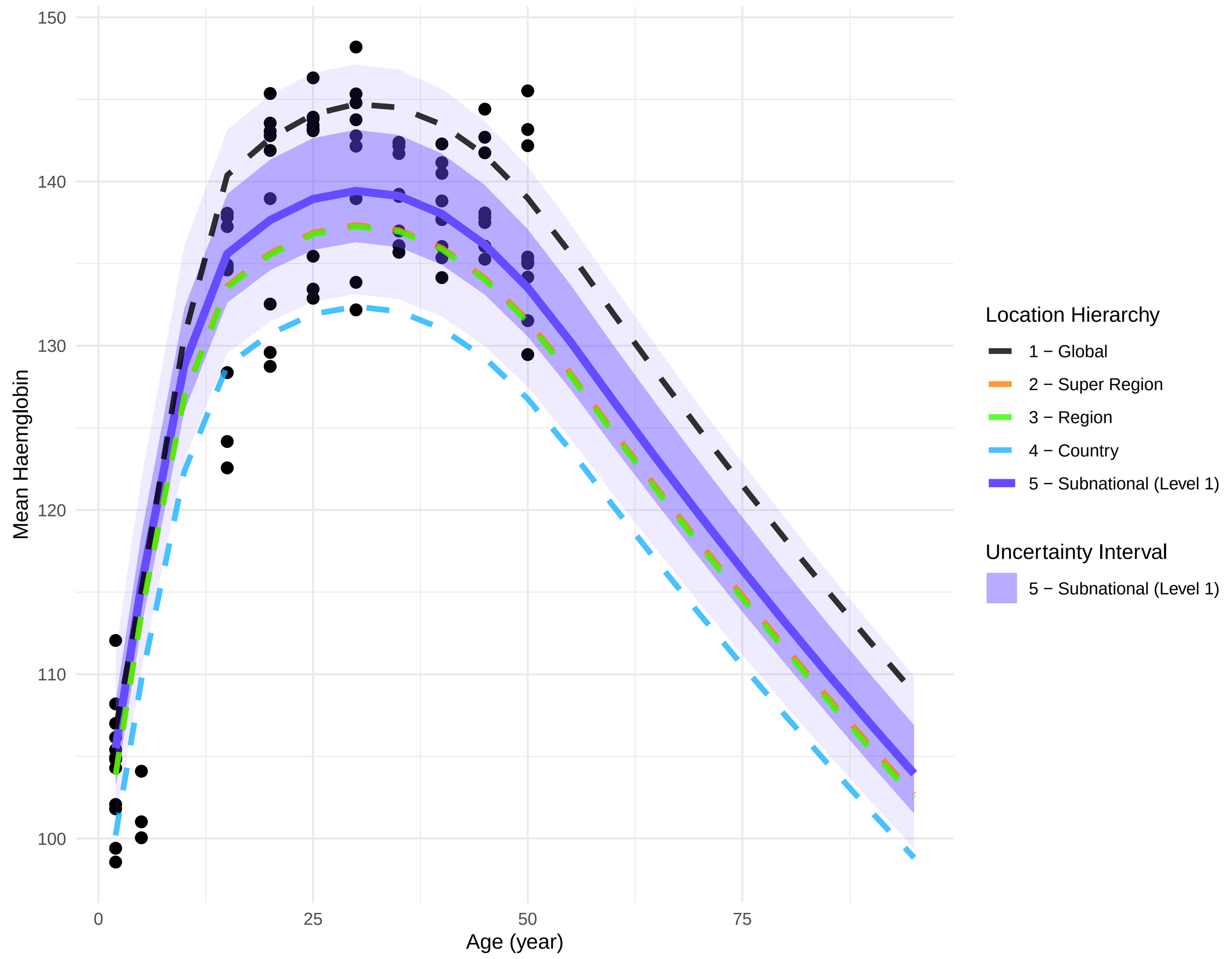

MR−BRT Age/Sex Fits − Males 2+ Years Old − Mean Haemglobin − Jammu & Kashmir and Ladakh, Rural
WHO−2001 Adjustment Method

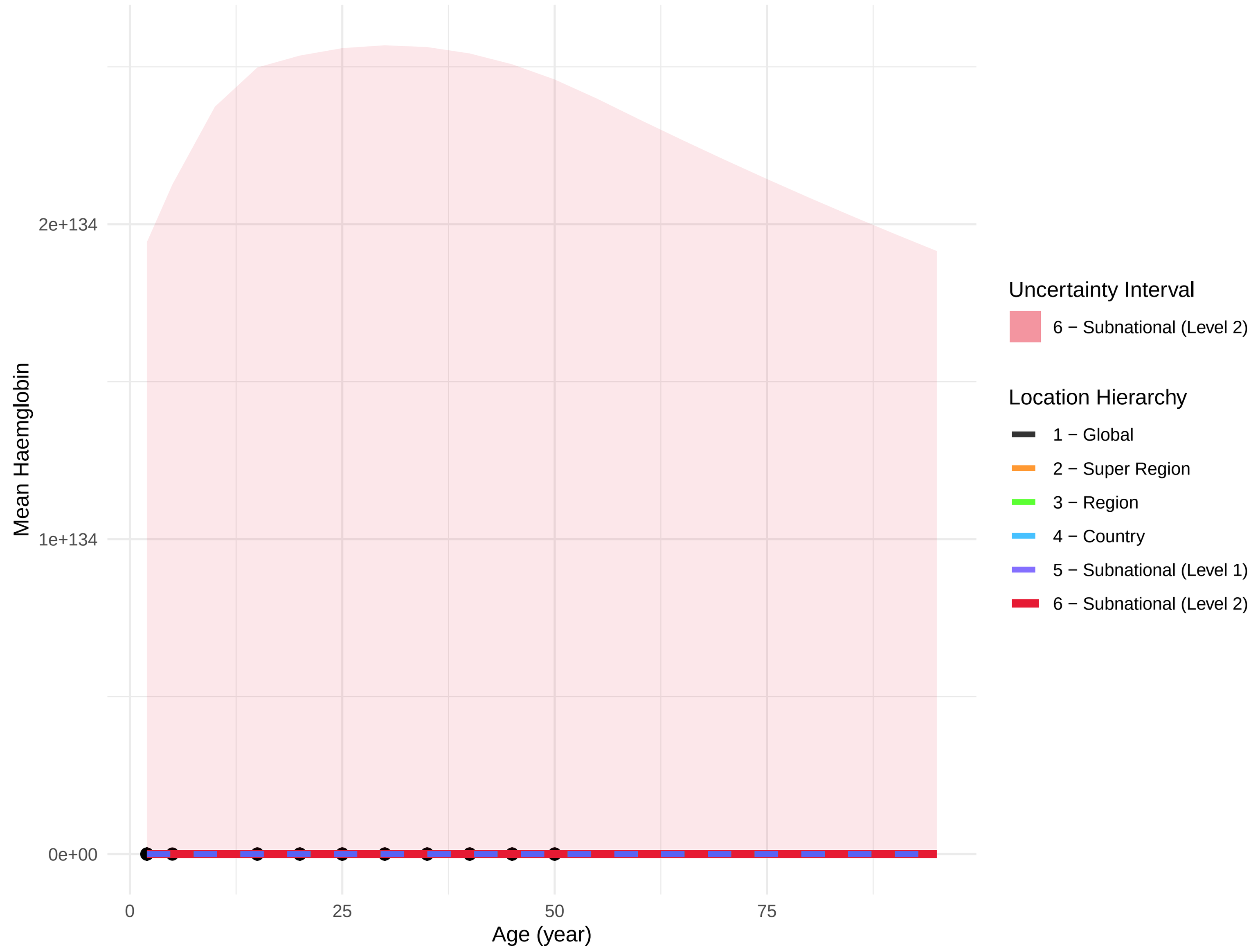

MR−BRT Age/Sex Fits − Males 2+ Years Old − Mean Haemglobin − Jammu & Kashmir and Ladakh, Urban
WHO−2001 Adjustment Method

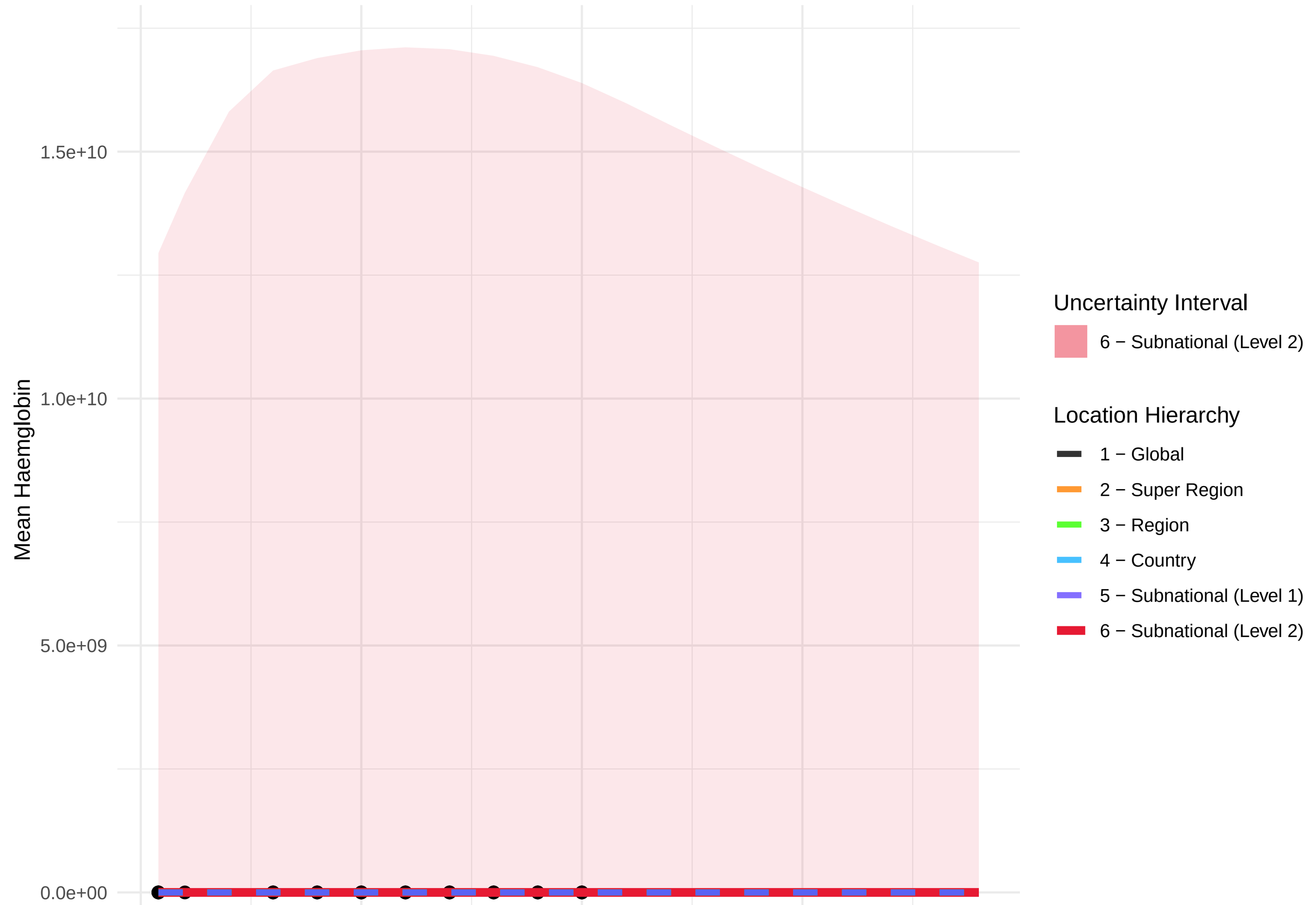

MR−BRT Age/Sex Fits − Males 2+ Years Old − Mean Haemoglobin − Jharkhand
WHO−2001 Adjustment Method

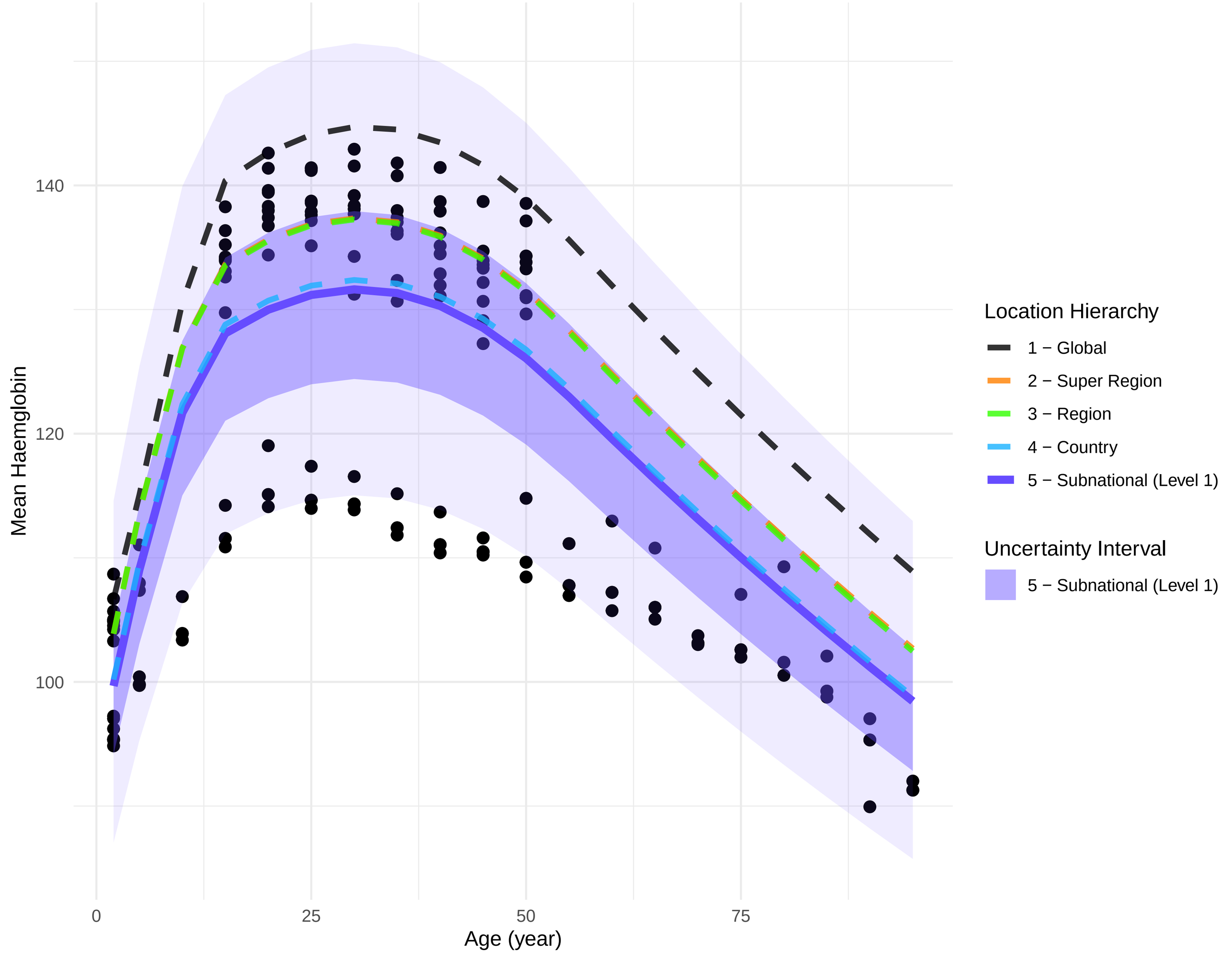

MR−BRT Age/Sex Fits − Males 2+ Years Old − Mean Haemoglobin − Jharkhand, Rural
WHO−2001 Adjustment Method

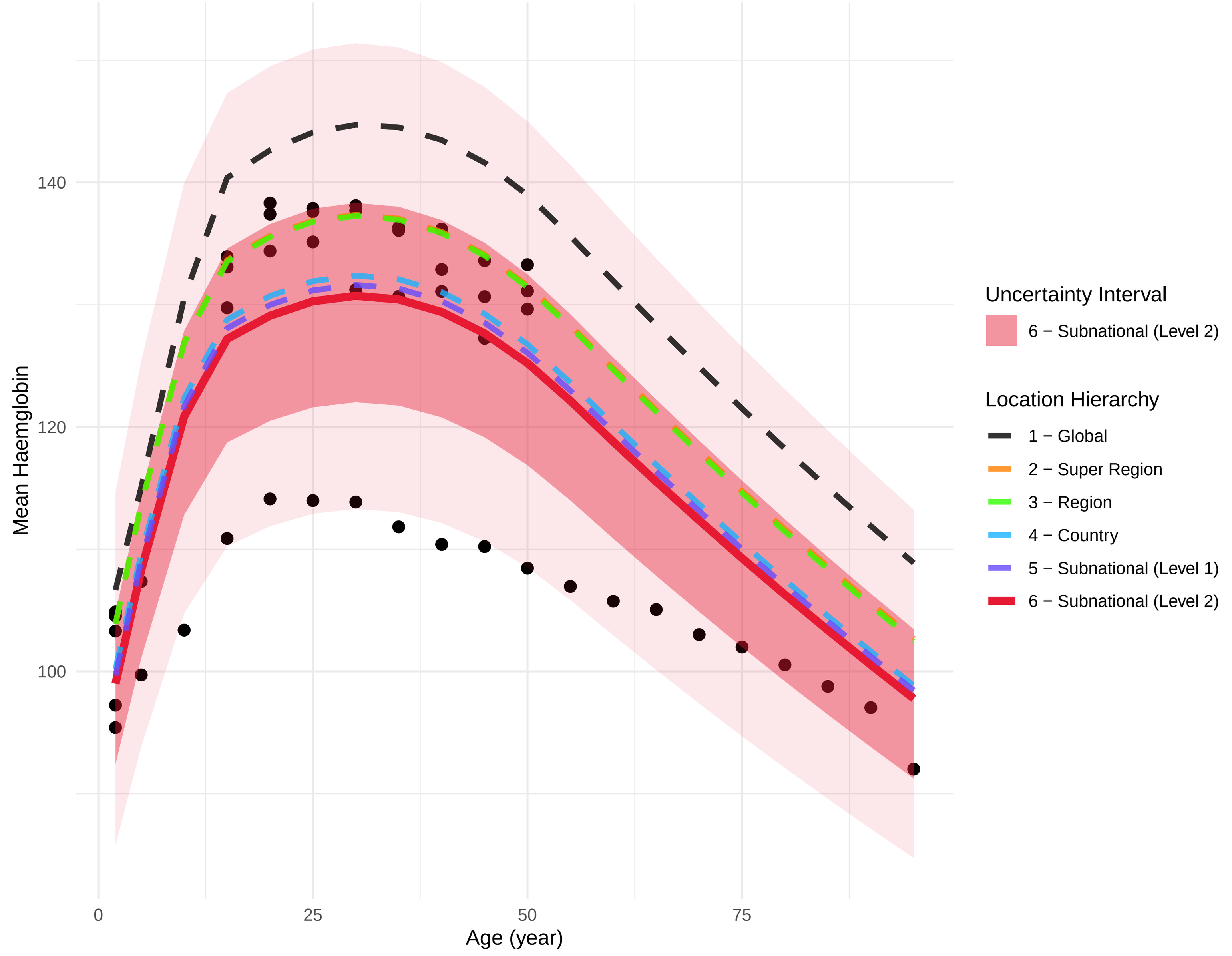

MR−BRT Age/Sex Fits − Males 2+ Years Old − Mean Haemoglobin − Jharkhand, Urban
WHO−2001 Adjustment Method

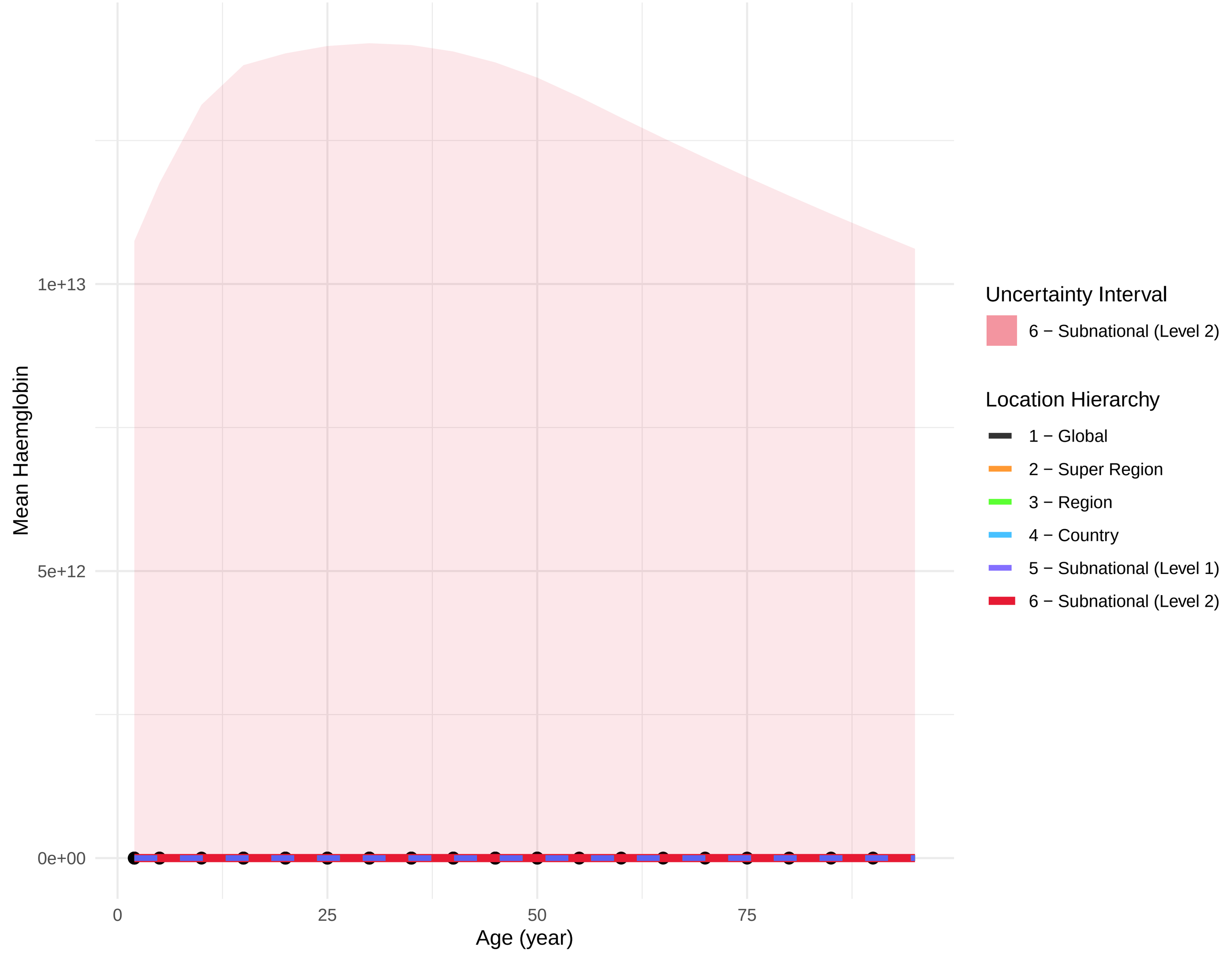

MR−BRT Age/Sex Fits − Males 2+ Years Old − Mean Haemoglobin − Karnataka
WHO−2001 Adjustment Method

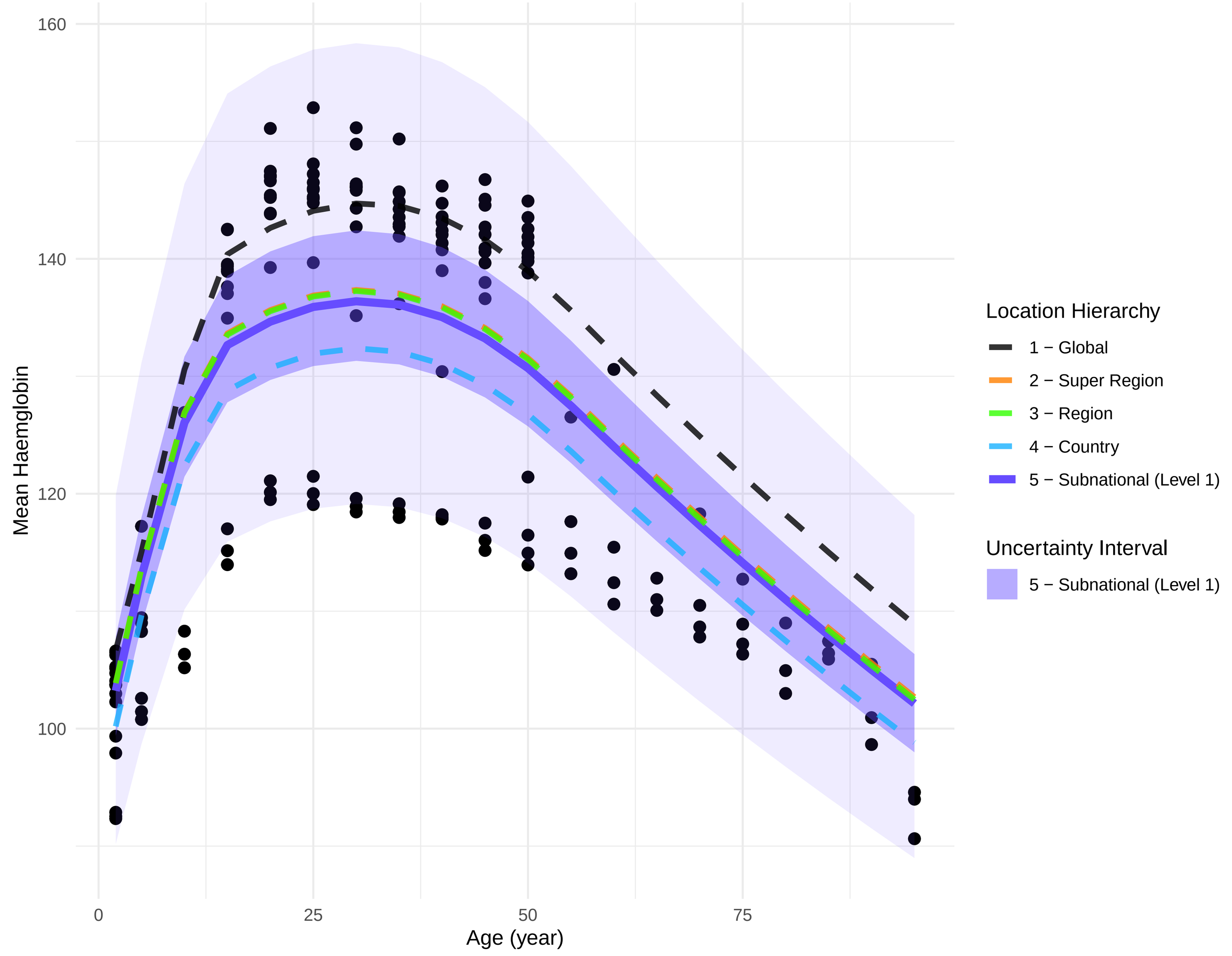

MR−BRT Age/Sex Fits − Males 2+ Years Old − Mean Haemoglobin − Karnataka, Rural
WHO−2001 Adjustment Method

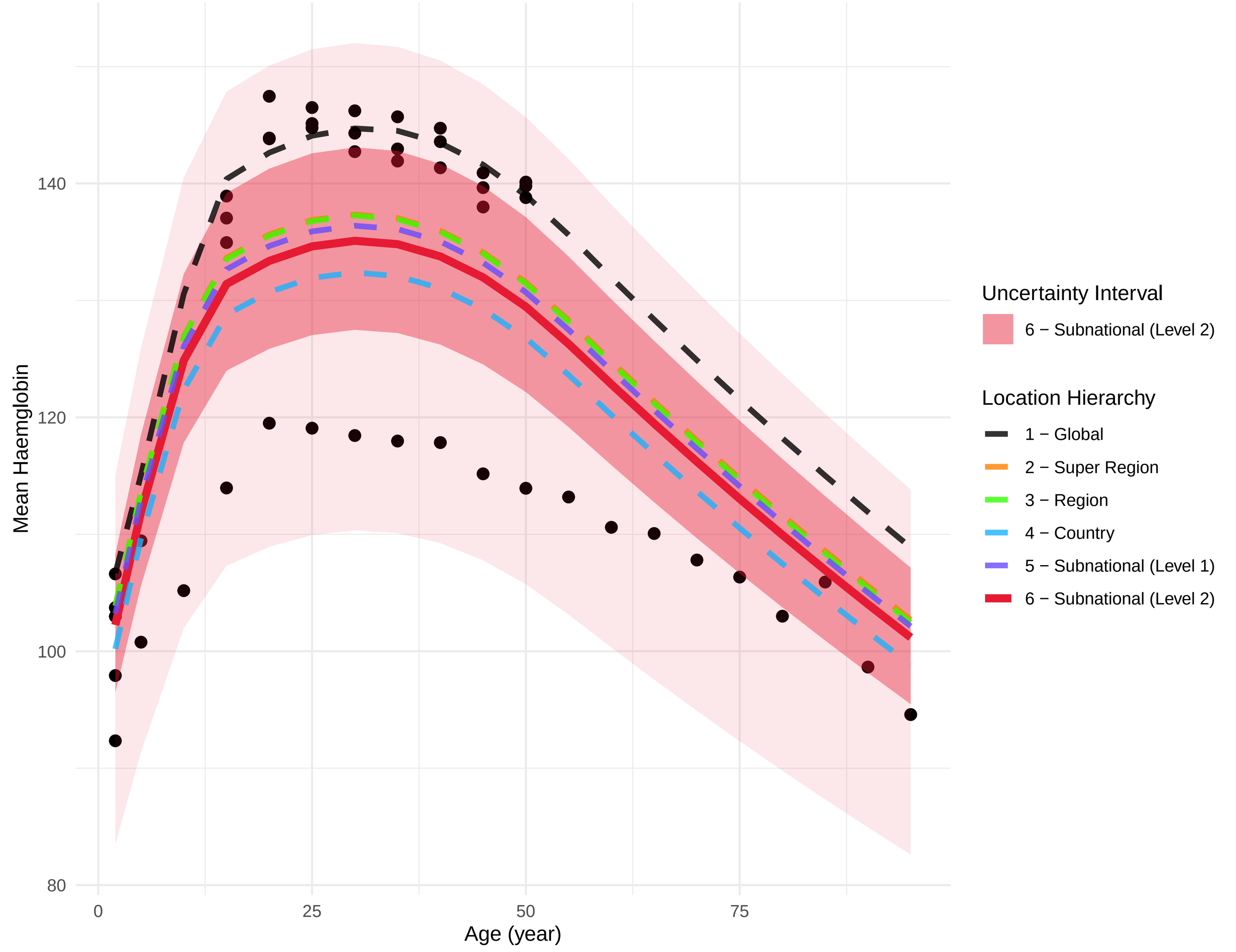

MR−BRT Age/Sex Fits − Males 2+ Years Old − Mean Haemoglobin − Karnataka, Urban
WHO−2001 Adjustment Method

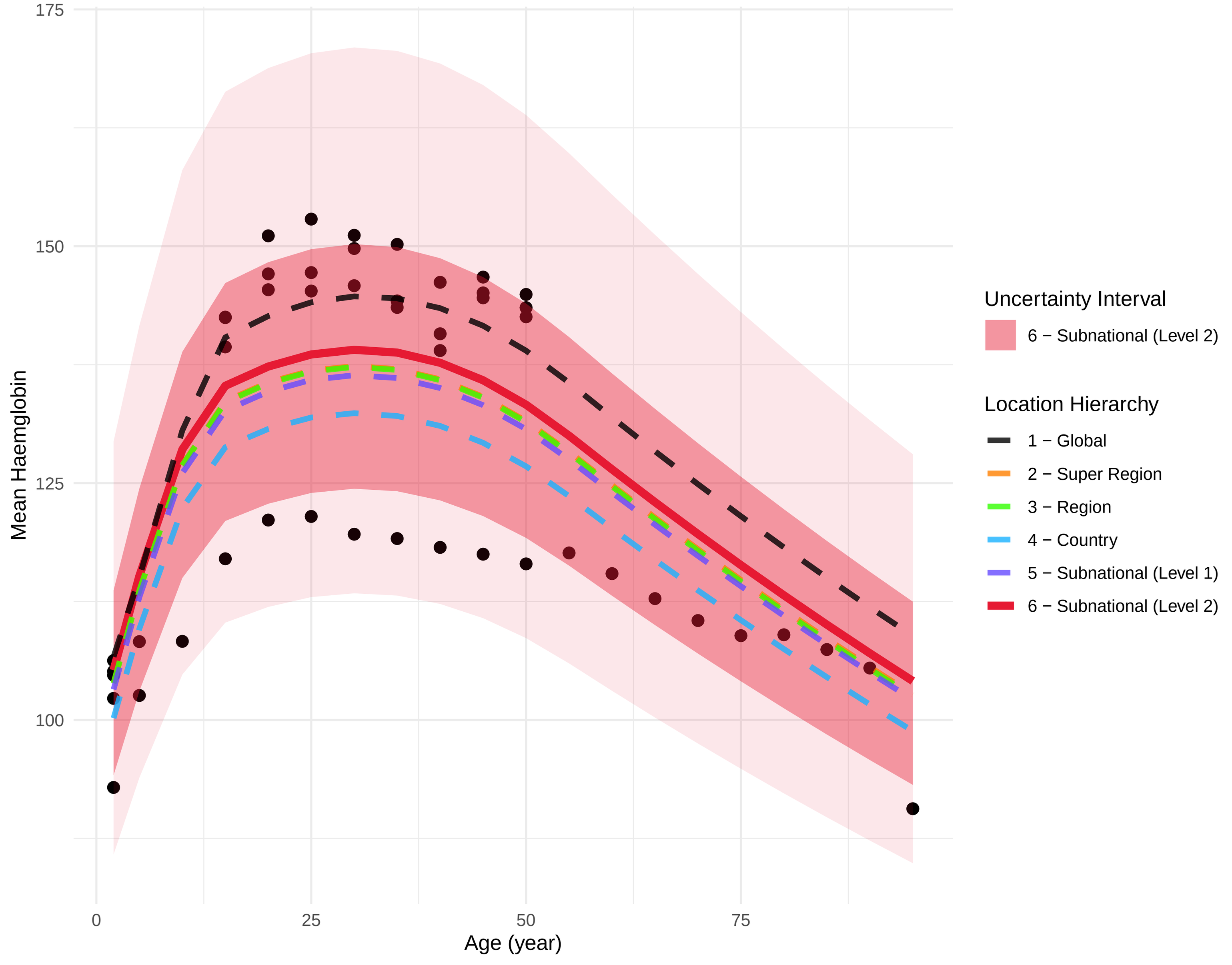

MR−BRT Age/Sex Fits − Males 2+ Years Old − Mean Haemoglobin − Kerala
WHO−2001 Adjustment Method

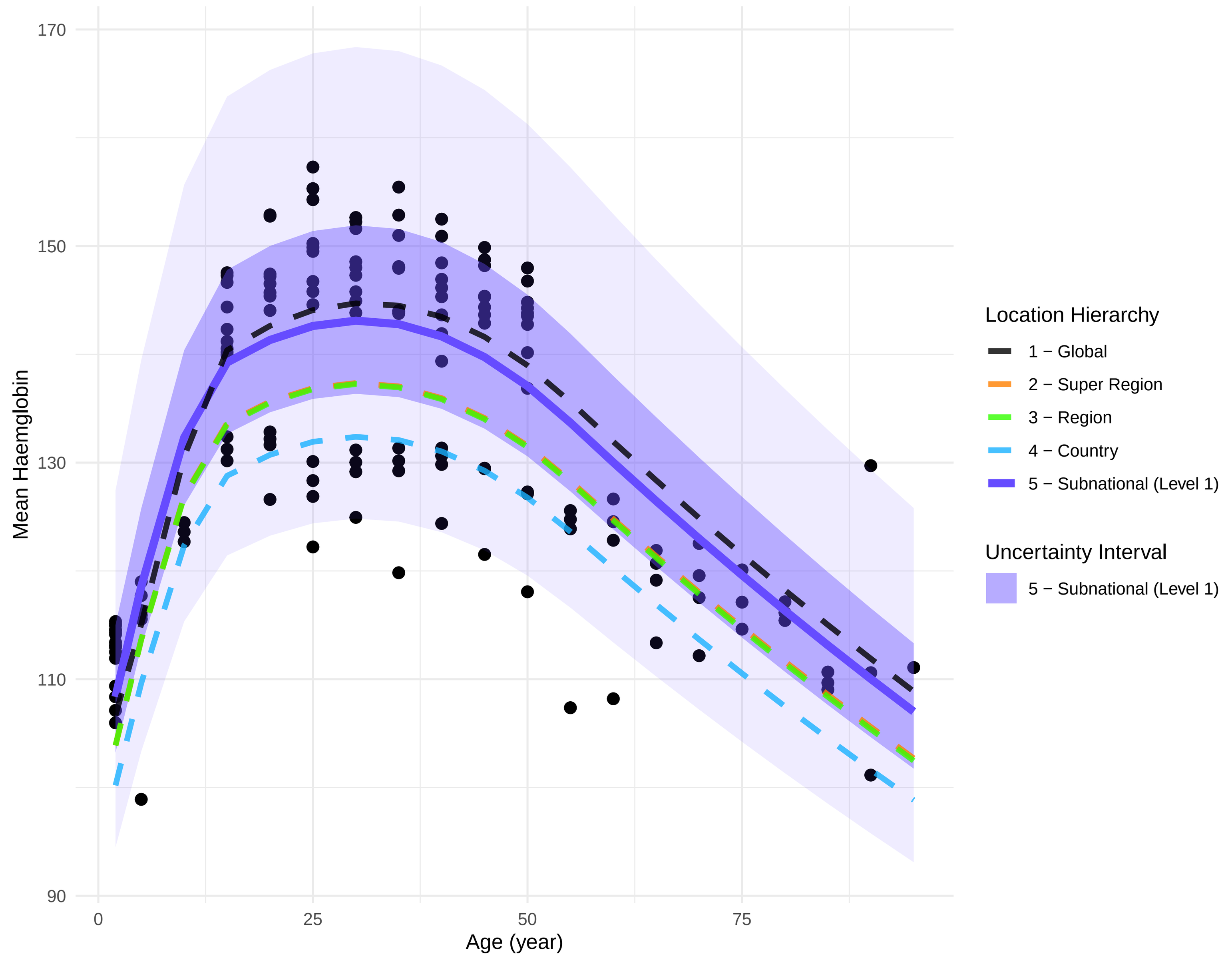

MR−BRT Age/Sex Fits − Males 2+ Years Old − Mean Haemoglobin − Kerala, Rural
WHO−2001 Adjustment Method

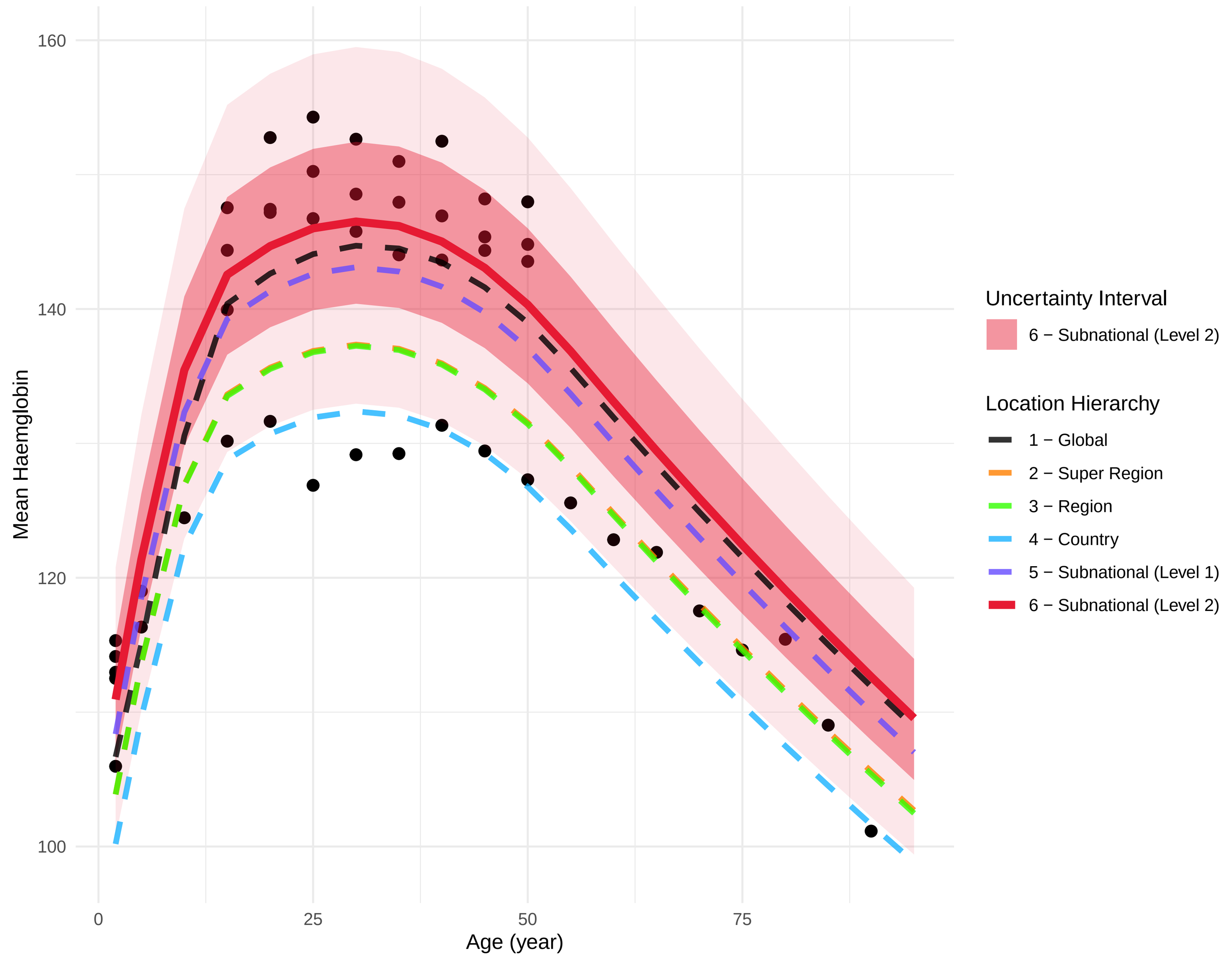

MR−BRT Age/Sex Fits − Males 2+ Years Old − Mean Haemoglobin − Kerala, Urban
WHO−2001 Adjustment Method

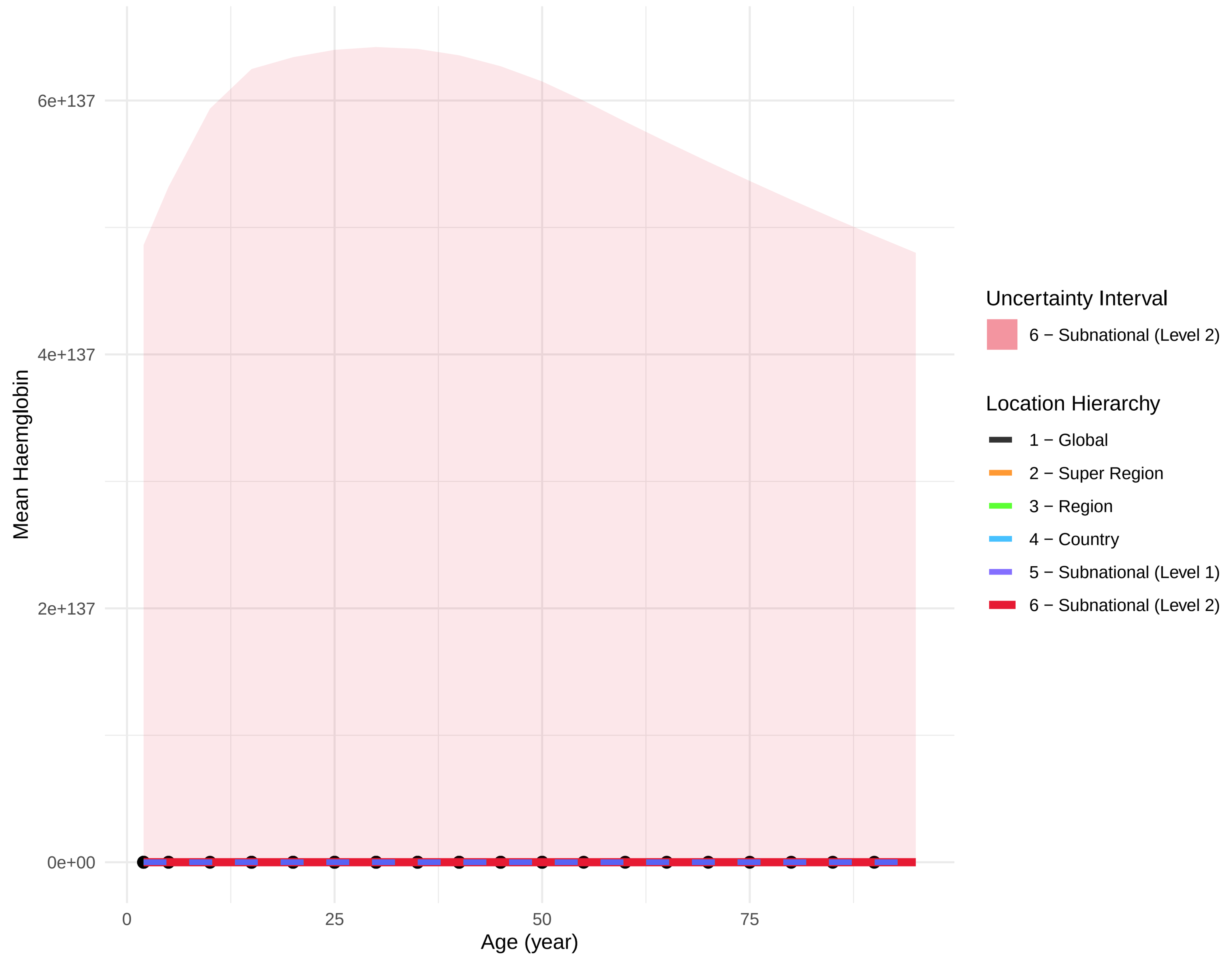

MR−BRT Age/Sex Fits − Males 2+ Years Old − Mean Haemoglobin − Madhya Pradesh
WHO−2001 Adjustment Method

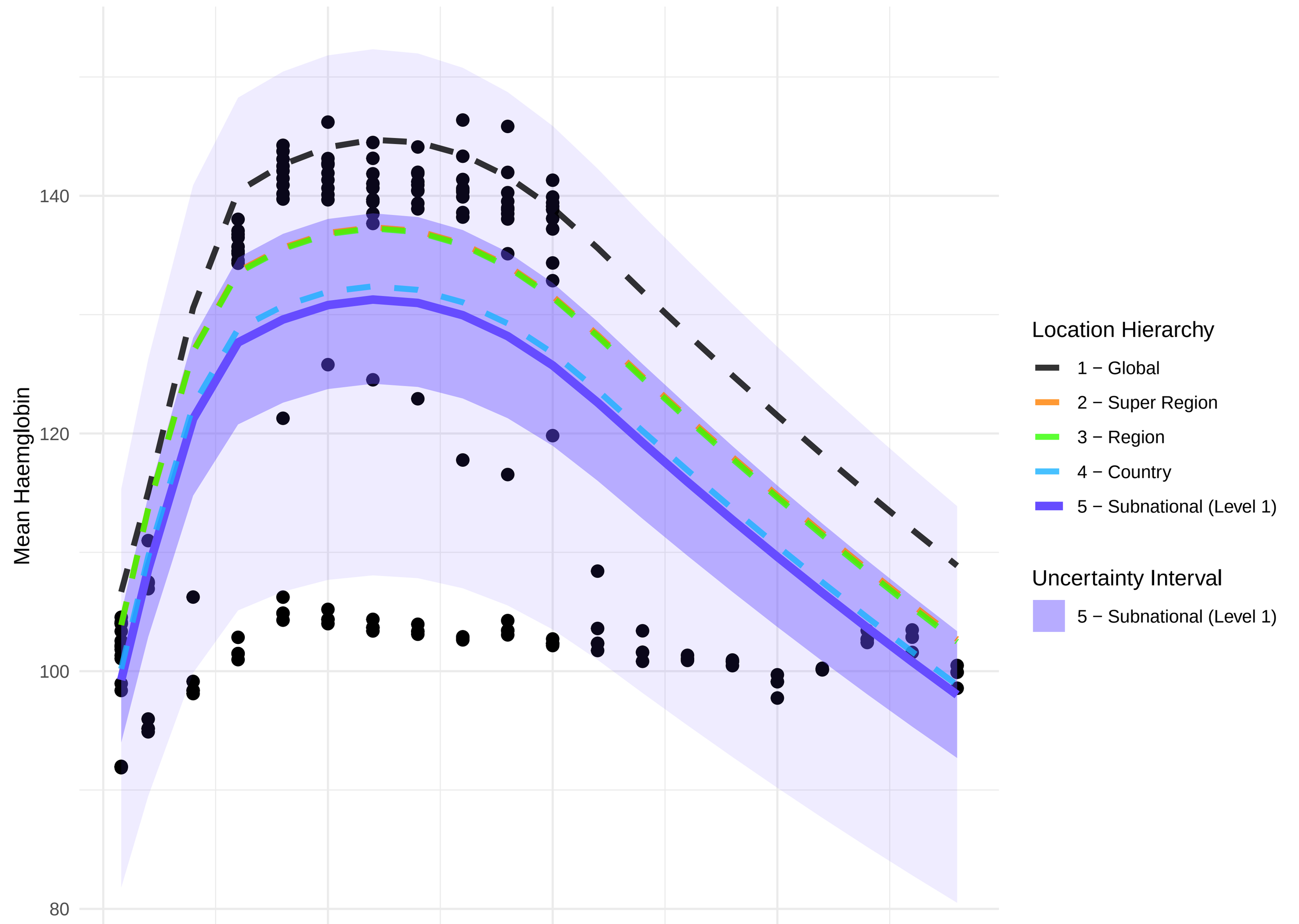

MR−BRT Age/Sex Fits − Males 2+ Years Old − Mean Haemoglobin − Madhya Pradesh, Rural
WHO−2001 Adjustment Method

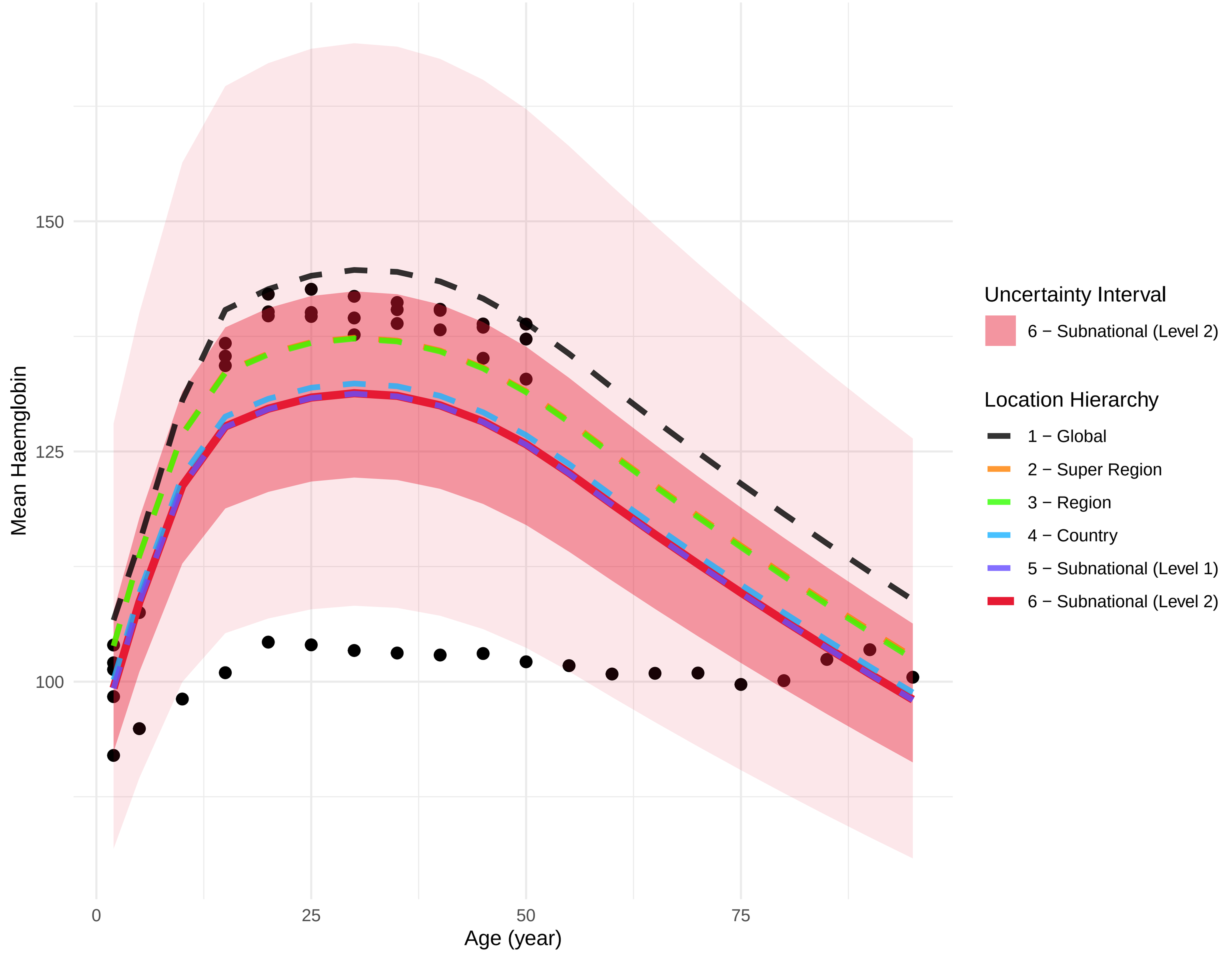

MR−BRT Age/Sex Fits − Males 2+ Years Old − Mean Haemoglobin − Madhya Pradesh, Urban
WHO−2001 Adjustment Method

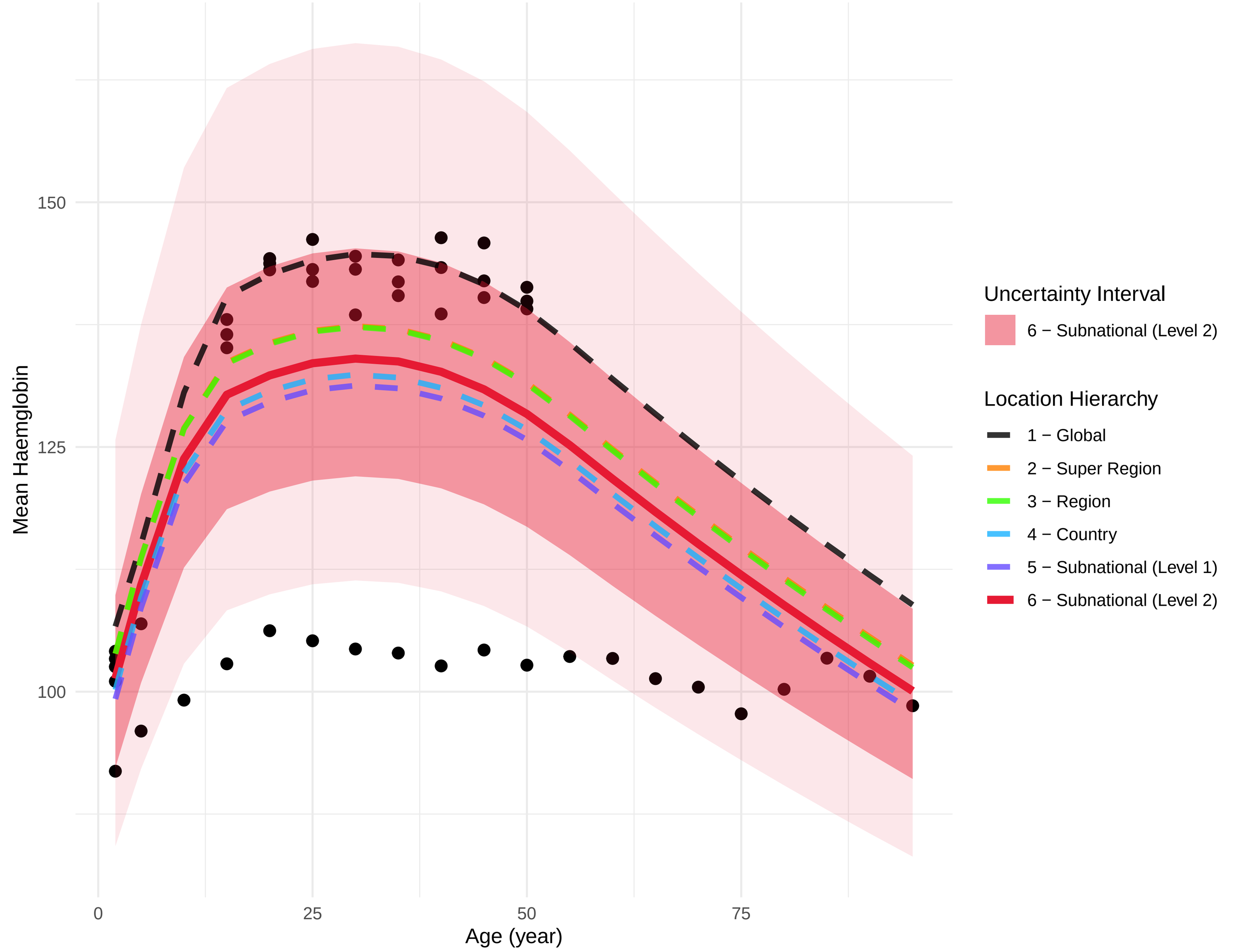

MR−BRT Age/Sex Fits − Males 2+ Years Old − Mean Haemoglobin − Maharashtra
WHO−2001 Adjustment Method

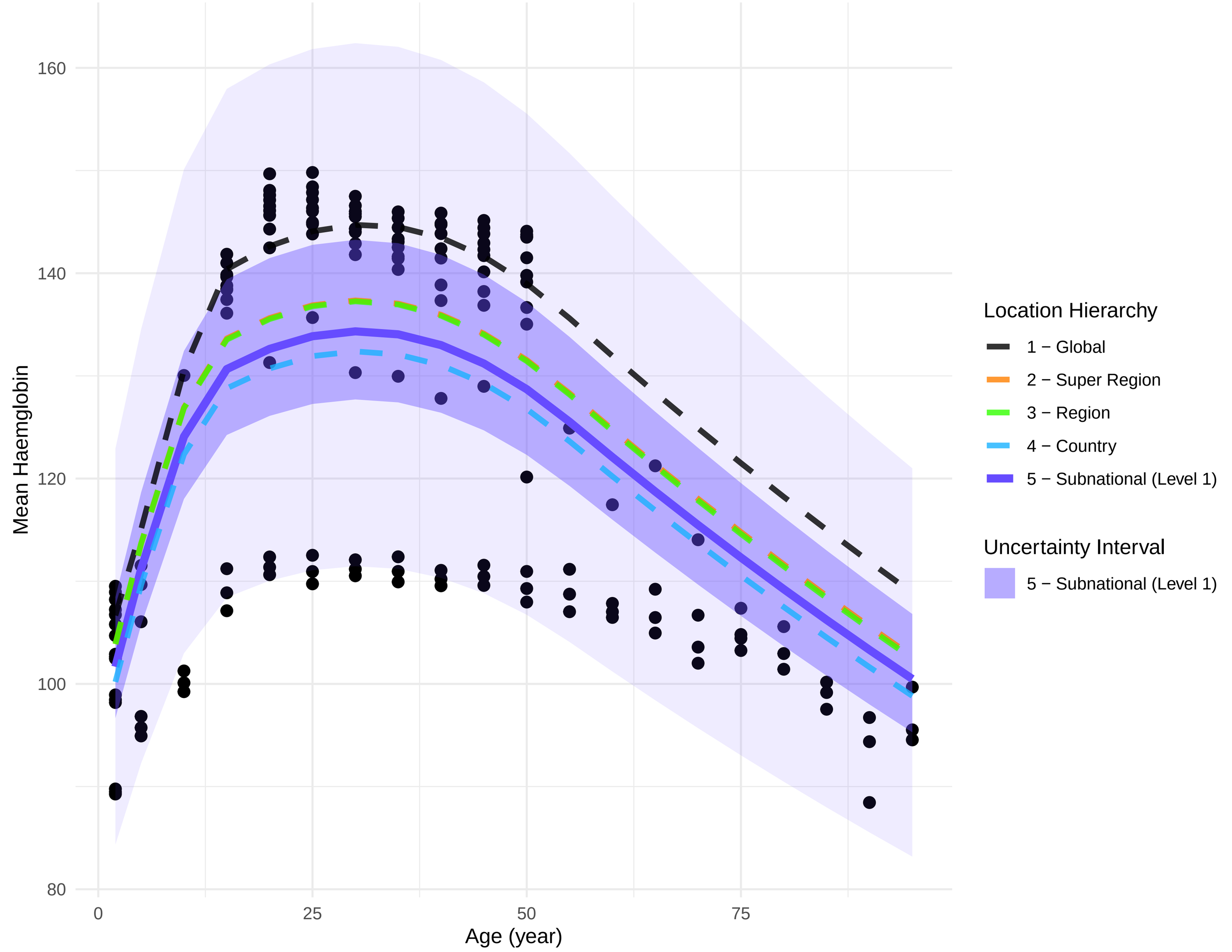

MR−BRT Age/Sex Fits − Males 2+ Years Old − Mean Haemoglobin − Maharashtra, Rural
WHO−2001 Adjustment Method

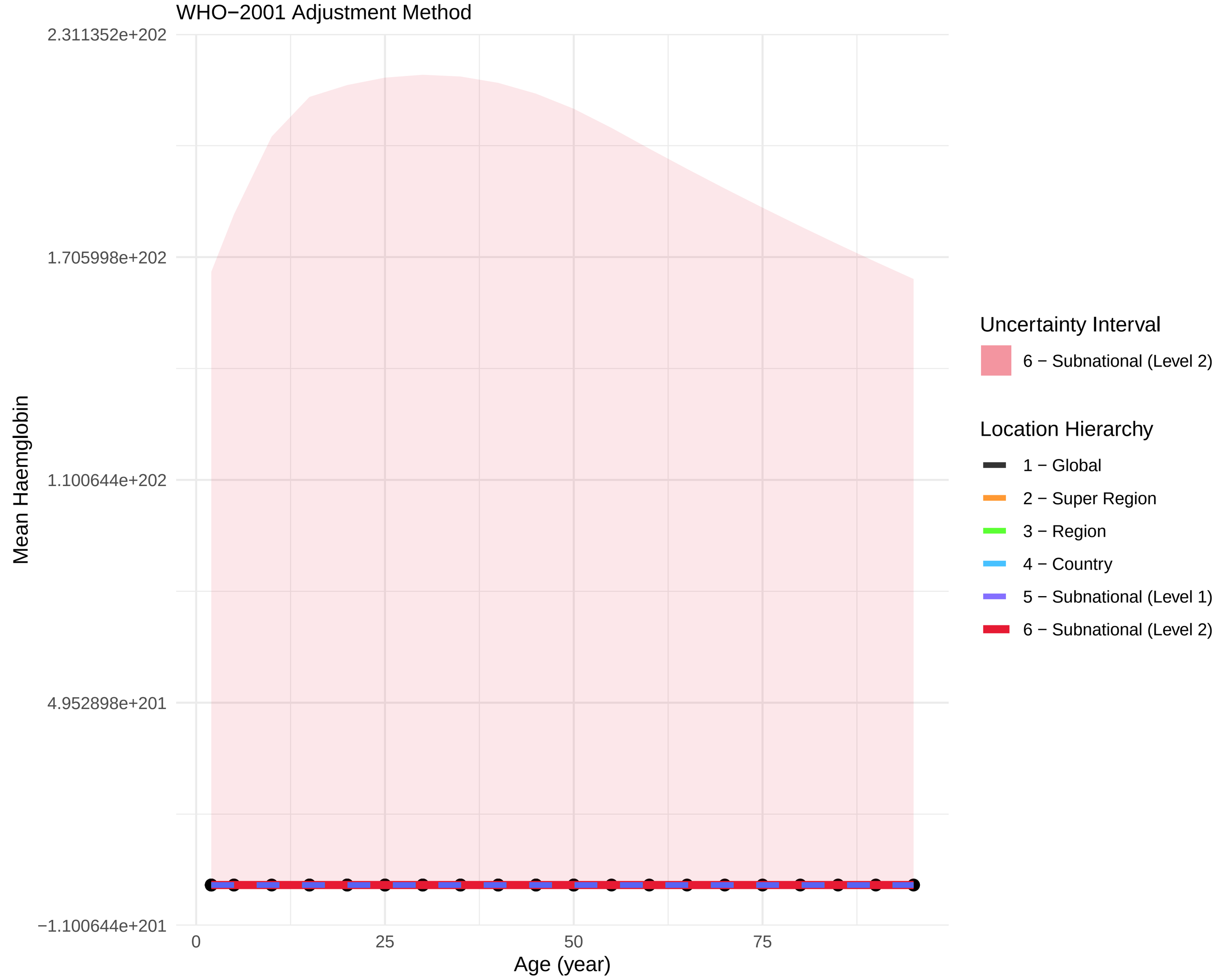

MR−BRT Age/Sex Fits − Males 2+ Years Old − Mean Haemoglobin − Maharashtra, Urban
WHO−2001 Adjustment Method

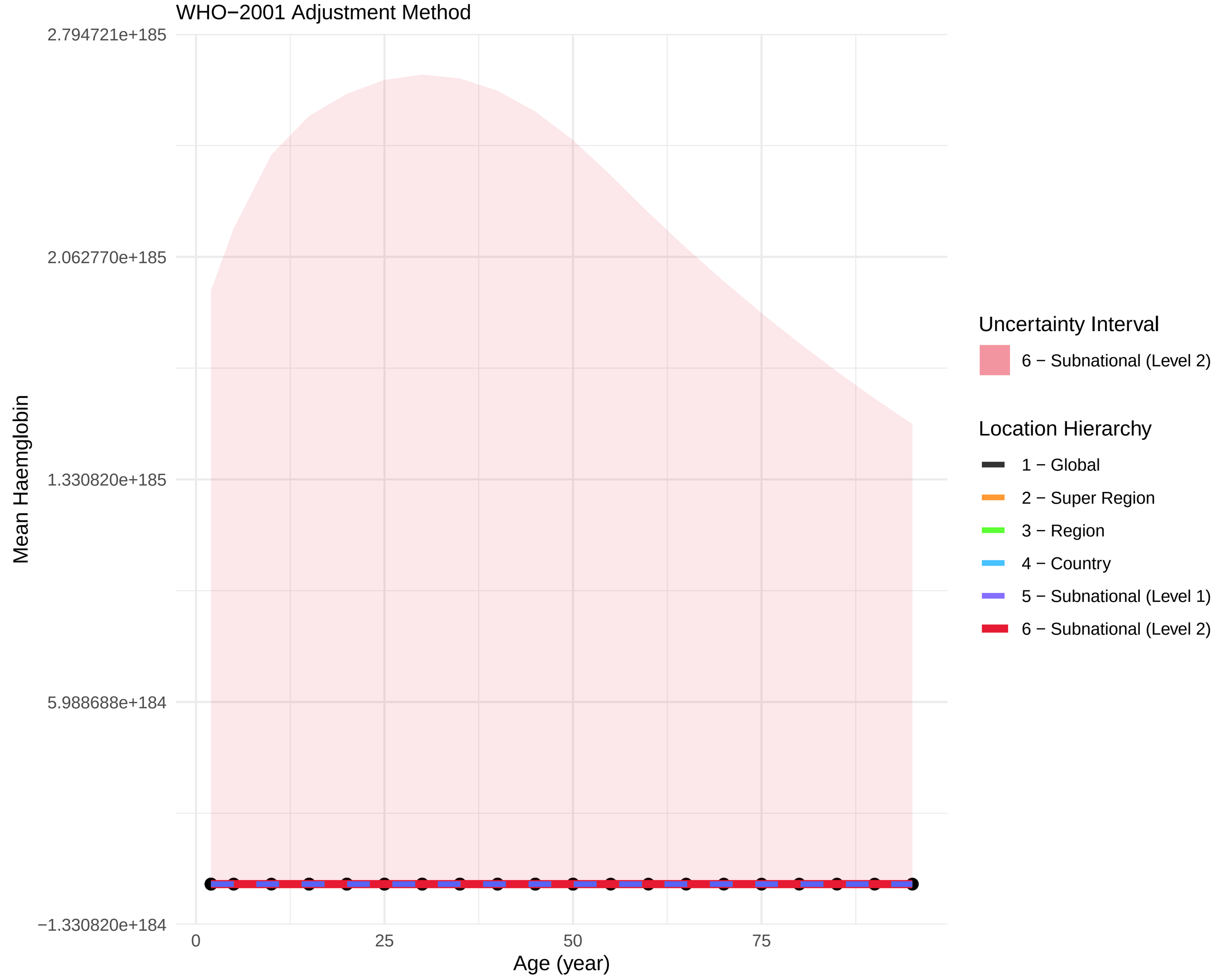

MR−BRT Age/Sex Fits − Males 2+ Years Old − Mean Haemoglobin − Manipur
WHO−2001 Adjustment Method

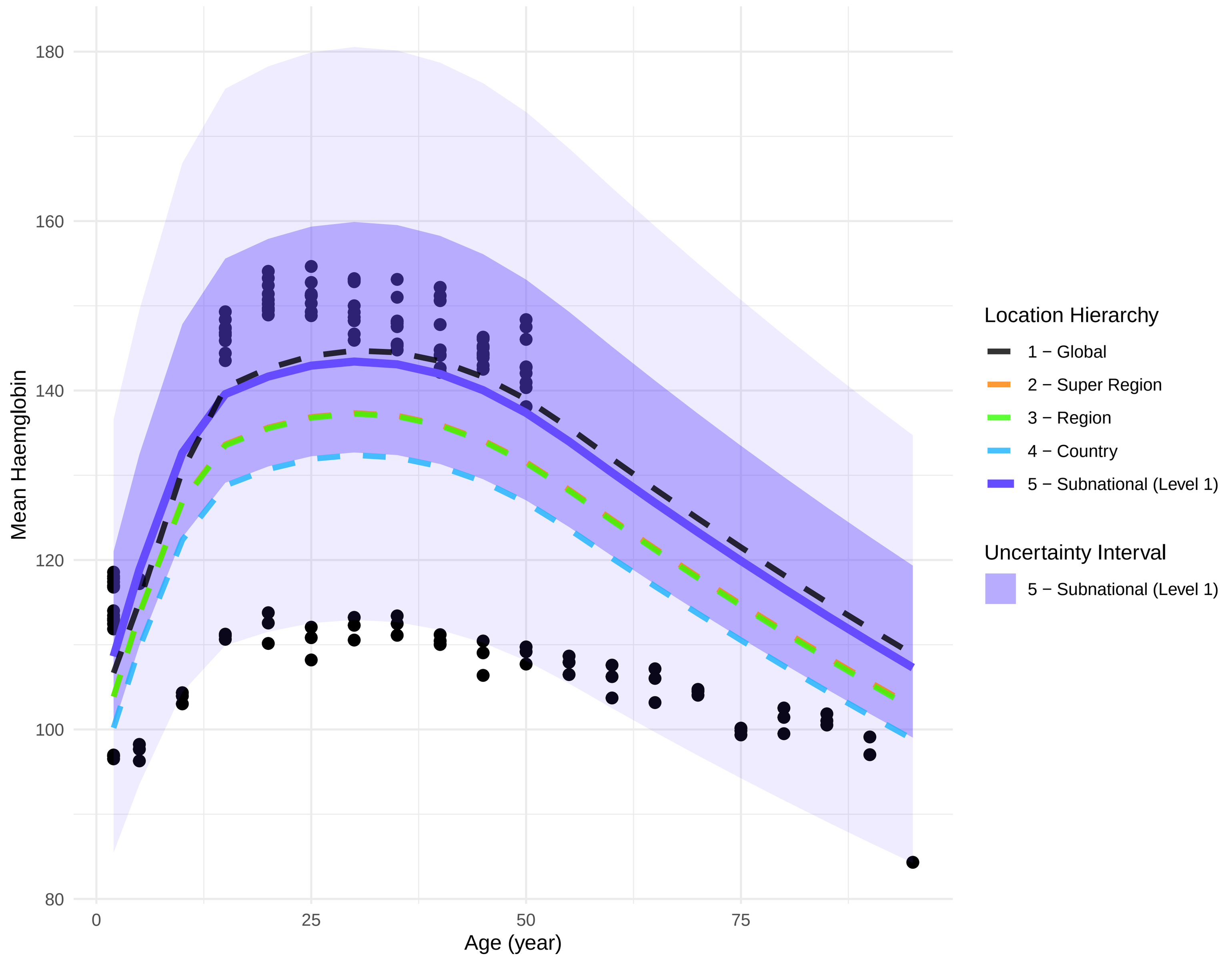

MR−BRT Age/Sex Fits − Males 2+ Years Old − Mean Haemoglobin − Manipur, Rural
WHO−2001 Adjustment Method

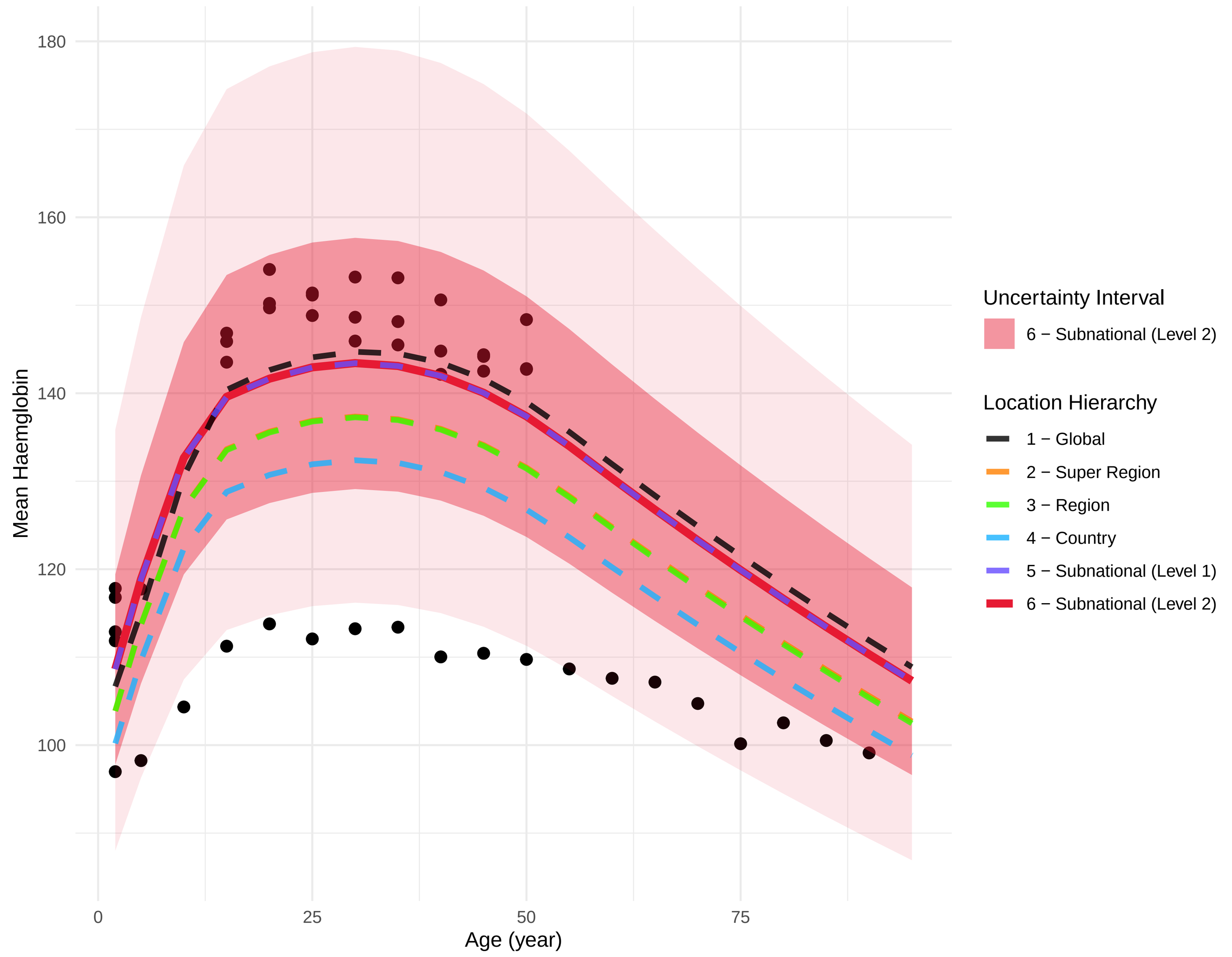

MR−BRT Age/Sex Fits − Males 2+ Years Old − Mean Haemoglobin − Manipur, Urban
WHO−2001 Adjustment Method

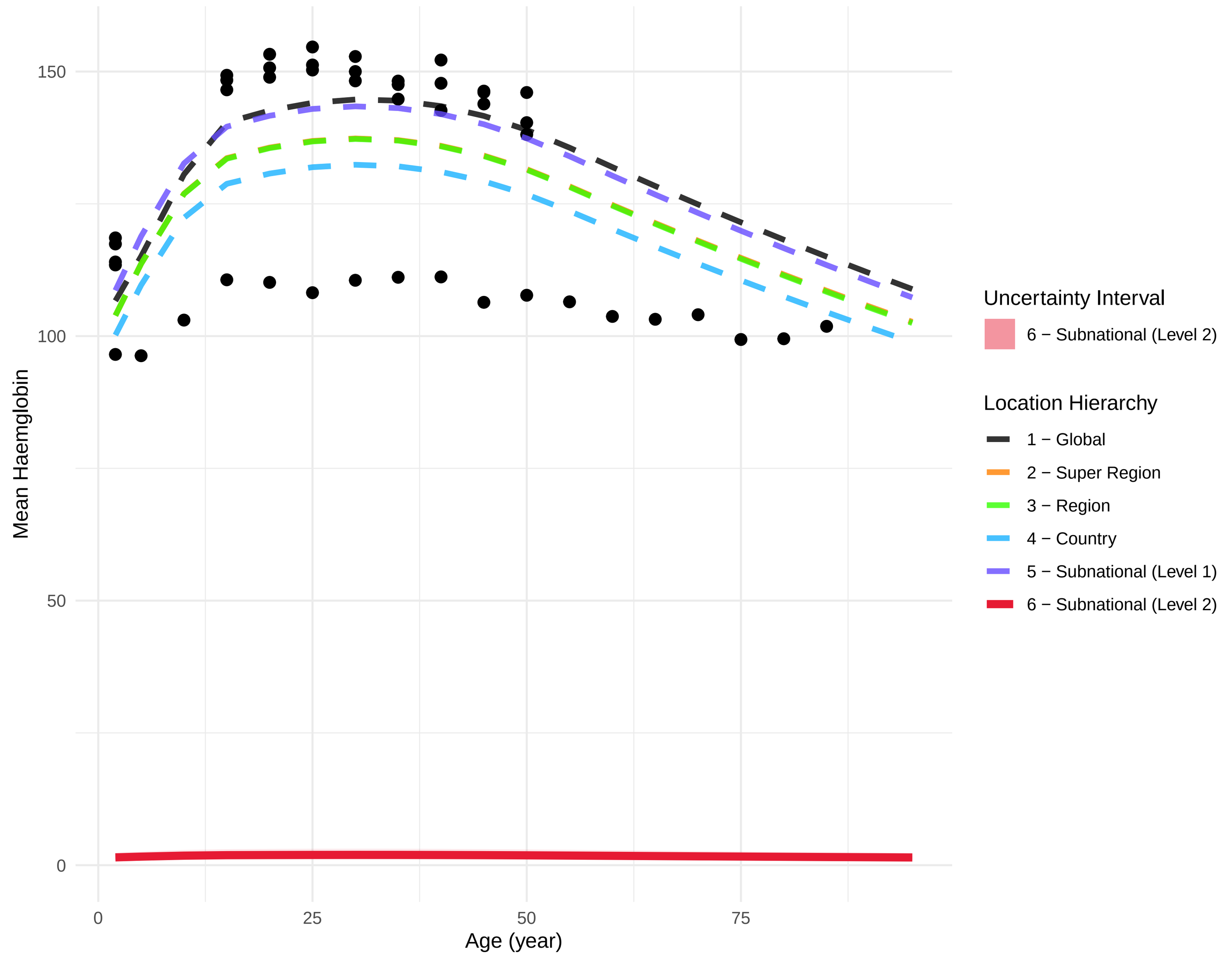

MR−BRT Age/Sex Fits − Males 2+ Years Old − Mean Haemoglobin − Meghalaya
WHO−2001 Adjustment Method

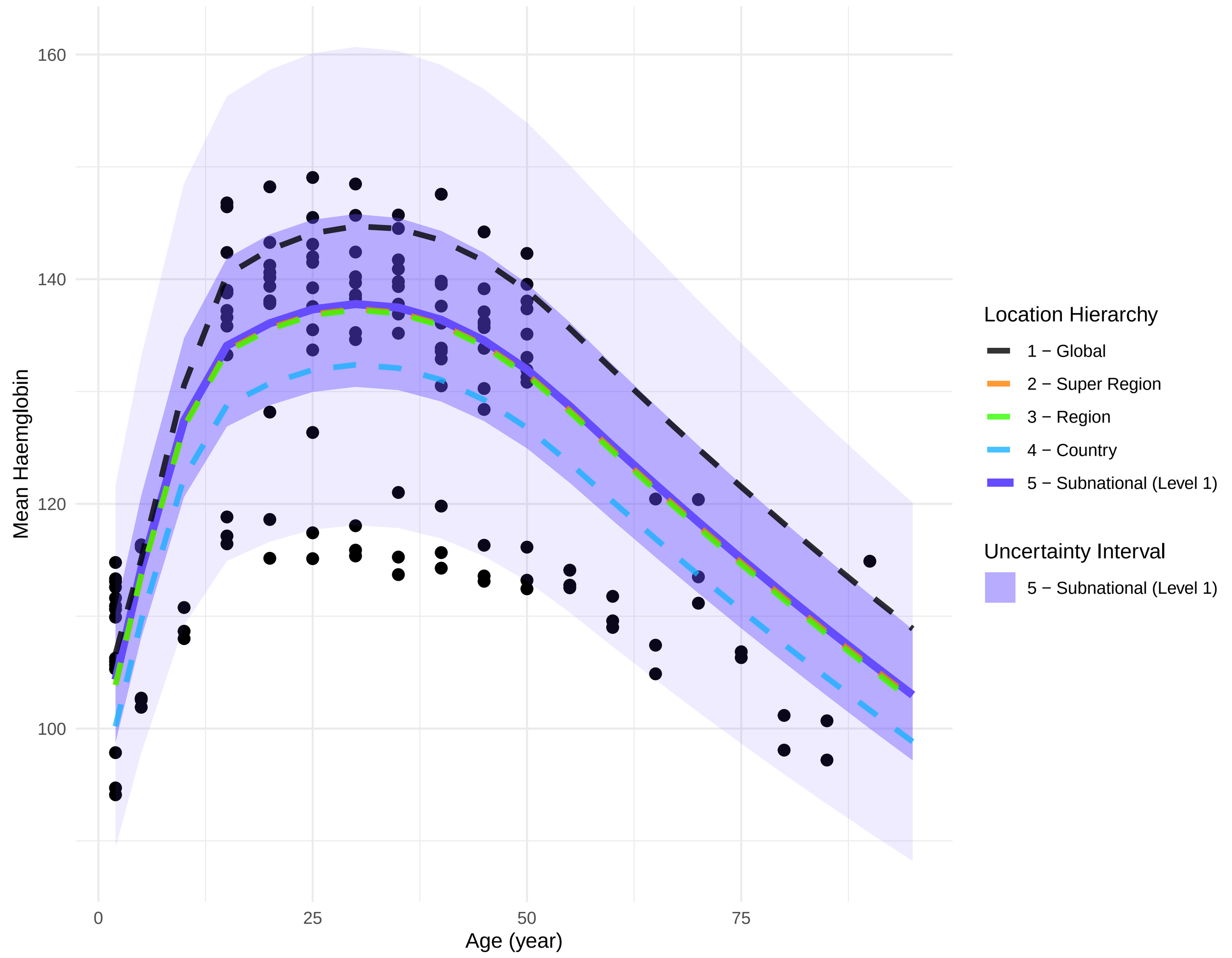

MR−BRT Age/Sex Fits − Males 2+ Years Old − Mean Haemoglobin − Meghalaya, Rural
WHO−2001 Adjustment Method

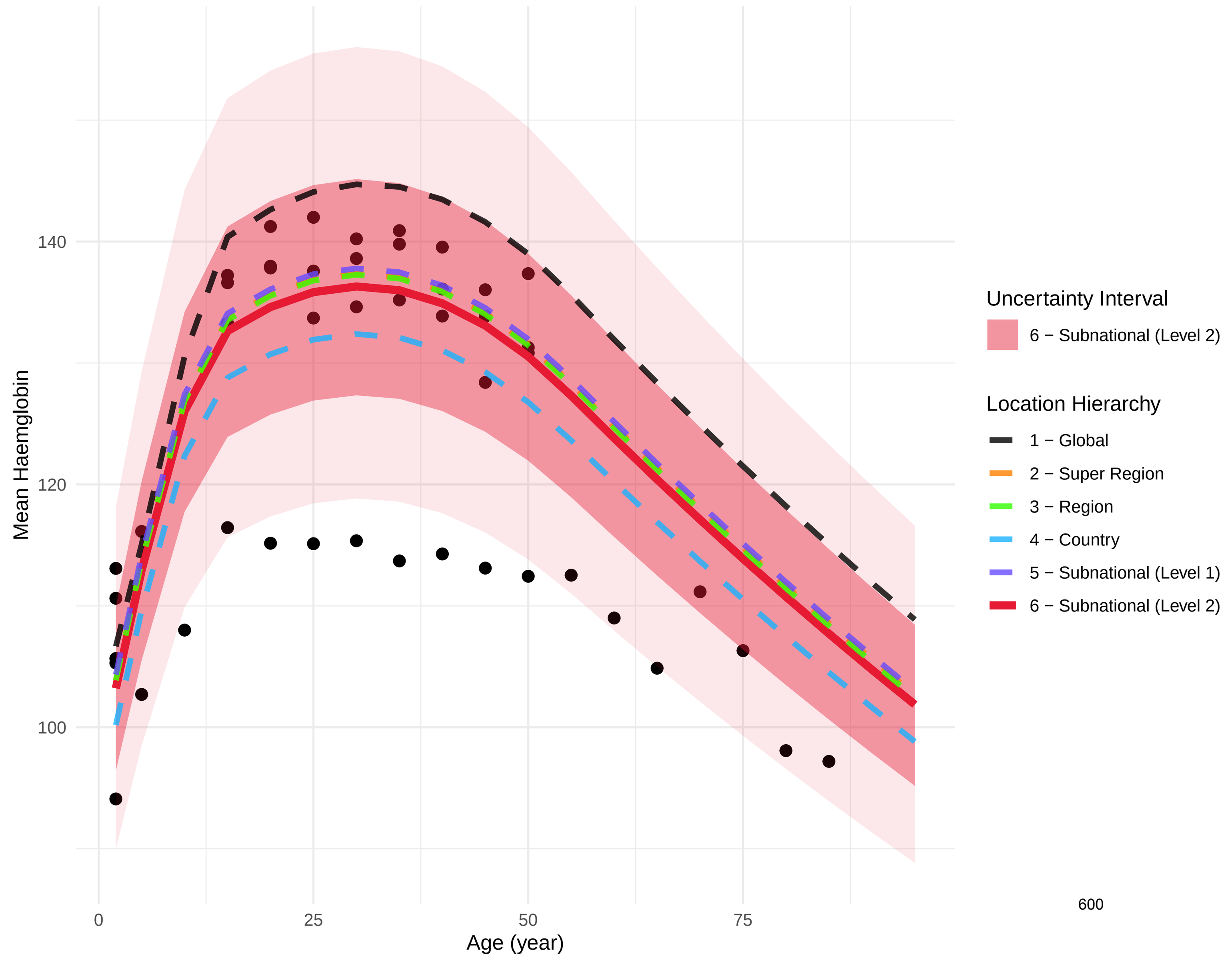

MR−BRT Age/Sex Fits − Males 2+ Years Old − Mean Haemglobin − Meghalaya, Urban
WHO−2001 Adjustment Method

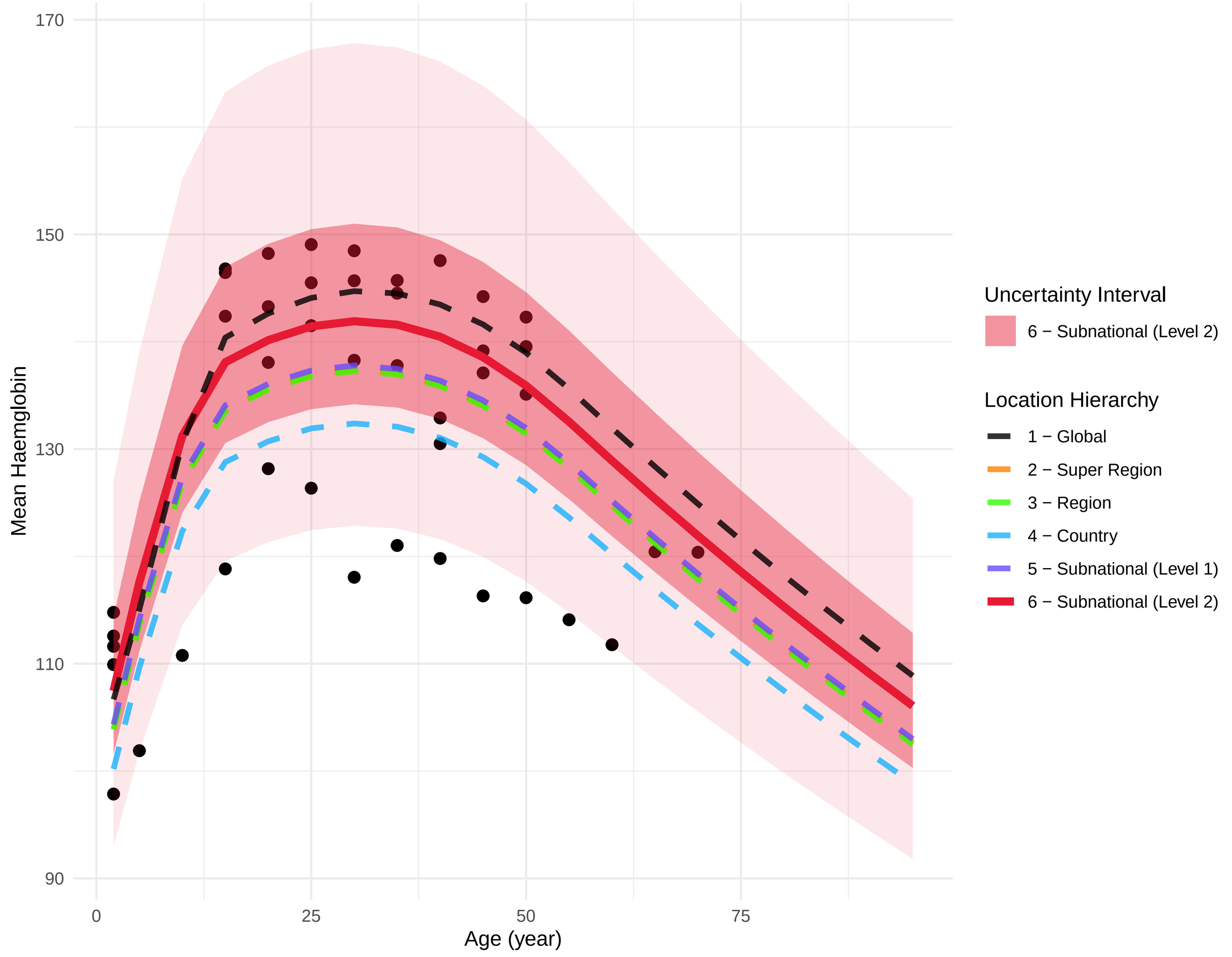

MR−BRT Age/Sex Fits − Males 2+ Years Old − Mean Haemglobin − Mizoram
WHO−2001 Adjustment Method

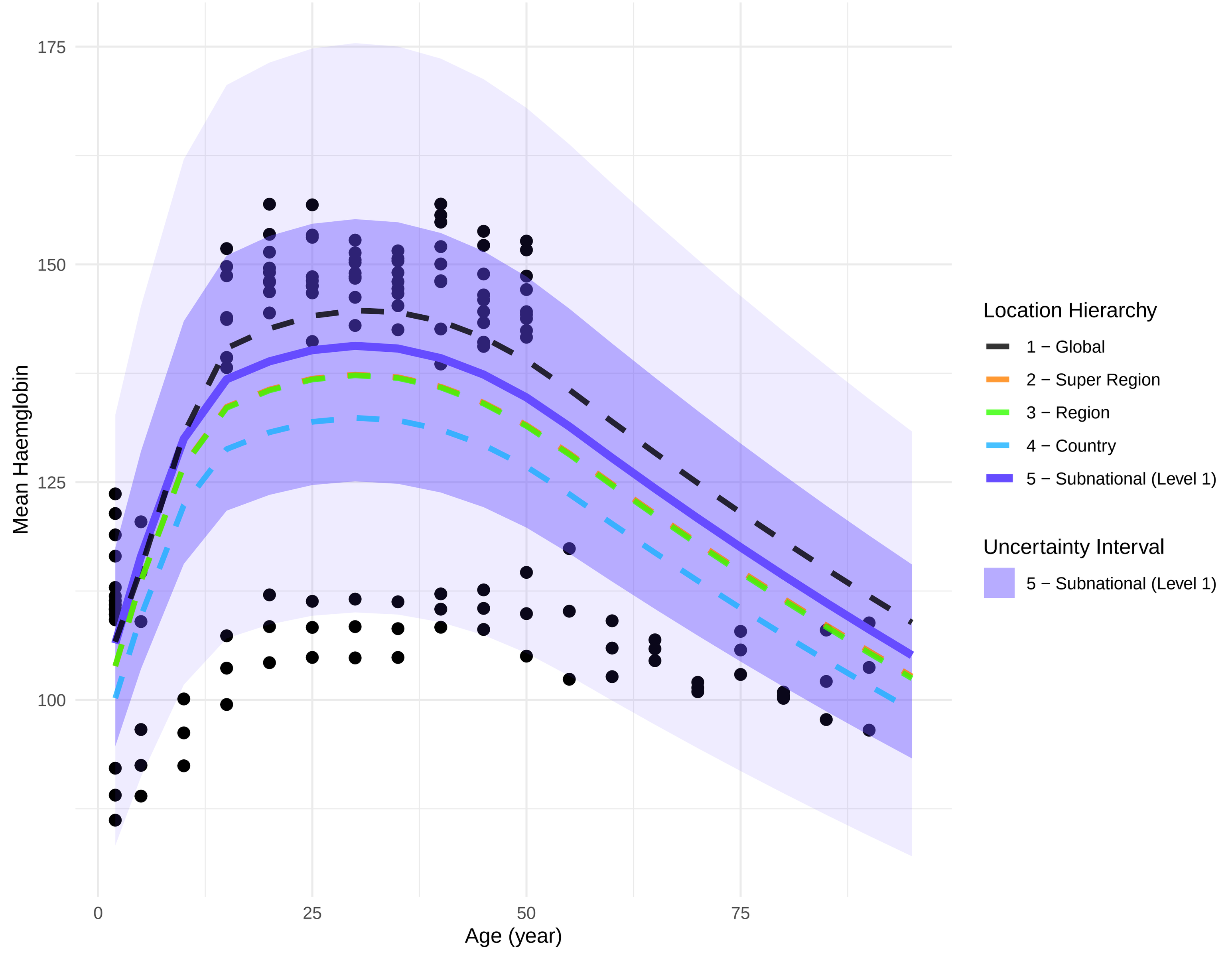

MR−BRT Age/Sex Fits − Males 2+ Years Old − Mean Haemglobin − Mizoram, Rural
WHO−2001 Adjustment Method

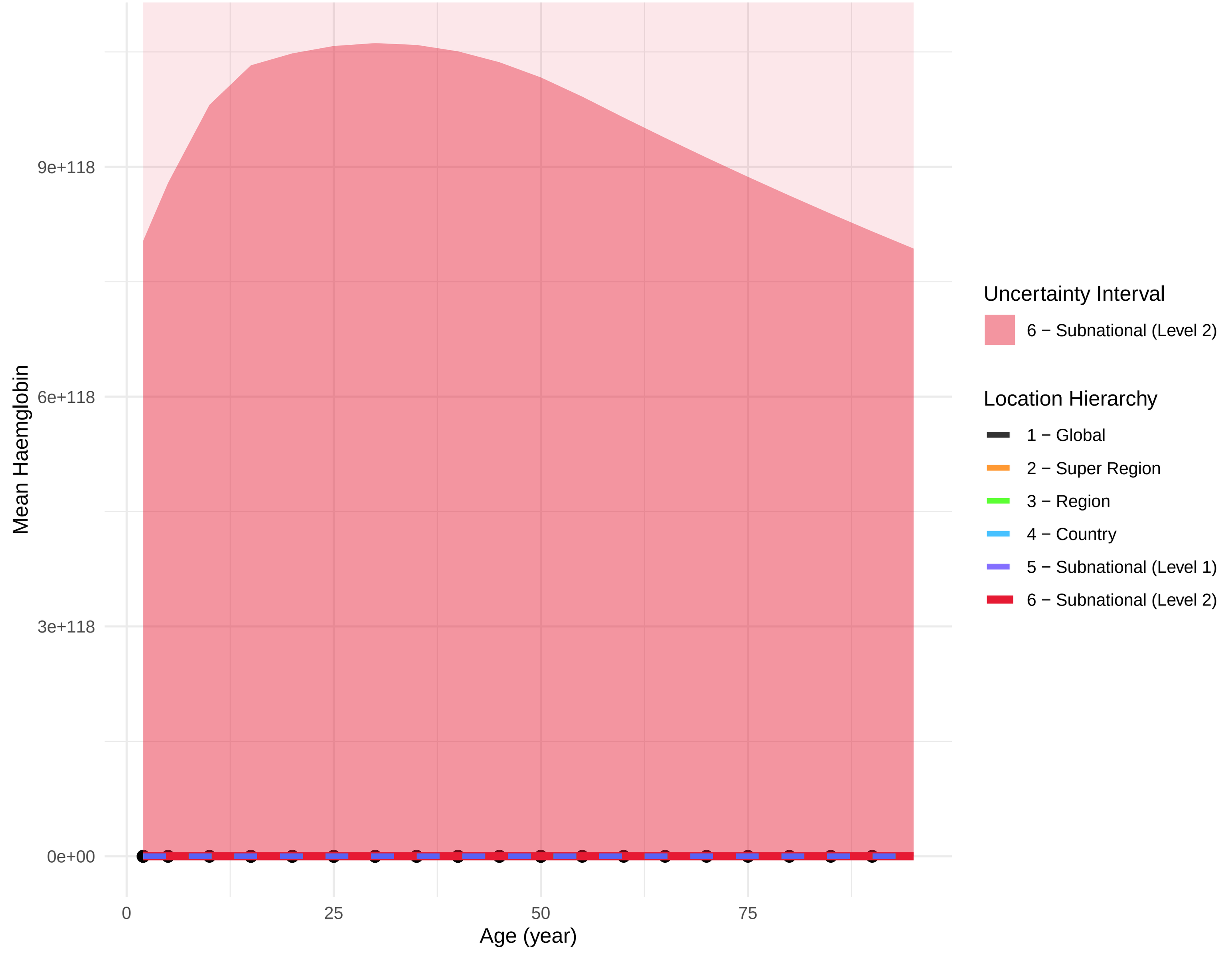

MR−BRT Age/Sex Fits − Males 2+ Years Old − Mean Haemglobin − Mizoram, Urban
WHO−2001 Adjustment Method

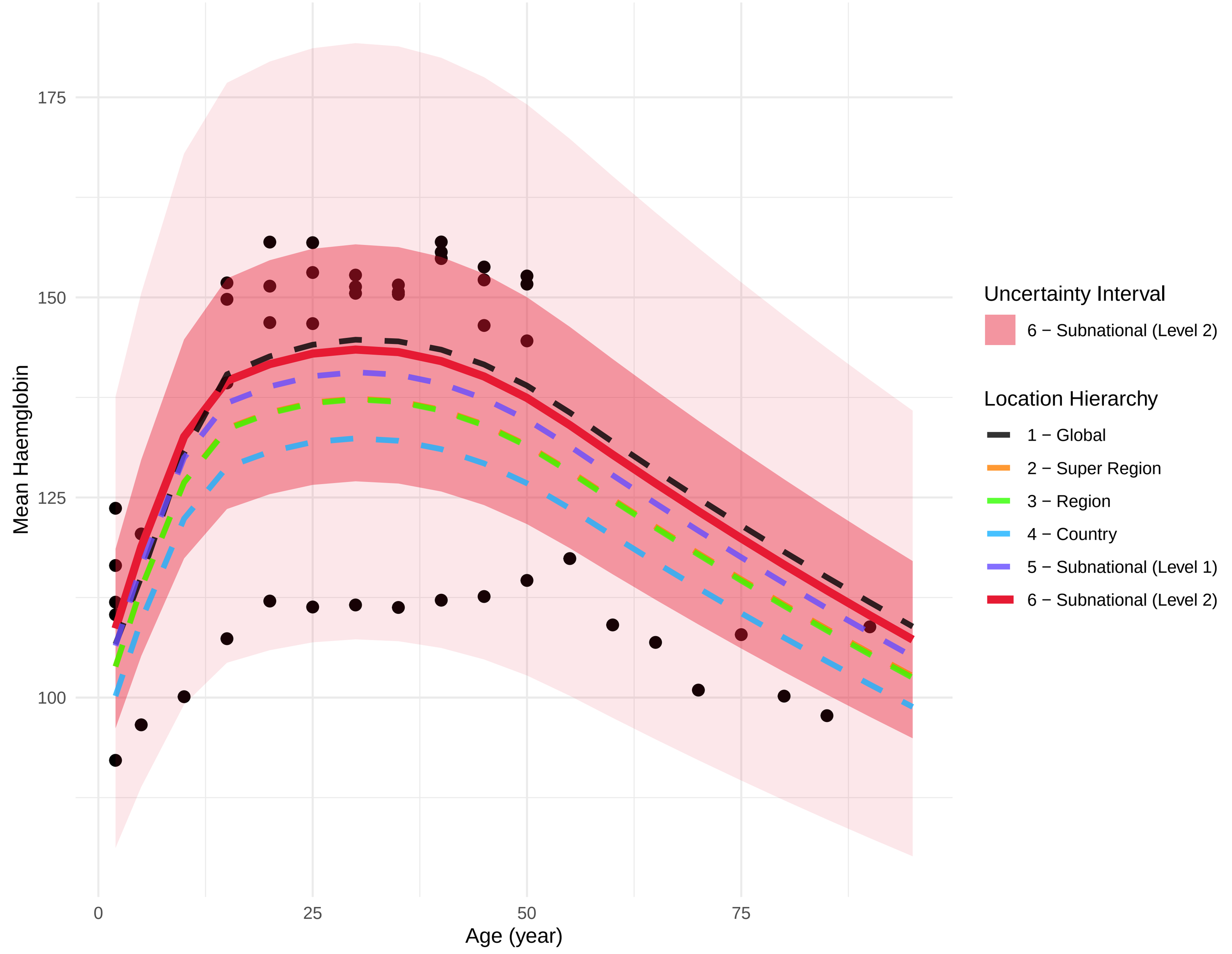

MR−BRT Age/Sex Fits − Males 2+ Years Old − Mean Haemglobin − Nagaland
WHO−2001 Adjustment Method

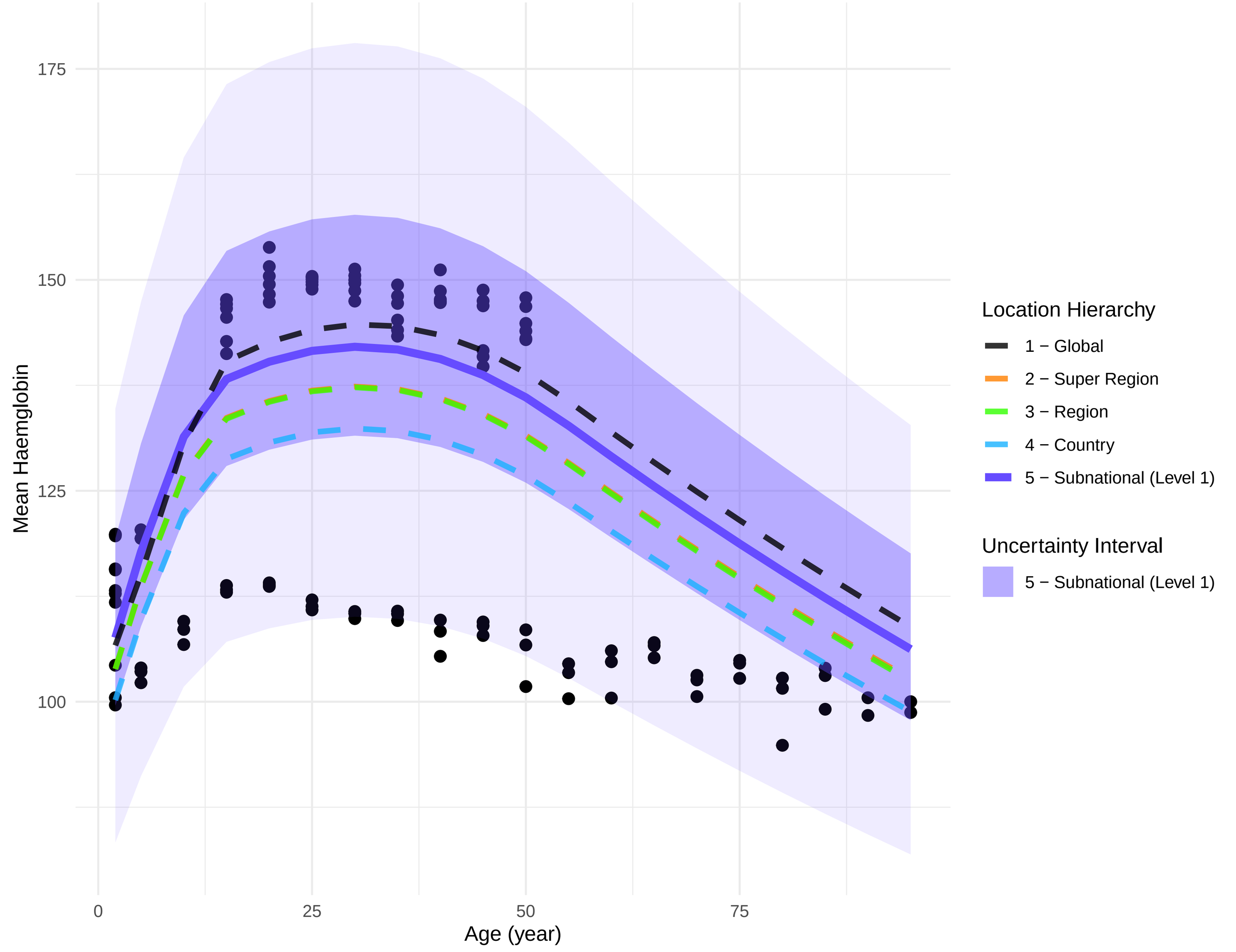

MR−BRT Age/Sex Fits − Males 2+ Years Old − Mean Haemglobin − Nagaland, Rural
WHO−2001 Adjustment Method

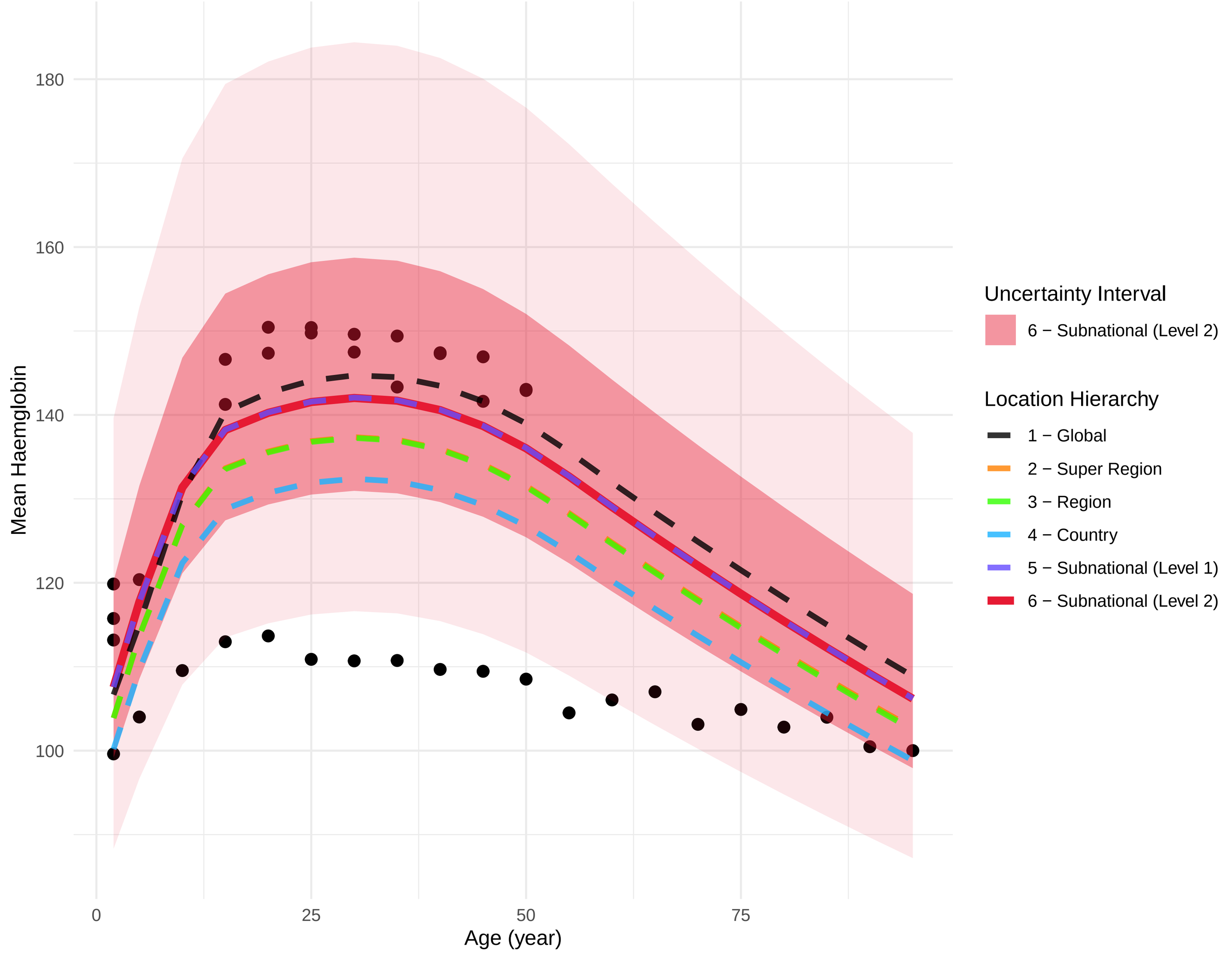

MR−BRT Age/Sex Fits − Males 2+ Years Old − Mean Haemglobin − Nagaland, Urban
WHO−2001 Adjustment Method

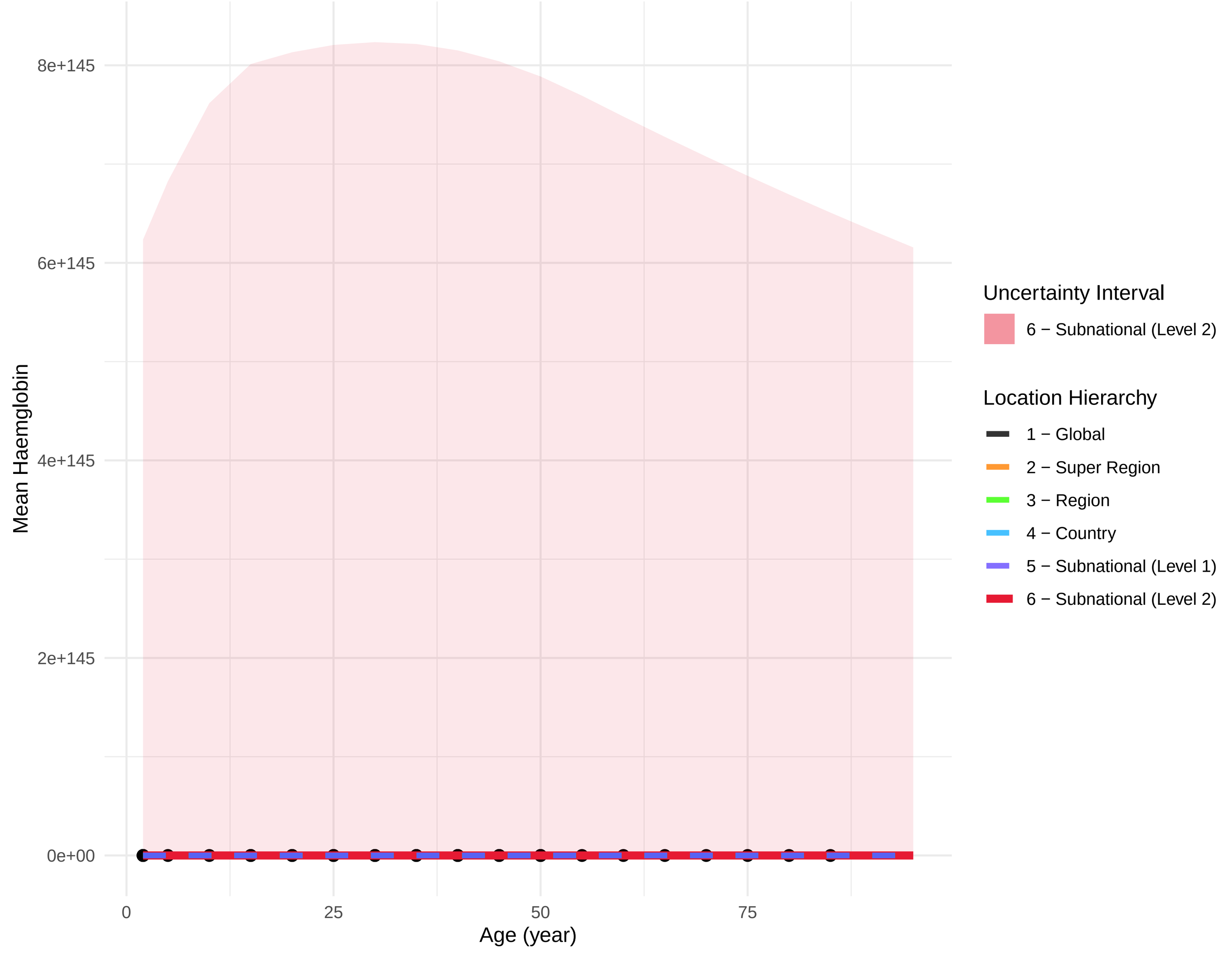

MR−BRT Age/Sex Fits − Males 2+ Years Old − Mean Haemglobin − Odisha
WHO−2001 Adjustment Method

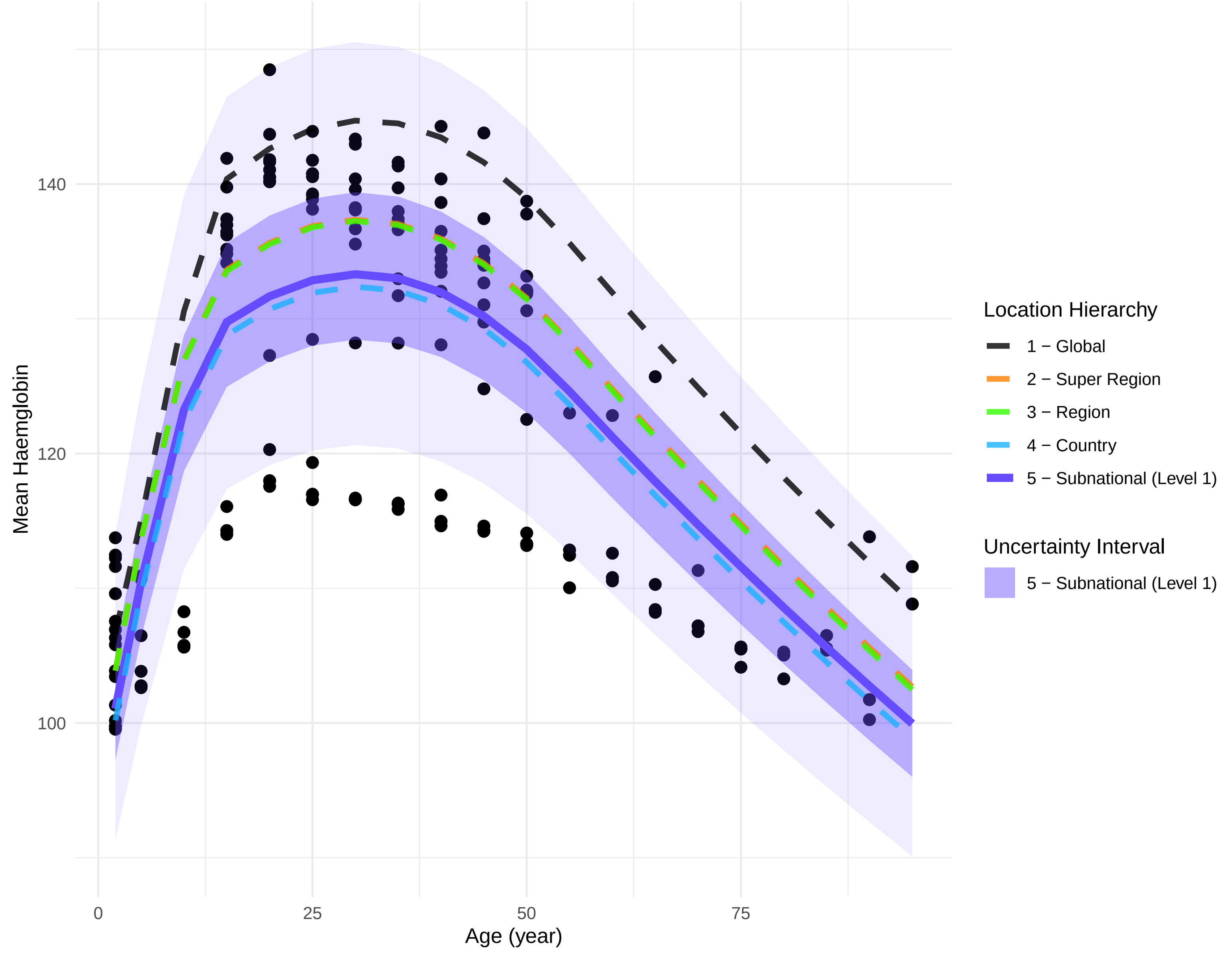

MR−BRT Age/Sex Fits − Males 2+ Years Old − Mean Haemglobin − Odisha, Rural
WHO−2001 Adjustment Method

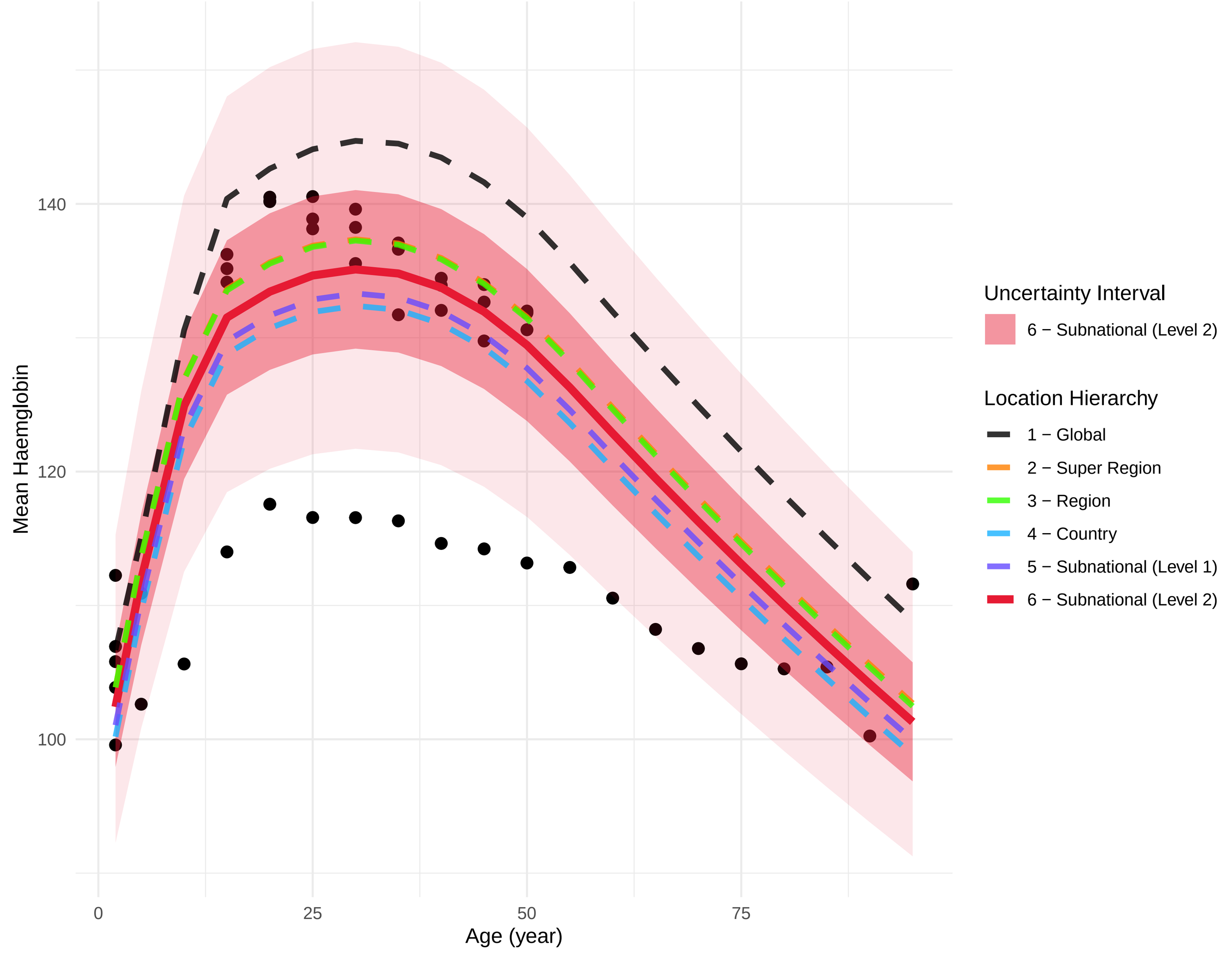

MR−BRT Age/Sex Fits − Males 2+ Years Old − Mean Haemglobin − Odisha, Urban
WHO−2001 Adjustment Method

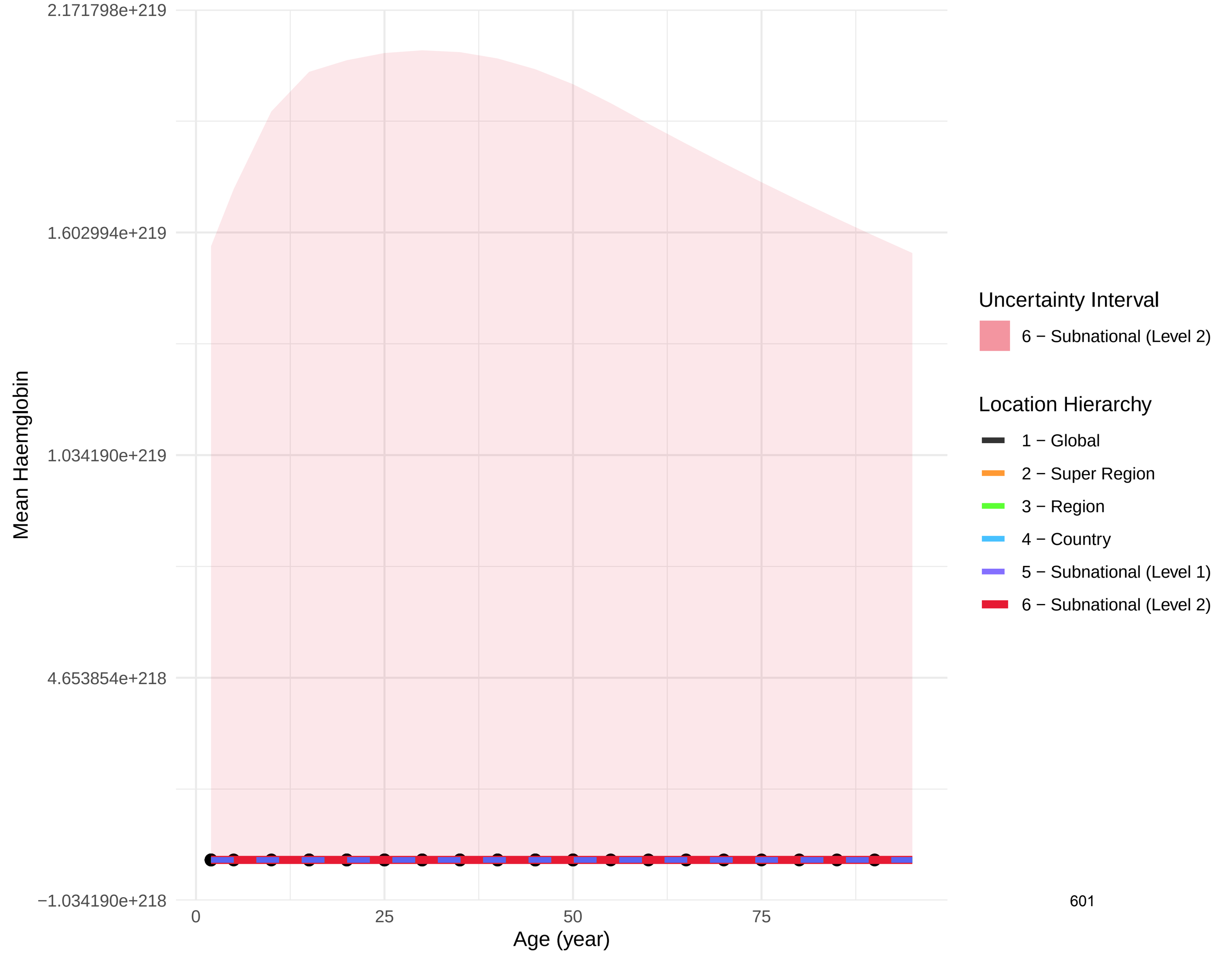

MR−BRT Age/Sex Fits − Males 2+ Years Old − Mean Haemglobin − Other Union Territories
WHO−2001 Adjustment Method

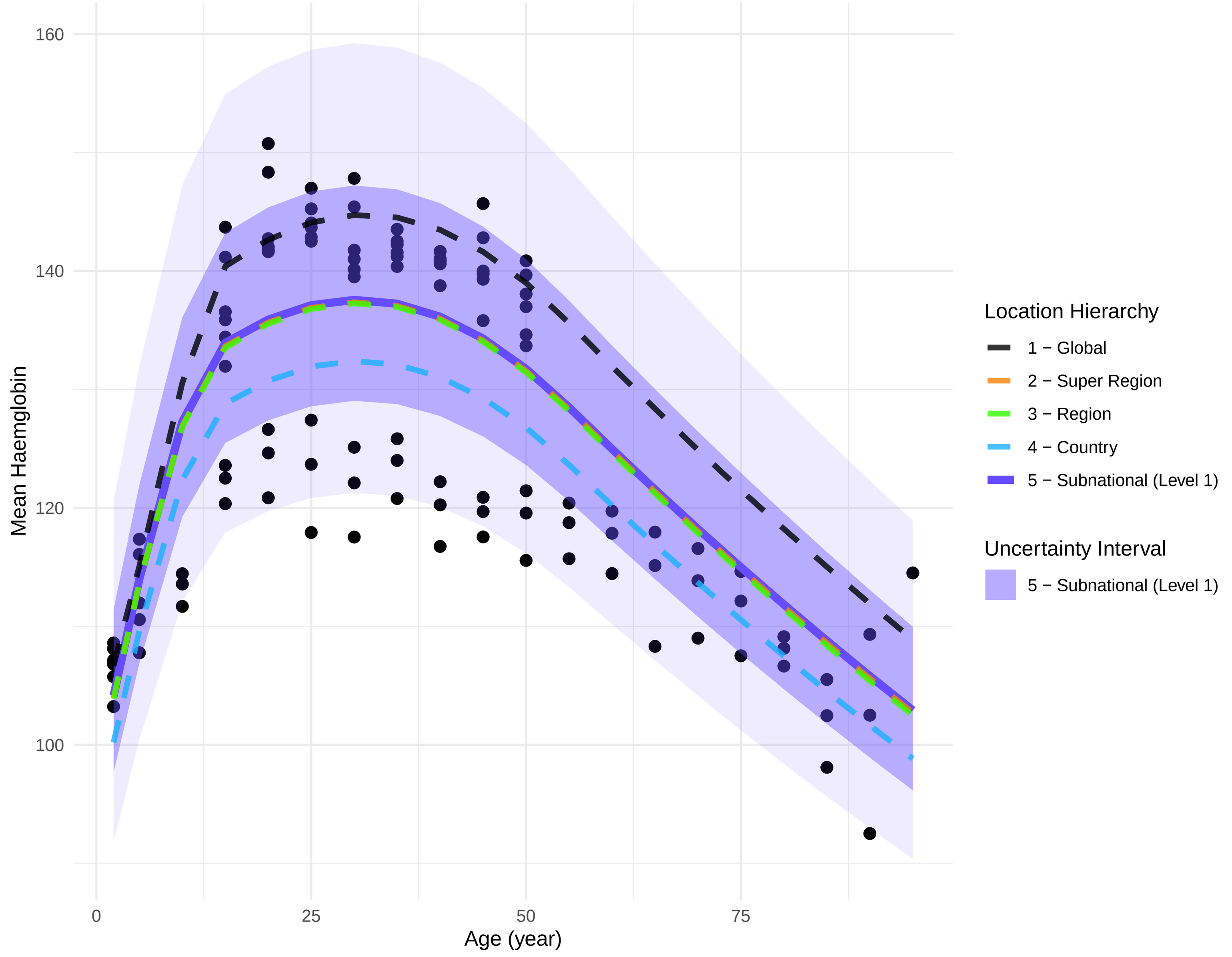

MR−BRT Age/Sex Fits − Males 2+ Years Old − Mean Haemglobin − Other Union Territories, Rural
WHO−2001 Adjustment Method

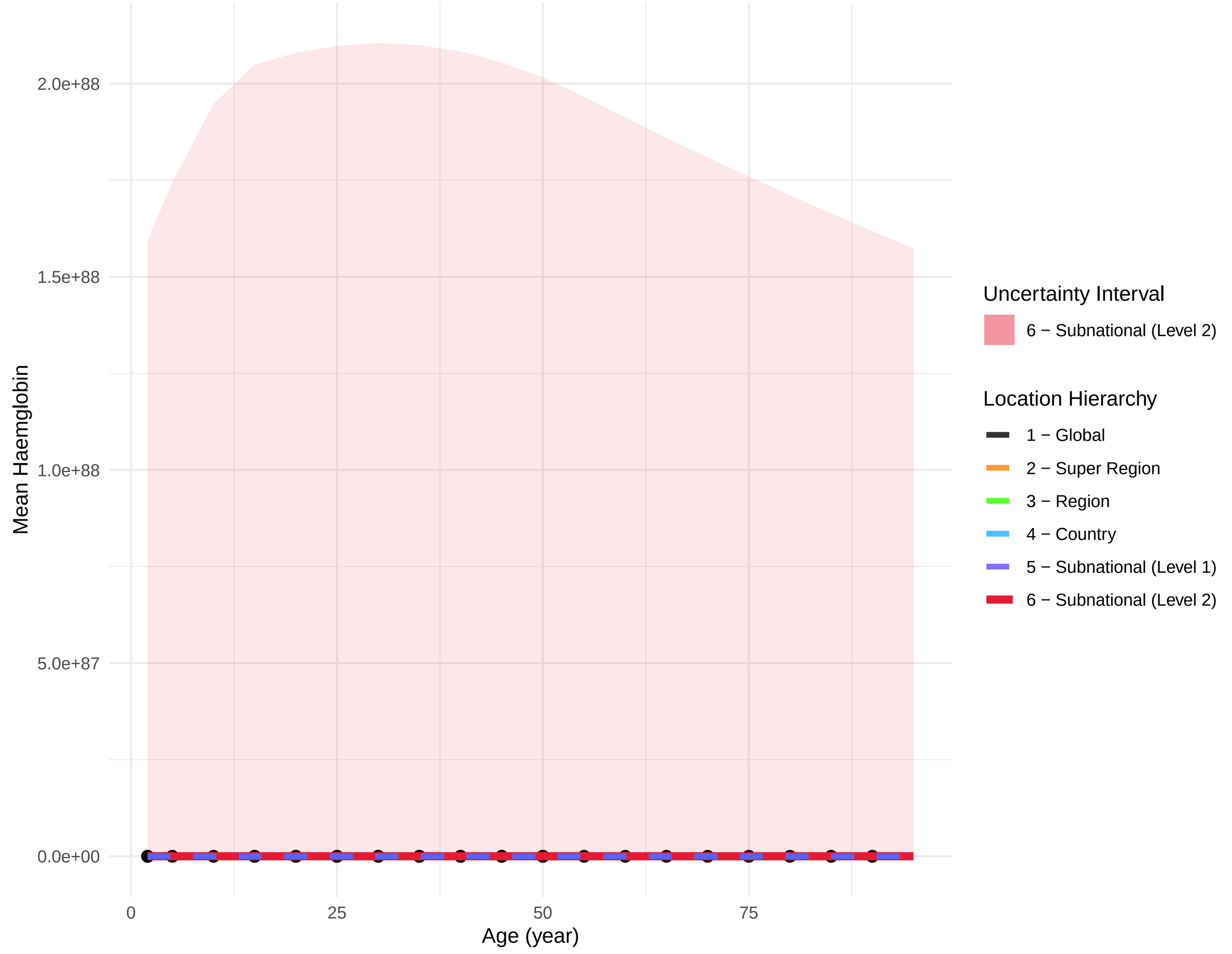

MR−BRT Age/Sex Fits − Males 2+ Years Old − Mean Haemglobin − Other Union Territories, Urban
WHO−2001 Adjustment Method

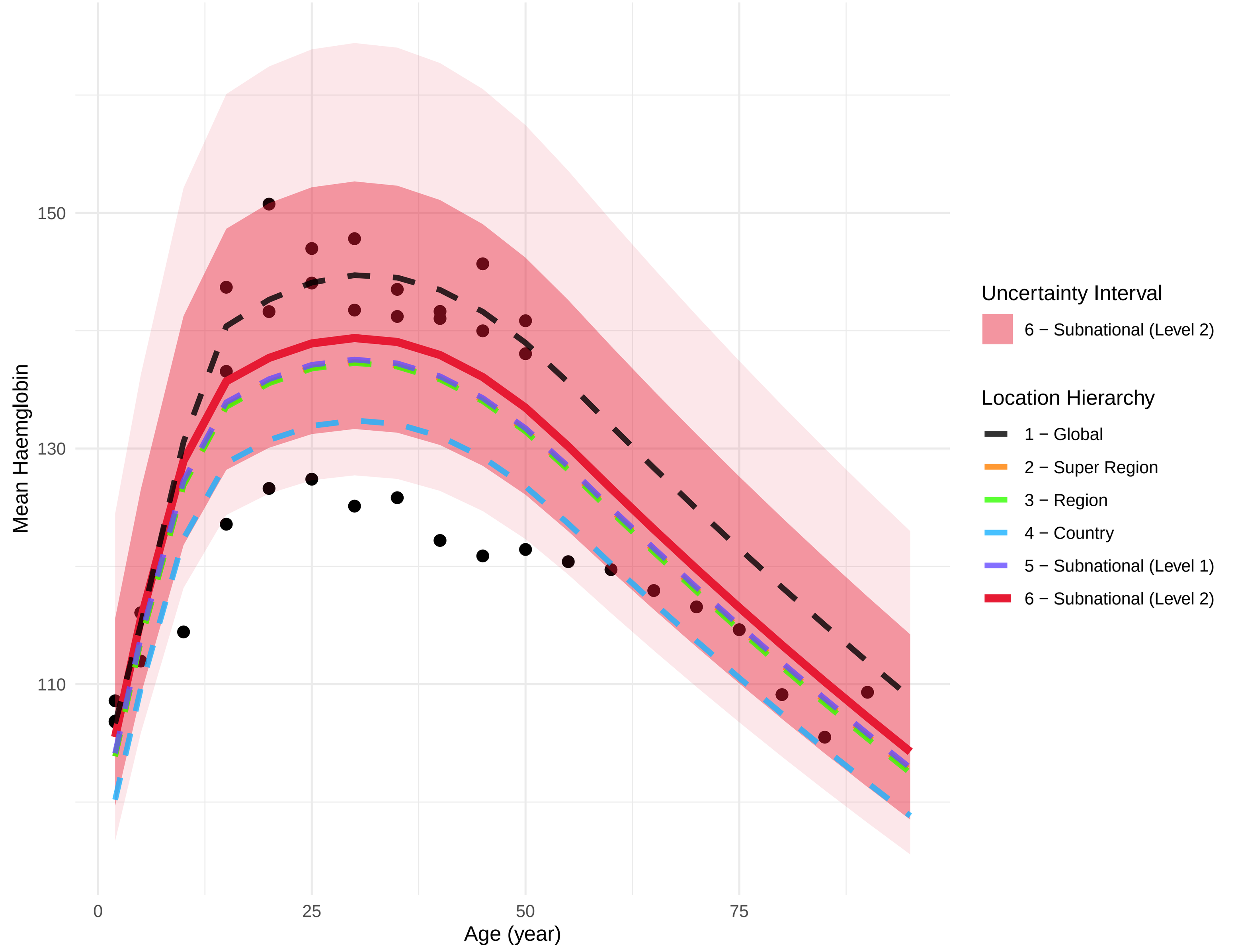

MR−BRT Age/Sex Fits − Males 2+ Years Old − Mean Haemglobin − Punjab
WHO−2001 Adjustment Method

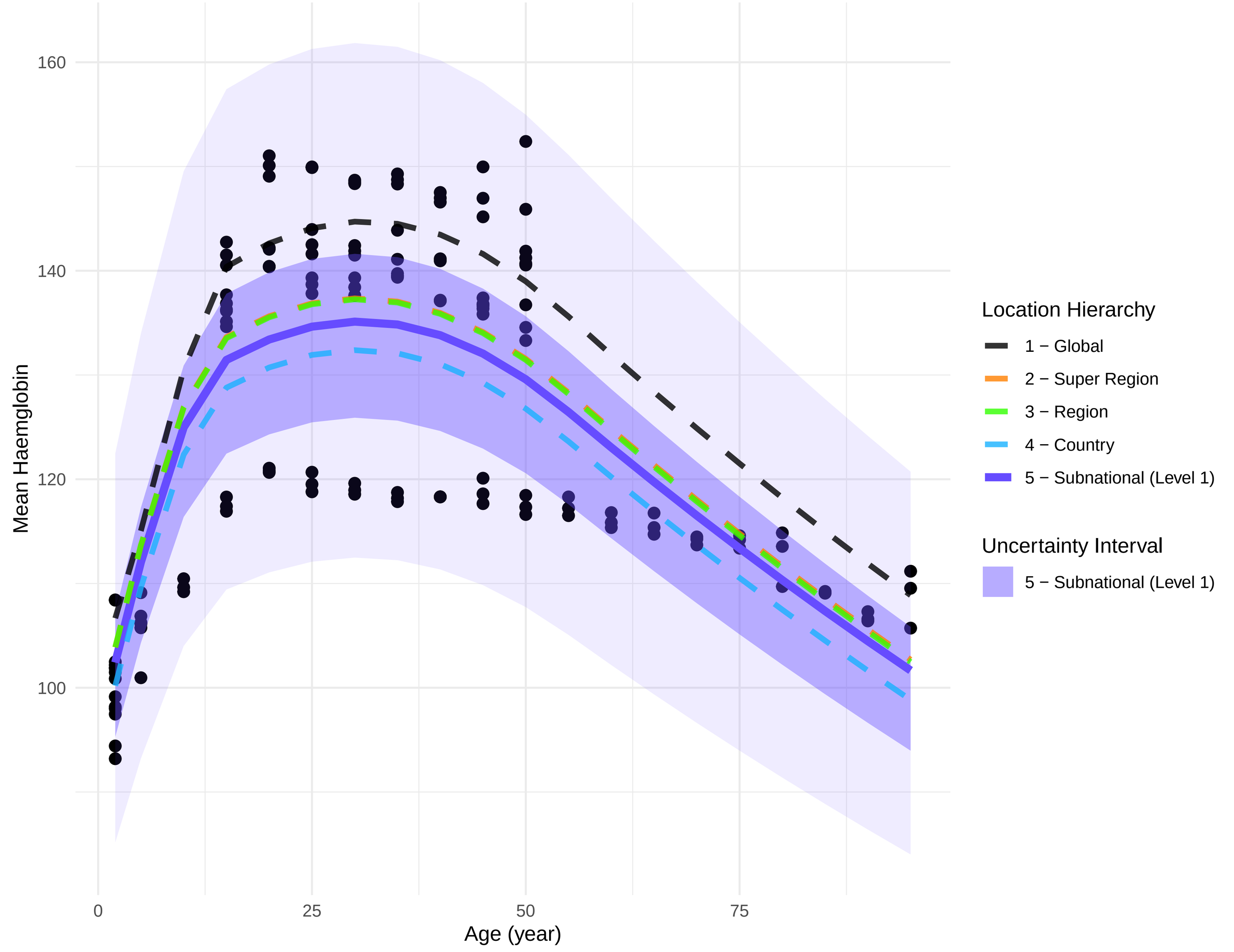

MR−BRT Age/Sex Fits − Males 2+ Years Old − Mean Haemglobin − Punjab, Rural
WHO−2001 Adjustment Method

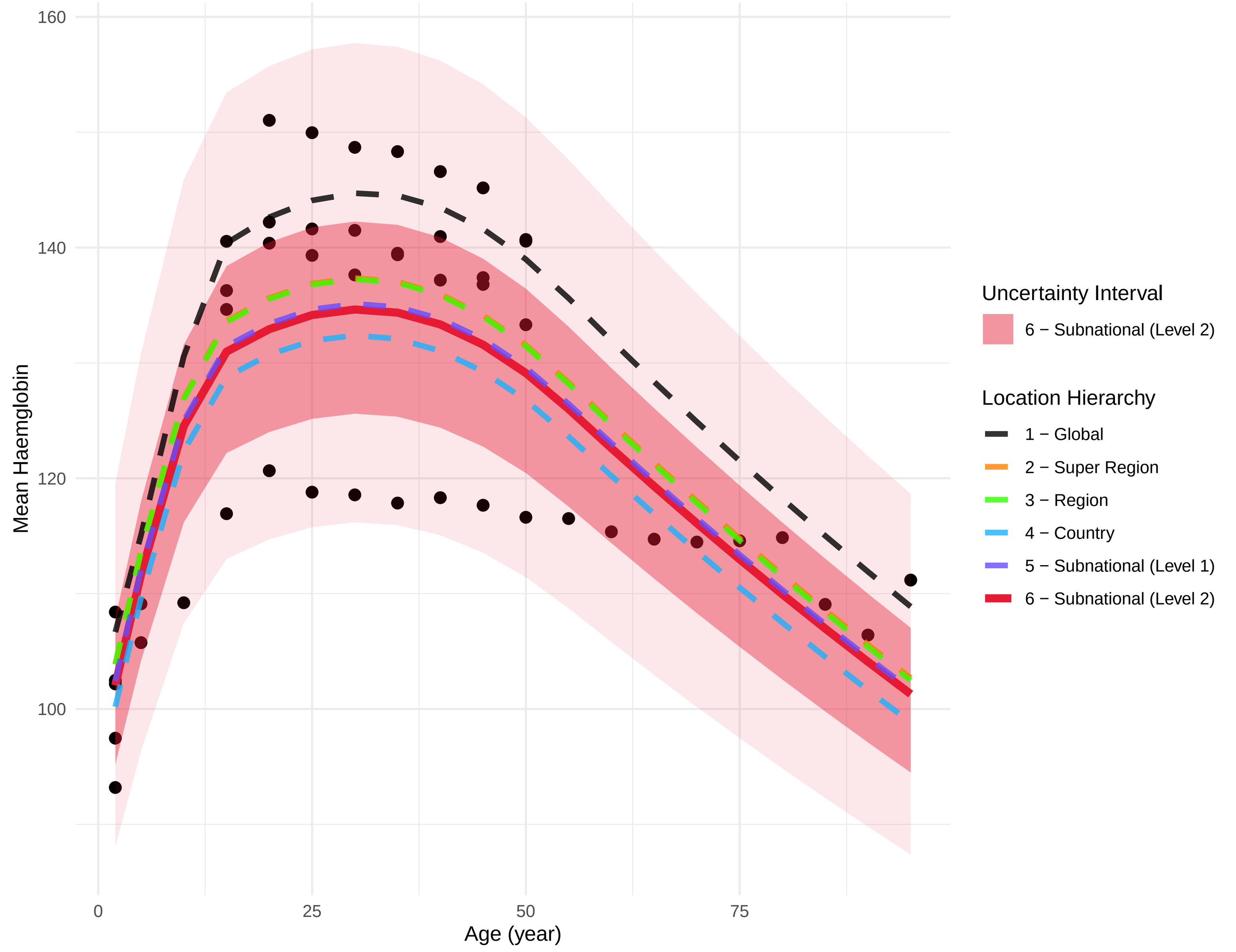

MR−BRT Age/Sex Fits − Males 2+ Years Old − Mean Haemglobin − Punjab, Urban
WHO−2001 Adjustment Method

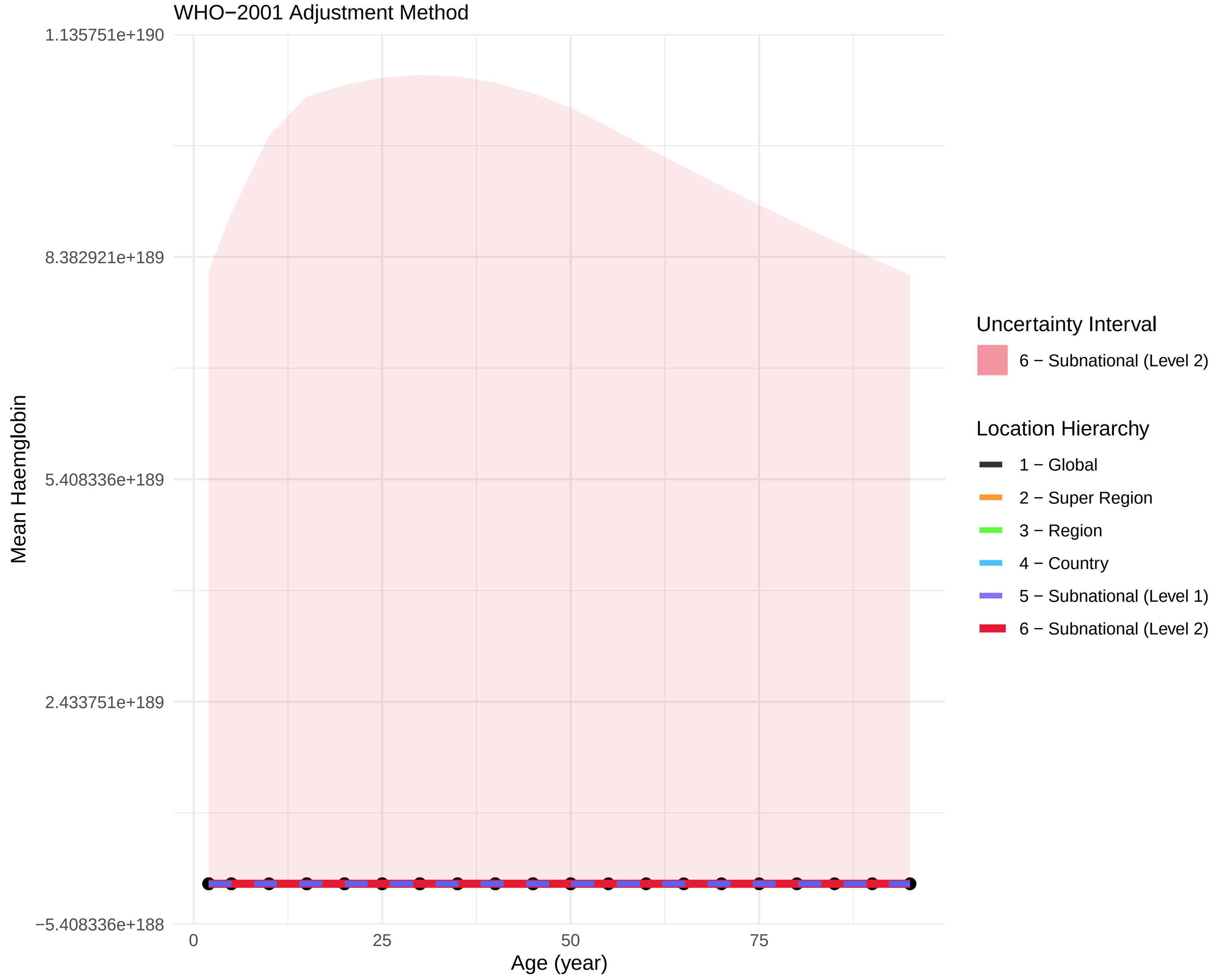

MR−BRT Age/Sex Fits − Males 2+ Years Old − Mean Haemglobin − Rajasthan
WHO−2001 Adjustment Method

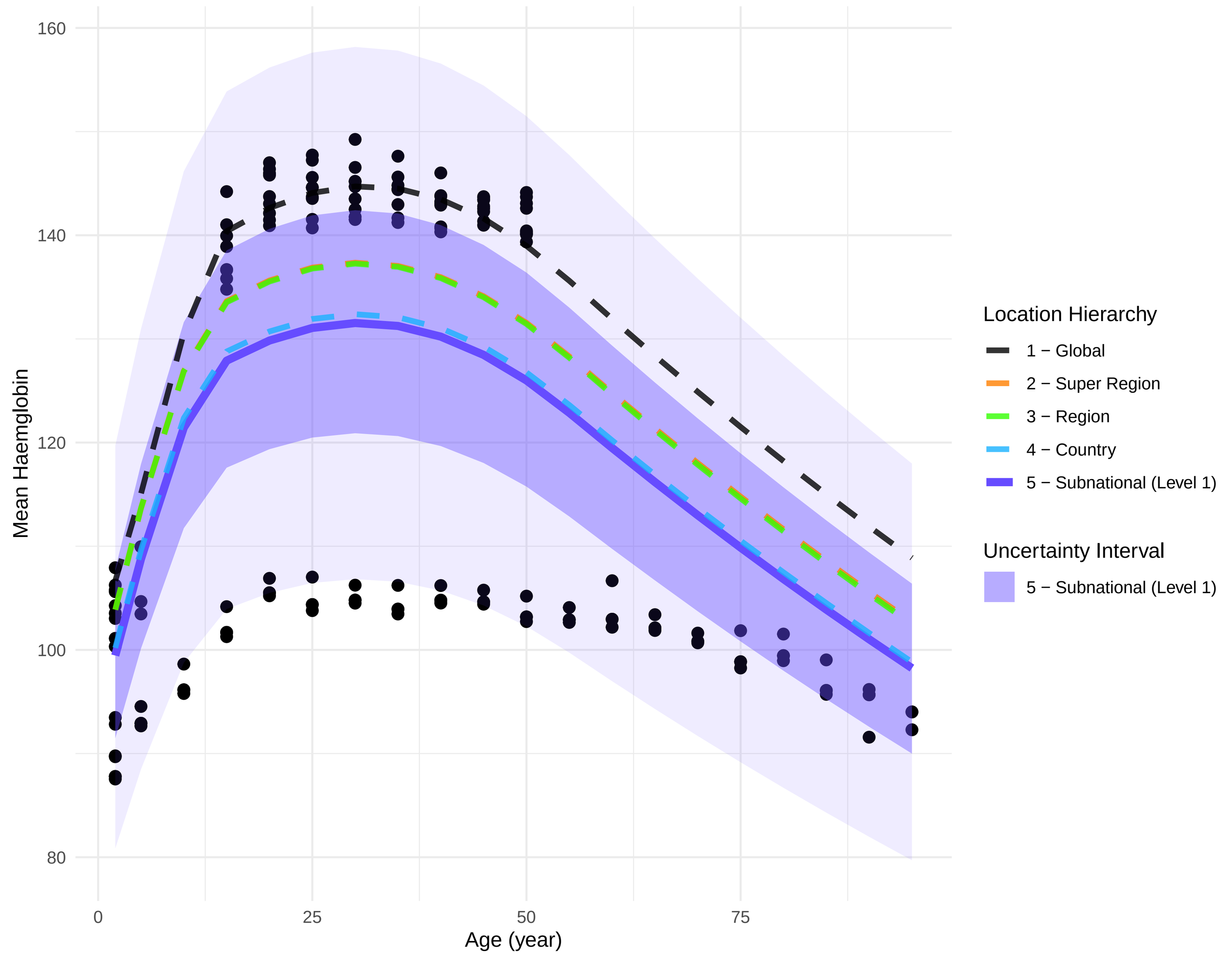

MR−BRT Age/Sex Fits − Males 2+ Years Old − Mean Haemglobin − Rajasthan, Rural
WHO−2001 Adjustment Method

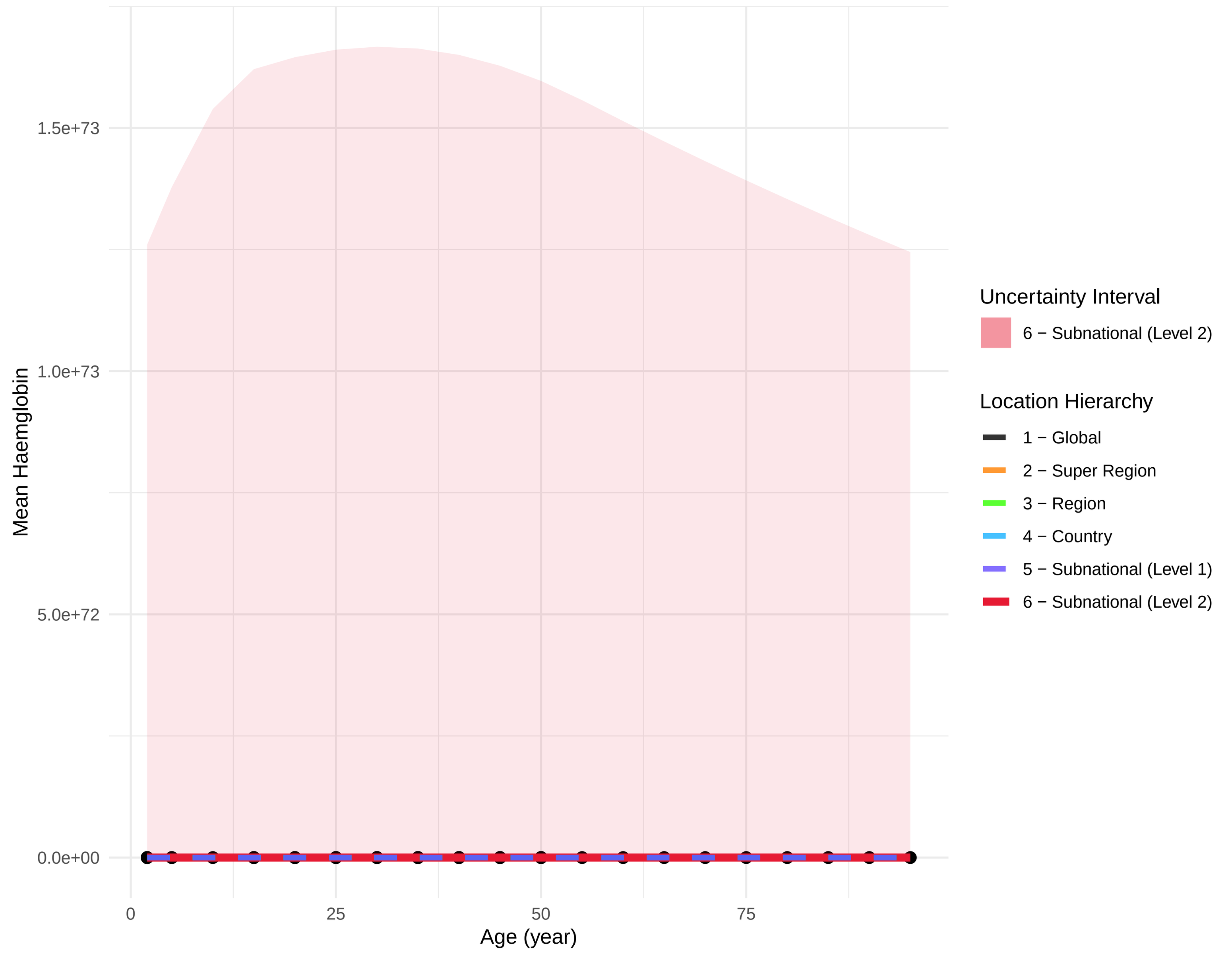

MR−BRT Age/Sex Fits − Males 2+ Years Old − Mean Haemglobin − Rajasthan, Urban
WHO−2001 Adjustment Method

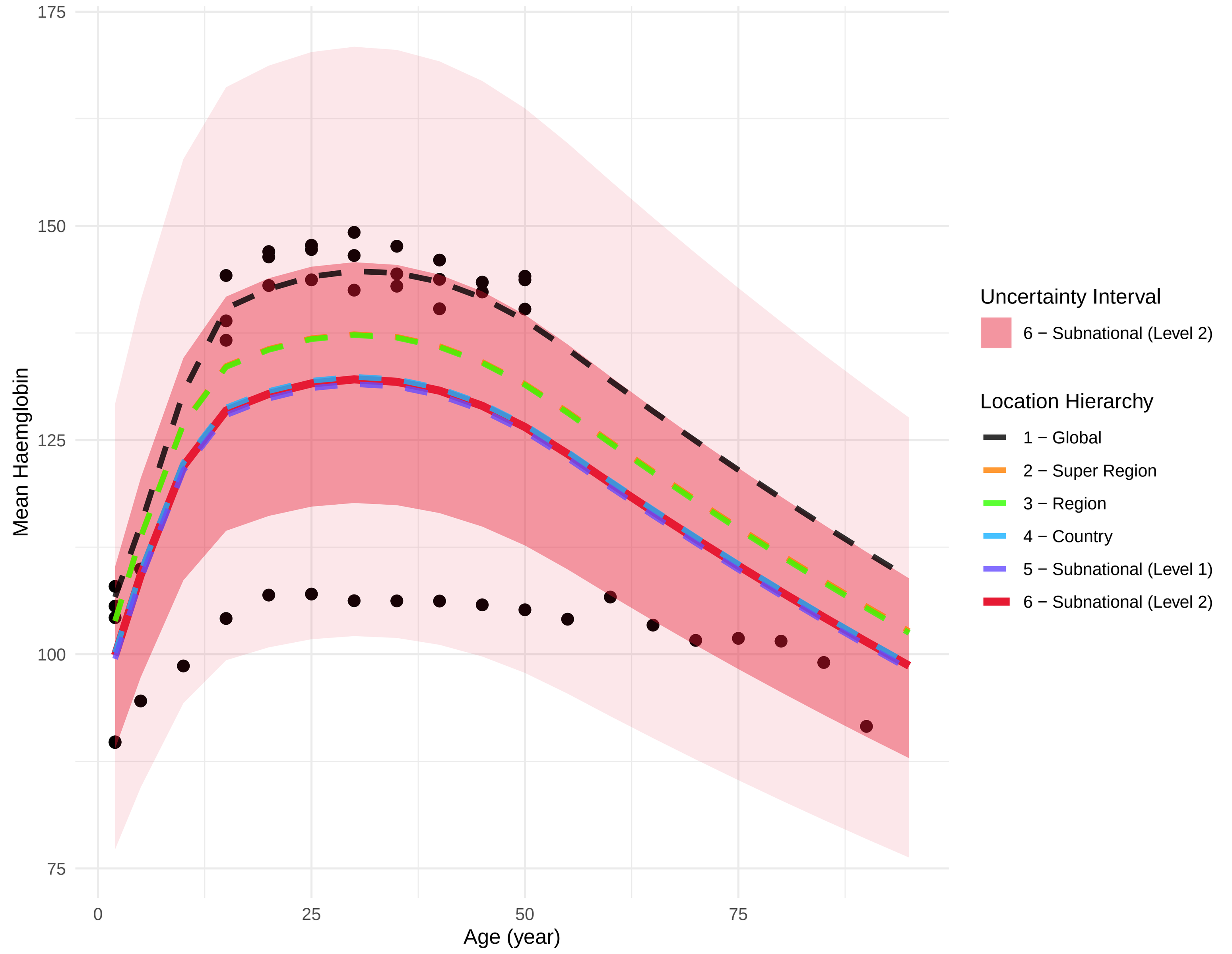

MR−BRT Age/Sex Fits − Males 2+ Years Old − Mean Haemglobin − Sikkim
WHO−2001 Adjustment Method

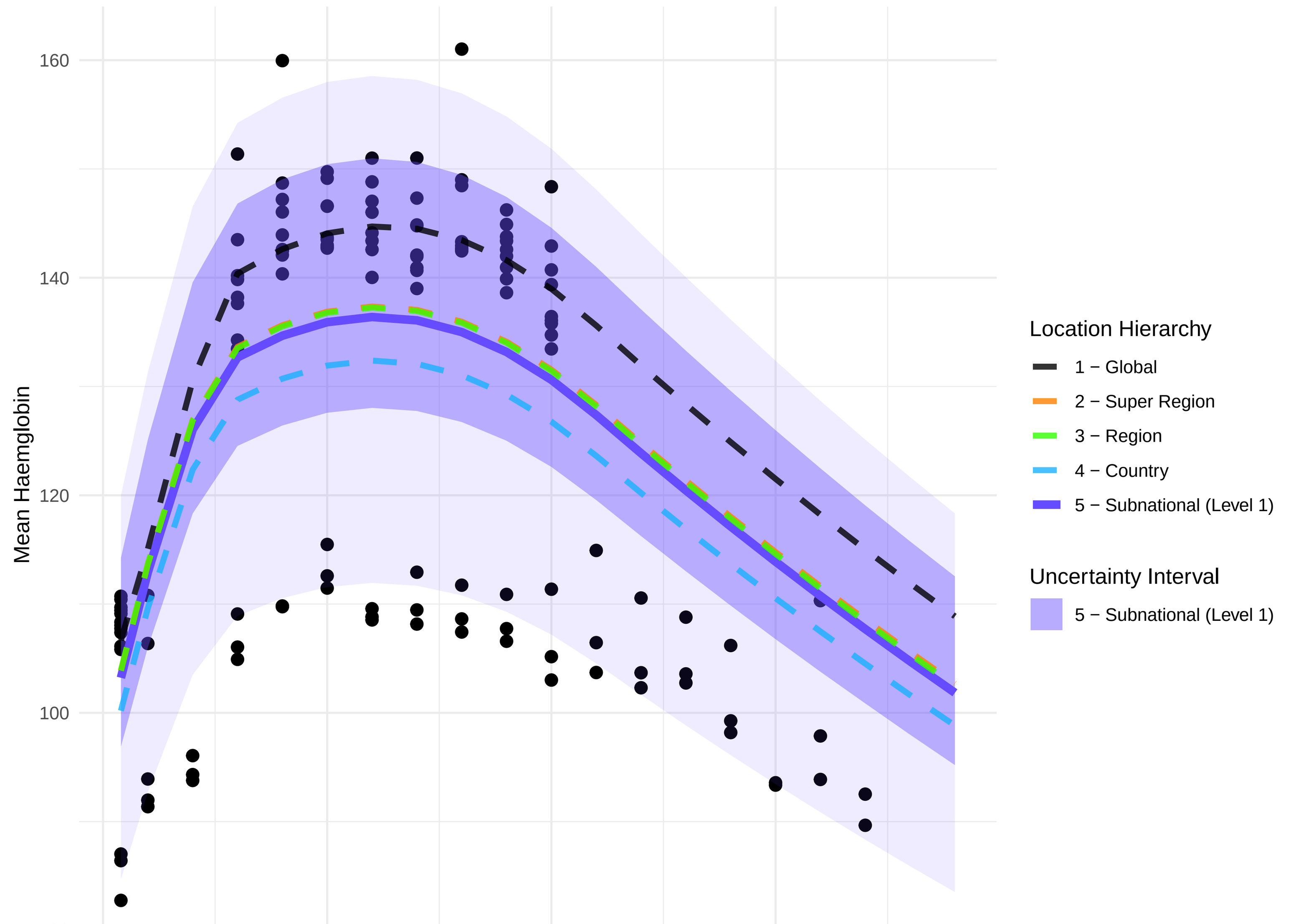

MR−BRT Age/Sex Fits − Males 2+ Years Old − Mean Haemglobin − Sikkim, Rural
WHO−2001 Adjustment Method

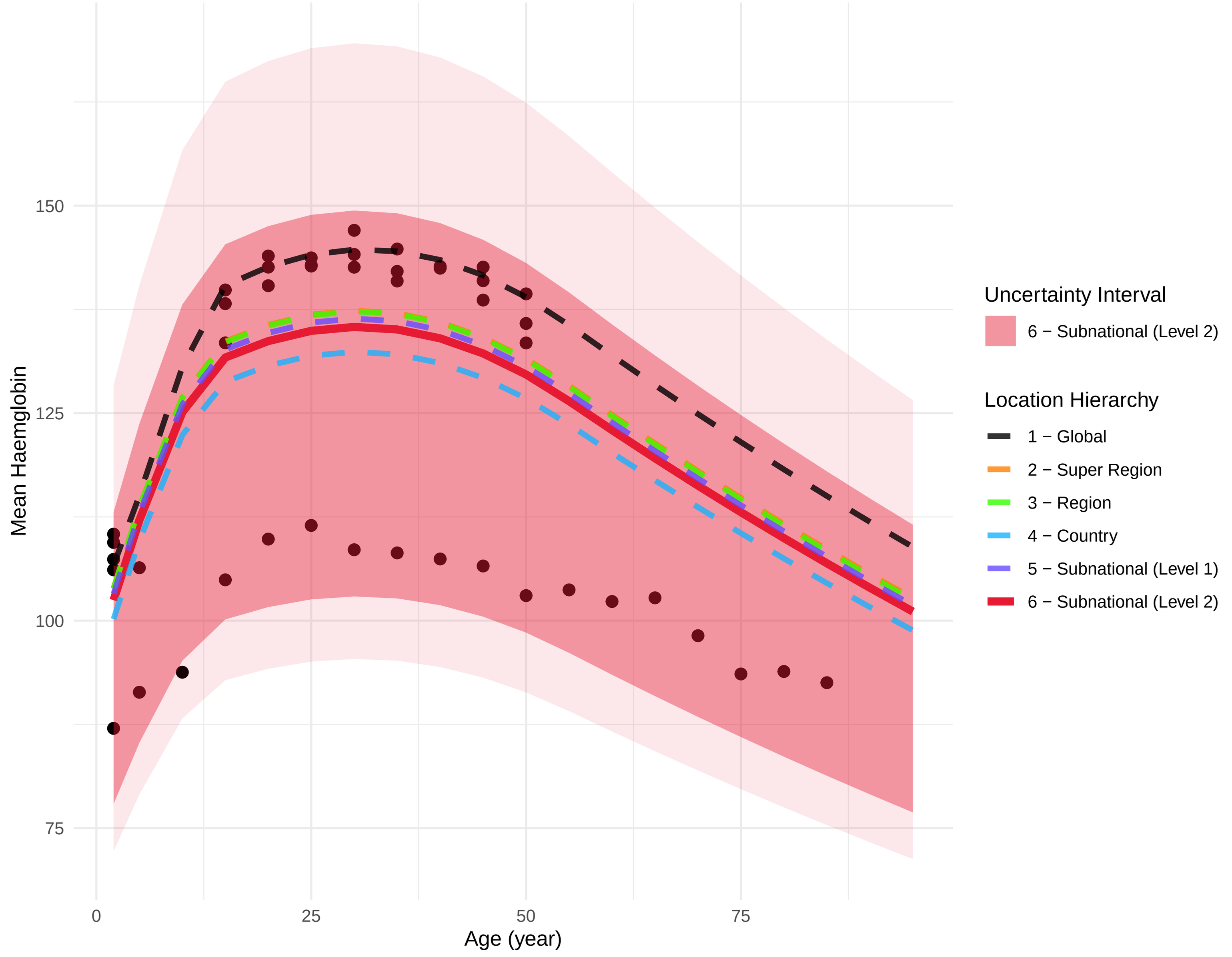

MR−BRT Age/Sex Fits − Males 2+ Years Old − Mean Haemglobin − Sikkim, Urban
WHO−2001 Adjustment Method

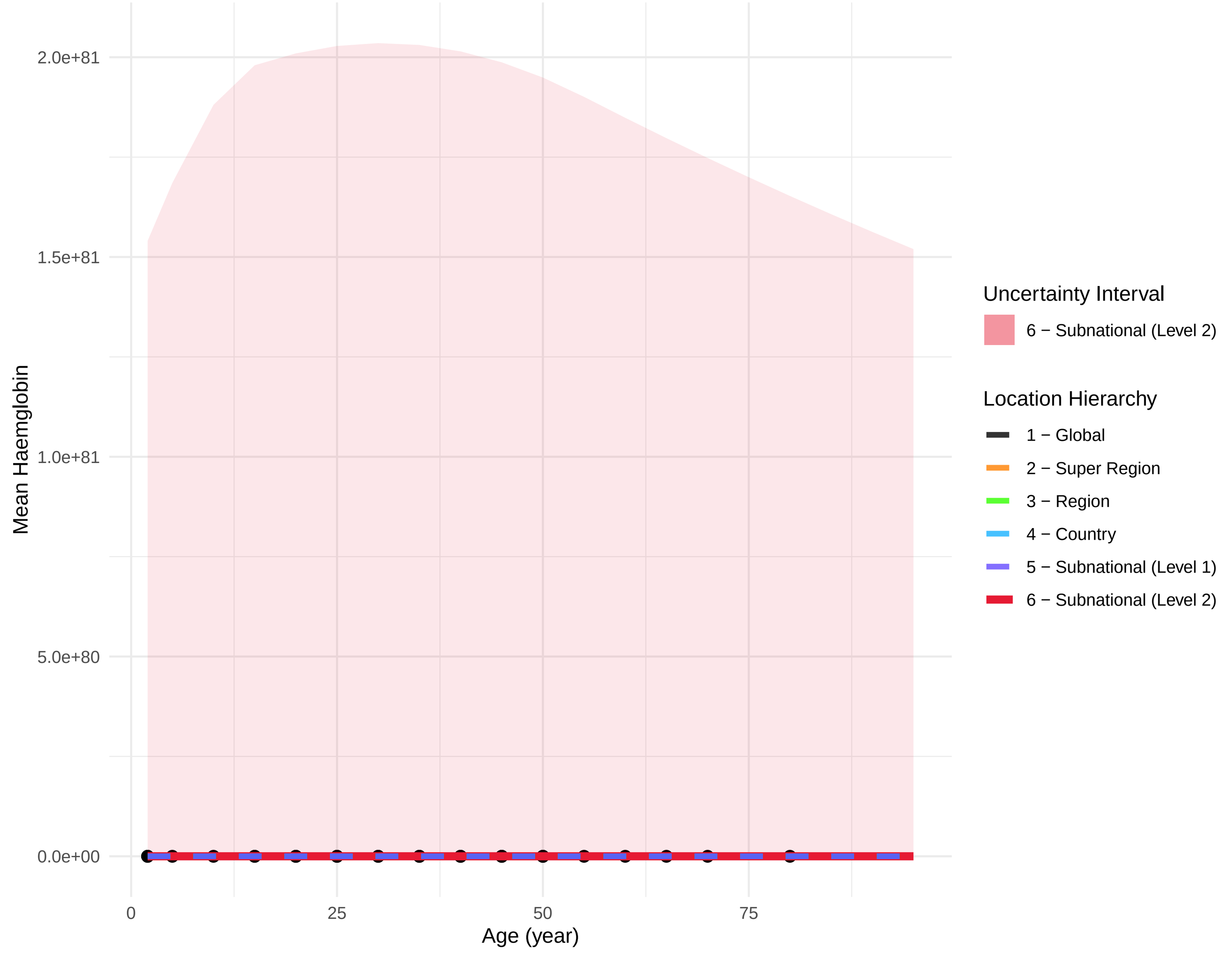

MR−BRT Age/Sex Fits − Males 2+ Years Old − Mean Haemglobin − Tamil Nadu
WHO−2001 Adjustment Method

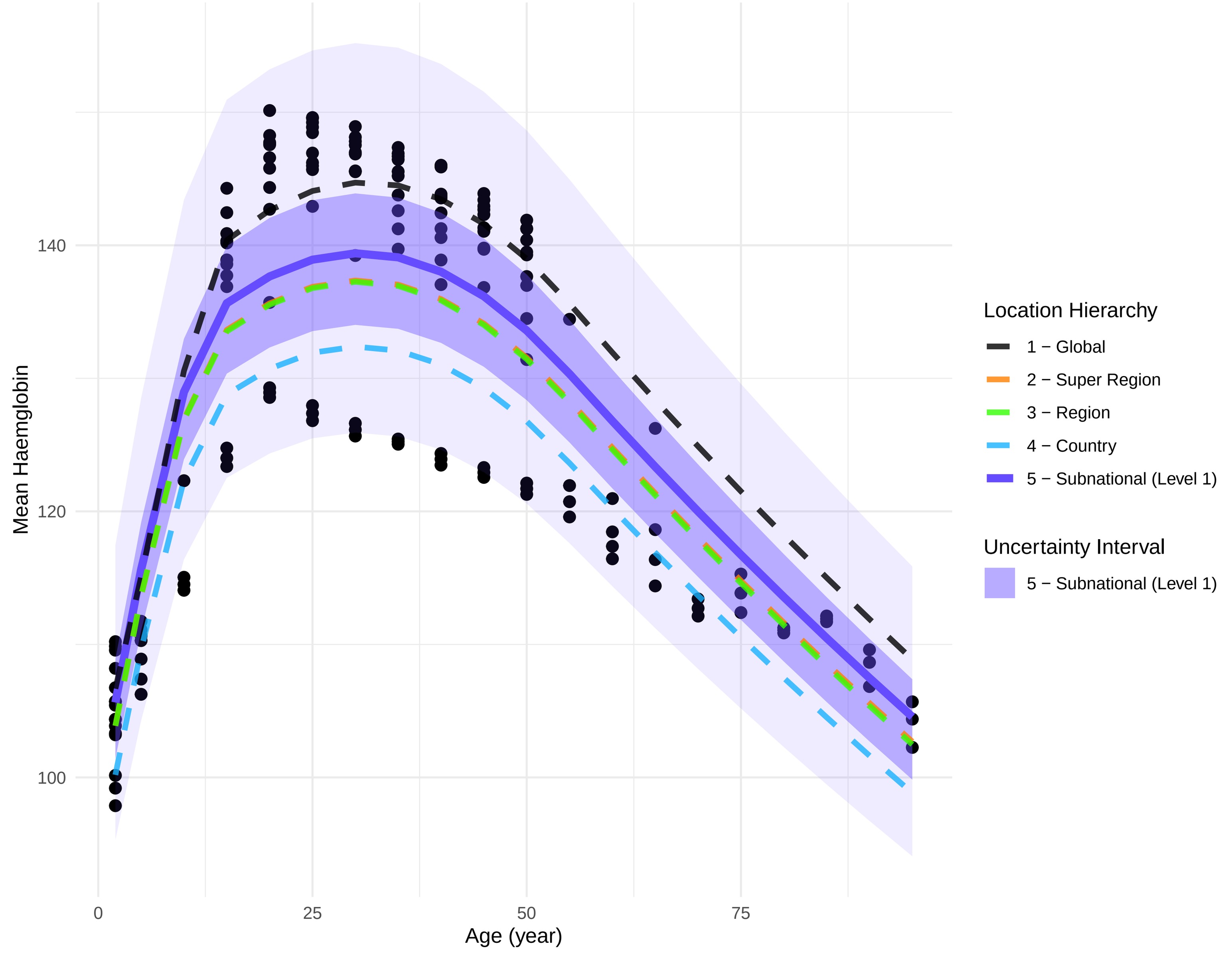

MR−BRT Age/Sex Fits − Males 2+ Years Old − Mean Haemglobin − Tamil Nadu, Rural
WHO−2001 Adjustment Method

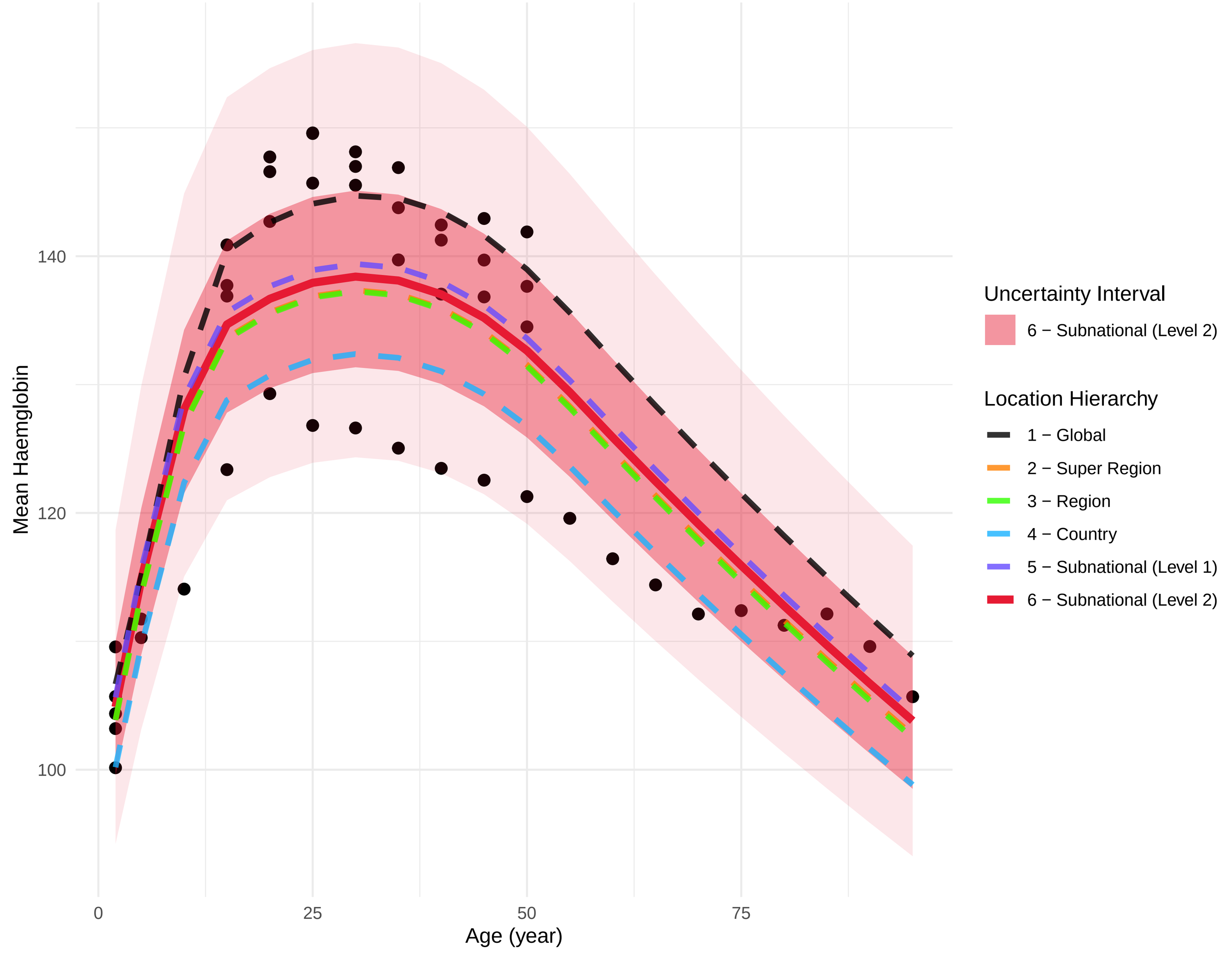

MR−BRT Age/Sex Fits − Males 2+ Years Old − Mean Haemglobin − Tamil Nadu, Urban
WHO−2001 Adjustment Method

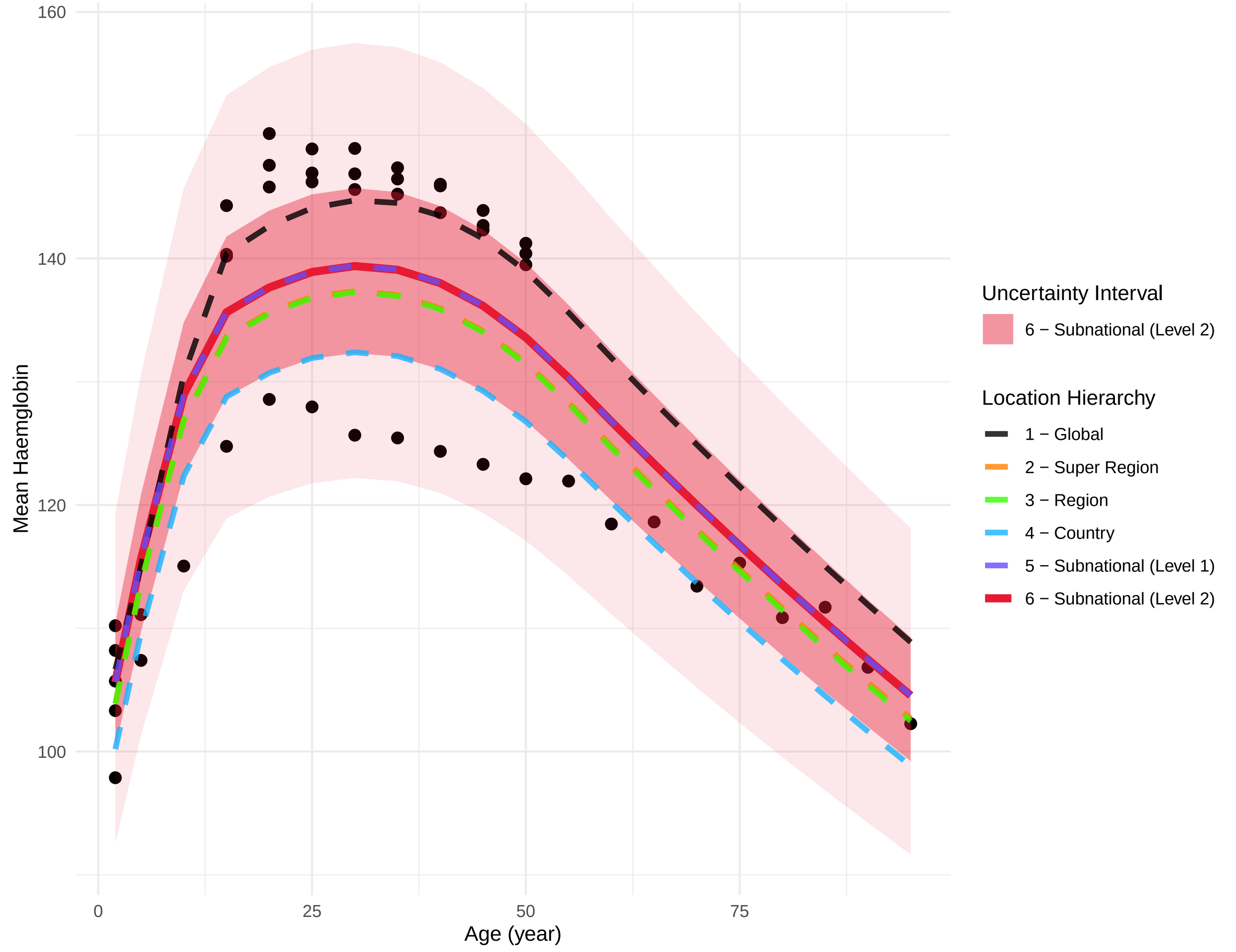

MR−BRT Age/Sex Fits − Males 2+ Years Old − Mean Haemglobin − Telangana
WHO−2001 Adjustment Method

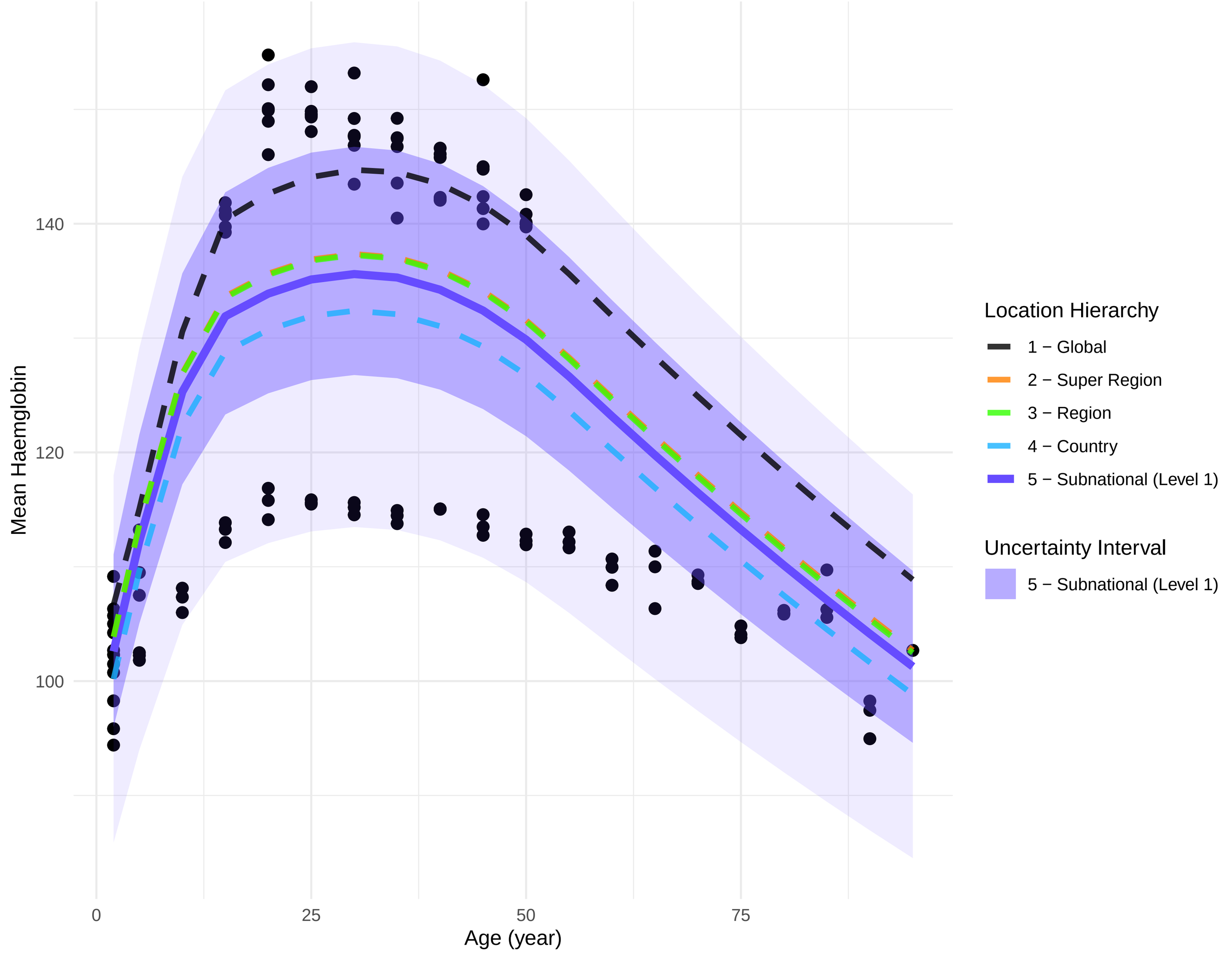

MR−BRT Age/Sex Fits − Males 2+ Years Old − Mean Haemglobin − Telangana, Rural
WHO−2001 Adjustment Method

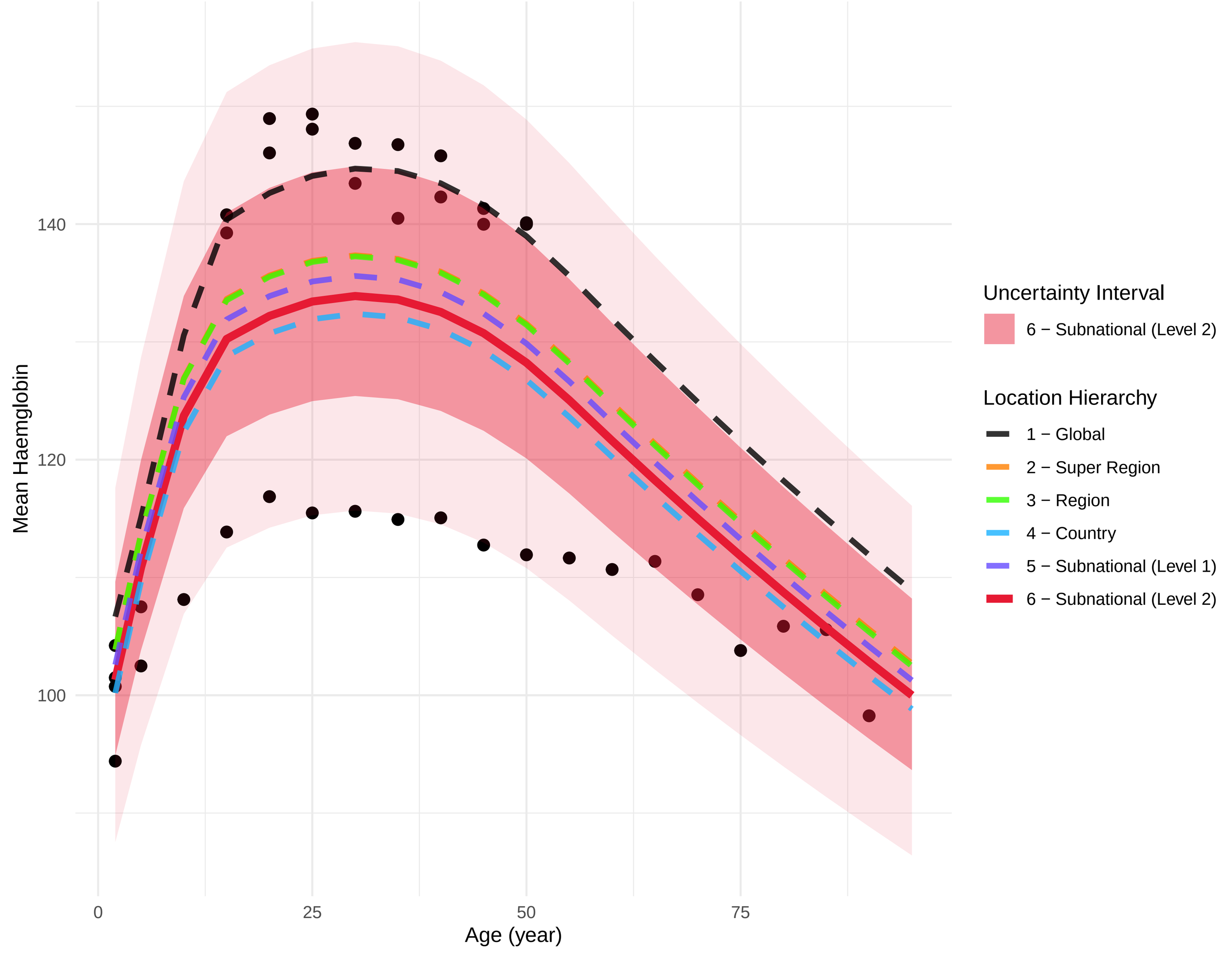

MR−BRT Age/Sex Fits − Males 2+ Years Old − Mean Haemglobin − Telangana, Urban
WHO−2001 Adjustment Method

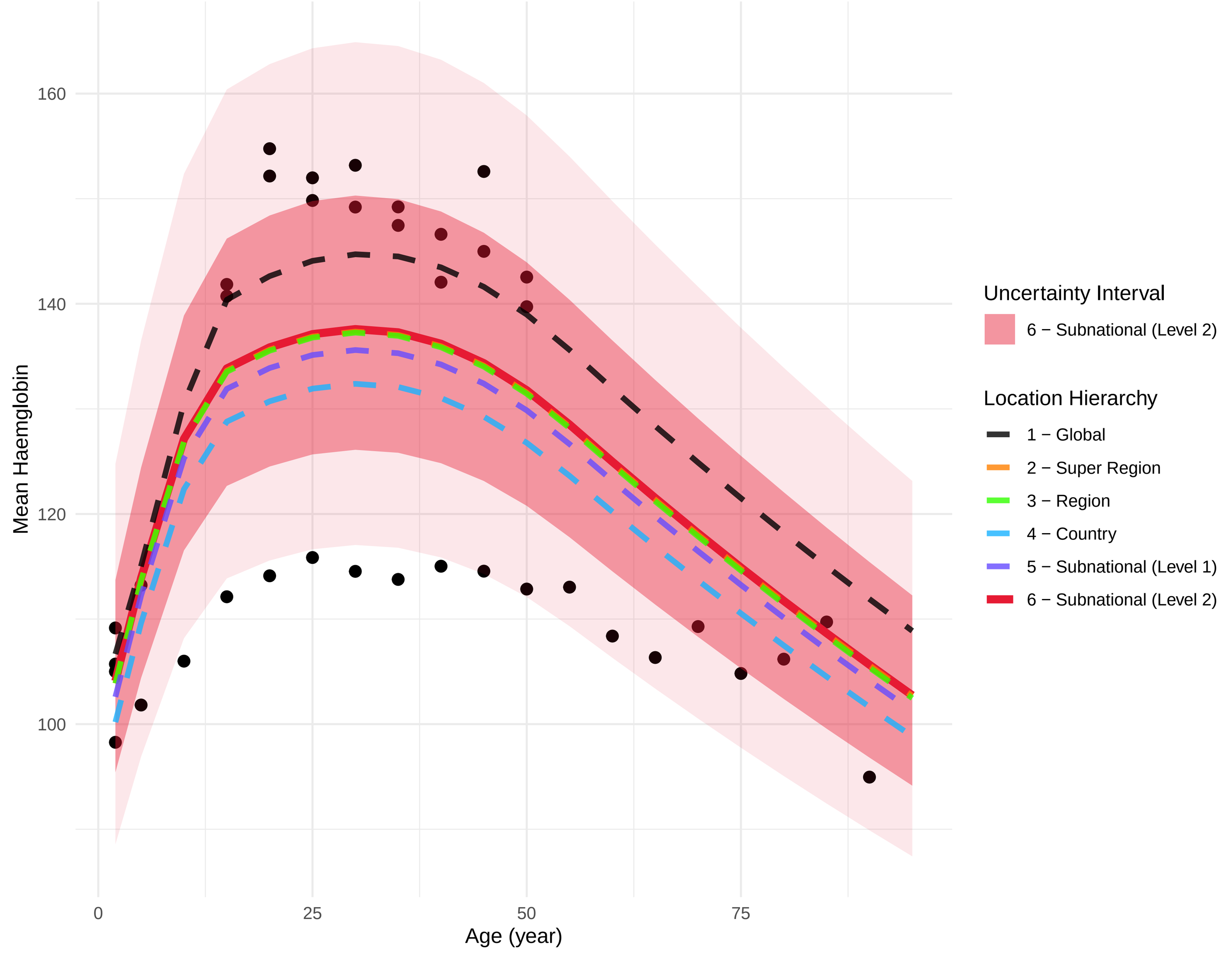

MR−BRT Age/Sex Fits − Males 2+ Years Old − Mean Haemglobin − Tripura
WHO−2001 Adjustment Method

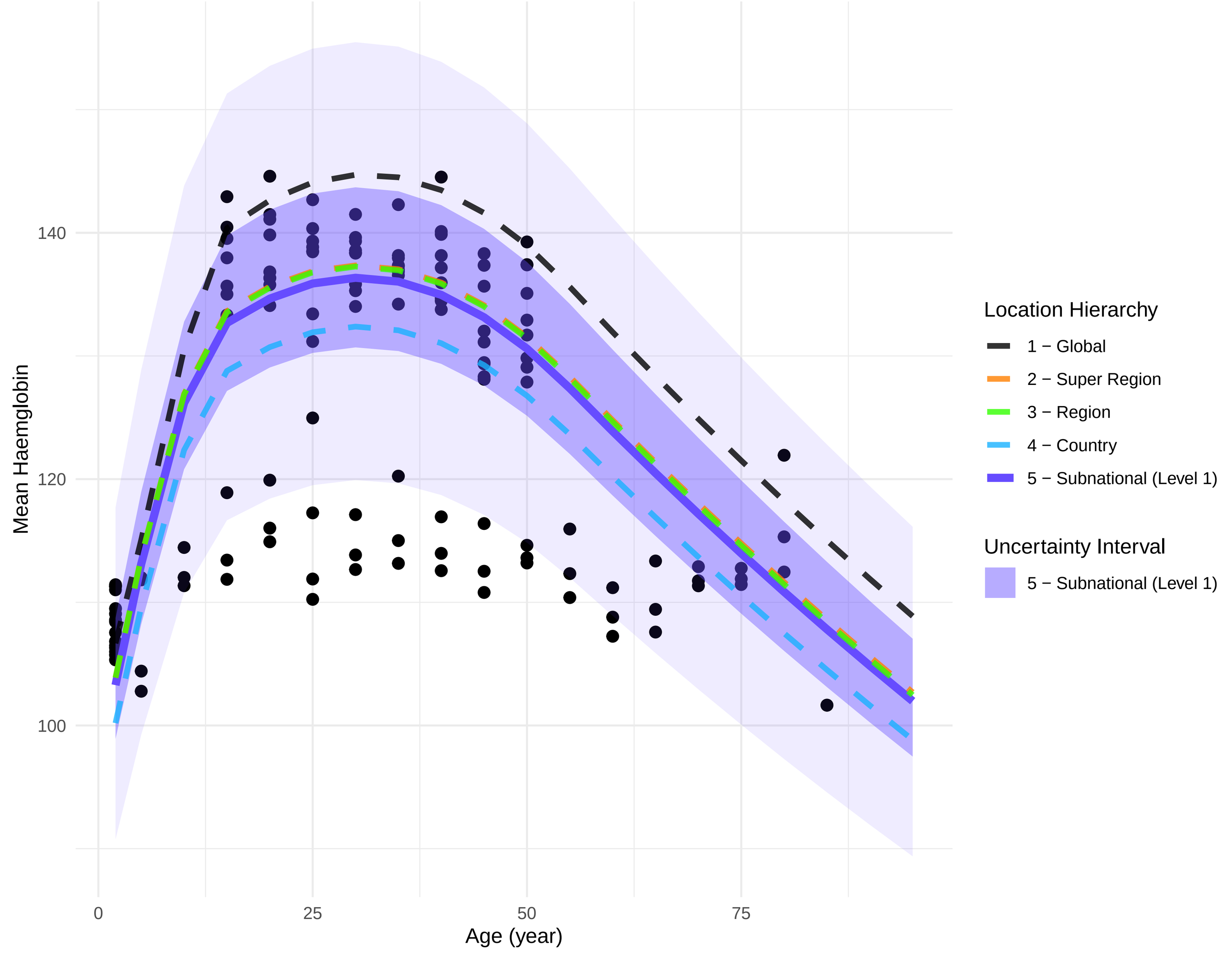

MR−BRT Age/Sex Fits − Males 2+ Years Old − Mean Haemglobin − Tripura, Rural
WHO−2001 Adjustment Method

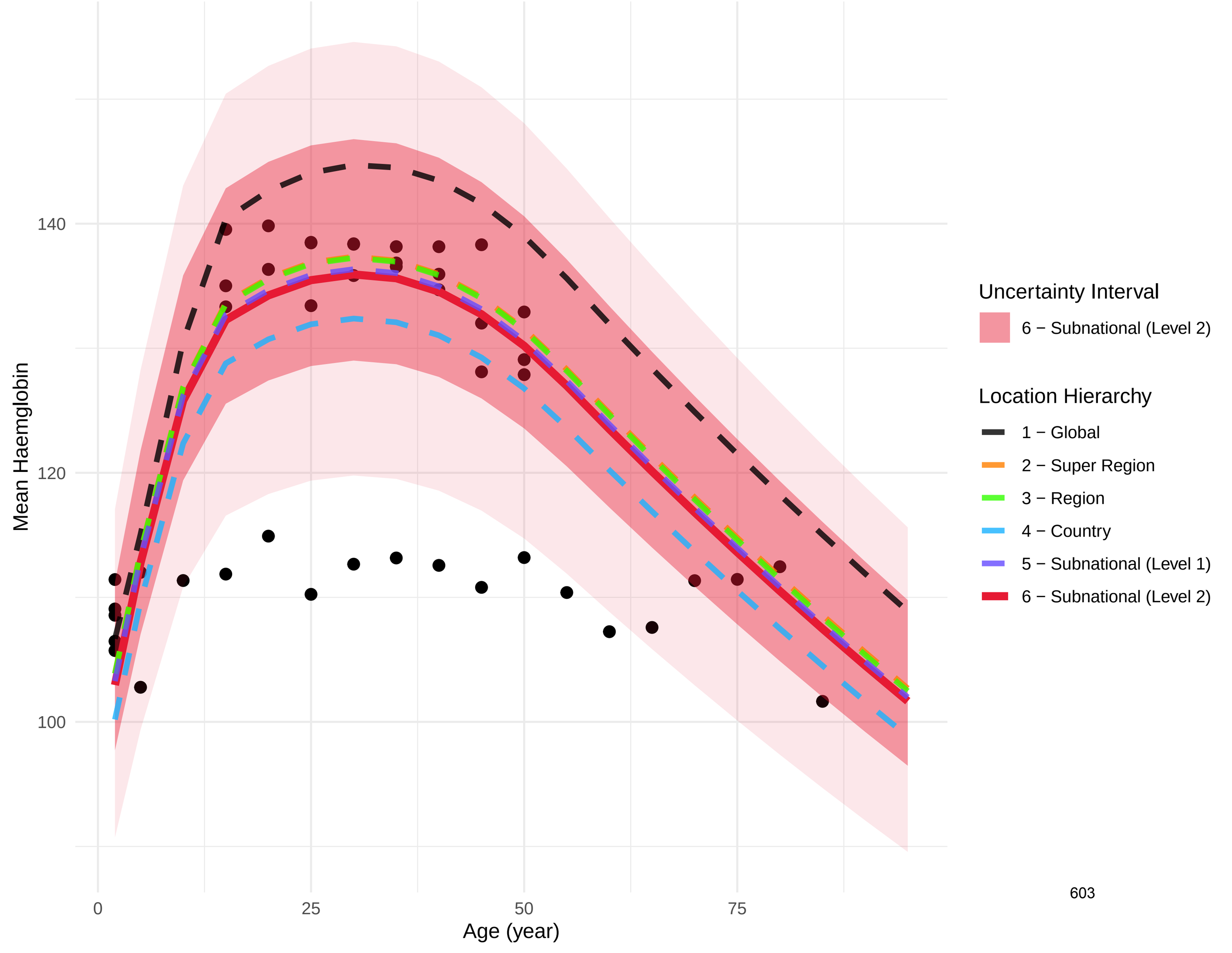

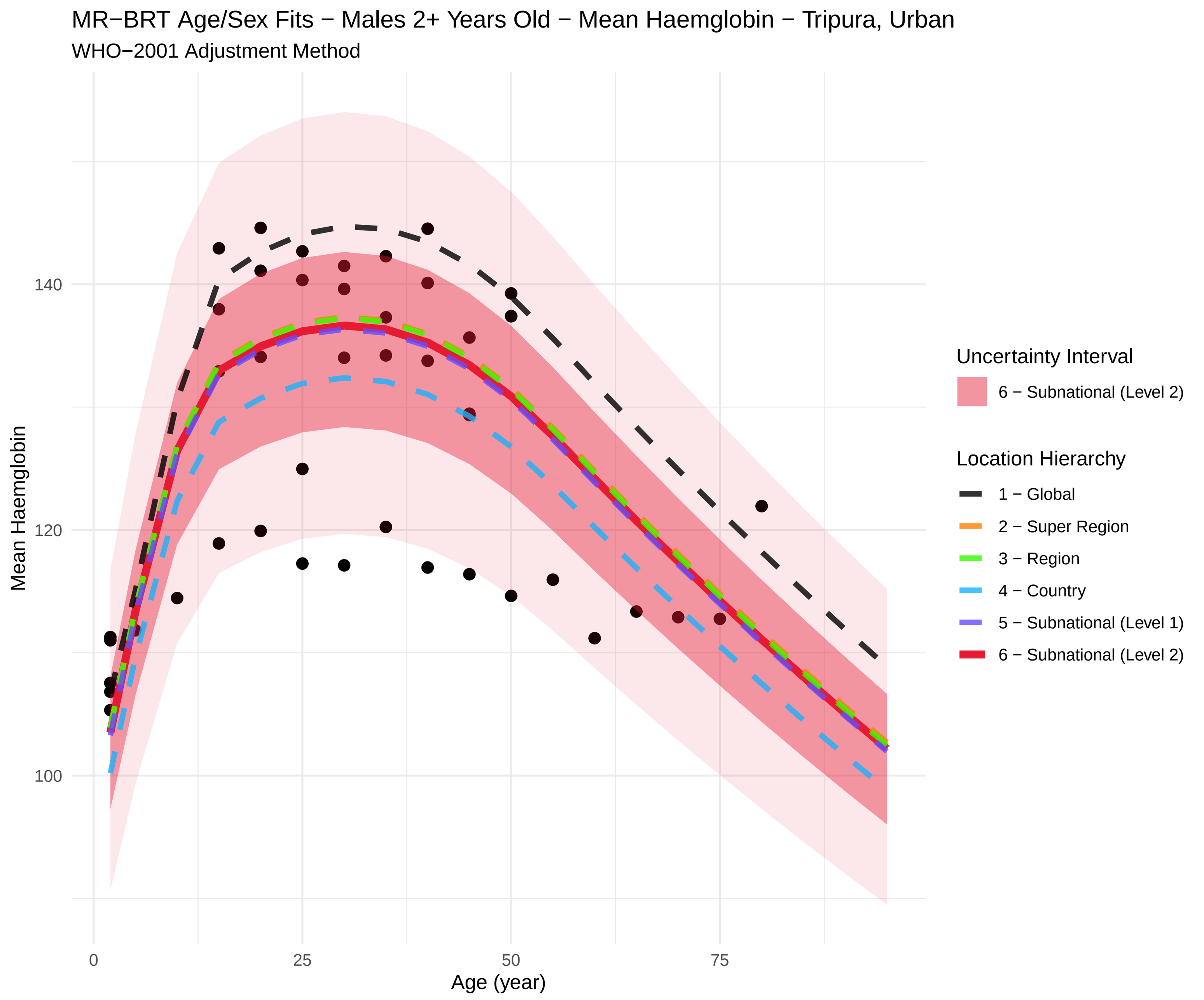
MR−BRT Age/Sex Fits − Males 2+ Years Old − Mean Haemglobin − Tripura, Urban
WHO−2001 Adjustment Method
Mean Haemglobin
Age (year)
Uncertainty Interval
6 − Subnational (Level 2)
Location Hierarchy
1 − Global
2 − Super Region
3 − Region
4 − Country
5 − Subnational (Level 1)
6 − Subnational (Level 2)

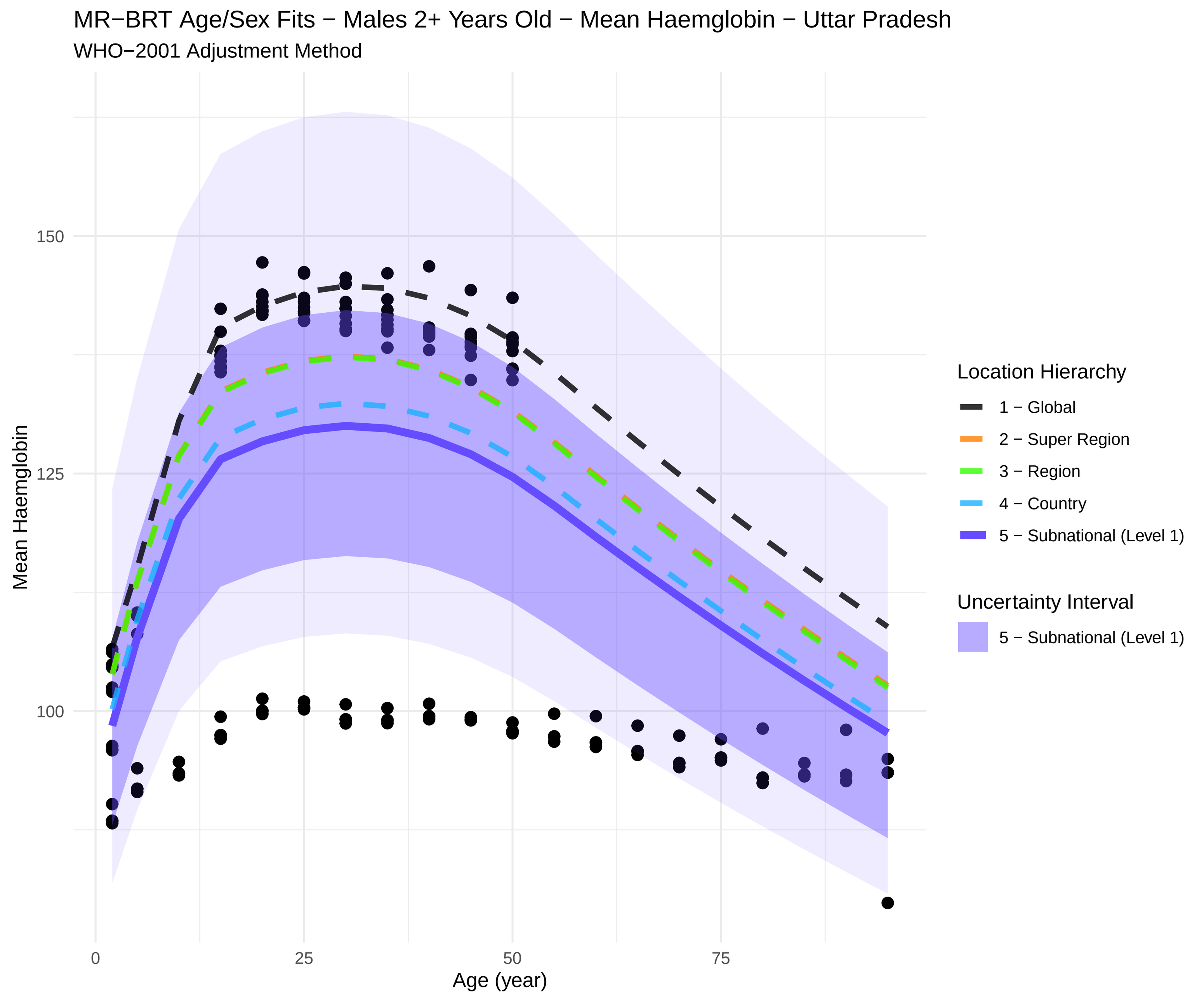
MR−BRT Age/Sex Fits − Males 2+ Years Old − Mean Haemglobin − Uttar Pradesh
WHO−2001 Adjustment Method
Mean Haemglobin
Age (year)
Location Hierarchy
1 − Global
2 − Super Region
3 − Region
4 − Country
5 − Subnational (Level 1)
Uncertainty Interval
5 − Subnational (Level 1)

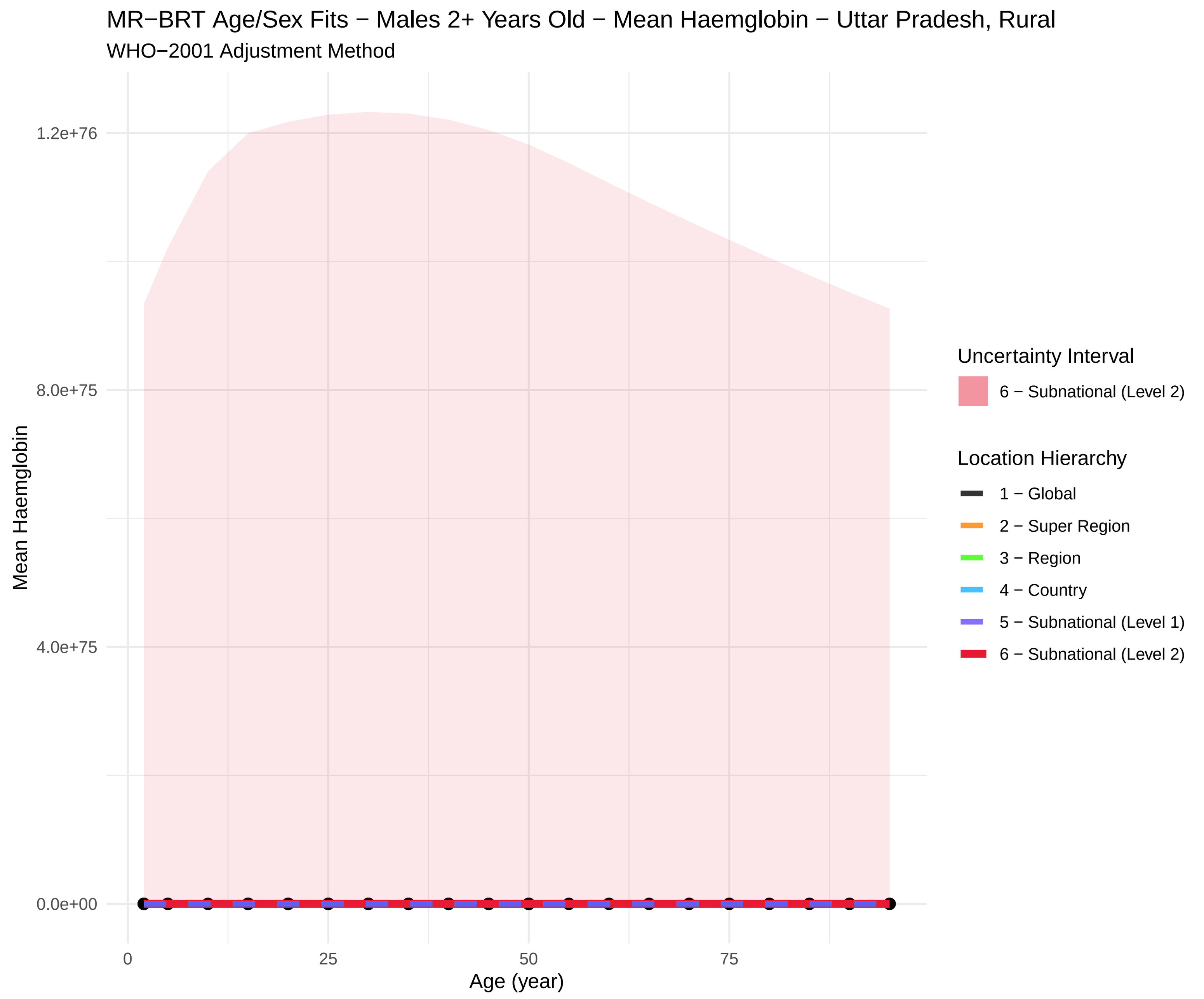
MR−BRT Age/Sex Fits − Males 2+ Years Old − Mean Haemglobin − Uttar Pradesh, Rural
WHO−2001 Adjustment Method
Mean Haemglobin
Age (year)
Uncertainty Interval
6 − Subnational (Level 2)
Location Hierarchy
1 − Global
2 − Super Region
3 − Region
4 − Country
5 − Subnational (Level 1)
6 − Subnational (Level 2)

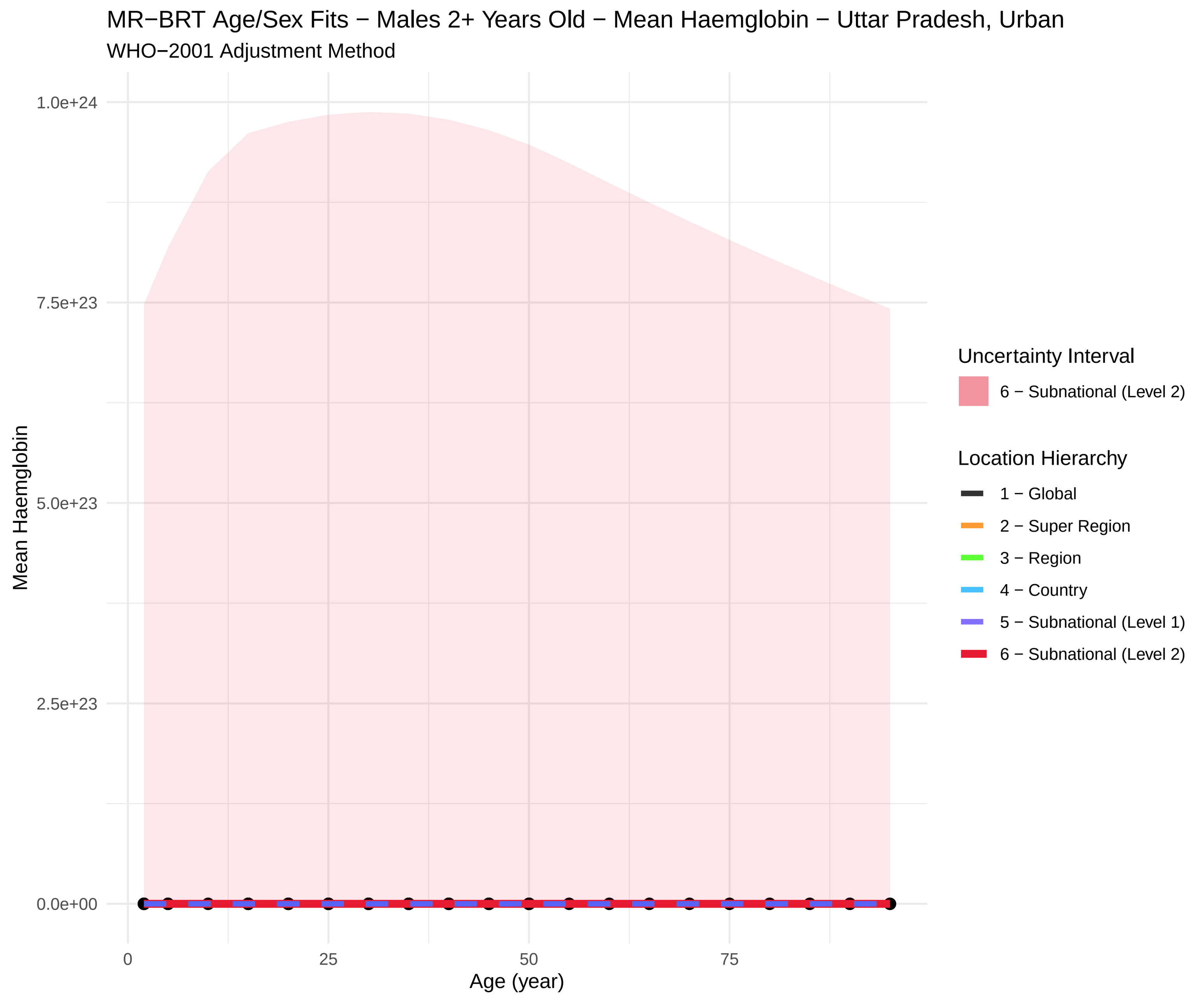
MR−BRT Age/Sex Fits − Males 2+ Years Old − Mean Haemglobin − Uttar Pradesh, Urban
WHO−2001 Adjustment Method
Mean Haemglobin
Age (year)
Uncertainty Interval
6 − Subnational (Level 2)
Location Hierarchy
1 − Global
2 − Super Region
3 − Region
4 − Country
5 − Subnational (Level 1)
6 − Subnational (Level 2)

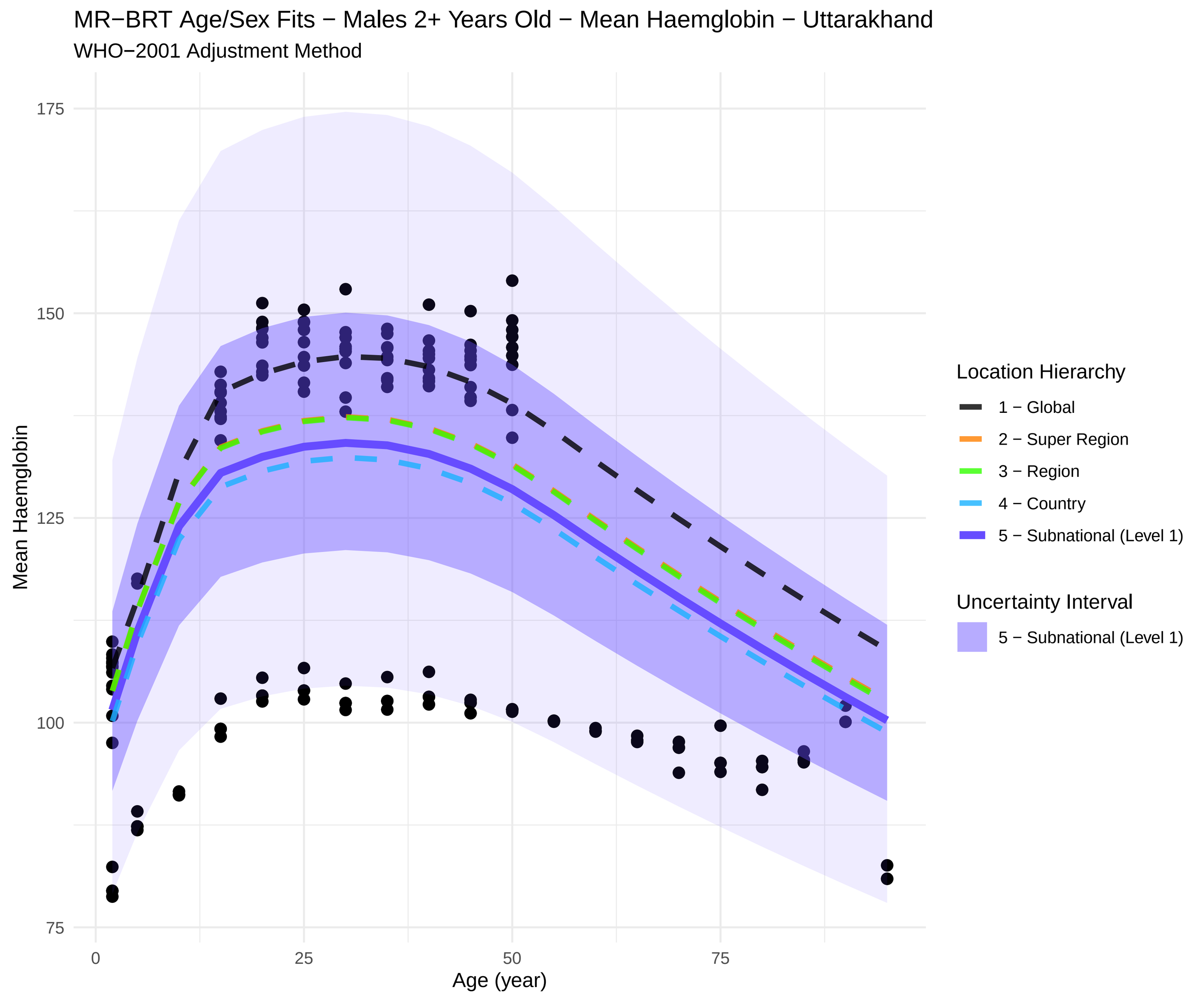
MR−BRT Age/Sex Fits − Males 2+ Years Old − Mean Haemglobin − Uttarakhand
WHO−2001 Adjustment Method
Mean Haemglobin
Age (year)
Location Hierarchy
1 − Global
2 − Super Region
3 − Region
4 − Country
5 − Subnational (Level 1)
Uncertainty Interval
5 − Subnational (Level 1)

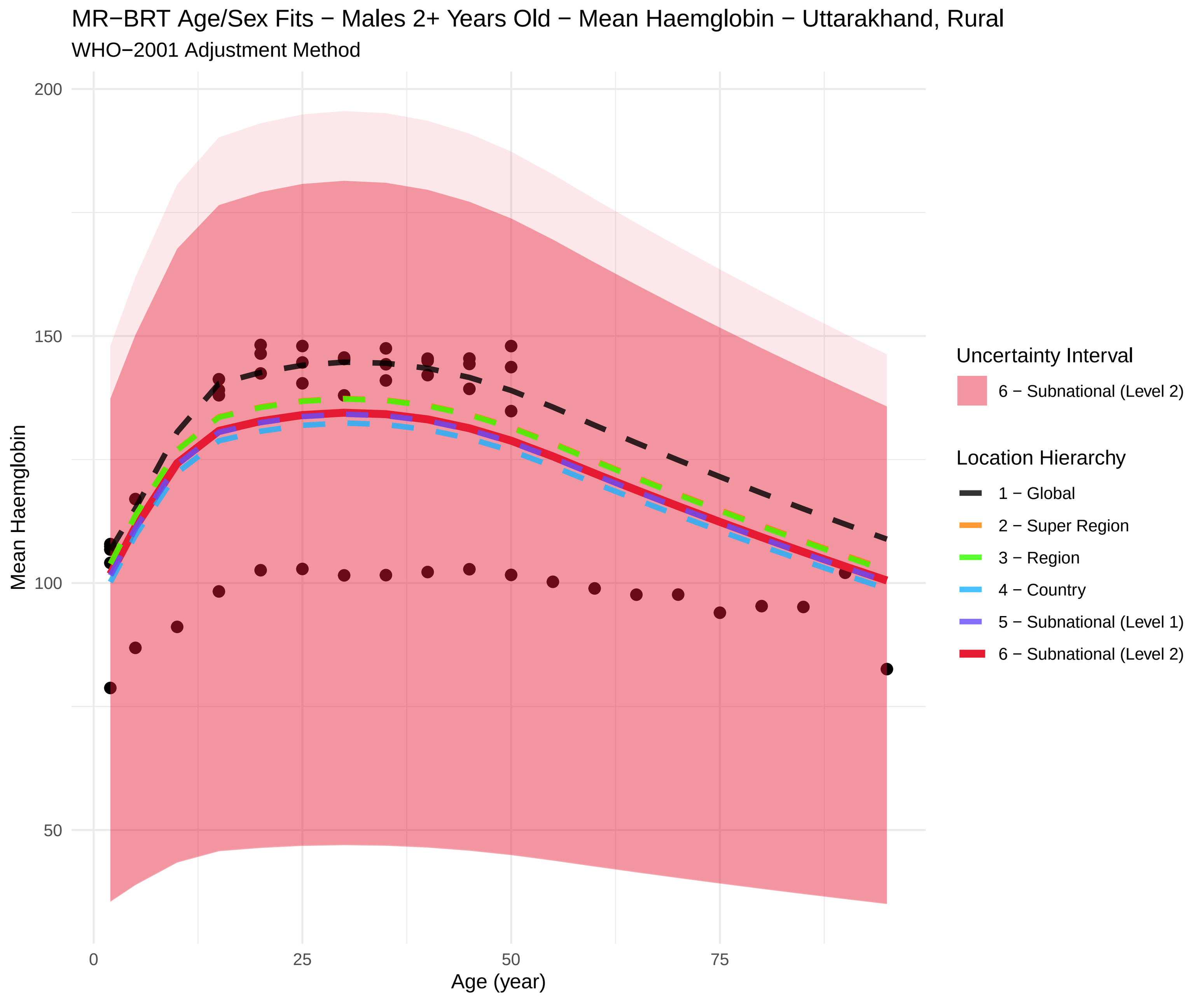
MR−BRT Age/Sex Fits − Males 2+ Years Old − Mean Haemglobin − Uttarakhand, Rural
WHO−2001 Adjustment Method
Mean Haemglobin
Age (year)
Uncertainty Interval
6 − Subnational (Level 2)
Location Hierarchy
1 − Global
2 − Super Region
3 − Region
4 − Country
5 − Subnational (Level 1)
6 − Subnational (Level 2)

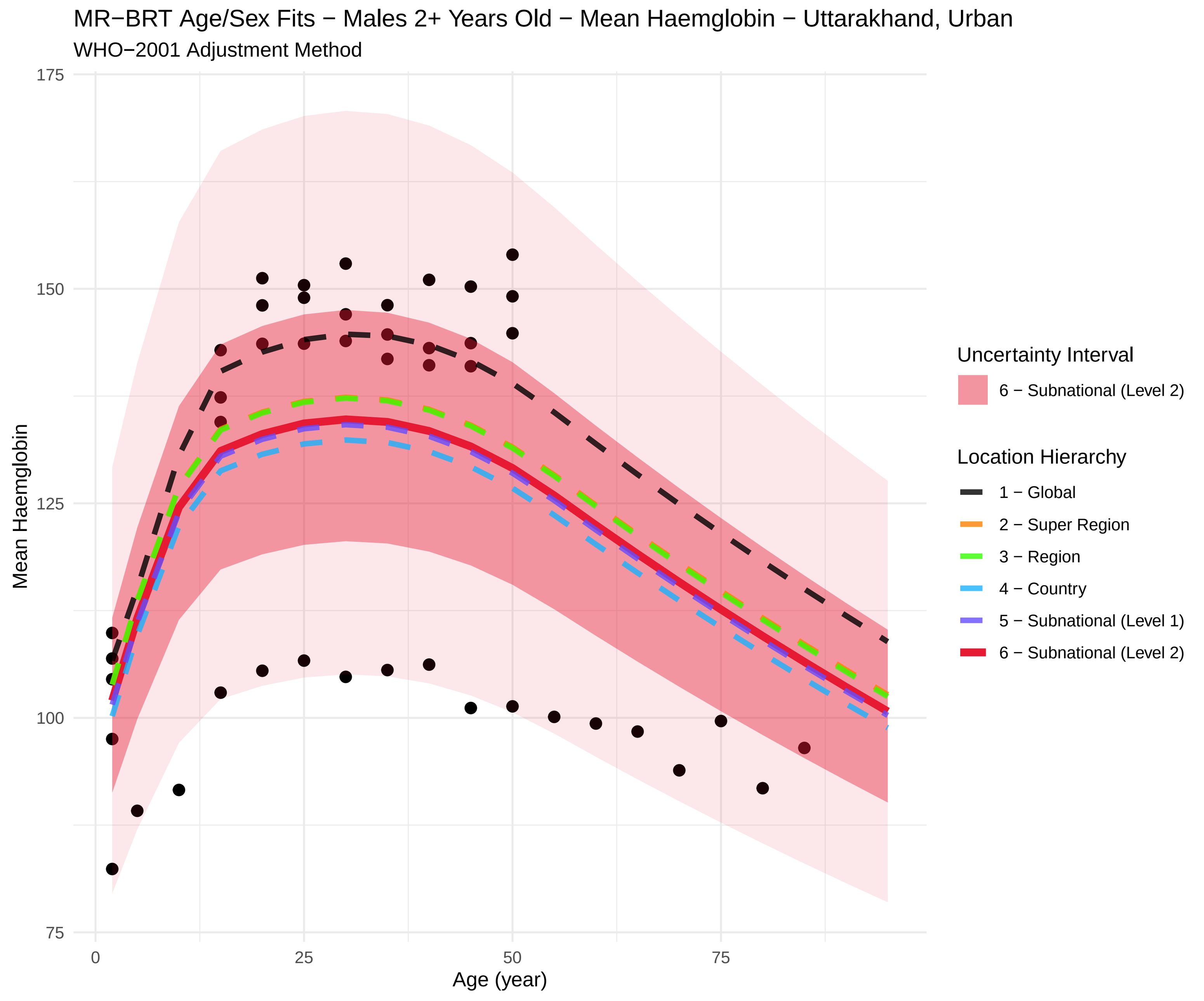
MR−BRT Age/Sex Fits − Males 2+ Years Old − Mean Haemglobin − Uttarakhand, Urban
WHO−2001 Adjustment Method
Mean Haemglobin
Age (year)
Uncertainty Interval
6 − Subnational (Level 2)
Location Hierarchy
1 − Global
2 − Super Region
3 − Region
4 − Country
5 − Subnational (Level 1)
6 − Subnational (Level 2)

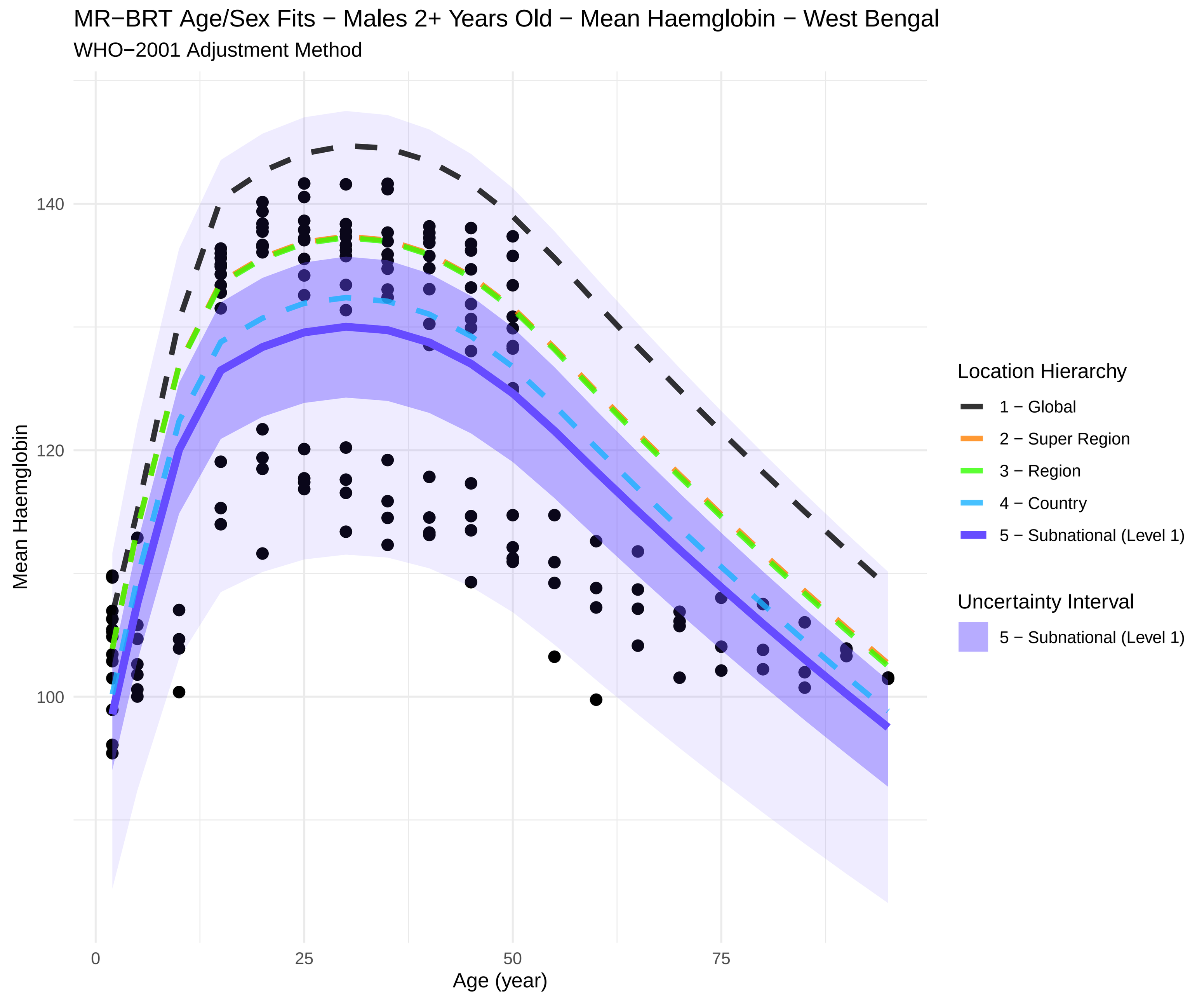
MR−BRT Age/Sex Fits − Males 2+ Years Old − Mean Haemglobin − West Bengal
WHO−2001 Adjustment Method
Mean Haemglobin
Age (year)
Location Hierarchy
1 − Global
2 − Super Region
3 − Region
4 − Country
5 − Subnational (Level 1)
Uncertainty Interval
5 − Subnational (Level 1)

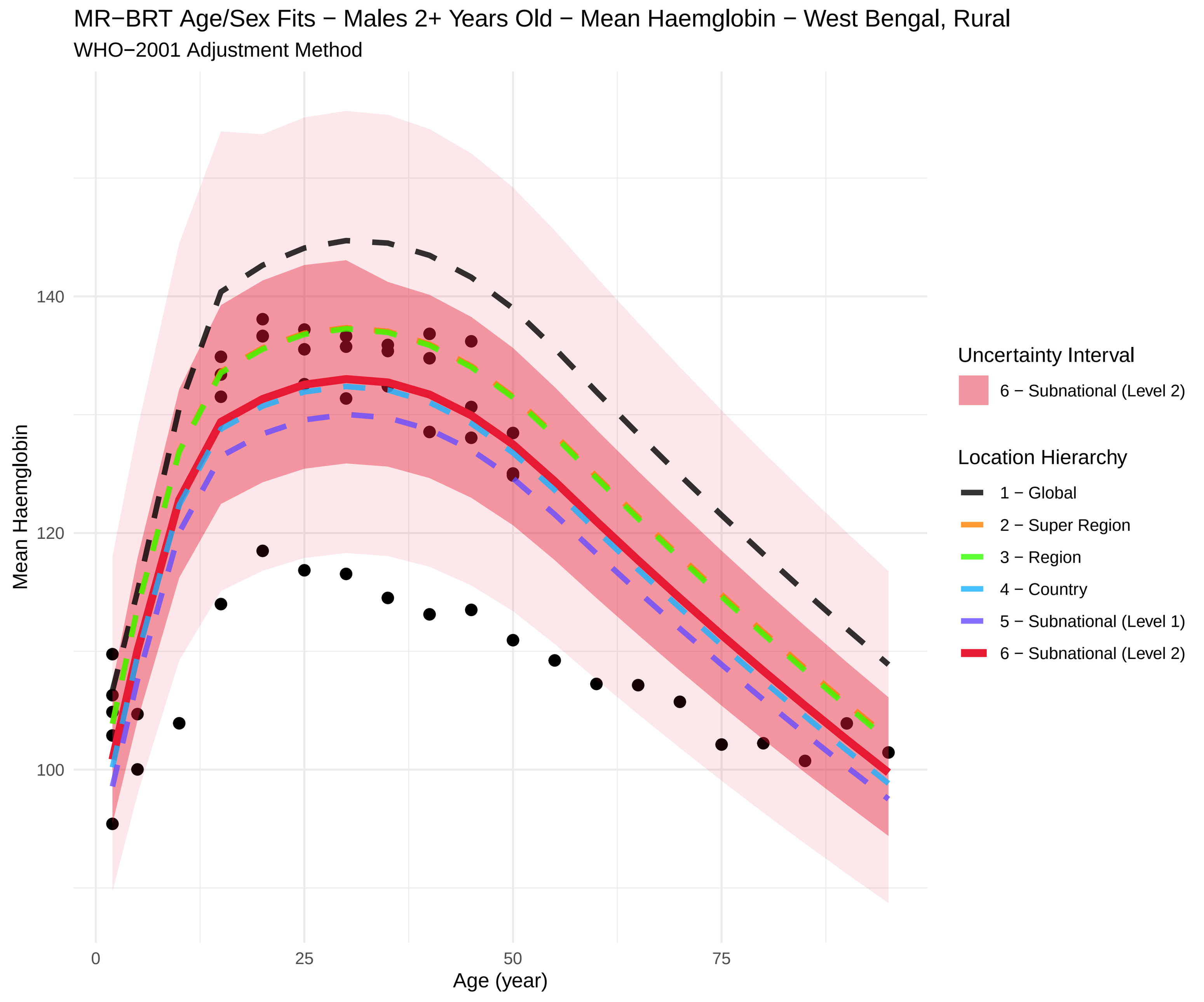
MR−BRT Age/Sex Fits − Males 2+ Years Old − Mean Haemglobin − West Bengal, Rural
WHO−2001 Adjustment Method
Mean Haemglobin
Age (year)
Uncertainty Interval
6 − Subnational (Level 2)
Location Hierarchy
1 − Global
2 − Super Region
3 − Region
4 − Country
5 − Subnational (Level 1)
6 − Subnational (Level 2)

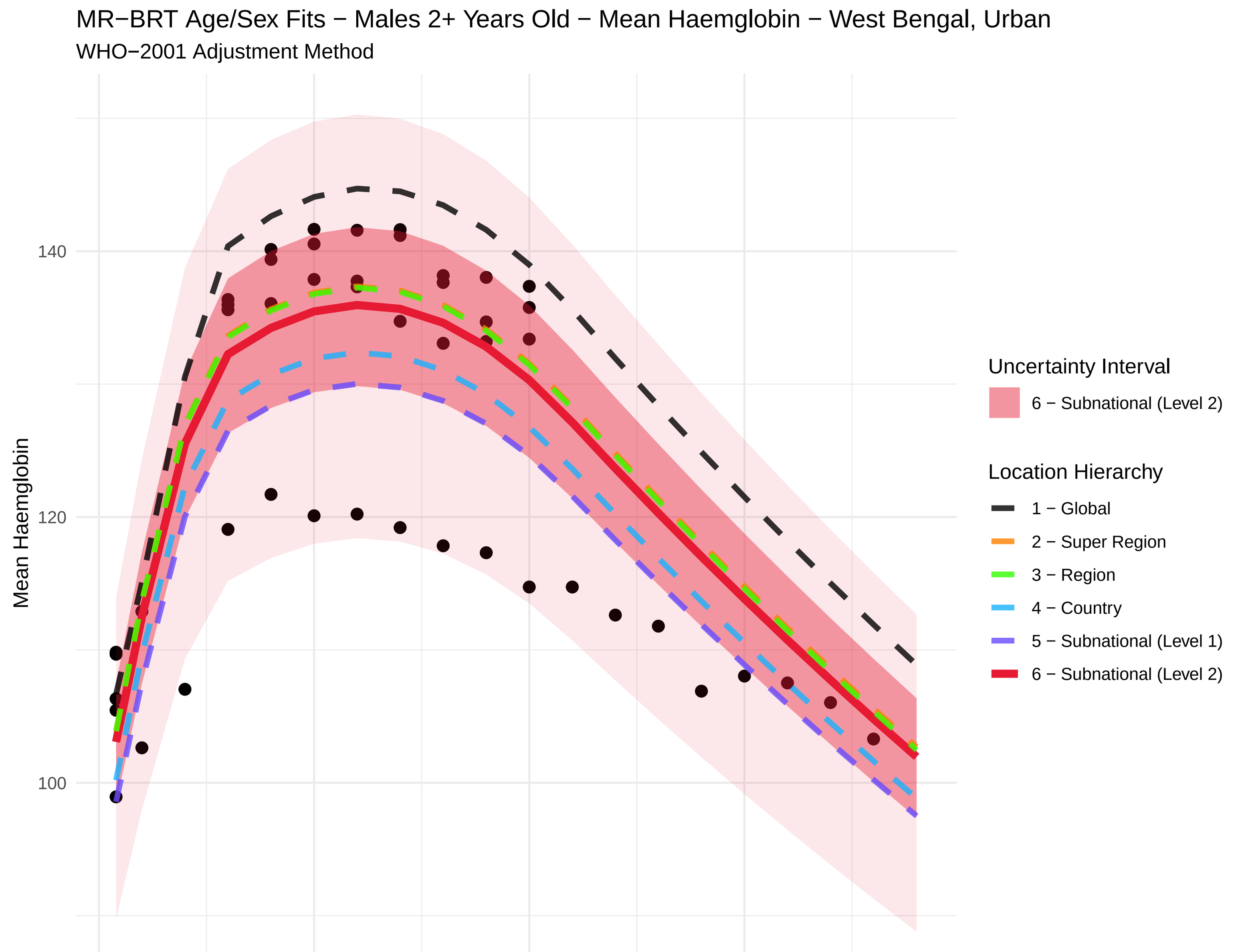
MR−BRT Age/Sex Fits − Males 2+ Years Old − Mean Haemglobin − West Bengal, Urban
WHO−2001 Adjustment Method
Mean Haemglobin
Age (year)
Uncertainty Interval
6 − Subnational (Level 2)
Location Hierarchy
1 − Global
2 − Super Region
3 − Region
4 − Country
5 − Subnational (Level 1)
6 − Subnational (Level 2)

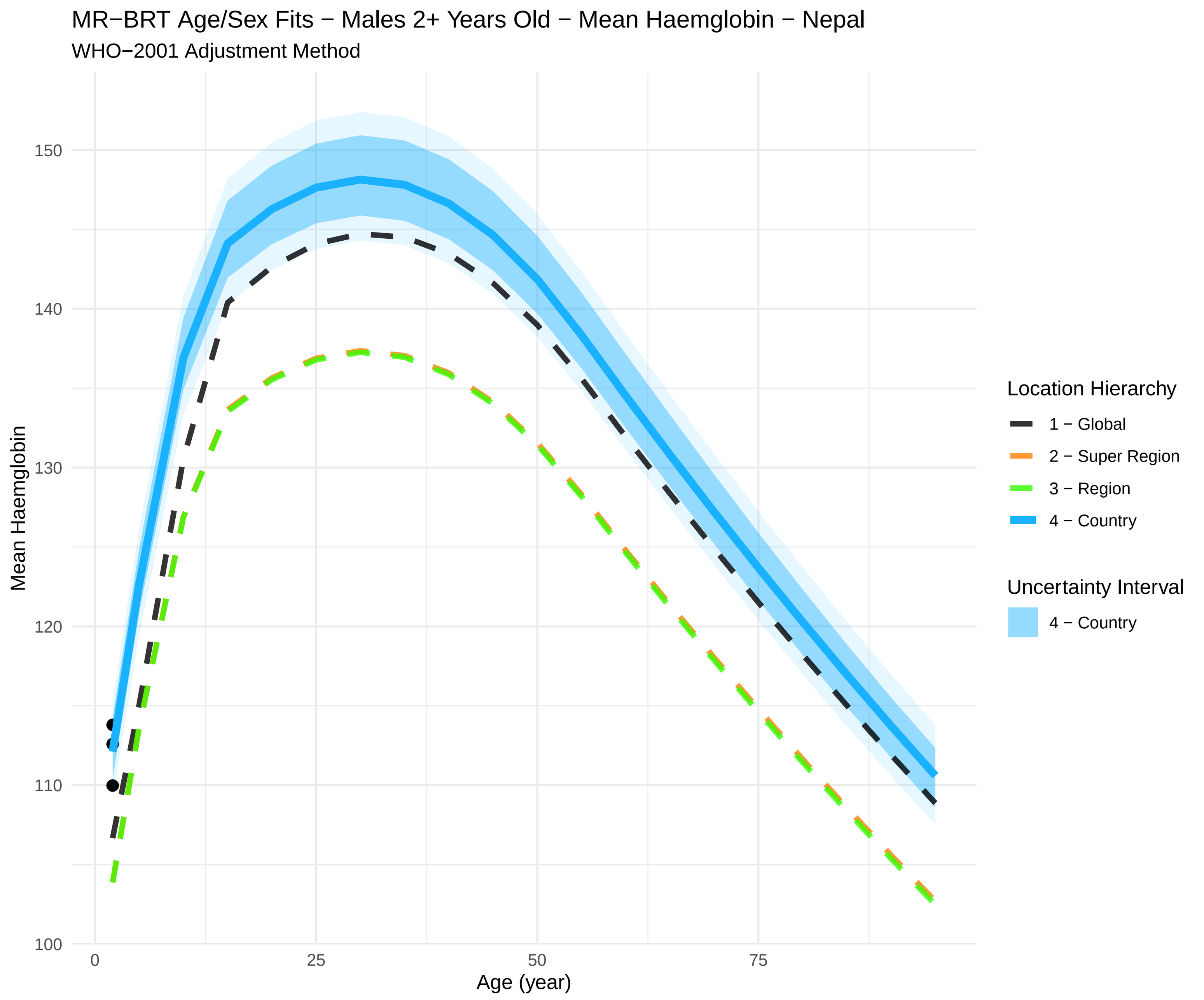
MR−BRT Age/Sex Fits − Males 2+ Years Old − Mean Haemglobin − Nepal
WHO−2001 Adjustment Method
Mean Haemglobin
Age (year)
Location Hierarchy
1 − Global
2 − Super Region
3 − Region
4 − Country
Uncertainty Interval
4 − Country

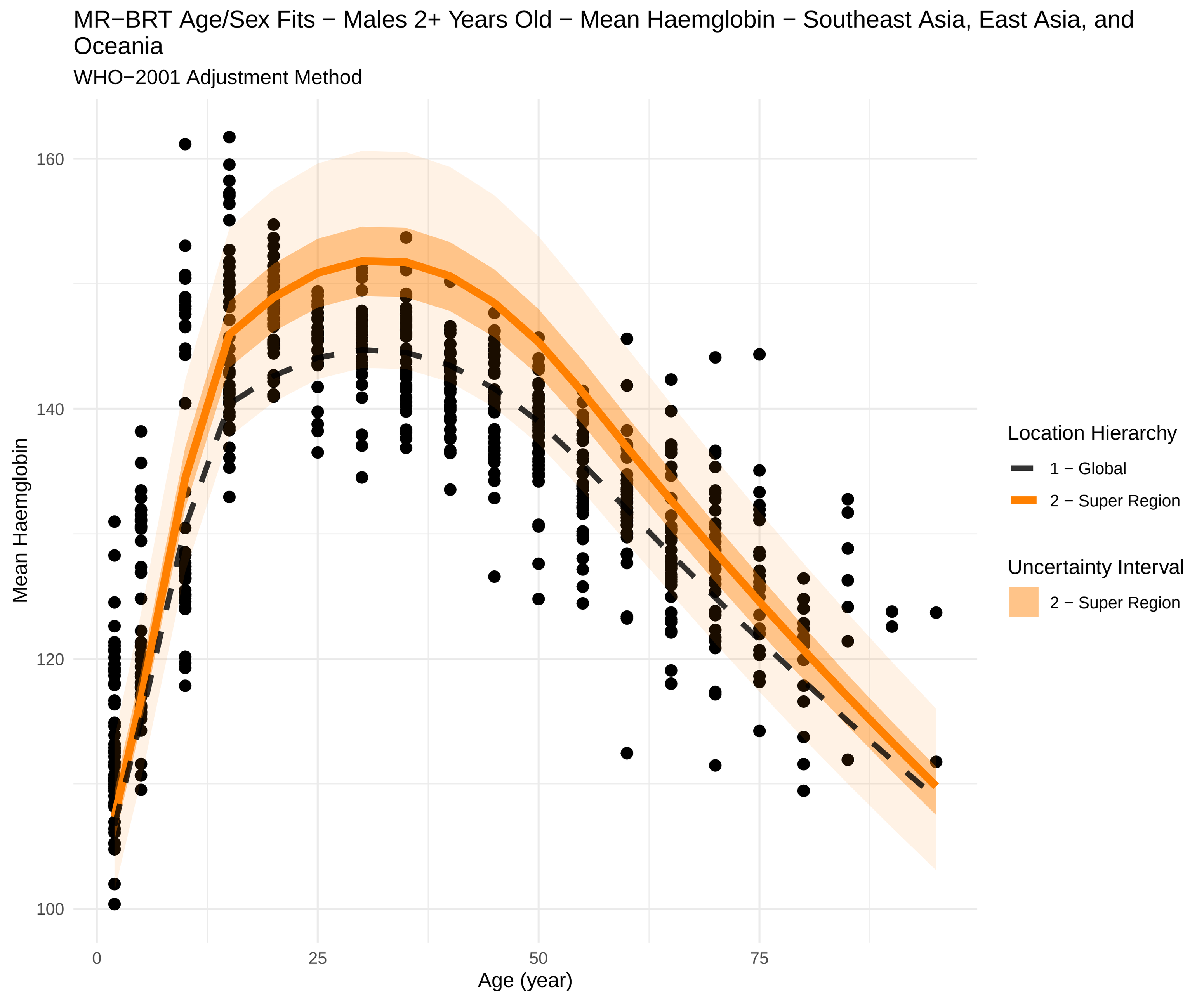
MR−BRT Age/Sex Fits − Males 2+ Years Old − Mean Haemglobin − Southeast Asia, East Asia, and Oceania
WHO−2001 Adjustment Method
Mean Haemglobin
Age (year)
Location Hierarchy
1 − Global
2 − Super Region
Uncertainty Interval
2 − Super Region

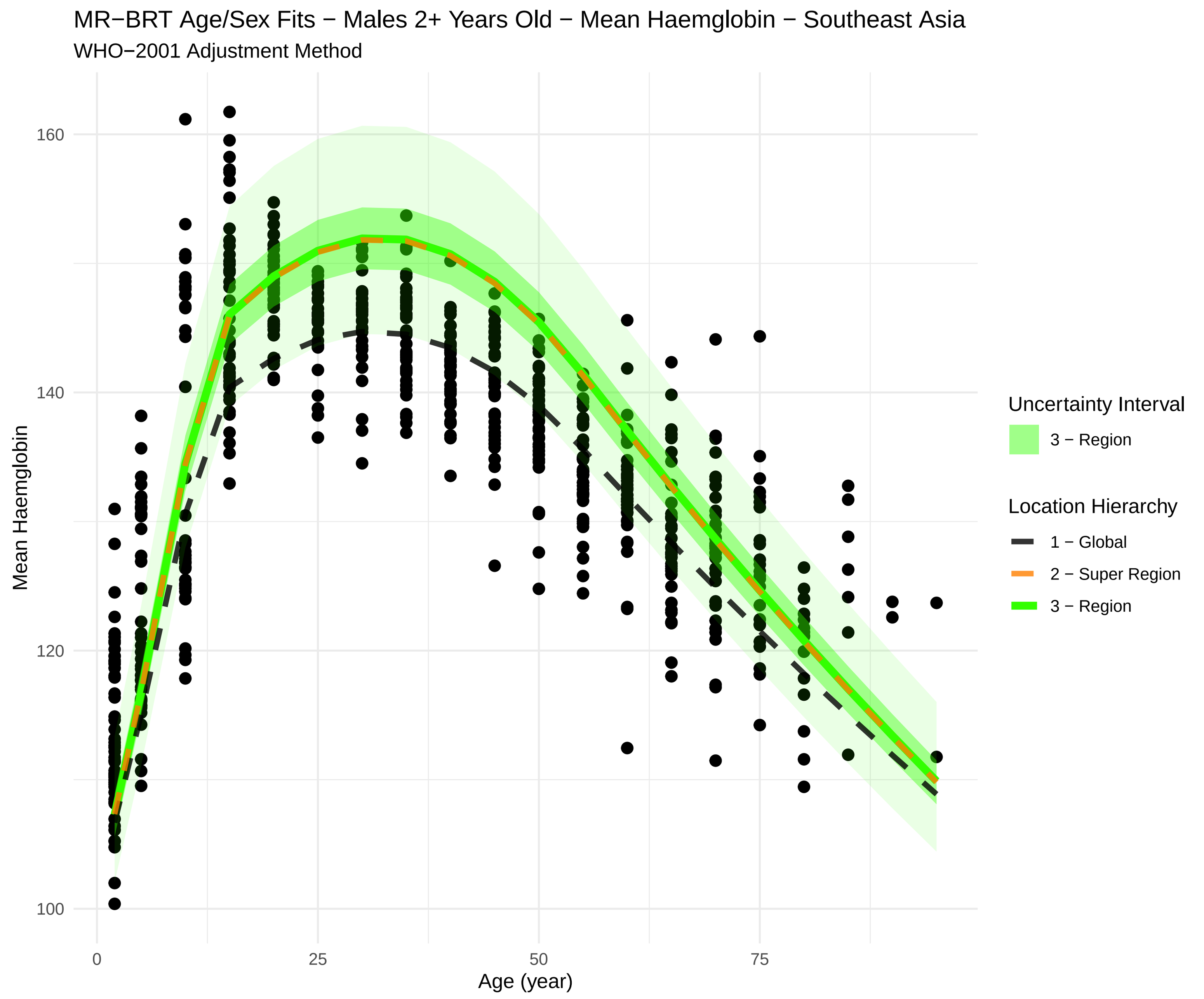
MR−BRT Age/Sex Fits − Males 2+ Years Old − Mean Haemglobin − Southeast Asia
WHO−2001 Adjustment Method
Mean Haemglobin
Age (year)
Uncertainty Interval
3 − Region
Location Hierarchy
1 − Global
2 − Super Region
3 − Region

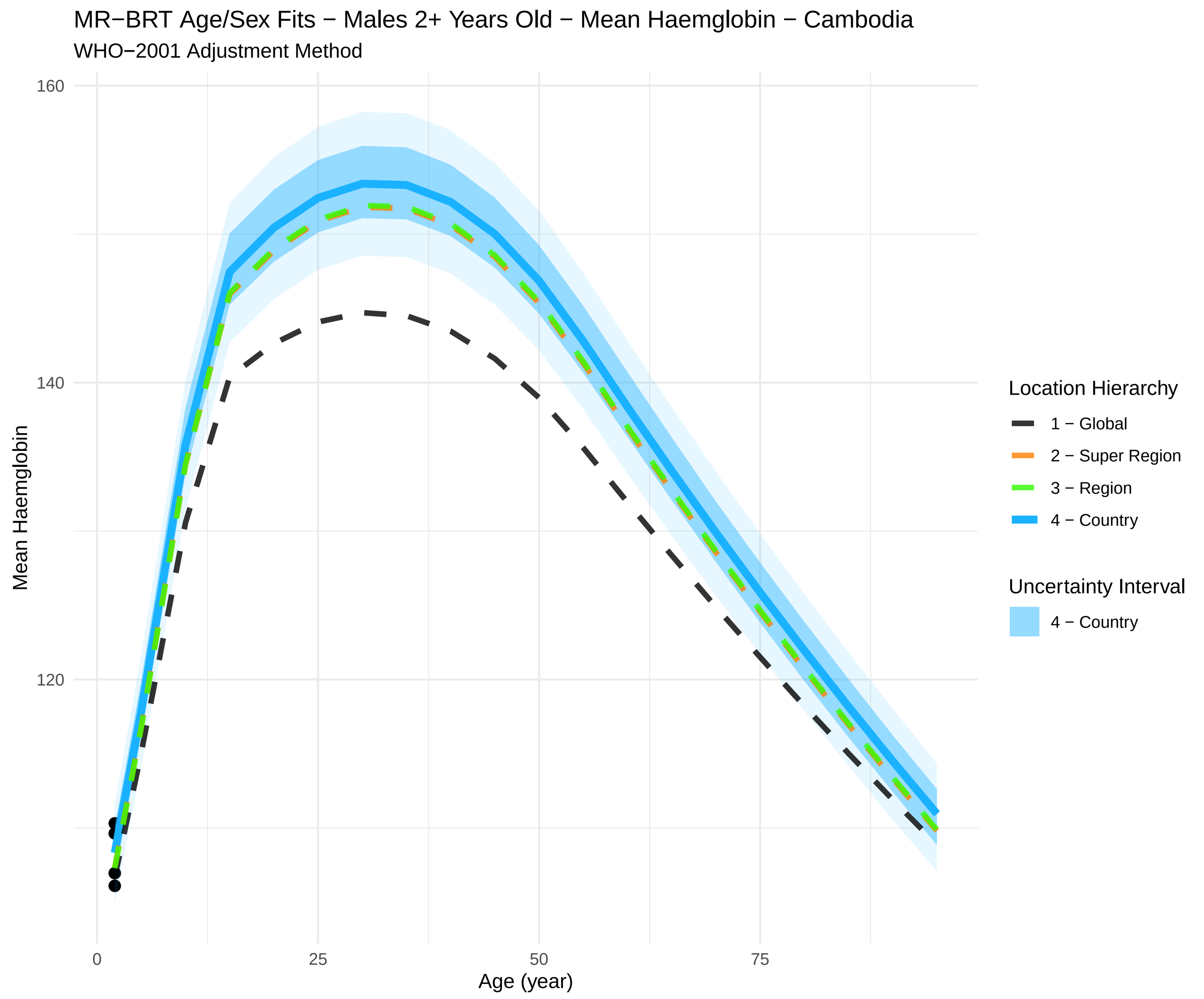
MR−BRT Age/Sex Fits − Males 2+ Years Old − Mean Haemglobin − Cambodia
WHO−2001 Adjustment Method
Mean Haemglobin
Age (year)
Location Hierarchy
1 − Global
2 − Super Region
3 − Region
4 − Country
Uncertainty Interval
4 − Country

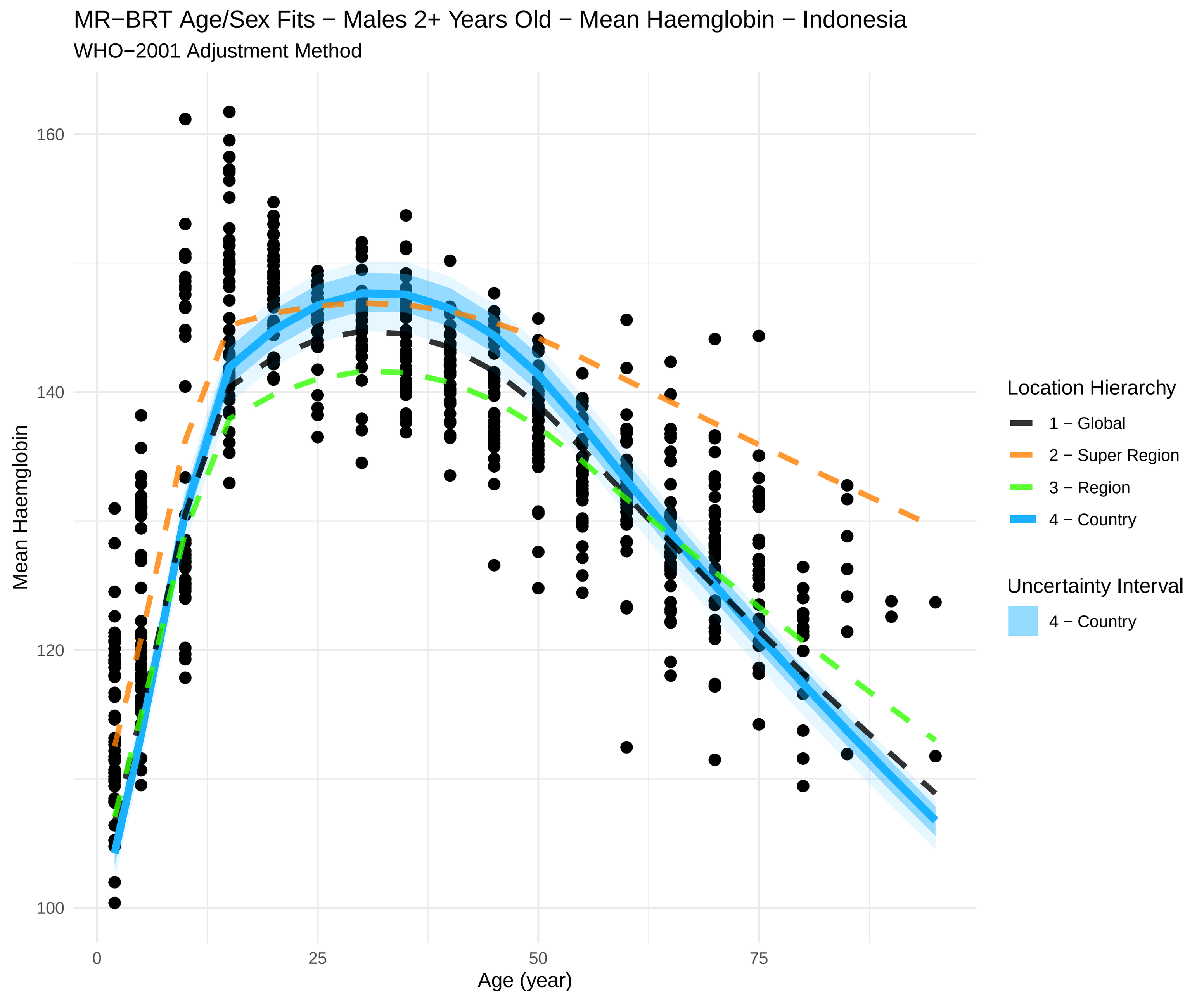
MR−BRT Age/Sex Fits − Males 2+ Years Old − Mean Haemglobin − Indonesia
WHO−2001 Adjustment Method
Mean Haemglobin
Age (year)
Location Hierarchy
1 − Global
2 − Super Region
3 − Region
4 − Country
Uncertainty Interval
4 − Country

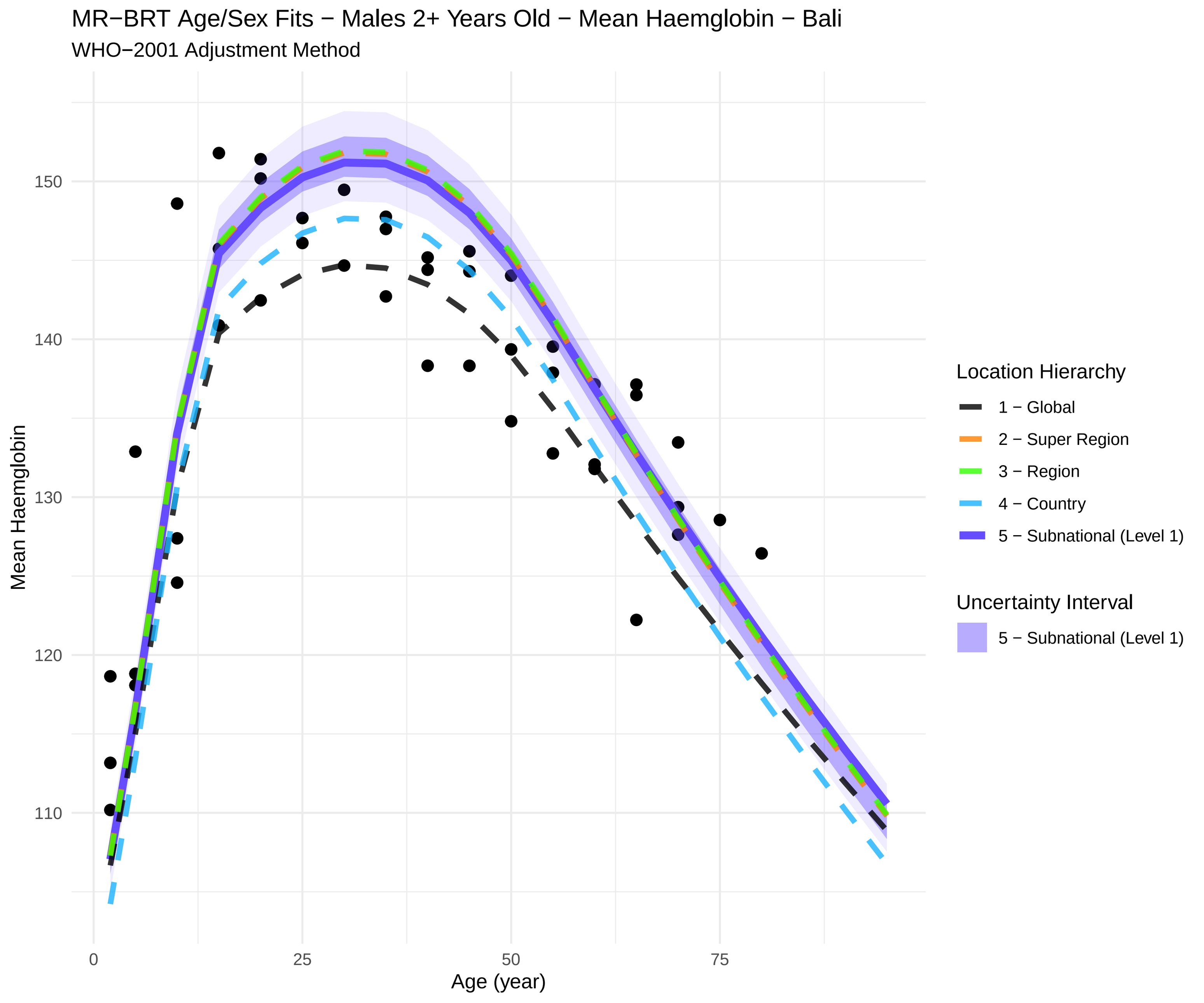
MR−BRT Age/Sex Fits − Males 2+ Years Old − Mean Haemglobin − Bali
WHO−2001 Adjustment Method
Mean Haemglobin
Age (year)
Location Hierarchy
1 − Global
2 − Super Region
3 − Region
4 − Country
5 − Subnational (Level 1)
Uncertainty Interval
5 − Subnational (Level 1)

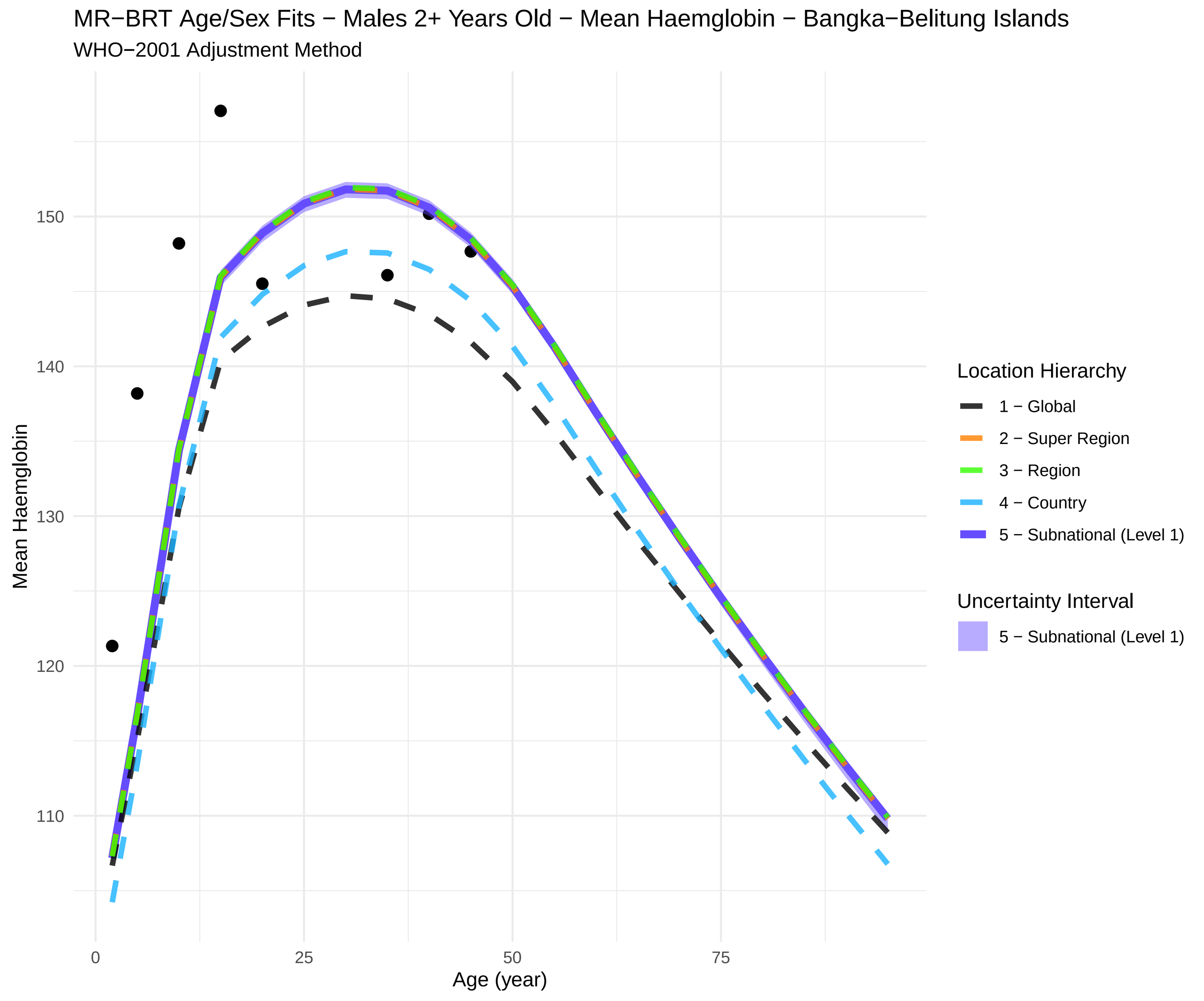
MR−BRT Age/Sex Fits − Males 2+ Years Old − Mean Haemglobin − Bangka−Belitung Islands
WHO−2001 Adjustment Method
Mean Haemglobin
Age (year)
Location Hierarchy
1 − Global
2 − Super Region
3 − Region
4 − Country
5 − Subnational (Level 1)
Uncertainty Interval
5 − Subnational (Level 1)

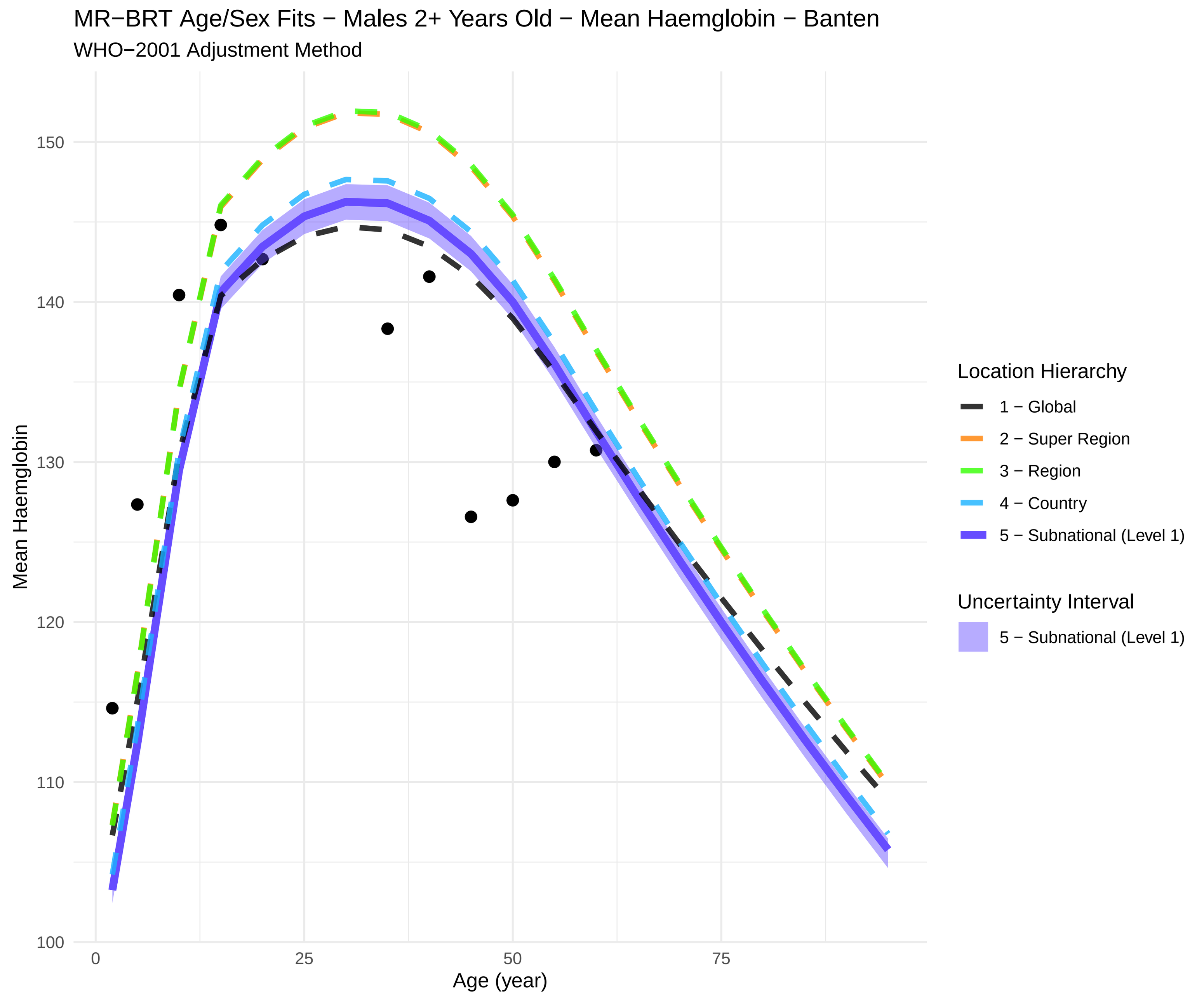
MR−BRT Age/Sex Fits − Males 2+ Years Old − Mean Haemglobin − Banten
WHO−2001 Adjustment Method
Mean Haemglobin
Age (year)
Location Hierarchy
1 − Global
2 − Super Region
3 − Region
4 − Country
5 − Subnational (Level 1)
Uncertainty Interval
5 − Subnational (Level 1)

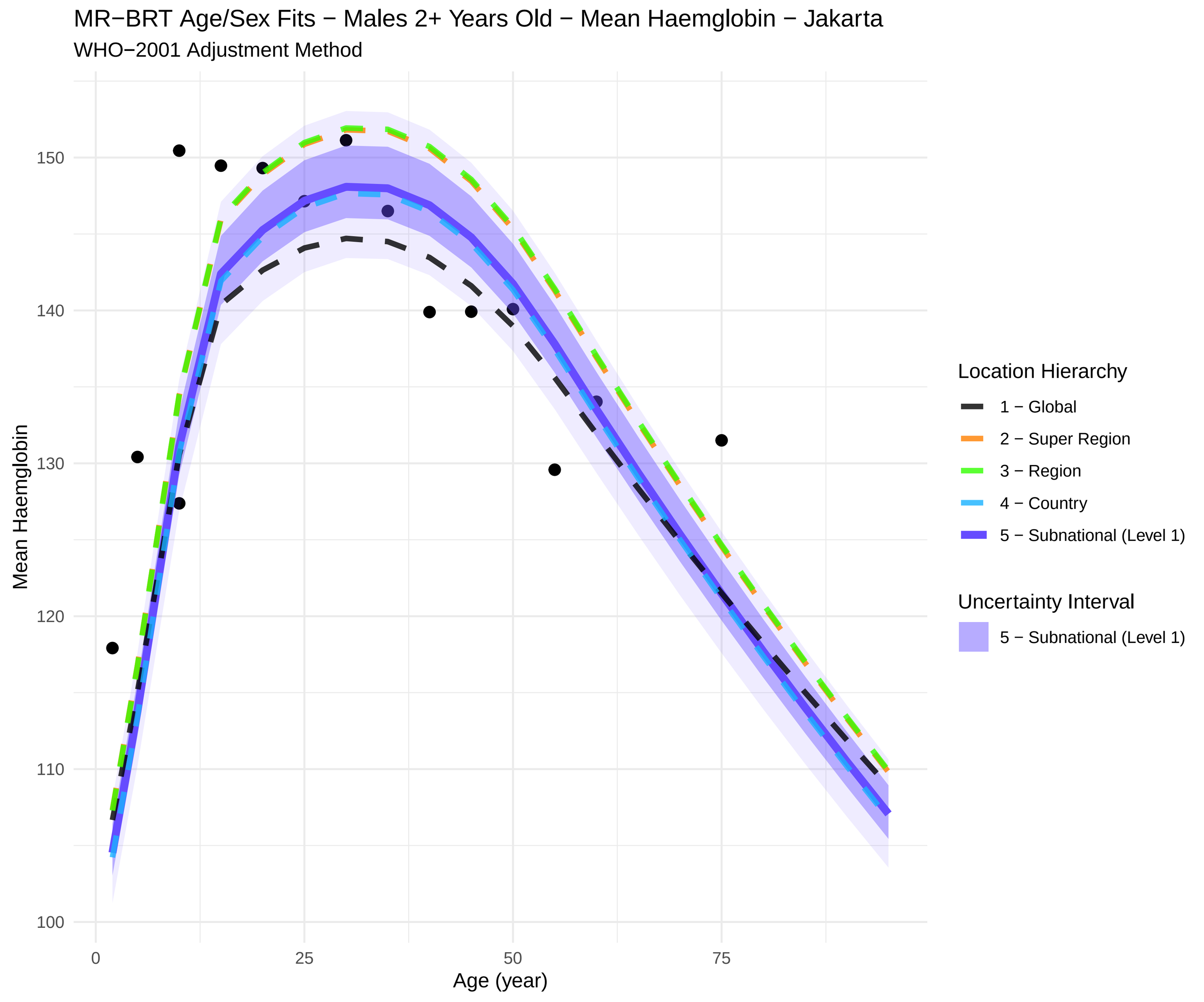
MR−BRT Age/Sex Fits − Males 2+ Years Old − Mean Haemglobin − Jakarta
WHO−2001 Adjustment Method
Mean Haemglobin
Age (year)
Location Hierarchy
1 − Global
2 − Super Region
3 − Region
4 − Country
5 − Subnational (Level 1)
Uncertainty Interval
5 − Subnational (Level 1)

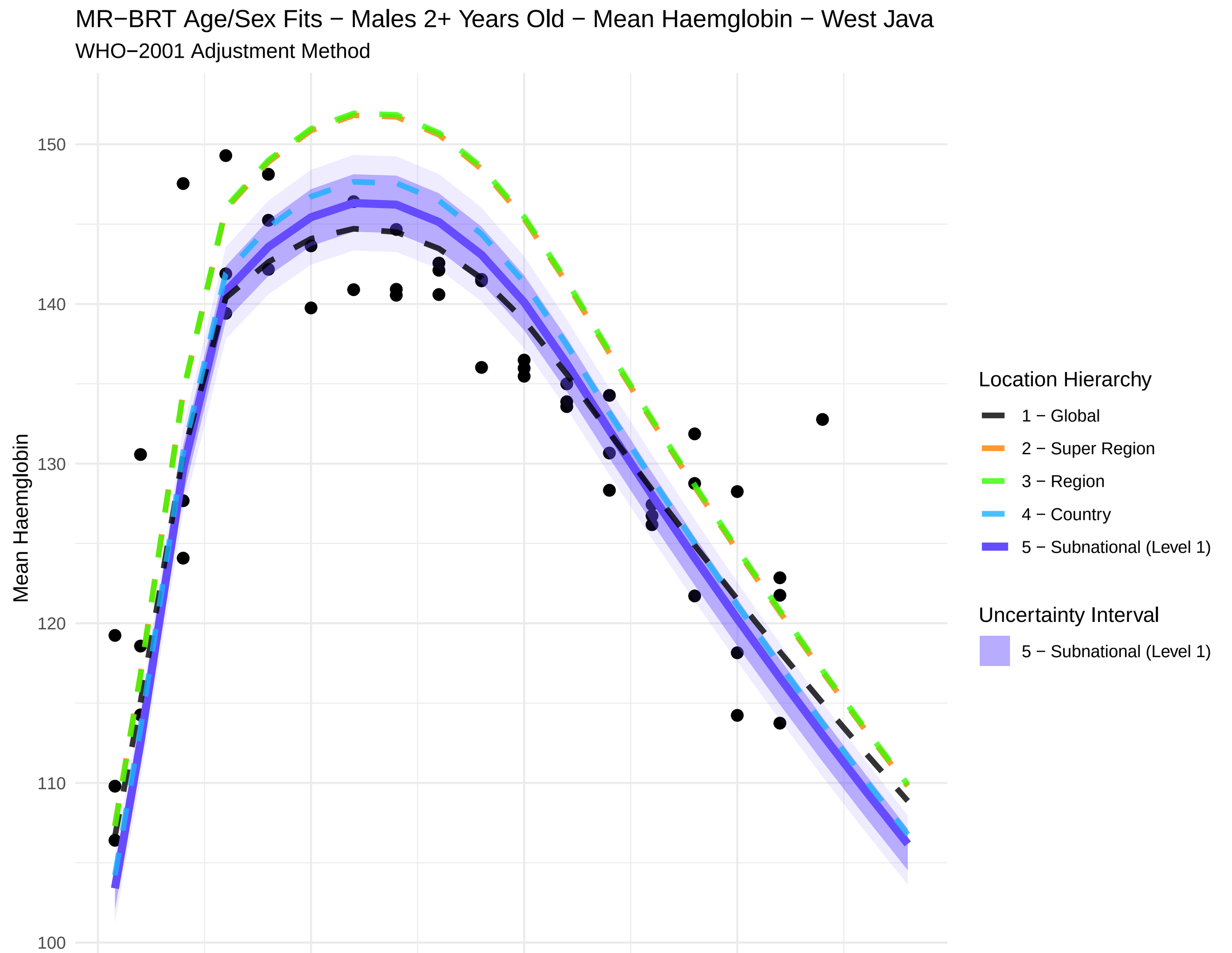
MR−BRT Age/Sex Fits − Males 2+ Years Old − Mean Haemglobin − West Java
WHO−2001 Adjustment Method
Mean Haemglobin
Age (year)
Location Hierarchy
1 − Global
2 − Super Region
3 − Region
4 − Country
5 − Subnational (Level 1)
Uncertainty Interval
5 − Subnational (Level 1)

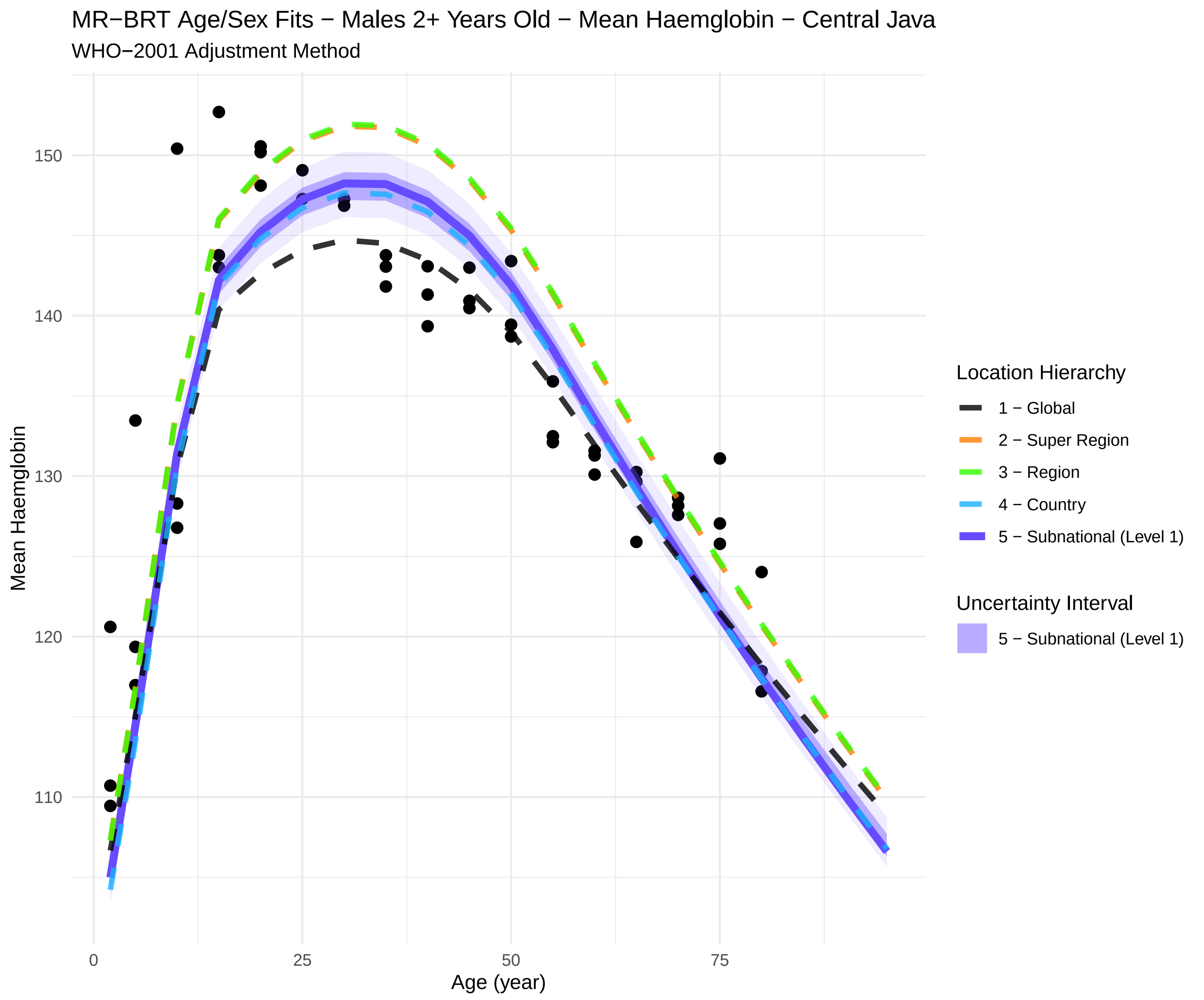
MR−BRT Age/Sex Fits − Males 2+ Years Old − Mean Haemglobin − Central Java
WHO−2001 Adjustment Method
Mean Haemglobin
Age (year)
Location Hierarchy
1 − Global
2 − Super Region
3 − Region
4 − Country
5 − Subnational (Level 1)
Uncertainty Interval
5 − Subnational (Level 1)

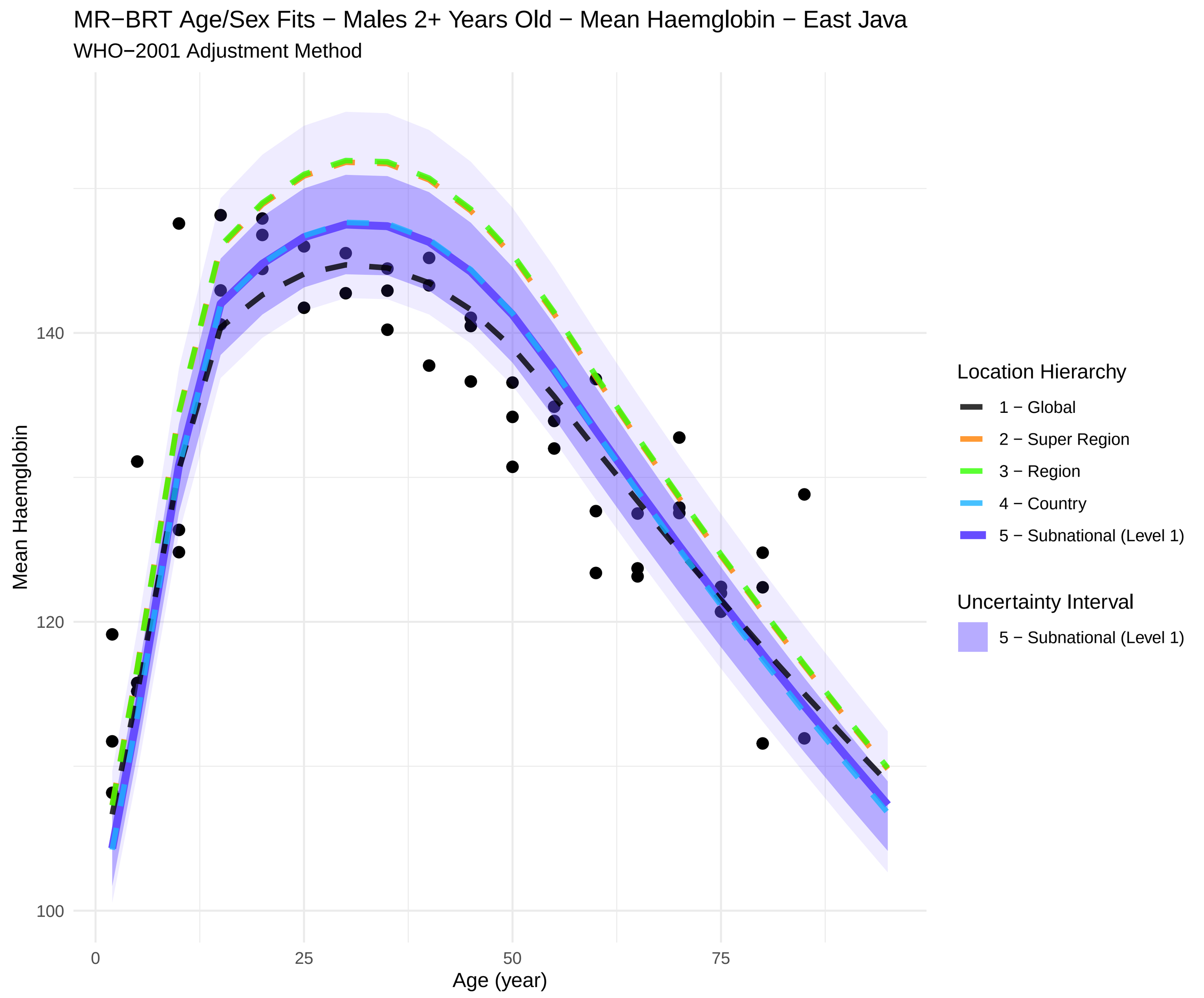
MR−BRT Age/Sex Fits − Males 2+ Years Old − Mean Haemglobin − East Java
WHO−2001 Adjustment Method
Mean Haemglobin
Age (year)
Location Hierarchy
1 − Global
2 − Super Region
3 − Region
4 − Country
5 − Subnational (Level 1)
Uncertainty Interval
5 − Subnational (Level 1)

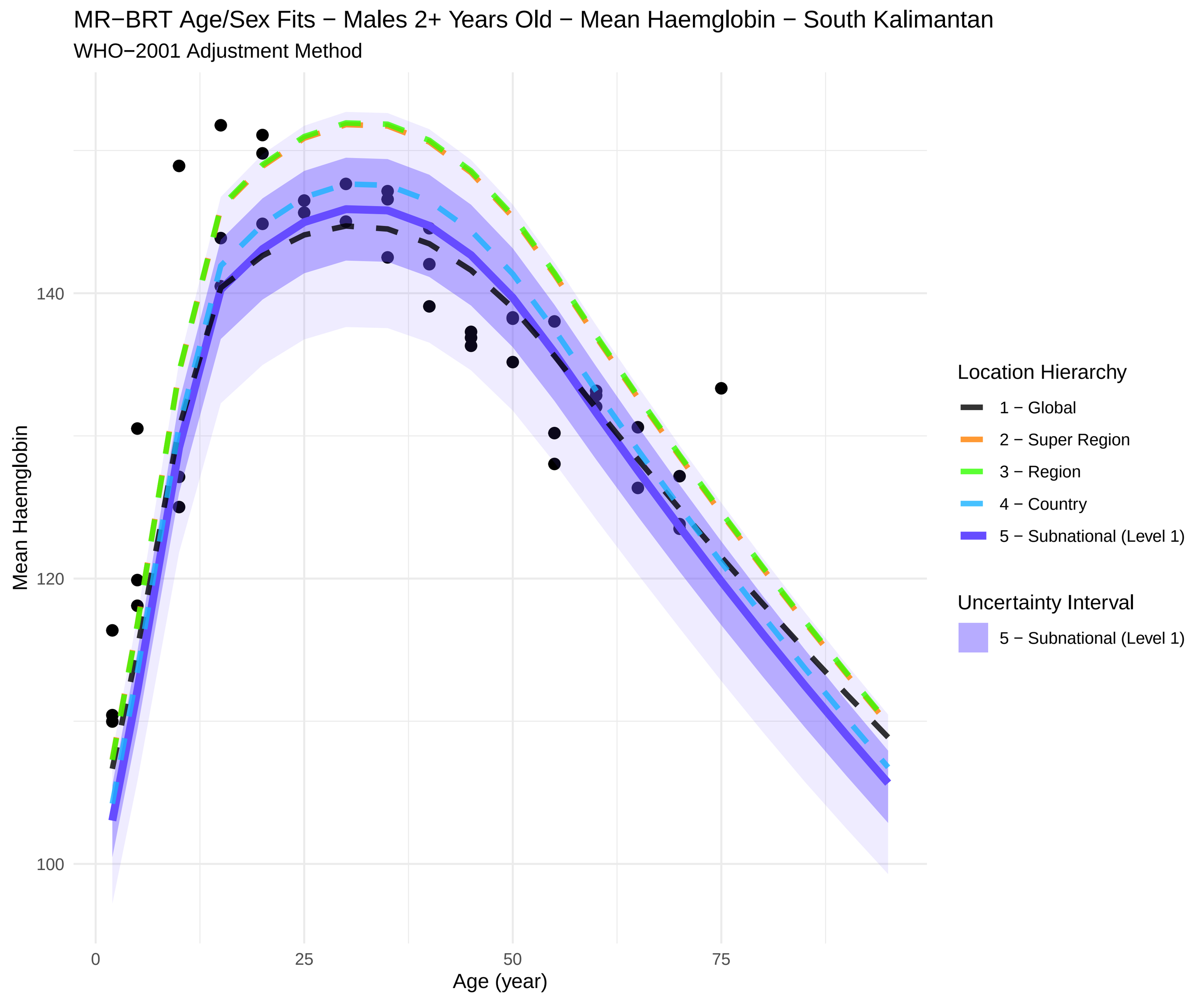
MR−BRT Age/Sex Fits − Males 2+ Years Old − Mean Haemglobin − South Kalimantan
WHO−2001 Adjustment Method
Mean Haemglobin
Age (year)
Location Hierarchy
1 − Global
2 − Super Region
3 − Region
4 − Country
5 − Subnational (Level 1)
Uncertainty Interval
5 − Subnational (Level 1)

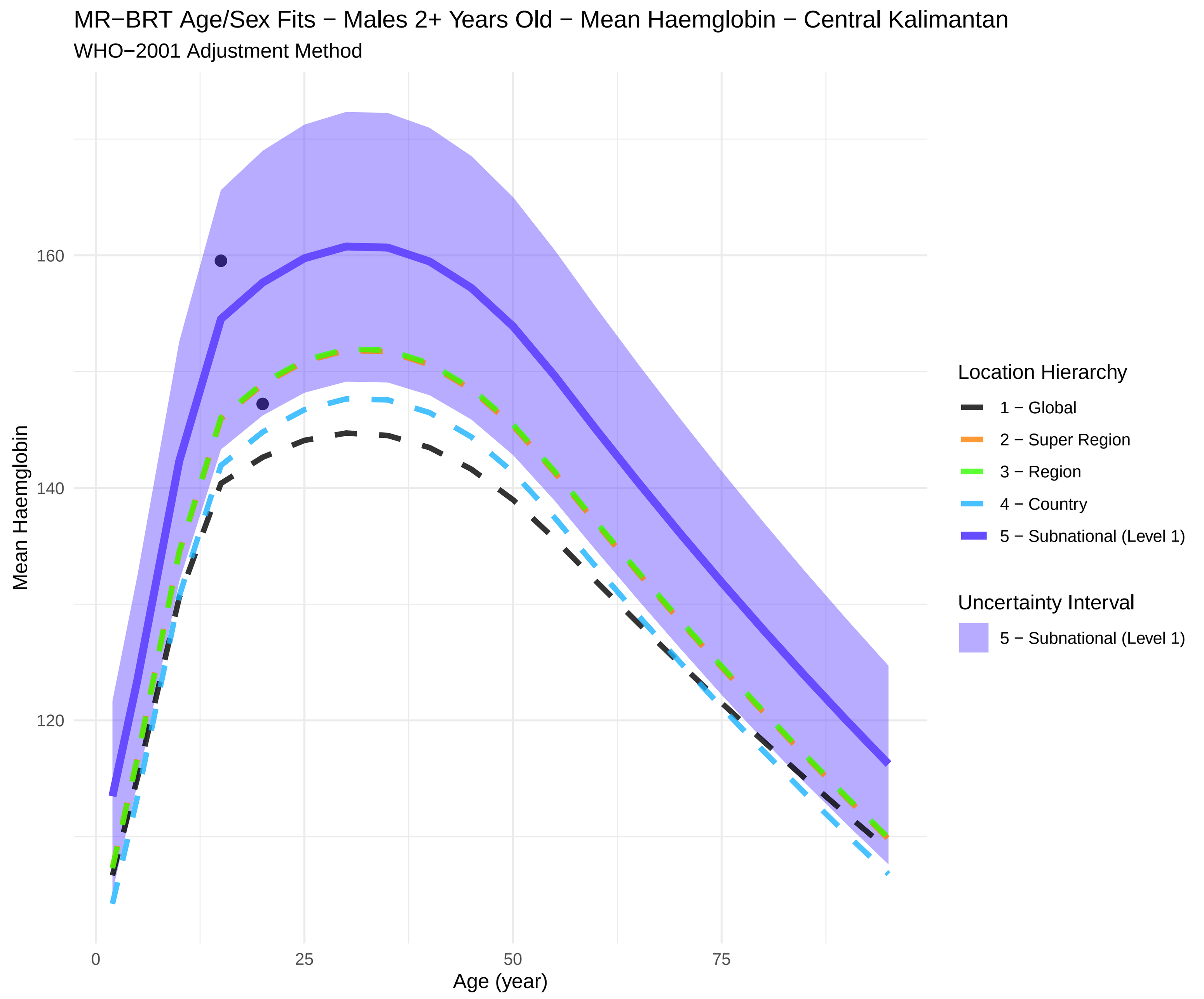
MR−BRT Age/Sex Fits − Males 2+ Years Old − Mean Haemglobin − Central Kalimantan
WHO−2001 Adjustment Method
Mean Haemglobin
Age (year)
Location Hierarchy
1 − Global
2 − Super Region
3 − Region
4 − Country
5 − Subnational (Level 1)
Uncertainty Interval
5 − Subnational (Level 1)

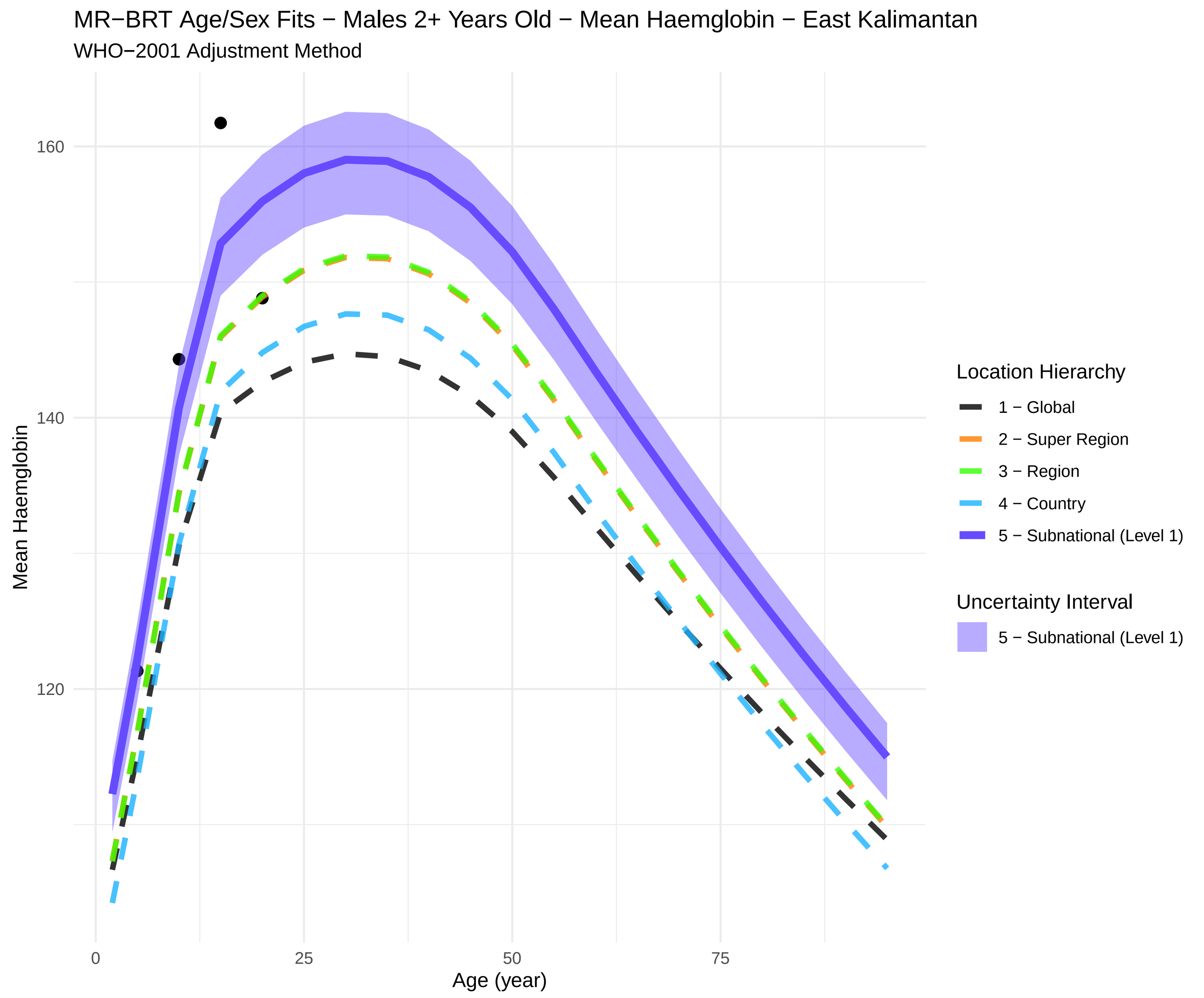
MR−BRT Age/Sex Fits − Males 2+ Years Old − Mean Haemglobin − East Kalimantan
WHO−2001 Adjustment Method
Mean Haemglobin
Age (year)
Location Hierarchy
1 − Global
2 − Super Region
3 − Region
4 − Country
5 − Subnational (Level 1)
Uncertainty Interval
5 − Subnational (Level 1)

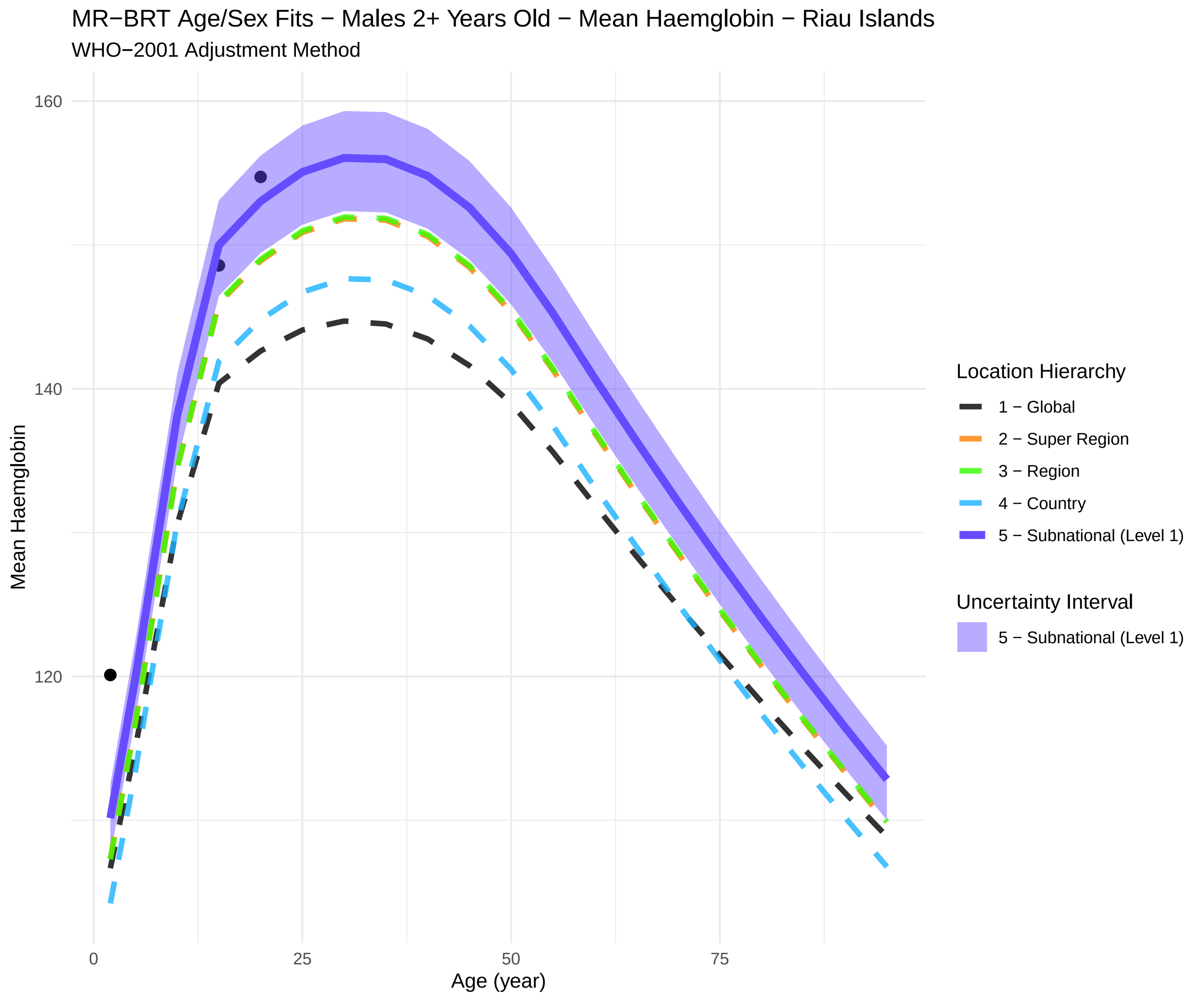
MR−BRT Age/Sex Fits − Males 2+ Years Old − Mean Haemglobin − Riau Islands
WHO−2001 Adjustment Method
Mean Haemglobin
Age (year)
Location Hierarchy
1 − Global
2 − Super Region
3 − Region
4 − Country
5 − Subnational (Level 1)
Uncertainty Interval
5 − Subnational (Level 1)

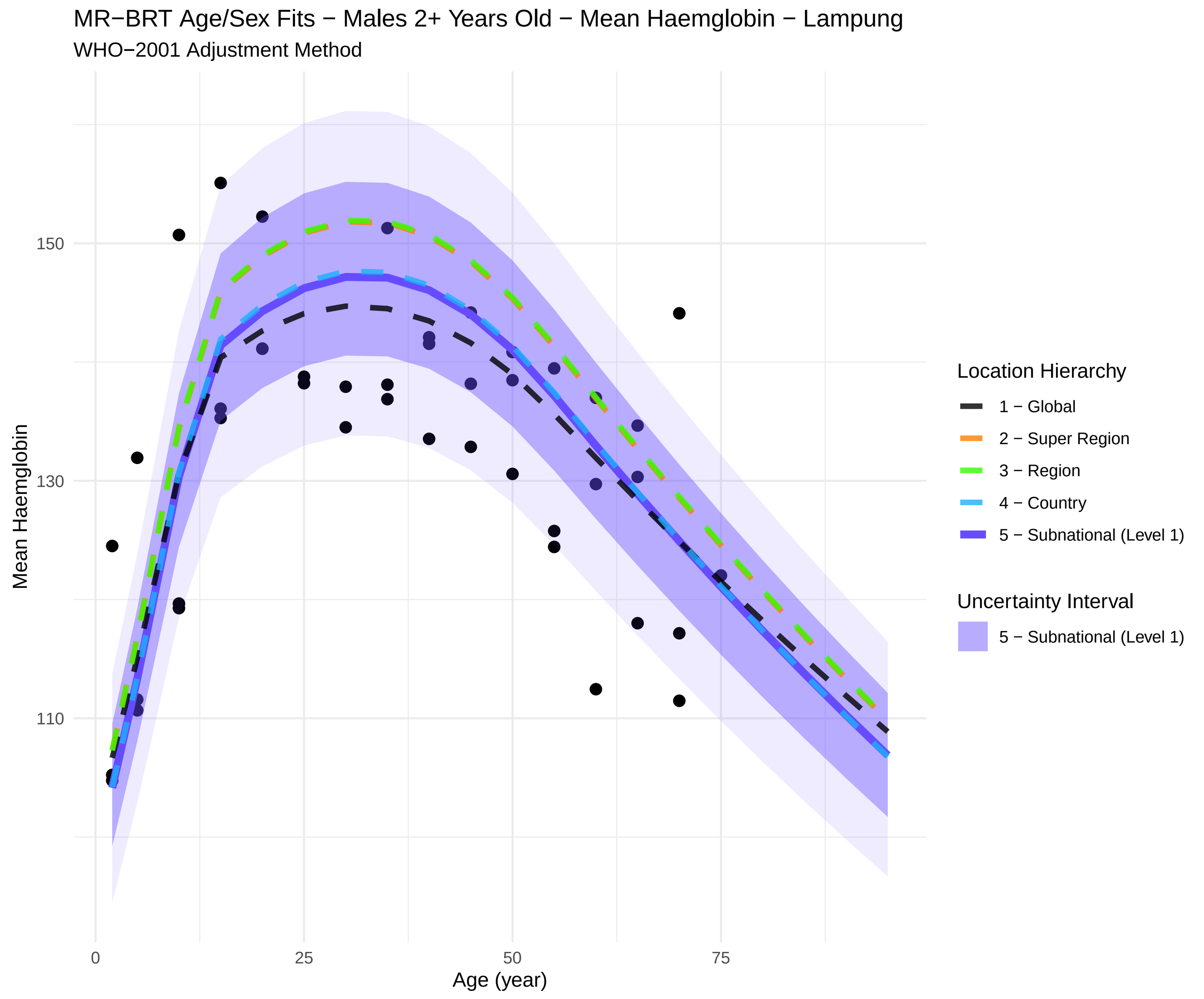
MR−BRT Age/Sex Fits − Males 2+ Years Old − Mean Haemglobin − Lampung
WHO−2001 Adjustment Method
Mean Haemglobin
Age (year)
Location Hierarchy
1 − Global
2 − Super Region
3 − Region
4 − Country
5 − Subnational (Level 1)
Uncertainty Interval
5 − Subnational (Level 1)

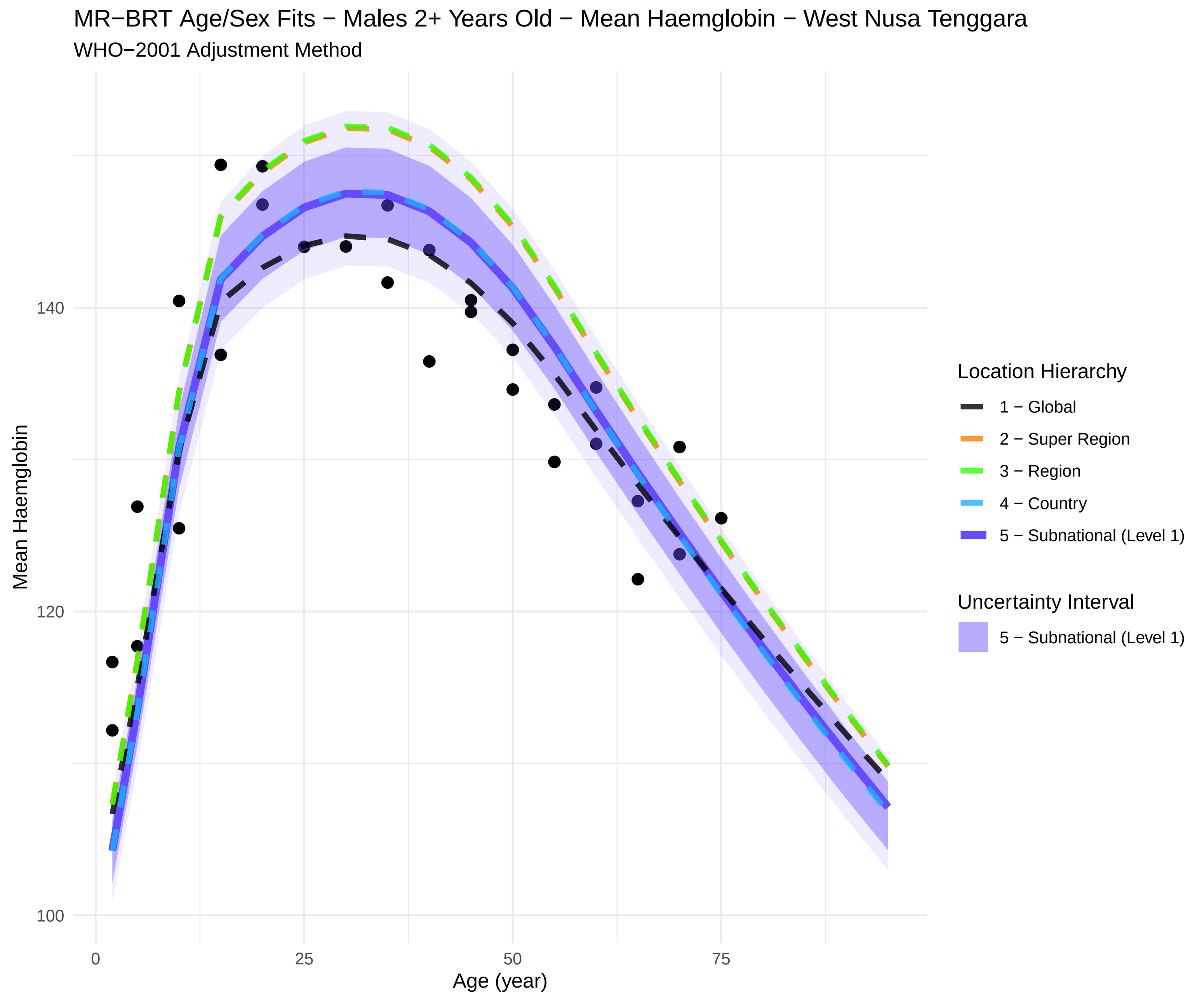
MR−BRT Age/Sex Fits − Males 2+ Years Old − Mean Haemglobin − West Nusa Tenggara
WHO−2001 Adjustment Method
Mean Haemglobin
Age (year)
Location Hierarchy
1 − Global
2 − Super Region
3 − Region
4 − Country
5 − Subnational (Level 1)
Uncertainty Interval
5 − Subnational (Level 1)

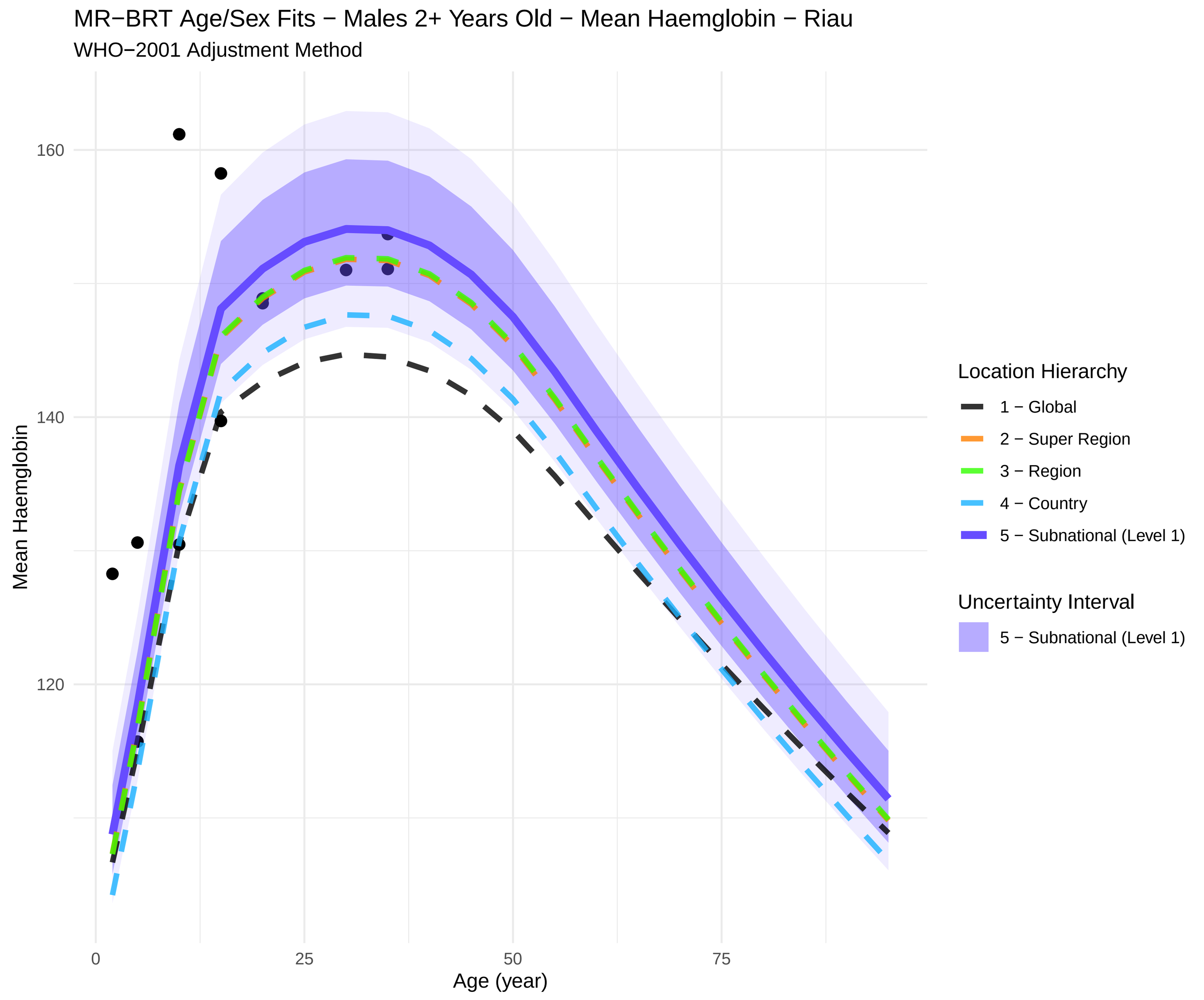
MR−BRT Age/Sex Fits − Males 2+ Years Old − Mean Haemglobin − Riau
WHO−2001 Adjustment Method
Mean Haemglobin
Age (year)
Location Hierarchy
1 − Global
2 − Super Region
3 − Region
4 − Country
5 − Subnational (Level 1)
Uncertainty Interval
5 − Subnational (Level 1)

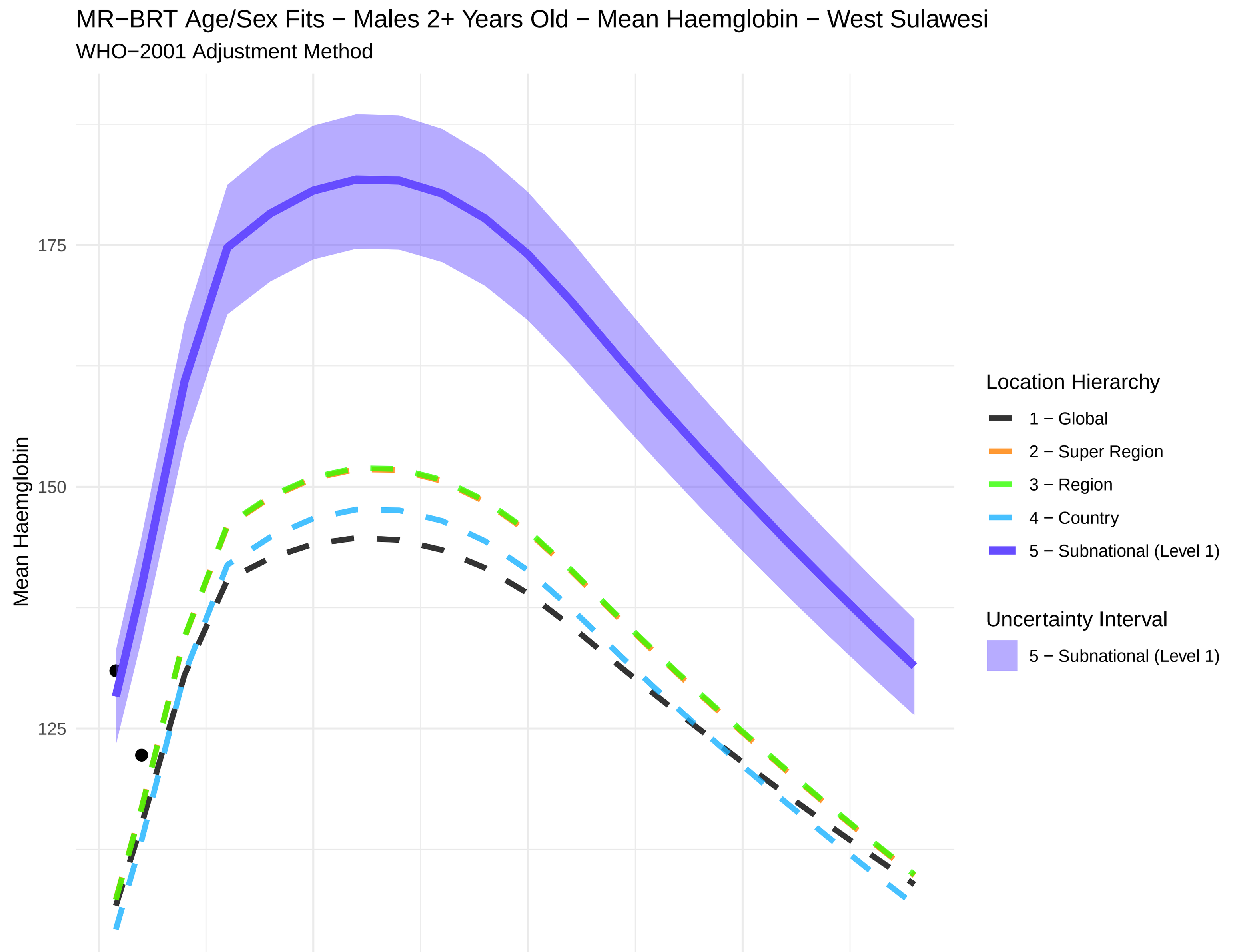
MR−BRT Age/Sex Fits − Males 2+ Years Old − Mean Haemglobin − West Sulawesi
WHO−2001 Adjustment Method
Mean Haemglobin
Age (year)
Location Hierarchy
1 − Global
2 − Super Region
3 − Region
4 − Country
5 − Subnational (Level 1)
Uncertainty Interval
5 − Subnational (Level 1)

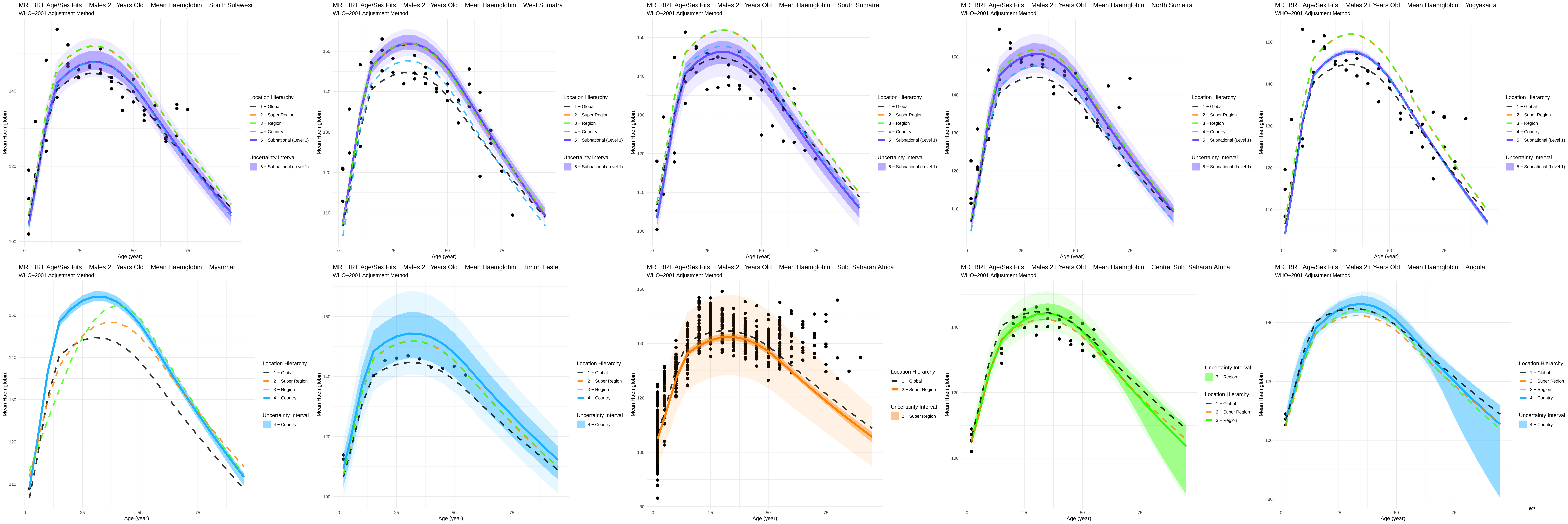

MR−BRT Age/Sex Fits − Males 2+ Years Old − Mean Haemglobin − South Sulawesi
WHO−2001 Adjustment Method
MR−BRT Age/Sex Fits − Males 2+ Years Old − Mean Haemglobin − West Sumatra
WHO−2001 Adjustment Method
MR−BRT Age/Sex Fits − Males 2+ Years Old − Mean Haemglobin − South Sumatra
WHO−2001 Adjustment Method
MR−BRT Age/Sex Fits − Males 2+ Years Old − Mean Haemglobin − North Sumatra
WHO−2001 Adjustment Method
MR−BRT Age/Sex Fits − Males 2+ Years Old − Mean Haemglobin − Yogyakarta
WHO−2001 Adjustment Method
Location Hierarchy
1 − Global
2 − Super Region
3 − Region
4 − Country
5 − Subnational (Level 1)
Uncertainty Interval
5 − Subnational (Level 1)
MR−BRT Age/Sex Fits − Males 2+ Years Old − Mean Haemglobin − Myanmar
WHO−2001 Adjustment Method
MR−BRT Age/Sex Fits − Males 2+ Years Old − Mean Haemglobin − Timor−Leste
WHO−2001 Adjustment Method
MR−BRT Age/Sex Fits − Males 2+ Years Old − Mean Haemglobin − Angola
WHO−2001 Adjustment Method
Location Hierarchy
1 − Global
2 − Super Region
3 − Region
4 − Country
Uncertainty Interval
4 − Country
MR−BRT Age/Sex Fits − Males 2+ Years Old − Mean Haemglobin − Sub−Saharan Africa
WHO−2001 Adjustment Method
Location Hierarchy
1 − Global
2 − Super Region
Uncertainty Interval
2 − Super Region
MR−BRT Age/Sex Fits − Males 2+ Years Old − Mean Haemglobin − Central Sub−Saharan Africa
WHO−2001 Adjustment Method
Uncertainty Interval
3 − Region
Location Hierarchy
1 − Global
2 − Super Region
3 − Region
Mean Haemglobin
Age (year)

MR−BRT Age/Sex Fits − Males 2+ Years Old − Mean Haemglobin − Democratic Republic of the Congo
WHO−2001 Adjustment Method

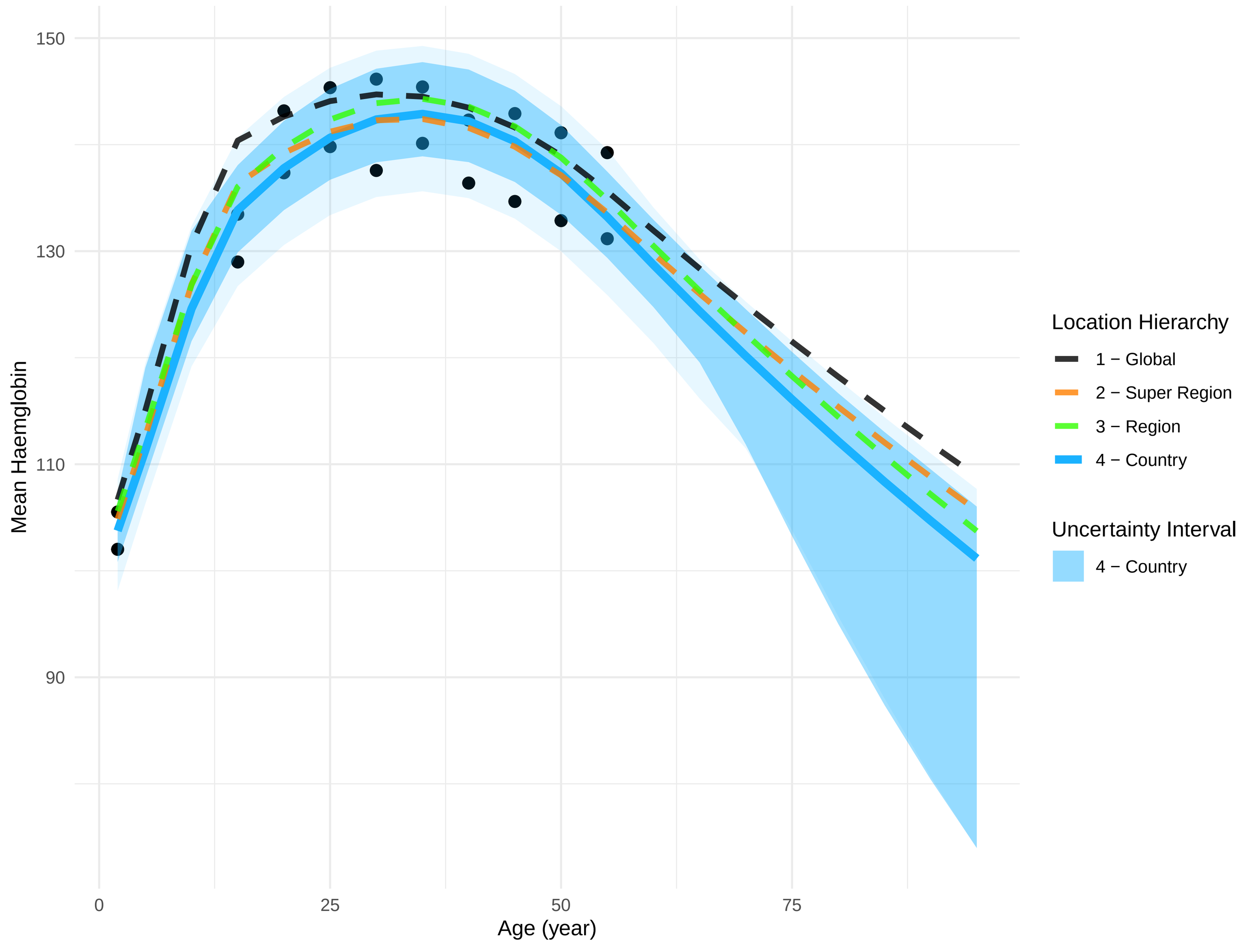

MR−BRT Age/Sex Fits − Males 2+ Years Old − Mean Haemglobin − Gabon
WHO−2001 Adjustment Method

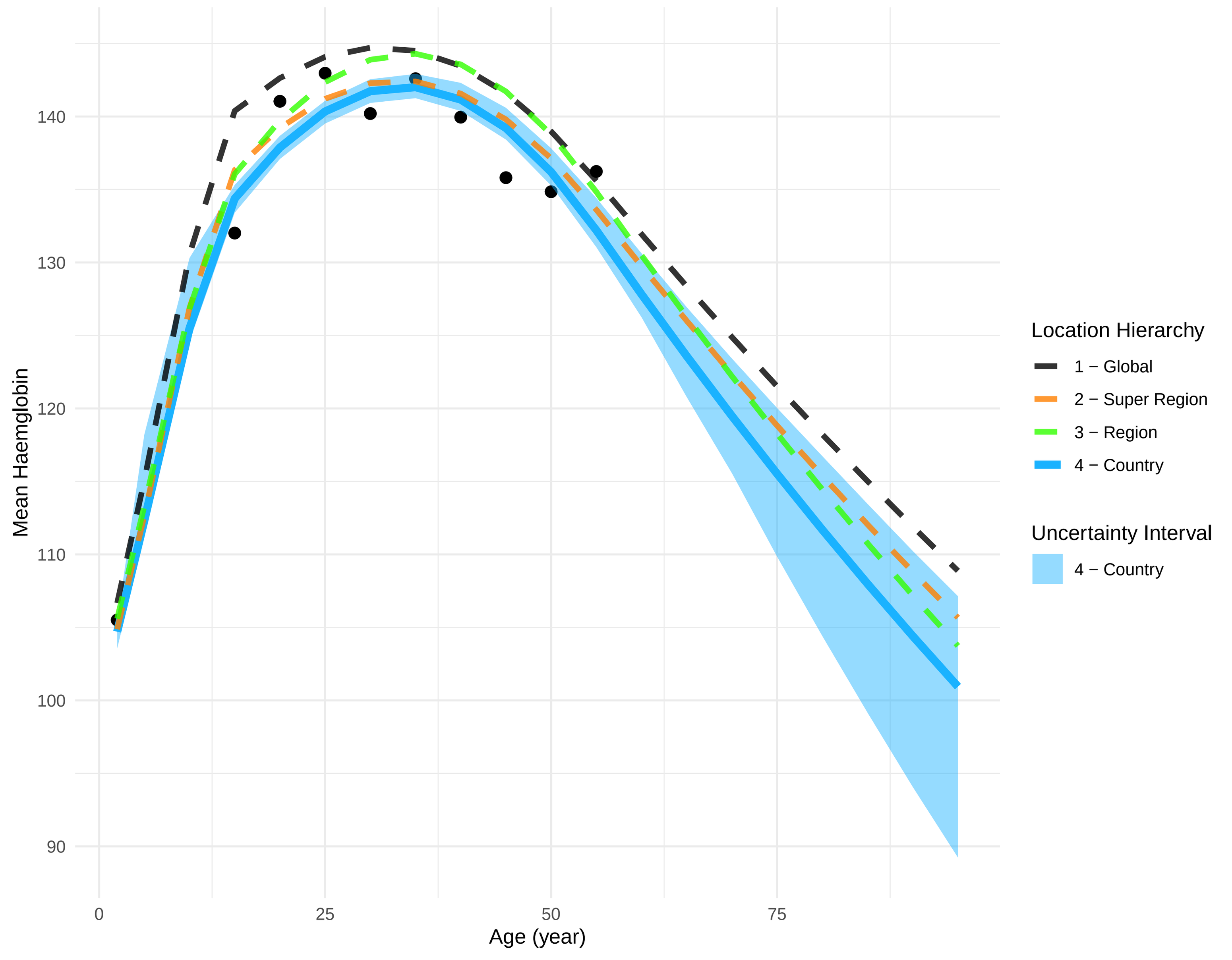

MR−BRT Age/Sex Fits − Males 2+ Years Old − Mean Haemglobin − Eastern Sub−Saharan Africa
WHO−2001 Adjustment Method

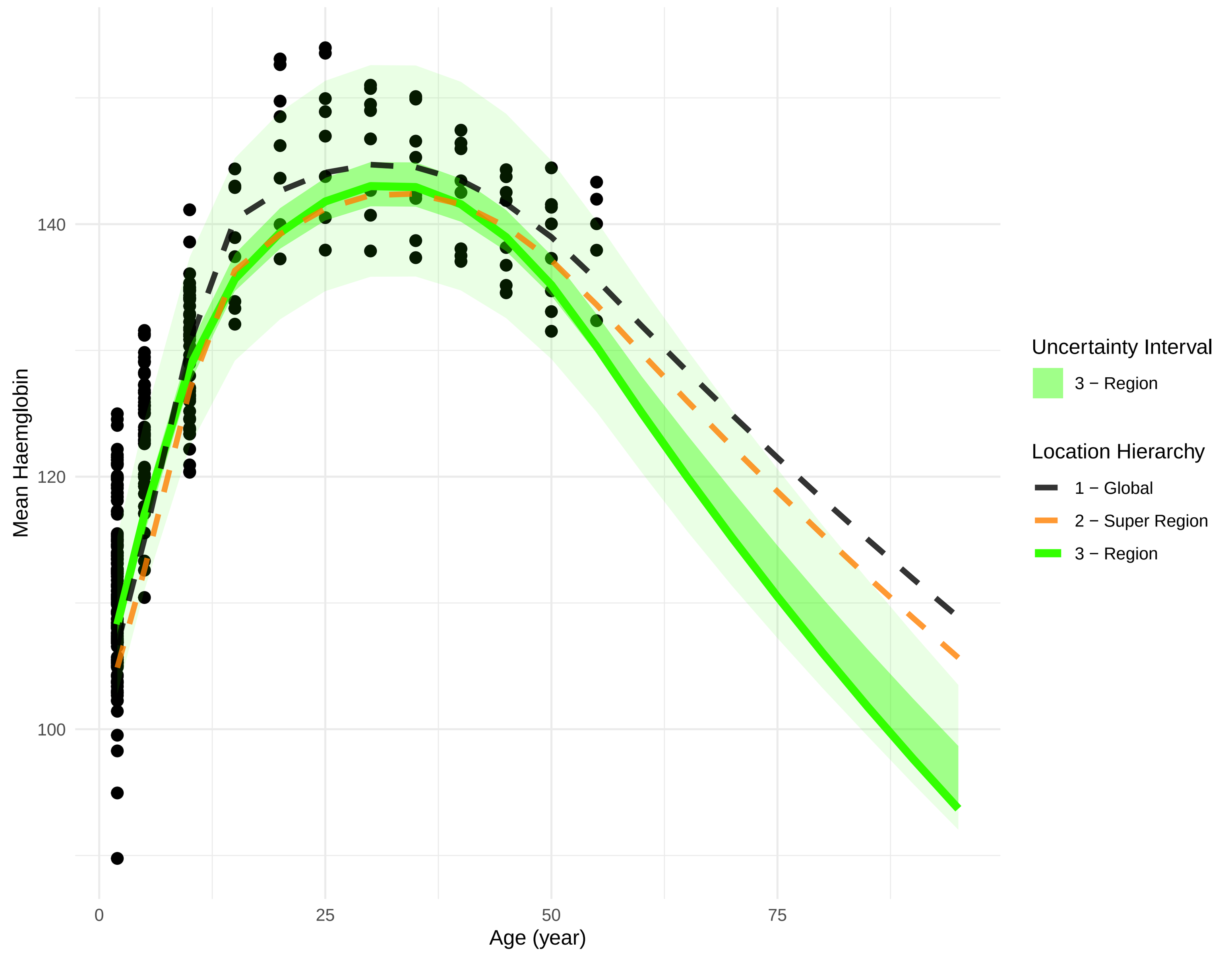

MR−BRT Age/Sex Fits − Males 2+ Years Old − Mean Haemglobin − Burundi
WHO−2001 Adjustment Method

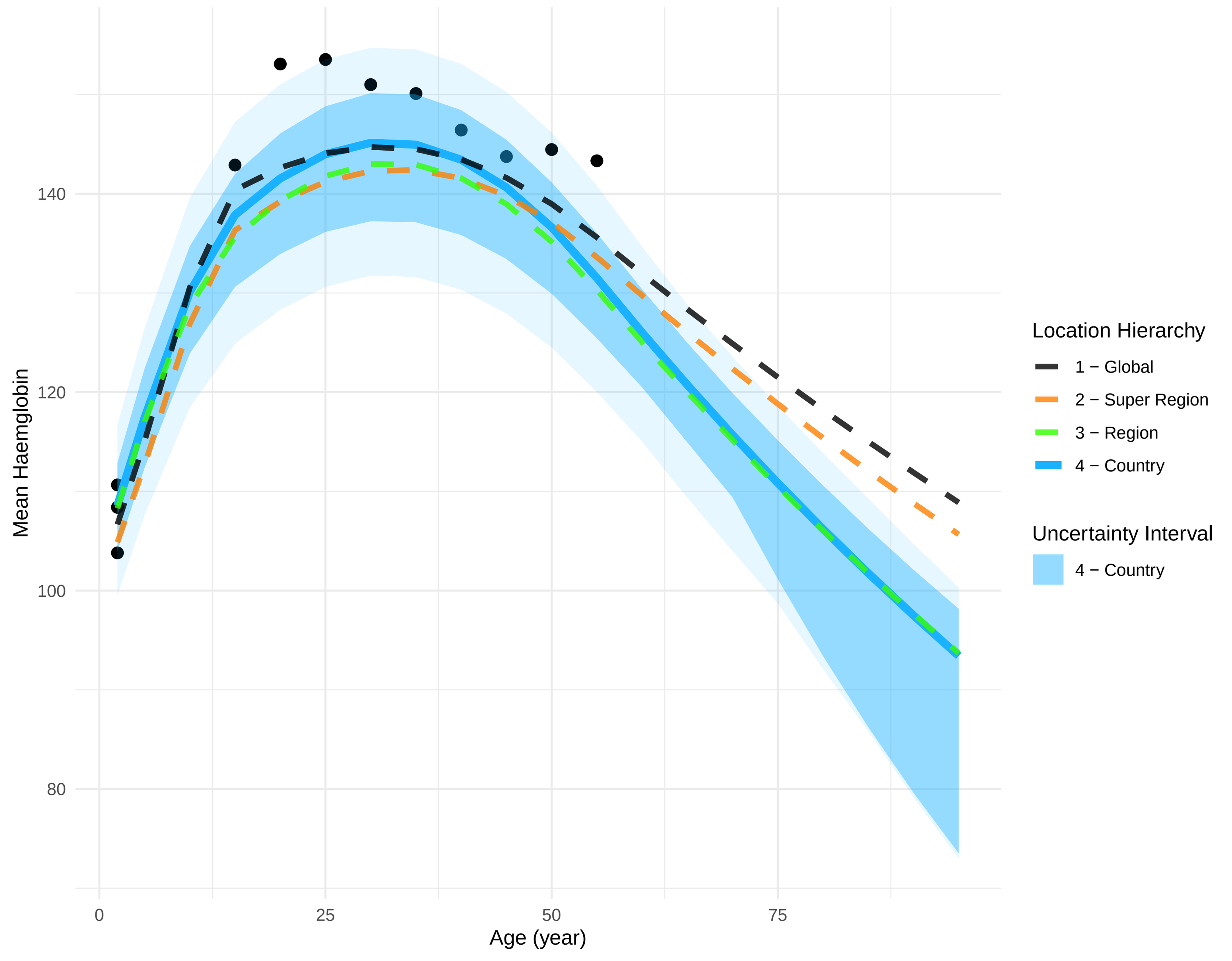

MR−BRT Age/Sex Fits − Males 2+ Years Old − Mean Haemglobin − Ethiopia
WHO−2001 Adjustment Method

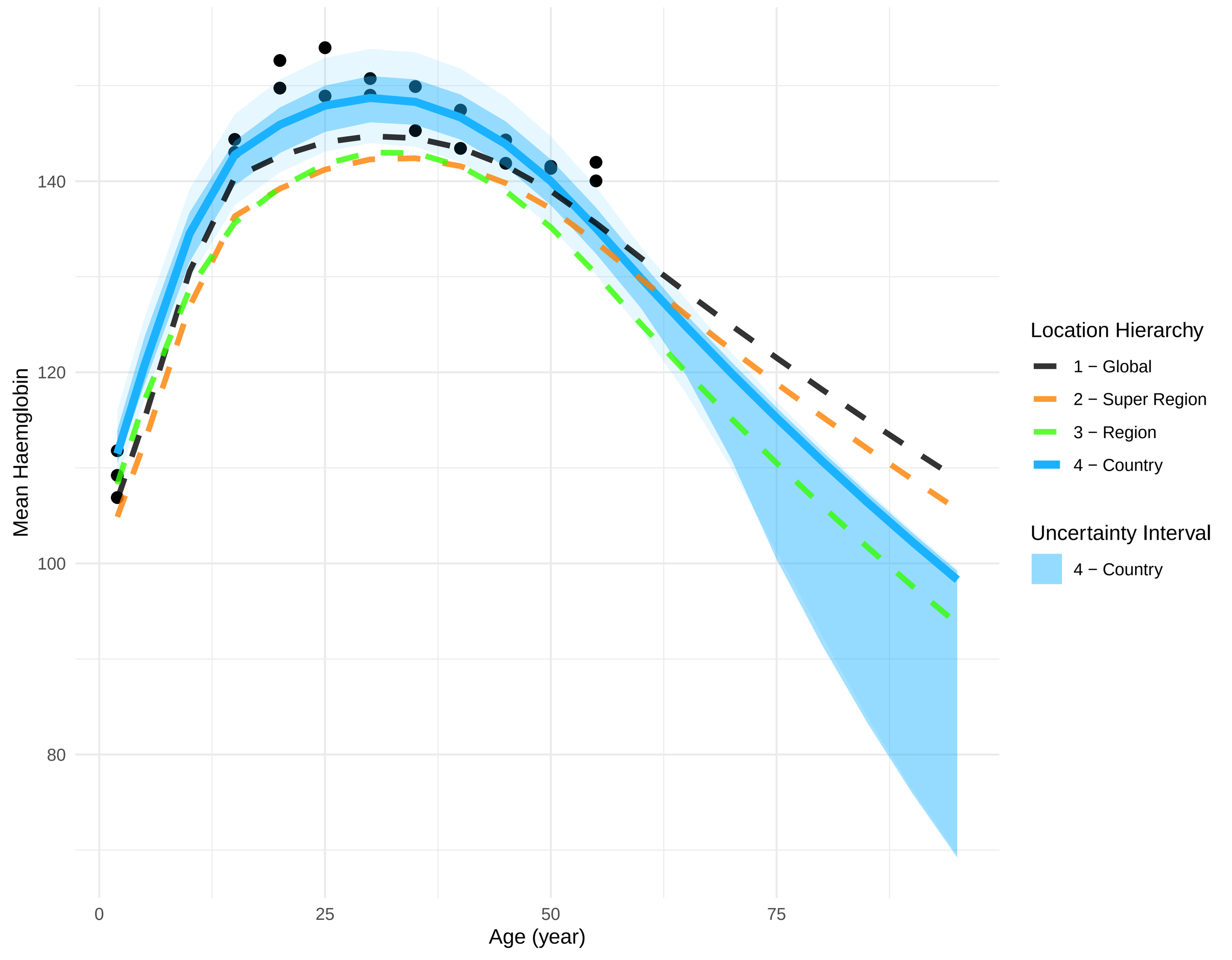

MR−BRT Age/Sex Fits − Males 2+ Years Old − Mean Haemglobin − Kenya
WHO−2001 Adjustment Method

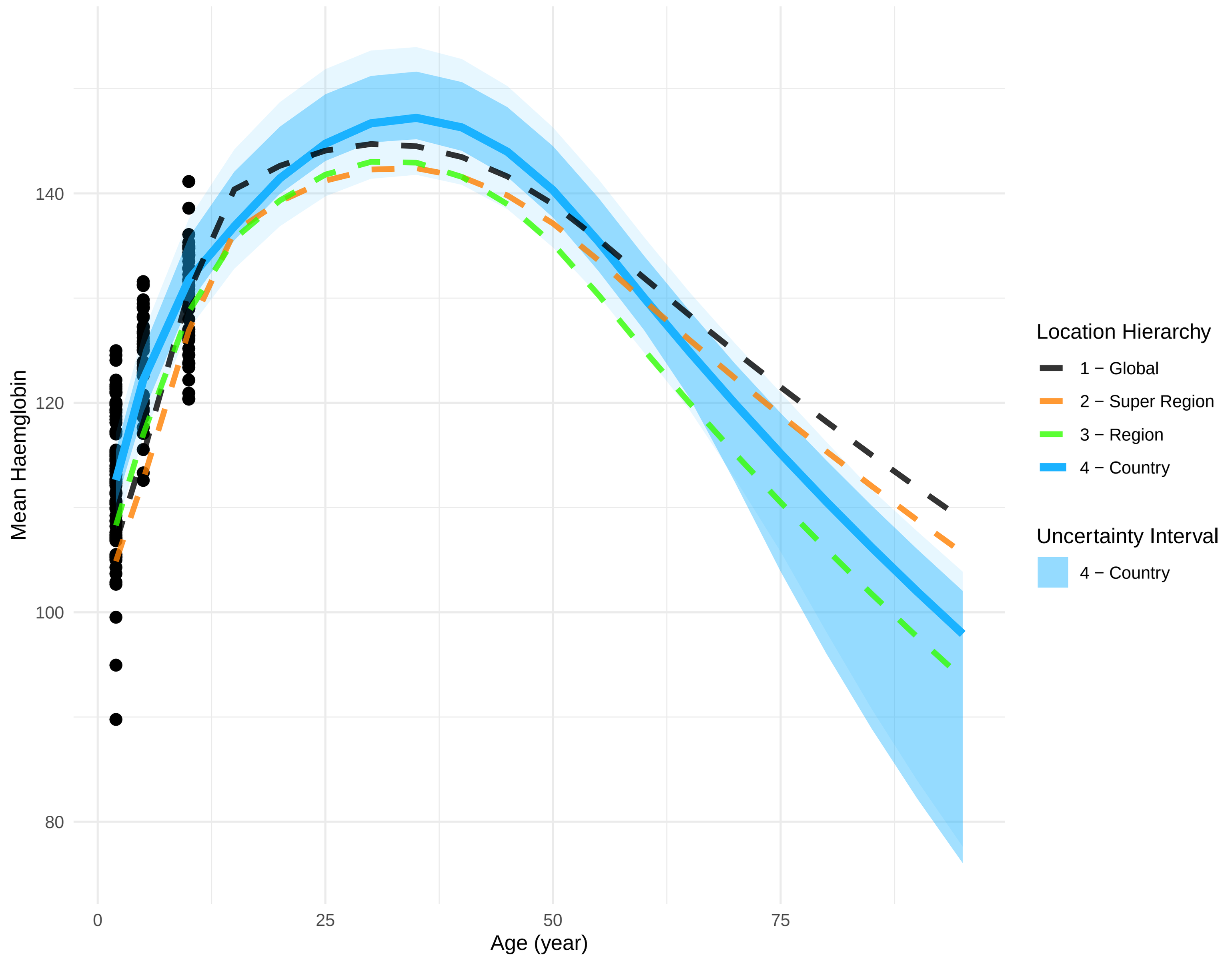

MR−BRT Age/Sex Fits − Males 2+ Years Old − Mean Haemglobin − Baringo
WHO−2001 Adjustment Method

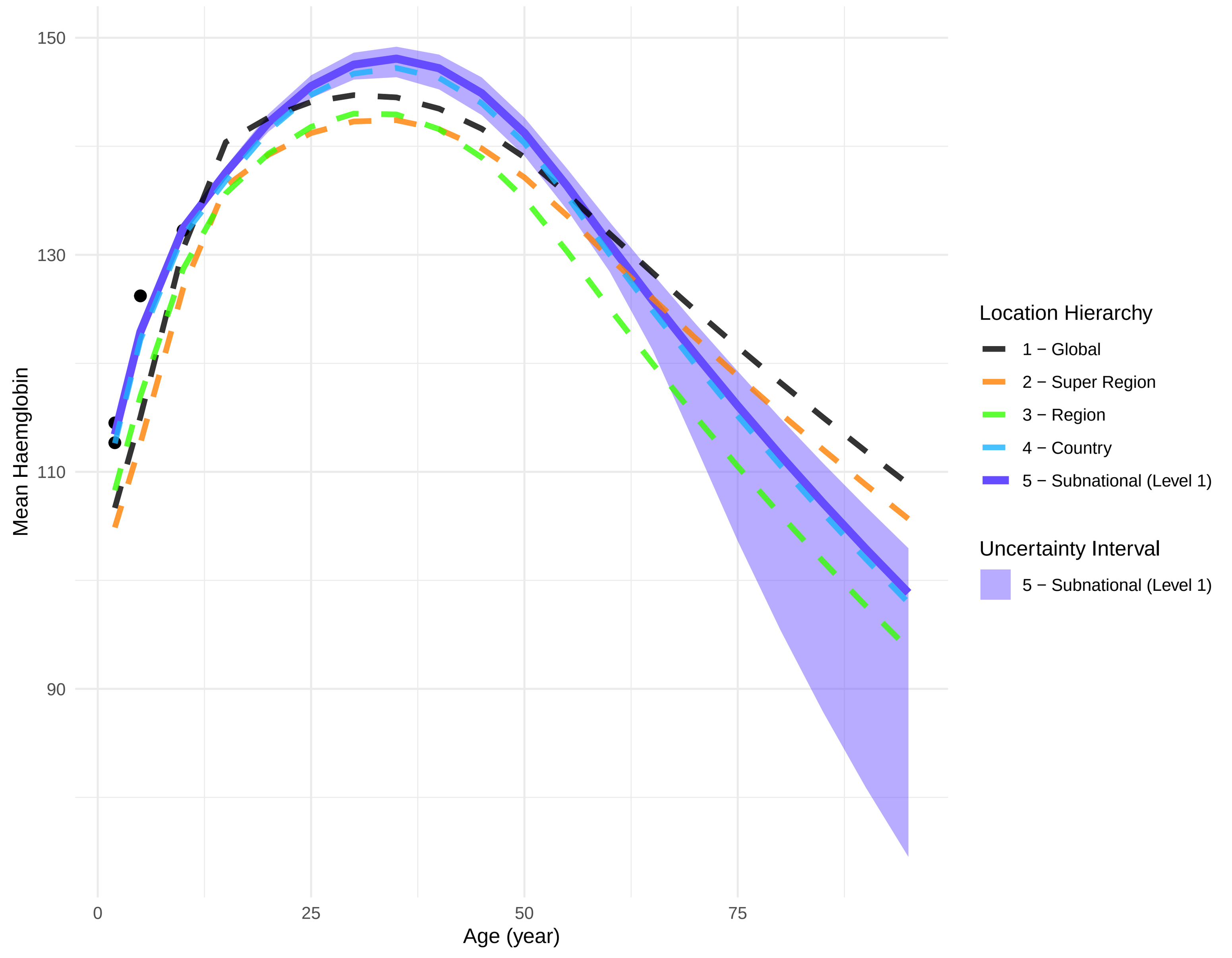

MR−BRT Age/Sex Fits − Males 2+ Years Old − Mean Haemglobin − Bomet
WHO−2001 Adjustment Method

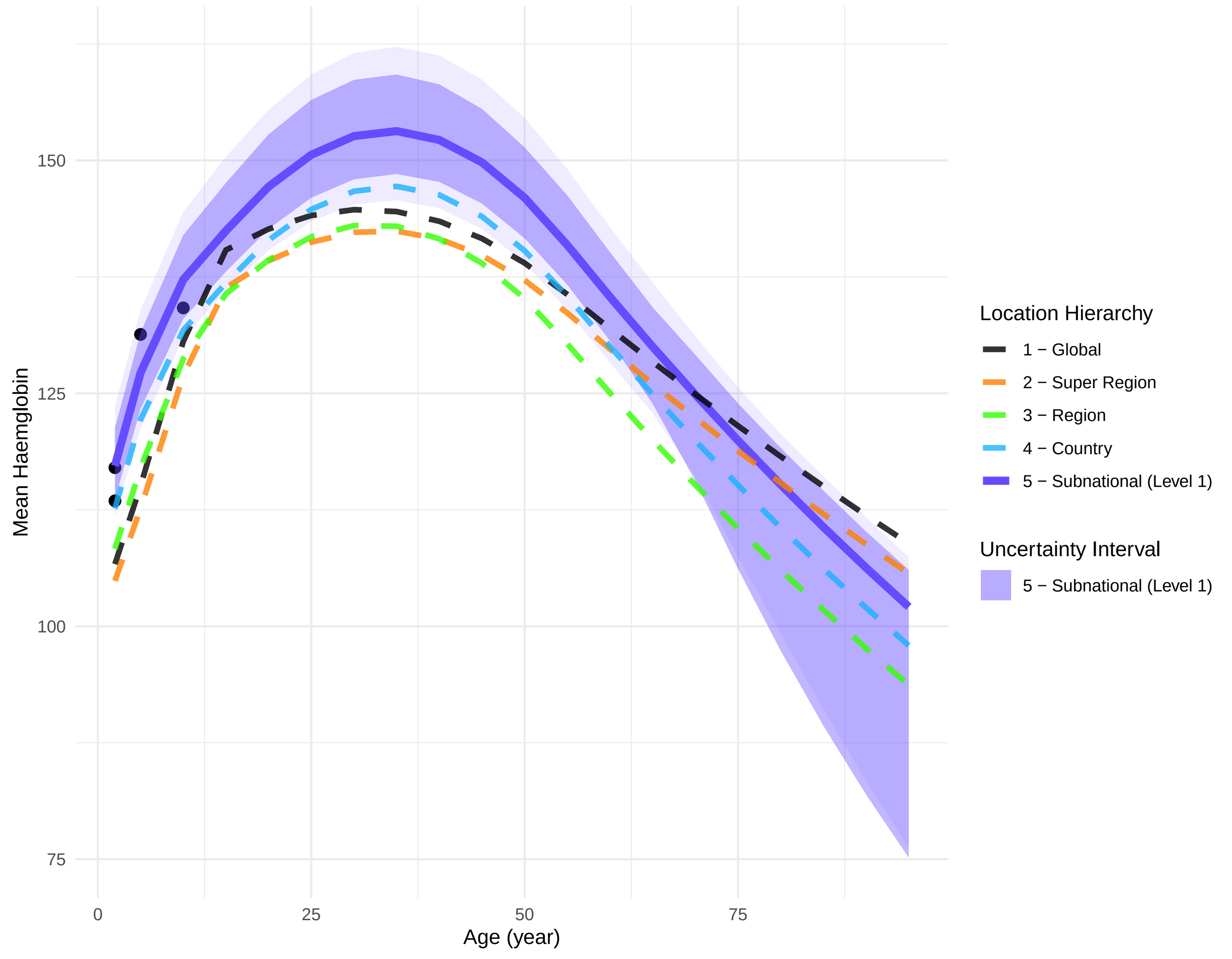

MR−BRT Age/Sex Fits − Males 2+ Years Old − Mean Haemglobin − Bungoma
WHO−2001 Adjustment Method

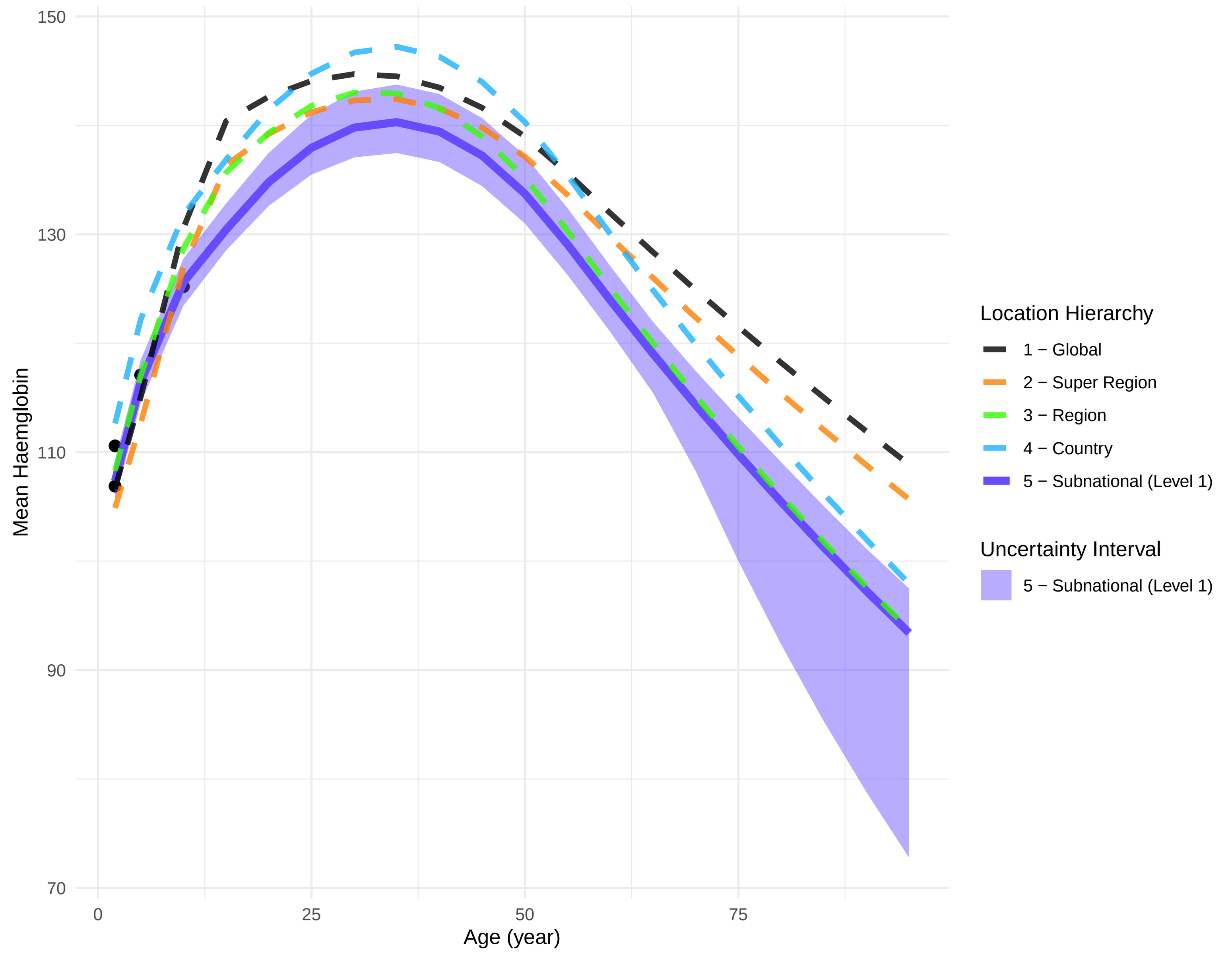

MR−BRT Age/Sex Fits − Males 2+ Years Old − Mean Haemglobin − Busia
WHO−2001 Adjustment Method

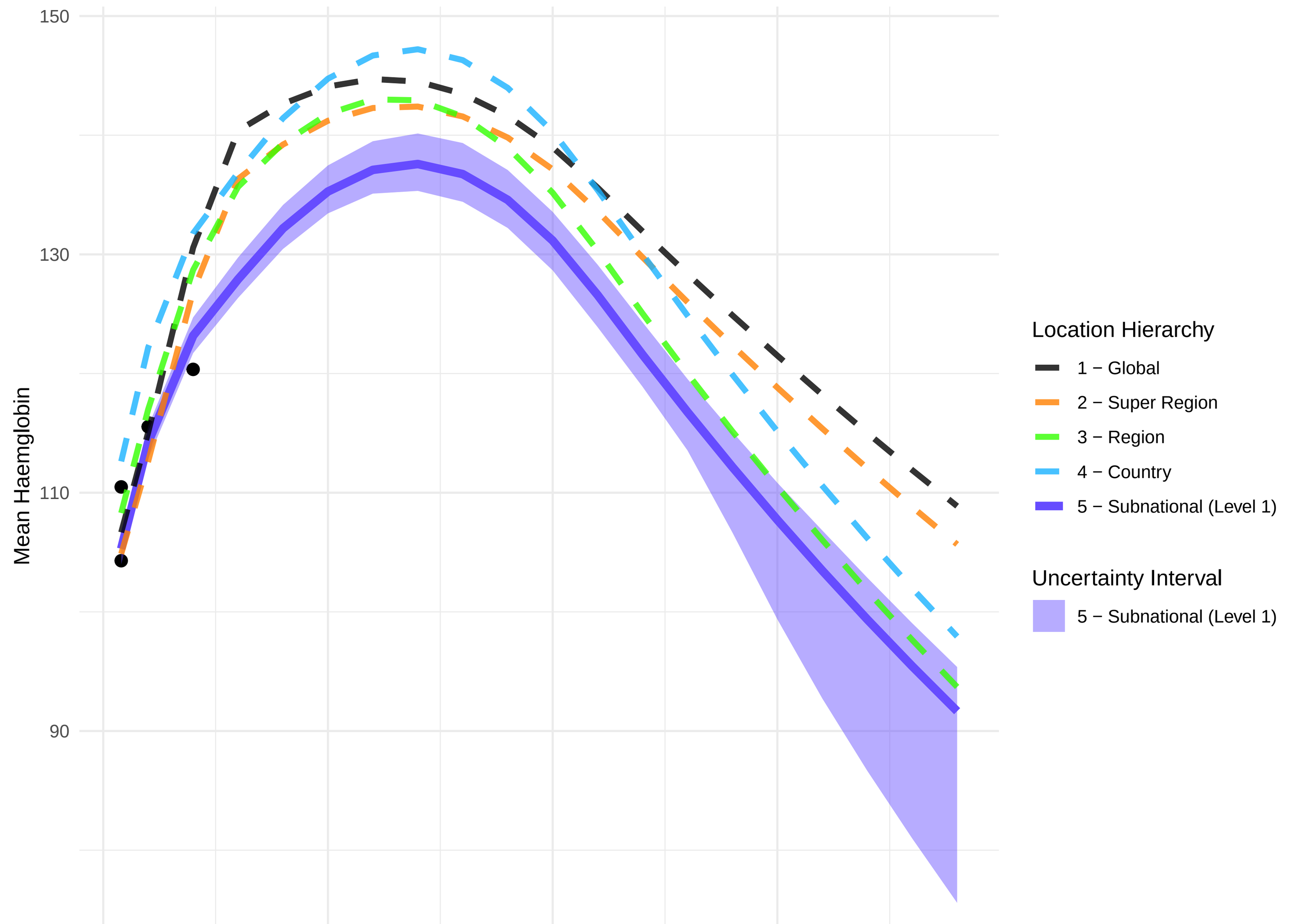

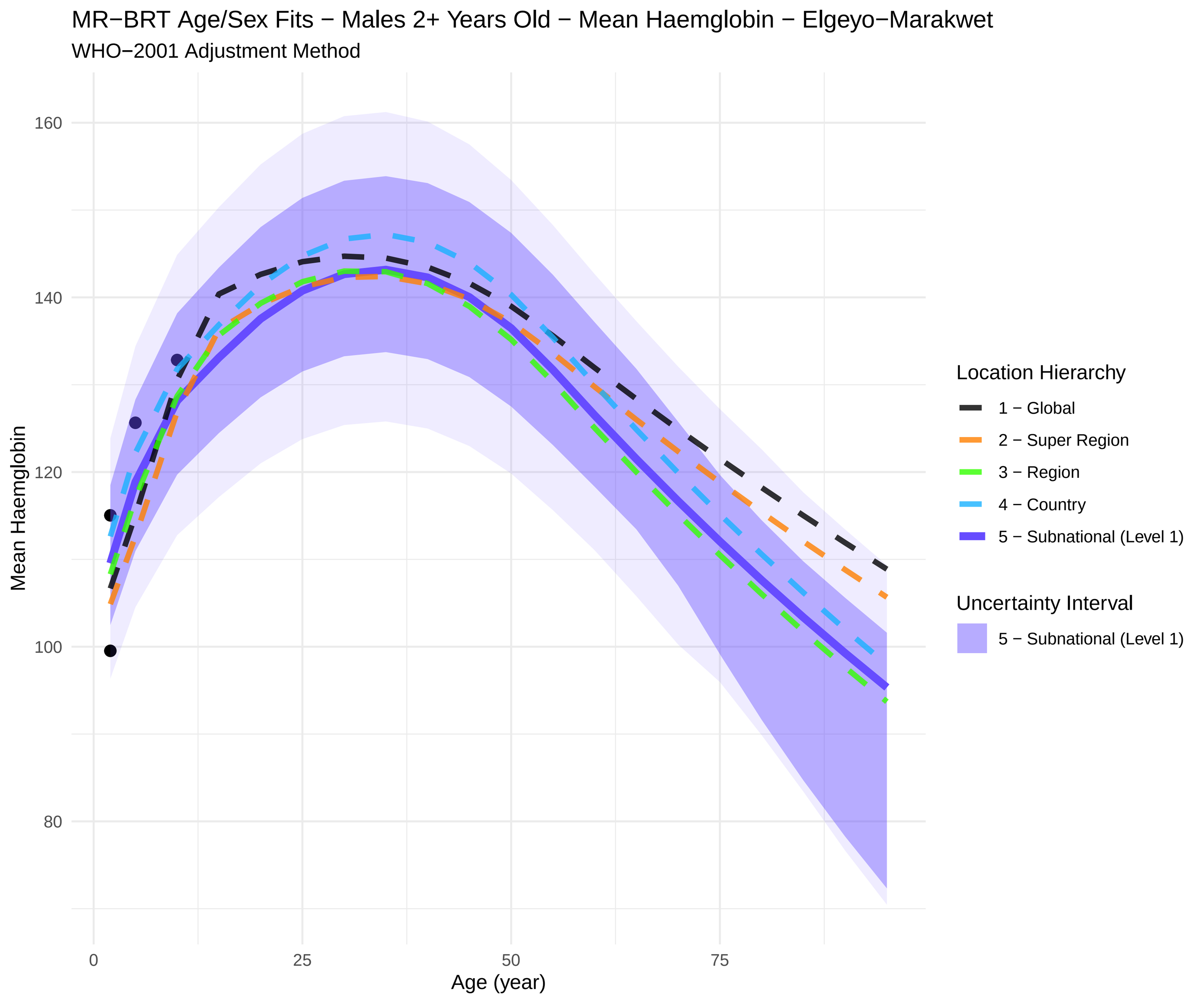
MR−BRT Age/Sex Fits − Males 2+ Years Old − Mean Haemglobin − Elgeyo−Marakwet
WHO−2001 Adjustment Method
Mean Haemglobin
Age (year)
Location Hierarchy
1 − Global
2 − Super Region
3 − Region
4 − Country
5 − Subnational (Level 1)
Uncertainty Interval
5 − Subnational (Level 1)

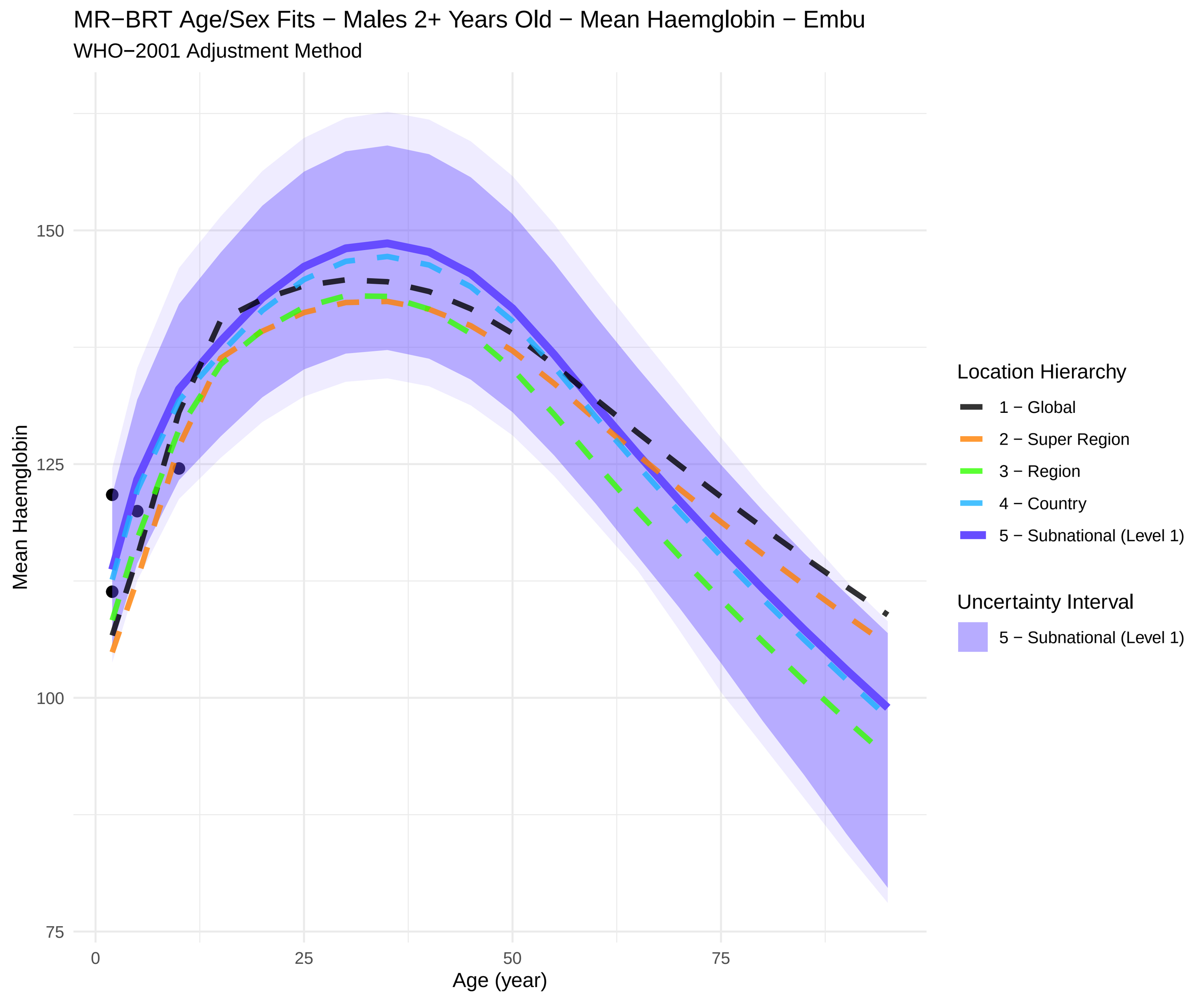
MR−BRT Age/Sex Fits − Males 2+ Years Old − Mean Haemglobin − Embu
WHO−2001 Adjustment Method
Mean Haemglobin
Age (year)
Location Hierarchy
1 − Global
2 − Super Region
3 − Region
4 − Country
5 − Subnational (Level 1)
Uncertainty Interval
5 − Subnational (Level 1)

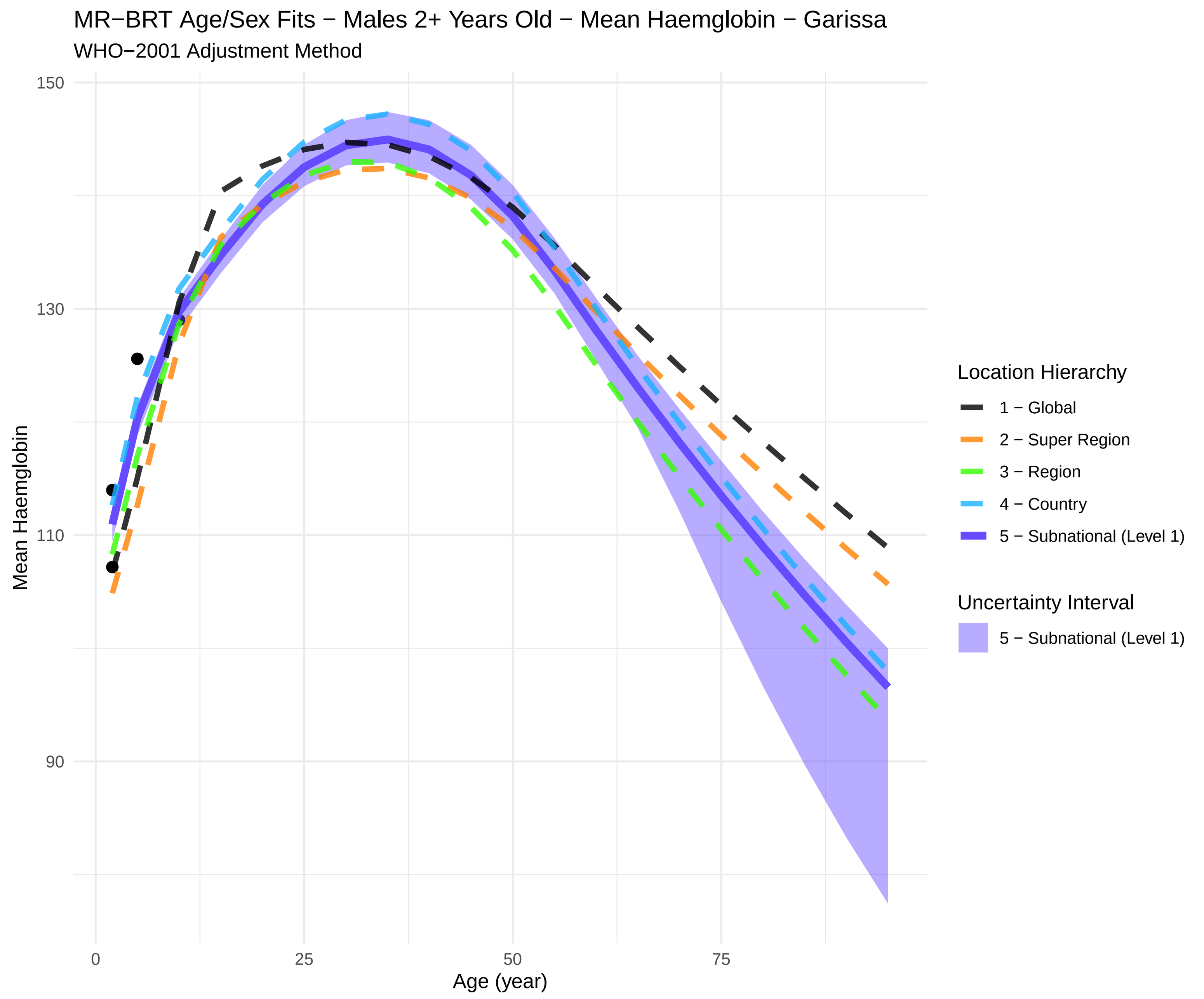
MR−BRT Age/Sex Fits − Males 2+ Years Old − Mean Haemglobin − Garissa
WHO−2001 Adjustment Method
Mean Haemglobin
Age (year)
Location Hierarchy
1 − Global
2 − Super Region
3 − Region
4 − Country
5 − Subnational (Level 1)
Uncertainty Interval
5 − Subnational (Level 1)

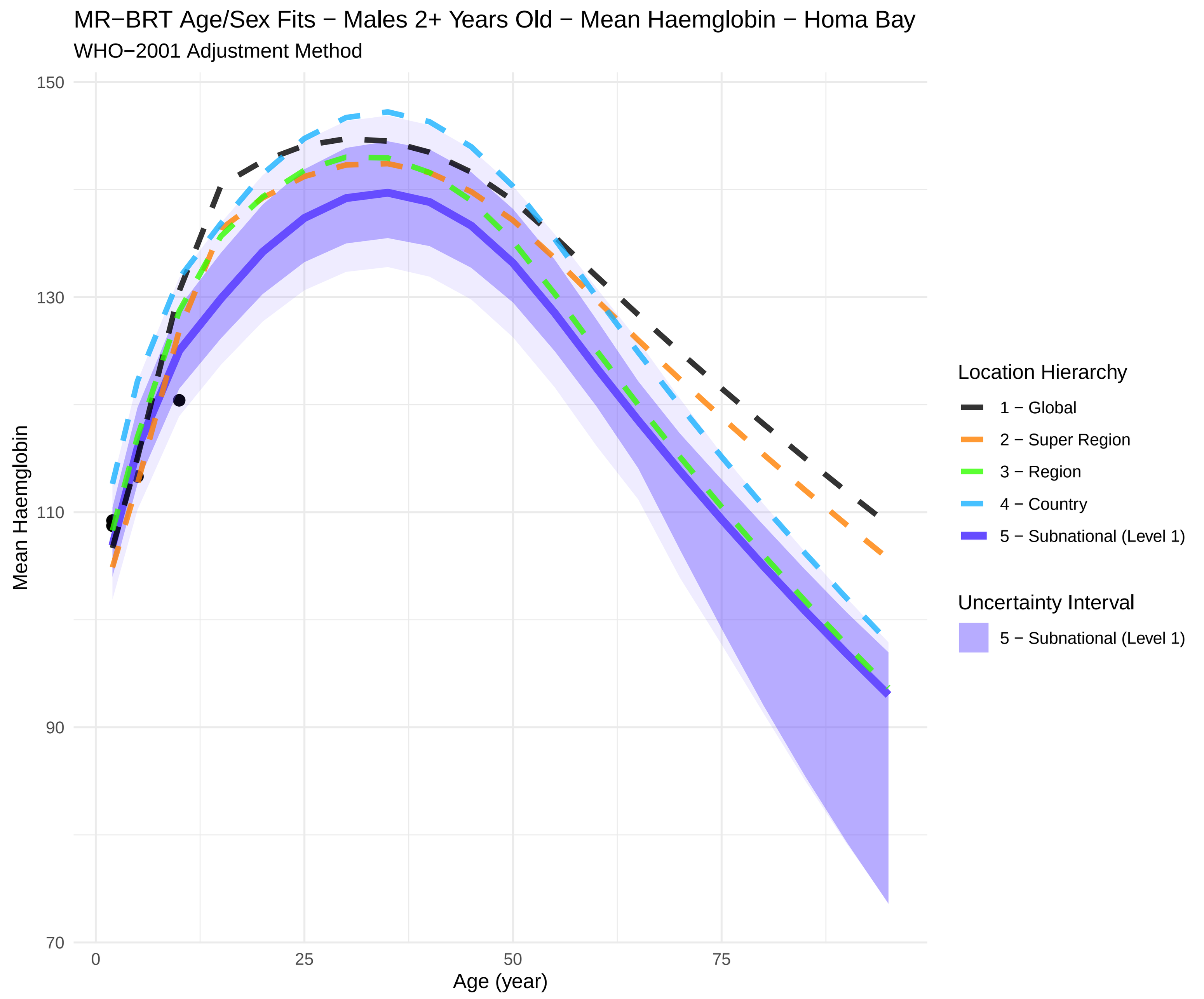
MR−BRT Age/Sex Fits − Males 2+ Years Old − Mean Haemglobin − Homa Bay
WHO−2001 Adjustment Method
Mean Haemglobin
Age (year)
Location Hierarchy
1 − Global
2 − Super Region
3 − Region
4 − Country
5 − Subnational (Level 1)
Uncertainty Interval
5 − Subnational (Level 1)

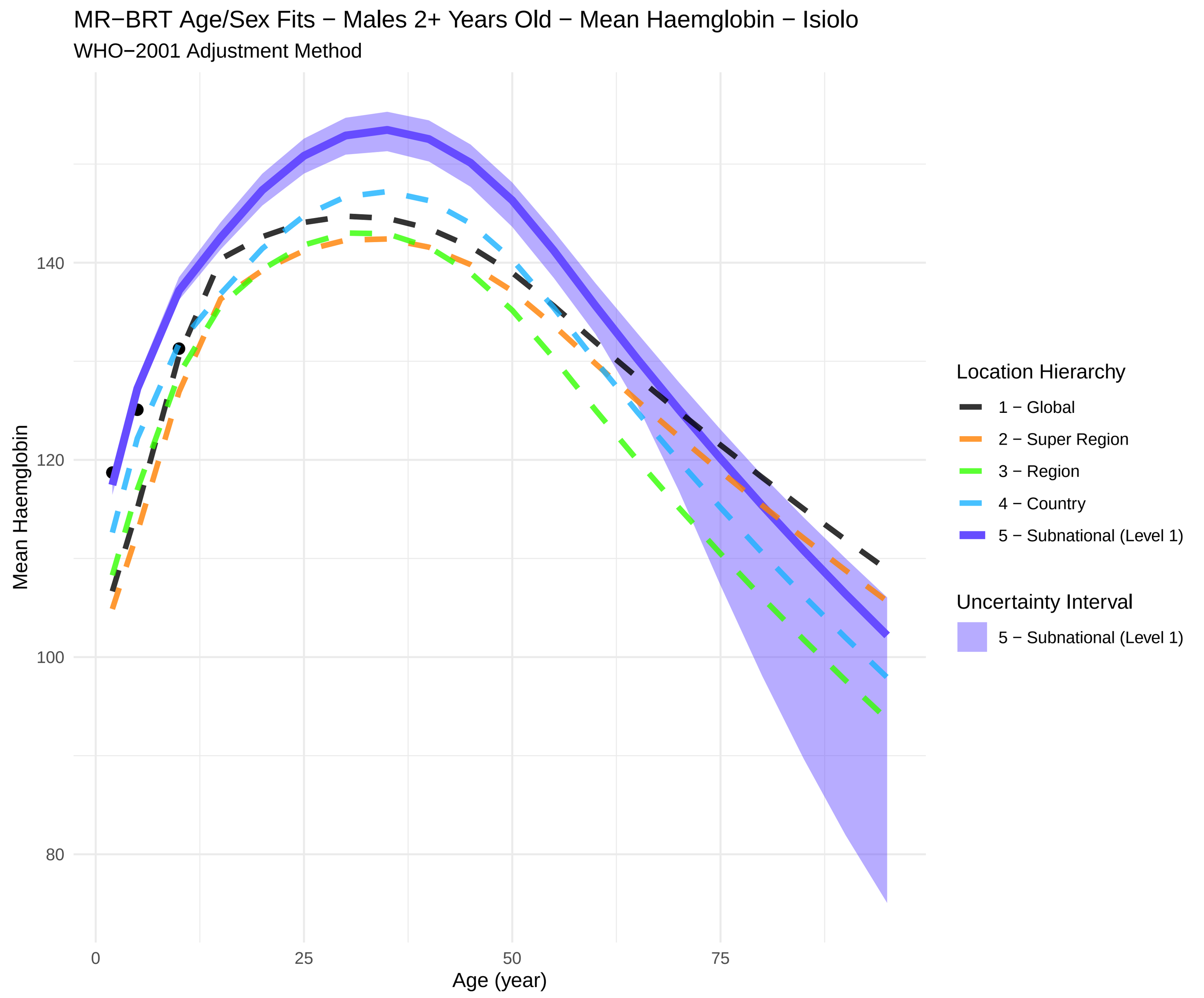
MR−BRT Age/Sex Fits − Males 2+ Years Old − Mean Haemglobin − Isiolo
WHO−2001 Adjustment Method
Mean Haemglobin
Age (year)
Location Hierarchy
1 − Global
2 − Super Region
3 − Region
4 − Country
5 − Subnational (Level 1)
Uncertainty Interval
5 − Subnational (Level 1)

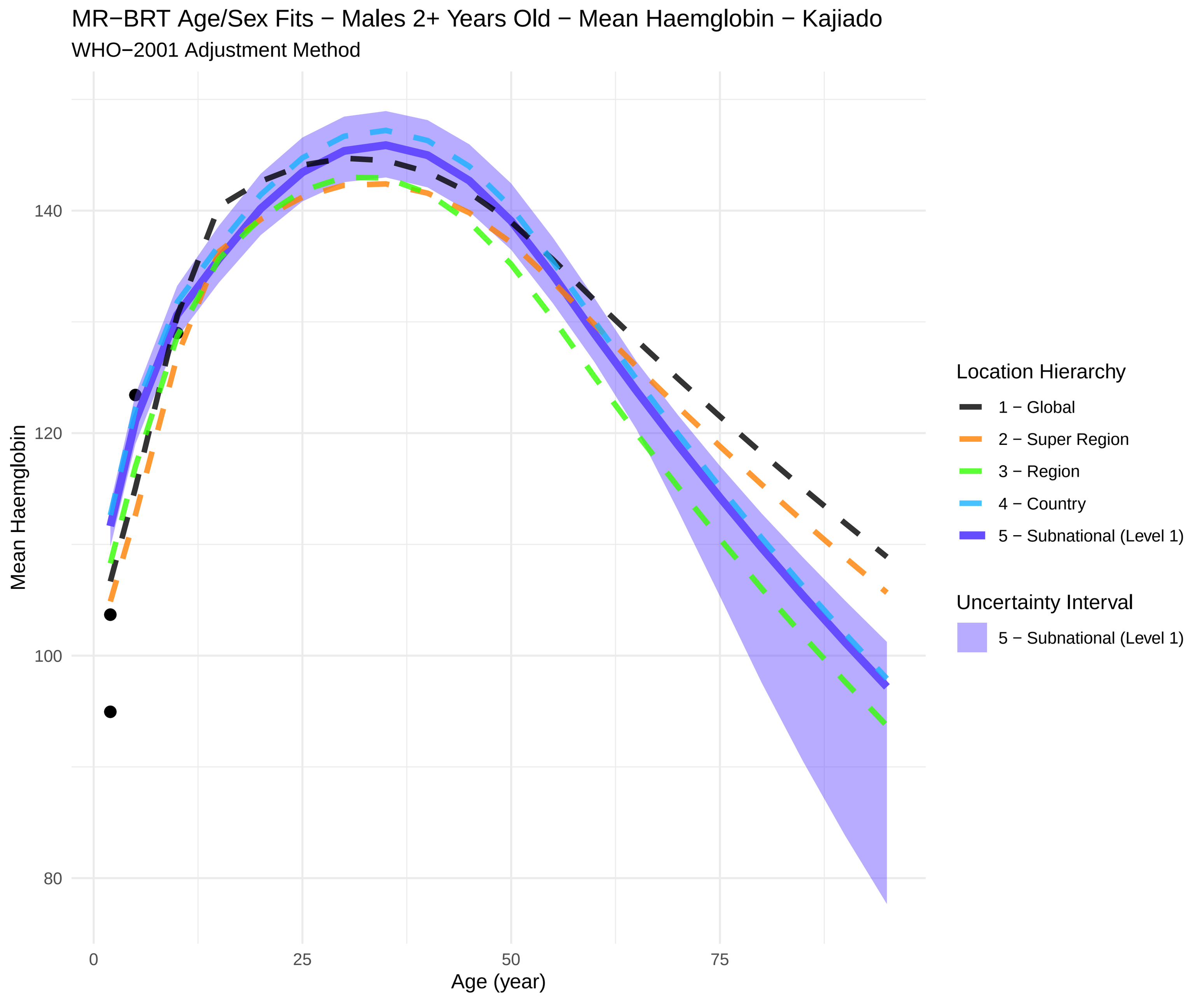
MR−BRT Age/Sex Fits − Males 2+ Years Old − Mean Haemglobin − Kajiado
WHO−2001 Adjustment Method
Mean Haemglobin
Age (year)
Location Hierarchy
1 − Global
2 − Super Region
3 − Region
4 − Country
5 − Subnational (Level 1)
Uncertainty Interval
5 − Subnational (Level 1)

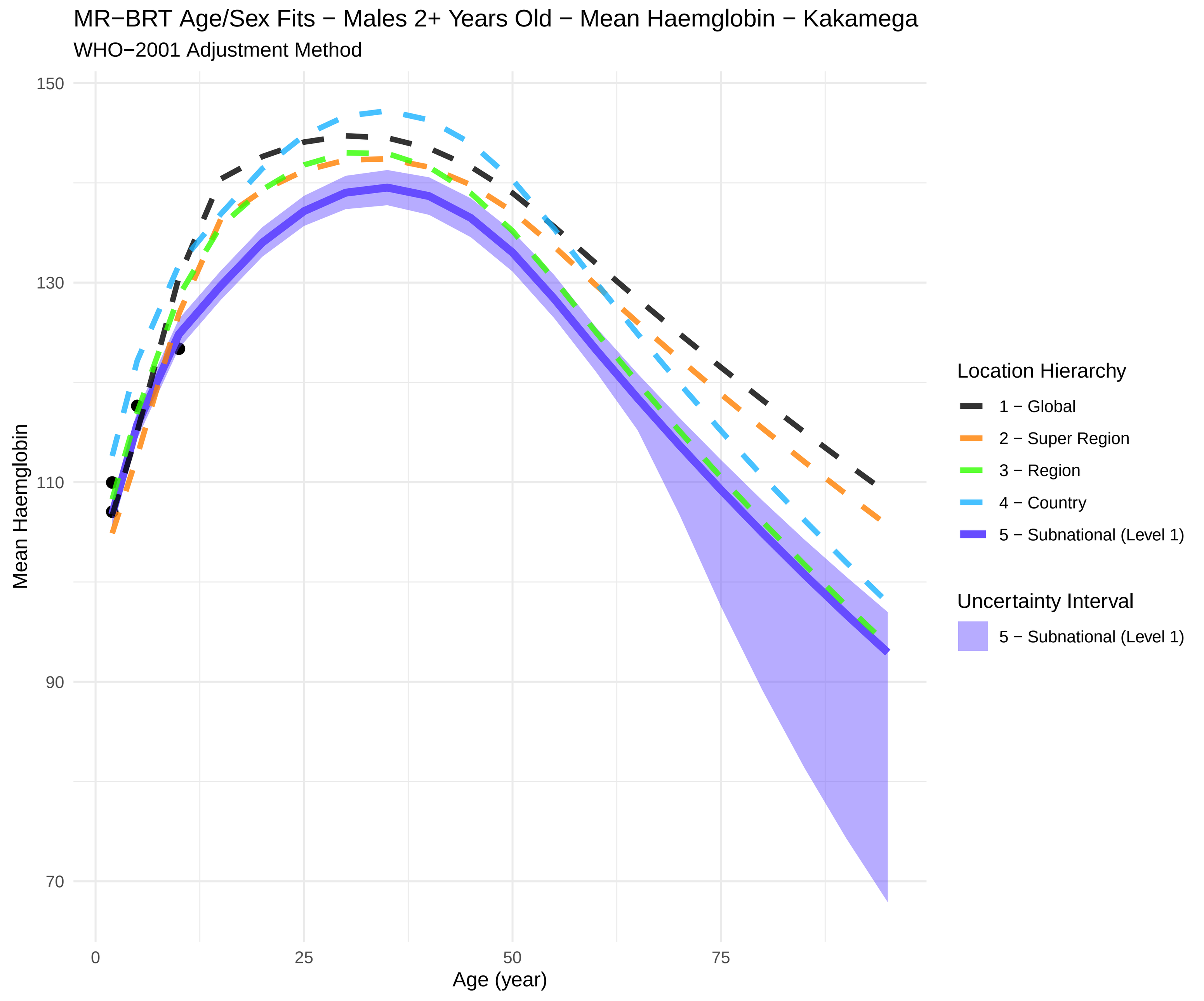
MR−BRT Age/Sex Fits − Males 2+ Years Old − Mean Haemglobin − Kakamega
WHO−2001 Adjustment Method
Mean Haemglobin
Age (year)
Location Hierarchy
1 − Global
2 − Super Region
3 − Region
4 − Country
5 − Subnational (Level 1)
Uncertainty Interval
5 − Subnational (Level 1)

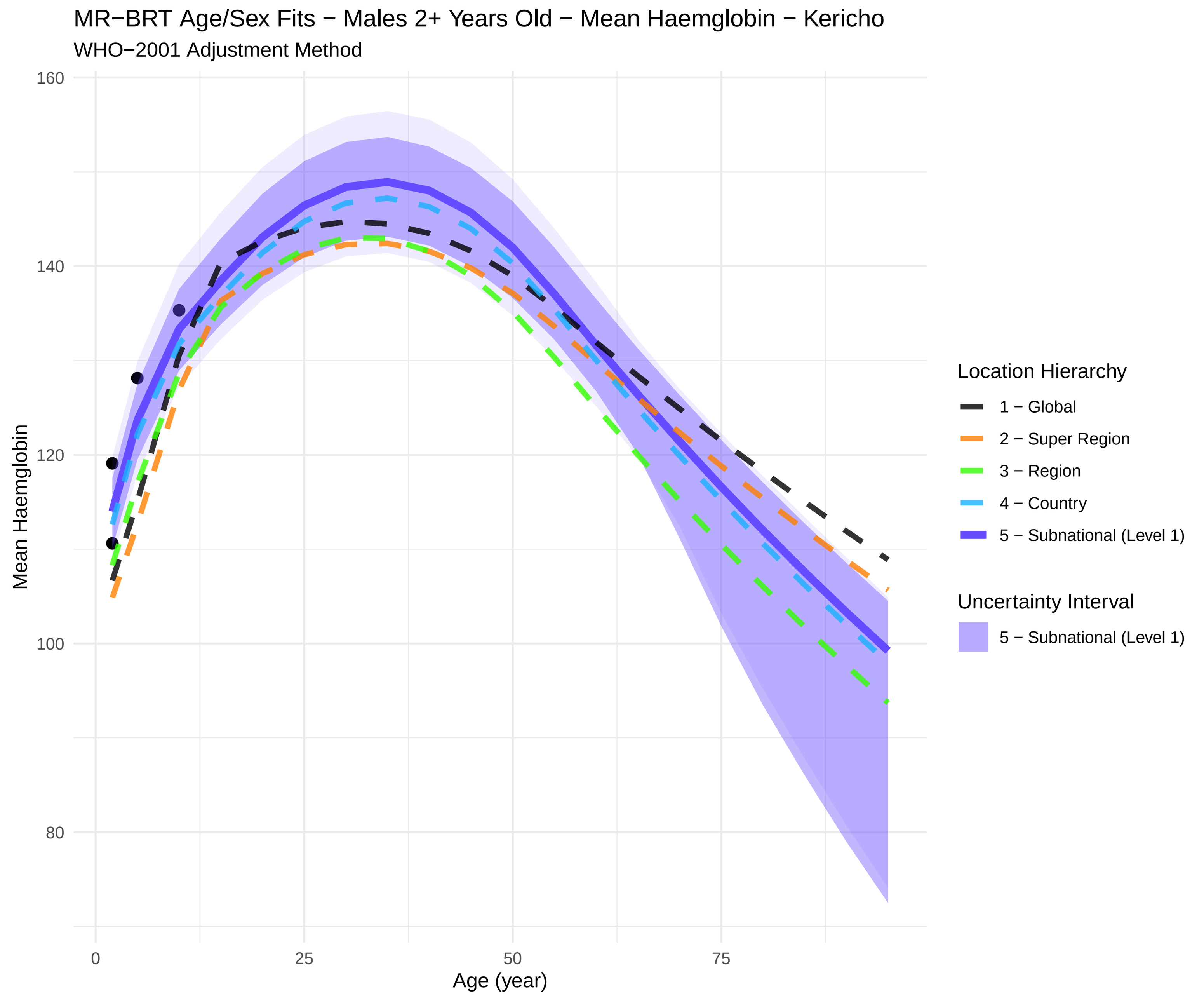
MR−BRT Age/Sex Fits − Males 2+ Years Old − Mean Haemglobin − Kericho
WHO−2001 Adjustment Method
Mean Haemglobin
Age (year)
Location Hierarchy
1 − Global
2 − Super Region
3 − Region
4 − Country
5 − Subnational (Level 1)
Uncertainty Interval
5 − Subnational (Level 1)

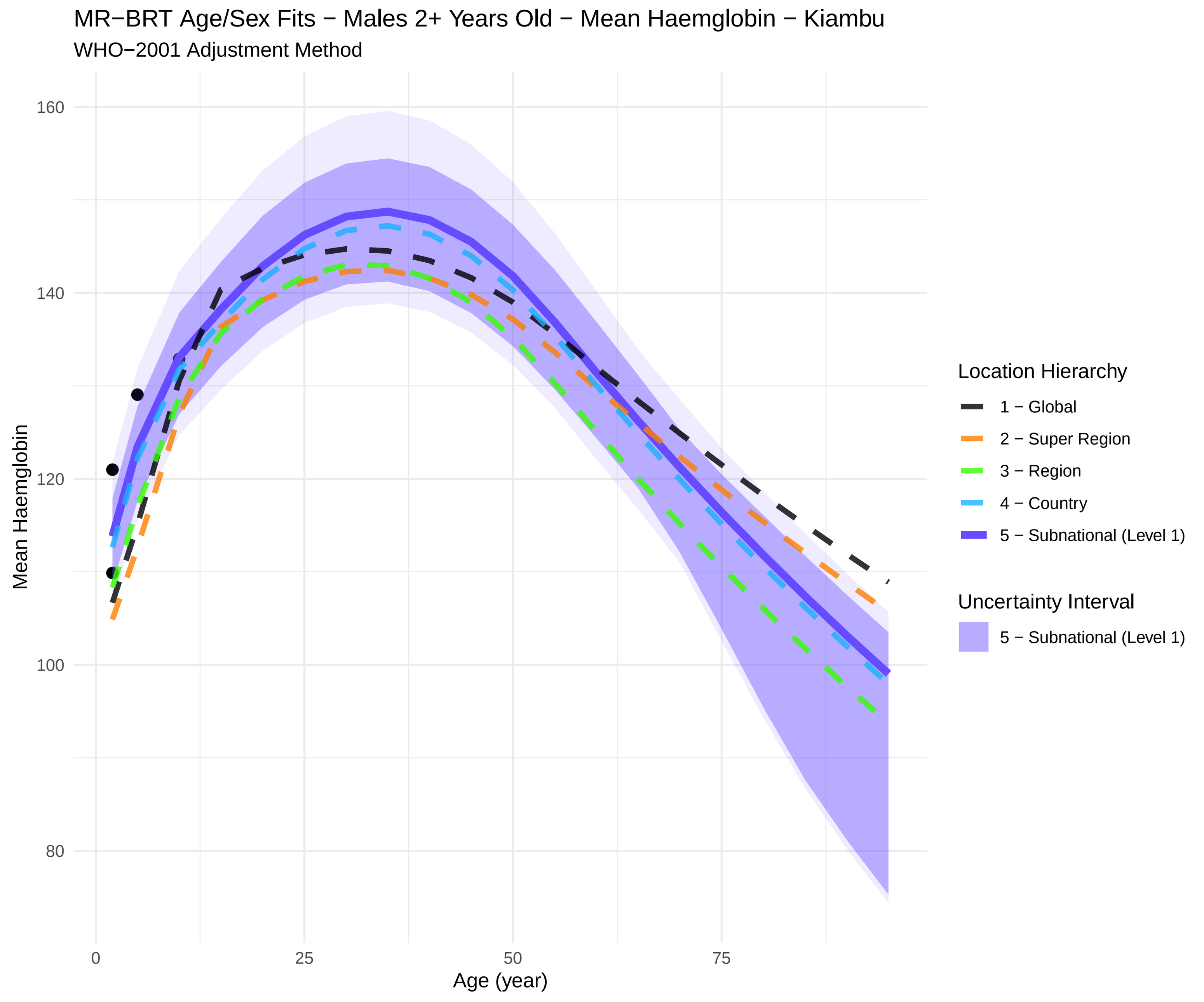
MR−BRT Age/Sex Fits − Males 2+ Years Old − Mean Haemglobin − Kiambu
WHO−2001 Adjustment Method
Mean Haemglobin
Age (year)
Location Hierarchy
1 − Global
2 − Super Region
3 − Region
4 − Country
5 − Subnational (Level 1)
Uncertainty Interval
5 − Subnational (Level 1)

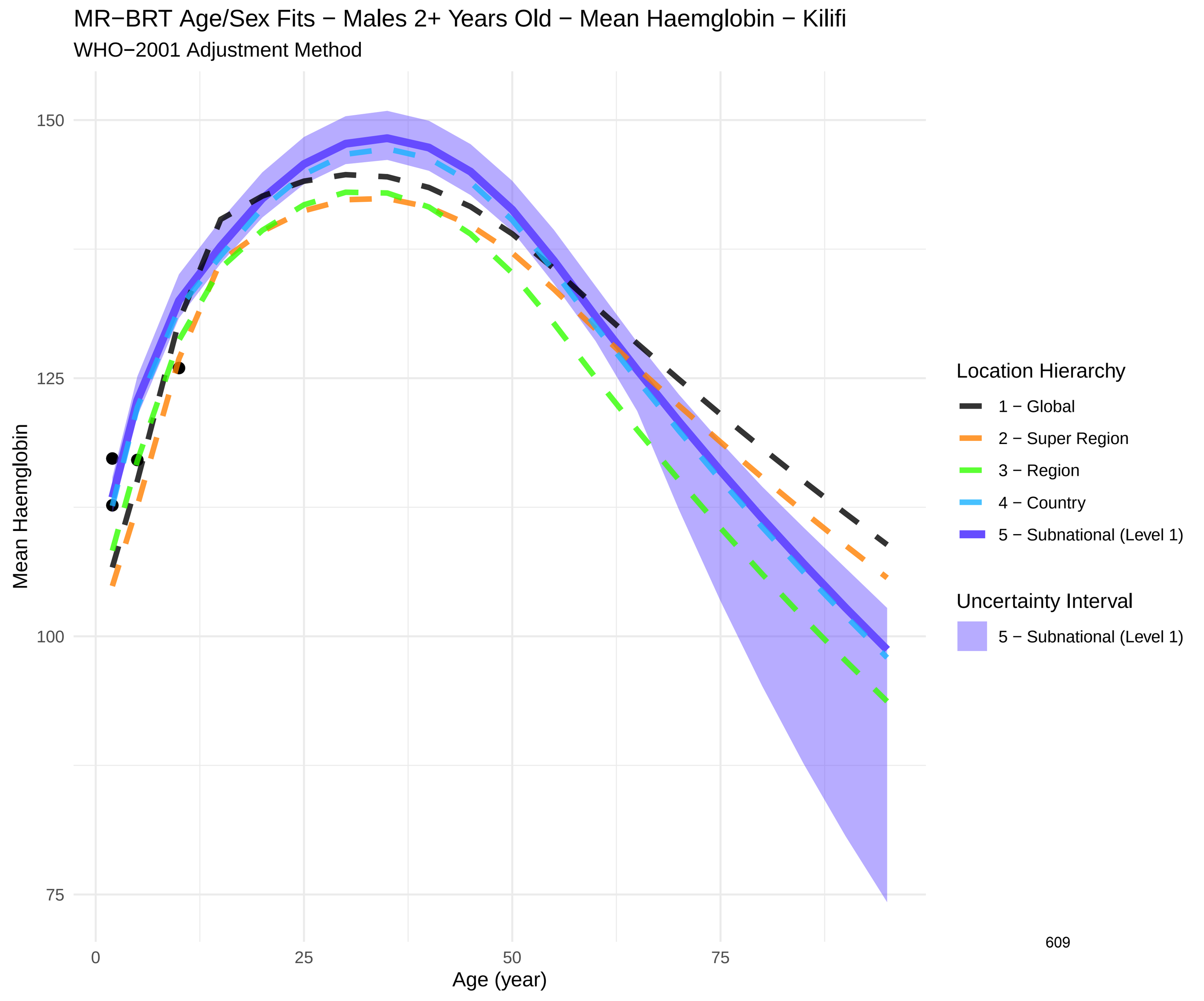
MR−BRT Age/Sex Fits − Males 2+ Years Old − Mean Haemglobin − Kilifi
WHO−2001 Adjustment Method
Mean Haemglobin
Age (year)
Location Hierarchy
1 − Global
2 − Super Region
3 − Region
4 − Country
5 − Subnational (Level 1)
Uncertainty Interval
5 − Subnational (Level 1)

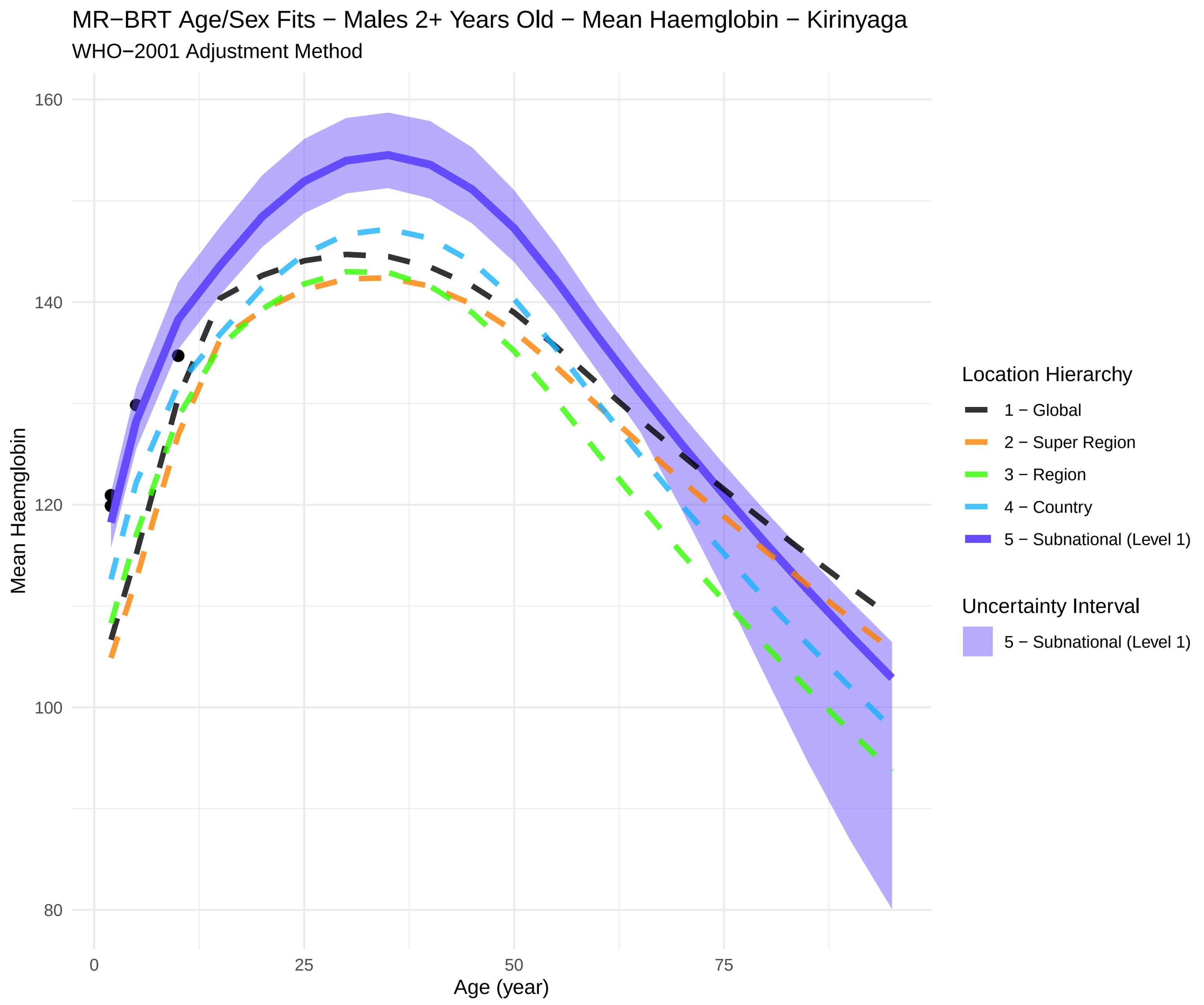
MR−BRT Age/Sex Fits − Males 2+ Years Old − Mean Haemglobin − Kirinyaga
WHO−2001 Adjustment Method
Mean Haemglobin
Age (year)
Location Hierarchy
1 − Global
2 − Super Region
3 − Region
4 − Country
5 − Subnational (Level 1)
Uncertainty Interval
5 − Subnational (Level 1)

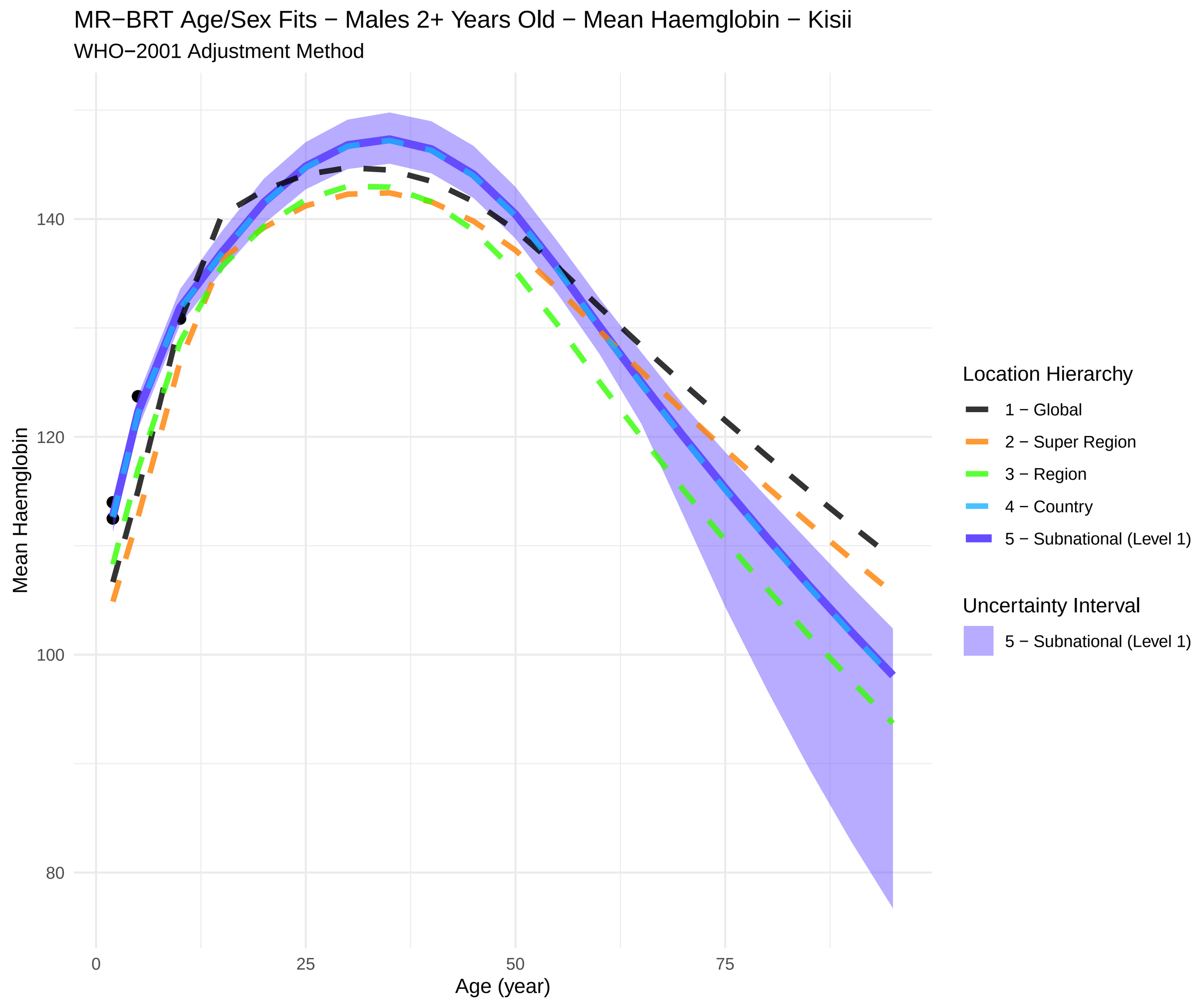
MR−BRT Age/Sex Fits − Males 2+ Years Old − Mean Haemglobin − Kisii
WHO−2001 Adjustment Method
Mean Haemglobin
Age (year)
Location Hierarchy
1 − Global
2 − Super Region
3 − Region
4 − Country
5 − Subnational (Level 1)
Uncertainty Interval
5 − Subnational (Level 1)

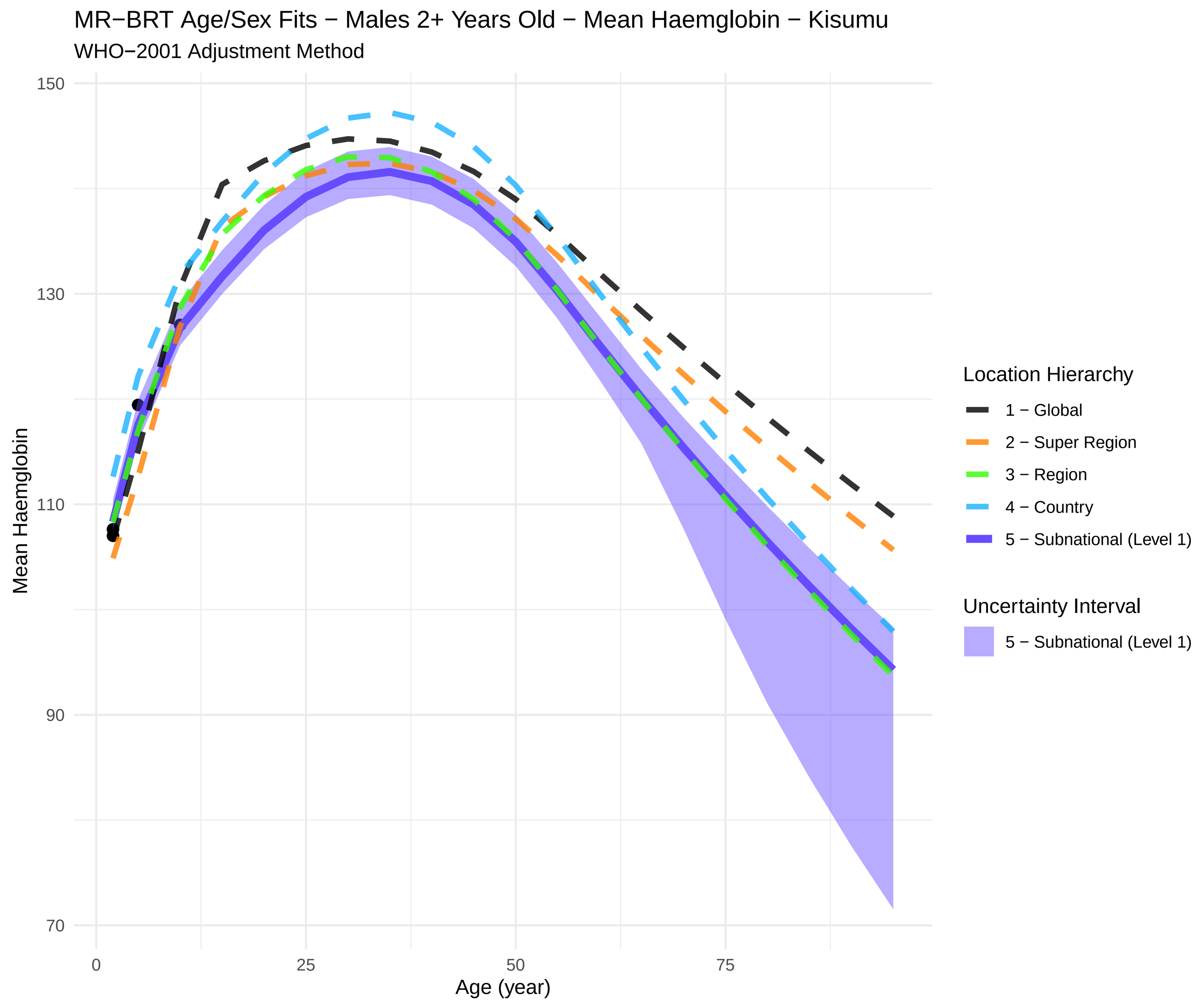
MR−BRT Age/Sex Fits − Males 2+ Years Old − Mean Haemglobin − Kisumu
WHO−2001 Adjustment Method
Mean Haemglobin
Age (year)
Location Hierarchy
1 − Global
2 − Super Region
3 − Region
4 − Country
5 − Subnational (Level 1)
Uncertainty Interval
5 − Subnational (Level 1)

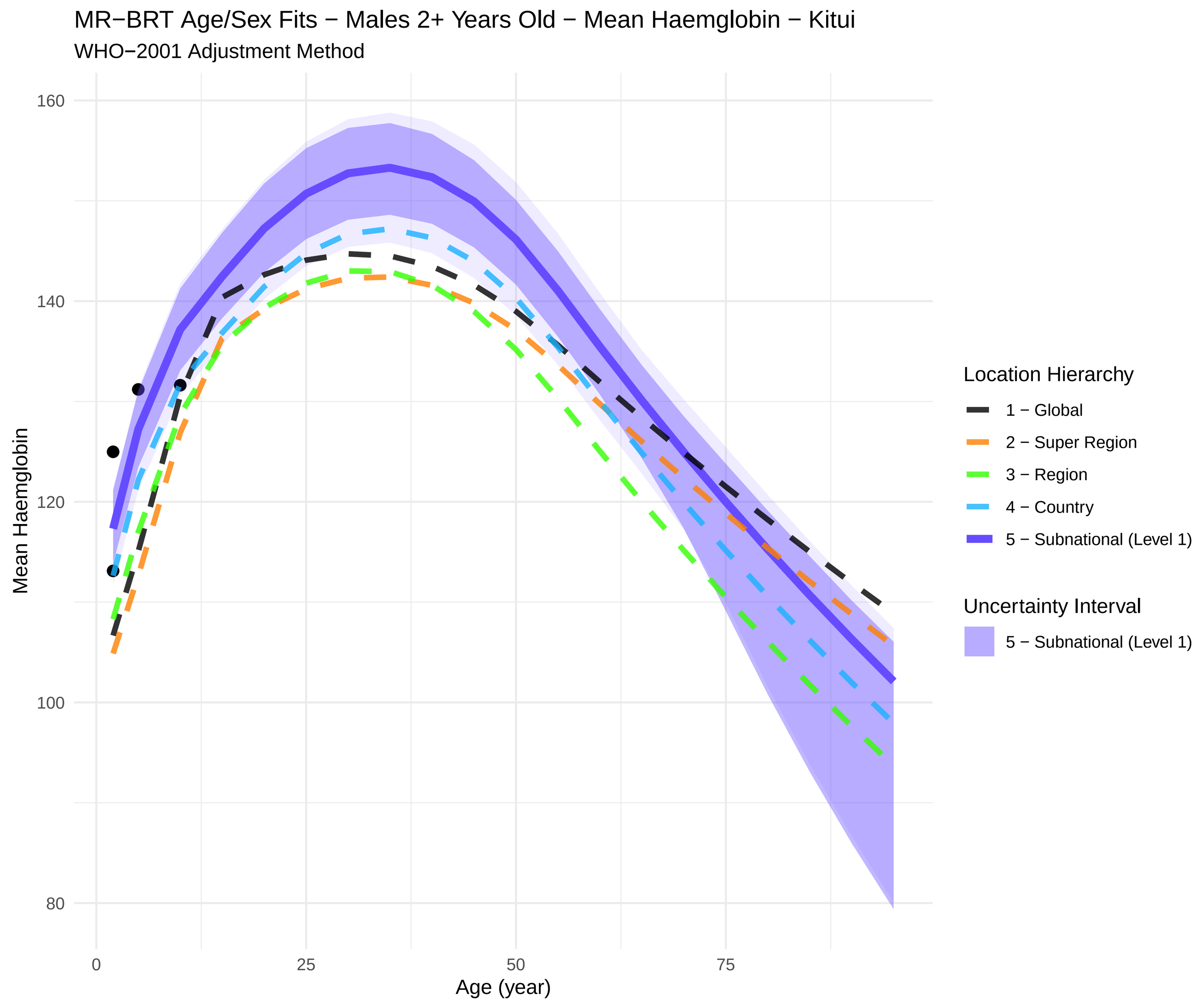
MR−BRT Age/Sex Fits − Males 2+ Years Old − Mean Haemglobin − Kitui
WHO−2001 Adjustment Method
Mean Haemglobin
Age (year)
Location Hierarchy
1 − Global
2 − Super Region
3 − Region
4 − Country
5 − Subnational (Level 1)
Uncertainty Interval
5 − Subnational (Level 1)

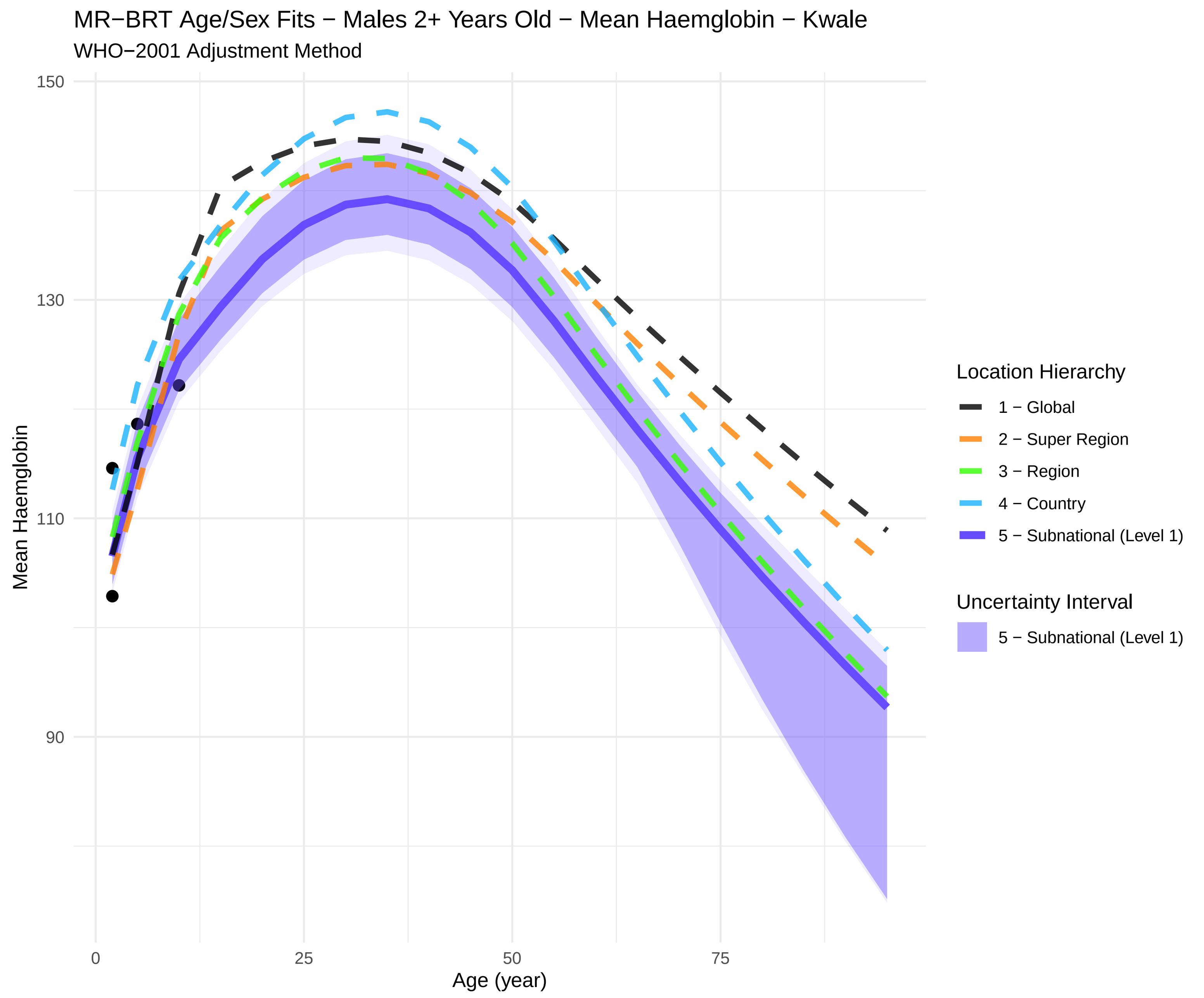
MR−BRT Age/Sex Fits − Males 2+ Years Old − Mean Haemglobin − Kwale
WHO−2001 Adjustment Method
Mean Haemglobin
Age (year)
Location Hierarchy
1 − Global
2 − Super Region
3 − Region
4 − Country
5 − Subnational (Level 1)
Uncertainty Interval
5 − Subnational (Level 1)

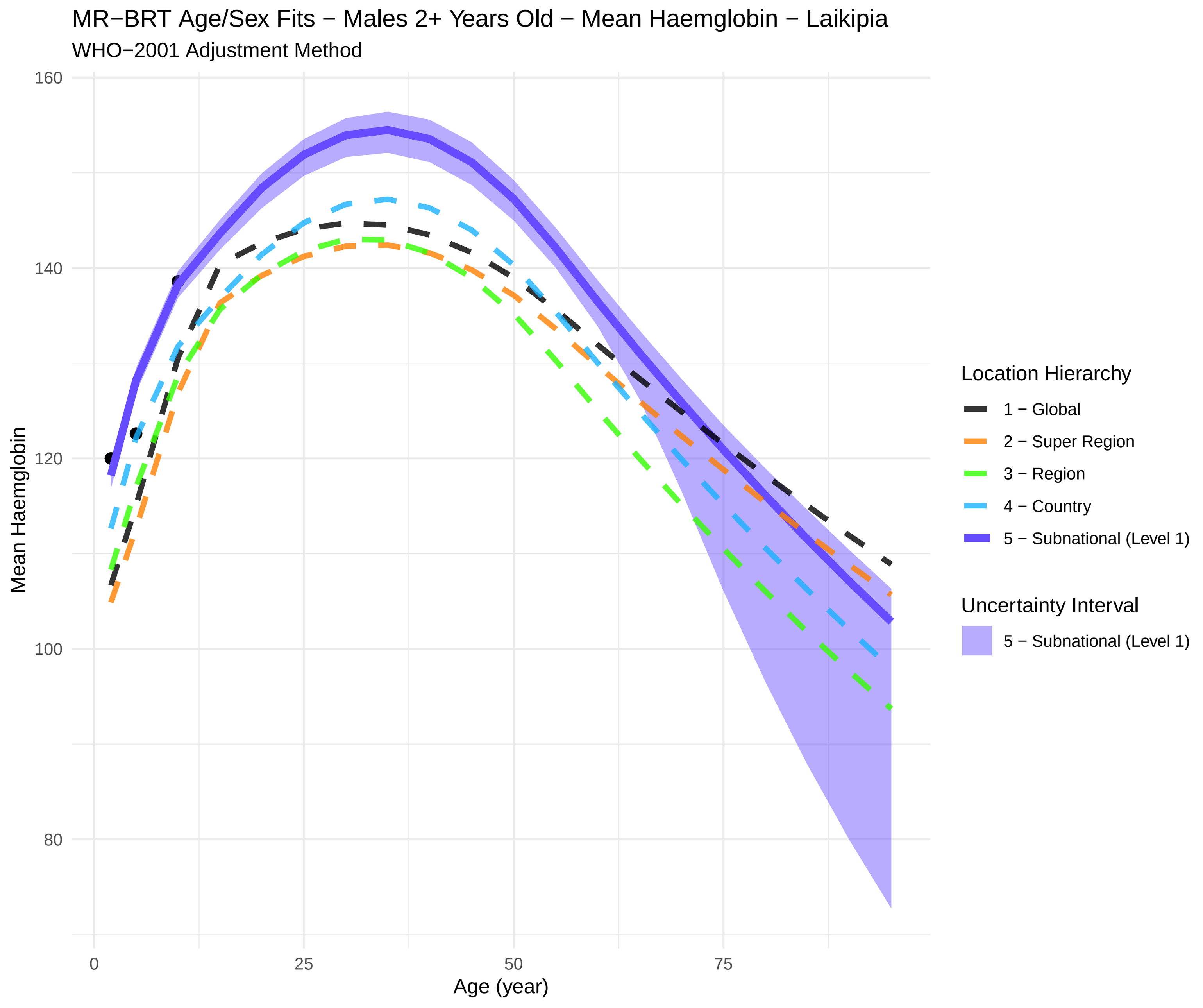
MR−BRT Age/Sex Fits − Males 2+ Years Old − Mean Haemglobin − Laikipia
WHO−2001 Adjustment Method
Mean Haemglobin
Age (year)
Location Hierarchy
1 − Global
2 − Super Region
3 − Region
4 − Country
5 − Subnational (Level 1)
Uncertainty Interval
5 − Subnational (Level 1)

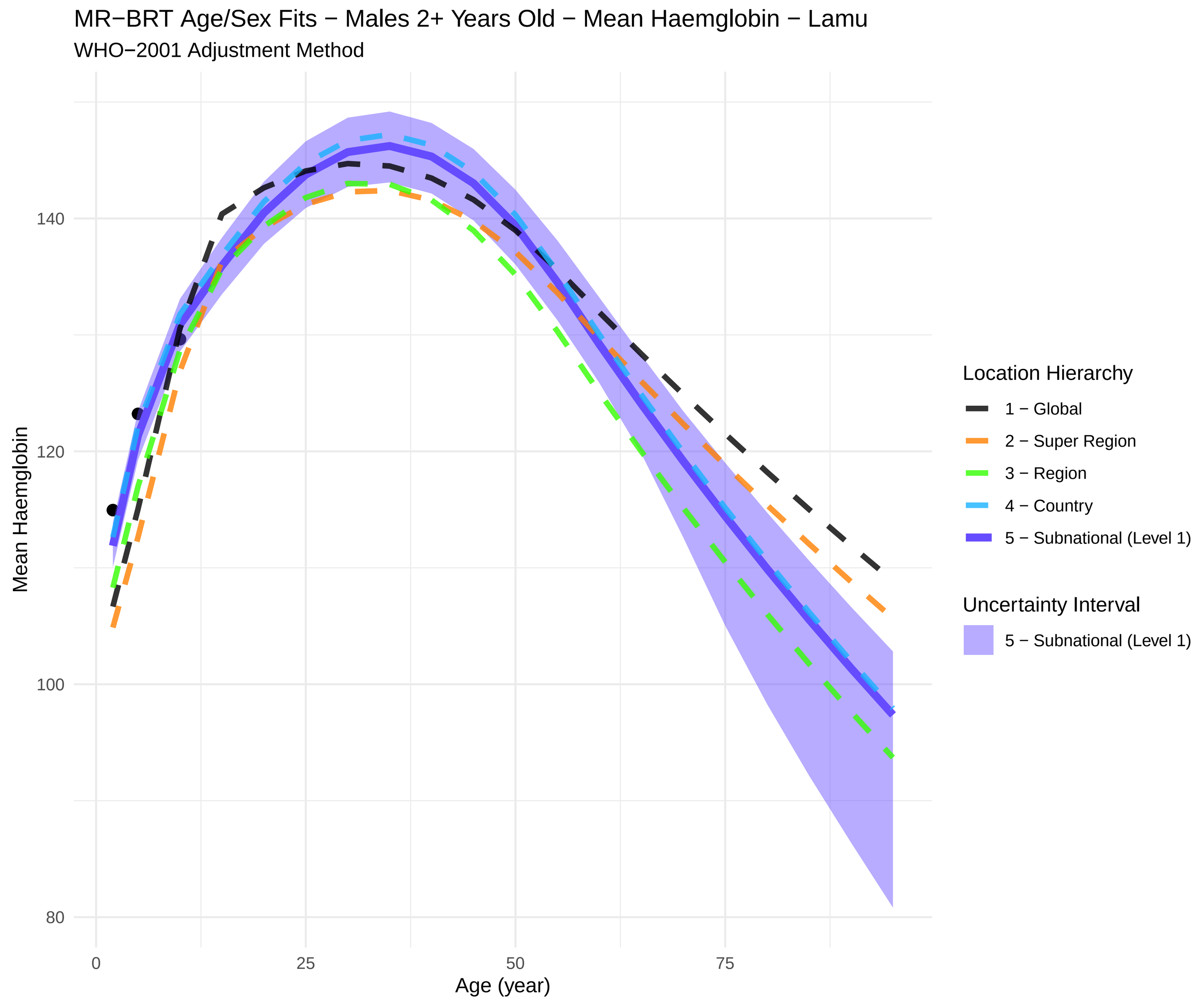
MR−BRT Age/Sex Fits − Males 2+ Years Old − Mean Haemglobin − Lamu
WHO−2001 Adjustment Method
Mean Haemglobin
Age (year)
Location Hierarchy
1 − Global
2 − Super Region
3 − Region
4 − Country
5 − Subnational (Level 1)
Uncertainty Interval
5 − Subnational (Level 1)

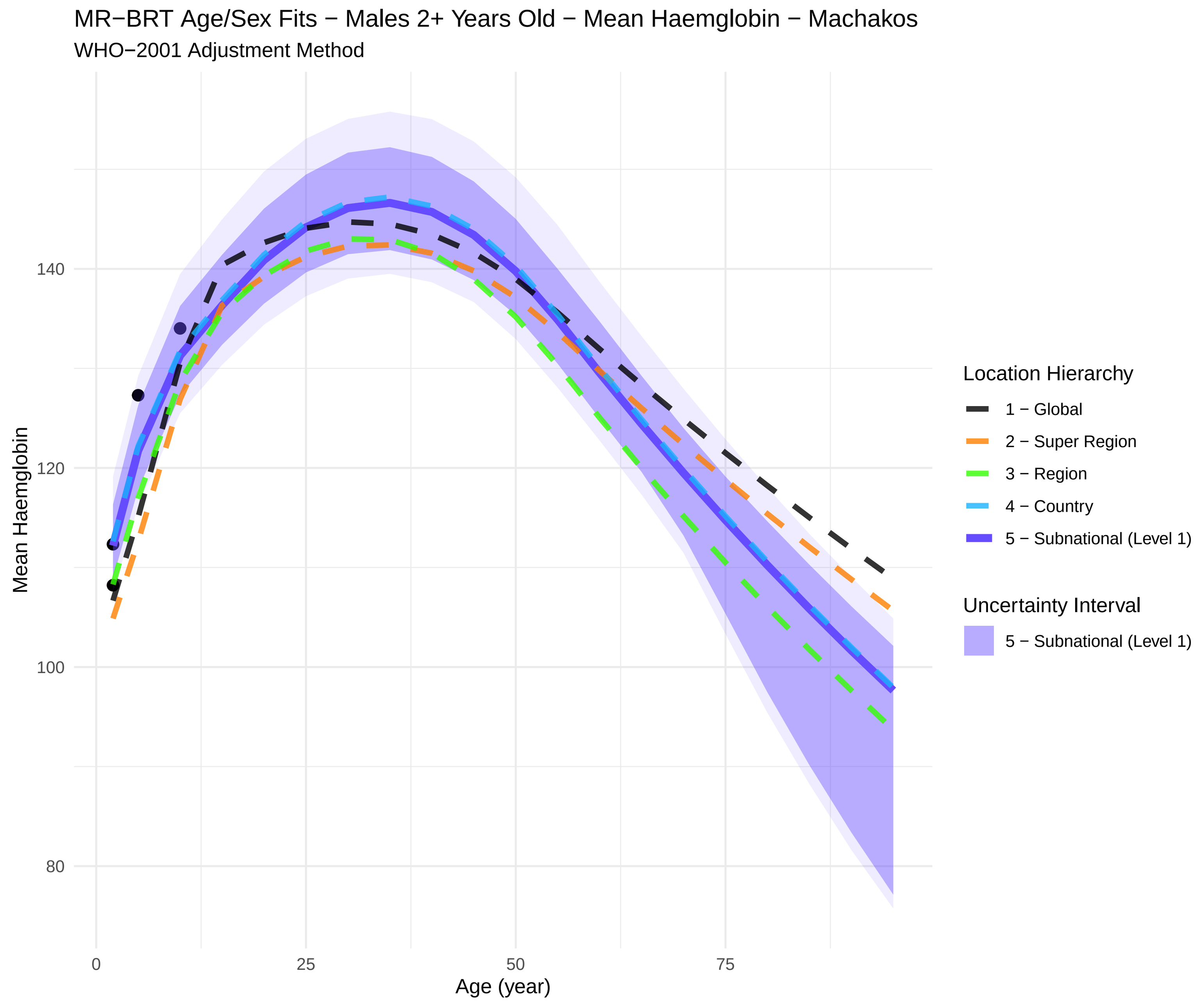
MR−BRT Age/Sex Fits − Males 2+ Years Old − Mean Haemglobin − Machakos
WHO−2001 Adjustment Method
Mean Haemglobin
Age (year)
Location Hierarchy
1 − Global
2 − Super Region
3 − Region
4 − Country
5 − Subnational (Level 1)
Uncertainty Interval
5 − Subnational (Level 1)

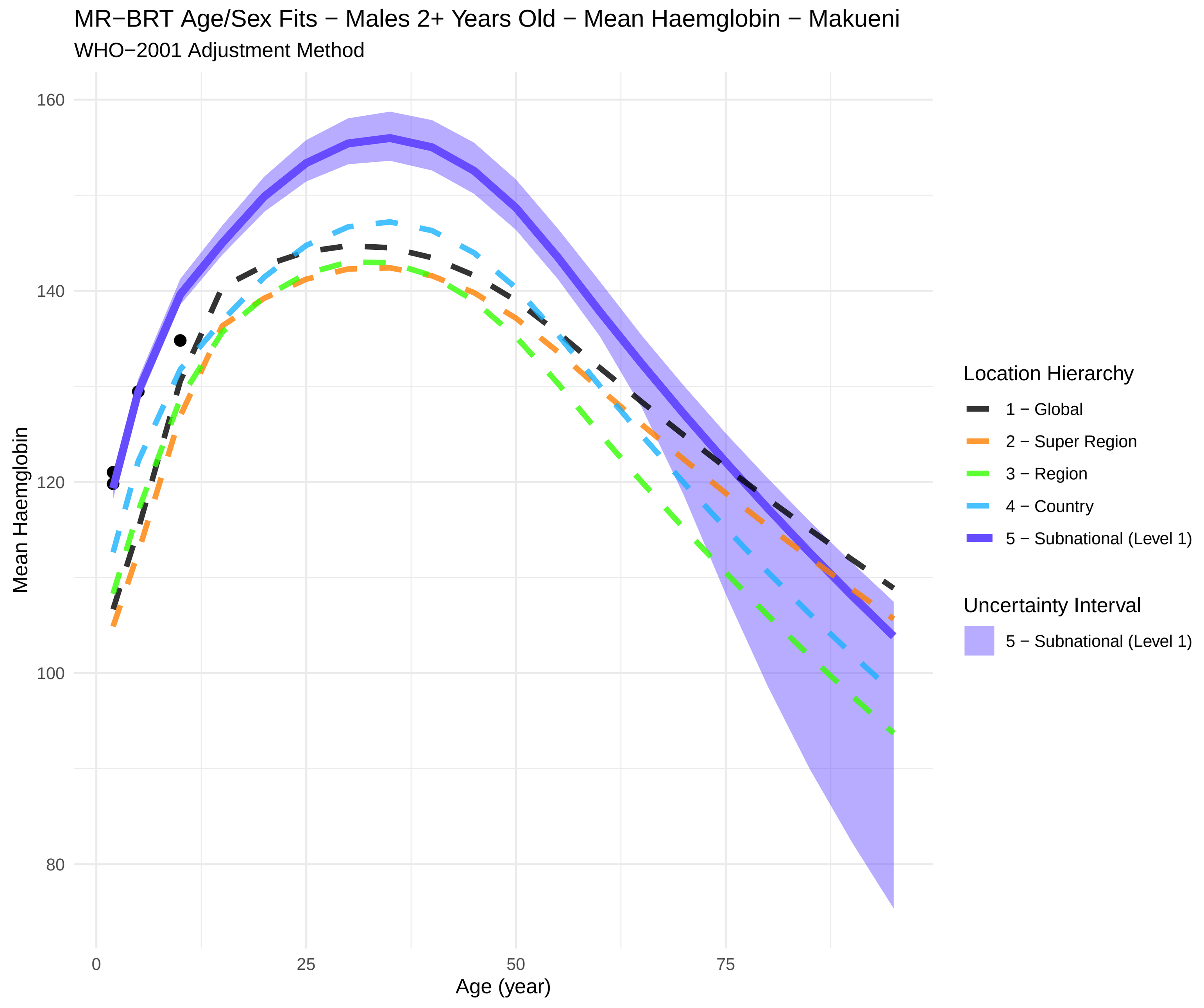
MR−BRT Age/Sex Fits − Males 2+ Years Old − Mean Haemglobin − Makueni
WHO−2001 Adjustment Method
Mean Haemglobin
Age (year)
Location Hierarchy
1 − Global
2 − Super Region
3 − Region
4 − Country
5 − Subnational (Level 1)
Uncertainty Interval
5 − Subnational (Level 1)

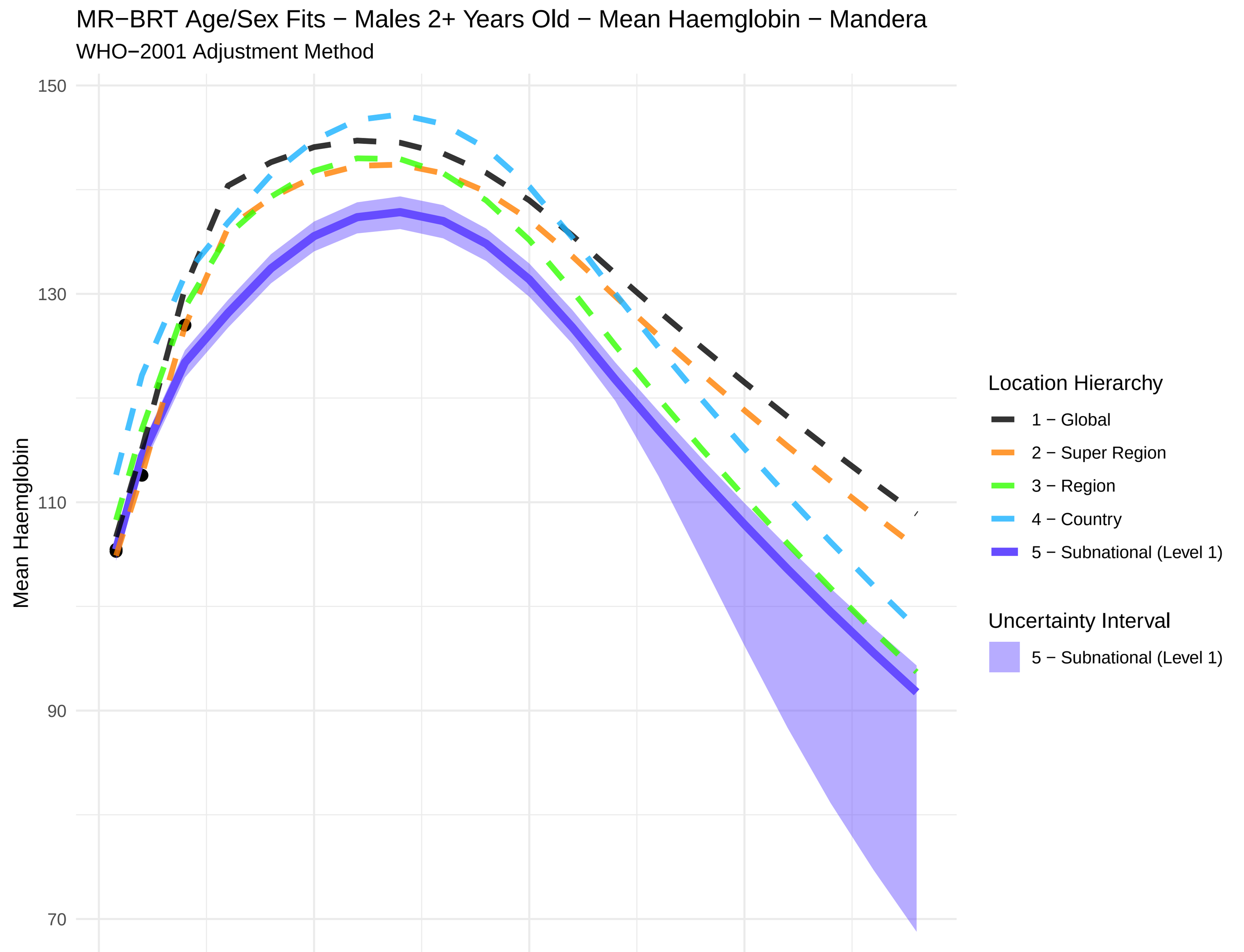
MR−BRT Age/Sex Fits − Males 2+ Years Old − Mean Haemglobin − Mandera
WHO−2001 Adjustment Method
Mean Haemglobin
Age (year)
Location Hierarchy
1 − Global
2 − Super Region
3 − Region
4 − Country
5 − Subnational (Level 1)
Uncertainty Interval
5 − Subnational (Level 1)

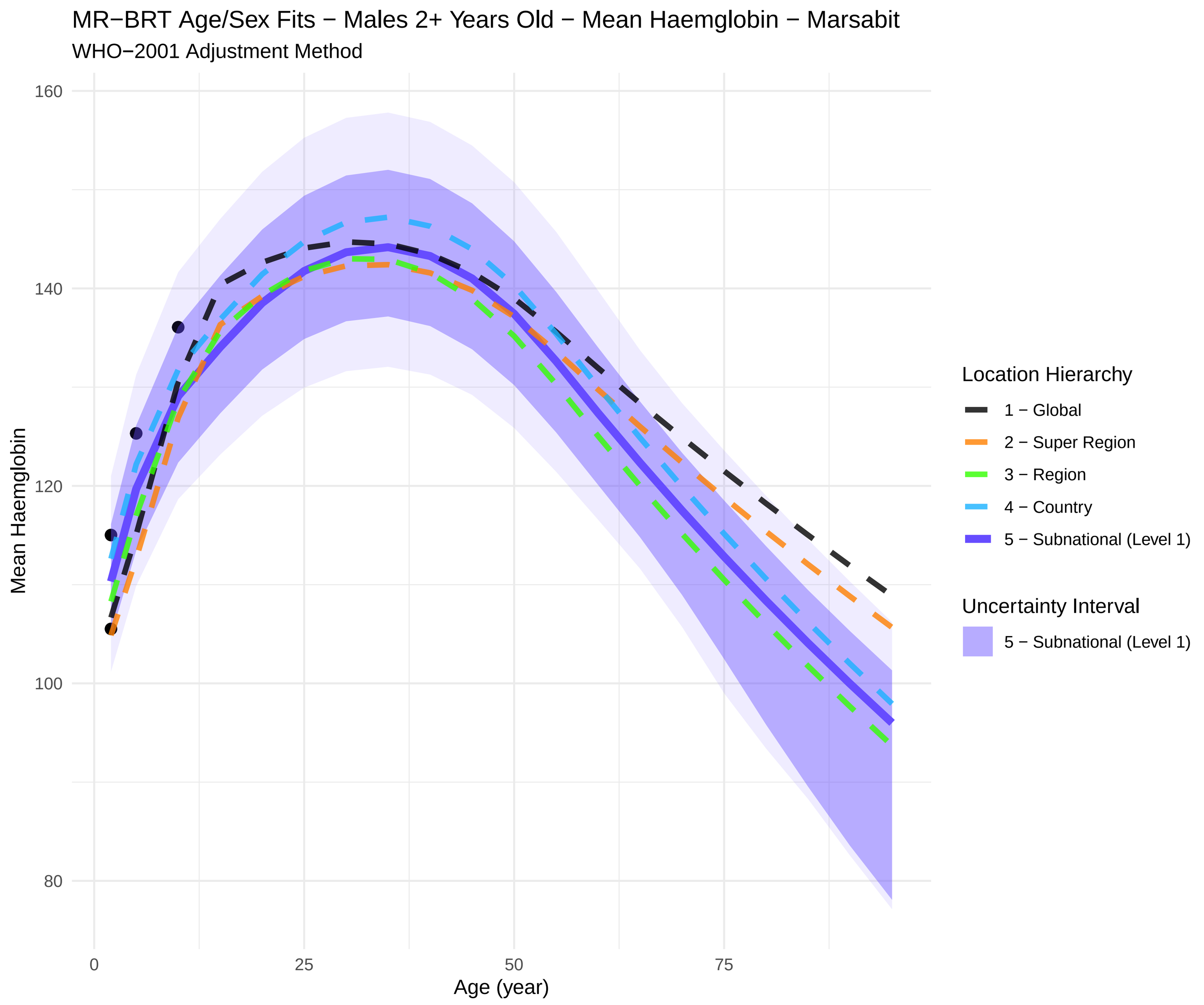
MR−BRT Age/Sex Fits − Males 2+ Years Old − Mean Haemglobin − Marsabit
WHO−2001 Adjustment Method
Mean Haemglobin
Age (year)
Location Hierarchy
1 − Global
2 − Super Region
3 − Region
4 − Country
5 − Subnational (Level 1)
Uncertainty Interval
5 − Subnational (Level 1)

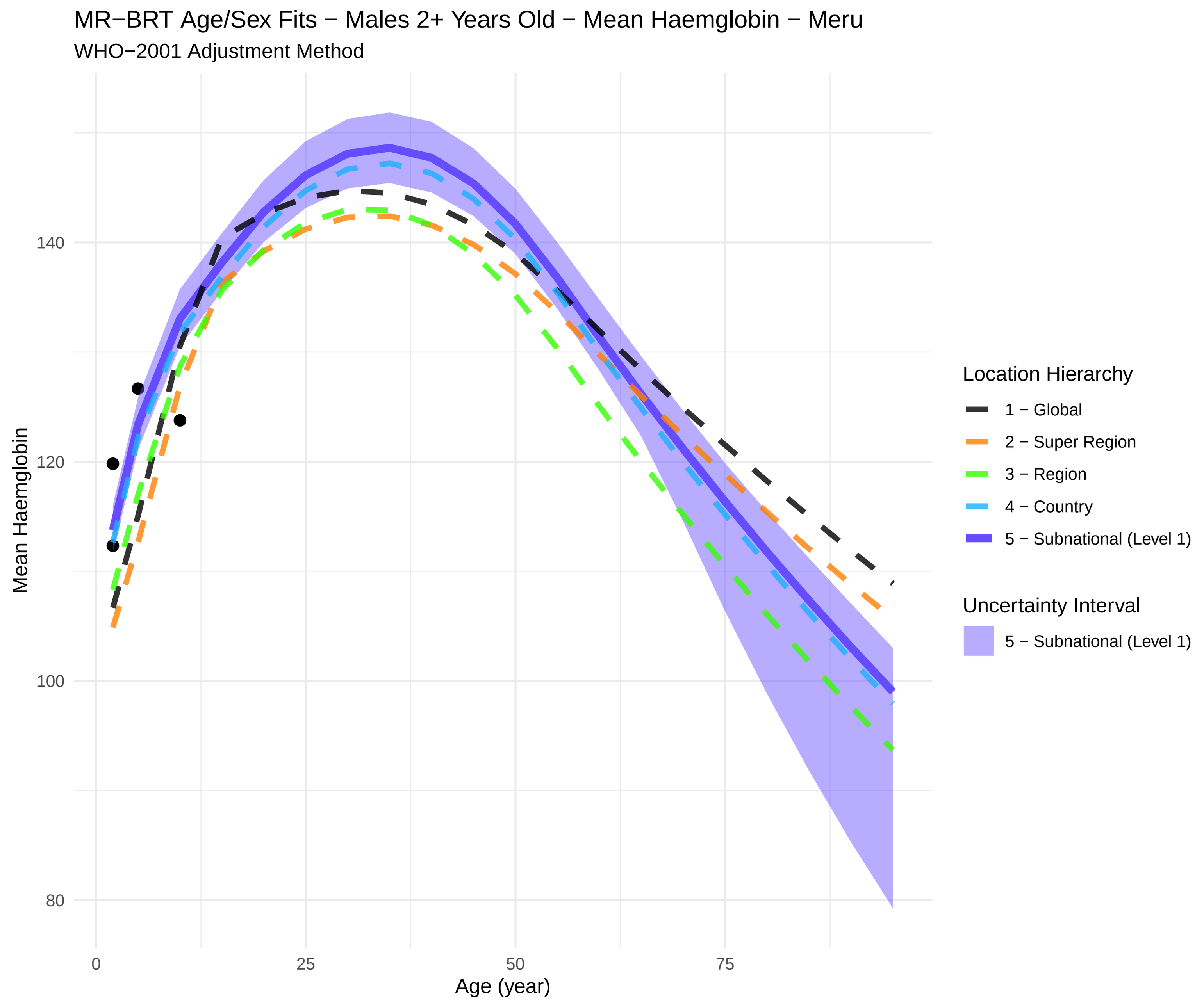
MR−BRT Age/Sex Fits − Males 2+ Years Old − Mean Haemglobin − Meru
WHO−2001 Adjustment Method
Mean Haemglobin
Age (year)
Location Hierarchy
1 − Global
2 − Super Region
3 − Region
4 − Country
5 − Subnational (Level 1)
Uncertainty Interval
5 − Subnational (Level 1)

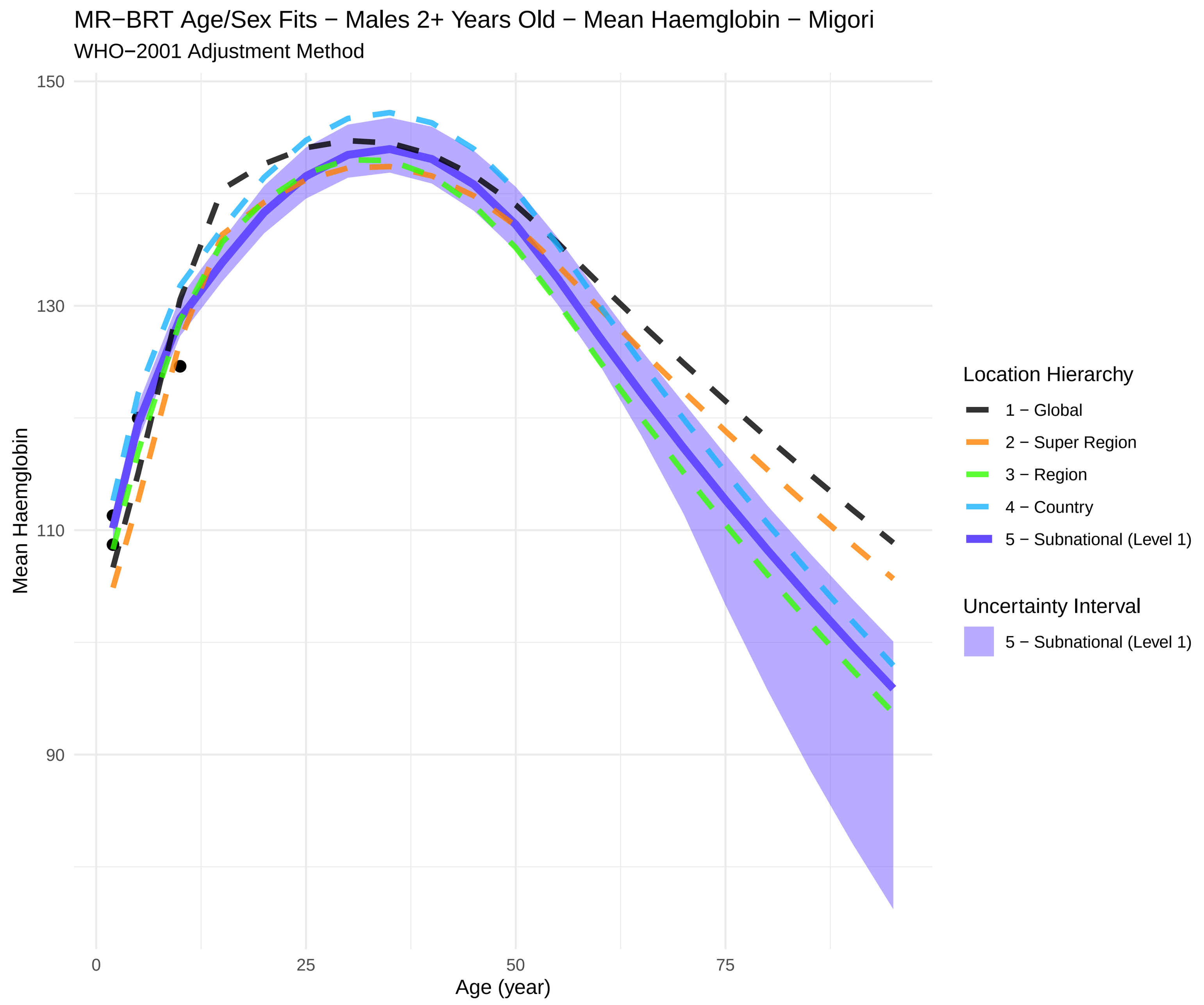
MR−BRT Age/Sex Fits − Males 2+ Years Old − Mean Haemglobin − Migori
WHO−2001 Adjustment Method
Mean Haemglobin
Age (year)
Location Hierarchy
1 − Global
2 − Super Region
3 − Region
4 − Country
5 − Subnational (Level 1)
Uncertainty Interval
5 − Subnational (Level 1)

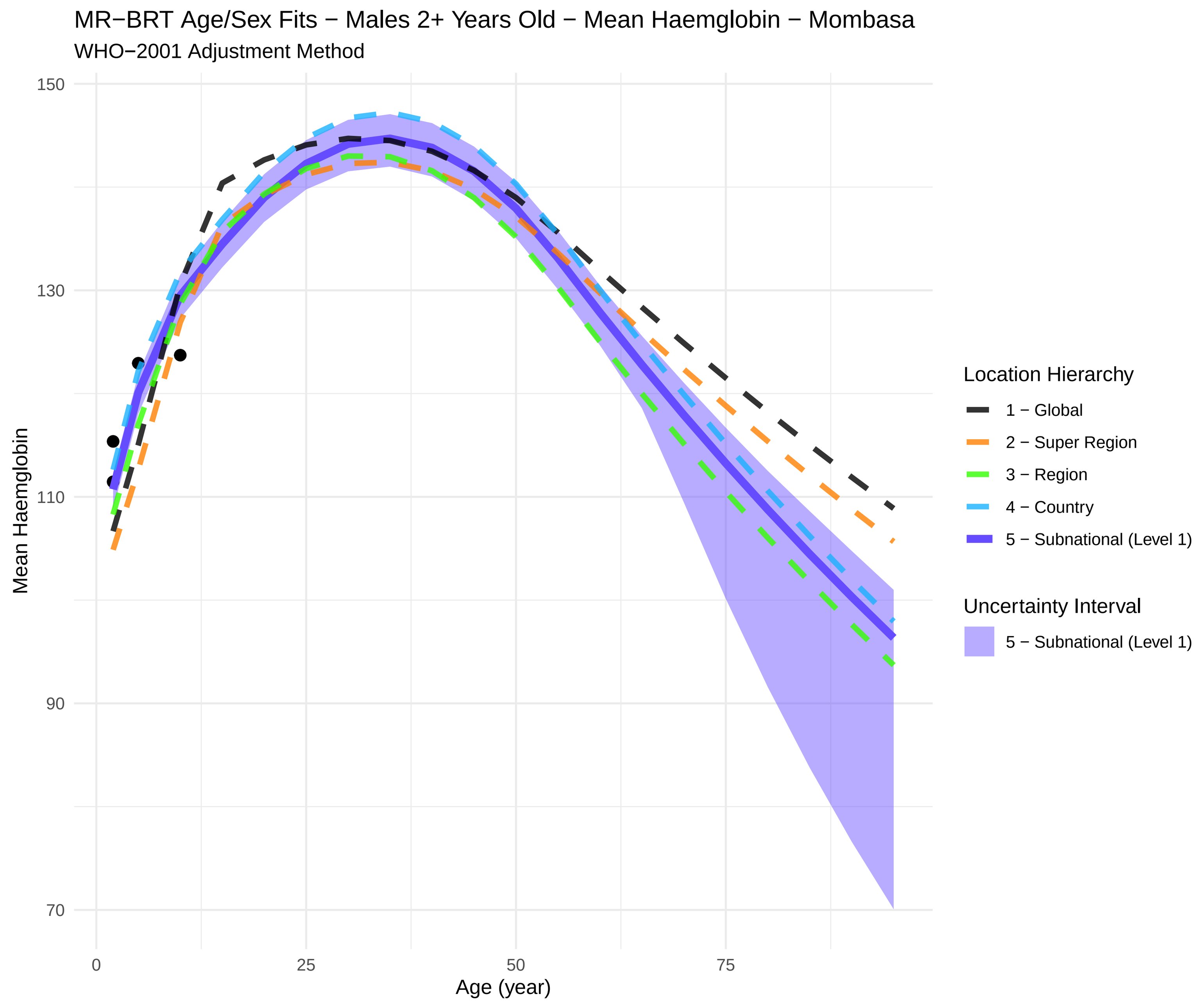
MR−BRT Age/Sex Fits − Males 2+ Years Old − Mean Haemglobin − Mombasa
WHO−2001 Adjustment Method
Mean Haemglobin
Age (year)
Location Hierarchy
1 − Global
2 − Super Region
3 − Region
4 − Country
5 − Subnational (Level 1)
Uncertainty Interval
5 − Subnational (Level 1)

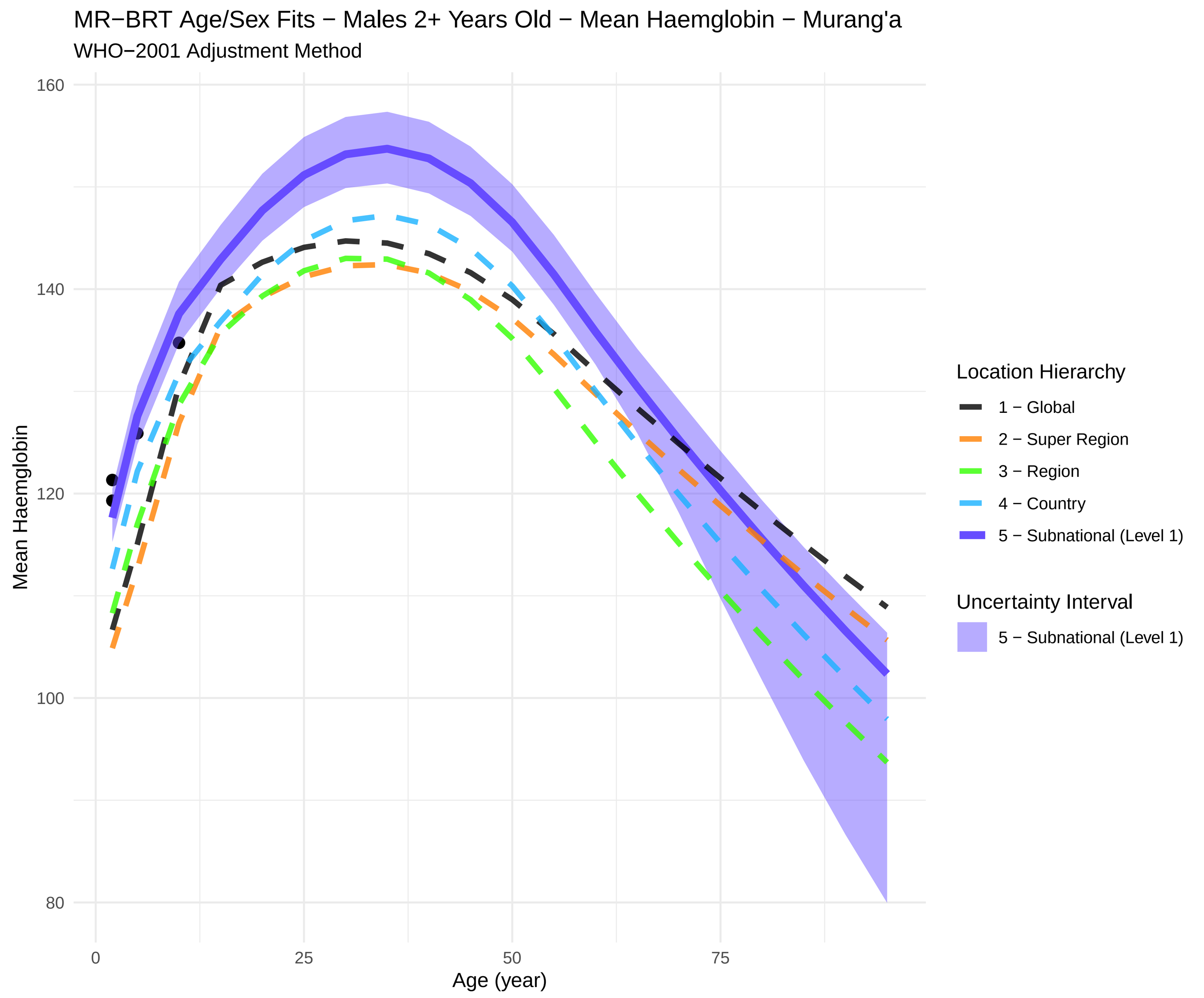
MR−BRT Age/Sex Fits − Males 2+ Years Old − Mean Haemglobin − Murang'a
WHO−2001 Adjustment Method
Mean Haemglobin
Age (year)
Location Hierarchy
1 − Global
2 − Super Region
3 − Region
4 − Country
5 − Subnational (Level 1)
Uncertainty Interval
5 − Subnational (Level 1)

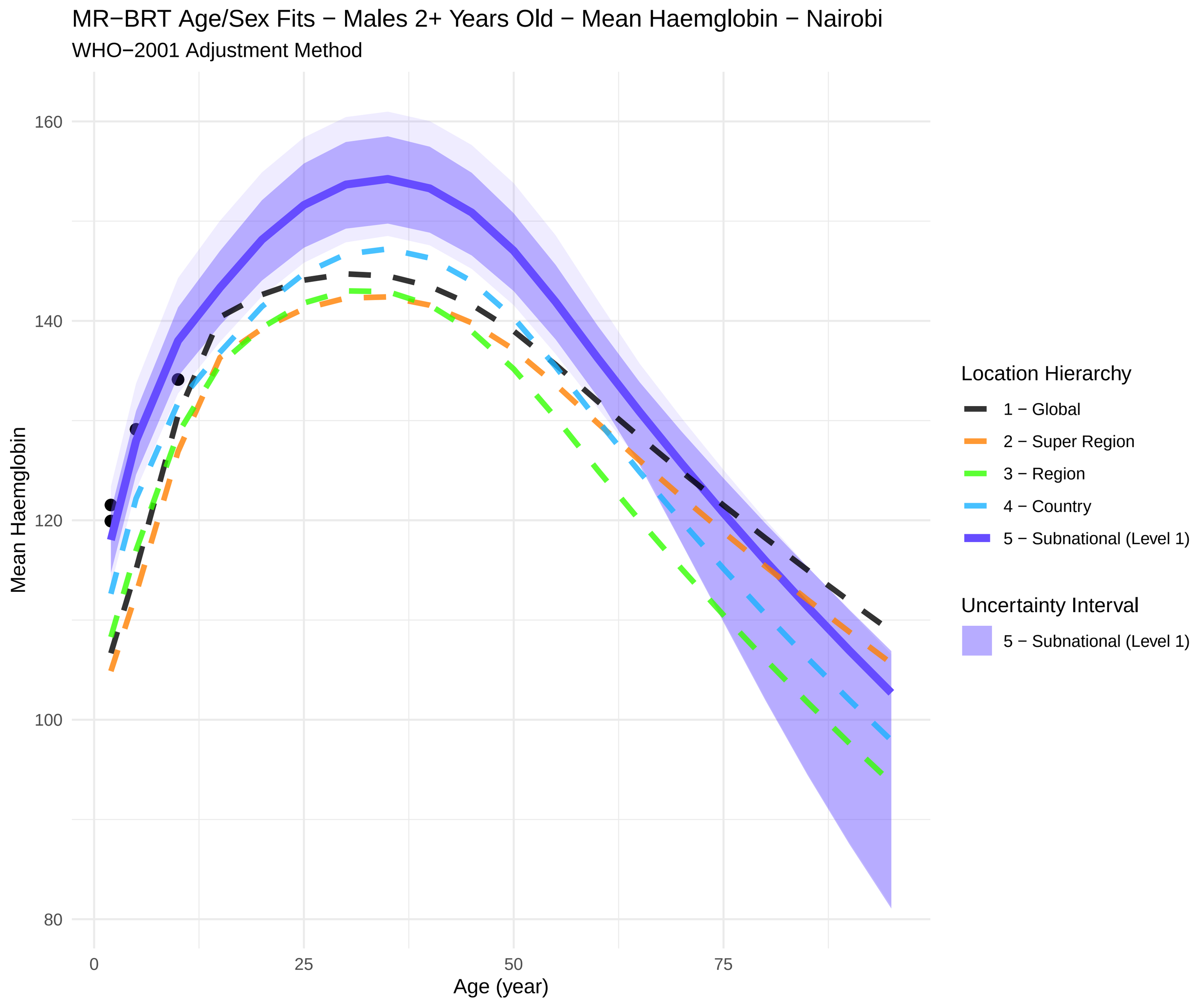
MR−BRT Age/Sex Fits − Males 2+ Years Old − Mean Haemglobin − Nairobi
WHO−2001 Adjustment Method
Mean Haemglobin
Age (year)
Location Hierarchy
1 − Global
2 − Super Region
3 − Region
4 − Country
5 − Subnational (Level 1)
Uncertainty Interval
5 − Subnational (Level 1)

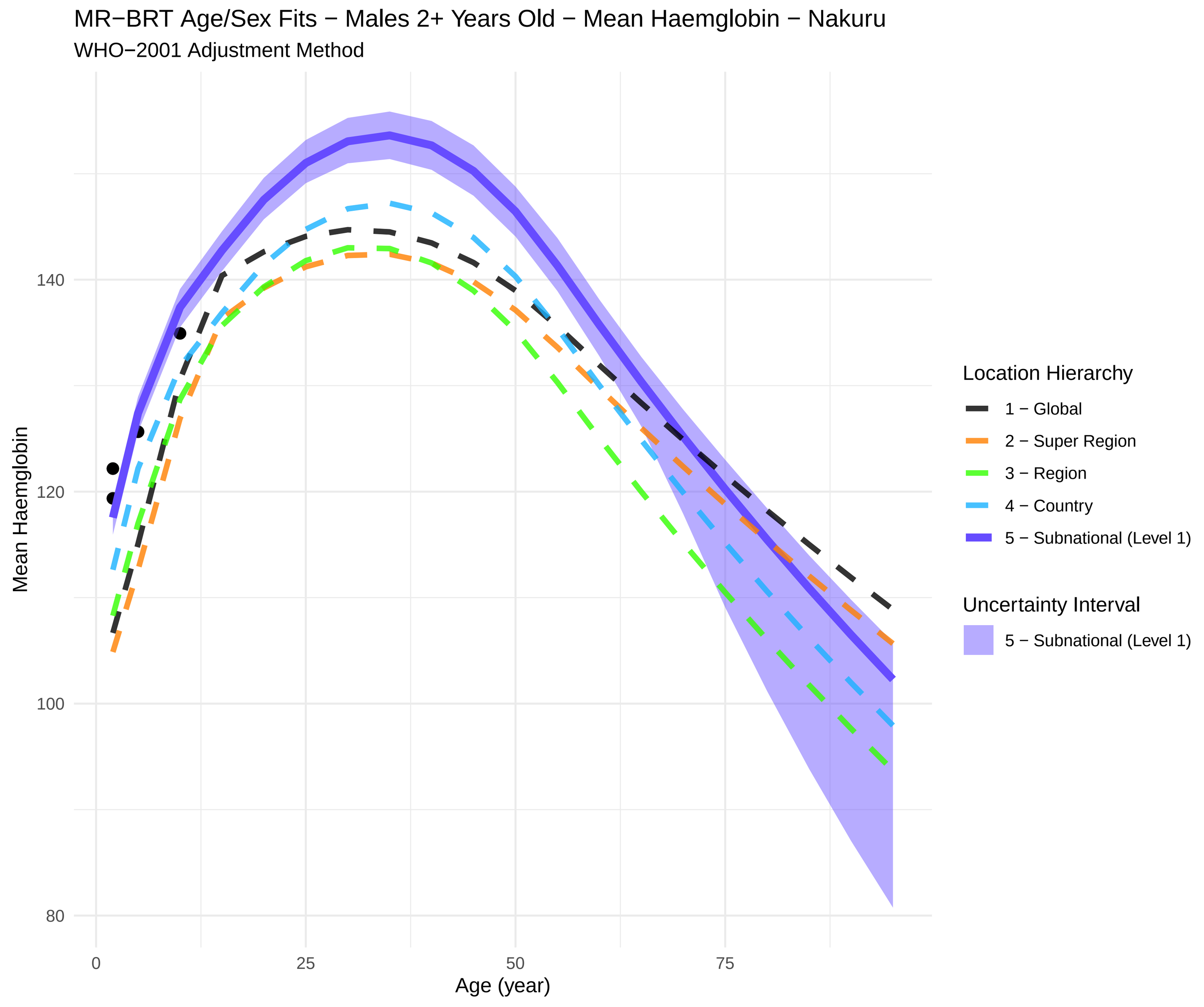
MR−BRT Age/Sex Fits − Males 2+ Years Old − Mean Haemglobin − Nakuru
WHO−2001 Adjustment Method
Mean Haemglobin
Age (year)
Location Hierarchy
1 − Global
2 − Super Region
3 − Region
4 − Country
5 − Subnational (Level 1)
Uncertainty Interval
5 − Subnational (Level 1)

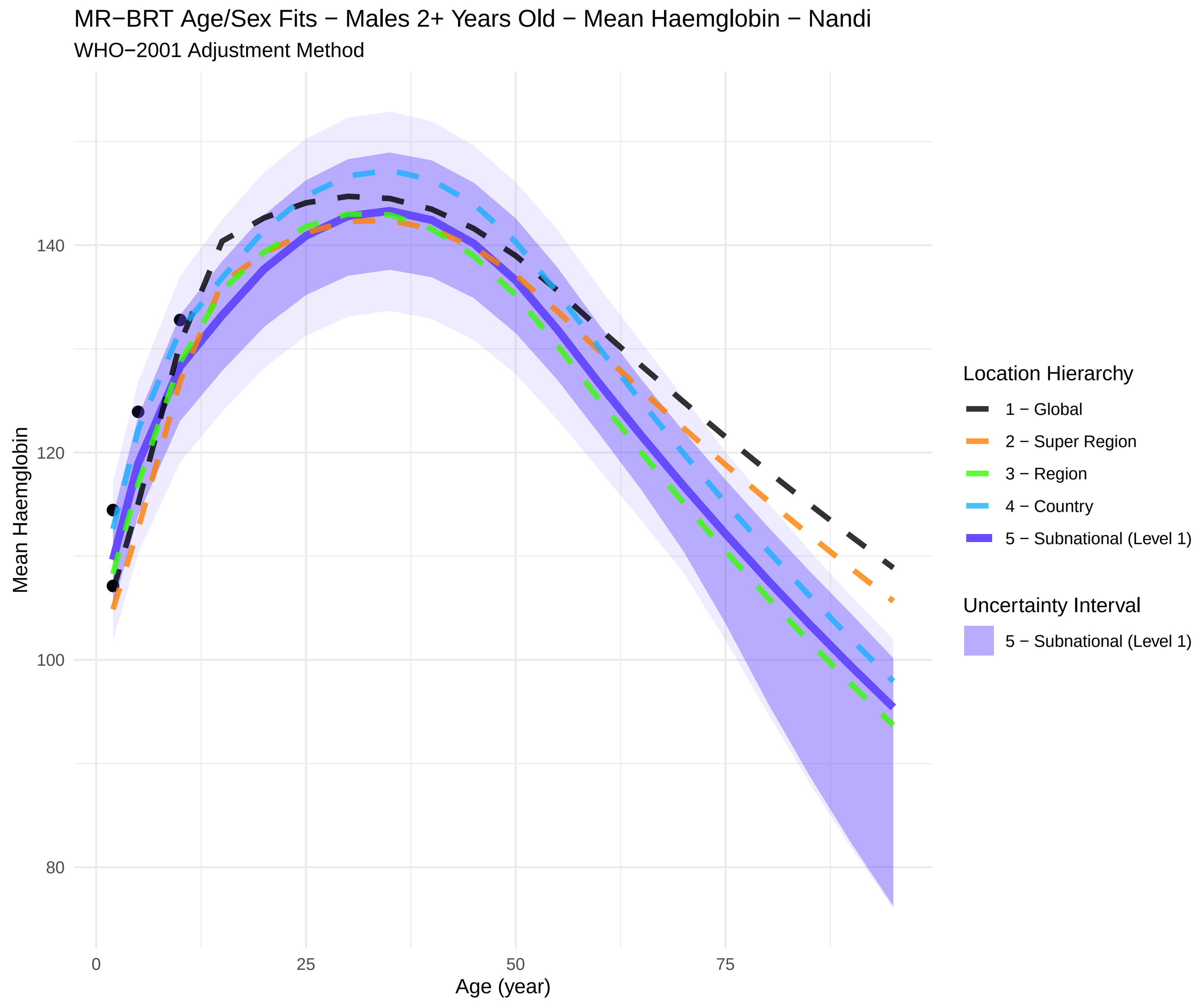
MR−BRT Age/Sex Fits − Males 2+ Years Old − Mean Haemglobin − Nandi
WHO−2001 Adjustment Method
Mean Haemglobin
Age (year)
Location Hierarchy
1 − Global
2 − Super Region
3 − Region
4 − Country
5 − Subnational (Level 1)
Uncertainty Interval
5 − Subnational (Level 1)

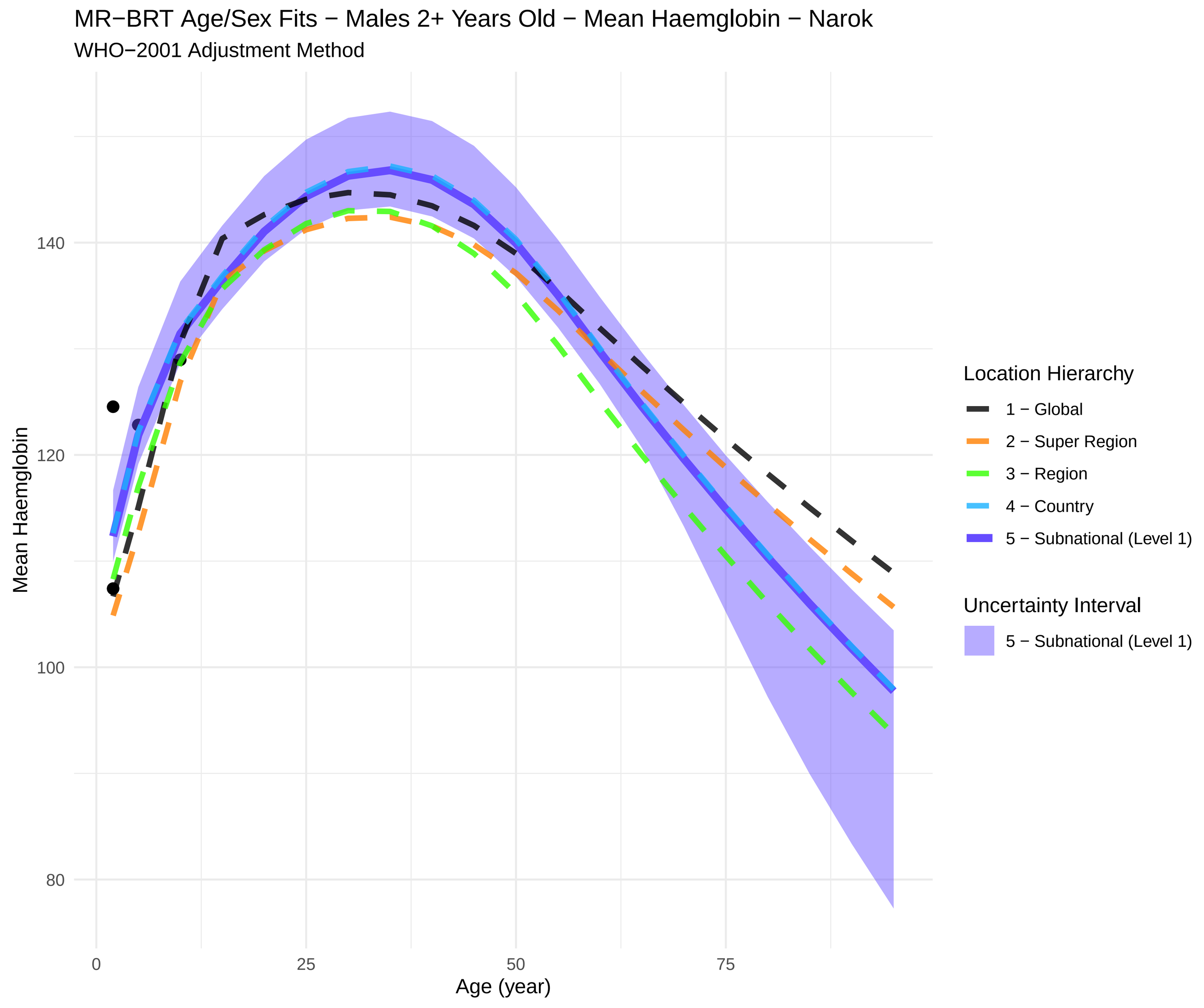
MR−BRT Age/Sex Fits − Males 2+ Years Old − Mean Haemglobin − Narok
WHO−2001 Adjustment Method
Mean Haemglobin
Age (year)
Location Hierarchy
1 − Global
2 − Super Region
3 − Region
4 − Country
5 − Subnational (Level 1)
Uncertainty Interval
5 − Subnational (Level 1)

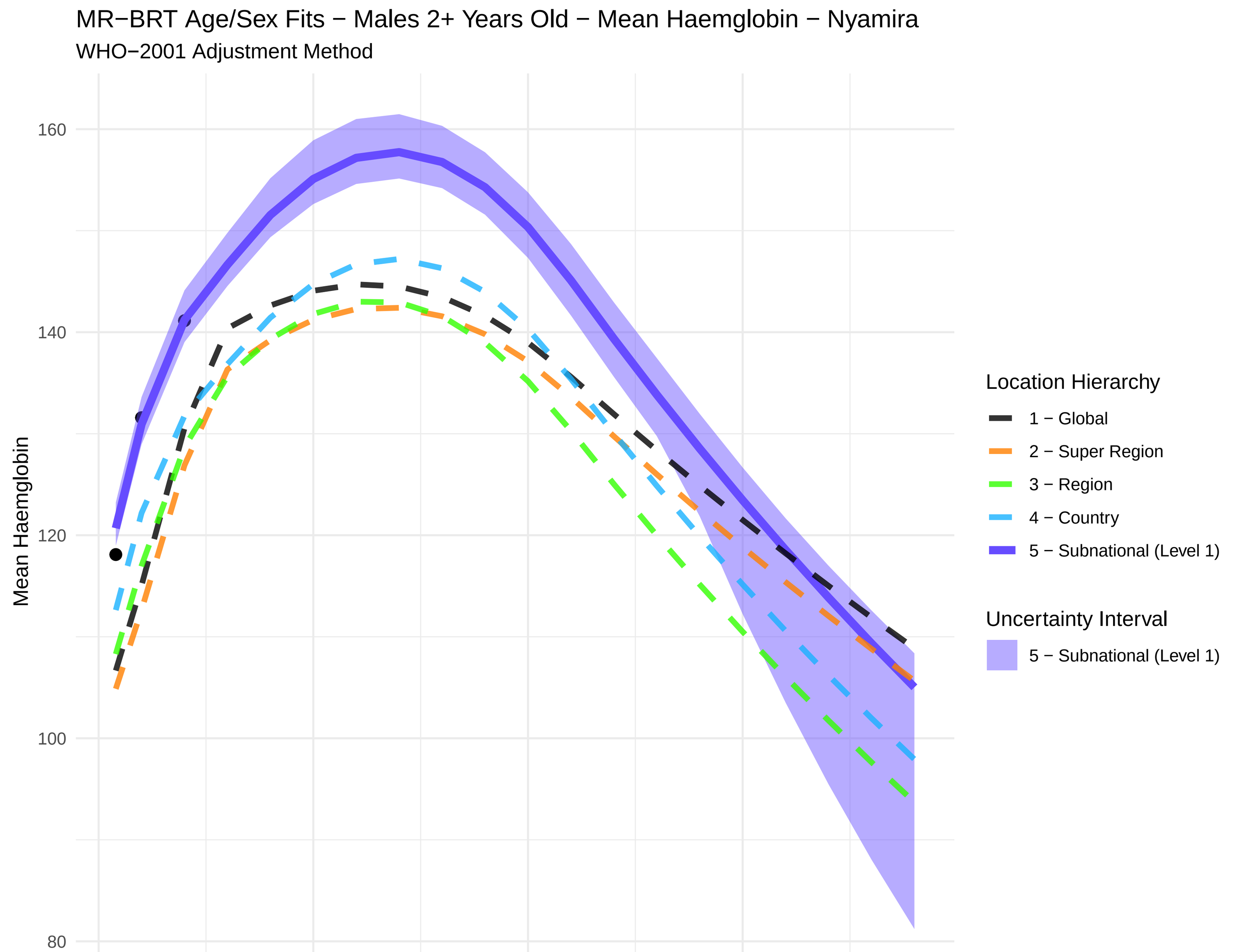
MR−BRT Age/Sex Fits − Males 2+ Years Old − Mean Haemglobin − Nyamira
WHO−2001 Adjustment Method
Mean Haemglobin
Age (year)
Location Hierarchy
1 − Global
2 − Super Region
3 − Region
4 − Country
5 − Subnational (Level 1)
Uncertainty Interval
5 − Subnational (Level 1)

MR−BRT Age/Sex Fits − Males 2+ Years Old − Mean Haemglobin − Nyandarua
WHO−2001 Adjustment Method

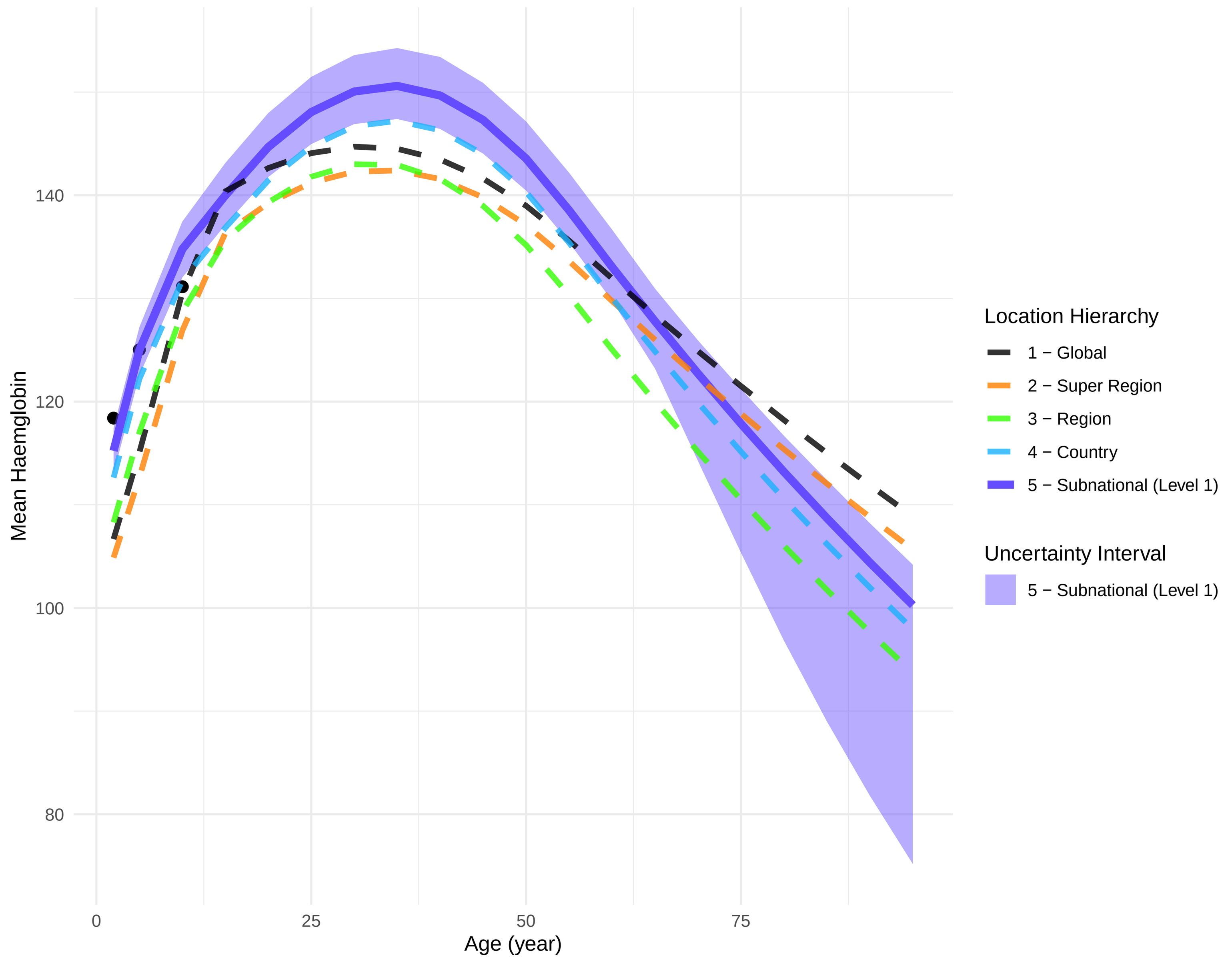

MR−BRT Age/Sex Fits − Males 2+ Years Old − Mean Haemglobin − Nyeri
WHO−2001 Adjustment Method

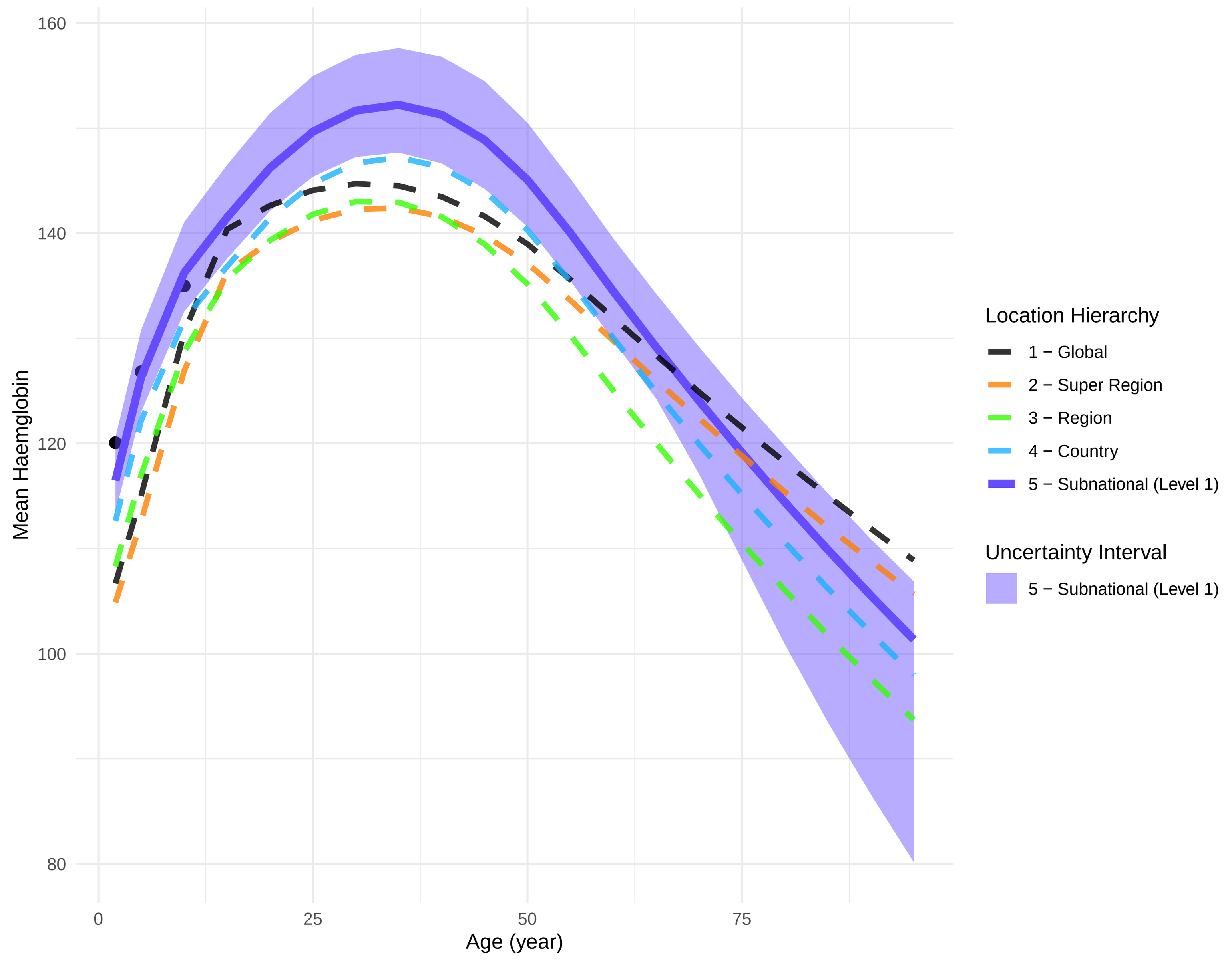

MR−BRT Age/Sex Fits − Males 2+ Years Old − Mean Haemglobin − Samburu
WHO−2001 Adjustment Method

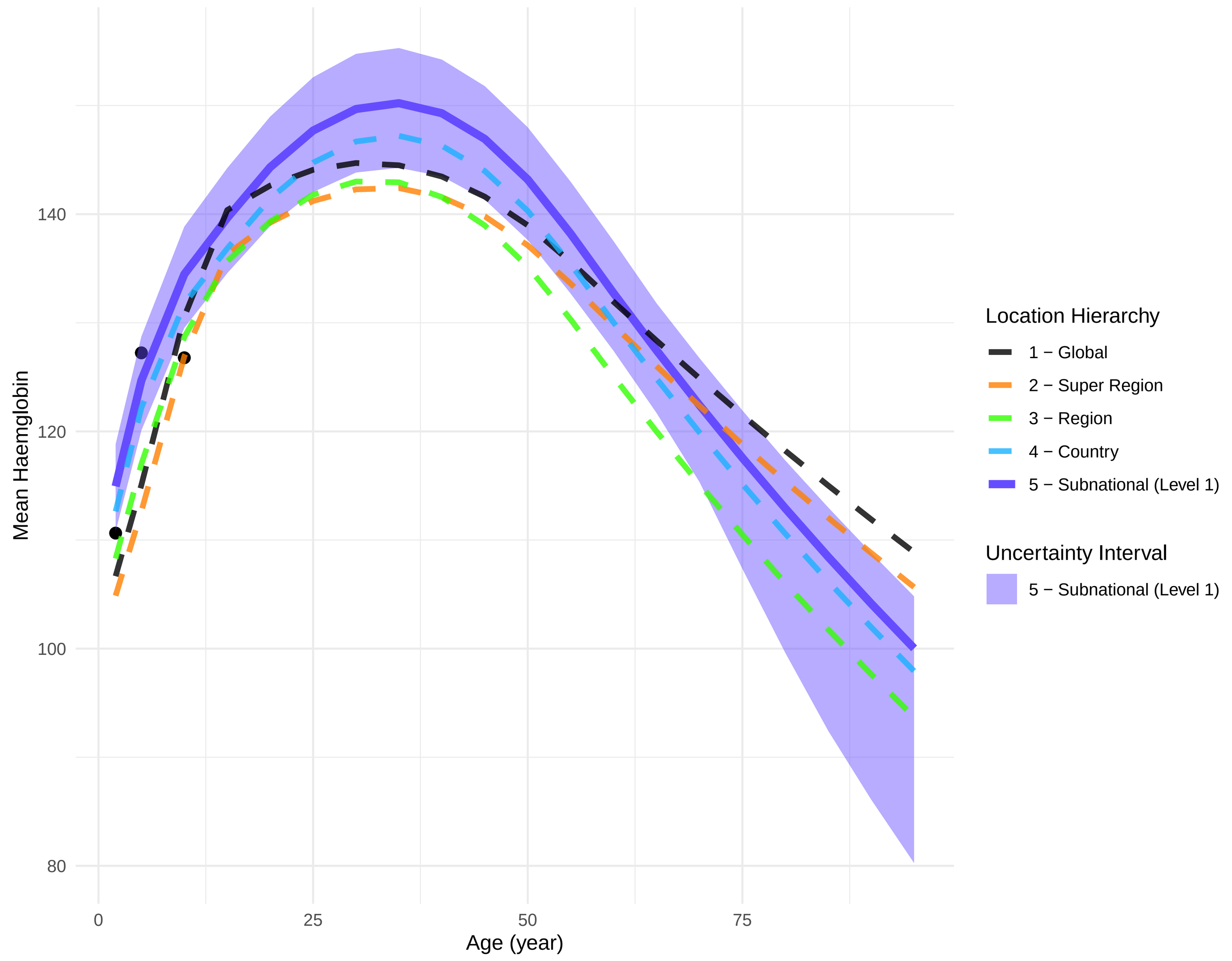

MR−BRT Age/Sex Fits − Males 2+ Years Old − Mean Haemglobin − Siaya
WHO−2001 Adjustment Method

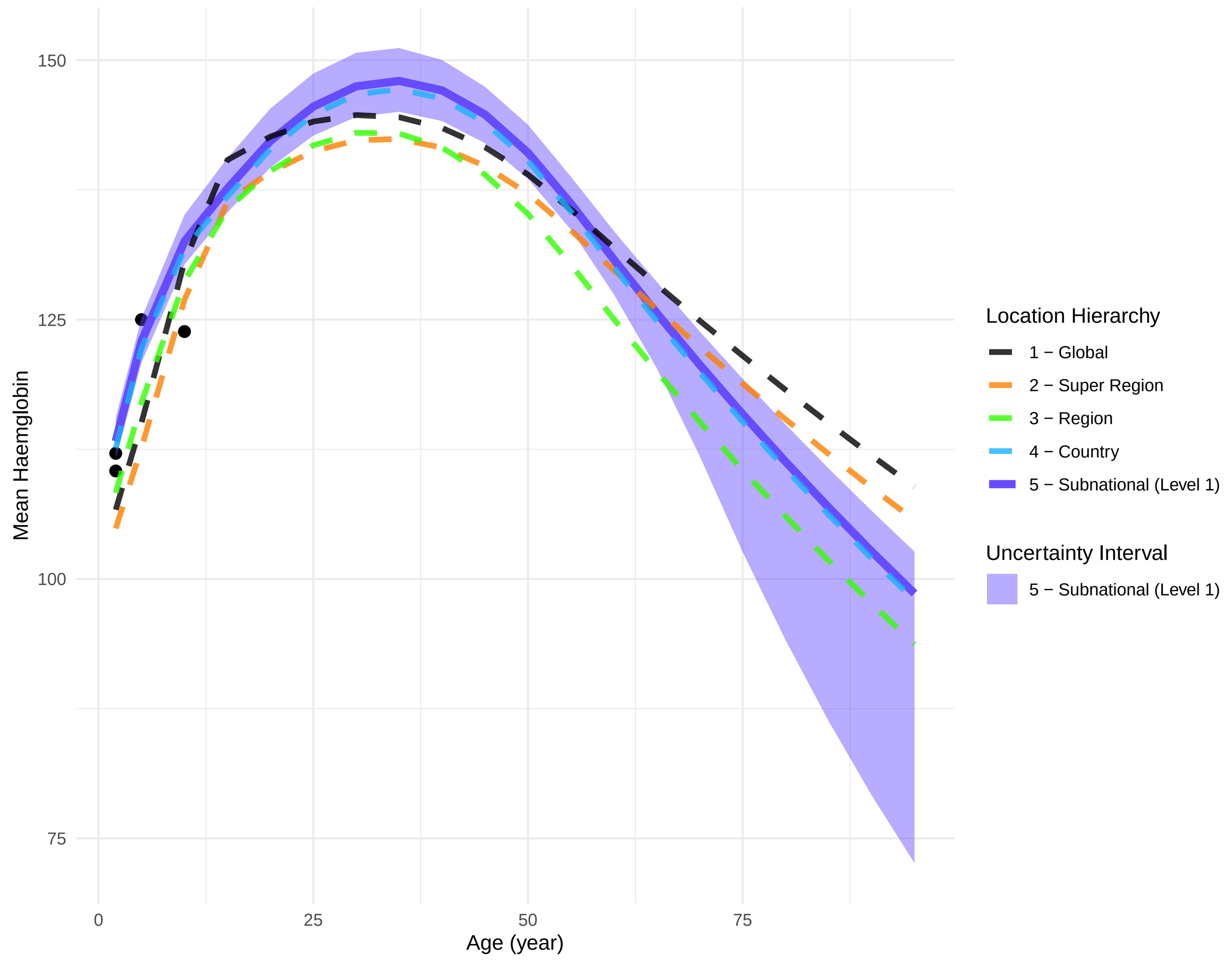

MR−BRT Age/Sex Fits − Males 2+ Years Old − Mean Haemglobin − Taita Taveta
WHO−2001 Adjustment Method

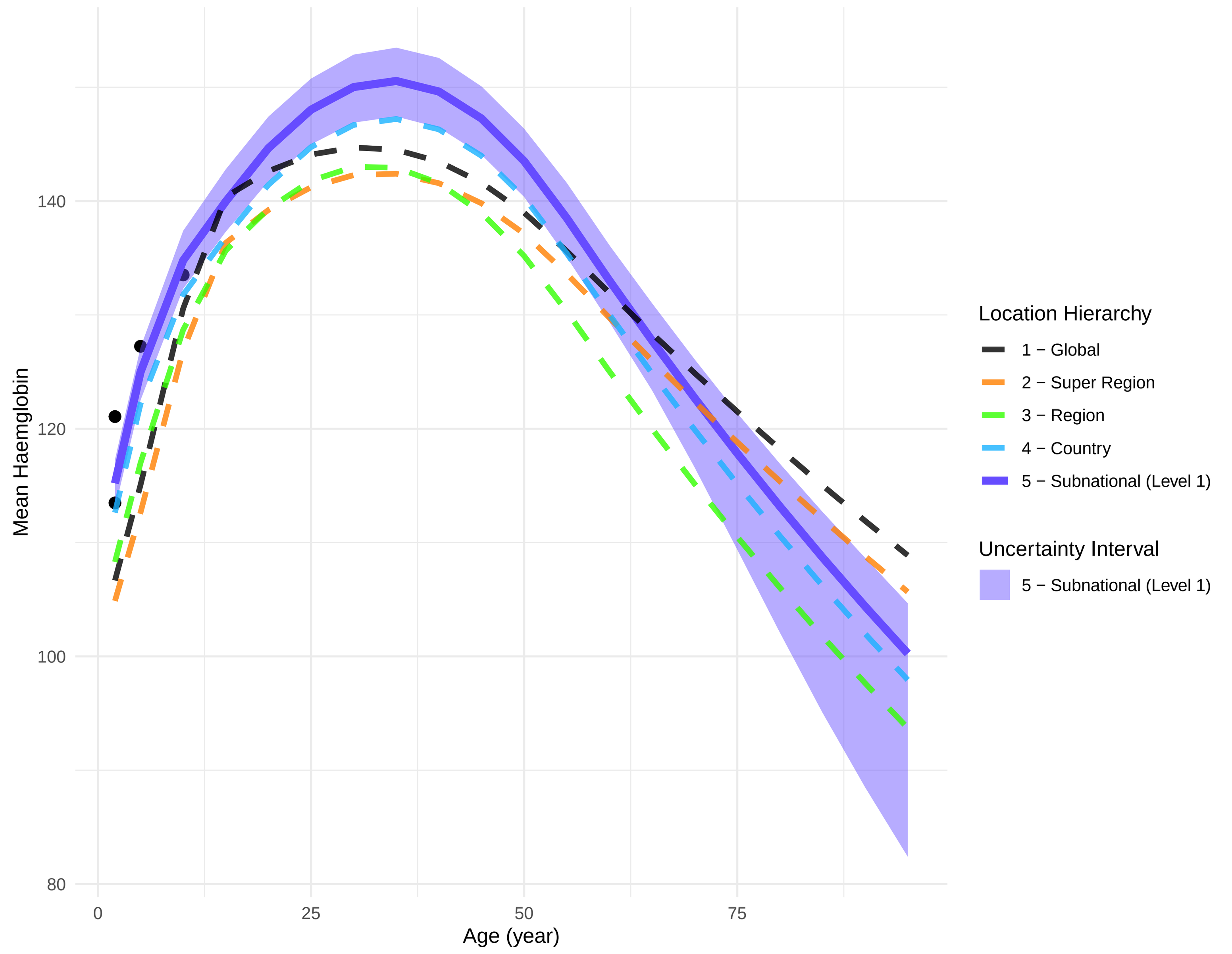

MR−BRT Age/Sex Fits − Males 2+ Years Old − Mean Haemglobin − Tana River
WHO−2001 Adjustment Method

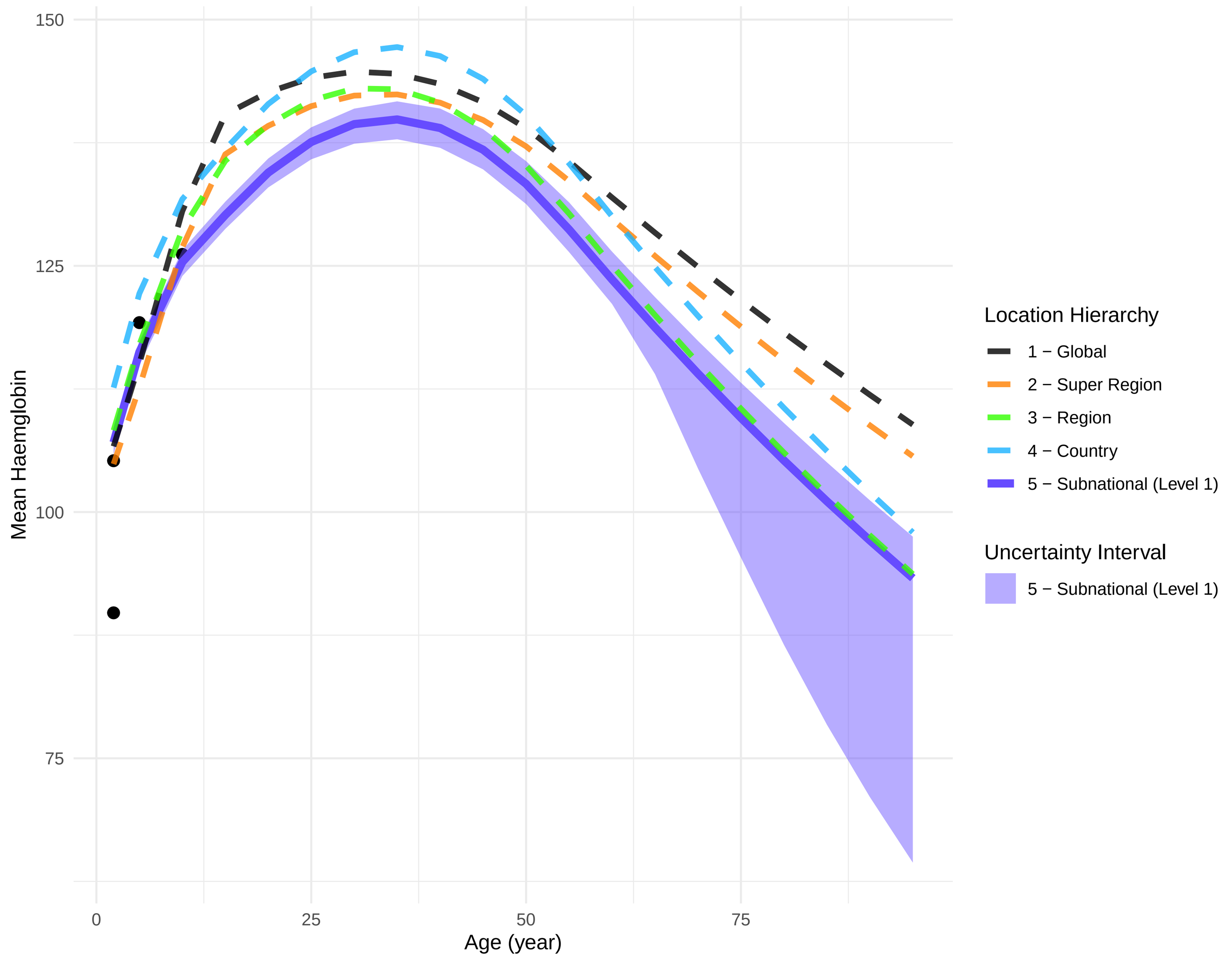

MR−BRT Age/Sex Fits − Males 2+ Years Old − Mean Haemglobin − Tharaka Nithi
WHO−2001 Adjustment Method

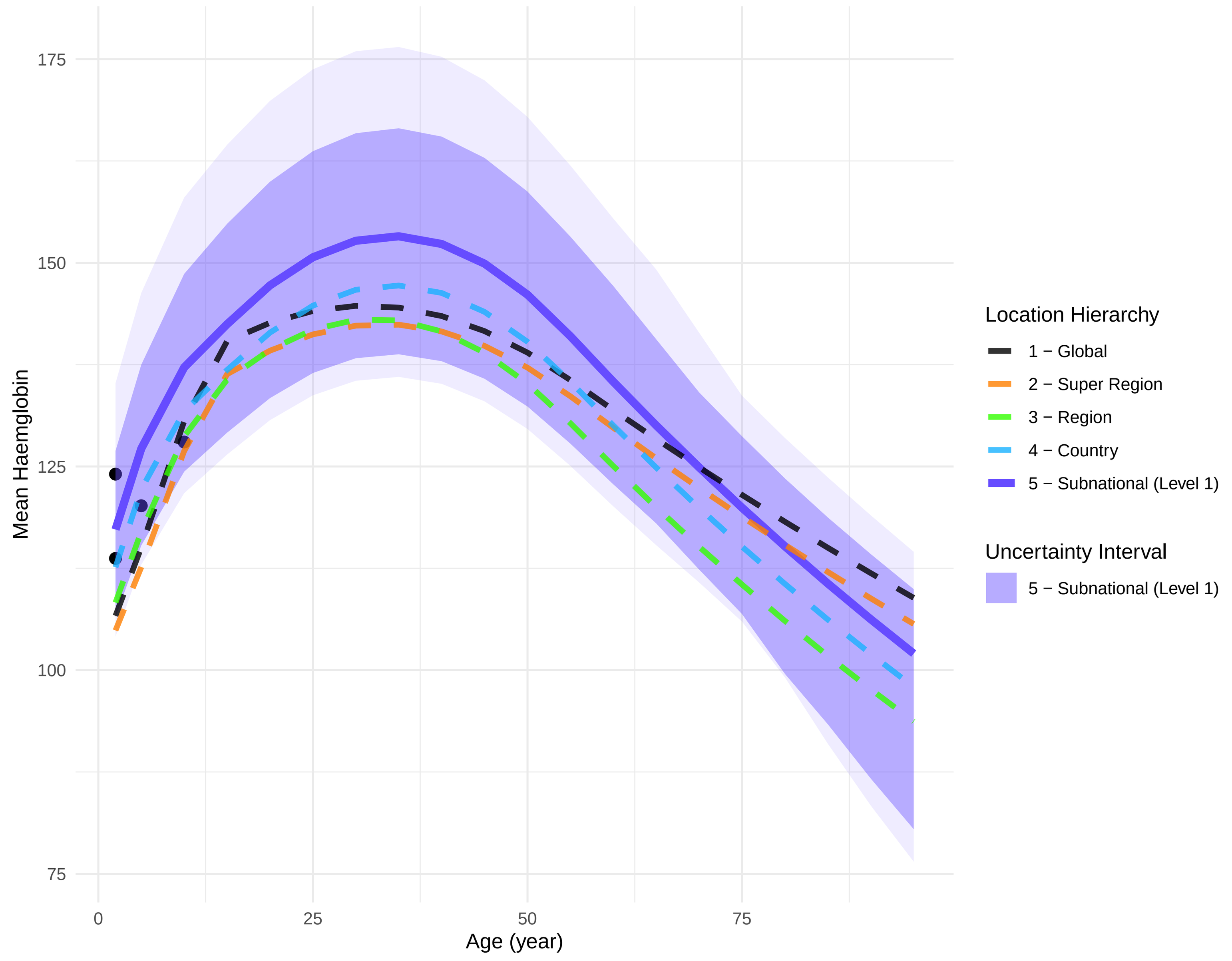

MR−BRT Age/Sex Fits − Males 2+ Years Old − Mean Haemglobin − Trans Nzoia
WHO−2001 Adjustment Method

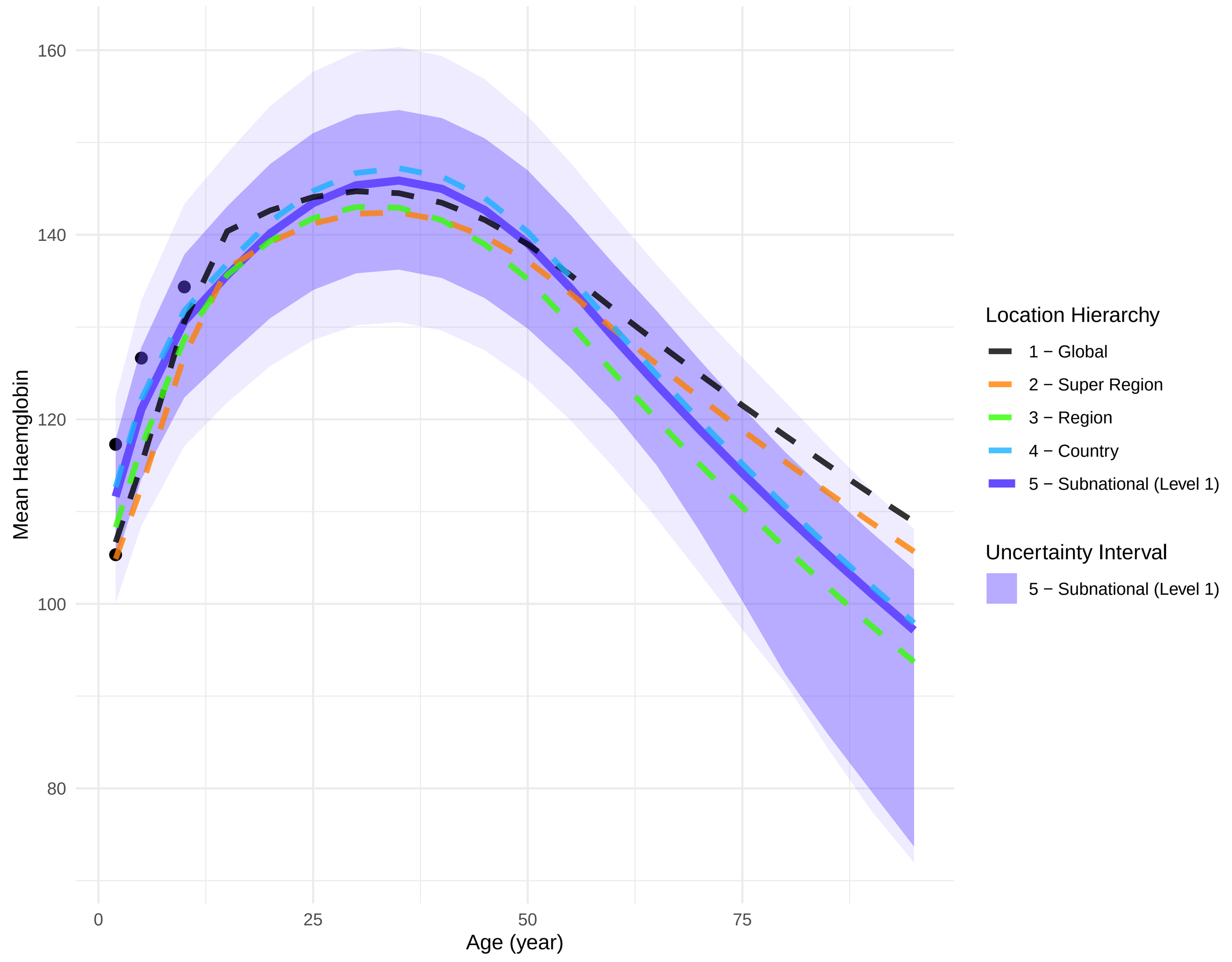

MR−BRT Age/Sex Fits − Males 2+ Years Old − Mean Haemglobin − Turkana
WHO−2001 Adjustment Method

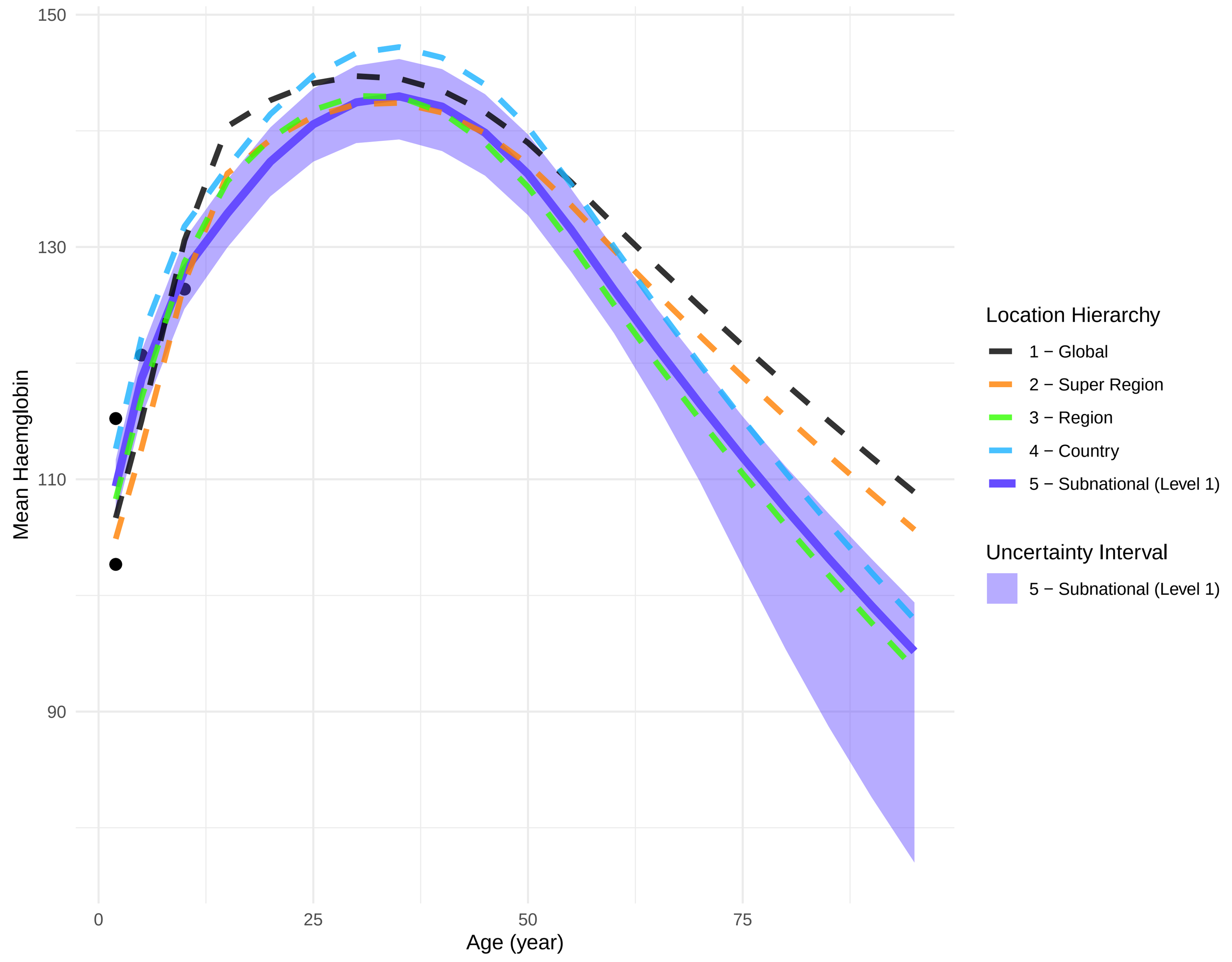

MR−BRT Age/Sex Fits − Males 2+ Years Old − Mean Haemglobin − Uasin Gishu
WHO−2001 Adjustment Method

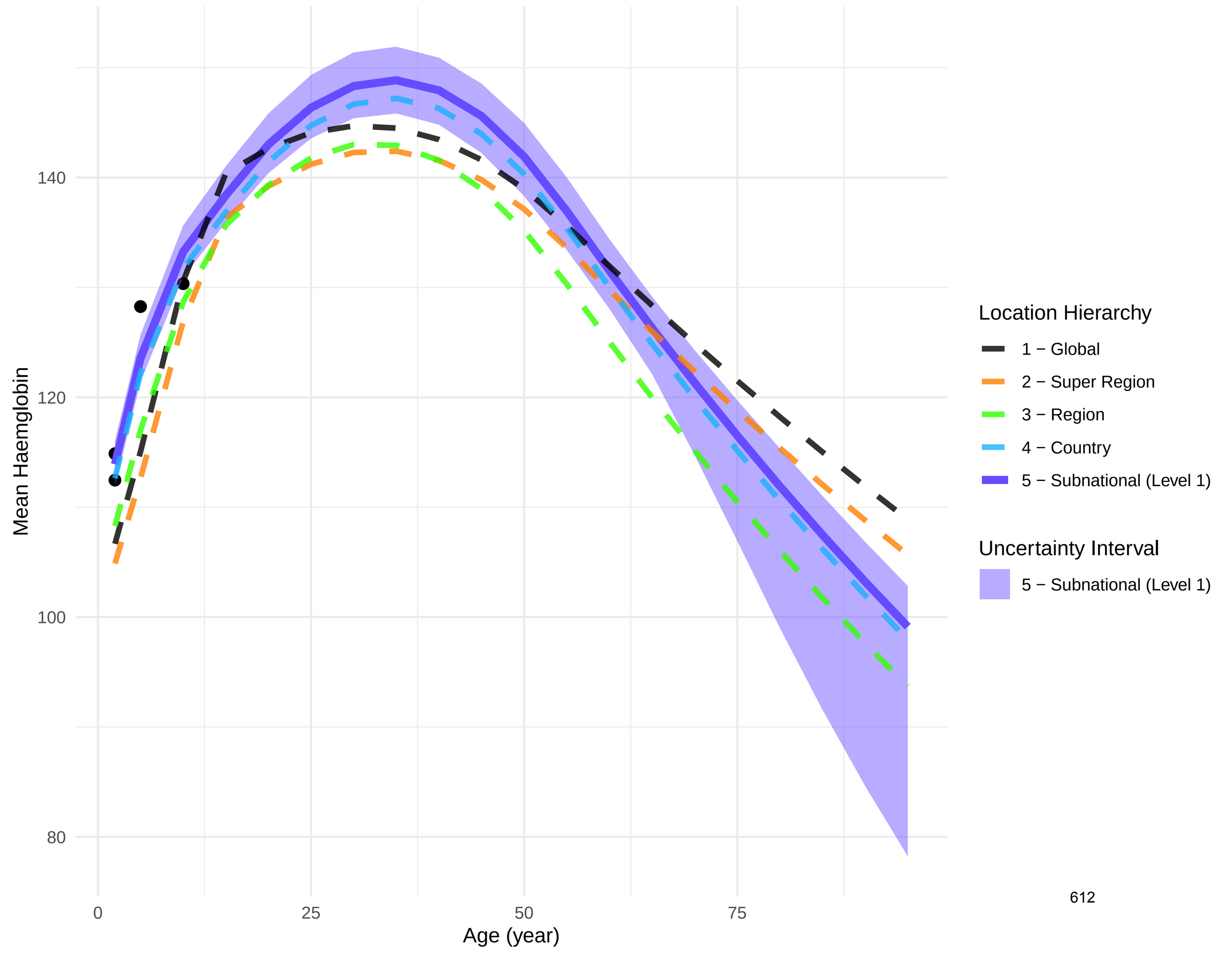

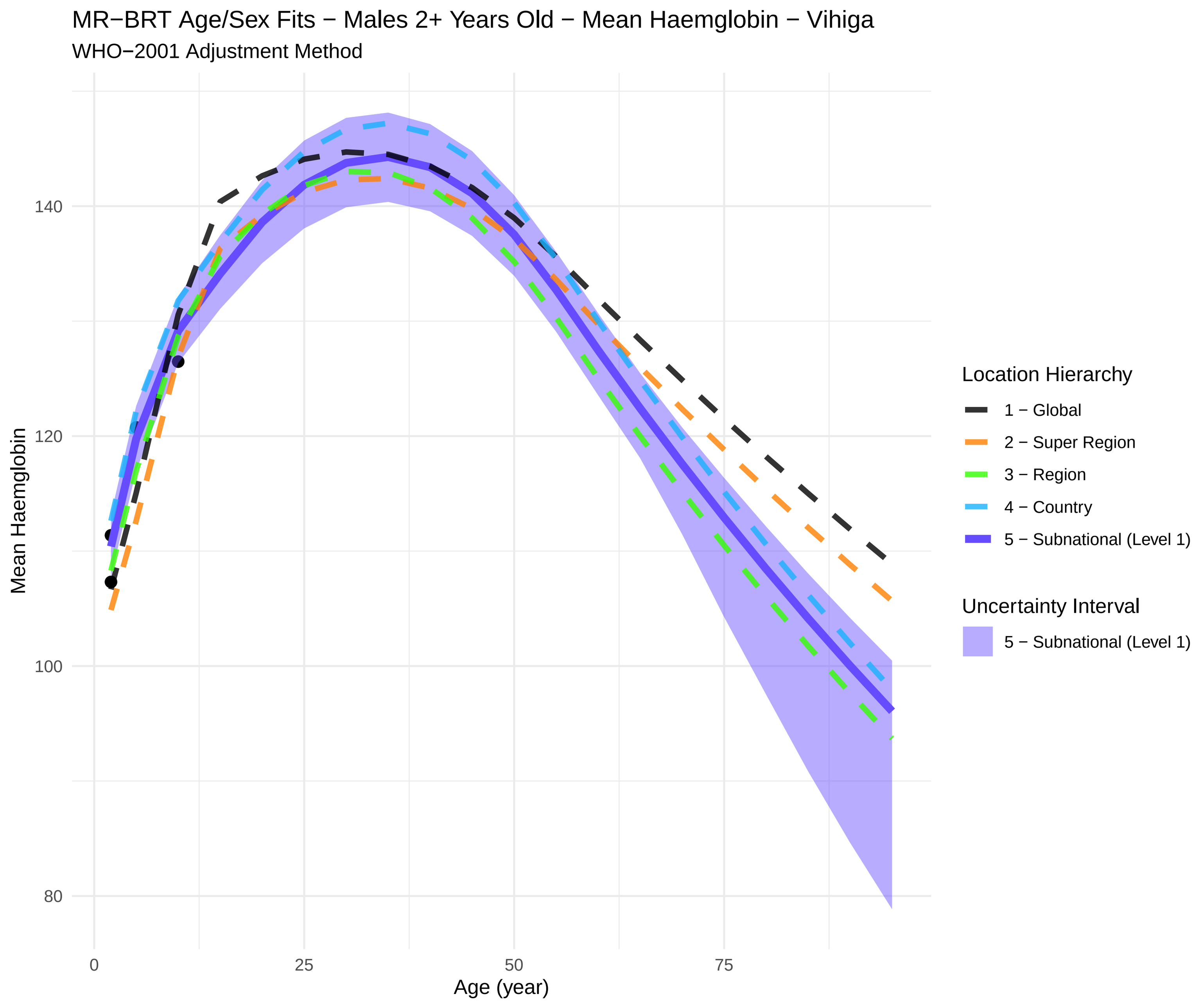
MR−BRT Age/Sex Fits − Males 2+ Years Old − Mean Haemglobin − Vihiga
WHO−2001 Adjustment Method
Mean Haemglobin
Age (year)
Location Hierarchy
1 − Global
2 − Super Region
3 − Region
4 − Country
5 − Subnational (Level 1)
Uncertainty Interval
5 − Subnational (Level 1)

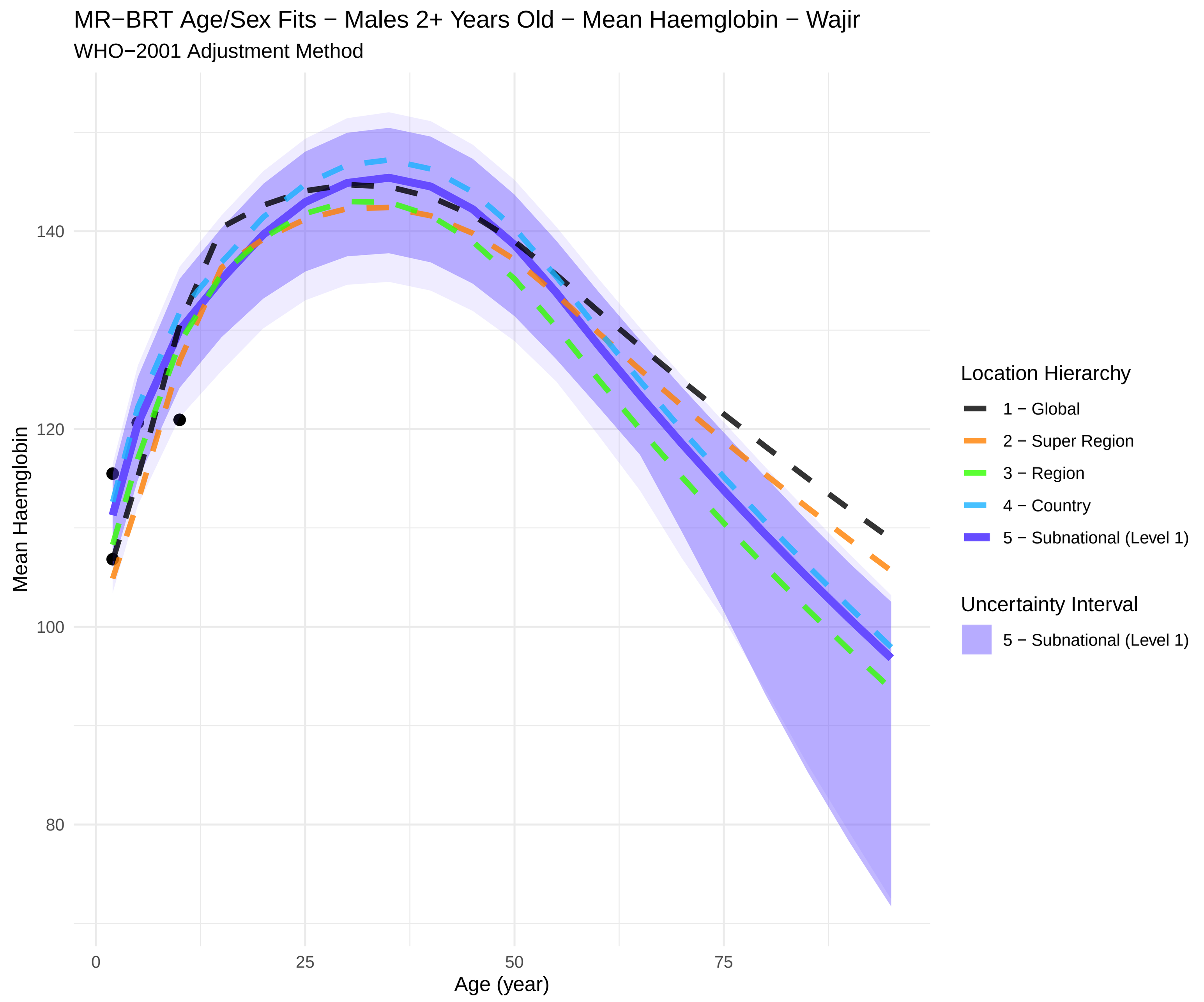
MR−BRT Age/Sex Fits − Males 2+ Years Old − Mean Haemglobin − Wajir
WHO−2001 Adjustment Method
Mean Haemglobin
Age (year)
Location Hierarchy
1 − Global
2 − Super Region
3 − Region
4 − Country
5 − Subnational (Level 1)
Uncertainty Interval
5 − Subnational (Level 1)

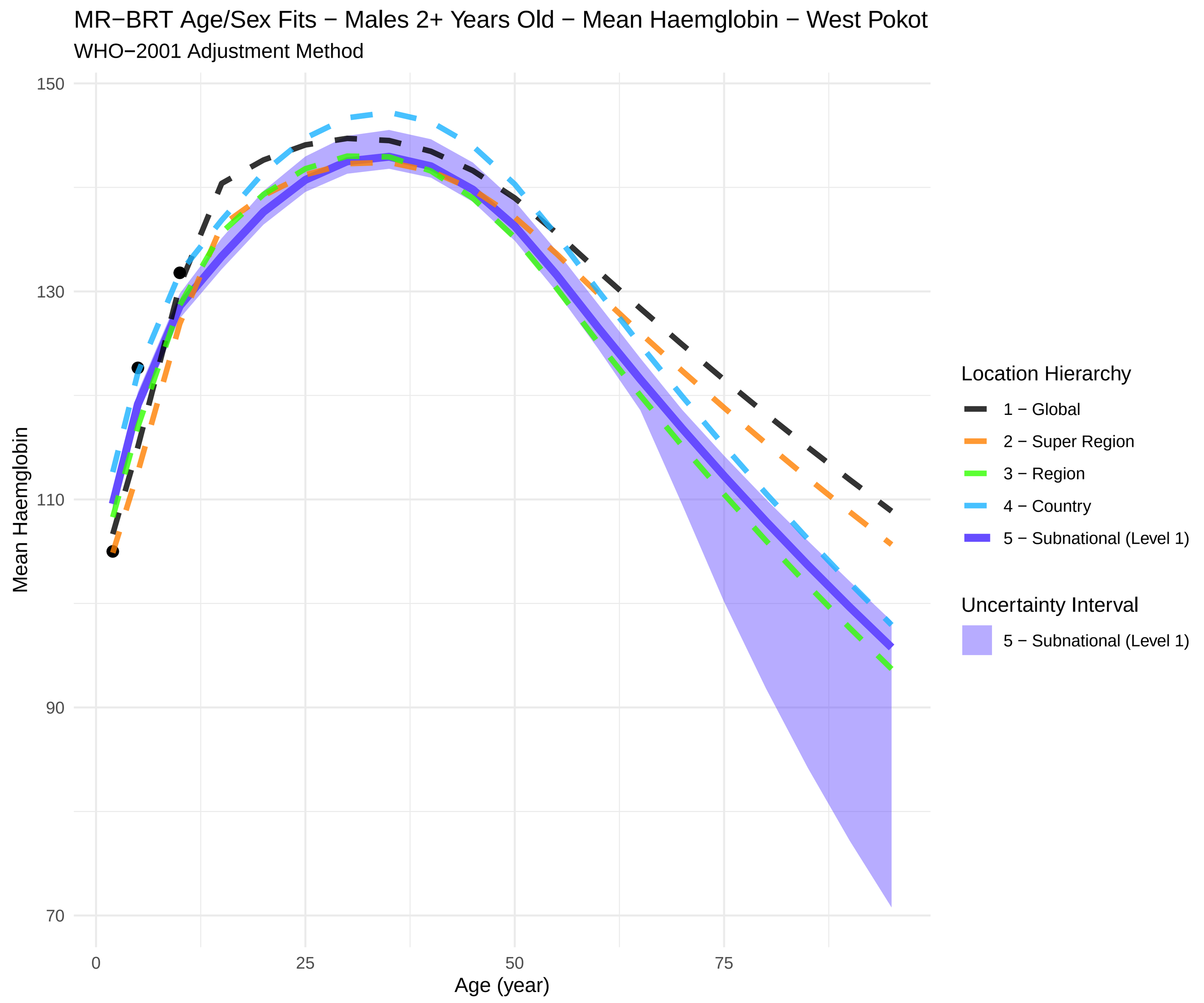
MR−BRT Age/Sex Fits − Males 2+ Years Old − Mean Haemglobin − West Pokot
WHO−2001 Adjustment Method
Mean Haemglobin
Age (year)
Location Hierarchy
1 − Global
2 − Super Region
3 − Region
4 − Country
5 − Subnational (Level 1)
Uncertainty Interval
5 − Subnational (Level 1)

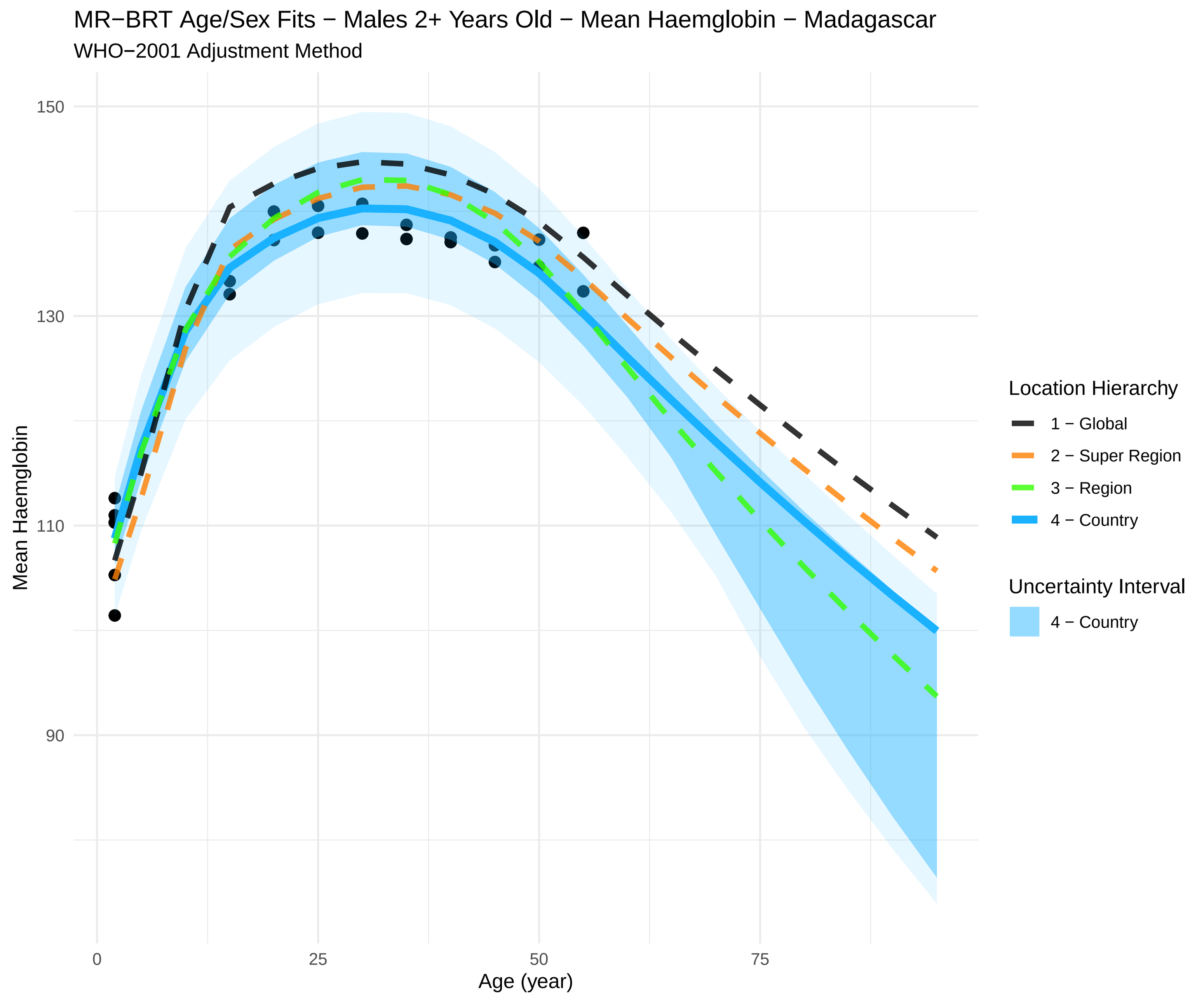
MR−BRT Age/Sex Fits − Males 2+ Years Old − Mean Haemglobin − Madagascar
WHO−2001 Adjustment Method
Mean Haemglobin
Age (year)
Location Hierarchy
1 − Global
2 − Super Region
3 − Region
4 − Country
Uncertainty Interval
4 − Country

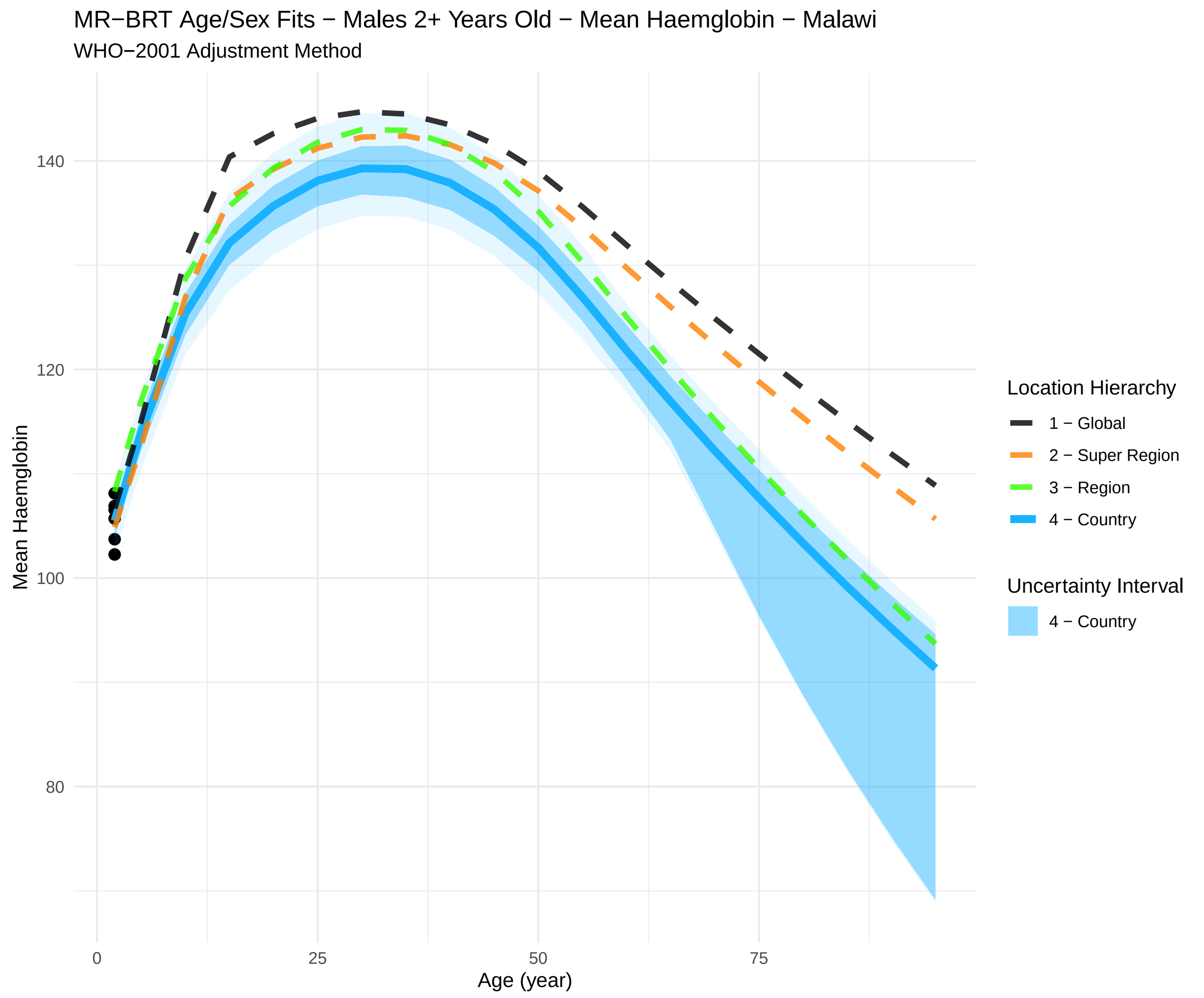
MR−BRT Age/Sex Fits − Males 2+ Years Old − Mean Haemglobin − Malawi
WHO−2001 Adjustment Method
Mean Haemglobin
Age (year)
Location Hierarchy
1 − Global
2 − Super Region
3 − Region
4 − Country
Uncertainty Interval
4 − Country

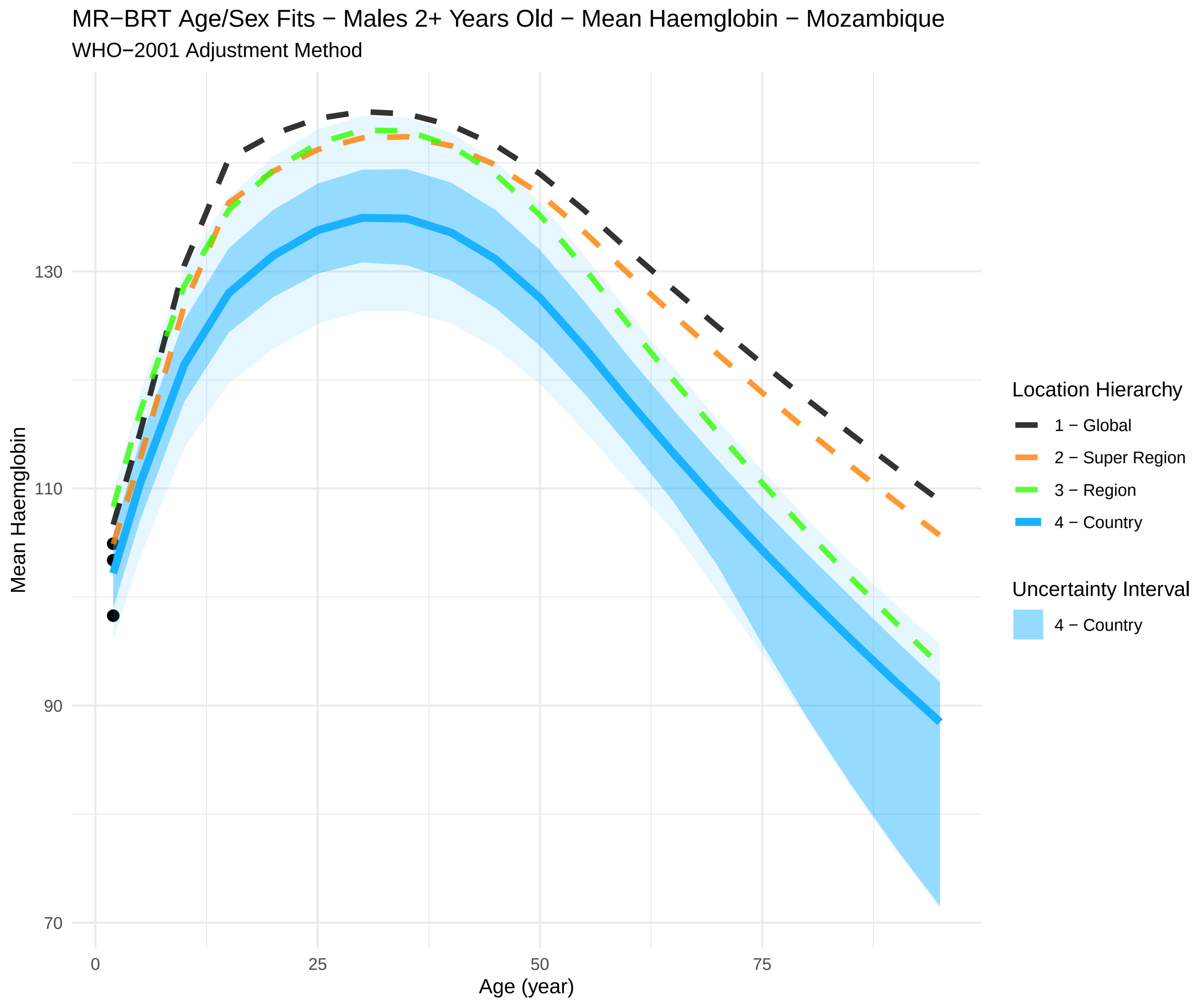
MR−BRT Age/Sex Fits − Males 2+ Years Old − Mean Haemglobin − Mozambique
WHO−2001 Adjustment Method
Mean Haemglobin
Age (year)
Location Hierarchy
1 − Global
2 − Super Region
3 − Region
4 − Country
Uncertainty Interval
4 − Country

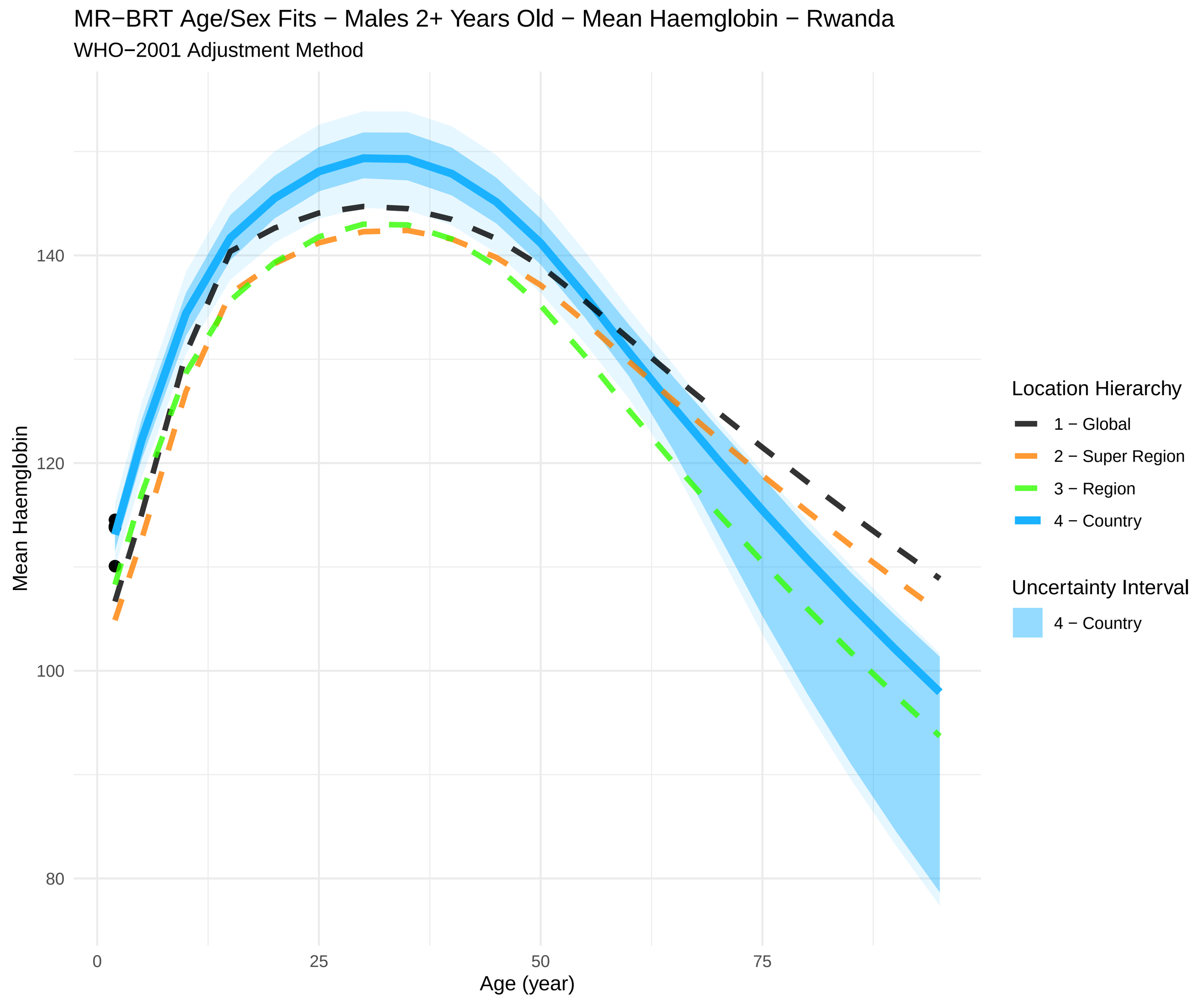
MR−BRT Age/Sex Fits − Males 2+ Years Old − Mean Haemglobin − Rwanda
WHO−2001 Adjustment Method
Mean Haemglobin
Age (year)
Location Hierarchy
1 − Global
2 − Super Region
3 − Region
4 − Country
Uncertainty Interval
4 − Country

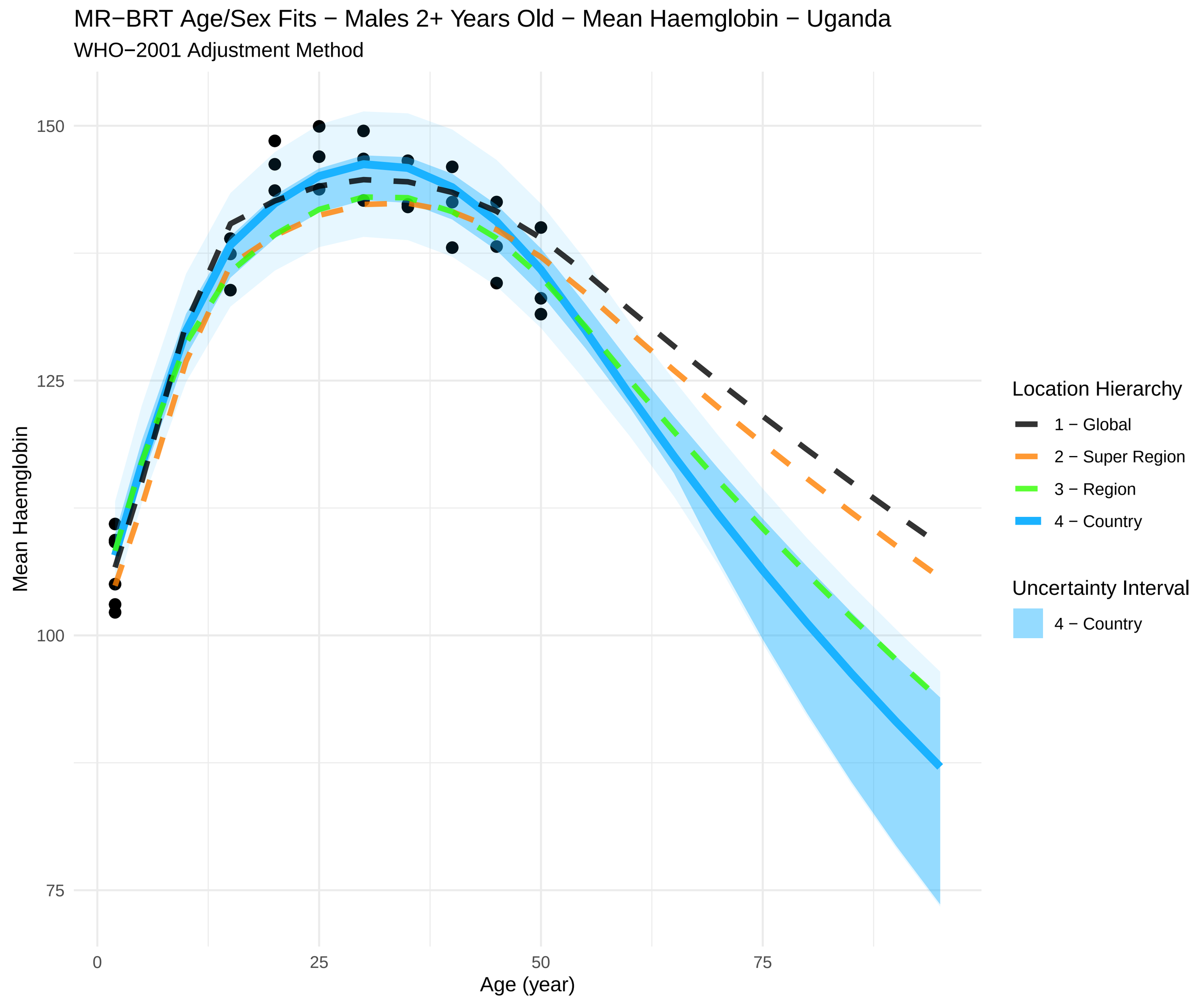
MR−BRT Age/Sex Fits − Males 2+ Years Old − Mean Haemglobin − Uganda
WHO−2001 Adjustment Method
Mean Haemglobin
Age (year)
Location Hierarchy
1 − Global
2 − Super Region
3 − Region
4 − Country
Uncertainty Interval
4 − Country

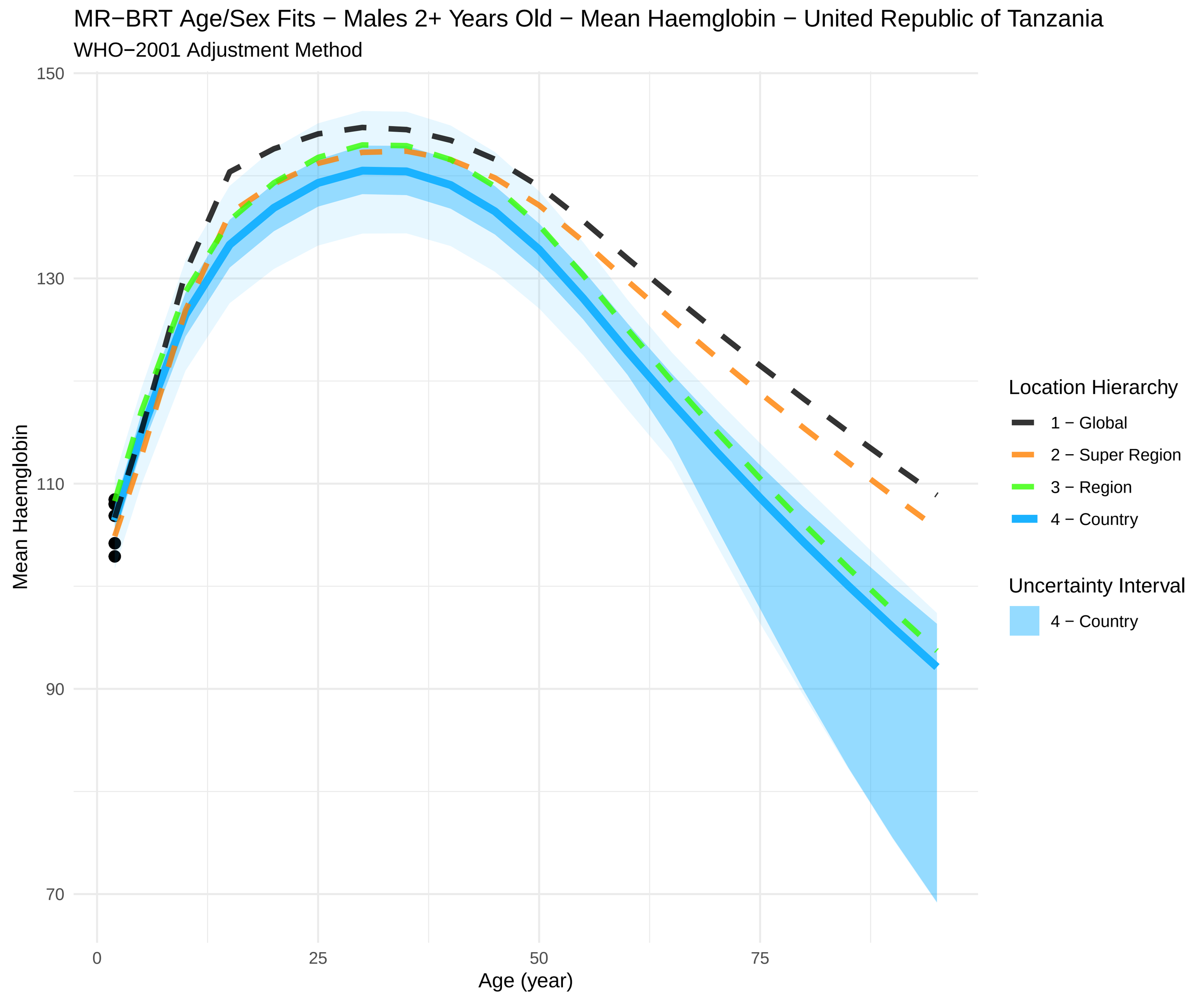
MR−BRT Age/Sex Fits − Males 2+ Years Old − Mean Haemglobin − United Republic of Tanzania
WHO−2001 Adjustment Method
Mean Haemglobin
Age (year)
Location Hierarchy
1 − Global
2 − Super Region
3 − Region
4 − Country
Uncertainty Interval
4 − Country

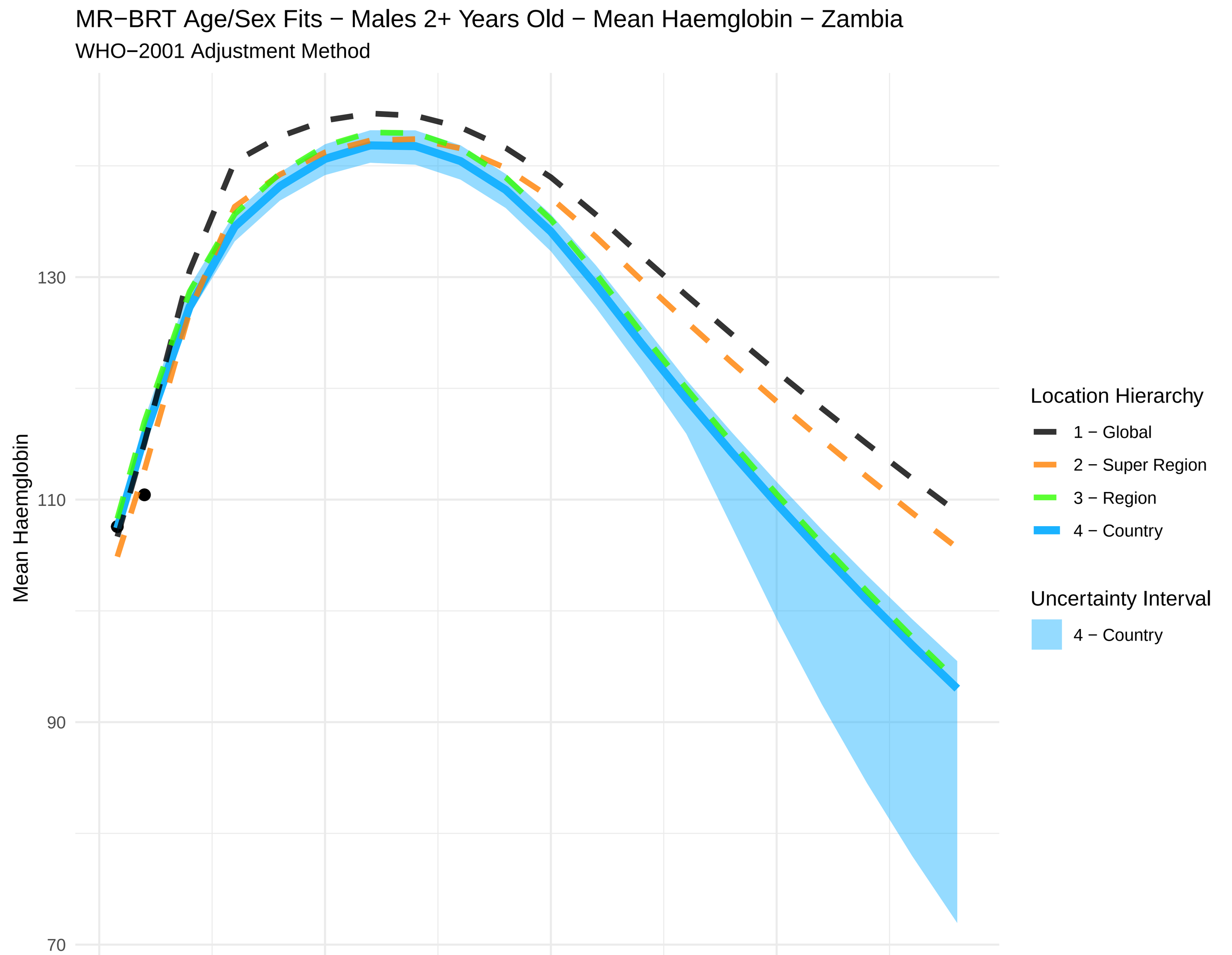
MR−BRT Age/Sex Fits − Males 2+ Years Old − Mean Haemglobin − Zambia
WHO−2001 Adjustment Method
Mean Haemglobin
Age (year)
Location Hierarchy
1 − Global
2 − Super Region
3 − Region
4 − Country
Uncertainty Interval
4 − Country

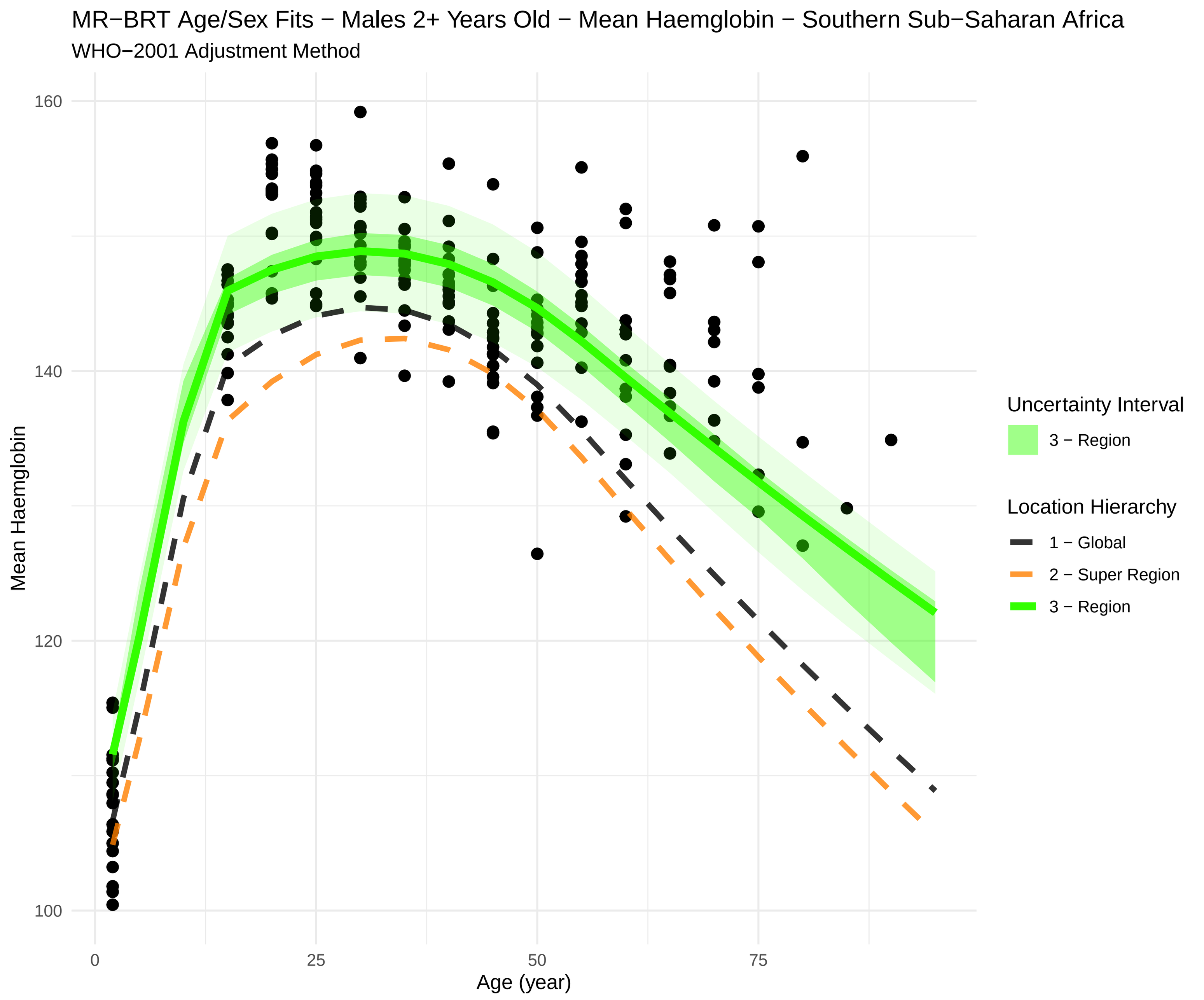
MR−BRT Age/Sex Fits − Males 2+ Years Old − Mean Haemglobin − Southern Sub−Saharan Africa
WHO−2001 Adjustment Method
Mean Haemglobin
Age (year)
Uncertainty Interval
3 − Region
Location Hierarchy
1 − Global
2 − Super Region
3 − Region

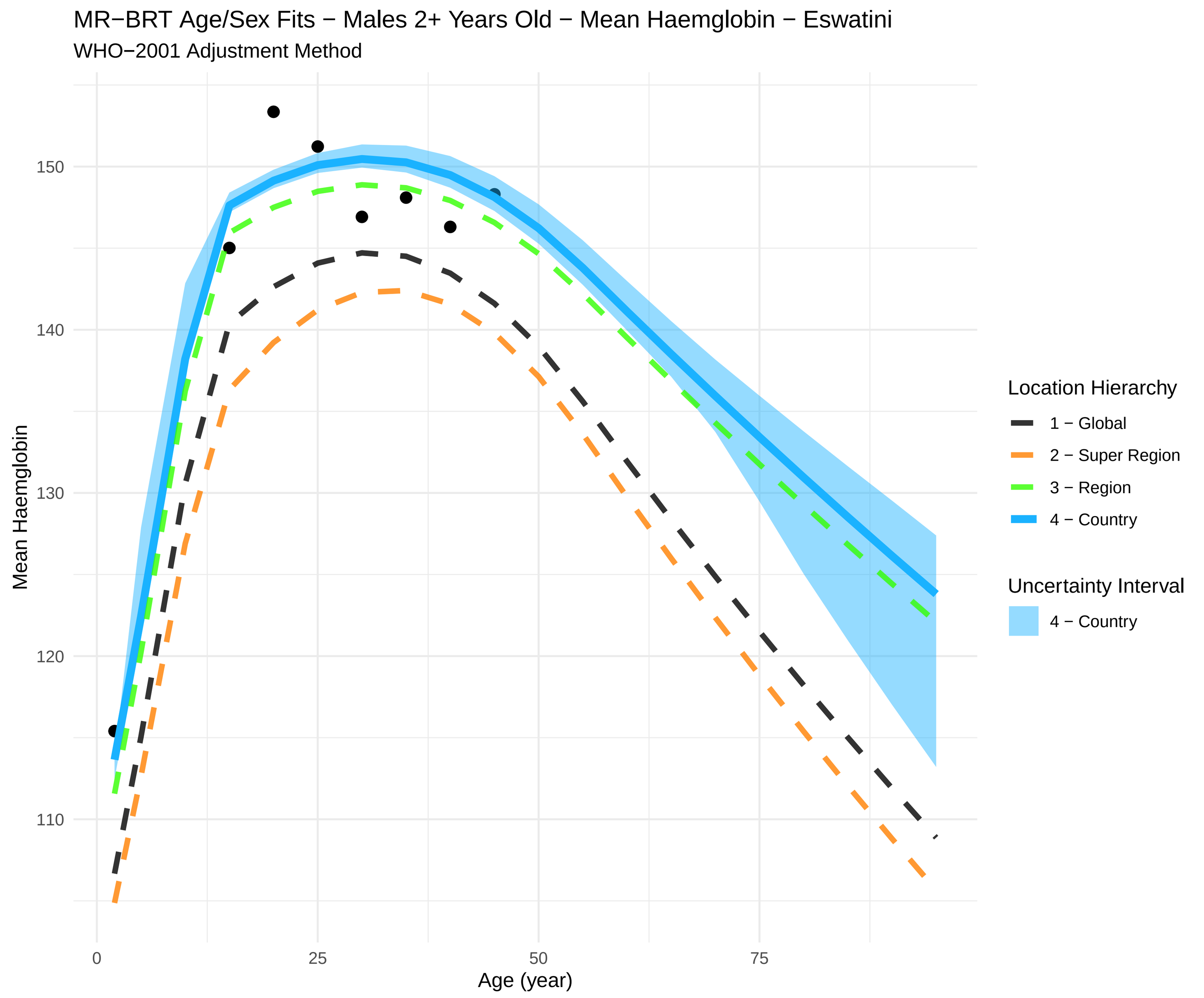
MR−BRT Age/Sex Fits − Males 2+ Years Old − Mean Haemglobin − Eswatini
WHO−2001 Adjustment Method
Mean Haemglobin
Age (year)
Location Hierarchy
1 − Global
2 − Super Region
3 − Region
4 − Country
Uncertainty Interval
4 − Country

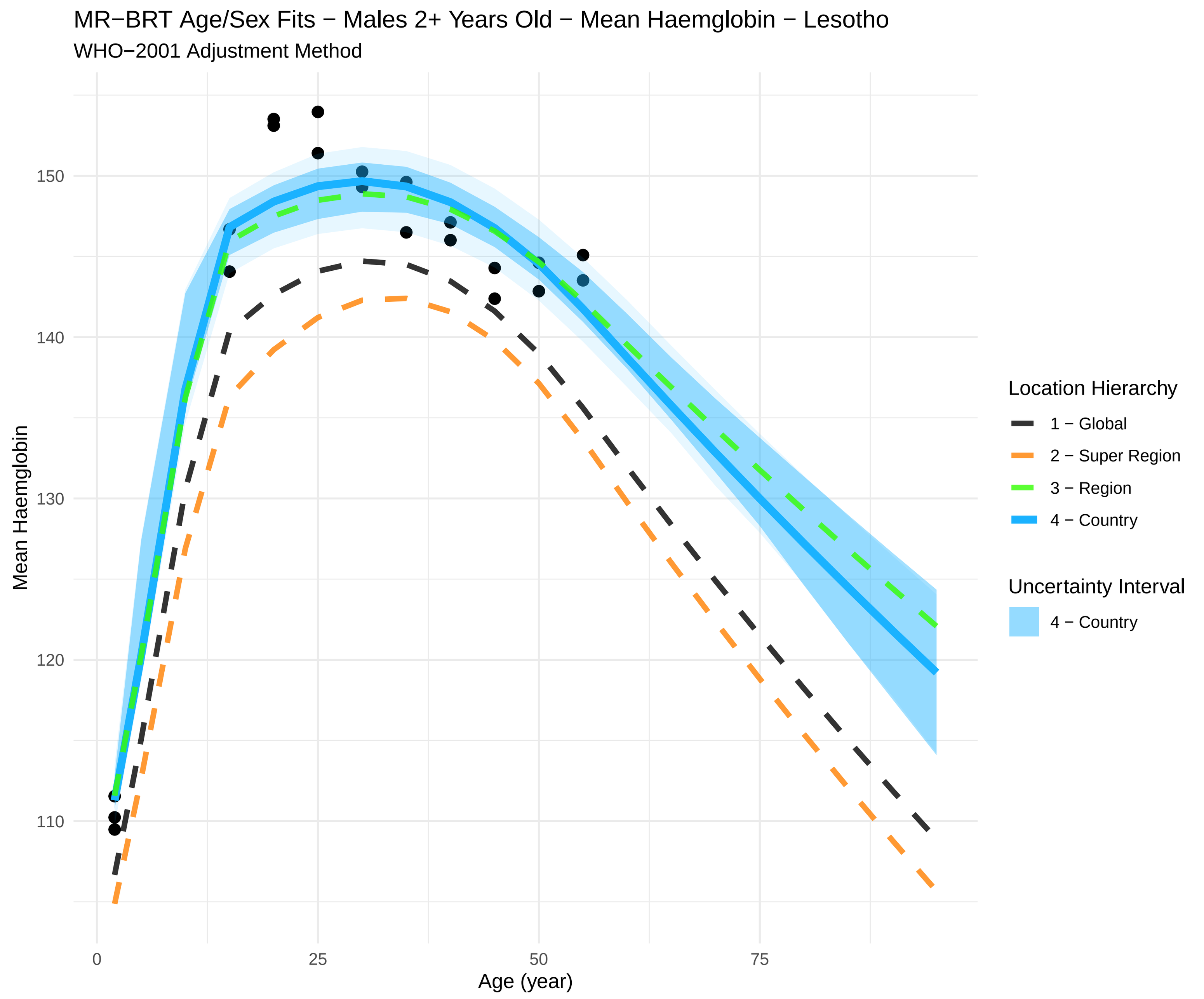
MR−BRT Age/Sex Fits − Males 2+ Years Old − Mean Haemglobin − Lesotho
WHO−2001 Adjustment Method
Mean Haemglobin
Age (year)
Location Hierarchy
1 − Global
2 − Super Region
3 − Region
4 − Country
Uncertainty Interval
4 − Country

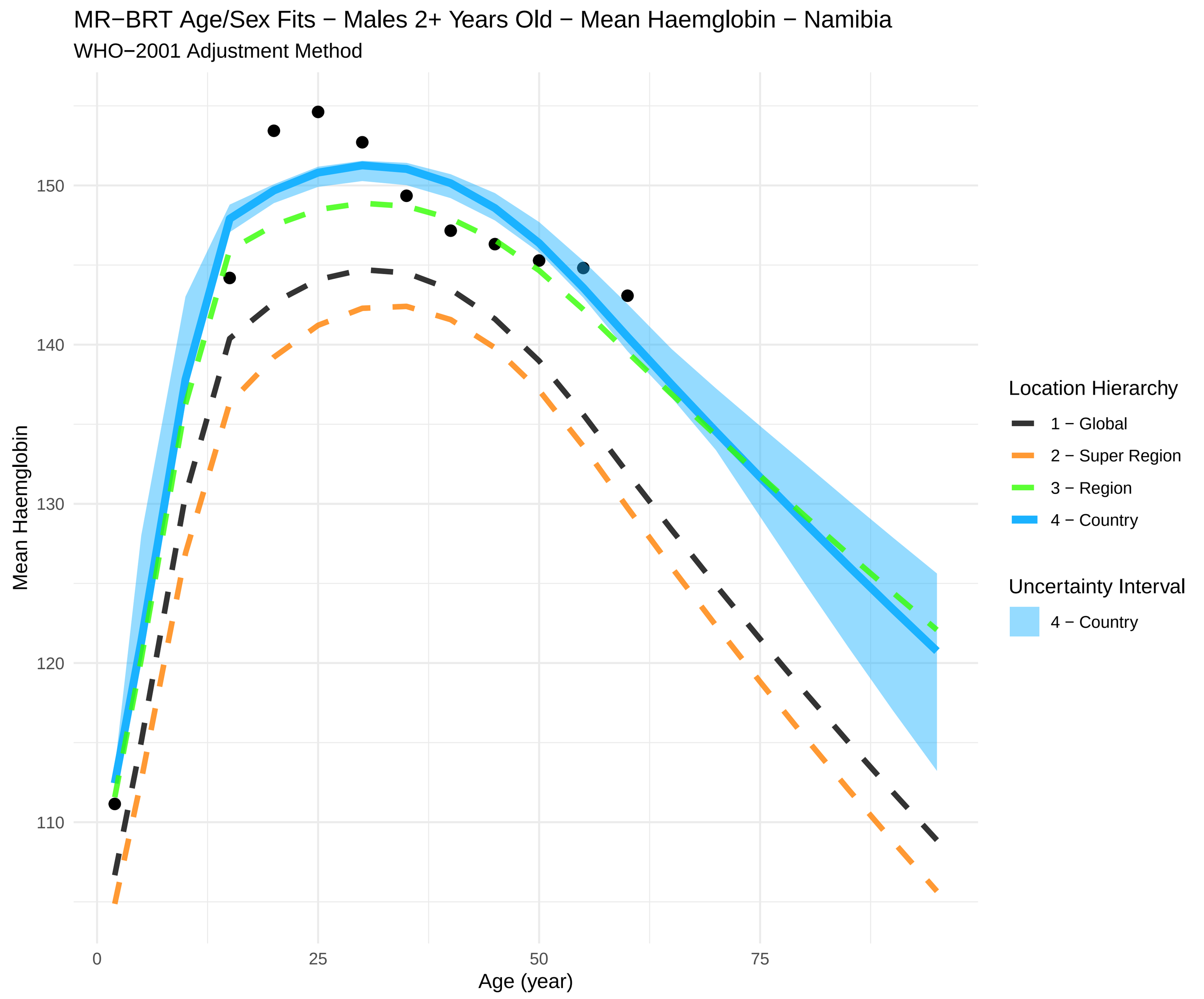
MR−BRT Age/Sex Fits − Males 2+ Years Old − Mean Haemglobin − Namibia
WHO−2001 Adjustment Method
Mean Haemglobin
Age (year)
Location Hierarchy
1 − Global
2 − Super Region
3 − Region
4 − Country
Uncertainty Interval
4 − Country

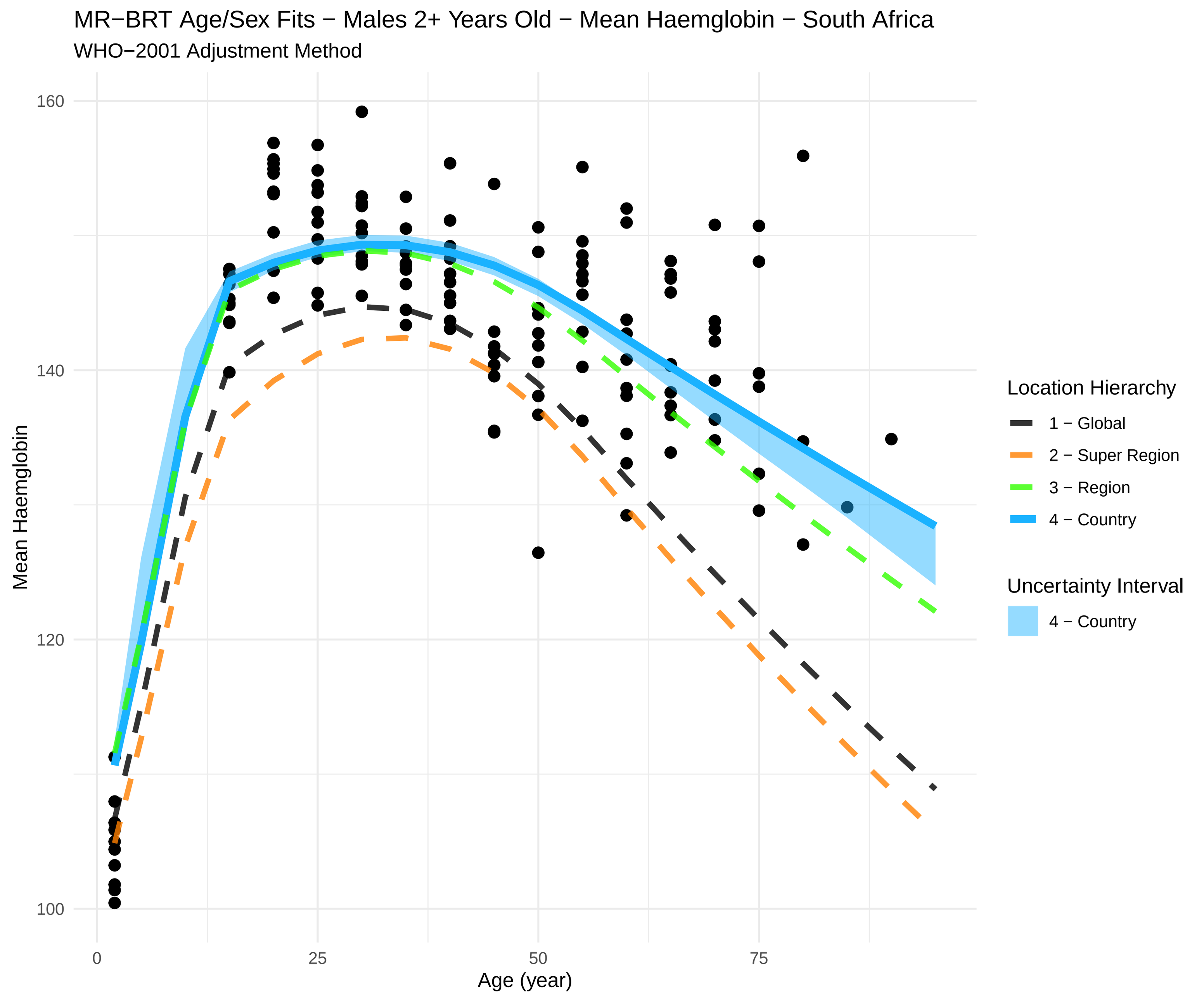
MR−BRT Age/Sex Fits − Males 2+ Years Old − Mean Haemglobin − South Africa
WHO−2001 Adjustment Method
Mean Haemglobin
Age (year)
Location Hierarchy
1 − Global
2 − Super Region
3 − Region
4 − Country
Uncertainty Interval
4 − Country

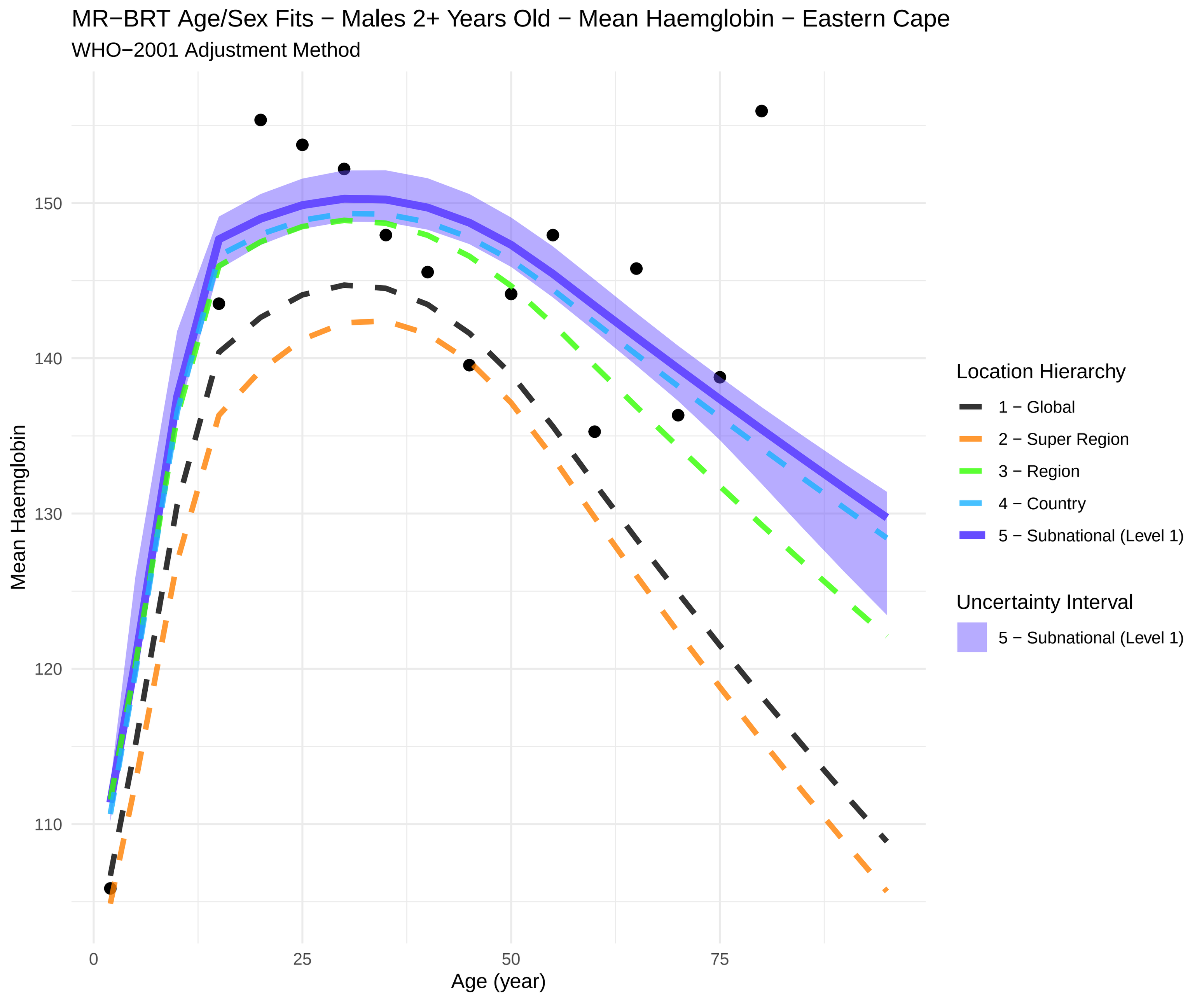
MR−BRT Age/Sex Fits − Males 2+ Years Old − Mean Haemglobin − Eastern Cape
WHO−2001 Adjustment Method
Mean Haemglobin
Age (year)
Location Hierarchy
1 − Global
2 − Super Region
3 − Region
4 − Country
5 − Subnational (Level 1)
Uncertainty Interval
5 − Subnational (Level 1)

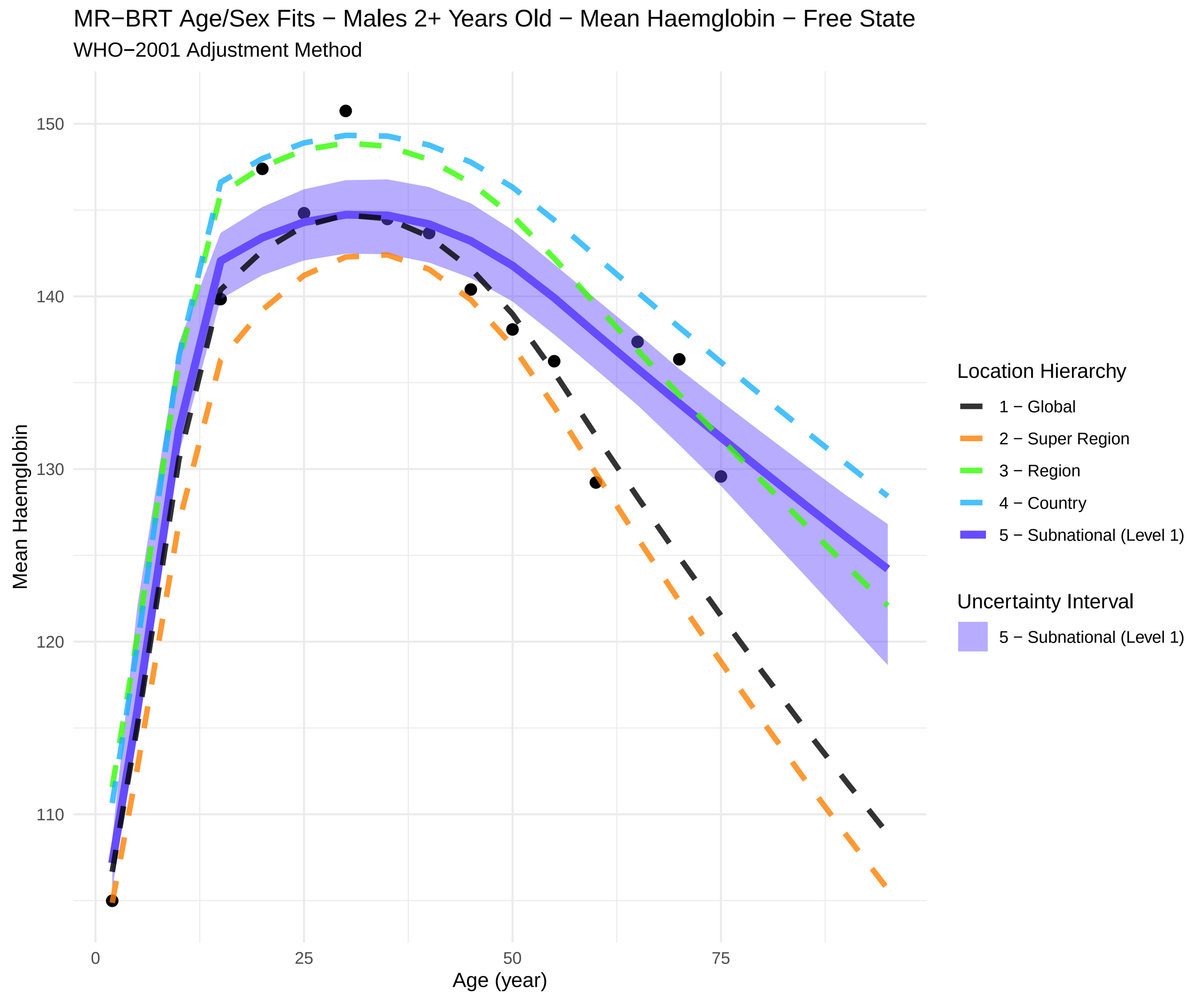
MR−BRT Age/Sex Fits − Males 2+ Years Old − Mean Haemglobin − Free State
WHO−2001 Adjustment Method
Mean Haemglobin
Age (year)
Location Hierarchy
1 − Global
2 − Super Region
3 − Region
4 − Country
5 − Subnational (Level 1)
Uncertainty Interval
5 − Subnational (Level 1)

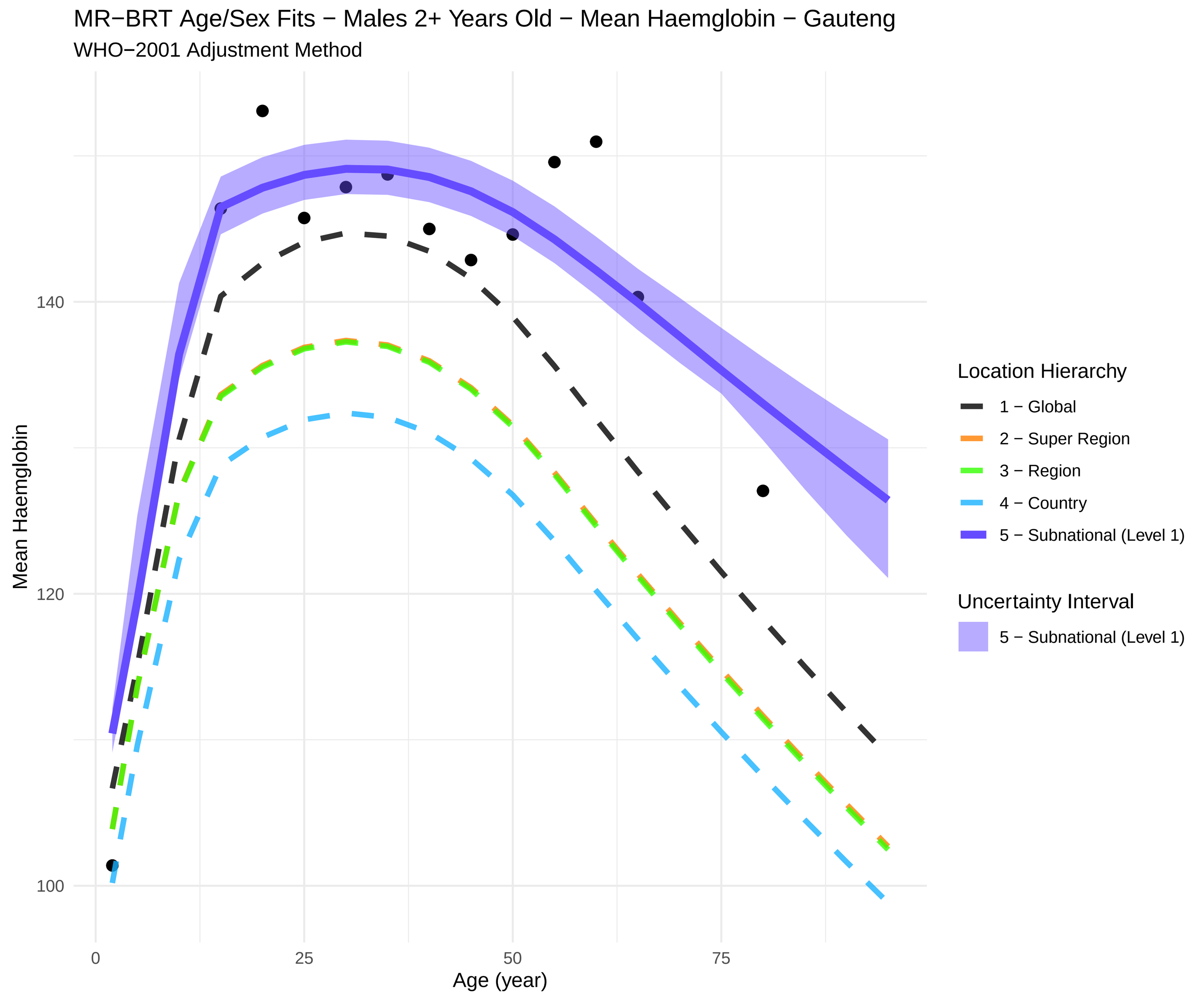
MR−BRT Age/Sex Fits − Males 2+ Years Old − Mean Haemglobin − Gauteng
WHO−2001 Adjustment Method
Mean Haemglobin
Age (year)
Location Hierarchy
1 − Global
2 − Super Region
3 − Region
4 − Country
5 − Subnational (Level 1)
Uncertainty Interval
5 − Subnational (Level 1)

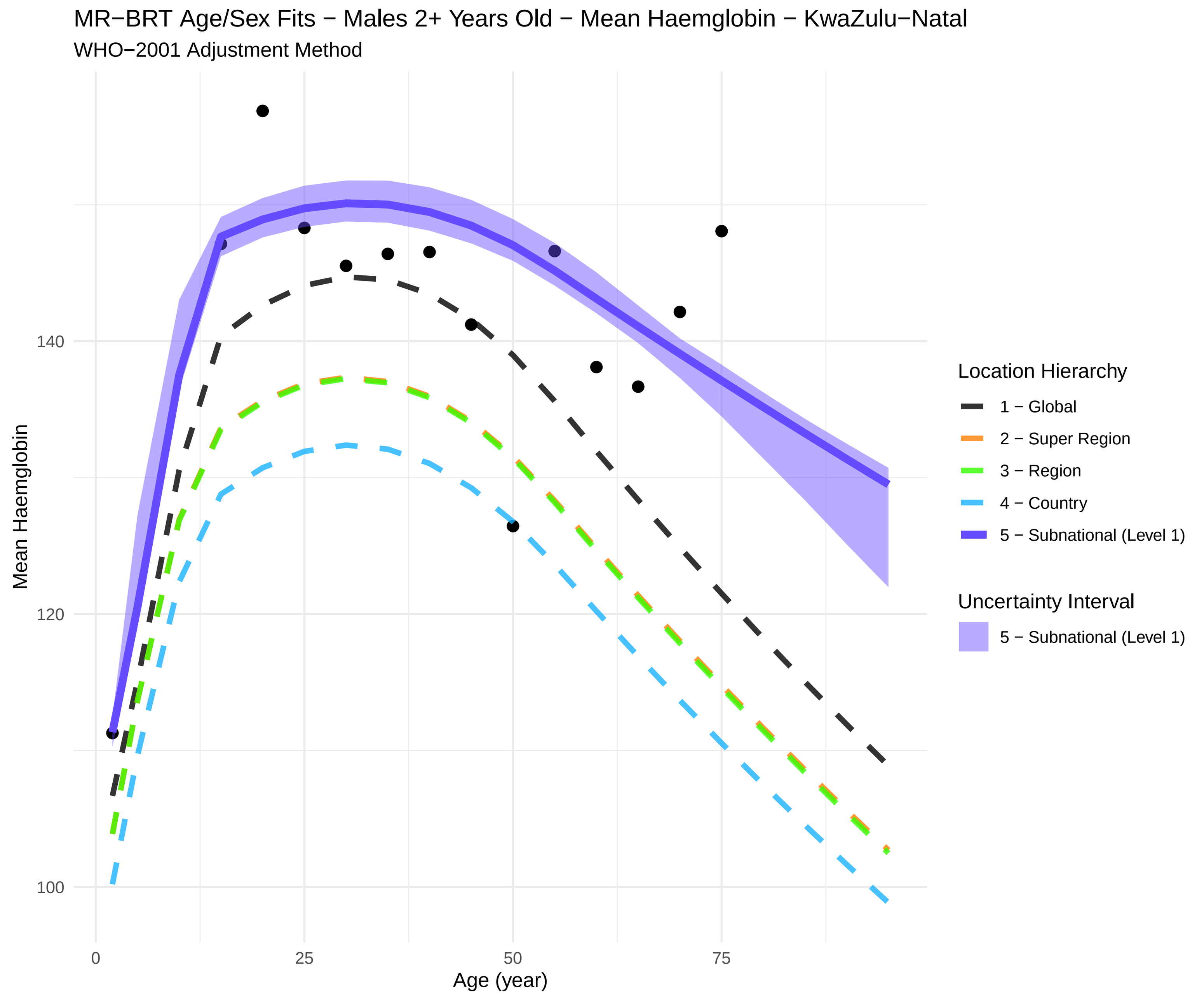
MR−BRT Age/Sex Fits − Males 2+ Years Old − Mean Haemglobin − KwaZulu−Natal
WHO−2001 Adjustment Method
Mean Haemglobin
Age (year)
Location Hierarchy
1 − Global
2 − Super Region
3 − Region
4 − Country
5 − Subnational (Level 1)
Uncertainty Interval
5 − Subnational (Level 1)

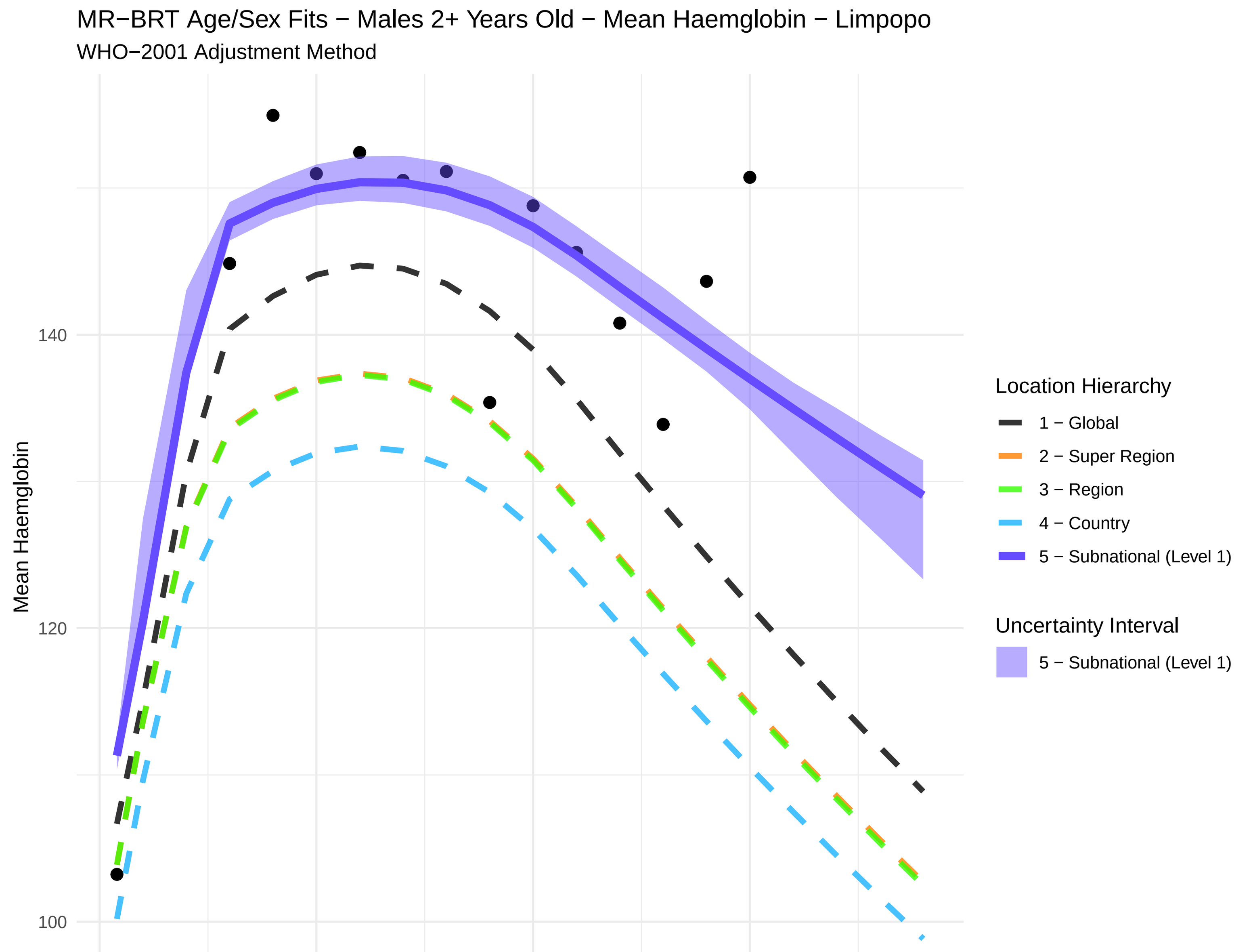
MR−BRT Age/Sex Fits − Males 2+ Years Old − Mean Haemglobin − Limpopo
WHO−2001 Adjustment Method
Mean Haemglobin
Age (year)
Location Hierarchy
1 − Global
2 − Super Region
3 − Region
4 − Country
5 − Subnational (Level 1)
Uncertainty Interval
5 − Subnational (Level 1)

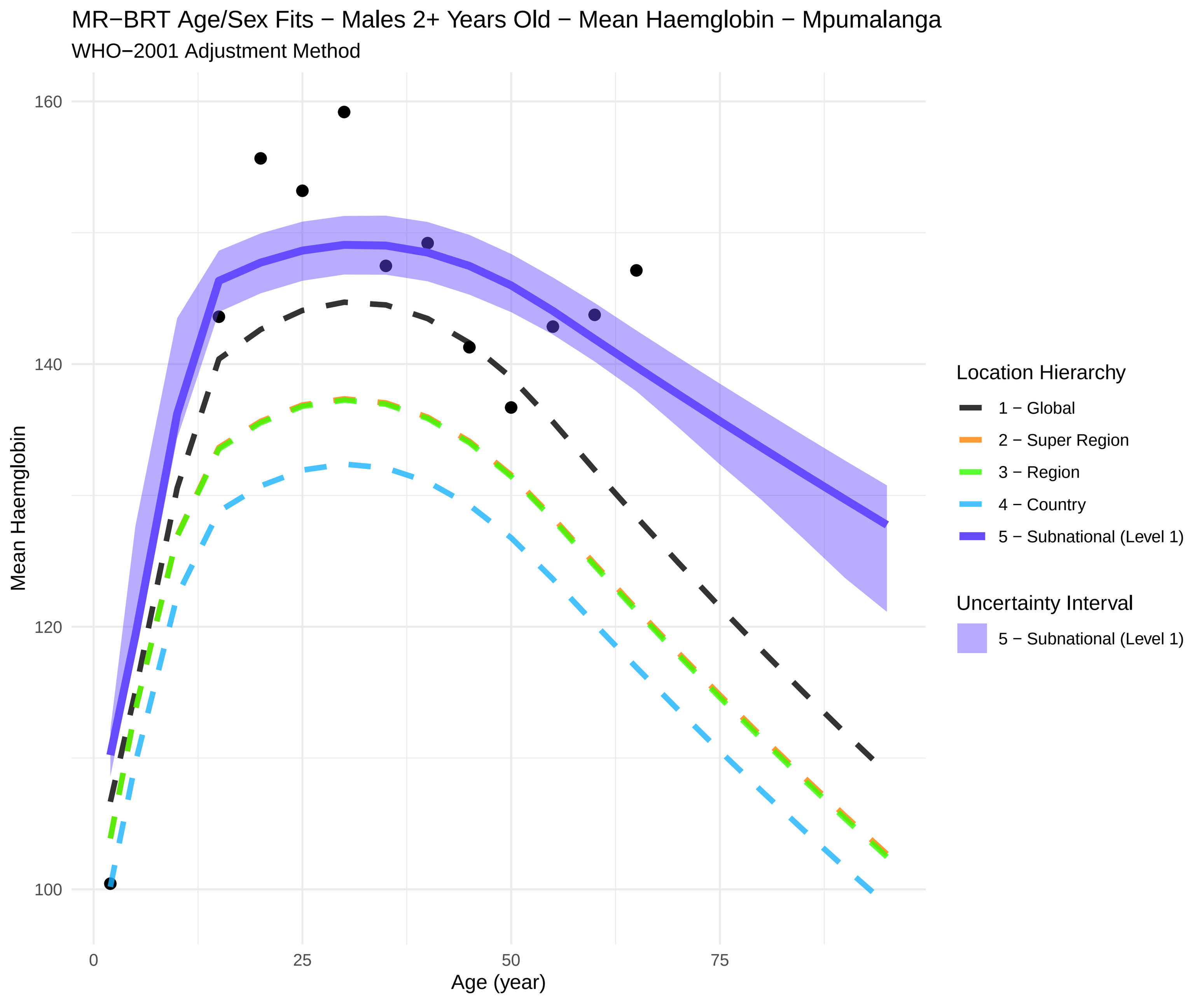
MR−BRT Age/Sex Fits − Males 2+ Years Old − Mean Haemglobin − Mpumalanga
WHO−2001 Adjustment Method
Location Hierarchy
1 − Global
2 − Super Region
3 − Region
4 − Country
5 − Subnational (Level 1)
Uncertainty Interval
5 − Subnational (Level 1)
Mean Haemglobin
Age (year)

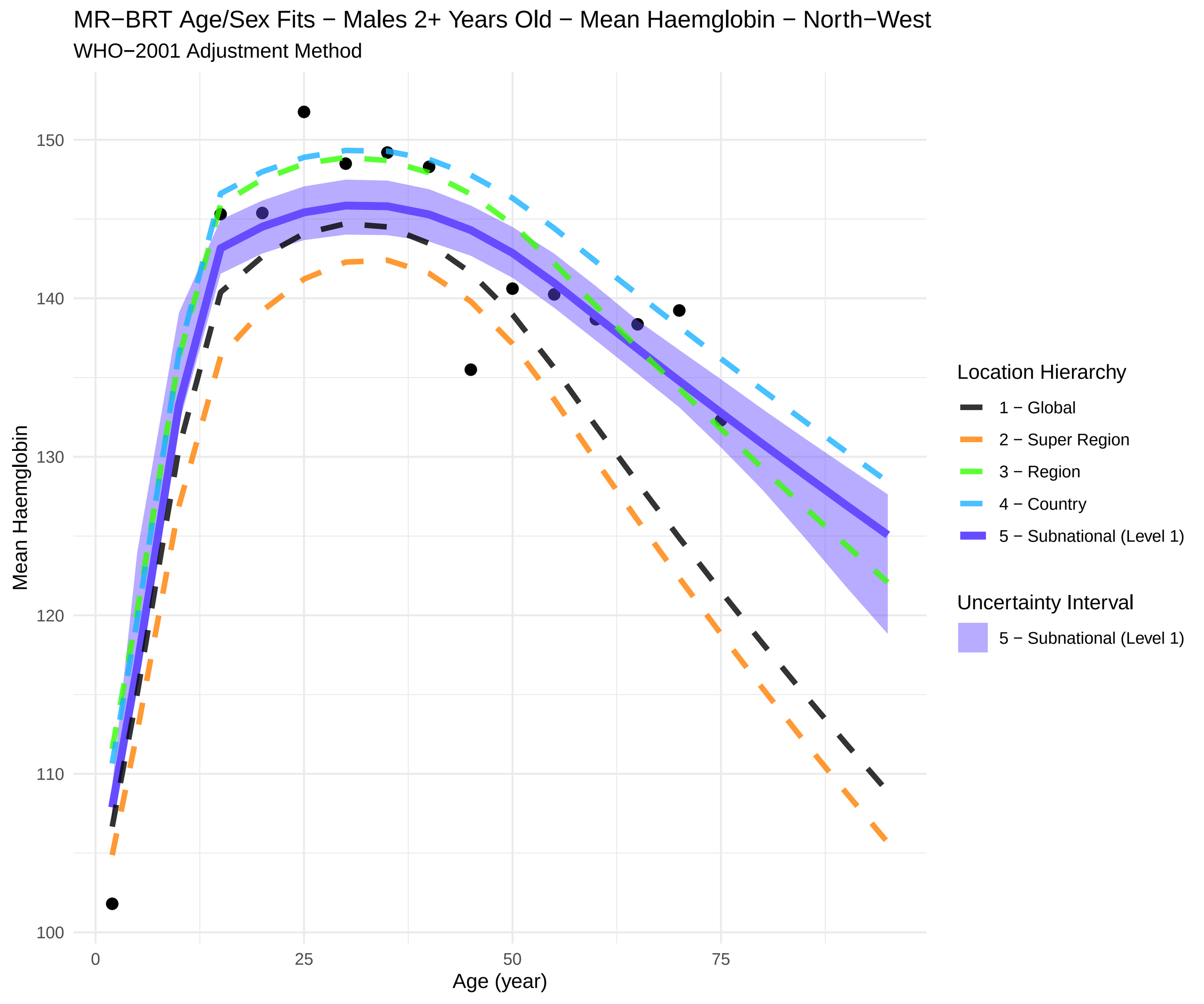
MR−BRT Age/Sex Fits − Males 2+ Years Old − Mean Haemglobin − North−West
WHO−2001 Adjustment Method
Location Hierarchy
1 − Global
2 − Super Region
3 − Region
4 − Country
5 − Subnational (Level 1)
Uncertainty Interval
5 − Subnational (Level 1)
Mean Haemglobin
Age (year)

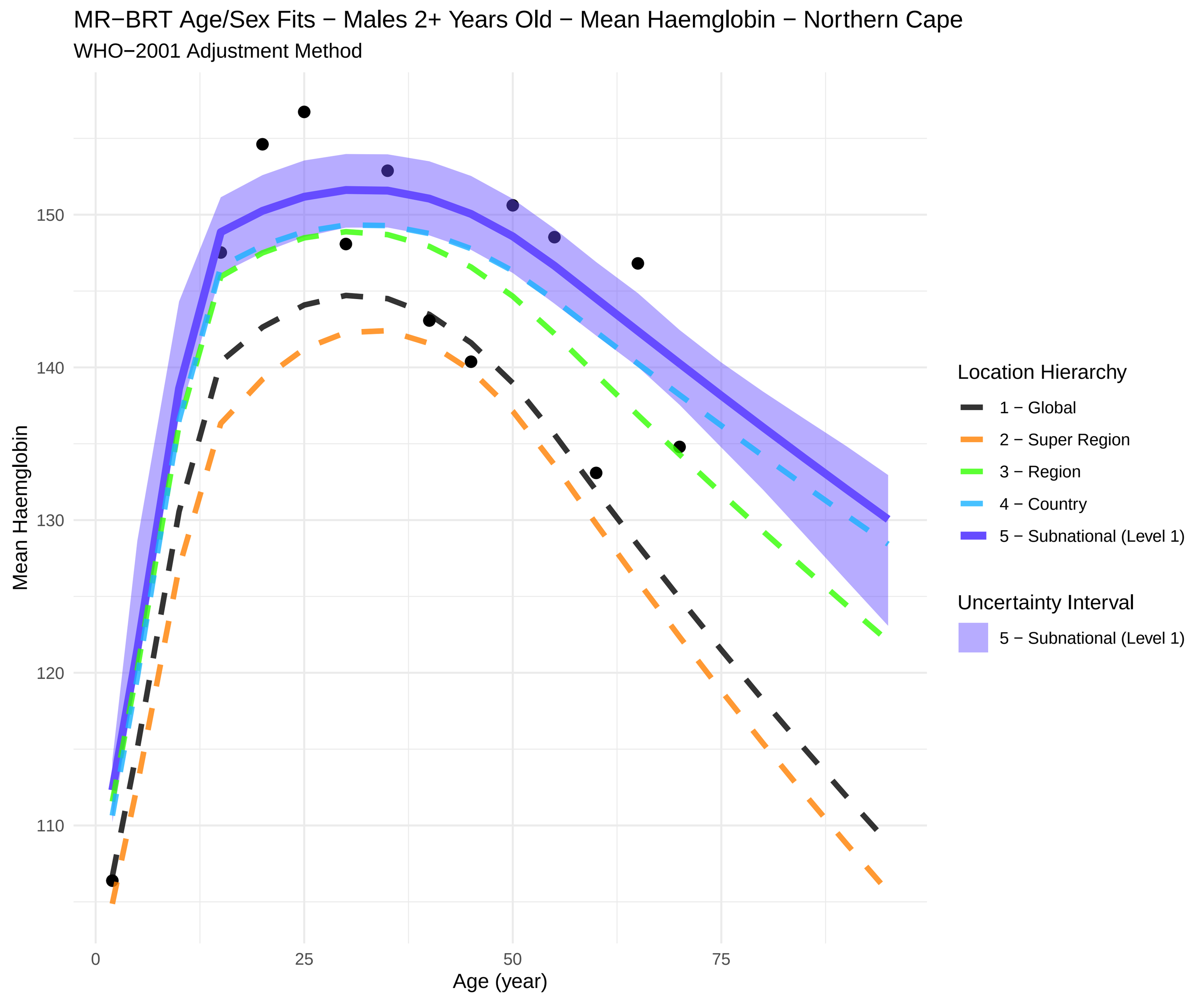
MR−BRT Age/Sex Fits − Males 2+ Years Old − Mean Haemglobin − Northern Cape
WHO−2001 Adjustment Method
Location Hierarchy
1 − Global
2 − Super Region
3 − Region
4 − Country
5 − Subnational (Level 1)
Uncertainty Interval
5 − Subnational (Level 1)
Mean Haemglobin
Age (year)

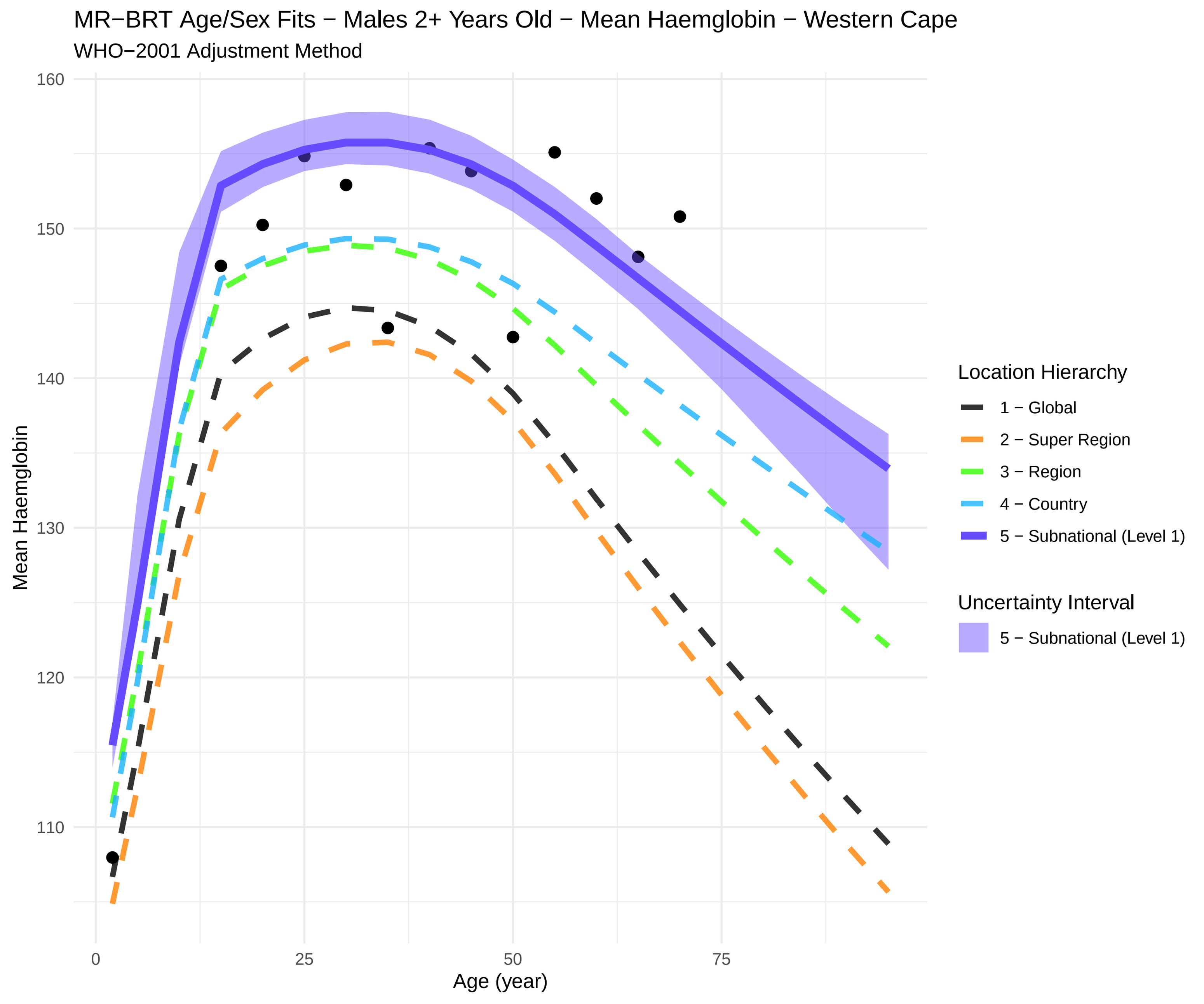
MR−BRT Age/Sex Fits − Males 2+ Years Old − Mean Haemglobin − Western Cape
WHO−2001 Adjustment Method
Location Hierarchy
1 − Global
2 − Super Region
3 − Region
4 − Country
5 − Subnational (Level 1)
Uncertainty Interval
5 − Subnational (Level 1)
Mean Haemglobin
Age (year)

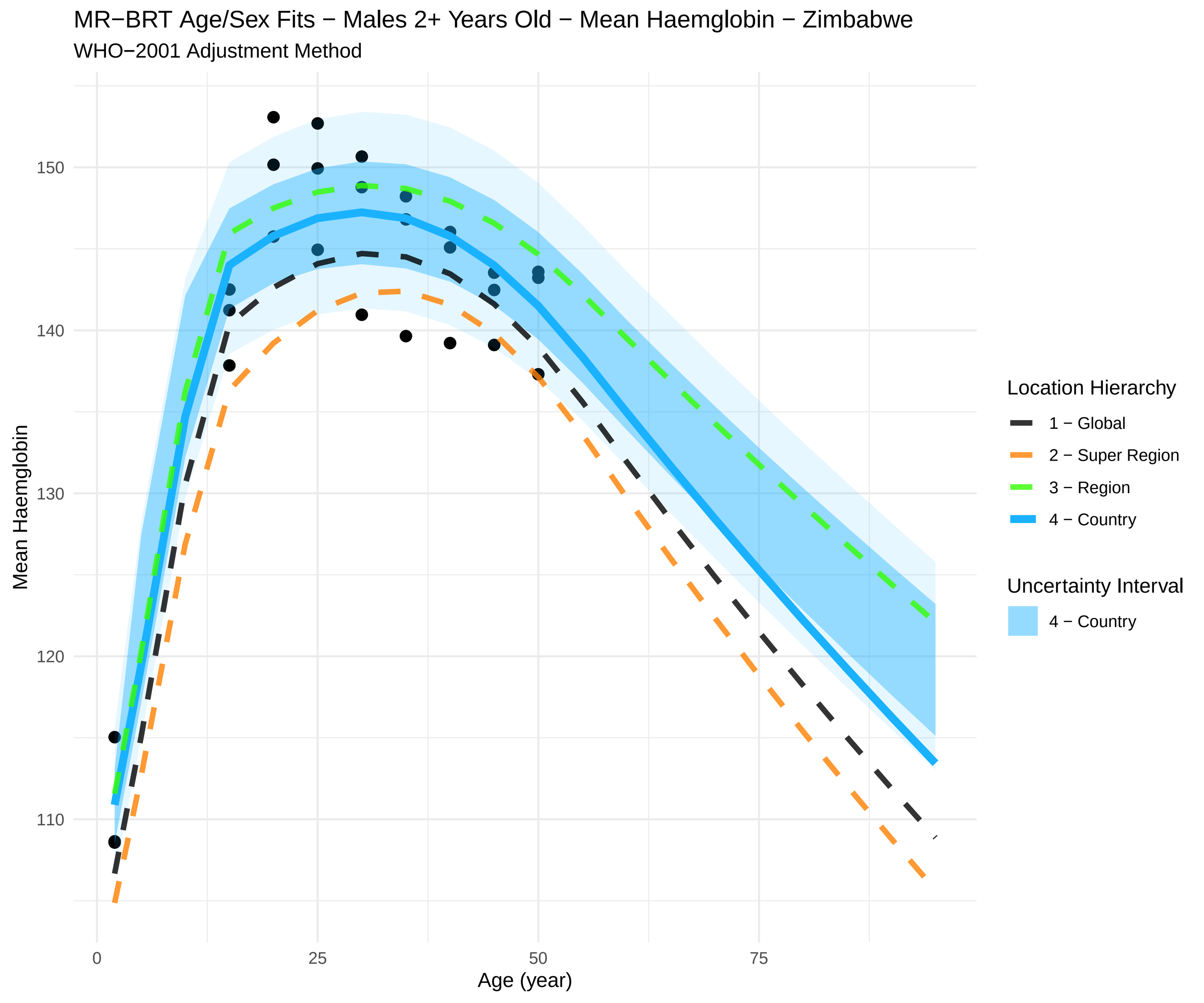
MR−BRT Age/Sex Fits − Males 2+ Years Old − Mean Haemglobin − Zimbabwe
WHO−2001 Adjustment Method
Location Hierarchy
1 − Global
2 − Super Region
3 − Region
4 − Country
Uncertainty Interval
4 − Country
Mean Haemglobin
Age (year)

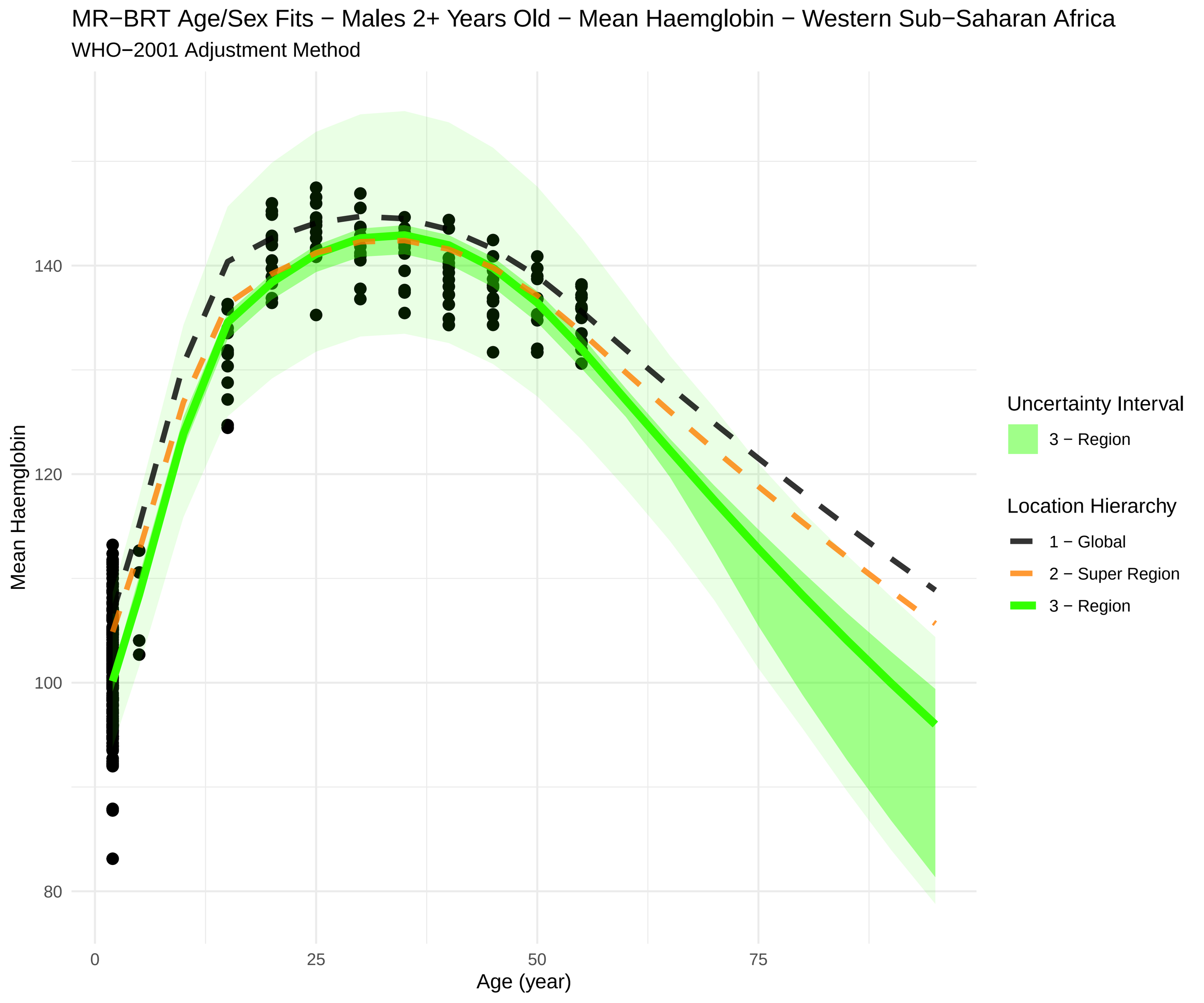
MR−BRT Age/Sex Fits − Males 2+ Years Old − Mean Haemglobin − Western Sub−Saharan Africa
WHO−2001 Adjustment Method
Uncertainty Interval
3 − Region
Location Hierarchy
1 − Global
2 − Super Region
3 − Region
Mean Haemglobin
Age (year)

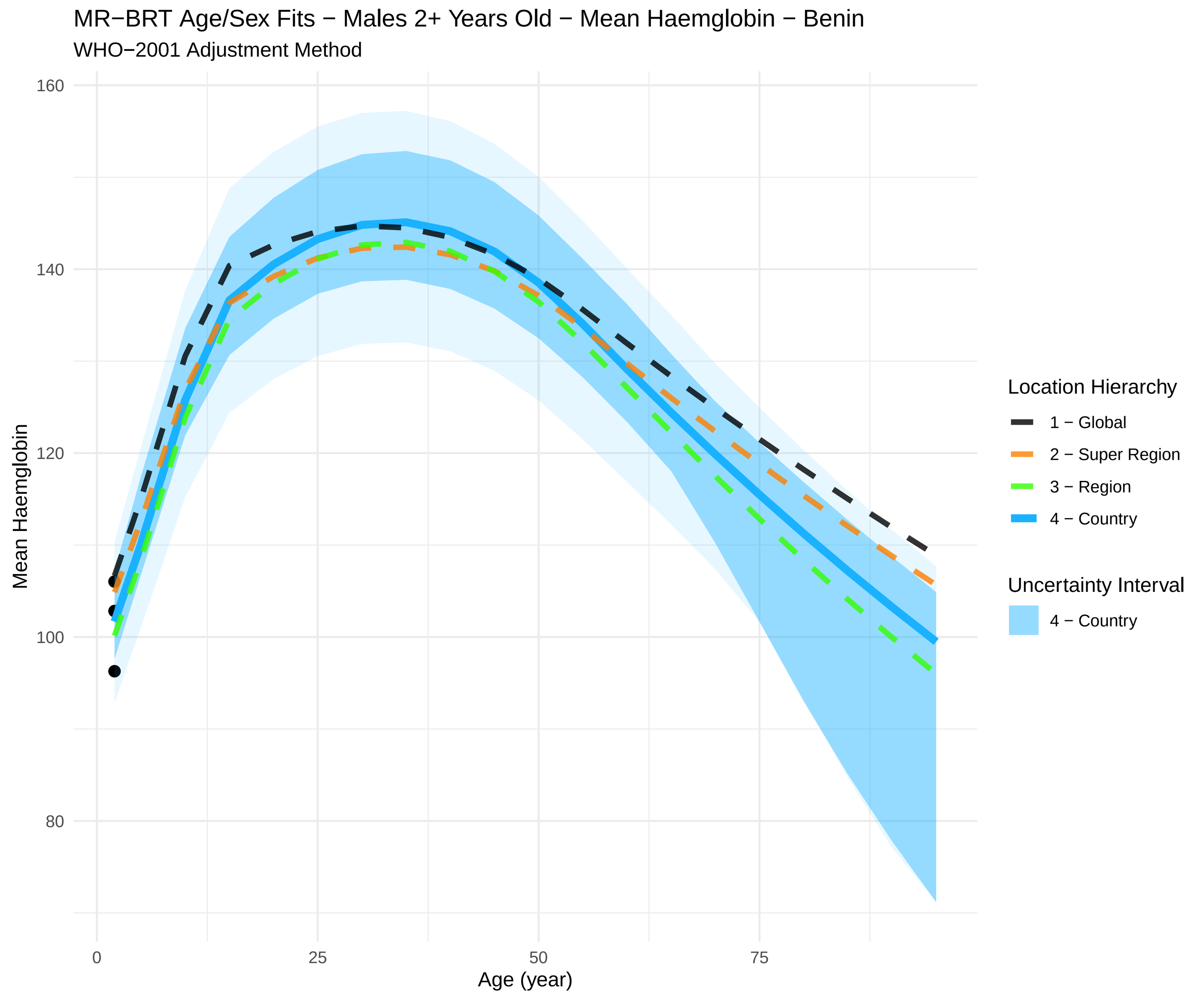
MR−BRT Age/Sex Fits − Males 2+ Years Old − Mean Haemglobin − Benin
WHO−2001 Adjustment Method
Location Hierarchy
1 − Global
2 − Super Region
3 − Region
4 − Country
Uncertainty Interval
4 − Country
Mean Haemglobin
Age (year)

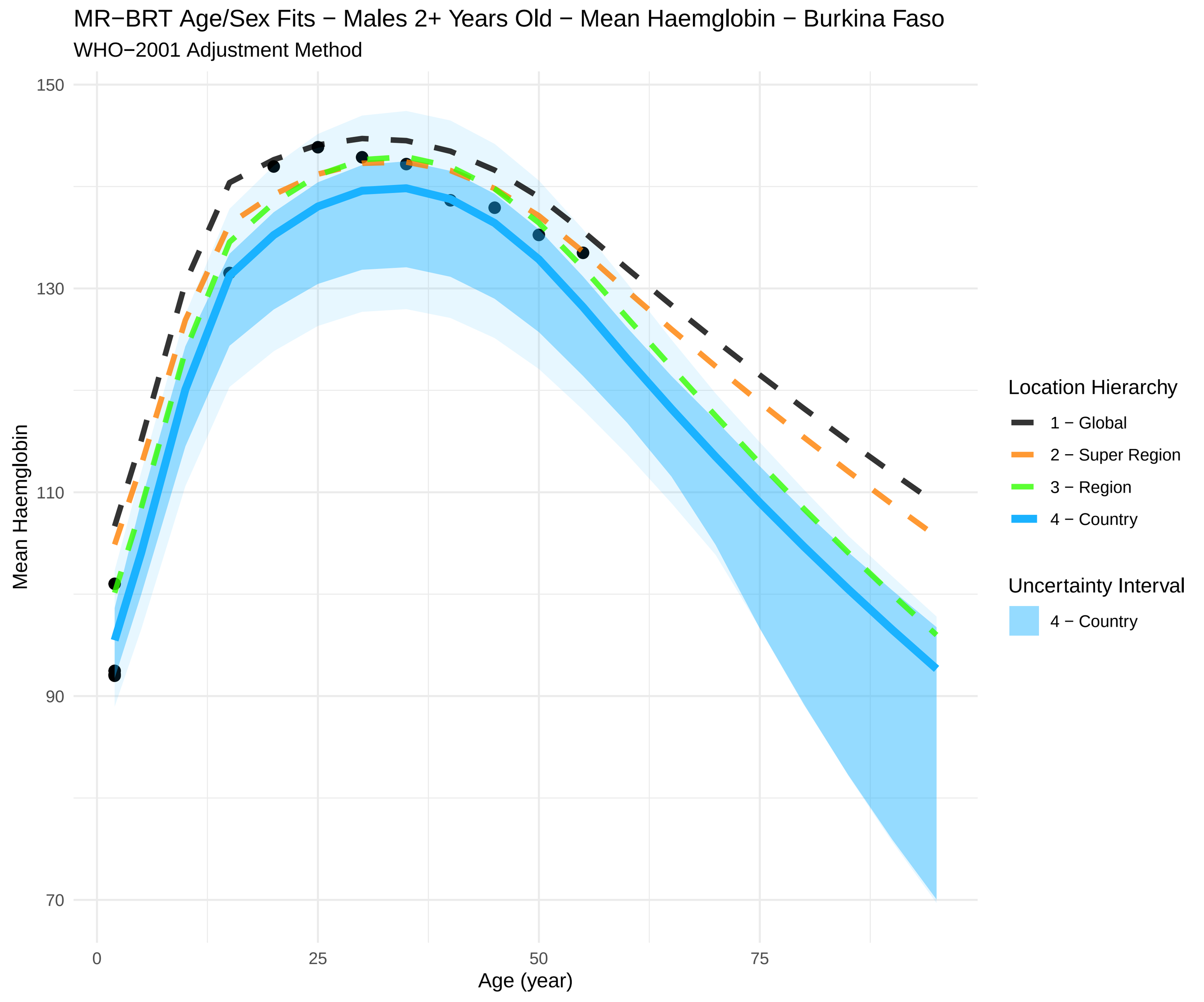
MR−BRT Age/Sex Fits − Males 2+ Years Old − Mean Haemglobin − Burkina Faso
WHO−2001 Adjustment Method
Location Hierarchy
1 − Global
2 − Super Region
3 − Region
4 − Country
Uncertainty Interval
4 − Country
Mean Haemglobin
Age (year)

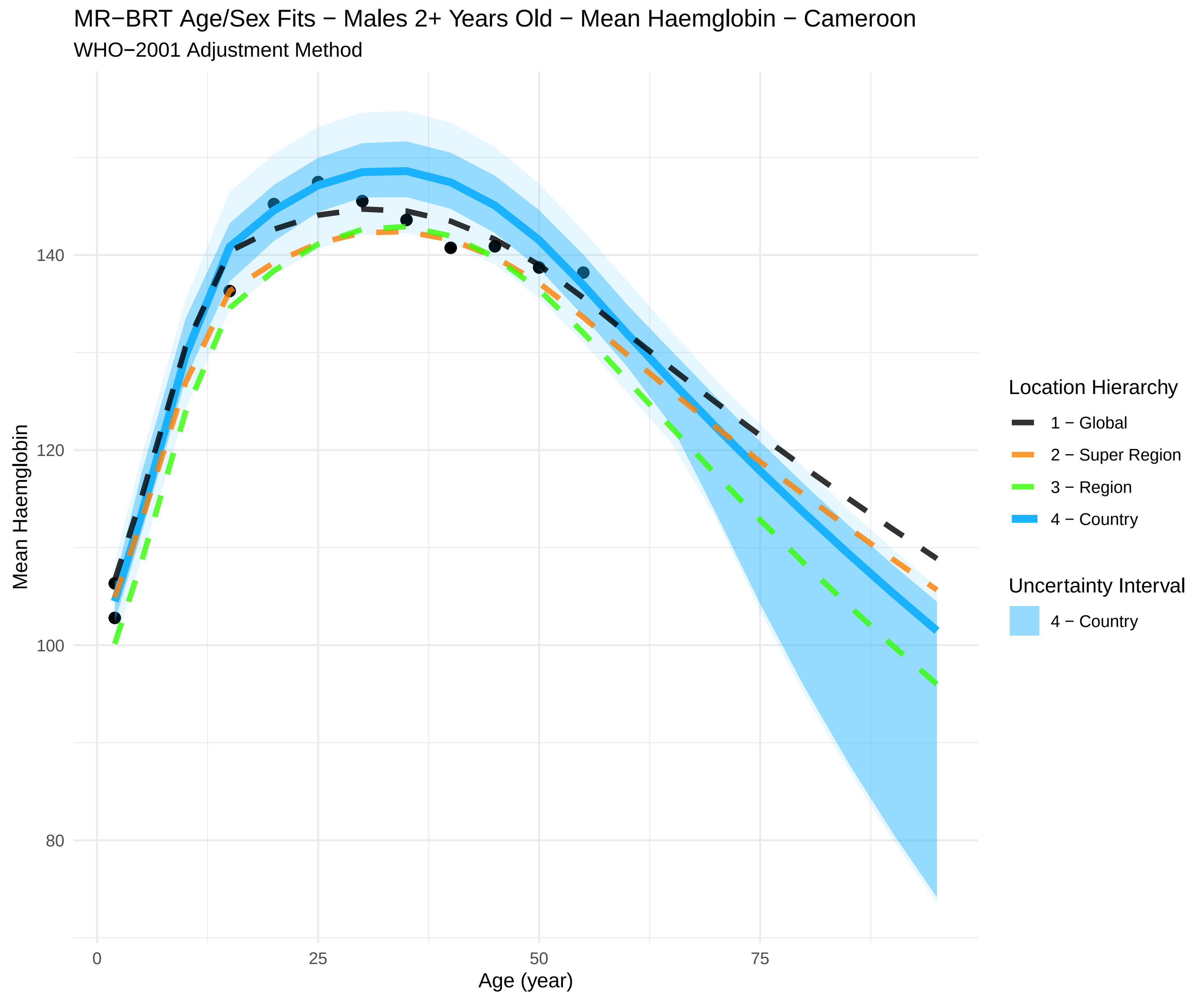
MR−BRT Age/Sex Fits − Males 2+ Years Old − Mean Haemglobin − Cameroon
WHO−2001 Adjustment Method
Location Hierarchy
1 − Global
2 − Super Region
3 − Region
4 − Country
Uncertainty Interval
4 − Country
Mean Haemglobin
Age (year)

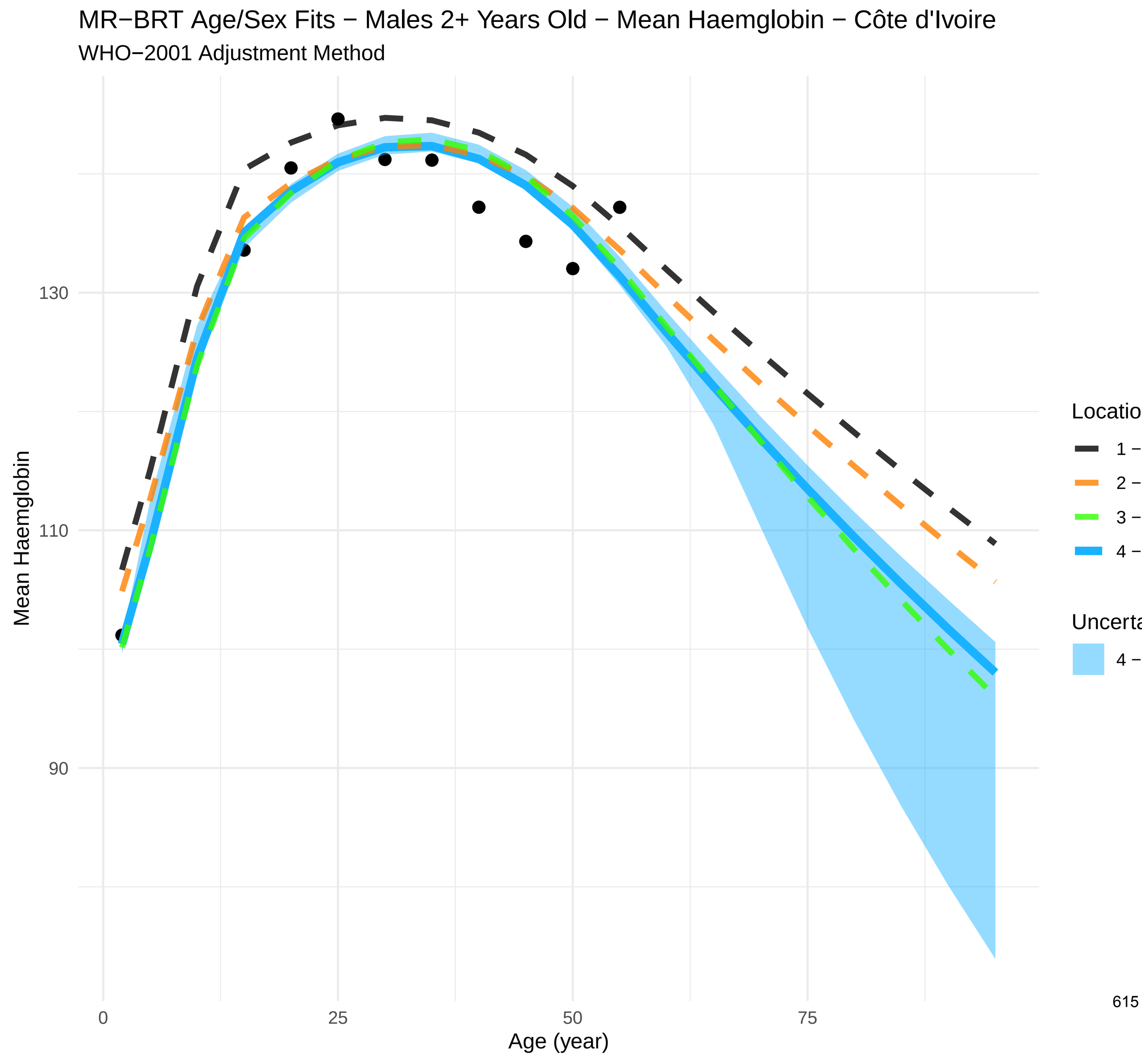
MR−BRT Age/Sex Fits − Males 2+ Years Old − Mean Haemglobin − Côte d'Ivoire
WHO−2001 Adjustment Method
Location Hierarchy
1 − Global
2 − Super Region
3 − Region
4 − Country
Uncertainty Interval
4 − Country
Mean Haemglobin
Age (year)

MR−BRT Age/Sex Fits − Males 2+ Years Old − Mean Haemoglobin − Gambia
WHO−2001 Adjustment Method

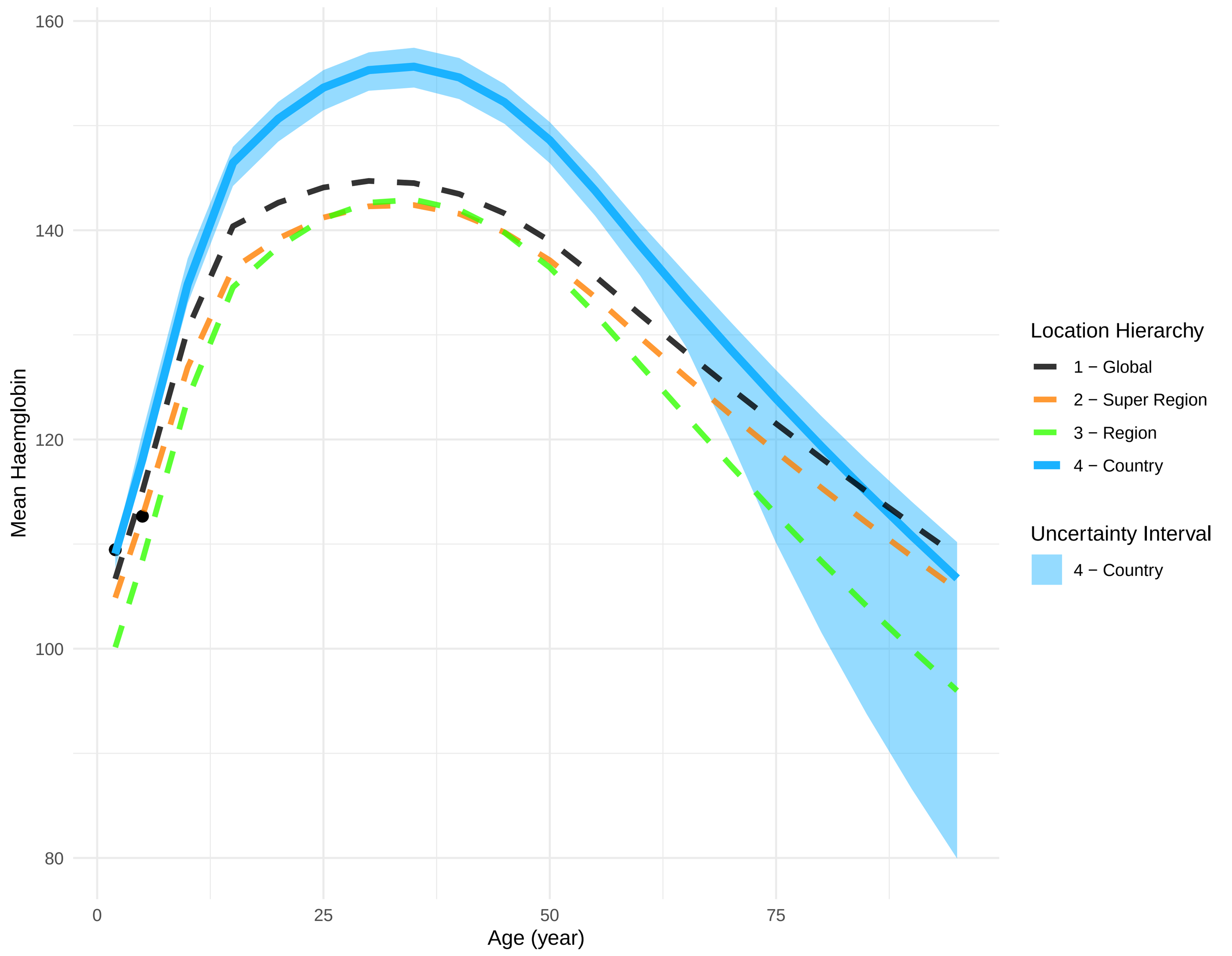

MR−BRT Age/Sex Fits − Males 2+ Years Old − Mean Haemoglobin − Ghana
WHO−2001 Adjustment Method

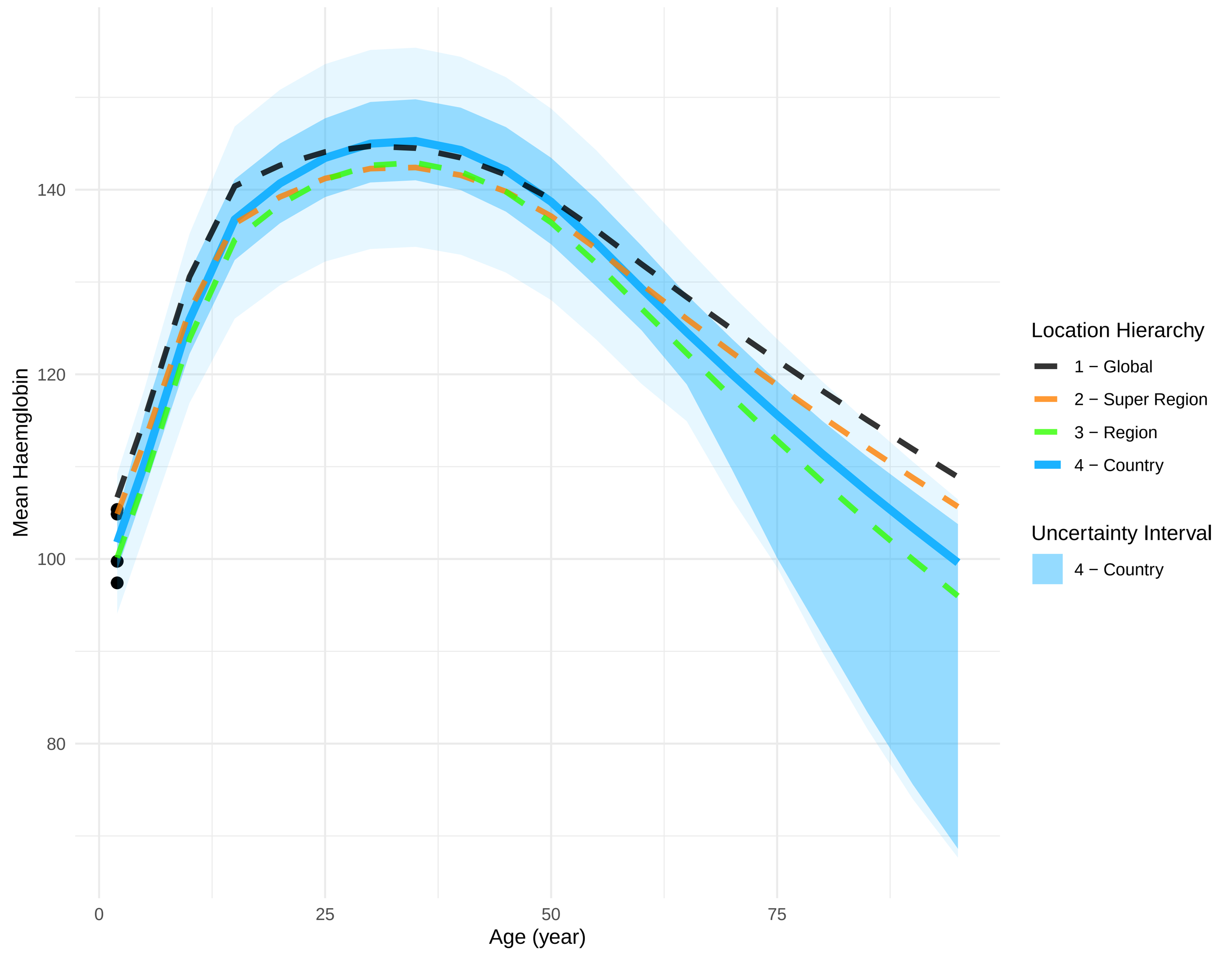

MR−BRT Age/Sex Fits − Males 2+ Years Old − Mean Haemoglobin − Guinea
WHO−2001 Adjustment Method

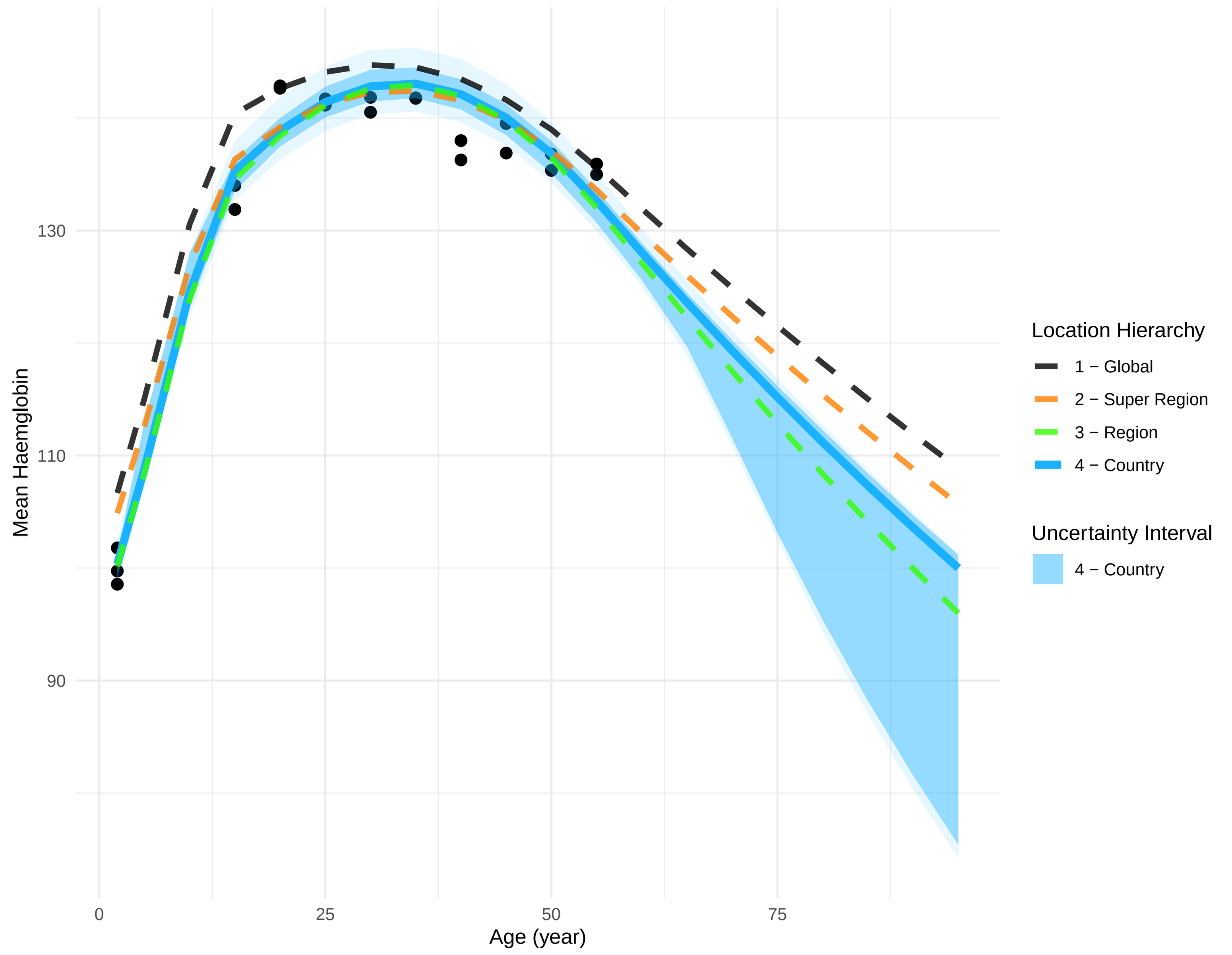

MR−BRT Age/Sex Fits − Males 2+ Years Old − Mean Haemoglobin − Liberia
WHO−2001 Adjustment Method

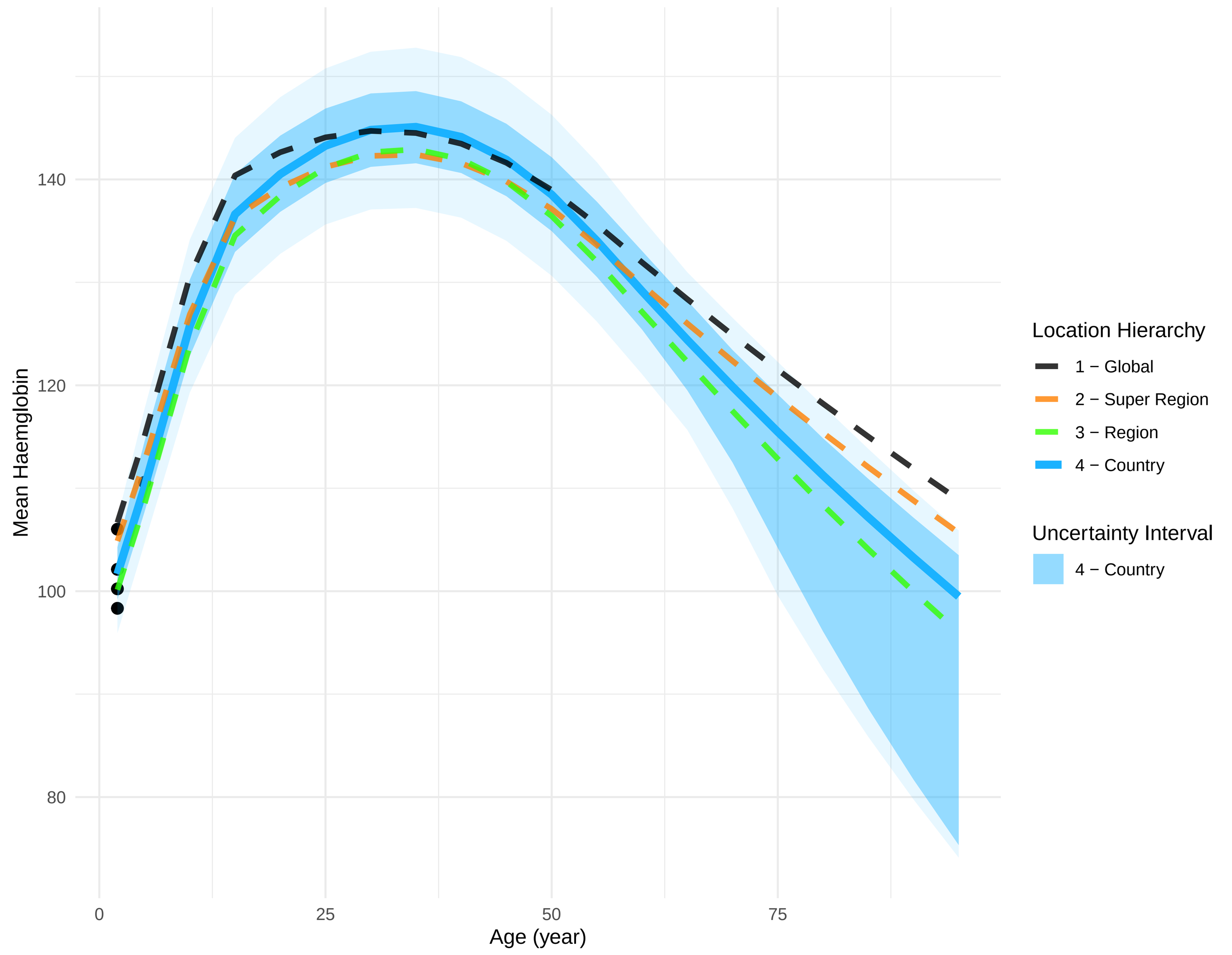

MR−BRT Age/Sex Fits − Males 2+ Years Old − Mean Haemoglobin − Mali
WHO−2001 Adjustment Method

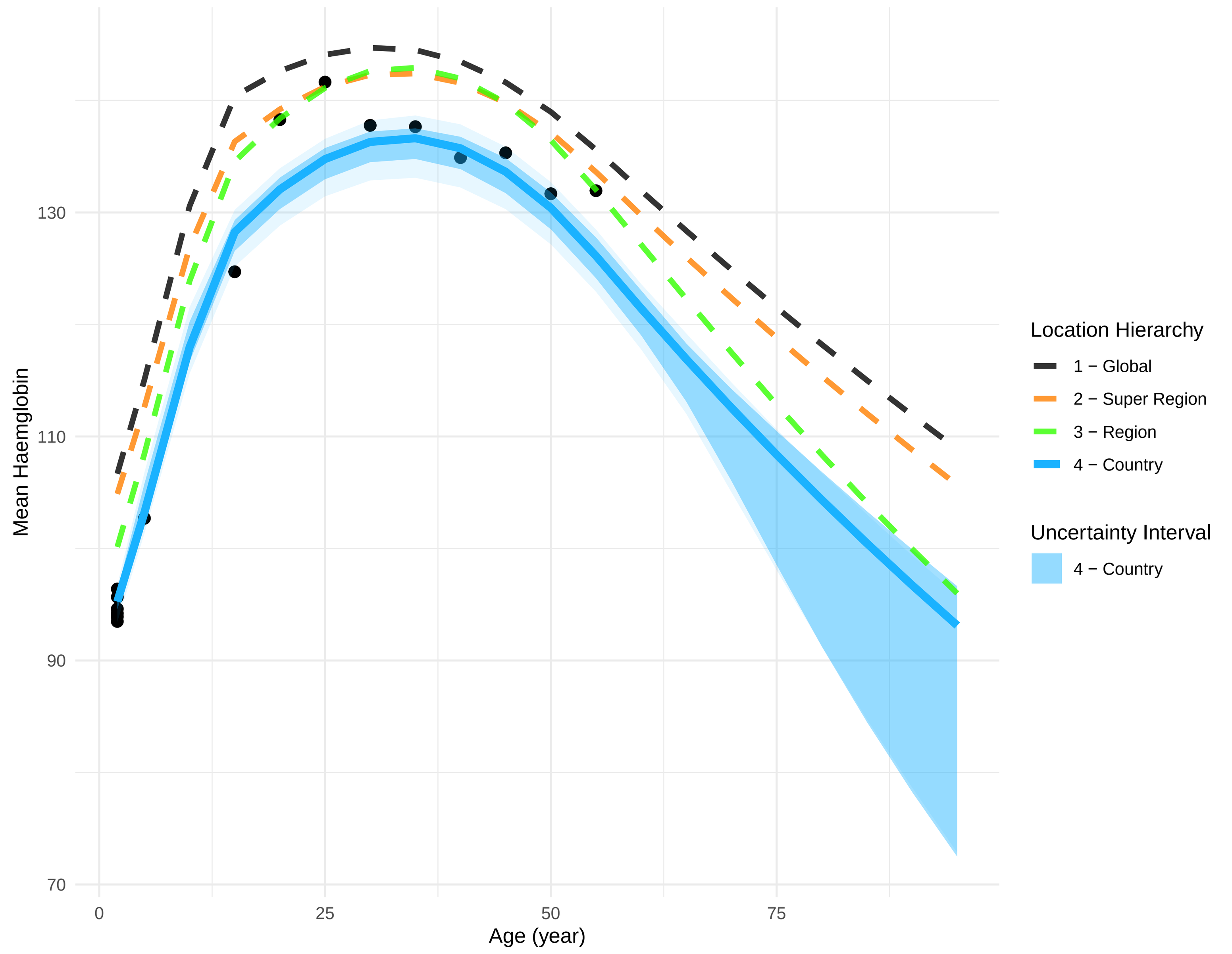

MR−BRT Age/Sex Fits − Males 2+ Years Old − Mean Haemoglobin − Niger
WHO−2001 Adjustment Method

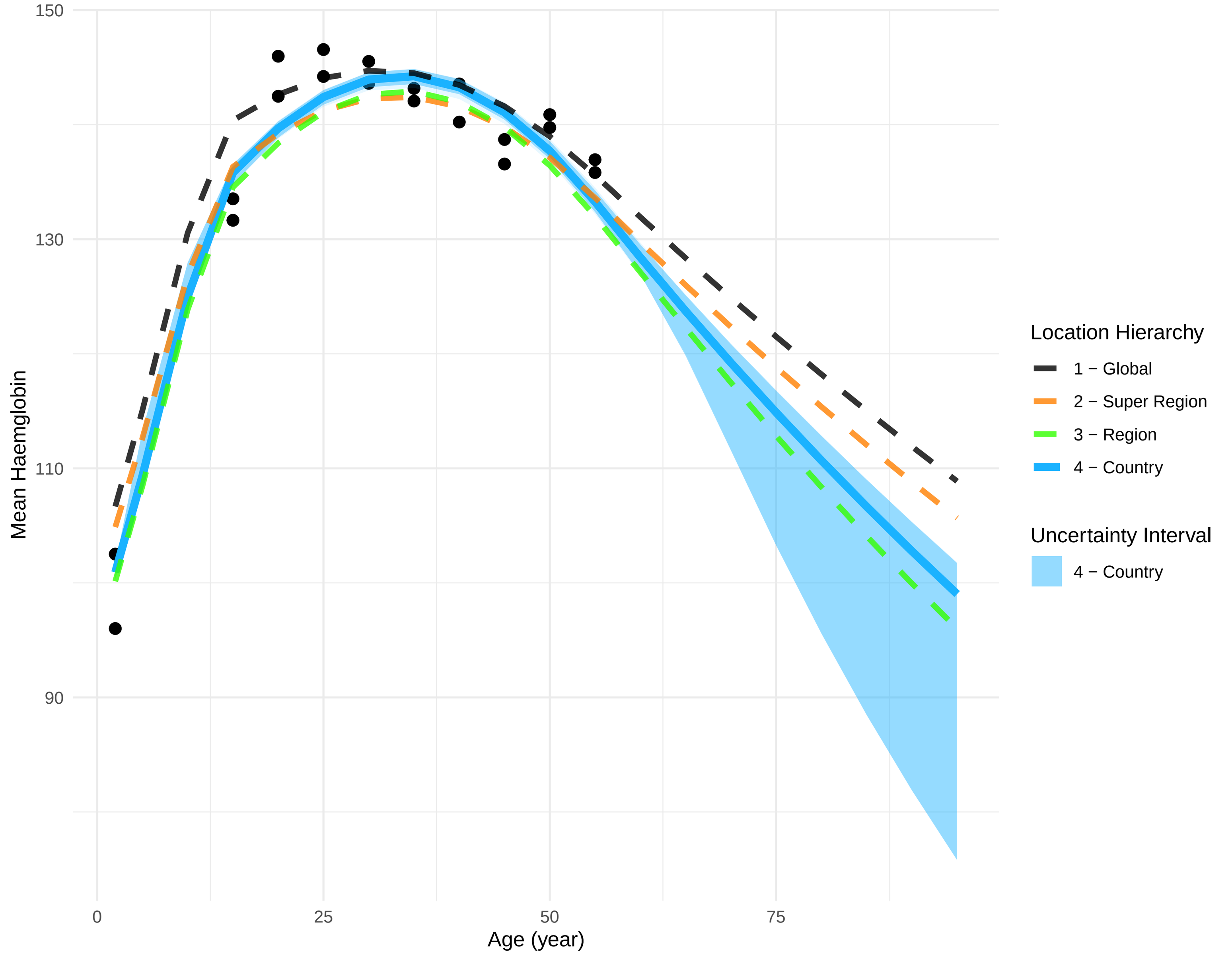

MR−BRT Age/Sex Fits − Males 2+ Years Old − Mean Haemoglobin − Nigeria
WHO−2001 Adjustment Method

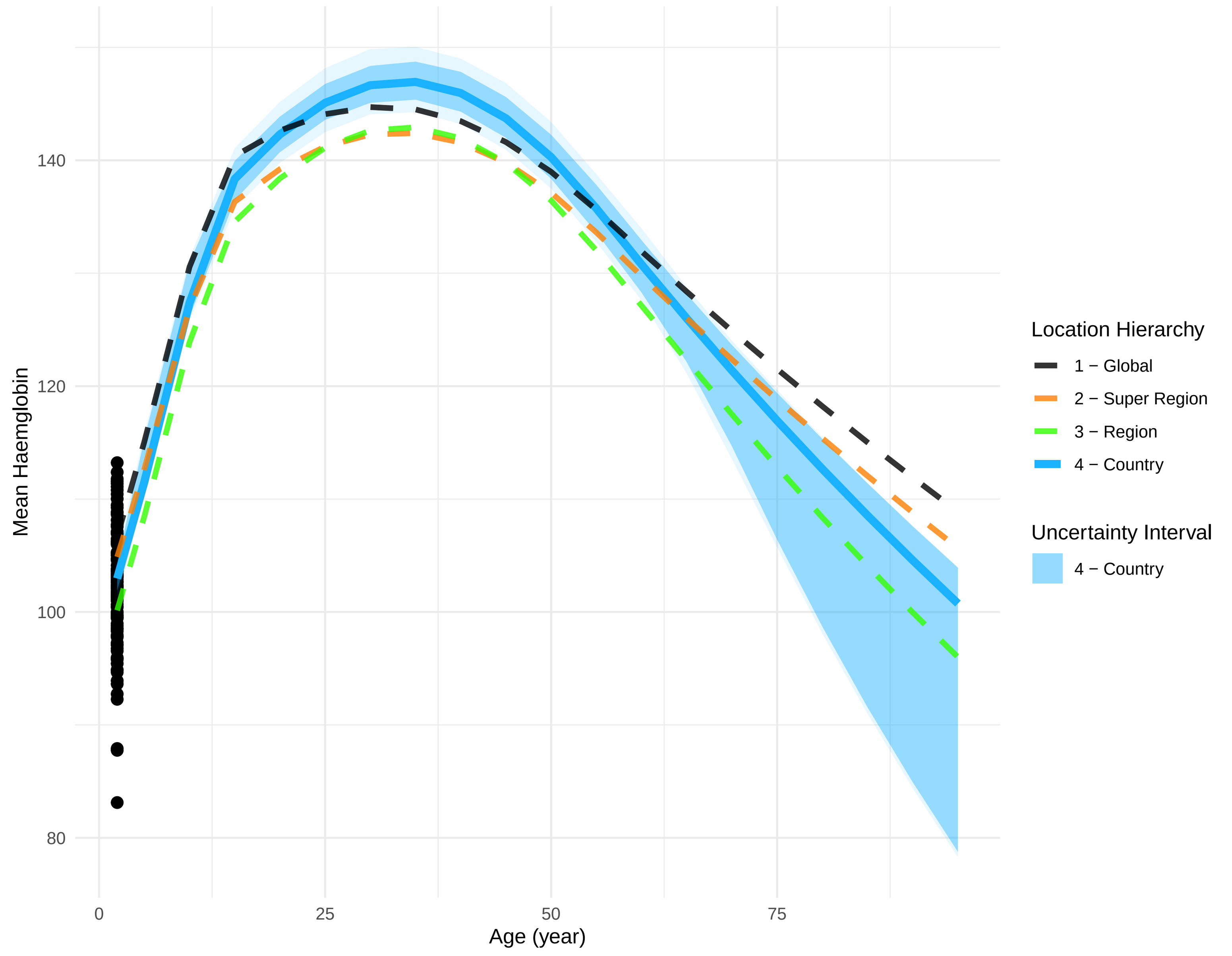

MR−BRT Age/Sex Fits − Males 2+ Years Old − Mean Haemoglobin − Abia
WHO−2001 Adjustment Method

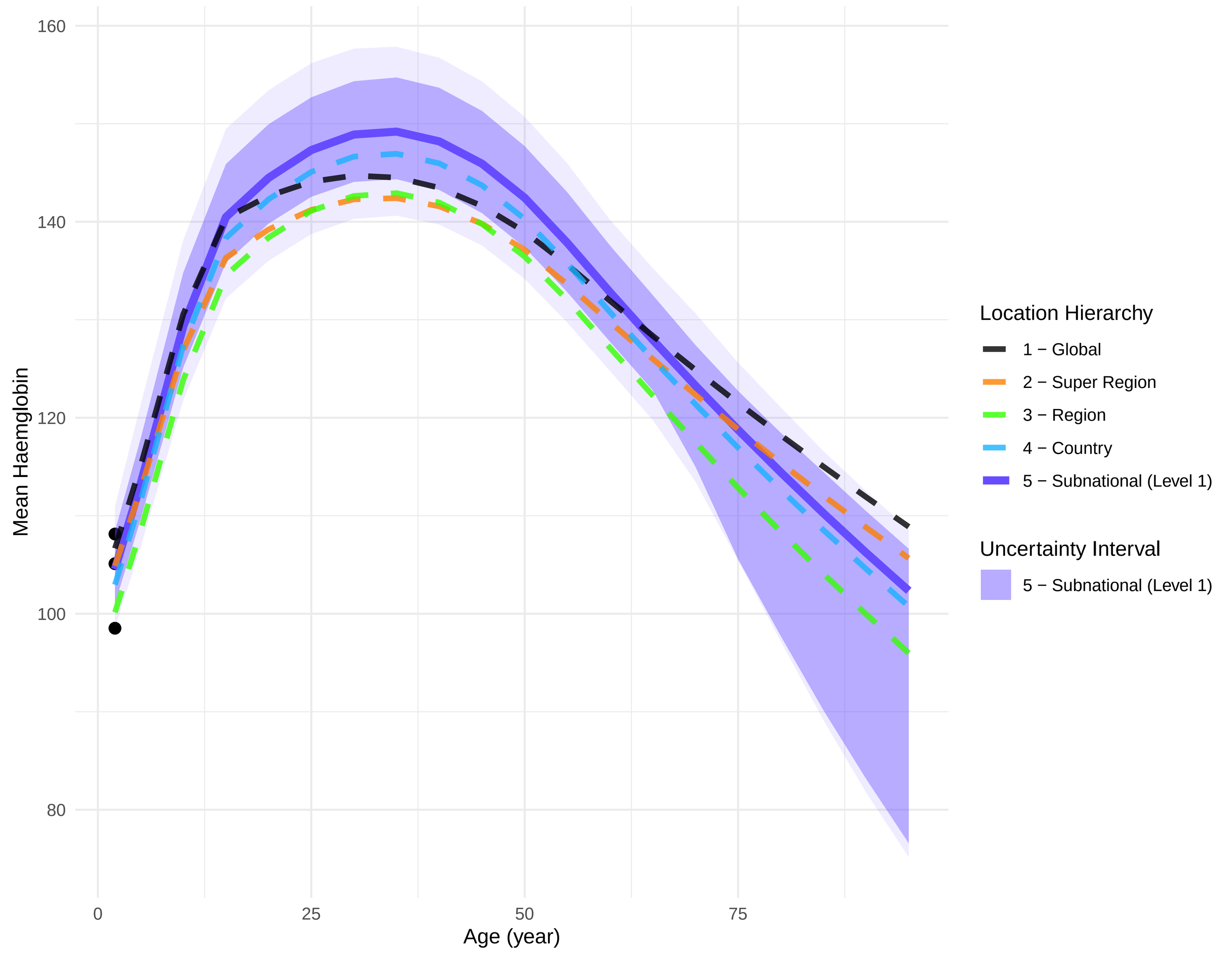

MR−BRT Age/Sex Fits − Males 2+ Years Old − Mean Haemoglobin − Adamawa
WHO−2001 Adjustment Method

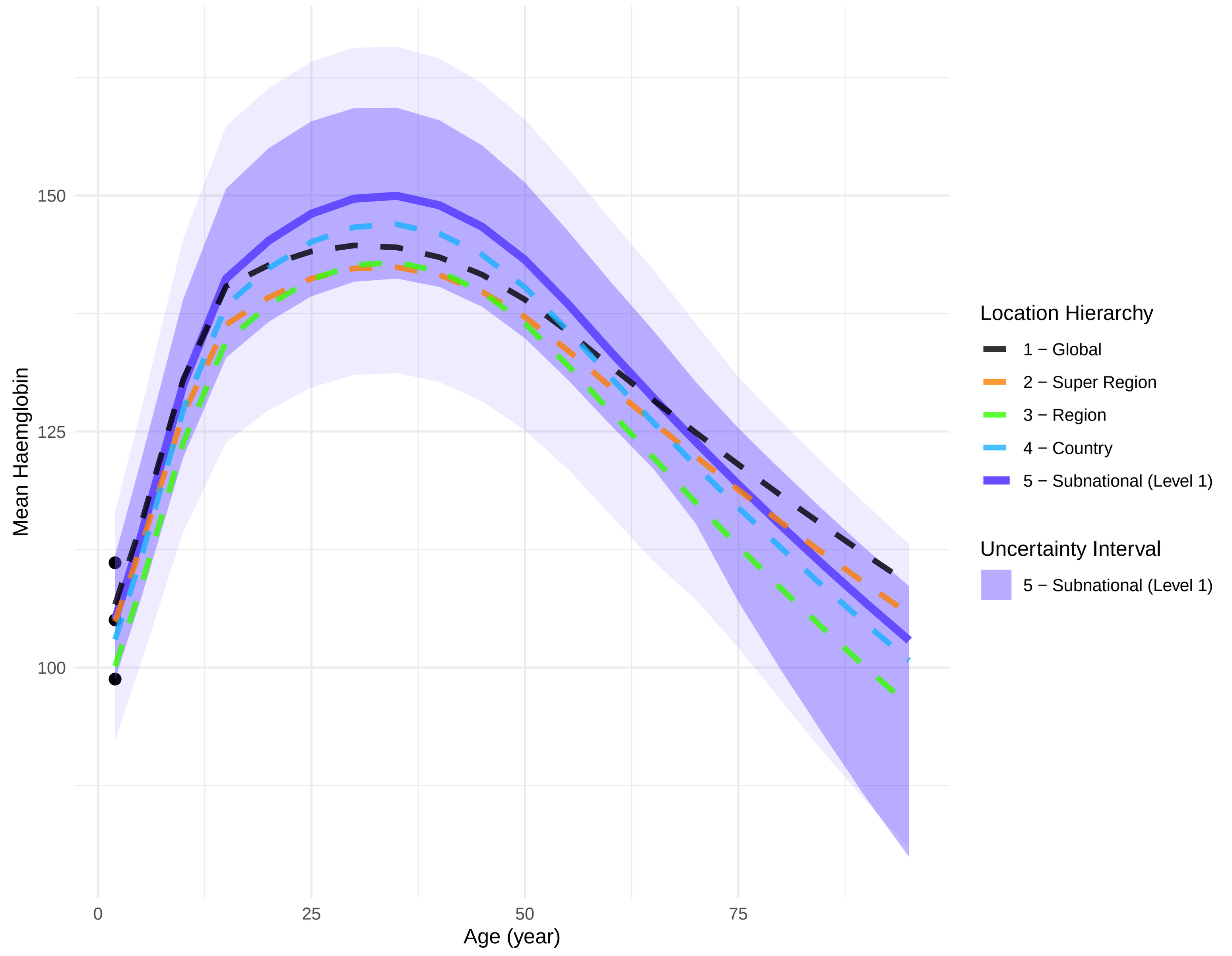

MR−BRT Age/Sex Fits − Males 2+ Years Old − Mean Haemoglobin − Akwa Ibom
WHO−2001 Adjustment Method

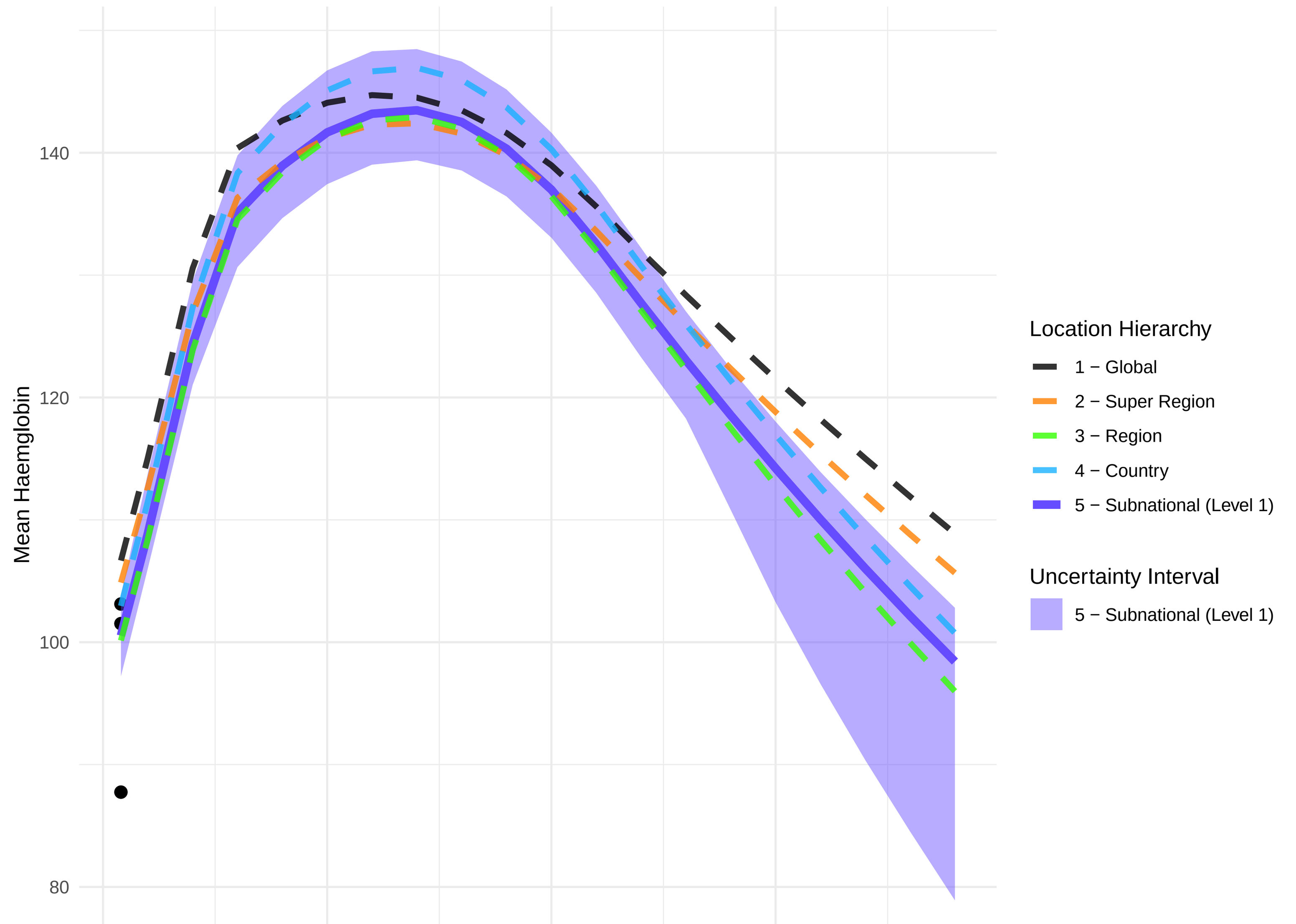

MR−BRT Age/Sex Fits − Males 2+ Years Old − Mean Haemglobin − Anambra
WHO−2001 Adjustment Method

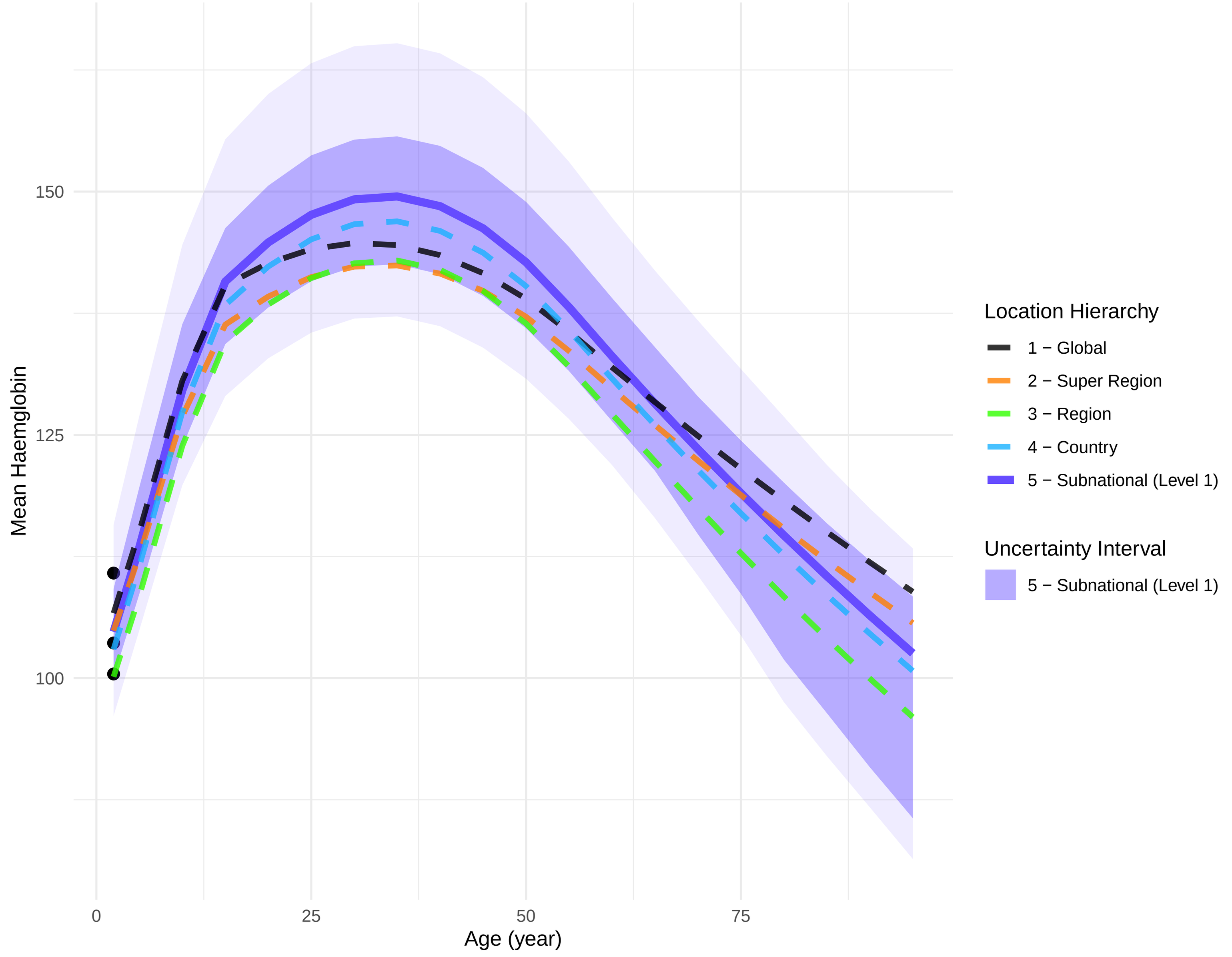

MR−BRT Age/Sex Fits − Males 2+ Years Old − Mean Haemglobin − Bauchi
WHO−2001 Adjustment Method

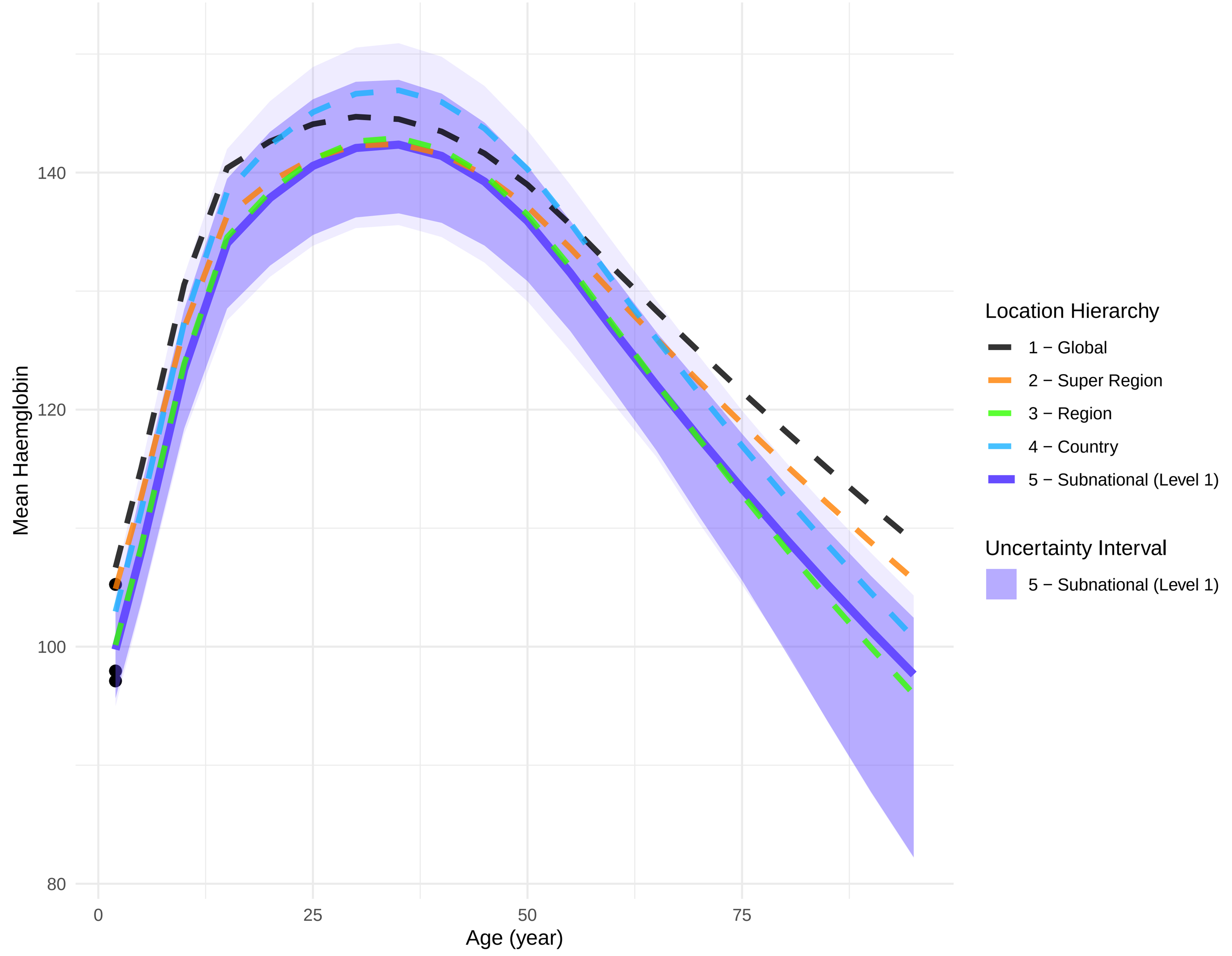

MR−BRT Age/Sex Fits − Males 2+ Years Old − Mean Haemglobin − Bayelsa
WHO−2001 Adjustment Method

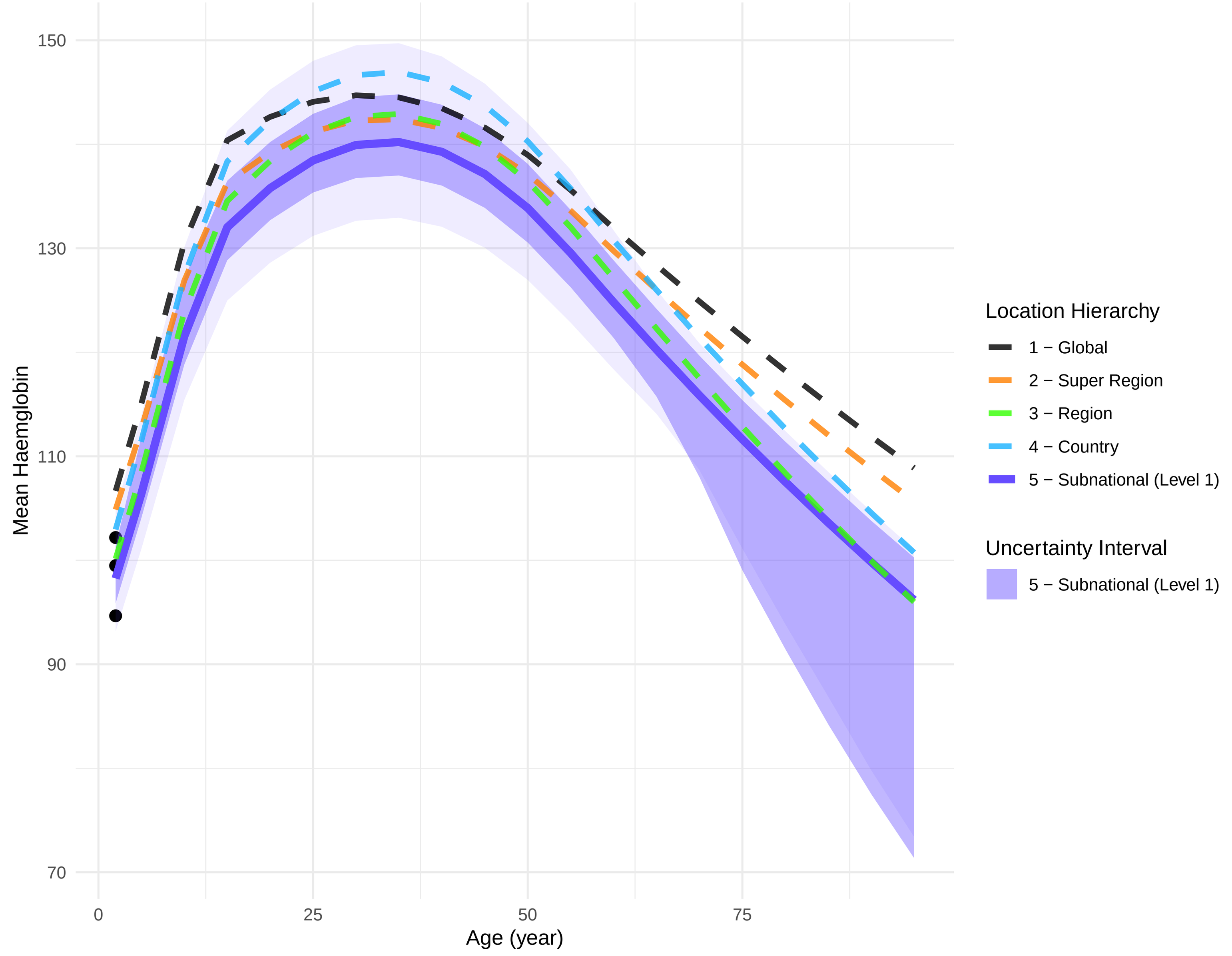

MR−BRT Age/Sex Fits − Males 2+ Years Old − Mean Haemglobin − Benue
WHO−2001 Adjustment Method

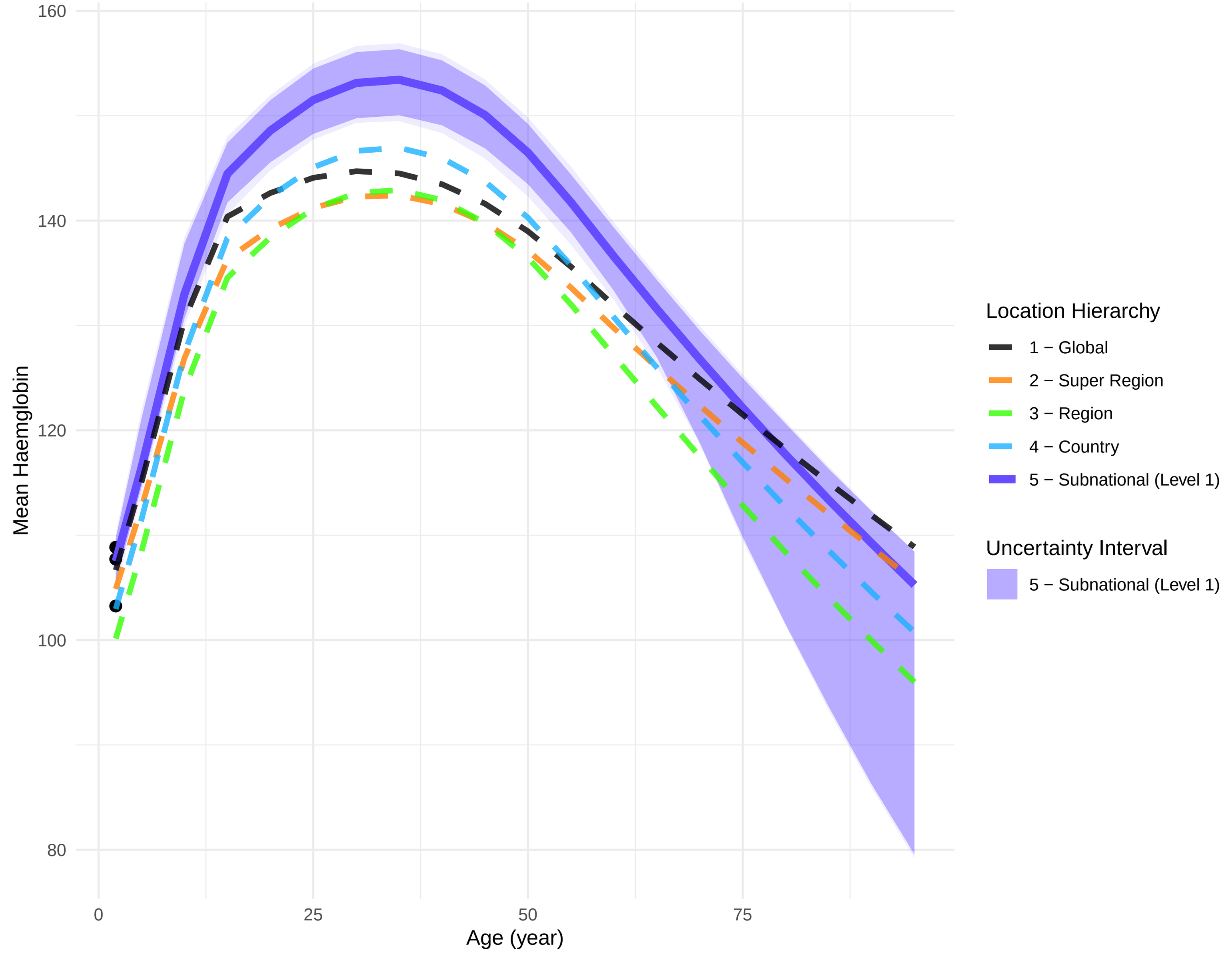

MR−BRT Age/Sex Fits − Males 2+ Years Old − Mean Haemglobin − Borno
WHO−2001 Adjustment Method

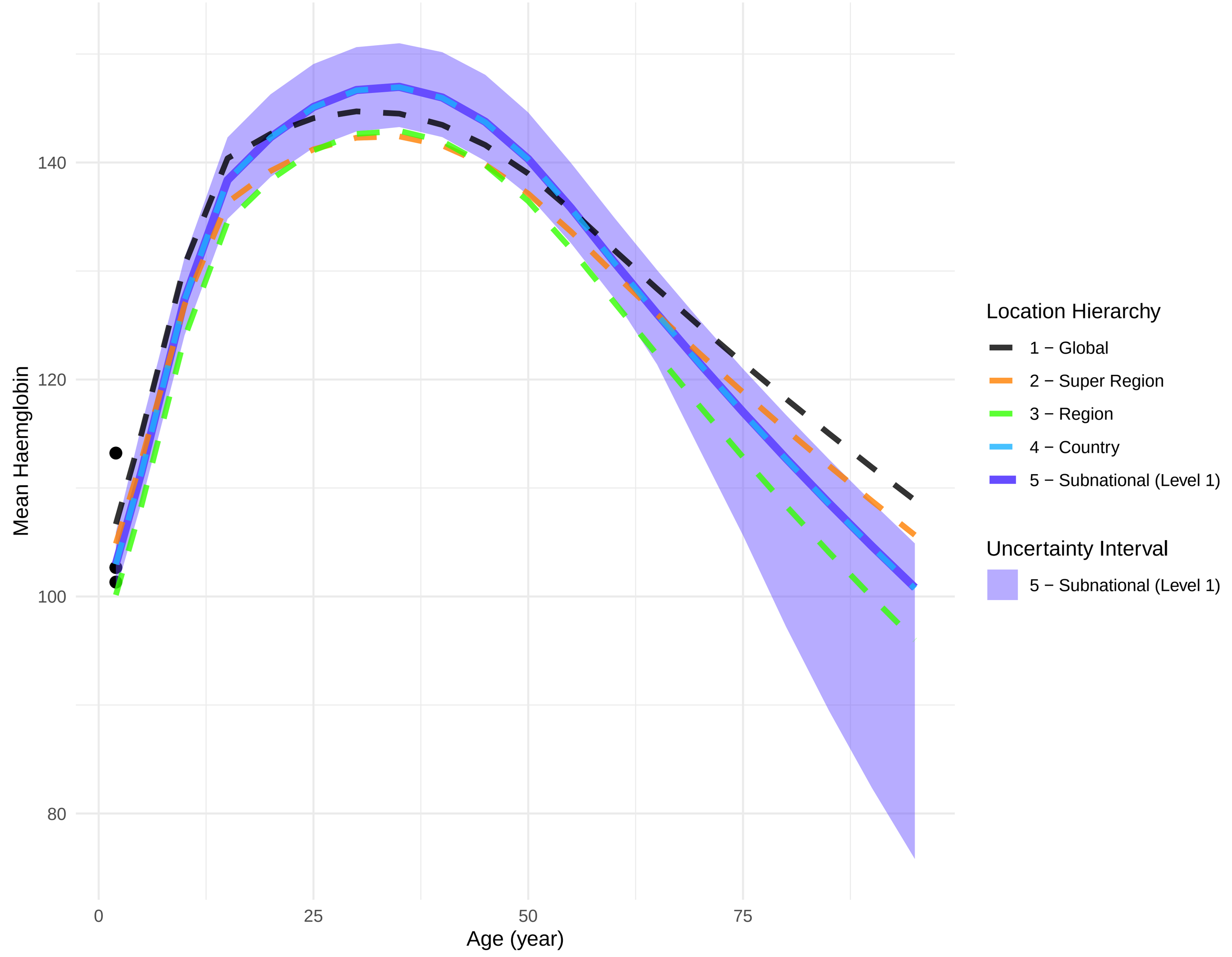

MR−BRT Age/Sex Fits − Males 2+ Years Old − Mean Haemglobin − Cross River
WHO−2001 Adjustment Method

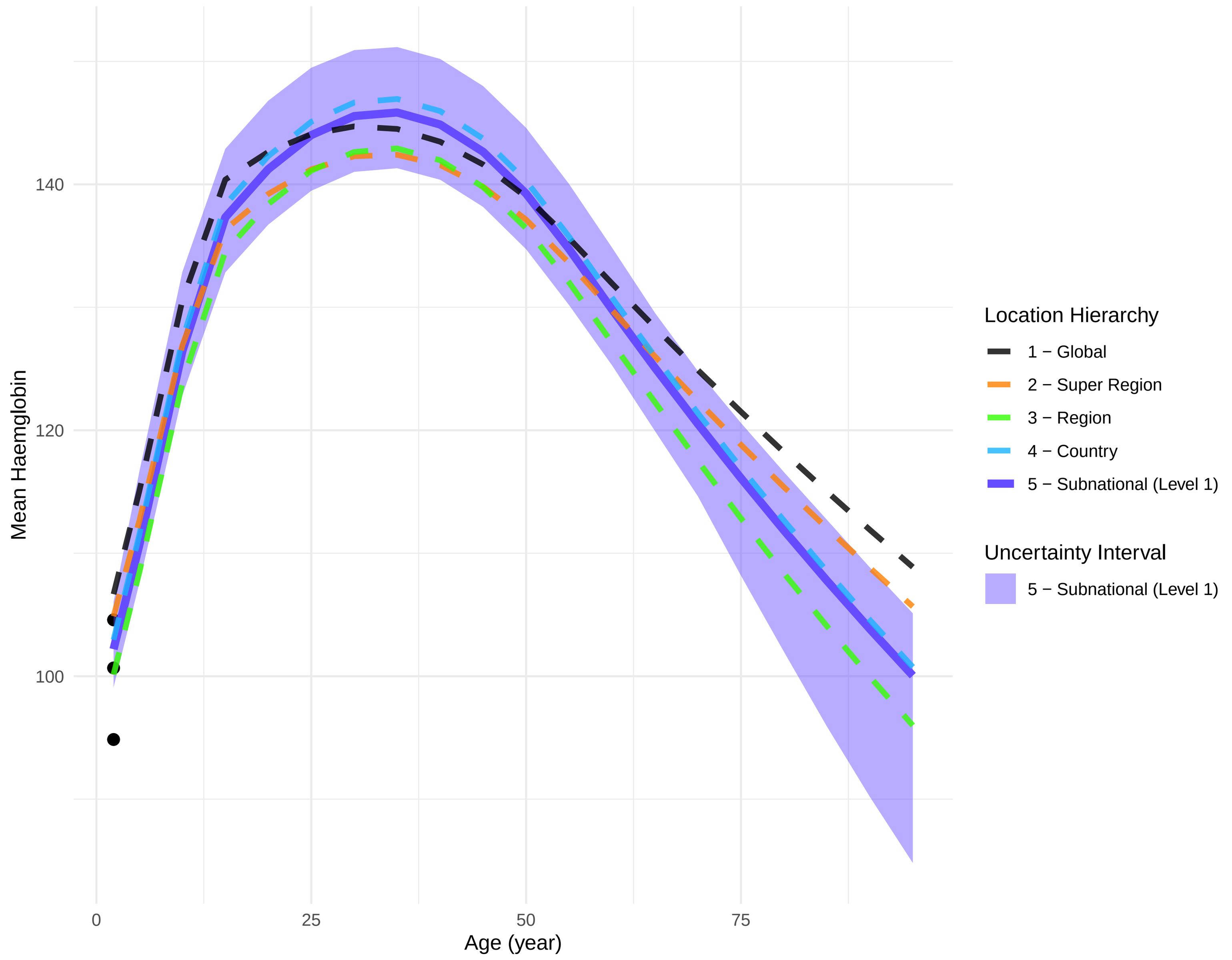

MR−BRT Age/Sex Fits − Males 2+ Years Old − Mean Haemglobin − Delta
WHO−2001 Adjustment Method

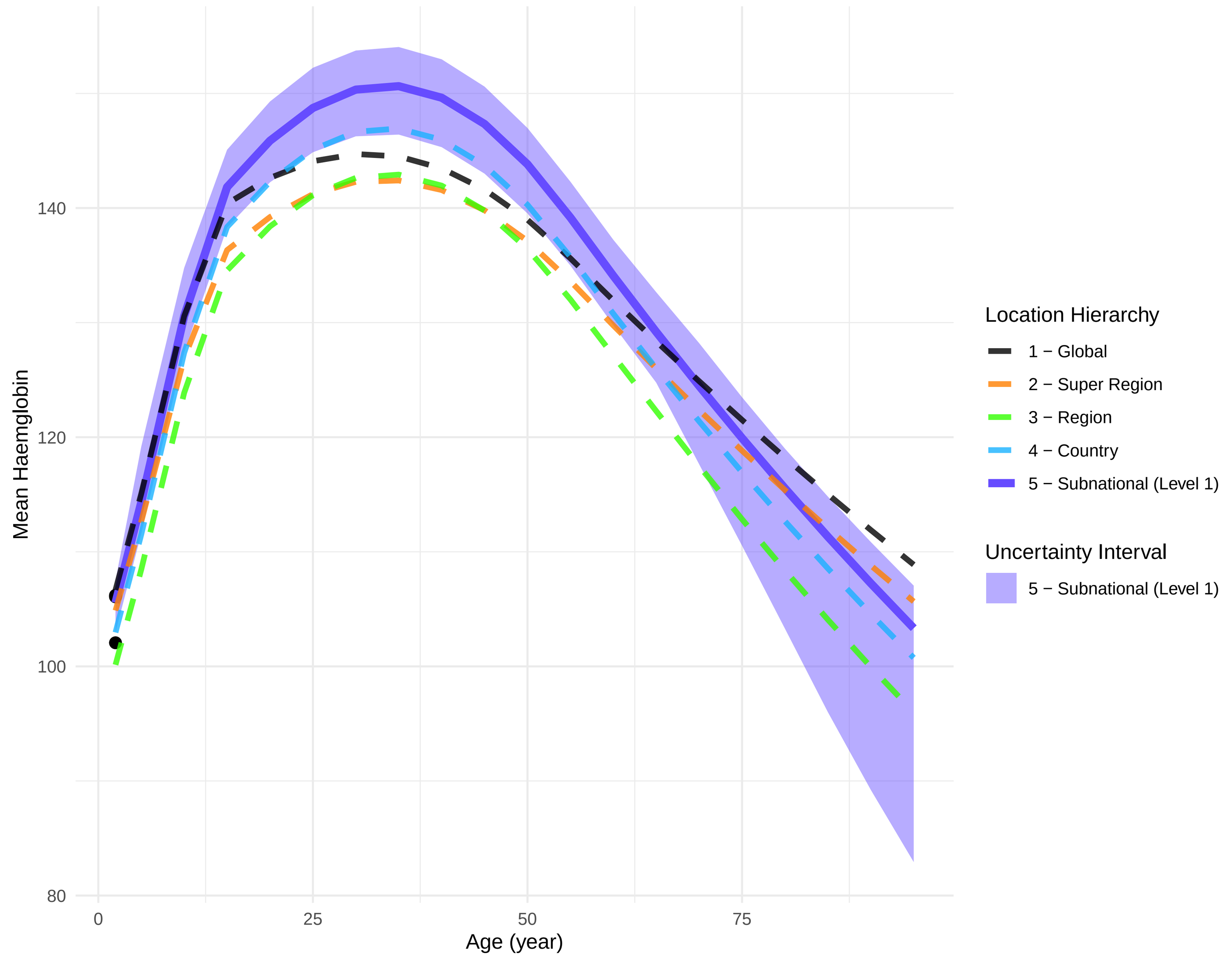

MR−BRT Age/Sex Fits − Males 2+ Years Old − Mean Haemglobin − Ebonyi
WHO−2001 Adjustment Method

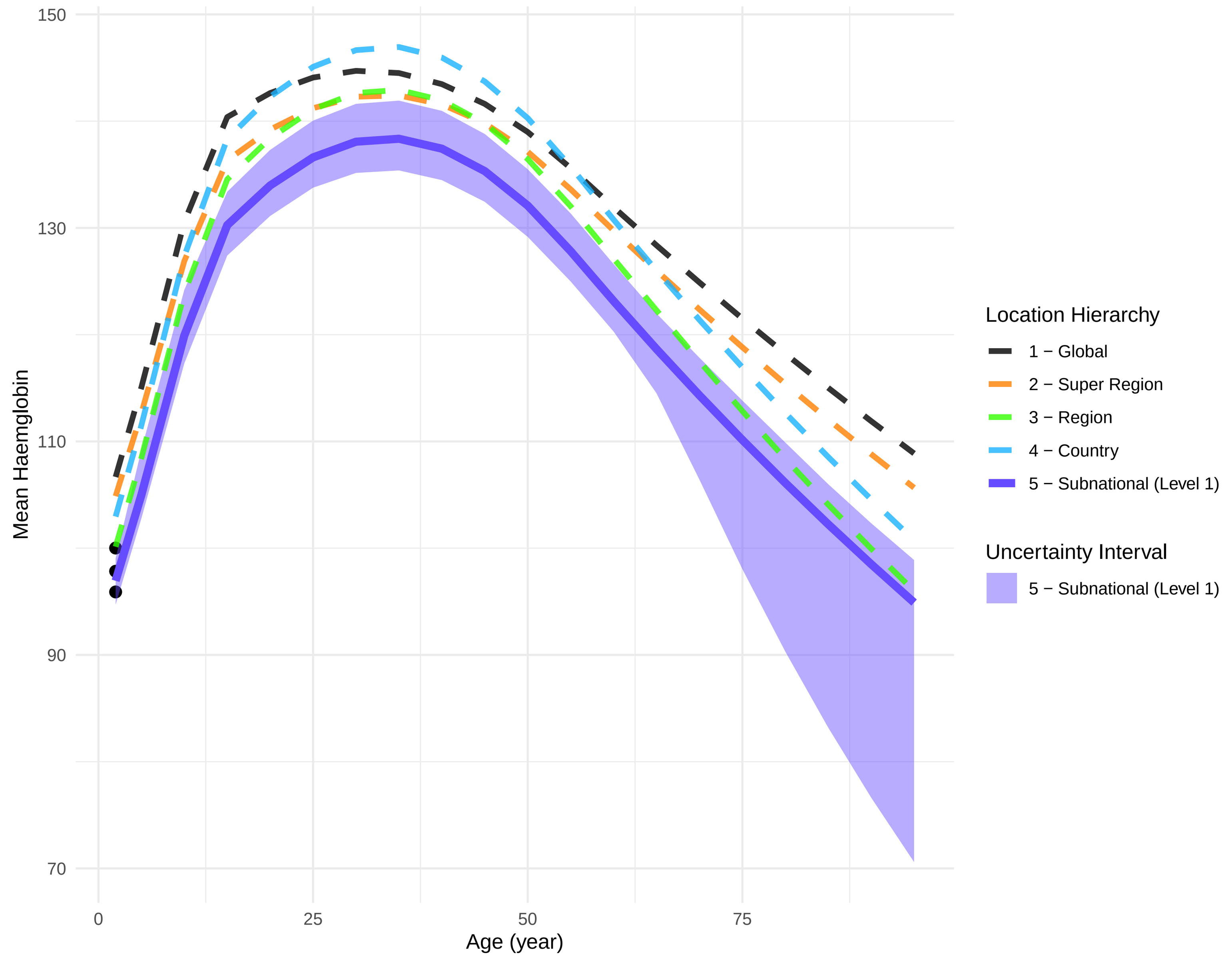

MR−BRT Age/Sex Fits − Males 2+ Years Old − Mean Haemglobin − Edo
WHO−2001 Adjustment Method

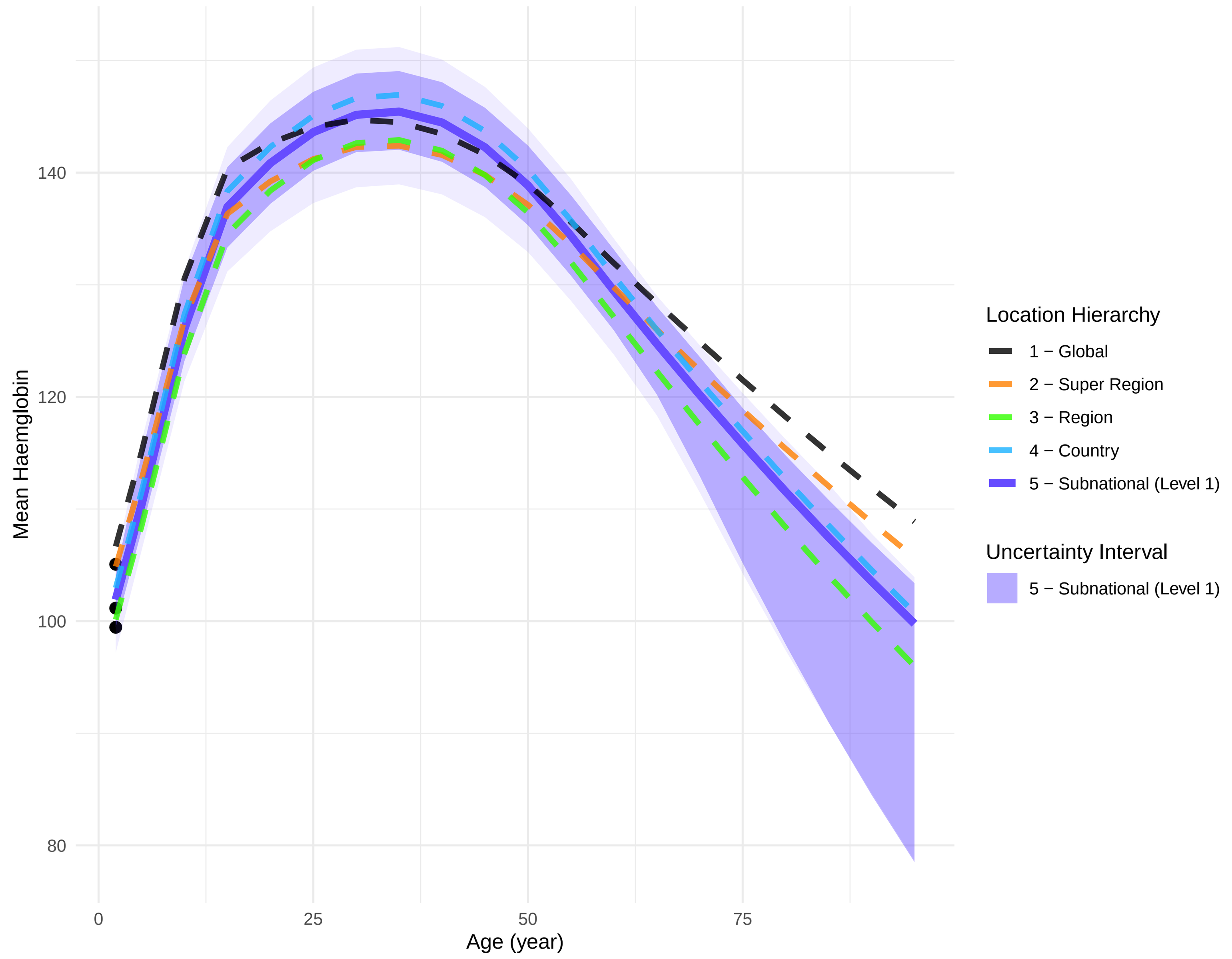

MR−BRT Age/Sex Fits − Males 2+ Years Old − Mean Haemglobin − Ekiti
WHO−2001 Adjustment Method

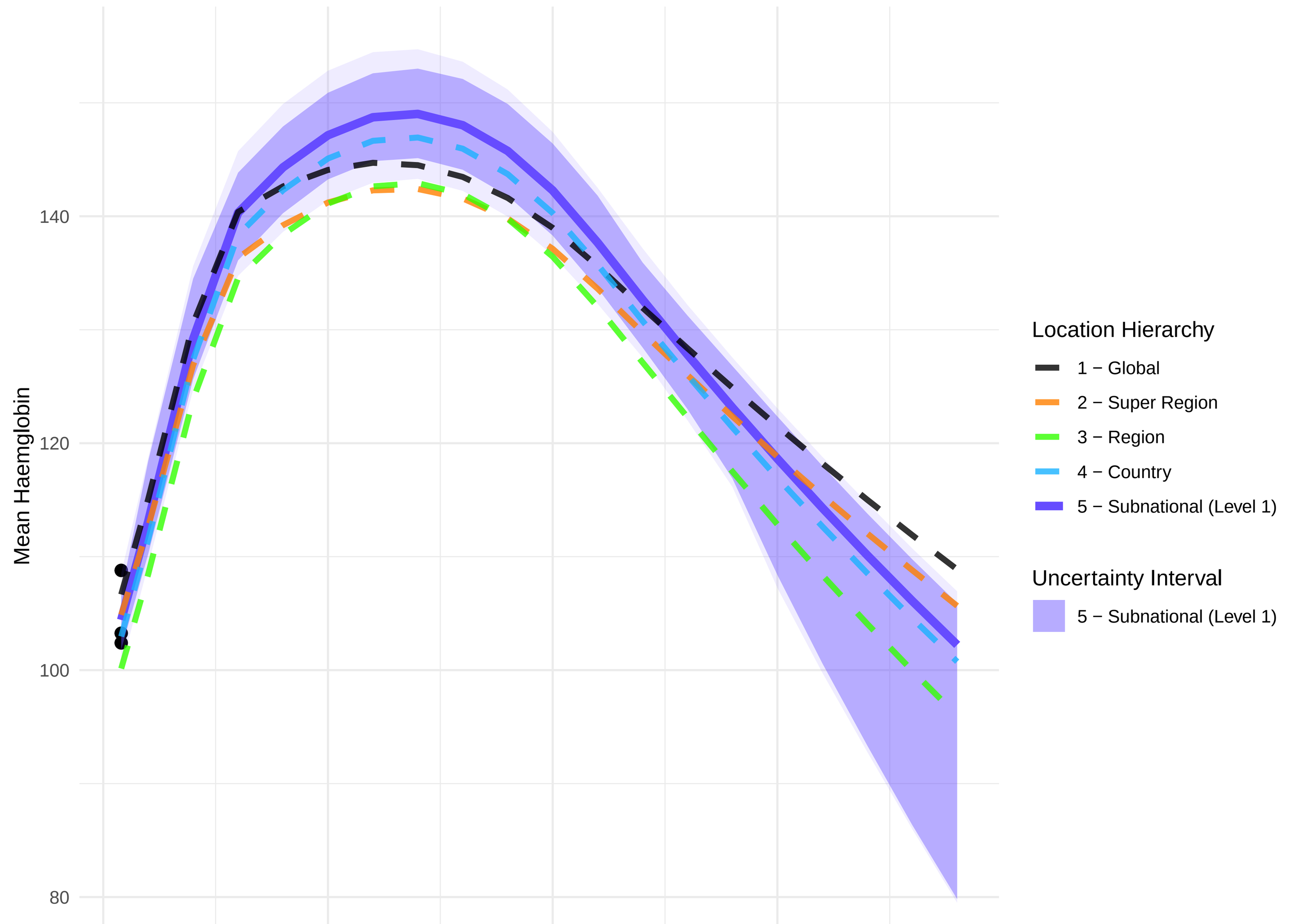

MR−BRT Age/Sex Fits − Males 2+ Years Old − Mean Haemglobin − Enugu
WHO−2001 Adjustment Method

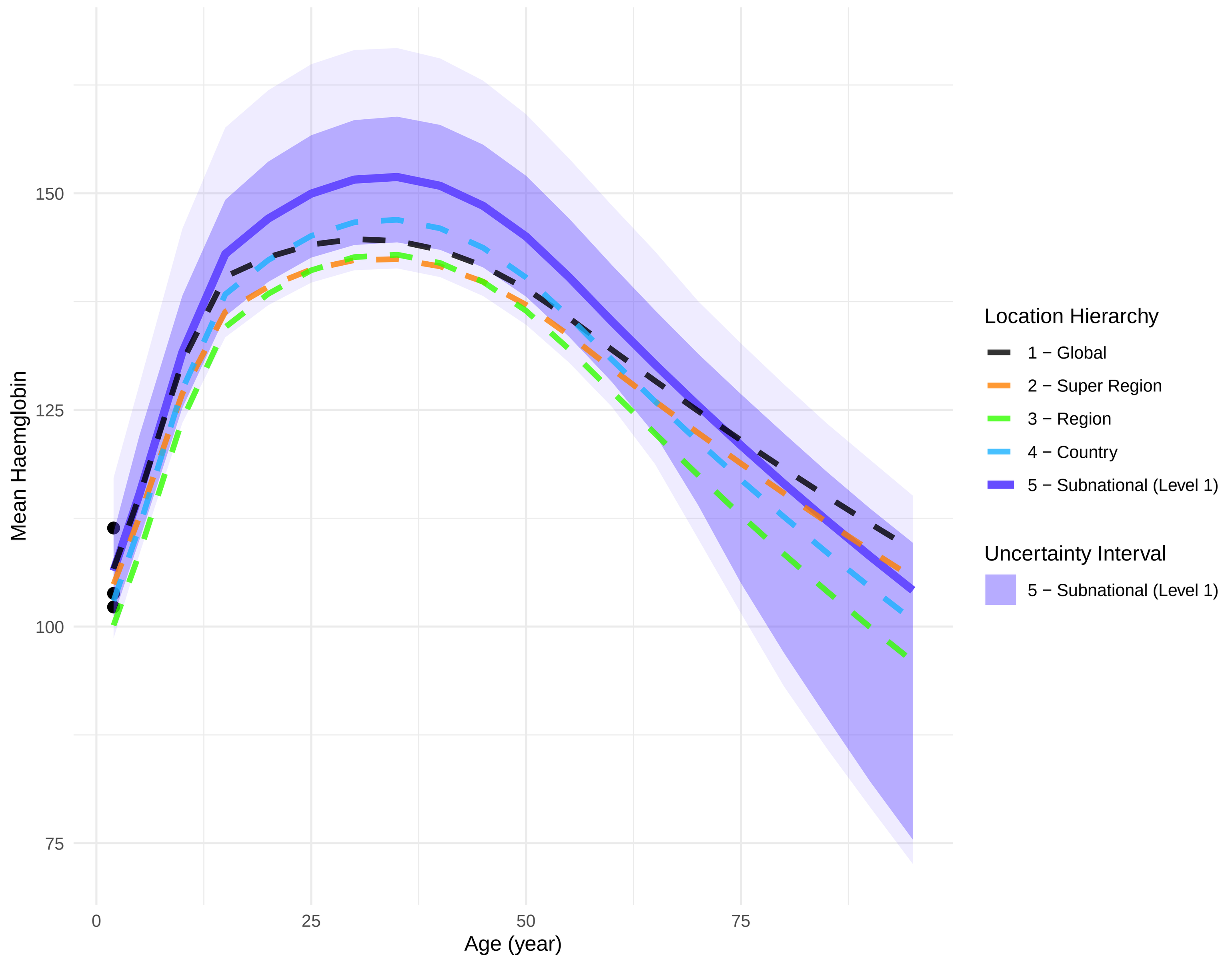

MR−BRT Age/Sex Fits − Males 2+ Years Old − Mean Haemglobin − FCT (Abuja)
WHO−2001 Adjustment Method

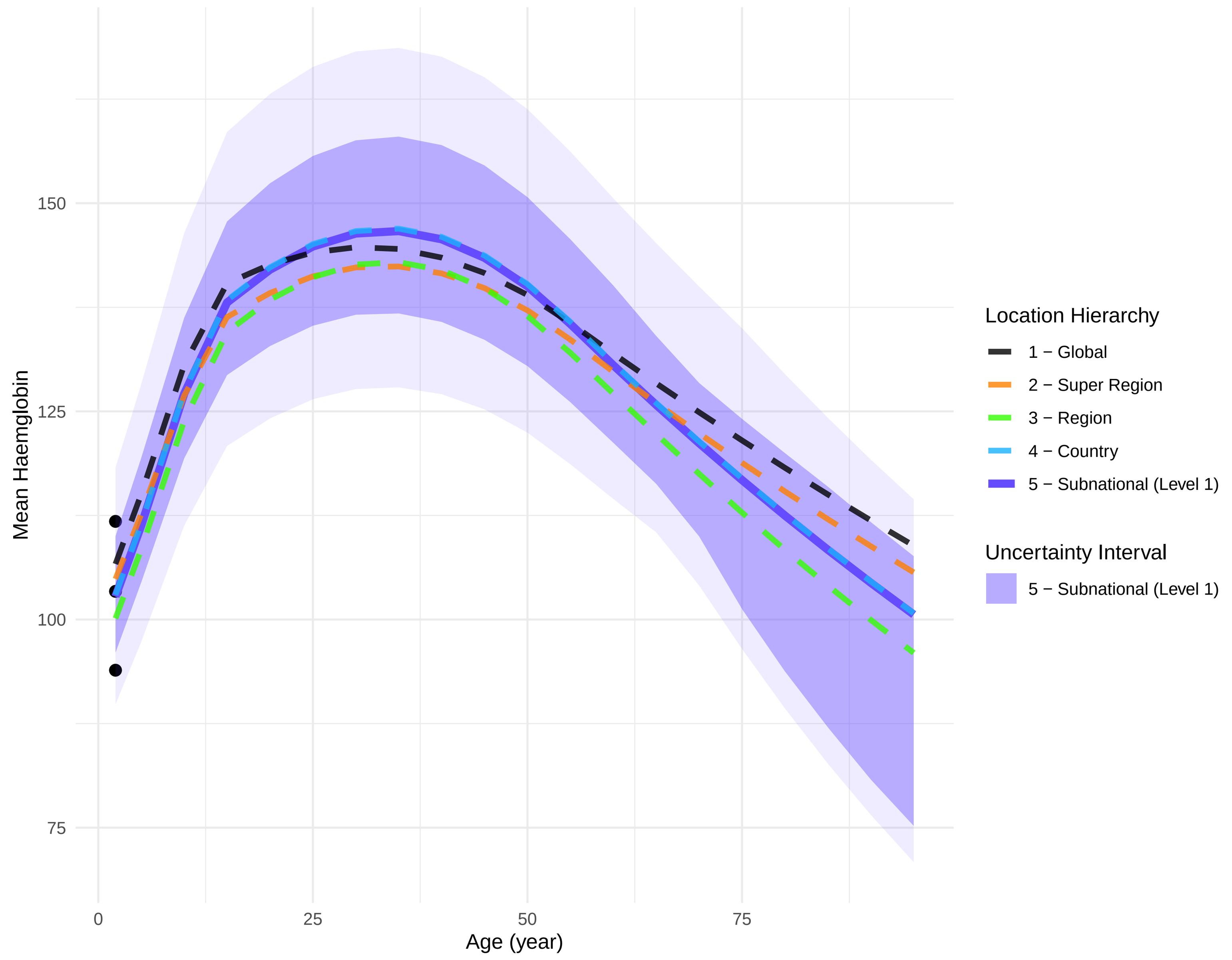

MR−BRT Age/Sex Fits − Males 2+ Years Old − Mean Haemglobin − Gombe
WHO−2001 Adjustment Method

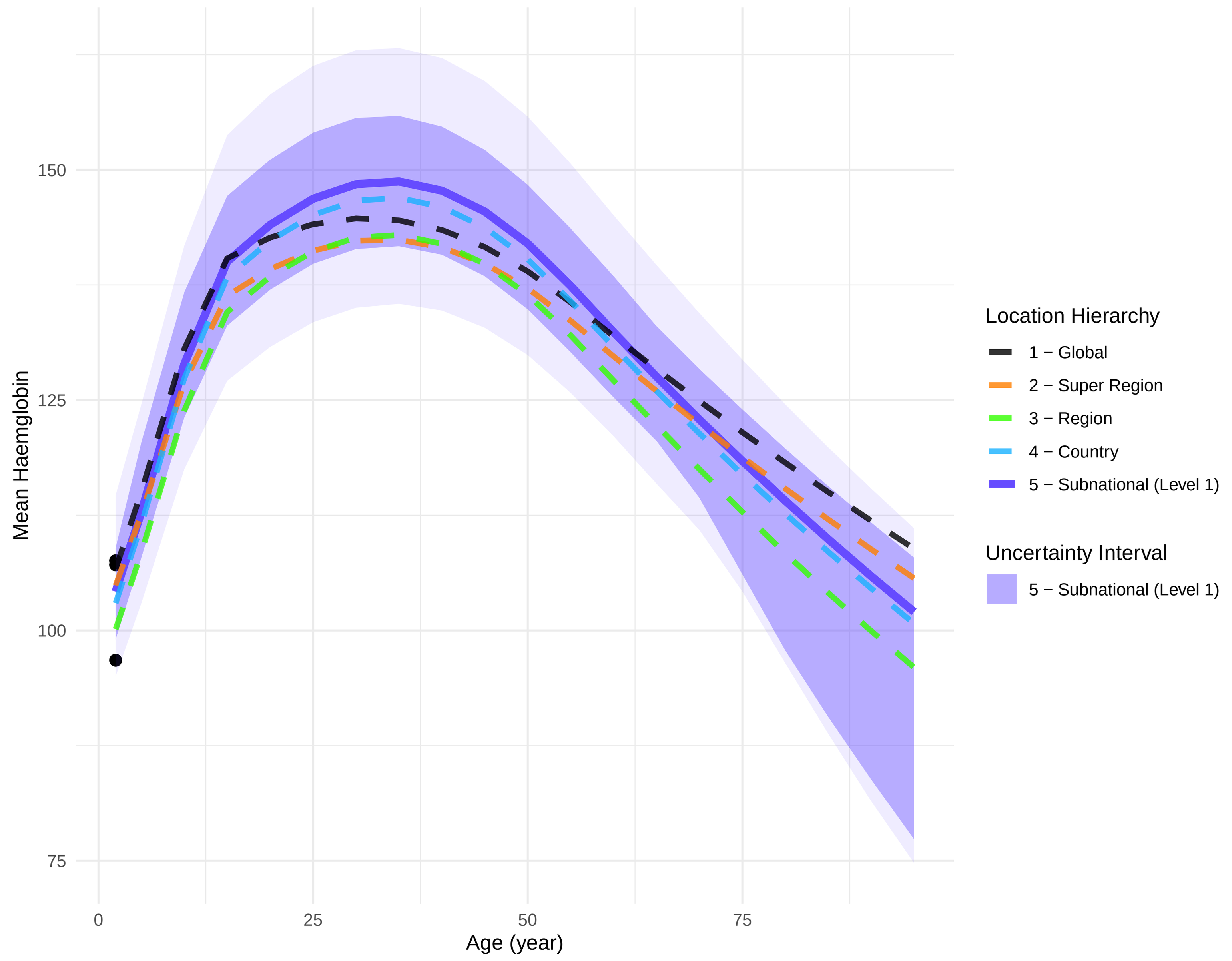

MR−BRT Age/Sex Fits − Males 2+ Years Old − Mean Haemglobin − Imo
WHO−2001 Adjustment Method

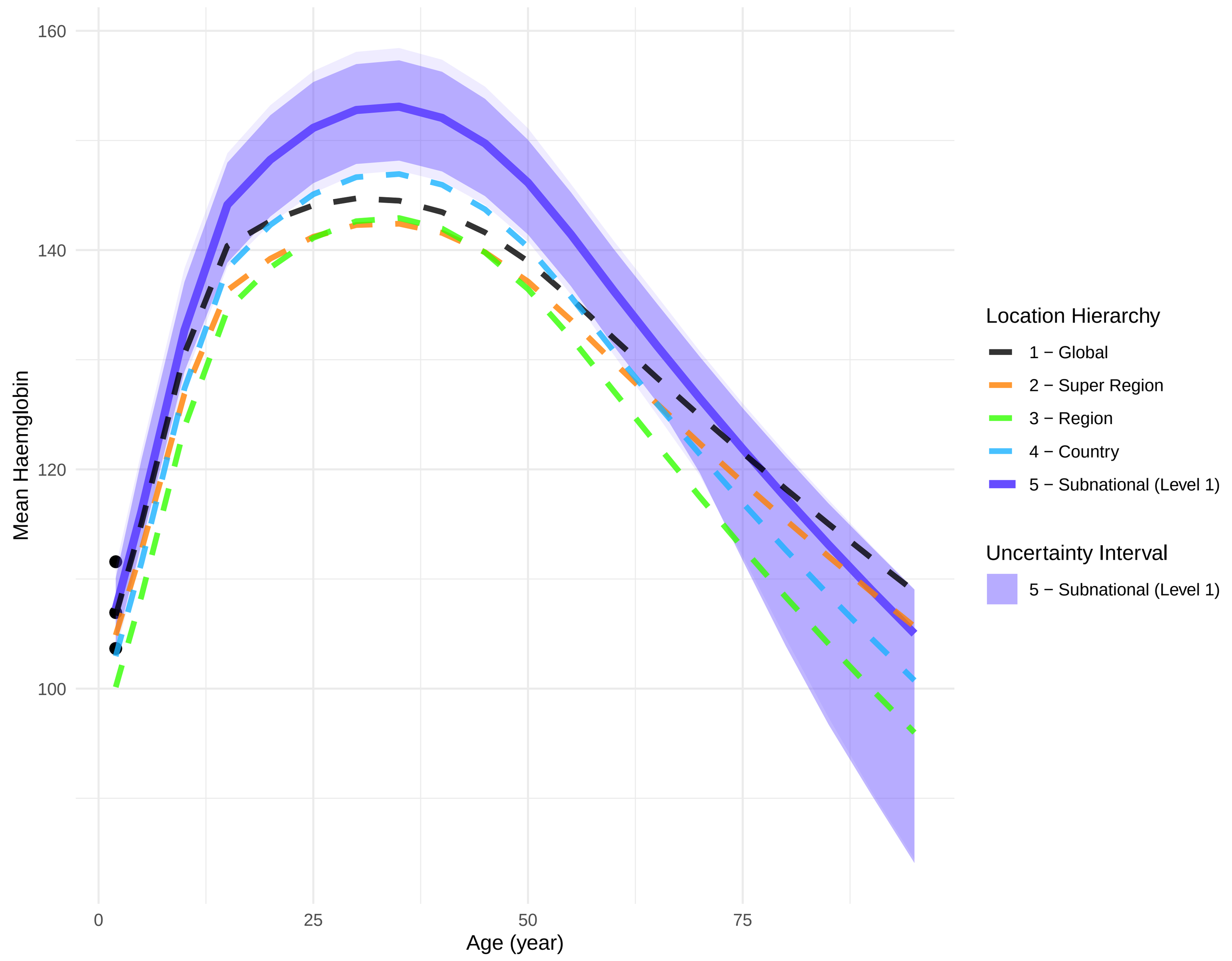

MR−BRT Age/Sex Fits − Males 2+ Years Old − Mean Haemglobin − Jigawa
WHO−2001 Adjustment Method

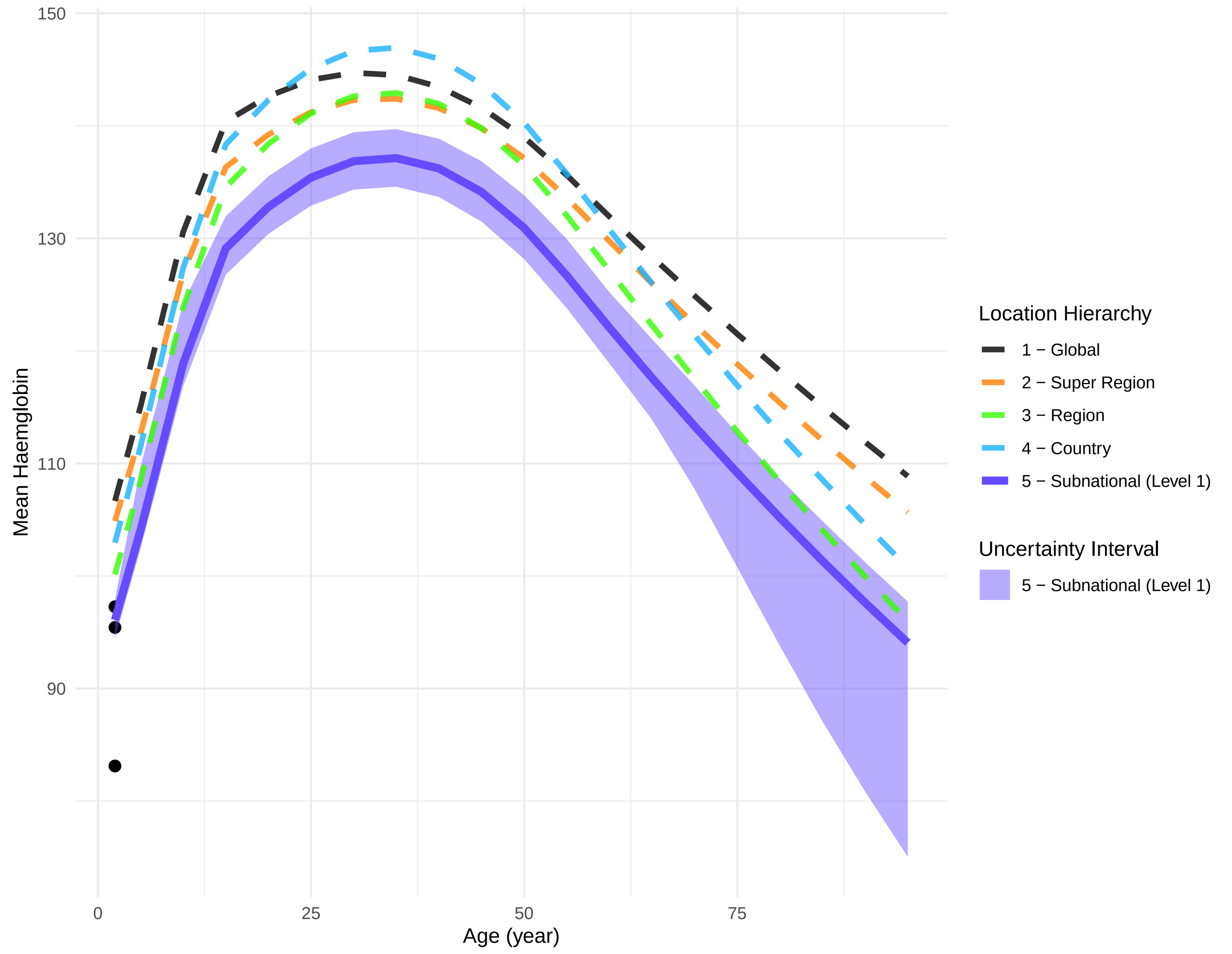

MR−BRT Age/Sex Fits − Males 2+ Years Old − Mean Haemglobin − Kaduna
WHO−2001 Adjustment Method

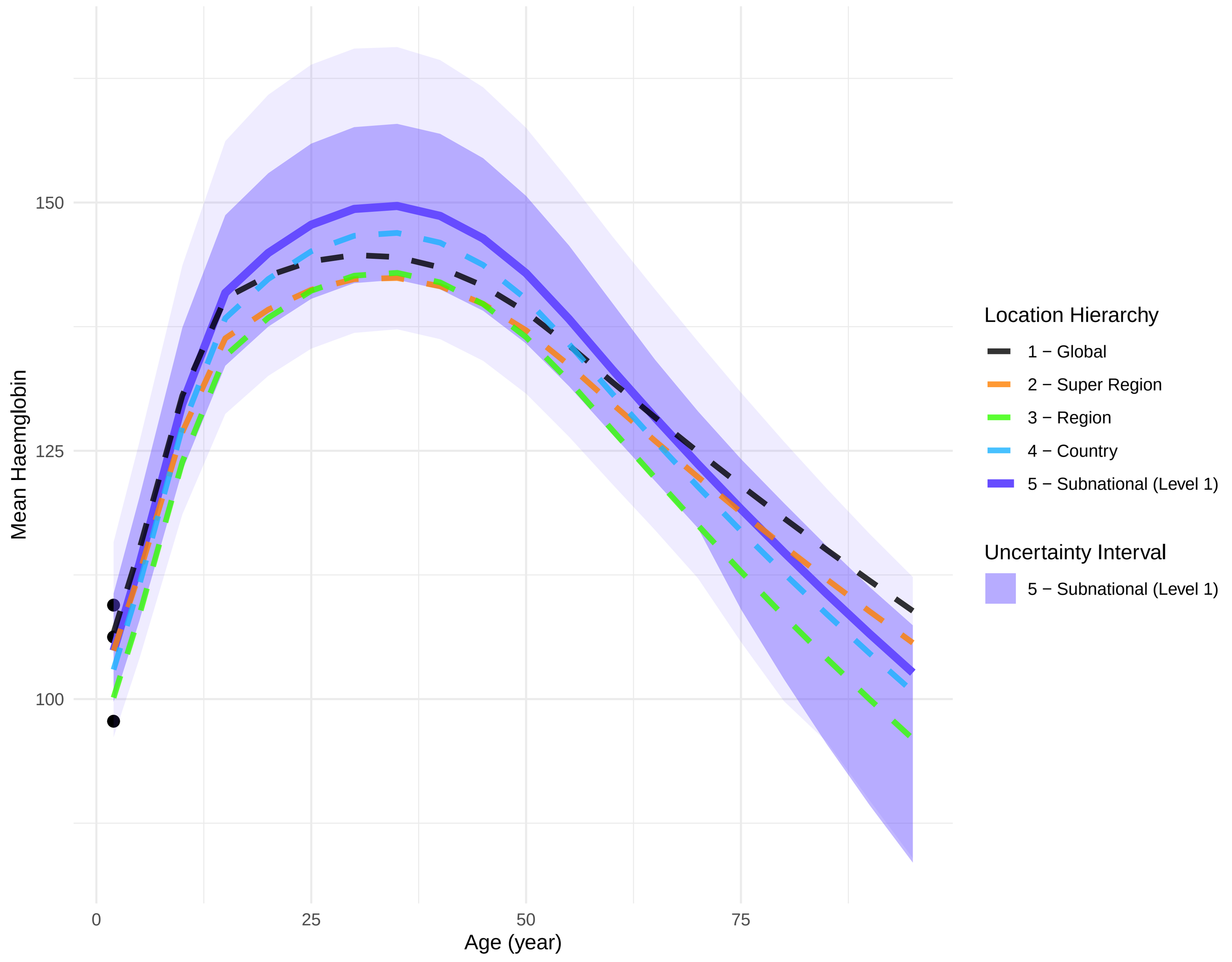

MR−BRT Age/Sex Fits − Males 2+ Years Old − Mean Haemglobin − Kano
WHO−2001 Adjustment Method

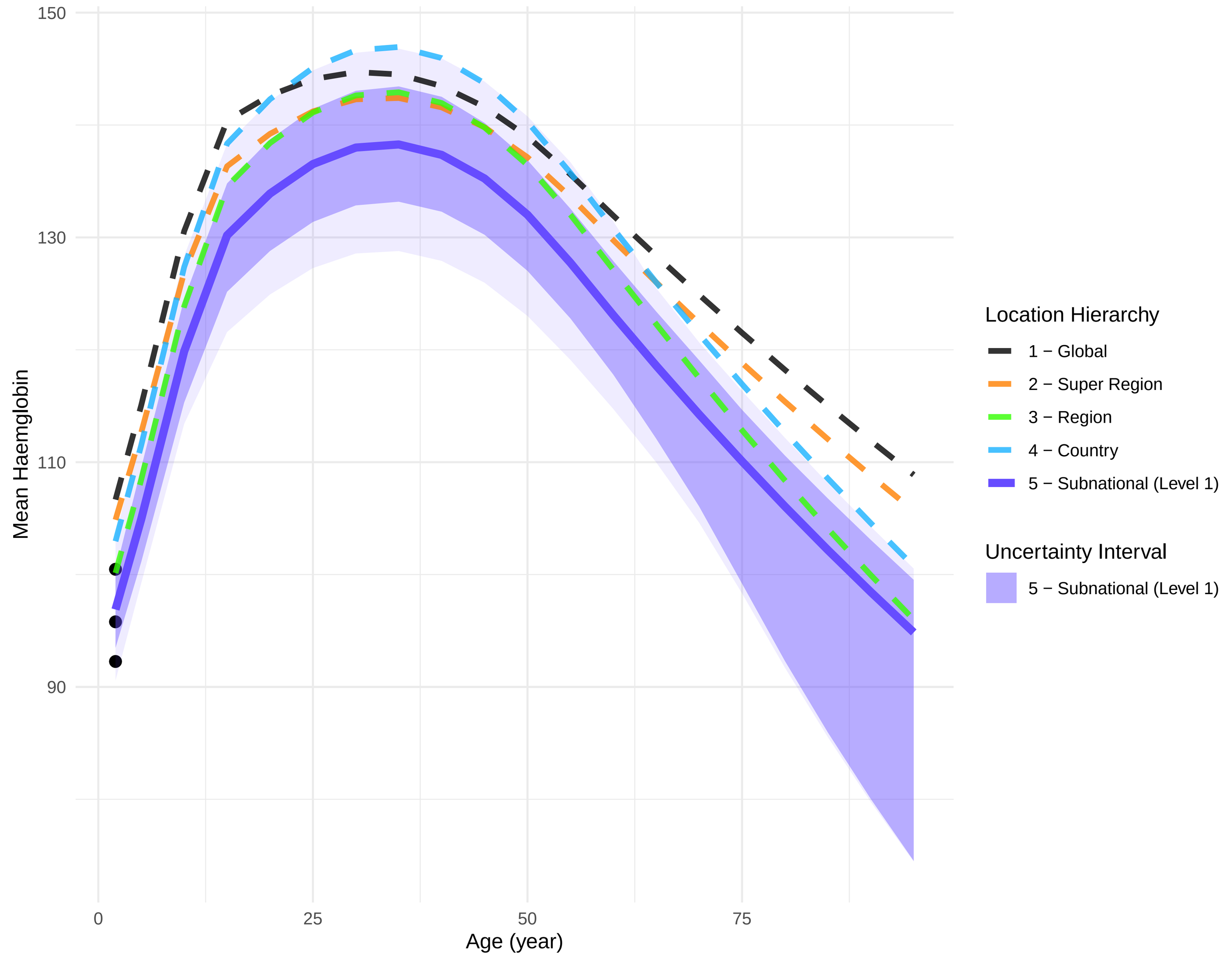

MR−BRT Age/Sex Fits − Males 2+ Years Old − Mean Haemglobin − Katsina
WHO−2001 Adjustment Method

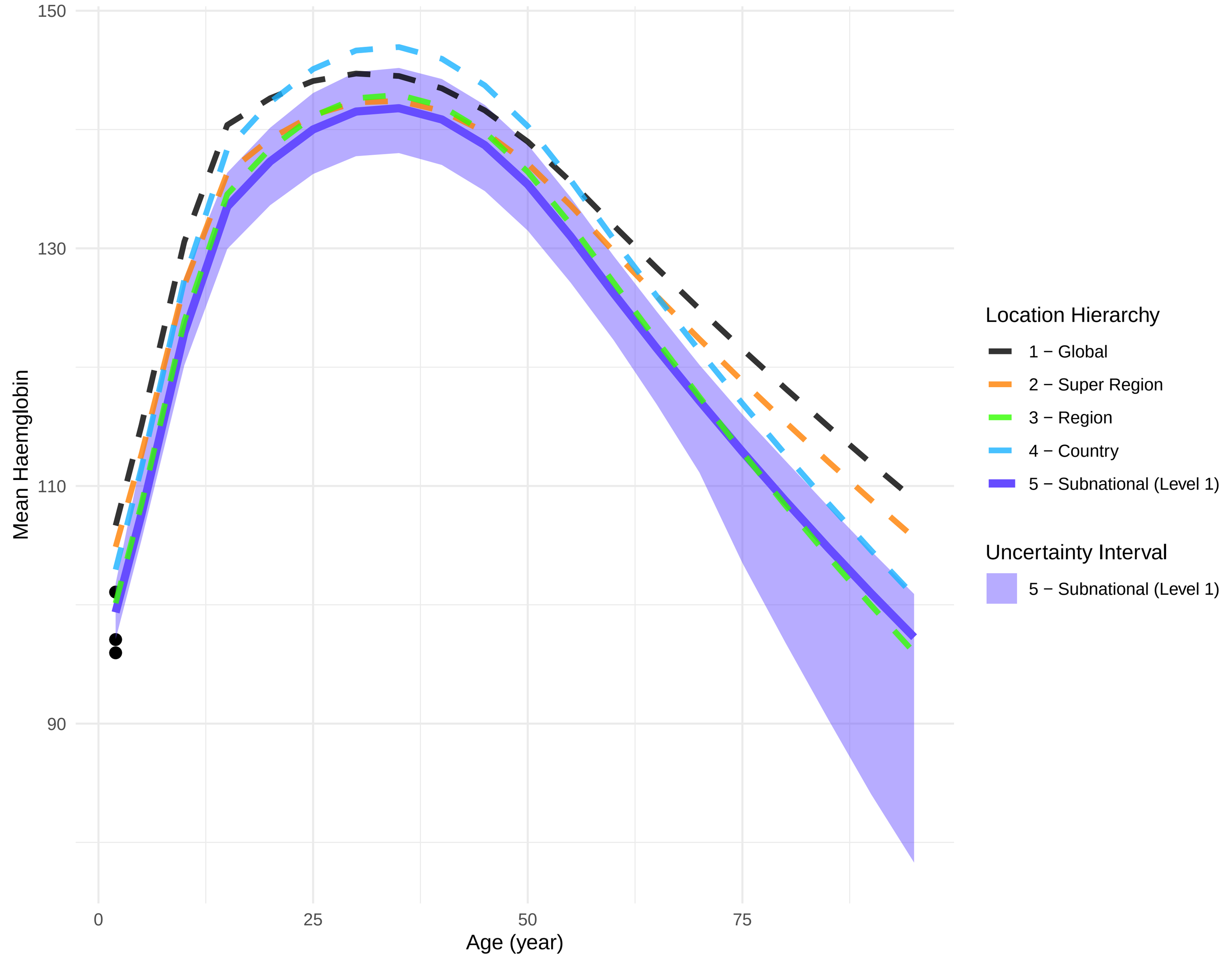

MR−BRT Age/Sex Fits − Males 2+ Years Old − Mean Haemglobin − Kebbi
WHO−2001 Adjustment Method

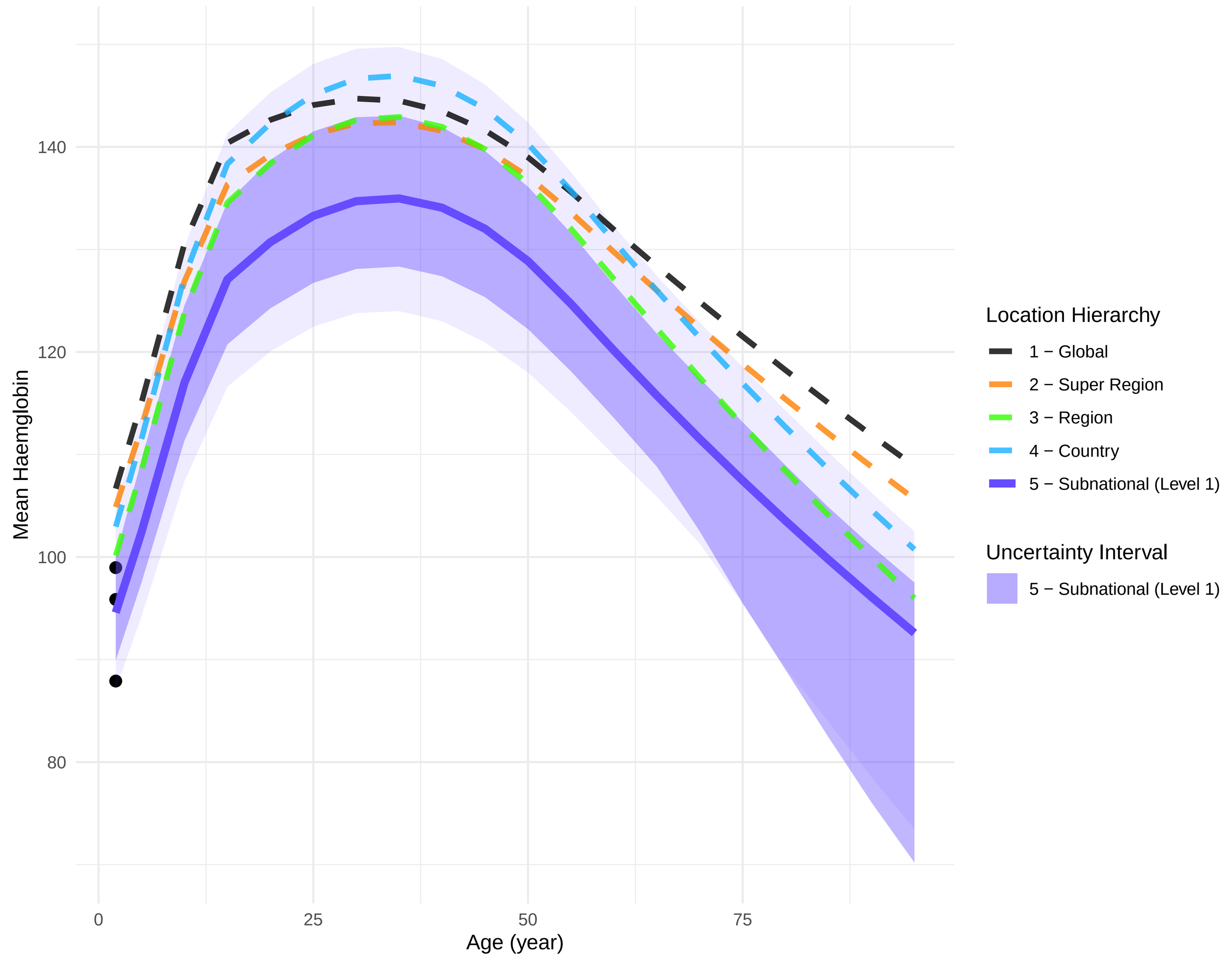

MR−BRT Age/Sex Fits − Males 2+ Years Old − Mean Haemglobin − Kogi
WHO−2001 Adjustment Method

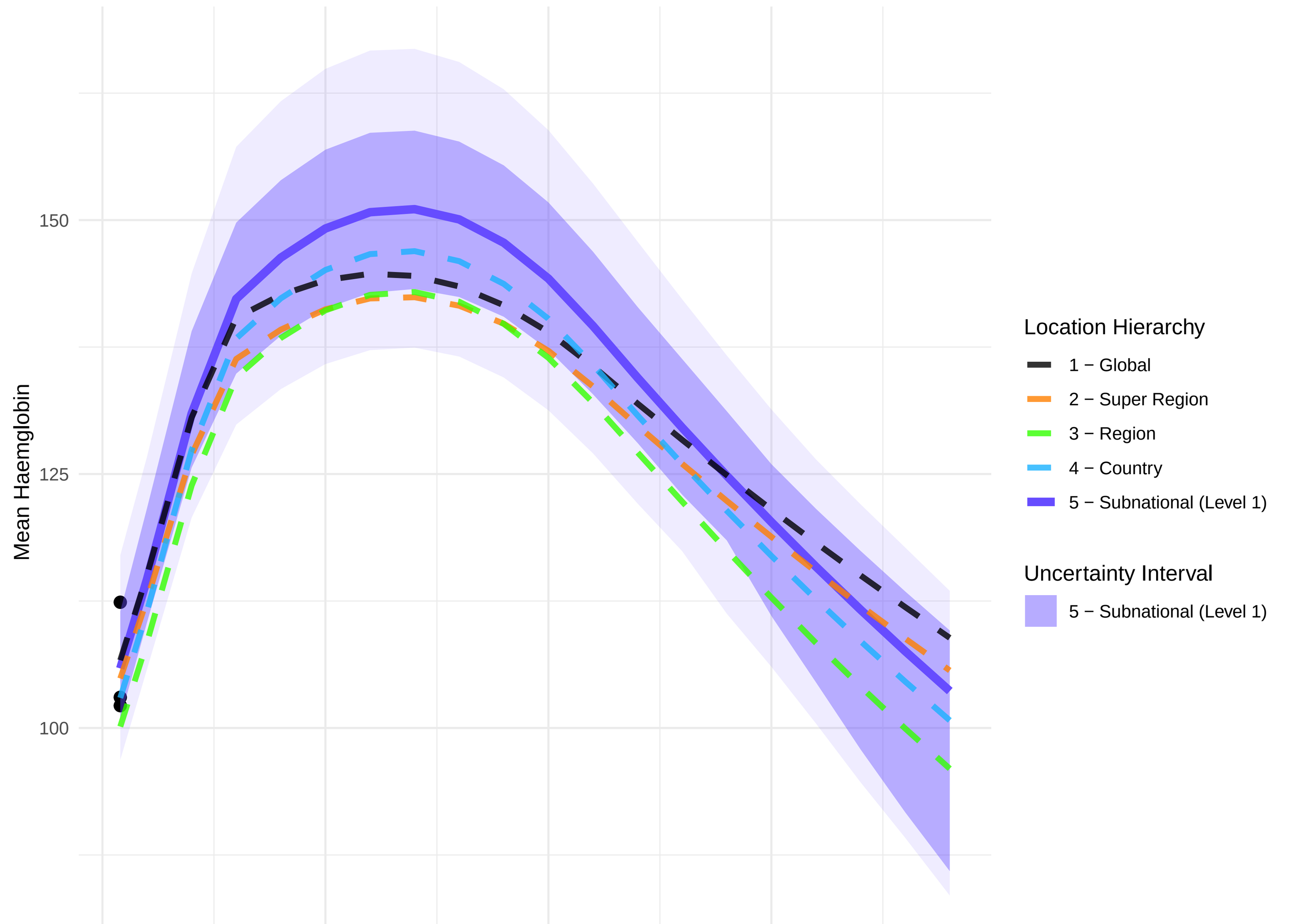

MR−BRT Age/Sex Fits − Males 2+ Years Old − Mean Haemglobin − Kwara
WHO−2001 Adjustment Method

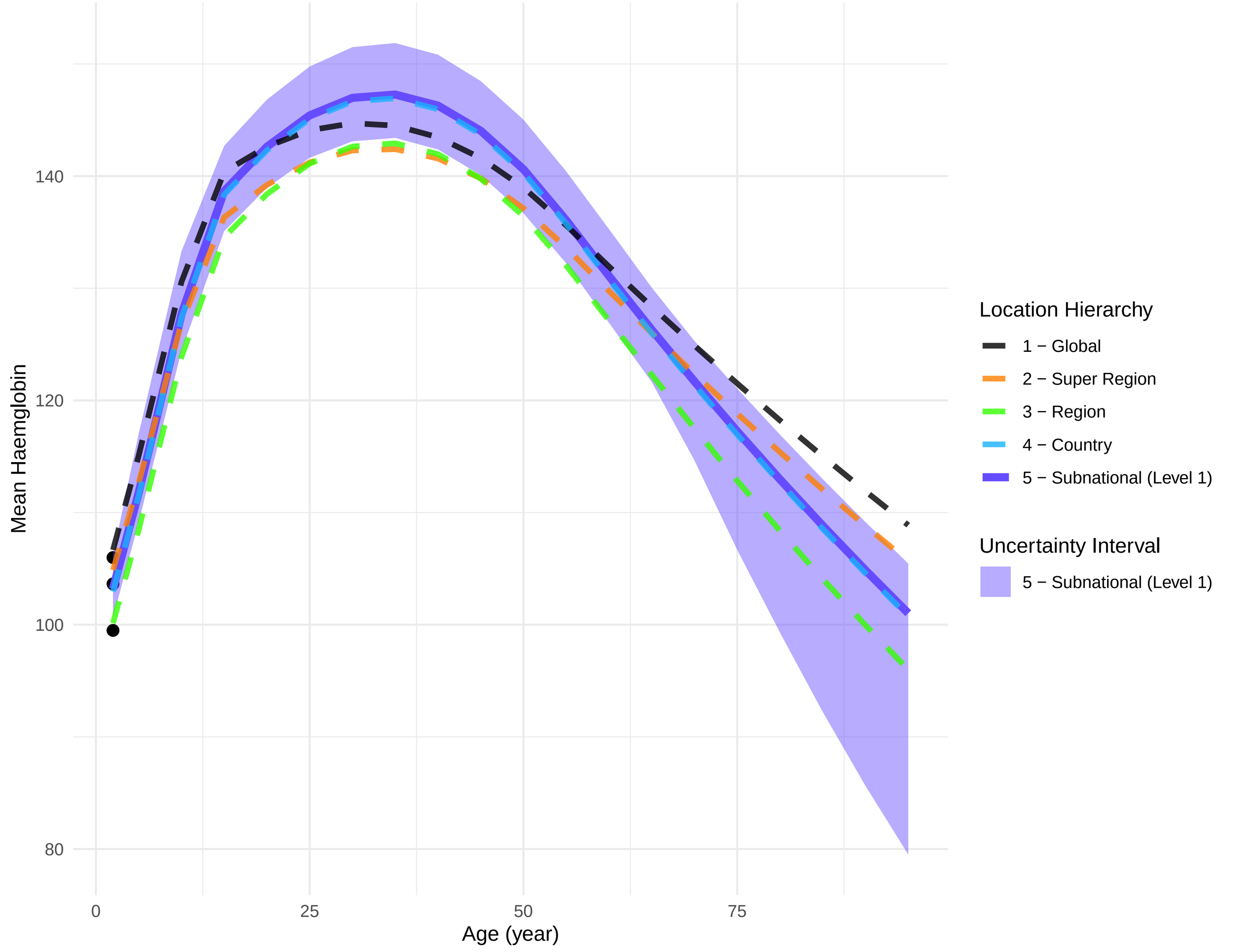

MR−BRT Age/Sex Fits − Males 2+ Years Old − Mean Haemglobin − Lagos
WHO−2001 Adjustment Method

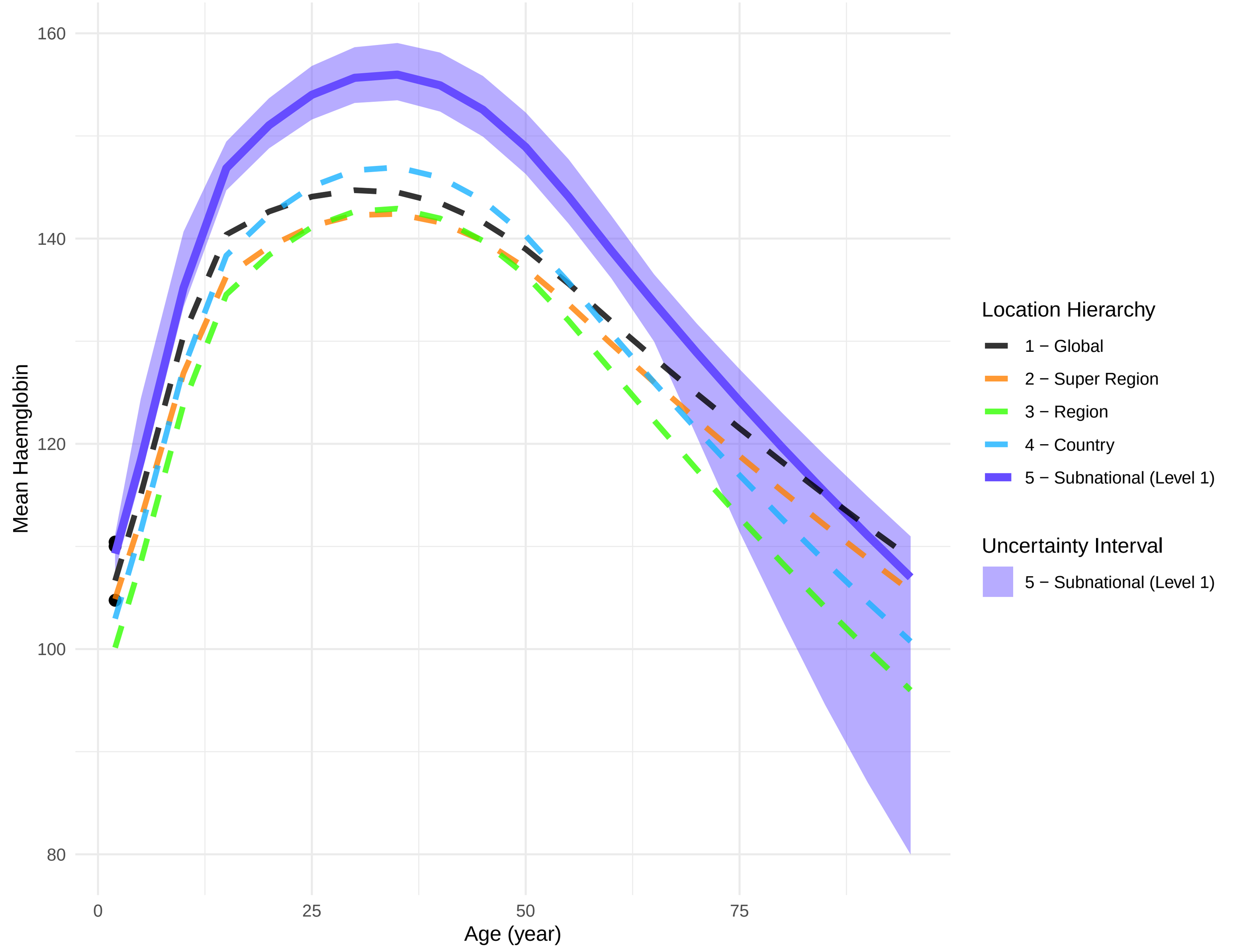

MR−BRT Age/Sex Fits − Males 2+ Years Old − Mean Haemglobin − Nasarawa
WHO−2001 Adjustment Method

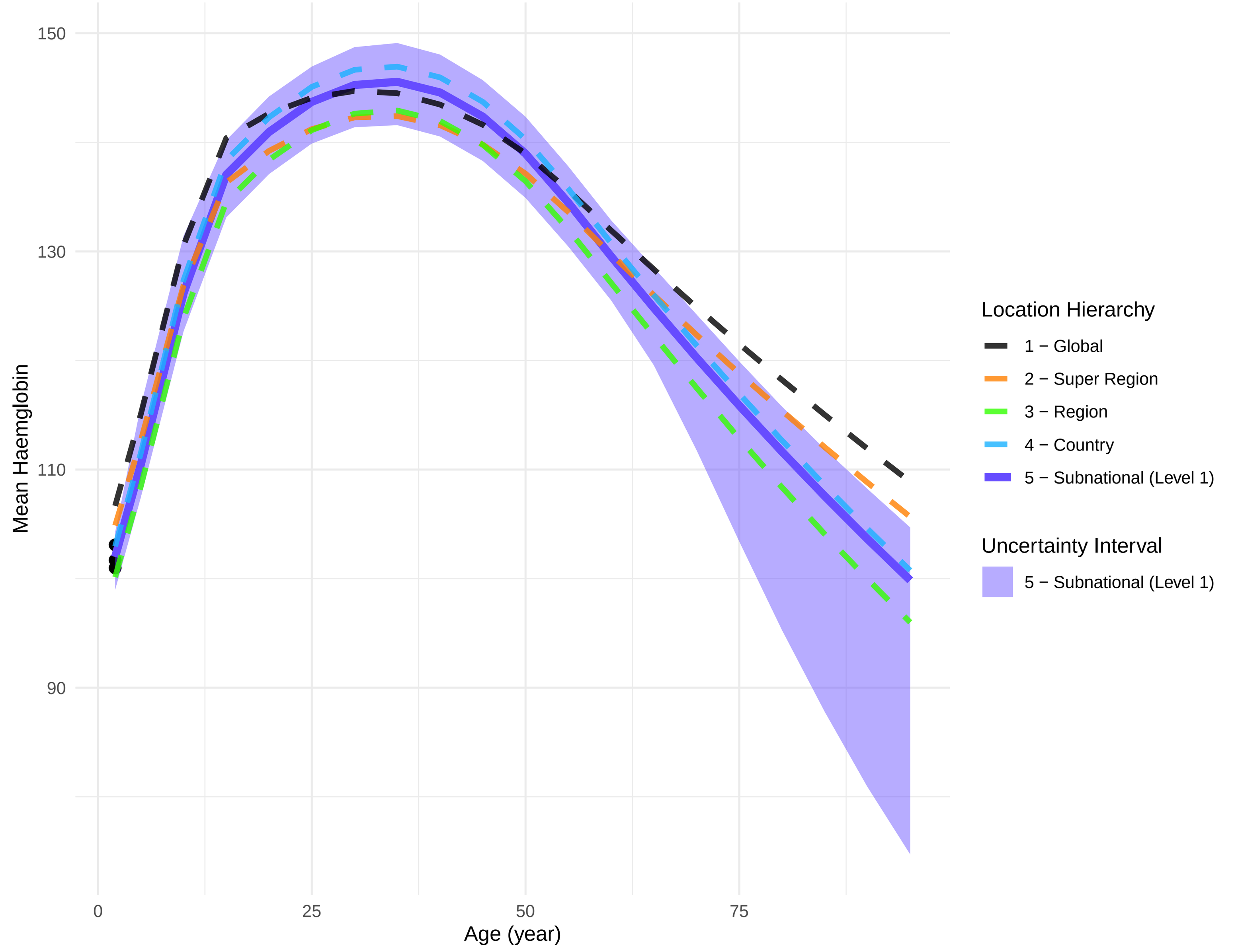

MR−BRT Age/Sex Fits − Males 2+ Years Old − Mean Haemglobin − Niger
WHO−2001 Adjustment Method

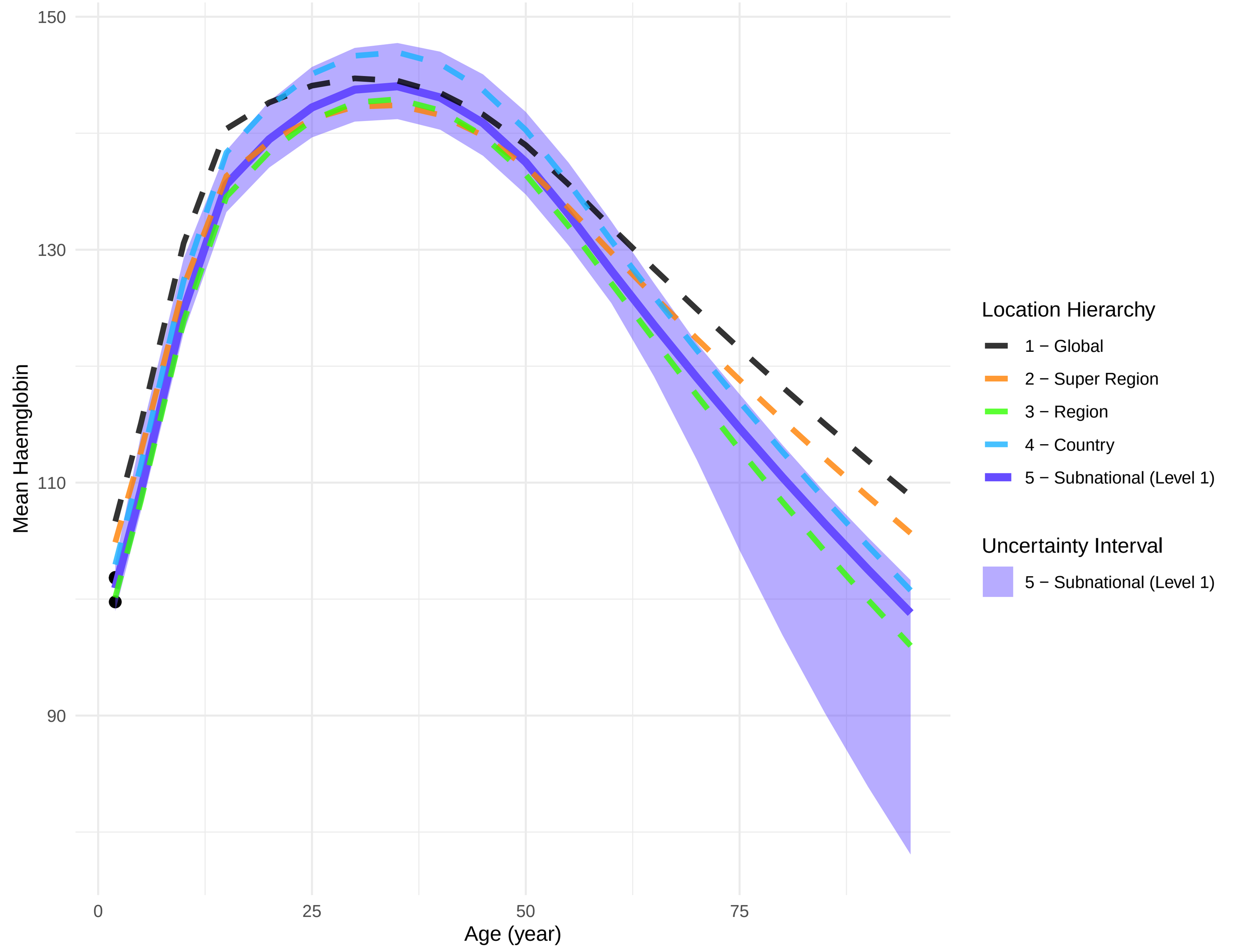

MR−BRT Age/Sex Fits − Males 2+ Years Old − Mean Haemglobin − Ogun
WHO−2001 Adjustment Method

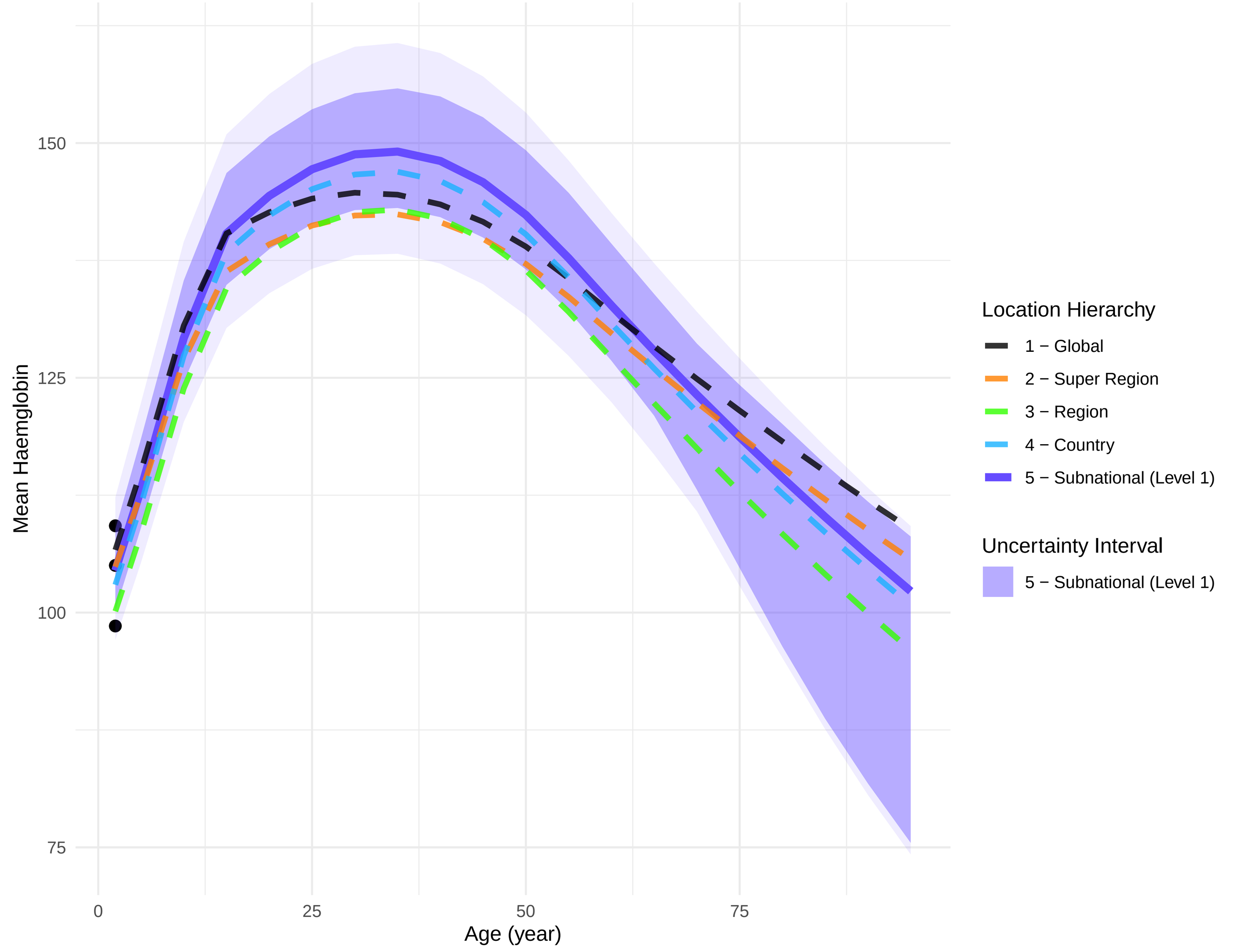

MR−BRT Age/Sex Fits − Males 2+ Years Old − Mean Haemglobin − Ondo
WHO−2001 Adjustment Method

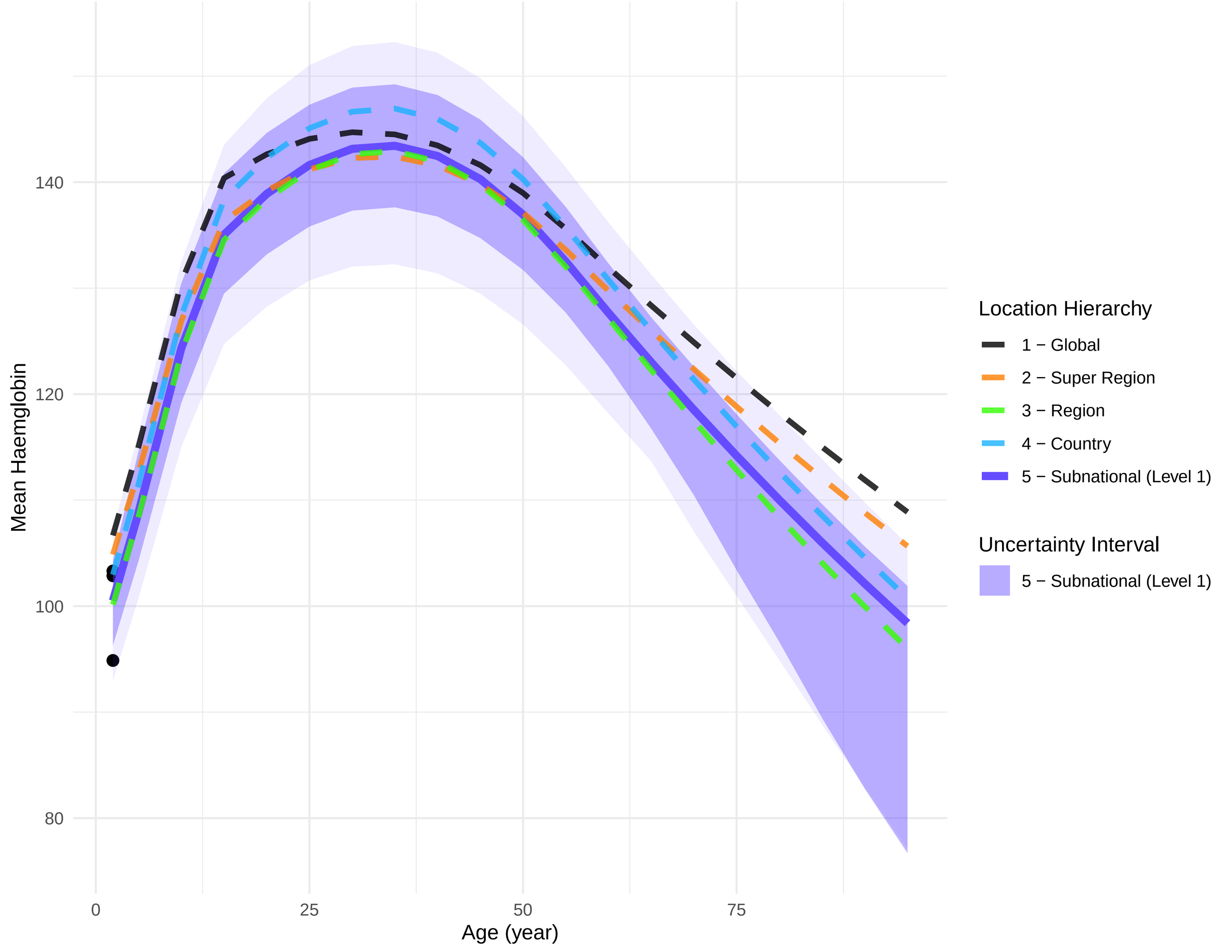

MR−BRT Age/Sex Fits − Males 2+ Years Old − Mean Haemglobin − Osun
WHO−2001 Adjustment Method

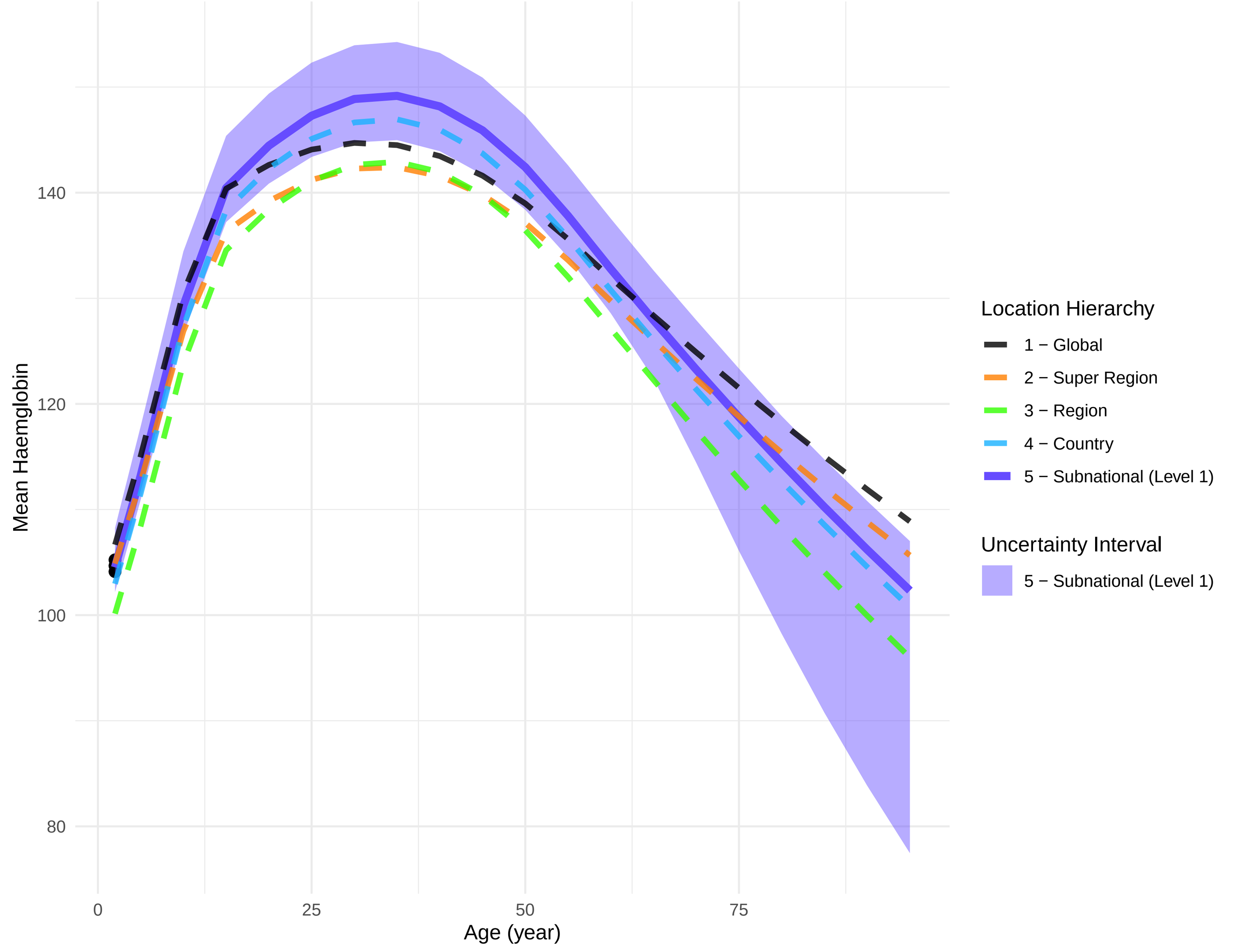

MR−BRT Age/Sex Fits − Males 2+ Years Old − Mean Haemglobin − Oyo
WHO−2001 Adjustment Method

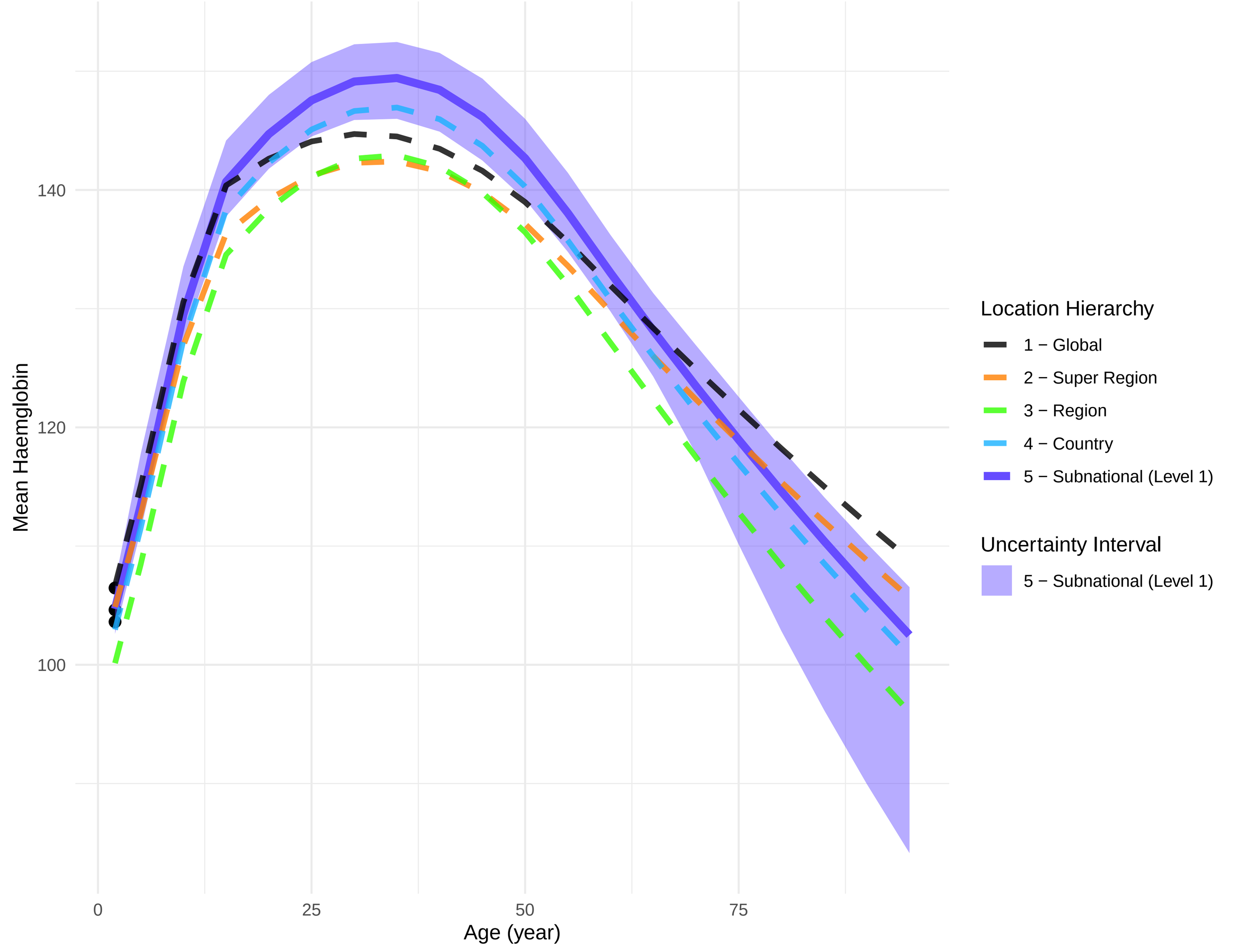

MR−BRT Age/Sex Fits − Males 2+ Years Old − Mean Haemglobin − Plateau
WHO−2001 Adjustment Method

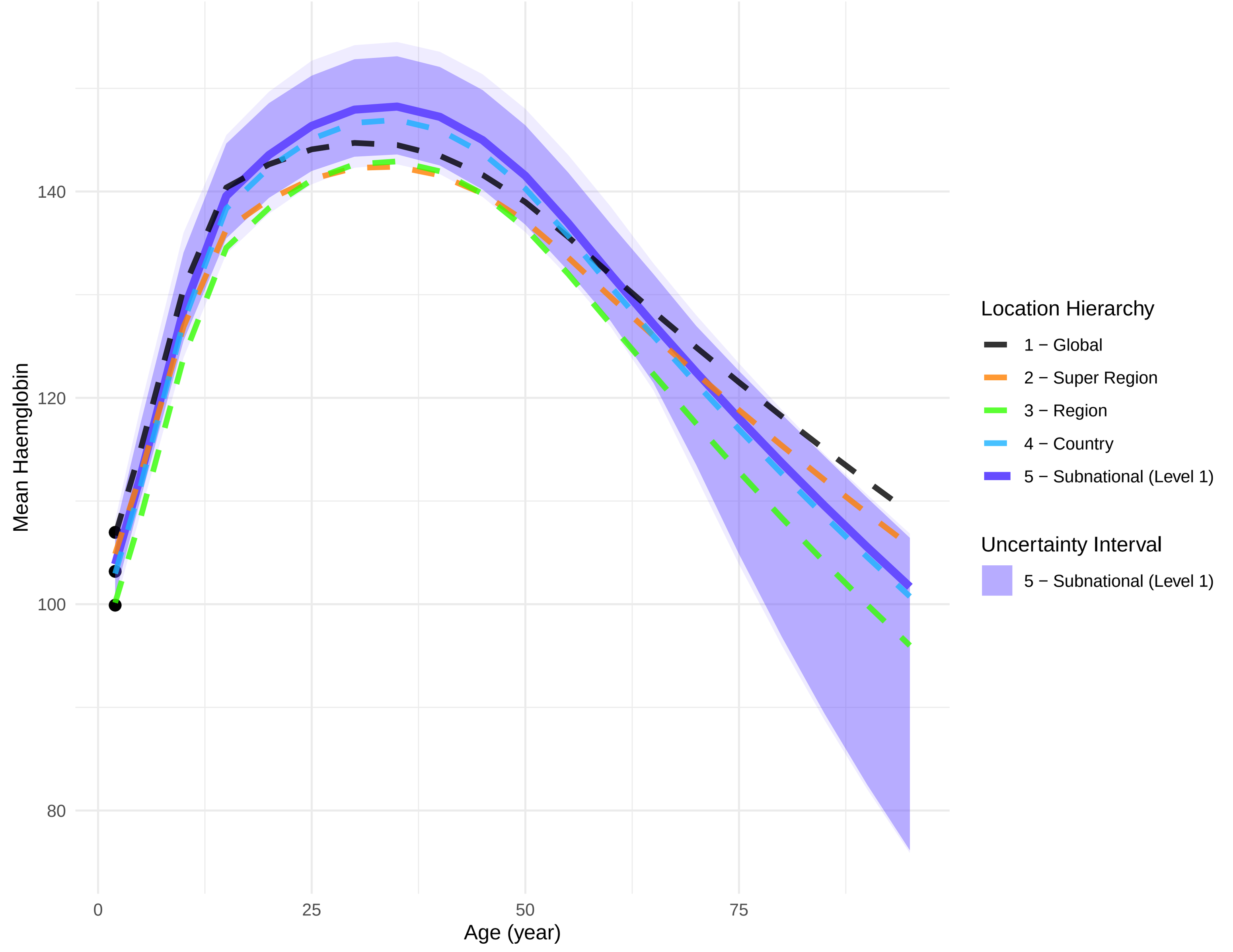

MR−BRT Age/Sex Fits − Males 2+ Years Old − Mean Haemglobin − Rivers
WHO−2001 Adjustment Method

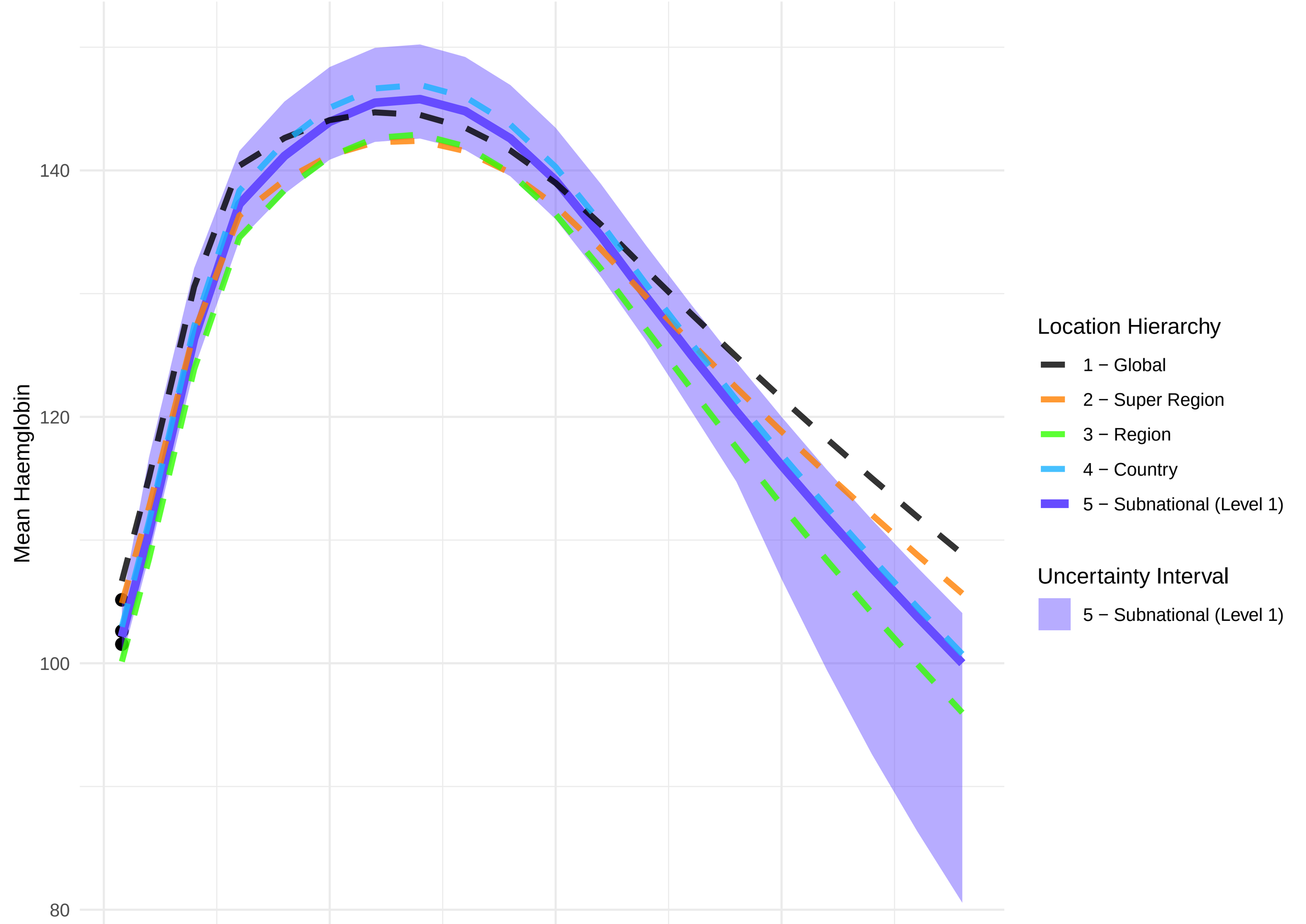

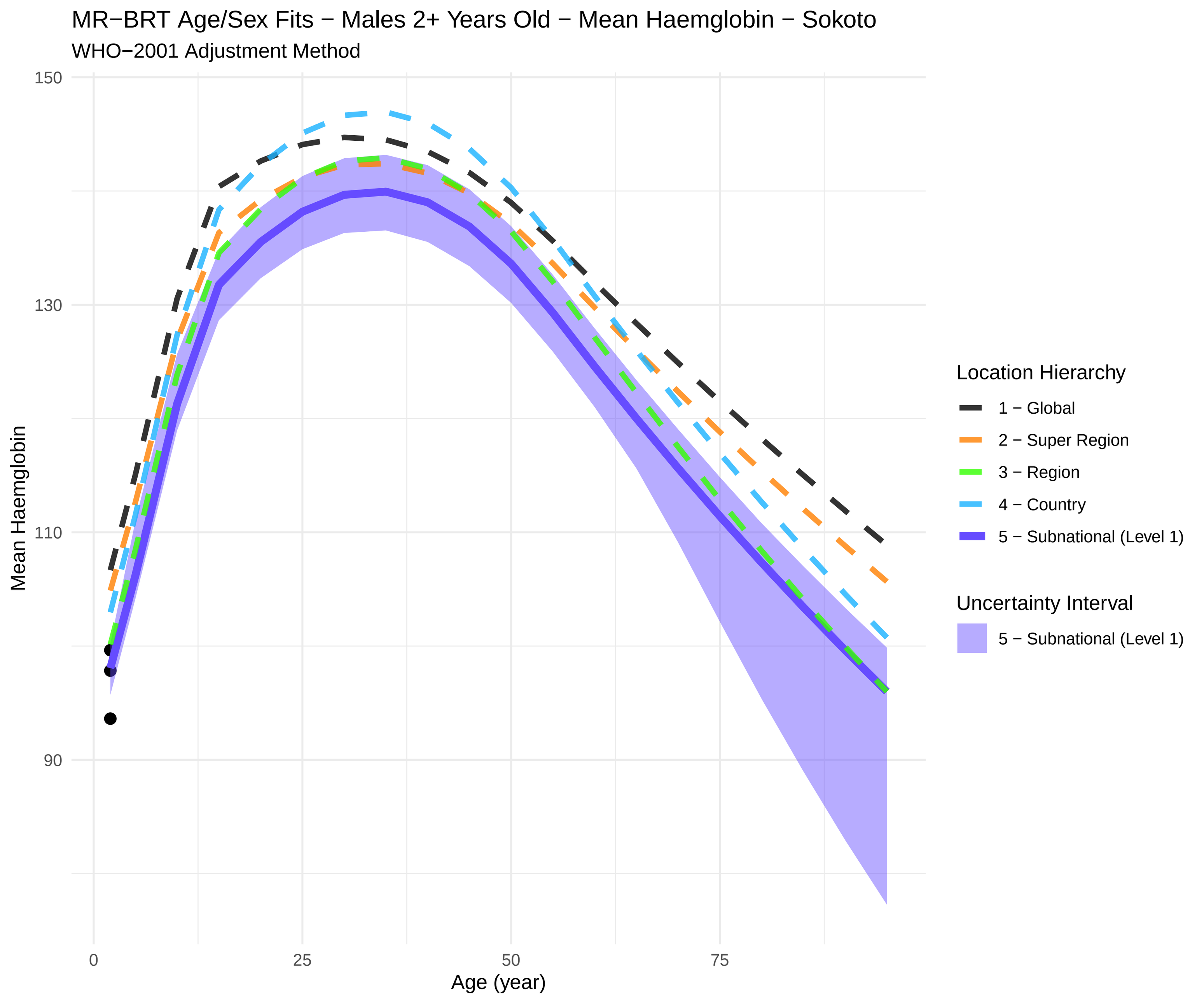
MR−BRT Age/Sex Fits − Males 2+ Years Old − Mean Haemglobin − Sokoto
WHO−2001 Adjustment Method
Mean Haemglobin
Age (year)
Location Hierarchy
1 − Global
2 − Super Region
3 − Region
4 − Country
5 − Subnational (Level 1)
Uncertainty Interval
5 − Subnational (Level 1)

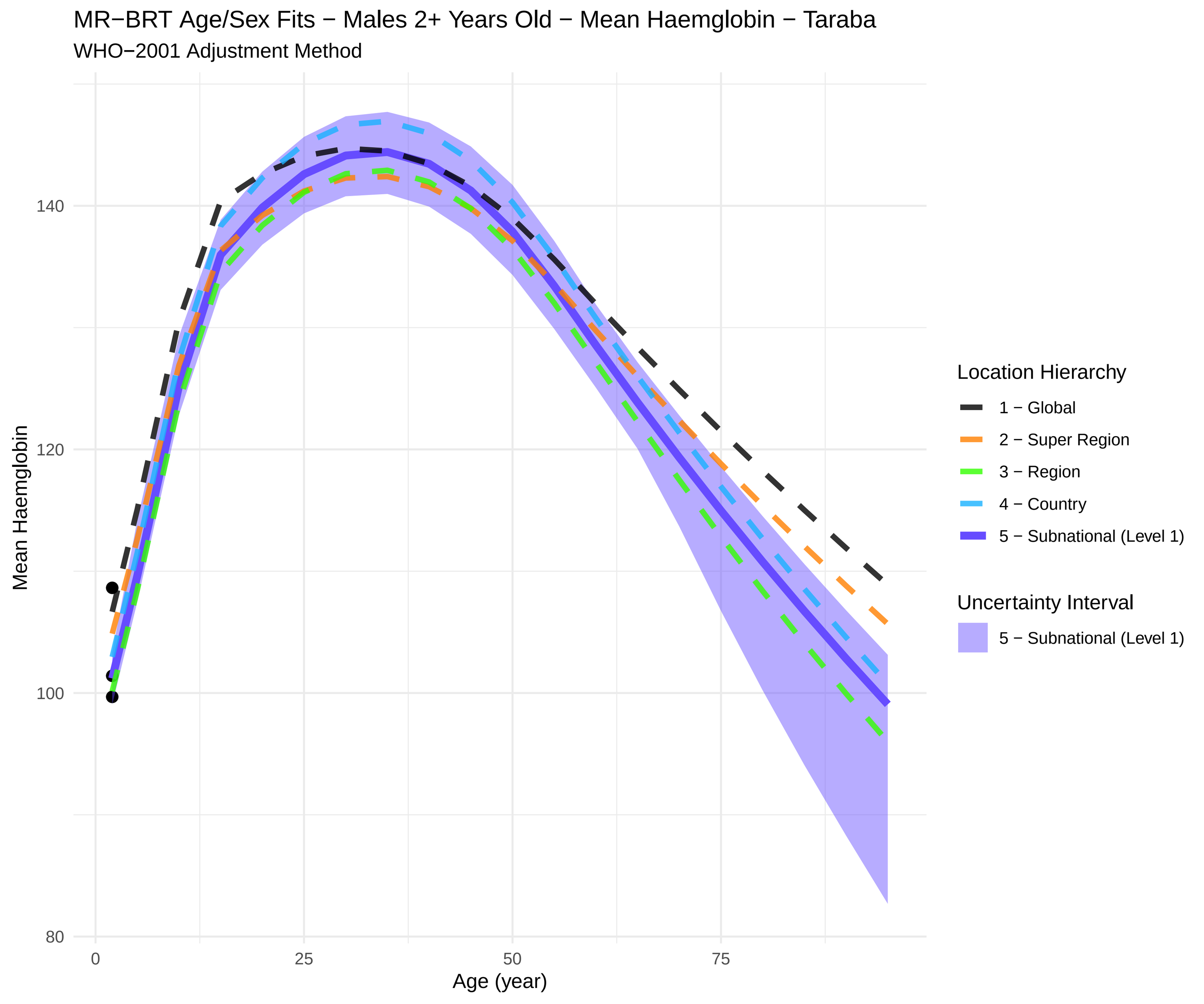
MR−BRT Age/Sex Fits − Males 2+ Years Old − Mean Haemglobin − Taraba
WHO−2001 Adjustment Method
Mean Haemglobin
Age (year)
Location Hierarchy
1 − Global
2 − Super Region
3 − Region
4 − Country
5 − Subnational (Level 1)
Uncertainty Interval
5 − Subnational (Level 1)

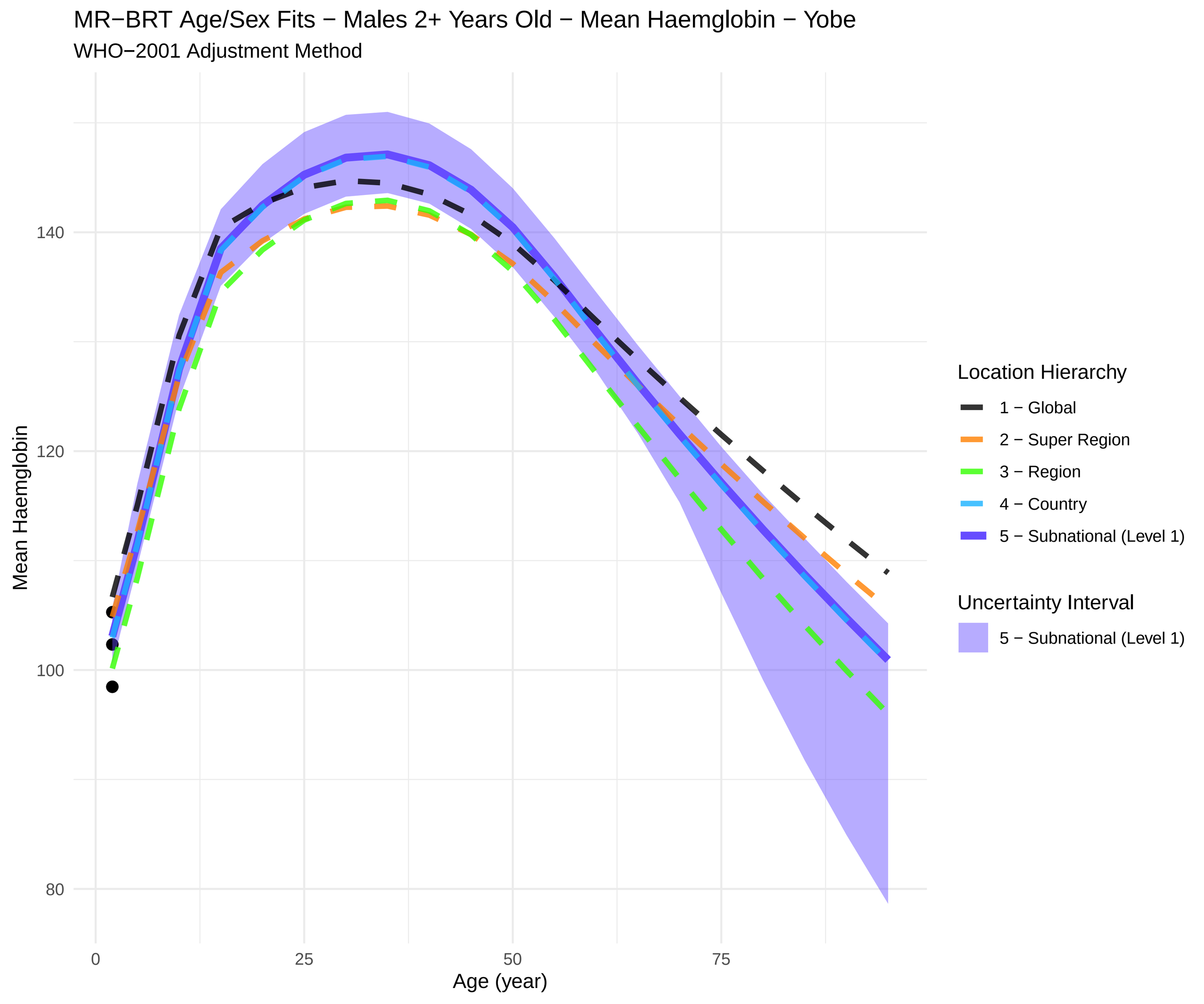
MR−BRT Age/Sex Fits − Males 2+ Years Old − Mean Haemglobin − Yobe
WHO−2001 Adjustment Method
Mean Haemglobin
Age (year)
Location Hierarchy
1 − Global
2 − Super Region
3 − Region
4 − Country
5 − Subnational (Level 1)
Uncertainty Interval
5 − Subnational (Level 1)

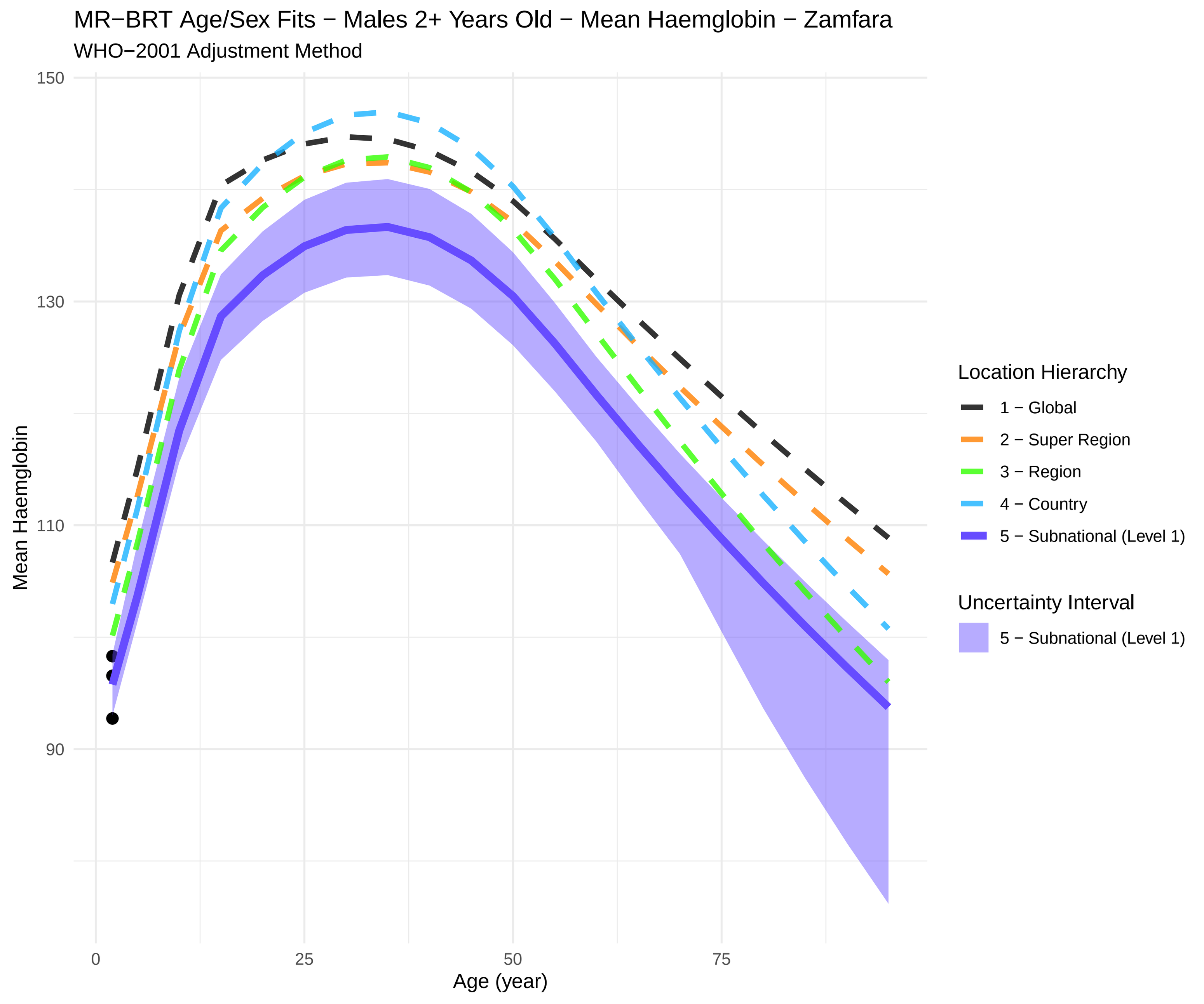
MR−BRT Age/Sex Fits − Males 2+ Years Old − Mean Haemglobin − Zamfara
WHO−2001 Adjustment Method
Mean Haemglobin
Age (year)
Location Hierarchy
1 − Global
2 − Super Region
3 − Region
4 − Country
5 − Subnational (Level 1)
Uncertainty Interval
5 − Subnational (Level 1)

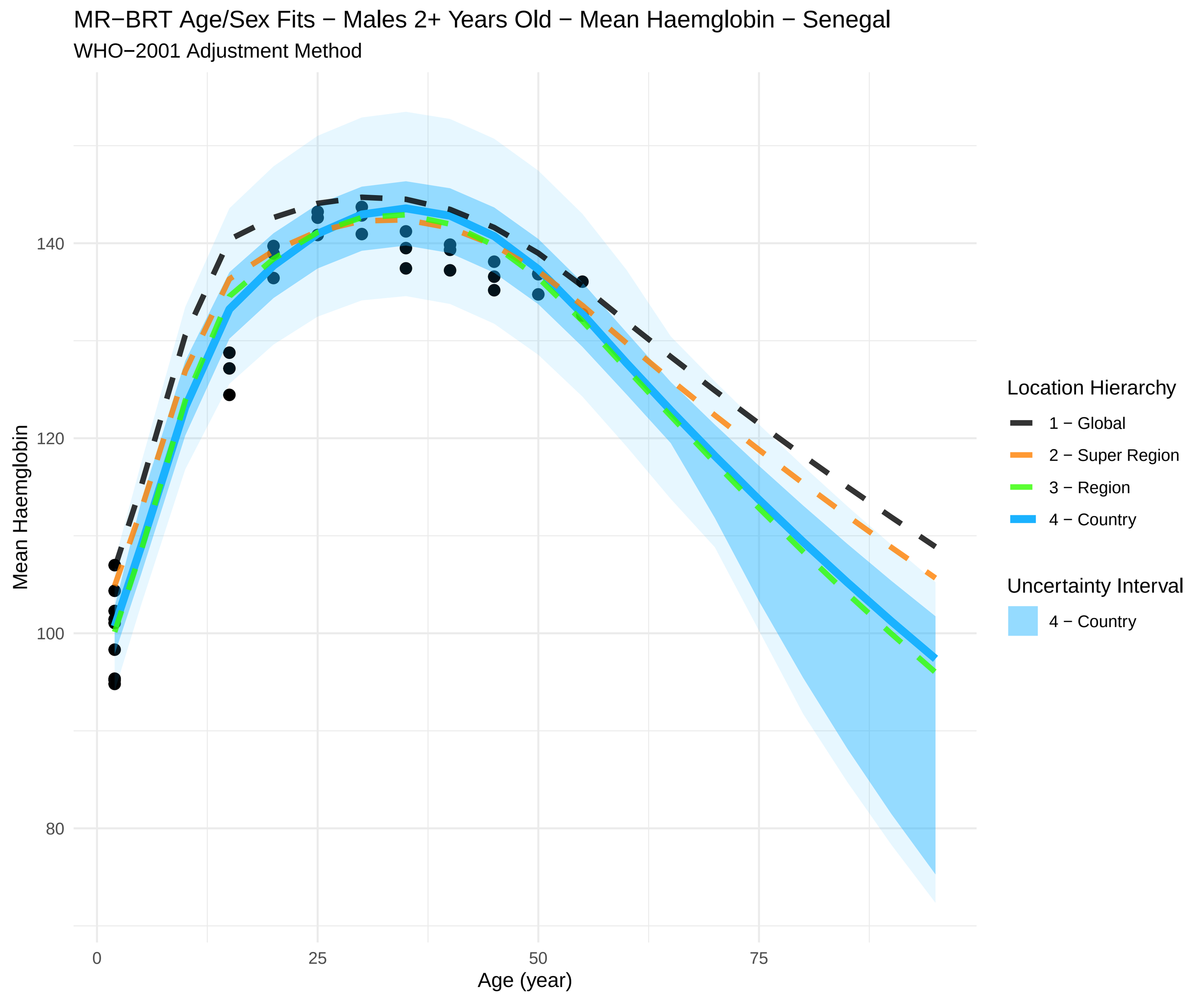
MR−BRT Age/Sex Fits − Males 2+ Years Old − Mean Haemglobin − Senegal
WHO−2001 Adjustment Method
Mean Haemglobin
Age (year)
Location Hierarchy
1 − Global
2 − Super Region
3 − Region
4 − Country
Uncertainty Interval
4 − Country

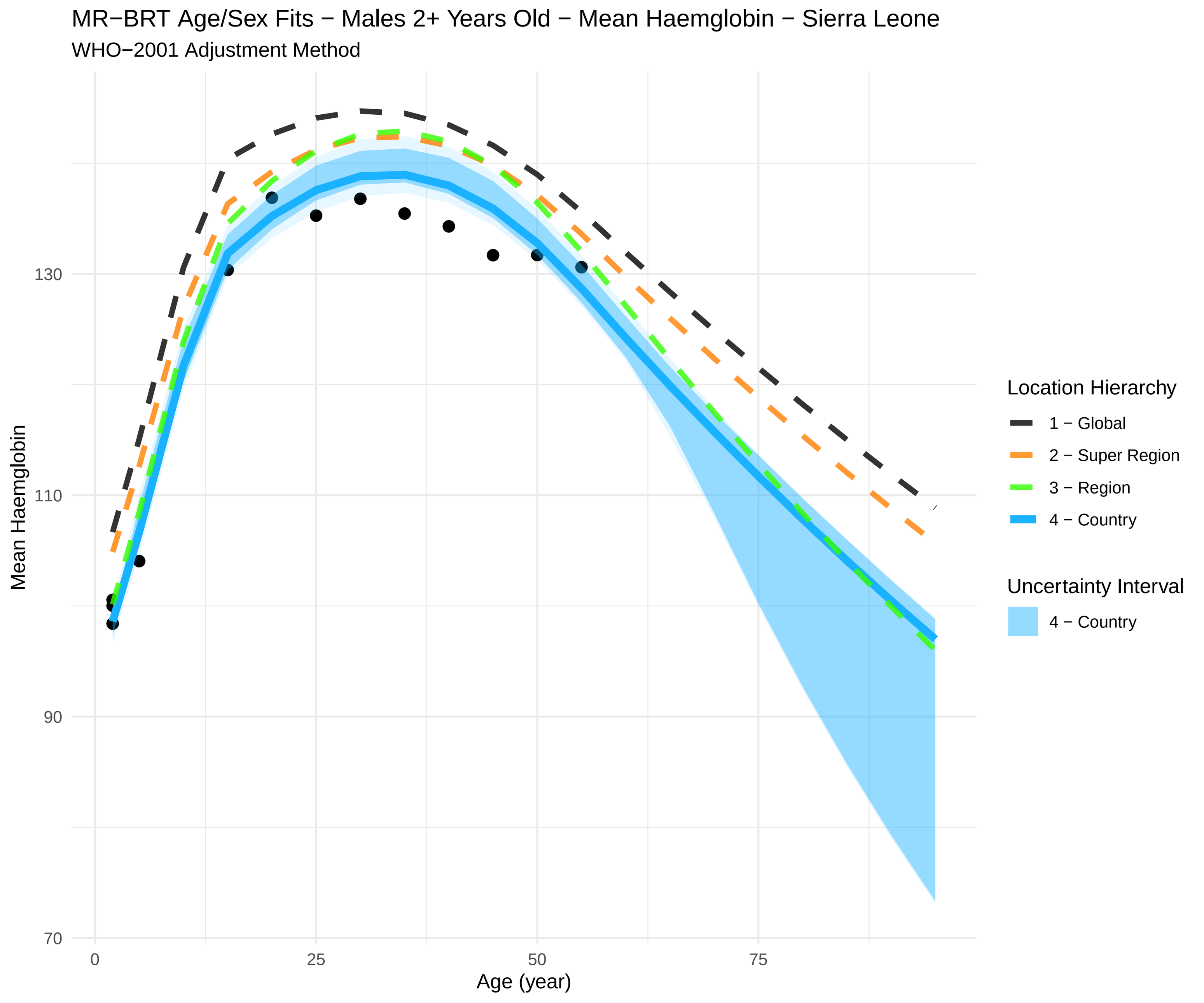
MR−BRT Age/Sex Fits − Males 2+ Years Old − Mean Haemglobin − Sierra Leone
WHO−2001 Adjustment Method
Mean Haemglobin
Age (year)
Location Hierarchy
1 − Global
2 − Super Region
3 − Region
4 − Country
Uncertainty Interval
4 − Country

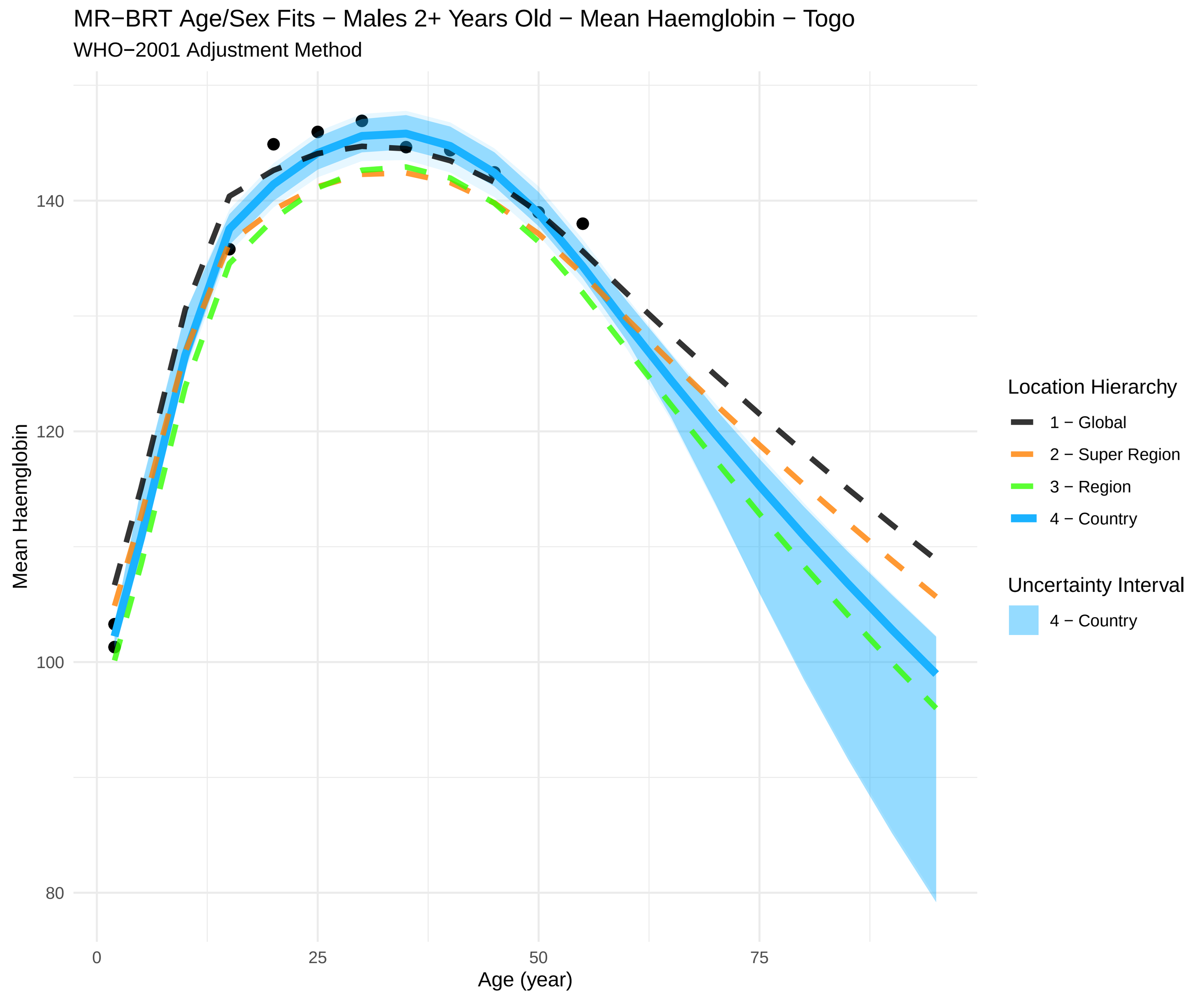
MR−BRT Age/Sex Fits − Males 2+ Years Old − Mean Haemglobin − Togo
WHO−2001 Adjustment Method
Mean Haemglobin
Age (year)
Location Hierarchy
1 − Global
2 − Super Region
3 − Region
4 − Country
Uncertainty Interval
4 − Country

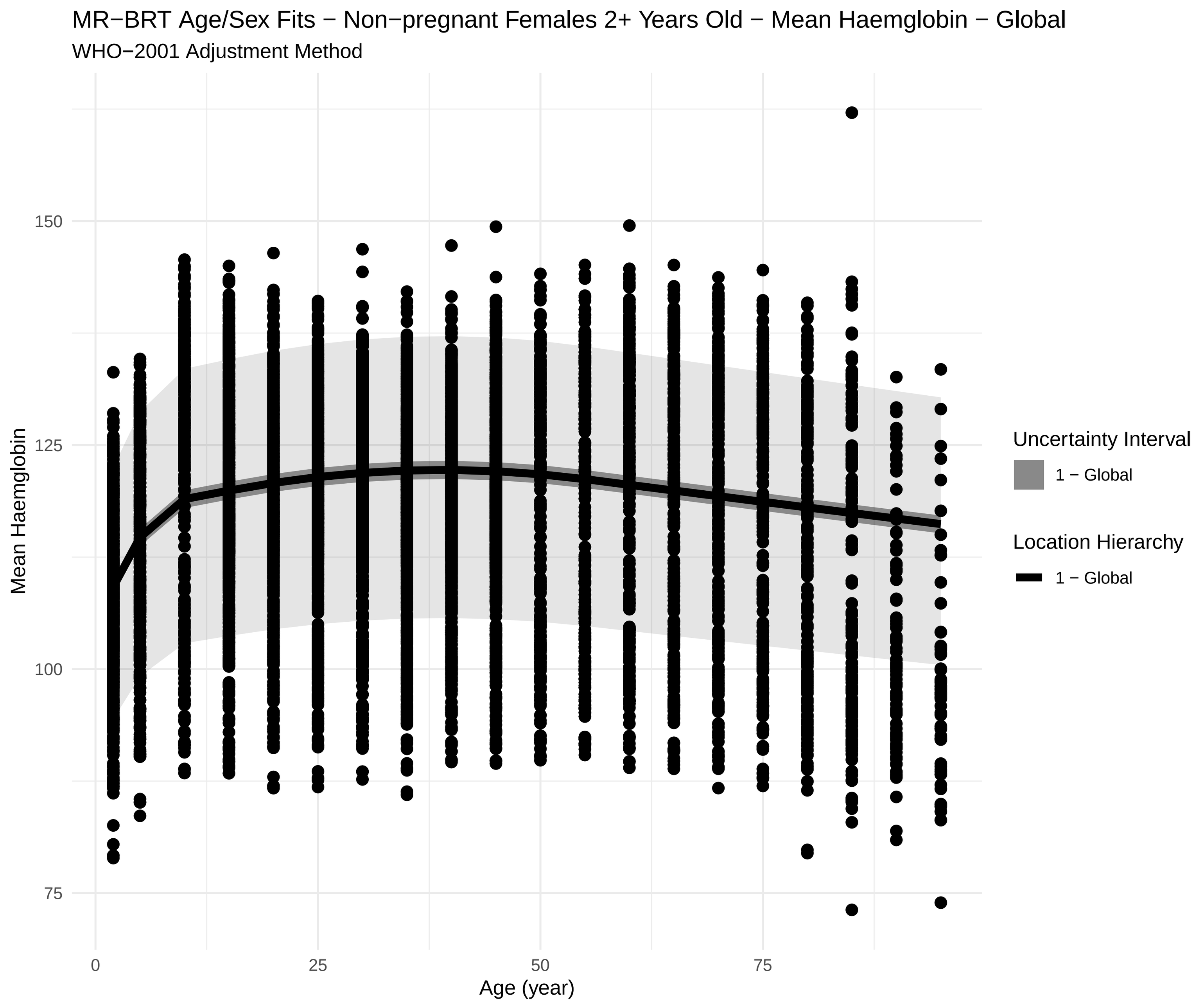
MR−BRT Age/Sex Fits − Non−pregnant Females 2+ Years Old − Mean Haemoglobin − Global
WHO−2001 Adjustment Method
Mean Haemoglobin
Age (year)
Uncertainty Interval
1 − Global
Location Hierarchy
1 − Global

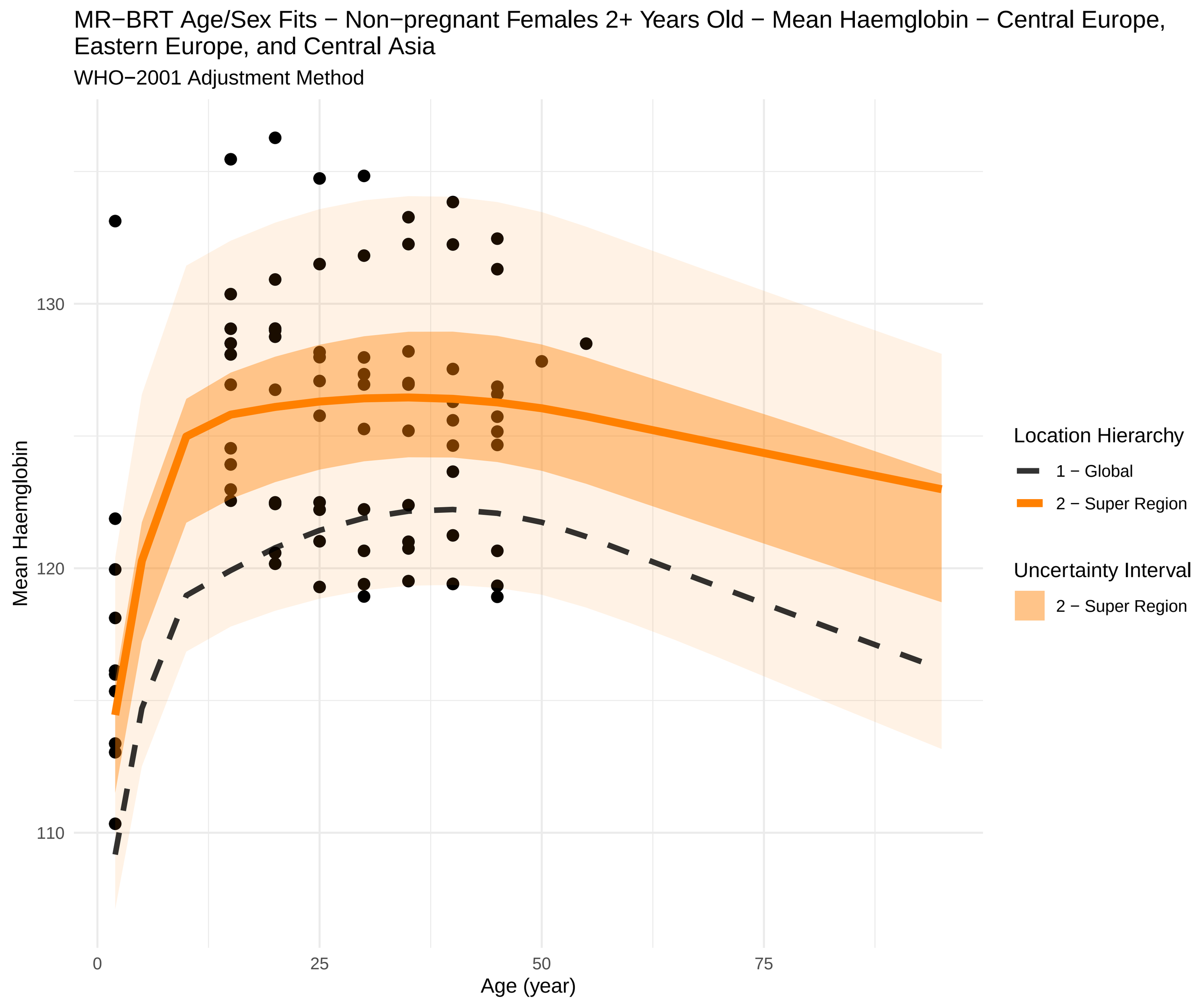
MR−BRT Age/Sex Fits − Non−pregnant Females 2+ Years Old − Mean Haemoglobin − Central Europe, Eastern Europe, and Central Asia
WHO−2001 Adjustment Method
Mean Haemoglobin
Age (year)
Location Hierarchy
1 − Global
2 − Super Region
Uncertainty Interval
2 − Super Region

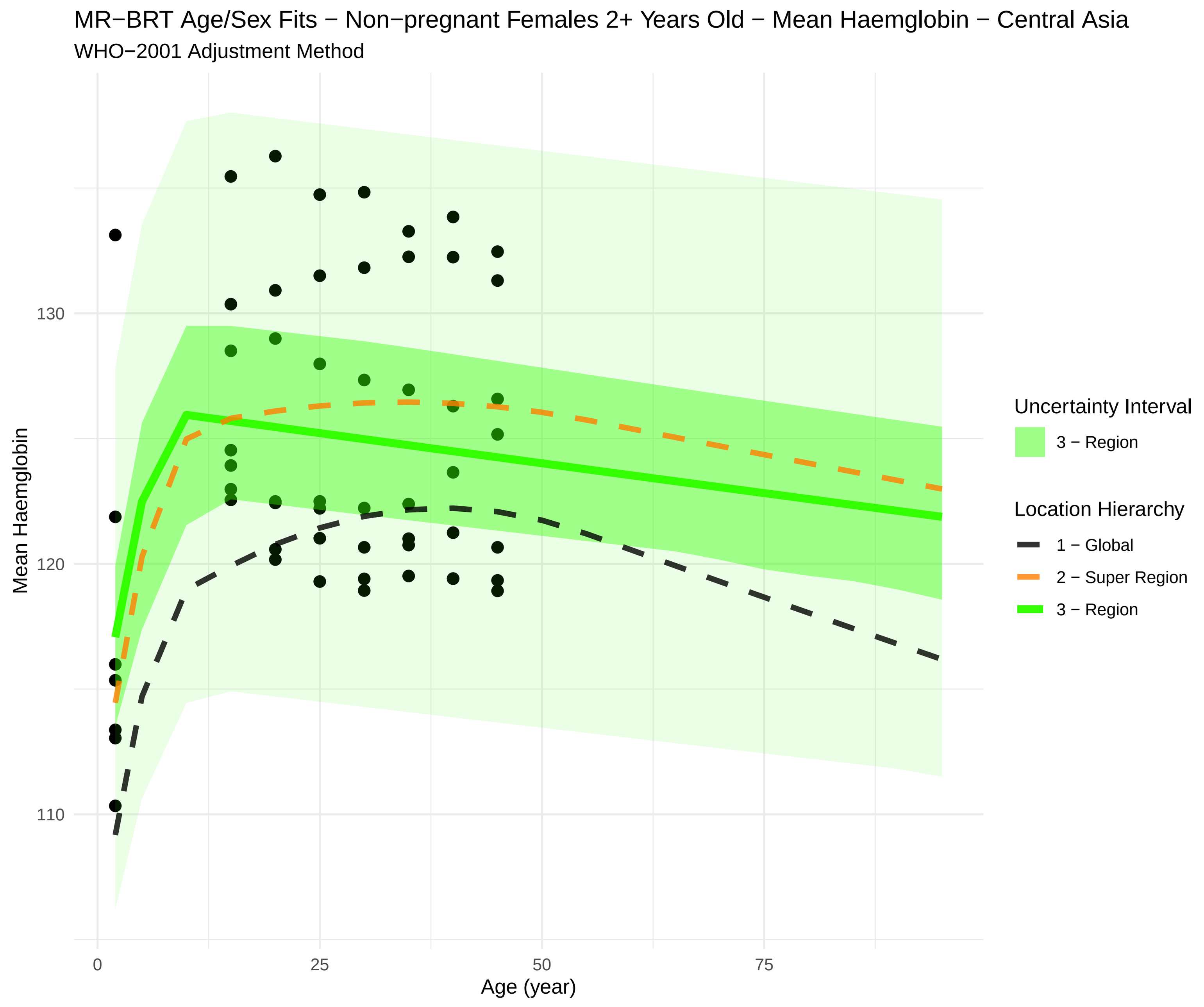
MR−BRT Age/Sex Fits − Non−pregnant Females 2+ Years Old − Mean Haemoglobin − Central Asia
WHO−2001 Adjustment Method
Mean Haemoglobin
Age (year)
Uncertainty Interval
3 − Region
Location Hierarchy
1 − Global
2 − Super Region
3 − Region

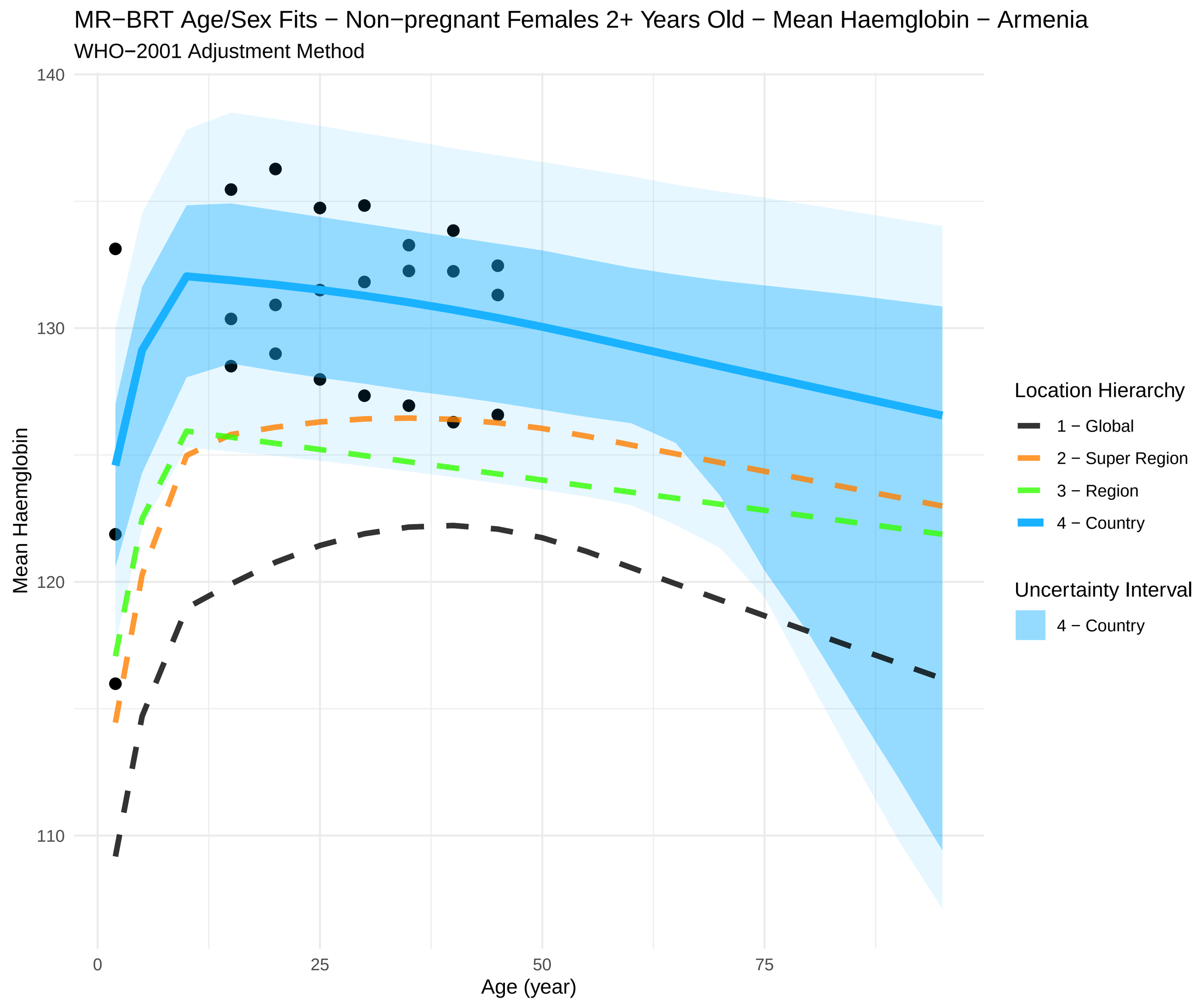
MR−BRT Age/Sex Fits − Non−pregnant Females 2+ Years Old − Mean Haemoglobin − Armenia
WHO−2001 Adjustment Method
Mean Haemoglobin
Age (year)
Location Hierarchy
1 − Global
2 − Super Region
3 − Region
4 − Country
Uncertainty Interval
4 − Country

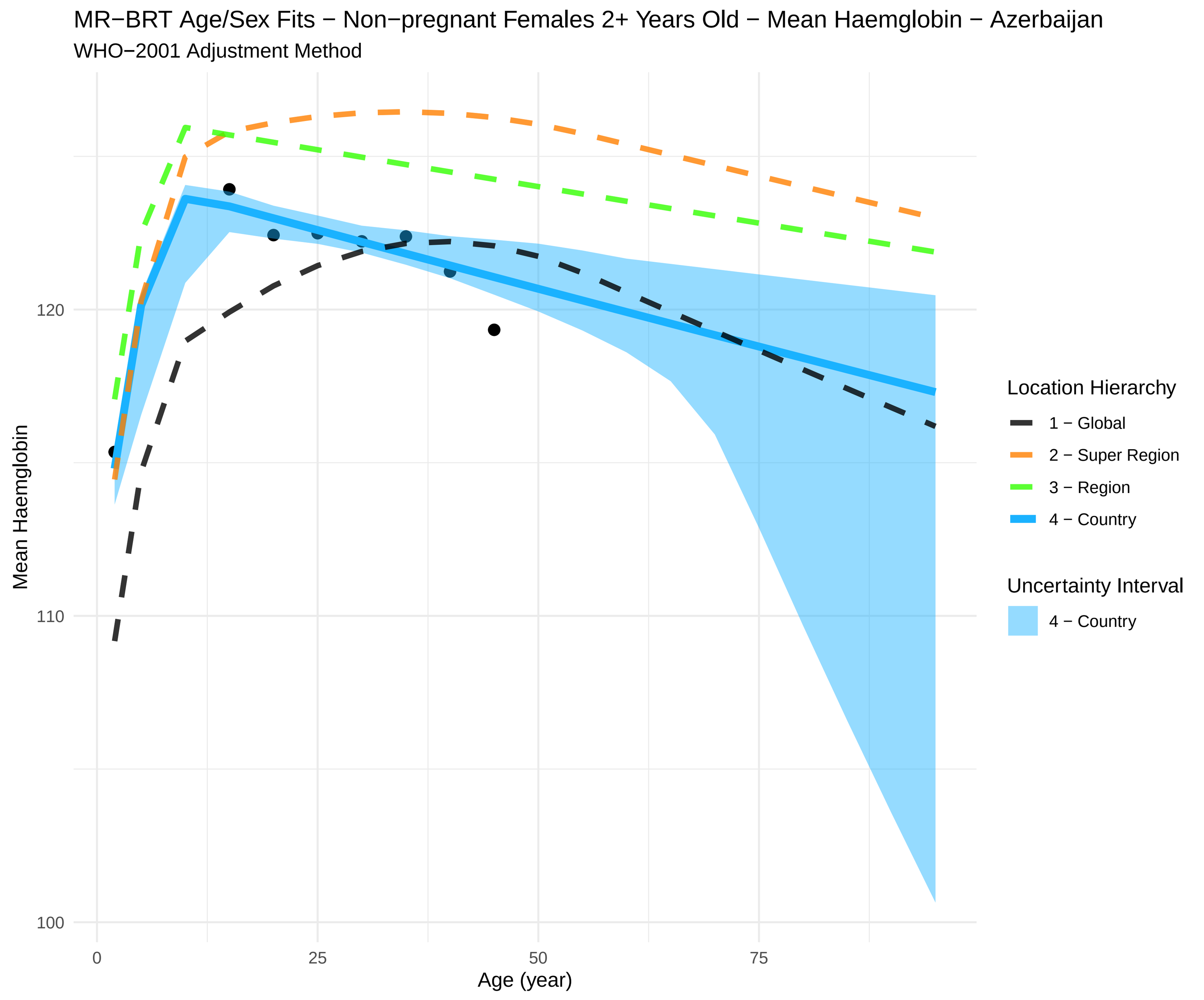
MR−BRT Age/Sex Fits − Non−pregnant Females 2+ Years Old − Mean Haemoglobin − Azerbaijan
WHO−2001 Adjustment Method
Mean Haemoglobin
Age (year)
Location Hierarchy
1 − Global
2 − Super Region
3 − Region
4 − Country
Uncertainty Interval
4 − Country

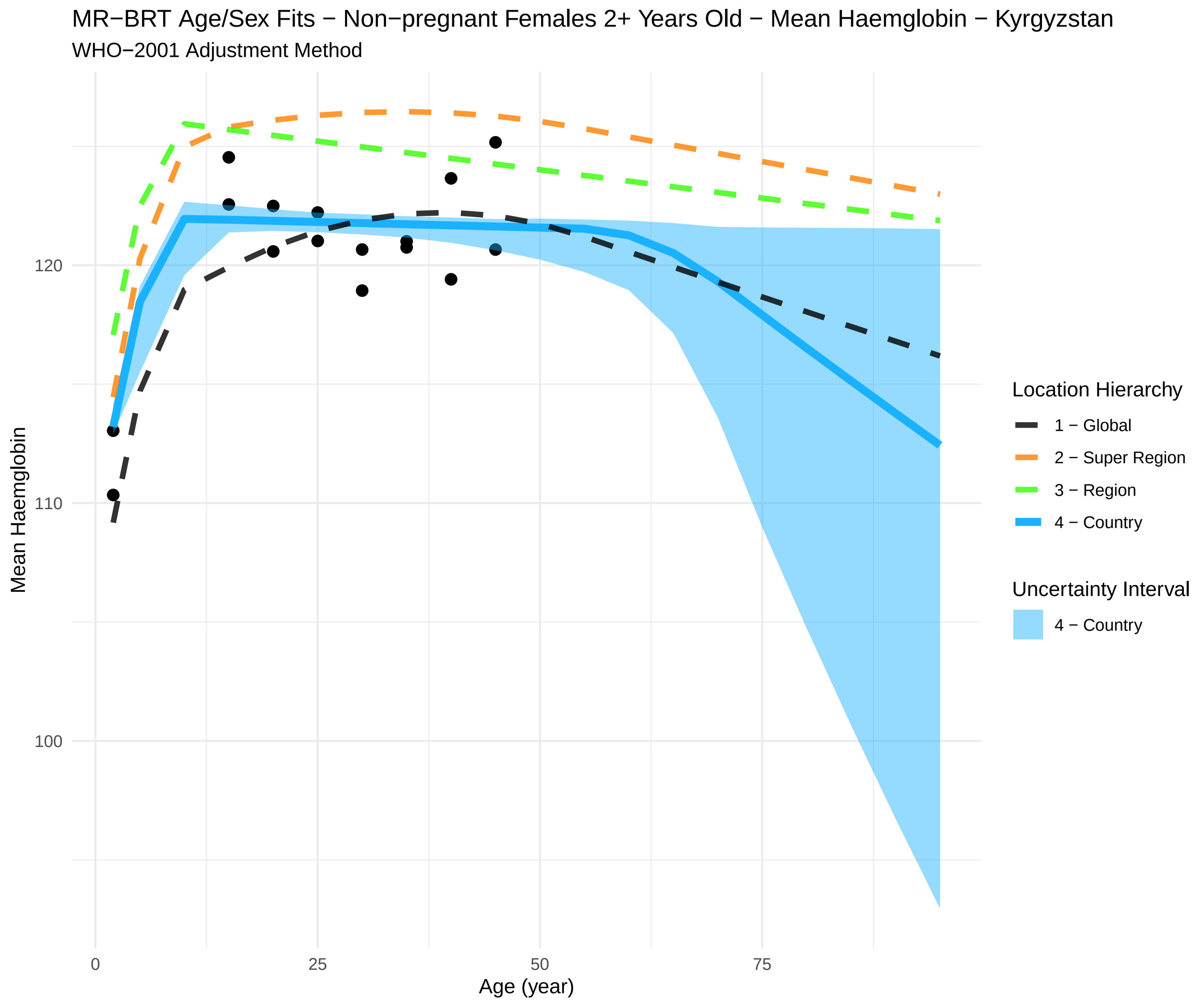
MR−BRT Age/Sex Fits − Non−pregnant Females 2+ Years Old − Mean Haemoglobin − Kyrgyzstan
WHO−2001 Adjustment Method
Mean Haemoglobin
Age (year)
Location Hierarchy
1 − Global
2 − Super Region
3 − Region
4 − Country
Uncertainty Interval
4 − Country

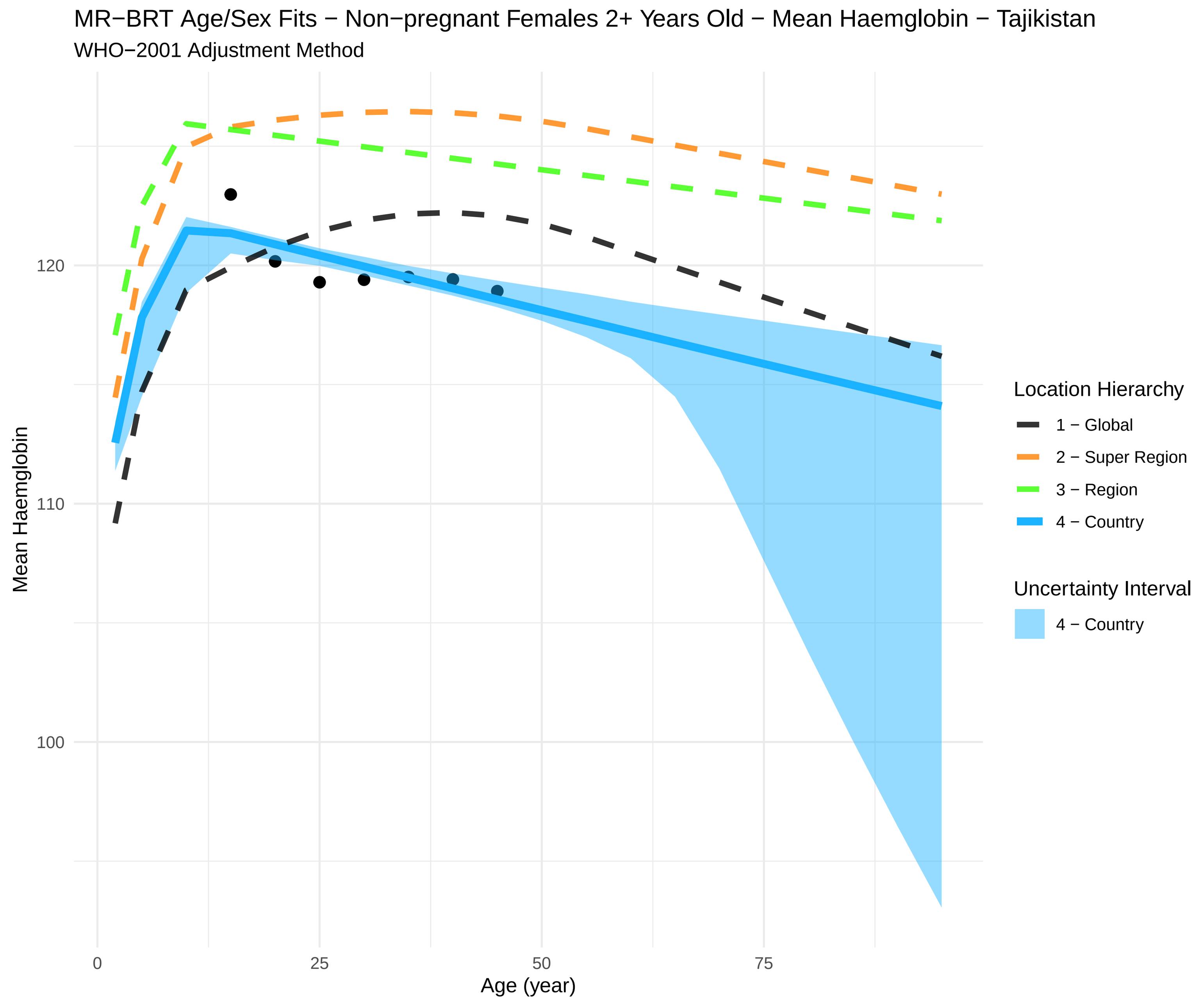
MR−BRT Age/Sex Fits − Non−pregnant Females 2+ Years Old − Mean Haemoglobin − Tajikistan
WHO−2001 Adjustment Method
Mean Haemoglobin
Age (year)
Location Hierarchy
1 − Global
2 − Super Region
3 − Region
4 − Country
Uncertainty Interval
4 − Country

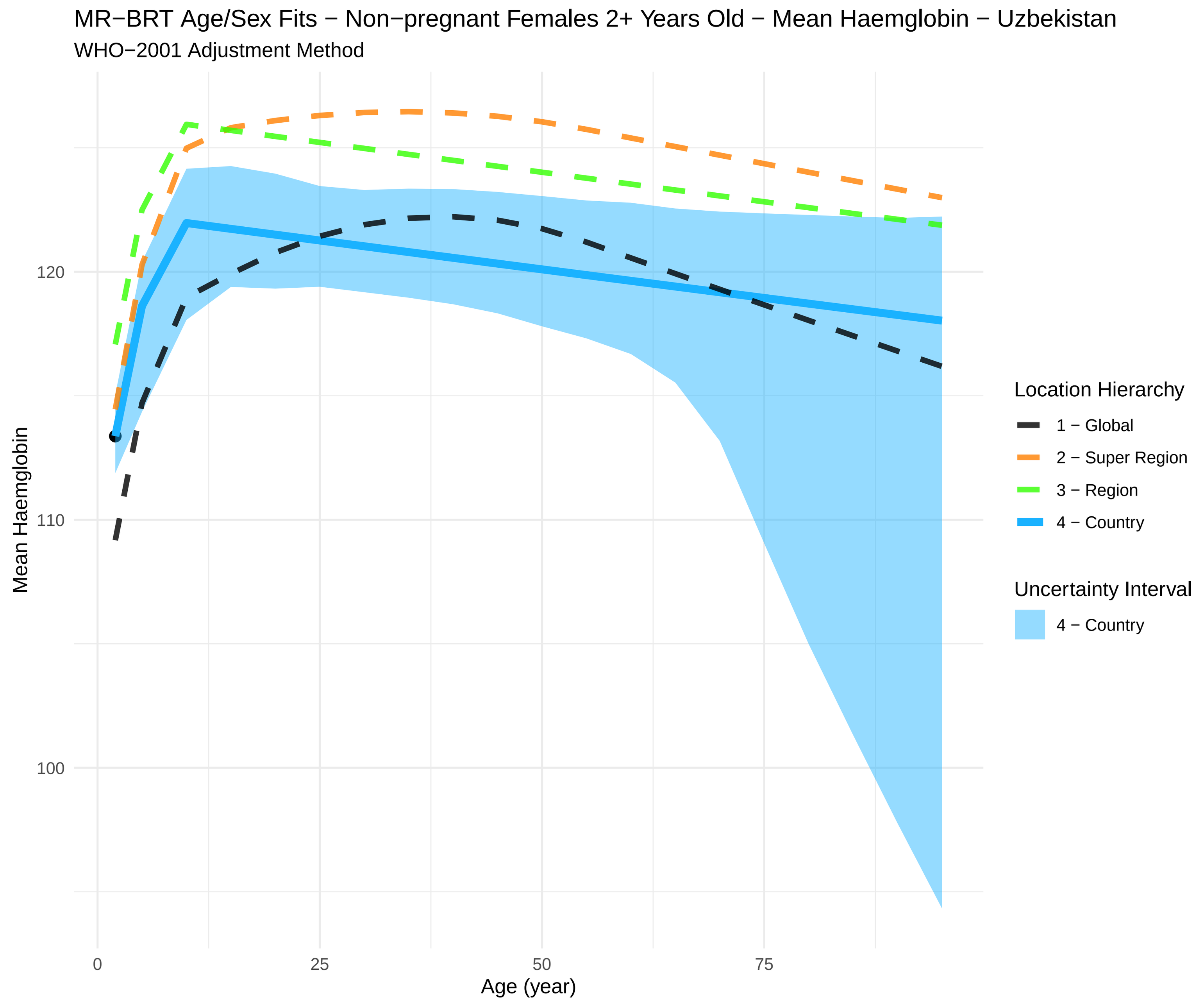
MR−BRT Age/Sex Fits − Non−pregnant Females 2+ Years Old − Mean Haemoglobin − Uzbekistan
WHO−2001 Adjustment Method
Mean Haemoglobin
Age (year)
Location Hierarchy
1 − Global
2 − Super Region
3 − Region
4 − Country
Uncertainty Interval
4 − Country

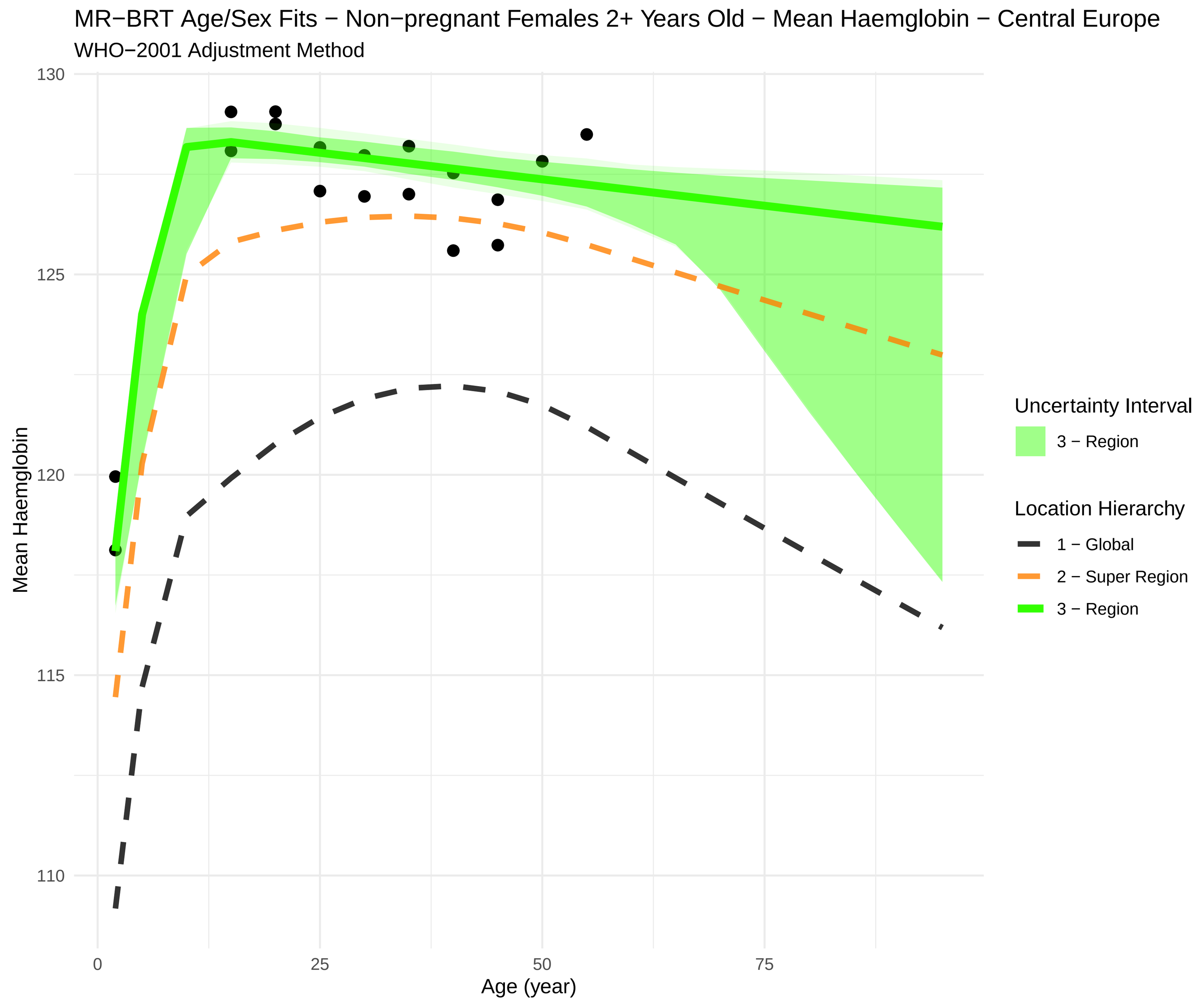
MR−BRT Age/Sex Fits − Non−pregnant Females 2+ Years Old − Mean Haemoglobin − Central Europe
WHO−2001 Adjustment Method
Mean Haemoglobin
Age (year)
Uncertainty Interval
3 − Region
Location Hierarchy
1 − Global
2 − Super Region
3 − Region

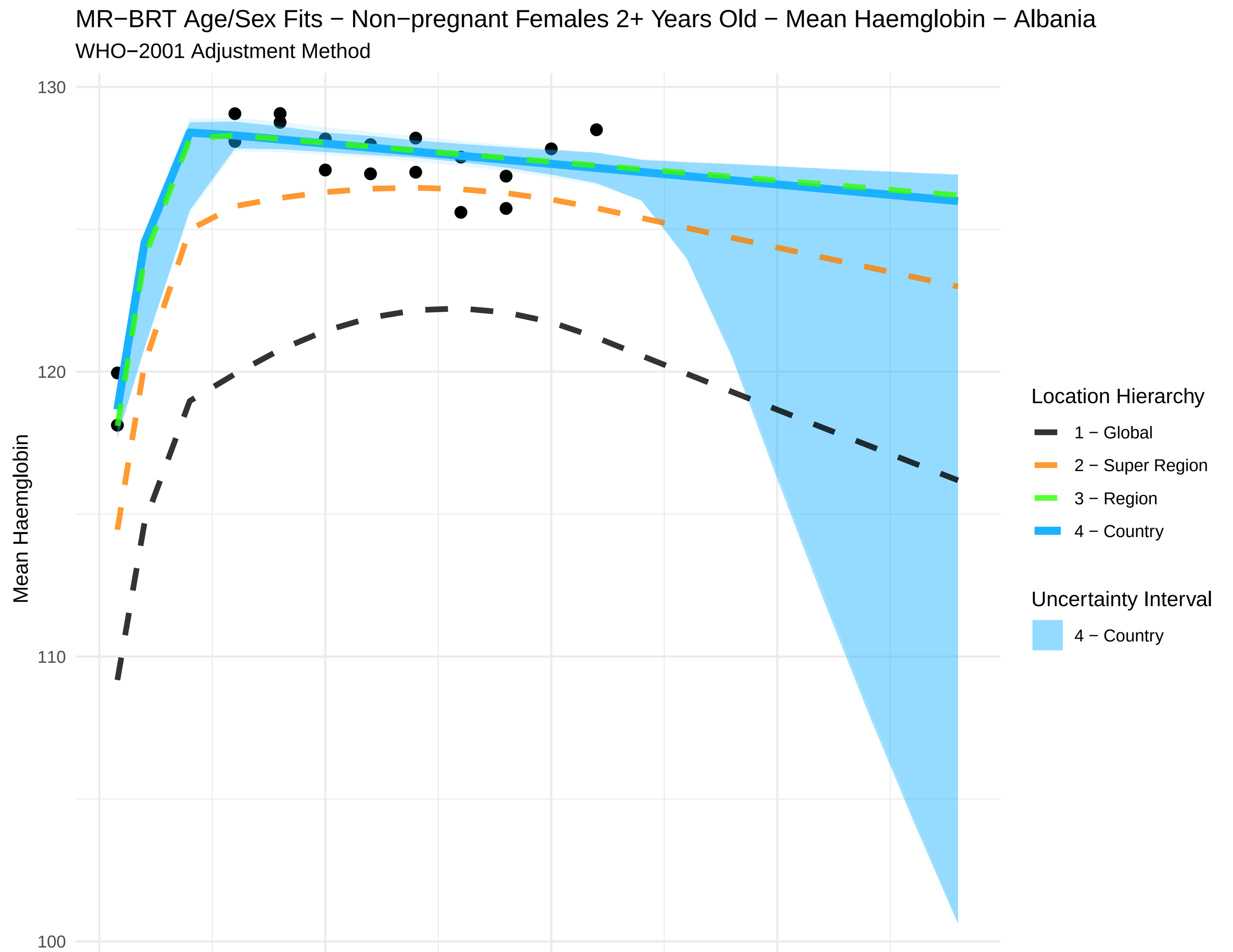
MR−BRT Age/Sex Fits − Non−pregnant Females 2+ Years Old − Mean Haemoglobin − Albania
WHO−2001 Adjustment Method
Mean Haemoglobin
Age (year)
Location Hierarchy
1 − Global
2 − Super Region
3 − Region
4 − Country
Uncertainty Interval
4 − Country

MR−BRT Age/Sex Fits − Non−pregnant Females 2+ Years Old − Mean Haemglobin − Eastern Europe
WHO−2001 Adjustment Method

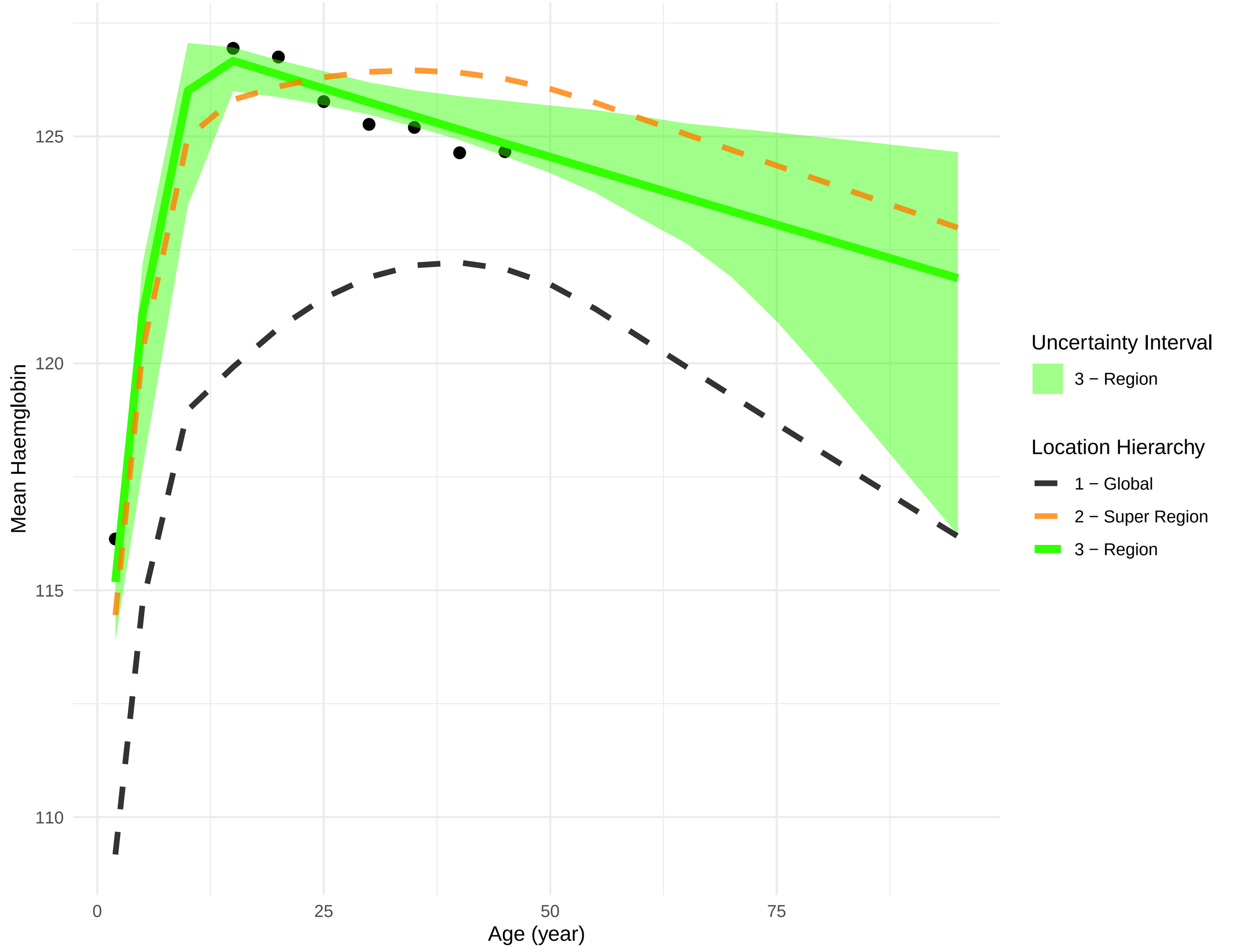

MR−BRT Age/Sex Fits − Non−pregnant Females 2+ Years Old − Mean Haemglobin − Republic of Moldova
WHO−2001 Adjustment Method

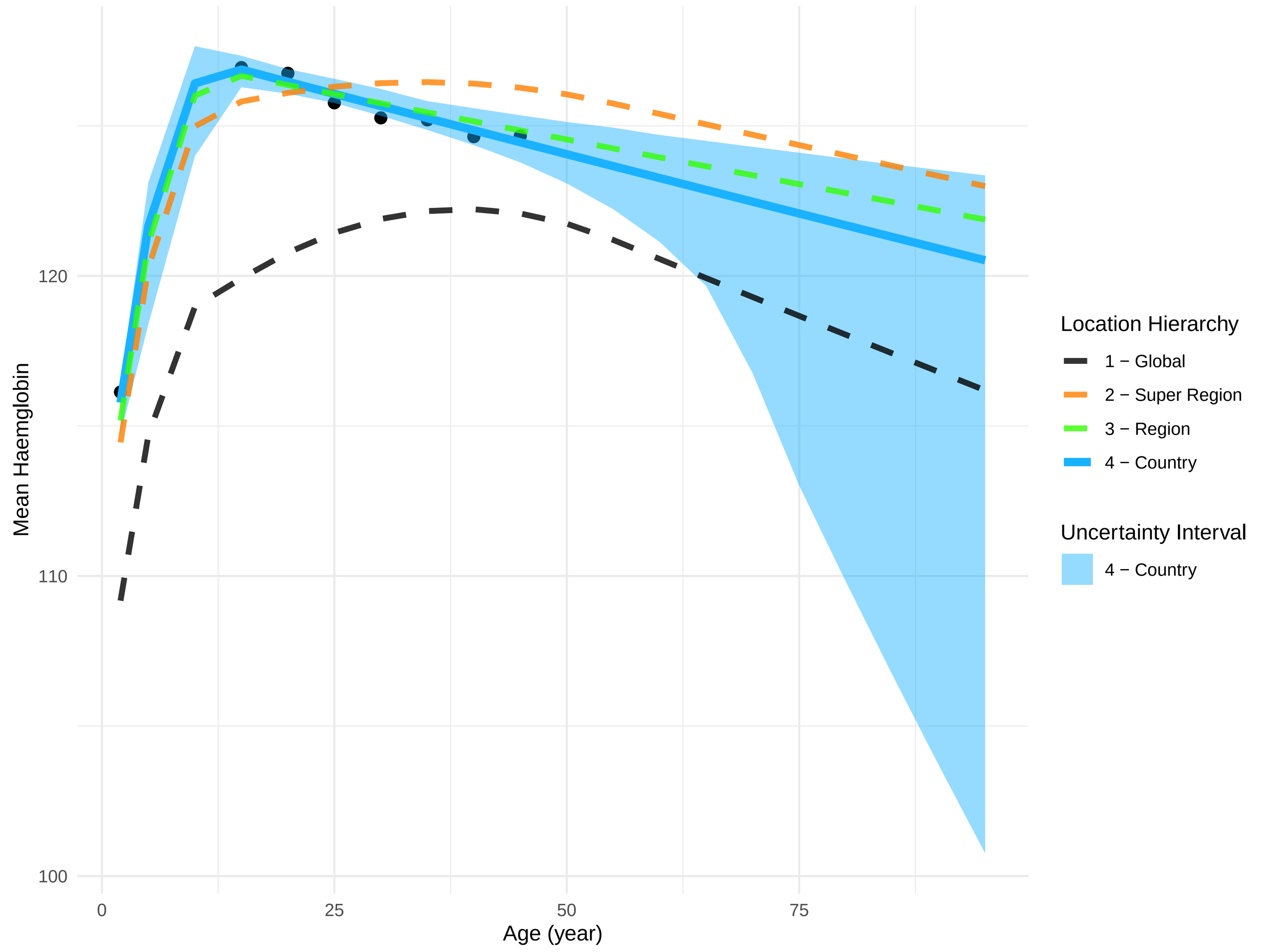

MR−BRT Age/Sex Fits − Non−pregnant Females 2+ Years Old − Mean Haemglobin − High−income
WHO−2001 Adjustment Method

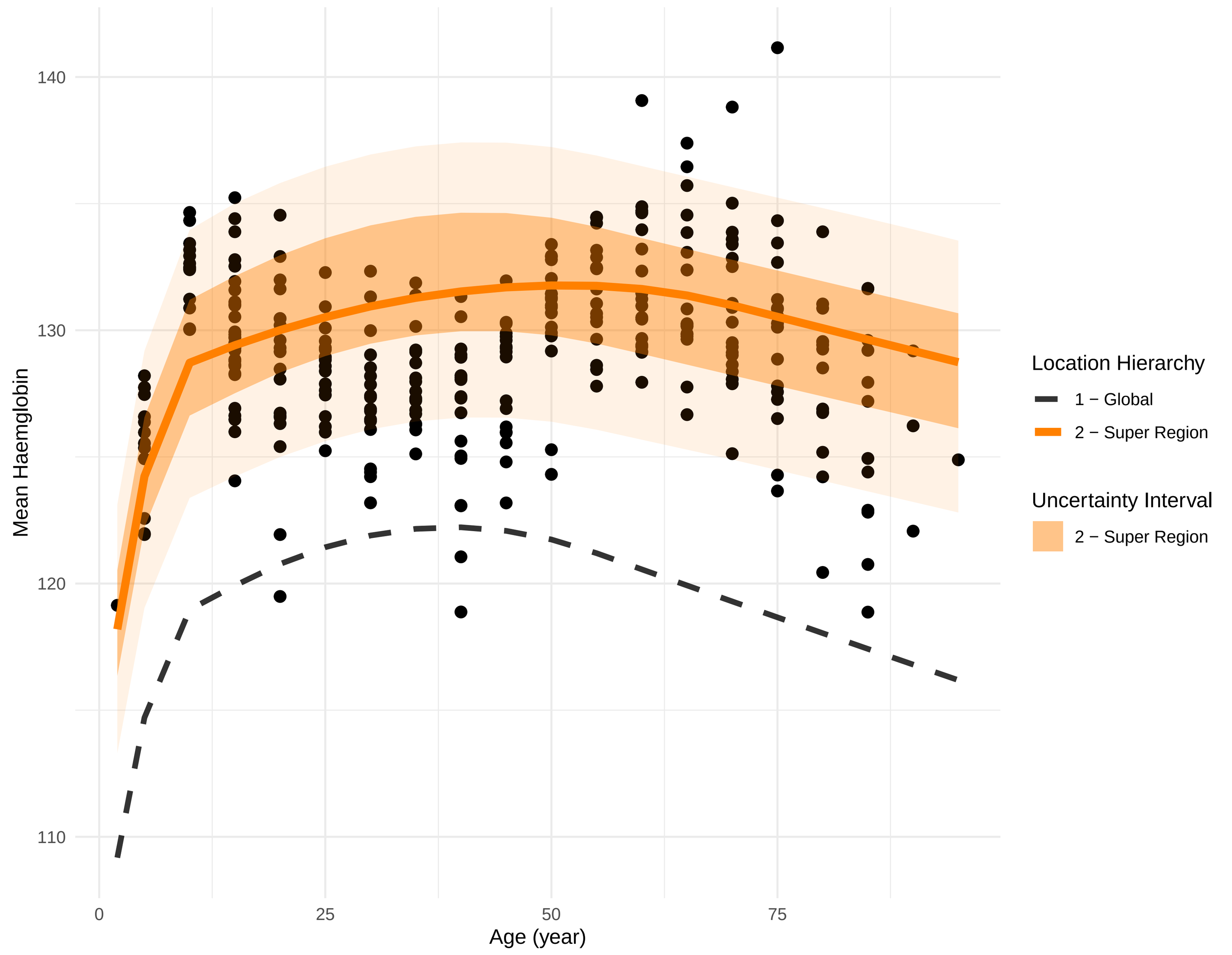

MR−BRT Age/Sex Fits − Non−pregnant Females 2+ Years Old − Mean Haemglobin − Western Europe
WHO−2001 Adjustment Method

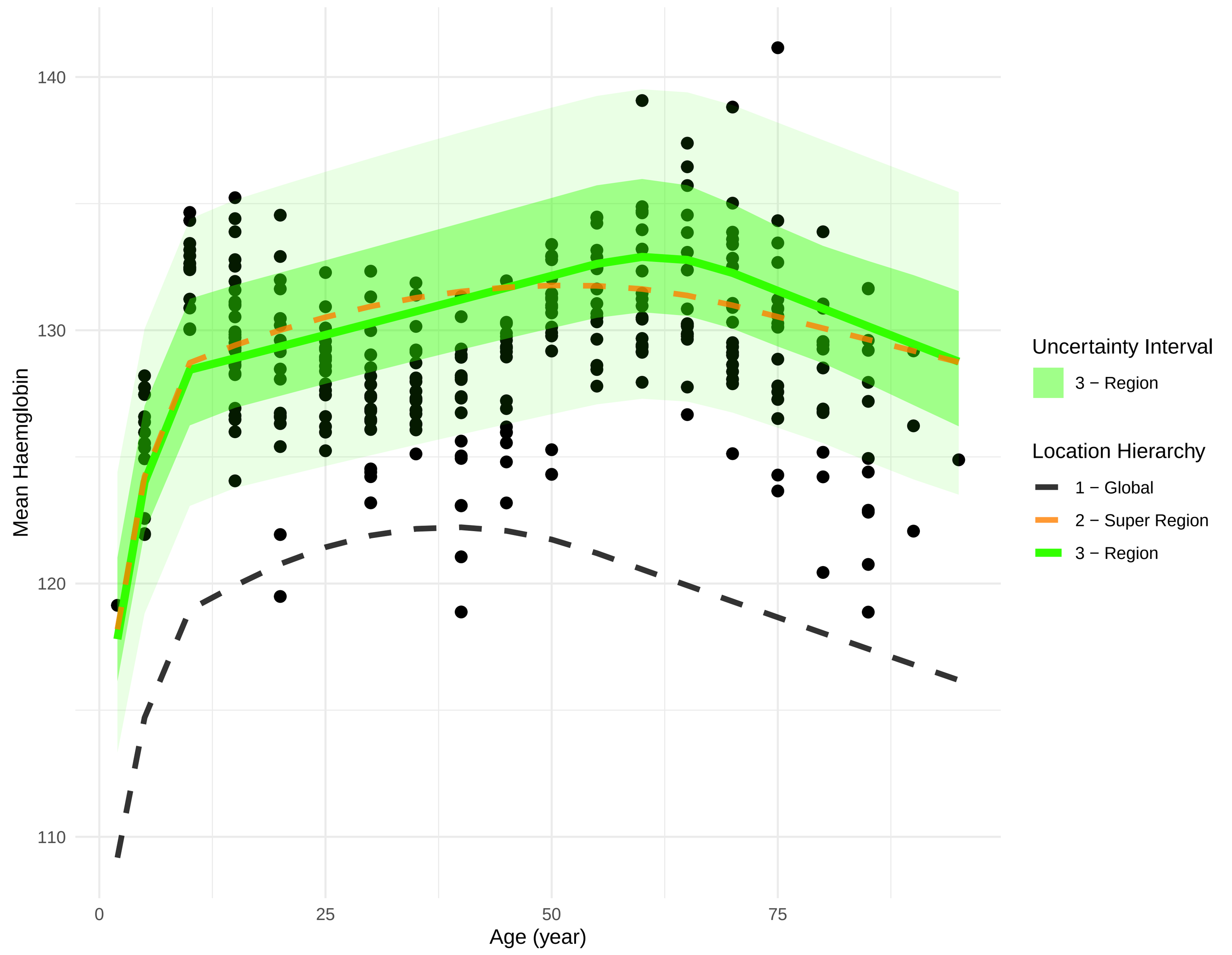

MR−BRT Age/Sex Fits − Non−pregnant Females 2+ Years Old − Mean Haemglobin − United Kingdom
WHO−2001 Adjustment Method

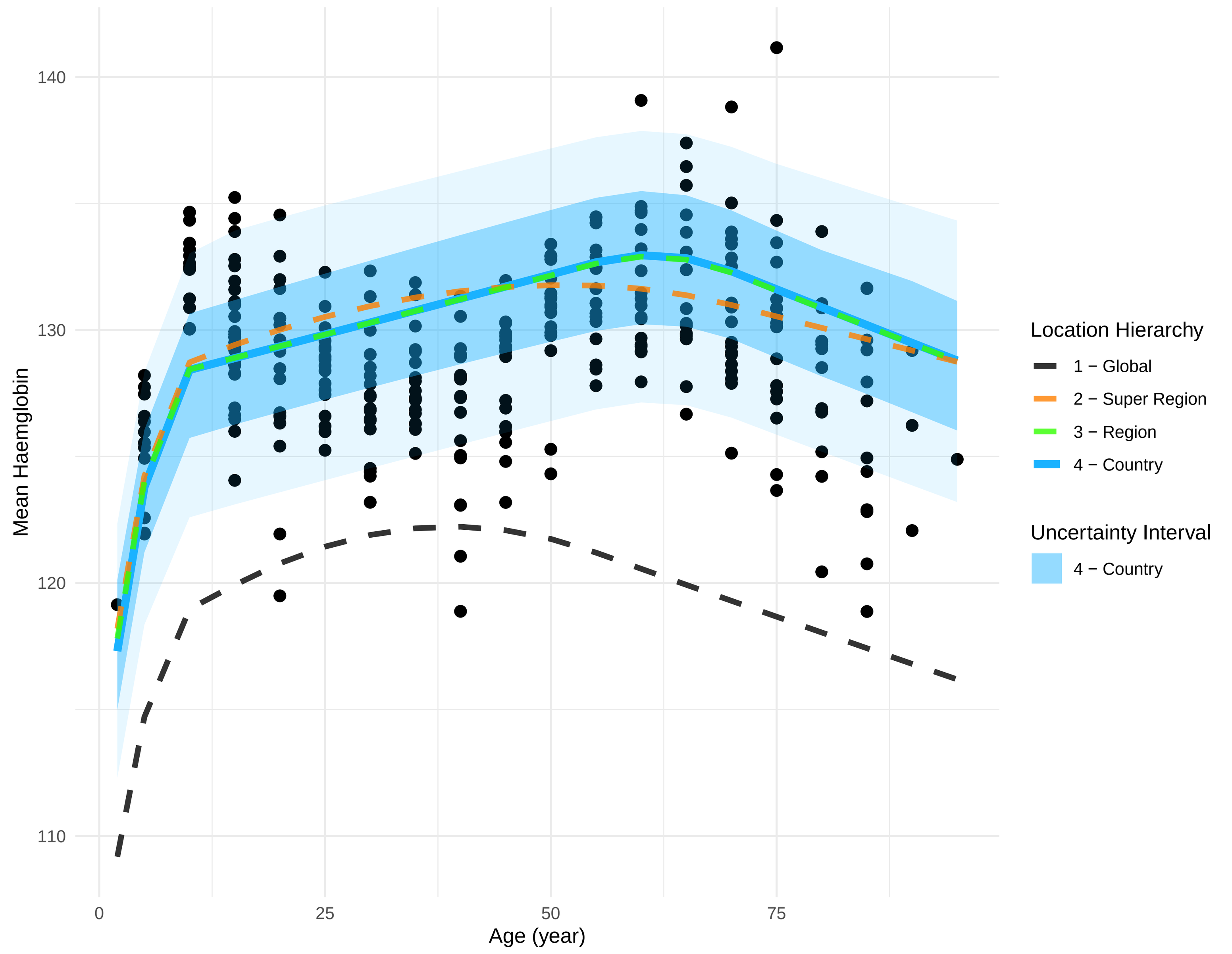

MR−BRT Age/Sex Fits − Non−pregnant Females 2+ Years Old − Mean Haemglobin − England
WHO−2001 Adjustment Method

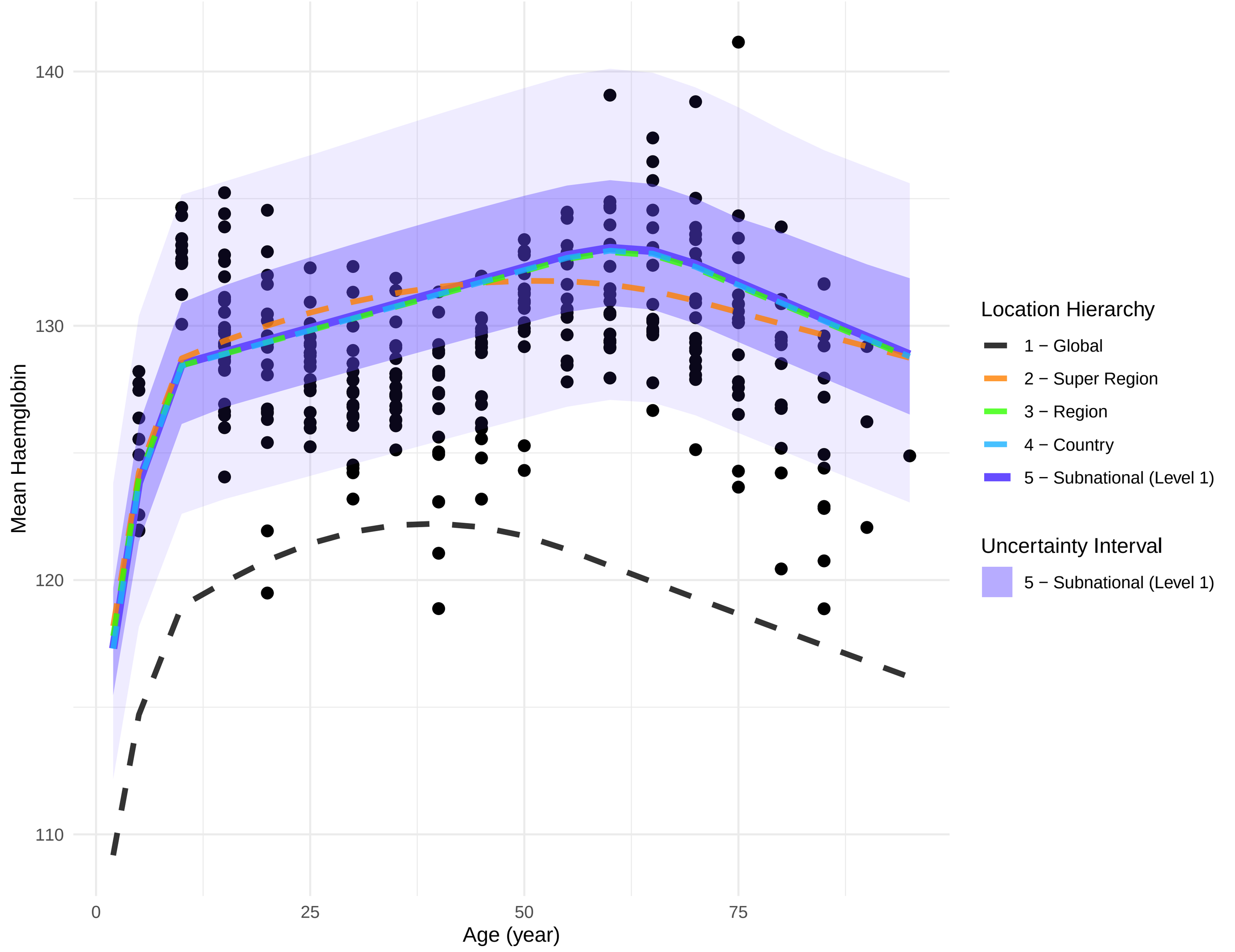

MR−BRT Age/Sex Fits − Non−pregnant Females 2+ Years Old − Mean Haemglobin − East Midlands
WHO−2001 Adjustment Method

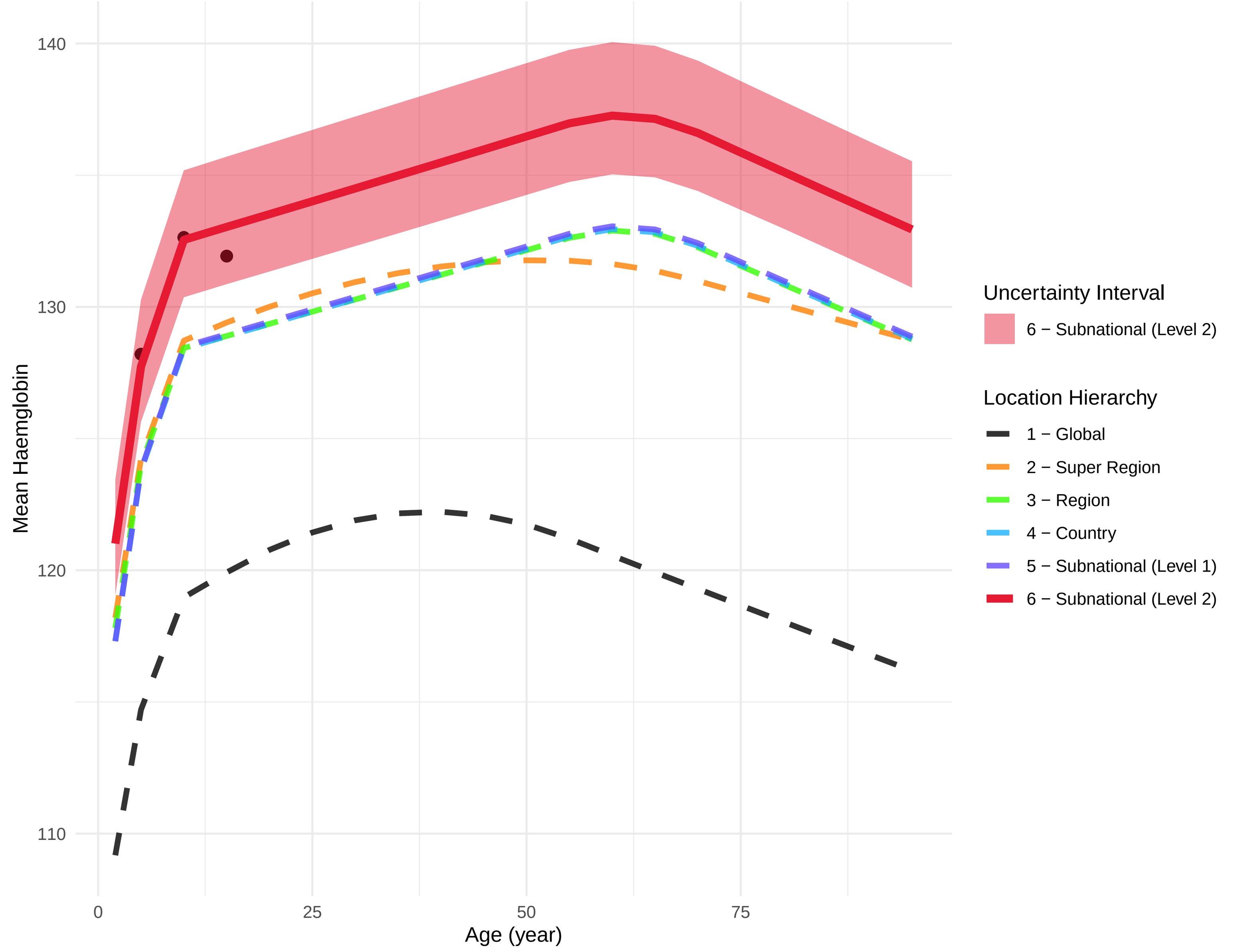

MR−BRT Age/Sex Fits − Non−pregnant Females 2+ Years Old − Mean Haemglobin − East of England
WHO−2001 Adjustment Method

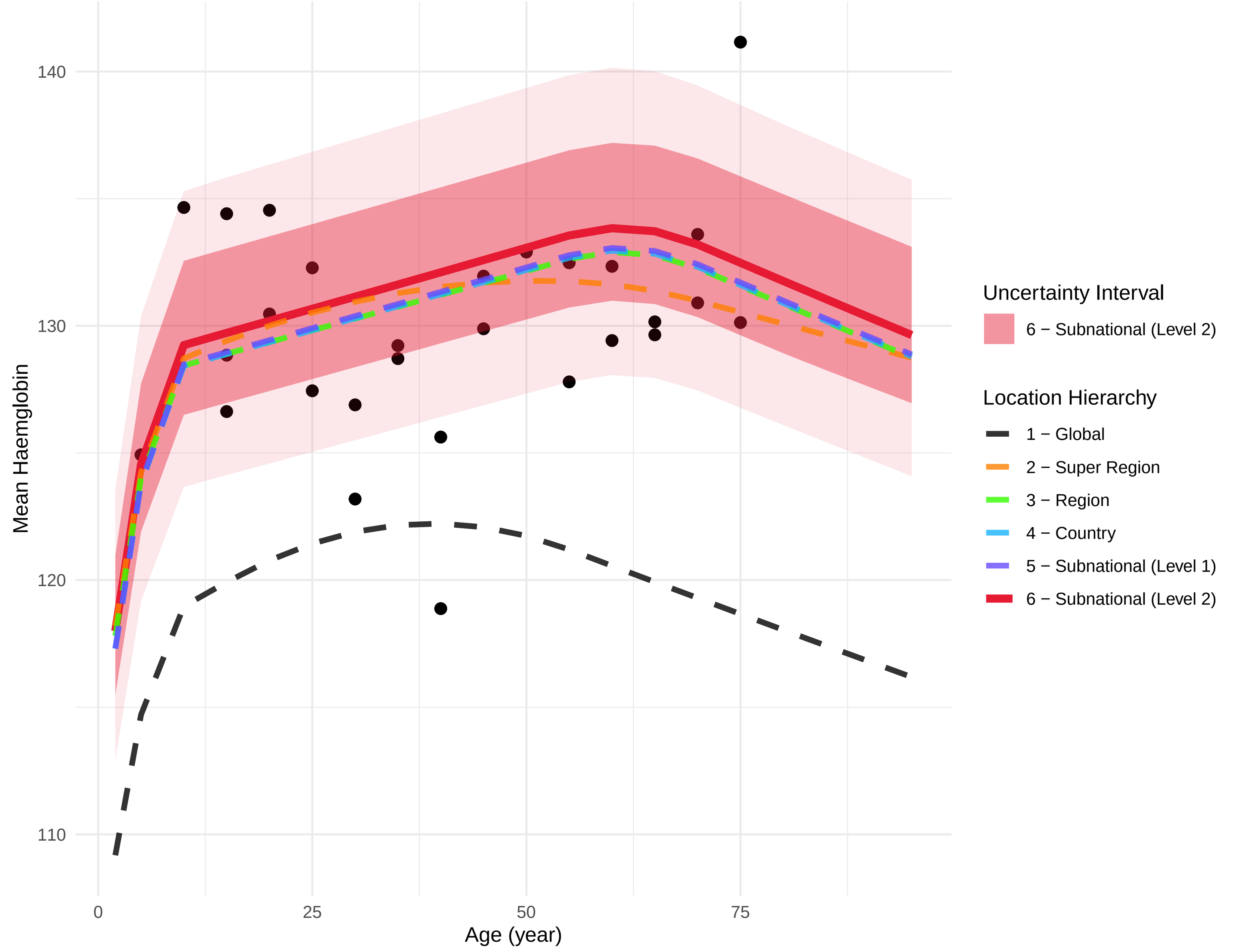

MR−BRT Age/Sex Fits − Non−pregnant Females 2+ Years Old − Mean Haemglobin − Greater London
WHO−2001 Adjustment Method

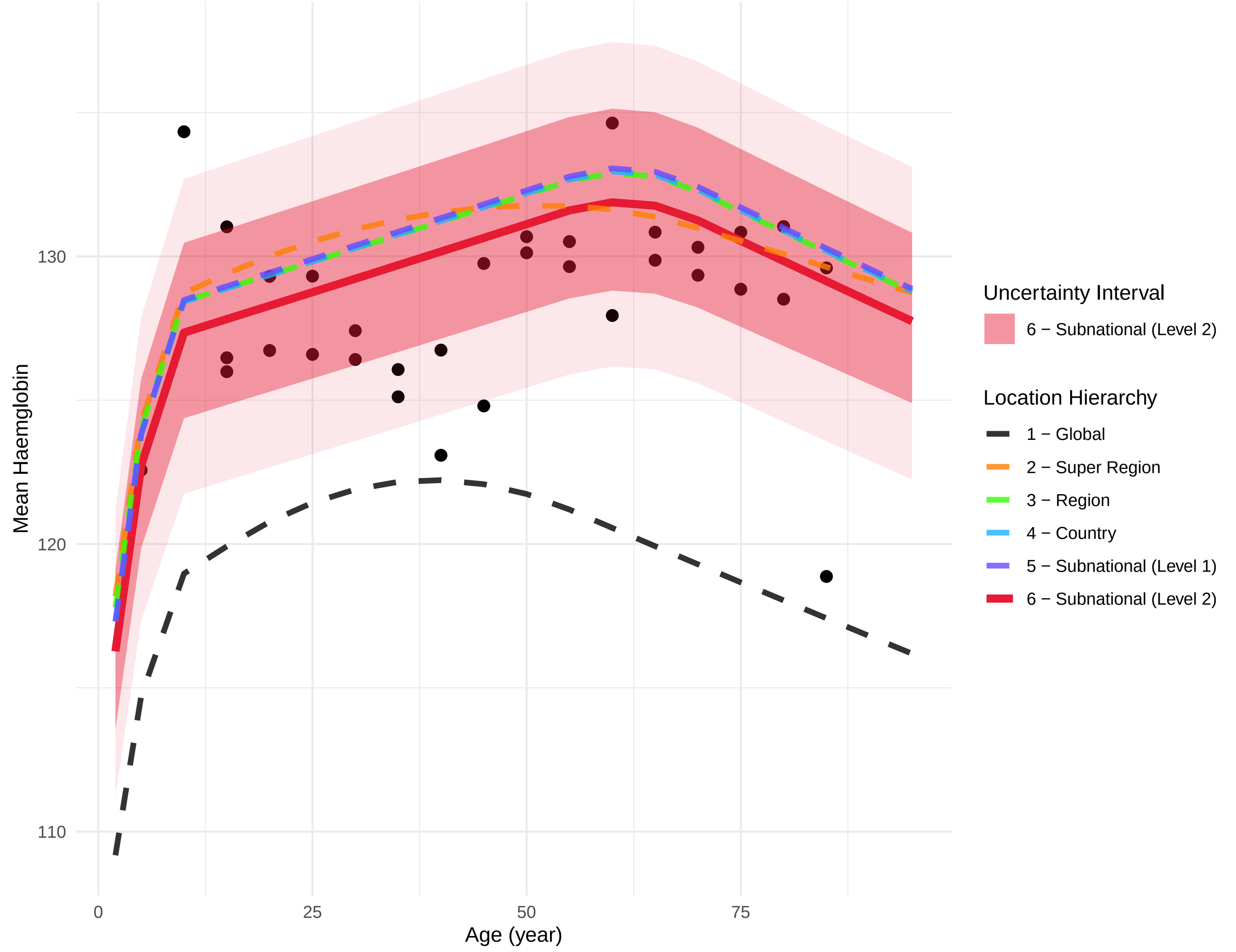

MR−BRT Age/Sex Fits − Non−pregnant Females 2+ Years Old − Mean Haemglobin − North East England
WHO−2001 Adjustment Method

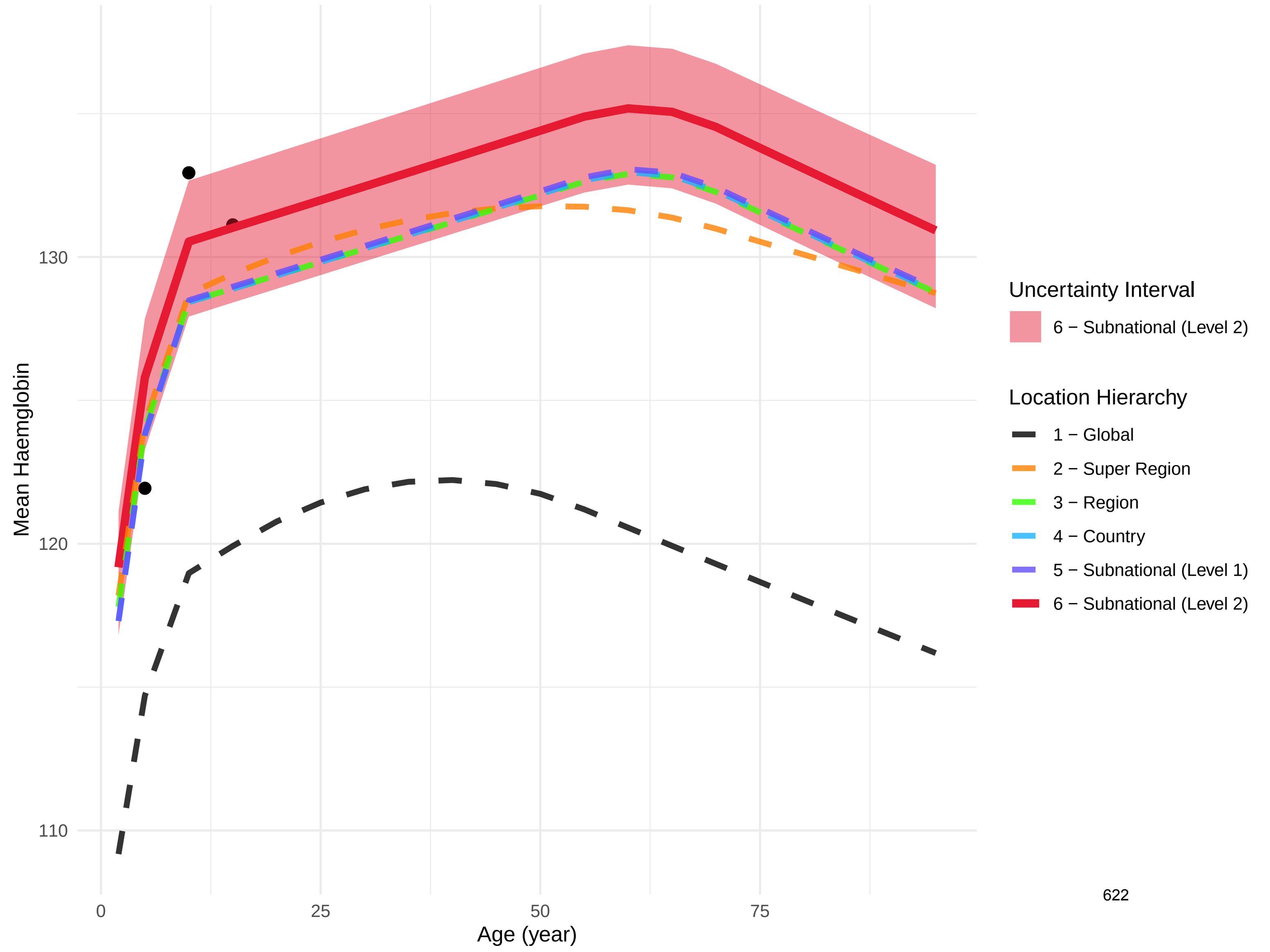

MR−BRT Age/Sex Fits − Non-pregnant Females 2+ Years Old − Mean Haemglobin − North West England

WHO−2001 Adjustment Method

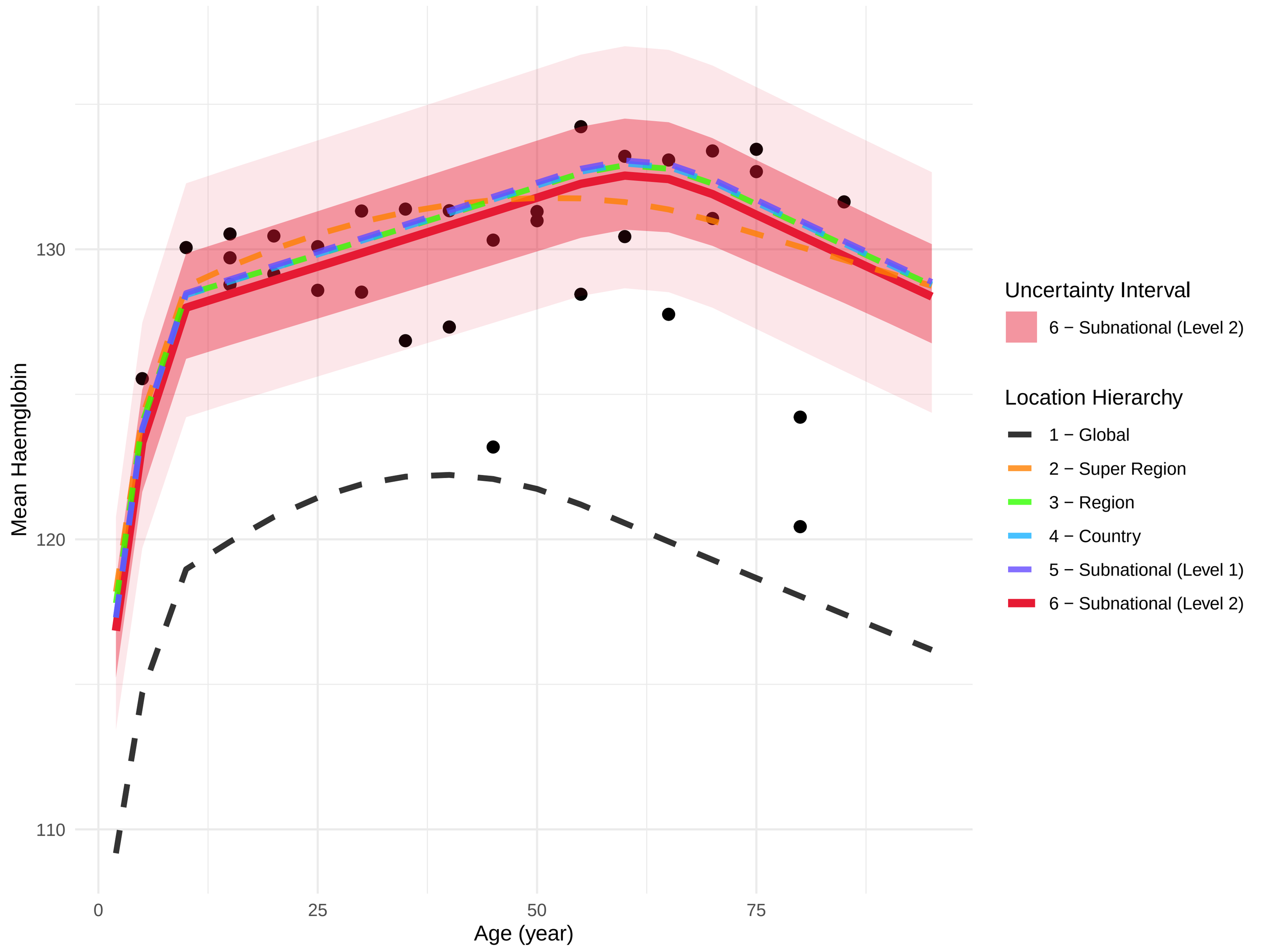

MR−BRT Age/Sex Fits − Non-pregnant Females 2+ Years Old − Mean Haemglobin − South East England

WHO−2001 Adjustment Method

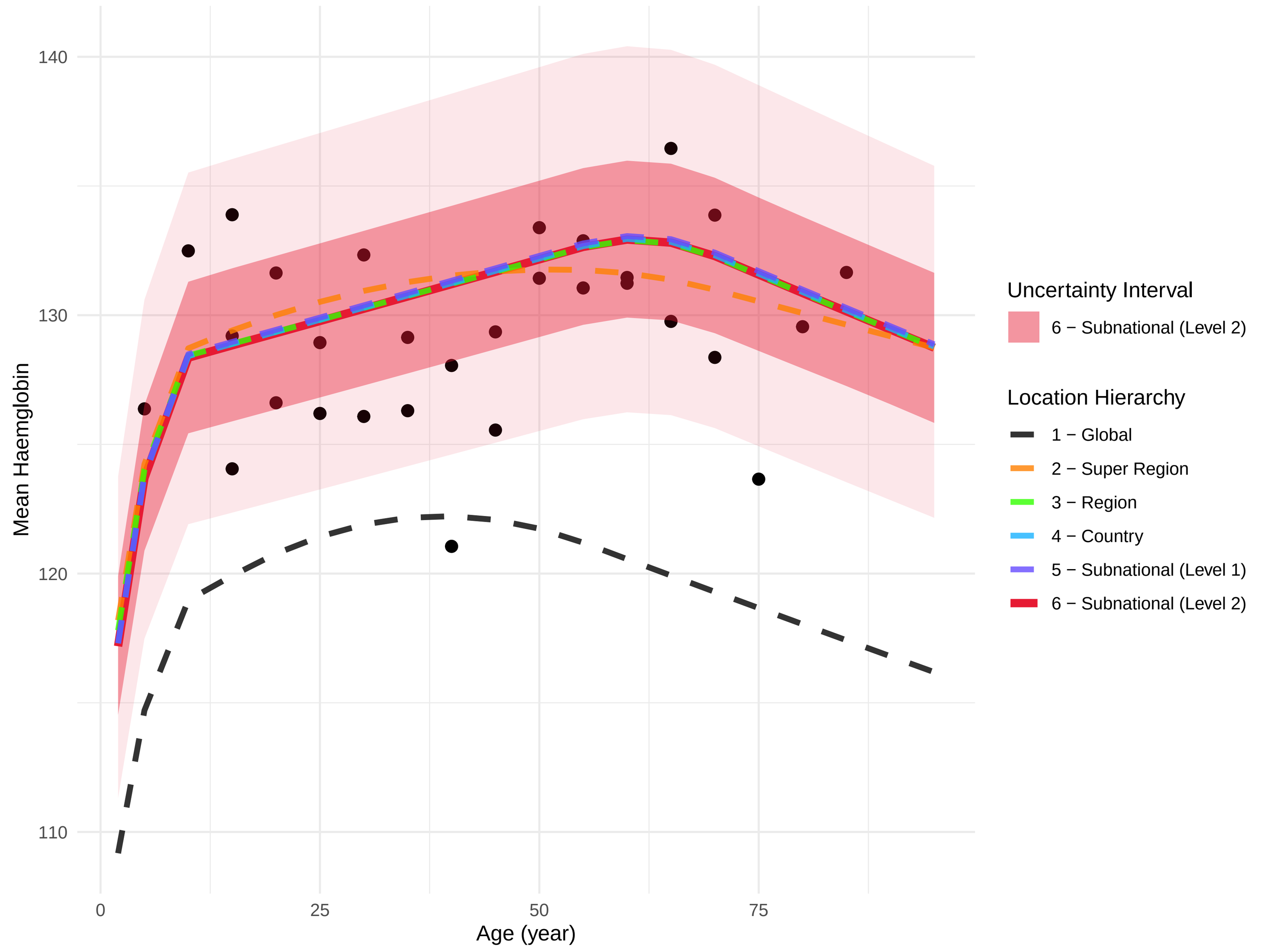

MR−BRT Age/Sex Fits − Non-pregnant Females 2+ Years Old − Mean Haemglobin − South West England

WHO−2001 Adjustment Method

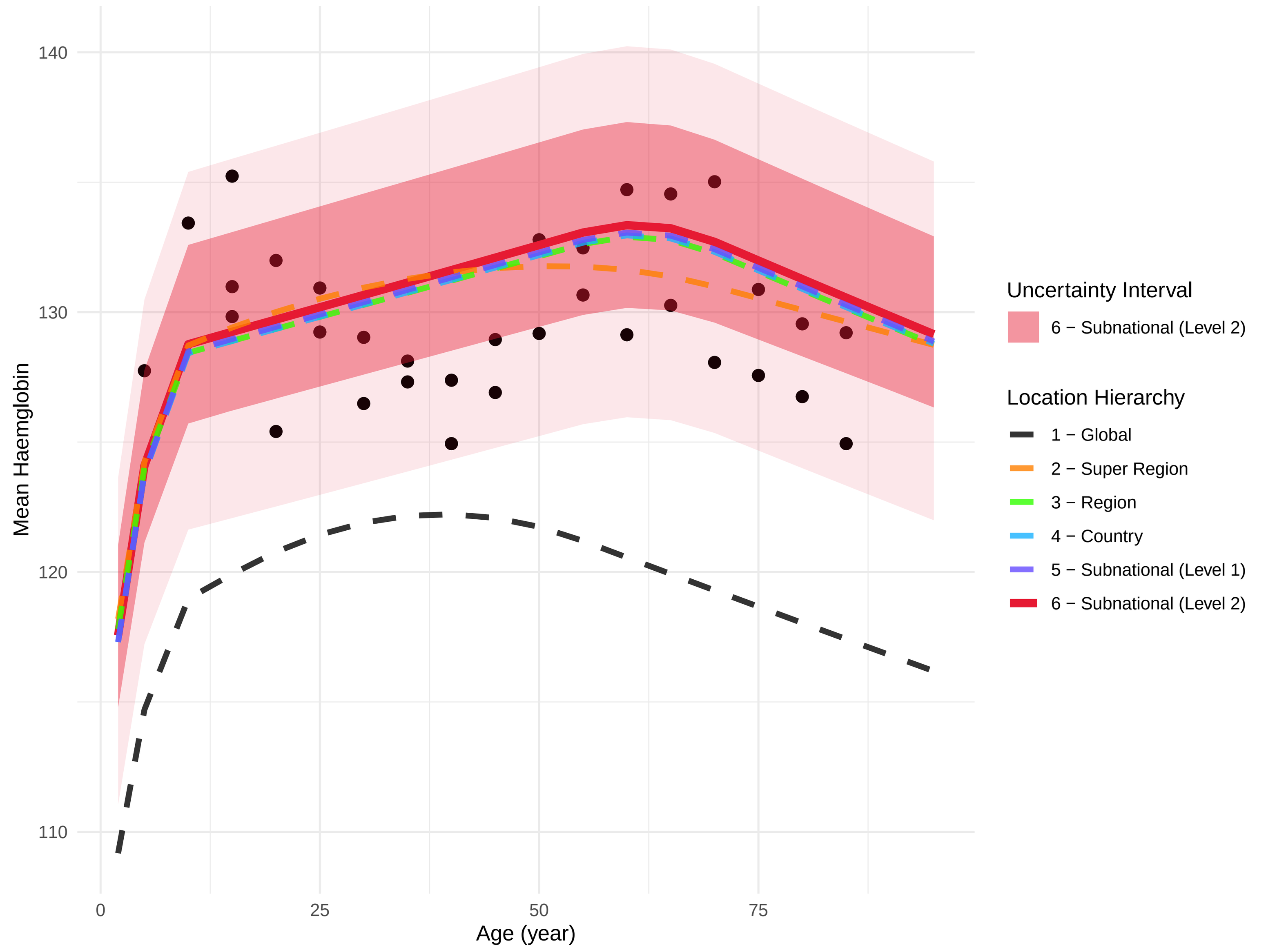

MR−BRT Age/Sex Fits − Non-pregnant Females 2+ Years Old − Mean Haemglobin − West Midlands

WHO−2001 Adjustment Method

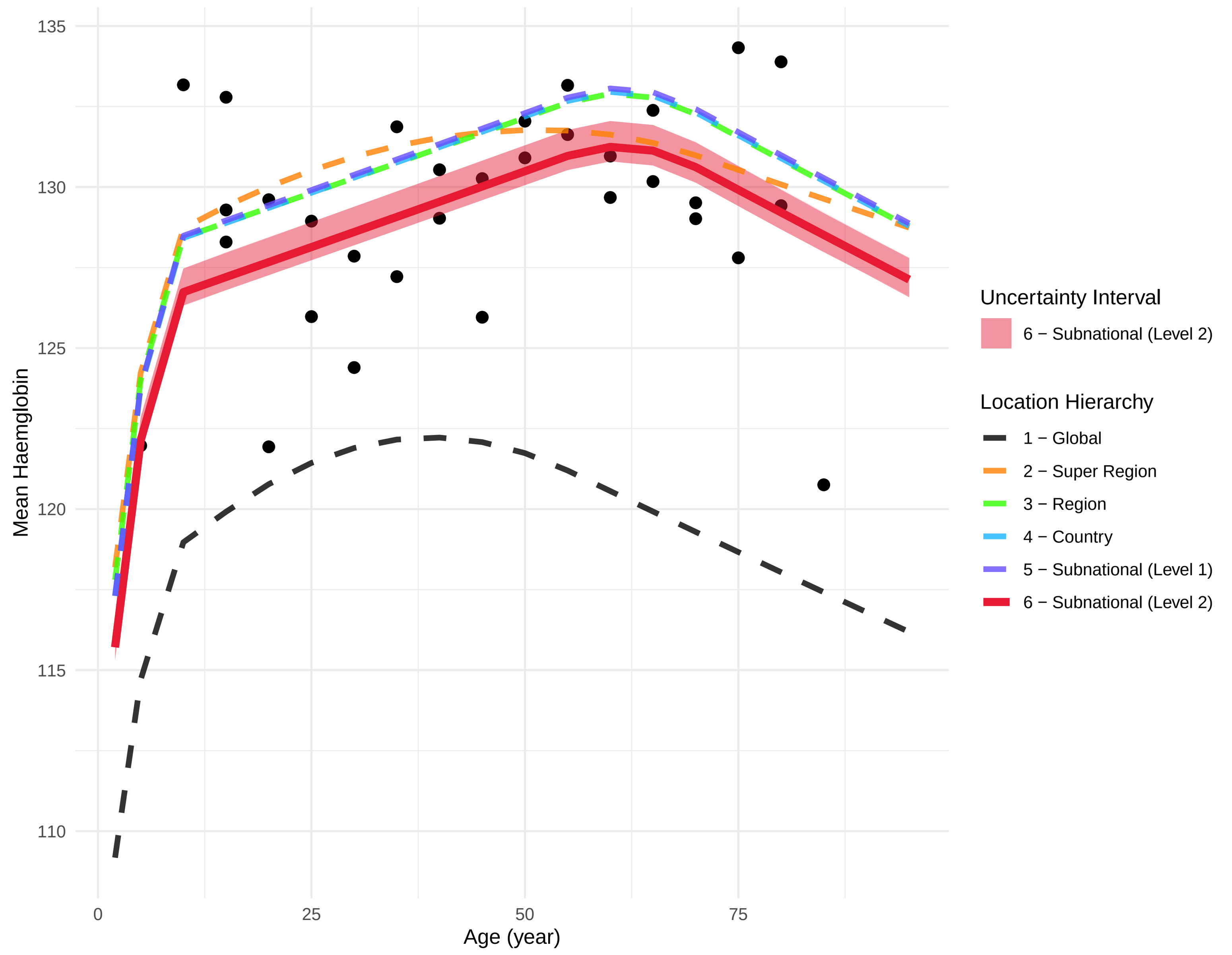

MR−BRT Age/Sex Fits − Non-pregnant Females 2+ Years Old − Mean Haemglobin − Yorkshire and the Humber

WHO−2001 Adjustment Method

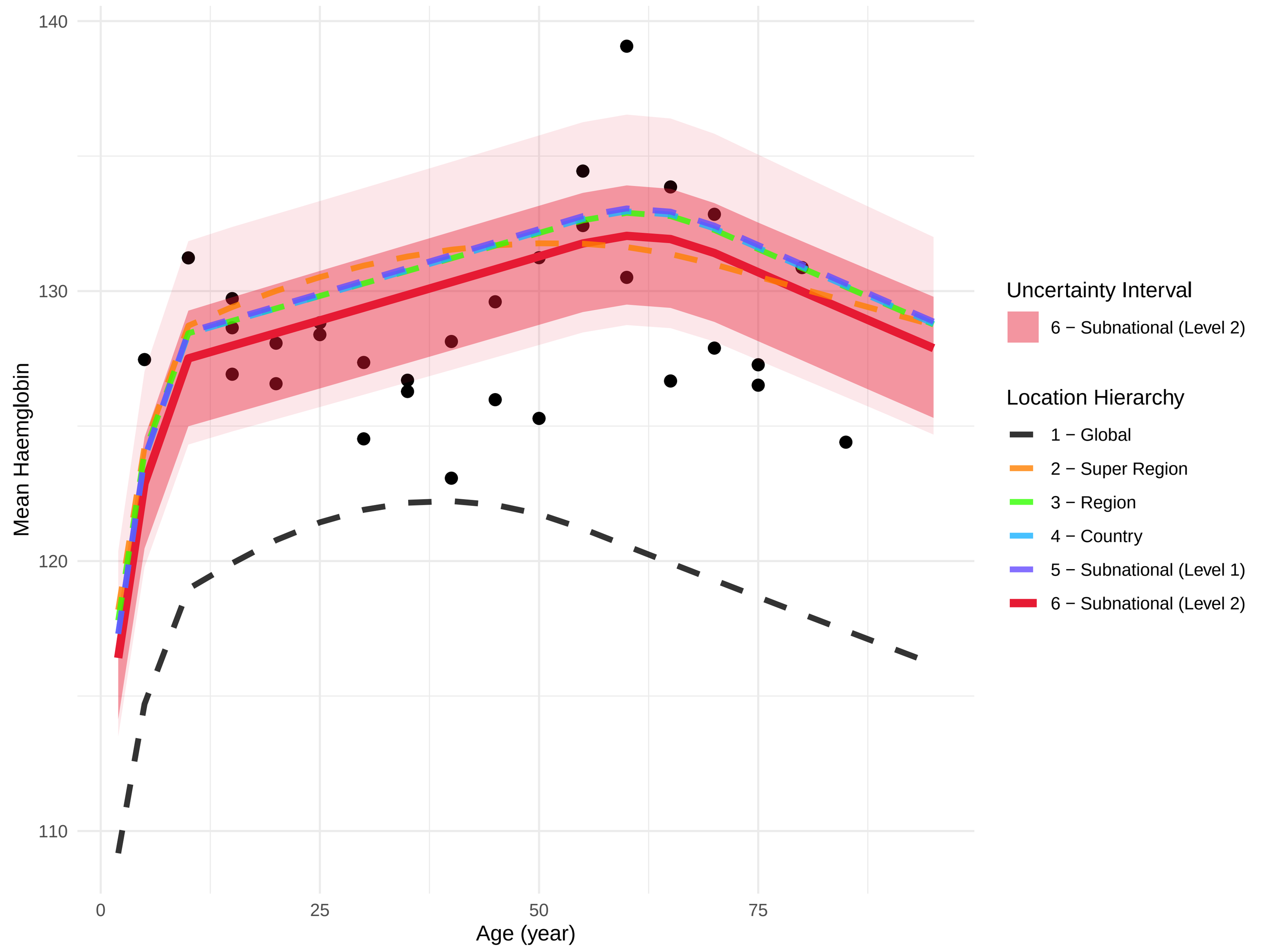

MR−BRT Age/Sex Fits − Non-pregnant Females 2+ Years Old − Mean Haemglobin − Scotland

WHO−2001 Adjustment Method

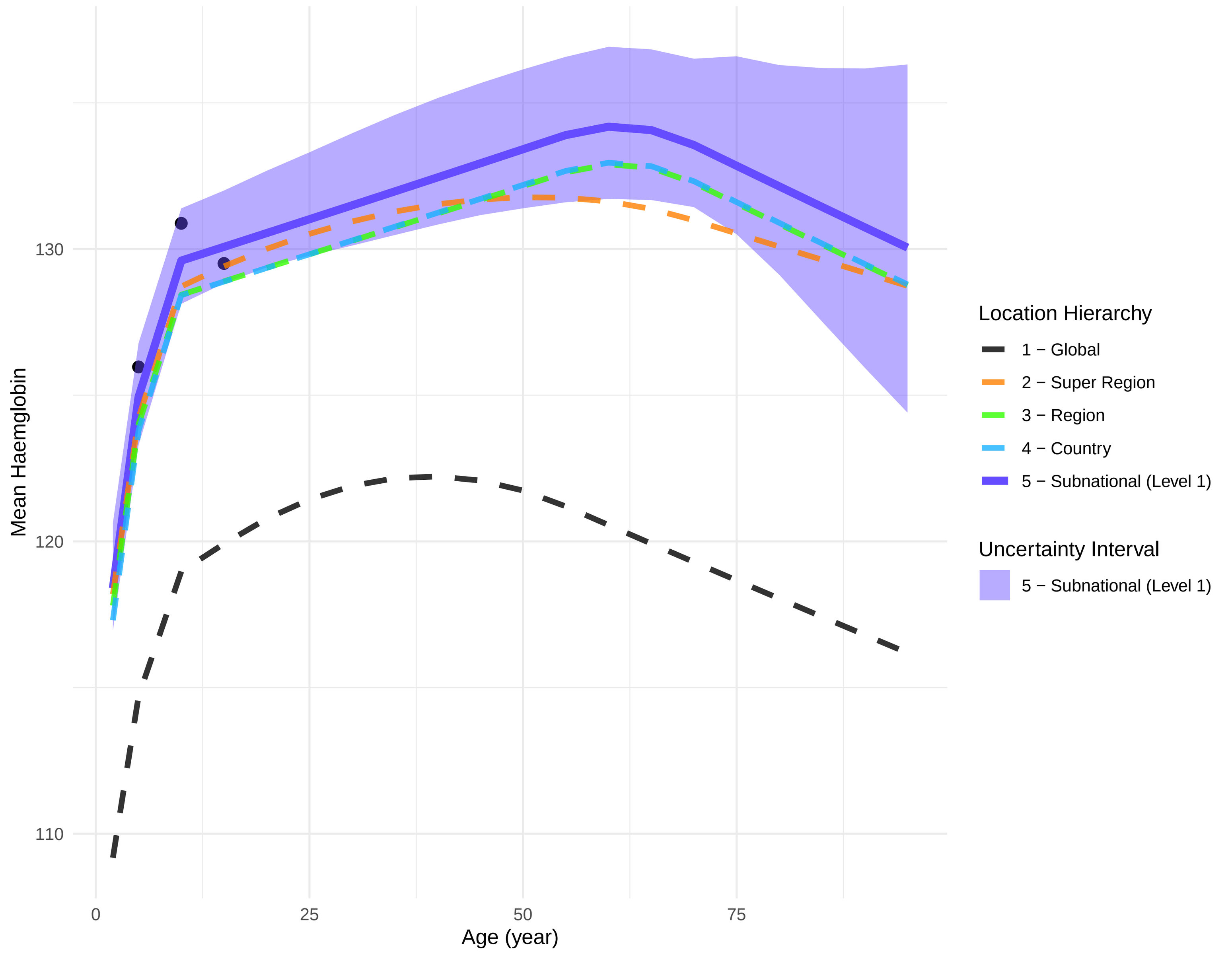

MR−BRT Age/Sex Fits − Non-pregnant Females 2+ Years Old − Mean Haemglobin − Wales

WHO−2001 Adjustment Method

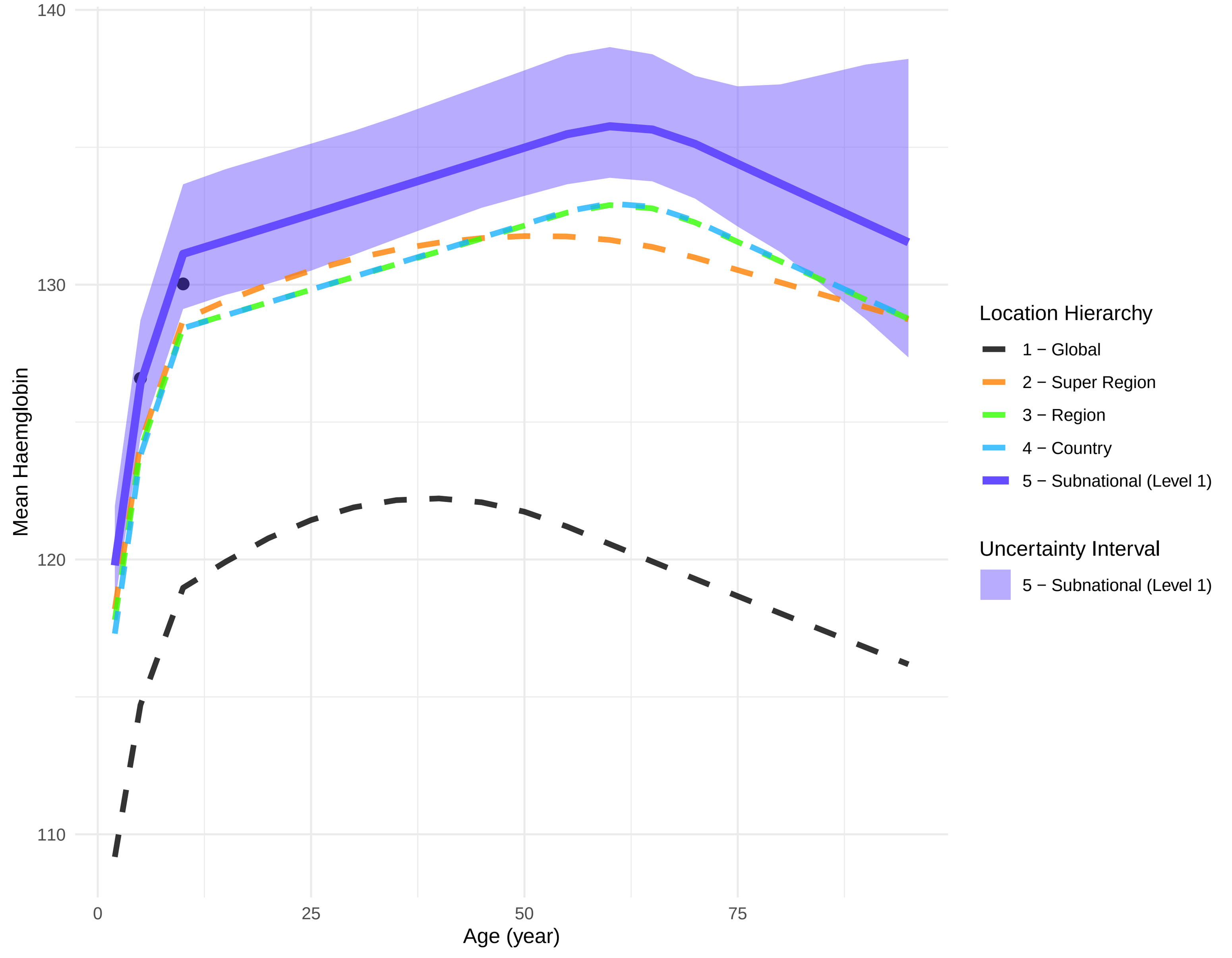

MR−BRT Age/Sex Fits − Non-pregnant Females 2+ Years Old − Mean Haemglobin − Latin America and Caribbean

WHO−2001 Adjustment Method

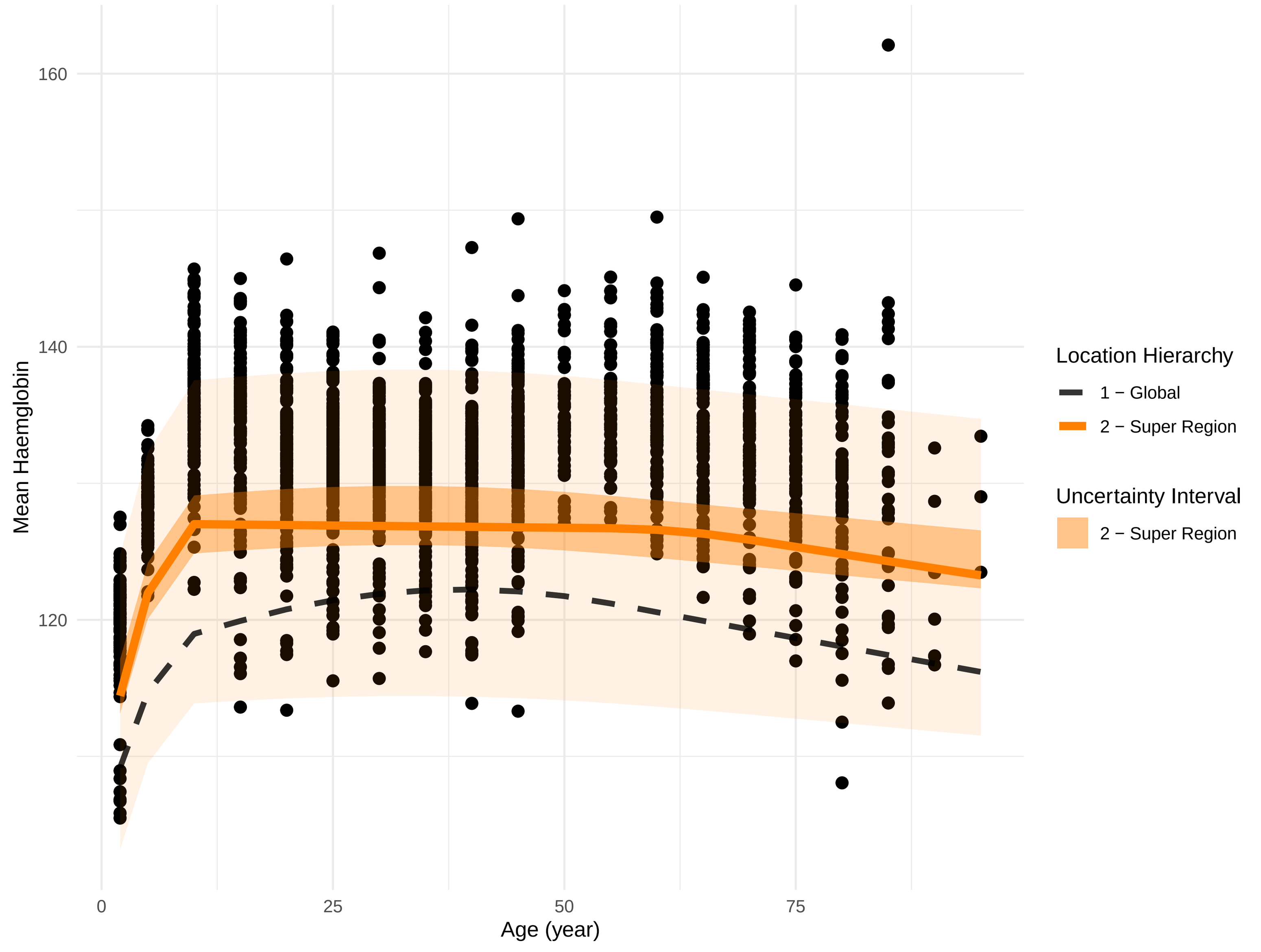

MR−BRT Age/Sex Fits − Non-pregnant Females 2+ Years Old − Mean Haemglobin − Andean Latin America

WHO−2001 Adjustment Method

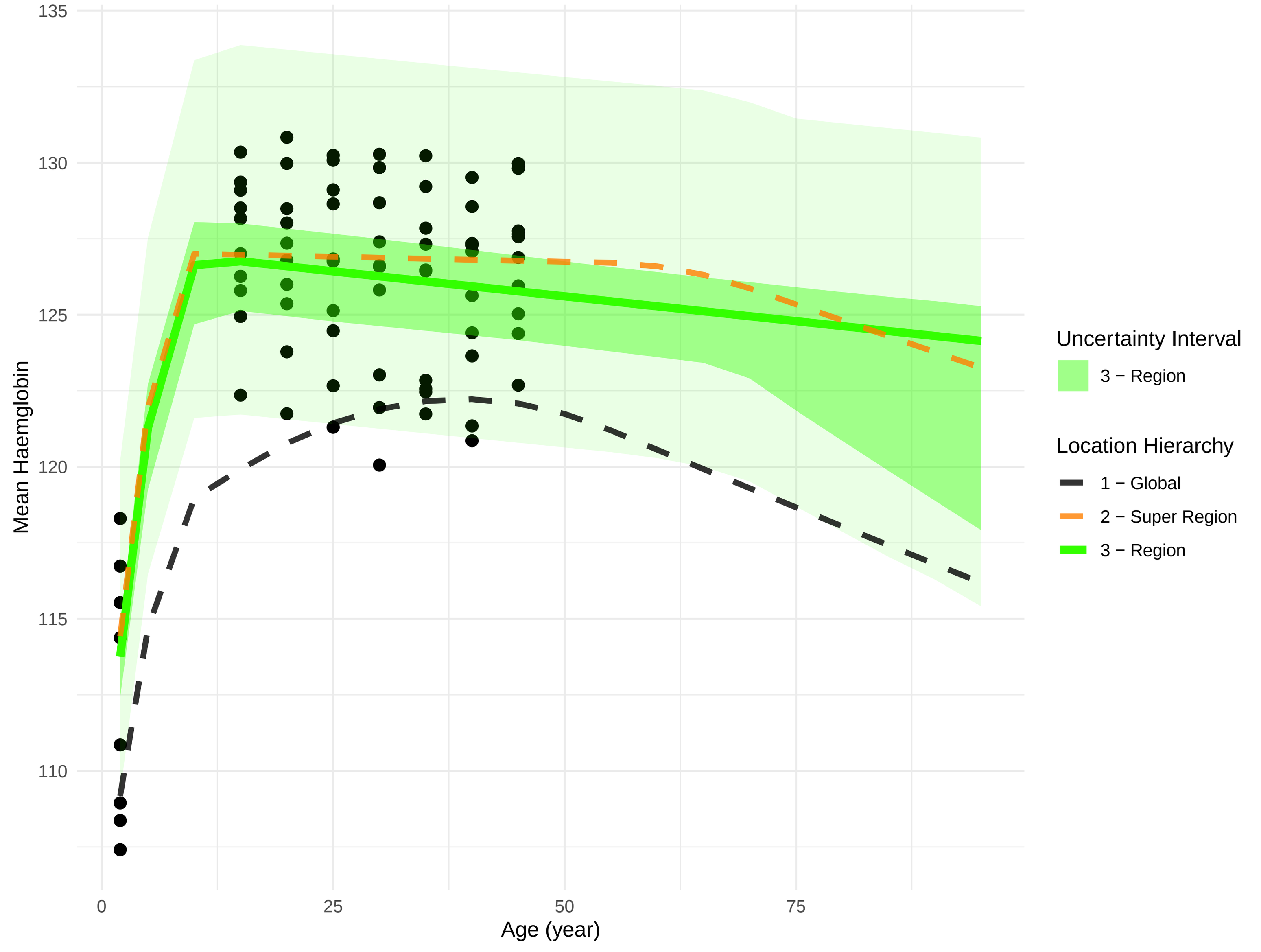

MR−BRT Age/Sex Fits − Non-pregnant Females 2+ Years Old − Mean Haemglobin − Bolivia (Plurinational State of)

WHO−2001 Adjustment Method

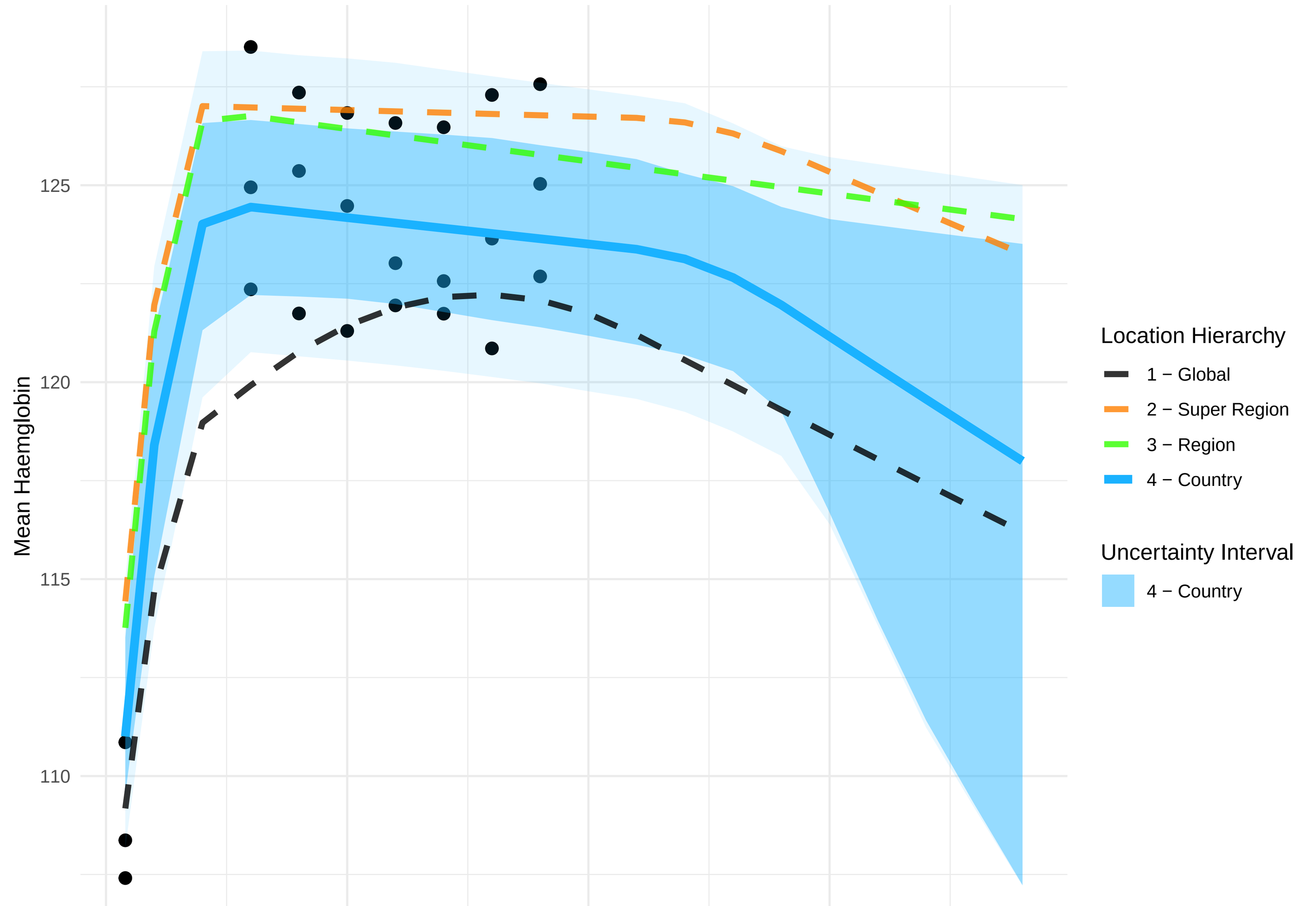

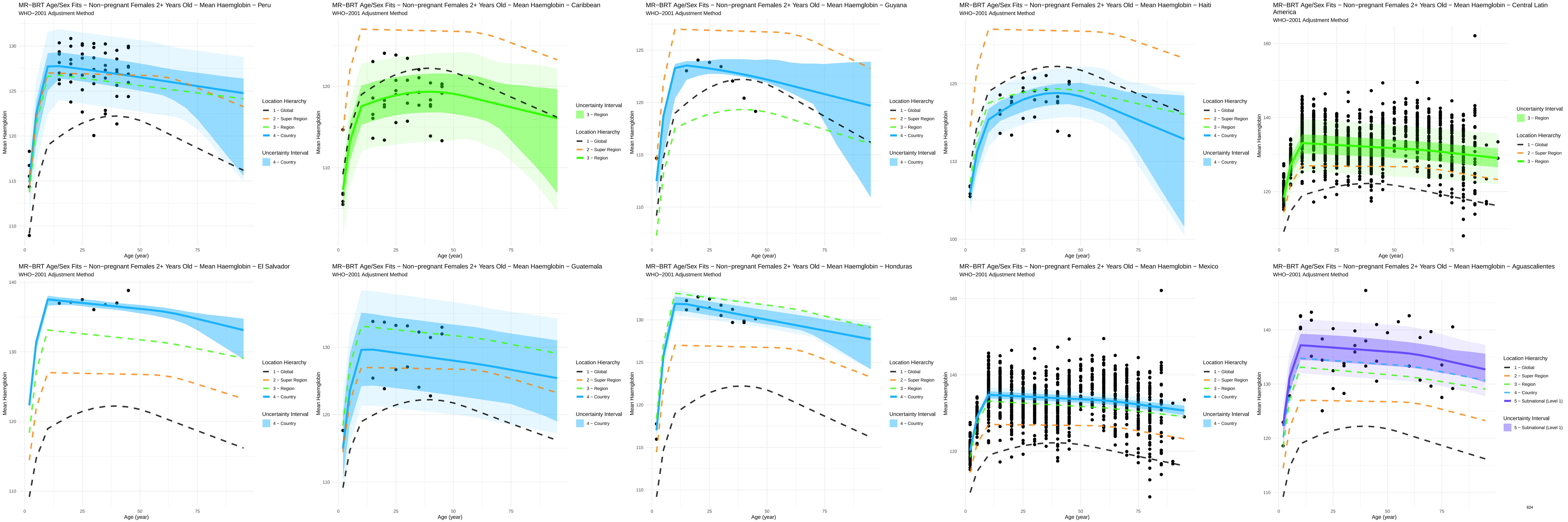

MR−BRT Age/Sex Fits − Non−pregnant Females 2+ Years Old − Mean Haemglobin − Peru
WHO−2001 Adjustment Method
Location Hierarchy
1 − Global
2 − Super Region
3 − Region
4 − Country
Uncertainty Interval
4 − Country
MR−BRT Age/Sex Fits − Non−pregnant Females 2+ Years Old − Mean Haemglobin − Caribbean
WHO−2001 Adjustment Method
Uncertainty Interval
3 − Region
Location Hierarchy
1 − Global
2 − Super Region
3 − Region
MR−BRT Age/Sex Fits − Non−pregnant Females 2+ Years Old − Mean Haemglobin − Guyana
WHO−2001 Adjustment Method
MR−BRT Age/Sex Fits − Non−pregnant Females 2+ Years Old − Mean Haemglobin − Haiti
WHO−2001 Adjustment Method
MR−BRT Age/Sex Fits − Non−pregnant Females 2+ Years Old − Mean Haemglobin − Central Latin America
WHO−2001 Adjustment Method
MR−BRT Age/Sex Fits − Non−pregnant Females 2+ Years Old − Mean Haemglobin − El Salvador
WHO−2001 Adjustment Method
MR−BRT Age/Sex Fits − Non−pregnant Females 2+ Years Old − Mean Haemglobin − Guatemala
WHO−2001 Adjustment Method
MR−BRT Age/Sex Fits − Non−pregnant Females 2+ Years Old − Mean Haemglobin − Honduras
WHO−2001 Adjustment Method
MR−BRT Age/Sex Fits − Non−pregnant Females 2+ Years Old − Mean Haemglobin − Mexico
WHO−2001 Adjustment Method
MR−BRT Age/Sex Fits − Non−pregnant Females 2+ Years Old − Mean Haemglobin − Aguascalientes
WHO−2001 Adjustment Method
Location Hierarchy
1 − Global
2 − Super Region
3 − Region
4 − Country
5 − Subnational (Level 1)
Uncertainty Interval
5 − Subnational (Level 1)
Mean Haemglobin
Age (year)

MR−BRT Age/Sex Fits − Non−pregnant Females 2+ Years Old − Mean Haemglobin − Baja California
WHO−2001 Adjustment Method

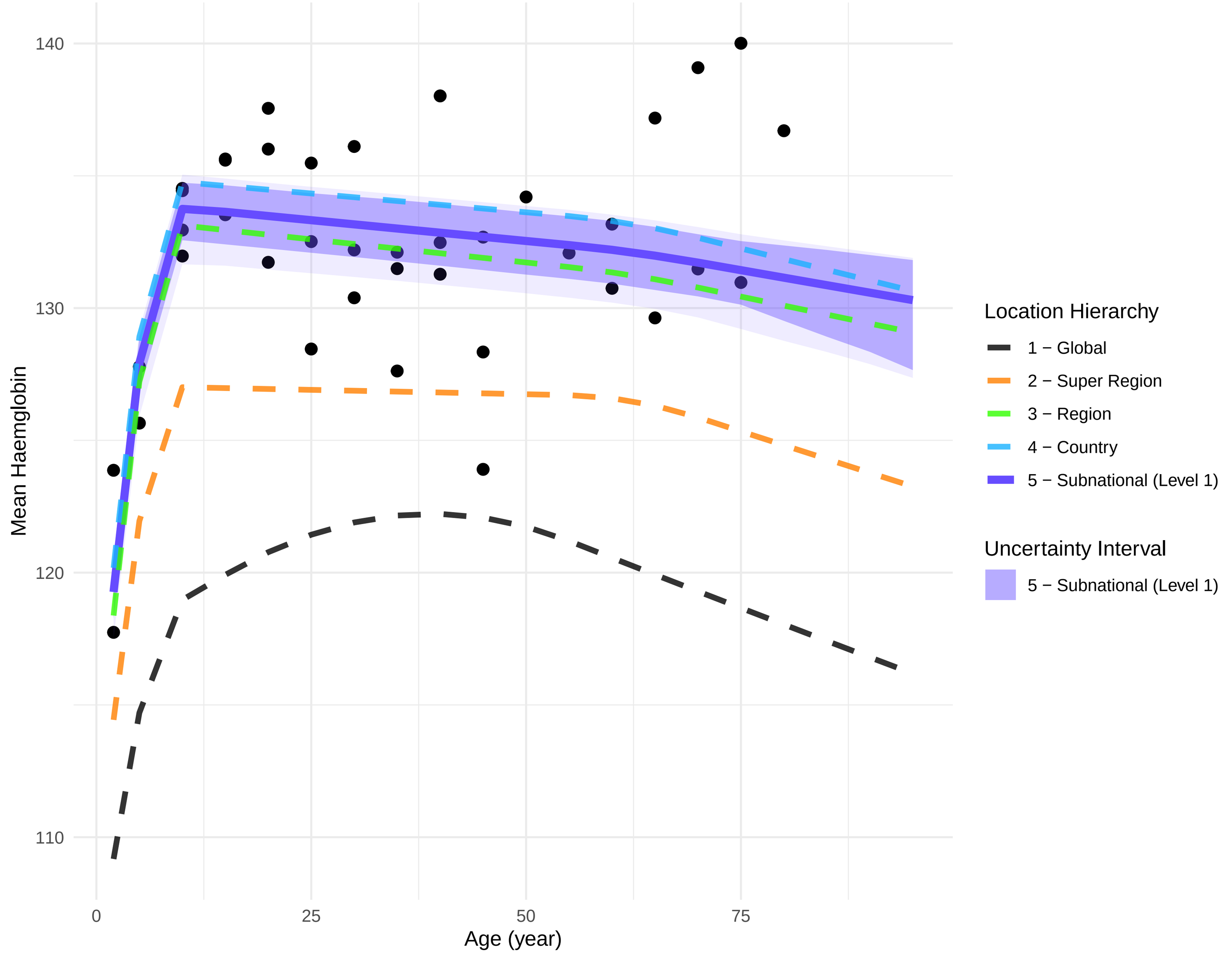

MR−BRT Age/Sex Fits − Non−pregnant Females 2+ Years Old − Mean Haemglobin − Baja California Sur
WHO−2001 Adjustment Method

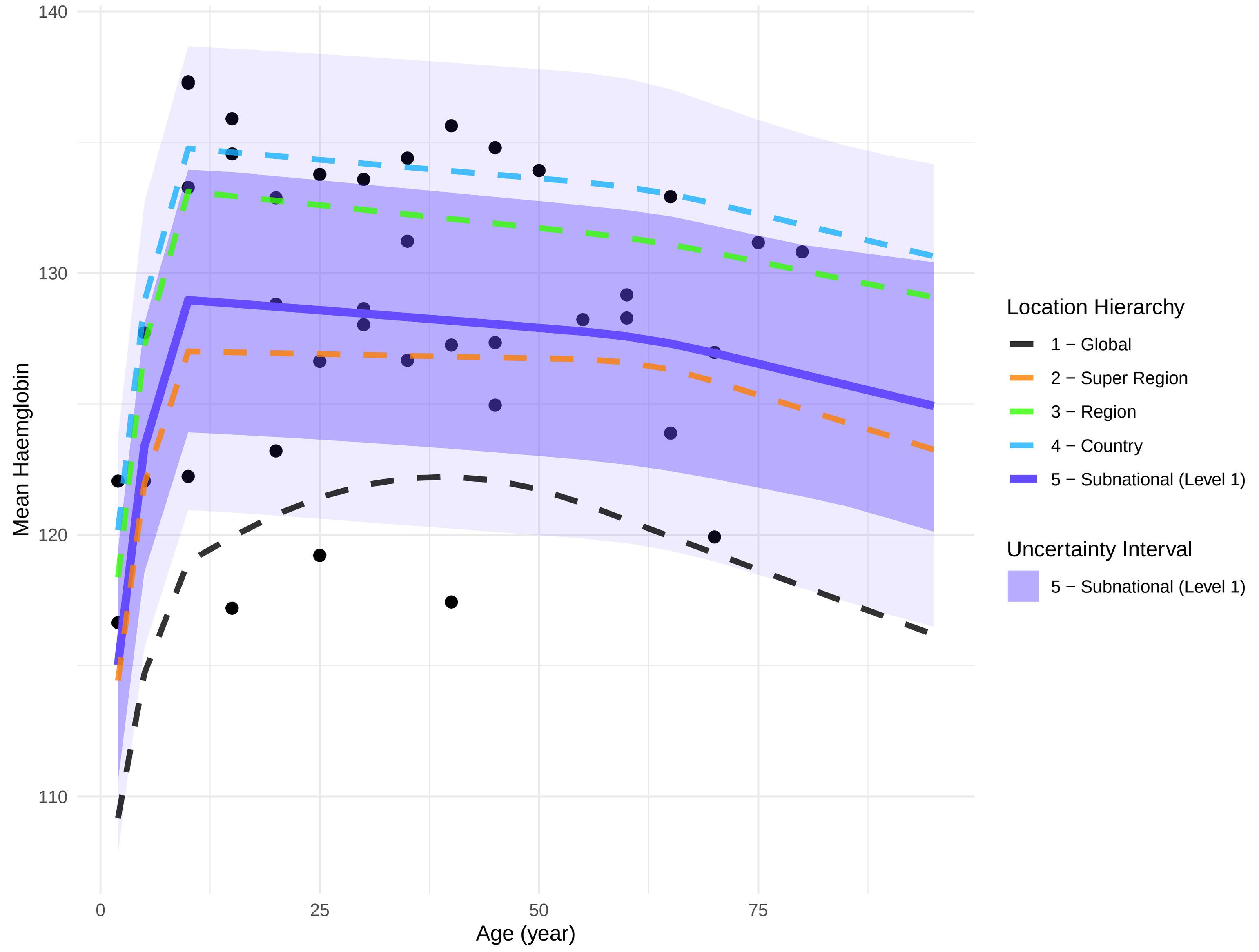

MR−BRT Age/Sex Fits − Non−pregnant Females 2+ Years Old − Mean Haemglobin − Campeche
WHO−2001 Adjustment Method

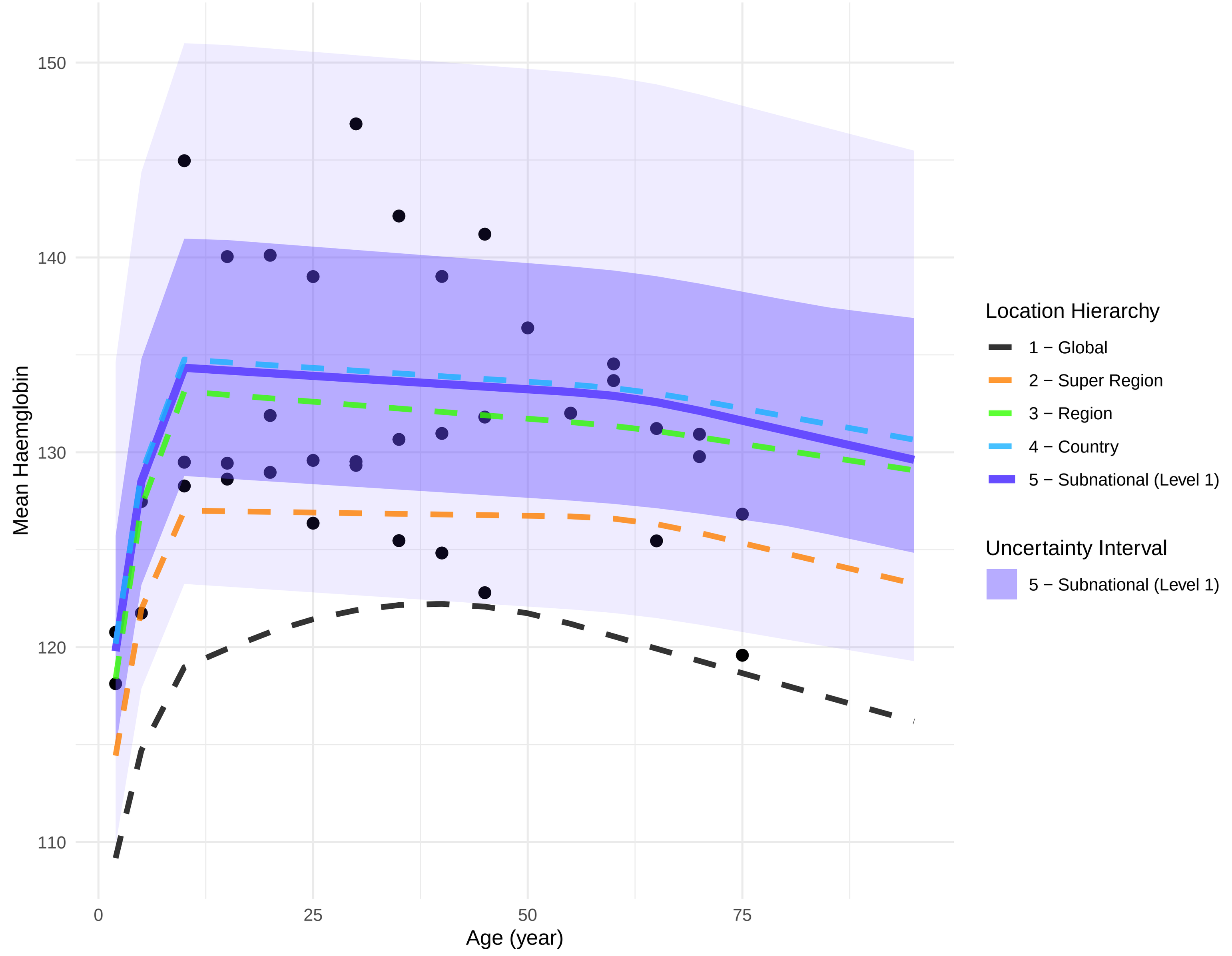

MR−BRT Age/Sex Fits − Non−pregnant Females 2+ Years Old − Mean Haemglobin − Chiapas
WHO−2001 Adjustment Method

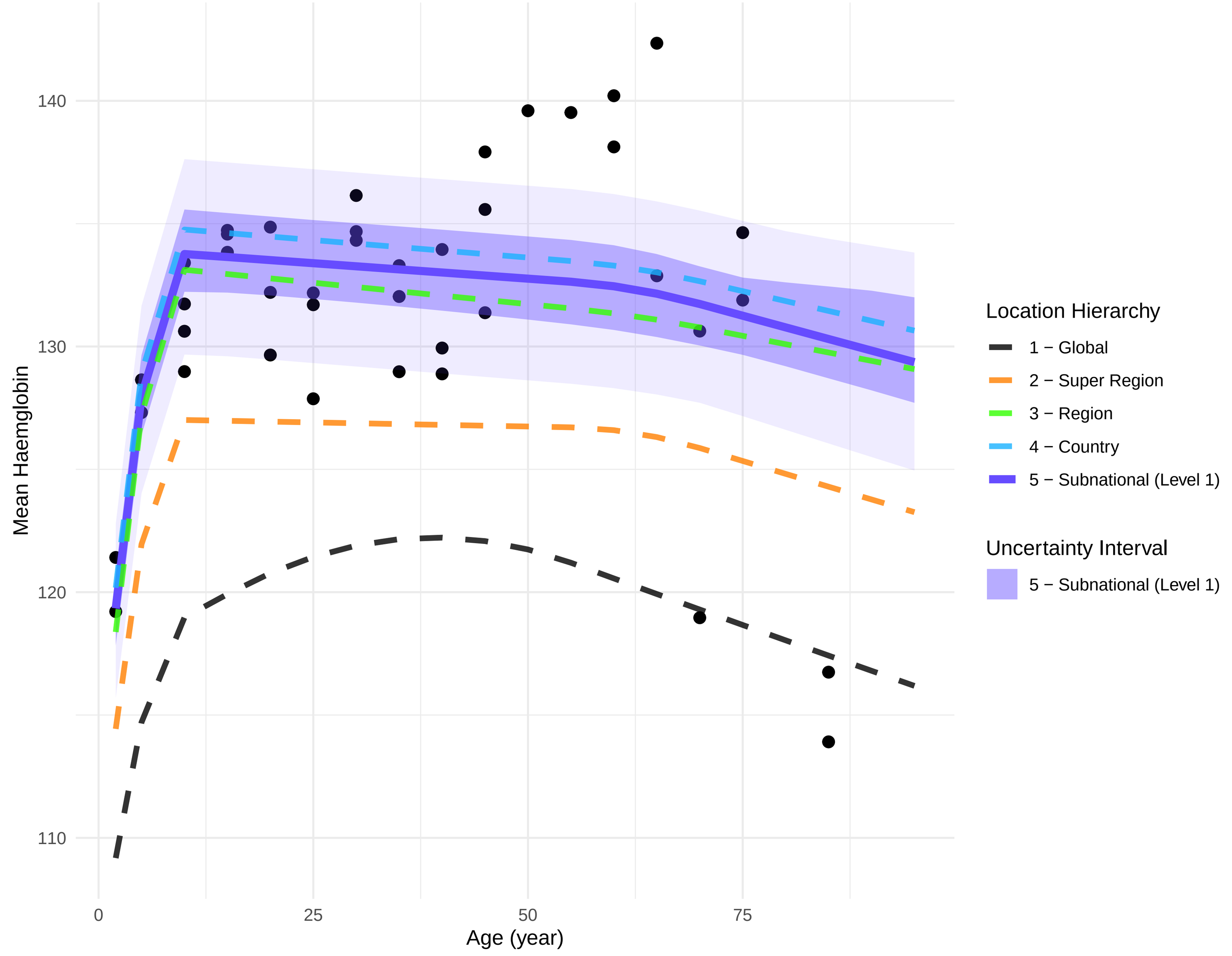

MR−BRT Age/Sex Fits − Non−pregnant Females 2+ Years Old − Mean Haemglobin − Chihuahua
WHO−2001 Adjustment Method

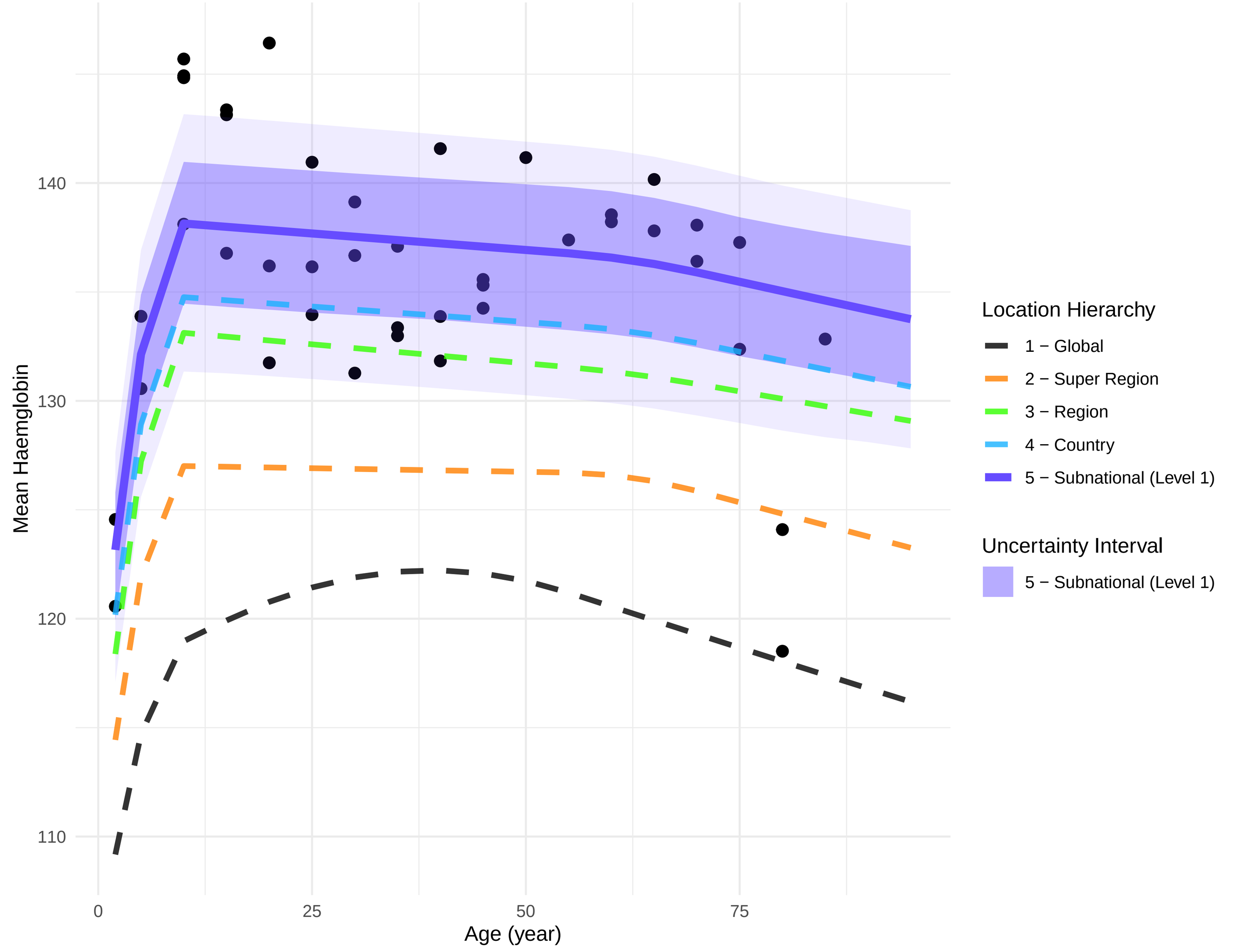

MR−BRT Age/Sex Fits − Non−pregnant Females 2+ Years Old − Mean Haemglobin − Coahuila
WHO−2001 Adjustment Method

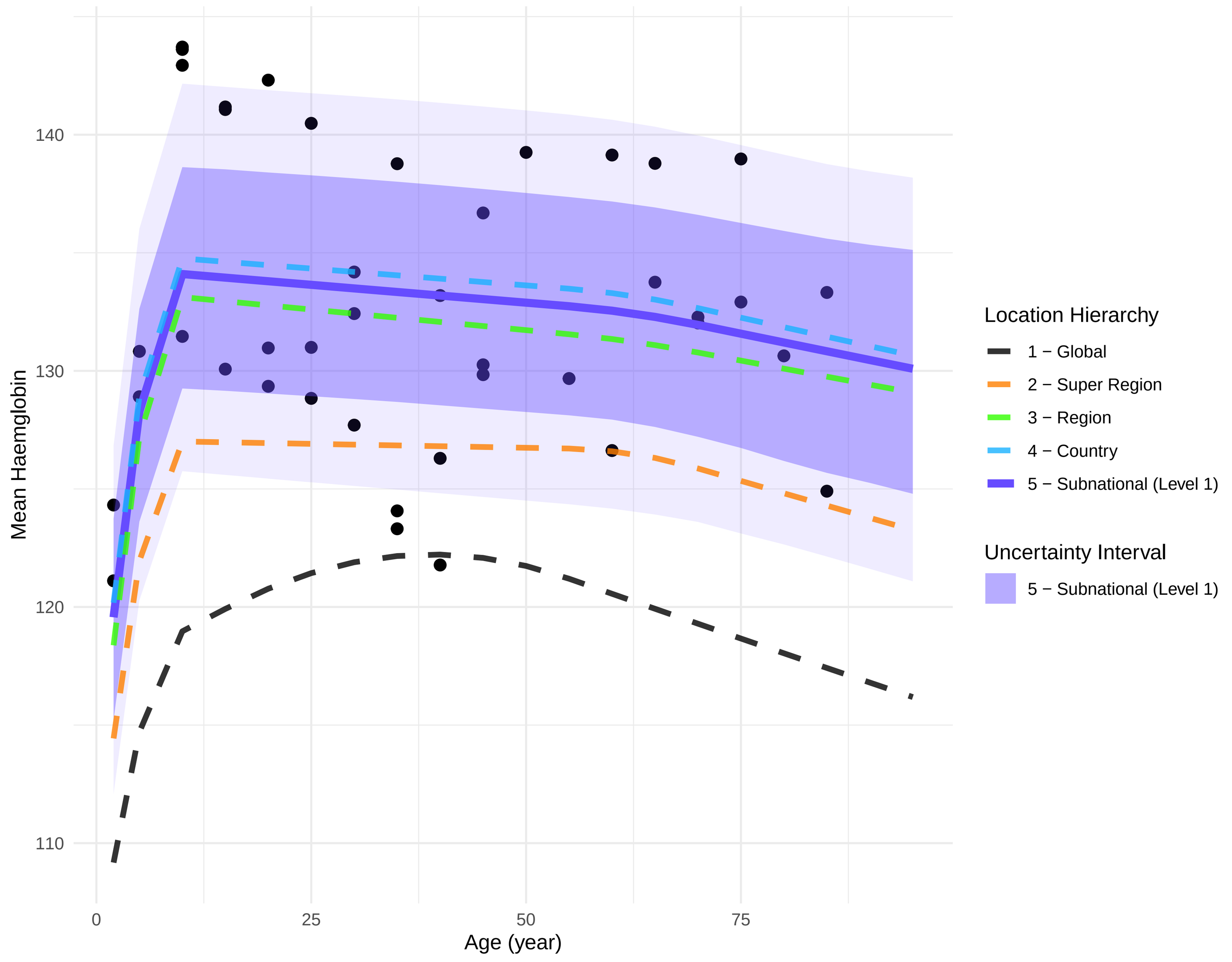

MR−BRT Age/Sex Fits − Non−pregnant Females 2+ Years Old − Mean Haemglobin − Colima
WHO−2001 Adjustment Method

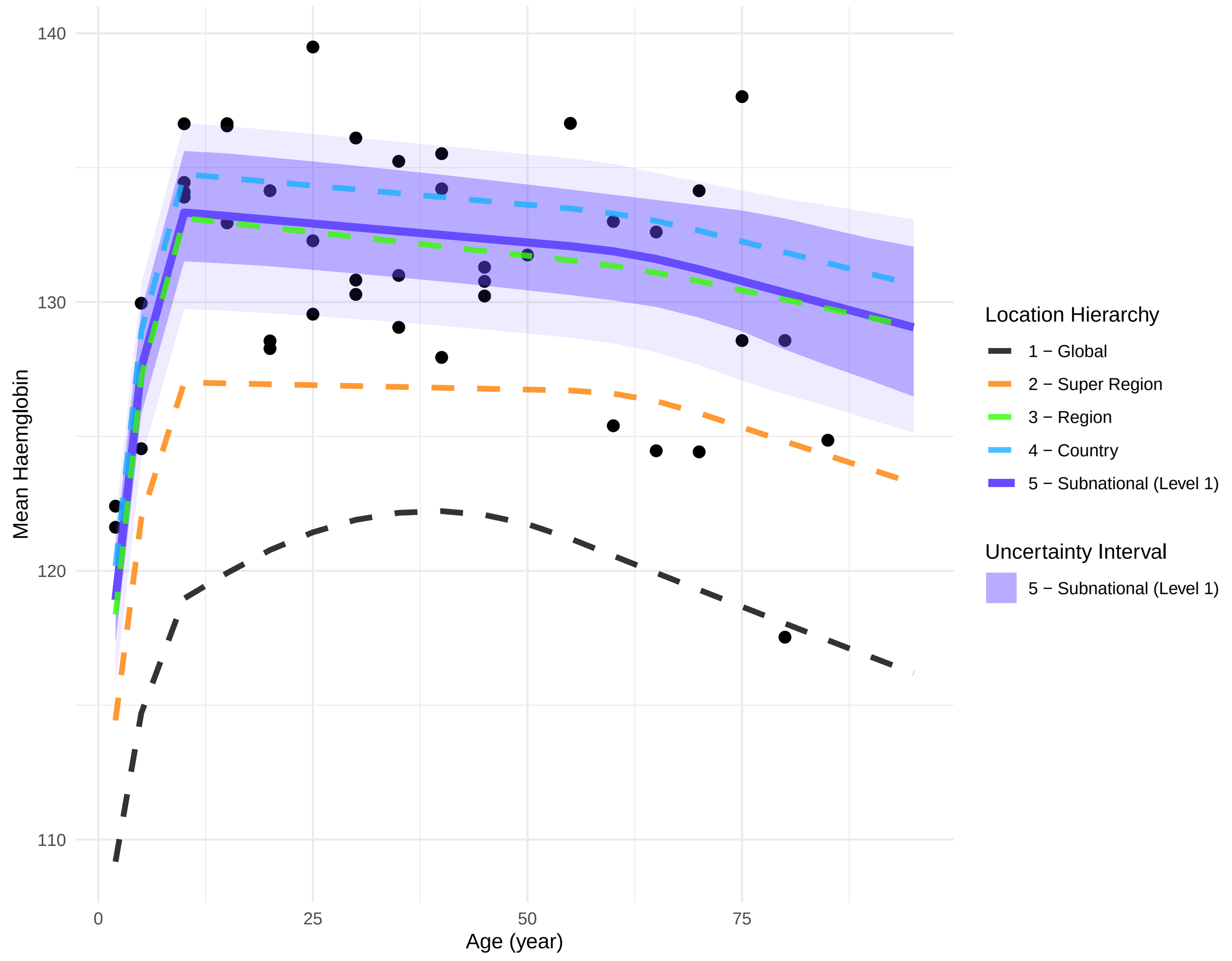

MR−BRT Age/Sex Fits − Non−pregnant Females 2+ Years Old − Mean Haemglobin − Durango
WHO−2001 Adjustment Method

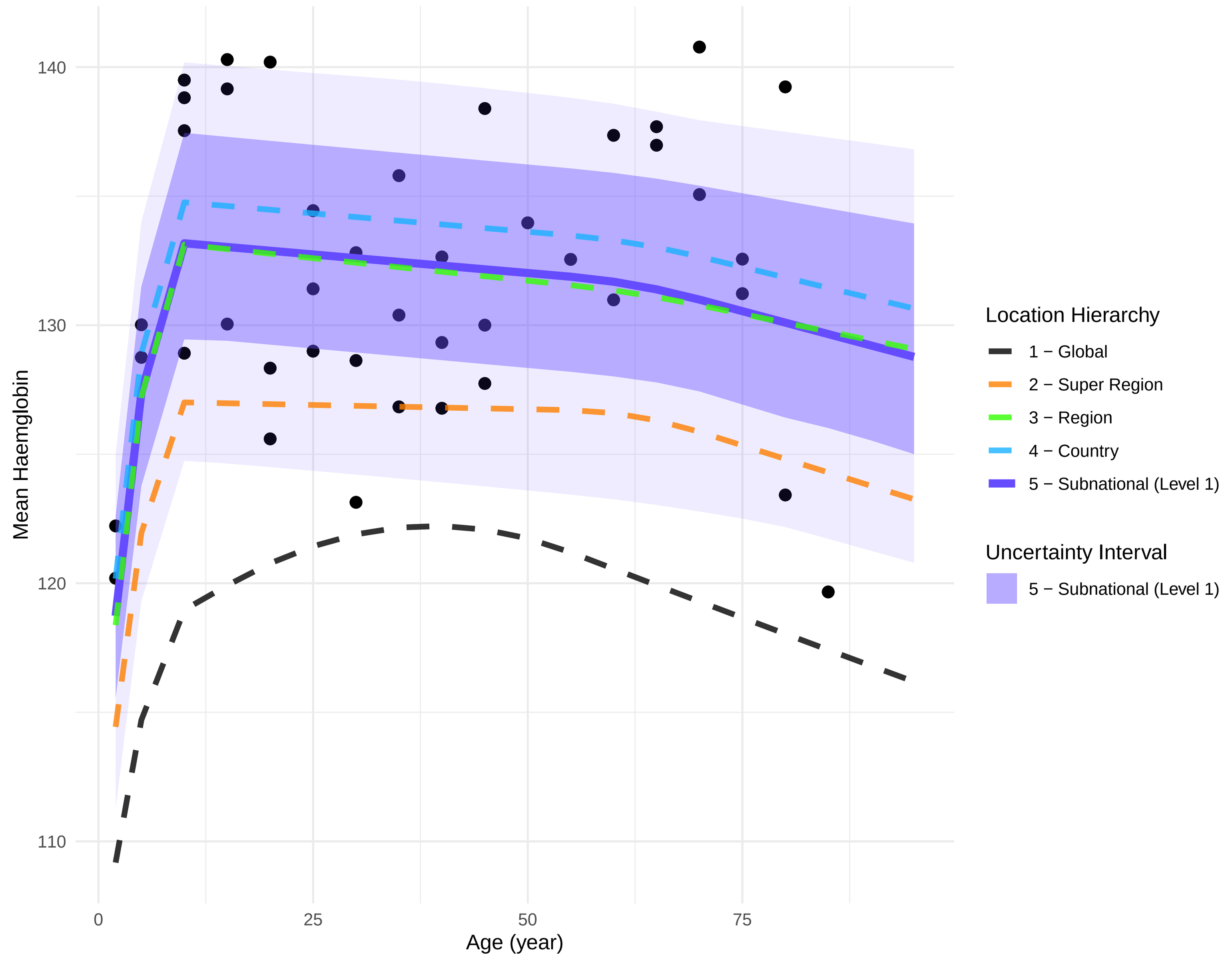

MR−BRT Age/Sex Fits − Non−pregnant Females 2+ Years Old − Mean Haemglobin − Guanajuato
WHO−2001 Adjustment Method

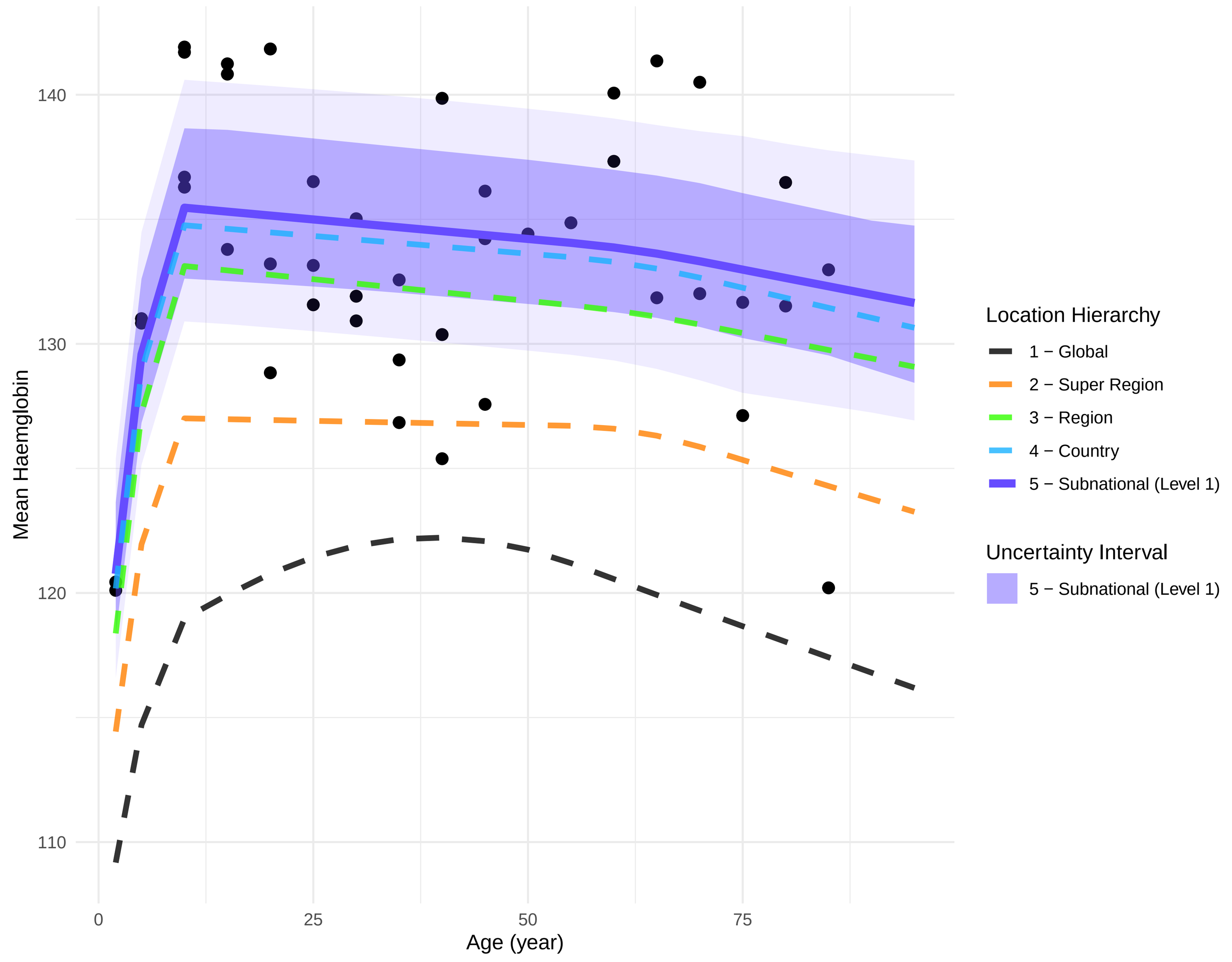

MR−BRT Age/Sex Fits − Non−pregnant Females 2+ Years Old − Mean Haemglobin − Guerrero
WHO−2001 Adjustment Method

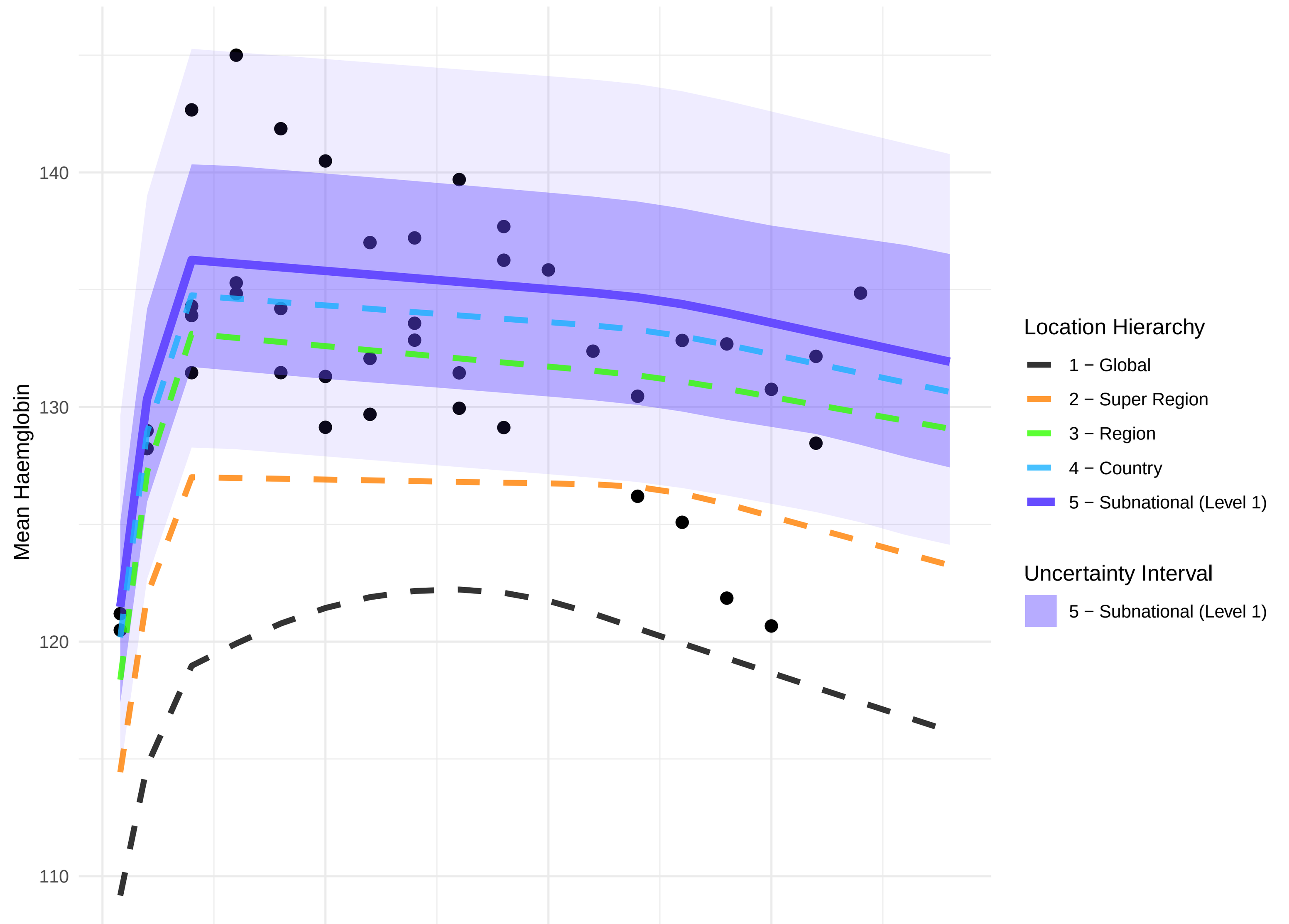

MR−BRT Age/Sex Fits − Non−pregnant Females 2+ Years Old − Mean Haemglobin − Hidalgo
WHO−2001 Adjustment Method

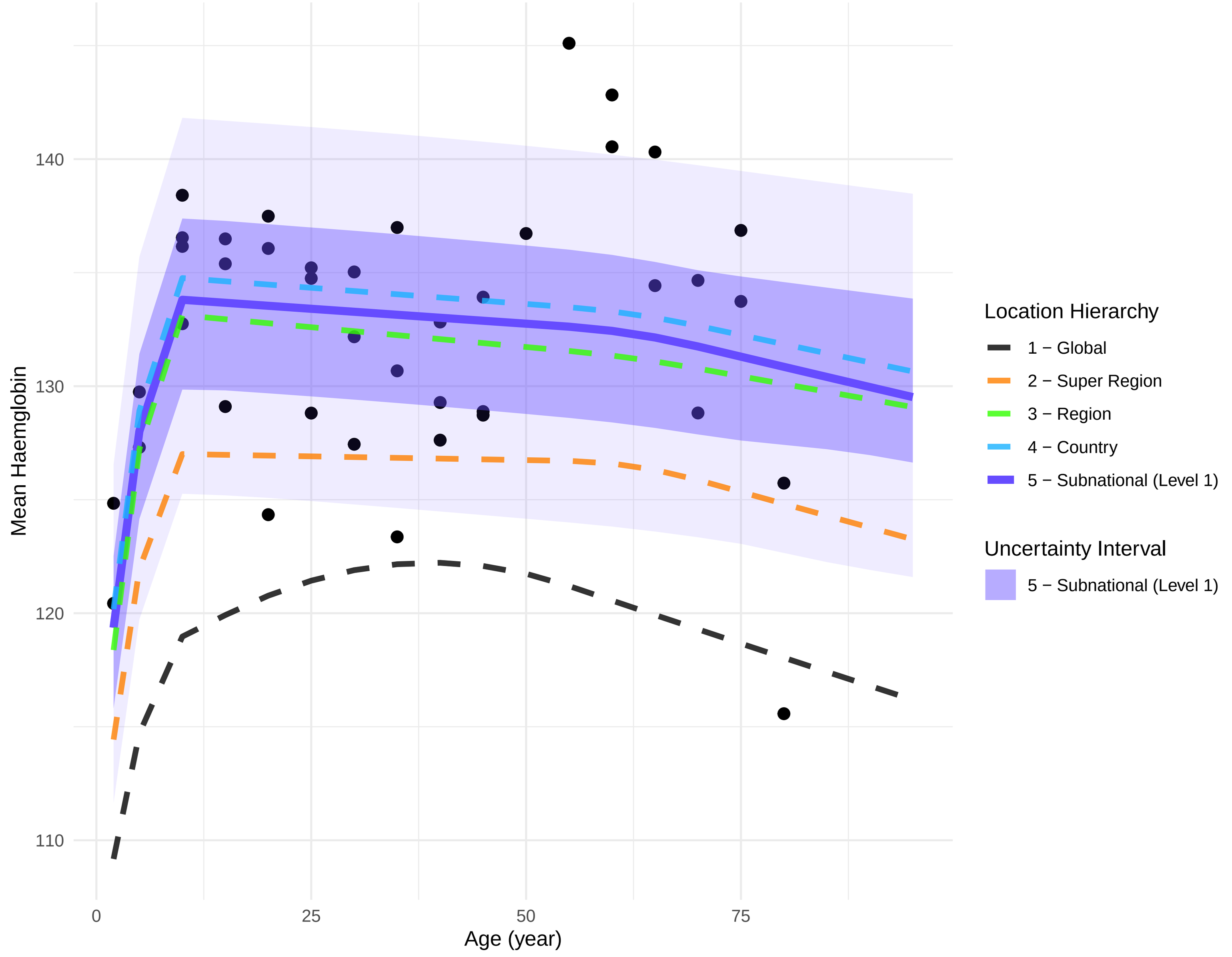

MR−BRT Age/Sex Fits − Non−pregnant Females 2+ Years Old − Mean Haemglobin − Jalisco
WHO−2001 Adjustment Method

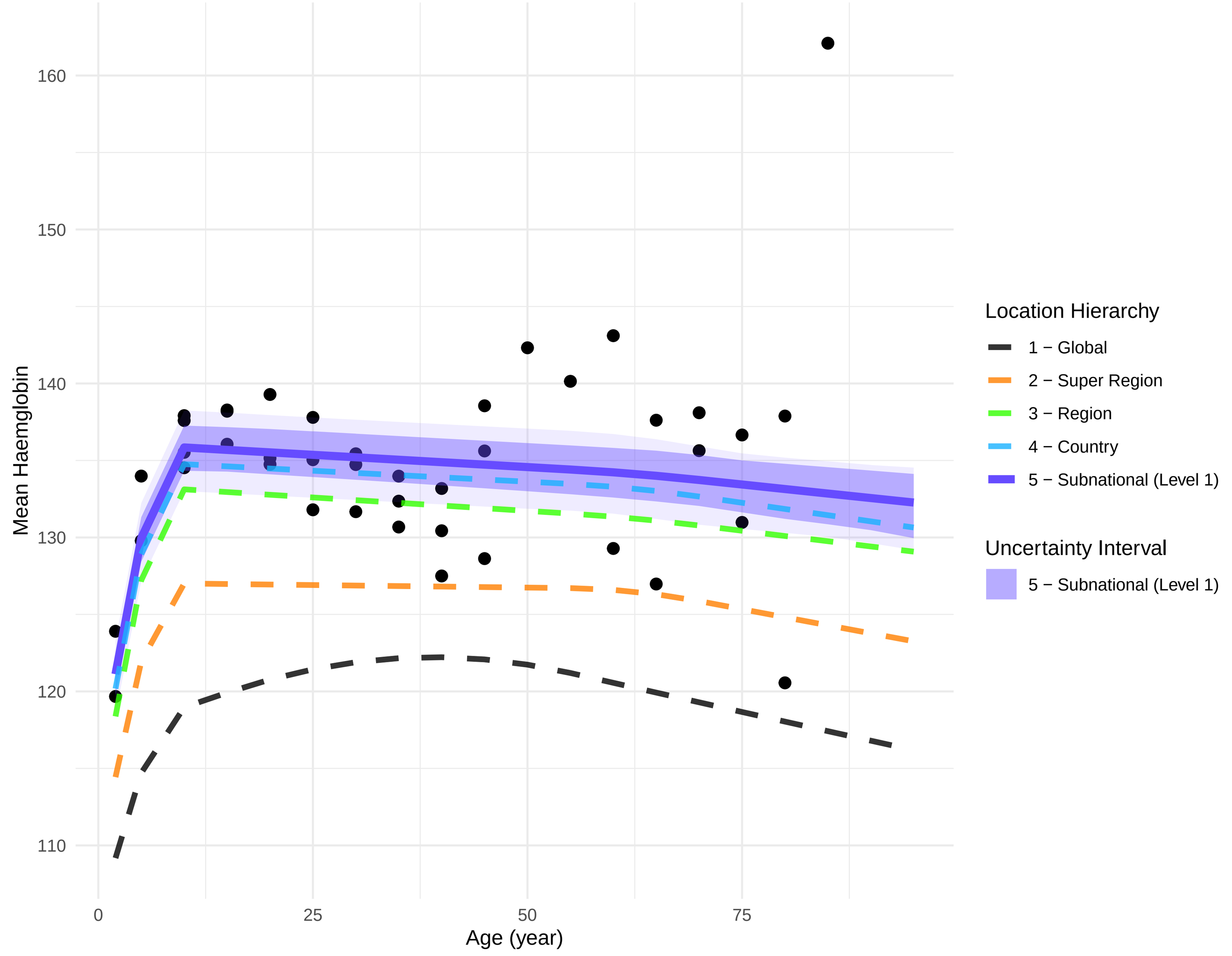

MR−BRT Age/Sex Fits − Non−pregnant Females 2+ Years Old − Mean Haemglobin − México
WHO−2001 Adjustment Method

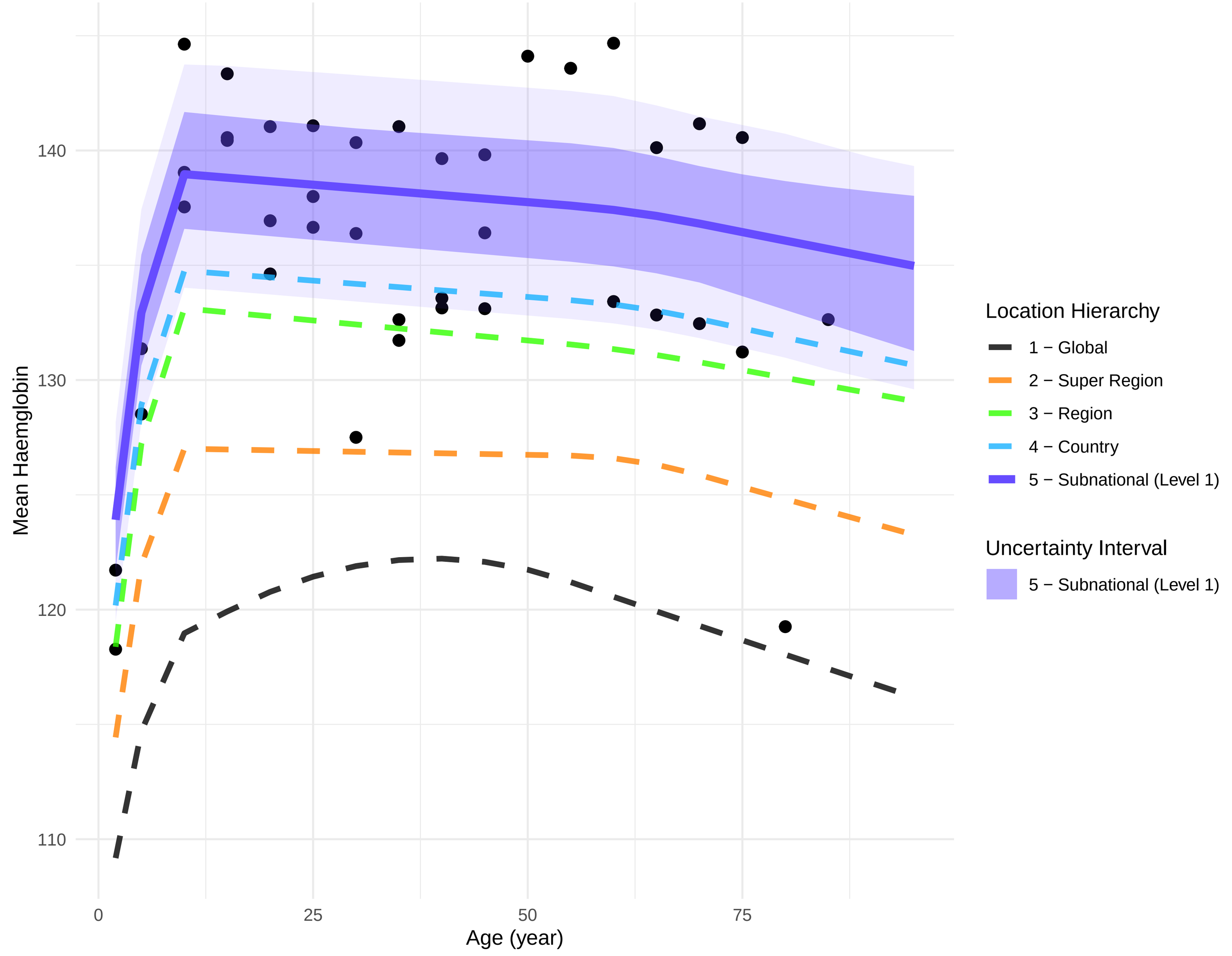

MR−BRT Age/Sex Fits − Non−pregnant Females 2+ Years Old − Mean Haemglobin − Mexico City
WHO−2001 Adjustment Method

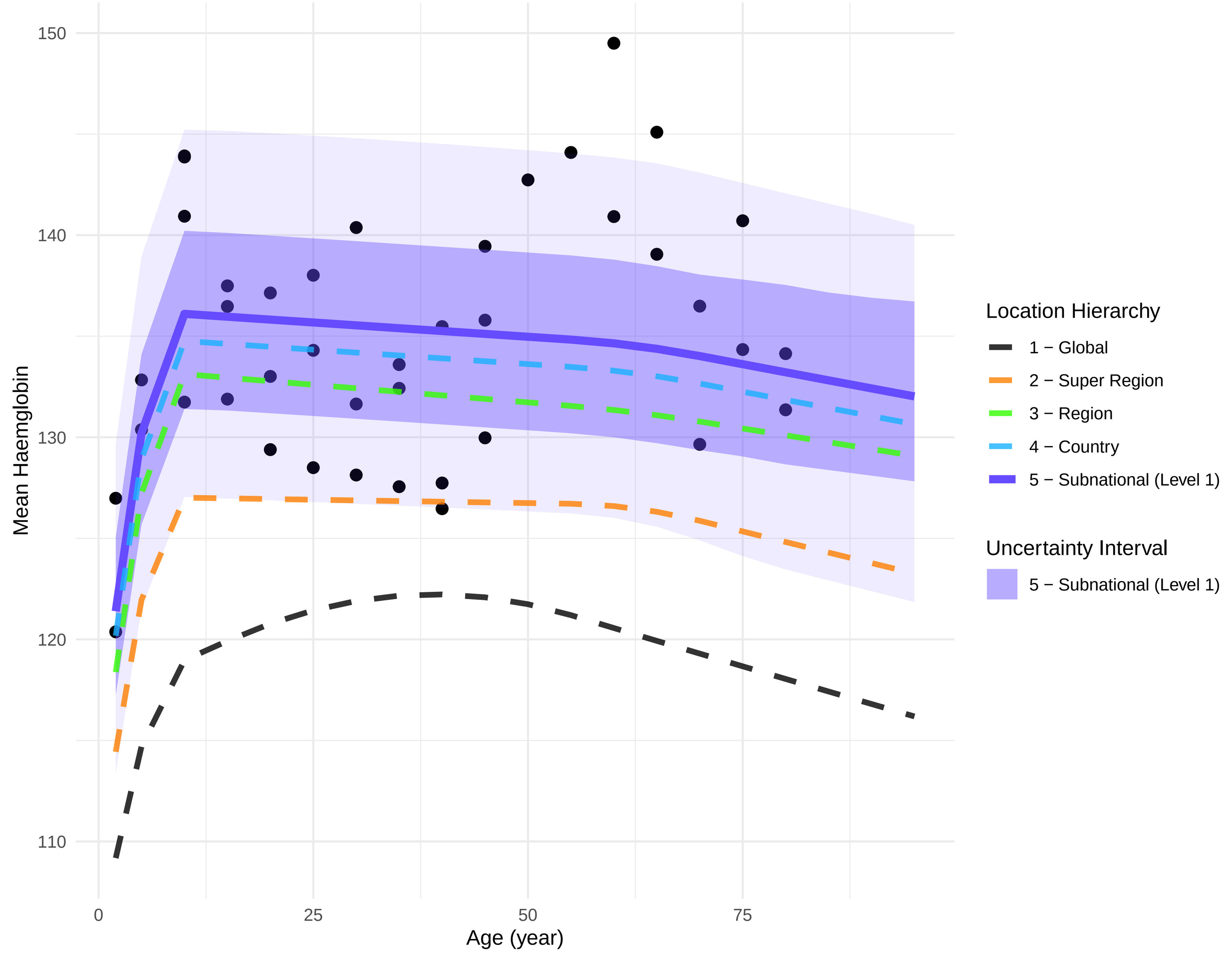

MR−BRT Age/Sex Fits − Non−pregnant Females 2+ Years Old − Mean Haemglobin − Michoacán de Ocampo
WHO−2001 Adjustment Method

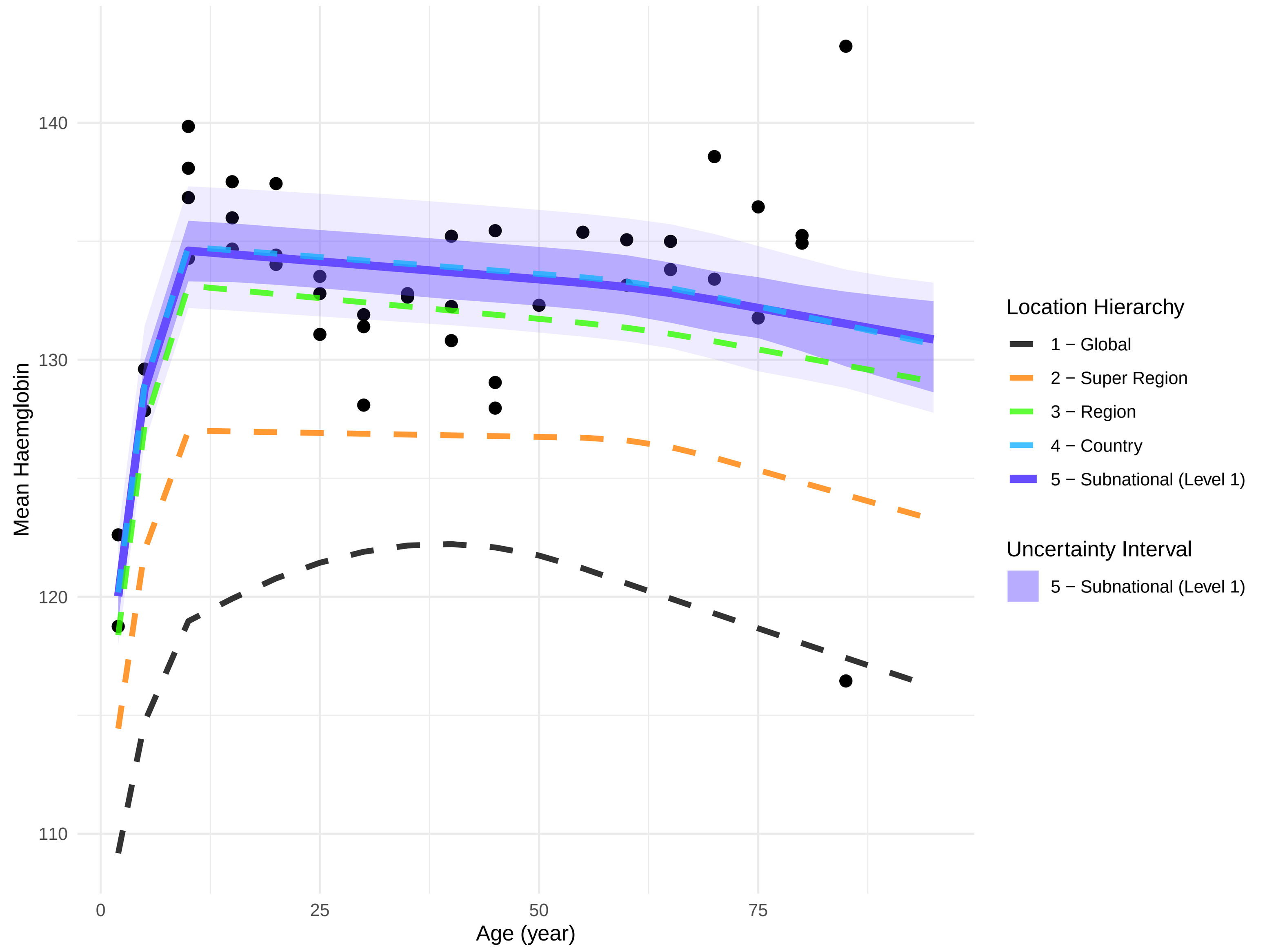

MR−BRT Age/Sex Fits − Non−pregnant Females 2+ Years Old − Mean Haemglobin − Morelos
WHO−2001 Adjustment Method

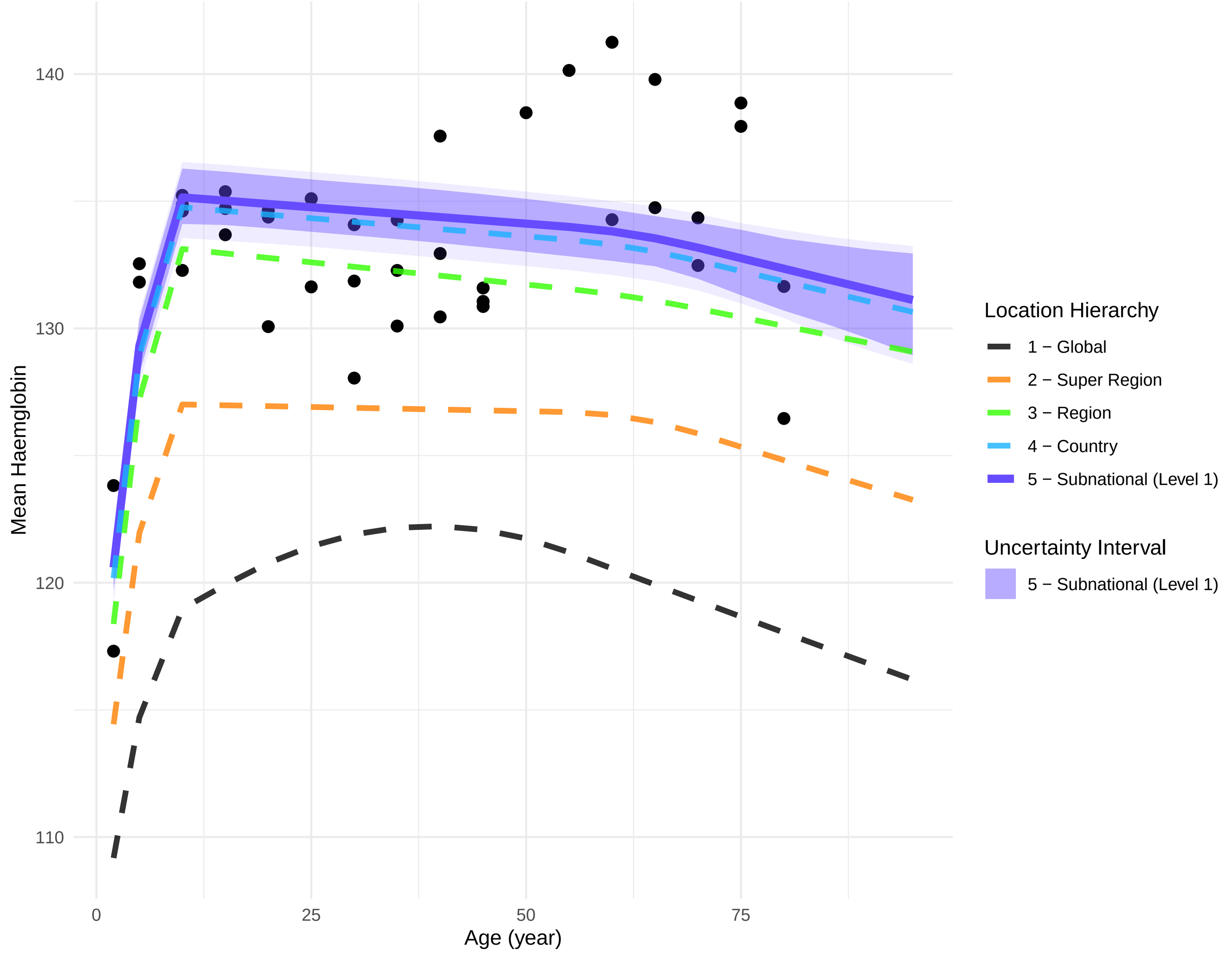

MR−BRT Age/Sex Fits − Non−pregnant Females 2+ Years Old − Mean Haemglobin − Nayarit
WHO−2001 Adjustment Method

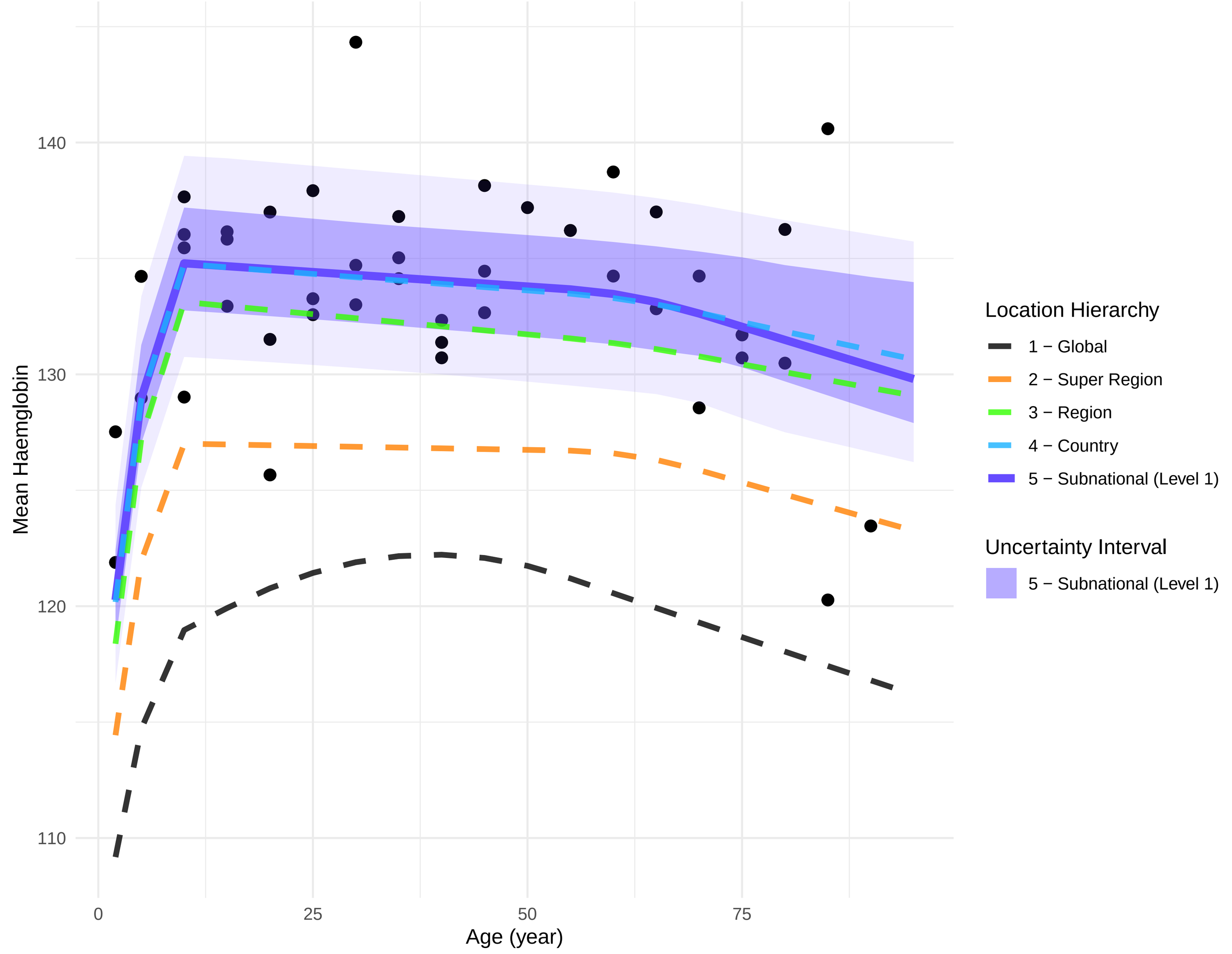

MR−BRT Age/Sex Fits − Non−pregnant Females 2+ Years Old − Mean Haemglobin − Nuevo León
WHO−2001 Adjustment Method

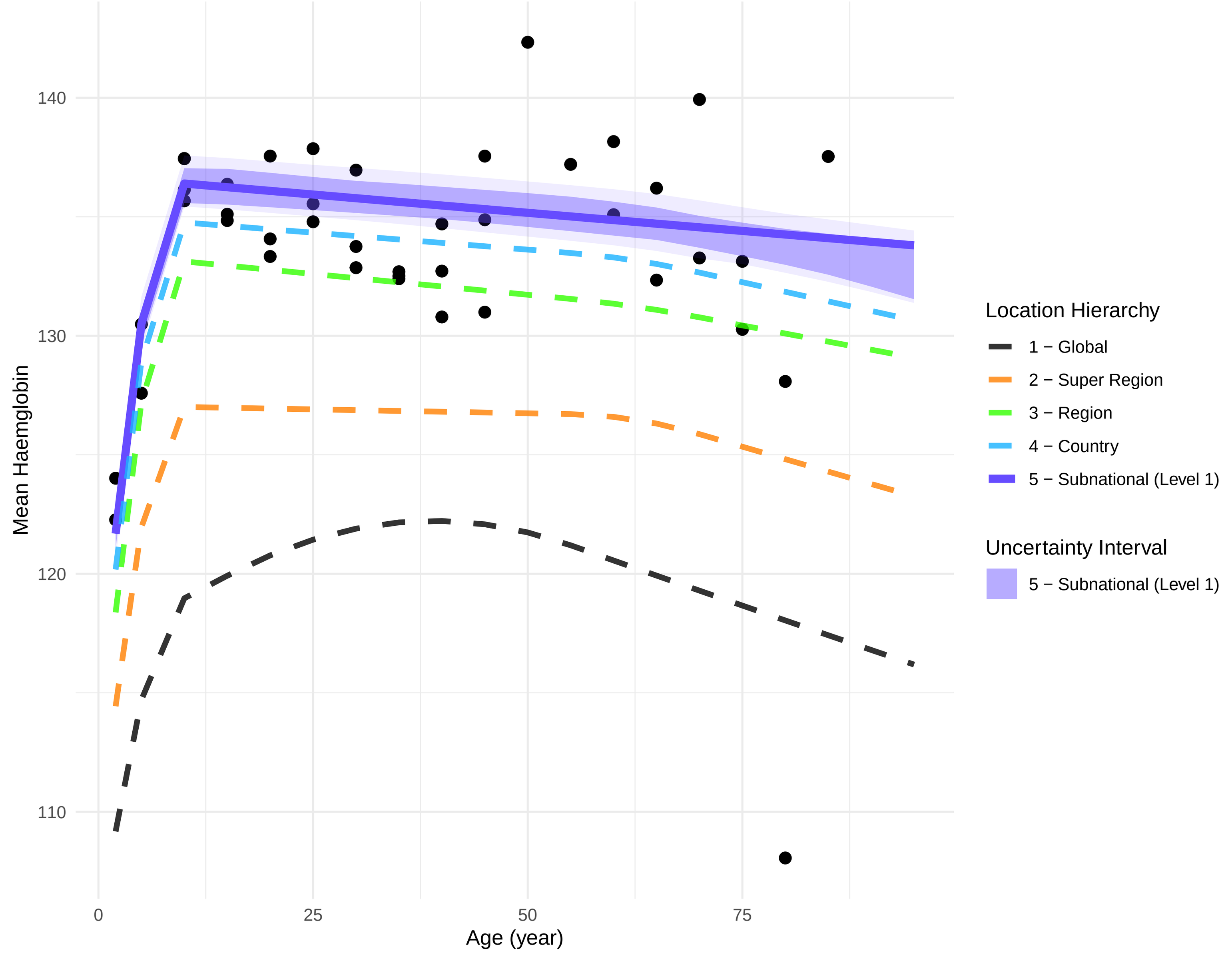

MR−BRT Age/Sex Fits − Non−pregnant Females 2+ Years Old − Mean Haemglobin − Oaxaca
WHO−2001 Adjustment Method

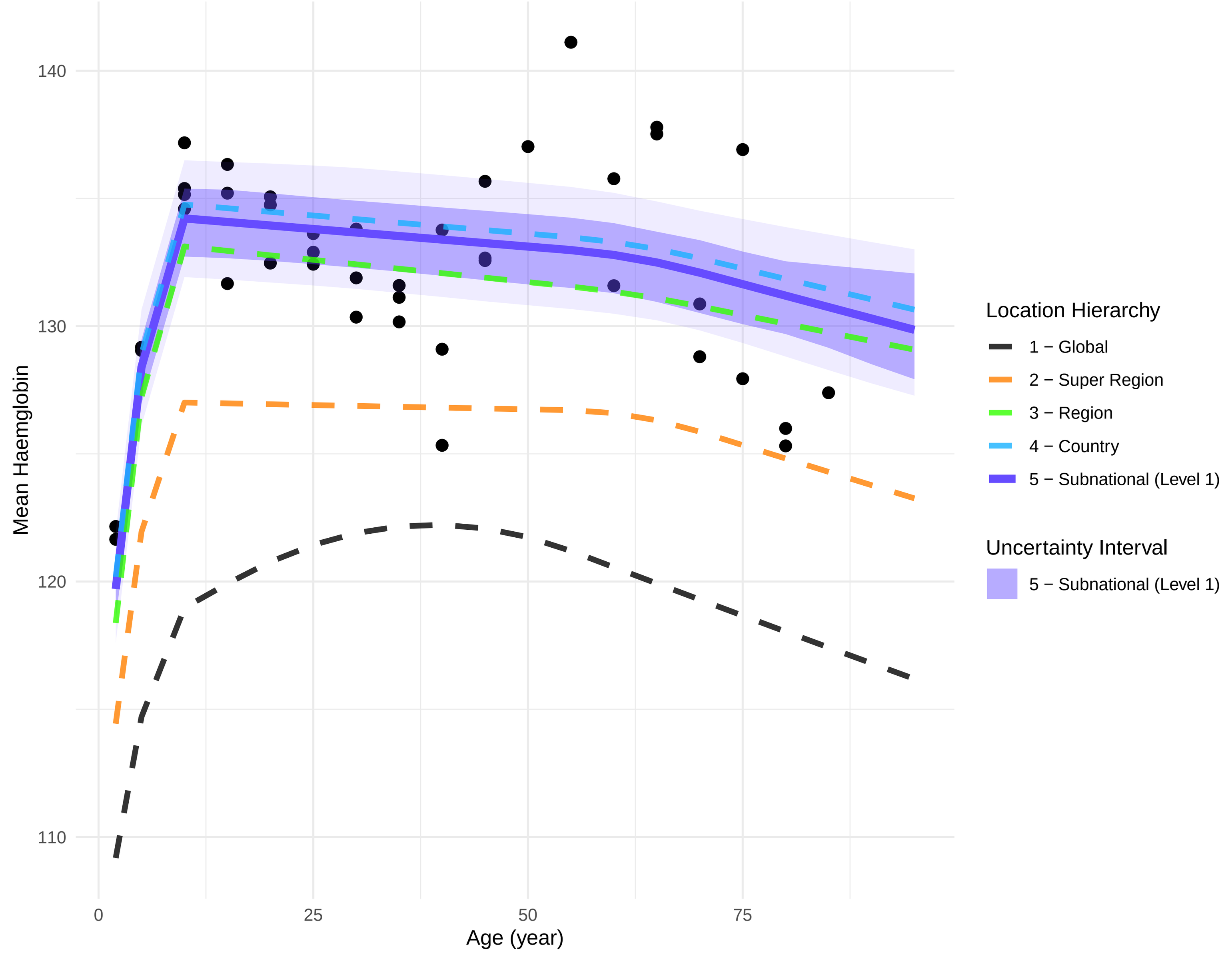

MR−BRT Age/Sex Fits − Non−pregnant Females 2+ Years Old − Mean Haemglobin − Puebla
WHO−2001 Adjustment Method

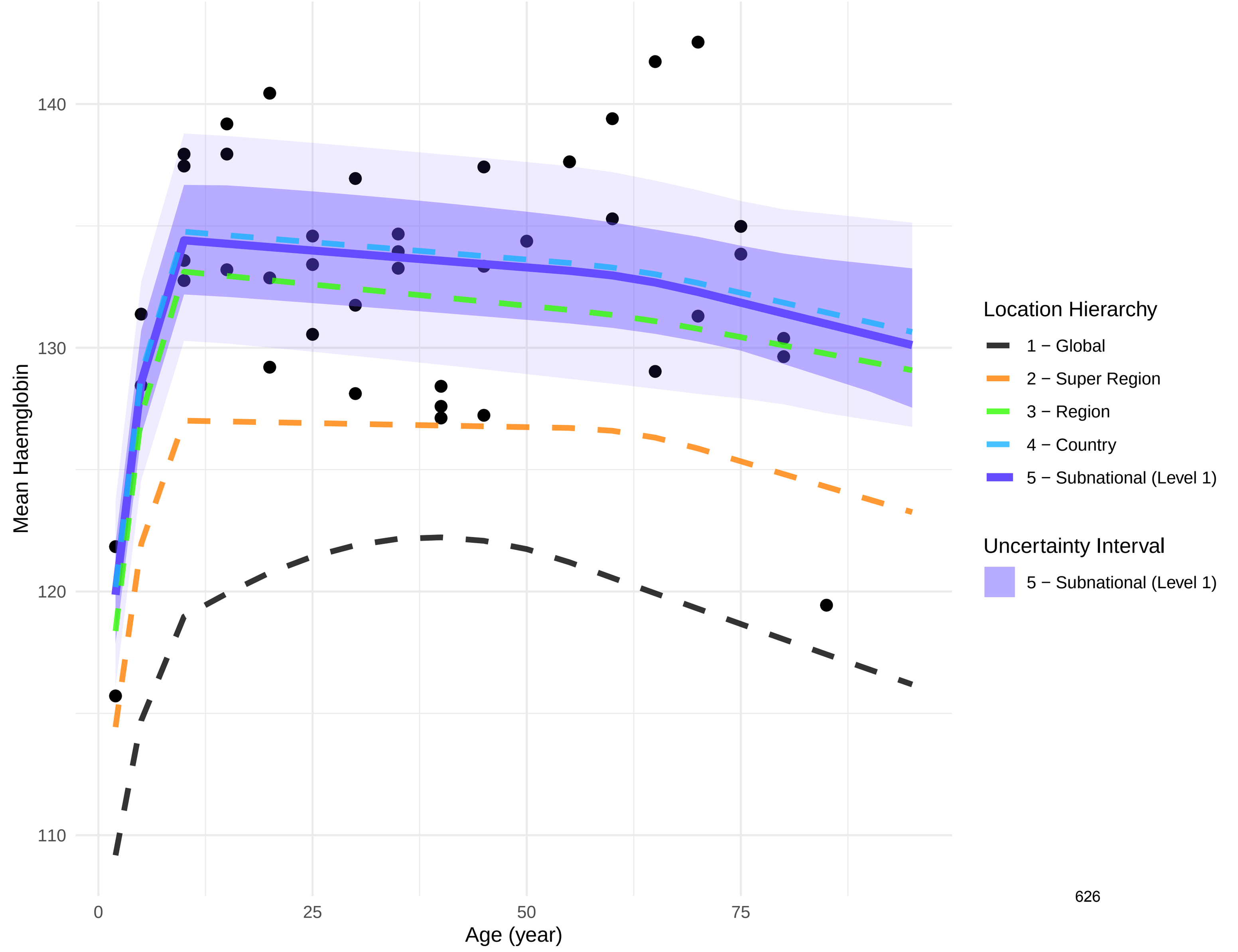

MR−BRT Age/Sex Fits − Non-pregnant Females 2+ Years Old − Mean Haemglobin − Querétaro
WHO−2001 Adjustment Method

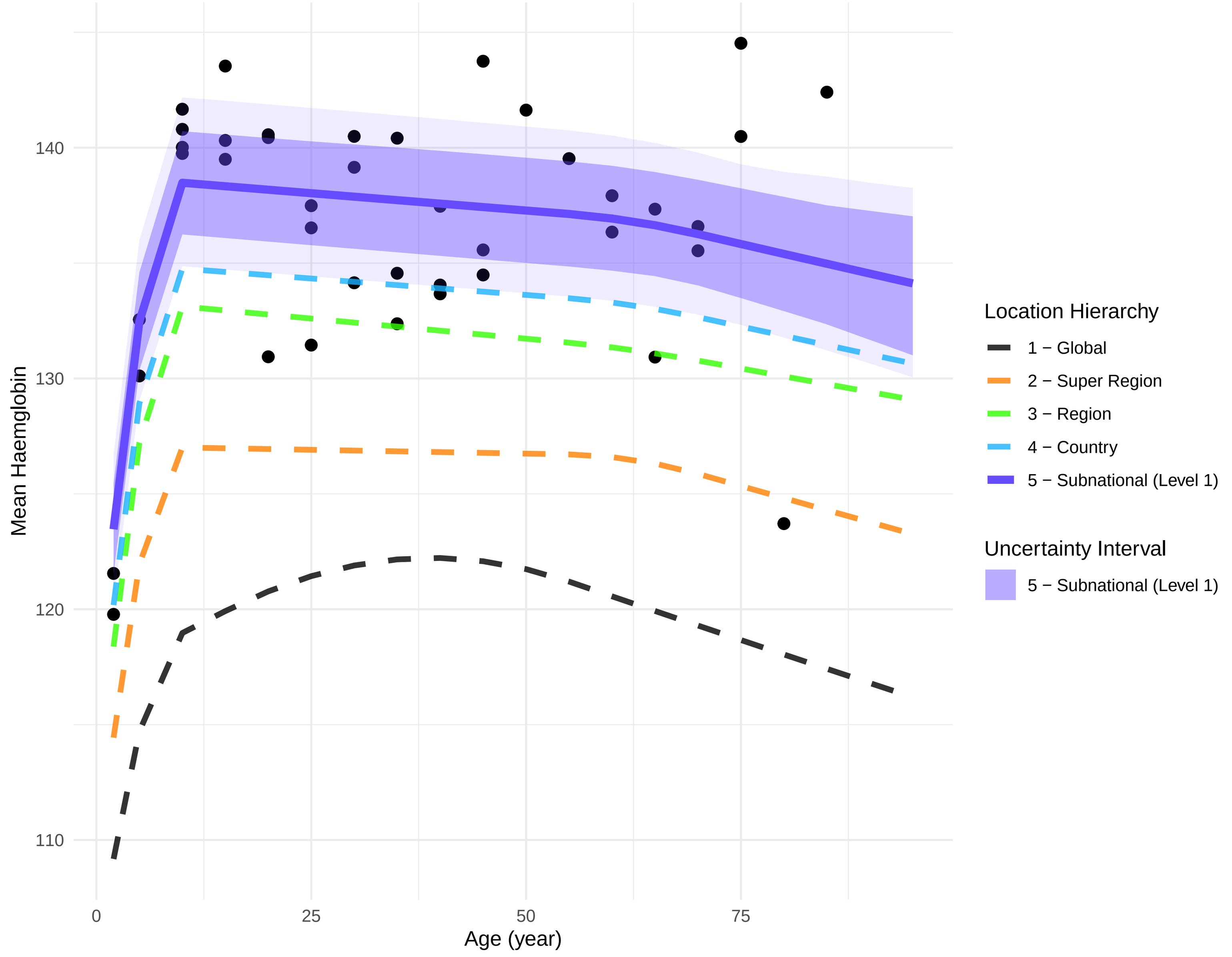

MR−BRT Age/Sex Fits − Non-pregnant Females 2+ Years Old − Mean Haemglobin − Quintana Roo
WHO−2001 Adjustment Method

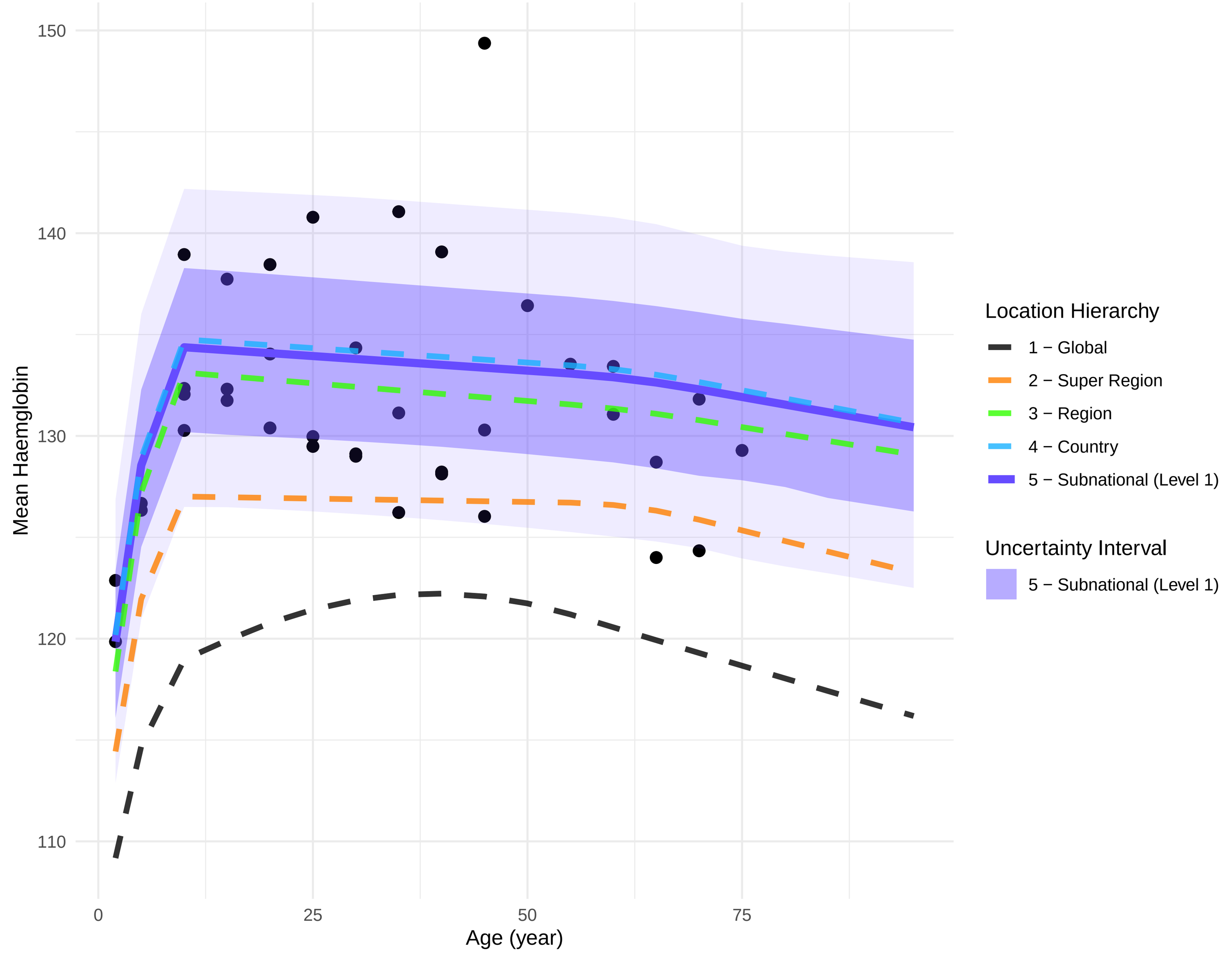

MR−BRT Age/Sex Fits − Non-pregnant Females 2+ Years Old − Mean Haemglobin − San Luis Potosí
WHO−2001 Adjustment Method

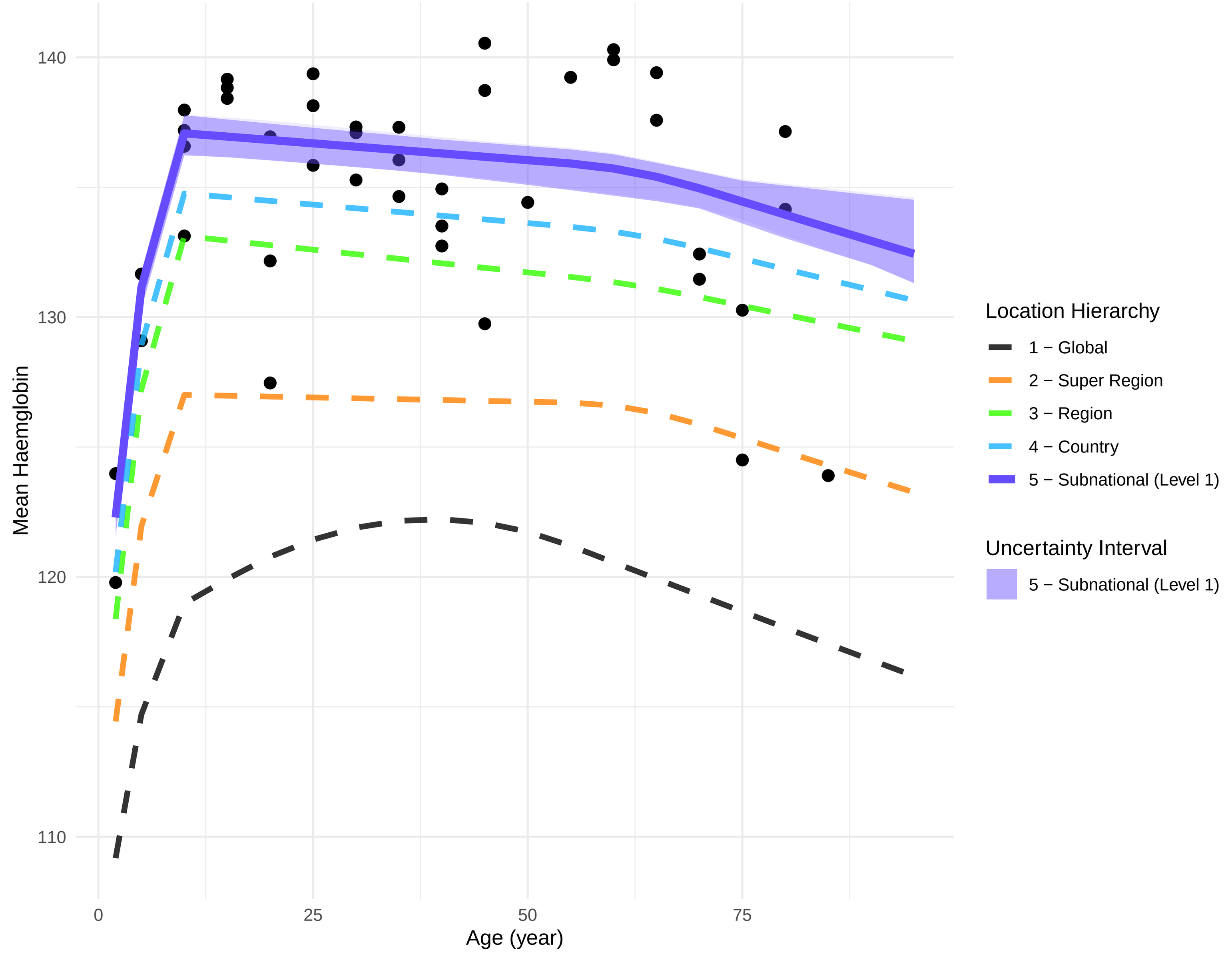

MR−BRT Age/Sex Fits − Non-pregnant Females 2+ Years Old − Mean Haemglobin − Sinaloa
WHO−2001 Adjustment Method

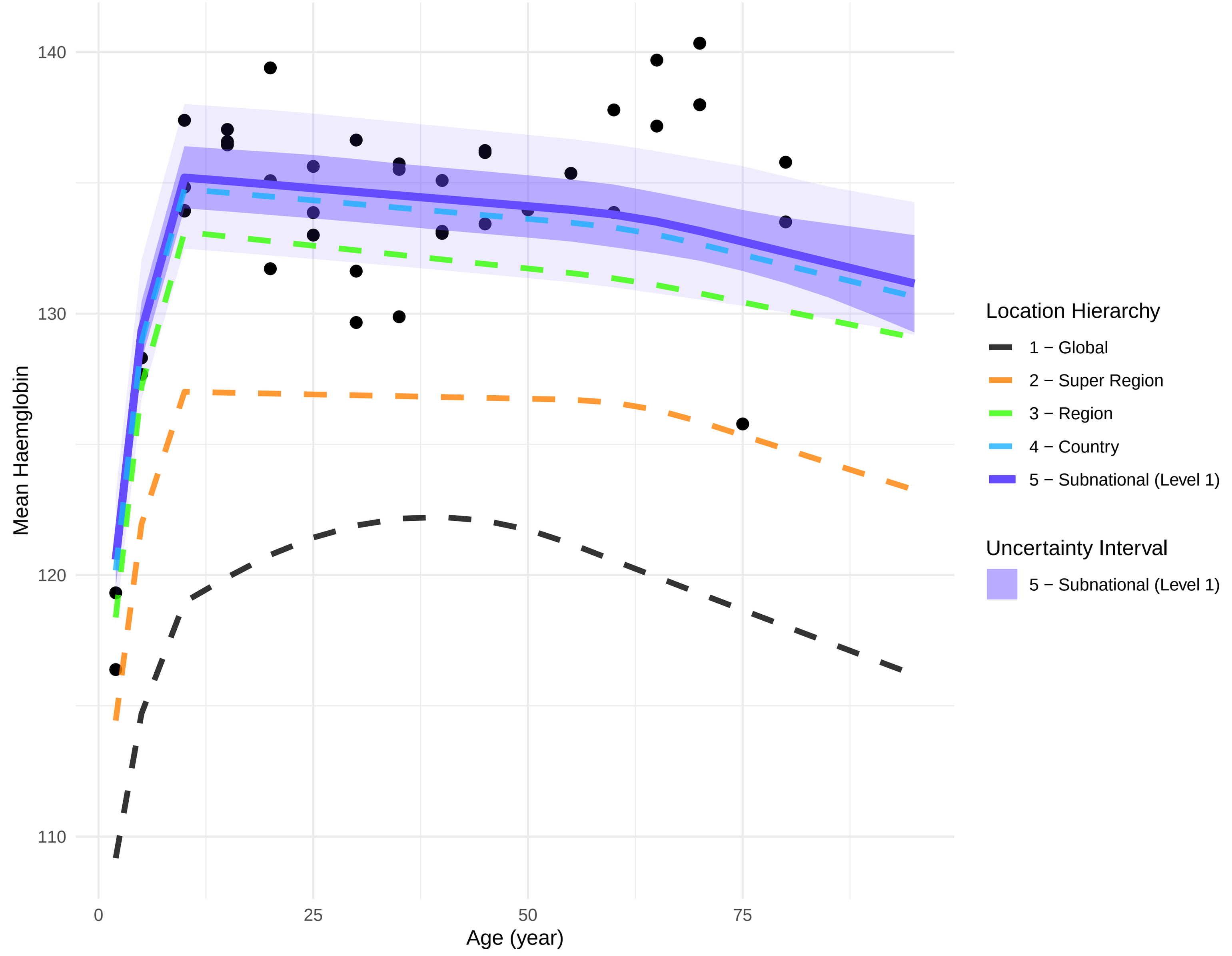

MR−BRT Age/Sex Fits − Non-pregnant Females 2+ Years Old − Mean Haemglobin − Sonora
WHO−2001 Adjustment Method

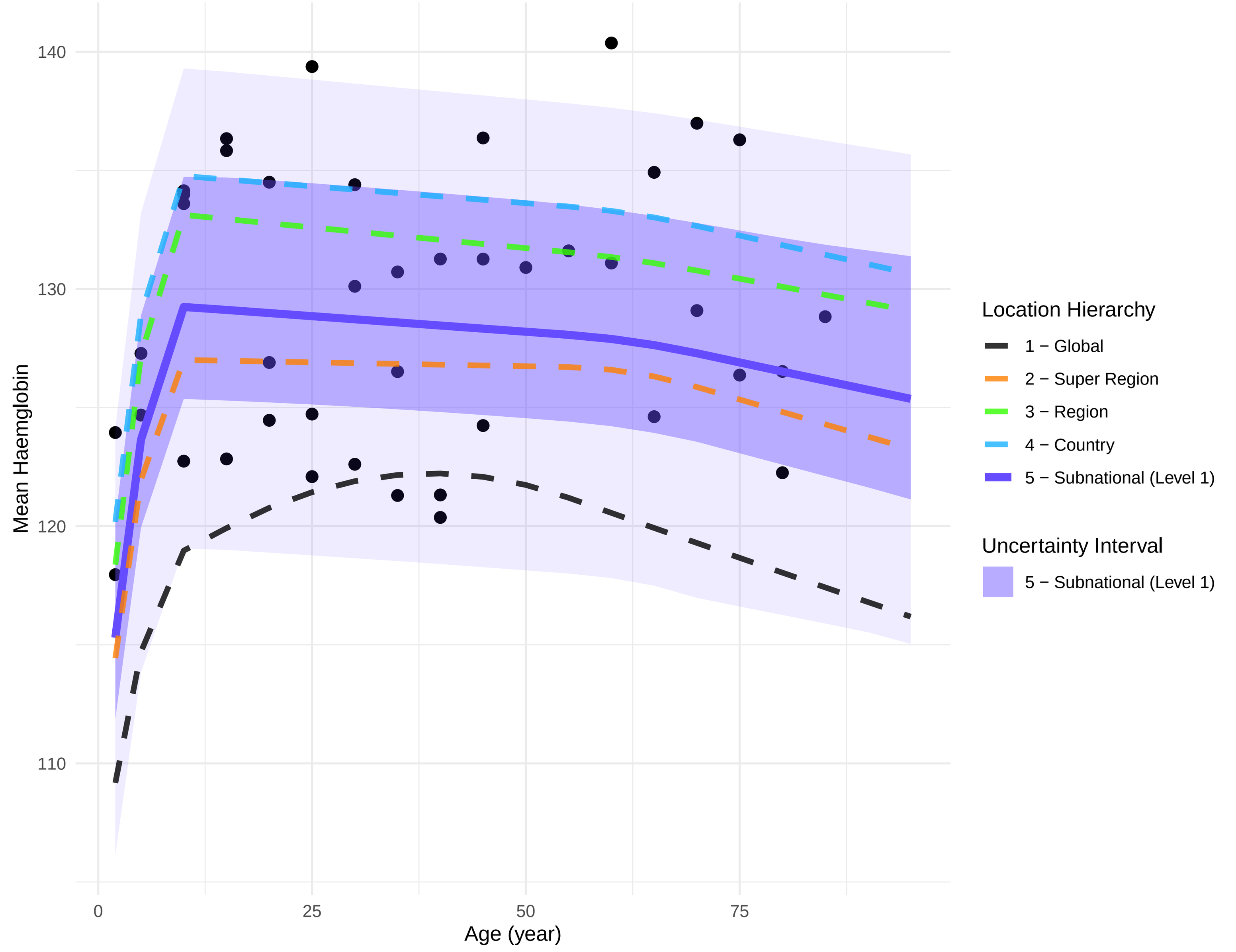

MR−BRT Age/Sex Fits − Non-pregnant Females 2+ Years Old − Mean Haemglobin − Tabasco
WHO−2001 Adjustment Method

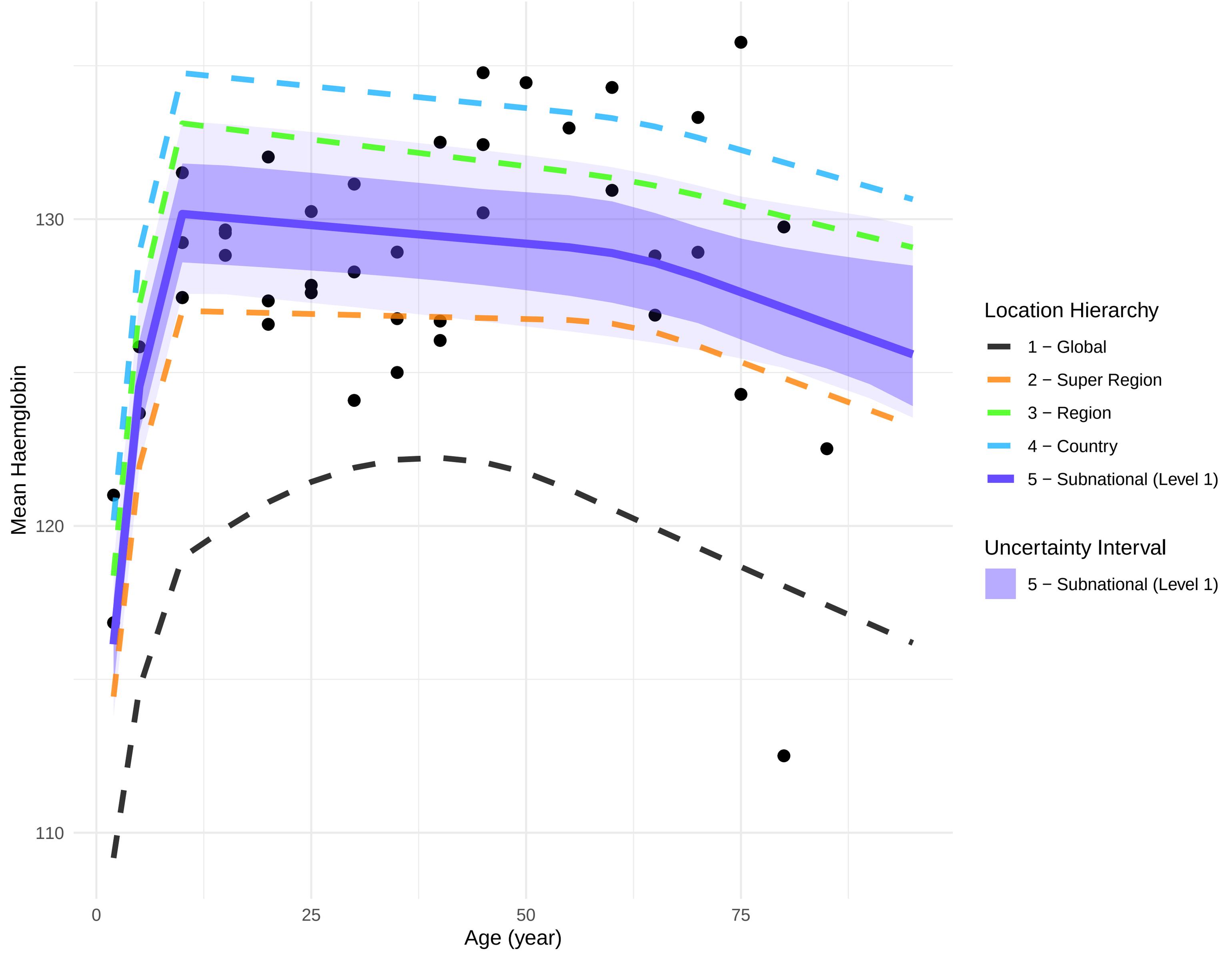

MR−BRT Age/Sex Fits − Non-pregnant Females 2+ Years Old − Mean Haemglobin − Tamaulipas
WHO−2001 Adjustment Method

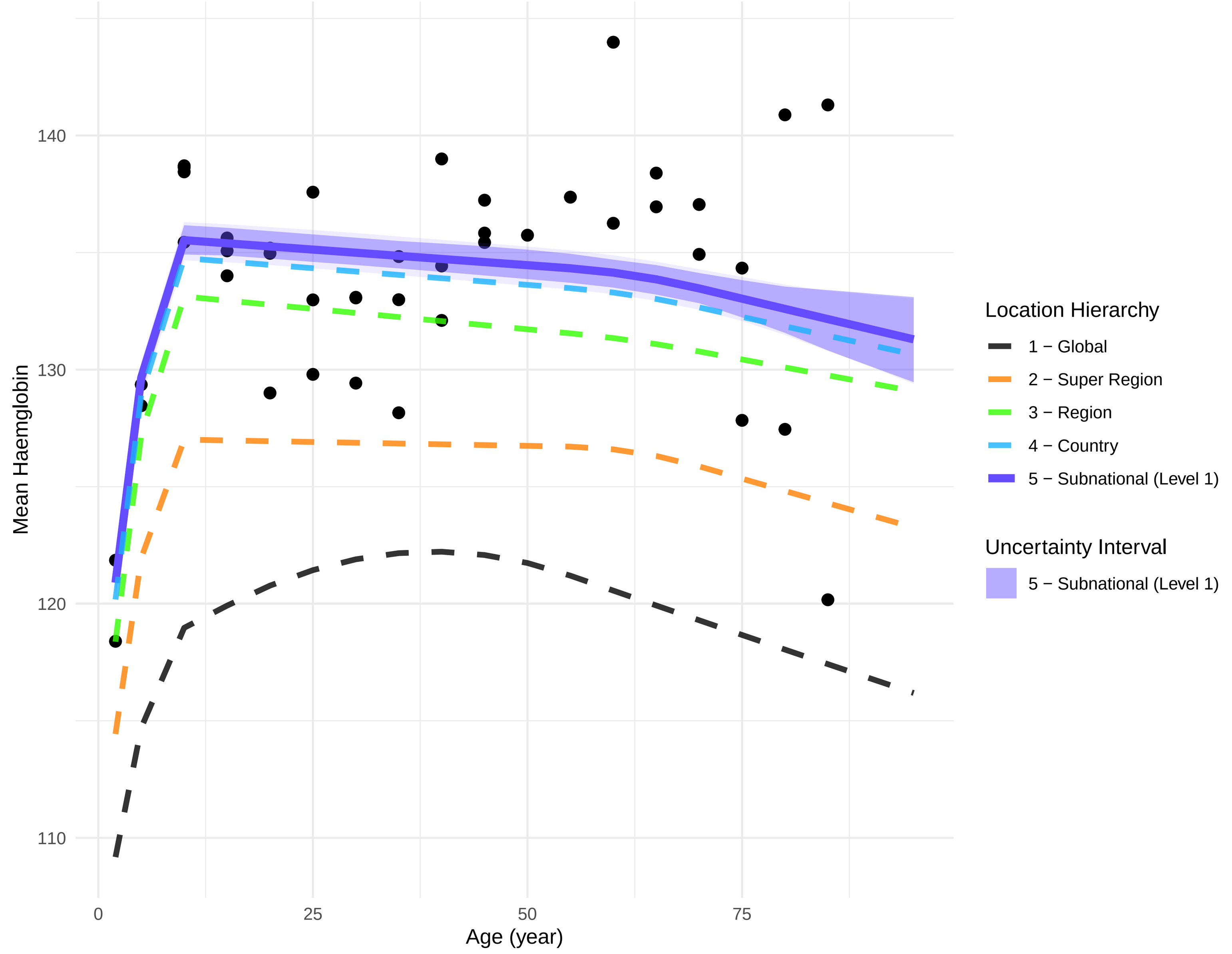

MR−BRT Age/Sex Fits − Non-pregnant Females 2+ Years Old − Mean Haemglobin − Tlaxcala
WHO−2001 Adjustment Method

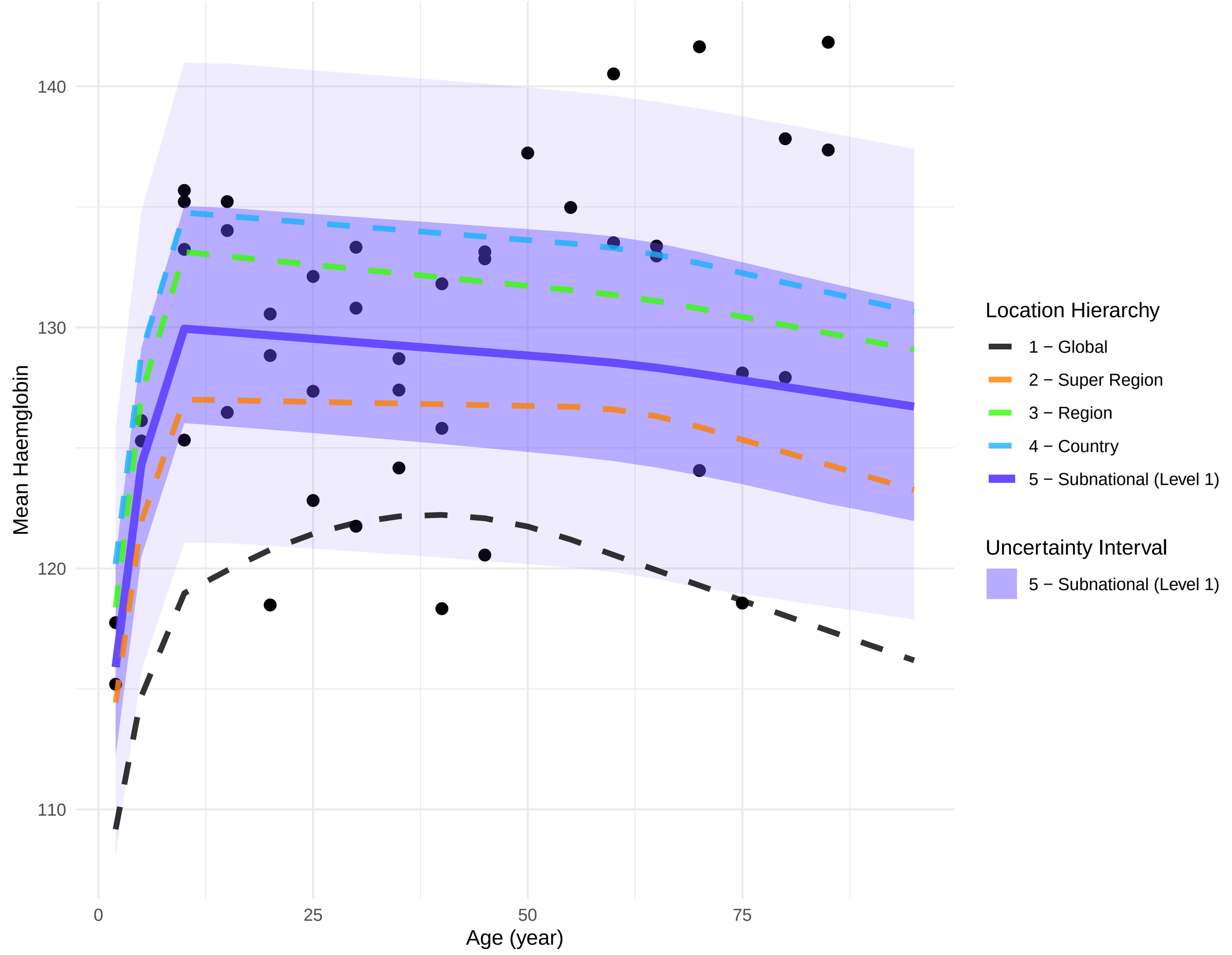

MR−BRT Age/Sex Fits − Non-pregnant Females 2+ Years Old − Mean Haemglobin − Veracruz de Ignacio de la Llave
WHO−2001 Adjustment Method

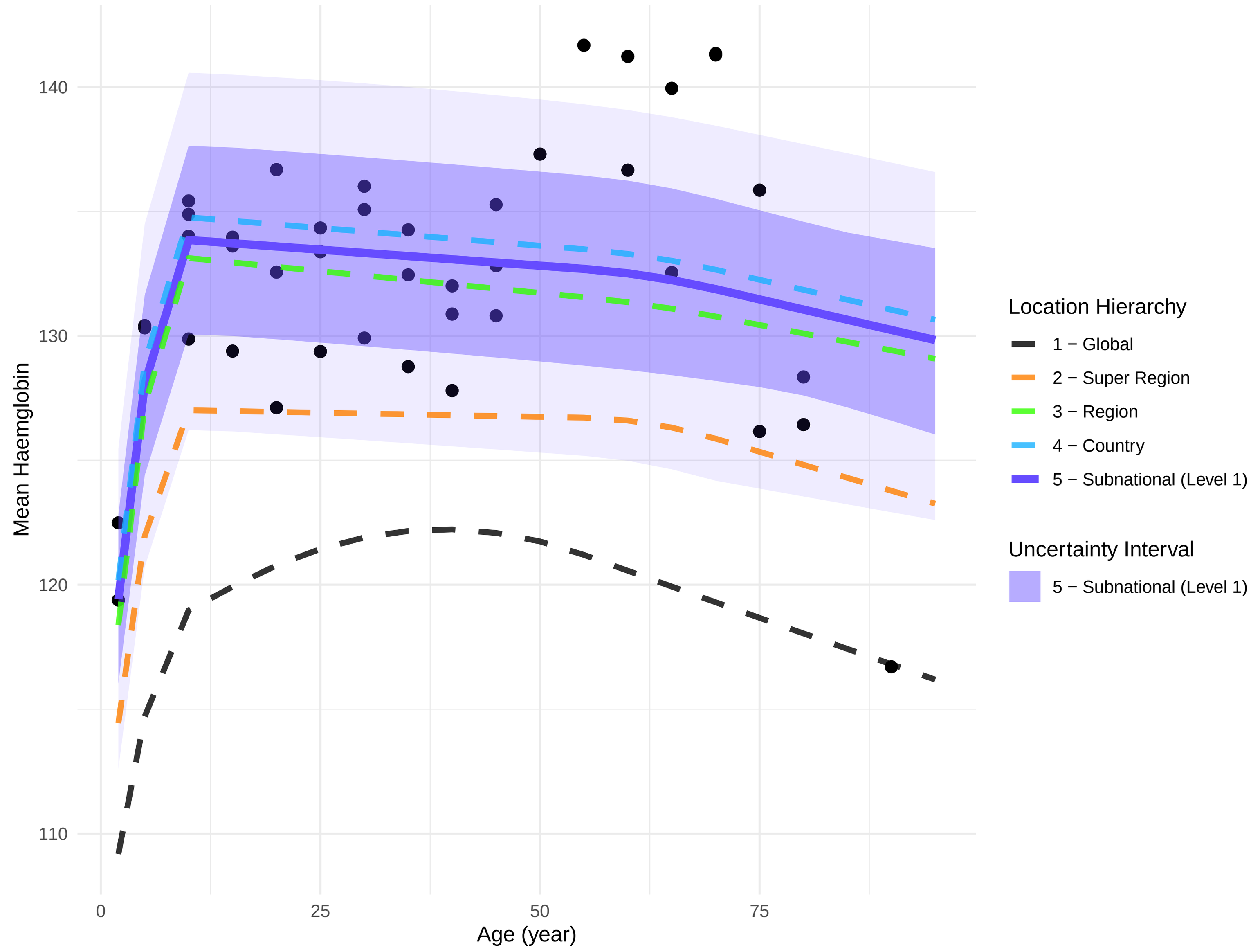

MR−BRT Age/Sex Fits − Non-pregnant Females 2+ Years Old − Mean Haemglobin − Yucatán
WHO−2001 Adjustment Method

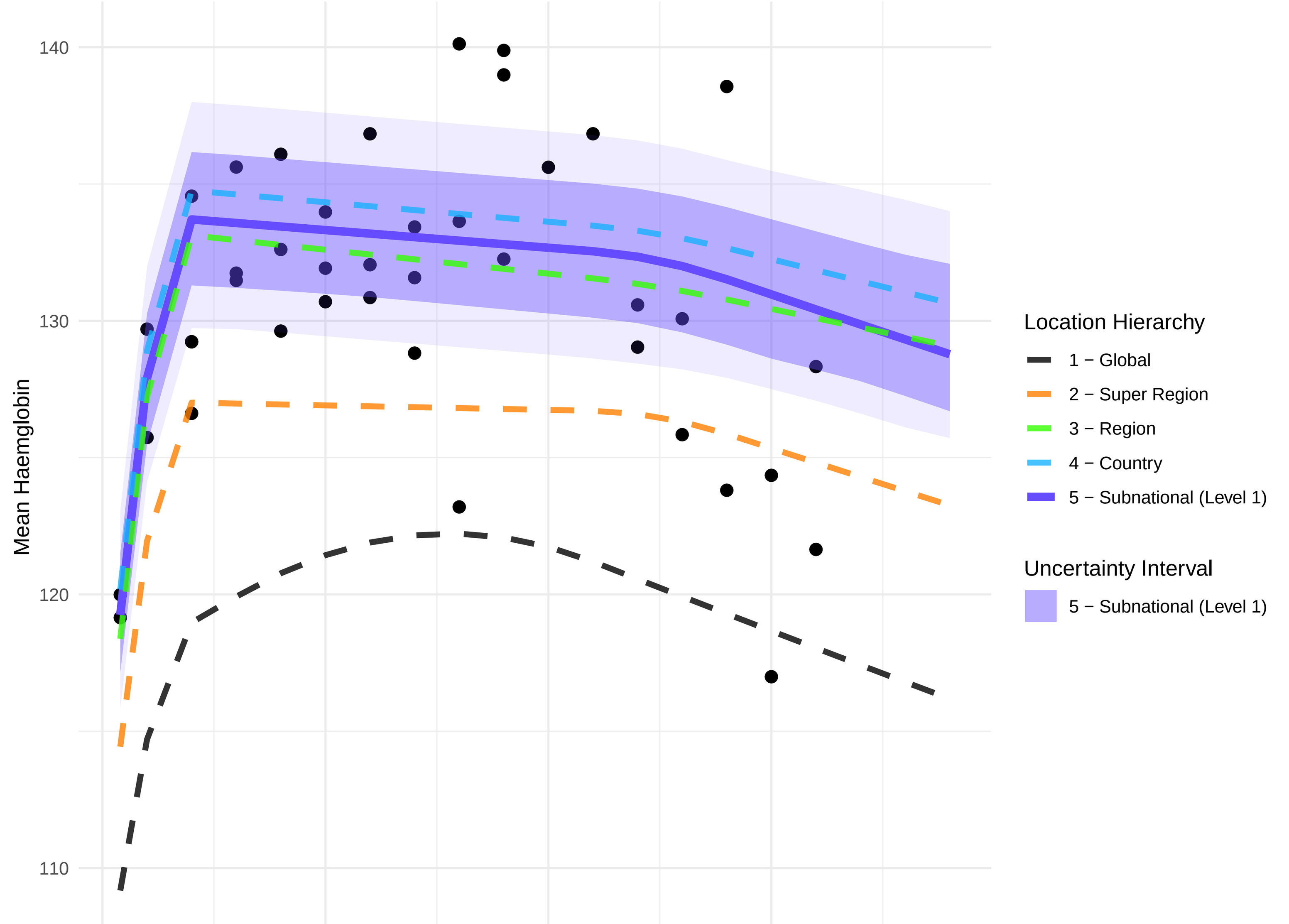

MR−BRT Age/Sex Fits − Non−pregnant Females 2+ Years Old − Mean Haemglobin − Zacatecas
WHO−2001 Adjustment Method

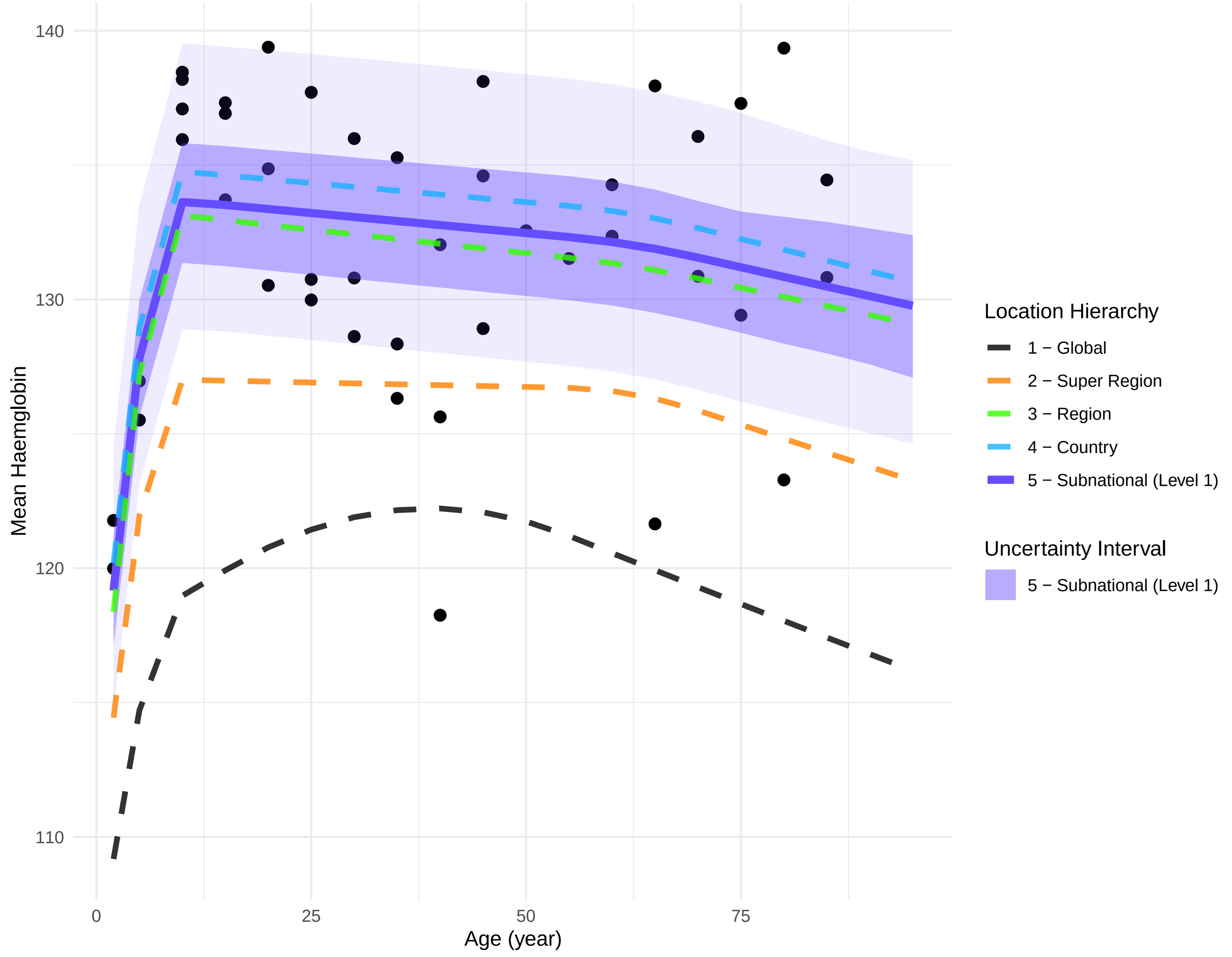

MR−BRT Age/Sex Fits − Non−pregnant Females 2+ Years Old − Mean Haemglobin − Tropical Latin America
WHO−2001 Adjustment Method

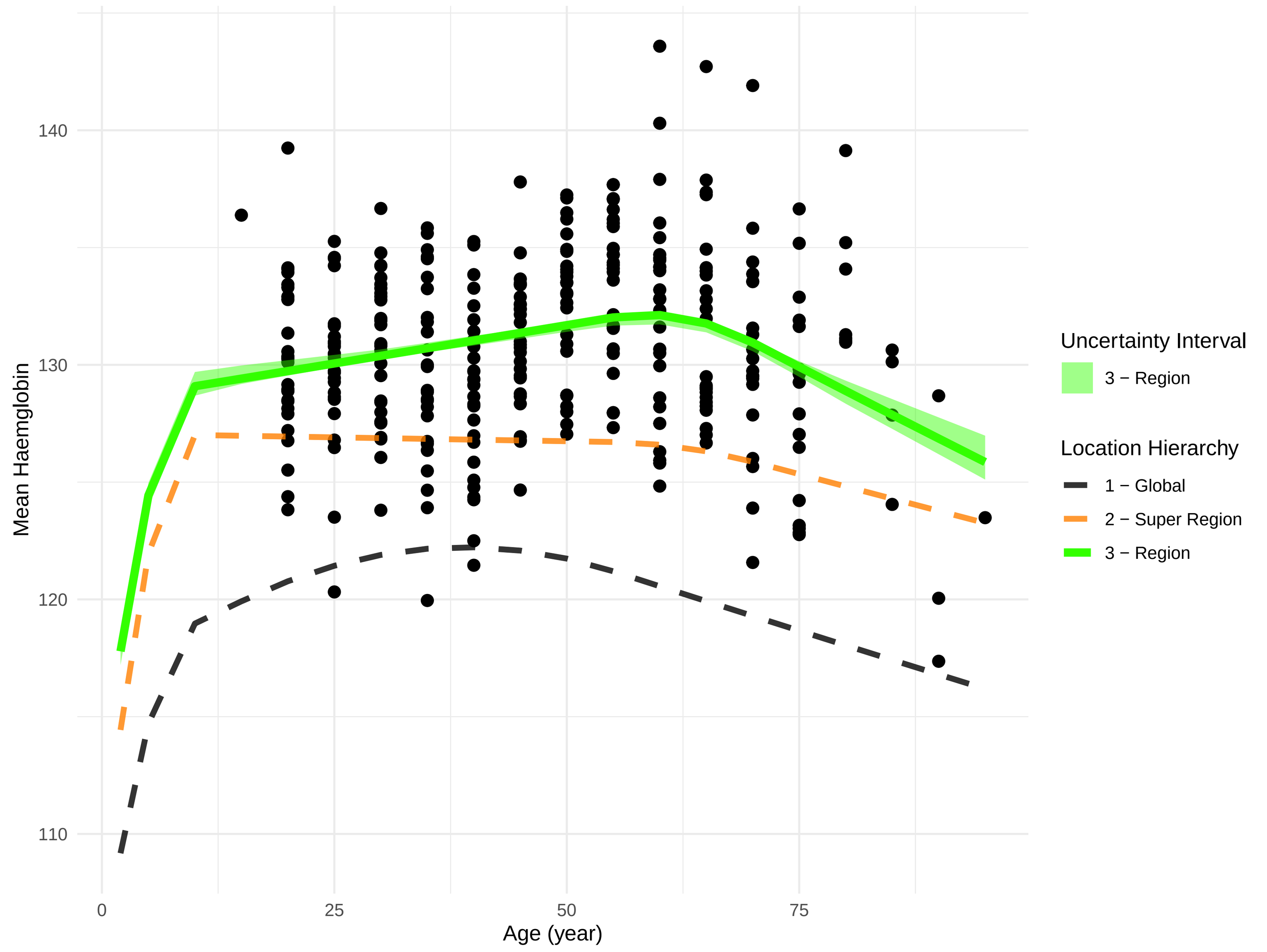

MR−BRT Age/Sex Fits − Non−pregnant Females 2+ Years Old − Mean Haemglobin − Brazil
WHO−2001 Adjustment Method

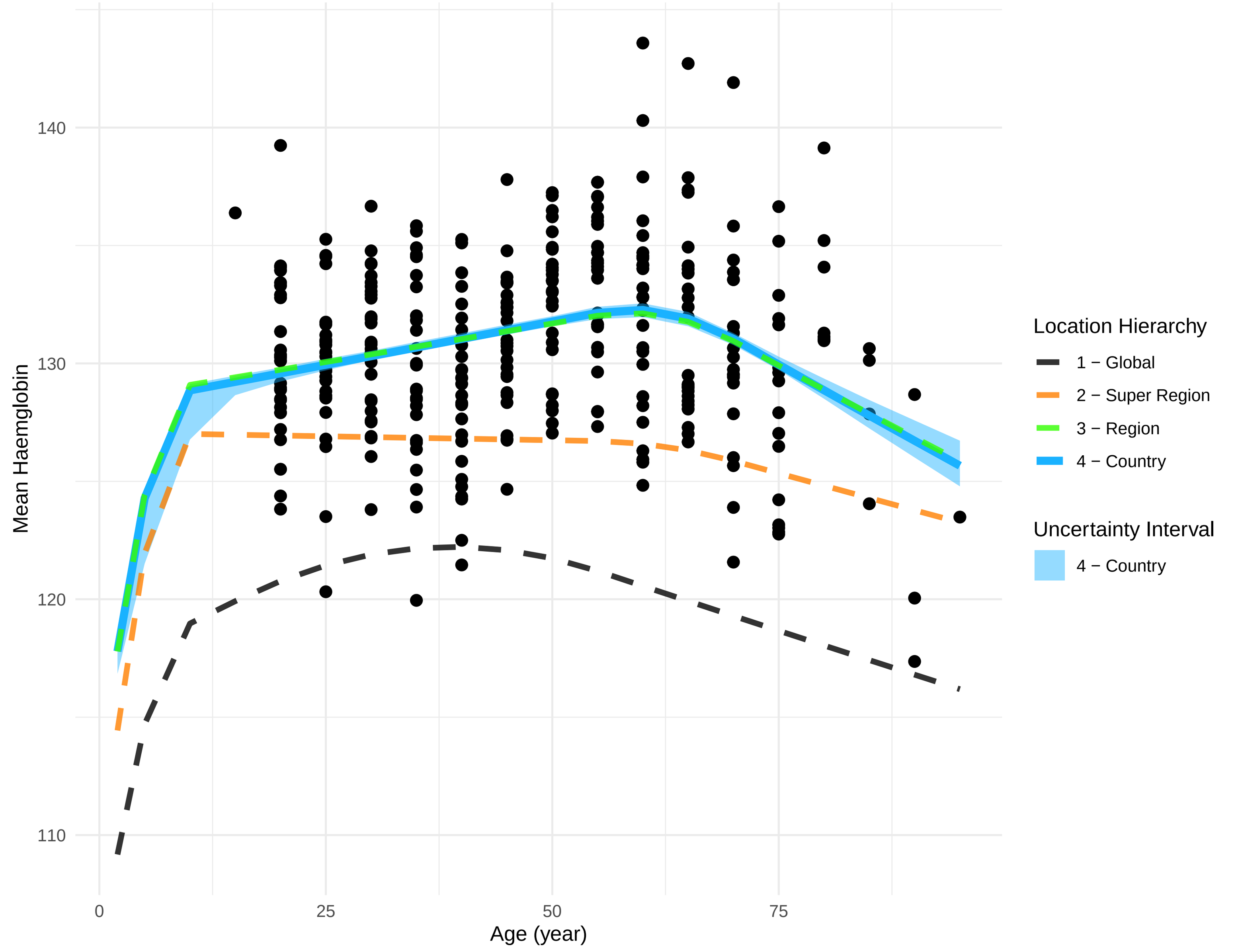

MR−BRT Age/Sex Fits − Non−pregnant Females 2+ Years Old − Mean Haemglobin − Acre
WHO−2001 Adjustment Method

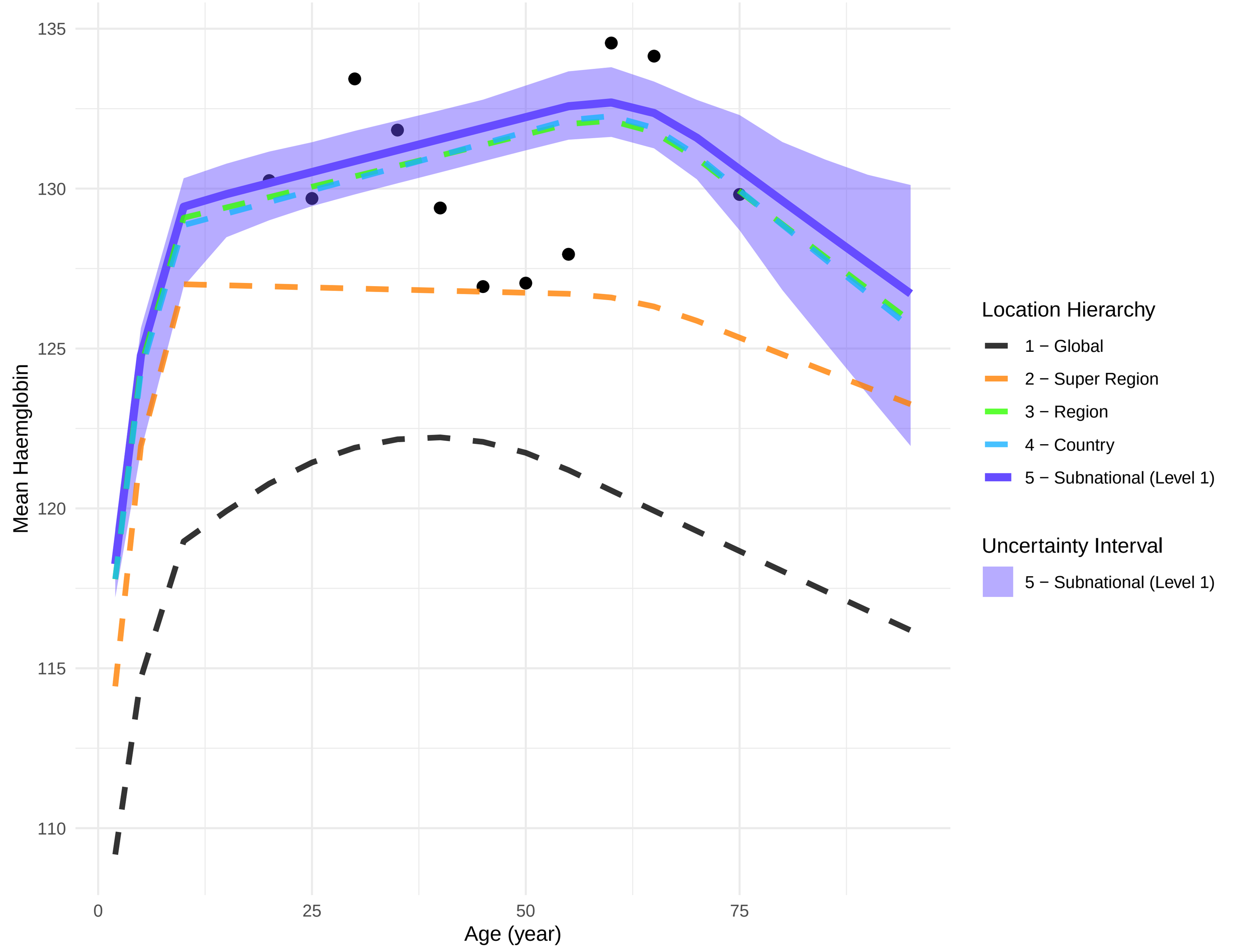

MR−BRT Age/Sex Fits − Non−pregnant Females 2+ Years Old − Mean Haemglobin − Alagoas
WHO−2001 Adjustment Method

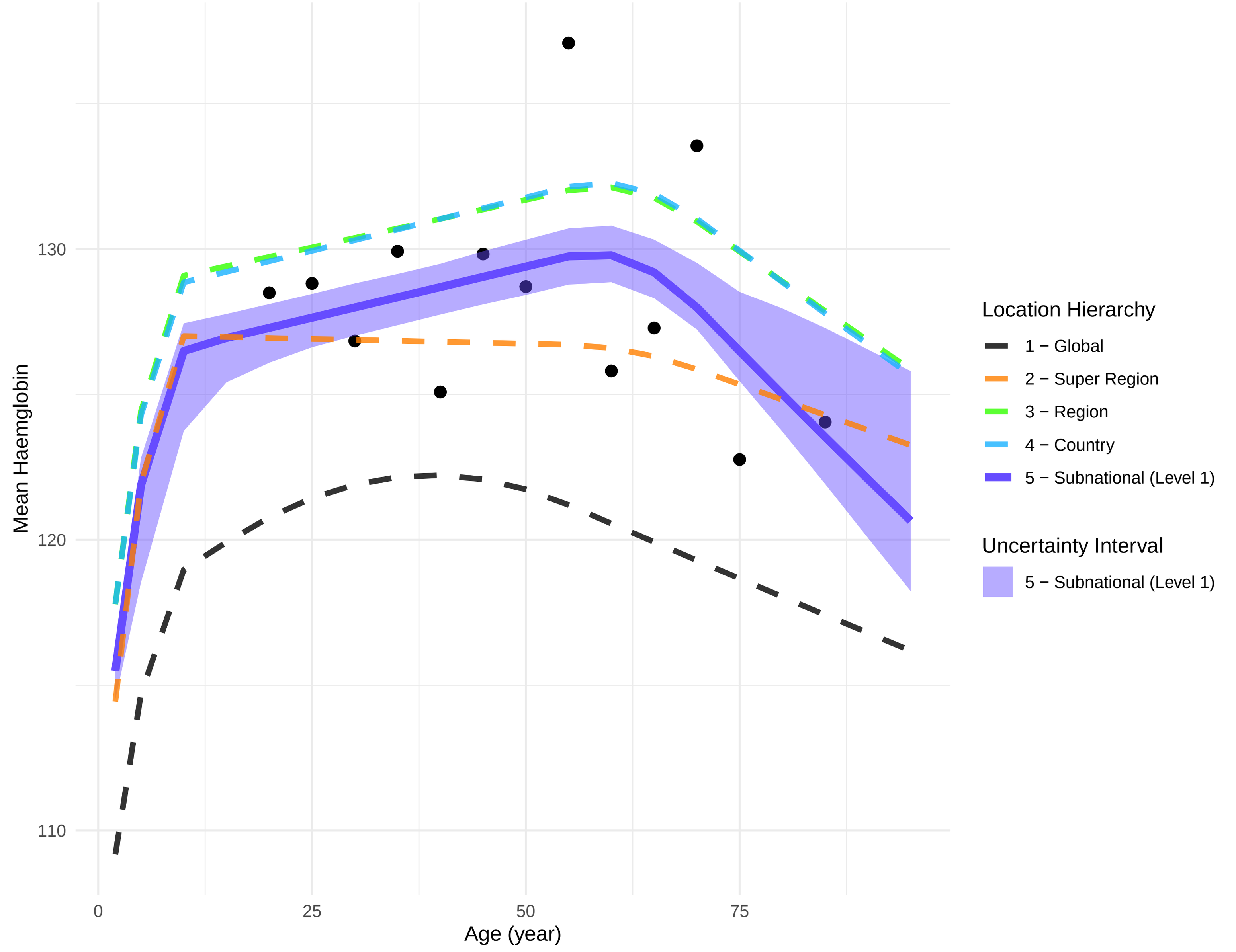

MR−BRT Age/Sex Fits − Non−pregnant Females 2+ Years Old − Mean Haemglobin − Amapá
WHO−2001 Adjustment Method

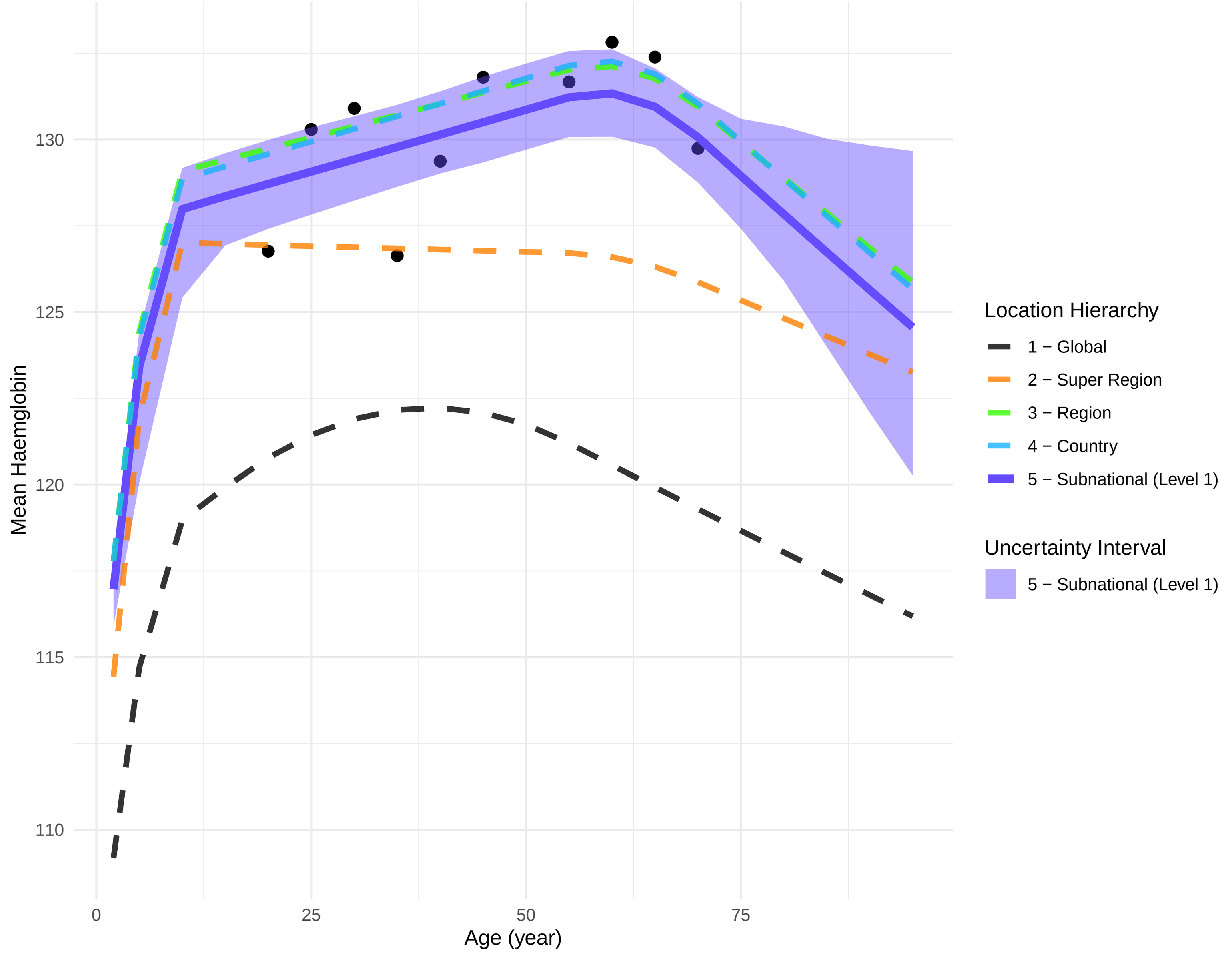

MR−BRT Age/Sex Fits − Non−pregnant Females 2+ Years Old − Mean Haemglobin − Amazonas
WHO−2001 Adjustment Method

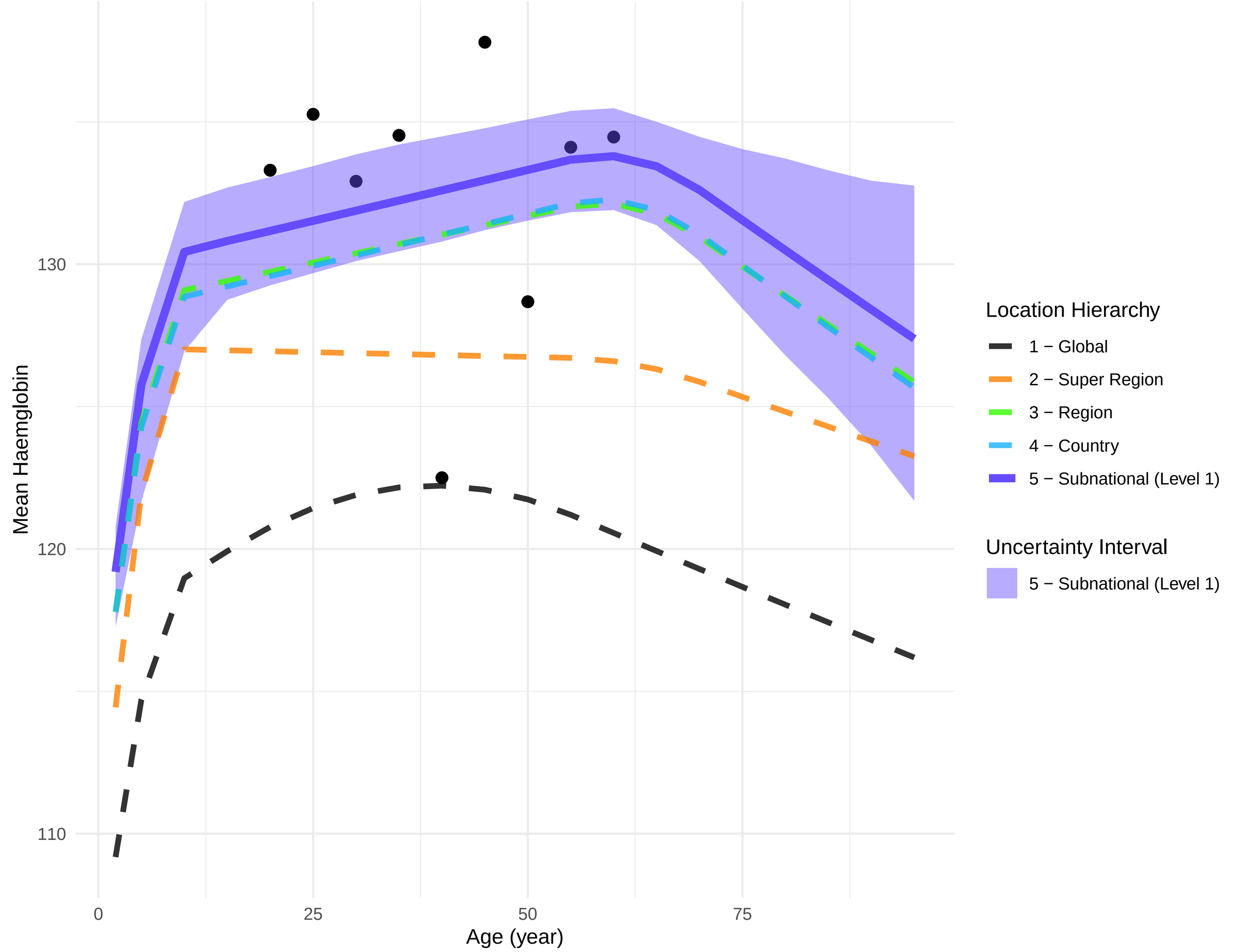

MR−BRT Age/Sex Fits − Non−pregnant Females 2+ Years Old − Mean Haemglobin − Bahia
WHO−2001 Adjustment Method

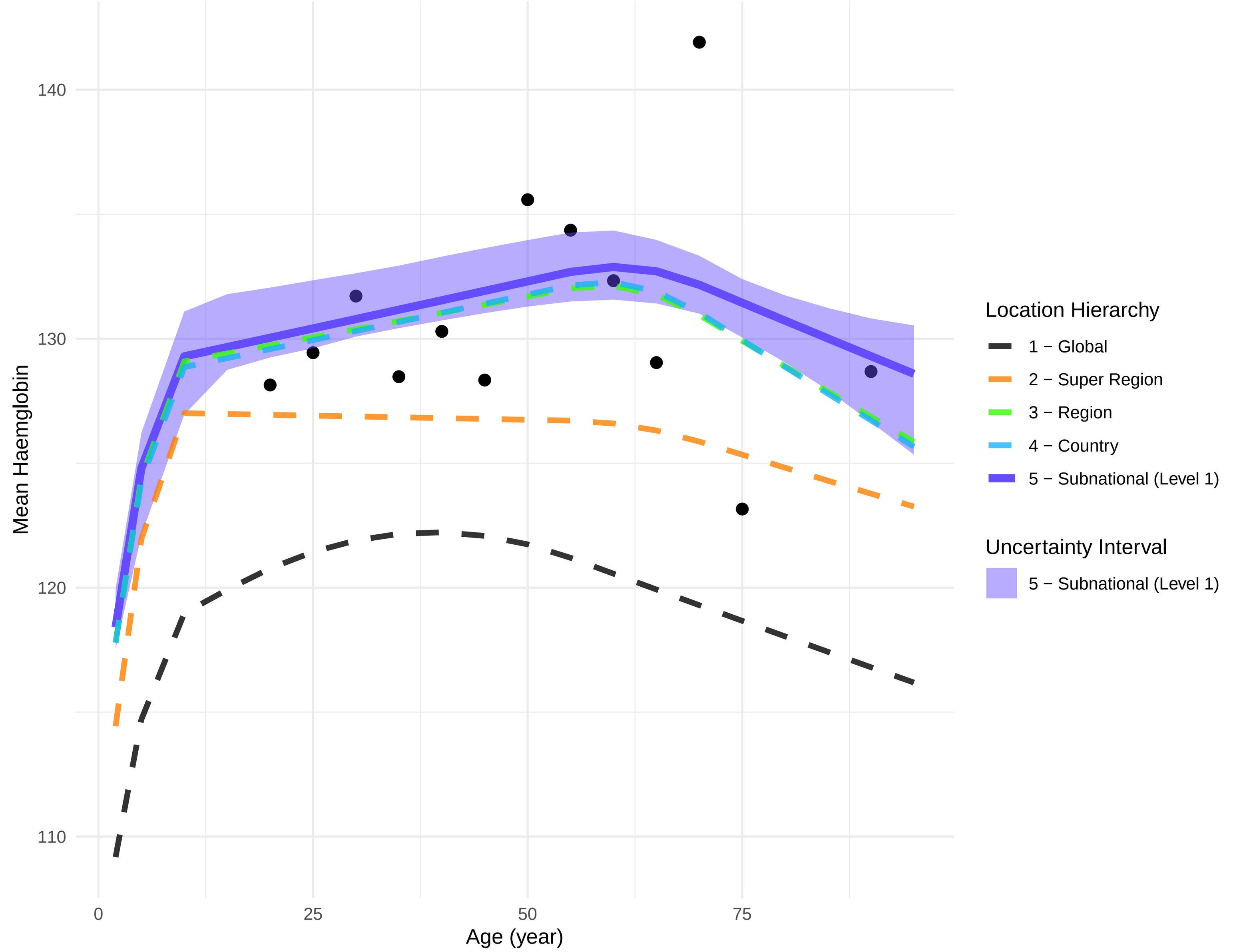

MR−BRT Age/Sex Fits − Non−pregnant Females 2+ Years Old − Mean Haemglobin − Ceará
WHO−2001 Adjustment Method

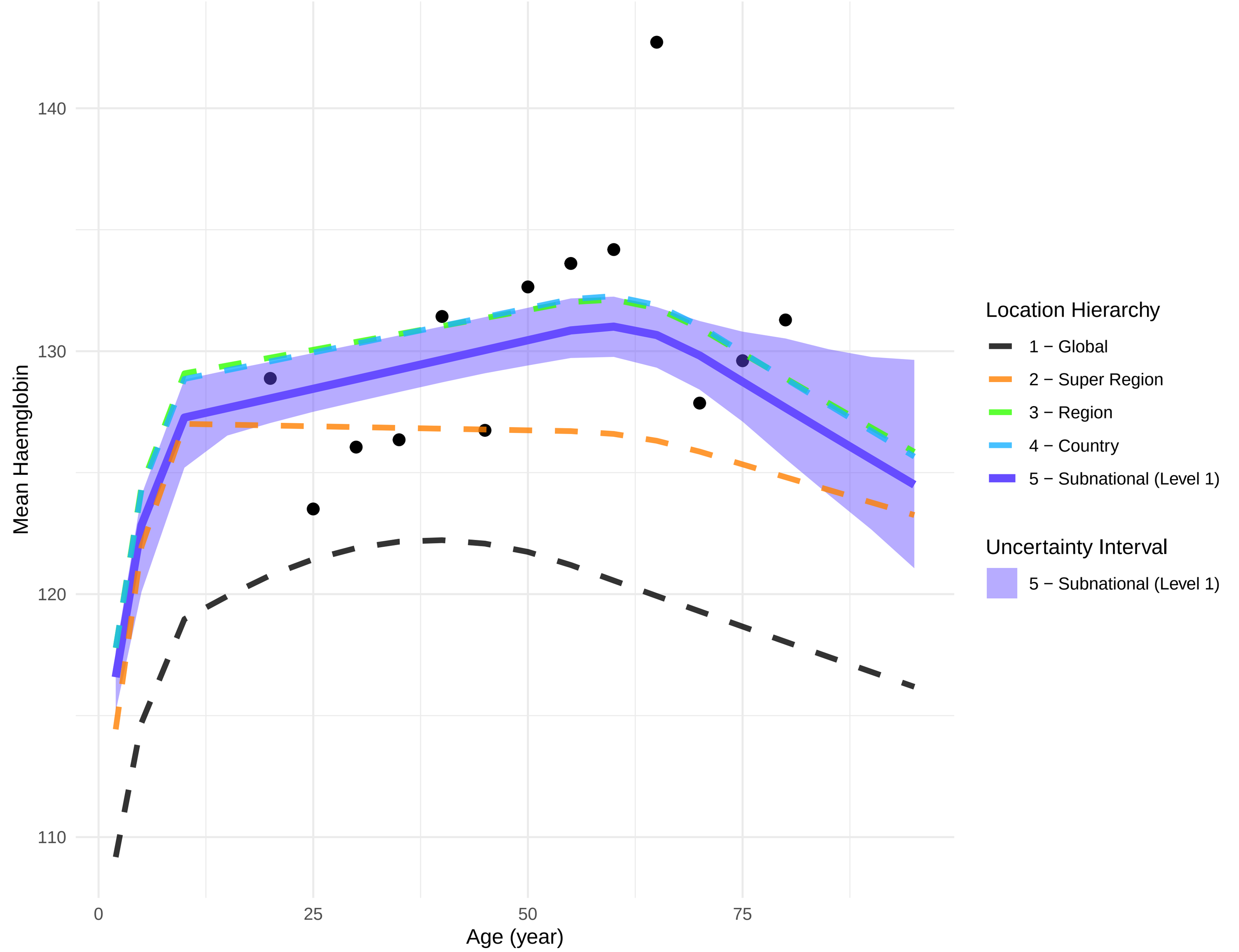

MR−BRT Age/Sex Fits − Non−pregnant Females 2+ Years Old − Mean Haemglobin − Distrito Federal
WHO−2001 Adjustment Method

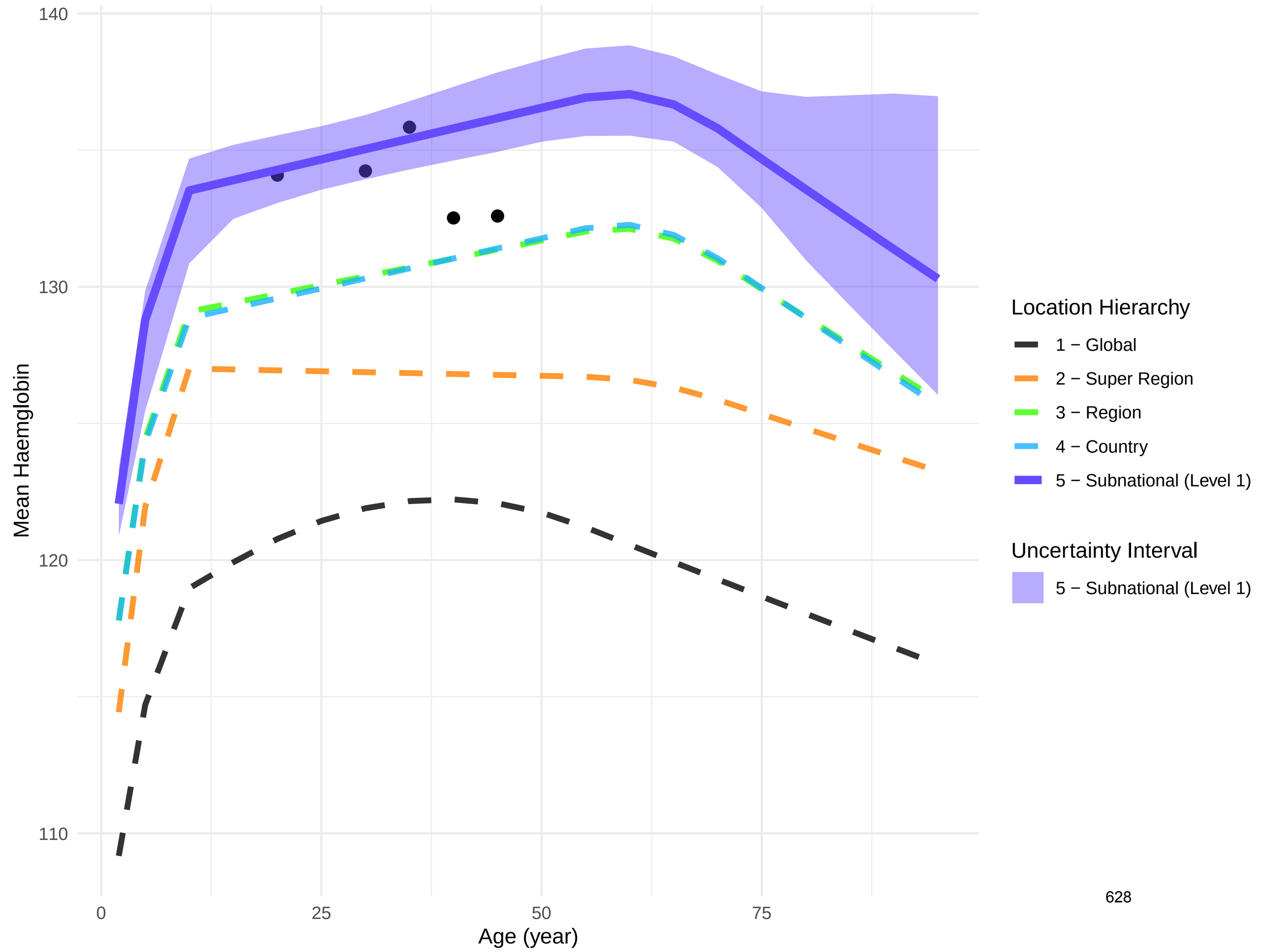

MR−BRT Age/Sex Fits − Non−pregnant Females 2+ Years Old − Mean Haemglobin − Espírito Santo
WHO−2001 Adjustment Method

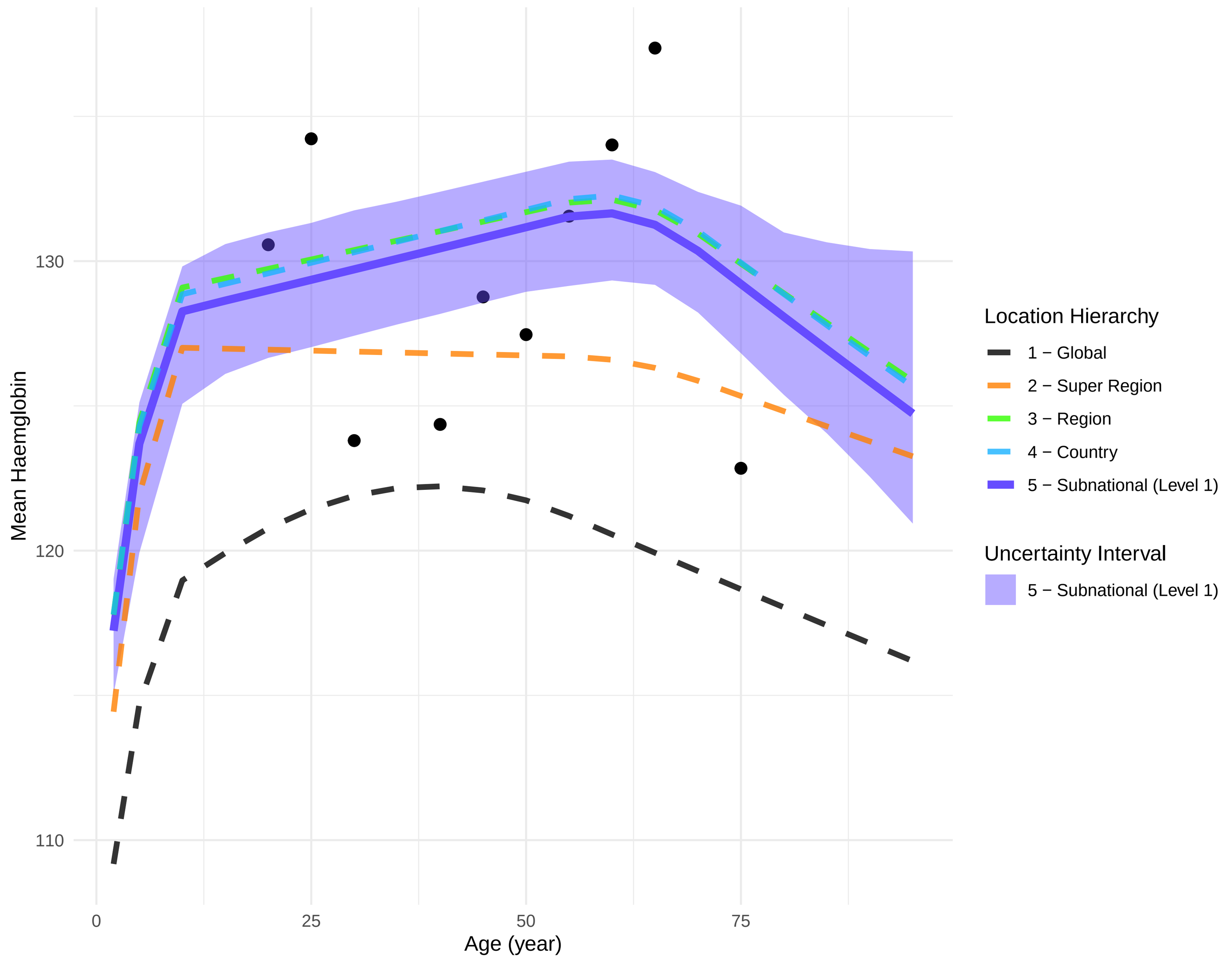

MR−BRT Age/Sex Fits − Non−pregnant Females 2+ Years Old − Mean Haemglobin − Goiás
WHO−2001 Adjustment Method

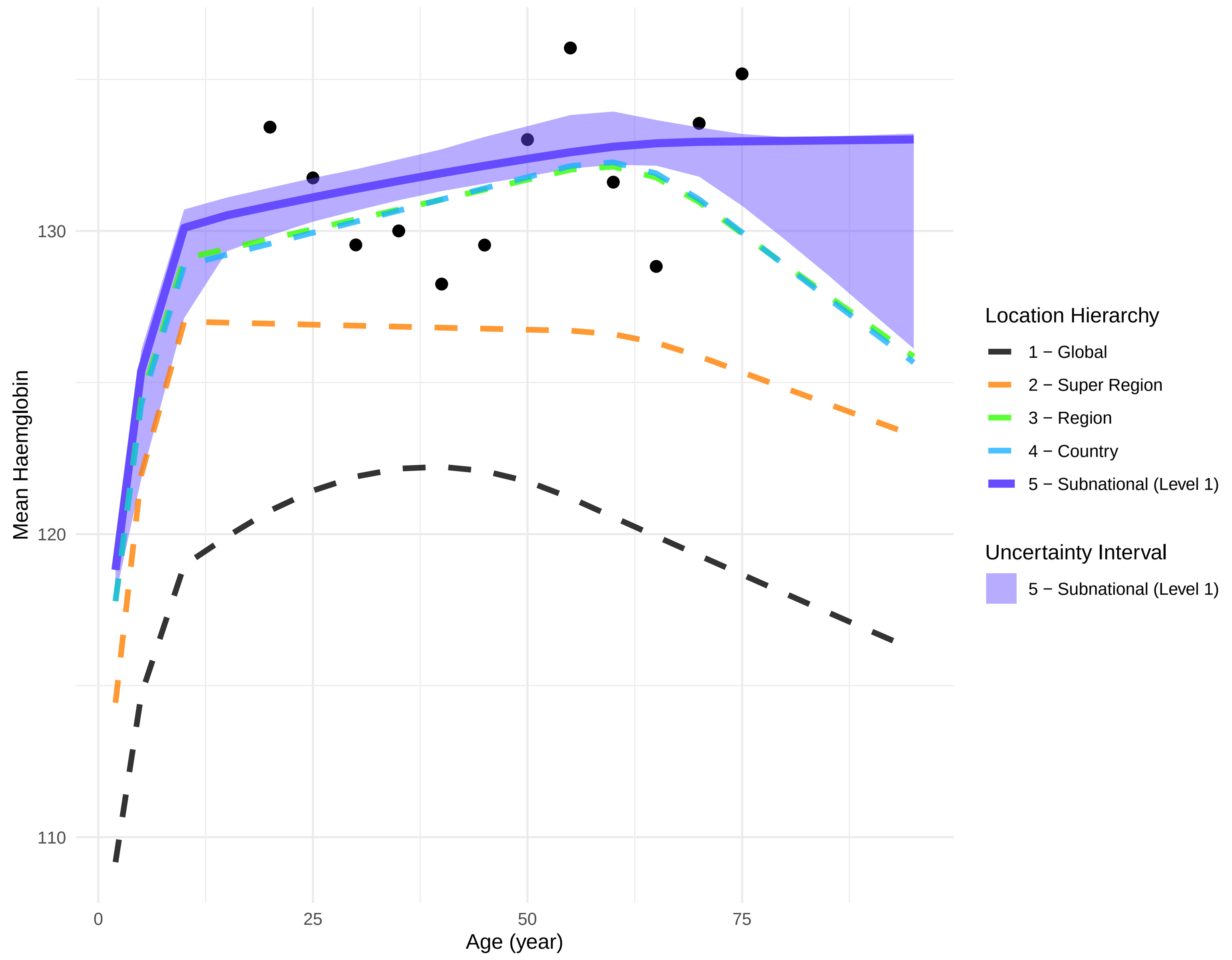

MR−BRT Age/Sex Fits − Non−pregnant Females 2+ Years Old − Mean Haemglobin − Maranhão
WHO−2001 Adjustment Method

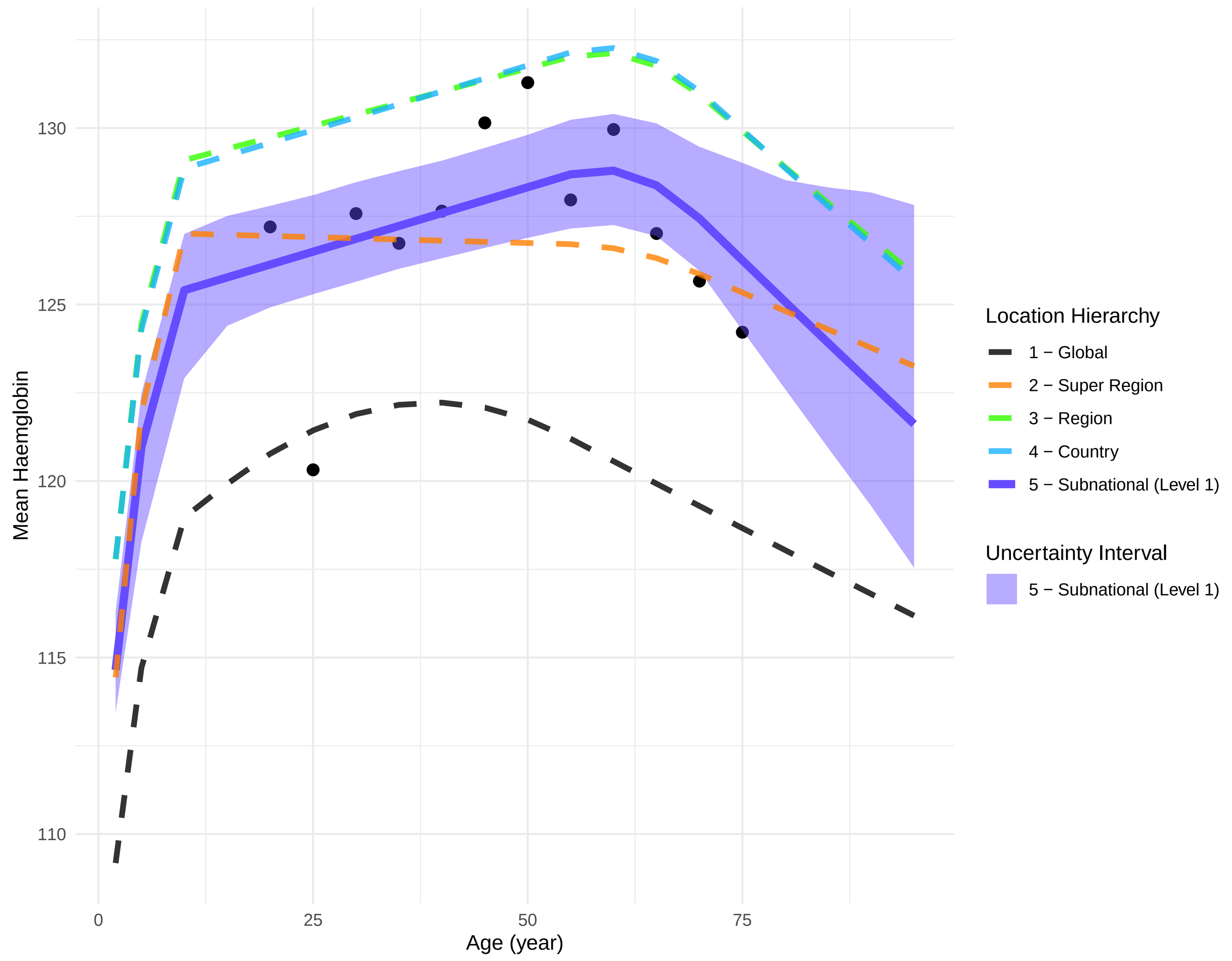

MR−BRT Age/Sex Fits − Non−pregnant Females 2+ Years Old − Mean Haemglobin − Mato Grosso
WHO−2001 Adjustment Method

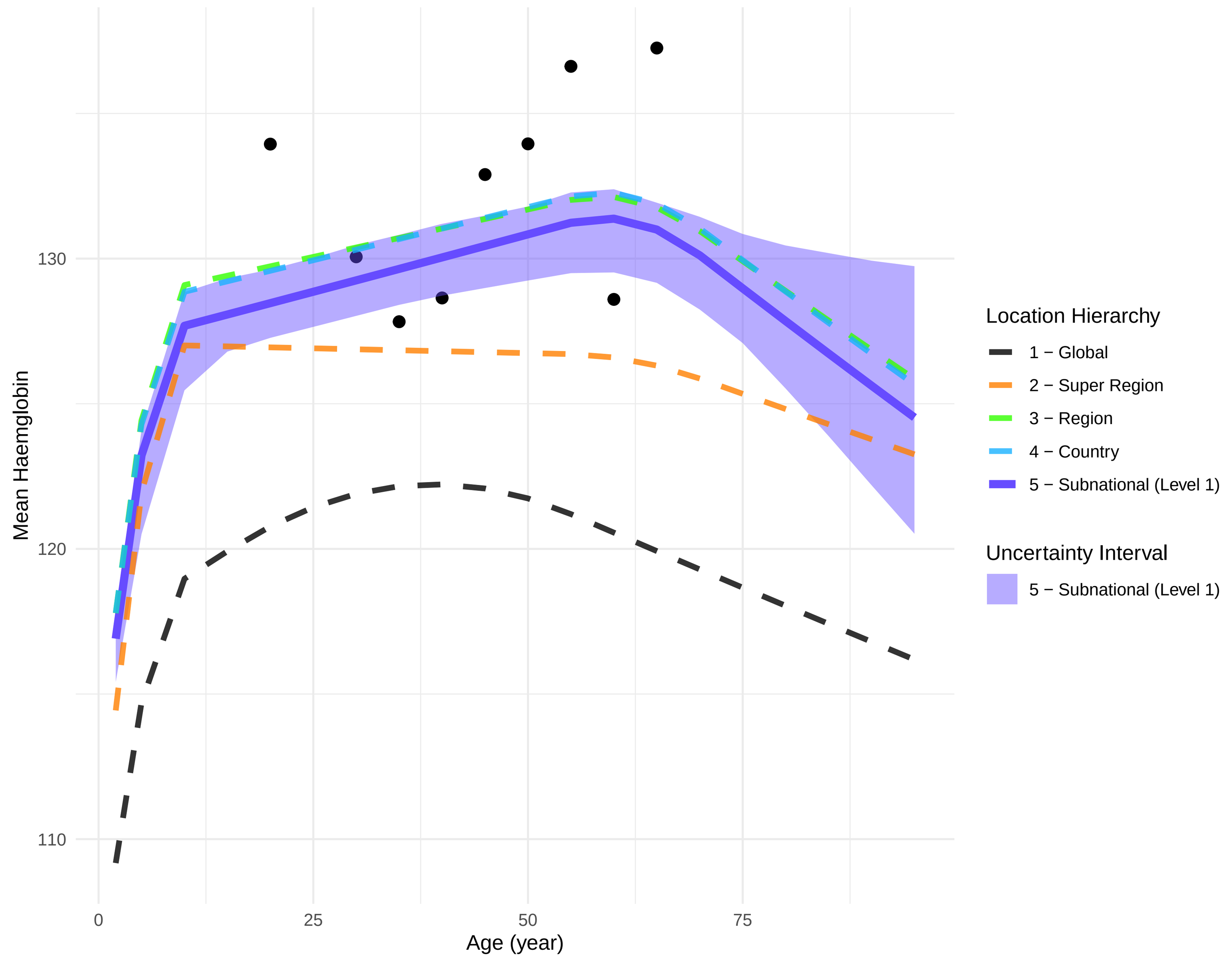

MR−BRT Age/Sex Fits − Non−pregnant Females 2+ Years Old − Mean Haemglobin − Mato Grosso do Sul
WHO−2001 Adjustment Method

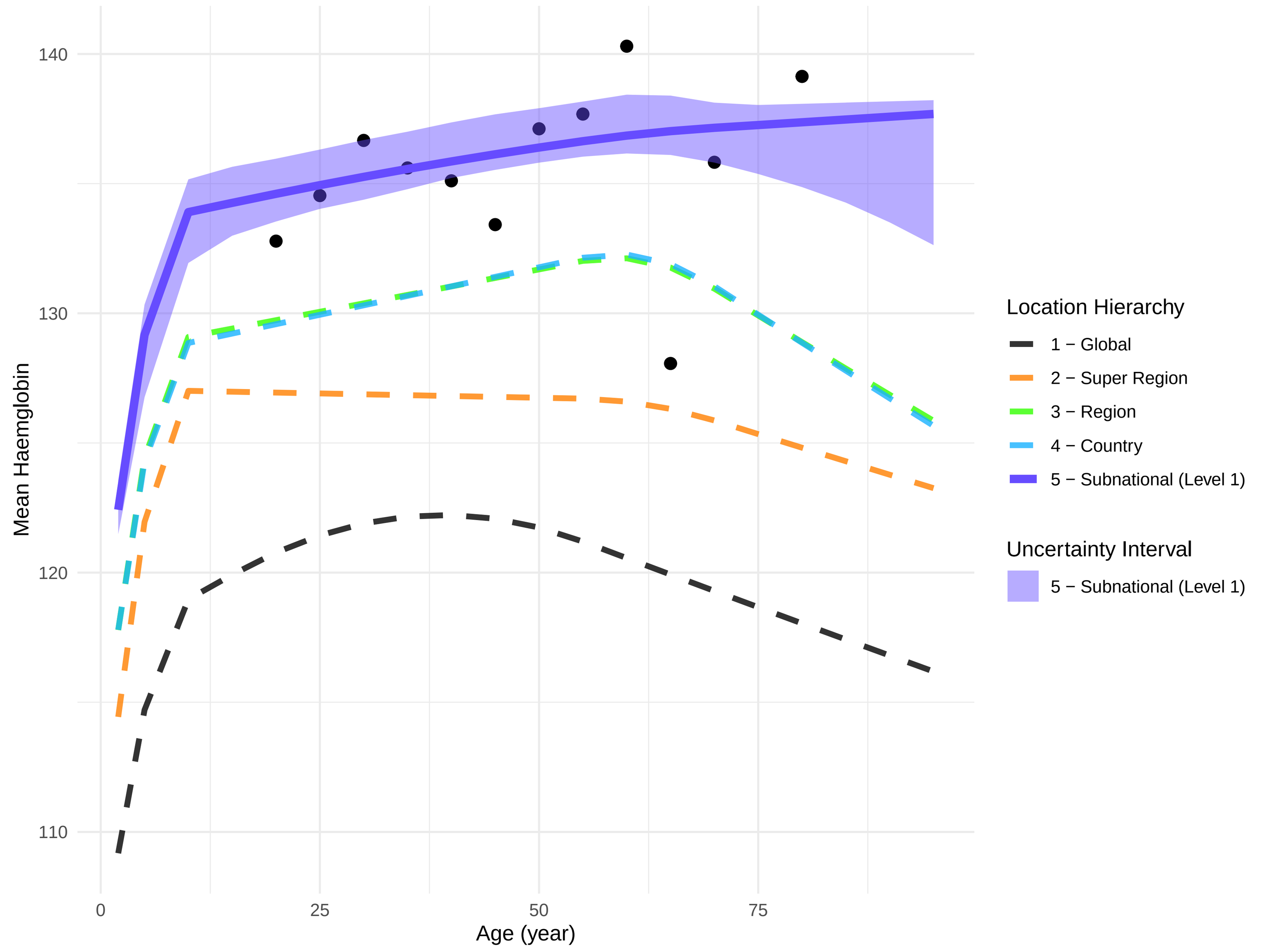

MR−BRT Age/Sex Fits − Non−pregnant Females 2+ Years Old − Mean Haemglobin − Minas Gerais
WHO−2001 Adjustment Method

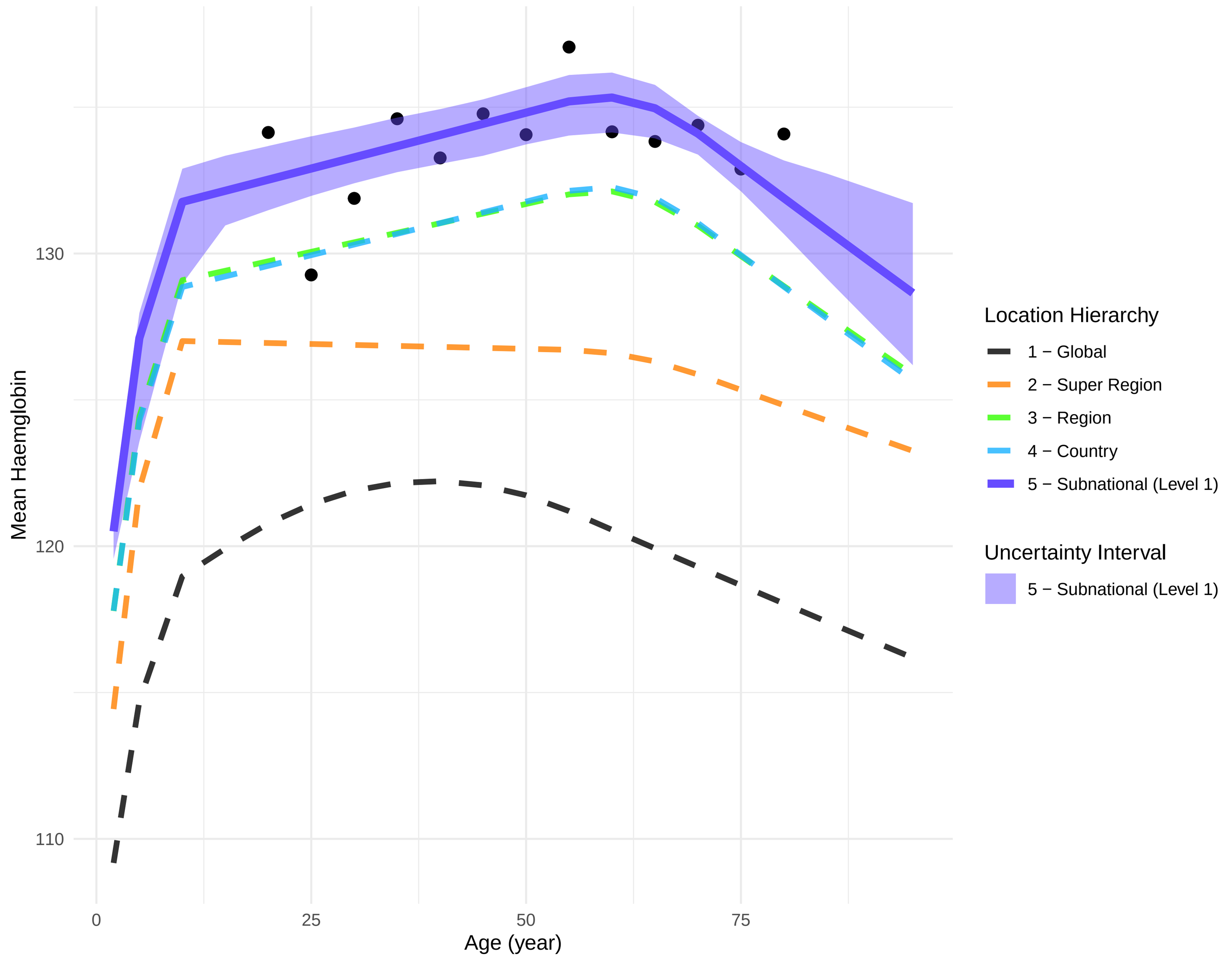

MR−BRT Age/Sex Fits − Non−pregnant Females 2+ Years Old − Mean Haemglobin − Pará
WHO−2001 Adjustment Method

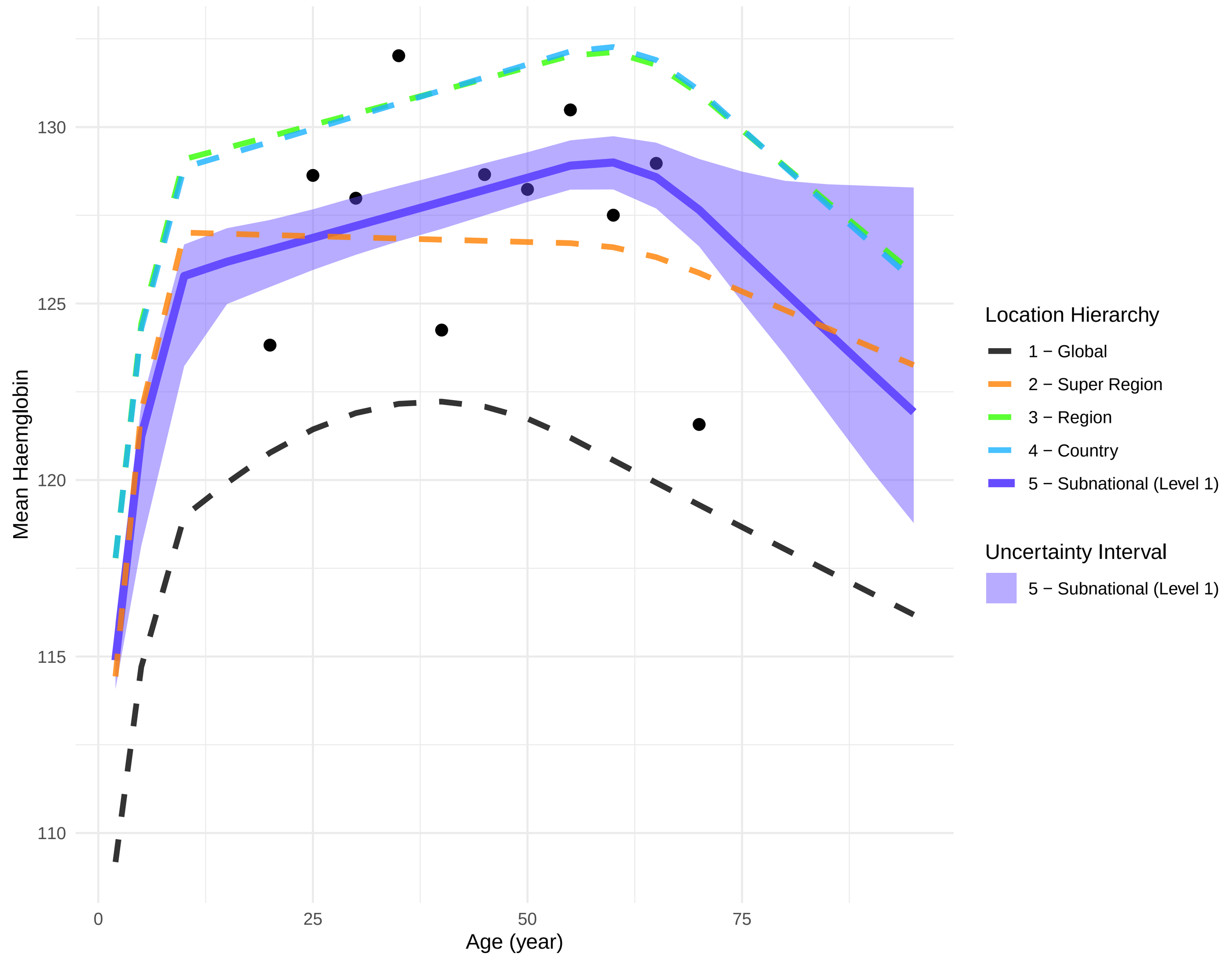

MR−BRT Age/Sex Fits − Non−pregnant Females 2+ Years Old − Mean Haemglobin − Paraíba
WHO−2001 Adjustment Method

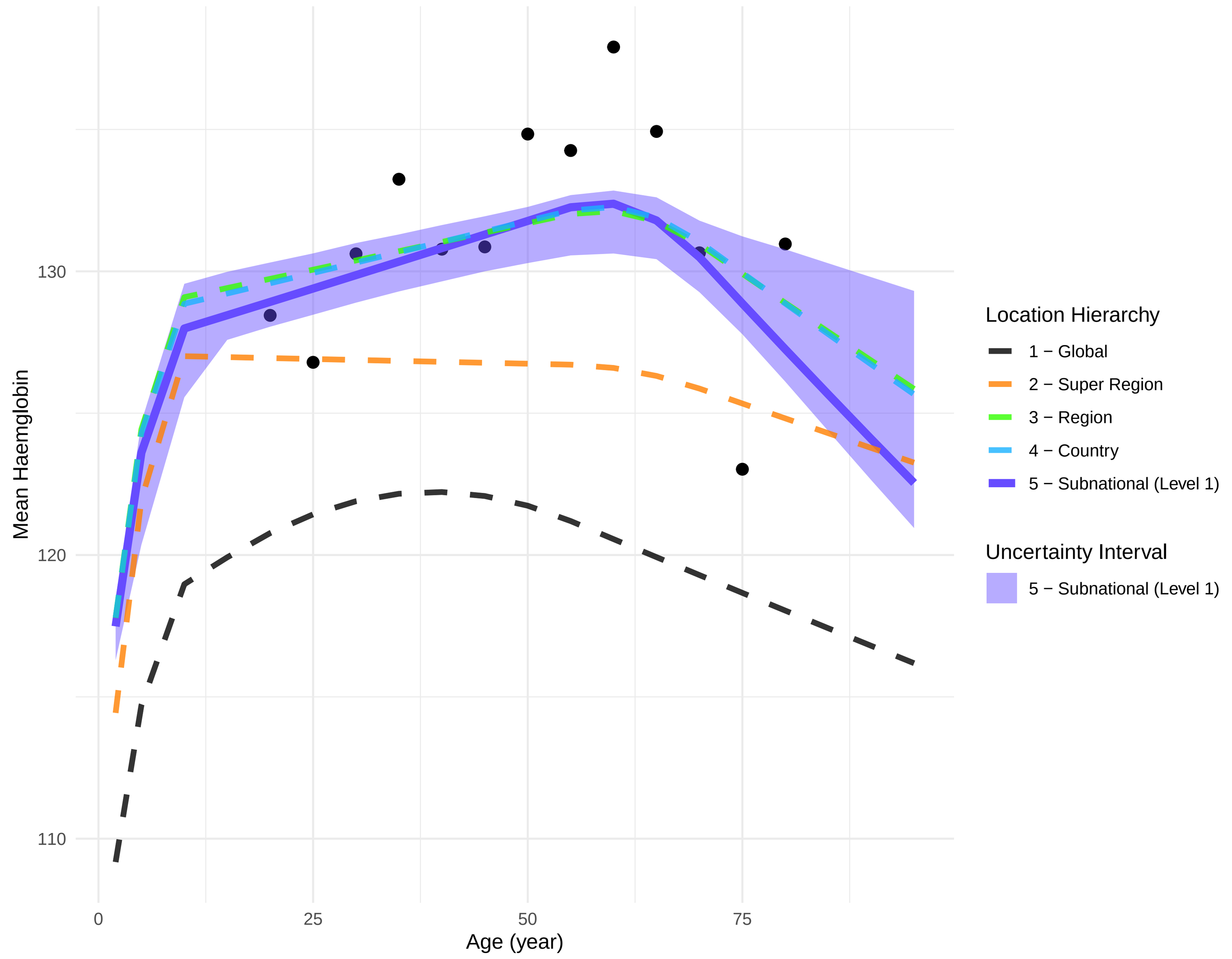

MR−BRT Age/Sex Fits − Non−pregnant Females 2+ Years Old − Mean Haemglobin − Paraná
WHO−2001 Adjustment Method

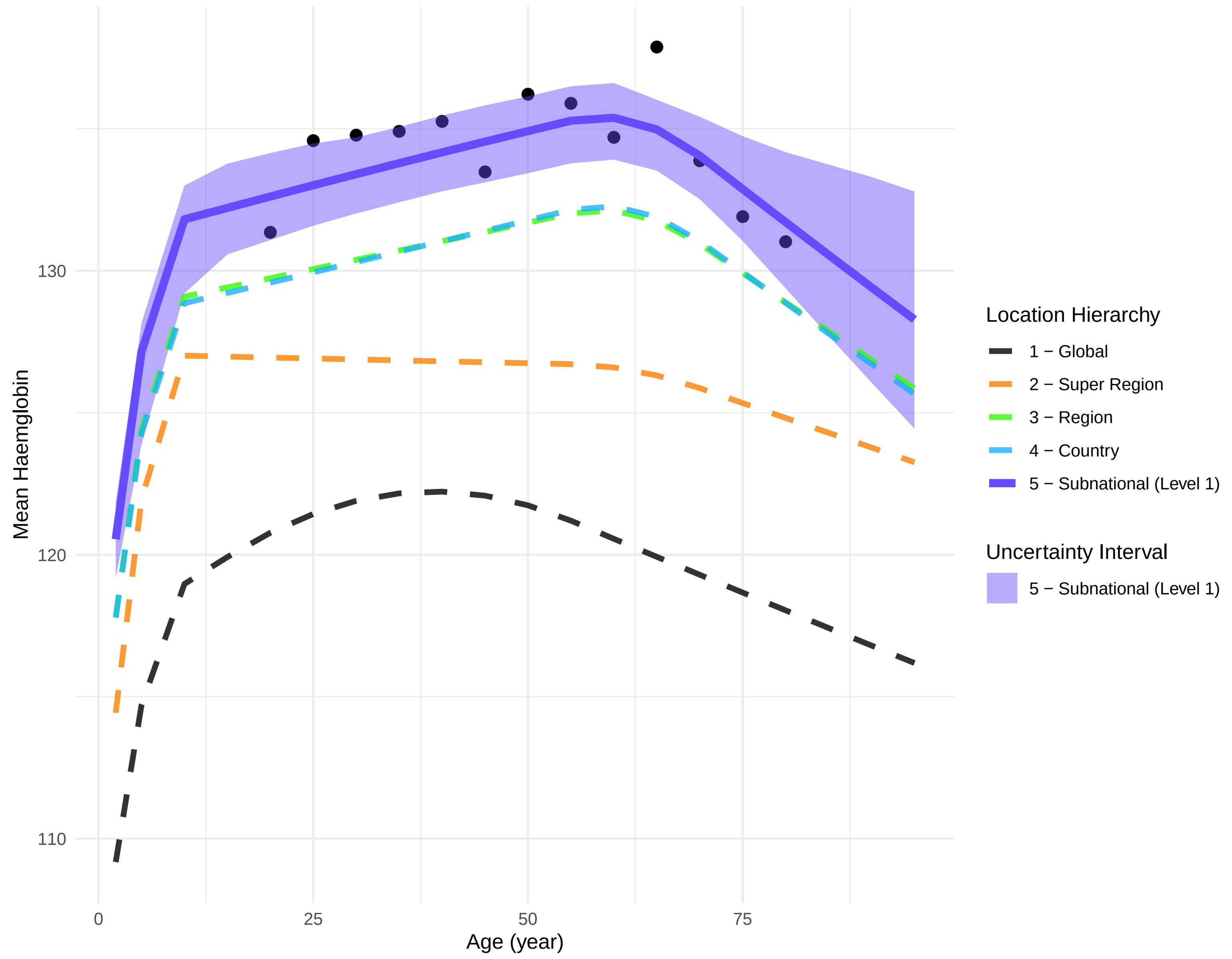

MR−BRT Age/Sex Fits − Non−pregnant Females 2+ Years Old − Mean Haemglobin − Pernambuco
WHO−2001 Adjustment Method

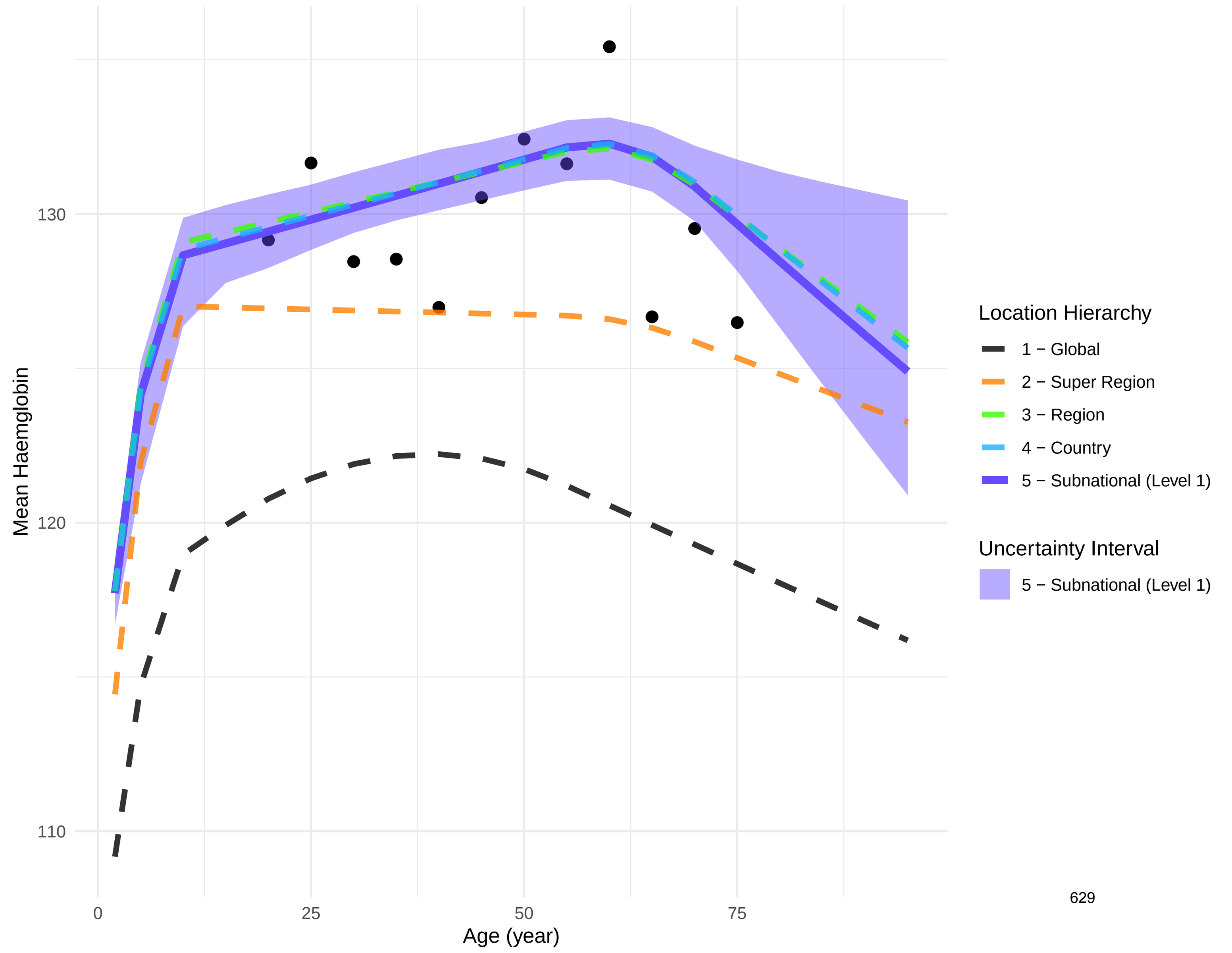

MR−BRT Age/Sex Fits − Non−pregnant Females 2+ Years Old − Mean Haemglobin − Piaui
WHO−2001 Adjustment Method

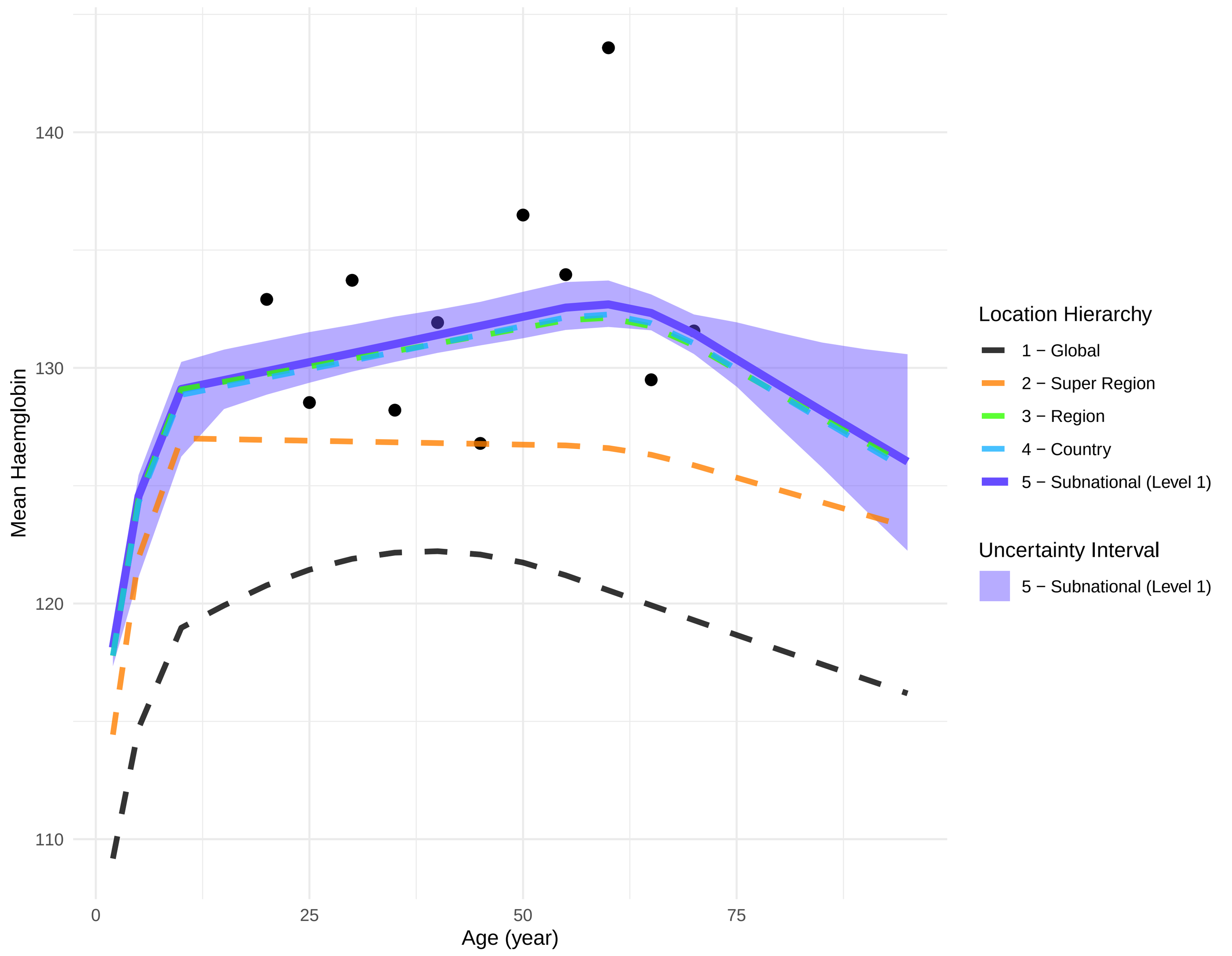

MR−BRT Age/Sex Fits − Non−pregnant Females 2+ Years Old − Mean Haemglobin − Rio de Janeiro
WHO−2001 Adjustment Method

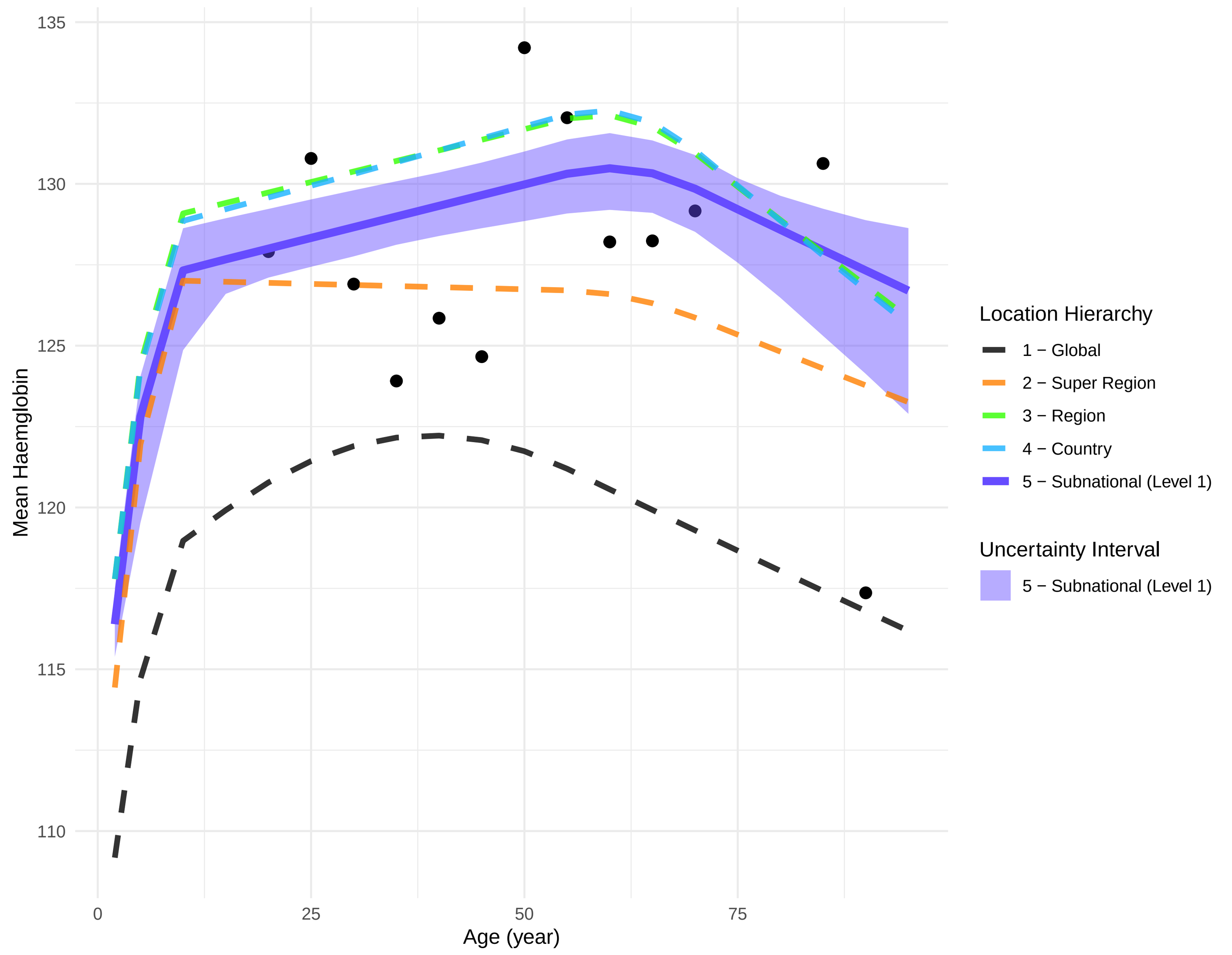

MR−BRT Age/Sex Fits − Non−pregnant Females 2+ Years Old − Mean Haemglobin − Rio Grande do Norte
WHO−2001 Adjustment Method

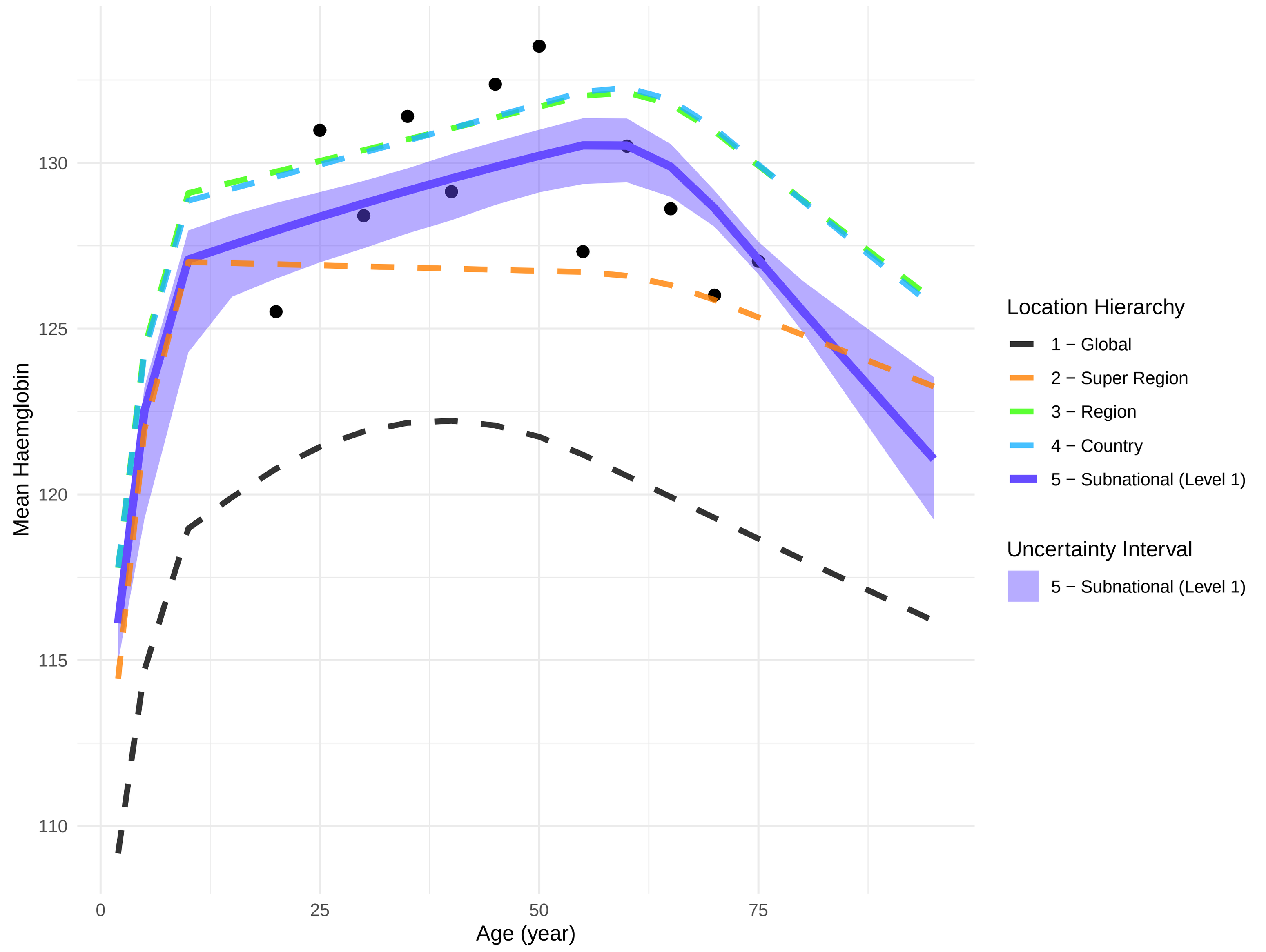

MR−BRT Age/Sex Fits − Non−pregnant Females 2+ Years Old − Mean Haemglobin − Rio Grande do Sul
WHO−2001 Adjustment Method

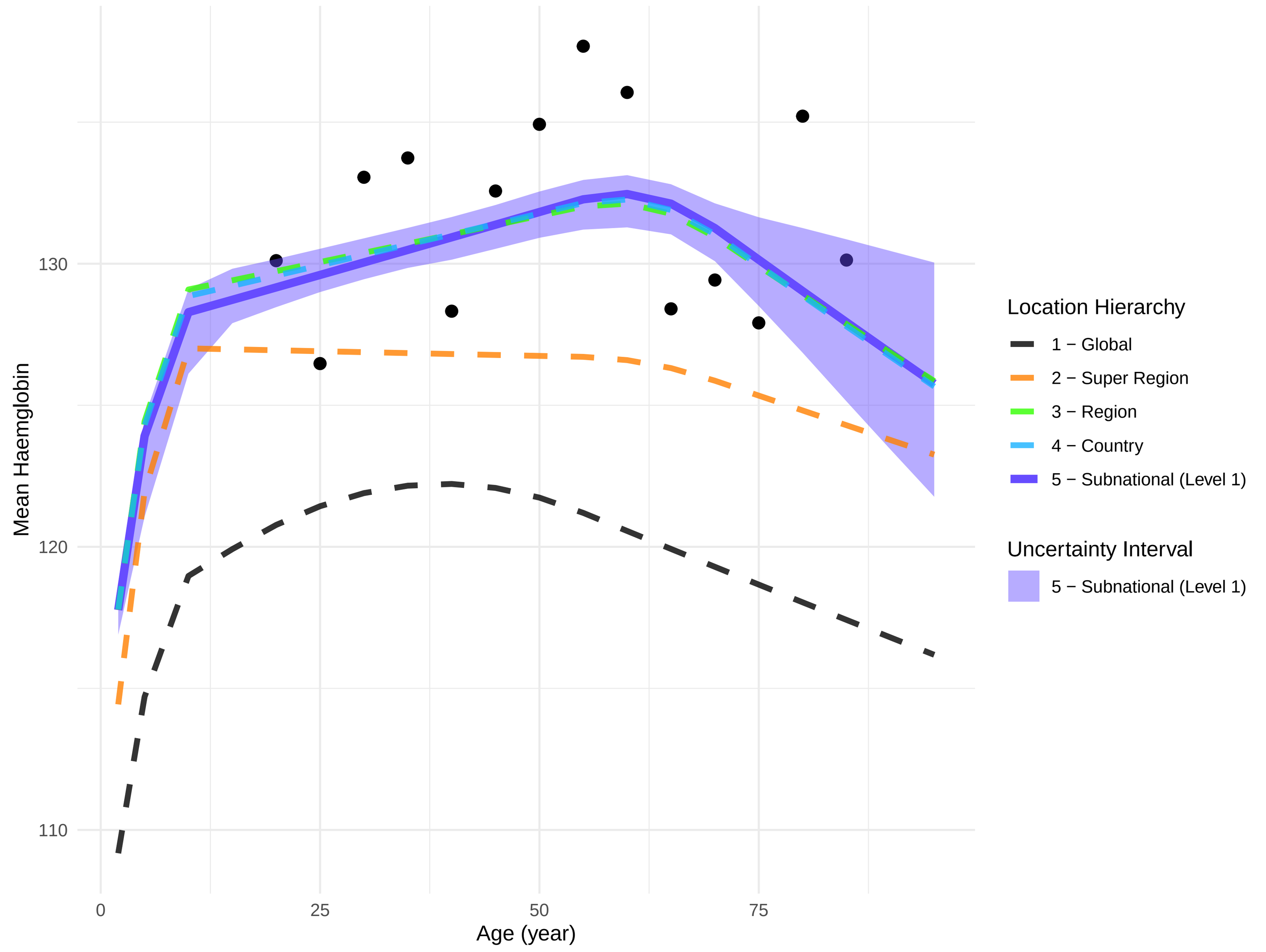

MR−BRT Age/Sex Fits − Non−pregnant Females 2+ Years Old − Mean Haemglobin − Rondônia
WHO−2001 Adjustment Method

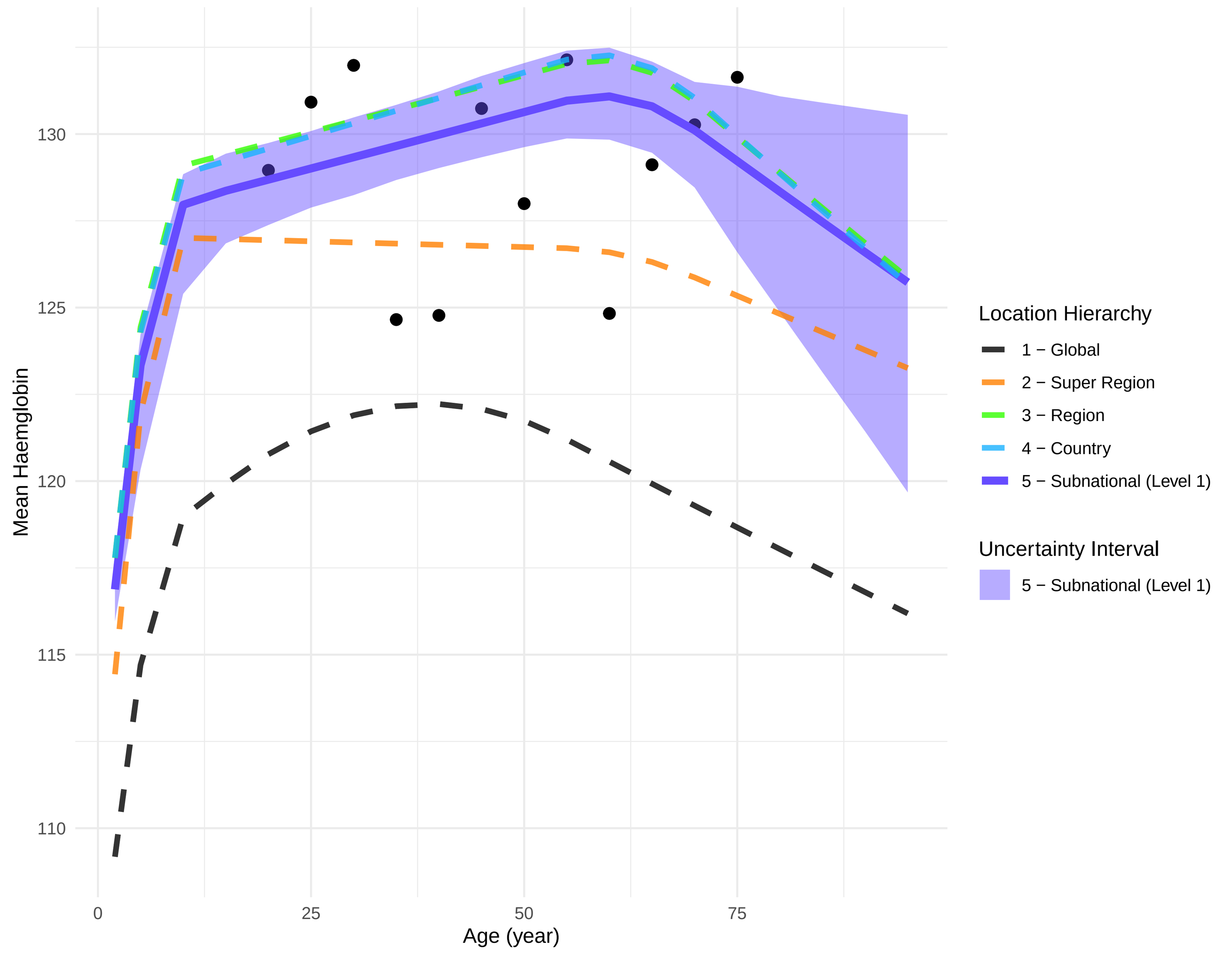

MR−BRT Age/Sex Fits − Non−pregnant Females 2+ Years Old − Mean Haemglobin − Roraima
WHO−2001 Adjustment Method

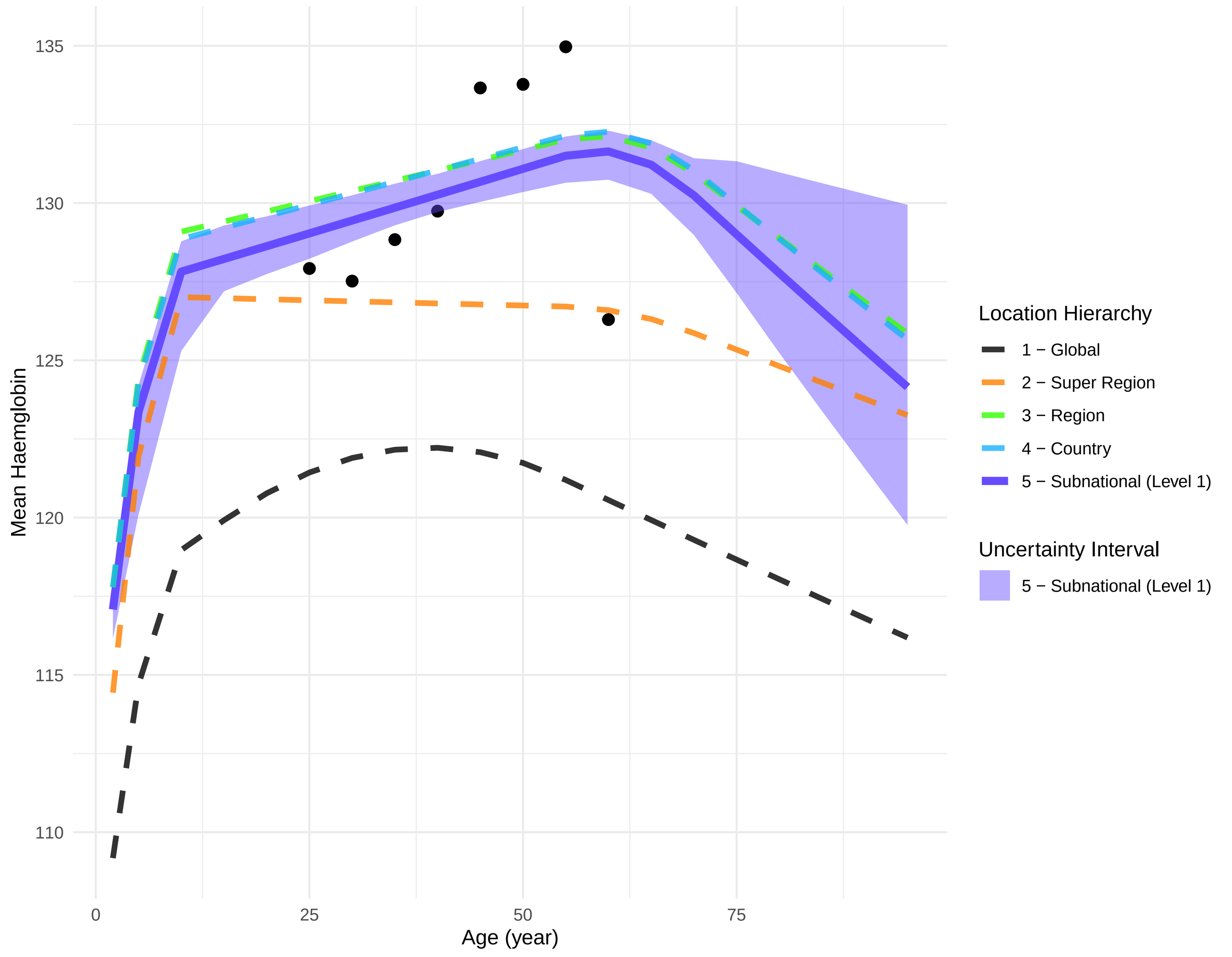

MR−BRT Age/Sex Fits − Non−pregnant Females 2+ Years Old − Mean Haemglobin − Santa Catarina
WHO−2001 Adjustment Method

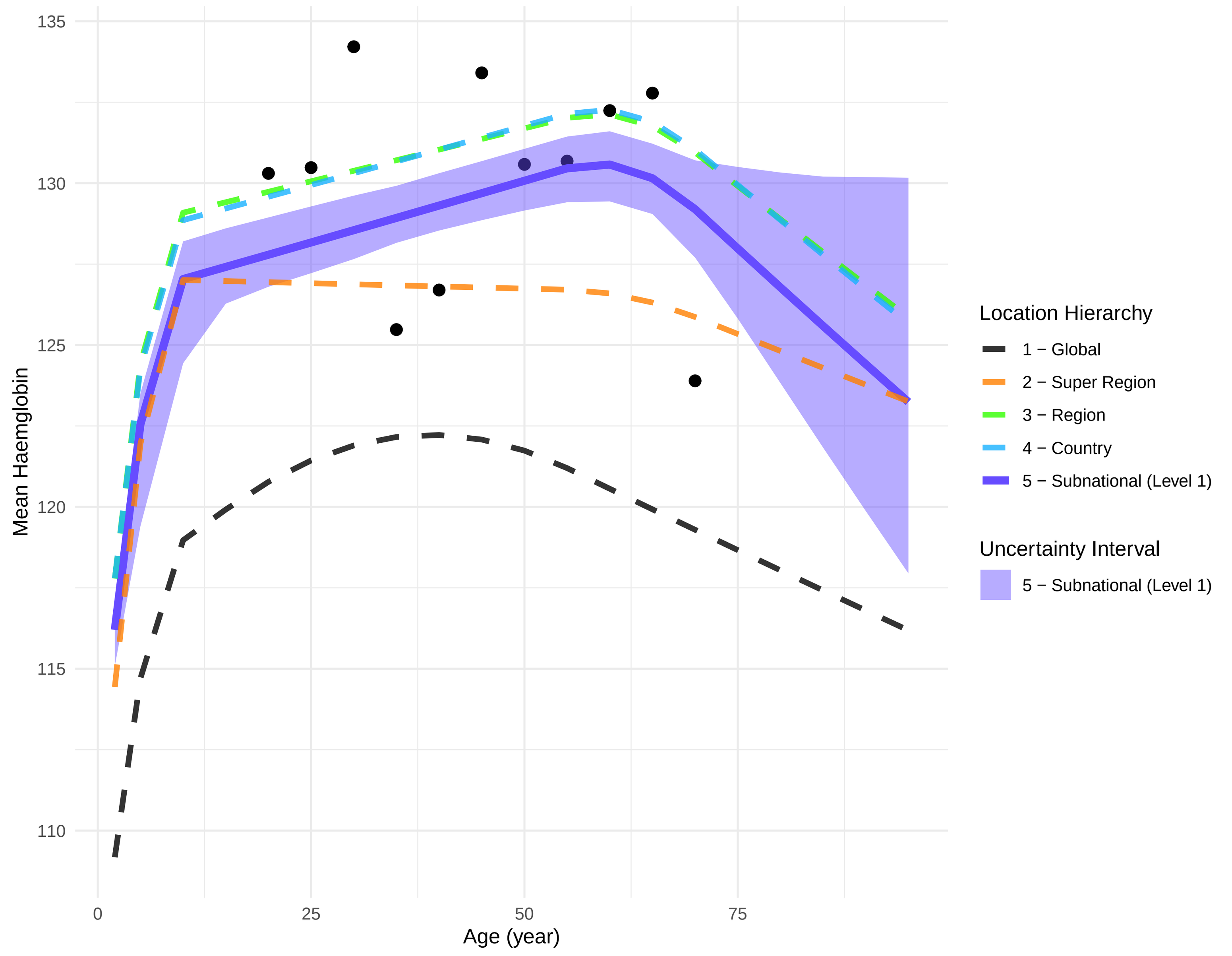

MR−BRT Age/Sex Fits − Non−pregnant Females 2+ Years Old − Mean Haemglobin − São Paulo
WHO−2001 Adjustment Method

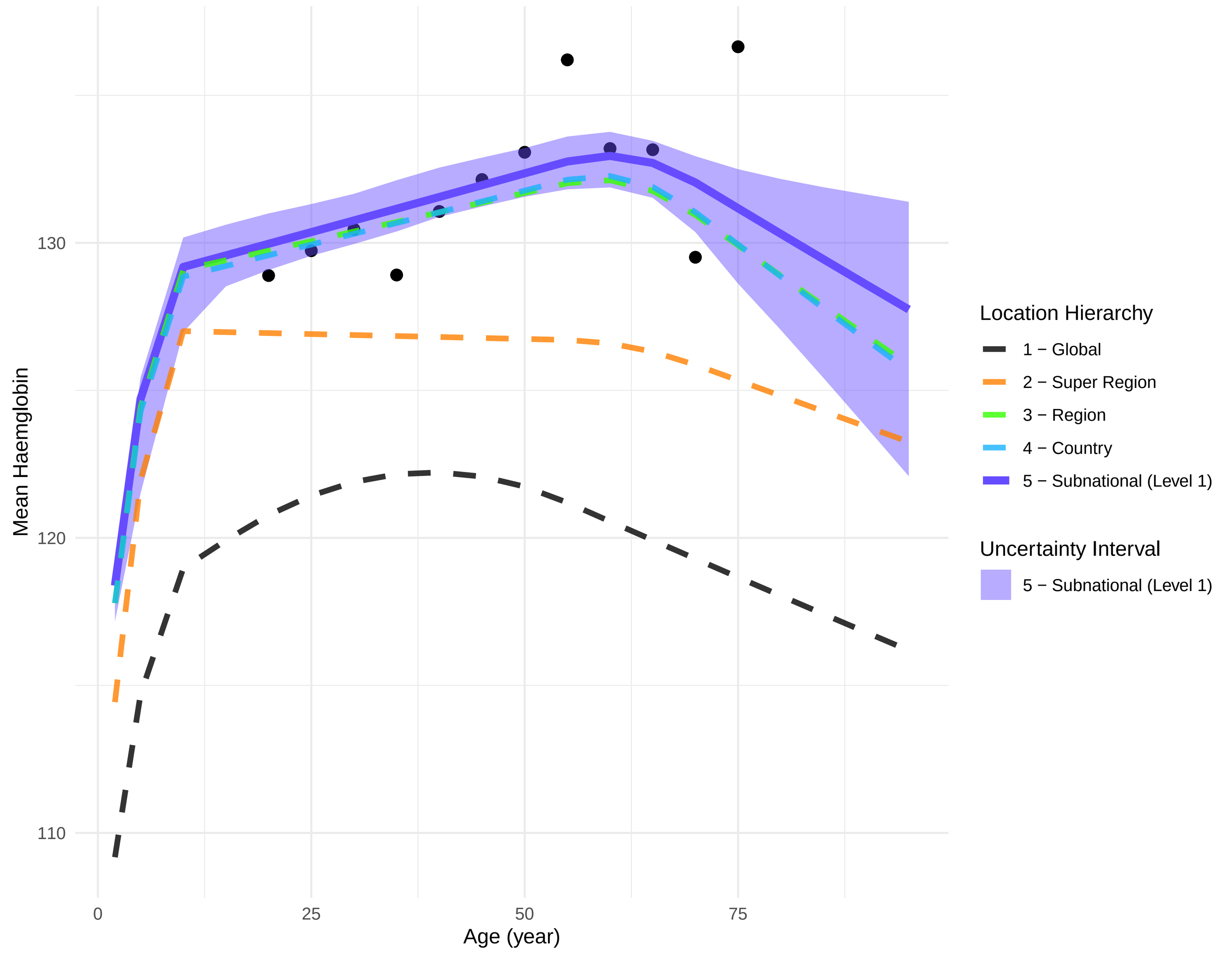

MR−BRT Age/Sex Fits − Non−pregnant Females 2+ Years Old − Mean Haemglobin − Sergipe
WHO−2001 Adjustment Method

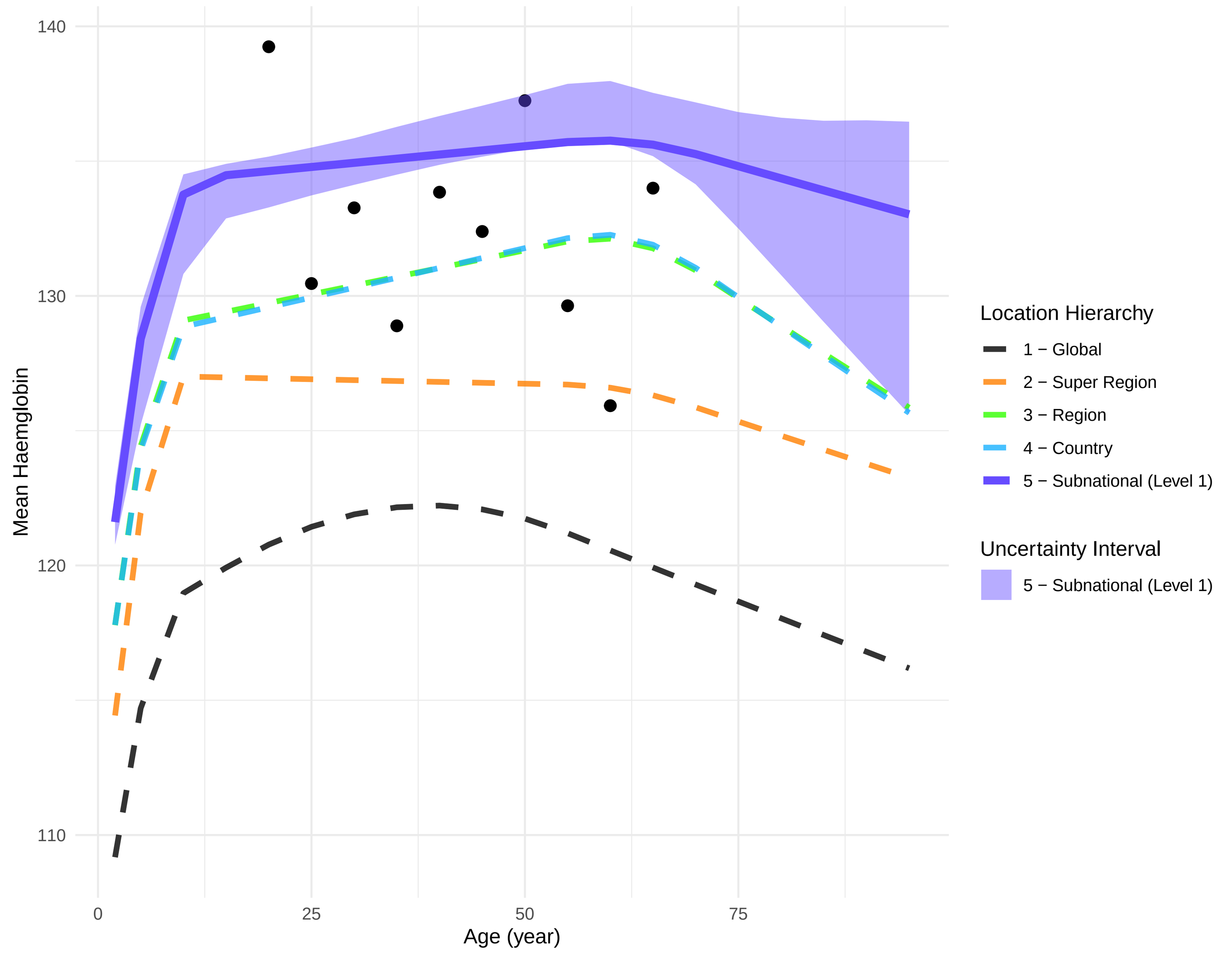

MR−BRT Age/Sex Fits − Non−pregnant Females 2+ Years Old − Mean Haemglobin − Tocantins
WHO−2001 Adjustment Method

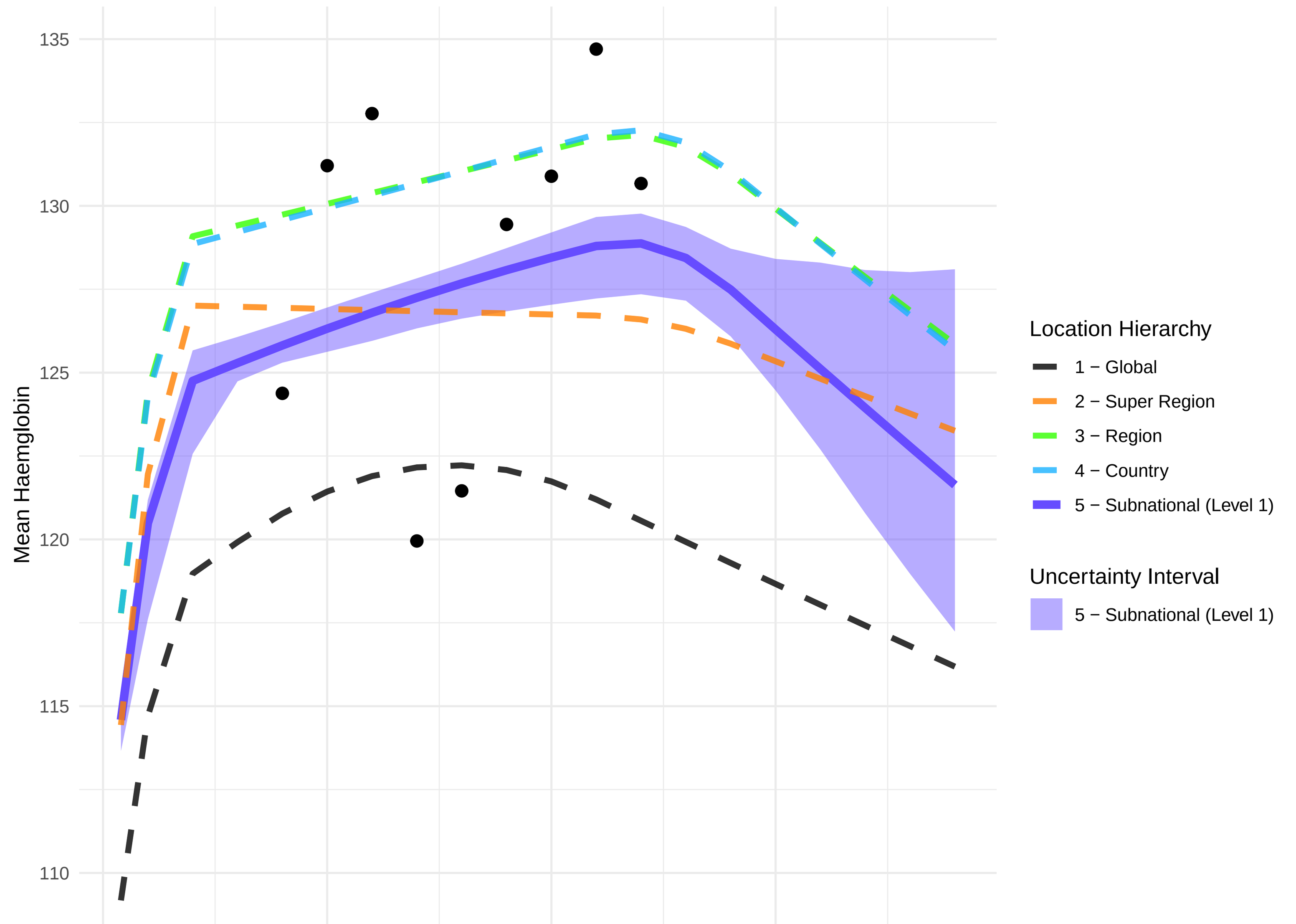

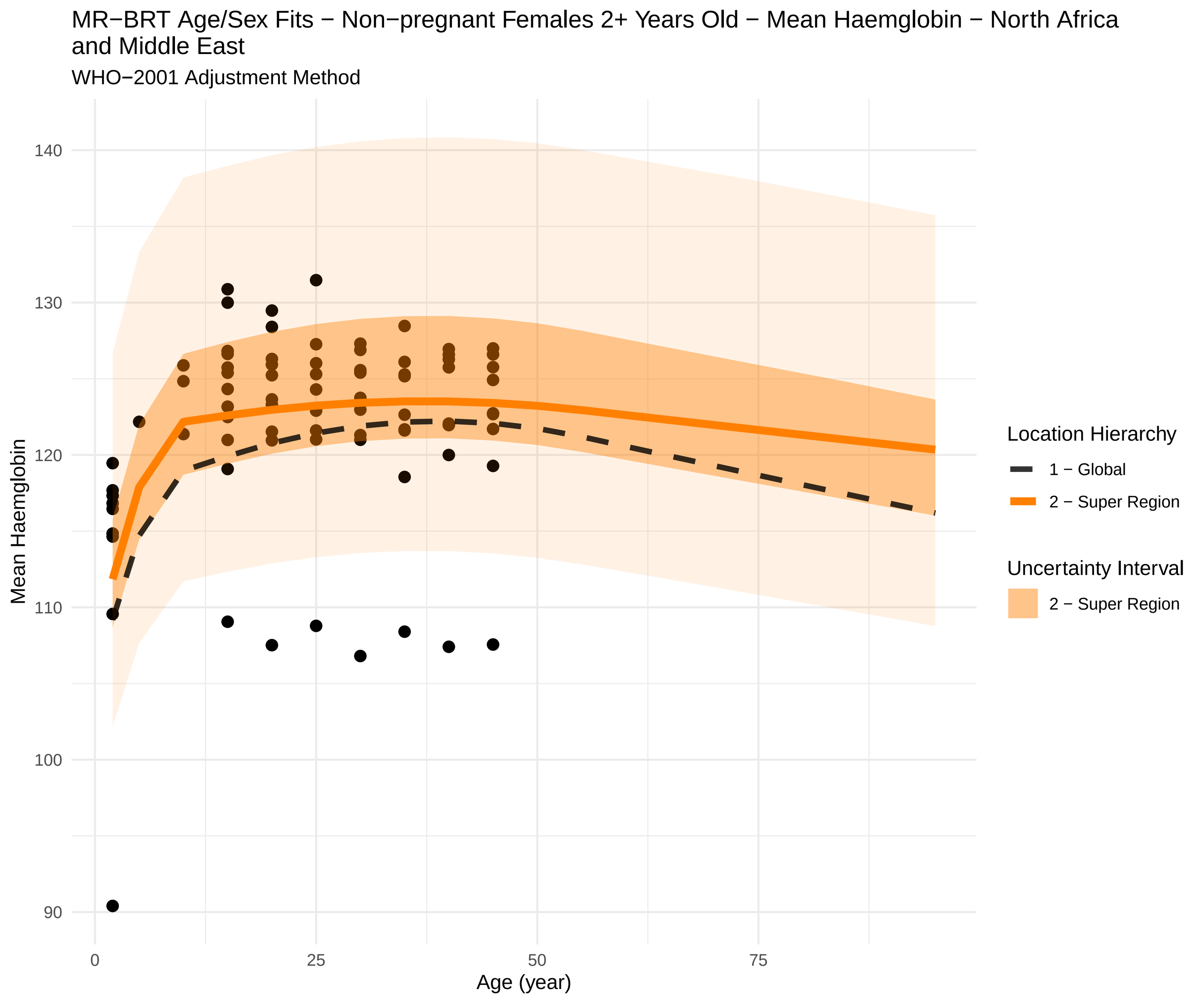
MR−BRT Age/Sex Fits − Non−pregnant Females 2+ Years Old − Mean Haemglobin − North Africa and Middle East
WHO−2001 Adjustment Method
Mean Haemglobin
Age (year)
Location Hierarchy
1 − Global
2 − Super Region
Uncertainty Interval
2 − Super Region

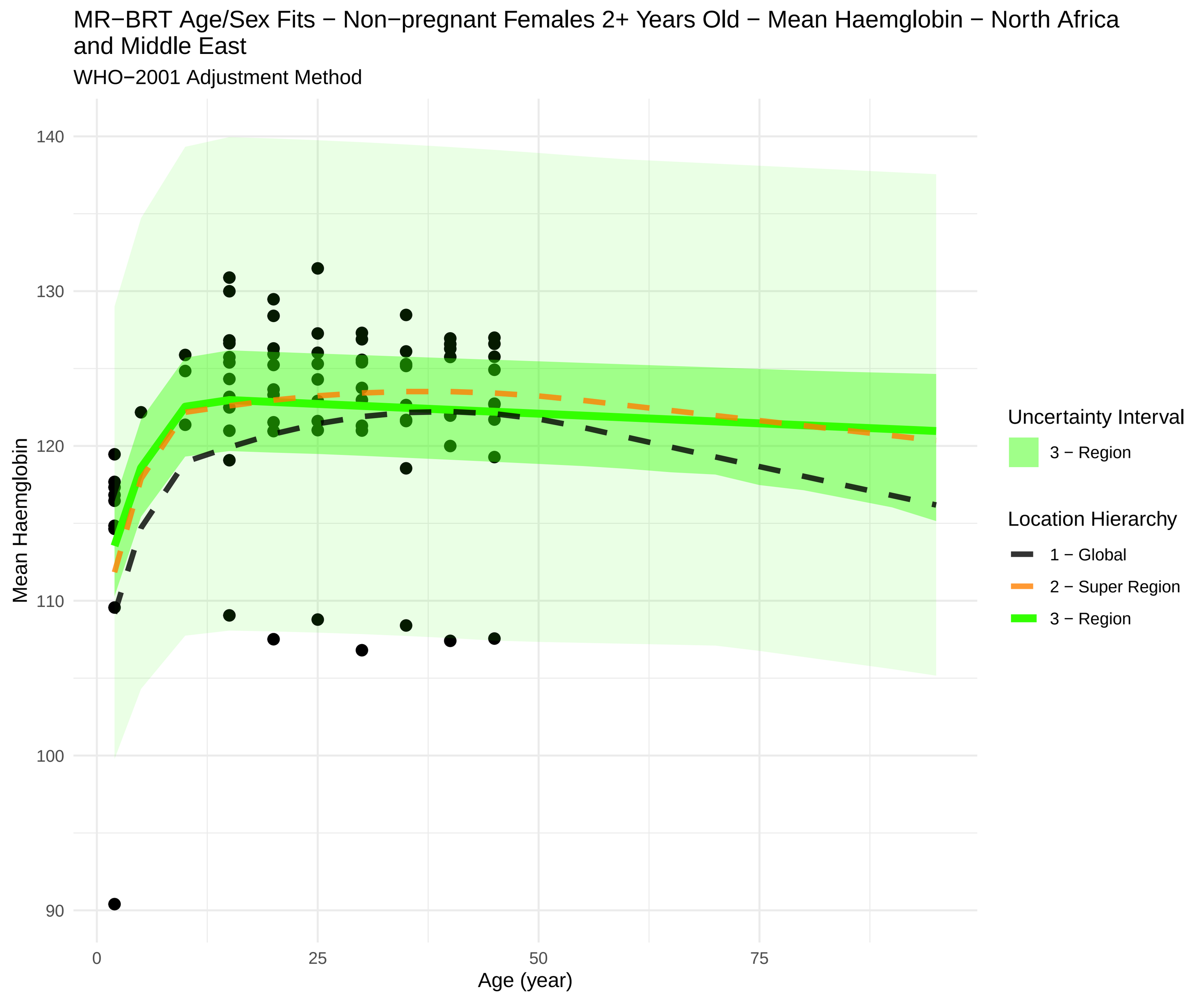
MR−BRT Age/Sex Fits − Non−pregnant Females 2+ Years Old − Mean Haemglobin − North Africa and Middle East
WHO−2001 Adjustment Method
Mean Haemglobin
Age (year)
Uncertainty Interval
3 − Region
Location Hierarchy
1 − Global
2 − Super Region
3 − Region

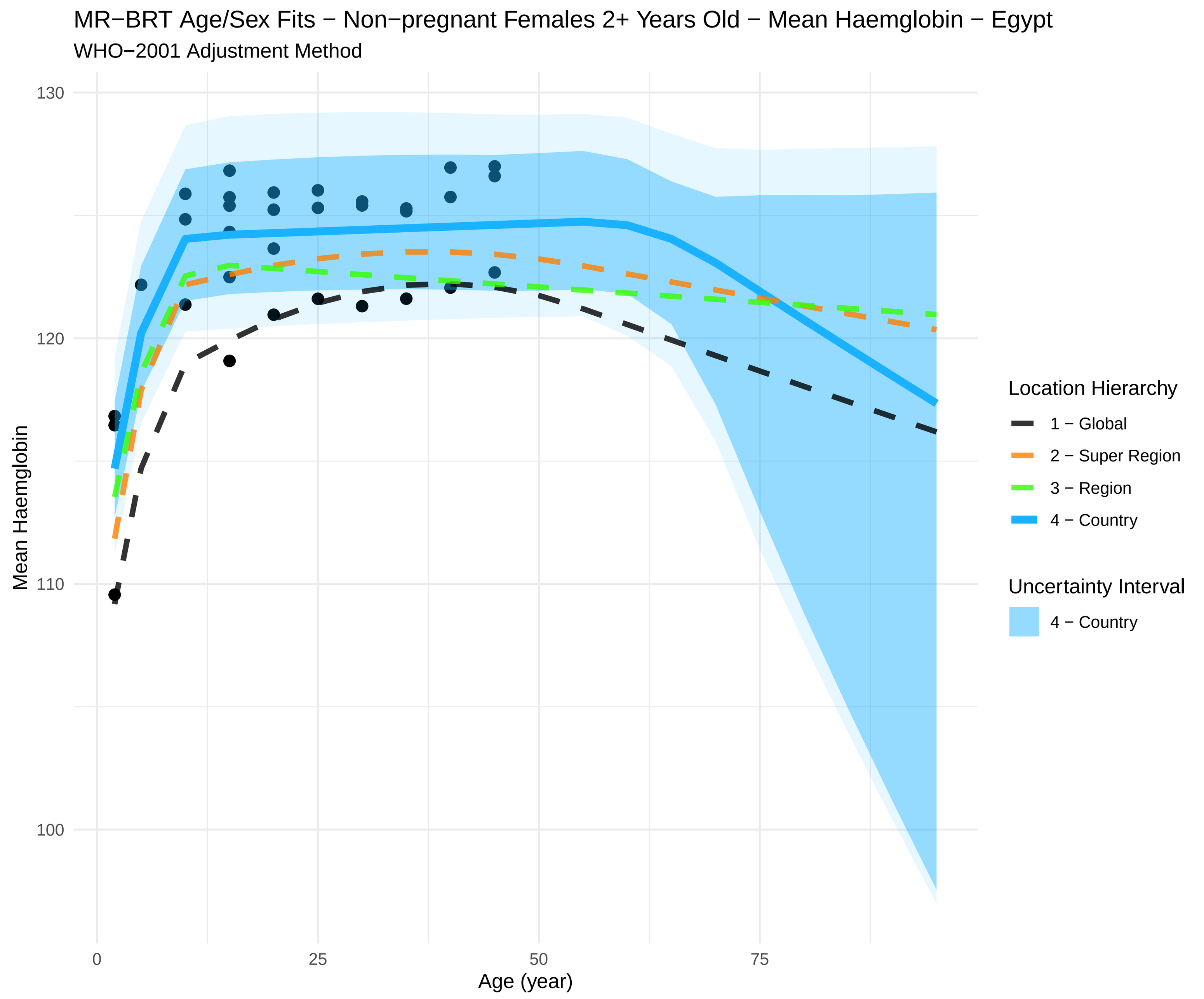
MR−BRT Age/Sex Fits − Non−pregnant Females 2+ Years Old − Mean Haemglobin − Egypt
WHO−2001 Adjustment Method
Mean Haemglobin
Age (year)
Location Hierarchy
1 − Global
2 − Super Region
3 − Region
4 − Country
Uncertainty Interval
4 − Country

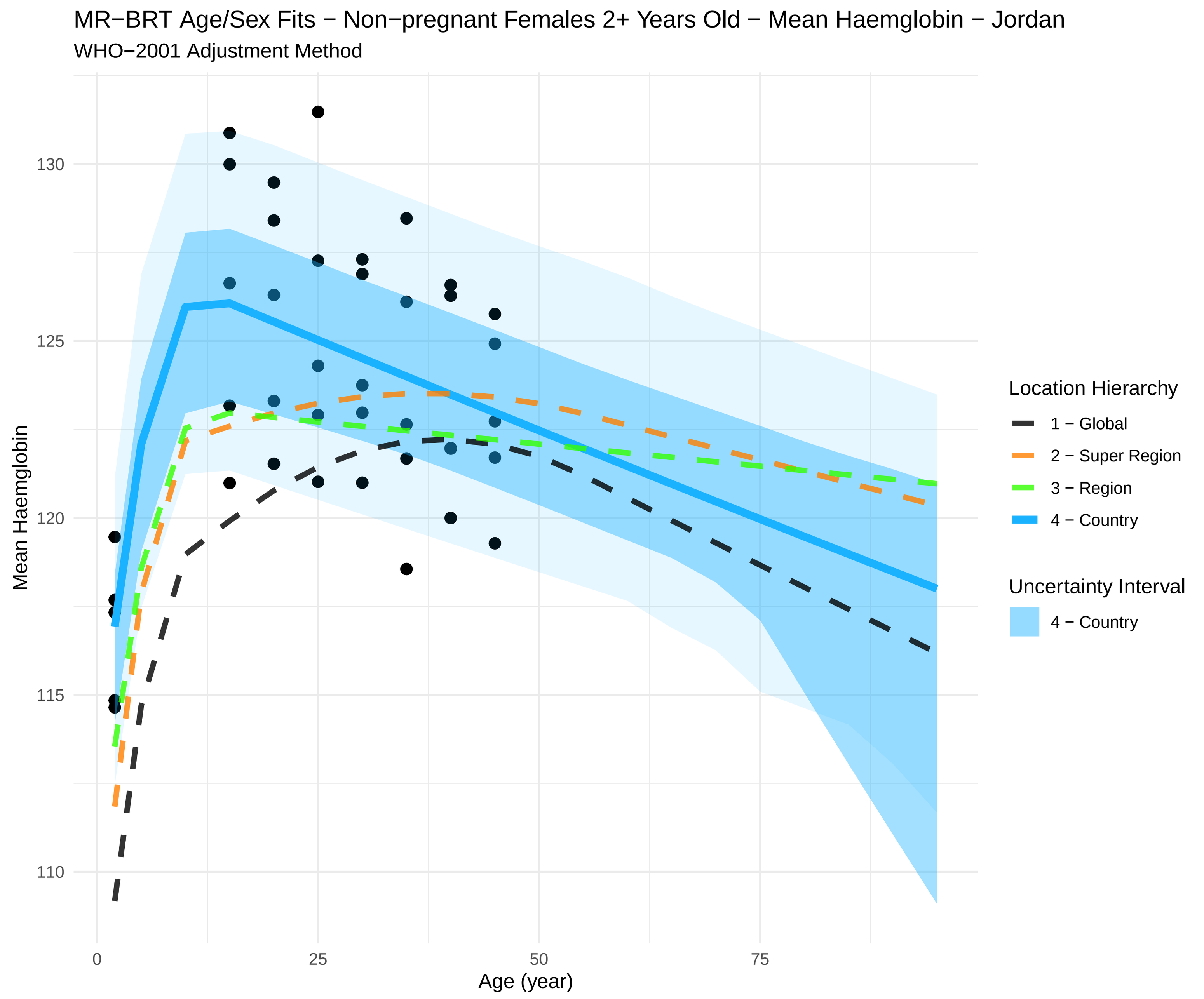
MR−BRT Age/Sex Fits − Non−pregnant Females 2+ Years Old − Mean Haemglobin − Jordan
WHO−2001 Adjustment Method
Mean Haemglobin
Age (year)
Location Hierarchy
1 − Global
2 − Super Region
3 − Region
4 − Country
Uncertainty Interval
4 − Country

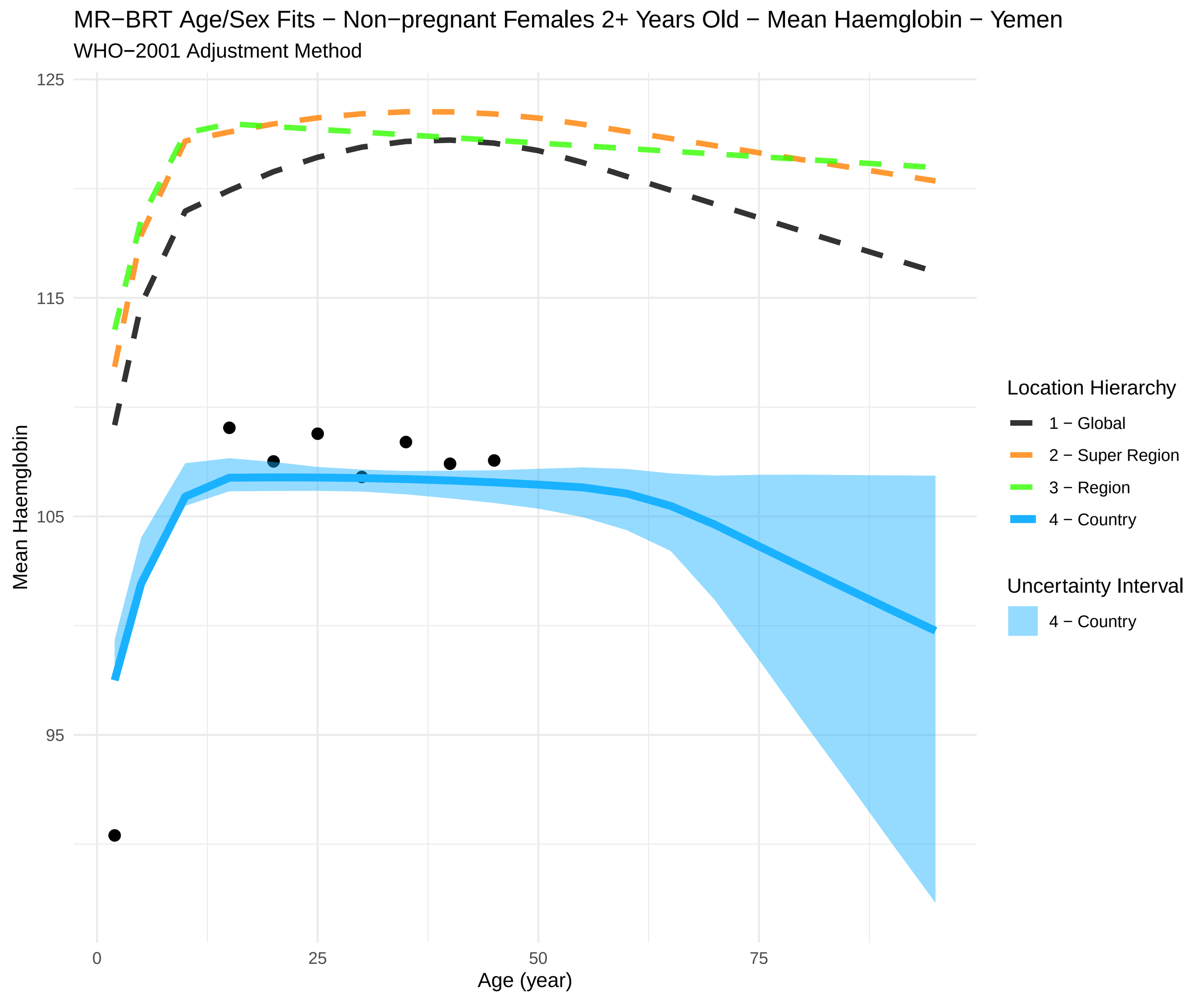
MR−BRT Age/Sex Fits − Non−pregnant Females 2+ Years Old − Mean Haemglobin − Yemen
WHO−2001 Adjustment Method
Mean Haemglobin
Age (year)
Location Hierarchy
1 − Global
2 − Super Region
3 − Region
4 − Country
Uncertainty Interval
4 − Country

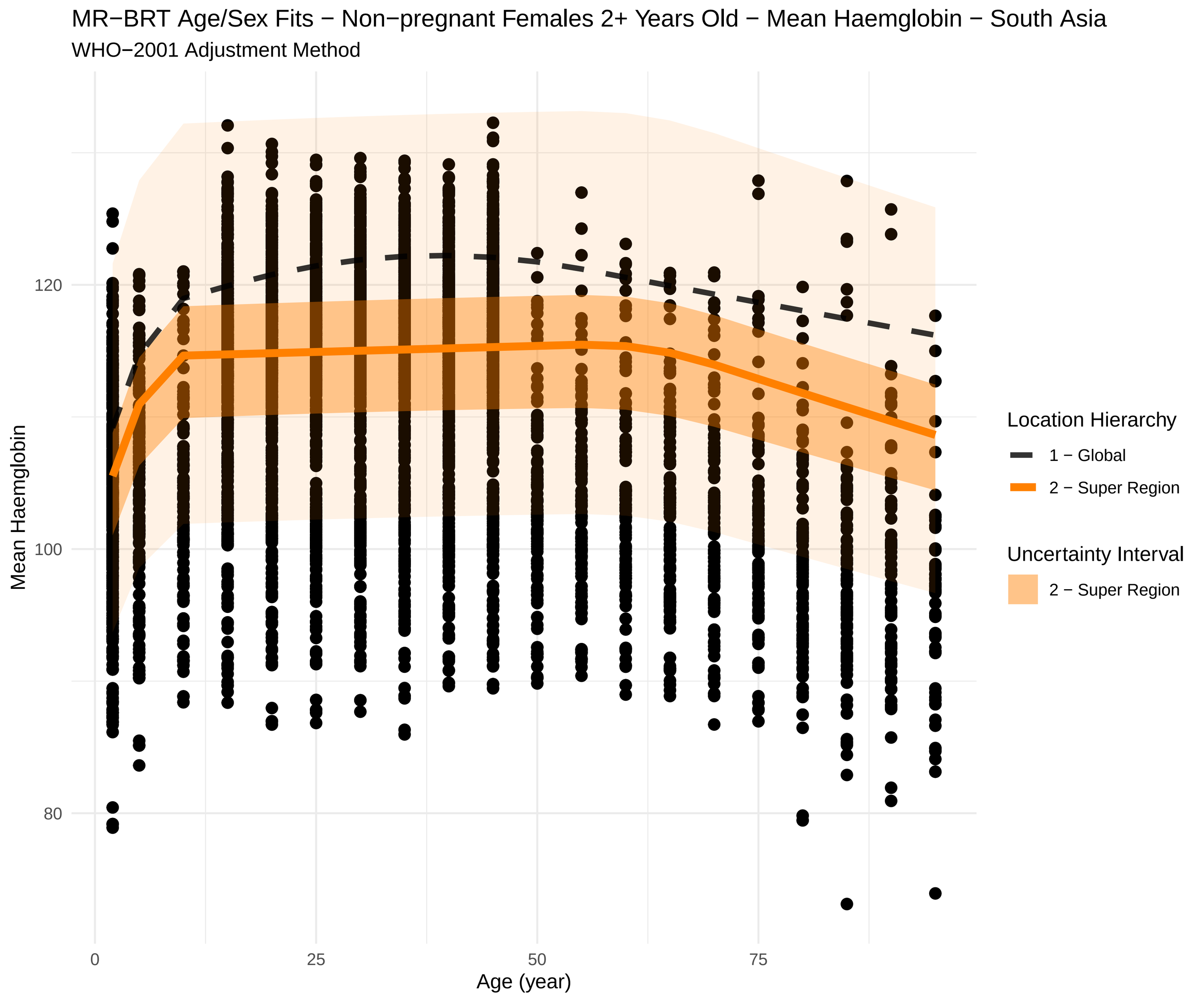
MR−BRT Age/Sex Fits − Non−pregnant Females 2+ Years Old − Mean Haemglobin − South Asia
WHO−2001 Adjustment Method
Mean Haemglobin
Age (year)
Location Hierarchy
1 − Global
2 − Super Region
Uncertainty Interval
2 − Super Region

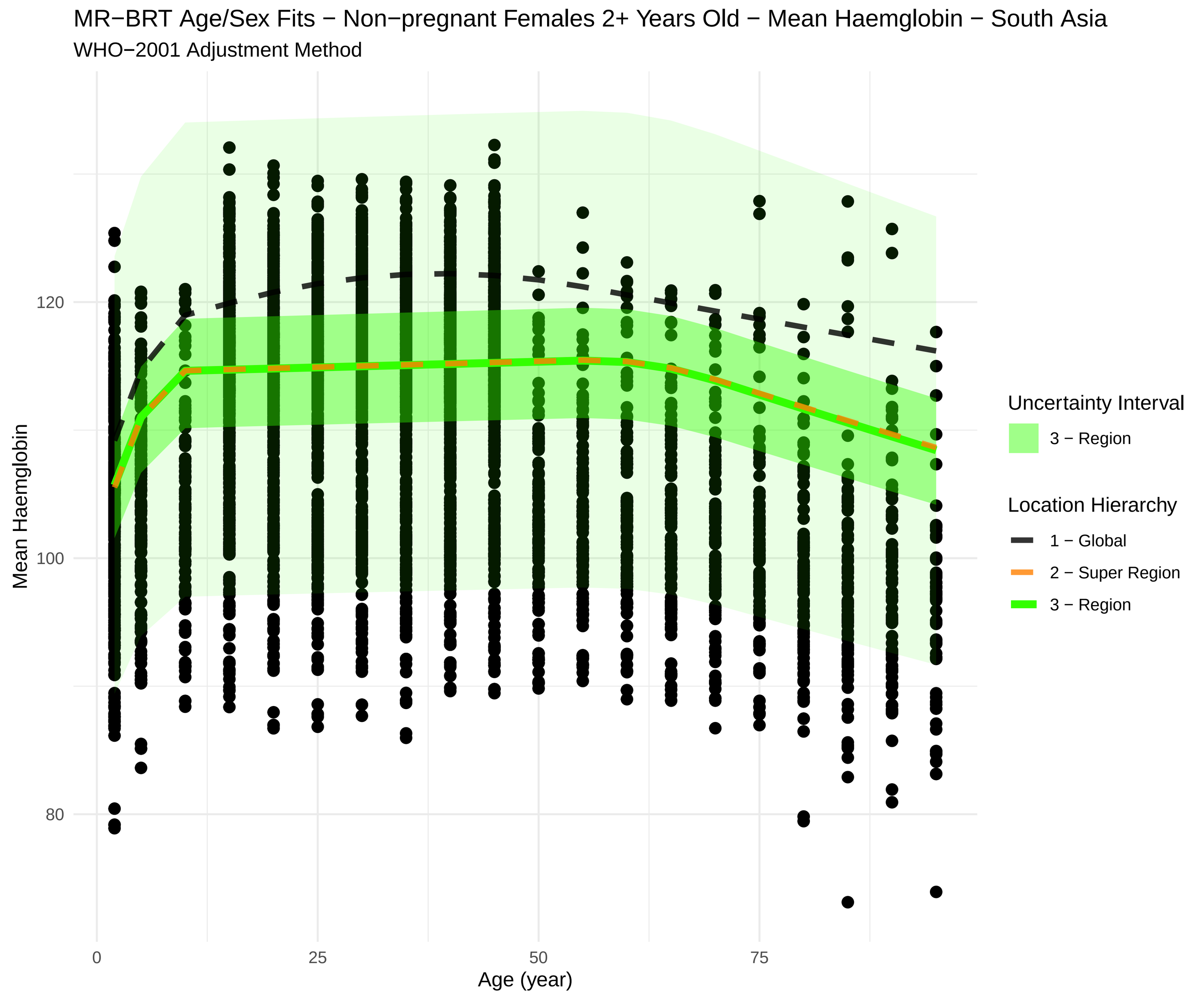
MR−BRT Age/Sex Fits − Non−pregnant Females 2+ Years Old − Mean Haemglobin − South Asia
WHO−2001 Adjustment Method
Mean Haemglobin
Age (year)
Uncertainty Interval
3 − Region
Location Hierarchy
1 − Global
2 − Super Region
3 − Region

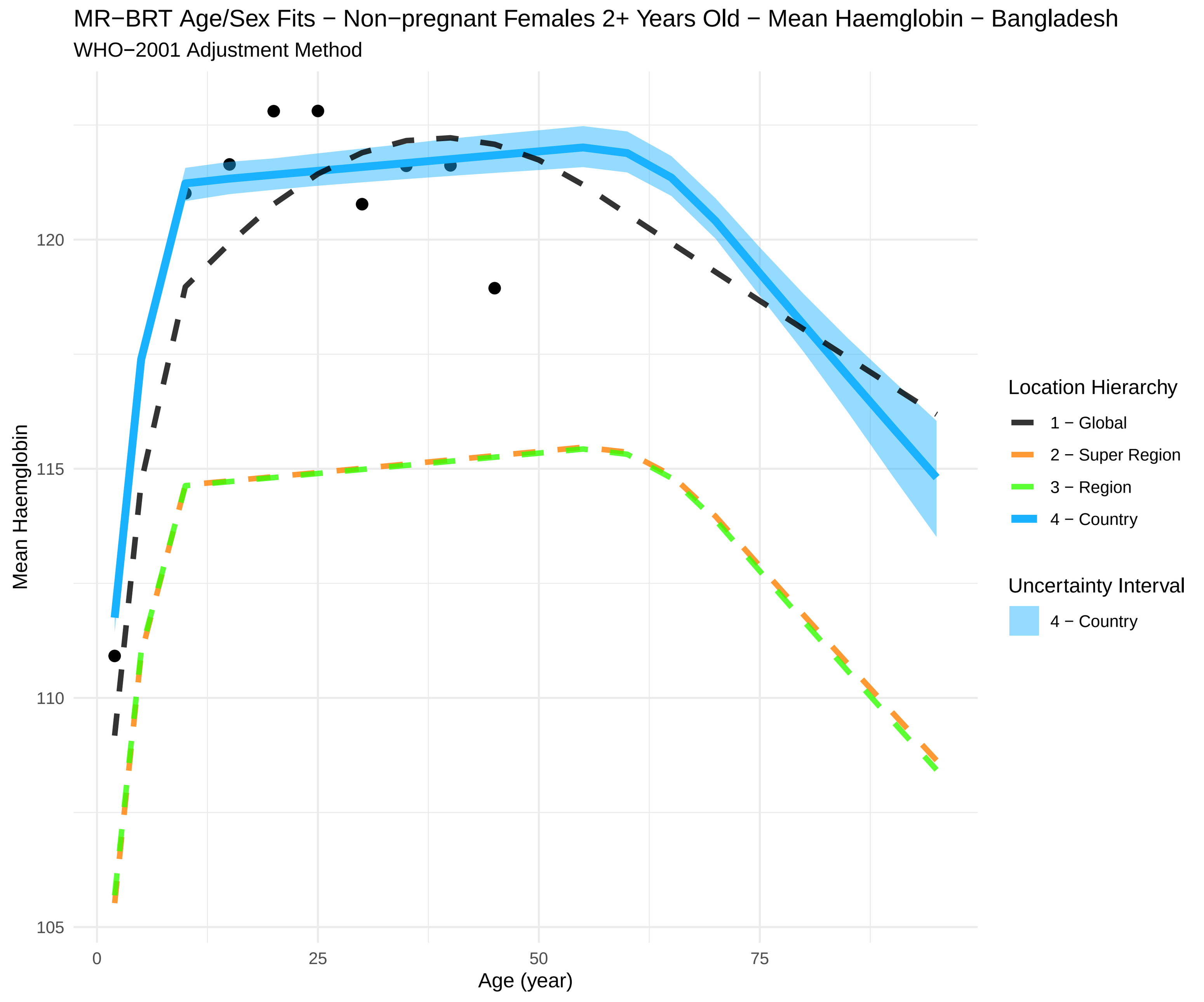
MR−BRT Age/Sex Fits − Non−pregnant Females 2+ Years Old − Mean Haemglobin − Bangladesh
WHO−2001 Adjustment Method
Mean Haemglobin
Age (year)
Location Hierarchy
1 − Global
2 − Super Region
3 − Region
4 − Country
Uncertainty Interval
4 − Country

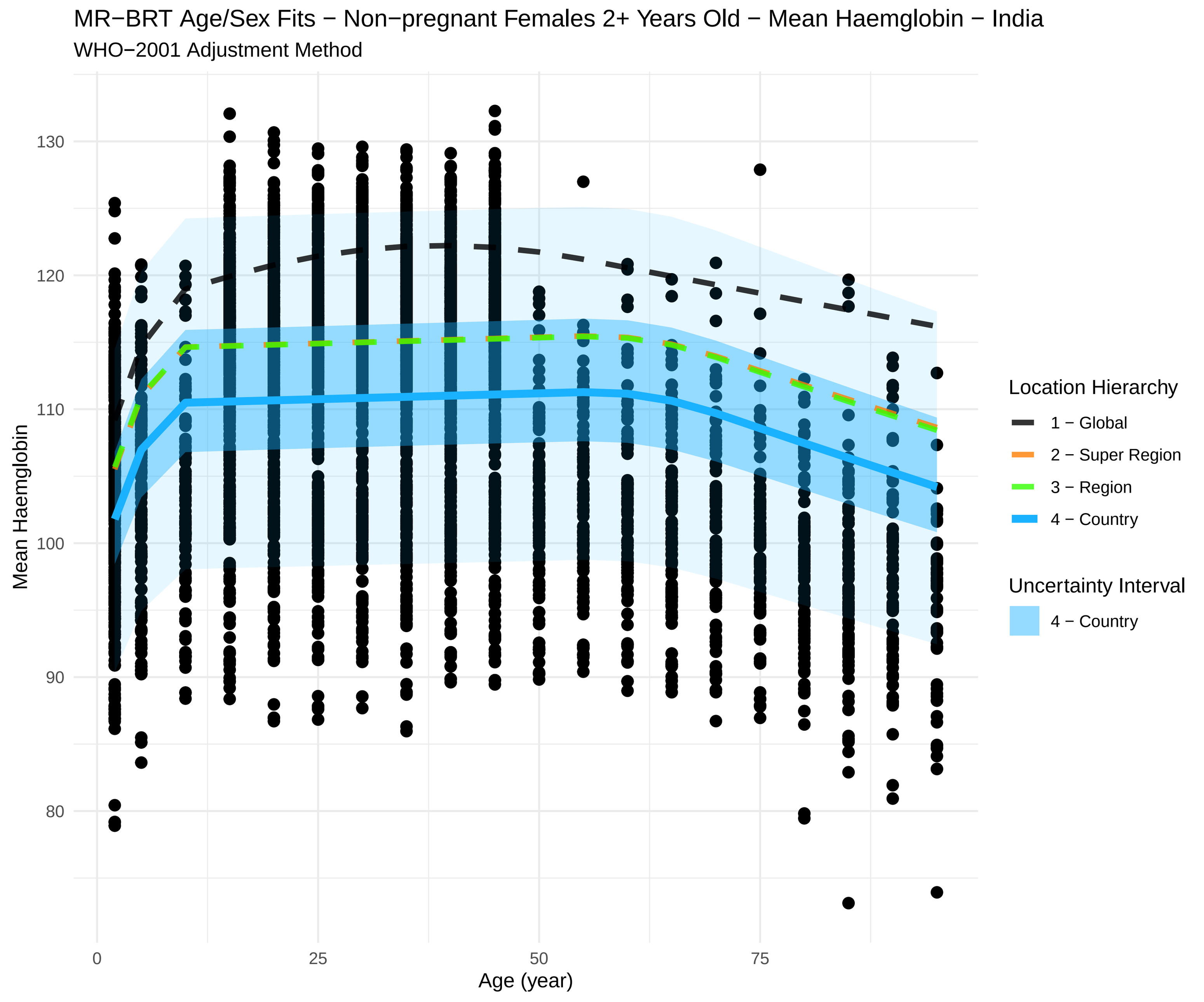
MR−BRT Age/Sex Fits − Non−pregnant Females 2+ Years Old − Mean Haemglobin − India
WHO−2001 Adjustment Method
Mean Haemglobin
Age (year)
Location Hierarchy
1 − Global
2 − Super Region
3 − Region
4 − Country
Uncertainty Interval
4 − Country

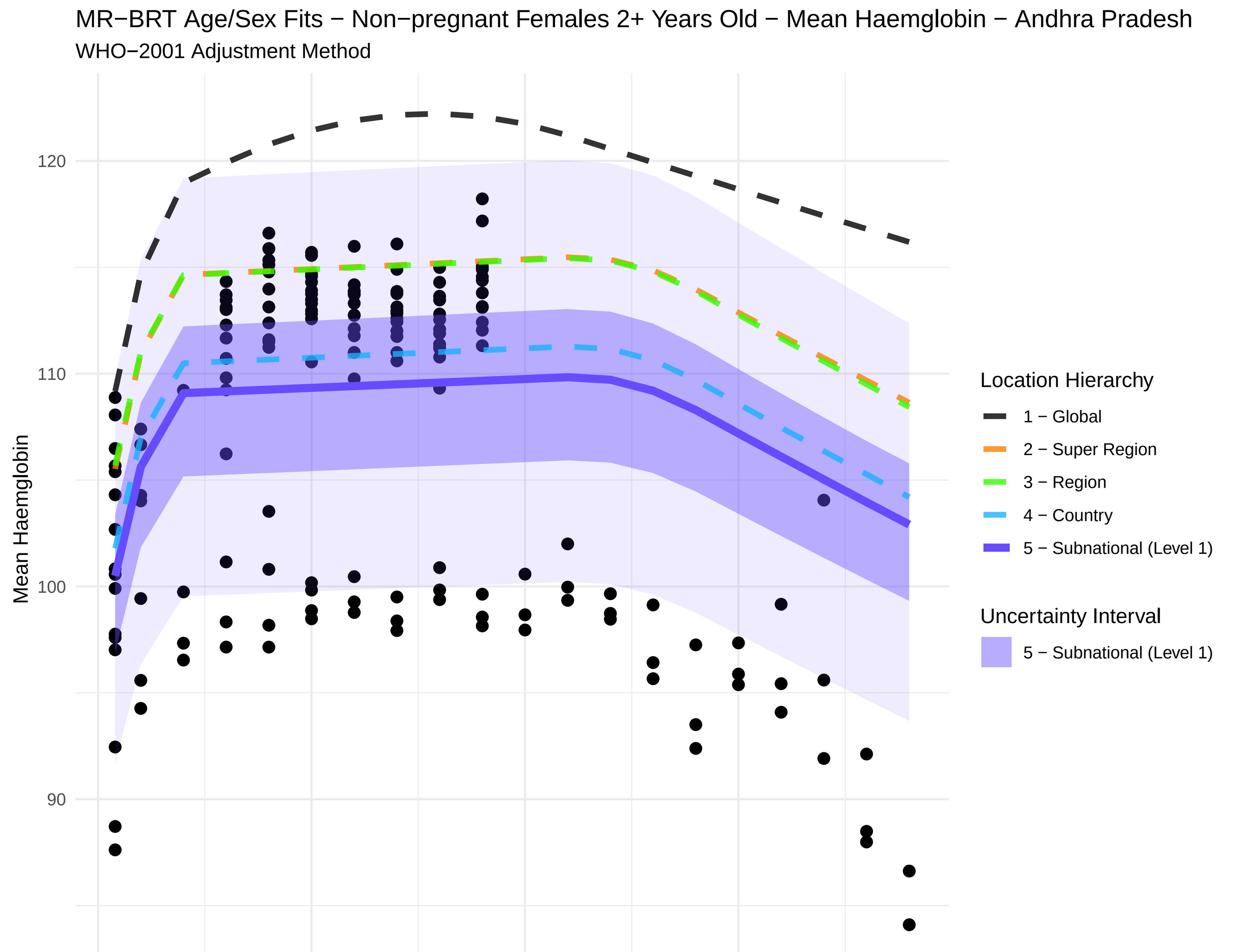
MR−BRT Age/Sex Fits − Non−pregnant Females 2+ Years Old − Mean Haemglobin − Andhra Pradesh
WHO−2001 Adjustment Method
Mean Haemglobin
Age (year)
Location Hierarchy
1 − Global
2 − Super Region
3 − Region
4 − Country
5 − Subnational (Level 1)
Uncertainty Interval
5 − Subnational (Level 1)

MR−BRT Age/Sex Fits − Non−pregnant Females 2+ Years Old − Mean Haemglobin − Andhra Pradesh, Rural
WHO−2001 Adjustment Method

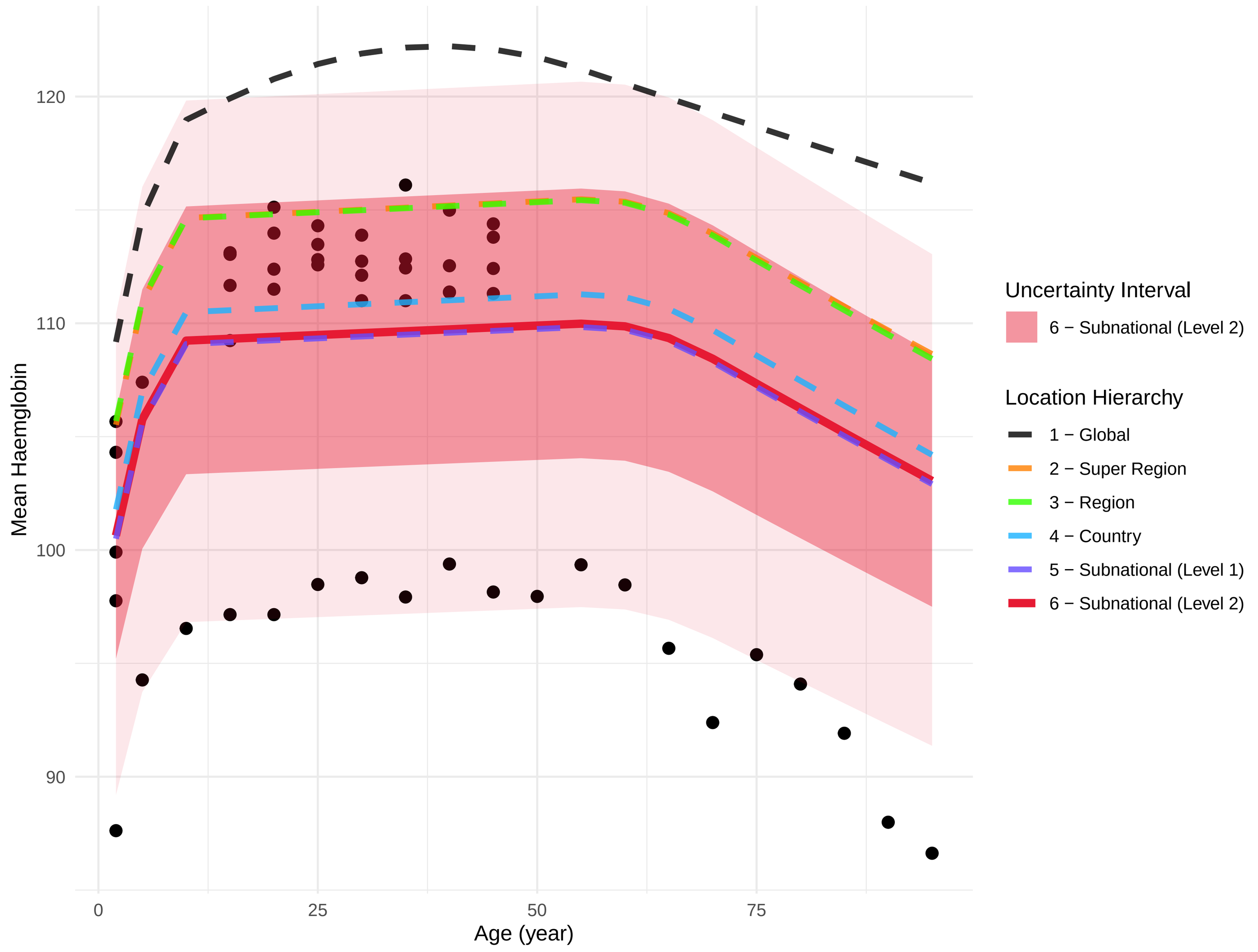

MR−BRT Age/Sex Fits − Non−pregnant Females 2+ Years Old − Mean Haemglobin − Andhra Pradesh, Urban
WHO−2001 Adjustment Method

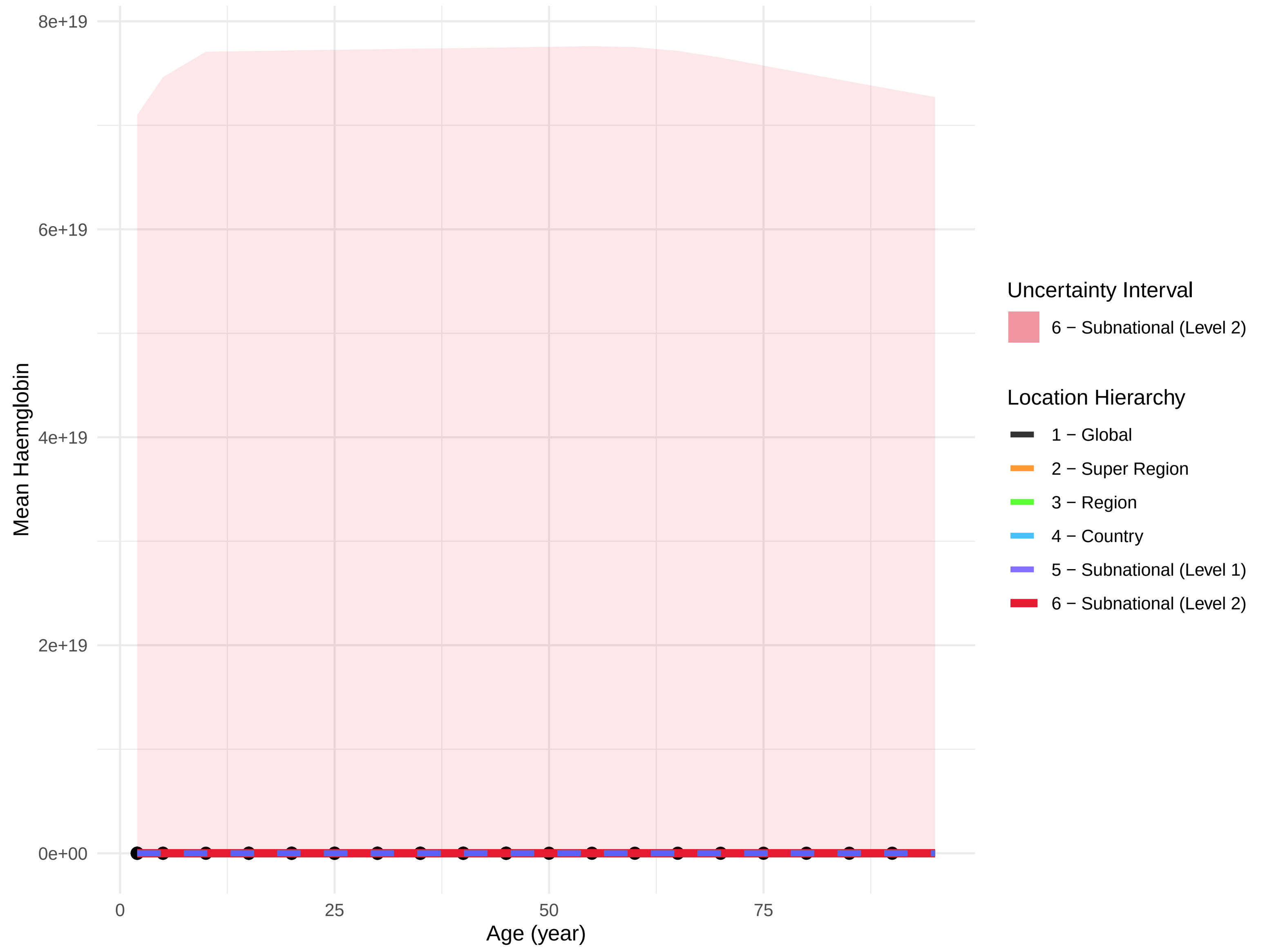

MR−BRT Age/Sex Fits − Non−pregnant Females 2+ Years Old − Mean Haemglobin − Arunachal Pradesh
WHO−2001 Adjustment Method

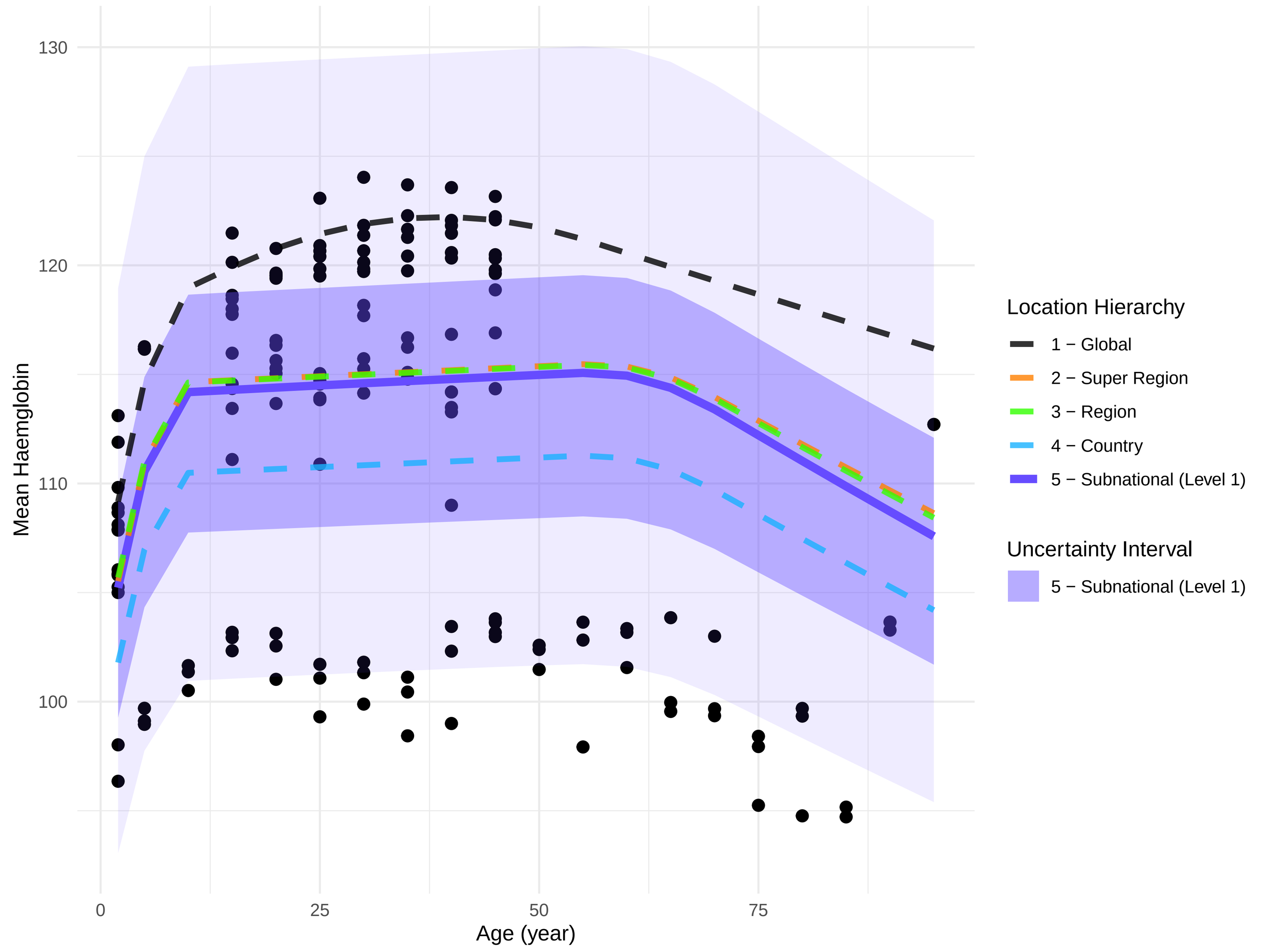

MR−BRT Age/Sex Fits − Non−pregnant Females 2+ Years Old − Mean Haemglobin − Arunachal Pradesh, Rural
WHO−2001 Adjustment Method

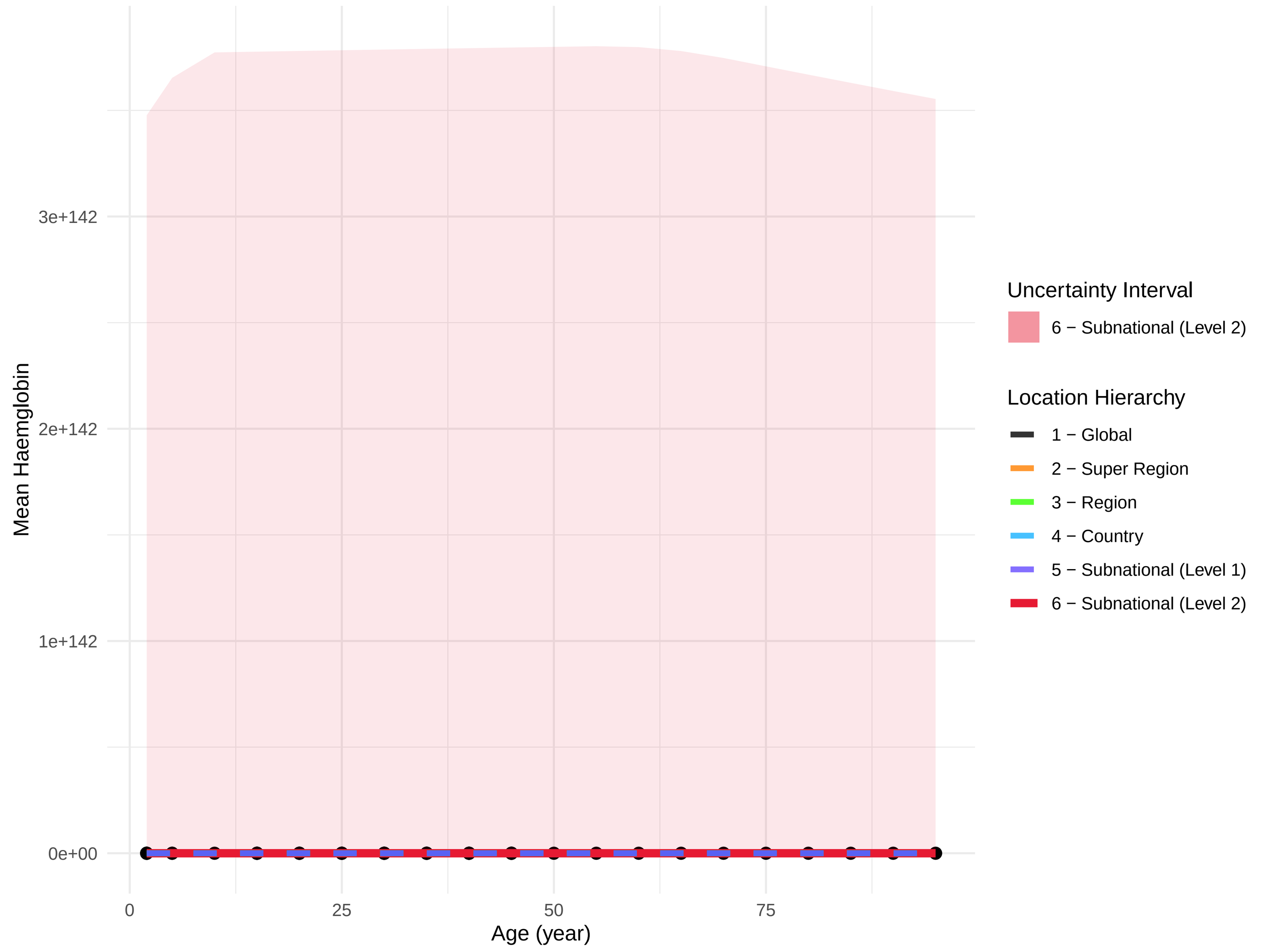

MR−BRT Age/Sex Fits − Non−pregnant Females 2+ Years Old − Mean Haemglobin − Arunachal Pradesh, Urban
WHO−2001 Adjustment Method

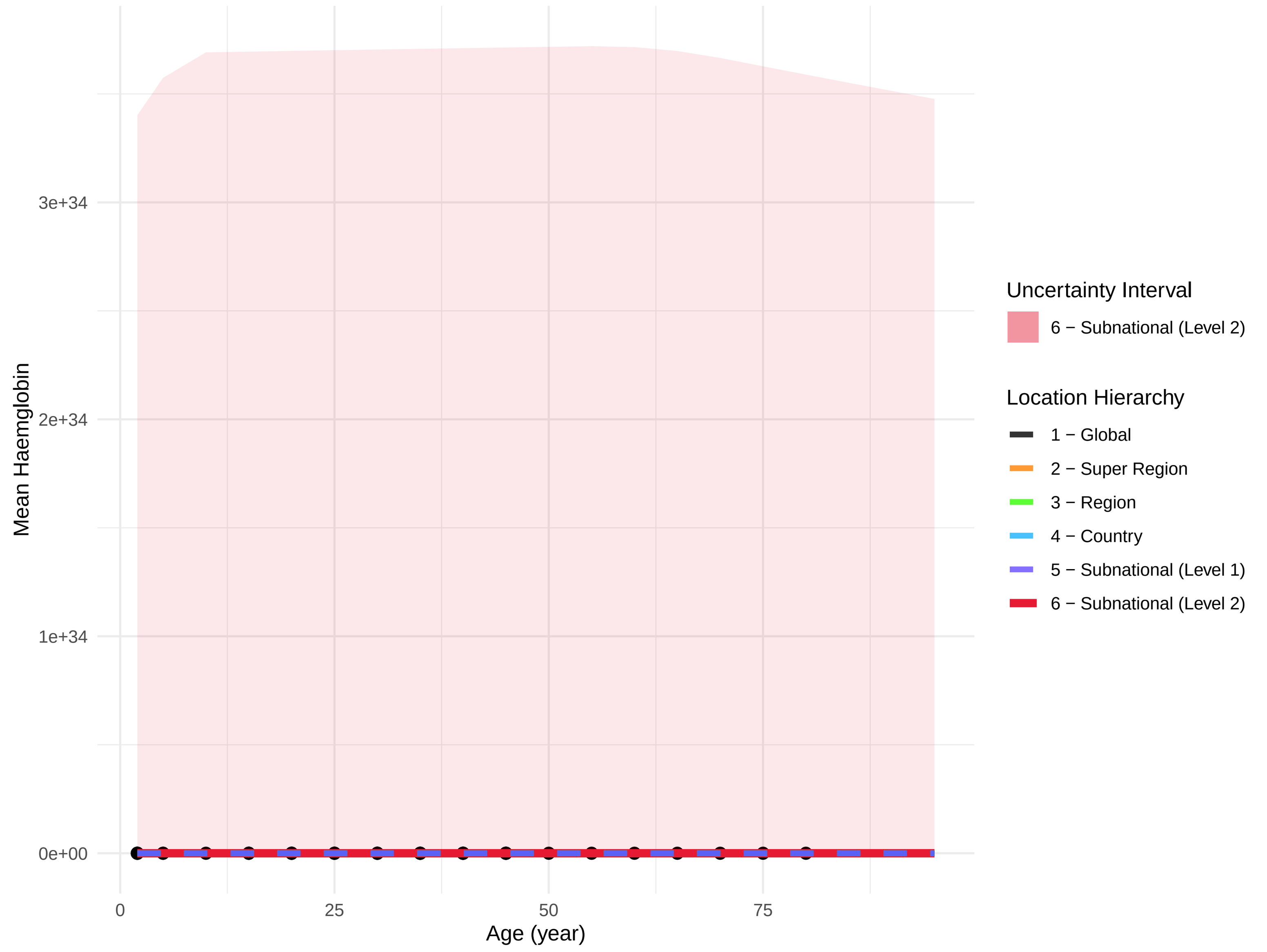

MR−BRT Age/Sex Fits − Non−pregnant Females 2+ Years Old − Mean Haemglobin − Assam
WHO−2001 Adjustment Method

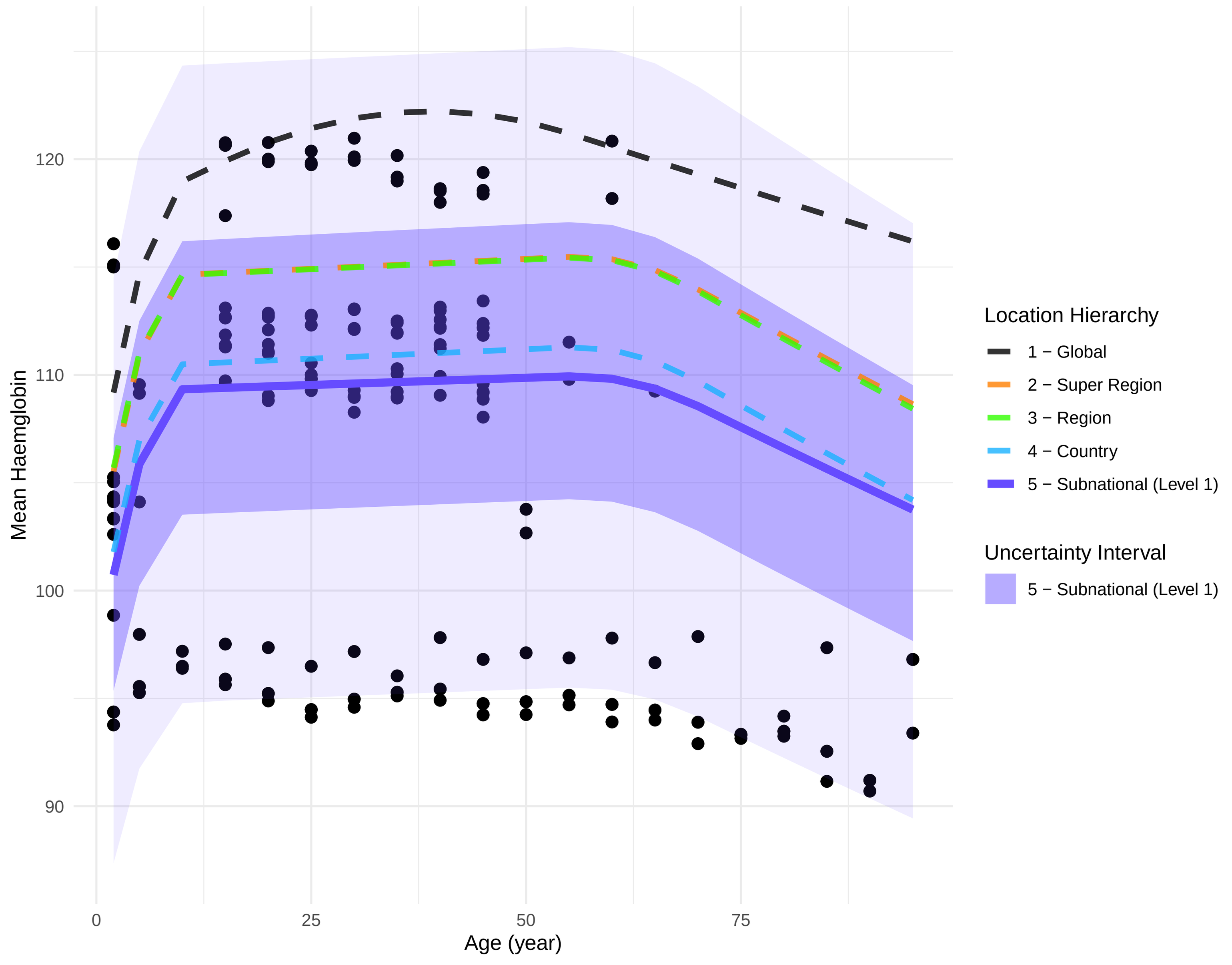

MR−BRT Age/Sex Fits − Non−pregnant Females 2+ Years Old − Mean Haemglobin − Assam, Rural
WHO−2001 Adjustment Method

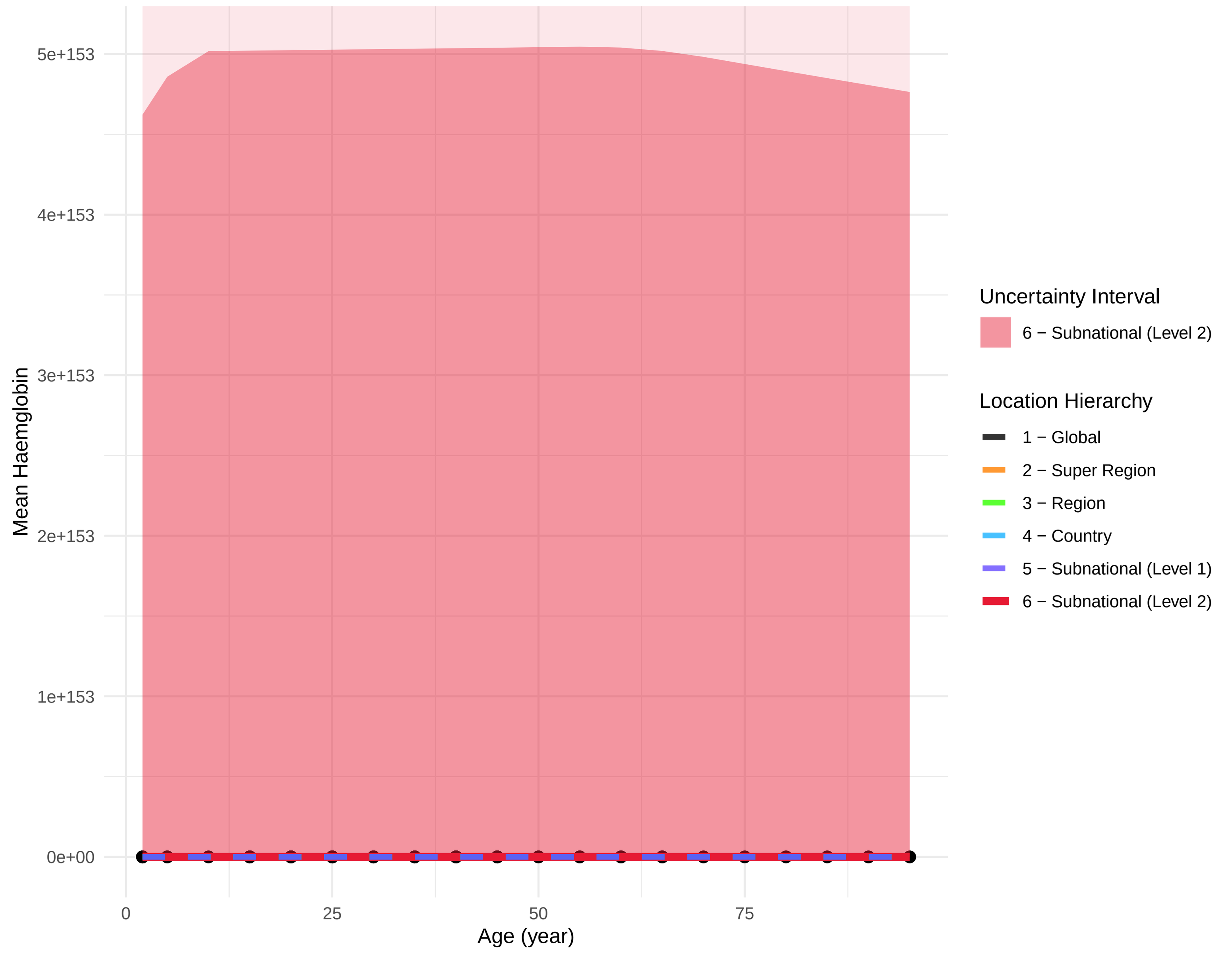

MR−BRT Age/Sex Fits − Non−pregnant Females 2+ Years Old − Mean Haemglobin − Assam, Urban
WHO−2001 Adjustment Method

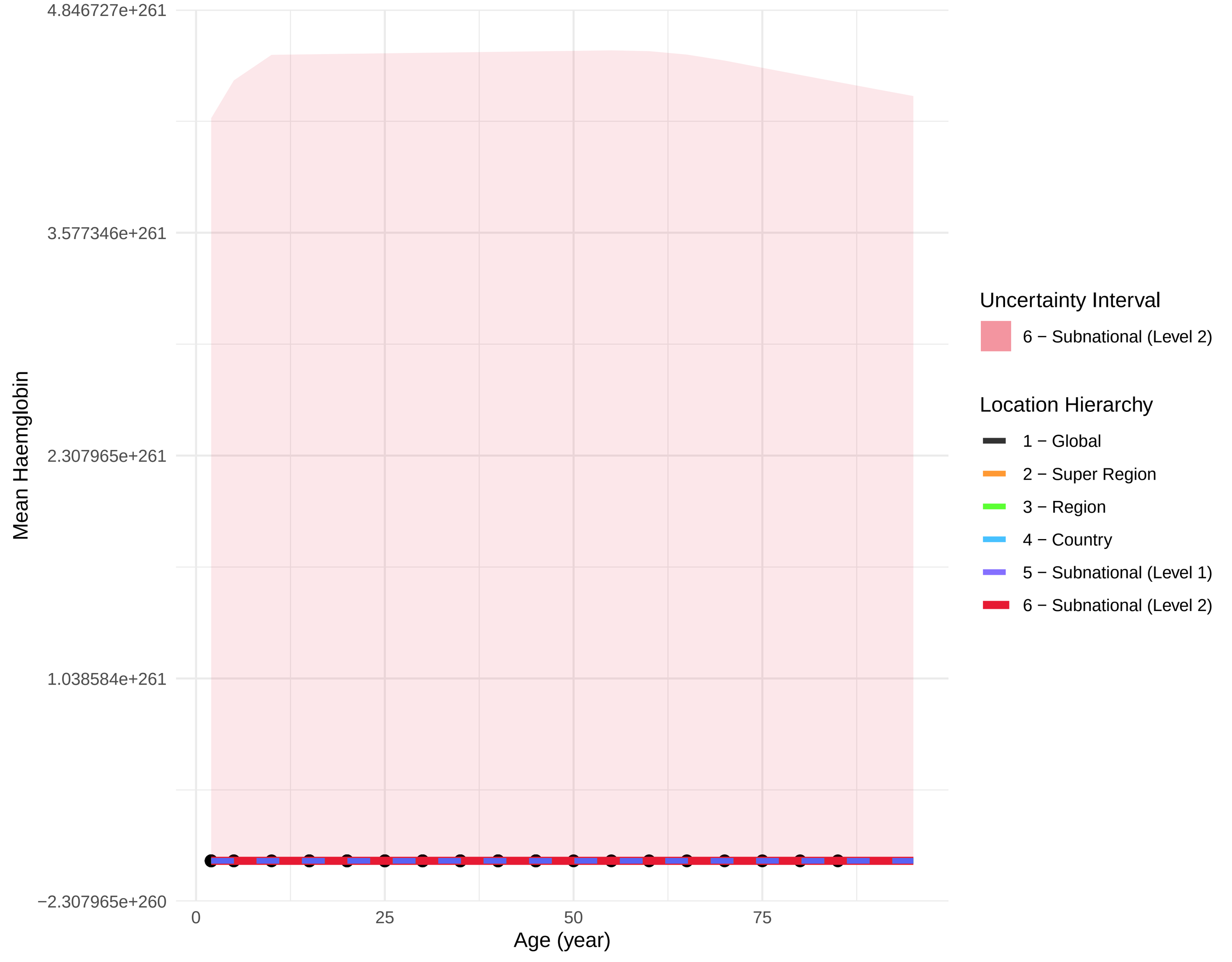

MR−BRT Age/Sex Fits − Non−pregnant Females 2+ Years Old − Mean Haemglobin − Bihar
WHO−2001 Adjustment Method

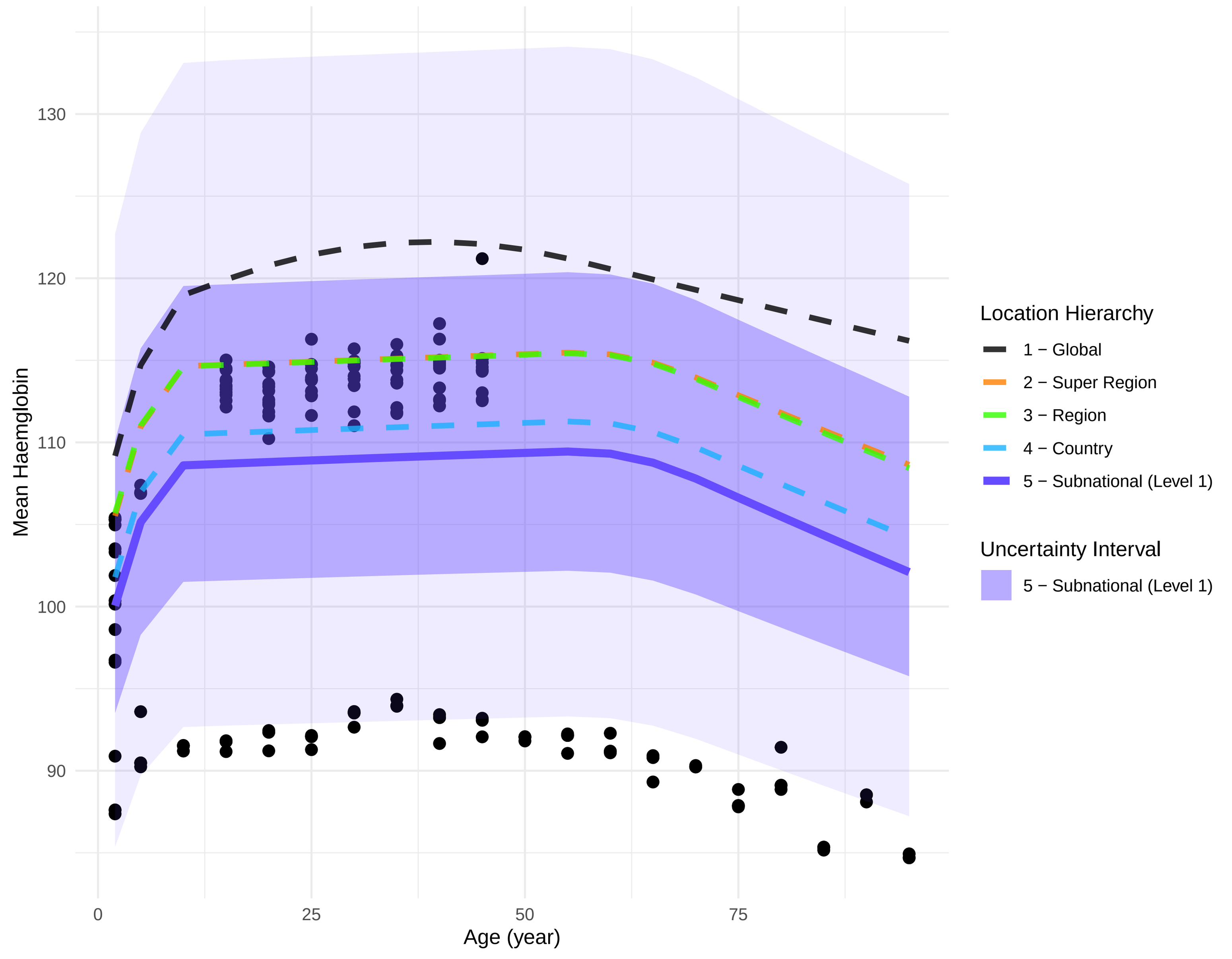

MR−BRT Age/Sex Fits − Non−pregnant Females 2+ Years Old − Mean Haemglobin − Bihar, Rural
WHO−2001 Adjustment Method

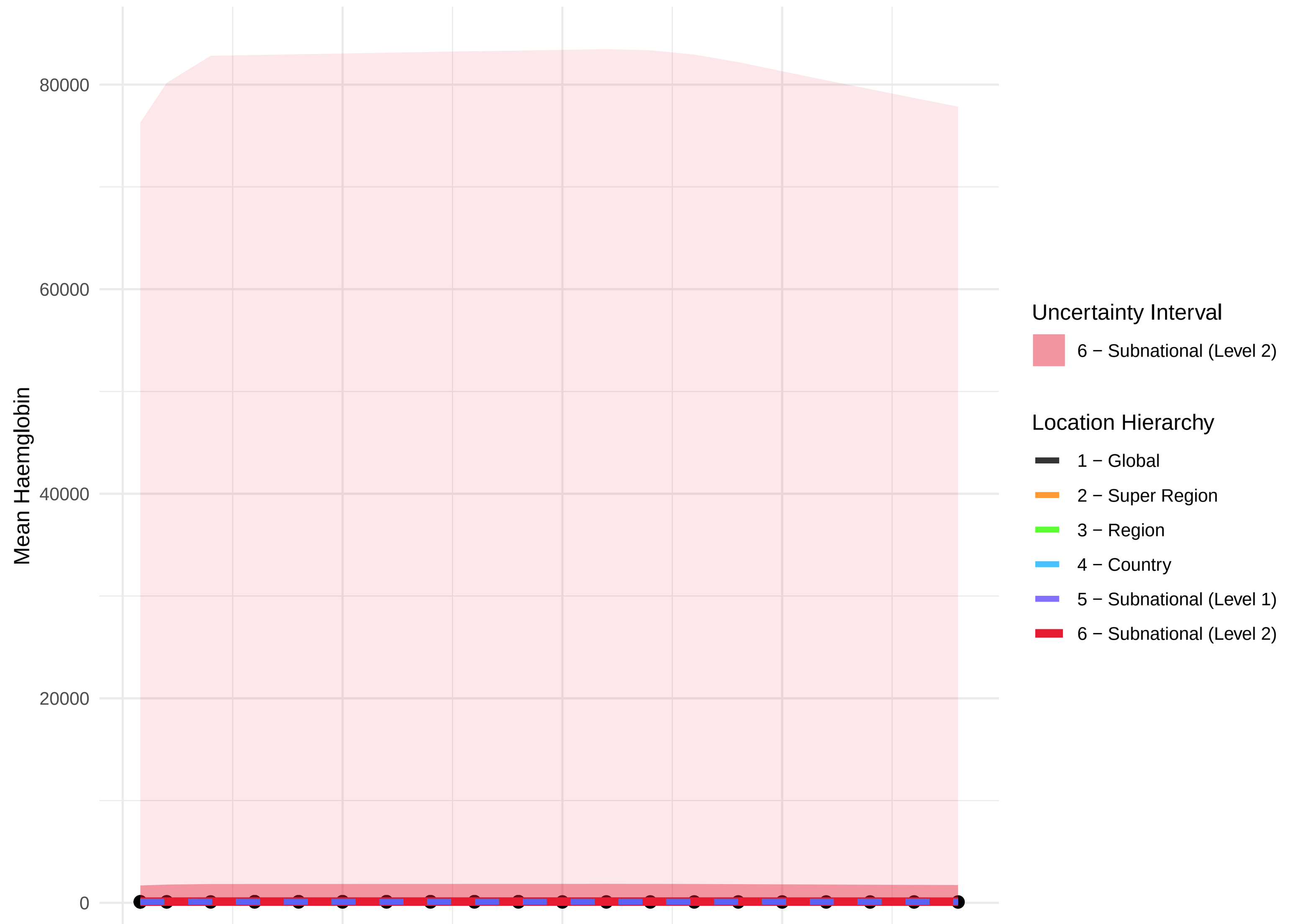

MR−BRT Age/Sex Fits − Non−pregnant Females 2+ Years Old − Mean Haemglobin − Bihar, Urban
WHO−2001 Adjustment Method

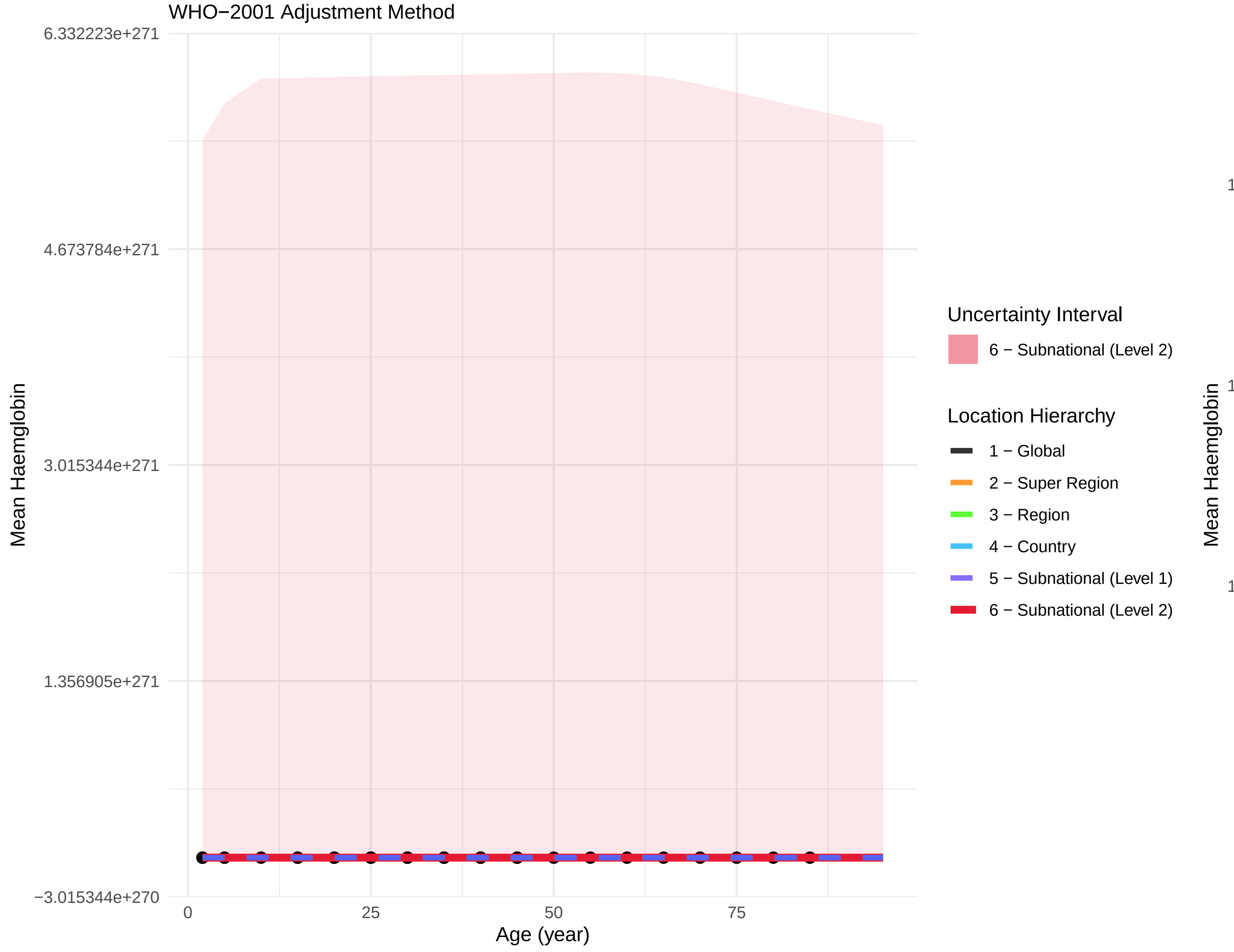

MR−BRT Age/Sex Fits − Non−pregnant Females 2+ Years Old − Mean Haemglobin − Chhattisgarh
WHO−2001 Adjustment Method

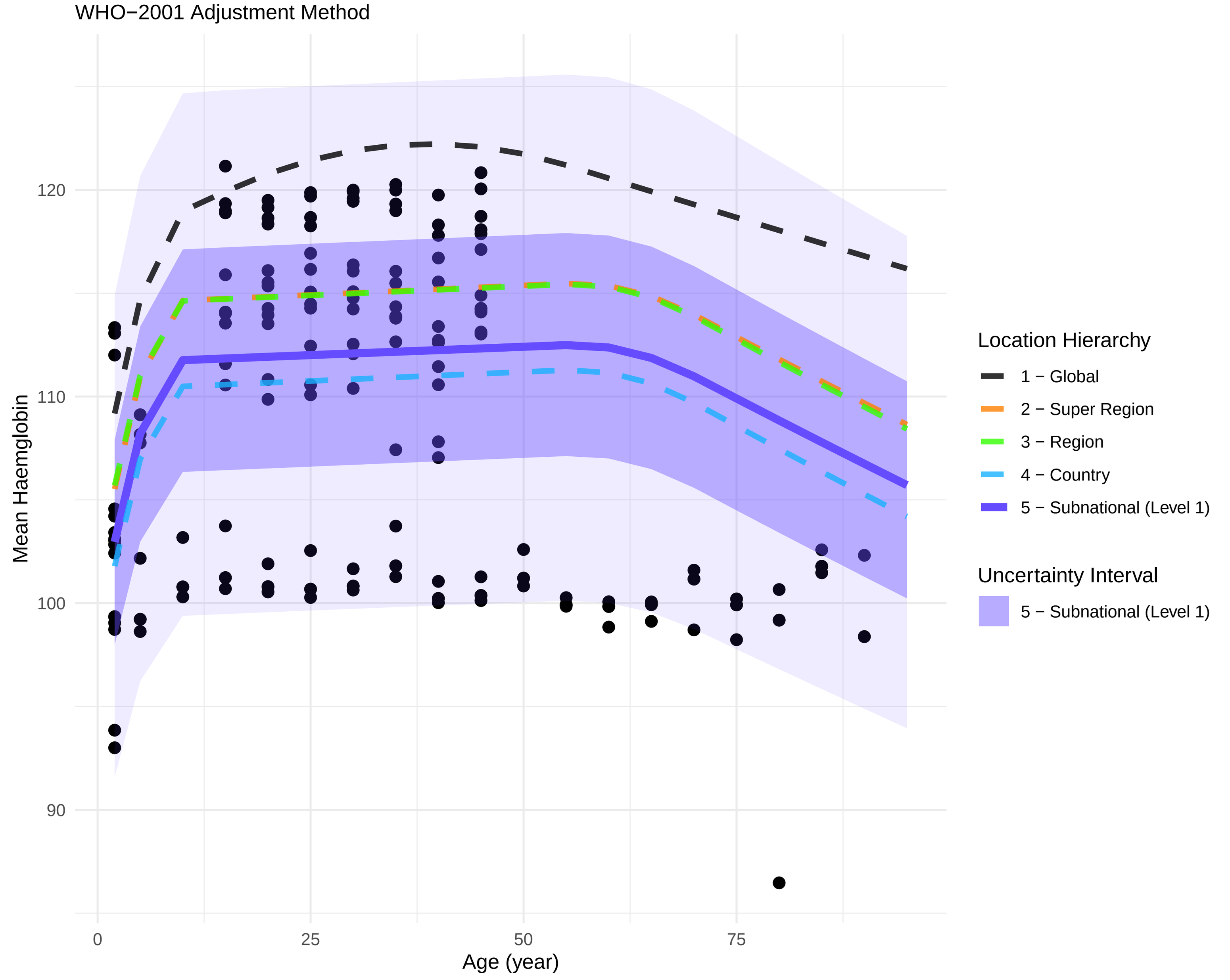

MR−BRT Age/Sex Fits − Non−pregnant Females 2+ Years Old − Mean Haemglobin − Chhattisgarh, Rural
WHO−2001 Adjustment Method

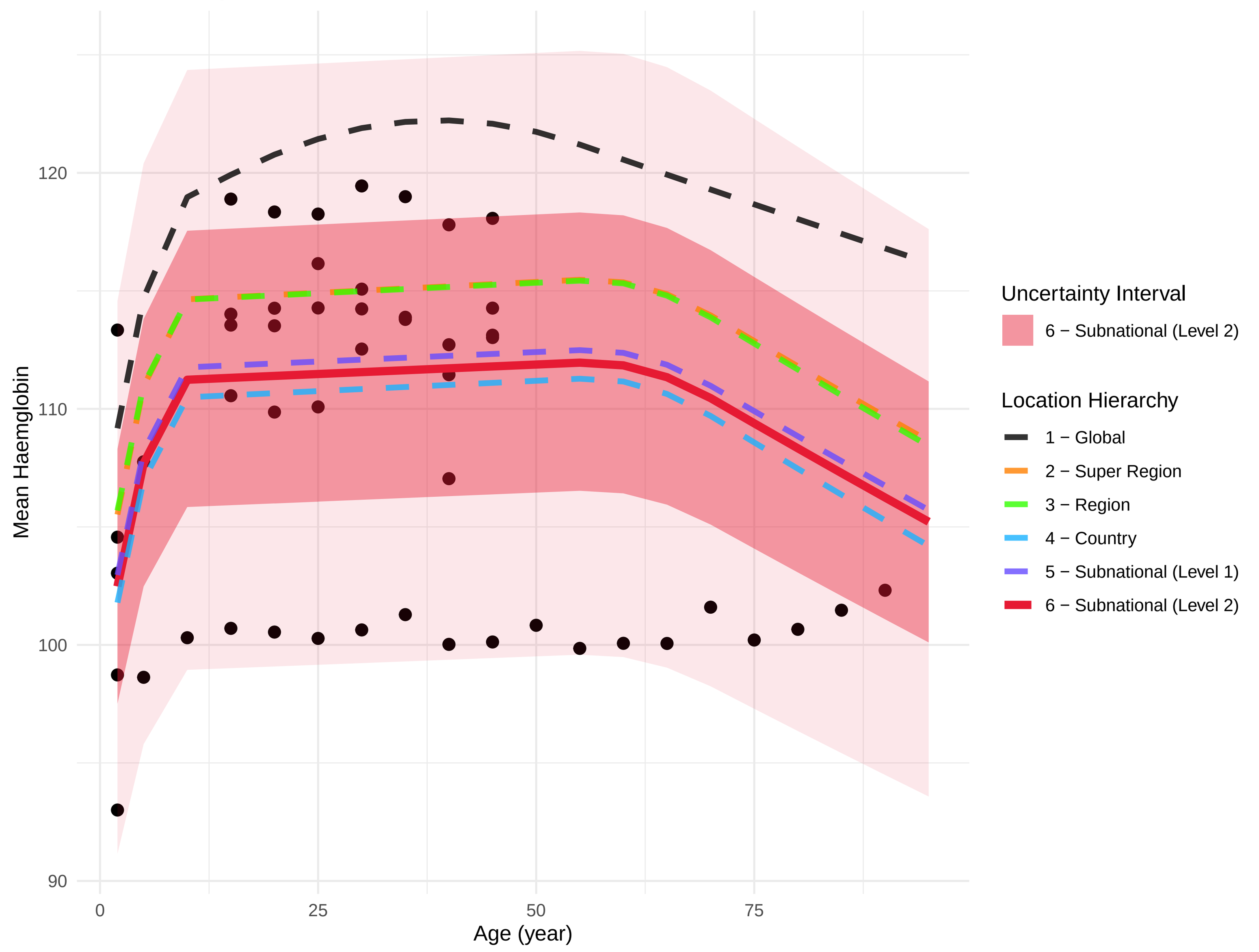

MR−BRT Age/Sex Fits − Non−pregnant Females 2+ Years Old − Mean Haemglobin − Chhattisgarh, Urban
WHO−2001 Adjustment Method

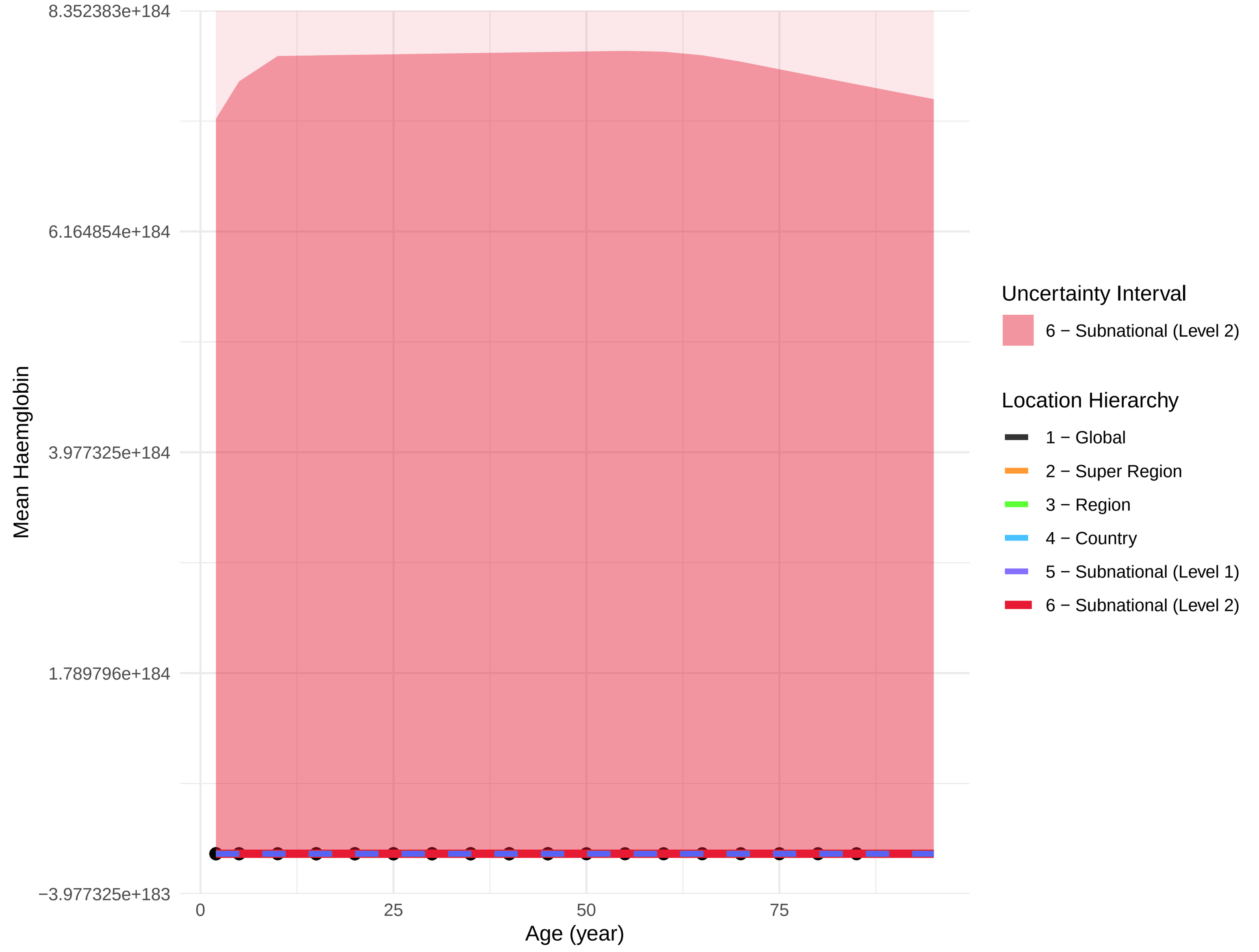

MR−BRT Age/Sex Fits − Non−pregnant Females 2+ Years Old − Mean Haemglobin − Delhi
WHO−2001 Adjustment Method

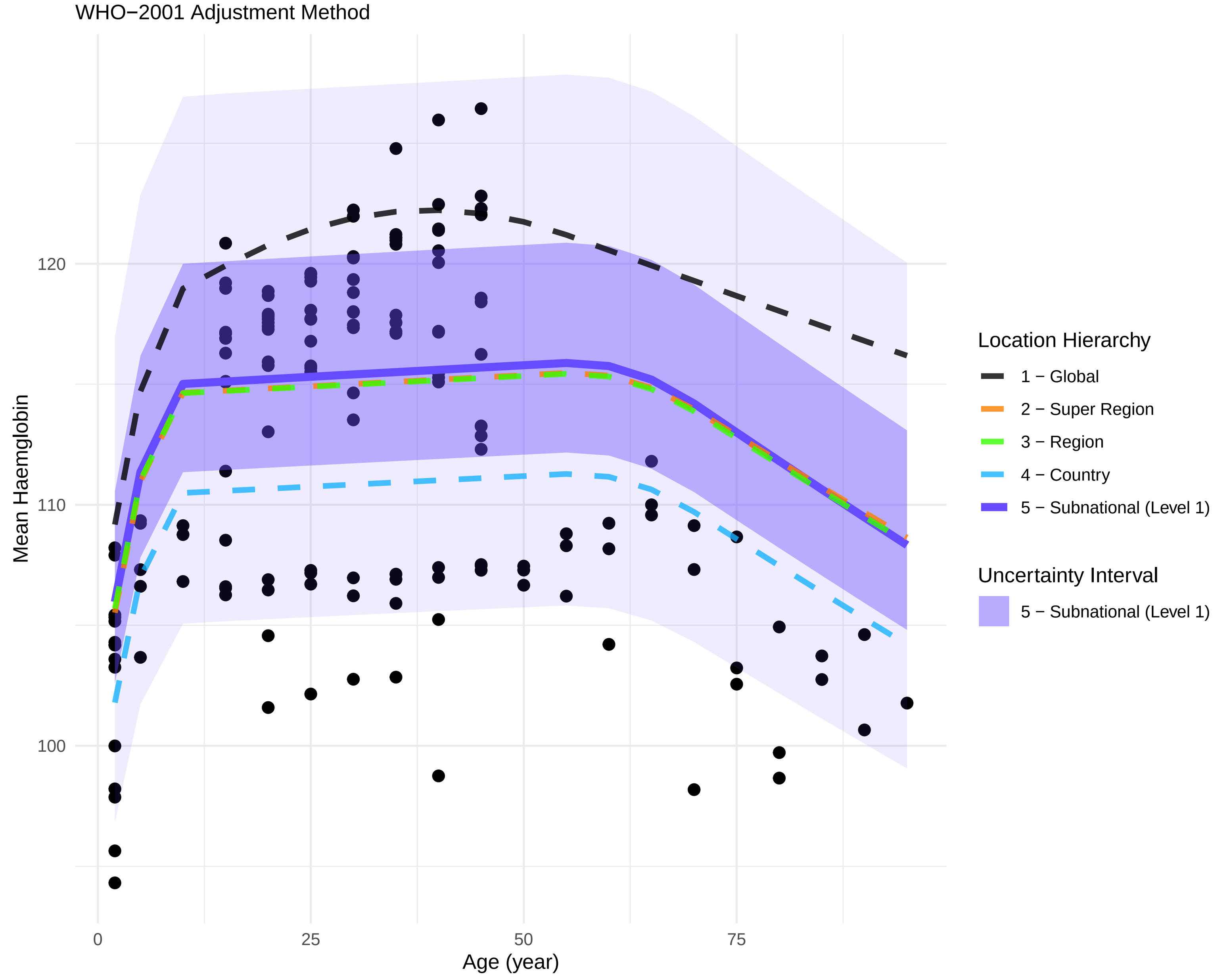

MR−BRT Age/Sex Fits − Non−pregnant Females 2+ Years Old − Mean Haemglobin − Delhi, Rural
WHO−2001 Adjustment Method

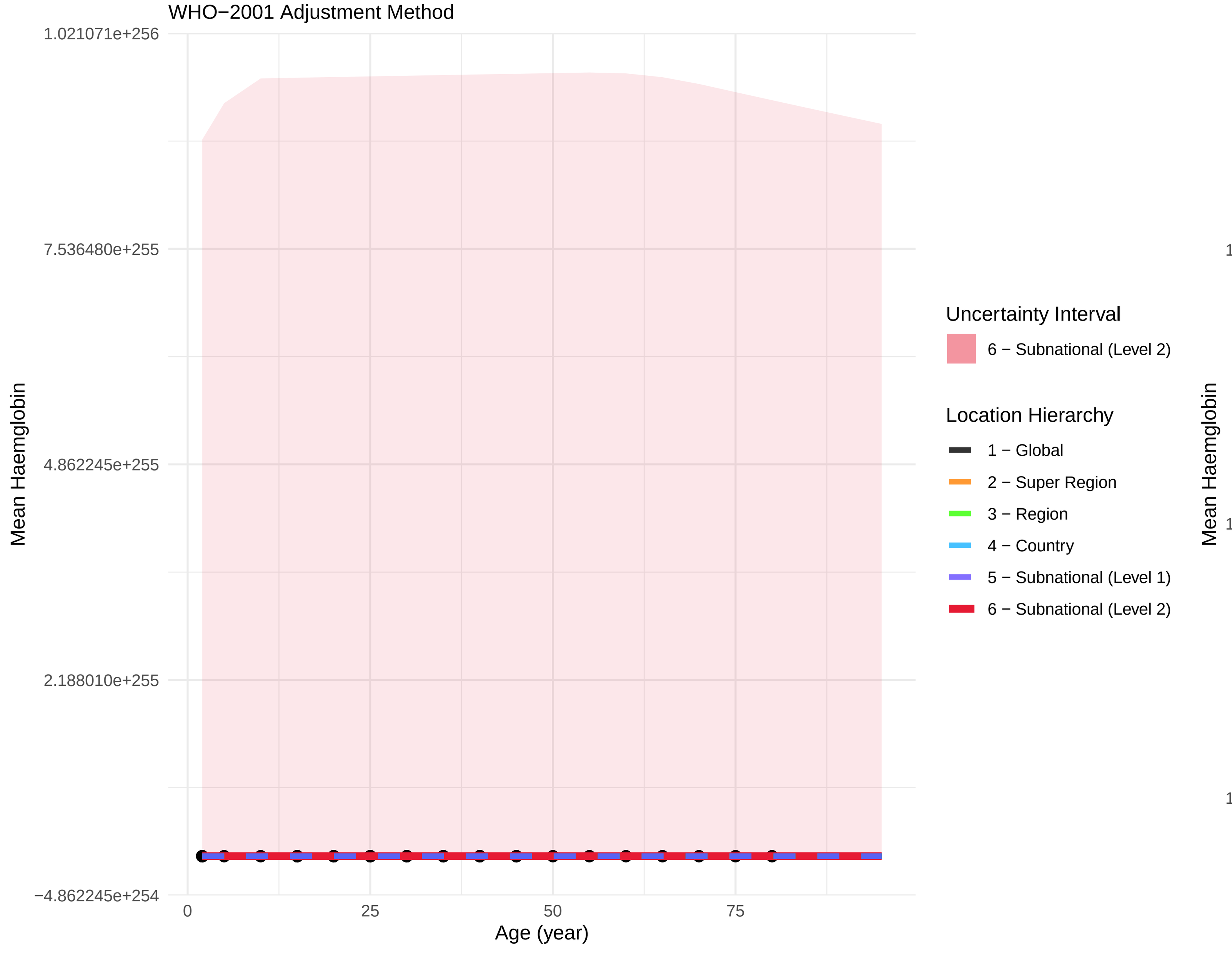

MR−BRT Age/Sex Fits − Non−pregnant Females 2+ Years Old − Mean Haemglobin − Delhi, Urban
WHO−2001 Adjustment Method

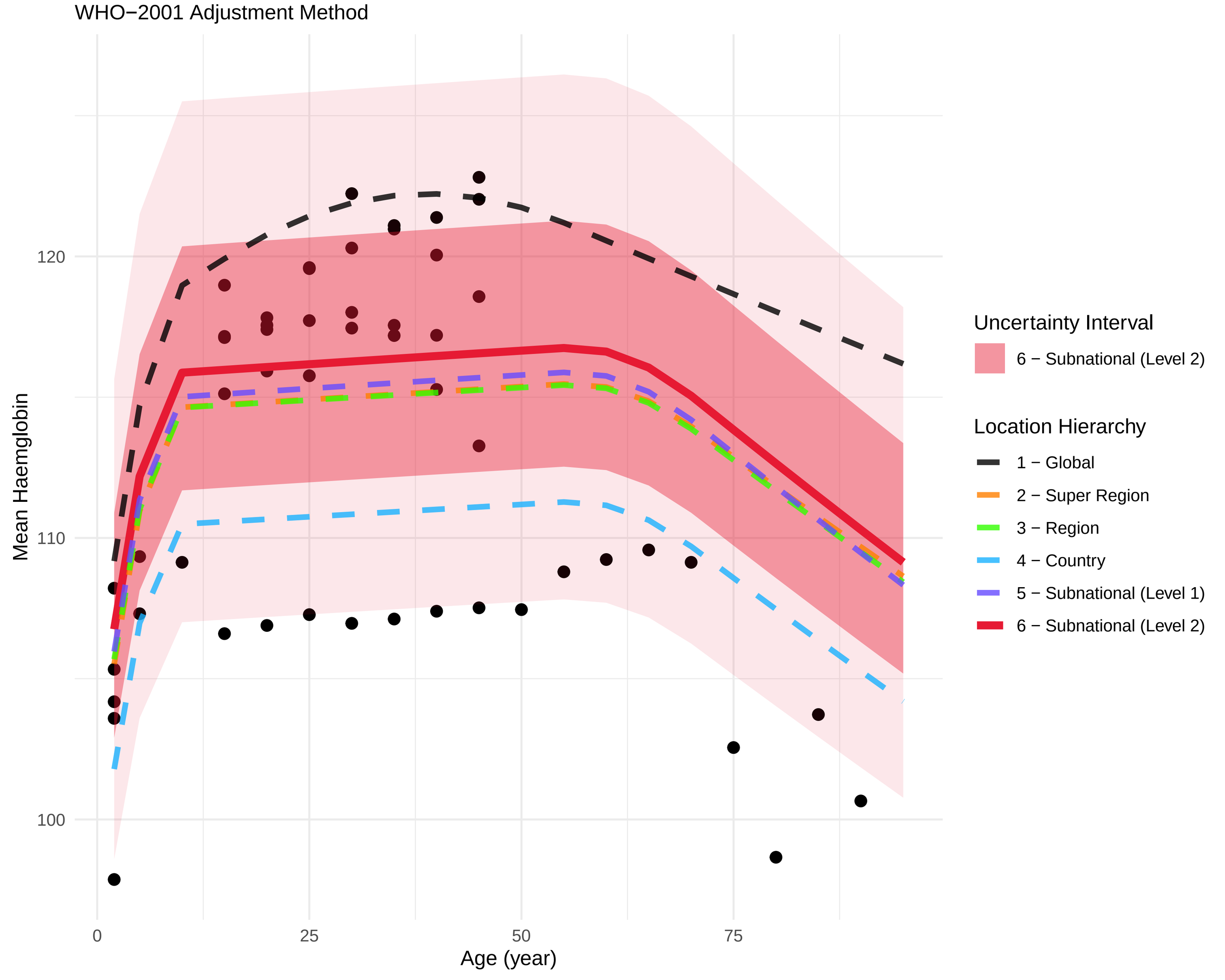

MR−BRT Age/Sex Fits − Non−pregnant Females 2+ Years Old − Mean Haemglobin − Goa
WHO−2001 Adjustment Method

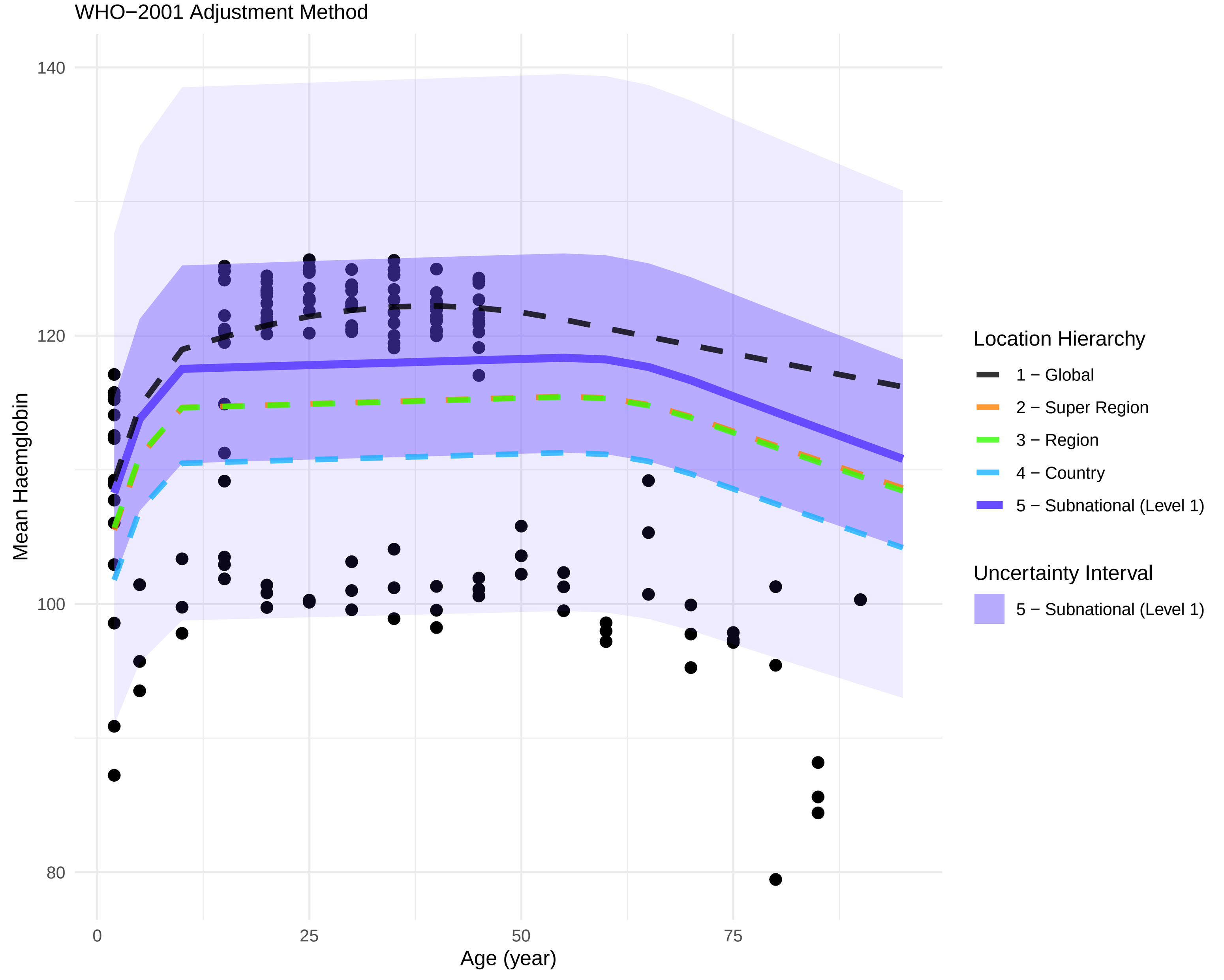

MR−BRT Age/Sex Fits − Non−pregnant Females 2+ Years Old − Mean Haemglobin − Goa, Rural
WHO−2001 Adjustment Method

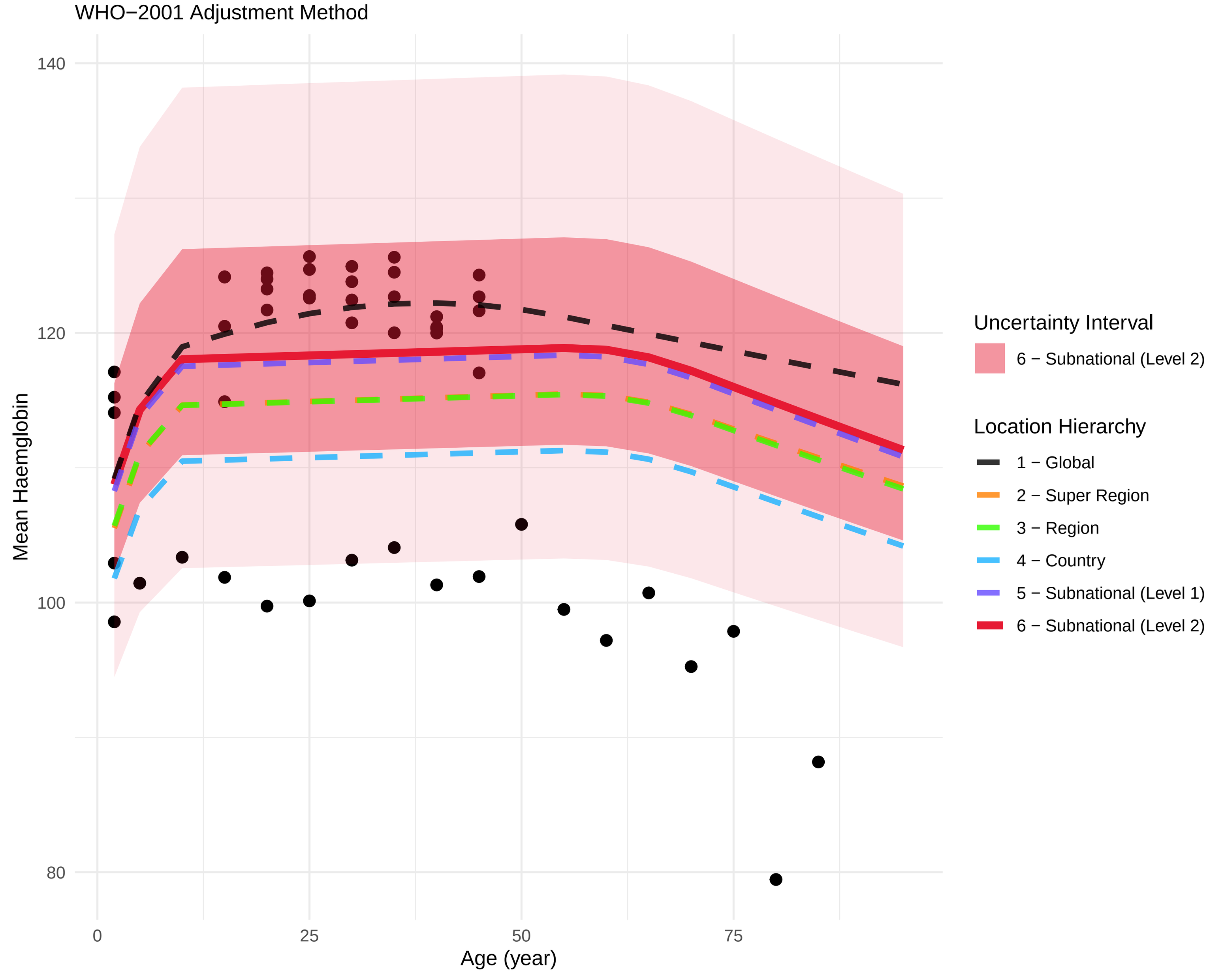

MR−BRT Age/Sex Fits − Non−pregnant Females 2+ Years Old − Mean Haemglobin − Goa, Urban
WHO−2001 Adjustment Method

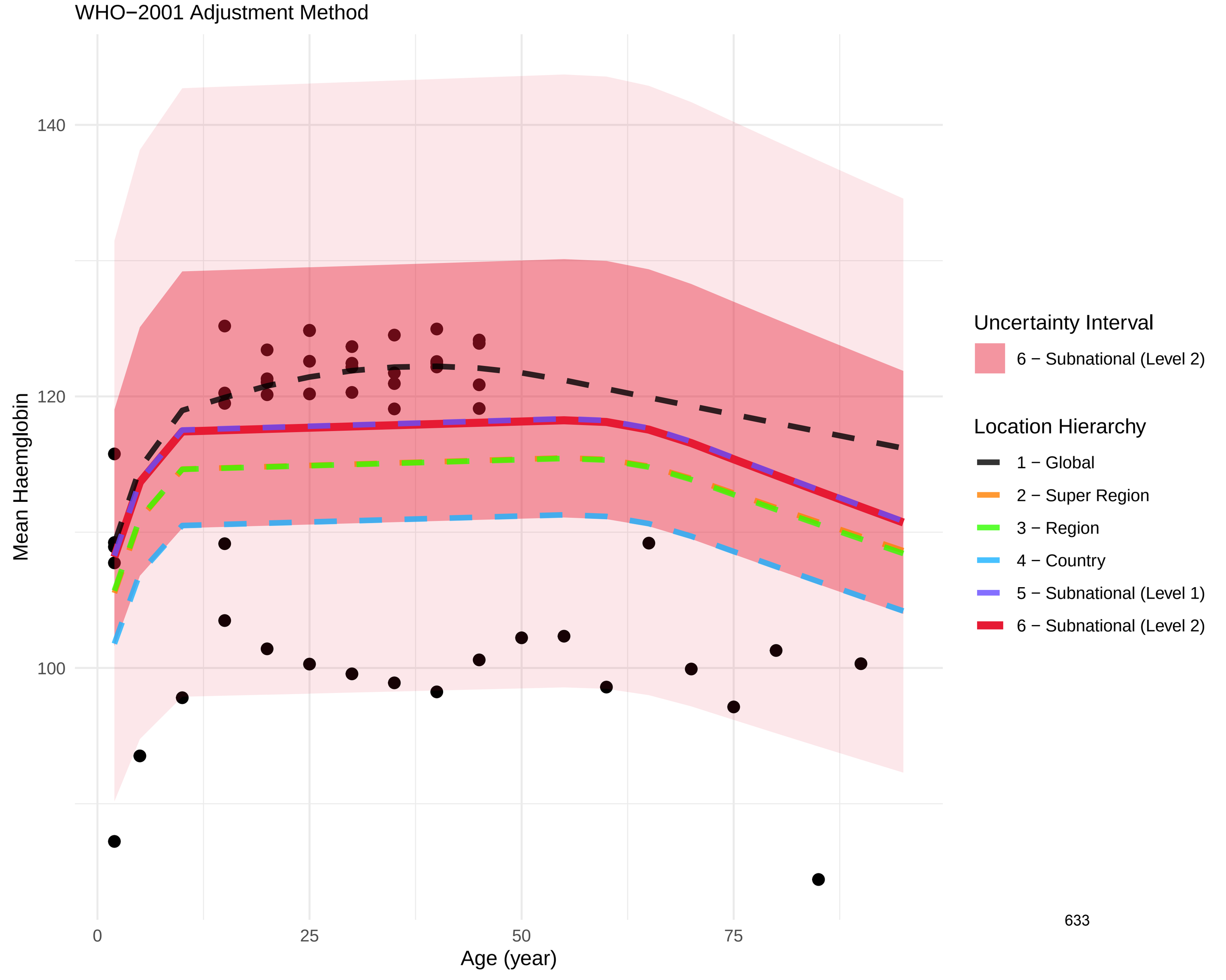

MR−BRT Age/Sex Fits − Non−pregnant Females 2+ Years Old − Mean Haemglobin − Gujarat
WHO−2001 Adjustment Method

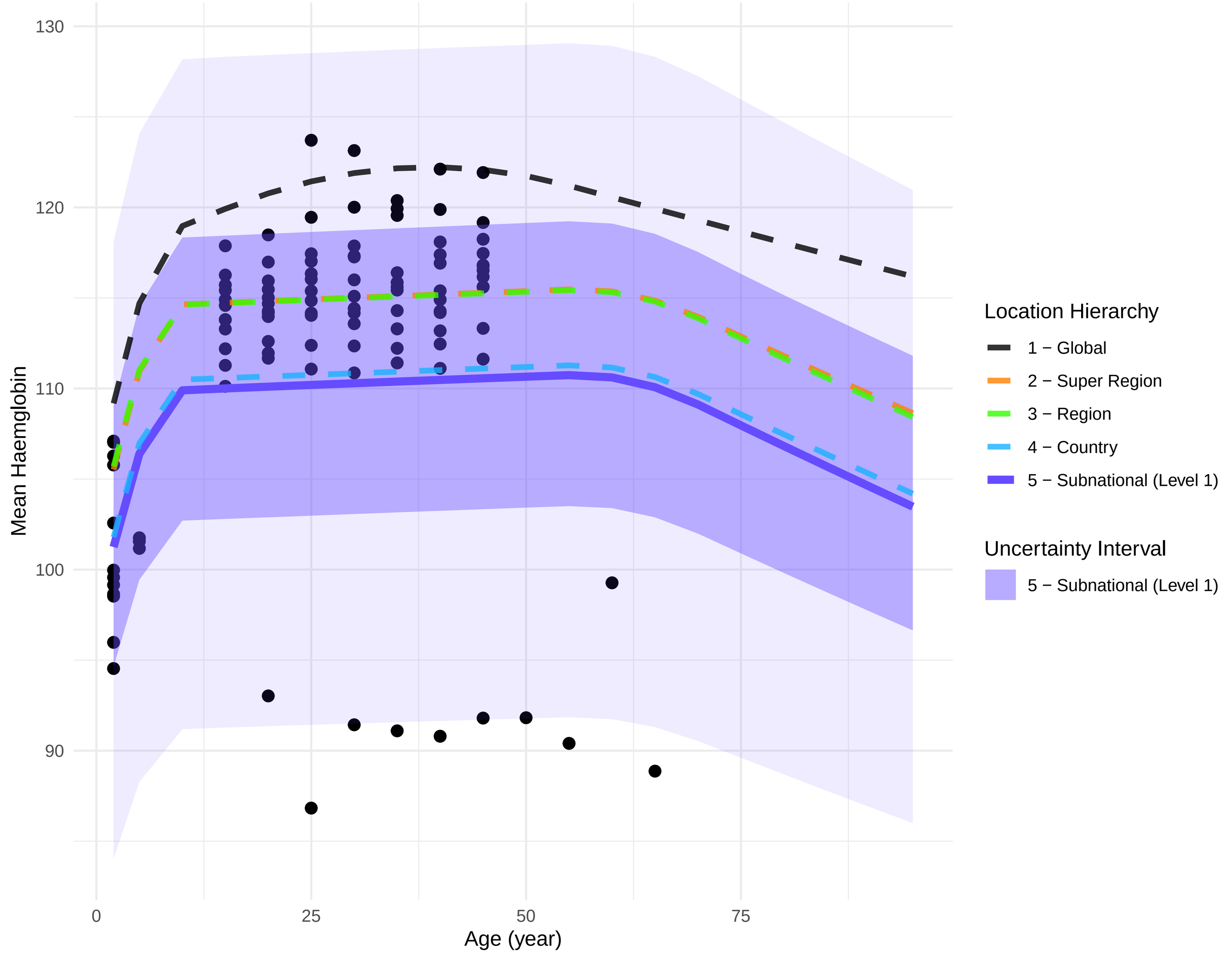

MR−BRT Age/Sex Fits − Non−pregnant Females 2+ Years Old − Mean Haemglobin − Gujarat, Rural
WHO−2001 Adjustment Method

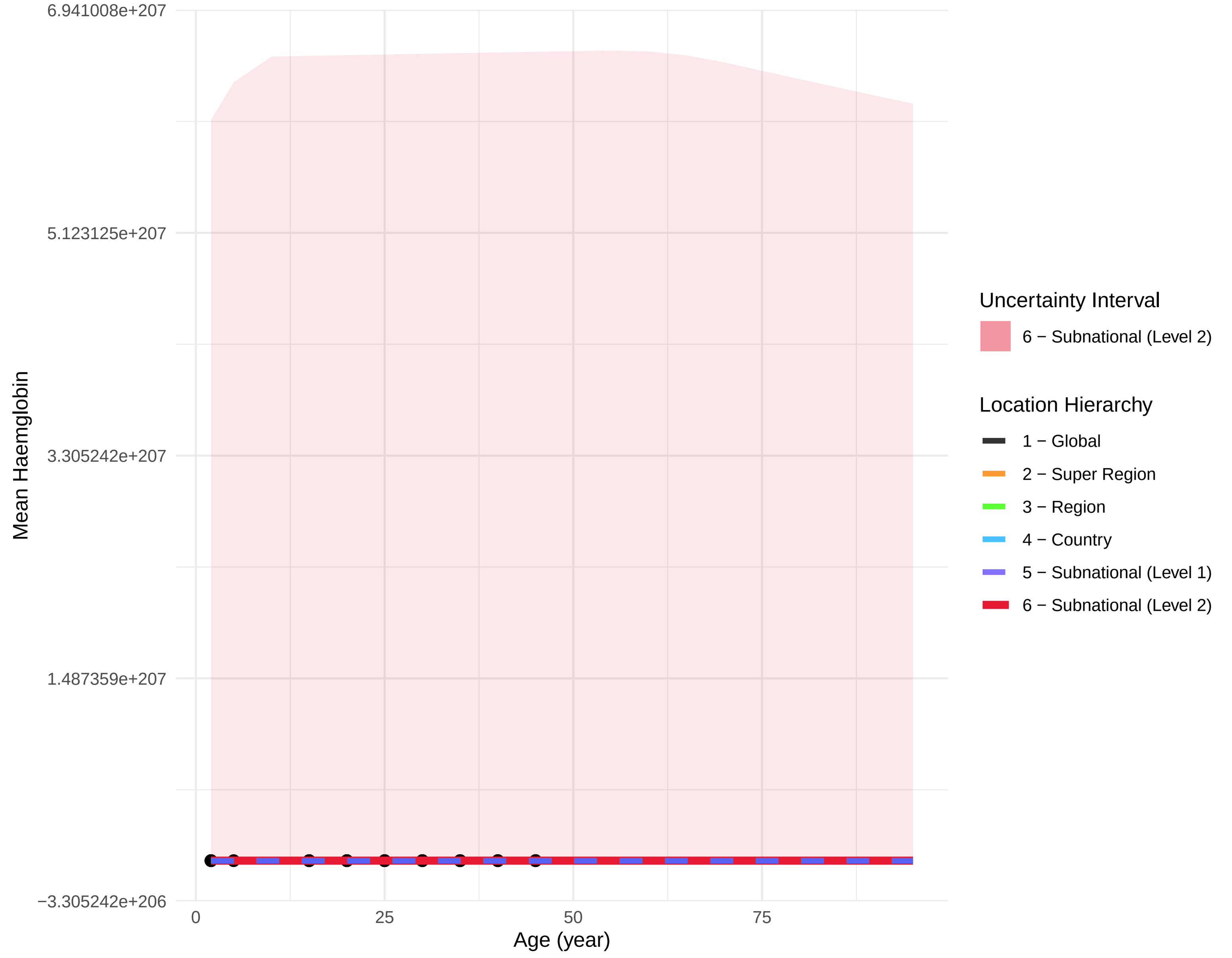

MR−BRT Age/Sex Fits − Non−pregnant Females 2+ Years Old − Mean Haemglobin − Gujarat, Urban
WHO−2001 Adjustment Method

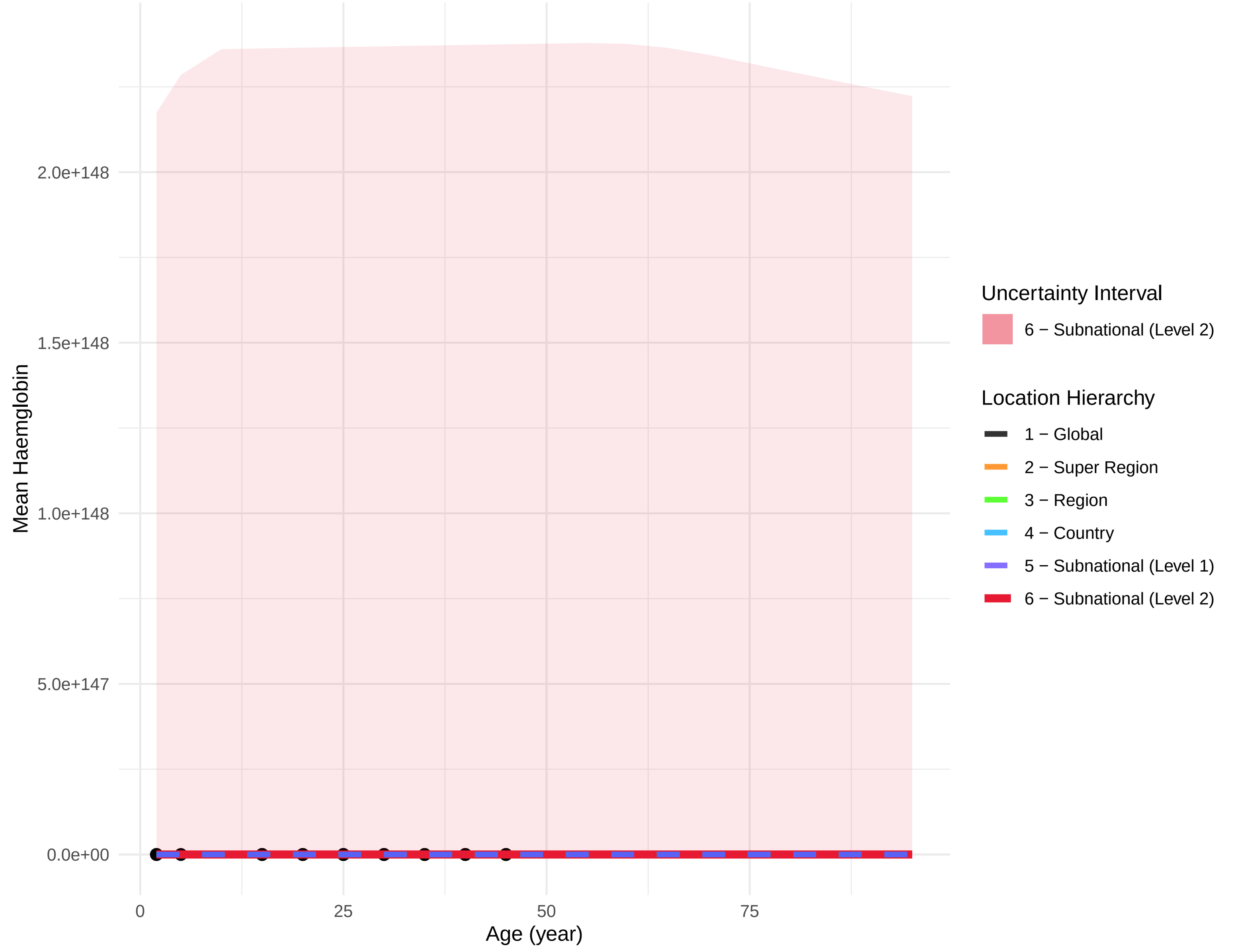

MR−BRT Age/Sex Fits − Non−pregnant Females 2+ Years Old − Mean Haemglobin − Haryana
WHO−2001 Adjustment Method

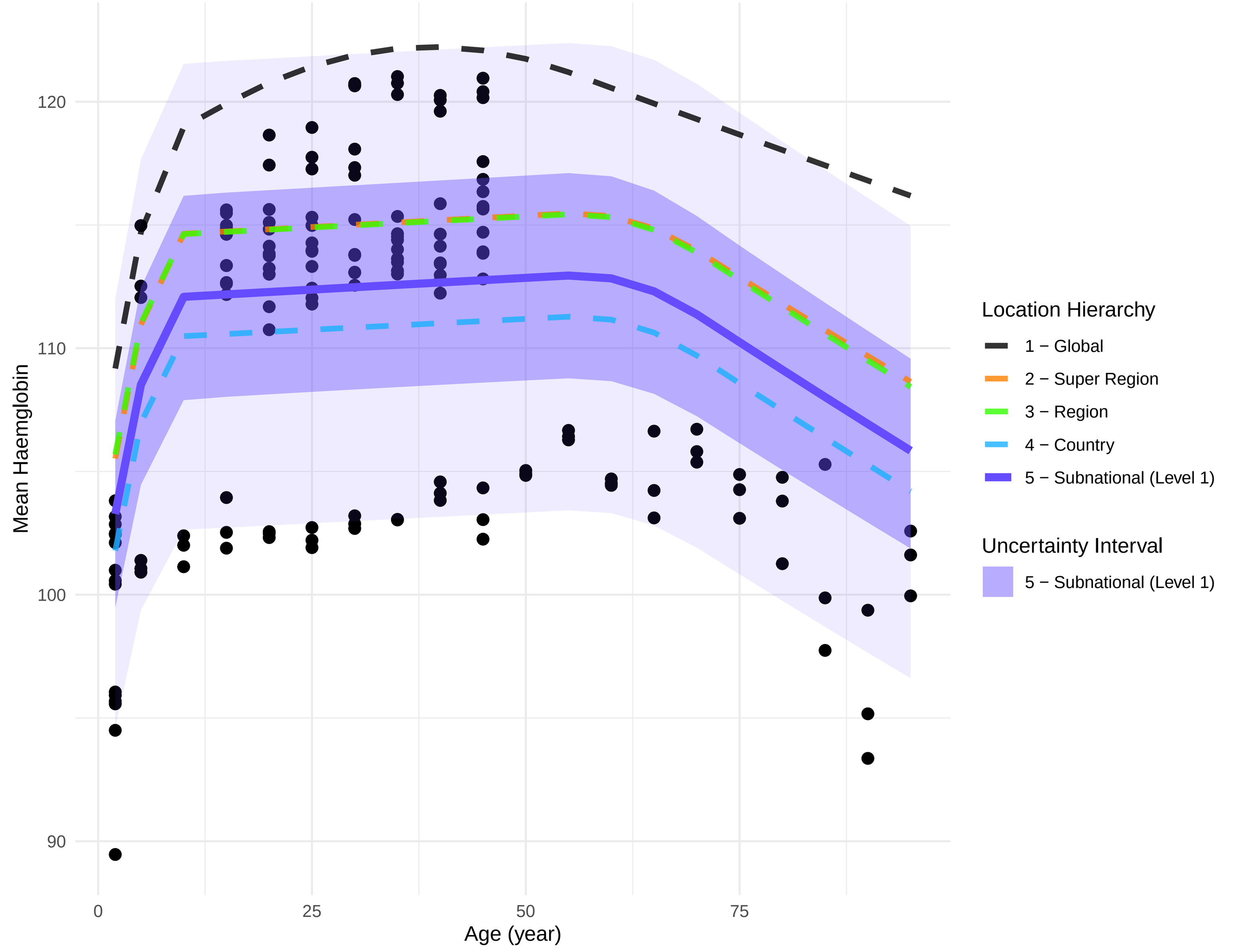

MR−BRT Age/Sex Fits − Non−pregnant Females 2+ Years Old − Mean Haemglobin − Haryana,
WHO−2001 Adjustment Method

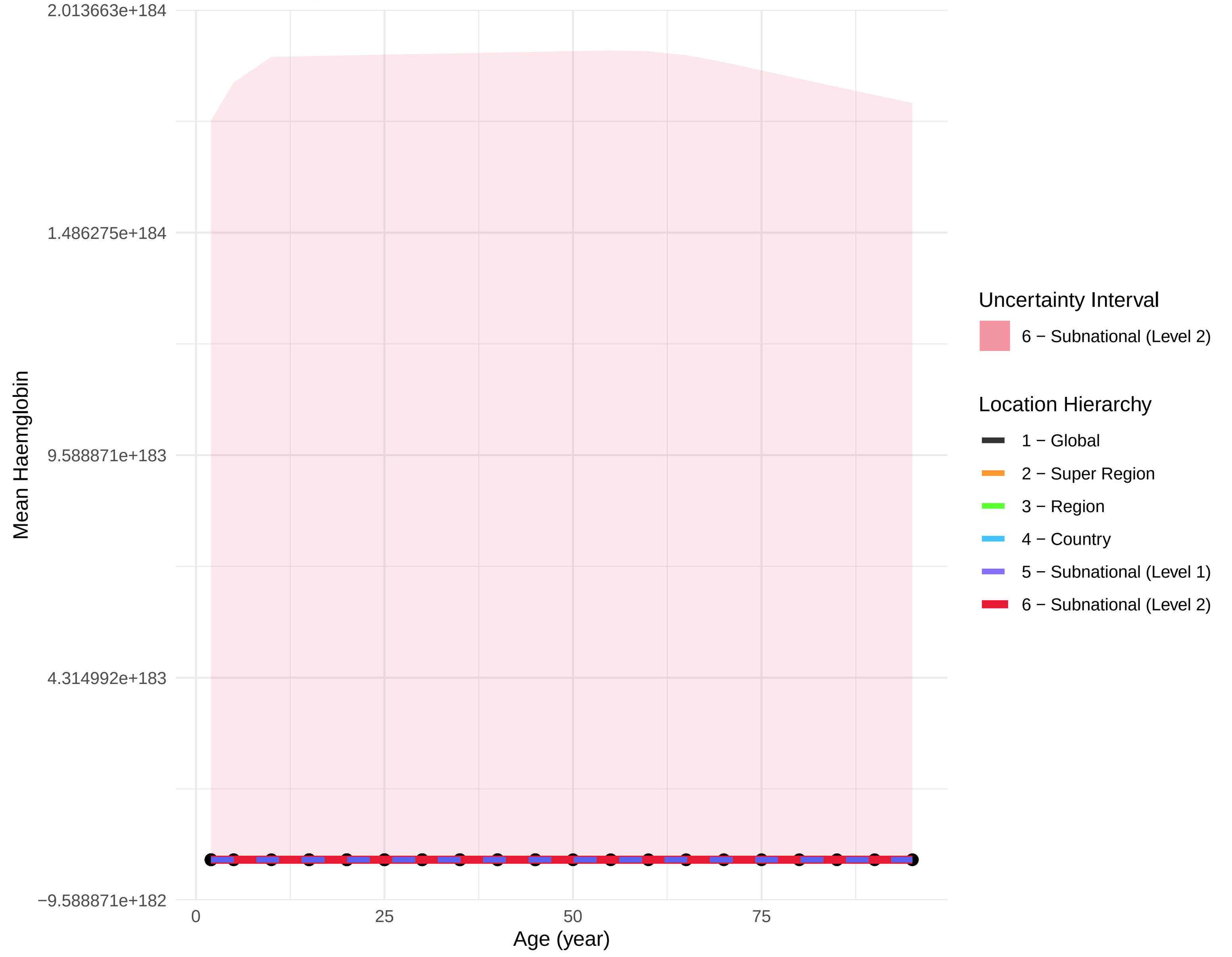

MR−BRT Age/Sex Fits − Non−pregnant Females 2+ Years Old − Mean Haemglobin − Haryana, Urban
WHO−2001 Adjustment Method

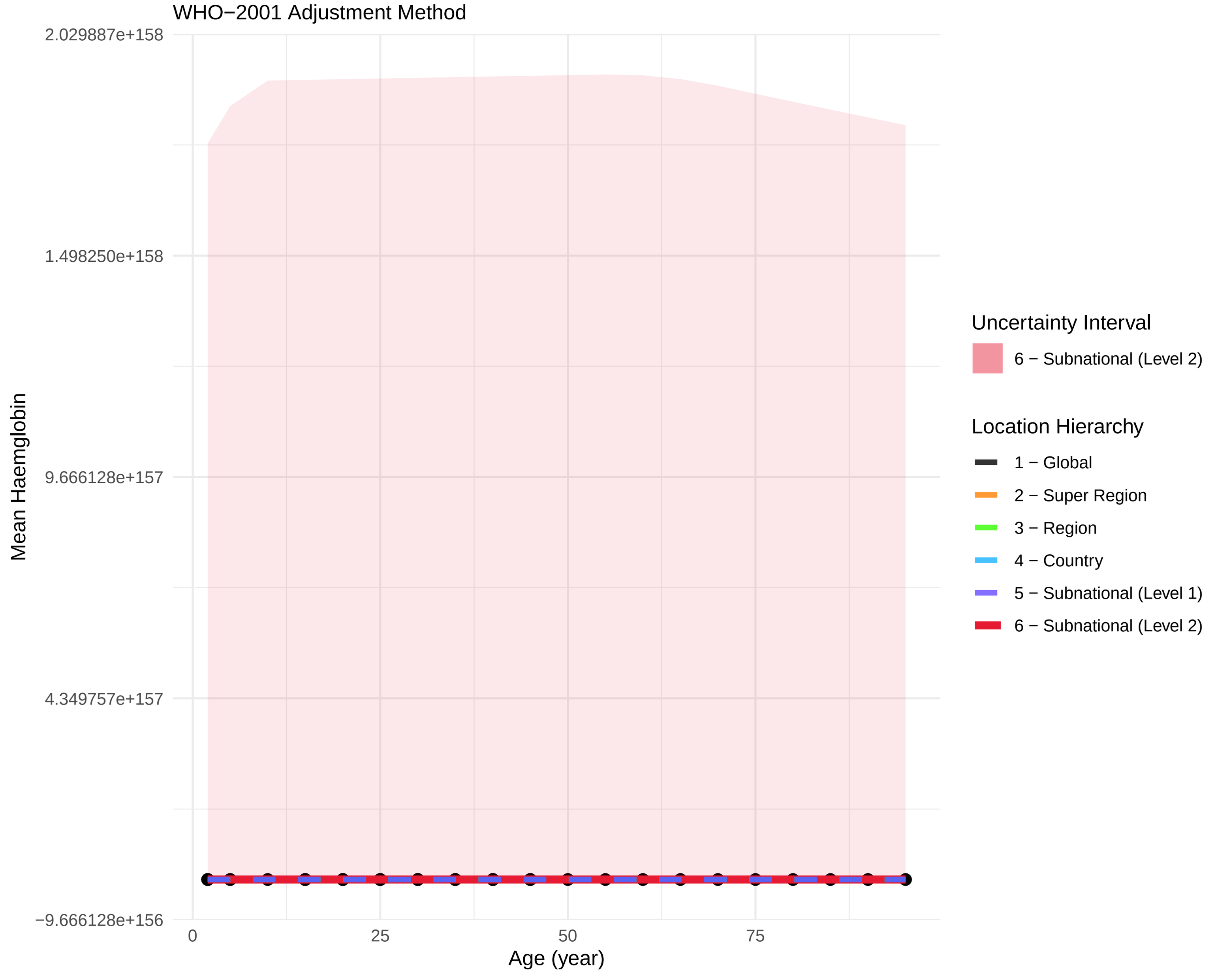

MR−BRT Age/Sex Fits − Non−pregnant Females 2+ Years Old − Mean Haemglobin − Himachal Pradesh
WHO−2001 Adjustment Method

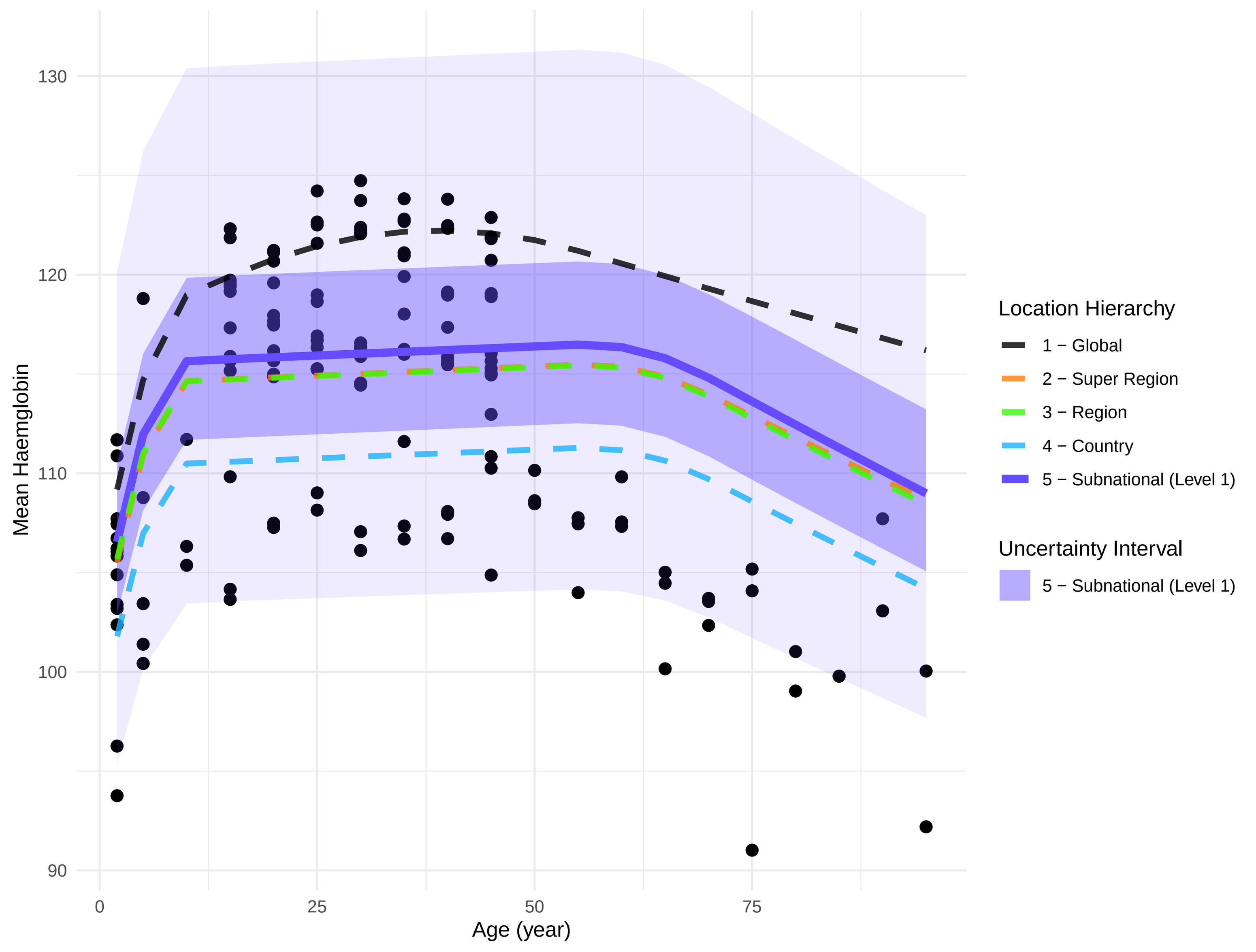

MR−BRT Age/Sex Fits − Non−pregnant Females 2+ Years Old − Mean Haemglobin − Himachal Pradesh, Rural
WHO−2001 Adjustment Method

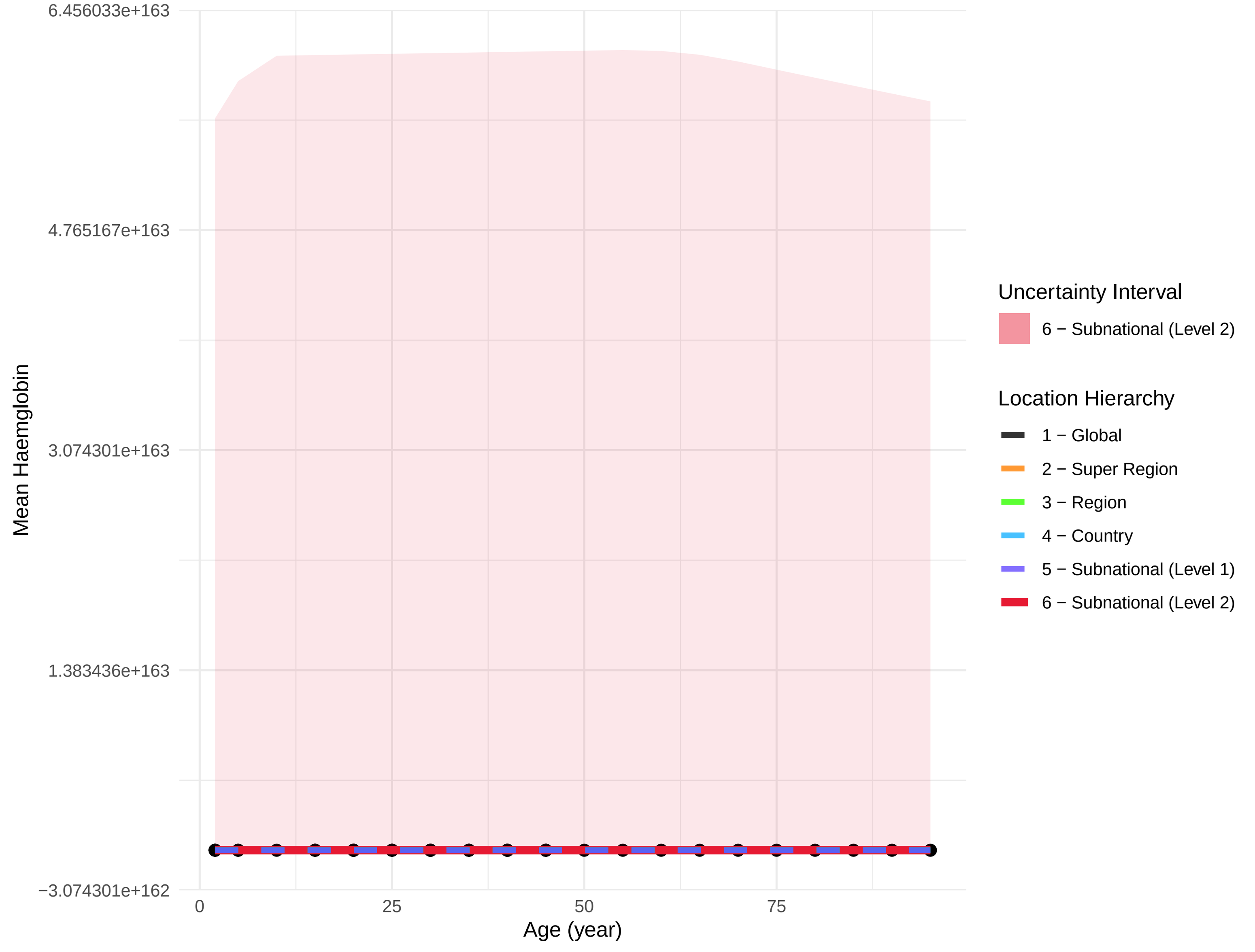

MR−BRT Age/Sex Fits − Non−pregnant Females 2+ Years Old − Mean Haemglobin − Himachal Pradesh, Urban
WHO−2001 Adjustment Method

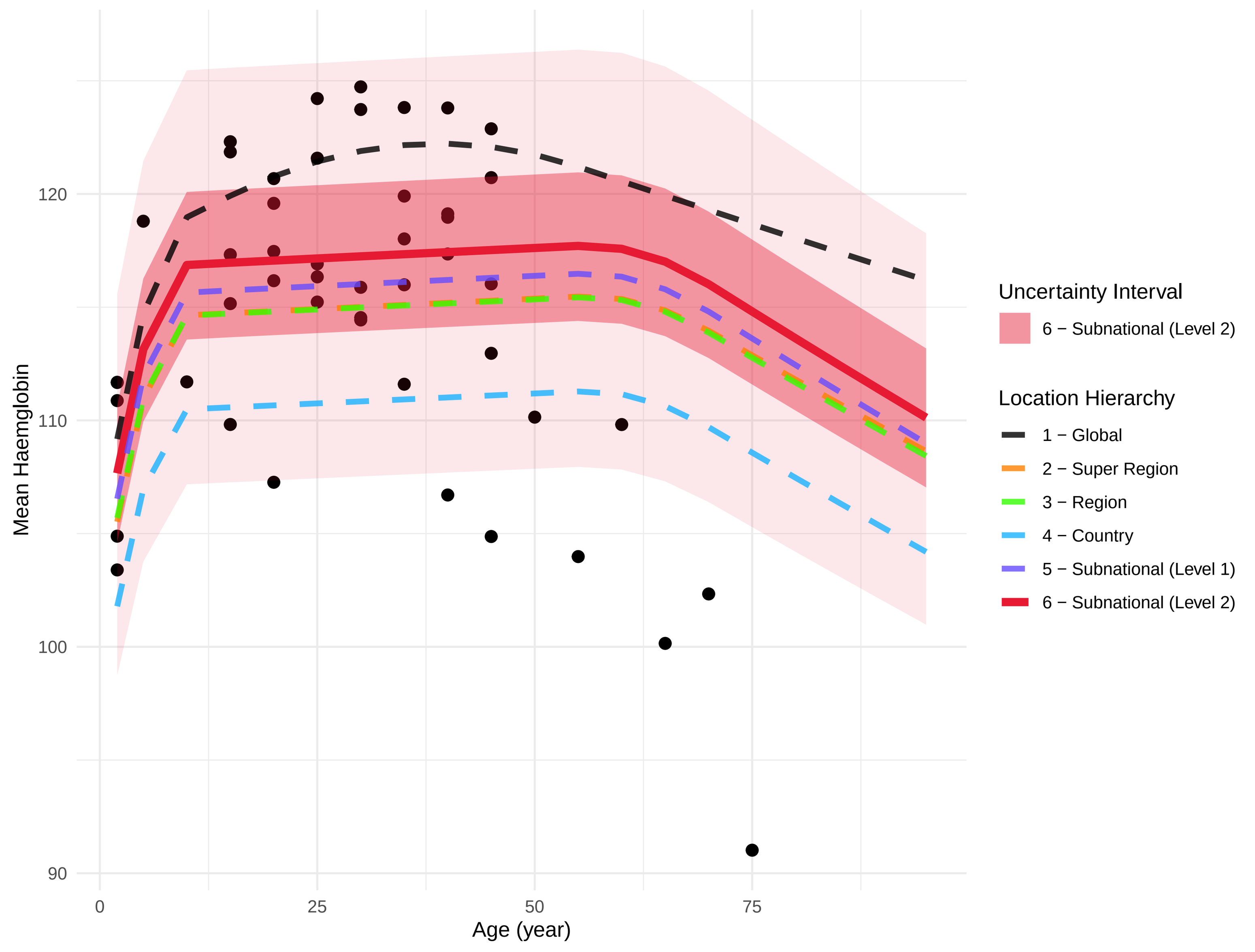

MR−BRT Age/Sex Fits − Non−pregnant Females 2+ Years Old − Mean Haemglobin − Jammu & Kashmir and Ladakh
WHO−2001 Adjustment Method

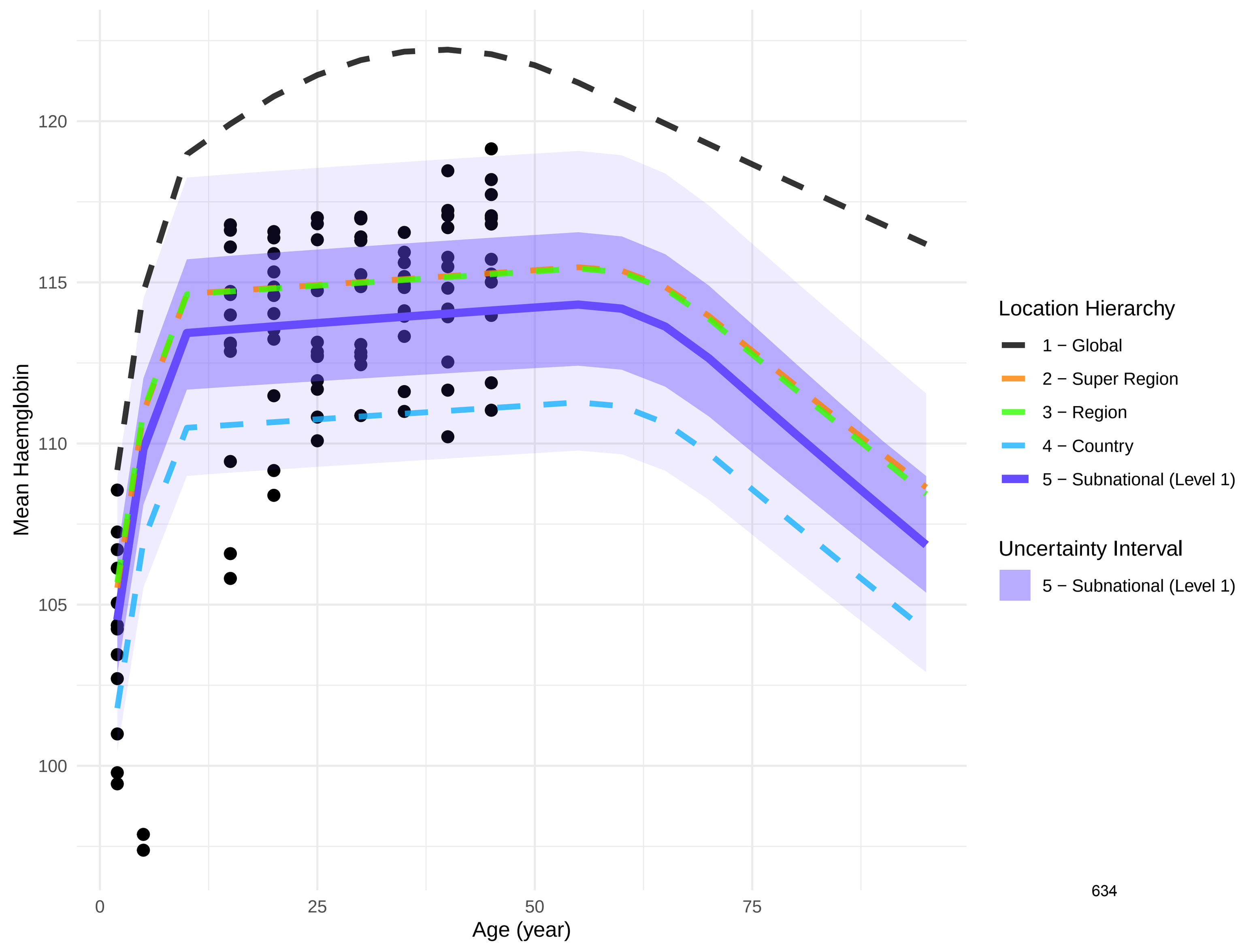

MR−BRT Age/Sex Fits − Non−pregnant Females 2+ Years Old − Mean Haemglobin − Jammu & Kashmir and Ladakh, Rural
WHO−2001 Adjustment Method

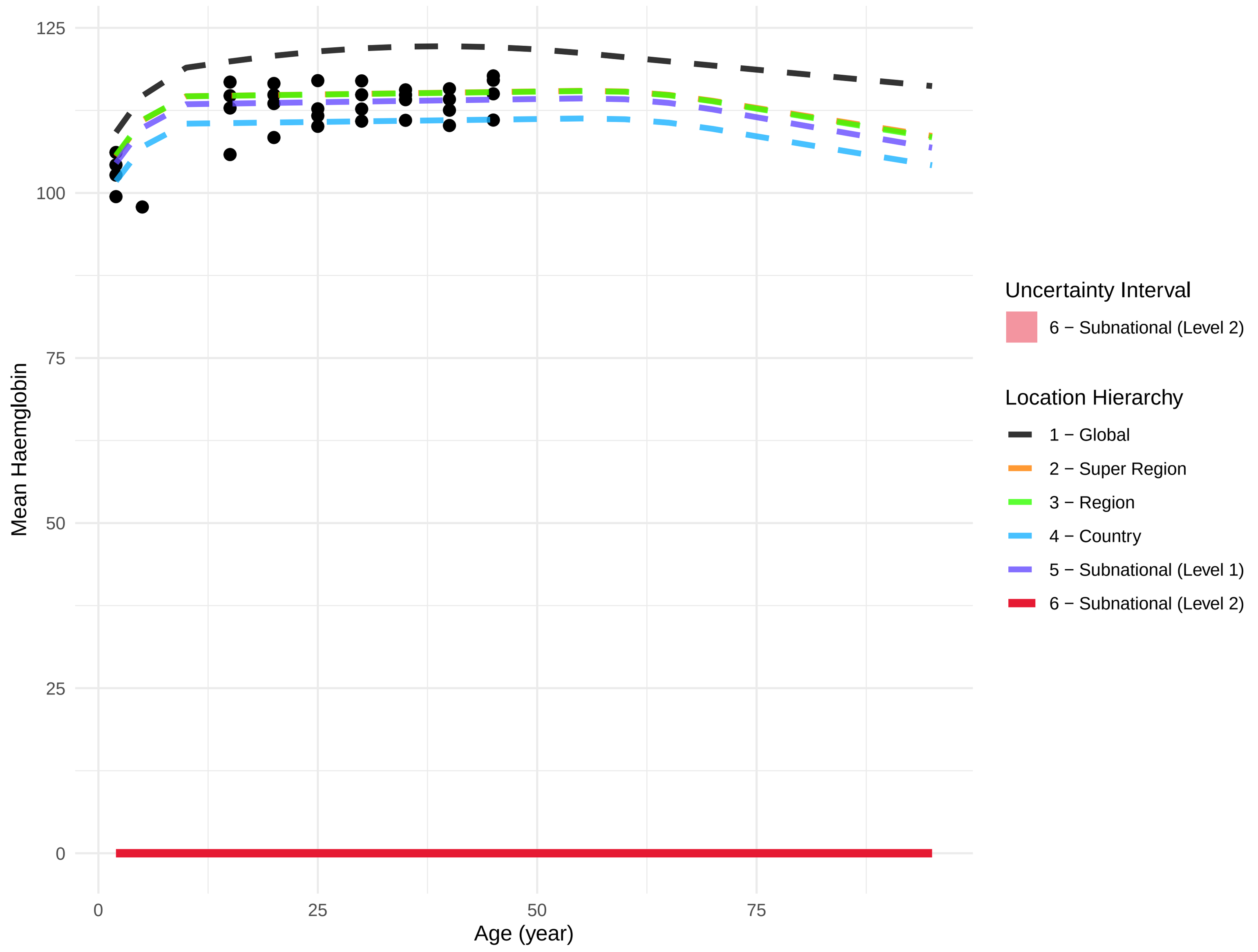

MR−BRT Age/Sex Fits − Non−pregnant Females 2+ Years Old − Mean Haemglobin − Jammu & Kashmir and Ladakh, Urban
WHO−2001 Adjustment Method

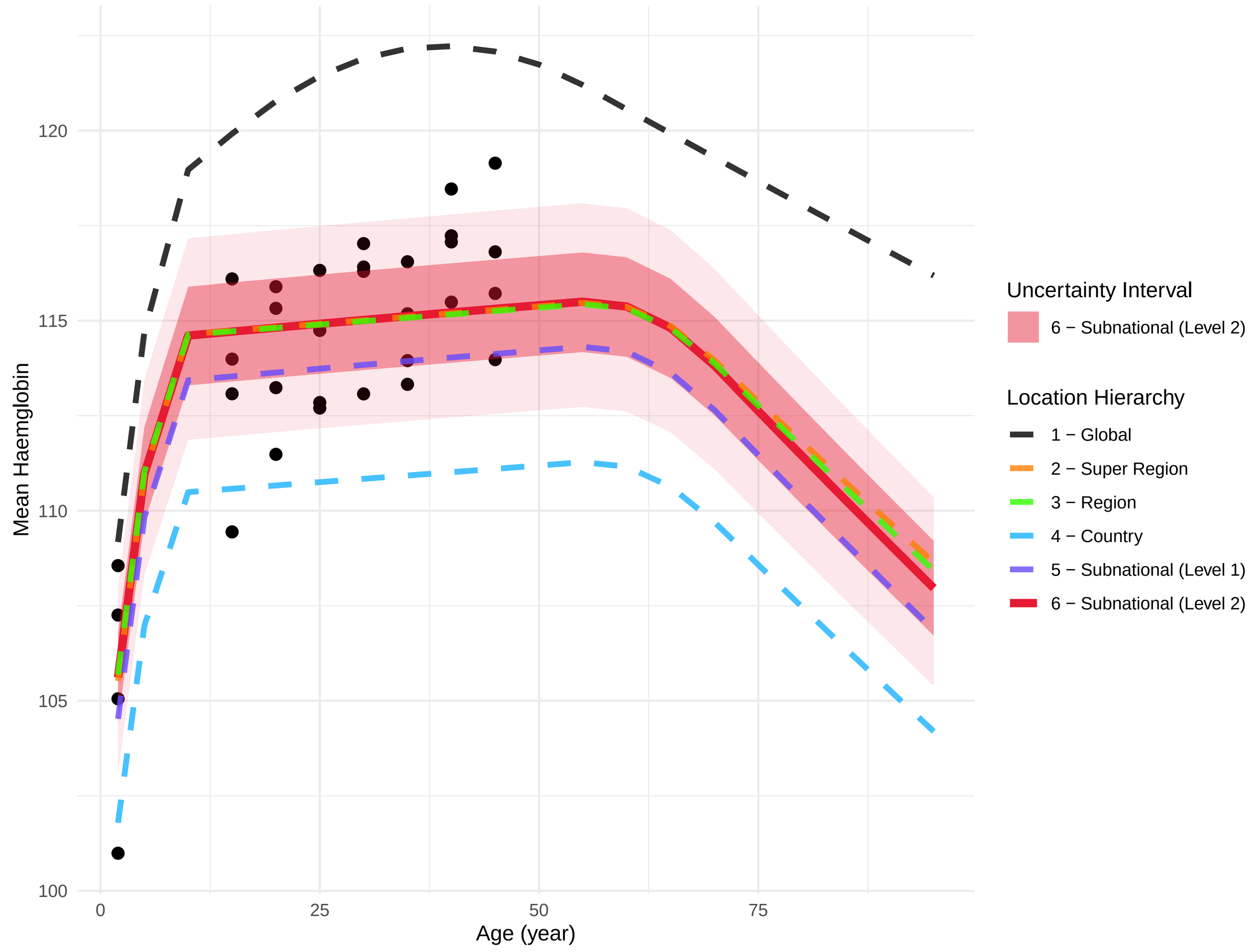

MR−BRT Age/Sex Fits − Non−pregnant Females 2+ Years Old − Mean Haemglobin − Jharkhand
WHO−2001 Adjustment Method

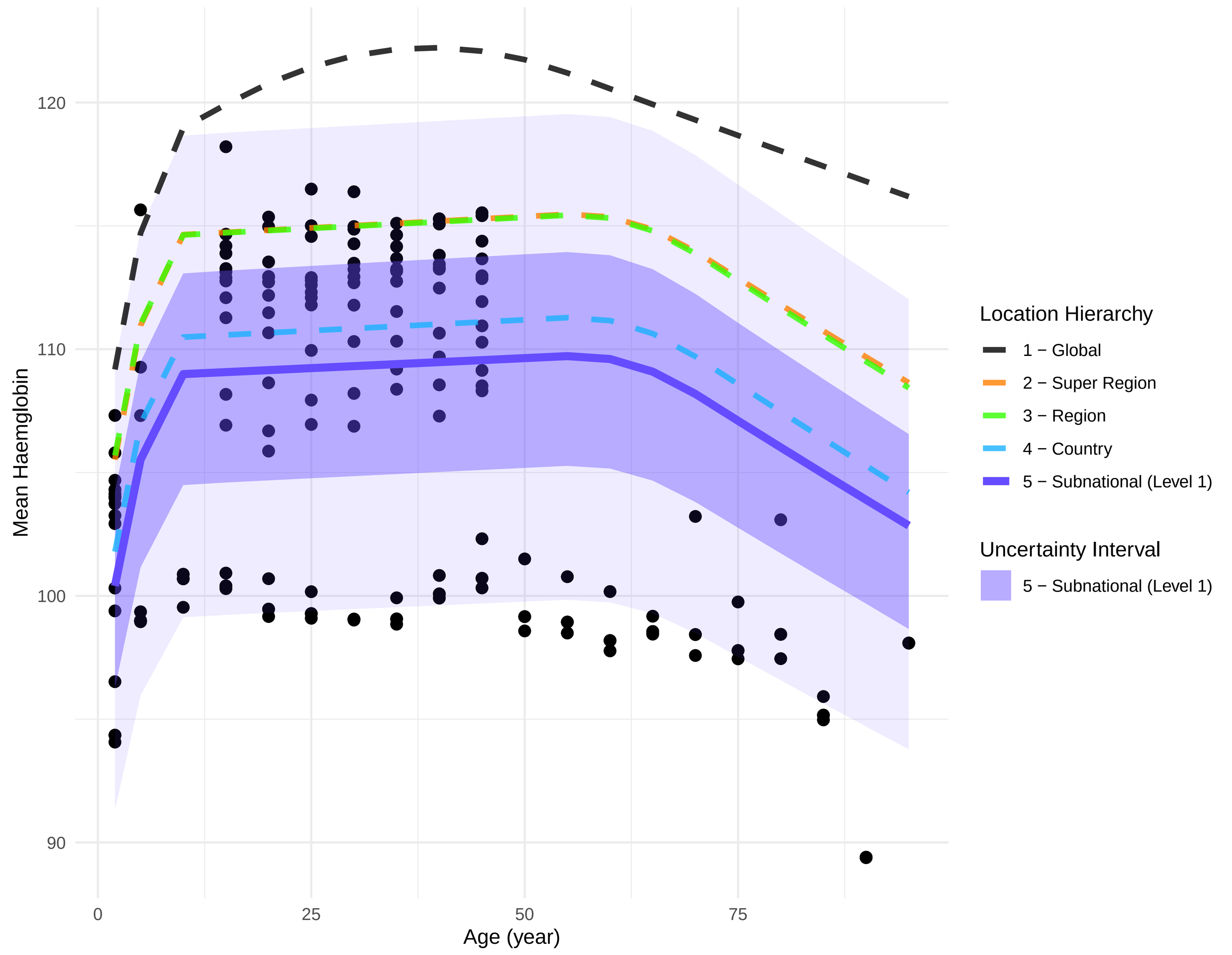

MR−BRT Age/Sex Fits − Non−pregnant Females 2+ Years Old − Mean Haemglobin − Jharkhand, Rural
WHO−2001 Adjustment Method

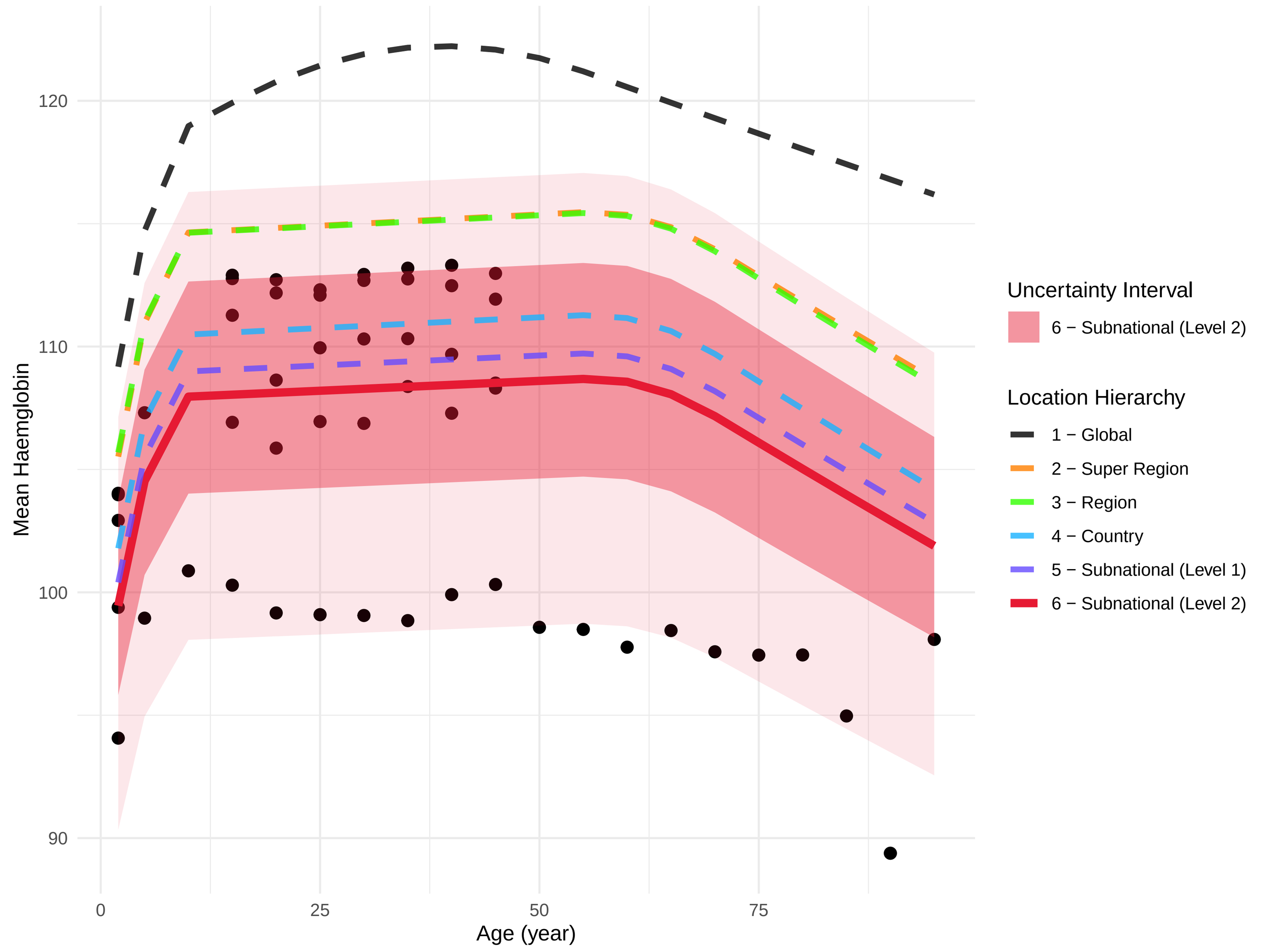

MR−BRT Age/Sex Fits − Non−pregnant Females 2+ Years Old − Mean Haemglobin − Jharkhand, Urban
WHO−2001 Adjustment Method

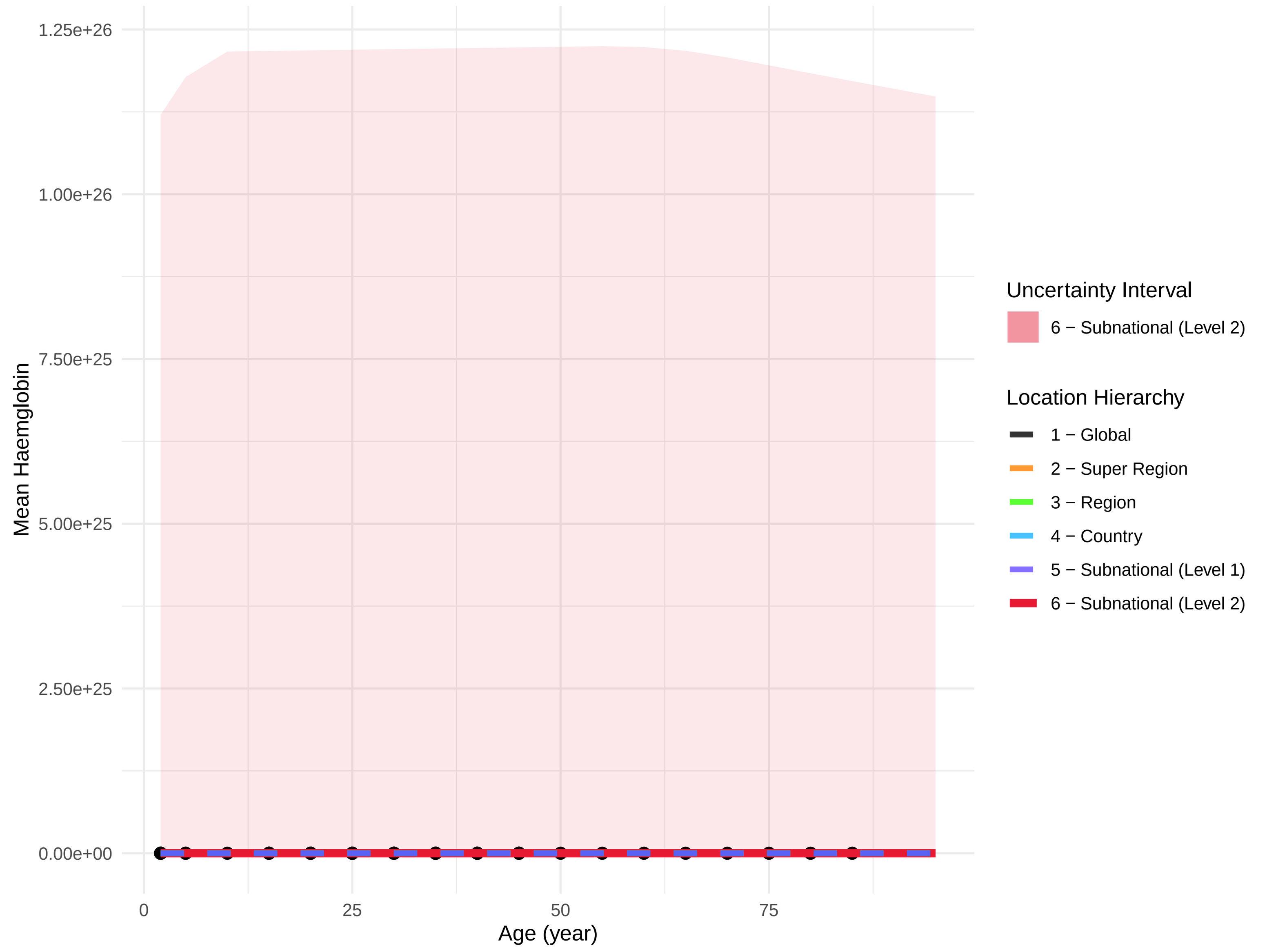

MR−BRT Age/Sex Fits − Non−pregnant Females 2+ Years Old − Mean Haemglobin − Karnataka
WHO−2001 Adjustment Method

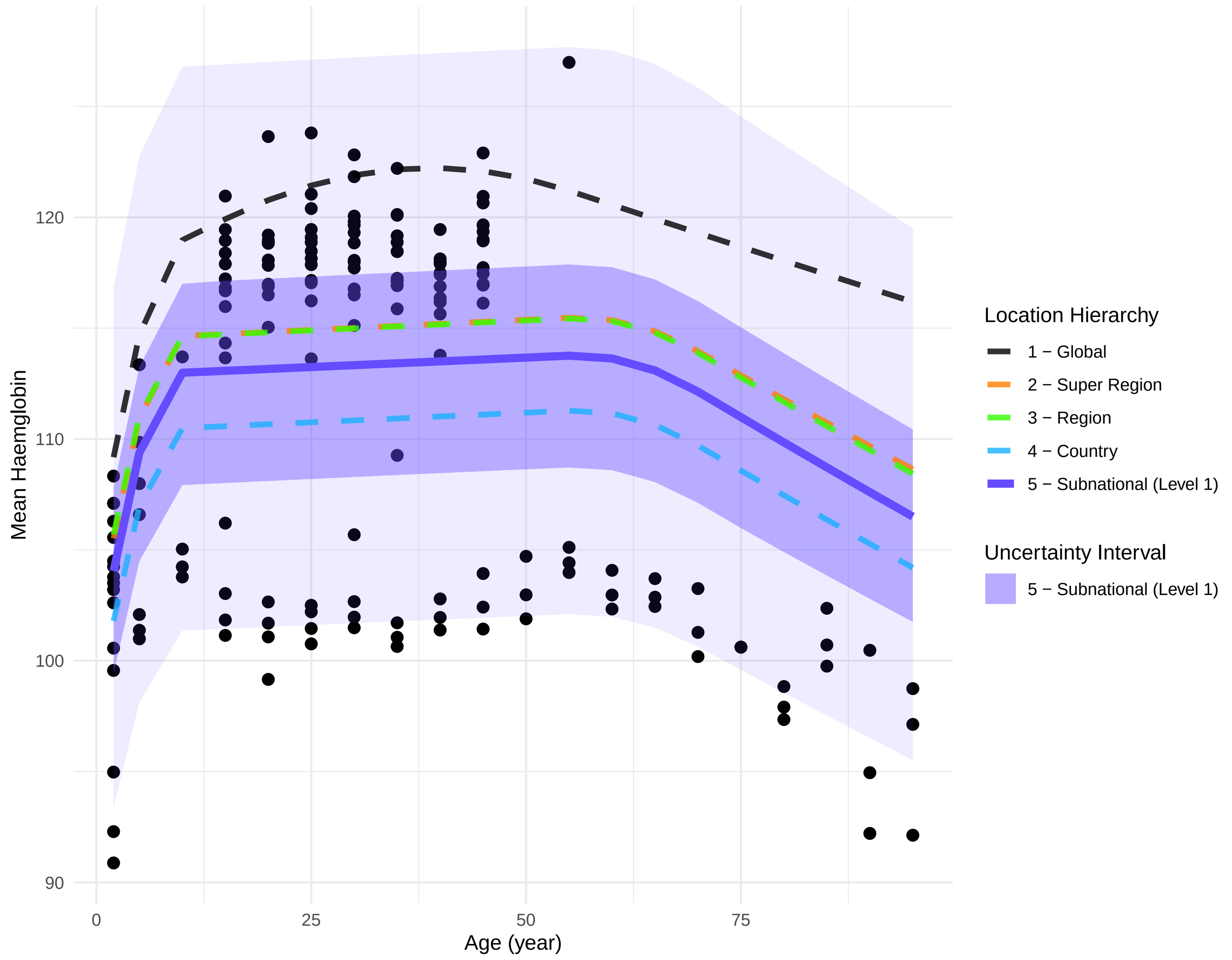

MR−BRT Age/Sex Fits − Non−pregnant Females 2+ Years Old − Mean Haemglobin − Karnataka, Rural
WHO−2001 Adjustment Method

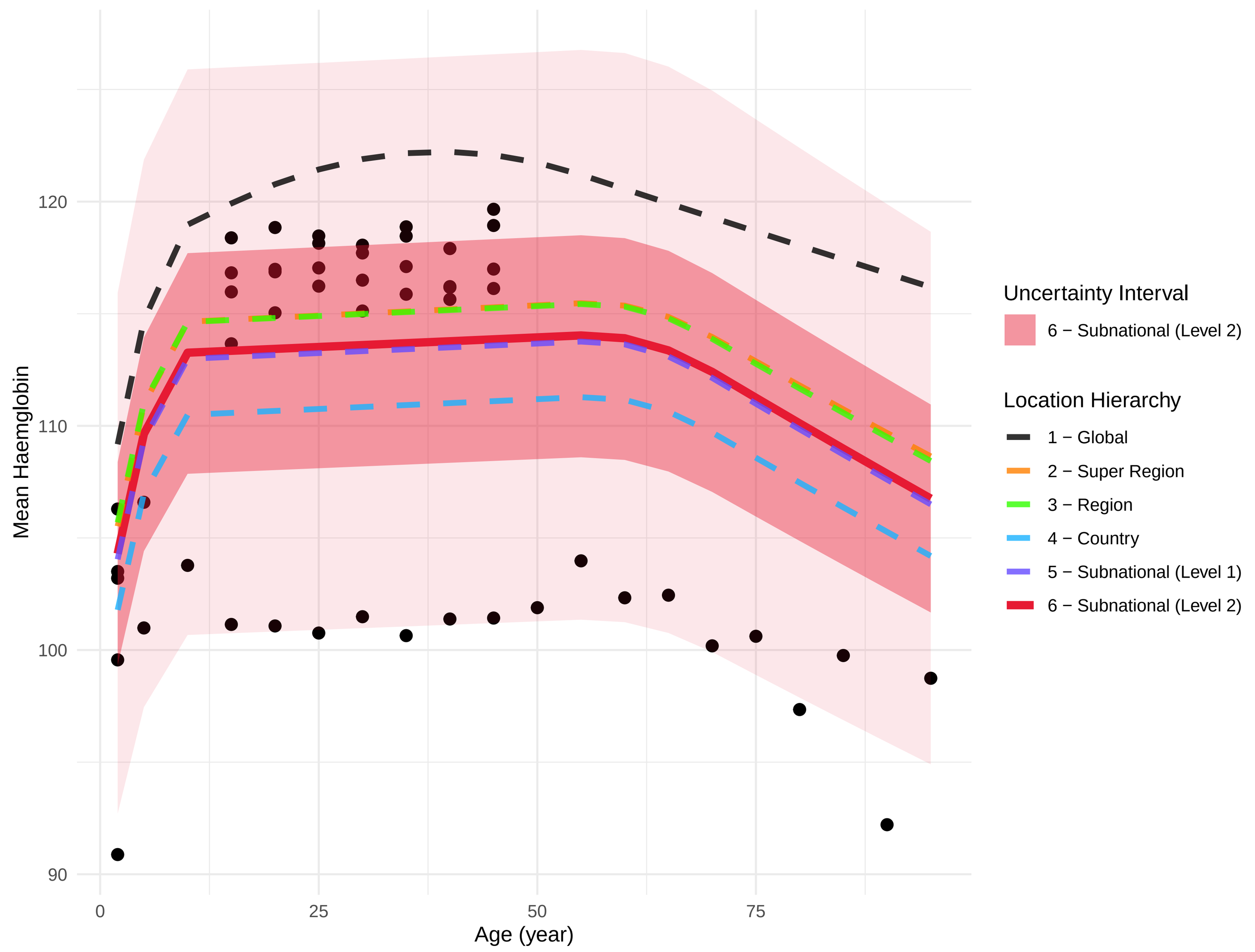

MR−BRT Age/Sex Fits − Non−pregnant Females 2+ Years Old − Mean Haemglobin − Karnataka, Urban
WHO−2001 Adjustment Method

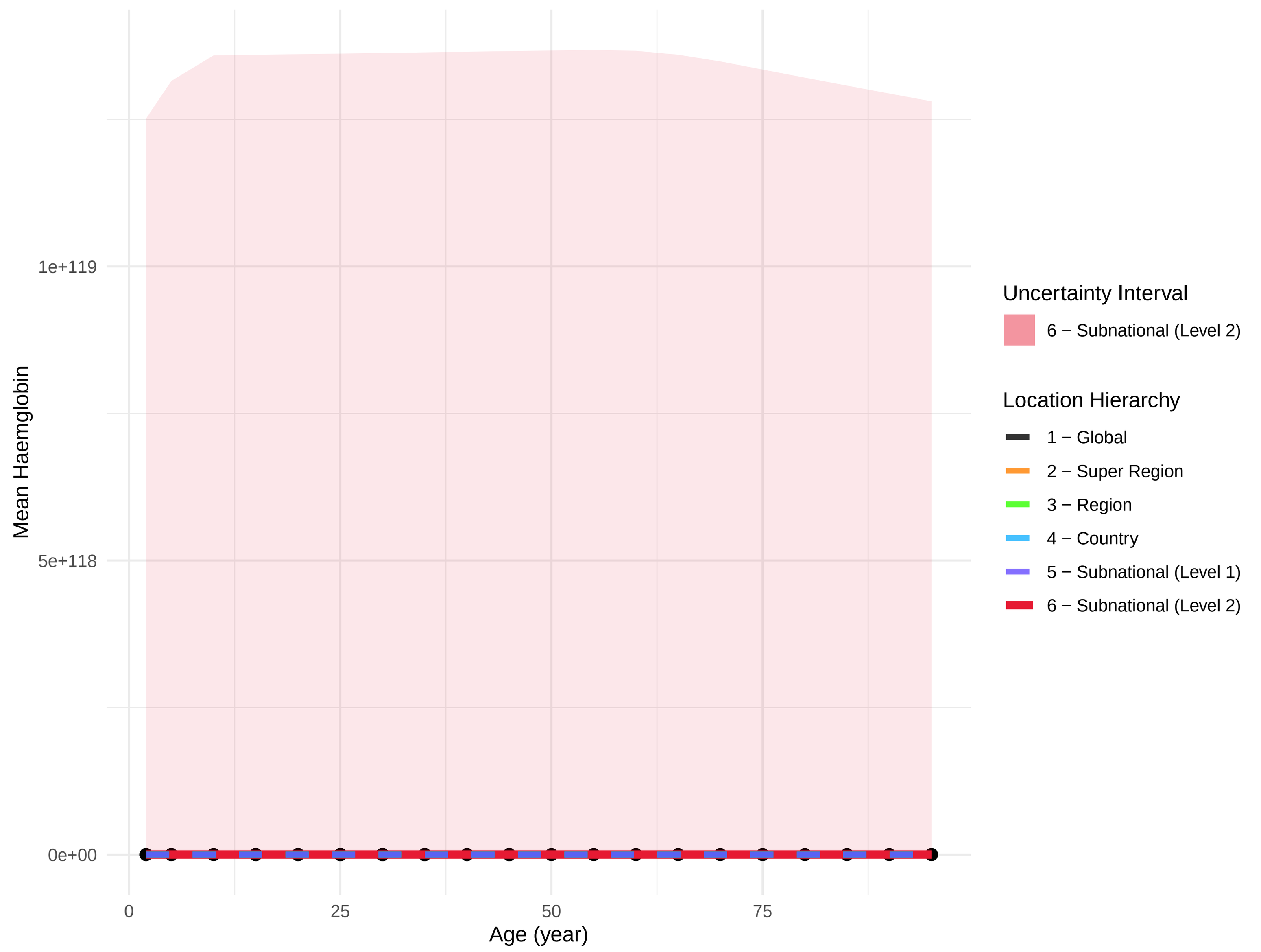

MR−BRT Age/Sex Fits − Non−pregnant Females 2+ Years Old − Mean Haemglobin − Kerala
WHO−2001 Adjustment Method

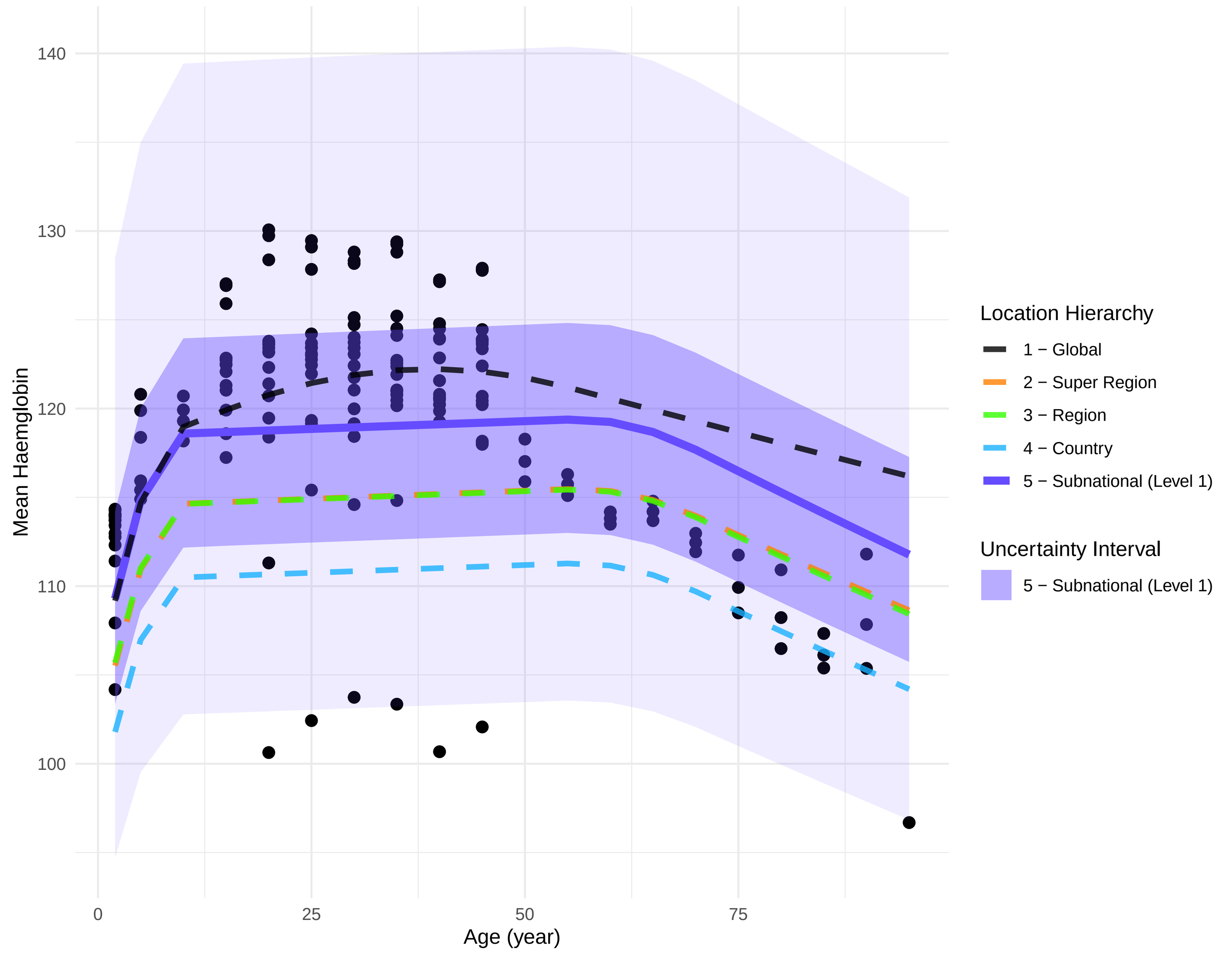

MR−BRT Age/Sex Fits − Non−pregnant Females 2+ Years Old − Mean Haemglobin − Kerala, Ru
WHO−2001 Adjustment Method

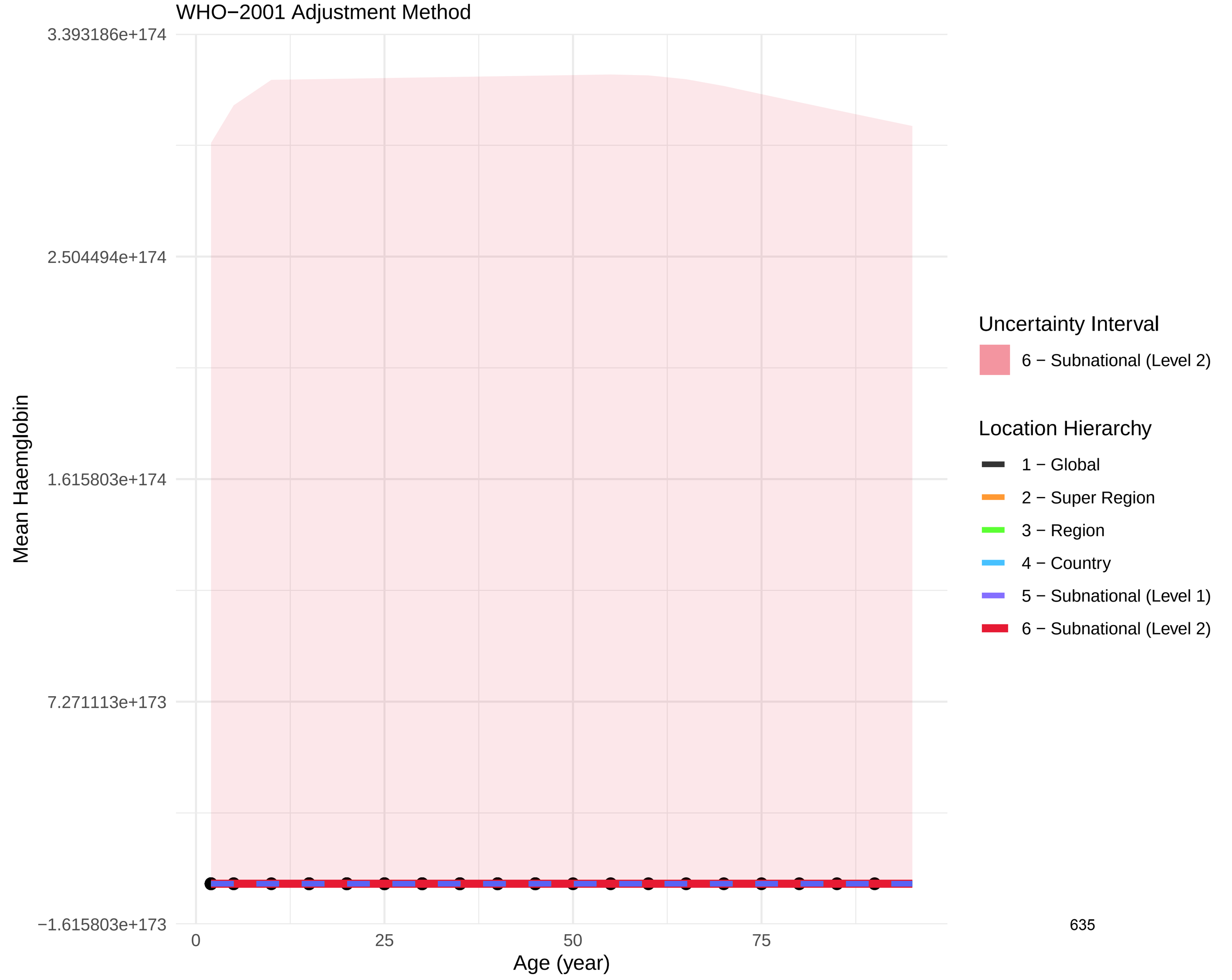

MR−BRT Age/Sex Fits − Non−pregnant Females 2+ Years Old − Mean Haemoglobin − Kerala, Urban
WHO−2001 Adjustment Method

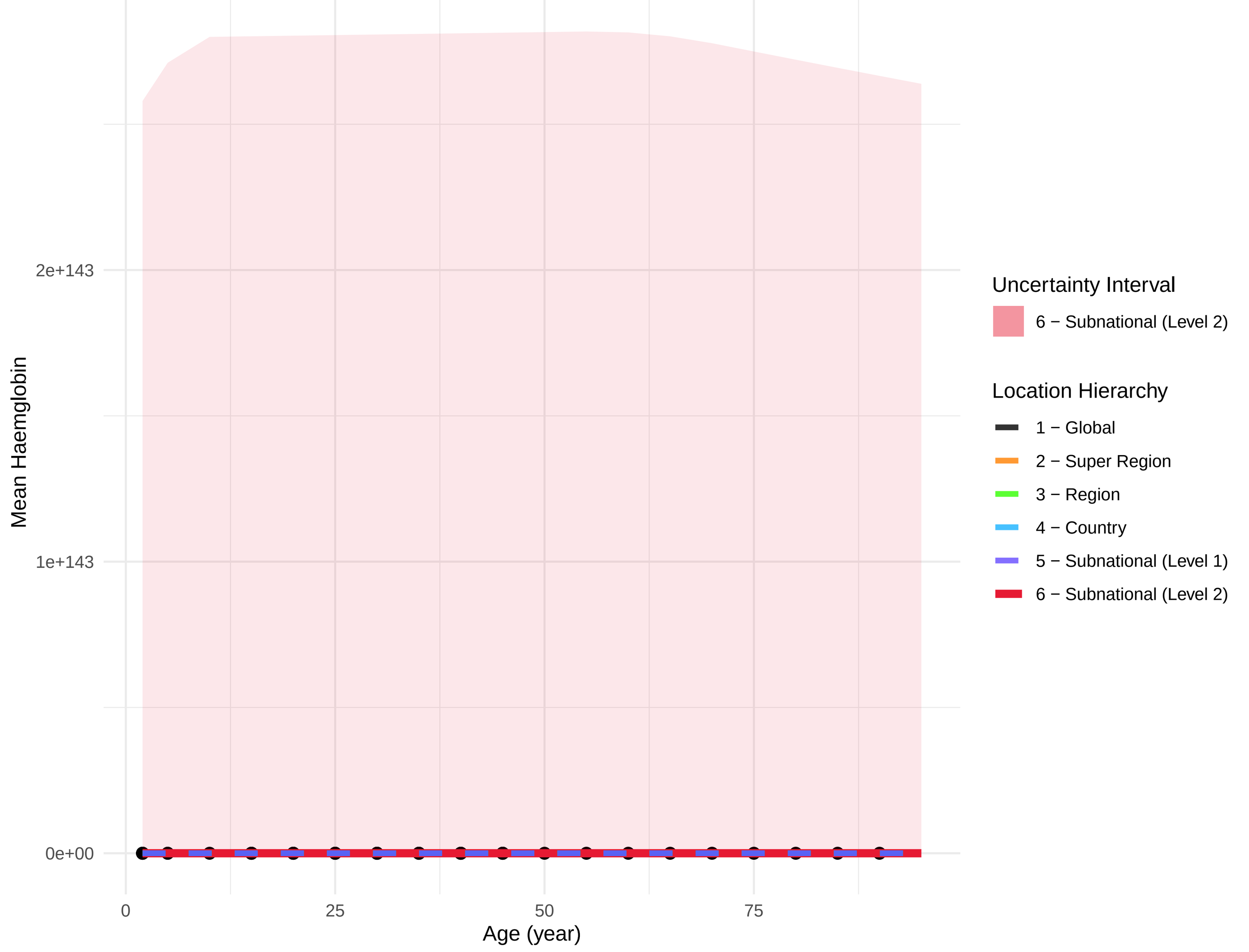

MR−BRT Age/Sex Fits − Non−pregnant Females 2+ Years Old − Mean Haemoglobin − Madhya Pradesh
WHO−2001 Adjustment Method

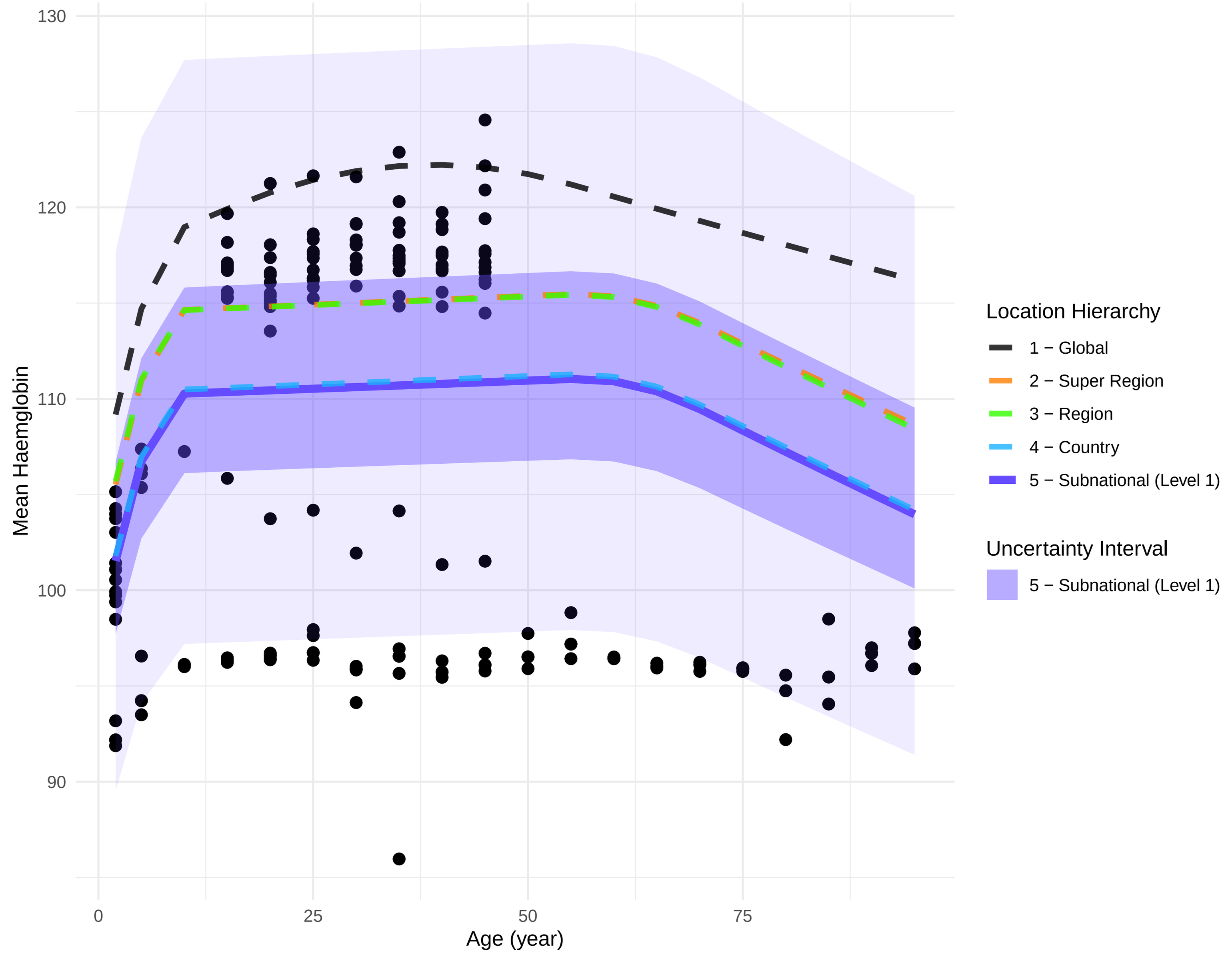

MR−BRT Age/Sex Fits − Non−pregnant Females 2+ Years Old − Mean Haemoglobin − Madhya Pradesh, Rural
WHO−2001 Adjustment Method

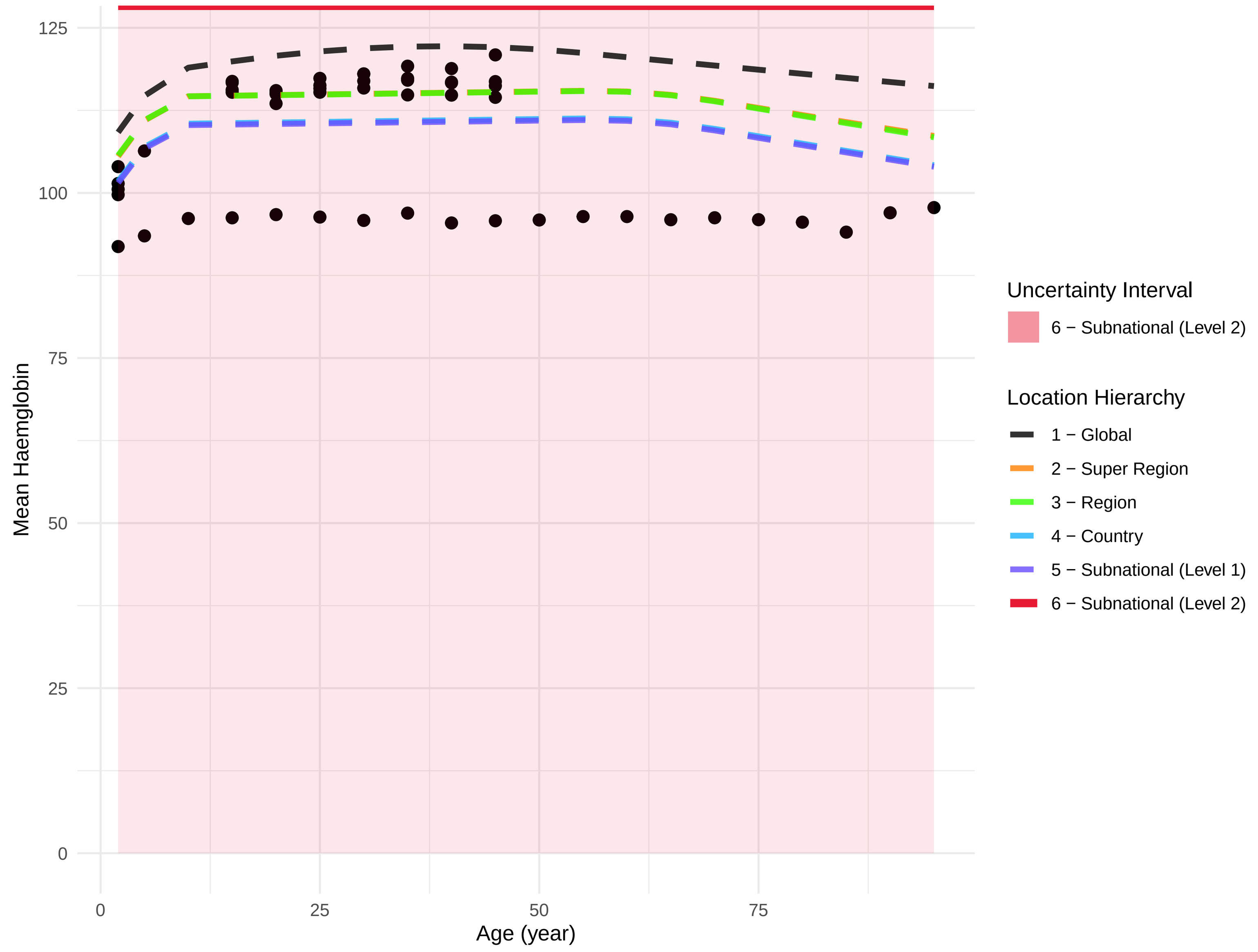

MR−BRT Age/Sex Fits − Non−pregnant Females 2+ Years Old − Mean Haemoglobin − Madhya Pradesh, Urban
WHO−2001 Adjustment Method

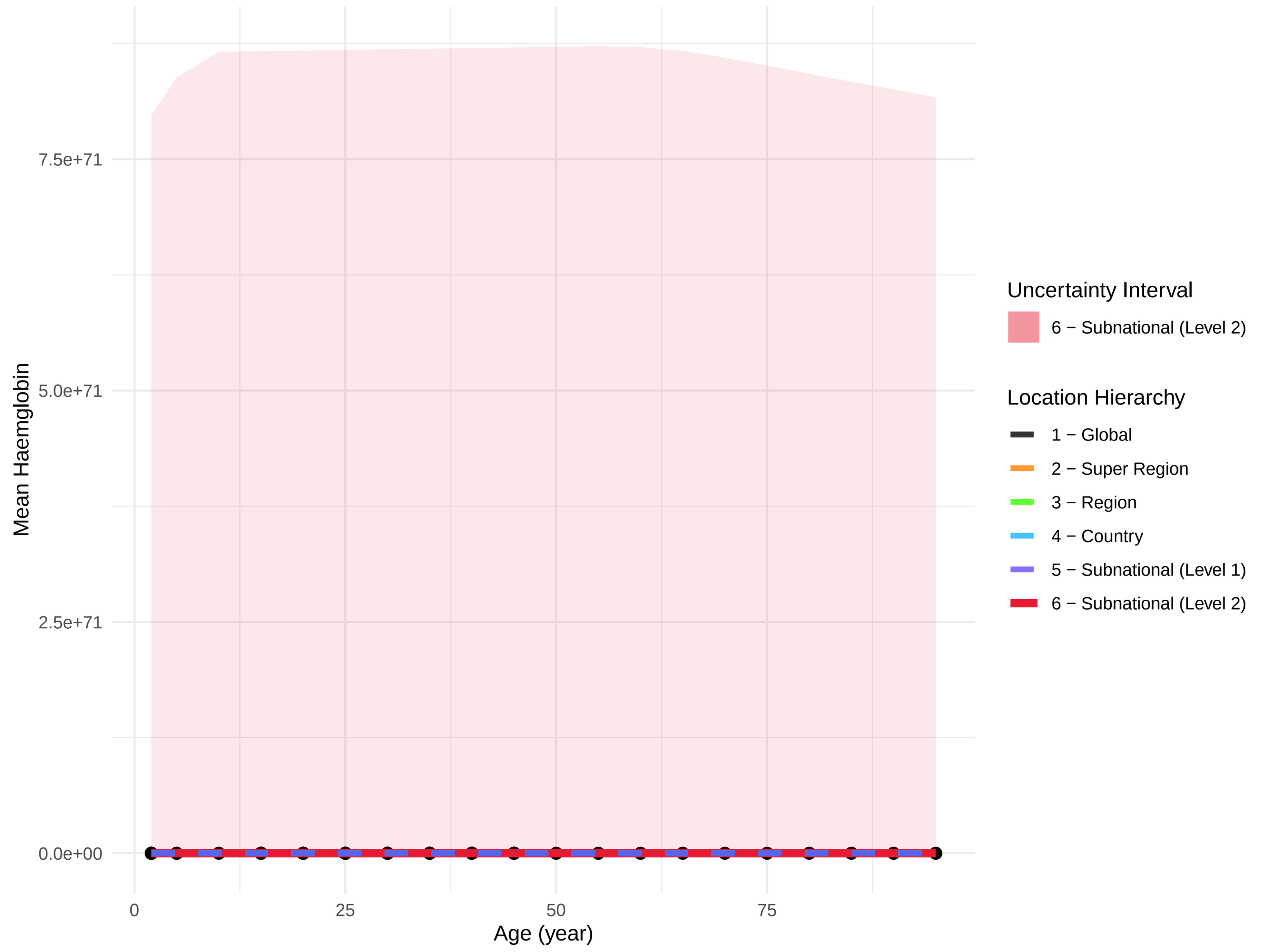

MR−BRT Age/Sex Fits − Non−pregnant Females 2+ Years Old − Mean Haemoglobin − Maharashtra
WHO−2001 Adjustment Method

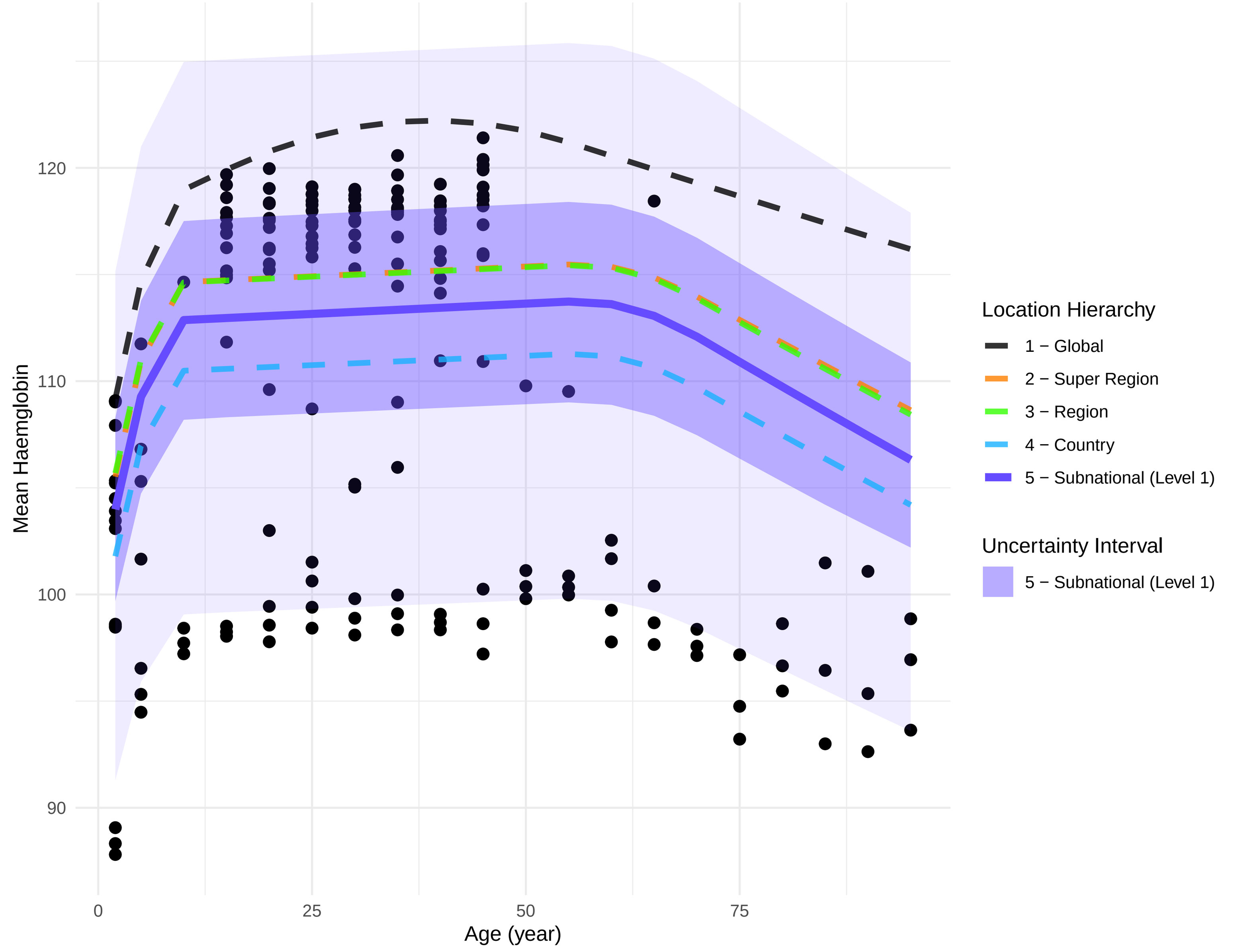

MR−BRT Age/Sex Fits − Non−pregnant Females 2+ Years Old − Mean Haemoglobin − Maharashtra, Rural
WHO−2001 Adjustment Method

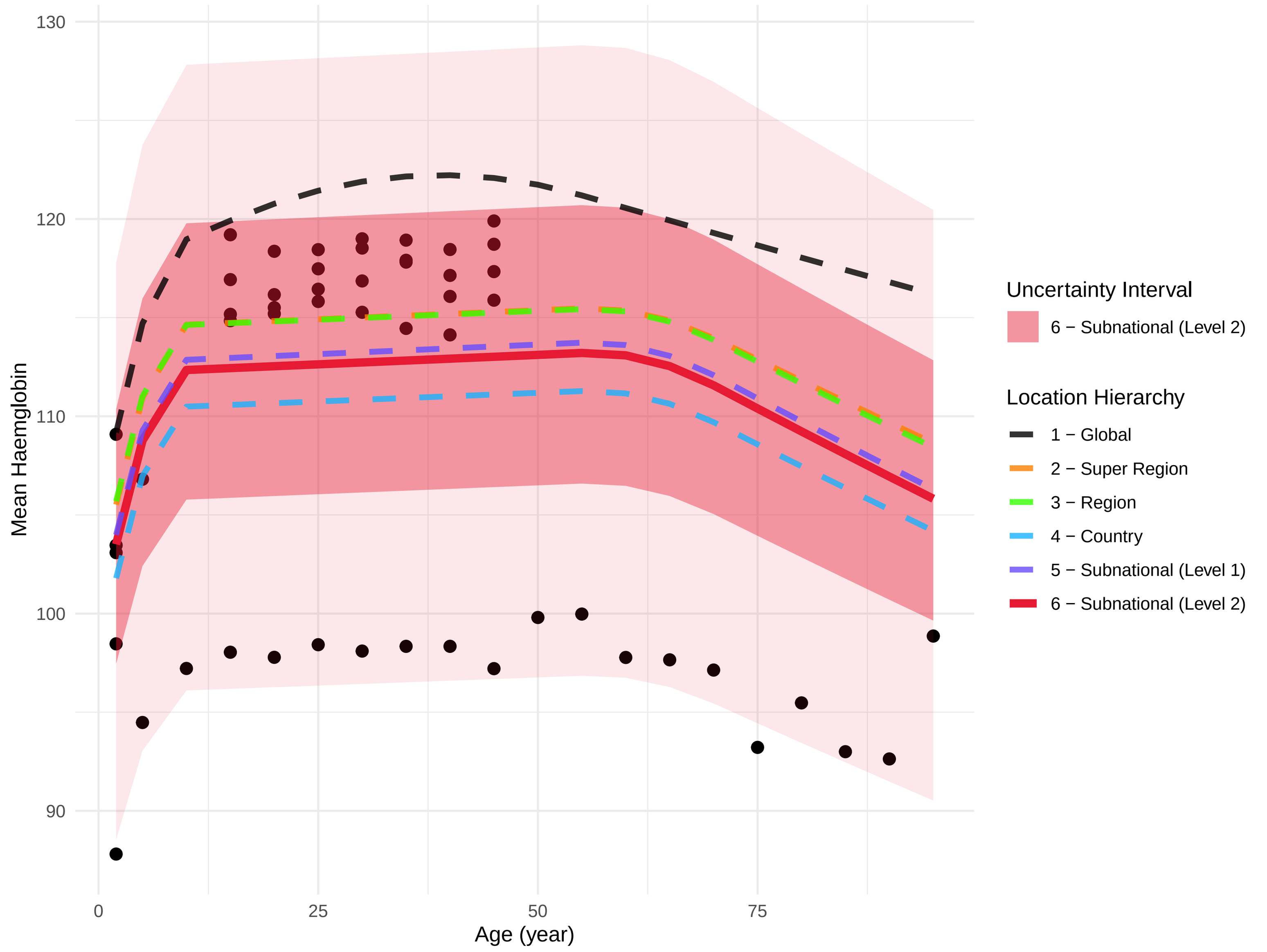

MR−BRT Age/Sex Fits − Non−pregnant Females 2+ Years Old − Mean Haemoglobin − Maharashtra, Urban
WHO−2001 Adjustment Method

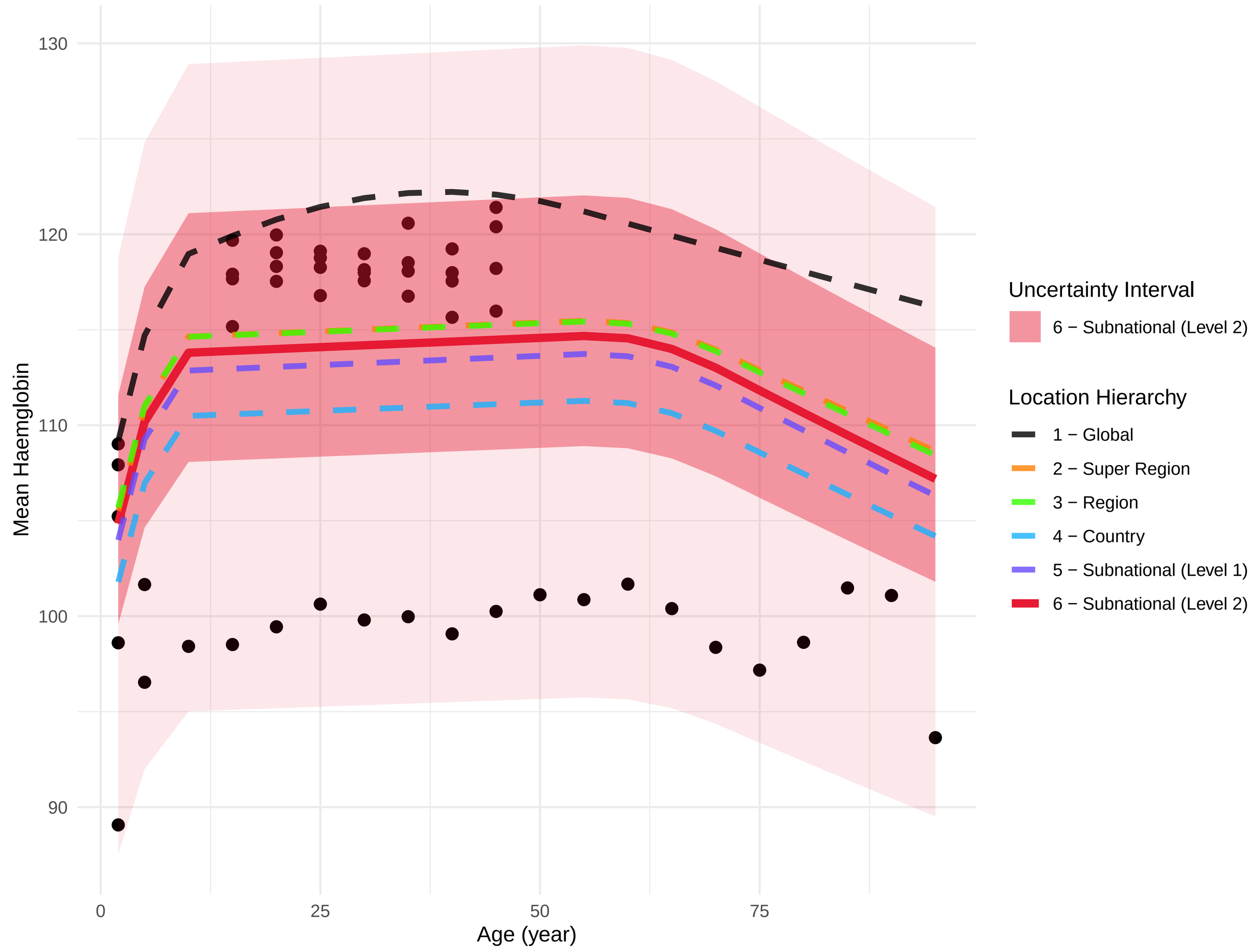

MR−BRT Age/Sex Fits − Non−pregnant Females 2+ Years Old − Mean Haemoglobin − Manipur
WHO−2001 Adjustment Method

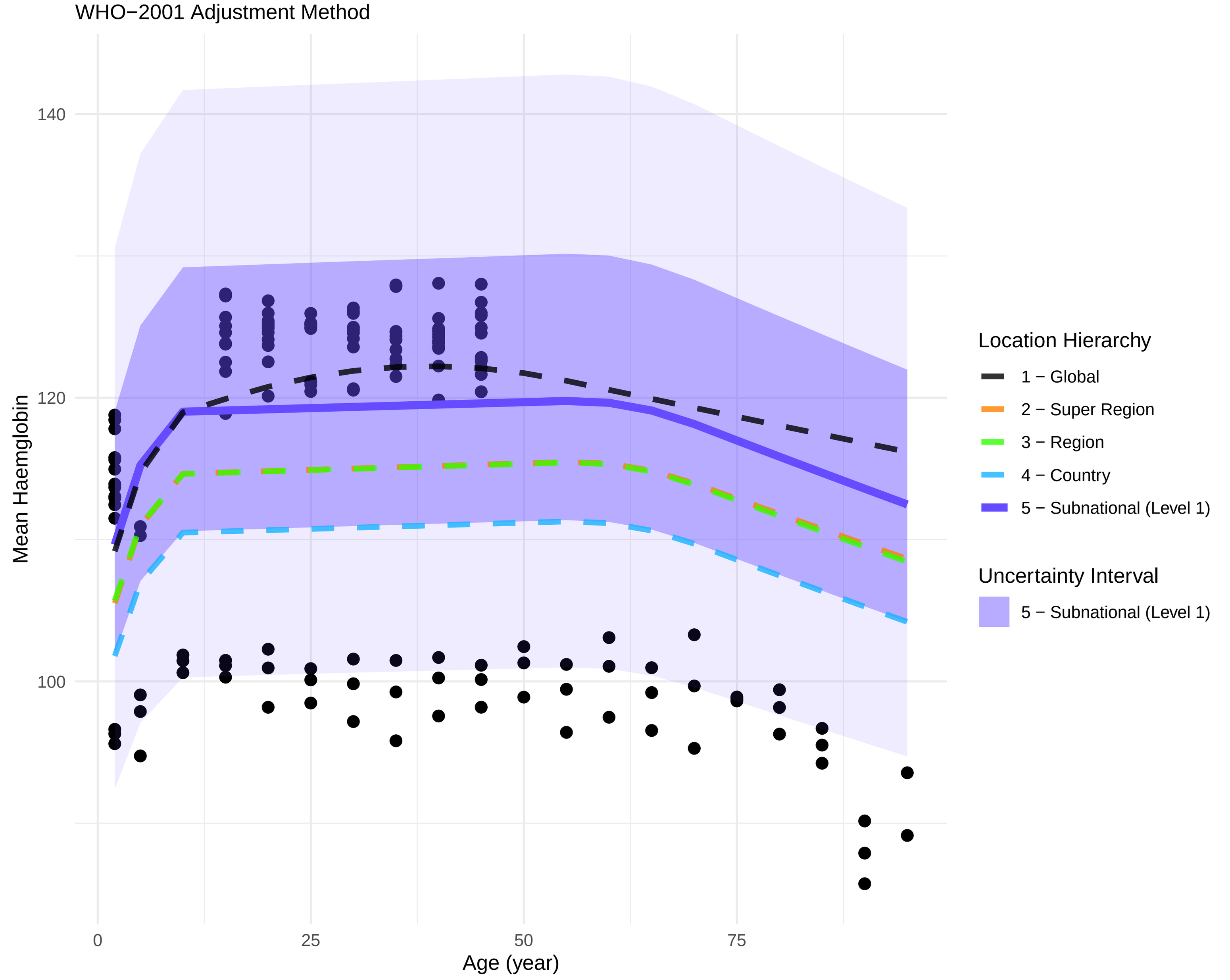

MR−BRT Age/Sex Fits − Non−pregnant Females 2+ Years Old − Mean Haemoglobin − Manipur, Rural
WHO−2001 Adjustment Method

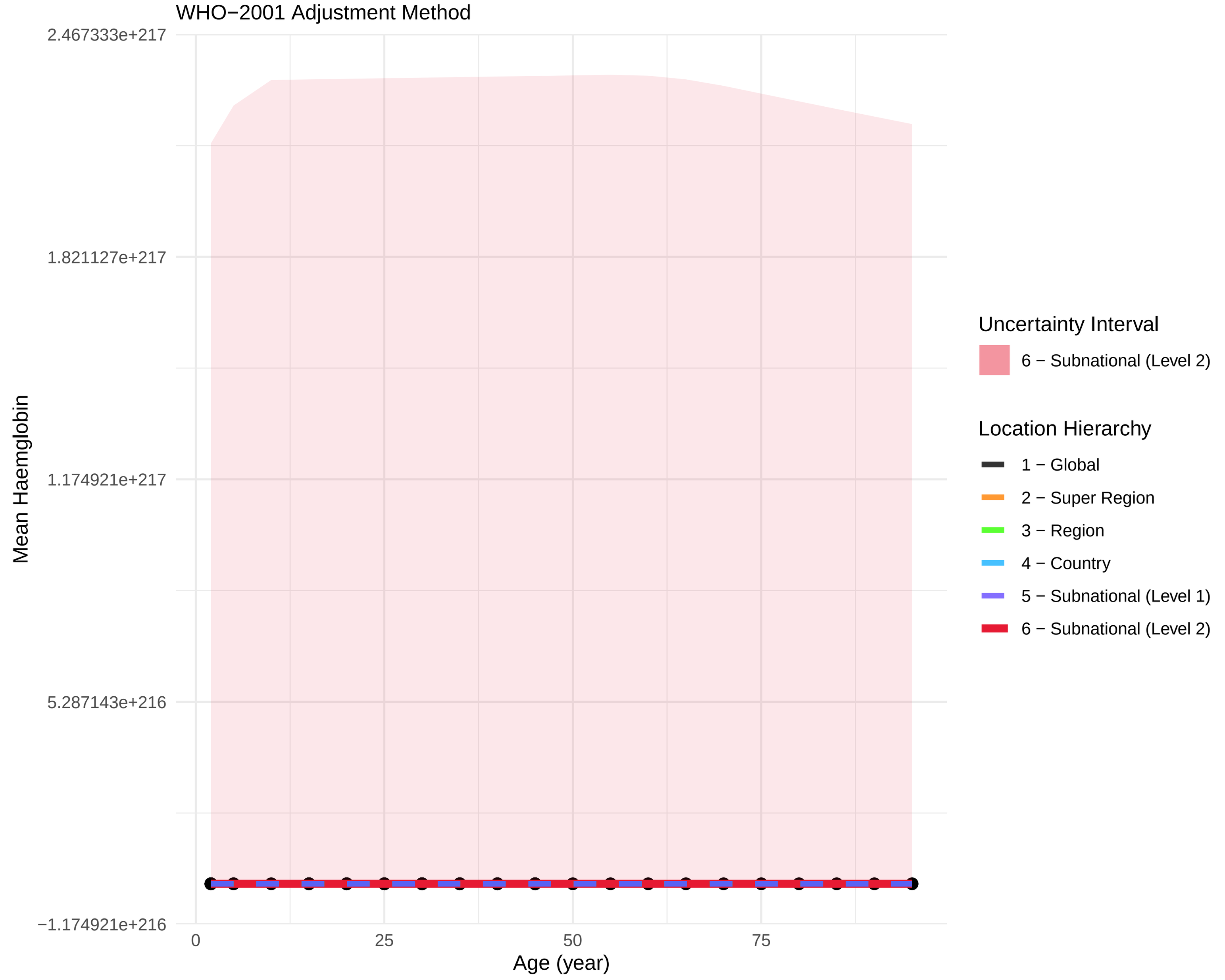

MR−BRT Age/Sex Fits − Non−pregnant Females 2+ Years Old − Mean Haemoglobin − Manipur, Urban
WHO−2001 Adjustment Method

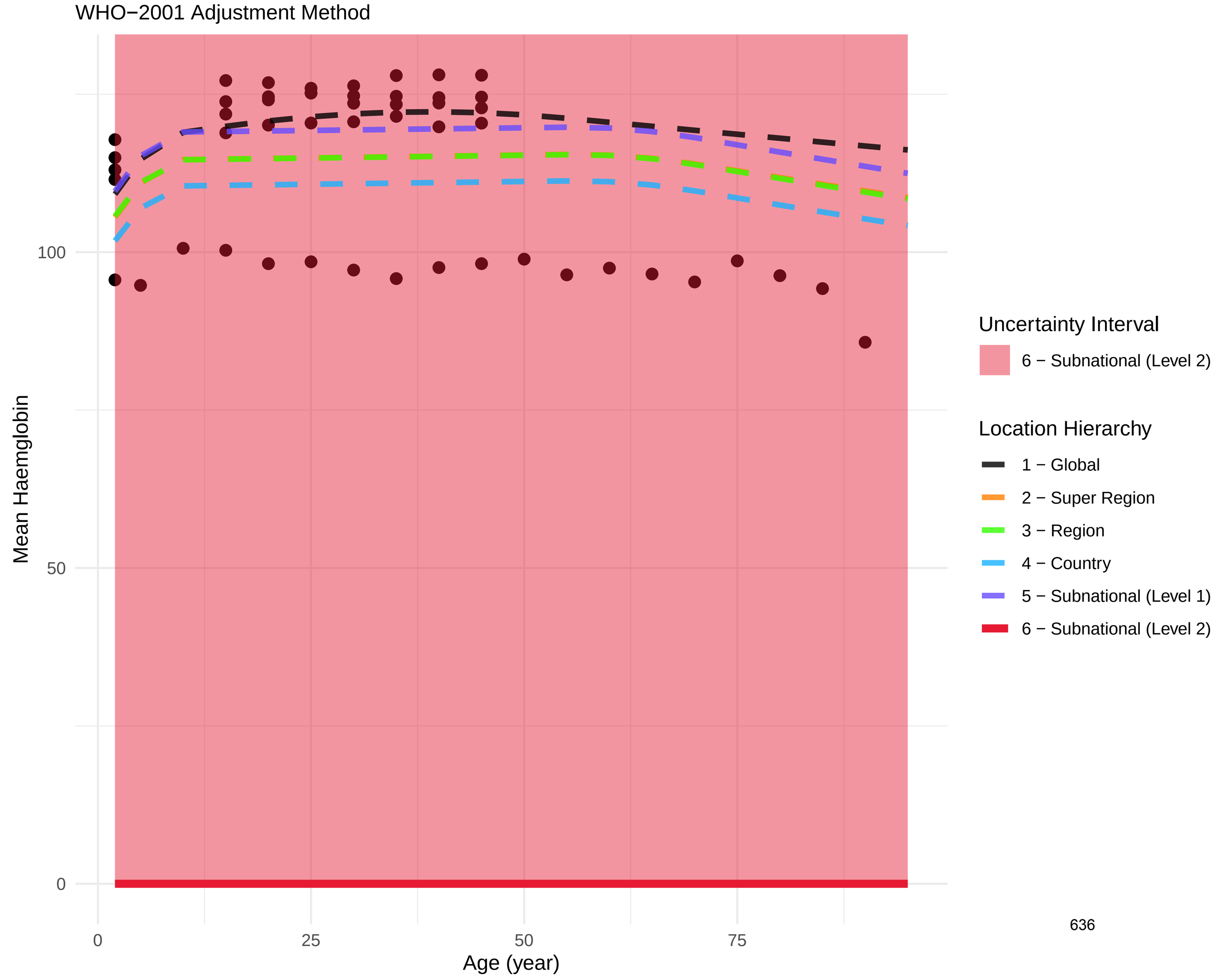

MR−BRT Age/Sex Fits − Non−pregnant Females 2+ Years Old − Mean Haemglobin − Meghalaya
WHO−2001 Adjustment Method

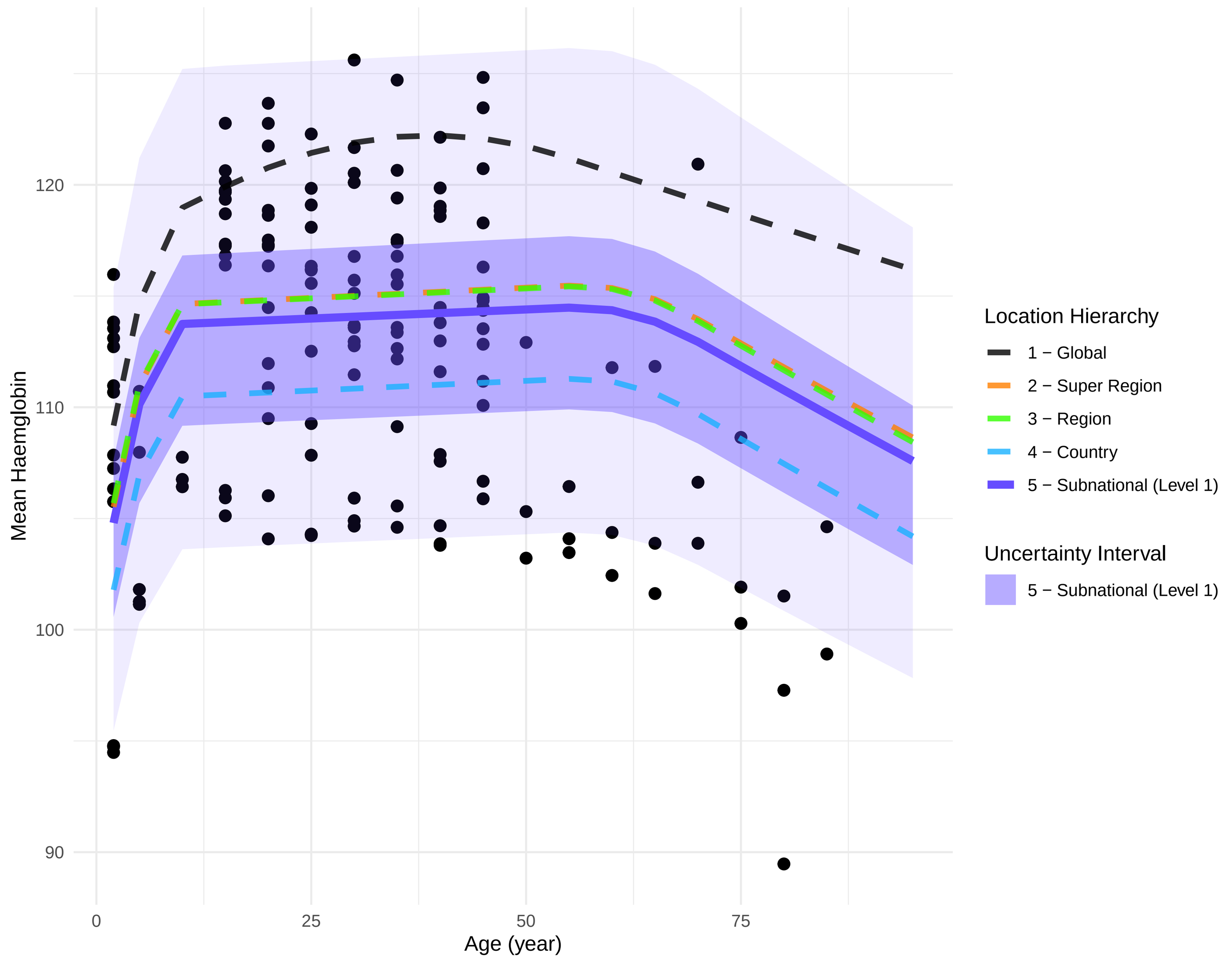

MR−BRT Age/Sex Fits − Non−pregnant Females 2+ Years Old − Mean Haemglobin − Meghalaya, Rural
WHO−2001 Adjustment Method

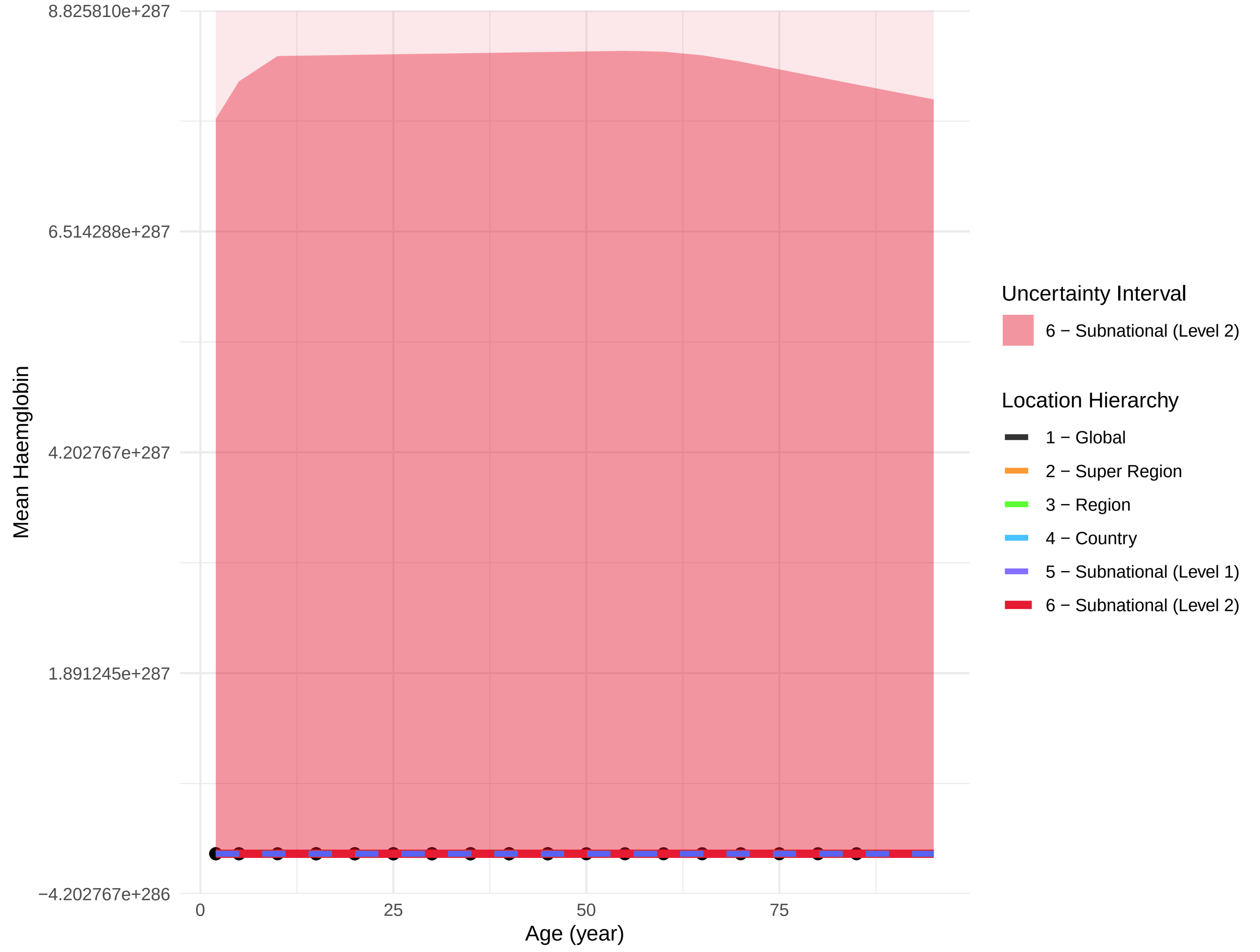

MR−BRT Age/Sex Fits − Non−pregnant Females 2+ Years Old − Mean Haemglobin − Meghalaya, Urban
WHO−2001 Adjustment Method

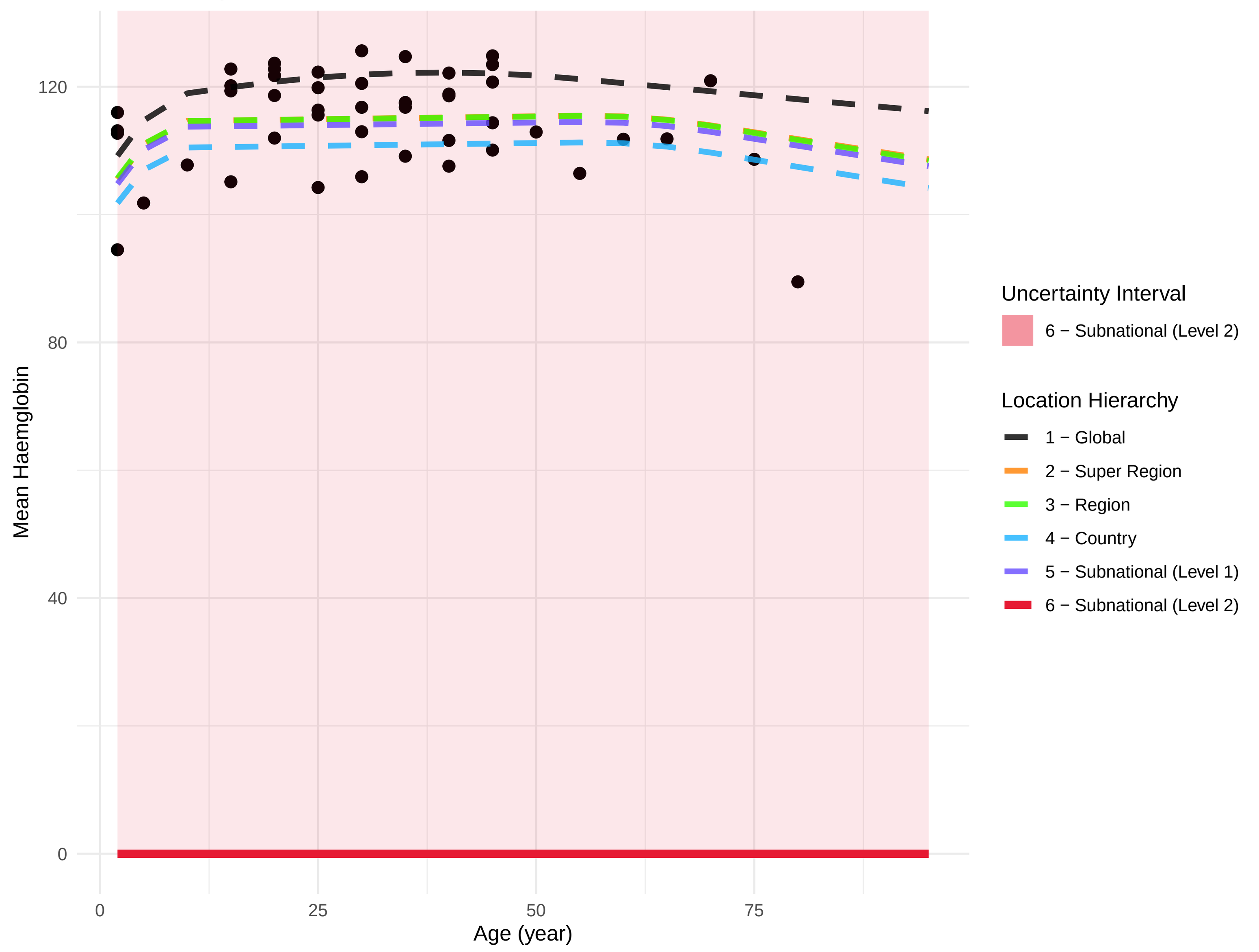

MR−BRT Age/Sex Fits − Non−pregnant Females 2+ Years Old − Mean Haemglobin − Mizoram
WHO−2001 Adjustment Method

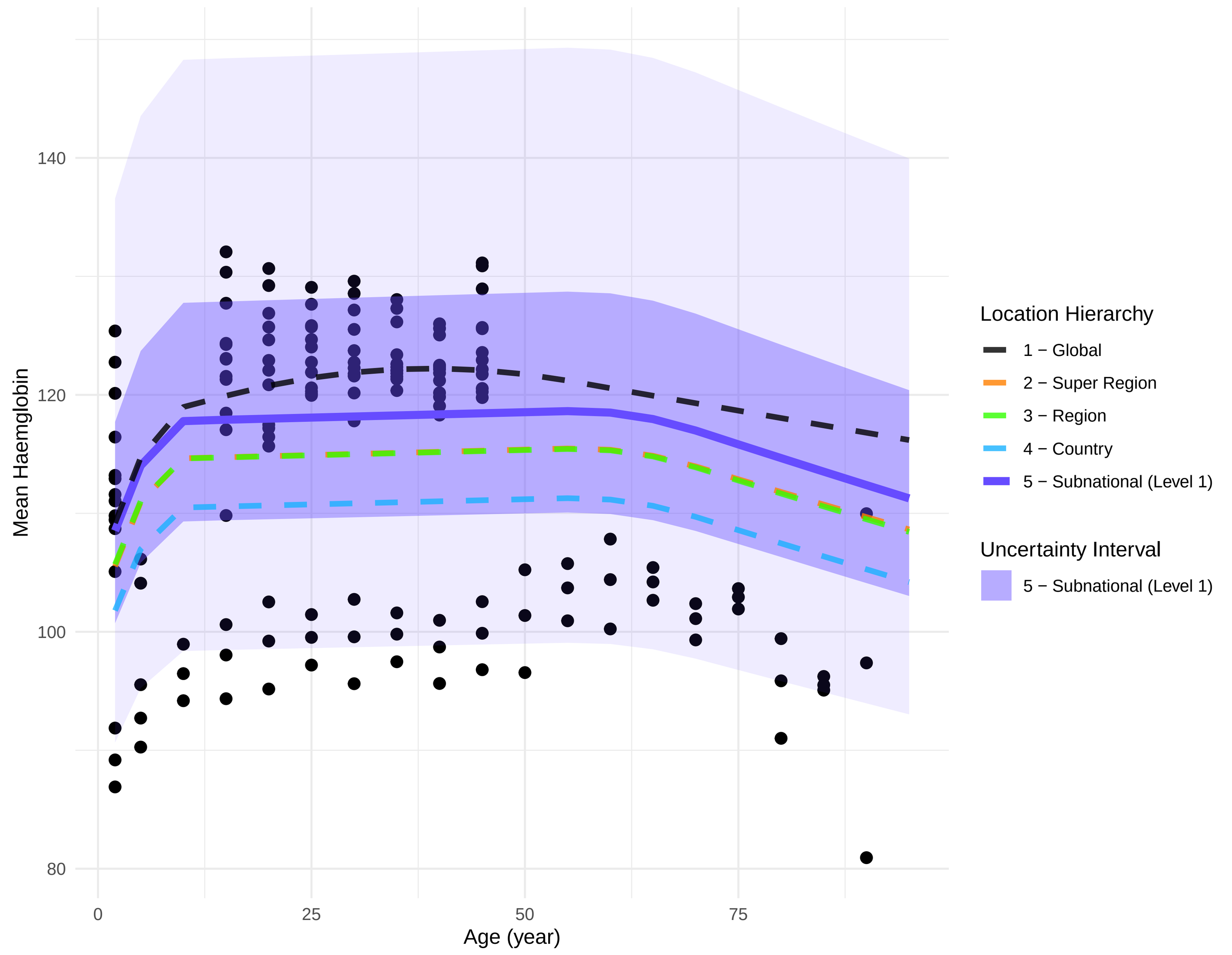

MR−BRT Age/Sex Fits − Non−pregnant Females 2+ Years Old − Mean Haemglobin − Mizoram, [illegible]
WHO−2001 Adjustment Method

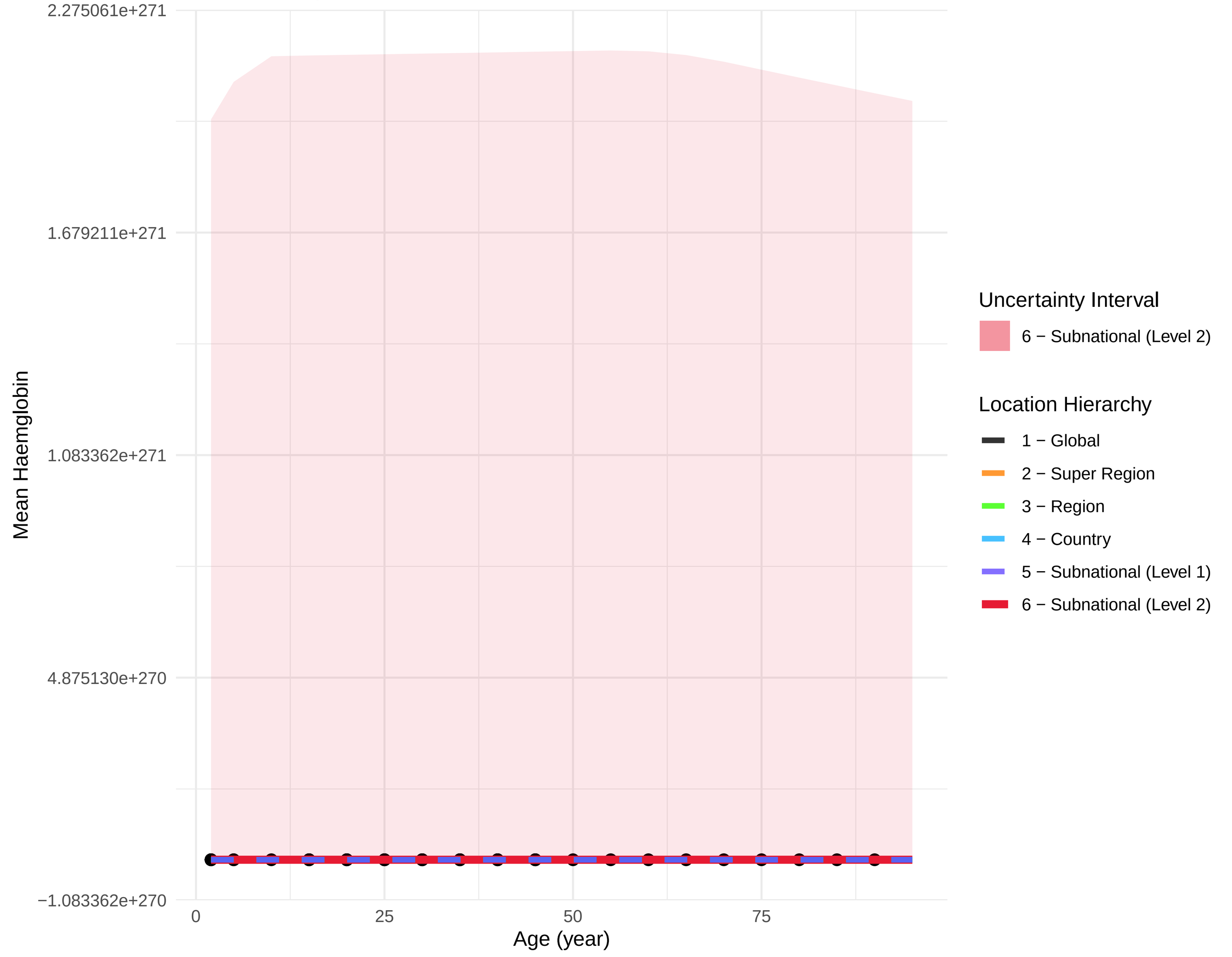

MR−BRT Age/Sex Fits − Non−pregnant Females 2+ Years Old − Mean Haemglobin − Mizoram, Urban
WHO−2001 Adjustment Method

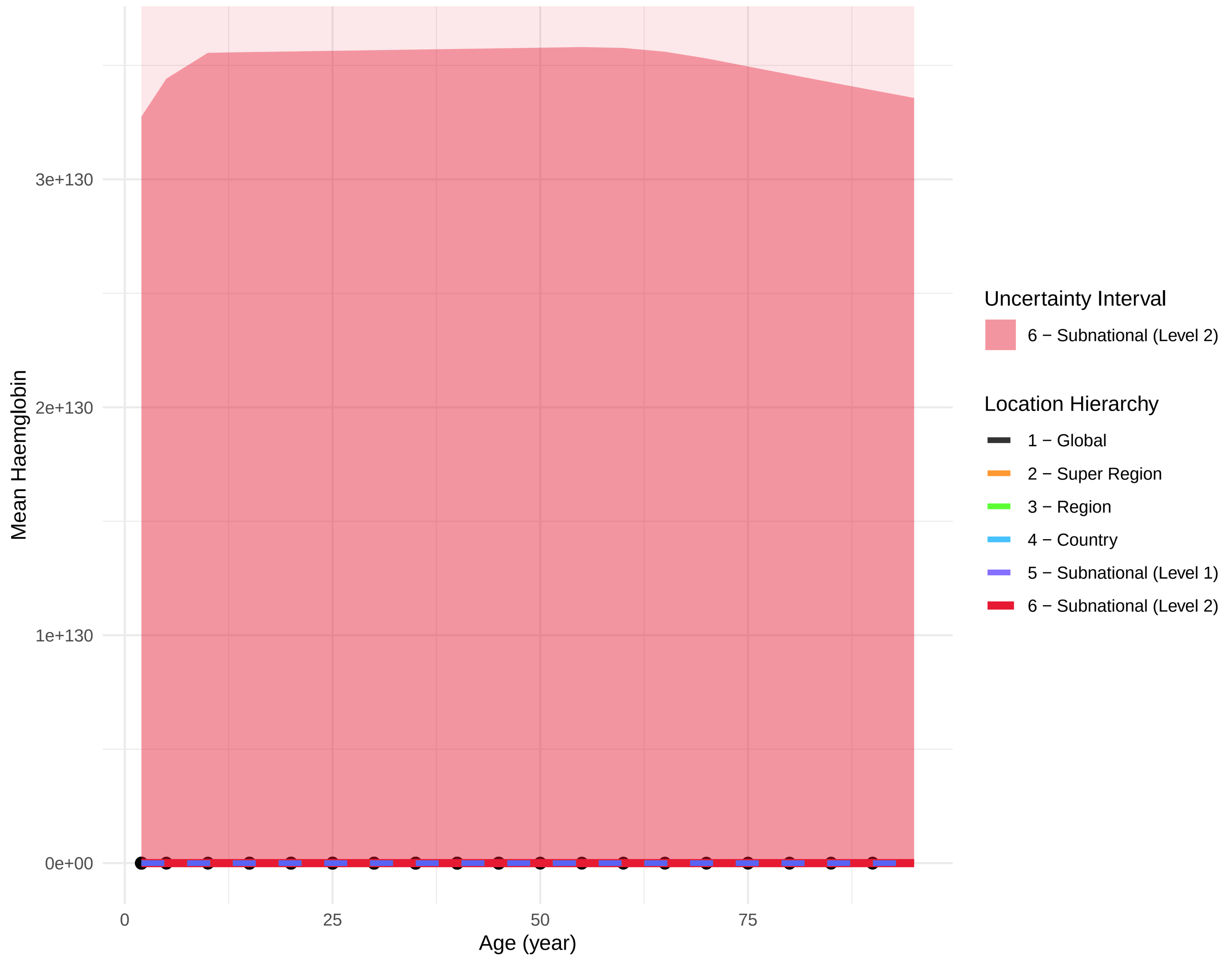

MR−BRT Age/Sex Fits − Non−pregnant Females 2+ Years Old − Mean Haemglobin − Nagaland
WHO−2001 Adjustment Method

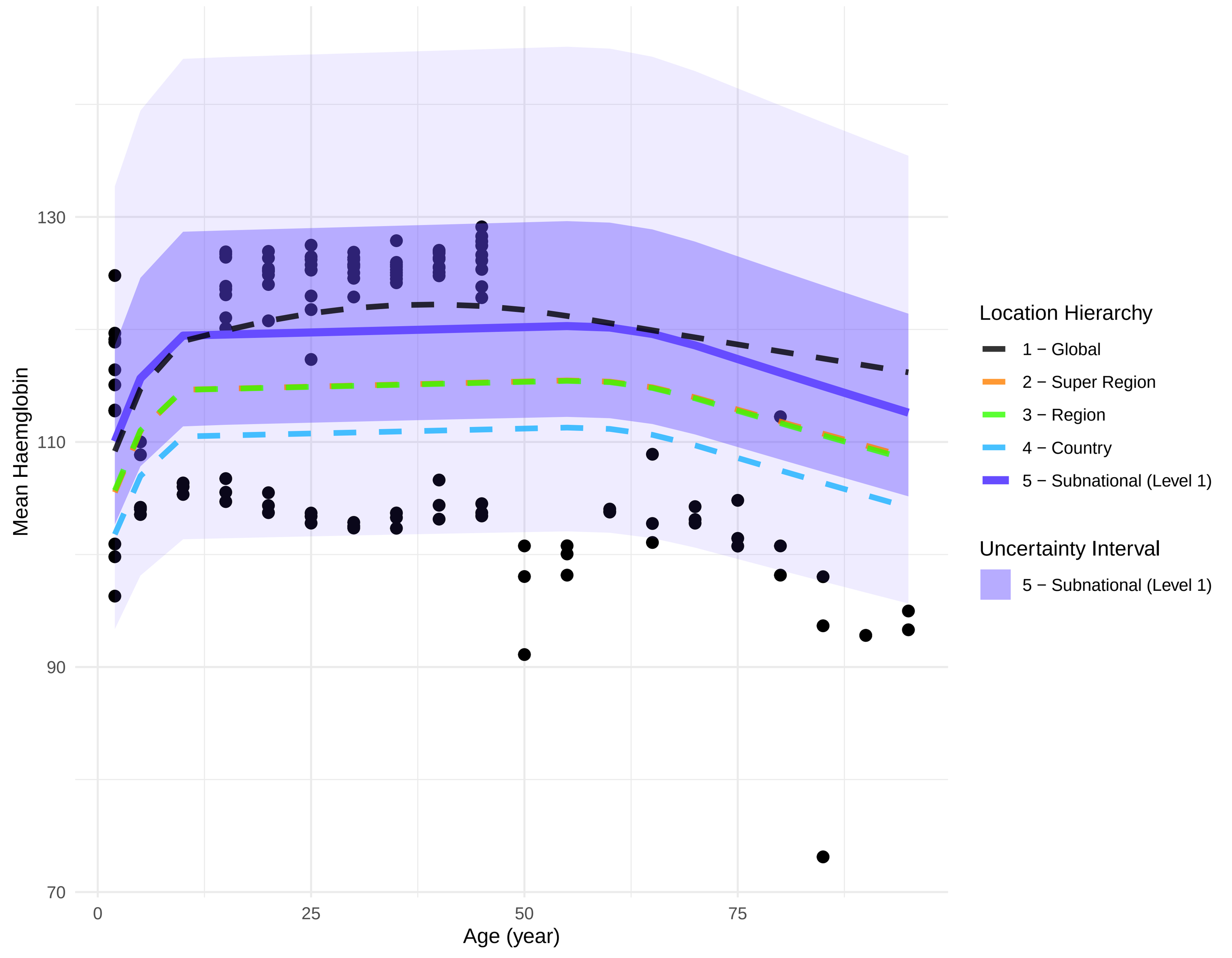

MR−BRT Age/Sex Fits − Non−pregnant Females 2+ Years Old − Mean Haemglobin − Nagaland, Rural
WHO−2001 Adjustment Method

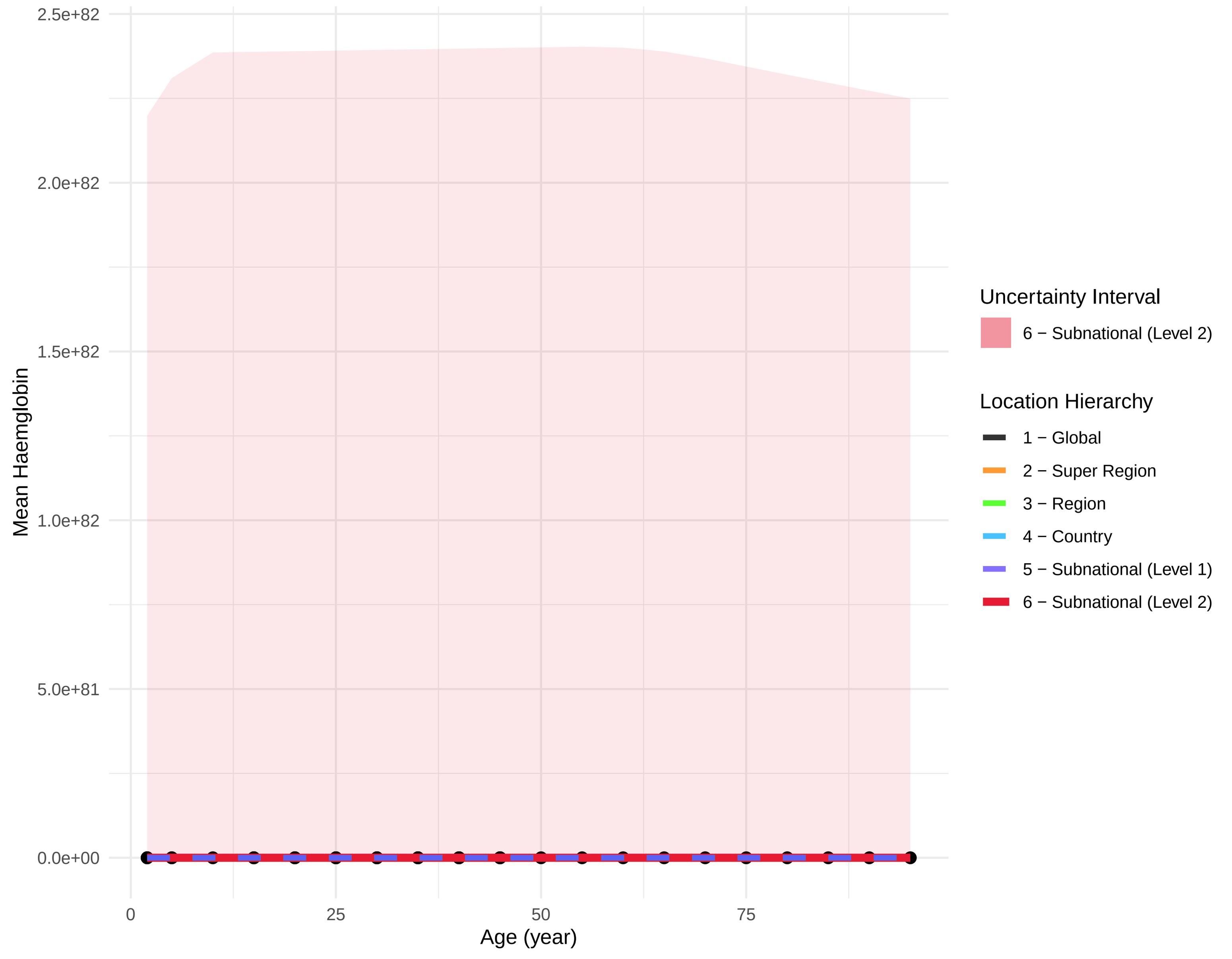

MR−BRT Age/Sex Fits − Non−pregnant Females 2+ Years Old − Mean Haemglobin − Nagaland, Urban
WHO−2001 Adjustment Method

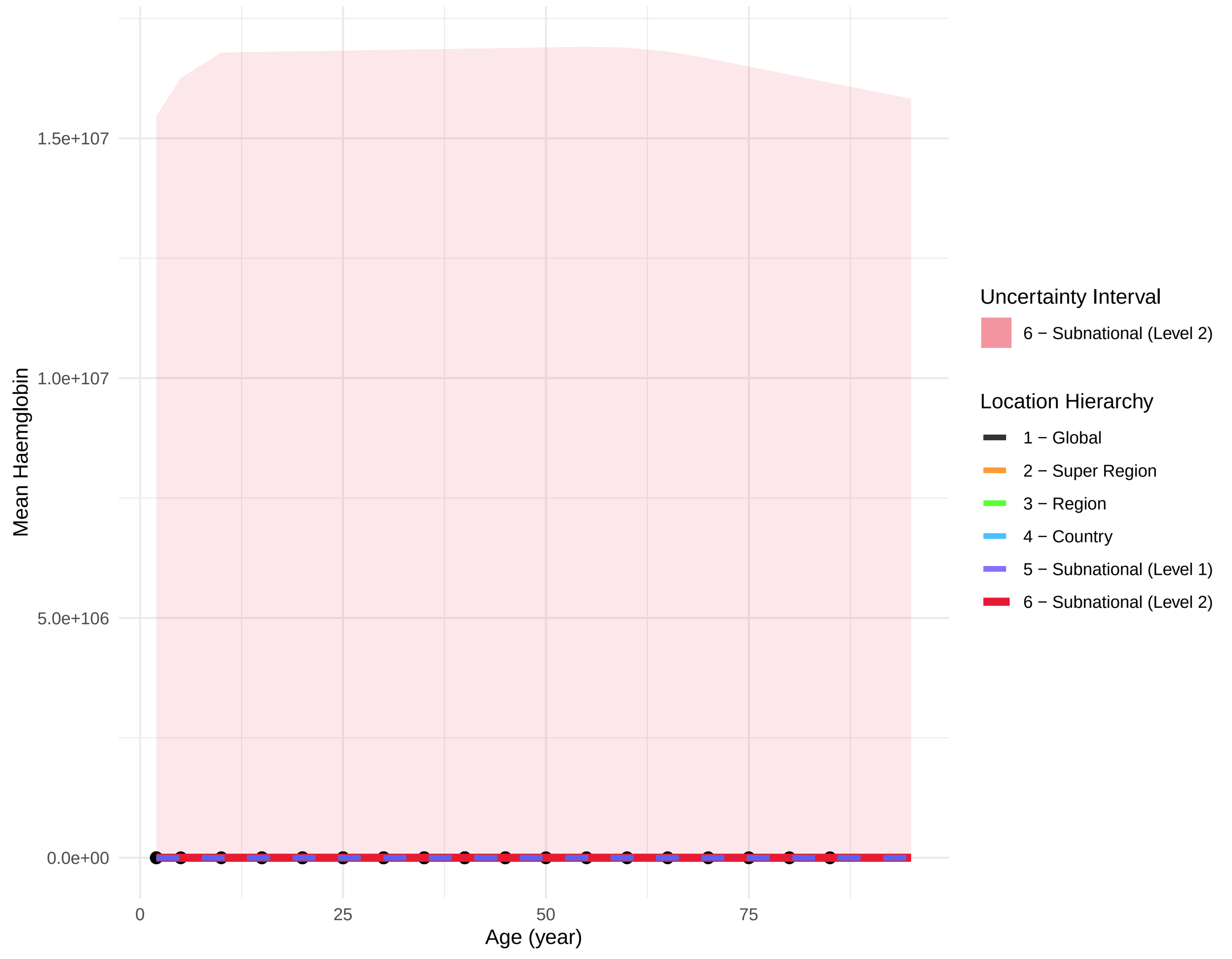

MR−BRT Age/Sex Fits − Non−pregnant Females 2+ Years Old − Mean Haemglobin − Odisha
WHO−2001 Adjustment Method

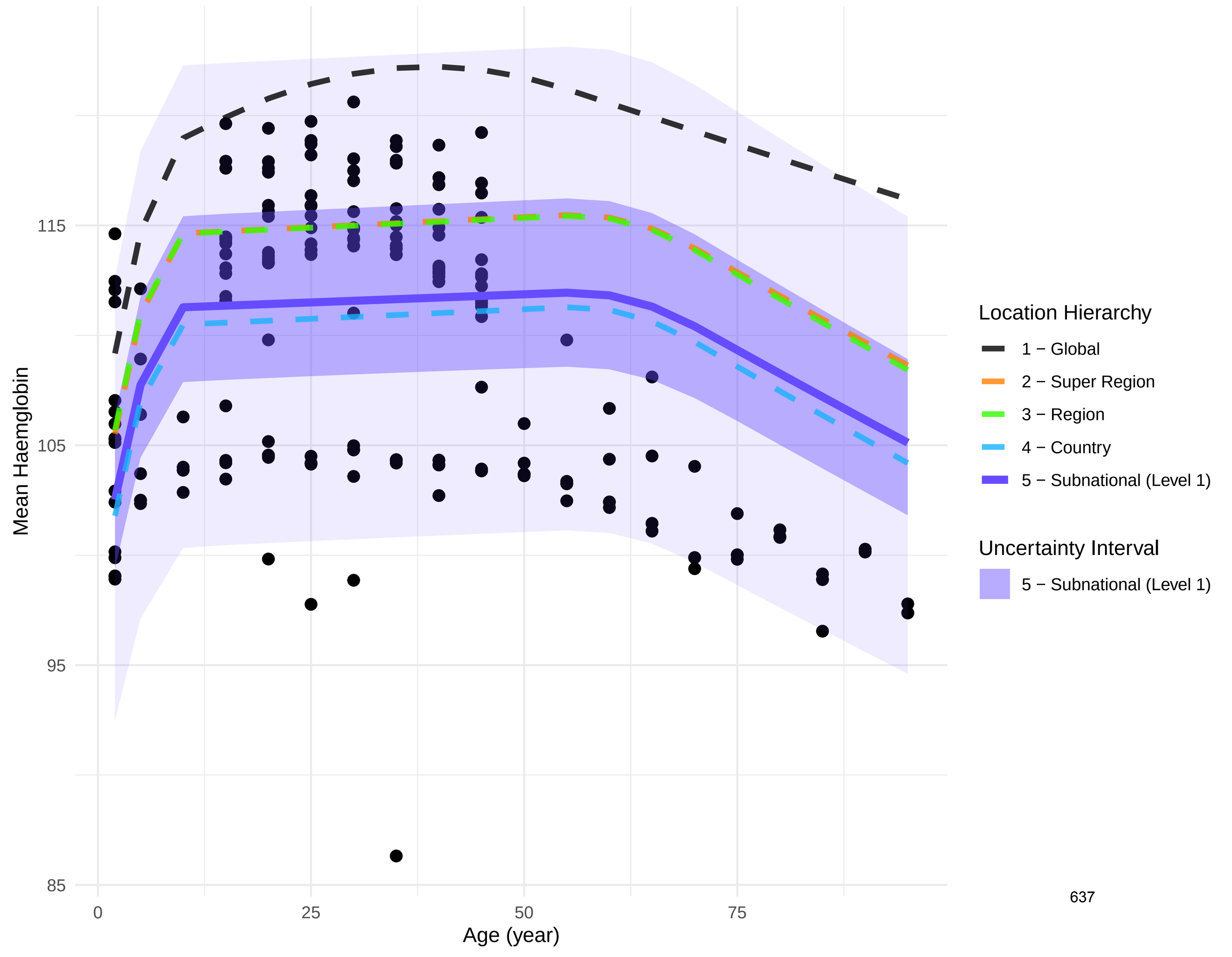

MR−BRT Age/Sex Fits − Non-pregnant Females 2+ Years Old − Mean Haemglobin − Odisha, Rural
WHO−2001 Adjustment Method

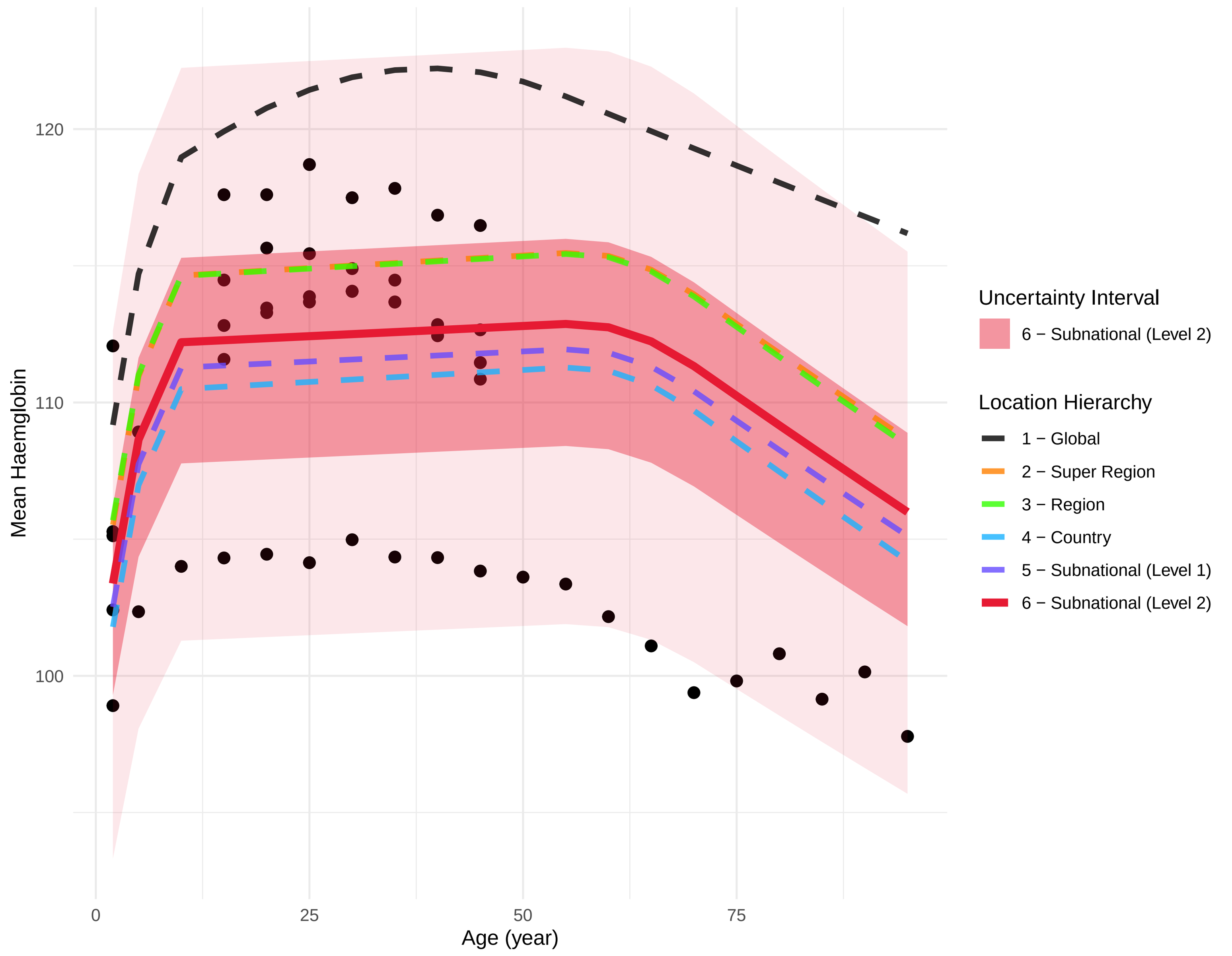

MR−BRT Age/Sex Fits − Non-pregnant Females 2+ Years Old − Mean Haemglobin − Odisha, Urban
WHO−2001 Adjustment Method

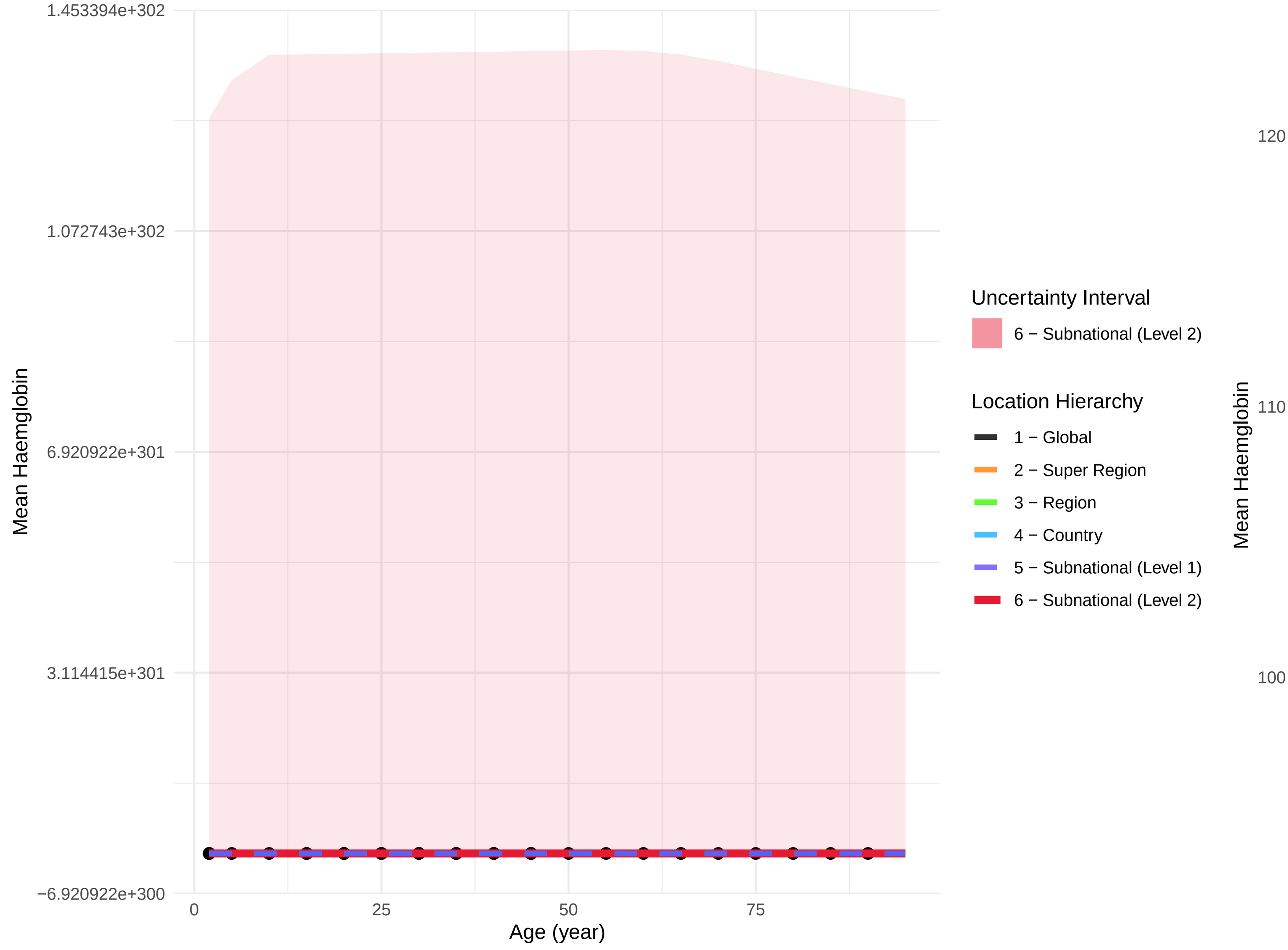

MR−BRT Age/Sex Fits − Non-pregnant Females 2+ Years Old − Mean Haemglobin − Other Union Territories
WHO−2001 Adjustment Method

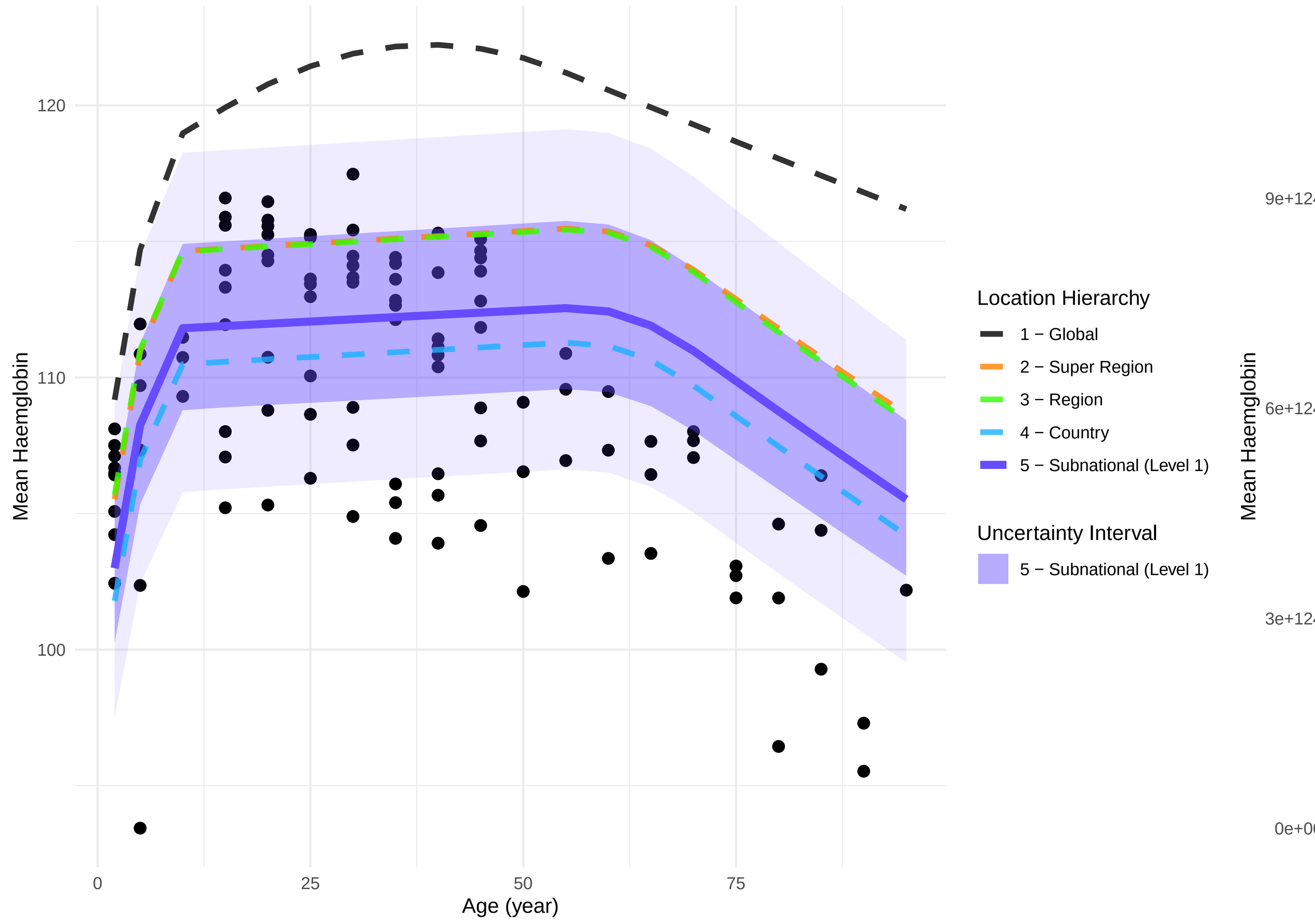

MR−BRT Age/Sex Fits − Non-pregnant Females 2+ Years Old − Mean Haemglobin − Other Union Territories, Rural
WHO−2001 Adjustment Method

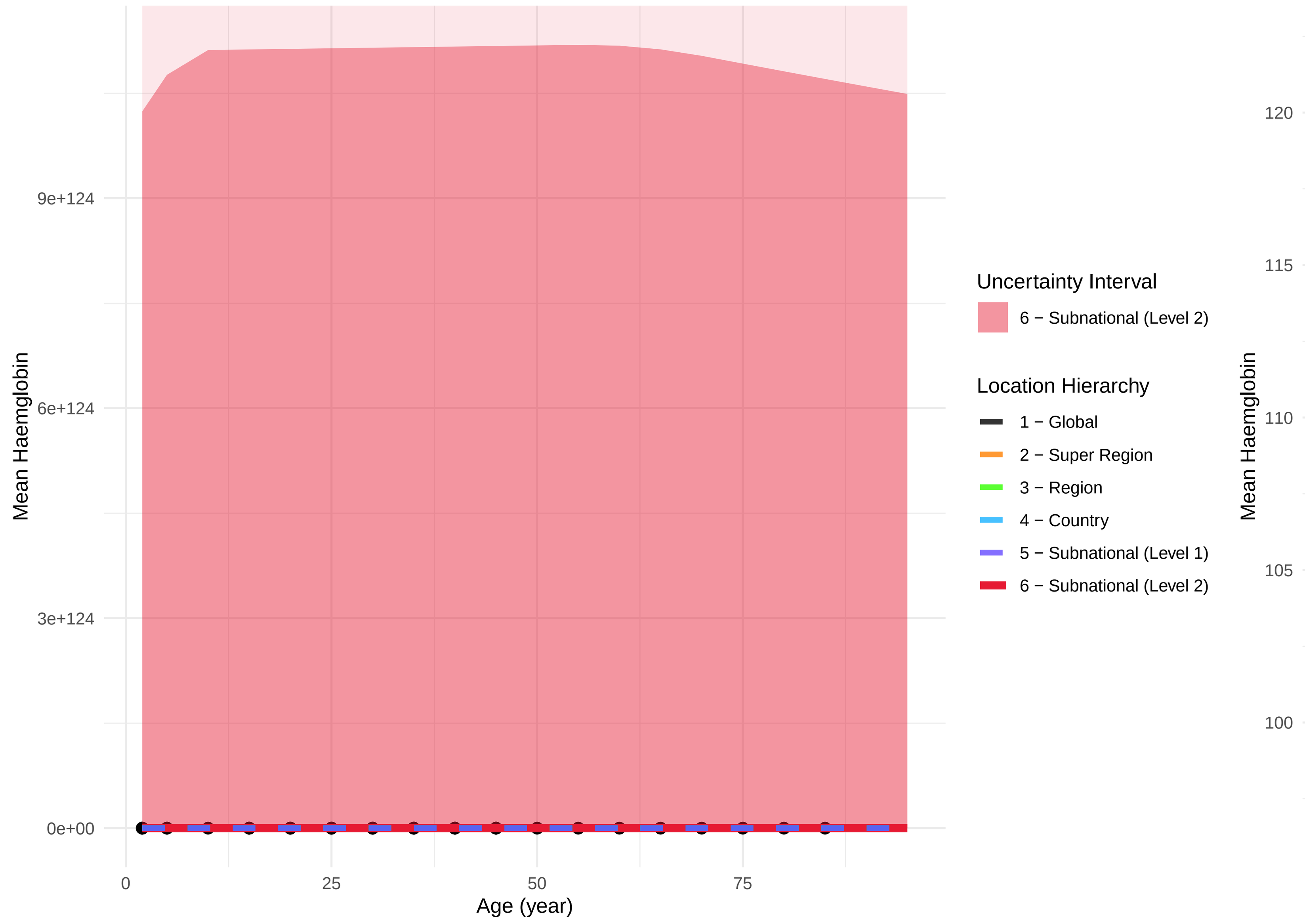

MR−BRT Age/Sex Fits − Non-pregnant Females 2+ Years Old − Mean Haemglobin − Other Union Territories, Urban
WHO−2001 Adjustment Method

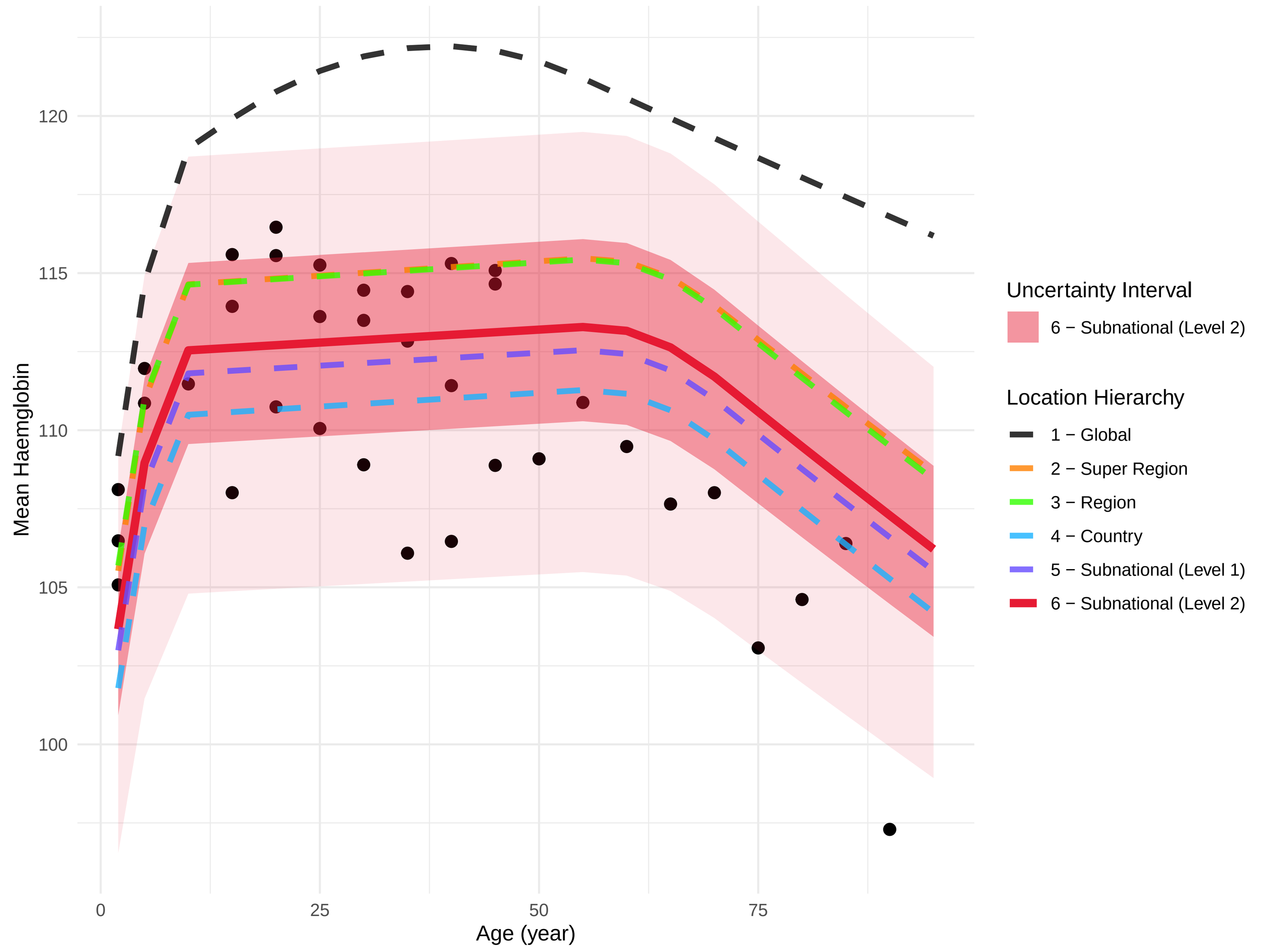

MR−BRT Age/Sex Fits − Non-pregnant Females 2+ Years Old − Mean Haemglobin − Punjab
WHO−2001 Adjustment Method

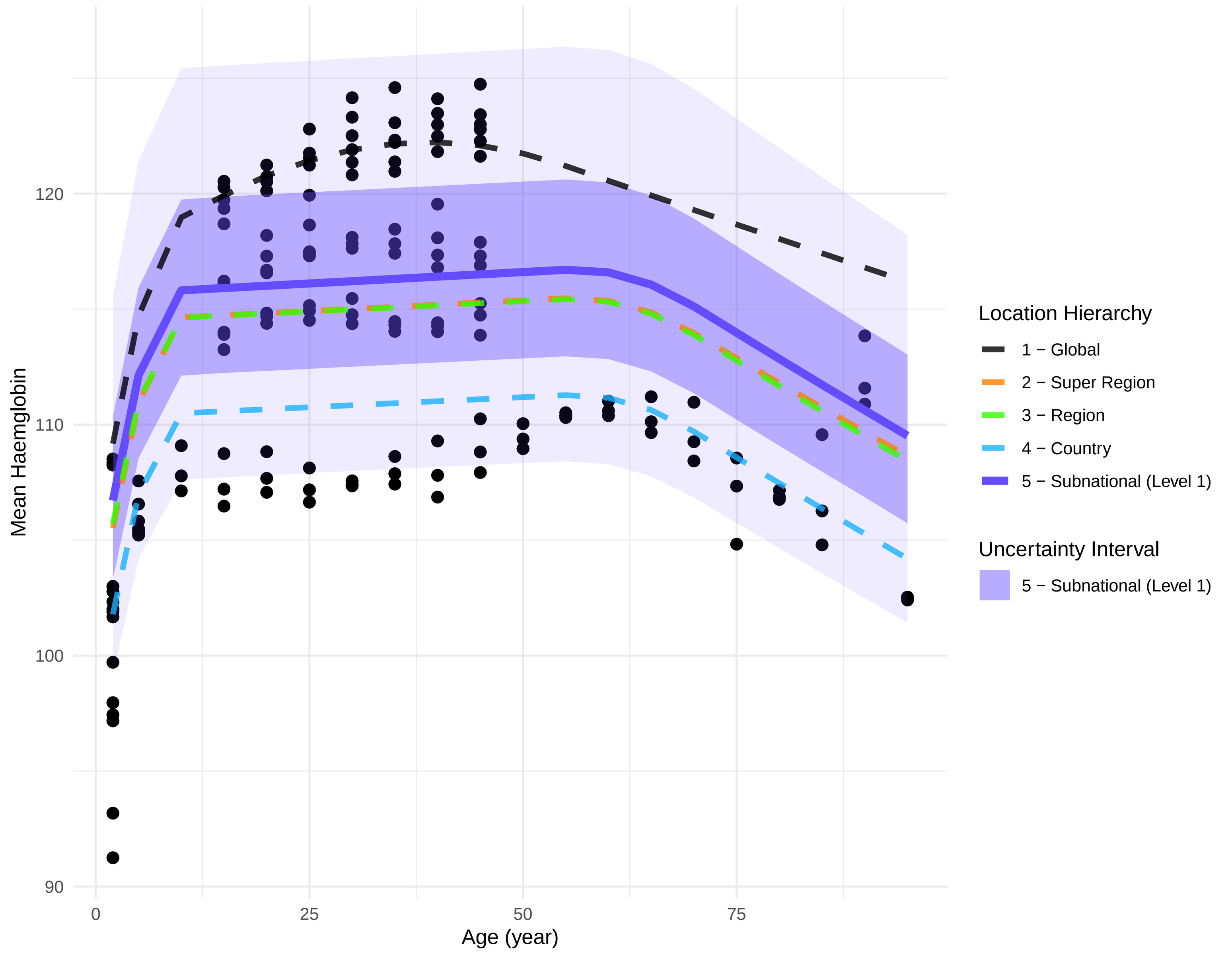

MR−BRT Age/Sex Fits − Non-pregnant Females 2+ Years Old − Mean Haemglobin − Punjab, Rural
WHO−2001 Adjustment Method

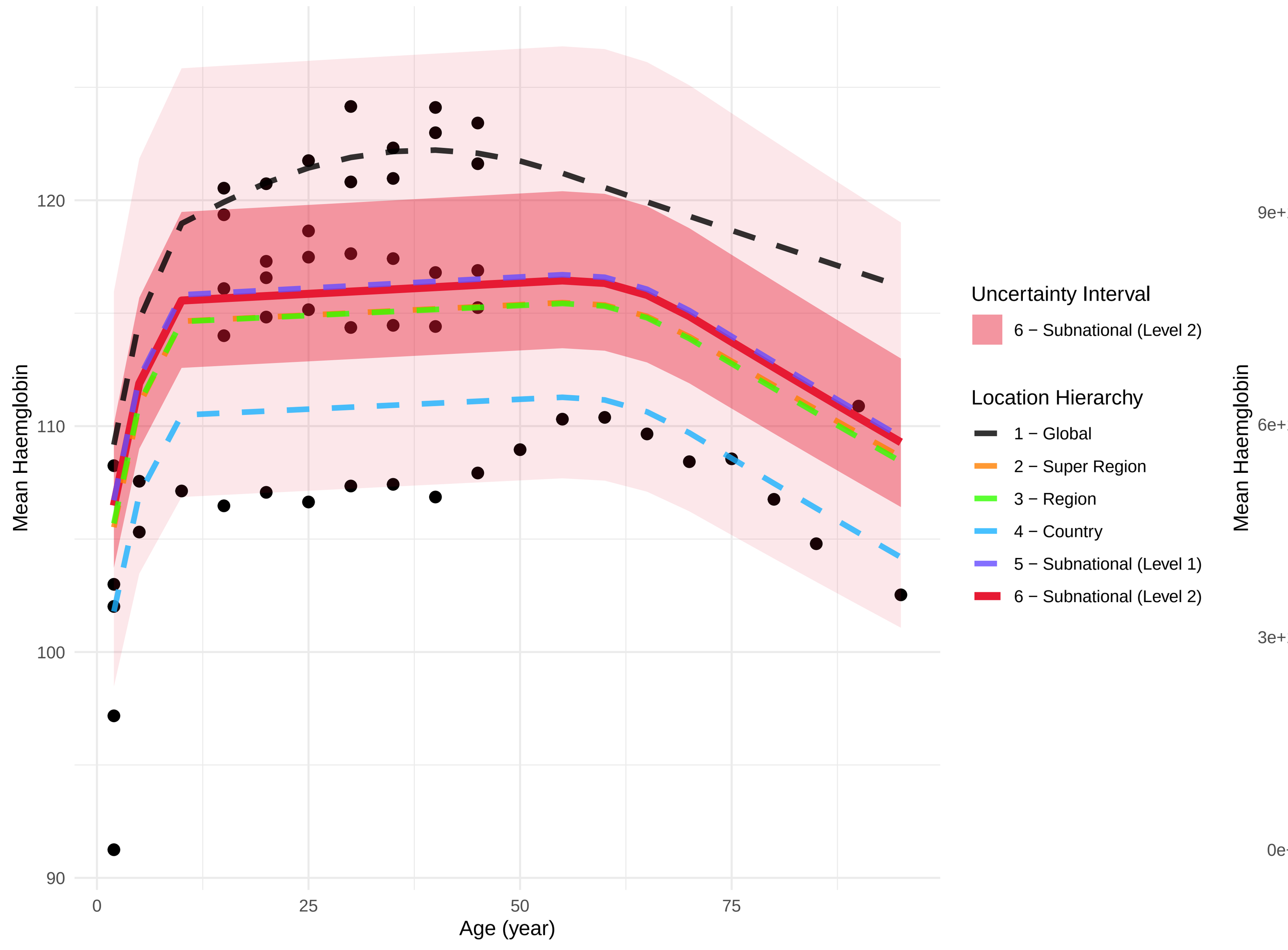

MR−BRT Age/Sex Fits − Non-pregnant Females 2+ Years Old − Mean Haemglobin − Punjab, Urban
WHO−2001 Adjustment Method

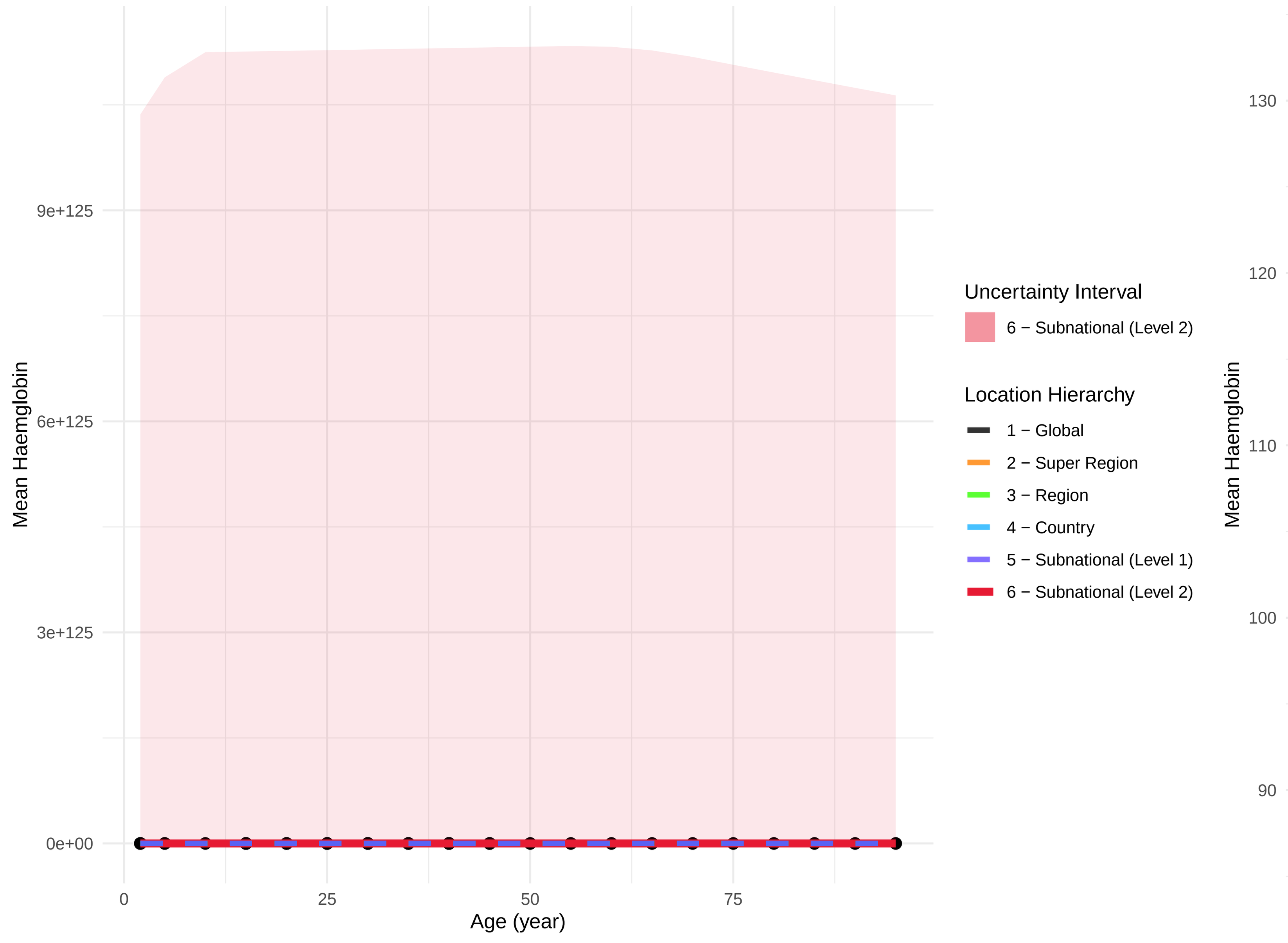

MR−BRT Age/Sex Fits − Non-pregnant Females 2+ Years Old − Mean Haemglobin − Rajasthan
WHO−2001 Adjustment Method

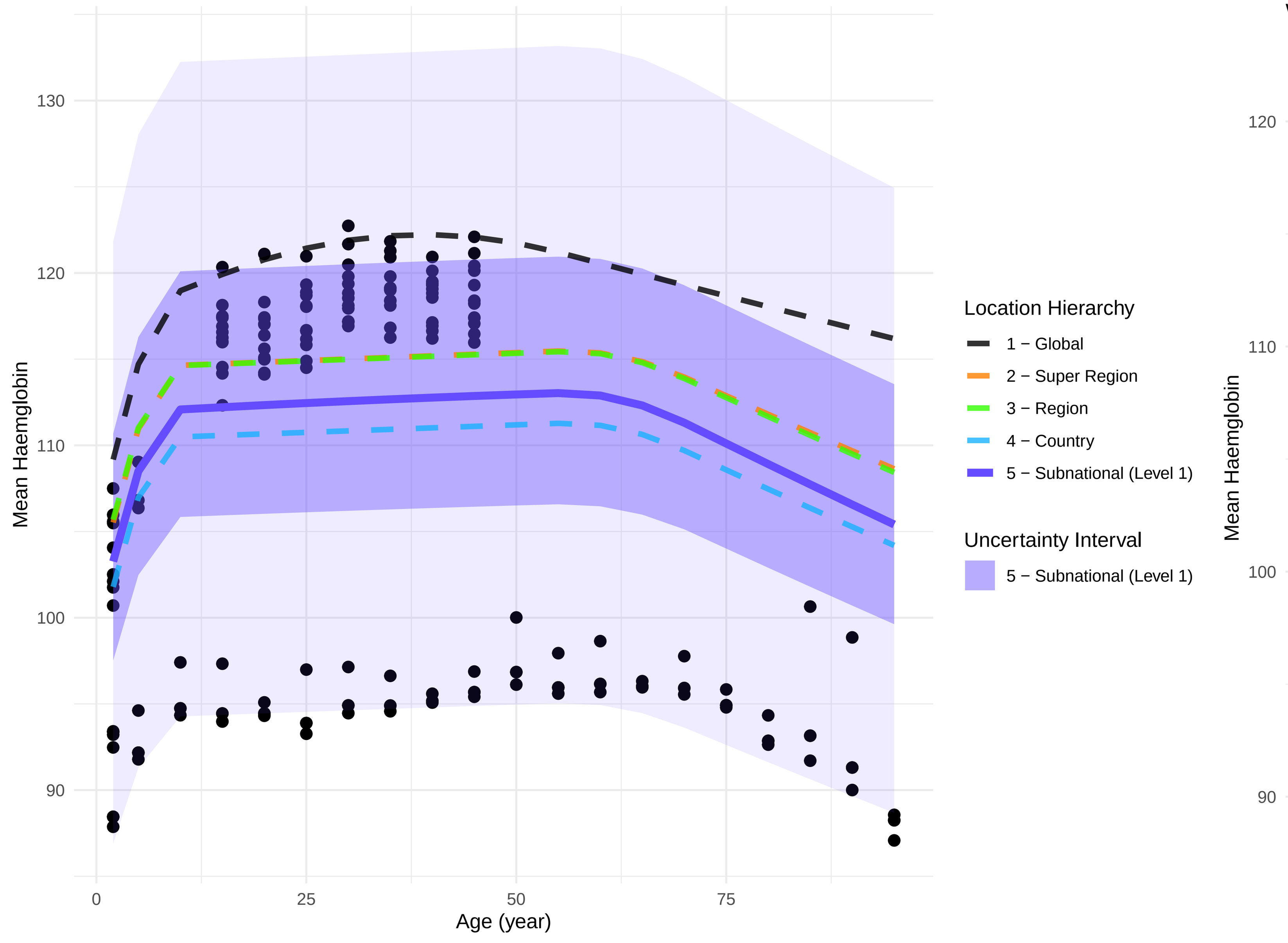

MR−BRT Age/Sex Fits − Non-pregnant Females 2+ Years Old − Mean Haemglobin − Rajasthan, Rural
WHO−2001 Adjustment Method

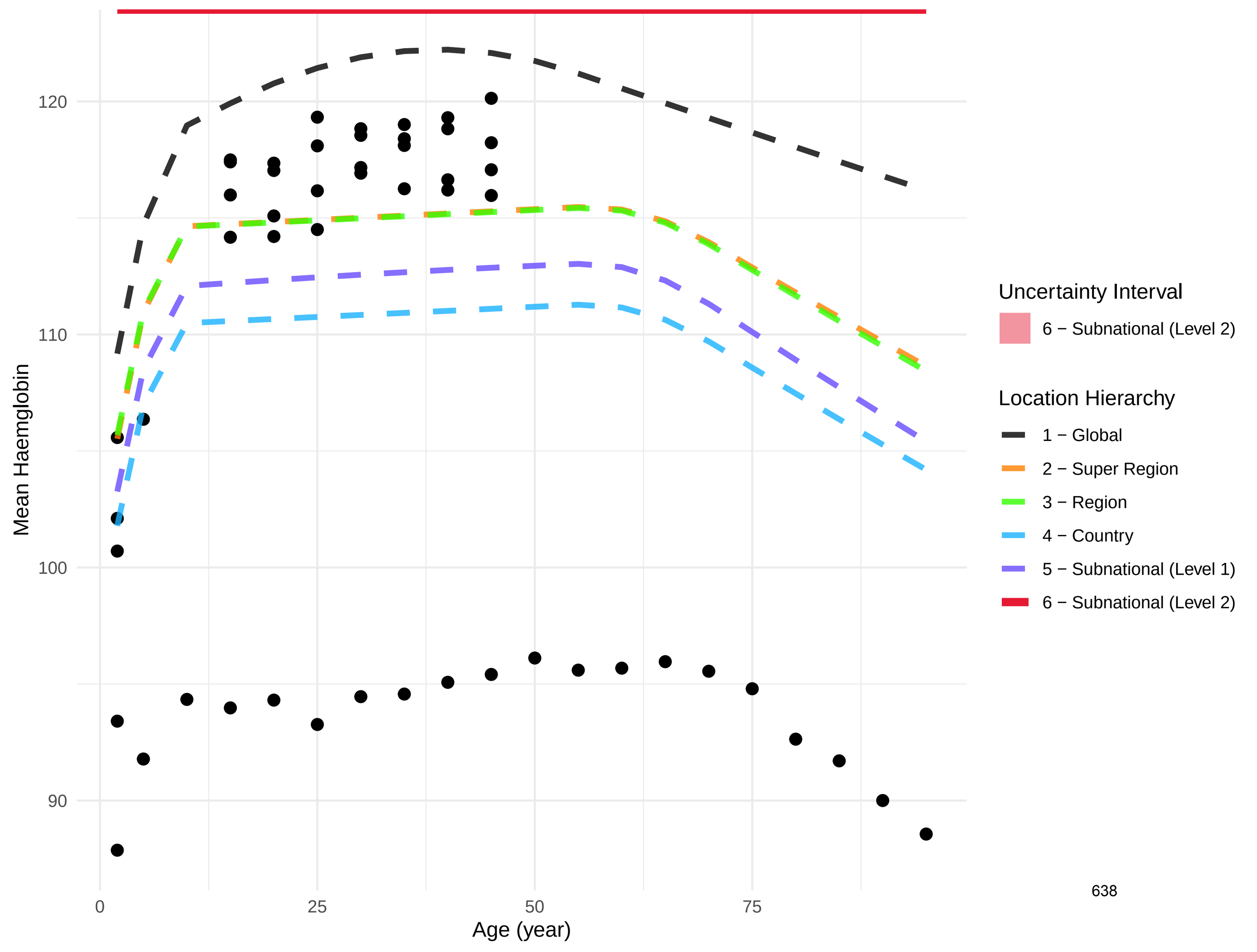

MR−BRT Age/Sex Fits − Non−pregnant Females 2+ Years Old − Mean Haemglobin − Rajasthan, Urban
WHO−2001 Adjustment Method

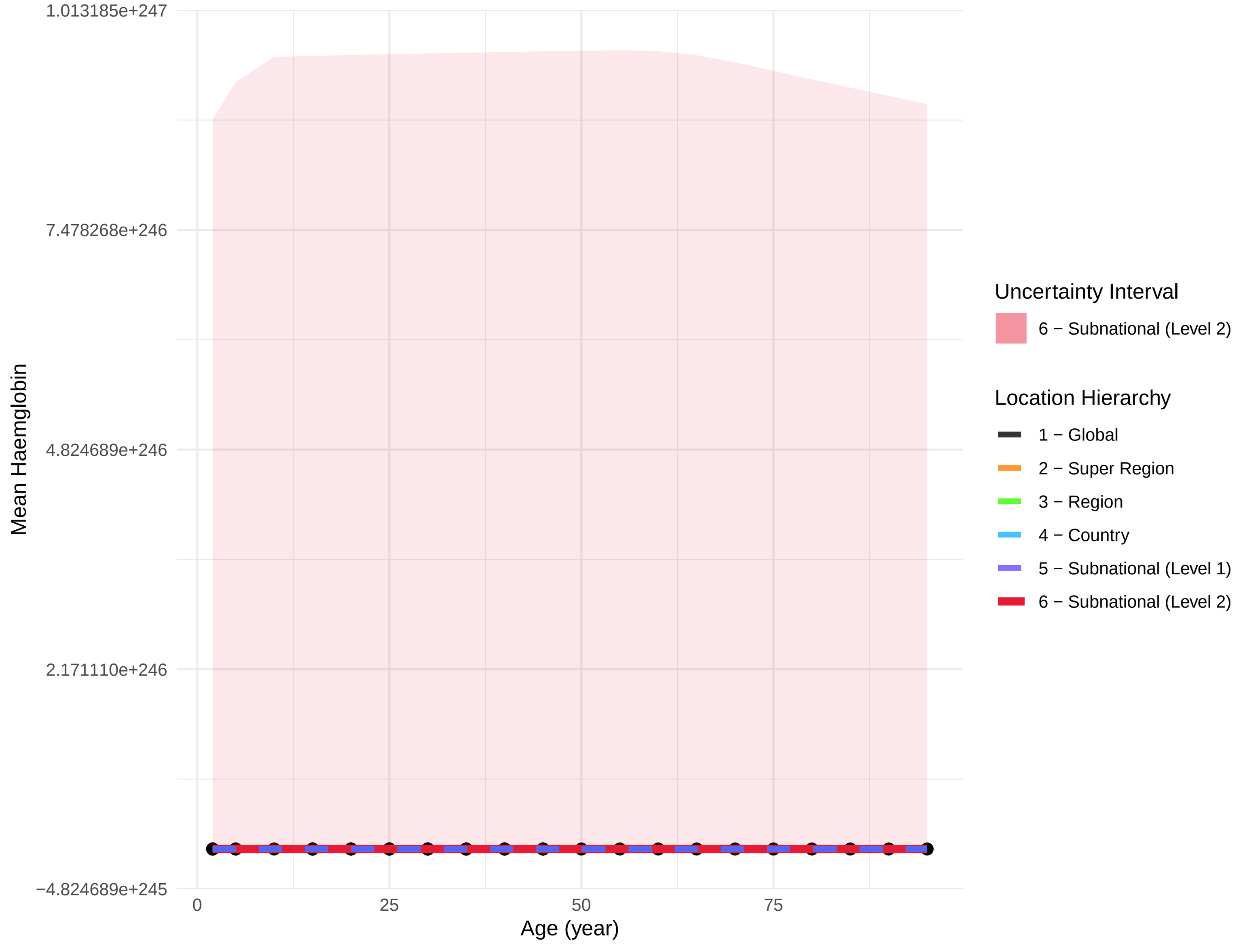

MR−BRT Age/Sex Fits − Non−pregnant Females 2+ Years Old − Mean Haemglobin − Sikkim
WHO−2001 Adjustment Method

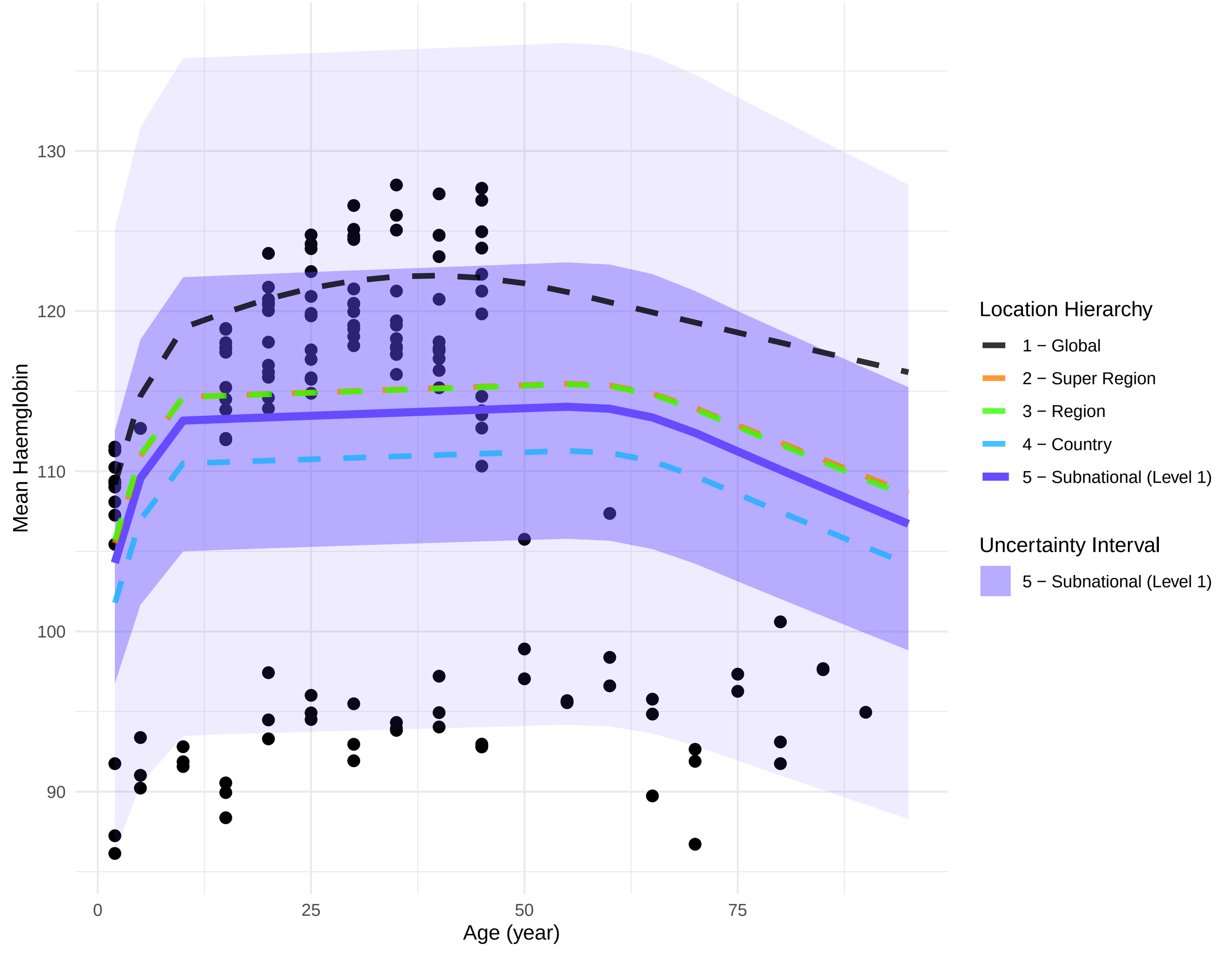

MR−BRT Age/Sex Fits − Non−pregnant Females 2+ Years Old − Mean Haemglobin − Sikkim, Rural
WHO−2001 Adjustment Method

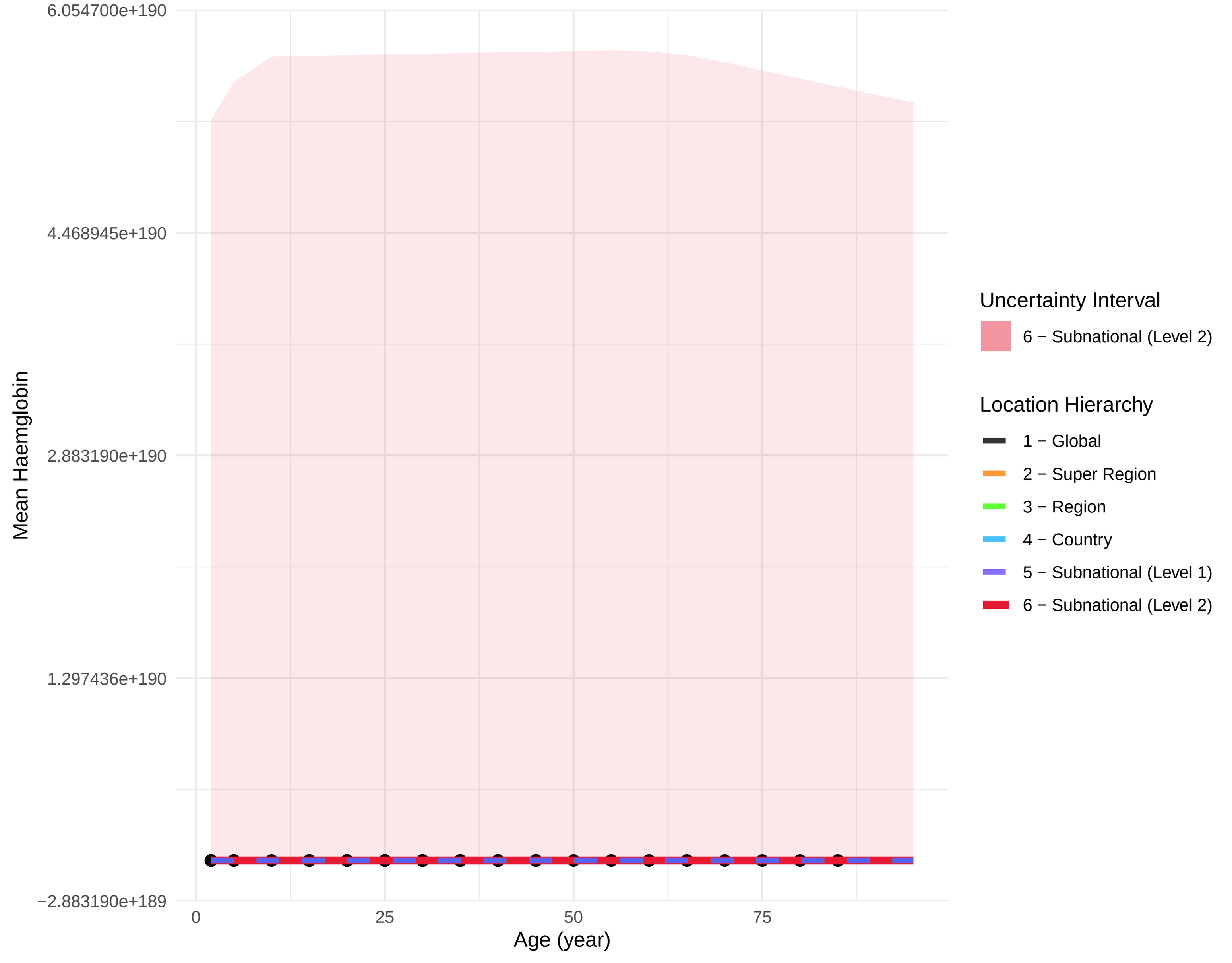

MR−BRT Age/Sex Fits − Non−pregnant Females 2+ Years Old − Mean Haemglobin − Sikkim, Urban
WHO−2001 Adjustment Method

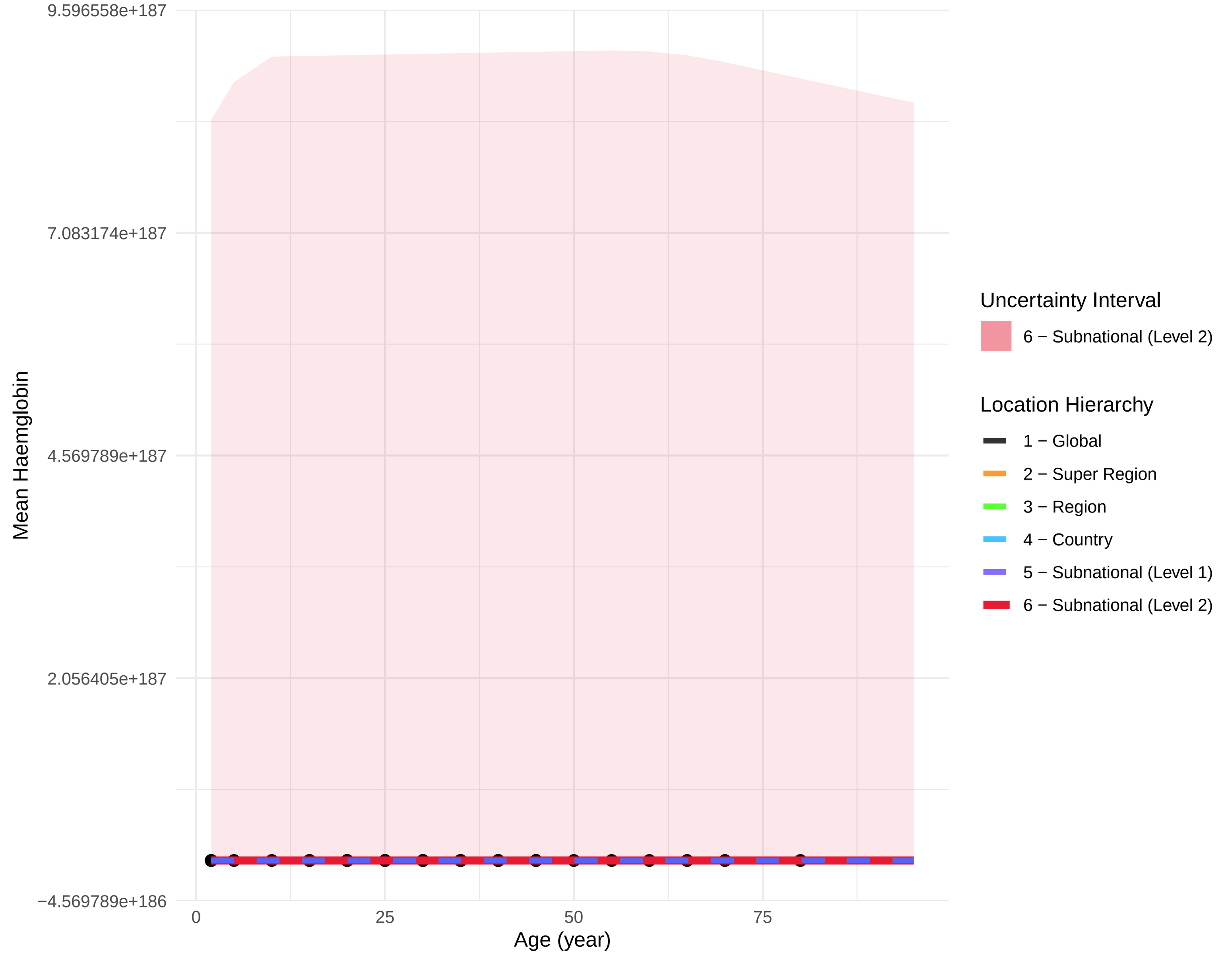

MR−BRT Age/Sex Fits − Non−pregnant Females 2+ Years Old − Mean Haemglobin − Tamil Nadu
WHO−2001 Adjustment Method

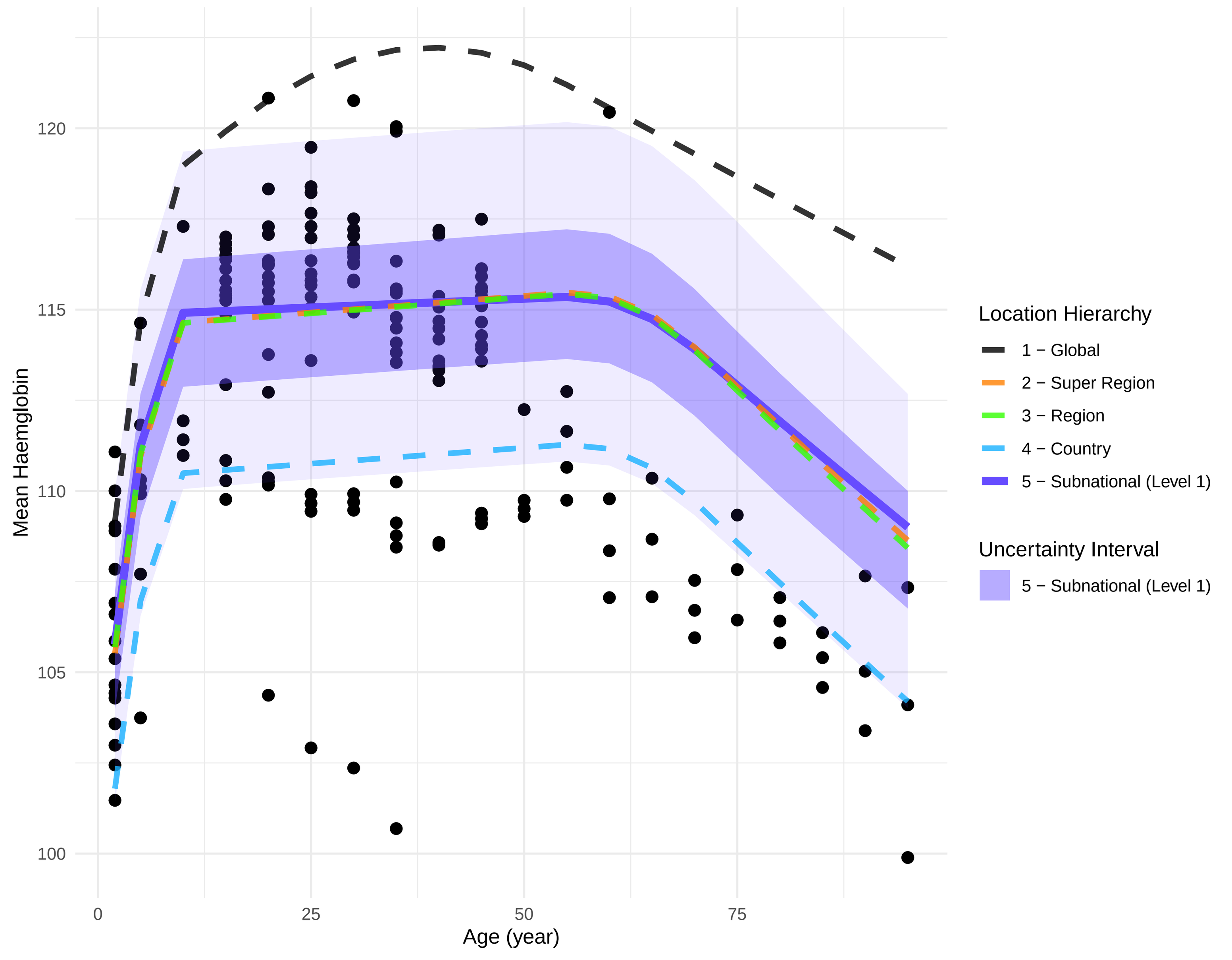

MR−BRT Age/Sex Fits − Non−pregnant Females 2+ Years Old − Mean Haemglobin − Tamil Nadu, Rural
WHO−2001 Adjustment Method

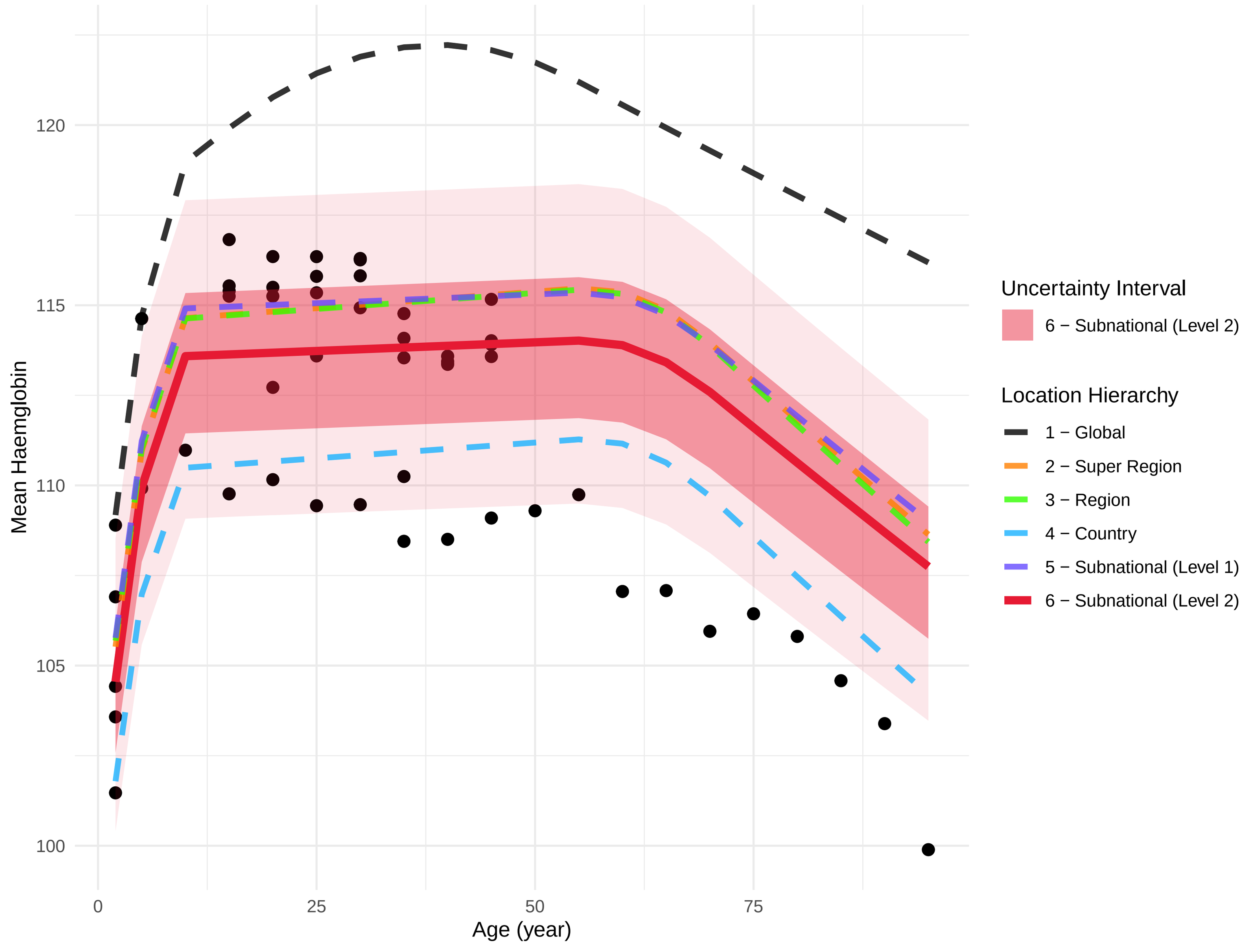

MR−BRT Age/Sex Fits − Non−pregnant Females 2+ Years Old − Mean Haemglobin − Tamil Nadu, Urban
WHO−2001 Adjustment Method

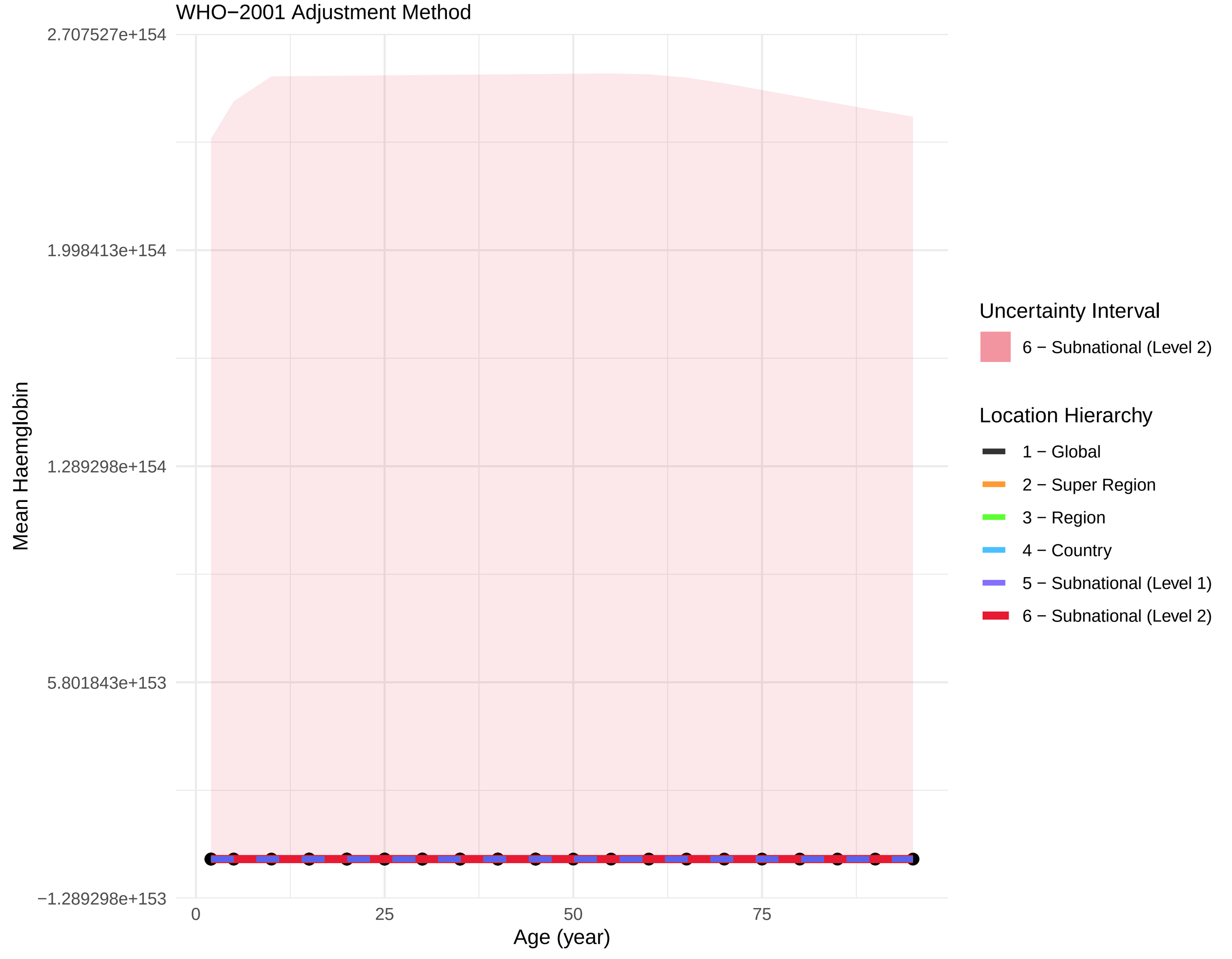

MR−BRT Age/Sex Fits − Non−pregnant Females 2+ Years Old − Mean Haemglobin − Telangana
WHO−2001 Adjustment Method

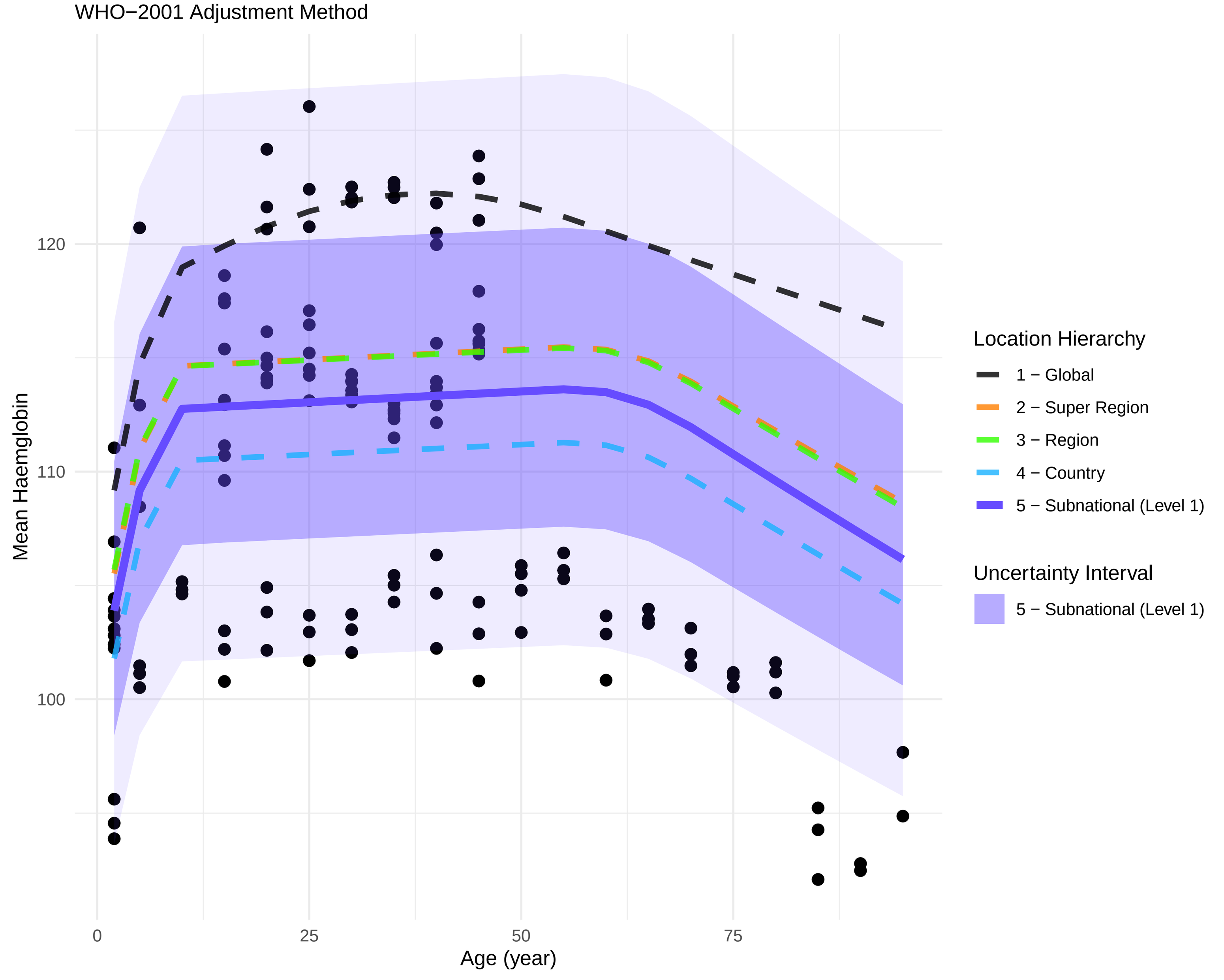

MR−BRT Age/Sex Fits − Non−pregnant Females 2+ Years Old − Mean Haemglobin − Telangana, Rural
WHO−2001 Adjustment Method

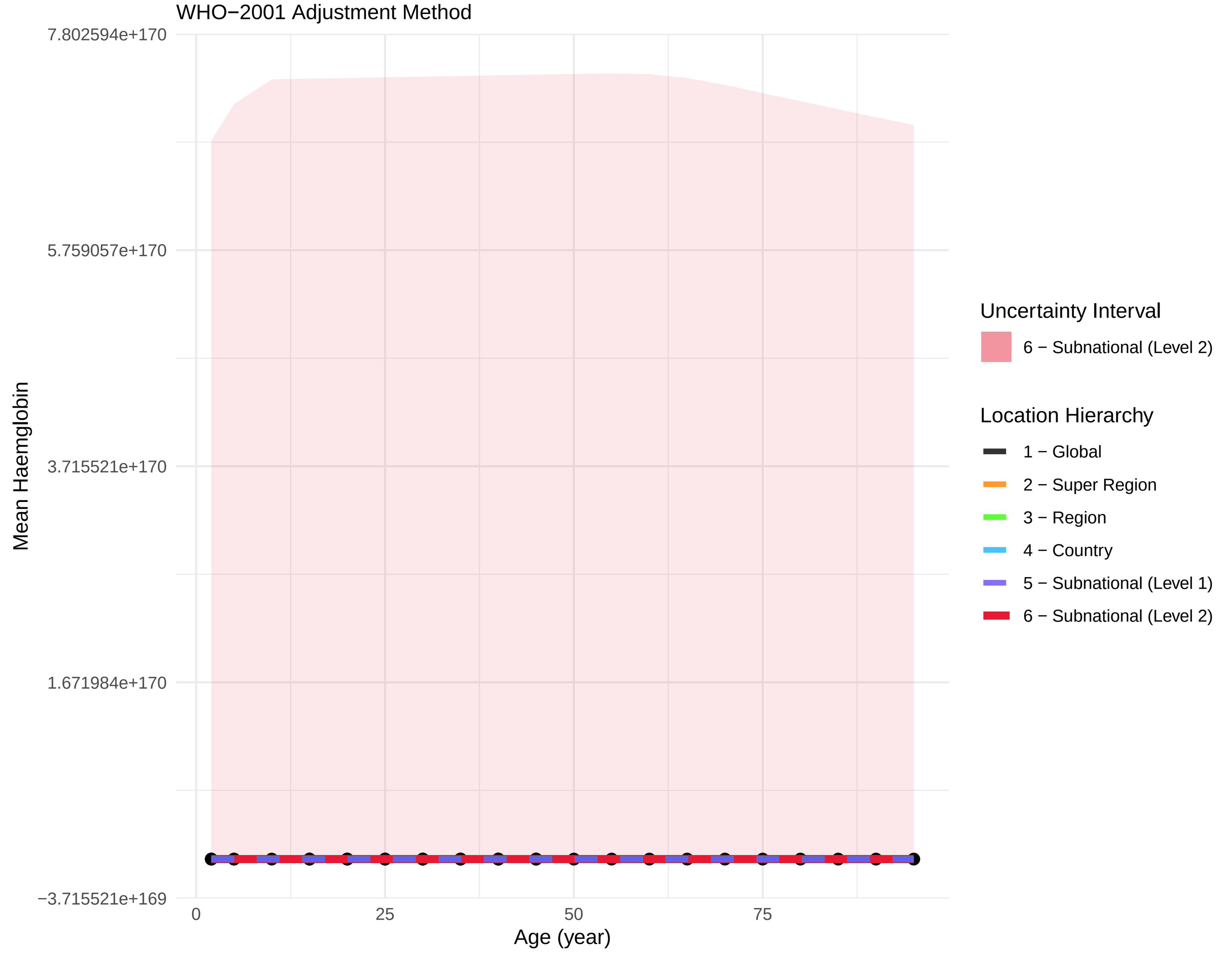

MR−BRT Age/Sex Fits − Non−pregnant Females 2+ Years Old − Mean Haemglobin − Telangana, Urban
WHO−2001 Adjustment Method

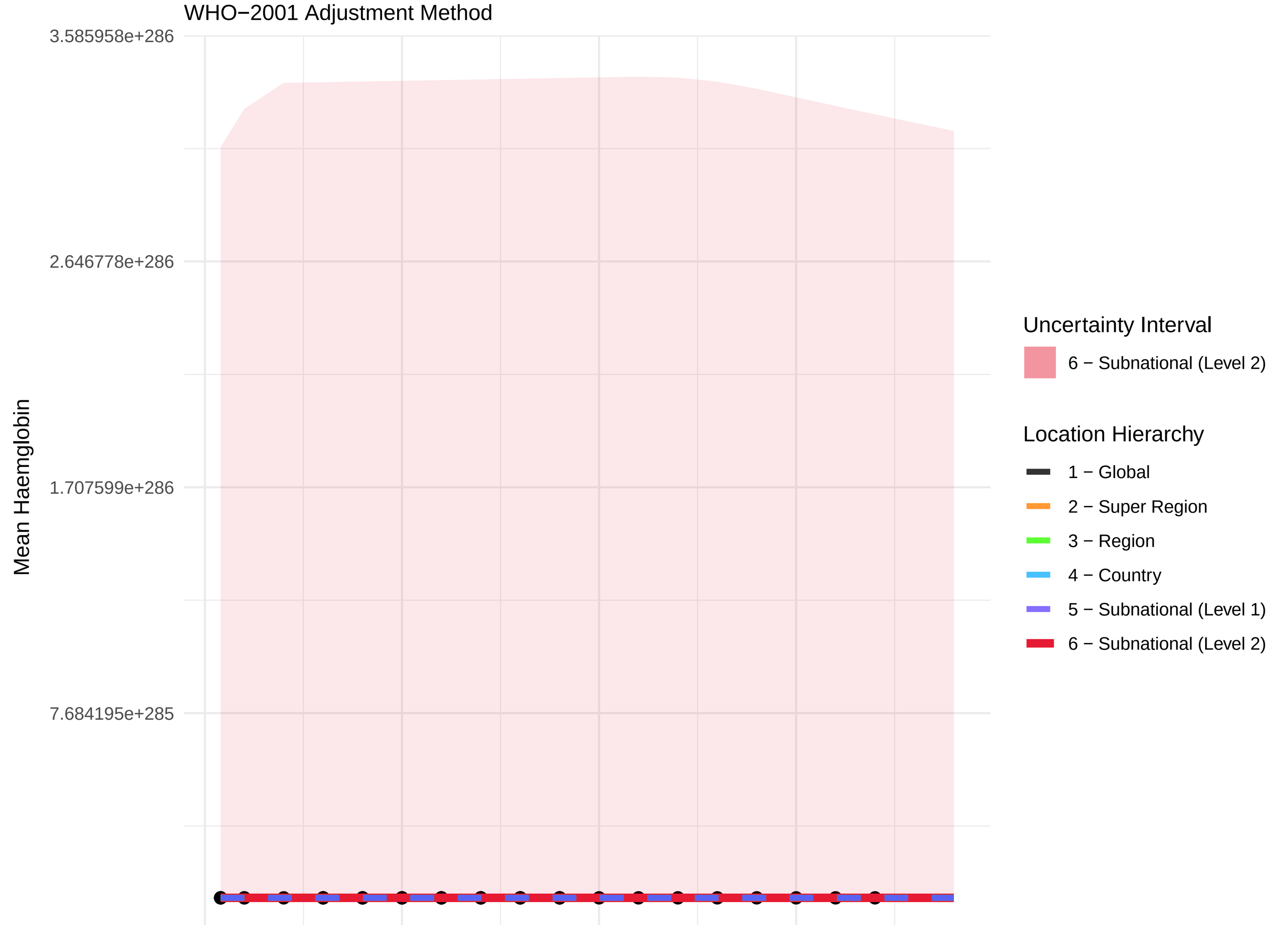

MR−BRT Age/Sex Fits − Non−pregnant Females 2+ Years Old − Mean Haemglobin − Tripura
WHO−2001 Adjustment Method

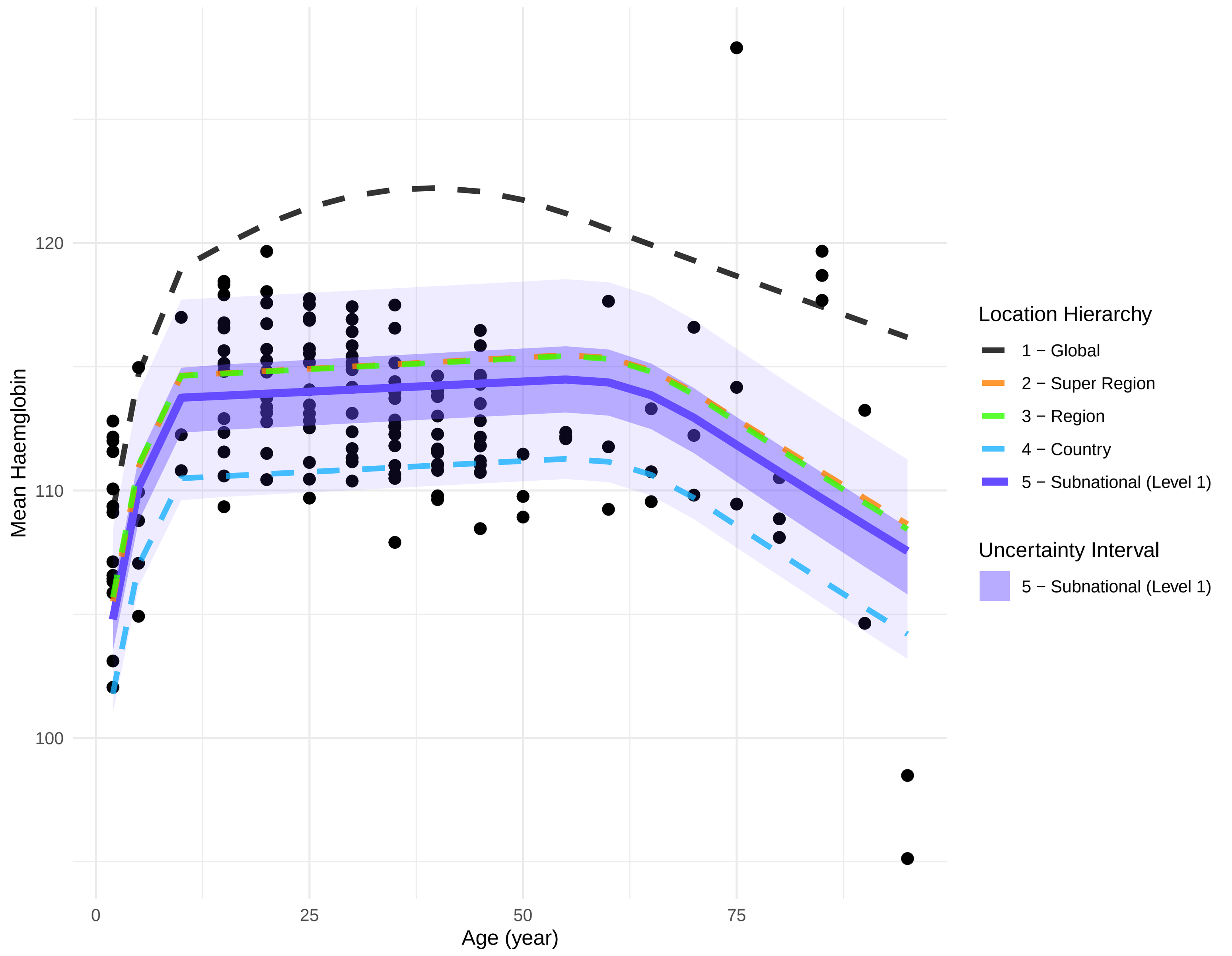

MR−BRT Age/Sex Fits − Non−pregnant Females 2+ Years Old − Mean Haemglobin − Tripura, Rural
WHO−2001 Adjustment Method

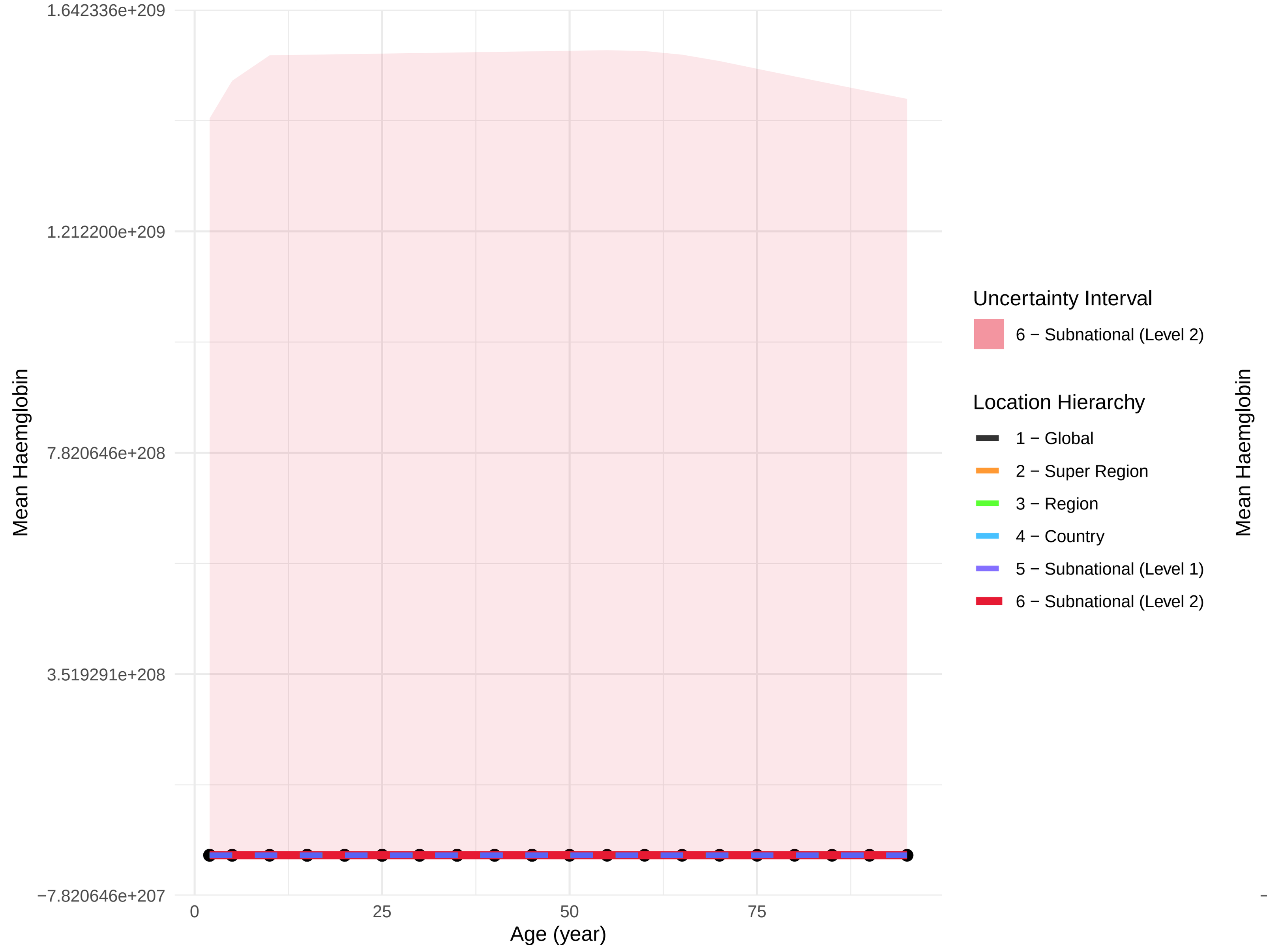

MR−BRT Age/Sex Fits − Non−pregnant Females 2+ Years Old − Mean Haemglobin − Tripura, Urban
WHO−2001 Adjustment Method

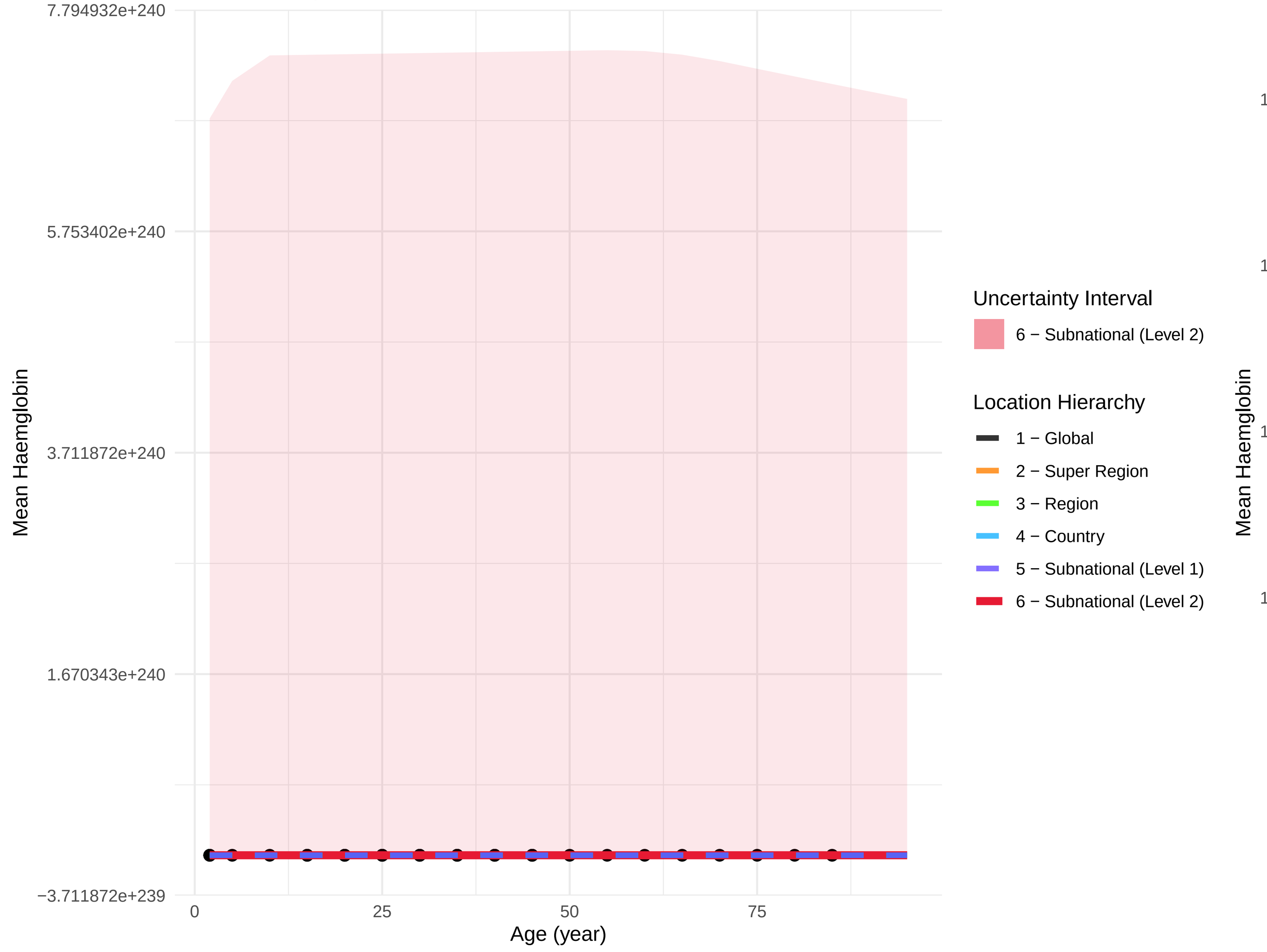

MR−BRT Age/Sex Fits − Non−pregnant Females 2+ Years Old − Mean Haemglobin − Uttar Pradesh
WHO−2001 Adjustment Method

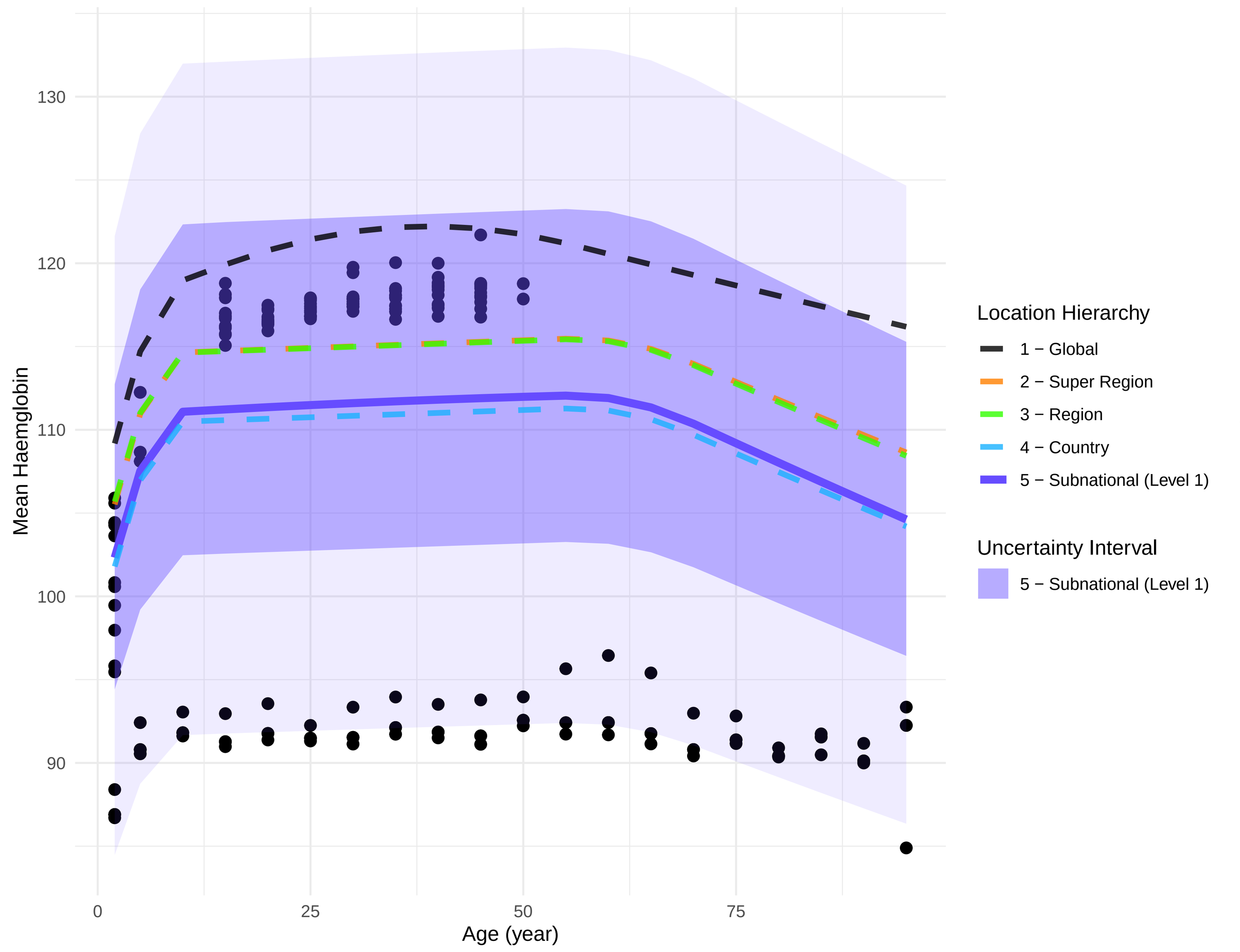

MR−BRT Age/Sex Fits − Non−pregnant Females 2+ Years Old − Mean Haemglobin − Uttar Pradesh, Rural
WHO−2001 Adjustment Method

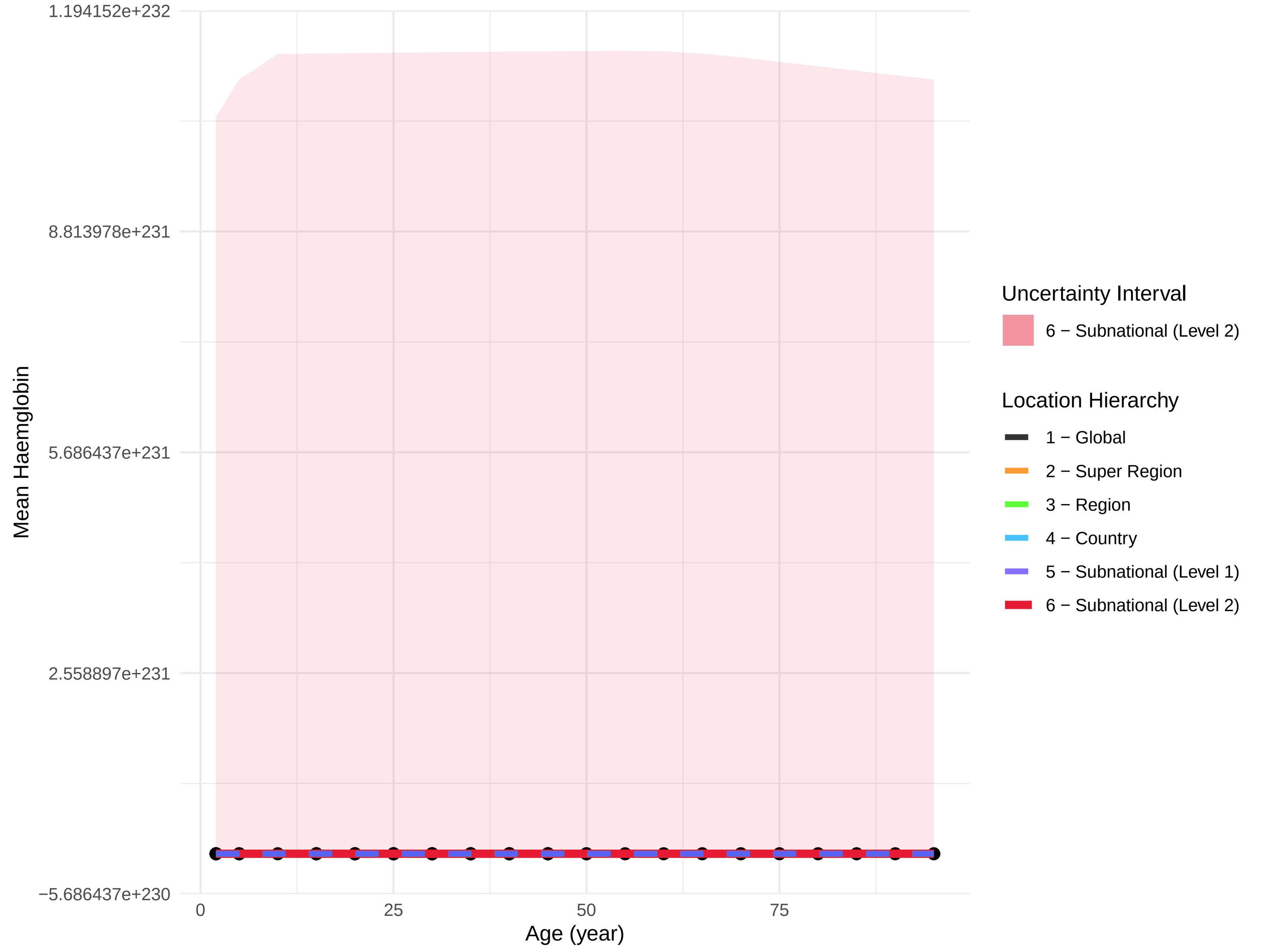

MR−BRT Age/Sex Fits − Non−pregnant Females 2+ Years Old − Mean Haemglobin − Uttar Pradesh, Urban
WHO−2001 Adjustment Method

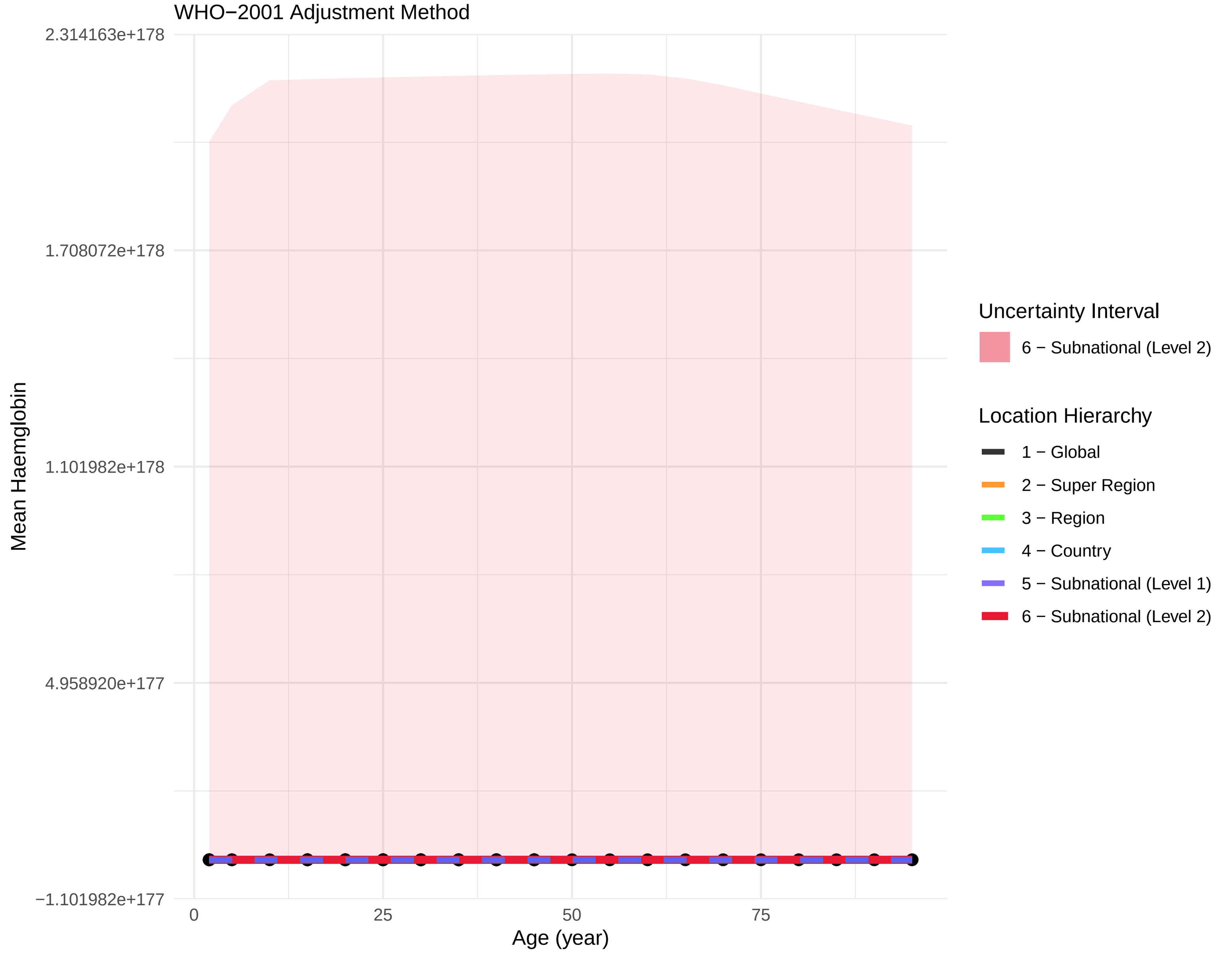

MR−BRT Age/Sex Fits − Non−pregnant Females 2+ Years Old − Mean Haemglobin − Uttarakhand
WHO−2001 Adjustment Method

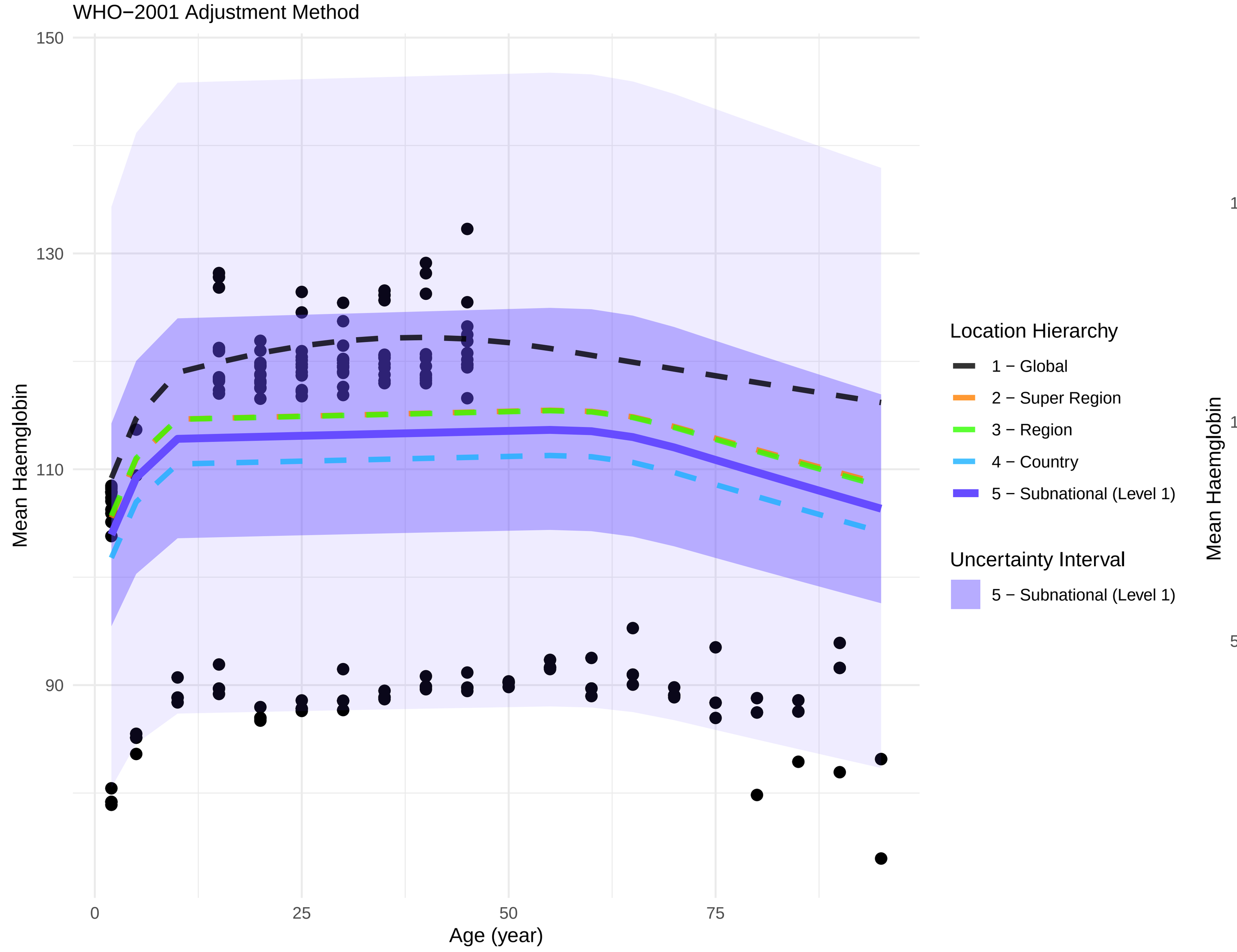

MR−BRT Age/Sex Fits − Non−pregnant Females 2+ Years Old − Mean Haemglobin − Uttarakhand, Rural
WHO−2001 Adjustment Method

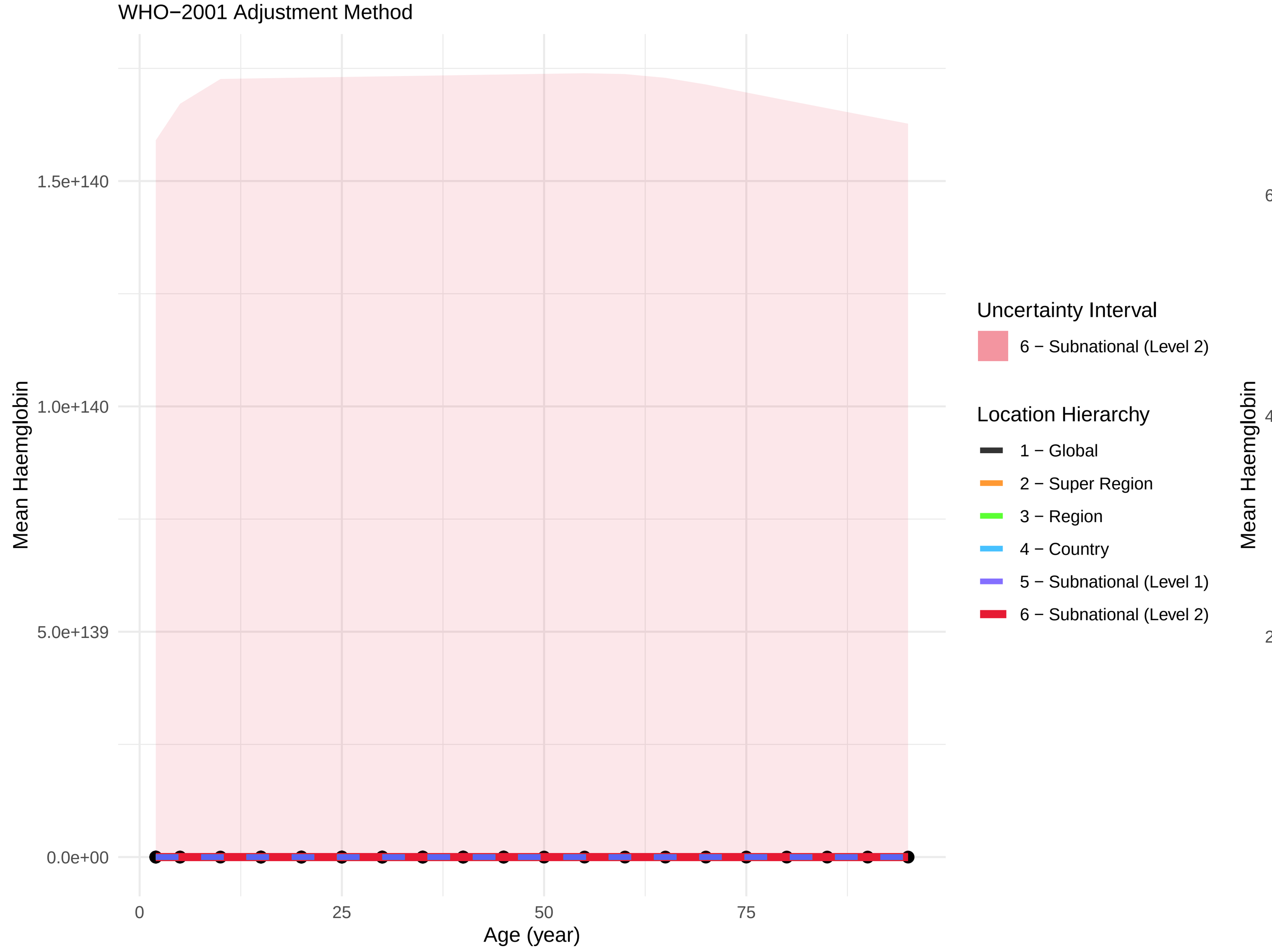

MR−BRT Age/Sex Fits − Non−pregnant Females 2+ Years Old − Mean Haemglobin − Uttarakhand, Urban
WHO−2001 Adjustment Method

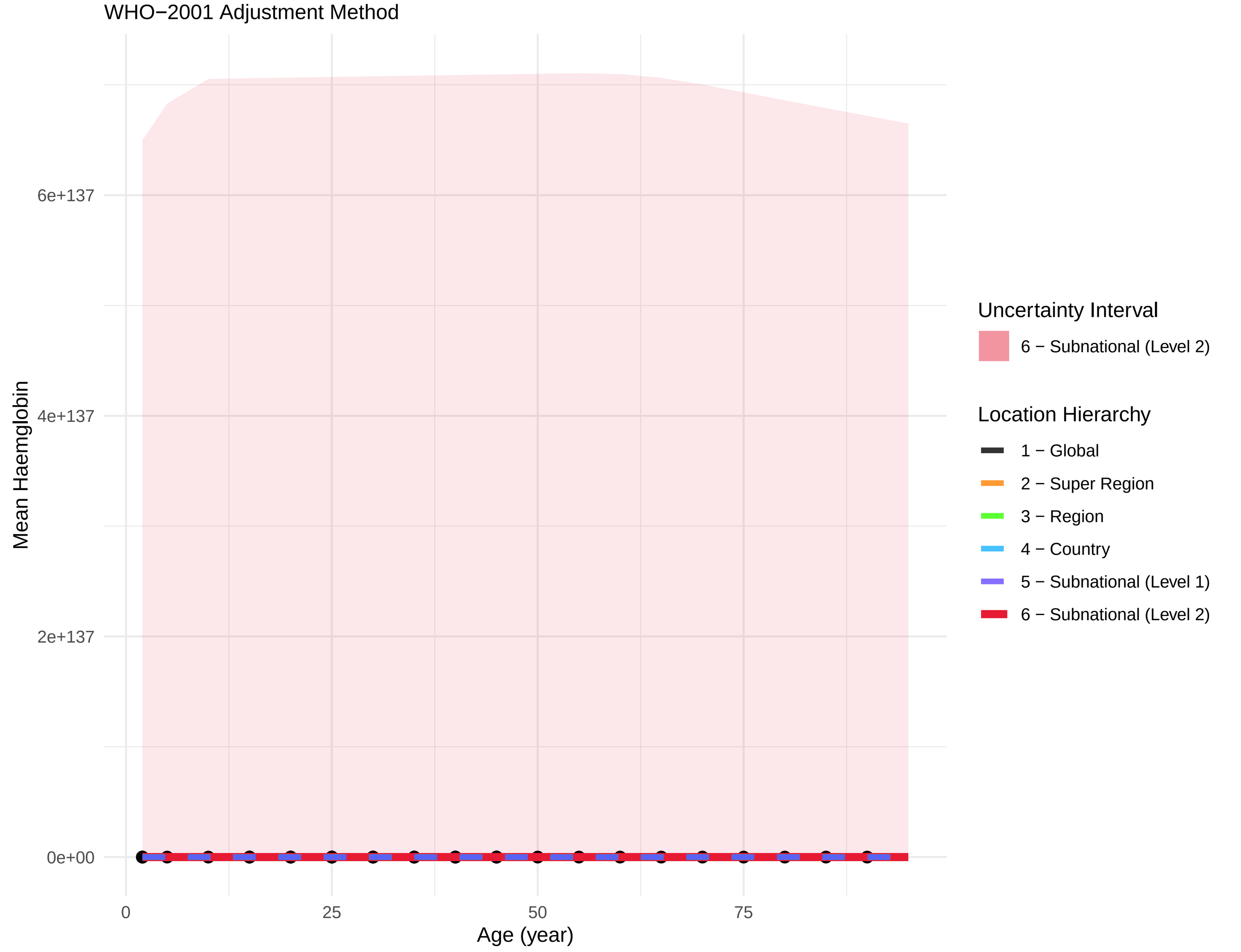

MR−BRT Age/Sex Fits − Non−pregnant Females 2+ Years Old − Mean Haemglobin − West Bengal
WHO−2001 Adjustment Method

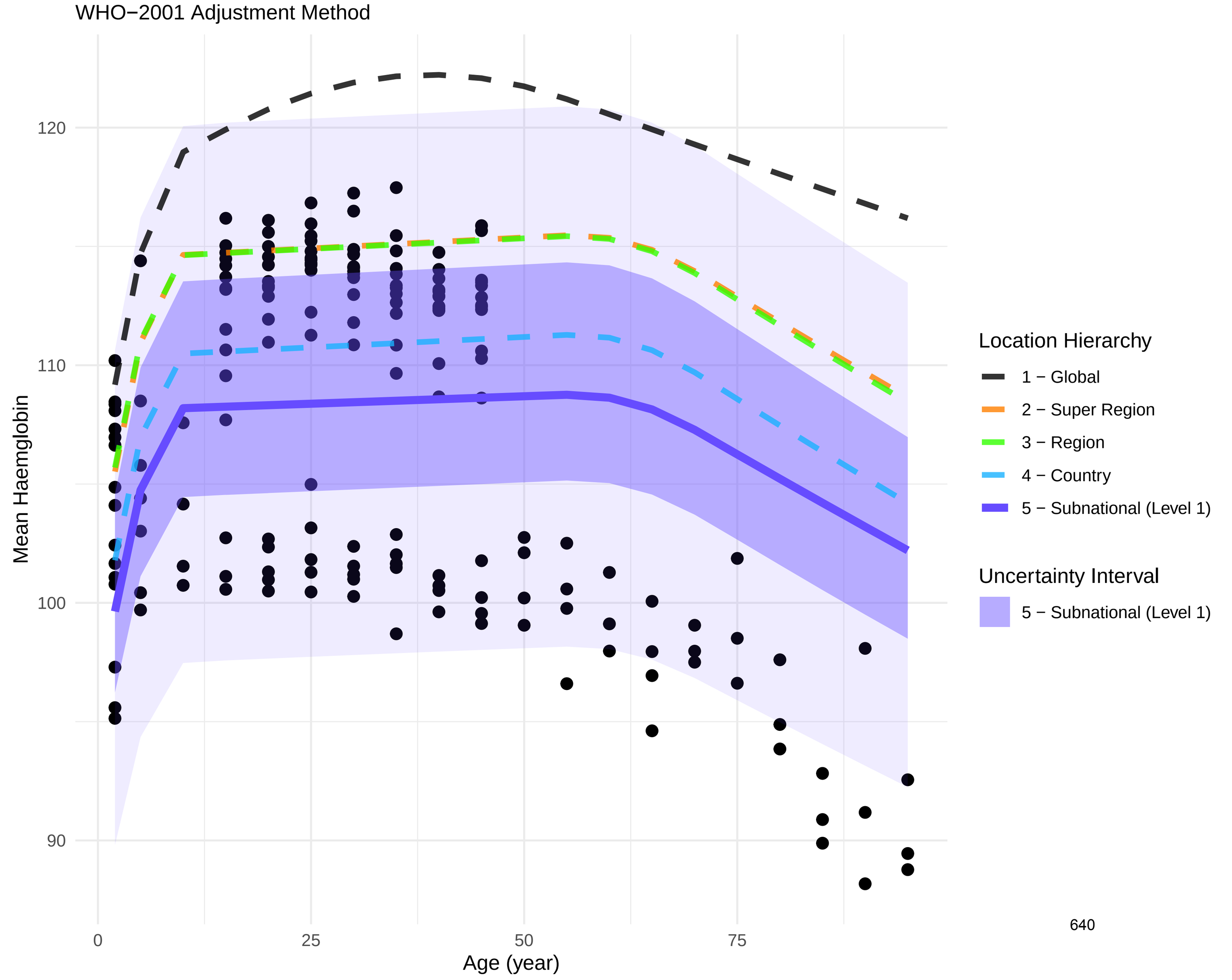

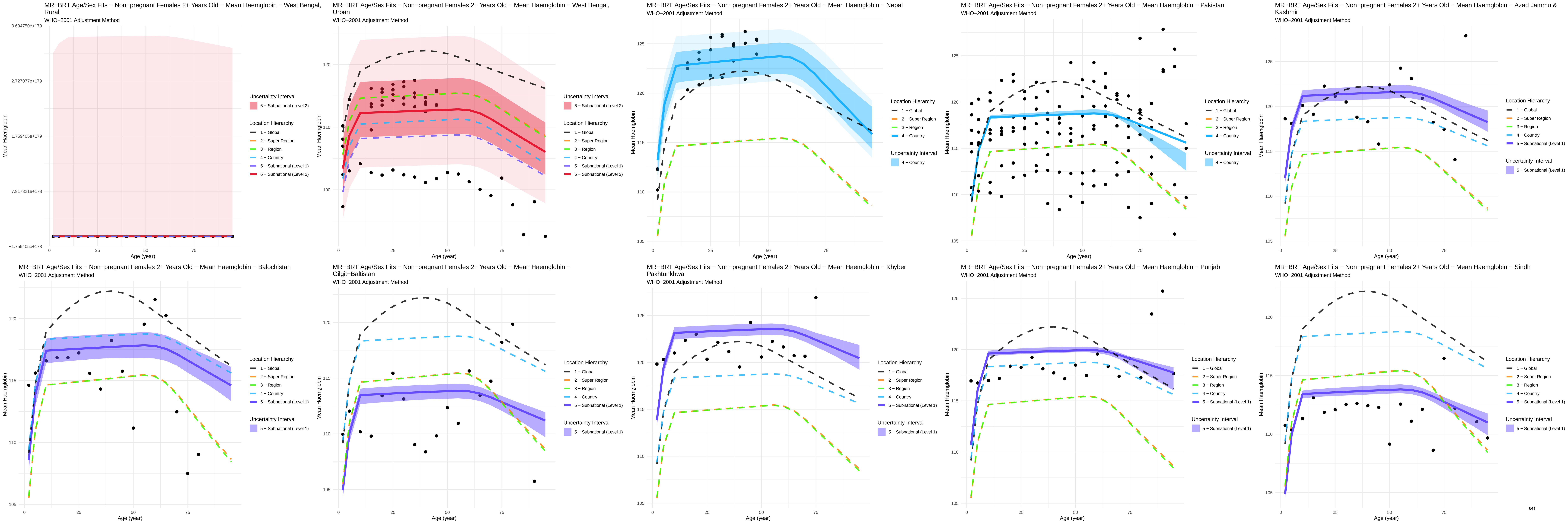
MR−BRT Age/Sex Fits − Non−pregnant Females 2+ Years Old − Mean Haemglobin − West Bengal, Rural
WHO−2001 Adjustment Method
Mean Haemglobin
Age (year)
Uncertainty Interval
6 − Subnational (Level 2)
Location Hierarchy
1 − Global
2 − Super Region
3 − Region
4 − Country
5 − Subnational (Level 1)
6 − Subnational (Level 2)
MR−BRT Age/Sex Fits − Non−pregnant Females 2+ Years Old − Mean Haemglobin − West Bengal, Urban
WHO−2001 Adjustment Method
Mean Haemglobin
Age (year)
Uncertainty Interval
6 − Subnational (Level 2)
Location Hierarchy
1 − Global
2 − Super Region
3 − Region
4 − Country
5 − Subnational (Level 1)
6 − Subnational (Level 2)
MR−BRT Age/Sex Fits − Non−pregnant Females 2+ Years Old − Mean Haemglobin − Nepal
WHO−2001 Adjustment Method
Mean Haemglobin
Age (year)
Location Hierarchy
1 − Global
2 − Super Region
3 − Region
4 − Country
Uncertainty Interval
4 − Country
MR−BRT Age/Sex Fits − Non−pregnant Females 2+ Years Old − Mean Haemglobin − Pakistan
WHO−2001 Adjustment Method
Mean Haemglobin
Age (year)
Location Hierarchy
1 − Global
2 − Super Region
3 − Region
4 − Country
Uncertainty Interval
4 − Country
MR−BRT Age/Sex Fits − Non−pregnant Females 2+ Years Old − Mean Haemglobin − Azad Jammu & Kashmir
WHO−2001 Adjustment Method
Mean Haemglobin
Age (year)
Location Hierarchy
1 − Global
2 − Super Region
3 − Region
4 − Country
5 − Subnational (Level 1)
Uncertainty Interval
5 − Subnational (Level 1)
MR−BRT Age/Sex Fits − Non−pregnant Females 2+ Years Old − Mean Haemglobin − Balochistan
WHO−2001 Adjustment Method
Mean Haemglobin
Age (year)
Location Hierarchy
1 − Global
2 − Super Region
3 − Region
4 − Country
5 − Subnational (Level 1)
Uncertainty Interval
5 − Subnational (Level 1)
MR−BRT Age/Sex Fits − Non−pregnant Females 2+ Years Old − Mean Haemglobin − Gilgit−Baltistan
WHO−2001 Adjustment Method
Mean Haemglobin
Age (year)
Location Hierarchy
1 − Global
2 − Super Region
3 − Region
4 − Country
5 − Subnational (Level 1)
Uncertainty Interval
5 − Subnational (Level 1)
MR−BRT Age/Sex Fits − Non−pregnant Females 2+ Years Old − Mean Haemglobin − Khyber Pakhtunkhwa
WHO−2001 Adjustment Method
Mean Haemglobin
Age (year)
Location Hierarchy
1 − Global
2 − Super Region
3 − Region
4 − Country
5 − Subnational (Level 1)
Uncertainty Interval
5 − Subnational (Level 1)
MR−BRT Age/Sex Fits − Non−pregnant Females 2+ Years Old − Mean Haemglobin − Punjab
WHO−2001 Adjustment Method
Mean Haemglobin
Age (year)
Location Hierarchy
1 − Global
2 − Super Region
3 − Region
4 − Country
5 − Subnational (Level 1)
Uncertainty Interval
5 − Subnational (Level 1)
MR−BRT Age/Sex Fits − Non−pregnant Females 2+ Years Old − Mean Haemglobin − Sindh
WHO−2001 Adjustment Method
Mean Haemglobin
Age (year)
Location Hierarchy
1 − Global
2 − Super Region
3 − Region
4 − Country
5 − Subnational (Level 1)
Uncertainty Interval
5 − Subnational (Level 1)

MR−BRT Age/Sex Fits − Non−pregnant Females 2+ Years Old − Mean Haemglobin − Southeast Asia, East Asia, and Oceania
WHO−2001 Adjustment Method

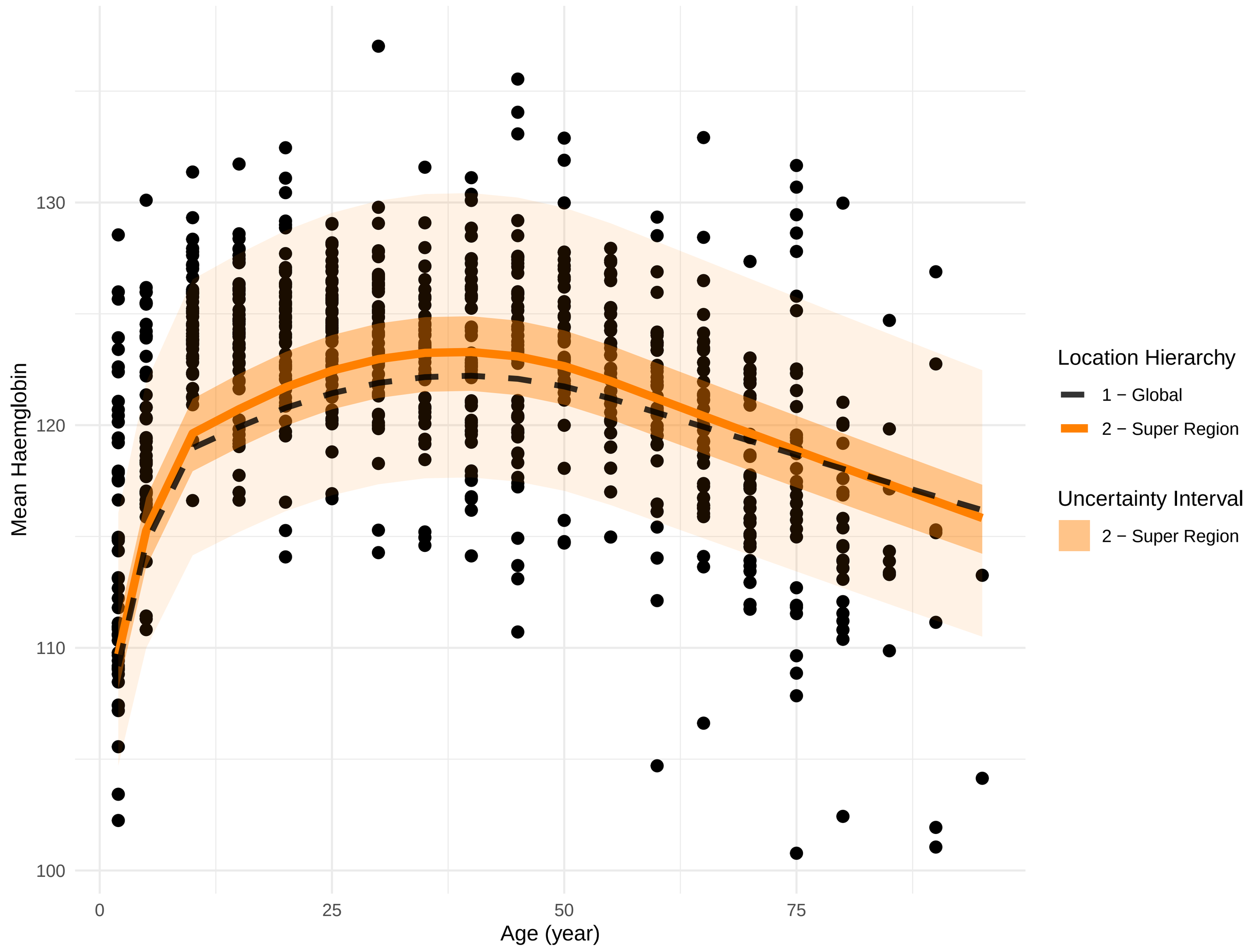

MR−BRT Age/Sex Fits − Non−pregnant Females 2+ Years Old − Mean Haemglobin − Oceania
WHO−2001 Adjustment Method

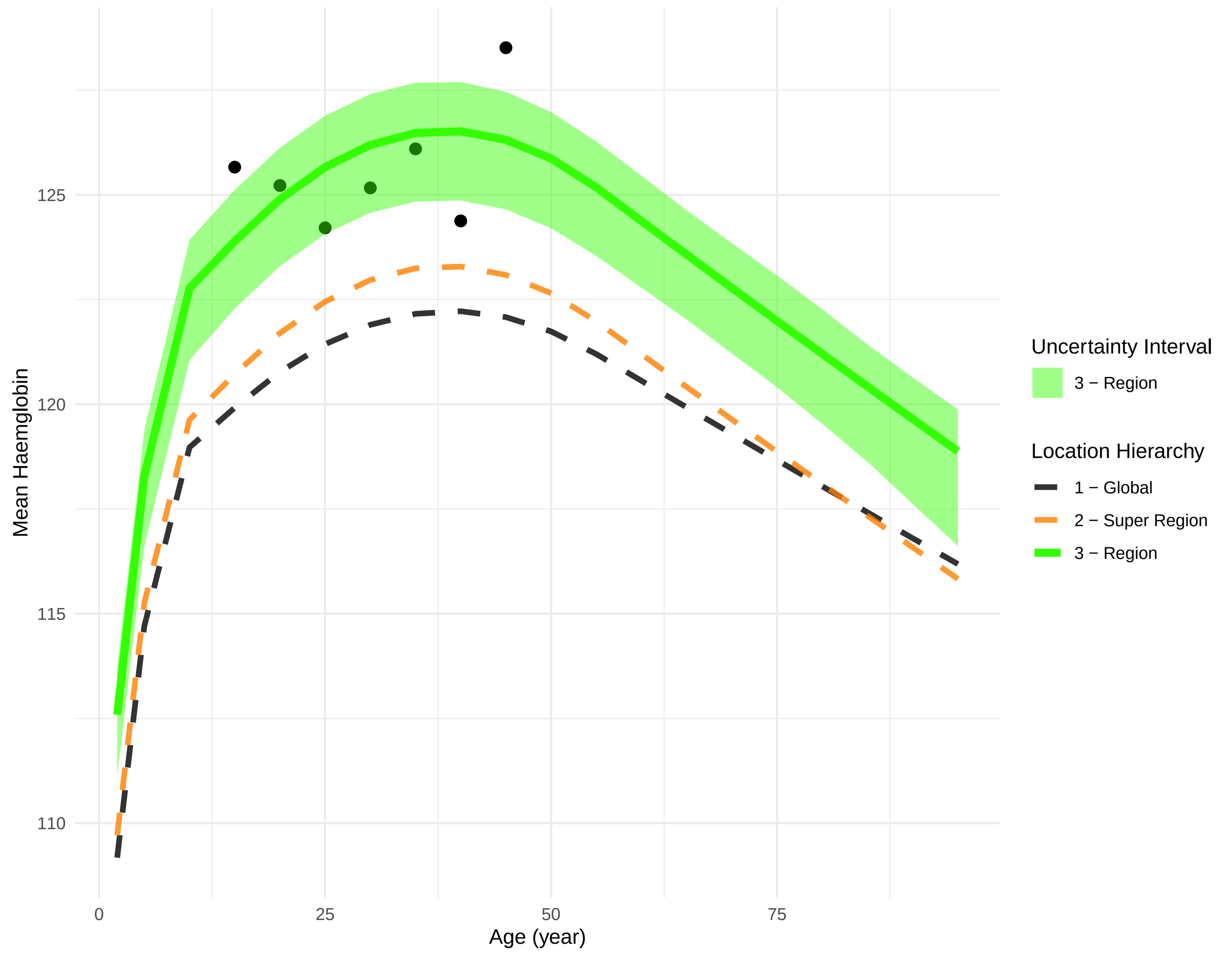

MR−BRT Age/Sex Fits − Non−pregnant Females 2+ Years Old − Mean Haemglobin − Papua New Guinea
WHO−2001 Adjustment Method

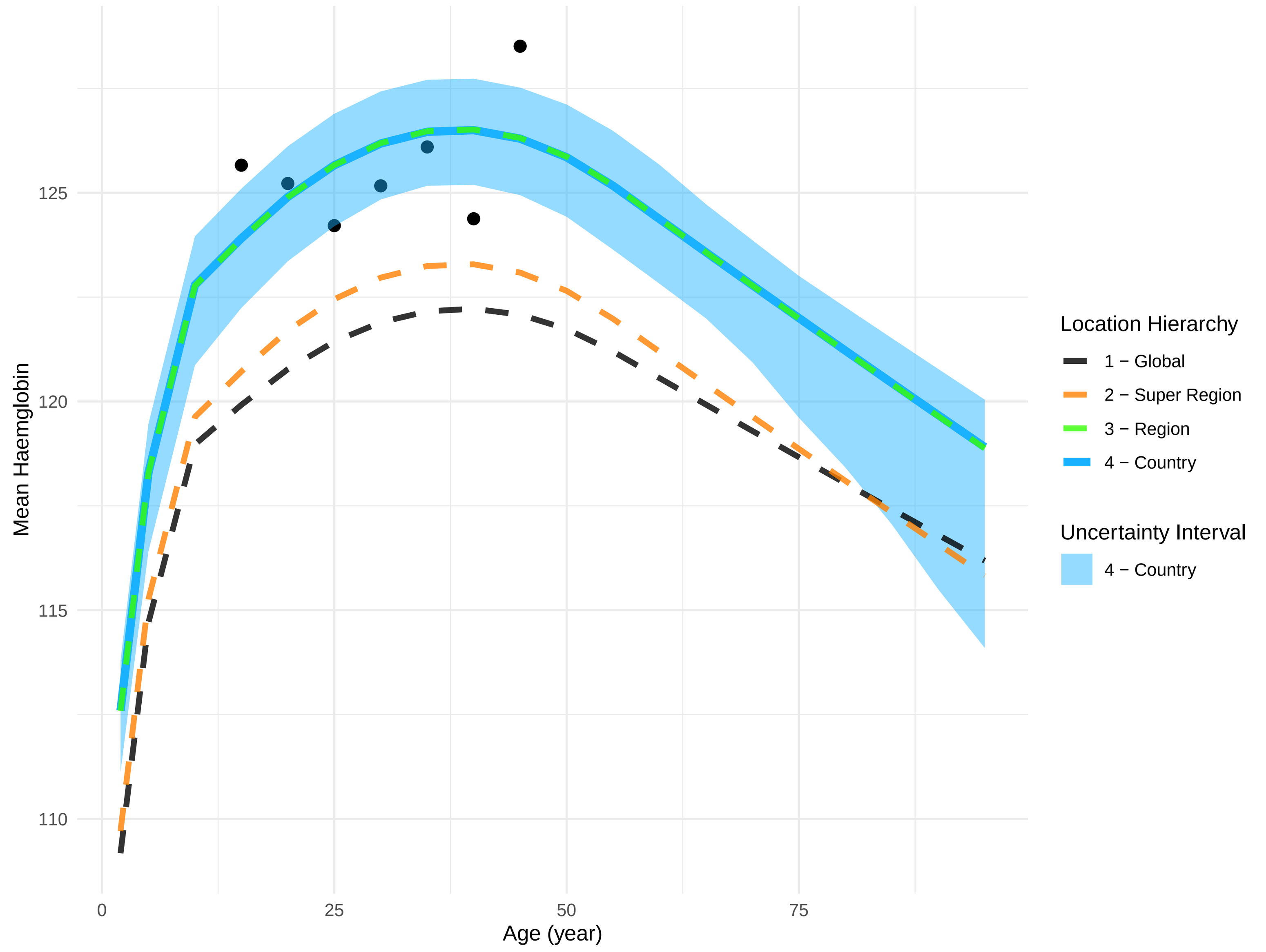

MR−BRT Age/Sex Fits − Non−pregnant Females 2+ Years Old − Mean Haemglobin − Southeast Asia
WHO−2001 Adjustment Method

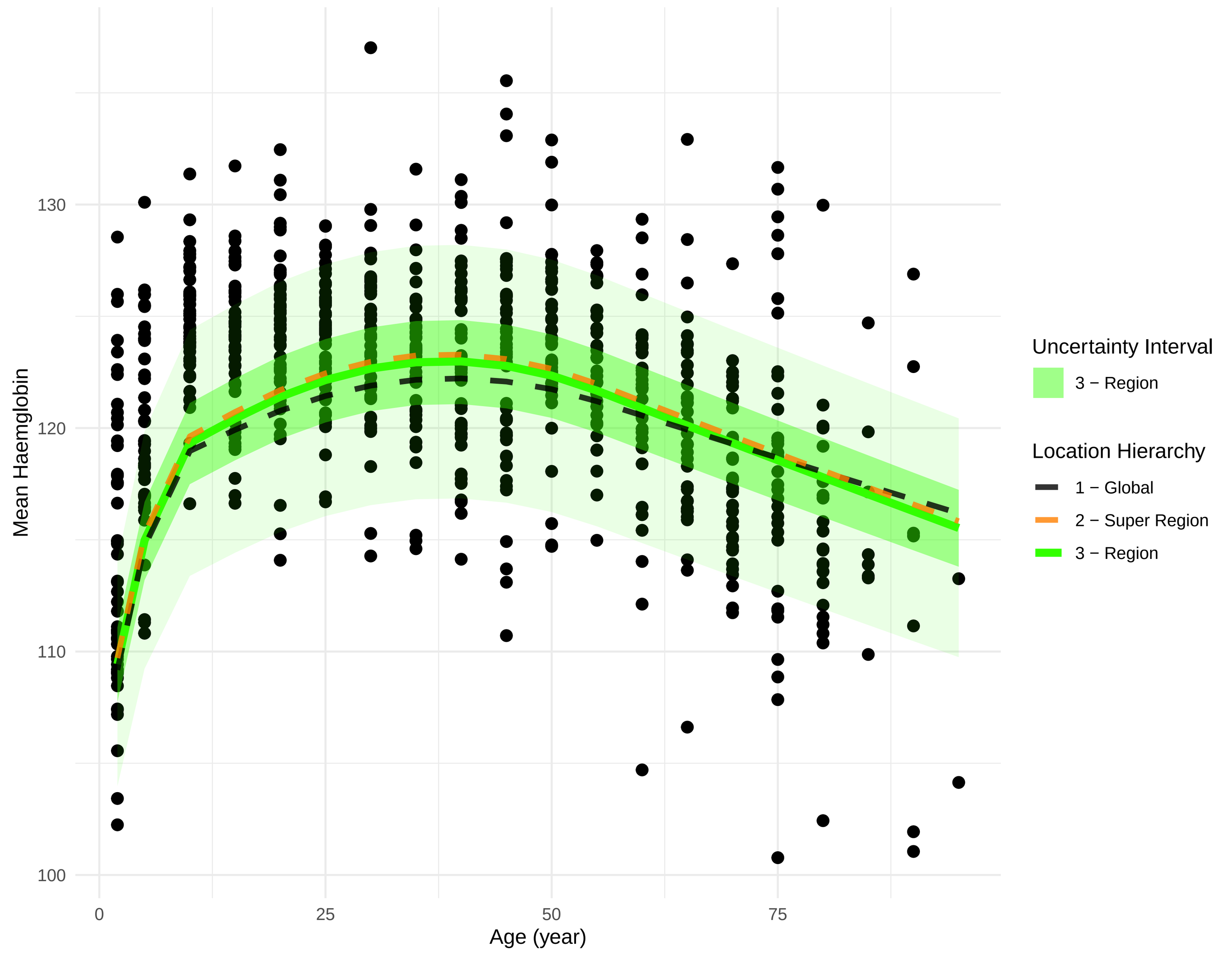

MR−BRT Age/Sex Fits − Non−pregnant Females 2+ Years Old − Mean Haemglobin − Cambodia
WHO−2001 Adjustment Method

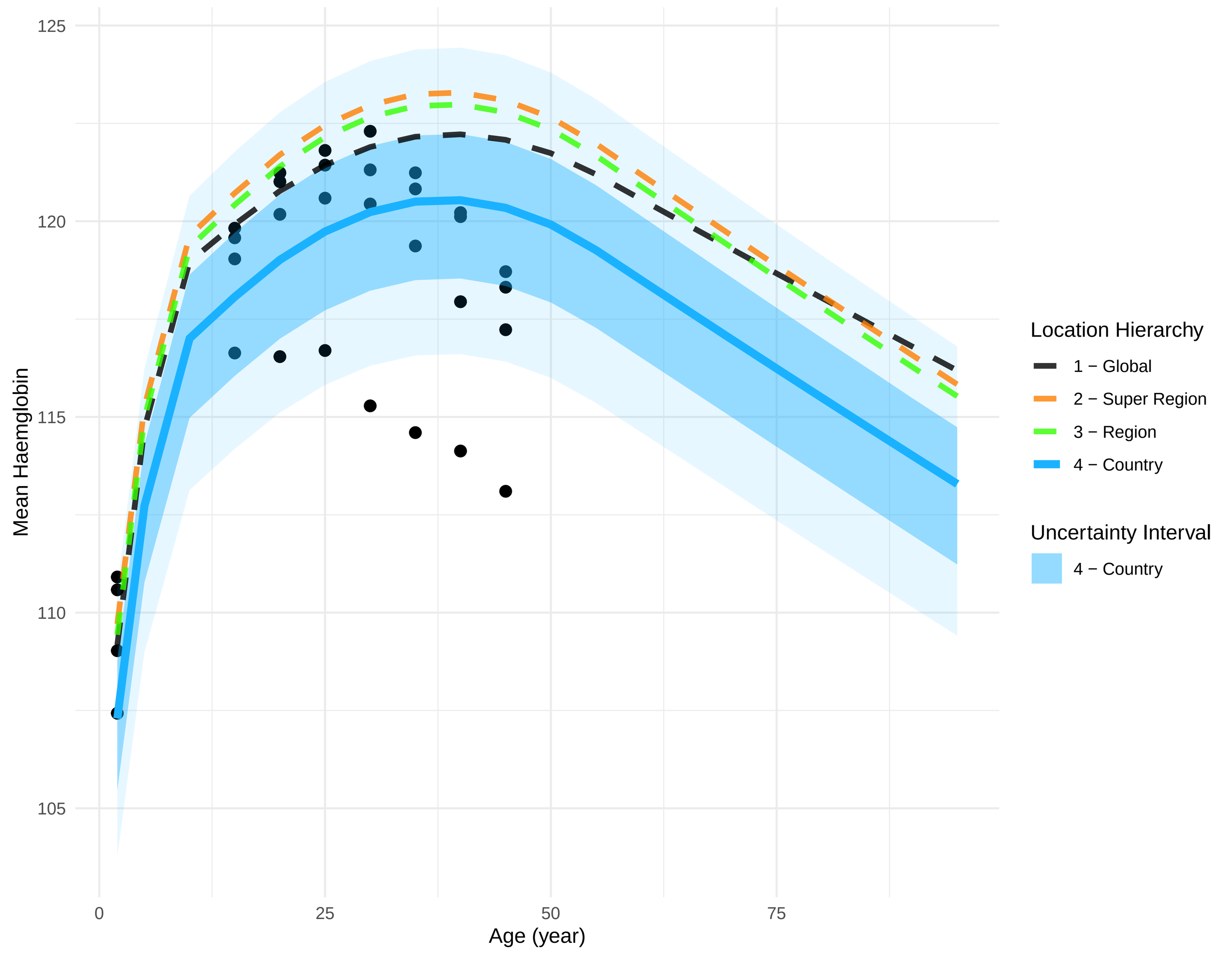

MR−BRT Age/Sex Fits − Non−pregnant Females 2+ Years Old − Mean Haemglobin − Indonesia
WHO−2001 Adjustment Method

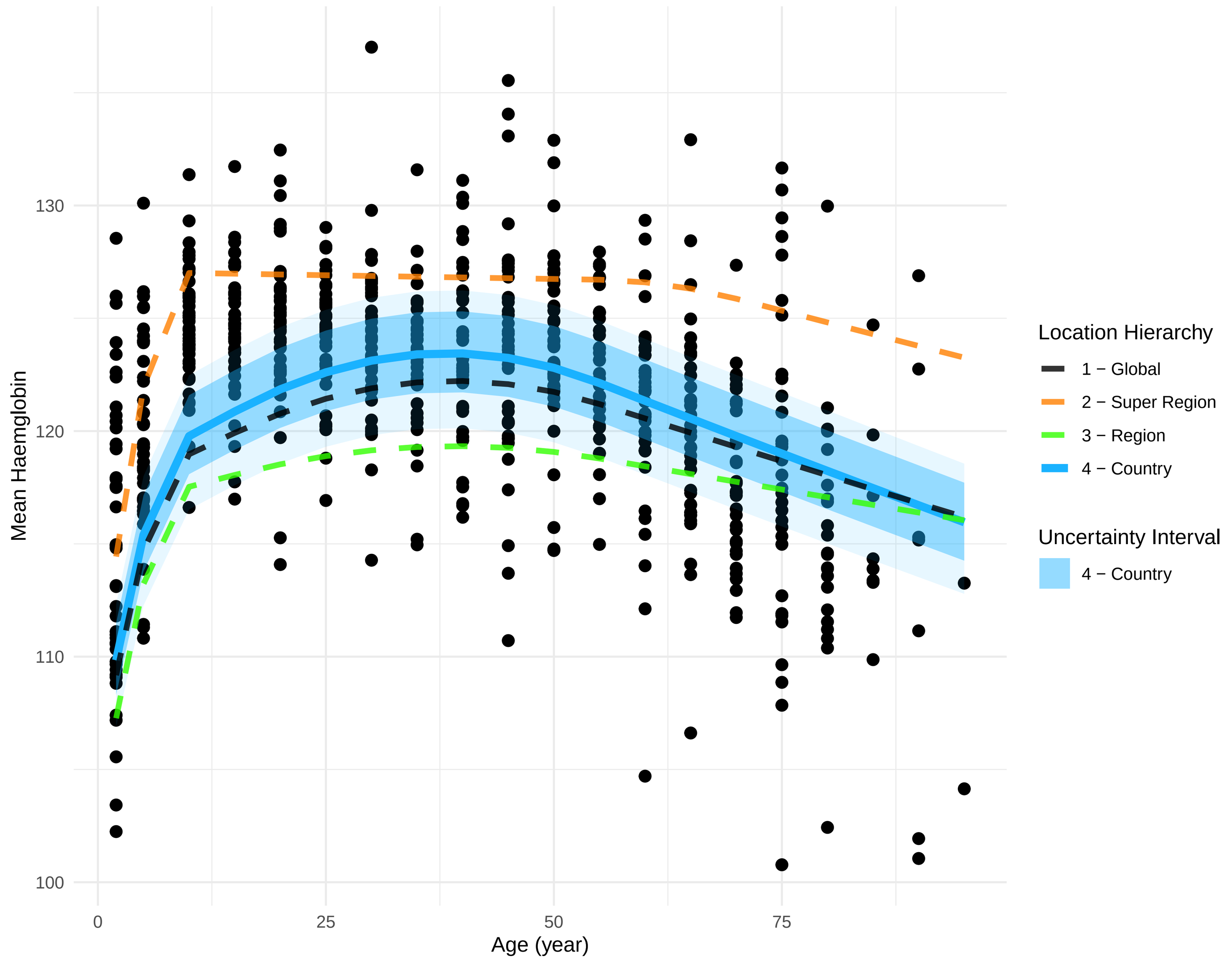

MR−BRT Age/Sex Fits − Non−pregnant Females 2+ Years Old − Mean Haemglobin − Bali
WHO−2001 Adjustment Method

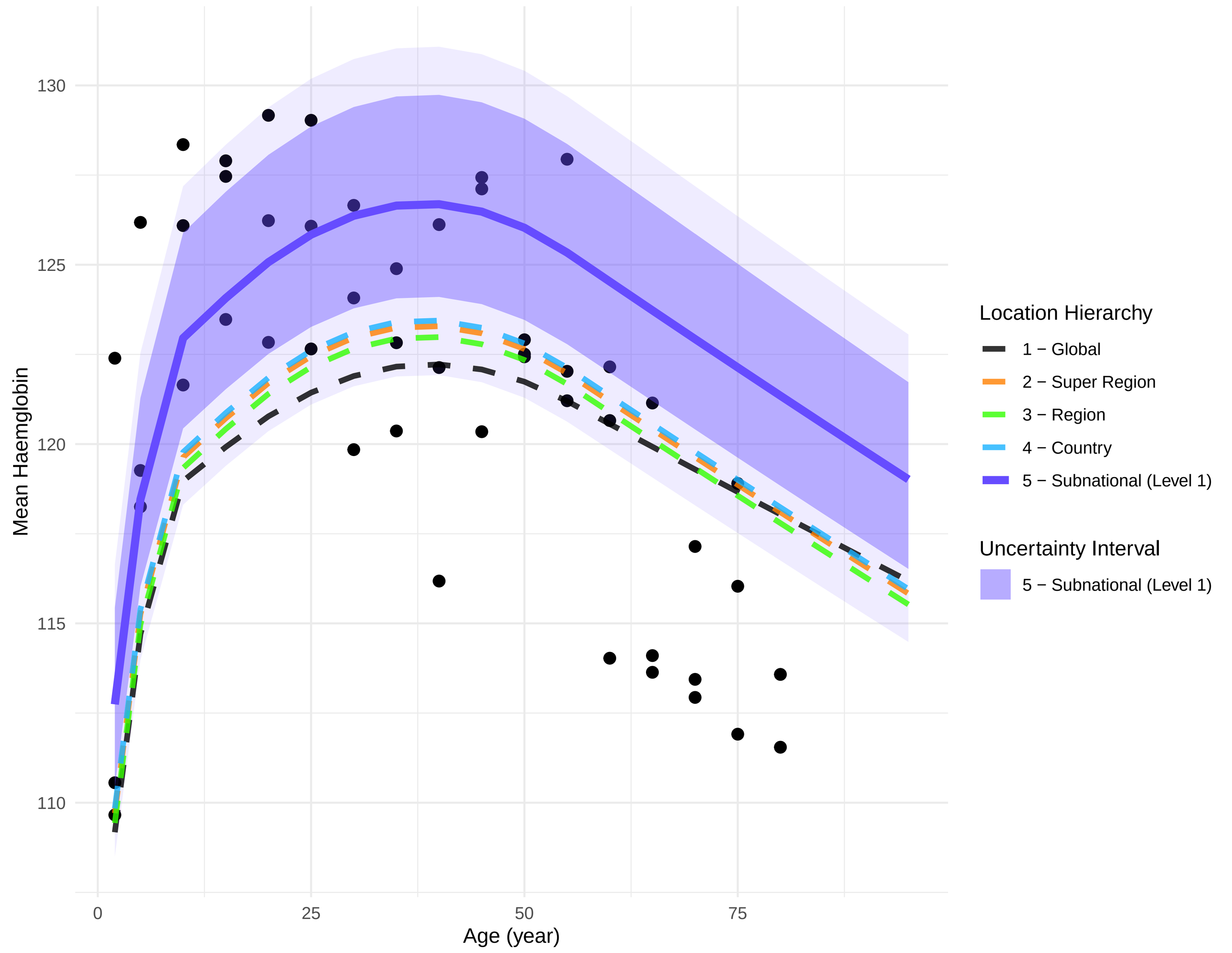

MR−BRT Age/Sex Fits − Non−pregnant Females 2+ Years Old − Mean Haemglobin − Bangka−Belitung Islands
WHO−2001 Adjustment Method

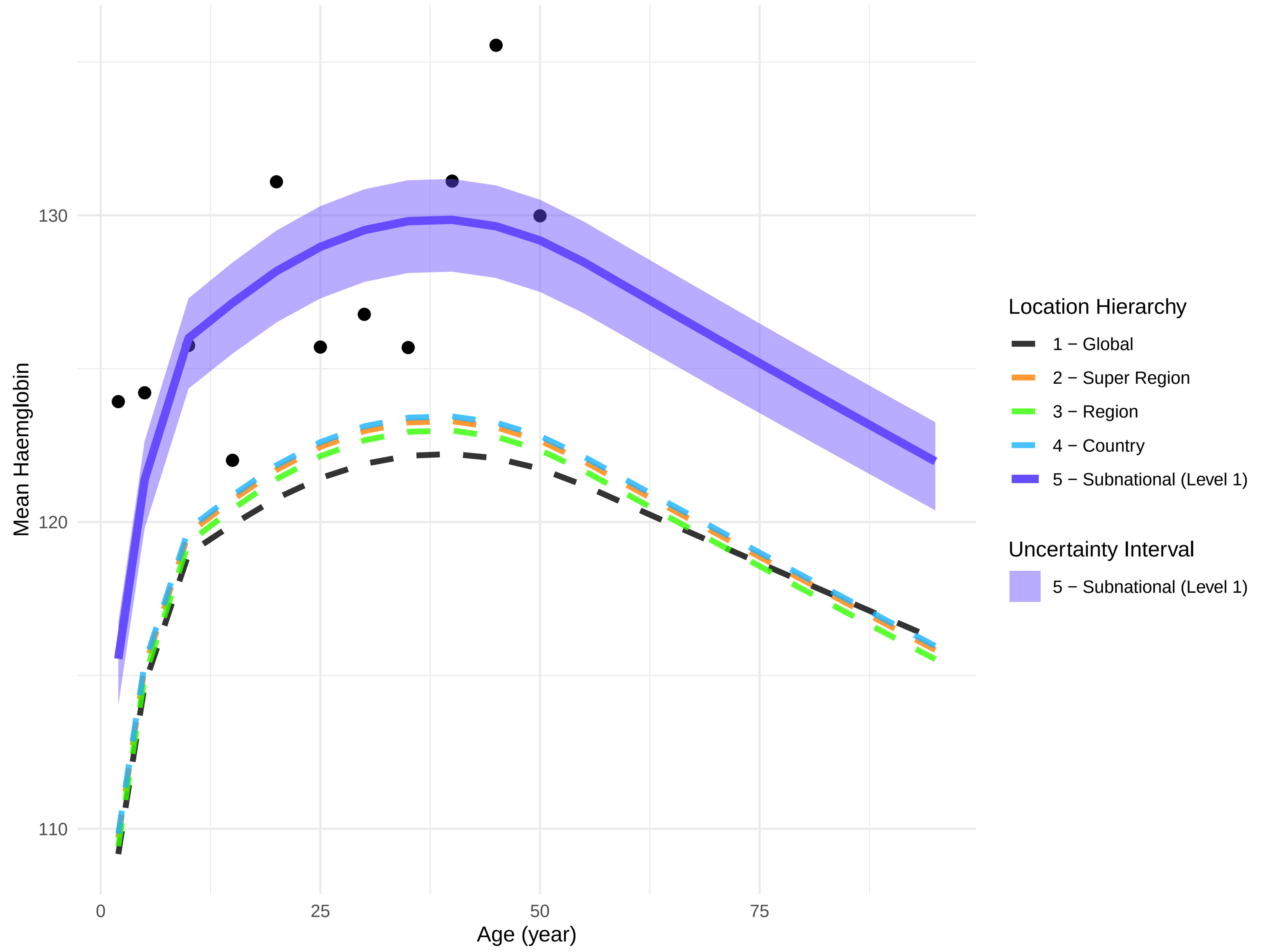

MR−BRT Age/Sex Fits − Non−pregnant Females 2+ Years Old − Mean Haemglobin − Banten
WHO−2001 Adjustment Method

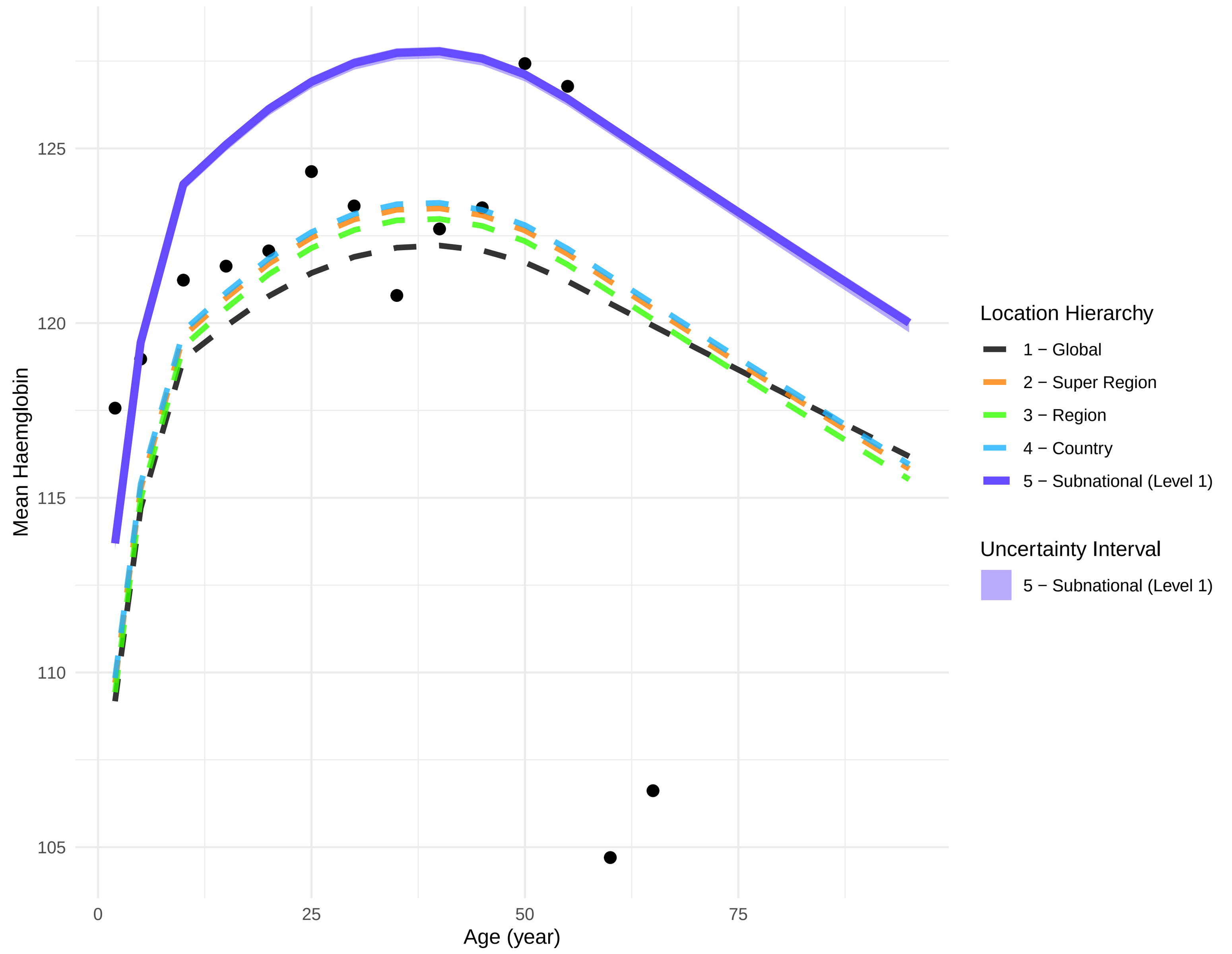

MR−BRT Age/Sex Fits − Non−pregnant Females 2+ Years Old − Mean Haemglobin − Jakarta
WHO−2001 Adjustment Method

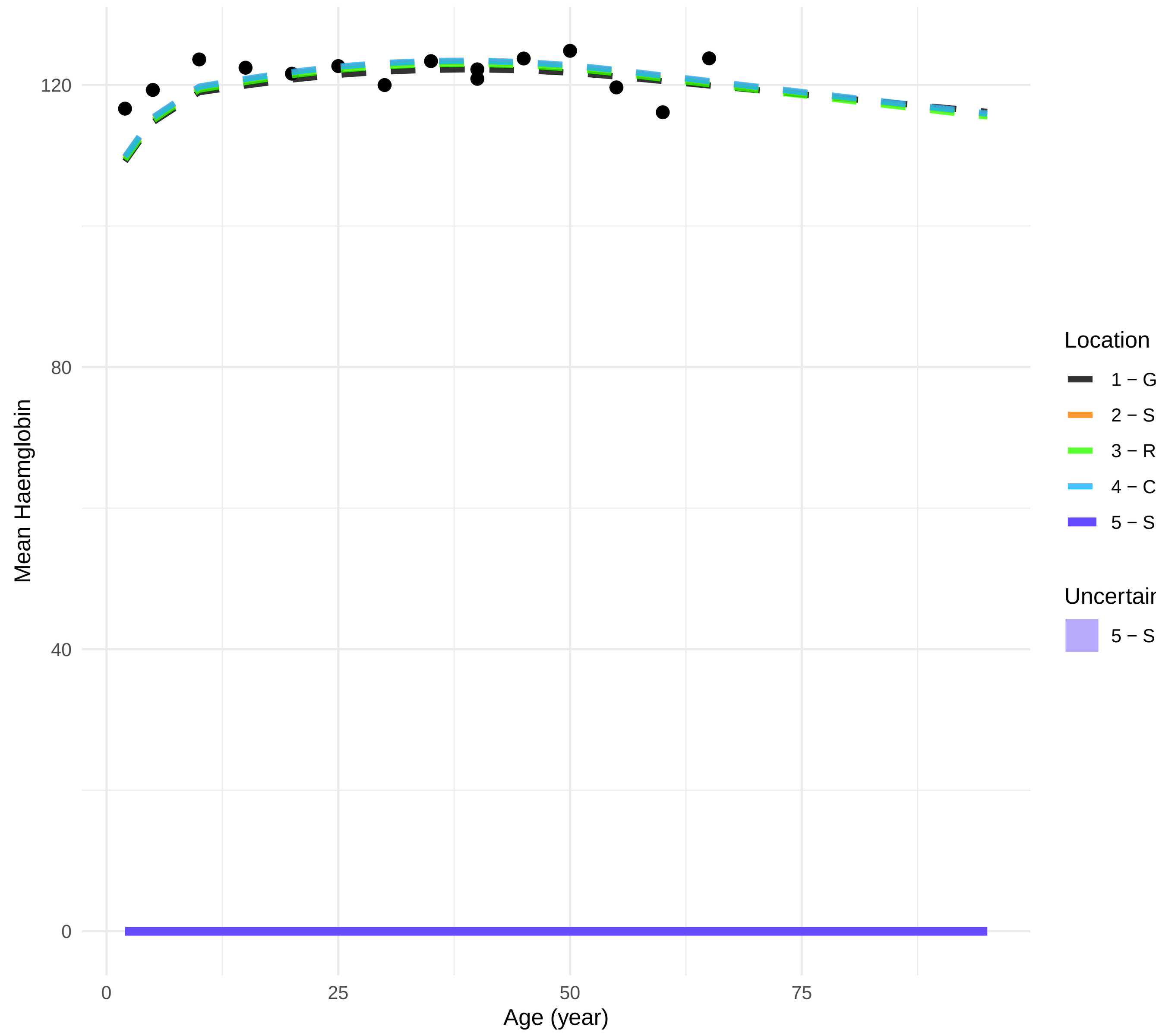

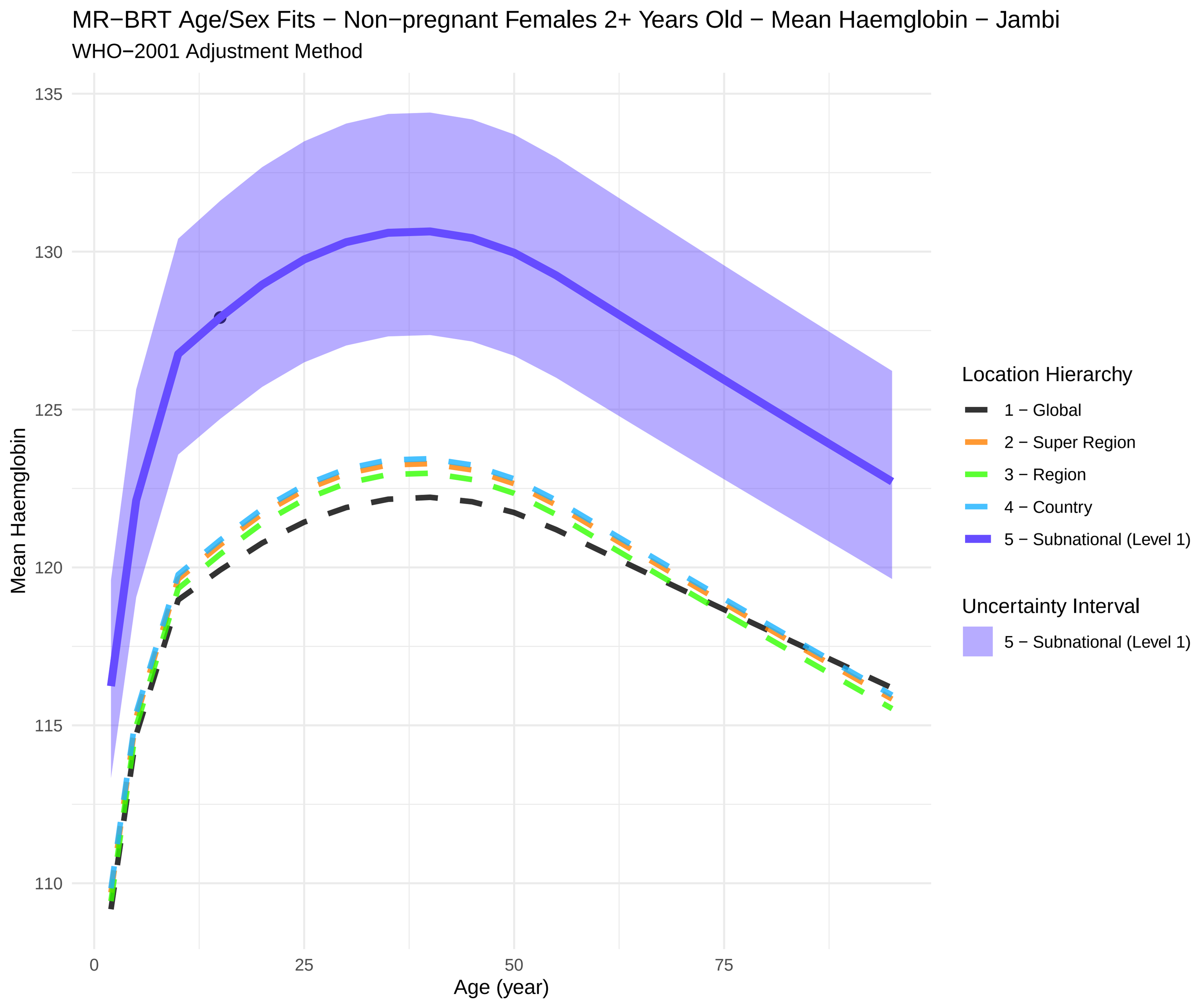
MR−BRT Age/Sex Fits − Non−pregnant Females 2+ Years Old − Mean Haemglobin − Jambi
WHO−2001 Adjustment Method
Mean Haemglobin
110
115
120
125
130
135
0
25
50
75
Age (year)
Location Hierarchy
1 − Global
2 − Super Region
3 − Region
4 − Country
5 − Subnational (Level 1)
Uncertainty Interval
5 − Subnational (Level 1)

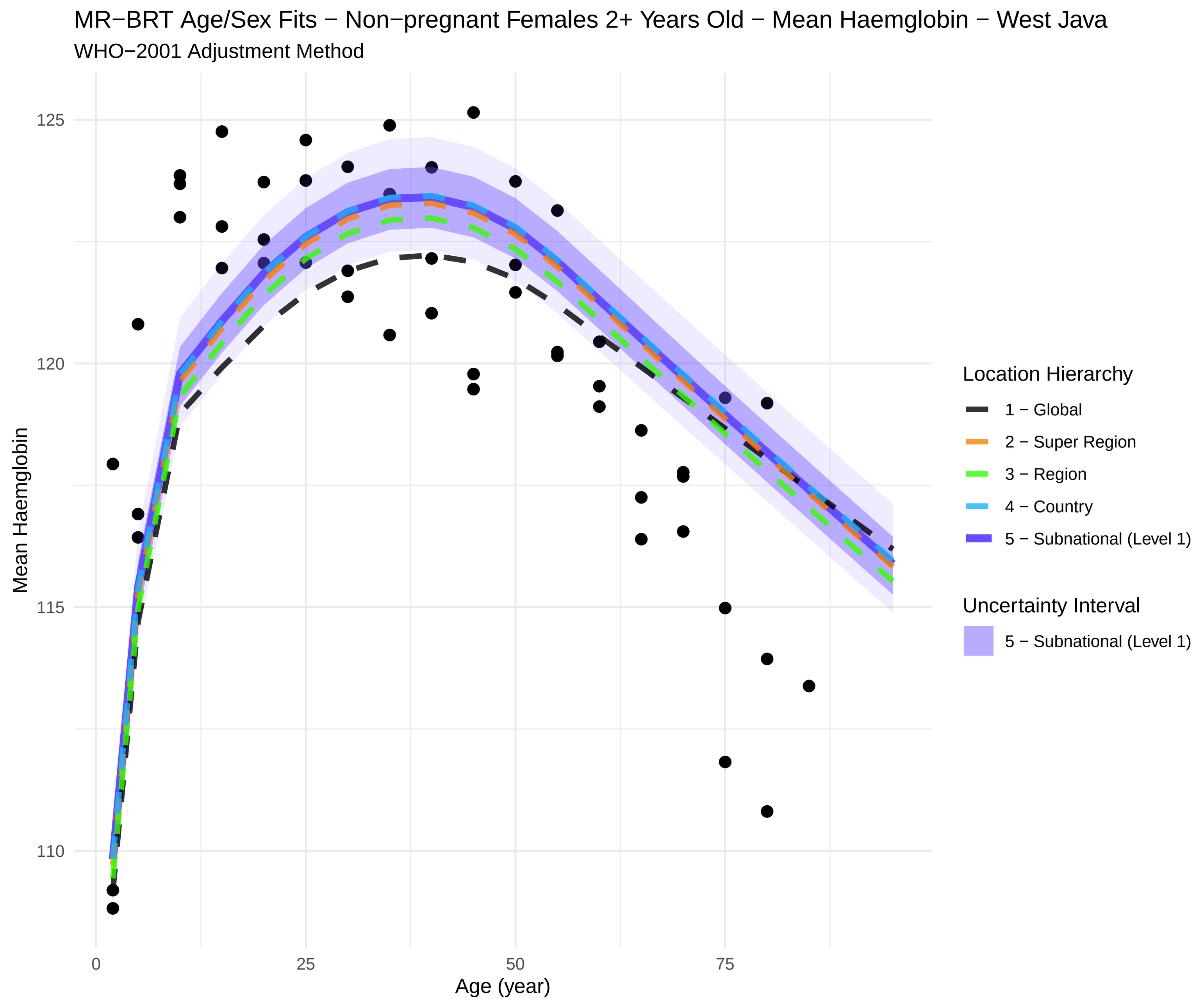
MR−BRT Age/Sex Fits − Non−pregnant Females 2+ Years Old − Mean Haemglobin − West Java
WHO−2001 Adjustment Method
Mean Haemglobin
110
115
120
125
0
25
50
75
Age (year)
Location Hierarchy
1 − Global
2 − Super Region
3 − Region
4 − Country
5 − Subnational (Level 1)
Uncertainty Interval
5 − Subnational (Level 1)

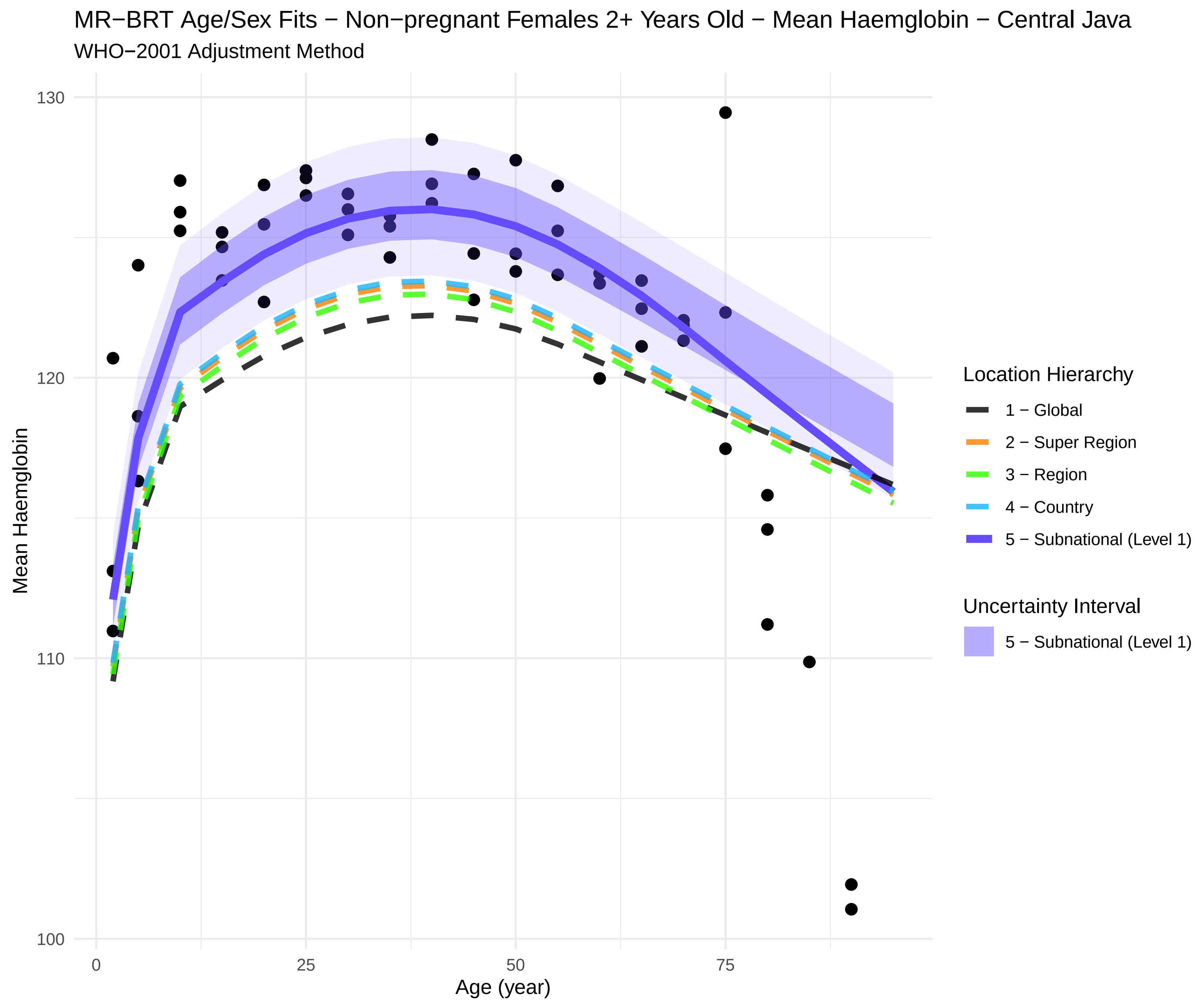
MR−BRT Age/Sex Fits − Non−pregnant Females 2+ Years Old − Mean Haemglobin − Central Java
WHO−2001 Adjustment Method
Mean Haemglobin
100
110
120
130
0
25
50
75
Age (year)
Location Hierarchy
1 − Global
2 − Super Region
3 − Region
4 − Country
5 − Subnational (Level 1)
Uncertainty Interval
5 − Subnational (Level 1)

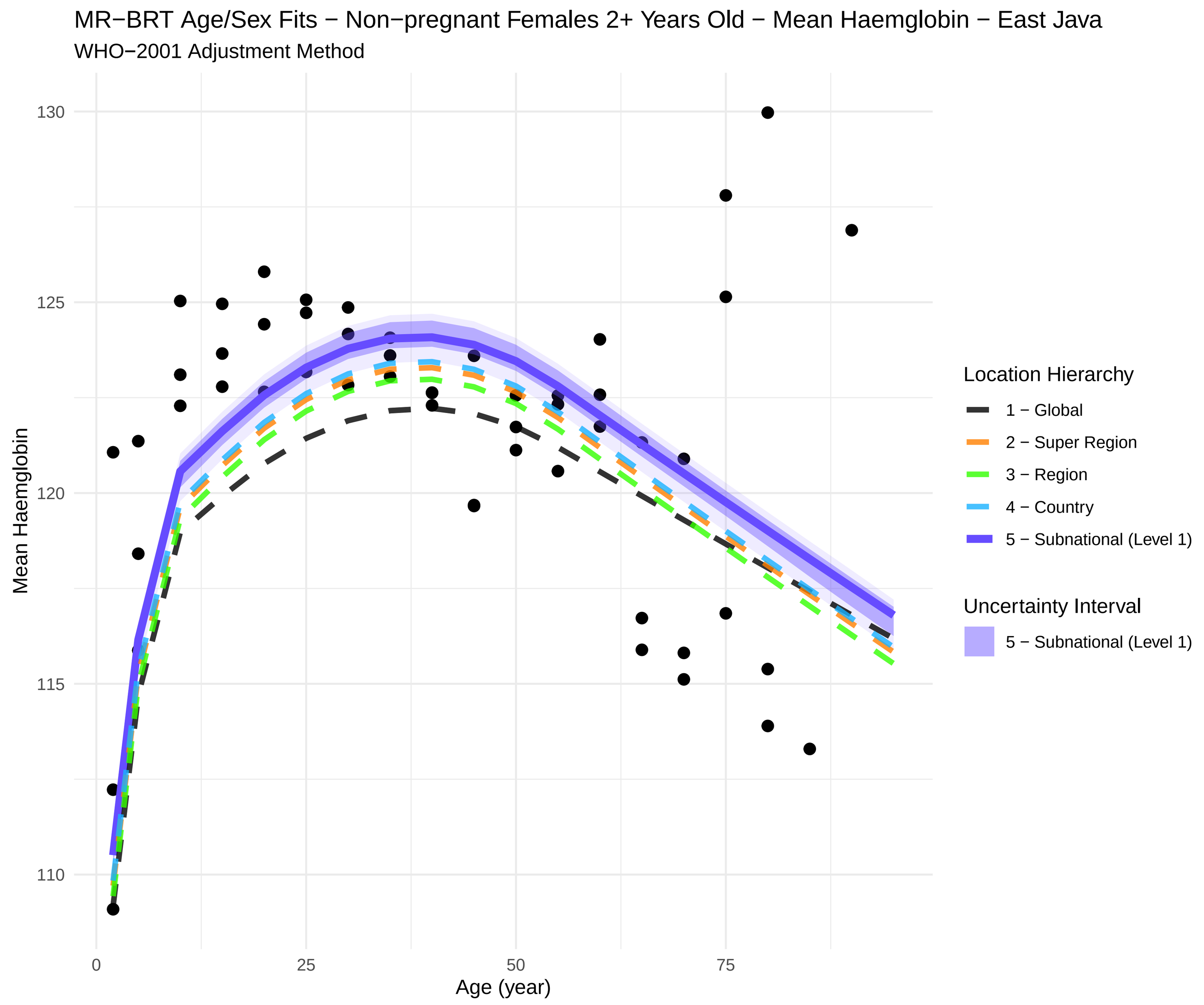
MR−BRT Age/Sex Fits − Non−pregnant Females 2+ Years Old − Mean Haemglobin − East Java
WHO−2001 Adjustment Method
Mean Haemglobin
110
115
120
125
130
0
25
50
75
Age (year)
Location Hierarchy
1 − Global
2 − Super Region
3 − Region
4 − Country
5 − Subnational (Level 1)
Uncertainty Interval
5 − Subnational (Level 1)

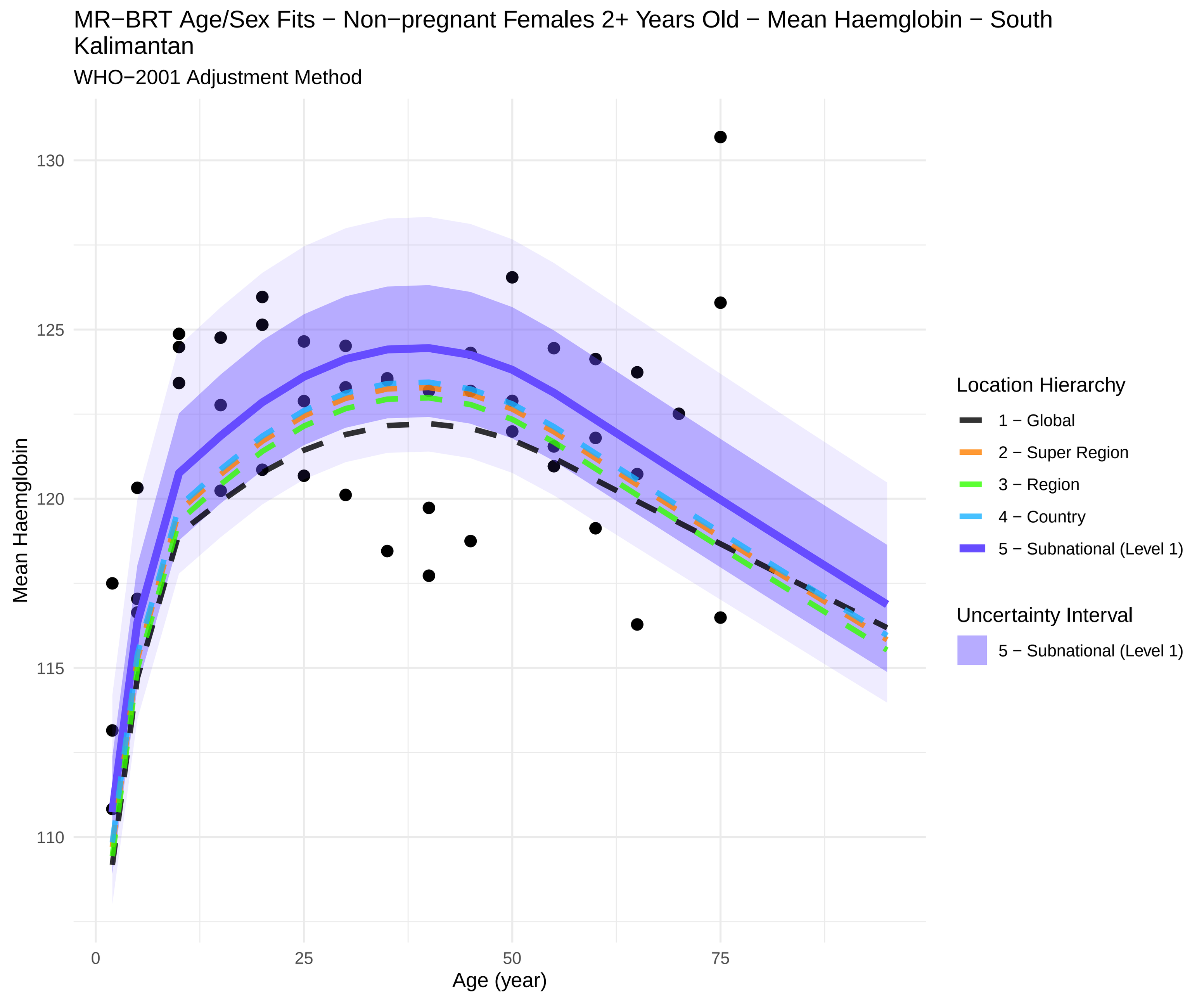
MR−BRT Age/Sex Fits − Non−pregnant Females 2+ Years Old − Mean Haemglobin − South Kalimantan
WHO−2001 Adjustment Method
Mean Haemglobin
110
115
120
125
130
0
25
50
75
Age (year)
Location Hierarchy
1 − Global
2 − Super Region
3 − Region
4 − Country
5 − Subnational (Level 1)
Uncertainty Interval
5 − Subnational (Level 1)

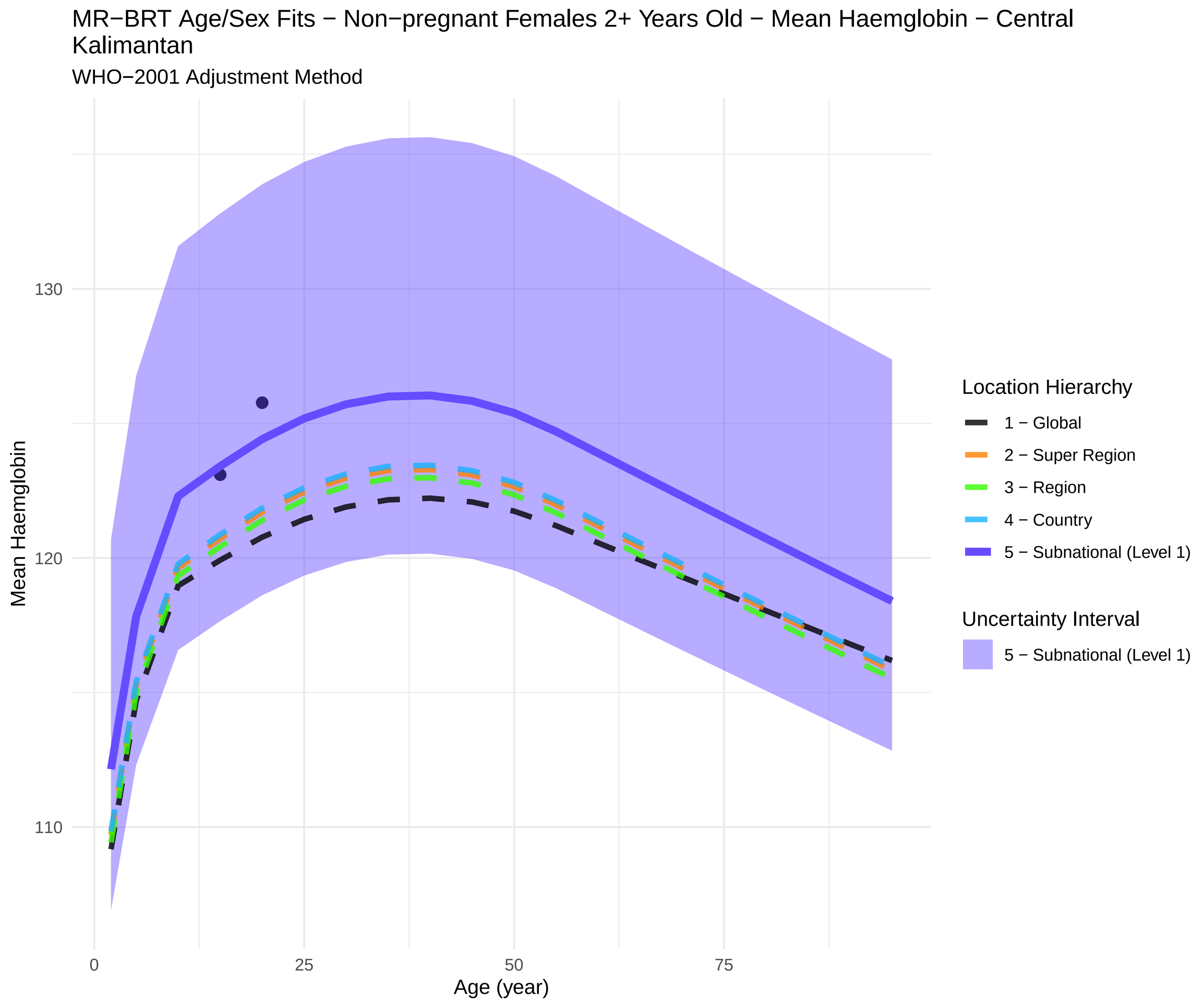
MR−BRT Age/Sex Fits − Non−pregnant Females 2+ Years Old − Mean Haemglobin − Central Kalimantan
WHO−2001 Adjustment Method
Mean Haemglobin
110
120
130
0
25
50
75
Age (year)
Location Hierarchy
1 − Global
2 − Super Region
3 − Region
4 − Country
5 − Subnational (Level 1)
Uncertainty Interval
5 − Subnational (Level 1)

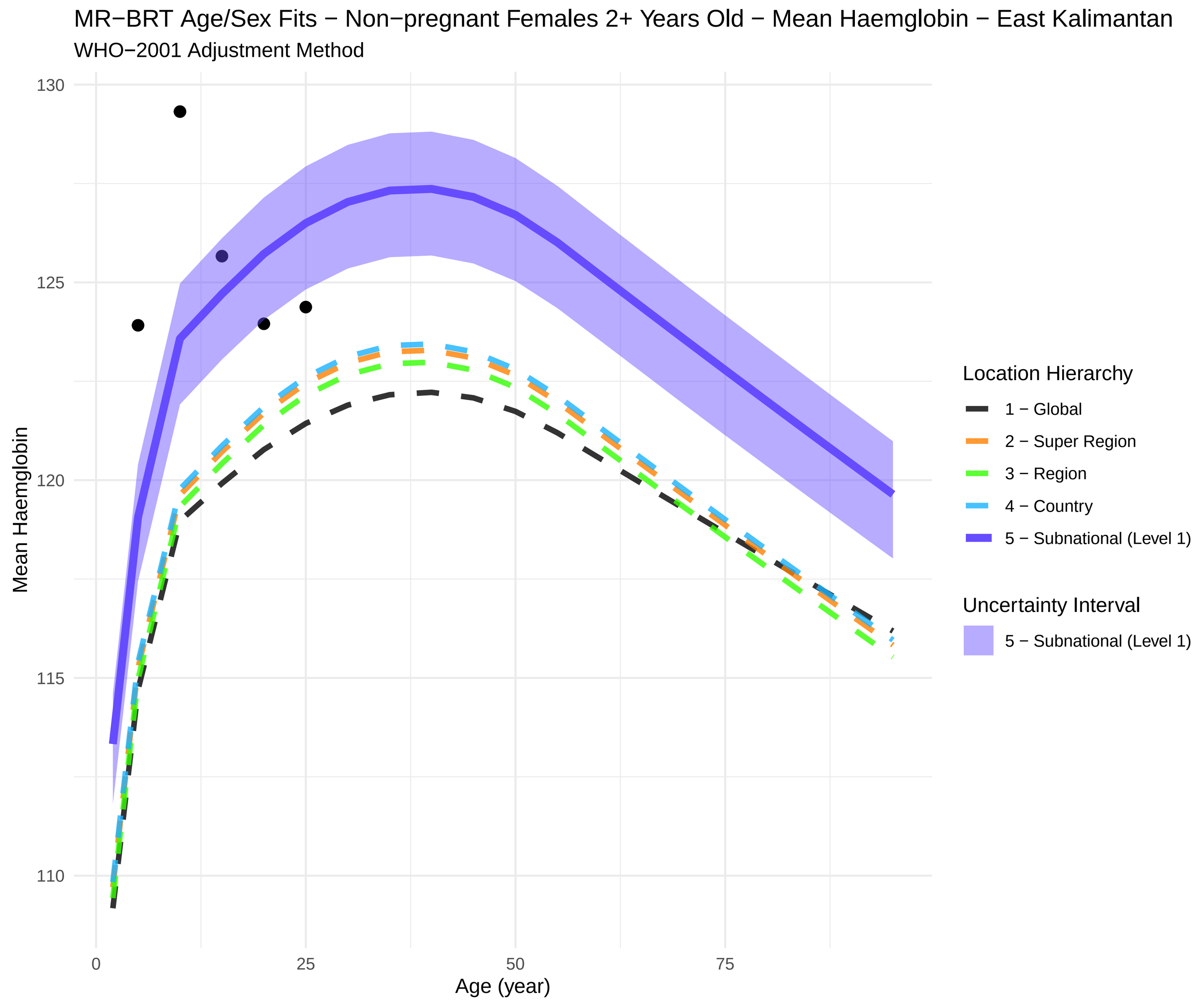
MR−BRT Age/Sex Fits − Non−pregnant Females 2+ Years Old − Mean Haemglobin − East Kalimantan
WHO−2001 Adjustment Method
Mean Haemglobin
110
115
120
125
130
0
25
50
75
Age (year)
Location Hierarchy
1 − Global
2 − Super Region
3 − Region
4 − Country
5 − Subnational (Level 1)
Uncertainty Interval
5 − Subnational (Level 1)

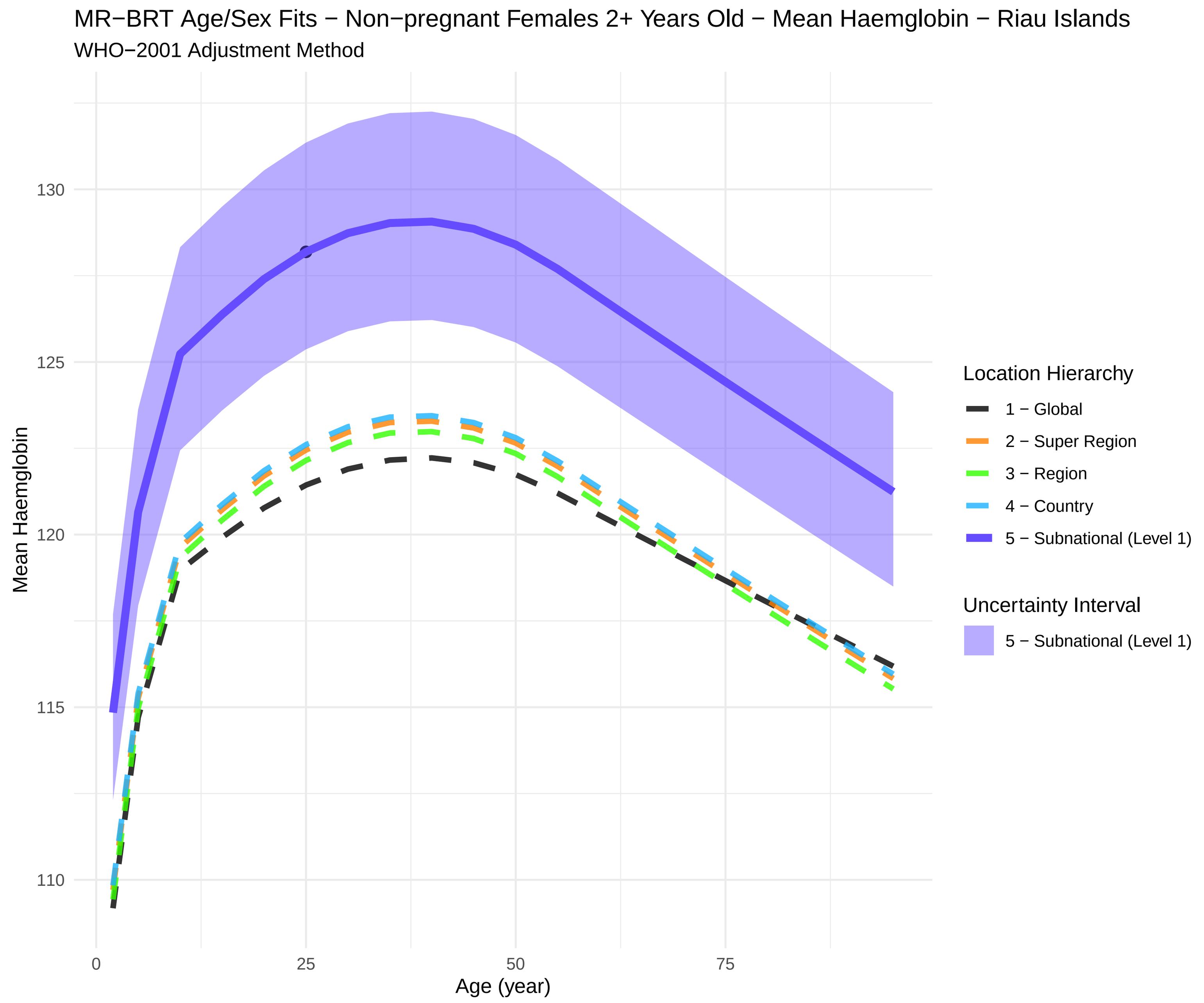
MR−BRT Age/Sex Fits − Non−pregnant Females 2+ Years Old − Mean Haemglobin − Riau Islands
WHO−2001 Adjustment Method
Mean Haemglobin
110
115
120
125
130
0
25
50
75
Age (year)
Location Hierarchy
1 − Global
2 − Super Region
3 − Region
4 − Country
5 − Subnational (Level 1)
Uncertainty Interval
5 − Subnational (Level 1)

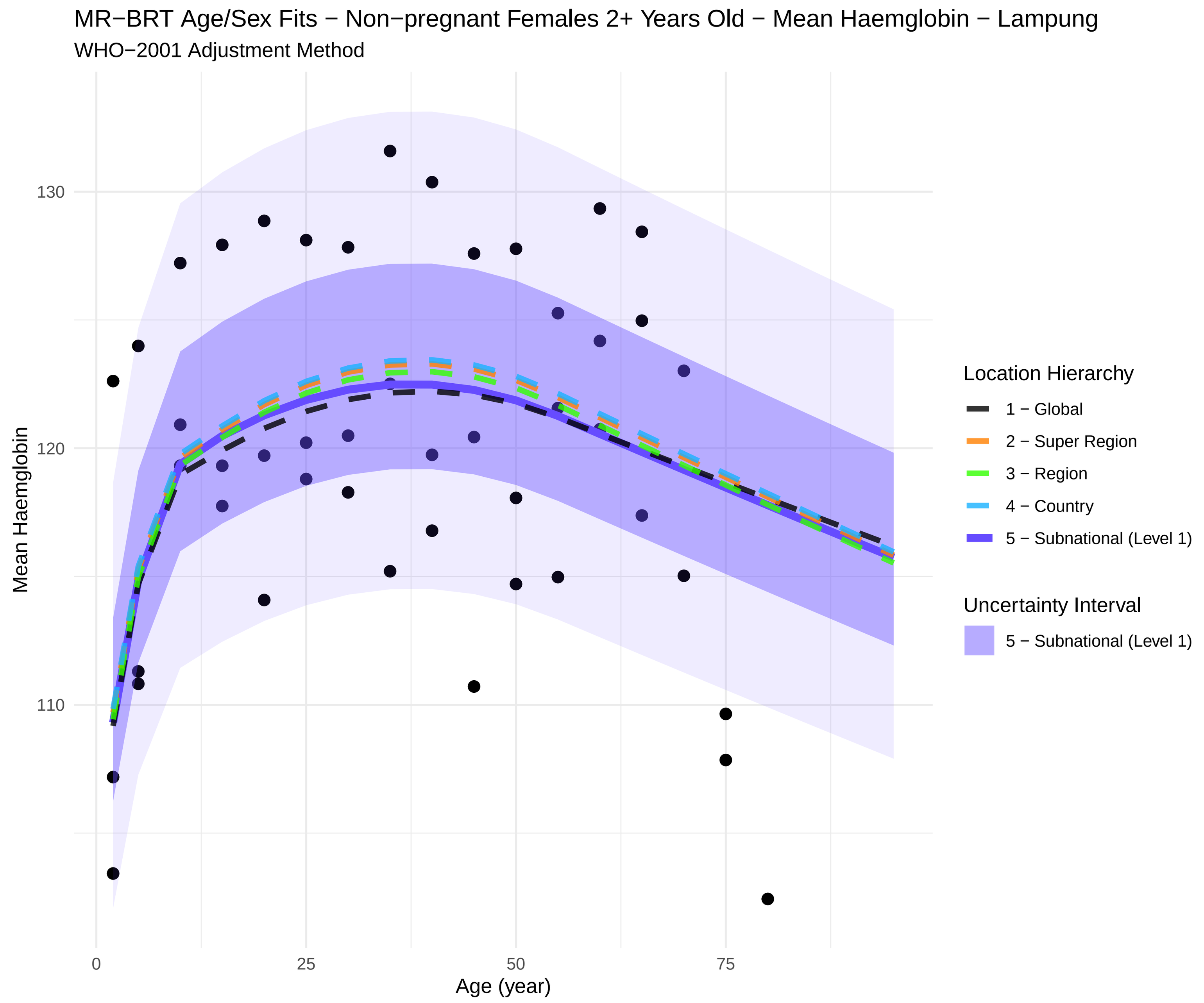
MR−BRT Age/Sex Fits − Non−pregnant Females 2+ Years Old − Mean Haemglobin − Lampung
WHO−2001 Adjustment Method
Mean Haemglobin
110
120
130
0
25
50
75
Age (year)
Location Hierarchy
1 − Global
2 − Super Region
3 − Region
4 − Country
5 − Subnational (Level 1)
Uncertainty Interval
5 − Subnational (Level 1)

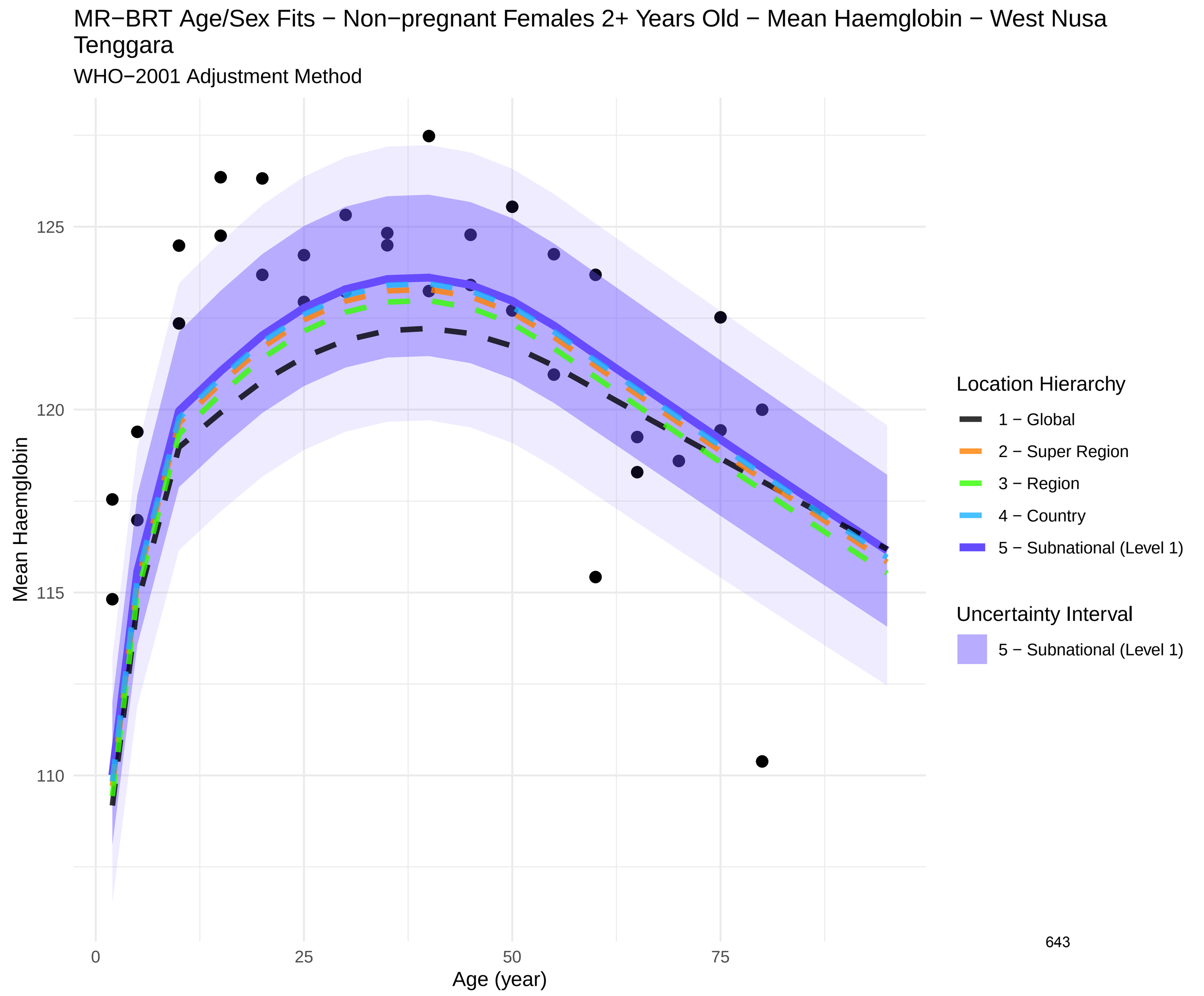
MR−BRT Age/Sex Fits − Non−pregnant Females 2+ Years Old − Mean Haemglobin − West Nusa Tenggara
WHO−2001 Adjustment Method
Mean Haemglobin
110
115
120
125
0
25
50
75
Age (year)
Location Hierarchy
1 − Global
2 − Super Region
3 − Region
4 − Country
5 − Subnational (Level 1)
Uncertainty Interval
5 − Subnational (Level 1)

MR−BRT Age/Sex Fits − Non−pregnant Females 2+ Years Old − Mean Haemglobin − Riau
WHO−2001 Adjustment Method

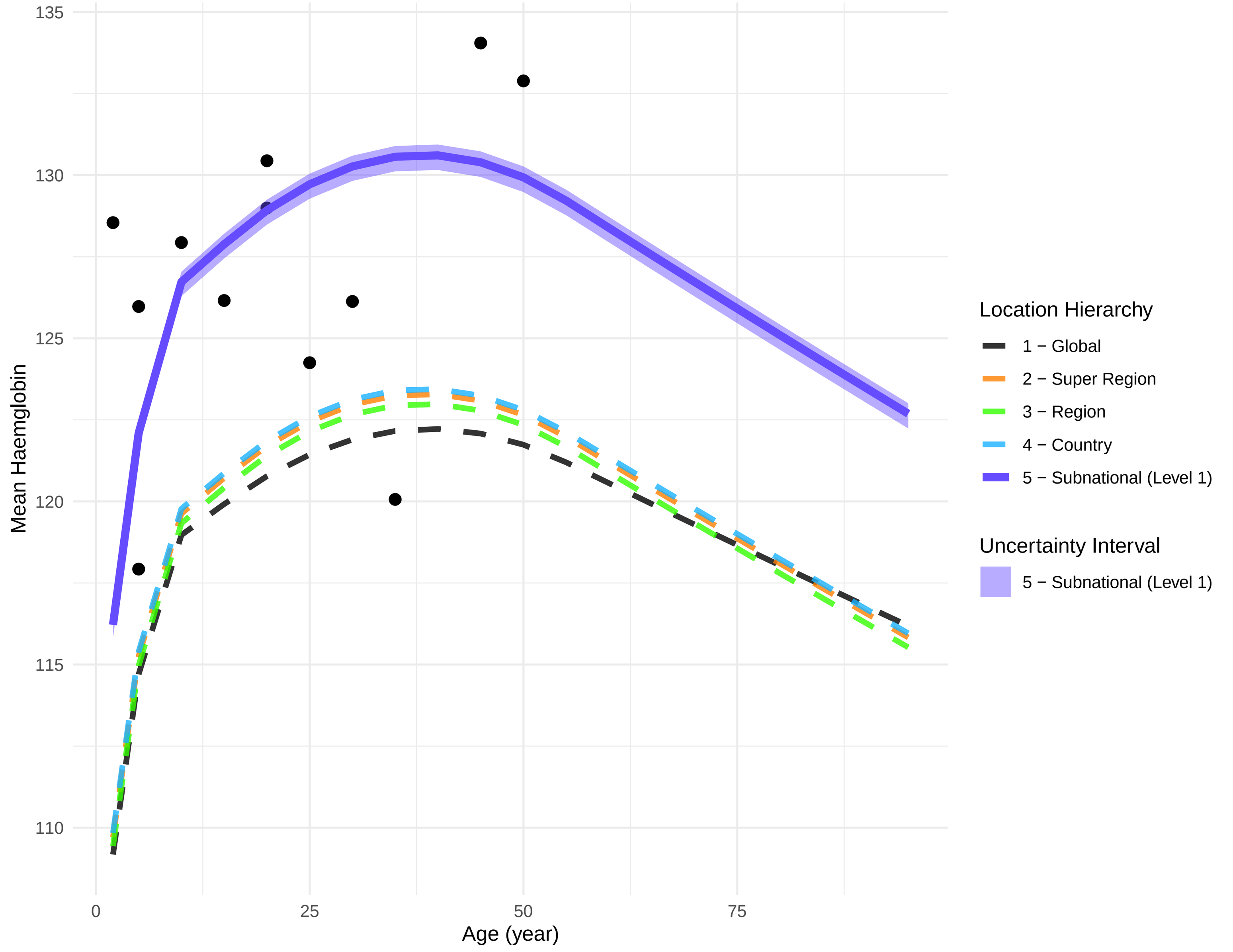

MR−BRT Age/Sex Fits − Non−pregnant Females 2+ Years Old − Mean Haemglobin − West Sulawesi
WHO−2001 Adjustment Method

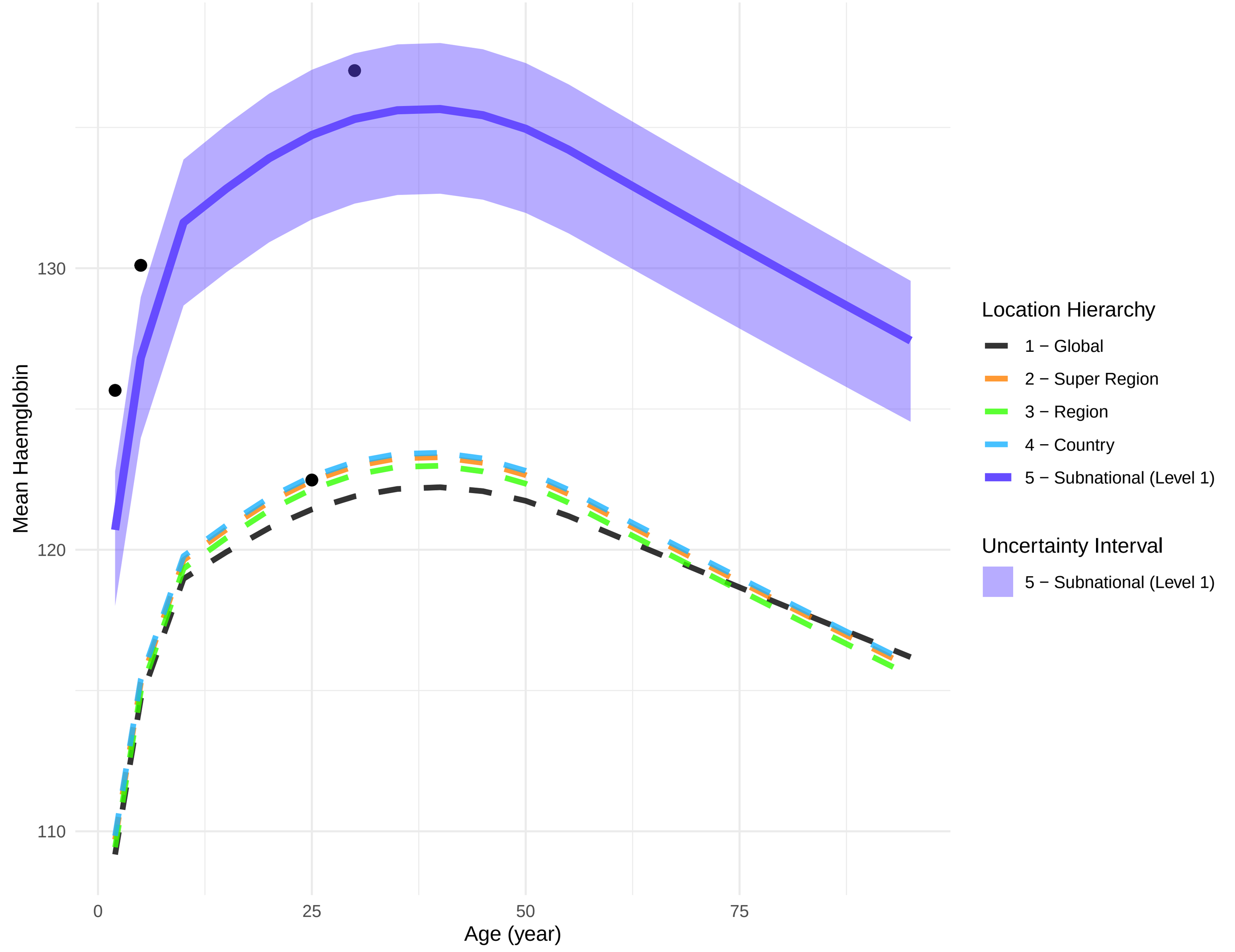

MR−BRT Age/Sex Fits − Non−pregnant Females 2+ Years Old − Mean Haemglobin − South Sulawesi
WHO−2001 Adjustment Method

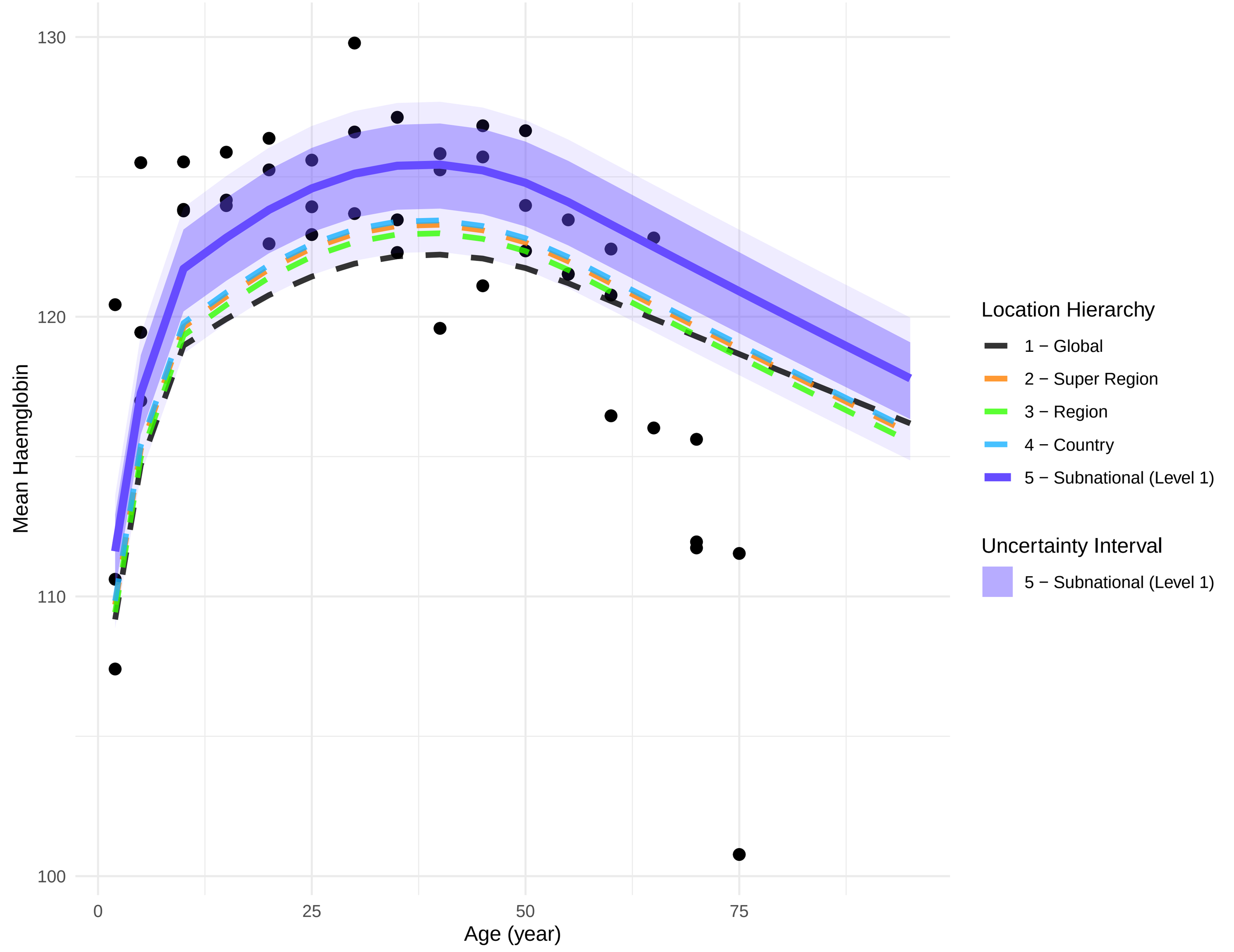

MR−BRT Age/Sex Fits − Non−pregnant Females 2+ Years Old − Mean Haemglobin − West Sumatra
WHO−2001 Adjustment Method

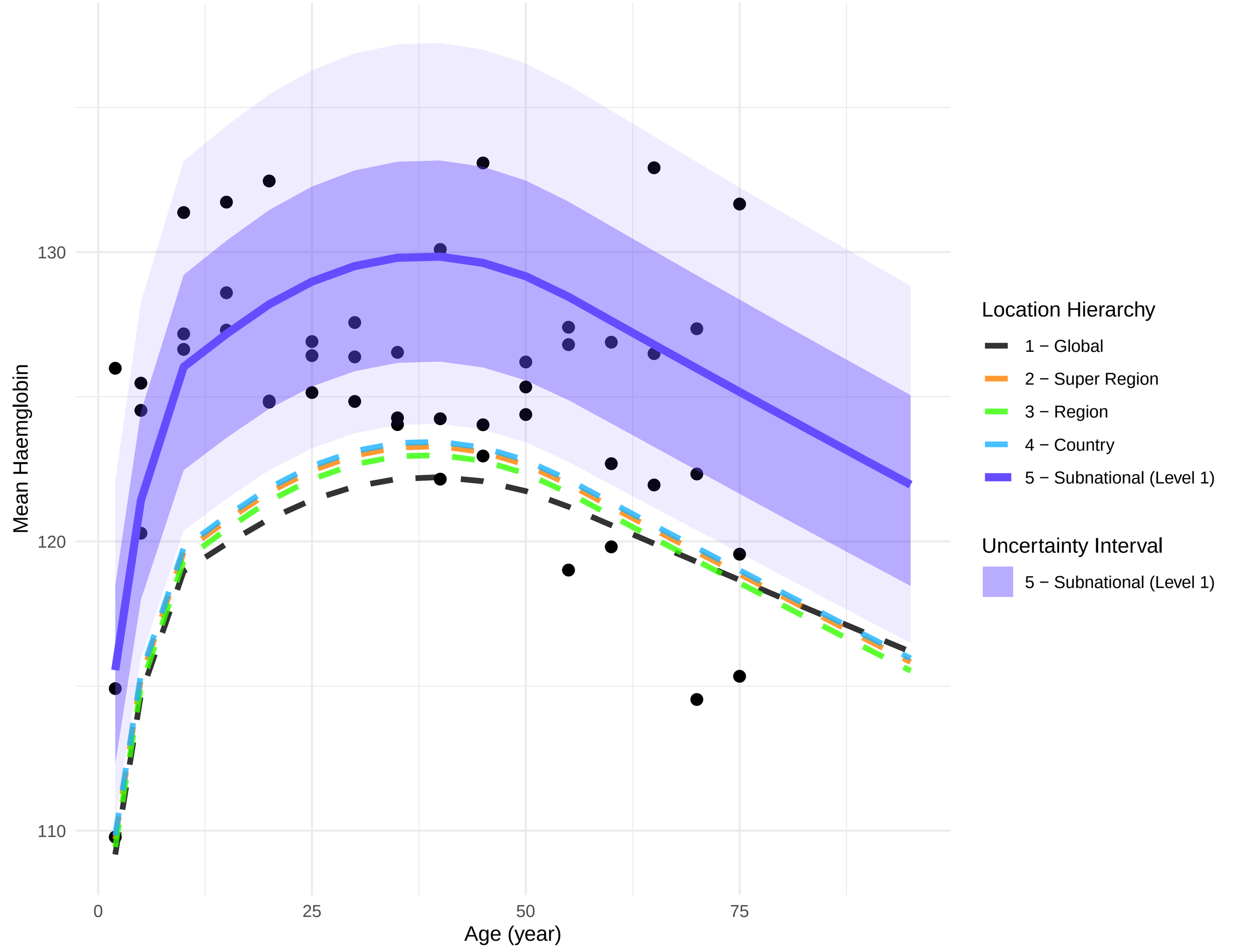

MR−BRT Age/Sex Fits − Non−pregnant Females 2+ Years Old − Mean Haemglobin − South Sumatra
WHO−2001 Adjustment Method

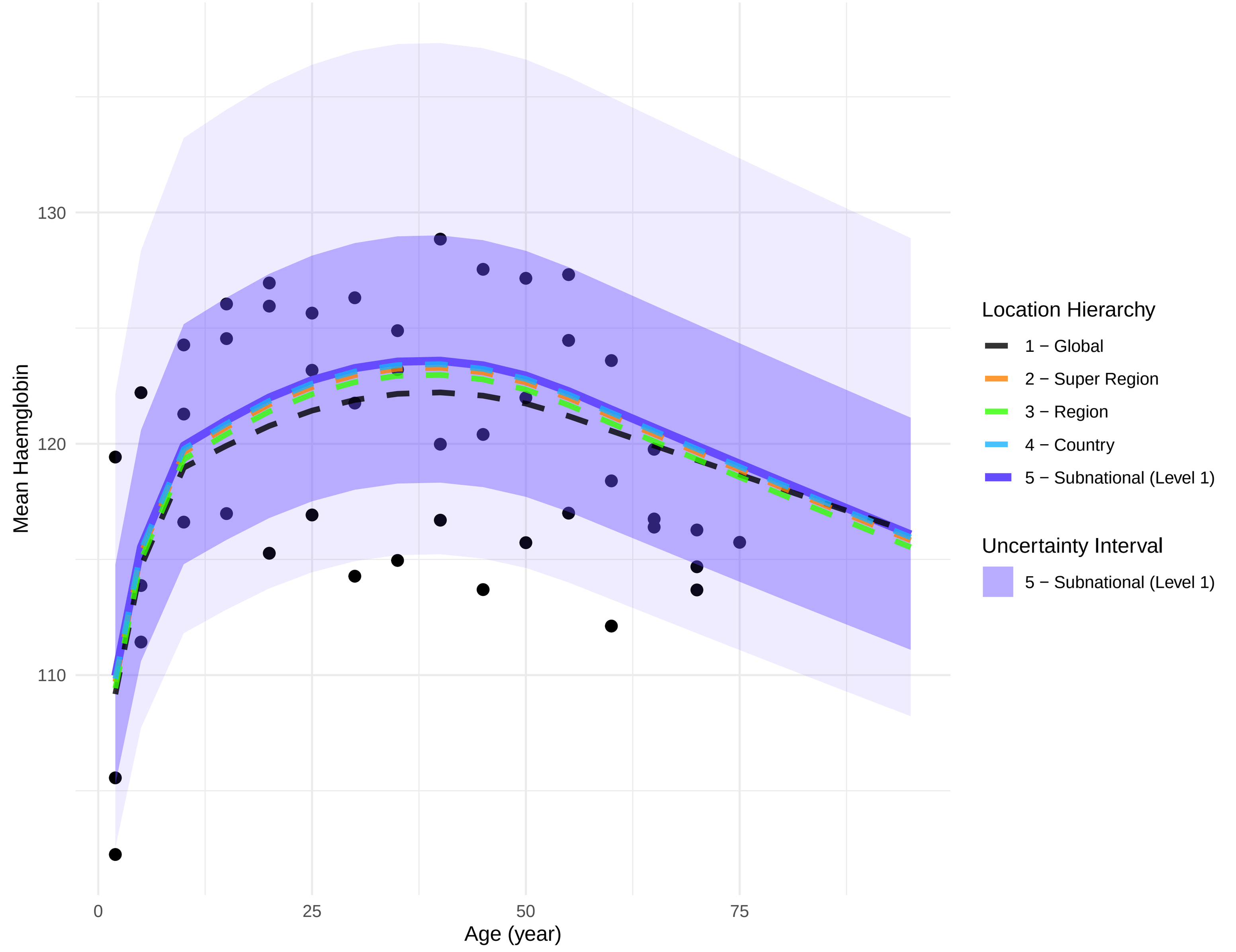

MR−BRT Age/Sex Fits − Non−pregnant Females 2+ Years Old − Mean Haemglobin − North Sumatra
WHO−2001 Adjustment Method

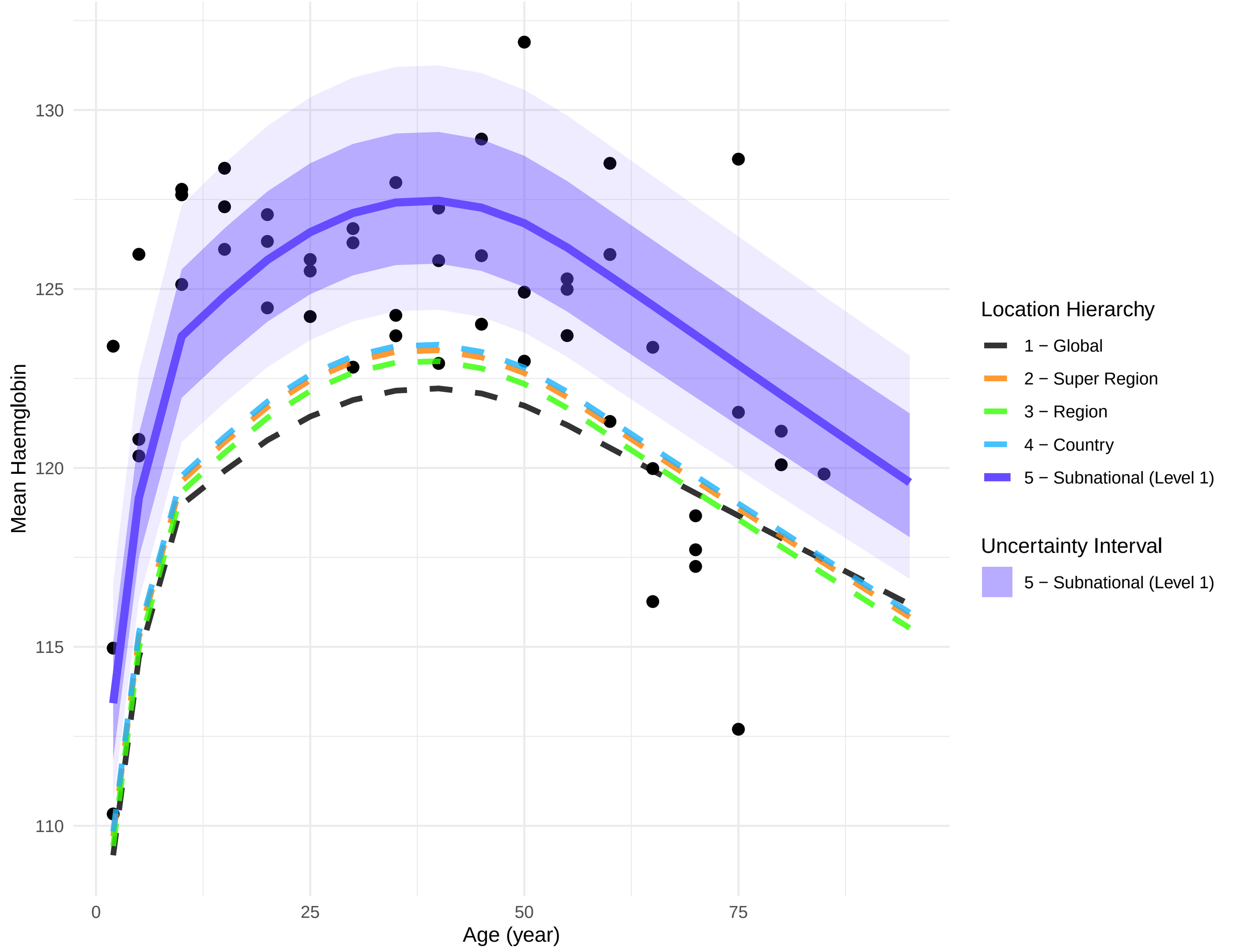

MR−BRT Age/Sex Fits − Non−pregnant Females 2+ Years Old − Mean Haemglobin − Yogyakarta
WHO−2001 Adjustment Method

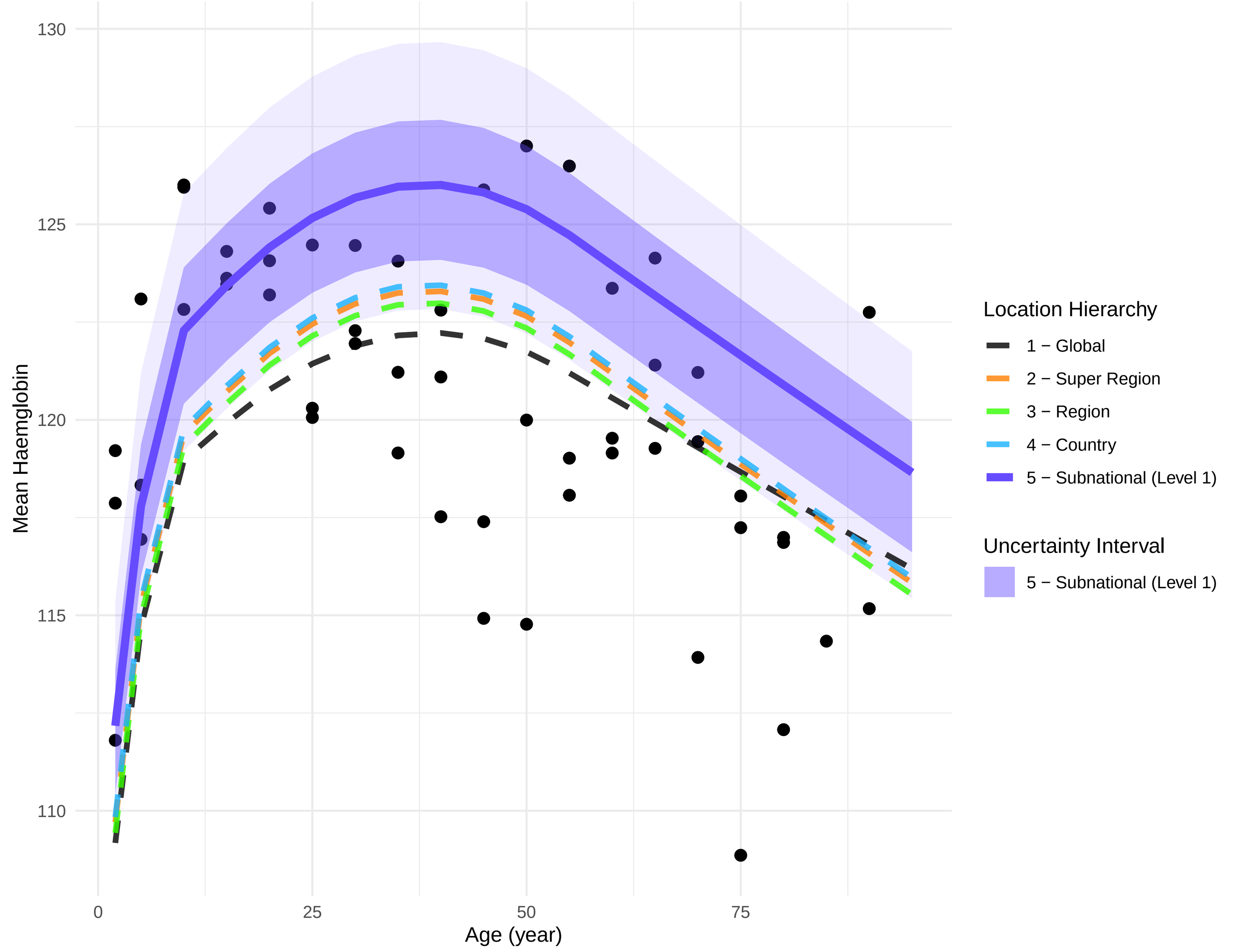

MR−BRT Age/Sex Fits − Non−pregnant Females 2+ Years Old − Mean Haemglobin − Myanmar
WHO−2001 Adjustment Method

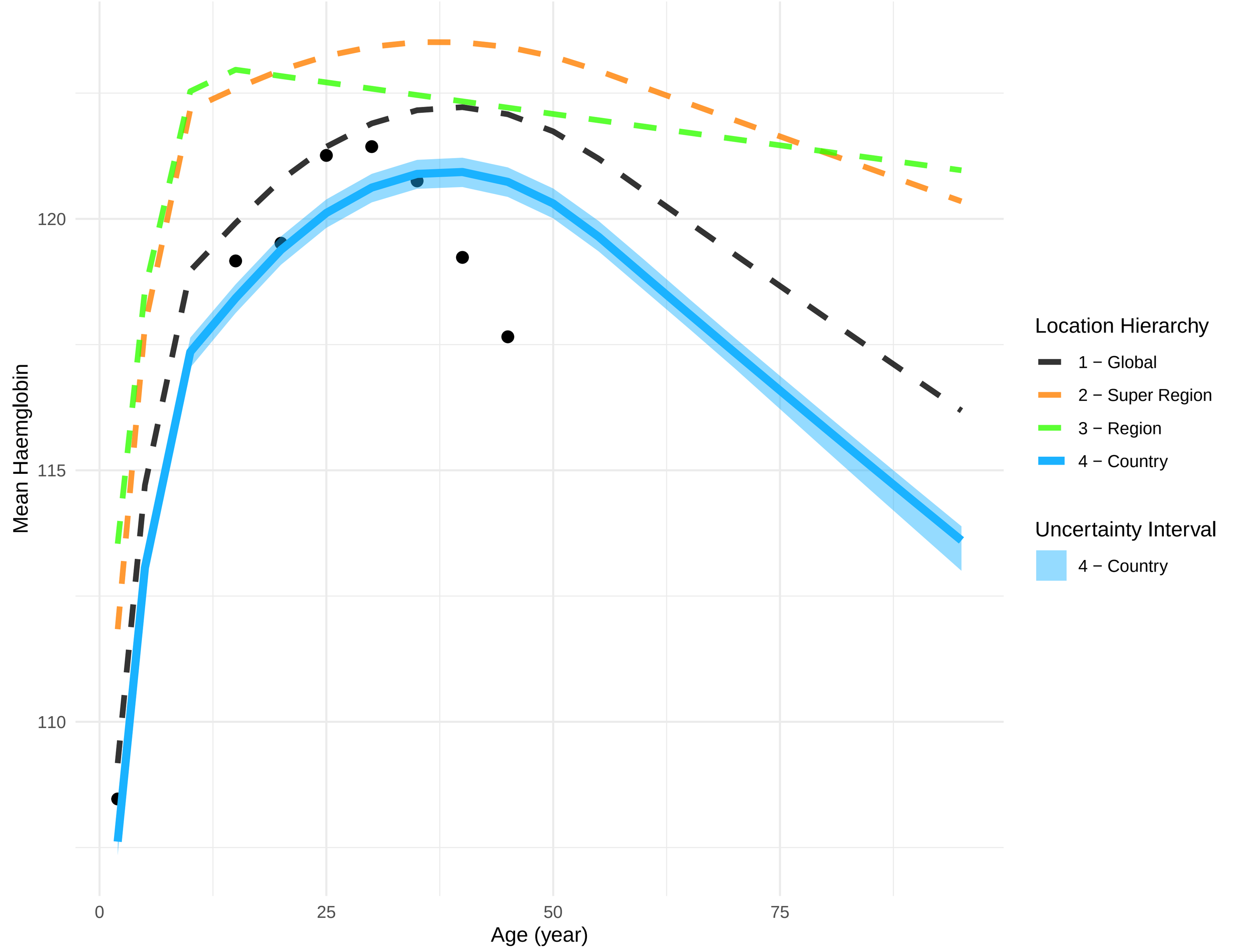

MR−BRT Age/Sex Fits − Non−pregnant Females 2+ Years Old − Mean Haemglobin − Timor−Leste
WHO−2001 Adjustment Method

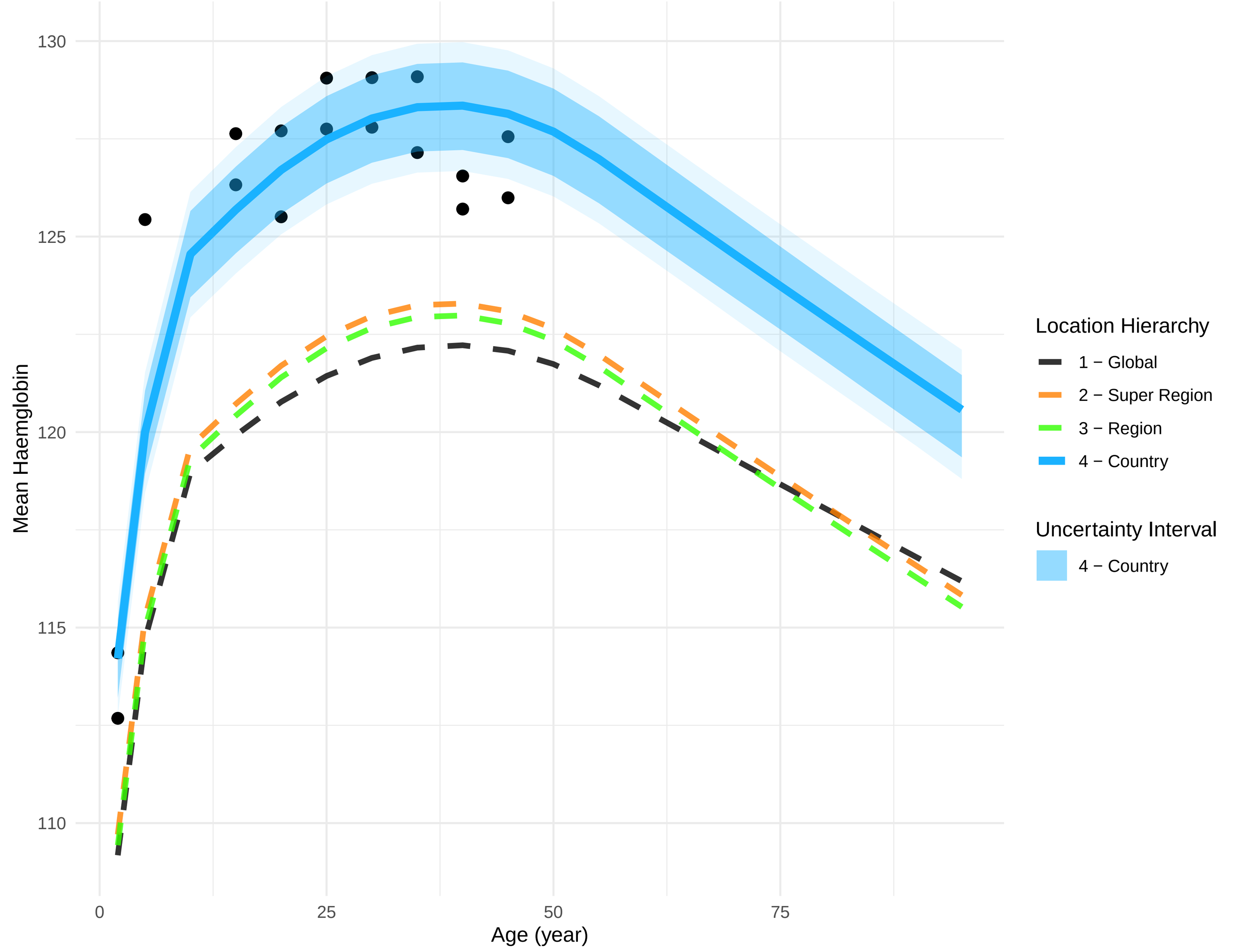

MR−BRT Age/Sex Fits − Non−pregnant Females 2+ Years Old − Mean Haemglobin − Sub−Saharan Africa
WHO−2001 Adjustment Method

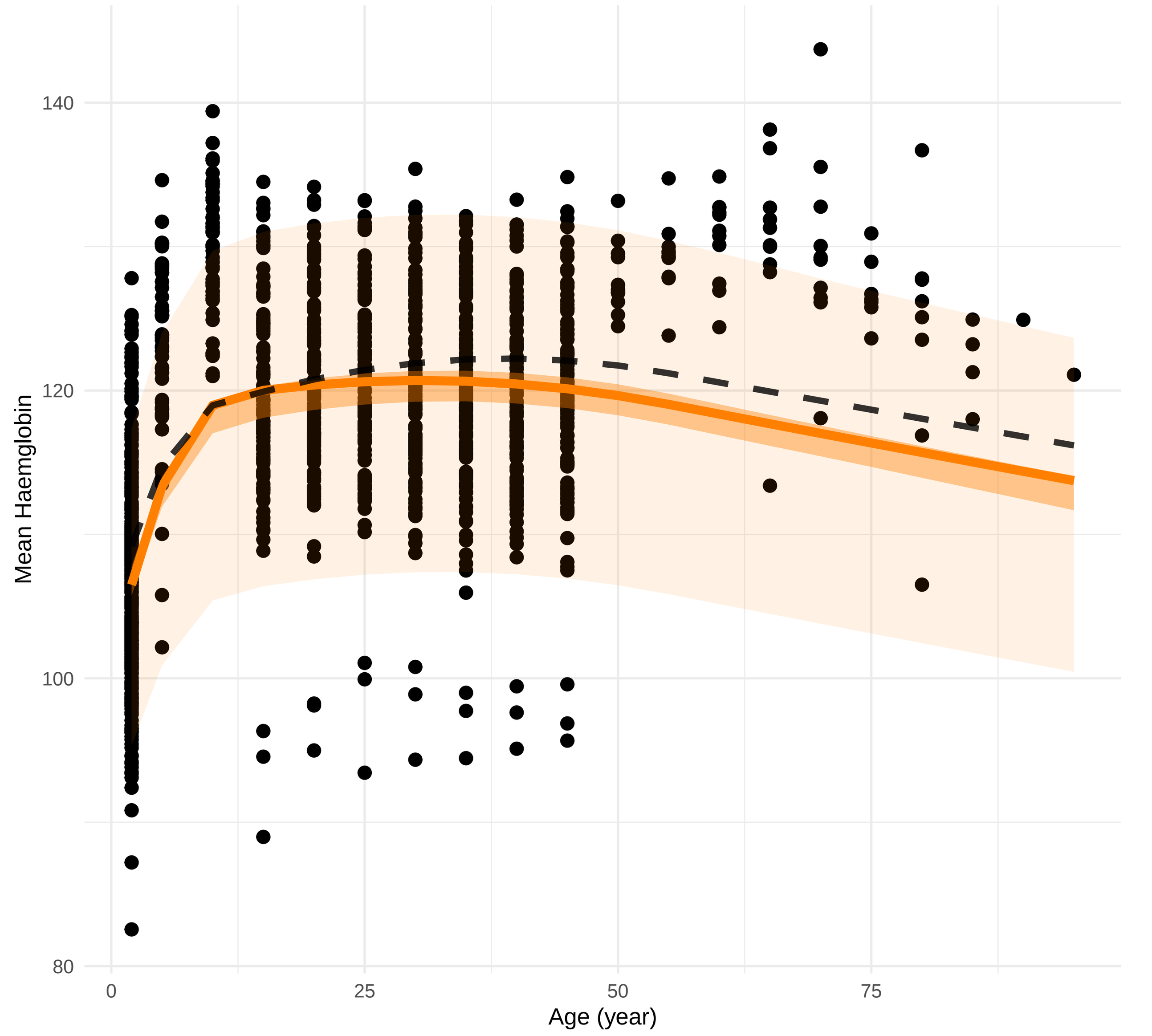

MR−BRT Age/Sex Fits − Non−pregnant Females 2+ Years Old − Mean Haemglobin − Central Sub−Saharan Africa
WHO−2001 Adjustment Method

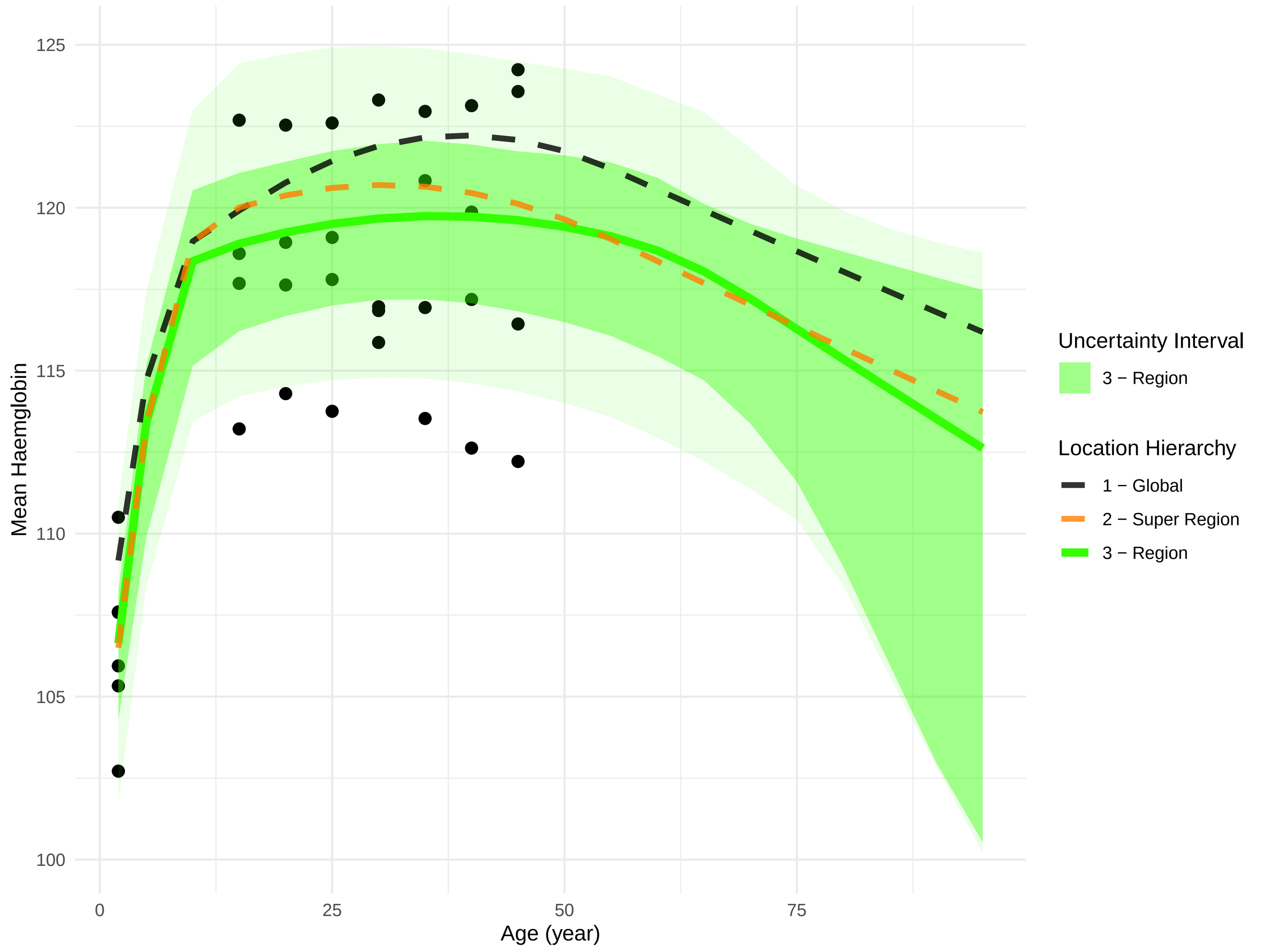

MR−BRT Age/Sex Fits − Non−pregnant Females 2+ Years Old − Mean Haemglobin − Angola
WHO−2001 Adjustment Method

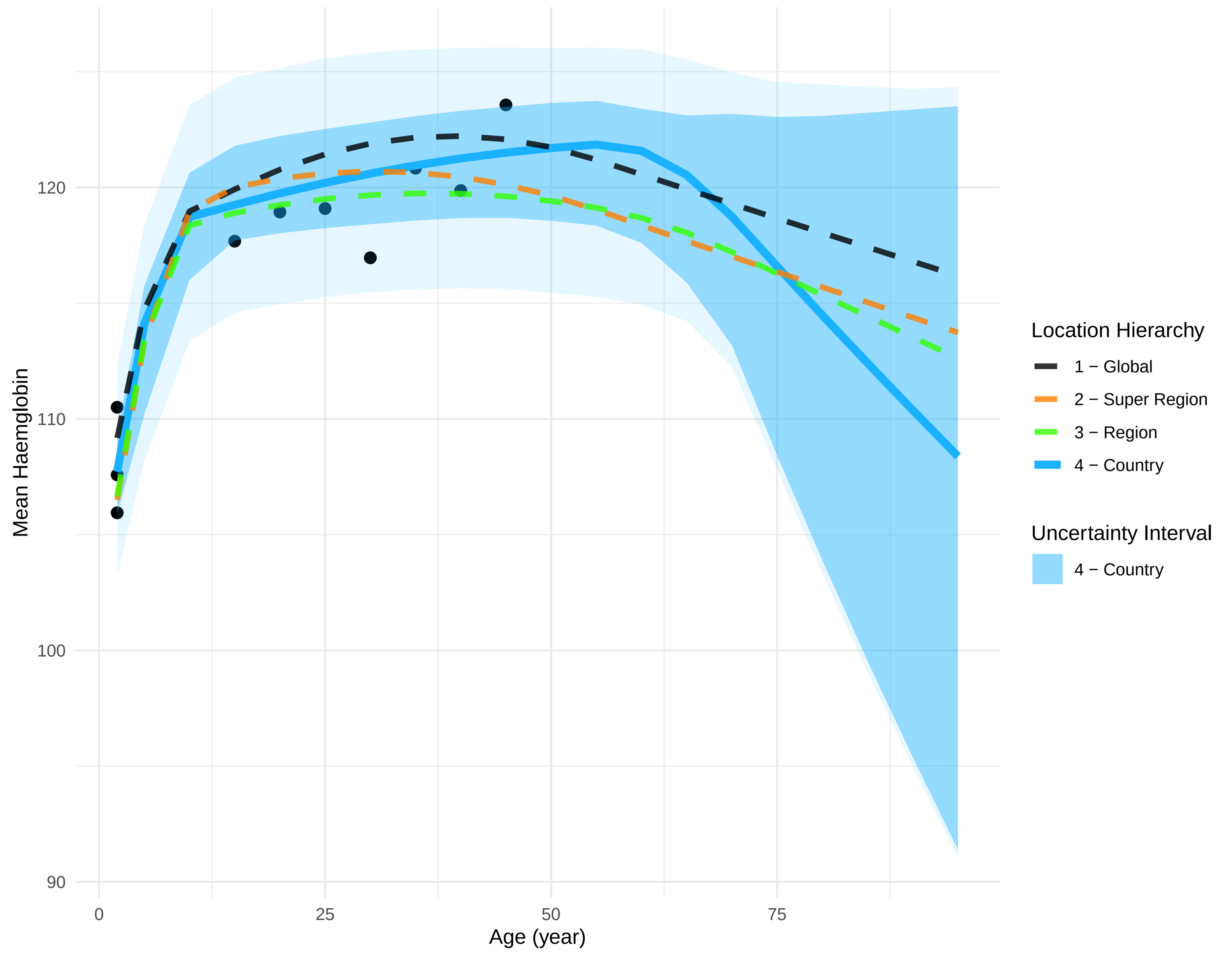

MR−BRT Age/Sex Fits − Non−pregnant Females 2+ Years Old − Mean Haemglobin − Democratic Republic of the Congo
WHO−2001 Adjustment Method

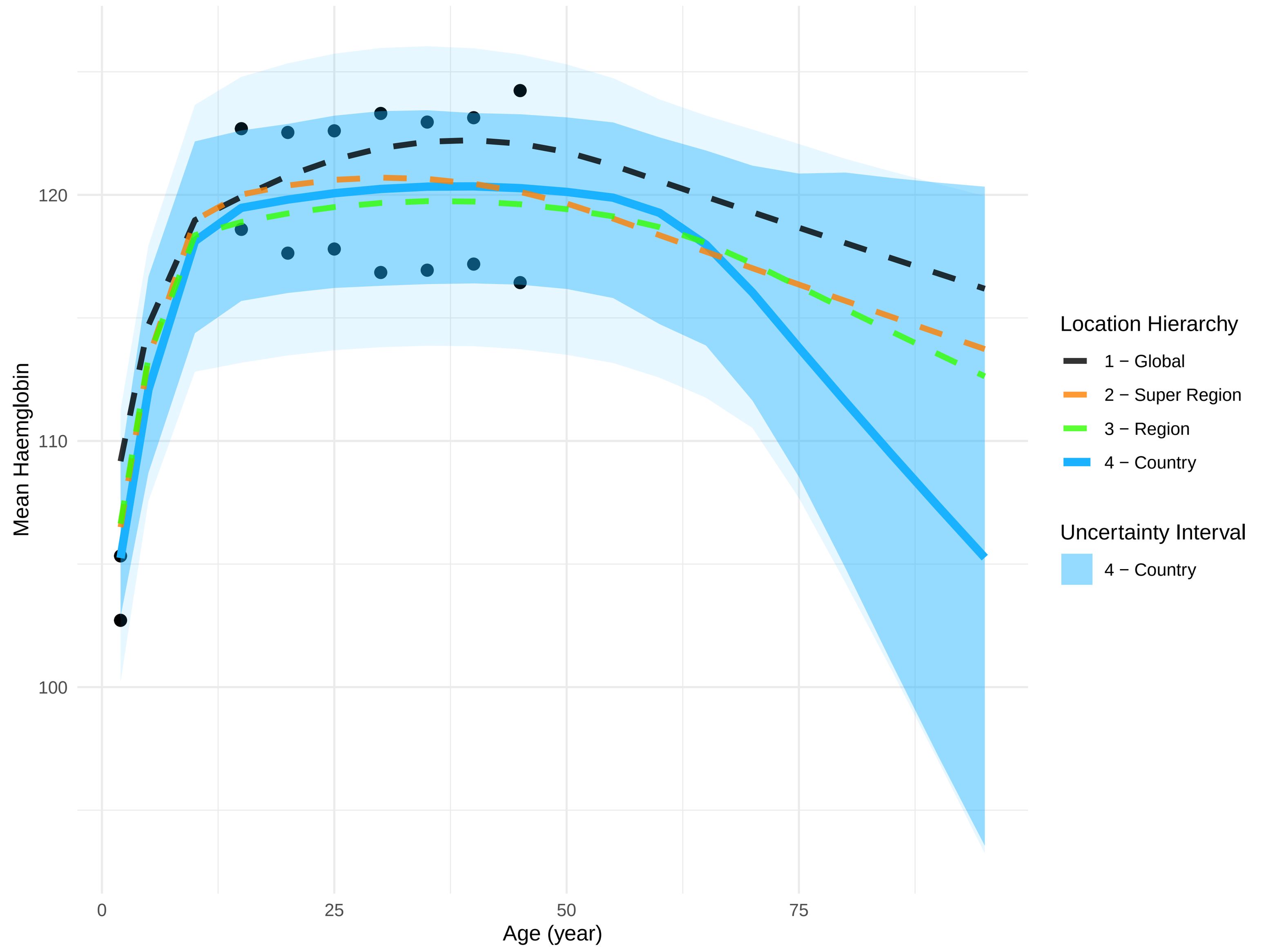

MR−BRT Age/Sex Fits − Non−pregnant Females 2+ Years Old − Mean Haemglobin − Gabon
WHO−2001 Adjustment Method

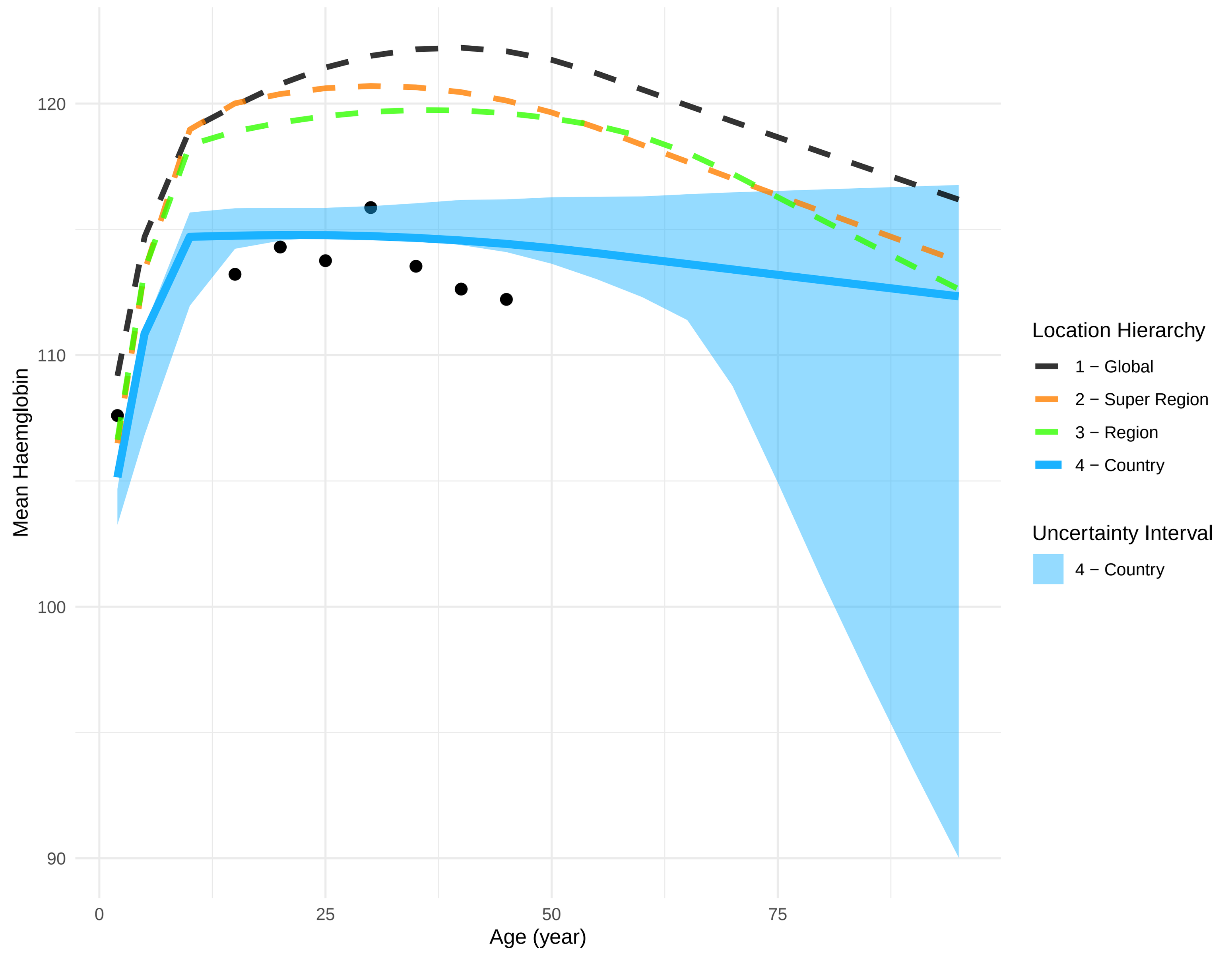

MR−BRT Age/Sex Fits − Non−pregnant Females 2+ Years Old − Mean Haemglobin − Eastern Sub−Saharan Africa
WHO−2001 Adjustment Method

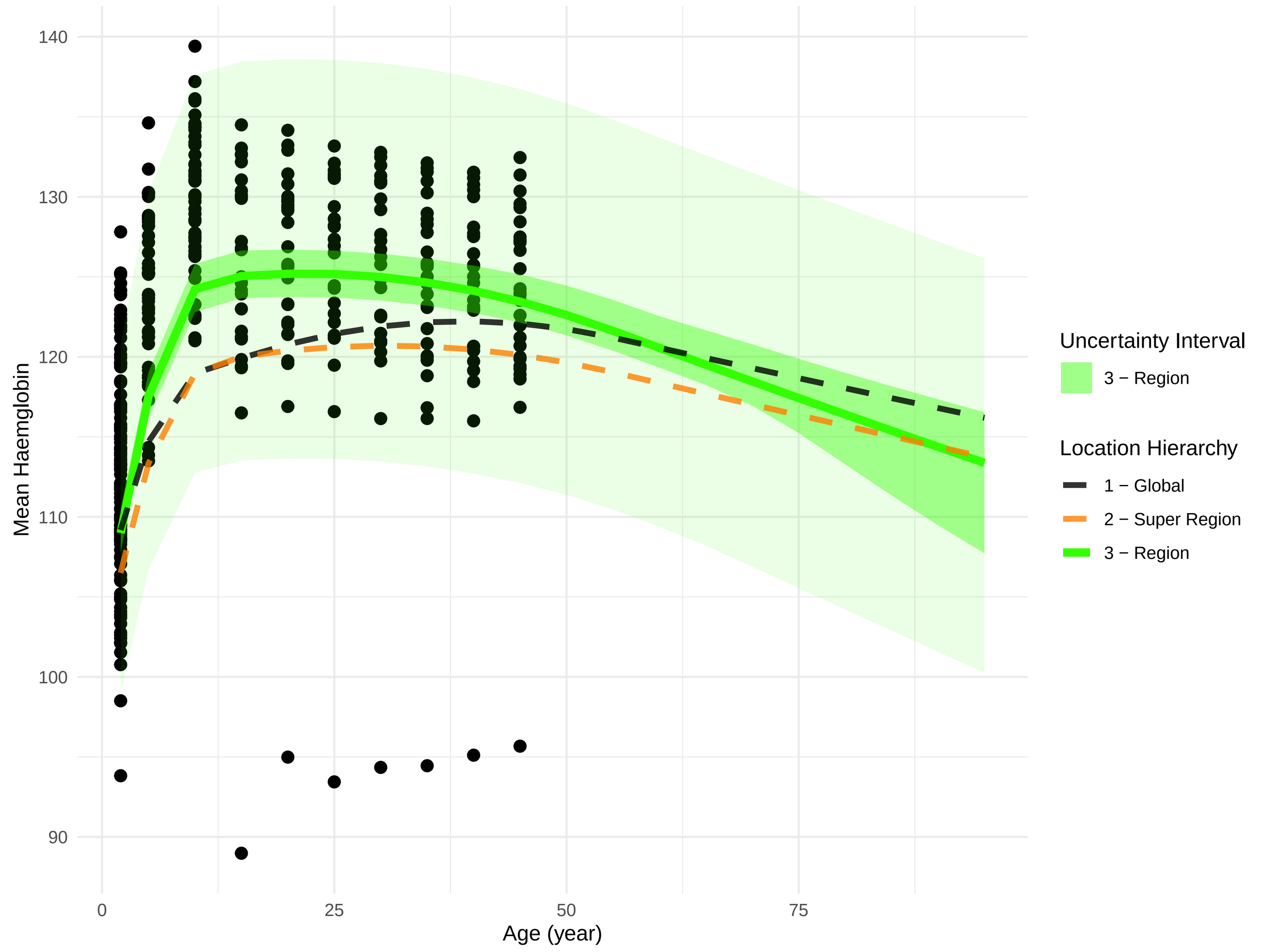

MR−BRT Age/Sex Fits − Non−pregnant Females 2+ Years Old − Mean Haemglobin − Burundi
WHO−2001 Adjustment Method

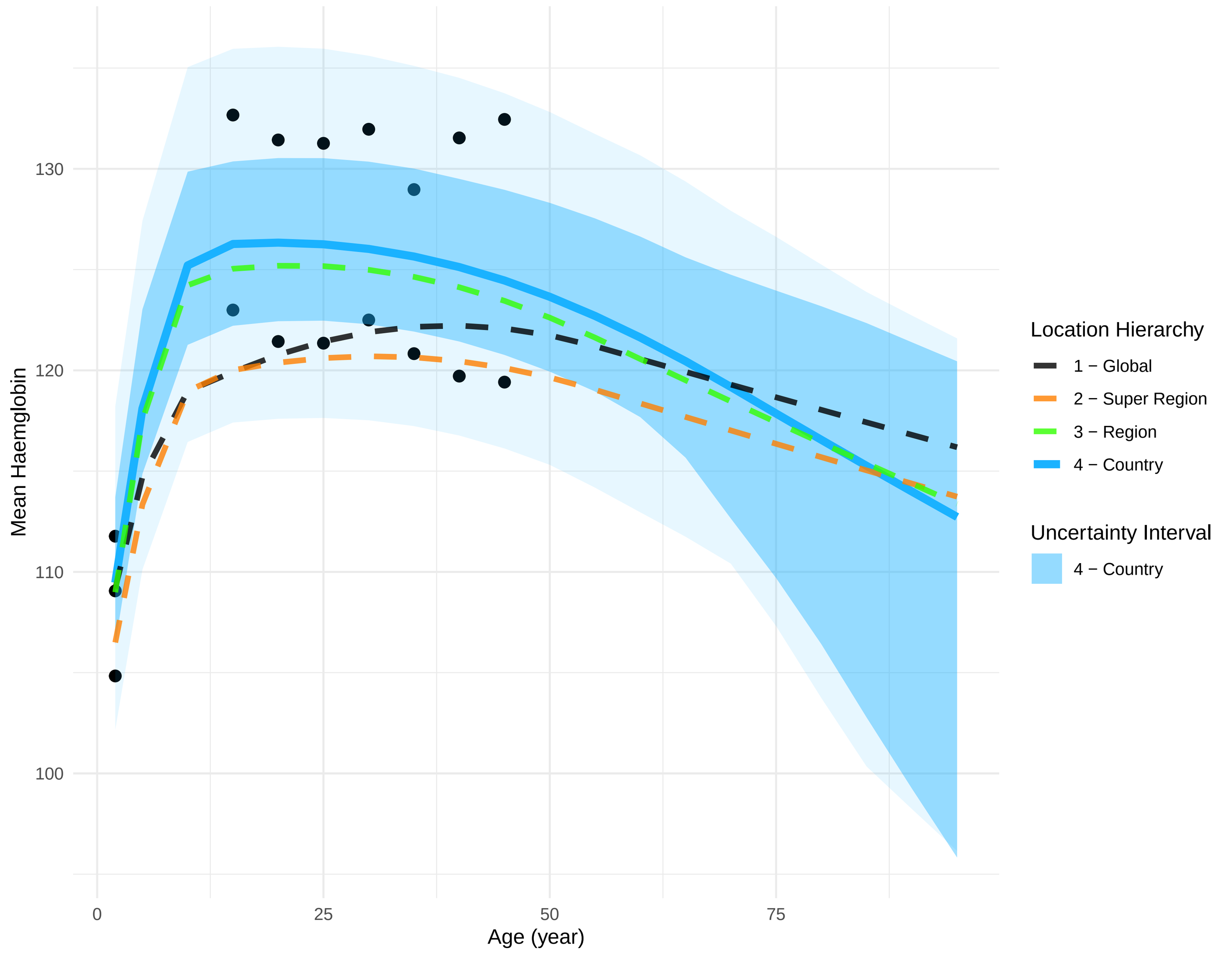

MR−BRT Age/Sex Fits − Non−pregnant Females 2+ Years Old − Mean Haemglobin − Ethiopia
WHO−2001 Adjustment Method

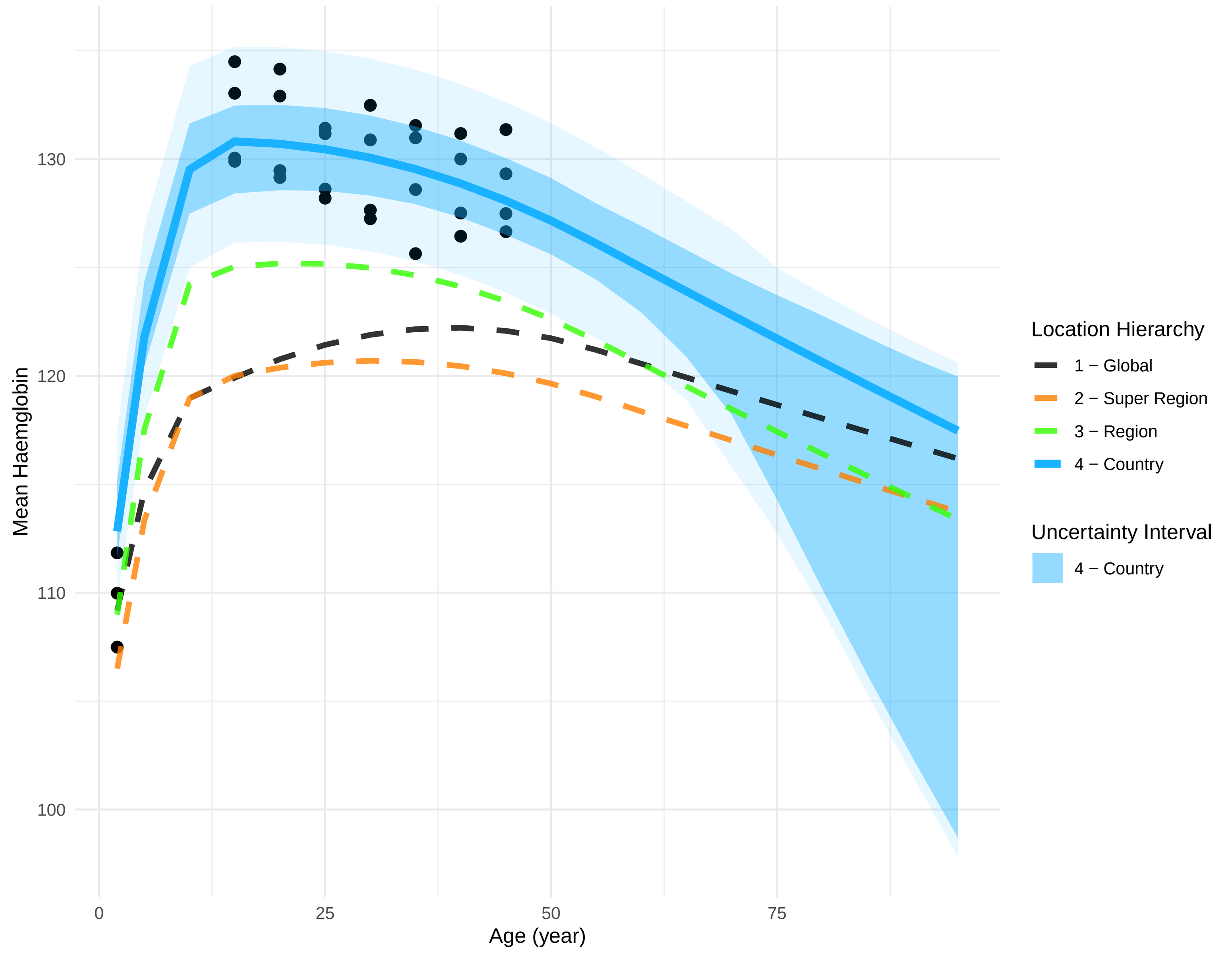

MR−BRT Age/Sex Fits − Non−pregnant Females 2+ Years Old − Mean Haemglobin − Kenya
WHO−2001 Adjustment Method

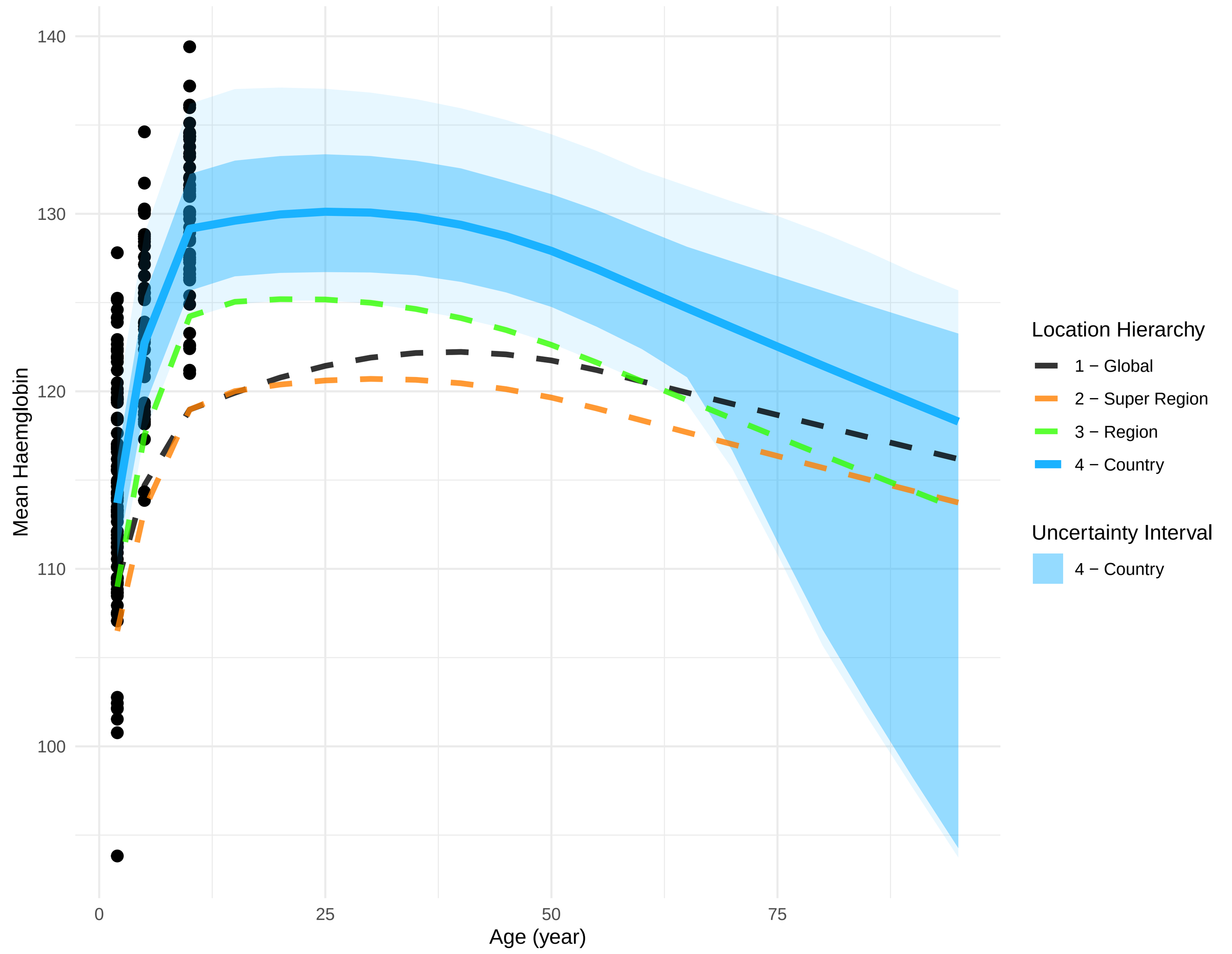

MR−BRT Age/Sex Fits − Non−pregnant Females 2+ Years Old − Mean Haemglobin − Baringo
WHO−2001 Adjustment Method

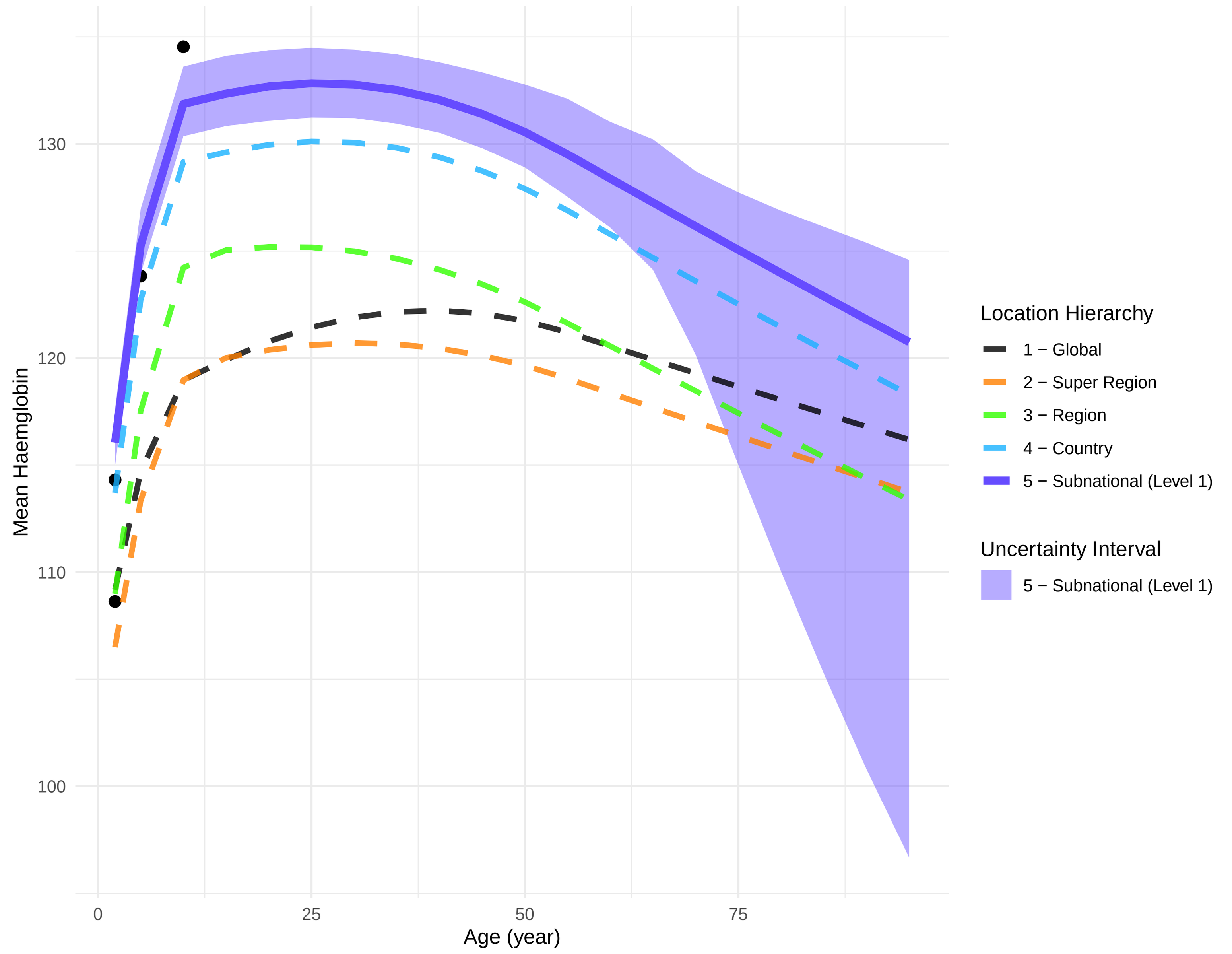

MR−BRT Age/Sex Fits − Non−pregnant Females 2+ Years Old − Mean Haemglobin − Bomet
WHO−2001 Adjustment Method

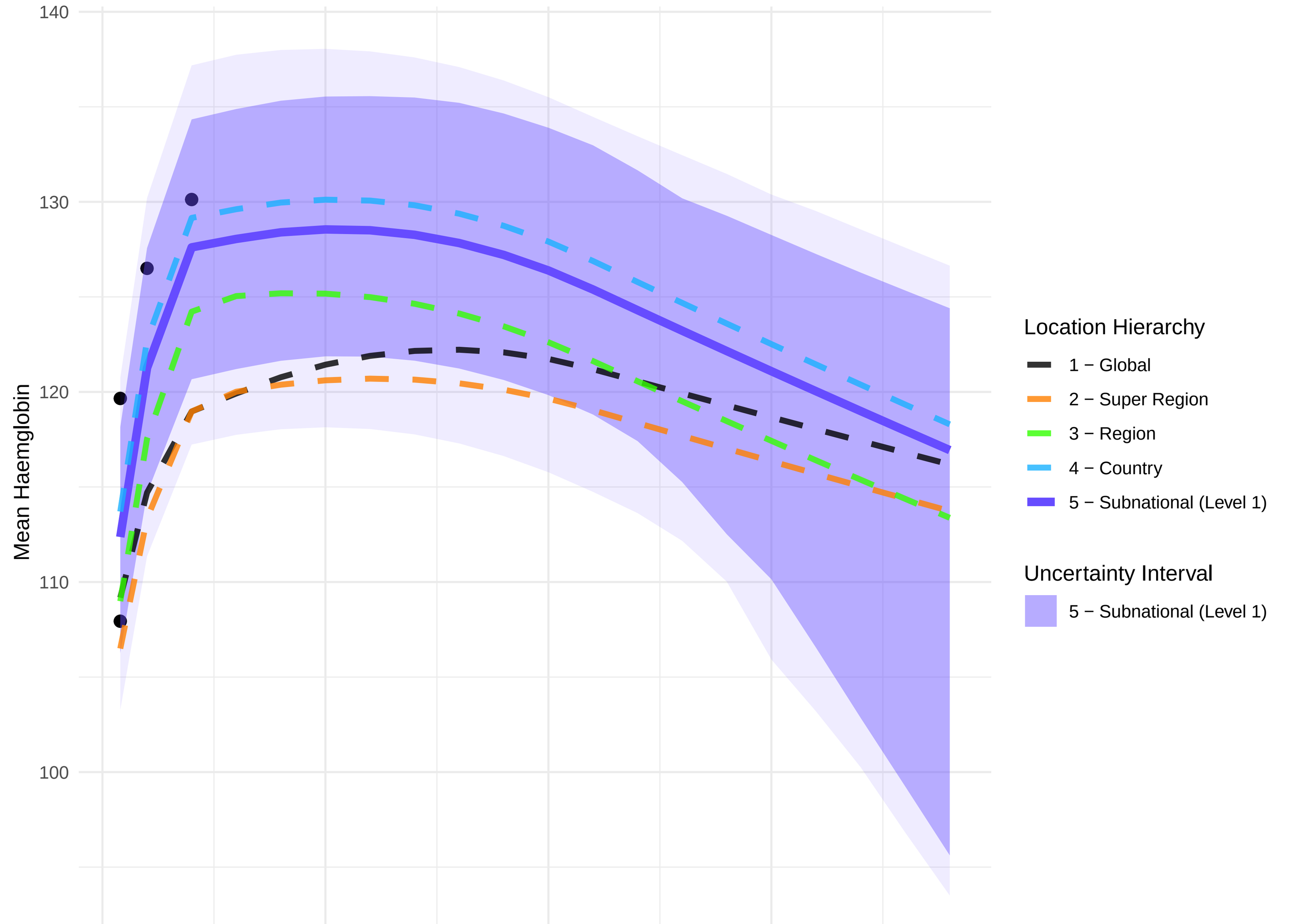

MR−BRT Age/Sex Fits − Non−pregnant Females 2+ Years Old − Mean Haemglobin − Bungoma
WHO−2001 Adjustment Method

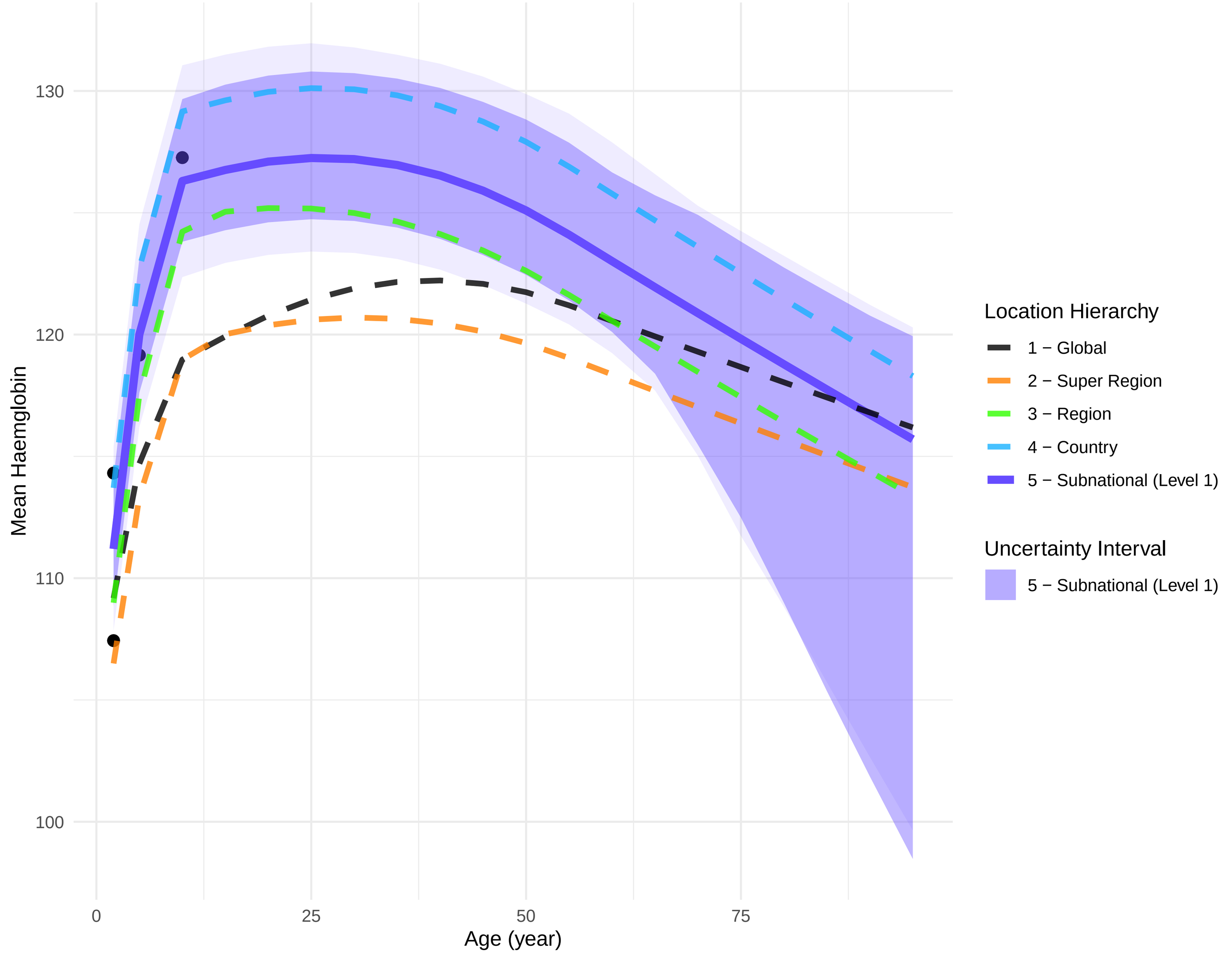

MR−BRT Age/Sex Fits − Non−pregnant Females 2+ Years Old − Mean Haemglobin − Busia
WHO−2001 Adjustment Method

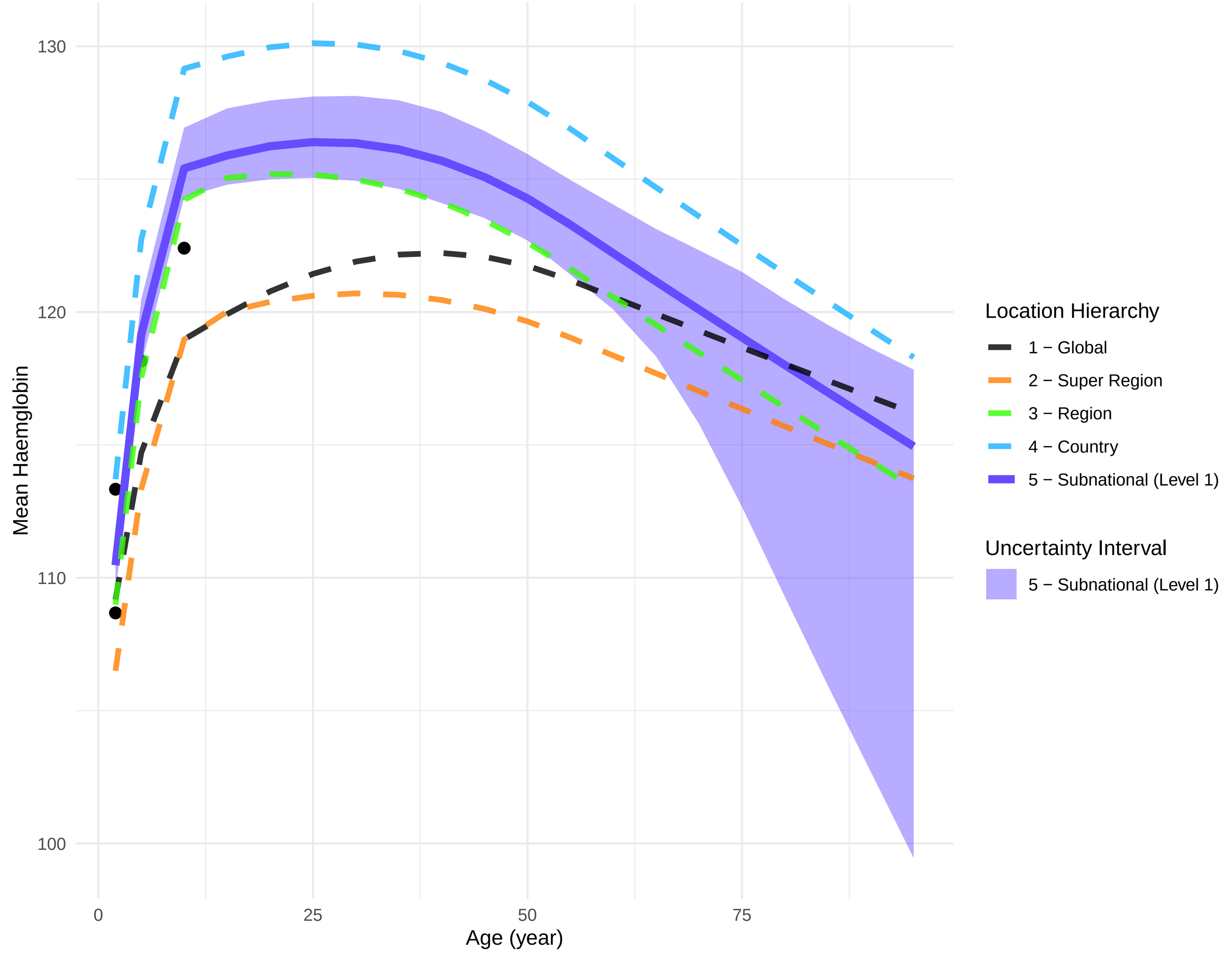

MR−BRT Age/Sex Fits − Non−pregnant Females 2+ Years Old − Mean Haemglobin − Elgeyo−Marakwet
WHO−2001 Adjustment Method

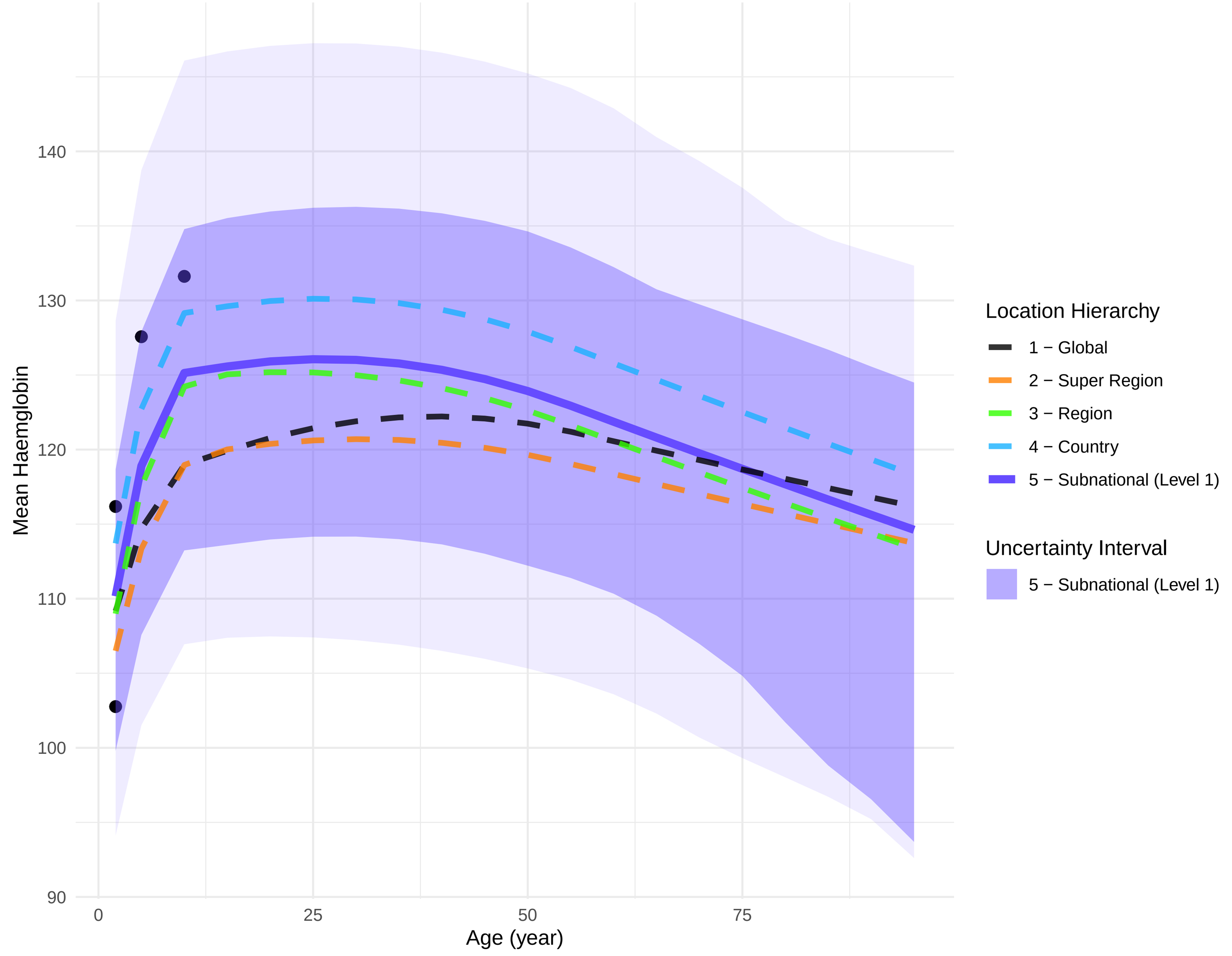

MR−BRT Age/Sex Fits − Non−pregnant Females 2+ Years Old − Mean Haemglobin − Embu
WHO−2001 Adjustment Method

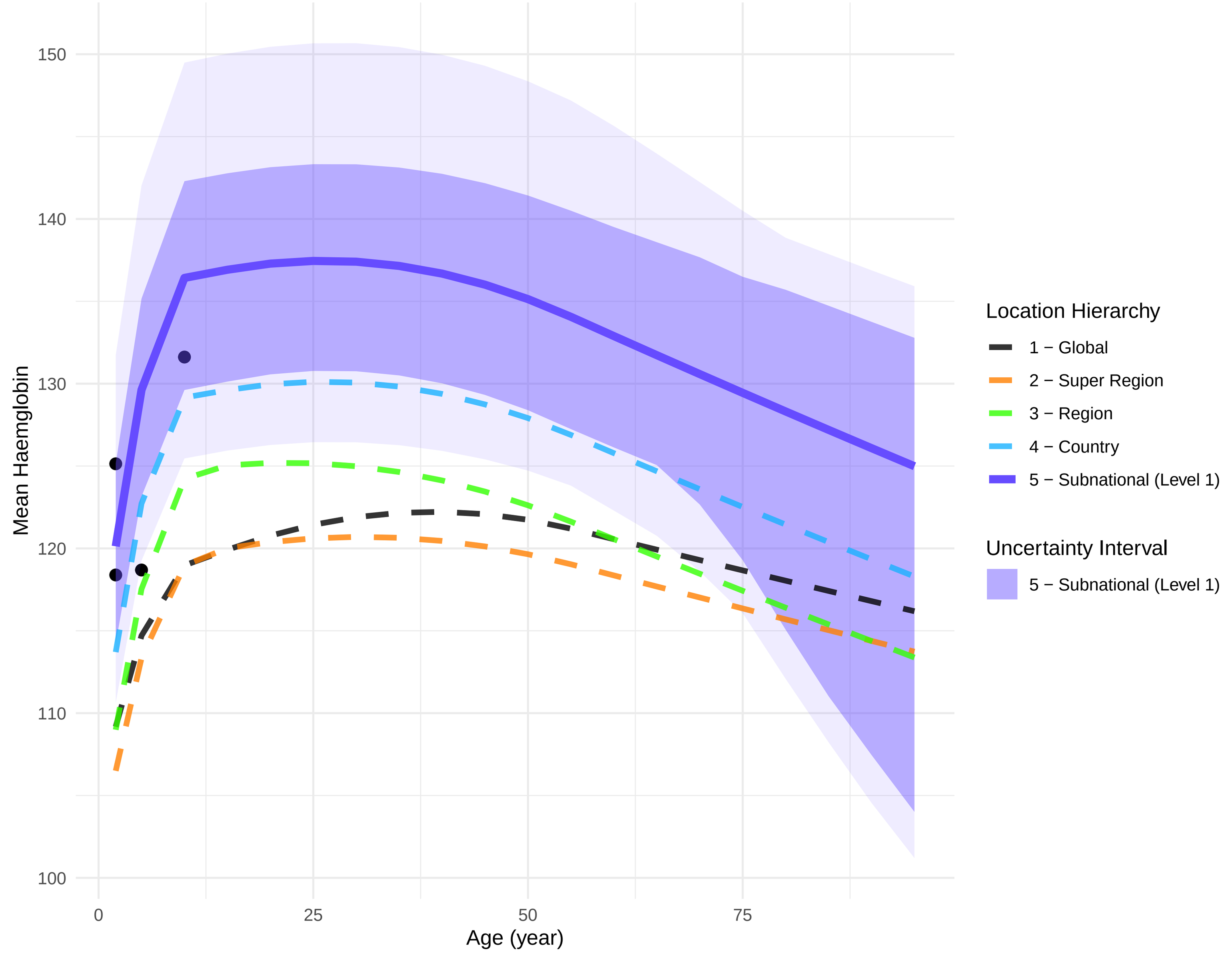

MR−BRT Age/Sex Fits − Non−pregnant Females 2+ Years Old − Mean Haemglobin − Garissa
WHO−2001 Adjustment Method

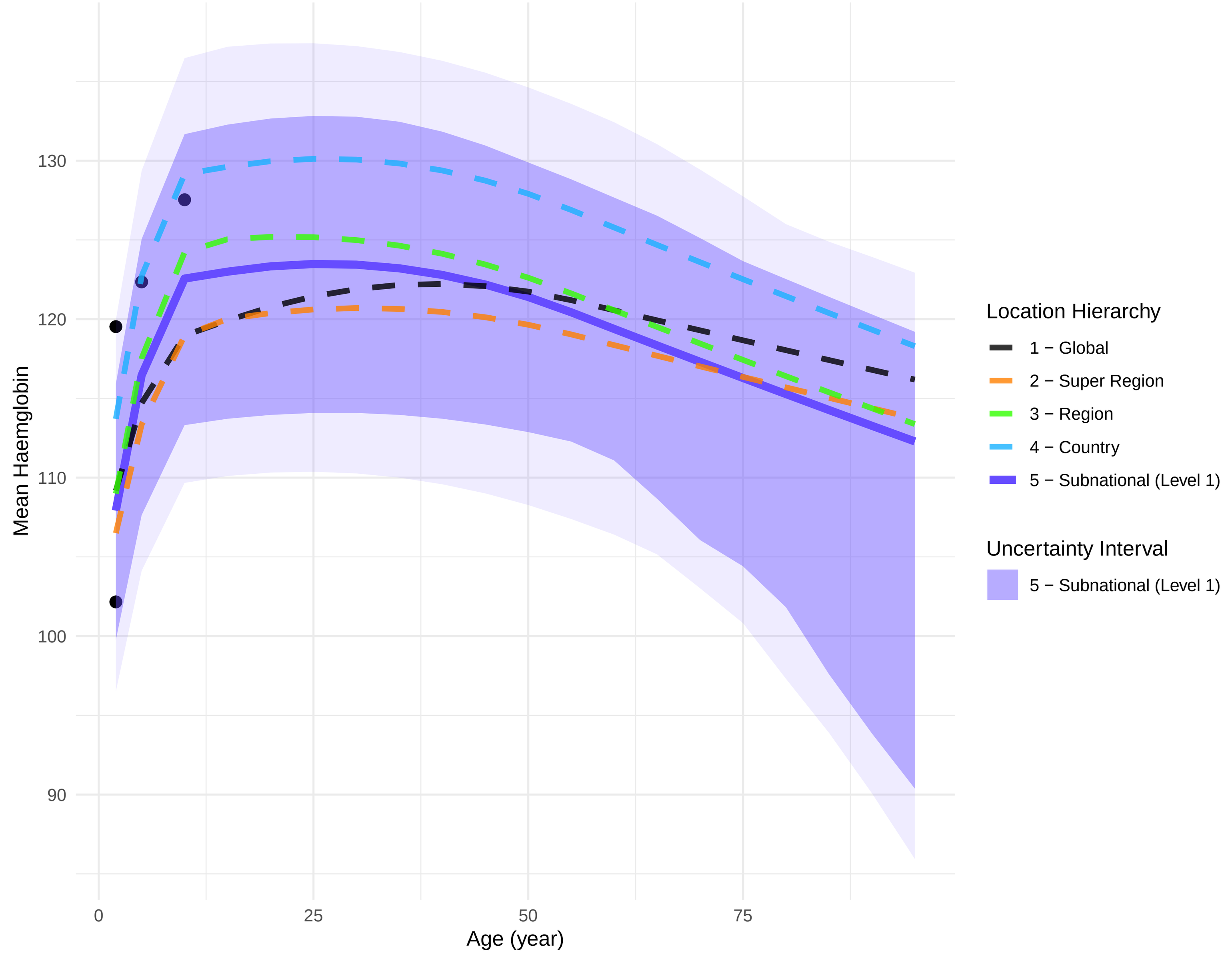

MR−BRT Age/Sex Fits − Non−pregnant Females 2+ Years Old − Mean Haemglobin − Homa Bay
WHO−2001 Adjustment Method

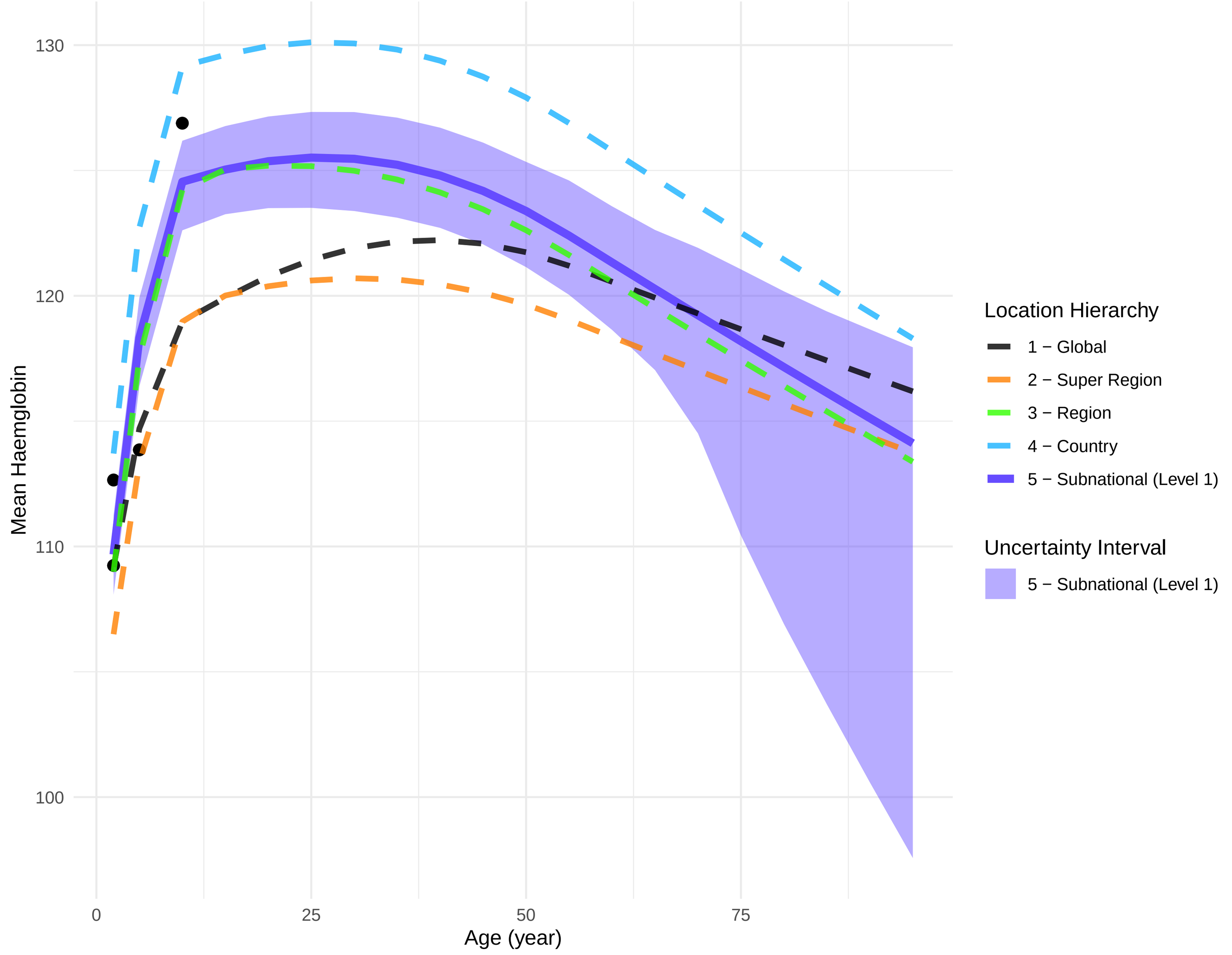

MR−BRT Age/Sex Fits − Non−pregnant Females 2+ Years Old − Mean Haemglobin − Isiolo
WHO−2001 Adjustment Method

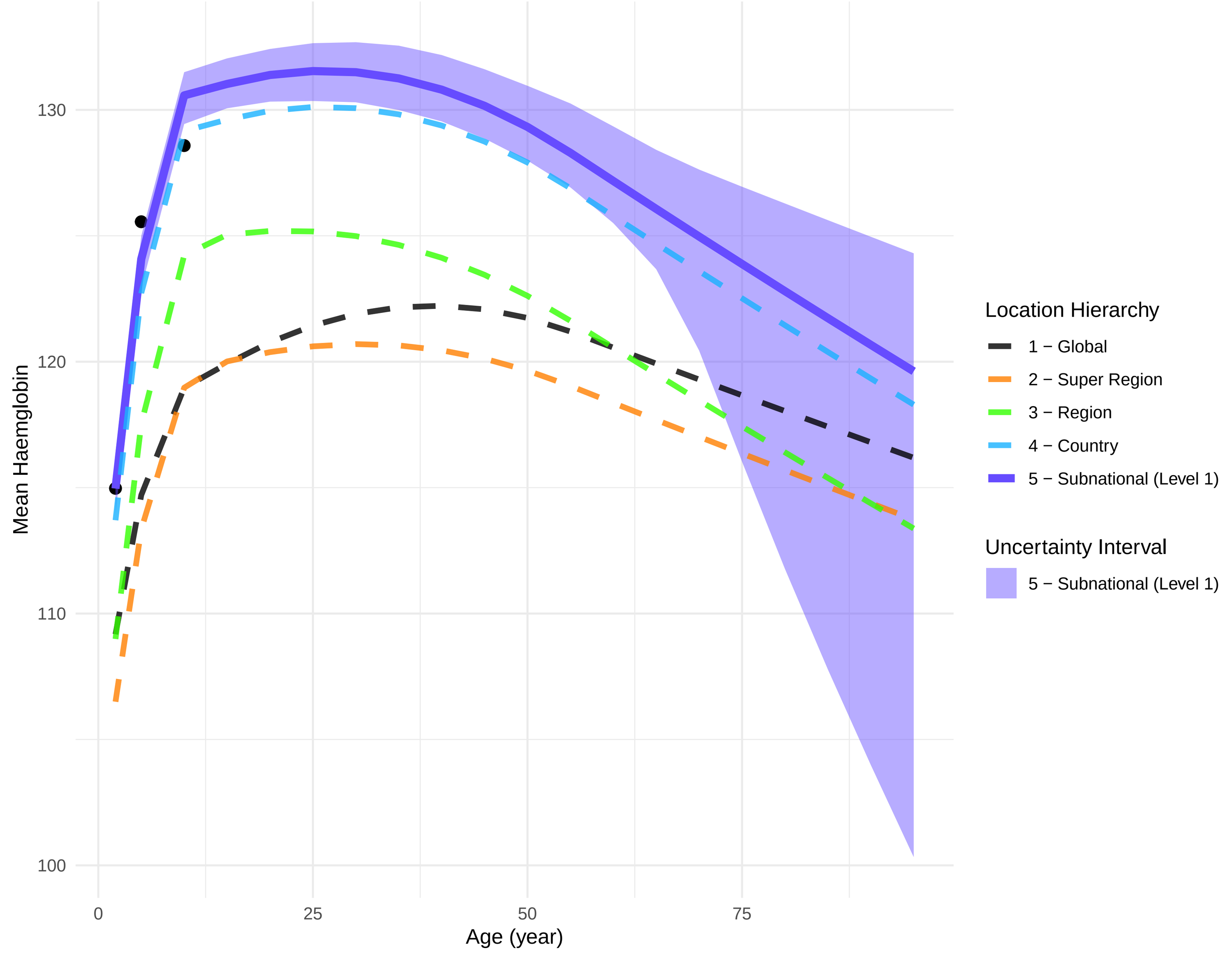

MR−BRT Age/Sex Fits − Non−pregnant Females 2+ Years Old − Mean Haemglobin − Kajiado
WHO−2001 Adjustment Method

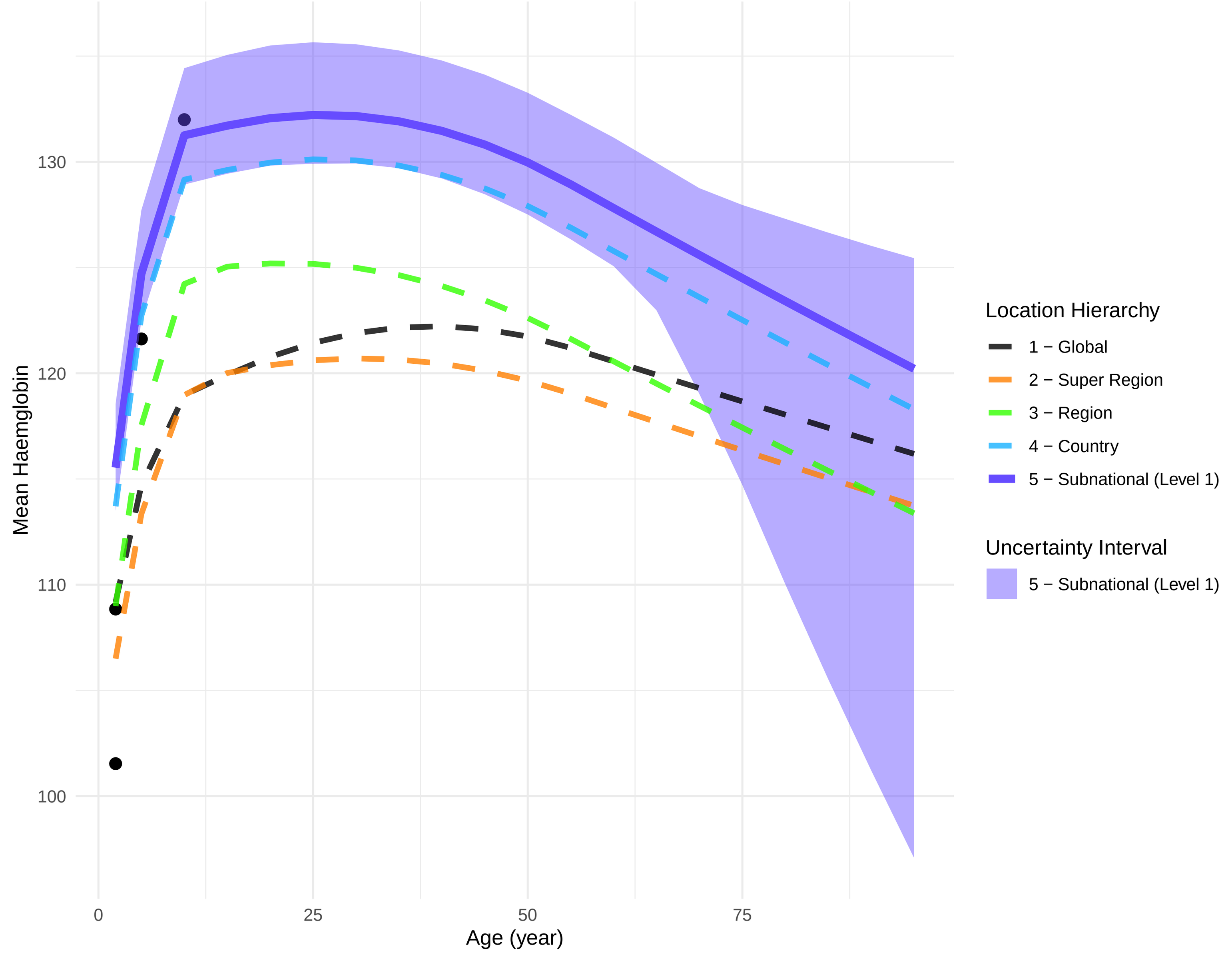

MR−BRT Age/Sex Fits − Non−pregnant Females 2+ Years Old − Mean Haemglobin − Kakamega
WHO−2001 Adjustment Method

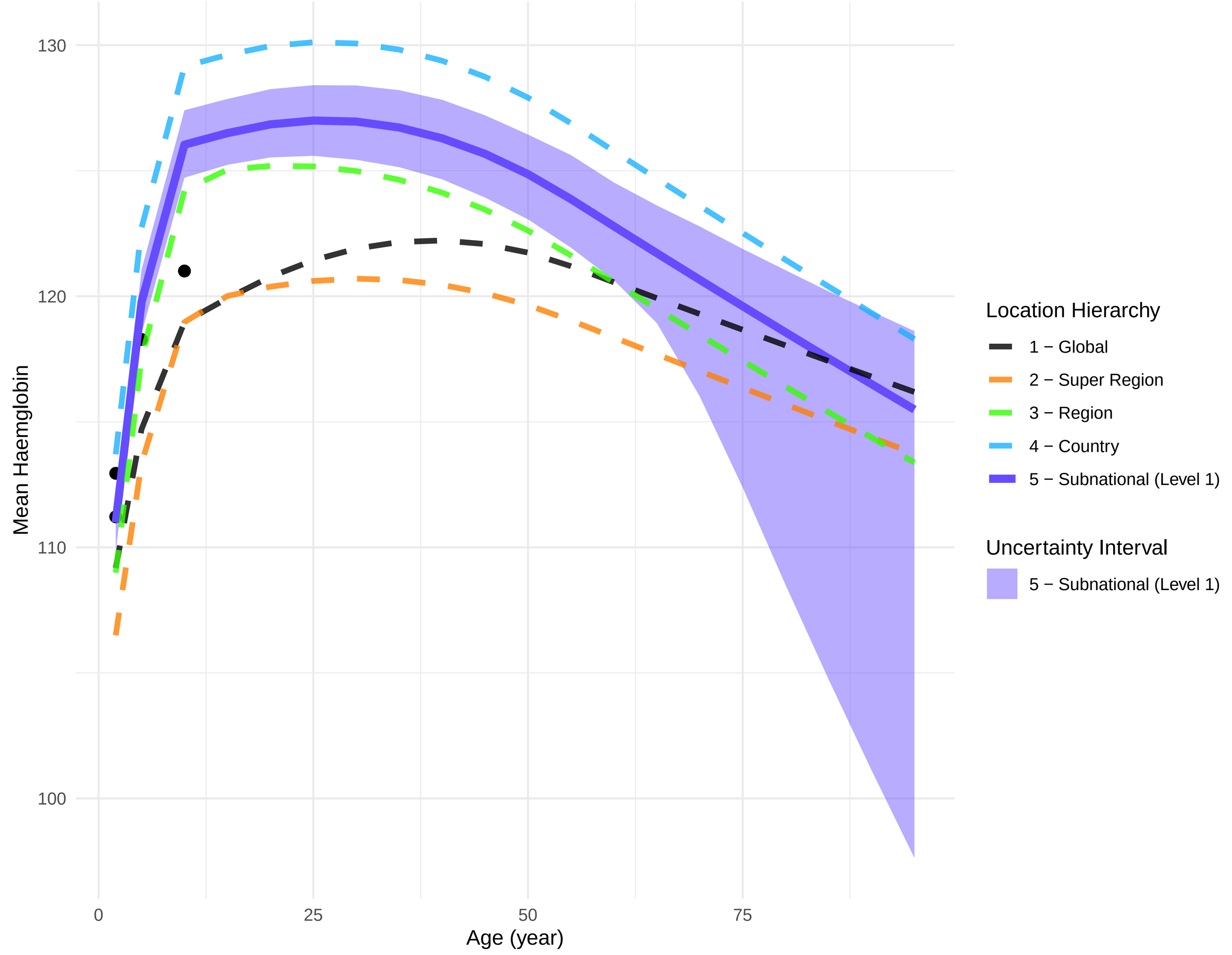

MR−BRT Age/Sex Fits − Non−pregnant Females 2+ Years Old − Mean Haemglobin − Kericho
WHO−2001 Adjustment Method

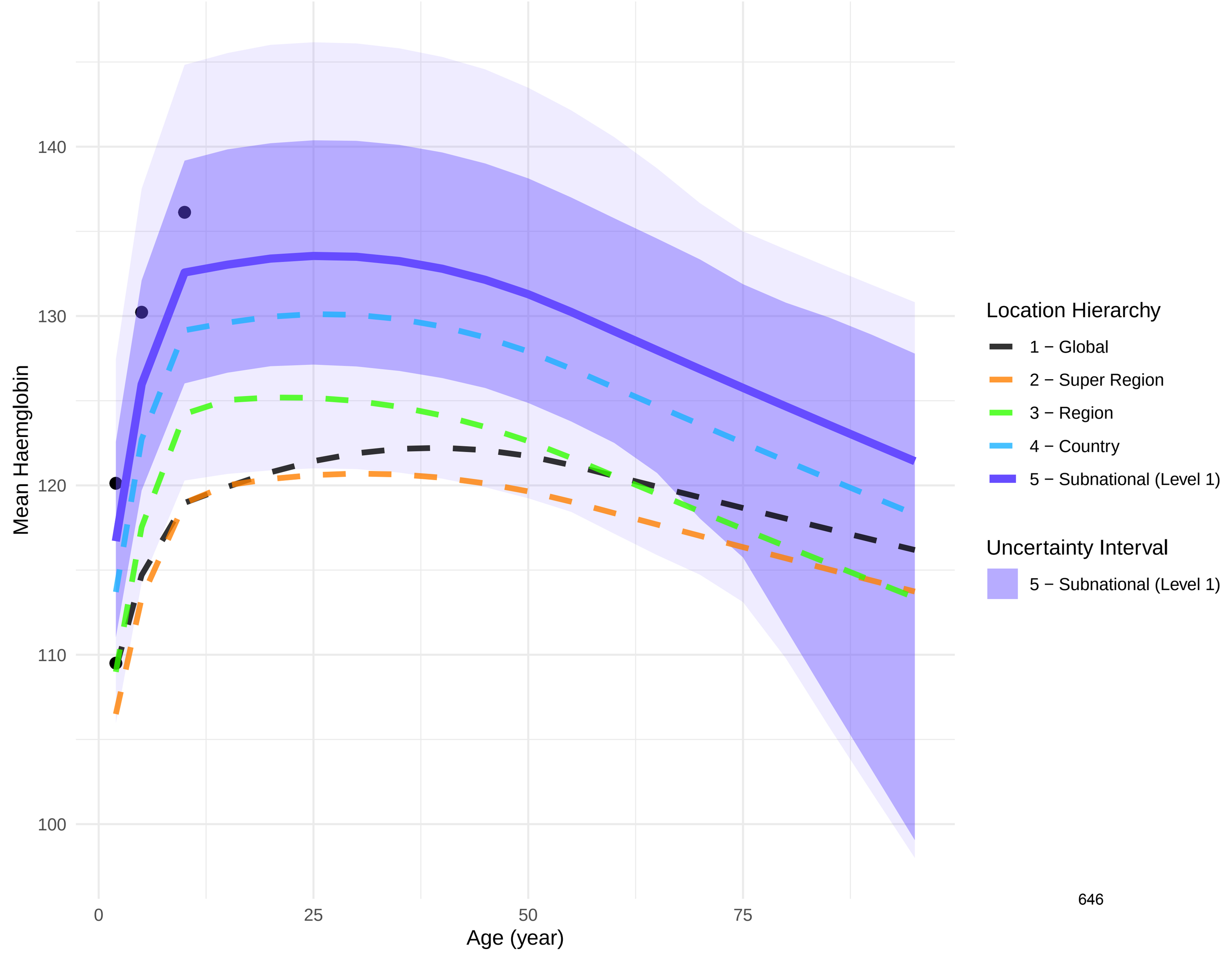

MR−BRT Age/Sex Fits − Non−pregnant Females 2+ Years Old − Mean Haemglobin − Kiambu
WHO−2001 Adjustment Method

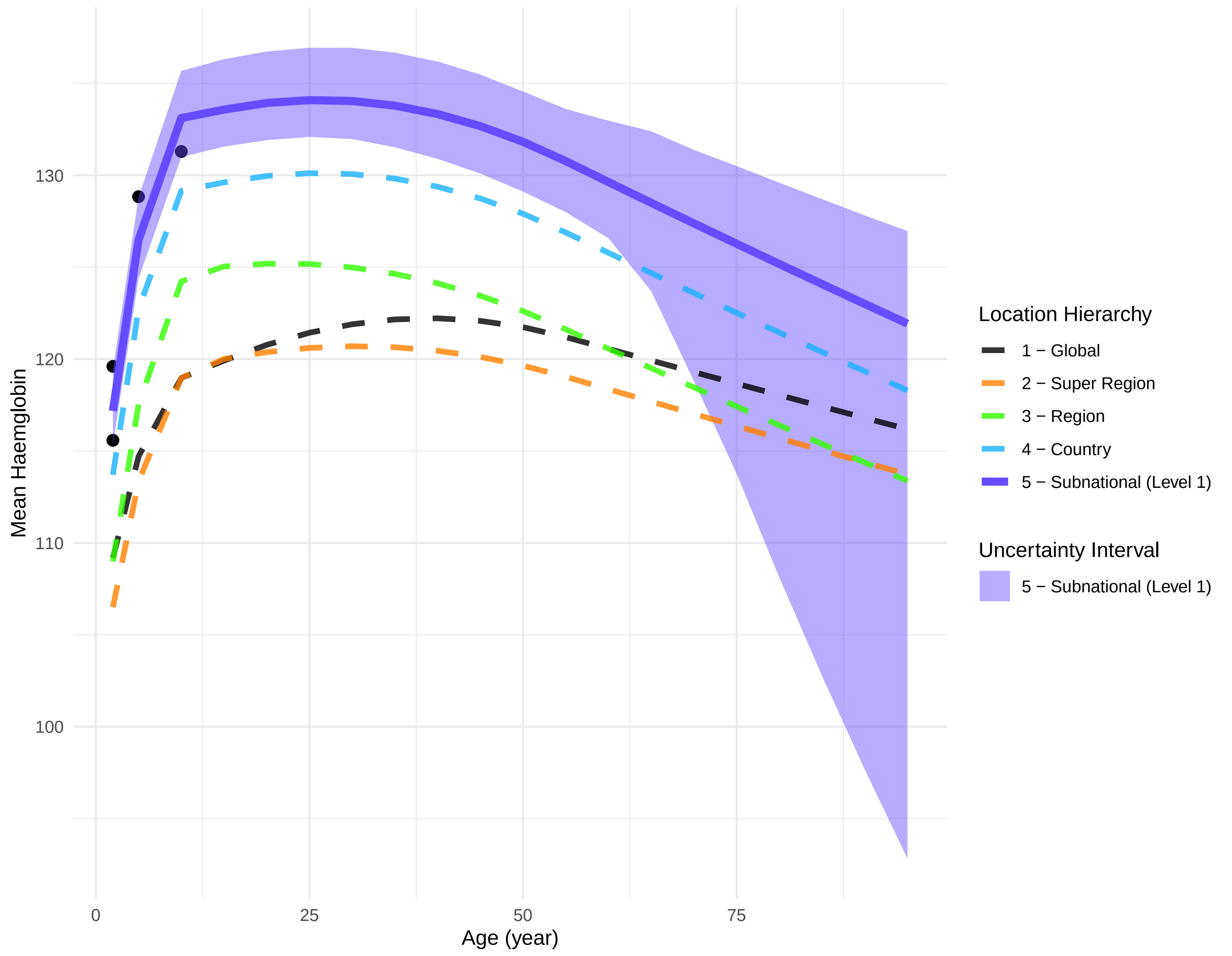

MR−BRT Age/Sex Fits − Non−pregnant Females 2+ Years Old − Mean Haemglobin − Kilifi
WHO−2001 Adjustment Method

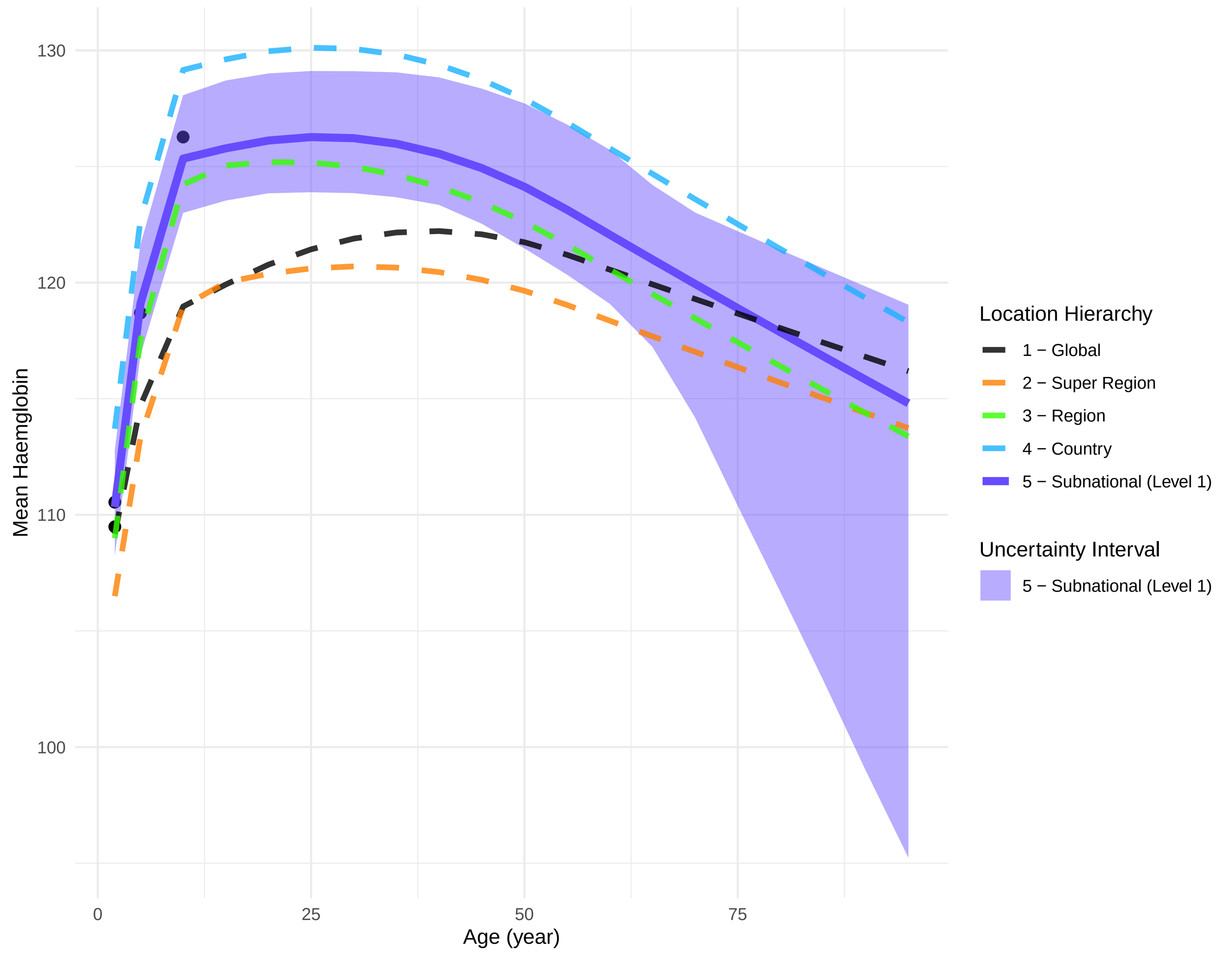

MR−BRT Age/Sex Fits − Non−pregnant Females 2+ Years Old − Mean Haemglobin − Kirinyaga
WHO−2001 Adjustment Method

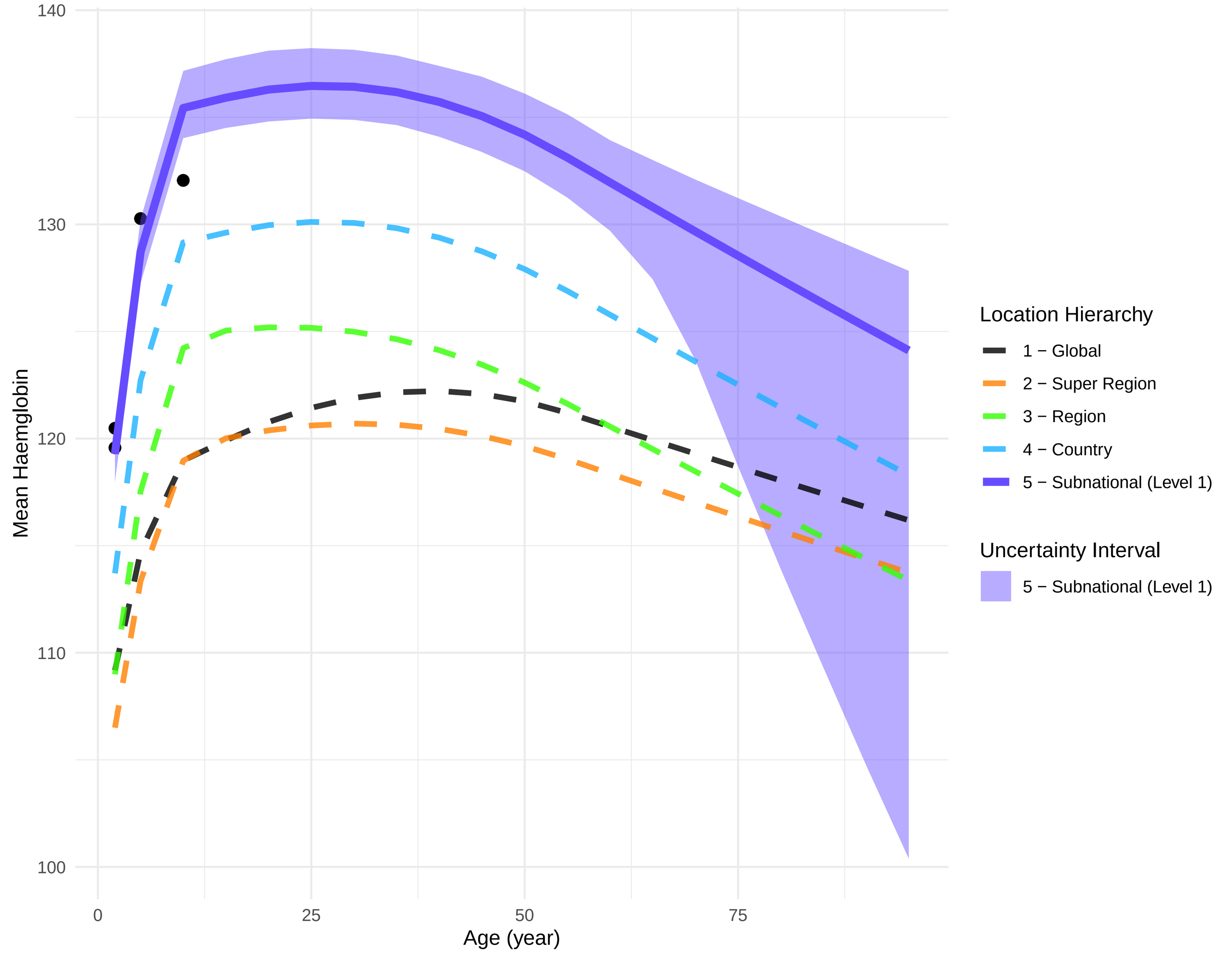

MR−BRT Age/Sex Fits − Non−pregnant Females 2+ Years Old − Mean Haemglobin − Kisii
WHO−2001 Adjustment Method

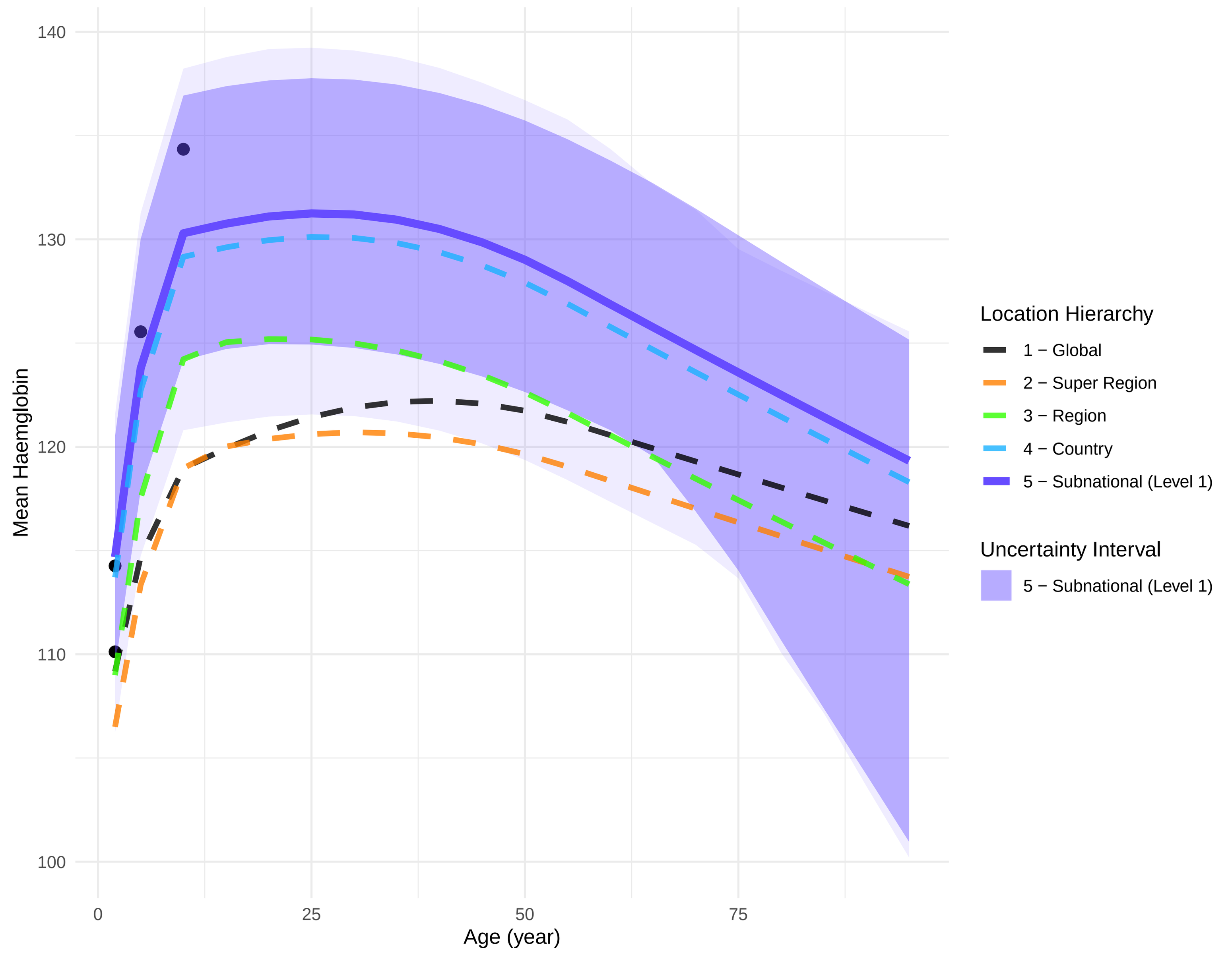

MR−BRT Age/Sex Fits − Non−pregnant Females 2+ Years Old − Mean Haemglobin − Kisumu
WHO−2001 Adjustment Method

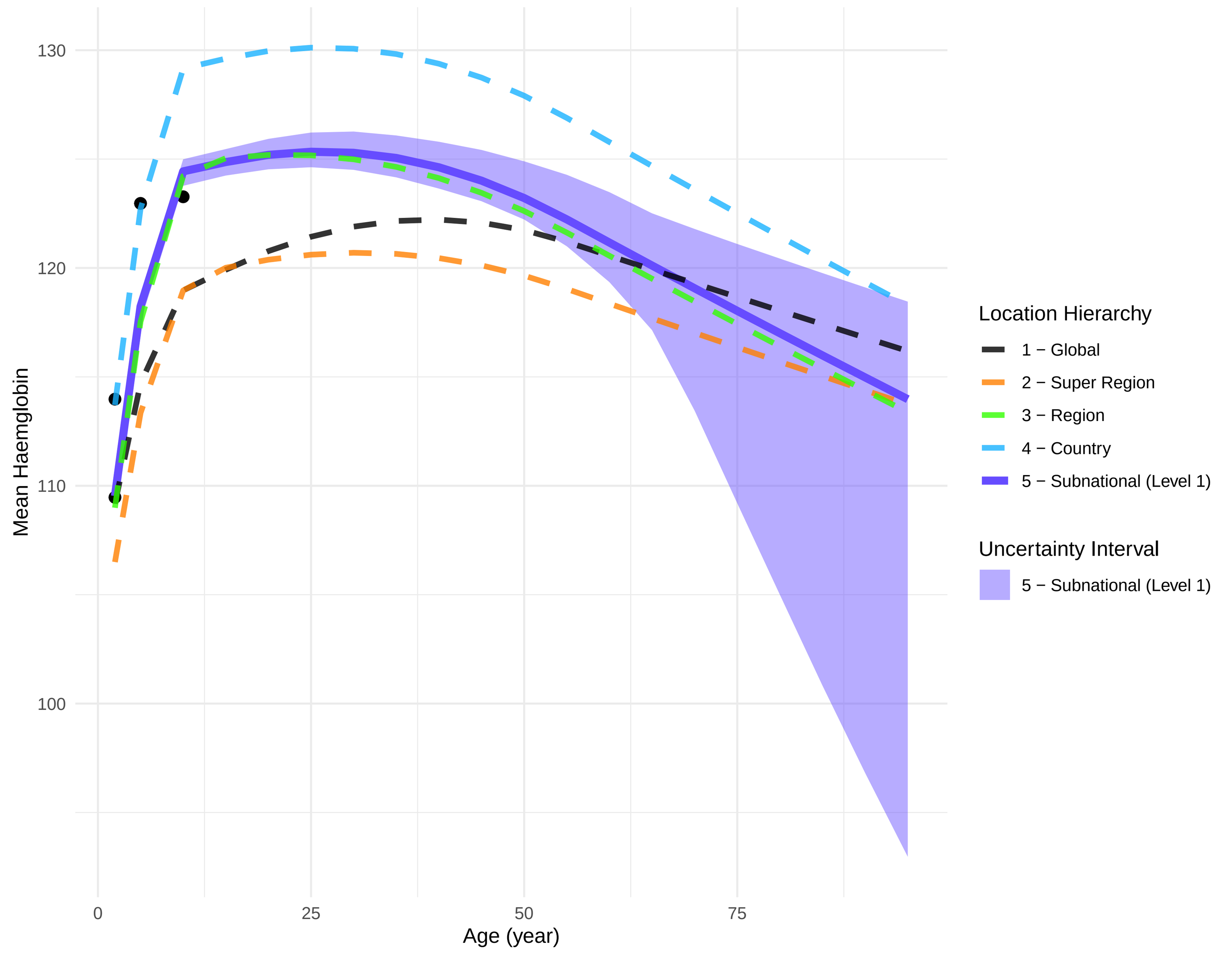

MR−BRT Age/Sex Fits − Non−pregnant Females 2+ Years Old − Mean Haemglobin − Kitui
WHO−2001 Adjustment Method

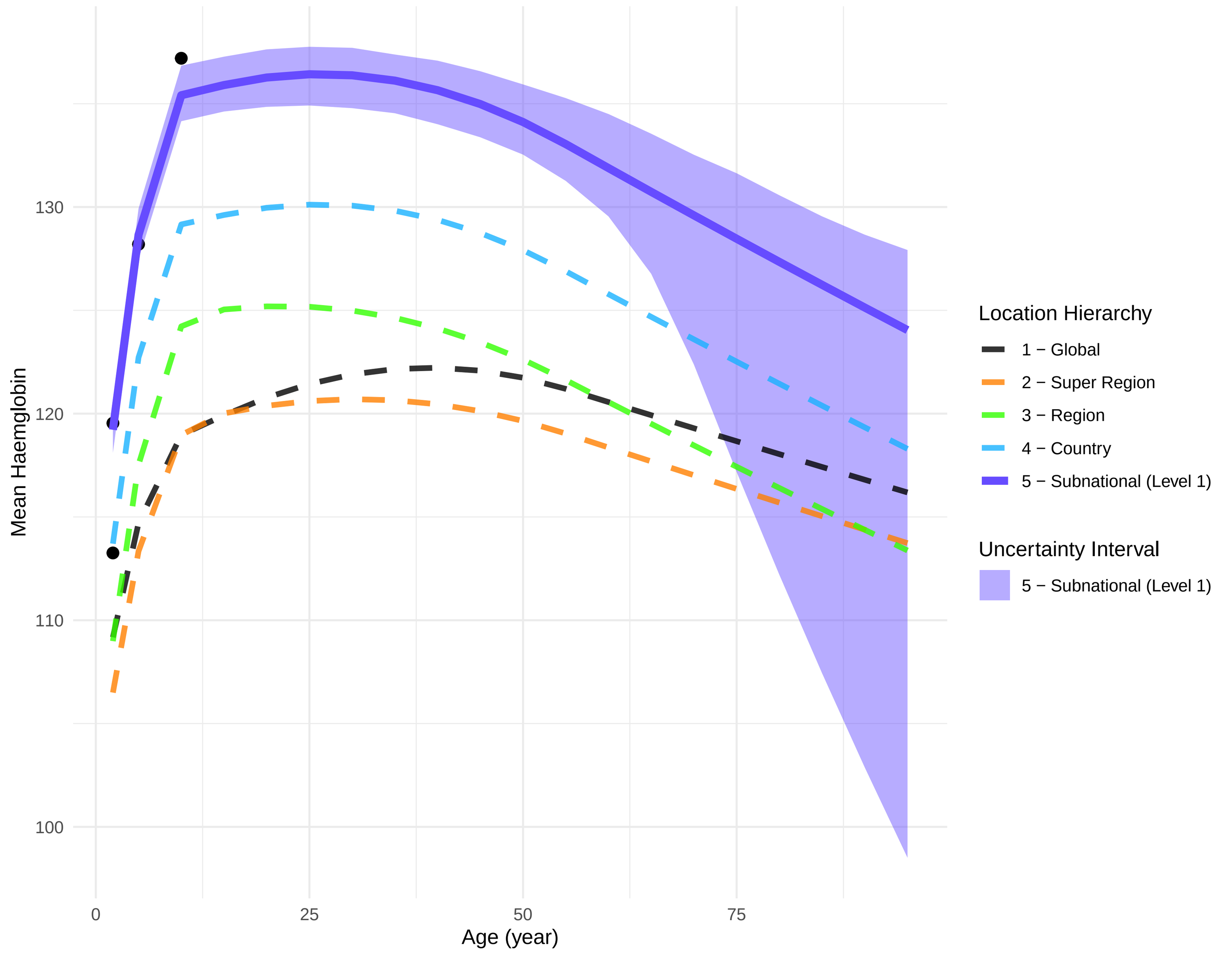

MR−BRT Age/Sex Fits − Non−pregnant Females 2+ Years Old − Mean Haemglobin − Kwale
WHO−2001 Adjustment Method

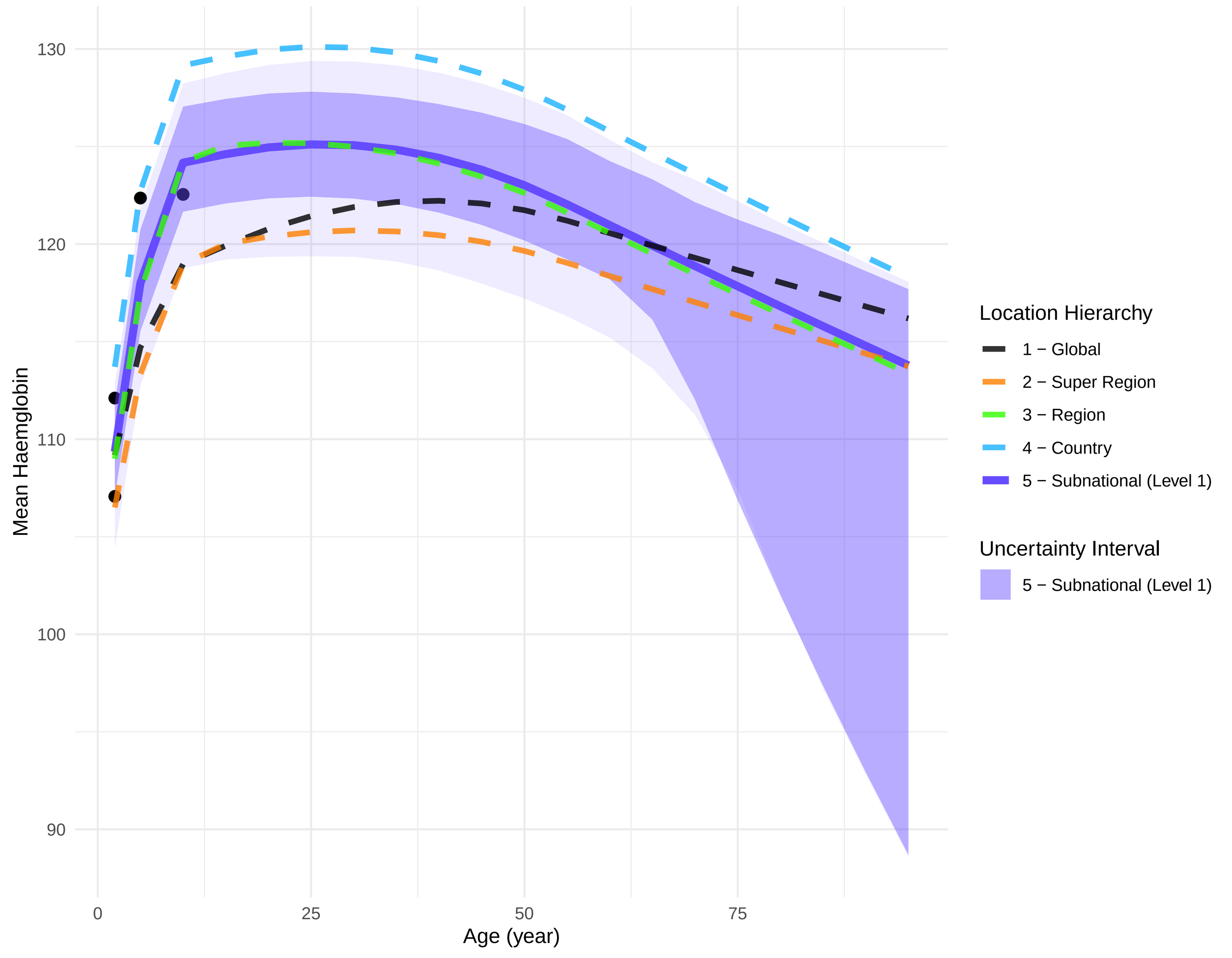

MR−BRT Age/Sex Fits − Non−pregnant Females 2+ Years Old − Mean Haemglobin − Laikipia
WHO−2001 Adjustment Method

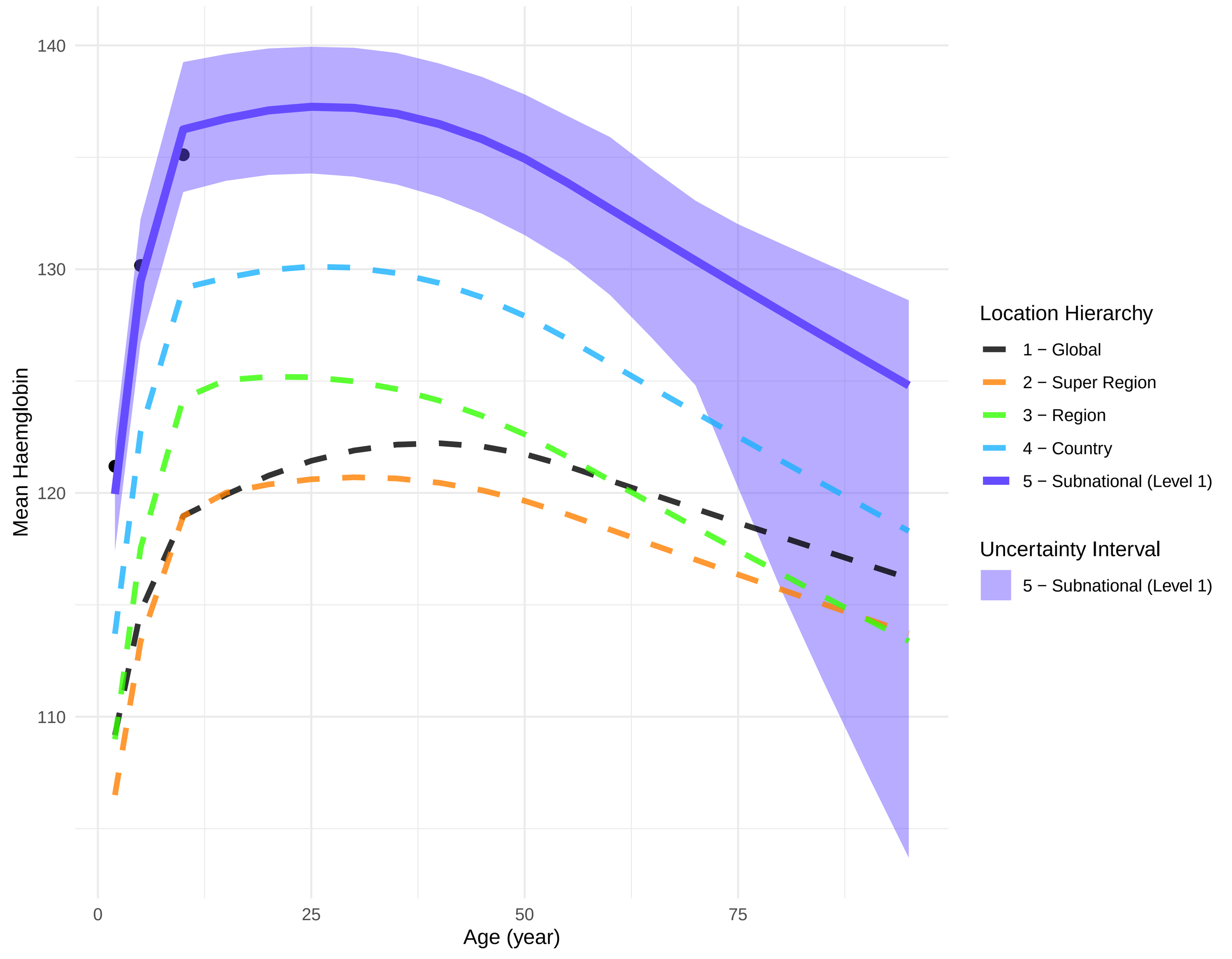

MR−BRT Age/Sex Fits − Non−pregnant Females 2+ Years Old − Mean Haemglobin − Lamu
WHO−2001 Adjustment Method

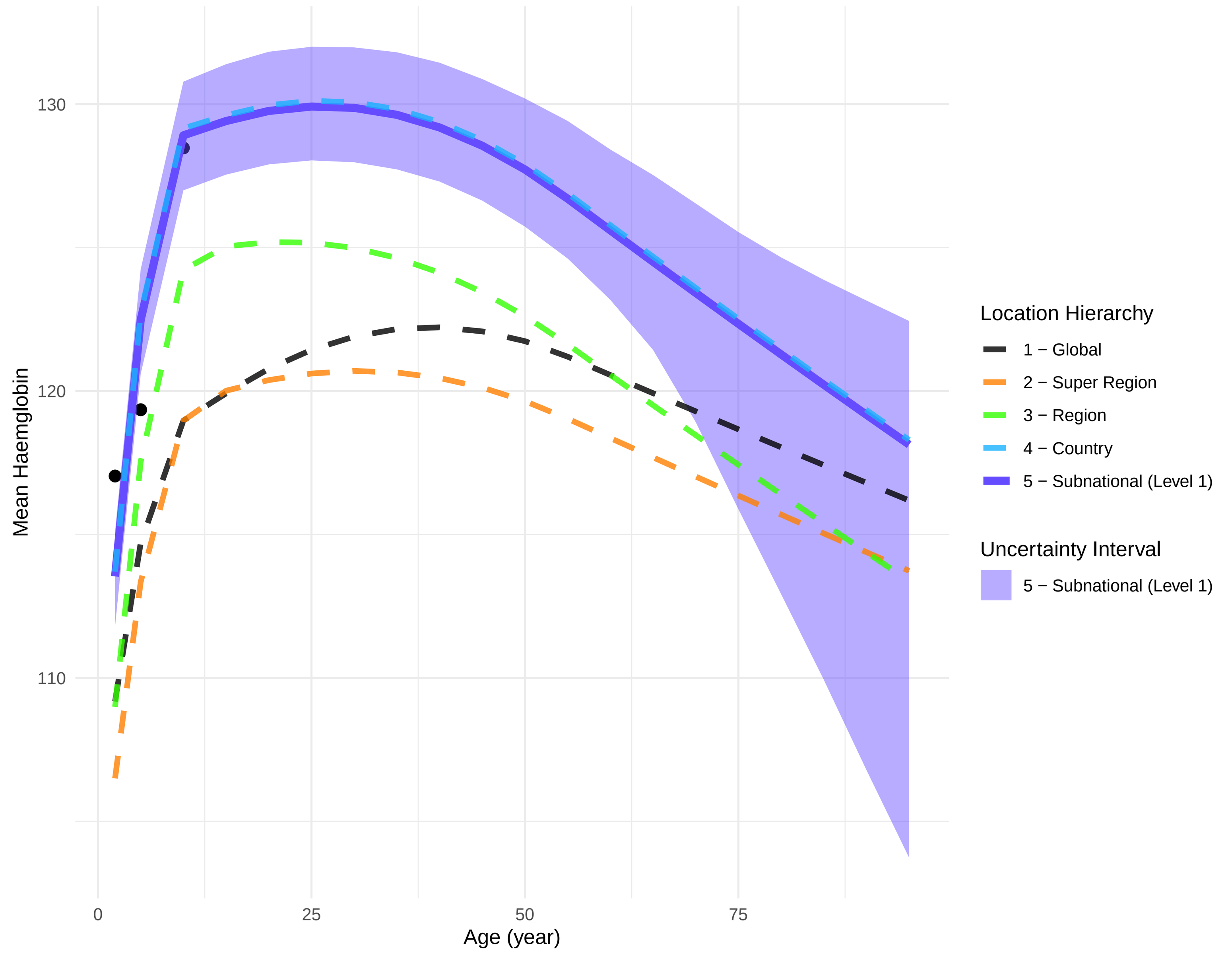

MR−BRT Age/Sex Fits − Non−pregnant Females 2+ Years Old − Mean Haemglobin − Machakos
WHO−2001 Adjustment Method

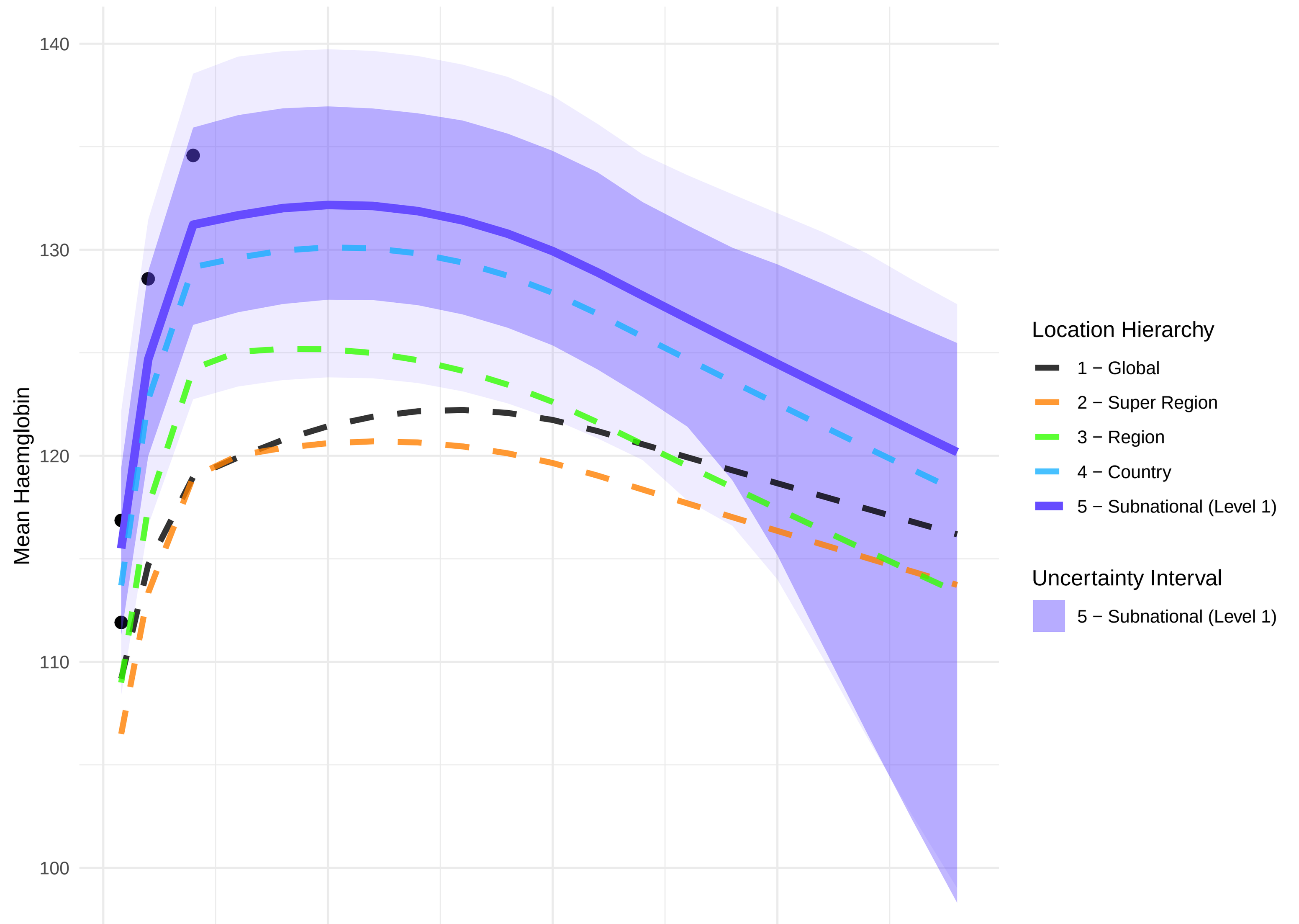

MR−BRT Age/Sex Fits − Non−pregnant Females 2+ Years Old − Mean Haemglobin − Makueni
WHO−2001 Adjustment Method

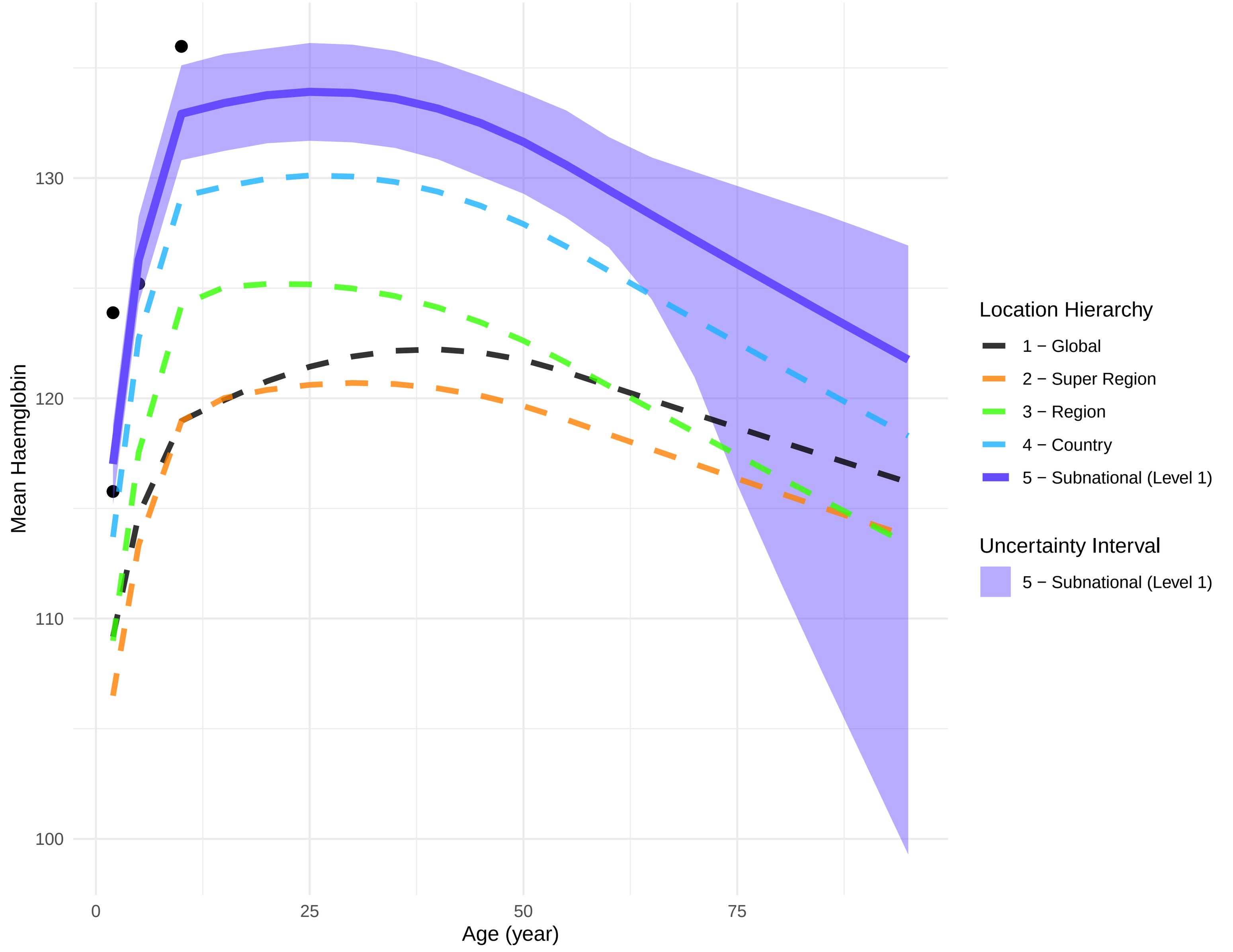

MR−BRT Age/Sex Fits − Non−pregnant Females 2+ Years Old − Mean Haemglobin − Mandera
WHO−2001 Adjustment Method

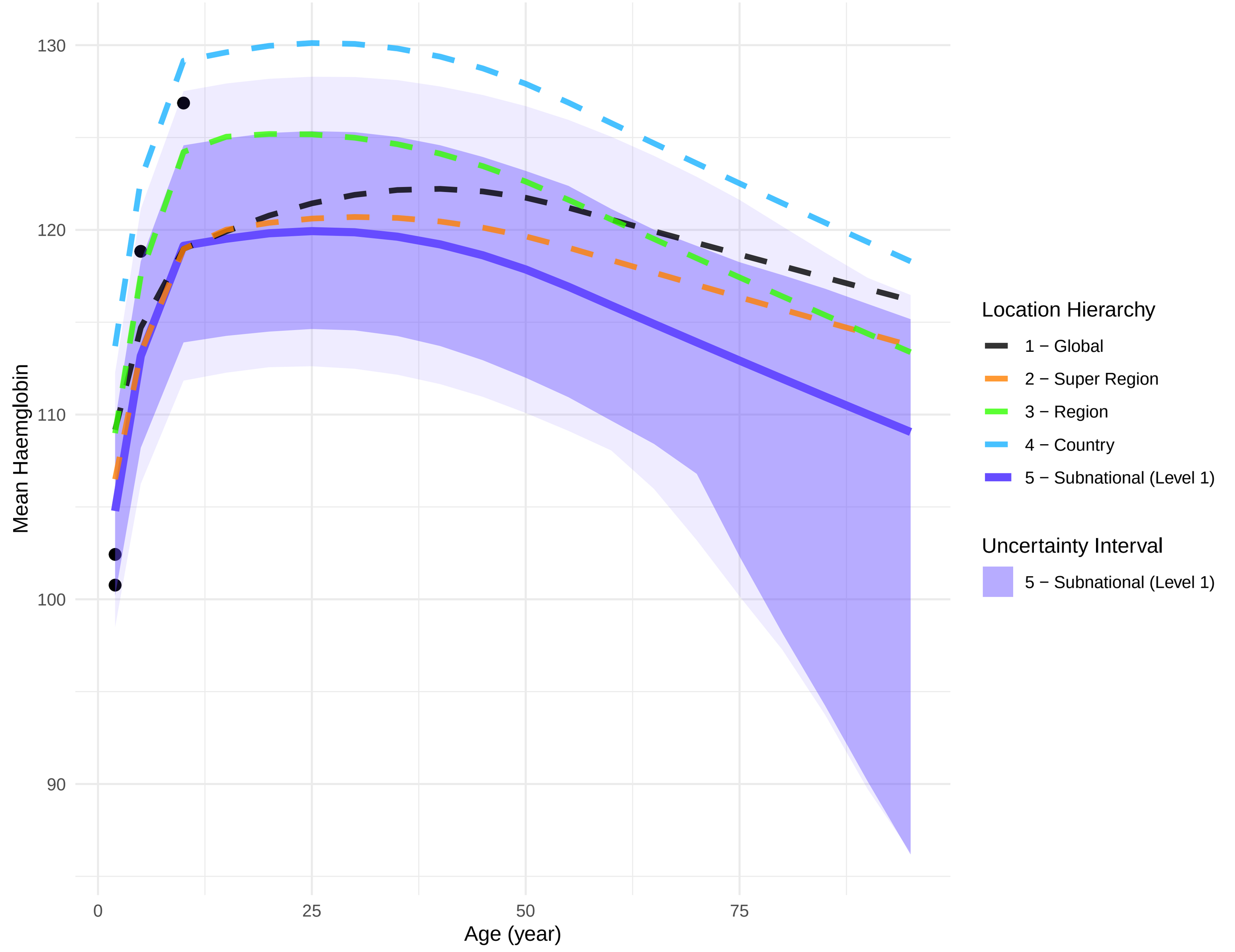

MR−BRT Age/Sex Fits − Non−pregnant Females 2+ Years Old − Mean Haemglobin − Marsabit
WHO−2001 Adjustment Method

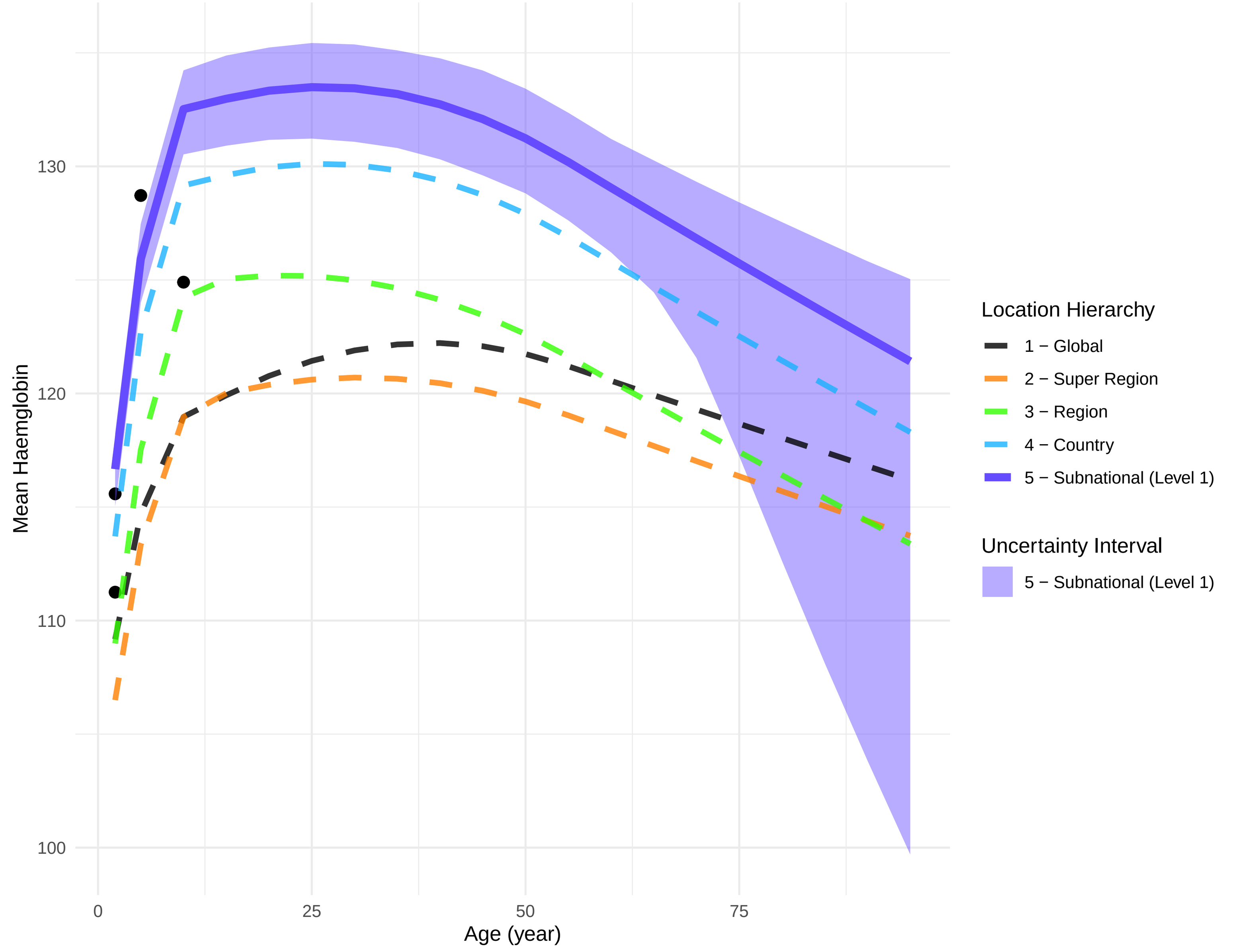

MR−BRT Age/Sex Fits − Non−pregnant Females 2+ Years Old − Mean Haemglobin − Meru
WHO−2001 Adjustment Method

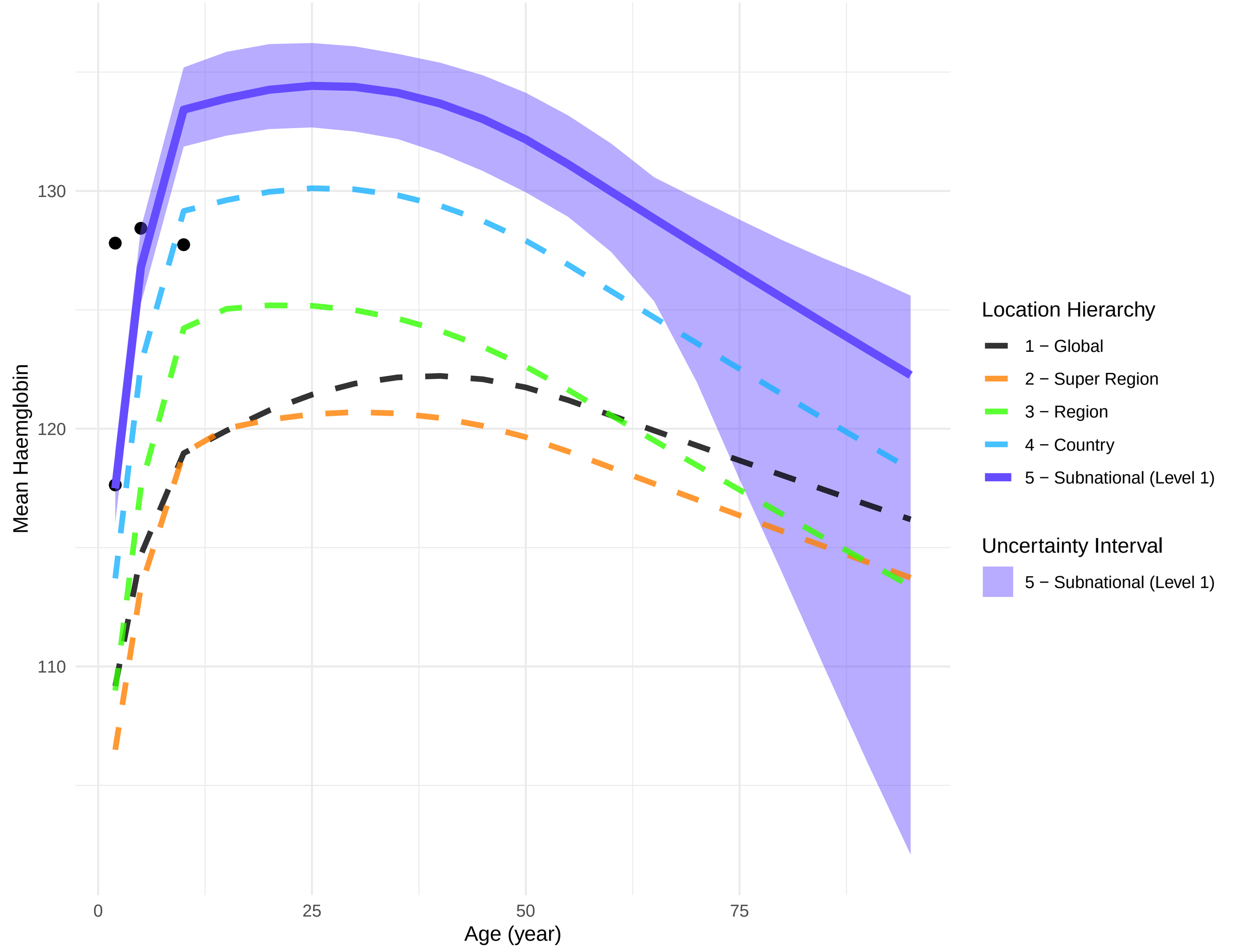

MR−BRT Age/Sex Fits − Non−pregnant Females 2+ Years Old − Mean Haemglobin − Migori
WHO−2001 Adjustment Method

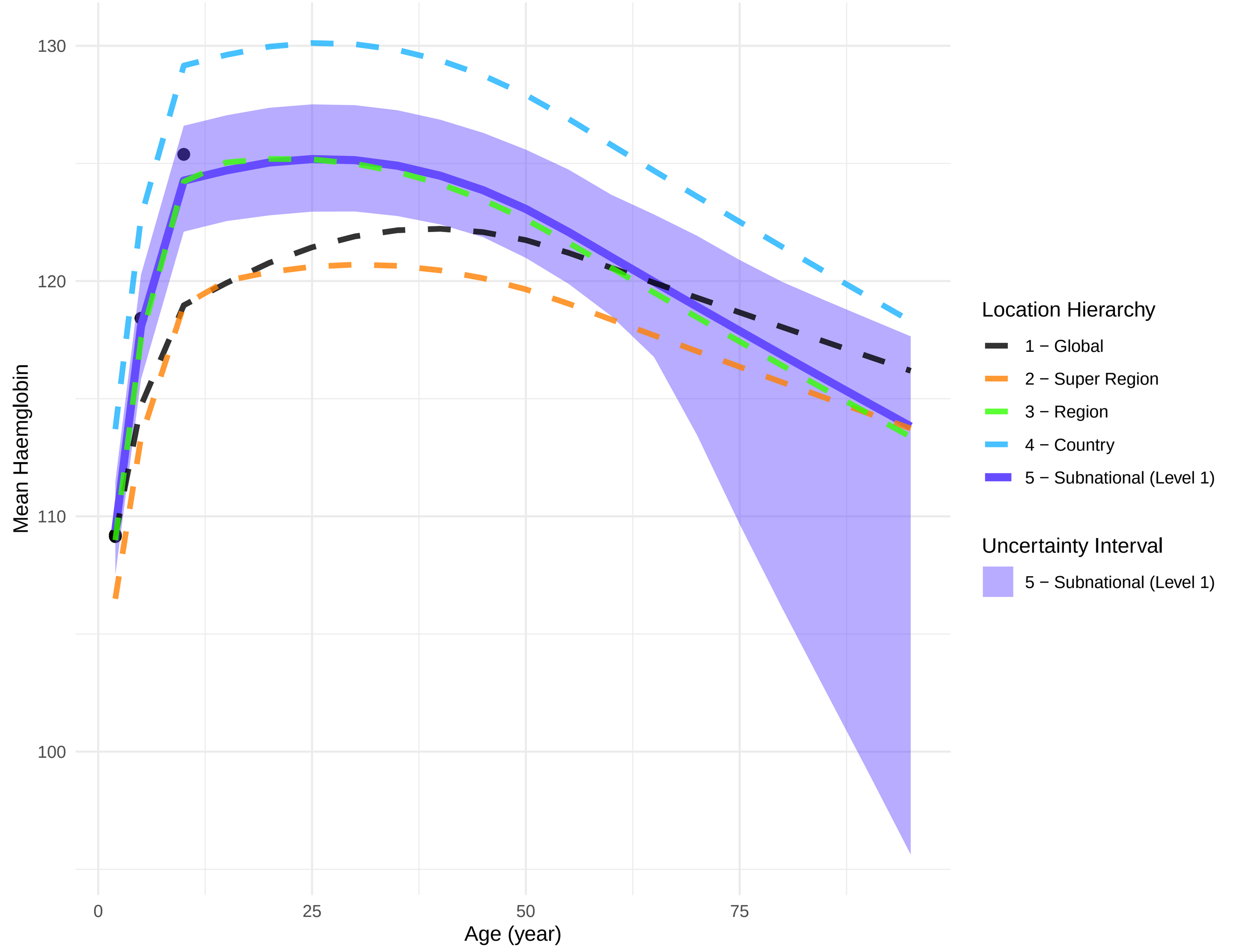

MR−BRT Age/Sex Fits − Non−pregnant Females 2+ Years Old − Mean Haemglobin − Mombasa
WHO−2001 Adjustment Method

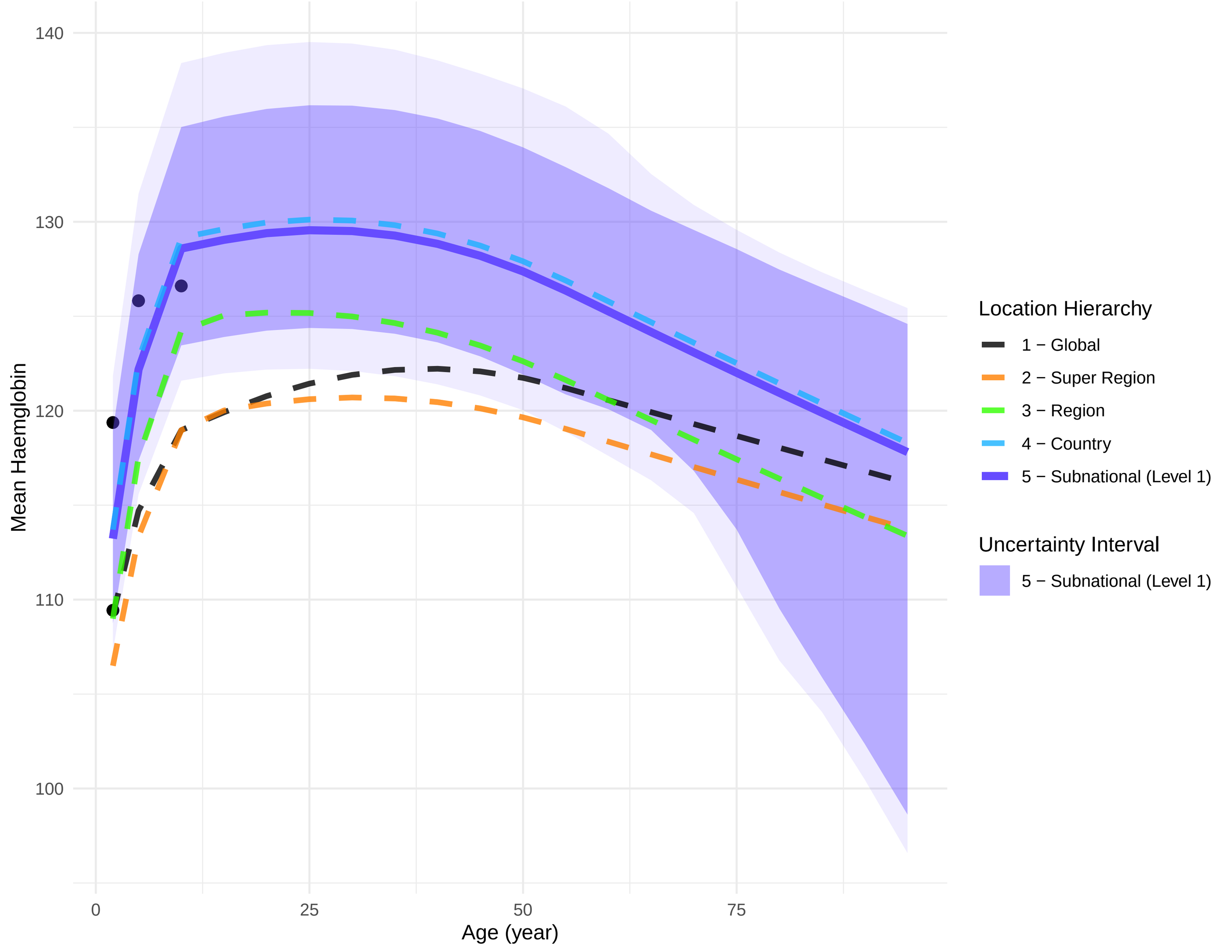

MR−BRT Age/Sex Fits − Non−pregnant Females 2+ Years Old − Mean Haemglobin − Murang'a
WHO−2001 Adjustment Method

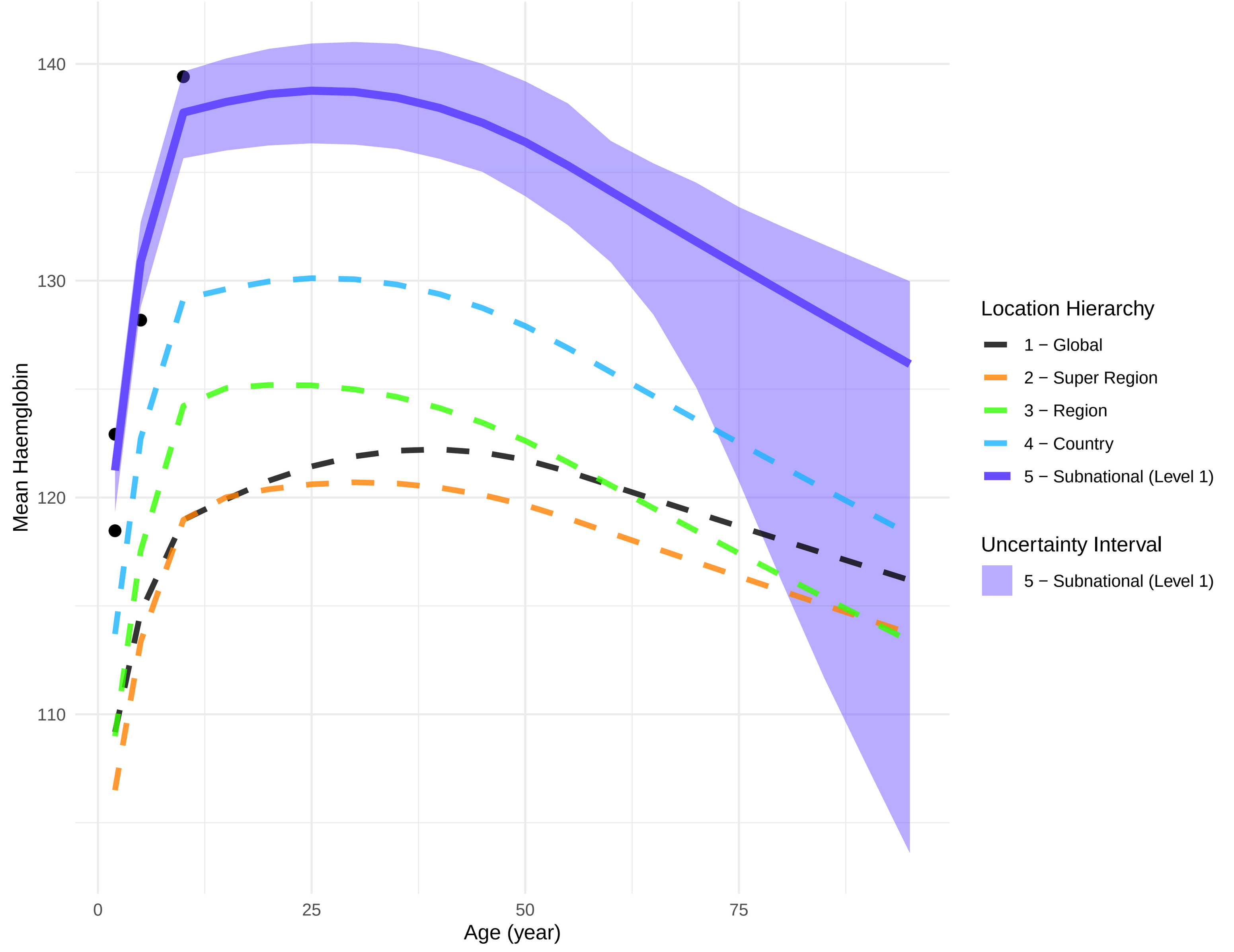

MR−BRT Age/Sex Fits − Non−pregnant Females 2+ Years Old − Mean Haemglobin − Nairobi
WHO−2001 Adjustment Method

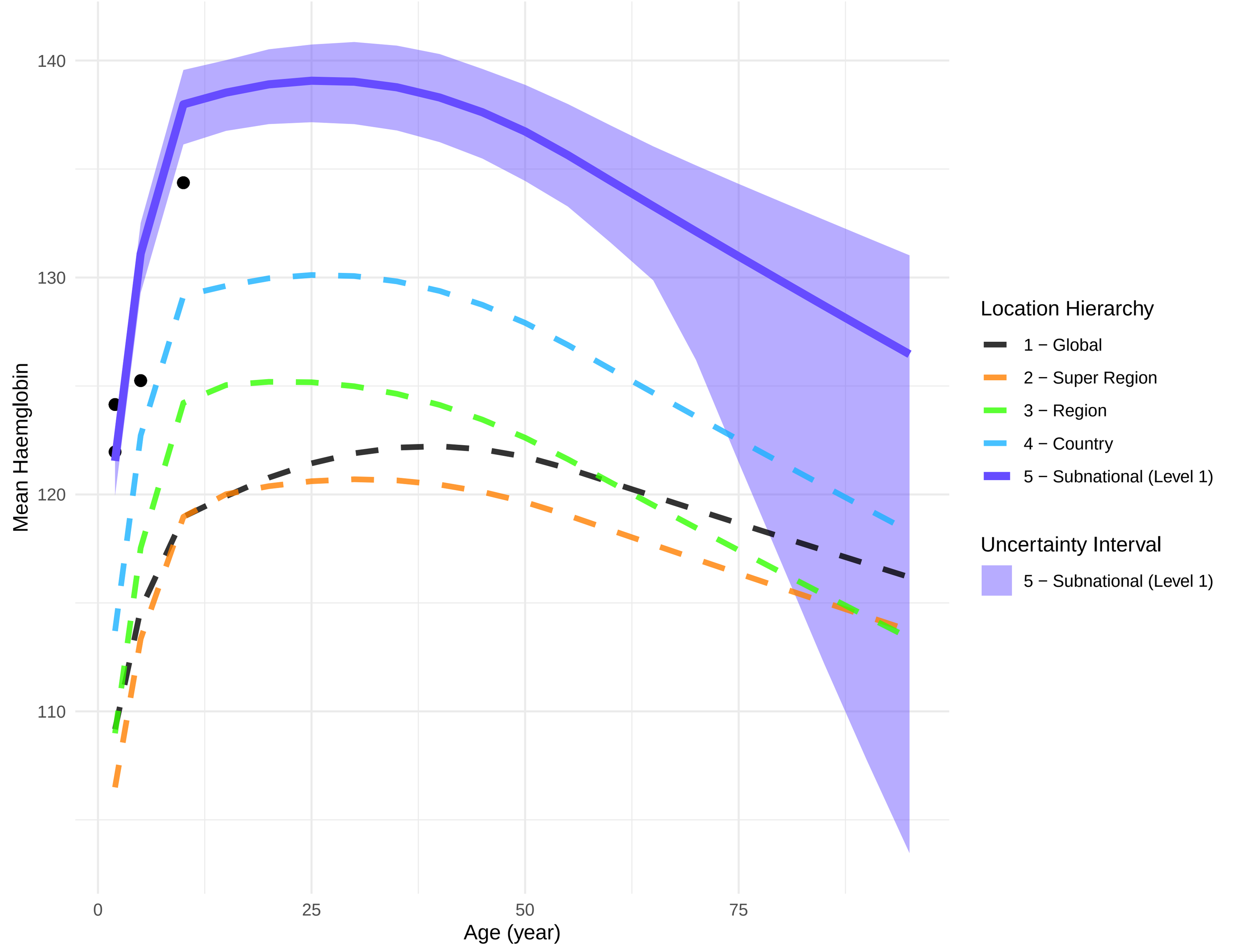

MR−BRT Age/Sex Fits − Non−pregnant Females 2+ Years Old − Mean Haemglobin − Nakuru
WHO−2001 Adjustment Method

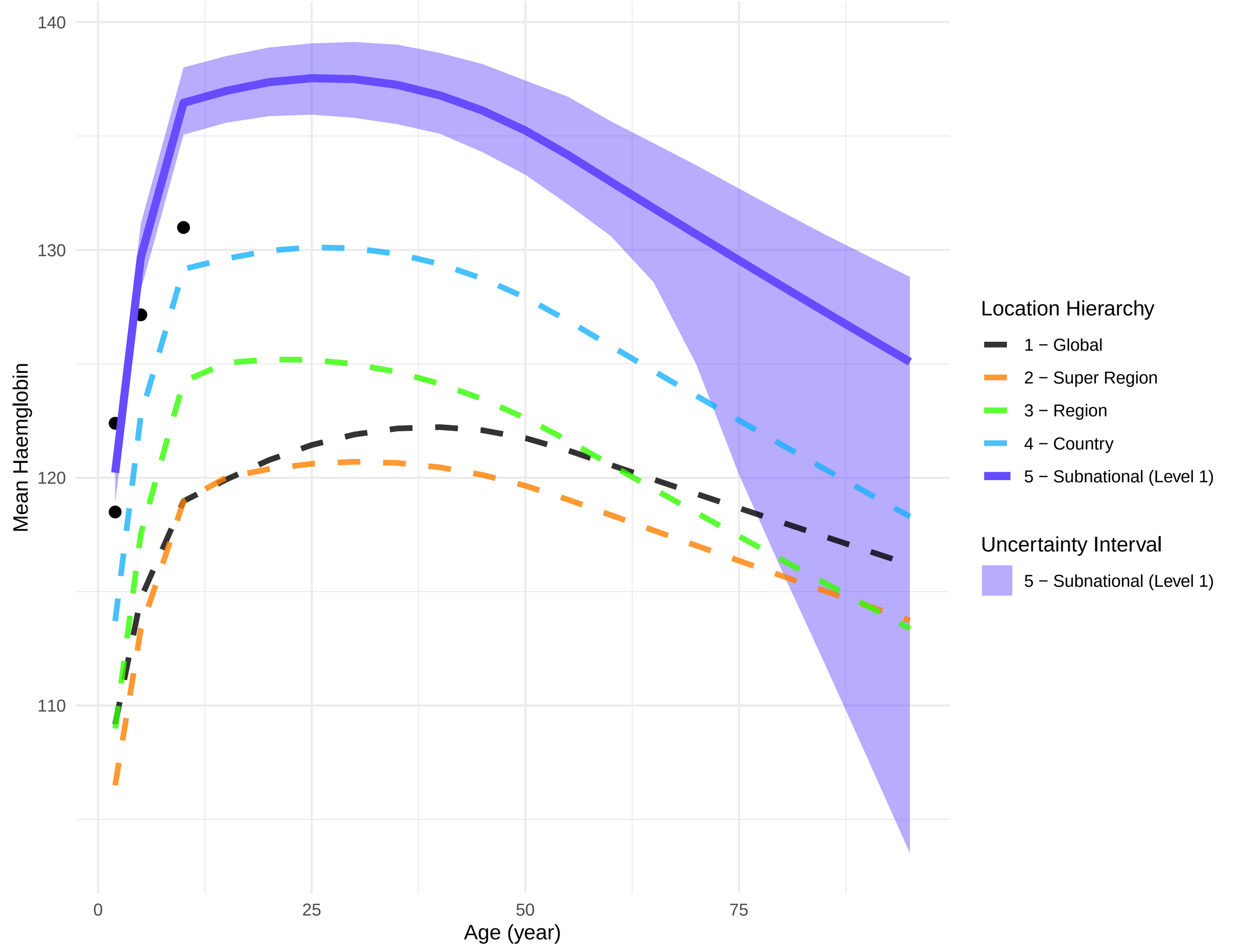

MR−BRT Age/Sex Fits − Non−pregnant Females 2+ Years Old − Mean Haemglobin − Nandi
WHO−2001 Adjustment Method

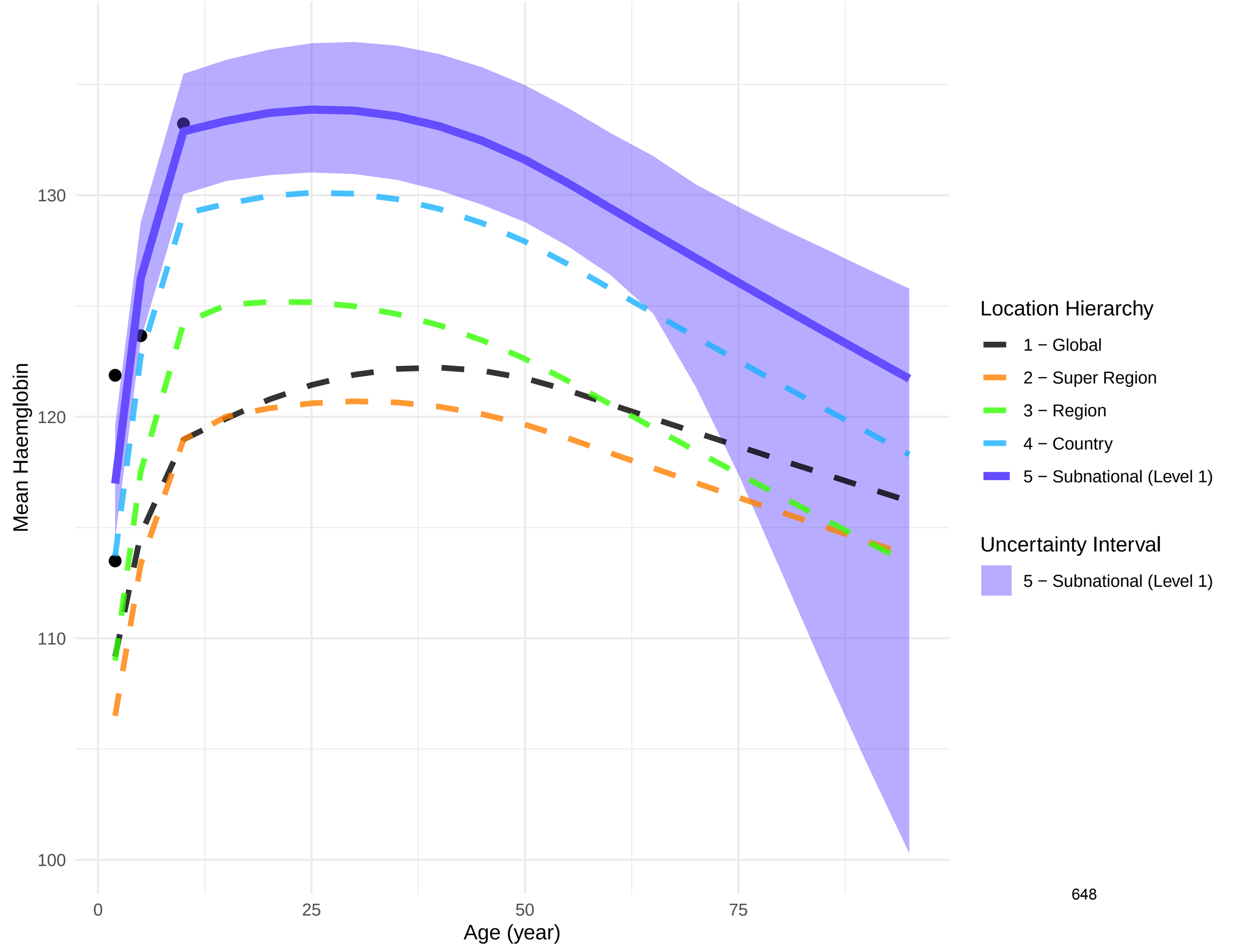

MR−BRT Age/Sex Fits − Non−pregnant Females 2+ Years Old − Mean Haemglobin − Narok
WHO−2001 Adjustment Method

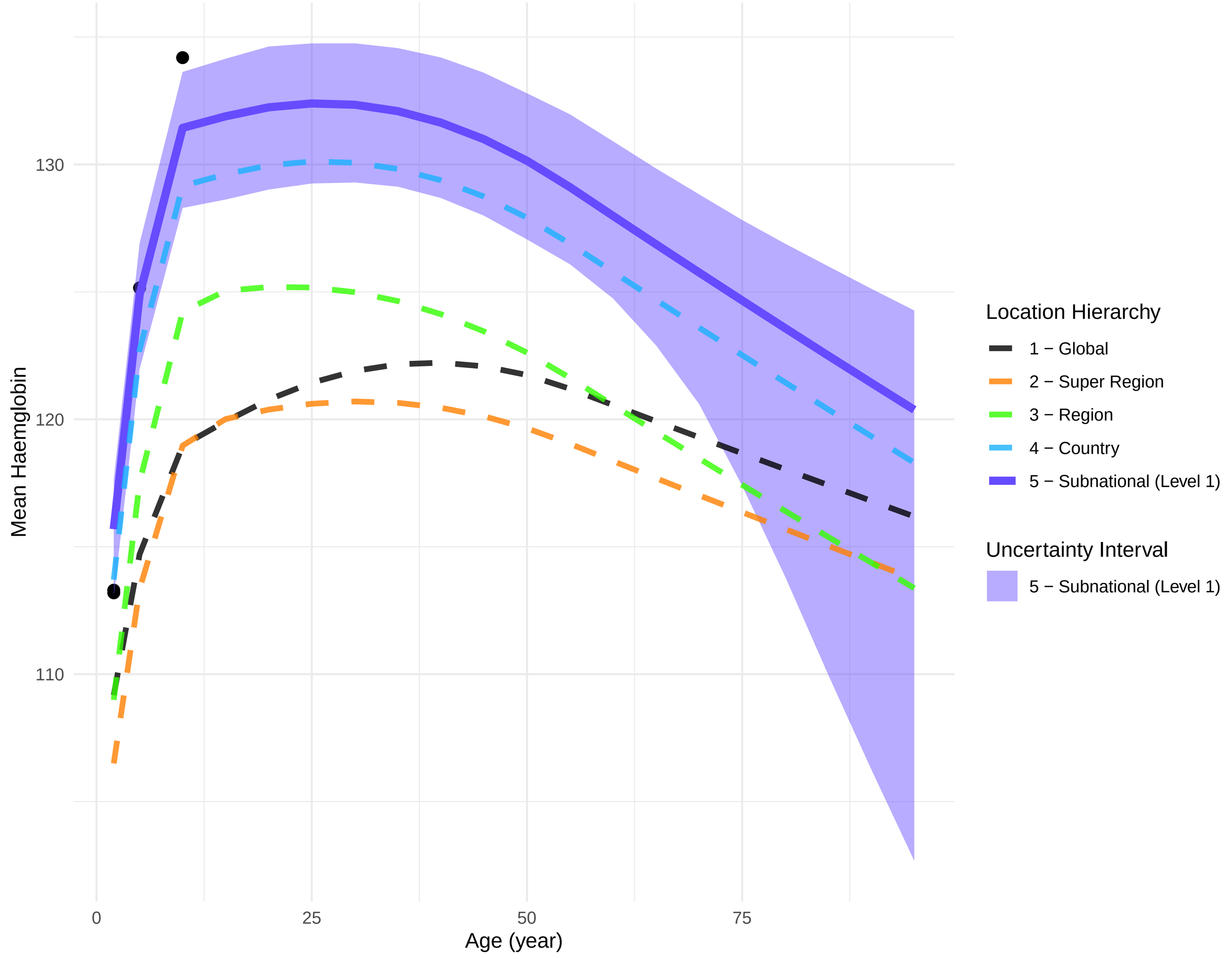

MR−BRT Age/Sex Fits − Non−pregnant Females 2+ Years Old − Mean Haemglobin − Nyamira
WHO−2001 Adjustment Method

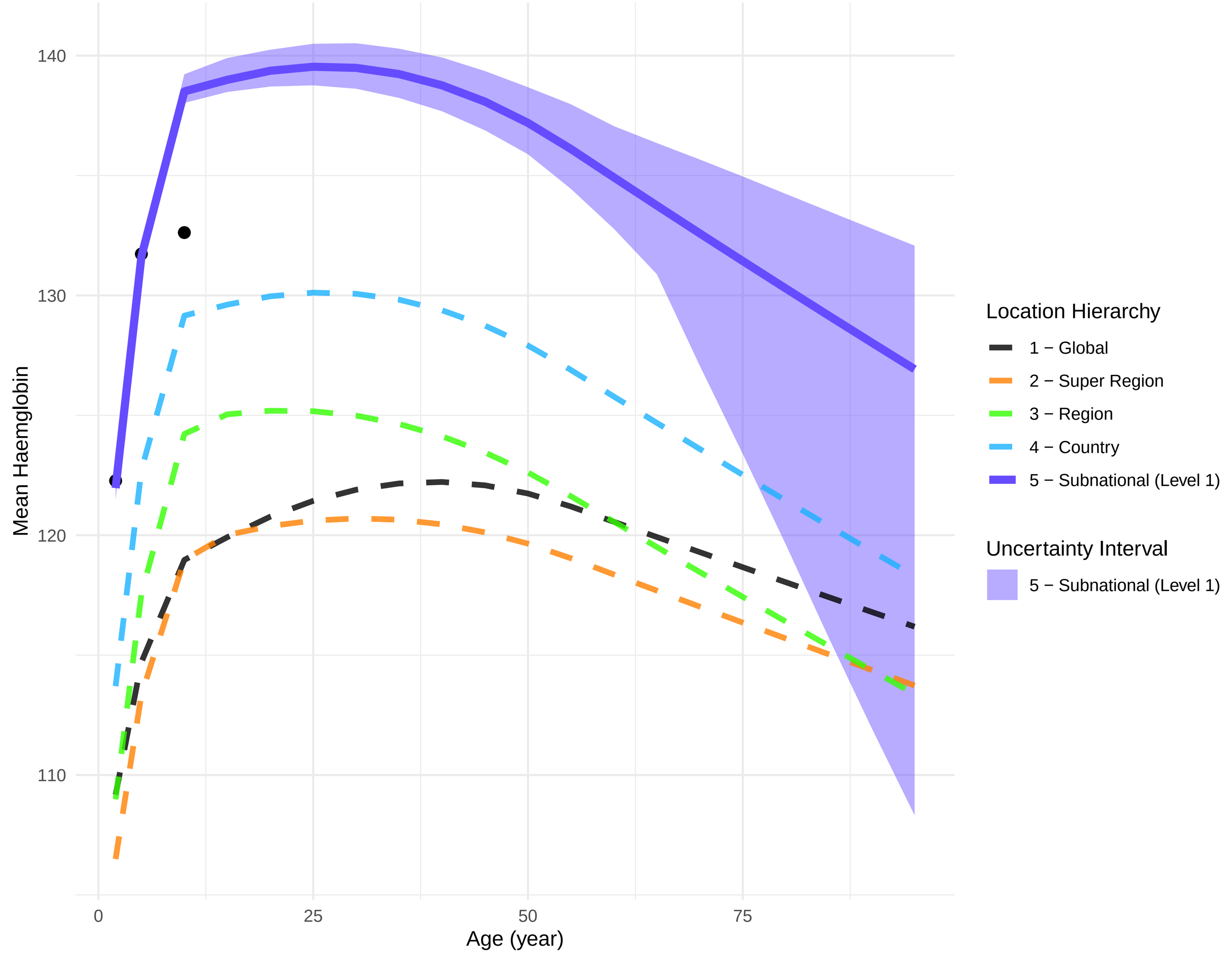

MR−BRT Age/Sex Fits − Non−pregnant Females 2+ Years Old − Mean Haemglobin − Nyandarua
WHO−2001 Adjustment Method

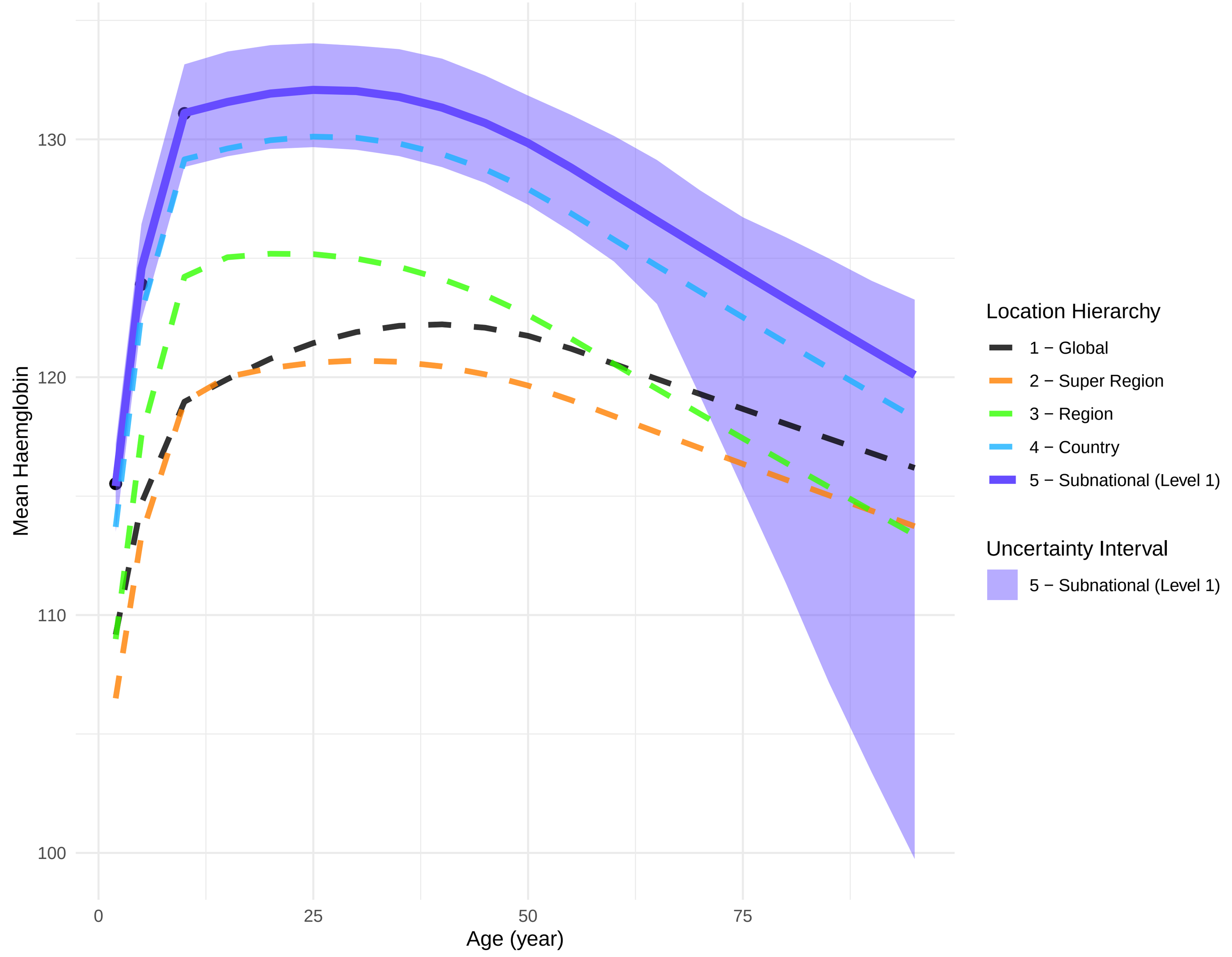

MR−BRT Age/Sex Fits − Non−pregnant Females 2+ Years Old − Mean Haemglobin − Nyeri
WHO−2001 Adjustment Method

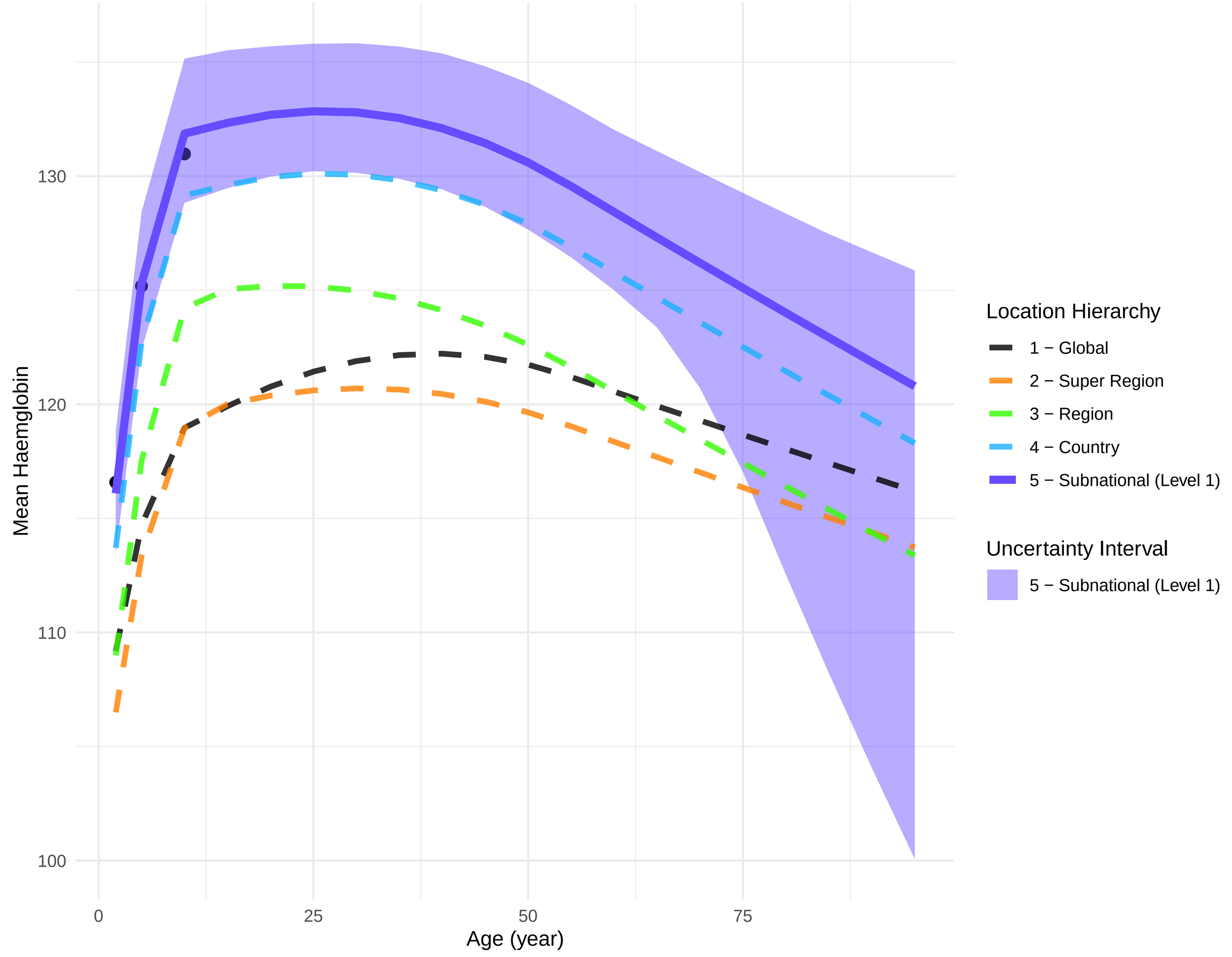

MR−BRT Age/Sex Fits − Non−pregnant Females 2+ Years Old − Mean Haemglobin − Samburu
WHO−2001 Adjustment Method

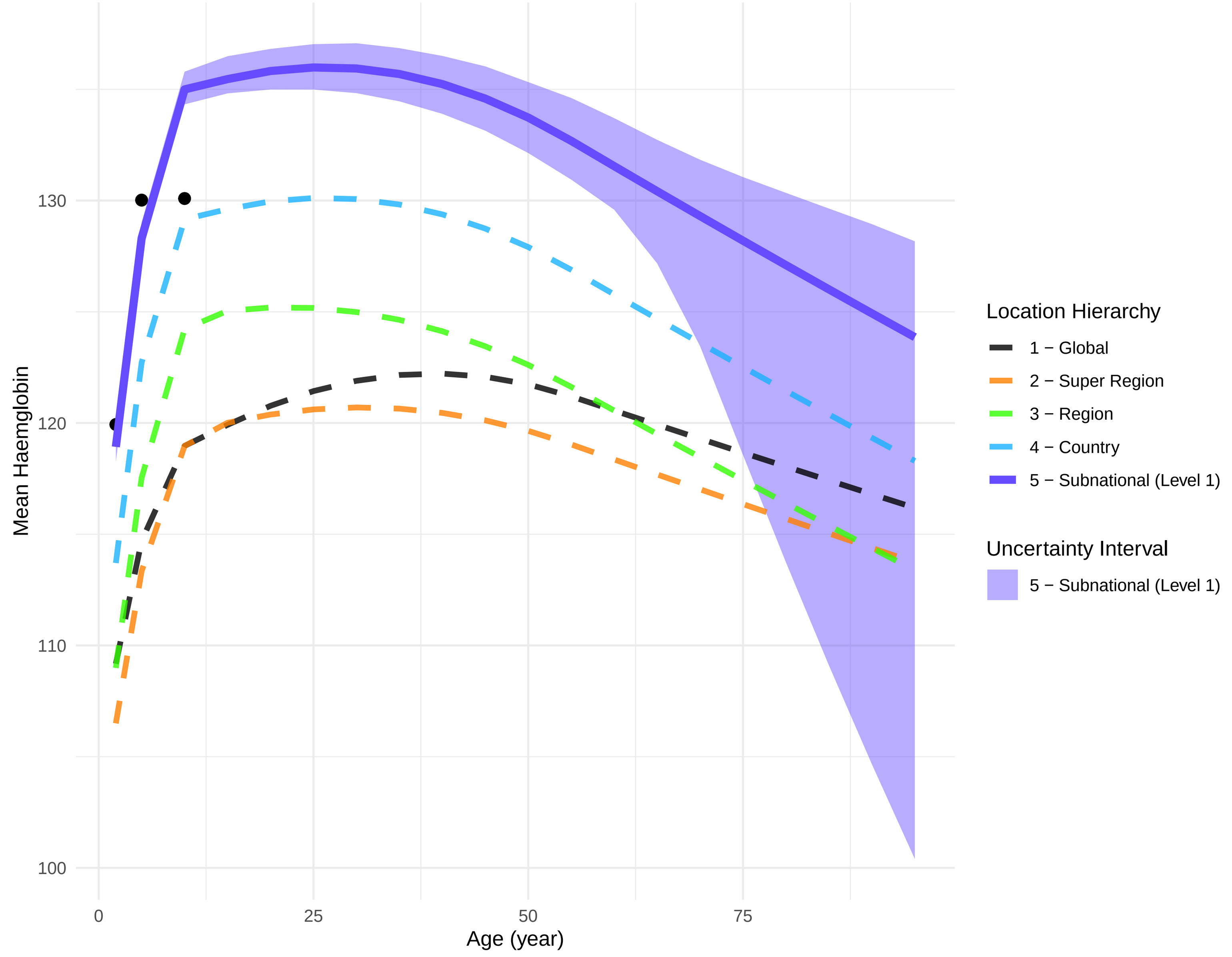

MR−BRT Age/Sex Fits − Non−pregnant Females 2+ Years Old − Mean Haemglobin − Siaya
WHO−2001 Adjustment Method

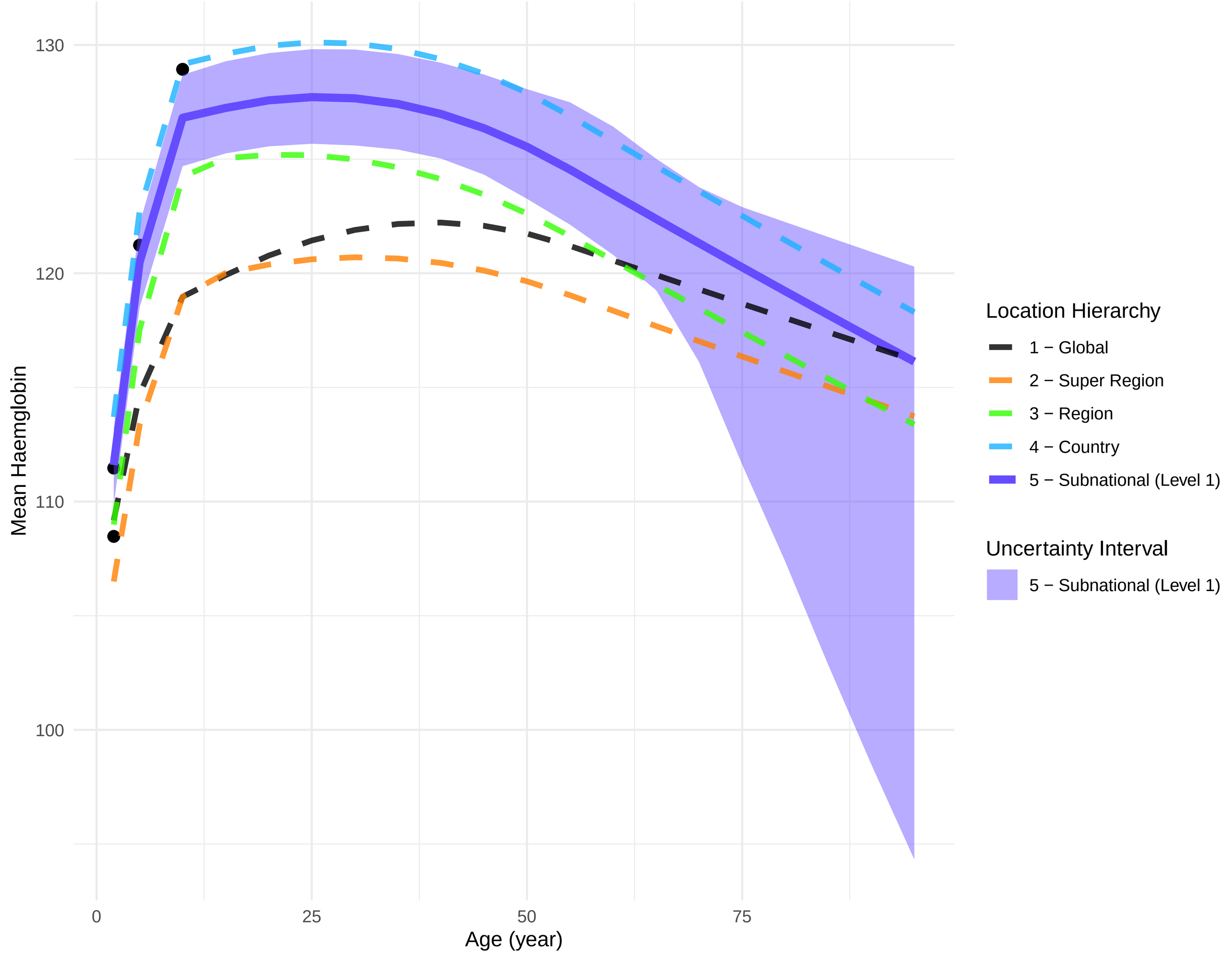

MR−BRT Age/Sex Fits − Non−pregnant Females 2+ Years Old − Mean Haemglobin − Taita Taveta
WHO−2001 Adjustment Method

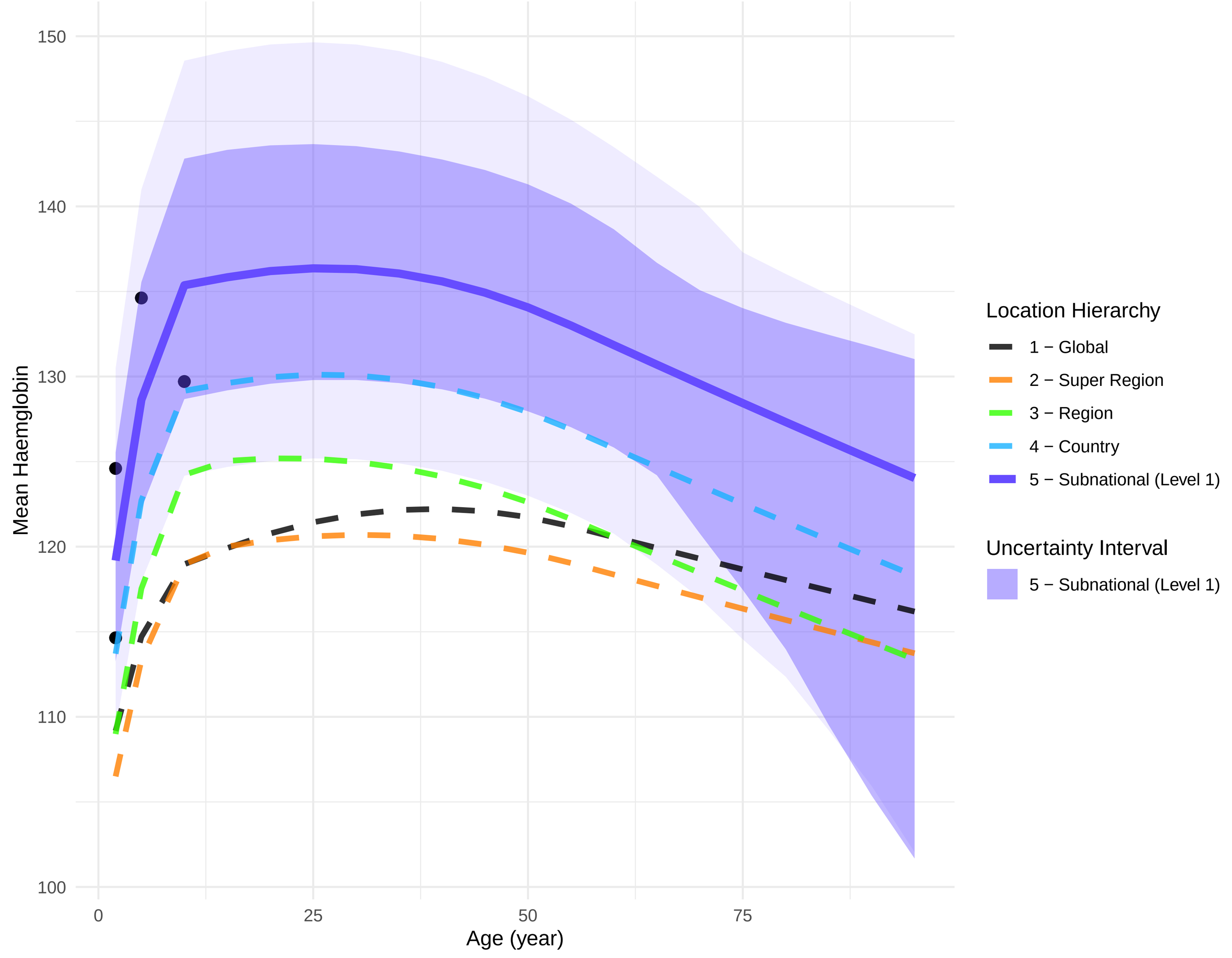

MR−BRT Age/Sex Fits − Non−pregnant Females 2+ Years Old − Mean Haemglobin − Tana River
WHO−2001 Adjustment Method

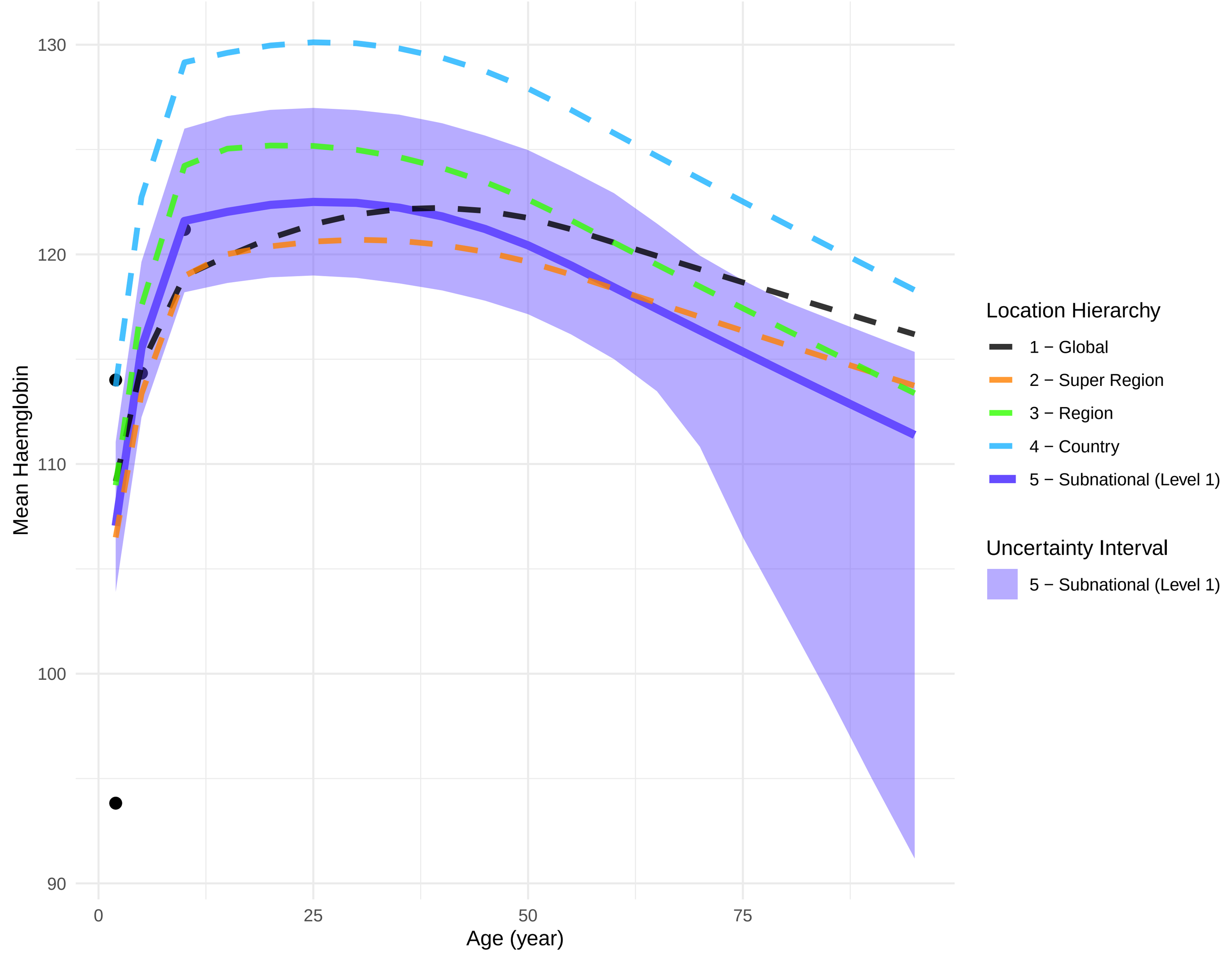

MR−BRT Age/Sex Fits − Non−pregnant Females 2+ Years Old − Mean Haemglobin − Tharaka Nithi
WHO−2001 Adjustment Method

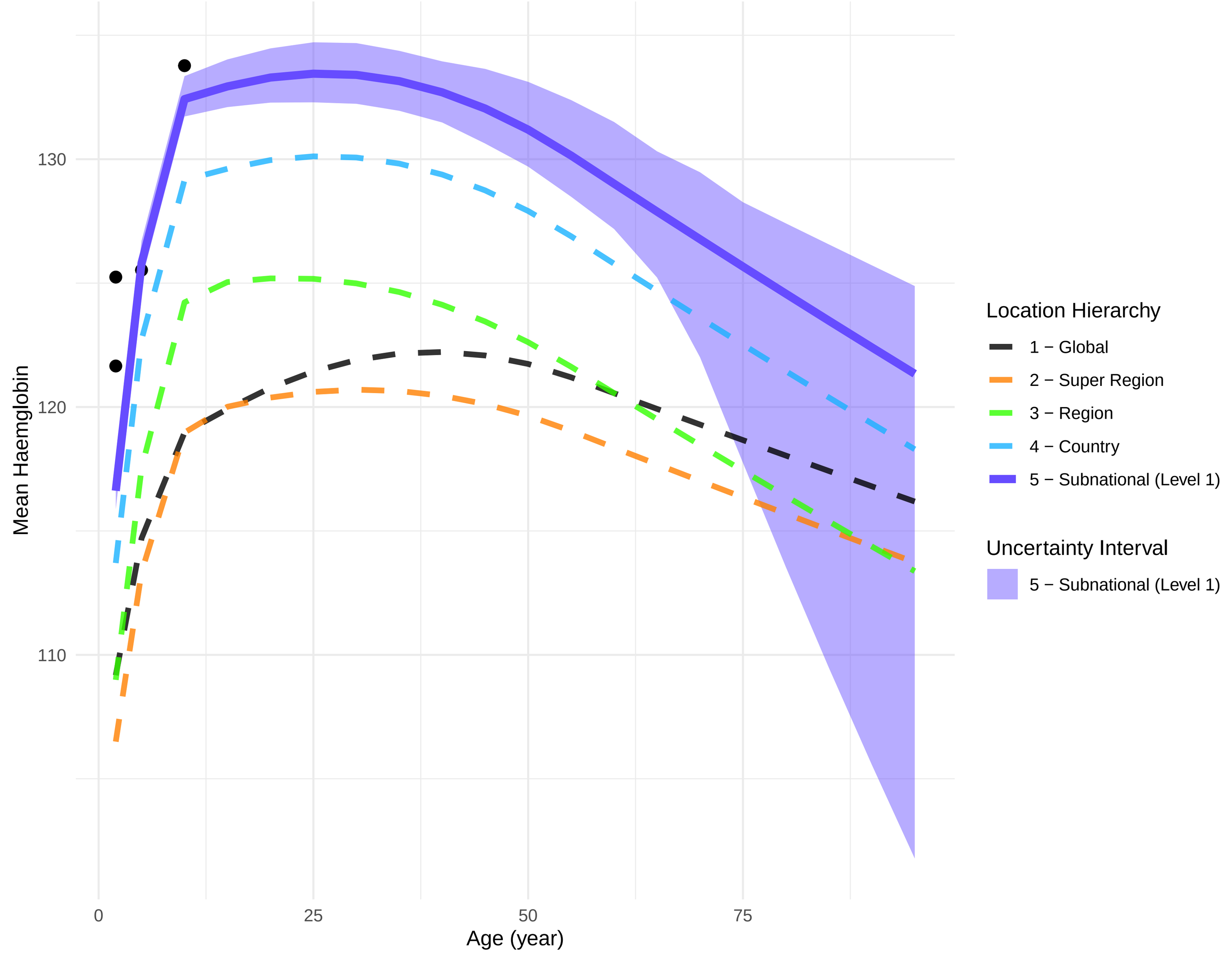

MR−BRT Age/Sex Fits − Non−pregnant Females 2+ Years Old − Mean Haemglobin − Trans Nzoia
WHO−2001 Adjustment Method

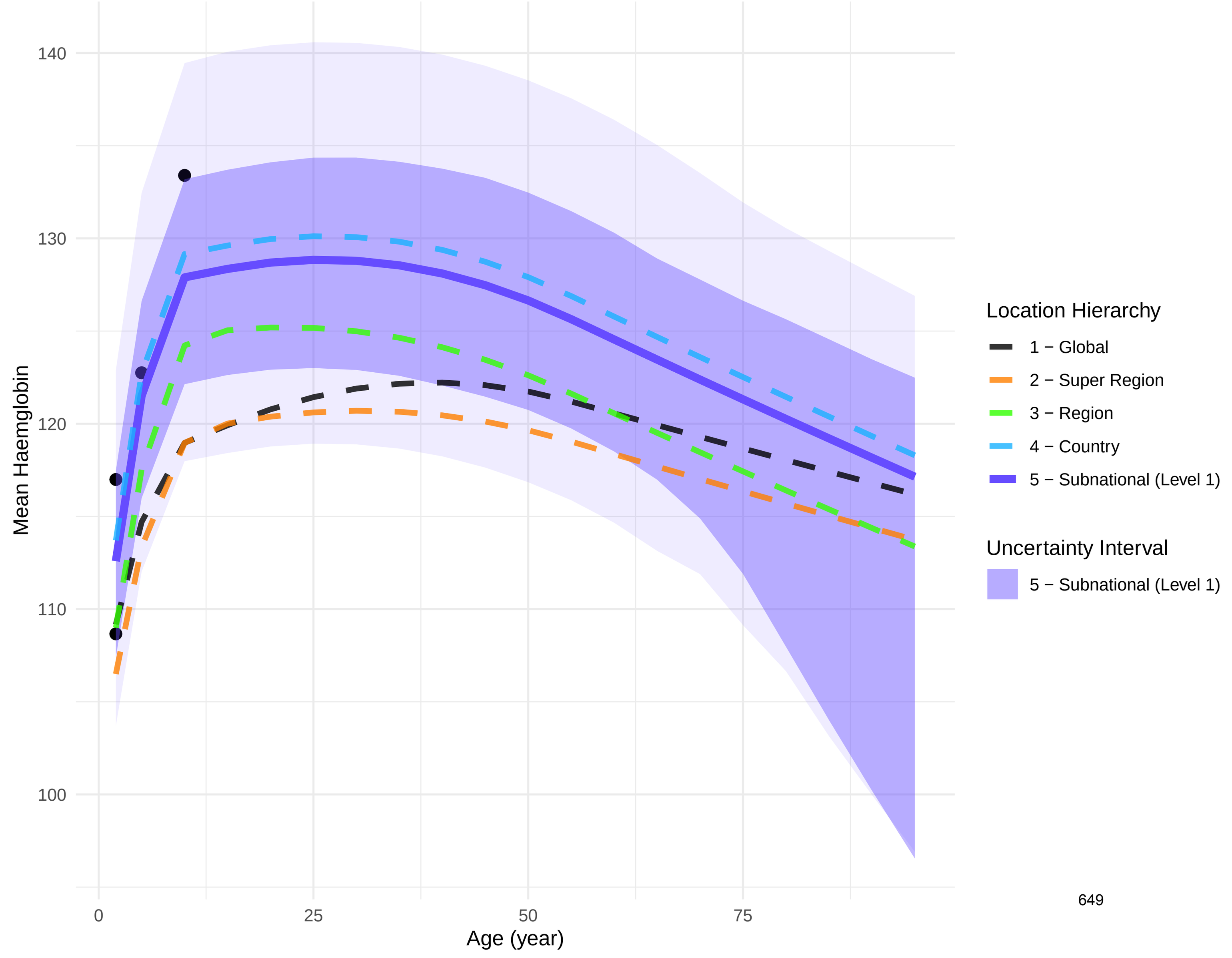

MR−BRT Age/Sex Fits − Non−pregnant Females 2+ Years Old − Mean Haemglobin − Turkana
WHO−2001 Adjustment Method

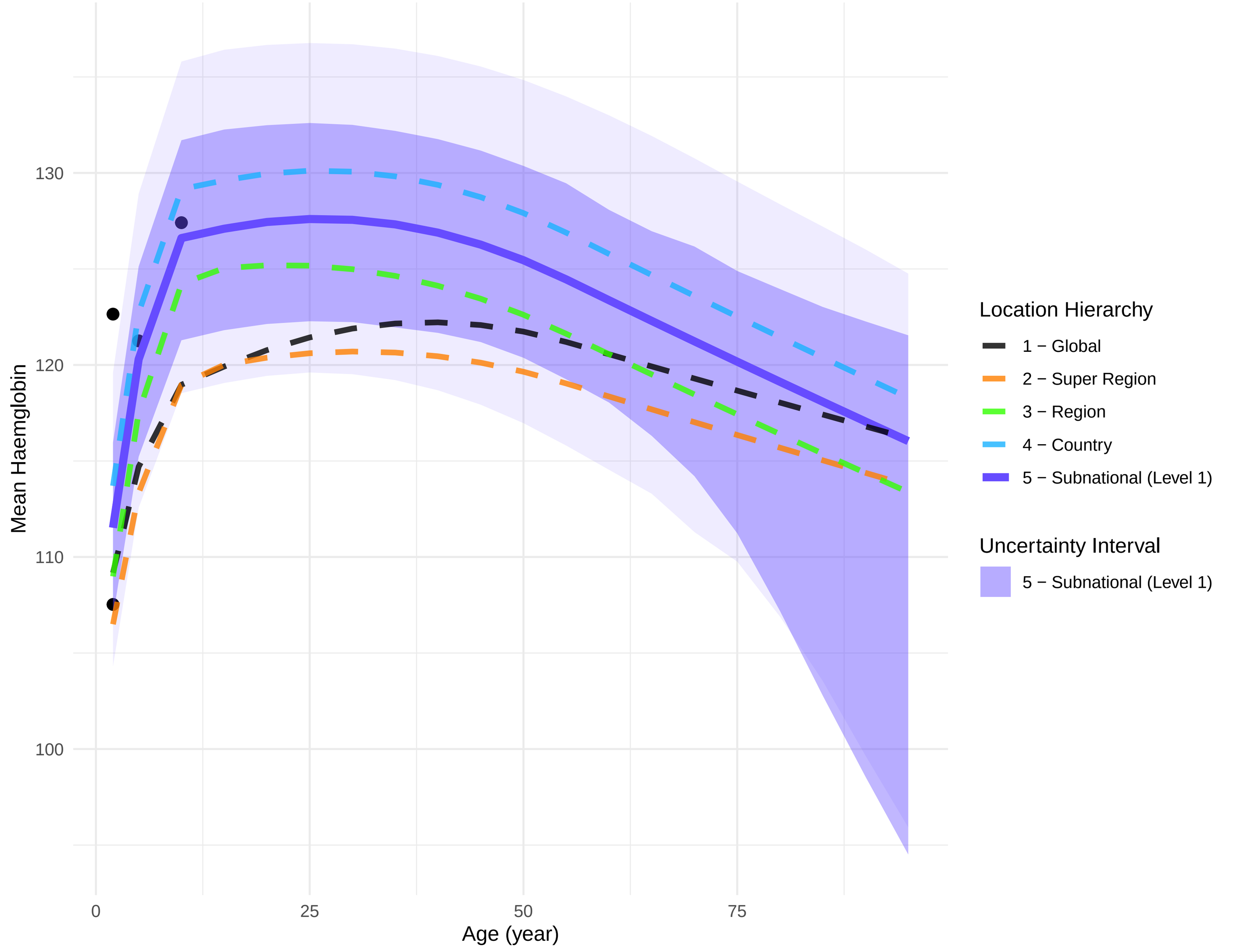

MR−BRT Age/Sex Fits − Non−pregnant Females 2+ Years Old − Mean Haemglobin − Uasin Gishu
WHO−2001 Adjustment Method

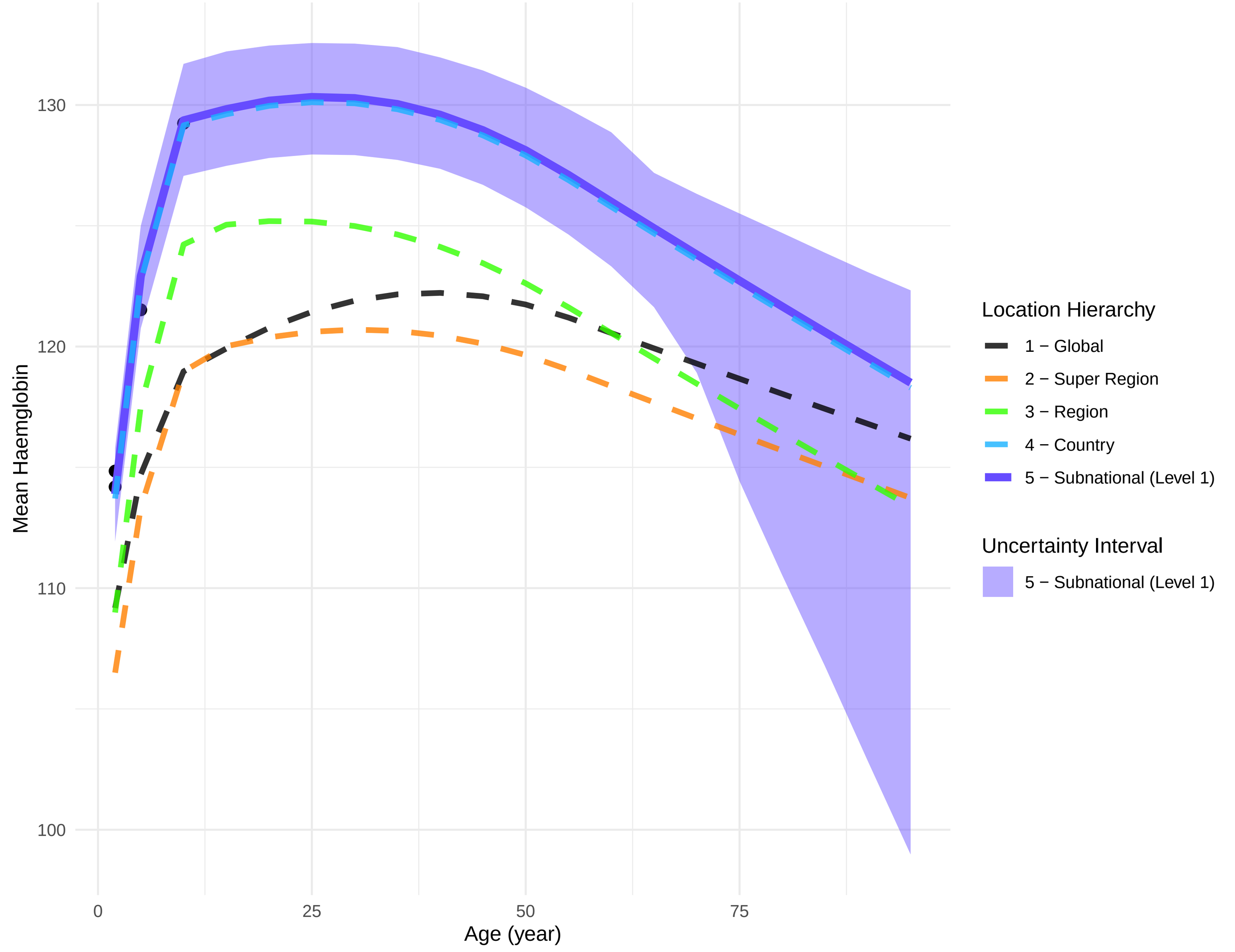

MR−BRT Age/Sex Fits − Non−pregnant Females 2+ Years Old − Mean Haemglobin − Vihiga
WHO−2001 Adjustment Method

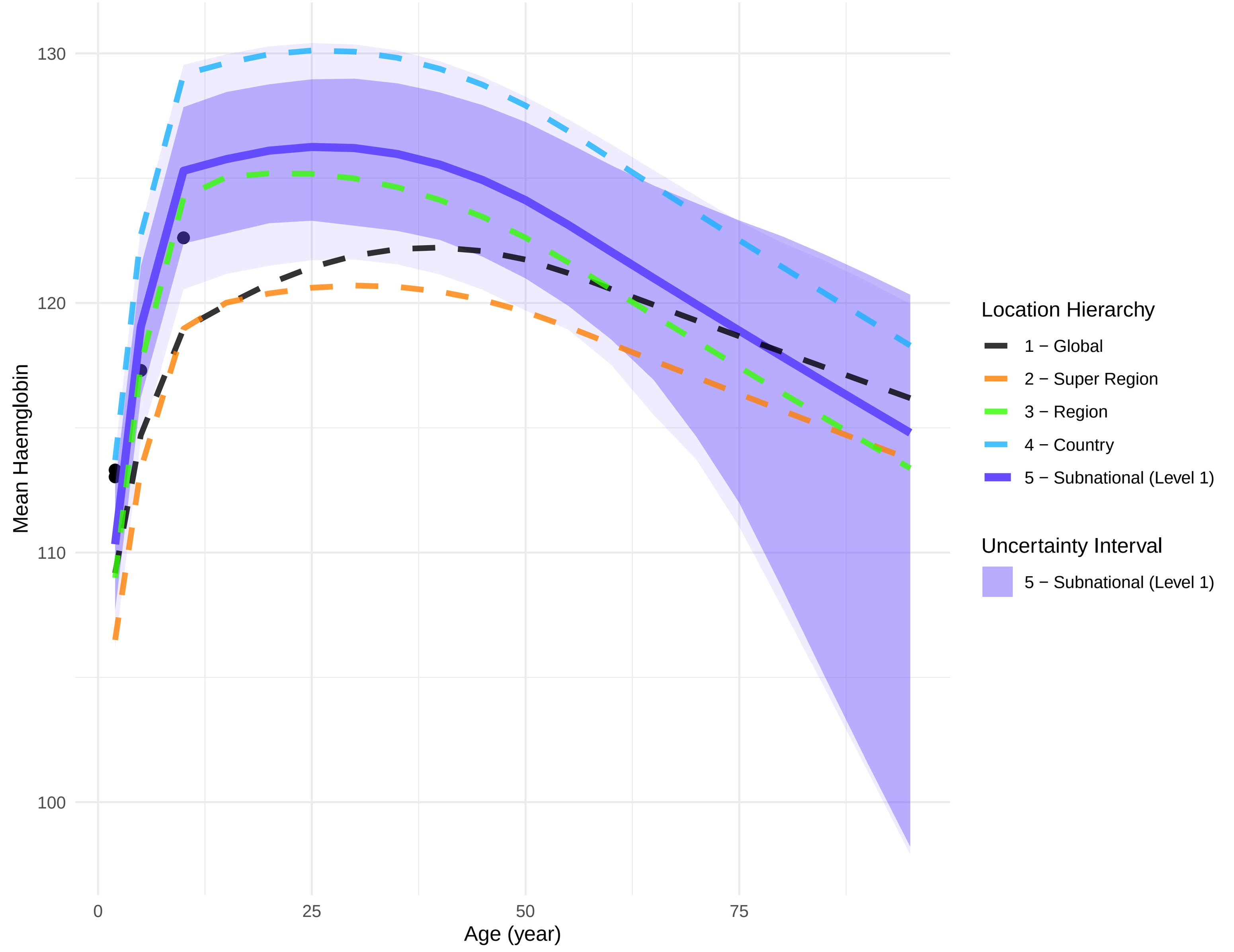

MR−BRT Age/Sex Fits − Non−pregnant Females 2+ Years Old − Mean Haemglobin − Wajir
WHO−2001 Adjustment Method

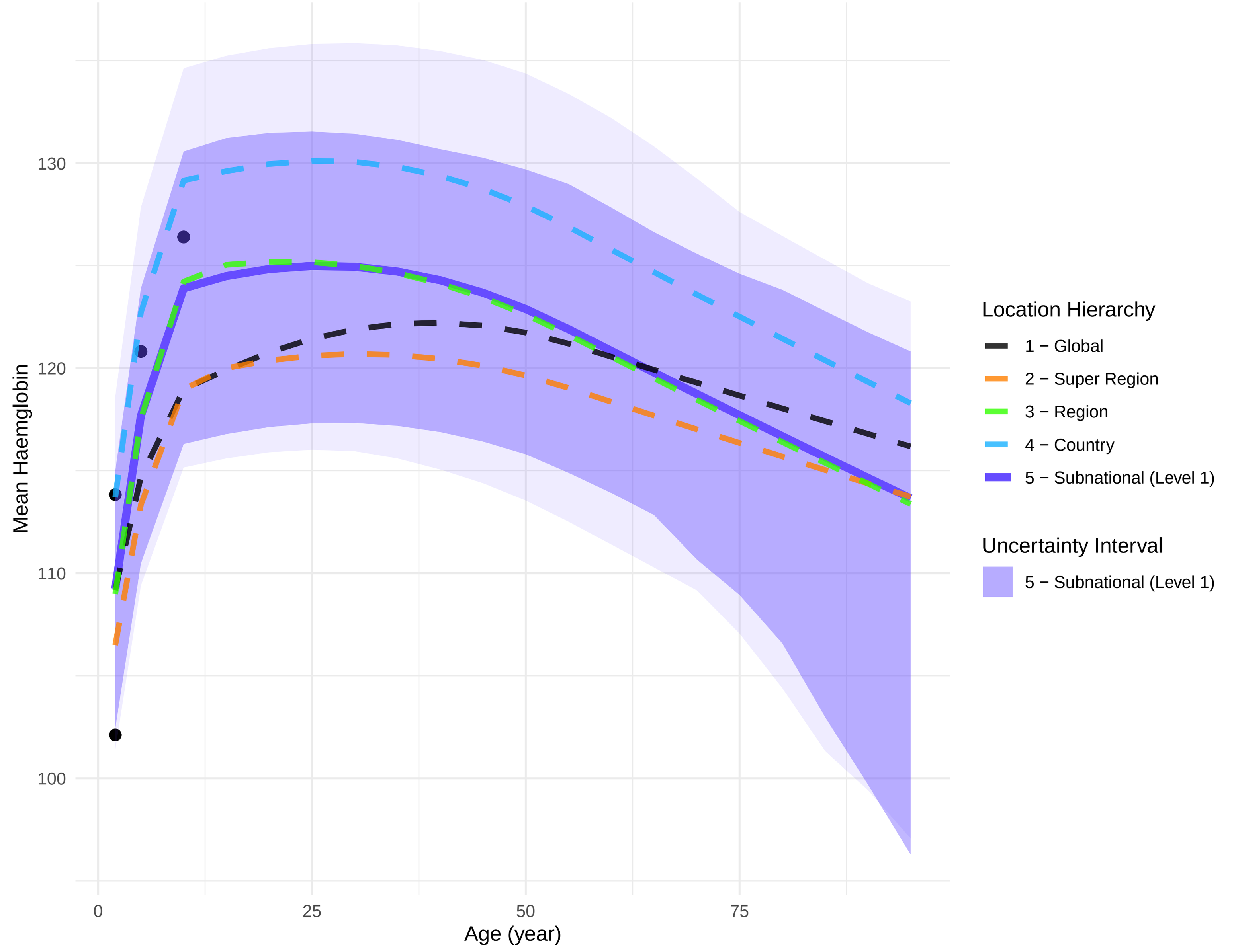

MR−BRT Age/Sex Fits − Non−pregnant Females 2+ Years Old − Mean Haemglobin − West Pokot
WHO−2001 Adjustment Method

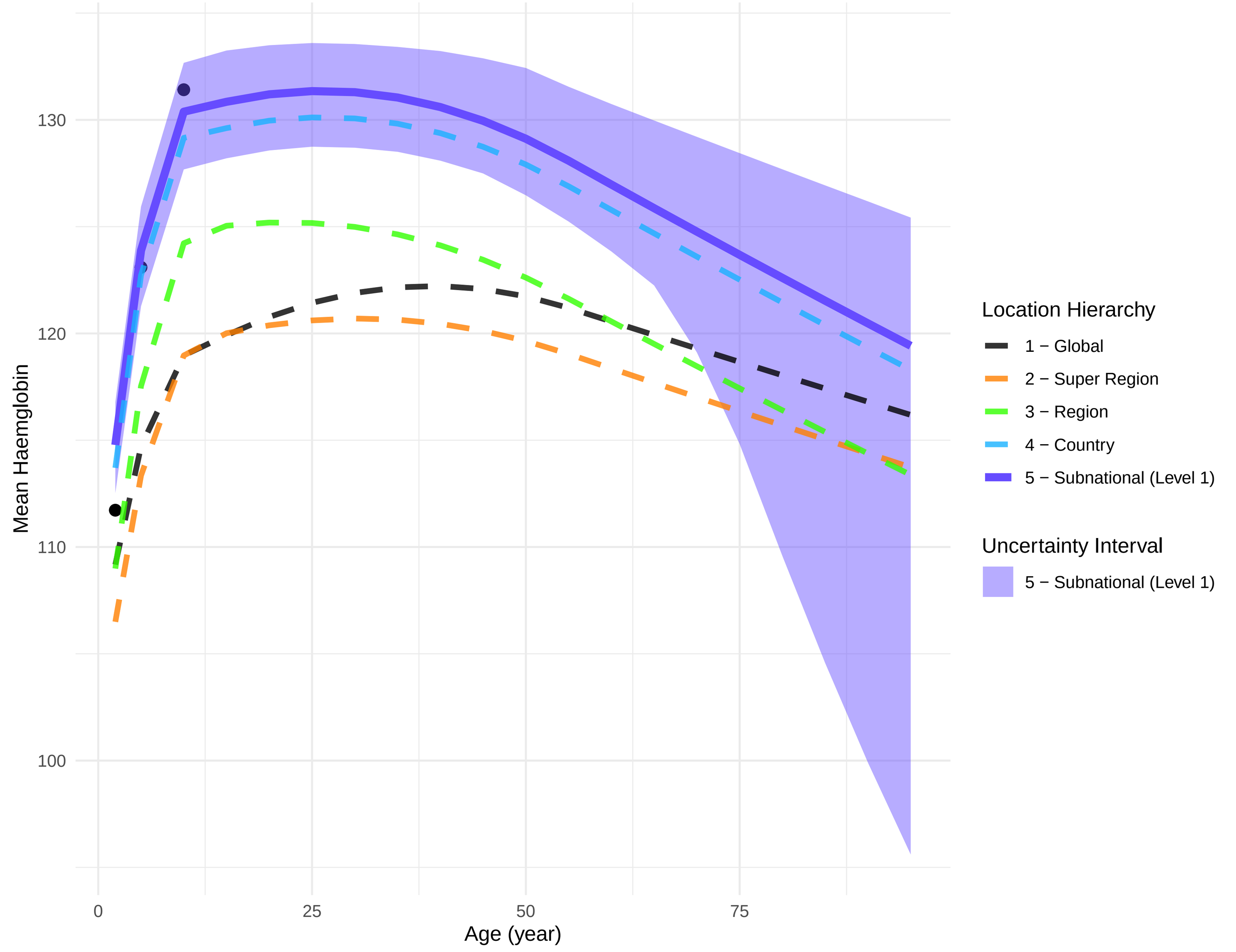

MR−BRT Age/Sex Fits − Non−pregnant Females 2+ Years Old − Mean Haemglobin − Madagascar
WHO−2001 Adjustment Method

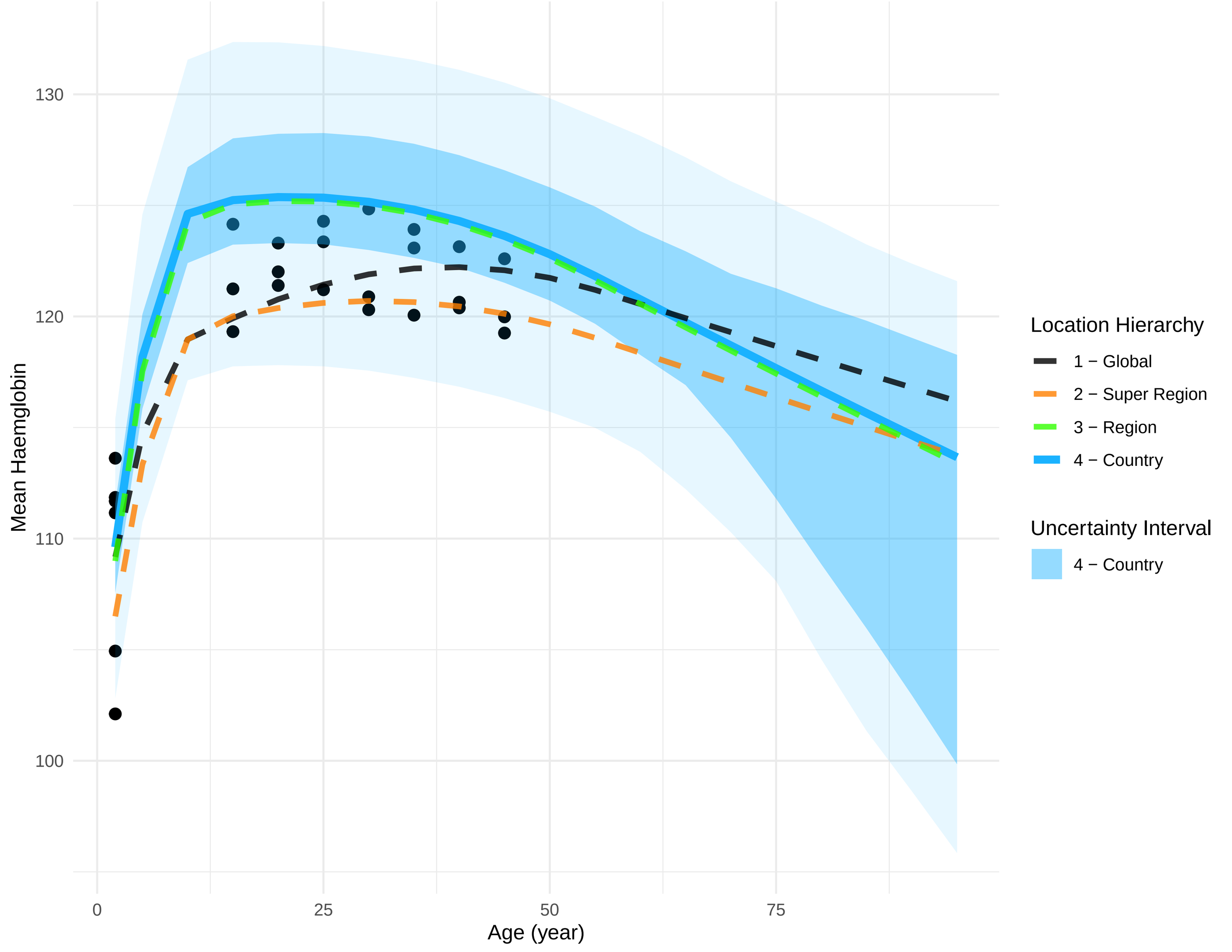

MR−BRT Age/Sex Fits − Non−pregnant Females 2+ Years Old − Mean Haemglobin − Malawi
WHO−2001 Adjustment Method

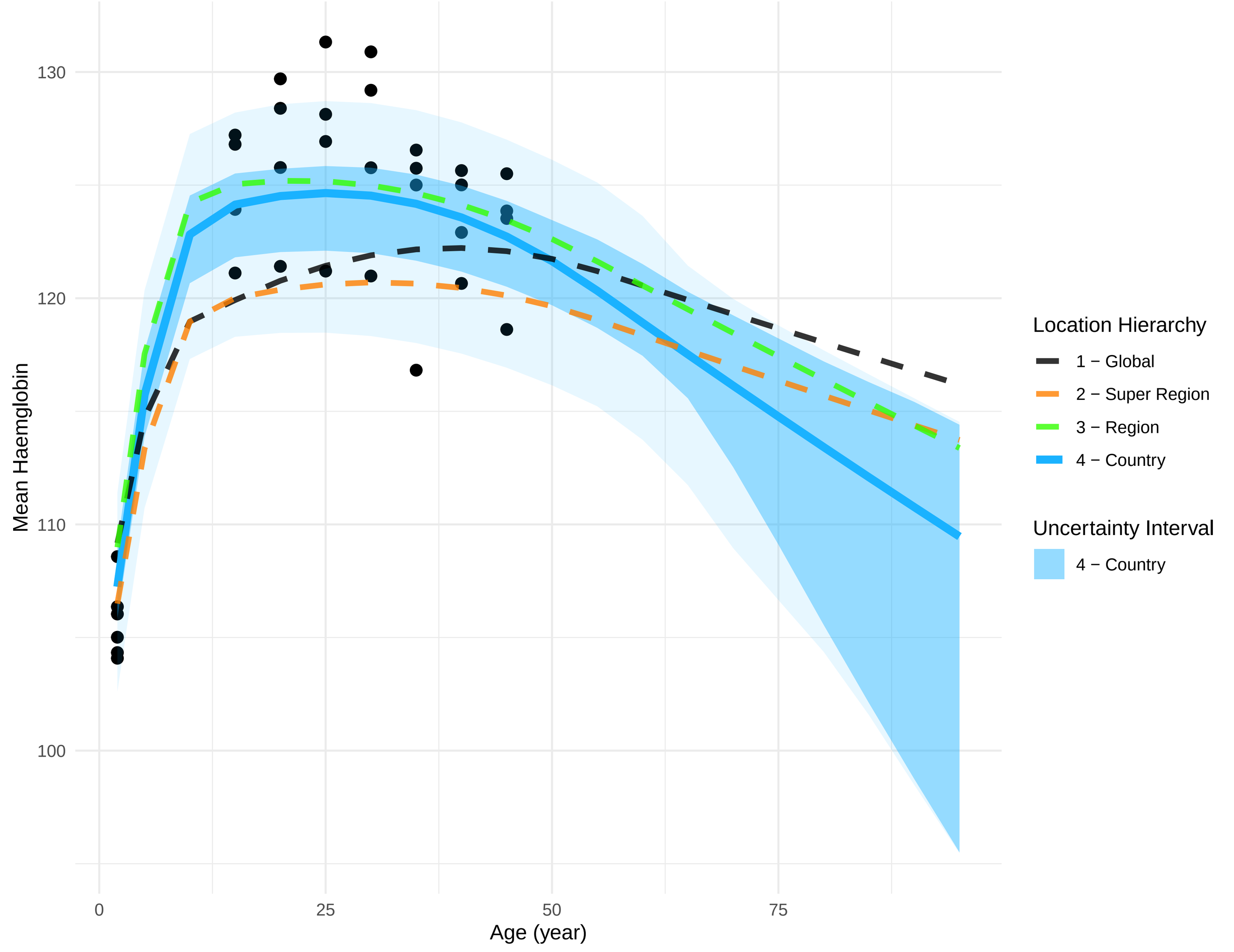

MR−BRT Age/Sex Fits − Non−pregnant Females 2+ Years Old − Mean Haemglobin − Mozambique
WHO−2001 Adjustment Method

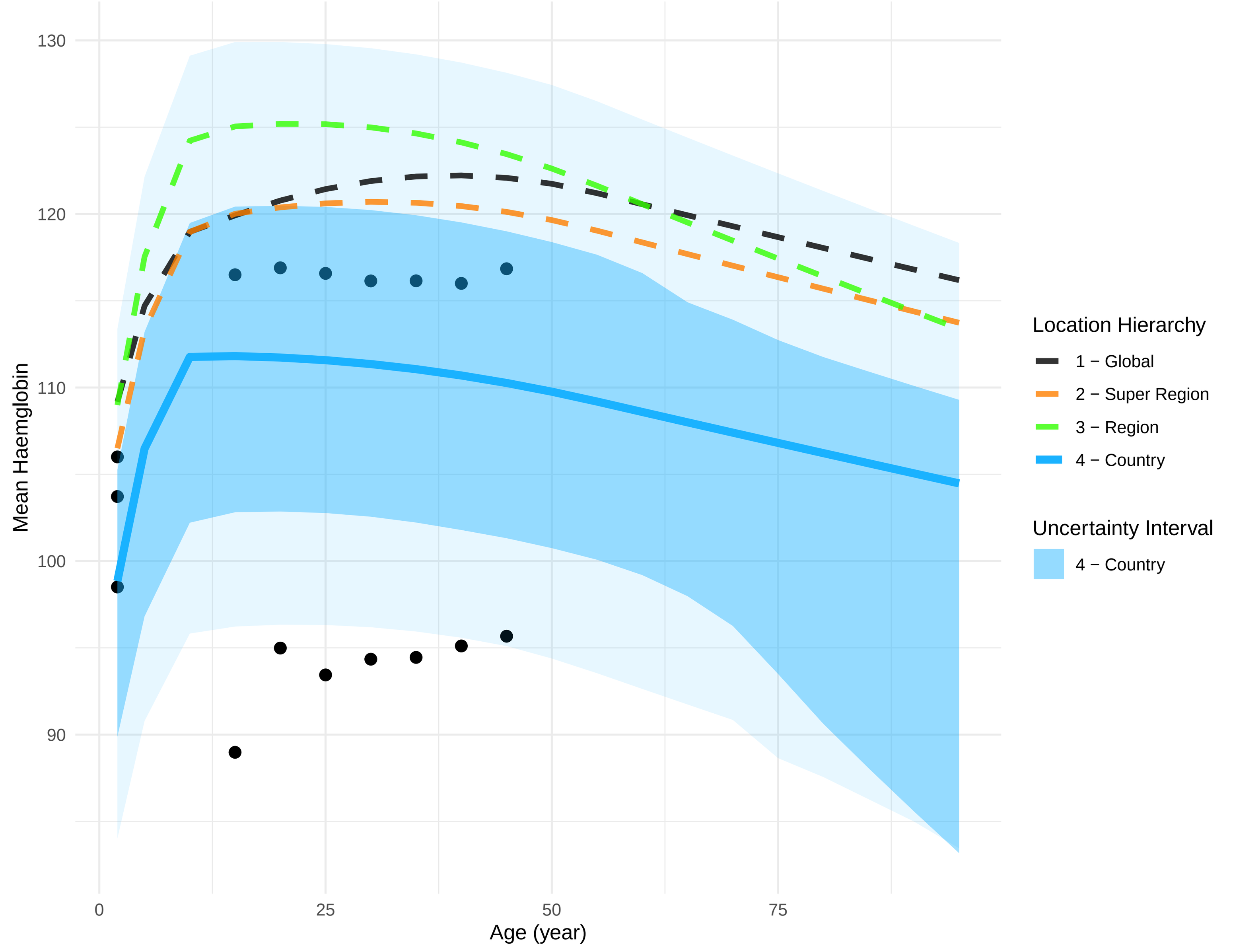

MR−BRT Age/Sex Fits − Non−pregnant Females 2+ Years Old − Mean Haemglobin − Rwanda
WHO−2001 Adjustment Method

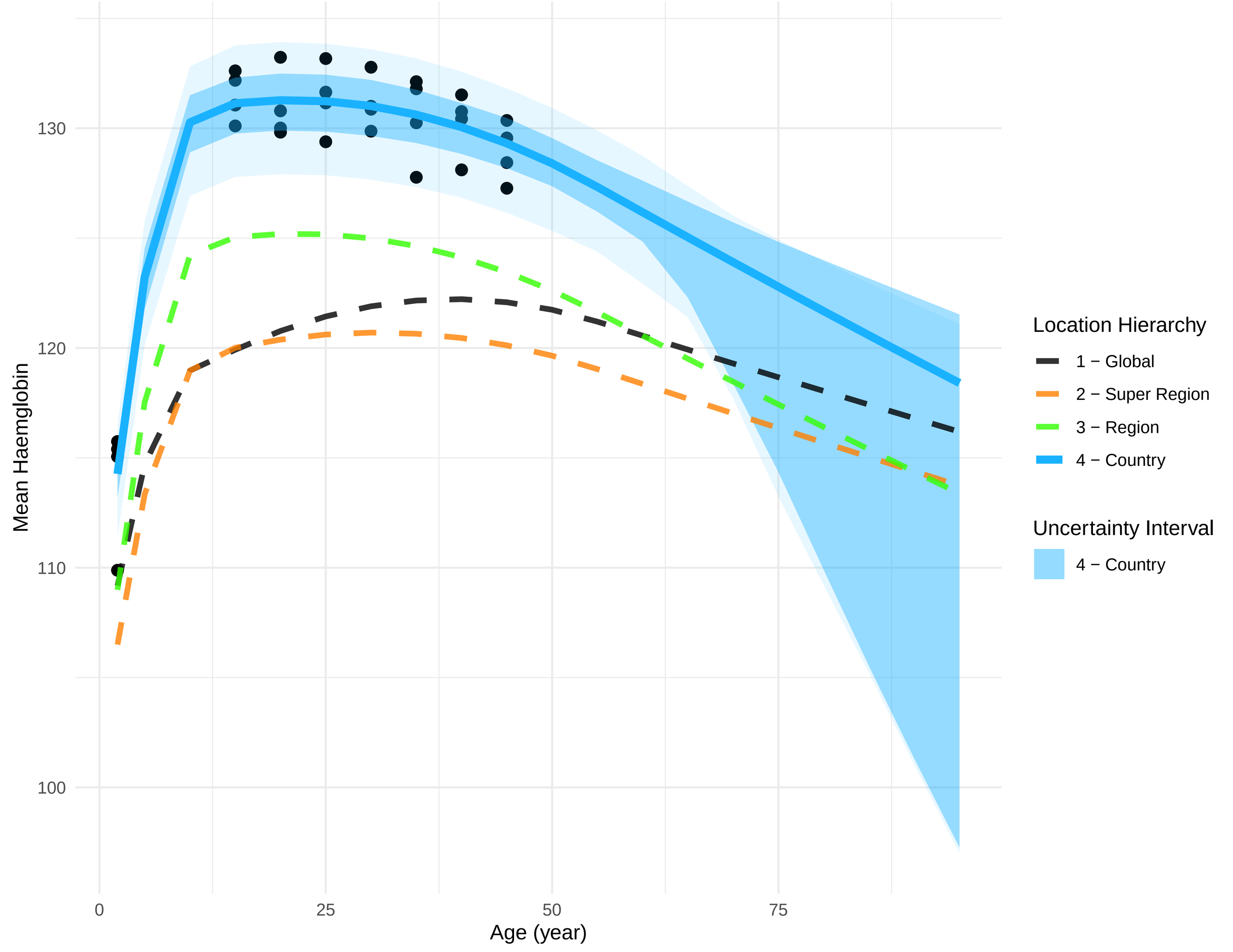

MR−BRT Age/Sex Fits − Non−pregnant Females 2+ Years Old − Mean Haemglobin − Uganda
WHO−2001 Adjustment Method

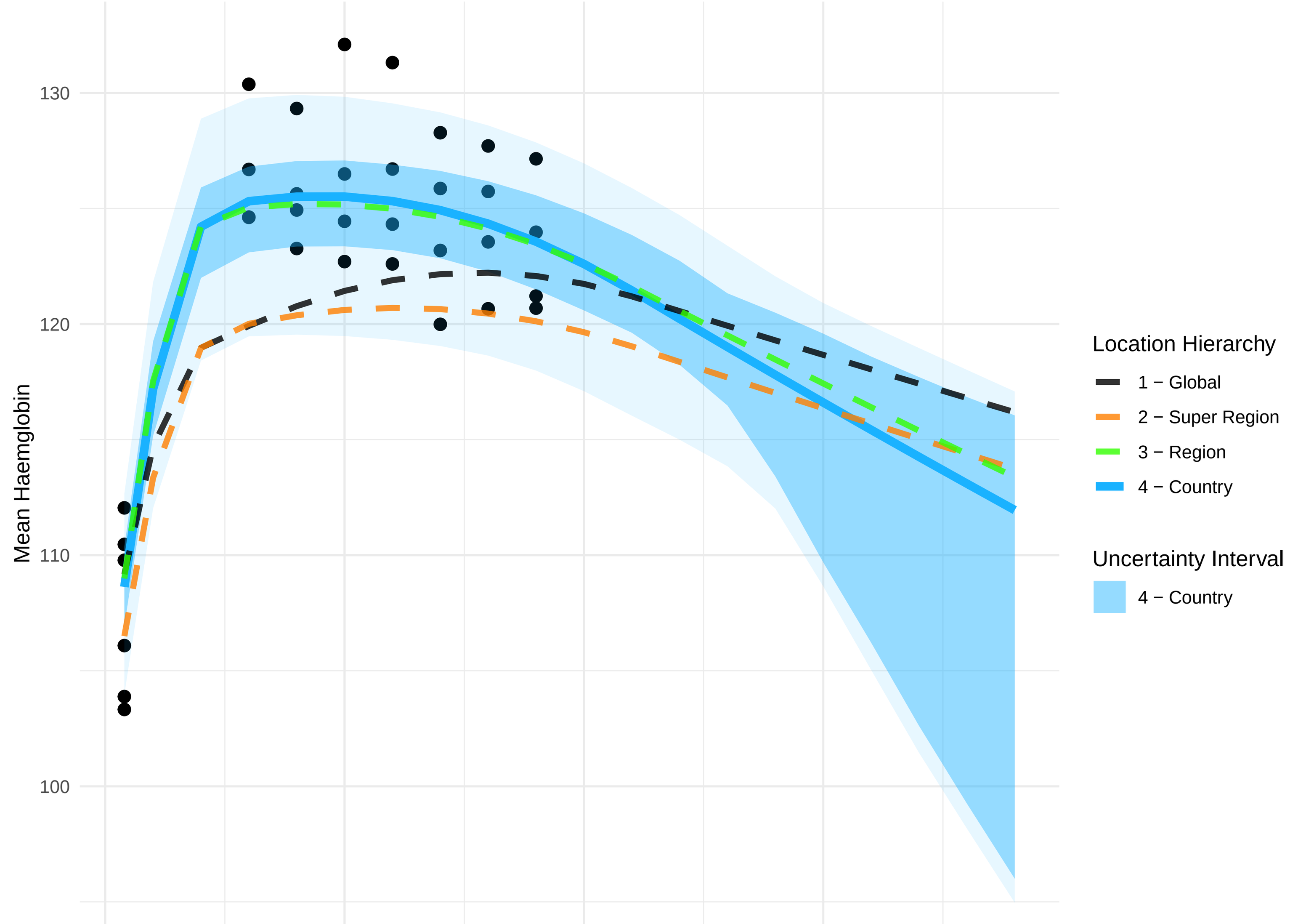

MR−BRT Age/Sex Fits − Non−pregnant Females 2+ Years Old − Mean Haemglobin − United Republic of Tanzania
WHO−2001 Adjustment Method

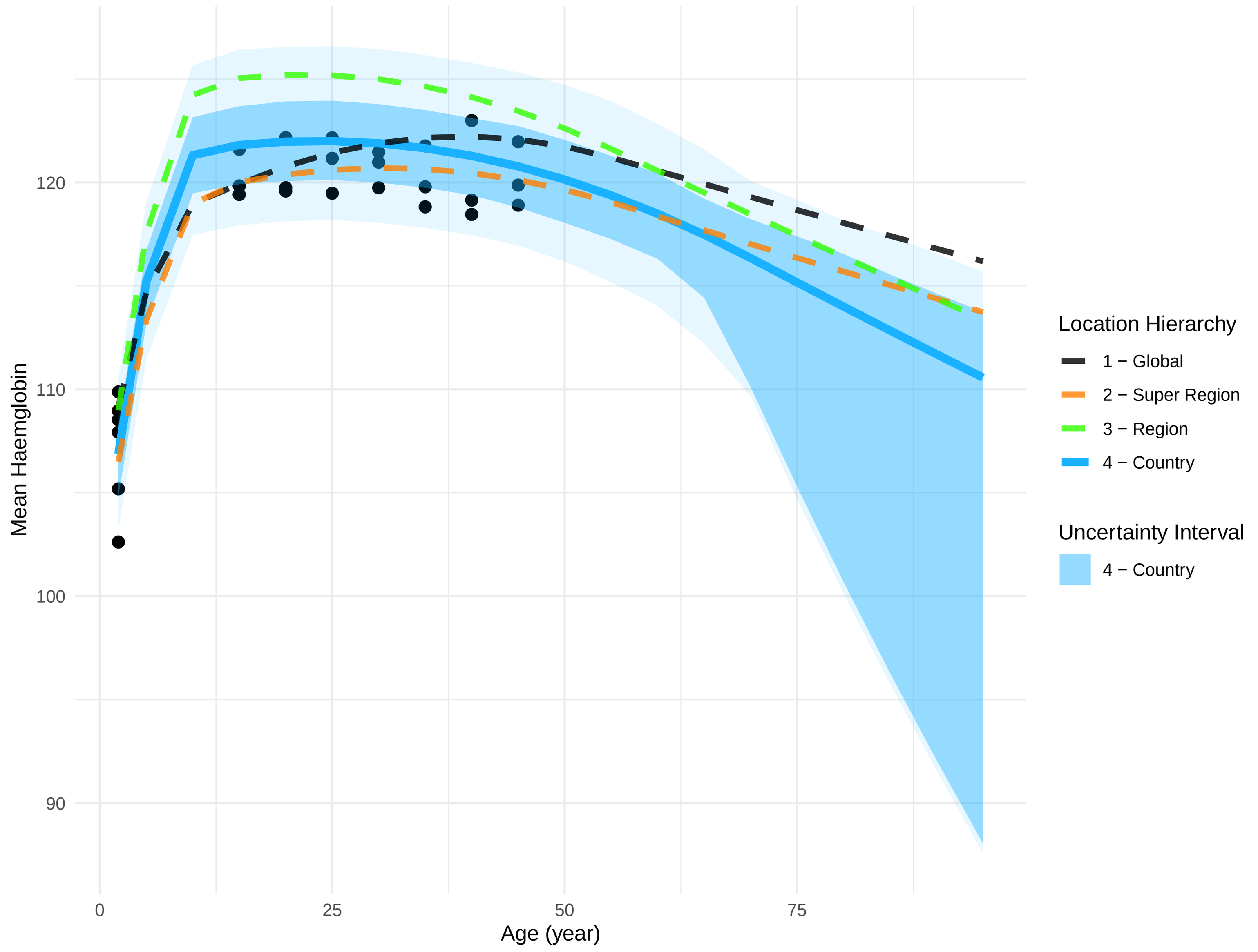

MR−BRT Age/Sex Fits − Non−pregnant Females 2+ Years Old − Mean Haemglobin − Zambia
WHO−2001 Adjustment Method

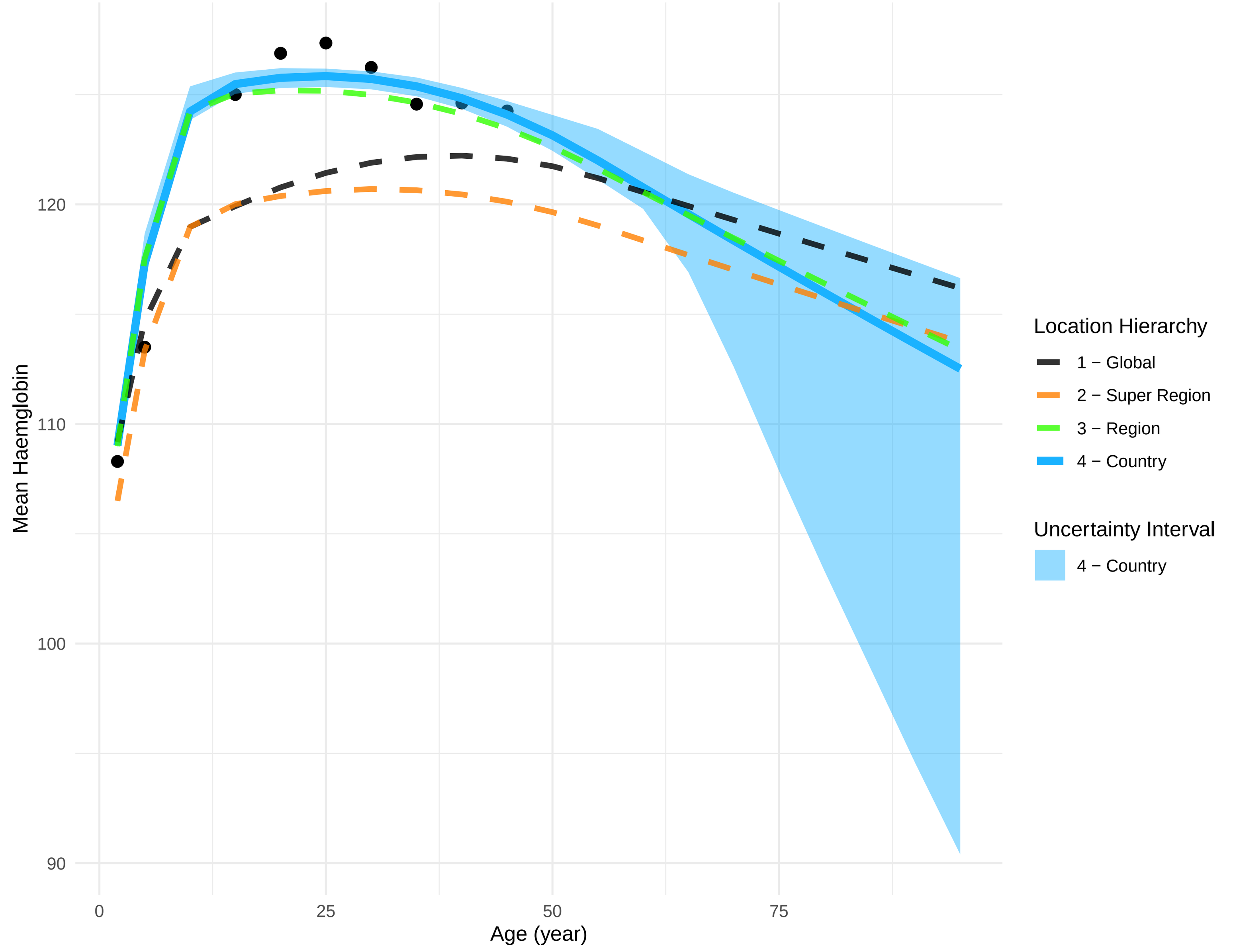

MR−BRT Age/Sex Fits − Non−pregnant Females 2+ Years Old − Mean Haemglobin − Southern Sub−Saharan Africa
WHO−2001 Adjustment Method

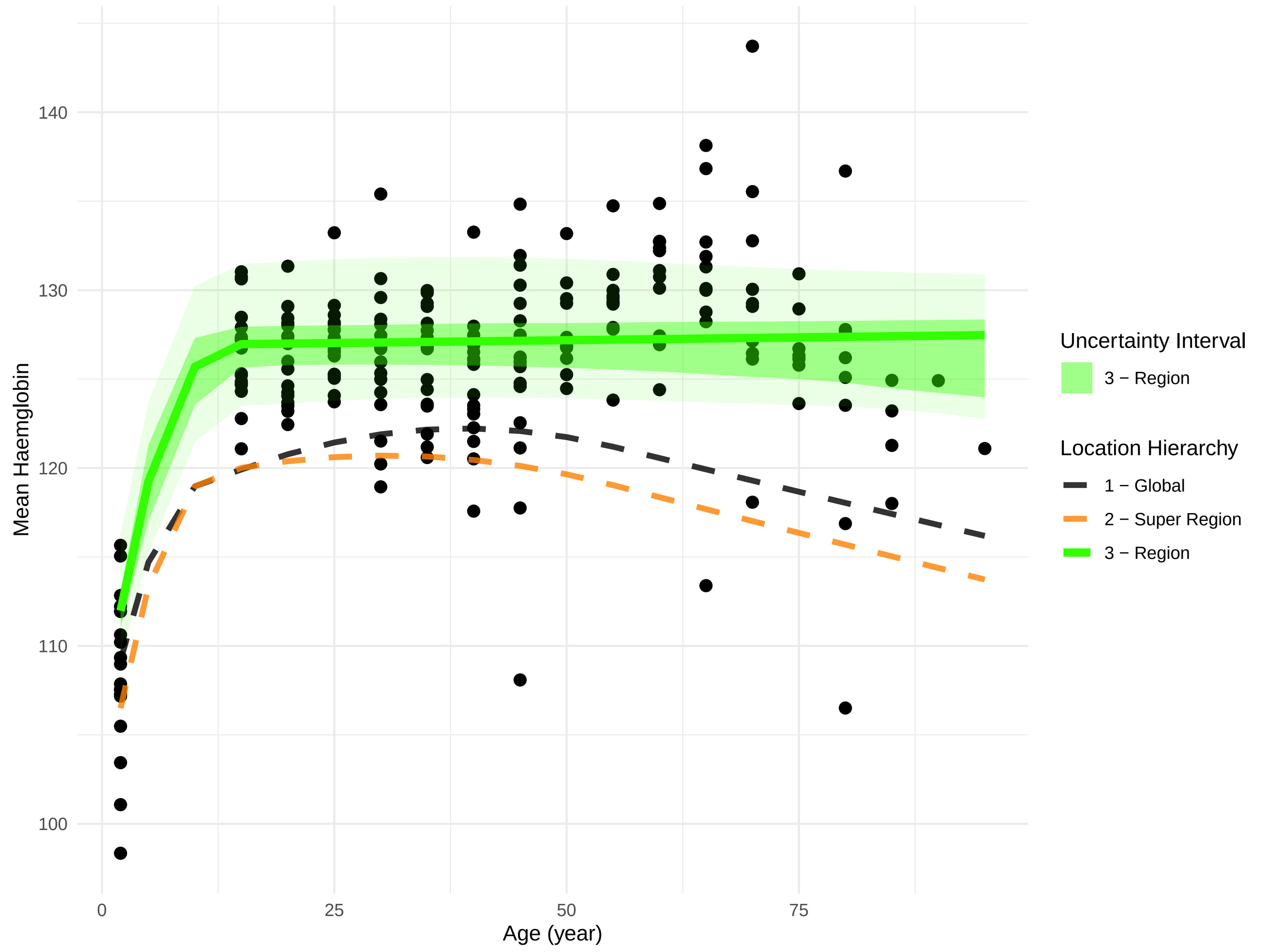

MR−BRT Age/Sex Fits − Non−pregnant Females 2+ Years Old − Mean Haemglobin − Eswatini
WHO−2001 Adjustment Method

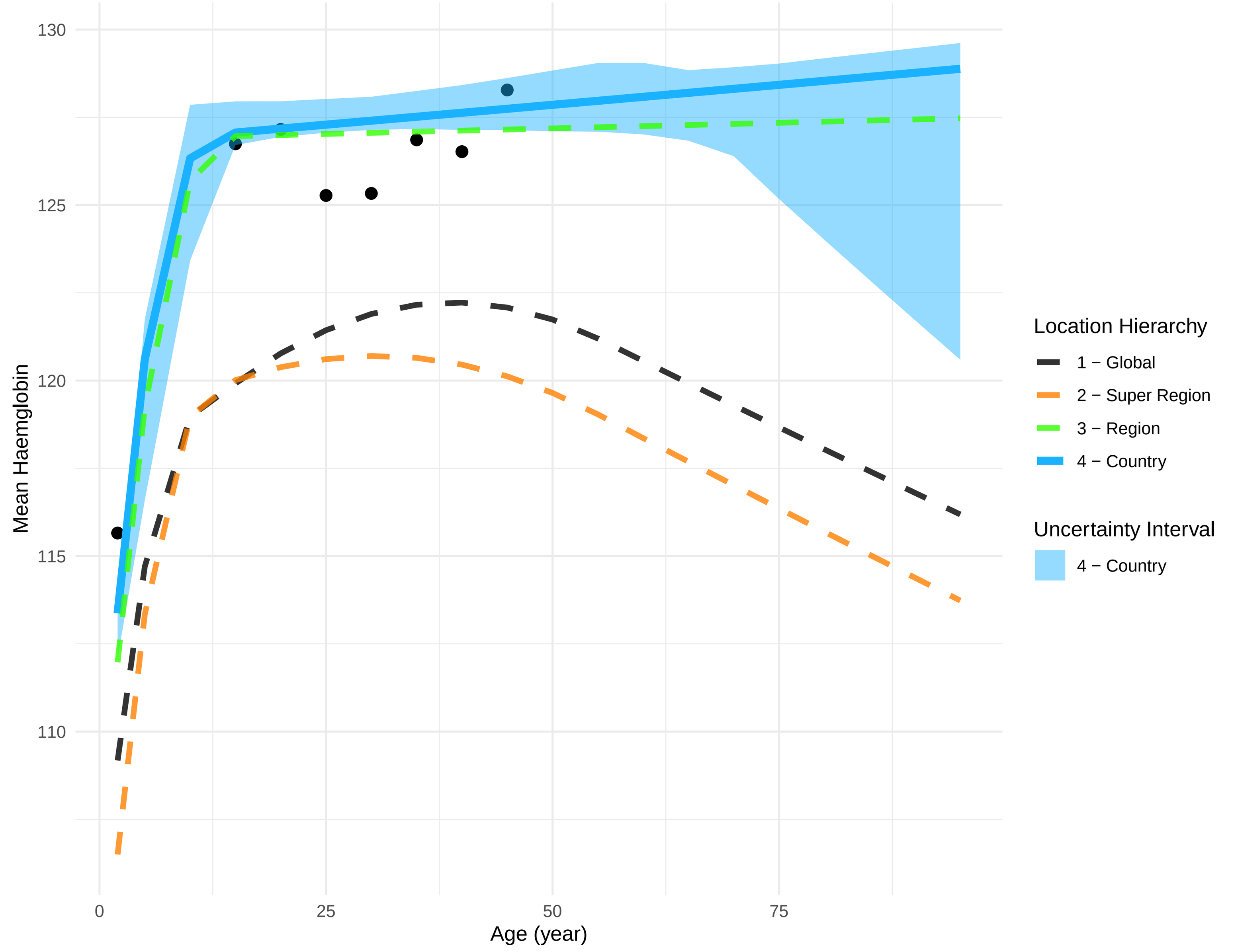

MR−BRT Age/Sex Fits − Non−pregnant Females 2+ Years Old − Mean Haemglobin − Lesotho
WHO−2001 Adjustment Method

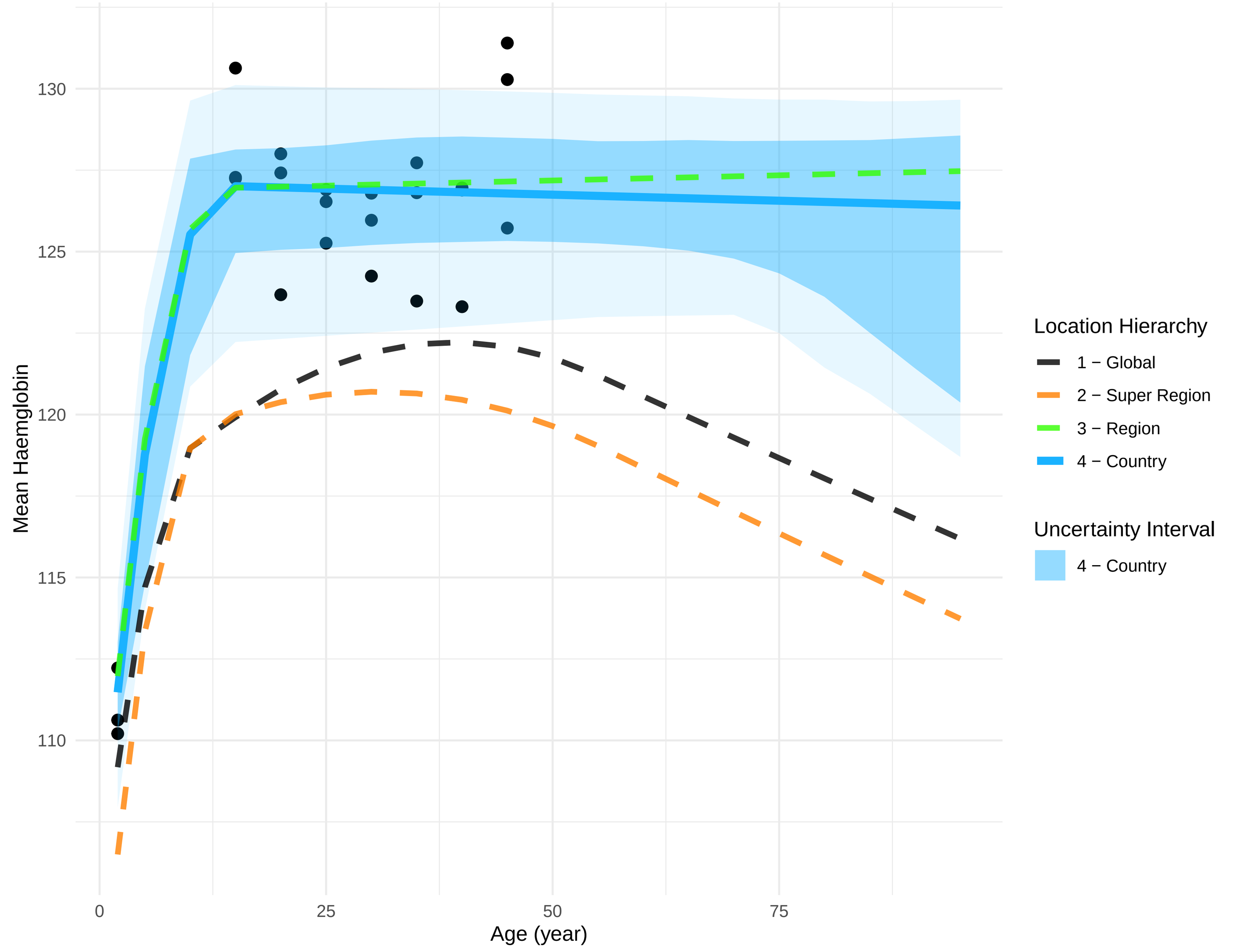

MR−BRT Age/Sex Fits − Non−pregnant Females 2+ Years Old − Mean Haemglobin − Namibia
WHO−2001 Adjustment Method

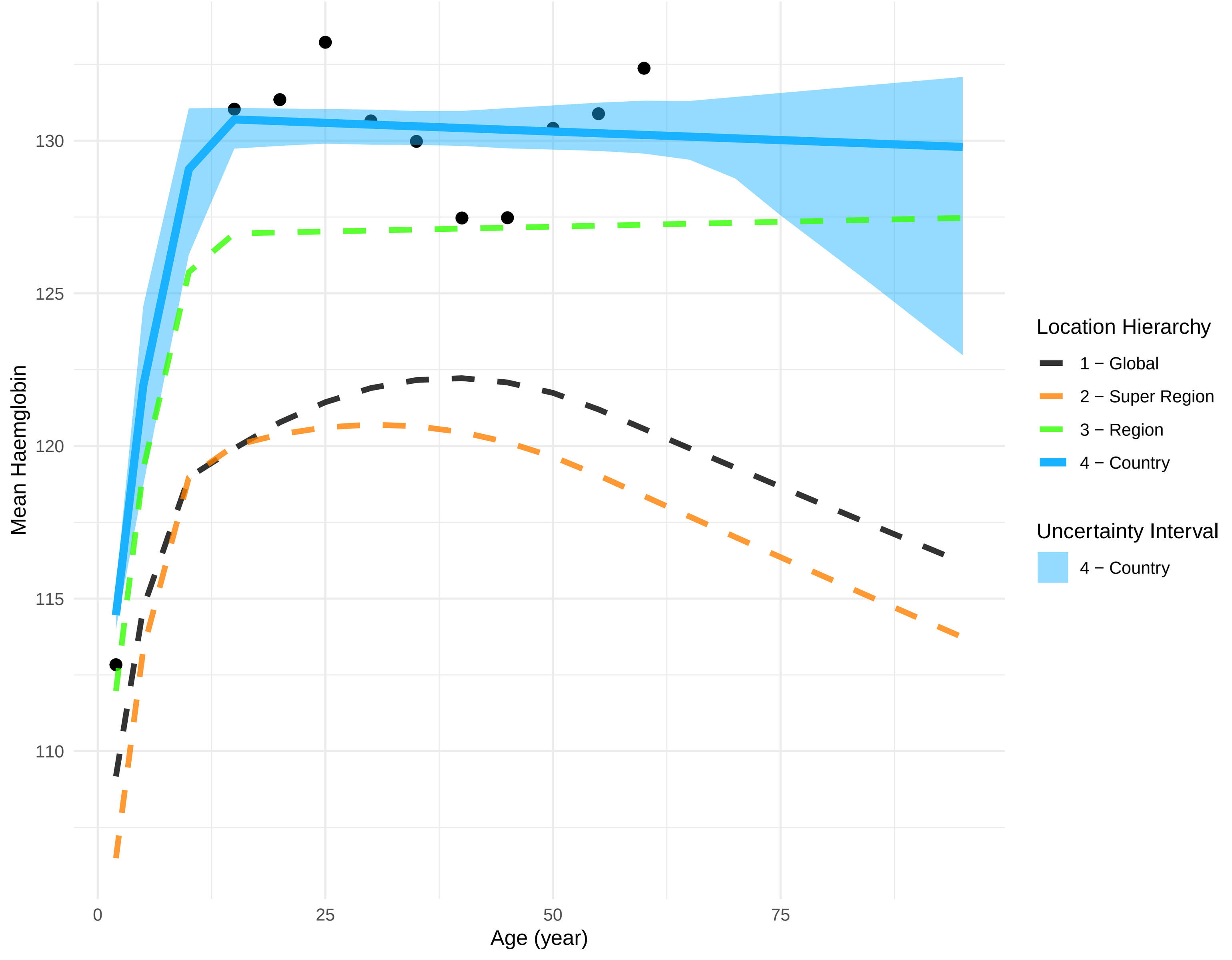

MR−BRT Age/Sex Fits − Non−pregnant Females 2+ Years Old − Mean Haemglobin − South Africa
WHO−2001 Adjustment Method

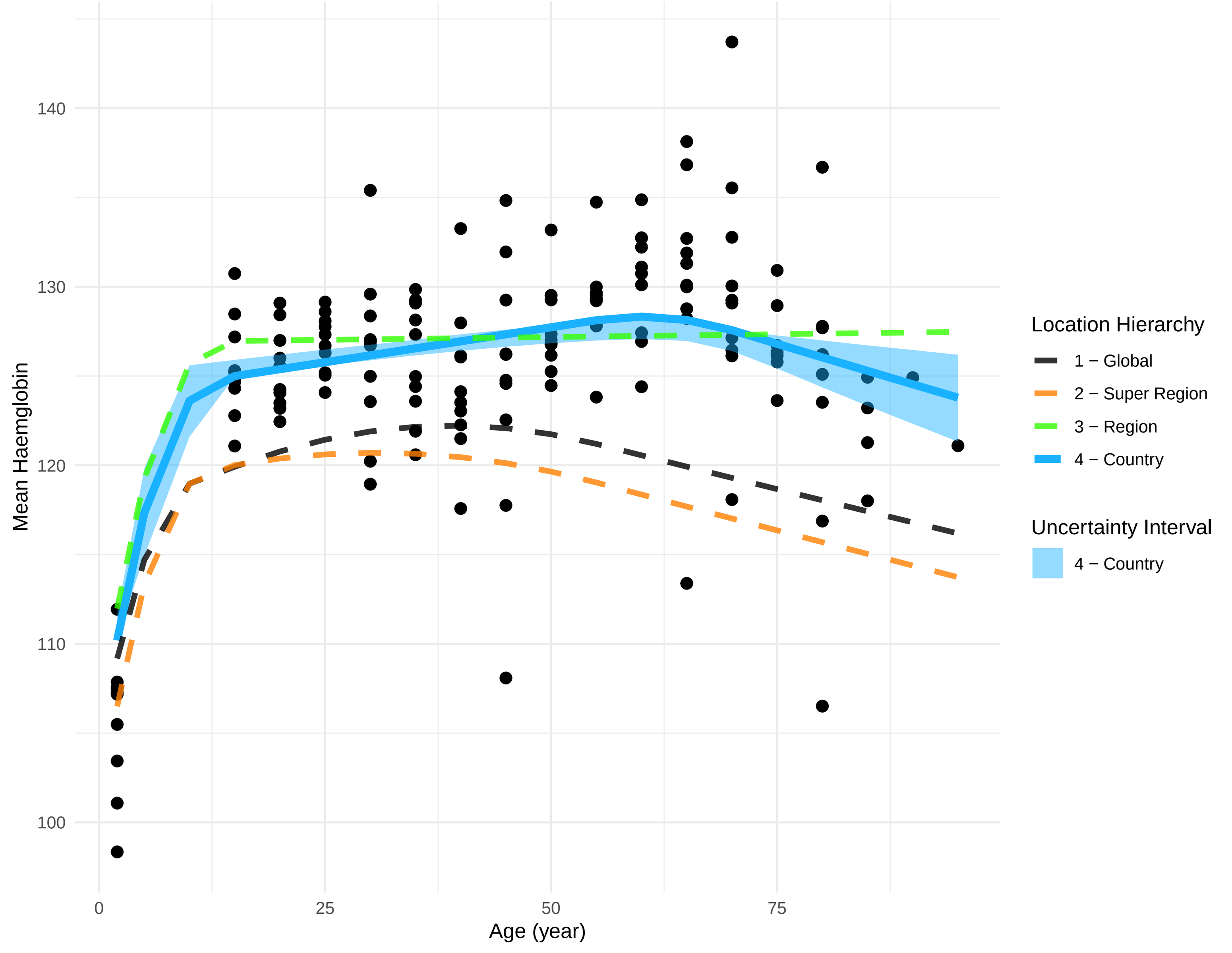

MR−BRT Age/Sex Fits − Non−pregnant Females 2+ Years Old − Mean Haemglobin − Eastern Cape
WHO−2001 Adjustment Method

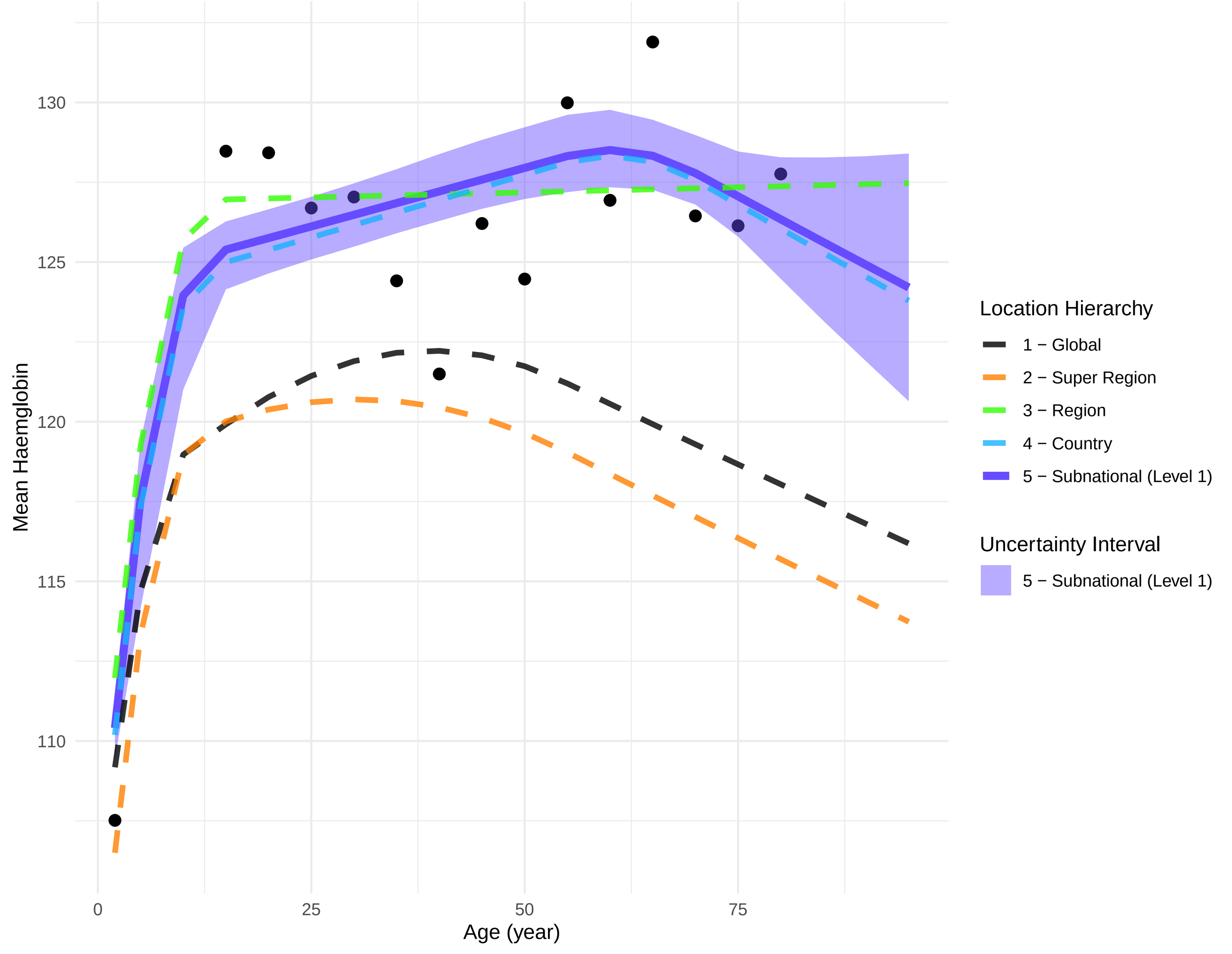

MR−BRT Age/Sex Fits − Non−pregnant Females 2+ Years Old − Mean Haemglobin − Free State
WHO−2001 Adjustment Method

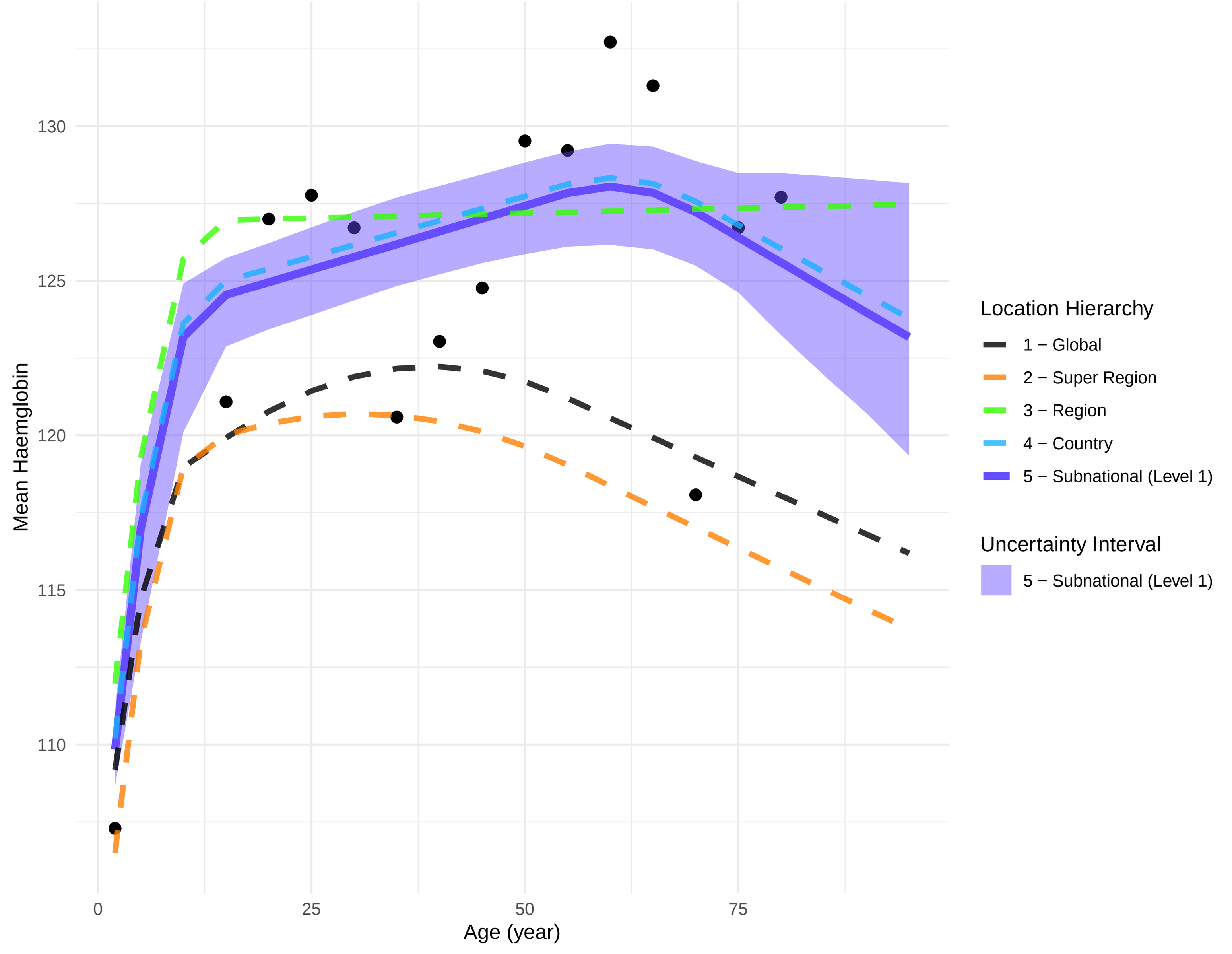

MR−BRT Age/Sex Fits − Non−pregnant Females 2+ Years Old − Mean Haemglobin − Gauteng
WHO−2001 Adjustment Method

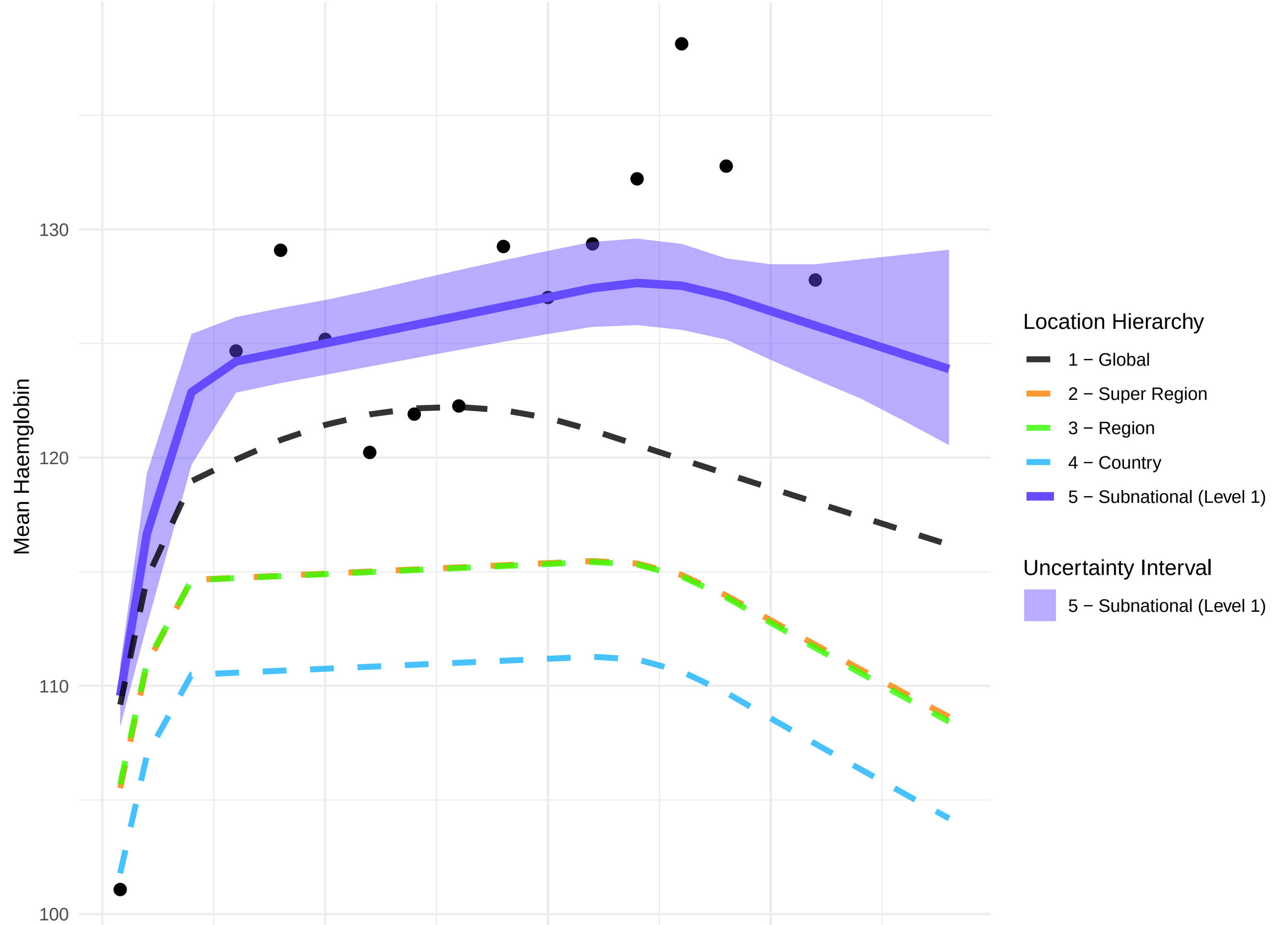

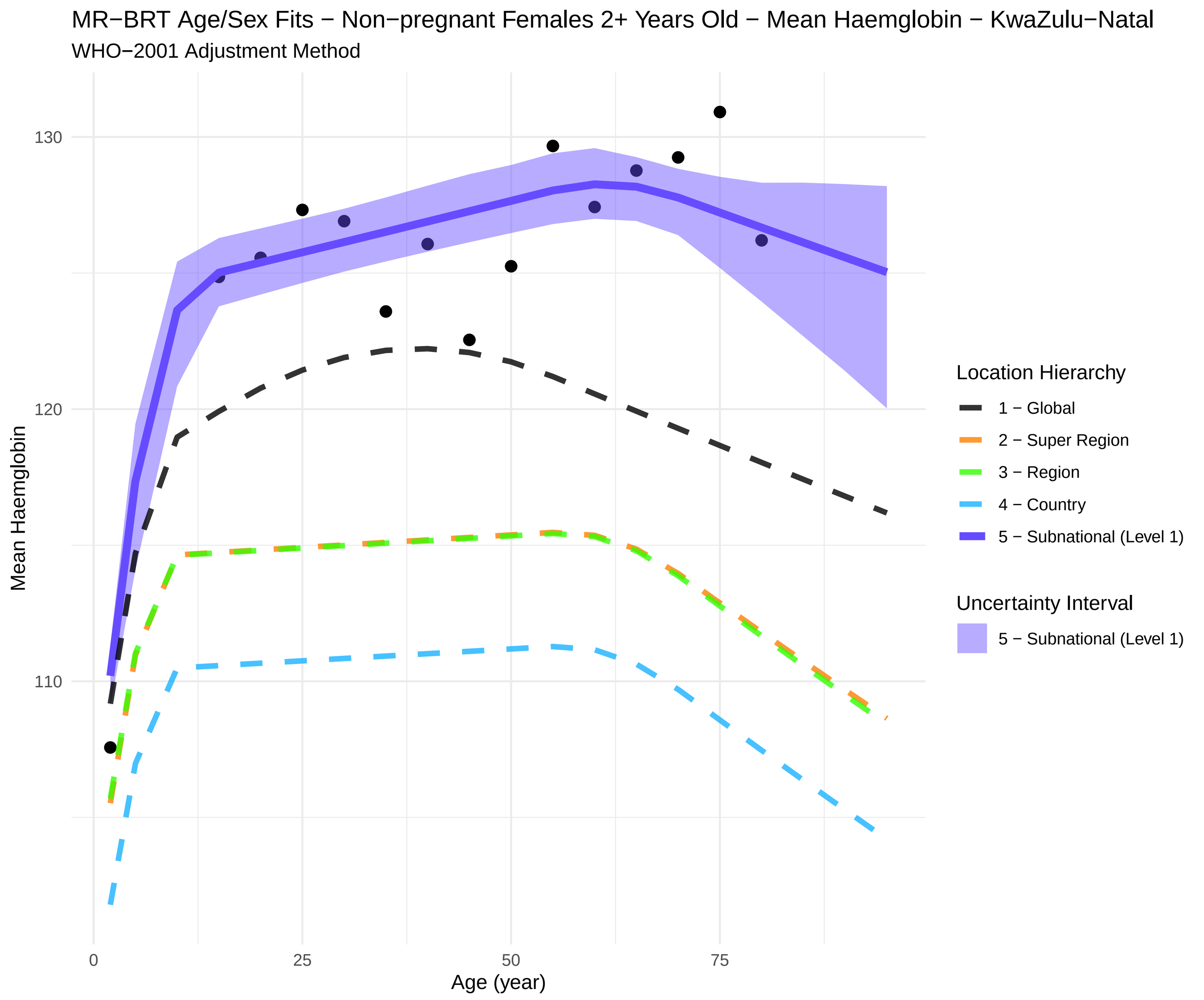
MR−BRT Age/Sex Fits − Non-pregnant Females 2+ Years Old − Mean Haemglobin − KwaZulu−Natal
WHO−2001 Adjustment Method
Mean Haemglobin
Age (year)
Location Hierarchy
1 − Global
2 − Super Region
3 − Region
4 − Country
5 − Subnational (Level 1)
Uncertainty Interval
5 − Subnational (Level 1)

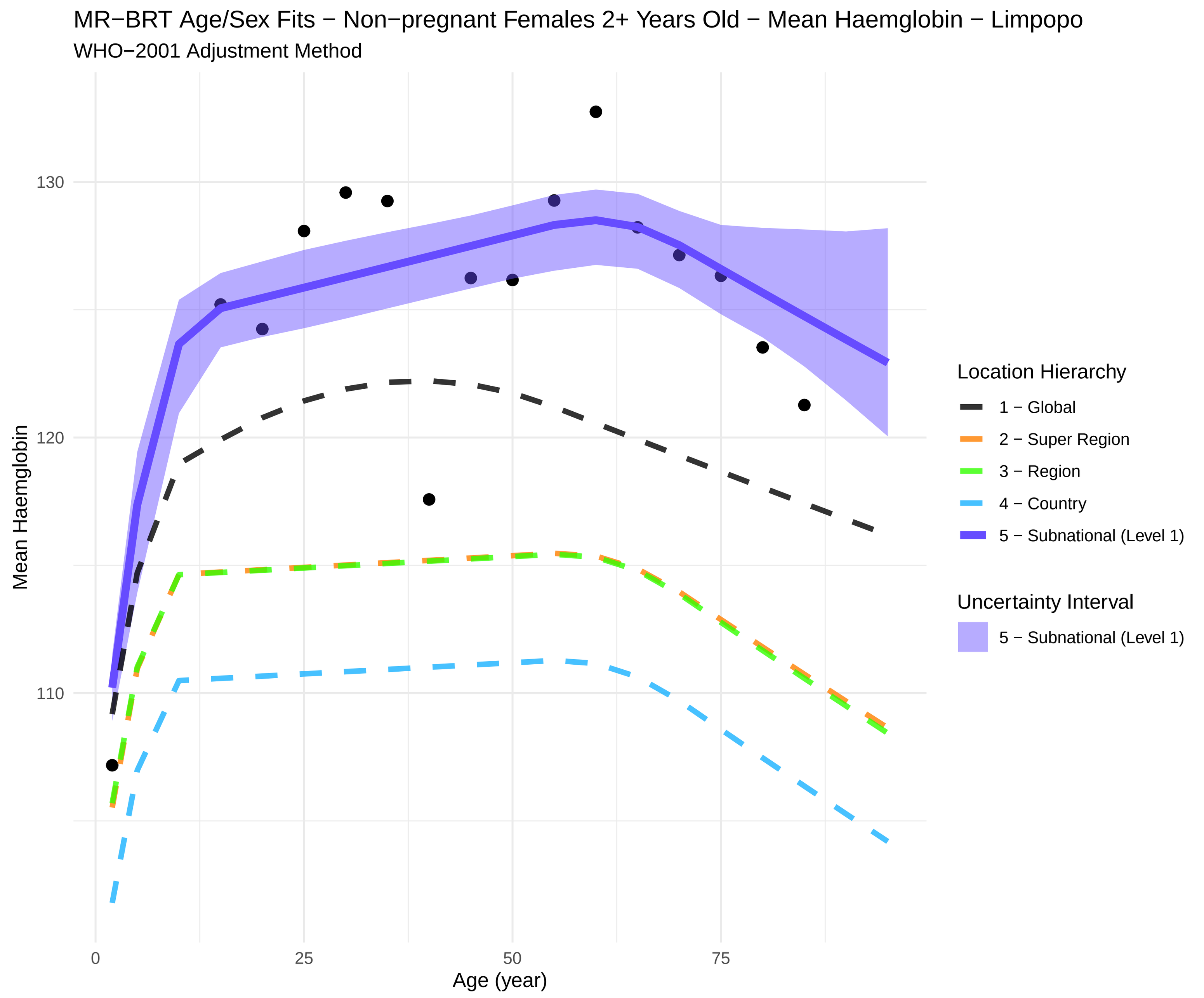
MR−BRT Age/Sex Fits − Non-pregnant Females 2+ Years Old − Mean Haemglobin − Limpopo
WHO−2001 Adjustment Method
Mean Haemglobin
Age (year)
Location Hierarchy
1 − Global
2 − Super Region
3 − Region
4 − Country
5 − Subnational (Level 1)
Uncertainty Interval
5 − Subnational (Level 1)

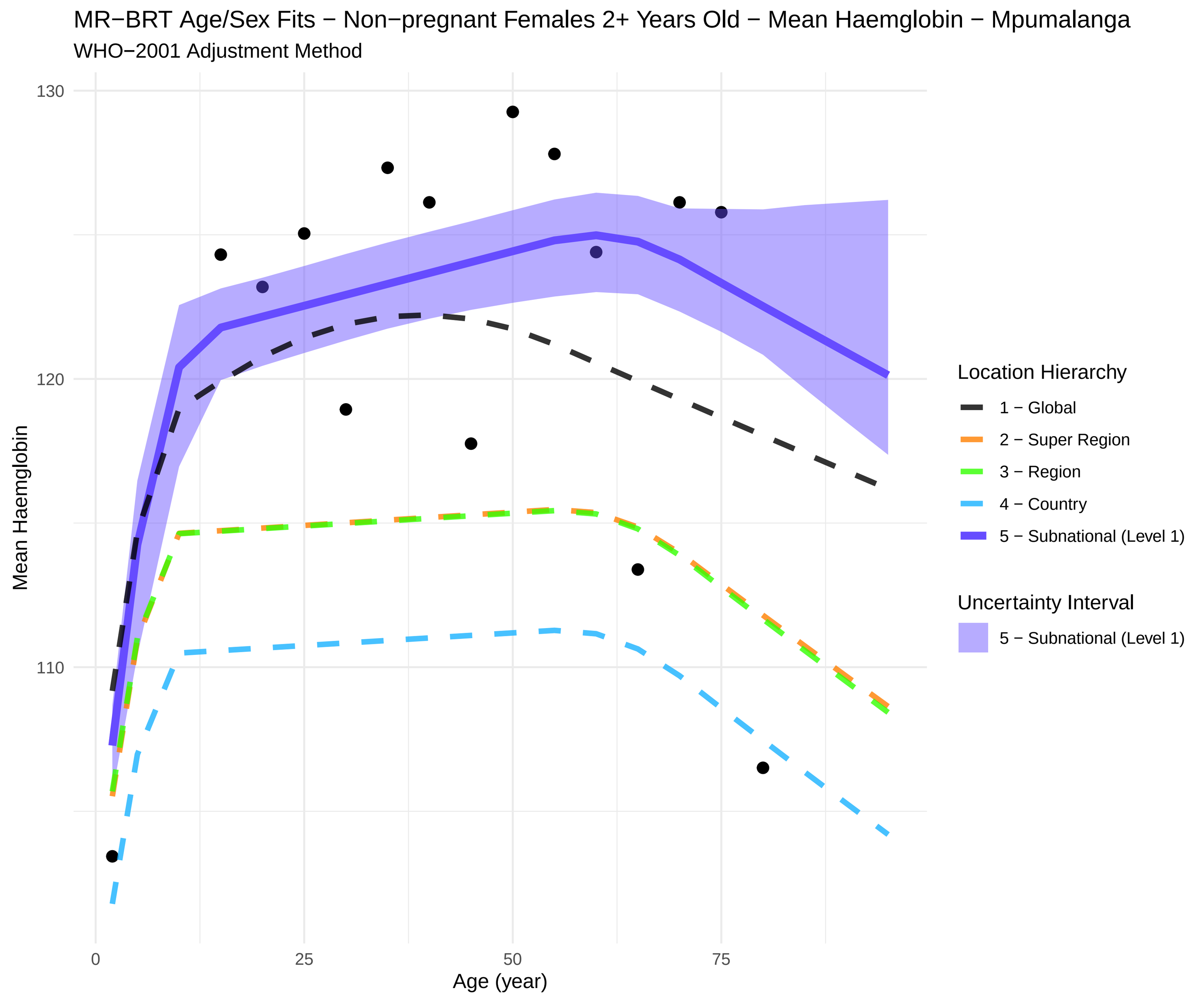
MR−BRT Age/Sex Fits − Non-pregnant Females 2+ Years Old − Mean Haemglobin − Mpumalanga
WHO−2001 Adjustment Method
Mean Haemglobin
Age (year)
Location Hierarchy
1 − Global
2 − Super Region
3 − Region
4 − Country
5 − Subnational (Level 1)
Uncertainty Interval
5 − Subnational (Level 1)

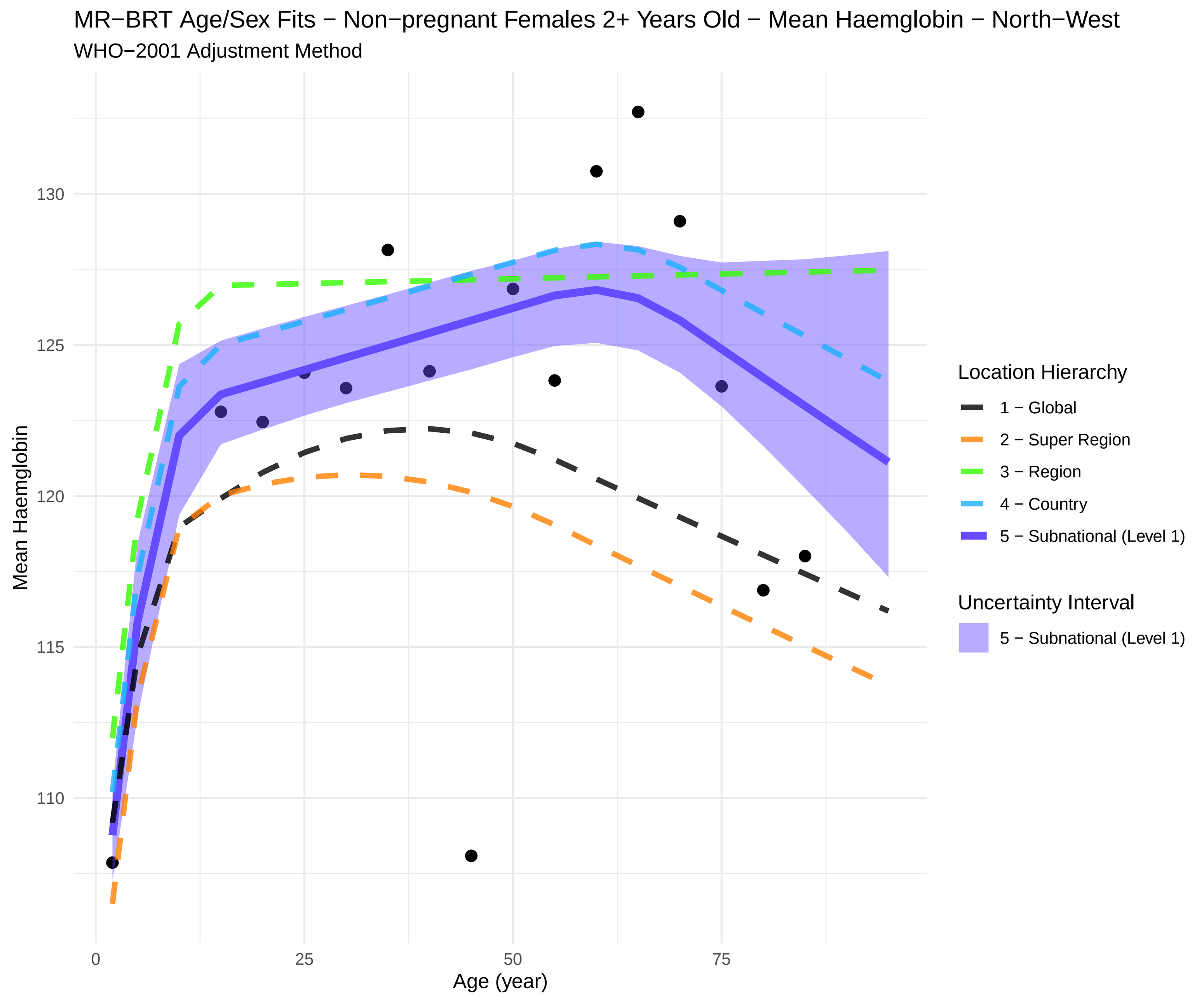
MR−BRT Age/Sex Fits − Non-pregnant Females 2+ Years Old − Mean Haemglobin − North−West
WHO−2001 Adjustment Method
Mean Haemglobin
Age (year)
Location Hierarchy
1 − Global
2 − Super Region
3 − Region
4 − Country
5 − Subnational (Level 1)
Uncertainty Interval
5 − Subnational (Level 1)

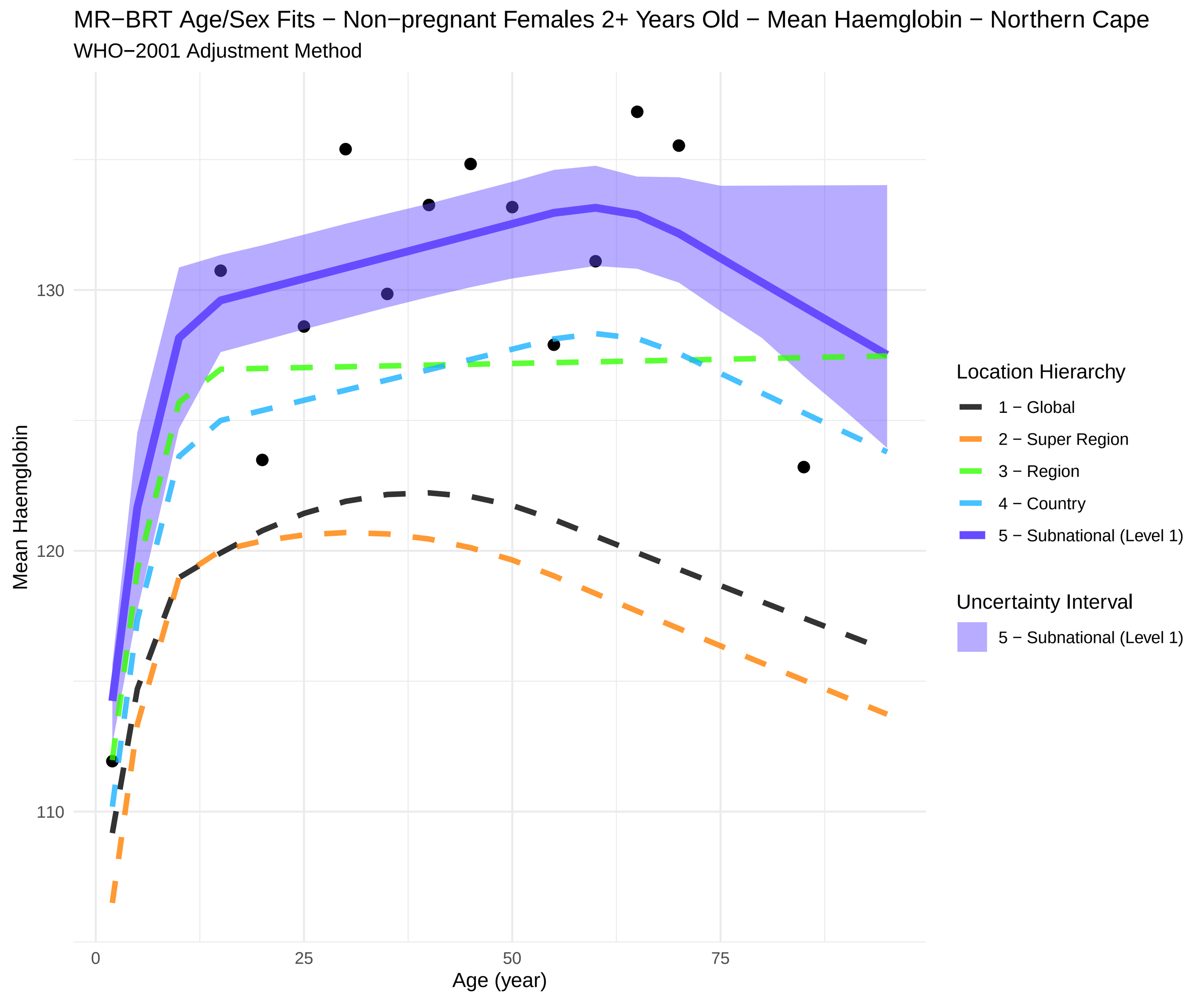
MR−BRT Age/Sex Fits − Non-pregnant Females 2+ Years Old − Mean Haemglobin − Northern Cape
WHO−2001 Adjustment Method
Mean Haemglobin
Age (year)
Location Hierarchy
1 − Global
2 − Super Region
3 − Region
4 − Country
5 − Subnational (Level 1)
Uncertainty Interval
5 − Subnational (Level 1)

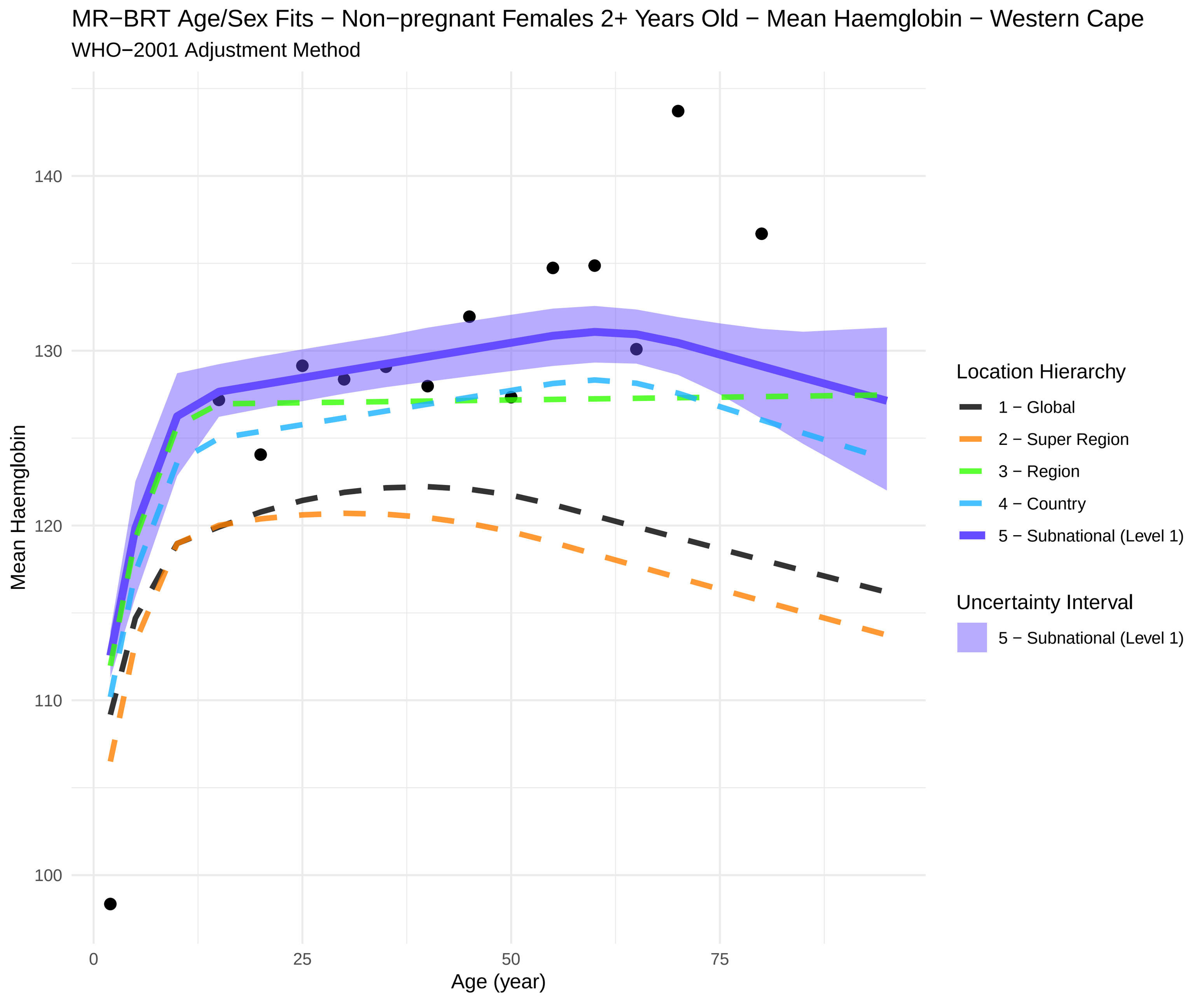
MR−BRT Age/Sex Fits − Non-pregnant Females 2+ Years Old − Mean Haemglobin − Western Cape
WHO−2001 Adjustment Method
Mean Haemglobin
Age (year)
Location Hierarchy
1 − Global
2 − Super Region
3 − Region
4 − Country
5 − Subnational (Level 1)
Uncertainty Interval
5 − Subnational (Level 1)

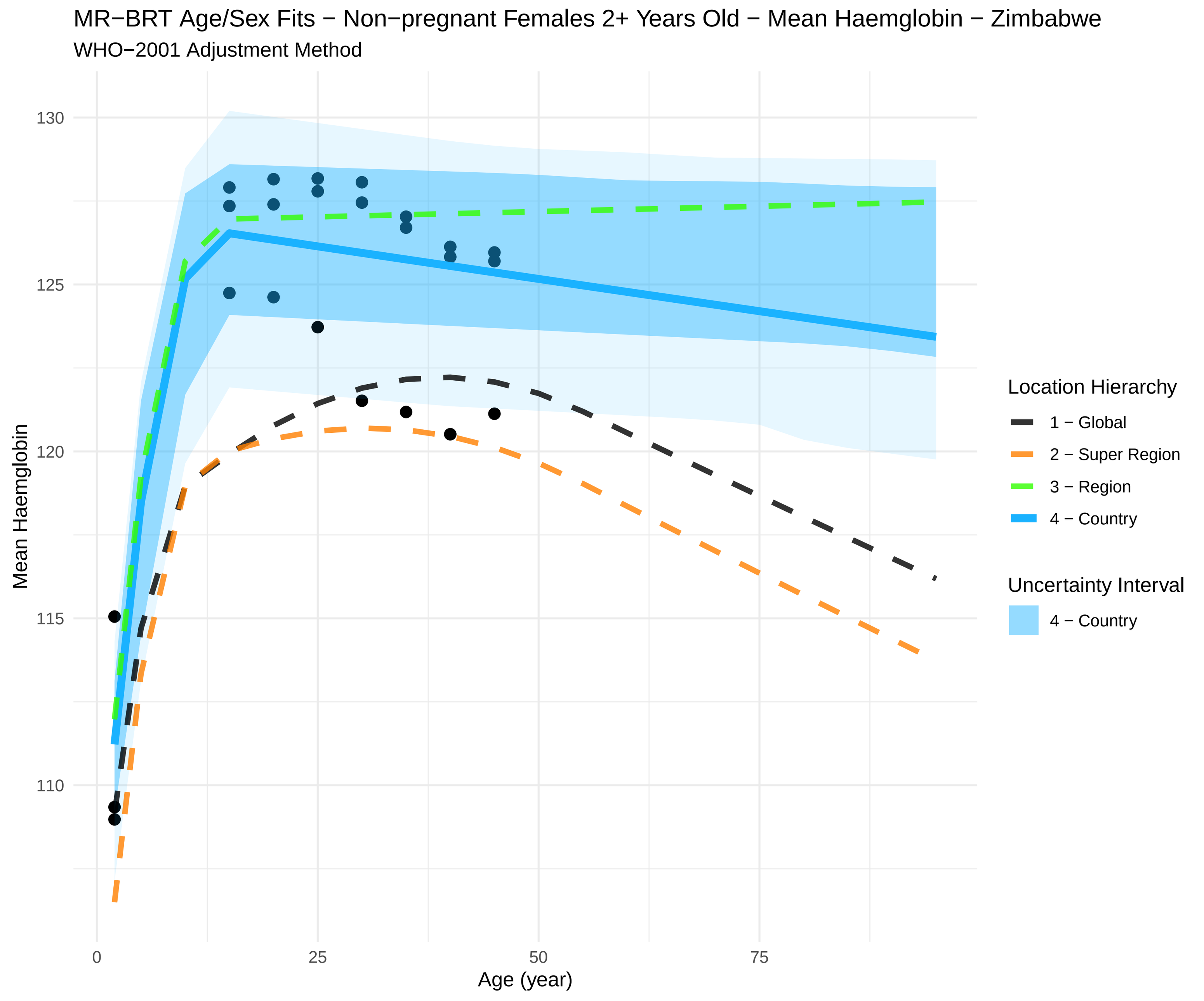
MR−BRT Age/Sex Fits − Non-pregnant Females 2+ Years Old − Mean Haemglobin − Zimbabwe
WHO−2001 Adjustment Method
Mean Haemglobin
Age (year)
Location Hierarchy
1 − Global
2 − Super Region
3 − Region
4 − Country
Uncertainty Interval
4 − Country

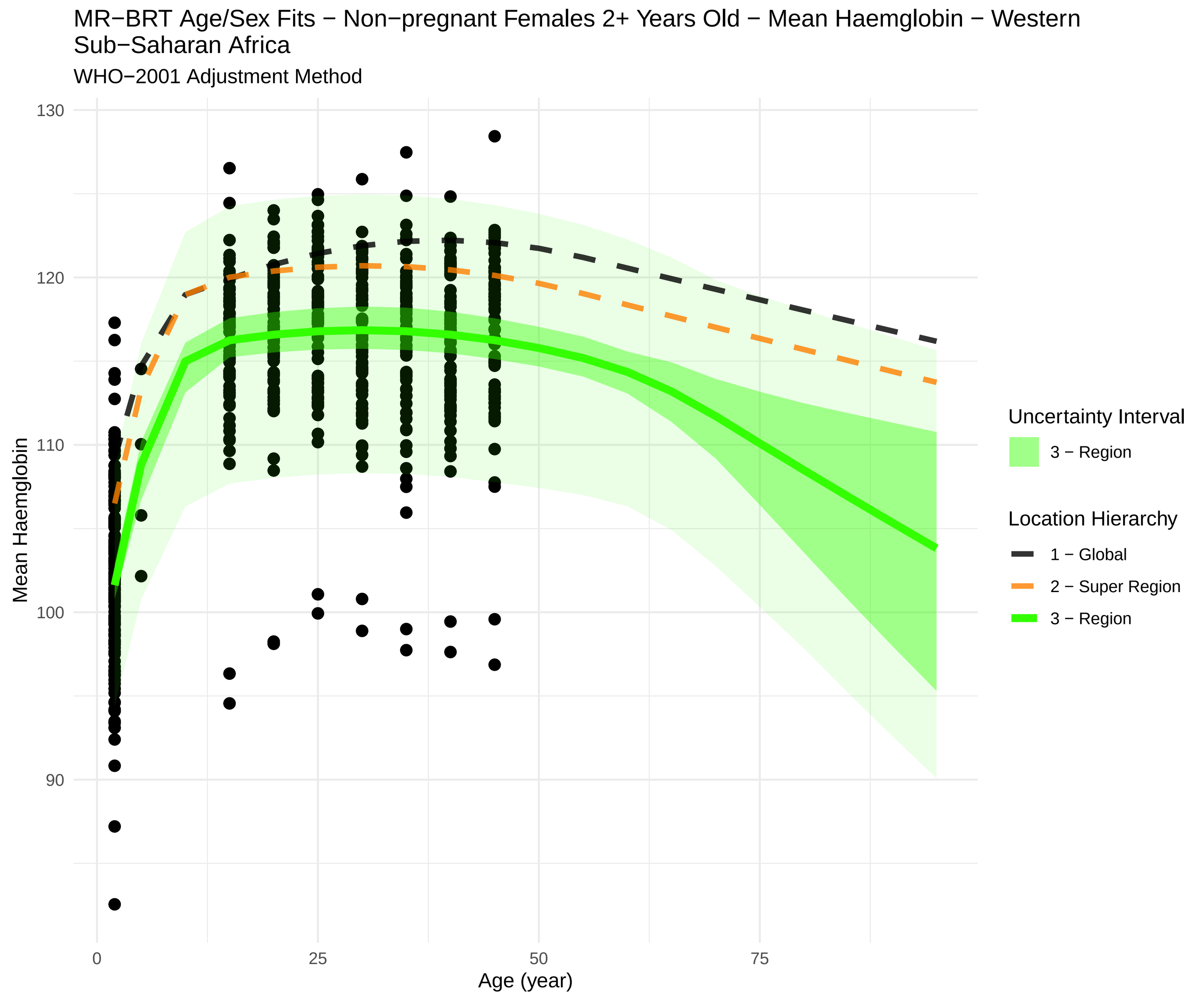
MR−BRT Age/Sex Fits − Non-pregnant Females 2+ Years Old − Mean Haemglobin − Western Sub−Saharan Africa
WHO−2001 Adjustment Method
Mean Haemglobin
Age (year)
Uncertainty Interval
3 − Region
Location Hierarchy
1 − Global
2 − Super Region
3 − Region

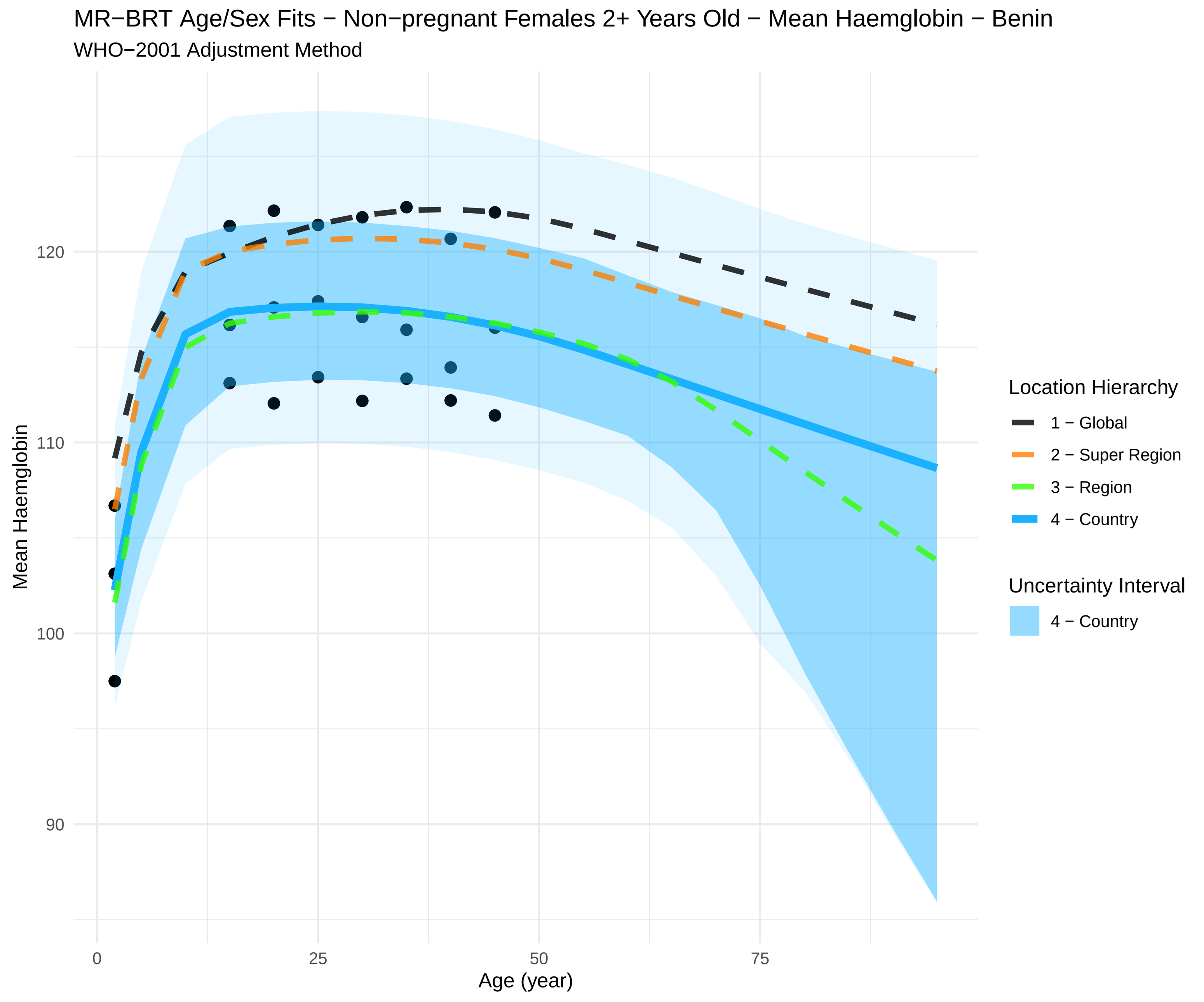
MR−BRT Age/Sex Fits − Non-pregnant Females 2+ Years Old − Mean Haemglobin − Benin
WHO−2001 Adjustment Method
Mean Haemglobin
Age (year)
Location Hierarchy
1 − Global
2 − Super Region
3 − Region
4 − Country
Uncertainty Interval
4 − Country

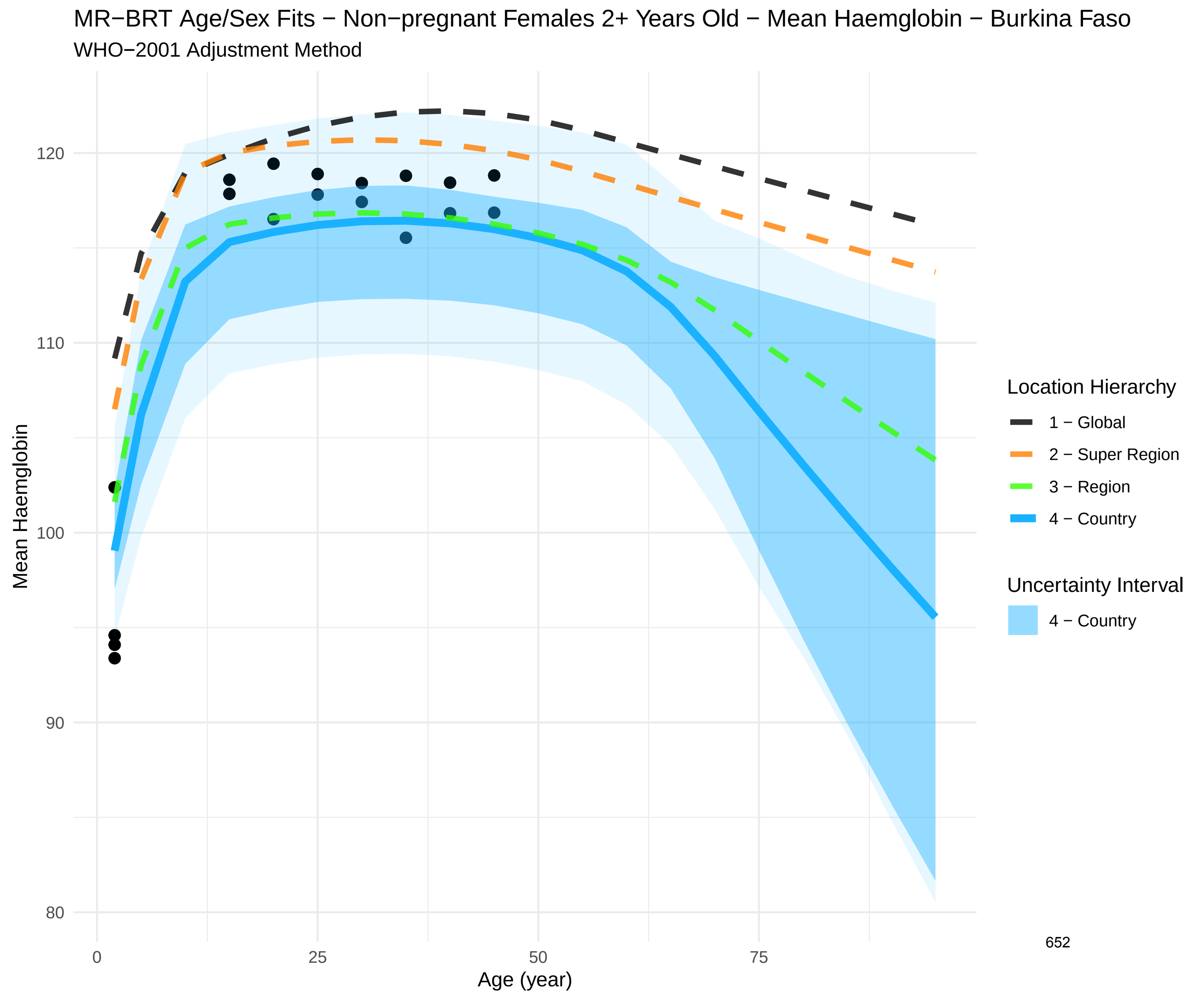
MR−BRT Age/Sex Fits − Non-pregnant Females 2+ Years Old − Mean Haemglobin − Burkina Faso
WHO−2001 Adjustment Method
Mean Haemglobin
Age (year)
Location Hierarchy
1 − Global
2 − Super Region
3 − Region
4 − Country
Uncertainty Interval
4 − Country

MR−BRT Age/Sex Fits − Non−pregnant Females 2+ Years Old − Mean Haemglobin − Cameroon
WHO−2001 Adjustment Method

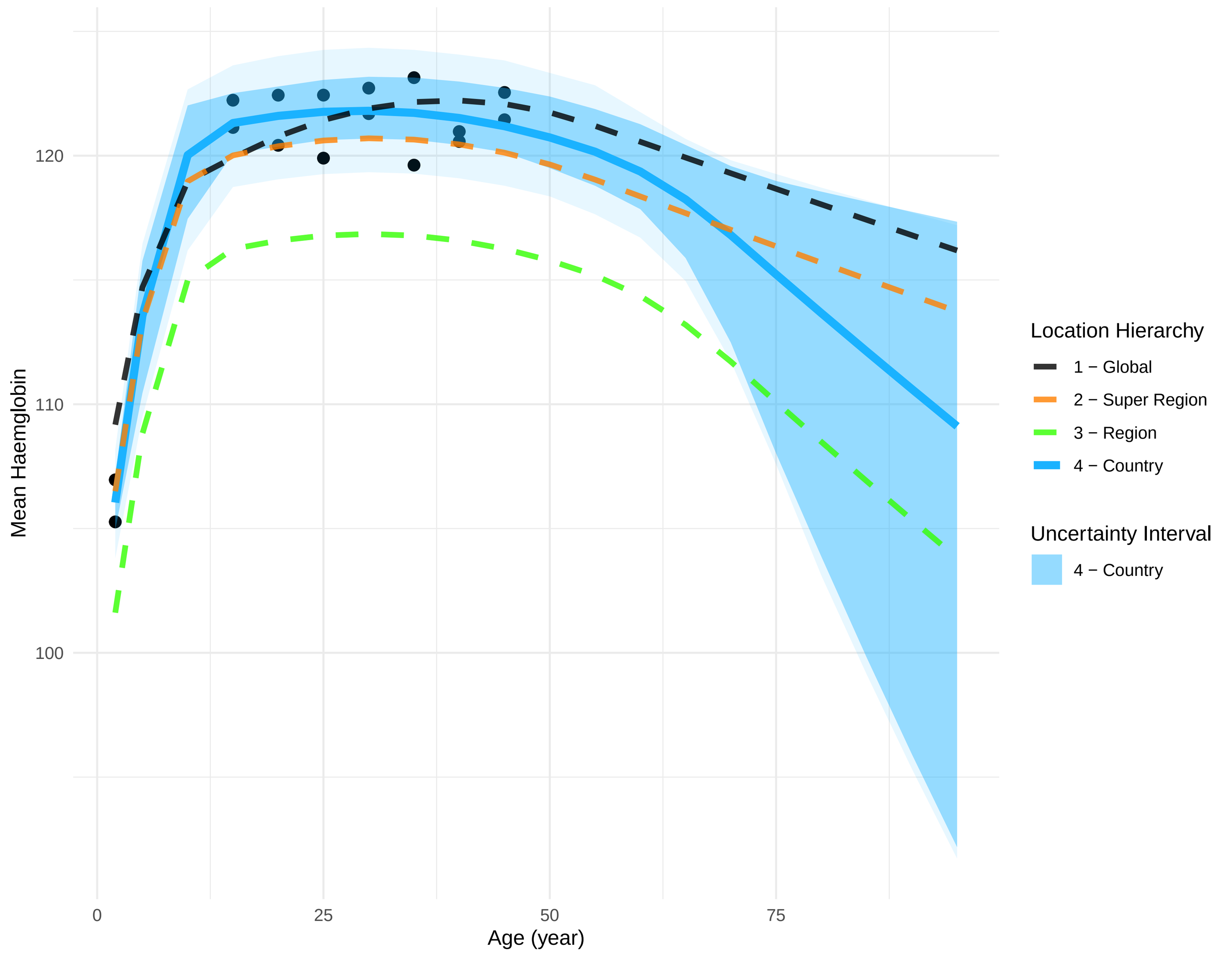

MR−BRT Age/Sex Fits − Non−pregnant Females 2+ Years Old − Mean Haemglobin − Côte d'Ivoire
WHO−2001 Adjustment Method

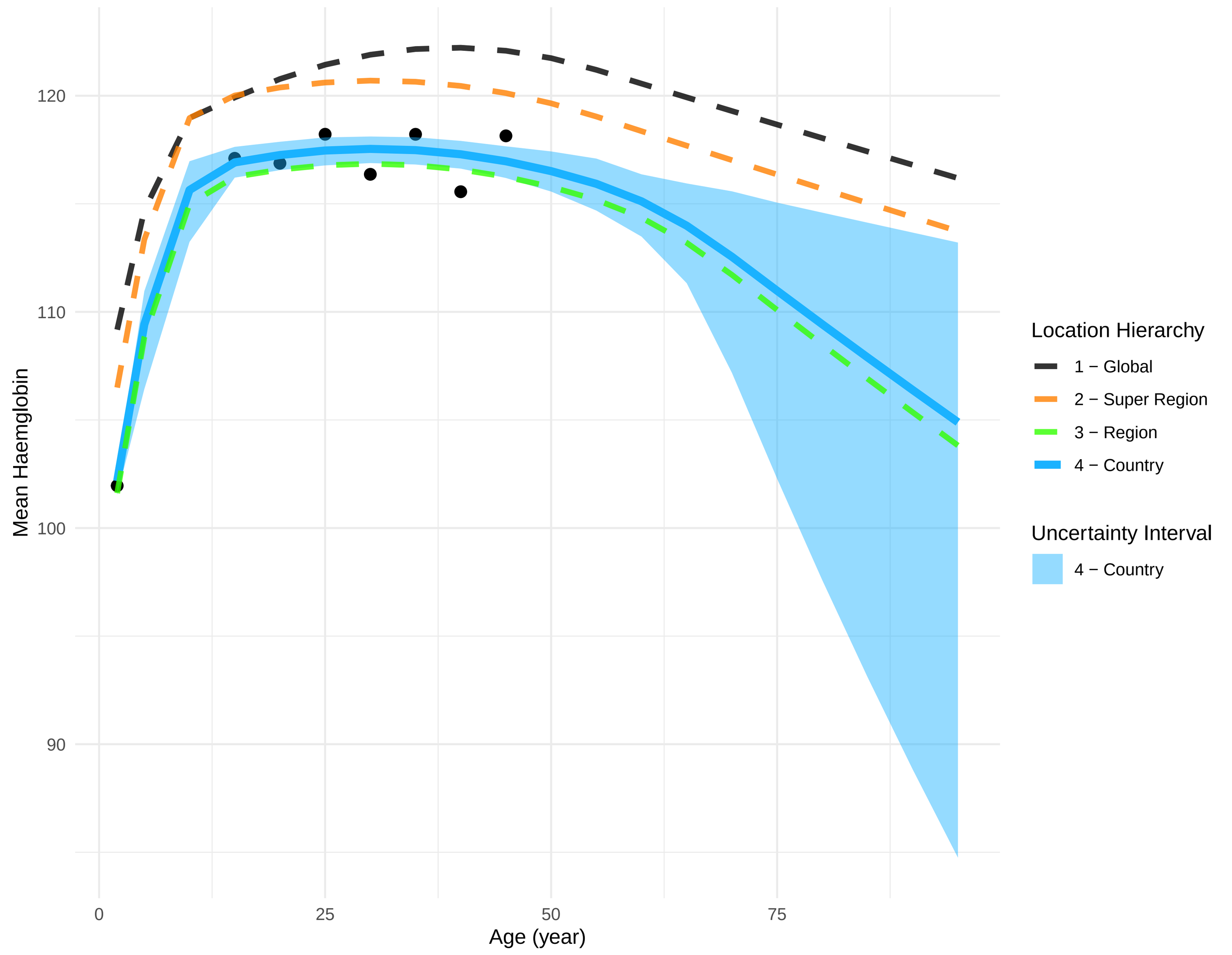

MR−BRT Age/Sex Fits − Non−pregnant Females 2+ Years Old − Mean Haemglobin − Gambia
WHO−2001 Adjustment Method

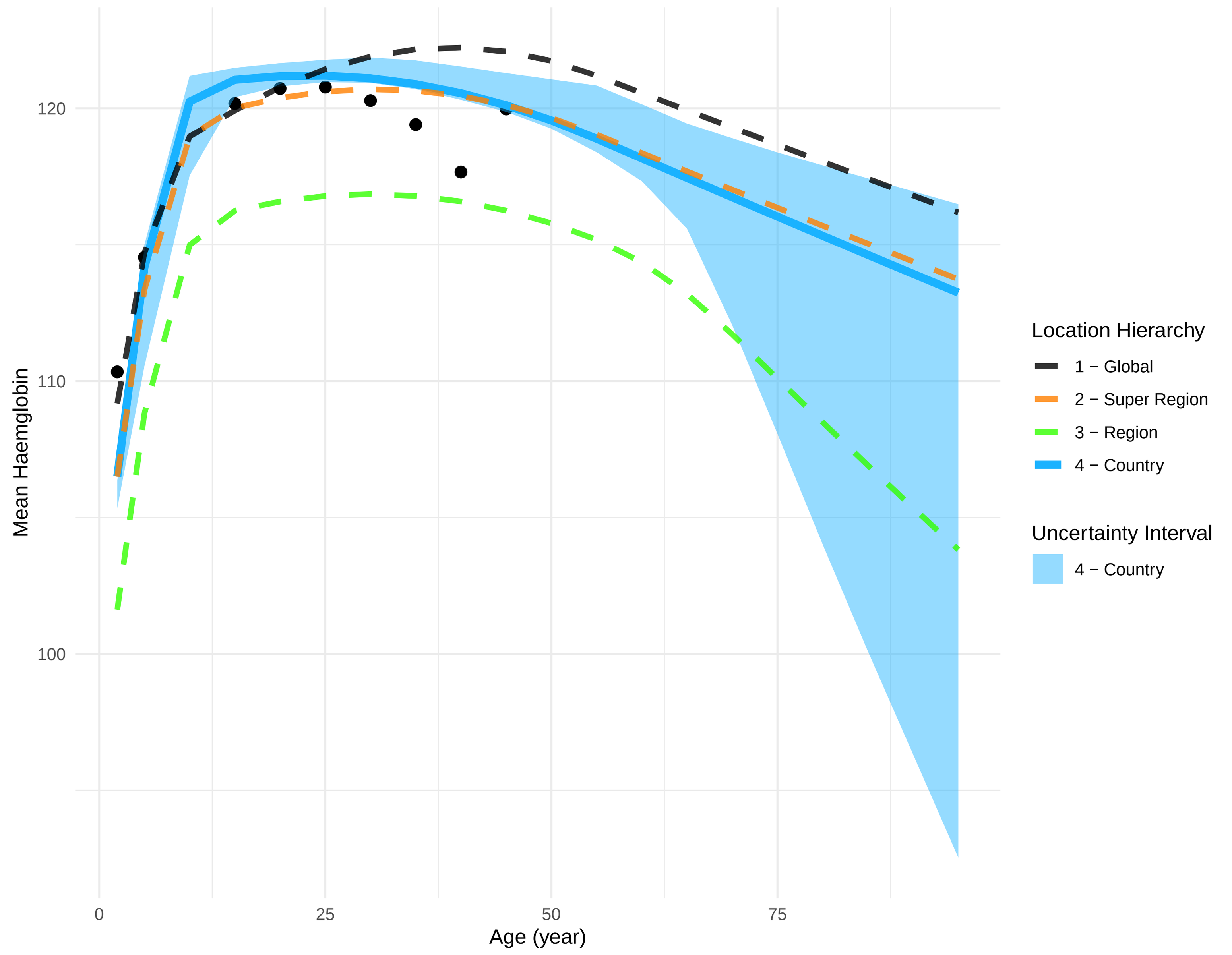

MR−BRT Age/Sex Fits − Non−pregnant Females 2+ Years Old − Mean Haemglobin − Ghana
WHO−2001 Adjustment Method

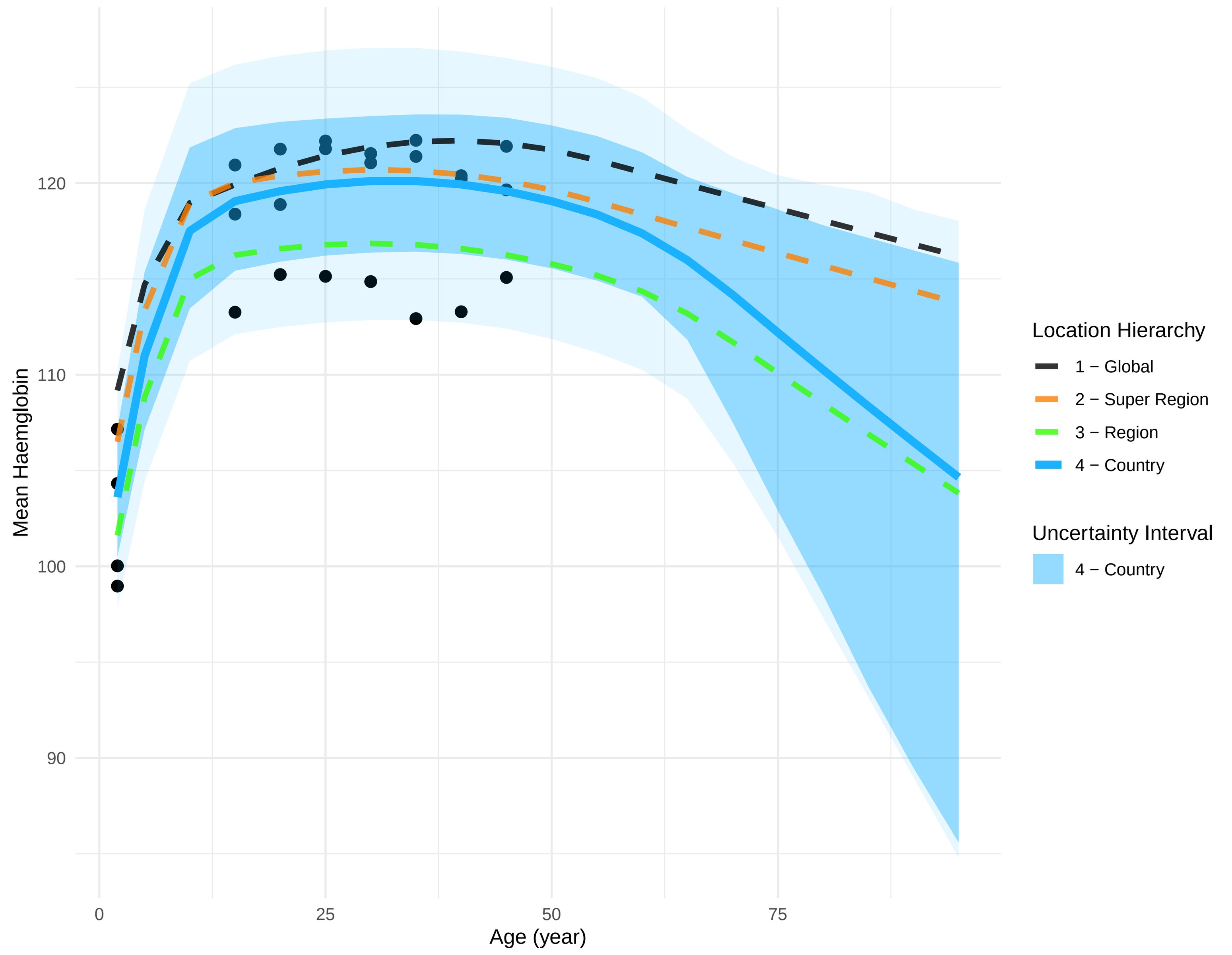

MR−BRT Age/Sex Fits − Non−pregnant Females 2+ Years Old − Mean Haemglobin − Guinea
WHO−2001 Adjustment Method

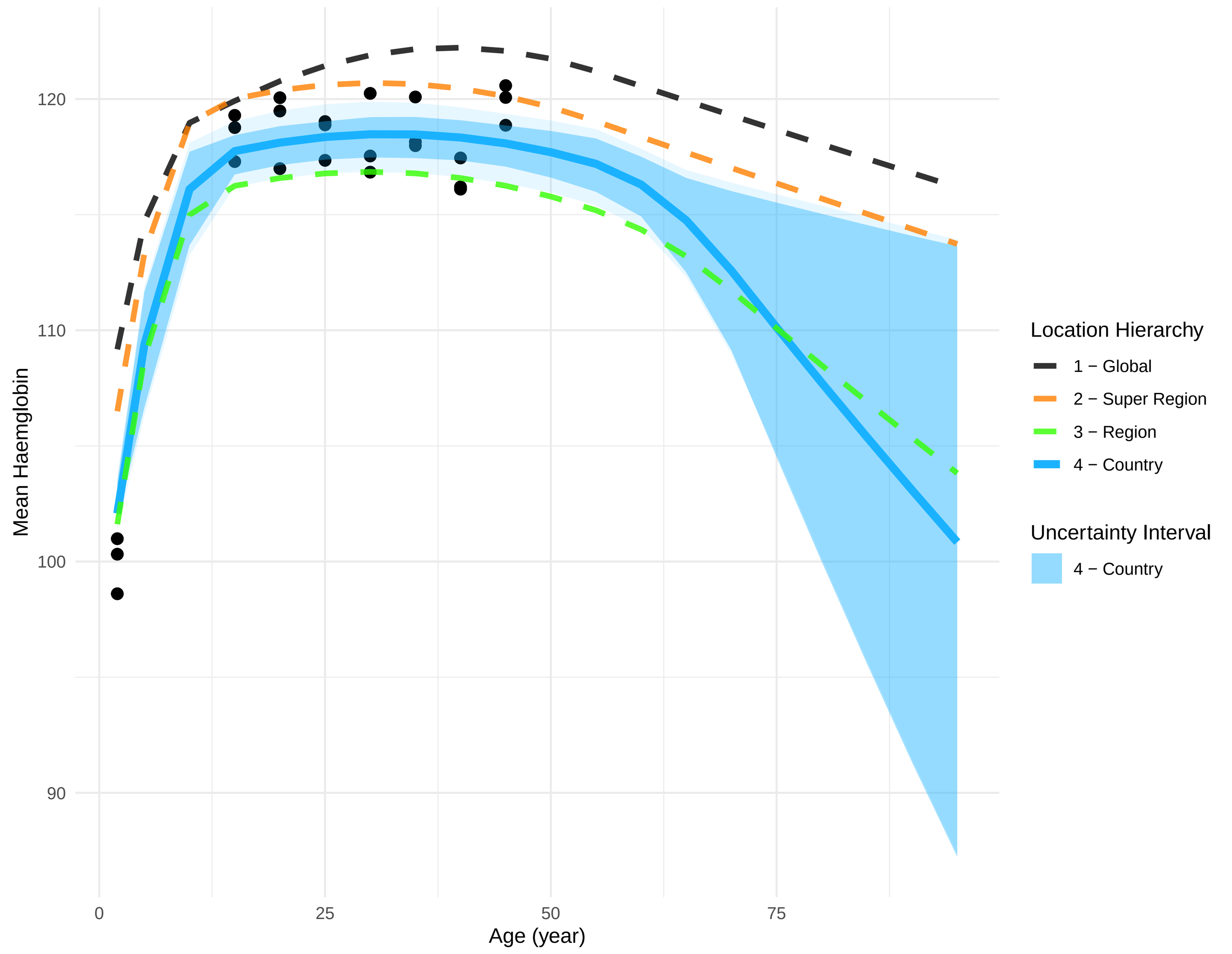

MR−BRT Age/Sex Fits − Non−pregnant Females 2+ Years Old − Mean Haemglobin − Liberia
WHO−2001 Adjustment Method

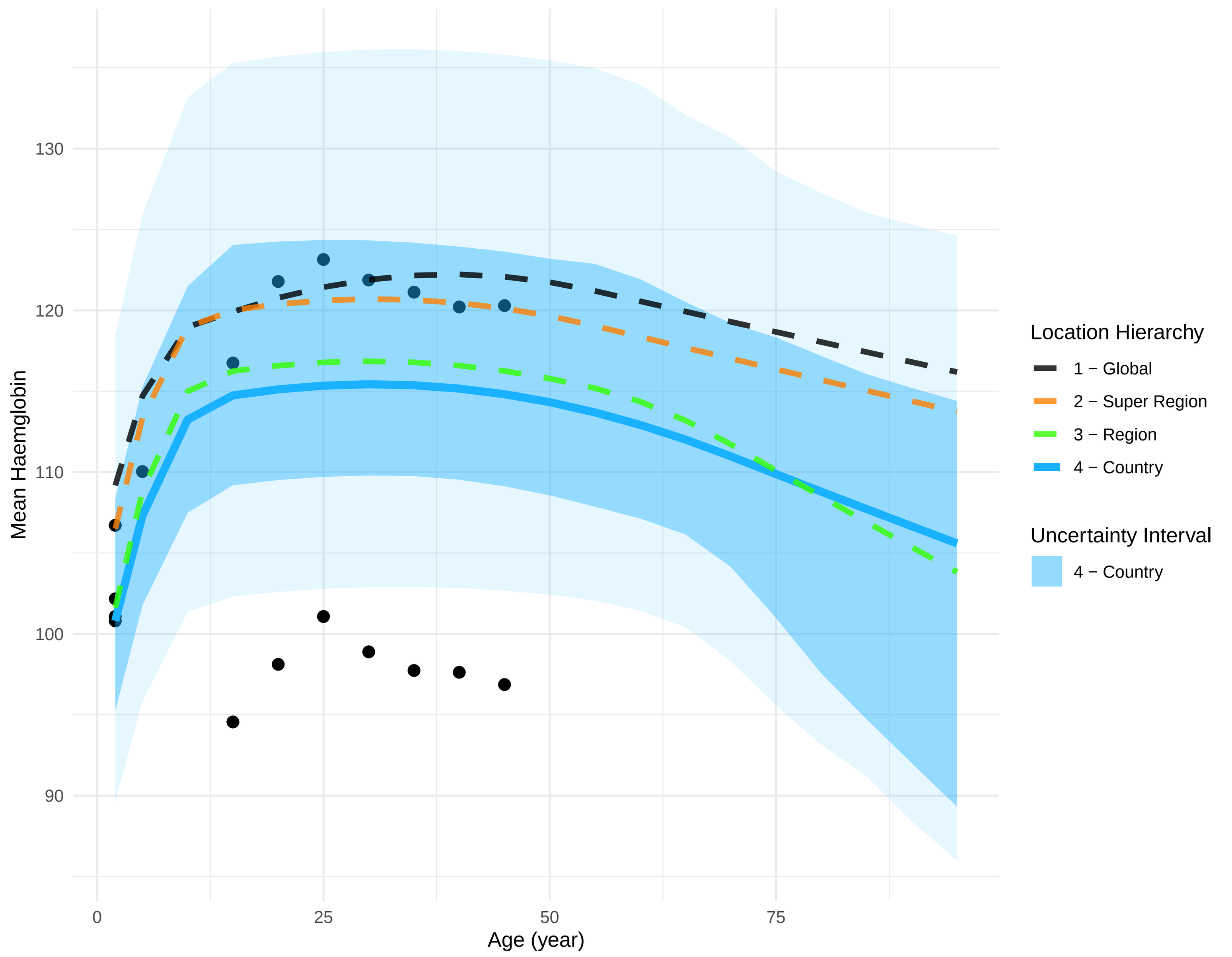

MR−BRT Age/Sex Fits − Non−pregnant Females 2+ Years Old − Mean Haemglobin − Mali
WHO−2001 Adjustment Method

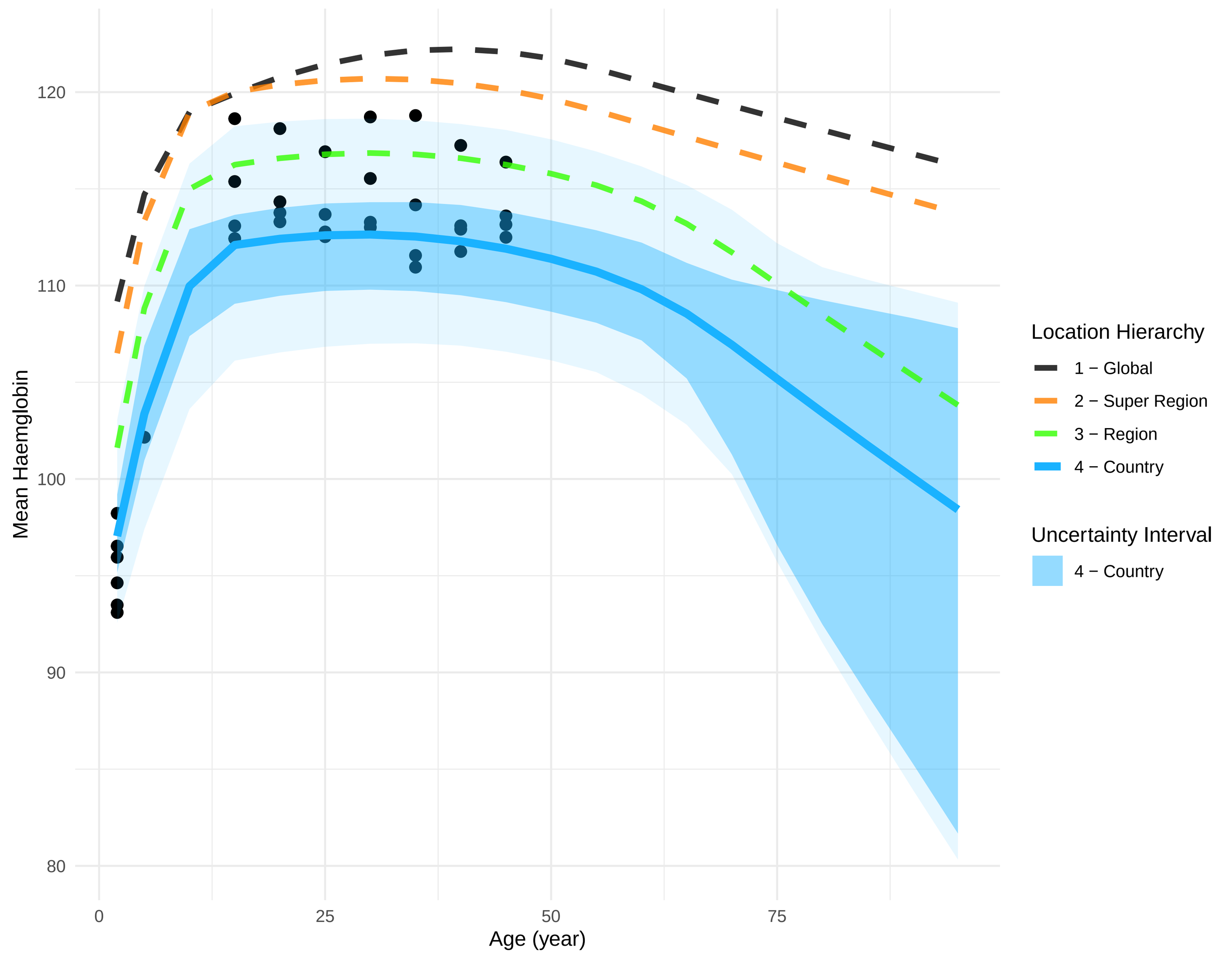

MR−BRT Age/Sex Fits − Non−pregnant Females 2+ Years Old − Mean Haemglobin − Niger
WHO−2001 Adjustment Method

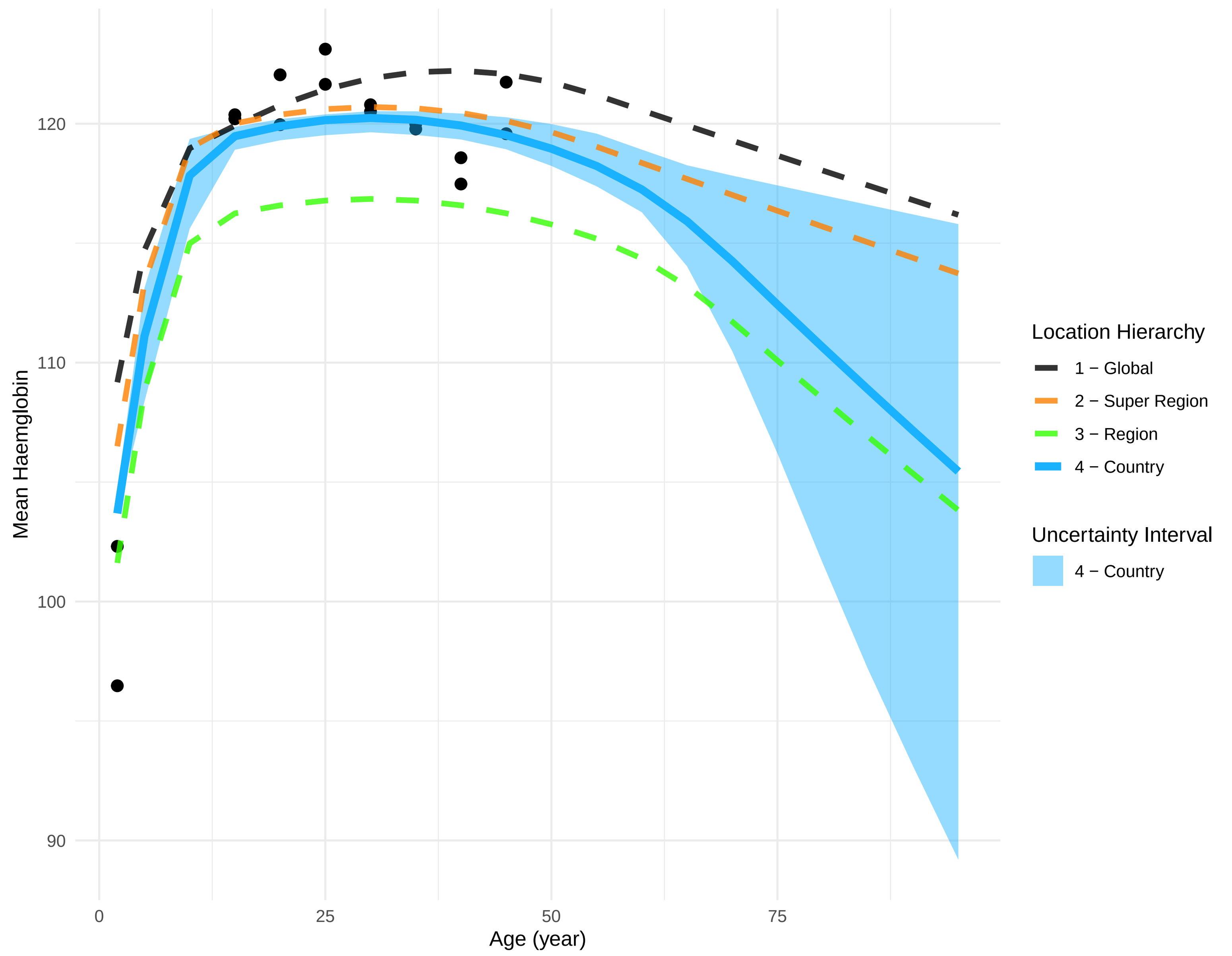

MR−BRT Age/Sex Fits − Non−pregnant Females 2+ Years Old − Mean Haemglobin − Nigeria
WHO−2001 Adjustment Method

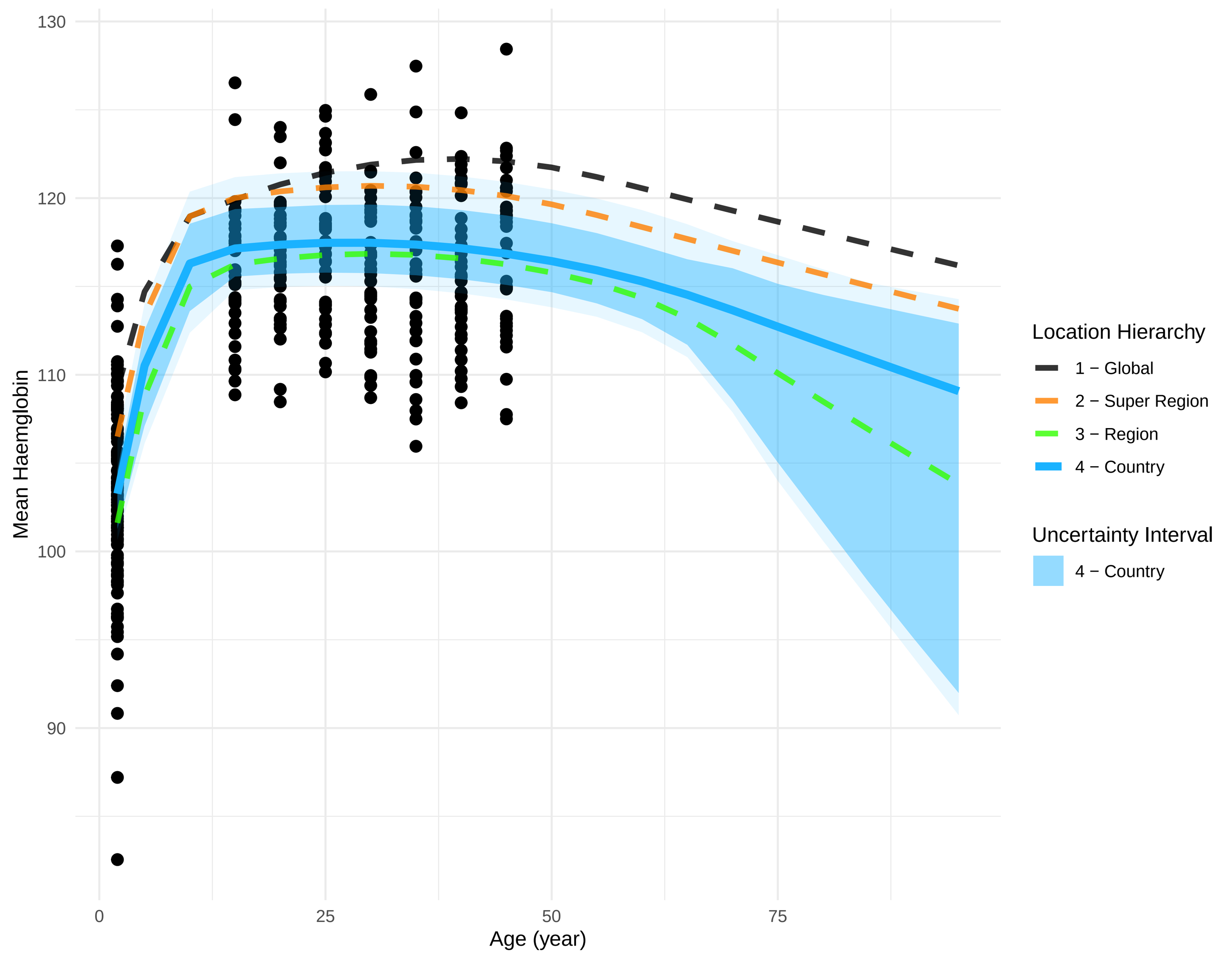

MR−BRT Age/Sex Fits − Non−pregnant Females 2+ Years Old − Mean Haemglobin − Abia
WHO−2001 Adjustment Method

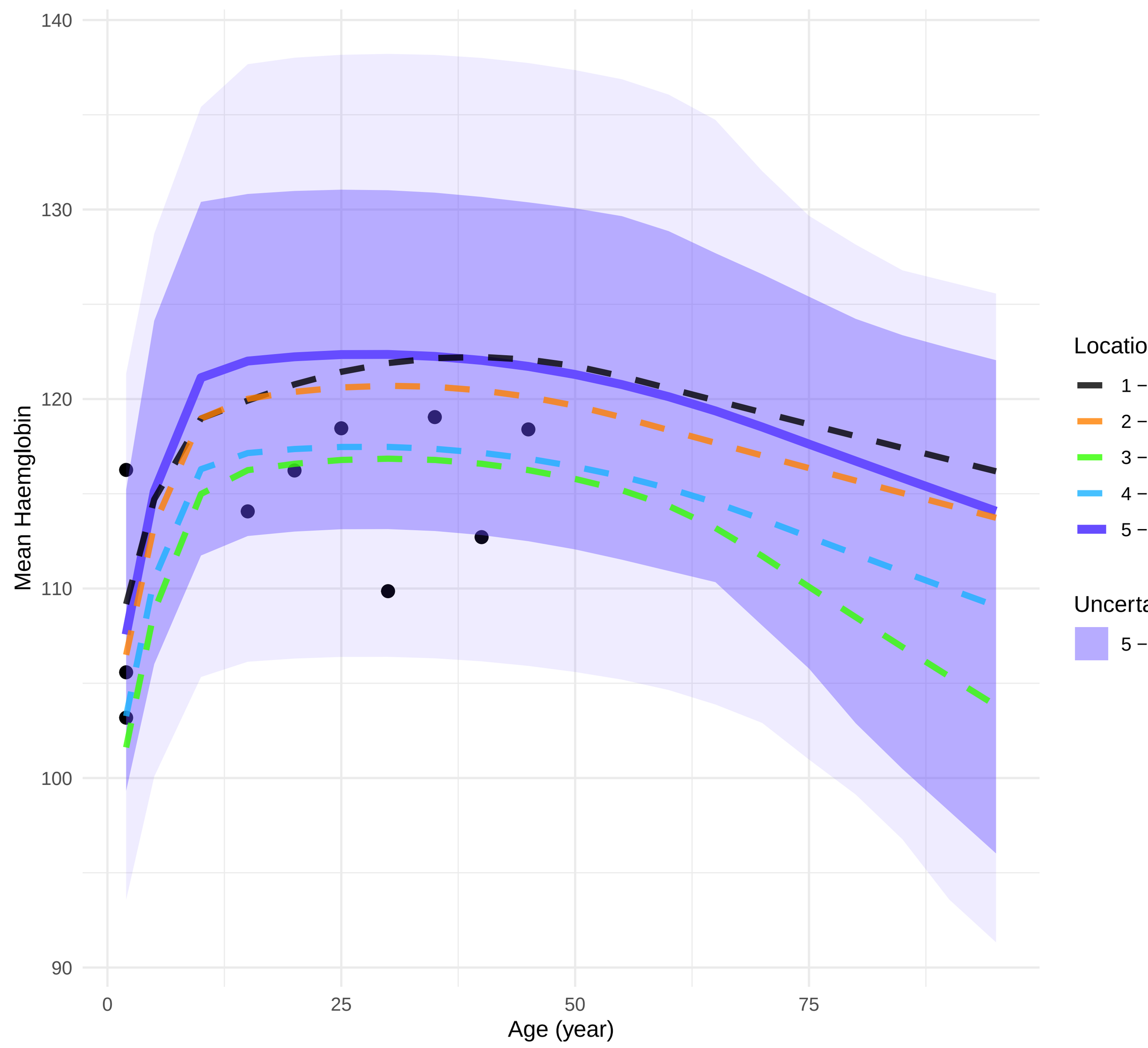

MR−BRT Age/Sex Fits − Non−pregnant Females 2+ Years Old − Mean Haemglobin − Adamawa
WHO−2001 Adjustment Method

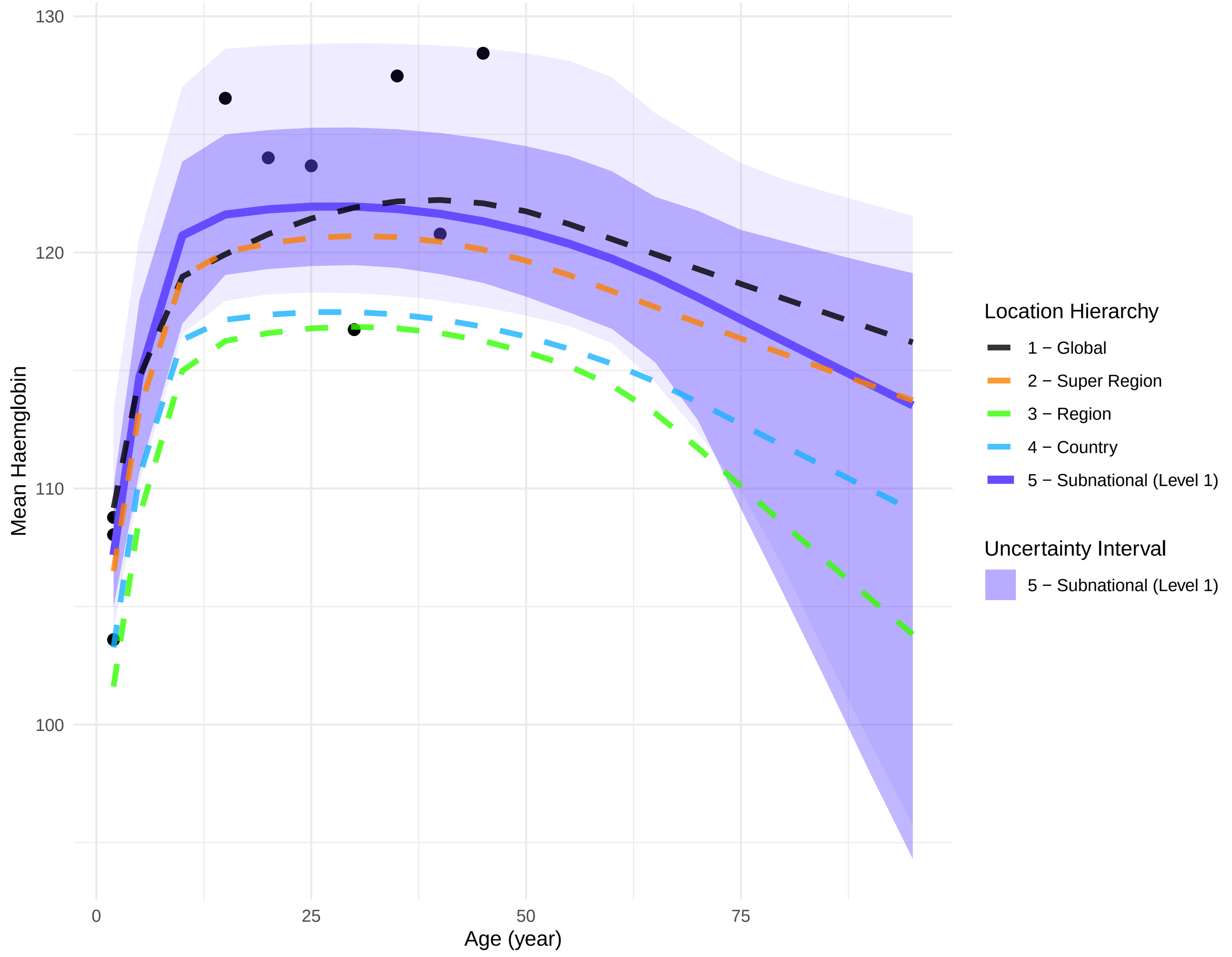

MR−BRT Age/Sex Fits − Non−pregnant Females 2+ Years Old − Mean Haemglobin − Akwa Ibom
WHO−2001 Adjustment Method

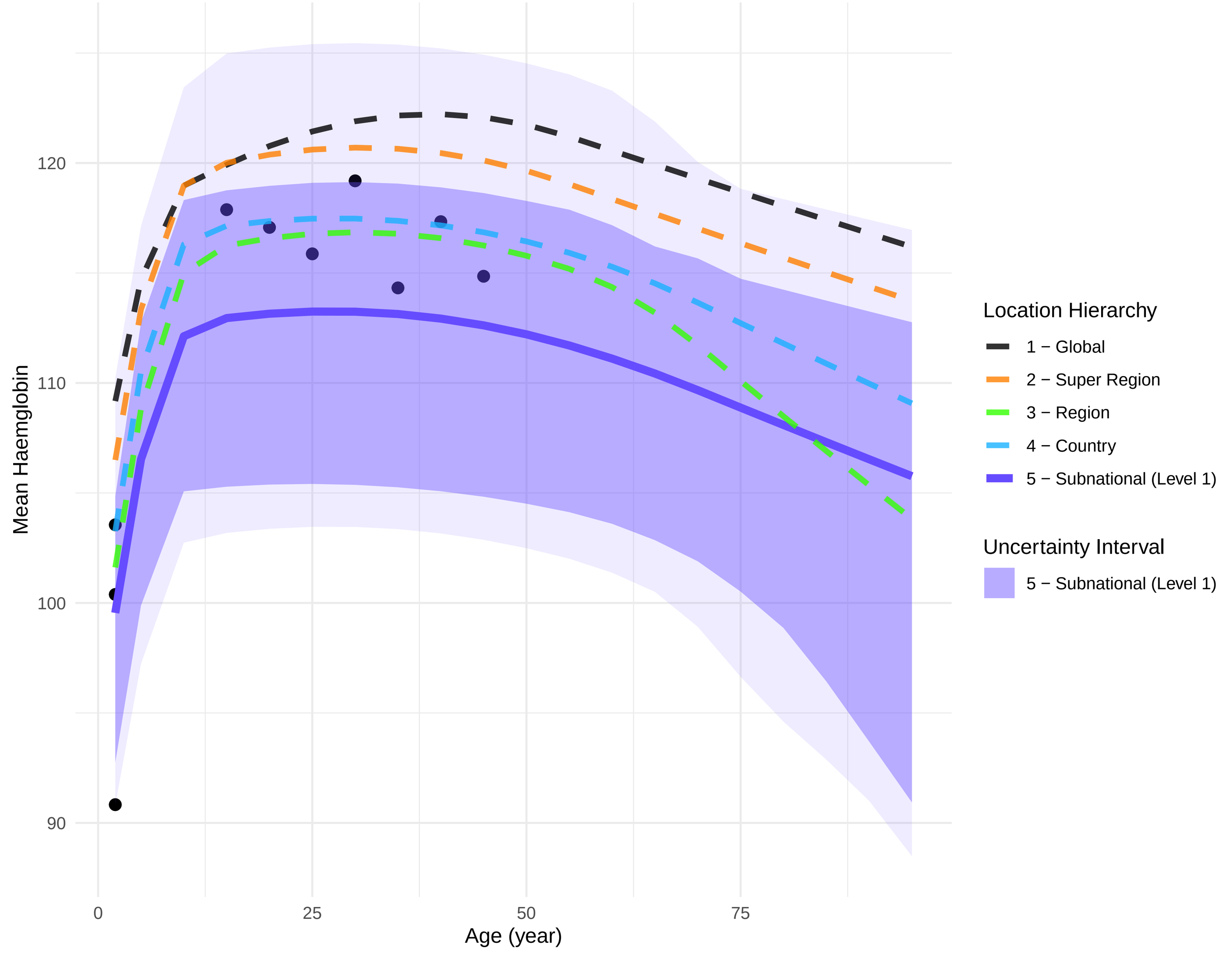

MR−BRT Age/Sex Fits − Non−pregnant Females 2+ Years Old − Mean Haemglobin − Anambra
WHO−2001 Adjustment Method

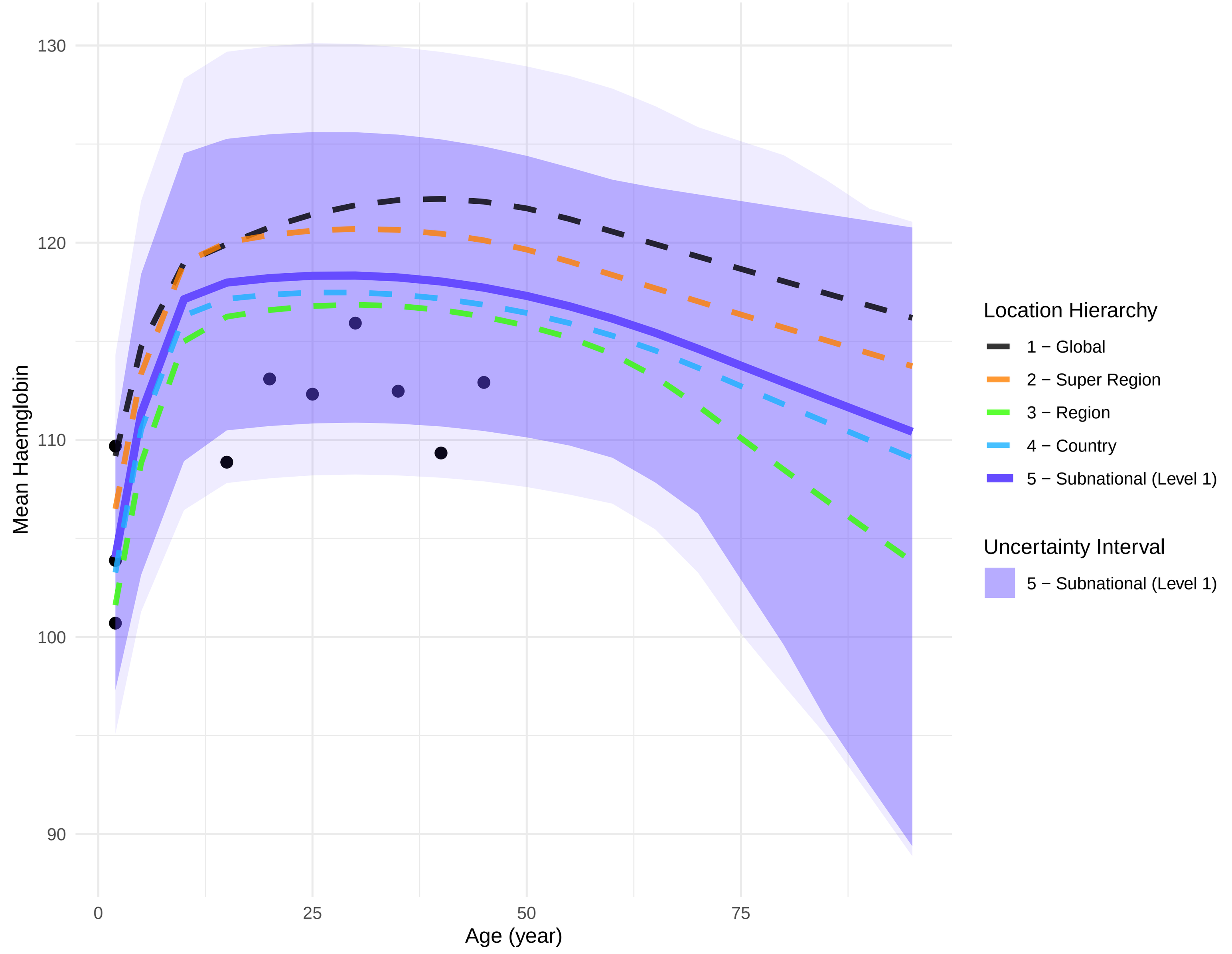

MR−BRT Age/Sex Fits − Non−pregnant Females 2+ Years Old − Mean Haemglobin − Bauchi
WHO−2001 Adjustment Method

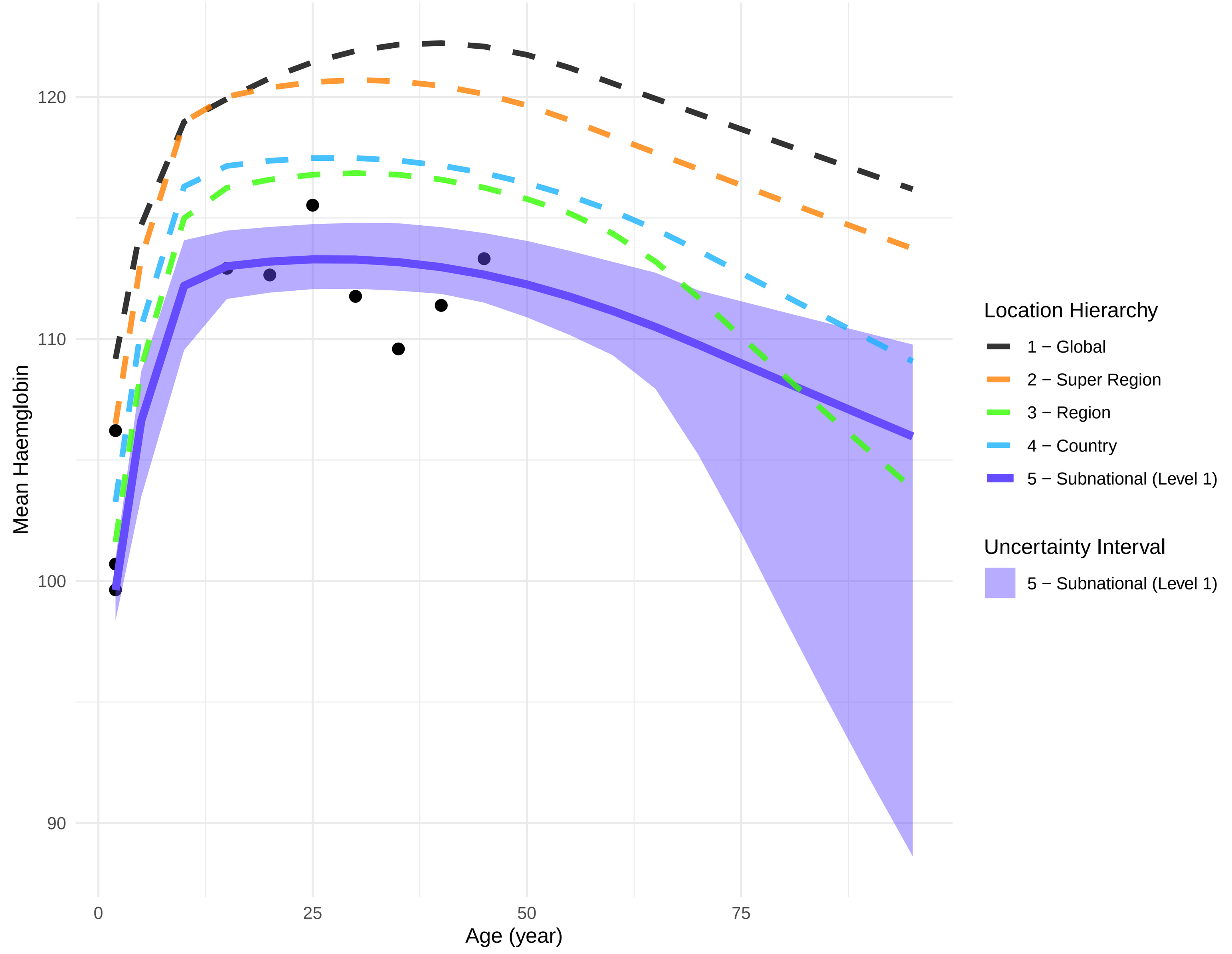

MR−BRT Age/Sex Fits − Non−pregnant Females 2+ Years Old − Mean Haemglobin − Bayelsa
WHO−2001 Adjustment Method

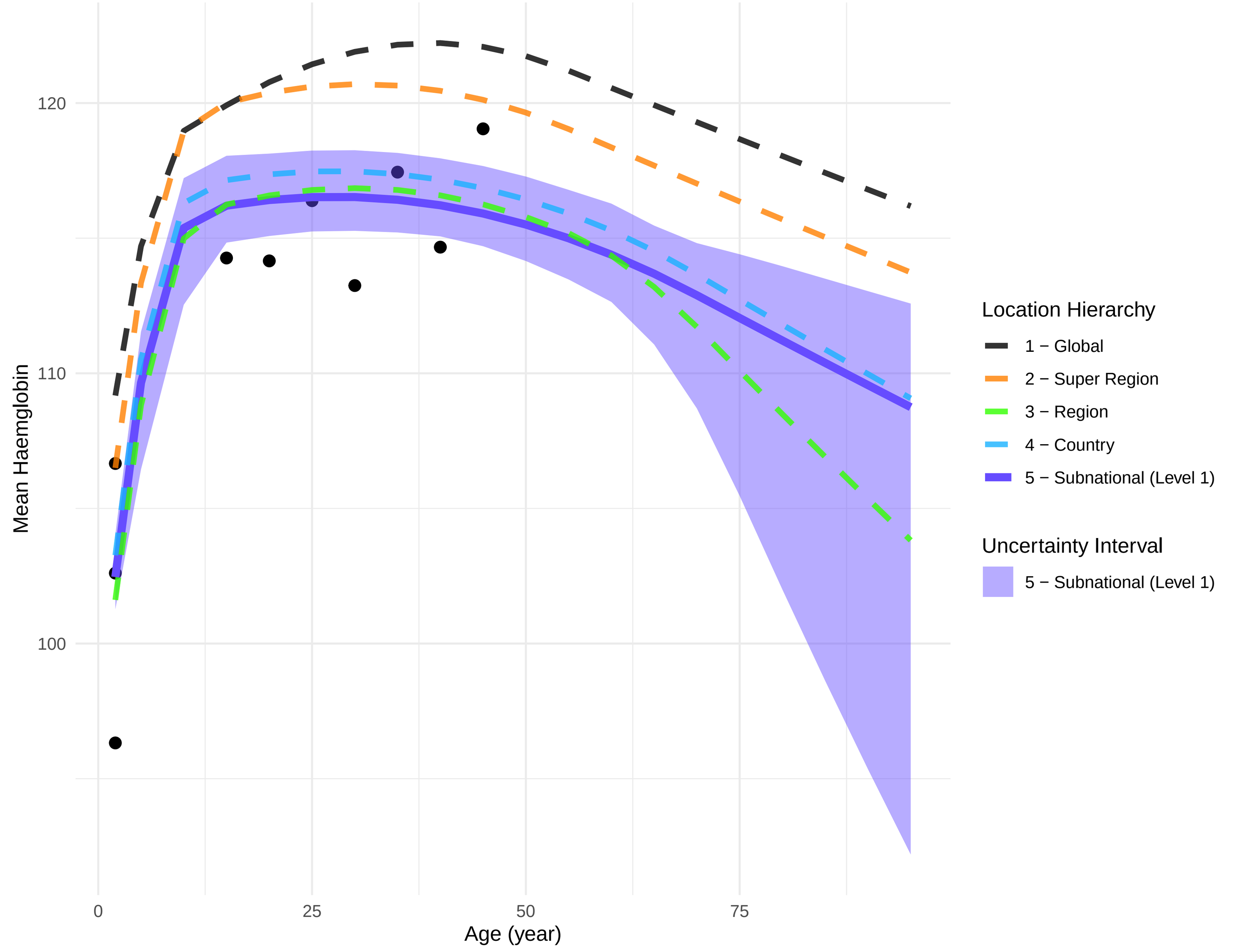

MR−BRT Age/Sex Fits − Non−pregnant Females 2+ Years Old − Mean Haemglobin − Benue
WHO−2001 Adjustment Method

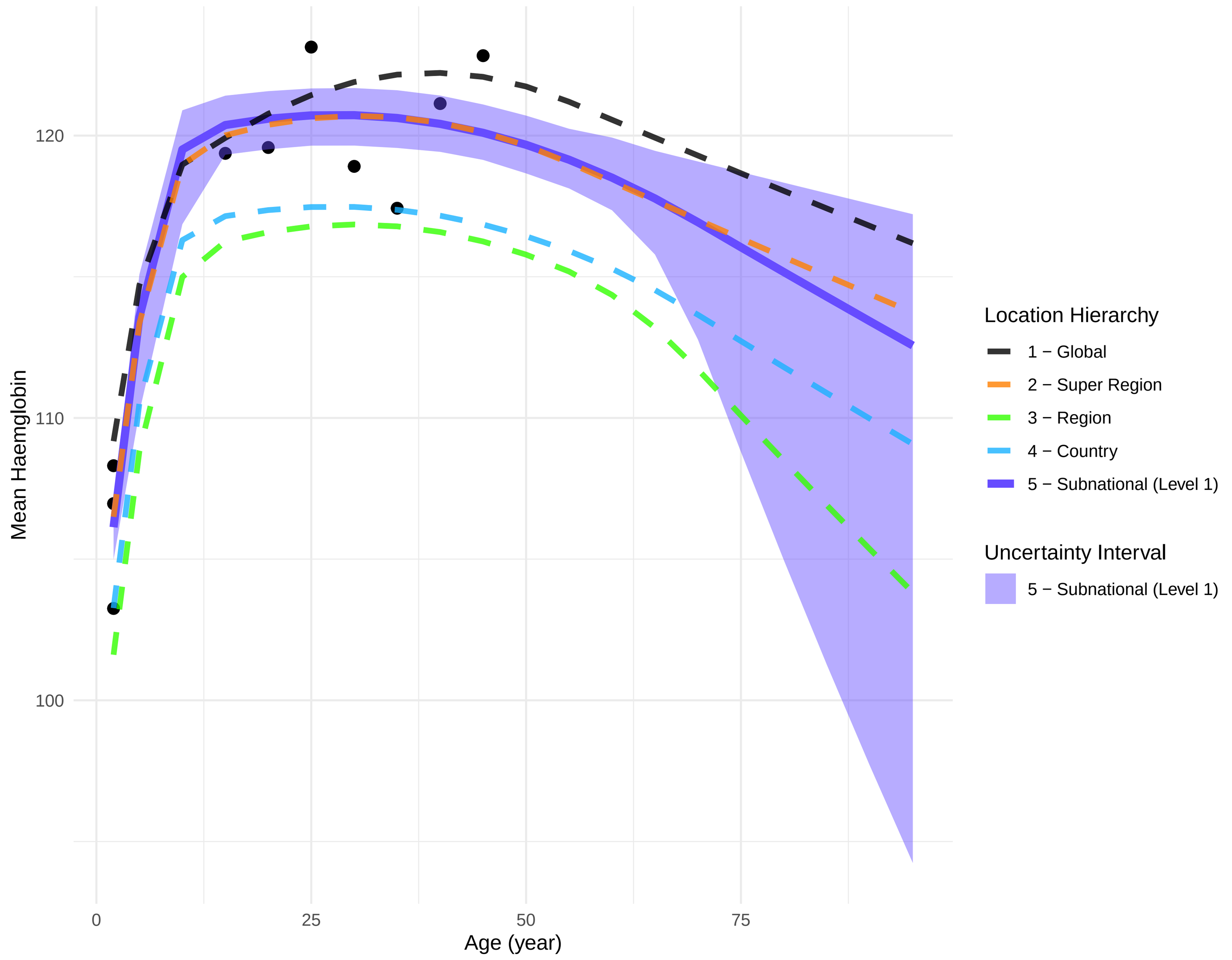

MR−BRT Age/Sex Fits − Non−pregnant Females 2+ Years Old − Mean Haemglobin − Borno
WHO−2001 Adjustment Method

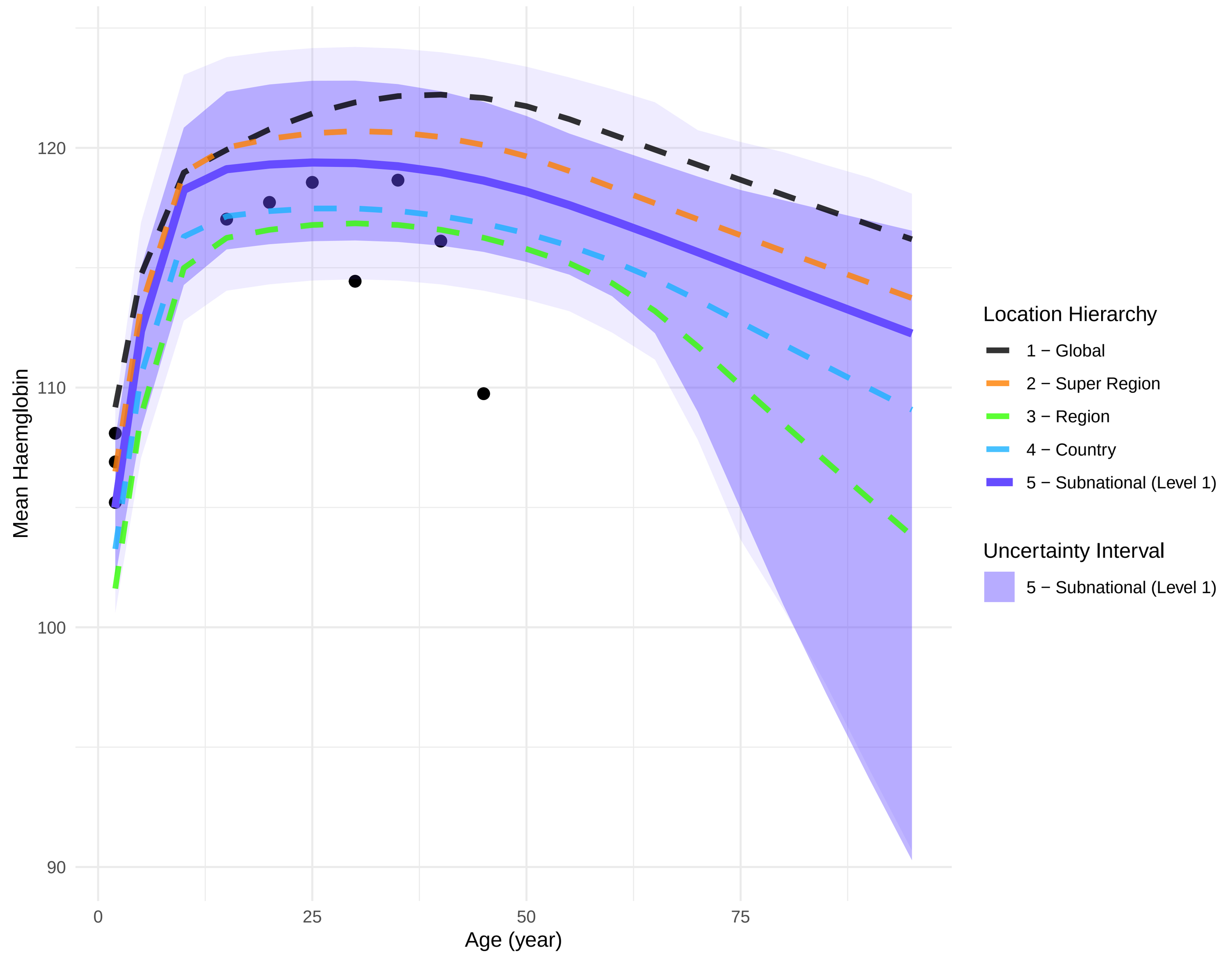

MR−BRT Age/Sex Fits − Non−pregnant Females 2+ Years Old − Mean Haemglobin − Cross River
WHO−2001 Adjustment Method

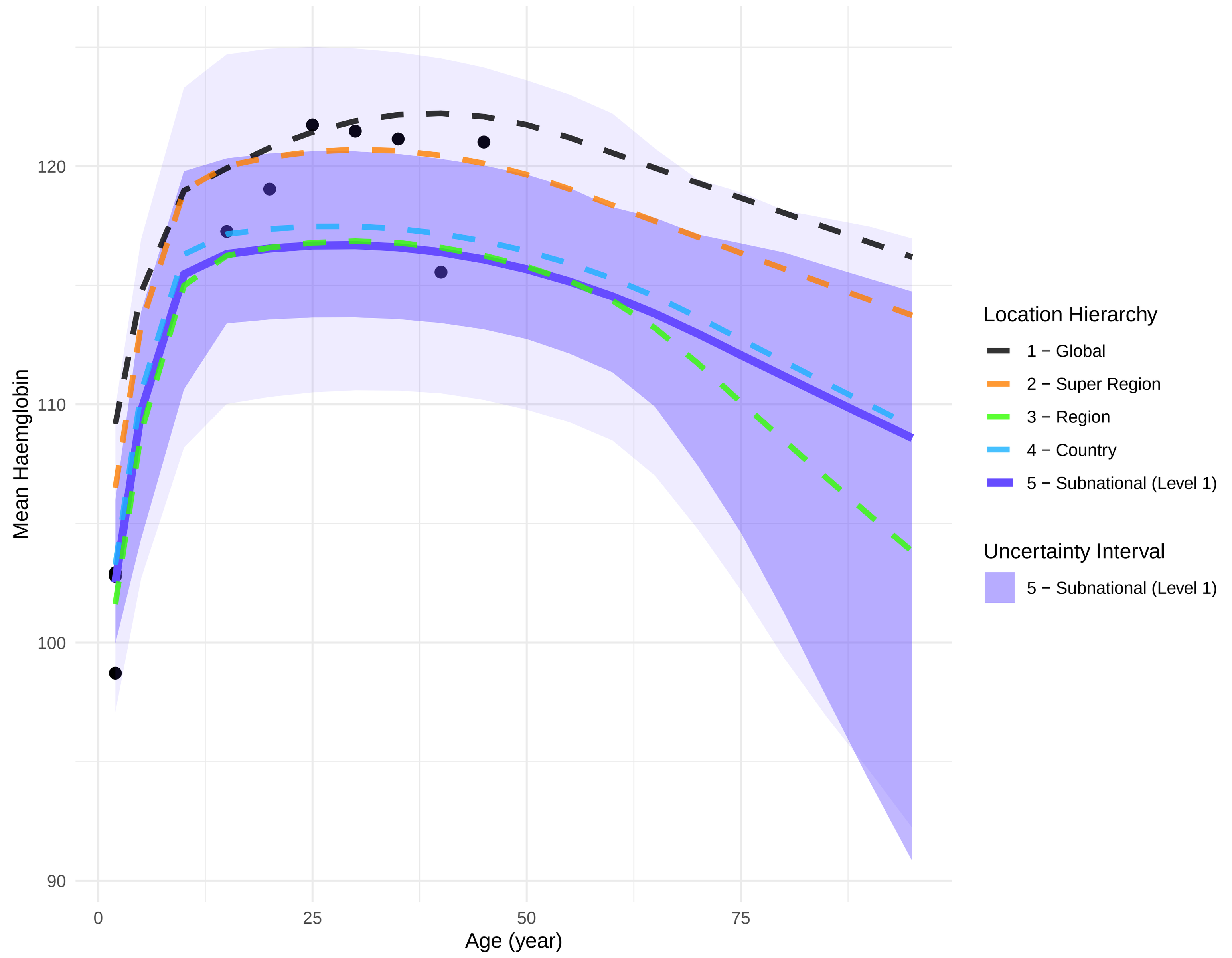

MR−BRT Age/Sex Fits − Non−pregnant Females 2+ Years Old − Mean Haemglobin − Delta
WHO−2001 Adjustment Method

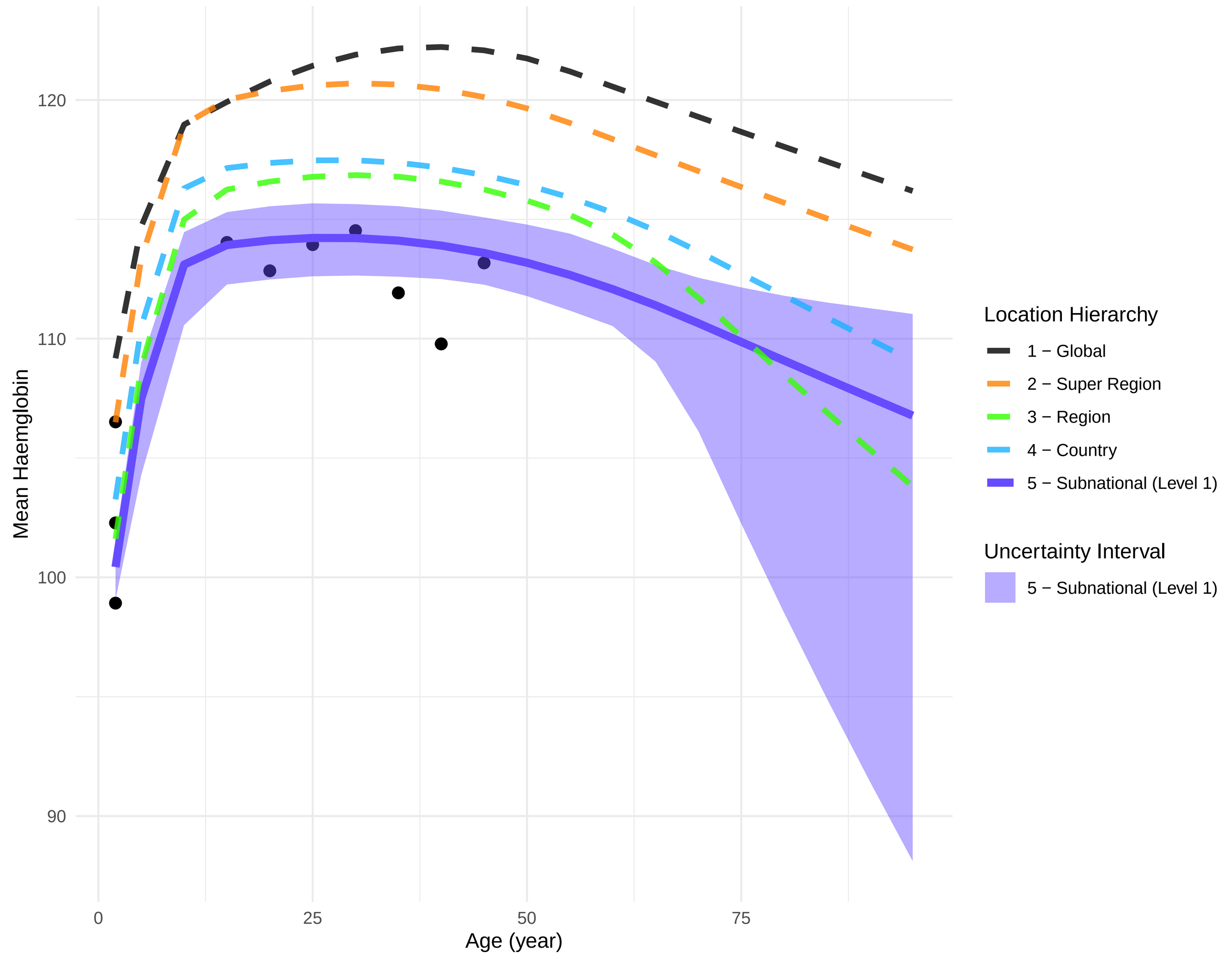

MR−BRT Age/Sex Fits − Non−pregnant Females 2+ Years Old − Mean Haemglobin − Ebonyi
WHO−2001 Adjustment Method

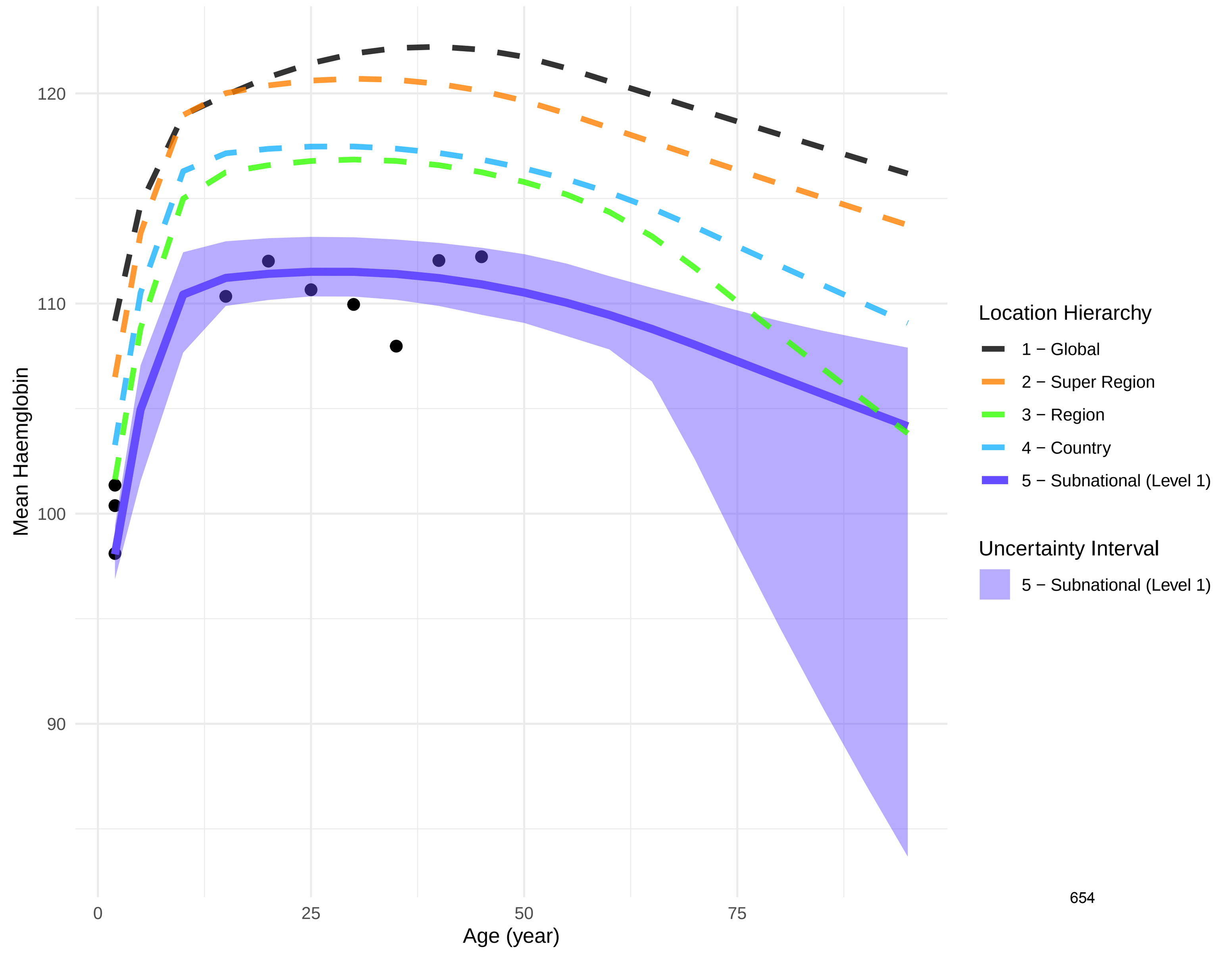

MR−BRT Age/Sex Fits − Non−pregnant Females 2+ Years Old − Mean Haemglobin − Edo
WHO−2001 Adjustment Method

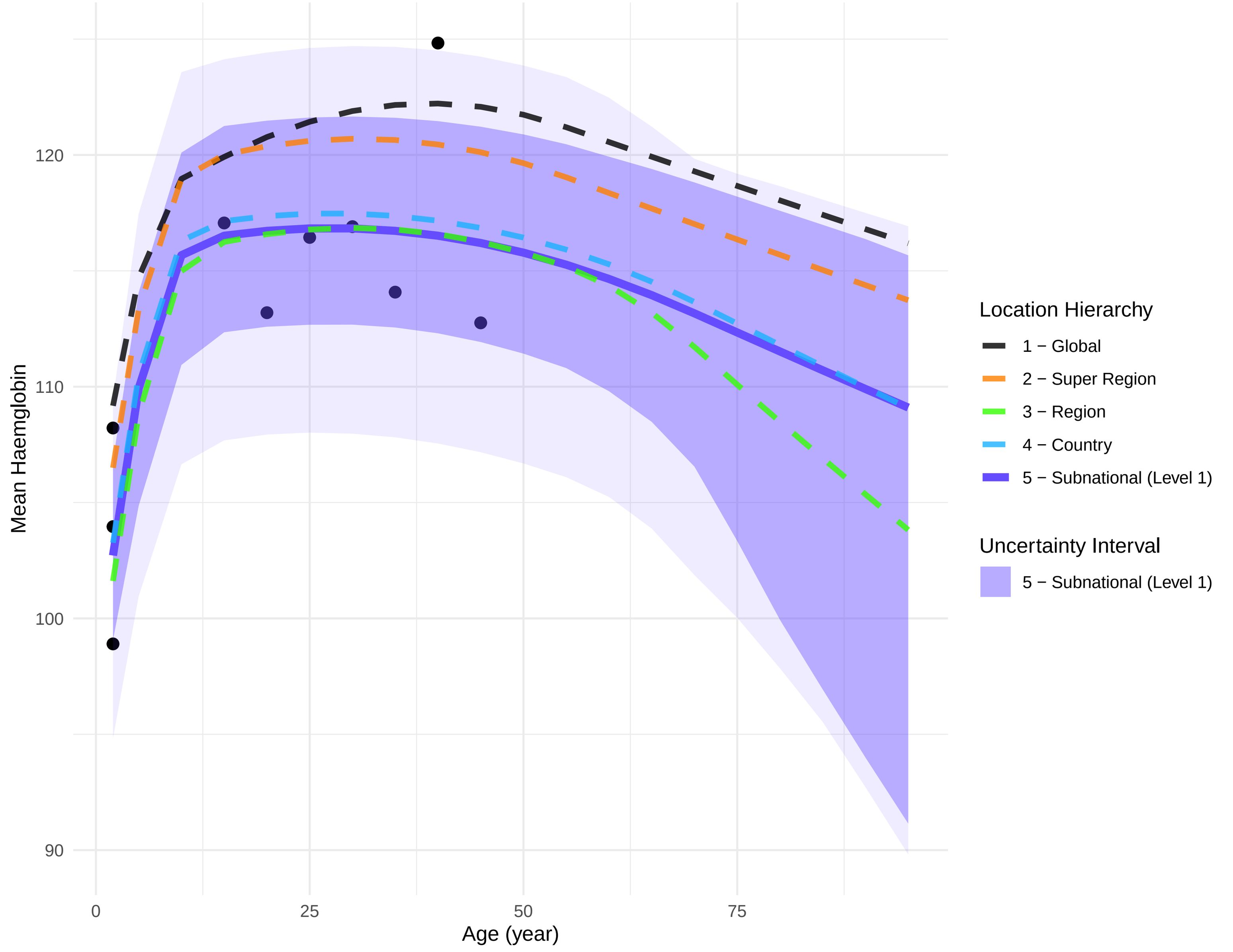

MR−BRT Age/Sex Fits − Non−pregnant Females 2+ Years Old − Mean Haemglobin − Ekiti
WHO−2001 Adjustment Method

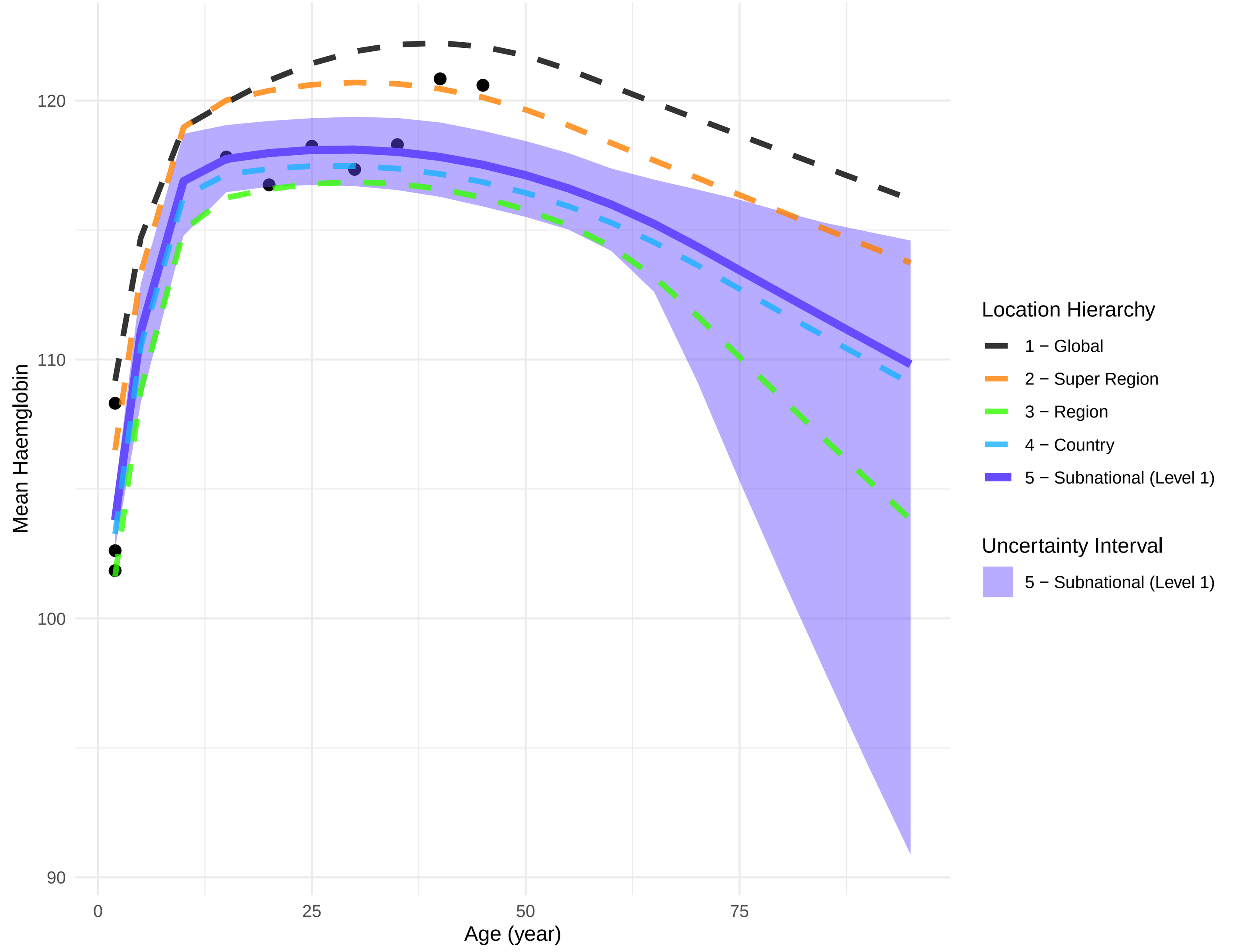

MR−BRT Age/Sex Fits − Non−pregnant Females 2+ Years Old − Mean Haemglobin − Enugu
WHO−2001 Adjustment Method

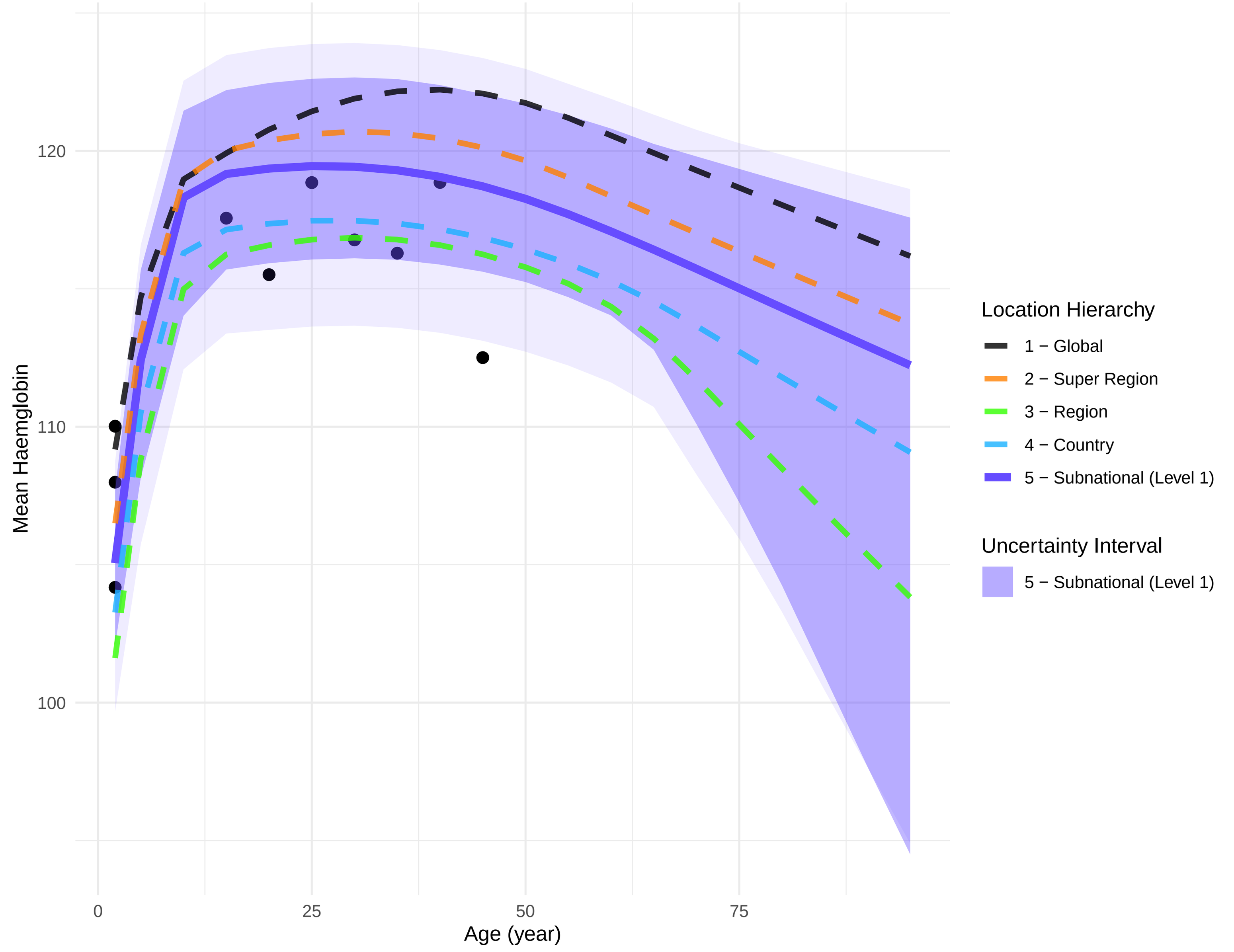

MR−BRT Age/Sex Fits − Non−pregnant Females 2+ Years Old − Mean Haemglobin − FCT (Abuja)
WHO−2001 Adjustment Method

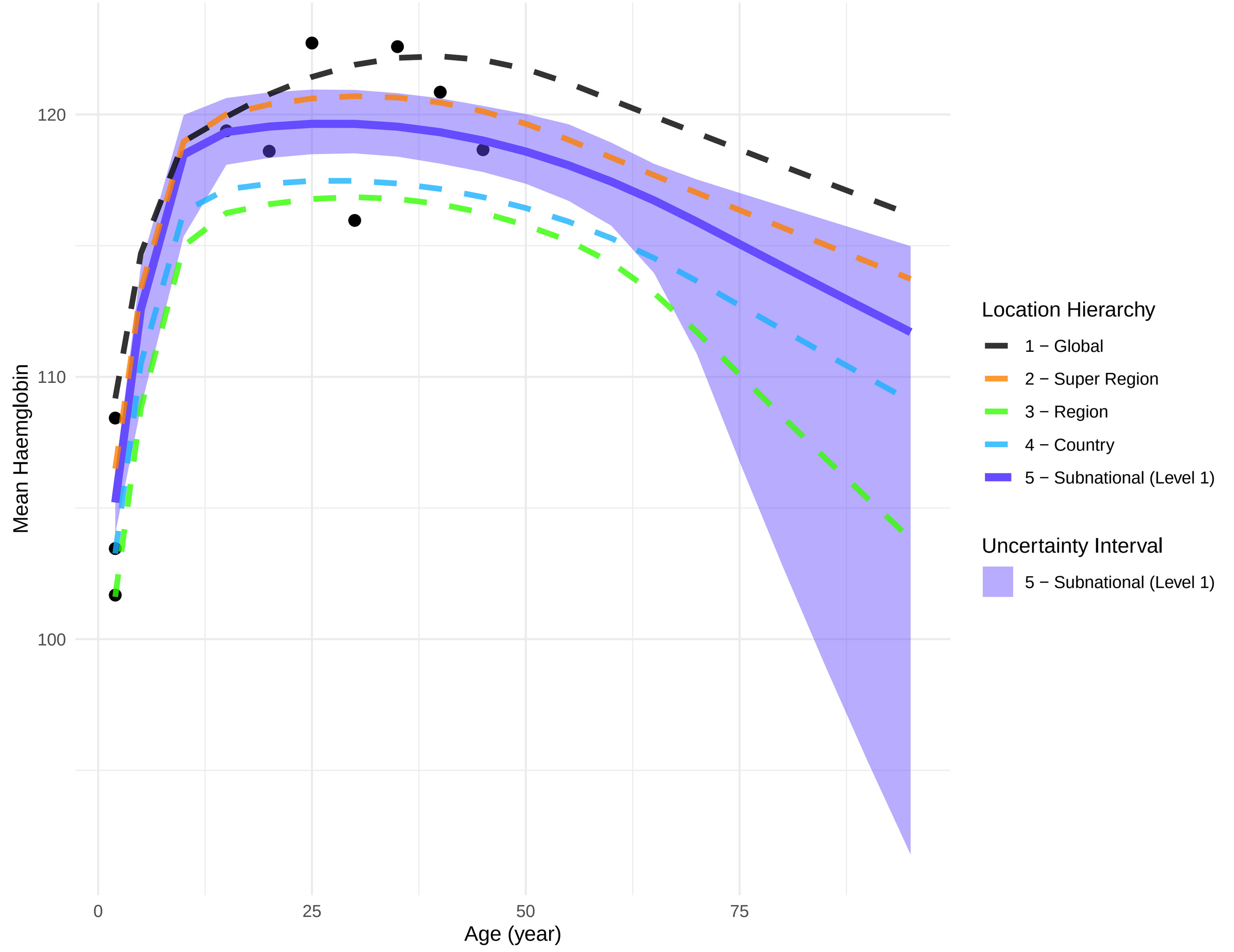

MR−BRT Age/Sex Fits − Non−pregnant Females 2+ Years Old − Mean Haemglobin − Gombe
WHO−2001 Adjustment Method

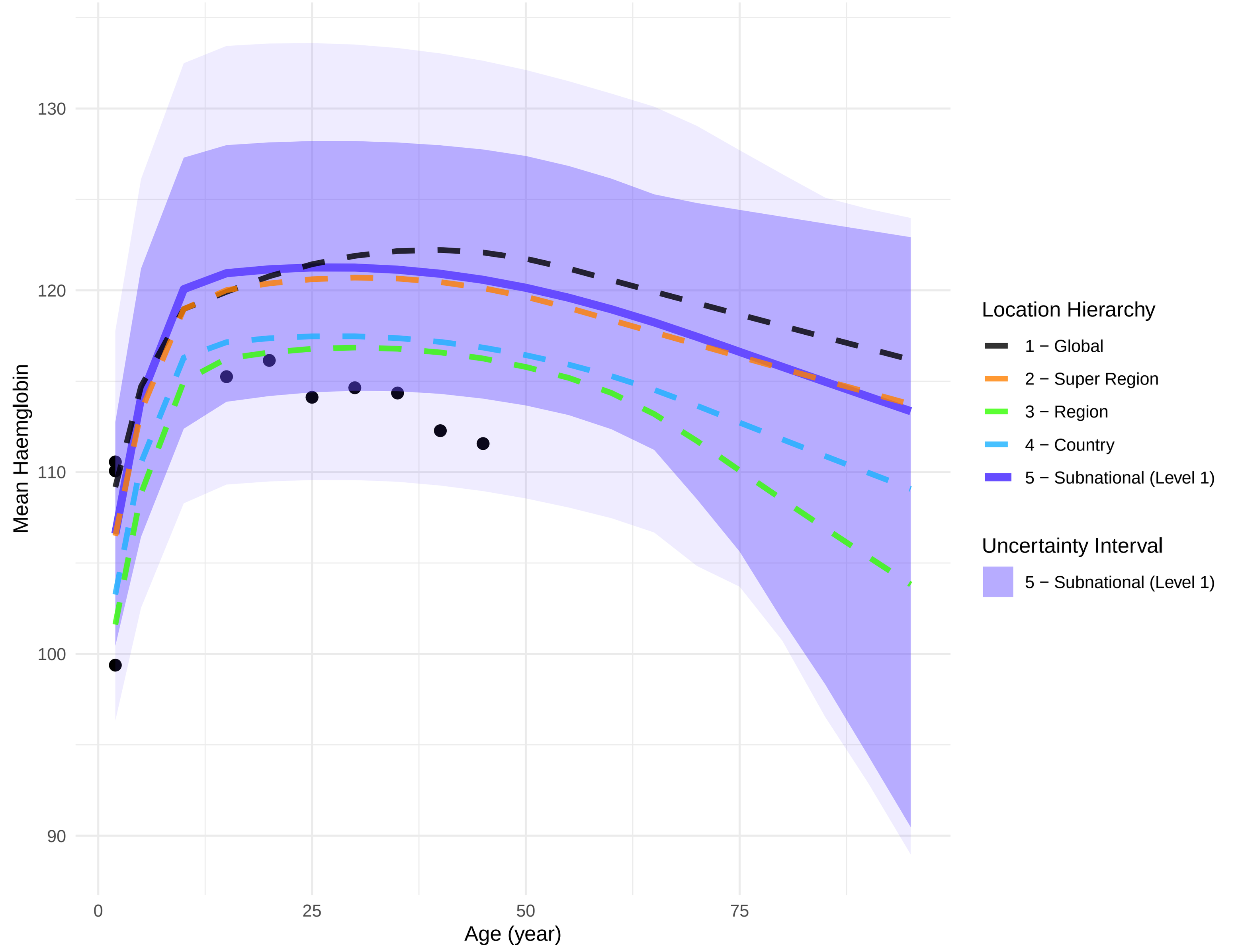

MR−BRT Age/Sex Fits − Non−pregnant Females 2+ Years Old − Mean Haemglobin − Imo
WHO−2001 Adjustment Method

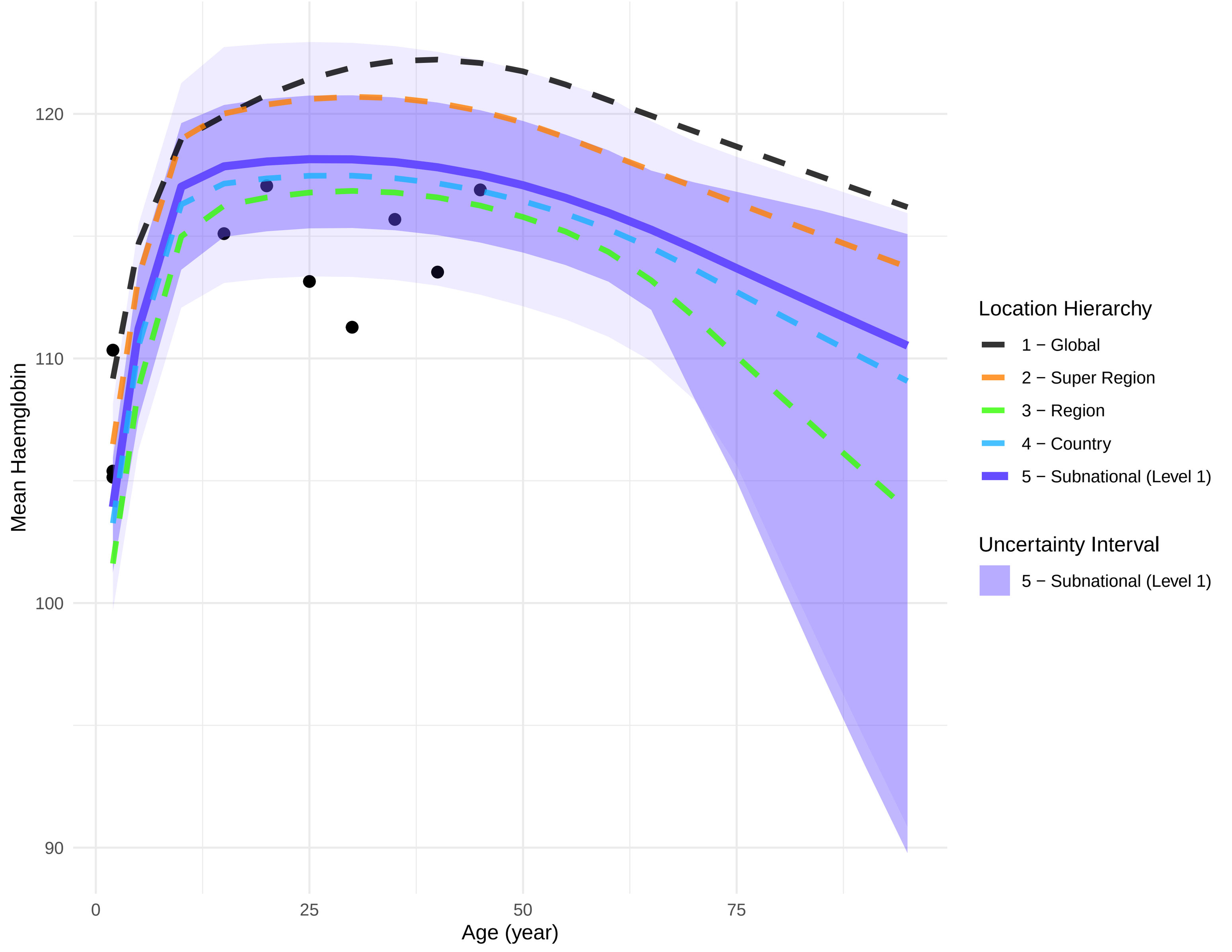

MR−BRT Age/Sex Fits − Non−pregnant Females 2+ Years Old − Mean Haemglobin − Jigawa
WHO−2001 Adjustment Method

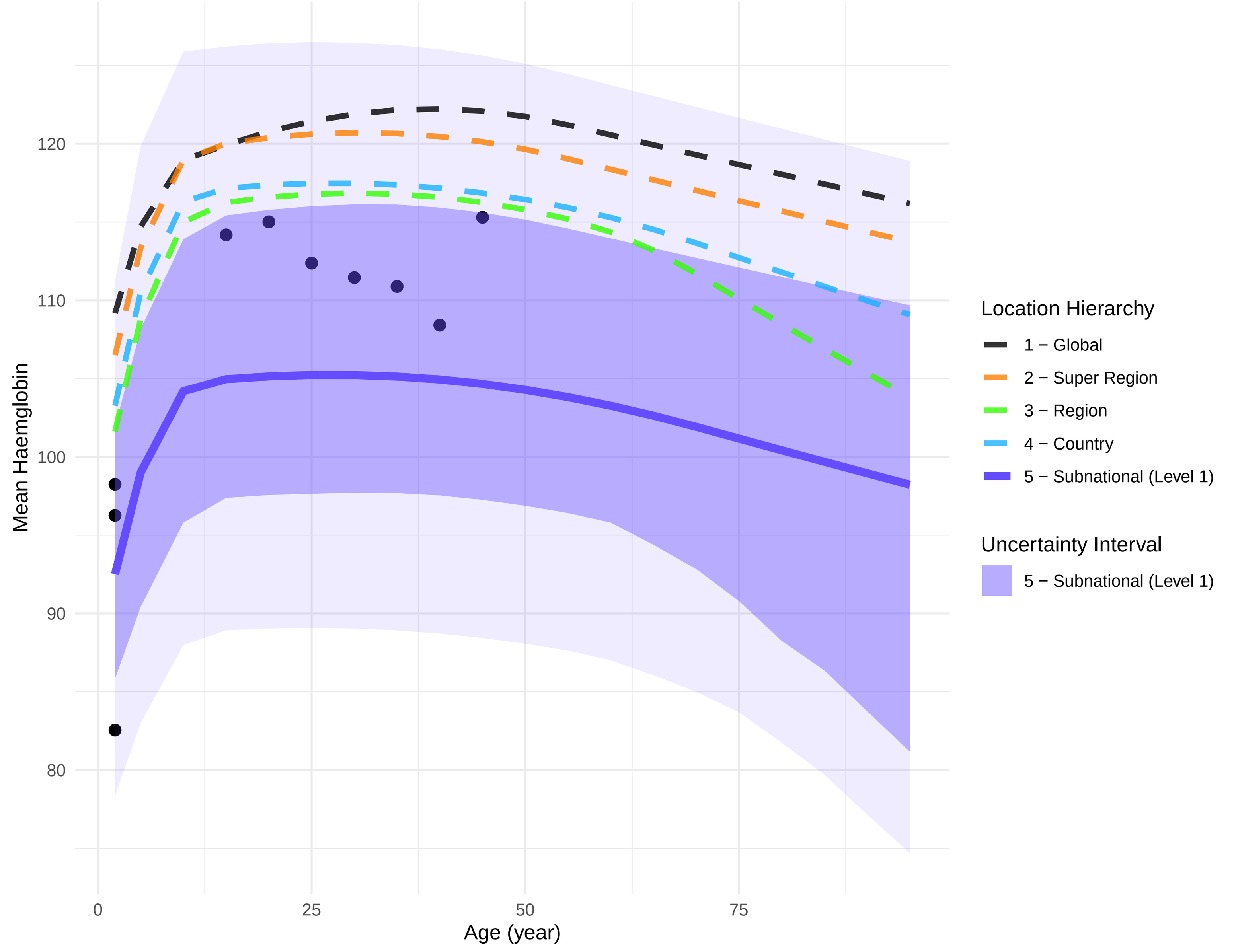

MR−BRT Age/Sex Fits − Non−pregnant Females 2+ Years Old − Mean Haemglobin − Kaduna
WHO−2001 Adjustment Method

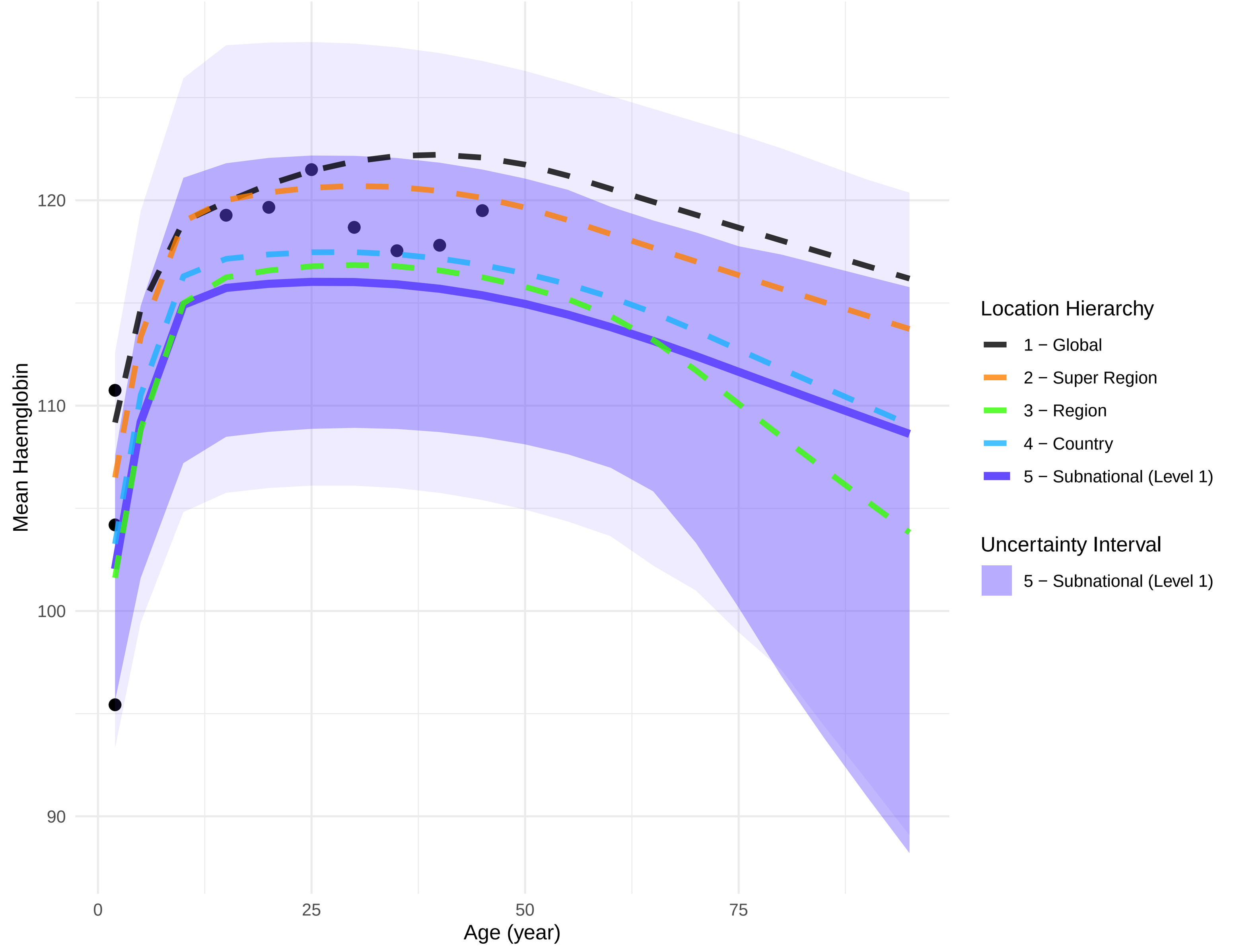

MR−BRT Age/Sex Fits − Non−pregnant Females 2+ Years Old − Mean Haemglobin − Kano
WHO−2001 Adjustment Method

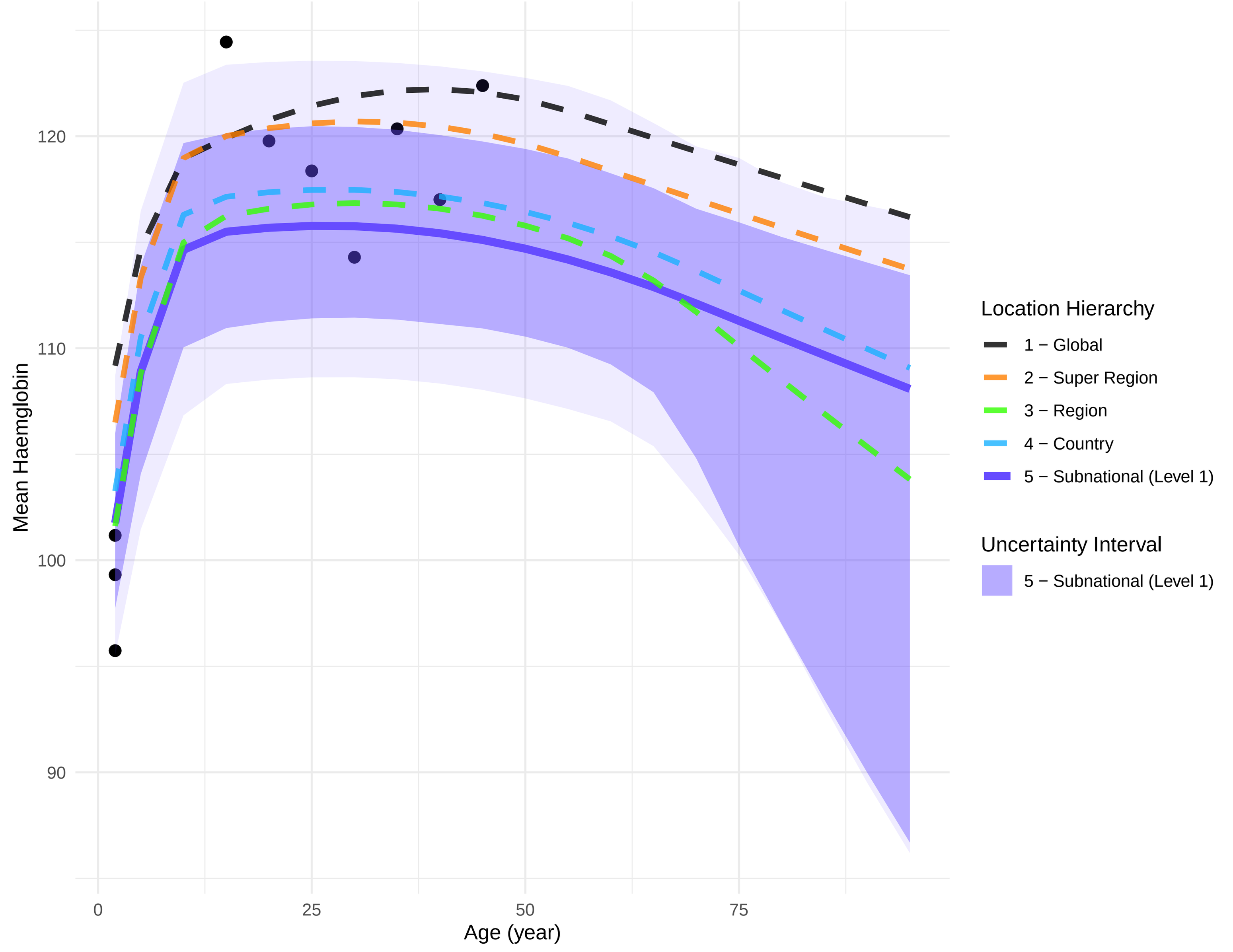

MR−BRT Age/Sex Fits − Non−pregnant Females 2+ Years Old − Mean Haemglobin − Katsina
WHO−2001 Adjustment Method

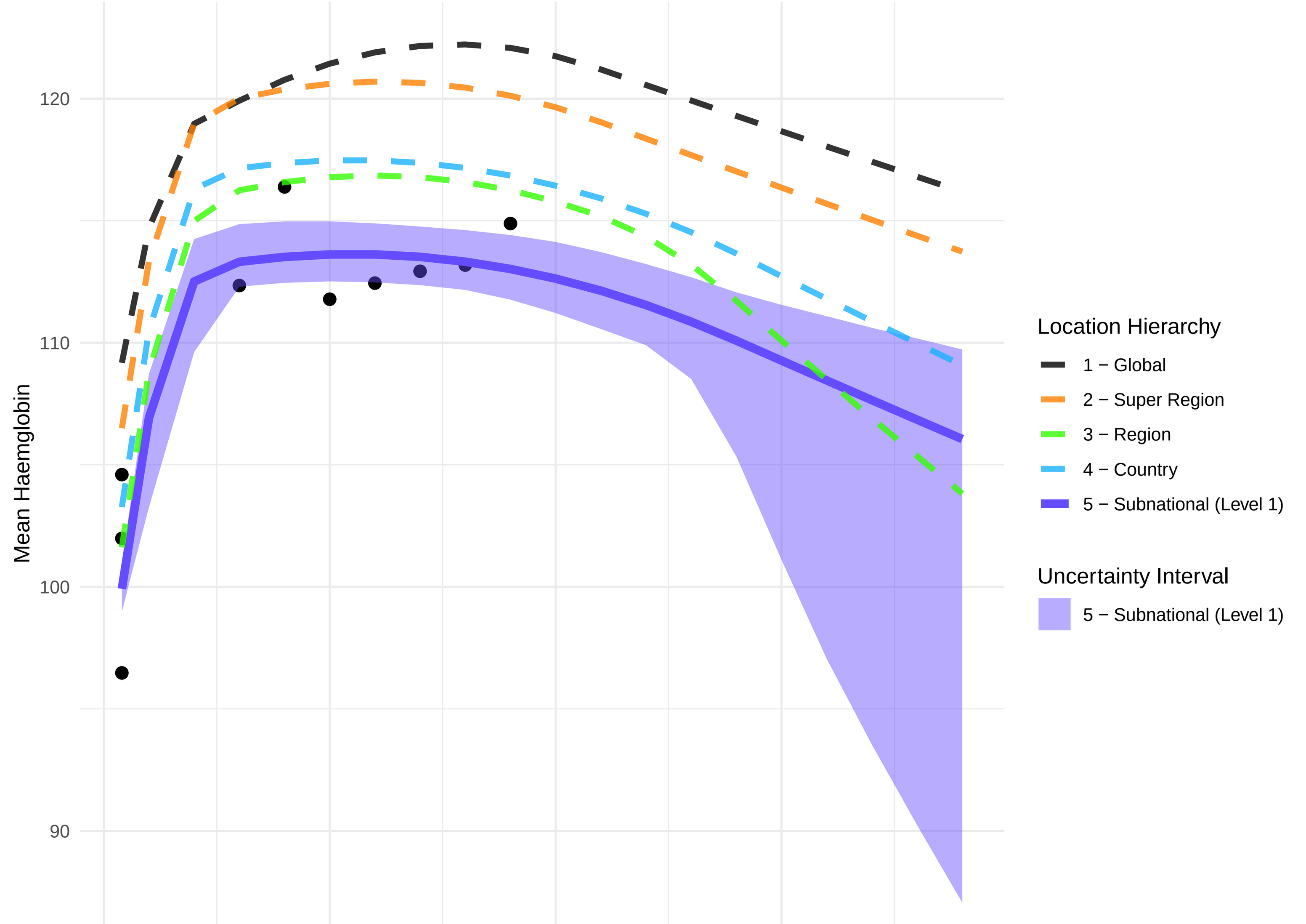

MR−BRT Age/Sex Fits − Non−pregnant Females 2+ Years Old − Mean Haemglobin − Kebbi
WHO−2001 Adjustment Method

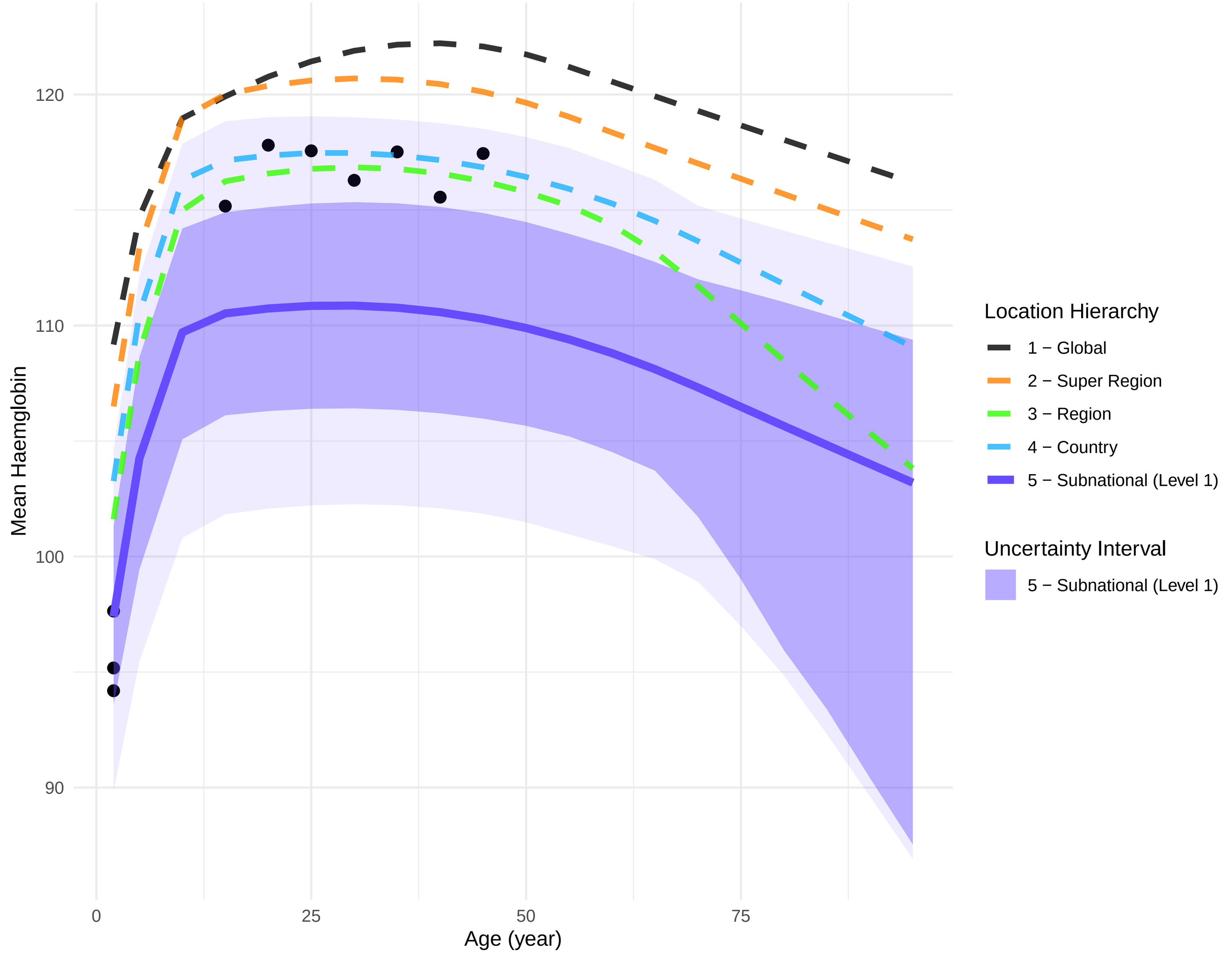

MR−BRT Age/Sex Fits − Non−pregnant Females 2+ Years Old − Mean Haemglobin − Kogi
WHO−2001 Adjustment Method

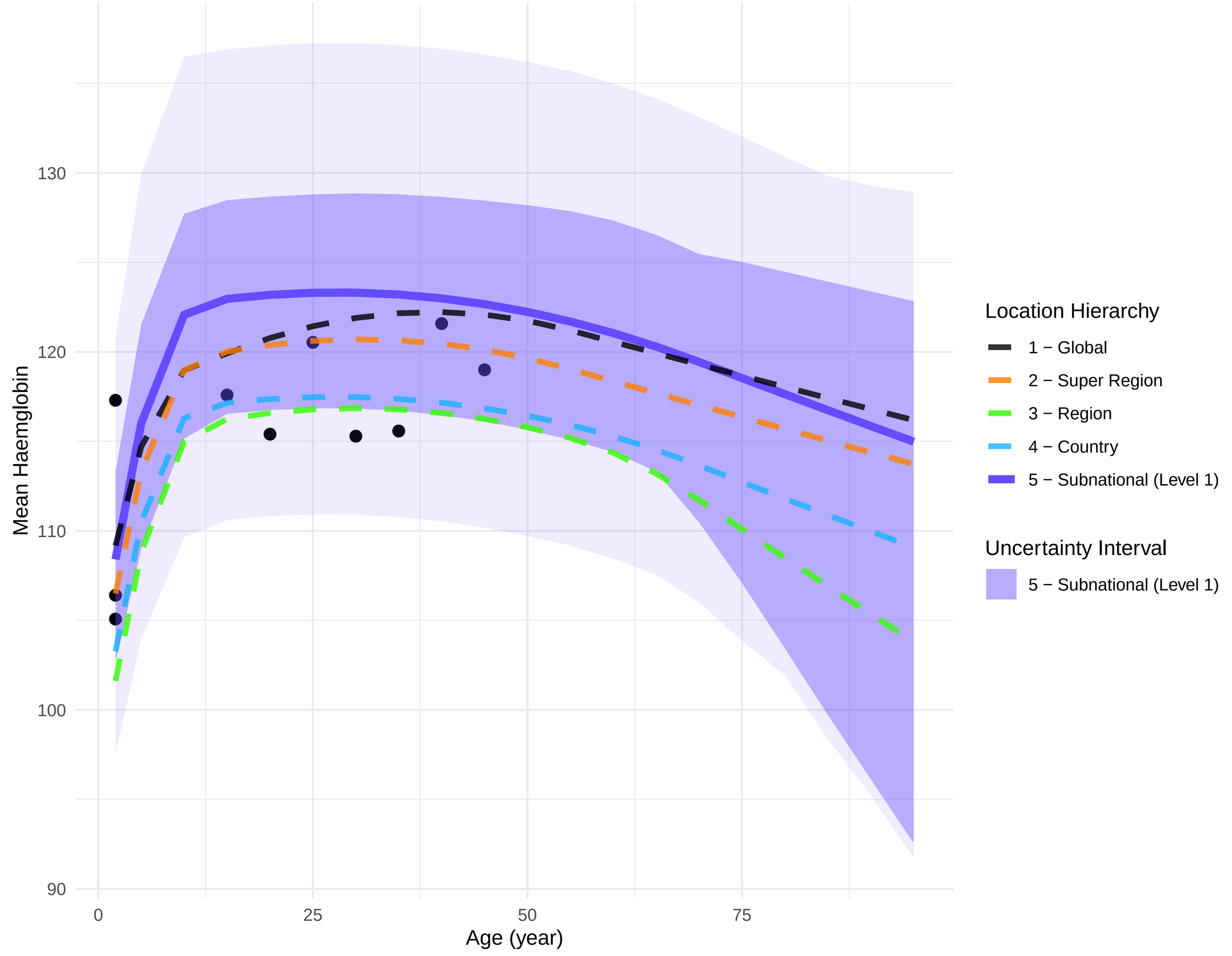

MR−BRT Age/Sex Fits − Non−pregnant Females 2+ Years Old − Mean Haemglobin − Kwara
WHO−2001 Adjustment Method

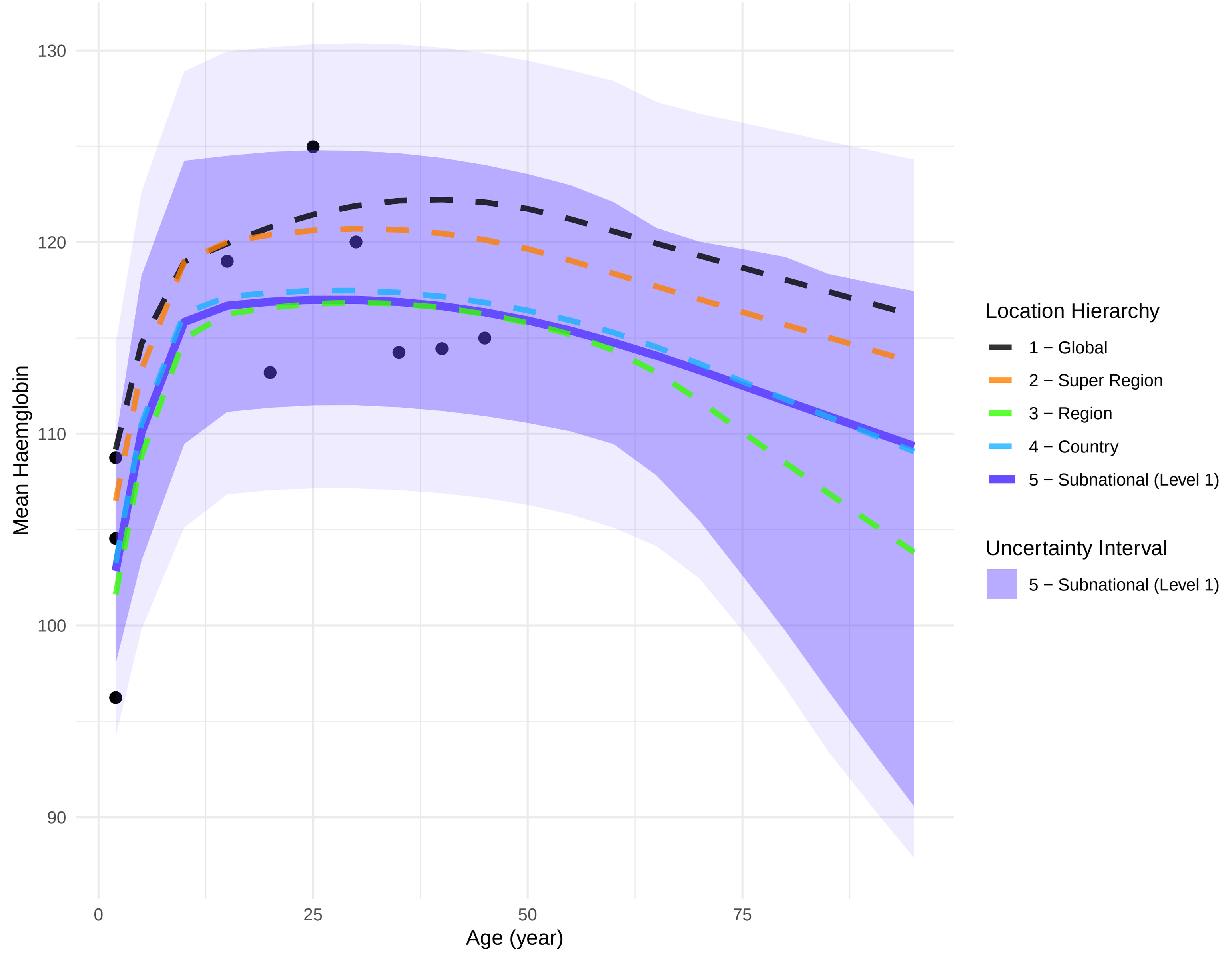

MR−BRT Age/Sex Fits − Non−pregnant Females 2+ Years Old − Mean Haemglobin − Lagos
WHO−2001 Adjustment Method

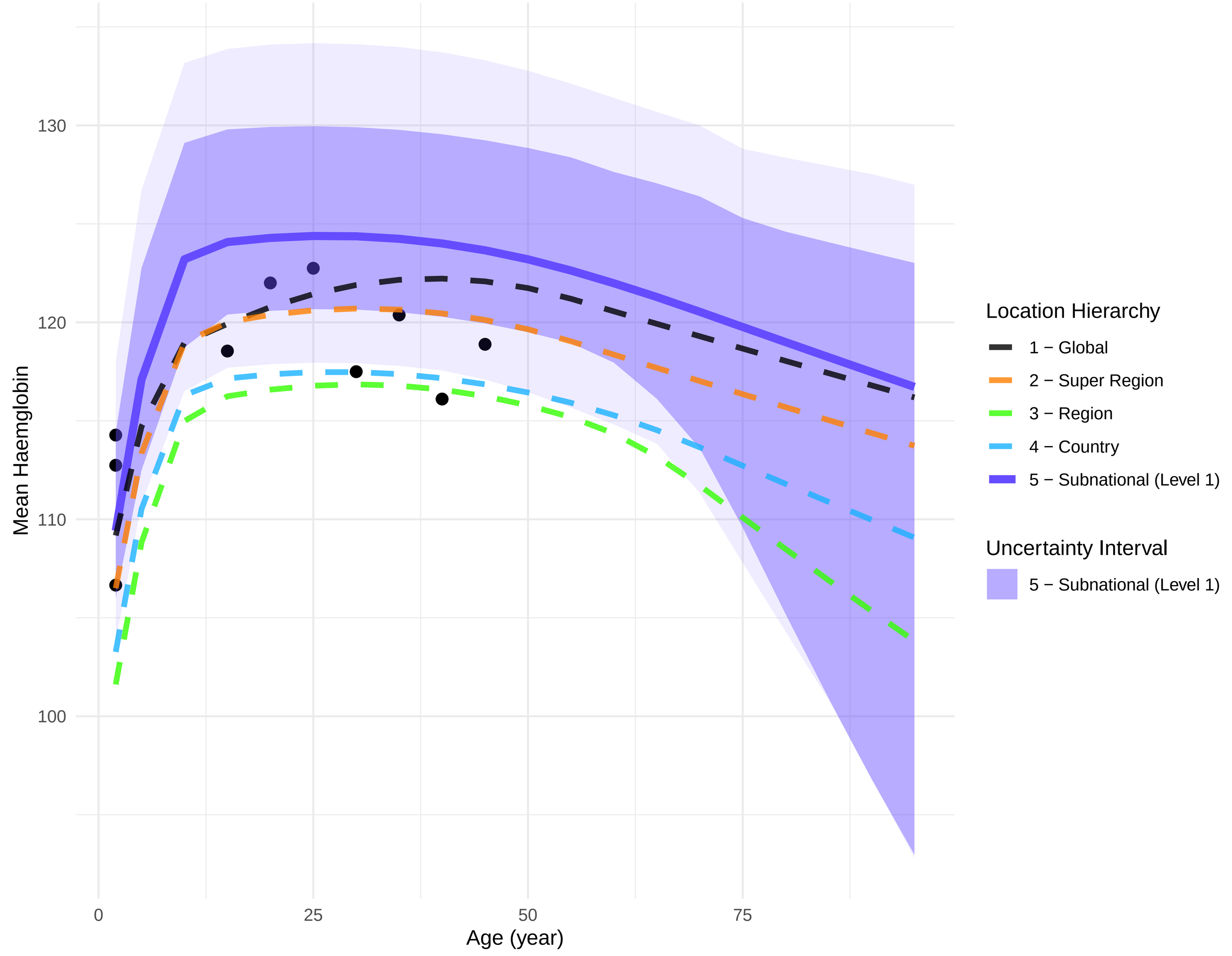

MR−BRT Age/Sex Fits − Non−pregnant Females 2+ Years Old − Mean Haemglobin − Nasarawa
WHO−2001 Adjustment Method

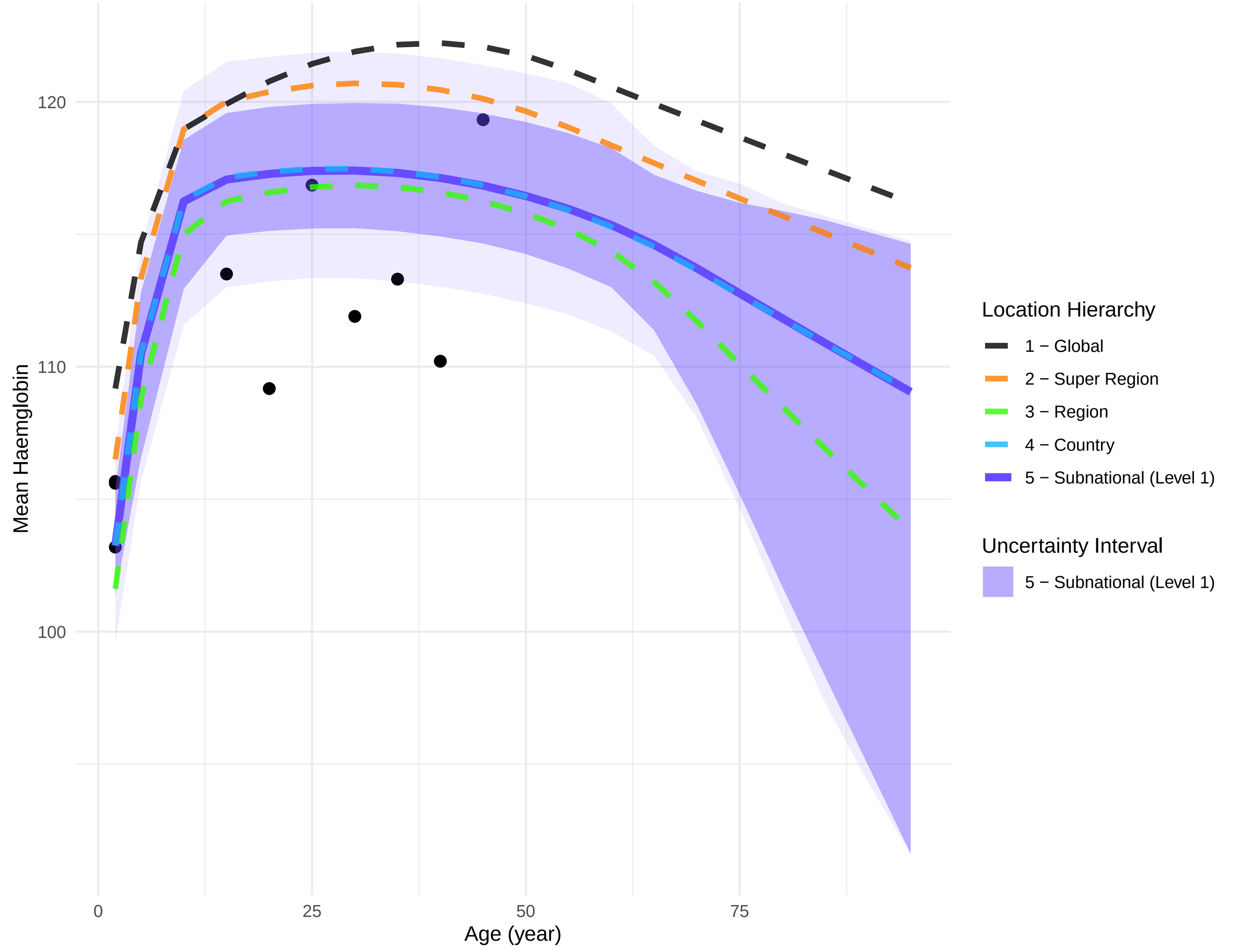

MR−BRT Age/Sex Fits − Non−pregnant Females 2+ Years Old − Mean Haemglobin − Niger
WHO−2001 Adjustment Method

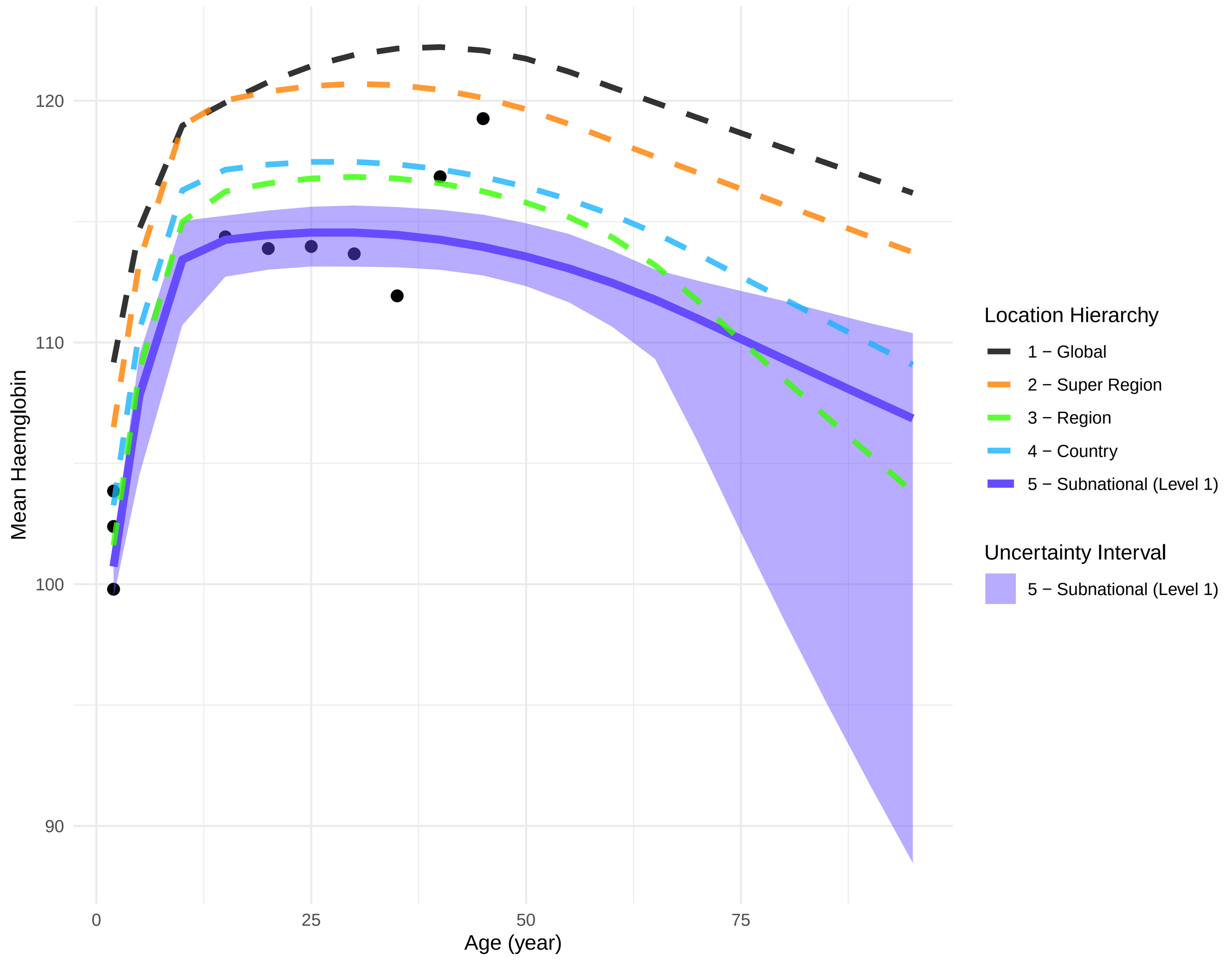

MR−BRT Age/Sex Fits − Non−pregnant Females 2+ Years Old − Mean Haemglobin − Ogun
WHO−2001 Adjustment Method

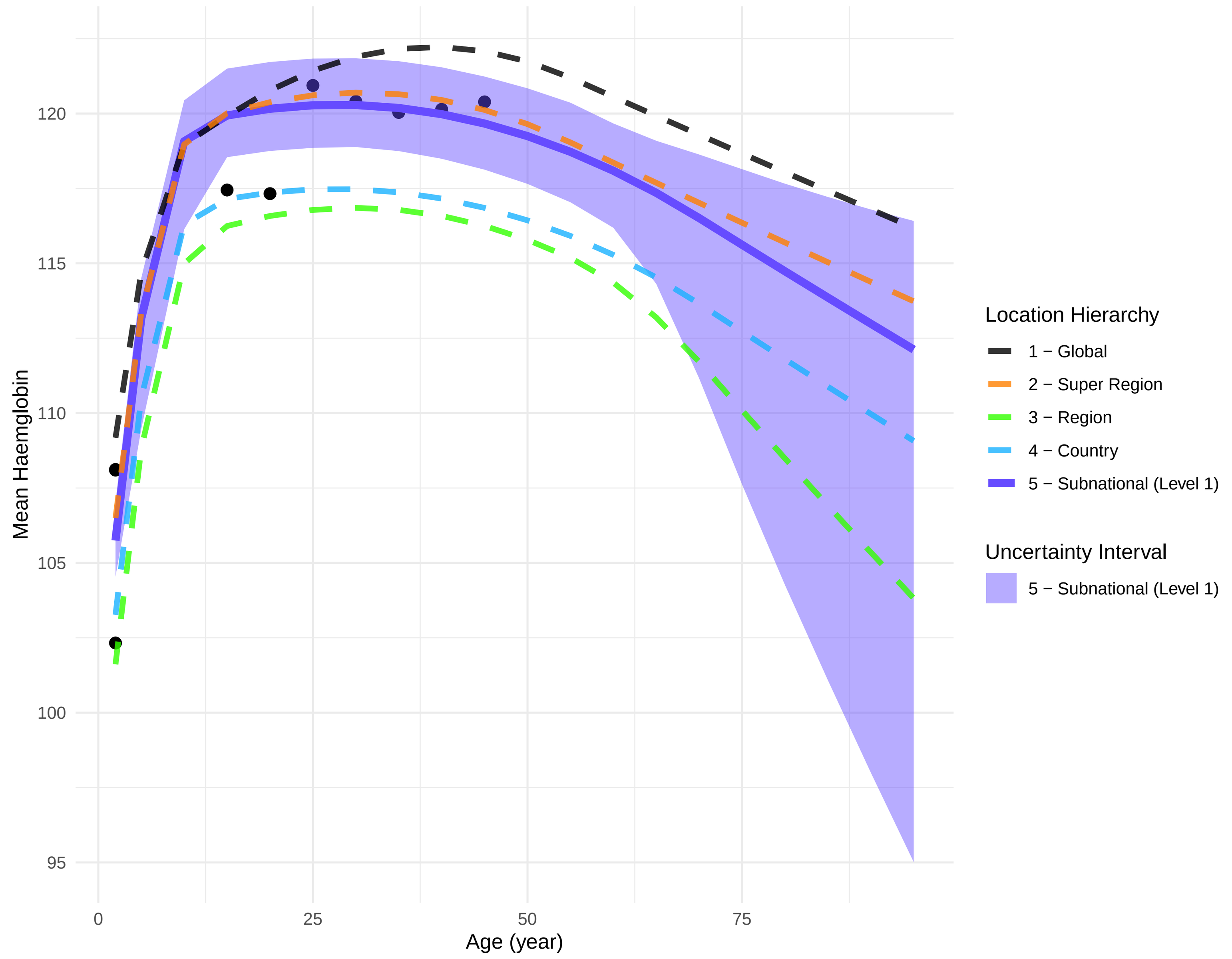

MR−BRT Age/Sex Fits − Non−pregnant Females 2+ Years Old − Mean Haemglobin − Ondo
WHO−2001 Adjustment Method

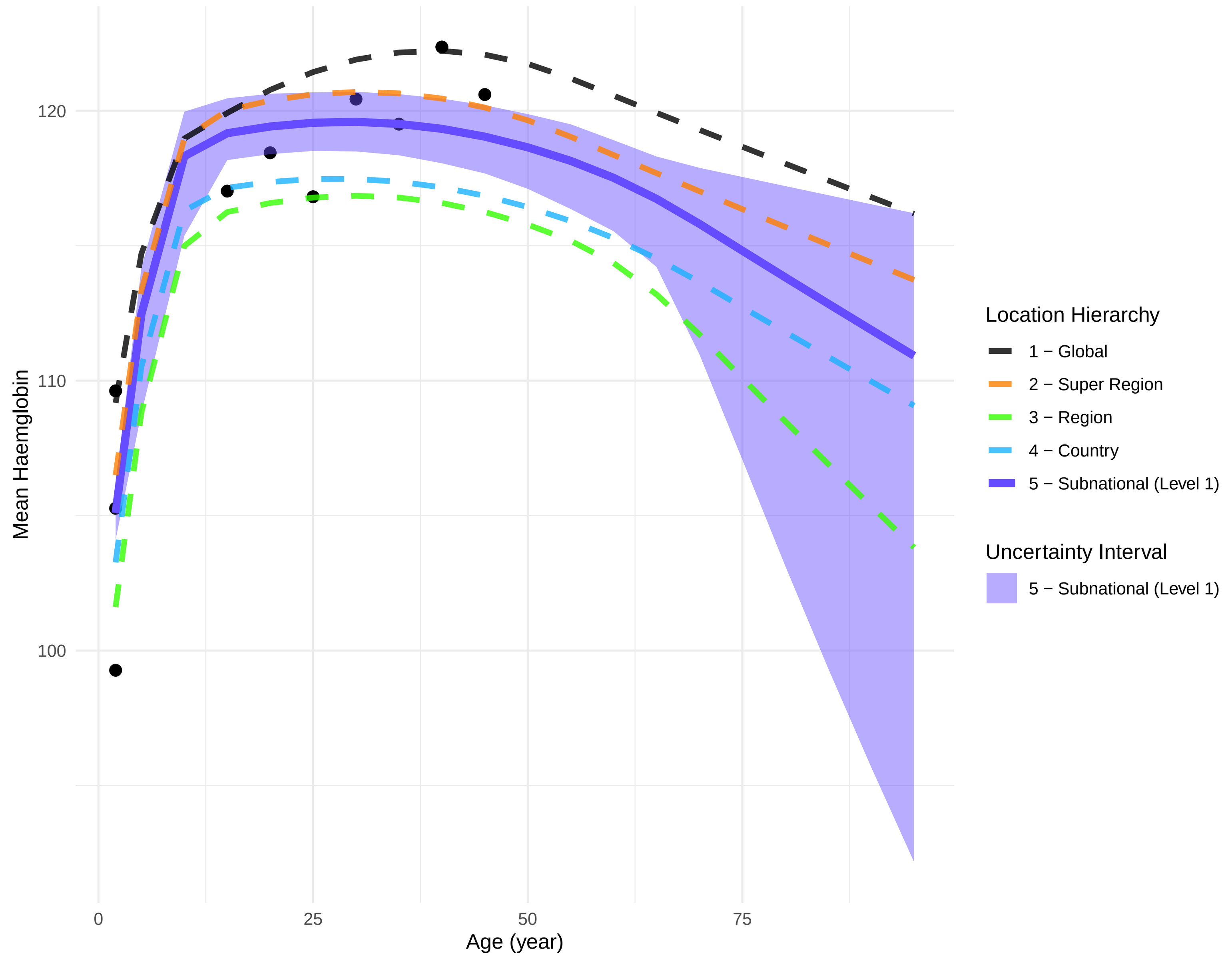

MR−BRT Age/Sex Fits − Non−pregnant Females 2+ Years Old − Mean Haemglobin − Osun
WHO−2001 Adjustment Method

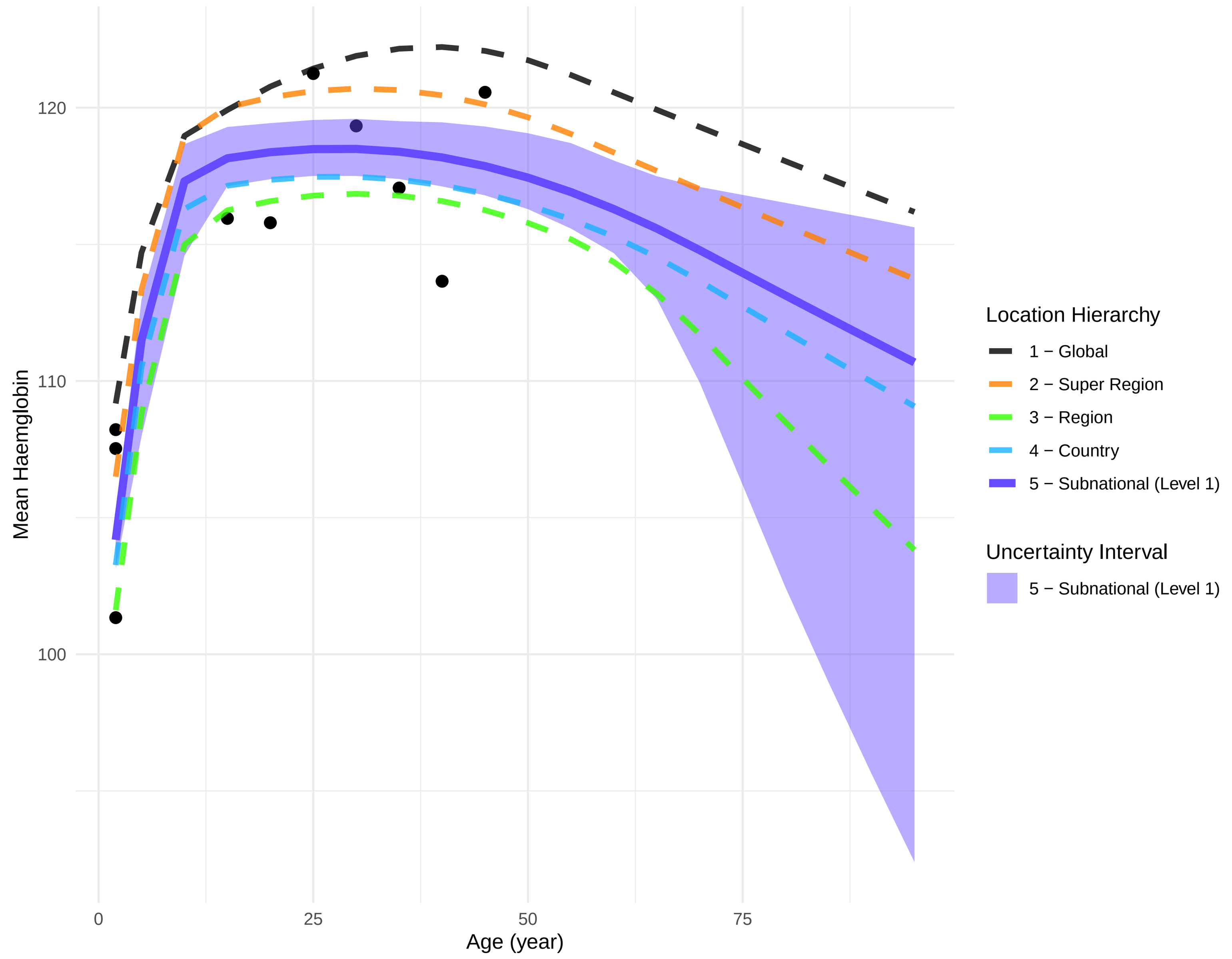

MR−BRT Age/Sex Fits − Non−pregnant Females 2+ Years Old − Mean Haemglobin − Oyo
WHO−2001 Adjustment Method

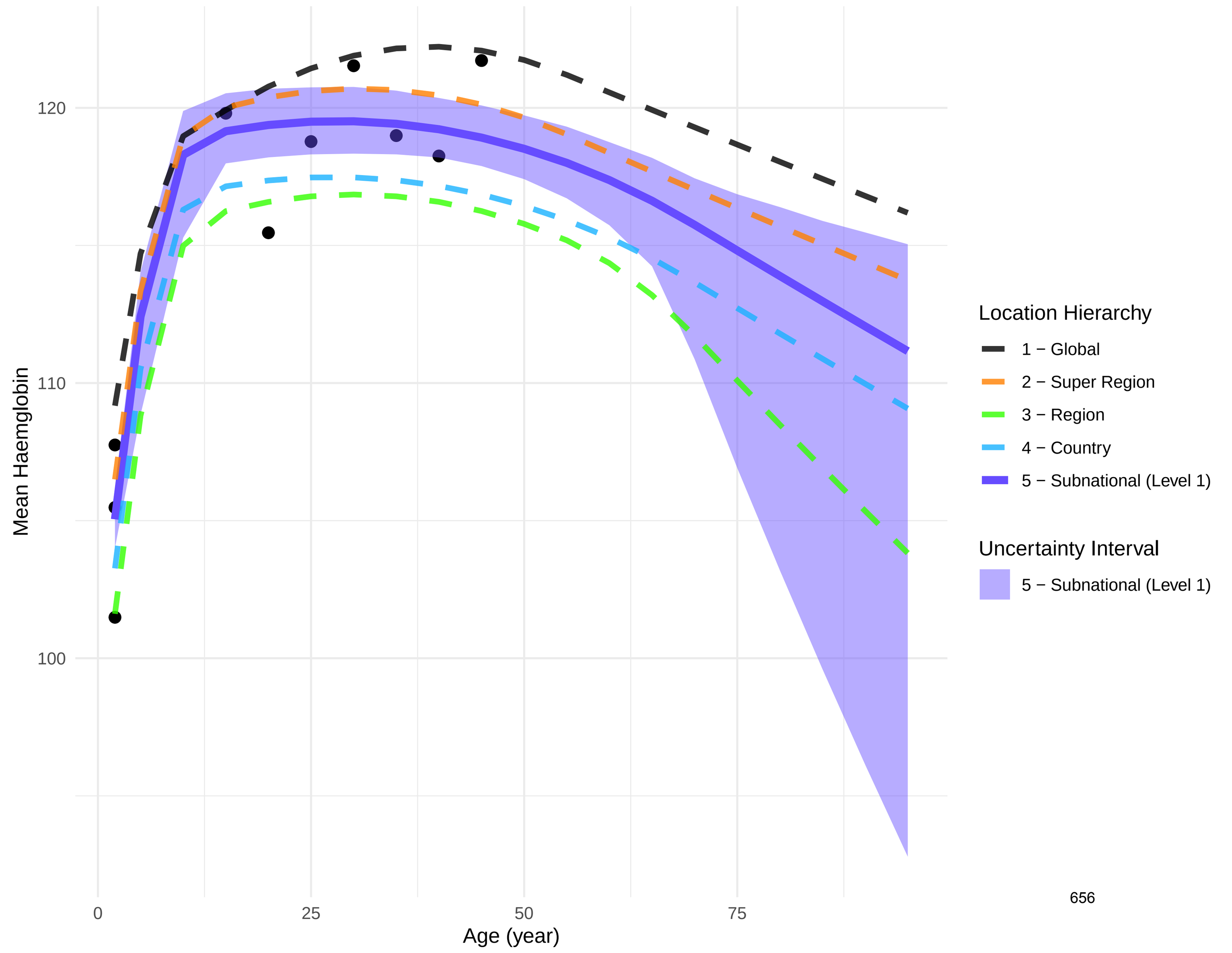

MR−BRT Age/Sex Fits − Non−pregnant Females 2+ Years Old − Mean Haemglobin − Plateau
WHO−2001 Adjustment Method

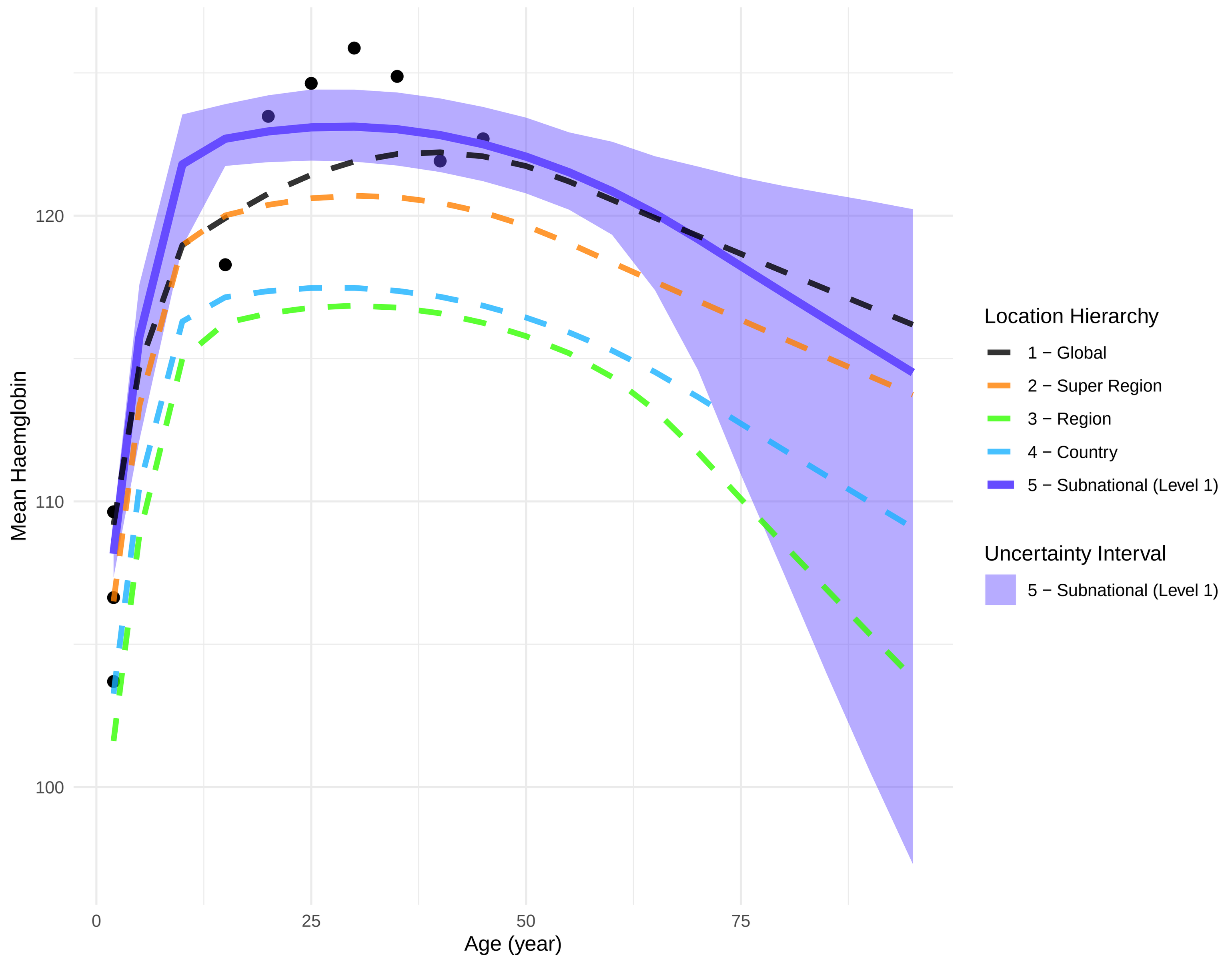

MR−BRT Age/Sex Fits − Non−pregnant Females 2+ Years Old − Mean Haemglobin − Rivers
WHO−2001 Adjustment Method

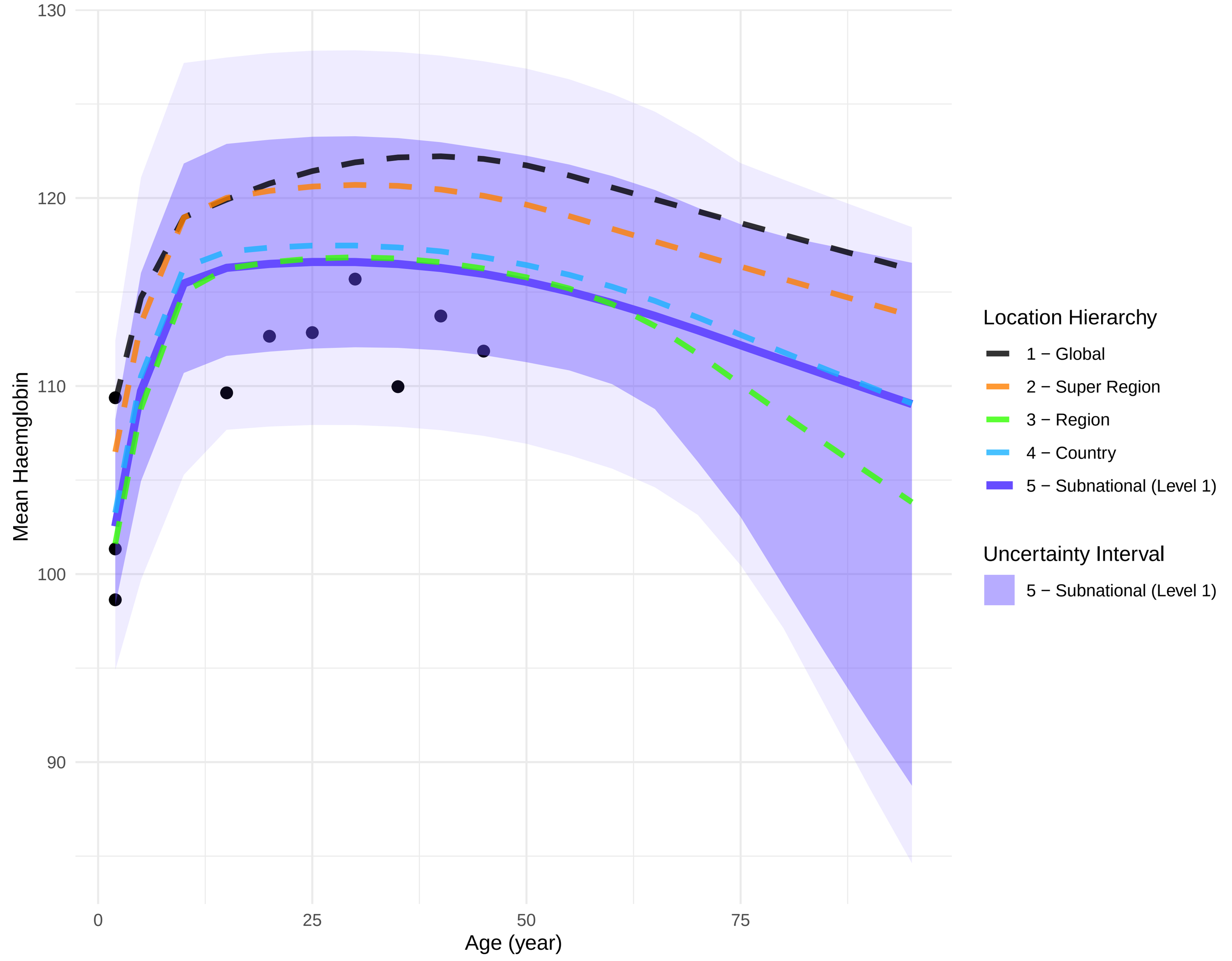

MR−BRT Age/Sex Fits − Non−pregnant Females 2+ Years Old − Mean Haemglobin − Sokoto
WHO−2001 Adjustment Method

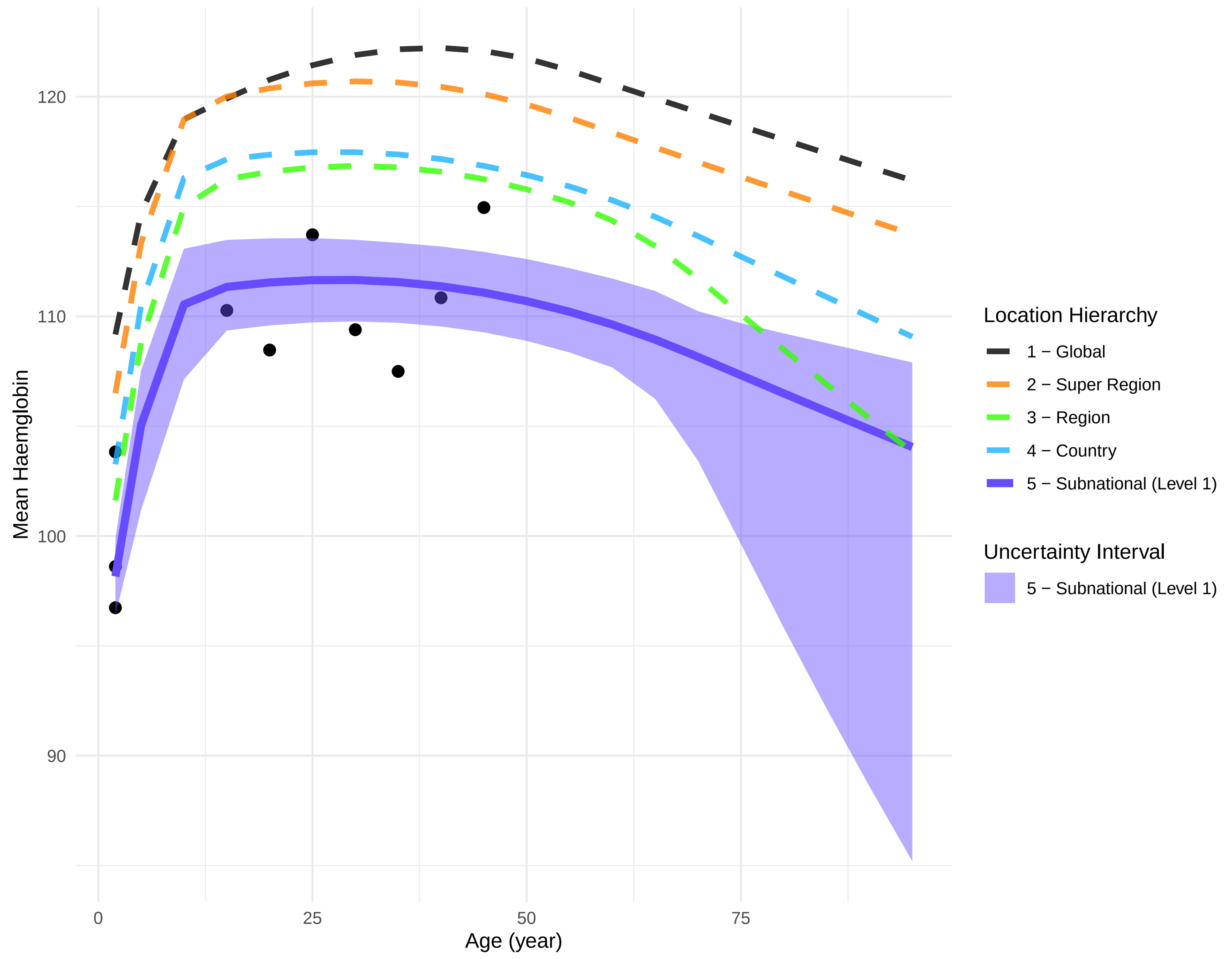

MR−BRT Age/Sex Fits − Non−pregnant Females 2+ Years Old − Mean Haemglobin − Taraba
WHO−2001 Adjustment Method

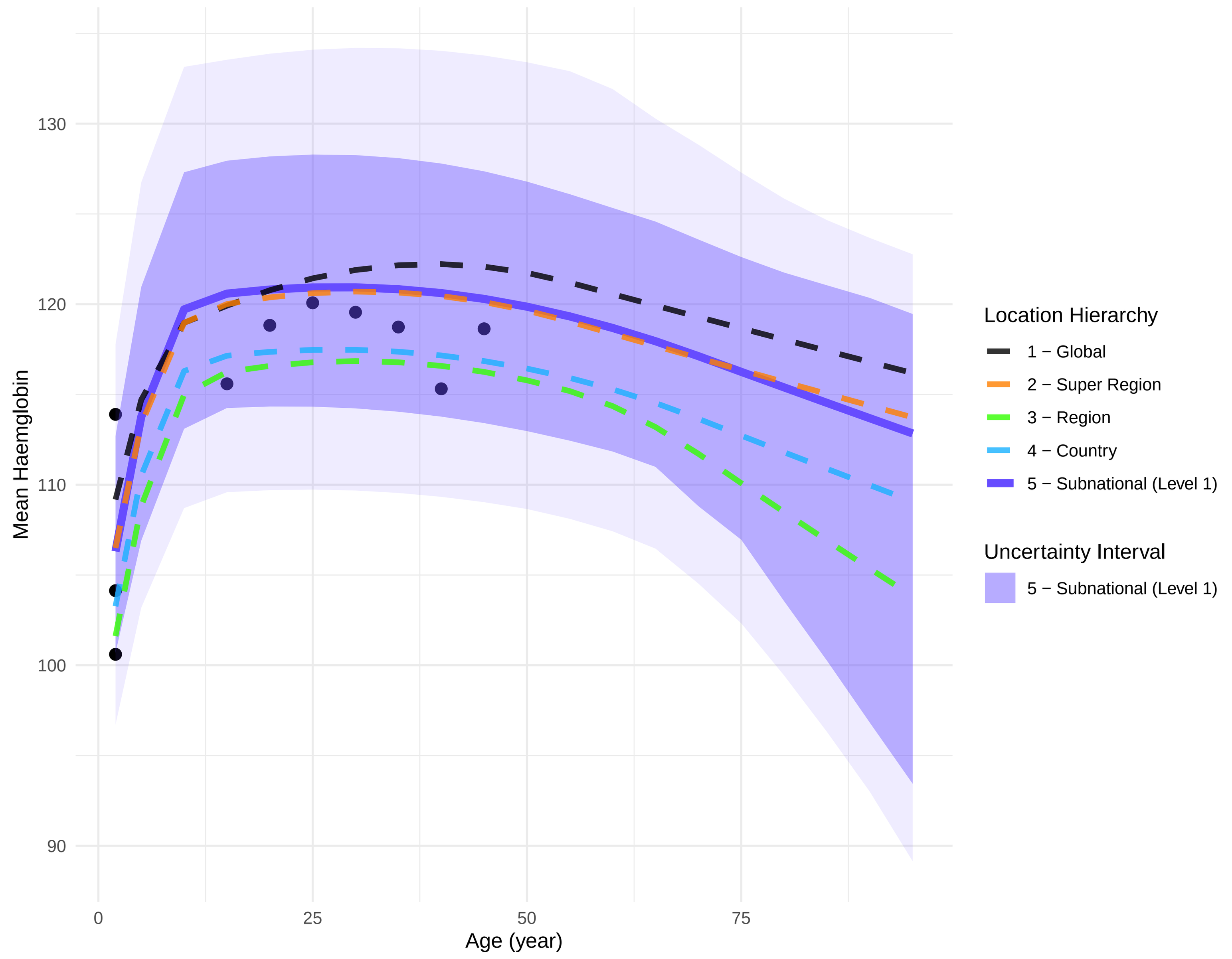

MR−BRT Age/Sex Fits − Non−pregnant Females 2+ Years Old − Mean Haemglobin − Yobe
WHO−2001 Adjustment Method

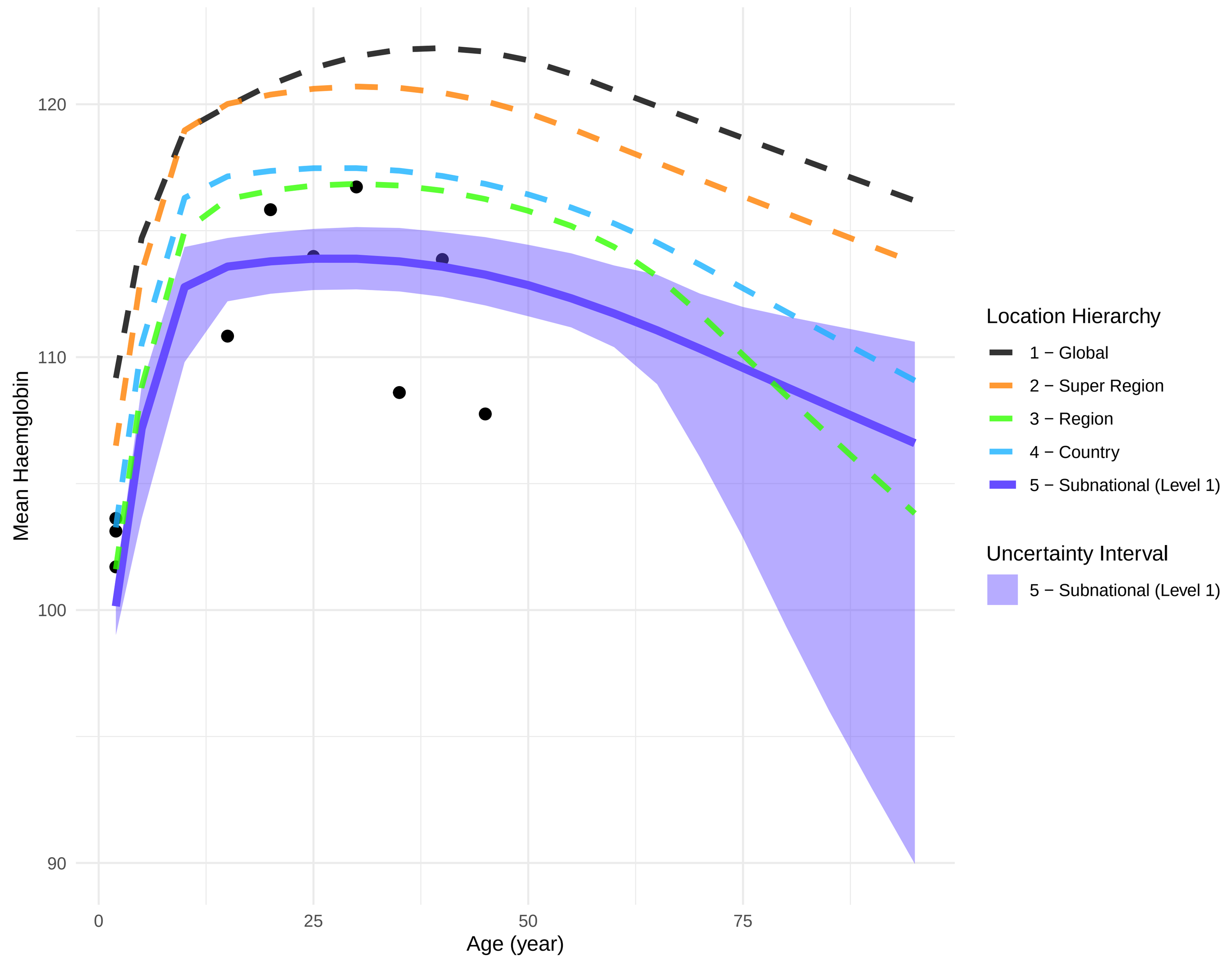

MR−BRT Age/Sex Fits − Non−pregnant Females 2+ Years Old − Mean Haemglobin − Zamfara
WHO−2001 Adjustment Method

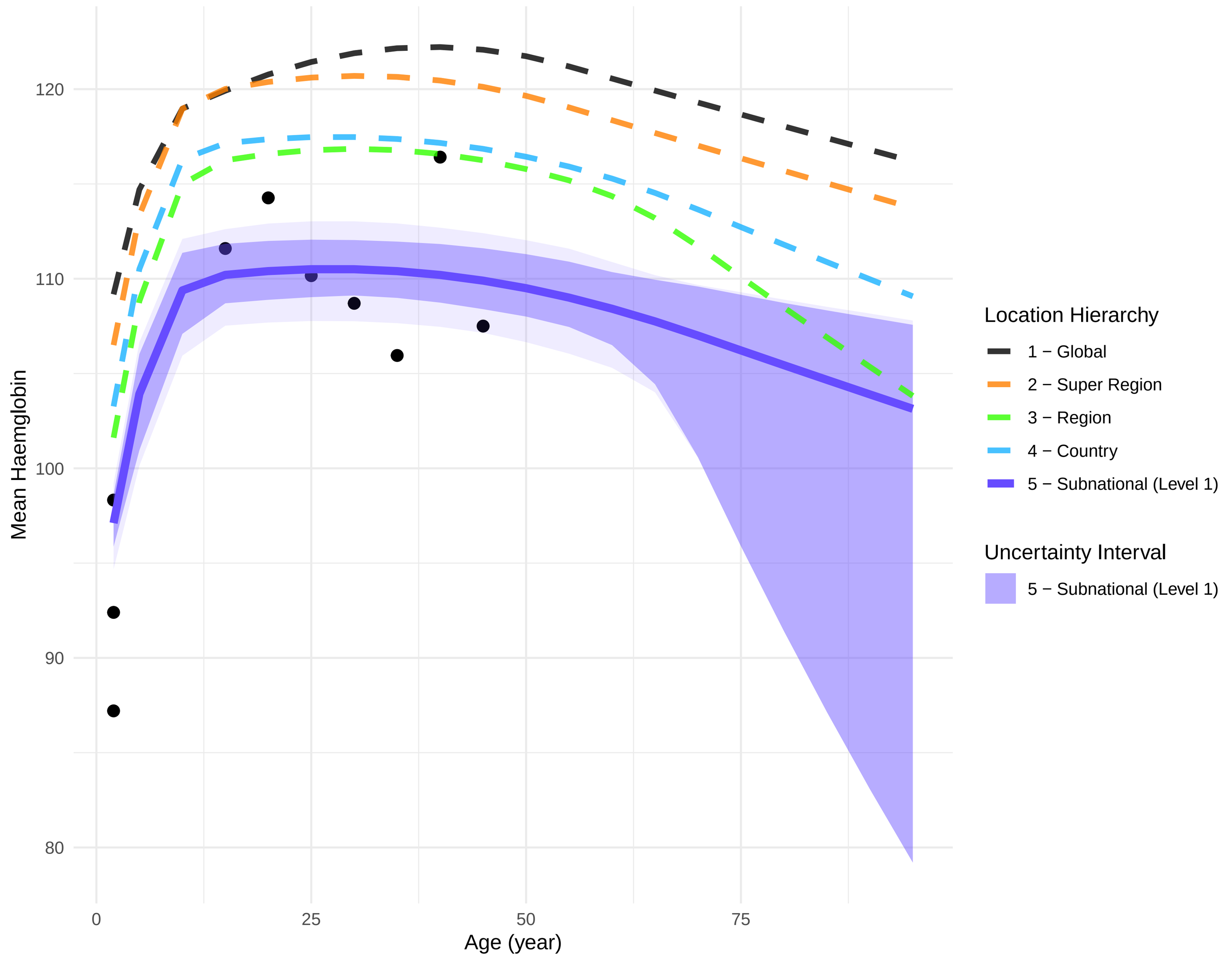

MR−BRT Age/Sex Fits − Non−pregnant Females 2+ Years Old − Mean Haemglobin − Senegal
WHO−2001 Adjustment Method

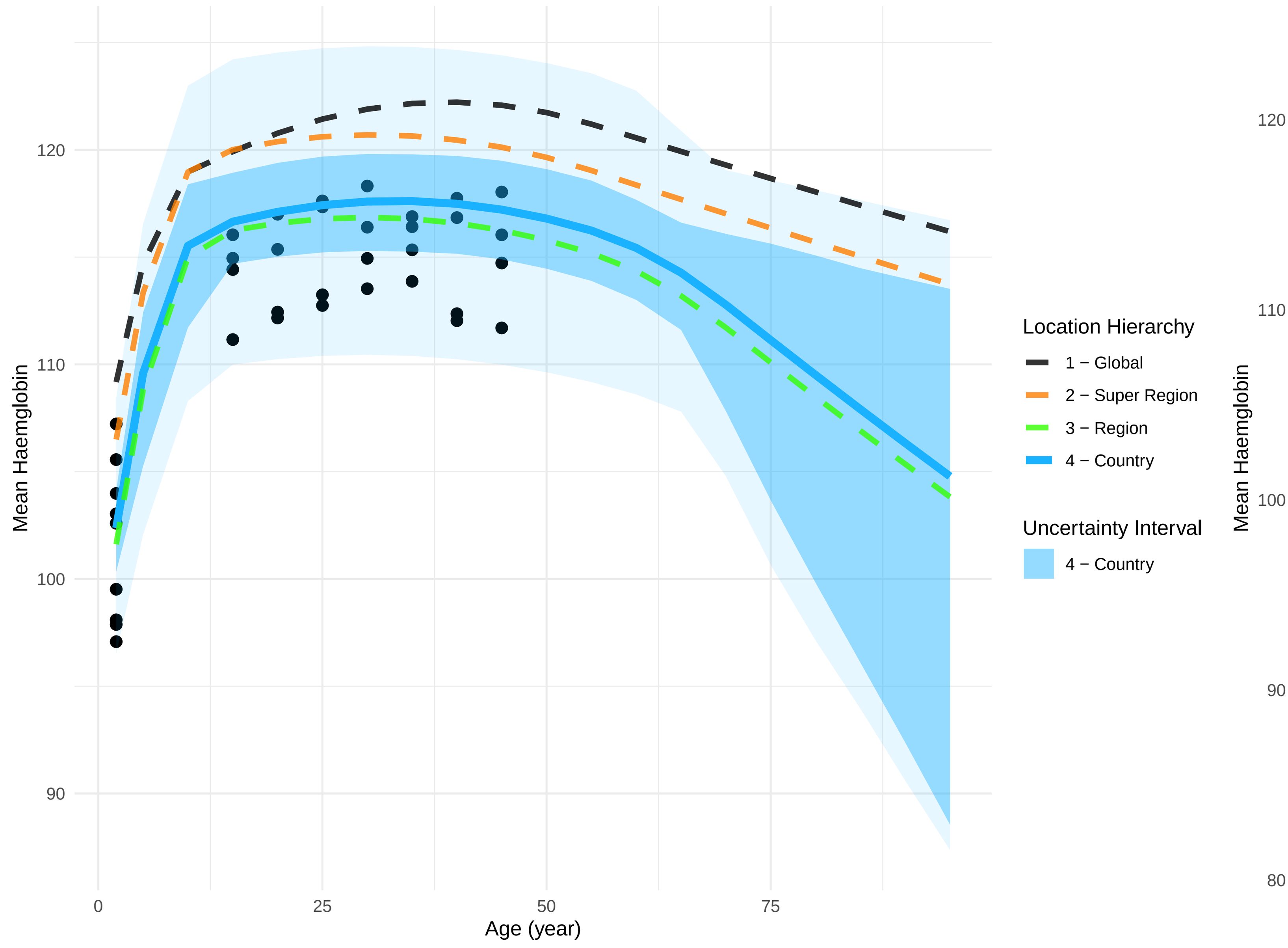

MR−BRT Age/Sex Fits − Non−pregnant Females 2+ Years Old − Mean Haemglobin − Sierra Leone
WHO−2001 Adjustment Method

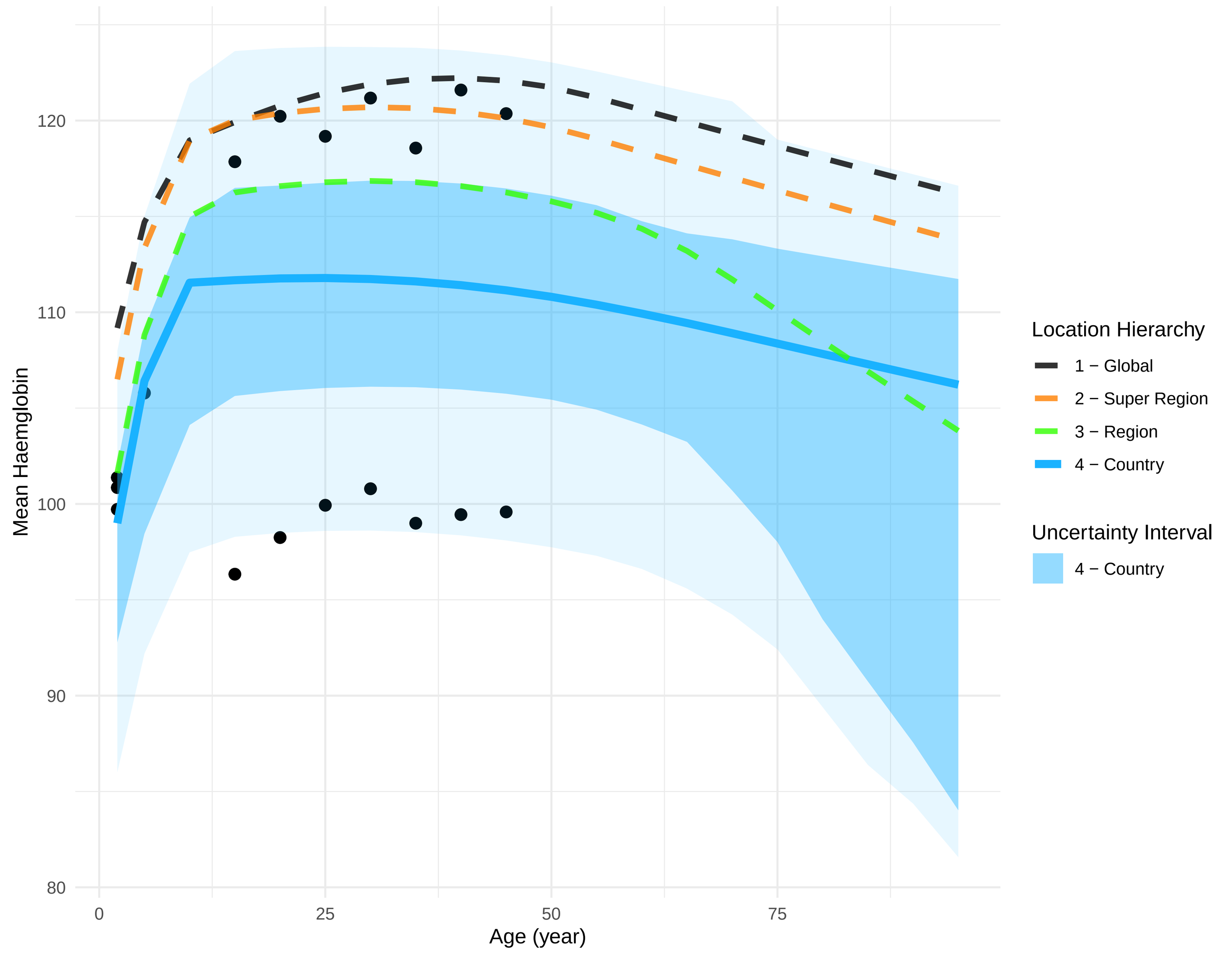

MR−BRT Age/Sex Fits − Non−pregnant Females 2+ Years Old − Mean Haemglobin − Togo
WHO−2001 Adjustment Method

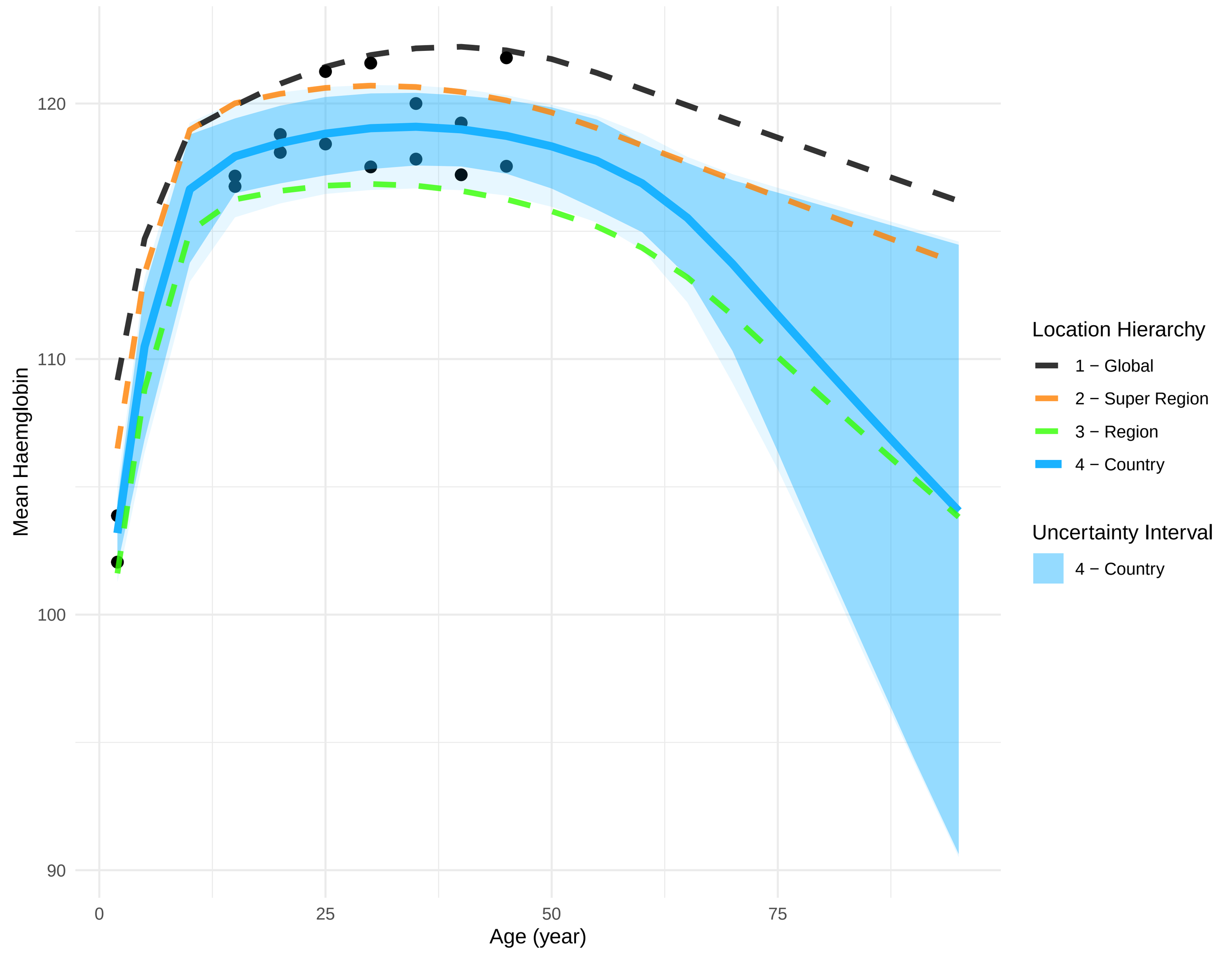

MR−BRT Age/Sex Fits − Males <2 Years Old − Total Anaemia Prevalence − Global
WHO−2024 Adjustment Method

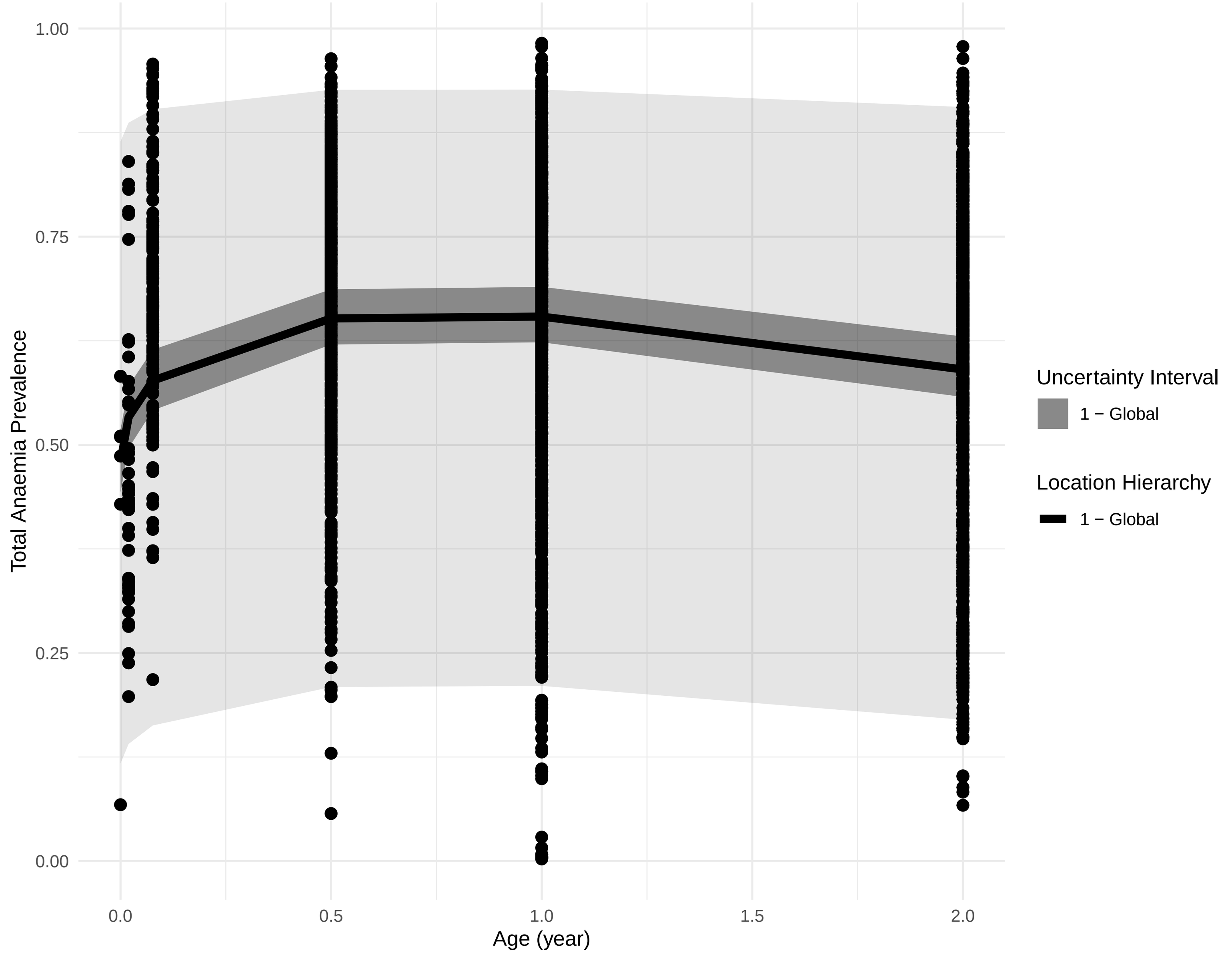

MR−BRT Age/Sex Fits − Males <2 Years Old − Total Anaemia Prevalence − Central Europe, Eastern Europe, and Central Asia
WHO−2024 Adjustment Method

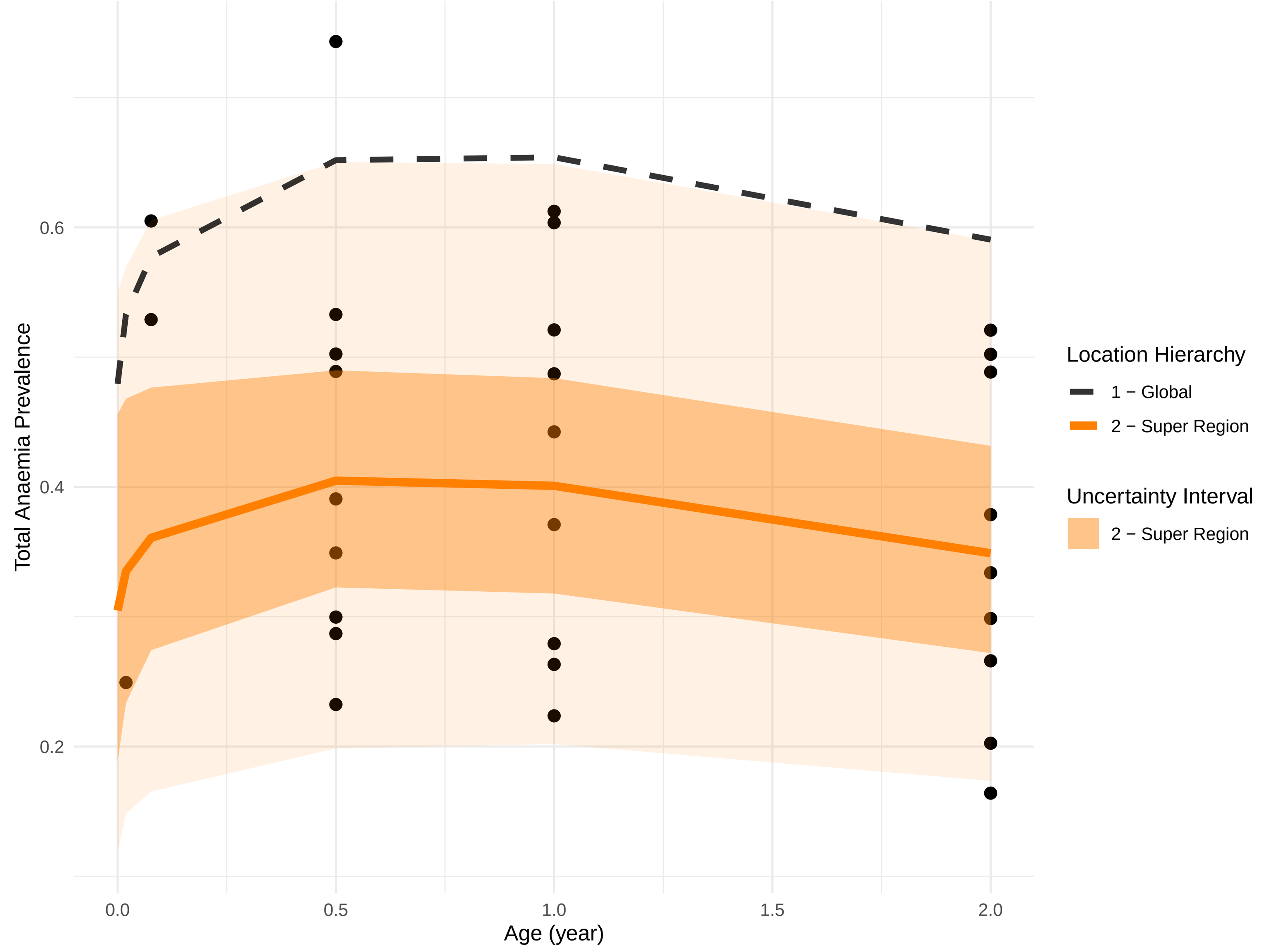

MR−BRT Age/Sex Fits − Males <2 Years Old − Total Anaemia Prevalence − Central Asia
WHO−2024 Adjustment Method

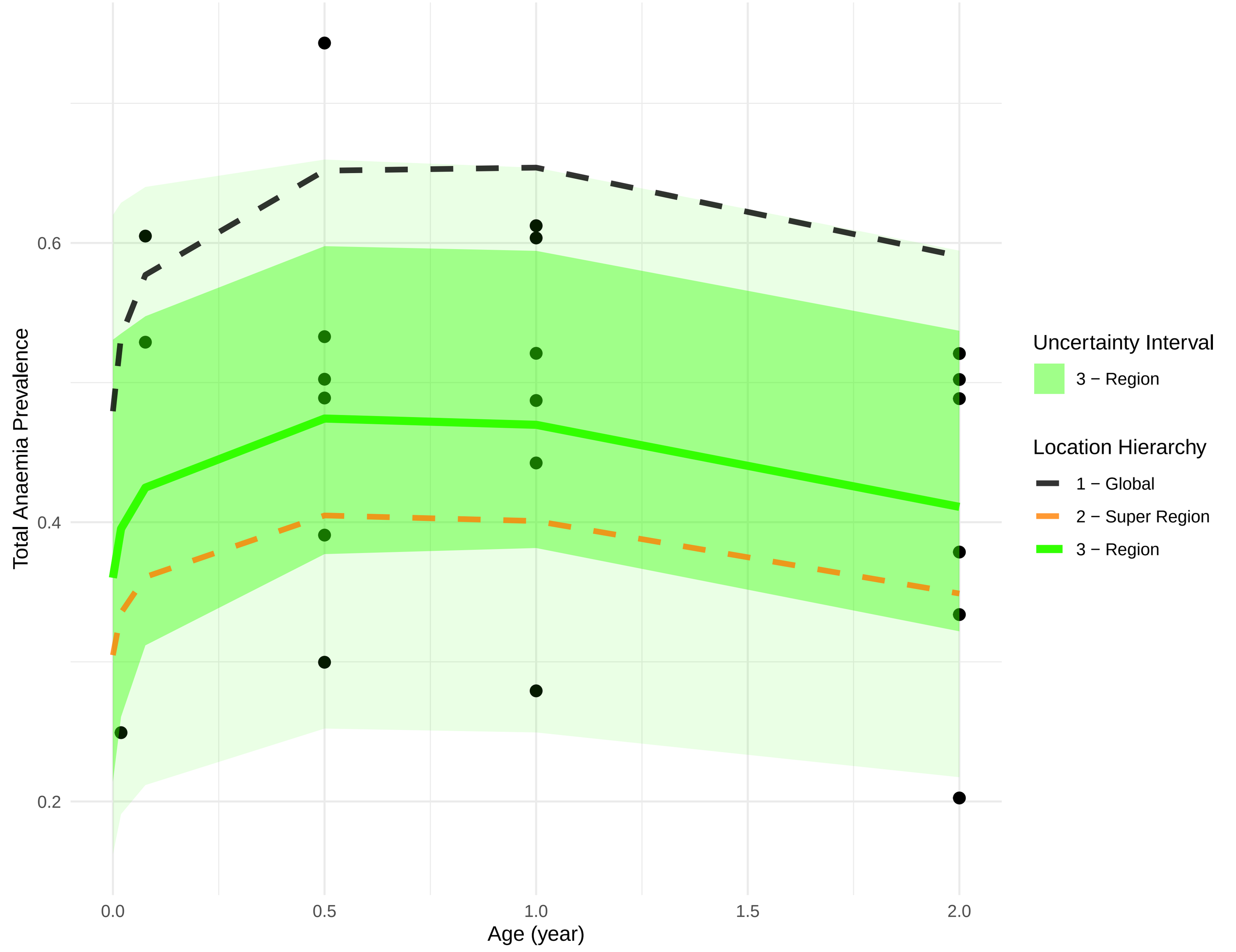

MR−BRT Age/Sex Fits − Males <2 Years Old − Total Anaemia Prevalence − Armenia
WHO−2024 Adjustment Method

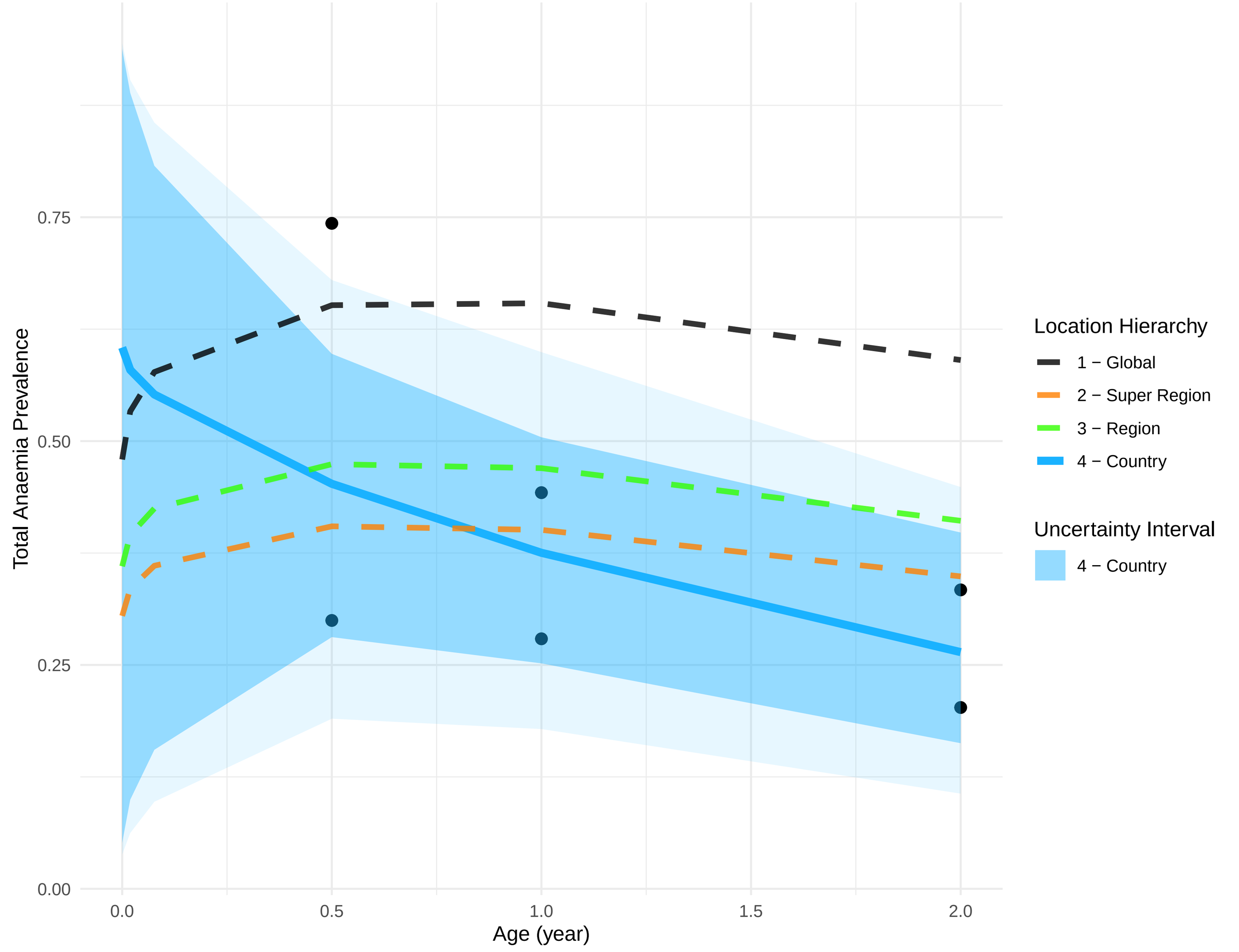

MR−BRT Age/Sex Fits − Males <2 Years Old − Total Anaemia Prevalence − Azerbaijan
WHO−2024 Adjustment Method

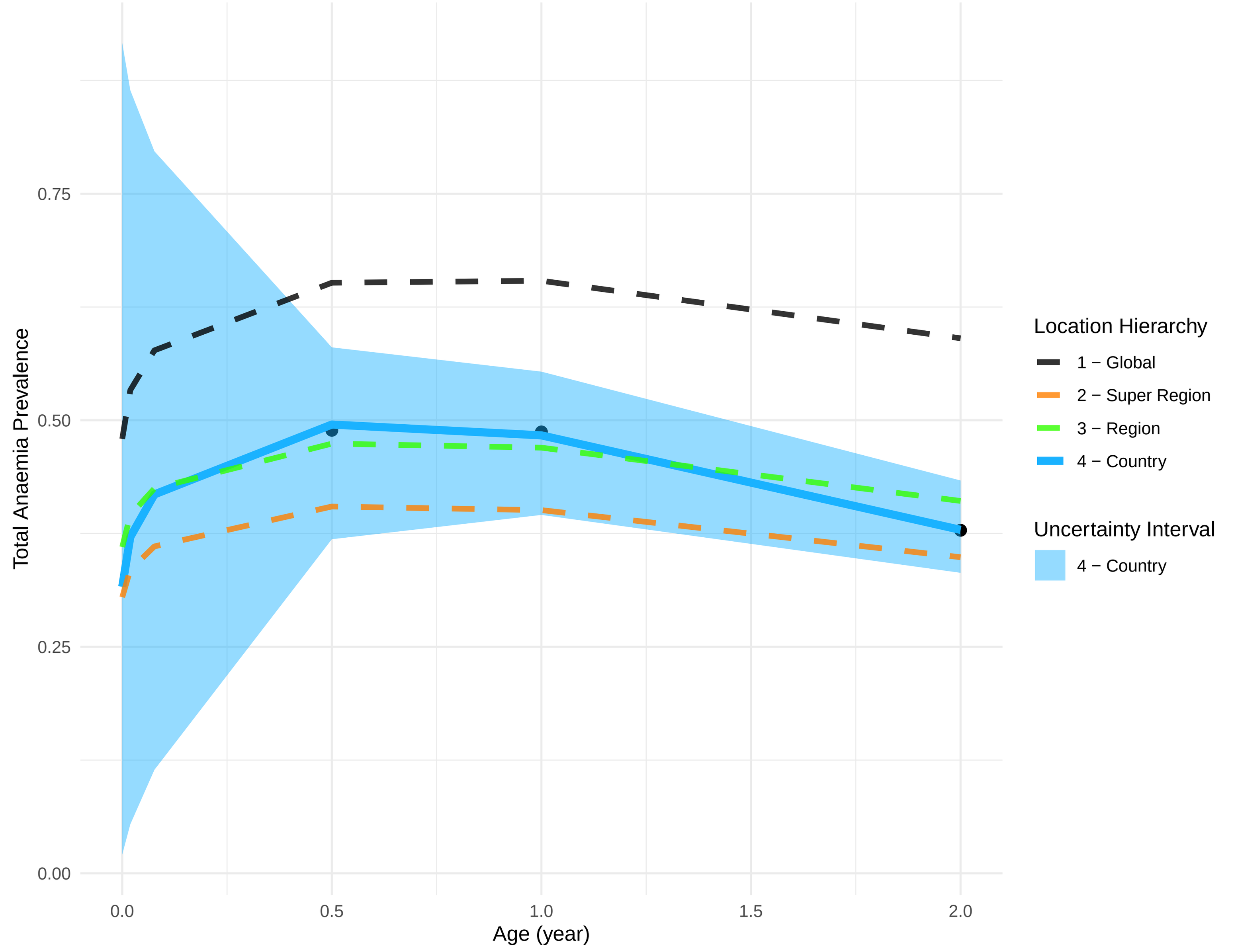

MR−BRT Age/Sex Fits − Males <2 Years Old − Total Anaemia Prevalence − Kyrgyzstan
WHO−2024 Adjustment Method

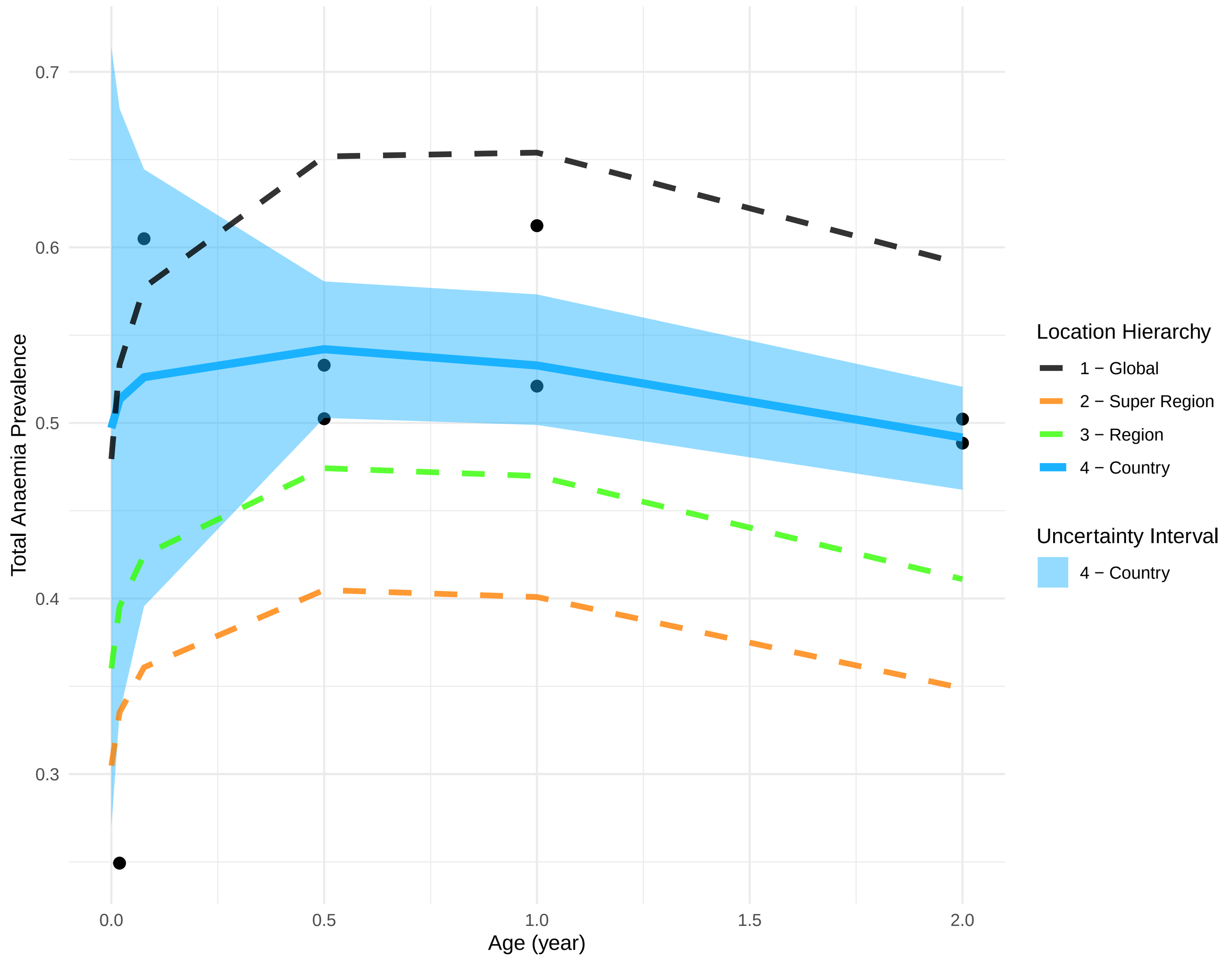

MR−BRT Age/Sex Fits − Males <2 Years Old − Total Anaemia Prevalence − Uzbekistan
WHO−2024 Adjustment Method

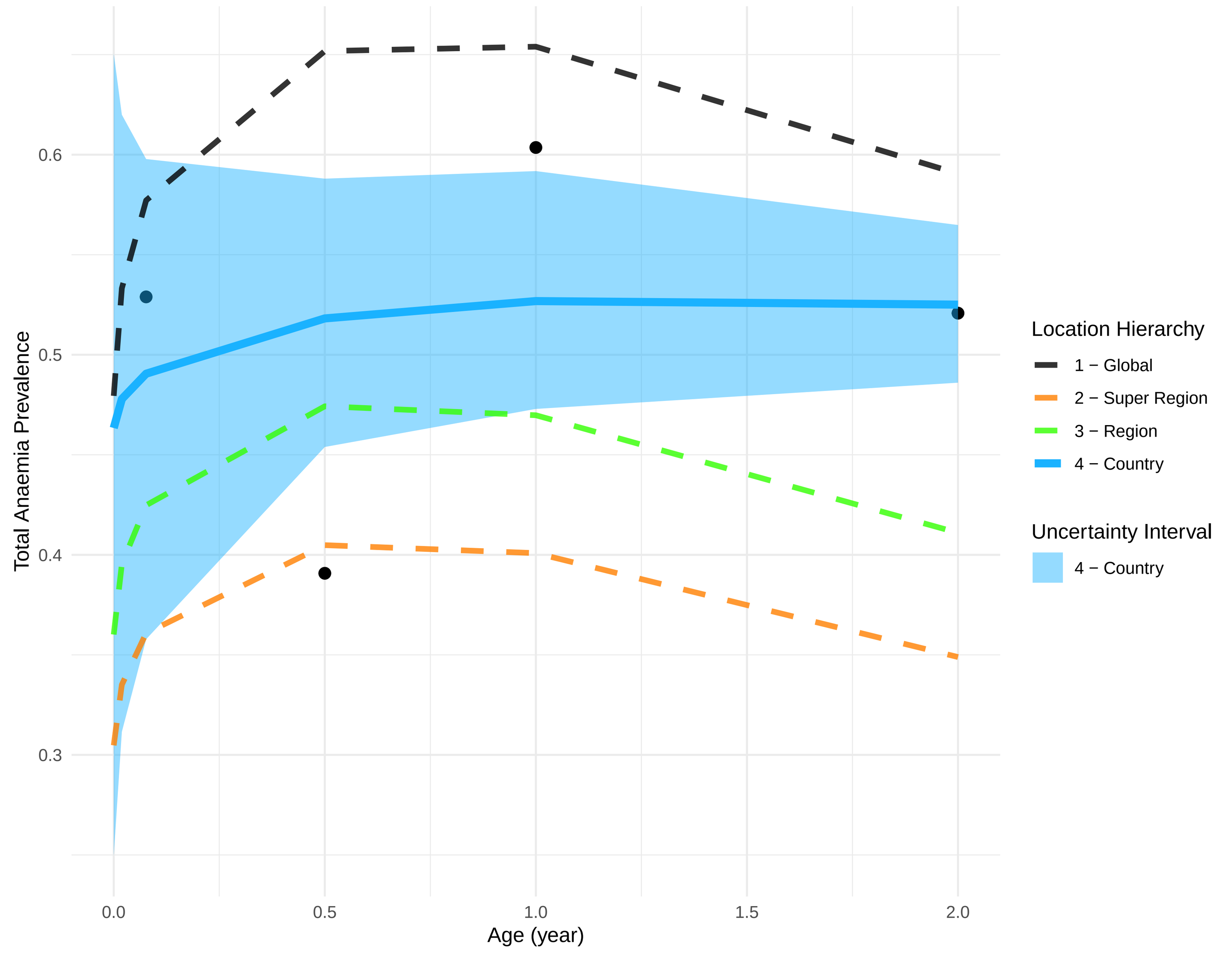

MR−BRT Age/Sex Fits − Males <2 Years Old − Total Anaemia Prevalence − Central Europe
WHO−2024 Adjustment Method

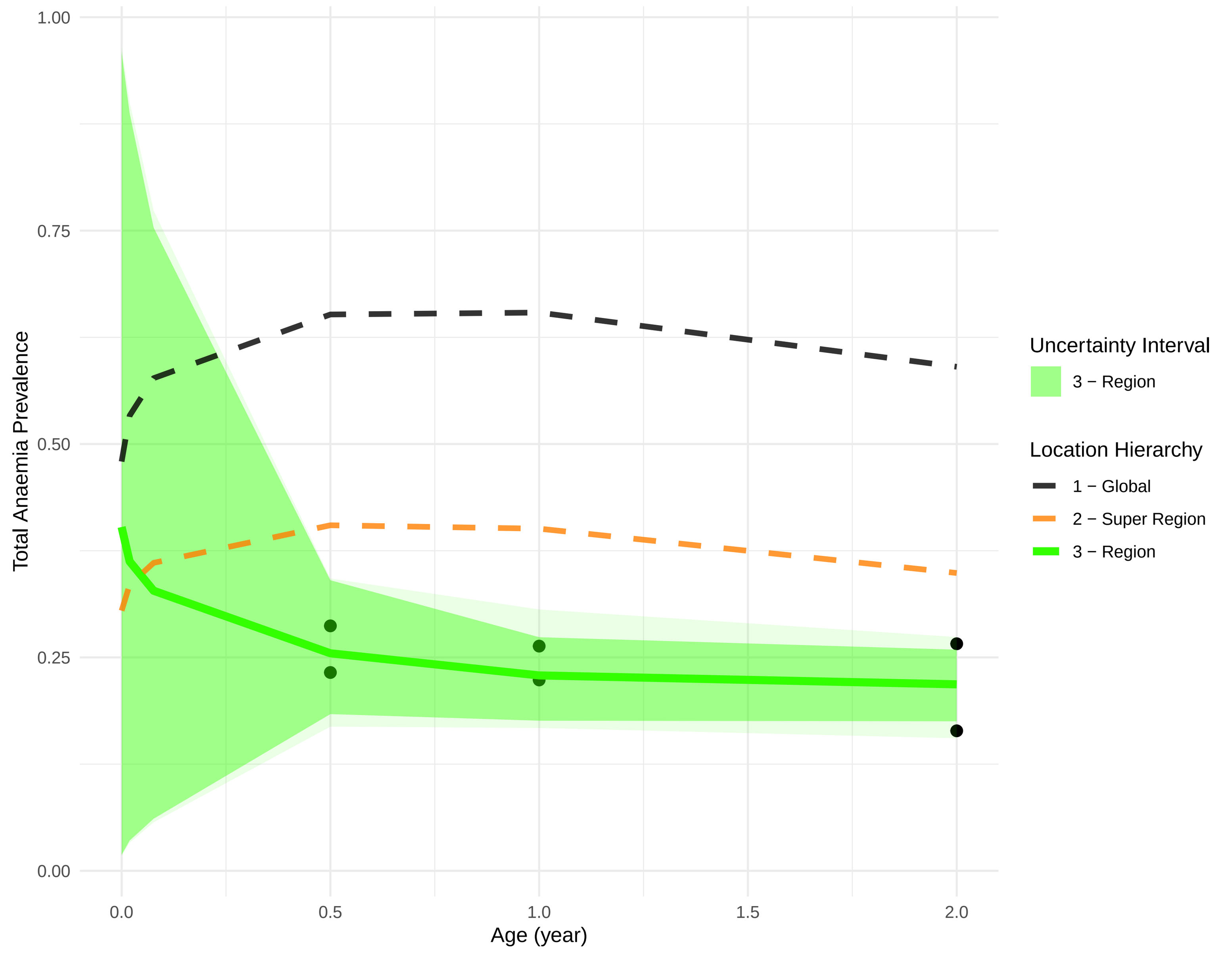

MR−BRT Age/Sex Fits − Males <2 Years Old − Total Anaemia Prevalence − Albania
WHO−2024 Adjustment Method

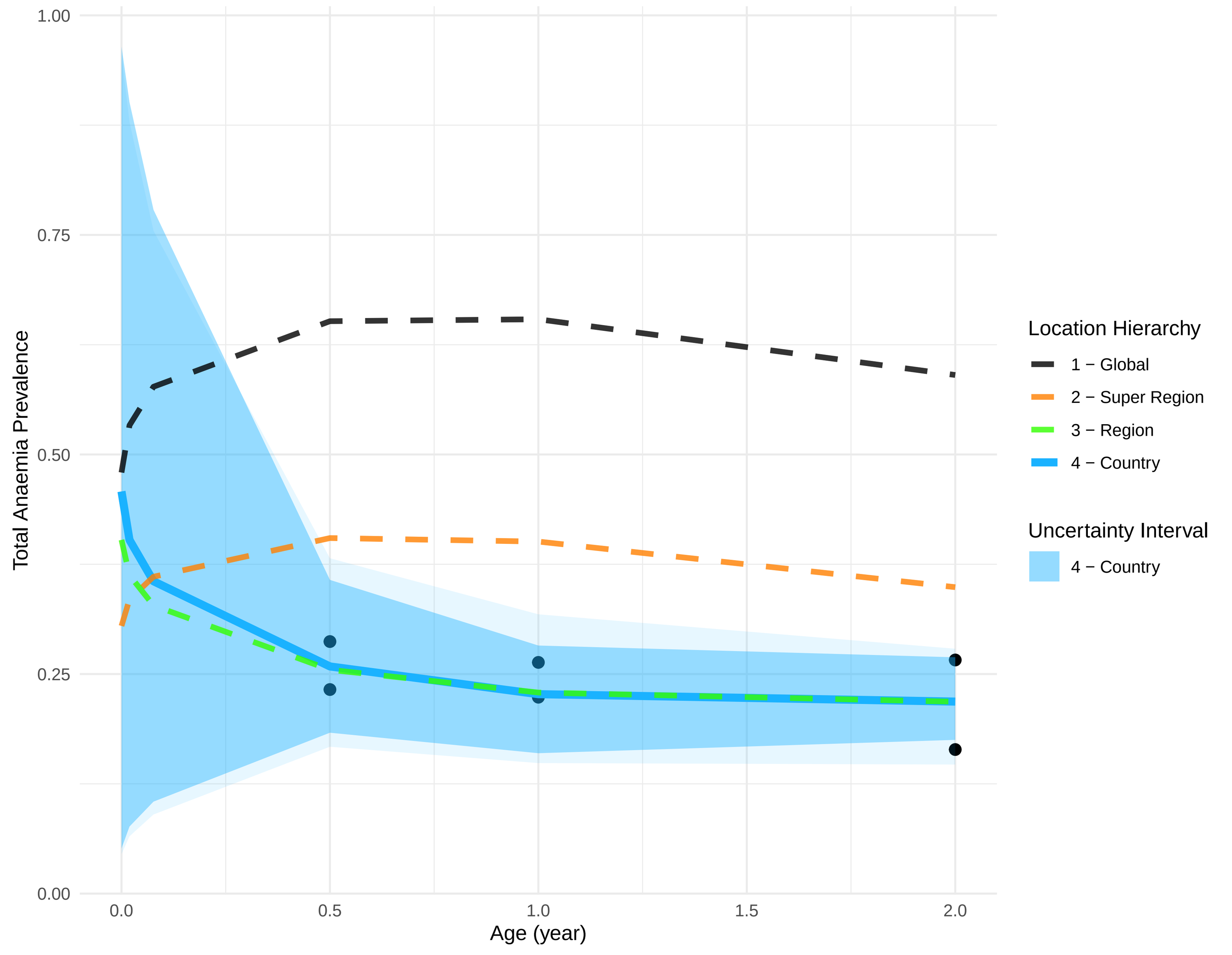

MR−BRT Age/Sex Fits − Males <2 Years Old − Total Anaemia Prevalence − Eastern Europe
WHO−2024 Adjustment Method

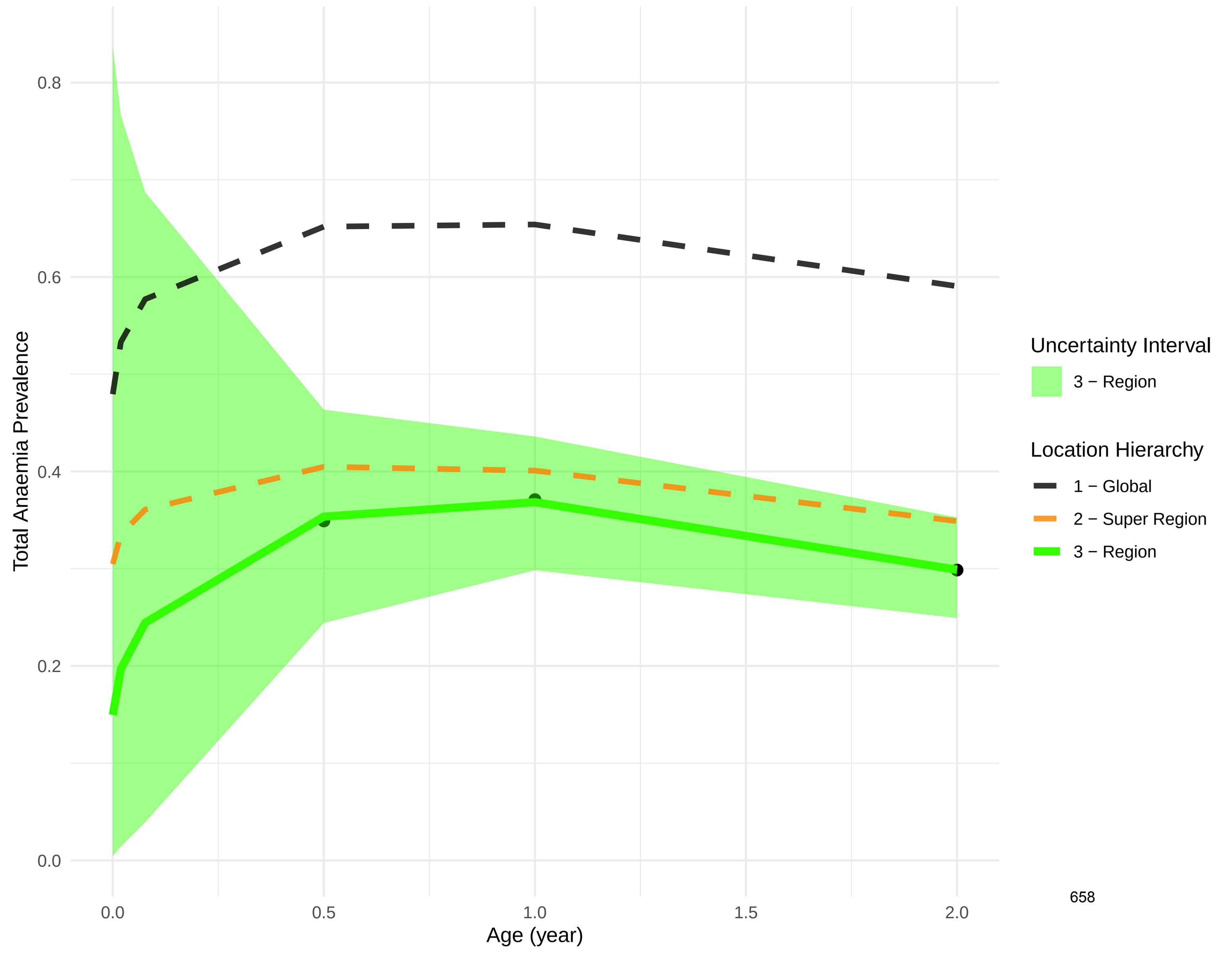

MR−BRT Age/Sex Fits − Males <2 Years Old − Total Anaemia Prevalence − Republic of Moldova
WHO−2024 Adjustment Method

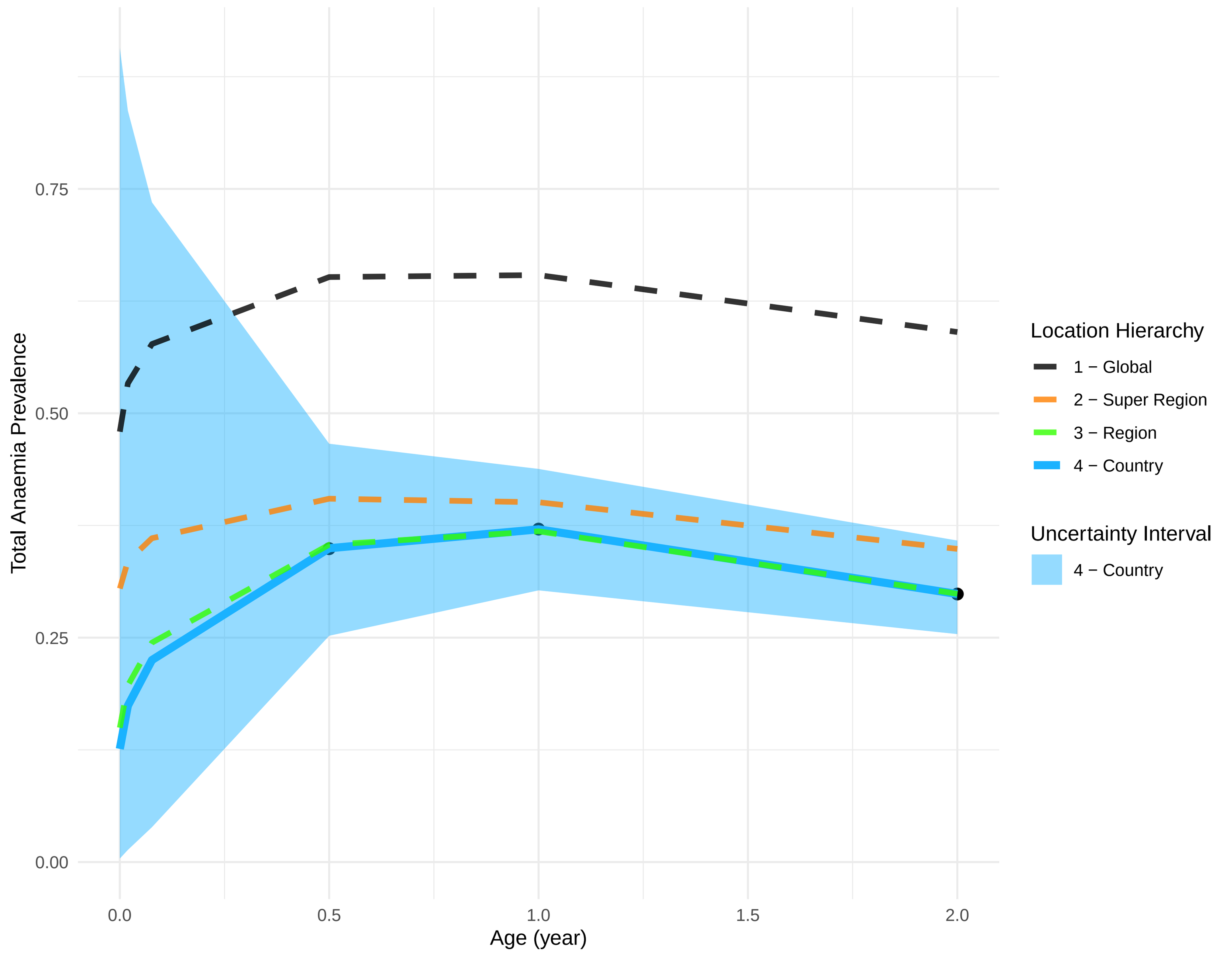

MR−BRT Age/Sex Fits − Males <2 Years Old − Total Anaemia Prevalence − High−income
WHO−2024 Adjustment Method

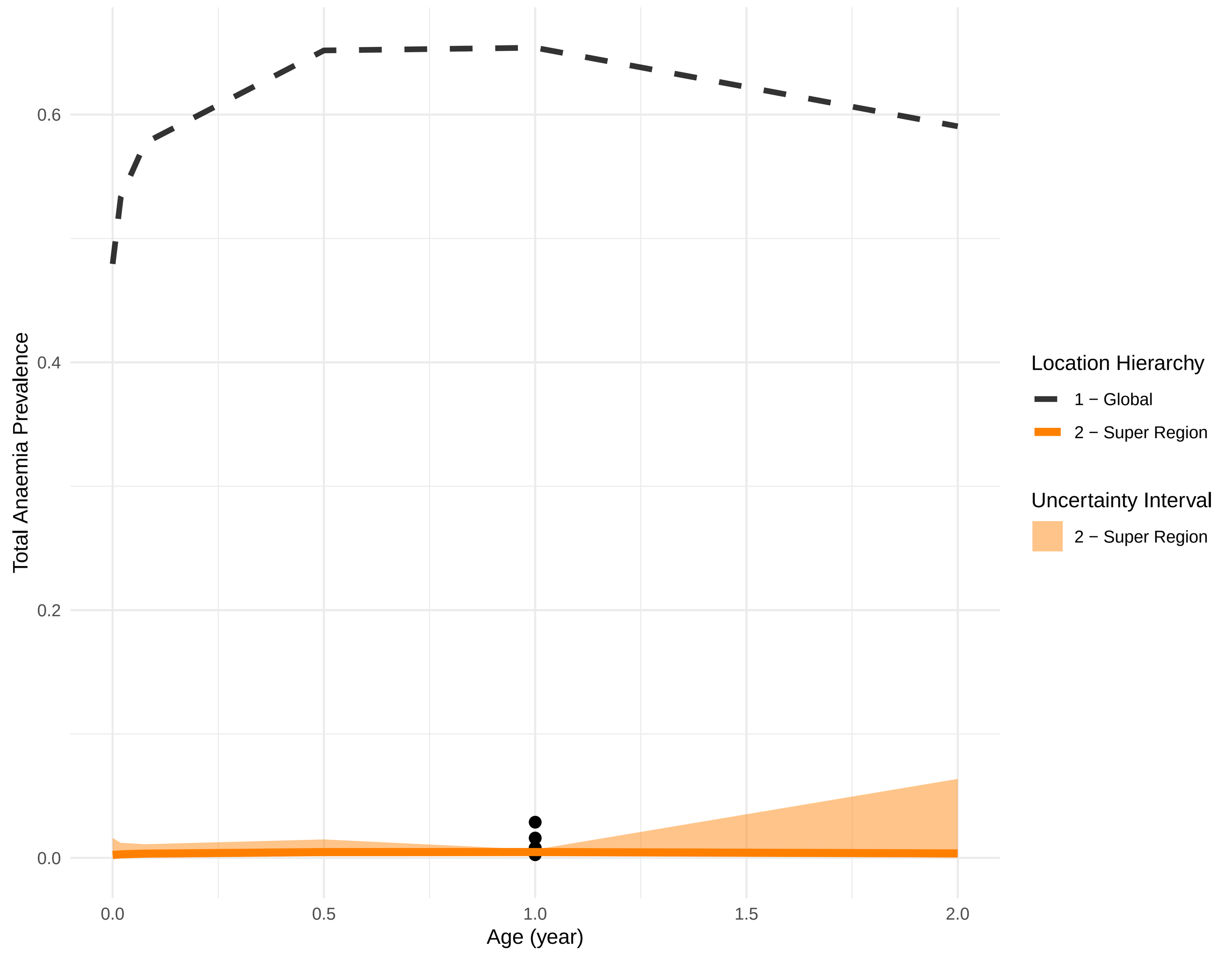

MR−BRT Age/Sex Fits − Males <2 Years Old − Total Anaemia Prevalence − Western Europe
WHO−2024 Adjustment Method

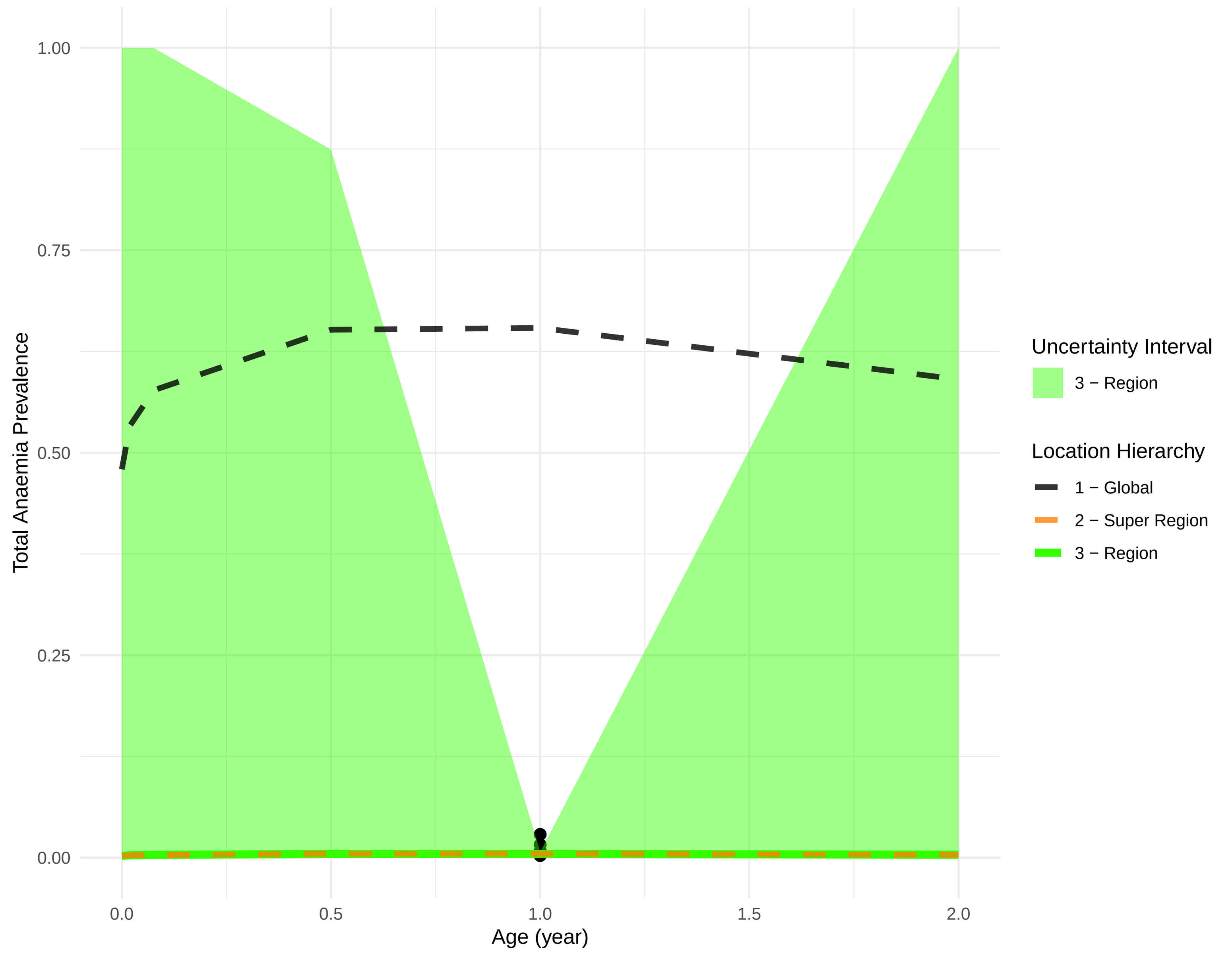

MR−BRT Age/Sex Fits − Males <2 Years Old − Total Anaemia Prevalence − United Kingdom
WHO−2024 Adjustment Method

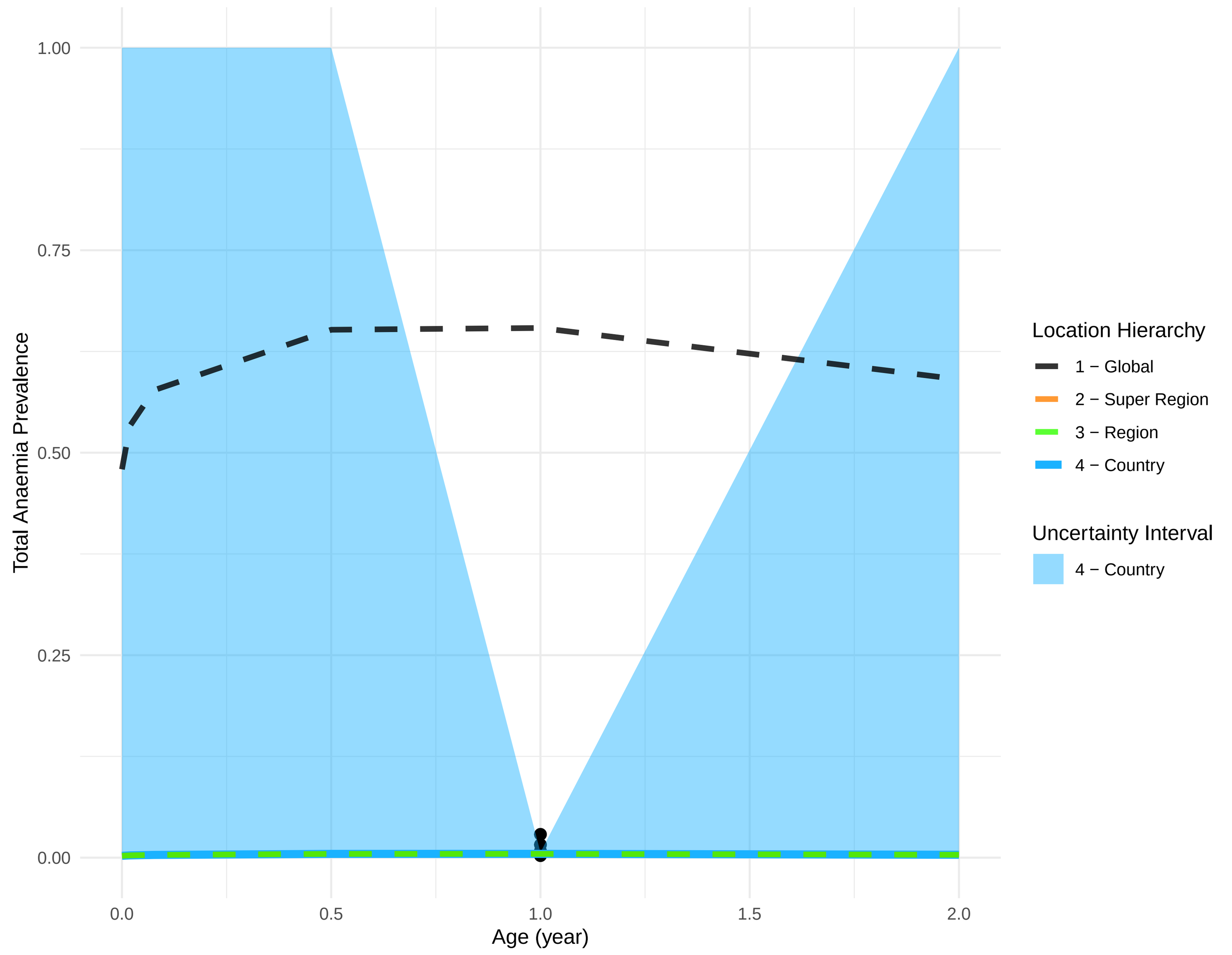

MR−BRT Age/Sex Fits − Males <2 Years Old − Total Anaemia Prevalence − England
WHO−2024 Adjustment Method

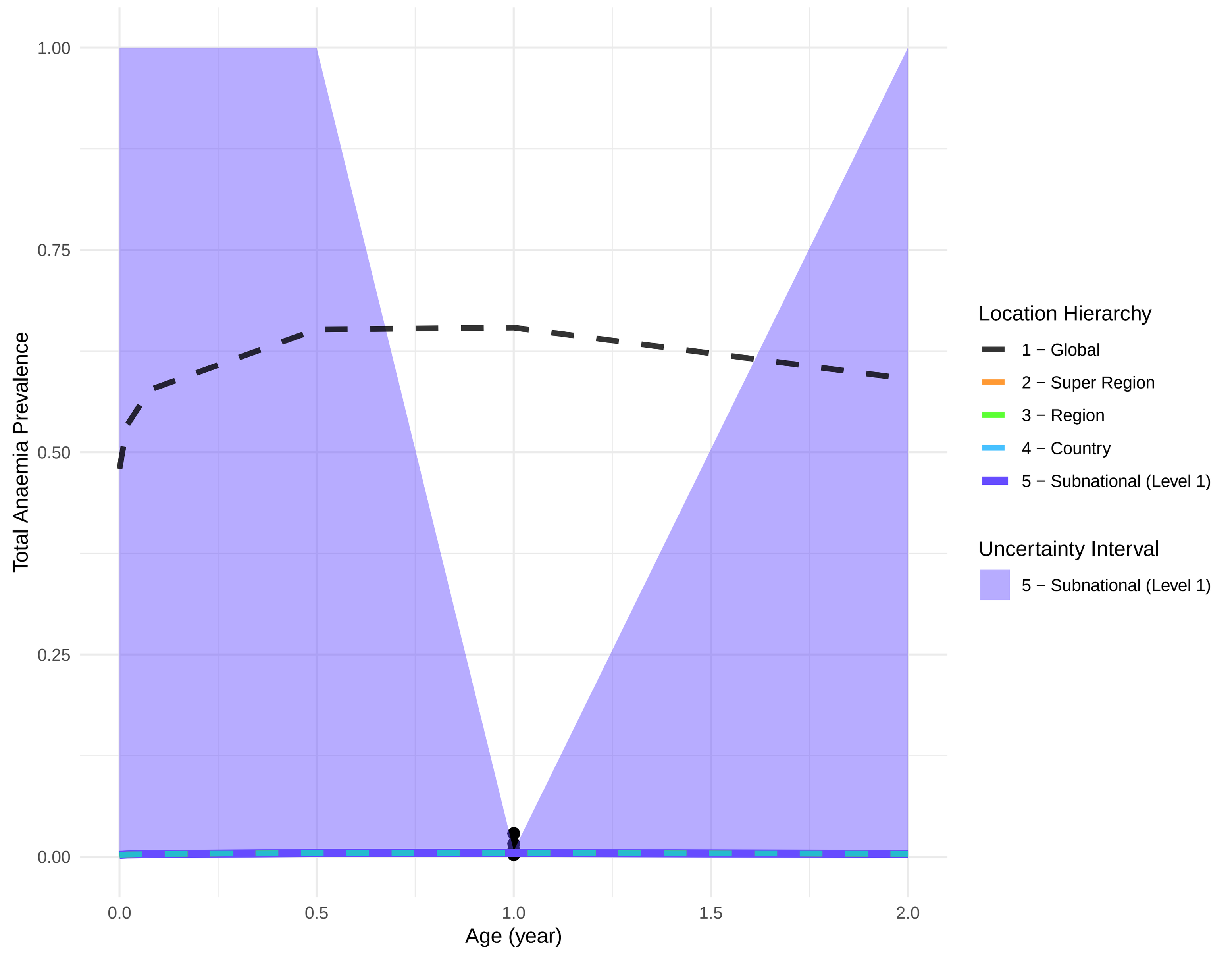

MR−BRT Age/Sex Fits − Males <2 Years Old − Total Anaemia Prevalence − East Midlands
WHO−2024 Adjustment Method

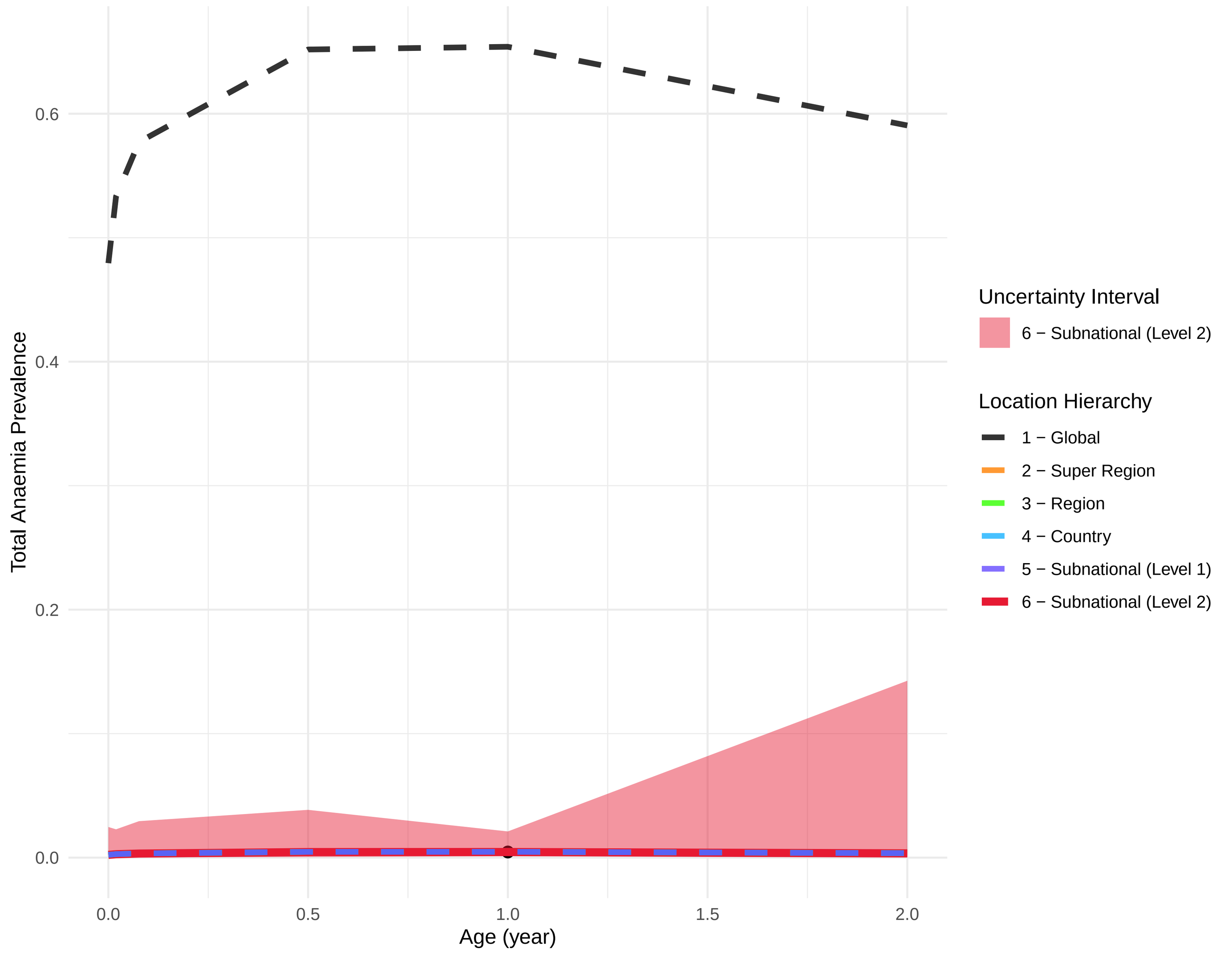

MR−BRT Age/Sex Fits − Males <2 Years Old − Total Anaemia Prevalence − Greater London
WHO−2024 Adjustment Method

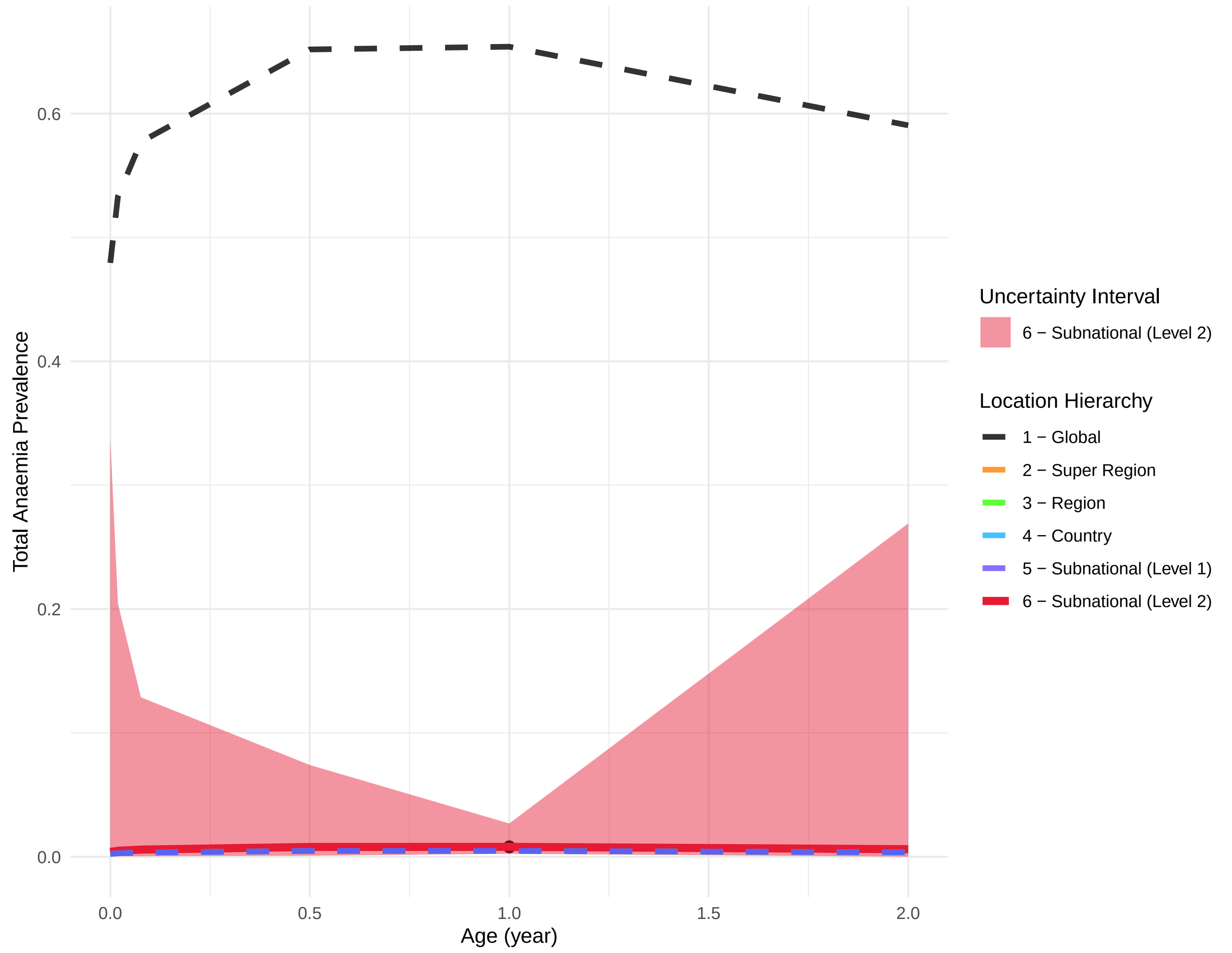

MR−BRT Age/Sex Fits − Males <2 Years Old − Total Anaemia Prevalence − North East England
WHO−2024 Adjustment Method

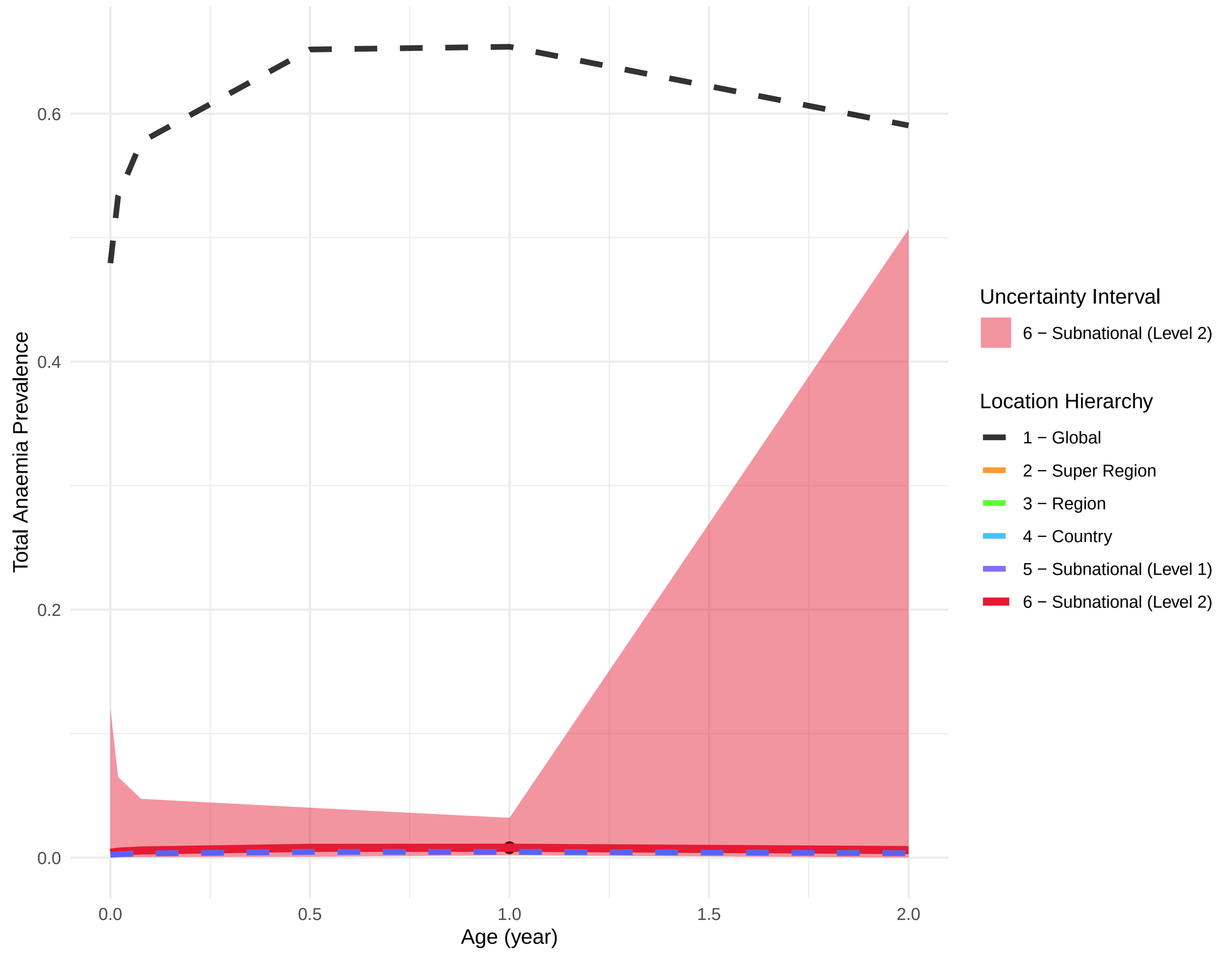

MR−BRT Age/Sex Fits − Males <2 Years Old − Total Anaemia Prevalence − North West England
WHO−2024 Adjustment Method

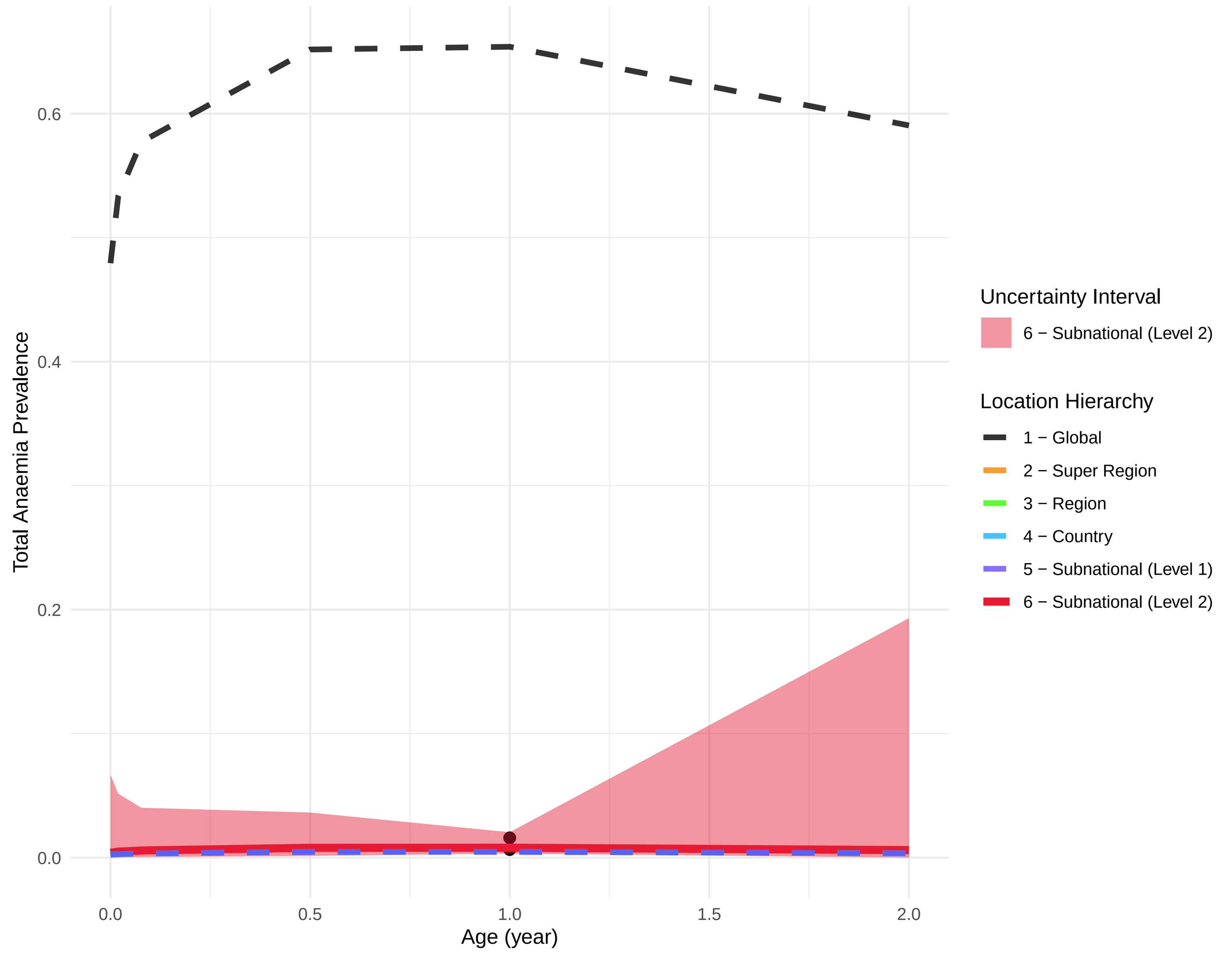

MR−BRT Age/Sex Fits − Males <2 Years Old − Total Anaemia Prevalence − South East England
WHO−2024 Adjustment Method

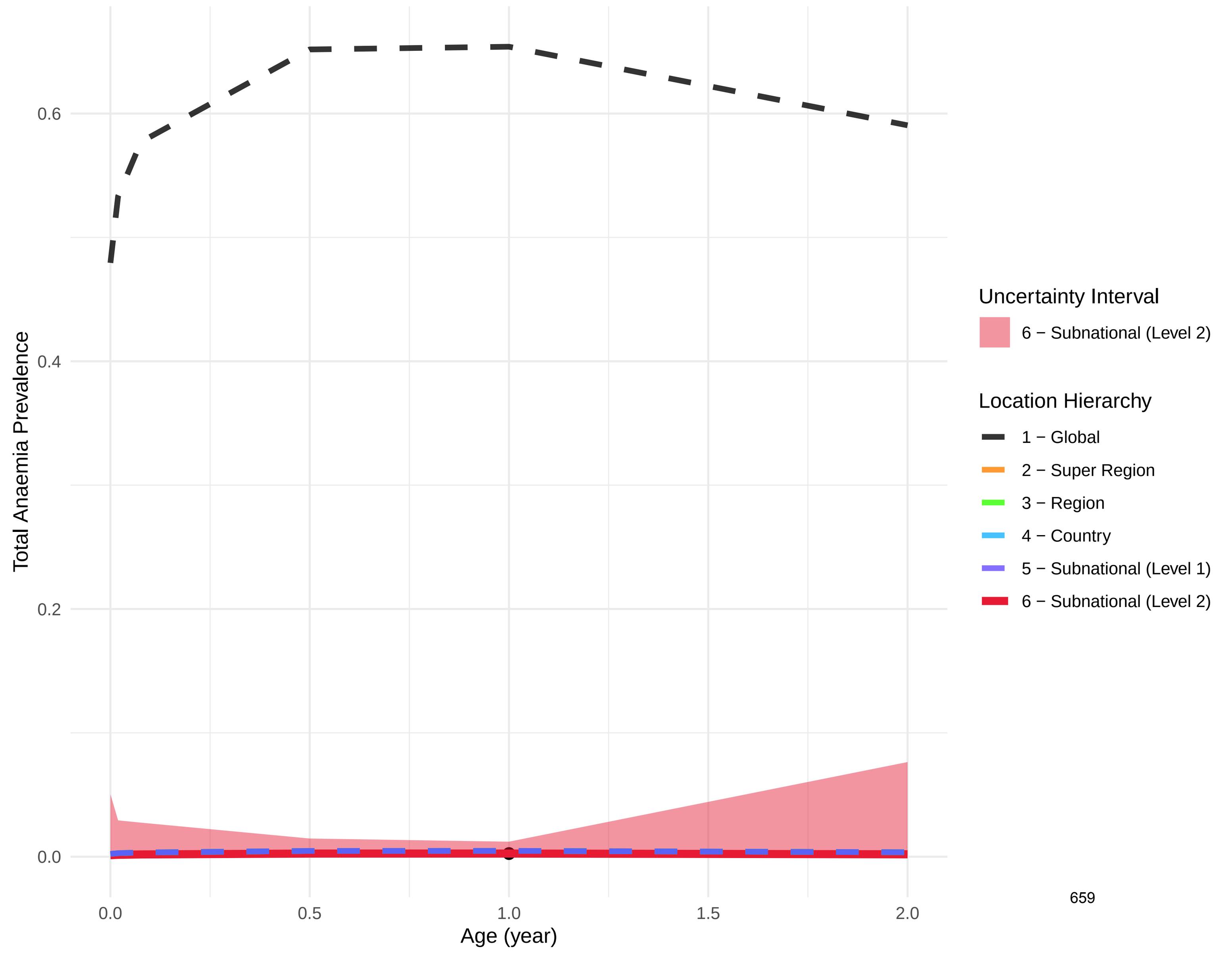

MR−BRT Age/Sex Fits − Males <2 Years Old − Total Anaemia Prevalence − South West England
WHO−2024 Adjustment Method

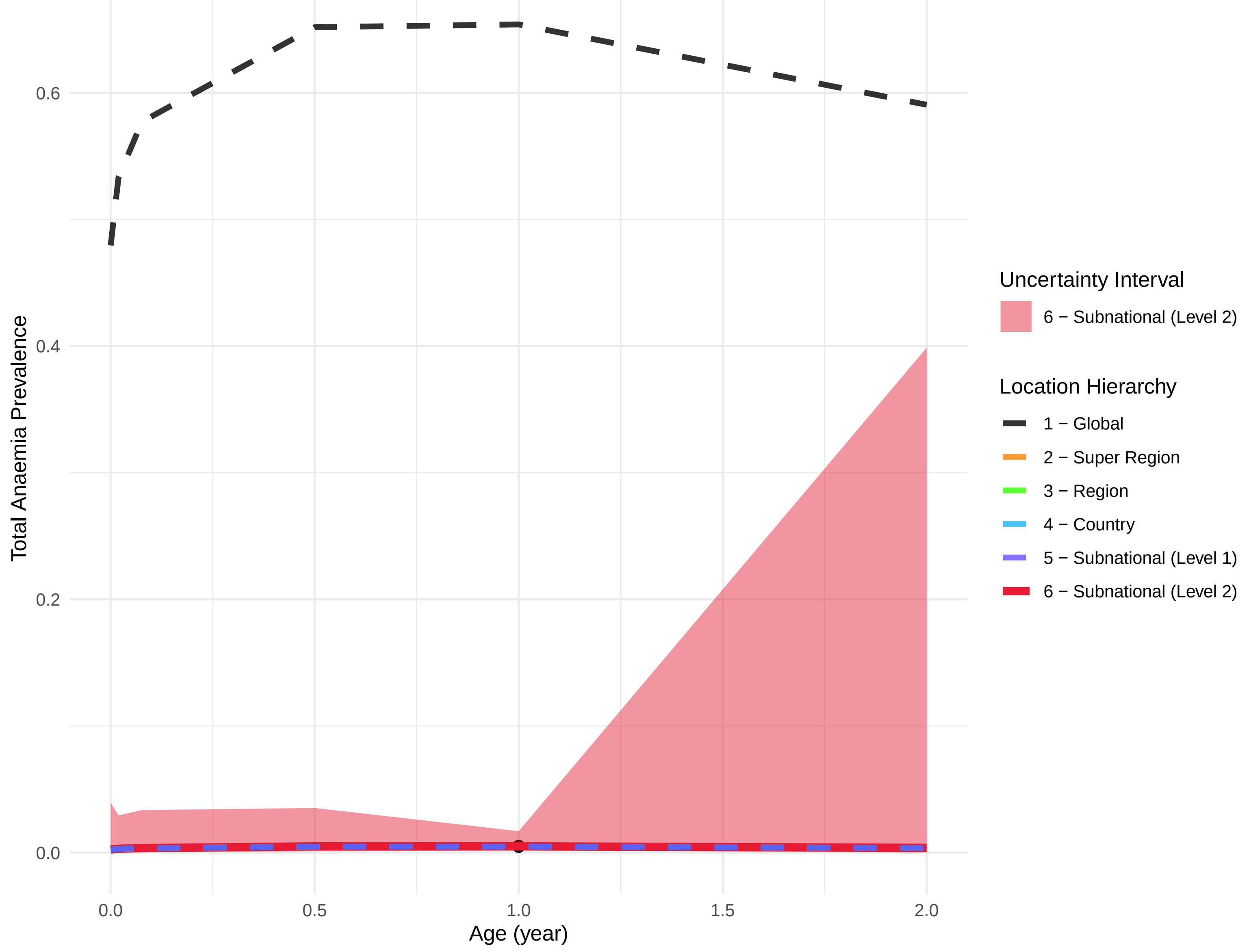

MR−BRT Age/Sex Fits − Males <2 Years Old − Total Anaemia Prevalence − West Midlands
WHO−2024 Adjustment Method

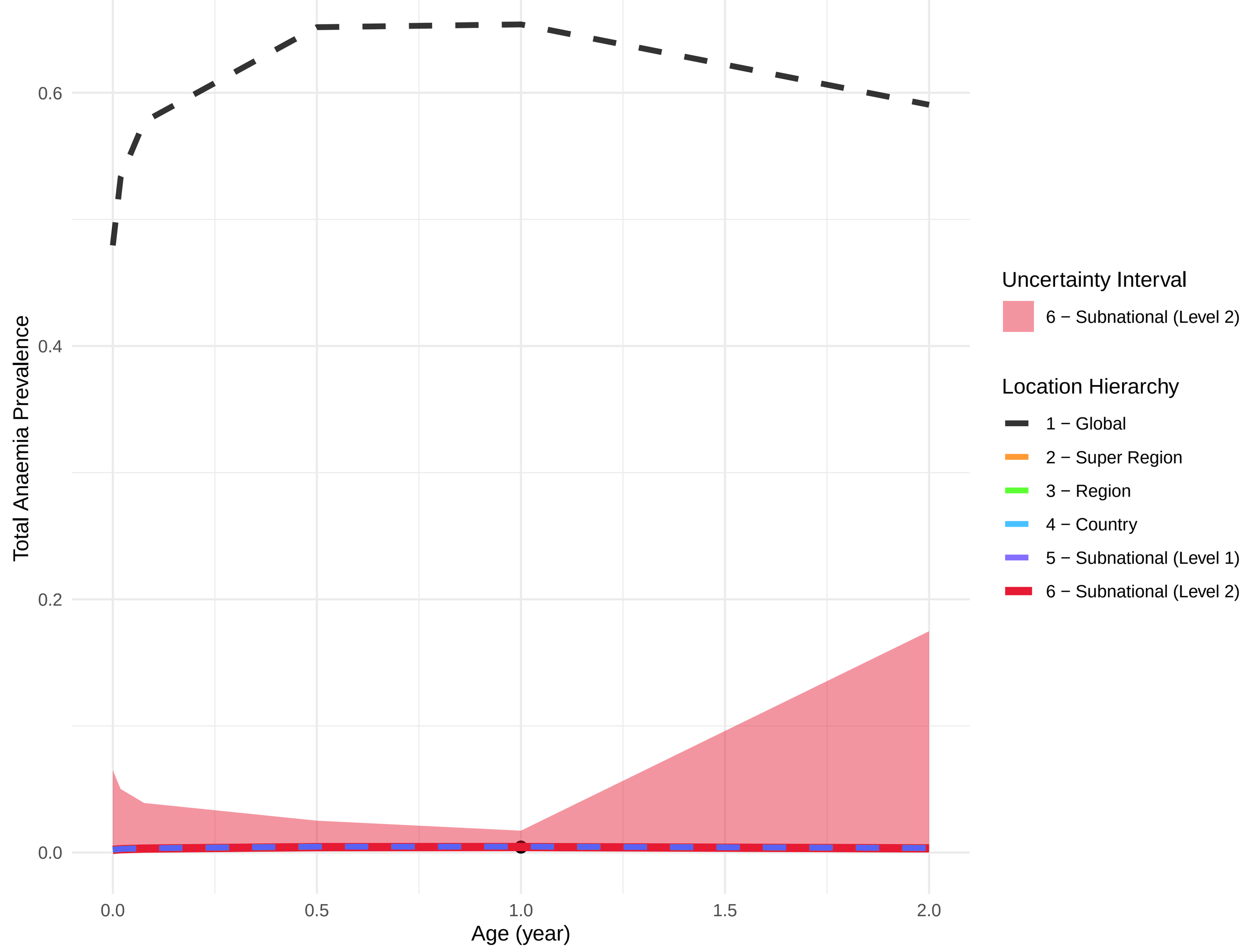

MR−BRT Age/Sex Fits − Males <2 Years Old − Total Anaemia Prevalence − Yorkshire and the Humber
WHO−2024 Adjustment Method

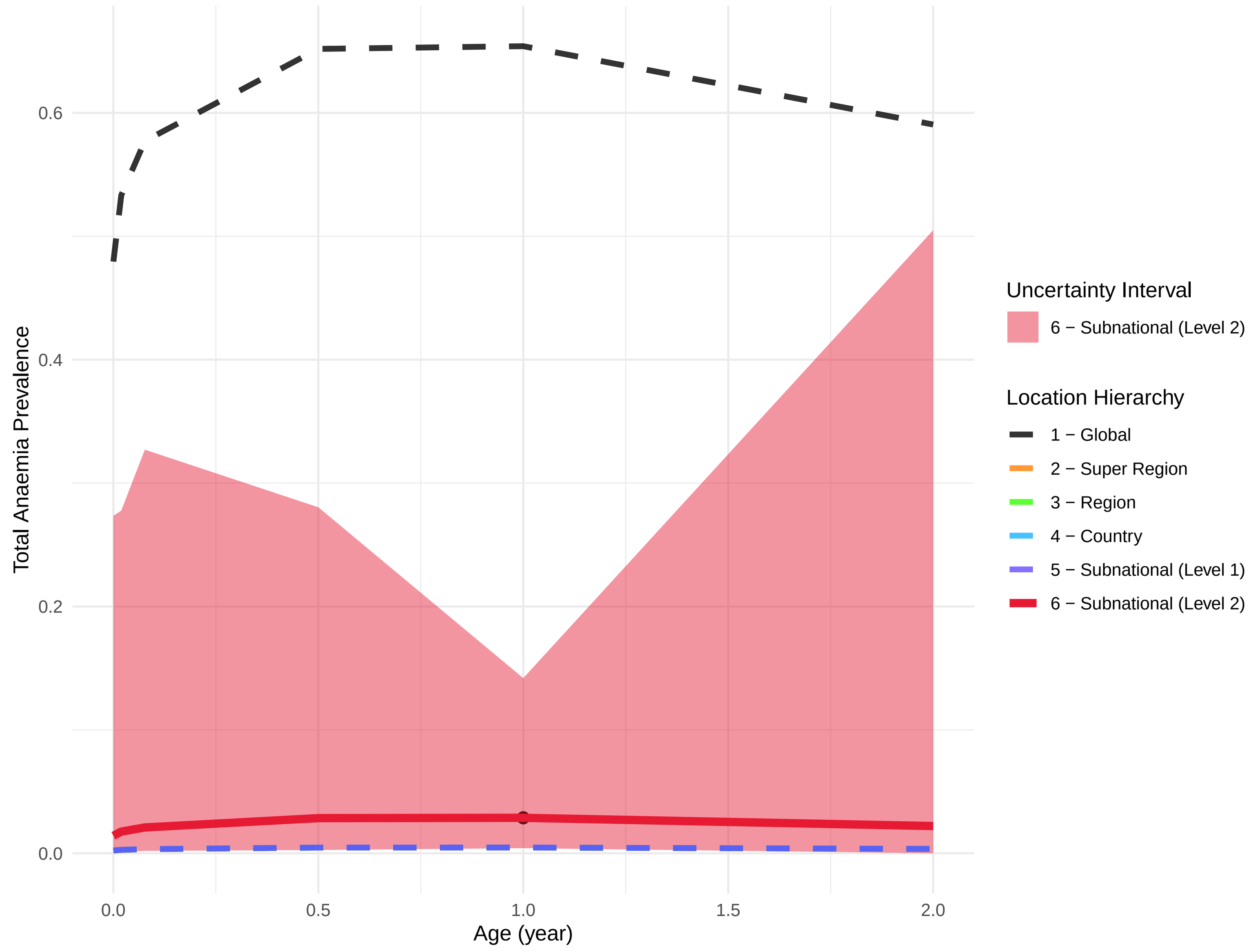

MR−BRT Age/Sex Fits − Males <2 Years Old − Total Anaemia Prevalence − Latin America and Caribbean
WHO−2024 Adjustment Method

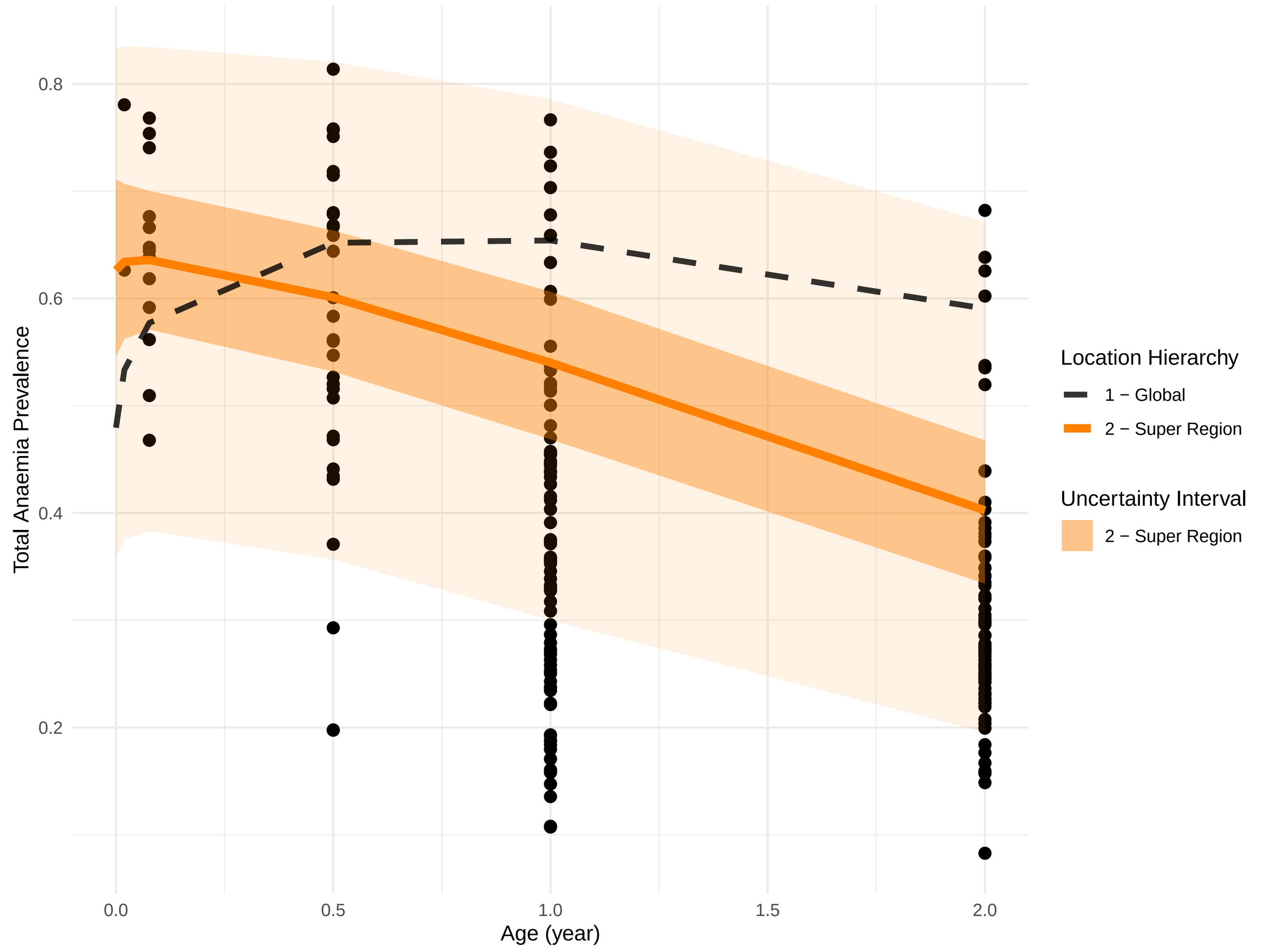

MR−BRT Age/Sex Fits − Males <2 Years Old − Total Anaemia Prevalence − Andean Latin America
WHO−2024 Adjustment Method

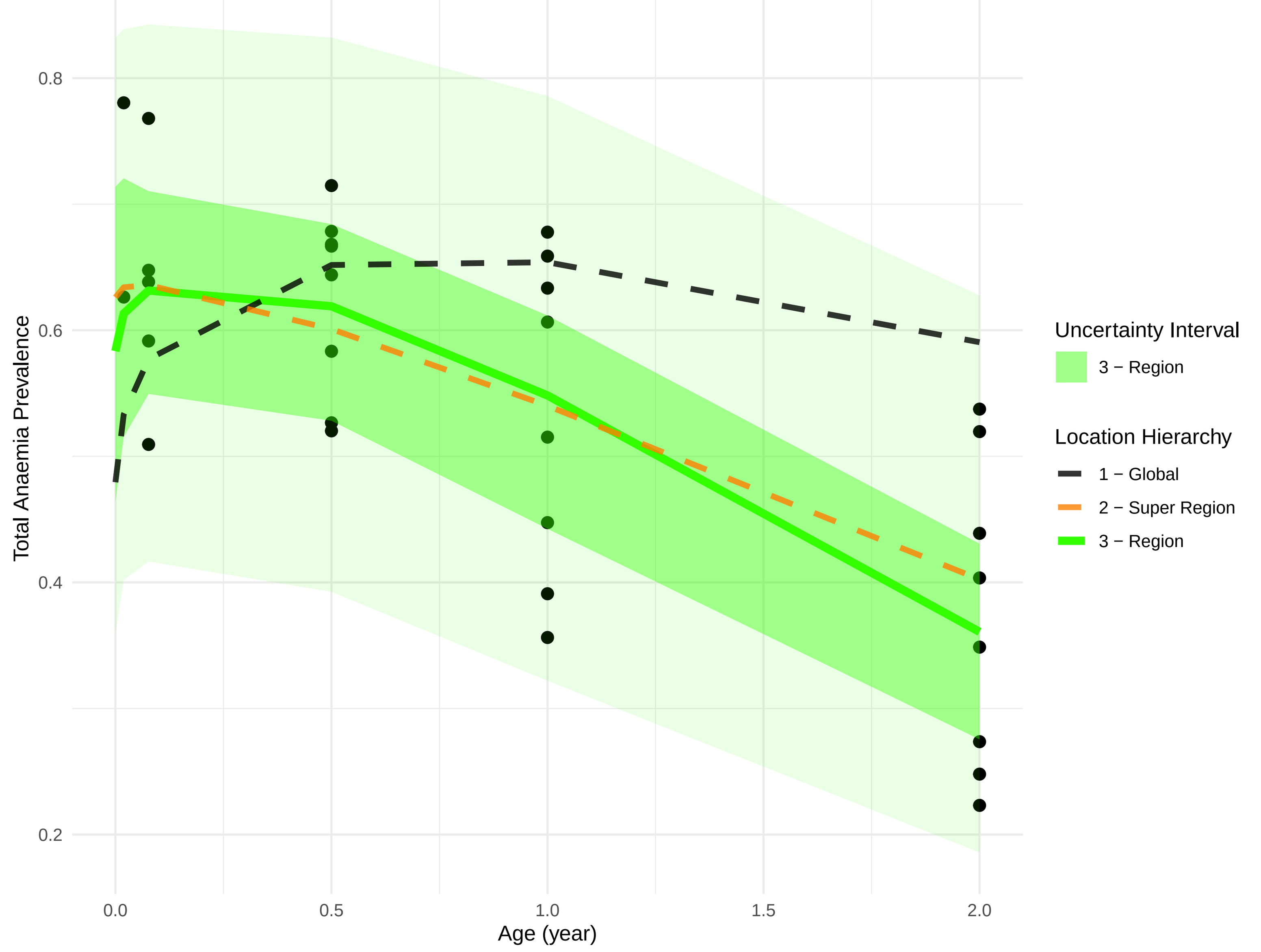

MR−BRT Age/Sex Fits − Males <2 Years Old − Total Anaemia Prevalence − Bolivia (Plurinational State of)
WHO−2024 Adjustment Method

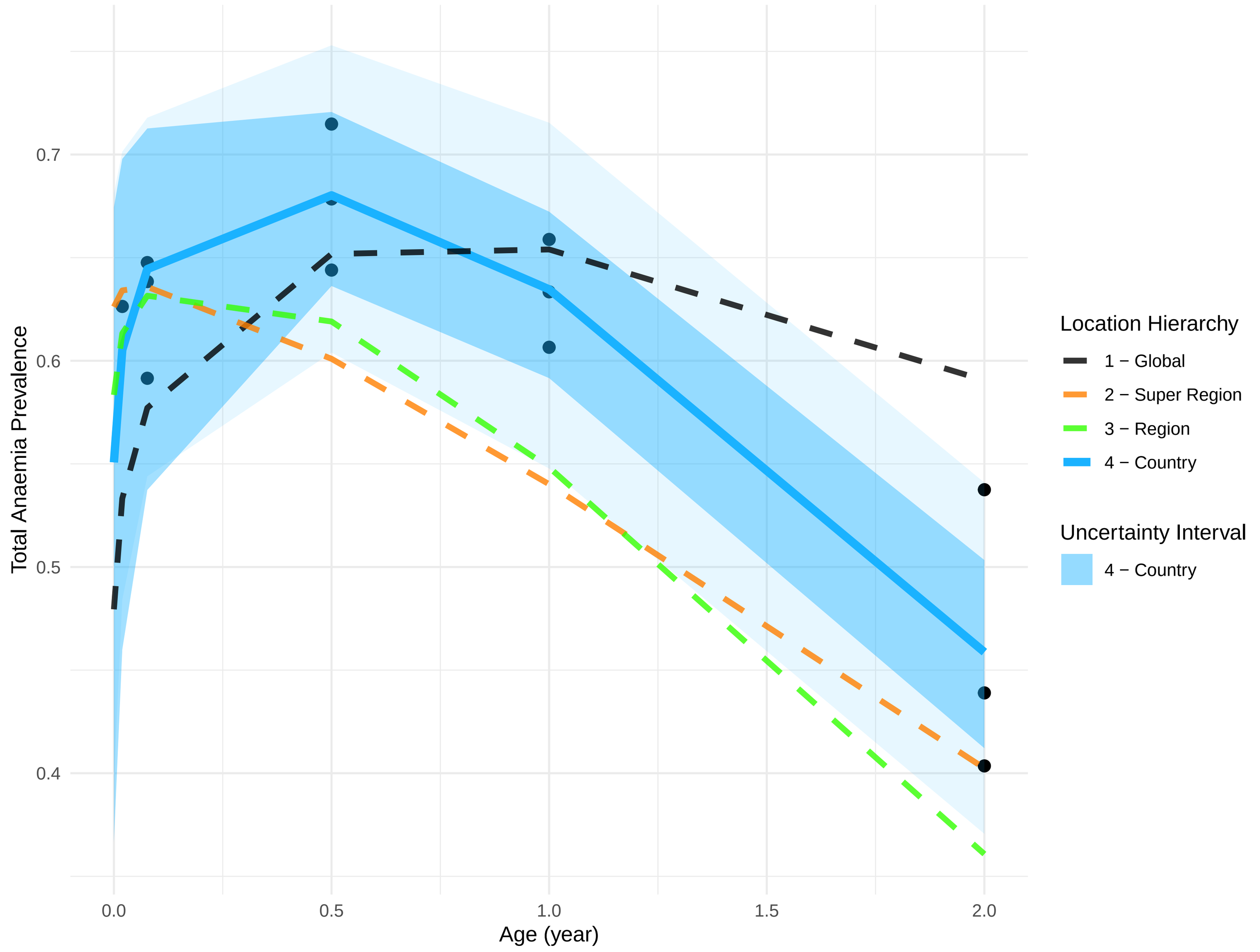

MR−BRT Age/Sex Fits − Males <2 Years Old − Total Anaemia Prevalence − Peru
WHO−2024 Adjustment Method

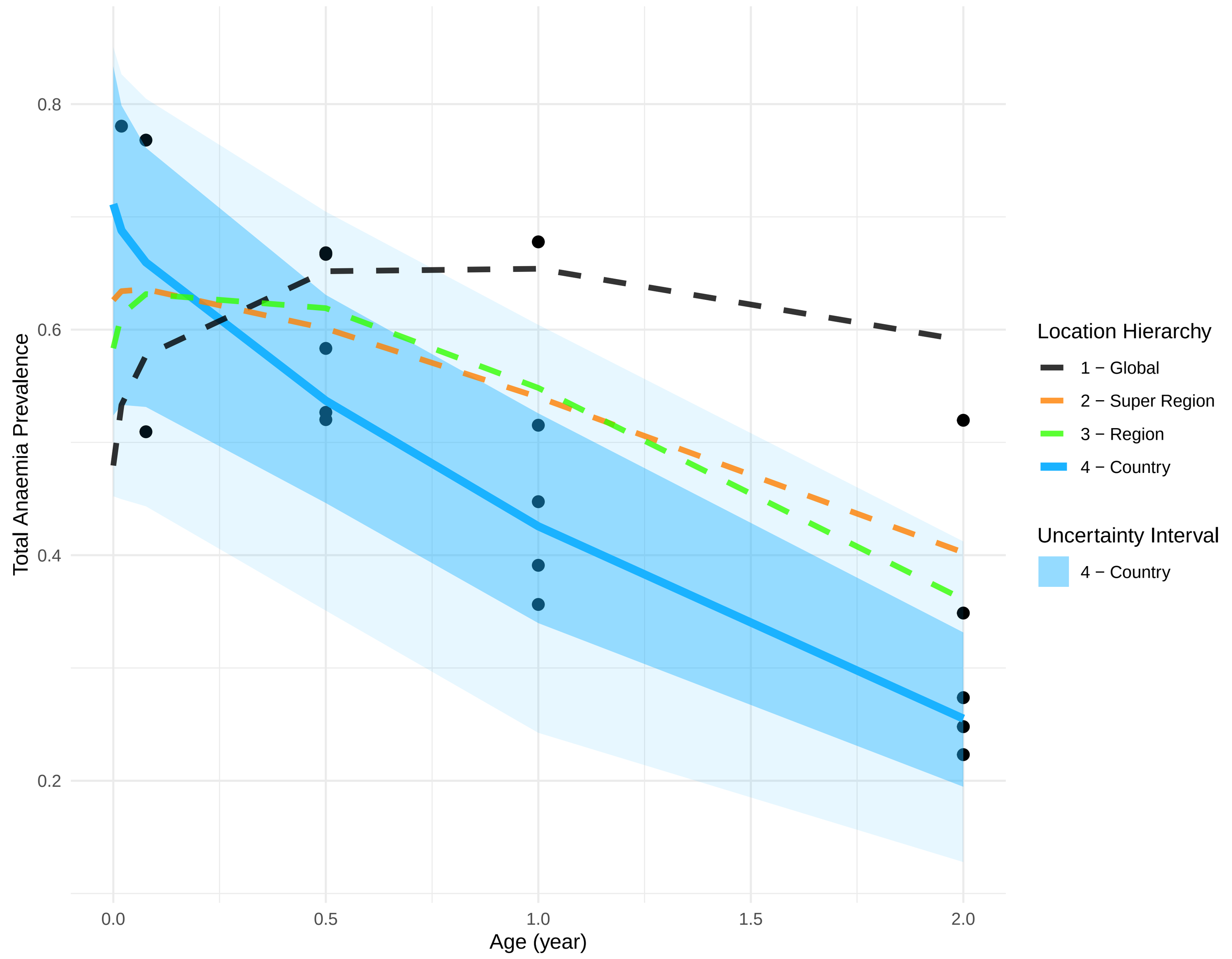

MR−BRT Age/Sex Fits − Males <2 Years Old − Total Anaemia Prevalence − Caribbean
WHO−2024 Adjustment Method

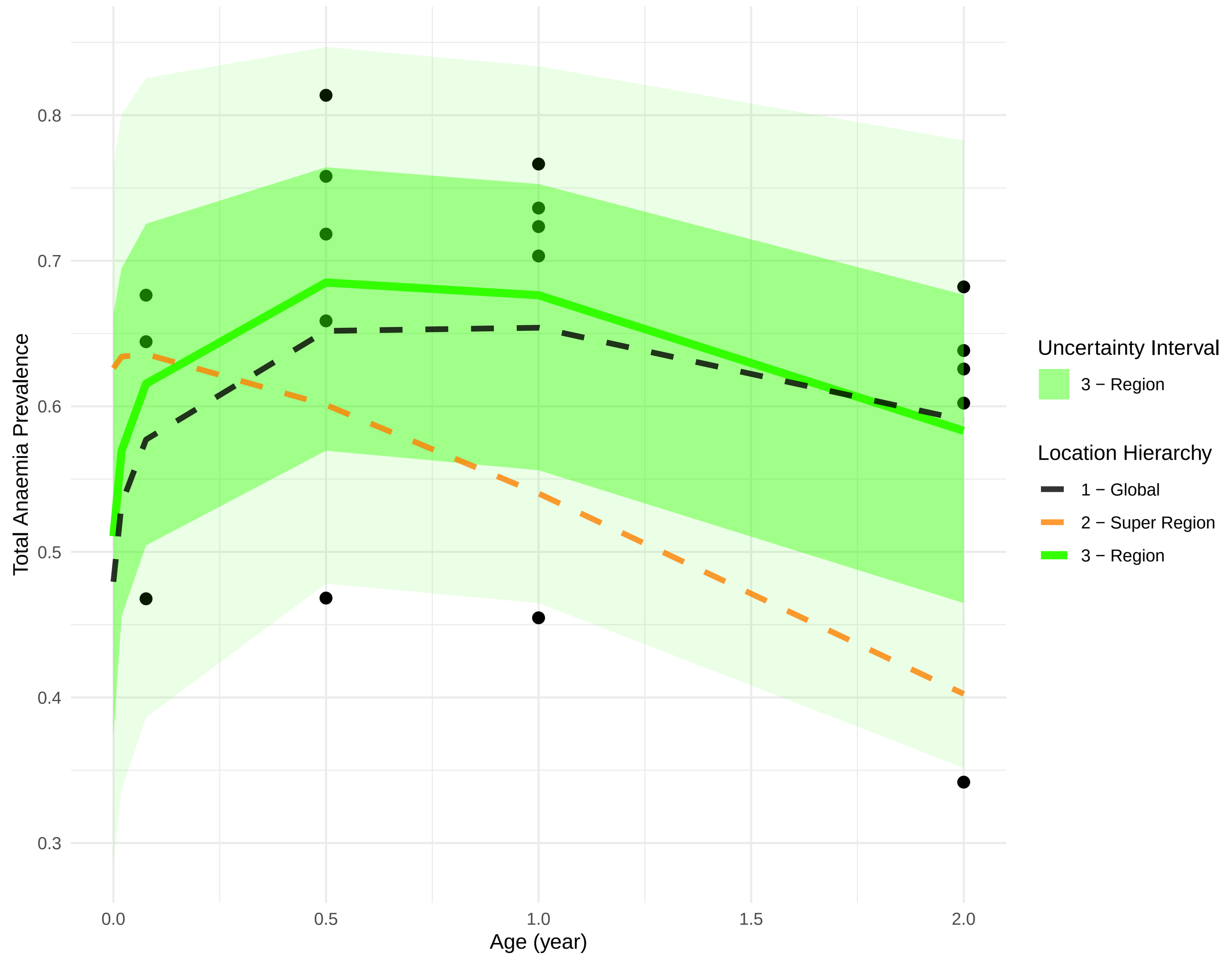

MR−BRT Age/Sex Fits − Males <2 Years Old − Total Anaemia Prevalence − Guyana
WHO−2024 Adjustment Method

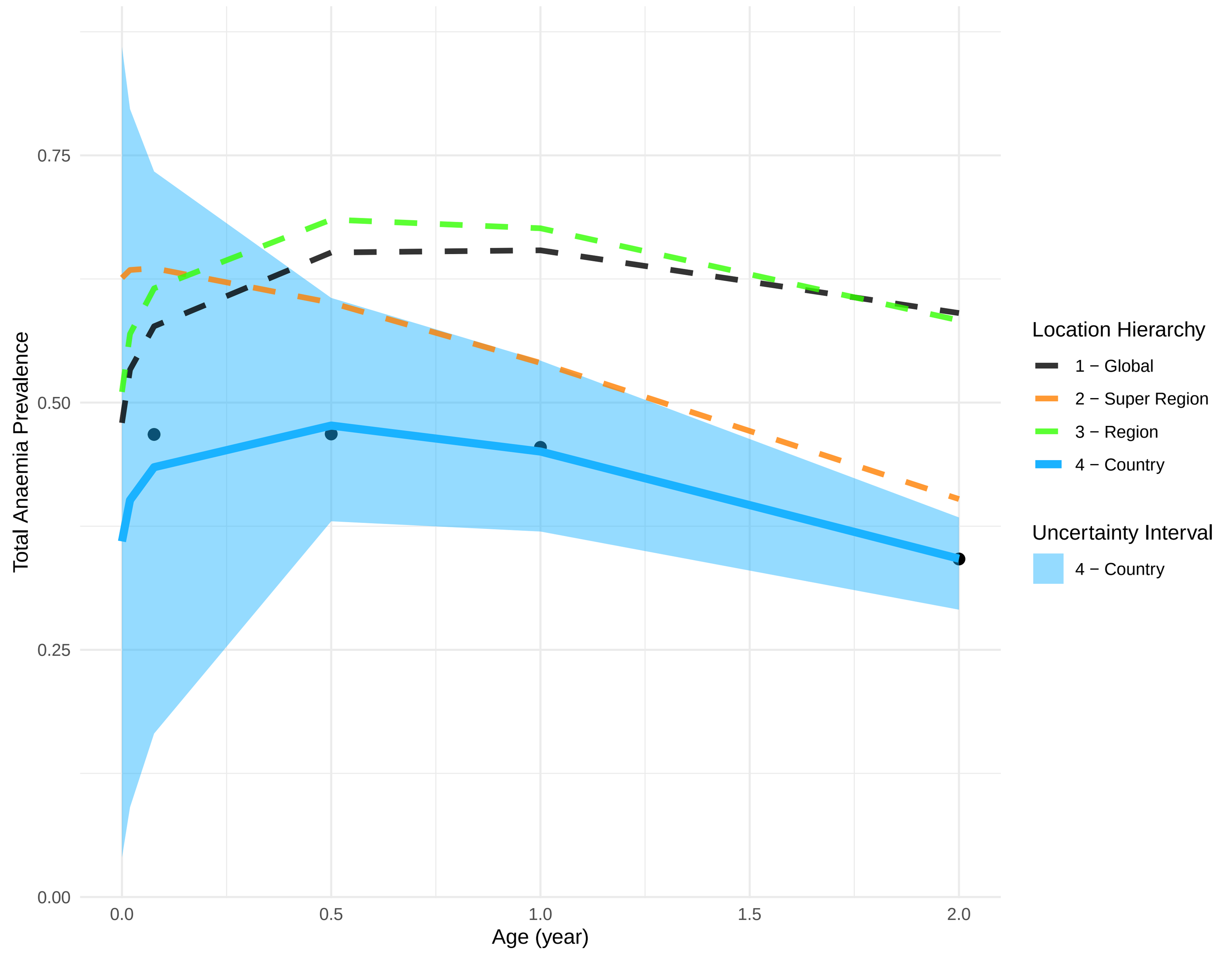

MR−BRT Age/Sex Fits − Males <2 Years Old − Total Anaemia Prevalence − Haiti
WHO−2024 Adjustment Method

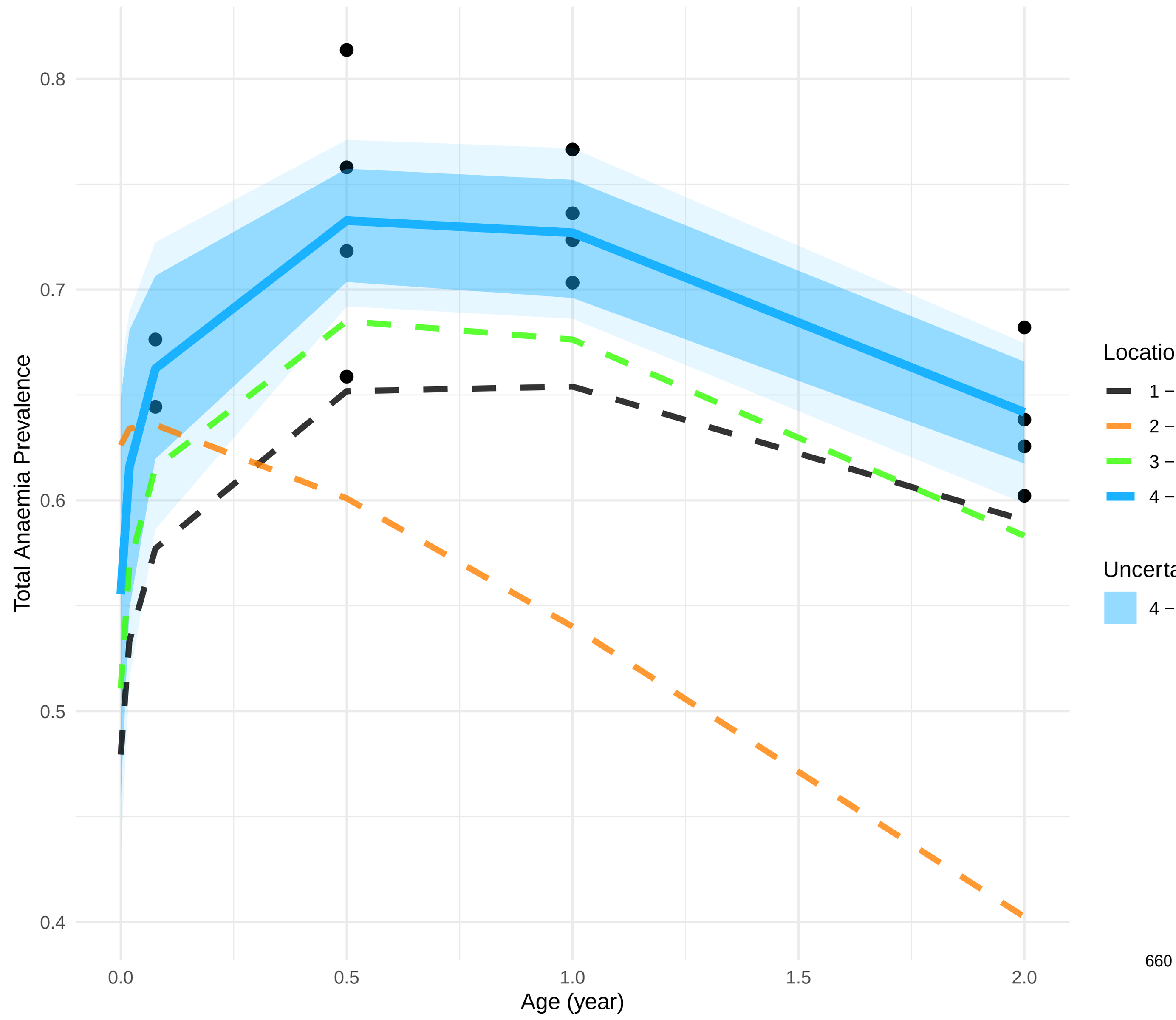

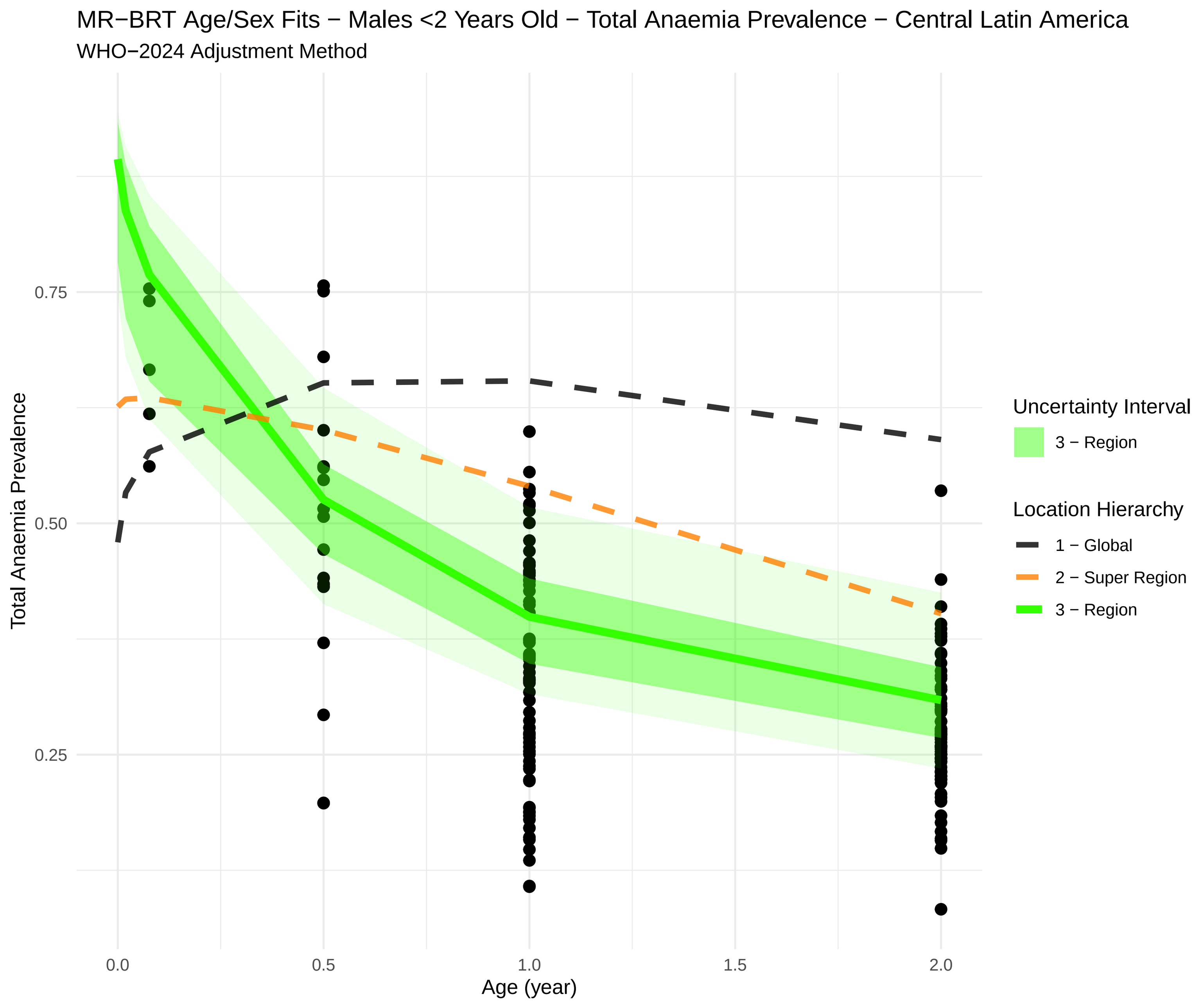
MR−BRT Age/Sex Fits − Males <2 Years Old − Total Anaemia Prevalence − Central Latin America
WHO−2024 Adjustment Method
Total Anaemia Prevalence
Age (year)
Uncertainty Interval
3 − Region
Location Hierarchy
1 − Global
2 − Super Region
3 − Region

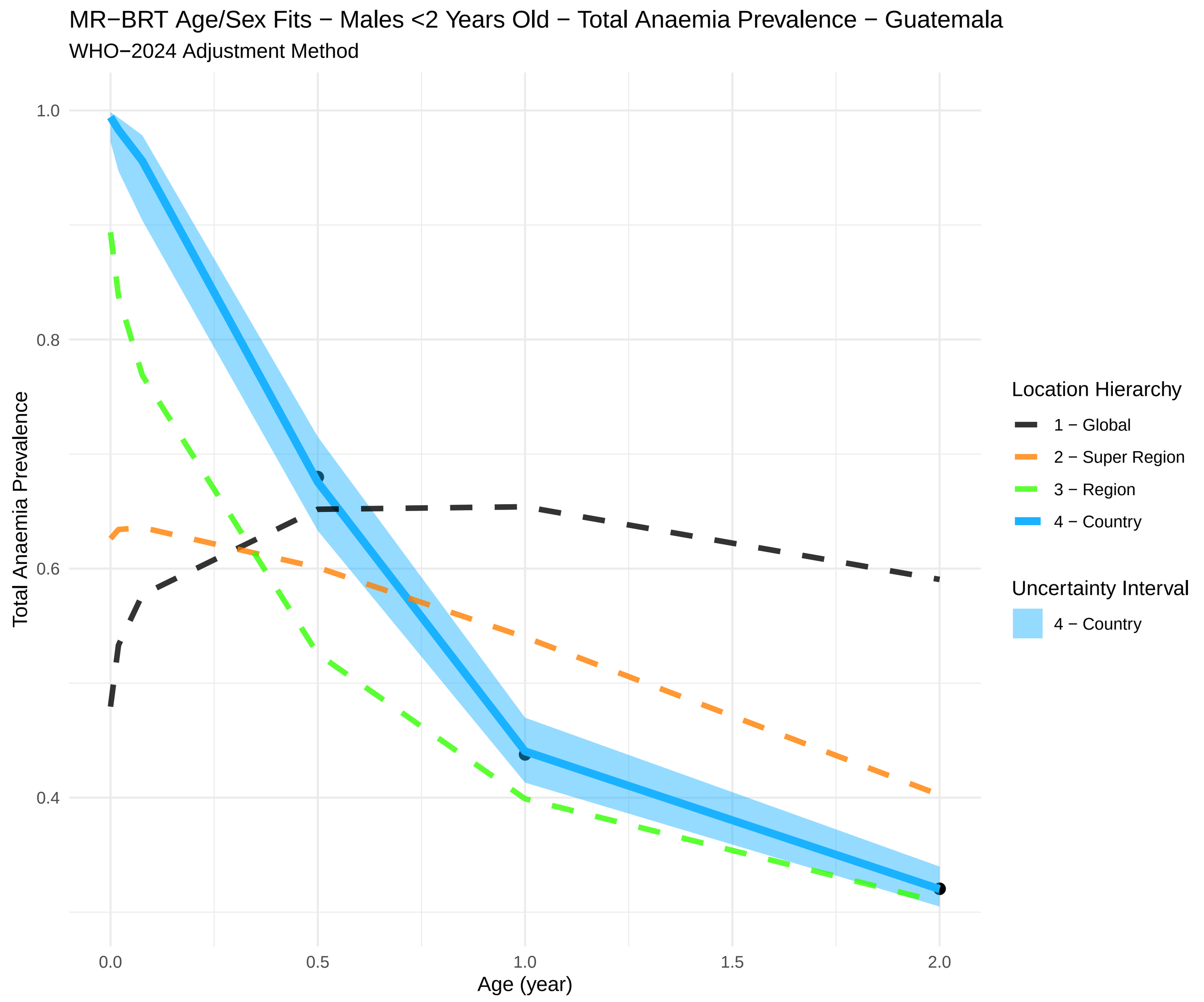
MR−BRT Age/Sex Fits − Males <2 Years Old − Total Anaemia Prevalence − Guatemala
WHO−2024 Adjustment Method
Total Anaemia Prevalence
Age (year)
Location Hierarchy
1 − Global
2 − Super Region
3 − Region
4 − Country
Uncertainty Interval
4 − Country

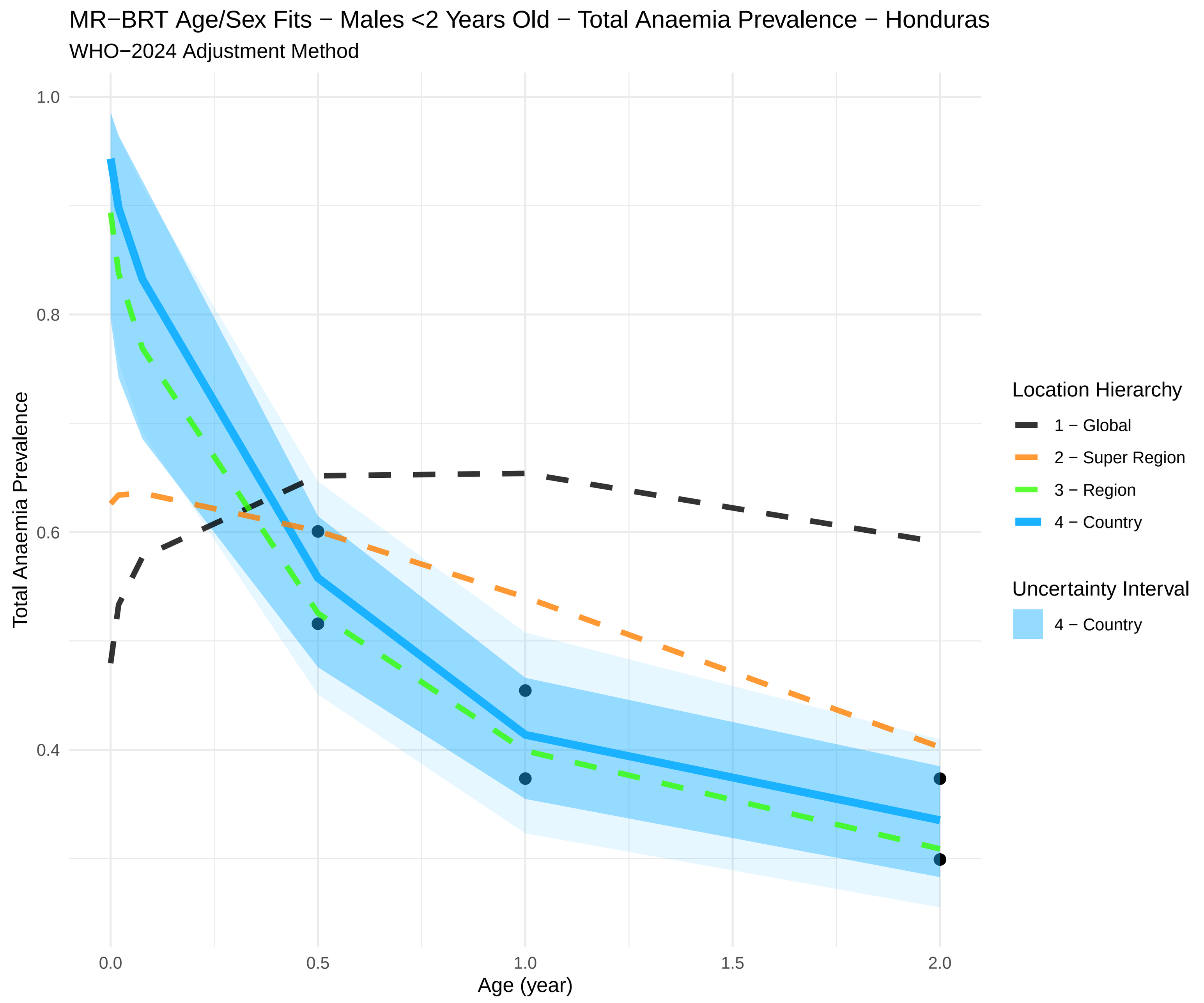
MR−BRT Age/Sex Fits − Males <2 Years Old − Total Anaemia Prevalence − Honduras
WHO−2024 Adjustment Method
Total Anaemia Prevalence
Age (year)
Location Hierarchy
1 − Global
2 − Super Region
3 − Region
4 − Country
Uncertainty Interval
4 − Country

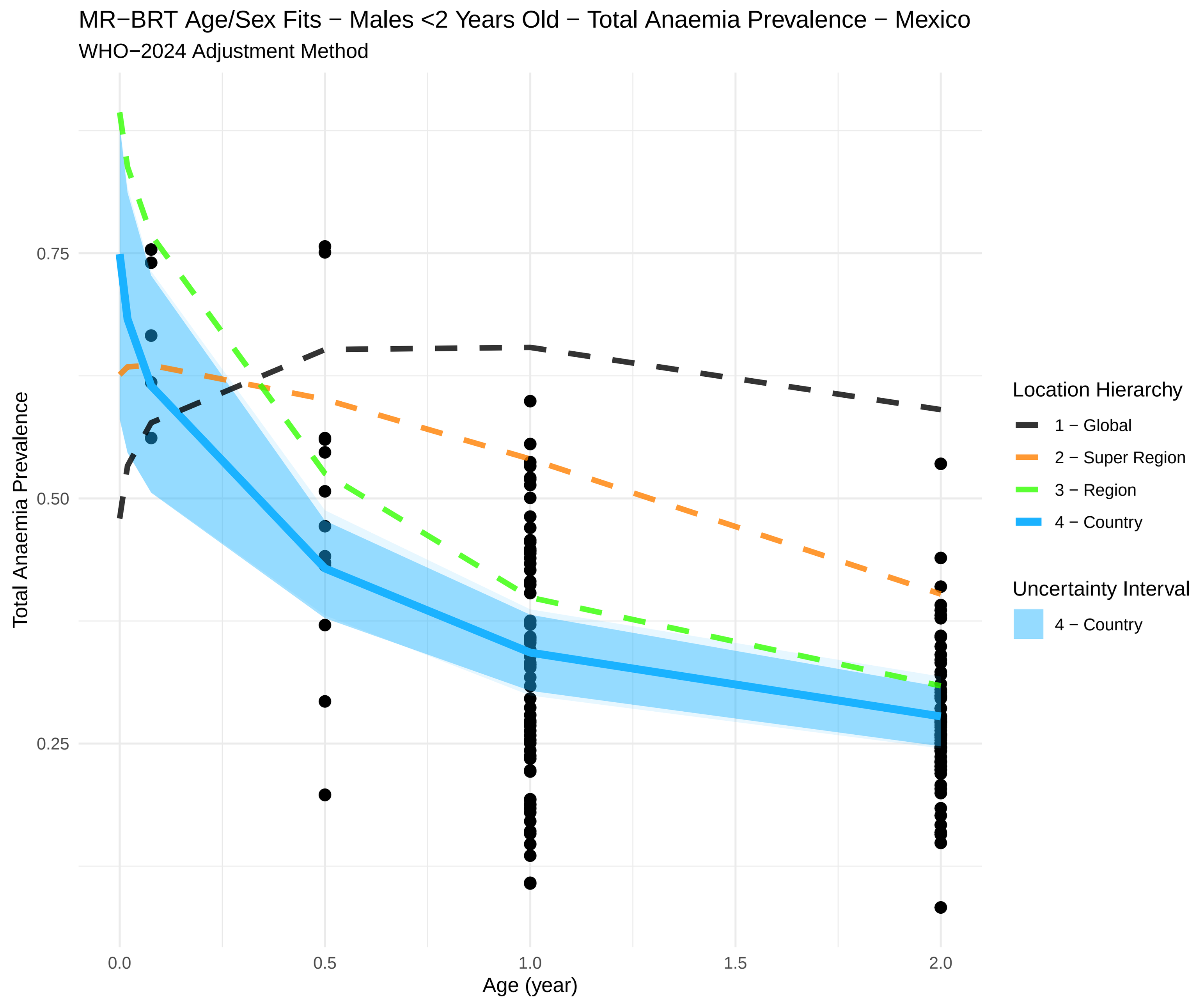
MR−BRT Age/Sex Fits − Males <2 Years Old − Total Anaemia Prevalence − Mexico
WHO−2024 Adjustment Method
Total Anaemia Prevalence
Age (year)
Location Hierarchy
1 − Global
2 − Super Region
3 − Region
4 − Country
Uncertainty Interval
4 − Country

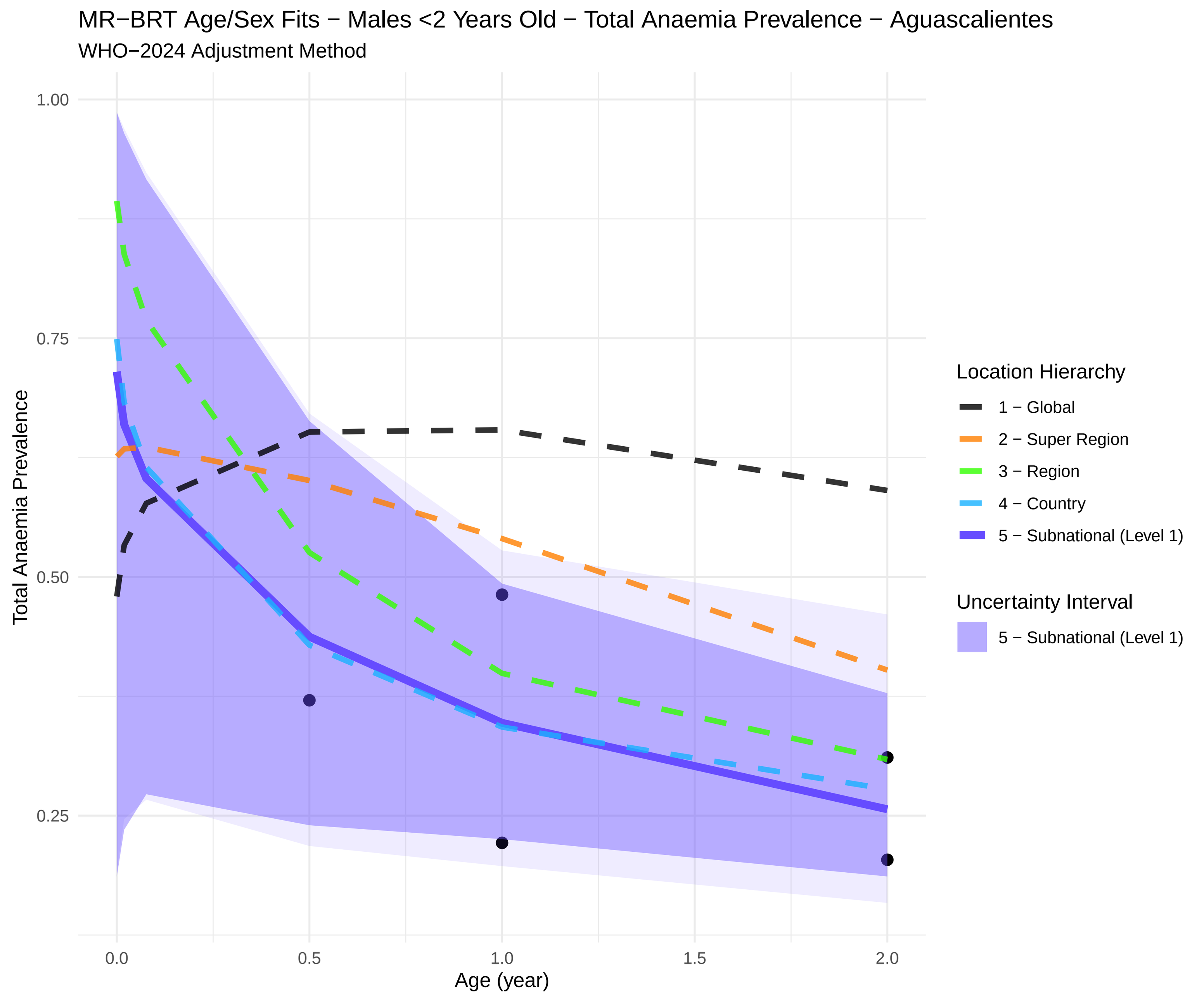
MR−BRT Age/Sex Fits − Males <2 Years Old − Total Anaemia Prevalence − Aguascalientes
WHO−2024 Adjustment Method
Total Anaemia Prevalence
Age (year)
Location Hierarchy
1 − Global
2 − Super Region
3 − Region
4 − Country
5 − Subnational (Level 1)
Uncertainty Interval
5 − Subnational (Level 1)

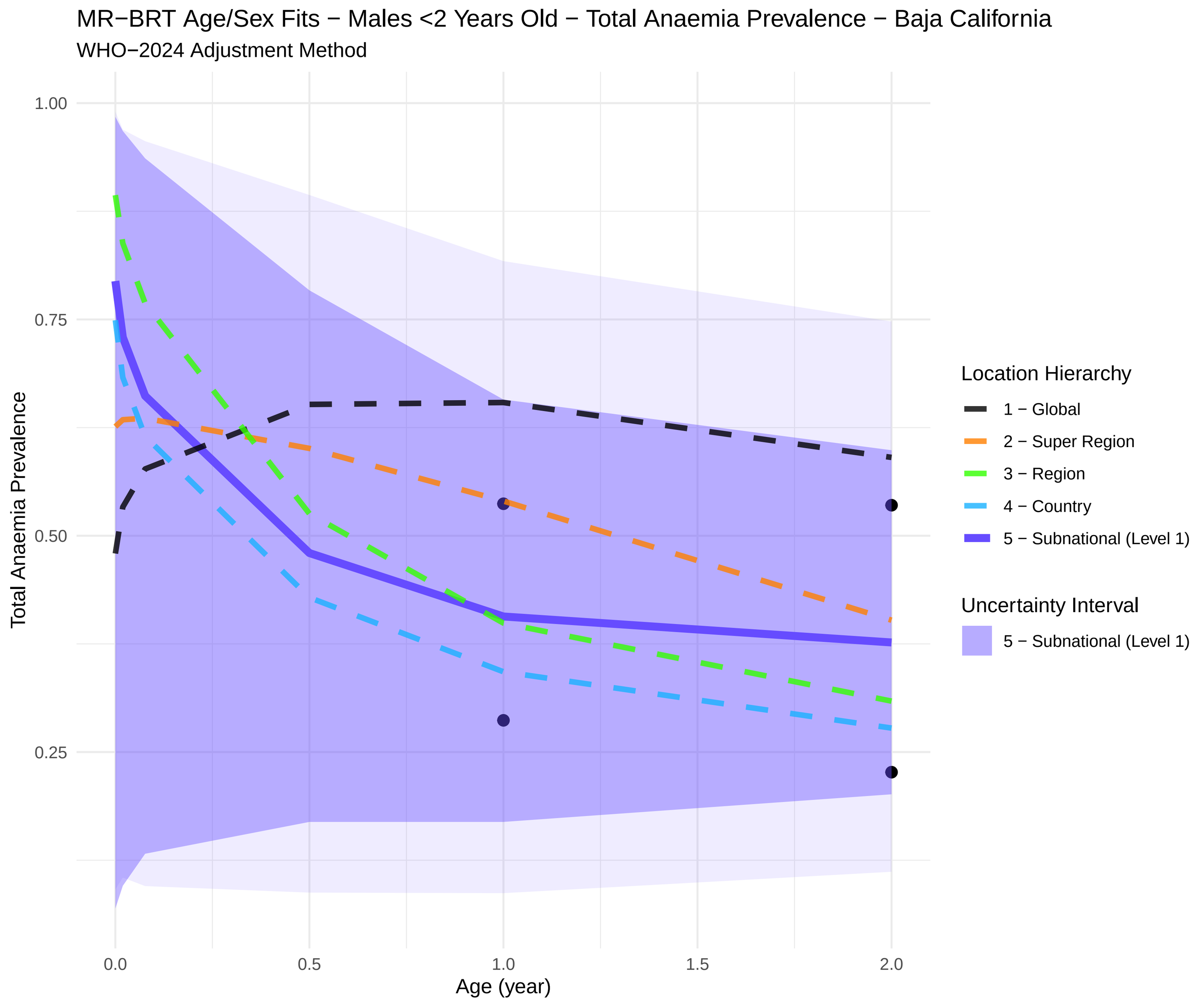
MR−BRT Age/Sex Fits − Males <2 Years Old − Total Anaemia Prevalence − Baja California
WHO−2024 Adjustment Method
Total Anaemia Prevalence
Age (year)
Location Hierarchy
1 − Global
2 − Super Region
3 − Region
4 − Country
5 − Subnational (Level 1)
Uncertainty Interval
5 − Subnational (Level 1)

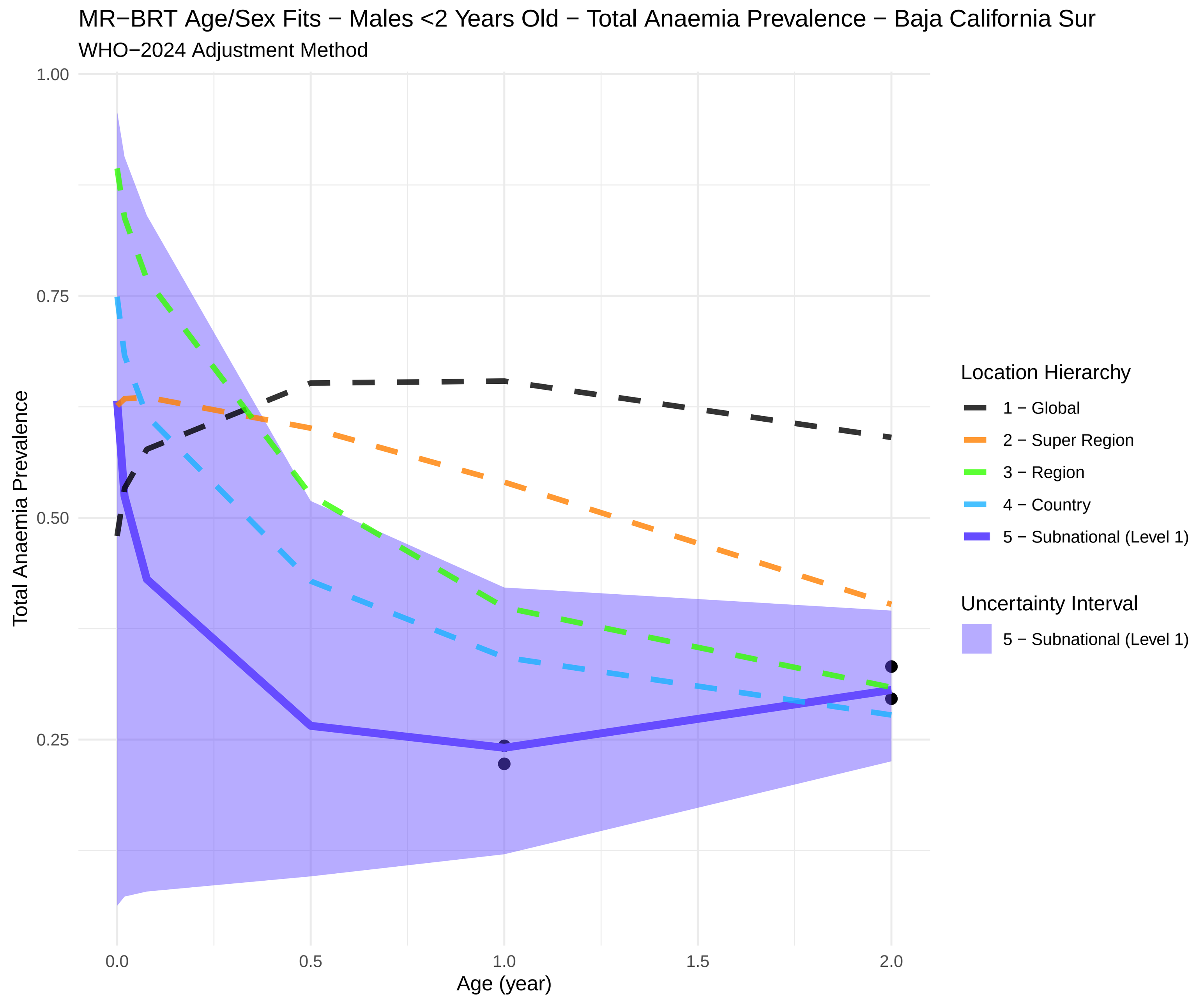
MR−BRT Age/Sex Fits − Males <2 Years Old − Total Anaemia Prevalence − Baja California Sur
WHO−2024 Adjustment Method
Total Anaemia Prevalence
Age (year)
Location Hierarchy
1 − Global
2 − Super Region
3 − Region
4 − Country
5 − Subnational (Level 1)
Uncertainty Interval
5 − Subnational (Level 1)

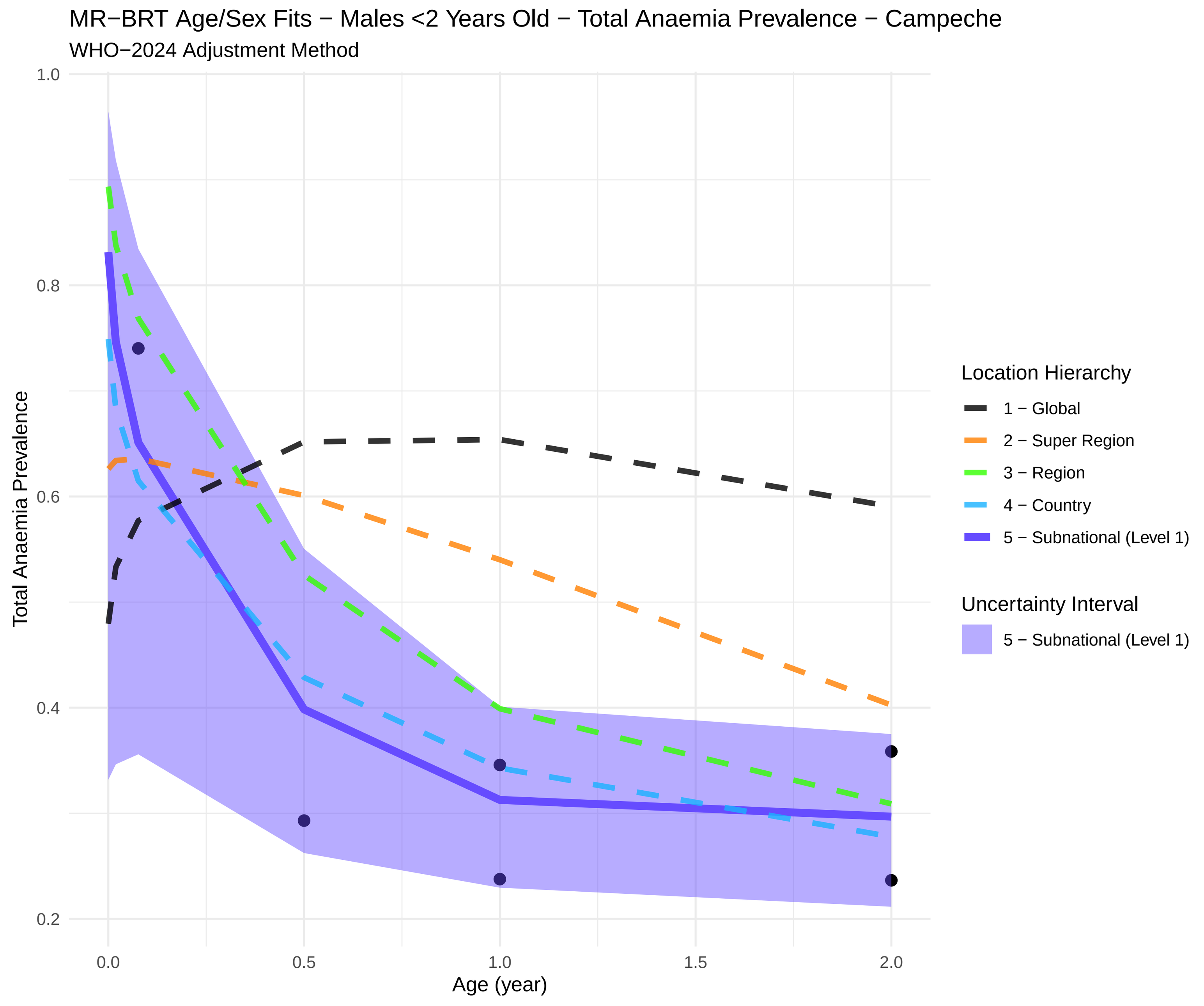
MR−BRT Age/Sex Fits − Males <2 Years Old − Total Anaemia Prevalence − Campeche
WHO−2024 Adjustment Method
Total Anaemia Prevalence
Age (year)
Location Hierarchy
1 − Global
2 − Super Region
3 − Region
4 − Country
5 − Subnational (Level 1)
Uncertainty Interval
5 − Subnational (Level 1)

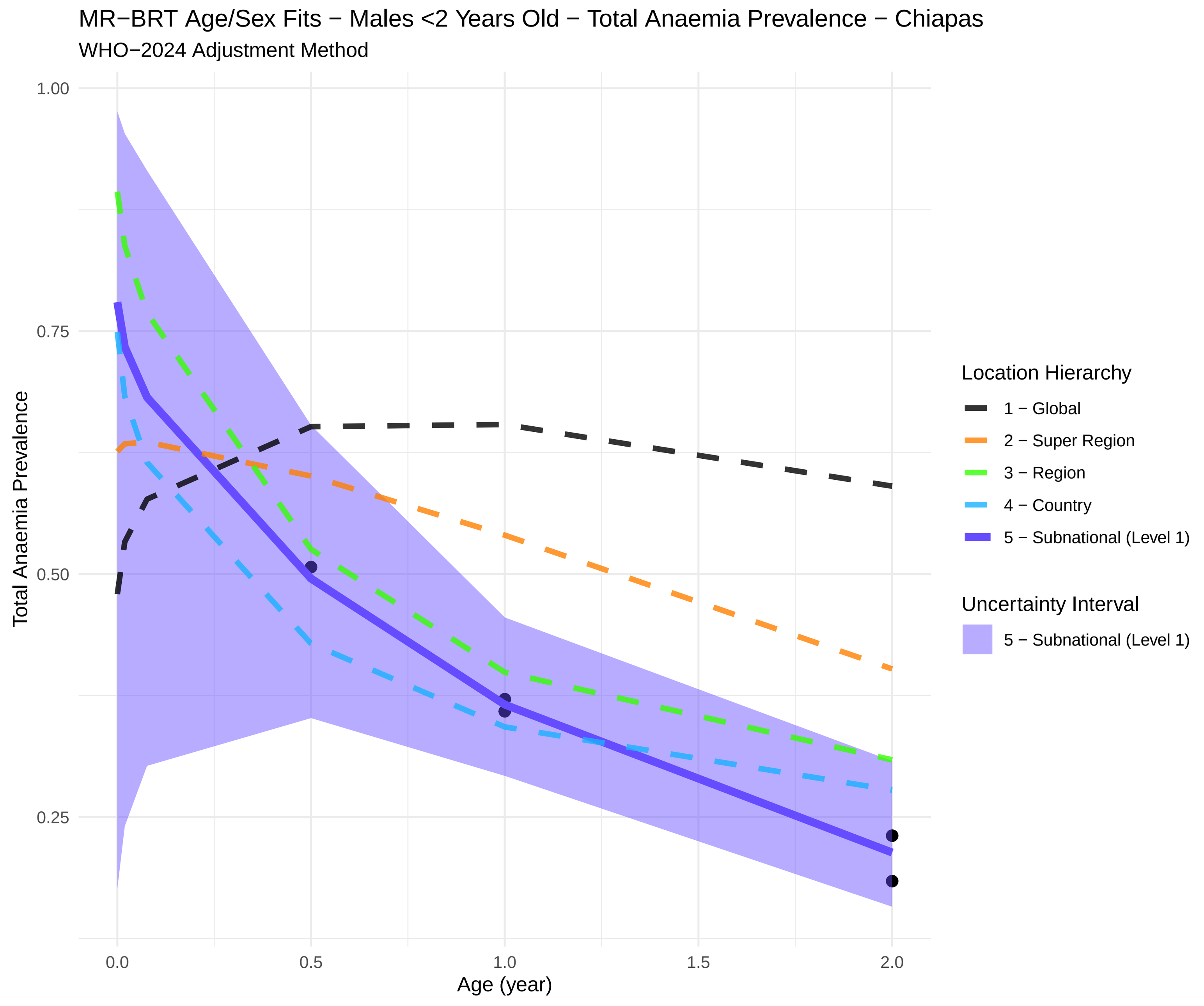
MR−BRT Age/Sex Fits − Males <2 Years Old − Total Anaemia Prevalence − Chiapas
WHO−2024 Adjustment Method
Total Anaemia Prevalence
Age (year)
Location Hierarchy
1 − Global
2 − Super Region
3 − Region
4 − Country
5 − Subnational (Level 1)
Uncertainty Interval
5 − Subnational (Level 1)

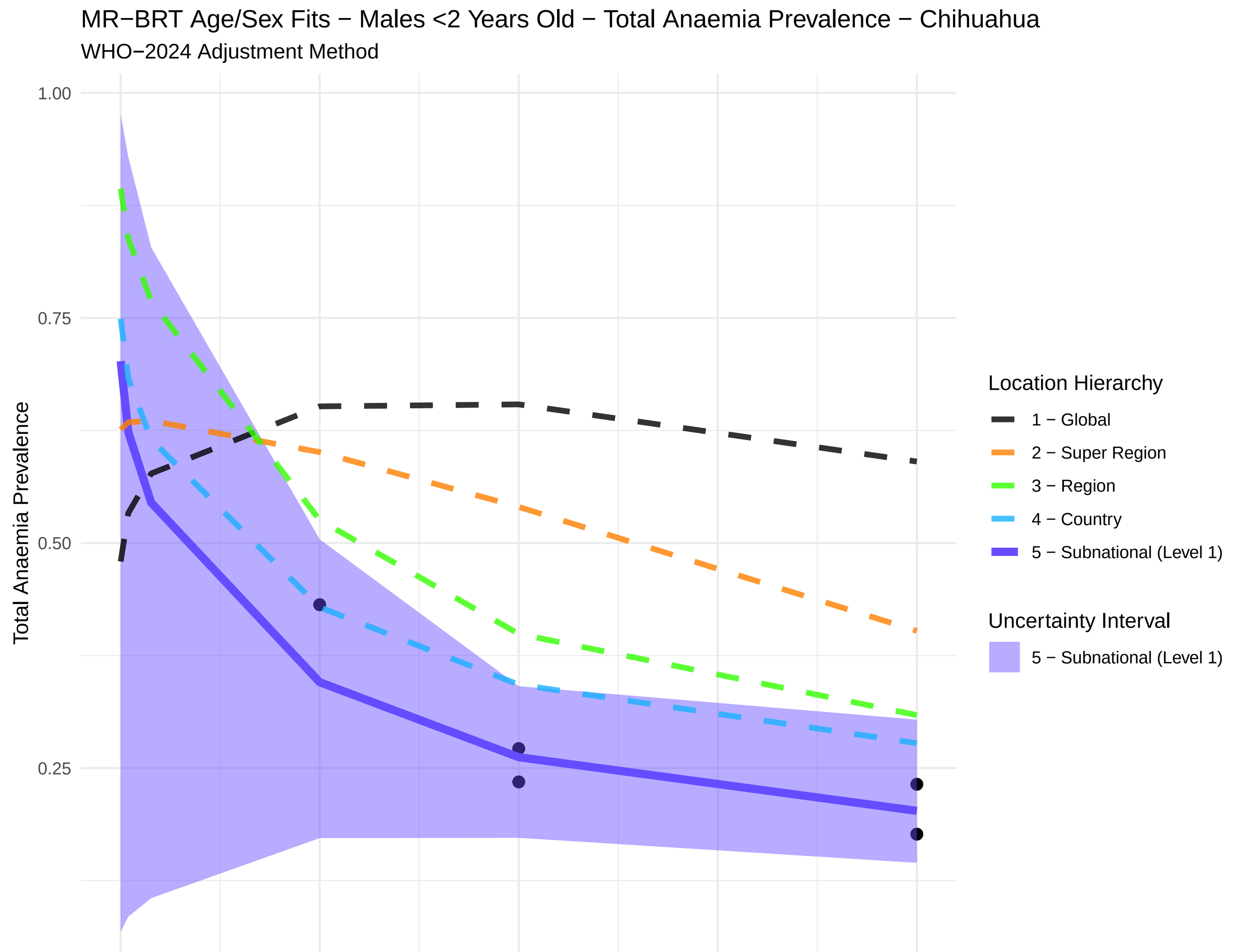
MR−BRT Age/Sex Fits − Males <2 Years Old − Total Anaemia Prevalence − Chihuahua
WHO−2024 Adjustment Method
Total Anaemia Prevalence
Age (year)
Location Hierarchy
1 − Global
2 − Super Region
3 − Region
4 − Country
5 − Subnational (Level 1)
Uncertainty Interval
5 − Subnational (Level 1)

MR−BRT Age/Sex Fits − Males <2 Years Old − Total Anaemia Prevalence − Coahuila
WHO−2024 Adjustment Method

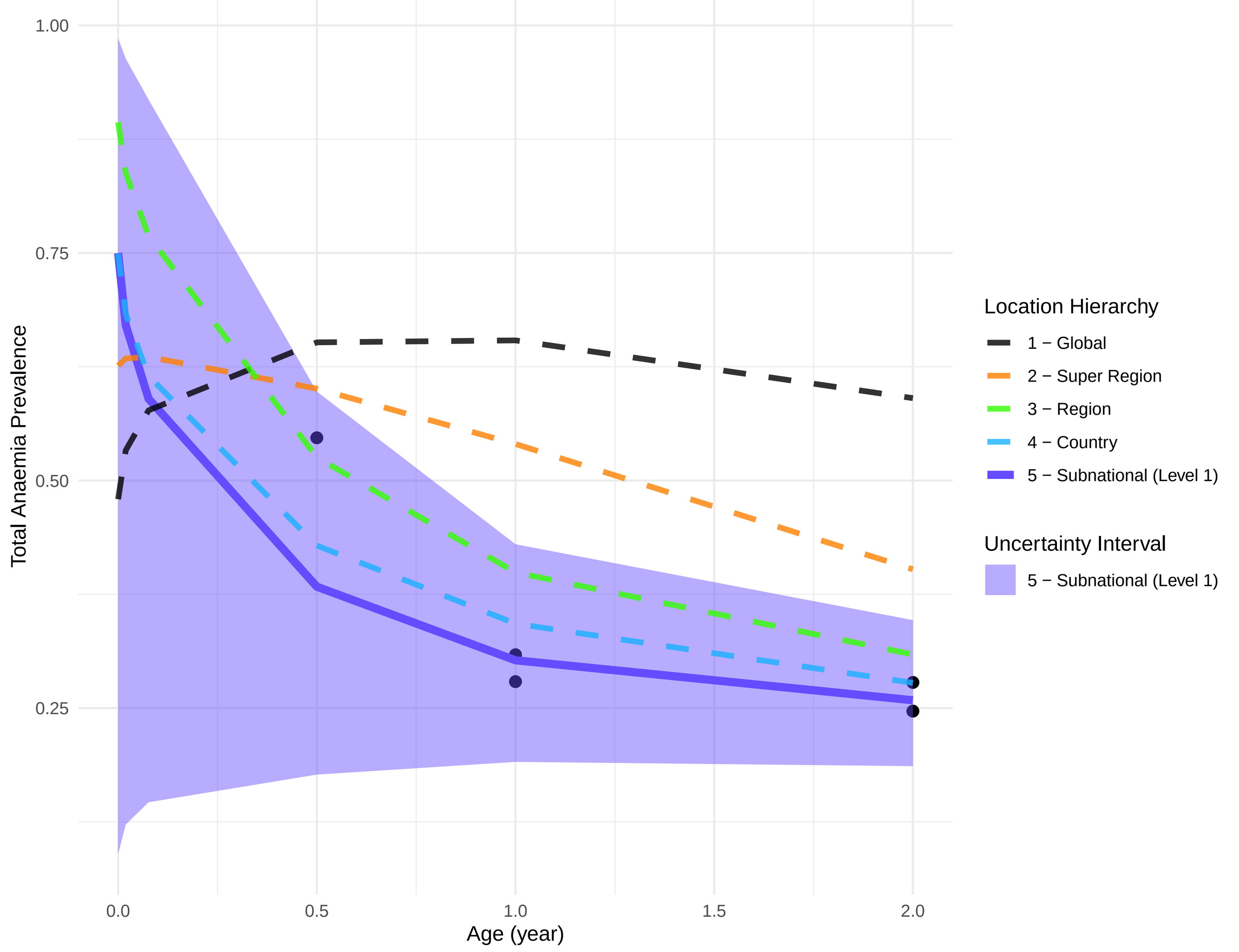

MR−BRT Age/Sex Fits − Males <2 Years Old − Total Anaemia Prevalence − Colima
WHO−2024 Adjustment Method

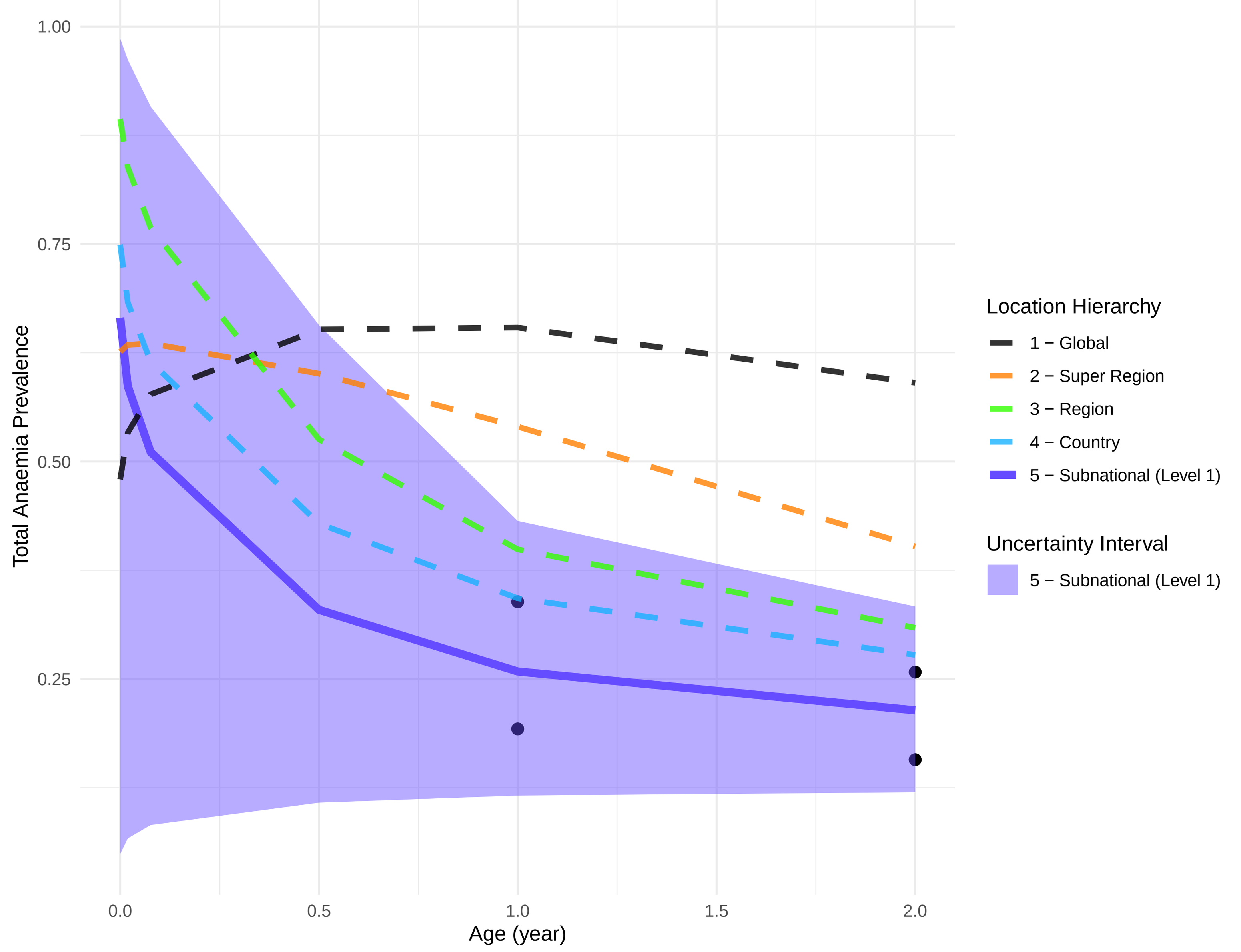

MR−BRT Age/Sex Fits − Males <2 Years Old − Total Anaemia Prevalence − Durango
WHO−2024 Adjustment Method

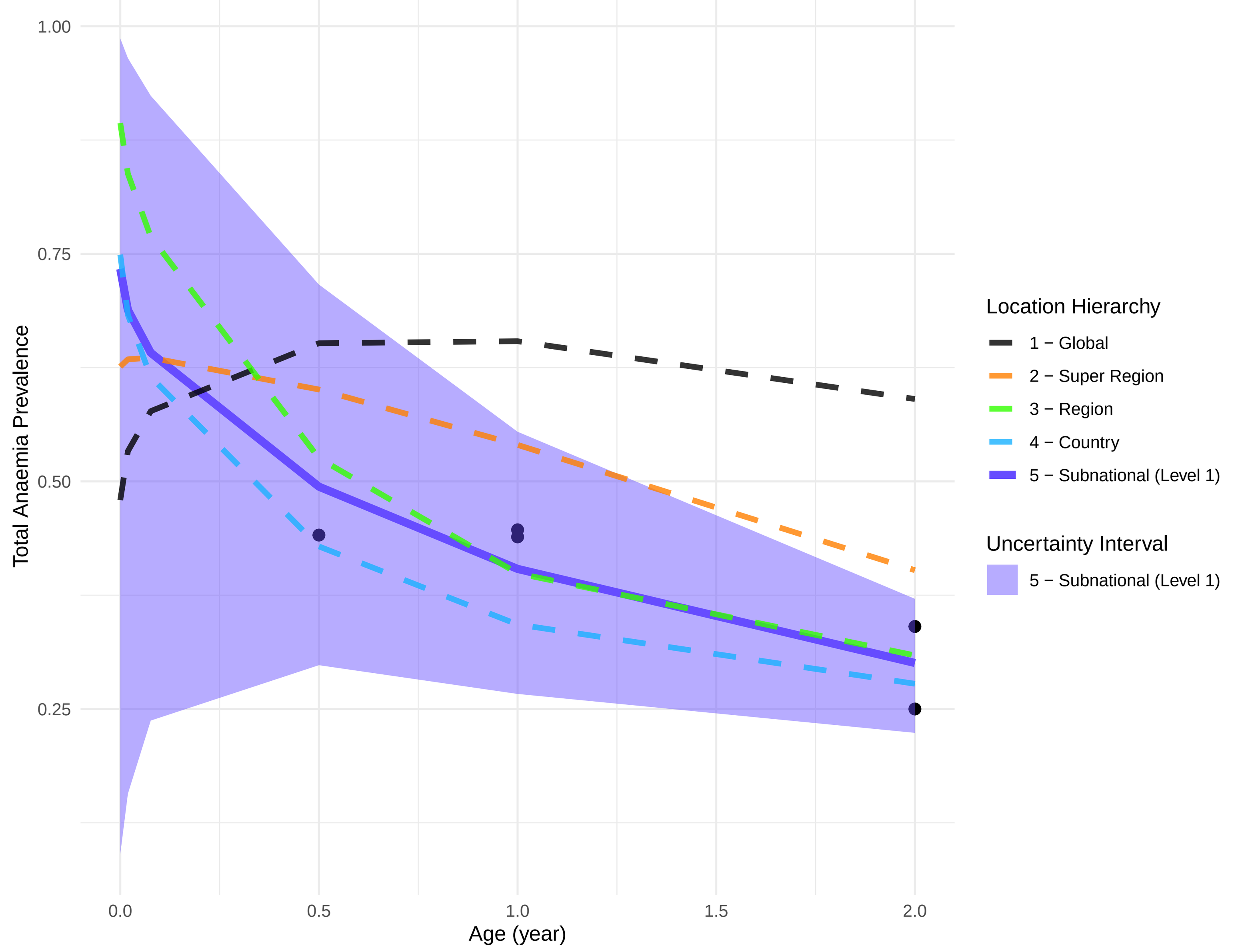

MR−BRT Age/Sex Fits − Males <2 Years Old − Total Anaemia Prevalence − Guanajuato
WHO−2024 Adjustment Method

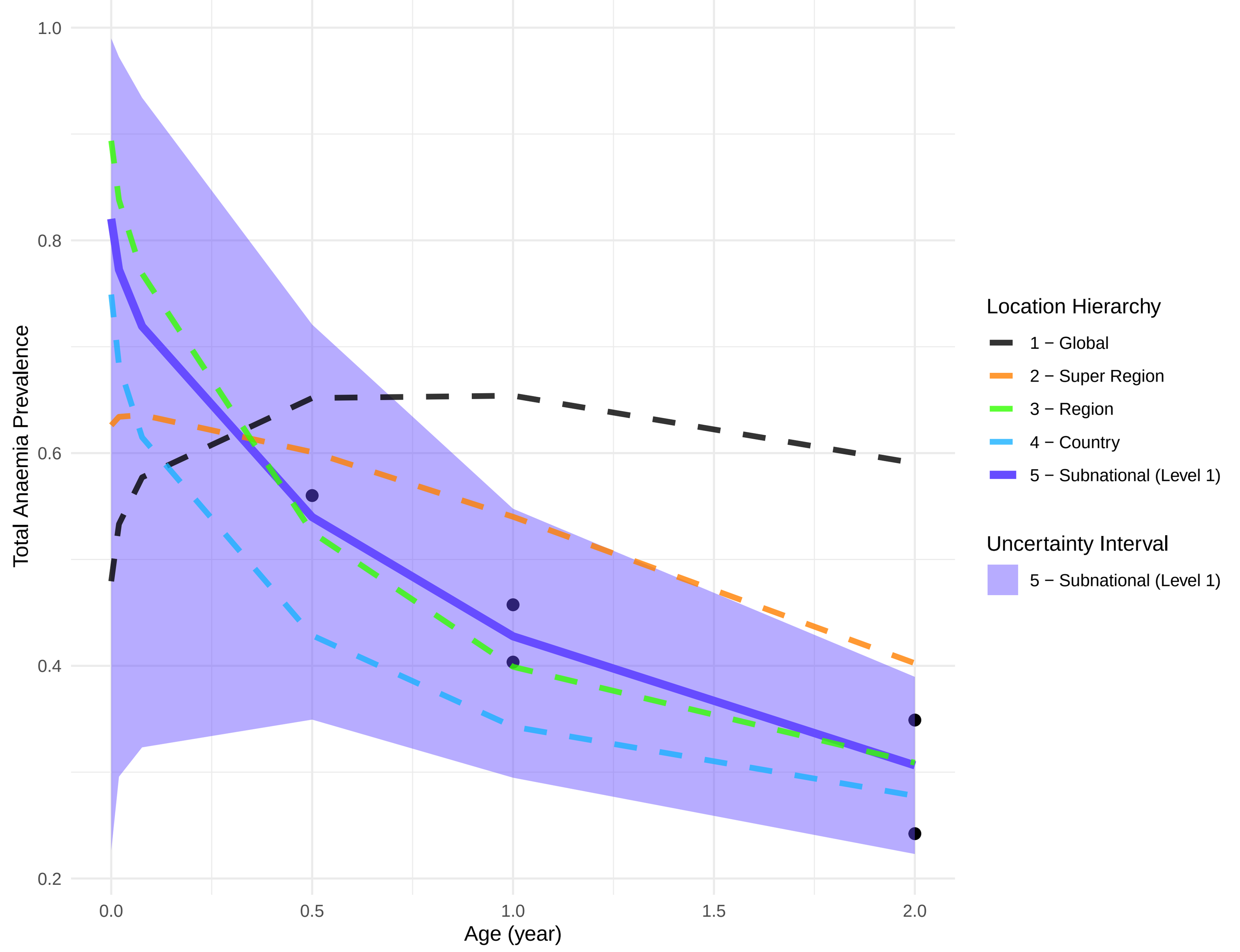

MR−BRT Age/Sex Fits − Males <2 Years Old − Total Anaemia Prevalence − Guerrero
WHO−2024 Adjustment Method

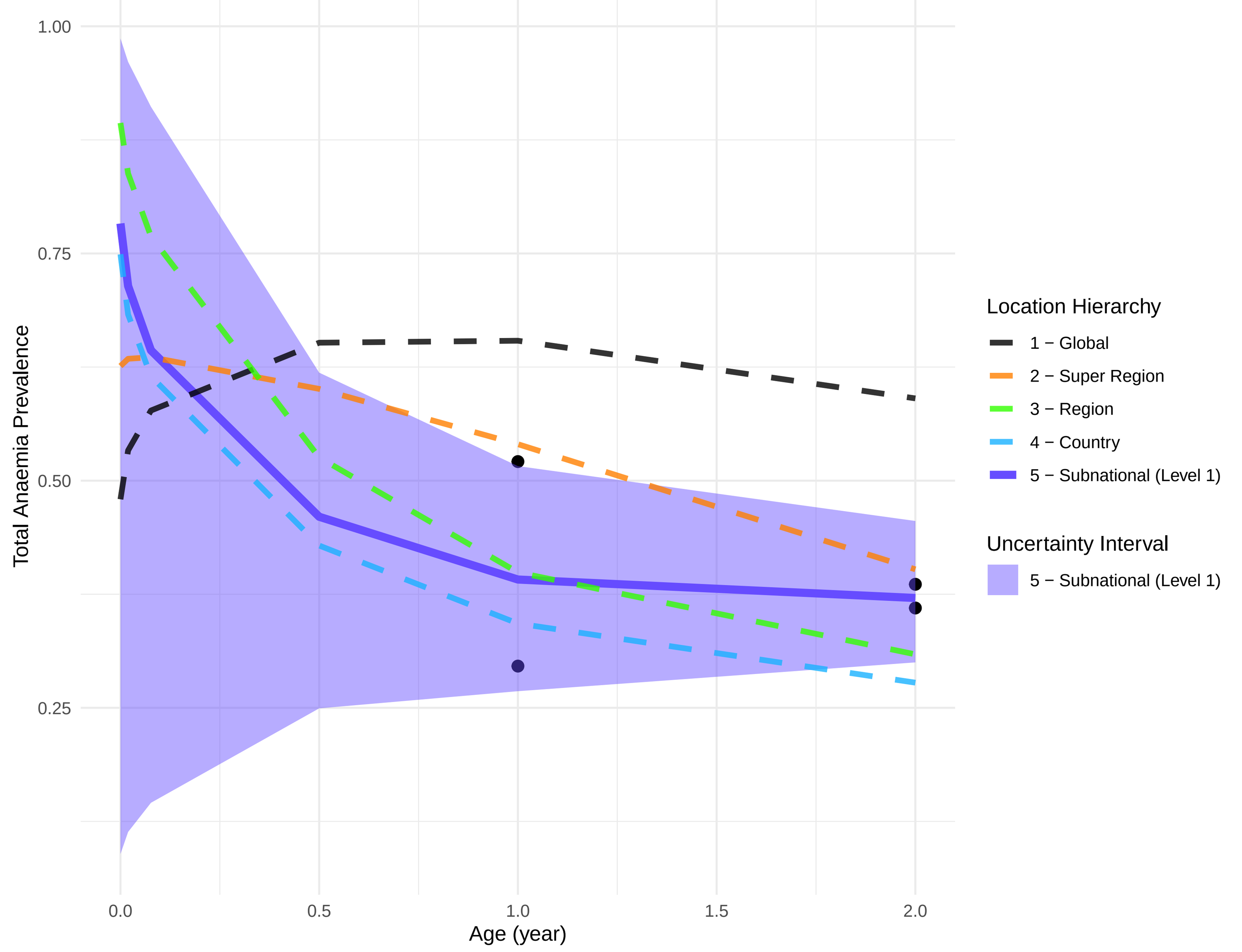

MR−BRT Age/Sex Fits − Males <2 Years Old − Total Anaemia Prevalence − Hidalgo
WHO−2024 Adjustment Method

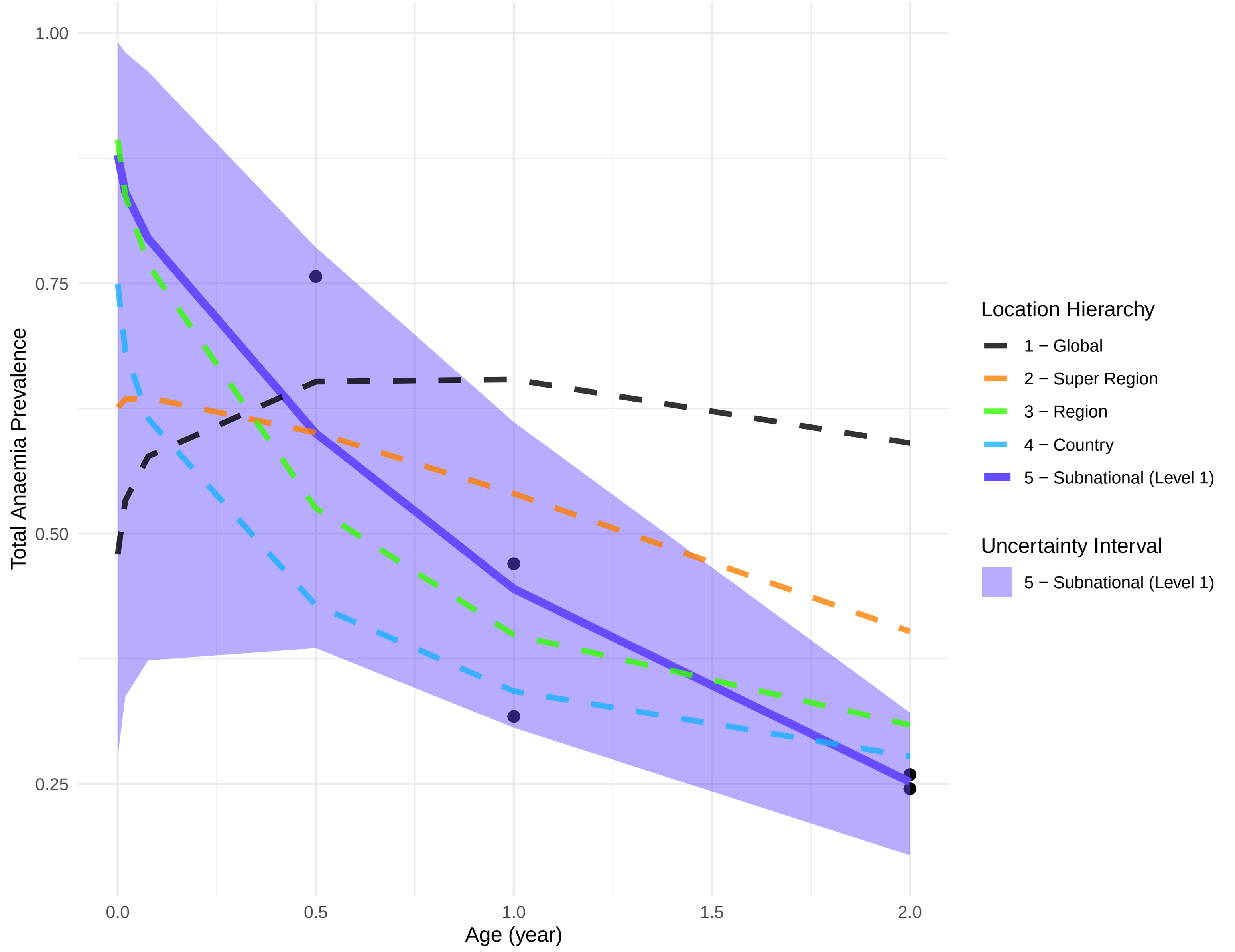

MR−BRT Age/Sex Fits − Males <2 Years Old − Total Anaemia Prevalence − Jalisco
WHO−2024 Adjustment Method

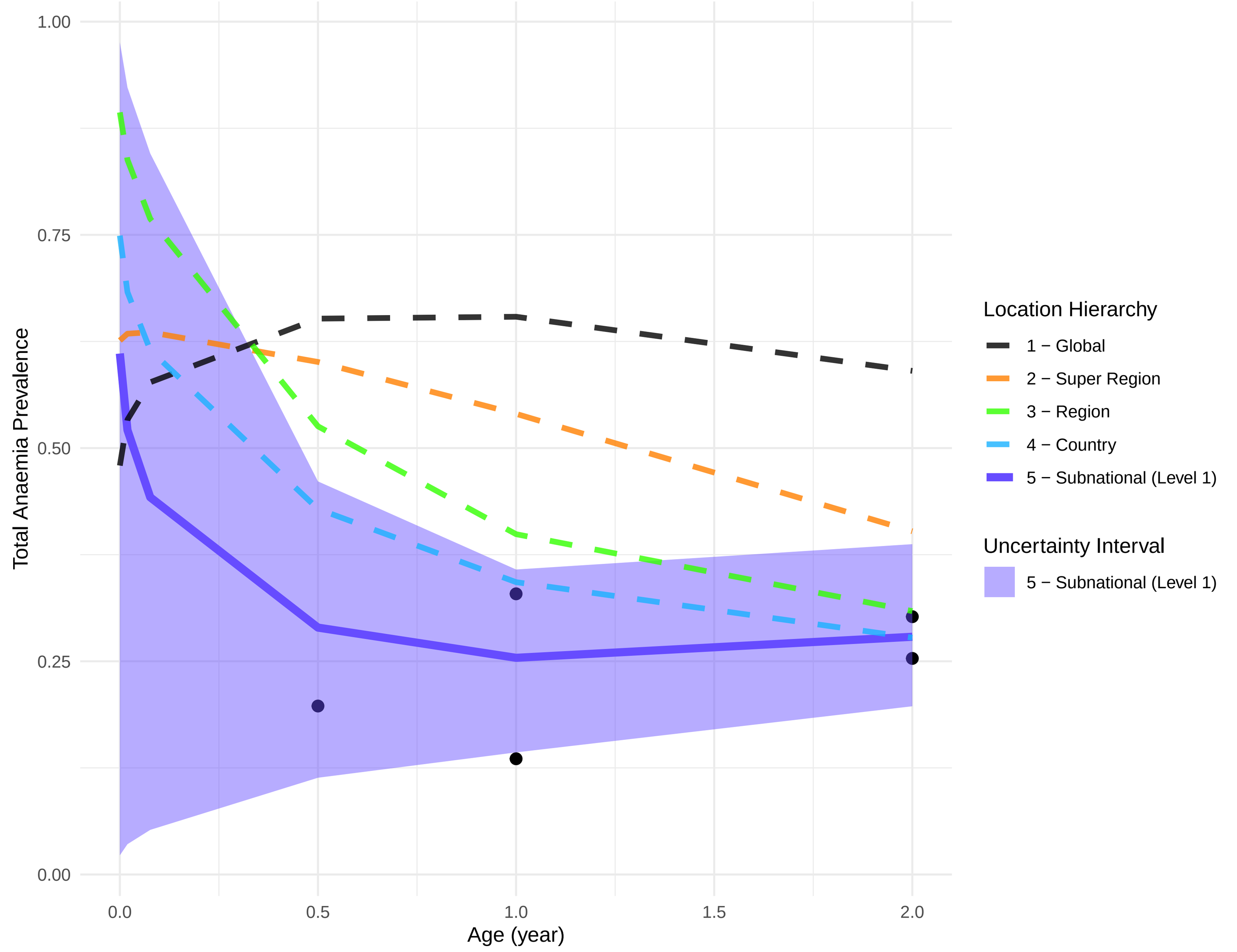

MR−BRT Age/Sex Fits − Males <2 Years Old − Total Anaemia Prevalence − México
WHO−2024 Adjustment Method

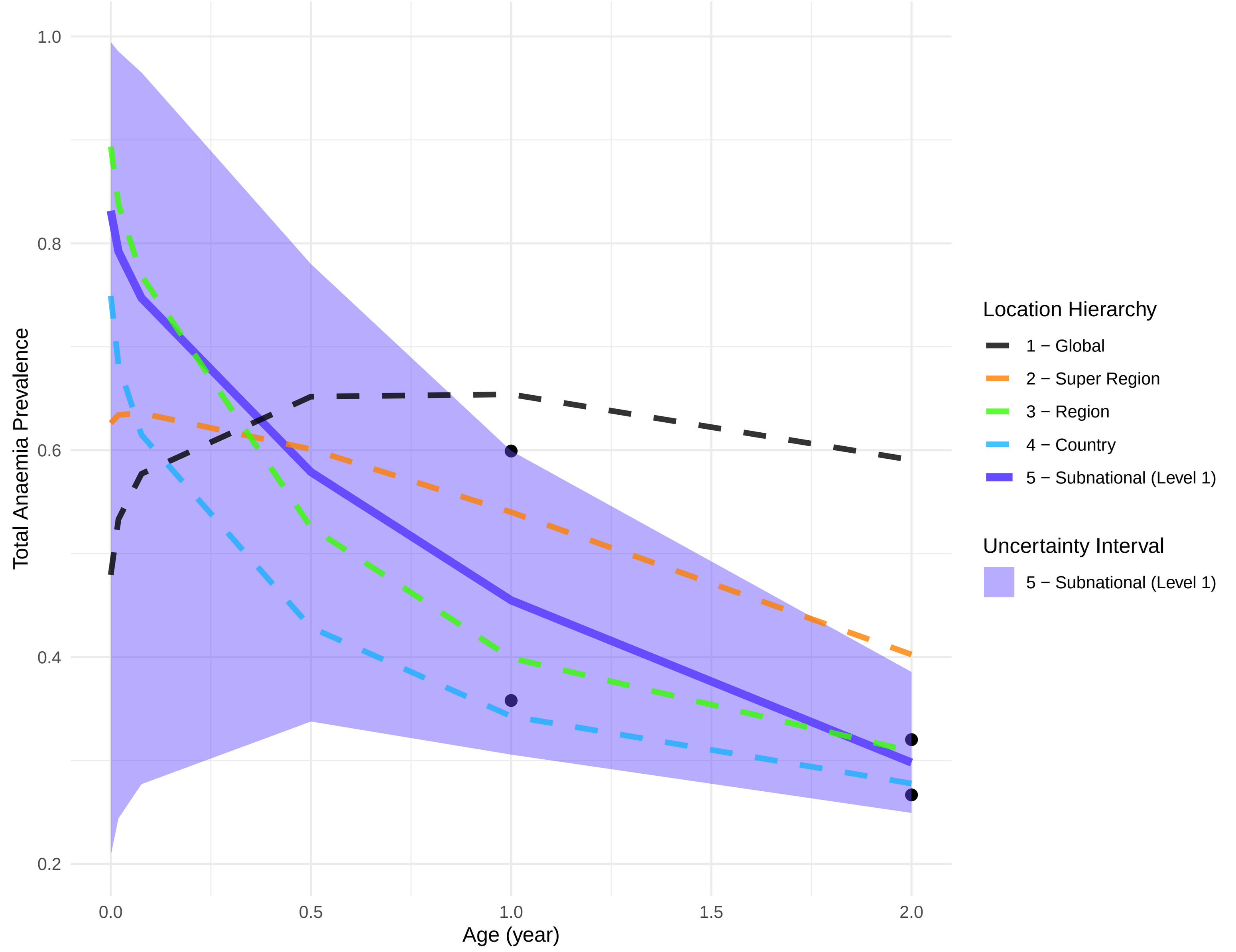

MR−BRT Age/Sex Fits − Males <2 Years Old − Total Anaemia Prevalence − Mexico City
WHO−2024 Adjustment Method

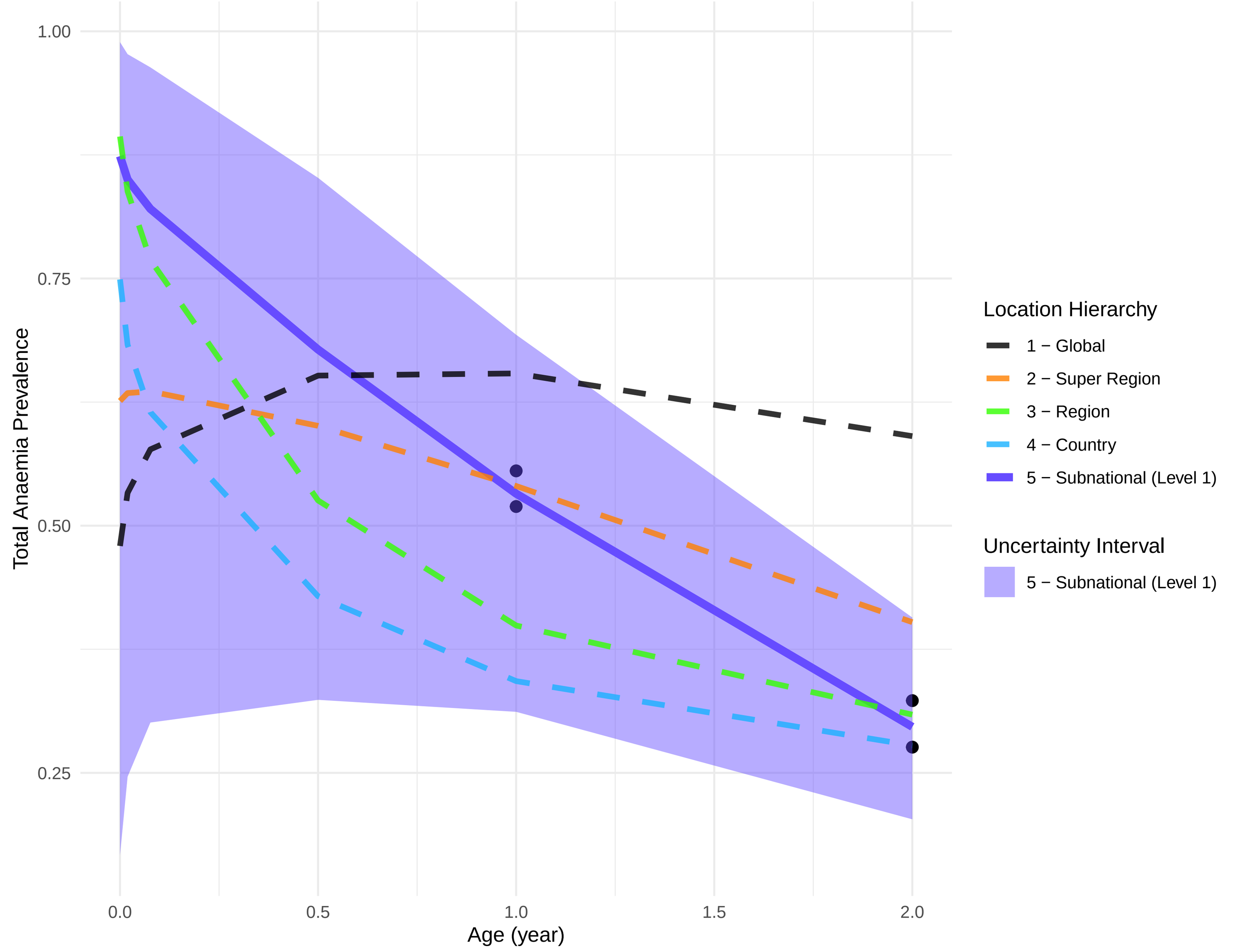

MR−BRT Age/Sex Fits − Males <2 Years Old − Total Anaemia Prevalence − Michoacán de Ocampo
WHO−2024 Adjustment Method

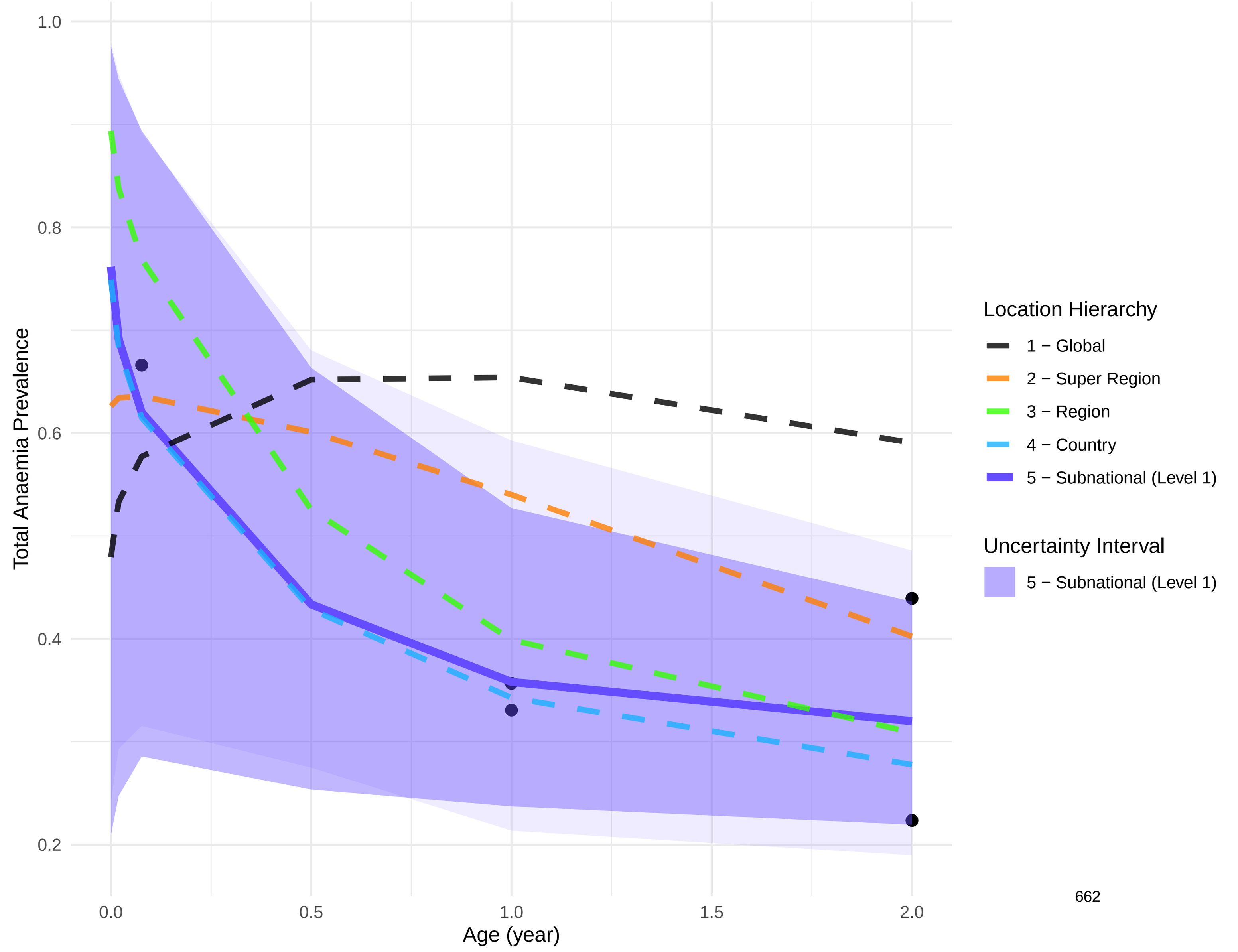

MR−BRT Age/Sex Fits − Males <2 Years Old − Total Anaemia Prevalence − Morelos
WHO−2024 Adjustment Method

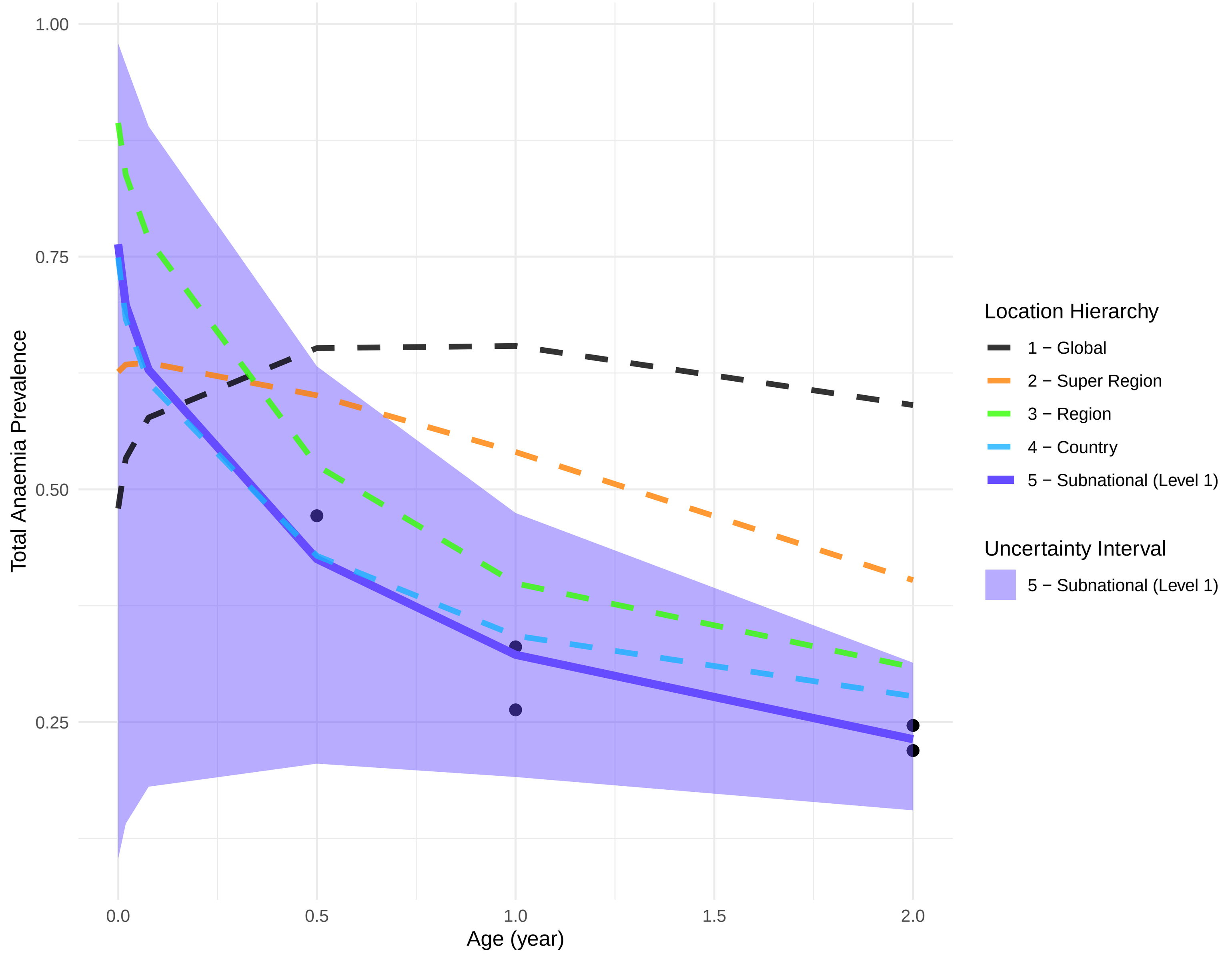

MR−BRT Age/Sex Fits − Males <2 Years Old − Total Anaemia Prevalence − Nayarit
WHO−2024 Adjustment Method

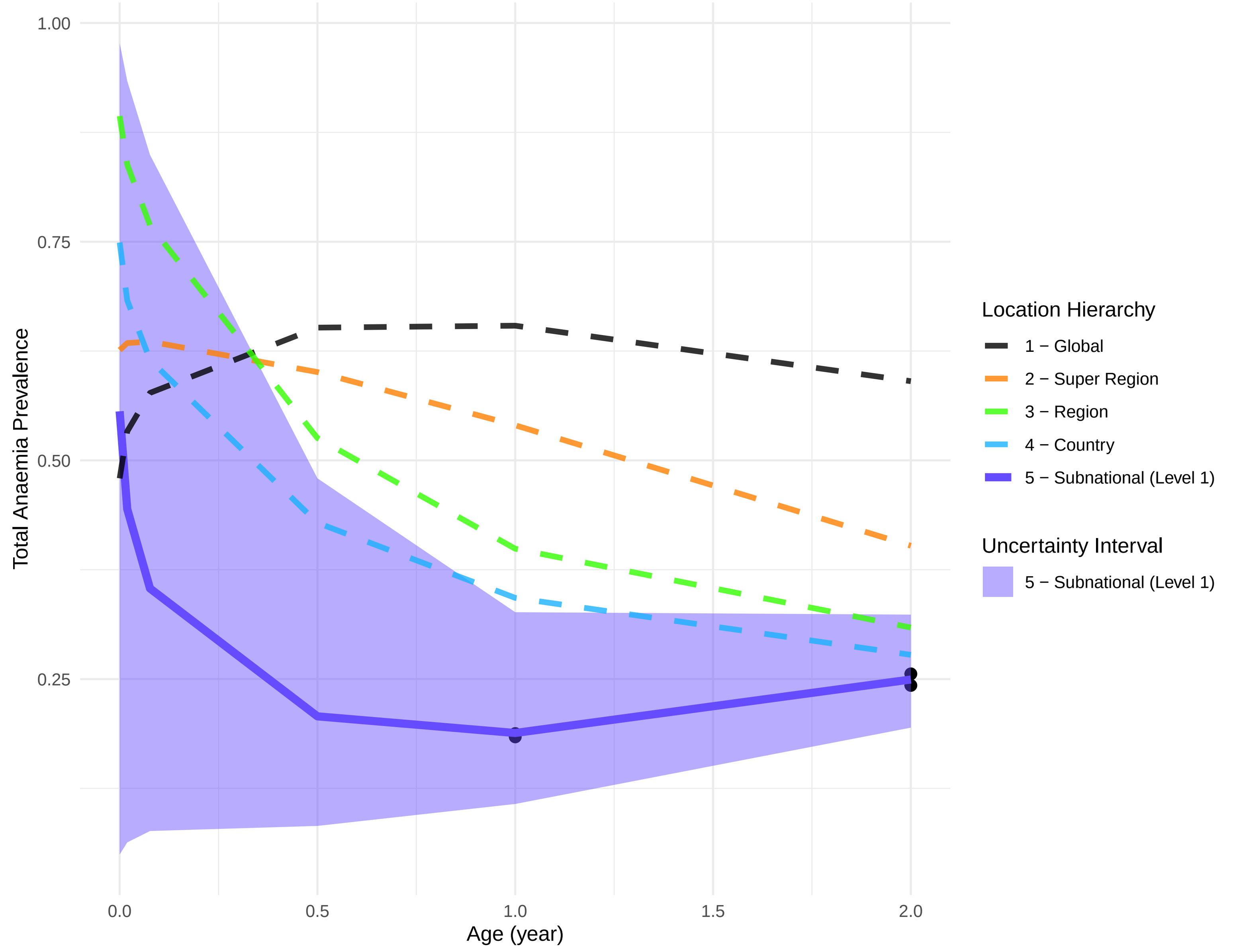

MR−BRT Age/Sex Fits − Males <2 Years Old − Total Anaemia Prevalence − Nuevo León
WHO−2024 Adjustment Method

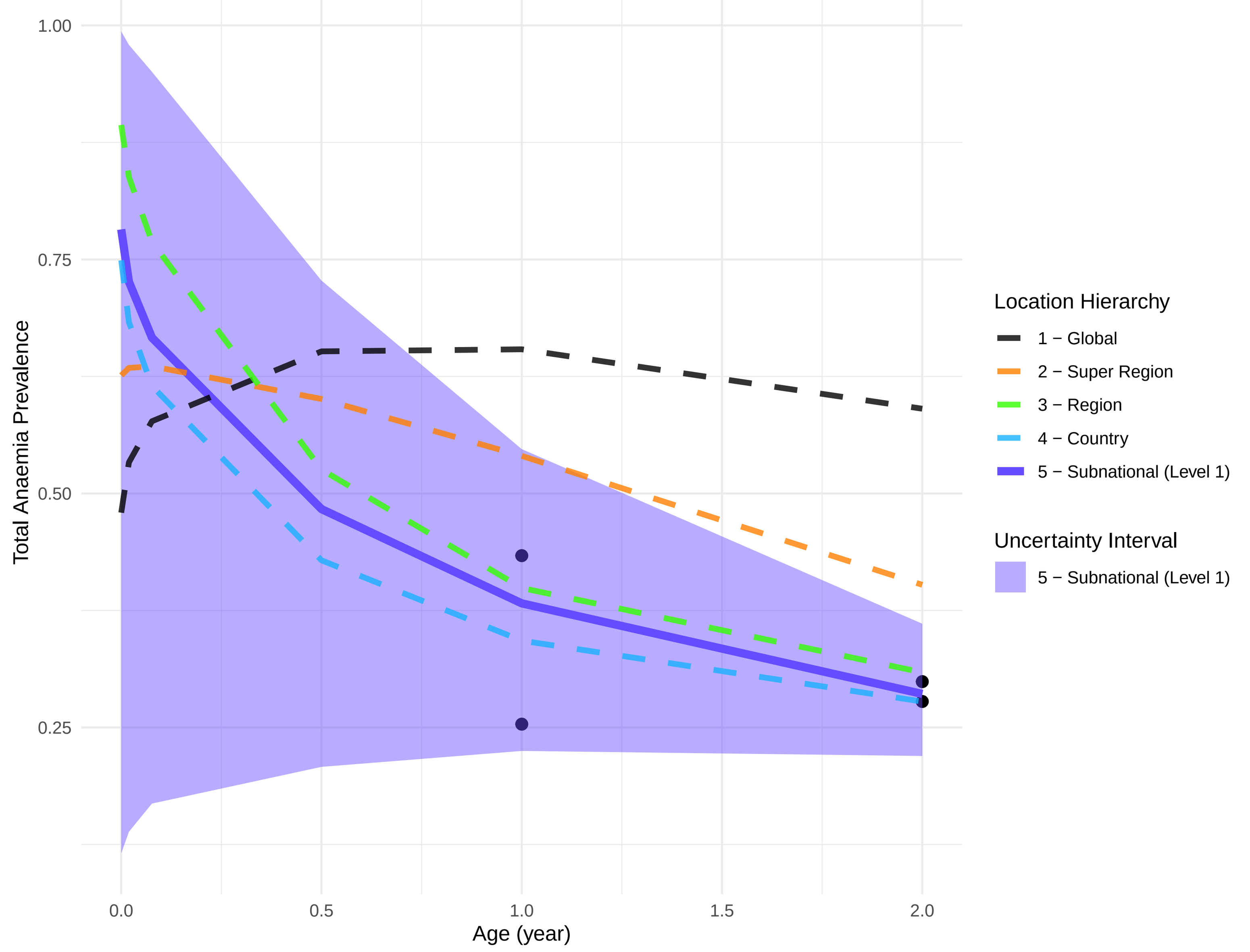

MR−BRT Age/Sex Fits − Males <2 Years Old − Total Anaemia Prevalence − Oaxaca
WHO−2024 Adjustment Method

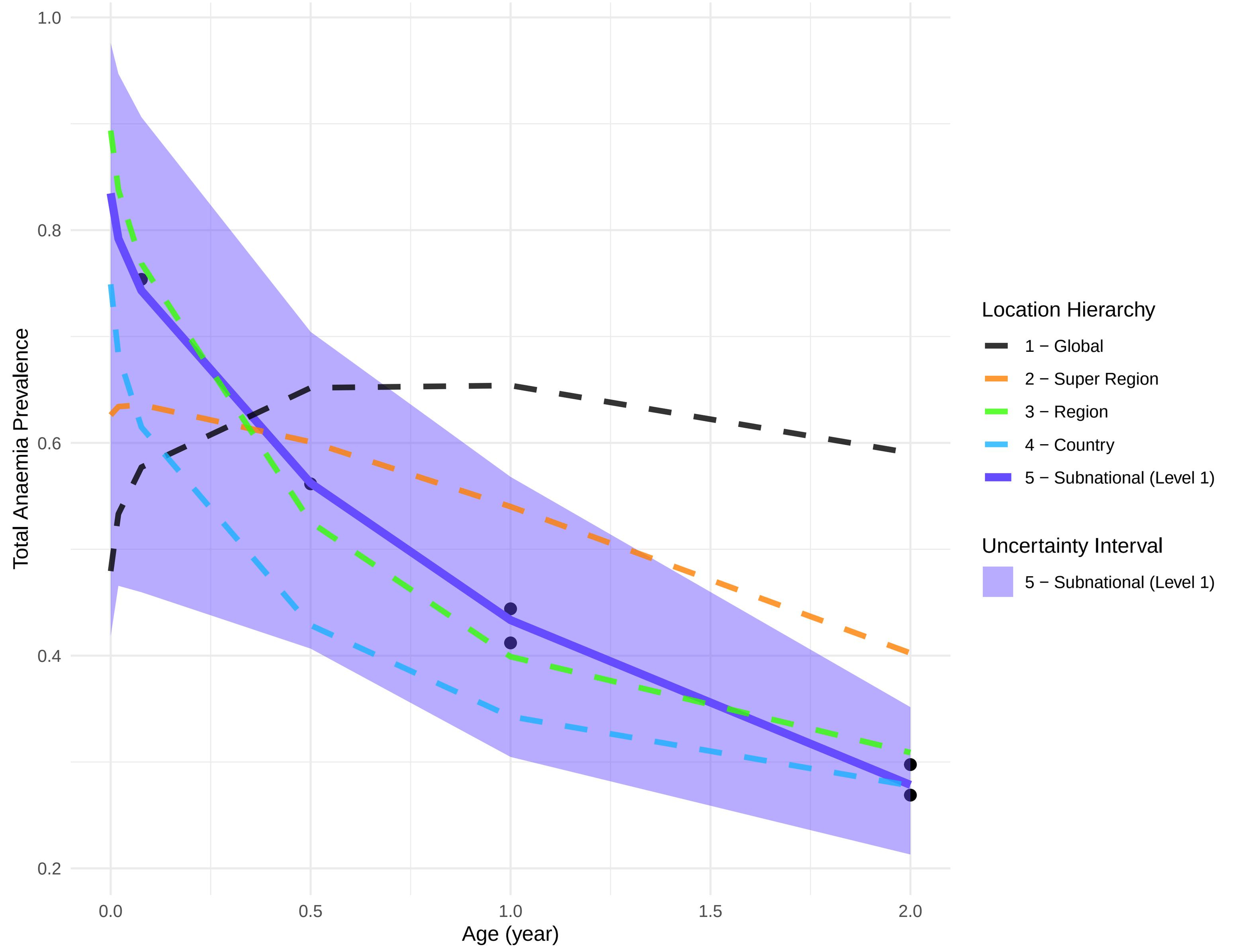

MR−BRT Age/Sex Fits − Males <2 Years Old − Total Anaemia Prevalence − Puebla
WHO−2024 Adjustment Method

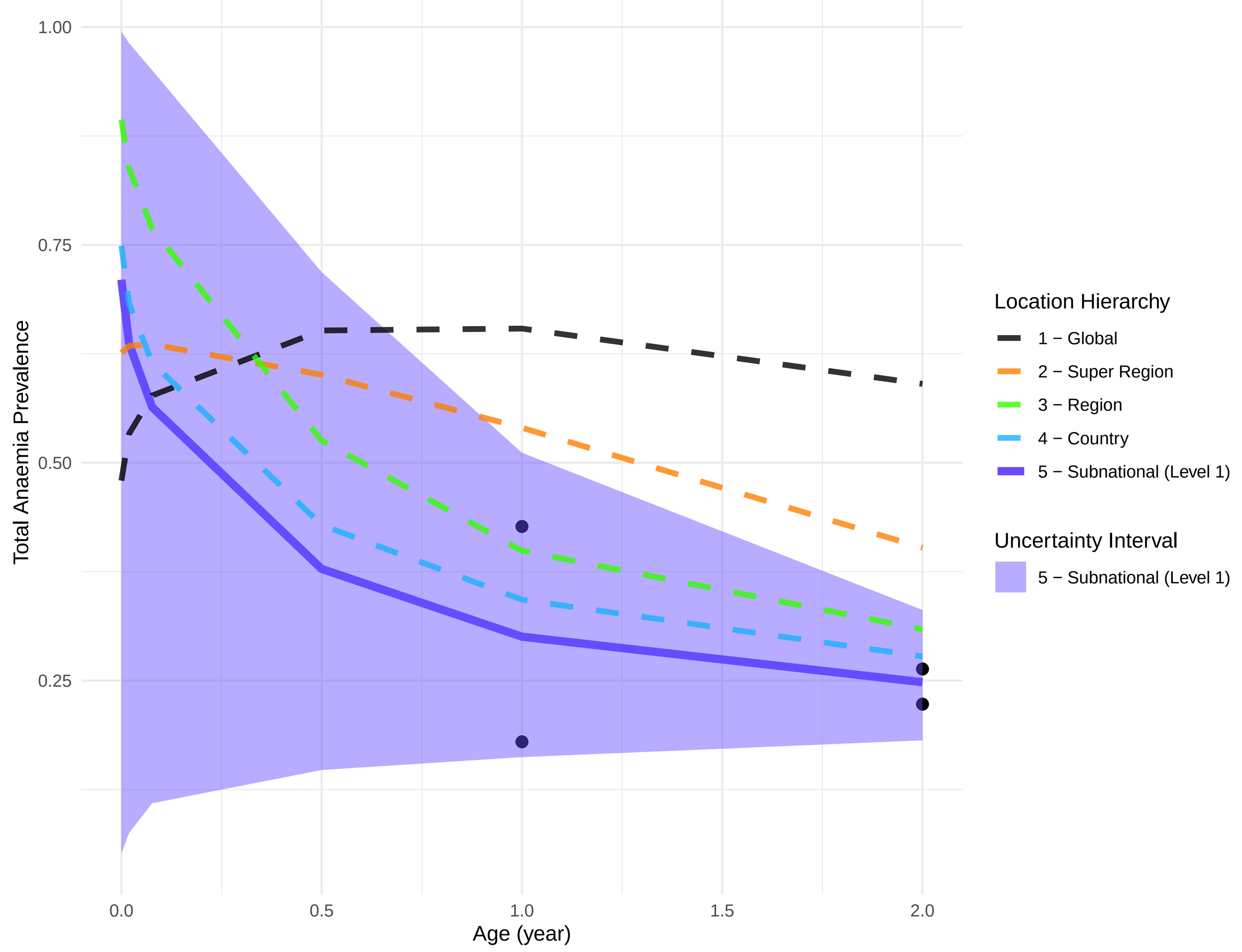

MR−BRT Age/Sex Fits − Males <2 Years Old − Total Anaemia Prevalence − Querétaro
WHO−2024 Adjustment Method

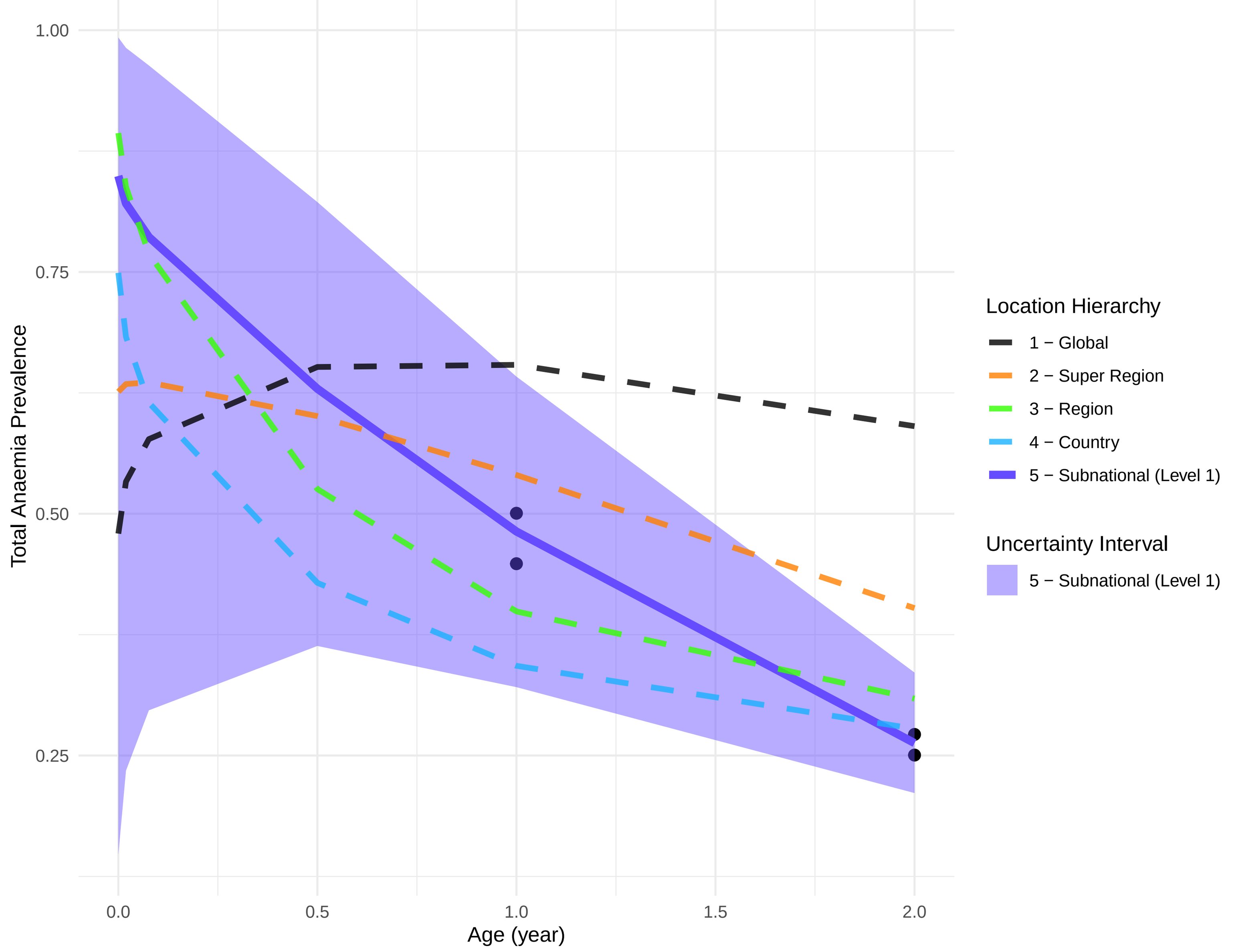

MR−BRT Age/Sex Fits − Males <2 Years Old − Total Anaemia Prevalence − Quintana Roo
WHO−2024 Adjustment Method

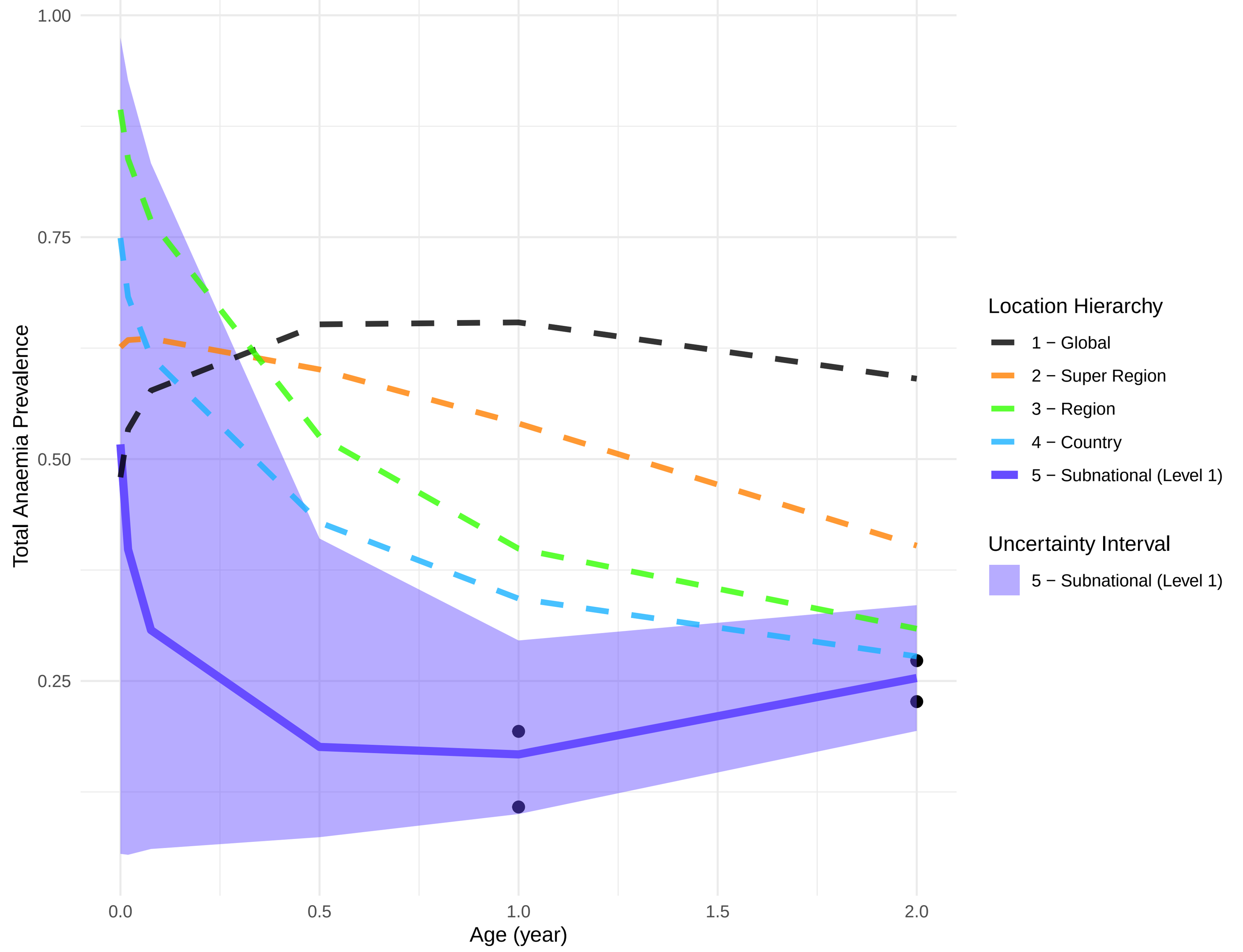

MR−BRT Age/Sex Fits − Males <2 Years Old − Total Anaemia Prevalence − San Luis Potosí
WHO−2024 Adjustment Method

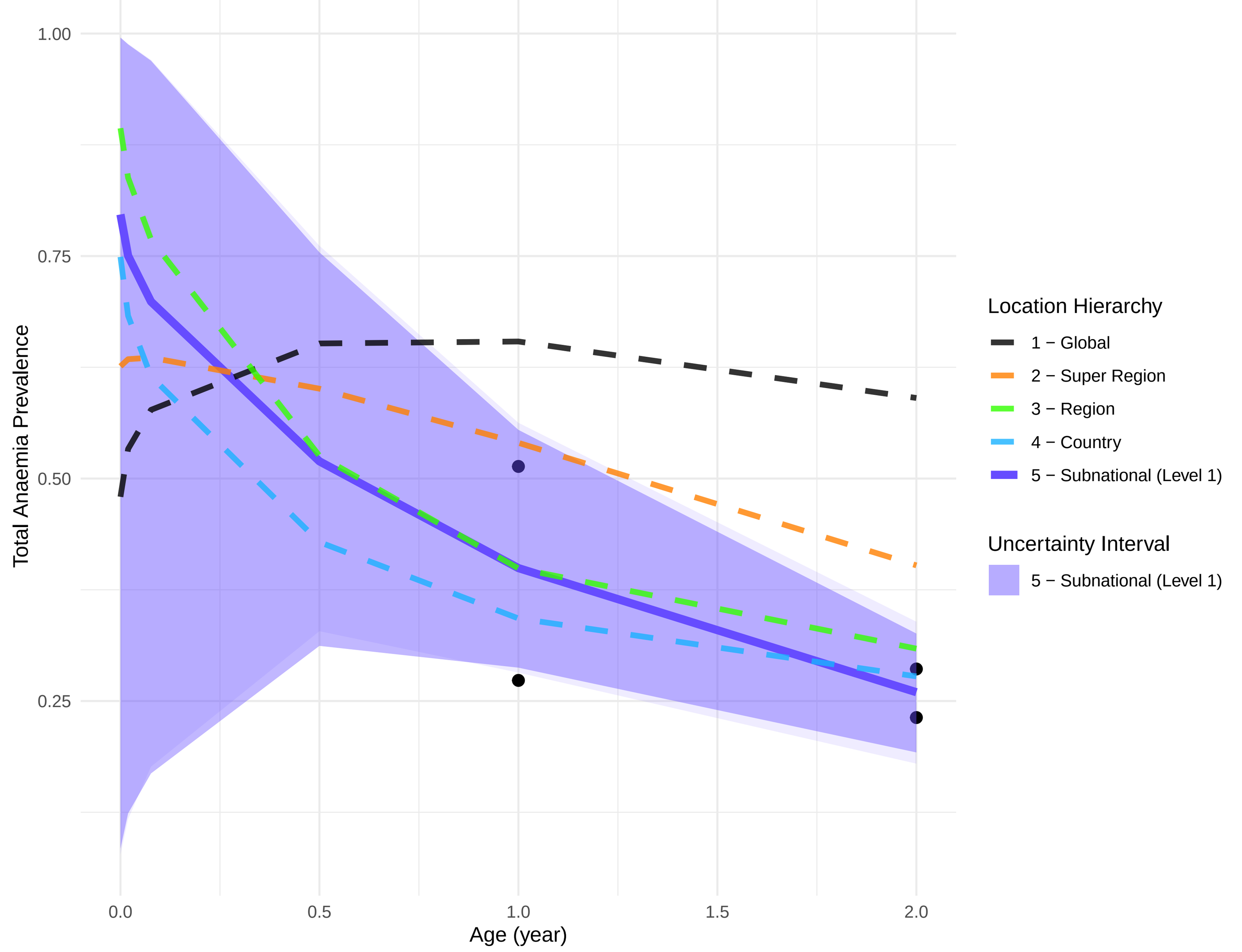

MR−BRT Age/Sex Fits − Males <2 Years Old − Total Anaemia Prevalence − Sinaloa
WHO−2024 Adjustment Method

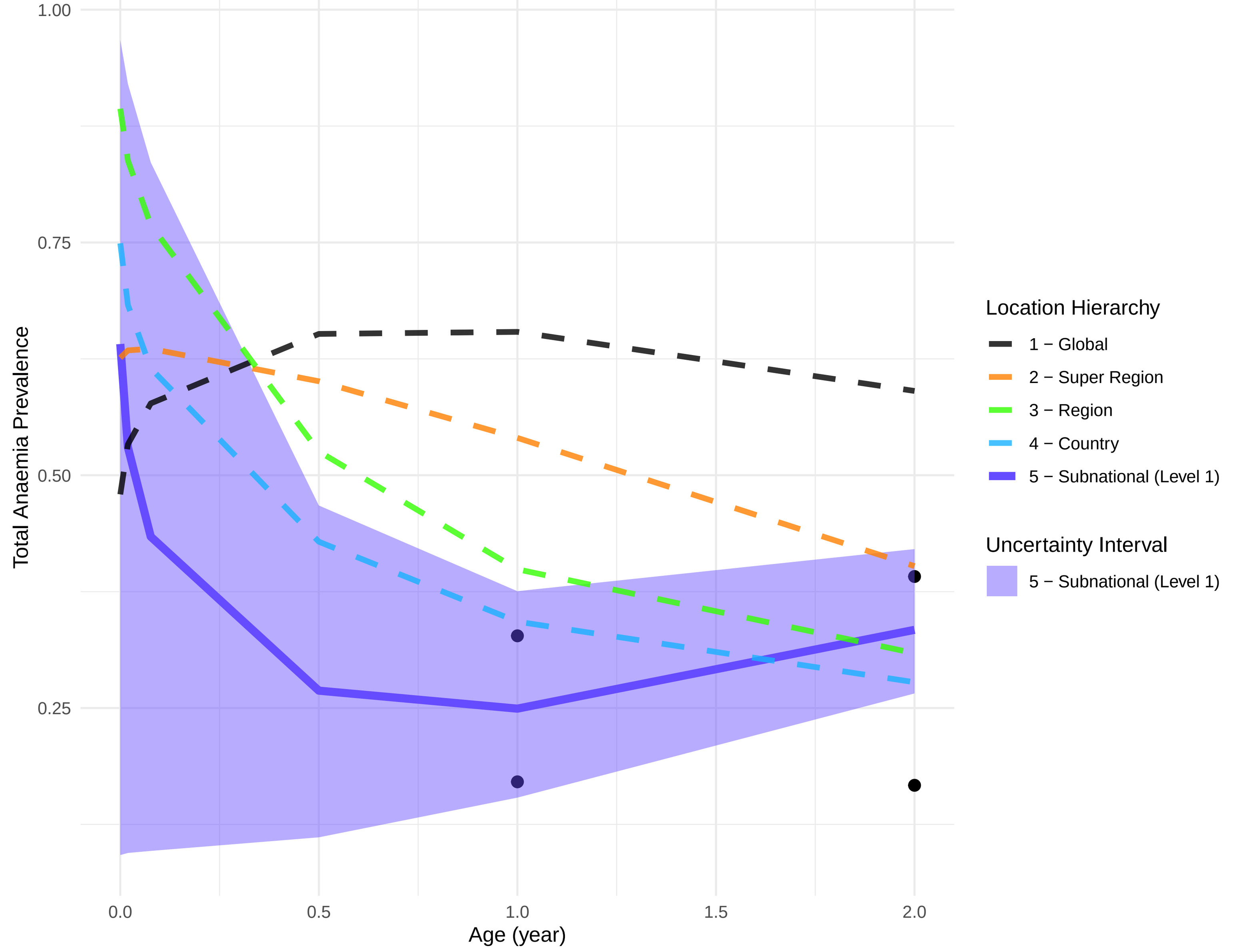

MR−BRT Age/Sex Fits − Males <2 Years Old − Total Anaemia Prevalence − Sonora
WHO−2024 Adjustment Method

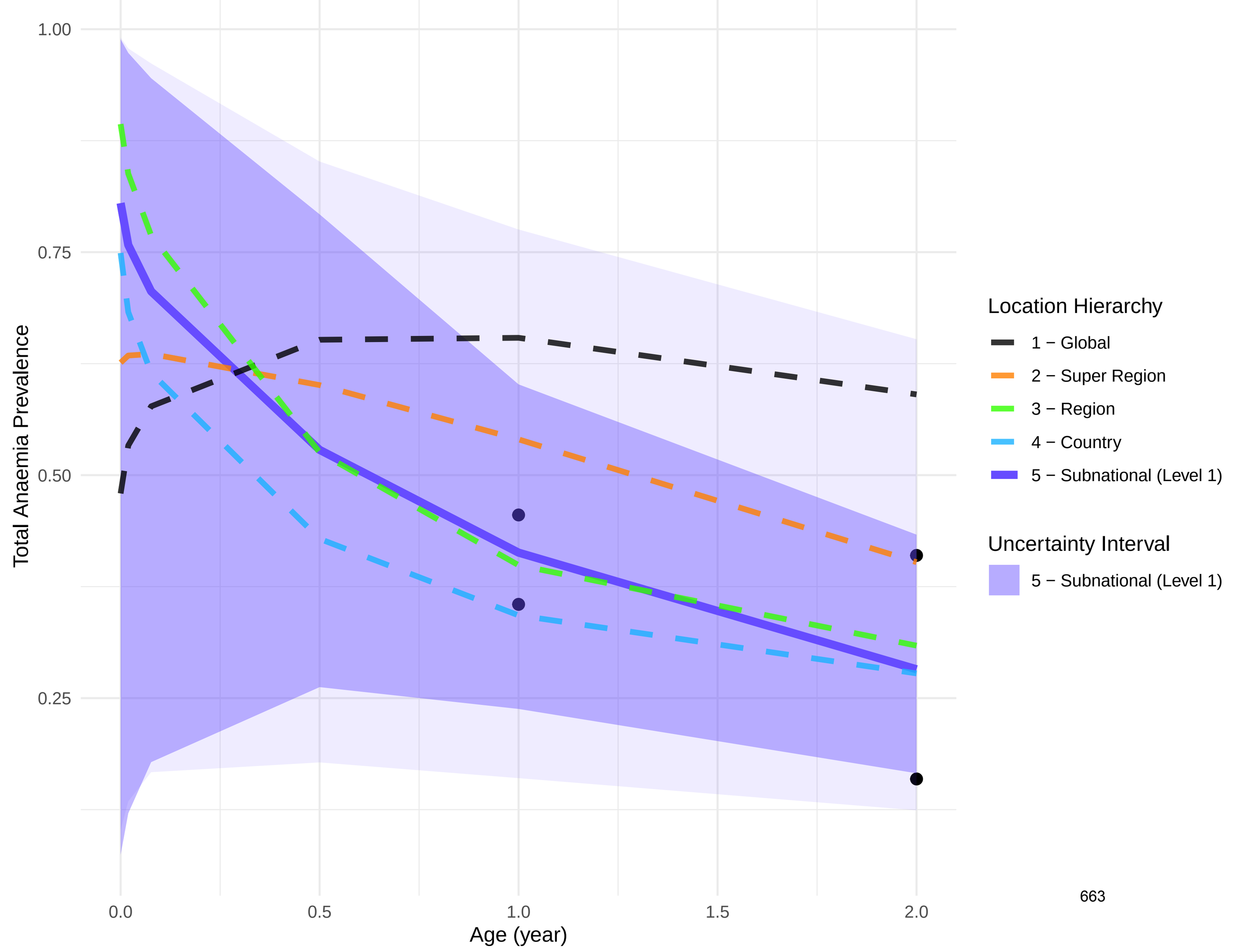

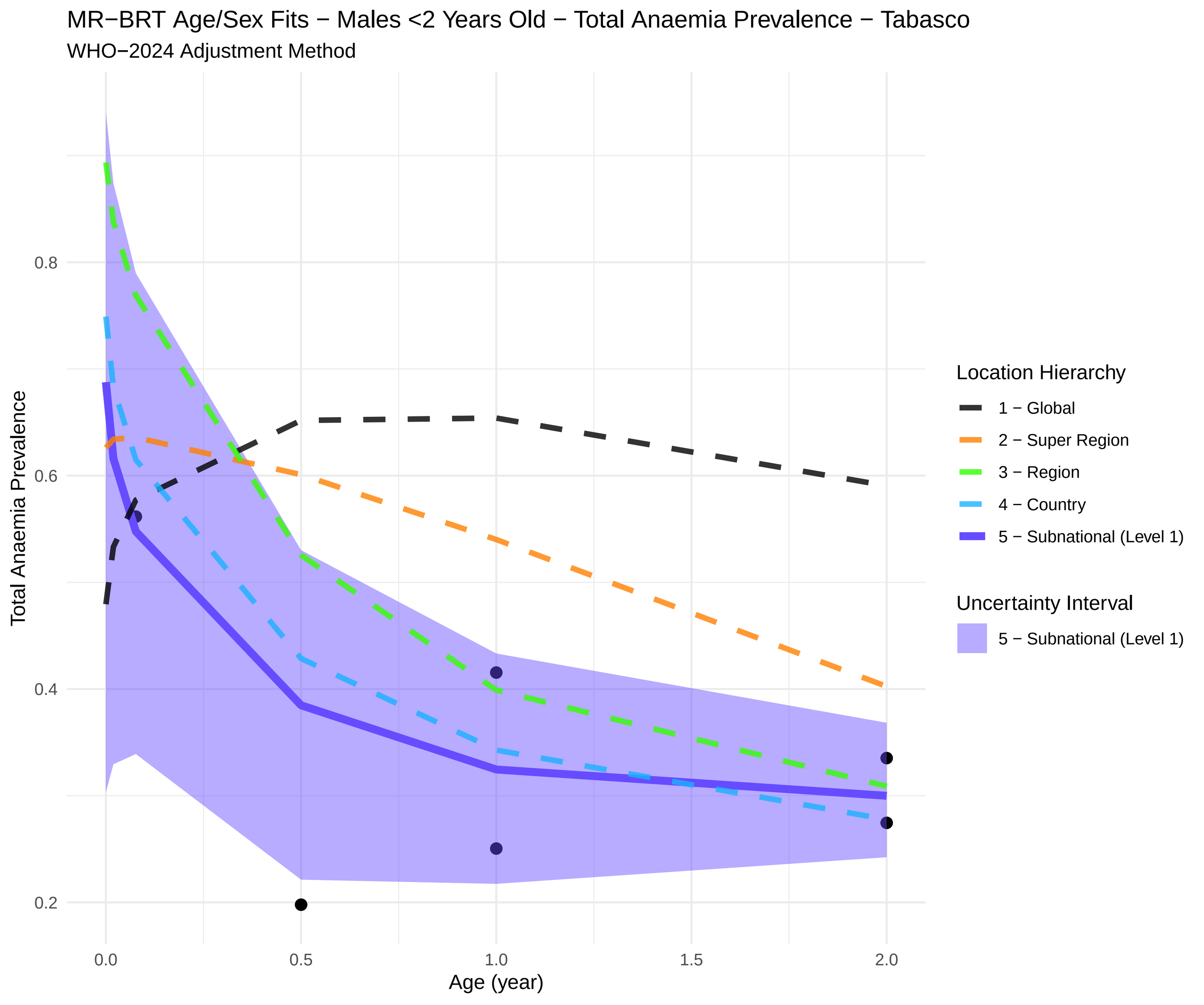
MR−BRT Age/Sex Fits − Males <2 Years Old − Total Anaemia Prevalence − Tabasco
WHO−2024 Adjustment Method
Total Anaemia Prevalence
Age (year)
Location Hierarchy
1 − Global
2 − Super Region
3 − Region
4 − Country
5 − Subnational (Level 1)
Uncertainty Interval
5 − Subnational (Level 1)

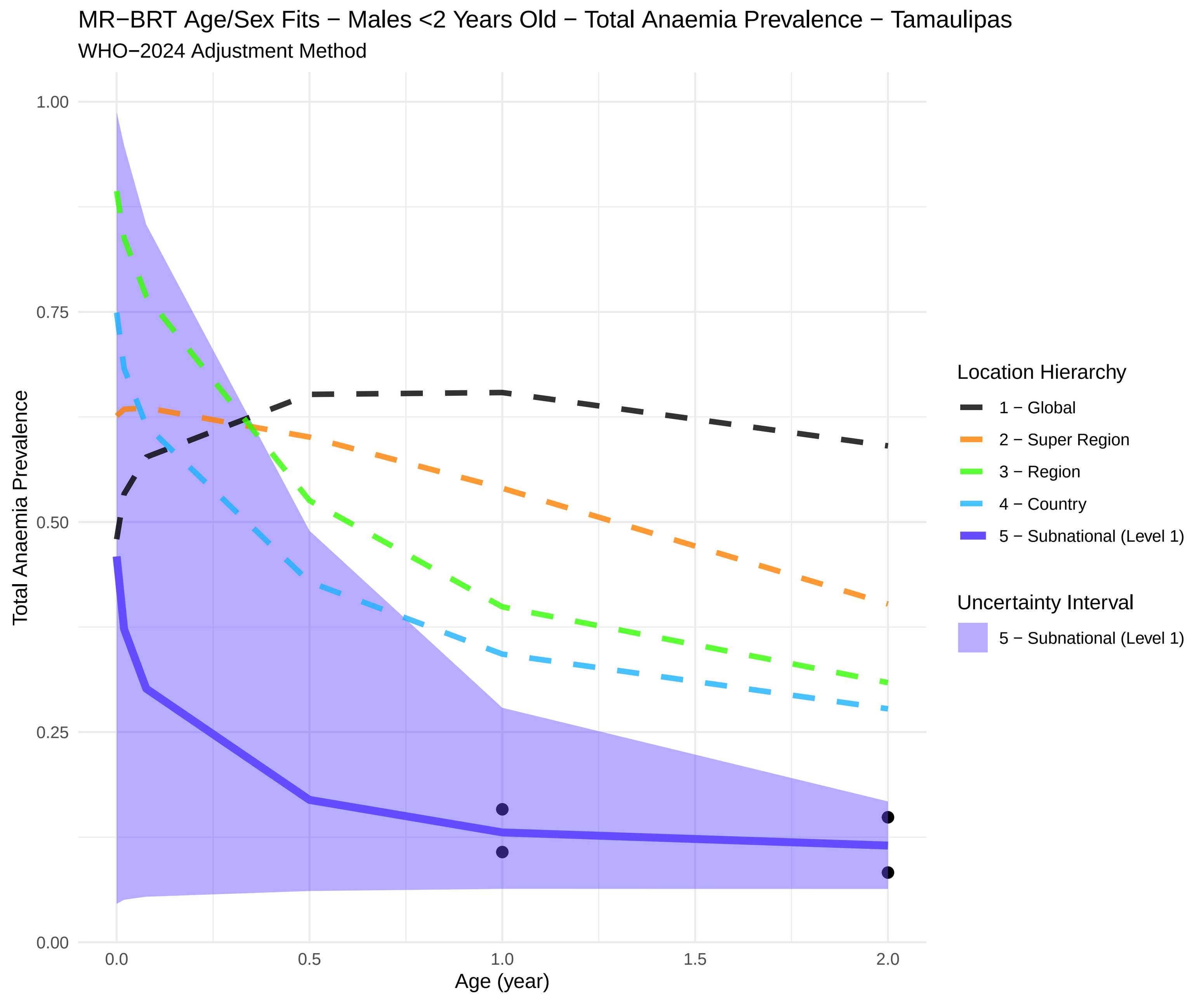
MR−BRT Age/Sex Fits − Males <2 Years Old − Total Anaemia Prevalence − Tamaulipas
WHO−2024 Adjustment Method
Total Anaemia Prevalence
Age (year)
Location Hierarchy
1 − Global
2 − Super Region
3 − Region
4 − Country
5 − Subnational (Level 1)
Uncertainty Interval
5 − Subnational (Level 1)

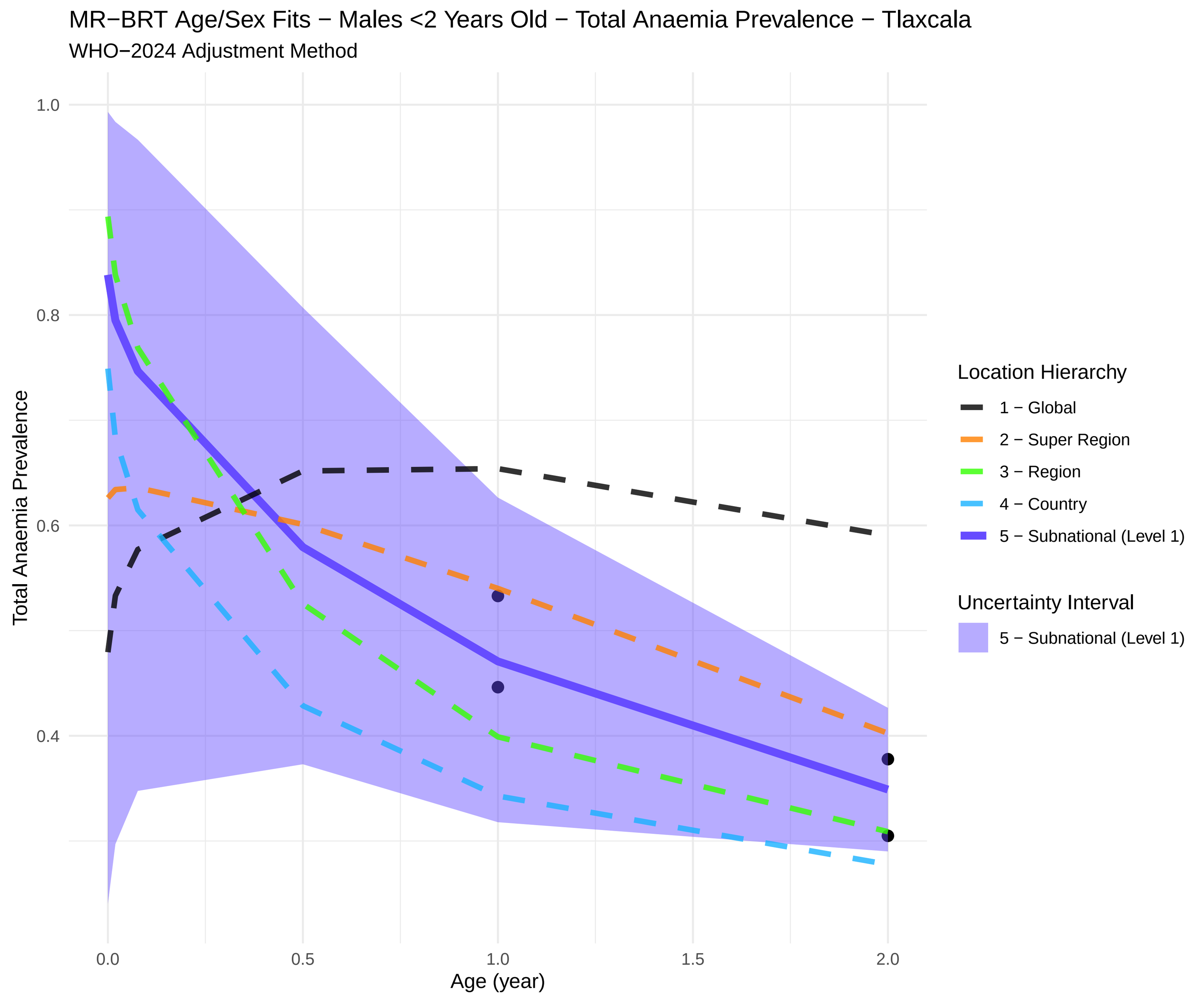
MR−BRT Age/Sex Fits − Males <2 Years Old − Total Anaemia Prevalence − Tlaxcala
WHO−2024 Adjustment Method
Total Anaemia Prevalence
Age (year)
Location Hierarchy
1 − Global
2 − Super Region
3 − Region
4 − Country
5 − Subnational (Level 1)
Uncertainty Interval
5 − Subnational (Level 1)

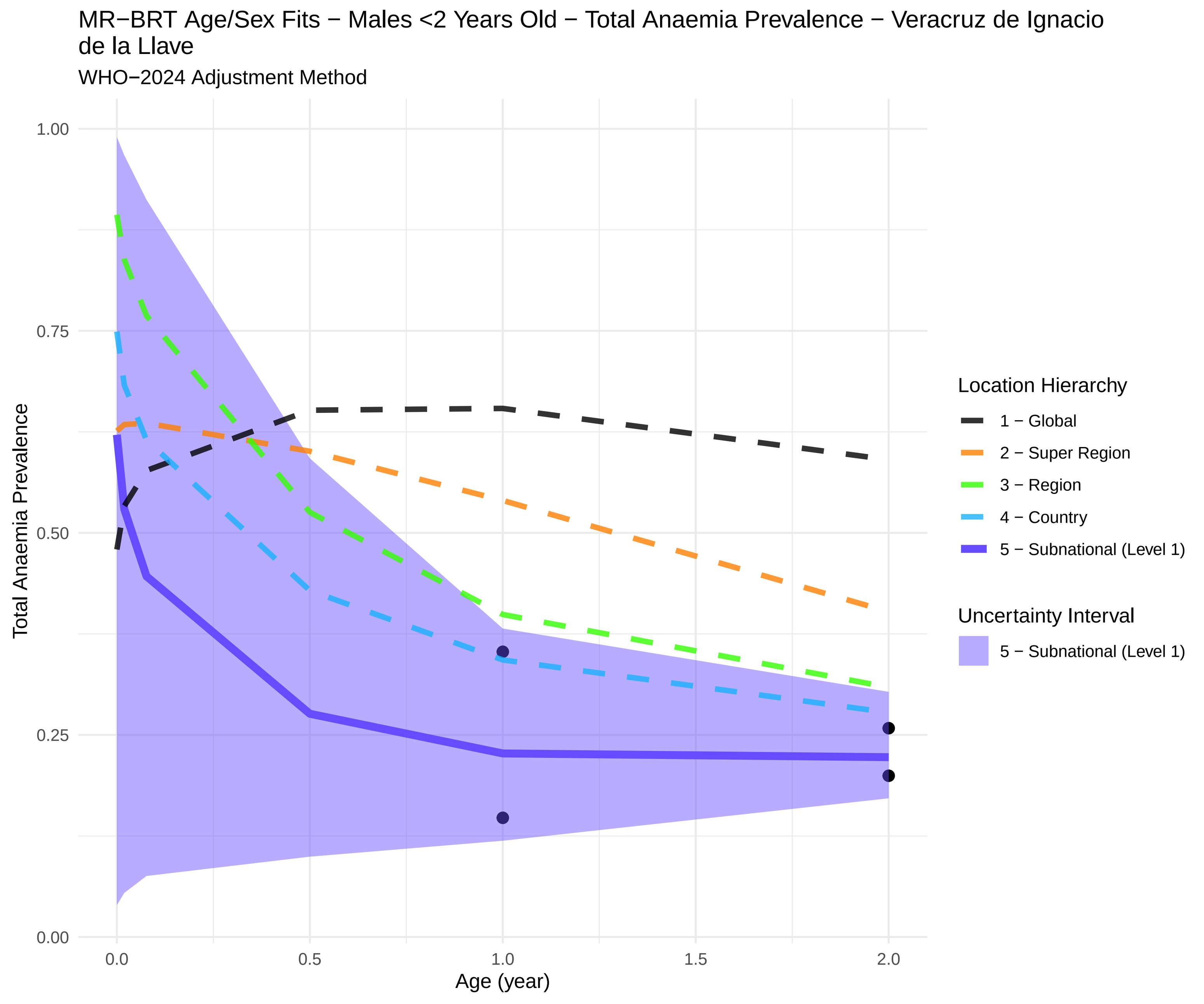
MR−BRT Age/Sex Fits − Males <2 Years Old − Total Anaemia Prevalence − Veracruz de Ignacio de la Llave
WHO−2024 Adjustment Method
Total Anaemia Prevalence
Age (year)
Location Hierarchy
1 − Global
2 − Super Region
3 − Region
4 − Country
5 − Subnational (Level 1)
Uncertainty Interval
5 − Subnational (Level 1)

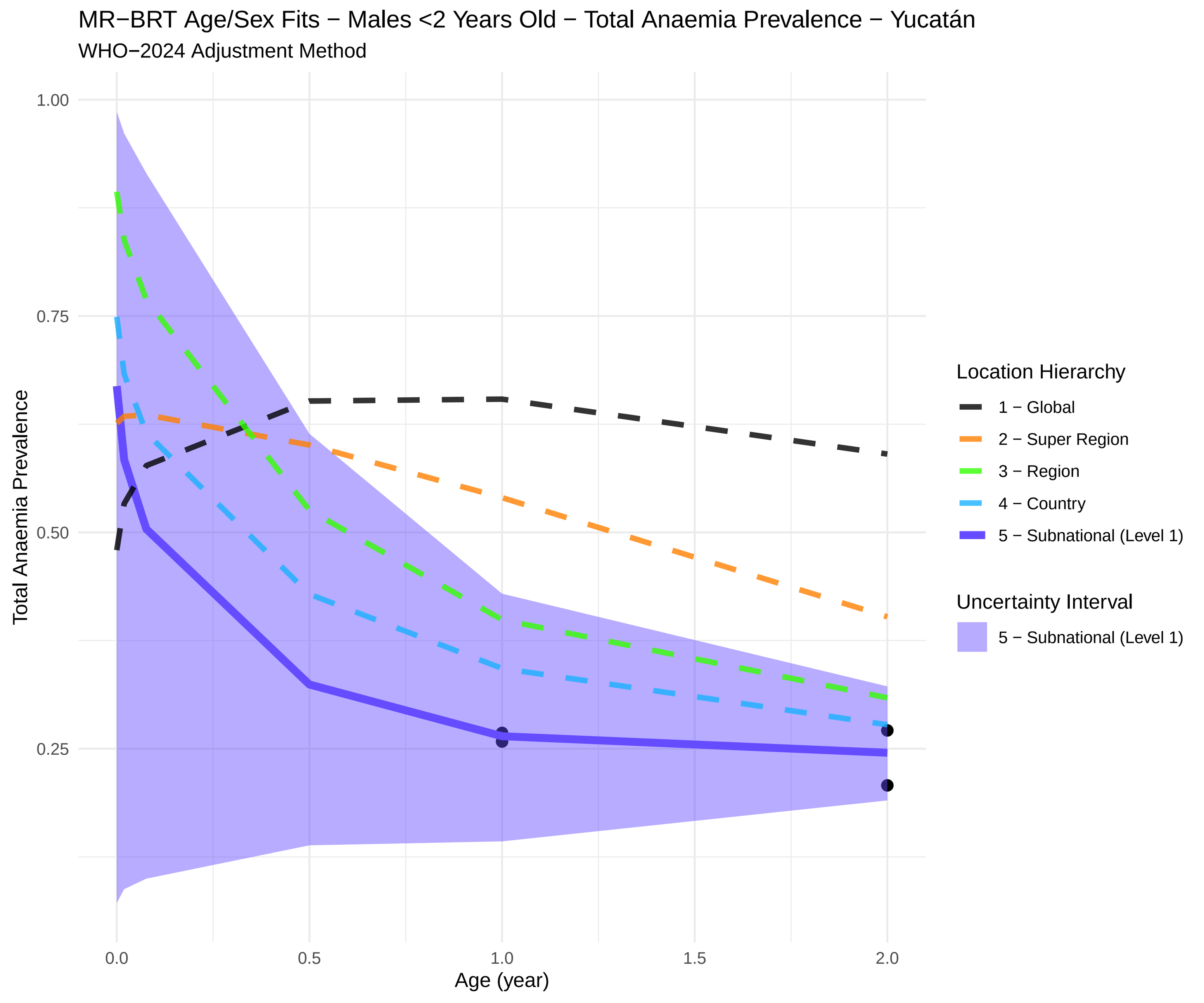
MR−BRT Age/Sex Fits − Males <2 Years Old − Total Anaemia Prevalence − Yucatán
WHO−2024 Adjustment Method
Total Anaemia Prevalence
Age (year)
Location Hierarchy
1 − Global
2 − Super Region
3 − Region
4 − Country
5 − Subnational (Level 1)
Uncertainty Interval
5 − Subnational (Level 1)

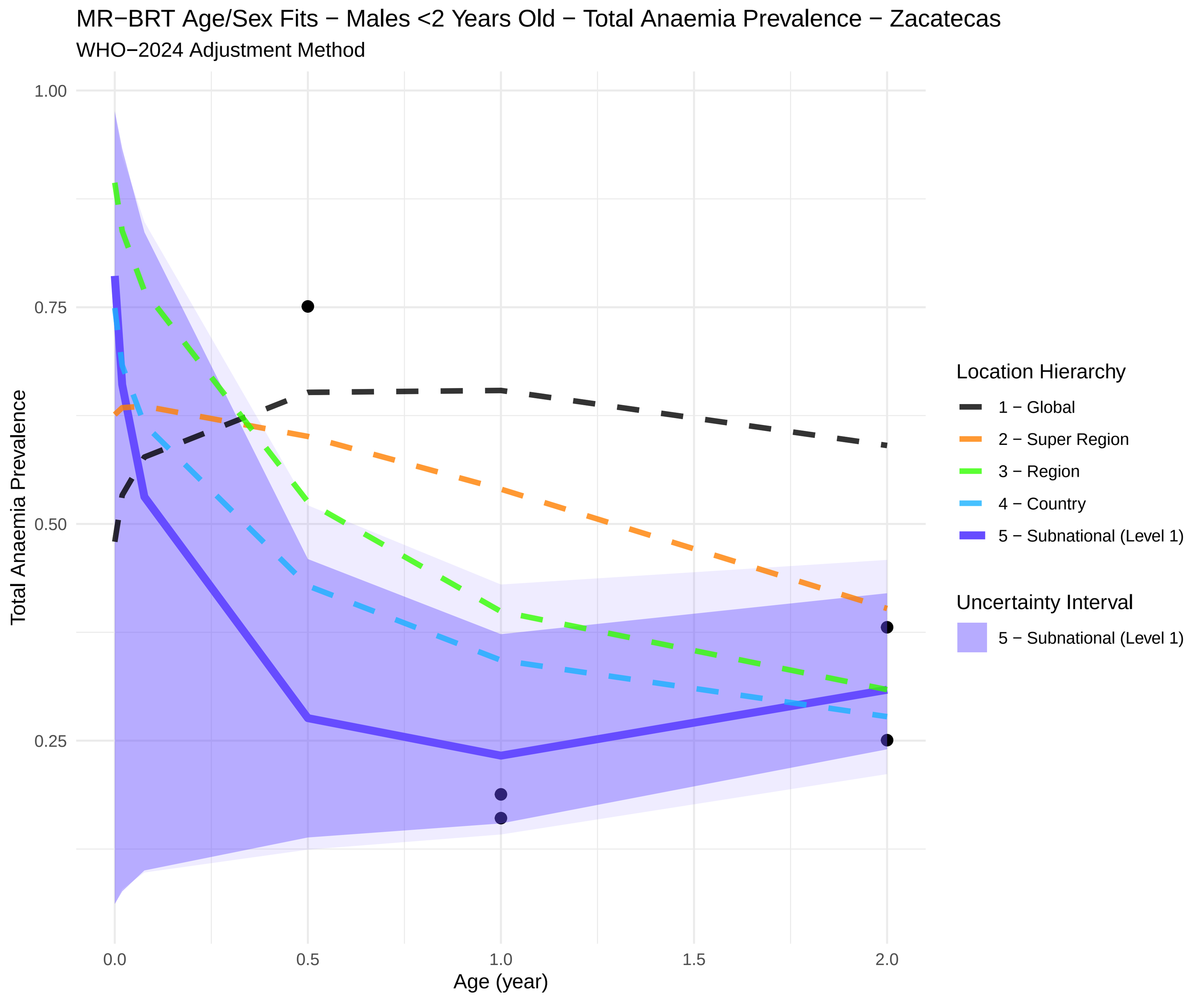
MR−BRT Age/Sex Fits − Males <2 Years Old − Total Anaemia Prevalence − Zacatecas
WHO−2024 Adjustment Method
Total Anaemia Prevalence
Age (year)
Location Hierarchy
1 − Global
2 − Super Region
3 − Region
4 − Country
5 − Subnational (Level 1)
Uncertainty Interval
5 − Subnational (Level 1)

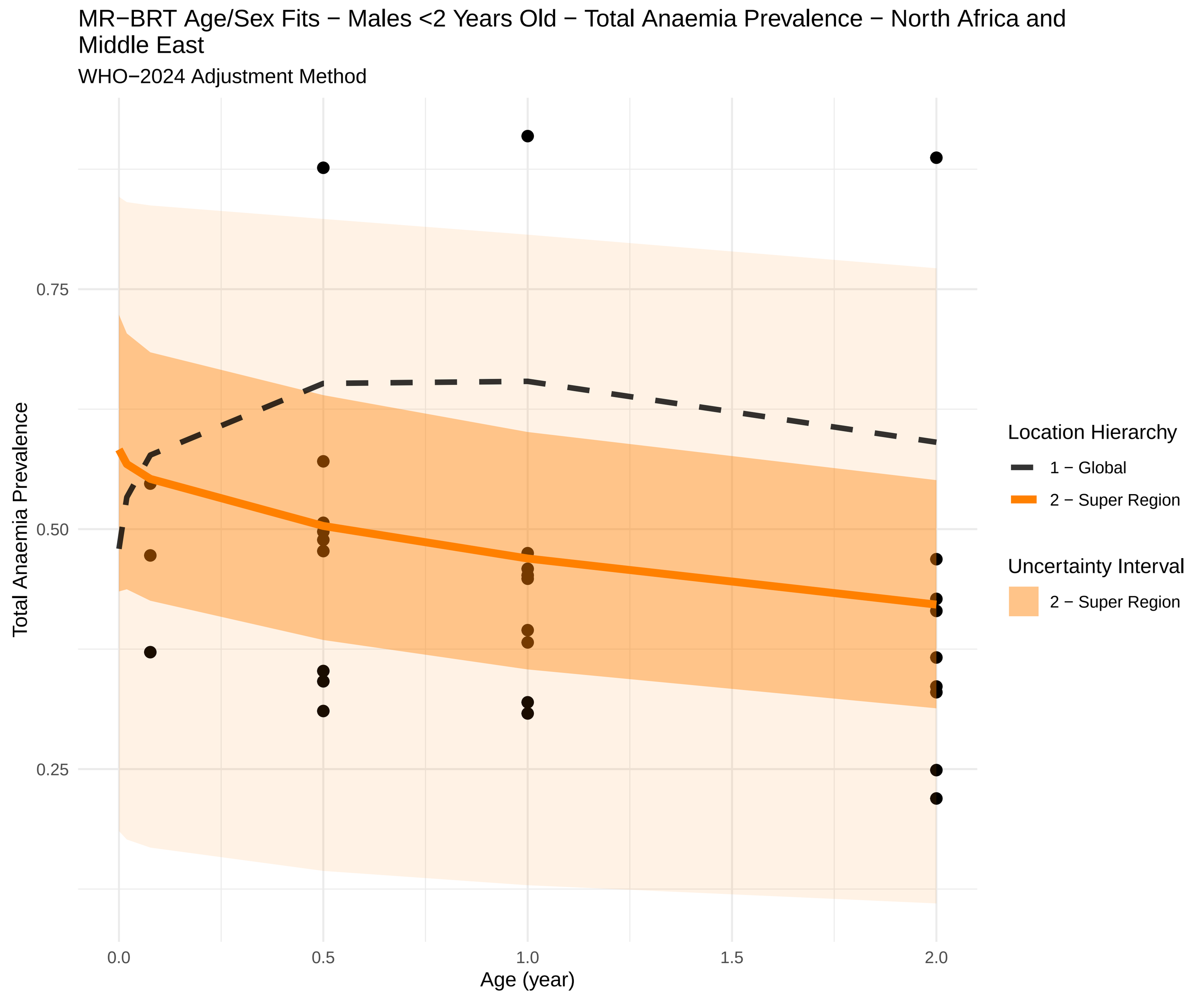
MR−BRT Age/Sex Fits − Males <2 Years Old − Total Anaemia Prevalence − North Africa and Middle East
WHO−2024 Adjustment Method
Total Anaemia Prevalence
Age (year)
Location Hierarchy
1 − Global
2 − Super Region
Uncertainty Interval
2 − Super Region

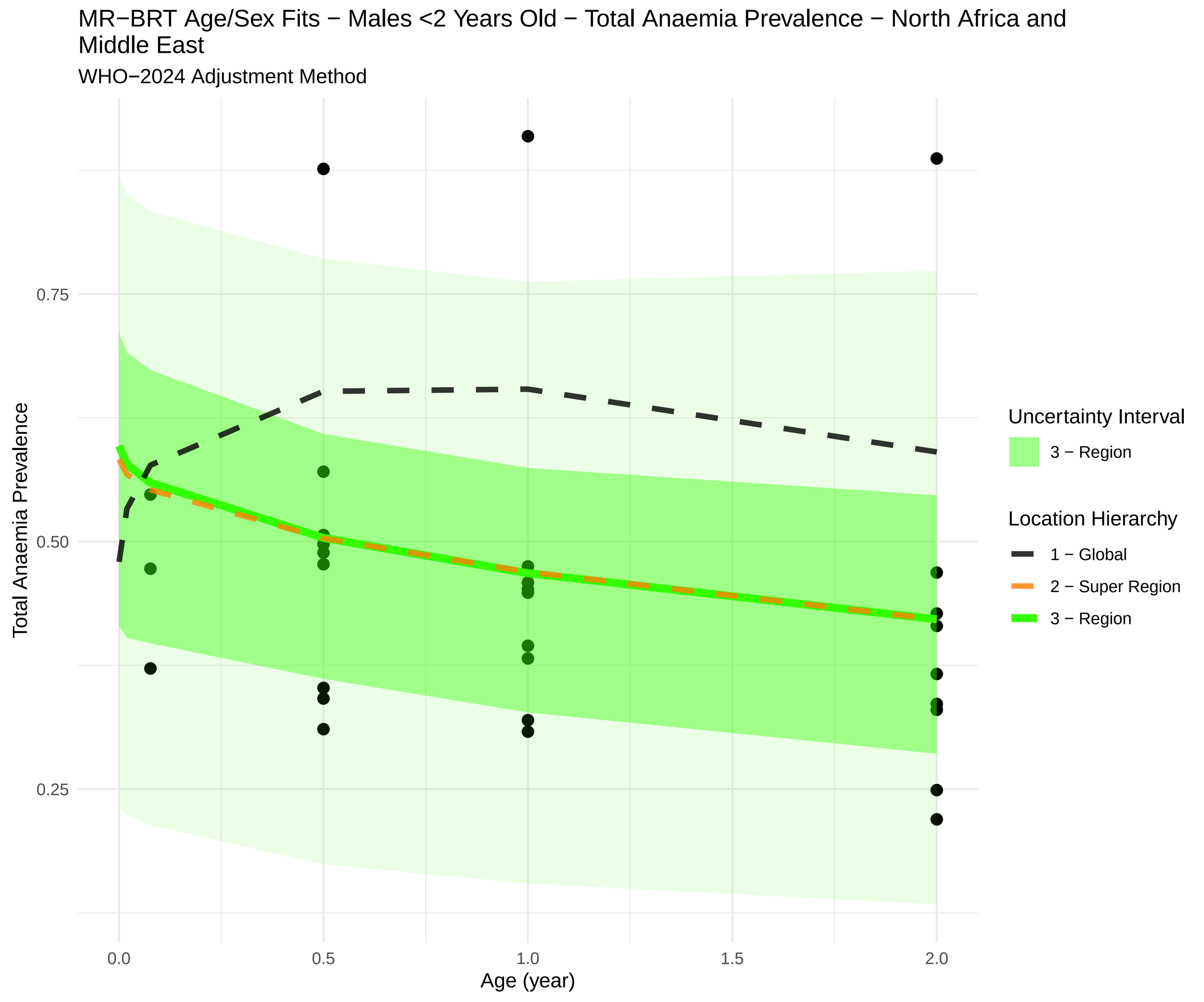
MR−BRT Age/Sex Fits − Males <2 Years Old − Total Anaemia Prevalence − North Africa and Middle East
WHO−2024 Adjustment Method
Total Anaemia Prevalence
Age (year)
Uncertainty Interval
3 − Region
Location Hierarchy
1 − Global
2 − Super Region
3 − Region

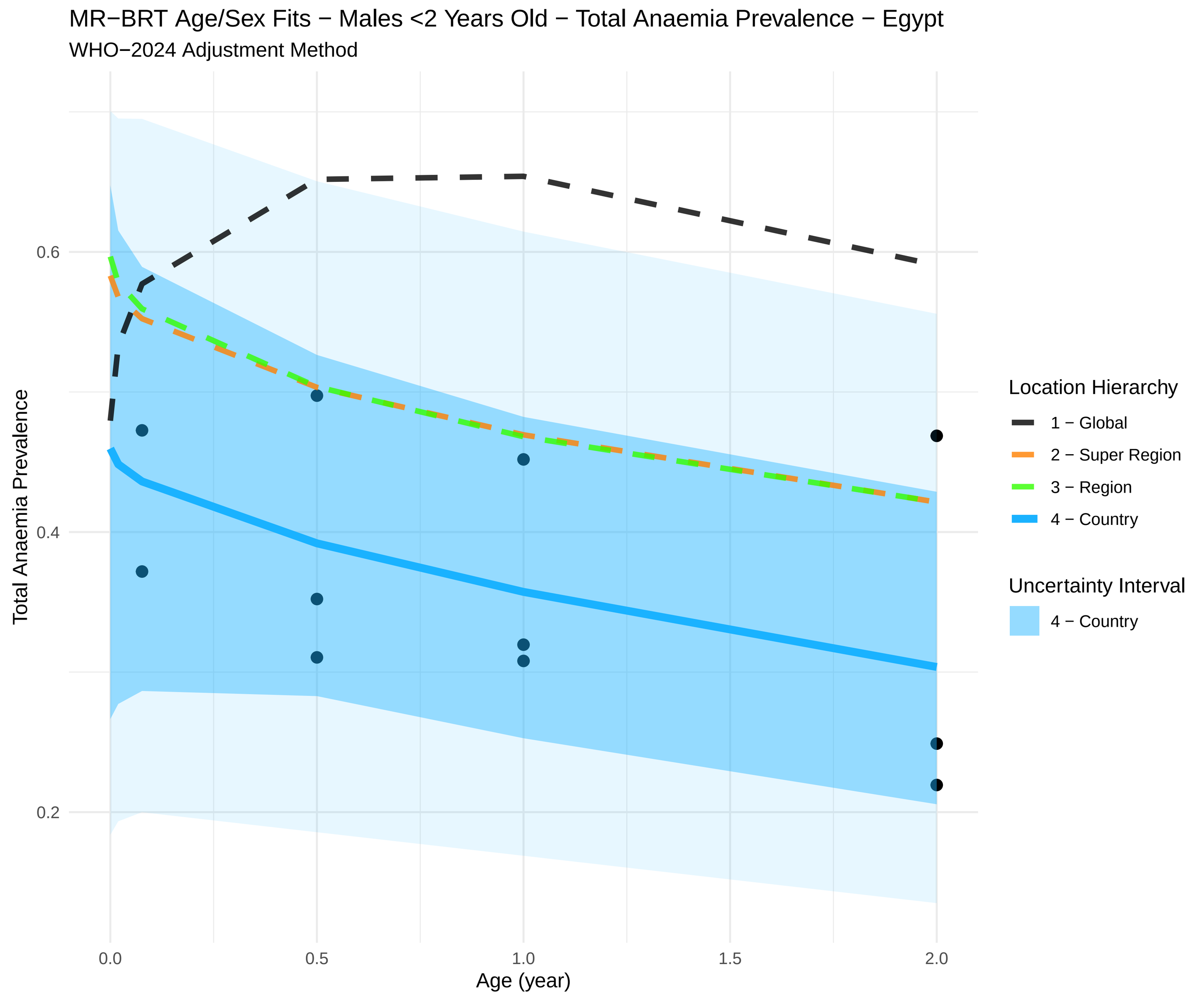
MR−BRT Age/Sex Fits − Males <2 Years Old − Total Anaemia Prevalence − Egypt
WHO−2024 Adjustment Method
Total Anaemia Prevalence
Age (year)
Location Hierarchy
1 − Global
2 − Super Region
3 − Region
4 − Country
Uncertainty Interval
4 − Country

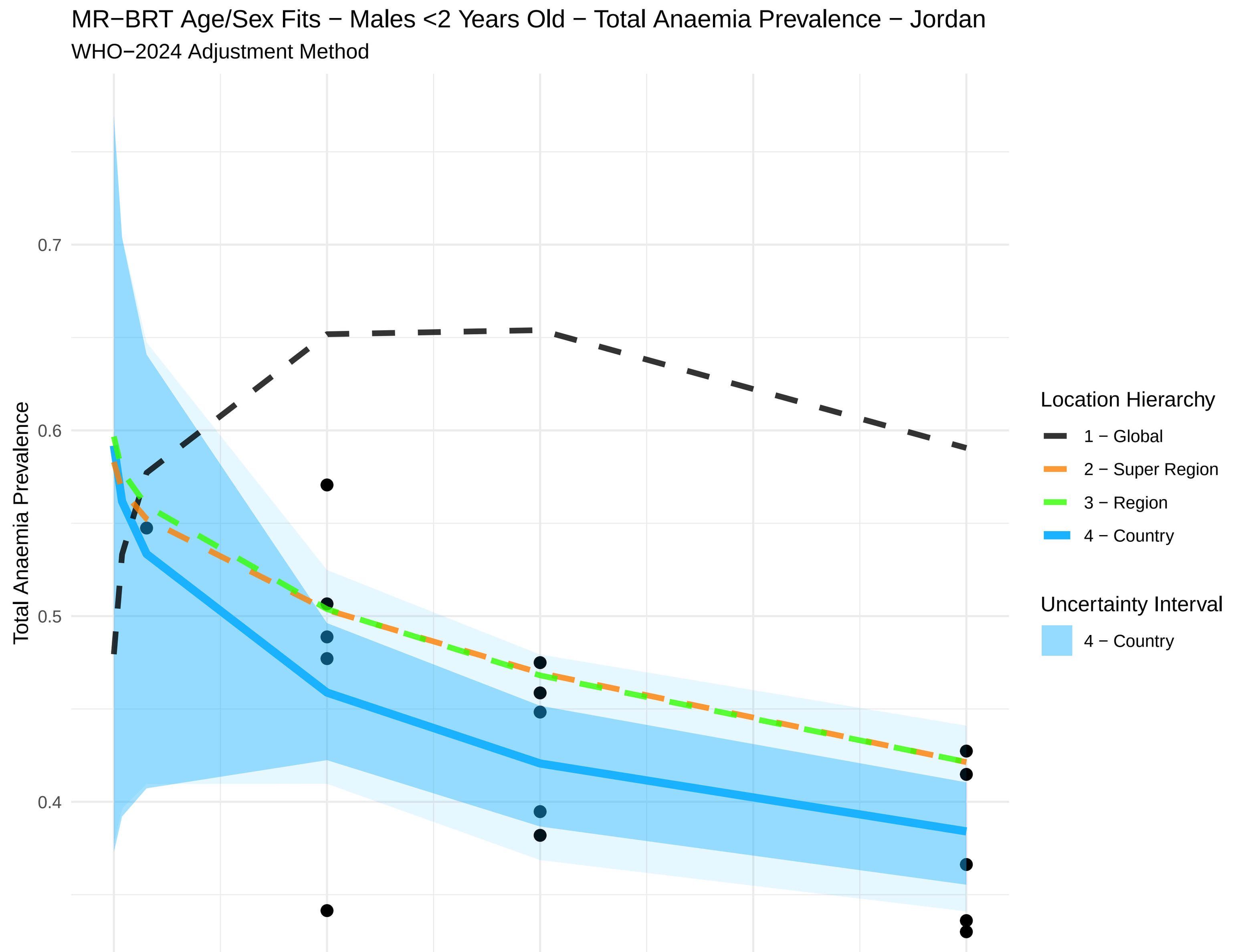
MR−BRT Age/Sex Fits − Males <2 Years Old − Total Anaemia Prevalence − Jordan
WHO−2024 Adjustment Method
Total Anaemia Prevalence
Age (year)
Location Hierarchy
1 − Global
2 − Super Region
3 − Region
4 − Country
Uncertainty Interval
4 − Country

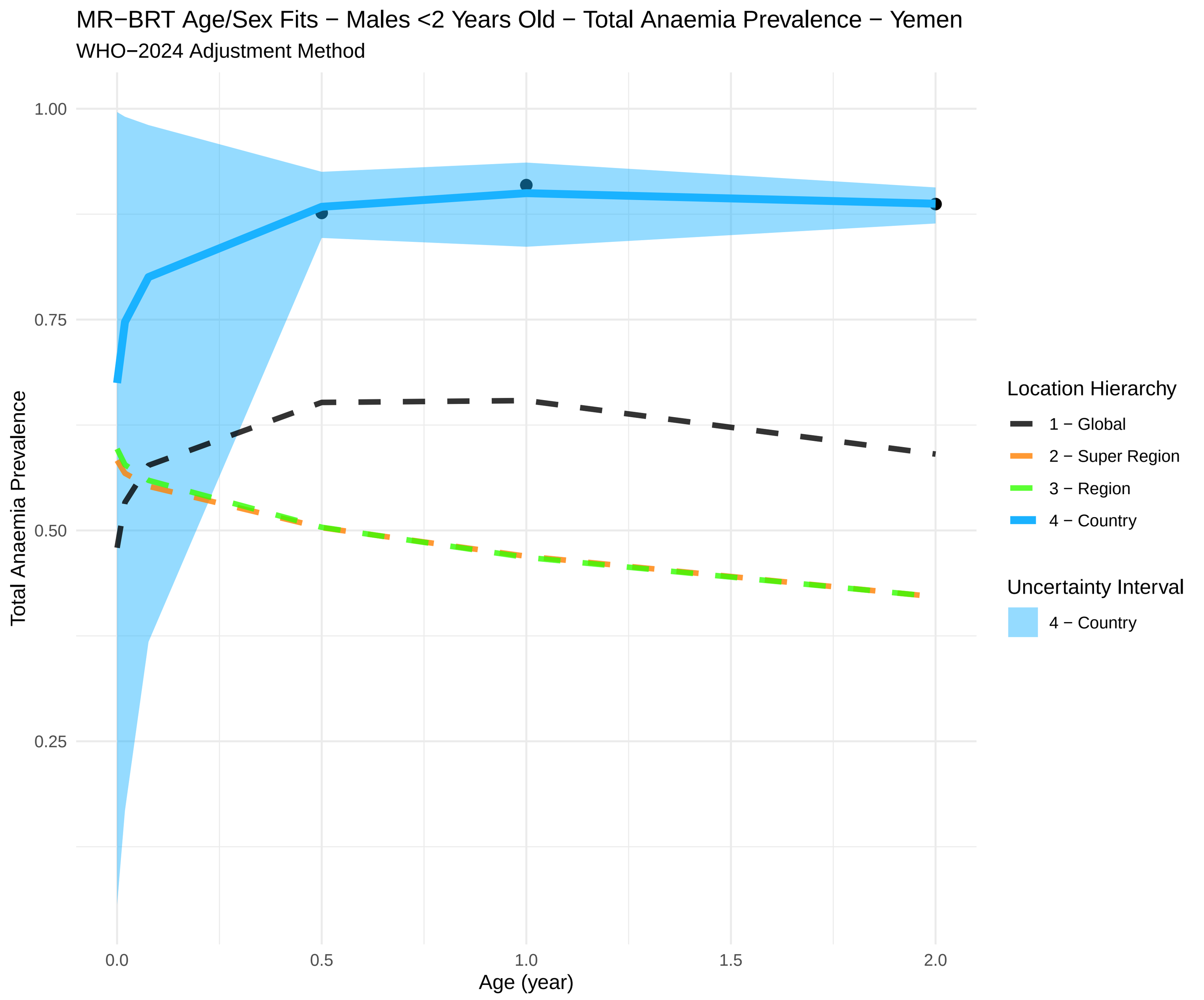
MR−BRT Age/Sex Fits − Males <2 Years Old − Total Anaemia Prevalence − Yemen
WHO−2024 Adjustment Method
Total Anaemia Prevalence
Age (year)
Location Hierarchy
1 − Global
2 − Super Region
3 − Region
4 − Country
Uncertainty Interval
4 − Country

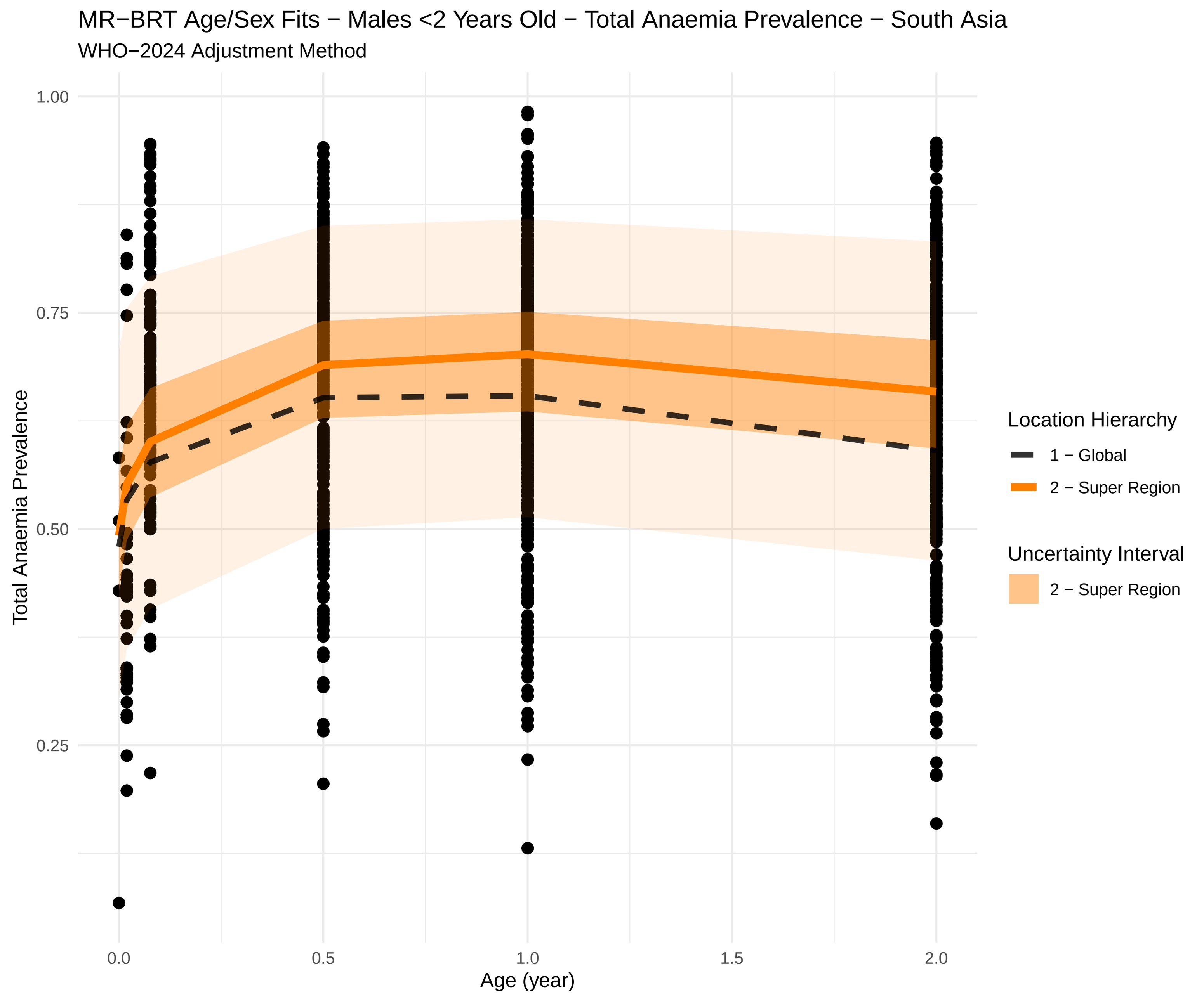
MR−BRT Age/Sex Fits − Males <2 Years Old − Total Anaemia Prevalence − South Asia
WHO−2024 Adjustment Method
Total Anaemia Prevalence
Age (year)
Location Hierarchy
1 − Global
2 − Super Region
Uncertainty Interval
2 − Super Region

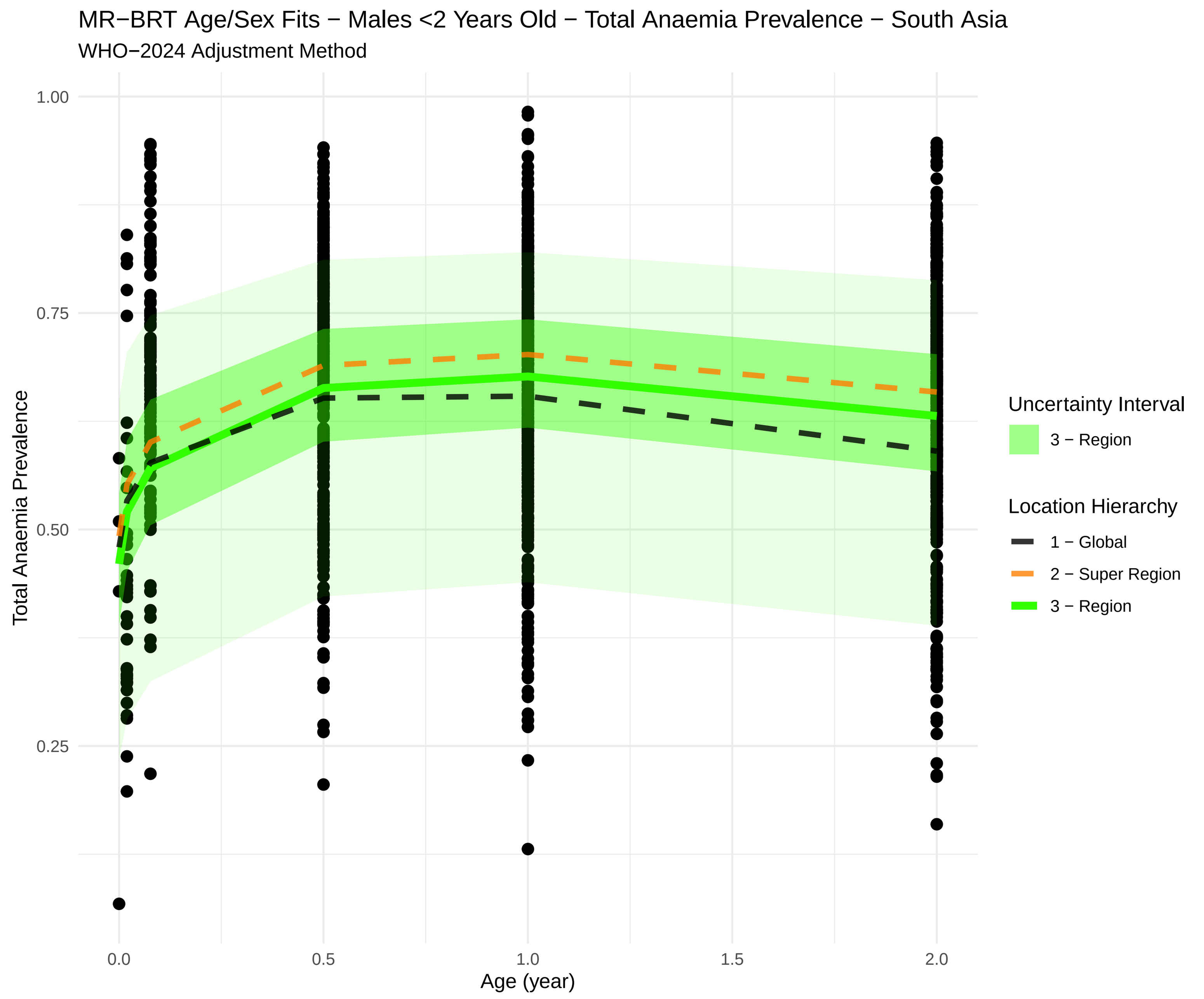
MR−BRT Age/Sex Fits − Males <2 Years Old − Total Anaemia Prevalence − South Asia
WHO−2024 Adjustment Method
Total Anaemia Prevalence
Age (year)
Uncertainty Interval
3 − Region
Location Hierarchy
1 − Global
2 − Super Region
3 − Region

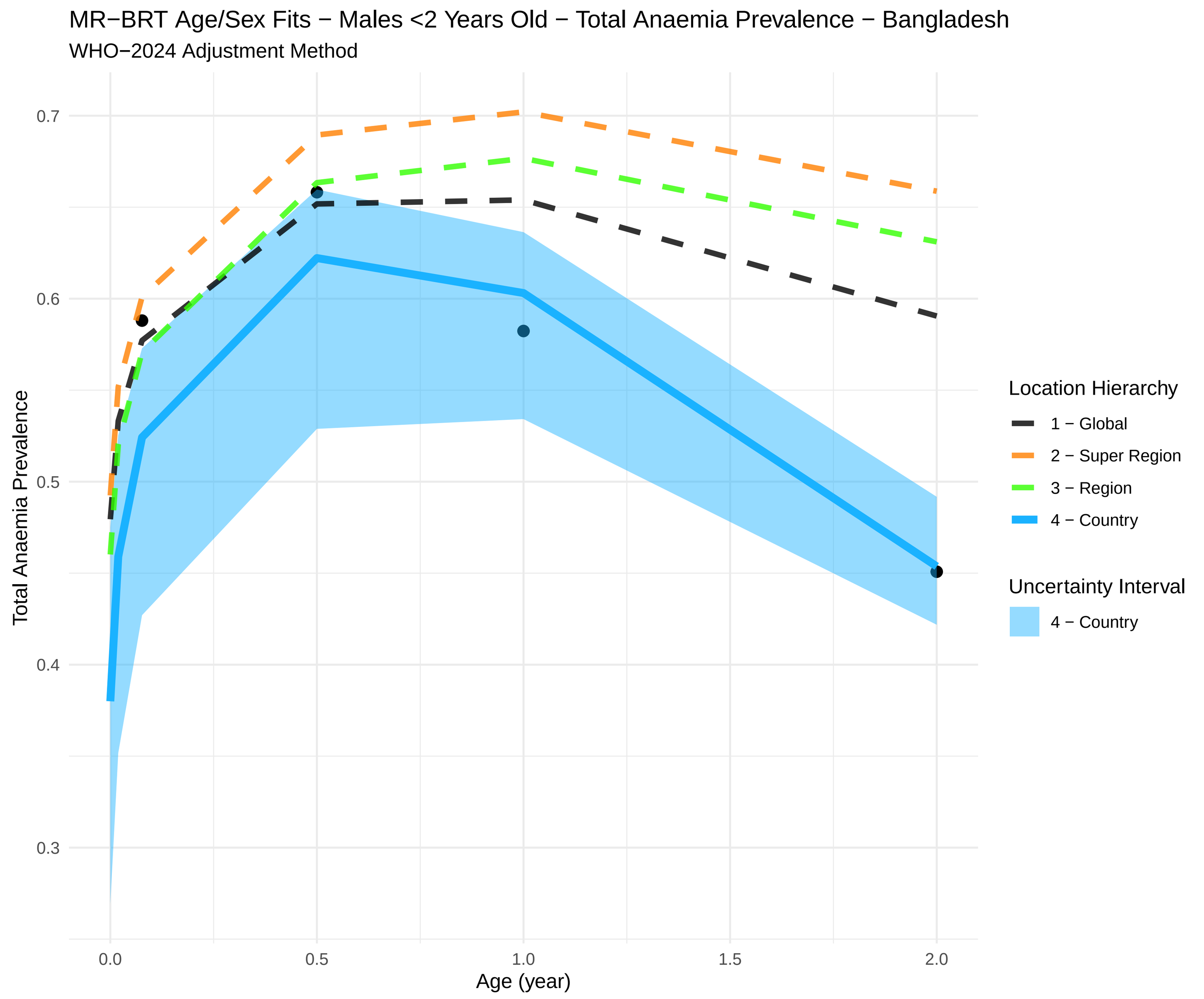
MR−BRT Age/Sex Fits − Males <2 Years Old − Total Anaemia Prevalence − Bangladesh
WHO−2024 Adjustment Method
Total Anaemia Prevalence
Age (year)
Location Hierarchy
1 − Global
2 − Super Region
3 − Region
4 − Country
Uncertainty Interval
4 − Country

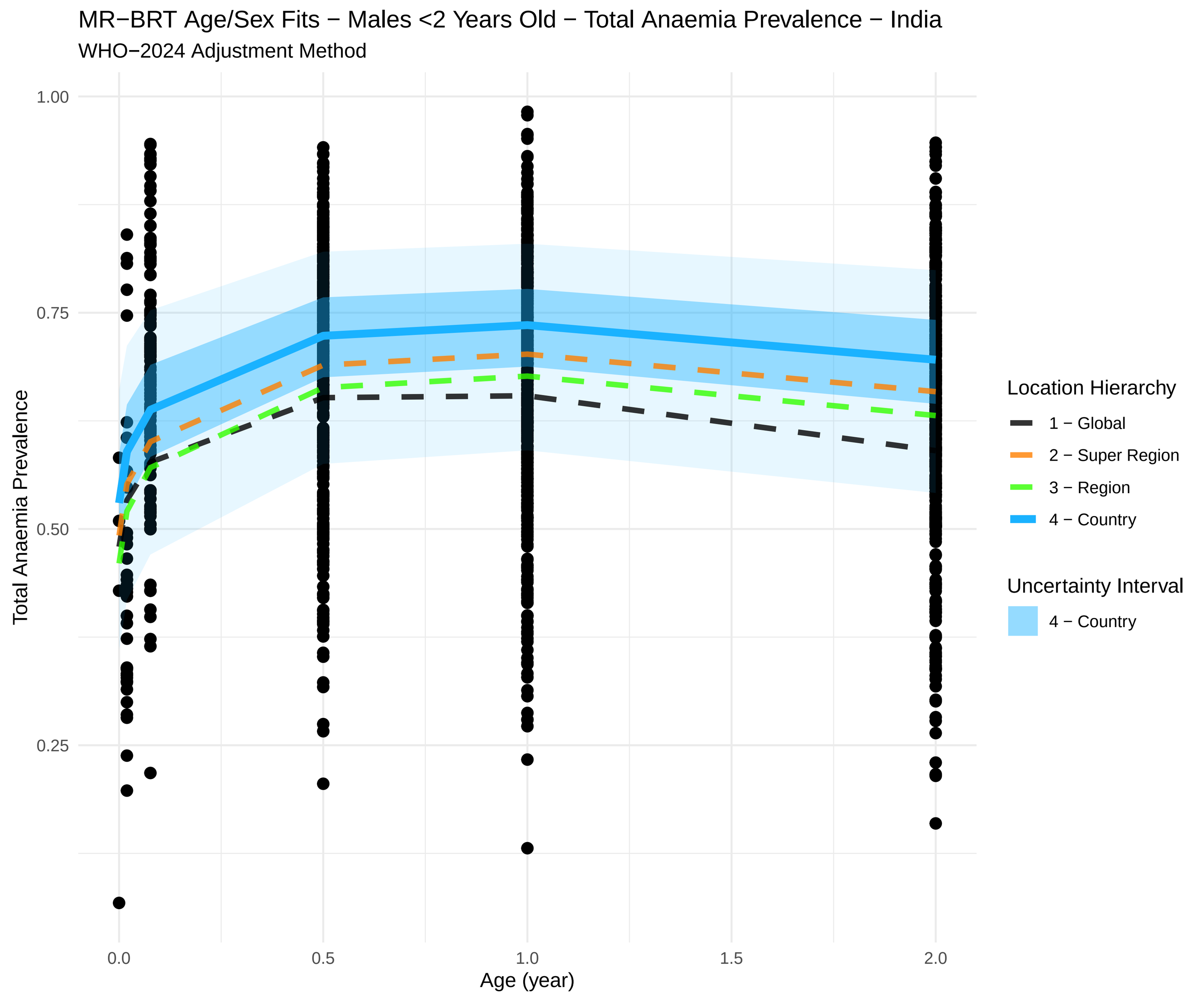
MR−BRT Age/Sex Fits − Males <2 Years Old − Total Anaemia Prevalence − India
WHO−2024 Adjustment Method
Total Anaemia Prevalence
Age (year)
Location Hierarchy
1 − Global
2 − Super Region
3 − Region
4 − Country
Uncertainty Interval
4 − Country

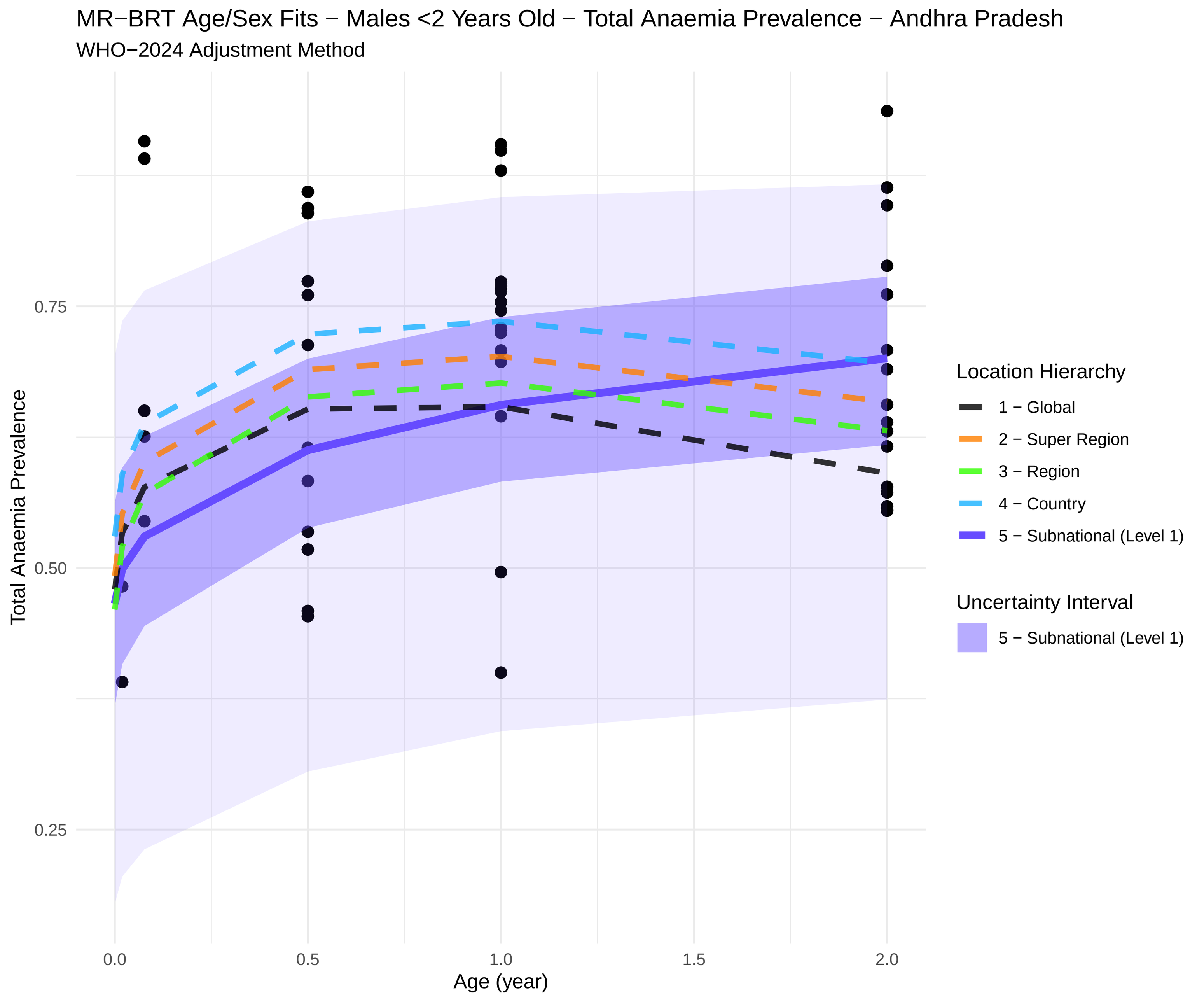
MR−BRT Age/Sex Fits − Males <2 Years Old − Total Anaemia Prevalence − Andhra Pradesh
WHO−2024 Adjustment Method
Total Anaemia Prevalence
Age (year)
Location Hierarchy
1 − Global
2 − Super Region
3 − Region
4 − Country
5 − Subnational (Level 1)
Uncertainty Interval
5 − Subnational (Level 1)

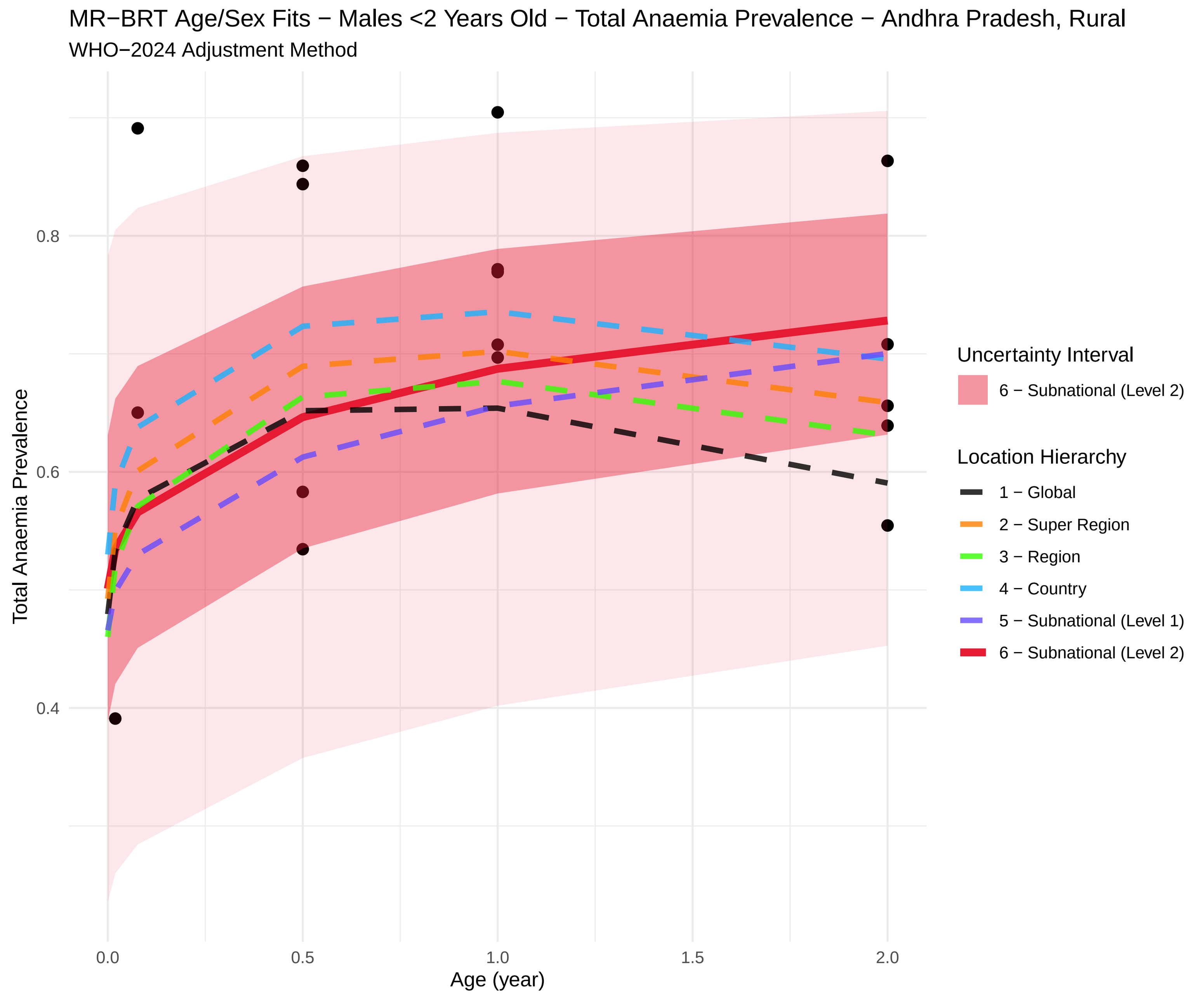
MR−BRT Age/Sex Fits − Males <2 Years Old − Total Anaemia Prevalence − Andhra Pradesh, Rural
WHO−2024 Adjustment Method
Total Anaemia Prevalence
Age (year)
Uncertainty Interval
6 − Subnational (Level 2)
Location Hierarchy
1 − Global
2 − Super Region
3 − Region
4 − Country
5 − Subnational (Level 1)
6 − Subnational (Level 2)

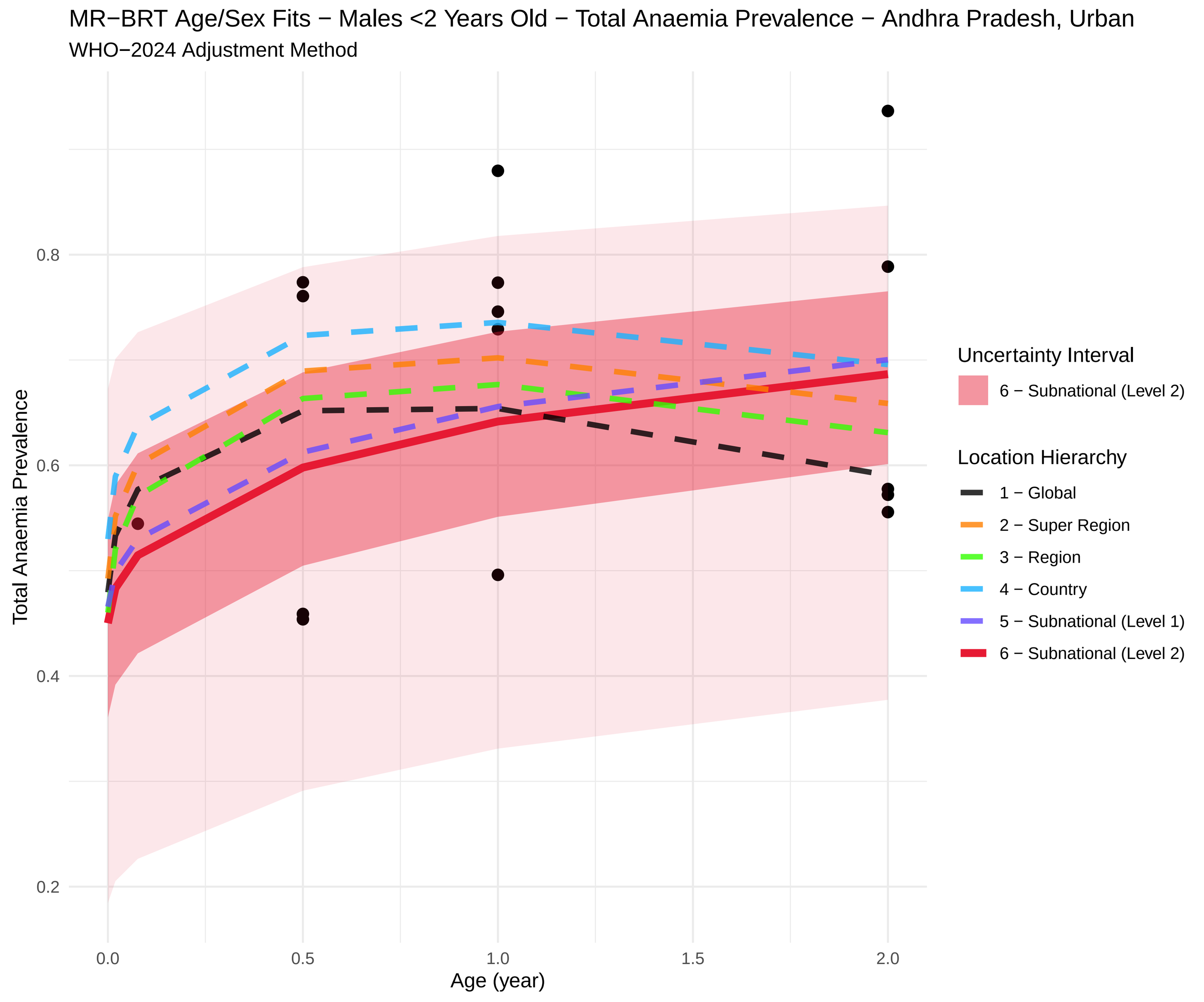
MR−BRT Age/Sex Fits − Males <2 Years Old − Total Anaemia Prevalence − Andhra Pradesh, Urban
WHO−2024 Adjustment Method
Total Anaemia Prevalence
Age (year)
Uncertainty Interval
6 − Subnational (Level 2)
Location Hierarchy
1 − Global
2 − Super Region
3 − Region
4 − Country
5 − Subnational (Level 1)
6 − Subnational (Level 2)

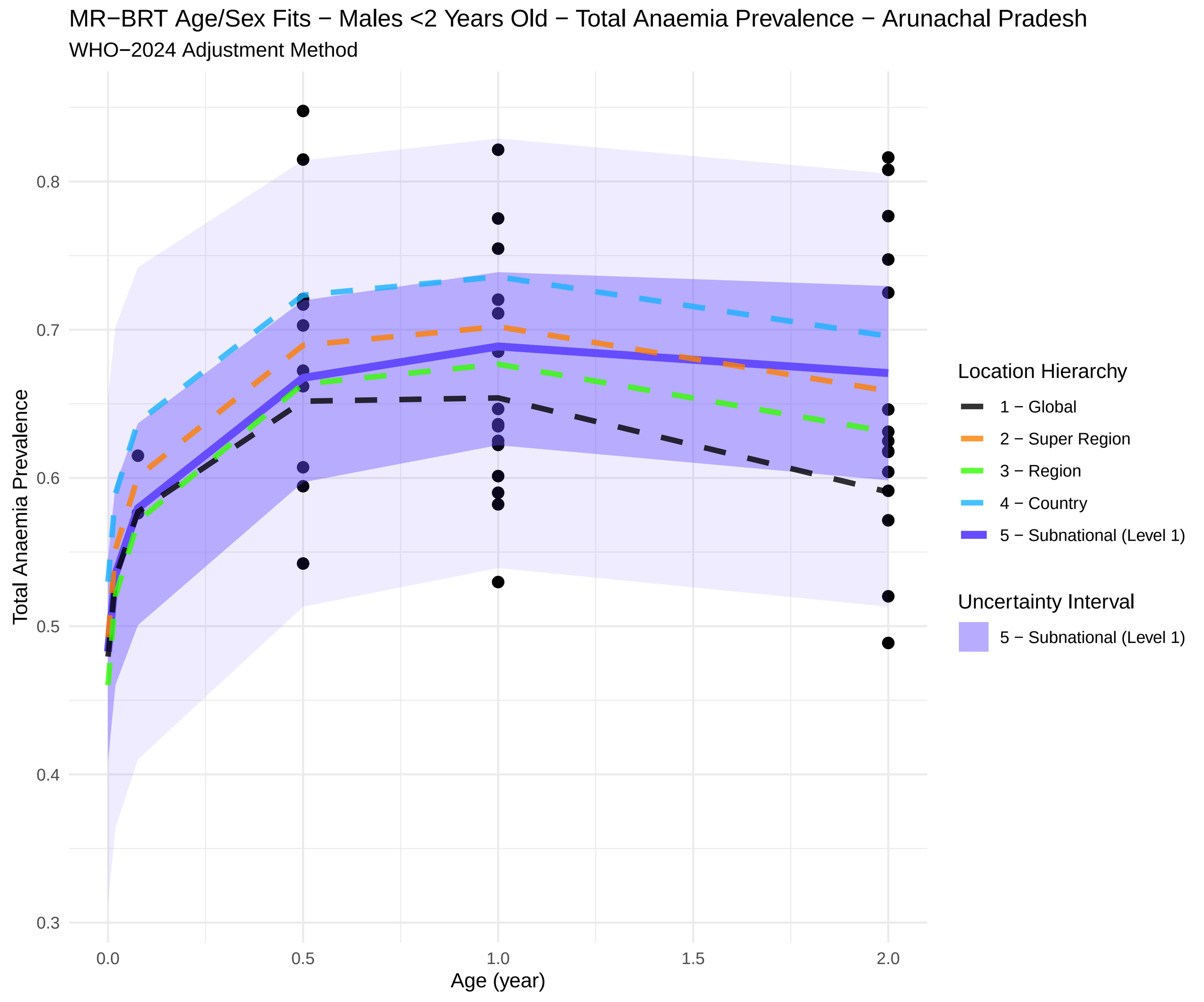
MR−BRT Age/Sex Fits − Males <2 Years Old − Total Anaemia Prevalence − Arunachal Pradesh
WHO−2024 Adjustment Method
Total Anaemia Prevalence
Age (year)
Location Hierarchy
1 − Global
2 − Super Region
3 − Region
4 − Country
5 − Subnational (Level 1)
Uncertainty Interval
5 − Subnational (Level 1)

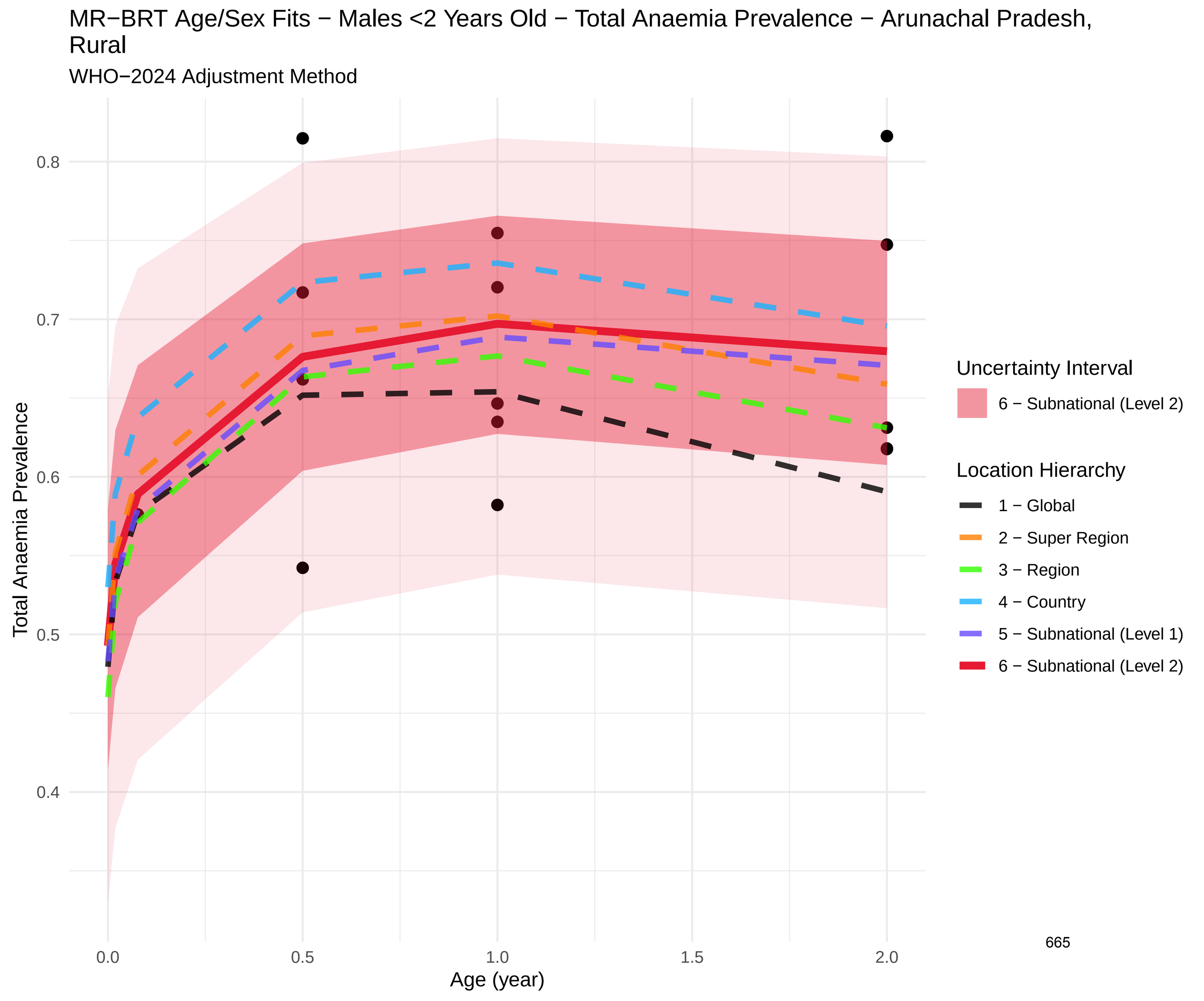
MR−BRT Age/Sex Fits − Males <2 Years Old − Total Anaemia Prevalence − Arunachal Pradesh, Rural
WHO−2024 Adjustment Method
Total Anaemia Prevalence
Age (year)
Uncertainty Interval
6 − Subnational (Level 2)
Location Hierarchy
1 − Global
2 − Super Region
3 − Region
4 − Country
5 − Subnational (Level 1)
6 − Subnational (Level 2)

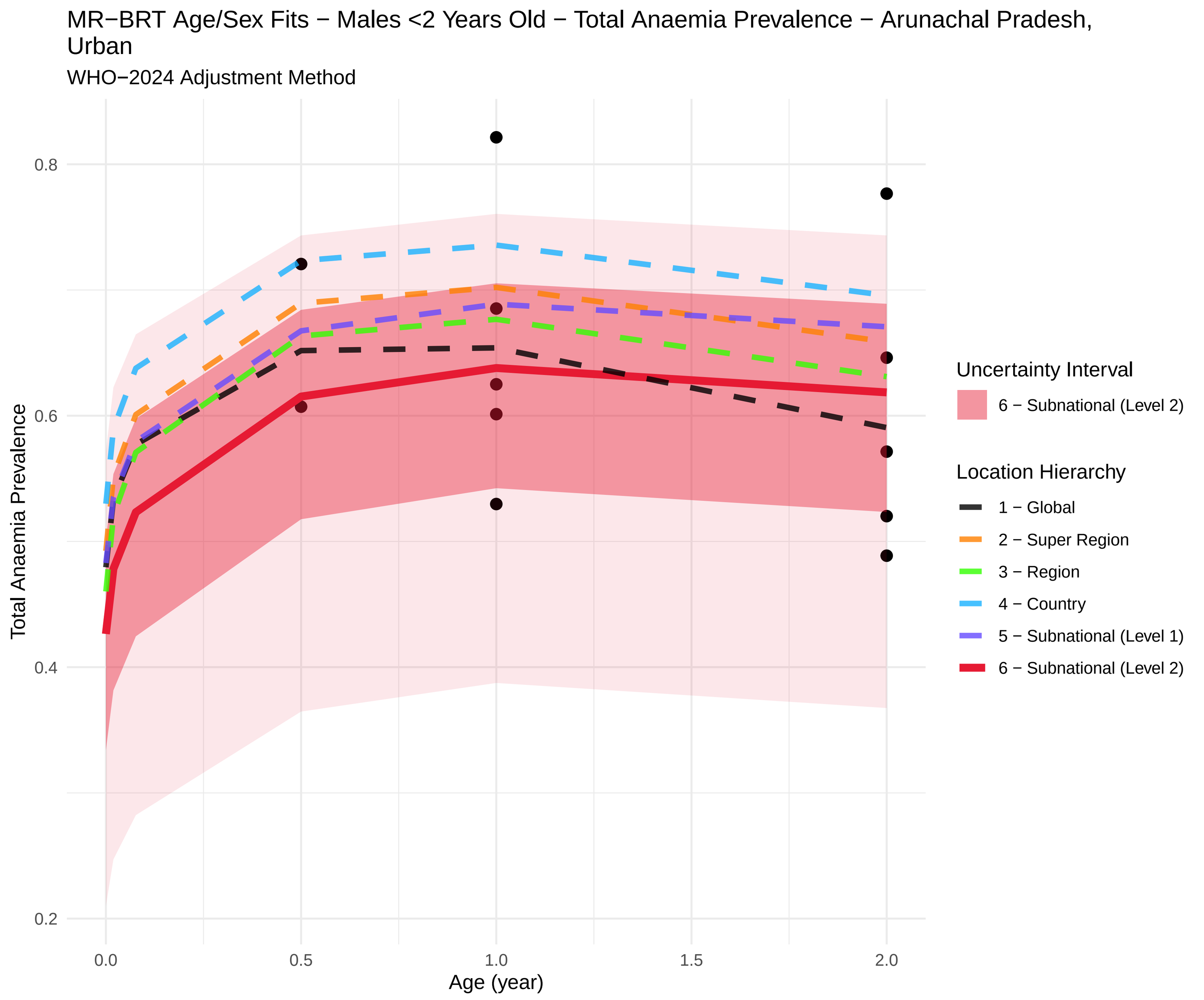
MR−BRT Age/Sex Fits − Males <2 Years Old − Total Anaemia Prevalence − Arunachal Pradesh, Urban
WHO−2024 Adjustment Method
Total Anaemia Prevalence
Age (year)
Uncertainty Interval
6 − Subnational (Level 2)
Location Hierarchy
1 − Global
2 − Super Region
3 − Region
4 − Country
5 − Subnational (Level 1)
6 − Subnational (Level 2)

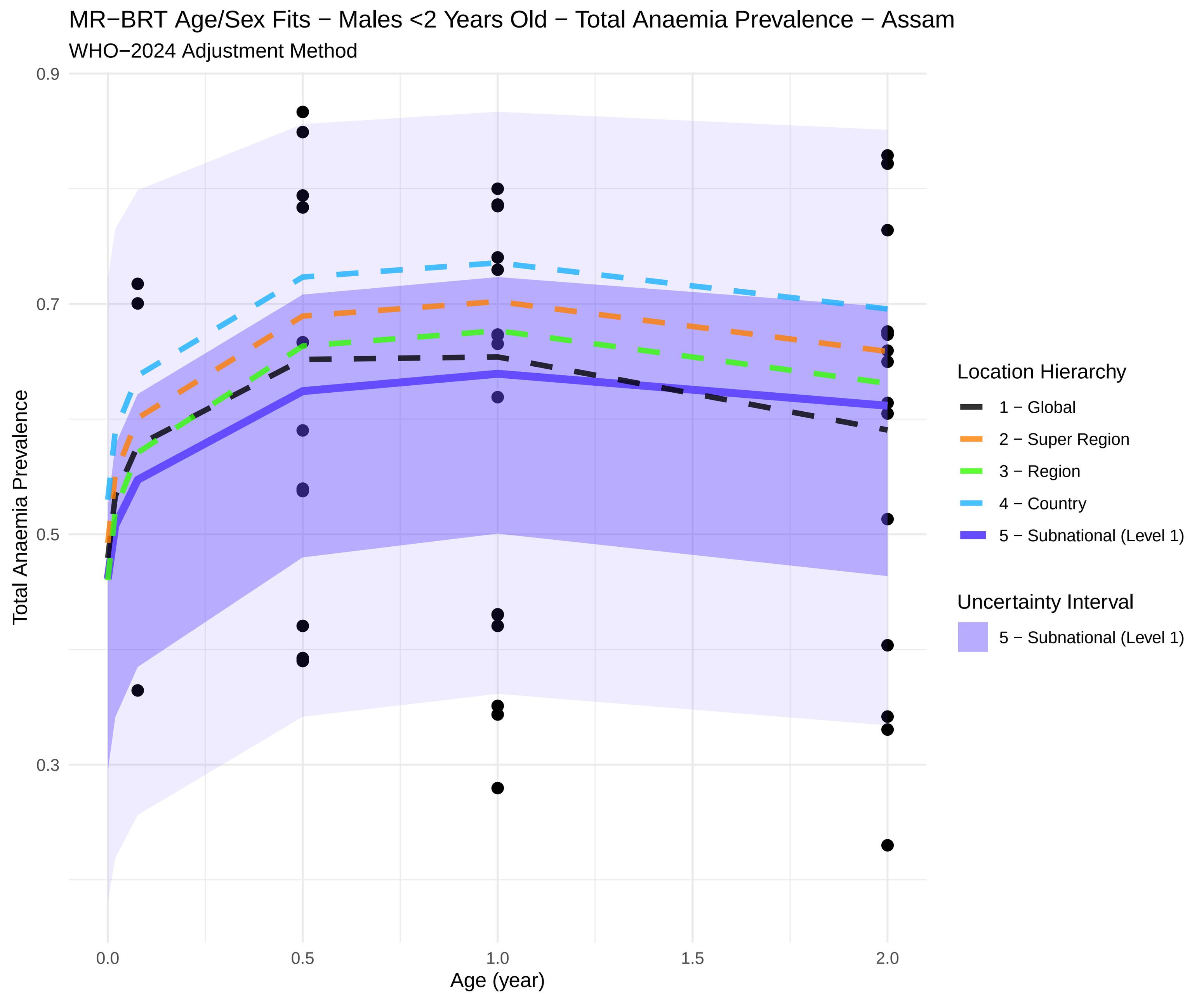
MR−BRT Age/Sex Fits − Males <2 Years Old − Total Anaemia Prevalence − Assam
WHO−2024 Adjustment Method
Total Anaemia Prevalence
Age (year)
Location Hierarchy
1 − Global
2 − Super Region
3 − Region
4 − Country
5 − Subnational (Level 1)
Uncertainty Interval
5 − Subnational (Level 1)

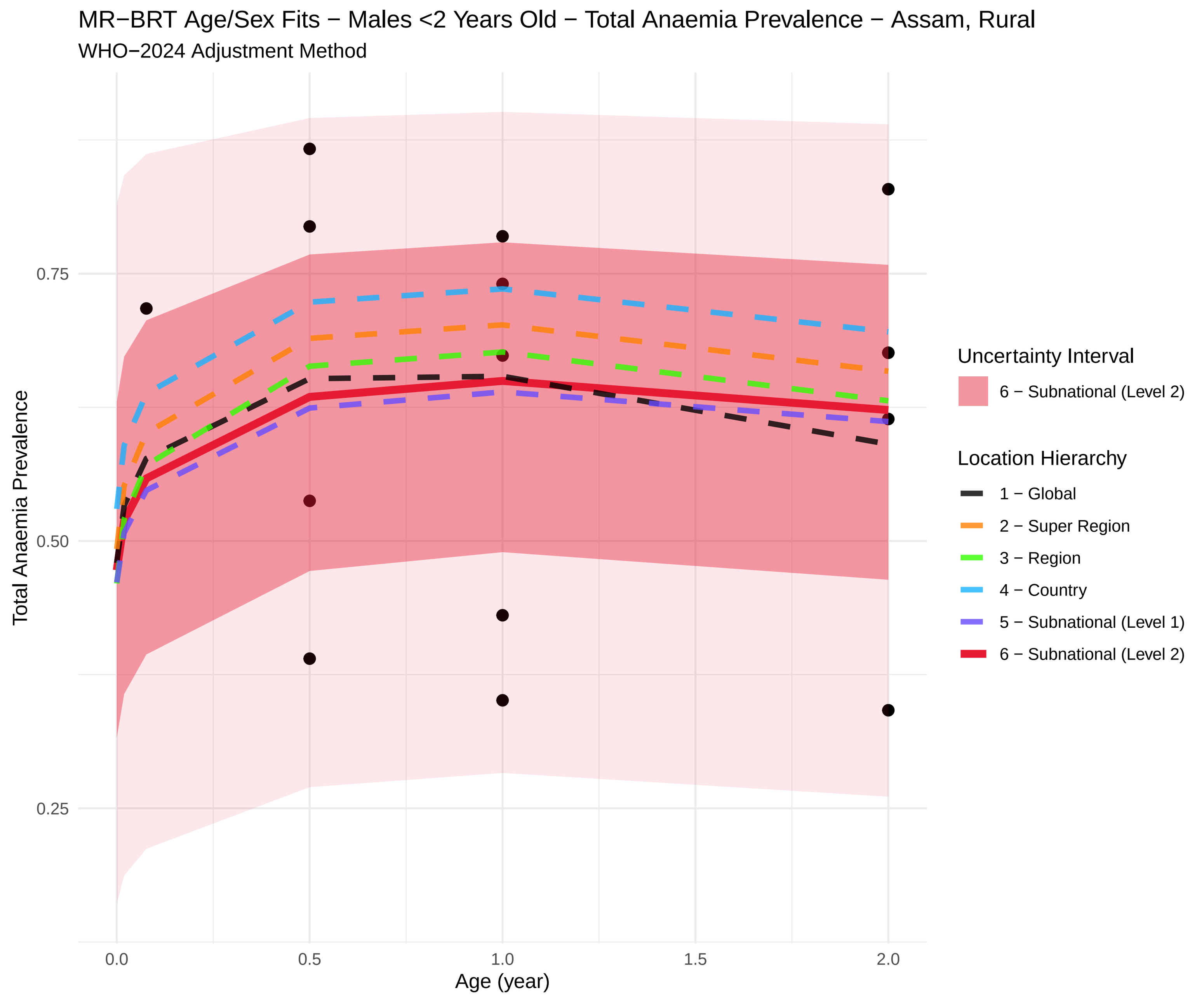
MR−BRT Age/Sex Fits − Males <2 Years Old − Total Anaemia Prevalence − Assam, Rural
WHO−2024 Adjustment Method
Total Anaemia Prevalence
Age (year)
Uncertainty Interval
6 − Subnational (Level 2)
Location Hierarchy
1 − Global
2 − Super Region
3 − Region
4 − Country
5 − Subnational (Level 1)
6 − Subnational (Level 2)

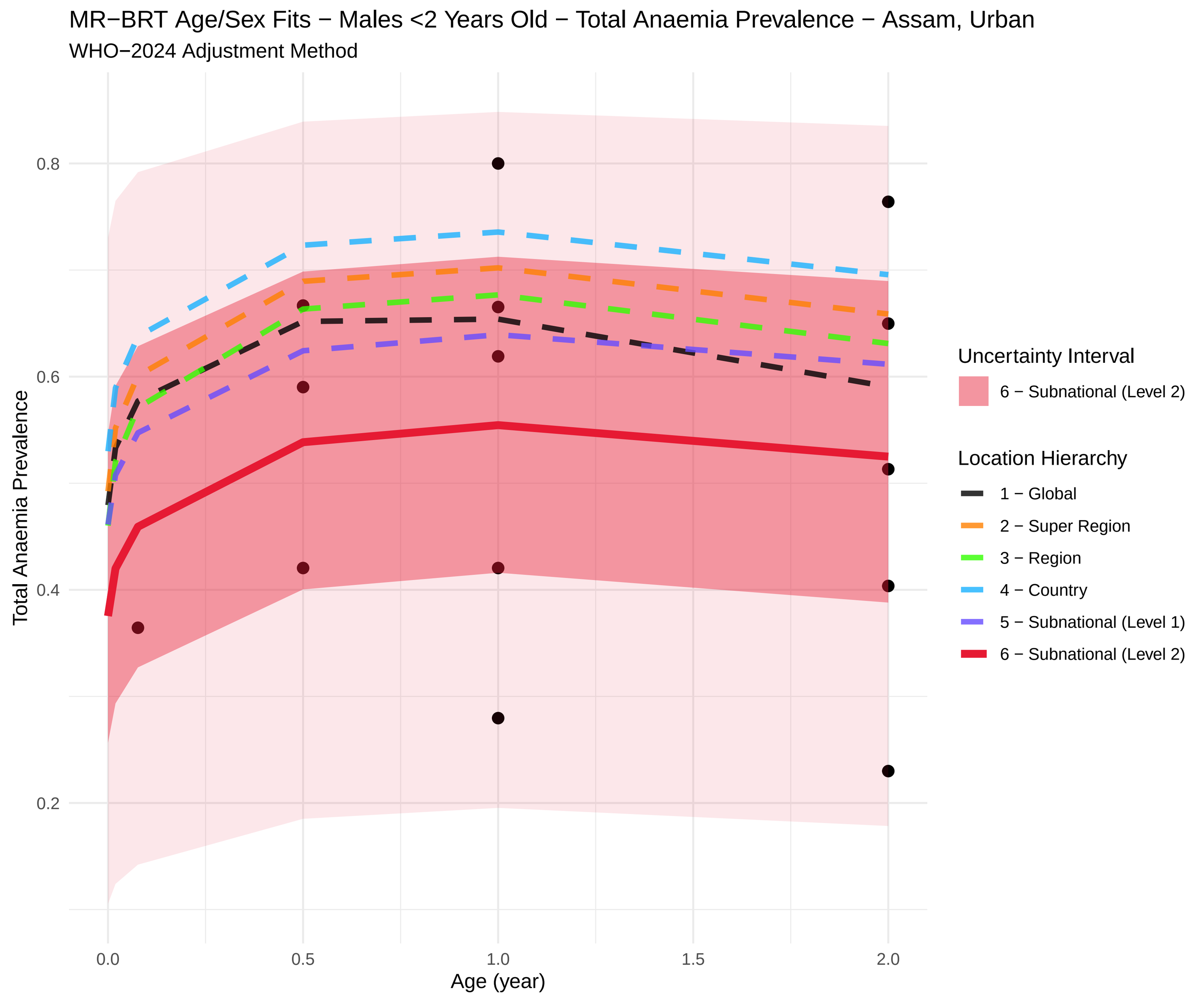
MR−BRT Age/Sex Fits − Males <2 Years Old − Total Anaemia Prevalence − Assam, Urban
WHO−2024 Adjustment Method
Total Anaemia Prevalence
Age (year)
Uncertainty Interval
6 − Subnational (Level 2)
Location Hierarchy
1 − Global
2 − Super Region
3 − Region
4 − Country
5 − Subnational (Level 1)
6 − Subnational (Level 2)

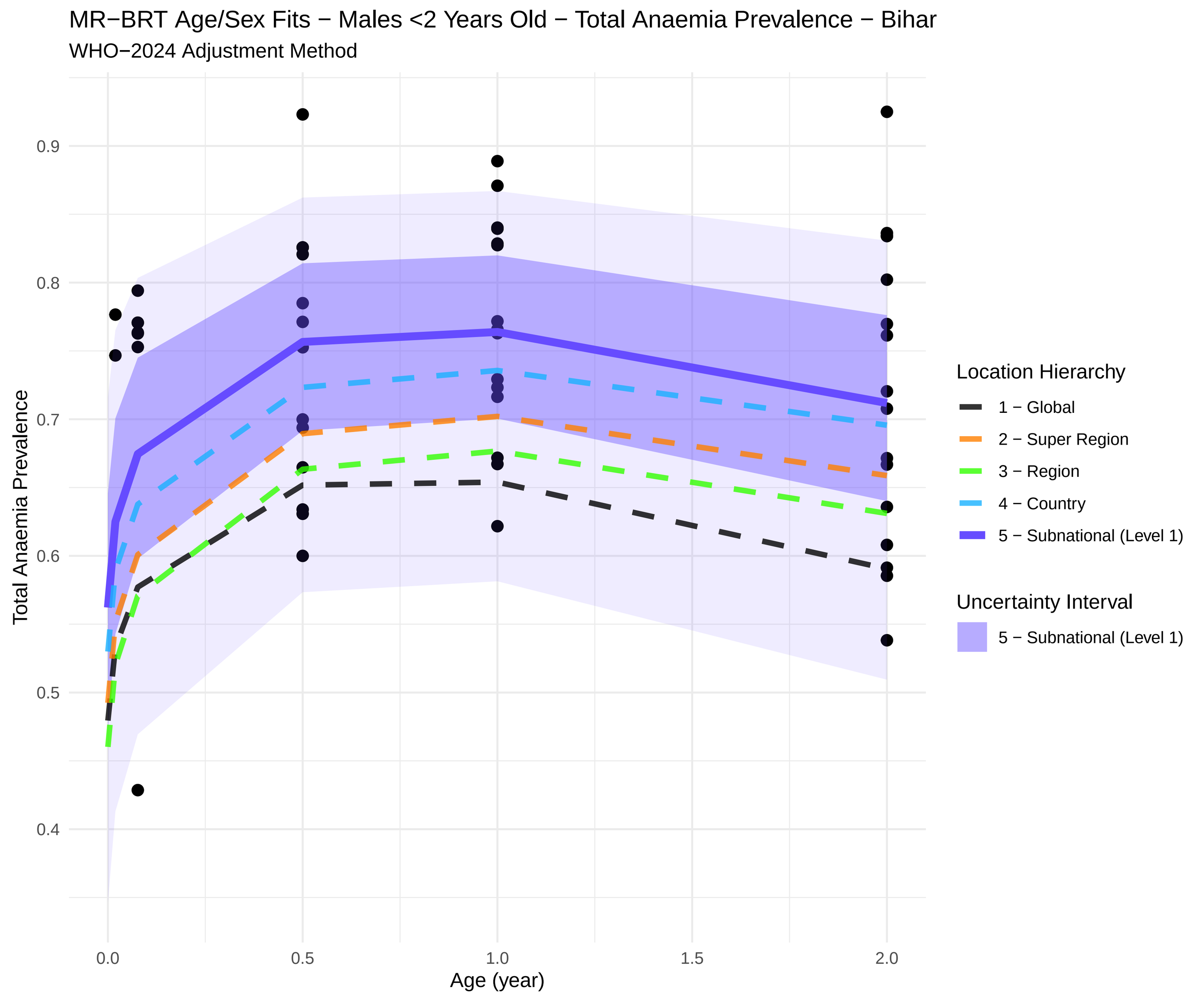
MR−BRT Age/Sex Fits − Males <2 Years Old − Total Anaemia Prevalence − Bihar
WHO−2024 Adjustment Method
Total Anaemia Prevalence
Age (year)
Location Hierarchy
1 − Global
2 − Super Region
3 − Region
4 − Country
5 − Subnational (Level 1)
Uncertainty Interval
5 − Subnational (Level 1)

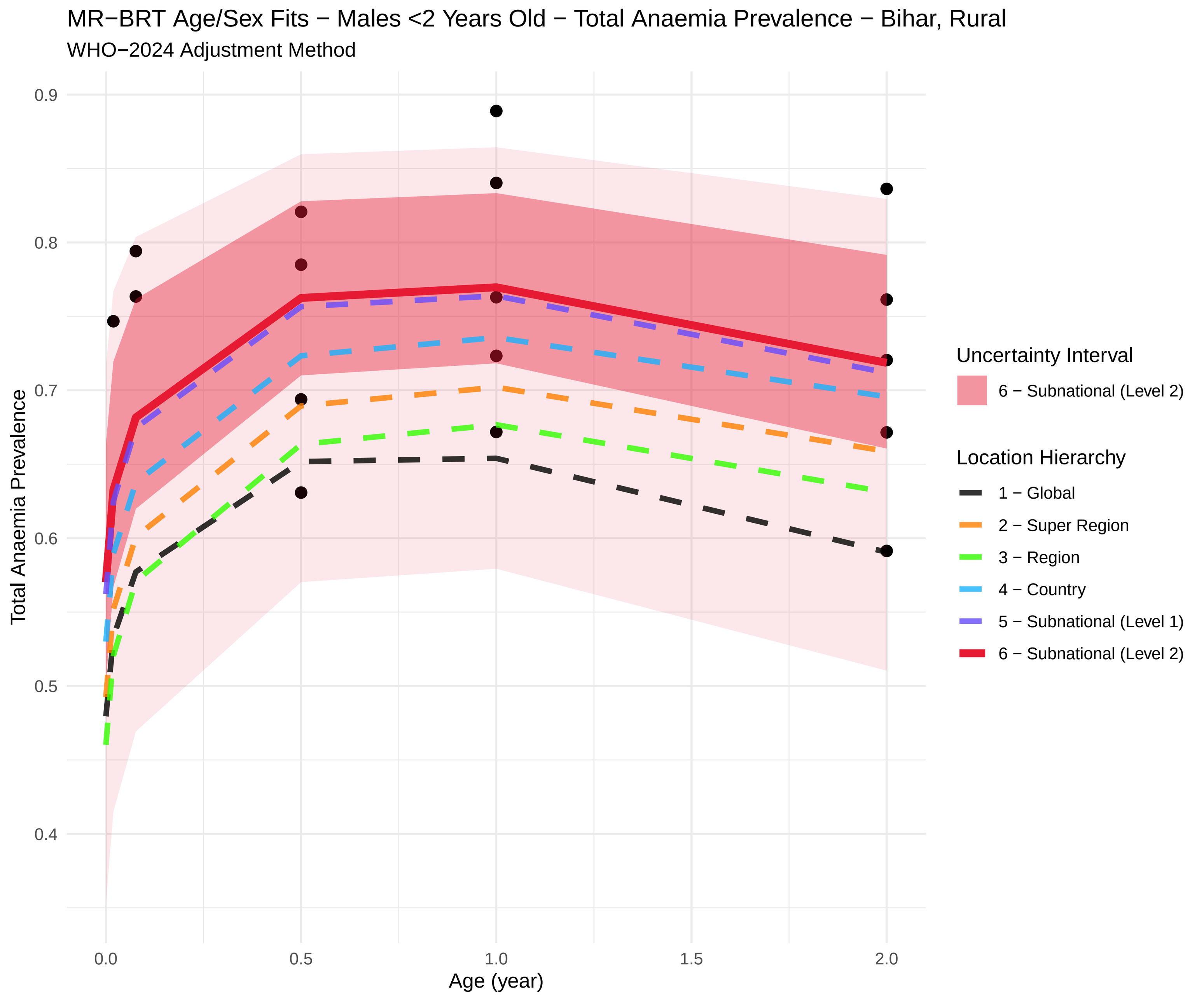
MR−BRT Age/Sex Fits − Males <2 Years Old − Total Anaemia Prevalence − Bihar, Rural
WHO−2024 Adjustment Method
Total Anaemia Prevalence
Age (year)
Uncertainty Interval
6 − Subnational (Level 2)
Location Hierarchy
1 − Global
2 − Super Region
3 − Region
4 − Country
5 − Subnational (Level 1)
6 − Subnational (Level 2)

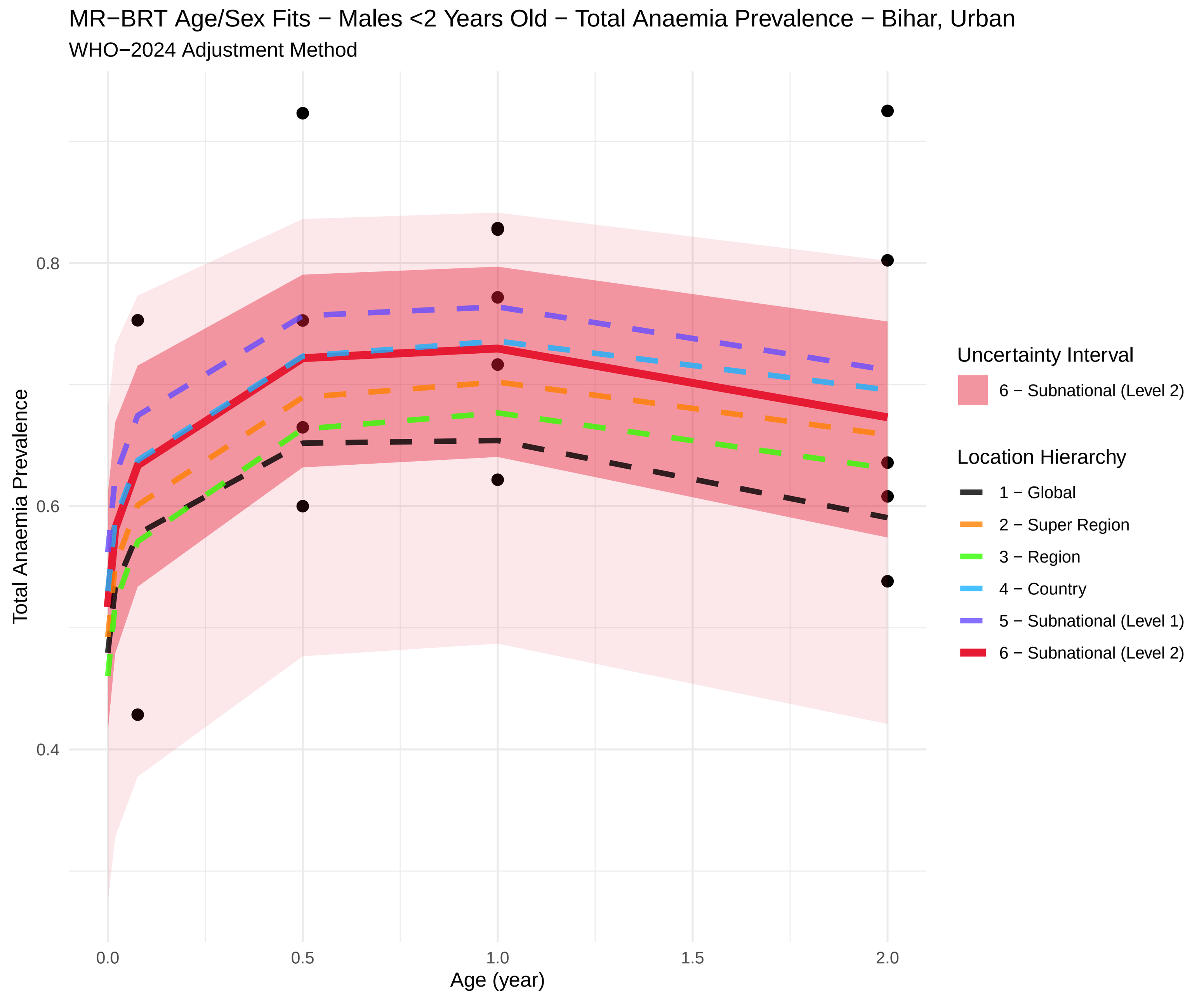
MR−BRT Age/Sex Fits − Males <2 Years Old − Total Anaemia Prevalence − Bihar, Urban
WHO−2024 Adjustment Method
Total Anaemia Prevalence
Age (year)
Uncertainty Interval
6 − Subnational (Level 2)
Location Hierarchy
1 − Global
2 − Super Region
3 − Region
4 − Country
5 − Subnational (Level 1)
6 − Subnational (Level 2)

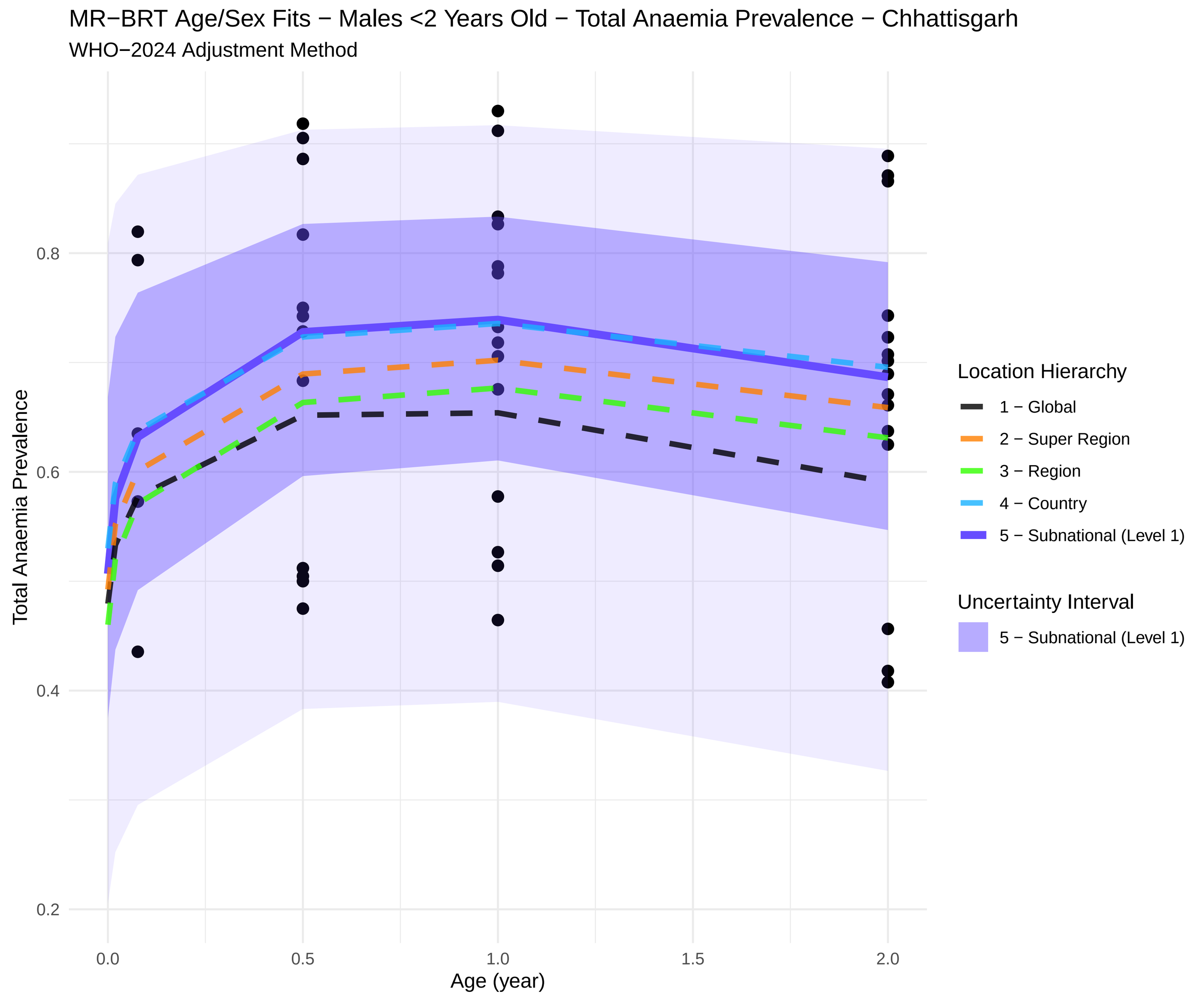
MR−BRT Age/Sex Fits − Males <2 Years Old − Total Anaemia Prevalence − Chhattisgarh
WHO−2024 Adjustment Method
Total Anaemia Prevalence
Age (year)
Location Hierarchy
1 − Global
2 − Super Region
3 − Region
4 − Country
5 − Subnational (Level 1)
Uncertainty Interval
5 − Subnational (Level 1)

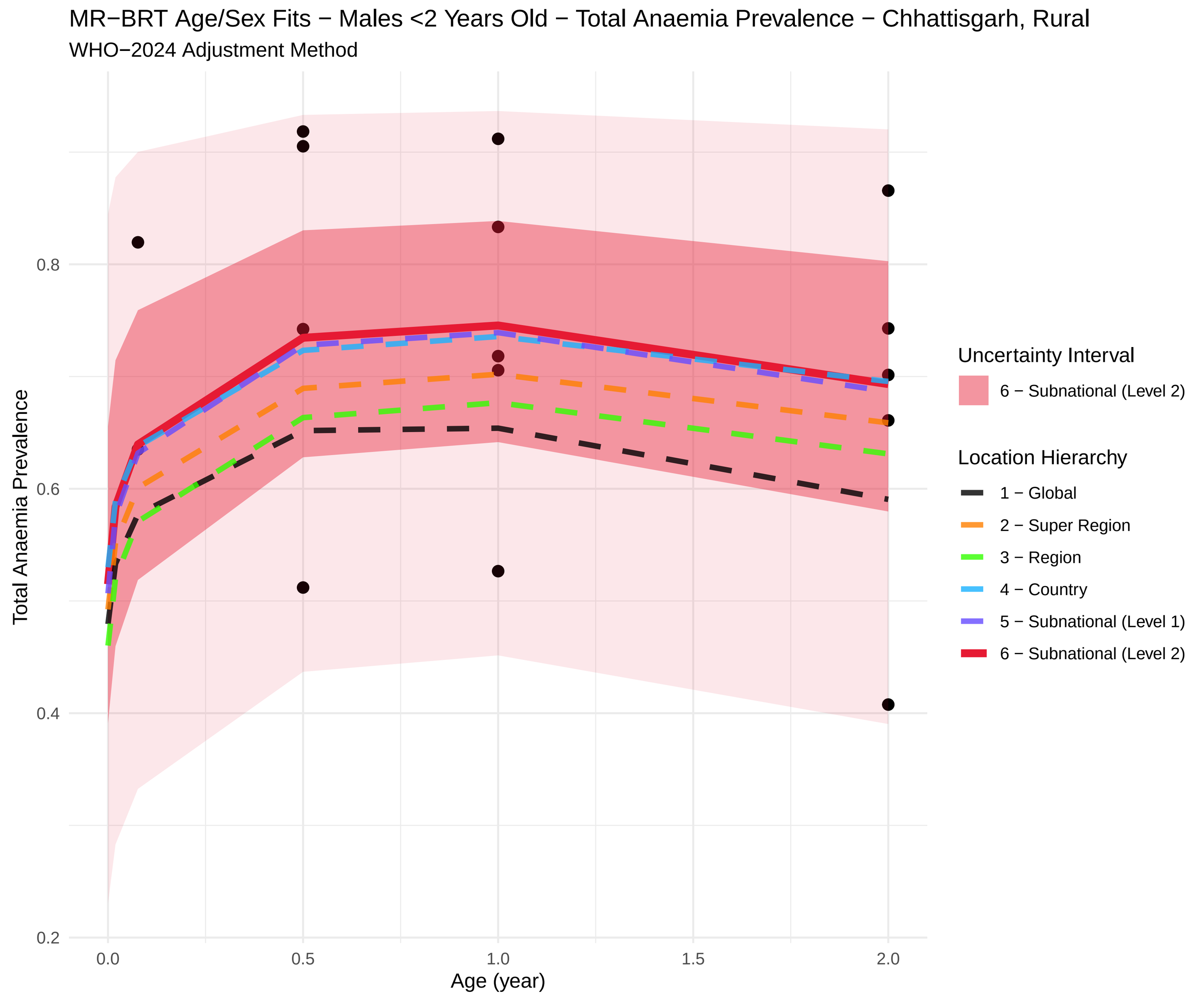
MR−BRT Age/Sex Fits − Males <2 Years Old − Total Anaemia Prevalence − Chhattisgarh, Rural
WHO−2024 Adjustment Method
Total Anaemia Prevalence
Age (year)
Uncertainty Interval
6 − Subnational (Level 2)
Location Hierarchy
1 − Global
2 − Super Region
3 − Region
4 − Country
5 − Subnational (Level 1)
6 − Subnational (Level 2)

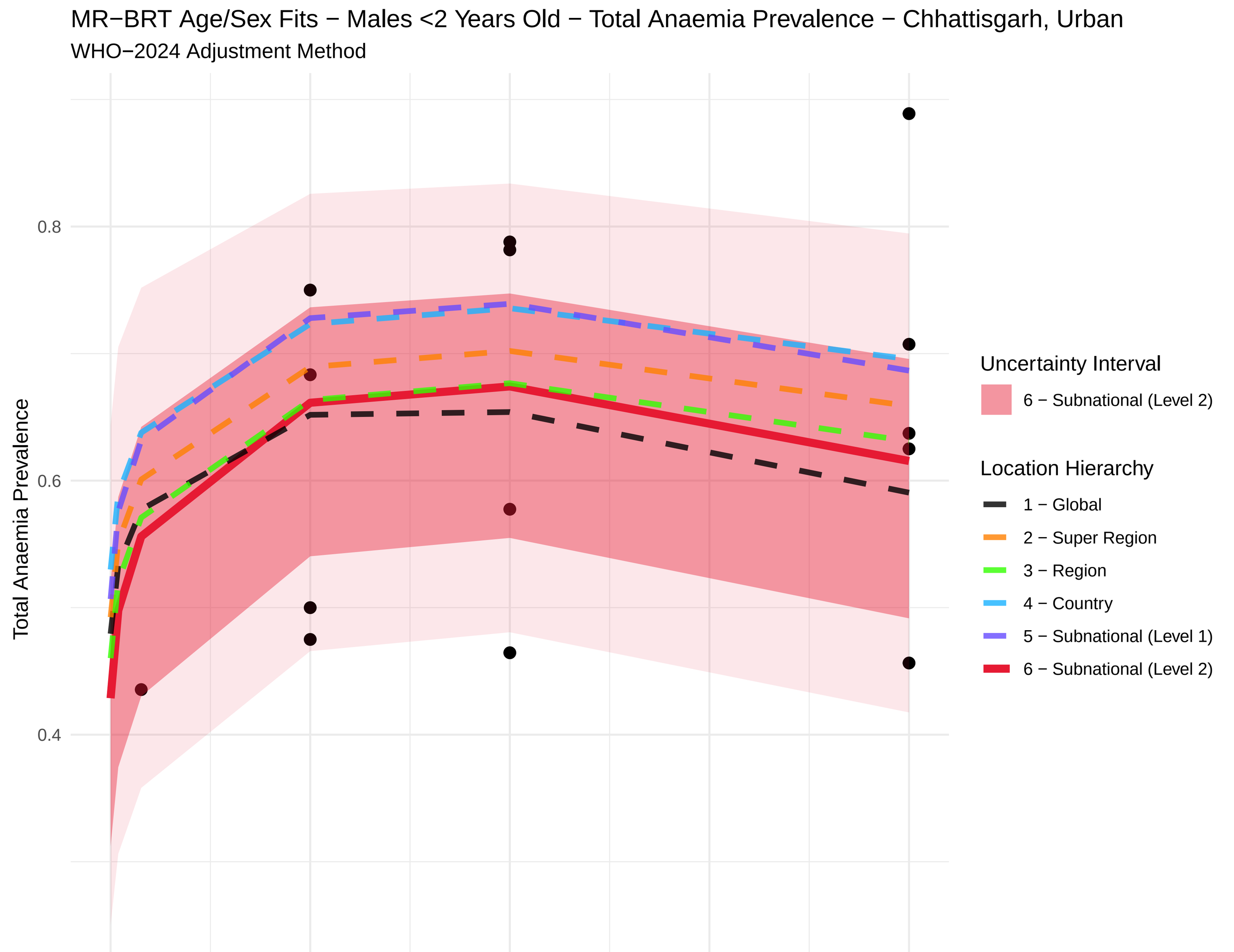
MR−BRT Age/Sex Fits − Males <2 Years Old − Total Anaemia Prevalence − Chhattisgarh, Urban
WHO−2024 Adjustment Method
Total Anaemia Prevalence
Age (year)
Uncertainty Interval
6 − Subnational (Level 2)
Location Hierarchy
1 − Global
2 − Super Region
3 − Region
4 − Country
5 − Subnational (Level 1)
6 − Subnational (Level 2)

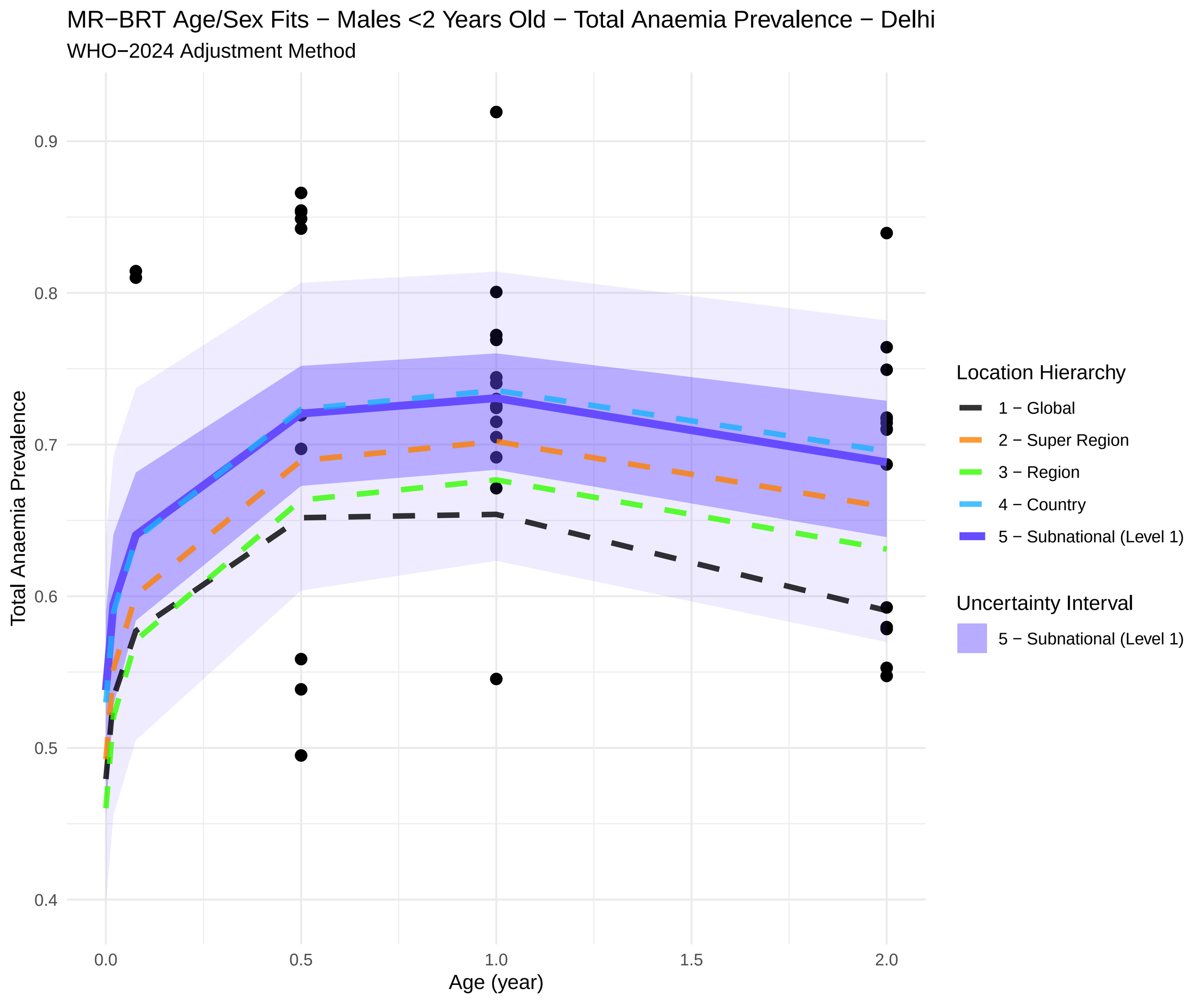
MR−BRT Age/Sex Fits − Males <2 Years Old − Total Anaemia Prevalence − Delhi
WHO−2024 Adjustment Method
Total Anaemia Prevalence
Age (year)
Location Hierarchy
1 − Global
2 − Super Region
3 − Region
4 − Country
5 − Subnational (Level 1)
Uncertainty Interval
5 − Subnational (Level 1)

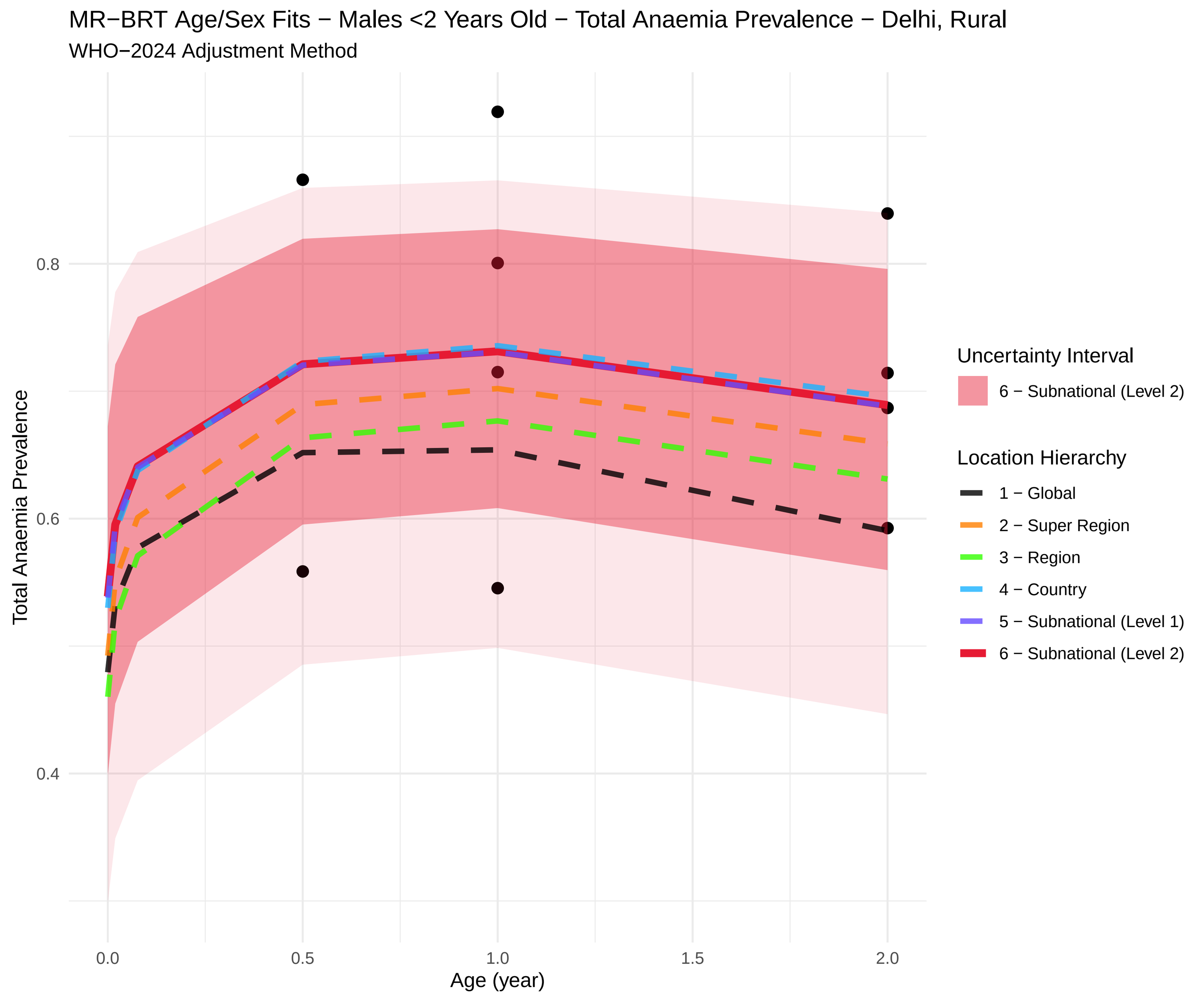
MR−BRT Age/Sex Fits − Males <2 Years Old − Total Anaemia Prevalence − Delhi, Rural
WHO−2024 Adjustment Method
Total Anaemia Prevalence
Age (year)
Uncertainty Interval
6 − Subnational (Level 2)
Location Hierarchy
1 − Global
2 − Super Region
3 − Region
4 − Country
5 − Subnational (Level 1)
6 − Subnational (Level 2)

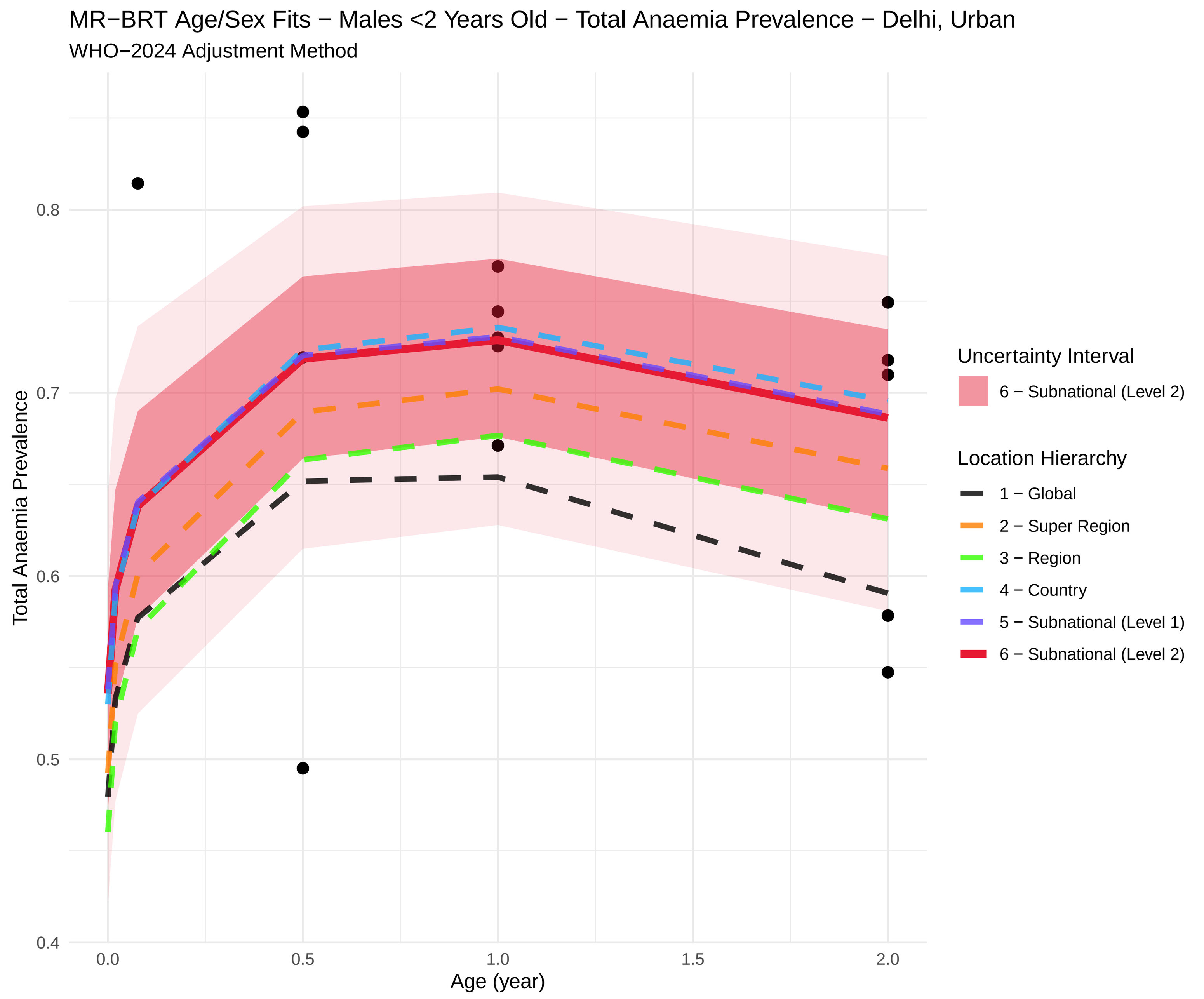
MR−BRT Age/Sex Fits − Males <2 Years Old − Total Anaemia Prevalence − Delhi, Urban
WHO−2024 Adjustment Method
Total Anaemia Prevalence
Age (year)
Uncertainty Interval
6 − Subnational (Level 2)
Location Hierarchy
1 − Global
2 − Super Region
3 − Region
4 − Country
5 − Subnational (Level 1)
6 − Subnational (Level 2)

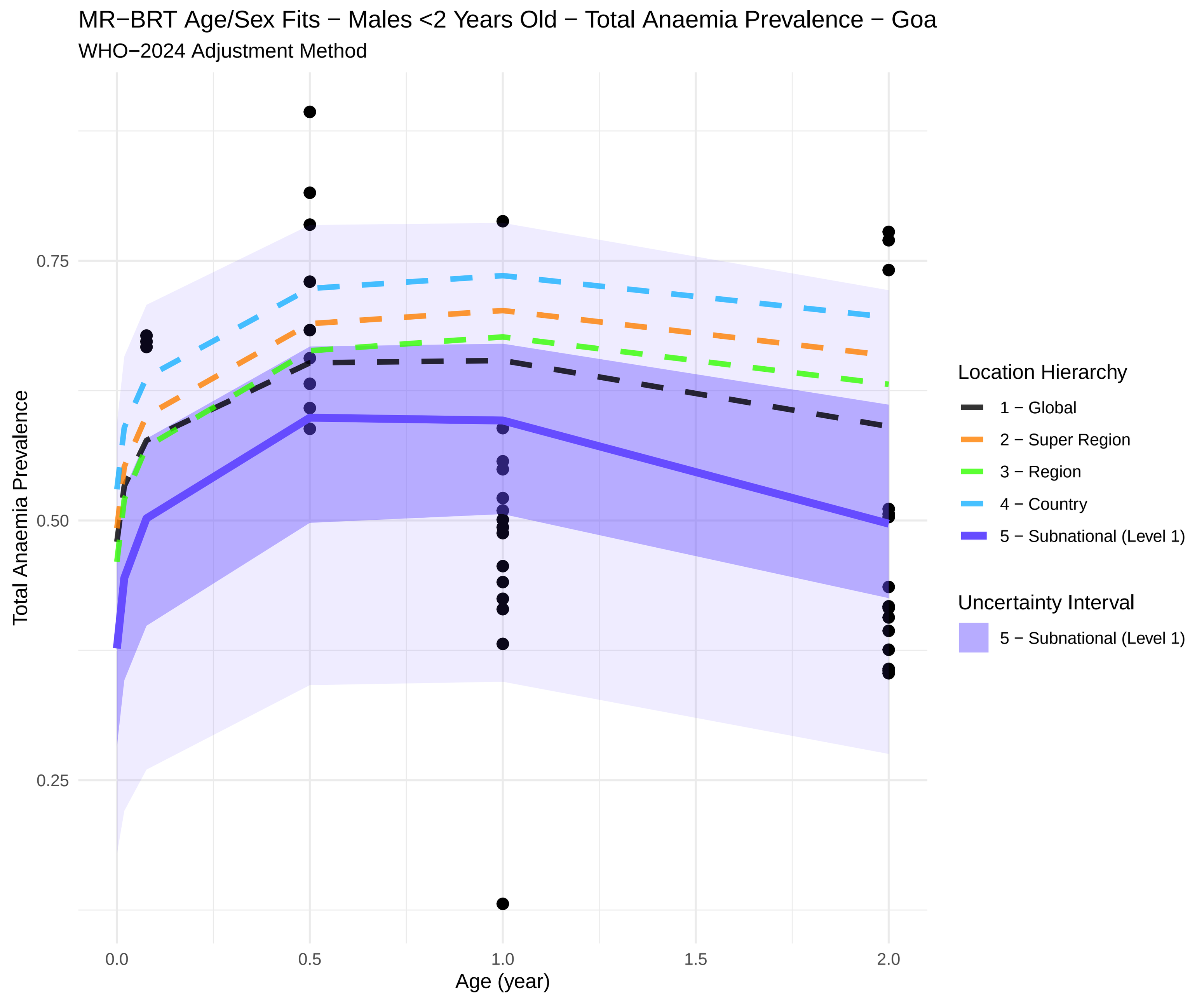
MR−BRT Age/Sex Fits − Males <2 Years Old − Total Anaemia Prevalence − Goa
WHO−2024 Adjustment Method
Total Anaemia Prevalence
Age (year)
Location Hierarchy
1 − Global
2 − Super Region
3 − Region
4 − Country
5 − Subnational (Level 1)
Uncertainty Interval
5 − Subnational (Level 1)

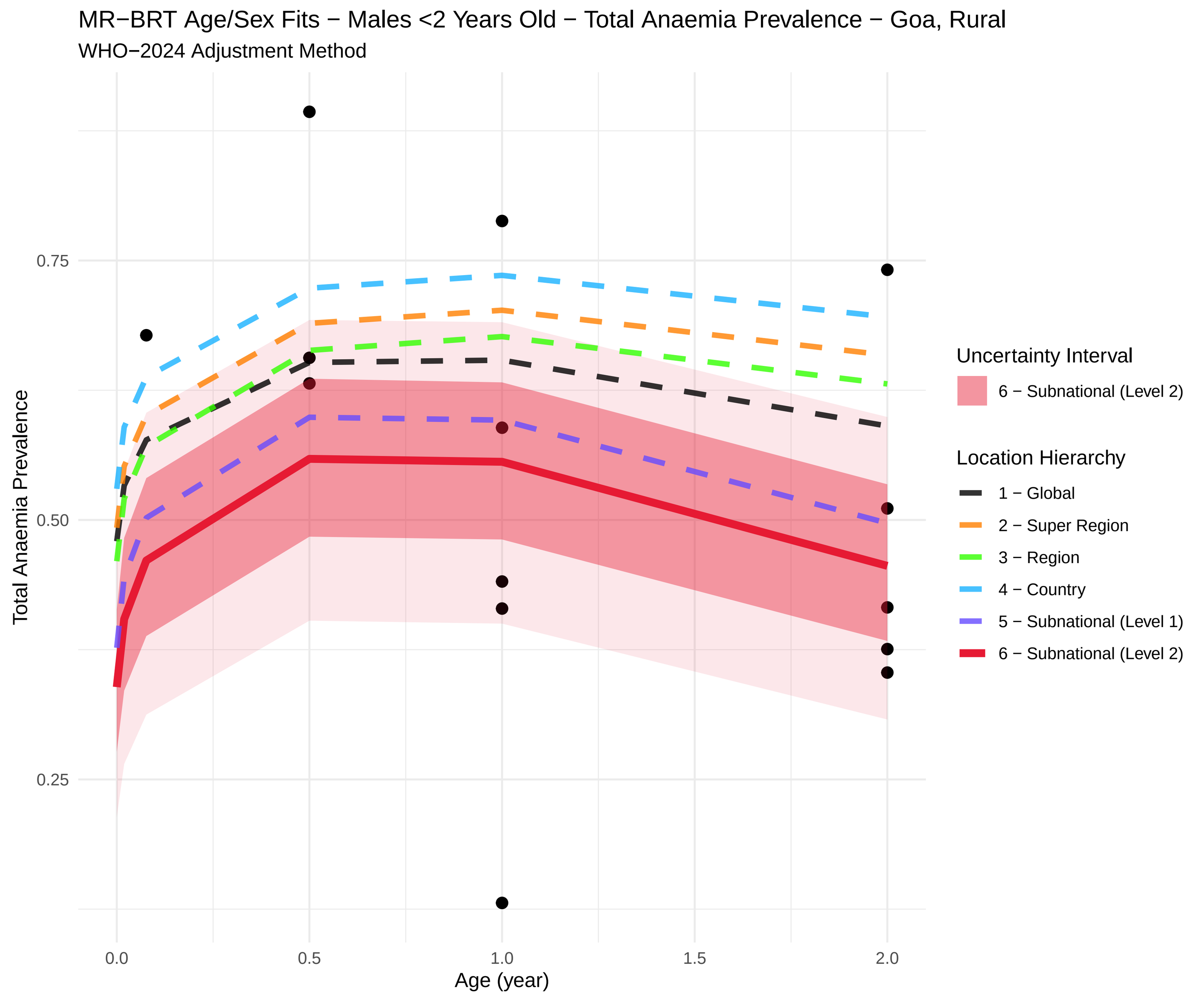
MR−BRT Age/Sex Fits − Males <2 Years Old − Total Anaemia Prevalence − Goa, Rural
WHO−2024 Adjustment Method
Total Anaemia Prevalence
Age (year)
Uncertainty Interval
6 − Subnational (Level 2)
Location Hierarchy
1 − Global
2 − Super Region
3 − Region
4 − Country
5 − Subnational (Level 1)
6 − Subnational (Level 2)

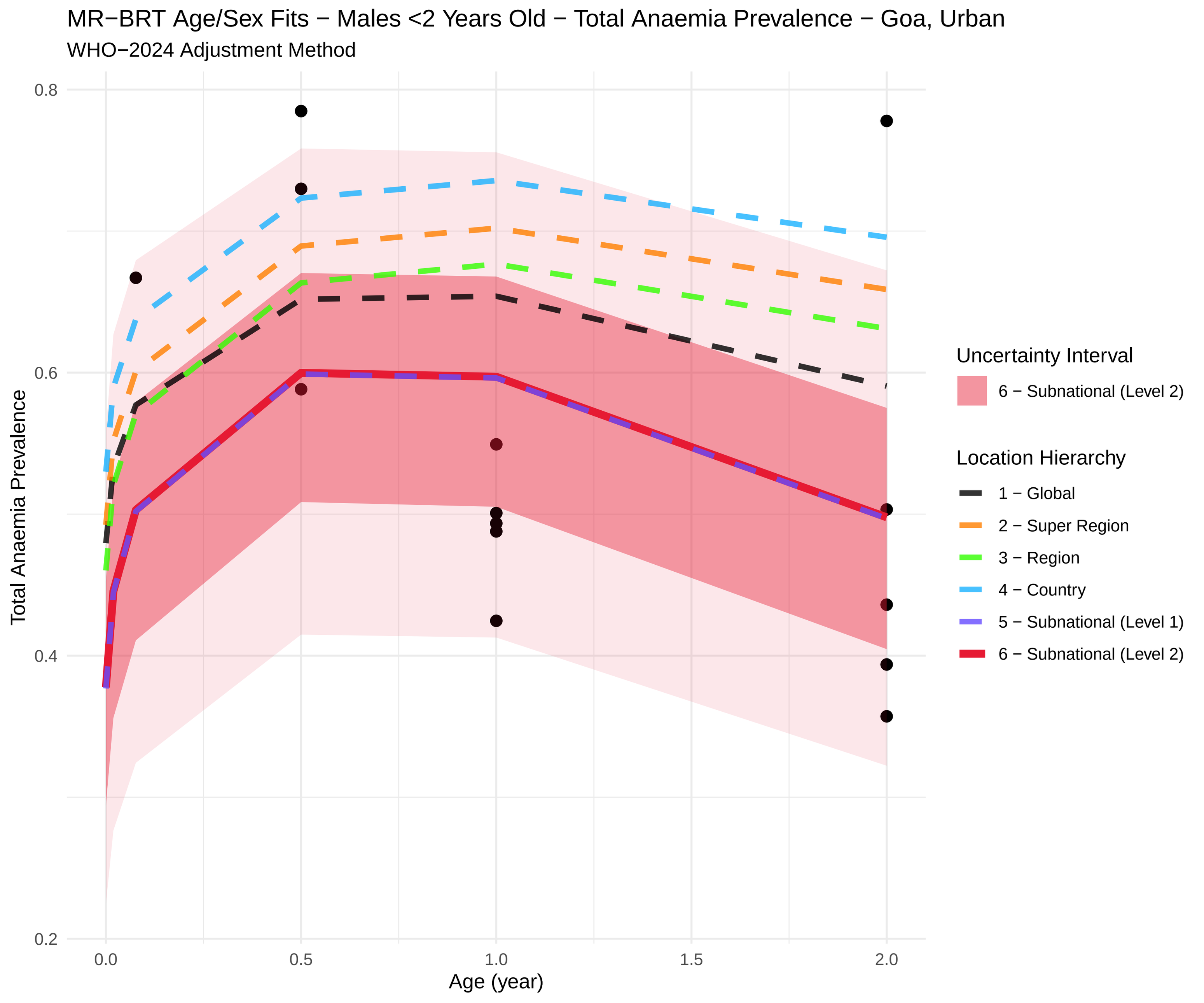
MR−BRT Age/Sex Fits − Males <2 Years Old − Total Anaemia Prevalence − Goa, Urban
WHO−2024 Adjustment Method
Total Anaemia Prevalence
Age (year)
Uncertainty Interval
6 − Subnational (Level 2)
Location Hierarchy
1 − Global
2 − Super Region
3 − Region
4 − Country
5 − Subnational (Level 1)
6 − Subnational (Level 2)

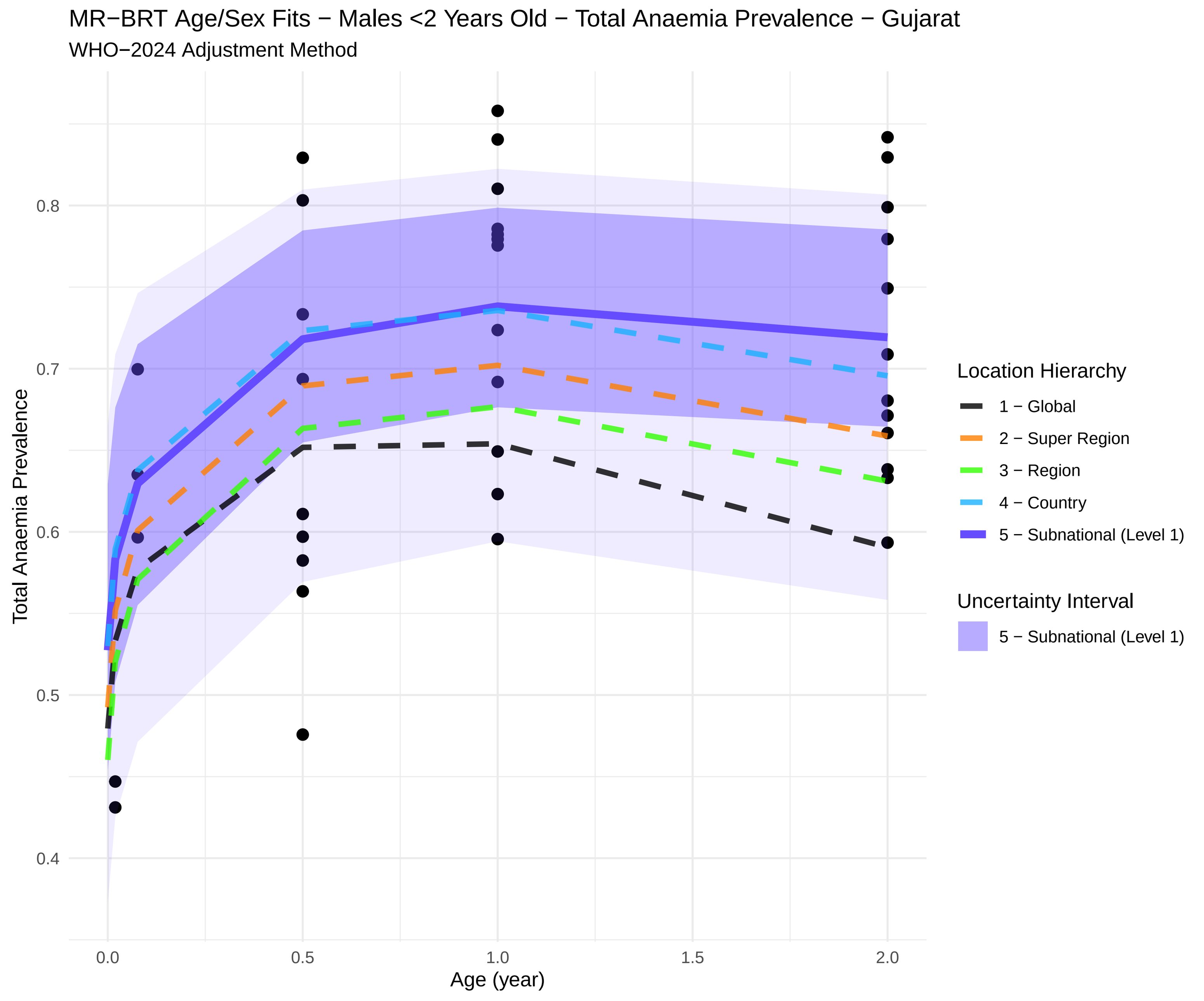
MR−BRT Age/Sex Fits − Males <2 Years Old − Total Anaemia Prevalence − Gujarat
WHO−2024 Adjustment Method
Total Anaemia Prevalence
Age (year)
Location Hierarchy
1 − Global
2 − Super Region
3 − Region
4 − Country
5 − Subnational (Level 1)
Uncertainty Interval
5 − Subnational (Level 1)

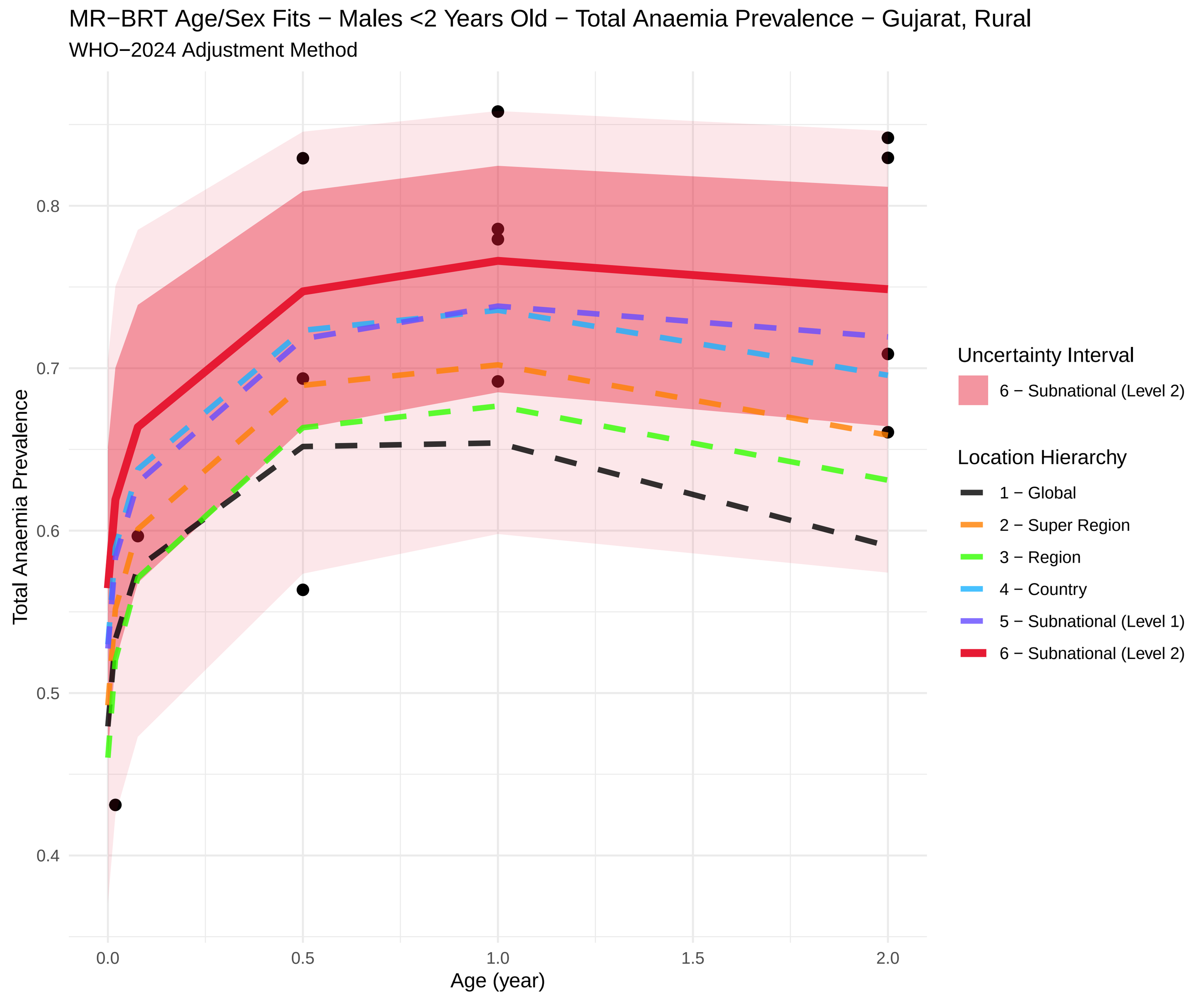
MR−BRT Age/Sex Fits − Males <2 Years Old − Total Anaemia Prevalence − Gujarat, Rural
WHO−2024 Adjustment Method
Total Anaemia Prevalence
Age (year)
Uncertainty Interval
6 − Subnational (Level 2)
Location Hierarchy
1 − Global
2 − Super Region
3 − Region
4 − Country
5 − Subnational (Level 1)
6 − Subnational (Level 2)

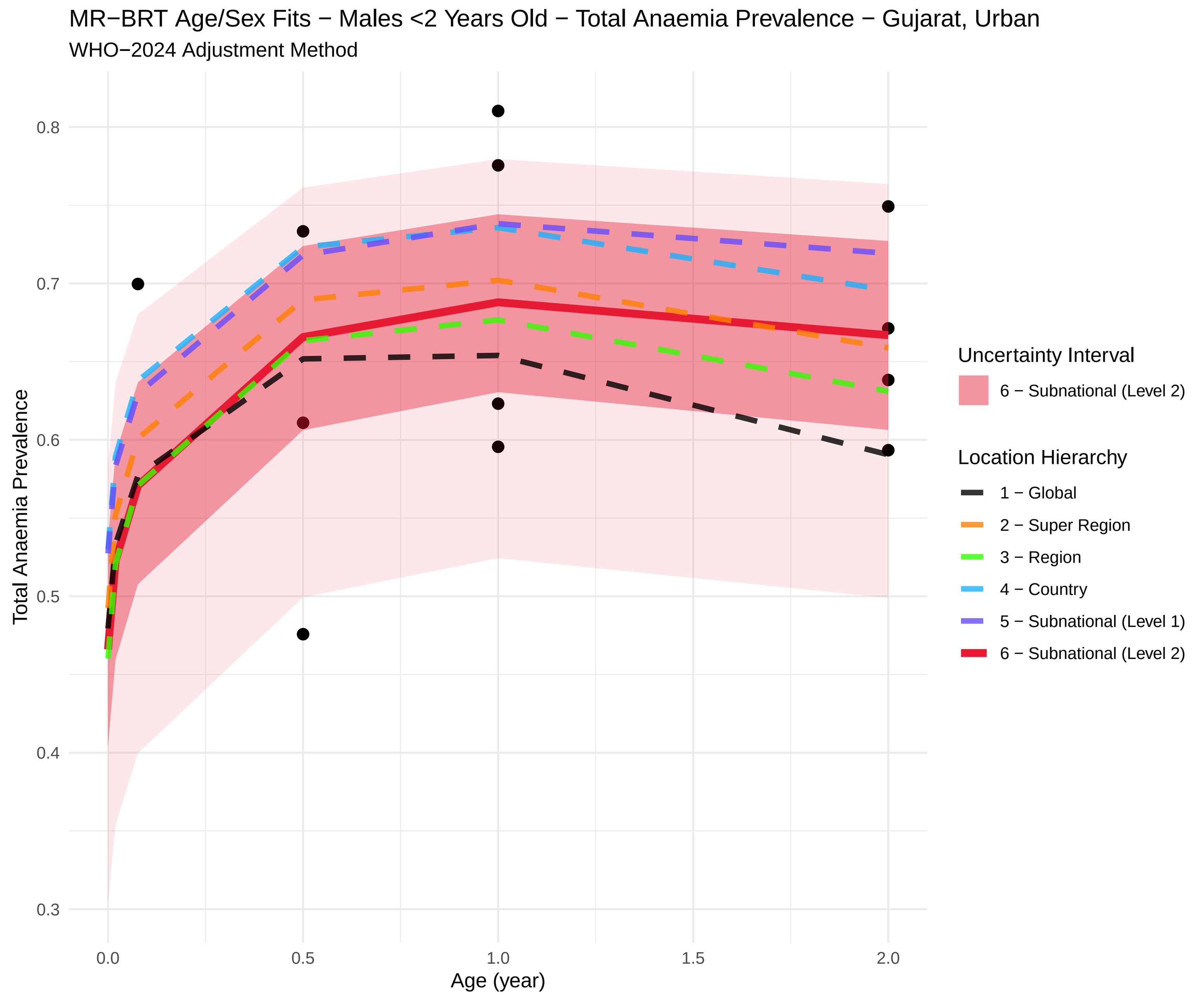
MR−BRT Age/Sex Fits − Males <2 Years Old − Total Anaemia Prevalence − Gujarat, Urban
WHO−2024 Adjustment Method
Total Anaemia Prevalence
Age (year)
Uncertainty Interval
6 − Subnational (Level 2)
Location Hierarchy
1 − Global
2 − Super Region
3 − Region
4 − Country
5 − Subnational (Level 1)
6 − Subnational (Level 2)

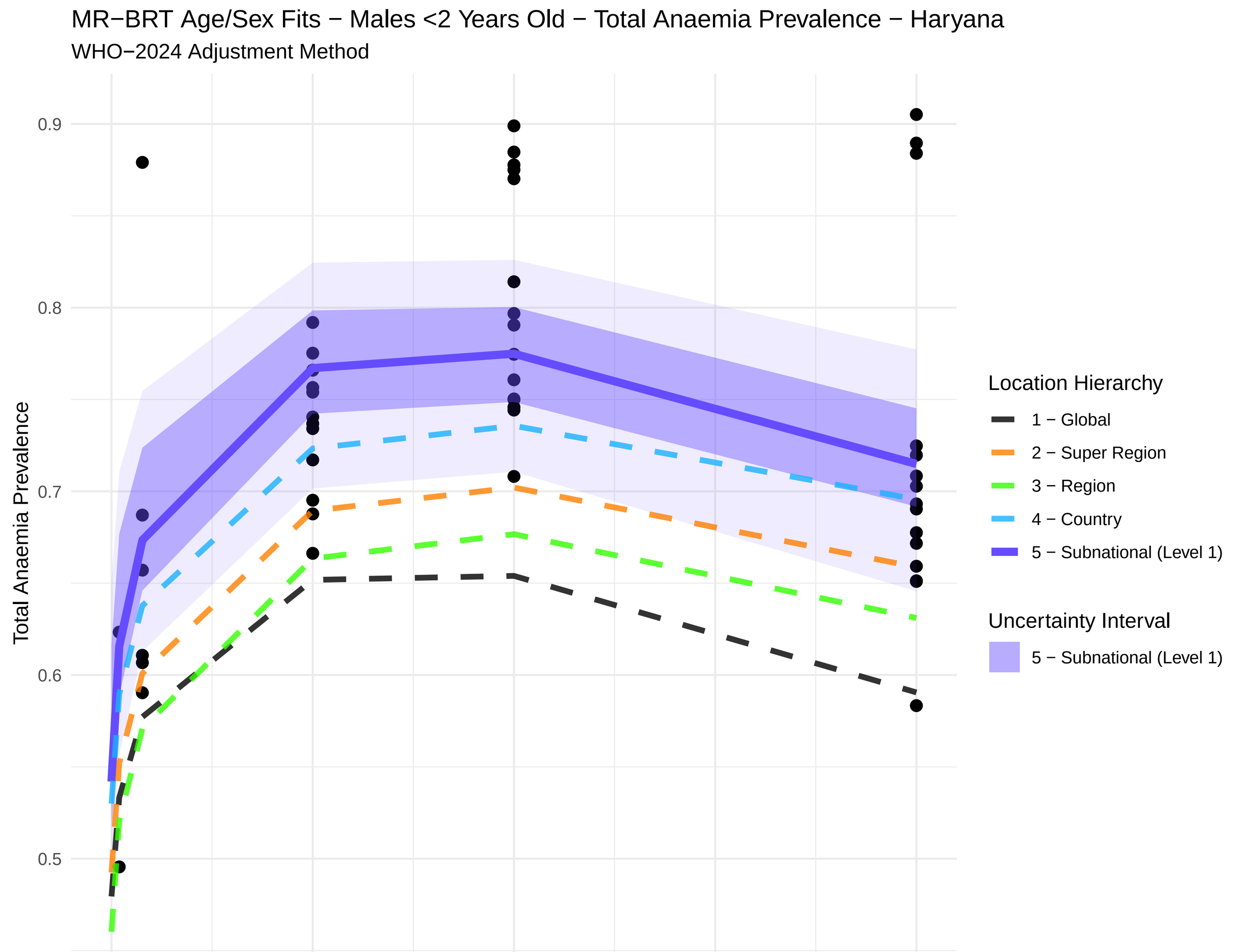
MR−BRT Age/Sex Fits − Males <2 Years Old − Total Anaemia Prevalence − Haryana
WHO−2024 Adjustment Method
Total Anaemia Prevalence
Age (year)
Location Hierarchy
1 − Global
2 − Super Region
3 − Region
4 − Country
5 − Subnational (Level 1)
Uncertainty Interval
5 − Subnational (Level 1)

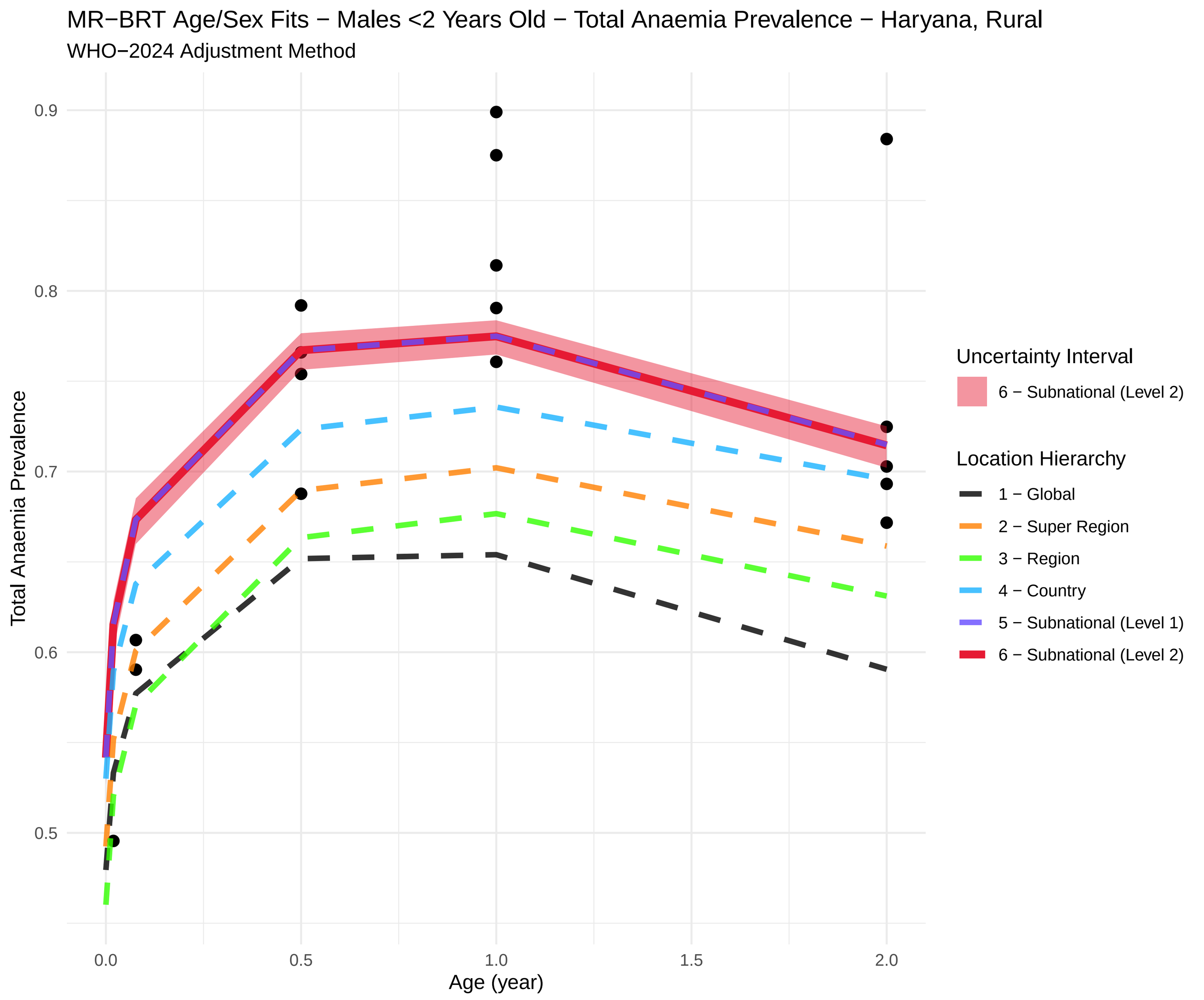
MR−BRT Age/Sex Fits − Males <2 Years Old − Total Anaemia Prevalence − Haryana, Rural
WHO−2024 Adjustment Method
Total Anaemia Prevalence
Age (year)
Uncertainty Interval
6 − Subnational (Level 2)
Location Hierarchy
1 − Global
2 − Super Region
3 − Region
4 − Country
5 − Subnational (Level 1)
6 − Subnational (Level 2)

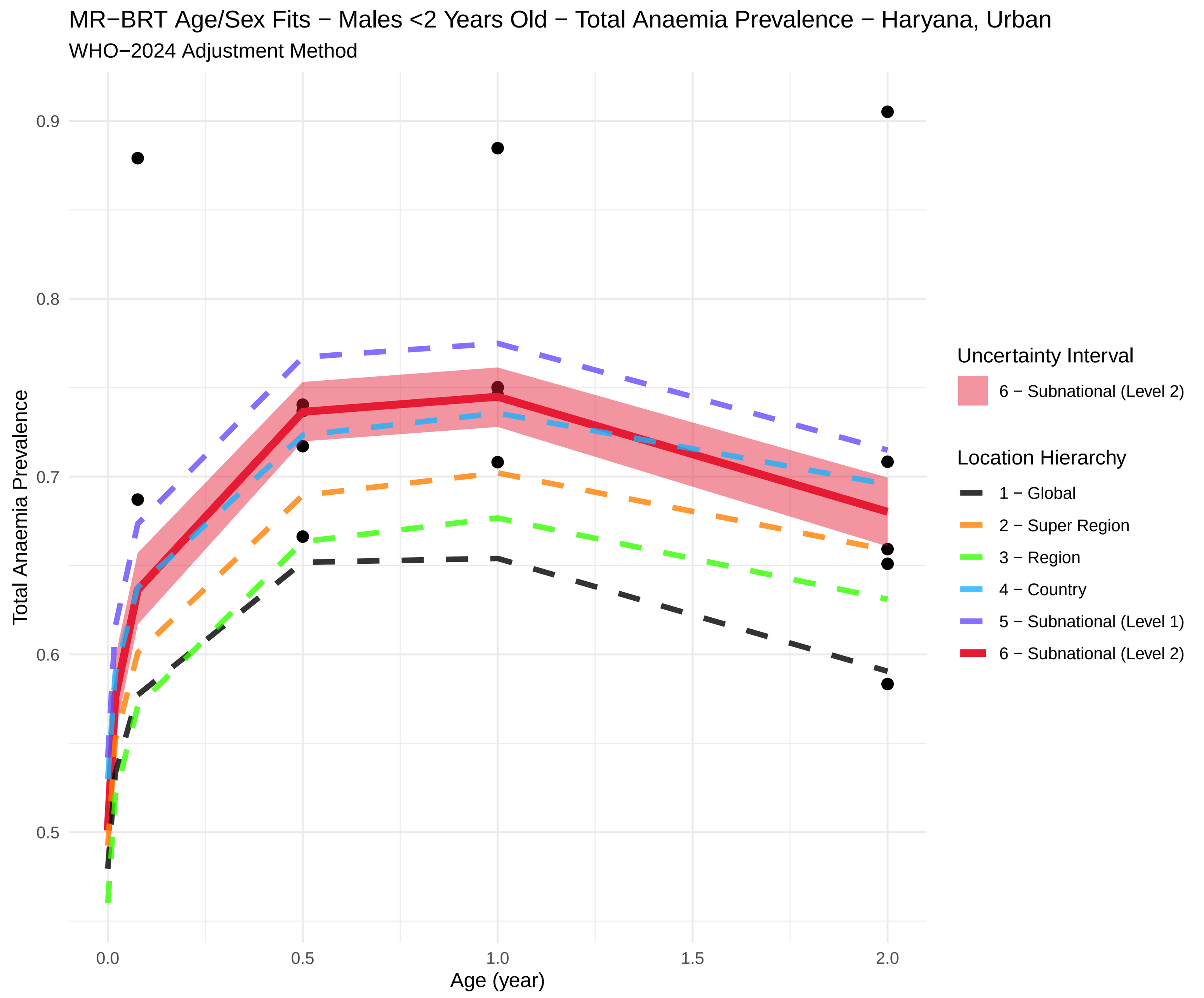
MR−BRT Age/Sex Fits − Males <2 Years Old − Total Anaemia Prevalence − Haryana, Urban
WHO−2024 Adjustment Method
Total Anaemia Prevalence
Age (year)
Uncertainty Interval
6 − Subnational (Level 2)
Location Hierarchy
1 − Global
2 − Super Region
3 − Region
4 − Country
5 − Subnational (Level 1)
6 − Subnational (Level 2)

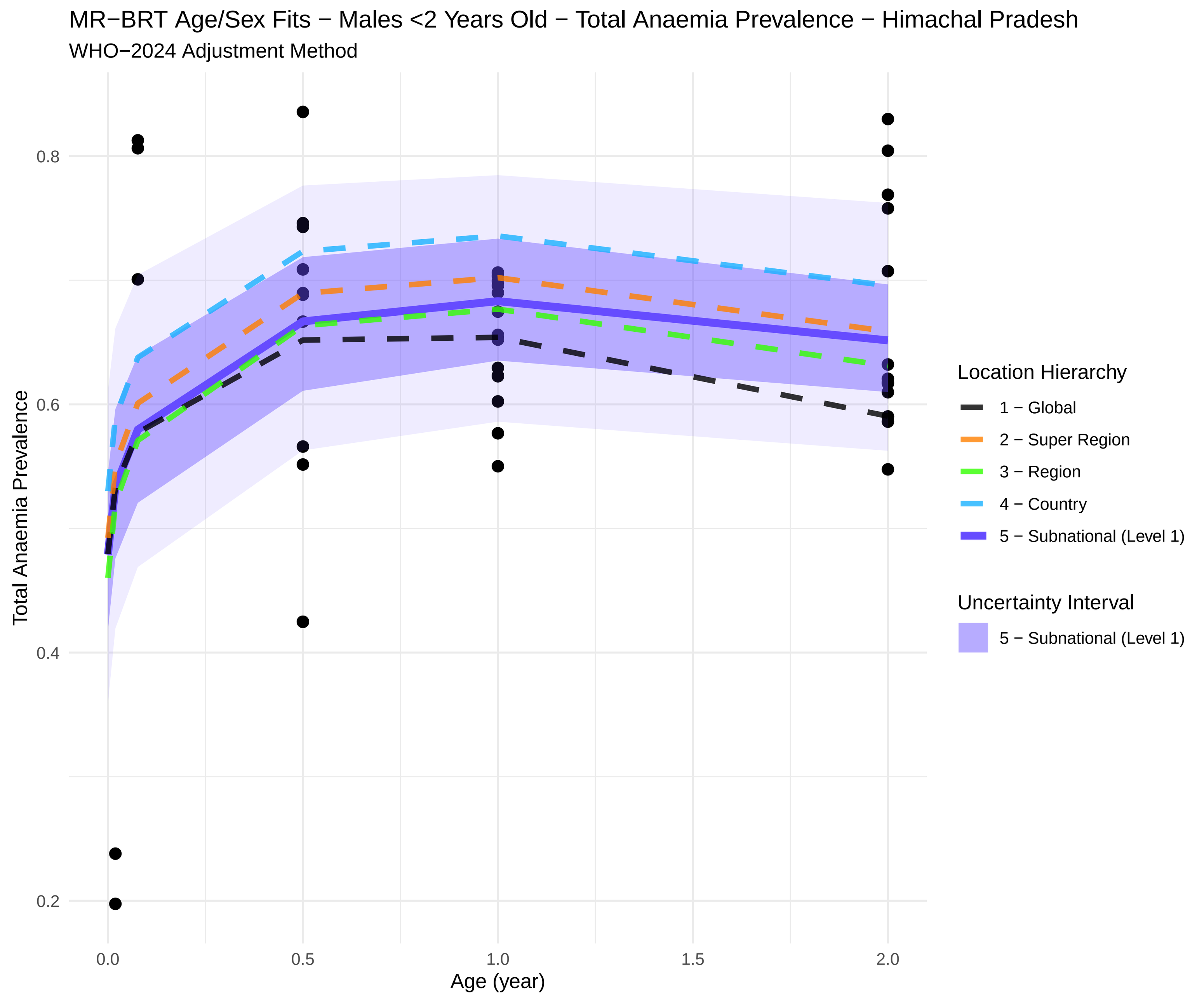
MR−BRT Age/Sex Fits − Males <2 Years Old − Total Anaemia Prevalence − Himachal Pradesh
WHO−2024 Adjustment Method
Total Anaemia Prevalence
Age (year)
Location Hierarchy
1 − Global
2 − Super Region
3 − Region
4 − Country
5 − Subnational (Level 1)
Uncertainty Interval
5 − Subnational (Level 1)

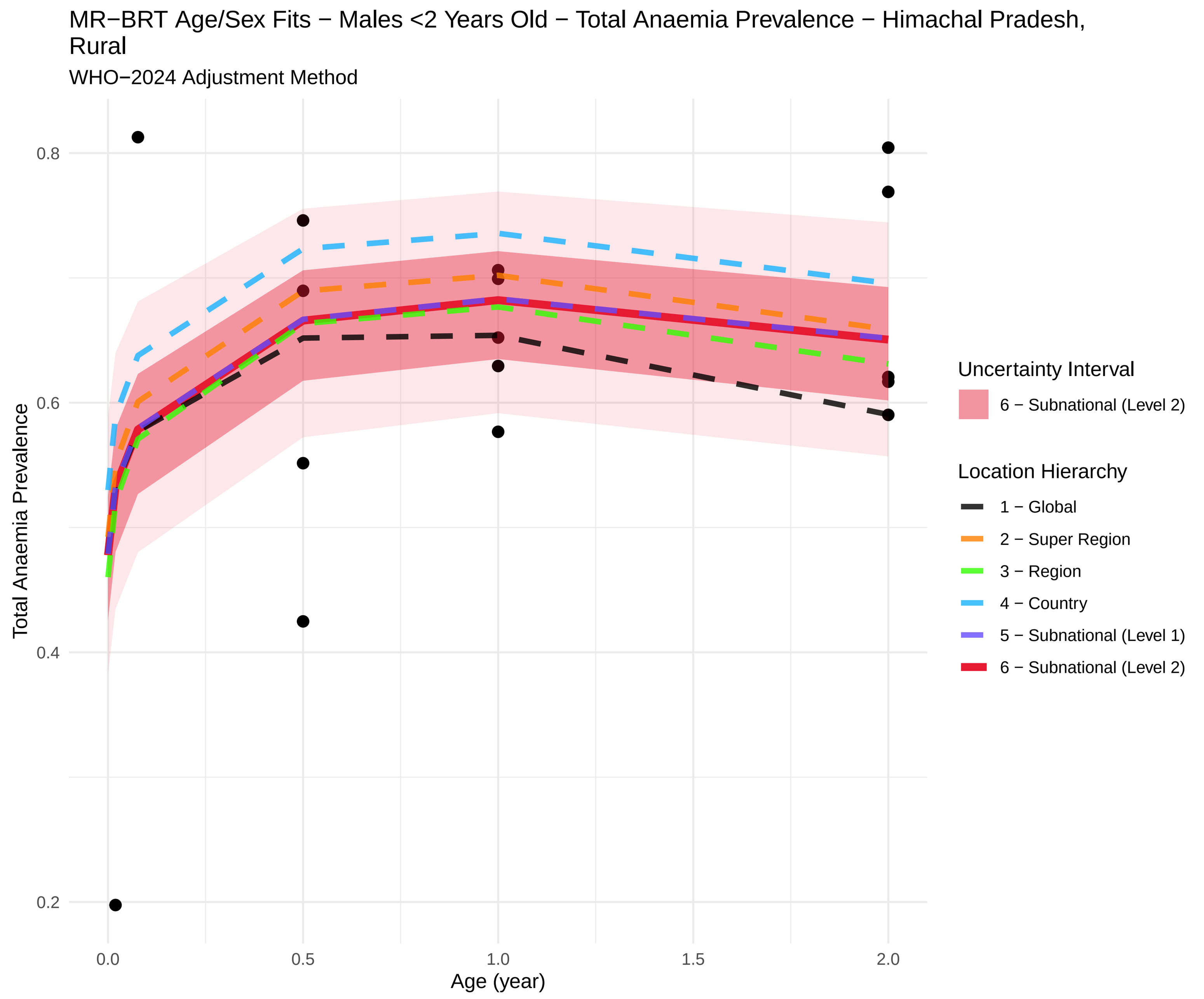
MR−BRT Age/Sex Fits − Males <2 Years Old − Total Anaemia Prevalence − Himachal Pradesh, Rural
WHO−2024 Adjustment Method
Total Anaemia Prevalence
Age (year)
Uncertainty Interval
6 − Subnational (Level 2)
Location Hierarchy
1 − Global
2 − Super Region
3 − Region
4 − Country
5 − Subnational (Level 1)
6 − Subnational (Level 2)

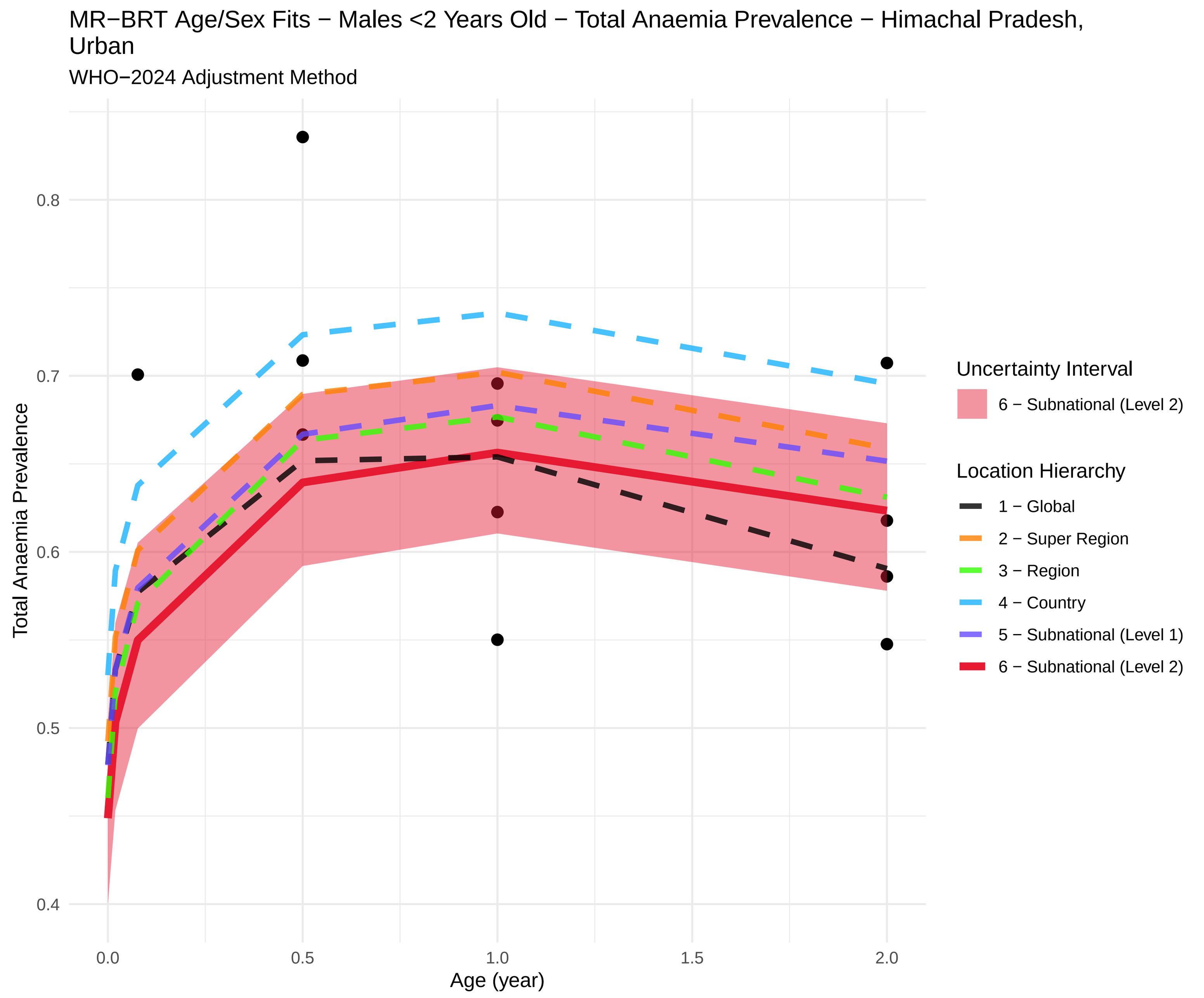
MR−BRT Age/Sex Fits − Males <2 Years Old − Total Anaemia Prevalence − Himachal Pradesh, Urban
WHO−2024 Adjustment Method
Total Anaemia Prevalence
Age (year)
Uncertainty Interval
6 − Subnational (Level 2)
Location Hierarchy
1 − Global
2 − Super Region
3 − Region
4 − Country
5 − Subnational (Level 1)
6 − Subnational (Level 2)

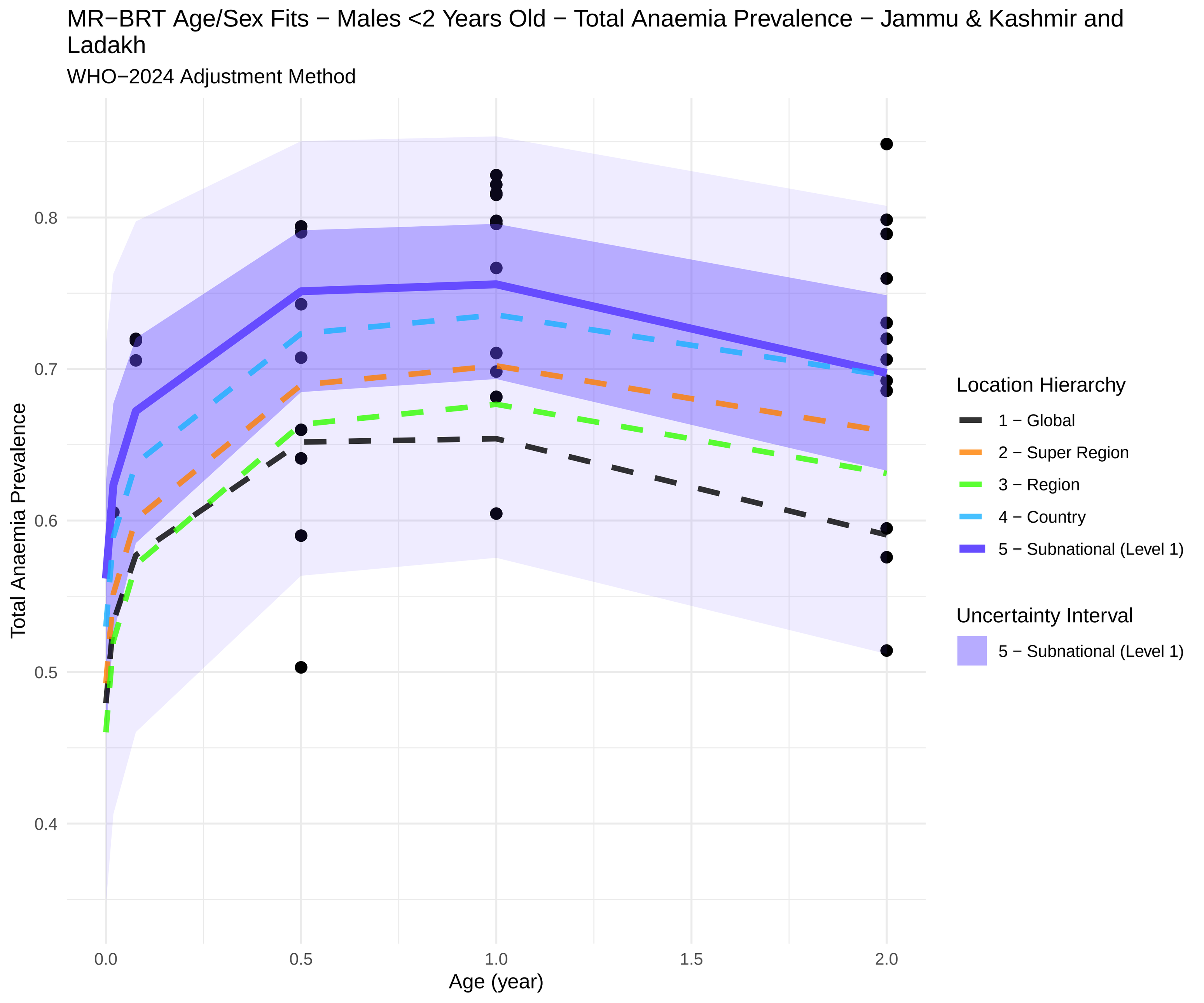
MR−BRT Age/Sex Fits − Males <2 Years Old − Total Anaemia Prevalence − Jammu & Kashmir and Ladakh
WHO−2024 Adjustment Method
Total Anaemia Prevalence
Age (year)
Location Hierarchy
1 − Global
2 − Super Region
3 − Region
4 − Country
5 − Subnational (Level 1)
Uncertainty Interval
5 − Subnational (Level 1)

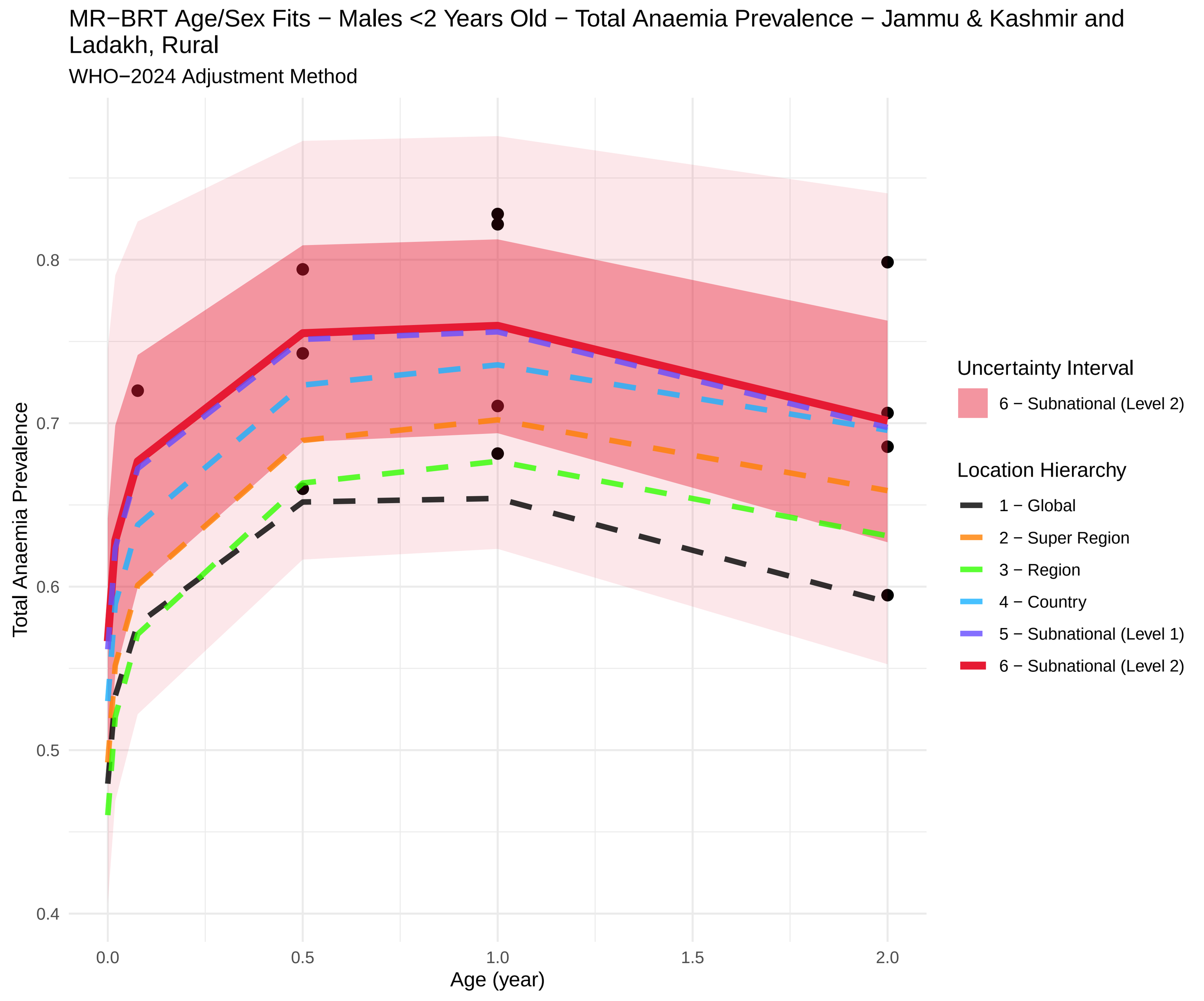
MR−BRT Age/Sex Fits − Males <2 Years Old − Total Anaemia Prevalence − Jammu & Kashmir and Ladakh, Rural
WHO−2024 Adjustment Method
Total Anaemia Prevalence
Age (year)
Uncertainty Interval
6 − Subnational (Level 2)
Location Hierarchy
1 − Global
2 − Super Region
3 − Region
4 − Country
5 − Subnational (Level 1)
6 − Subnational (Level 2)

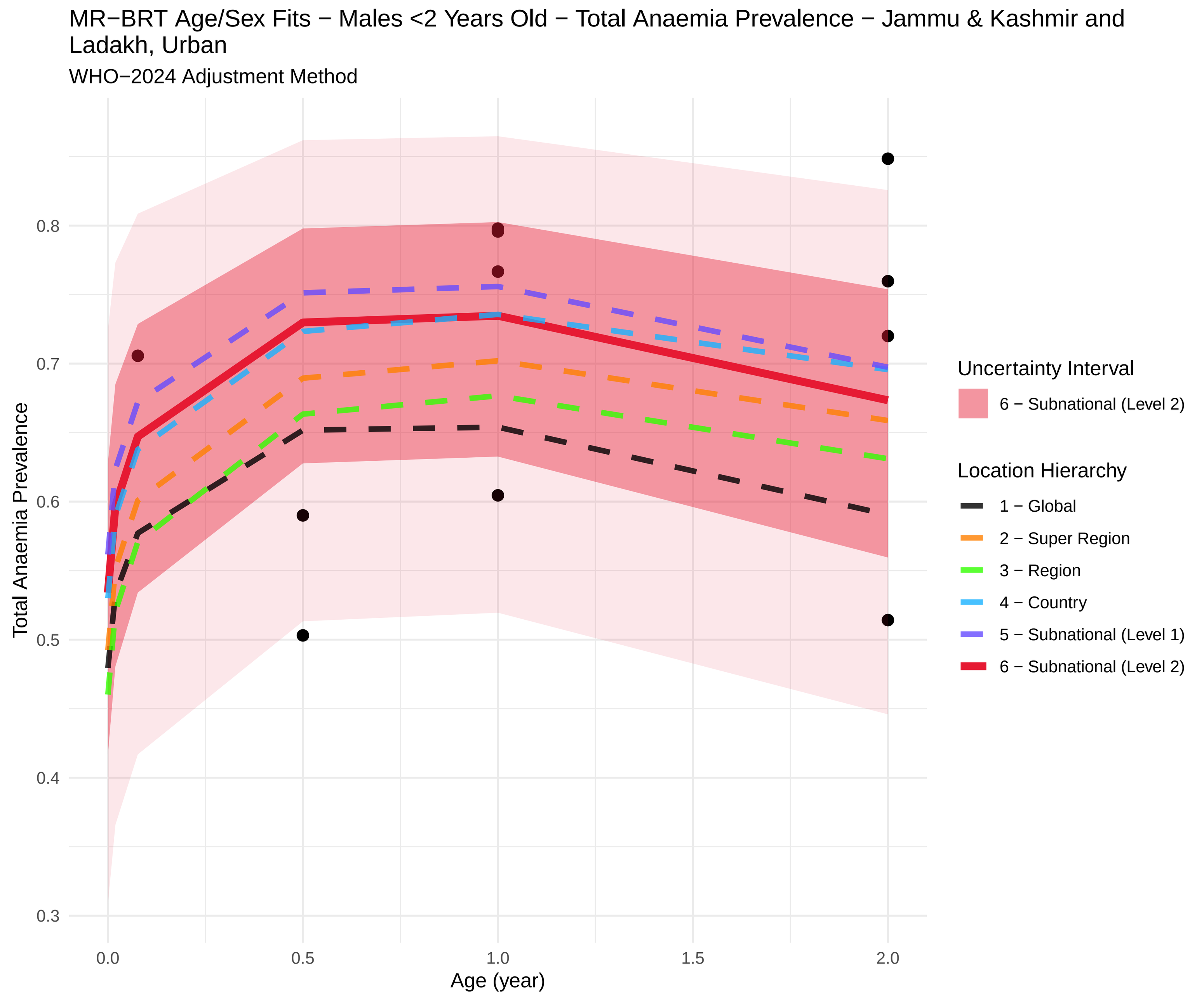
MR−BRT Age/Sex Fits − Males <2 Years Old − Total Anaemia Prevalence − Jammu & Kashmir and Ladakh, Urban
WHO−2024 Adjustment Method
Total Anaemia Prevalence
Age (year)
Uncertainty Interval
6 − Subnational (Level 2)
Location Hierarchy
1 − Global
2 − Super Region
3 − Region
4 − Country
5 − Subnational (Level 1)
6 − Subnational (Level 2)

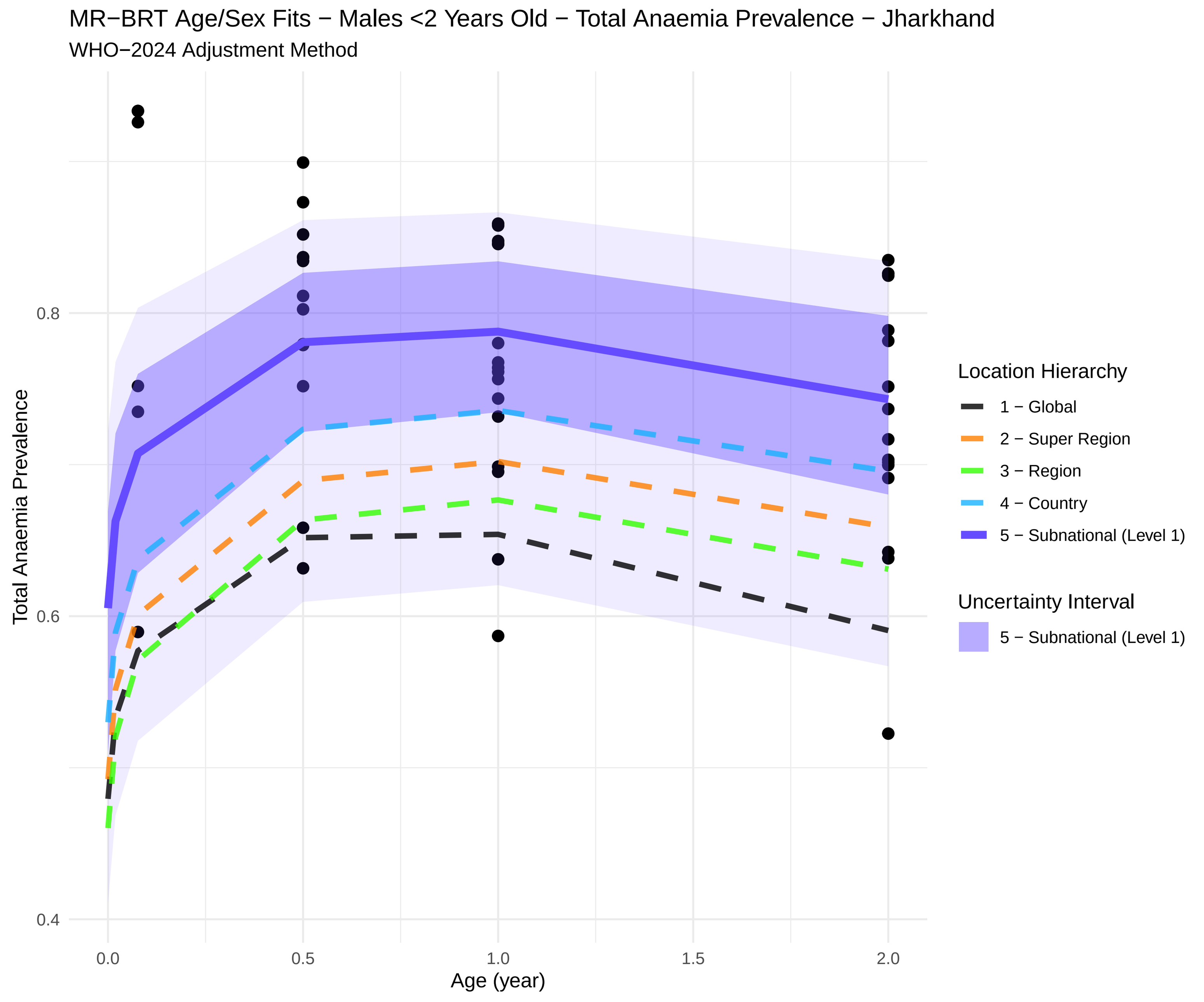
MR−BRT Age/Sex Fits − Males <2 Years Old − Total Anaemia Prevalence − Jharkhand
WHO−2024 Adjustment Method
Total Anaemia Prevalence
Age (year)
Location Hierarchy
1 − Global
2 − Super Region
3 − Region
4 − Country
5 − Subnational (Level 1)
Uncertainty Interval
5 − Subnational (Level 1)

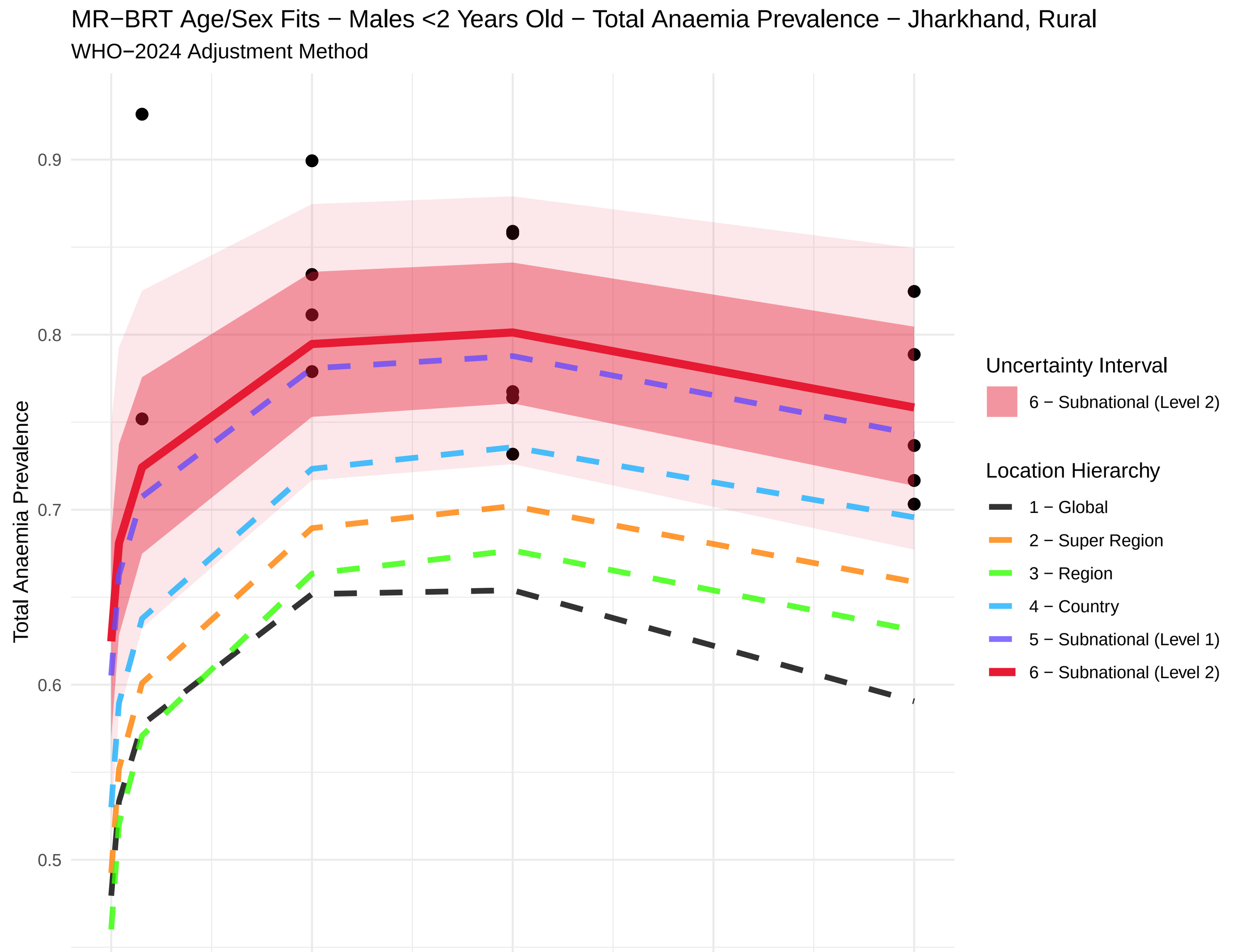
MR−BRT Age/Sex Fits − Males <2 Years Old − Total Anaemia Prevalence − Jharkhand, Rural
WHO−2024 Adjustment Method
Total Anaemia Prevalence
Age (year)
Uncertainty Interval
6 − Subnational (Level 2)
Location Hierarchy
1 − Global
2 − Super Region
3 − Region
4 − Country
5 − Subnational (Level 1)
6 − Subnational (Level 2)

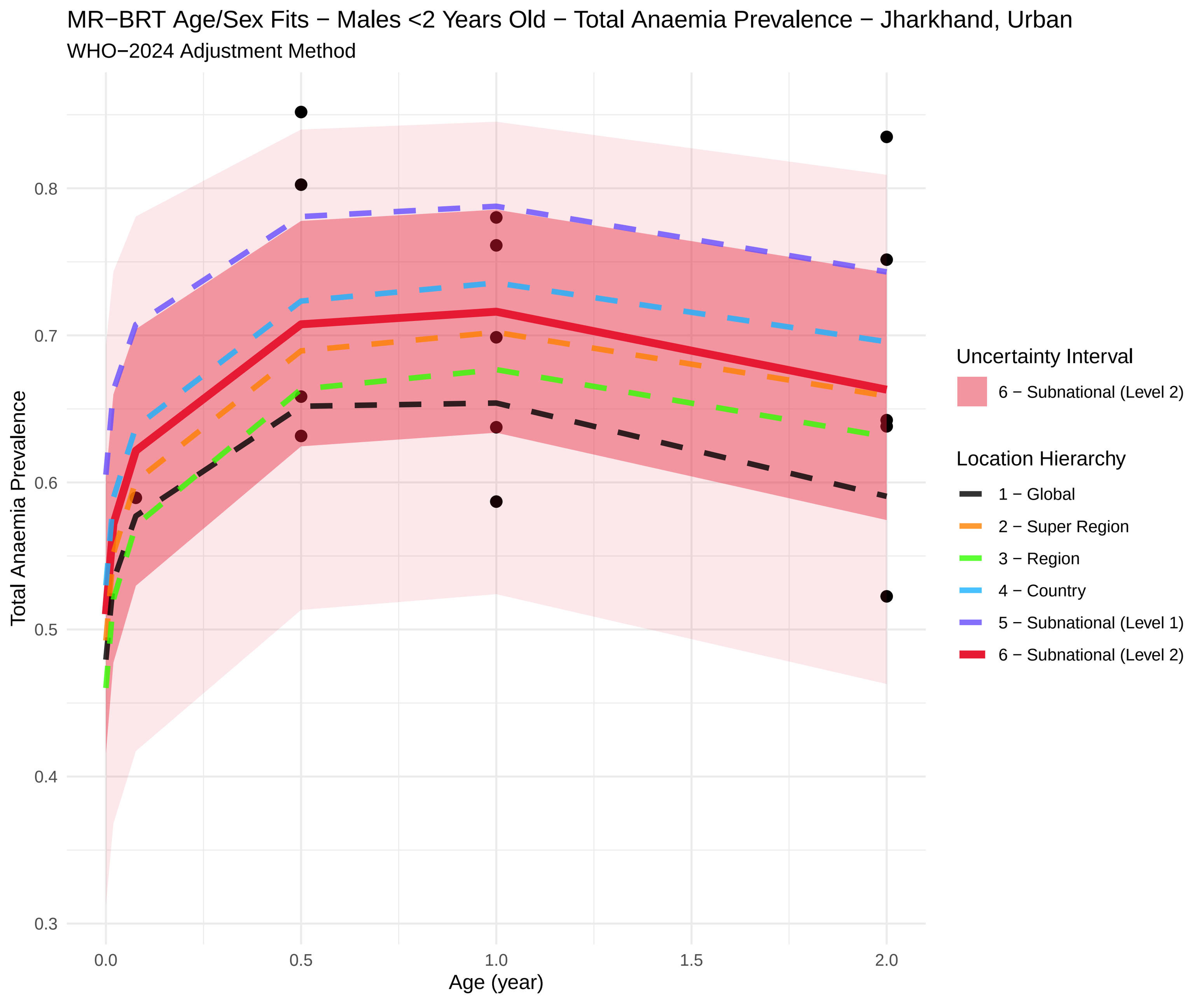
MR−BRT Age/Sex Fits − Males <2 Years Old − Total Anaemia Prevalence − Jharkhand, Urban
WHO−2024 Adjustment Method
Total Anaemia Prevalence
Age (year)
Uncertainty Interval
6 − Subnational (Level 2)
Location Hierarchy
1 − Global
2 − Super Region
3 − Region
4 − Country
5 − Subnational (Level 1)
6 − Subnational (Level 2)

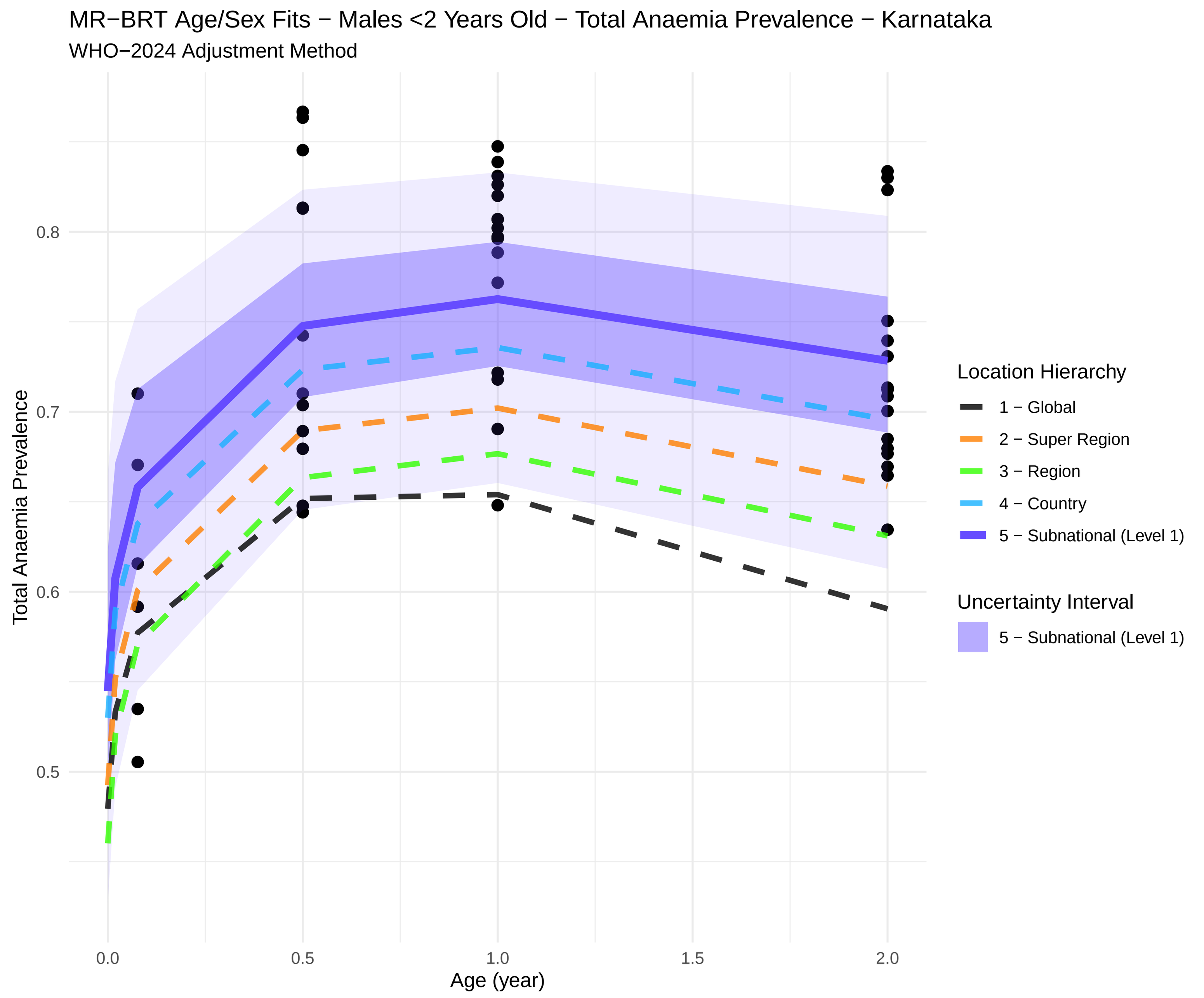
MR−BRT Age/Sex Fits − Males <2 Years Old − Total Anaemia Prevalence − Karnataka
WHO−2024 Adjustment Method
Total Anaemia Prevalence
Age (year)
Location Hierarchy
1 − Global
2 − Super Region
3 − Region
4 − Country
5 − Subnational (Level 1)
Uncertainty Interval
5 − Subnational (Level 1)

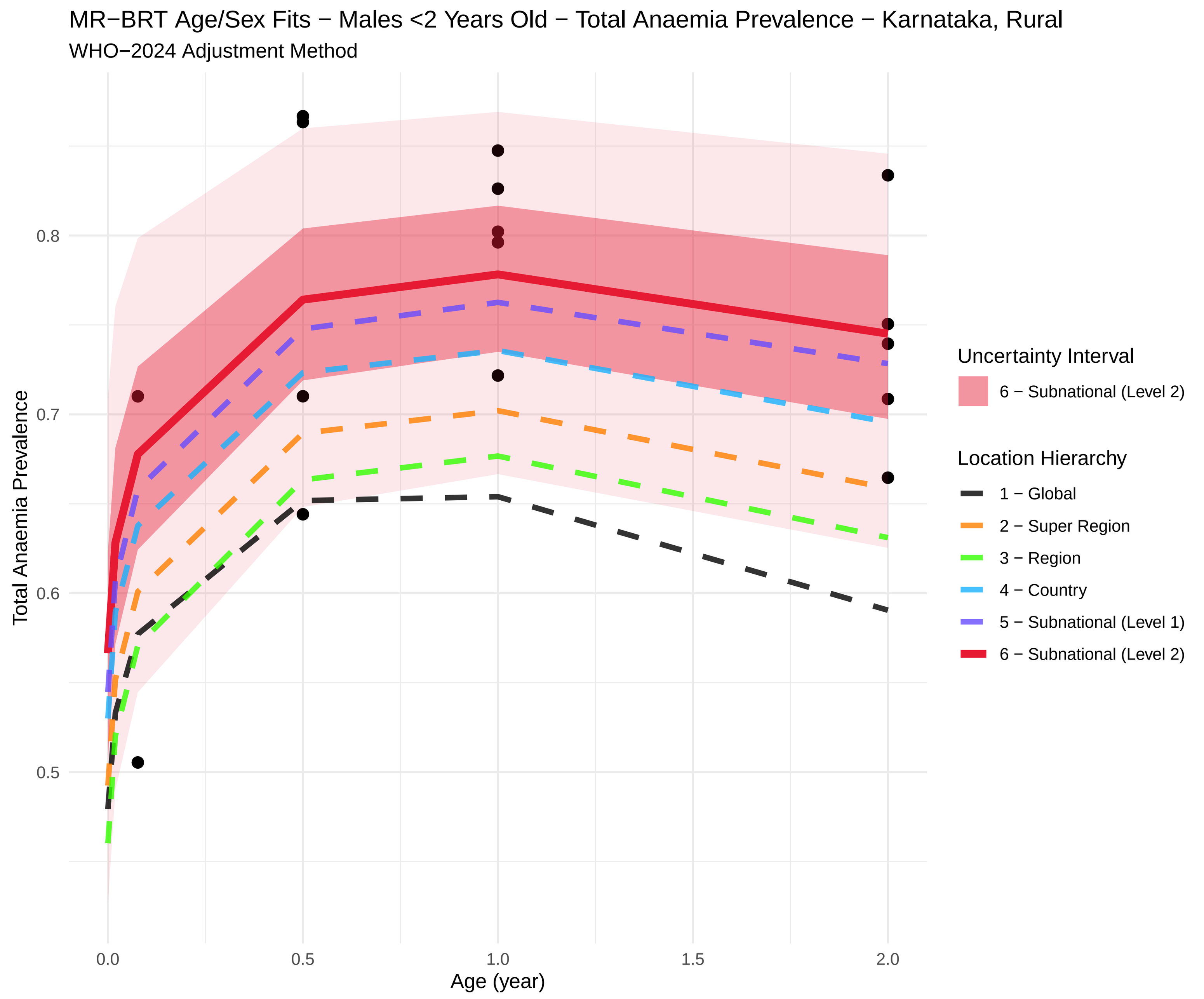
MR−BRT Age/Sex Fits − Males <2 Years Old − Total Anaemia Prevalence − Karnataka, Rural
WHO−2024 Adjustment Method
Total Anaemia Prevalence
Age (year)
Uncertainty Interval
6 − Subnational (Level 2)
Location Hierarchy
1 − Global
2 − Super Region
3 − Region
4 − Country
5 − Subnational (Level 1)
6 − Subnational (Level 2)

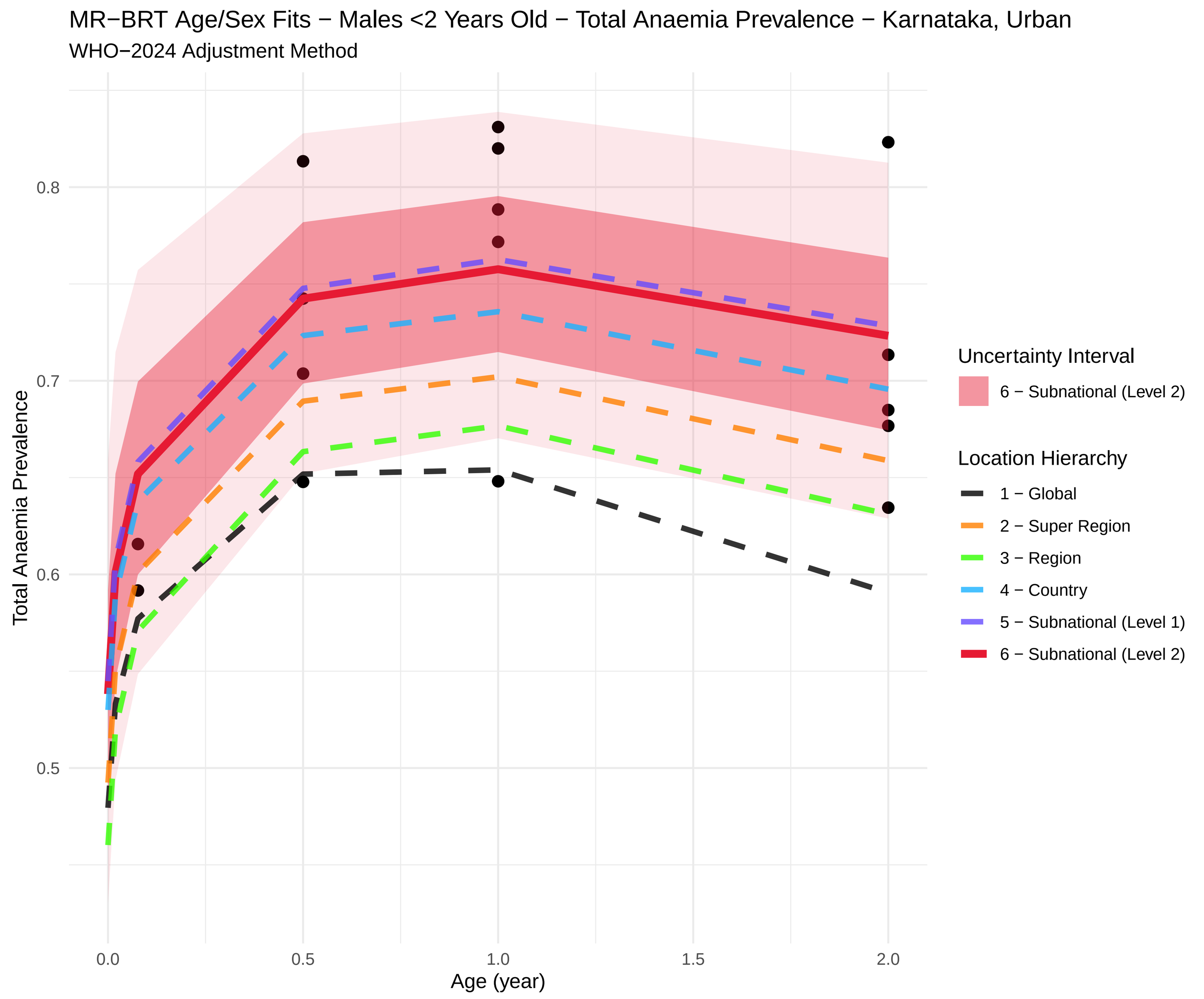
MR−BRT Age/Sex Fits − Males <2 Years Old − Total Anaemia Prevalence − Karnataka, Urban
WHO−2024 Adjustment Method
Total Anaemia Prevalence
Age (year)
Uncertainty Interval
6 − Subnational (Level 2)
Location Hierarchy
1 − Global
2 − Super Region
3 − Region
4 − Country
5 − Subnational (Level 1)
6 − Subnational (Level 2)

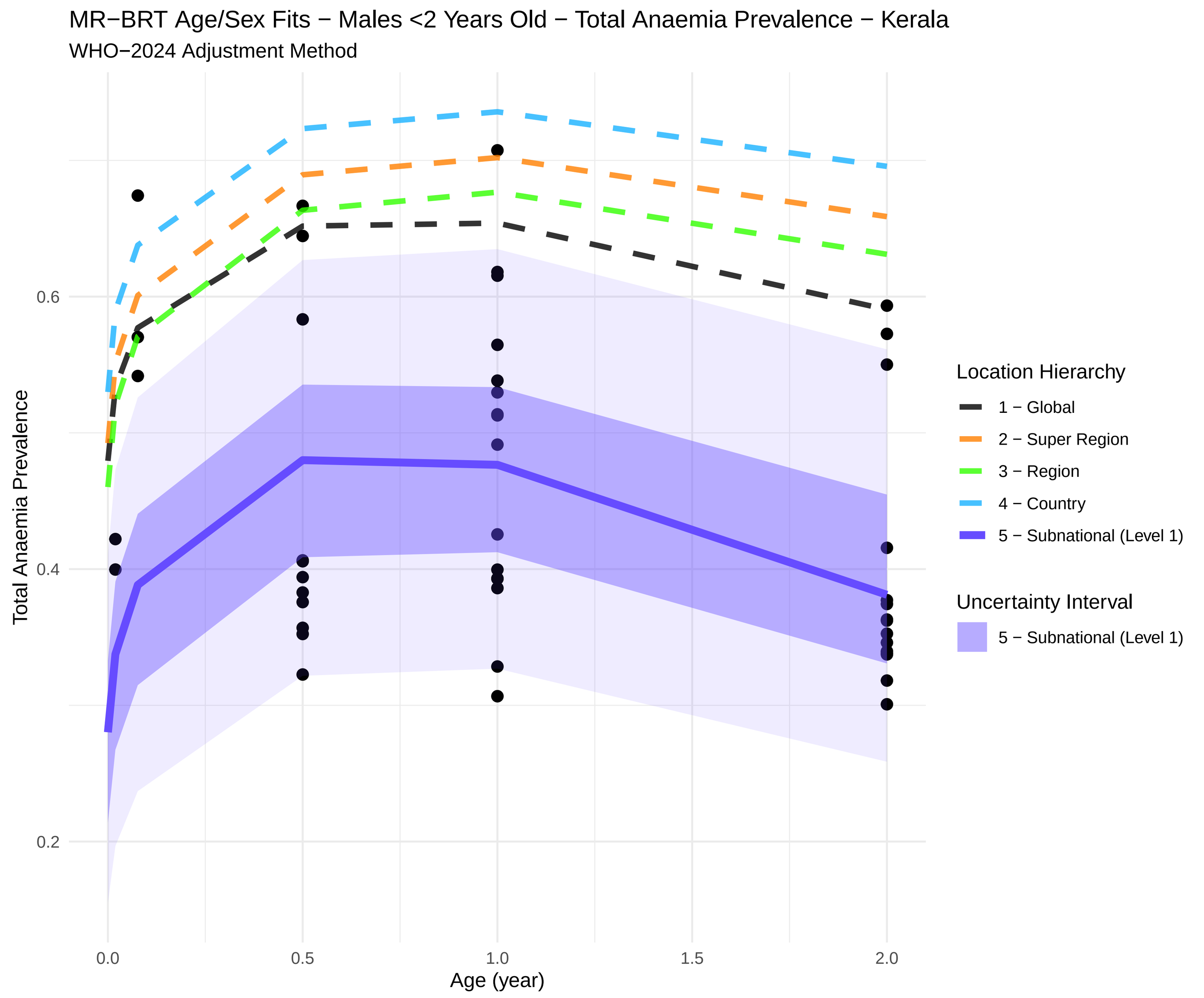
MR−BRT Age/Sex Fits − Males <2 Years Old − Total Anaemia Prevalence − Kerala
WHO−2024 Adjustment Method
Total Anaemia Prevalence
Age (year)
Location Hierarchy
1 − Global
2 − Super Region
3 − Region
4 − Country
5 − Subnational (Level 1)
Uncertainty Interval
5 − Subnational (Level 1)

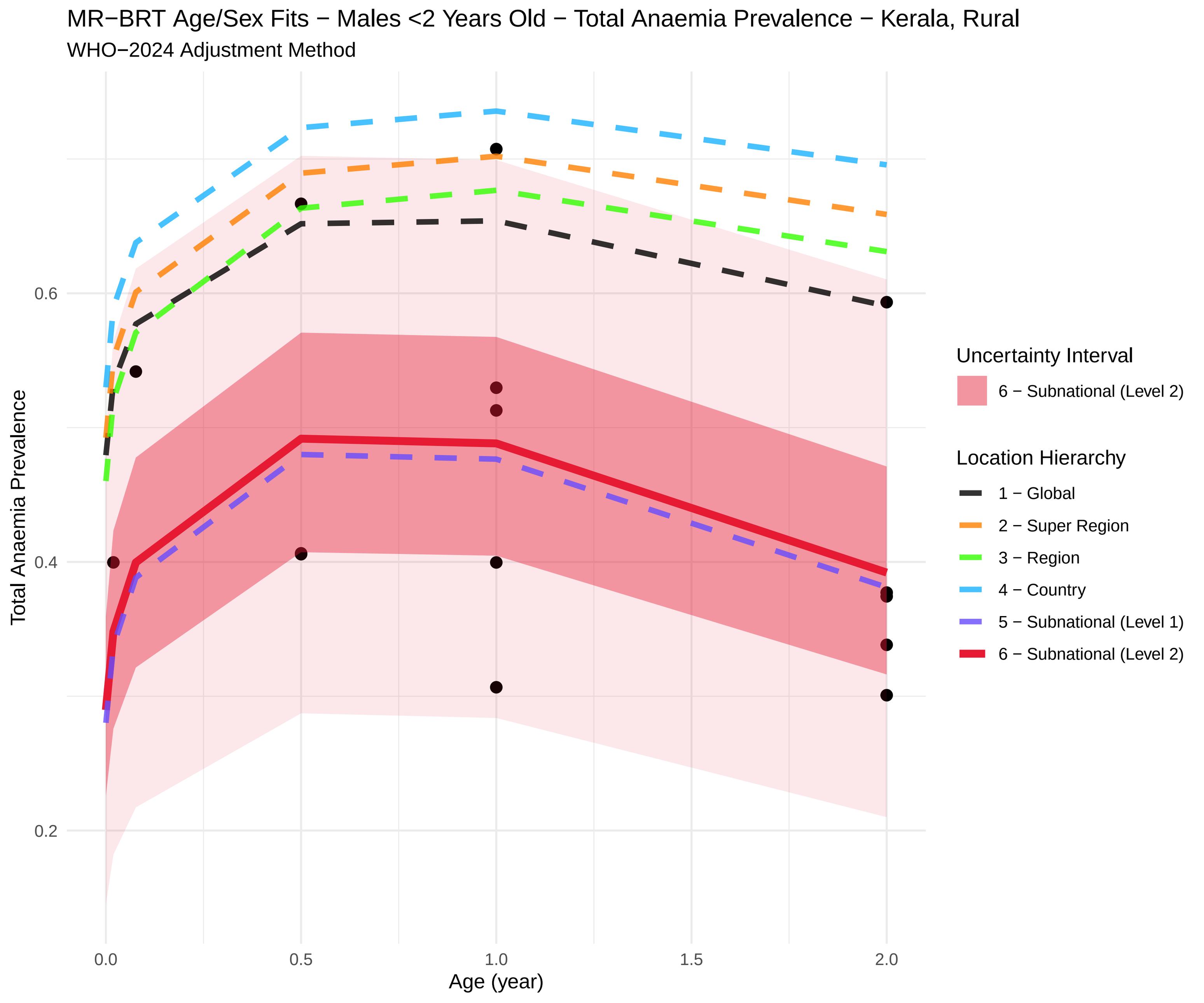
MR−BRT Age/Sex Fits − Males <2 Years Old − Total Anaemia Prevalence − Kerala, Rural
WHO−2024 Adjustment Method
Total Anaemia Prevalence
Age (year)
Uncertainty Interval
6 − Subnational (Level 2)
Location Hierarchy
1 − Global
2 − Super Region
3 − Region
4 − Country
5 − Subnational (Level 1)
6 − Subnational (Level 2)

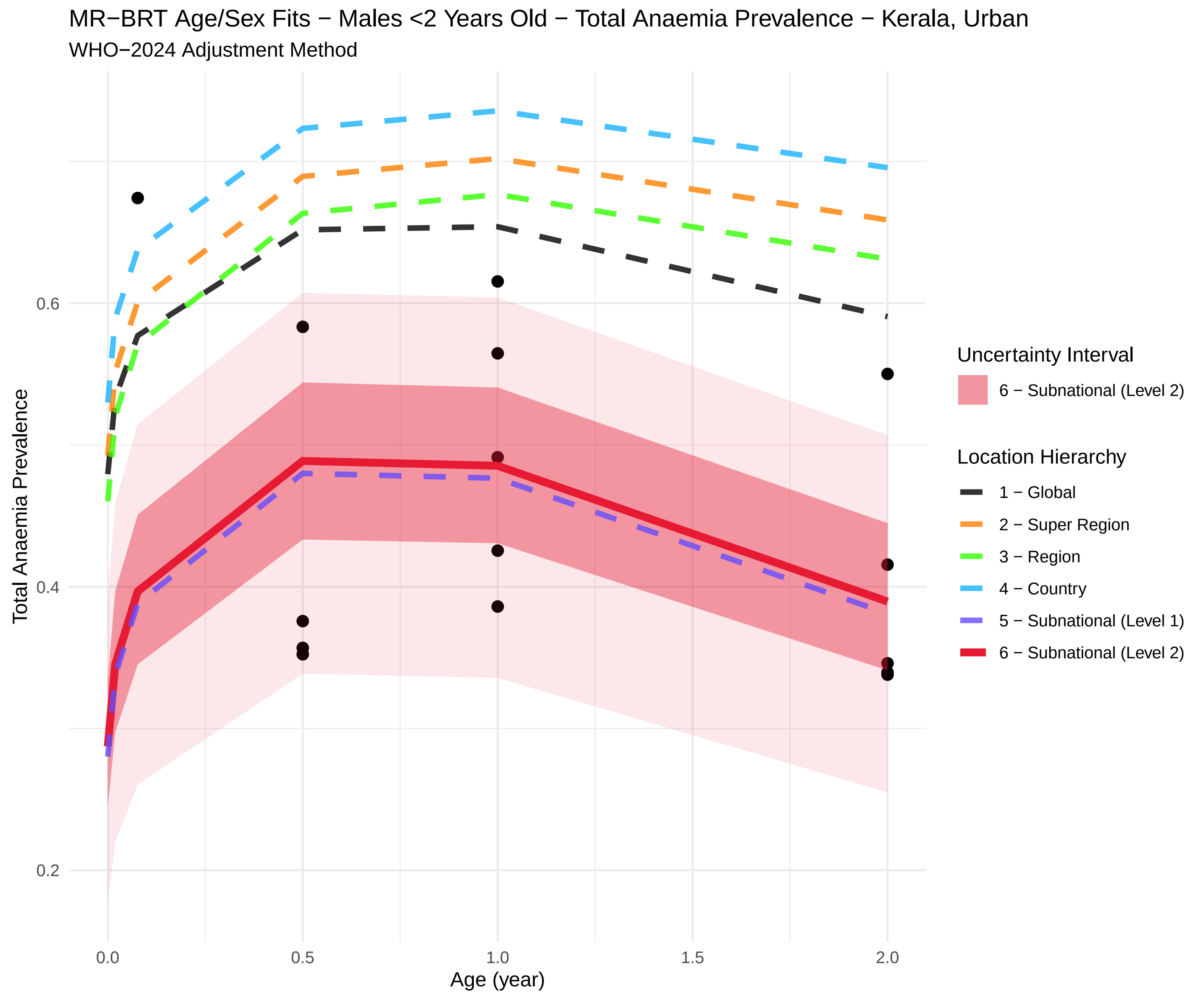
MR−BRT Age/Sex Fits − Males <2 Years Old − Total Anaemia Prevalence − Kerala, Urban
WHO−2024 Adjustment Method
Total Anaemia Prevalence
Age (year)
Uncertainty Interval
6 − Subnational (Level 2)
Location Hierarchy
1 − Global
2 − Super Region
3 − Region
4 − Country
5 − Subnational (Level 1)
6 − Subnational (Level 2)

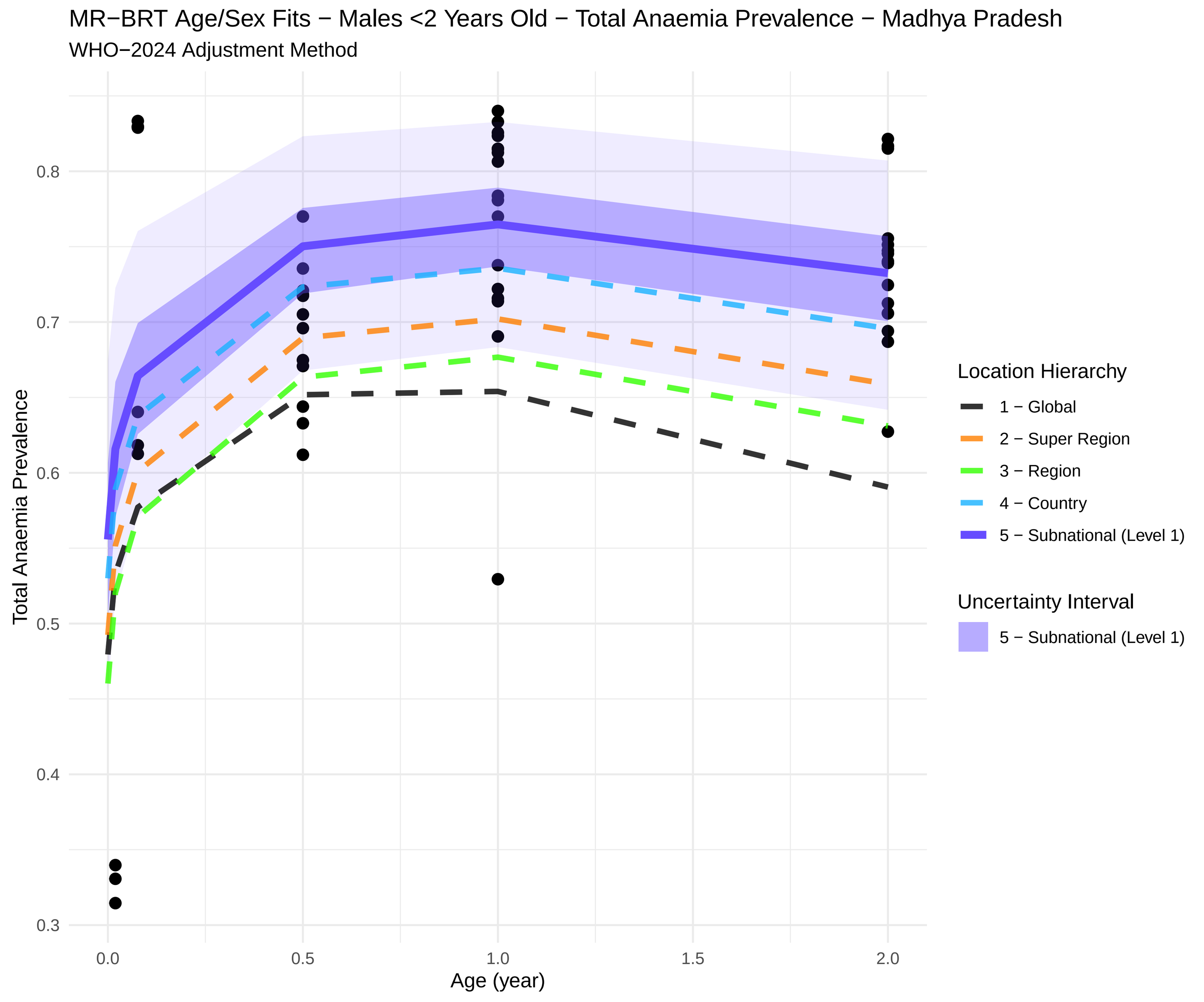
MR−BRT Age/Sex Fits − Males <2 Years Old − Total Anaemia Prevalence − Madhya Pradesh
WHO−2024 Adjustment Method
Total Anaemia Prevalence
Age (year)
Location Hierarchy
1 − Global
2 − Super Region
3 − Region
4 − Country
5 − Subnational (Level 1)
Uncertainty Interval
5 − Subnational (Level 1)

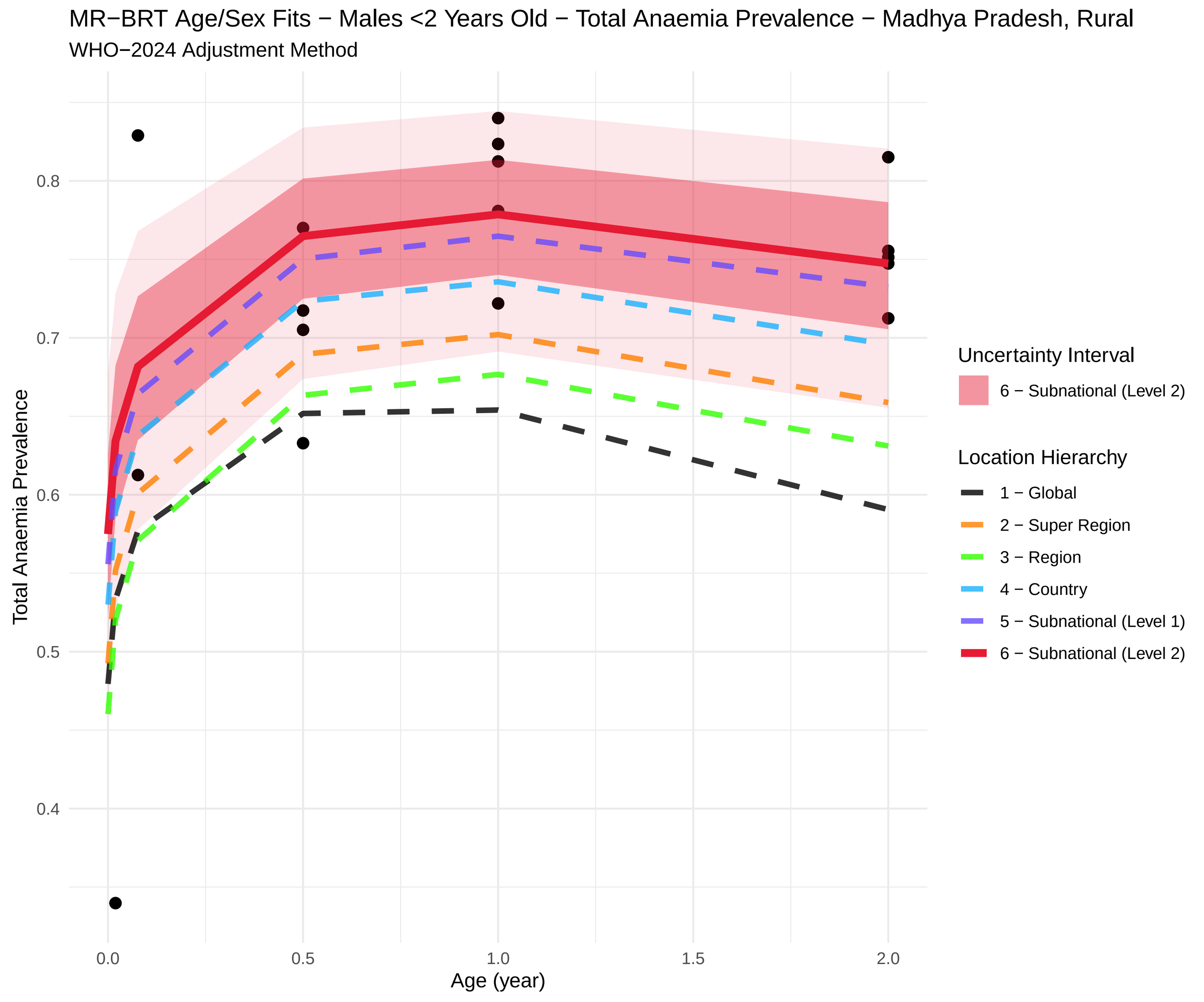
MR−BRT Age/Sex Fits − Males <2 Years Old − Total Anaemia Prevalence − Madhya Pradesh, Rural
WHO−2024 Adjustment Method
Total Anaemia Prevalence
Age (year)
Uncertainty Interval
6 − Subnational (Level 2)
Location Hierarchy
1 − Global
2 − Super Region
3 − Region
4 − Country
5 − Subnational (Level 1)
6 − Subnational (Level 2)

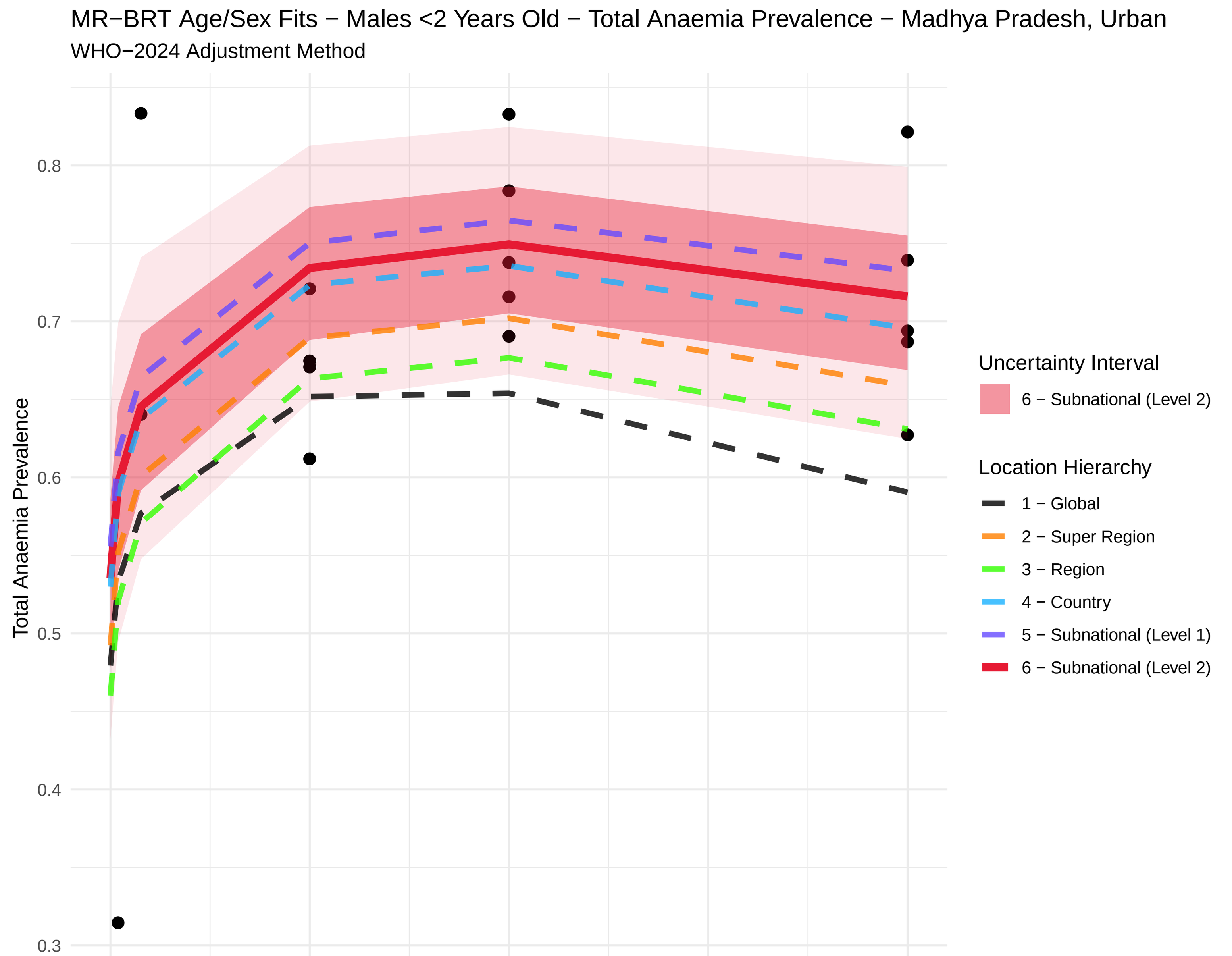
MR−BRT Age/Sex Fits − Males <2 Years Old − Total Anaemia Prevalence − Madhya Pradesh, Urban
WHO−2024 Adjustment Method
Total Anaemia Prevalence
Age (year)
Uncertainty Interval
6 − Subnational (Level 2)
Location Hierarchy
1 − Global
2 − Super Region
3 − Region
4 − Country
5 − Subnational (Level 1)
6 − Subnational (Level 2)

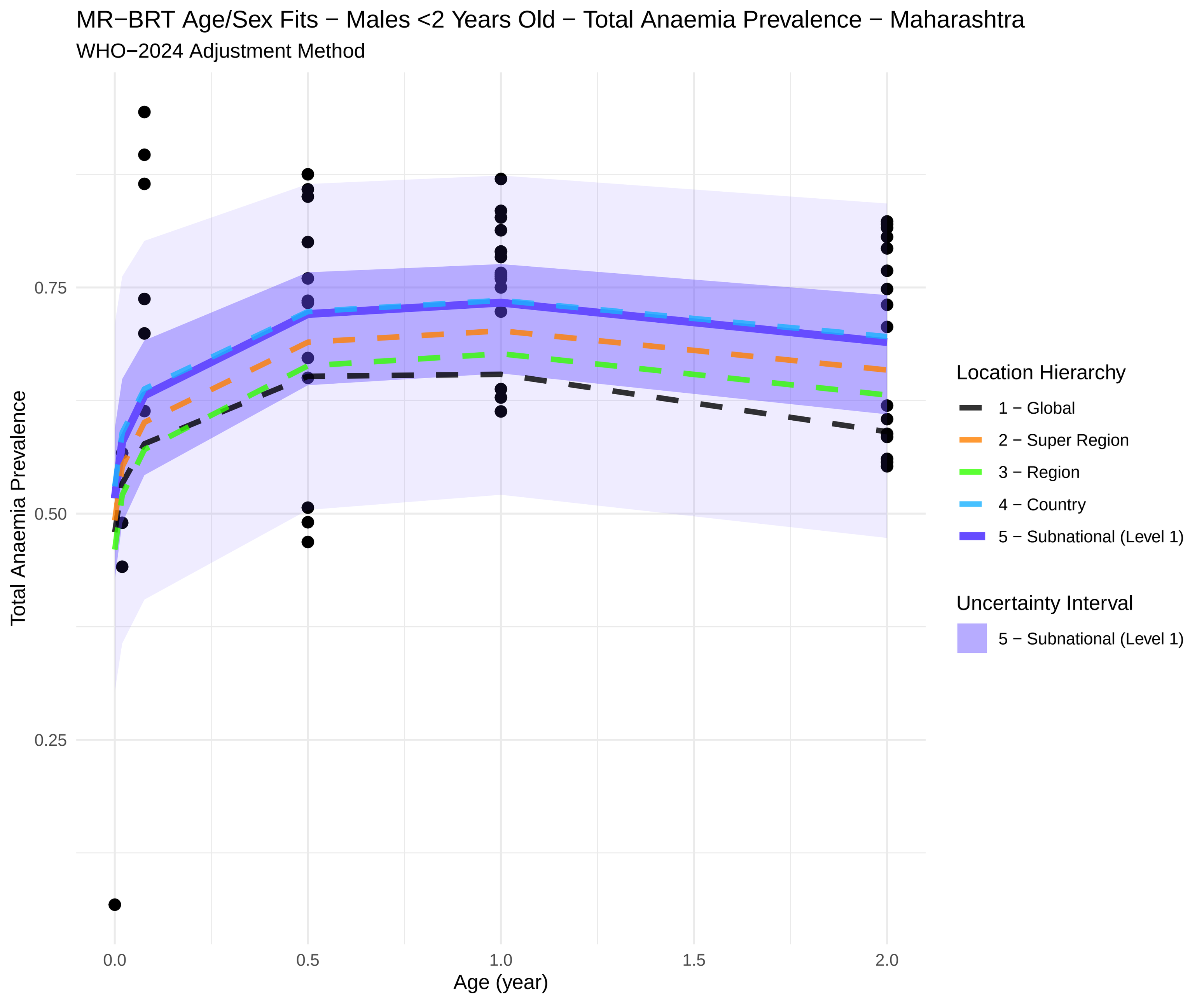
MR−BRT Age/Sex Fits − Males <2 Years Old − Total Anaemia Prevalence − Maharashtra
WHO−2024 Adjustment Method
Total Anaemia Prevalence
Age (year)
Location Hierarchy
1 − Global
2 − Super Region
3 − Region
4 − Country
5 − Subnational (Level 1)
Uncertainty Interval
5 − Subnational (Level 1)

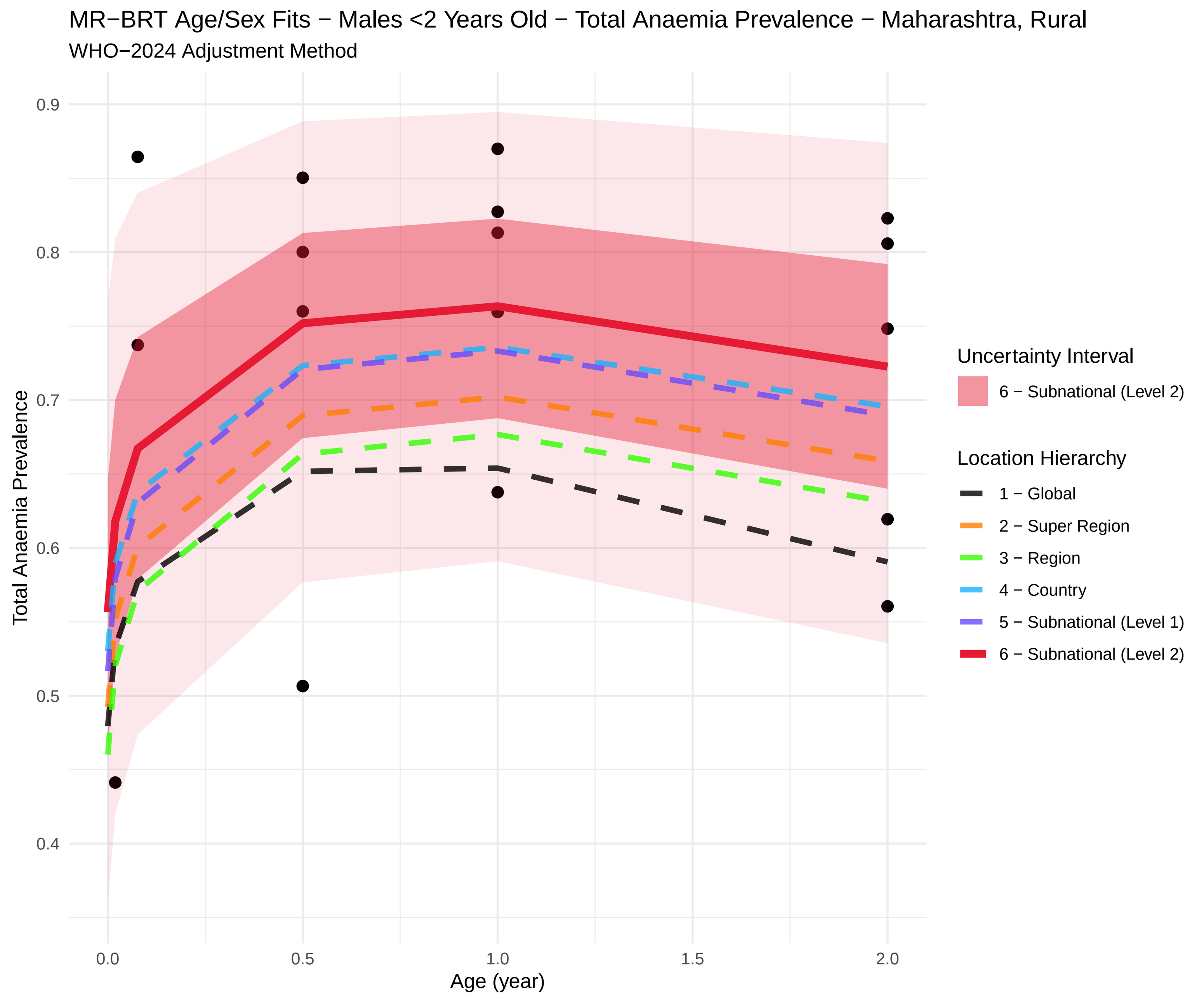
MR−BRT Age/Sex Fits − Males <2 Years Old − Total Anaemia Prevalence − Maharashtra, Rural
WHO−2024 Adjustment Method
Total Anaemia Prevalence
Age (year)
Uncertainty Interval
6 − Subnational (Level 2)
Location Hierarchy
1 − Global
2 − Super Region
3 − Region
4 − Country
5 − Subnational (Level 1)
6 − Subnational (Level 2)

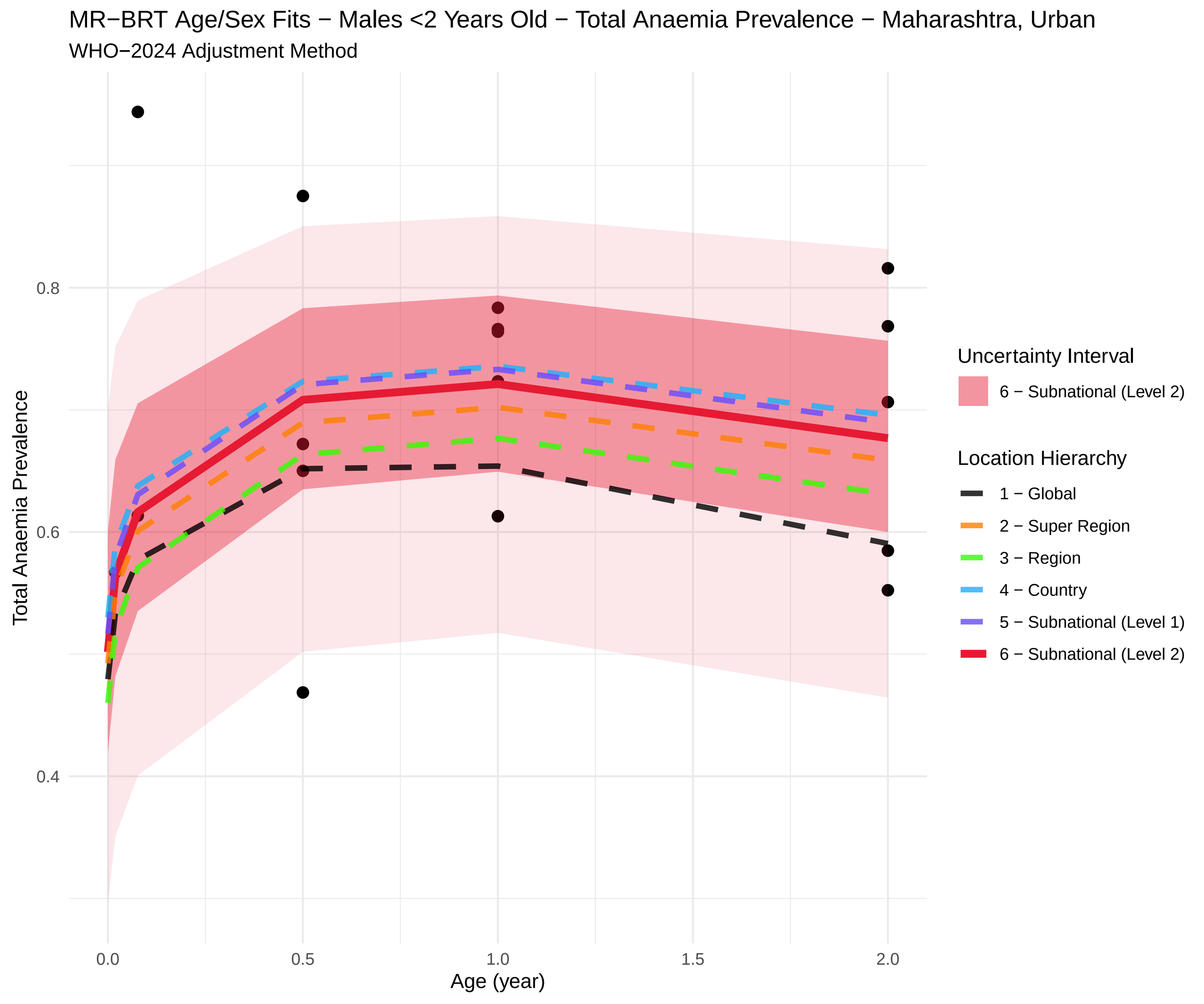
MR−BRT Age/Sex Fits − Males <2 Years Old − Total Anaemia Prevalence − Maharashtra, Urban
WHO−2024 Adjustment Method
Total Anaemia Prevalence
Age (year)
Uncertainty Interval
6 − Subnational (Level 2)
Location Hierarchy
1 − Global
2 − Super Region
3 − Region
4 − Country
5 − Subnational (Level 1)
6 − Subnational (Level 2)

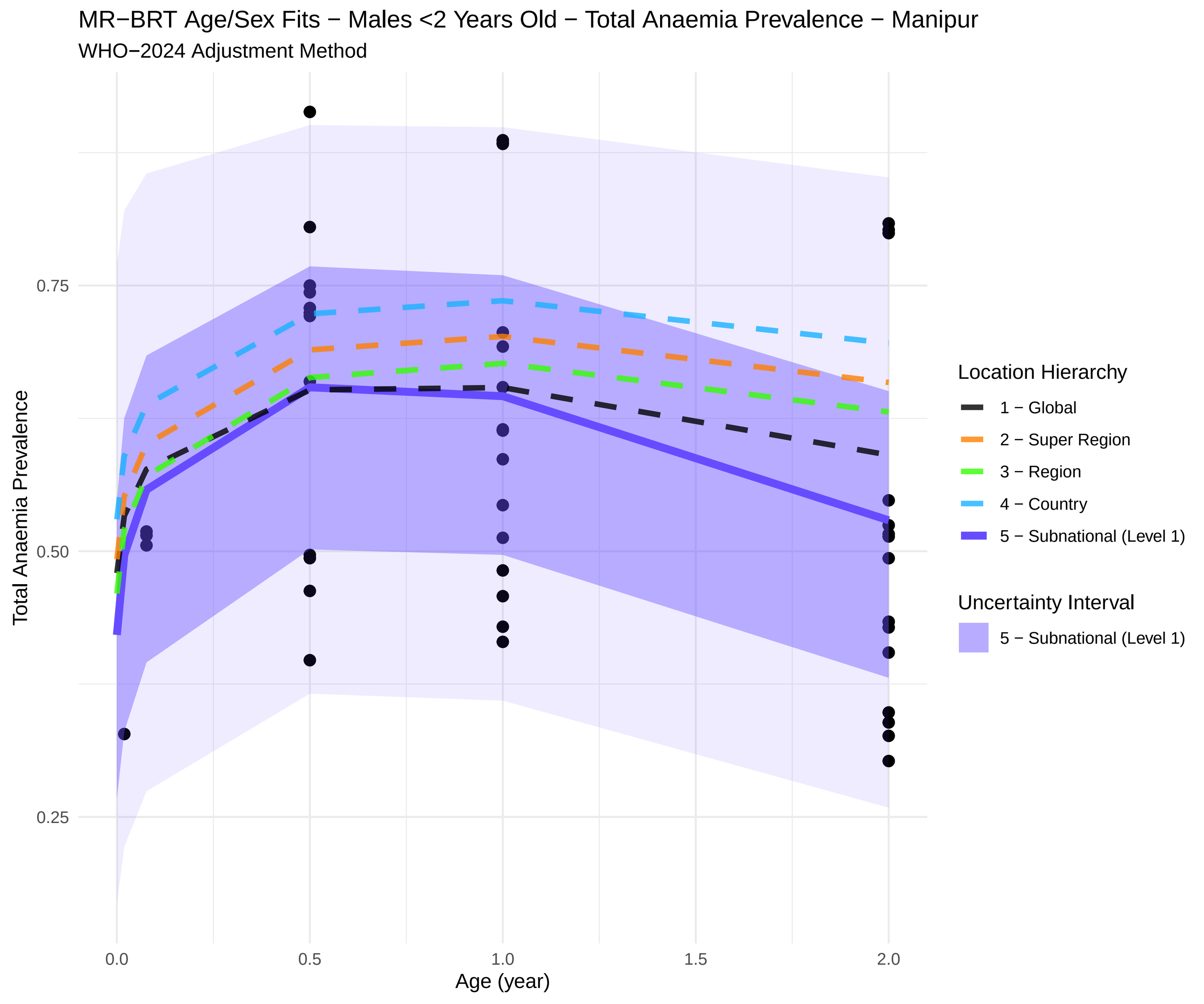
MR−BRT Age/Sex Fits − Males <2 Years Old − Total Anaemia Prevalence − Manipur
WHO−2024 Adjustment Method
Total Anaemia Prevalence
Age (year)
Location Hierarchy
1 − Global
2 − Super Region
3 − Region
4 − Country
5 − Subnational (Level 1)
Uncertainty Interval
5 − Subnational (Level 1)

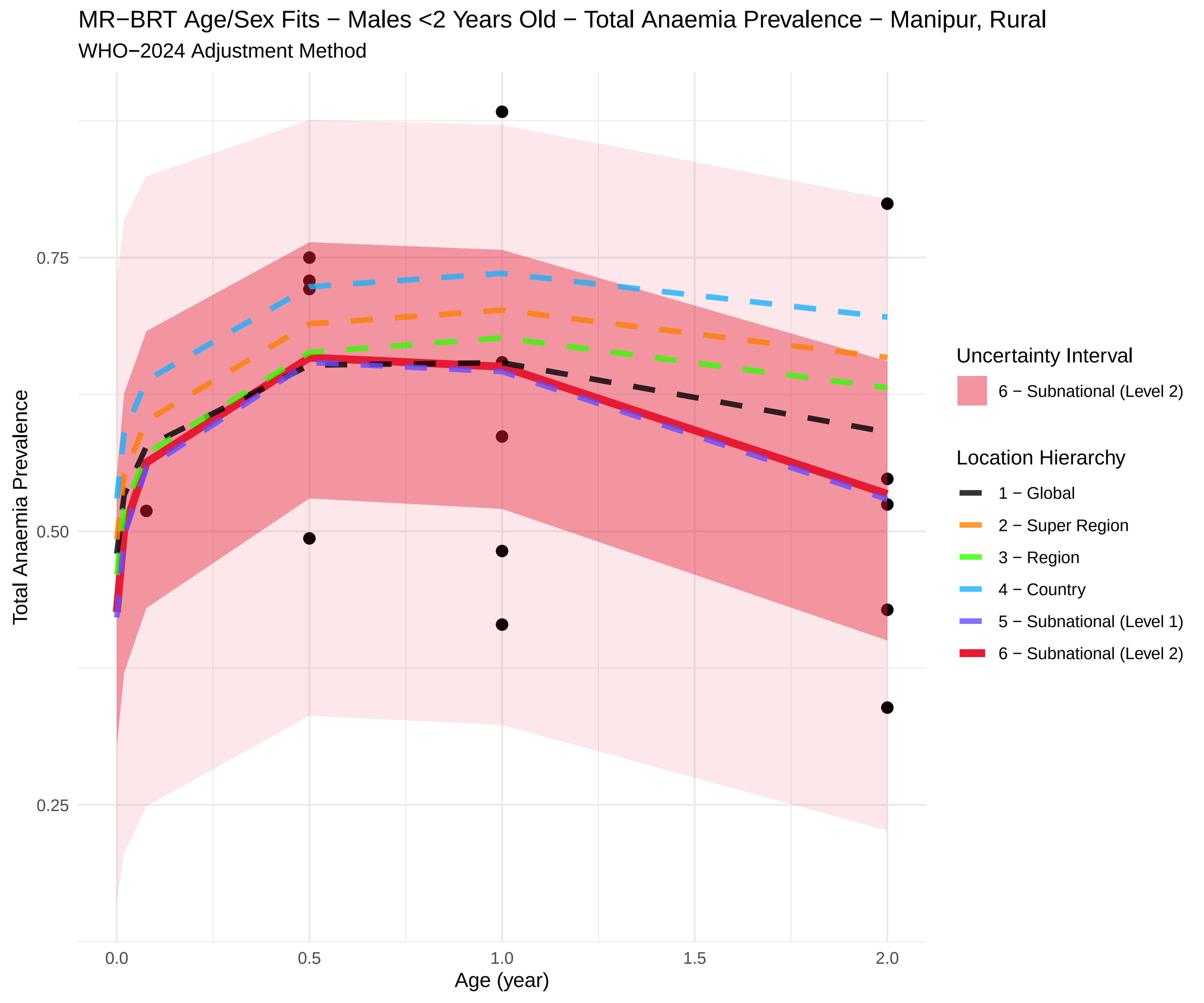
MR−BRT Age/Sex Fits − Males <2 Years Old − Total Anaemia Prevalence − Manipur, Rural
WHO−2024 Adjustment Method
Total Anaemia Prevalence
Age (year)
Uncertainty Interval
6 − Subnational (Level 2)
Location Hierarchy
1 − Global
2 − Super Region
3 − Region
4 − Country
5 − Subnational (Level 1)
6 − Subnational (Level 2)

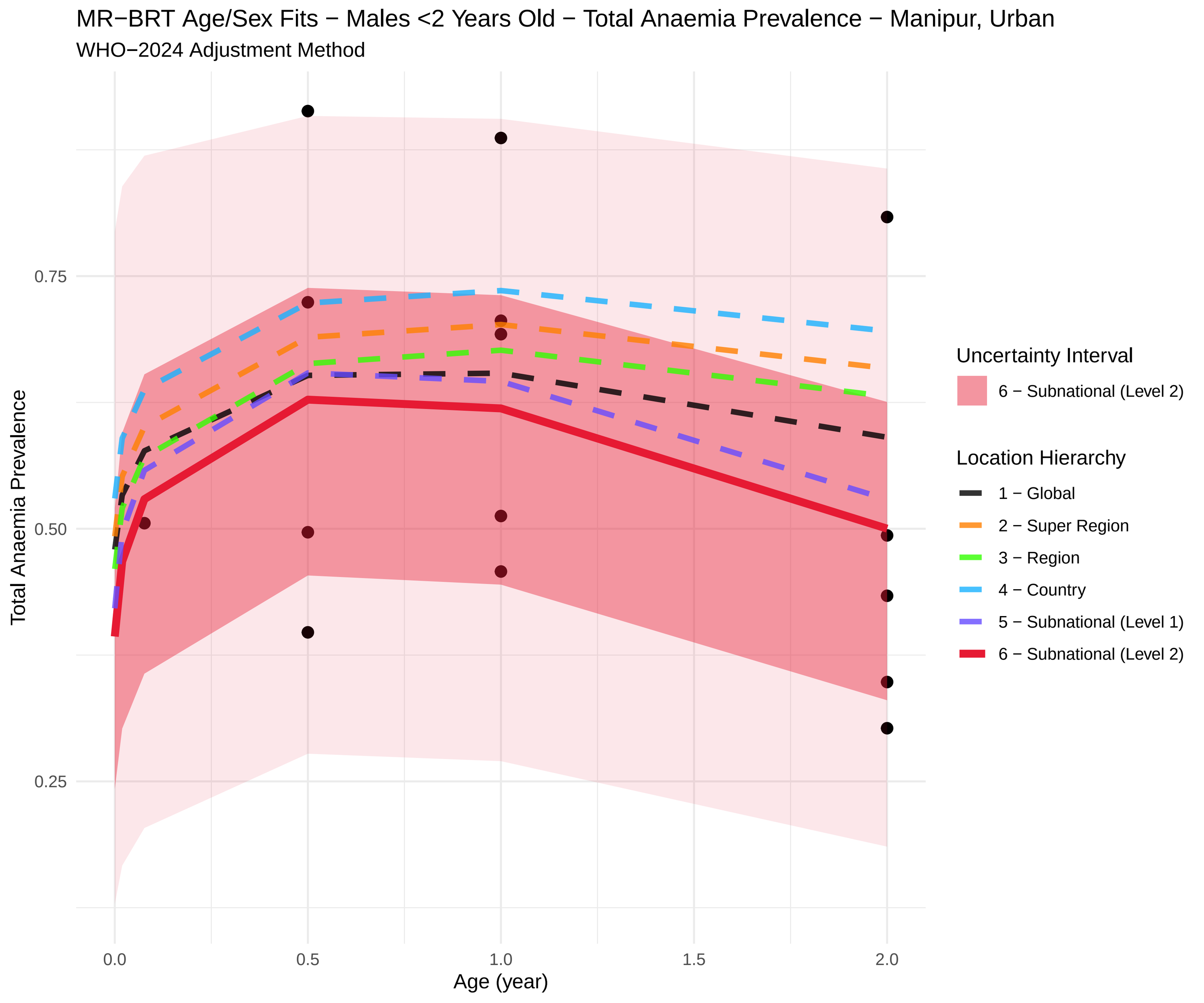
MR−BRT Age/Sex Fits − Males <2 Years Old − Total Anaemia Prevalence − Manipur, Urban
WHO−2024 Adjustment Method
Total Anaemia Prevalence
Age (year)
Uncertainty Interval
6 − Subnational (Level 2)
Location Hierarchy
1 − Global
2 − Super Region
3 − Region
4 − Country
5 − Subnational (Level 1)
6 − Subnational (Level 2)

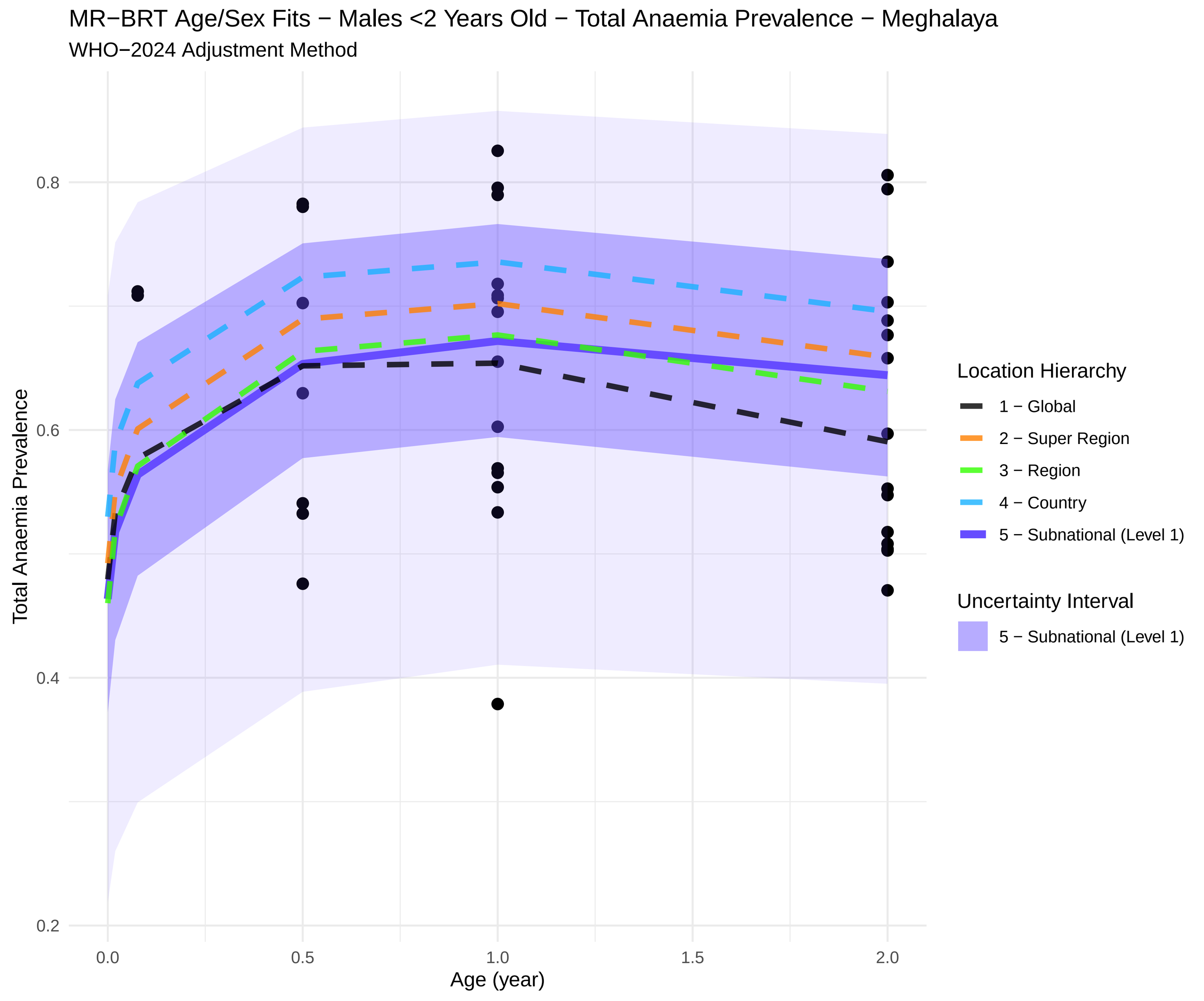
MR−BRT Age/Sex Fits − Males <2 Years Old − Total Anaemia Prevalence − Meghalaya
WHO−2024 Adjustment Method
Total Anaemia Prevalence
Age (year)
Location Hierarchy
1 − Global
2 − Super Region
3 − Region
4 − Country
5 − Subnational (Level 1)
Uncertainty Interval
5 − Subnational (Level 1)

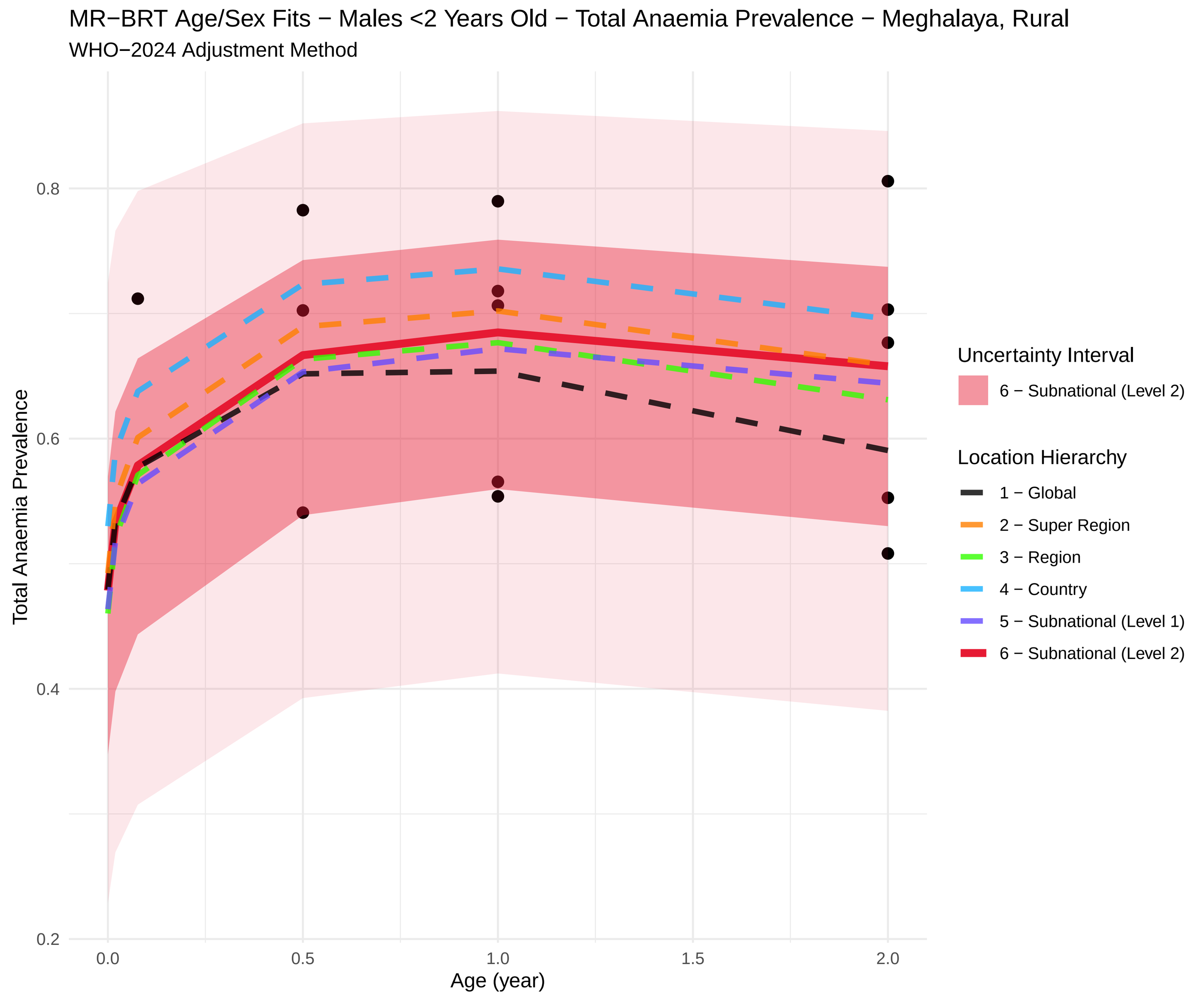
MR−BRT Age/Sex Fits − Males <2 Years Old − Total Anaemia Prevalence − Meghalaya, Rural
WHO−2024 Adjustment Method
Total Anaemia Prevalence
Age (year)
Uncertainty Interval
6 − Subnational (Level 2)
Location Hierarchy
1 − Global
2 − Super Region
3 − Region
4 − Country
5 − Subnational (Level 1)
6 − Subnational (Level 2)

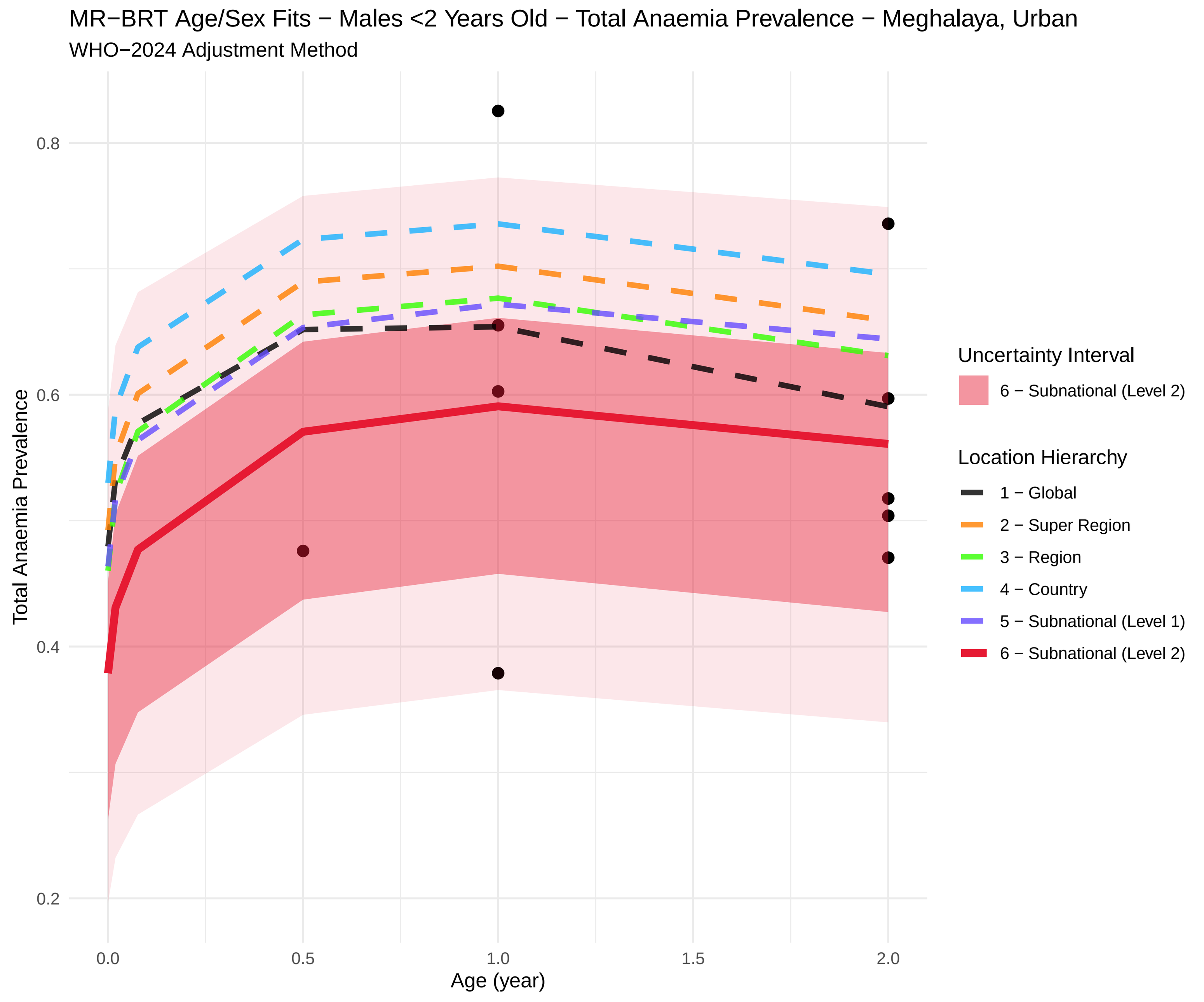
MR−BRT Age/Sex Fits − Males <2 Years Old − Total Anaemia Prevalence − Meghalaya, Urban
WHO−2024 Adjustment Method
Total Anaemia Prevalence
Age (year)
Uncertainty Interval
6 − Subnational (Level 2)
Location Hierarchy
1 − Global
2 − Super Region
3 − Region
4 − Country
5 − Subnational (Level 1)
6 − Subnational (Level 2)

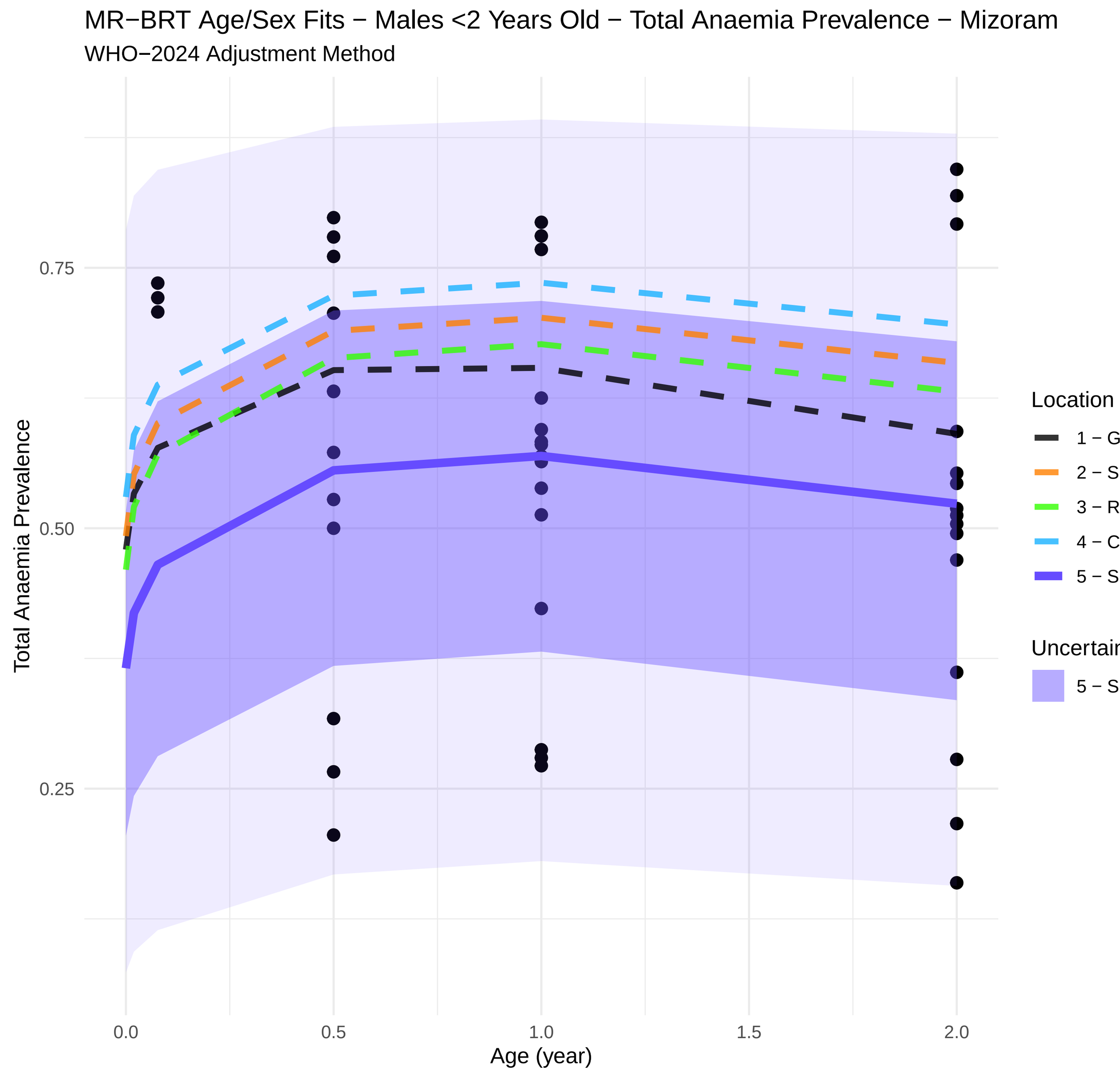
MR−BRT Age/Sex Fits − Males <2 Years Old − Total Anaemia Prevalence − Mizoram
WHO−2024 Adjustment Method
Total Anaemia Prevalence
Age (year)
Location Hierarchy
1 − Global
2 − Super Region
3 − Region
4 − Country
5 − Subnational (Level 1)
Uncertainty Interval
5 − Subnational (Level 1)

MR−BRT Age/Sex Fits − Males <2 Years Old − Total Anaemia Prevalence − Mizoram, Rural
WHO−2024 Adjustment Method

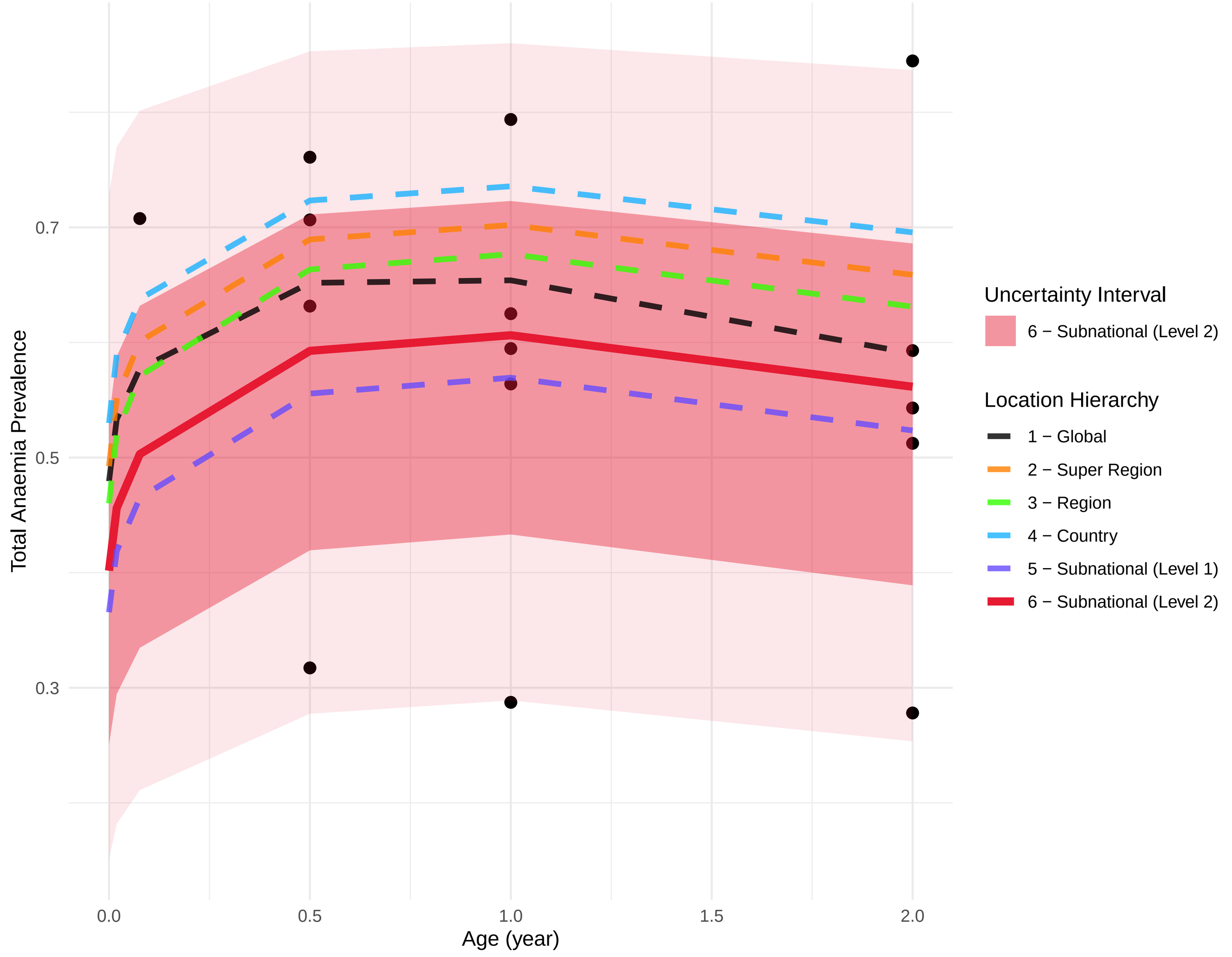

MR−BRT Age/Sex Fits − Males <2 Years Old − Total Anaemia Prevalence − Mizoram, Urban
WHO−2024 Adjustment Method

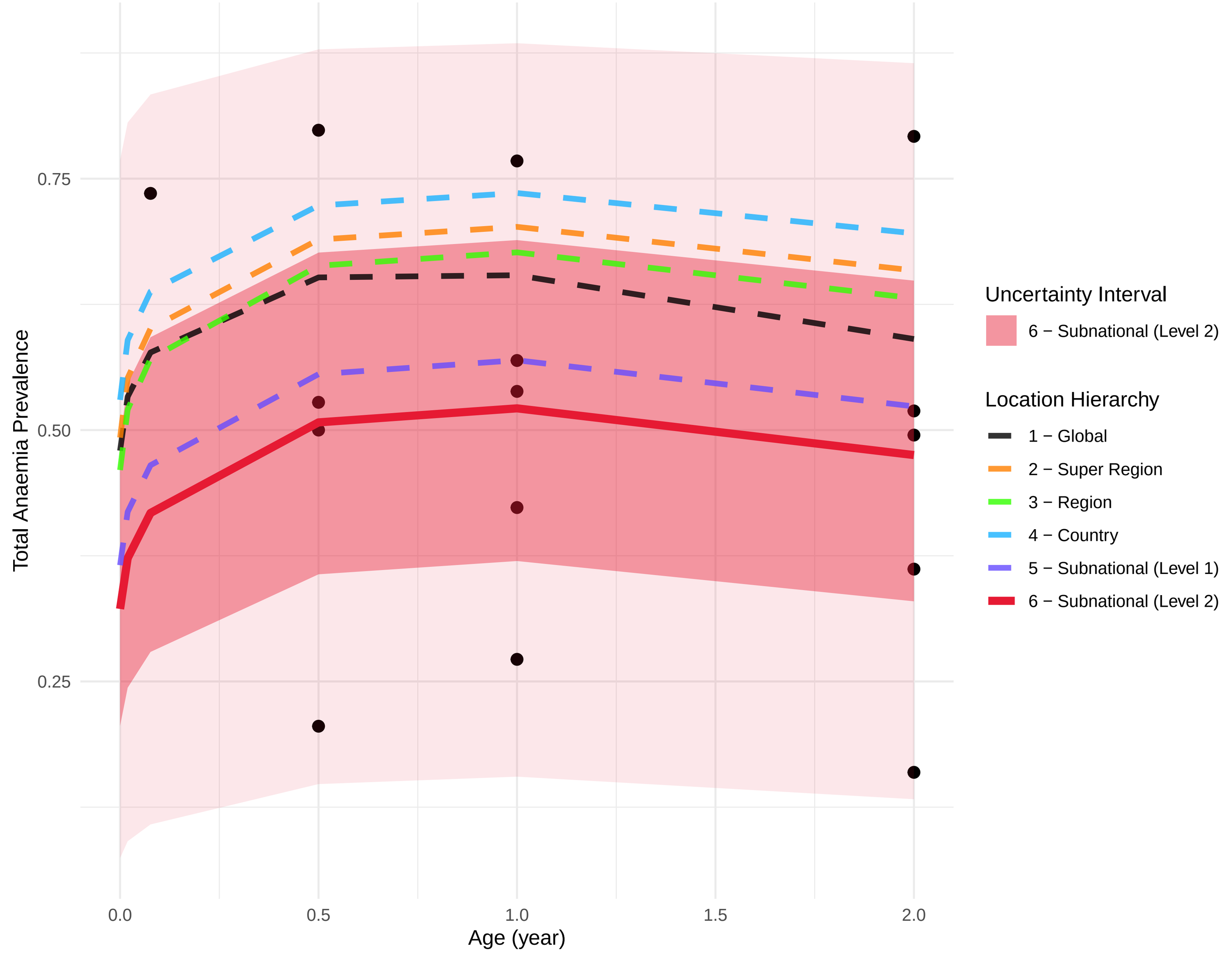

MR−BRT Age/Sex Fits − Males <2 Years Old − Total Anaemia Prevalence − Nagaland
WHO−2024 Adjustment Method

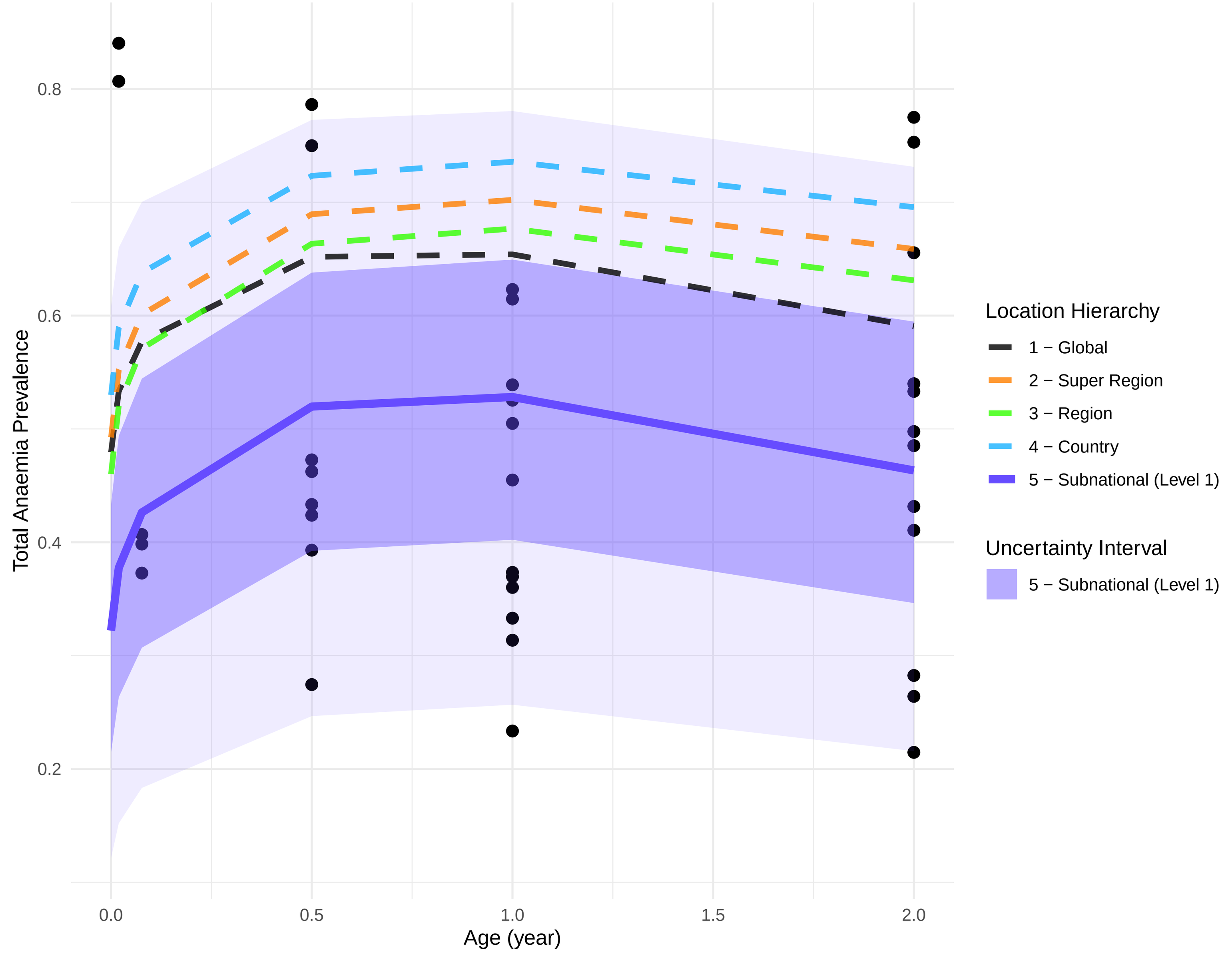

MR−BRT Age/Sex Fits − Males <2 Years Old − Total Anaemia Prevalence − Nagaland, Rural
WHO−2024 Adjustment Method

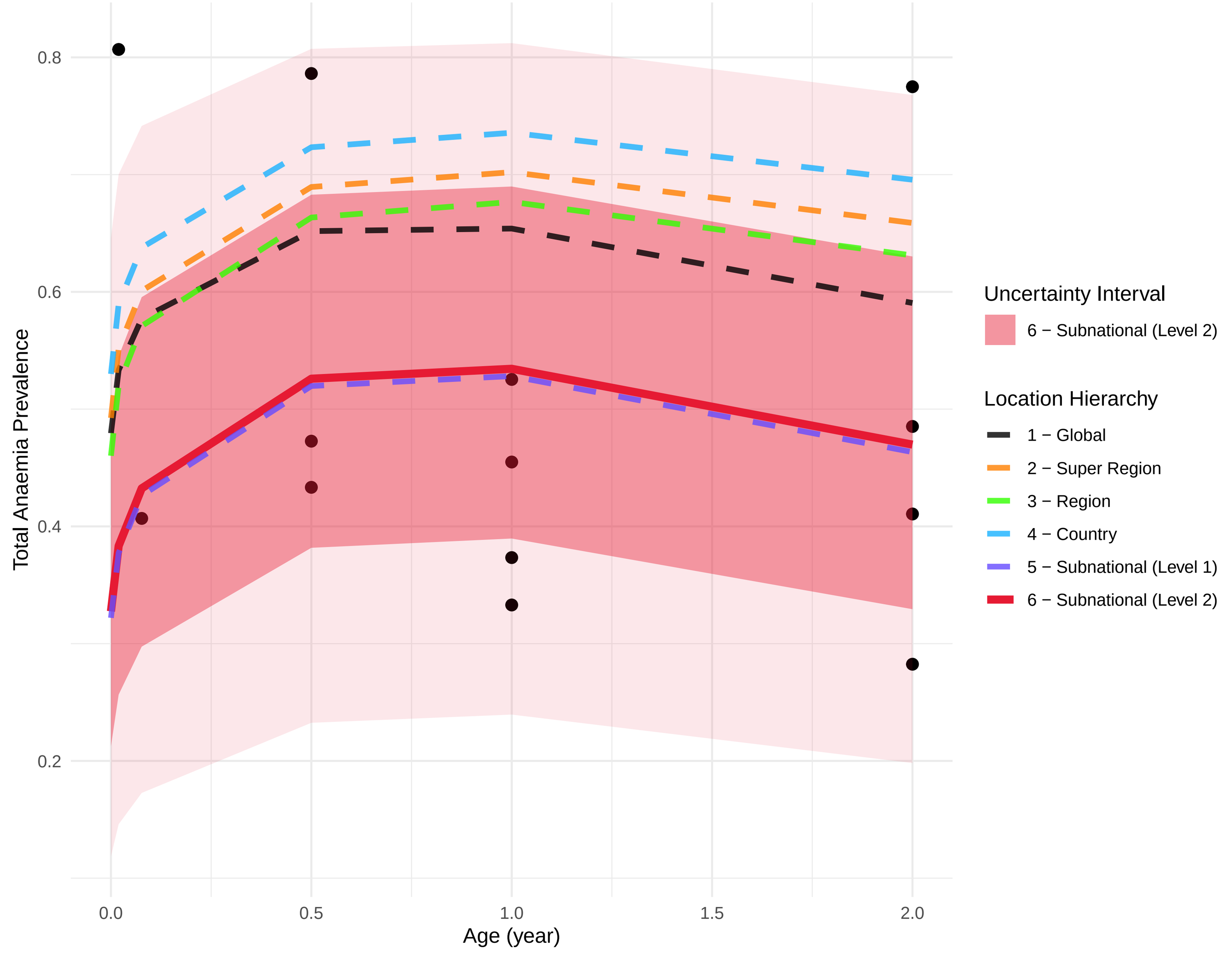

MR−BRT Age/Sex Fits − Males <2 Years Old − Total Anaemia Prevalence − Nagaland, Urban
WHO−2024 Adjustment Method

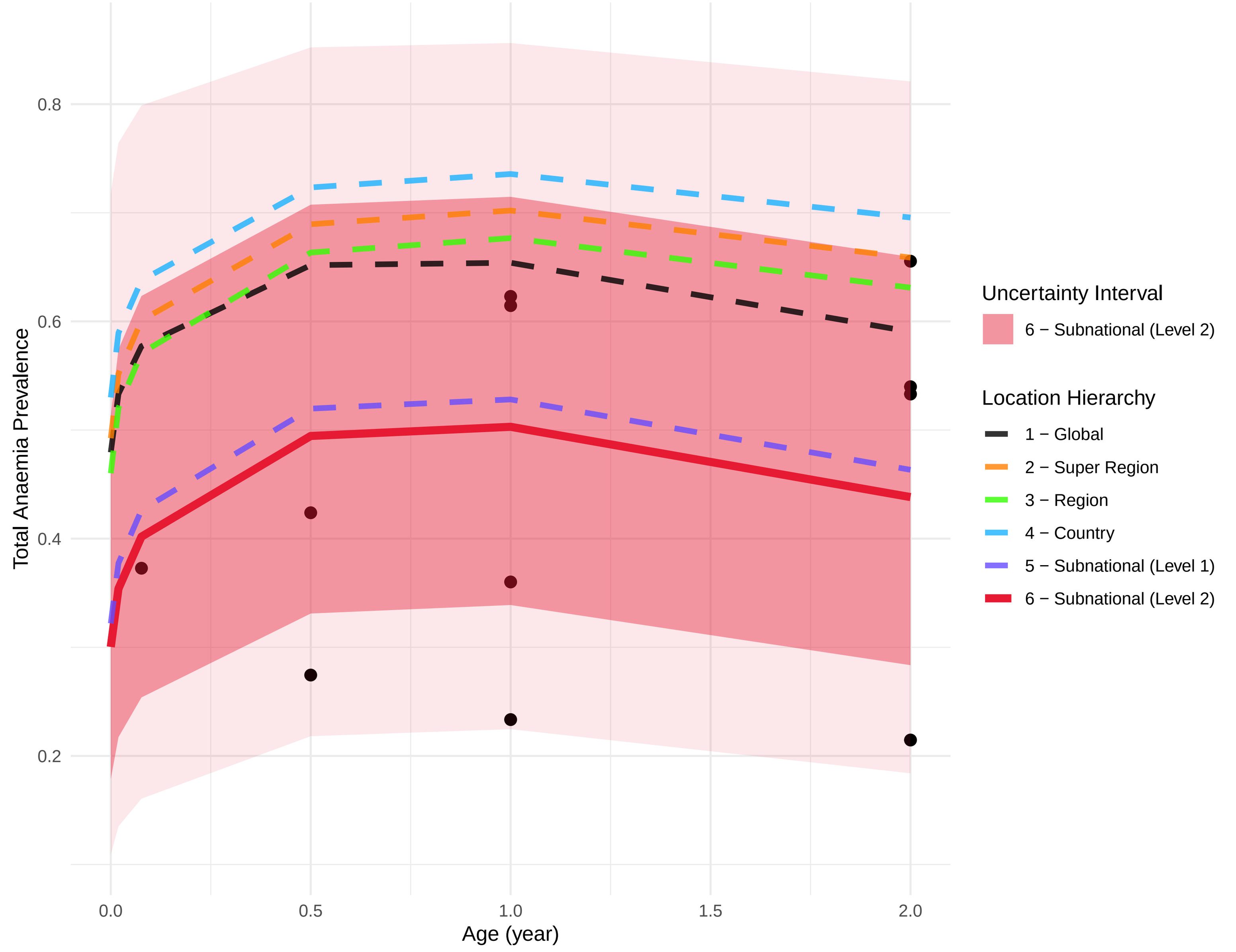

MR−BRT Age/Sex Fits − Males <2 Years Old − Total Anaemia Prevalence − Odisha
WHO−2024 Adjustment Method

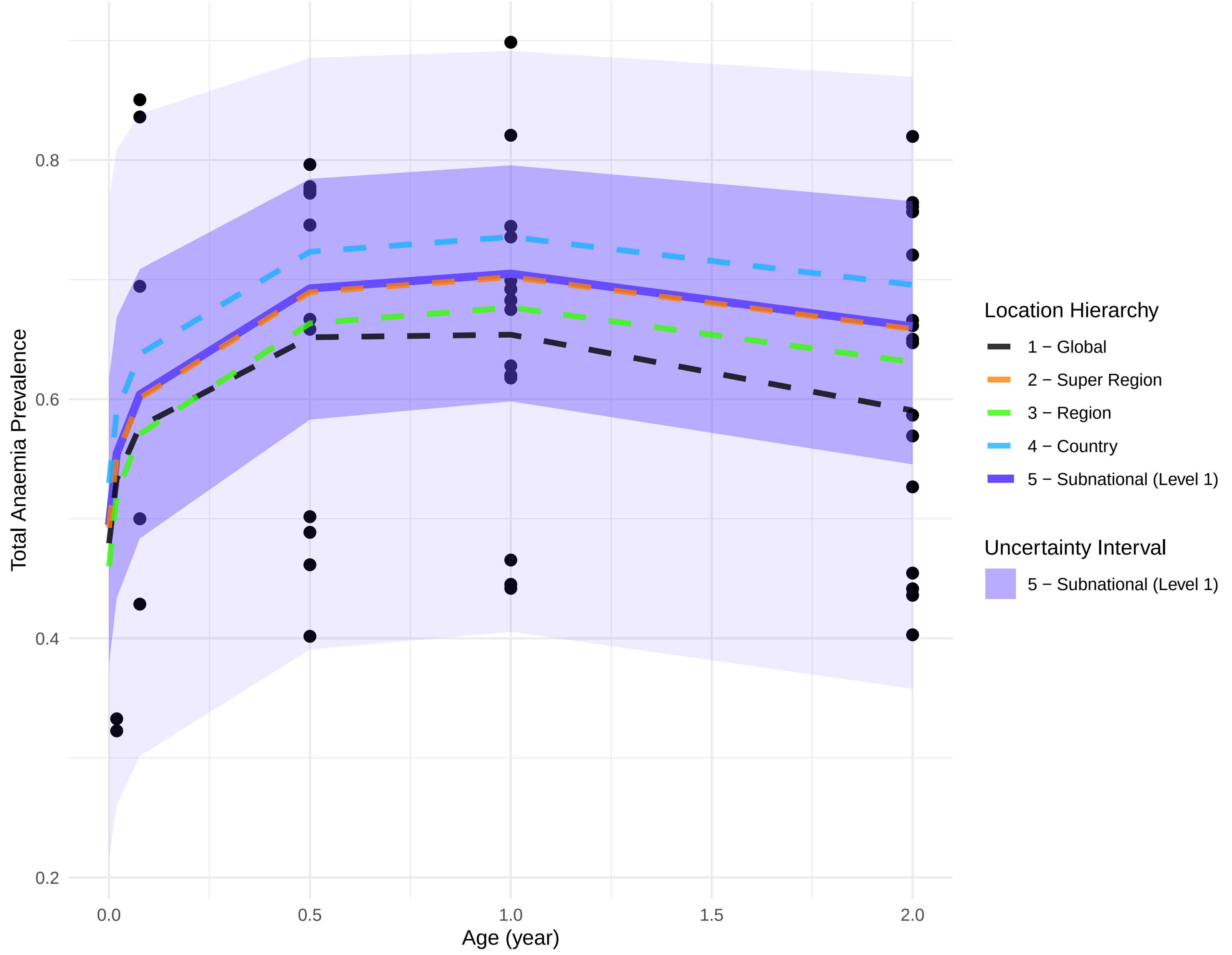

MR−BRT Age/Sex Fits − Males <2 Years Old − Total Anaemia Prevalence − Odisha, Rural
WHO−2024 Adjustment Method

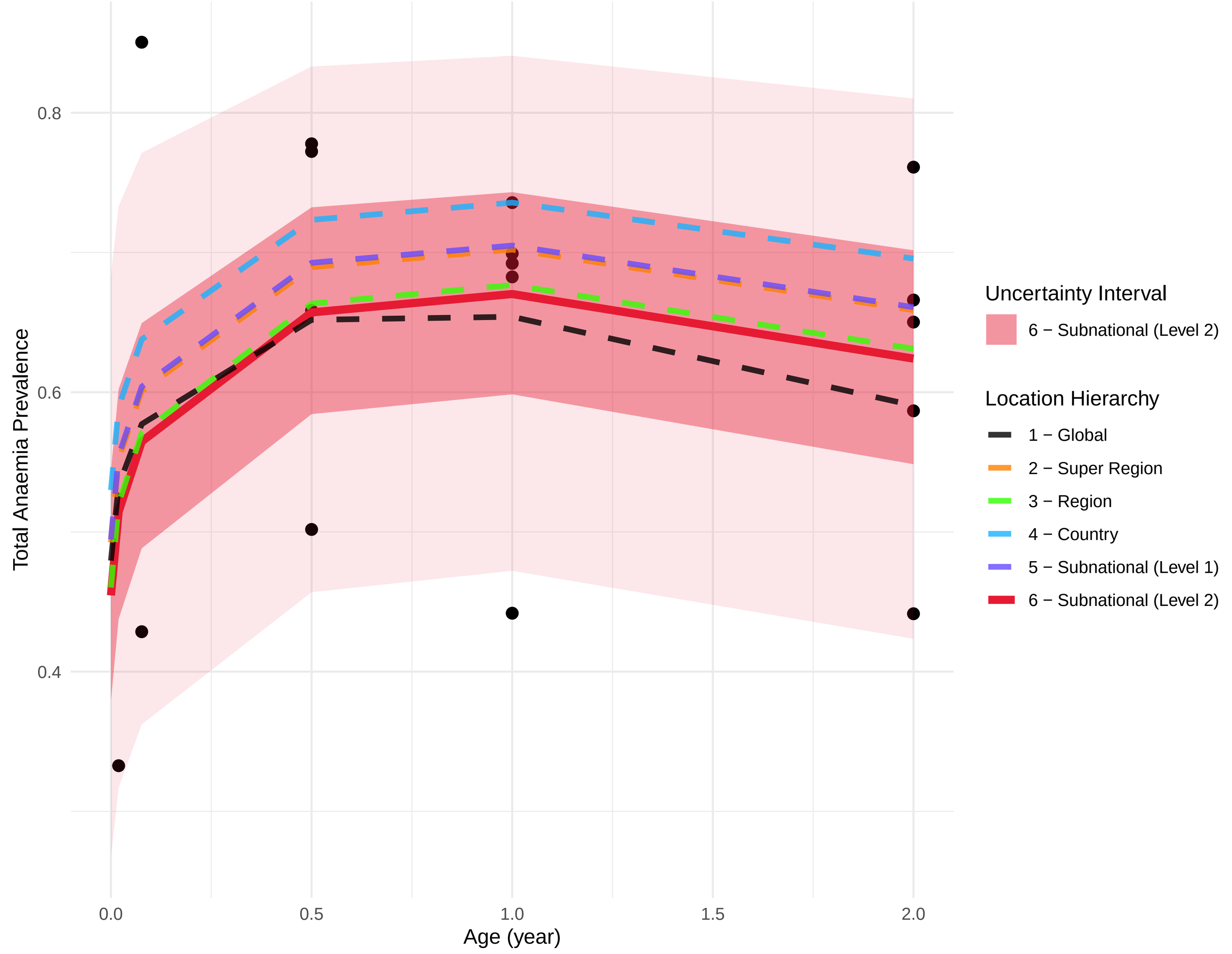

MR−BRT Age/Sex Fits − Males <2 Years Old − Total Anaemia Prevalence − Odisha, Urban
WHO−2024 Adjustment Method

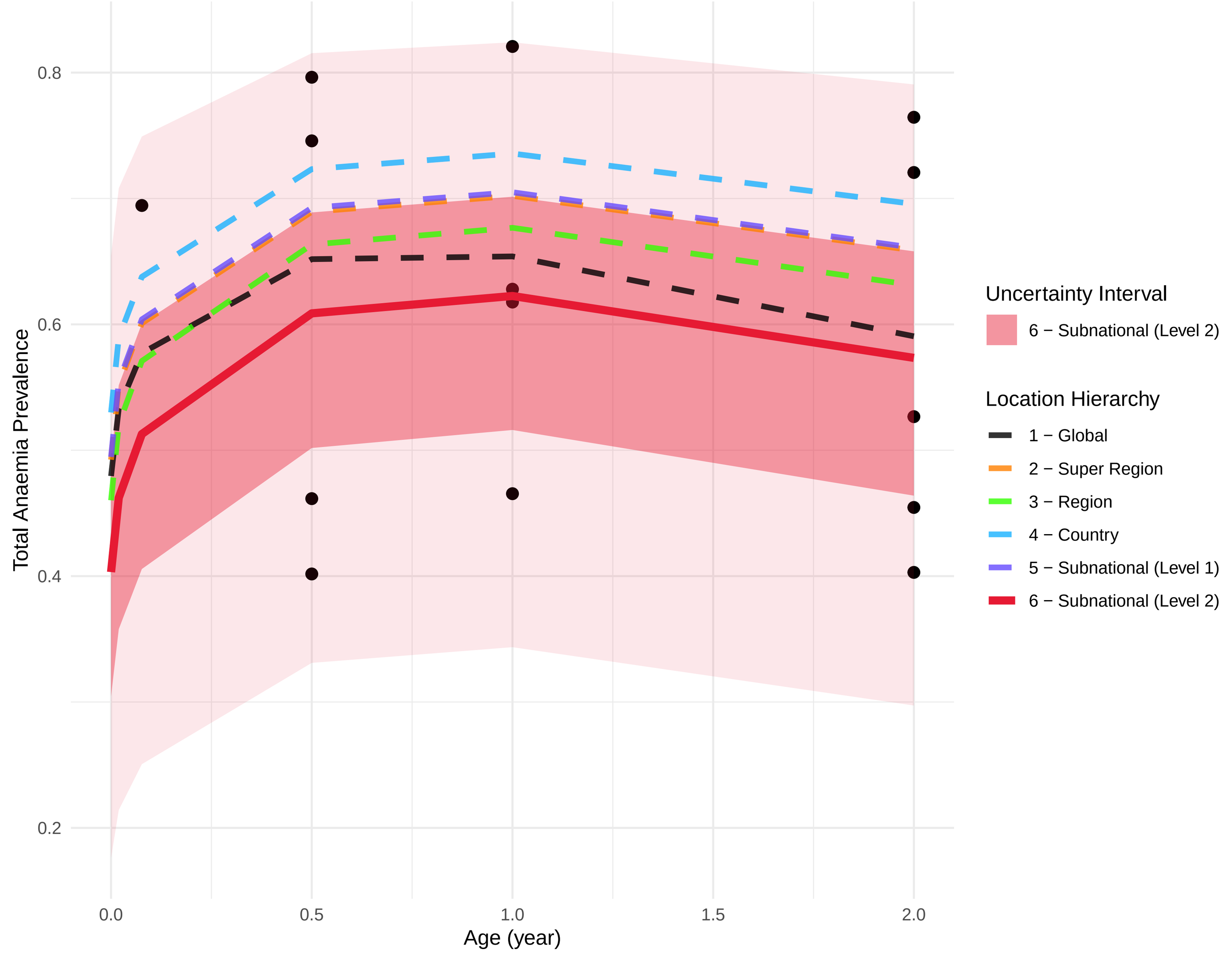

MR−BRT Age/Sex Fits − Males <2 Years Old − Total Anaemia Prevalence − Other Union Territories
WHO−2024 Adjustment Method

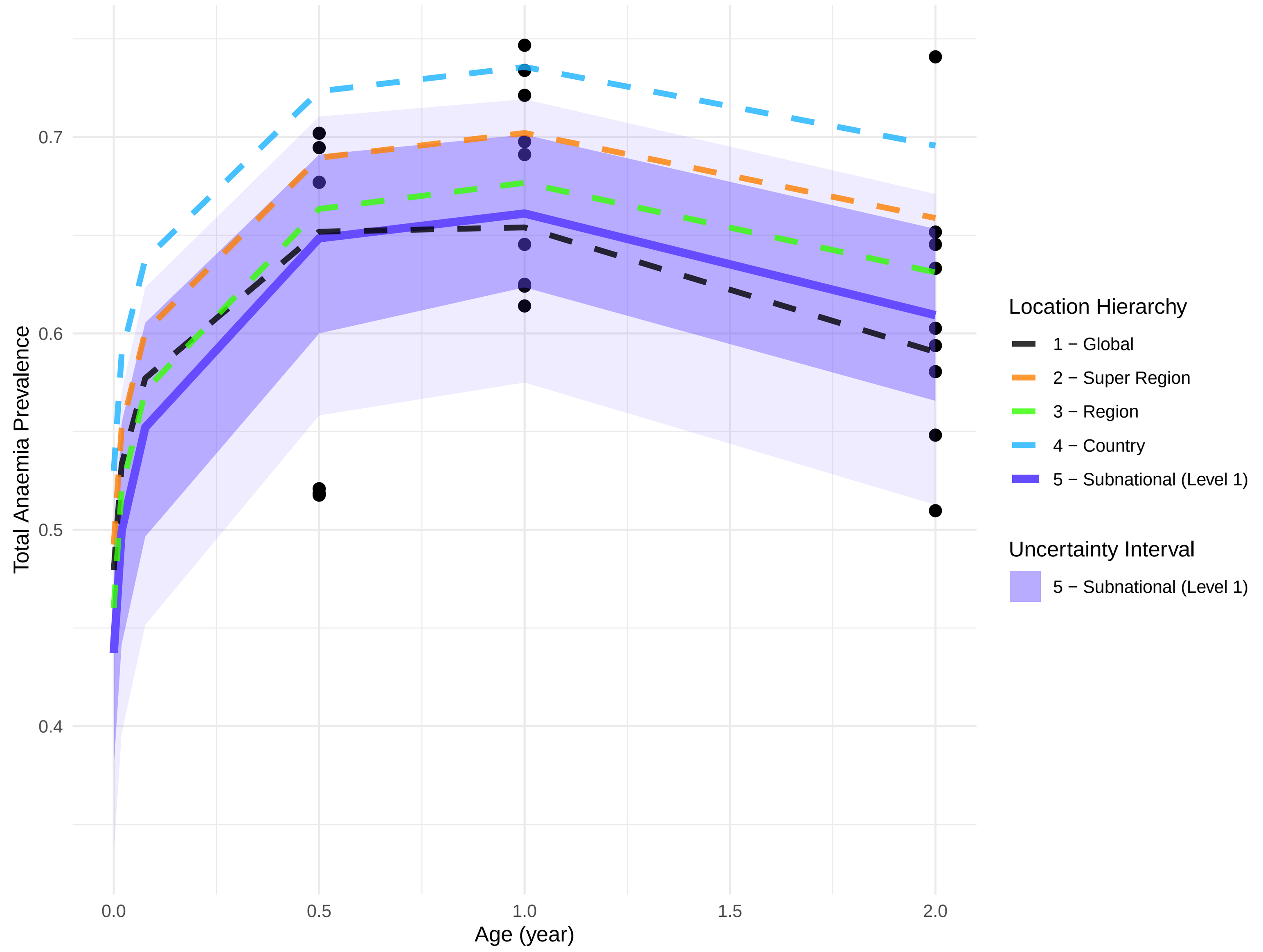

MR−BRT Age/Sex Fits − Males <2 Years Old − Total Anaemia Prevalence − Other Union Territories, Rural
WHO−2024 Adjustment Method

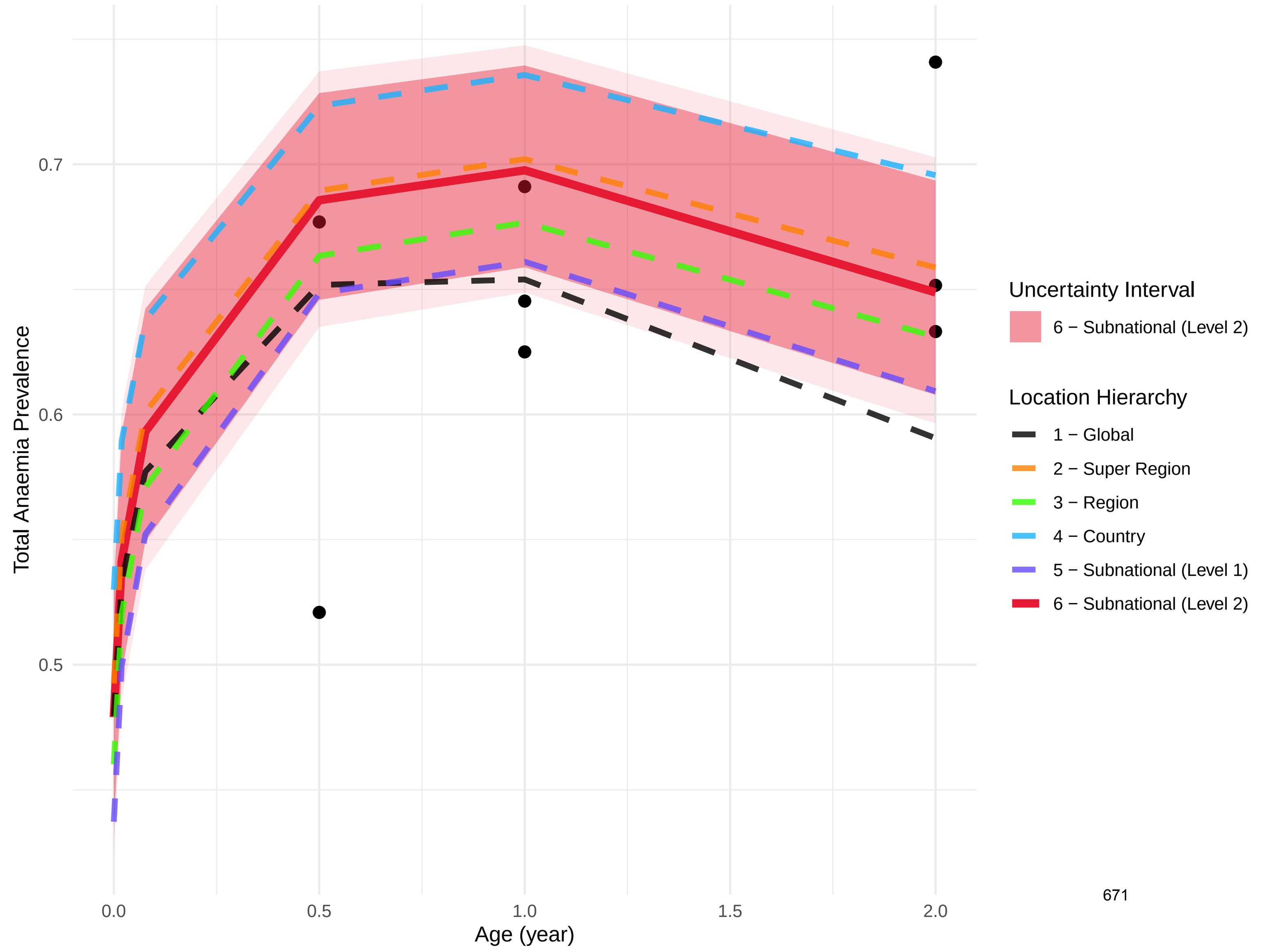

MR−BRT Age/Sex Fits − Males <2 Years Old − Total Anaemia Prevalence − Other Union Territories, Urban
WHO−2024 Adjustment Method

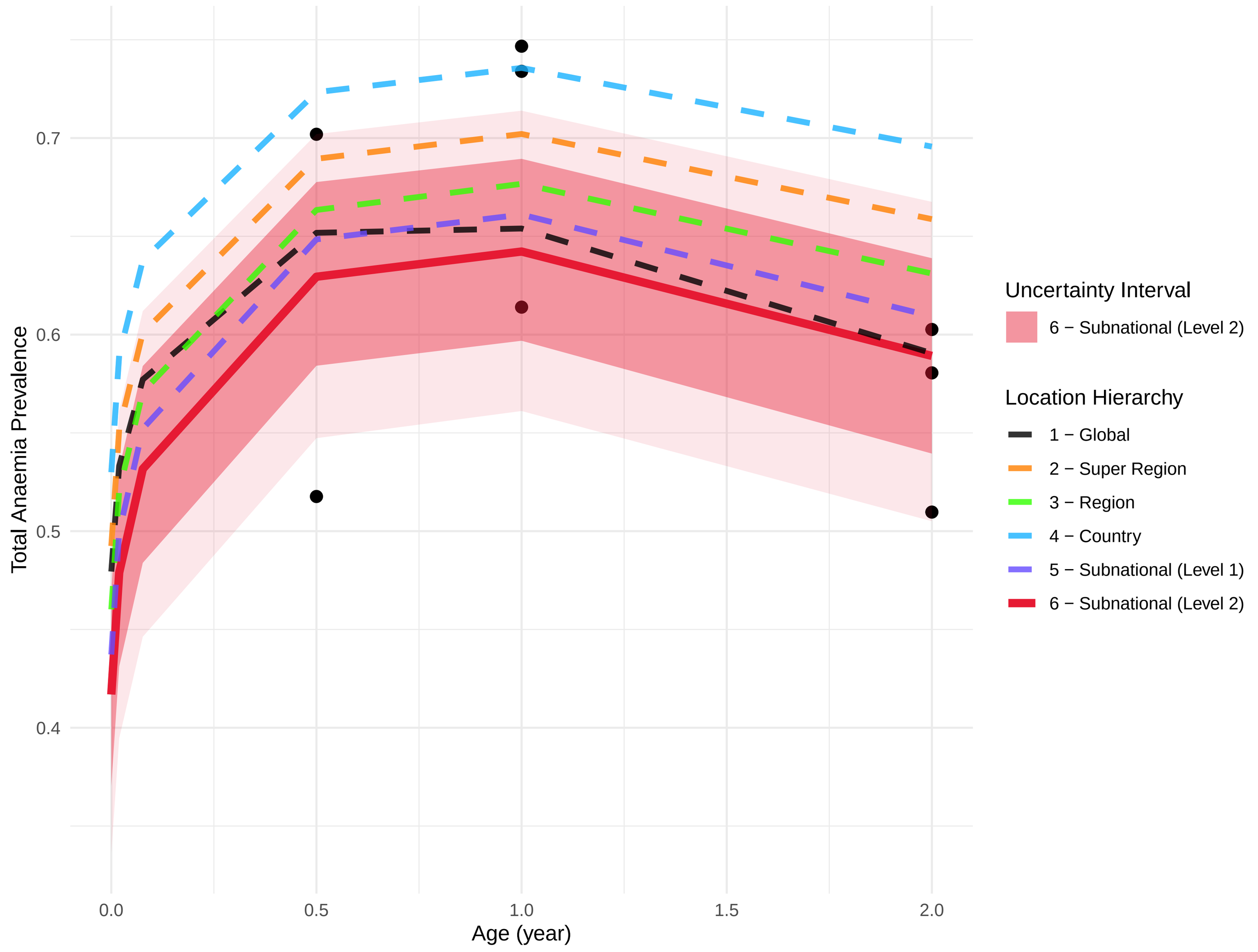

MR−BRT Age/Sex Fits − Males <2 Years Old − Total Anaemia Prevalence − Punjab
WHO−2024 Adjustment Method

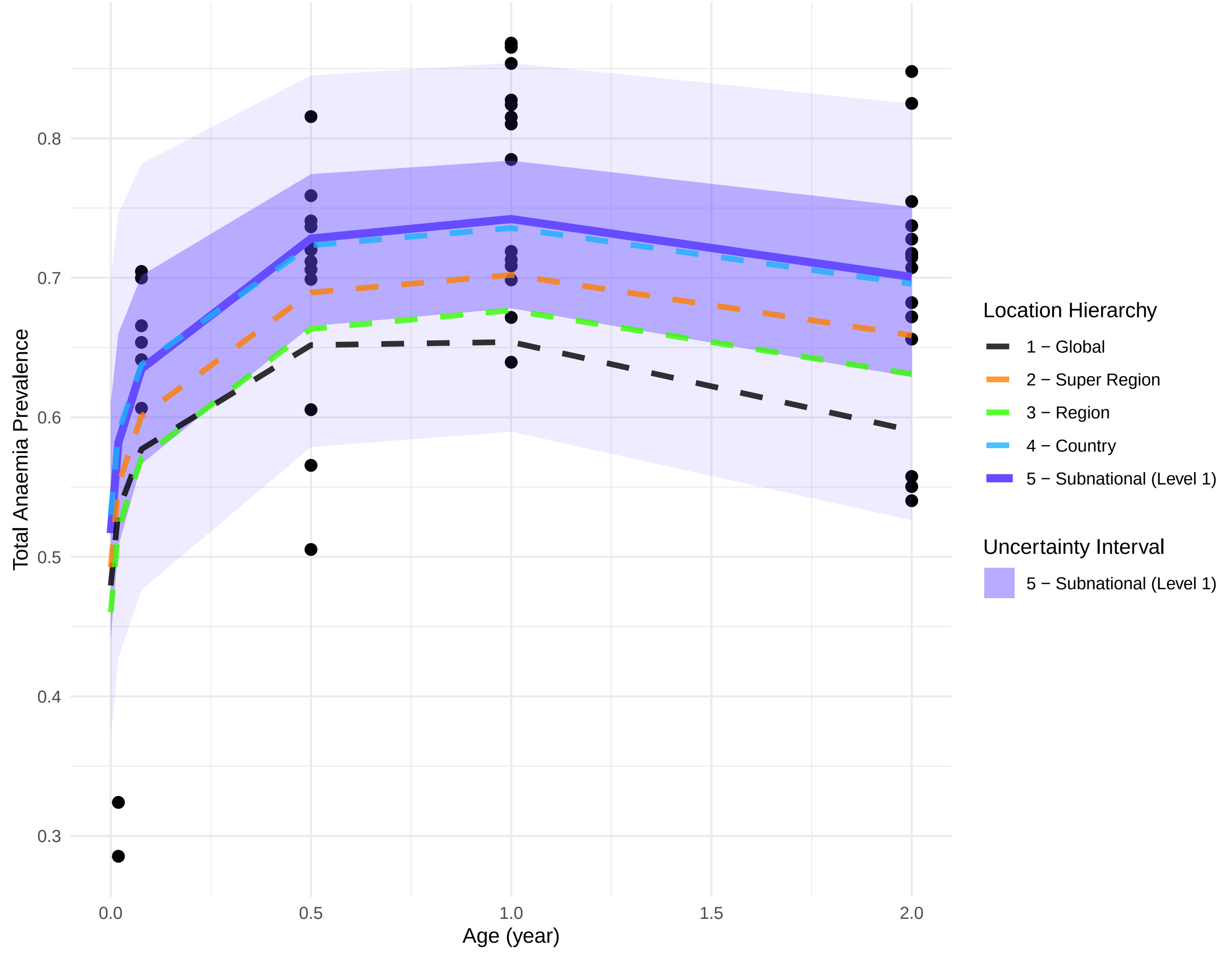

MR−BRT Age/Sex Fits − Males <2 Years Old − Total Anaemia Prevalence − Punjab, Rural
WHO−2024 Adjustment Method

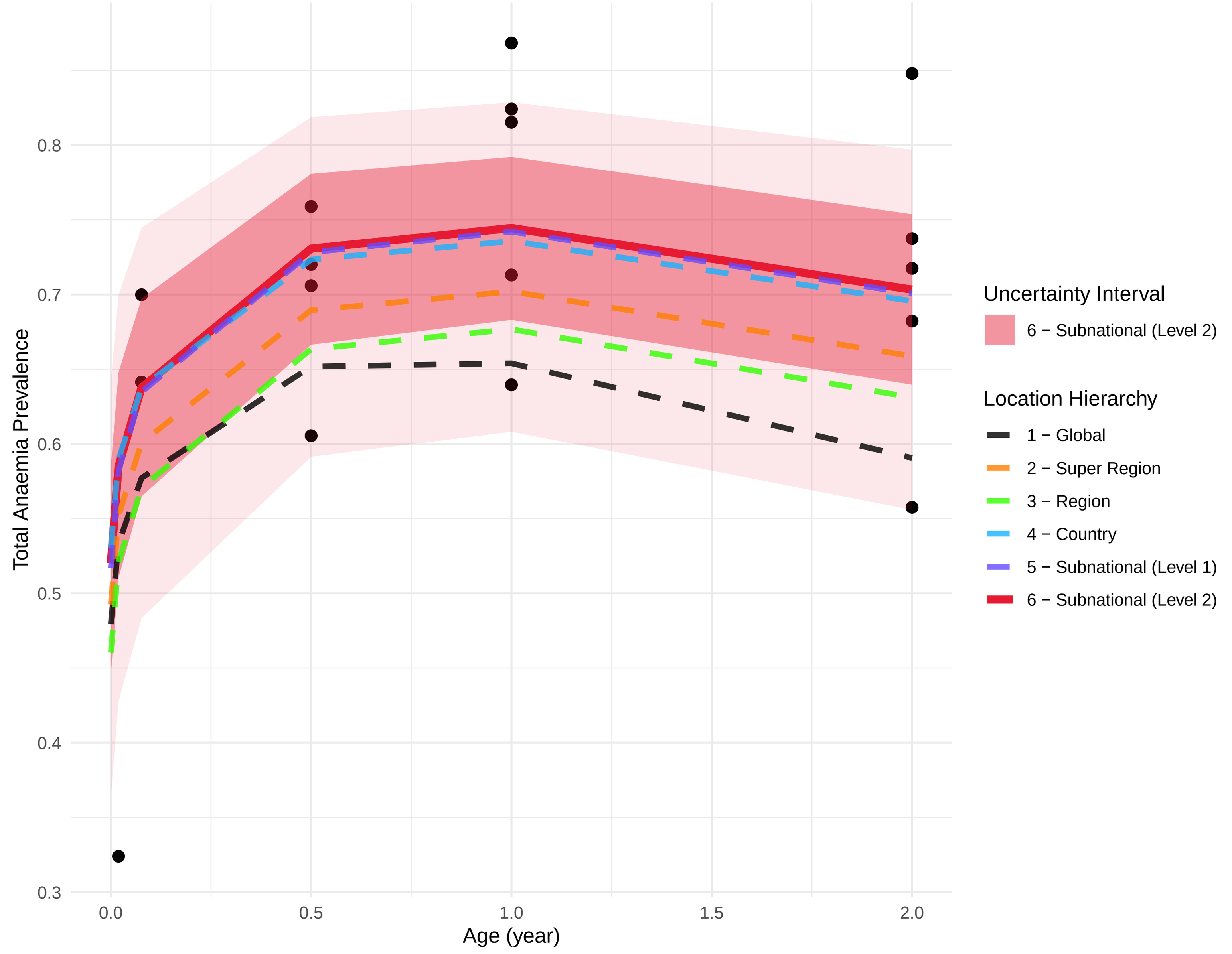

MR−BRT Age/Sex Fits − Males <2 Years Old − Total Anaemia Prevalence − Punjab, Urban
WHO−2024 Adjustment Method

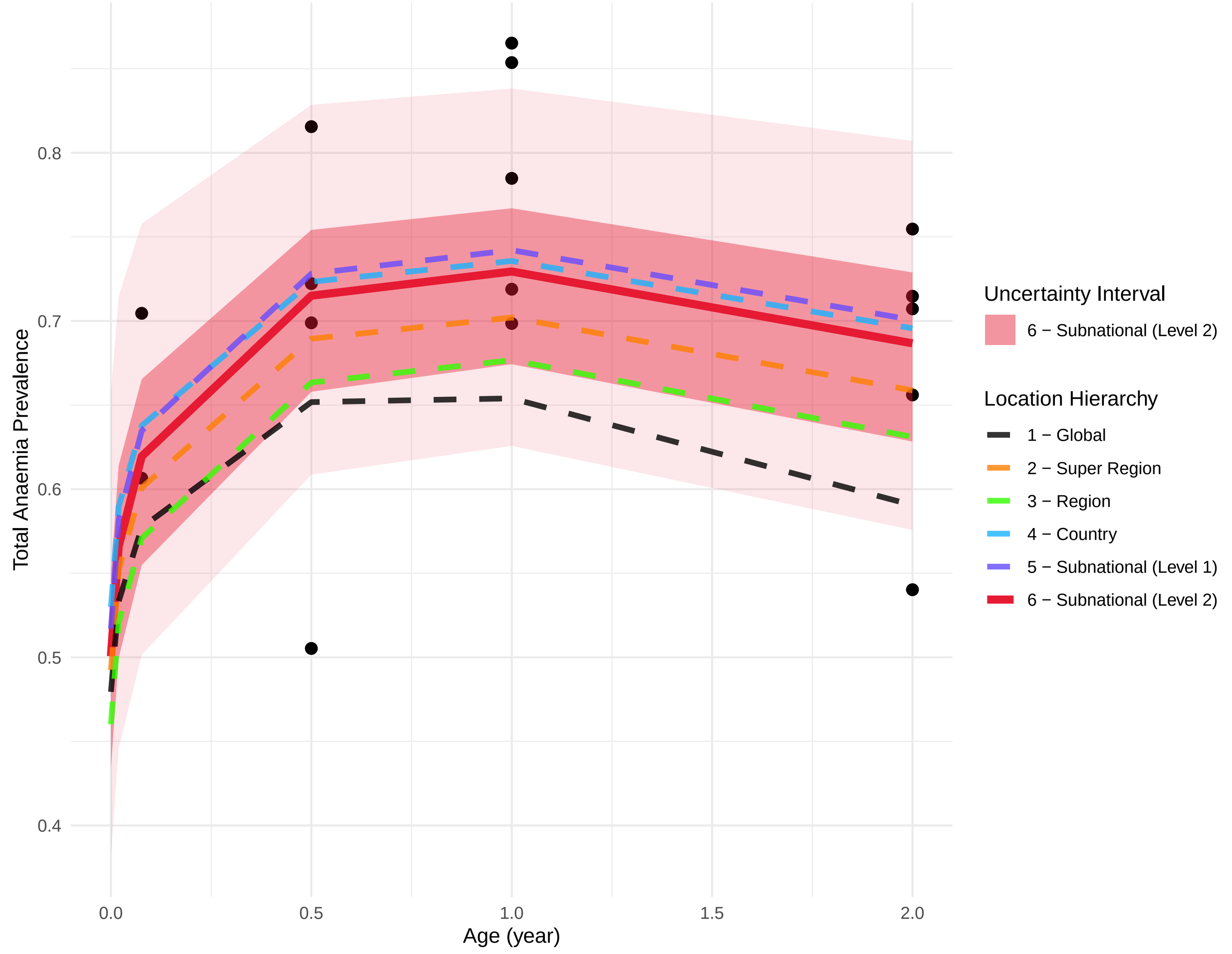

MR−BRT Age/Sex Fits − Males <2 Years Old − Total Anaemia Prevalence − Rajasthan
WHO−2024 Adjustment Method

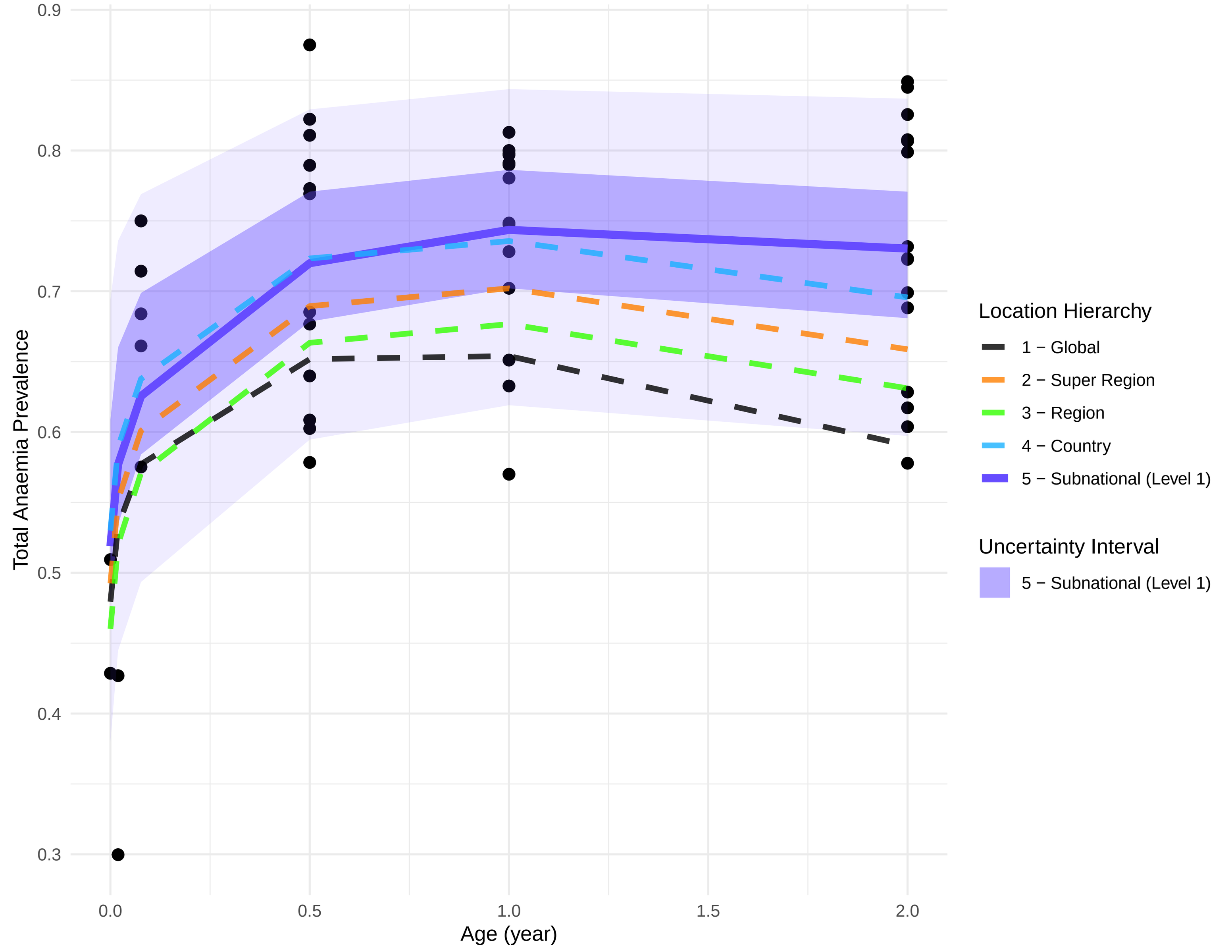

MR−BRT Age/Sex Fits − Males <2 Years Old − Total Anaemia Prevalence − Rajasthan, Rural
WHO−2024 Adjustment Method

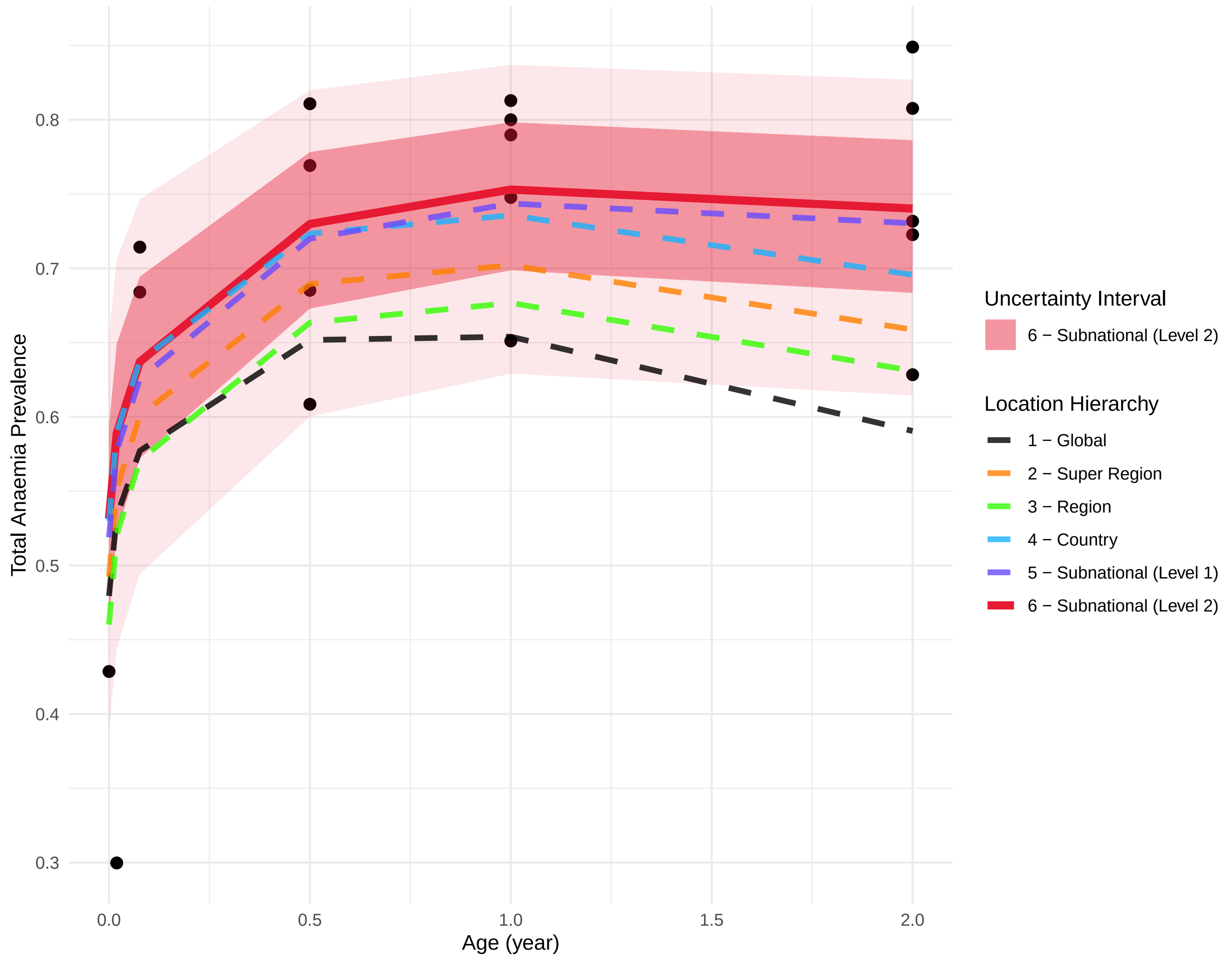

MR−BRT Age/Sex Fits − Males <2 Years Old − Total Anaemia Prevalence − Rajasthan, Urban
WHO−2024 Adjustment Method

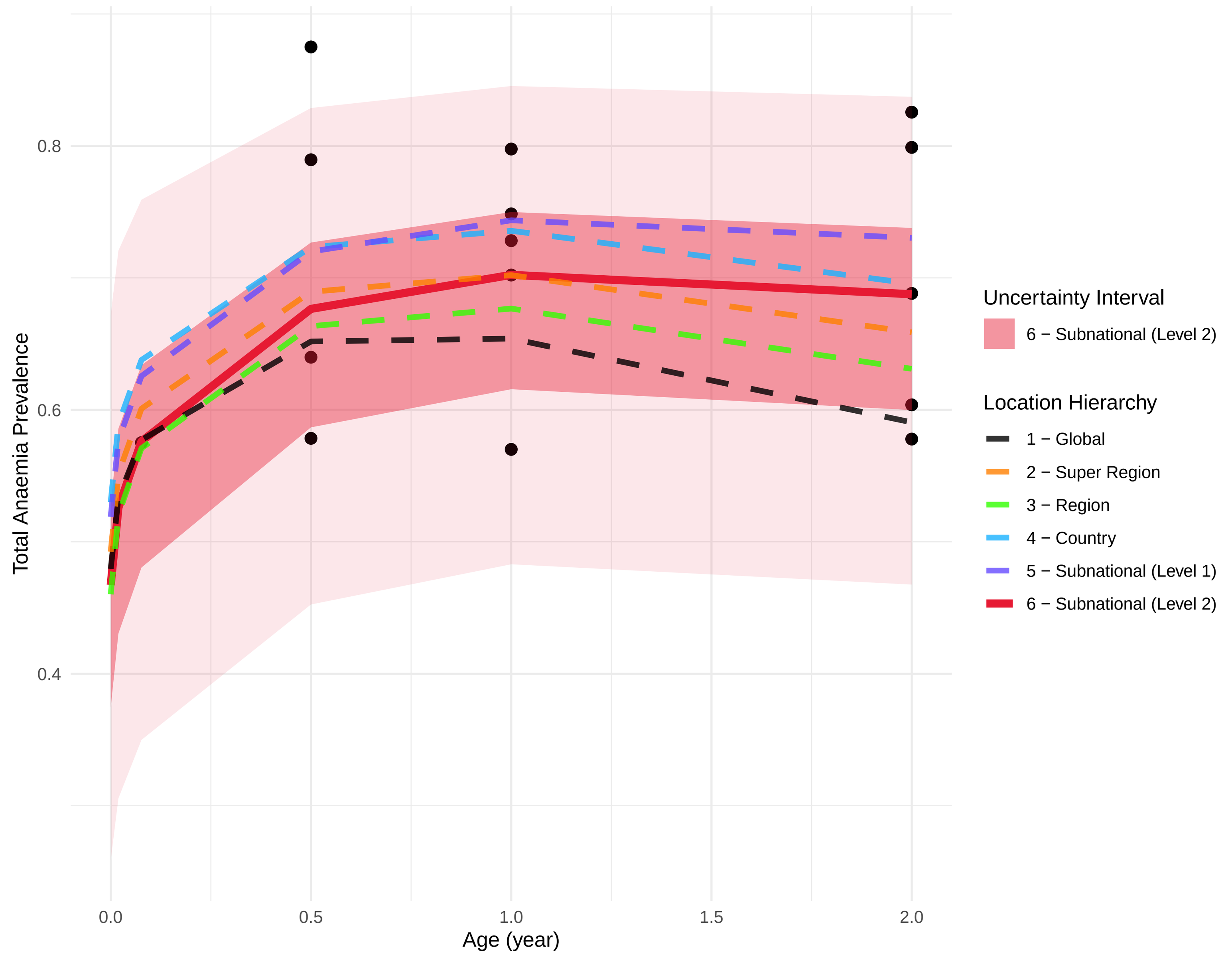

MR−BRT Age/Sex Fits − Males <2 Years Old − Total Anaemia Prevalence − Sikkim
WHO−2024 Adjustment Method

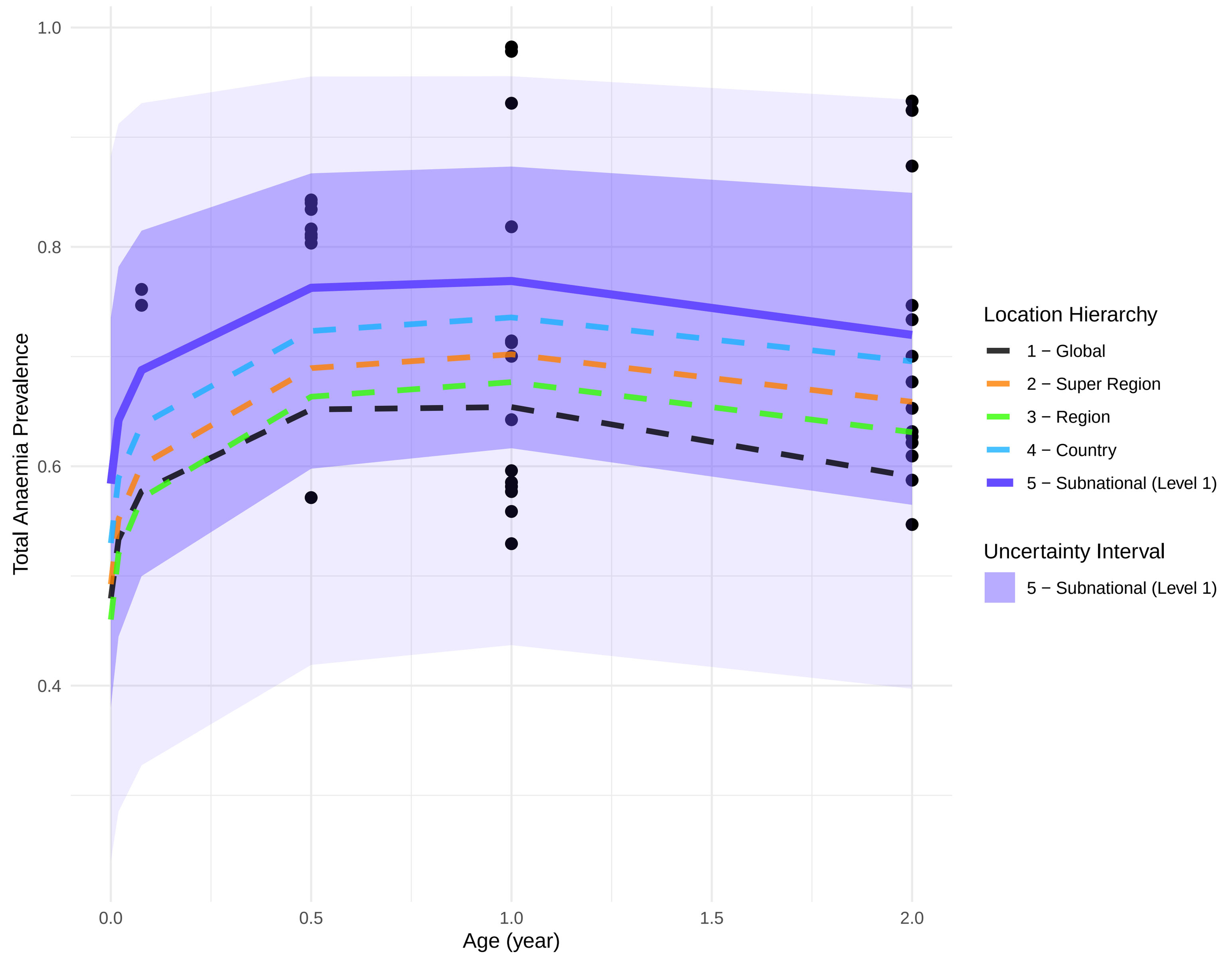

MR−BRT Age/Sex Fits − Males <2 Years Old − Total Anaemia Prevalence − Sikkim, Rural
WHO−2024 Adjustment Method

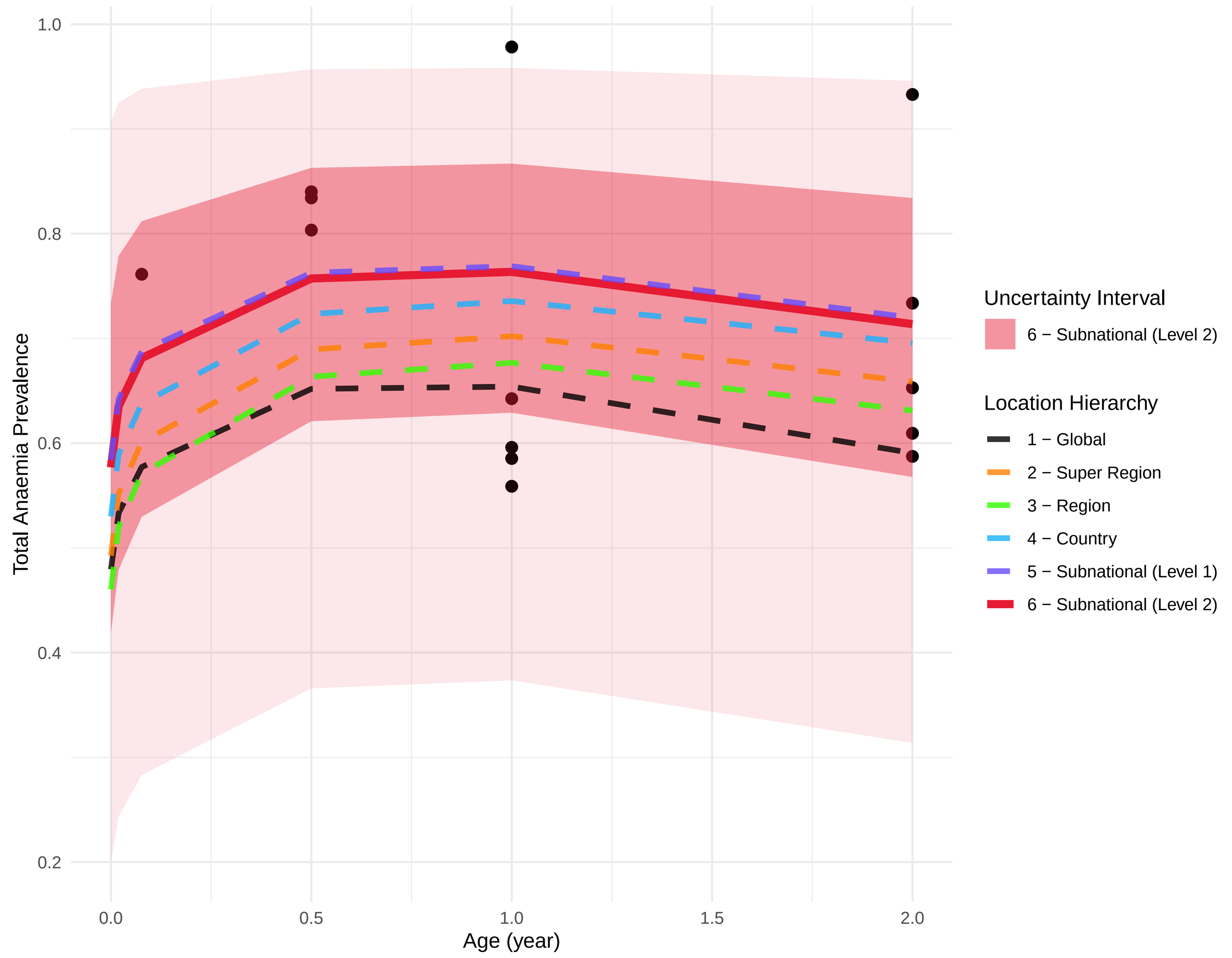

MR−BRT Age/Sex Fits − Males <2 Years Old − Total Anaemia Prevalence − Sikkim, Urban
WHO−2024 Adjustment Method

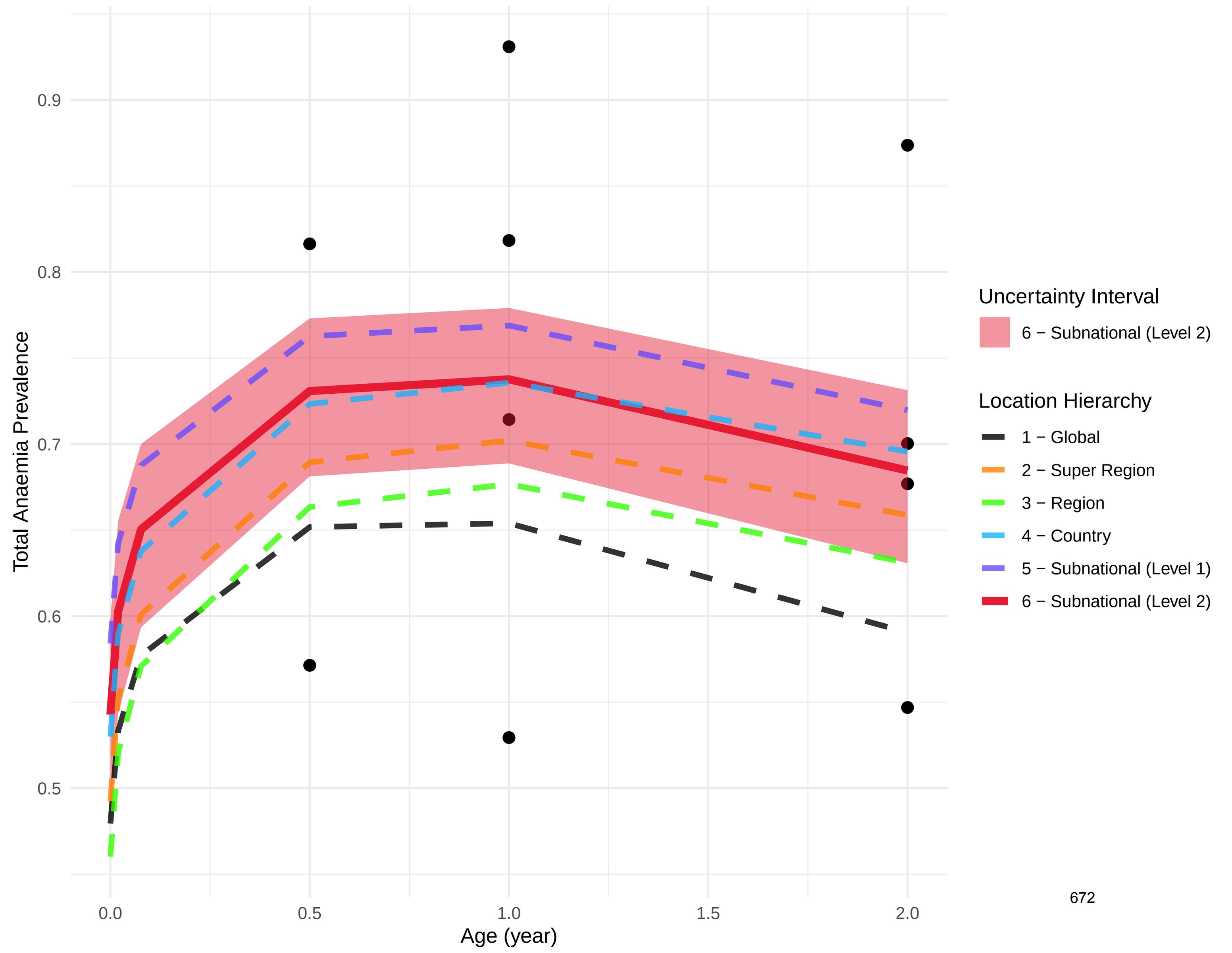

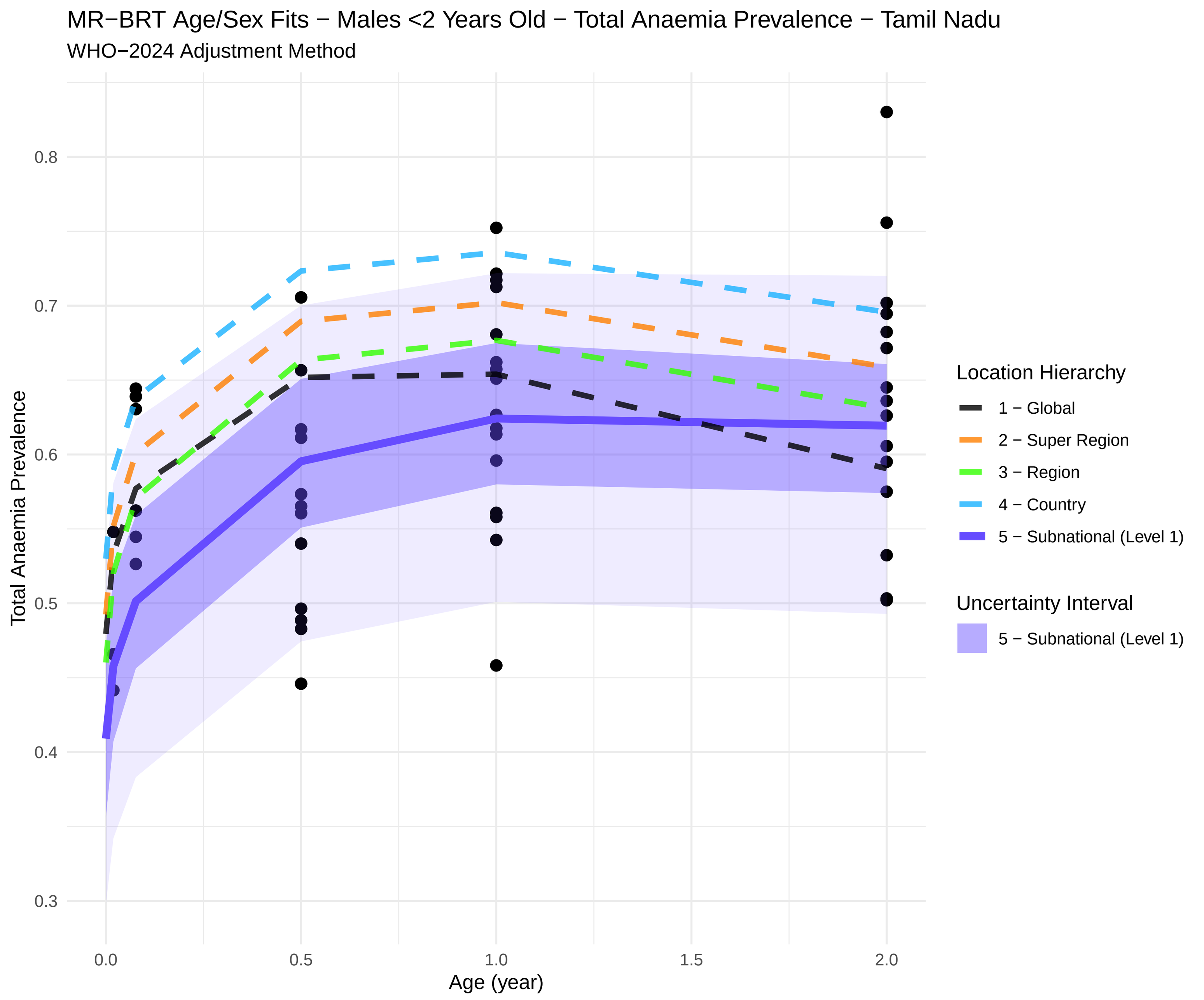
MR−BRT Age/Sex Fits − Males <2 Years Old − Total Anaemia Prevalence − Tamil Nadu
WHO−2024 Adjustment Method
Total Anaemia Prevalence
Age (year)
Location Hierarchy
1 − Global
2 − Super Region
3 − Region
4 − Country
5 − Subnational (Level 1)
Uncertainty Interval
5 − Subnational (Level 1)

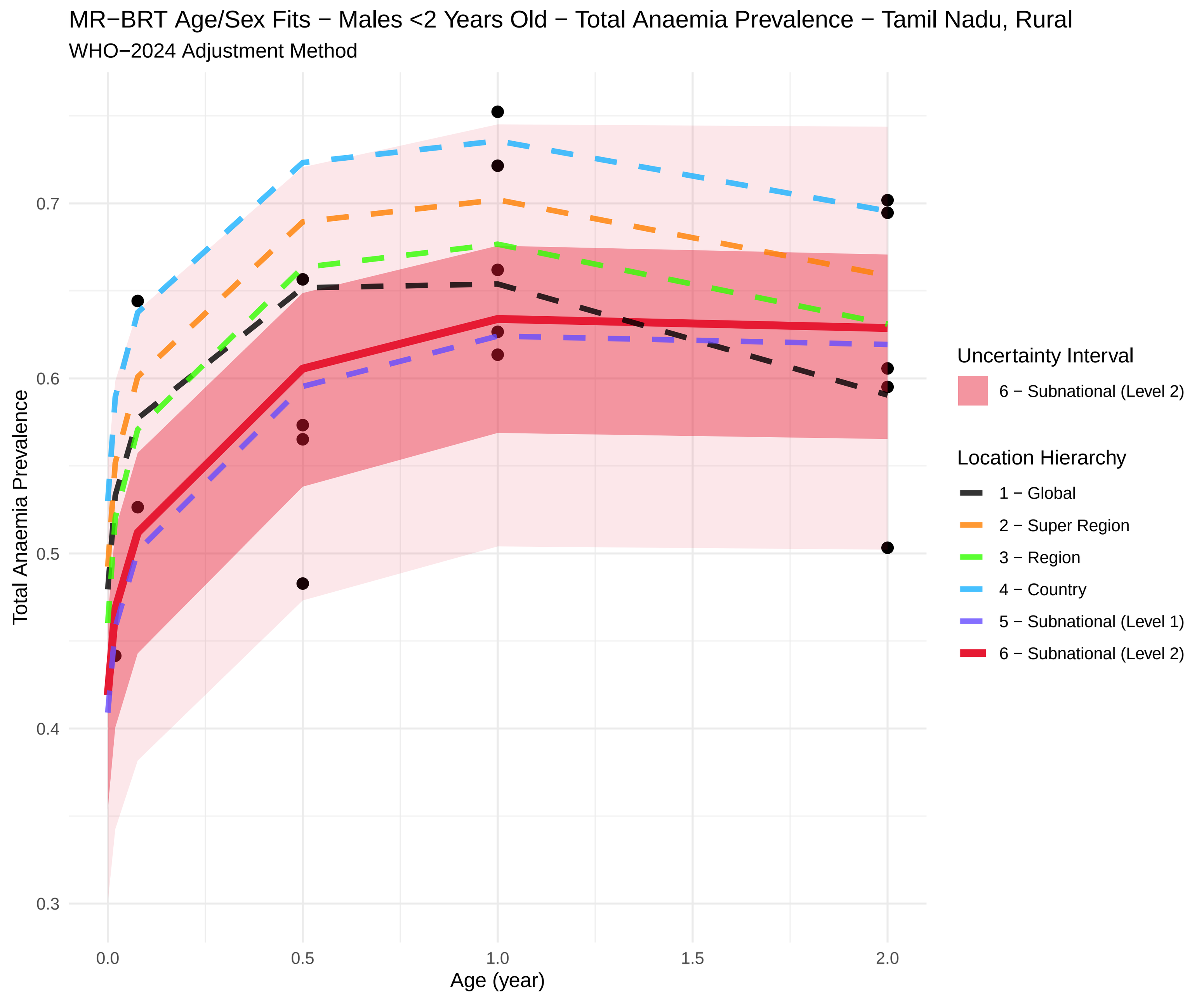
MR−BRT Age/Sex Fits − Males <2 Years Old − Total Anaemia Prevalence − Tamil Nadu, Rural
WHO−2024 Adjustment Method
Total Anaemia Prevalence
Age (year)
Uncertainty Interval
6 − Subnational (Level 2)
Location Hierarchy
1 − Global
2 − Super Region
3 − Region
4 − Country
5 − Subnational (Level 1)
6 − Subnational (Level 2)

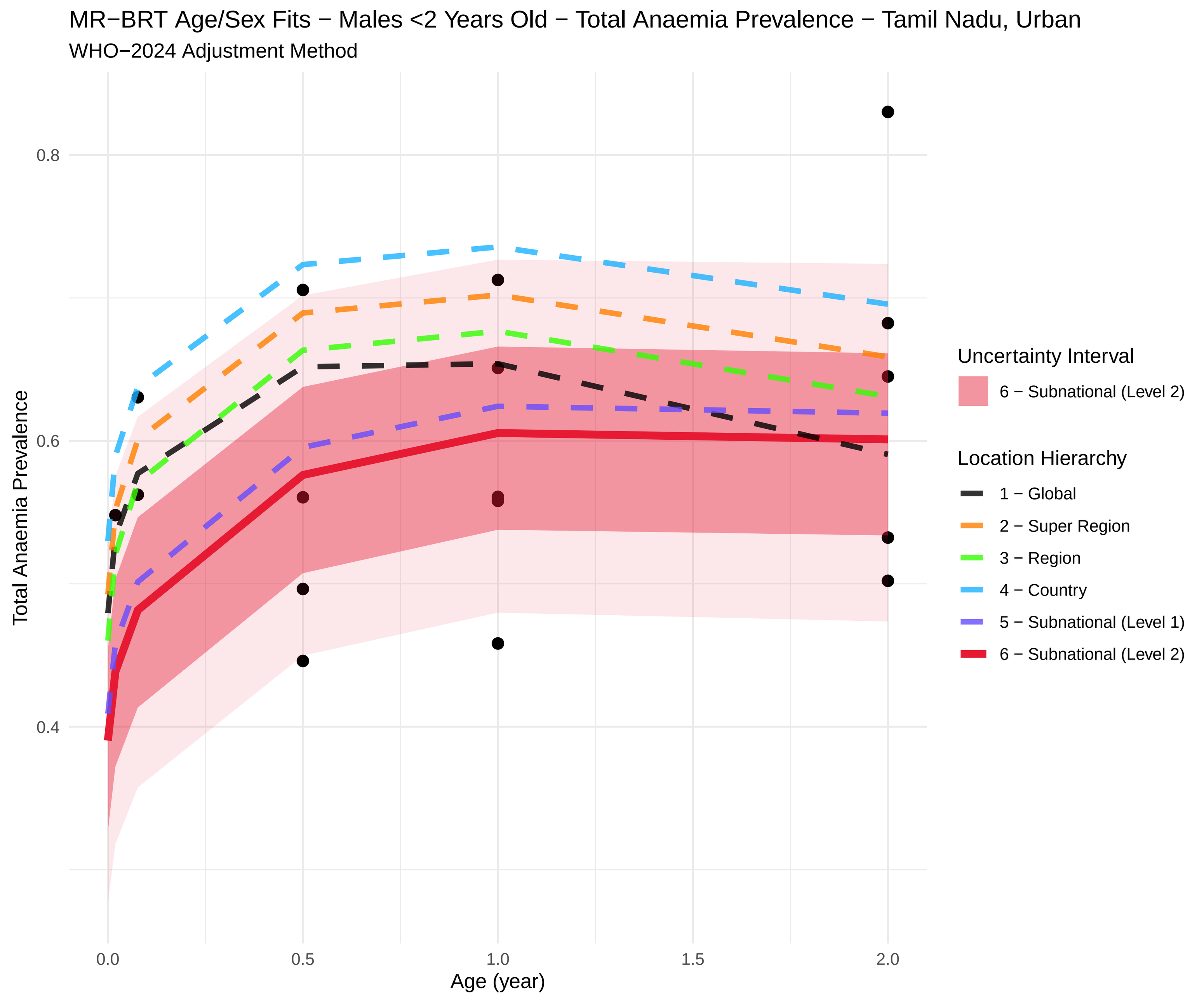
MR−BRT Age/Sex Fits − Males <2 Years Old − Total Anaemia Prevalence − Tamil Nadu, Urban
WHO−2024 Adjustment Method
Total Anaemia Prevalence
Age (year)
Uncertainty Interval
6 − Subnational (Level 2)
Location Hierarchy
1 − Global
2 − Super Region
3 − Region
4 − Country
5 − Subnational (Level 1)
6 − Subnational (Level 2)

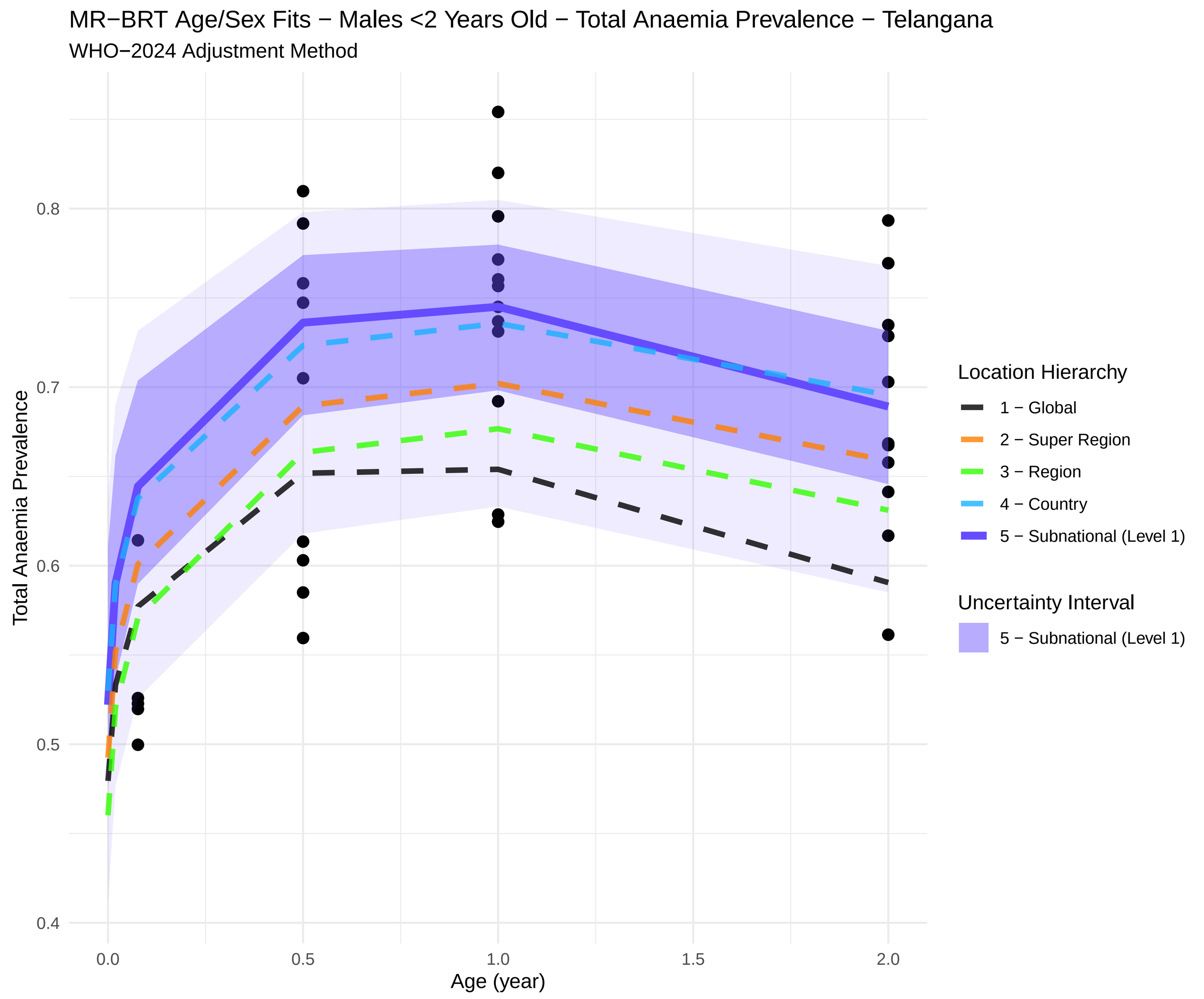
MR−BRT Age/Sex Fits − Males <2 Years Old − Total Anaemia Prevalence − Telangana
WHO−2024 Adjustment Method
Total Anaemia Prevalence
Age (year)
Location Hierarchy
1 − Global
2 − Super Region
3 − Region
4 − Country
5 − Subnational (Level 1)
Uncertainty Interval
5 − Subnational (Level 1)

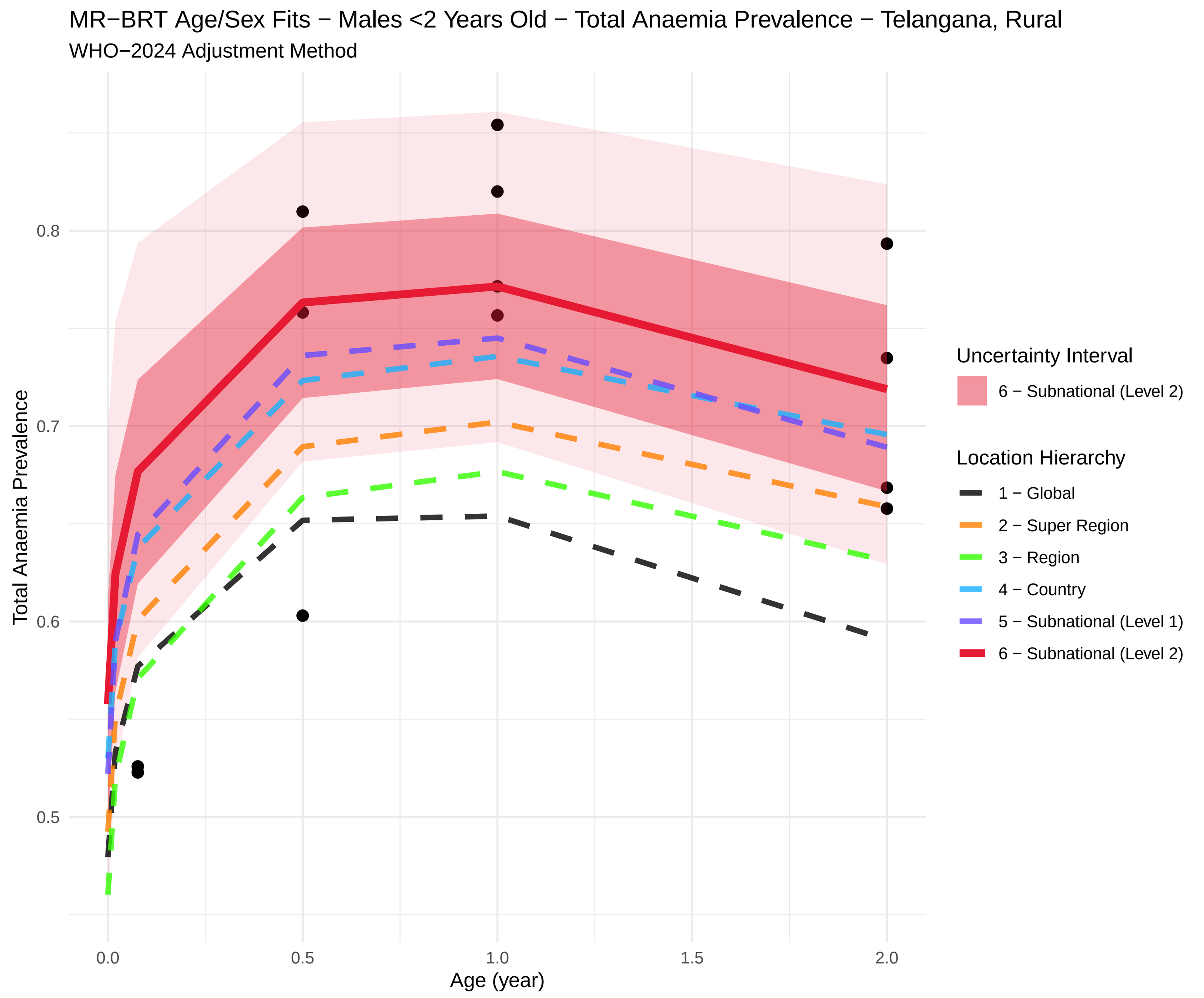
MR−BRT Age/Sex Fits − Males <2 Years Old − Total Anaemia Prevalence − Telangana, Rural
WHO−2024 Adjustment Method
Total Anaemia Prevalence
Age (year)
Uncertainty Interval
6 − Subnational (Level 2)
Location Hierarchy
1 − Global
2 − Super Region
3 − Region
4 − Country
5 − Subnational (Level 1)
6 − Subnational (Level 2)

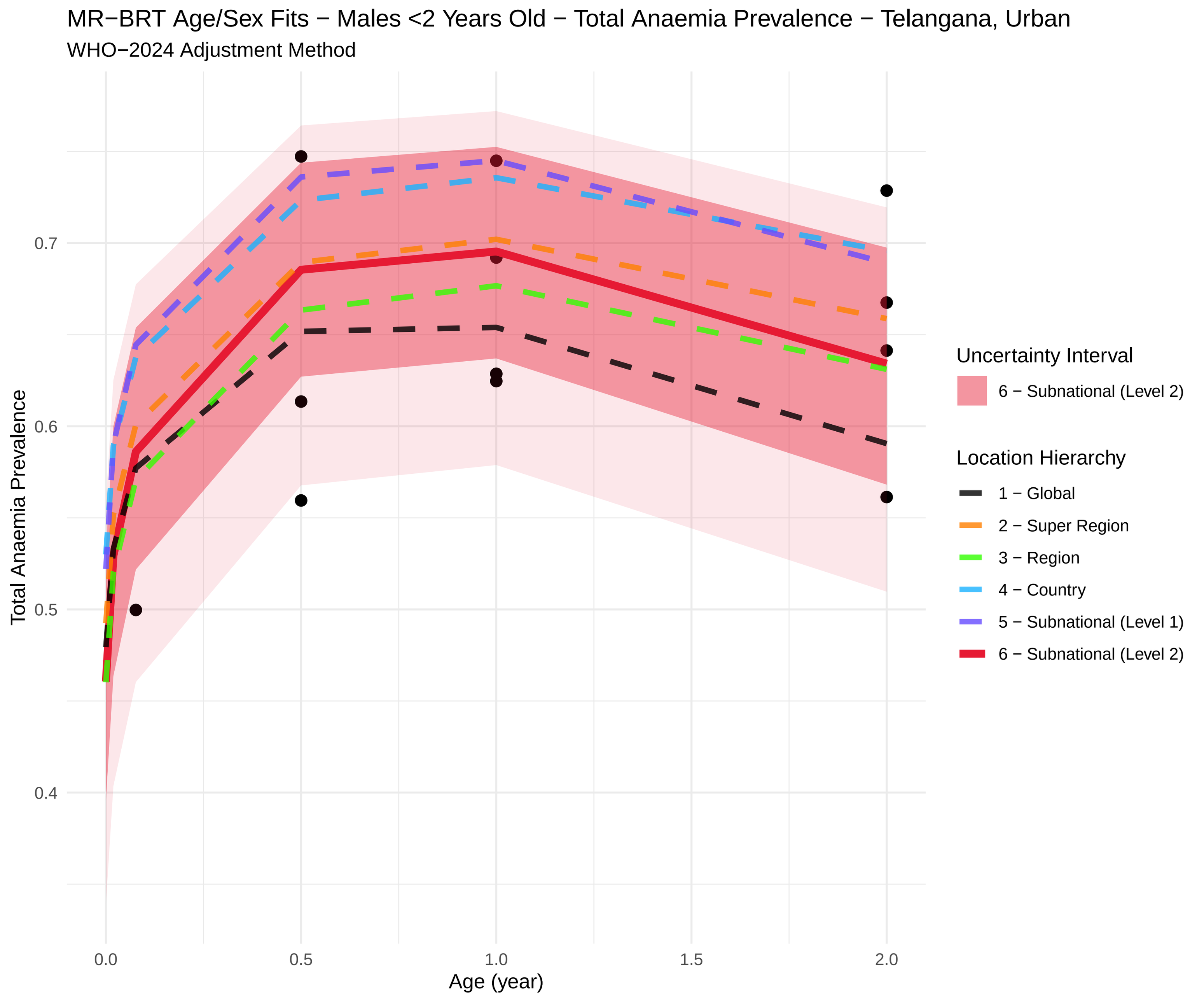
MR−BRT Age/Sex Fits − Males <2 Years Old − Total Anaemia Prevalence − Telangana, Urban
WHO−2024 Adjustment Method
Total Anaemia Prevalence
Age (year)
Uncertainty Interval
6 − Subnational (Level 2)
Location Hierarchy
1 − Global
2 − Super Region
3 − Region
4 − Country
5 − Subnational (Level 1)
6 − Subnational (Level 2)

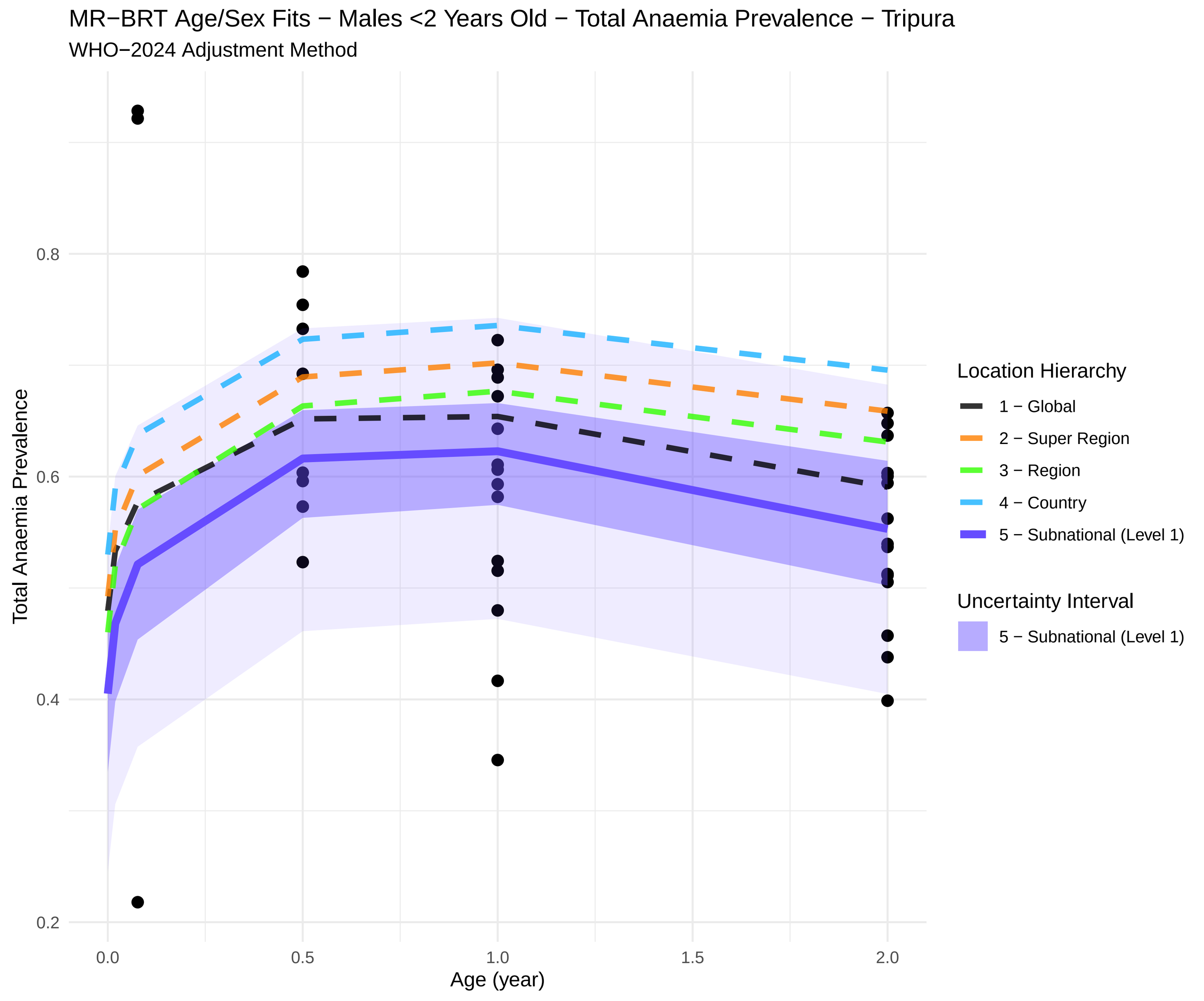
MR−BRT Age/Sex Fits − Males <2 Years Old − Total Anaemia Prevalence − Tripura
WHO−2024 Adjustment Method
Total Anaemia Prevalence
Age (year)
Location Hierarchy
1 − Global
2 − Super Region
3 − Region
4 − Country
5 − Subnational (Level 1)
Uncertainty Interval
5 − Subnational (Level 1)

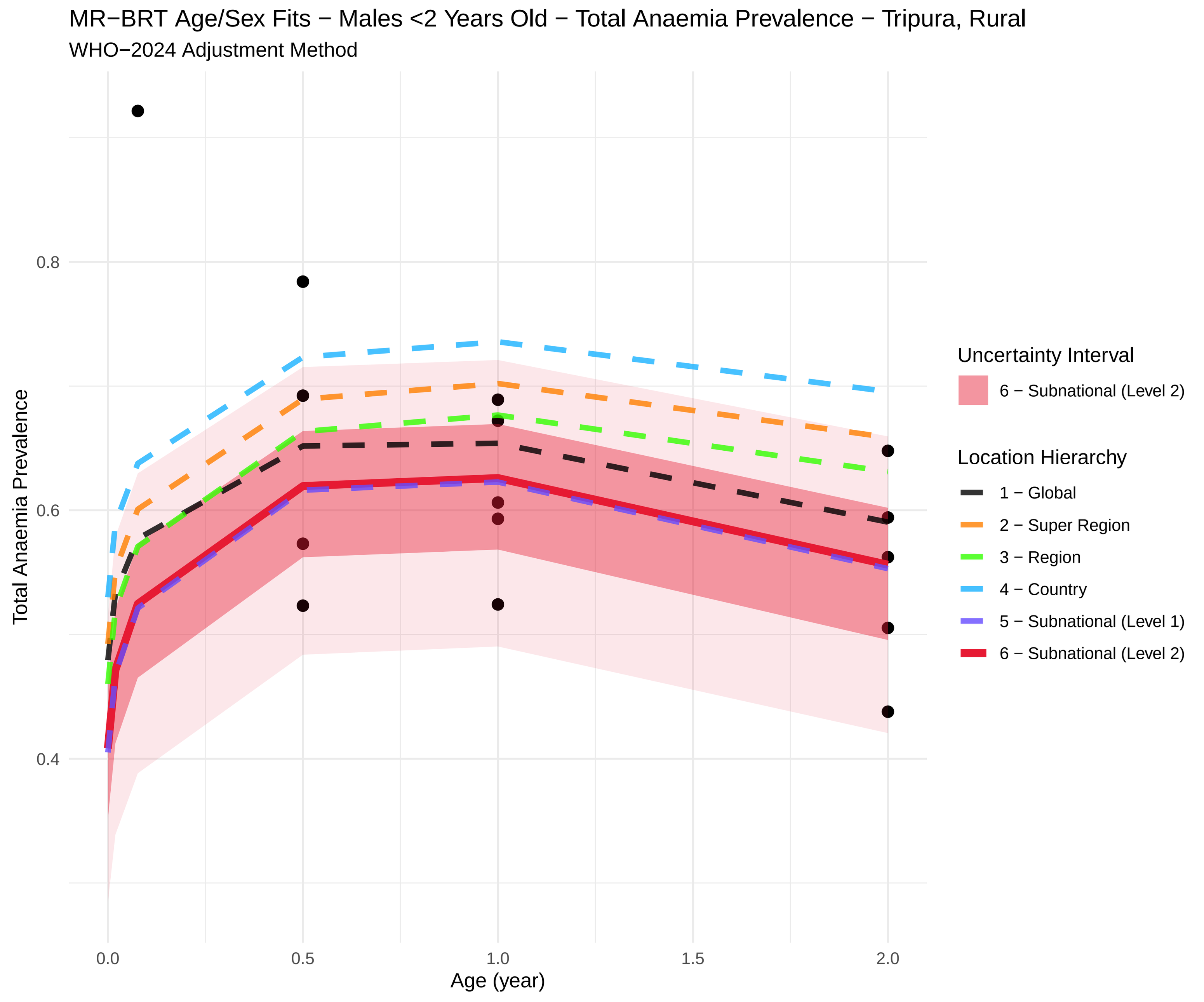
MR−BRT Age/Sex Fits − Males <2 Years Old − Total Anaemia Prevalence − Tripura, Rural
WHO−2024 Adjustment Method
Total Anaemia Prevalence
Age (year)
Uncertainty Interval
6 − Subnational (Level 2)
Location Hierarchy
1 − Global
2 − Super Region
3 − Region
4 − Country
5 − Subnational (Level 1)
6 − Subnational (Level 2)

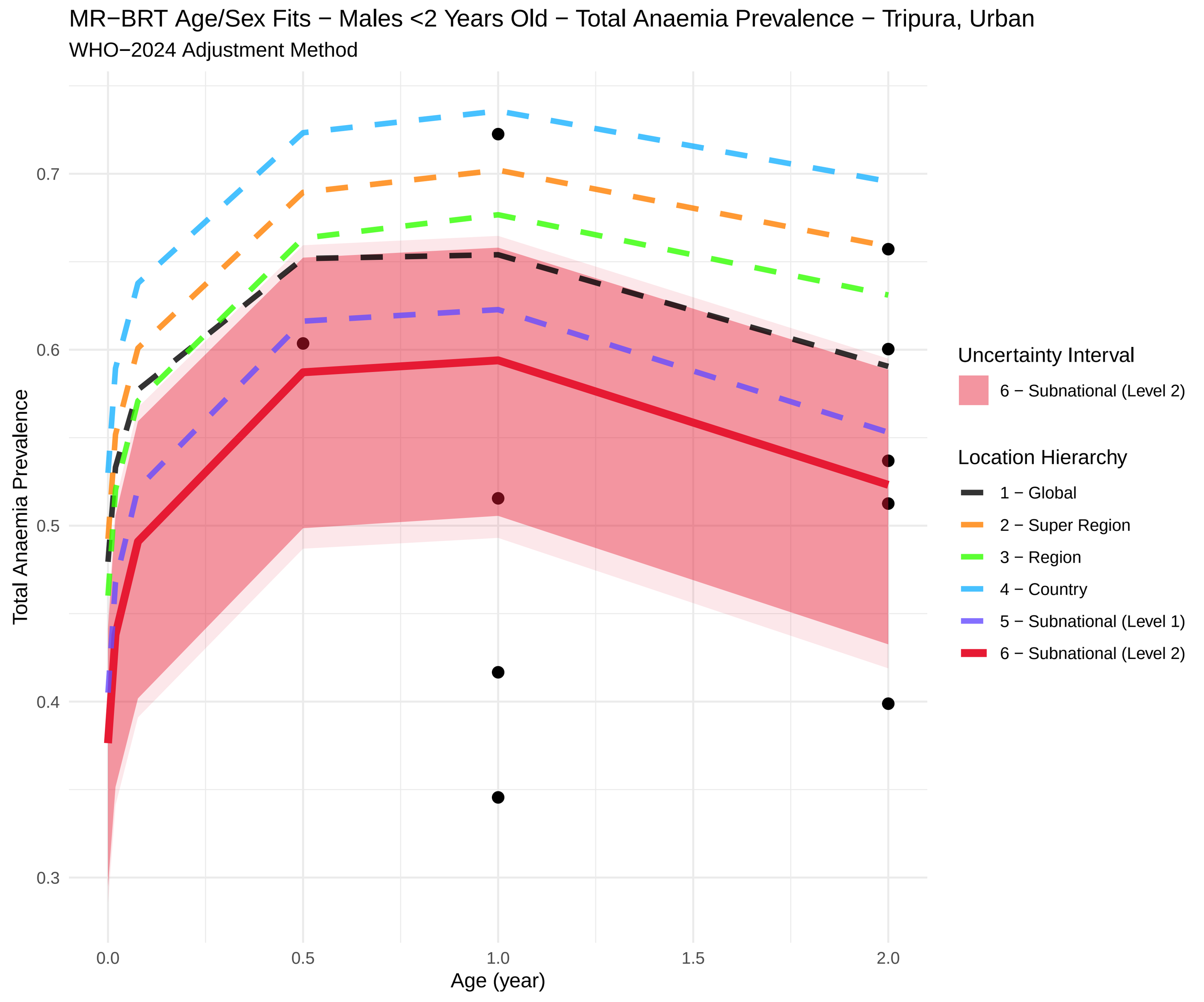
MR−BRT Age/Sex Fits − Males <2 Years Old − Total Anaemia Prevalence − Tripura, Urban
WHO−2024 Adjustment Method
Total Anaemia Prevalence
Age (year)
Uncertainty Interval
6 − Subnational (Level 2)
Location Hierarchy
1 − Global
2 − Super Region
3 − Region
4 − Country
5 − Subnational (Level 1)
6 − Subnational (Level 2)

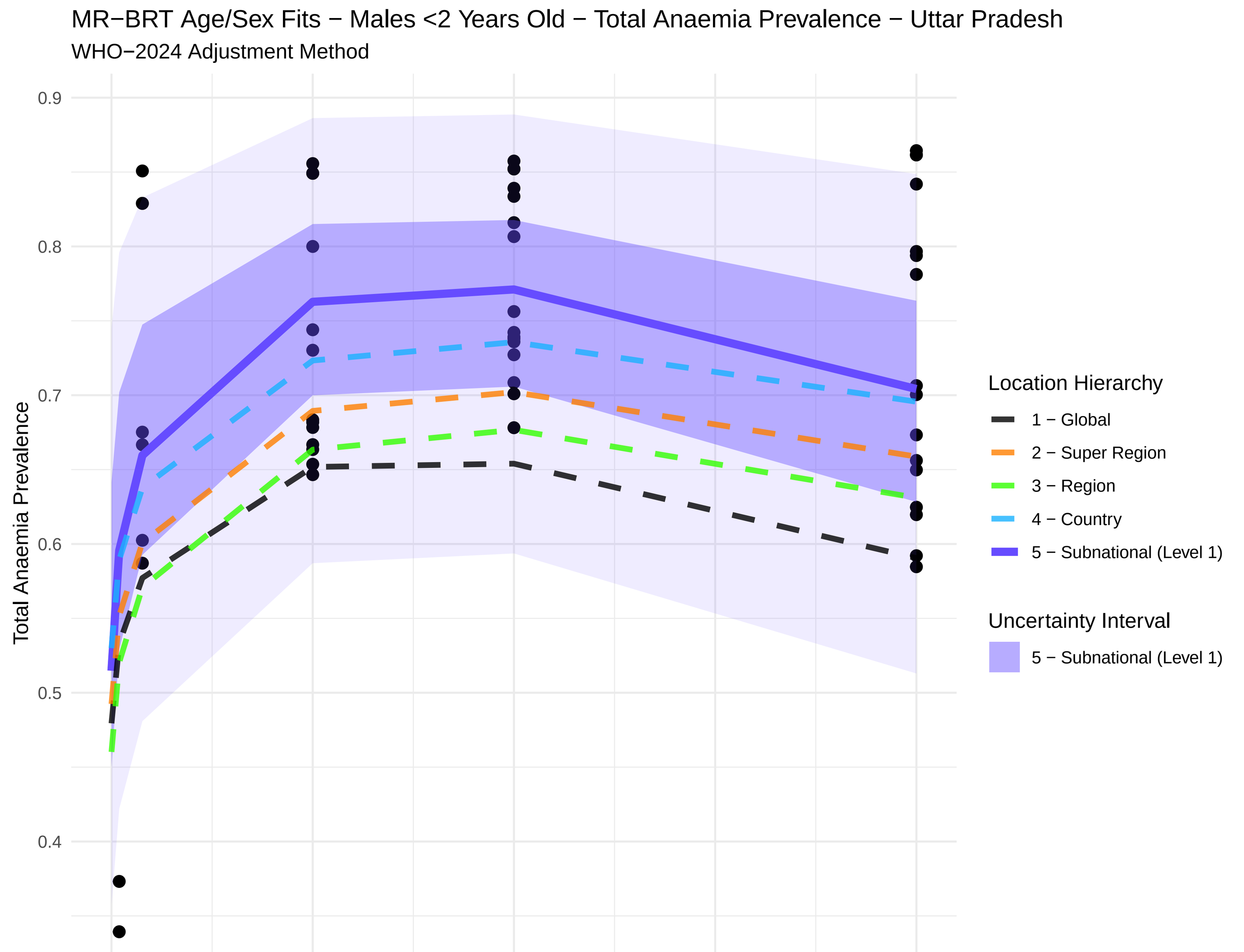
MR−BRT Age/Sex Fits − Males <2 Years Old − Total Anaemia Prevalence − Uttar Pradesh
WHO−2024 Adjustment Method
Total Anaemia Prevalence
Age (year)
Location Hierarchy
1 − Global
2 − Super Region
3 − Region
4 − Country
5 − Subnational (Level 1)
Uncertainty Interval
5 − Subnational (Level 1)

MR−BRT Age/Sex Fits − Males <2 Years Old − Total Anaemia Prevalence − Uttar Pradesh, Rural
WHO−2024 Adjustment Method

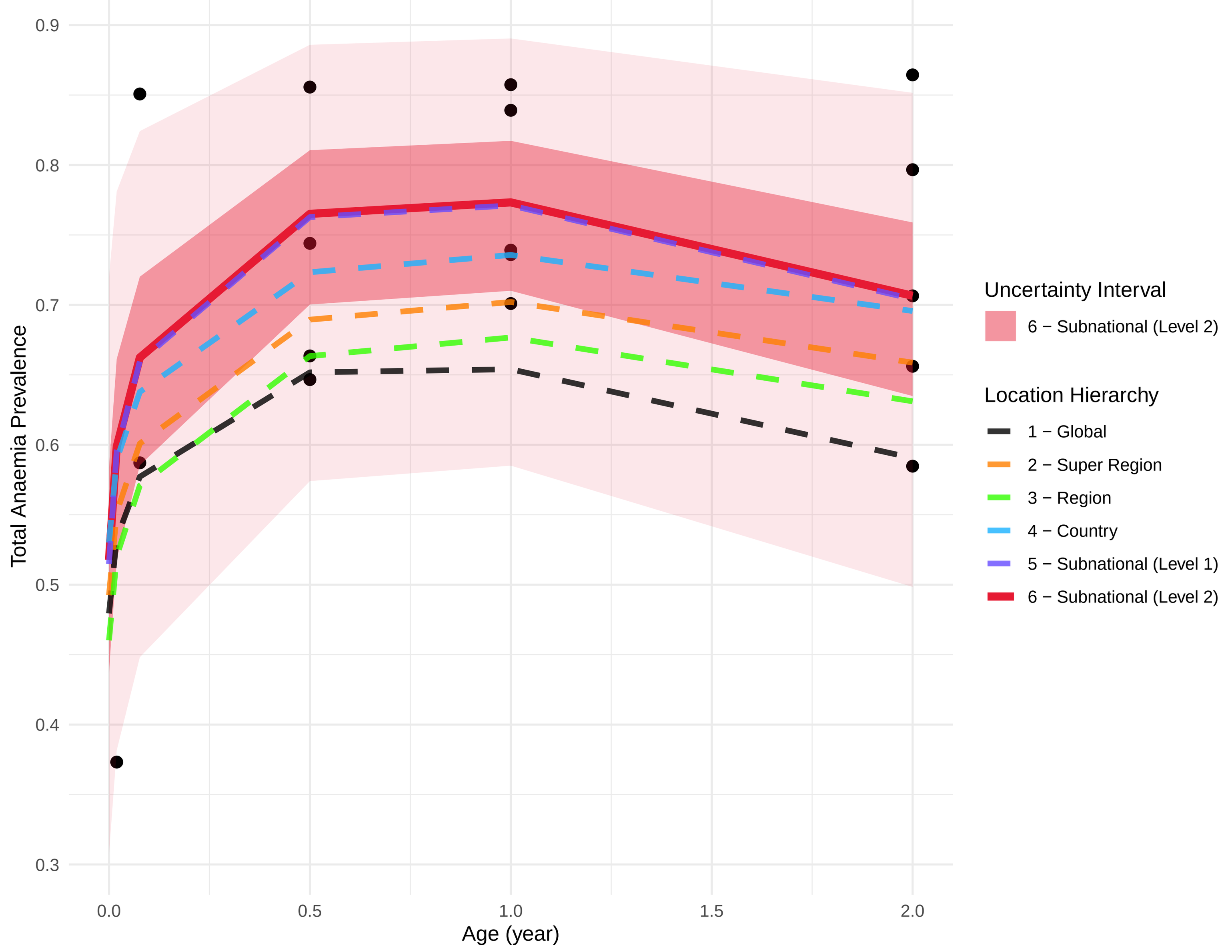

MR−BRT Age/Sex Fits − Males <2 Years Old − Total Anaemia Prevalence − Uttar Pradesh, Urban
WHO−2024 Adjustment Method

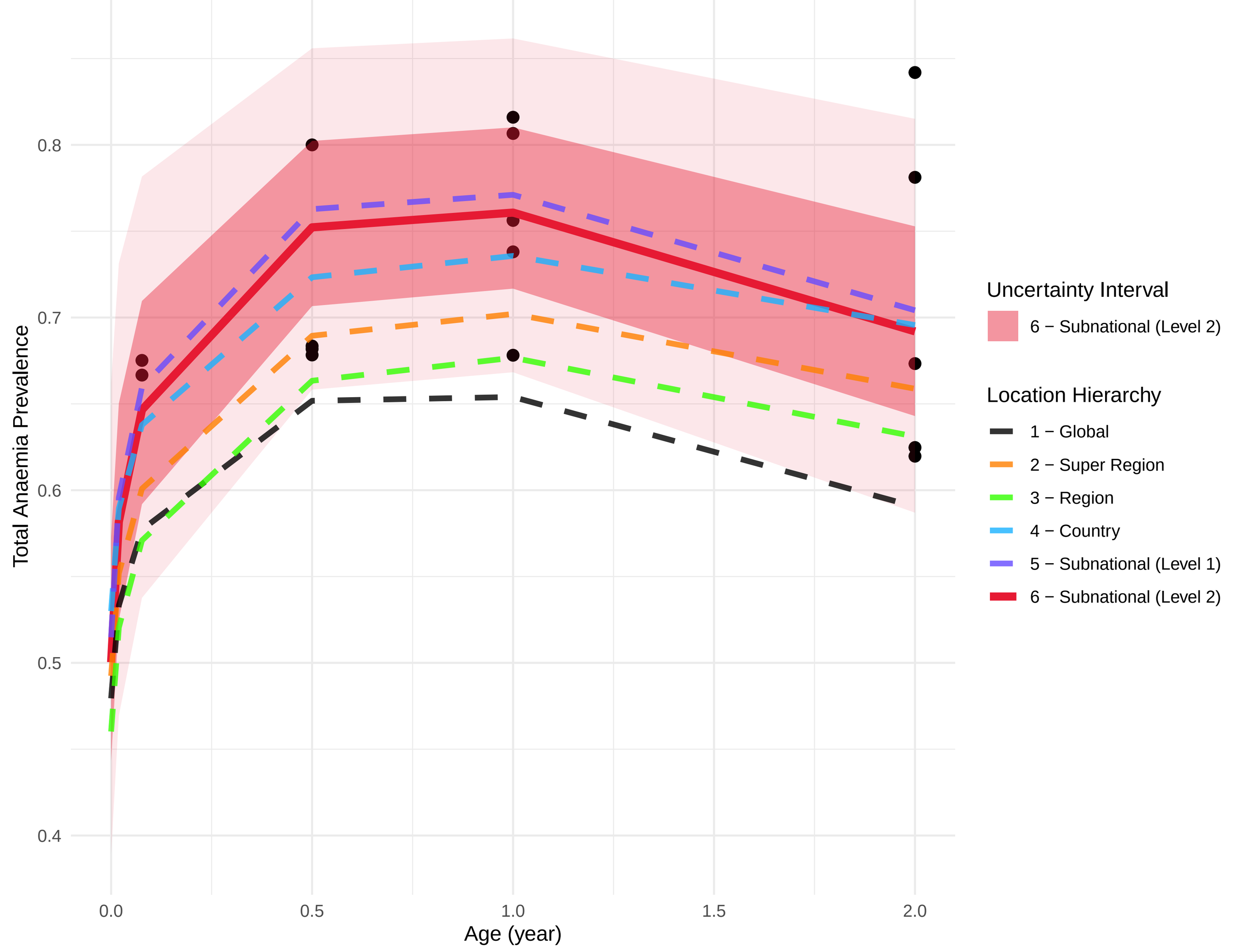

MR−BRT Age/Sex Fits − Males <2 Years Old − Total Anaemia Prevalence − Uttarakhand
WHO−2024 Adjustment Method

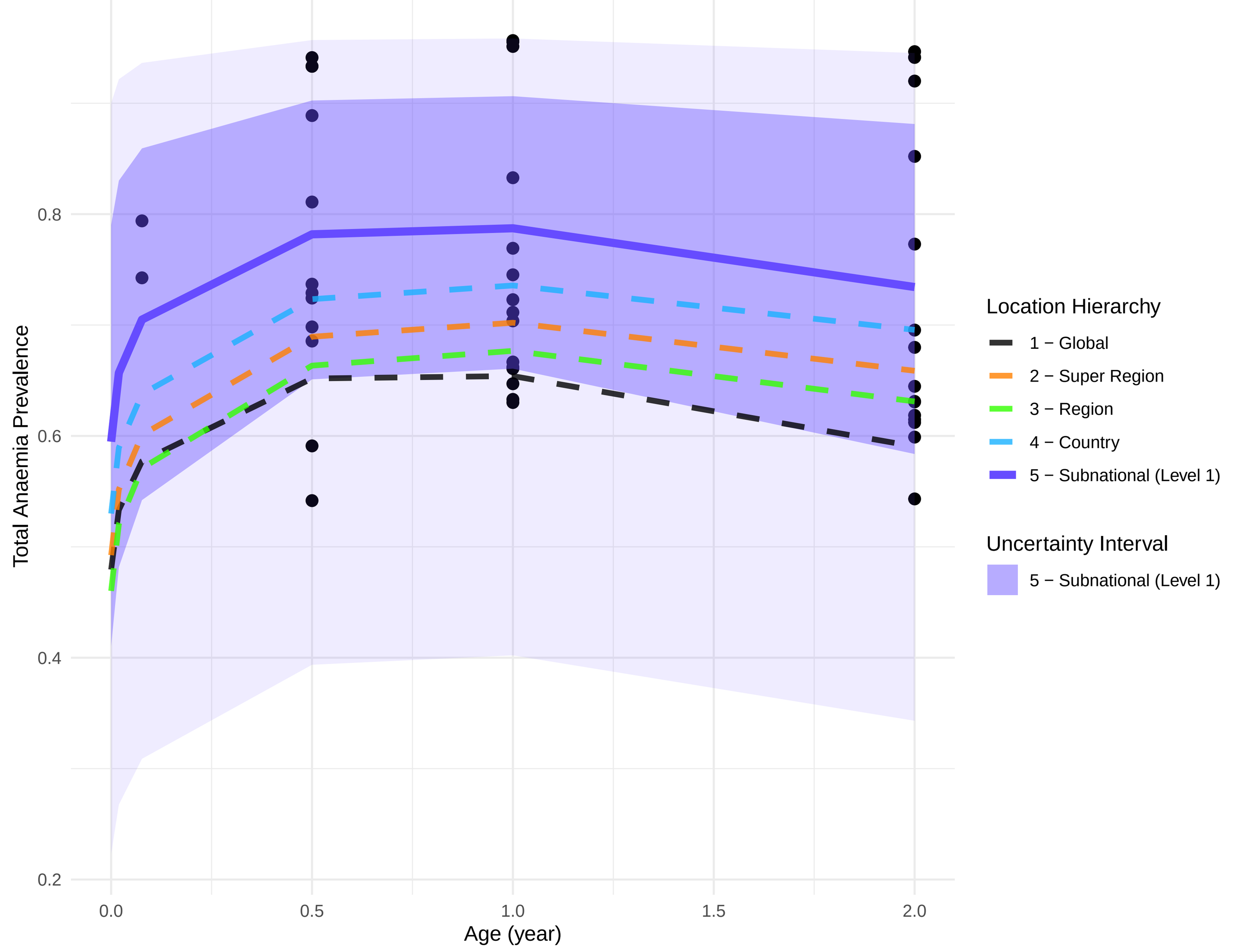

MR−BRT Age/Sex Fits − Males <2 Years Old − Total Anaemia Prevalence − Uttarakhand, Rural
WHO−2024 Adjustment Method

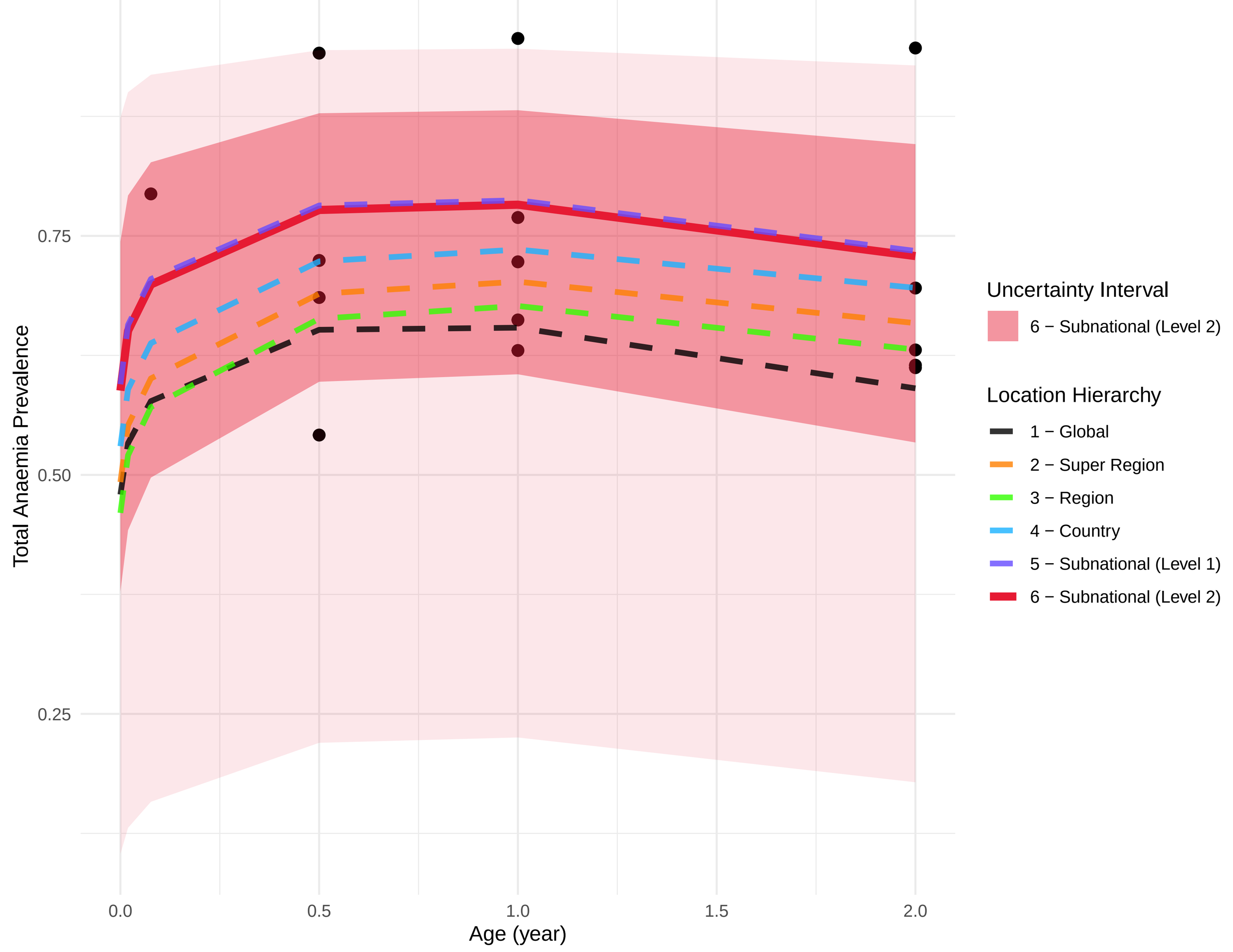

MR−BRT Age/Sex Fits − Males <2 Years Old − Total Anaemia Prevalence − Uttarakhand, Urban
WHO−2024 Adjustment Method

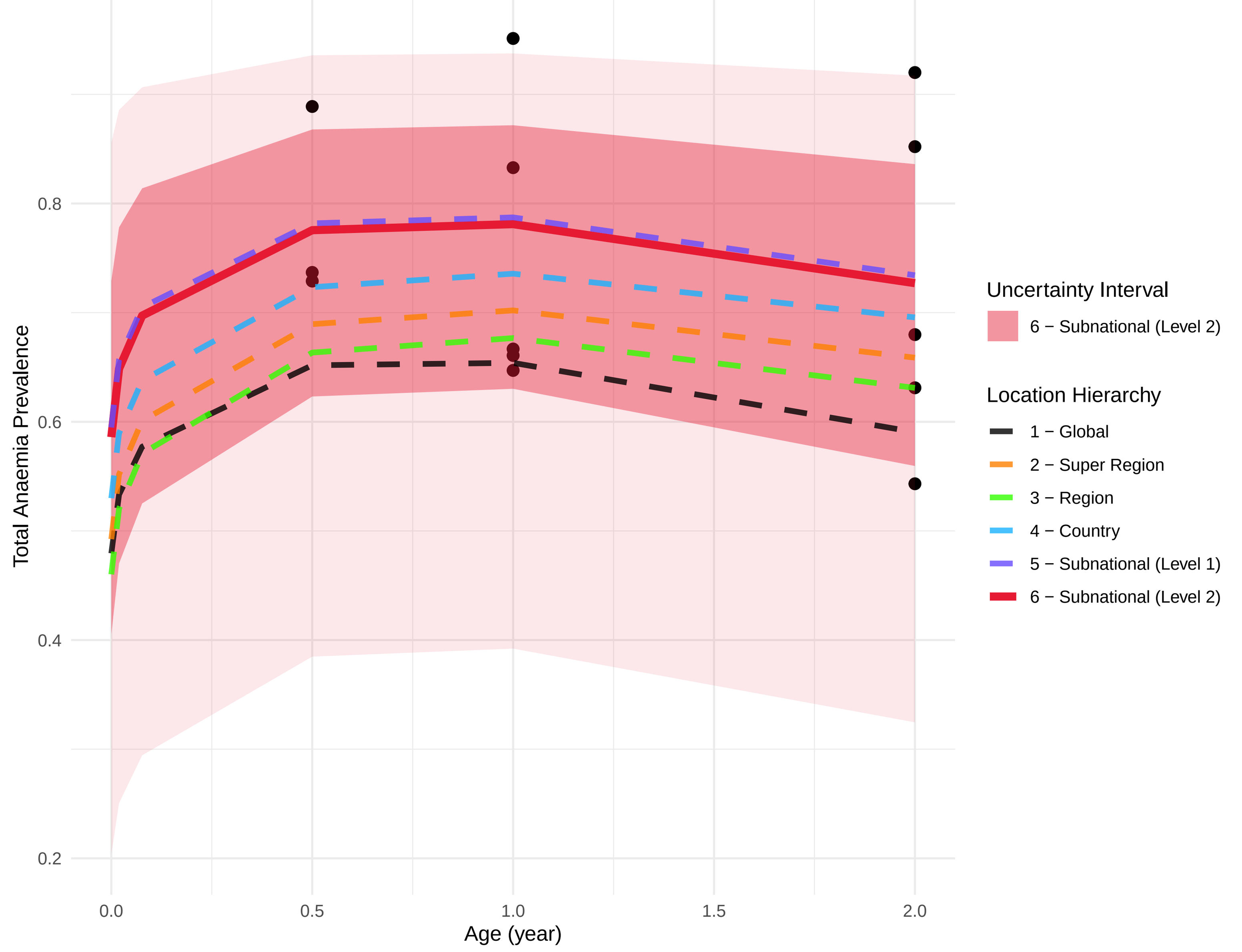

MR−BRT Age/Sex Fits − Males <2 Years Old − Total Anaemia Prevalence − West Bengal
WHO−2024 Adjustment Method

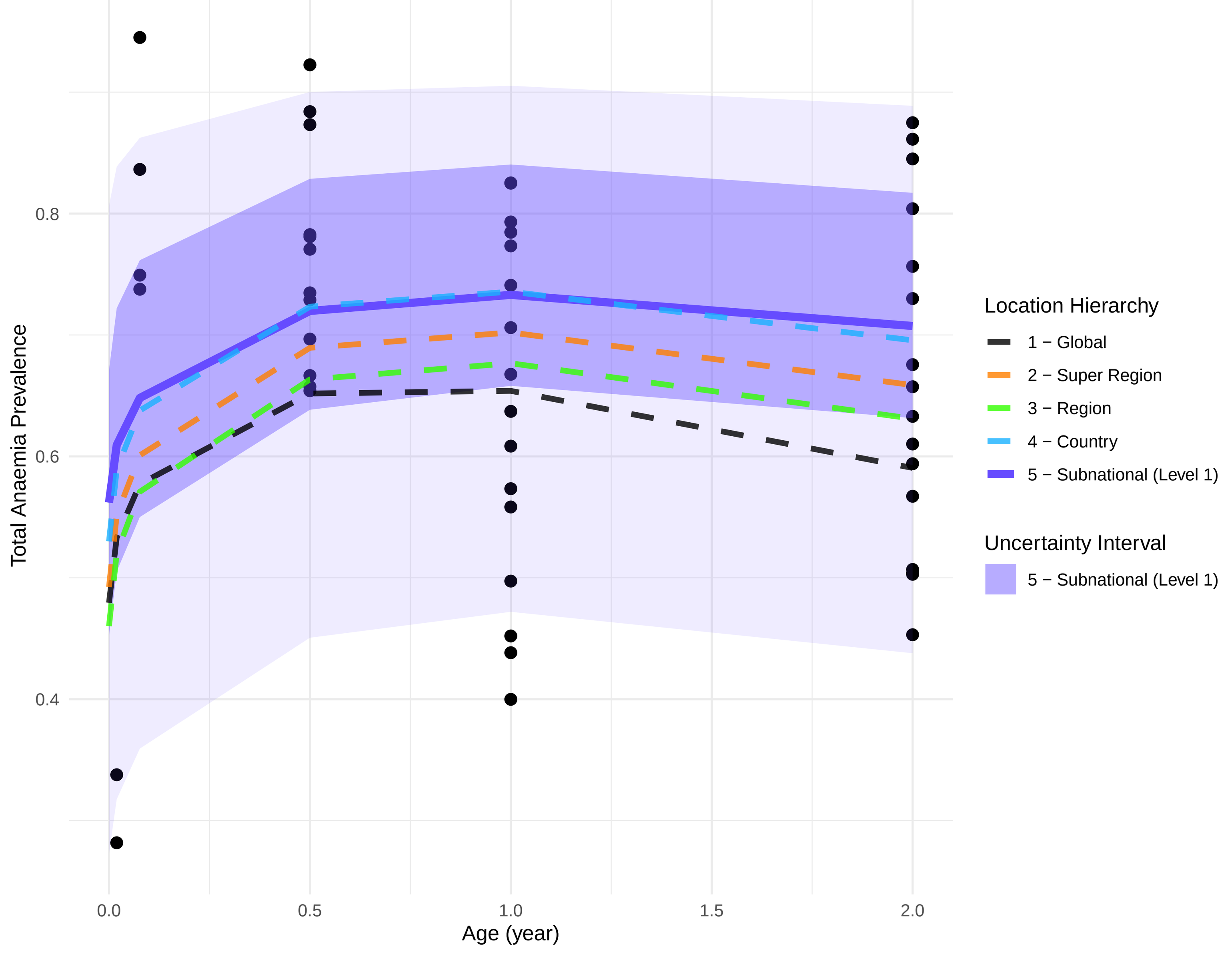

MR−BRT Age/Sex Fits − Males <2 Years Old − Total Anaemia Prevalence − West Bengal, Rural
WHO−2024 Adjustment Method

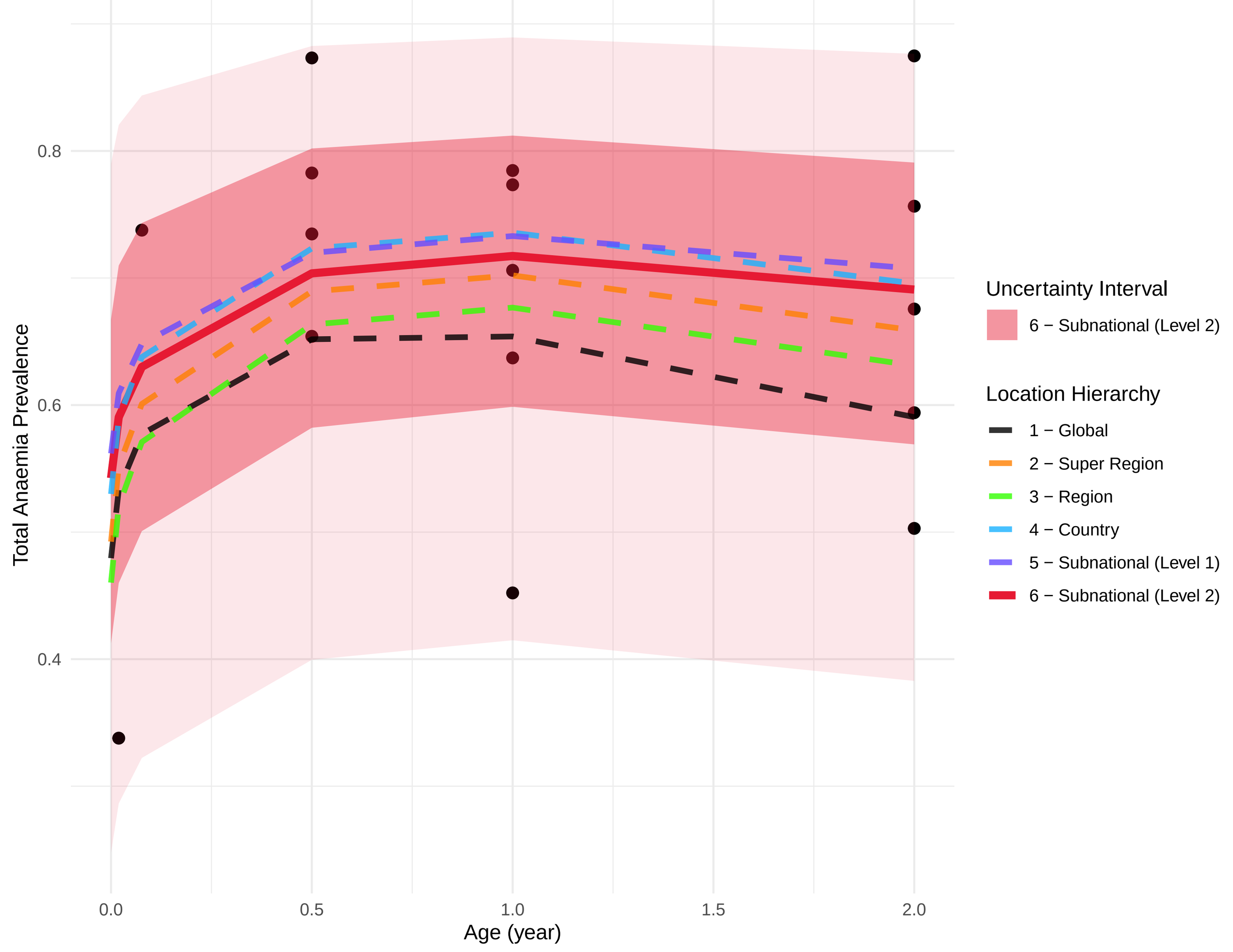

MR−BRT Age/Sex Fits − Males <2 Years Old − Total Anaemia Prevalence − West Bengal, Urban
WHO−2024 Adjustment Method

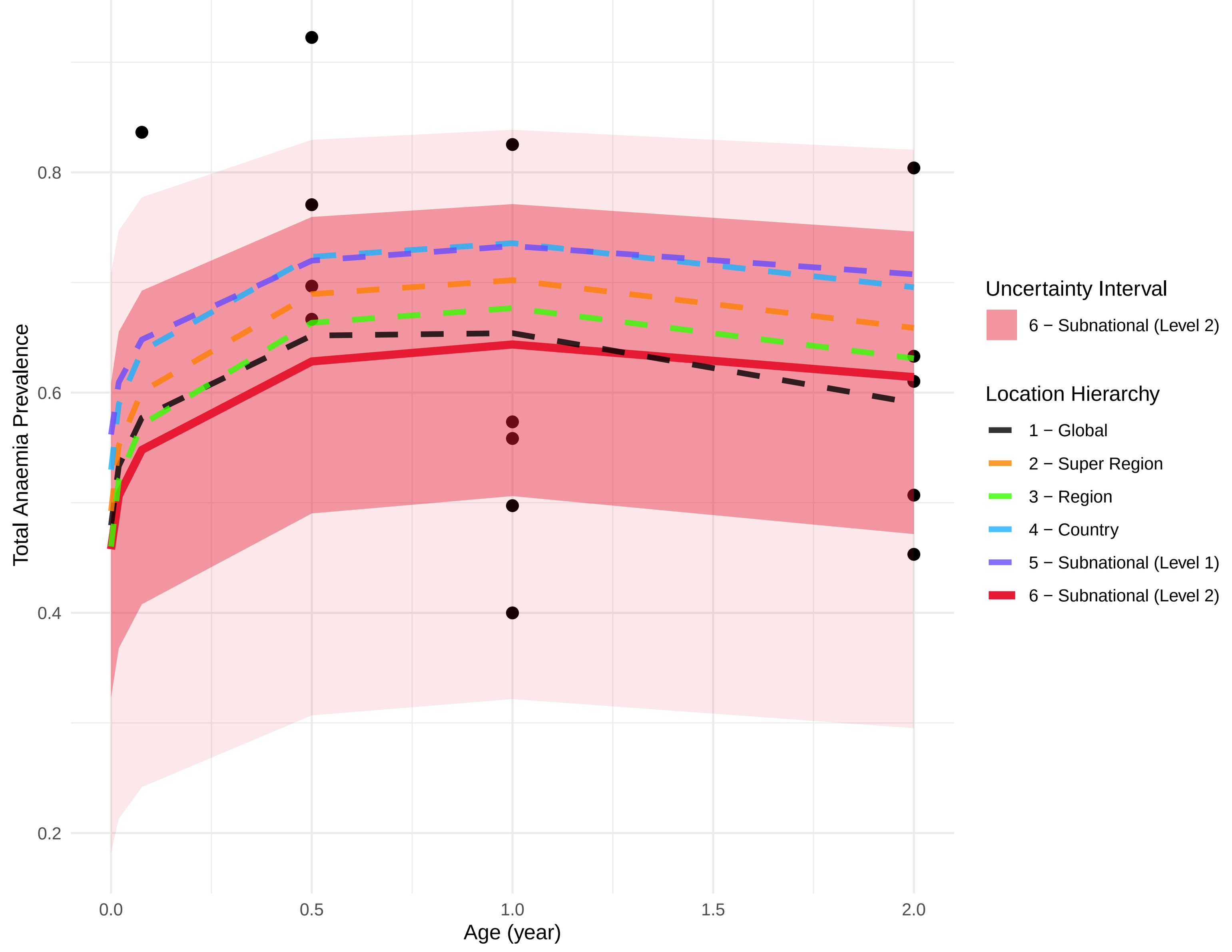

MR−BRT Age/Sex Fits − Males <2 Years Old − Total Anaemia Prevalence − Nepal
WHO−2024 Adjustment Method

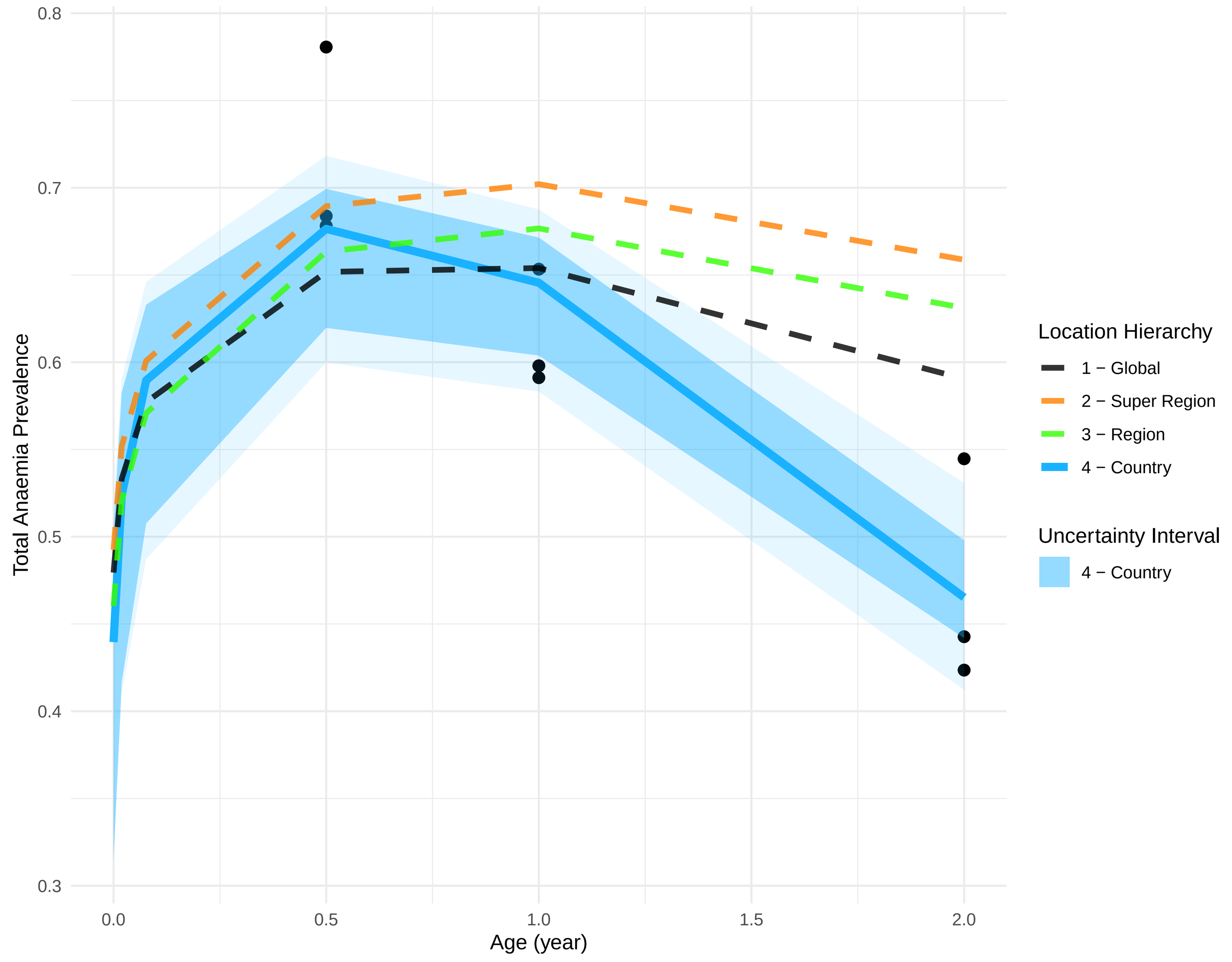

MR−BRT Age/Sex Fits − Males <2 Years Old − Total Anaemia Prevalence − Southeast Asia, East Asia, and Oceania
WHO−2024 Adjustment Method

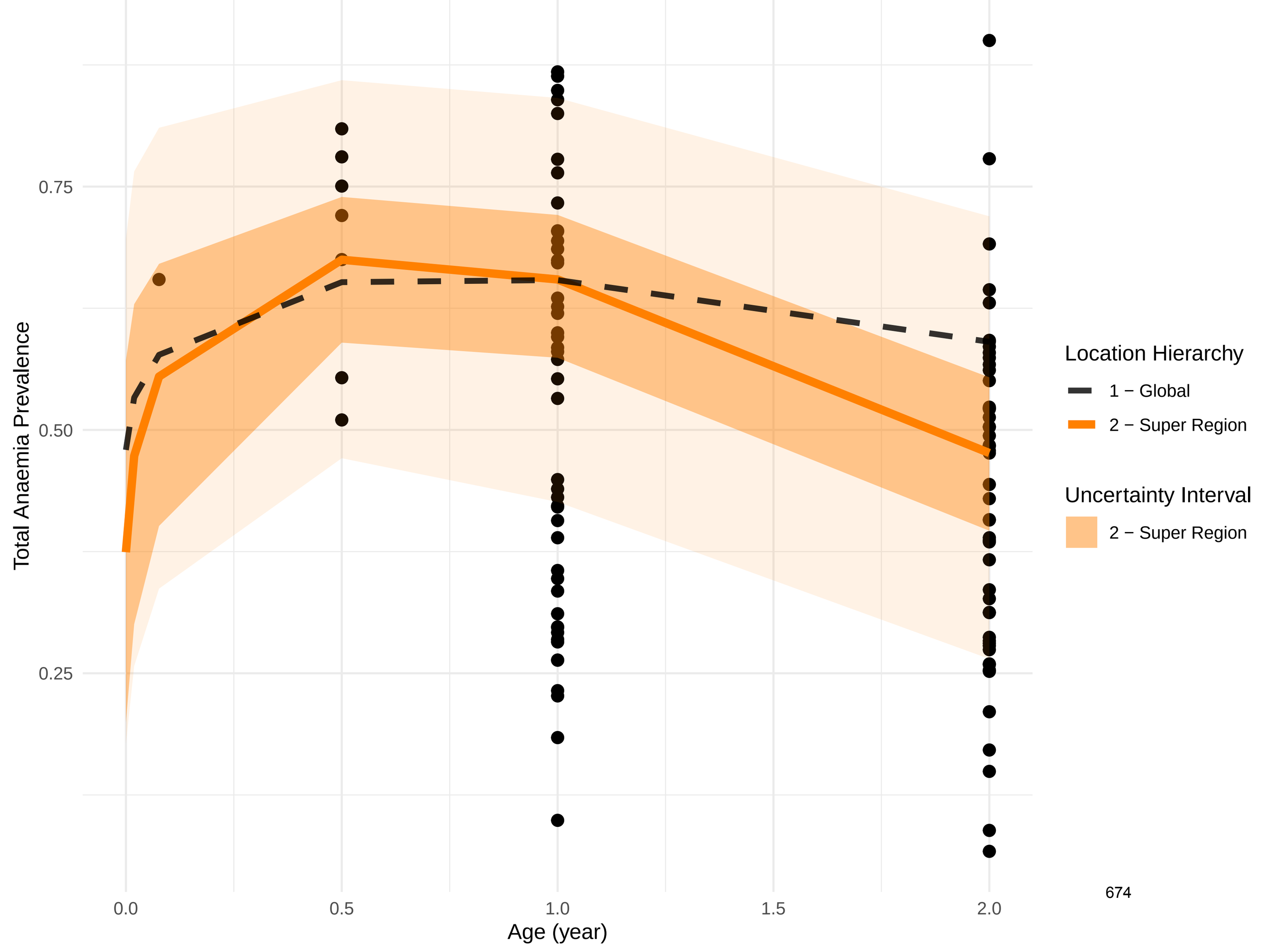

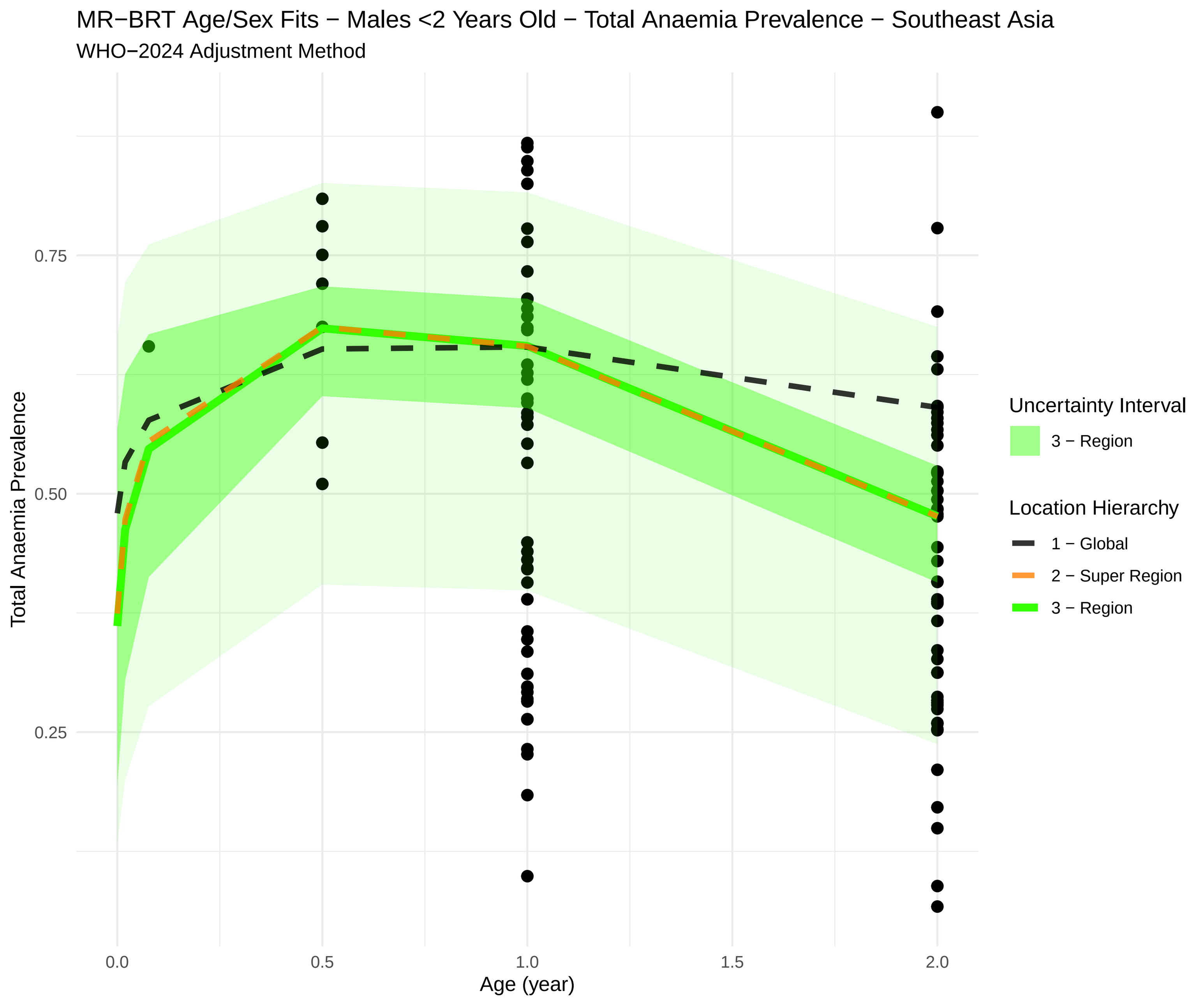
MR−BRT Age/Sex Fits − Males <2 Years Old − Total Anaemia Prevalence − Southeast Asia
WHO−2024 Adjustment Method
Total Anaemia Prevalence
Age (year)
Uncertainty Interval
3 − Region
Location Hierarchy
1 − Global
2 − Super Region
3 − Region

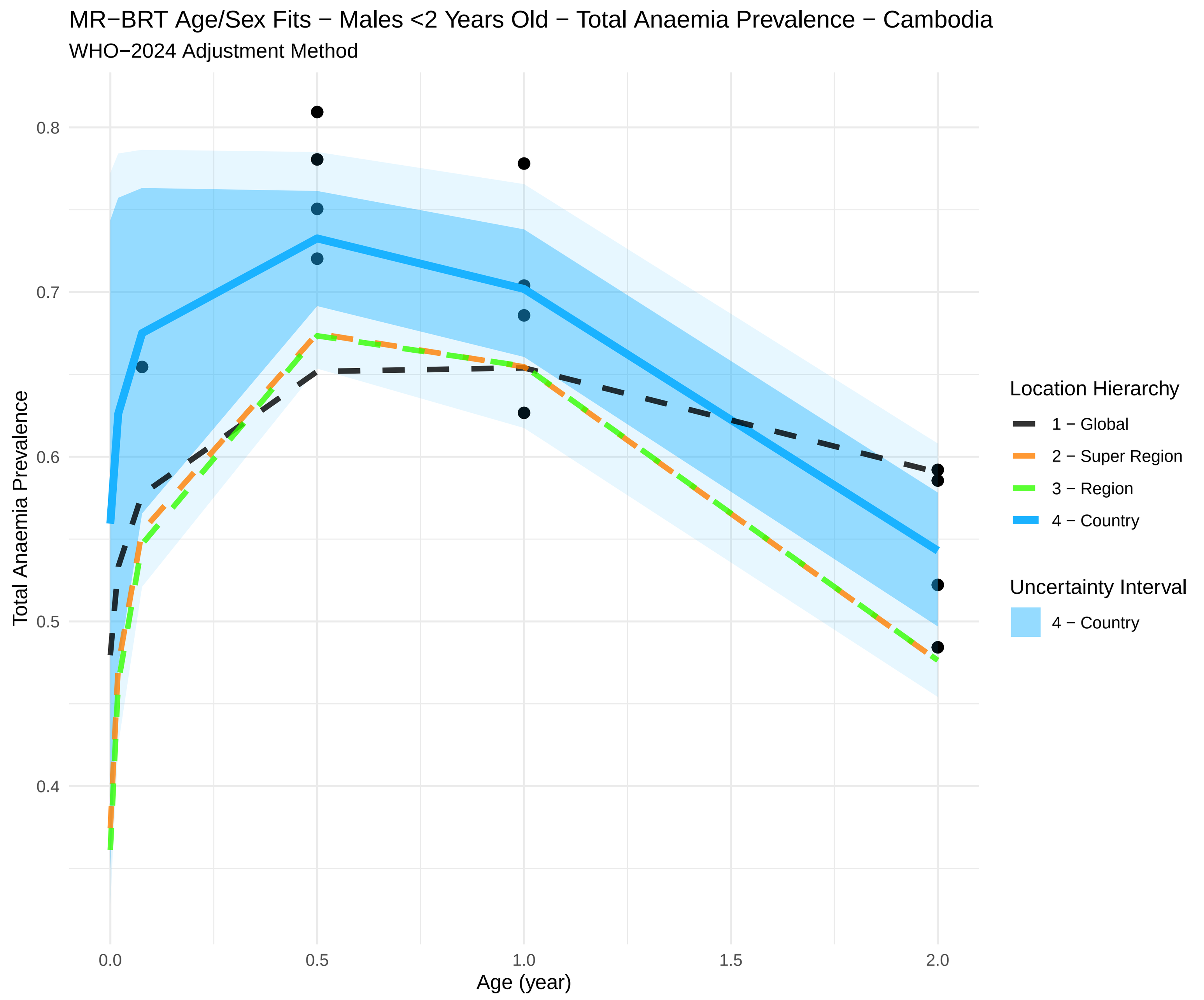
MR−BRT Age/Sex Fits − Males <2 Years Old − Total Anaemia Prevalence − Cambodia
WHO−2024 Adjustment Method
Total Anaemia Prevalence
Age (year)
Location Hierarchy
1 − Global
2 − Super Region
3 − Region
4 − Country
Uncertainty Interval
4 − Country

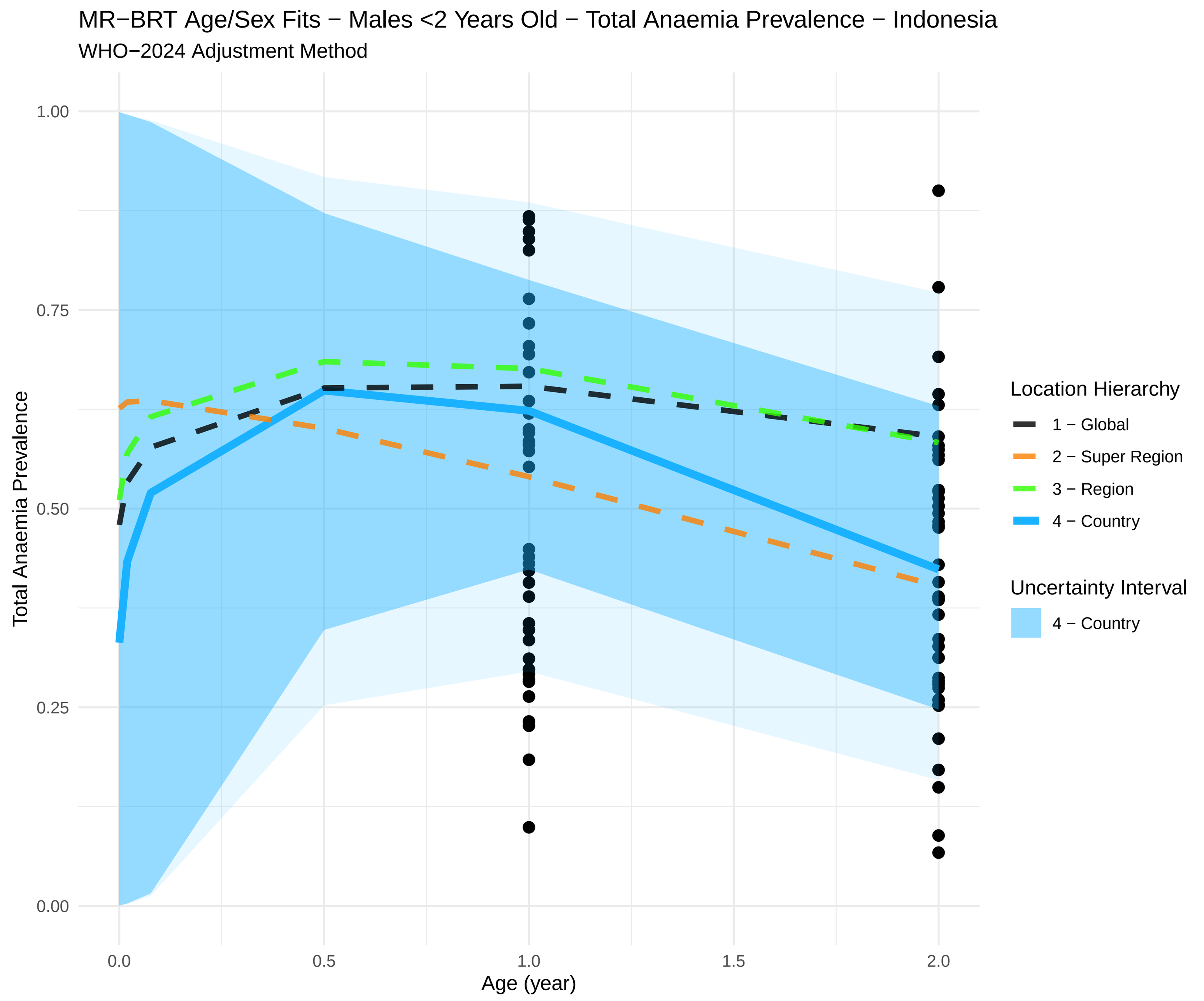
MR−BRT Age/Sex Fits − Males <2 Years Old − Total Anaemia Prevalence − Indonesia
WHO−2024 Adjustment Method
Total Anaemia Prevalence
Age (year)
Location Hierarchy
1 − Global
2 − Super Region
3 − Region
4 − Country
Uncertainty Interval
4 − Country

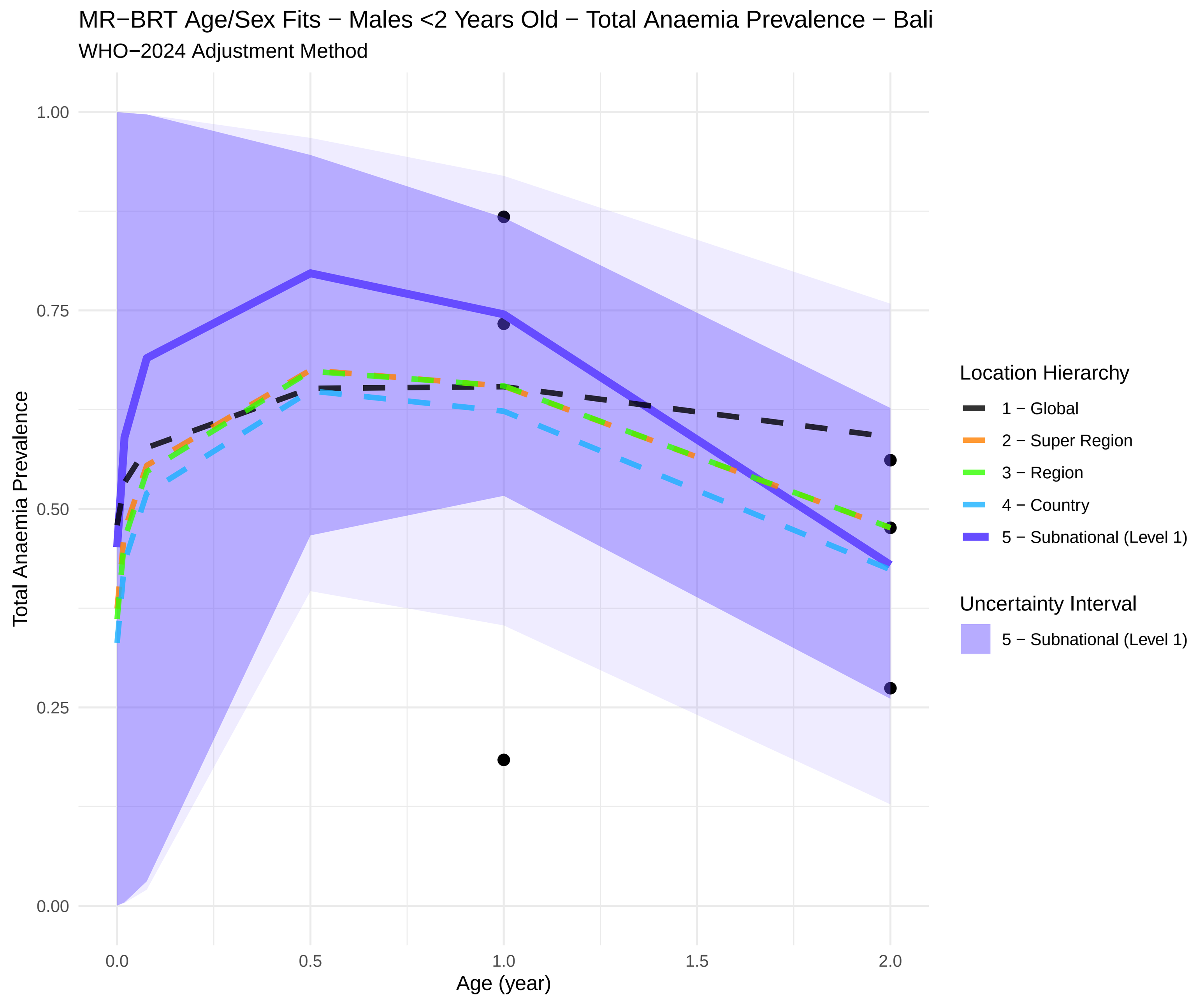
MR−BRT Age/Sex Fits − Males <2 Years Old − Total Anaemia Prevalence − Bali
WHO−2024 Adjustment Method
Total Anaemia Prevalence
Age (year)
Location Hierarchy
1 − Global
2 − Super Region
3 − Region
4 − Country
5 − Subnational (Level 1)
Uncertainty Interval
5 − Subnational (Level 1)

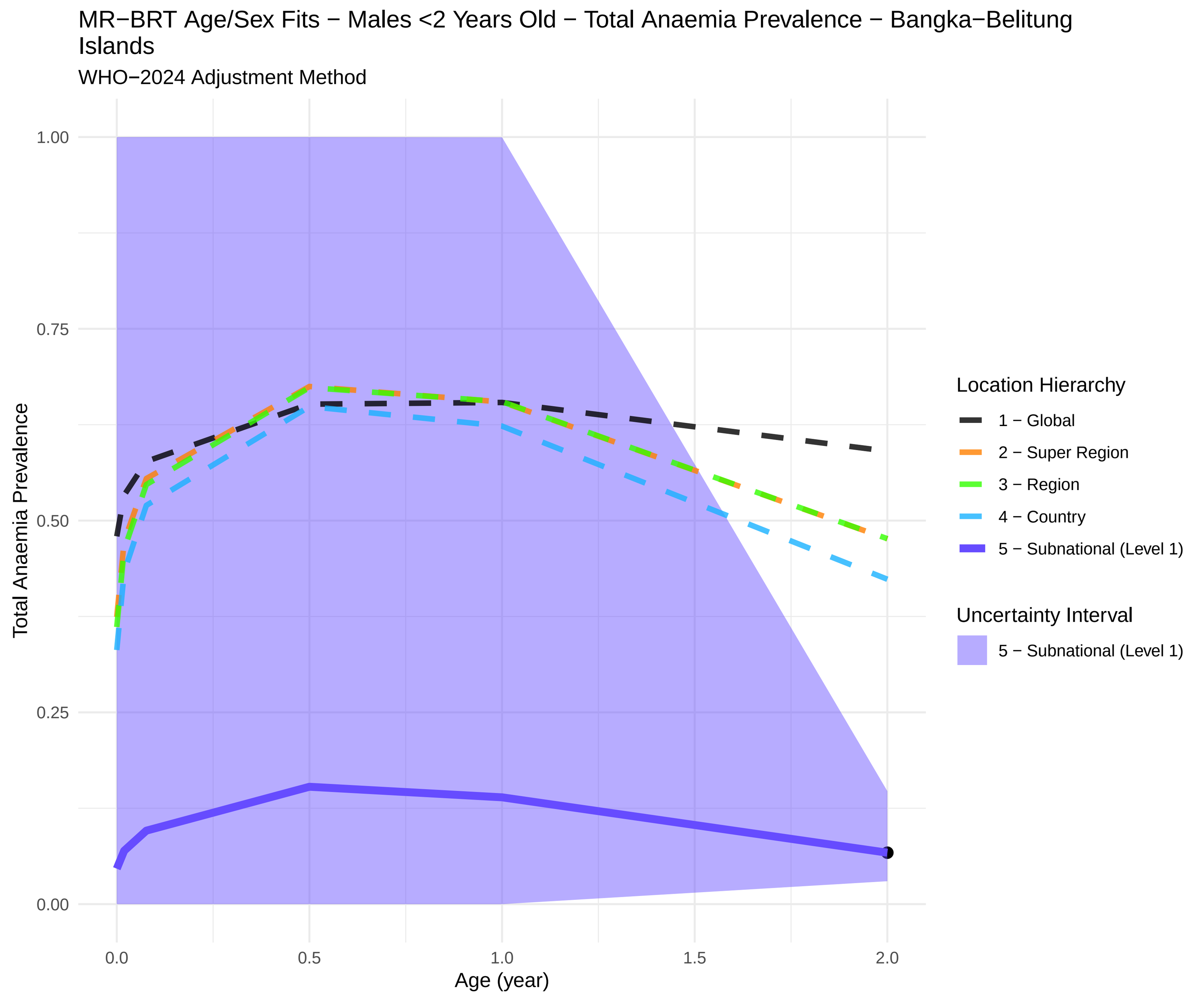
MR−BRT Age/Sex Fits − Males <2 Years Old − Total Anaemia Prevalence − Bangka−Belitung Islands
WHO−2024 Adjustment Method
Total Anaemia Prevalence
Age (year)
Location Hierarchy
1 − Global
2 − Super Region
3 − Region
4 − Country
5 − Subnational (Level 1)
Uncertainty Interval
5 − Subnational (Level 1)

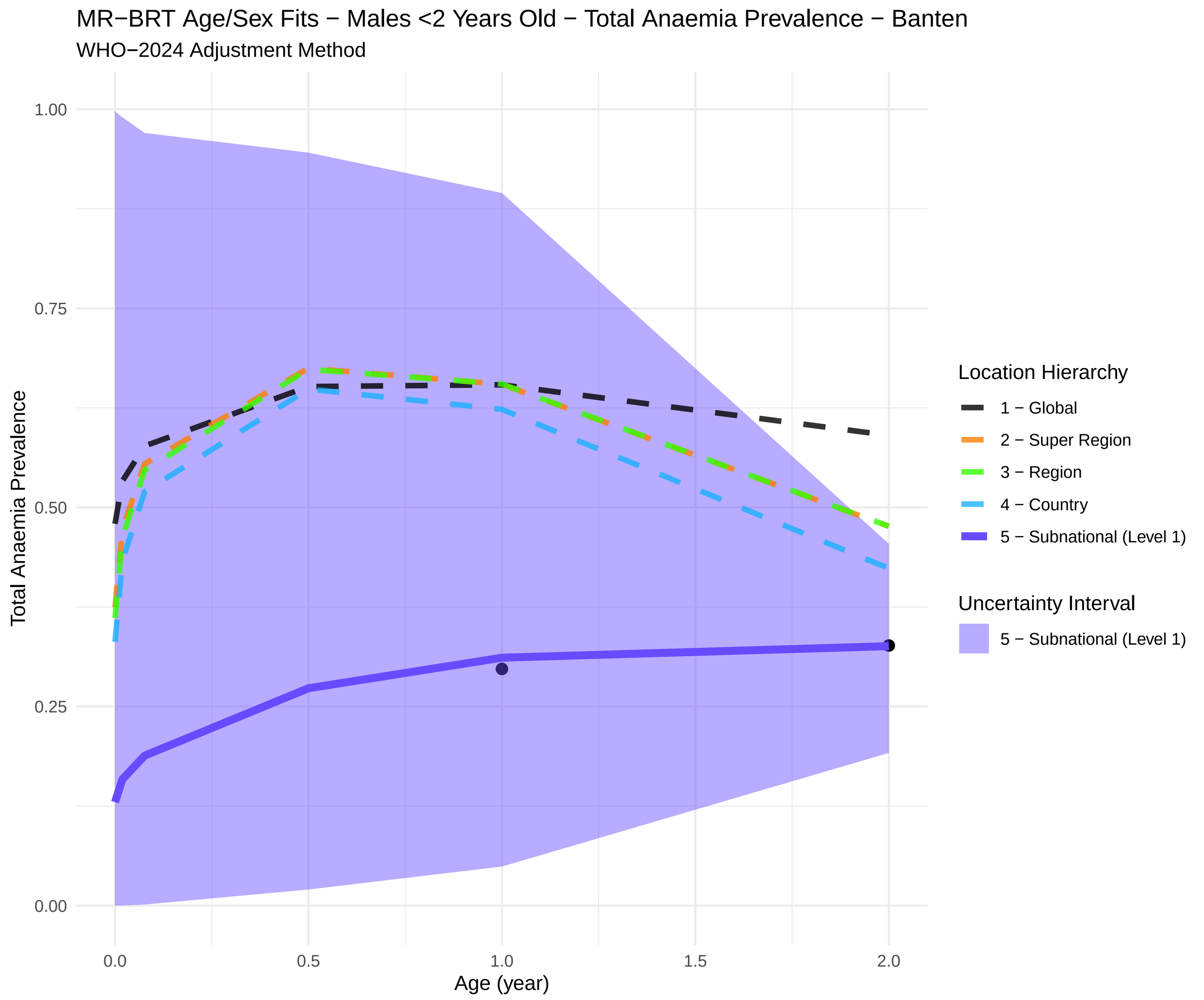
MR−BRT Age/Sex Fits − Males <2 Years Old − Total Anaemia Prevalence − Banten
WHO−2024 Adjustment Method
Total Anaemia Prevalence
Age (year)
Location Hierarchy
1 − Global
2 − Super Region
3 − Region
4 − Country
5 − Subnational (Level 1)
Uncertainty Interval
5 − Subnational (Level 1)

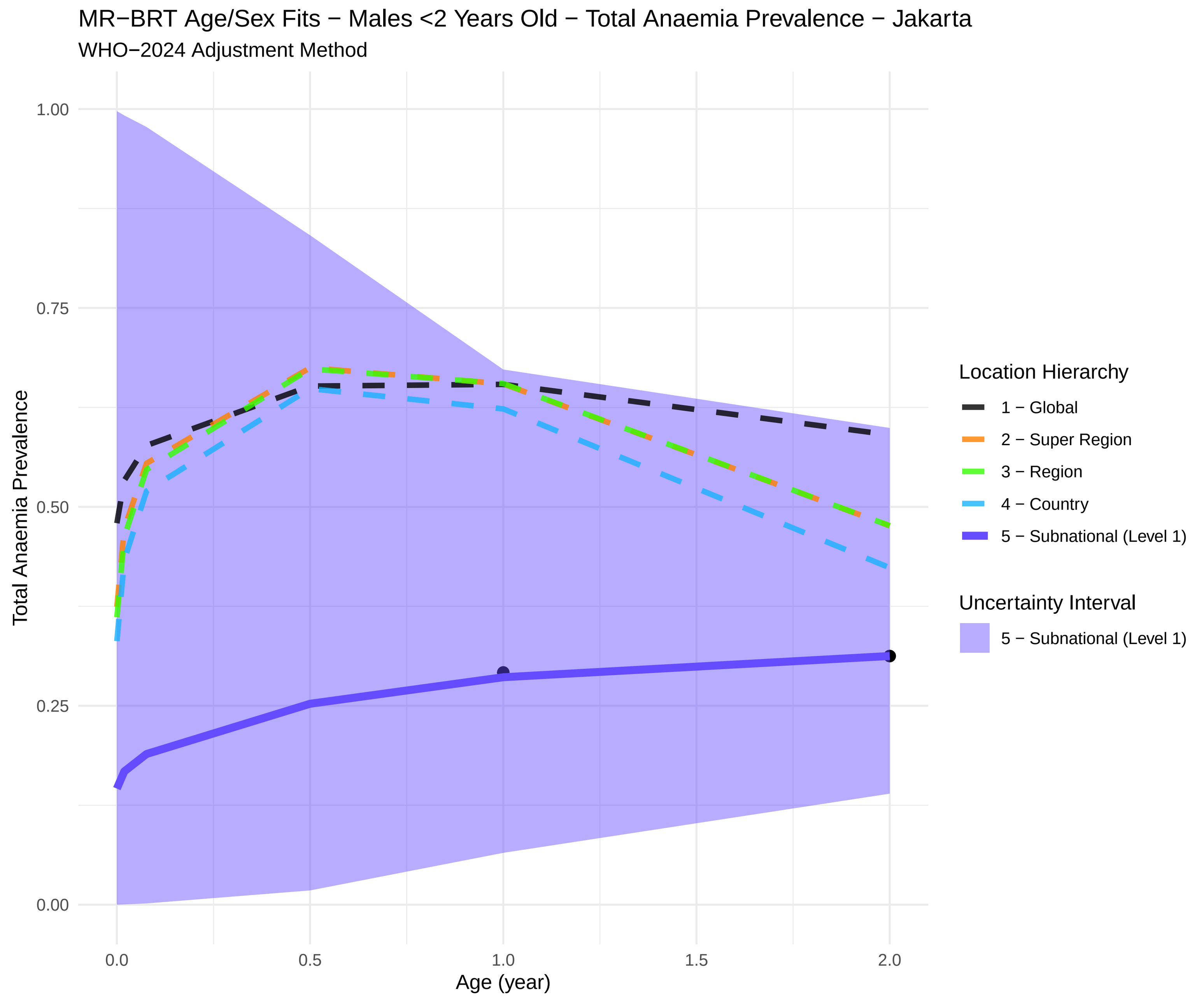
MR−BRT Age/Sex Fits − Males <2 Years Old − Total Anaemia Prevalence − Jakarta
WHO−2024 Adjustment Method
Total Anaemia Prevalence
Age (year)
Location Hierarchy
1 − Global
2 − Super Region
3 − Region
4 − Country
5 − Subnational (Level 1)
Uncertainty Interval
5 − Subnational (Level 1)

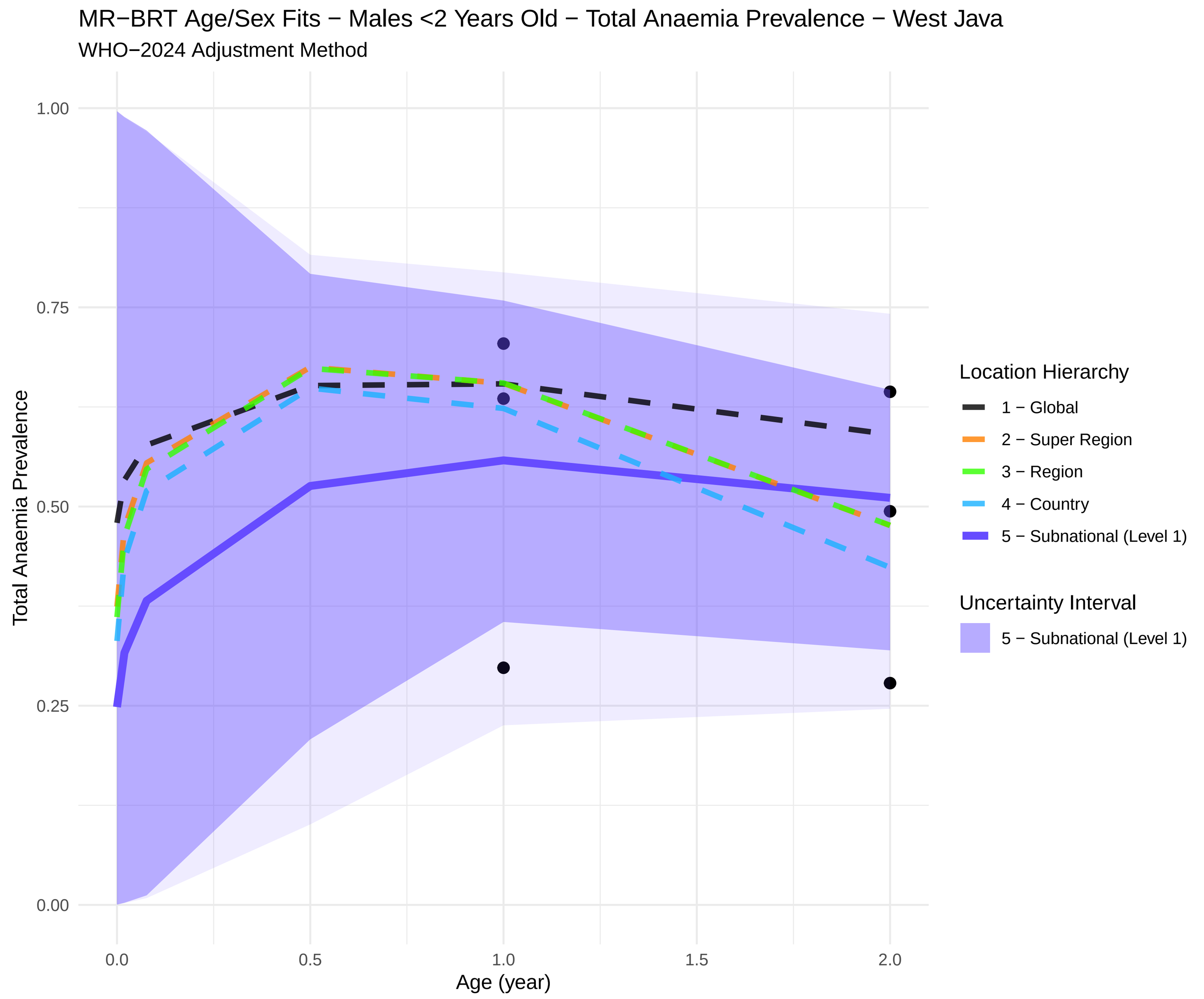
MR−BRT Age/Sex Fits − Males <2 Years Old − Total Anaemia Prevalence − West Java
WHO−2024 Adjustment Method
Total Anaemia Prevalence
Age (year)
Location Hierarchy
1 − Global
2 − Super Region
3 − Region
4 − Country
5 − Subnational (Level 1)
Uncertainty Interval
5 − Subnational (Level 1)

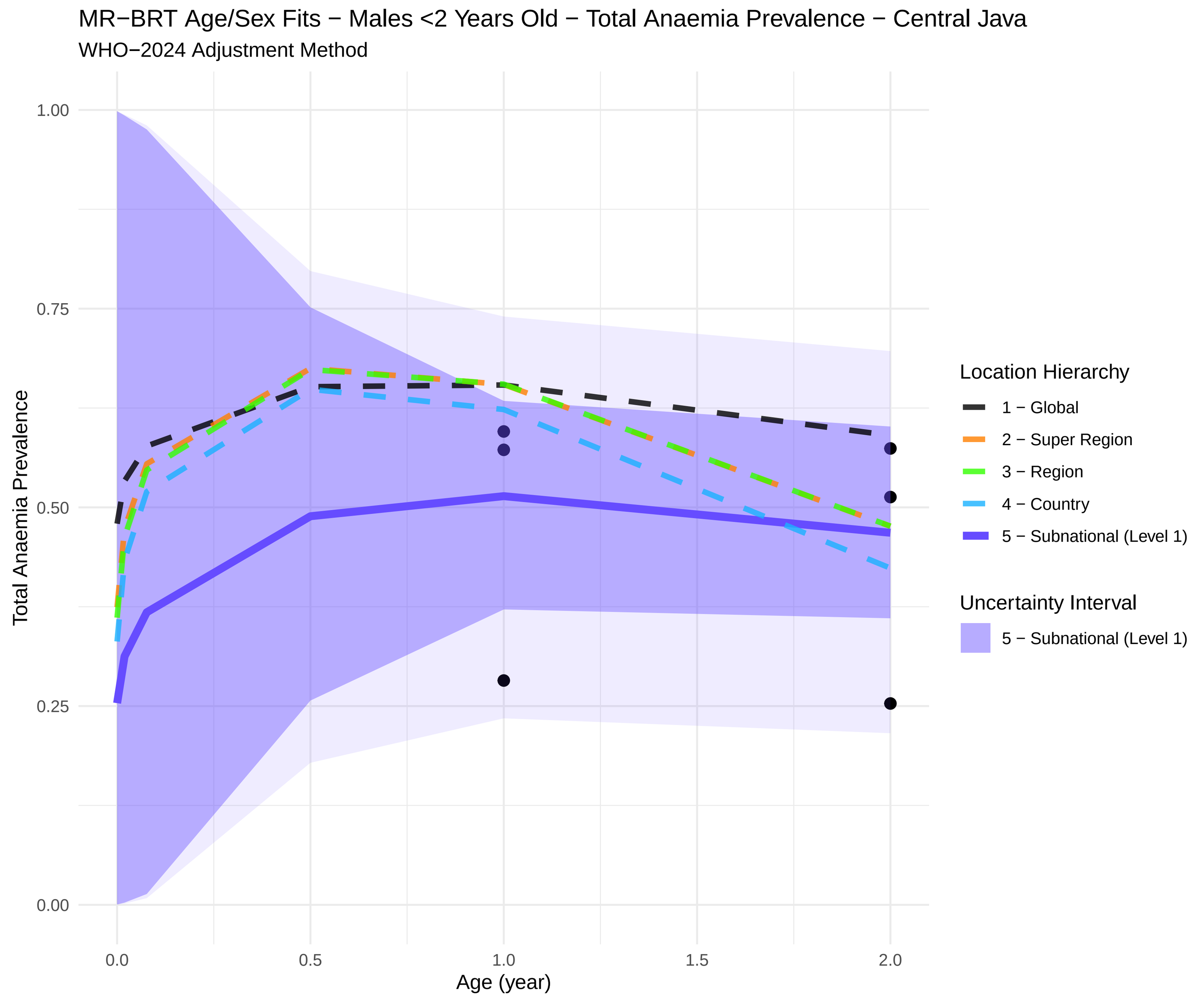
MR−BRT Age/Sex Fits − Males <2 Years Old − Total Anaemia Prevalence − Central Java
WHO−2024 Adjustment Method
Total Anaemia Prevalence
Age (year)
Location Hierarchy
1 − Global
2 − Super Region
3 − Region
4 − Country
5 − Subnational (Level 1)
Uncertainty Interval
5 − Subnational (Level 1)

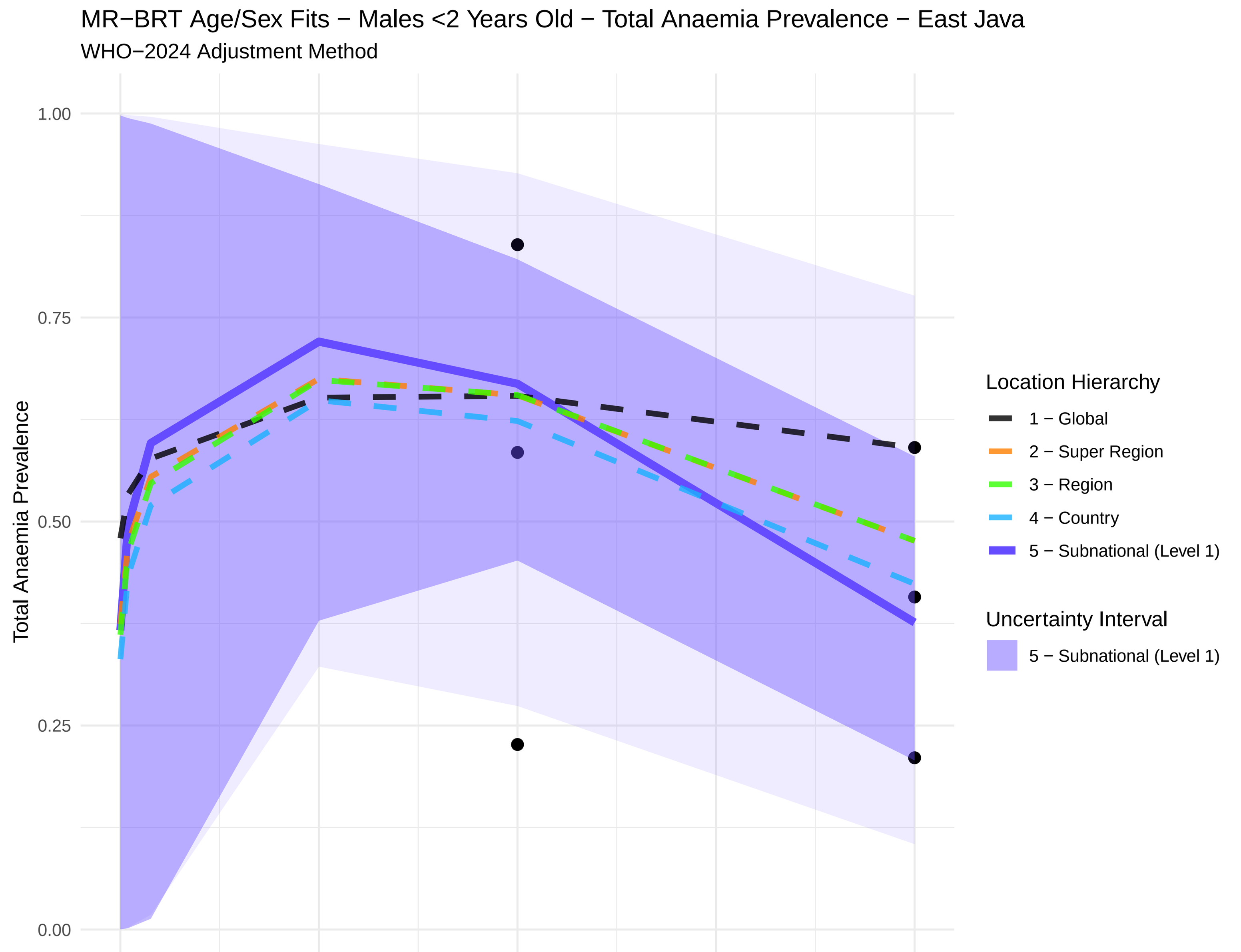
MR−BRT Age/Sex Fits − Males <2 Years Old − Total Anaemia Prevalence − East Java
WHO−2024 Adjustment Method
Total Anaemia Prevalence
Age (year)
Location Hierarchy
1 − Global
2 − Super Region
3 − Region
4 − Country
5 − Subnational (Level 1)
Uncertainty Interval
5 − Subnational (Level 1)

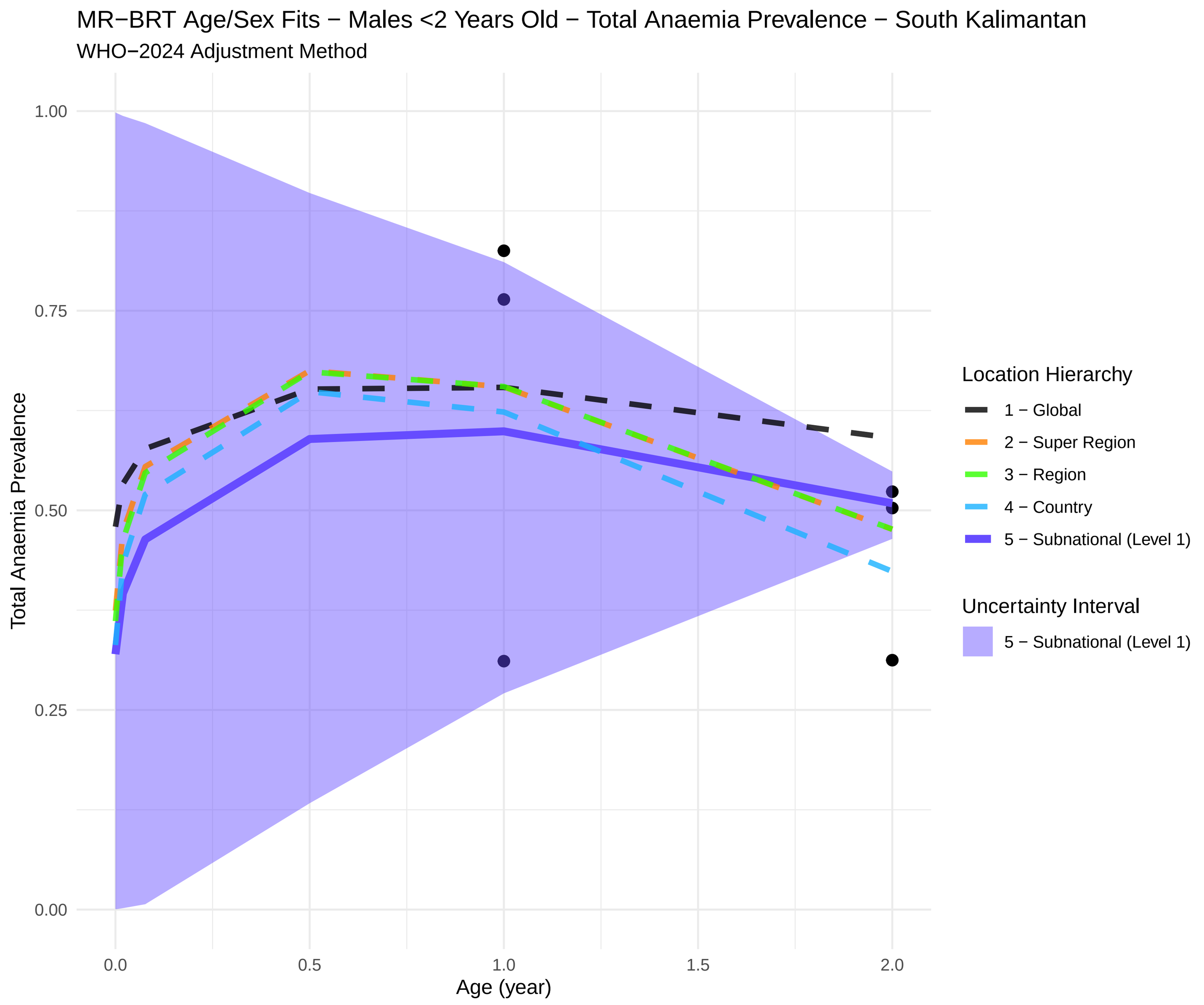
MR−BRT Age/Sex Fits − Males <2 Years Old − Total Anaemia Prevalence − South Kalimantan
WHO−2024 Adjustment Method
Total Anaemia Prevalence
Age (year)
Location Hierarchy
1 − Global
2 − Super Region
3 − Region
4 − Country
5 − Subnational (Level 1)
Uncertainty Interval
5 − Subnational (Level 1)

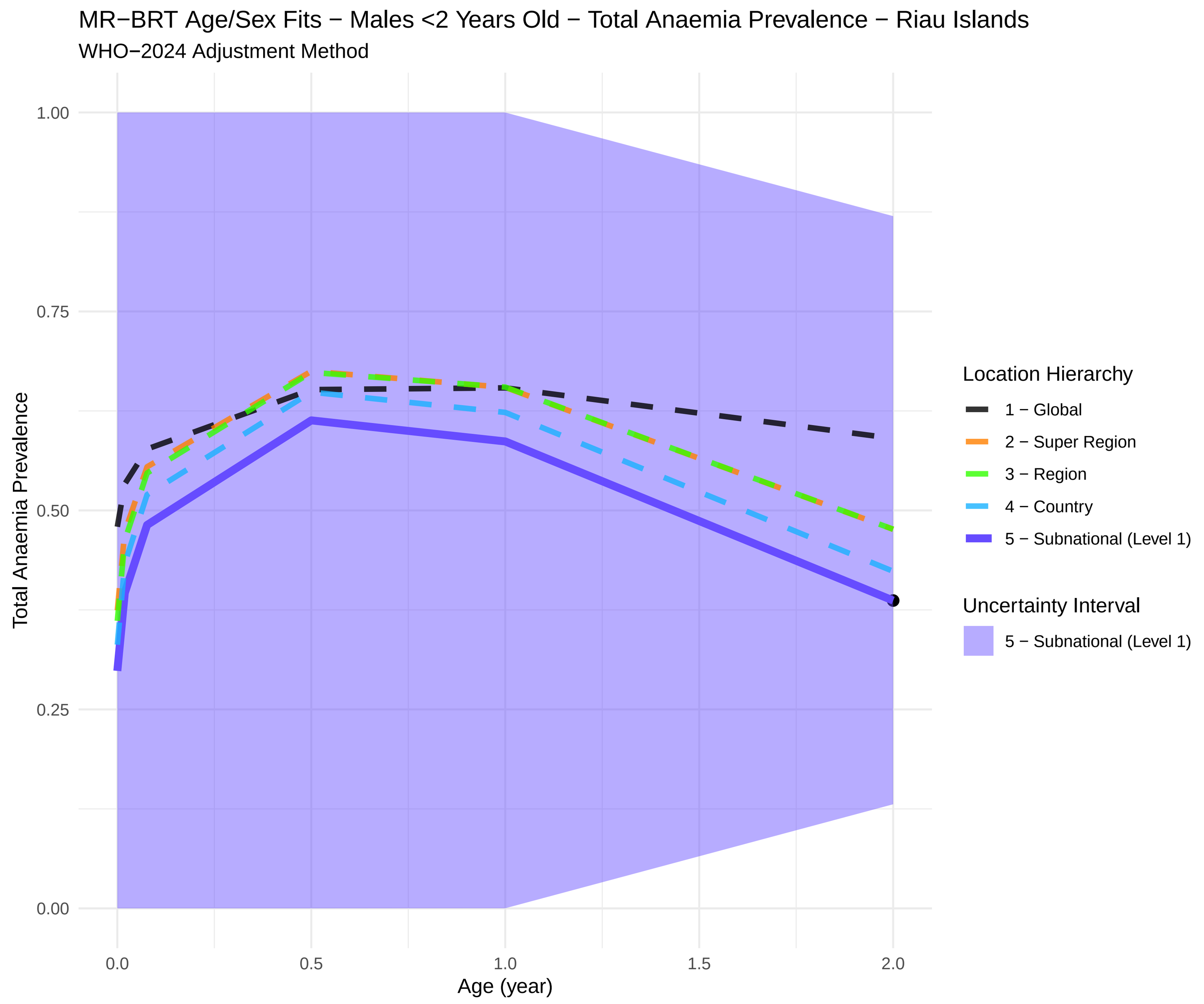
MR−BRT Age/Sex Fits − Males <2 Years Old − Total Anaemia Prevalence − Riau Islands
WHO−2024 Adjustment Method
Total Anaemia Prevalence
Age (year)
Location Hierarchy
1 − Global
2 − Super Region
3 − Region
4 − Country
5 − Subnational (Level 1)
Uncertainty Interval
5 − Subnational (Level 1)

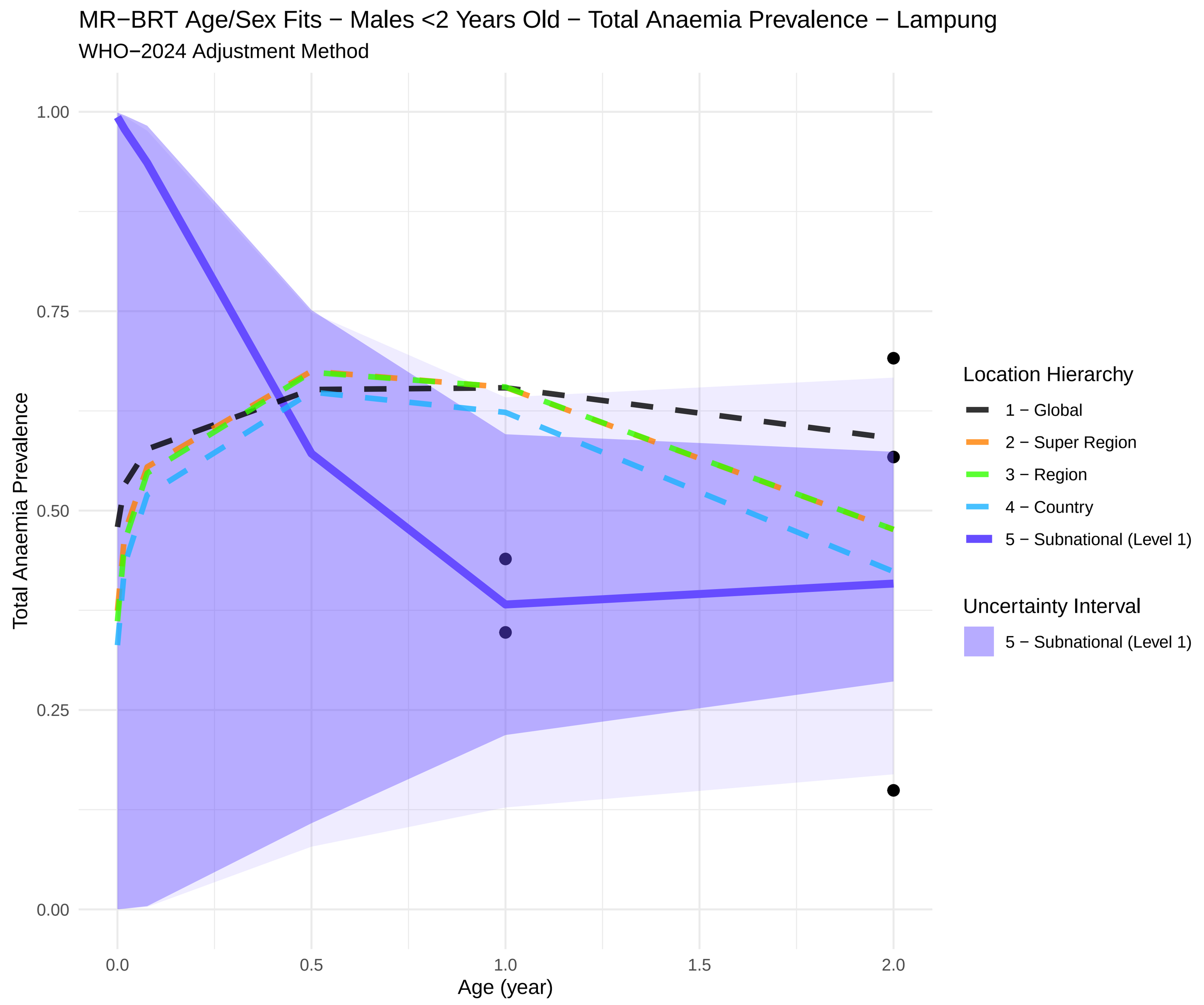
MR−BRT Age/Sex Fits − Males <2 Years Old − Total Anaemia Prevalence − Lampung
WHO−2024 Adjustment Method
Total Anaemia Prevalence
Age (year)
Location Hierarchy
1 − Global
2 − Super Region
3 − Region
4 − Country
5 − Subnational (Level 1)
Uncertainty Interval
5 − Subnational (Level 1)

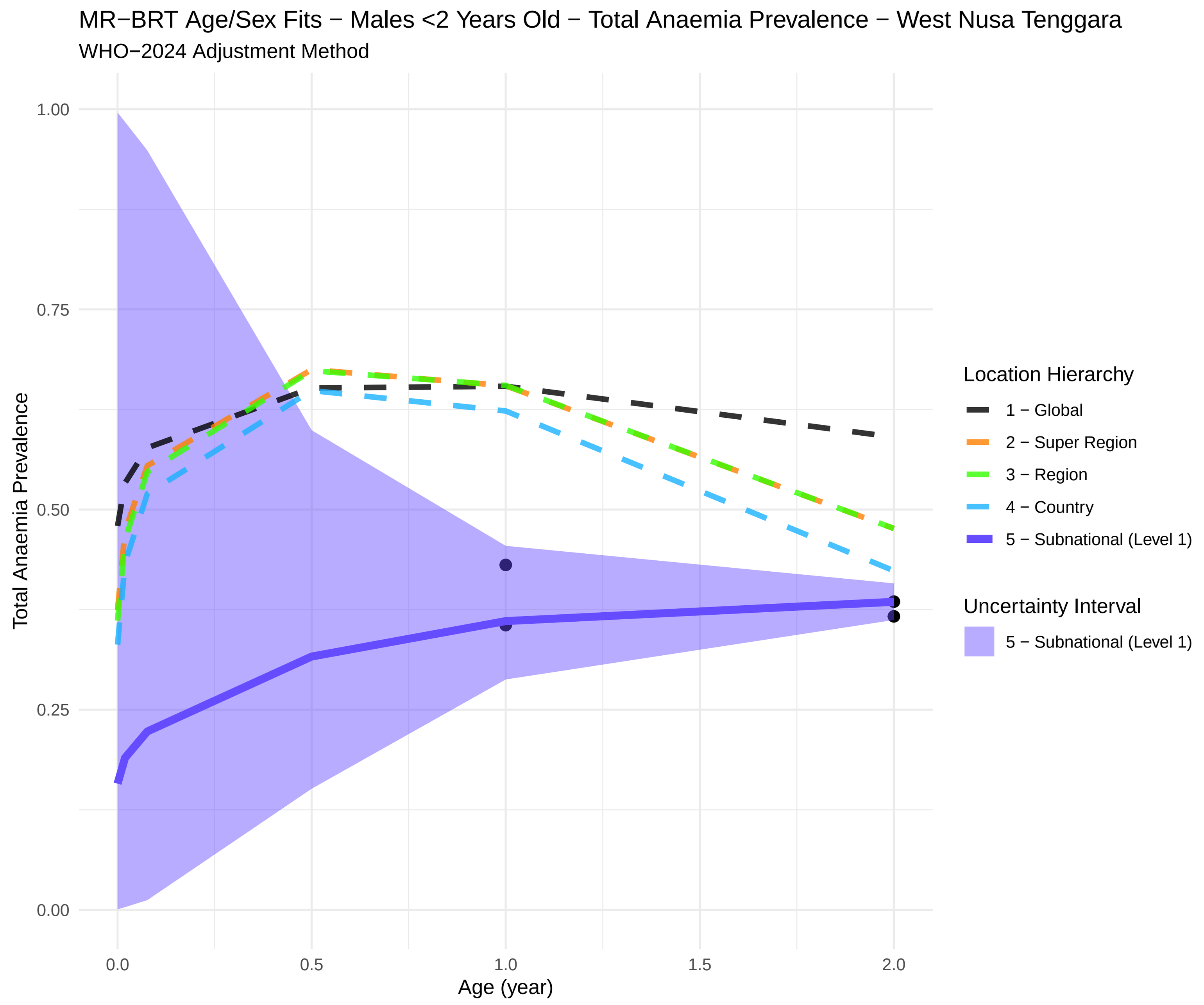
MR−BRT Age/Sex Fits − Males <2 Years Old − Total Anaemia Prevalence − West Nusa Tenggara
WHO−2024 Adjustment Method
Total Anaemia Prevalence
Age (year)
Location Hierarchy
1 − Global
2 − Super Region
3 − Region
4 − Country
5 − Subnational (Level 1)
Uncertainty Interval
5 − Subnational (Level 1)

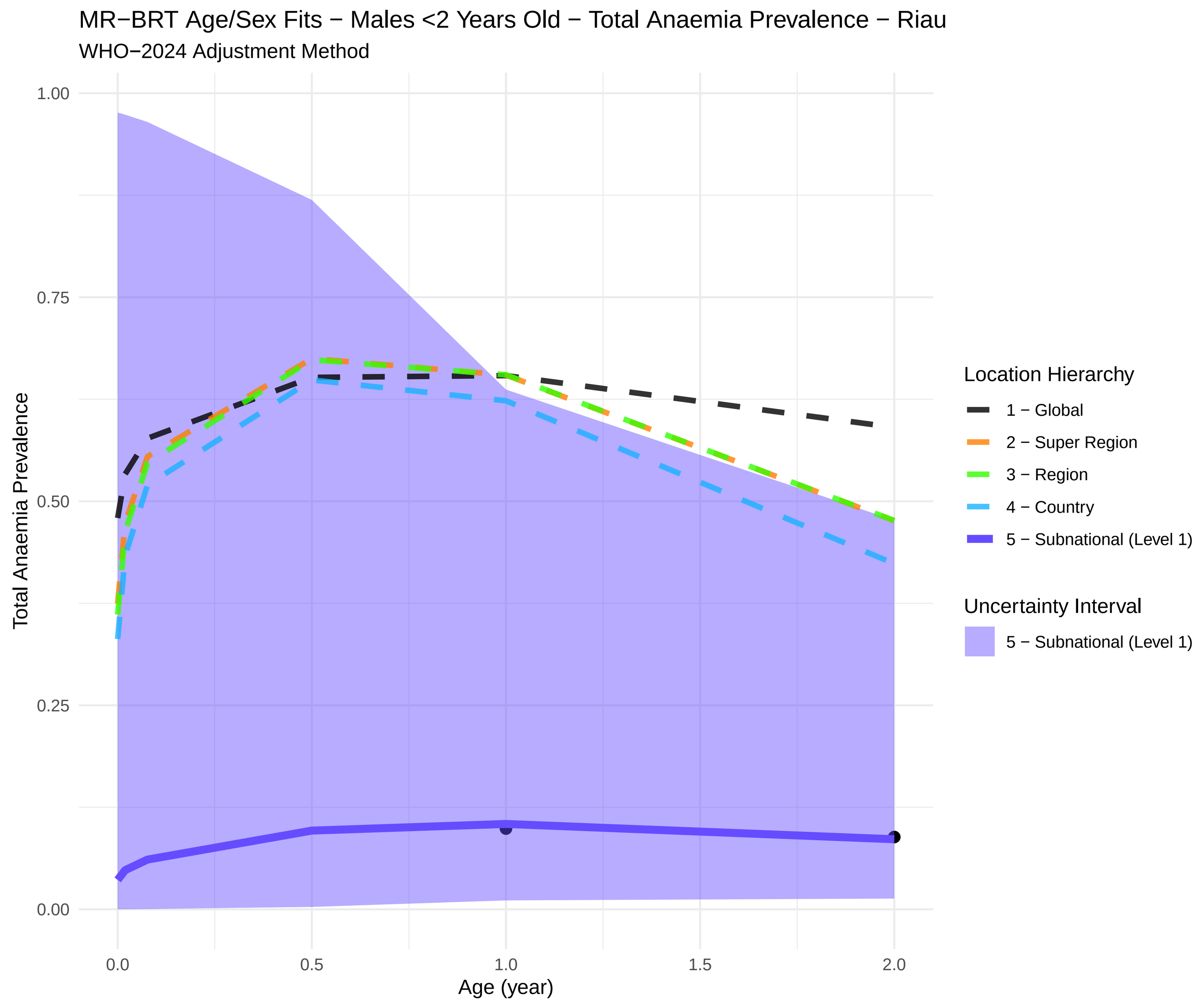
MR−BRT Age/Sex Fits − Males <2 Years Old − Total Anaemia Prevalence − Riau
WHO−2024 Adjustment Method
Total Anaemia Prevalence
Age (year)
Location Hierarchy
1 − Global
2 − Super Region
3 − Region
4 − Country
5 − Subnational (Level 1)
Uncertainty Interval
5 − Subnational (Level 1)

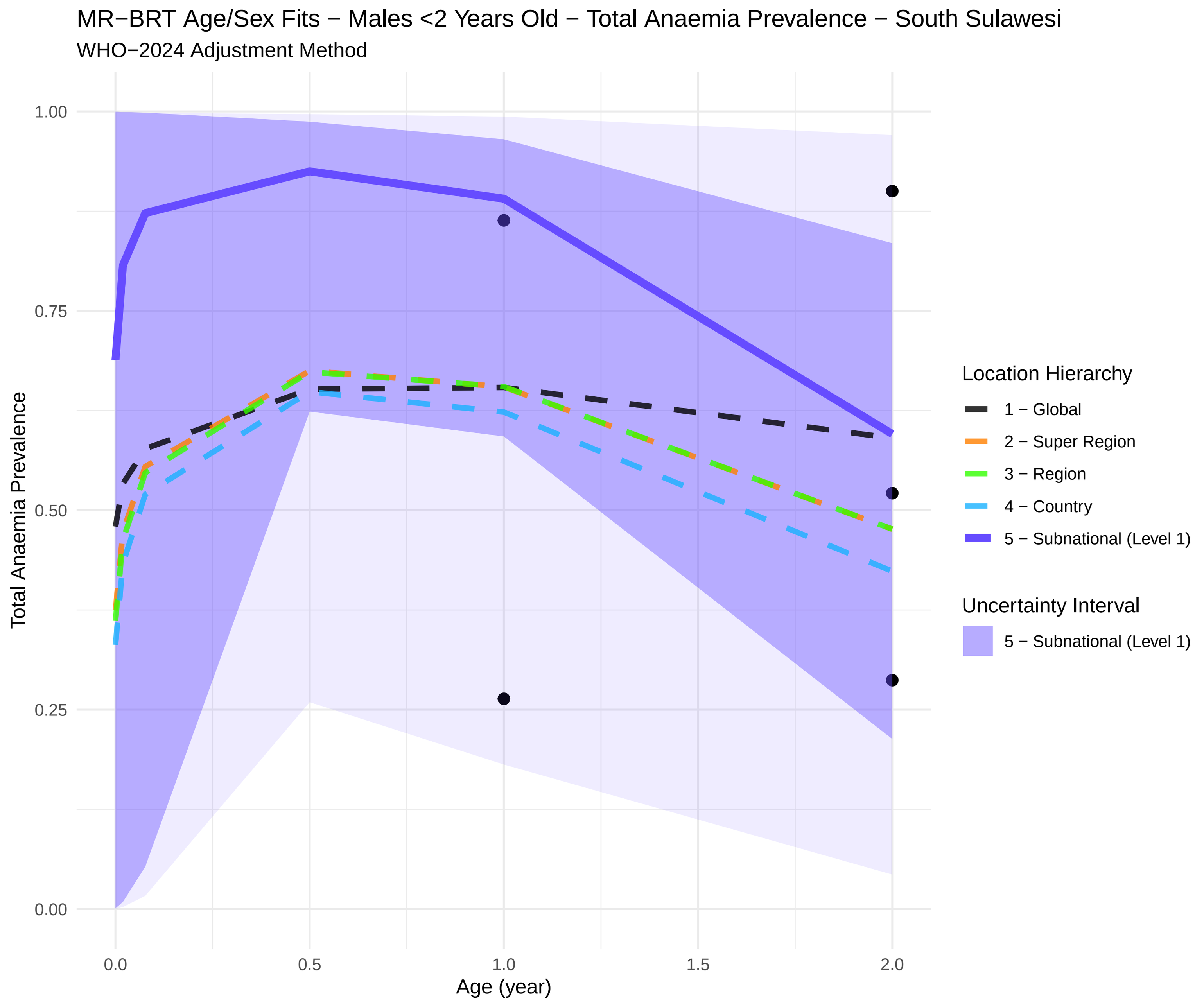
MR−BRT Age/Sex Fits − Males <2 Years Old − Total Anaemia Prevalence − South Sulawesi
WHO−2024 Adjustment Method
Total Anaemia Prevalence
Age (year)
Location Hierarchy
1 − Global
2 − Super Region
3 − Region
4 − Country
5 − Subnational (Level 1)
Uncertainty Interval
5 − Subnational (Level 1)

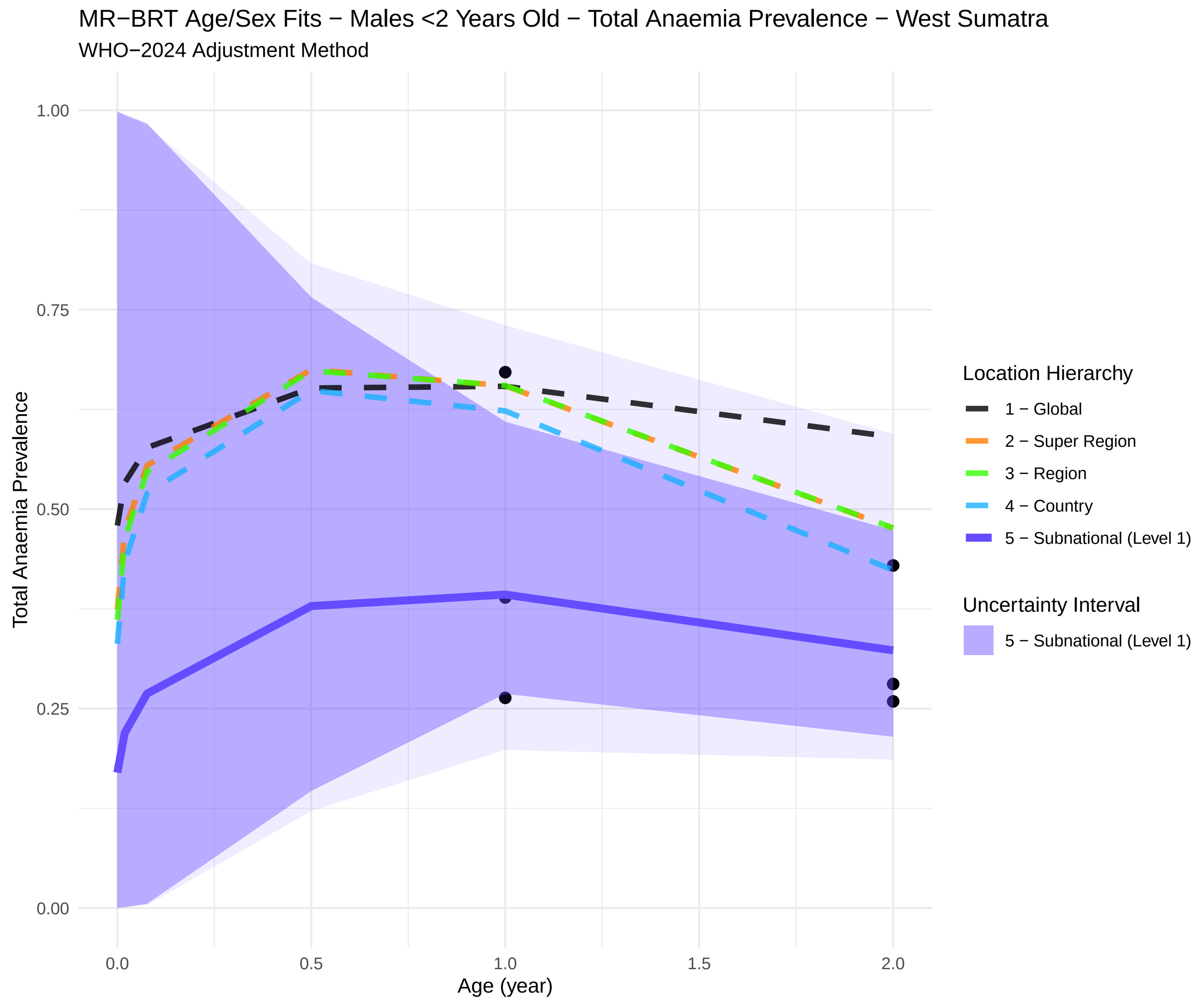
MR−BRT Age/Sex Fits − Males <2 Years Old − Total Anaemia Prevalence − West Sumatra
WHO−2024 Adjustment Method
Total Anaemia Prevalence
Age (year)
Location Hierarchy
1 − Global
2 − Super Region
3 − Region
4 − Country
5 − Subnational (Level 1)
Uncertainty Interval
5 − Subnational (Level 1)

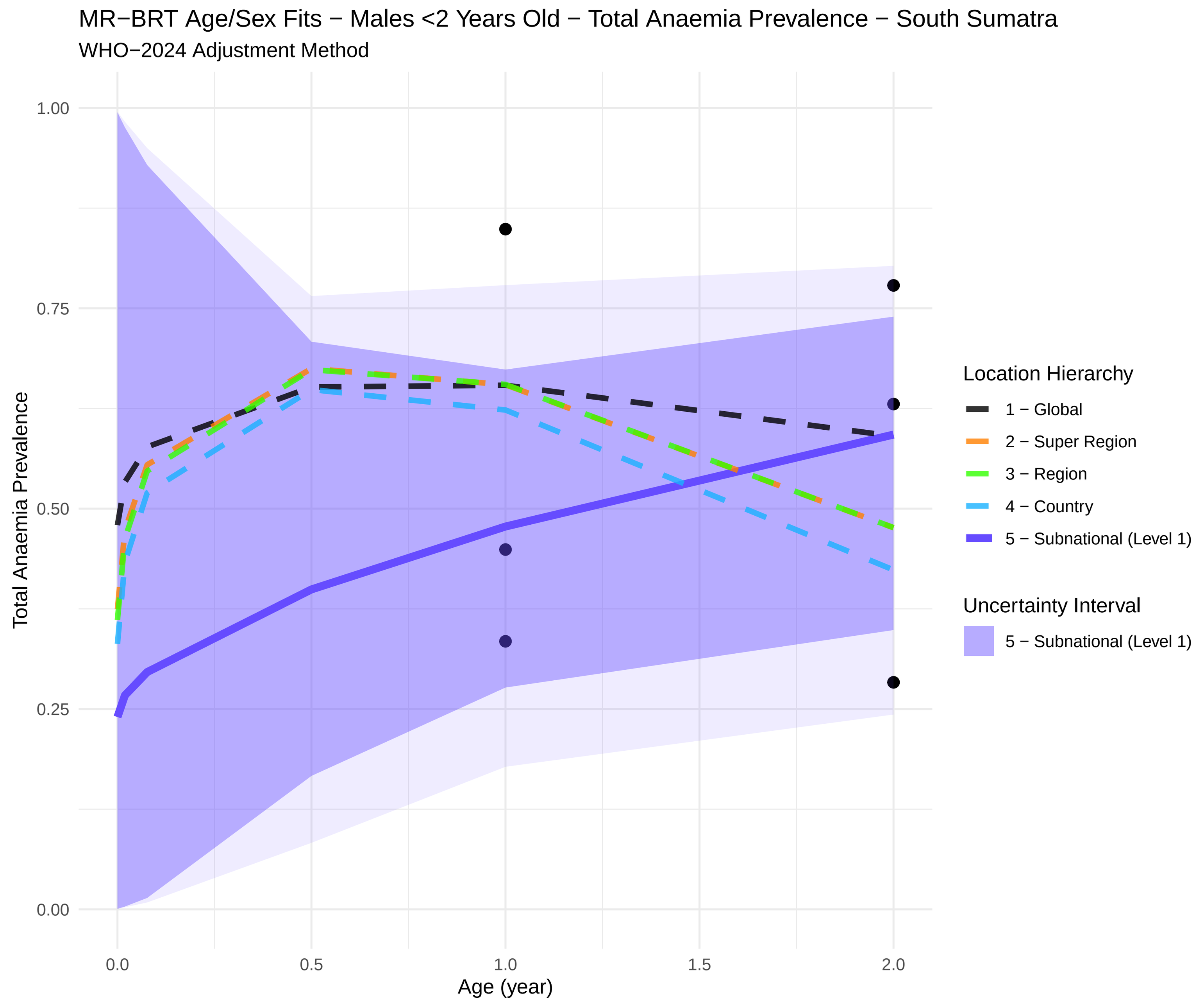
MR−BRT Age/Sex Fits − Males <2 Years Old − Total Anaemia Prevalence − South Sumatra
WHO−2024 Adjustment Method
Total Anaemia Prevalence
Age (year)
Location Hierarchy
1 − Global
2 − Super Region
3 − Region
4 − Country
5 − Subnational (Level 1)
Uncertainty Interval
5 − Subnational (Level 1)

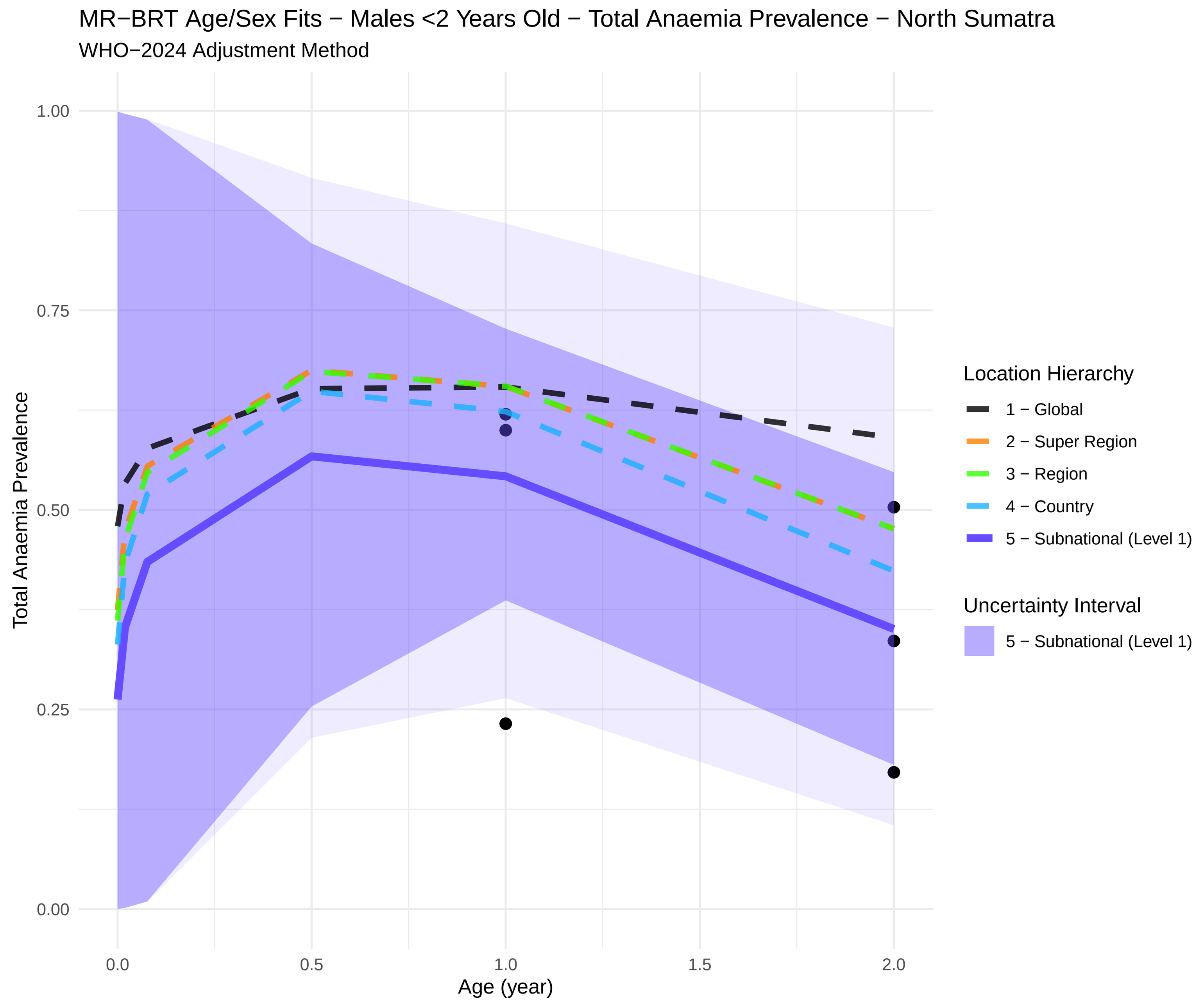
MR−BRT Age/Sex Fits − Males <2 Years Old − Total Anaemia Prevalence − North Sumatra
WHO−2024 Adjustment Method
Total Anaemia Prevalence
Age (year)
Location Hierarchy
1 − Global
2 − Super Region
3 − Region
4 − Country
5 − Subnational (Level 1)
Uncertainty Interval
5 − Subnational (Level 1)

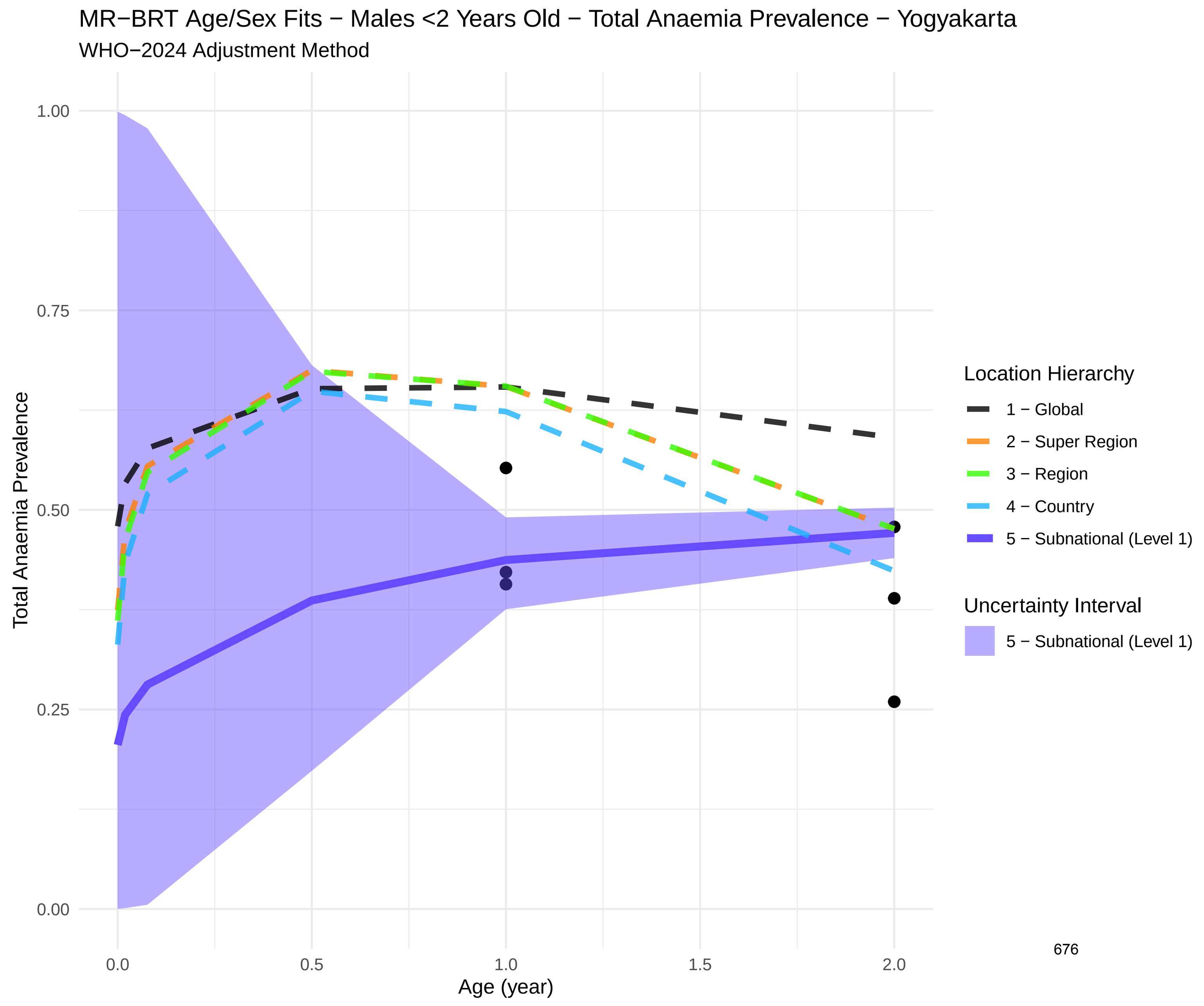
MR−BRT Age/Sex Fits − Males <2 Years Old − Total Anaemia Prevalence − Yogyakarta
WHO−2024 Adjustment Method
Total Anaemia Prevalence
Age (year)
Location Hierarchy
1 − Global
2 − Super Region
3 − Region
4 − Country
5 − Subnational (Level 1)
Uncertainty Interval
5 − Subnational (Level 1)

MR−BRT Age/Sex Fits − Males <2 Years Old − Total Anaemia Prevalence − Myanmar
WHO−2024 Adjustment Method

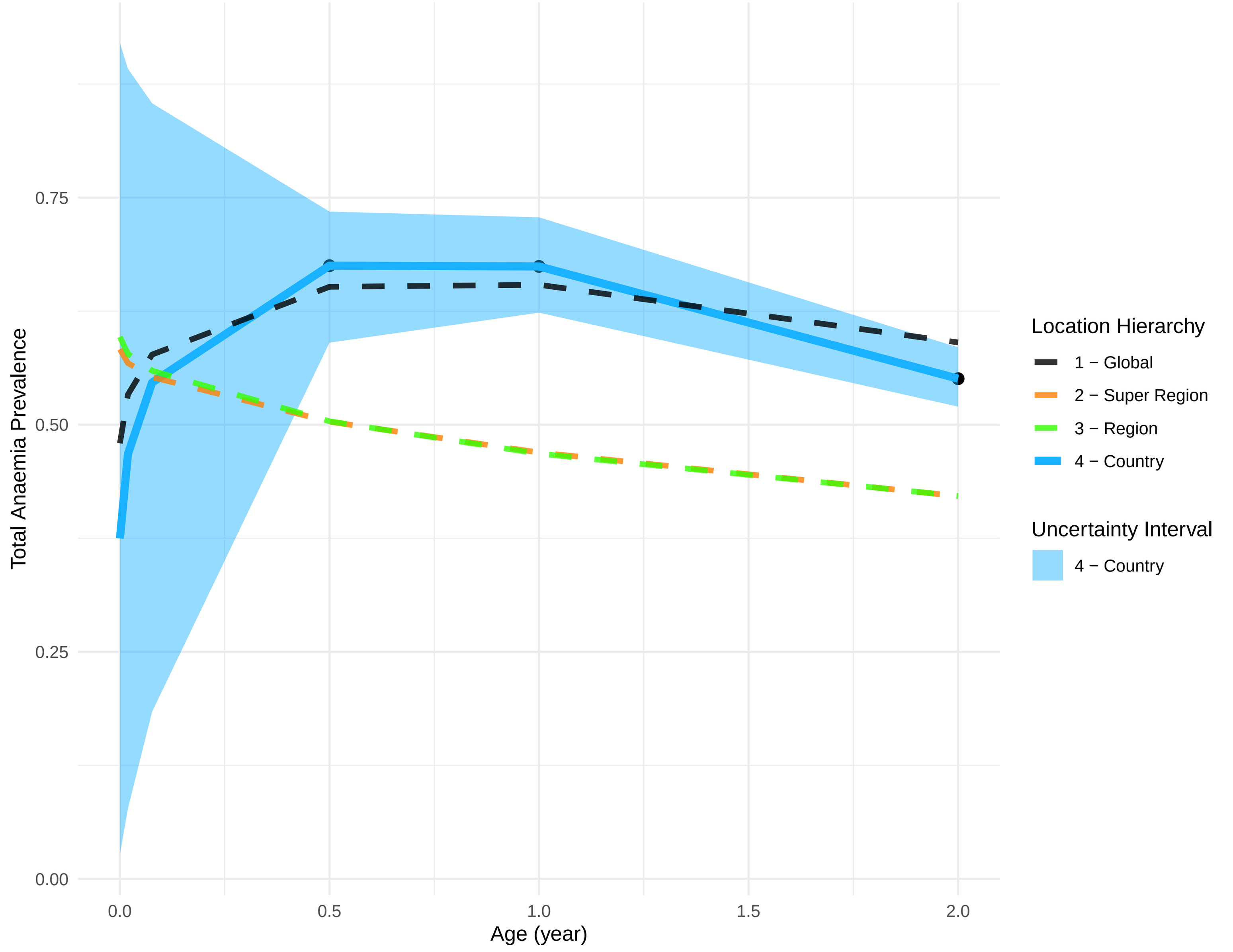

MR−BRT Age/Sex Fits − Males <2 Years Old − Total Anaemia Prevalence − Timor−Leste
WHO−2024 Adjustment Method

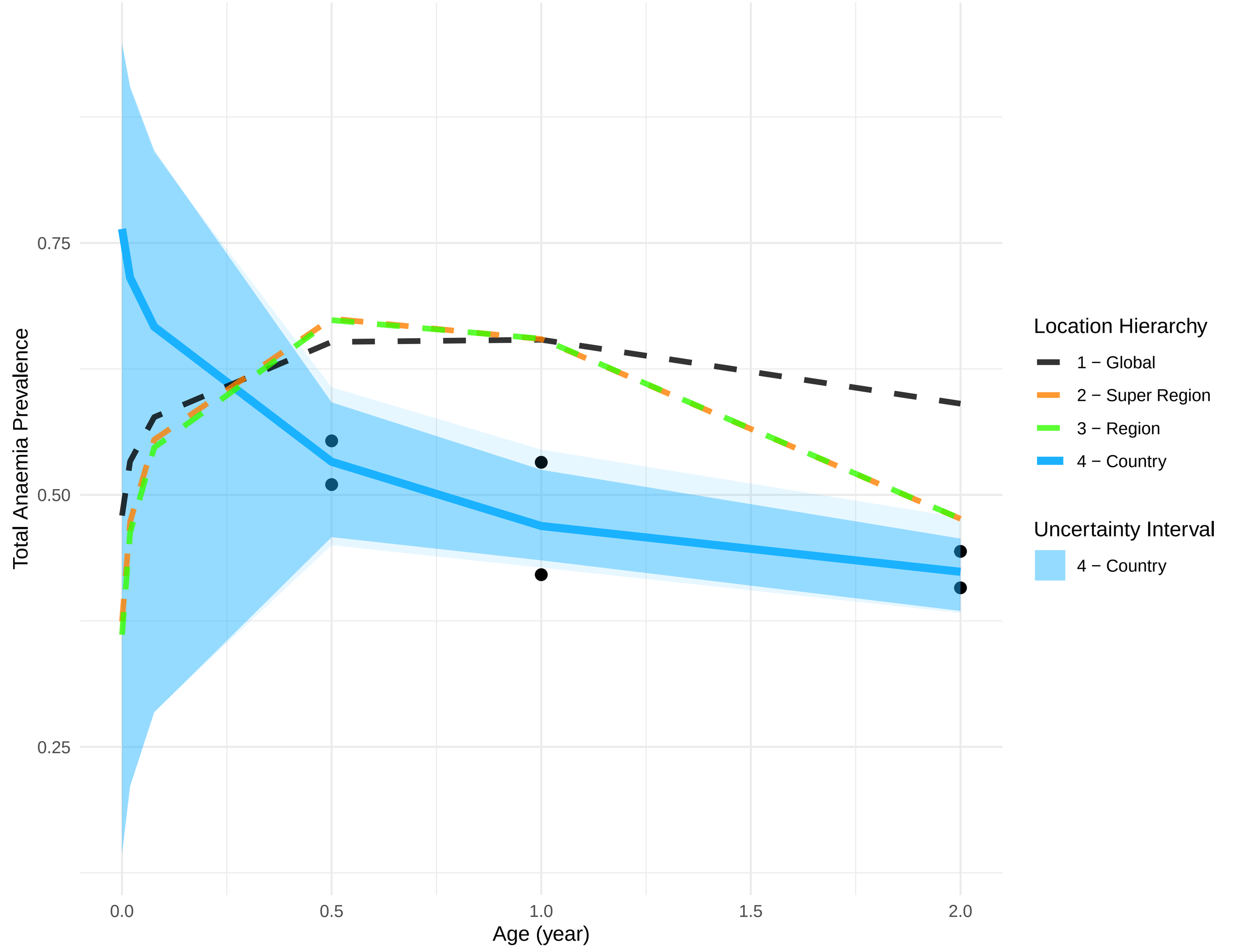

MR−BRT Age/Sex Fits − Males <2 Years Old − Total Anaemia Prevalence − Sub−Saharan Africa
WHO−2024 Adjustment Method

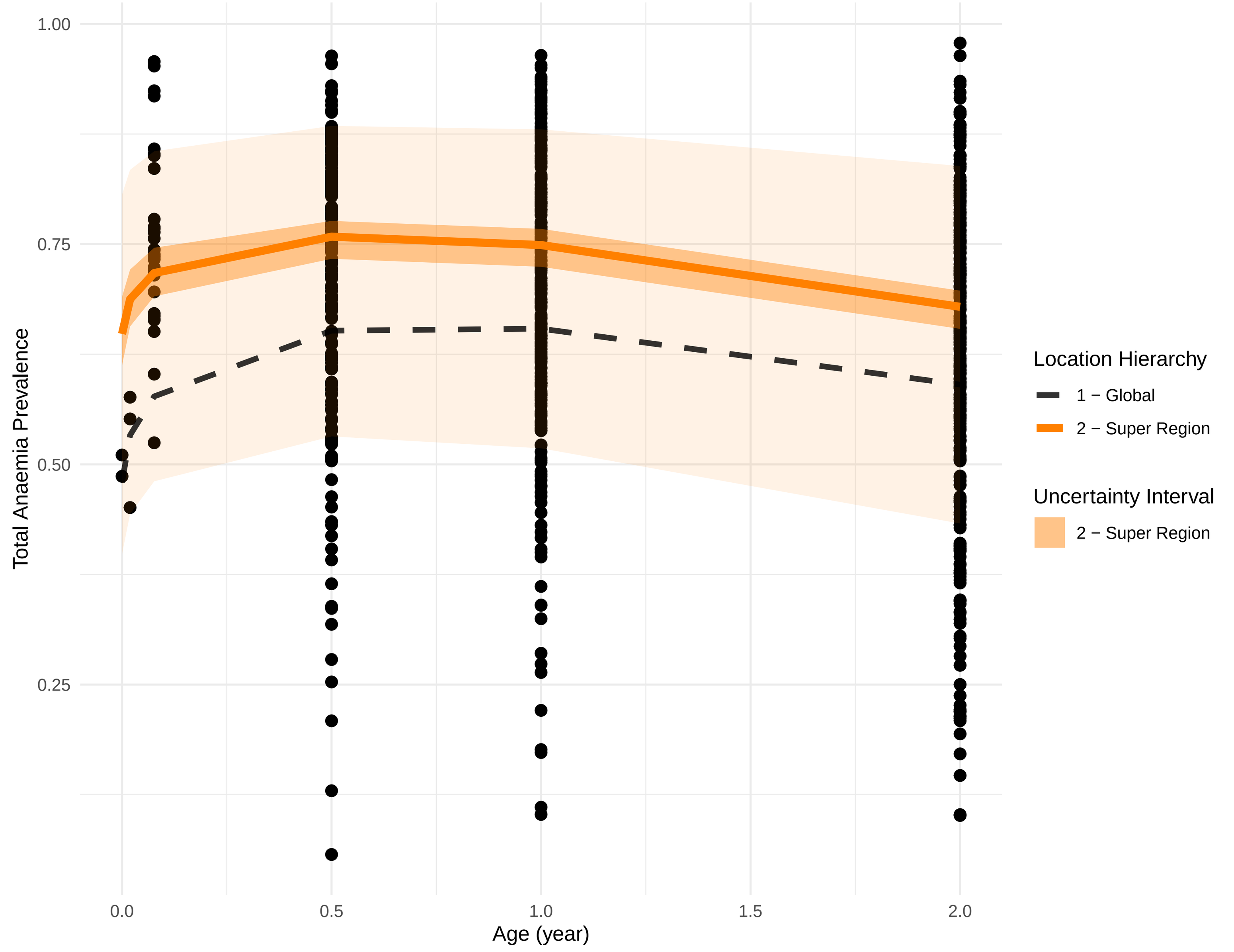

MR−BRT Age/Sex Fits − Males <2 Years Old − Total Anaemia Prevalence − Central Sub−Saharan Africa
WHO−2024 Adjustment Method

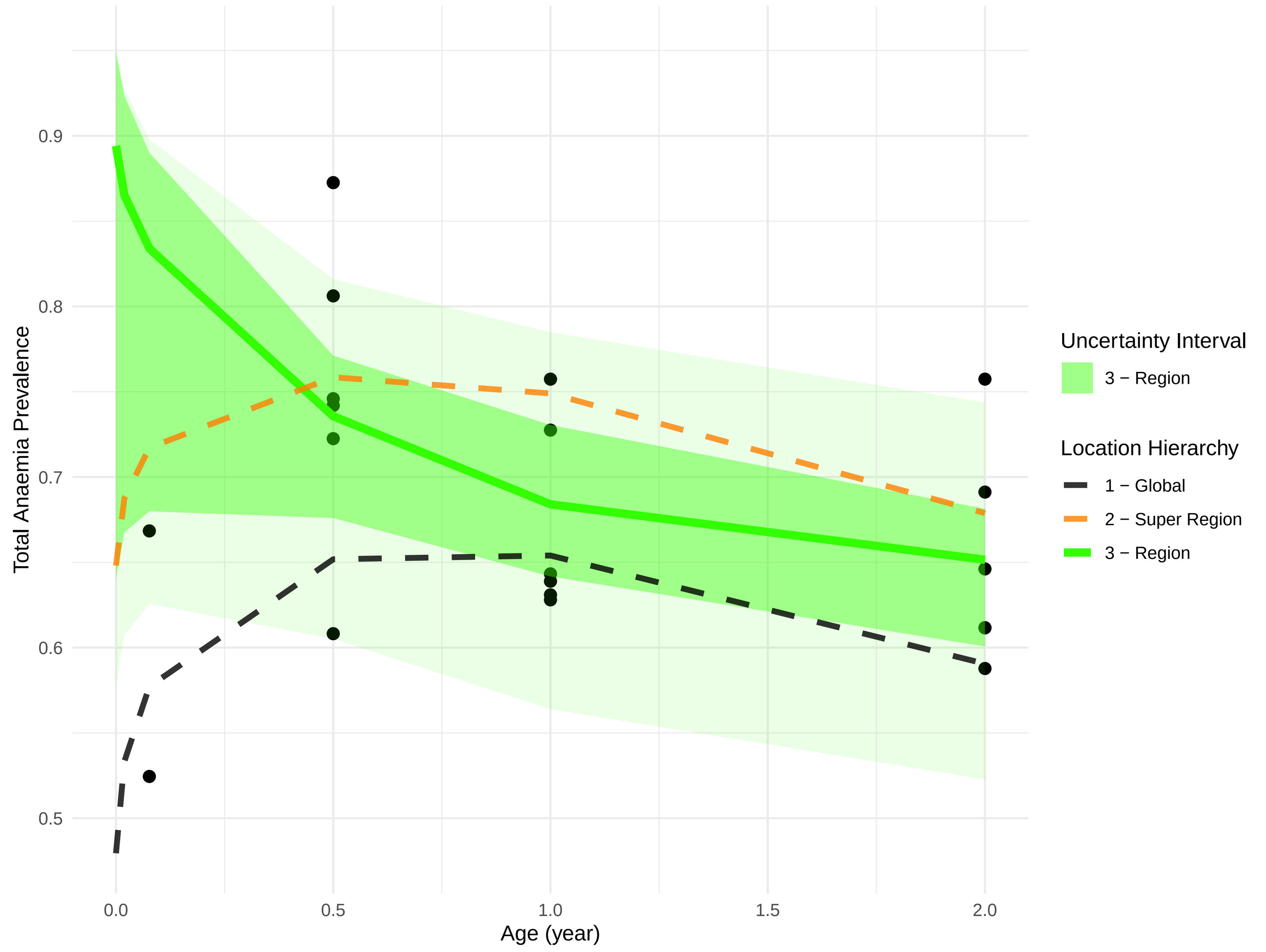

MR−BRT Age/Sex Fits − Males <2 Years Old − Total Anaemia Prevalence − Angola
WHO−2024 Adjustment Method

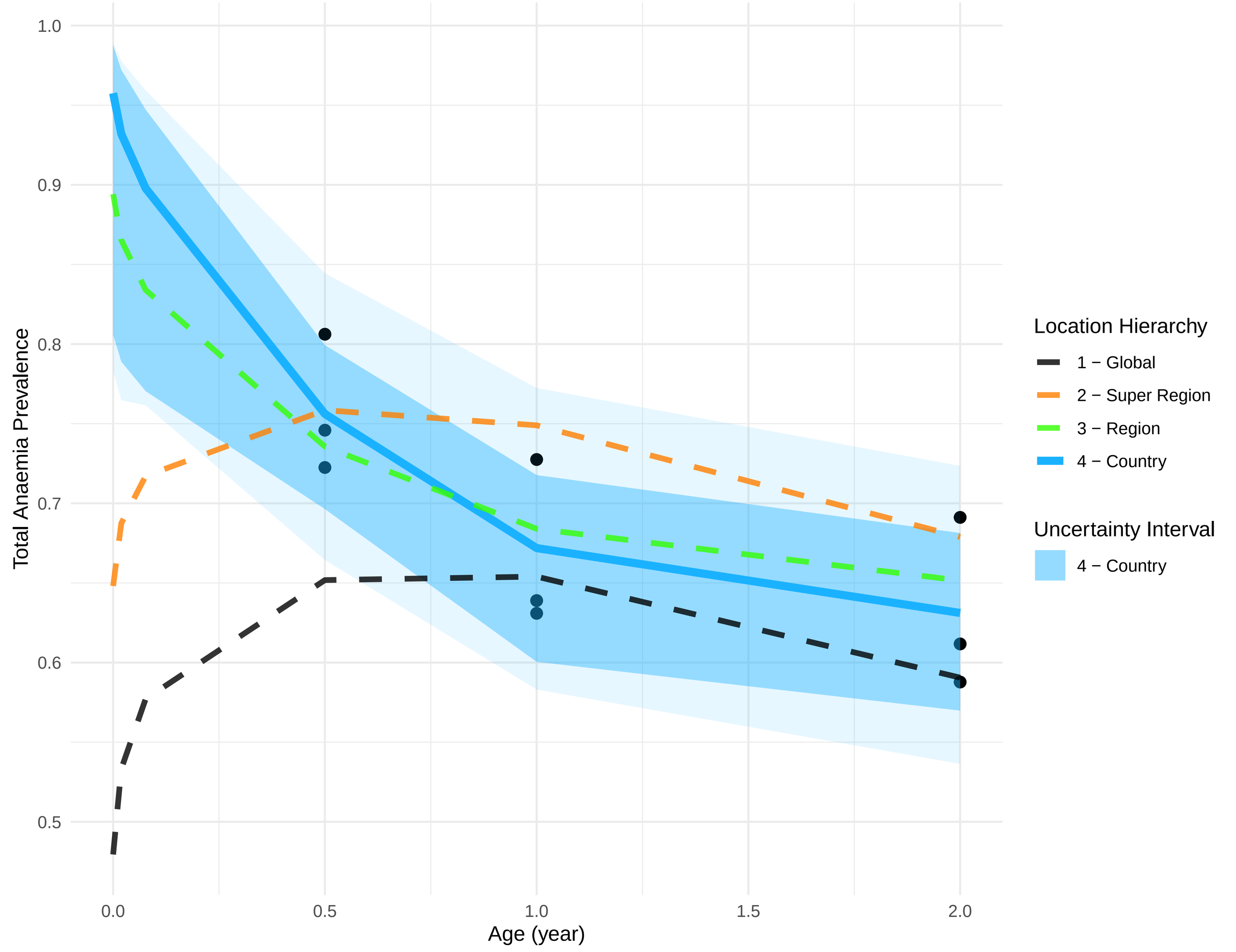

MR−BRT Age/Sex Fits − Males <2 Years Old − Total Anaemia Prevalence − Democratic Republic of the Congo
WHO−2024 Adjustment Method

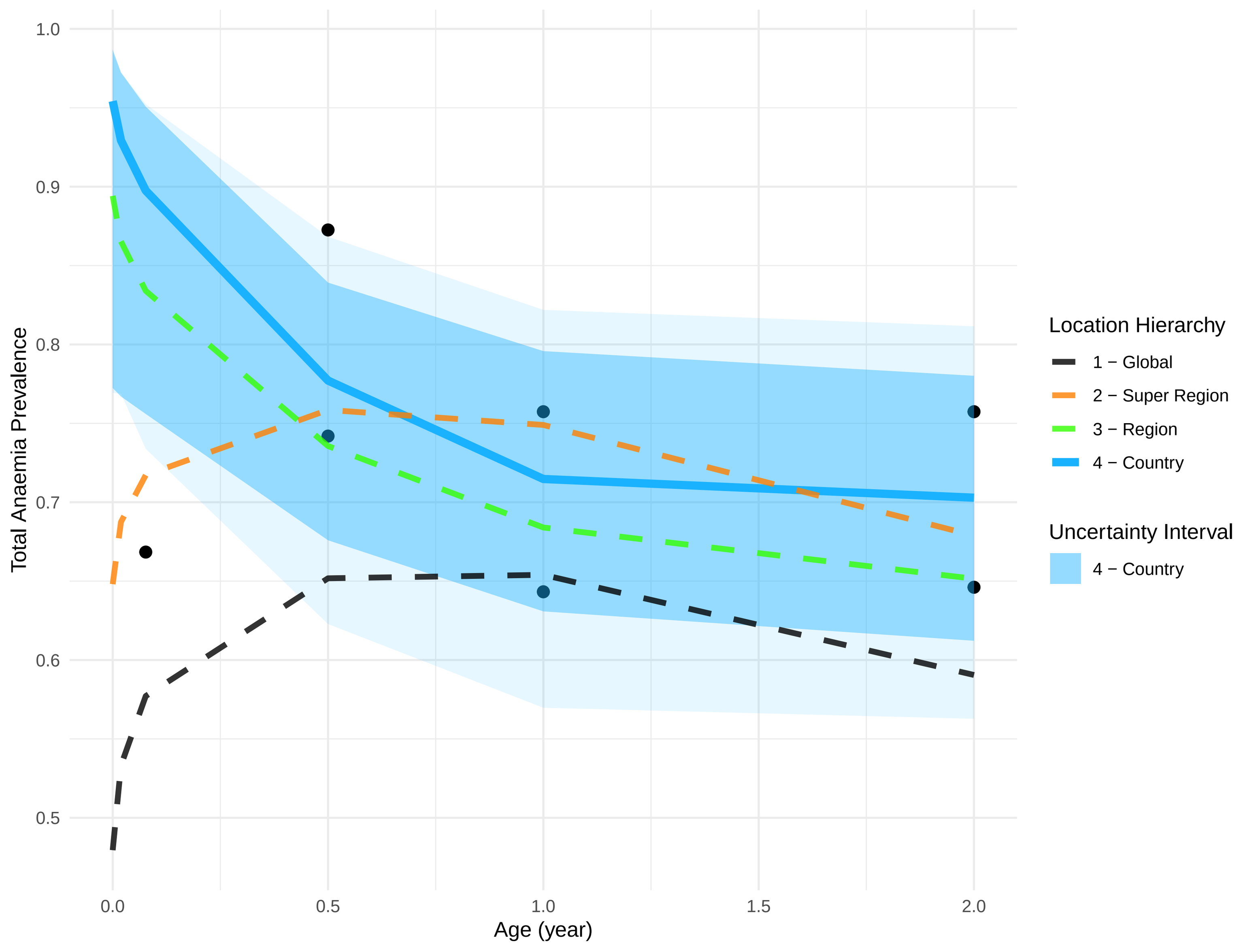

MR−BRT Age/Sex Fits − Males <2 Years Old − Total Anaemia Prevalence − Gabon
WHO−2024 Adjustment Method

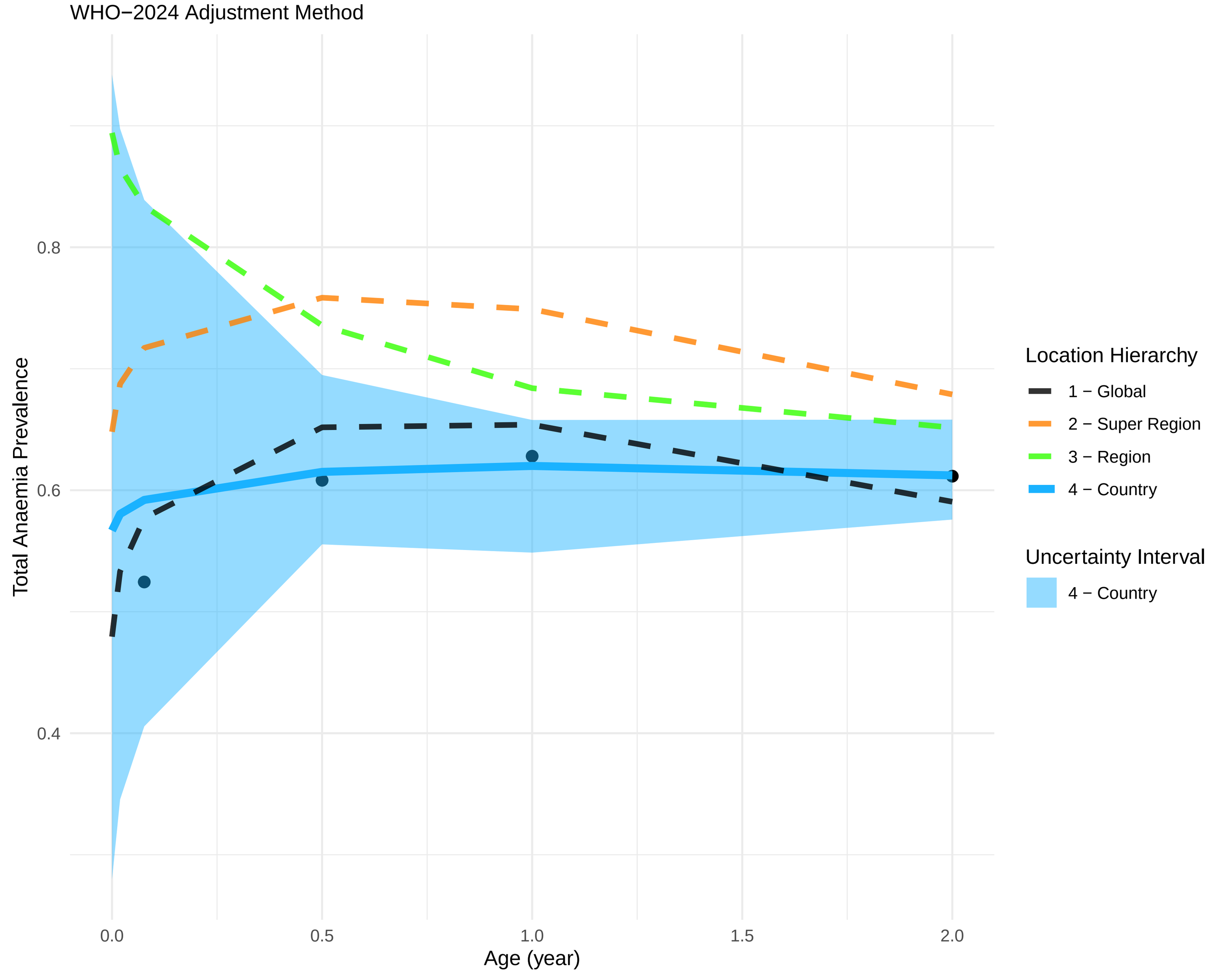

MR−BRT Age/Sex Fits − Males <2 Years Old − Total Anaemia Prevalence − Eastern Sub−Saharan Africa
WHO−2024 Adjustment Method

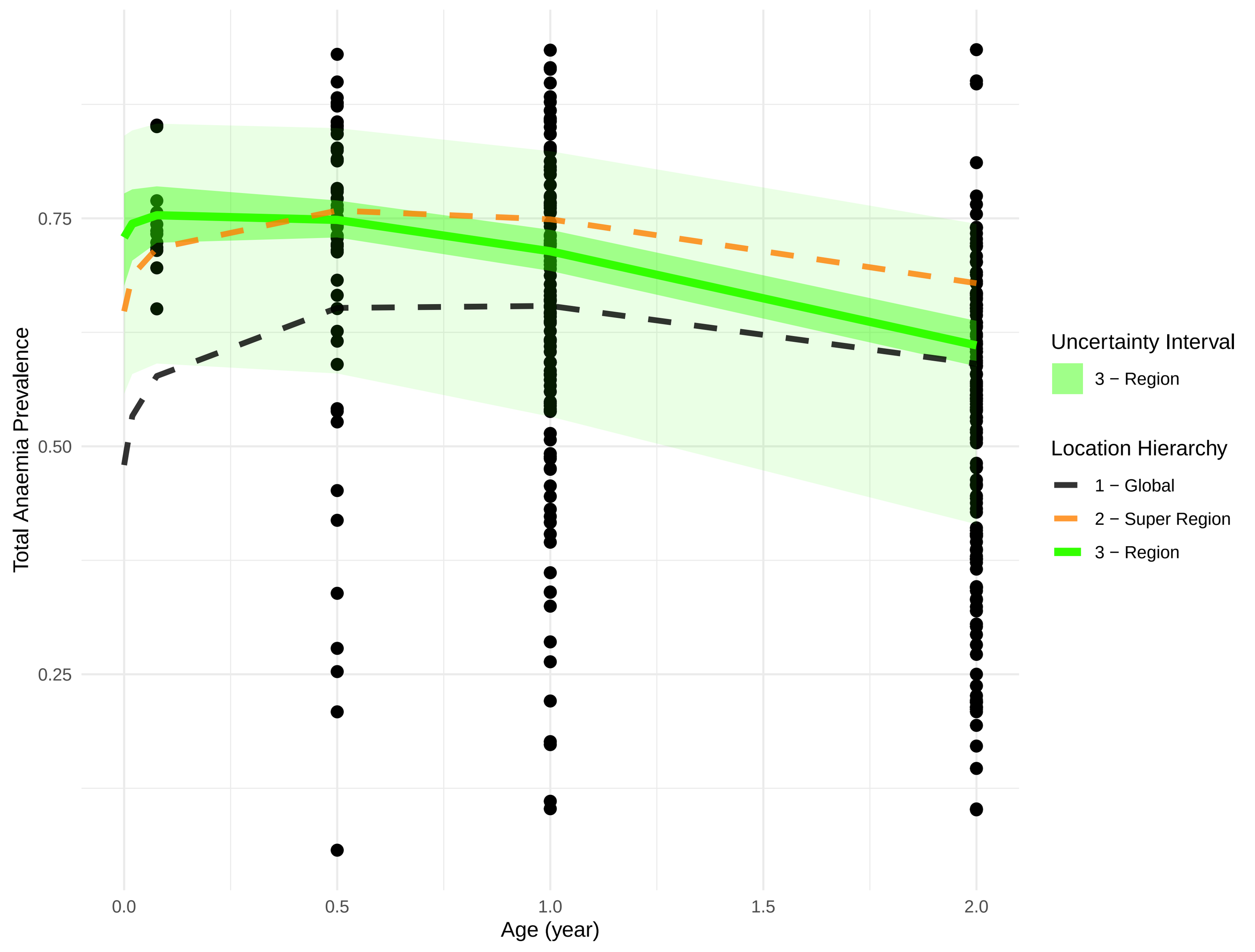

MR−BRT Age/Sex Fits − Males <2 Years Old − Total Anaemia Prevalence − Burundi
WHO−2024 Adjustment Method

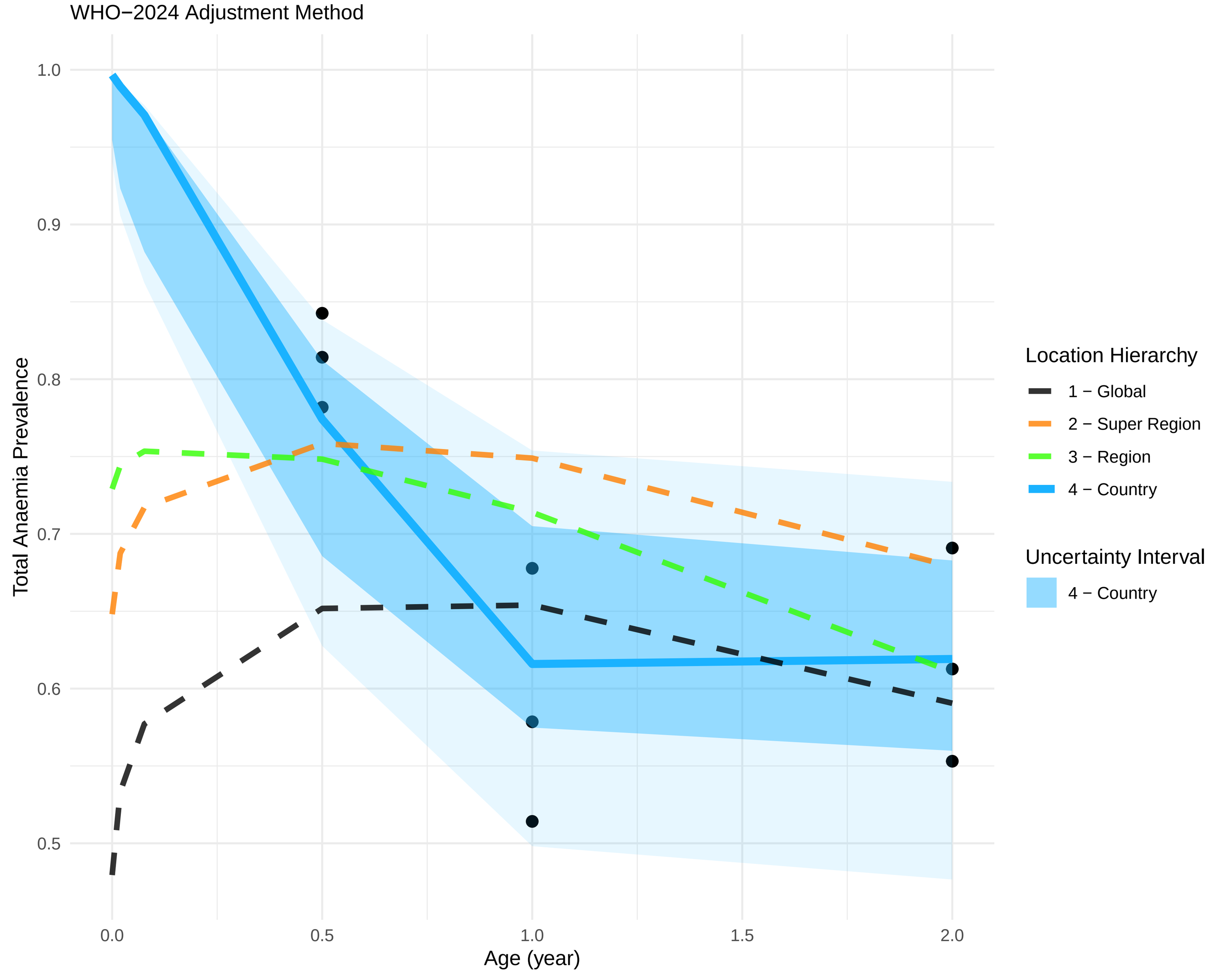

MR−BRT Age/Sex Fits − Males <2 Years Old − Total Anaemia Prevalence − Ethiopia
WHO−2024 Adjustment Method

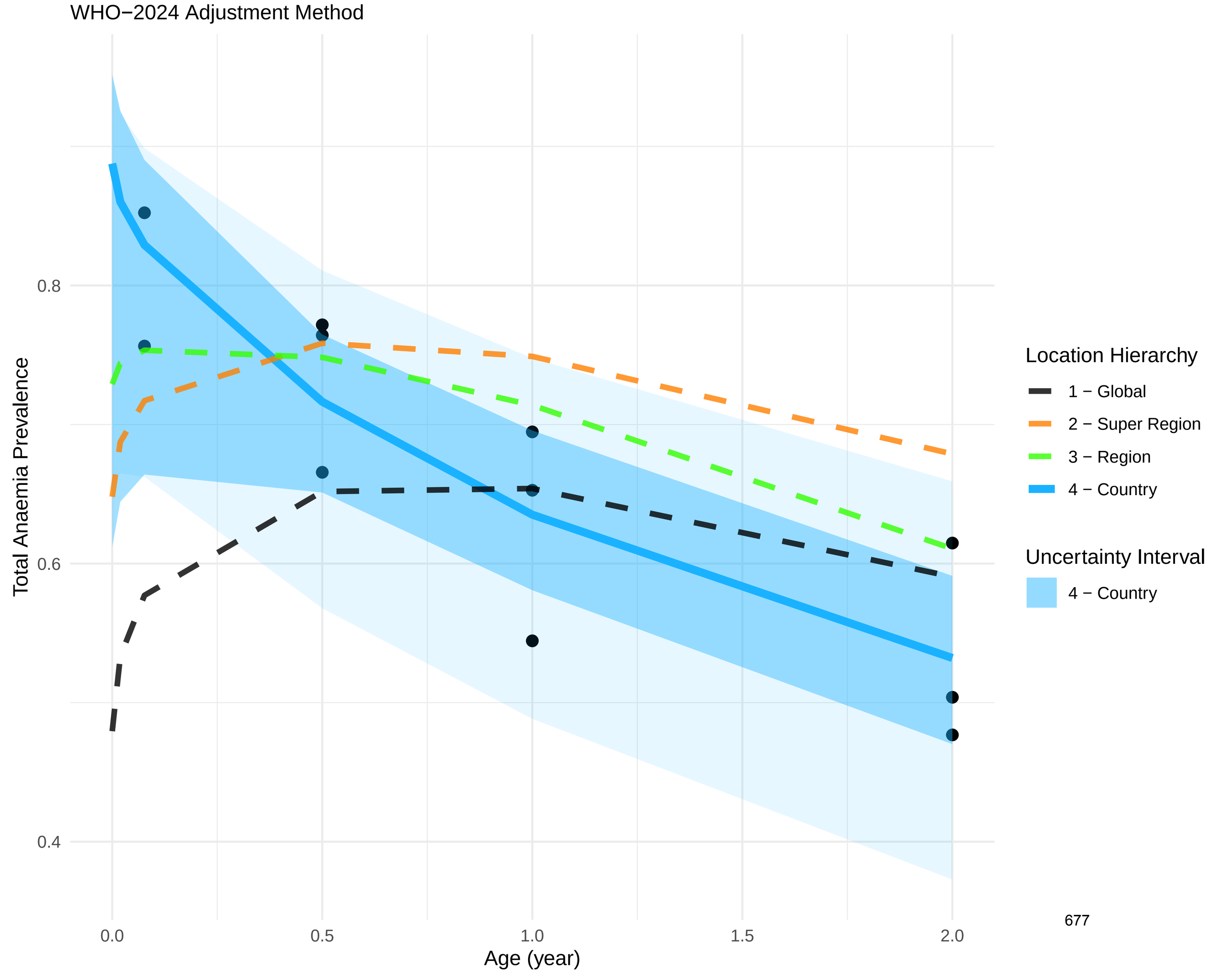

MR−BRT Age/Sex Fits − Males <2 Years Old − Total Anaemia Prevalence − Kenya
WHO−2024 Adjustment Method

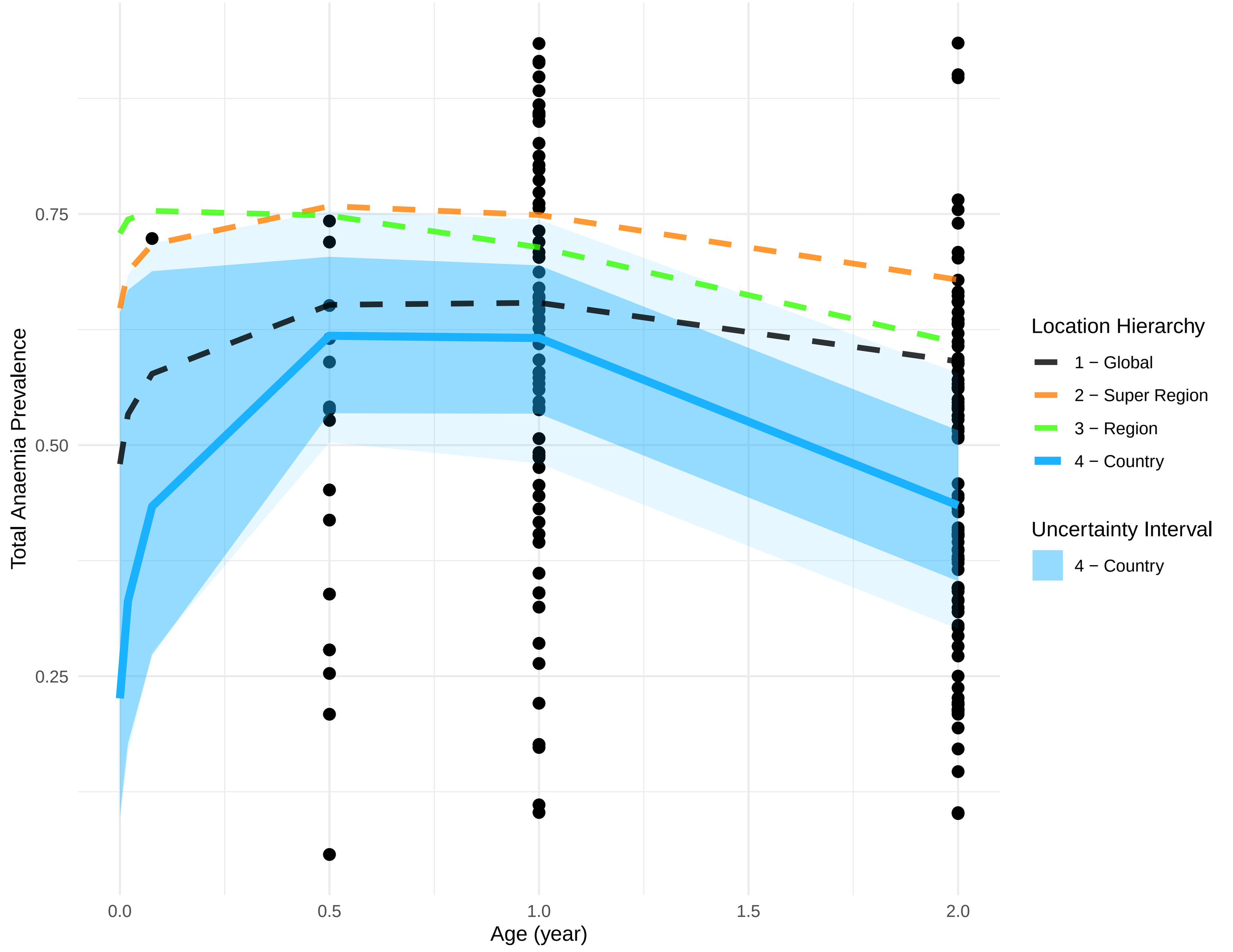

MR−BRT Age/Sex Fits − Males <2 Years Old − Total Anaemia Prevalence − Baringo
WHO−2024 Adjustment Method

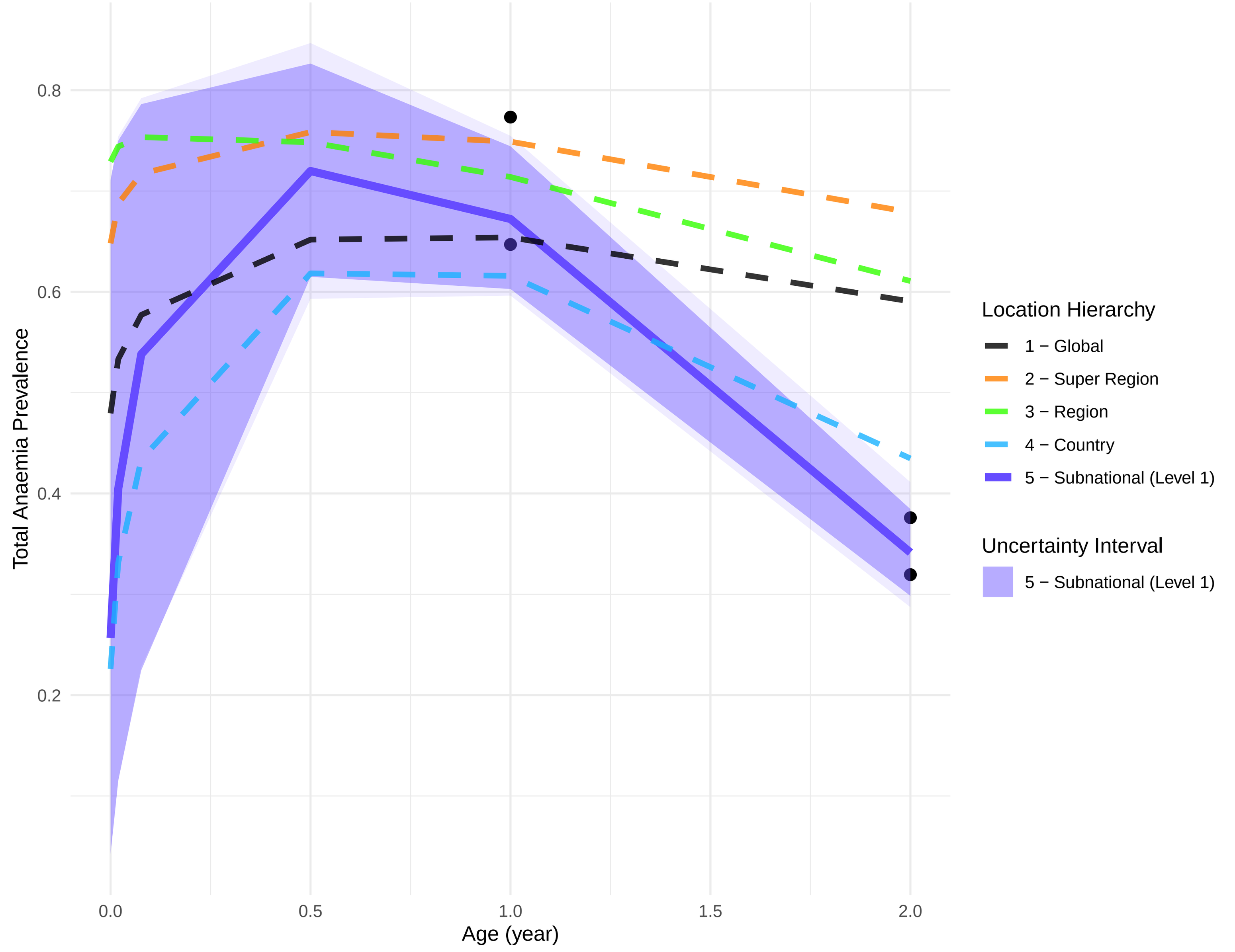

MR−BRT Age/Sex Fits − Males <2 Years Old − Total Anaemia Prevalence − Bomet
WHO−2024 Adjustment Method

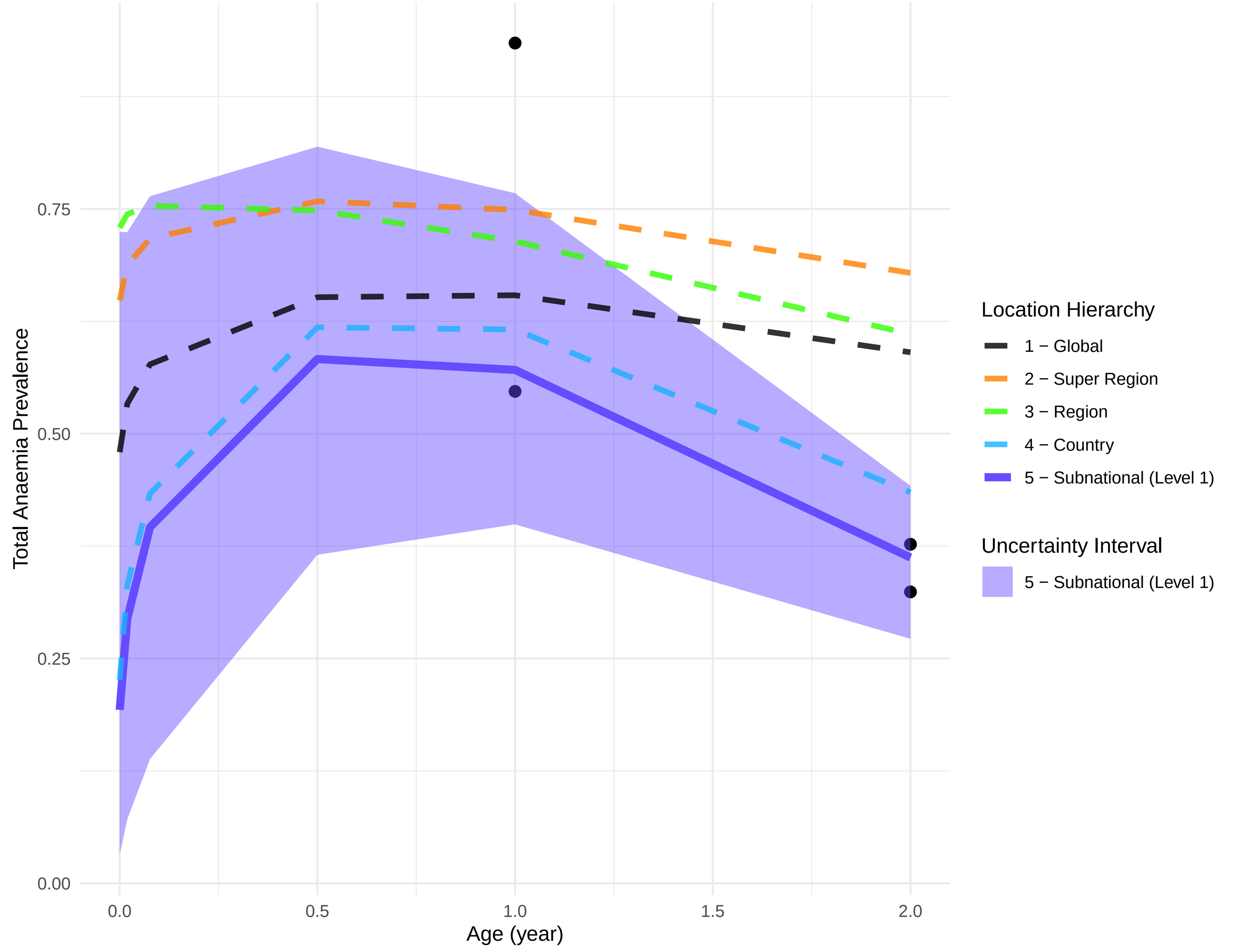

MR−BRT Age/Sex Fits − Males <2 Years Old − Total Anaemia Prevalence − Bungoma
WHO−2024 Adjustment Method

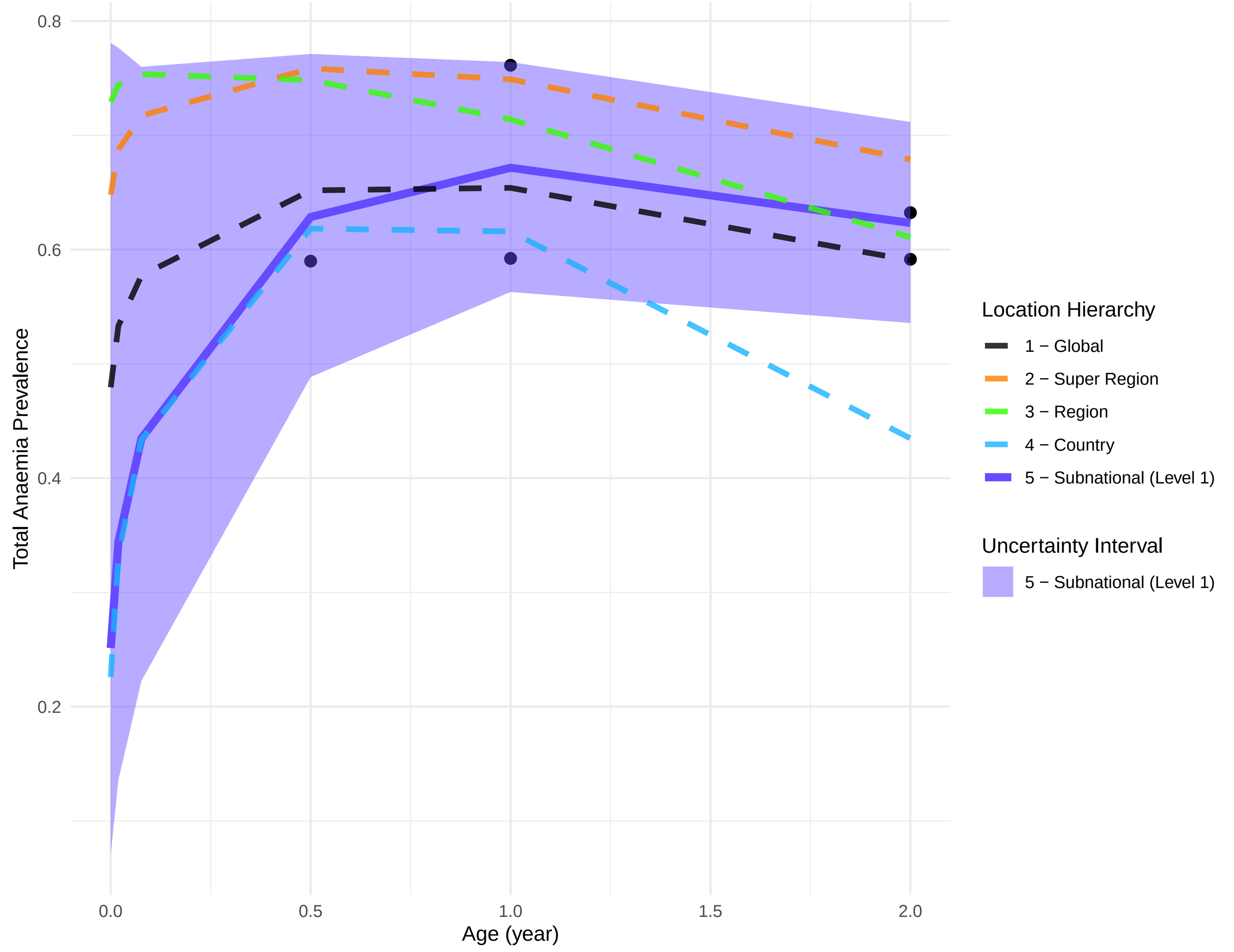

MR−BRT Age/Sex Fits − Males <2 Years Old − Total Anaemia Prevalence − Busia
WHO−2024 Adjustment Method

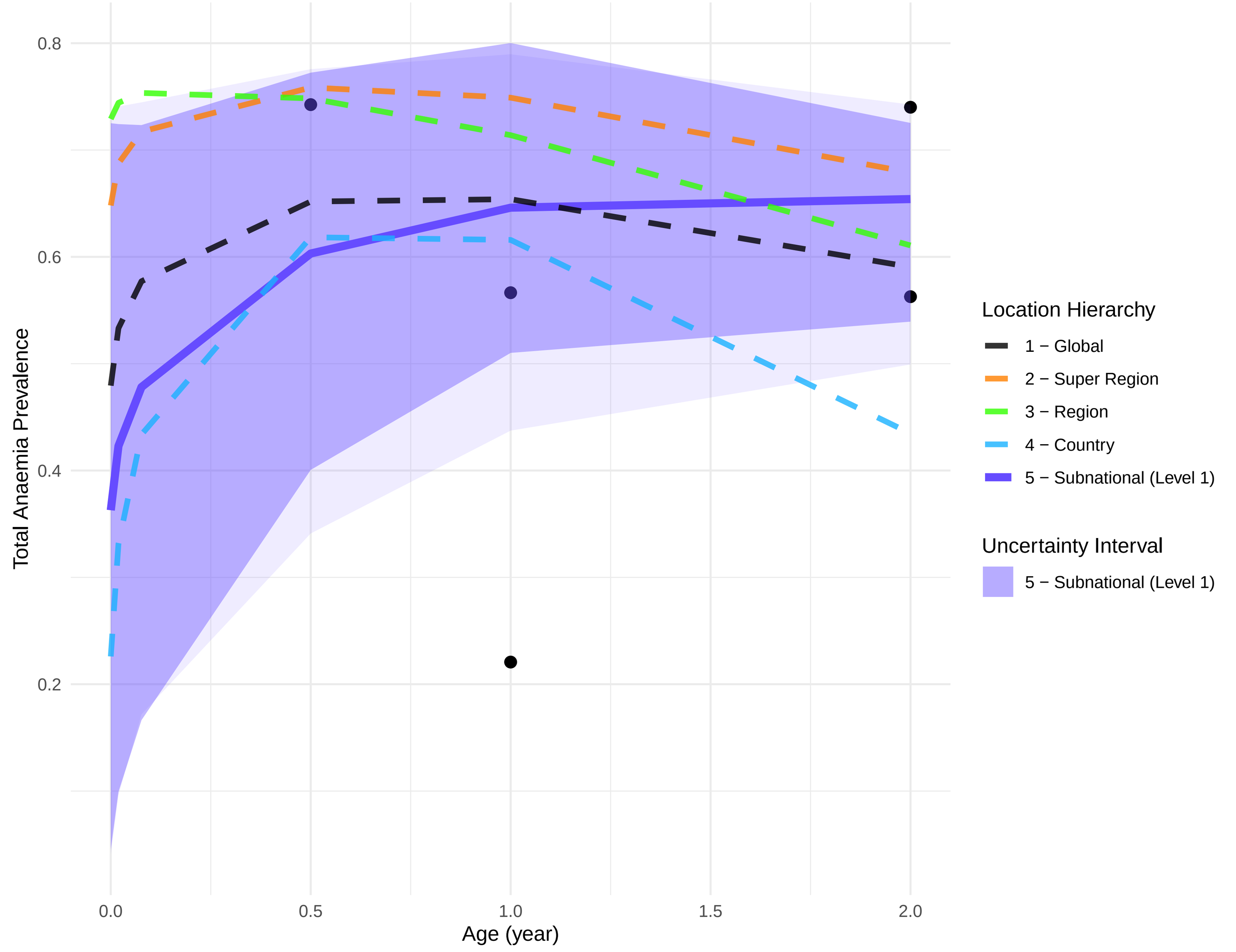

MR−BRT Age/Sex Fits − Males <2 Years Old − Total Anaemia Prevalence − Elgeyo−Marakwet
WHO−2024 Adjustment Method

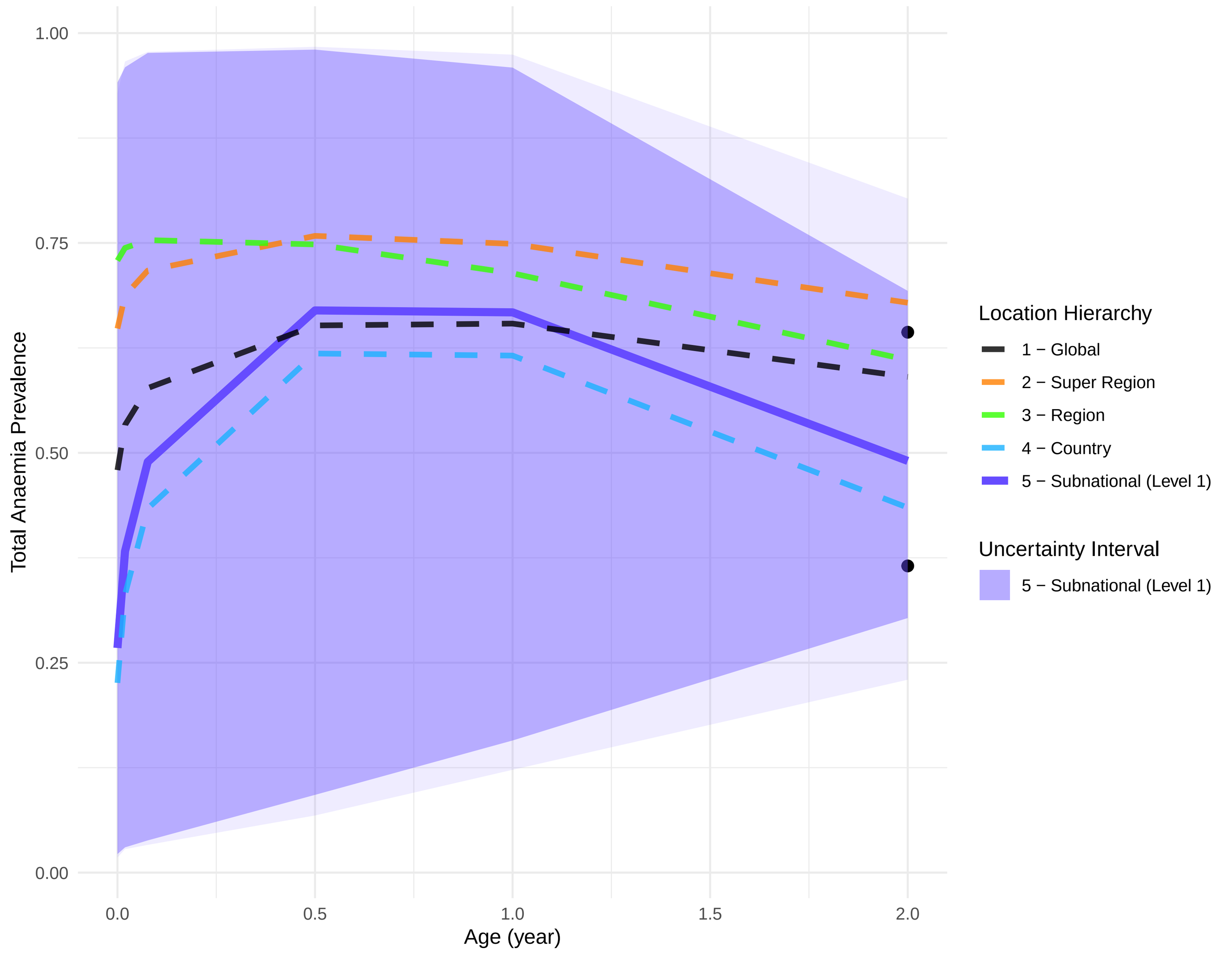

MR−BRT Age/Sex Fits − Males <2 Years Old − Total Anaemia Prevalence − Embu
WHO−2024 Adjustment Method

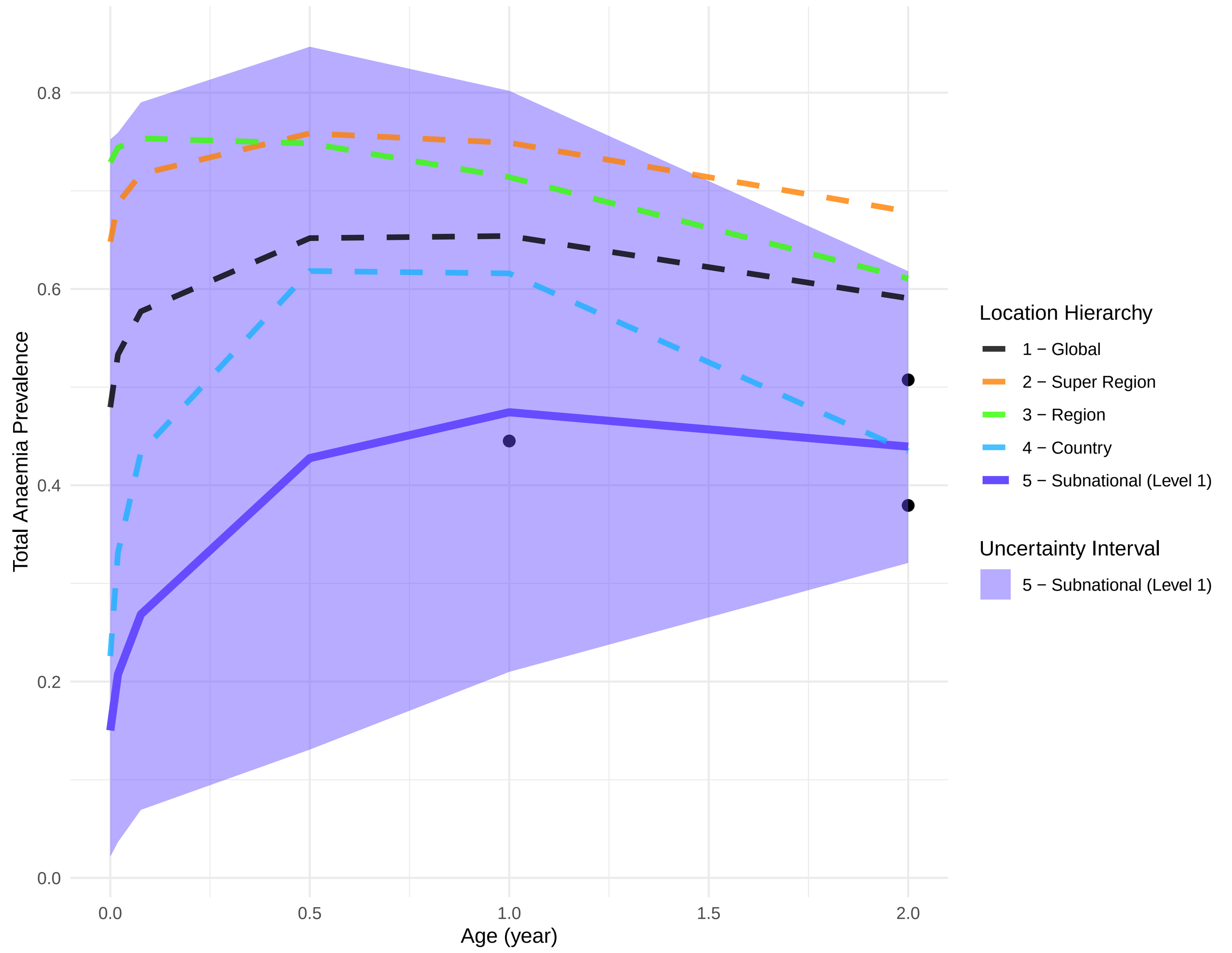

MR−BRT Age/Sex Fits − Males <2 Years Old − Total Anaemia Prevalence − Garissa
WHO−2024 Adjustment Method

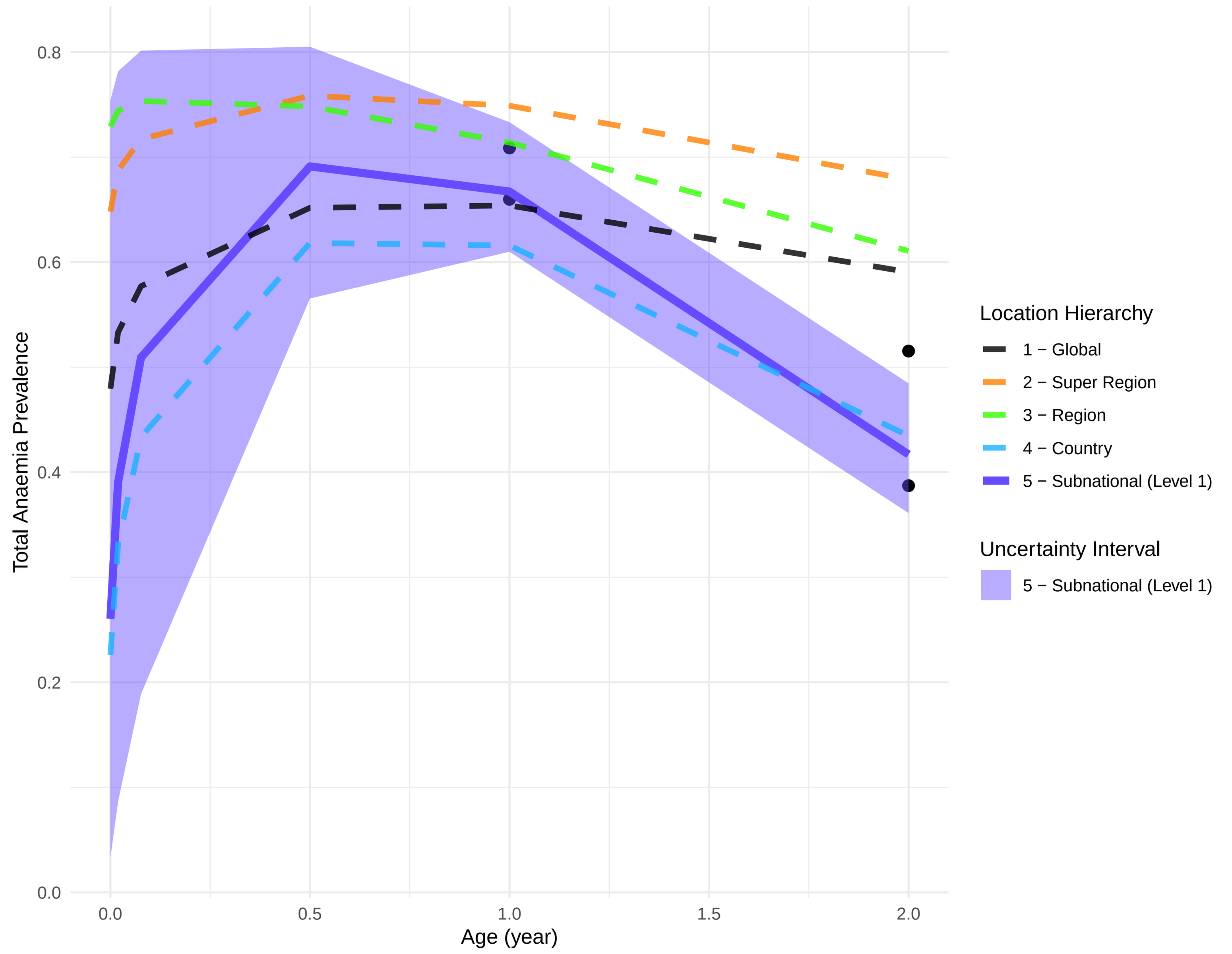

MR−BRT Age/Sex Fits − Males <2 Years Old − Total Anaemia Prevalence − Homa Bay
WHO−2024 Adjustment Method

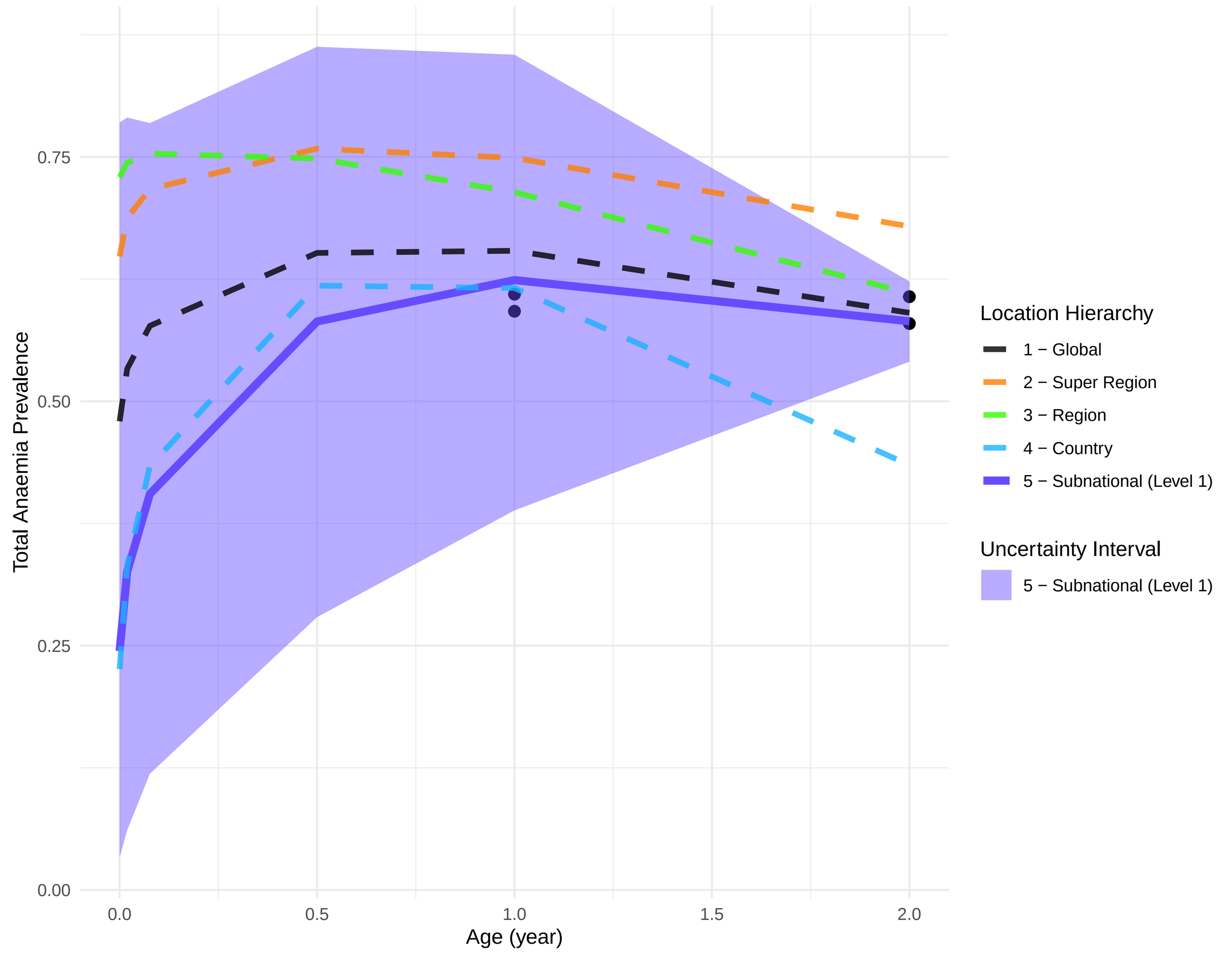

MR−BRT Age/Sex Fits − Males <2 Years Old − Total Anaemia Prevalence − Isiolo
WHO−2024 Adjustment Method

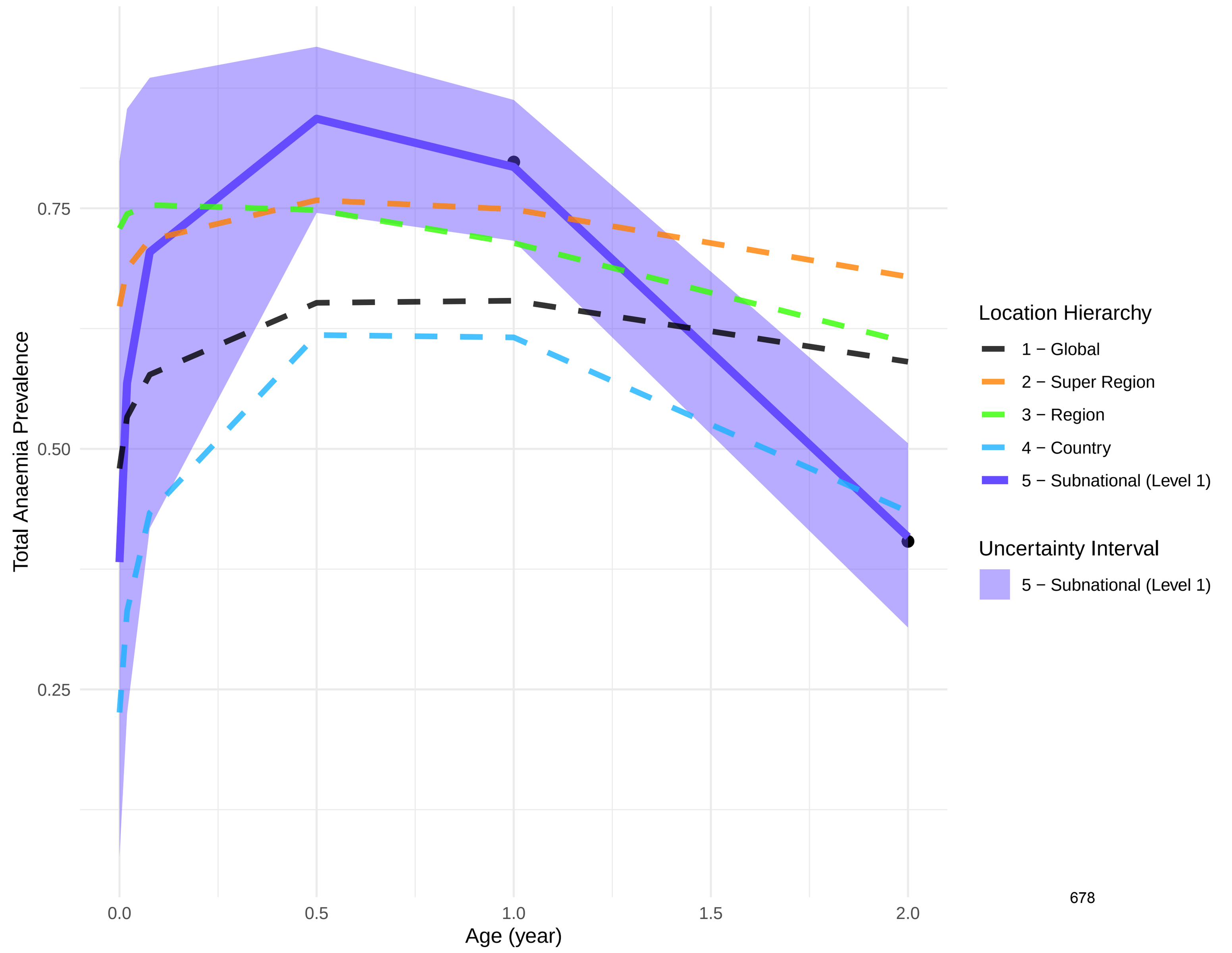

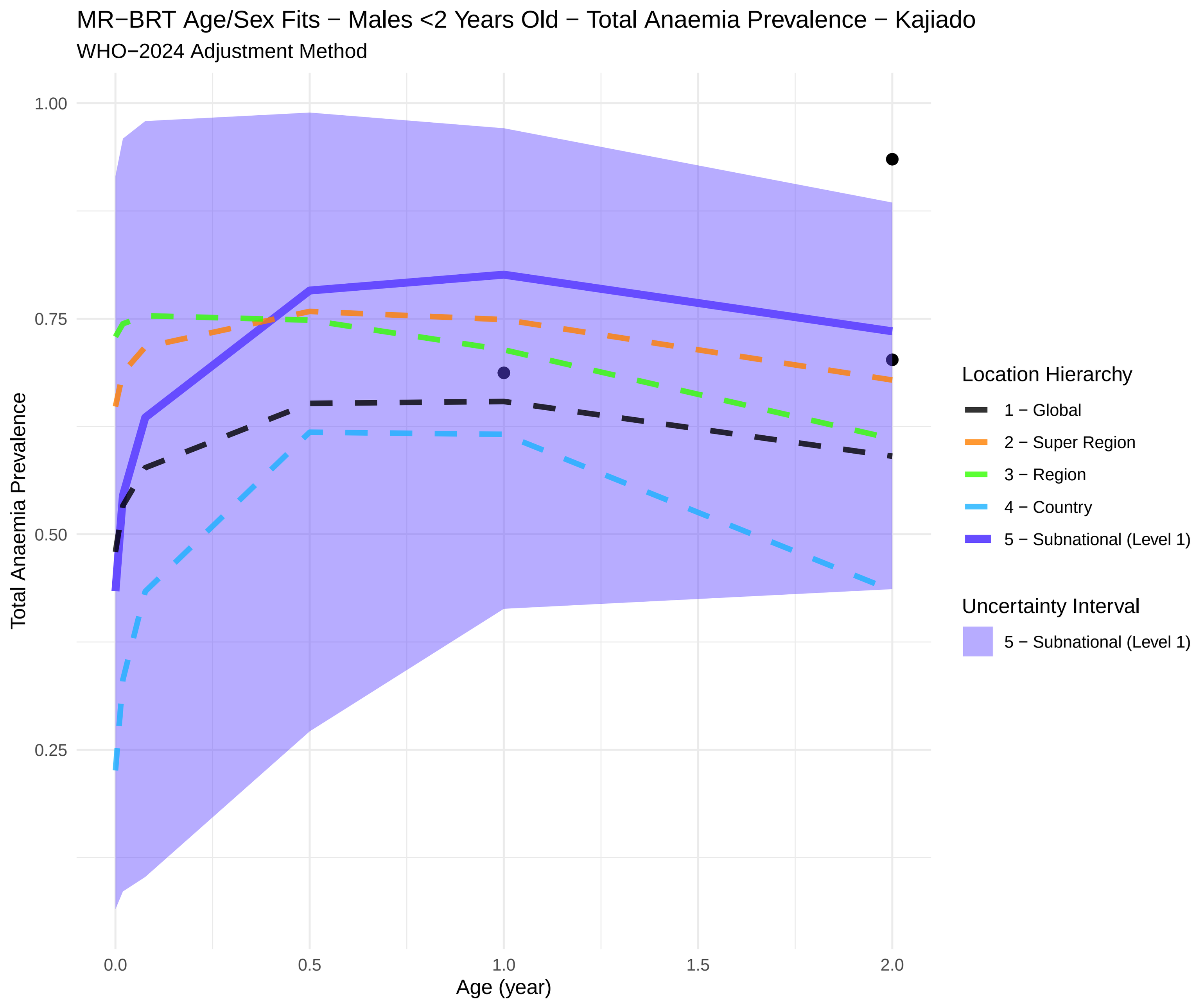
MR−BRT Age/Sex Fits − Males <2 Years Old − Total Anaemia Prevalence − Kajiado
WHO−2024 Adjustment Method
Total Anaemia Prevalence
Age (year)
Location Hierarchy
1 − Global
2 − Super Region
3 − Region
4 − Country
5 − Subnational (Level 1)
Uncertainty Interval
5 − Subnational (Level 1)

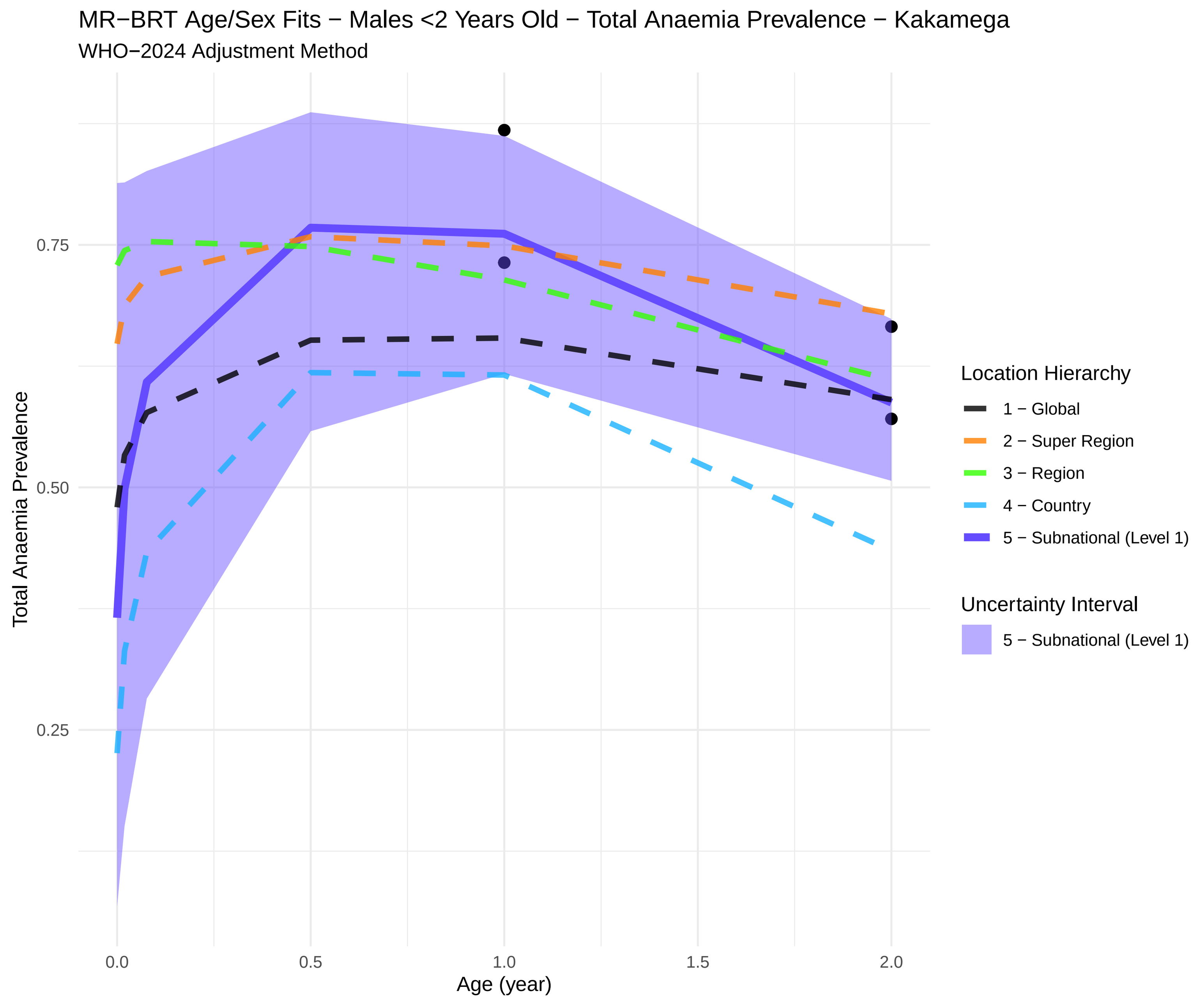
MR−BRT Age/Sex Fits − Males <2 Years Old − Total Anaemia Prevalence − Kakamega
WHO−2024 Adjustment Method
Total Anaemia Prevalence
Age (year)
Location Hierarchy
1 − Global
2 − Super Region
3 − Region
4 − Country
5 − Subnational (Level 1)
Uncertainty Interval
5 − Subnational (Level 1)

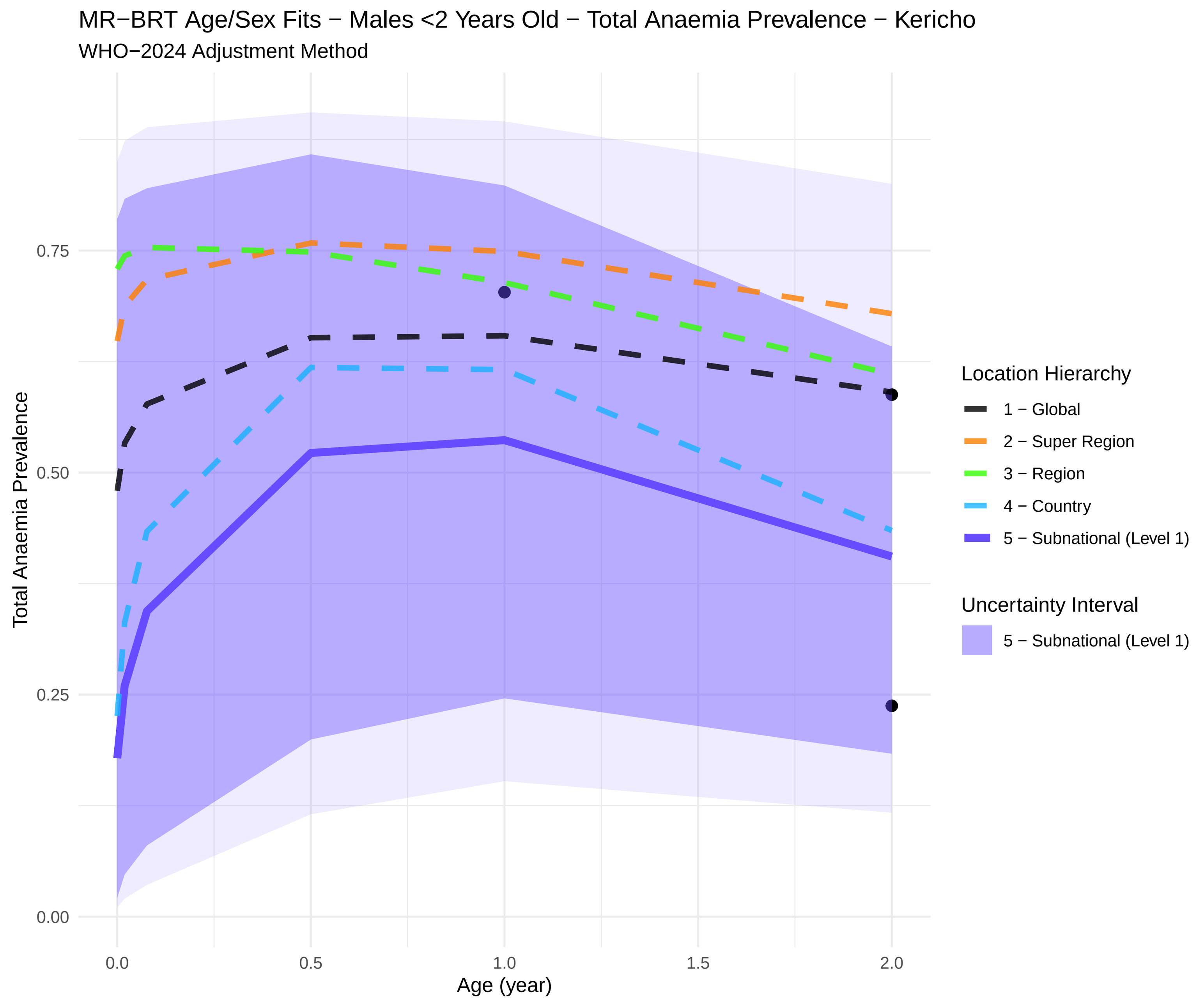
MR−BRT Age/Sex Fits − Males <2 Years Old − Total Anaemia Prevalence − Kericho
WHO−2024 Adjustment Method
Total Anaemia Prevalence
Age (year)
Location Hierarchy
1 − Global
2 − Super Region
3 − Region
4 − Country
5 − Subnational (Level 1)
Uncertainty Interval
5 − Subnational (Level 1)

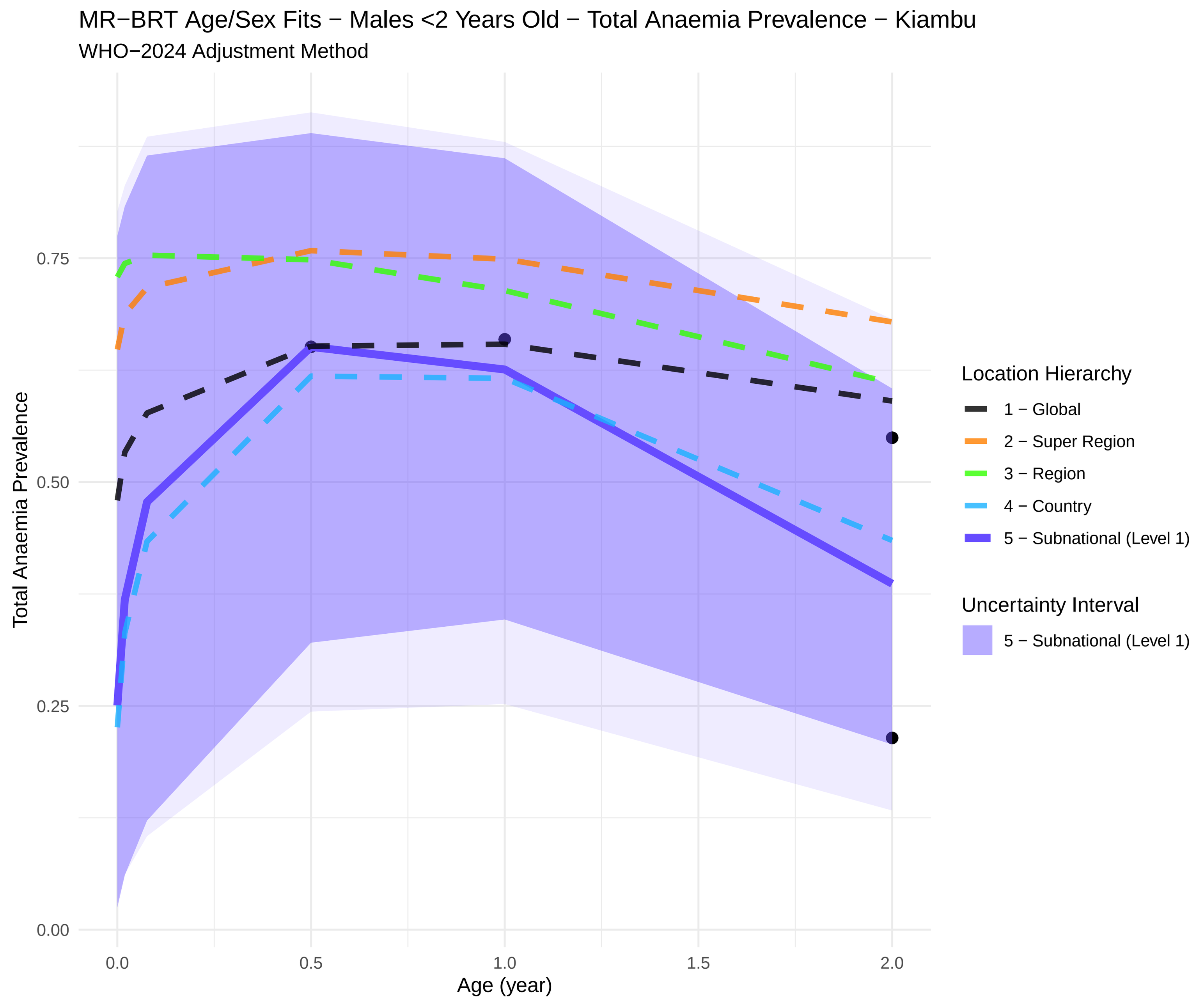
MR−BRT Age/Sex Fits − Males <2 Years Old − Total Anaemia Prevalence − Kiambu
WHO−2024 Adjustment Method
Total Anaemia Prevalence
Age (year)
Location Hierarchy
1 − Global
2 − Super Region
3 − Region
4 − Country
5 − Subnational (Level 1)
Uncertainty Interval
5 − Subnational (Level 1)

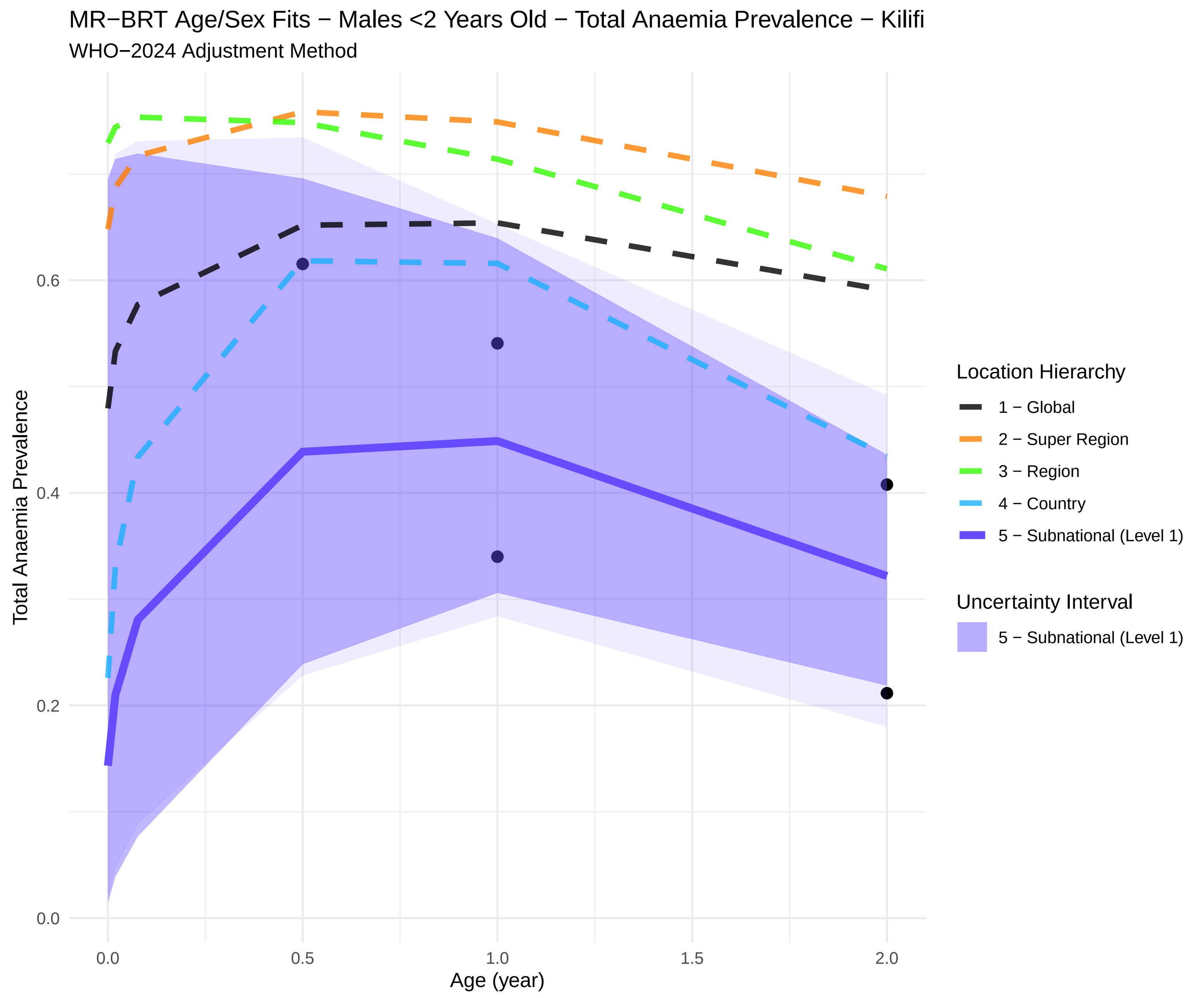
MR−BRT Age/Sex Fits − Males <2 Years Old − Total Anaemia Prevalence − Kilifi
WHO−2024 Adjustment Method
Total Anaemia Prevalence
Age (year)
Location Hierarchy
1 − Global
2 − Super Region
3 − Region
4 − Country
5 − Subnational (Level 1)
Uncertainty Interval
5 − Subnational (Level 1)

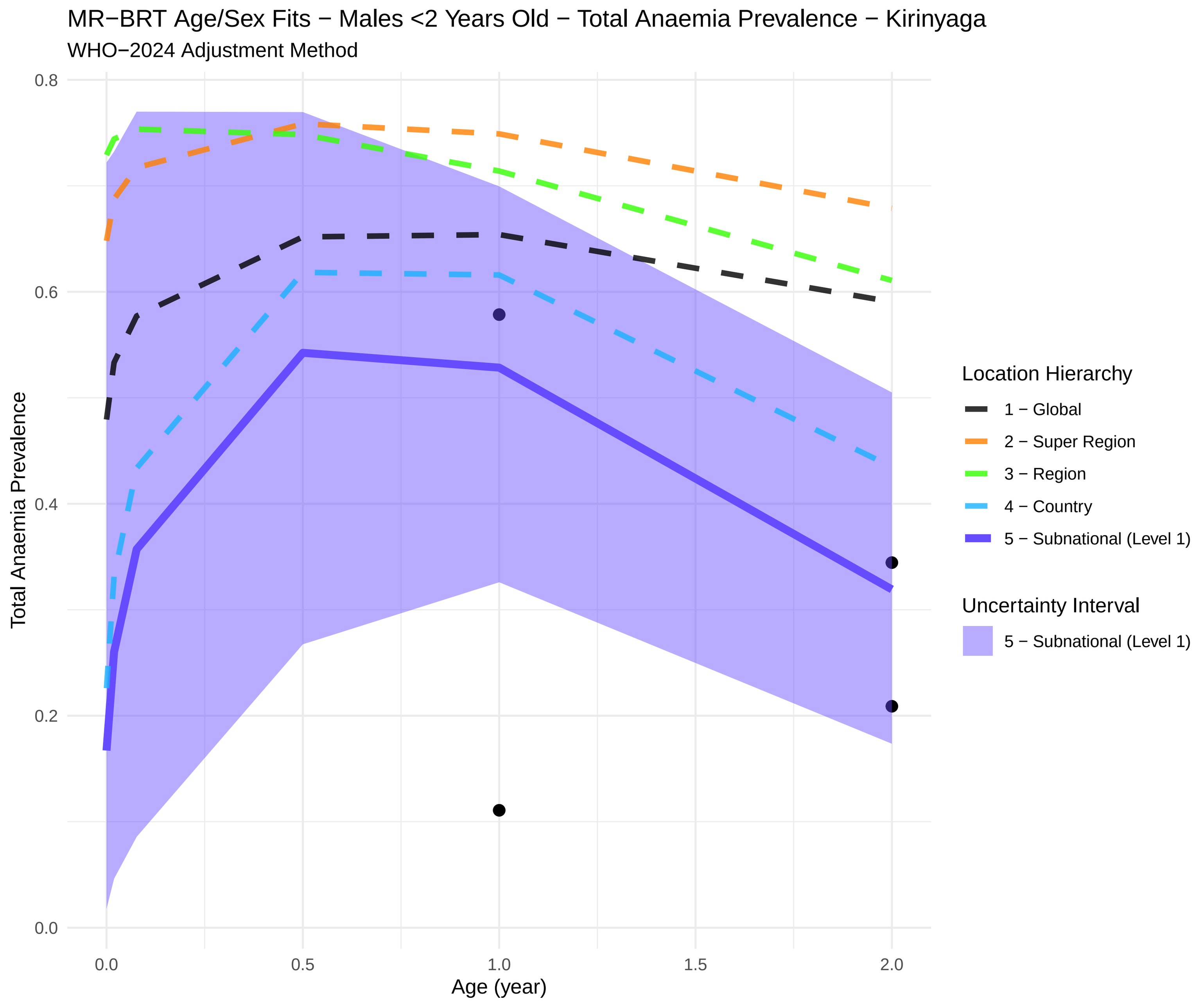
MR−BRT Age/Sex Fits − Males <2 Years Old − Total Anaemia Prevalence − Kirinyaga
WHO−2024 Adjustment Method
Total Anaemia Prevalence
Age (year)
Location Hierarchy
1 − Global
2 − Super Region
3 − Region
4 − Country
5 − Subnational (Level 1)
Uncertainty Interval
5 − Subnational (Level 1)

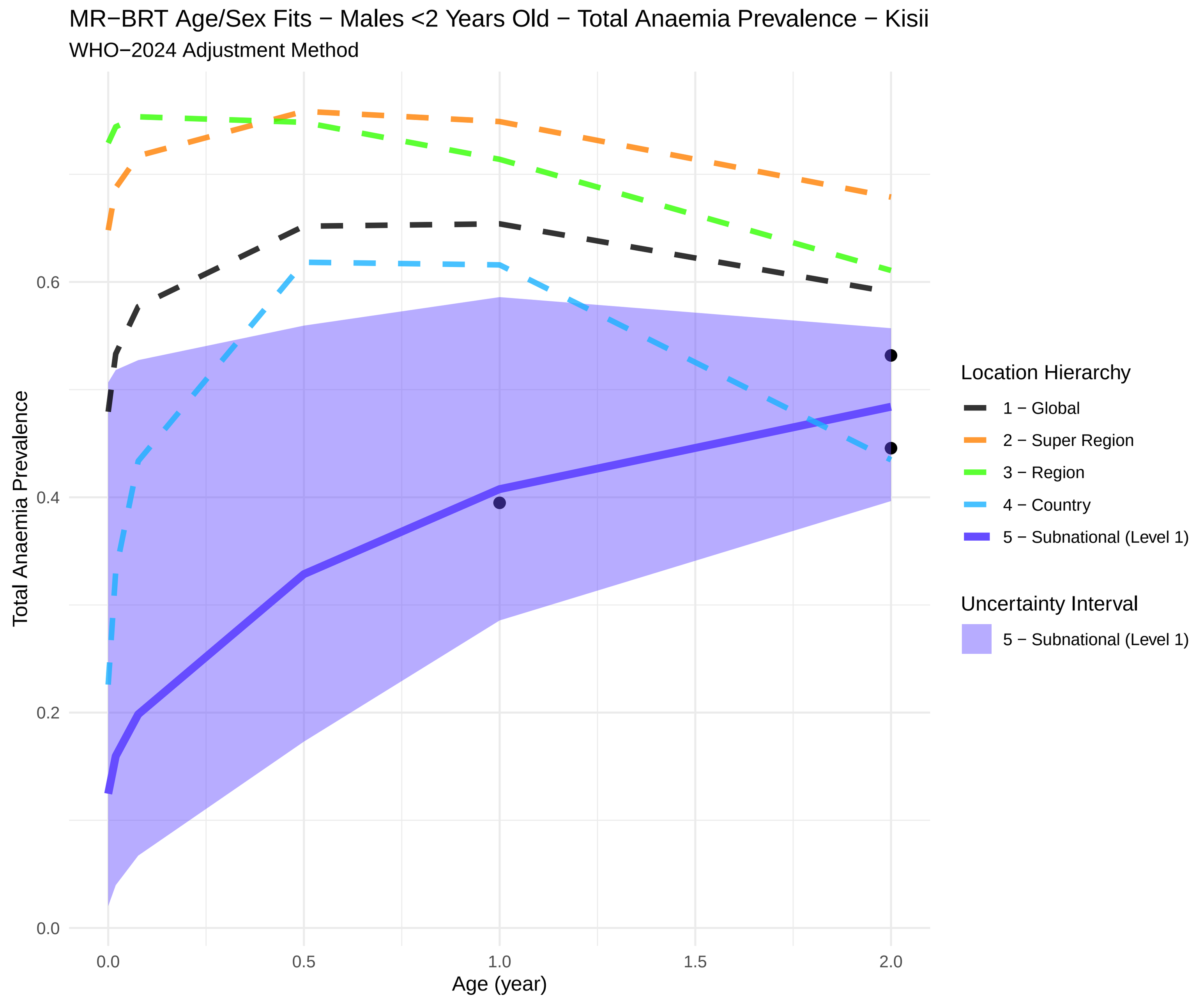
MR−BRT Age/Sex Fits − Males <2 Years Old − Total Anaemia Prevalence − Kisii
WHO−2024 Adjustment Method
Total Anaemia Prevalence
Age (year)
Location Hierarchy
1 − Global
2 − Super Region
3 − Region
4 − Country
5 − Subnational (Level 1)
Uncertainty Interval
5 − Subnational (Level 1)

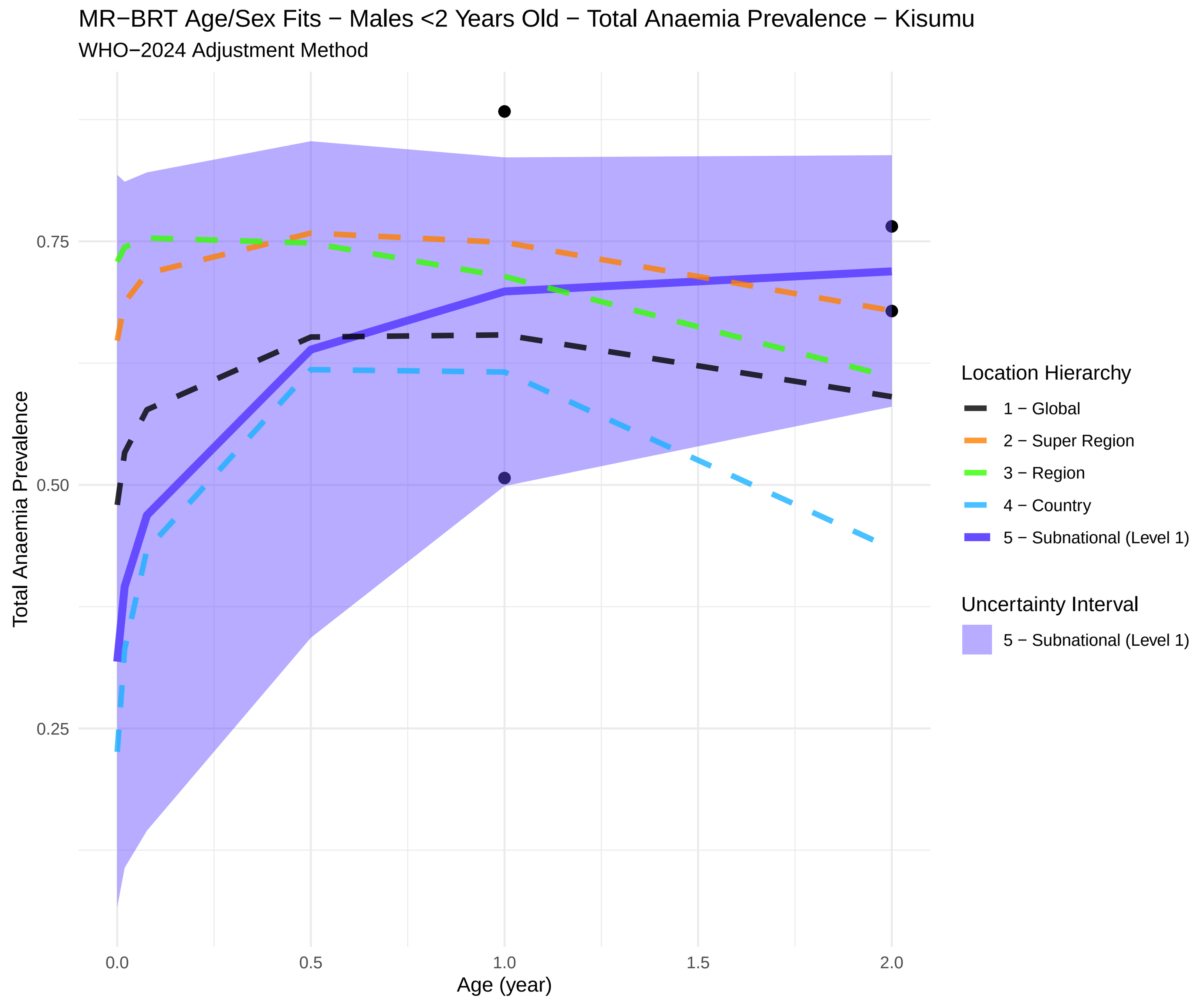
MR−BRT Age/Sex Fits − Males <2 Years Old − Total Anaemia Prevalence − Kisumu
WHO−2024 Adjustment Method
Total Anaemia Prevalence
Age (year)
Location Hierarchy
1 − Global
2 − Super Region
3 − Region
4 − Country
5 − Subnational (Level 1)
Uncertainty Interval
5 − Subnational (Level 1)

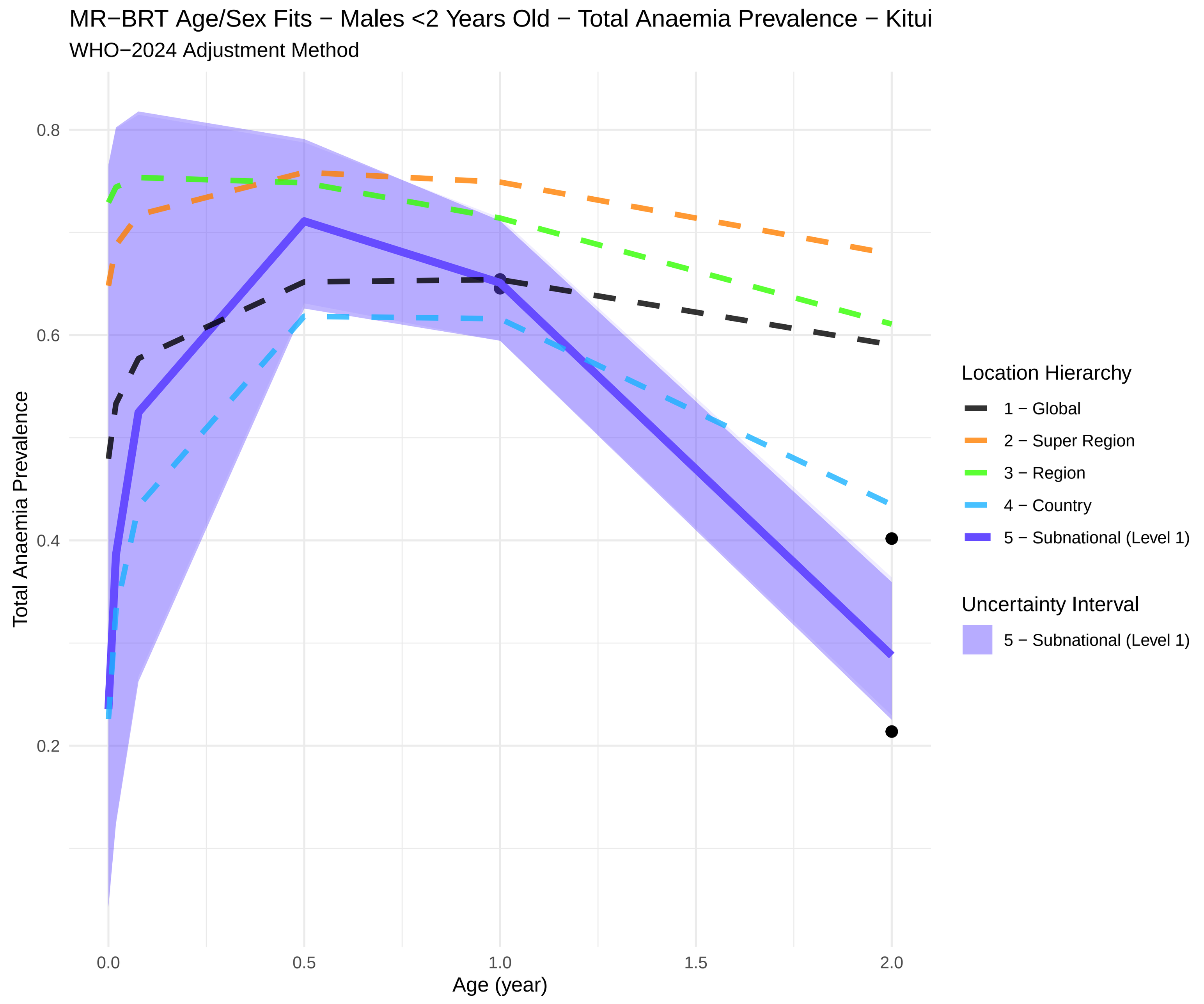
MR−BRT Age/Sex Fits − Males <2 Years Old − Total Anaemia Prevalence − Kitui
WHO−2024 Adjustment Method
Total Anaemia Prevalence
Age (year)
Location Hierarchy
1 − Global
2 − Super Region
3 − Region
4 − Country
5 − Subnational (Level 1)
Uncertainty Interval
5 − Subnational (Level 1)

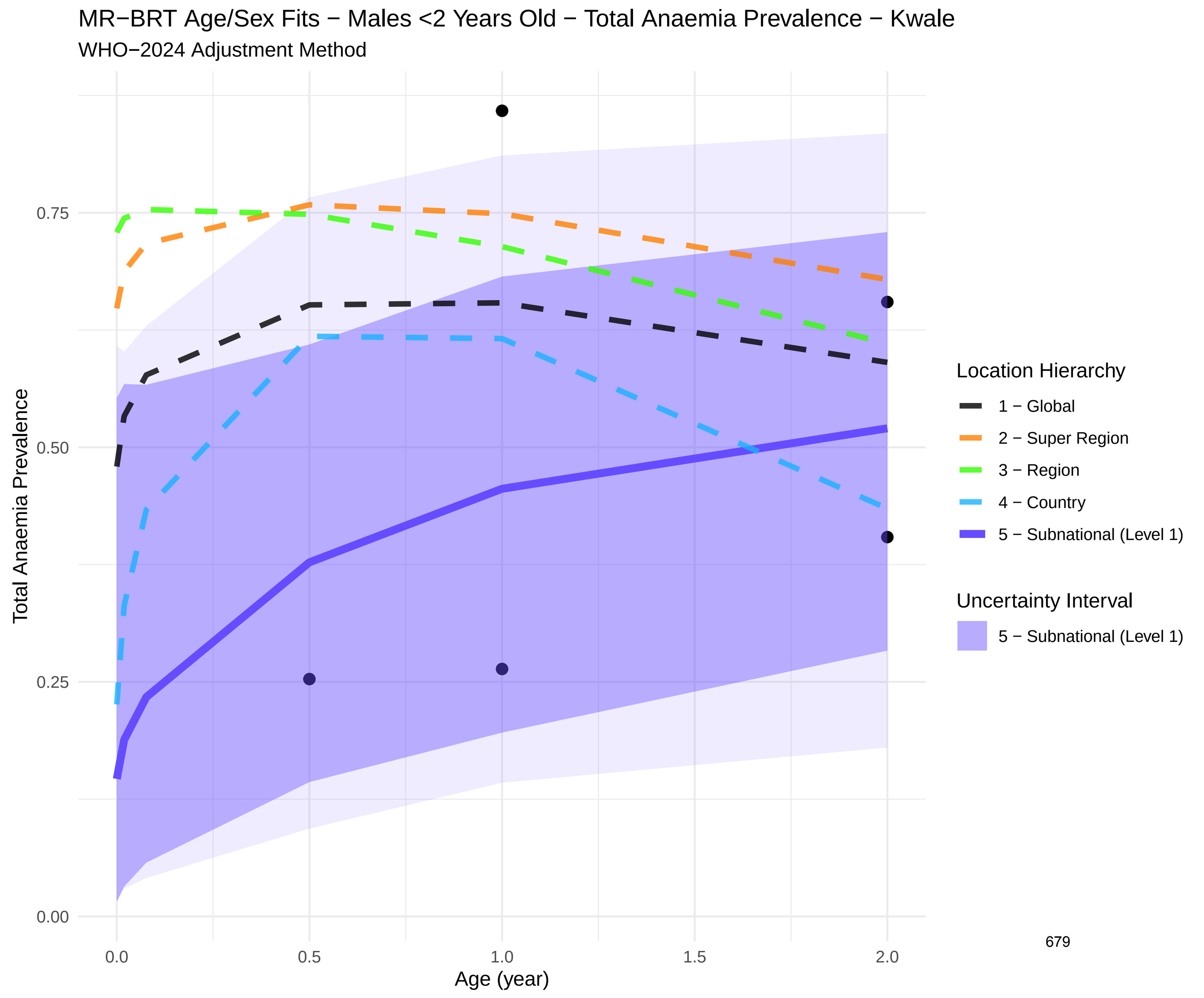
MR−BRT Age/Sex Fits − Males <2 Years Old − Total Anaemia Prevalence − Kwale
WHO−2024 Adjustment Method
Total Anaemia Prevalence
Age (year)
Location Hierarchy
1 − Global
2 − Super Region
3 − Region
4 − Country
5 − Subnational (Level 1)
Uncertainty Interval
5 − Subnational (Level 1)

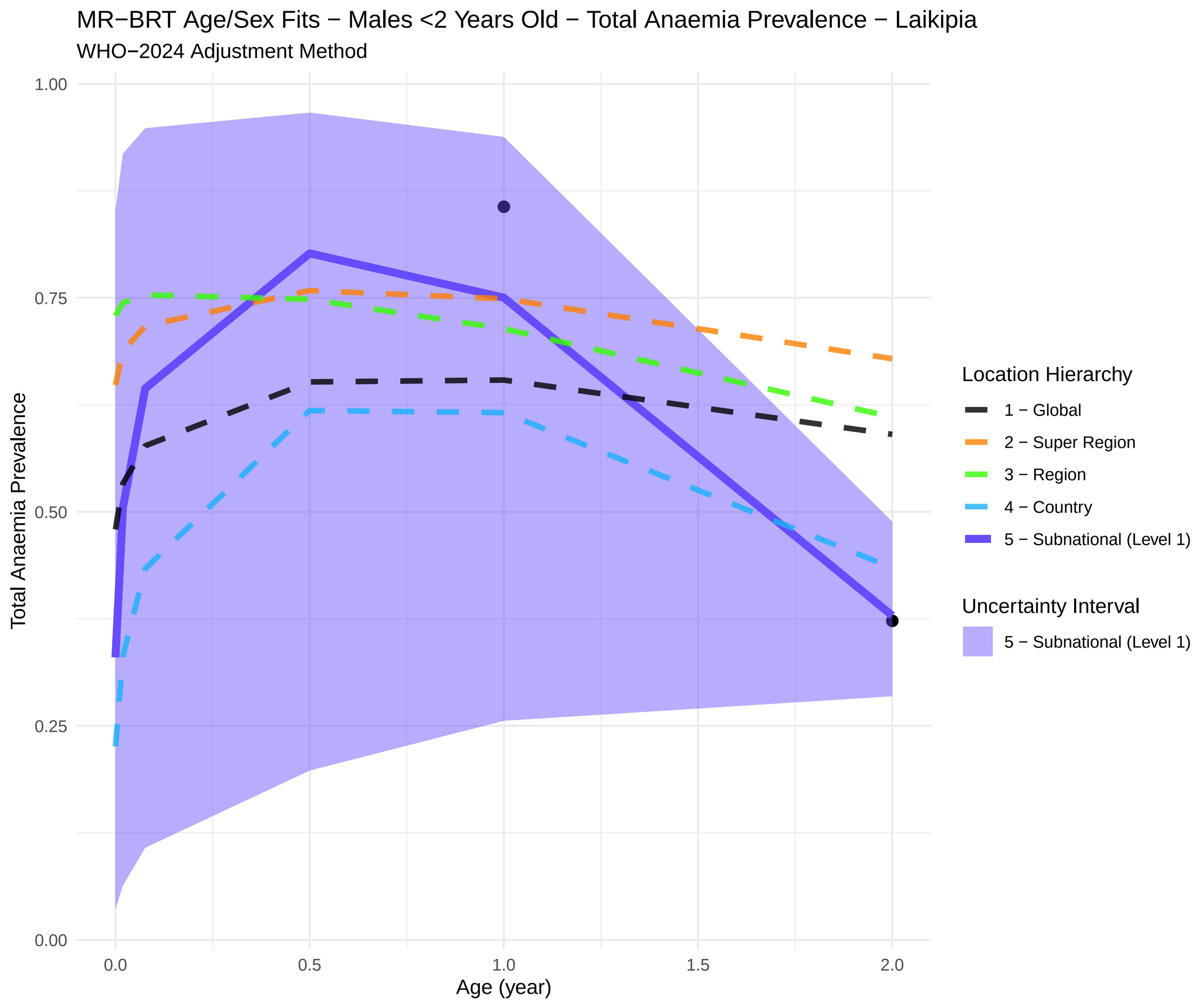
MR−BRT Age/Sex Fits − Males <2 Years Old − Total Anaemia Prevalence − Laikipia
WHO−2024 Adjustment Method
Total Anaemia Prevalence
Age (year)
Location Hierarchy
1 − Global
2 − Super Region
3 − Region
4 − Country
5 − Subnational (Level 1)
Uncertainty Interval
5 − Subnational (Level 1)

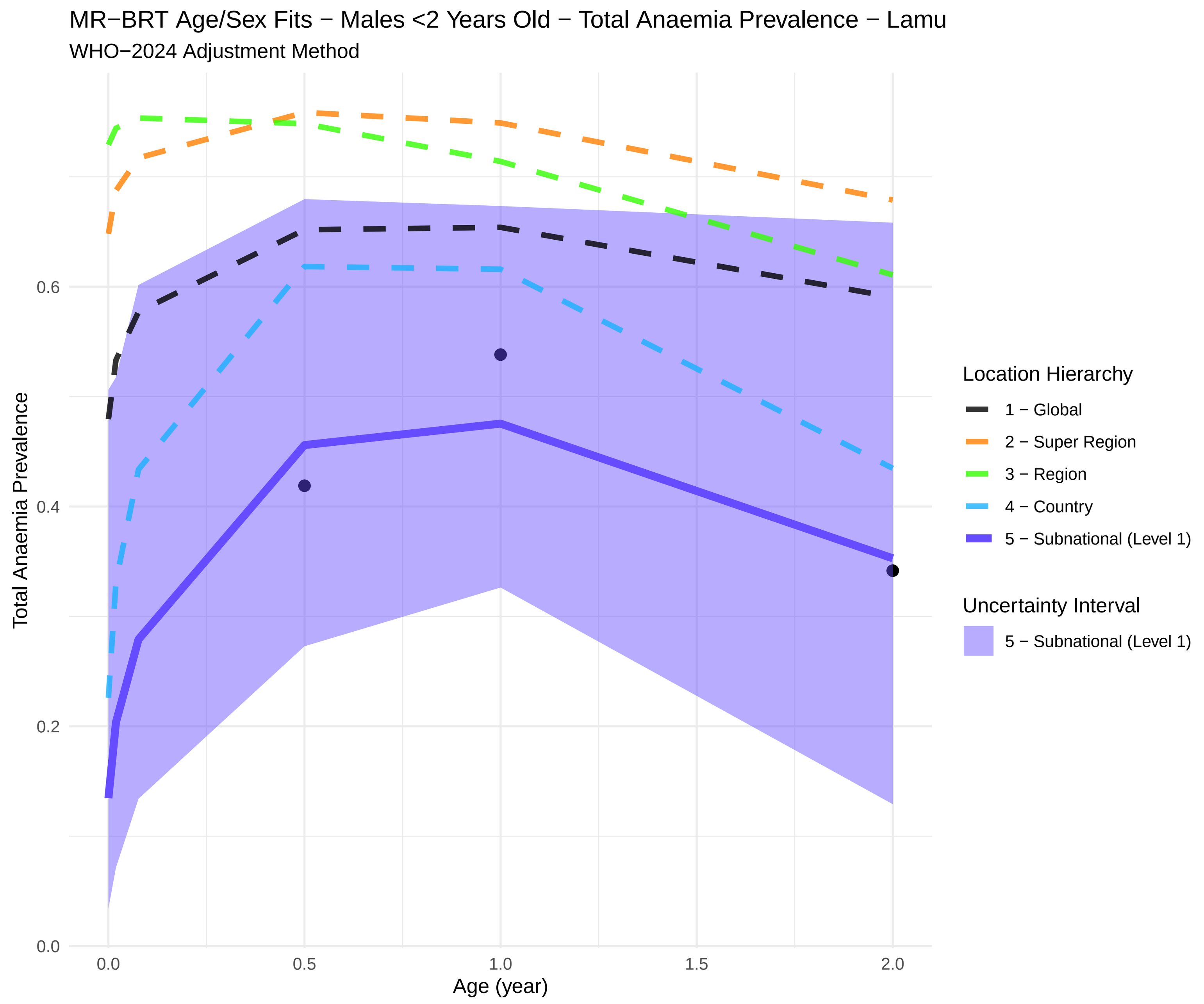
MR−BRT Age/Sex Fits − Males <2 Years Old − Total Anaemia Prevalence − Lamu
WHO−2024 Adjustment Method
Total Anaemia Prevalence
Age (year)
Location Hierarchy
1 − Global
2 − Super Region
3 − Region
4 − Country
5 − Subnational (Level 1)
Uncertainty Interval
5 − Subnational (Level 1)

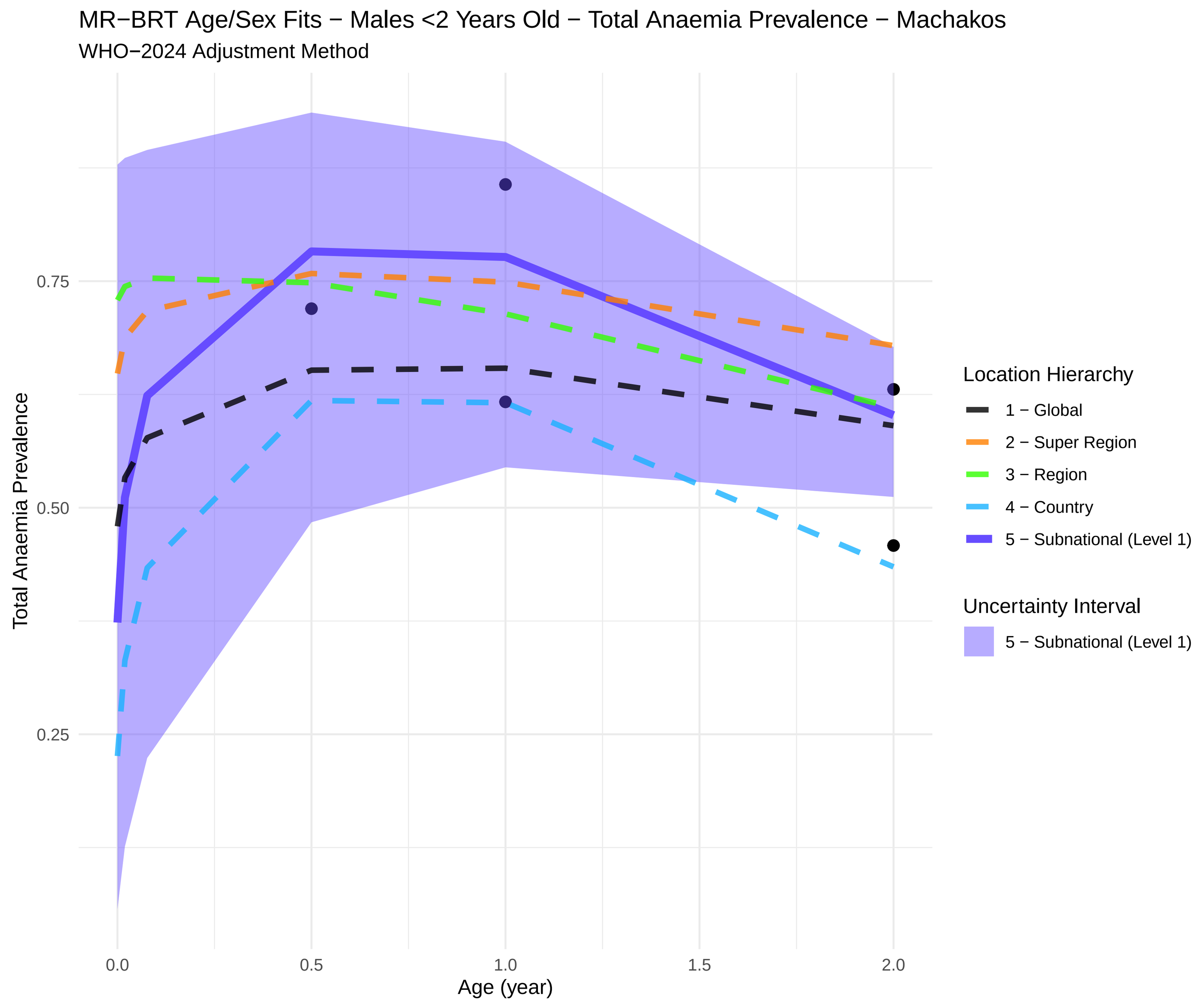
MR−BRT Age/Sex Fits − Males <2 Years Old − Total Anaemia Prevalence − Machakos
WHO−2024 Adjustment Method
Total Anaemia Prevalence
Age (year)
Location Hierarchy
1 − Global
2 − Super Region
3 − Region
4 − Country
5 − Subnational (Level 1)
Uncertainty Interval
5 − Subnational (Level 1)

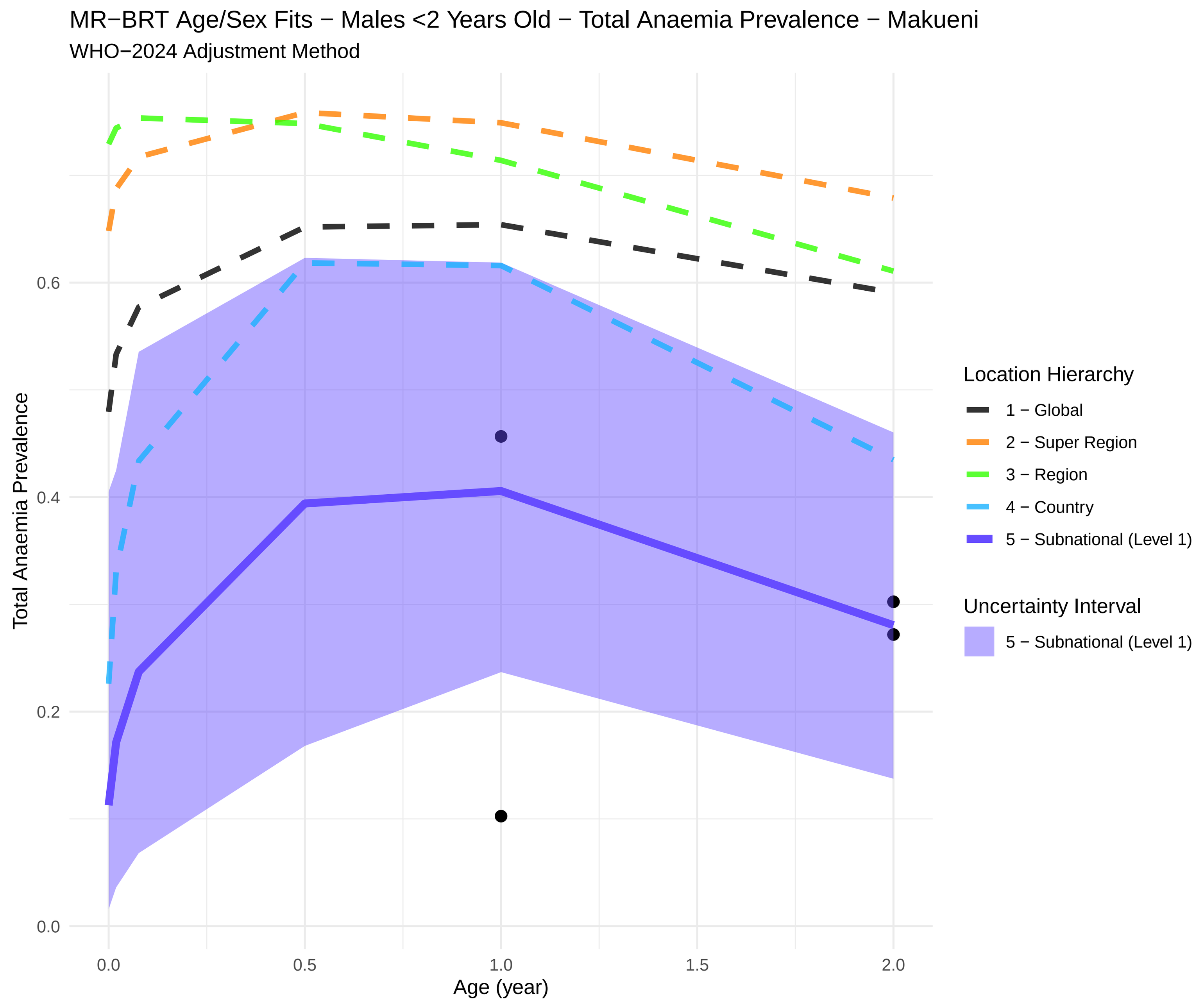
MR−BRT Age/Sex Fits − Males <2 Years Old − Total Anaemia Prevalence − Makueni
WHO−2024 Adjustment Method
Total Anaemia Prevalence
Age (year)
Location Hierarchy
1 − Global
2 − Super Region
3 − Region
4 − Country
5 − Subnational (Level 1)
Uncertainty Interval
5 − Subnational (Level 1)

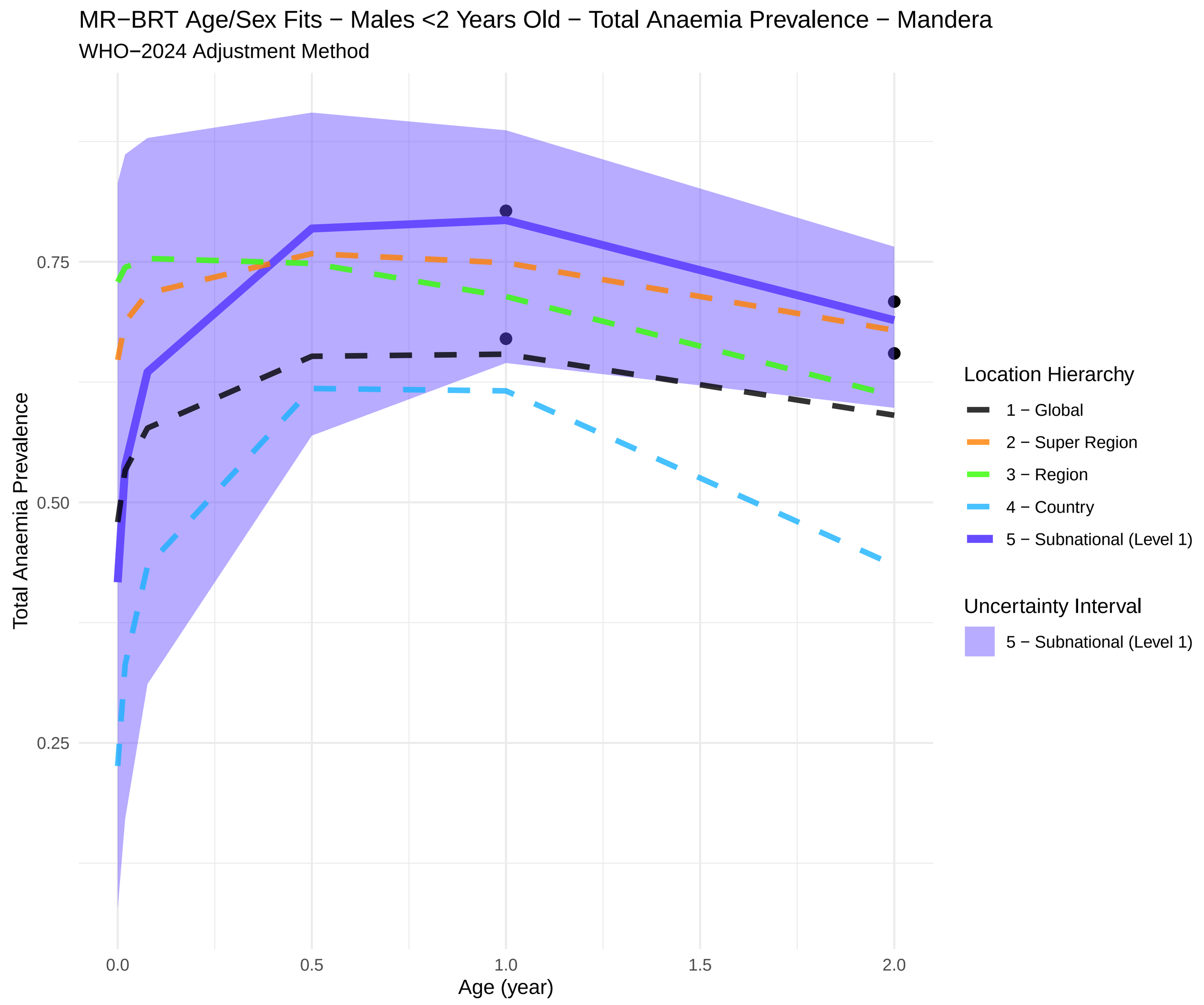
MR−BRT Age/Sex Fits − Males <2 Years Old − Total Anaemia Prevalence − Mandera
WHO−2024 Adjustment Method
Total Anaemia Prevalence
Age (year)
Location Hierarchy
1 − Global
2 − Super Region
3 − Region
4 − Country
5 − Subnational (Level 1)
Uncertainty Interval
5 − Subnational (Level 1)

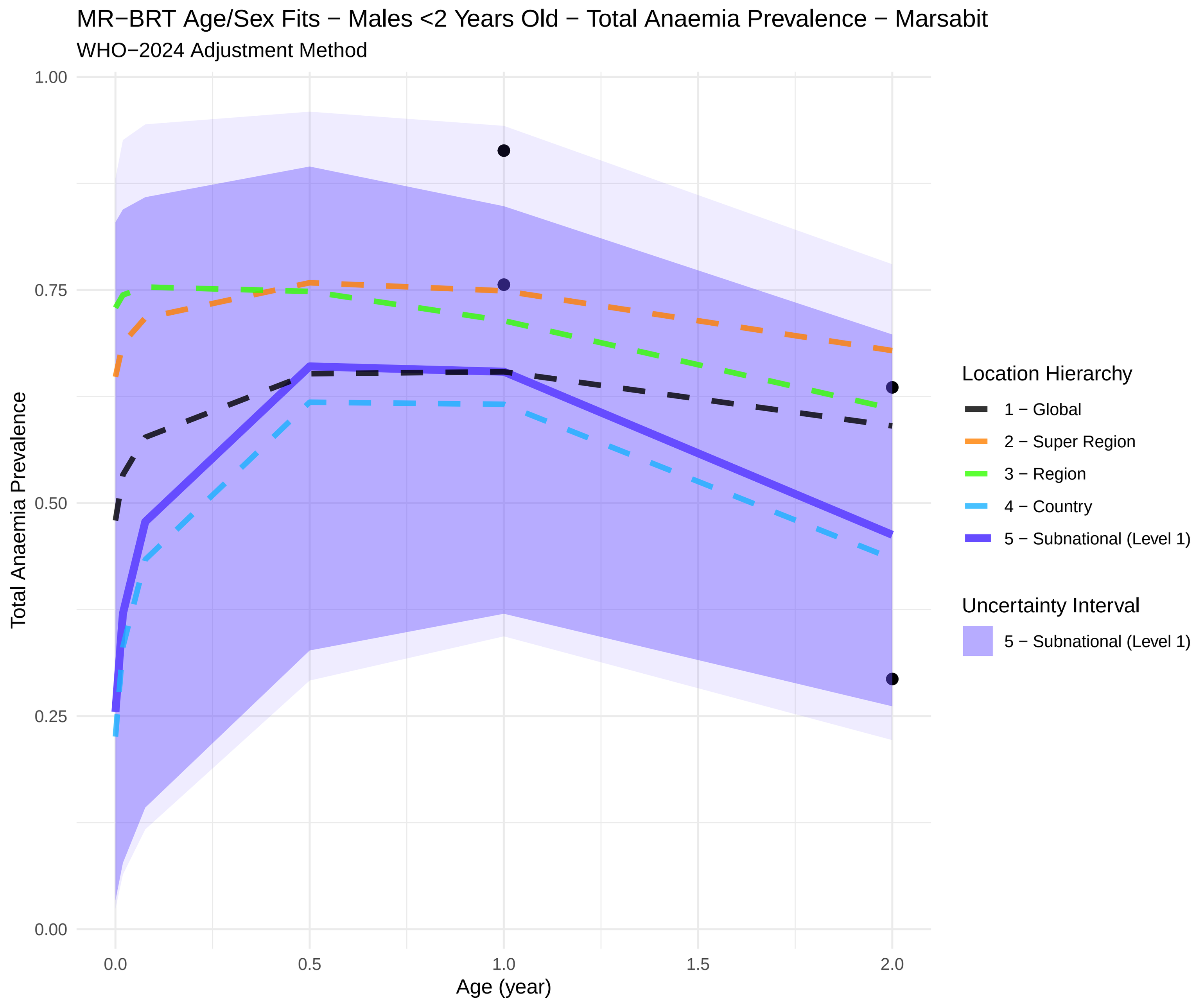
MR−BRT Age/Sex Fits − Males <2 Years Old − Total Anaemia Prevalence − Marsabit
WHO−2024 Adjustment Method
Total Anaemia Prevalence
Age (year)
Location Hierarchy
1 − Global
2 − Super Region
3 − Region
4 − Country
5 − Subnational (Level 1)
Uncertainty Interval
5 − Subnational (Level 1)

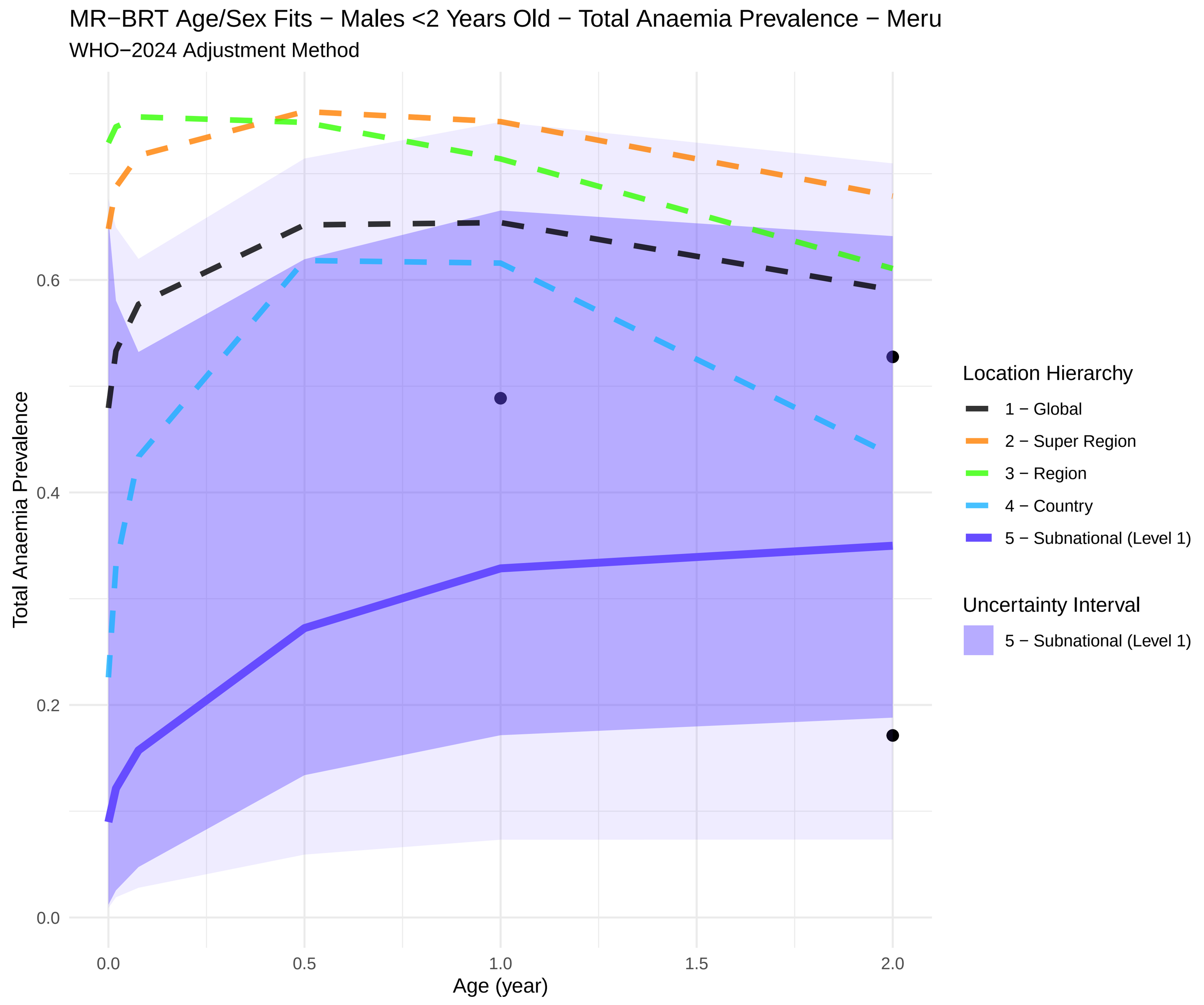
MR−BRT Age/Sex Fits − Males <2 Years Old − Total Anaemia Prevalence − Meru
WHO−2024 Adjustment Method
Total Anaemia Prevalence
Age (year)
Location Hierarchy
1 − Global
2 − Super Region
3 − Region
4 − Country
5 − Subnational (Level 1)
Uncertainty Interval
5 − Subnational (Level 1)

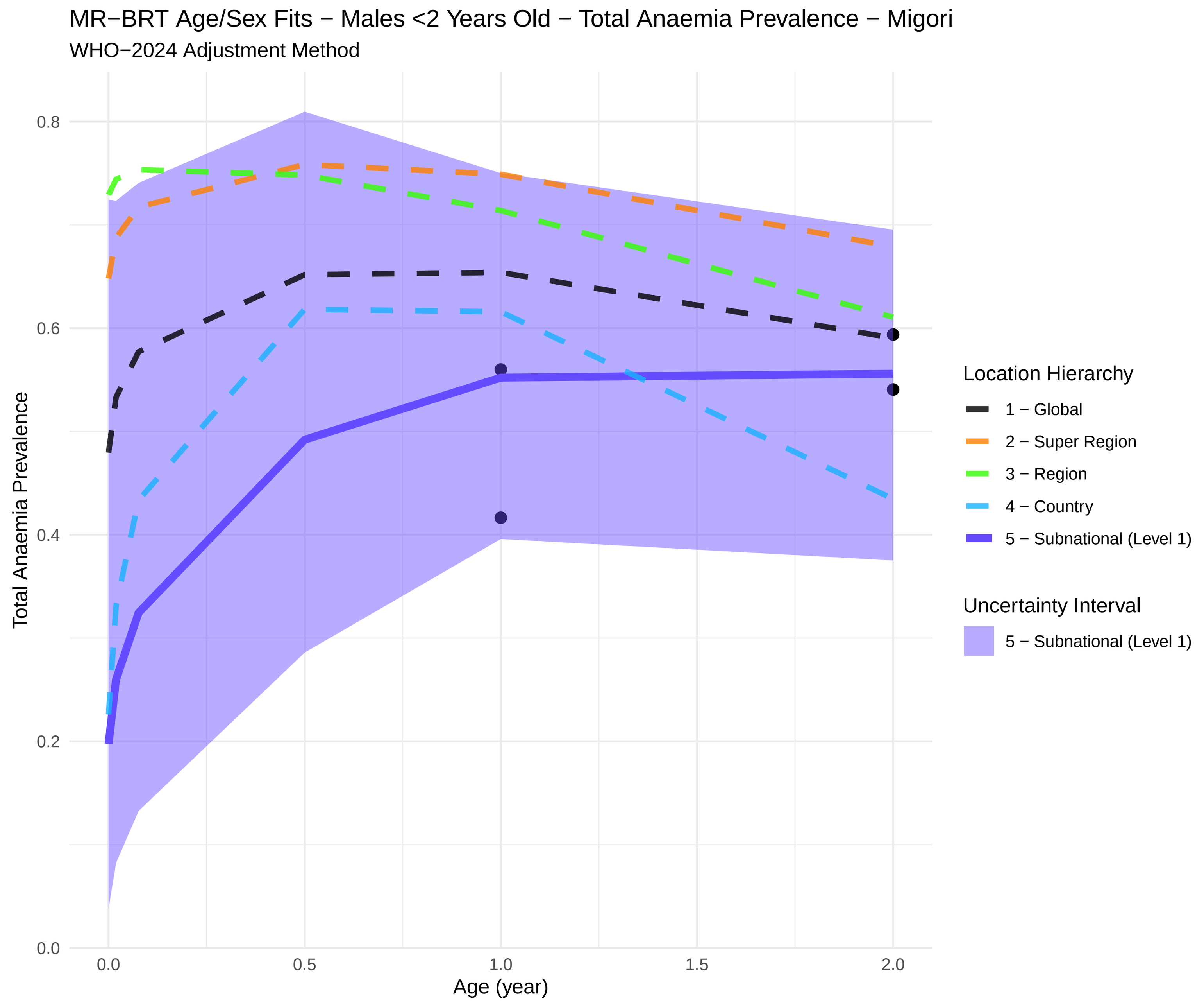
MR−BRT Age/Sex Fits − Males <2 Years Old − Total Anaemia Prevalence − Migori
WHO−2024 Adjustment Method
Total Anaemia Prevalence
Age (year)
Location Hierarchy
1 − Global
2 − Super Region
3 − Region
4 − Country
5 − Subnational (Level 1)
Uncertainty Interval
5 − Subnational (Level 1)

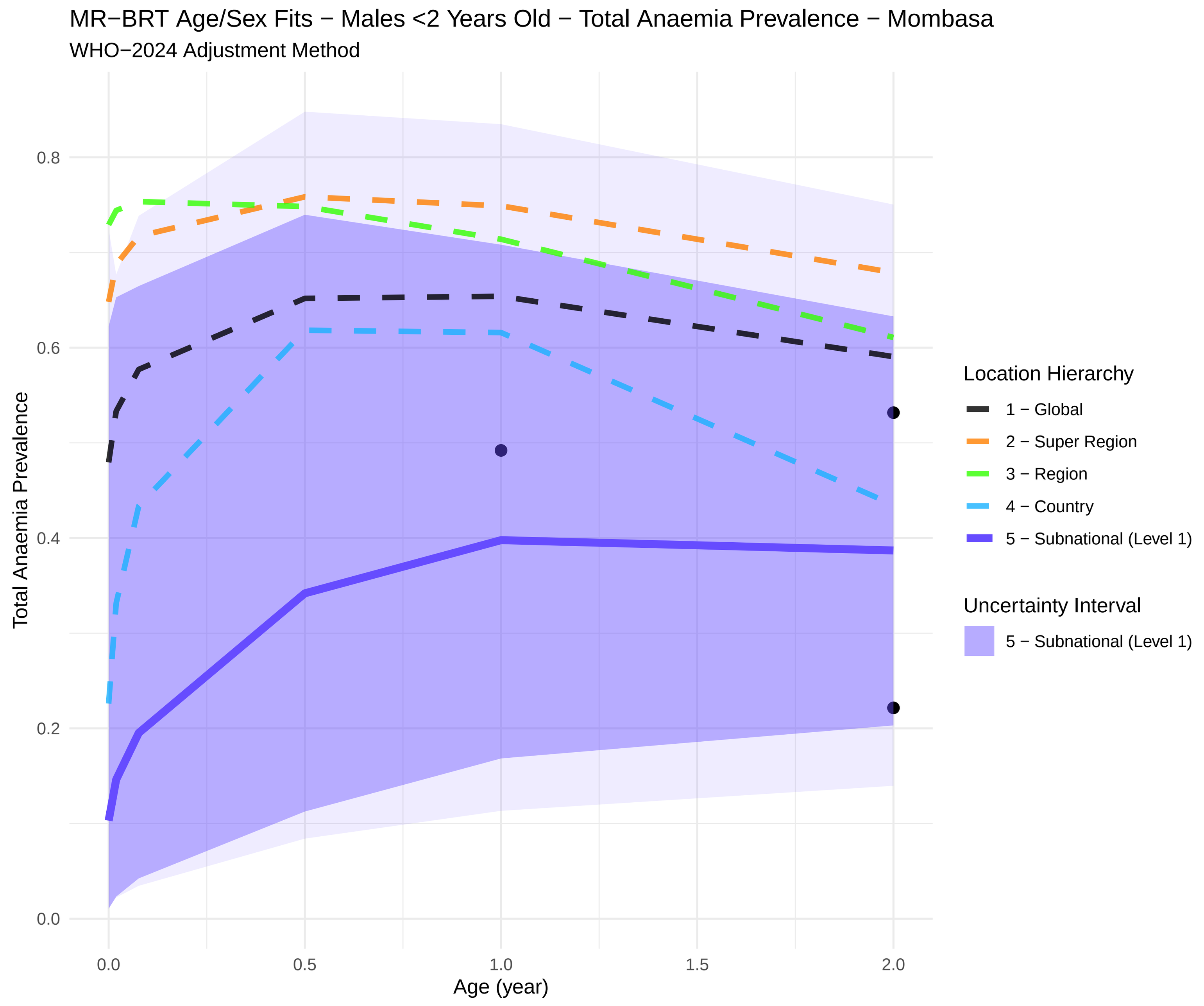
MR−BRT Age/Sex Fits − Males <2 Years Old − Total Anaemia Prevalence − Mombasa
WHO−2024 Adjustment Method
Total Anaemia Prevalence
Age (year)
Location Hierarchy
1 − Global
2 − Super Region
3 − Region
4 − Country
5 − Subnational (Level 1)
Uncertainty Interval
5 − Subnational (Level 1)

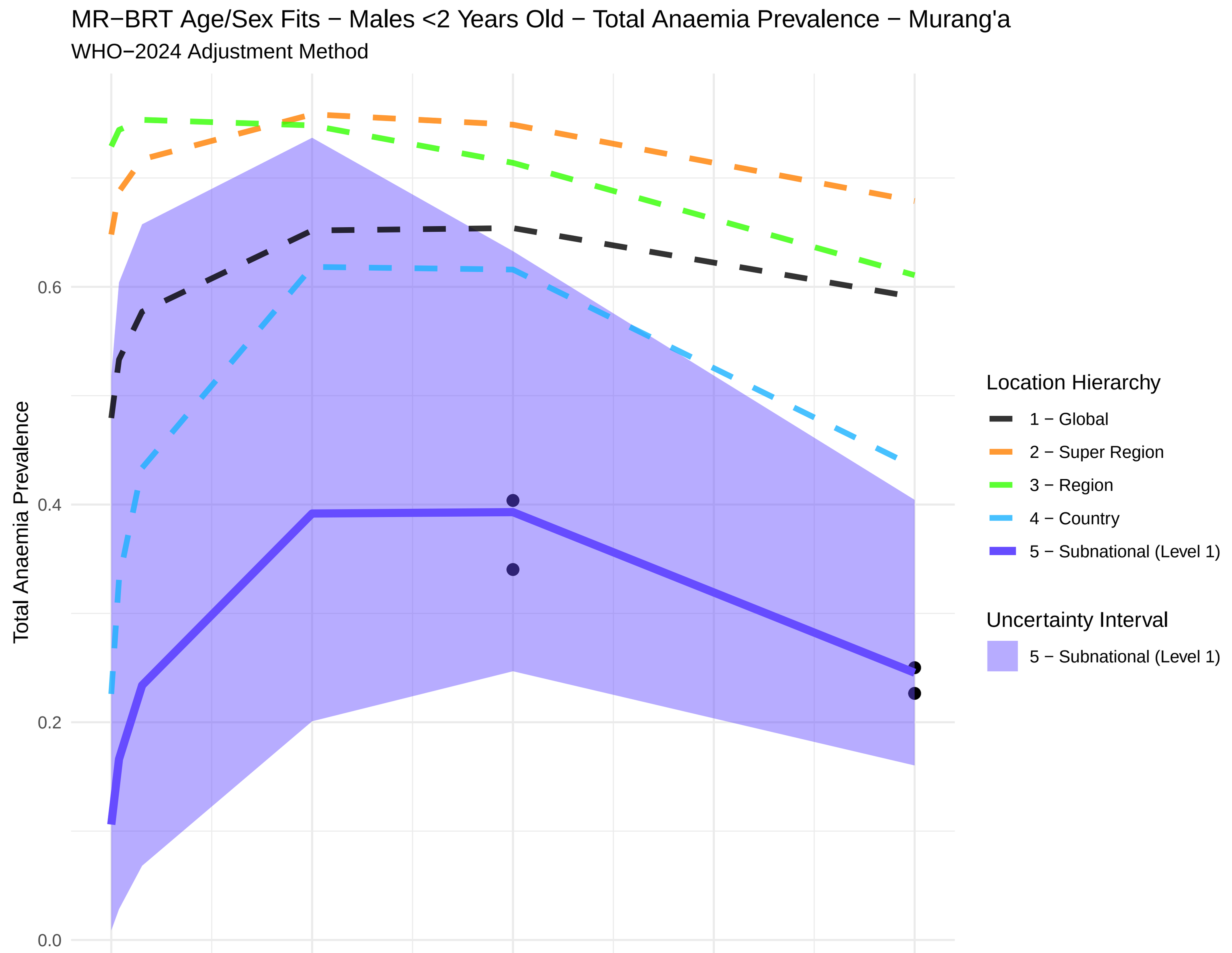
MR−BRT Age/Sex Fits − Males <2 Years Old − Total Anaemia Prevalence − Murang'a
WHO−2024 Adjustment Method
Total Anaemia Prevalence
Age (year)
Location Hierarchy
1 − Global
2 − Super Region
3 − Region
4 − Country
5 − Subnational (Level 1)
Uncertainty Interval
5 − Subnational (Level 1)

MR−BRT Age/Sex Fits − Males <2 Years Old − Total Anaemia Prevalence − Nairobi
WHO−2024 Adjustment Method

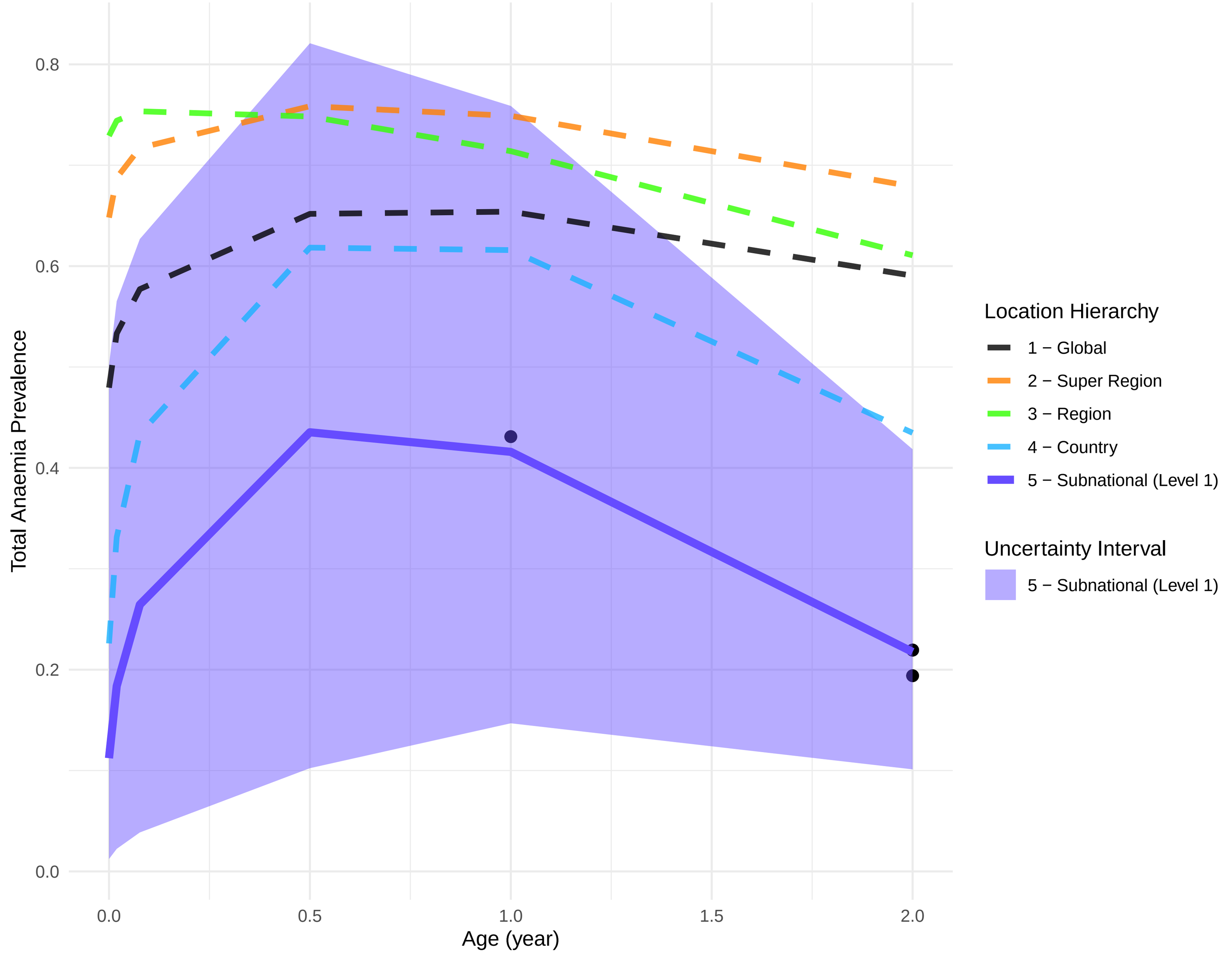

MR−BRT Age/Sex Fits − Males <2 Years Old − Total Anaemia Prevalence − Nakuru
WHO−2024 Adjustment Method

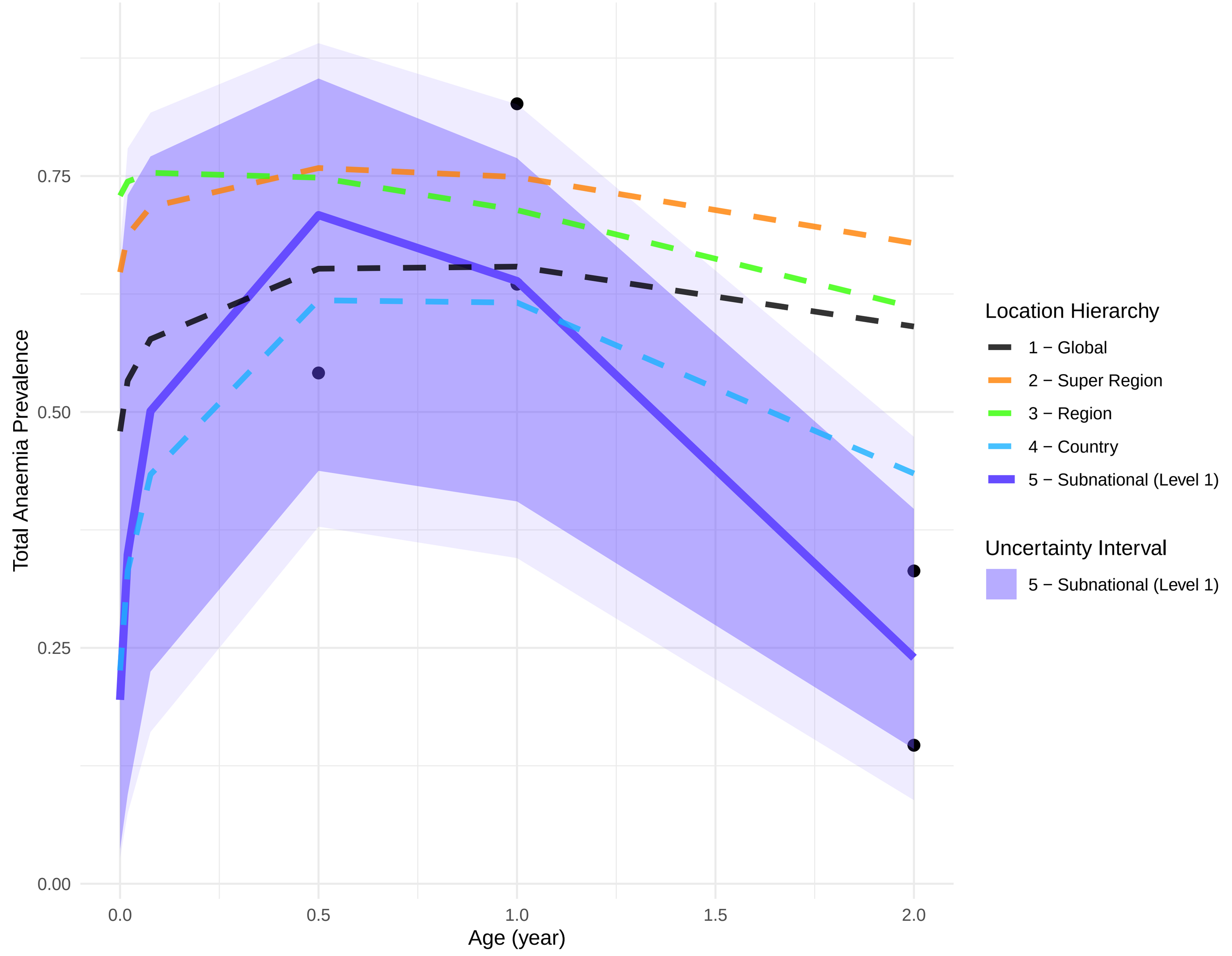

MR−BRT Age/Sex Fits − Males <2 Years Old − Total Anaemia Prevalence − Nandi
WHO−2024 Adjustment Method

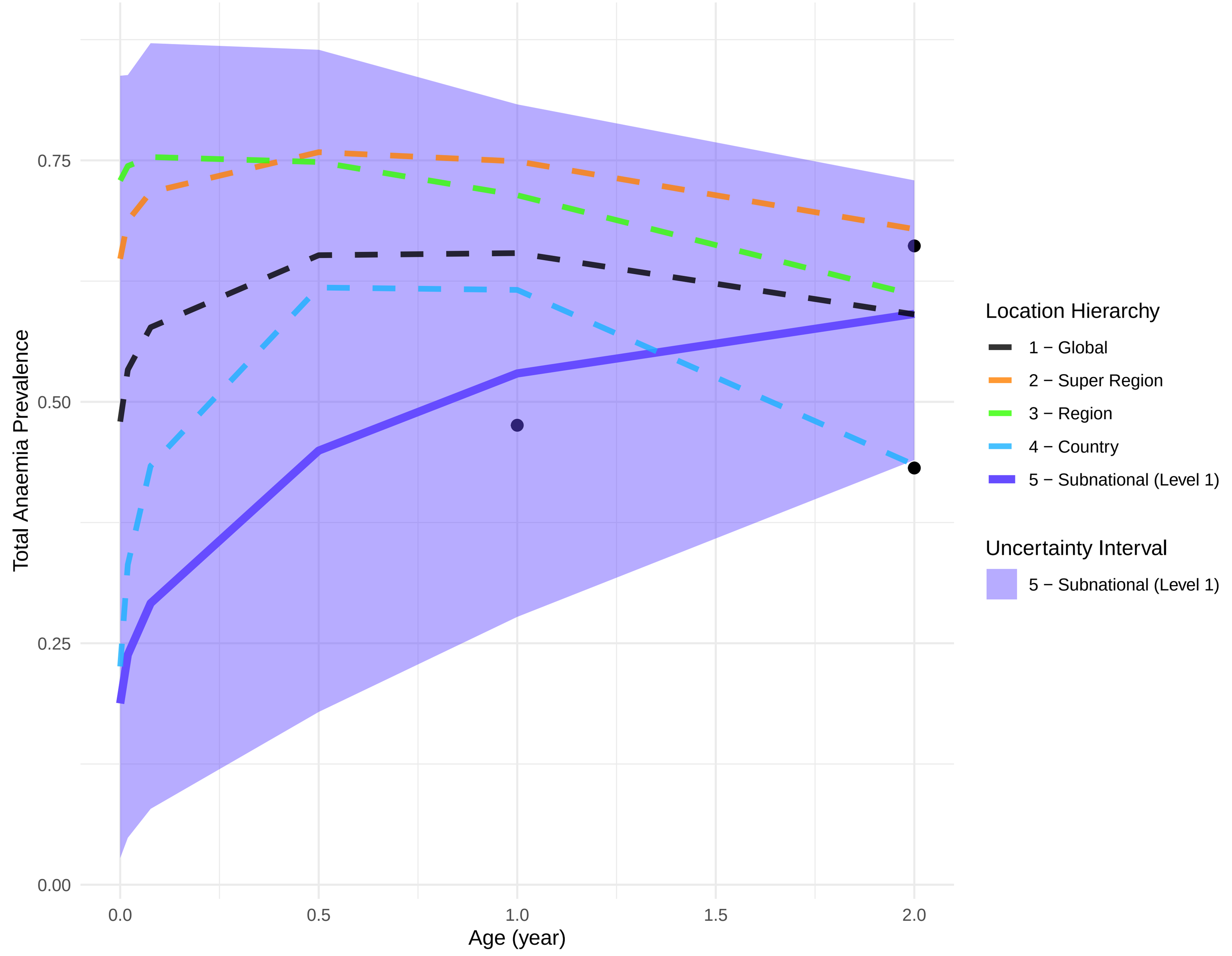

MR−BRT Age/Sex Fits − Males <2 Years Old − Total Anaemia Prevalence − Narok
WHO−2024 Adjustment Method

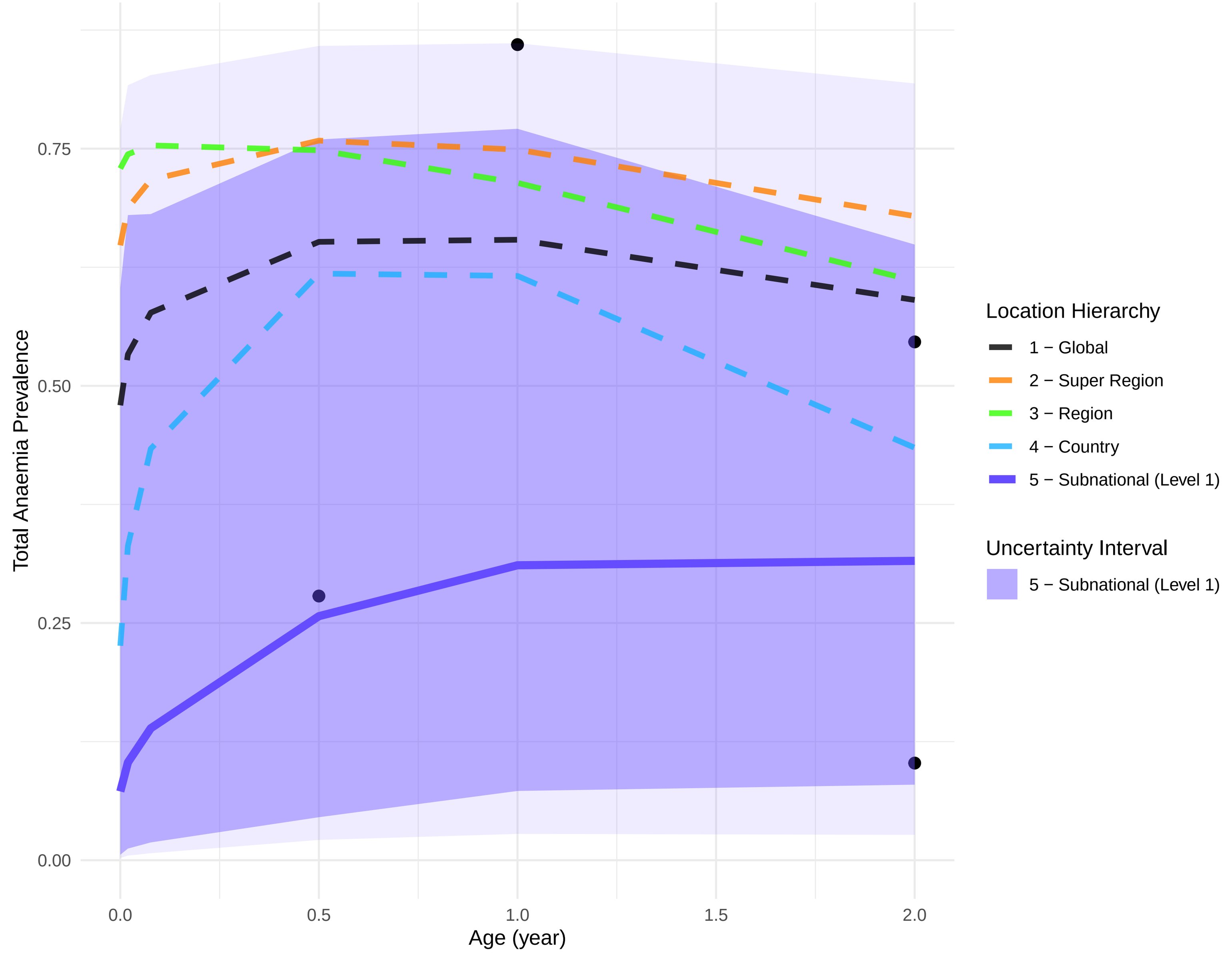

MR−BRT Age/Sex Fits − Males <2 Years Old − Total Anaemia Prevalence − Nyamira
WHO−2024 Adjustment Method

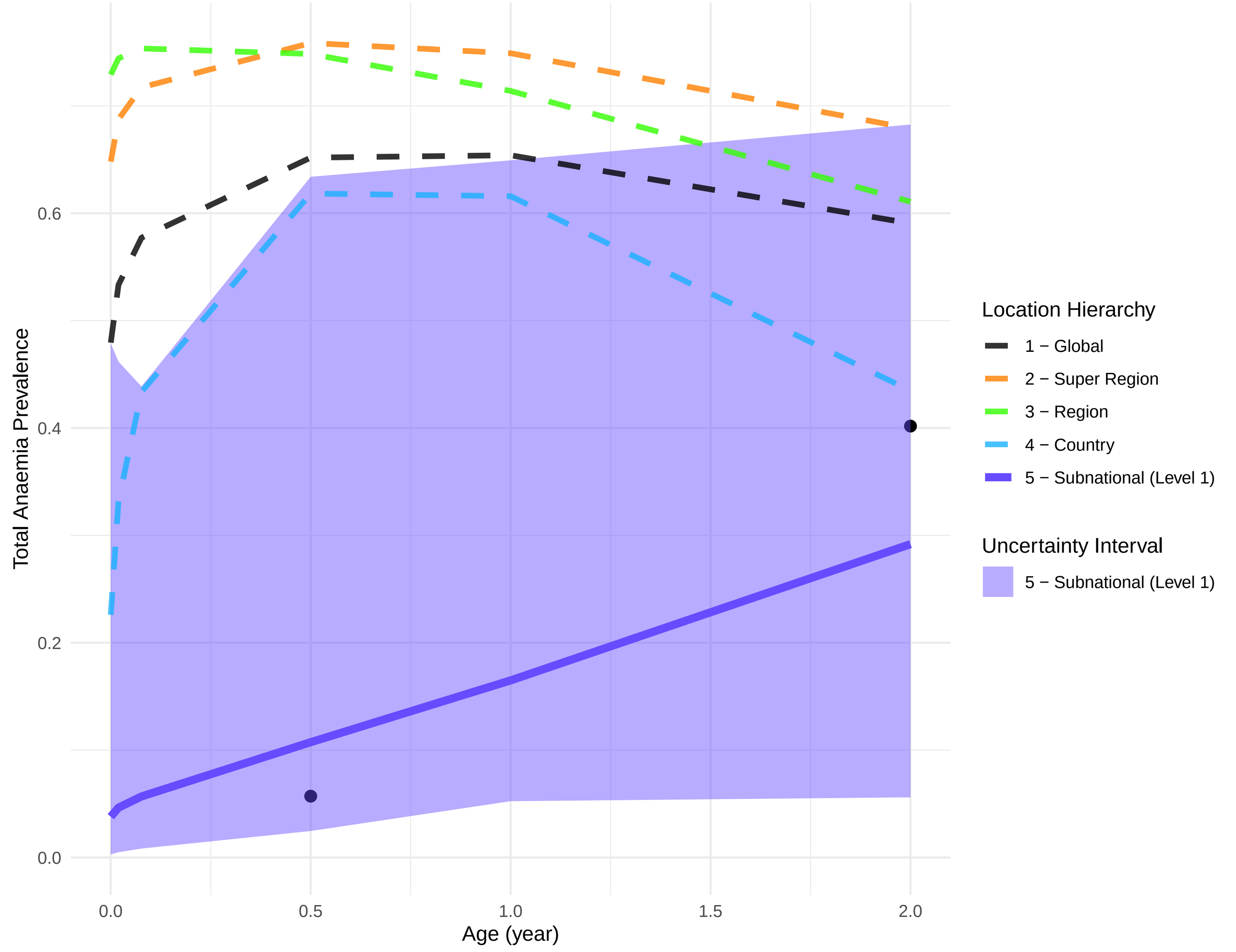

MR−BRT Age/Sex Fits − Males <2 Years Old − Total Anaemia Prevalence − Nyandarua
WHO−2024 Adjustment Method

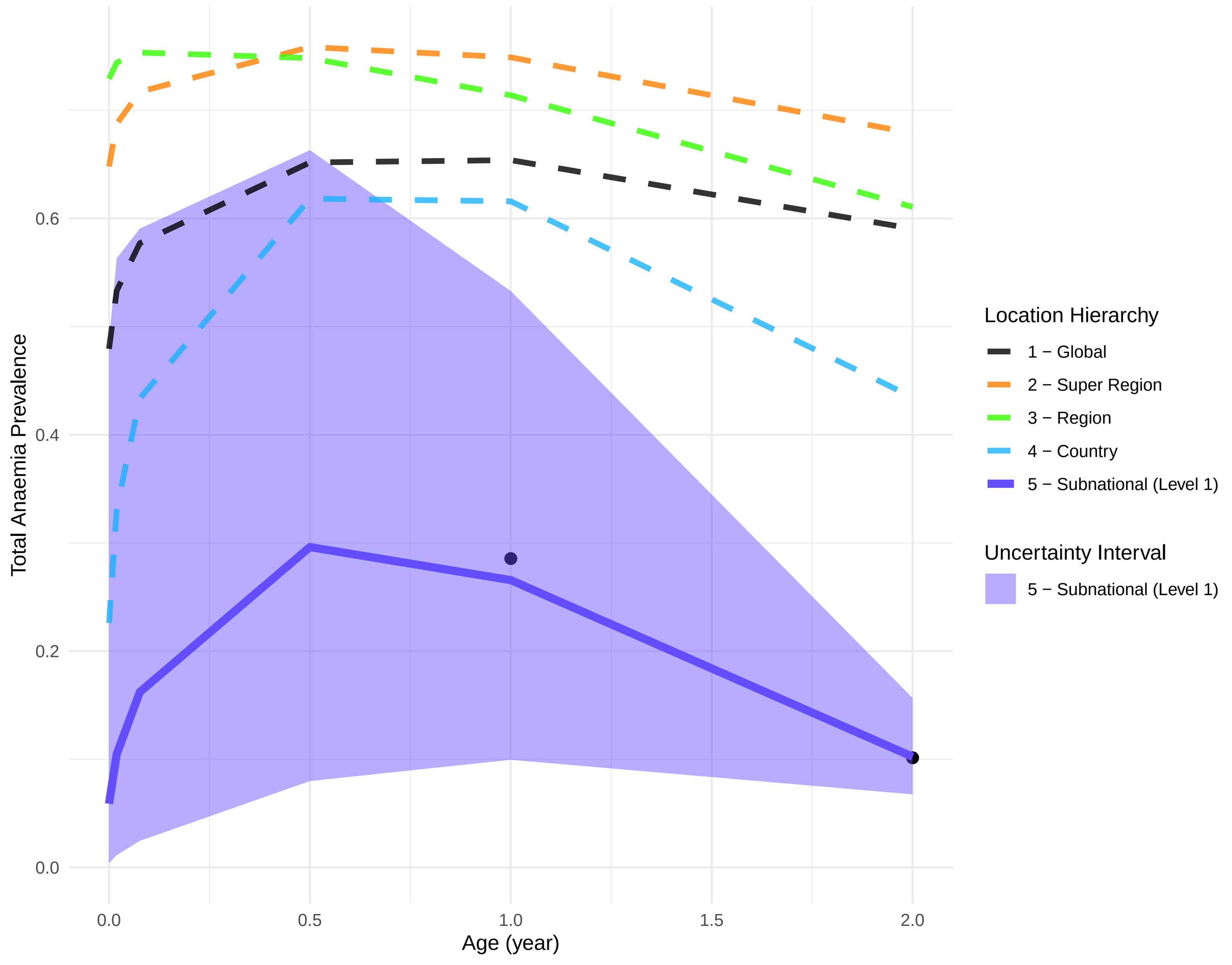

MR−BRT Age/Sex Fits − Males <2 Years Old − Total Anaemia Prevalence − Nyeri
WHO−2024 Adjustment Method

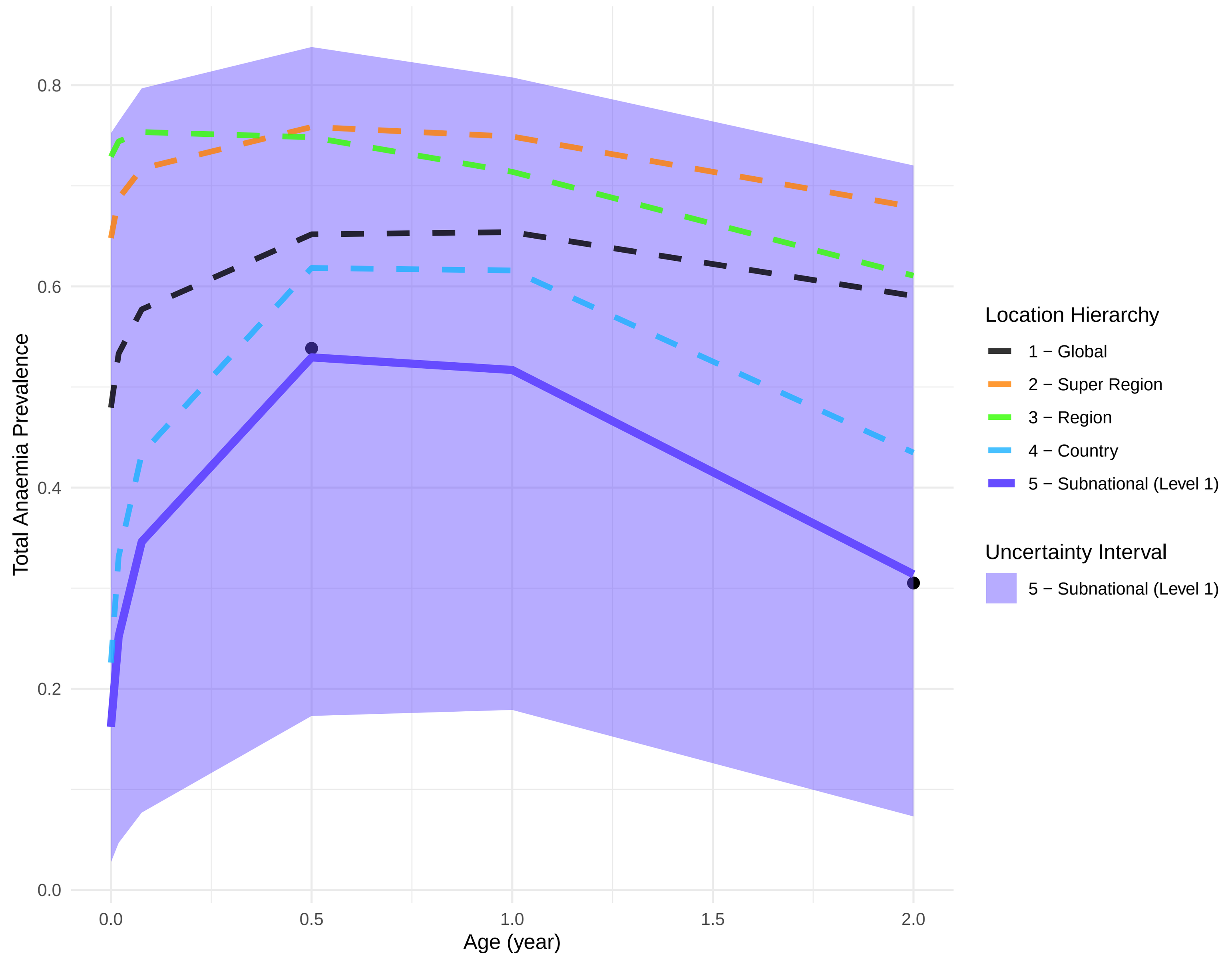

MR−BRT Age/Sex Fits − Males <2 Years Old − Total Anaemia Prevalence − Samburu
WHO−2024 Adjustment Method

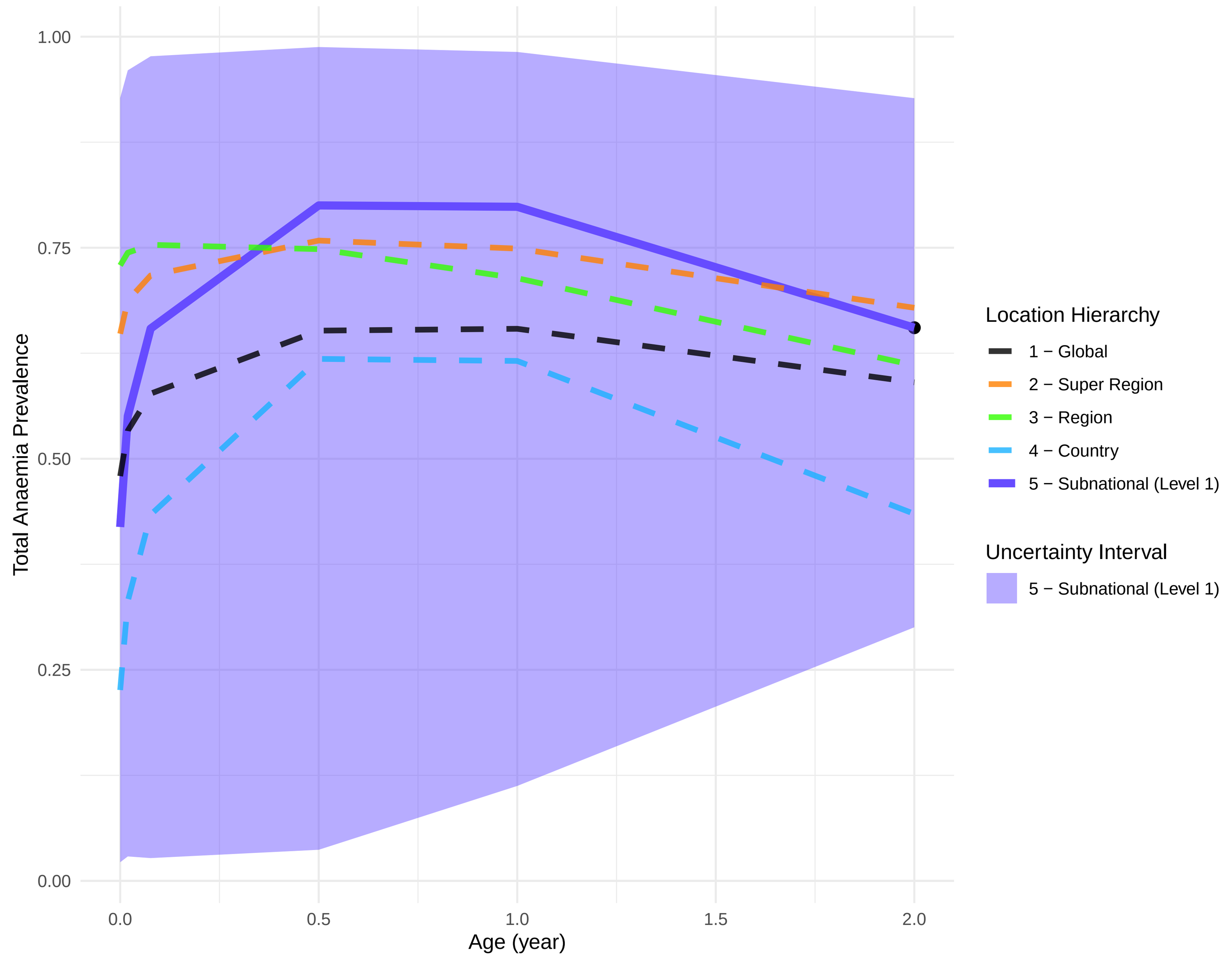

MR−BRT Age/Sex Fits − Males <2 Years Old − Total Anaemia Prevalence − Siaya
WHO−2024 Adjustment Method

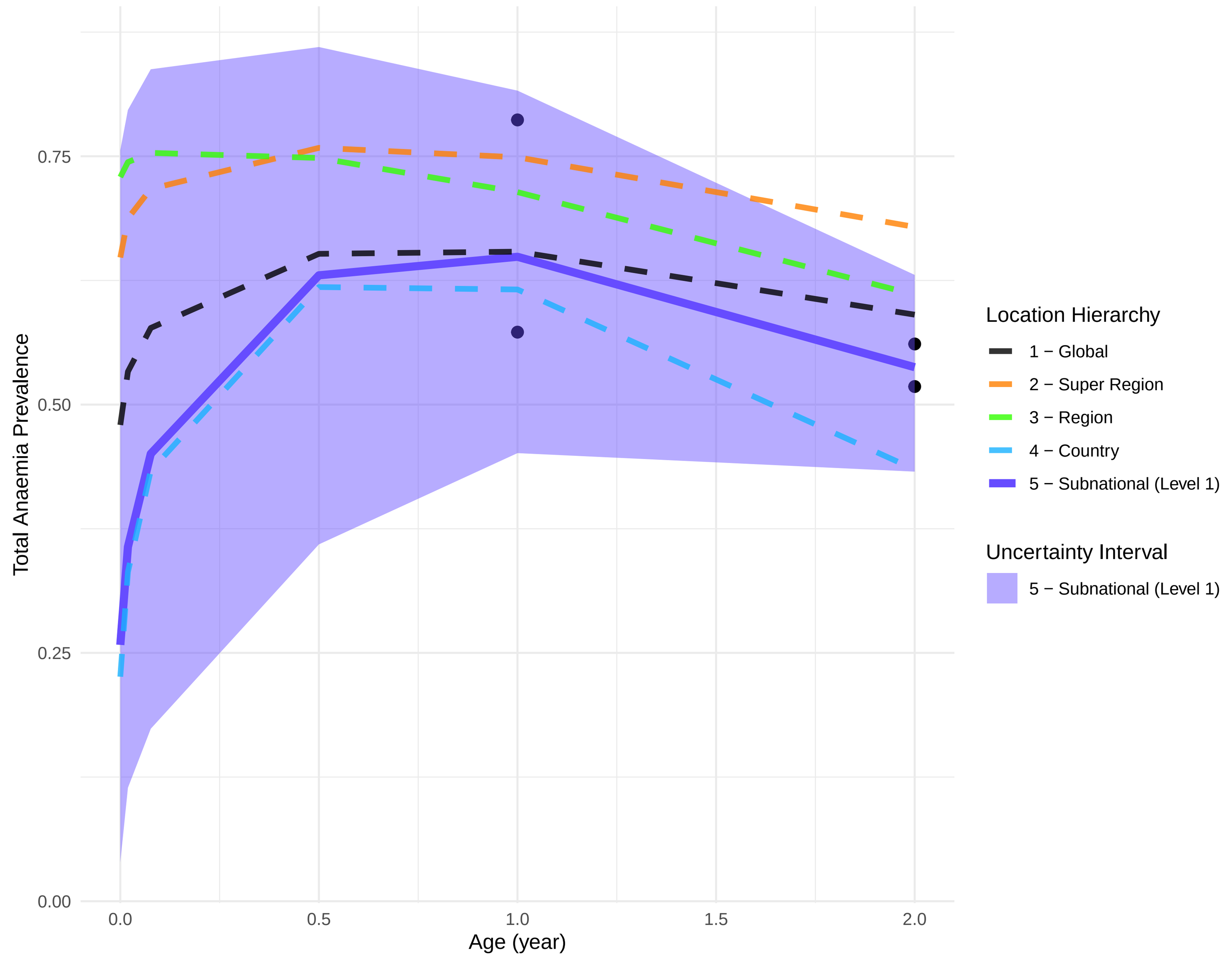

MR−BRT Age/Sex Fits − Males <2 Years Old − Total Anaemia Prevalence − Taita Taveta
WHO−2024 Adjustment Method

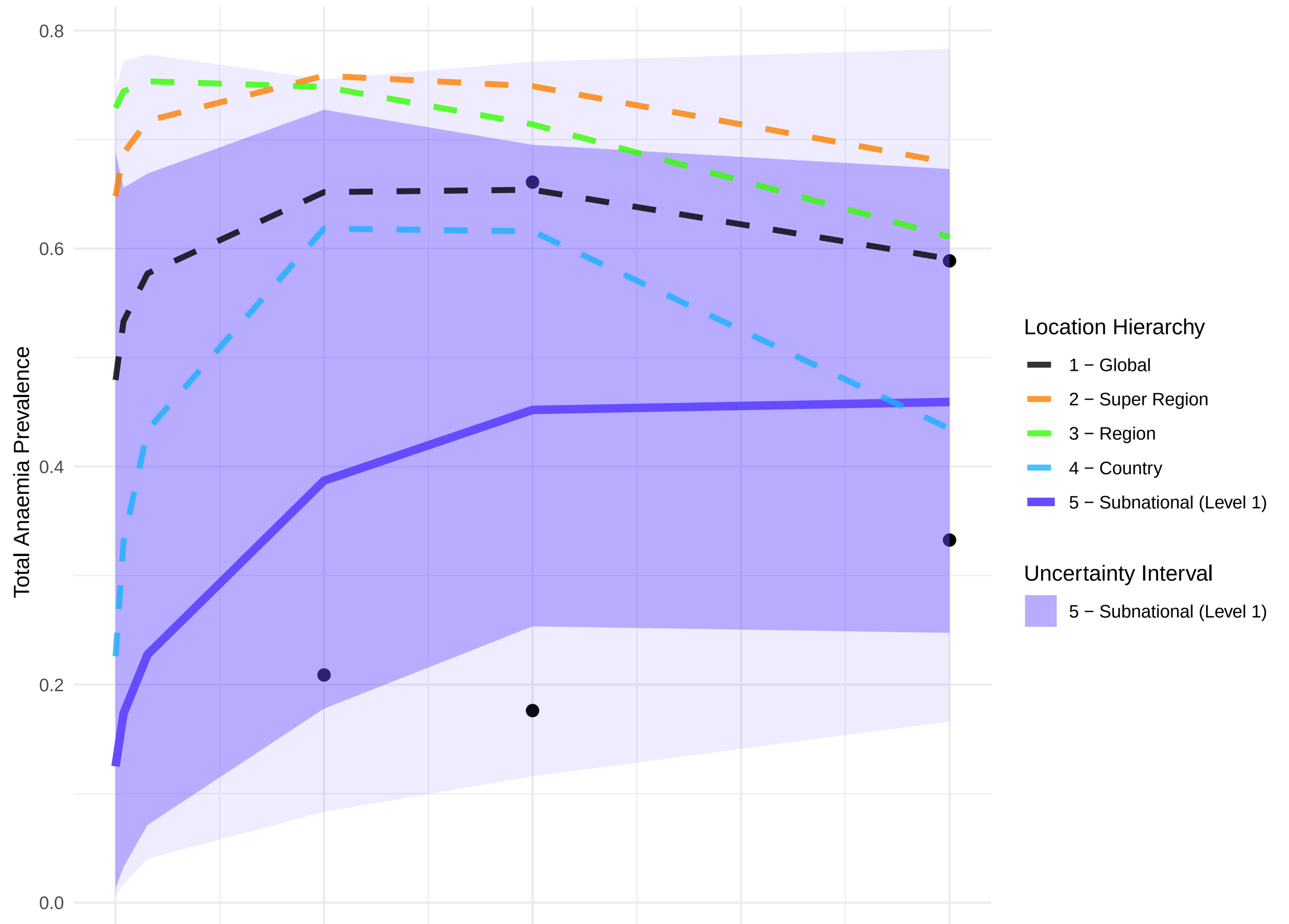

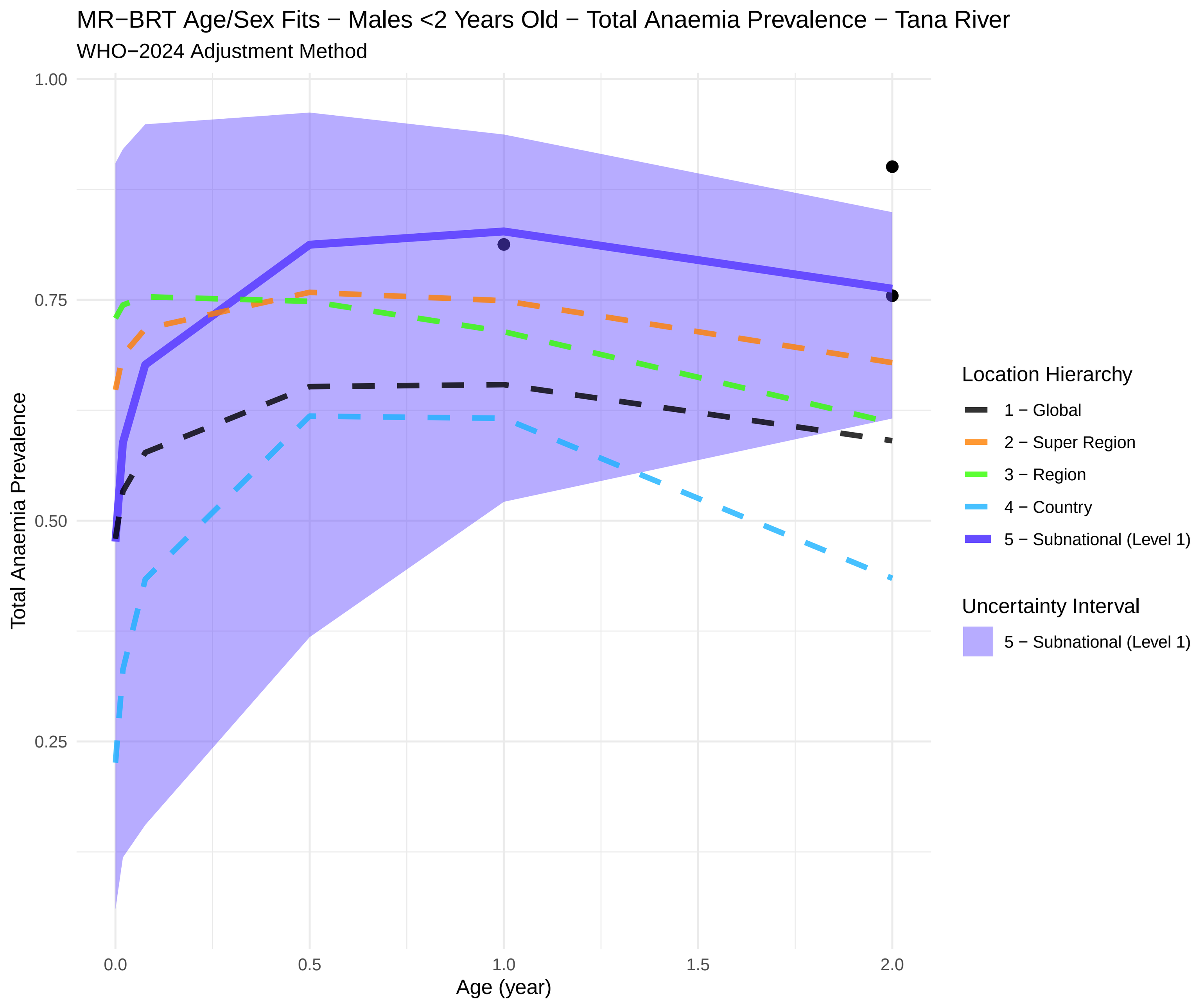
MR−BRT Age/Sex Fits − Males <2 Years Old − Total Anaemia Prevalence − Tana River
WHO−2024 Adjustment Method
Total Anaemia Prevalence
0.25
0.50
0.75
1.00
Age (year)
0.0
0.5
1.0
1.5
2.0
Location Hierarchy
1 − Global
2 − Super Region
3 − Region
4 − Country
5 − Subnational (Level 1)
Uncertainty Interval
5 − Subnational (Level 1)

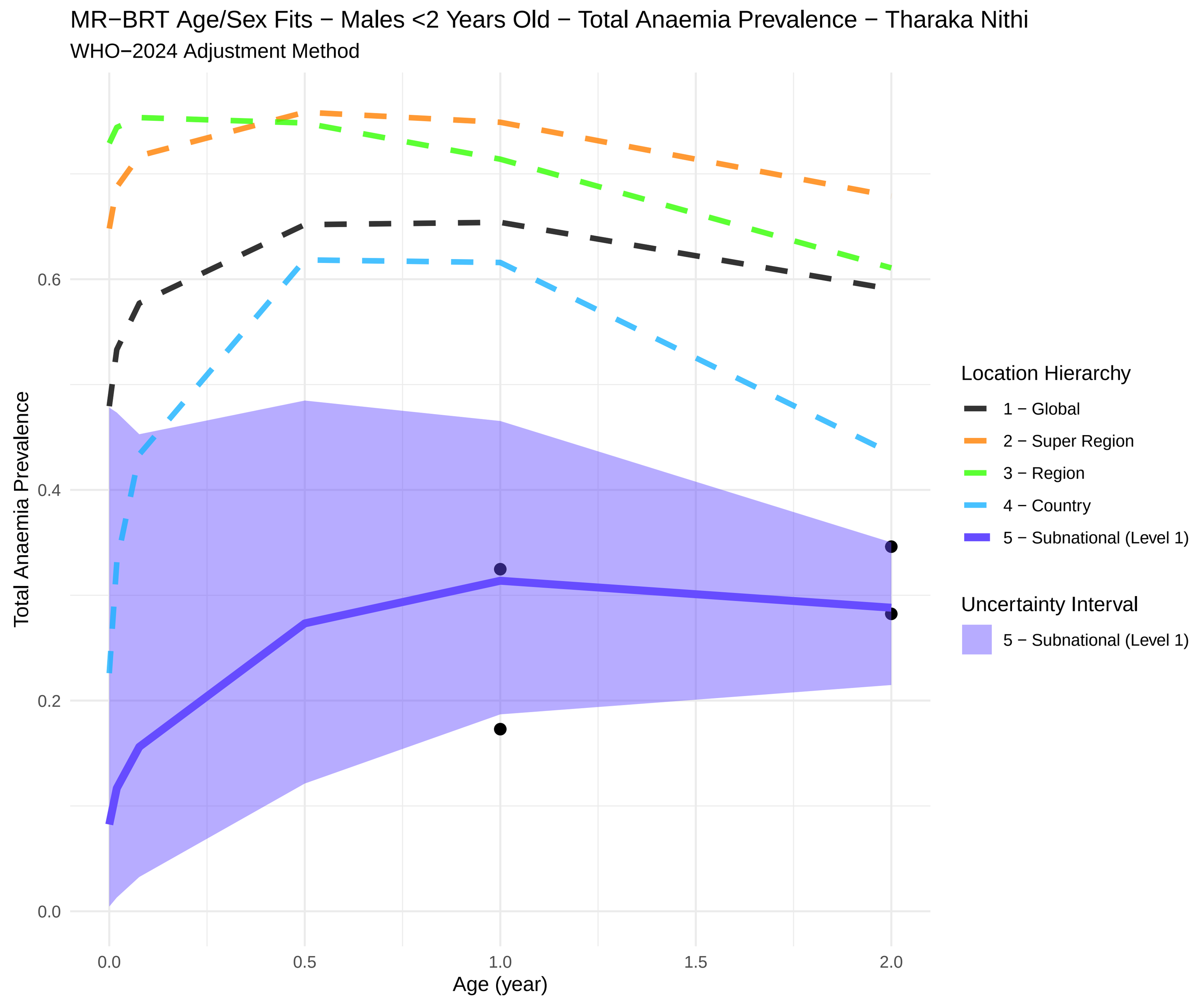
MR−BRT Age/Sex Fits − Males <2 Years Old − Total Anaemia Prevalence − Tharaka Nithi
WHO−2024 Adjustment Method
Total Anaemia Prevalence
0.0
0.2
0.4
0.6
Age (year)
0.0
0.5
1.0
1.5
2.0
Location Hierarchy
1 − Global
2 − Super Region
3 − Region
4 − Country
5 − Subnational (Level 1)
Uncertainty Interval
5 − Subnational (Level 1)

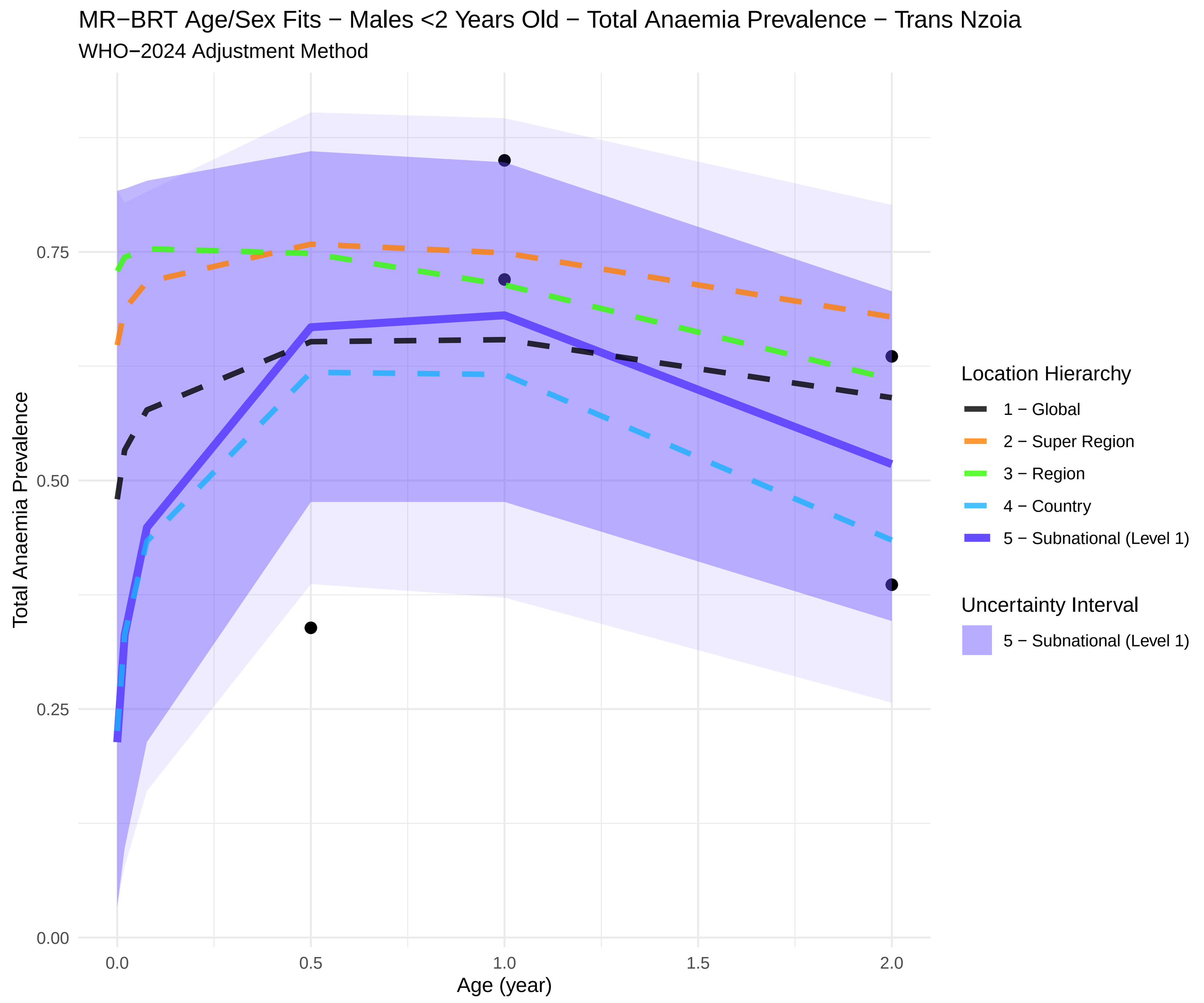
MR−BRT Age/Sex Fits − Males <2 Years Old − Total Anaemia Prevalence − Trans Nzoia
WHO−2024 Adjustment Method
Total Anaemia Prevalence
0.00
0.25
0.50
0.75
Age (year)
0.0
0.5
1.0
1.5
2.0
Location Hierarchy
1 − Global
2 − Super Region
3 − Region
4 − Country
5 − Subnational (Level 1)
Uncertainty Interval
5 − Subnational (Level 1)

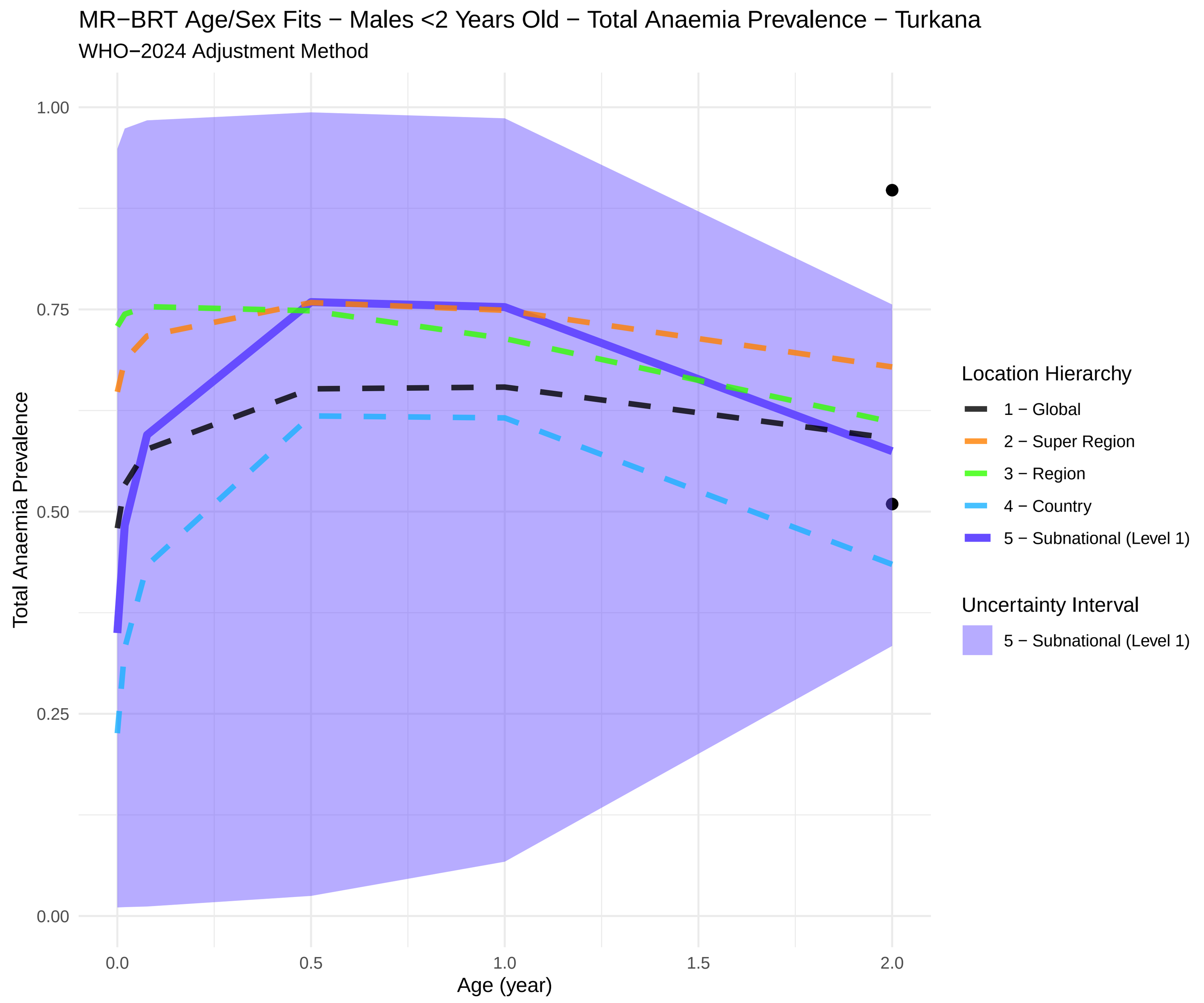
MR−BRT Age/Sex Fits − Males <2 Years Old − Total Anaemia Prevalence − Turkana
WHO−2024 Adjustment Method
Total Anaemia Prevalence
0.00
0.25
0.50
0.75
1.00
Age (year)
0.0
0.5
1.0
1.5
2.0
Location Hierarchy
1 − Global
2 − Super Region
3 − Region
4 − Country
5 − Subnational (Level 1)
Uncertainty Interval
5 − Subnational (Level 1)

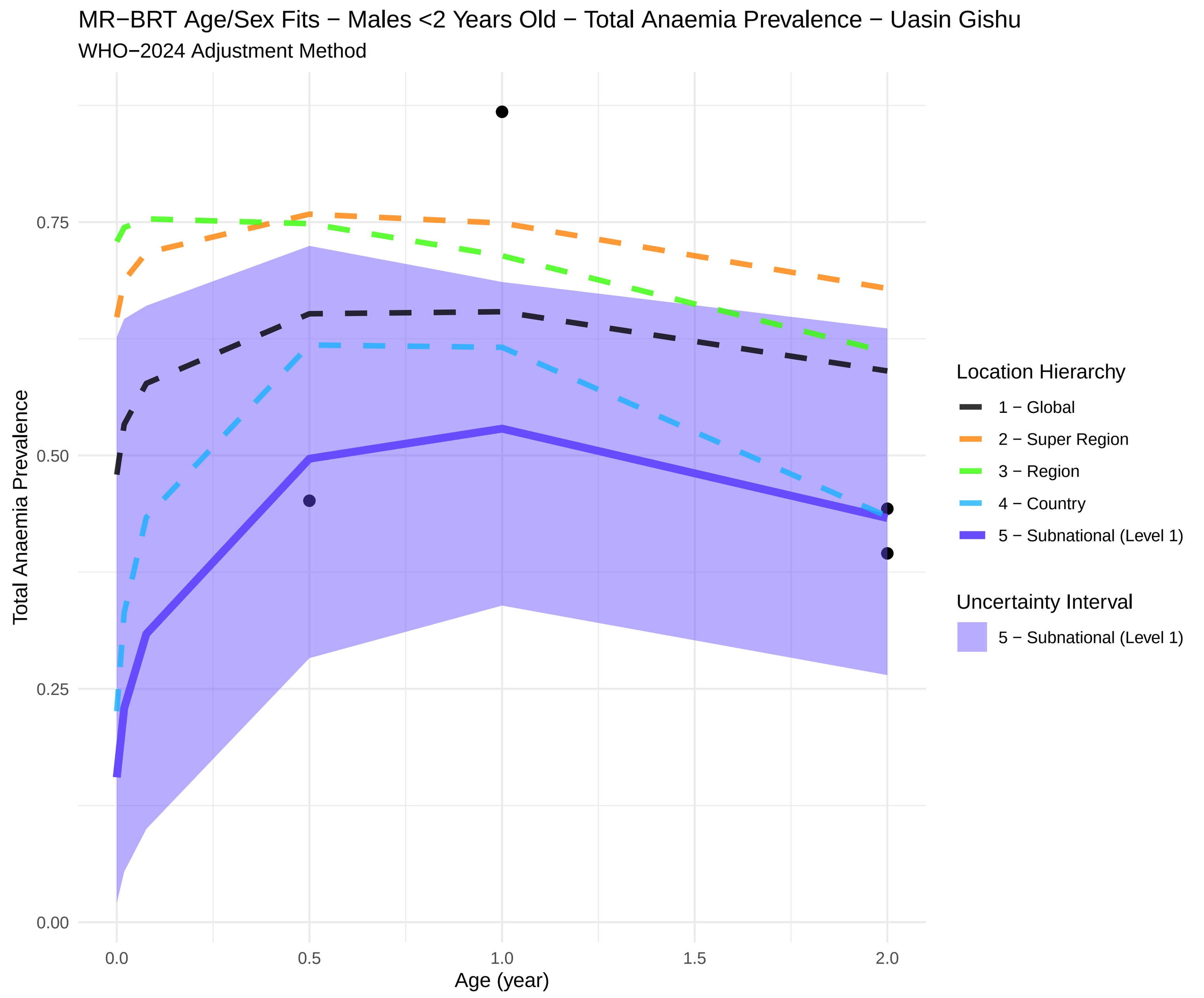
MR−BRT Age/Sex Fits − Males <2 Years Old − Total Anaemia Prevalence − Uasin Gishu
WHO−2024 Adjustment Method
Total Anaemia Prevalence
0.00
0.25
0.50
0.75
Age (year)
0.0
0.5
1.0
1.5
2.0
Location Hierarchy
1 − Global
2 − Super Region
3 − Region
4 − Country
5 − Subnational (Level 1)
Uncertainty Interval
5 − Subnational (Level 1)

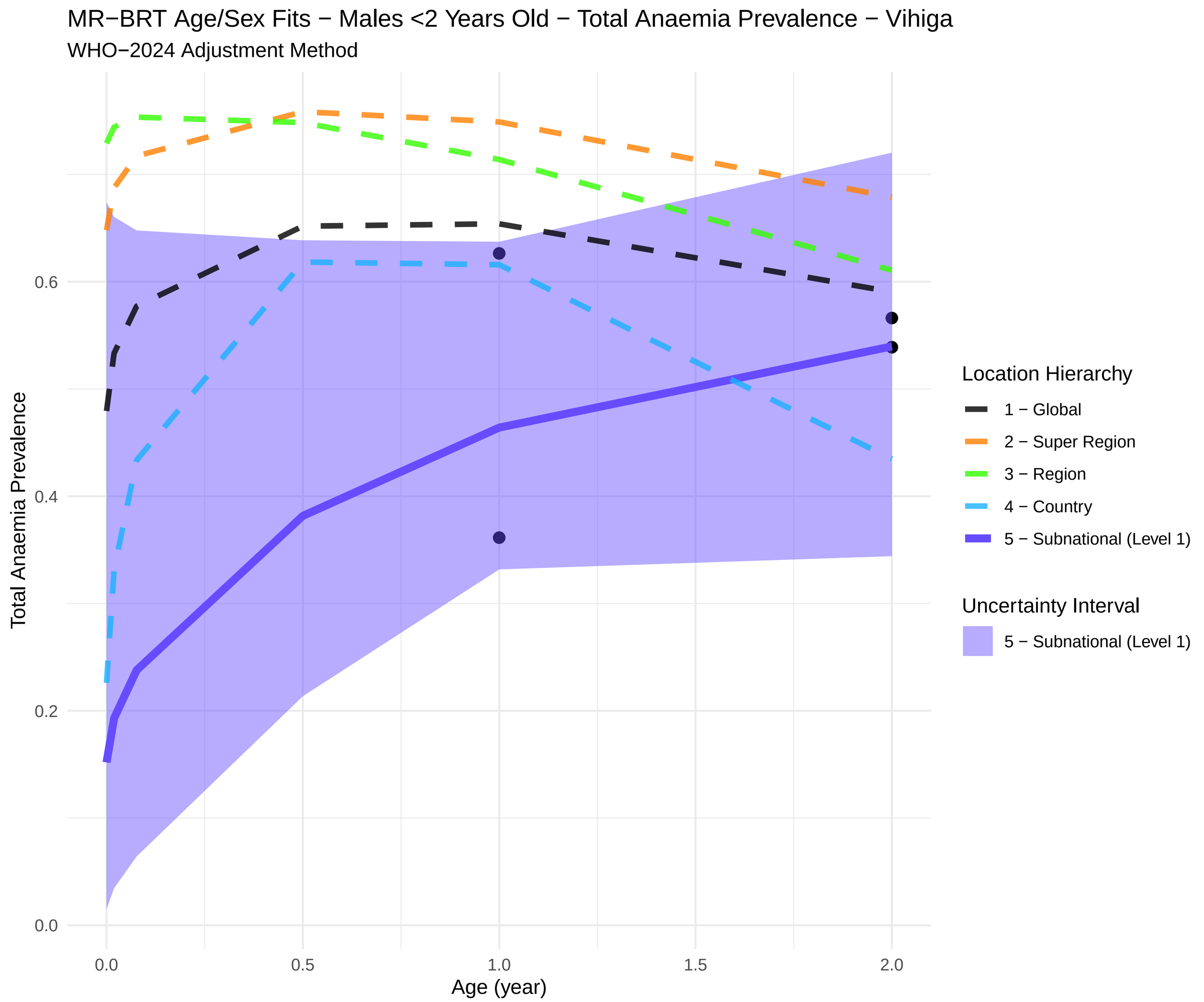
MR−BRT Age/Sex Fits − Males <2 Years Old − Total Anaemia Prevalence − Vihiga
WHO−2024 Adjustment Method
Total Anaemia Prevalence
0.0
0.2
0.4
0.6
Age (year)
0.0
0.5
1.0
1.5
2.0
Location Hierarchy
1 − Global
2 − Super Region
3 − Region
4 − Country
5 − Subnational (Level 1)
Uncertainty Interval
5 − Subnational (Level 1)

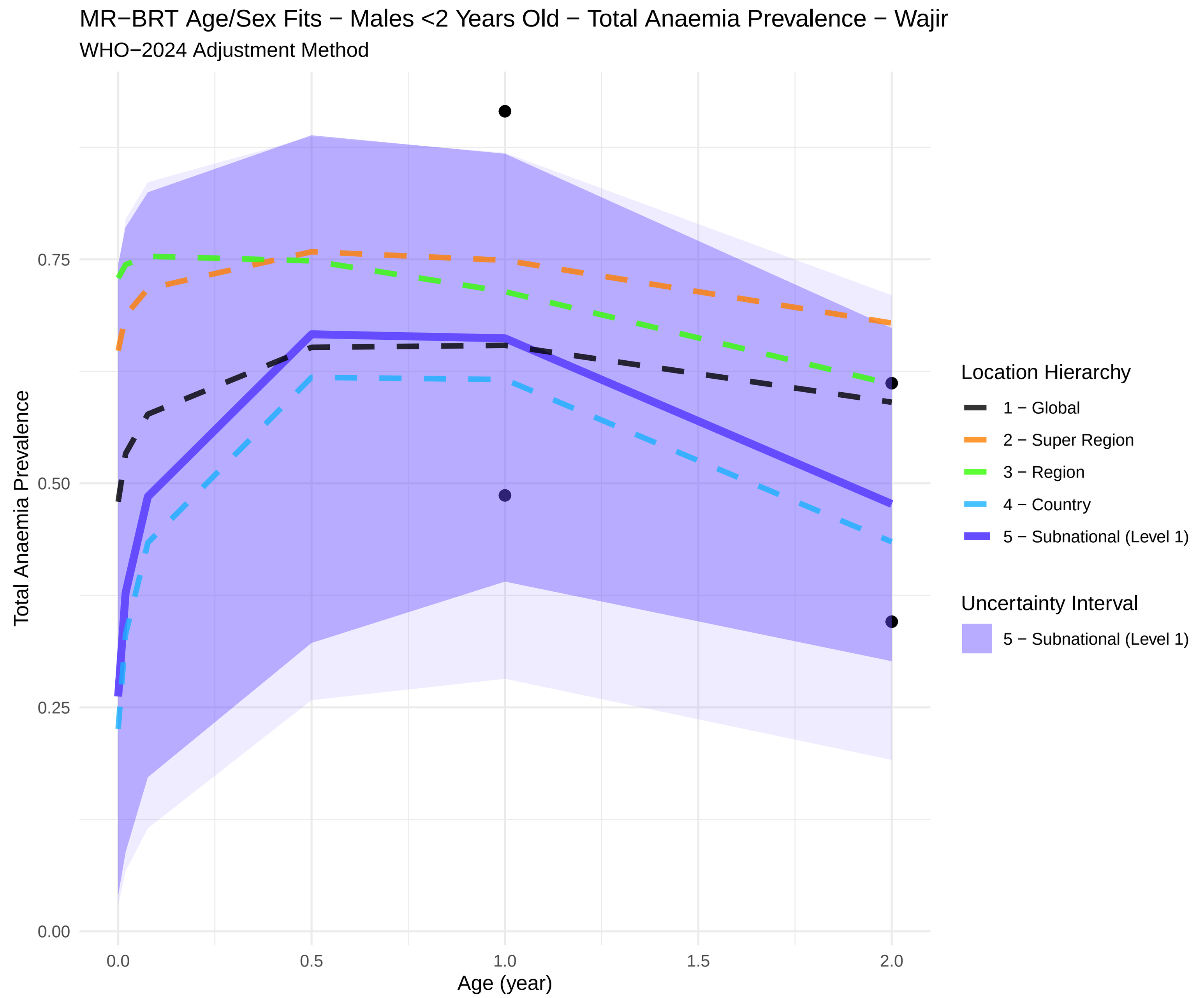
MR−BRT Age/Sex Fits − Males <2 Years Old − Total Anaemia Prevalence − Wajir
WHO−2024 Adjustment Method
Total Anaemia Prevalence
0.00
0.25
0.50
0.75
Age (year)
0.0
0.5
1.0
1.5
2.0
Location Hierarchy
1 − Global
2 − Super Region
3 − Region
4 − Country
5 − Subnational (Level 1)
Uncertainty Interval
5 − Subnational (Level 1)

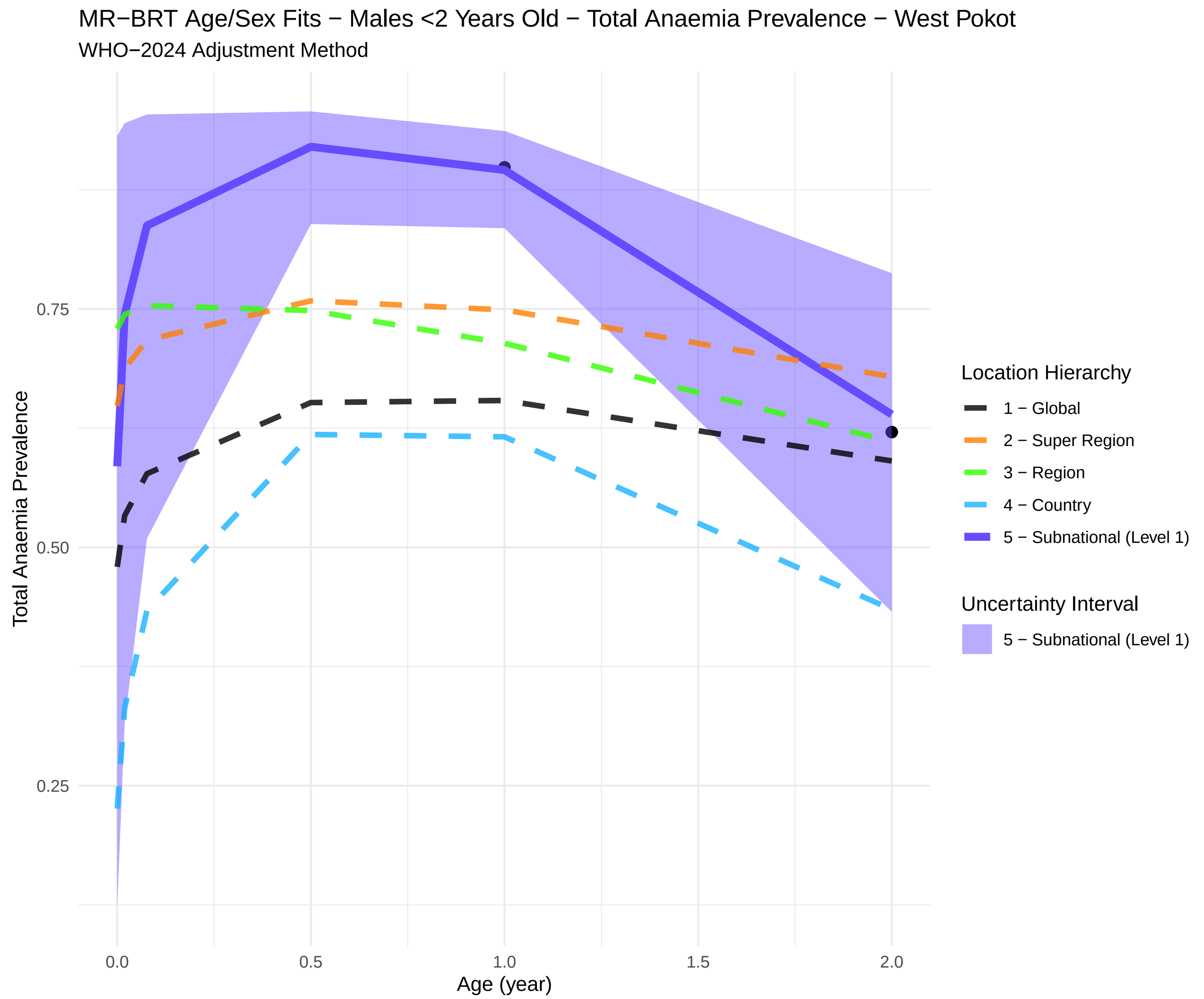
MR−BRT Age/Sex Fits − Males <2 Years Old − Total Anaemia Prevalence − West Pokot
WHO−2024 Adjustment Method
Total Anaemia Prevalence
0.25
0.50
0.75
Age (year)
0.0
0.5
1.0
1.5
2.0
Location Hierarchy
1 − Global
2 − Super Region
3 − Region
4 − Country
5 − Subnational (Level 1)
Uncertainty Interval
5 − Subnational (Level 1)

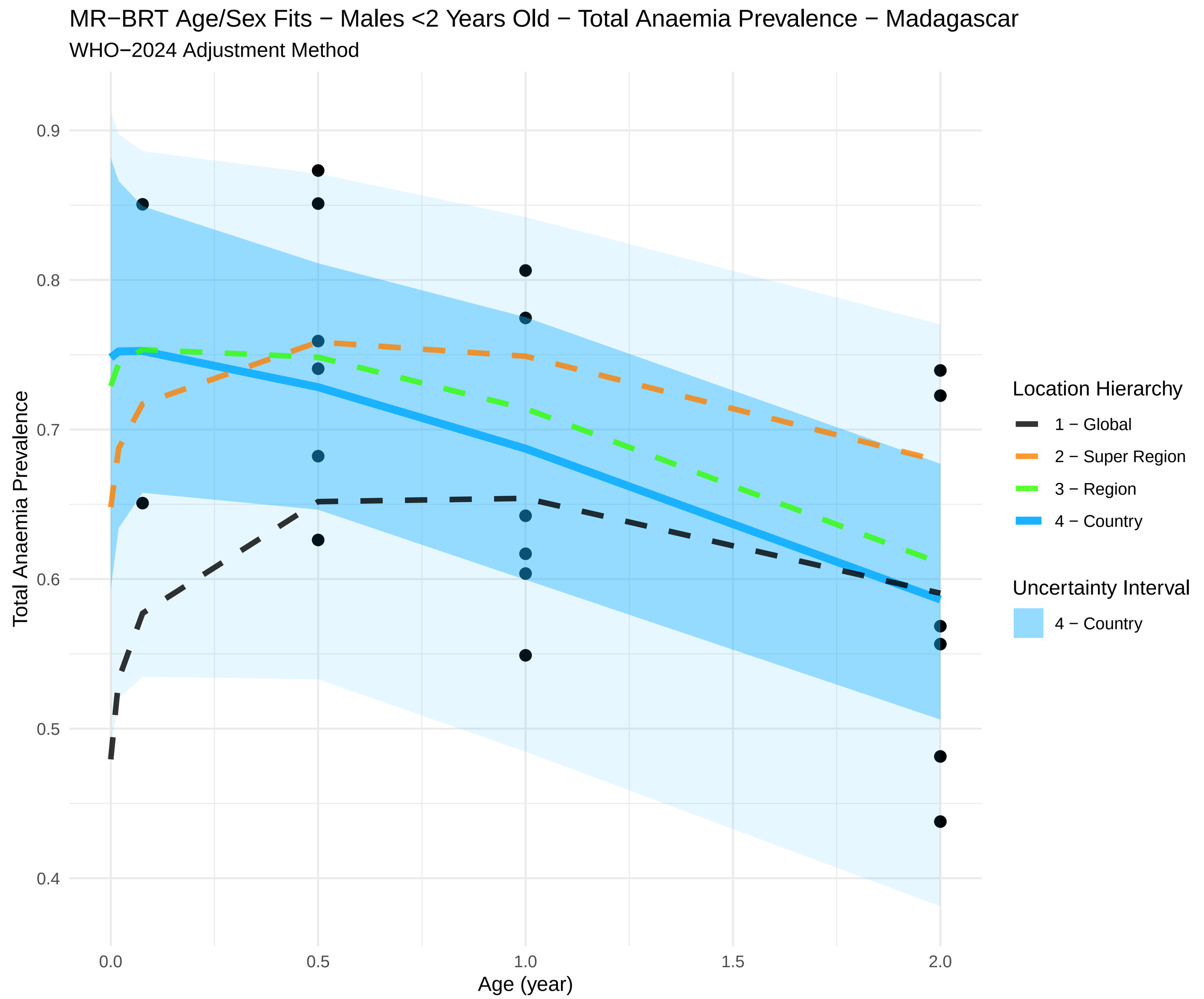
MR−BRT Age/Sex Fits − Males <2 Years Old − Total Anaemia Prevalence − Madagascar
WHO−2024 Adjustment Method
Total Anaemia Prevalence
0.4
0.5
0.6
0.7
0.8
0.9
Age (year)
0.0
0.5
1.0
1.5
2.0
Location Hierarchy
1 − Global
2 − Super Region
3 − Region
4 − Country
Uncertainty Interval
4 − Country

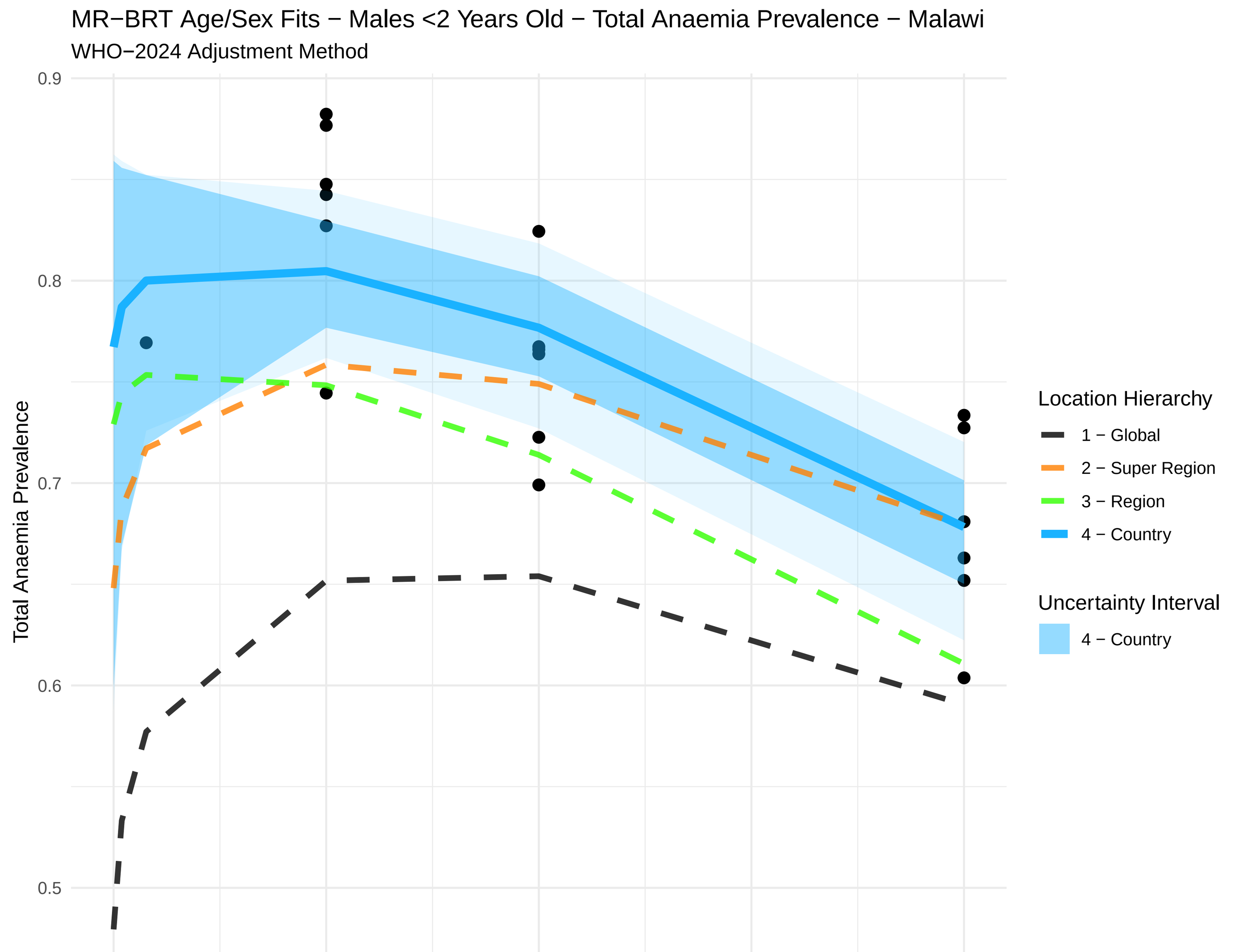
MR−BRT Age/Sex Fits − Males <2 Years Old − Total Anaemia Prevalence − Malawi
WHO−2024 Adjustment Method
Total Anaemia Prevalence
0.5
0.6
0.7
0.8
0.9
Age (year)
0.0
0.5
1.0
1.5
2.0
Location Hierarchy
1 − Global
2 − Super Region
3 − Region
4 − Country
Uncertainty Interval
4 − Country

MR−BRT Age/Sex Fits − Males <2 Years Old − Total Anaemia Prevalence − Mozambique
WHO−2024 Adjustment Method

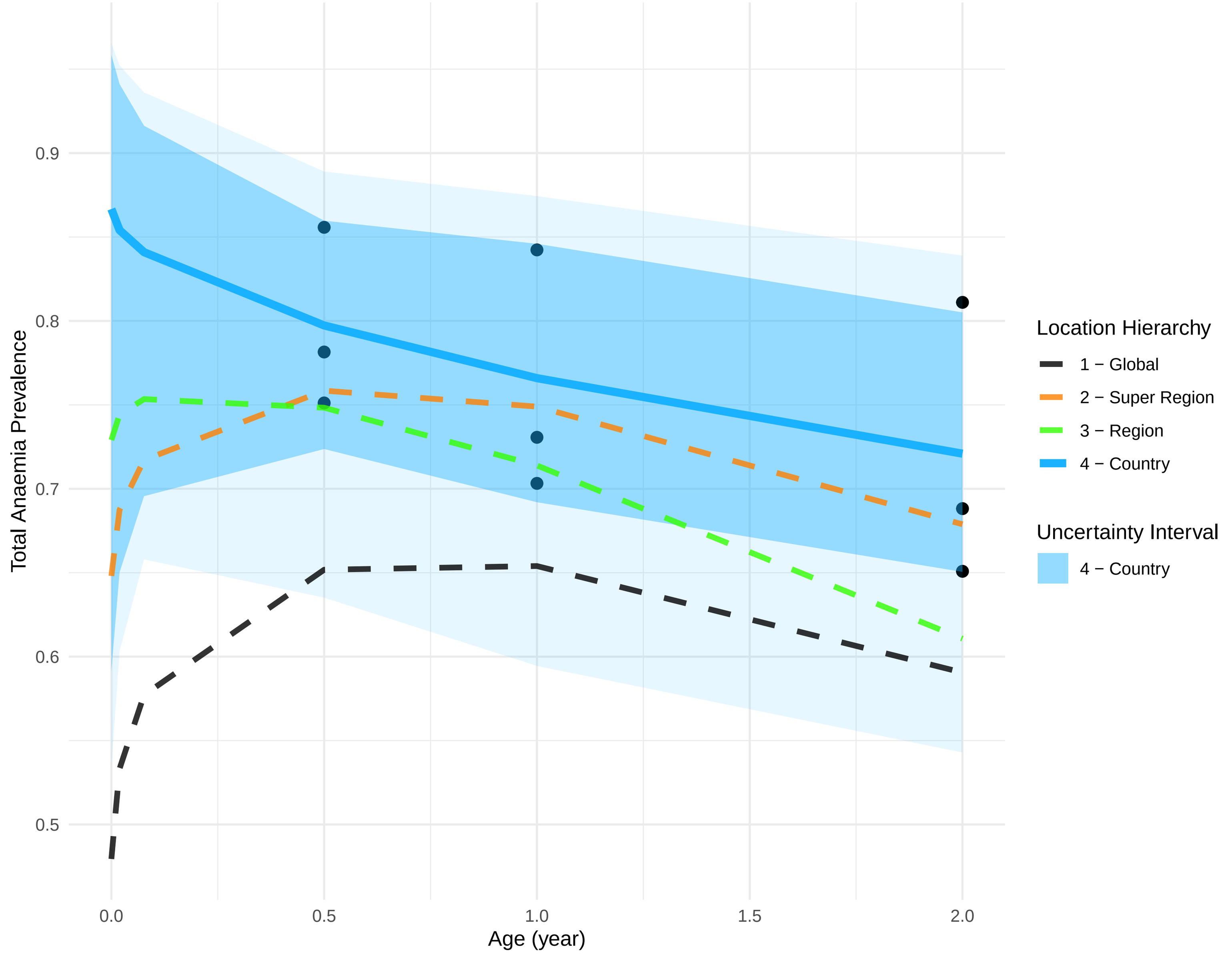

MR−BRT Age/Sex Fits − Males <2 Years Old − Total Anaemia Prevalence − Rwanda
WHO−2024 Adjustment Method

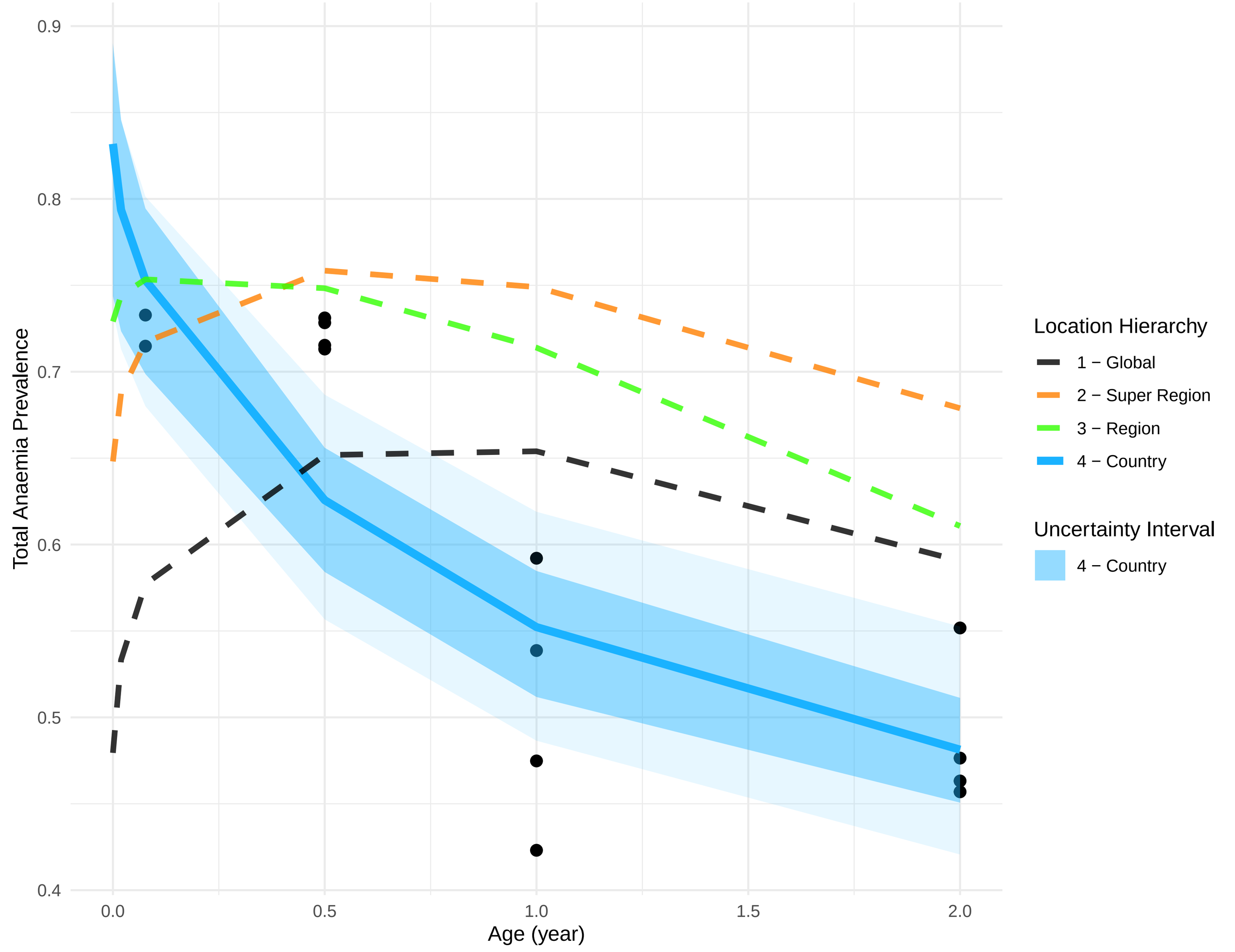

MR−BRT Age/Sex Fits − Males <2 Years Old − Total Anaemia Prevalence − Uganda
WHO−2024 Adjustment Method

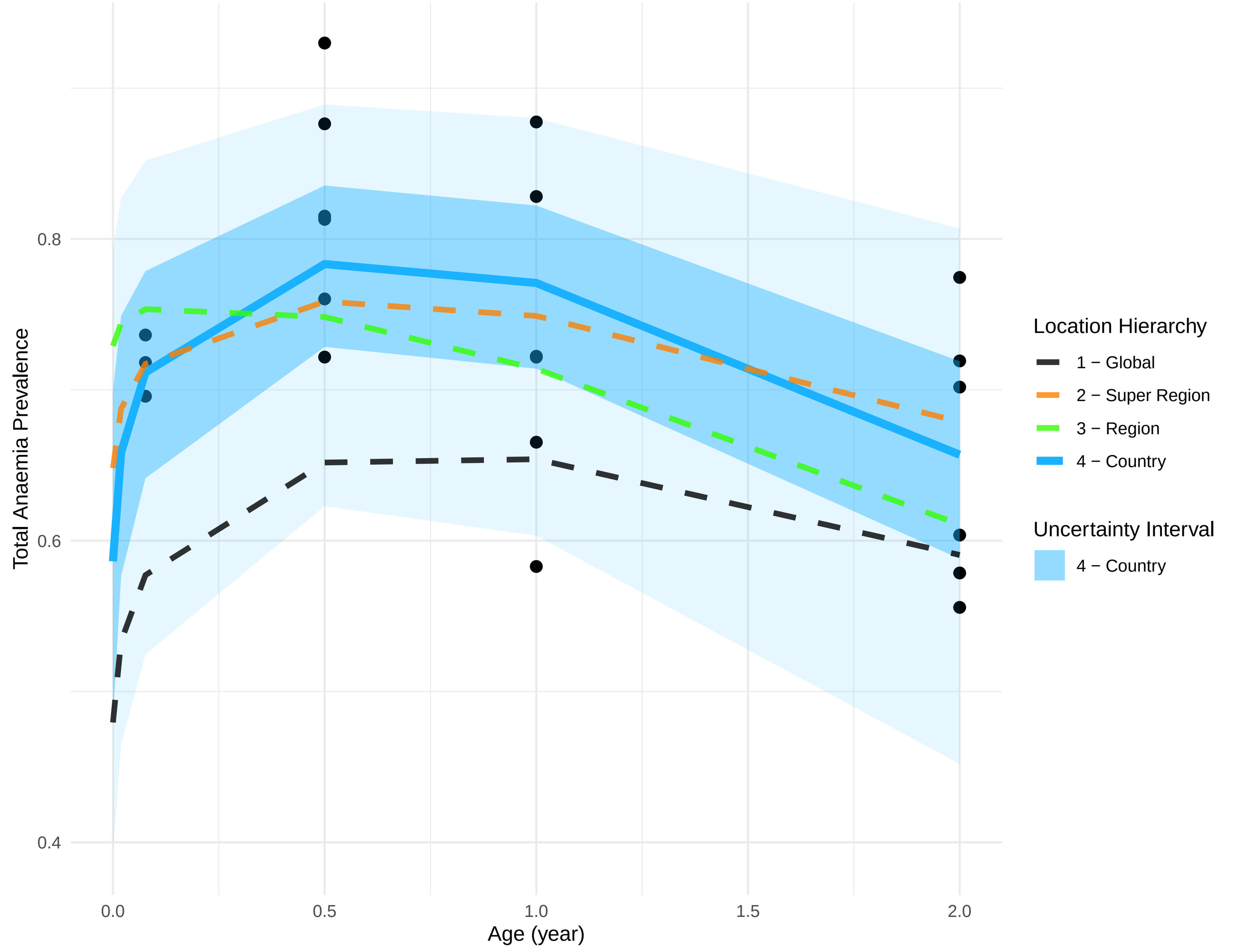

MR−BRT Age/Sex Fits − Males <2 Years Old − Total Anaemia Prevalence − United Republic of Tanzania
WHO−2024 Adjustment Method

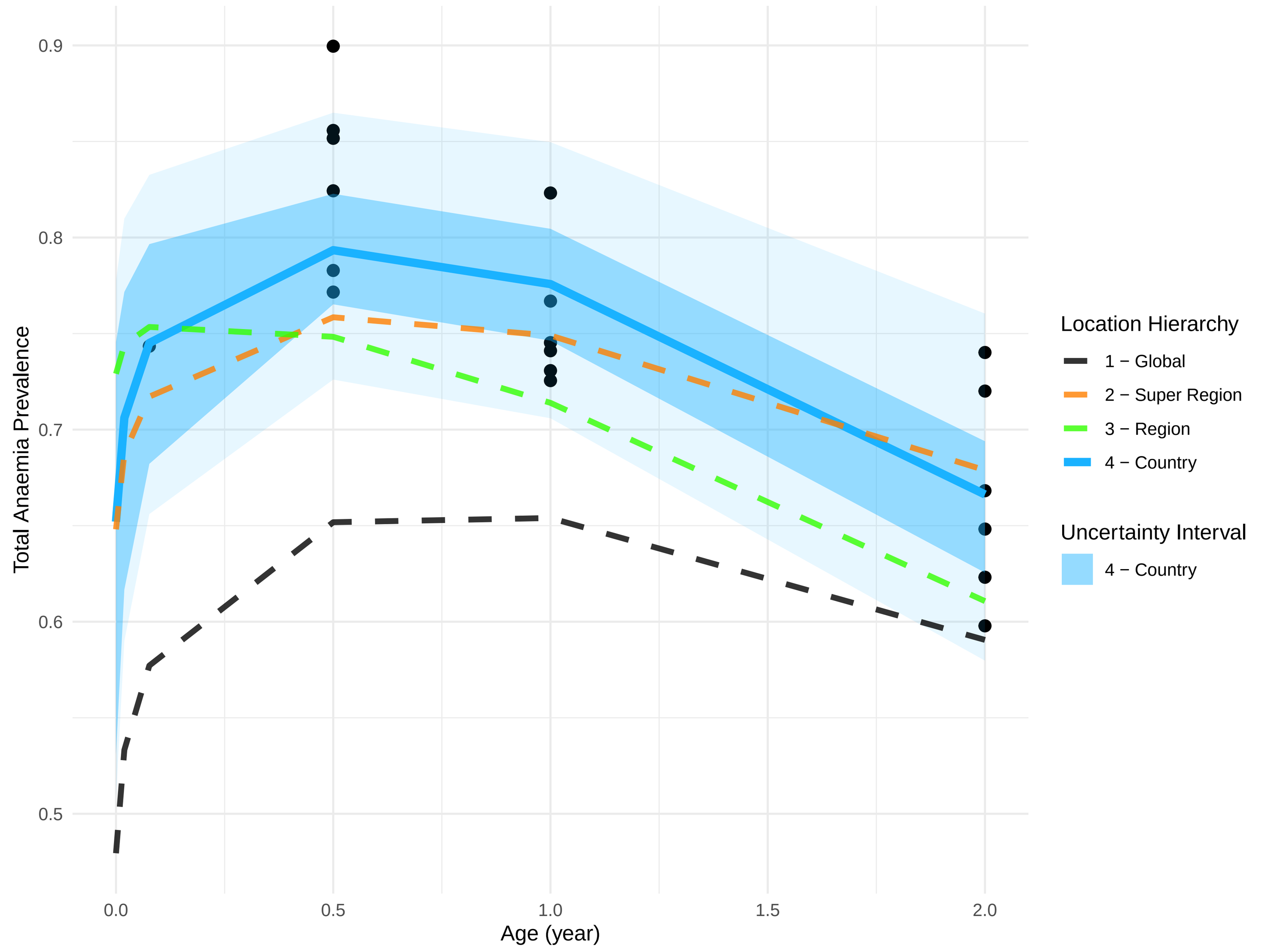

MR−BRT Age/Sex Fits − Males <2 Years Old − Total Anaemia Prevalence − Zambia
WHO−2024 Adjustment Method

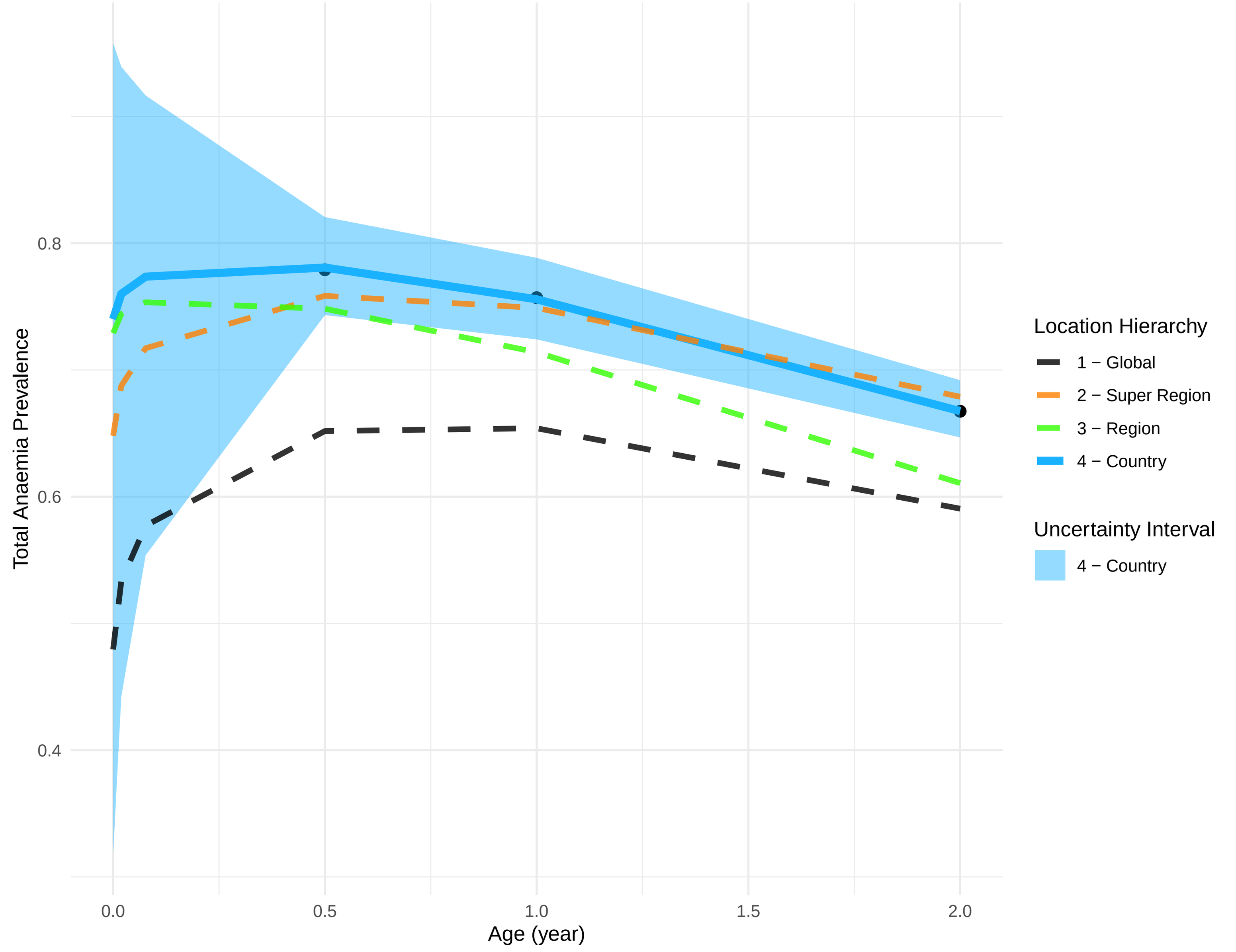

MR−BRT Age/Sex Fits − Males <2 Years Old − Total Anaemia Prevalence − Southern Sub−Saharan Africa
WHO−2024 Adjustment Method

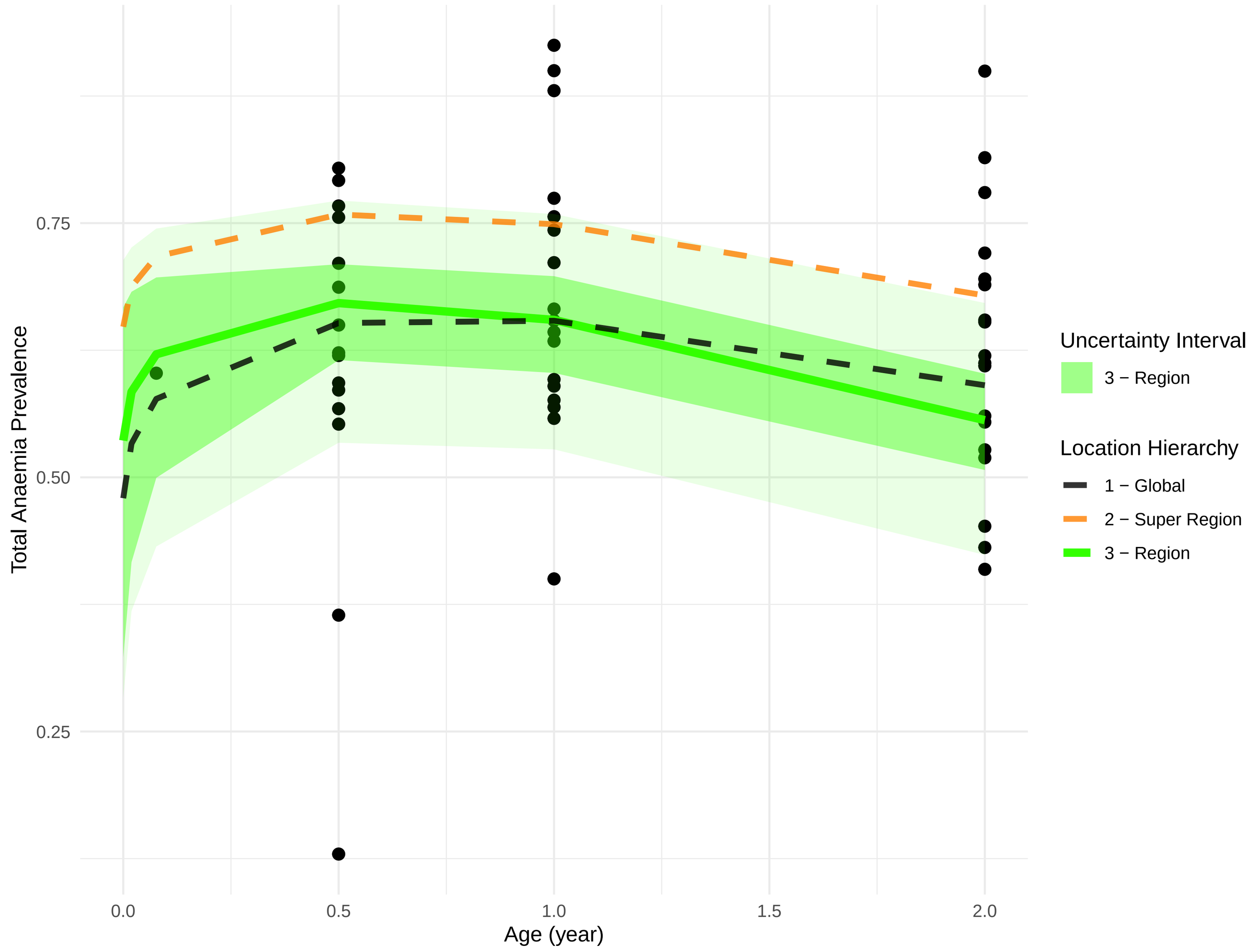

MR−BRT Age/Sex Fits − Males <2 Years Old − Total Anaemia Prevalence − Eswatini
WHO−2024 Adjustment Method

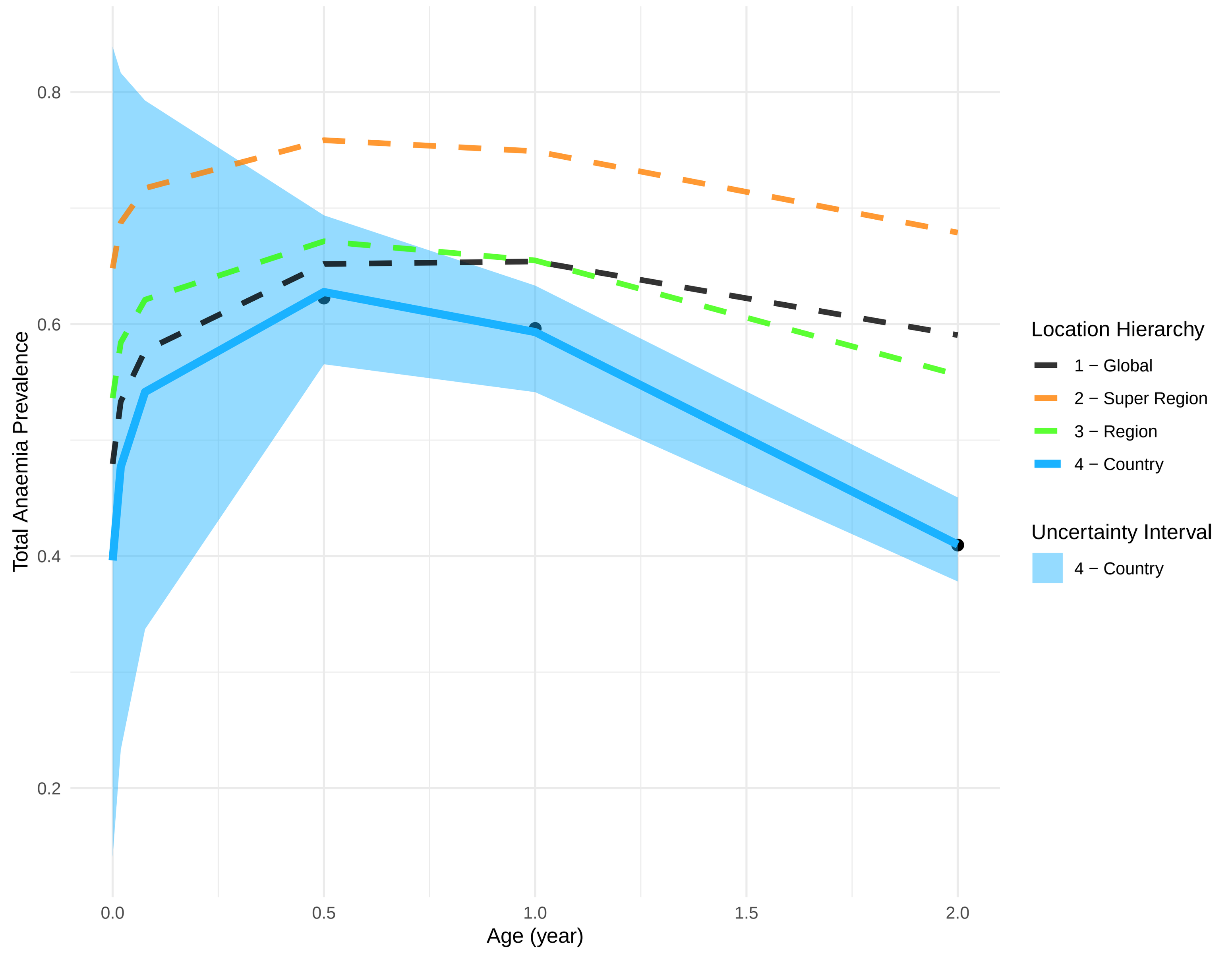

MR−BRT Age/Sex Fits − Males <2 Years Old − Total Anaemia Prevalence − Lesotho
WHO−2024 Adjustment Method

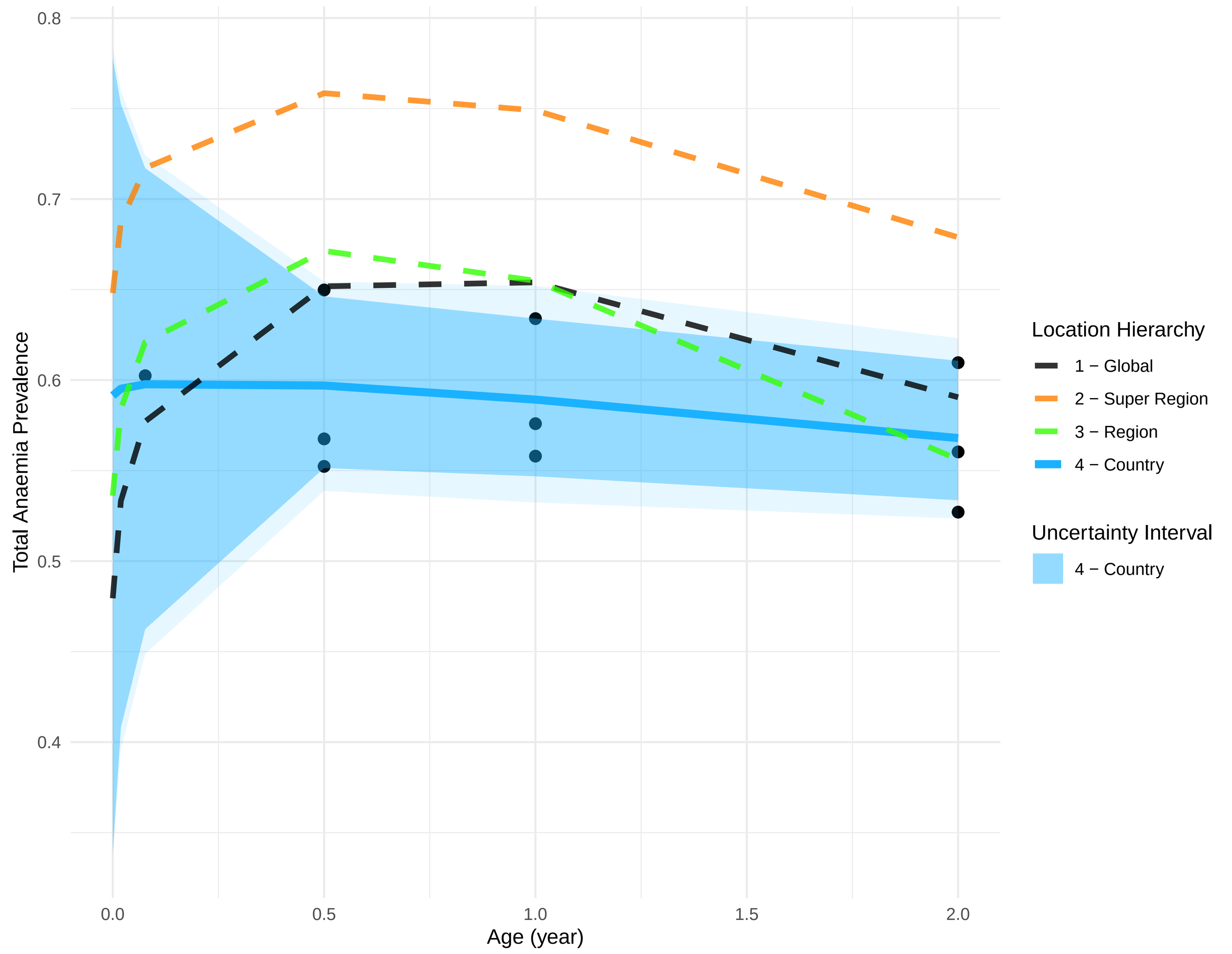

MR−BRT Age/Sex Fits − Males <2 Years Old − Total Anaemia Prevalence − Namibia
WHO−2024 Adjustment Method

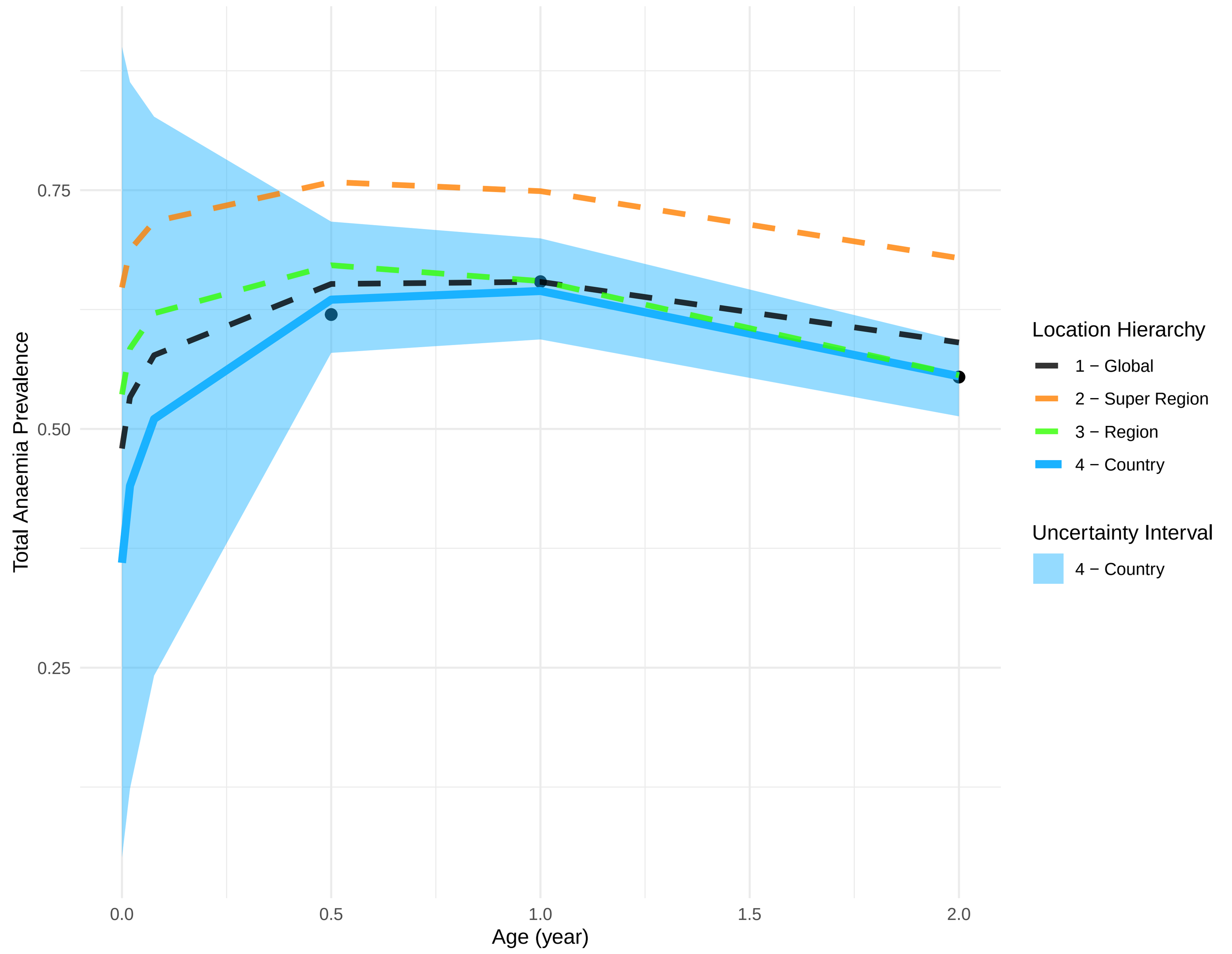

MR−BRT Age/Sex Fits − Males <2 Years Old − Total Anaemia Prevalence − South Africa
WHO−2024 Adjustment Method

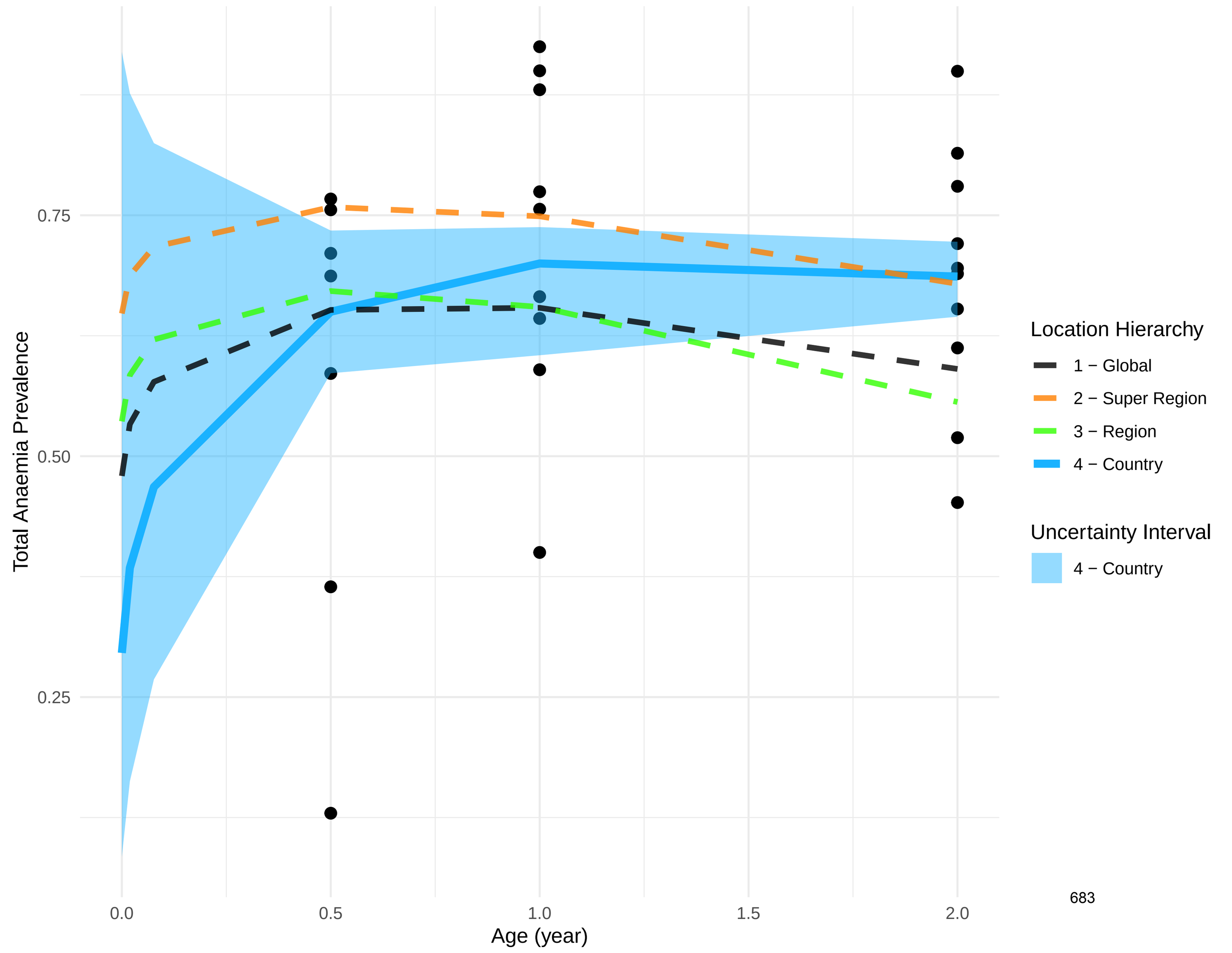

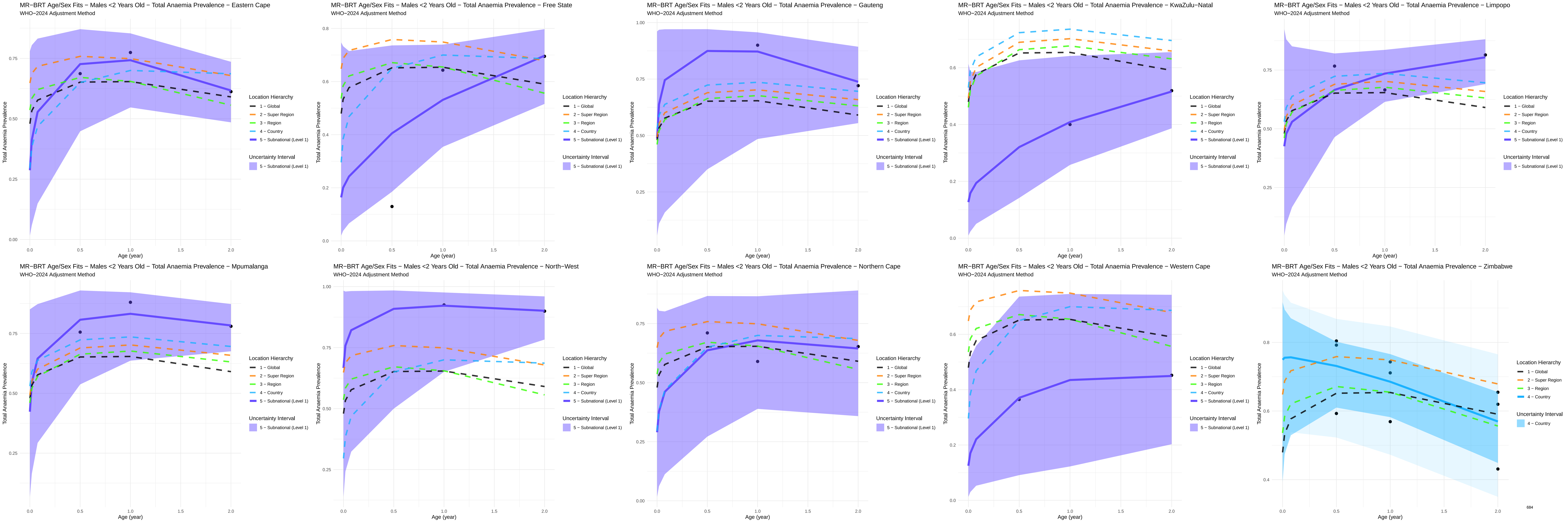
MR−BRT Age/Sex Fits − Males <2 Years Old − Total Anaemia Prevalence − Eastern Cape
WHO−2024 Adjustment Method
MR−BRT Age/Sex Fits − Males <2 Years Old − Total Anaemia Prevalence − Free State
WHO−2024 Adjustment Method
MR−BRT Age/Sex Fits − Males <2 Years Old − Total Anaemia Prevalence − Gauteng
WHO−2024 Adjustment Method
MR−BRT Age/Sex Fits − Males <2 Years Old − Total Anaemia Prevalence − KwaZulu−Natal
WHO−2024 Adjustment Method
MR−BRT Age/Sex Fits − Males <2 Years Old − Total Anaemia Prevalence − Limpopo
WHO−2024 Adjustment Method
MR−BRT Age/Sex Fits − Males <2 Years Old − Total Anaemia Prevalence − Mpumalanga
WHO−2024 Adjustment Method
MR−BRT Age/Sex Fits − Males <2 Years Old − Total Anaemia Prevalence − North−West
WHO−2024 Adjustment Method
MR−BRT Age/Sex Fits − Males <2 Years Old − Total Anaemia Prevalence − Northern Cape
WHO−2024 Adjustment Method
MR−BRT Age/Sex Fits − Males <2 Years Old − Total Anaemia Prevalence − Western Cape
WHO−2024 Adjustment Method
MR−BRT Age/Sex Fits − Males <2 Years Old − Total Anaemia Prevalence − Zimbabwe
WHO−2024 Adjustment Method
Total Anaemia Prevalence
Age (year)
Location Hierarchy
1 − Global
2 − Super Region
3 − Region
4 − Country
5 − Subnational (Level 1)
Uncertainty Interval
5 − Subnational (Level 1)
4 − Country

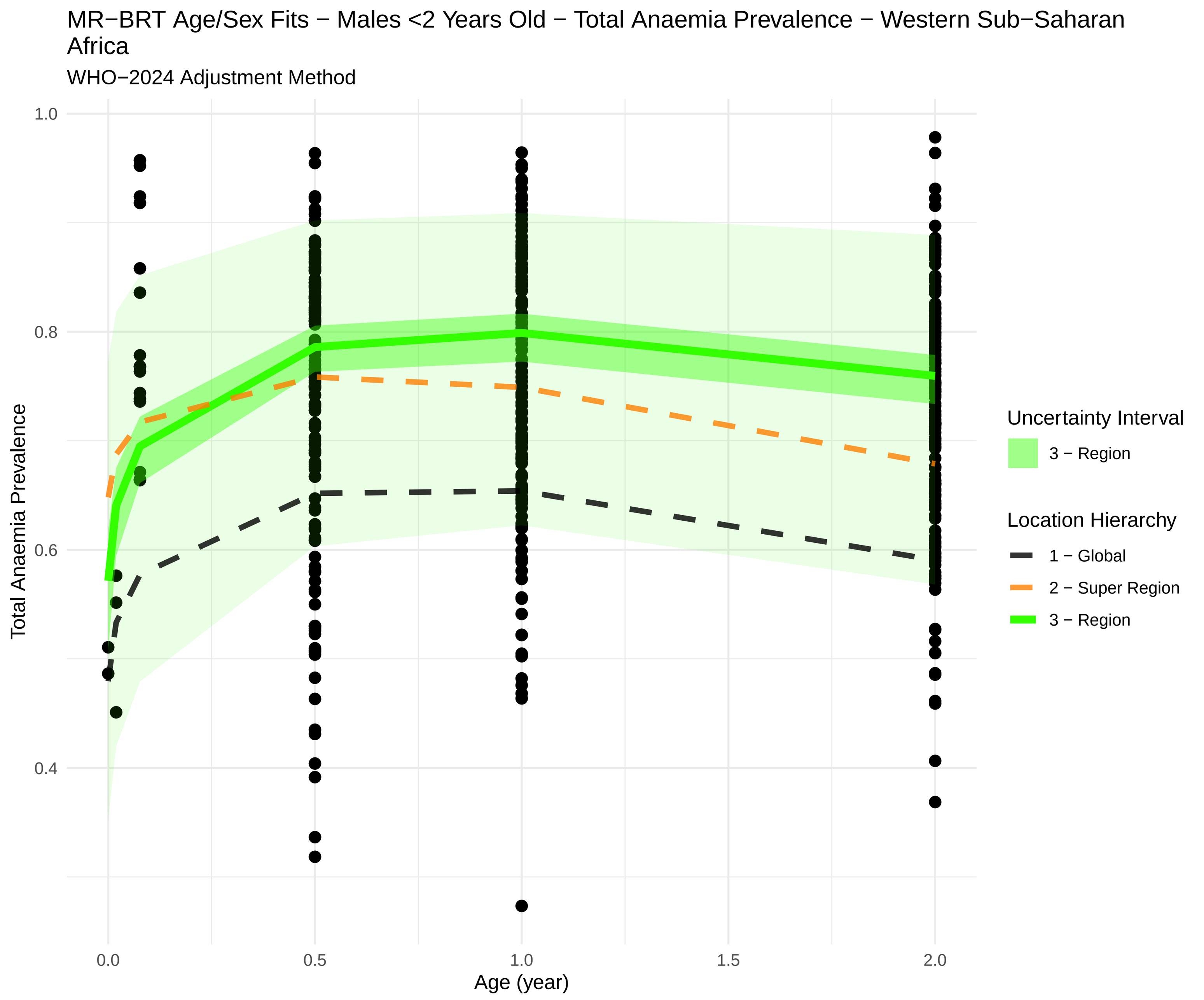
MR−BRT Age/Sex Fits − Males <2 Years Old − Total Anaemia Prevalence − Western Sub−Saharan Africa
WHO−2024 Adjustment Method
Total Anaemia Prevalence
Age (year)
Uncertainty Interval
3 − Region
Location Hierarchy
1 − Global
2 − Super Region
3 − Region

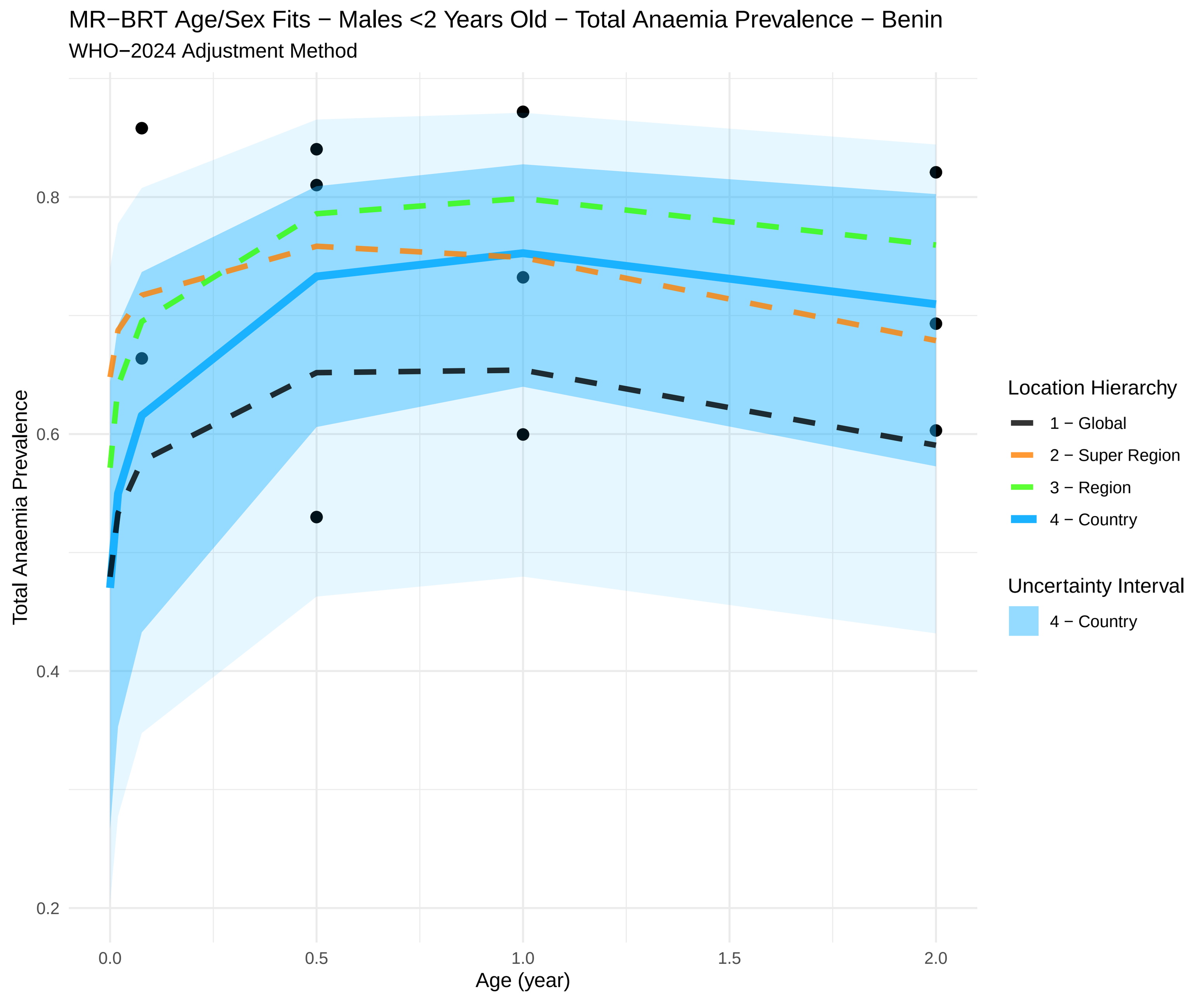
MR−BRT Age/Sex Fits − Males <2 Years Old − Total Anaemia Prevalence − Benin
WHO−2024 Adjustment Method
Total Anaemia Prevalence
Age (year)
Location Hierarchy
1 − Global
2 − Super Region
3 − Region
4 − Country
Uncertainty Interval
4 − Country

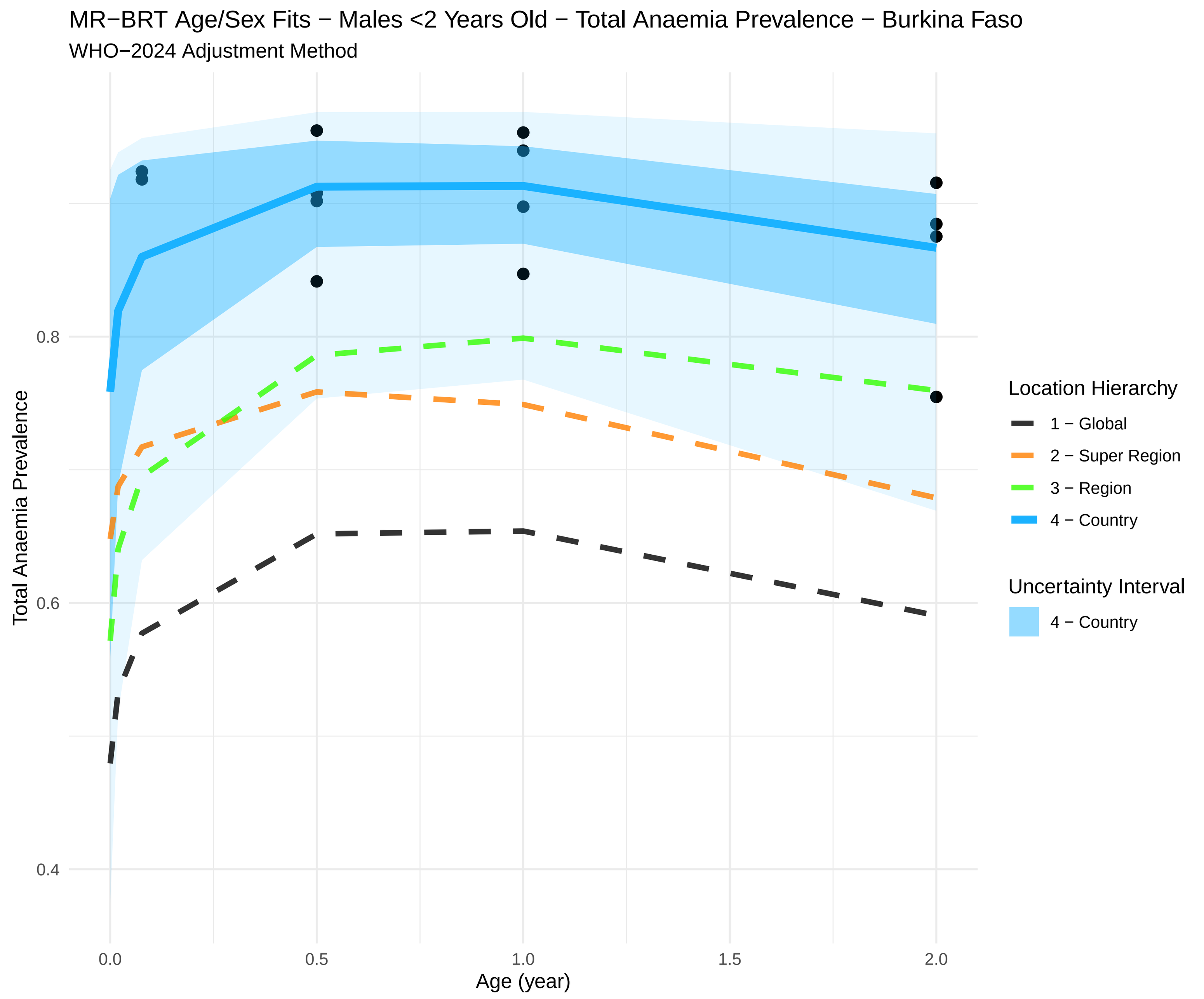
MR−BRT Age/Sex Fits − Males <2 Years Old − Total Anaemia Prevalence − Burkina Faso
WHO−2024 Adjustment Method
Total Anaemia Prevalence
Age (year)
Location Hierarchy
1 − Global
2 − Super Region
3 − Region
4 − Country
Uncertainty Interval
4 − Country

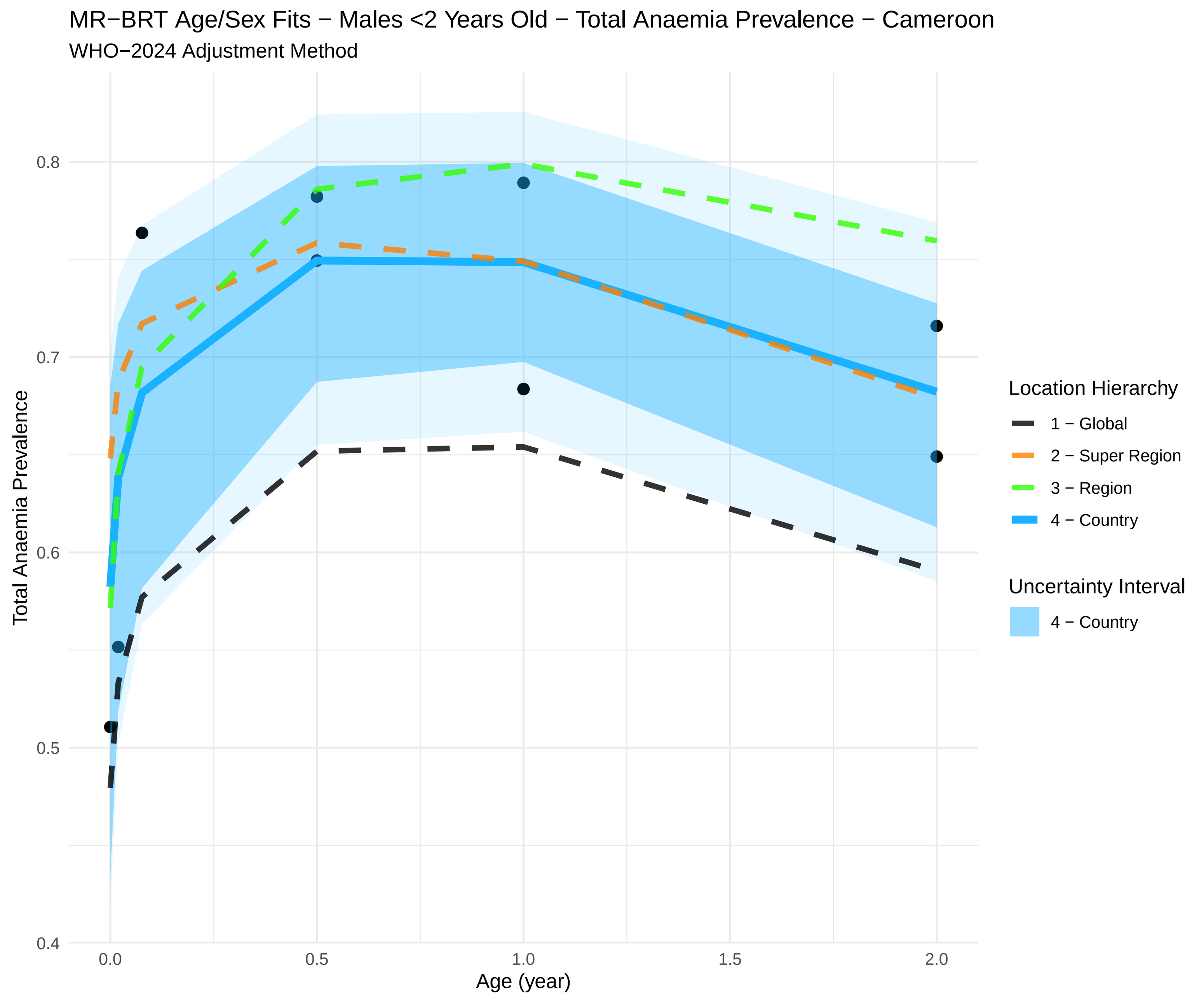
MR−BRT Age/Sex Fits − Males <2 Years Old − Total Anaemia Prevalence − Cameroon
WHO−2024 Adjustment Method
Total Anaemia Prevalence
Age (year)
Location Hierarchy
1 − Global
2 − Super Region
3 − Region
4 − Country
Uncertainty Interval
4 − Country

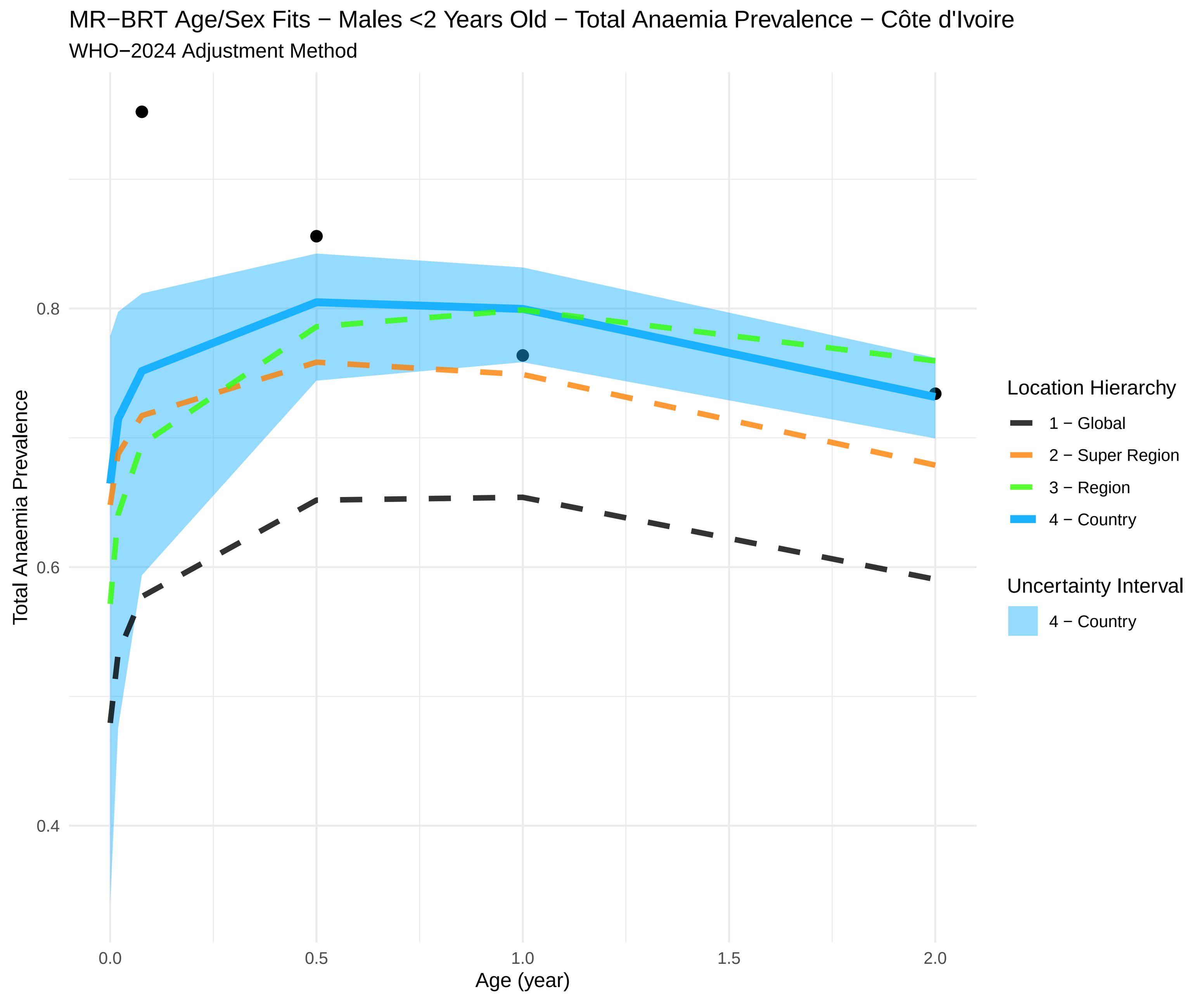
MR−BRT Age/Sex Fits − Males <2 Years Old − Total Anaemia Prevalence − Côte d'Ivoire
WHO−2024 Adjustment Method
Total Anaemia Prevalence
Age (year)
Location Hierarchy
1 − Global
2 − Super Region
3 − Region
4 − Country
Uncertainty Interval
4 − Country

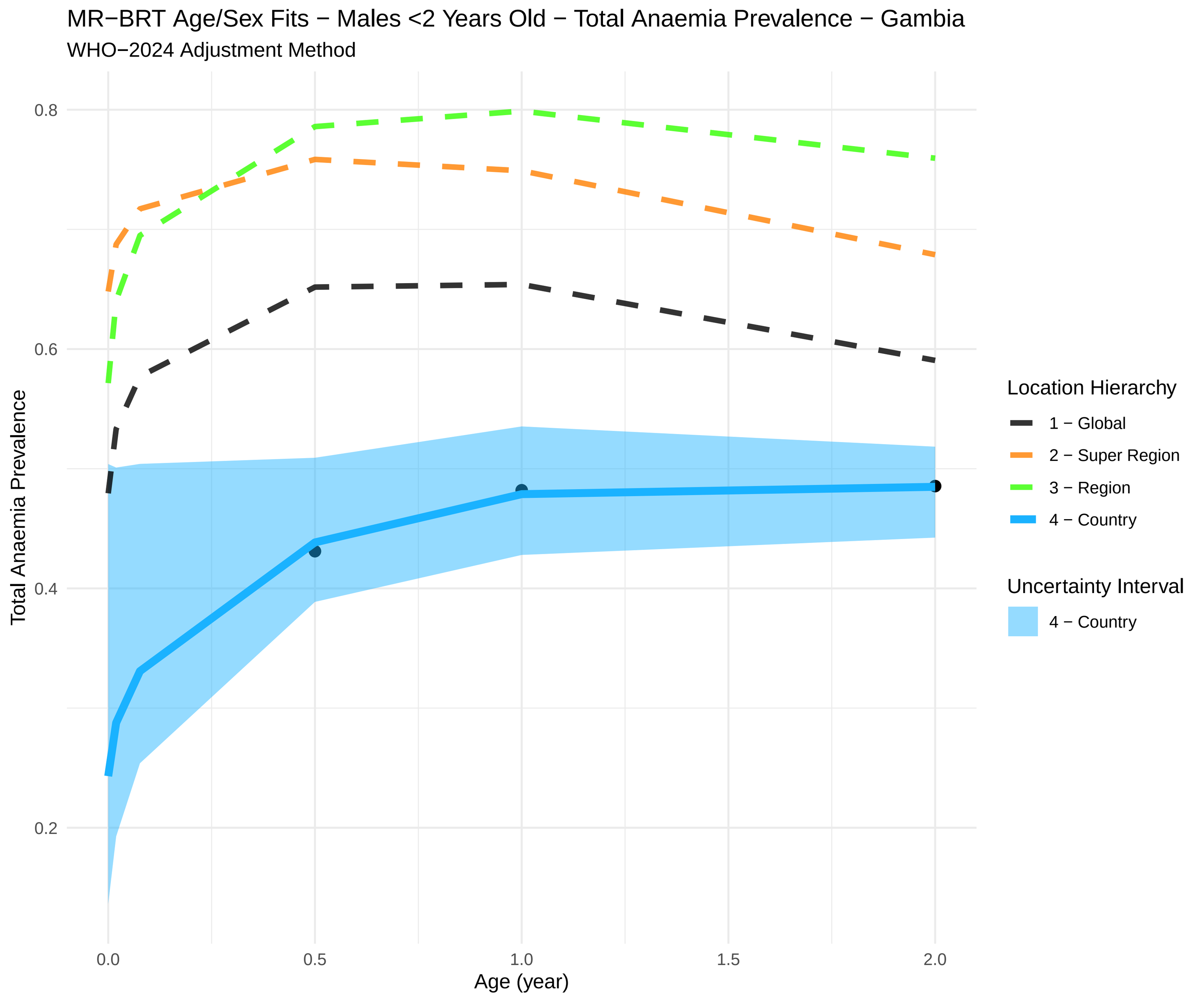
MR−BRT Age/Sex Fits − Males <2 Years Old − Total Anaemia Prevalence − Gambia
WHO−2024 Adjustment Method
Total Anaemia Prevalence
Age (year)
Location Hierarchy
1 − Global
2 − Super Region
3 − Region
4 − Country
Uncertainty Interval
4 − Country

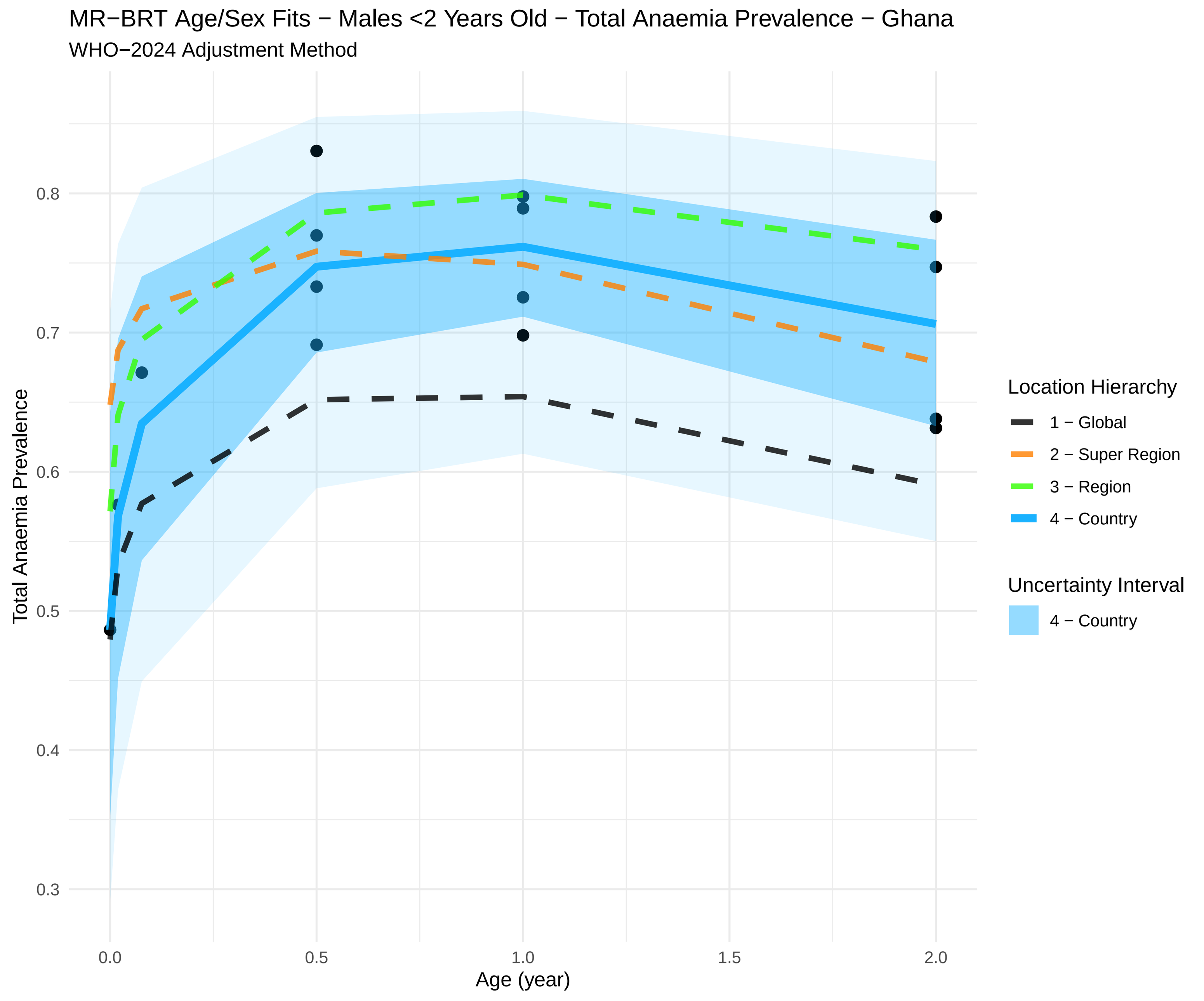
MR−BRT Age/Sex Fits − Males <2 Years Old − Total Anaemia Prevalence − Ghana
WHO−2024 Adjustment Method
Total Anaemia Prevalence
Age (year)
Location Hierarchy
1 − Global
2 − Super Region
3 − Region
4 − Country
Uncertainty Interval
4 − Country

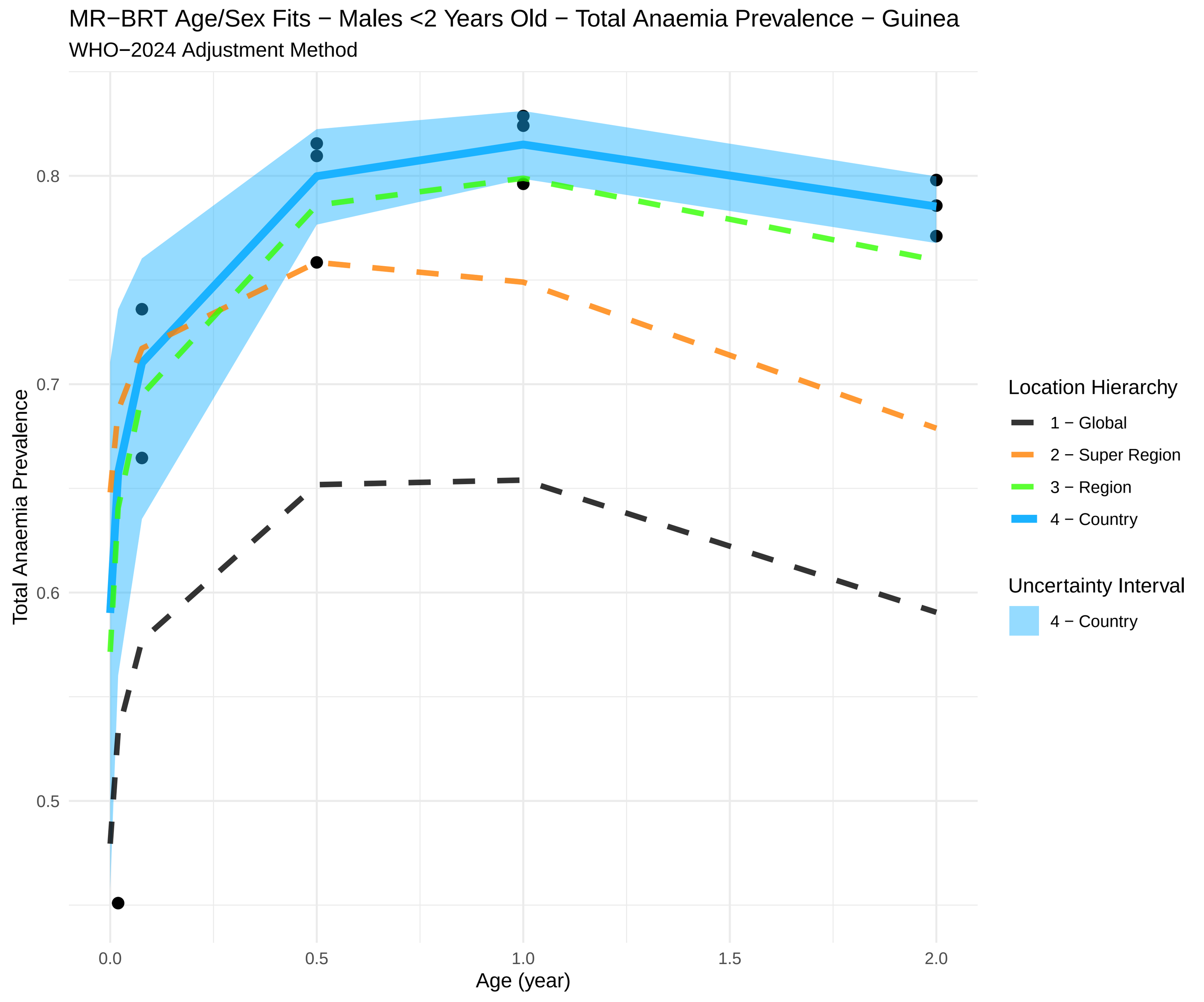
MR−BRT Age/Sex Fits − Males <2 Years Old − Total Anaemia Prevalence − Guinea
WHO−2024 Adjustment Method
Total Anaemia Prevalence
Age (year)
Location Hierarchy
1 − Global
2 − Super Region
3 − Region
4 − Country
Uncertainty Interval
4 − Country

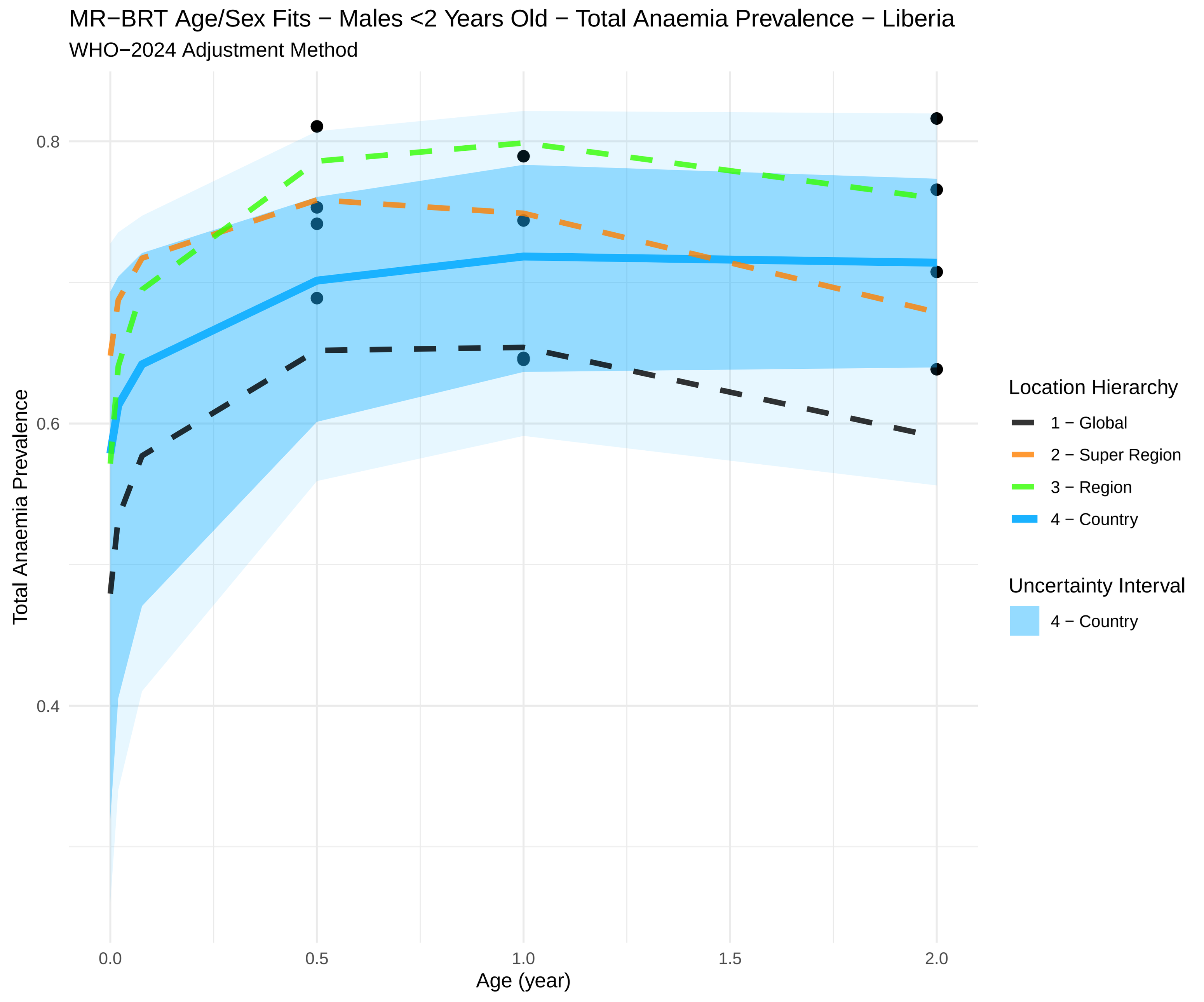
MR−BRT Age/Sex Fits − Males <2 Years Old − Total Anaemia Prevalence − Liberia
WHO−2024 Adjustment Method
Total Anaemia Prevalence
Age (year)
Location Hierarchy
1 − Global
2 − Super Region
3 − Region
4 − Country
Uncertainty Interval
4 − Country

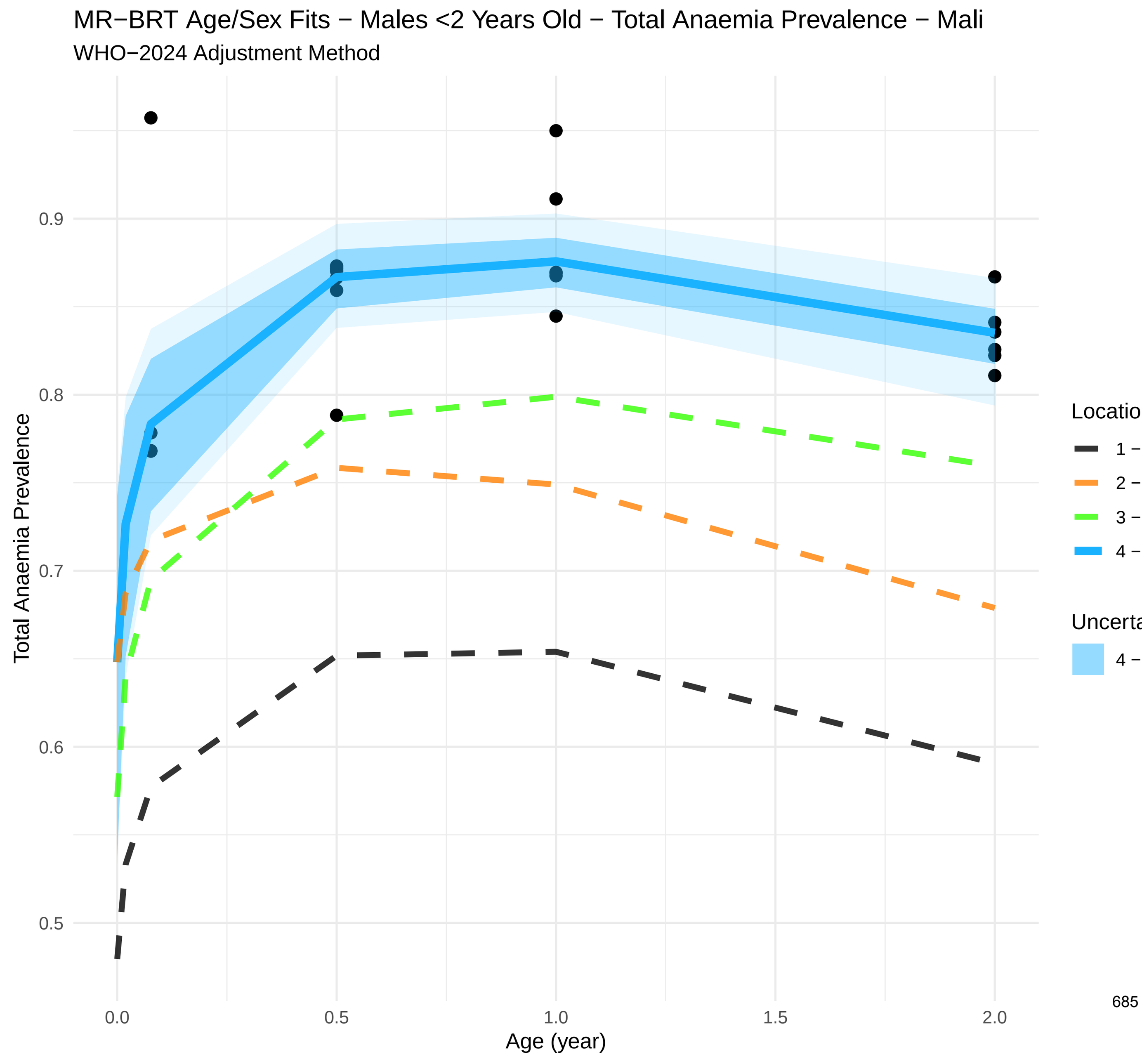
MR−BRT Age/Sex Fits − Males <2 Years Old − Total Anaemia Prevalence − Mali
WHO−2024 Adjustment Method
Total Anaemia Prevalence
Age (year)
Location Hierarchy
1 − Global
2 − Super Region
3 − Region
4 − Country
Uncertainty Interval
4 − Country

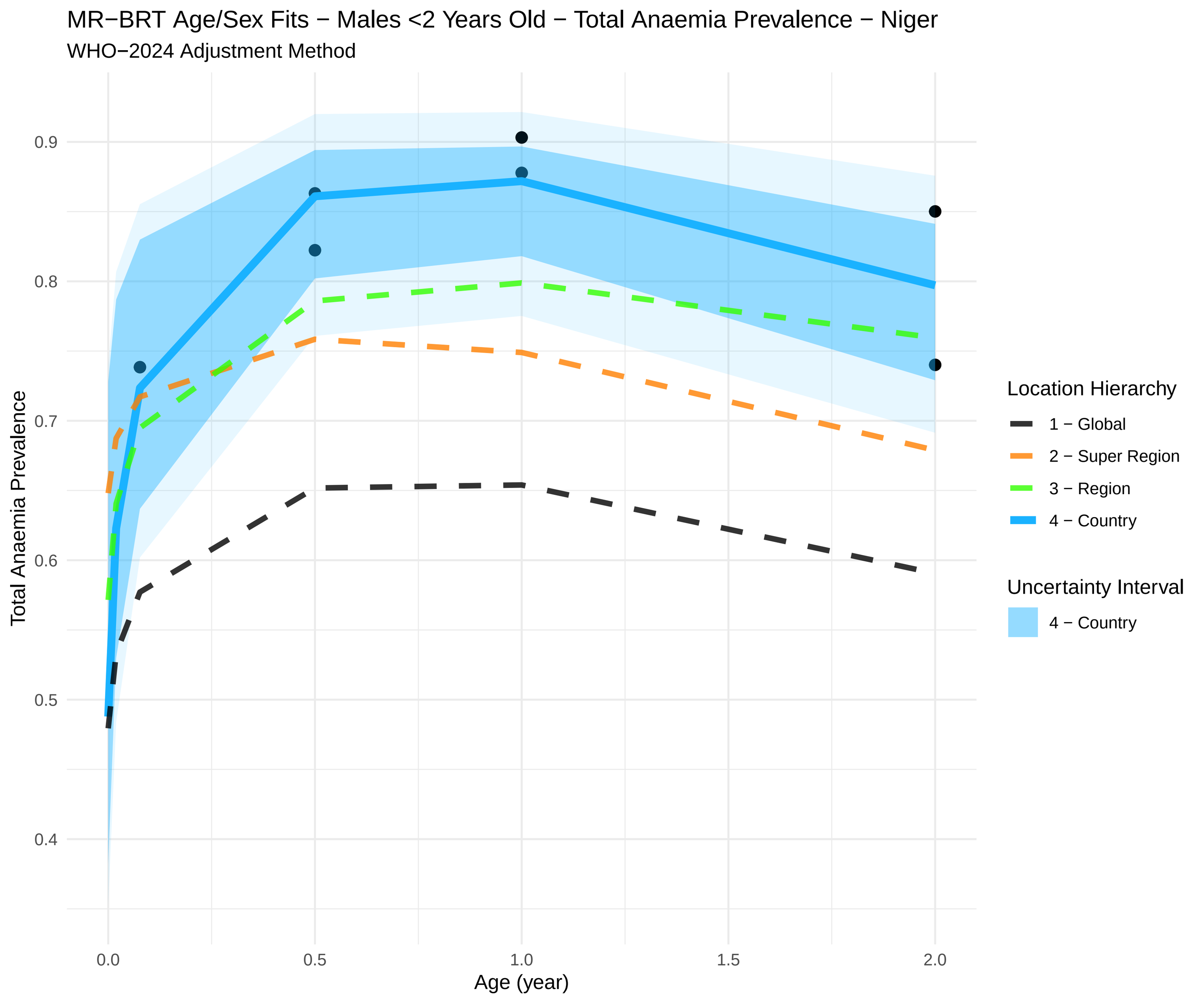
MR−BRT Age/Sex Fits − Males <2 Years Old − Total Anaemia Prevalence − Niger
WHO−2024 Adjustment Method
Total Anaemia Prevalence
Age (year)
Location Hierarchy
1 − Global
2 − Super Region
3 − Region
4 − Country
Uncertainty Interval
4 − Country

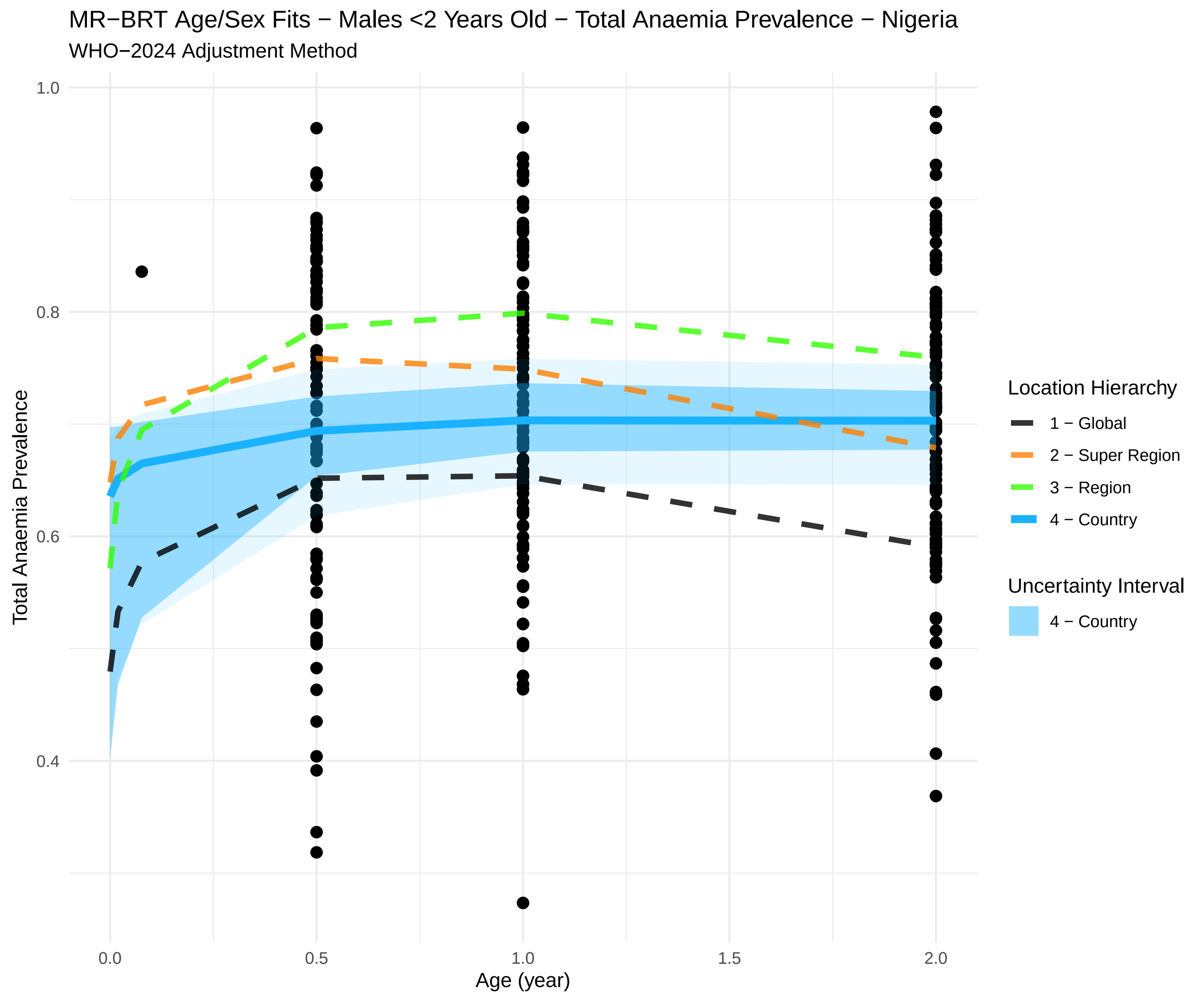
MR−BRT Age/Sex Fits − Males <2 Years Old − Total Anaemia Prevalence − Nigeria
WHO−2024 Adjustment Method
Total Anaemia Prevalence
Age (year)
Location Hierarchy
1 − Global
2 − Super Region
3 − Region
4 − Country
Uncertainty Interval
4 − Country

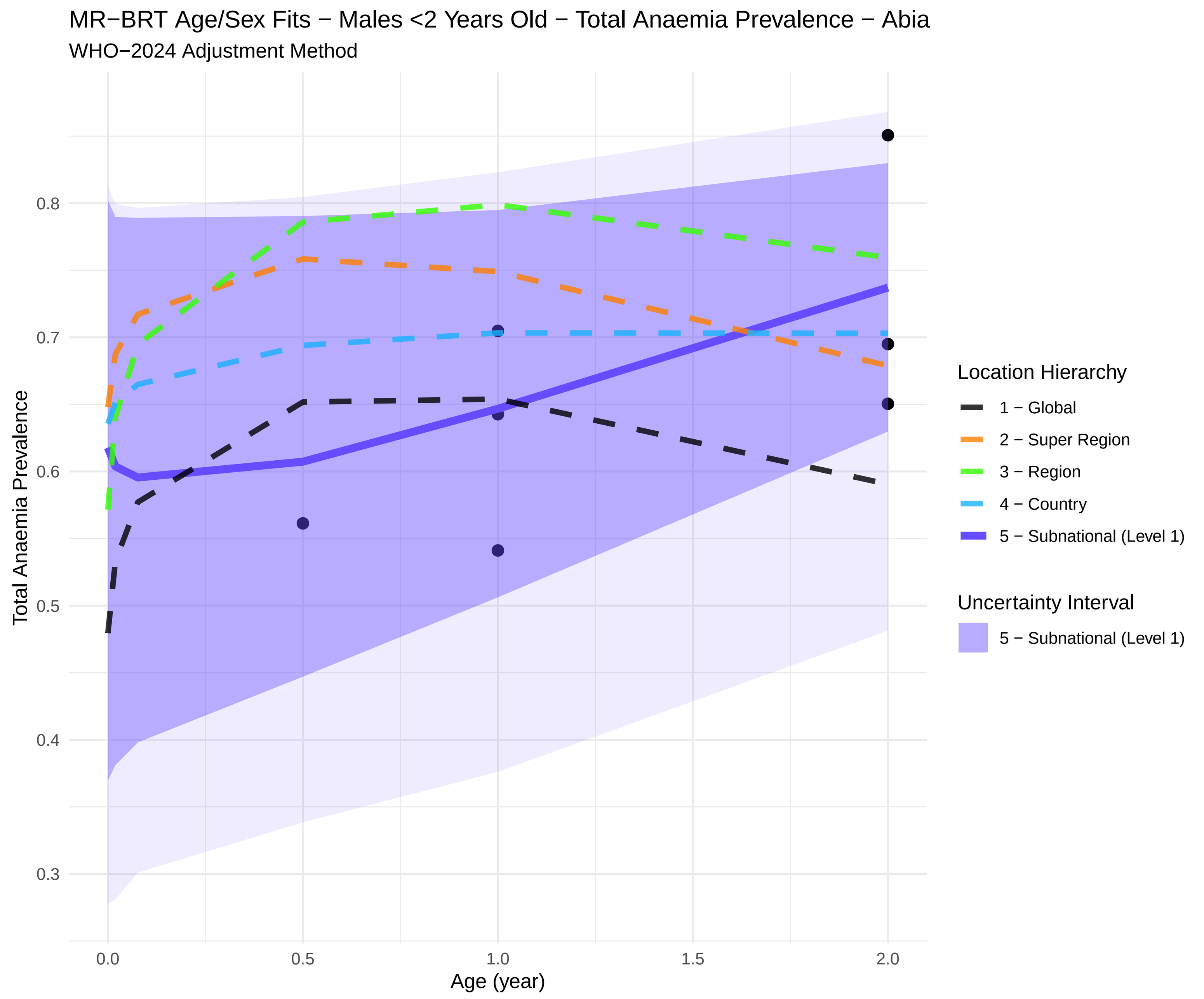
MR−BRT Age/Sex Fits − Males <2 Years Old − Total Anaemia Prevalence − Abia
WHO−2024 Adjustment Method
Total Anaemia Prevalence
Age (year)
Location Hierarchy
1 − Global
2 − Super Region
3 − Region
4 − Country
5 − Subnational (Level 1)
Uncertainty Interval
5 − Subnational (Level 1)

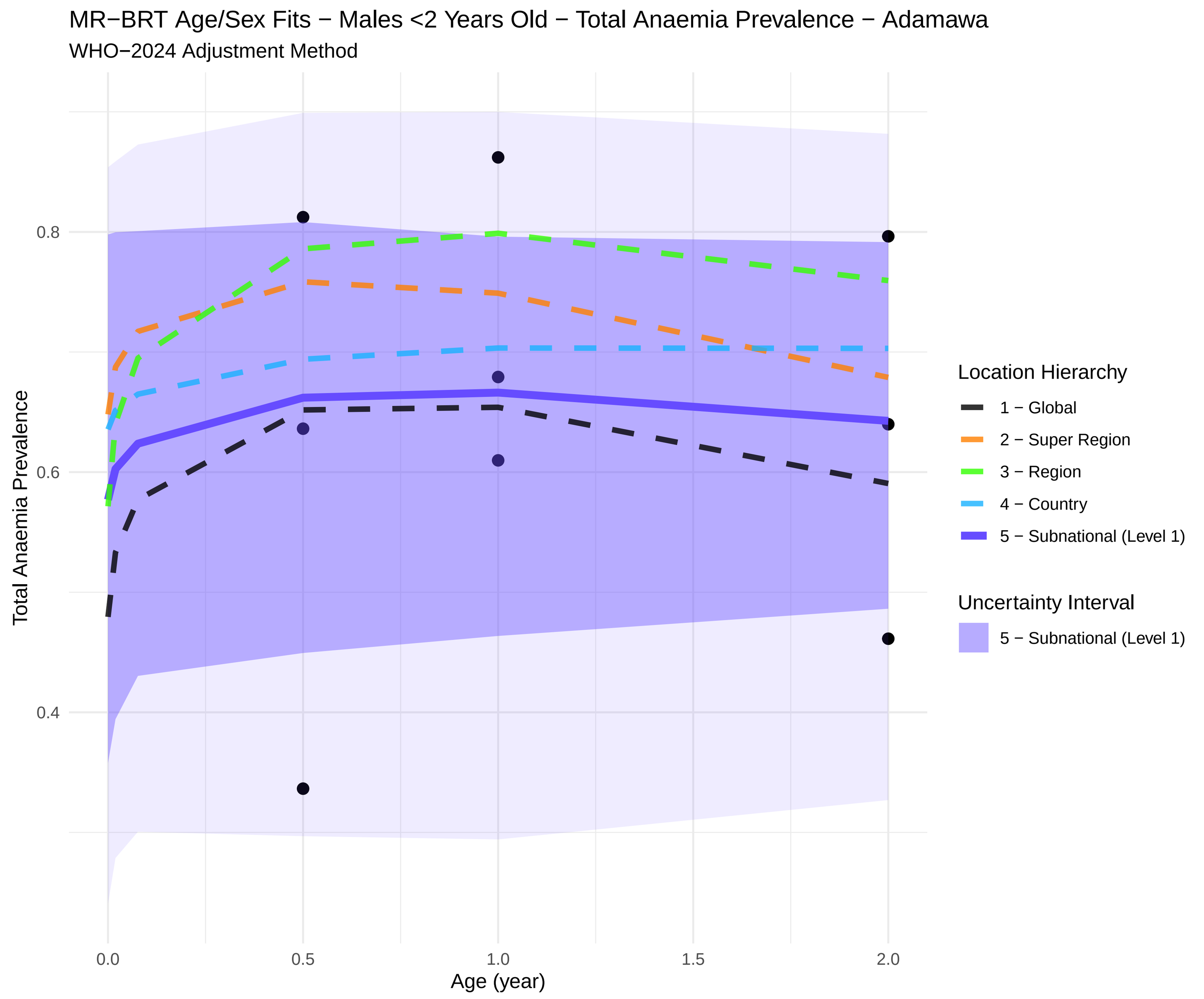
MR−BRT Age/Sex Fits − Males <2 Years Old − Total Anaemia Prevalence − Adamawa
WHO−2024 Adjustment Method
Total Anaemia Prevalence
Age (year)
Location Hierarchy
1 − Global
2 − Super Region
3 − Region
4 − Country
5 − Subnational (Level 1)
Uncertainty Interval
5 − Subnational (Level 1)

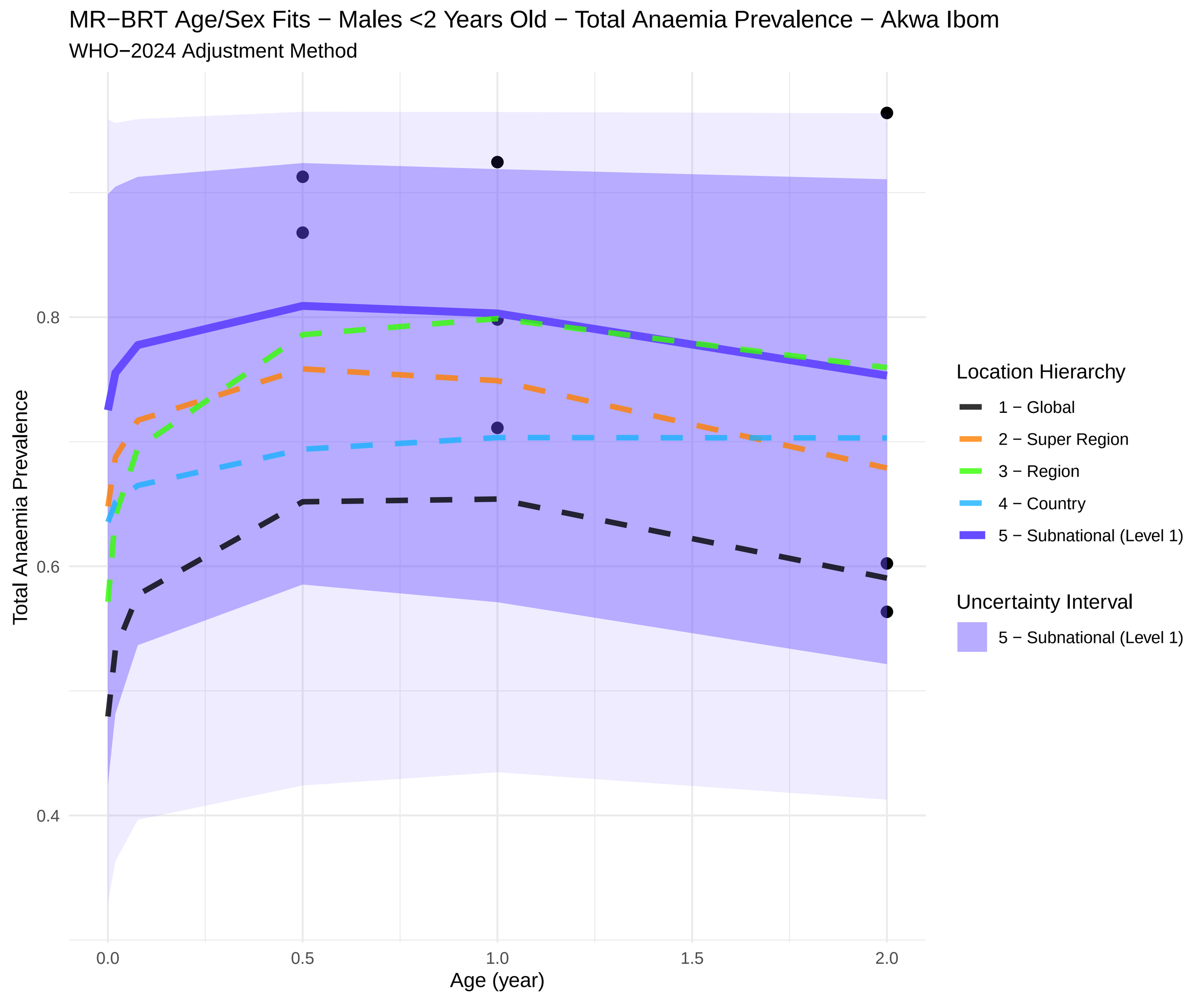
MR−BRT Age/Sex Fits − Males <2 Years Old − Total Anaemia Prevalence − Akwa Ibom
WHO−2024 Adjustment Method
Total Anaemia Prevalence
Age (year)
Location Hierarchy
1 − Global
2 − Super Region
3 − Region
4 − Country
5 − Subnational (Level 1)
Uncertainty Interval
5 − Subnational (Level 1)

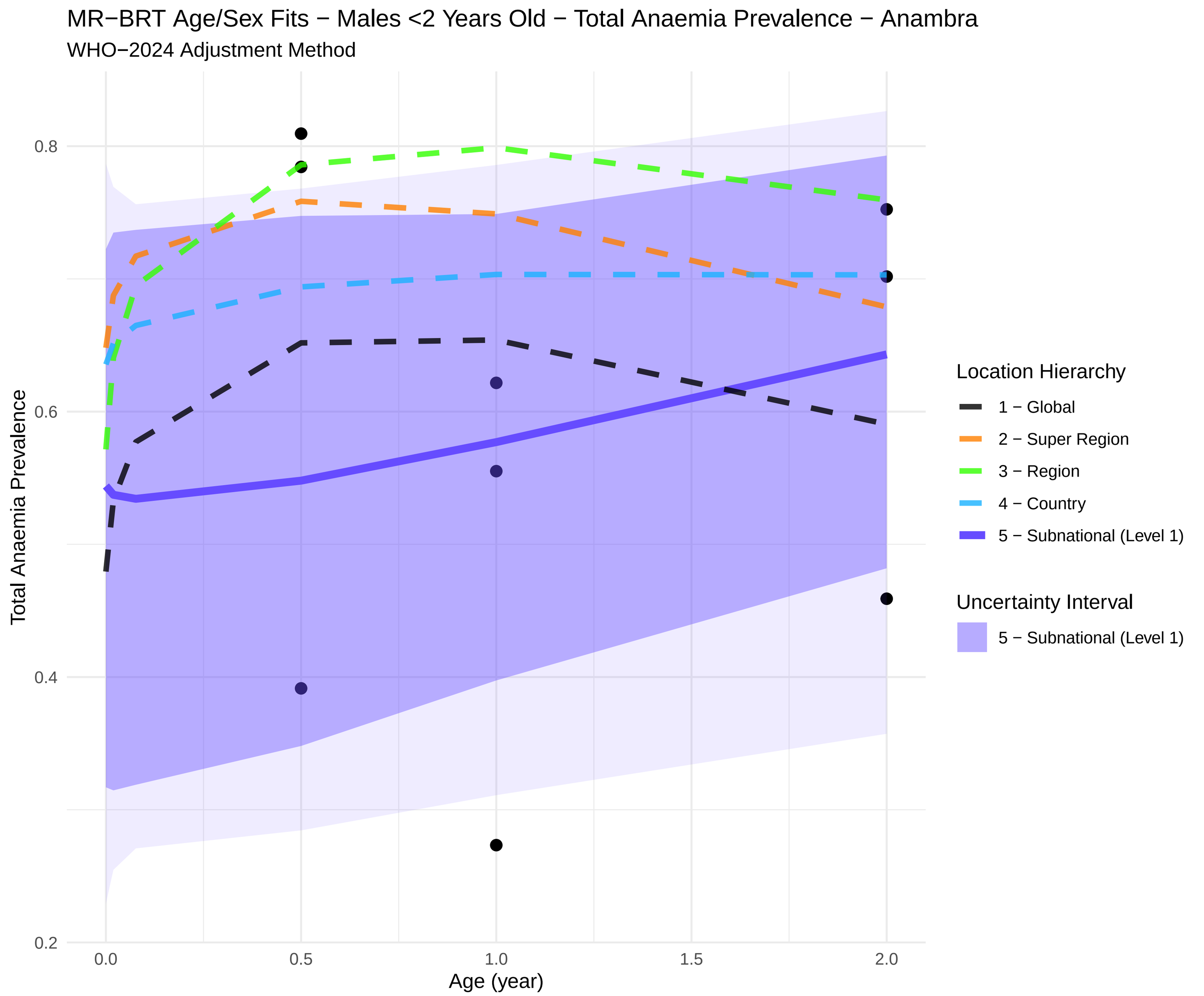
MR−BRT Age/Sex Fits − Males <2 Years Old − Total Anaemia Prevalence − Anambra
WHO−2024 Adjustment Method
Total Anaemia Prevalence
Age (year)
Location Hierarchy
1 − Global
2 − Super Region
3 − Region
4 − Country
5 − Subnational (Level 1)
Uncertainty Interval
5 − Subnational (Level 1)

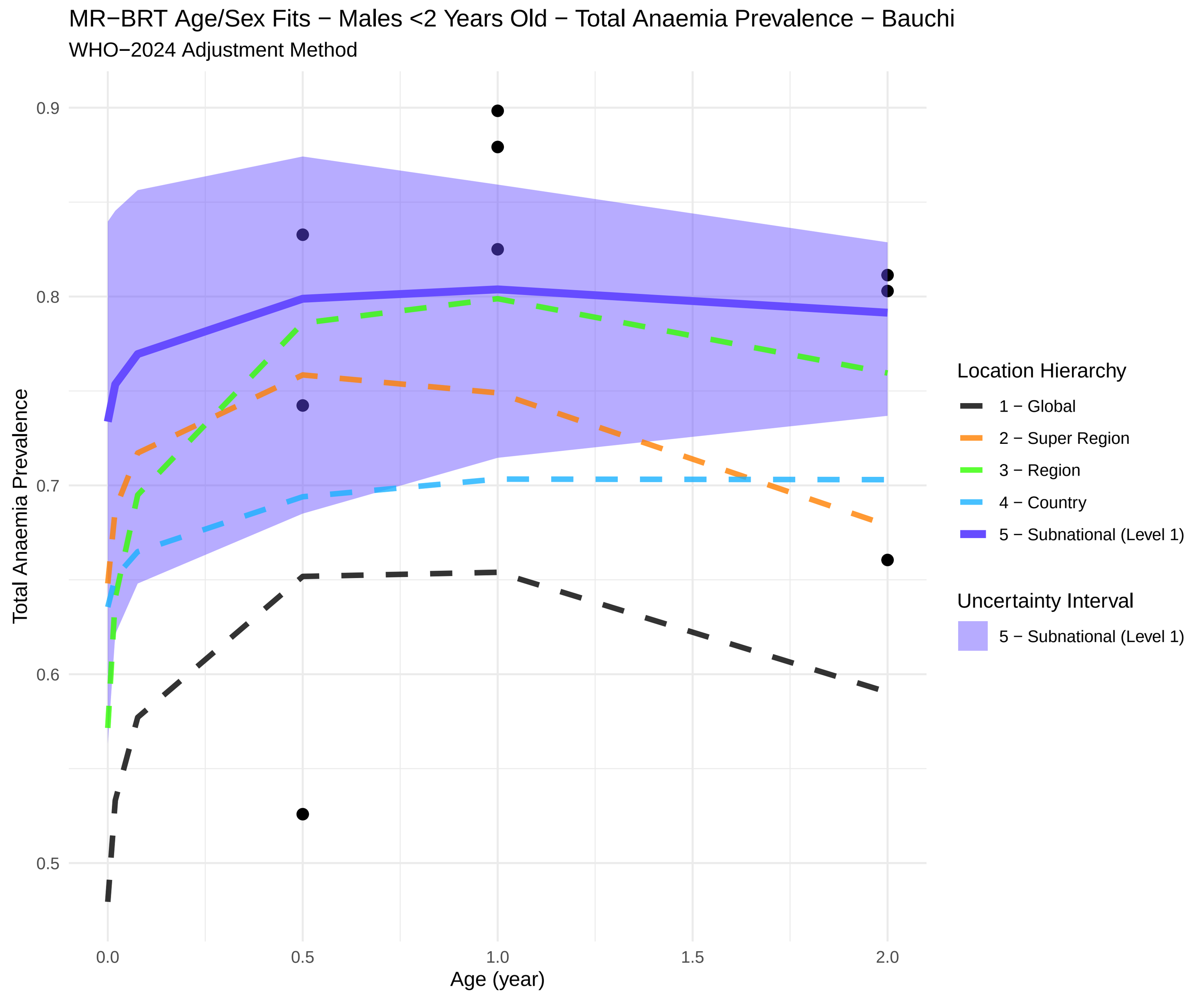
MR−BRT Age/Sex Fits − Males <2 Years Old − Total Anaemia Prevalence − Bauchi
WHO−2024 Adjustment Method
Total Anaemia Prevalence
Age (year)
Location Hierarchy
1 − Global
2 − Super Region
3 − Region
4 − Country
5 − Subnational (Level 1)
Uncertainty Interval
5 − Subnational (Level 1)

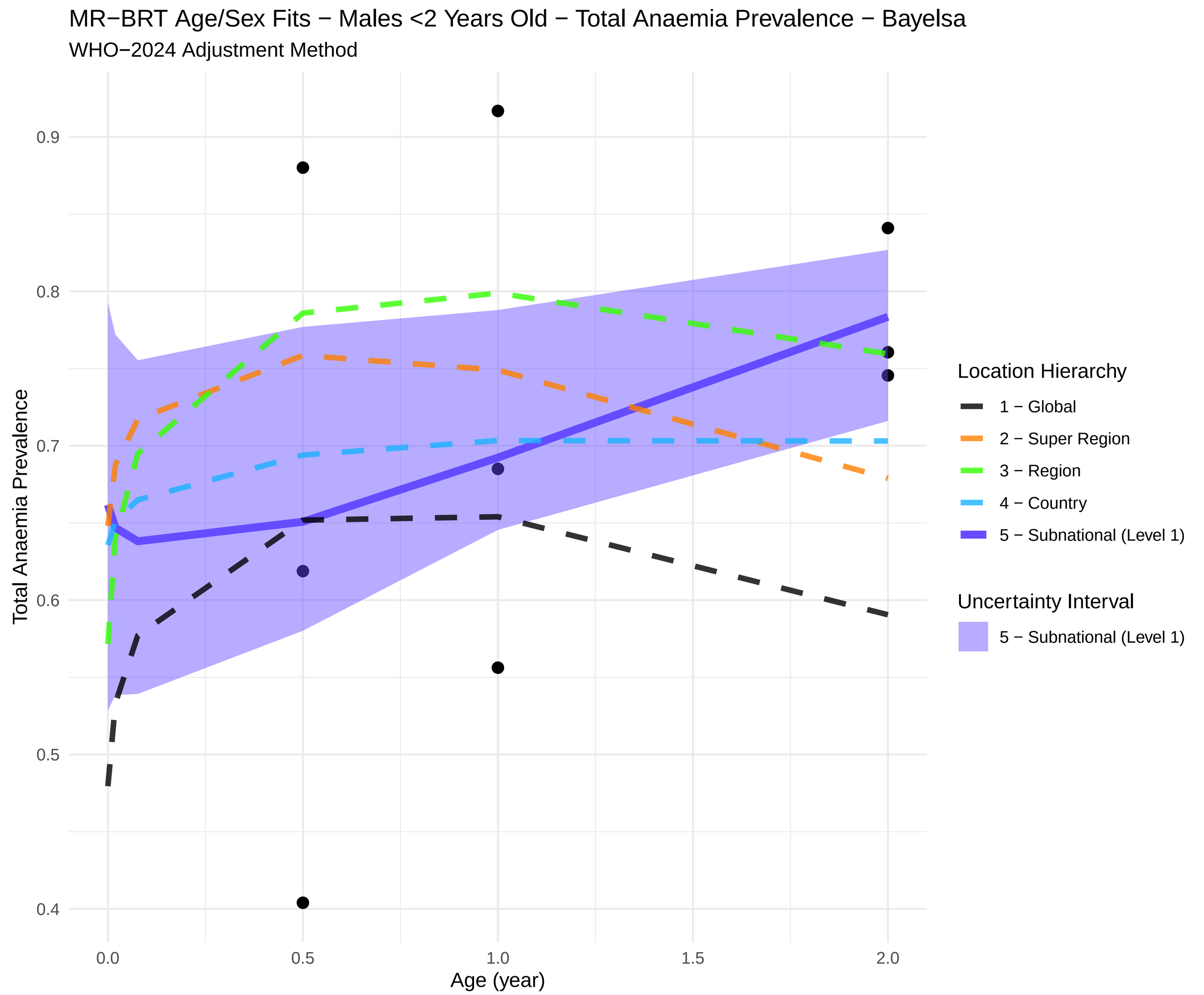
MR−BRT Age/Sex Fits − Males <2 Years Old − Total Anaemia Prevalence − Bayelsa
WHO−2024 Adjustment Method
Total Anaemia Prevalence
Age (year)
Location Hierarchy
1 − Global
2 − Super Region
3 − Region
4 − Country
5 − Subnational (Level 1)
Uncertainty Interval
5 − Subnational (Level 1)

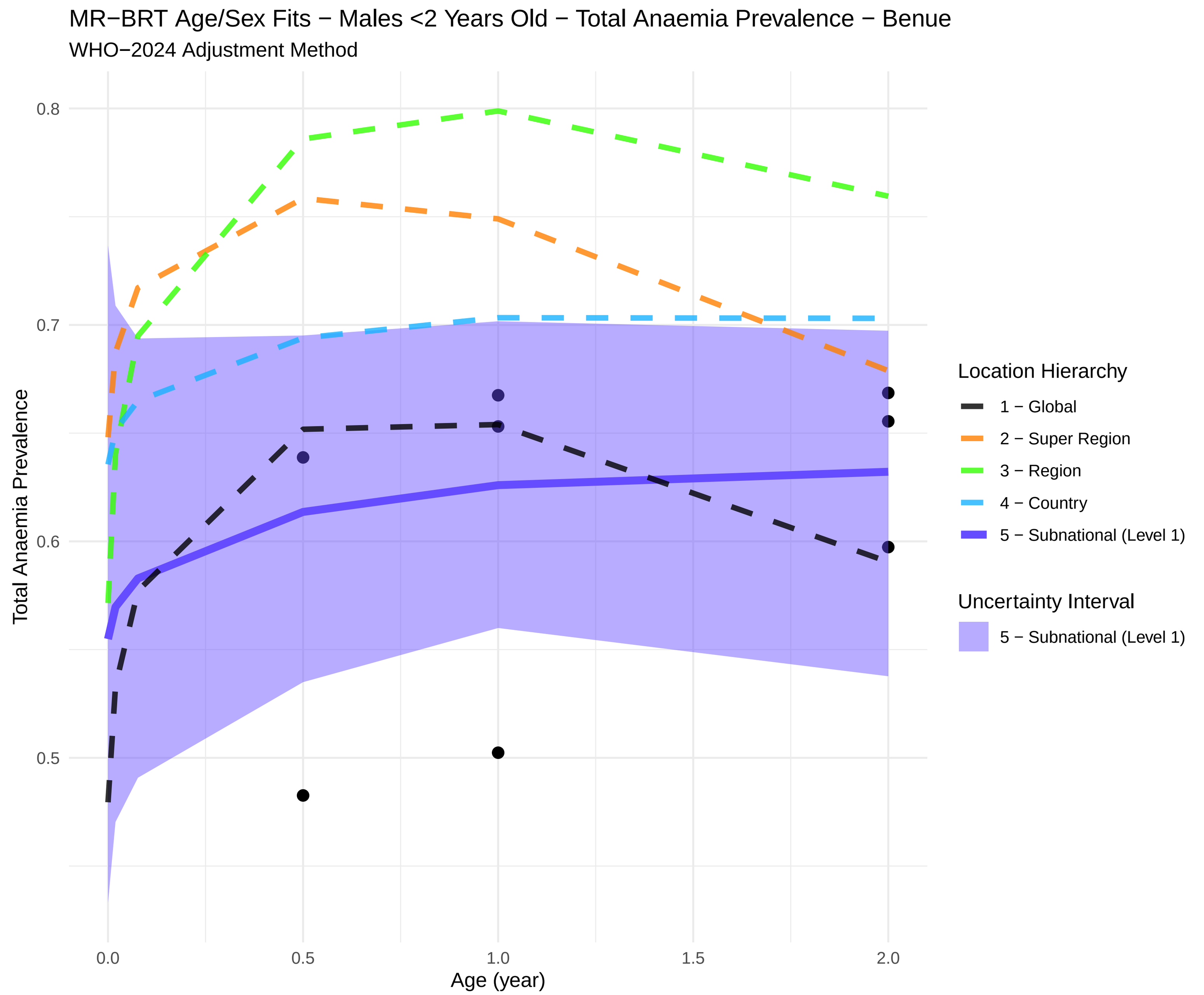
MR−BRT Age/Sex Fits − Males <2 Years Old − Total Anaemia Prevalence − Benue
WHO−2024 Adjustment Method
Total Anaemia Prevalence
Age (year)
Location Hierarchy
1 − Global
2 − Super Region
3 − Region
4 − Country
5 − Subnational (Level 1)
Uncertainty Interval
5 − Subnational (Level 1)

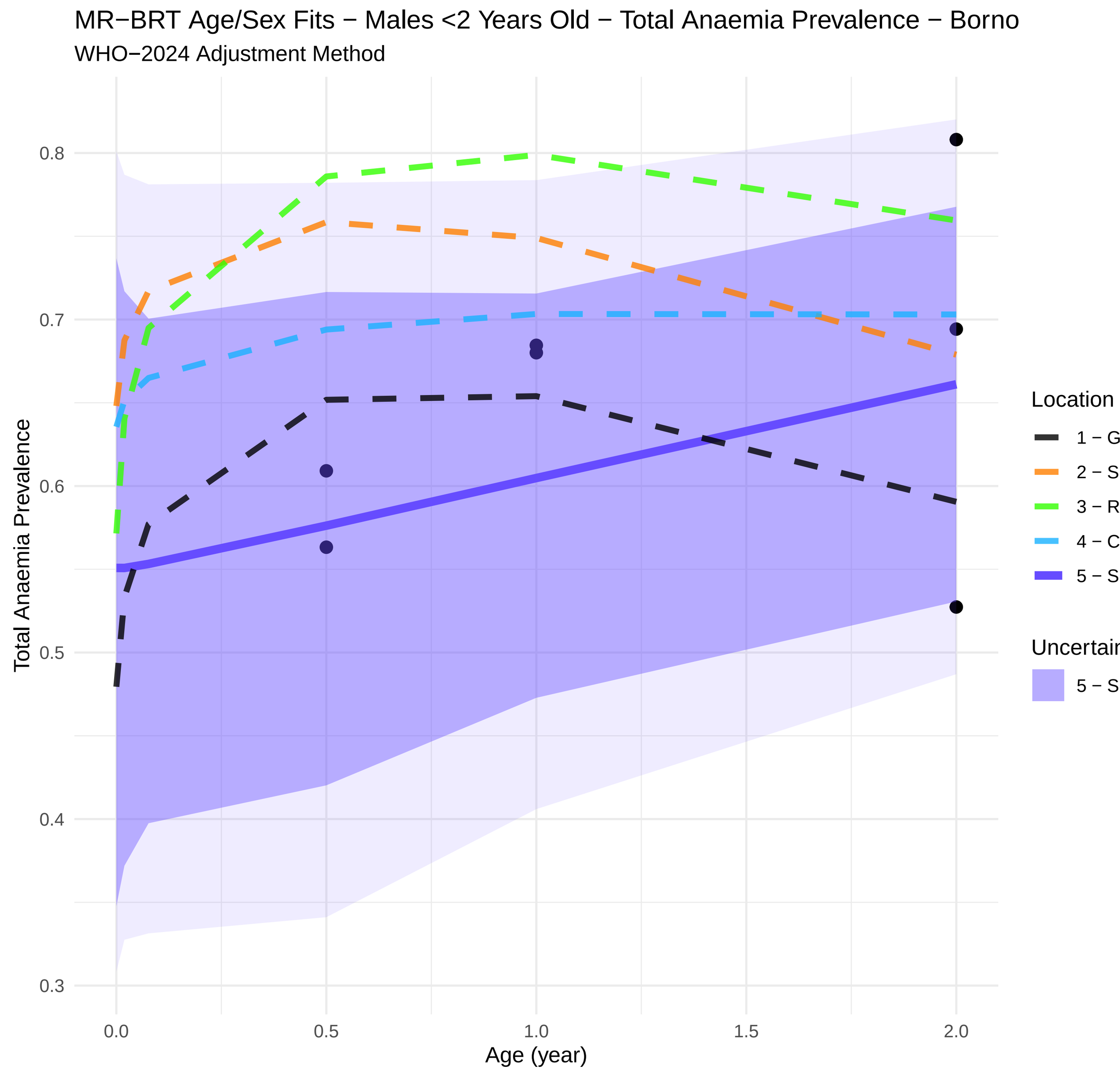
MR−BRT Age/Sex Fits − Males <2 Years Old − Total Anaemia Prevalence − Borno
WHO−2024 Adjustment Method
Total Anaemia Prevalence
Age (year)
Location Hierarchy
1 − Global
2 − Super Region
3 − Region
4 − Country
5 − Subnational (Level 1)
Uncertainty Interval
5 − Subnational (Level 1)

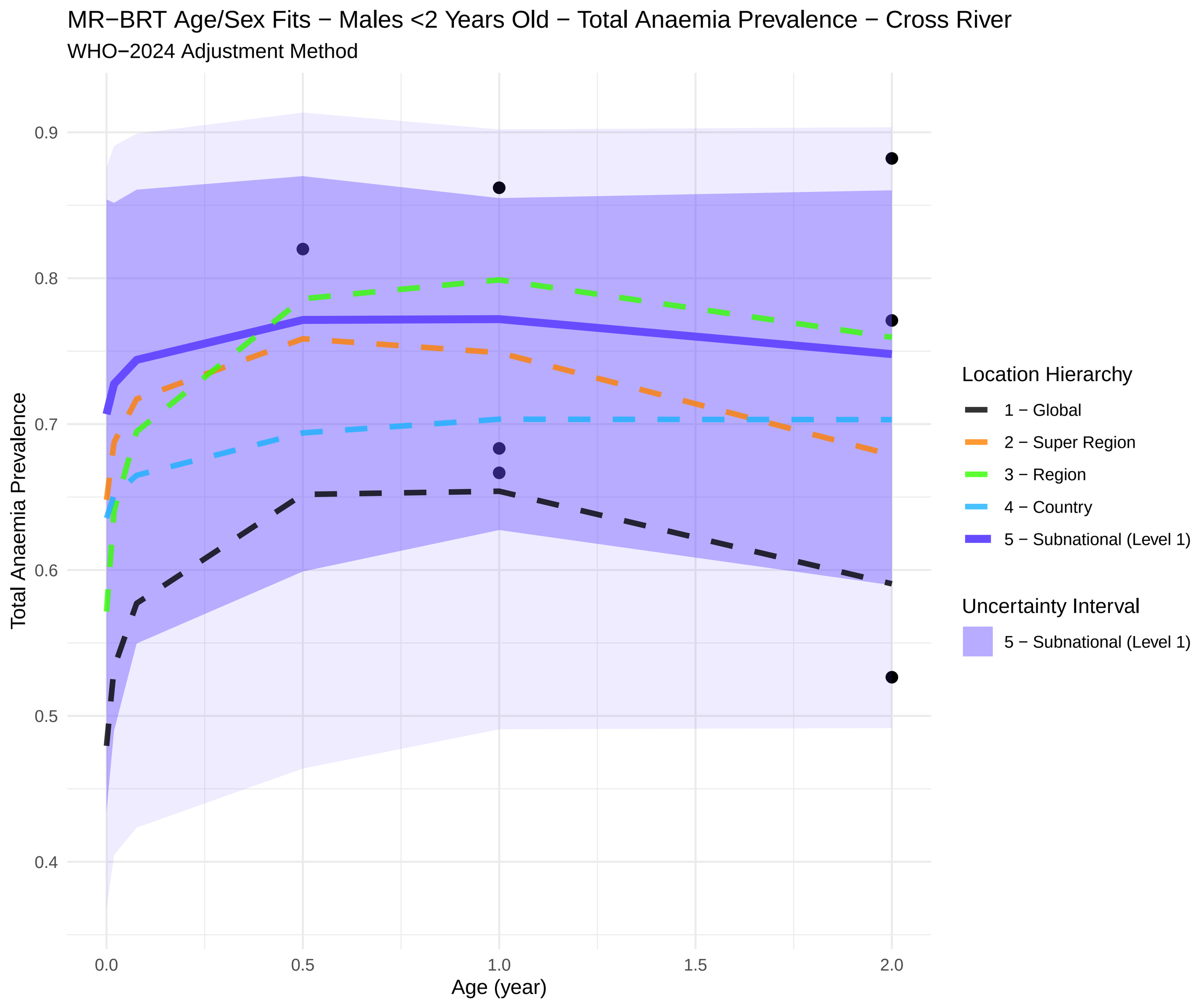
MR−BRT Age/Sex Fits − Males <2 Years Old − Total Anaemia Prevalence − Cross River
WHO−2024 Adjustment Method
Total Anaemia Prevalence
Age (year)
Location Hierarchy
1 − Global
2 − Super Region
3 − Region
4 − Country
5 − Subnational (Level 1)
Uncertainty Interval
5 − Subnational (Level 1)

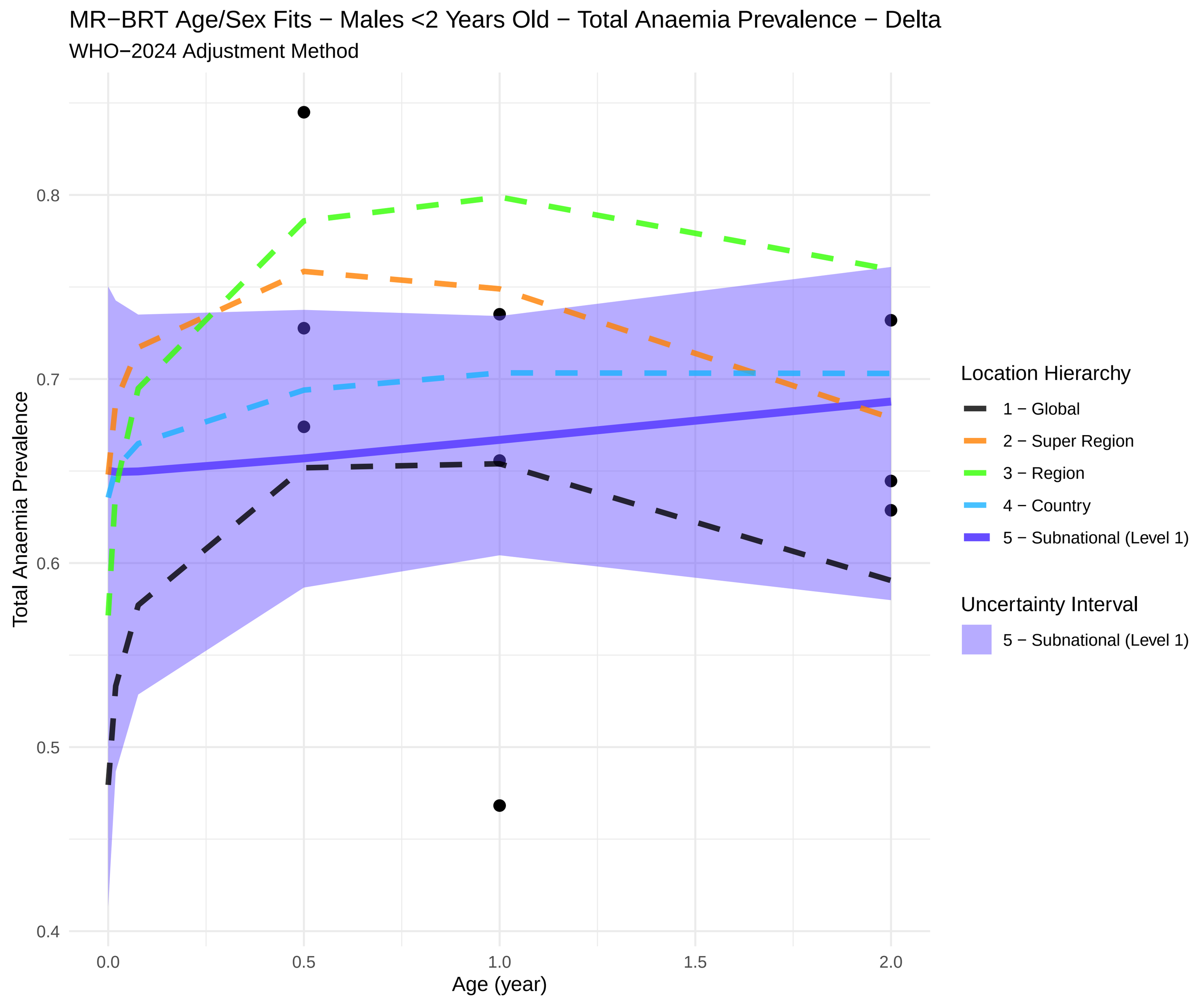
MR−BRT Age/Sex Fits − Males <2 Years Old − Total Anaemia Prevalence − Delta
WHO−2024 Adjustment Method
Total Anaemia Prevalence
Age (year)
Location Hierarchy
1 − Global
2 − Super Region
3 − Region
4 − Country
5 − Subnational (Level 1)
Uncertainty Interval
5 − Subnational (Level 1)

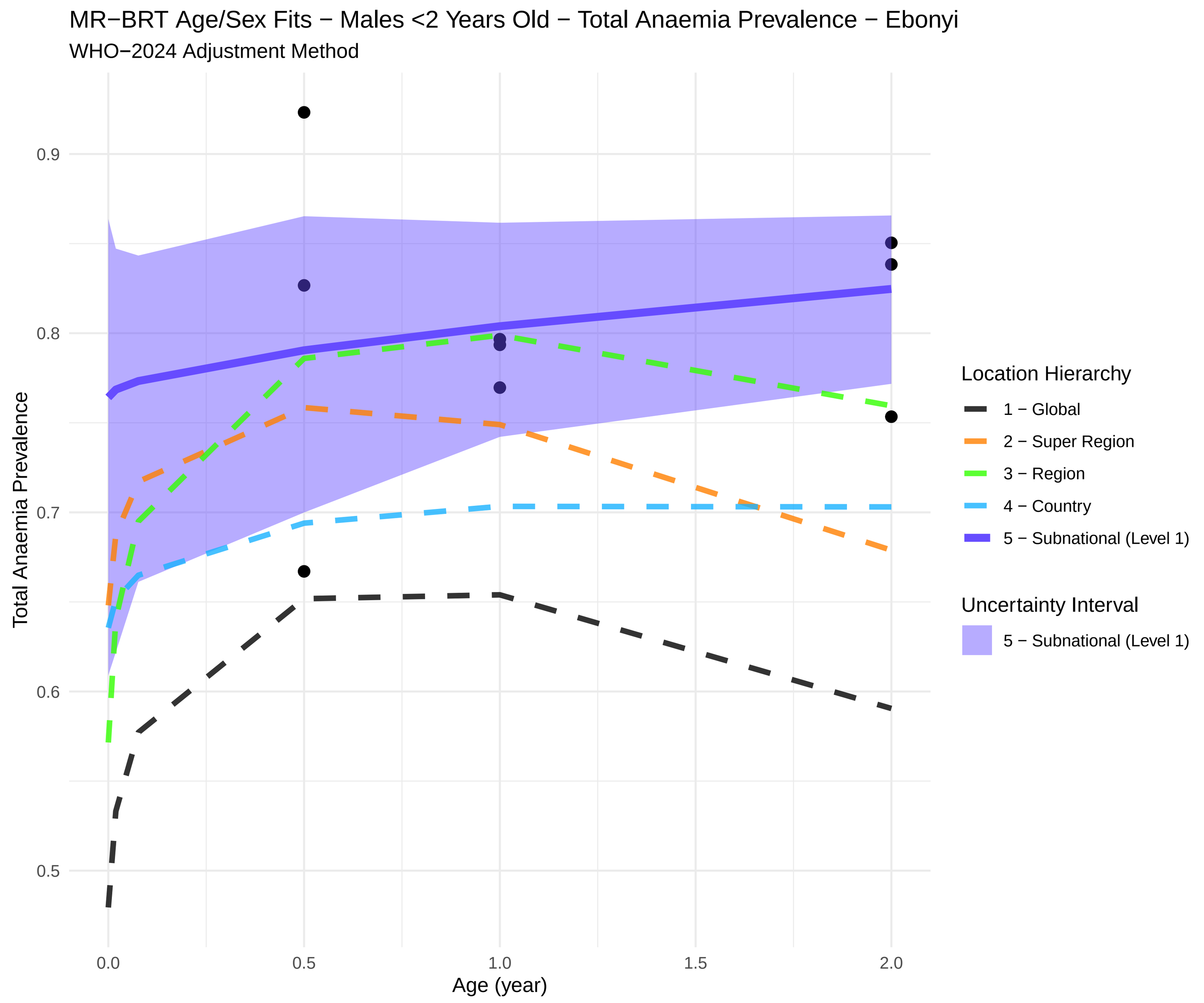
MR−BRT Age/Sex Fits − Males <2 Years Old − Total Anaemia Prevalence − Ebonyi
WHO−2024 Adjustment Method
Total Anaemia Prevalence
Age (year)
Location Hierarchy
1 − Global
2 − Super Region
3 − Region
4 − Country
5 − Subnational (Level 1)
Uncertainty Interval
5 − Subnational (Level 1)

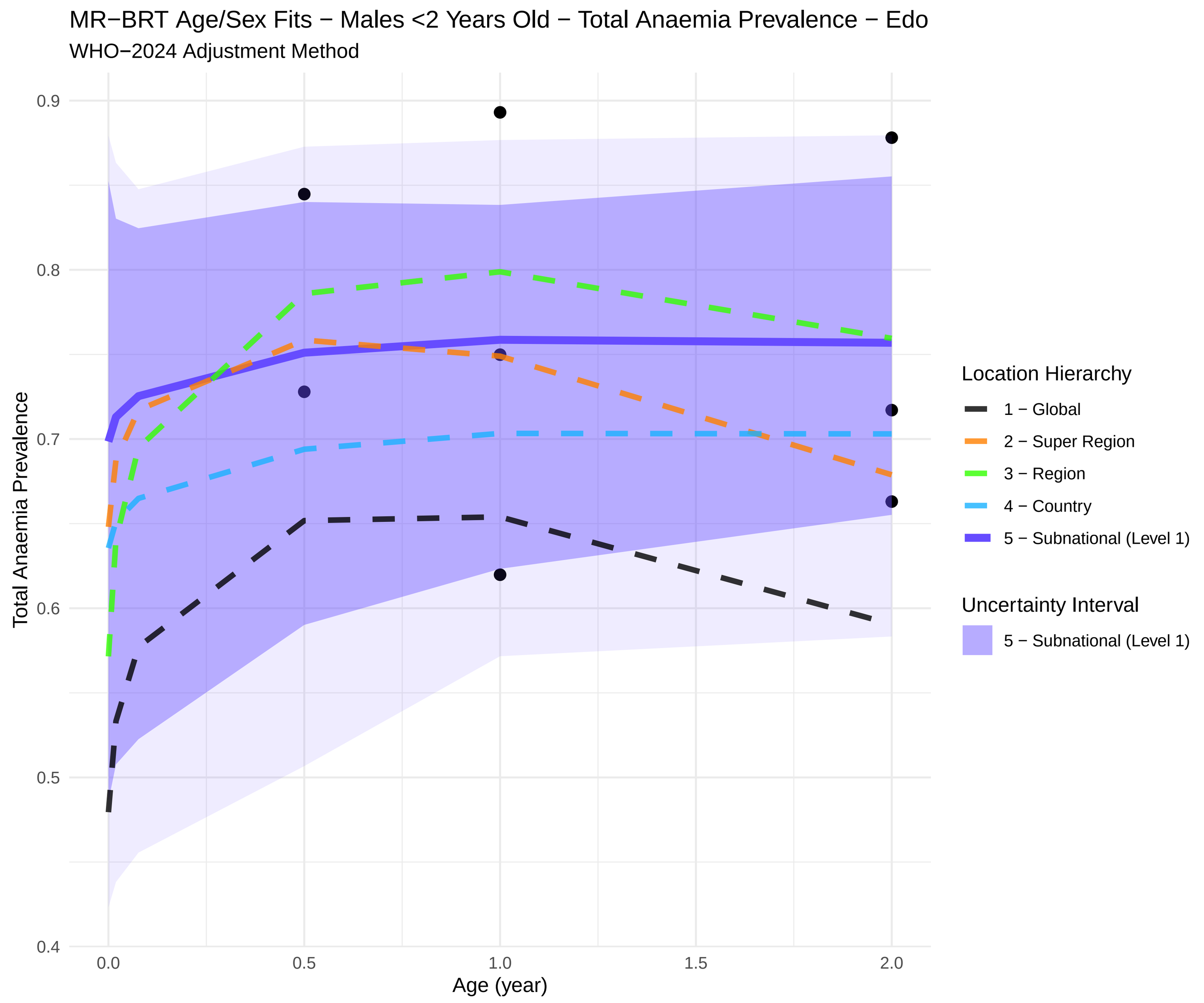
MR−BRT Age/Sex Fits − Males <2 Years Old − Total Anaemia Prevalence − Edo
WHO−2024 Adjustment Method
Total Anaemia Prevalence
Age (year)
Location Hierarchy
1 − Global
2 − Super Region
3 − Region
4 − Country
5 − Subnational (Level 1)
Uncertainty Interval
5 − Subnational (Level 1)

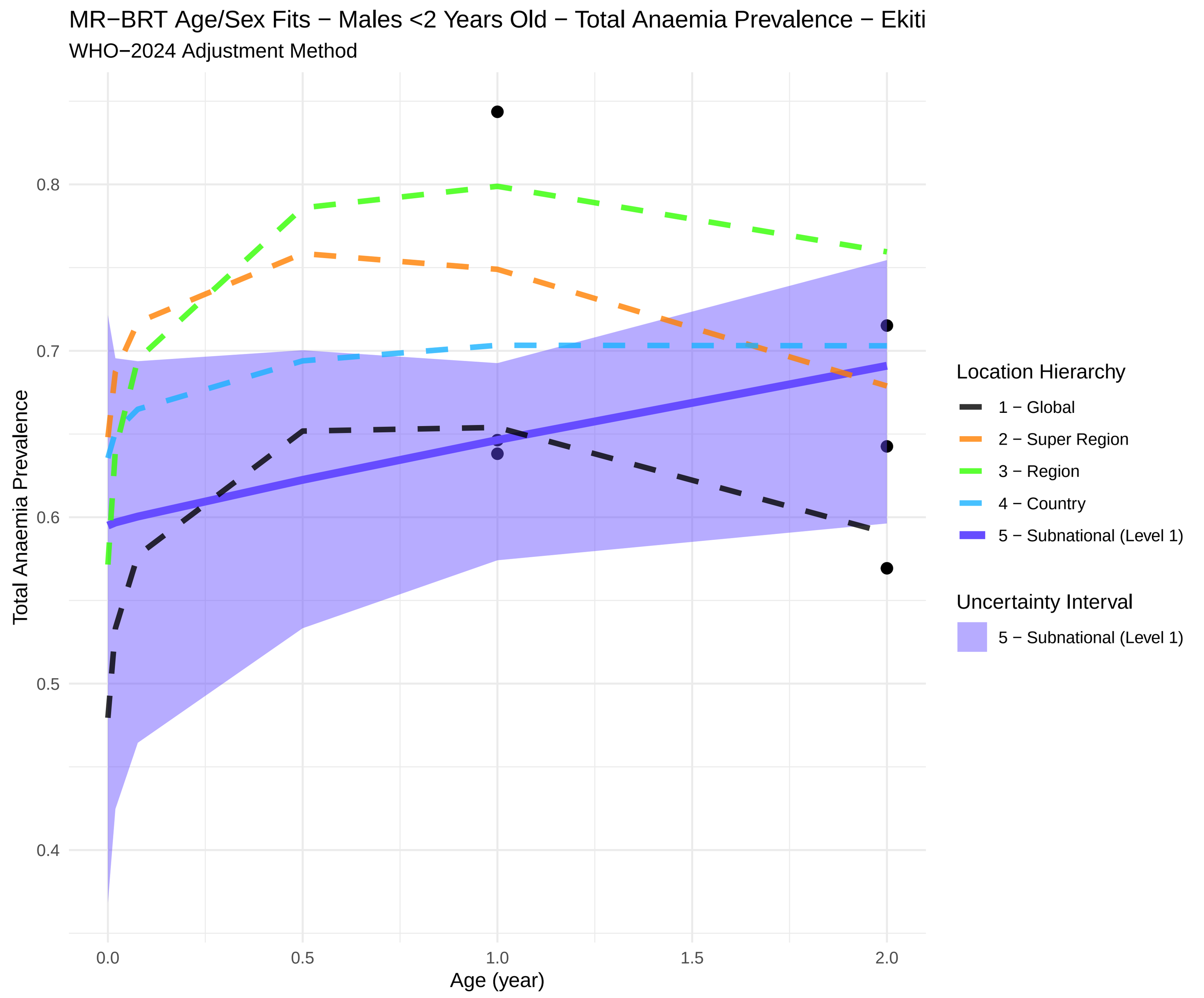
MR−BRT Age/Sex Fits − Males <2 Years Old − Total Anaemia Prevalence − Ekiti
WHO−2024 Adjustment Method
Total Anaemia Prevalence
Age (year)
Location Hierarchy
1 − Global
2 − Super Region
3 − Region
4 − Country
5 − Subnational (Level 1)
Uncertainty Interval
5 − Subnational (Level 1)

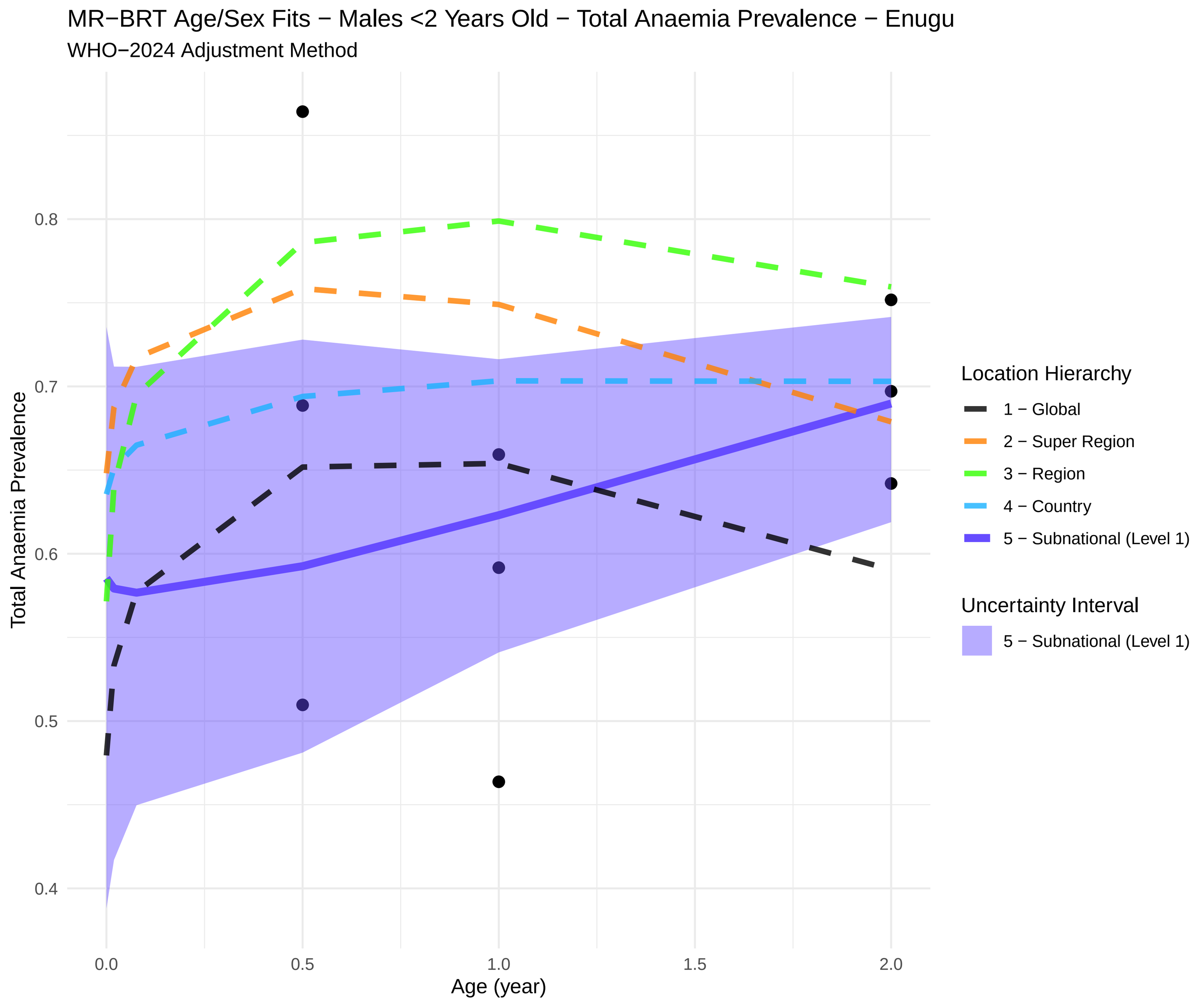
MR−BRT Age/Sex Fits − Males <2 Years Old − Total Anaemia Prevalence − Enugu
WHO−2024 Adjustment Method
Total Anaemia Prevalence
Age (year)
Location Hierarchy
1 − Global
2 − Super Region
3 − Region
4 − Country
5 − Subnational (Level 1)
Uncertainty Interval
5 − Subnational (Level 1)

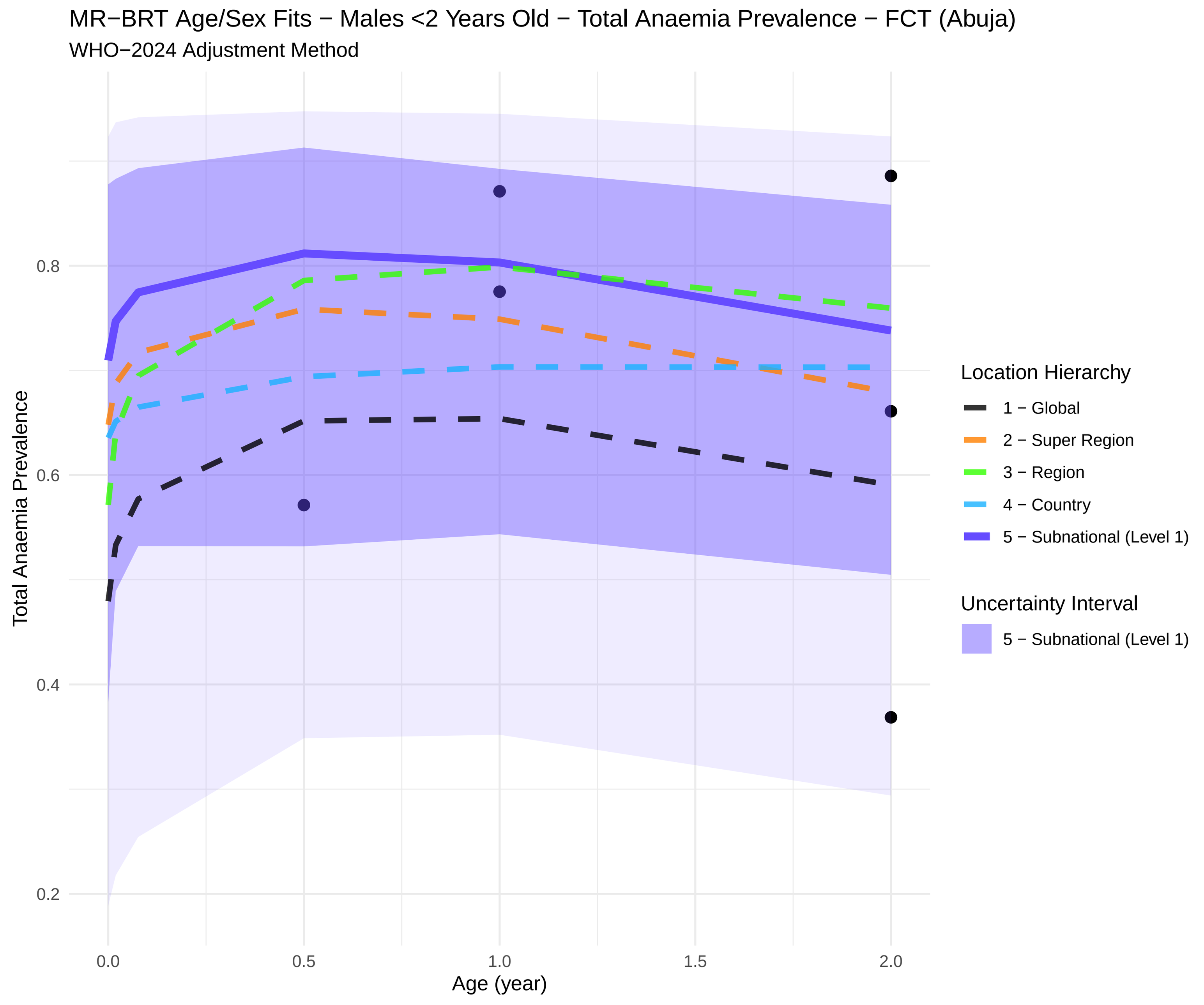
MR−BRT Age/Sex Fits − Males <2 Years Old − Total Anaemia Prevalence − FCT (Abuja)
WHO−2024 Adjustment Method
Total Anaemia Prevalence
Age (year)
Location Hierarchy
1 − Global
2 − Super Region
3 − Region
4 − Country
5 − Subnational (Level 1)
Uncertainty Interval
5 − Subnational (Level 1)

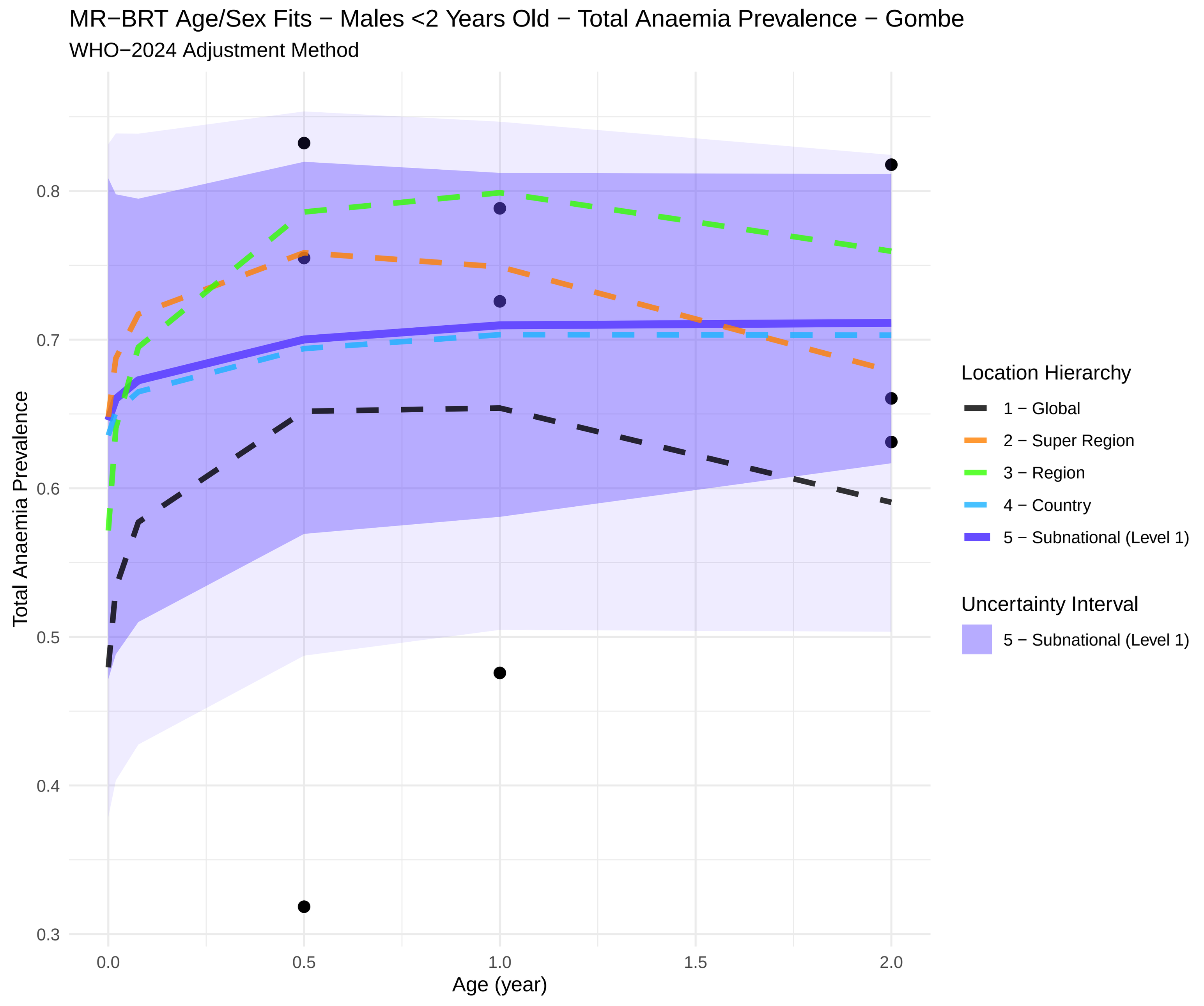
MR−BRT Age/Sex Fits − Males <2 Years Old − Total Anaemia Prevalence − Gombe
WHO−2024 Adjustment Method
Total Anaemia Prevalence
Age (year)
Location Hierarchy
1 − Global
2 − Super Region
3 − Region
4 − Country
5 − Subnational (Level 1)
Uncertainty Interval
5 − Subnational (Level 1)

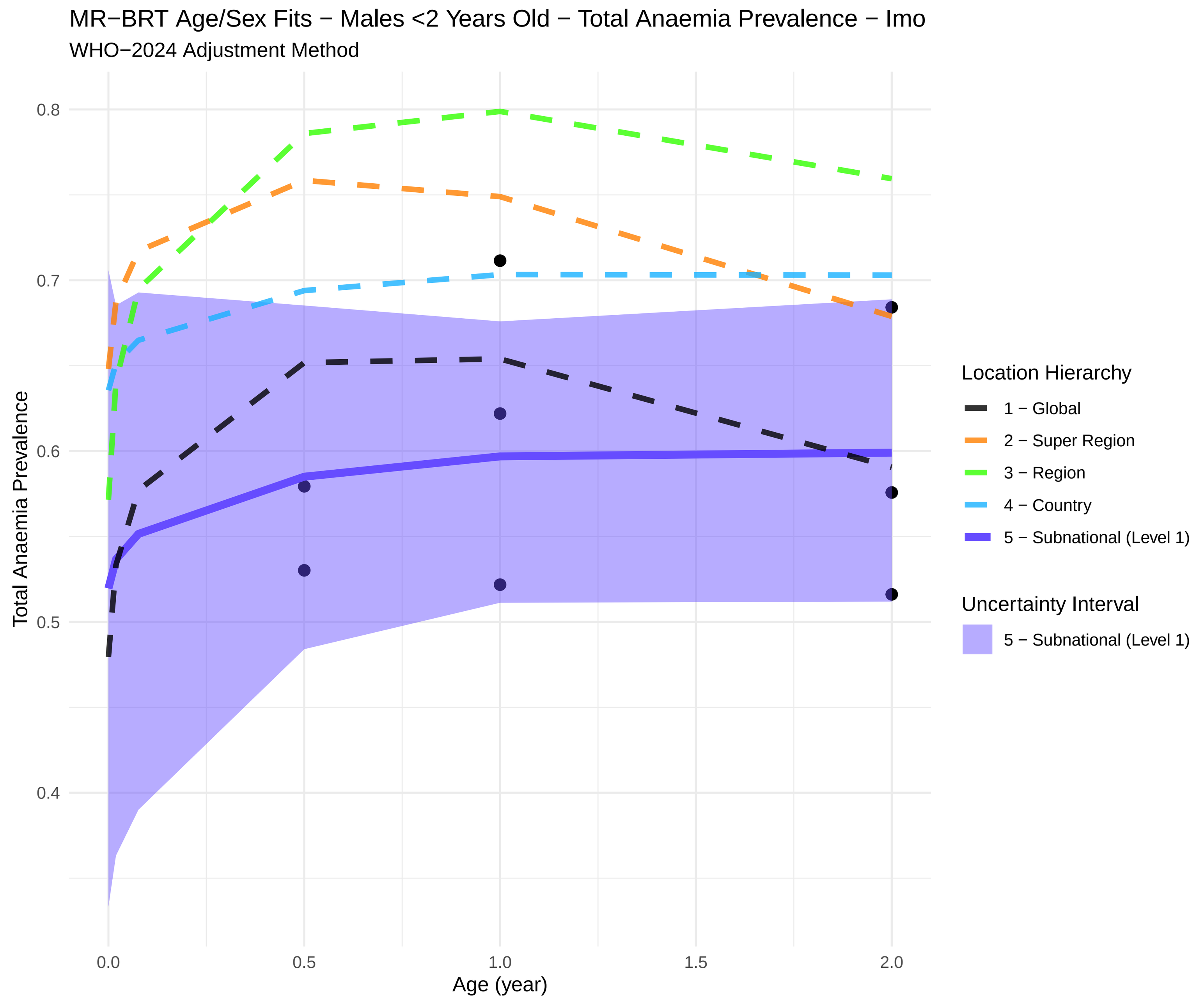
MR−BRT Age/Sex Fits − Males <2 Years Old − Total Anaemia Prevalence − Imo
WHO−2024 Adjustment Method
Total Anaemia Prevalence
Age (year)
Location Hierarchy
1 − Global
2 − Super Region
3 − Region
4 − Country
5 − Subnational (Level 1)
Uncertainty Interval
5 − Subnational (Level 1)

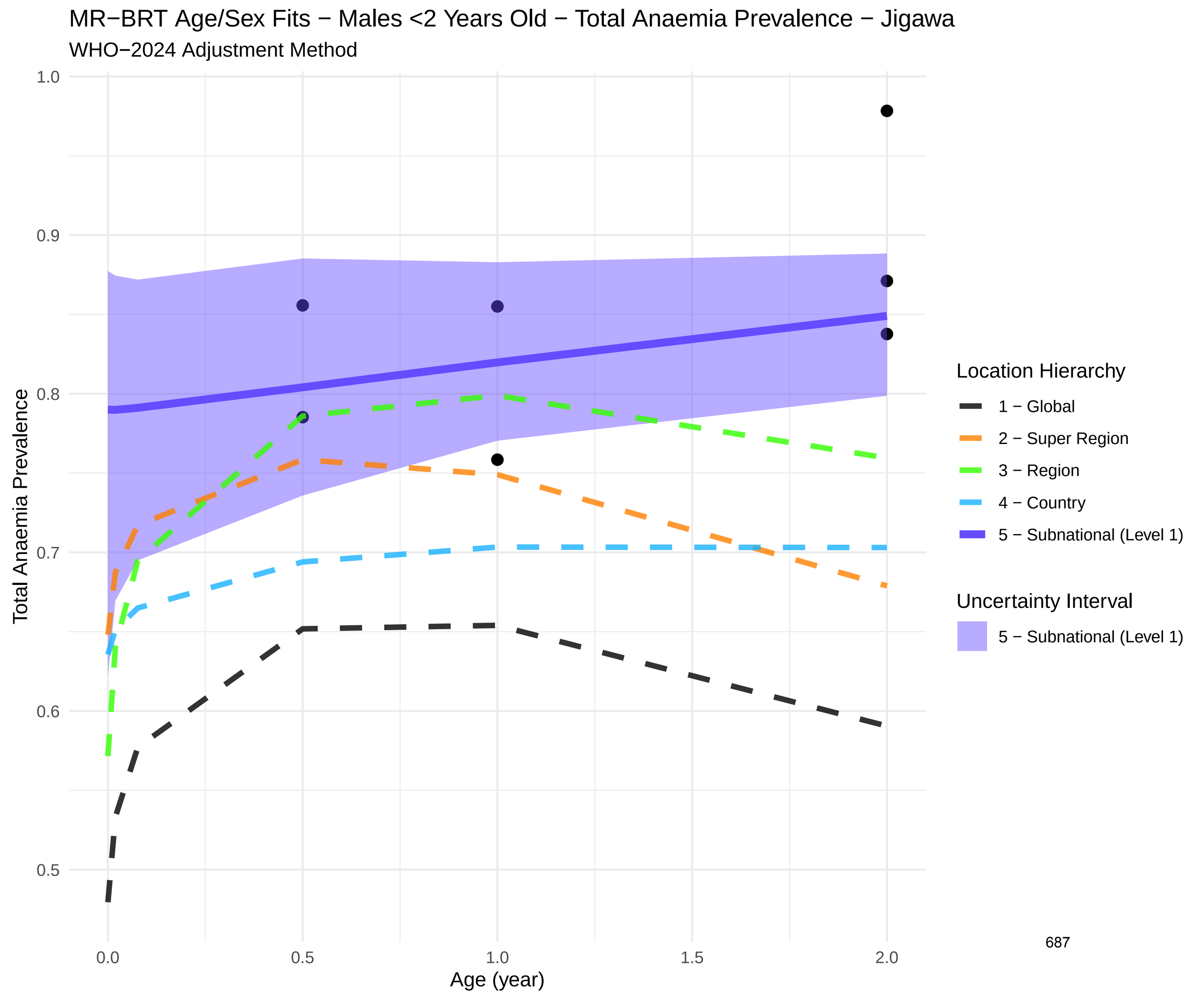
MR−BRT Age/Sex Fits − Males <2 Years Old − Total Anaemia Prevalence − Jigawa
WHO−2024 Adjustment Method
Total Anaemia Prevalence
Age (year)
Location Hierarchy
1 − Global
2 − Super Region
3 − Region
4 − Country
5 − Subnational (Level 1)
Uncertainty Interval
5 − Subnational (Level 1)

MR−BRT Age/Sex Fits − Males <2 Years Old − Total Anaemia Prevalence − Kaduna

WHO−2024 Adjustment Method

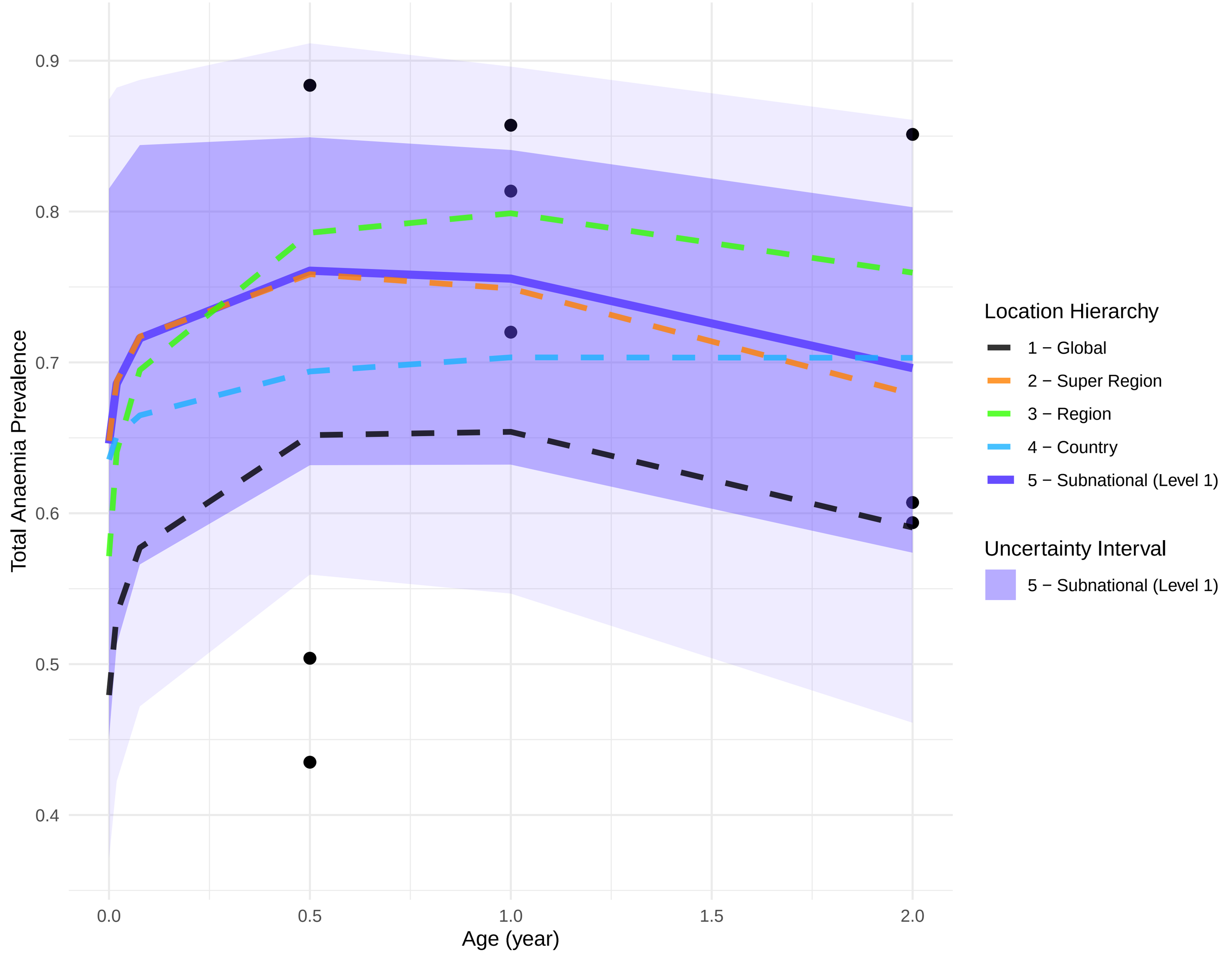

MR−BRT Age/Sex Fits − Males <2 Years Old − Total Anaemia Prevalence − Kano

WHO−2024 Adjustment Method

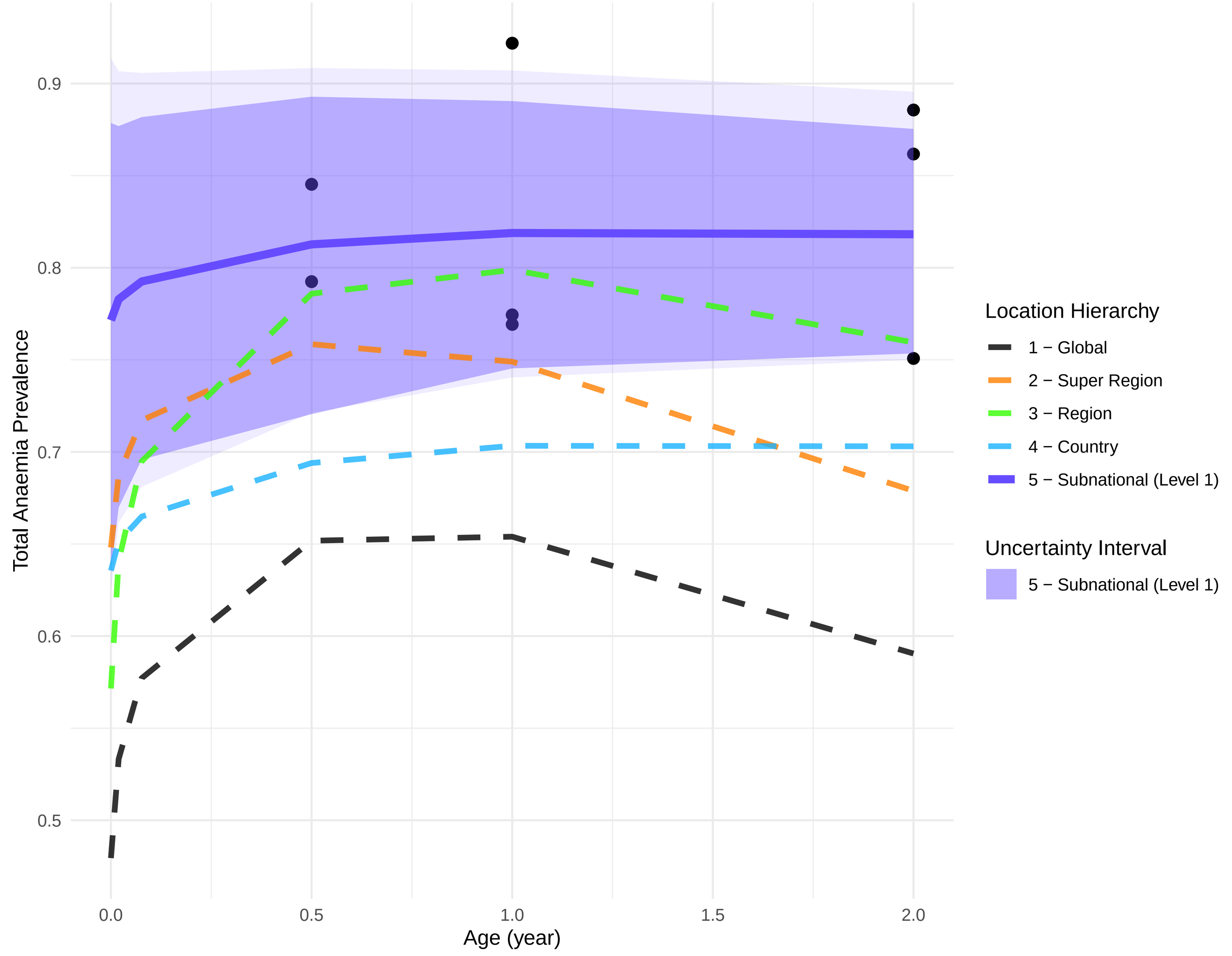

MR−BRT Age/Sex Fits − Males <2 Years Old − Total Anaemia Prevalence − Katsina

WHO−2024 Adjustment Method

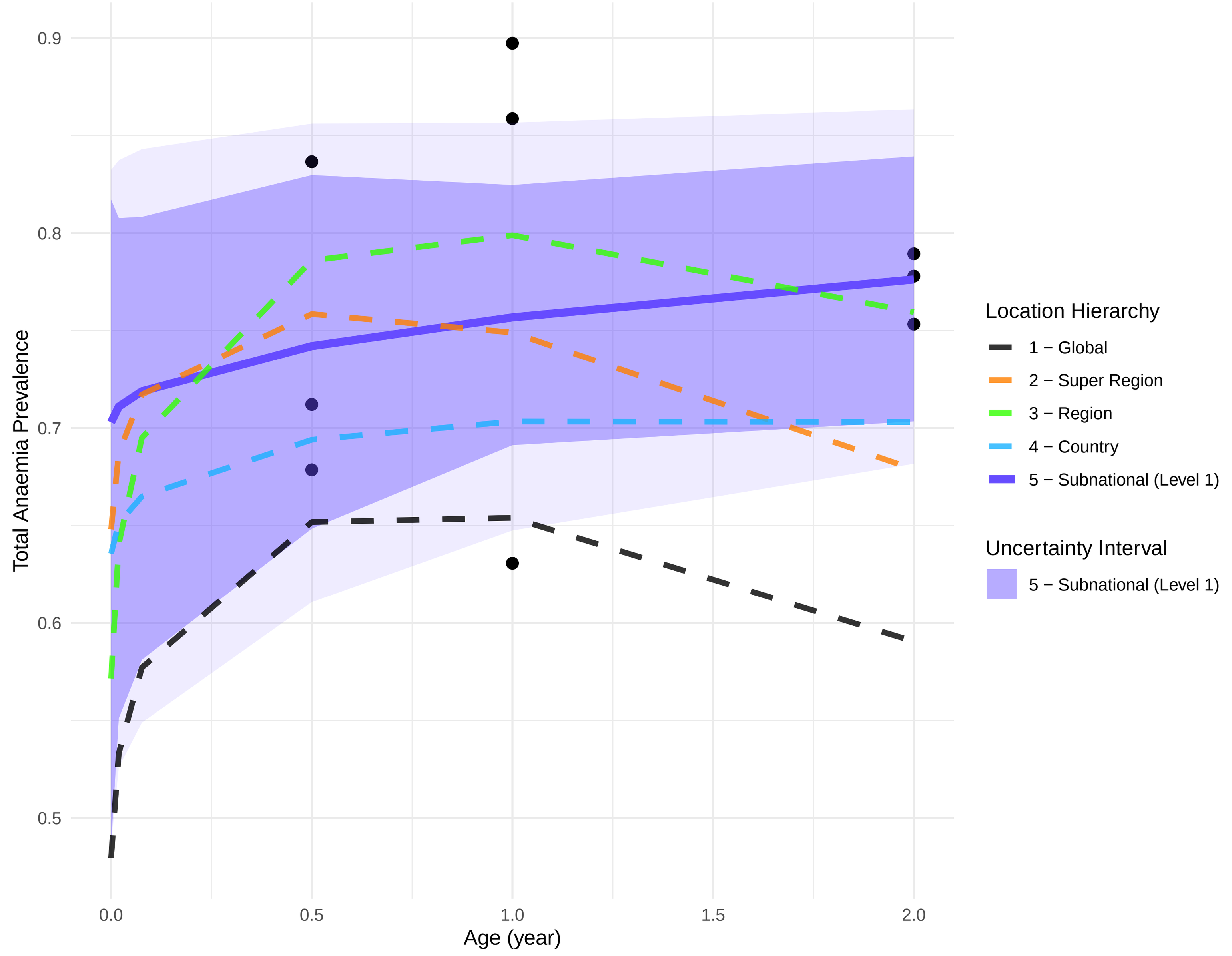

MR−BRT Age/Sex Fits − Males <2 Years Old − Total Anaemia Prevalence − Kebbi

WHO−2024 Adjustment Method

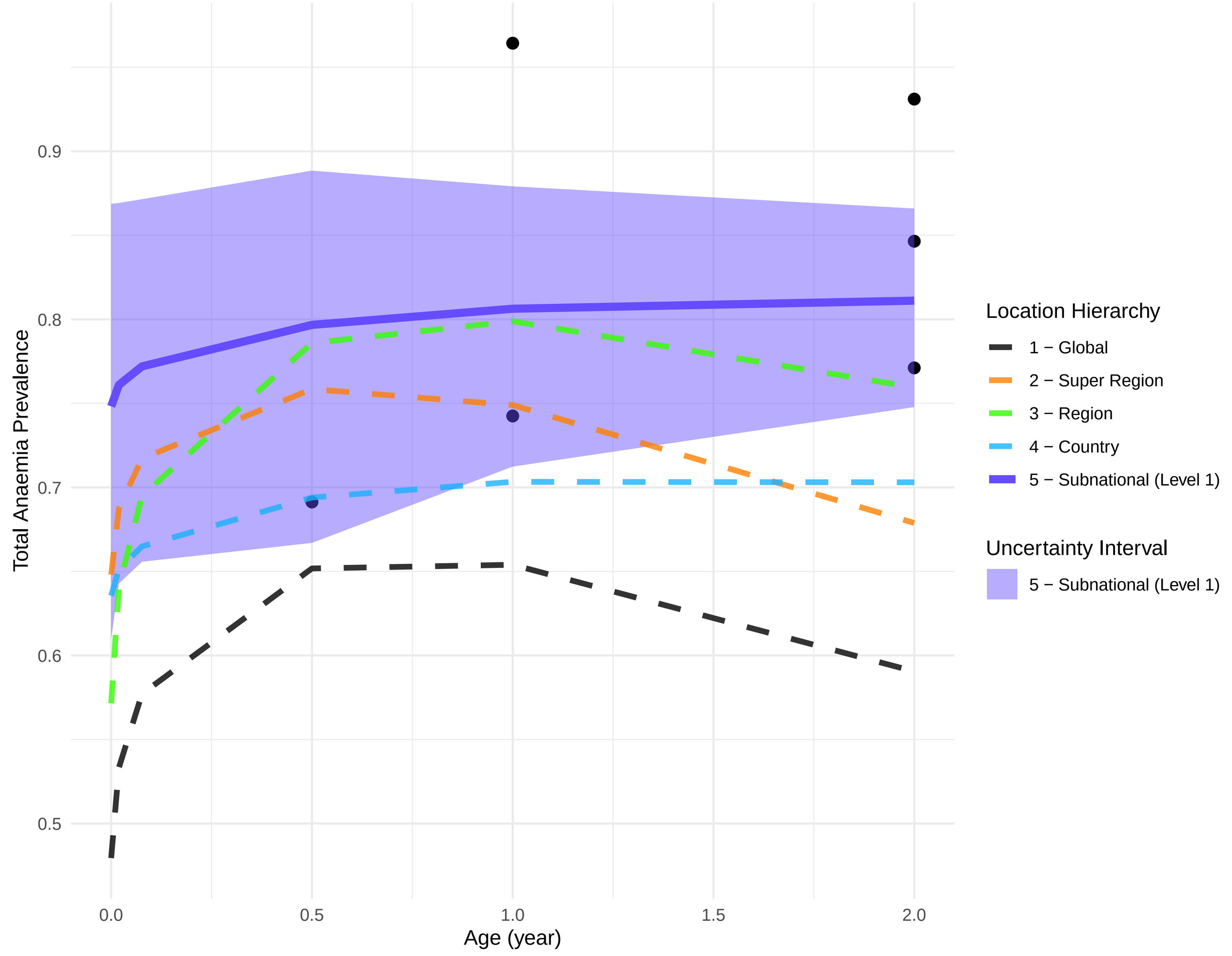

MR−BRT Age/Sex Fits − Males <2 Years Old − Total Anaemia Prevalence − Kogi

WHO−2024 Adjustment Method

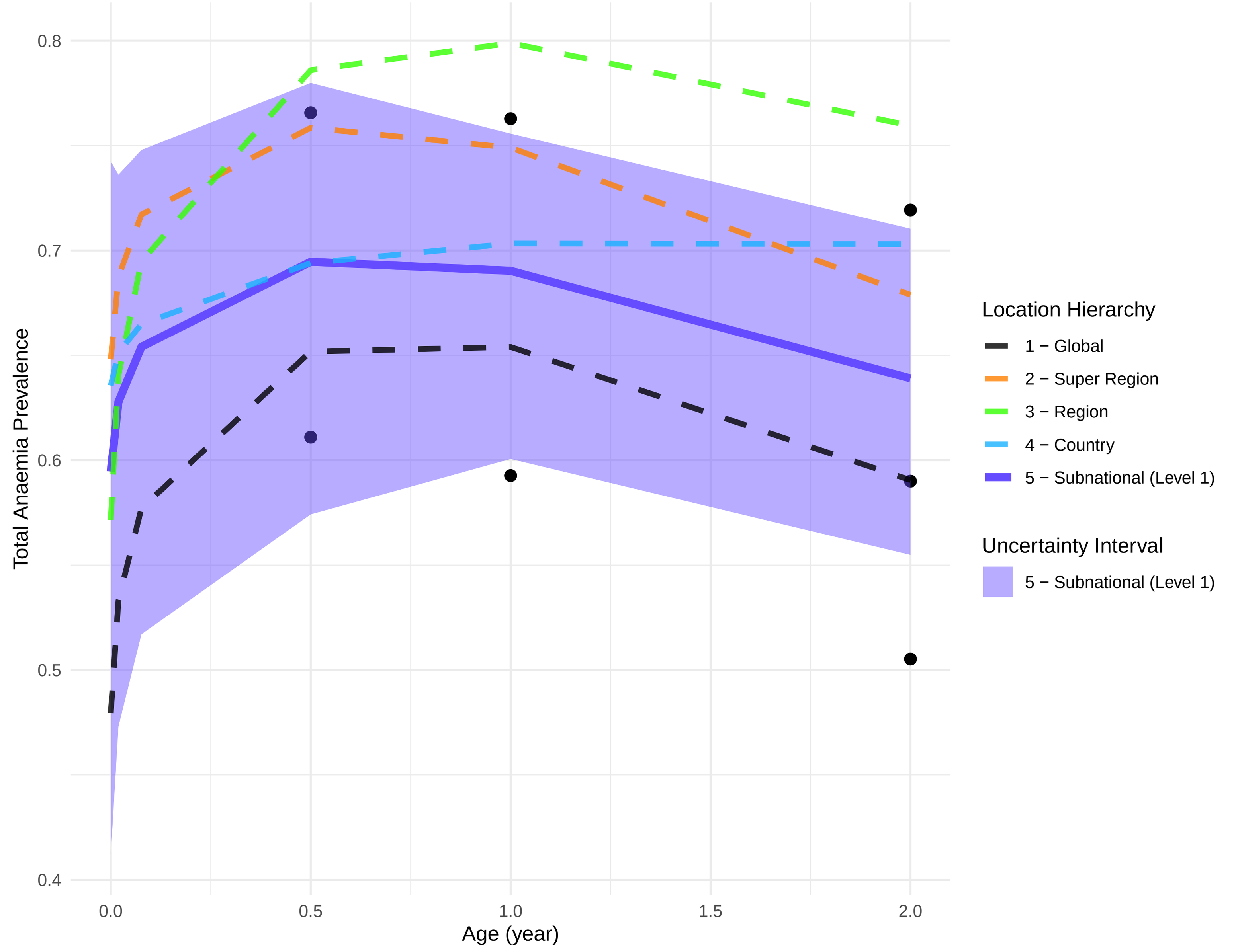

MR−BRT Age/Sex Fits − Males <2 Years Old − Total Anaemia Prevalence − Kwara

WHO−2024 Adjustment Method

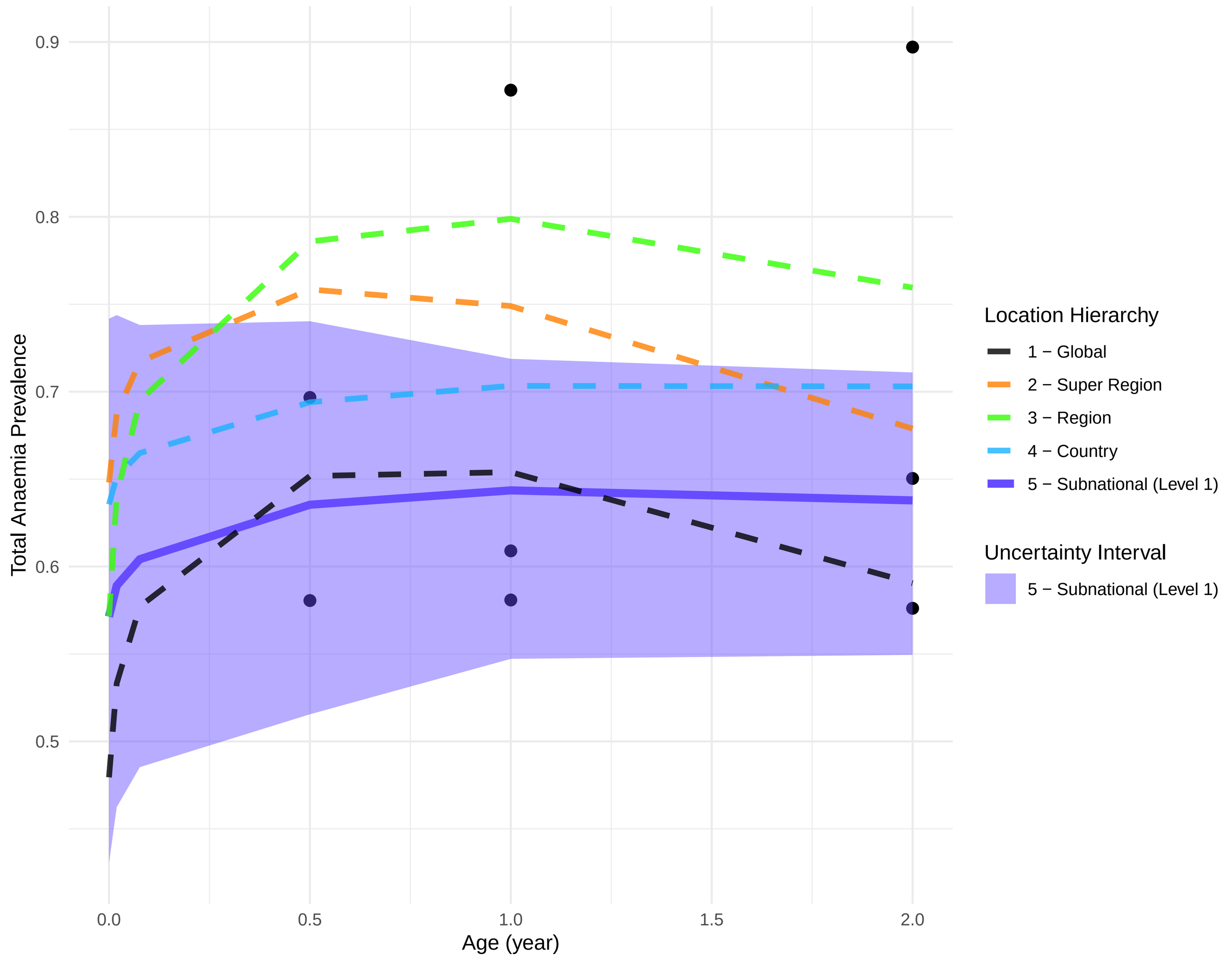

MR−BRT Age/Sex Fits − Males <2 Years Old − Total Anaemia Prevalence − Lagos

WHO−2024 Adjustment Method

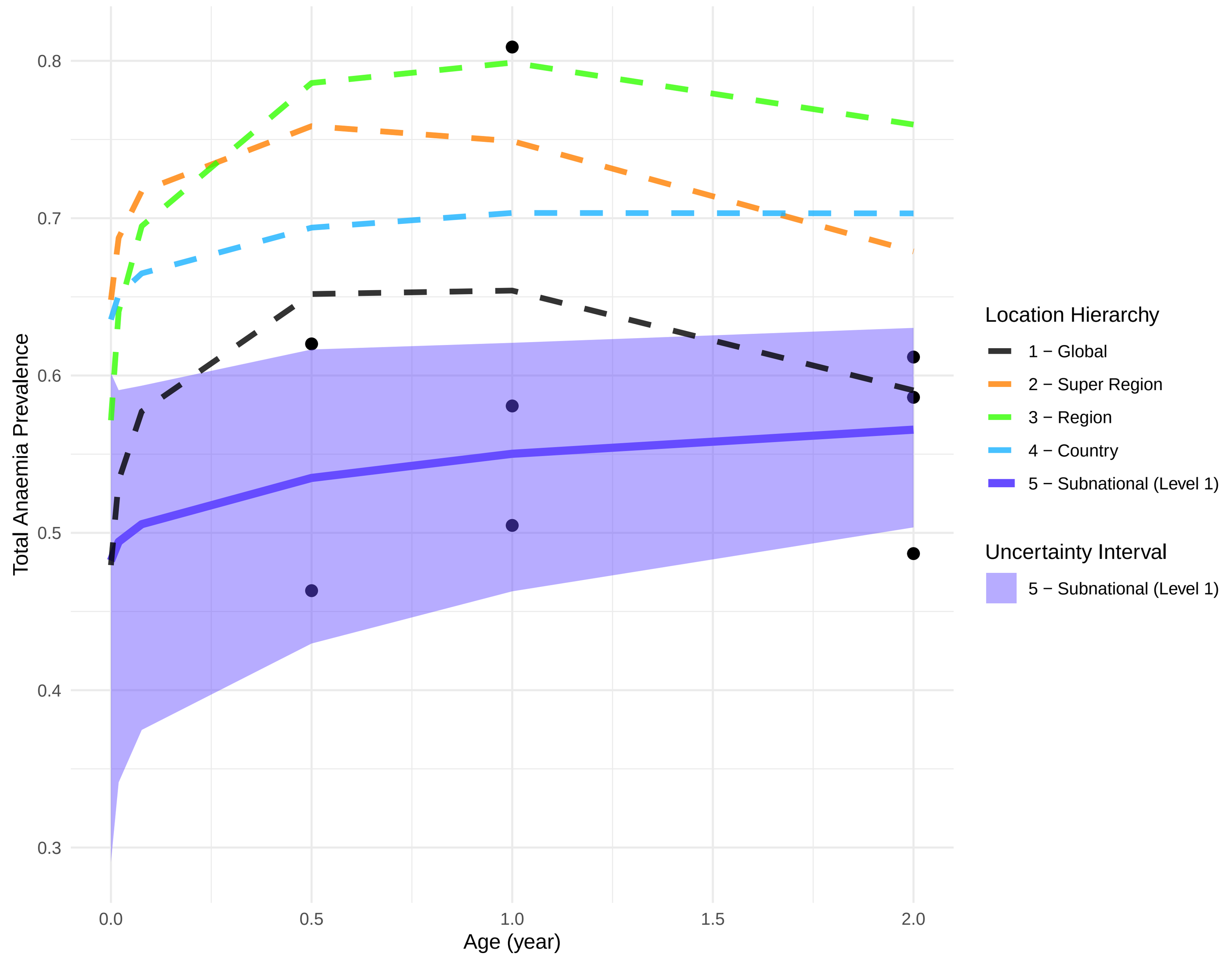

MR−BRT Age/Sex Fits − Males <2 Years Old − Total Anaemia Prevalence − Nasarawa

WHO−2024 Adjustment Method

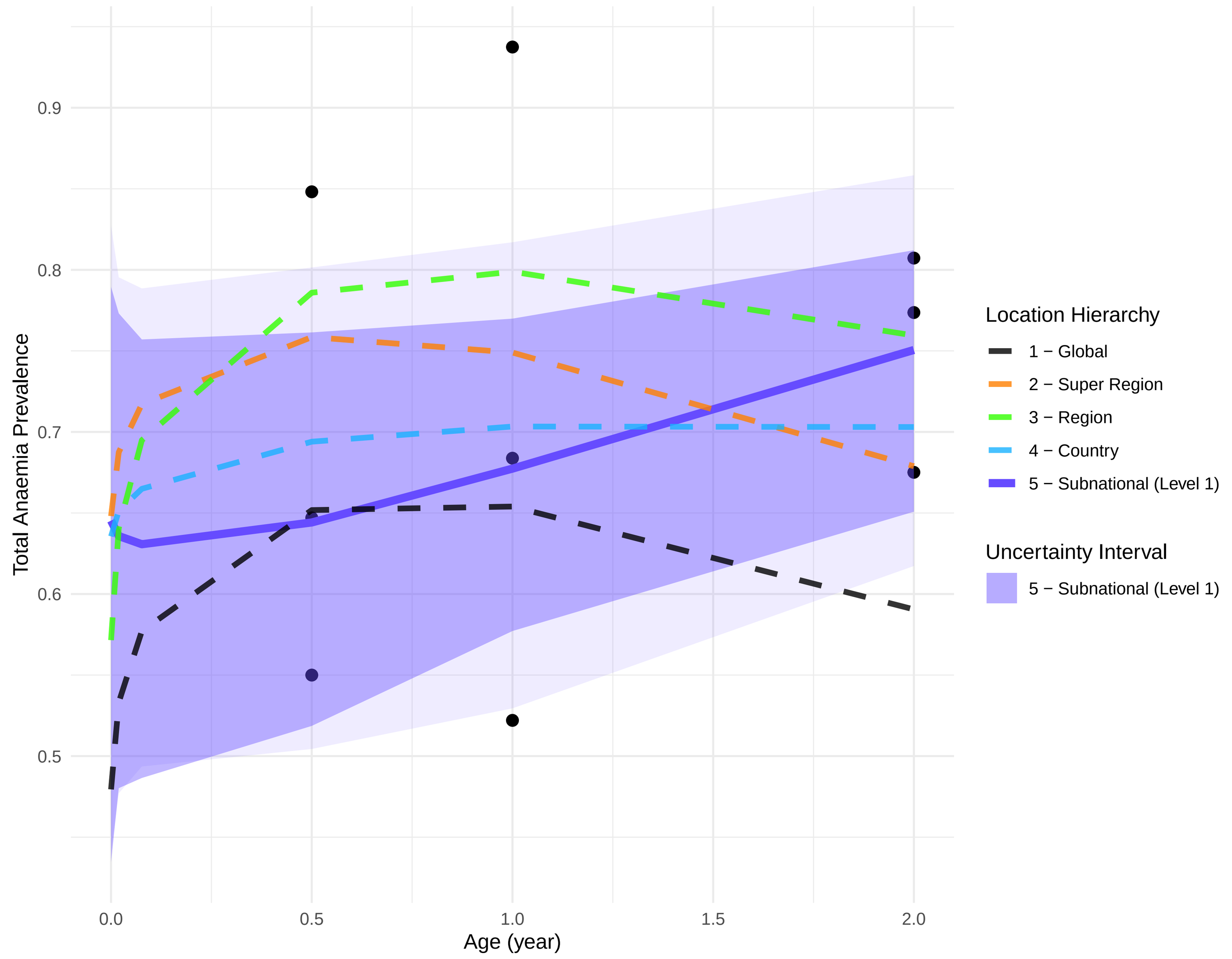

MR−BRT Age/Sex Fits − Males <2 Years Old − Total Anaemia Prevalence − Niger

WHO−2024 Adjustment Method

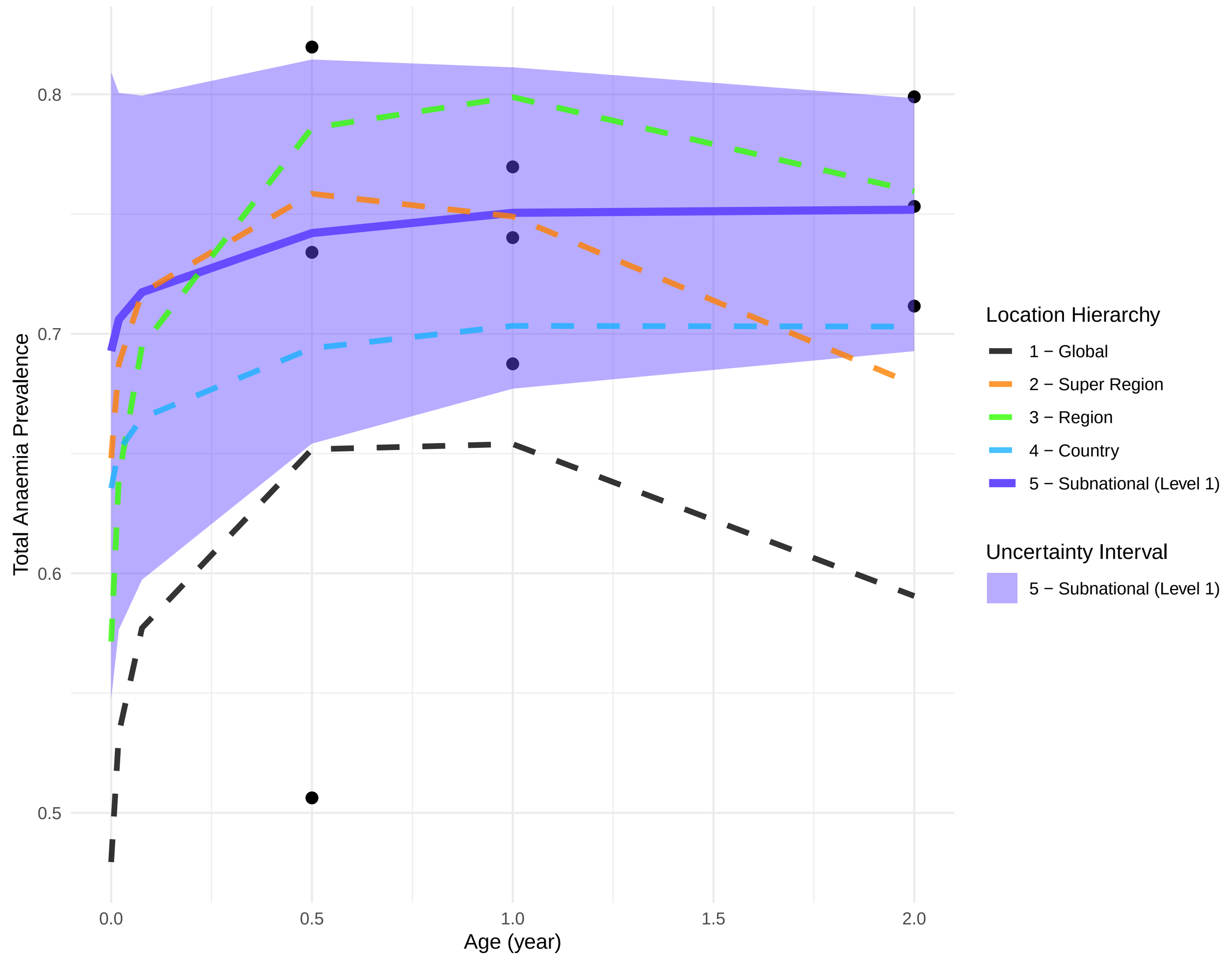

MR−BRT Age/Sex Fits − Males <2 Years Old − Total Anaemia Prevalence − Ogun

WHO−2024 Adjustment Method

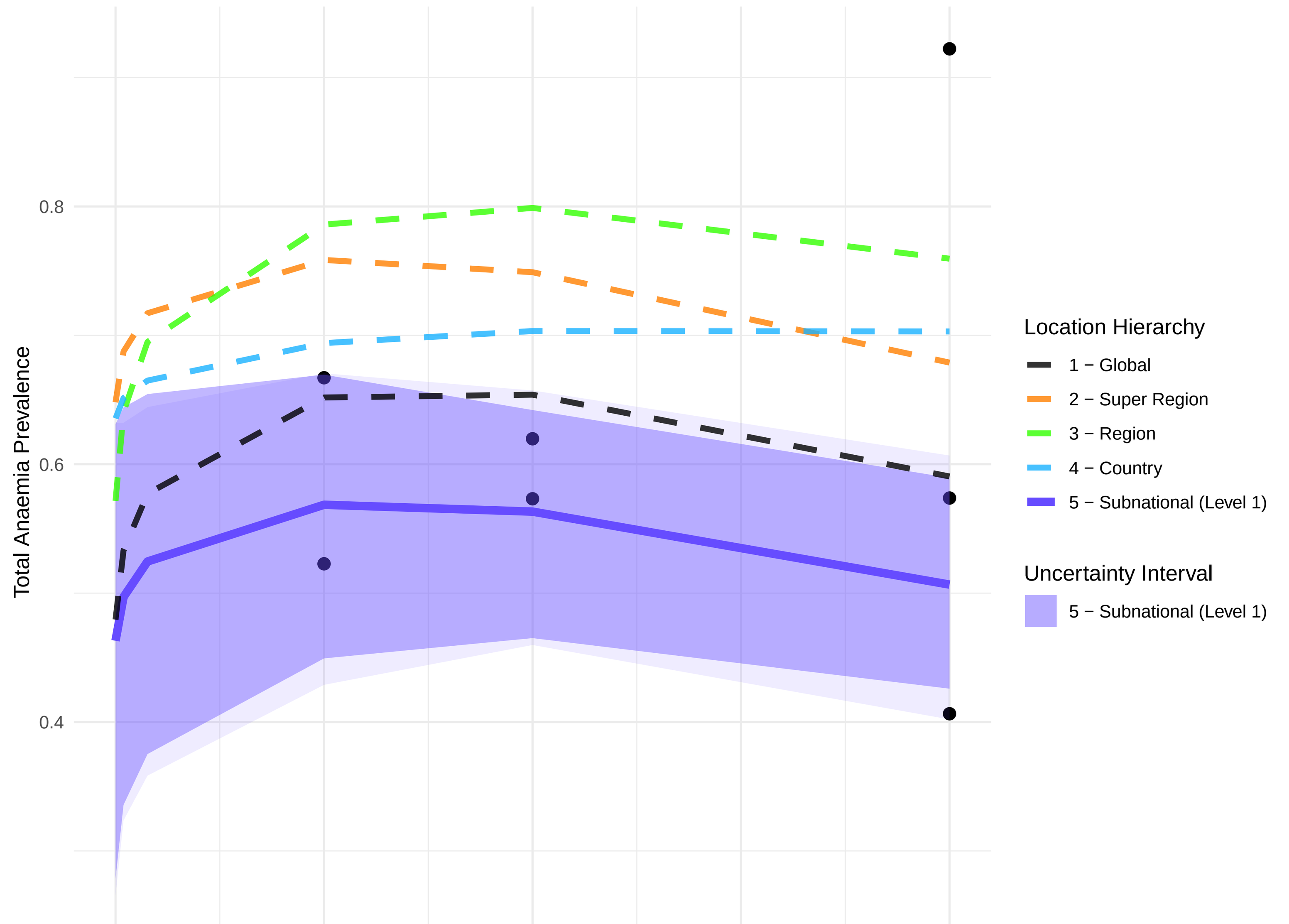

MR−BRT Age/Sex Fits − Males <2 Years Old − Total Anaemia Prevalence − Ondo
WHO−2024 Adjustment Method

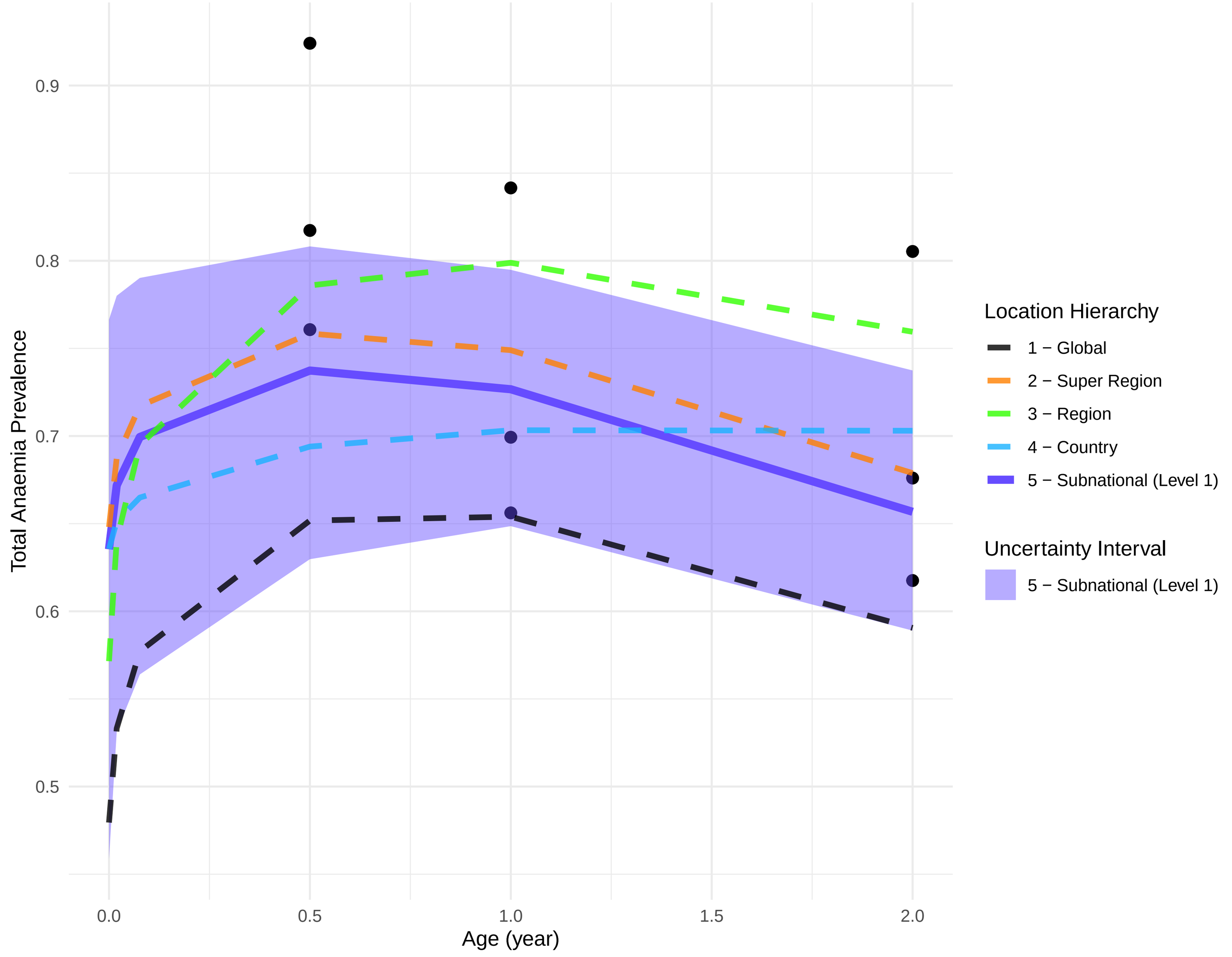

MR−BRT Age/Sex Fits − Males <2 Years Old − Total Anaemia Prevalence − Osun
WHO−2024 Adjustment Method

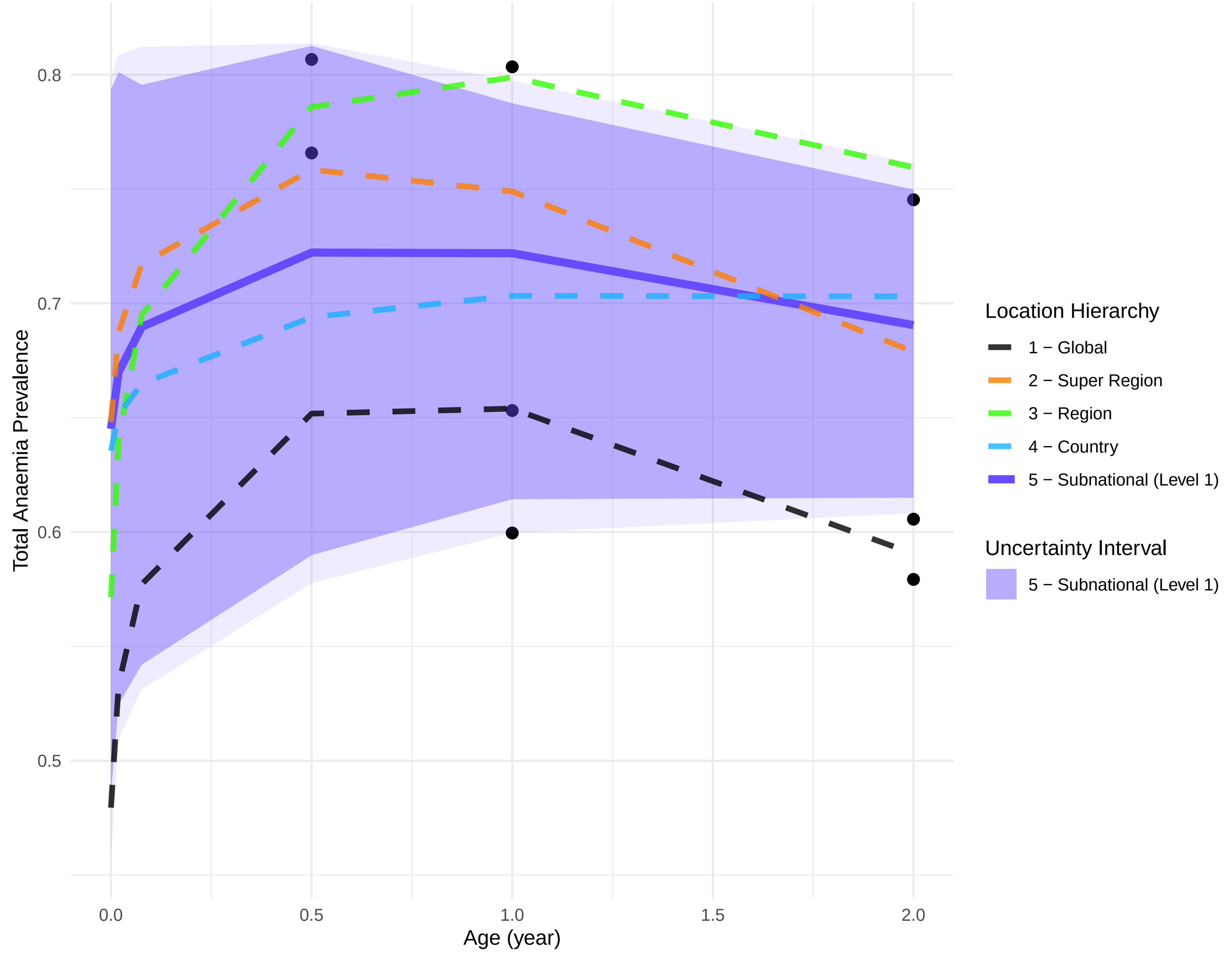

MR−BRT Age/Sex Fits − Males <2 Years Old − Total Anaemia Prevalence − Oyo
WHO−2024 Adjustment Method

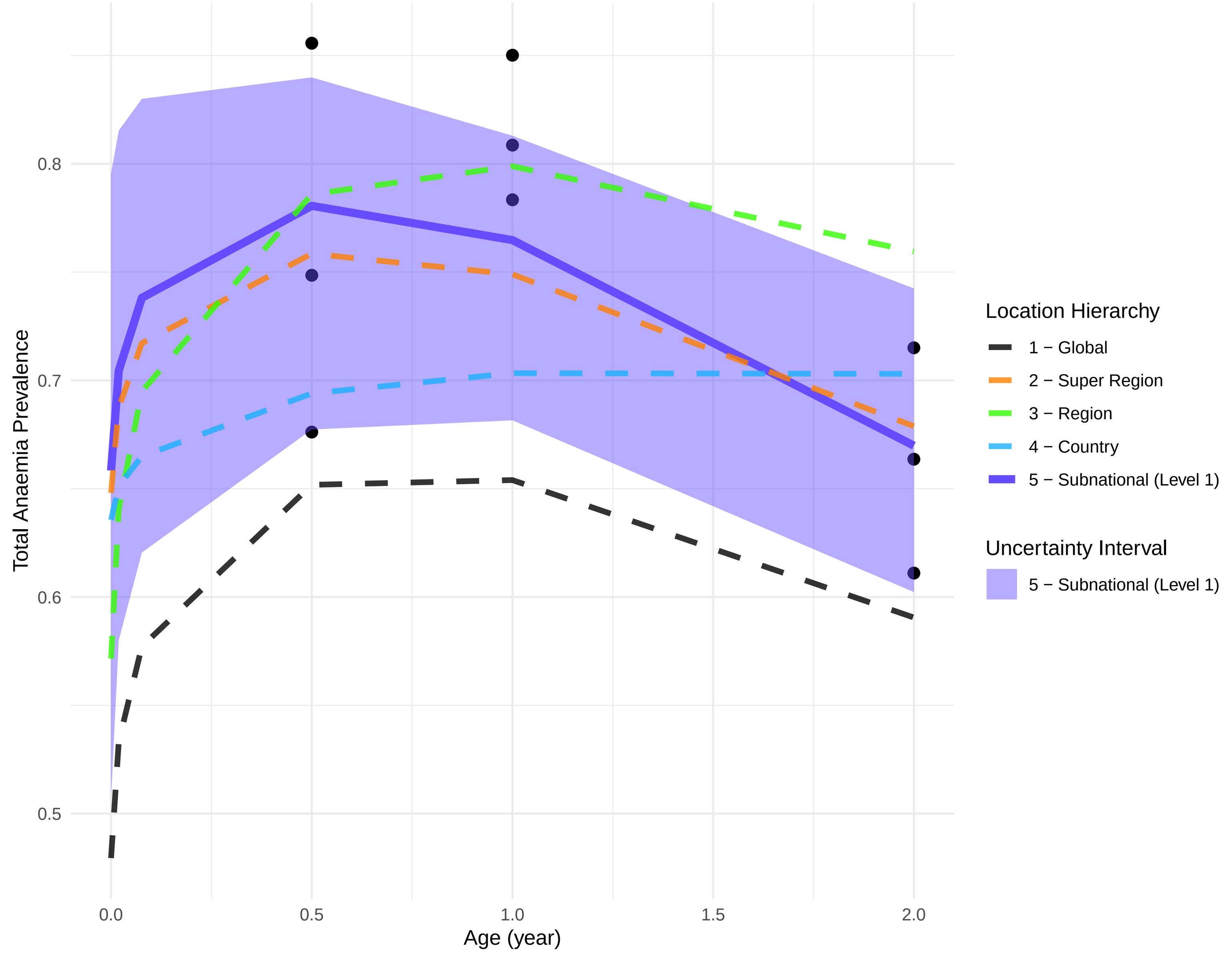

MR−BRT Age/Sex Fits − Males <2 Years Old − Total Anaemia Prevalence − Plateau
WHO−2024 Adjustment Method

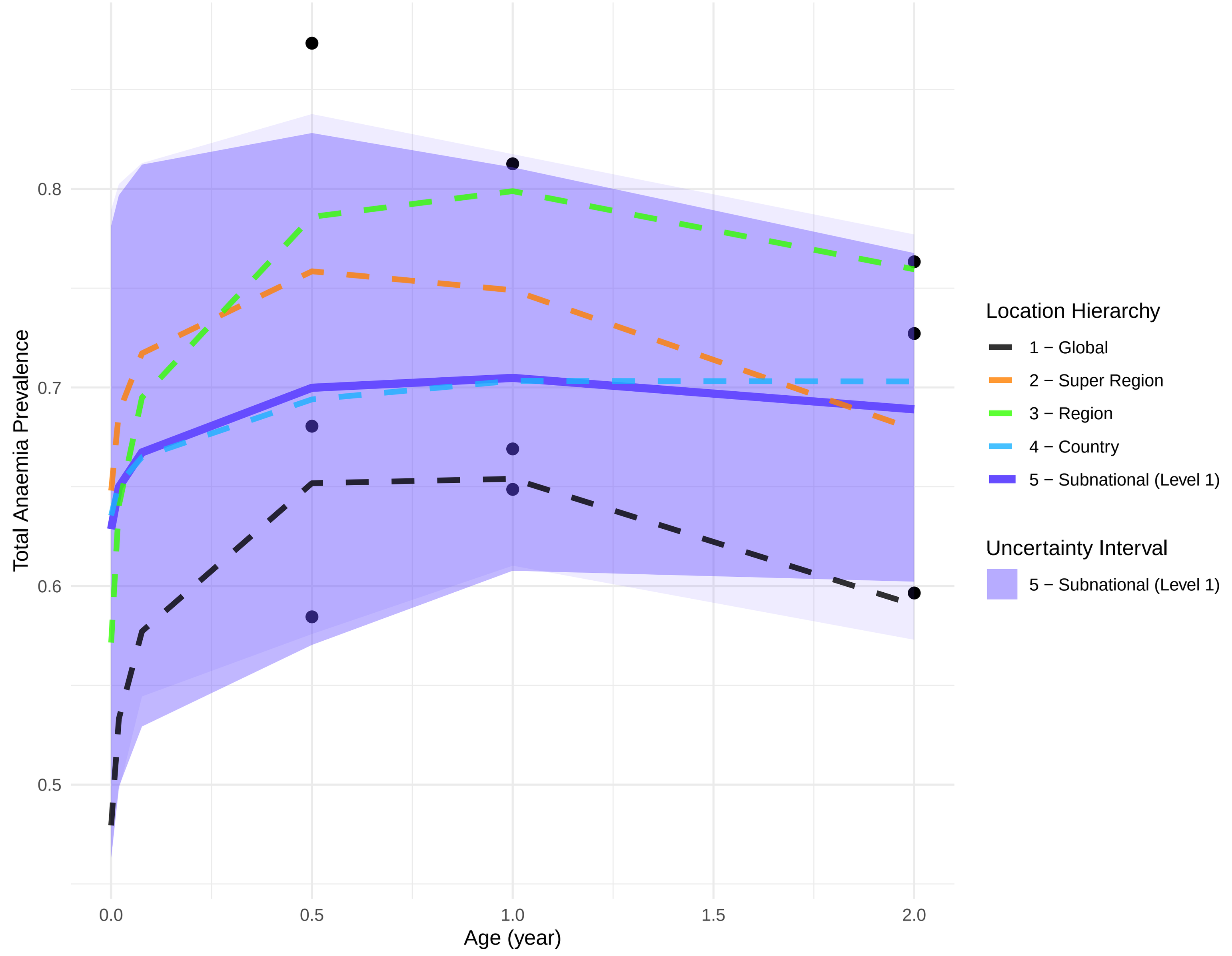

MR−BRT Age/Sex Fits − Males <2 Years Old − Total Anaemia Prevalence − Rivers
WHO−2024 Adjustment Method

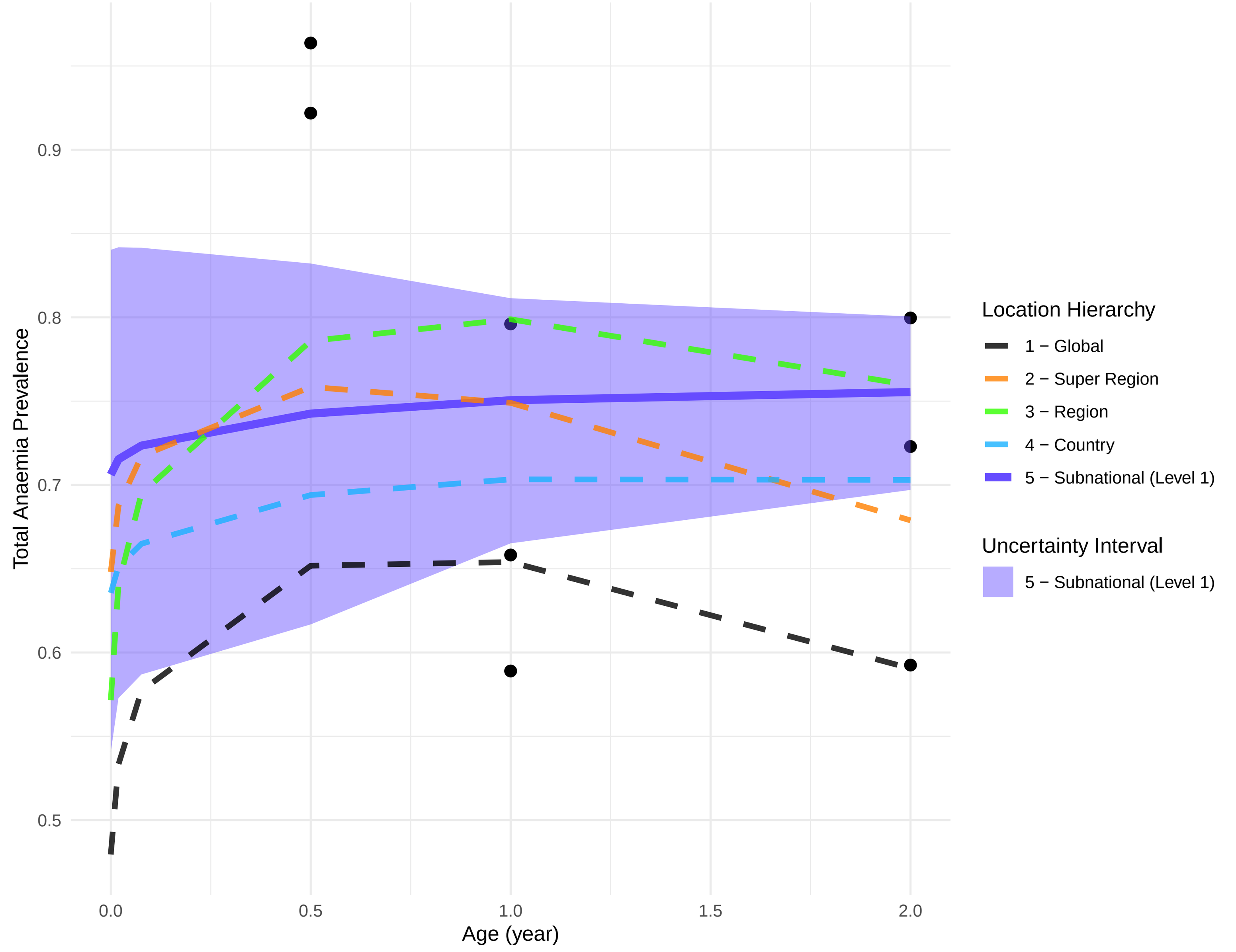

MR−BRT Age/Sex Fits − Males <2 Years Old − Total Anaemia Prevalence − Sokoto
WHO−2024 Adjustment Method

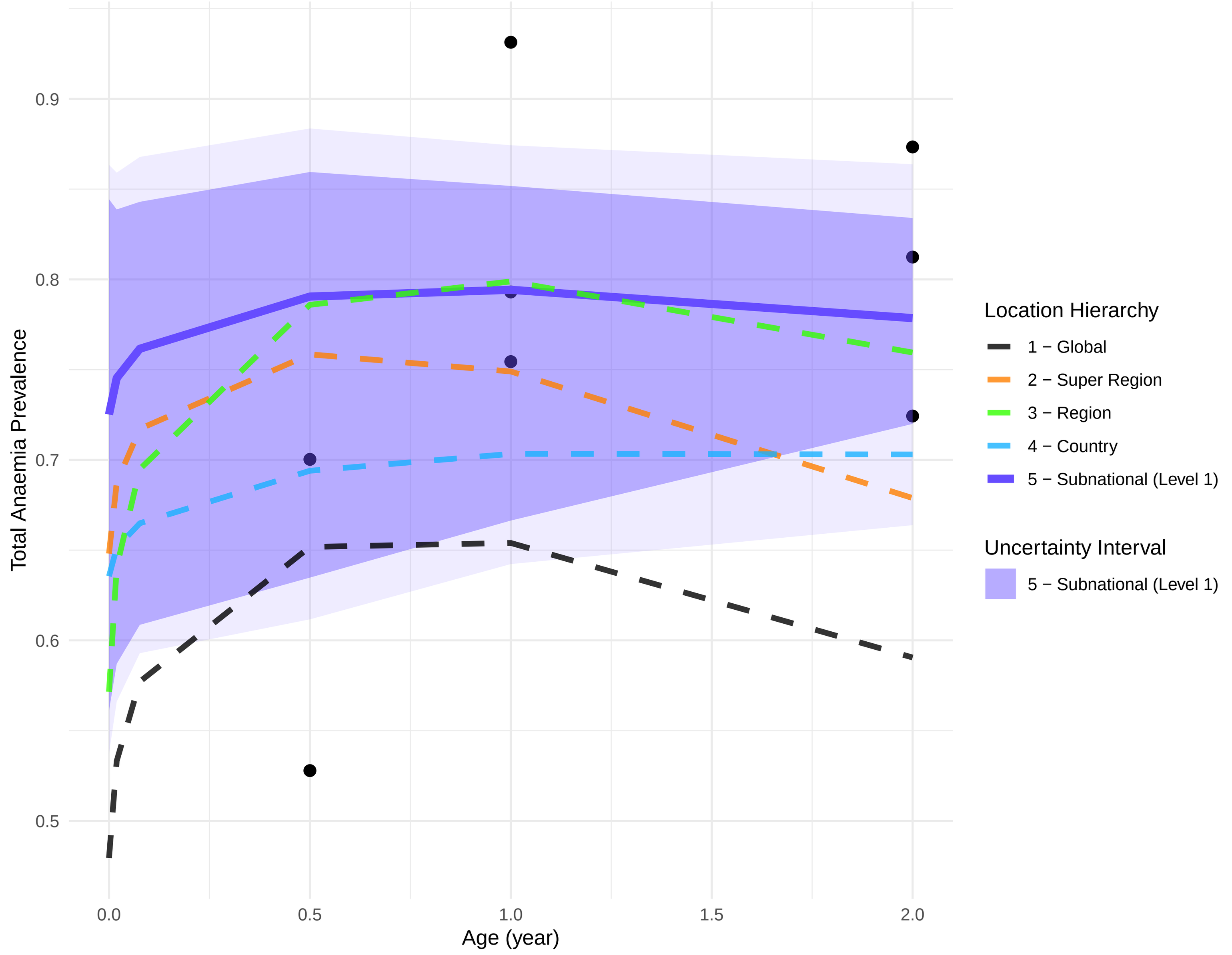

MR−BRT Age/Sex Fits − Males <2 Years Old − Total Anaemia Prevalence − Taraba
WHO−2024 Adjustment Method

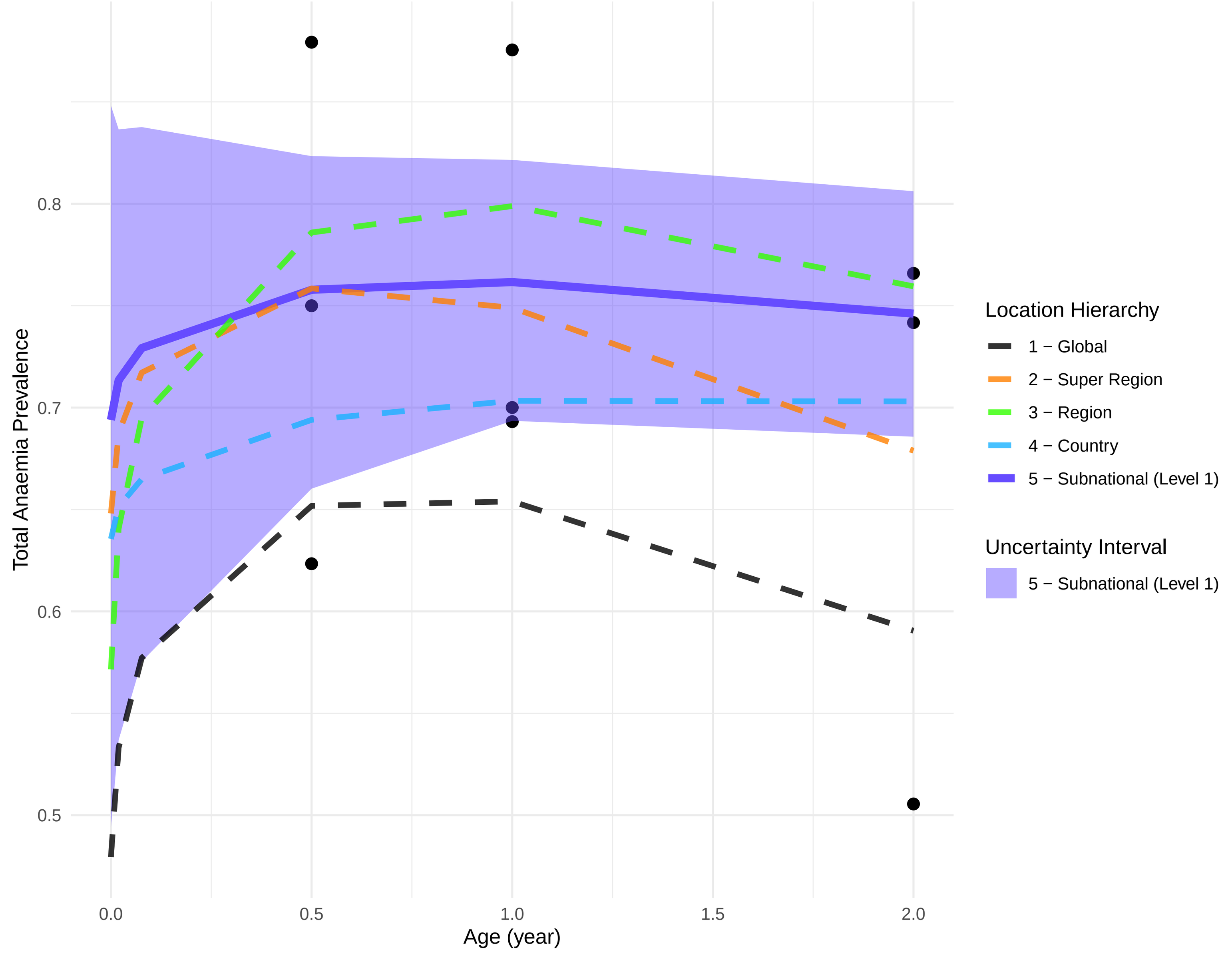

MR−BRT Age/Sex Fits − Males <2 Years Old − Total Anaemia Prevalence − Yobe
WHO−2024 Adjustment Method

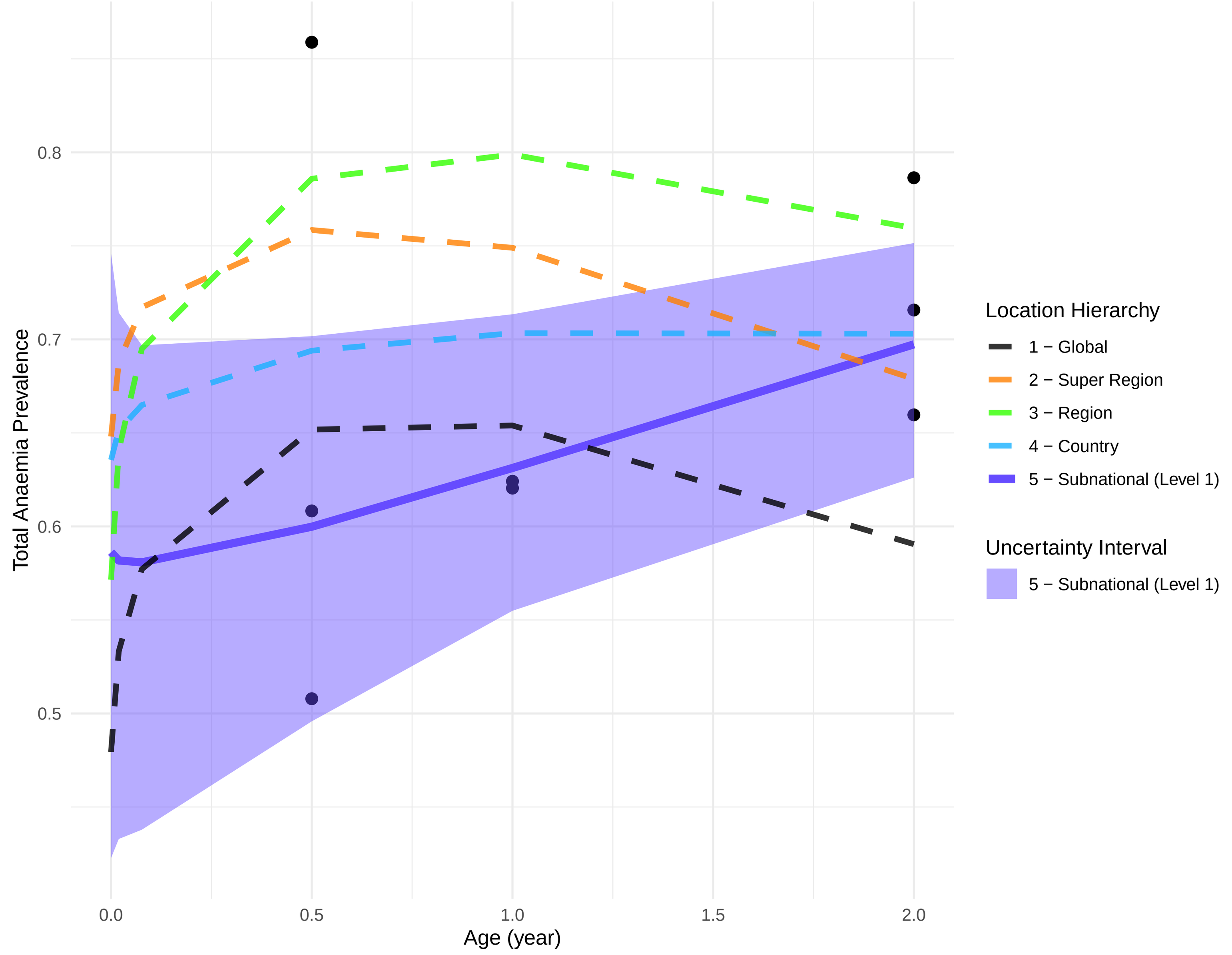

MR−BRT Age/Sex Fits − Males <2 Years Old − Total Anaemia Prevalence − Zamfara
WHO−2024 Adjustment Method

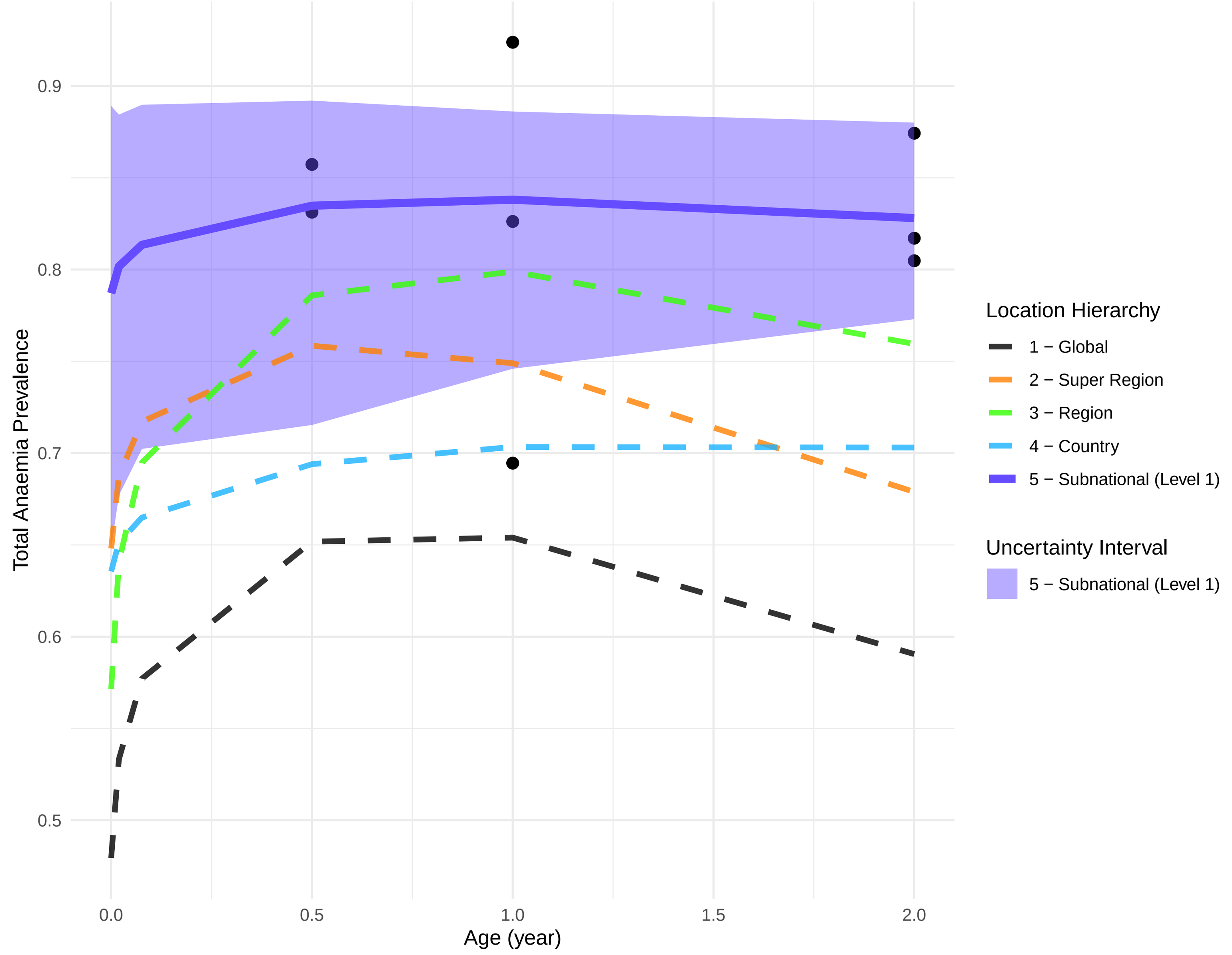

MR−BRT Age/Sex Fits − Males <2 Years Old − Total Anaemia Prevalence − Senegal
WHO−2024 Adjustment Method

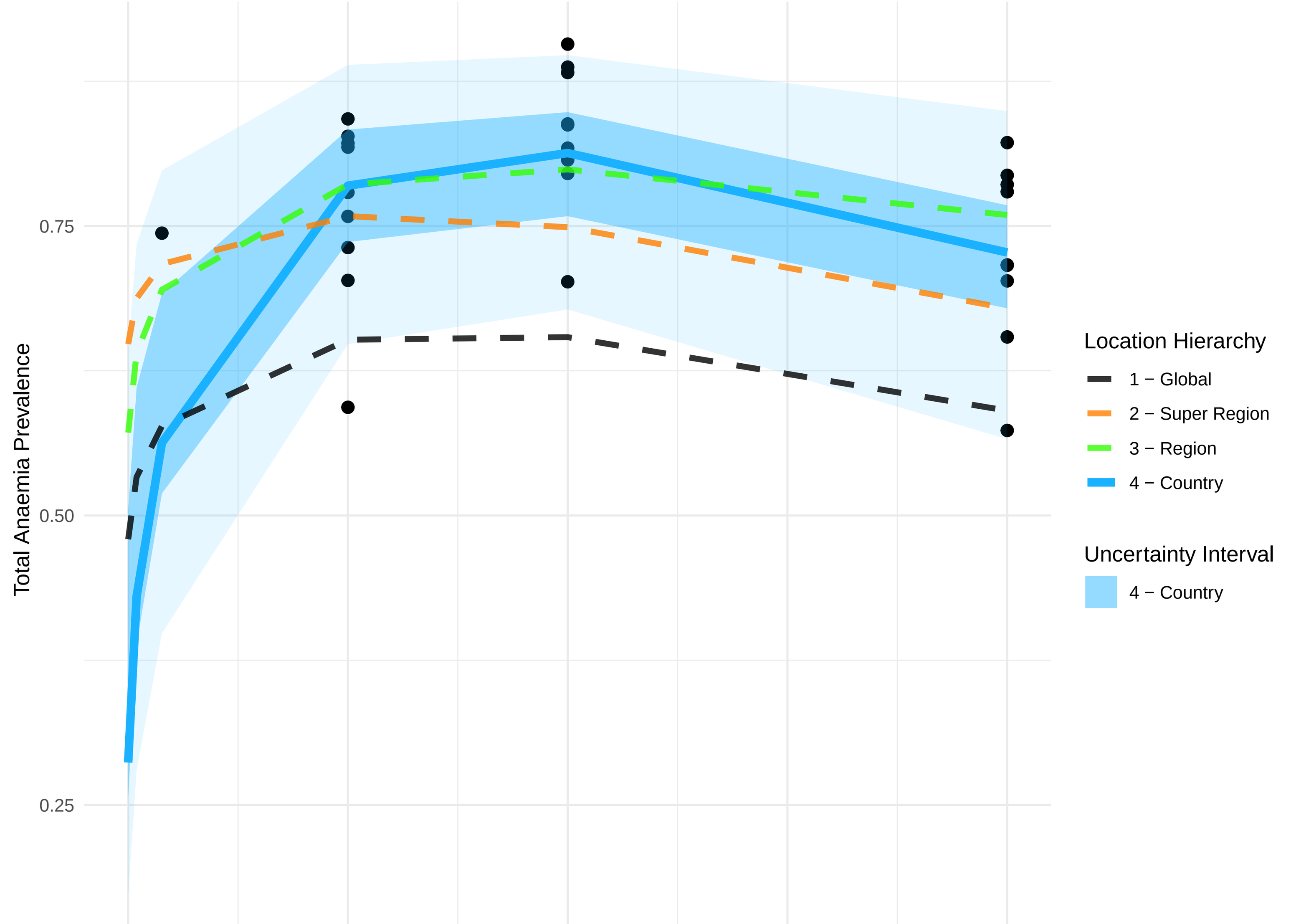

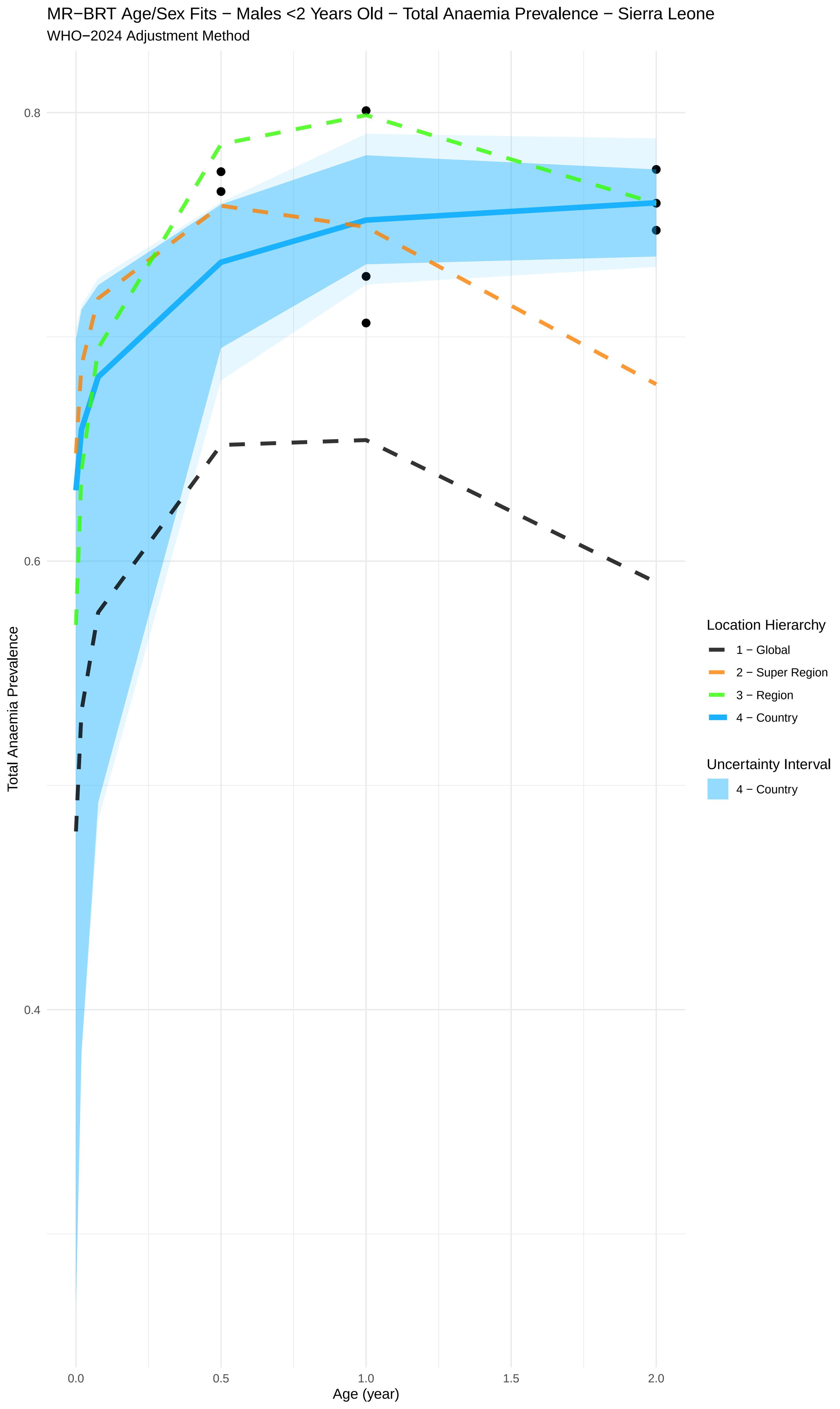
MR−BRT Age/Sex Fits − Males <2 Years Old − Total Anaemia Prevalence − Sierra Leone
WHO−2024 Adjustment Method
Total Anaemia Prevalence
Age (year)
Location Hierarchy
1 − Global
2 − Super Region
3 − Region
4 − Country
Uncertainty Interval
4 − Country

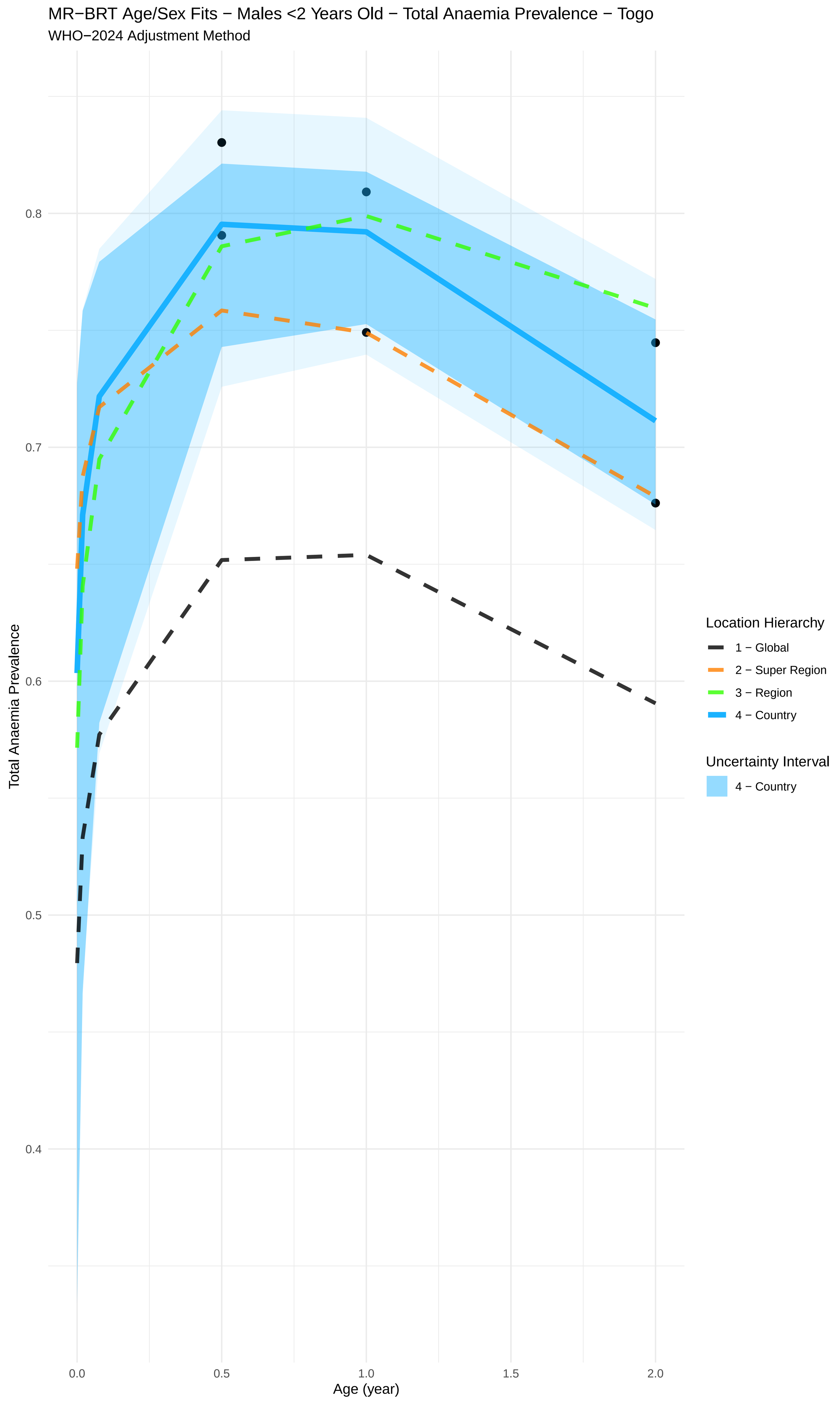
MR−BRT Age/Sex Fits − Males <2 Years Old − Total Anaemia Prevalence − Togo
WHO−2024 Adjustment Method
Total Anaemia Prevalence
Age (year)
Location Hierarchy
1 − Global
2 − Super Region
3 − Region
4 − Country
Uncertainty Interval
4 − Country

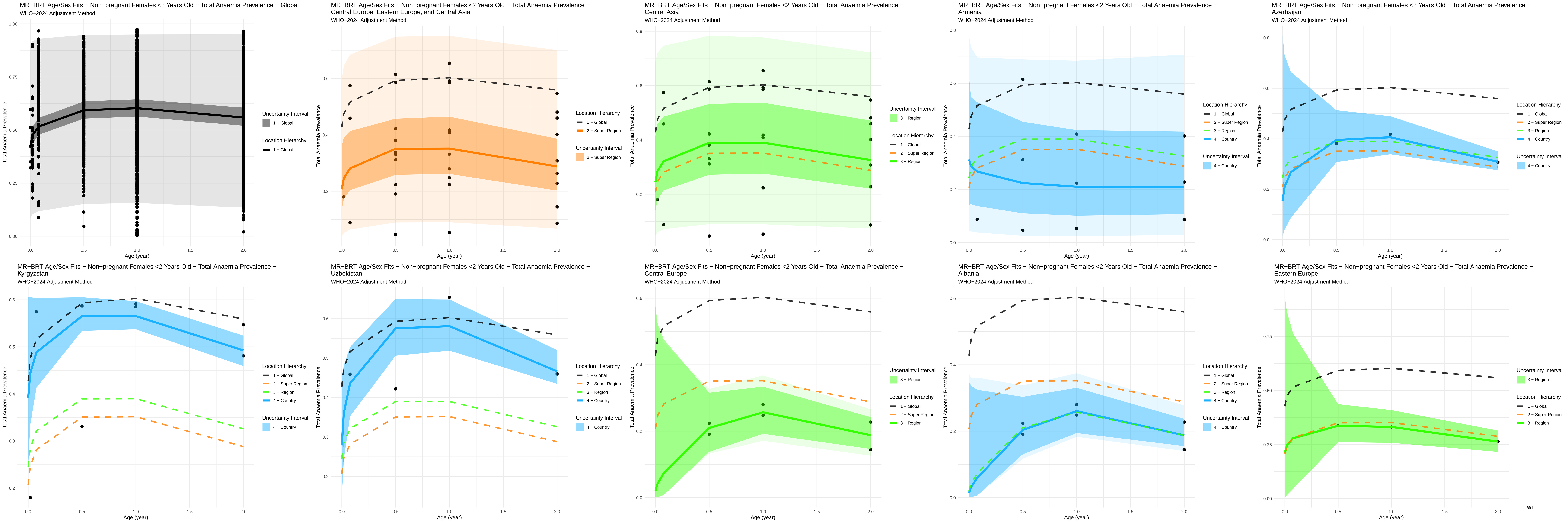
MR−BRT Age/Sex Fits − Non−pregnant Females <2 Years Old − Total Anaemia Prevalence − Global
WHO−2024 Adjustment Method
Uncertainty Interval
1 − Global
Location Hierarchy
1 − Global
MR−BRT Age/Sex Fits − Non−pregnant Females <2 Years Old − Total Anaemia Prevalence − Central Europe, Eastern Europe, and Central Asia
WHO−2024 Adjustment Method
Location Hierarchy
1 − Global
2 − Super Region
Uncertainty Interval
2 − Super Region
MR−BRT Age/Sex Fits − Non−pregnant Females <2 Years Old − Total Anaemia Prevalence − Central Asia
WHO−2024 Adjustment Method
Uncertainty Interval
3 − Region
Location Hierarchy
1 − Global
2 − Super Region
3 − Region
MR−BRT Age/Sex Fits − Non−pregnant Females <2 Years Old − Total Anaemia Prevalence − Armenia
WHO−2024 Adjustment Method
Location Hierarchy
1 − Global
2 − Super Region
3 − Region
4 − Country
Uncertainty Interval
4 − Country
MR−BRT Age/Sex Fits − Non−pregnant Females <2 Years Old − Total Anaemia Prevalence − Azerbaijan
WHO−2024 Adjustment Method
Location Hierarchy
1 − Global
2 − Super Region
3 − Region
4 − Country
Uncertainty Interval
4 − Country
MR−BRT Age/Sex Fits − Non−pregnant Females <2 Years Old − Total Anaemia Prevalence − Kyrgyzstan
WHO−2024 Adjustment Method
Location Hierarchy
1 − Global
2 − Super Region
3 − Region
4 − Country
Uncertainty Interval
4 − Country
MR−BRT Age/Sex Fits − Non−pregnant Females <2 Years Old − Total Anaemia Prevalence − Uzbekistan
WHO−2024 Adjustment Method
Location Hierarchy
1 − Global
2 − Super Region
3 − Region
4 − Country
Uncertainty Interval
4 − Country
MR−BRT Age/Sex Fits − Non−pregnant Females <2 Years Old − Total Anaemia Prevalence − Central Europe
WHO−2024 Adjustment Method
Uncertainty Interval
3 − Region
Location Hierarchy
1 − Global
2 − Super Region
3 − Region
MR−BRT Age/Sex Fits − Non−pregnant Females <2 Years Old − Total Anaemia Prevalence − Albania
WHO−2024 Adjustment Method
Location Hierarchy
1 − Global
2 − Super Region
3 − Region
4 − Country
Uncertainty Interval
4 − Country
MR−BRT Age/Sex Fits − Non−pregnant Females <2 Years Old − Total Anaemia Prevalence − Eastern Europe
WHO−2024 Adjustment Method
Uncertainty Interval
3 − Region
Location Hierarchy
1 − Global
2 − Super Region
3 − Region
Total Anaemia Prevalence
Age (year)

MR−BRT Age/Sex Fits − Non−pregnant Females <2 Years Old − Total Anaemia Prevalence − Republic of Moldova
WHO−2024 Adjustment Method

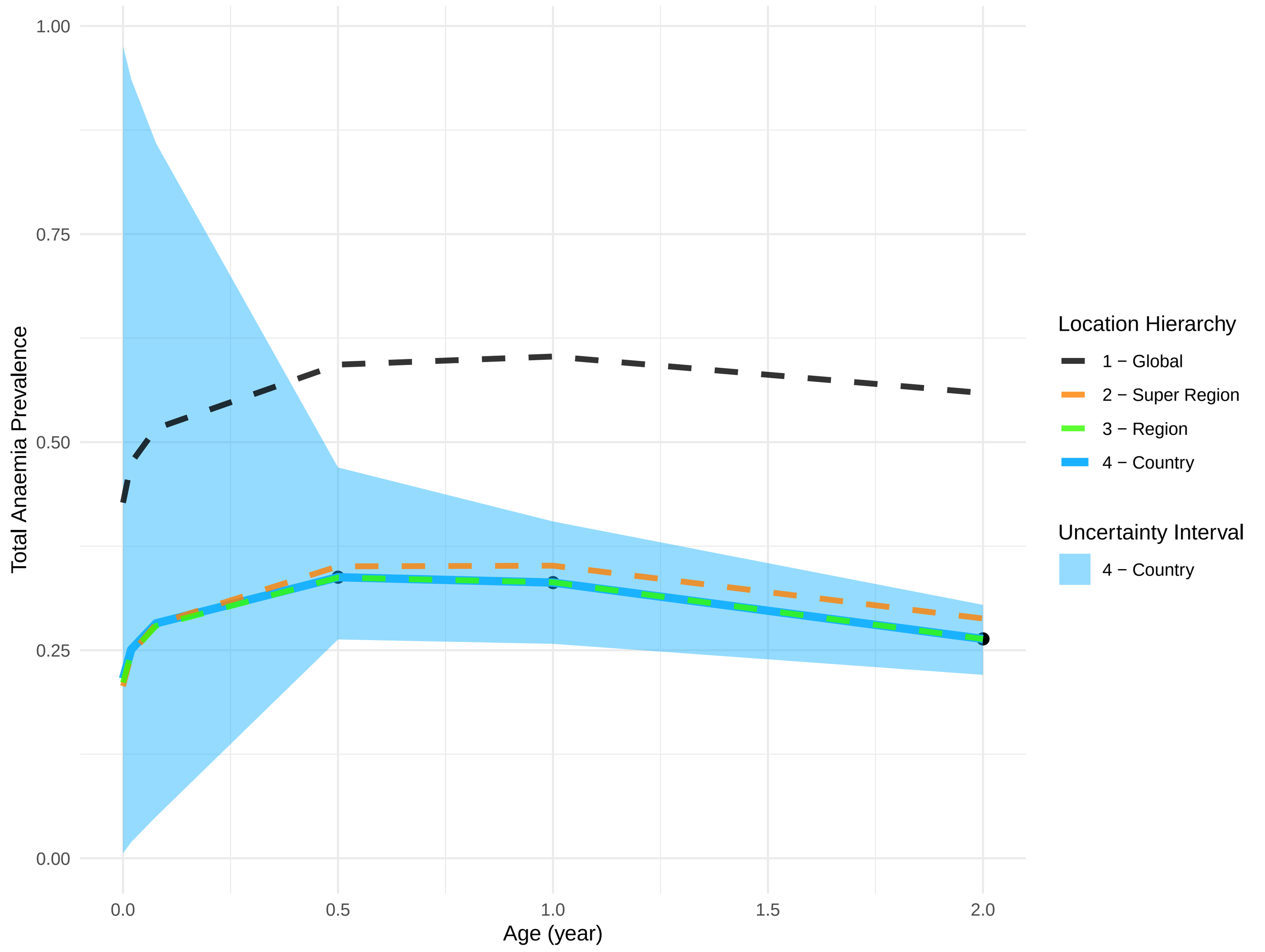

MR−BRT Age/Sex Fits − Non−pregnant Females <2 Years Old − Total Anaemia Prevalence − High−income
WHO−2024 Adjustment Method

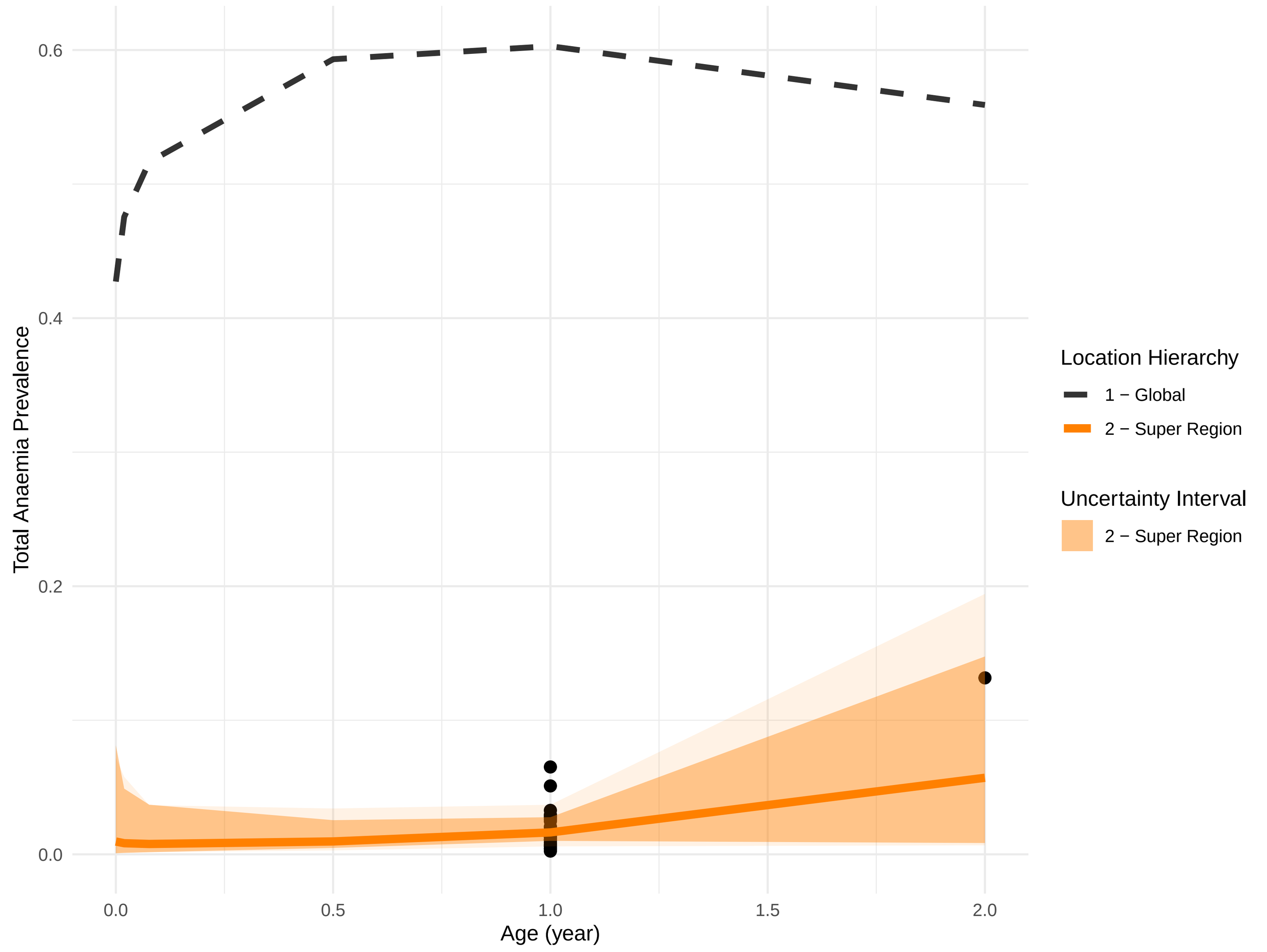

MR−BRT Age/Sex Fits − Non−pregnant Females <2 Years Old − Total Anaemia Prevalence − Western Europe
WHO−2024 Adjustment Method

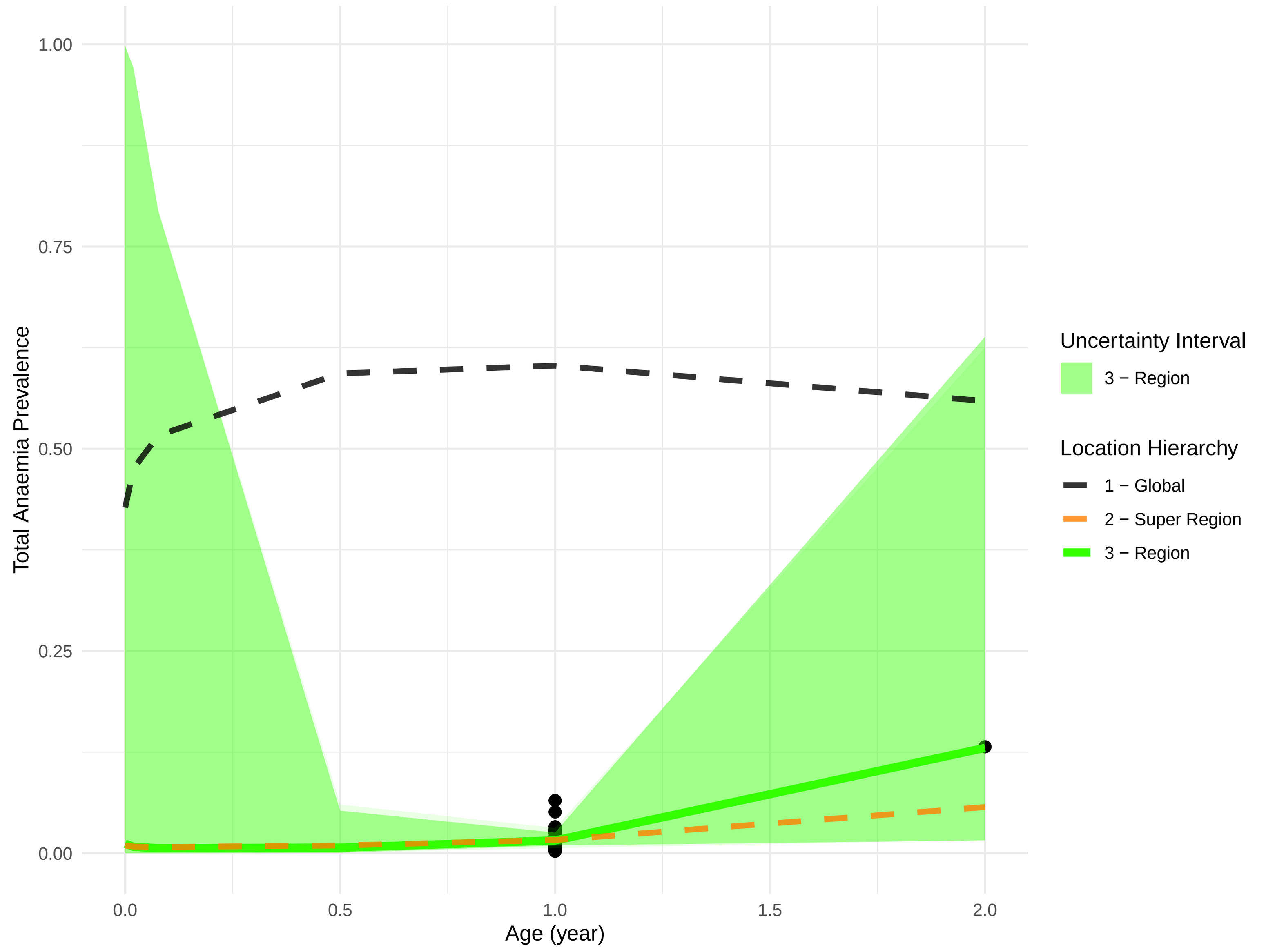

MR−BRT Age/Sex Fits − Non−pregnant Females <2 Years Old − Total Anaemia Prevalence − United Kingdom
WHO−2024 Adjustment Method

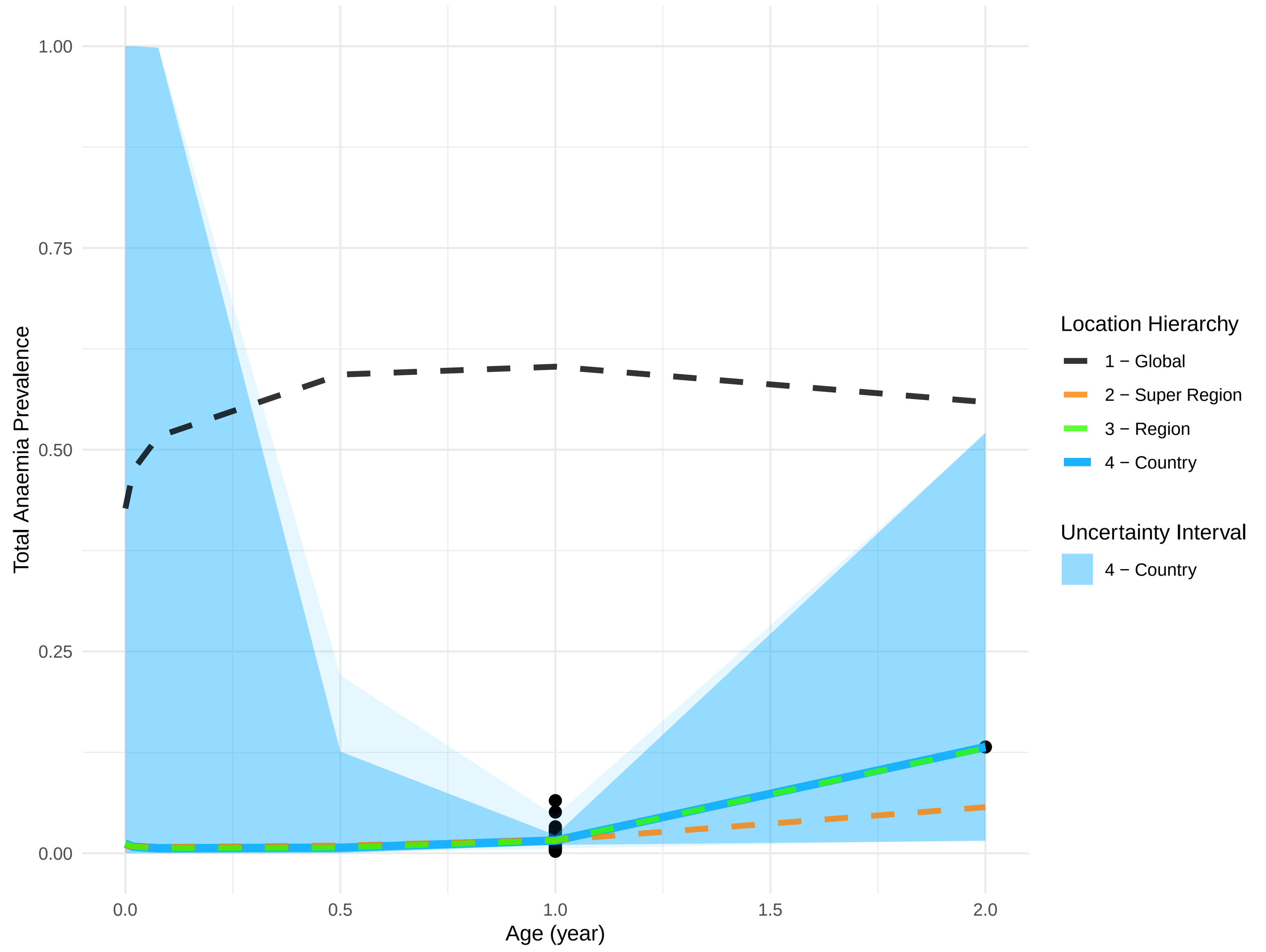

MR−BRT Age/Sex Fits − Non−pregnant Females <2 Years Old − Total Anaemia Prevalence − England
WHO−2024 Adjustment Method

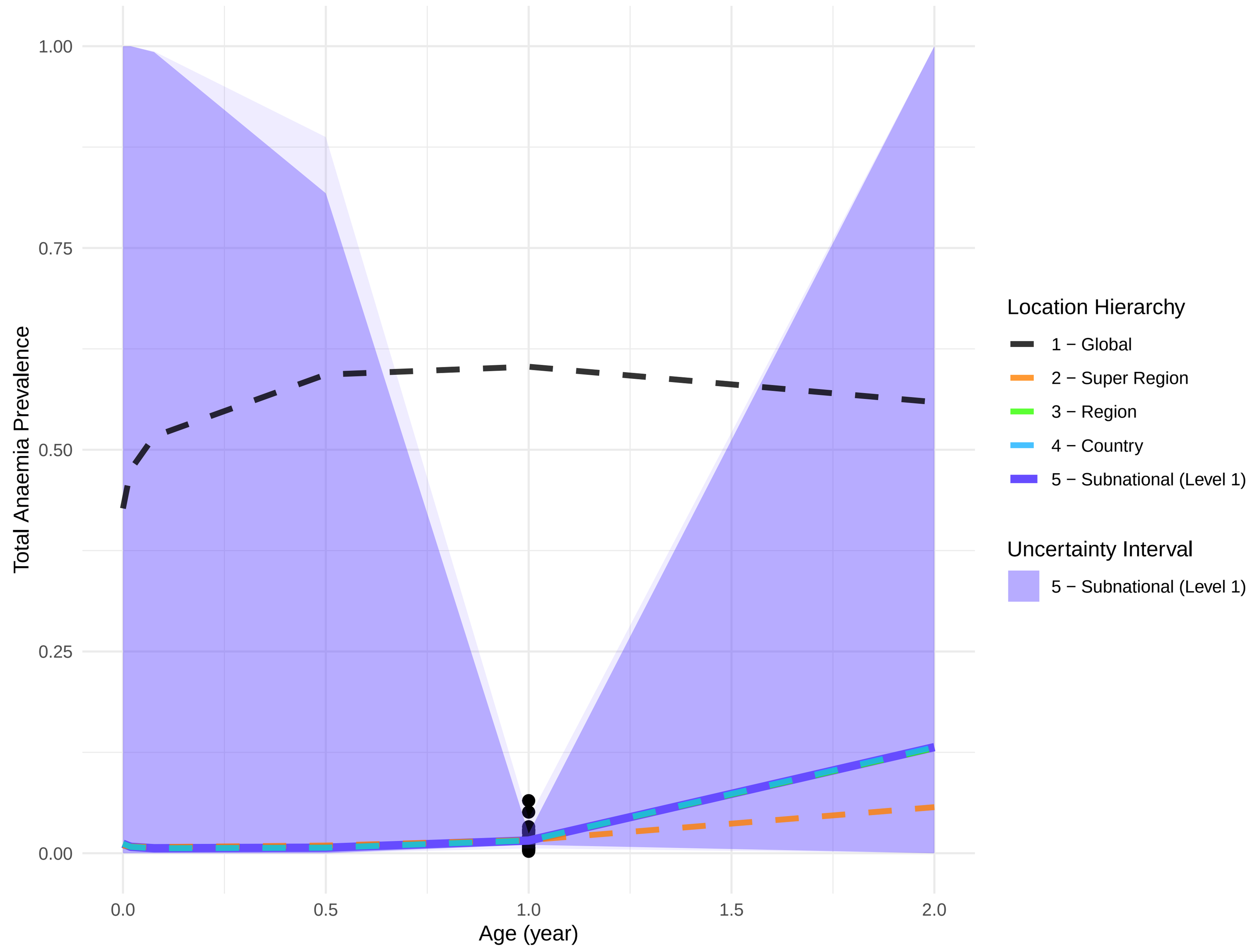

MR−BRT Age/Sex Fits − Non−pregnant Females <2 Years Old − Total Anaemia Prevalence − East Midlands
WHO−2024 Adjustment Method

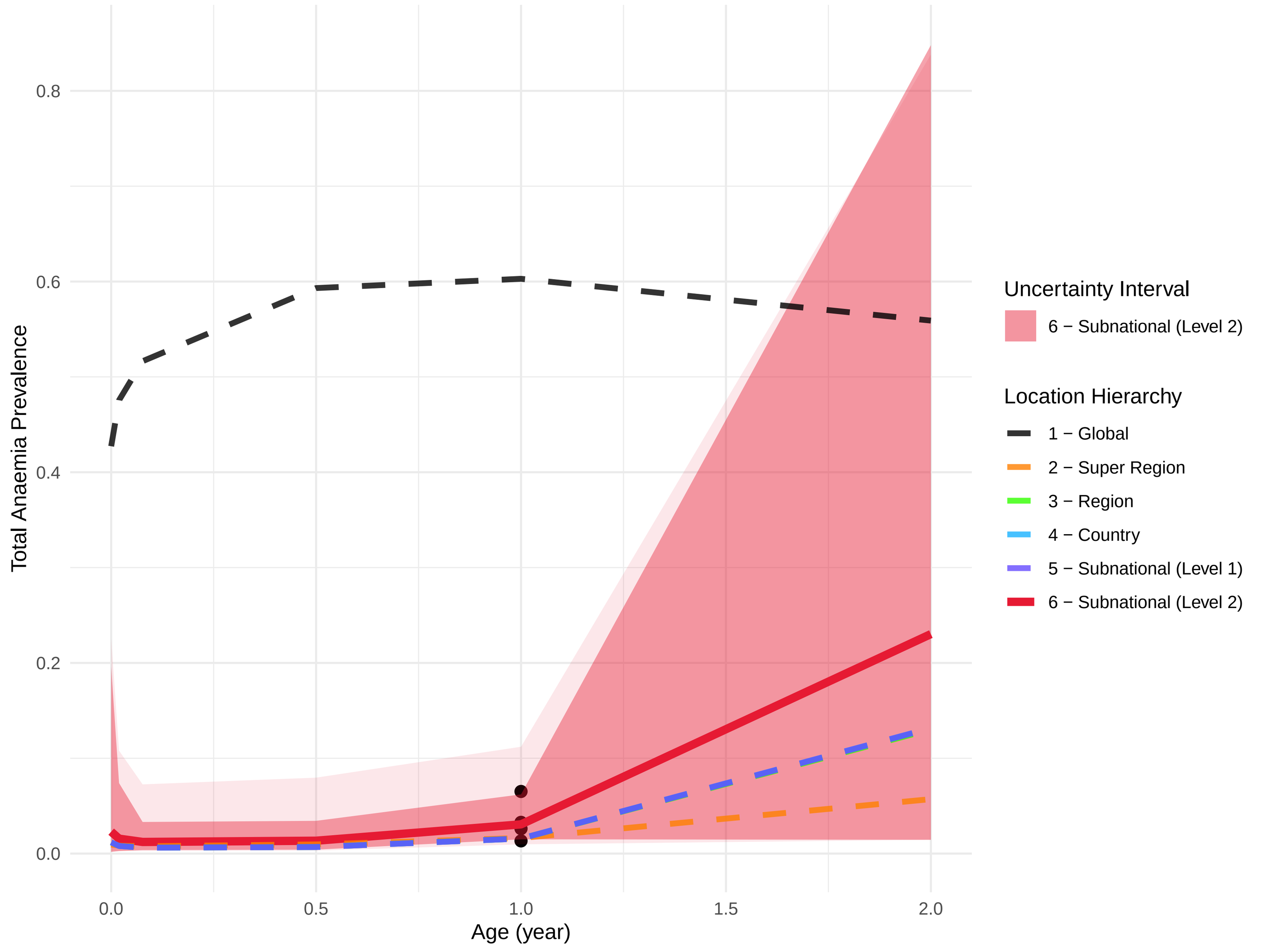

MR−BRT Age/Sex Fits − Non−pregnant Females <2 Years Old − Total Anaemia Prevalence − East of England
WHO−2024 Adjustment Method

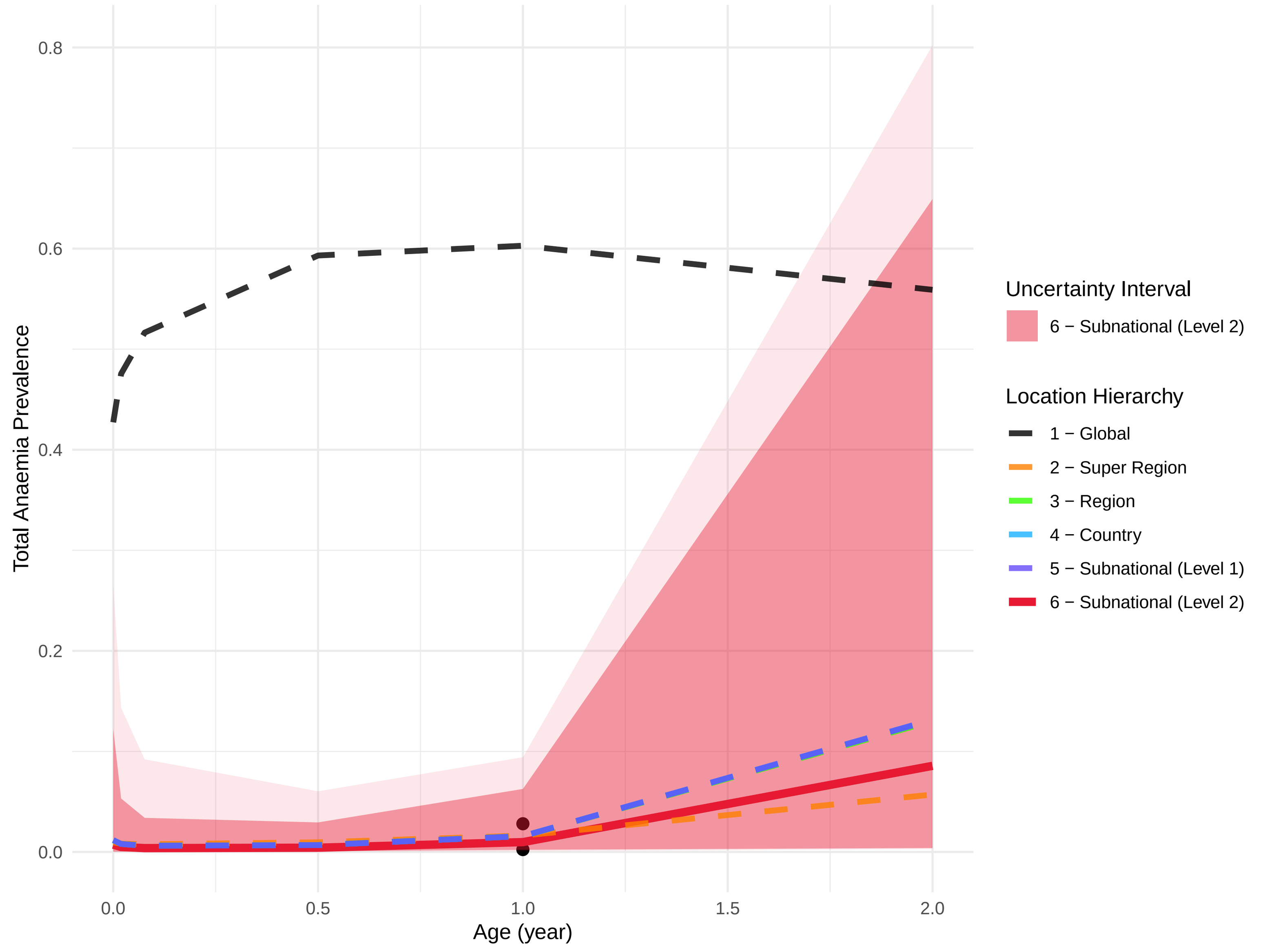

MR−BRT Age/Sex Fits − Non−pregnant Females <2 Years Old − Total Anaemia Prevalence − Greater London
WHO−2024 Adjustment Method

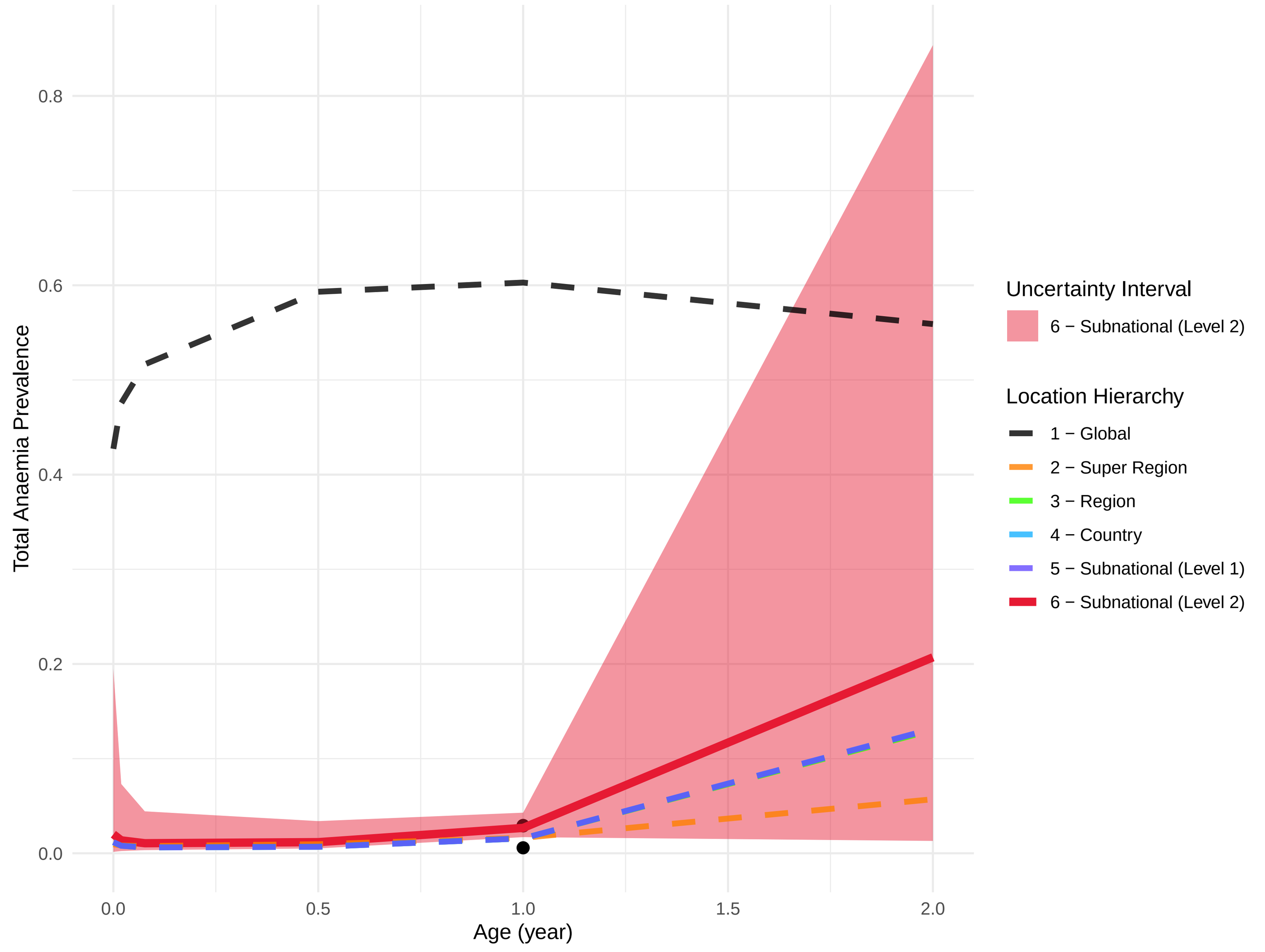

MR−BRT Age/Sex Fits − Non−pregnant Females <2 Years Old − Total Anaemia Prevalence − North East England
WHO−2024 Adjustment Method

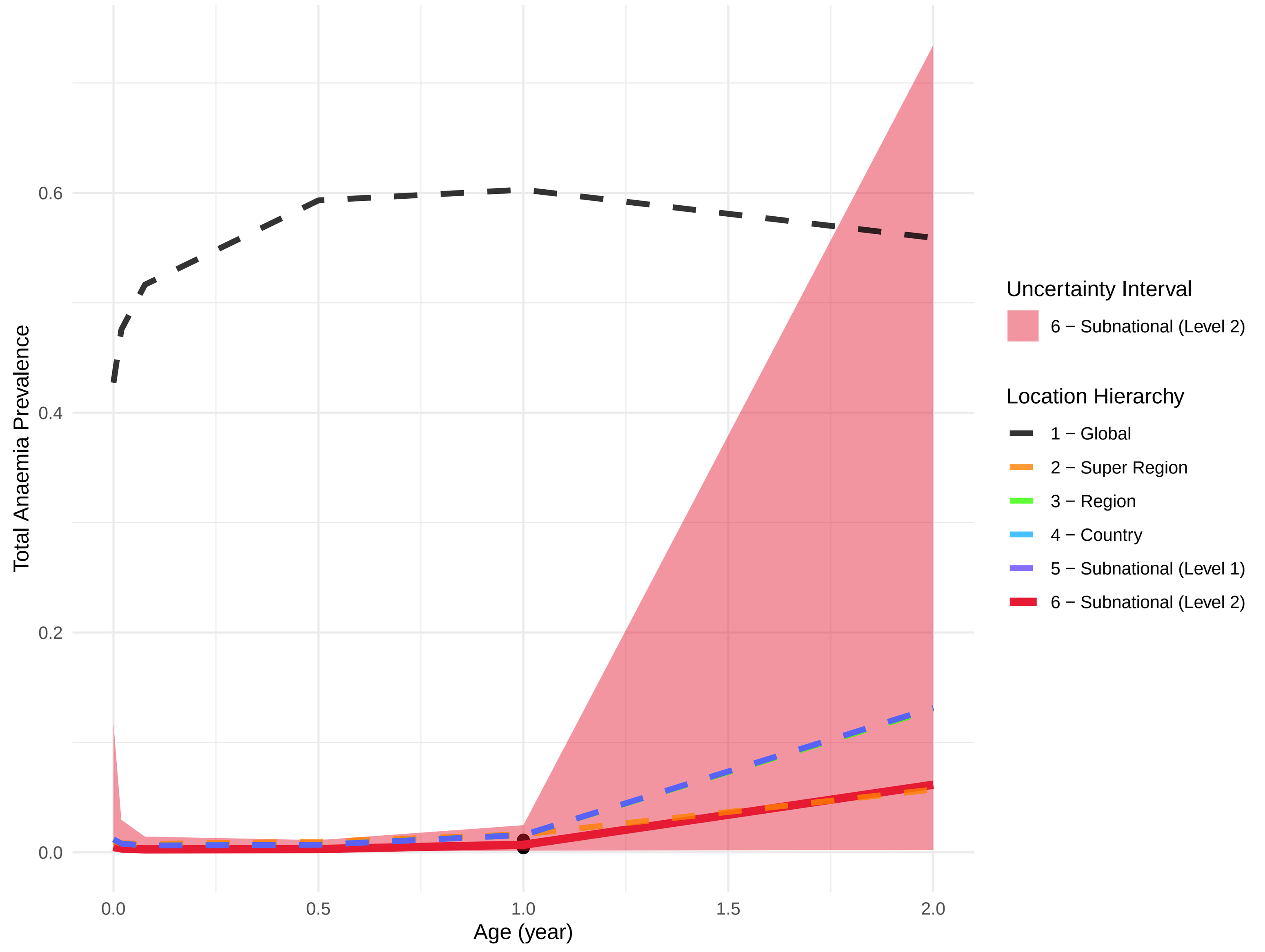

MR−BRT Age/Sex Fits − Non−pregnant Females <2 Years Old − Total Anaemia Prevalence − North West England
WHO−2024 Adjustment Method

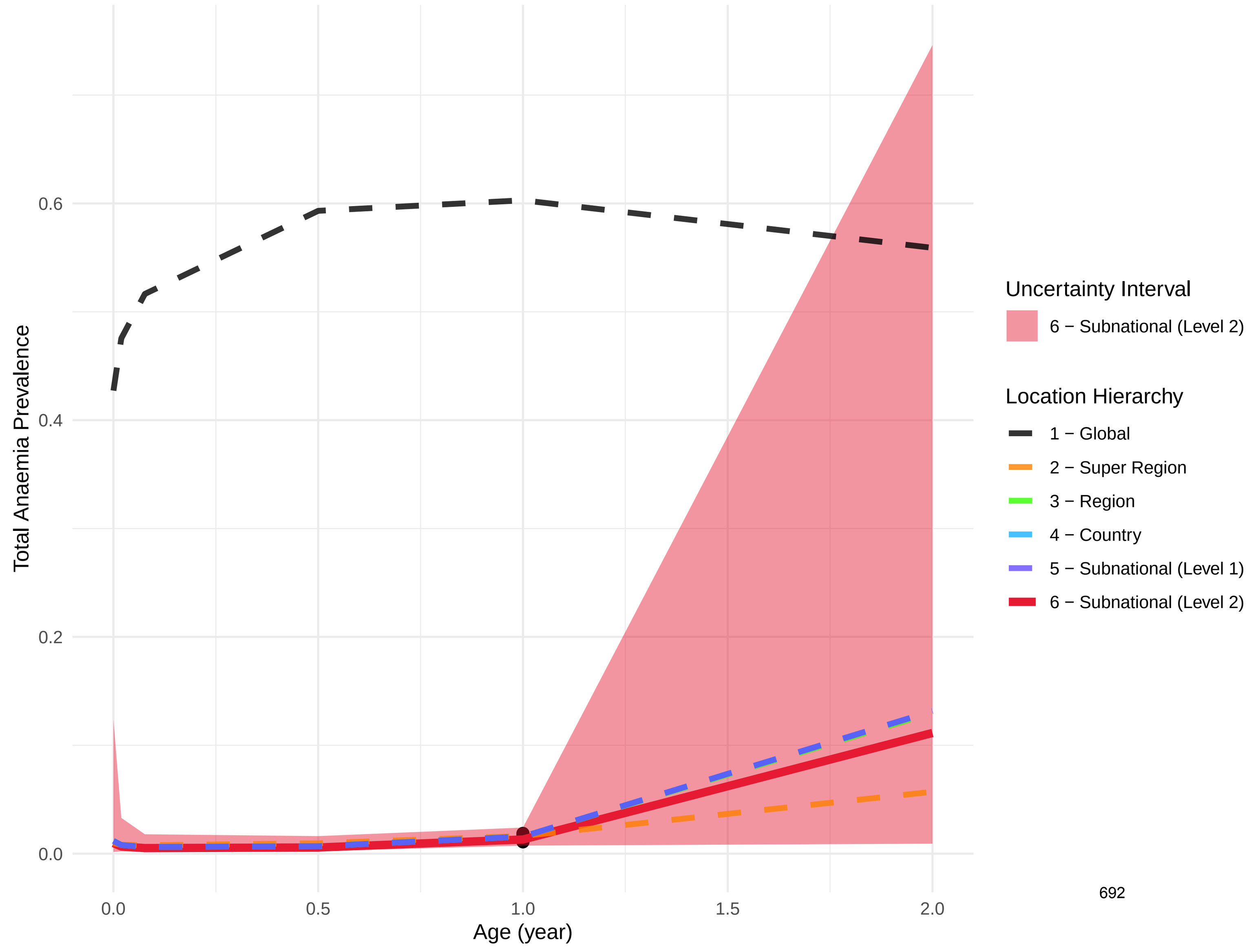

MR−BRT Age/Sex Fits − Non−pregnant Females <2 Years Old − Total Anaemia Prevalence − South East England
WHO−2024 Adjustment Method

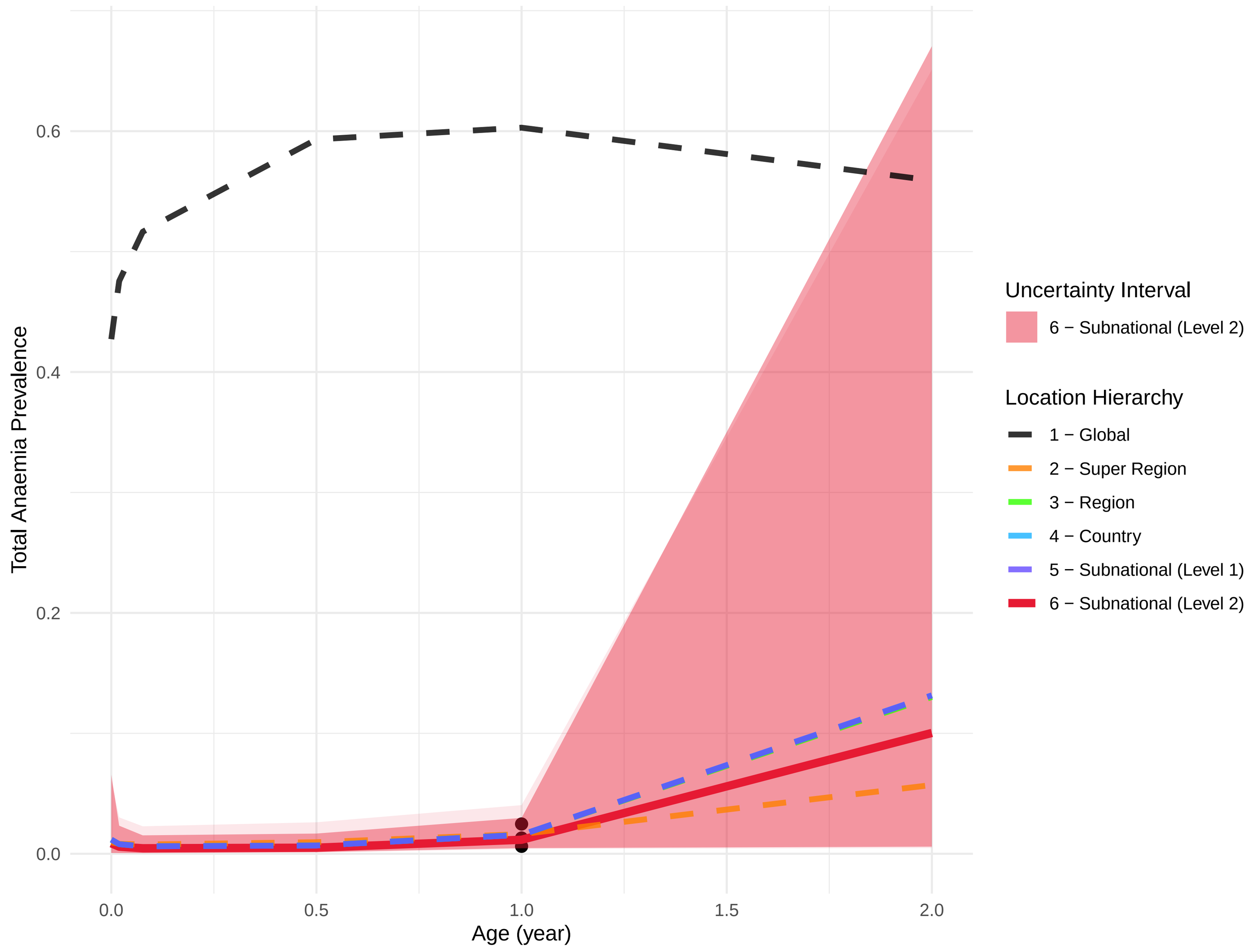

MR−BRT Age/Sex Fits − Non−pregnant Females <2 Years Old − Total Anaemia Prevalence − South West England
WHO−2024 Adjustment Method

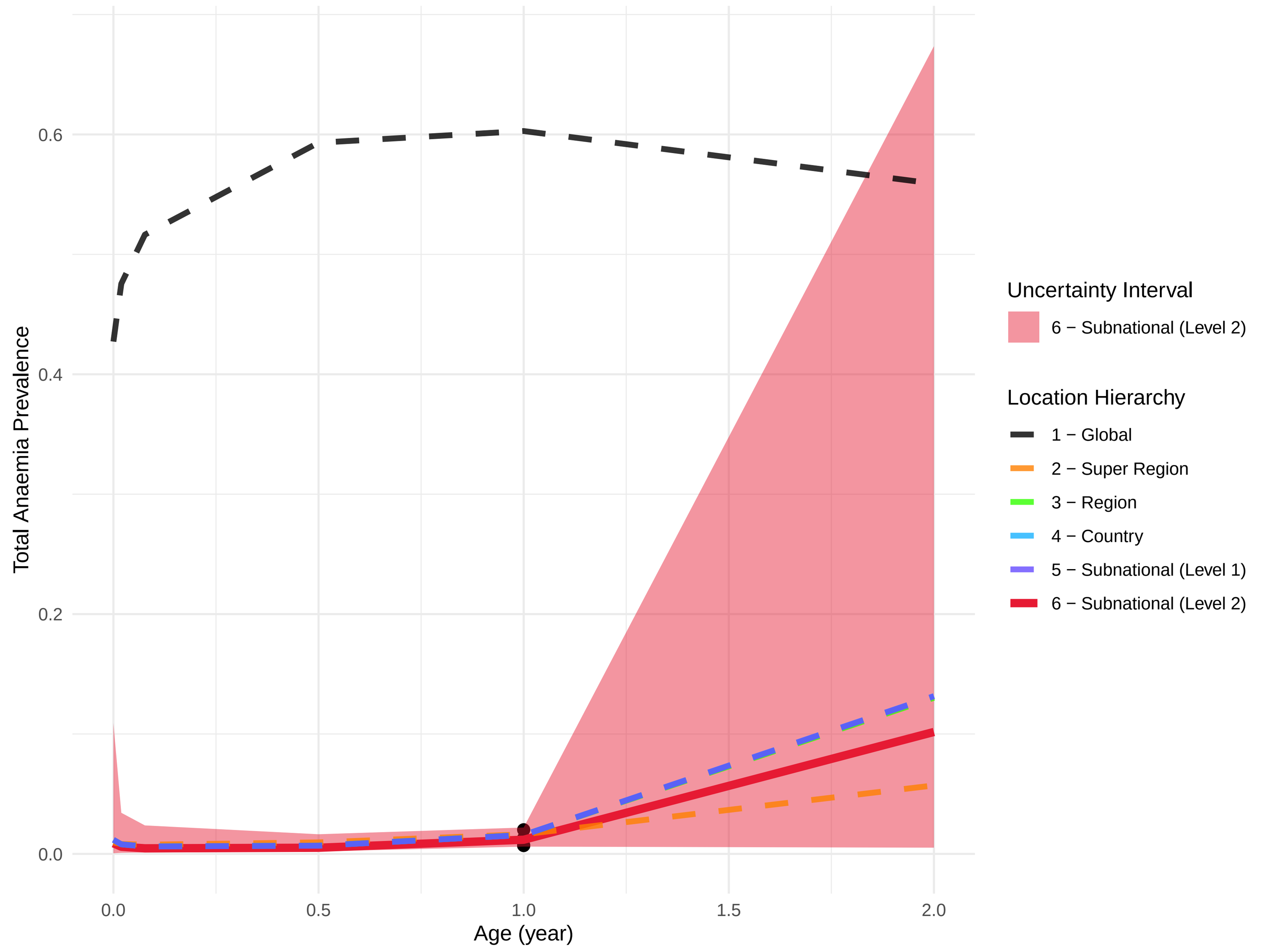

MR−BRT Age/Sex Fits − Non−pregnant Females <2 Years Old − Total Anaemia Prevalence − West Midlands
WHO−2024 Adjustment Method

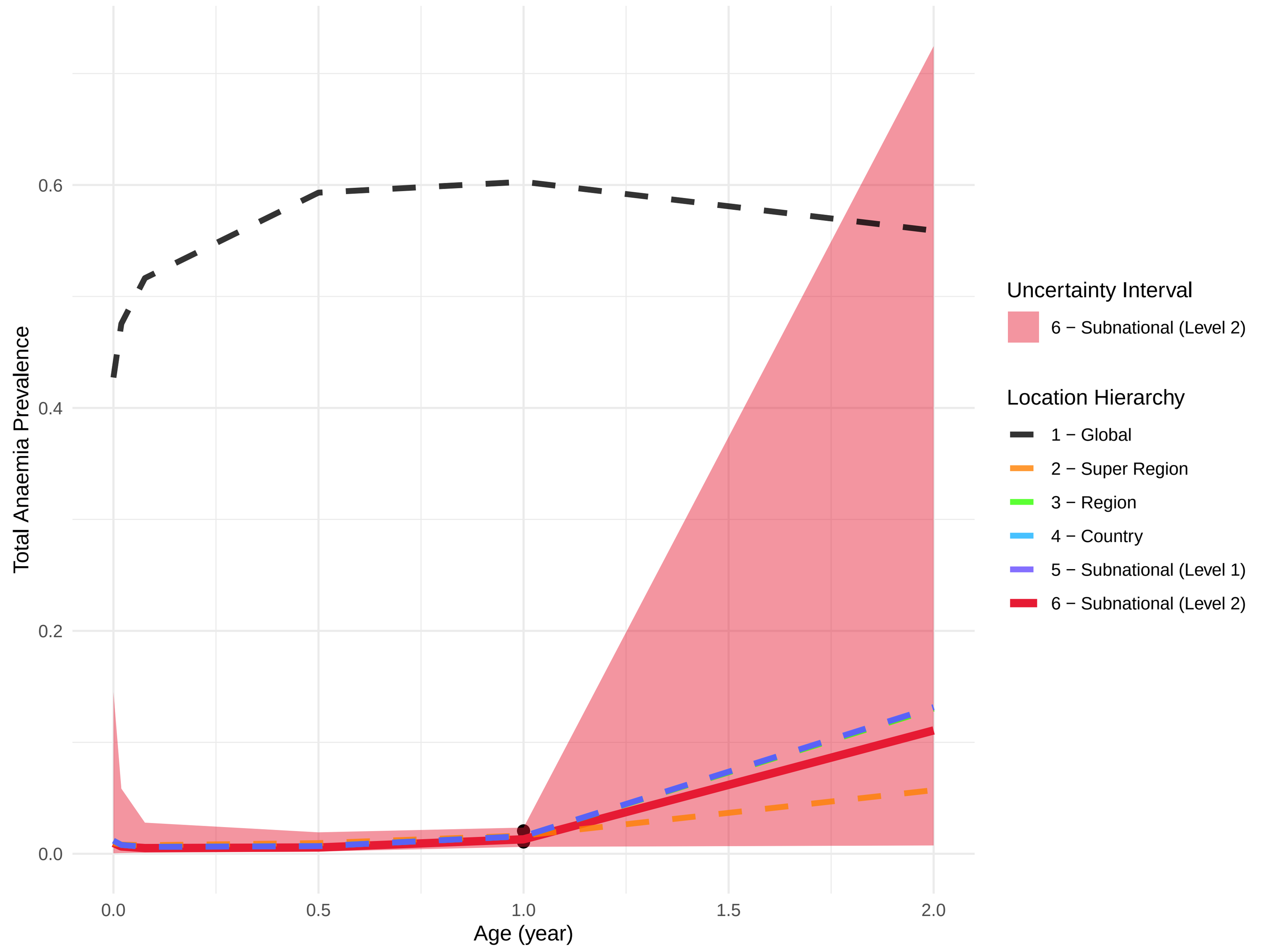

MR−BRT Age/Sex Fits − Non−pregnant Females <2 Years Old − Total Anaemia Prevalence − Yorkshire and the Humber
WHO−2024 Adjustment Method

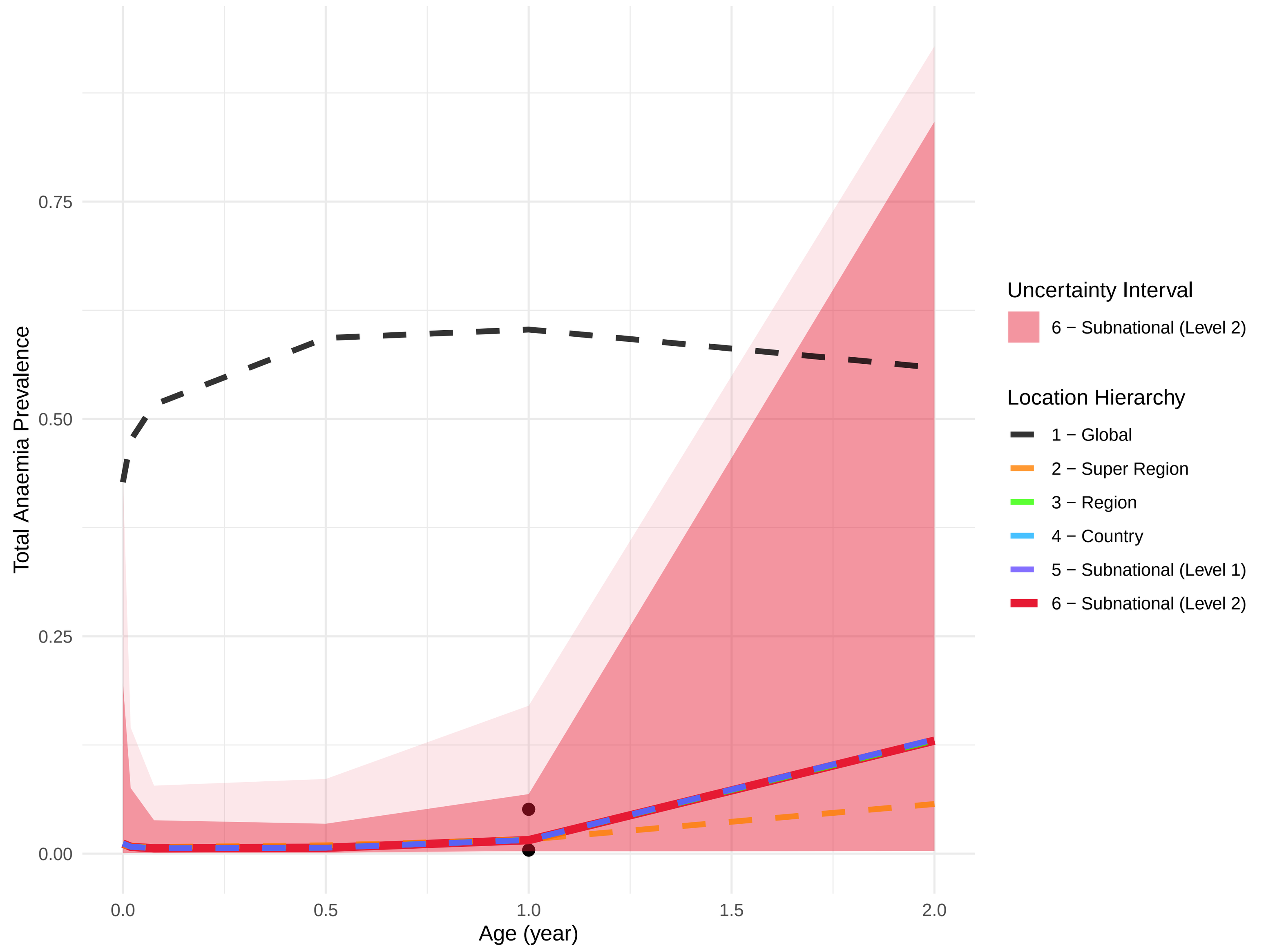

MR−BRT Age/Sex Fits − Non−pregnant Females <2 Years Old − Total Anaemia Prevalence − Latin America and Caribbean
WHO−2024 Adjustment Method

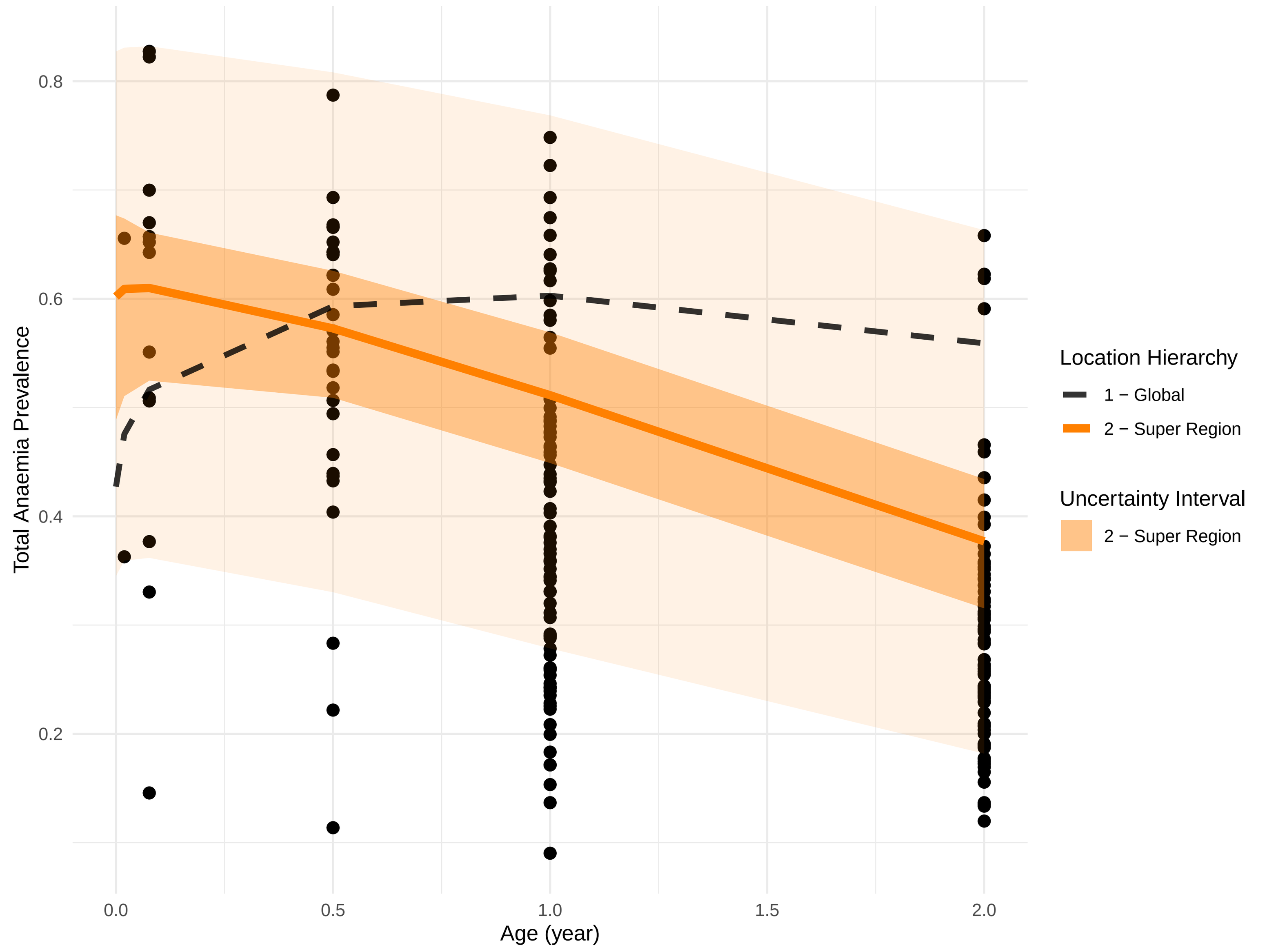

MR−BRT Age/Sex Fits − Non−pregnant Females <2 Years Old − Total Anaemia Prevalence − Andean Latin America
WHO−2024 Adjustment Method

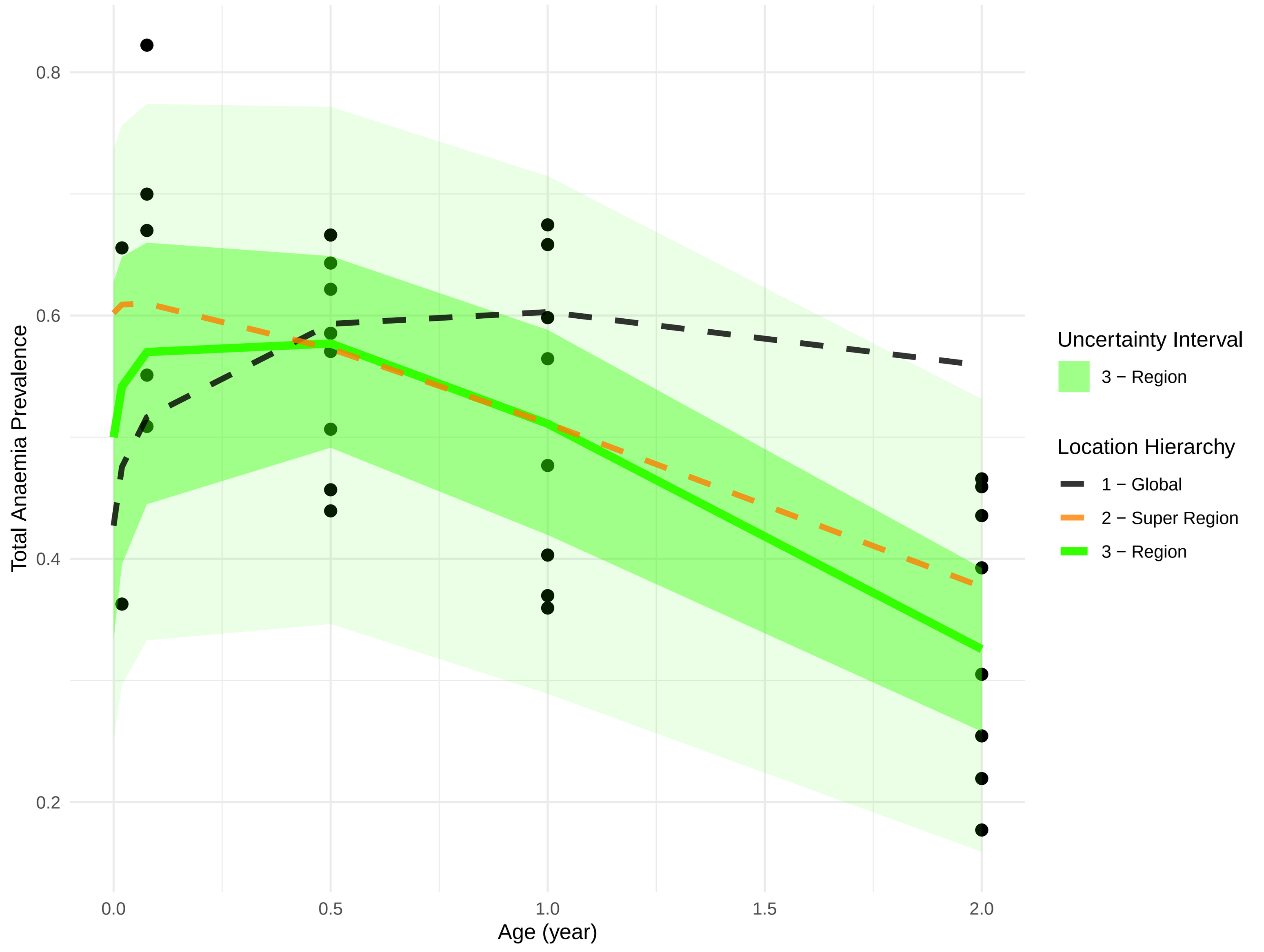

MR−BRT Age/Sex Fits − Non−pregnant Females <2 Years Old − Total Anaemia Prevalence − Bolivia (Plurinational State of)
WHO−2024 Adjustment Method

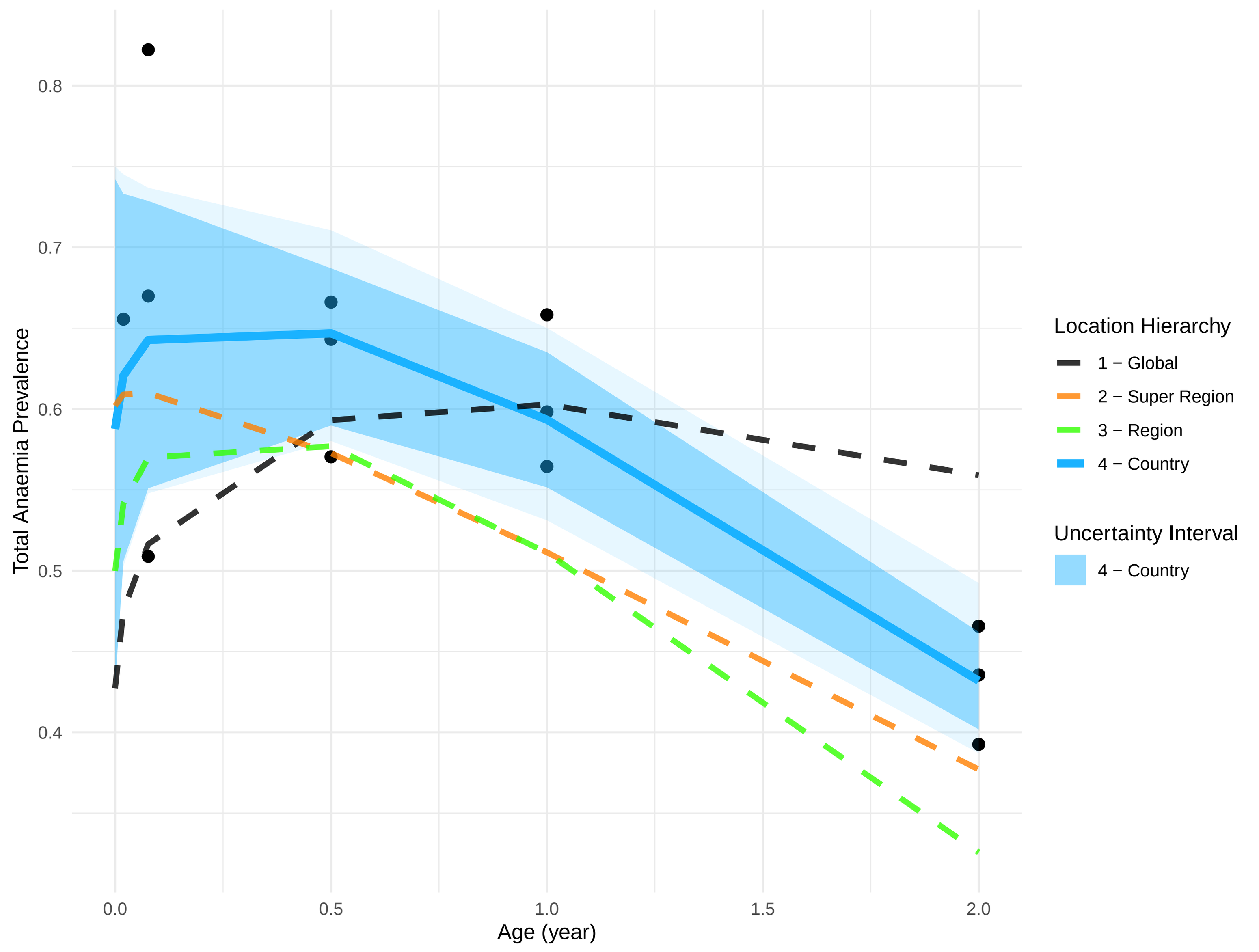

MR−BRT Age/Sex Fits − Non−pregnant Females <2 Years Old − Total Anaemia Prevalence − Peru
WHO−2024 Adjustment Method

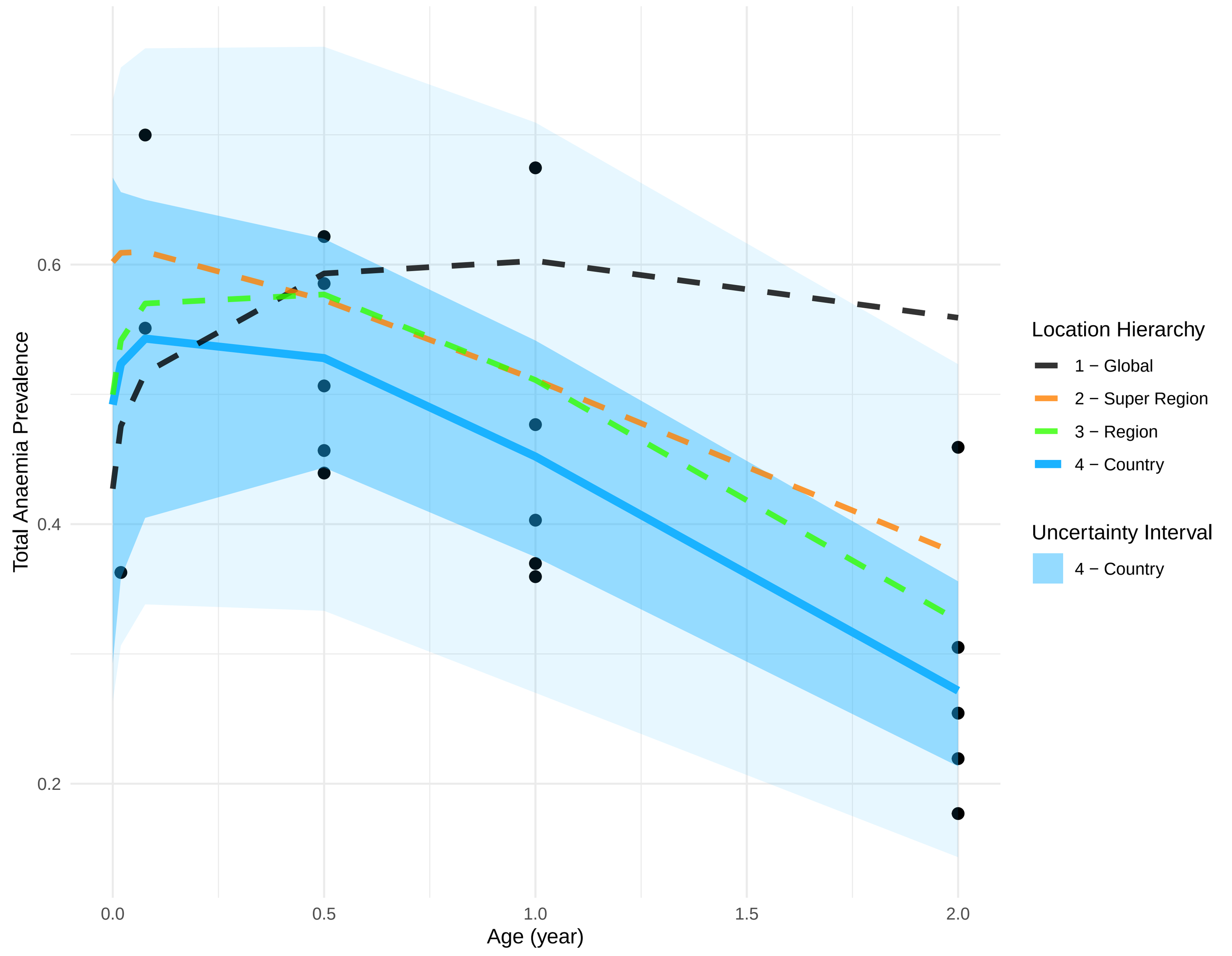

MR−BRT Age/Sex Fits − Non−pregnant Females <2 Years Old − Total Anaemia Prevalence − Caribbean
WHO−2024 Adjustment Method

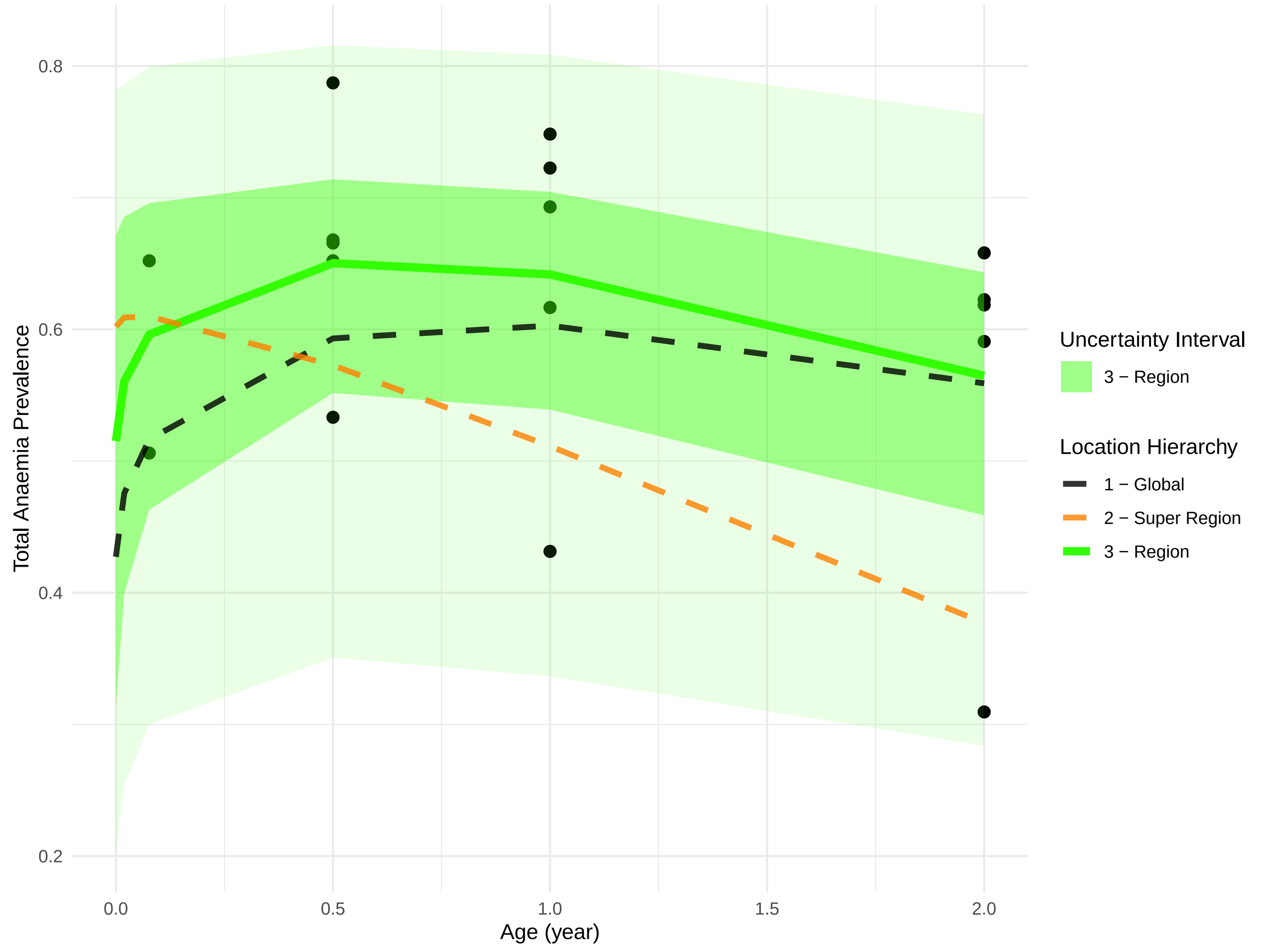

MR−BRT Age/Sex Fits − Non−pregnant Females <2 Years Old − Total Anaemia Prevalence − Guyana
WHO−2024 Adjustment Method

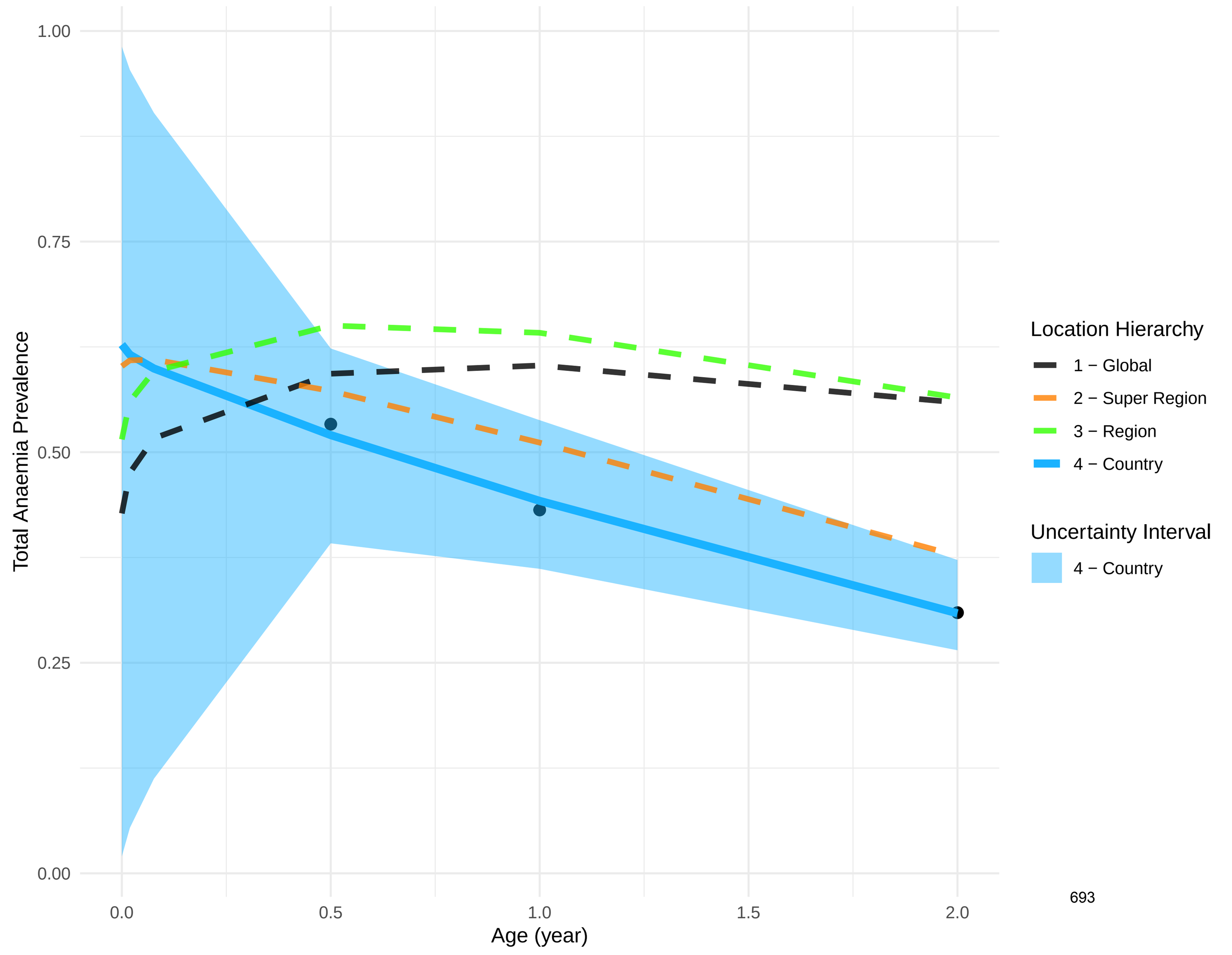

MR−BRT Age/Sex Fits − Non−pregnant Females <2 Years Old − Total Anaemia Prevalence − Haiti
WHO−2024 Adjustment Method

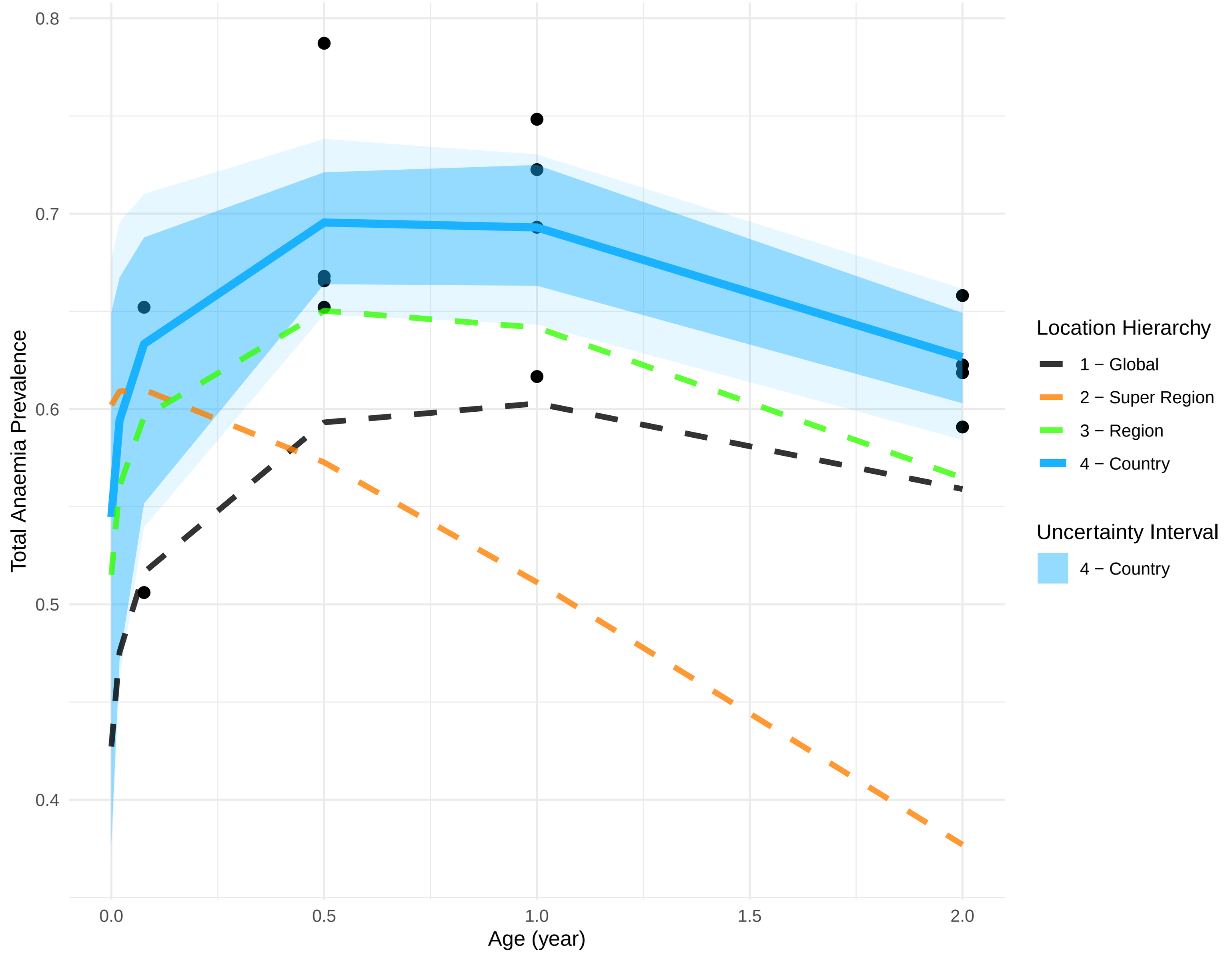

MR−BRT Age/Sex Fits − Non−pregnant Females <2 Years Old − Total Anaemia Prevalence − Central Latin America
WHO−2024 Adjustment Method

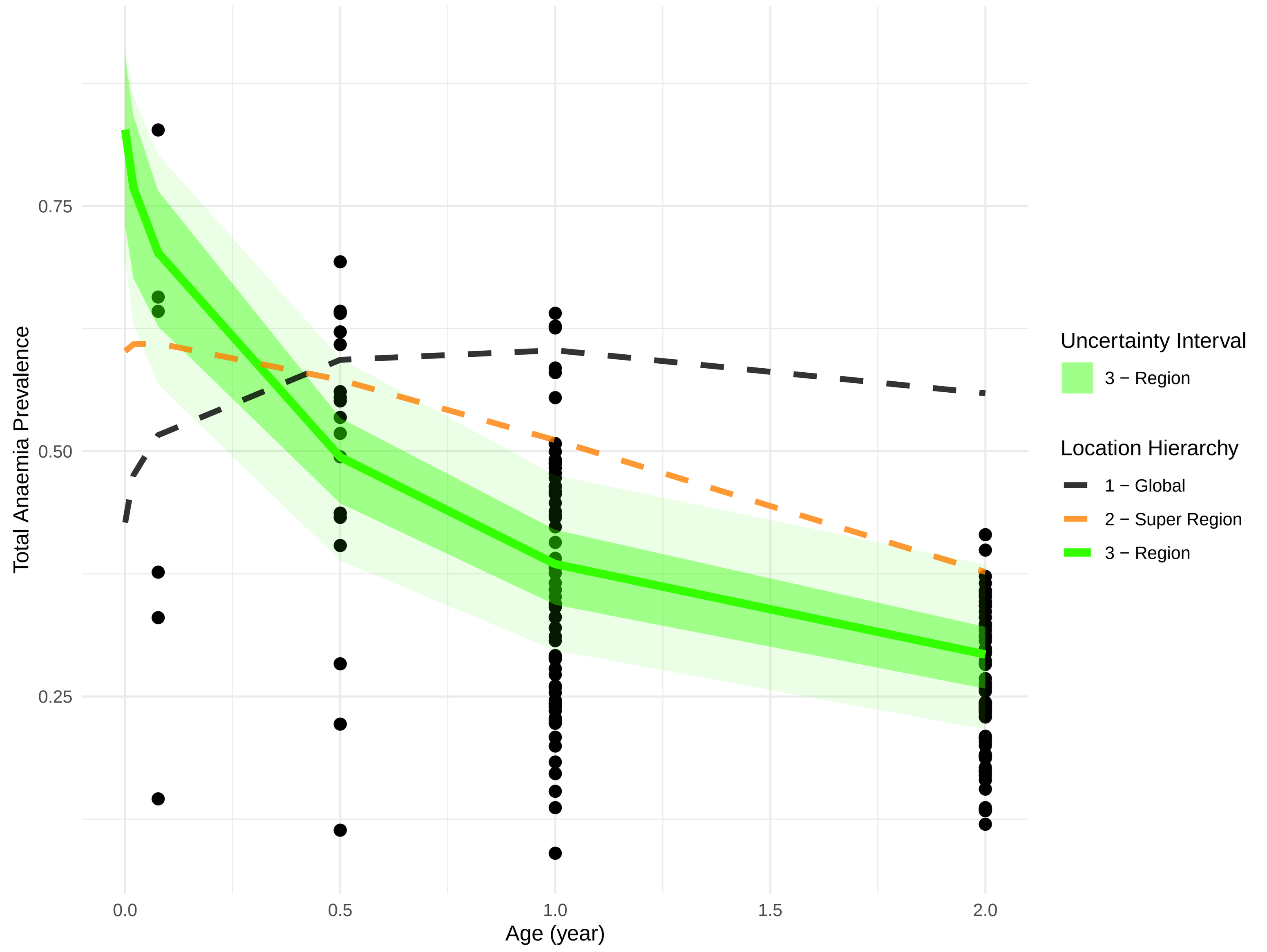

MR−BRT Age/Sex Fits − Non−pregnant Females <2 Years Old − Total Anaemia Prevalence − Guatemala
WHO−2024 Adjustment Method

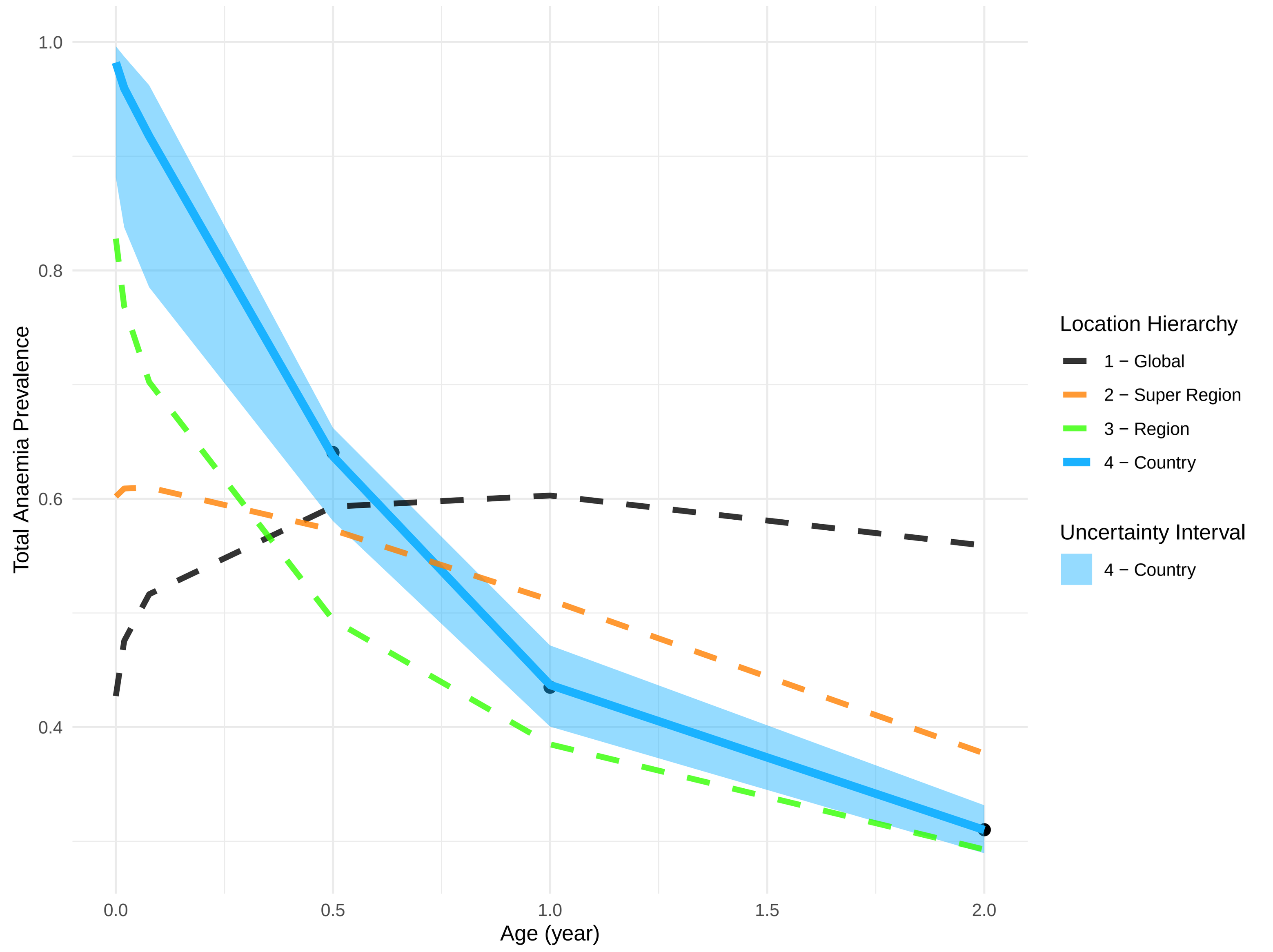

MR−BRT Age/Sex Fits − Non−pregnant Females <2 Years Old − Total Anaemia Prevalence − Honduras
WHO−2024 Adjustment Method

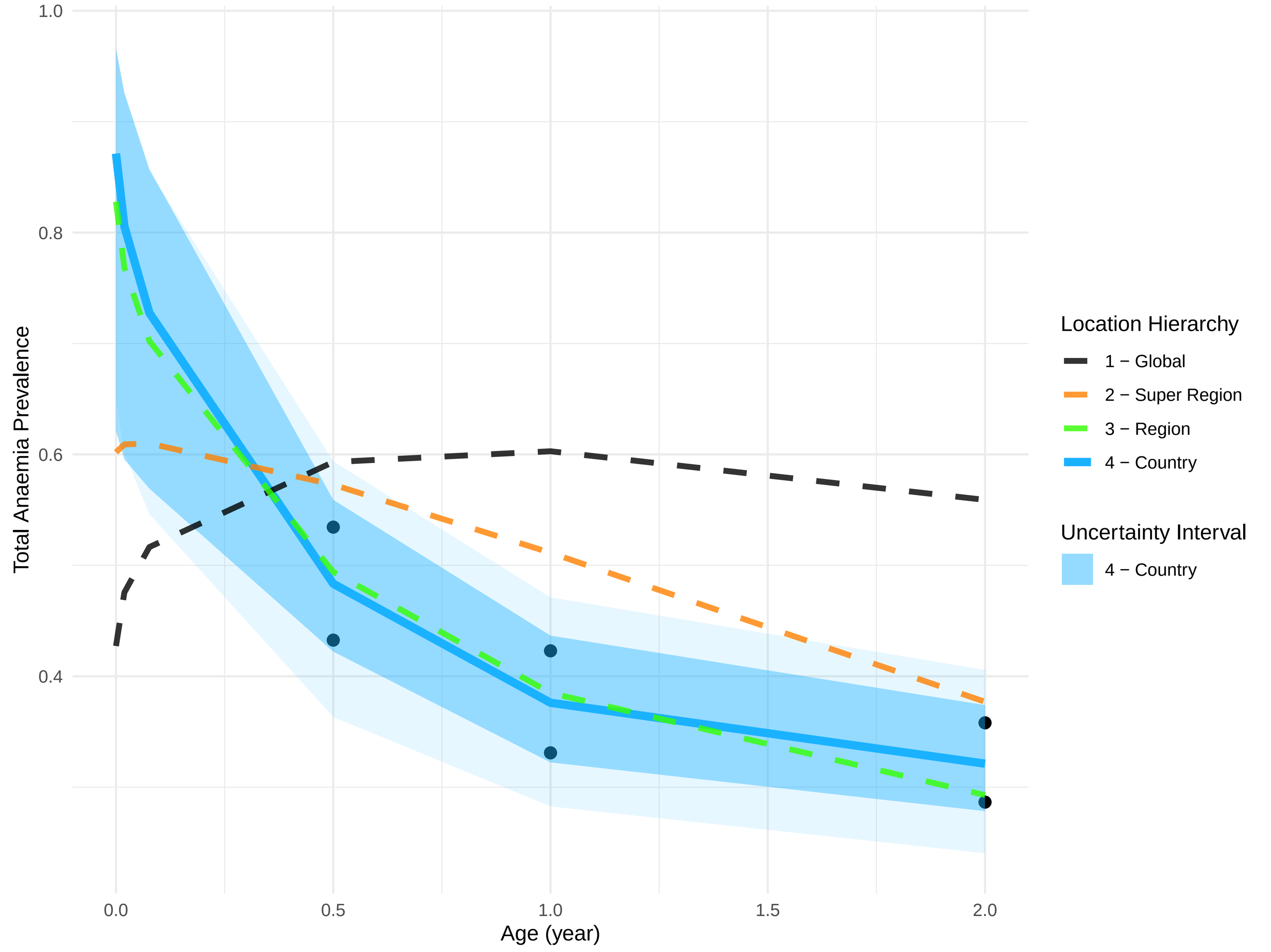

MR−BRT Age/Sex Fits − Non−pregnant Females <2 Years Old − Total Anaemia Prevalence − Mexico
WHO−2024 Adjustment Method

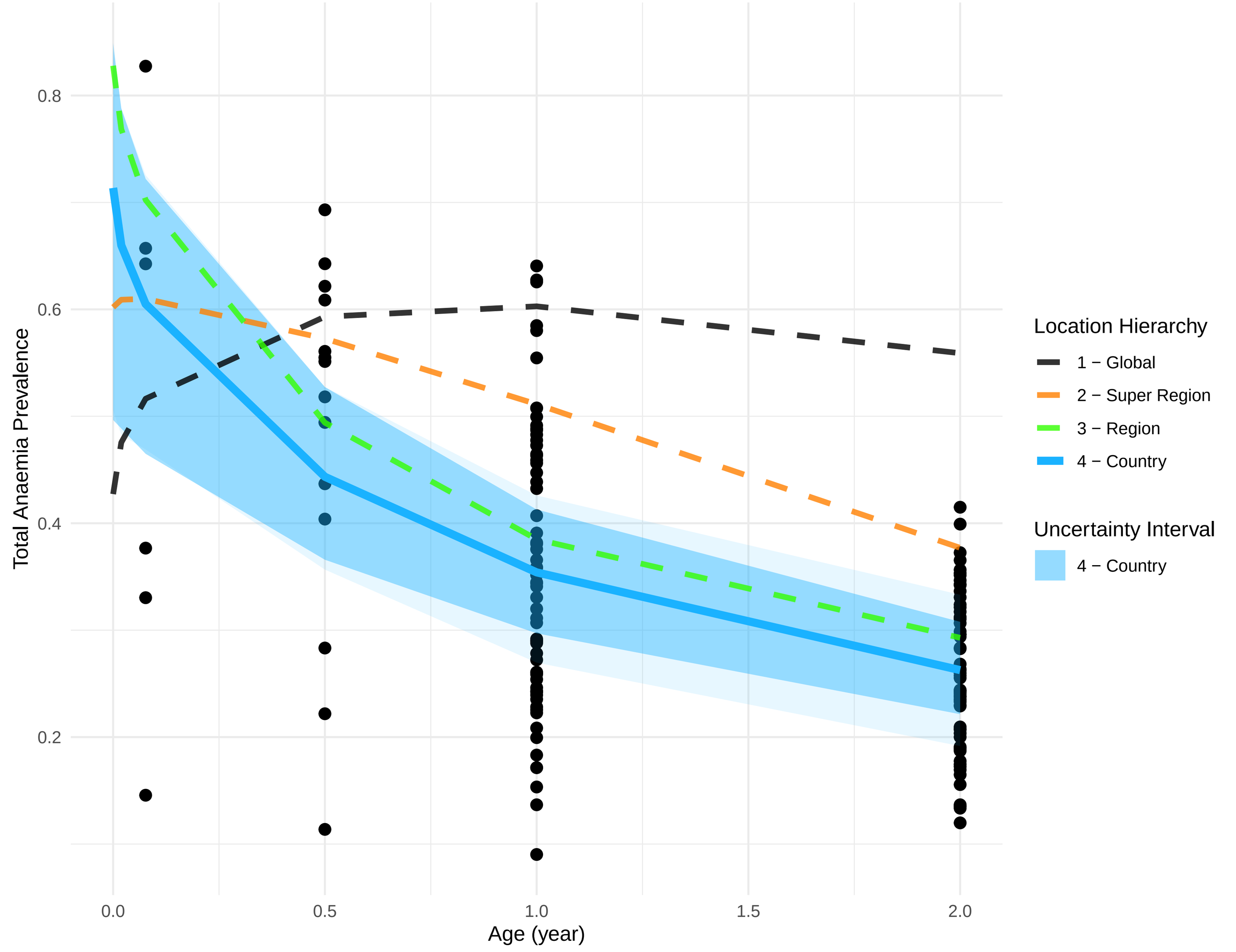

MR−BRT Age/Sex Fits − Non−pregnant Females <2 Years Old − Total Anaemia Prevalence − Aguascalientes
WHO−2024 Adjustment Method

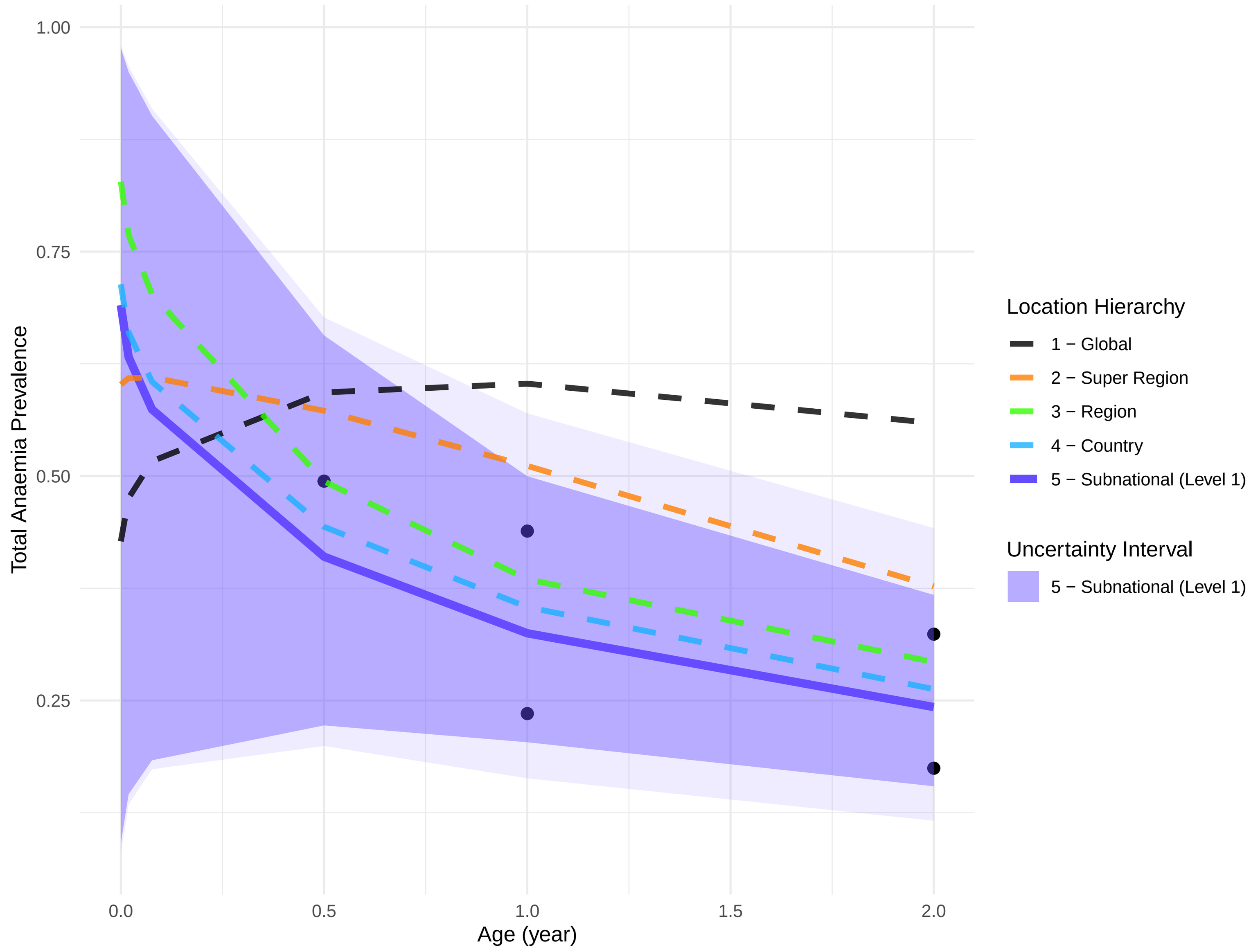

MR−BRT Age/Sex Fits − Non−pregnant Females <2 Years Old − Total Anaemia Prevalence − Baja California
WHO−2024 Adjustment Method

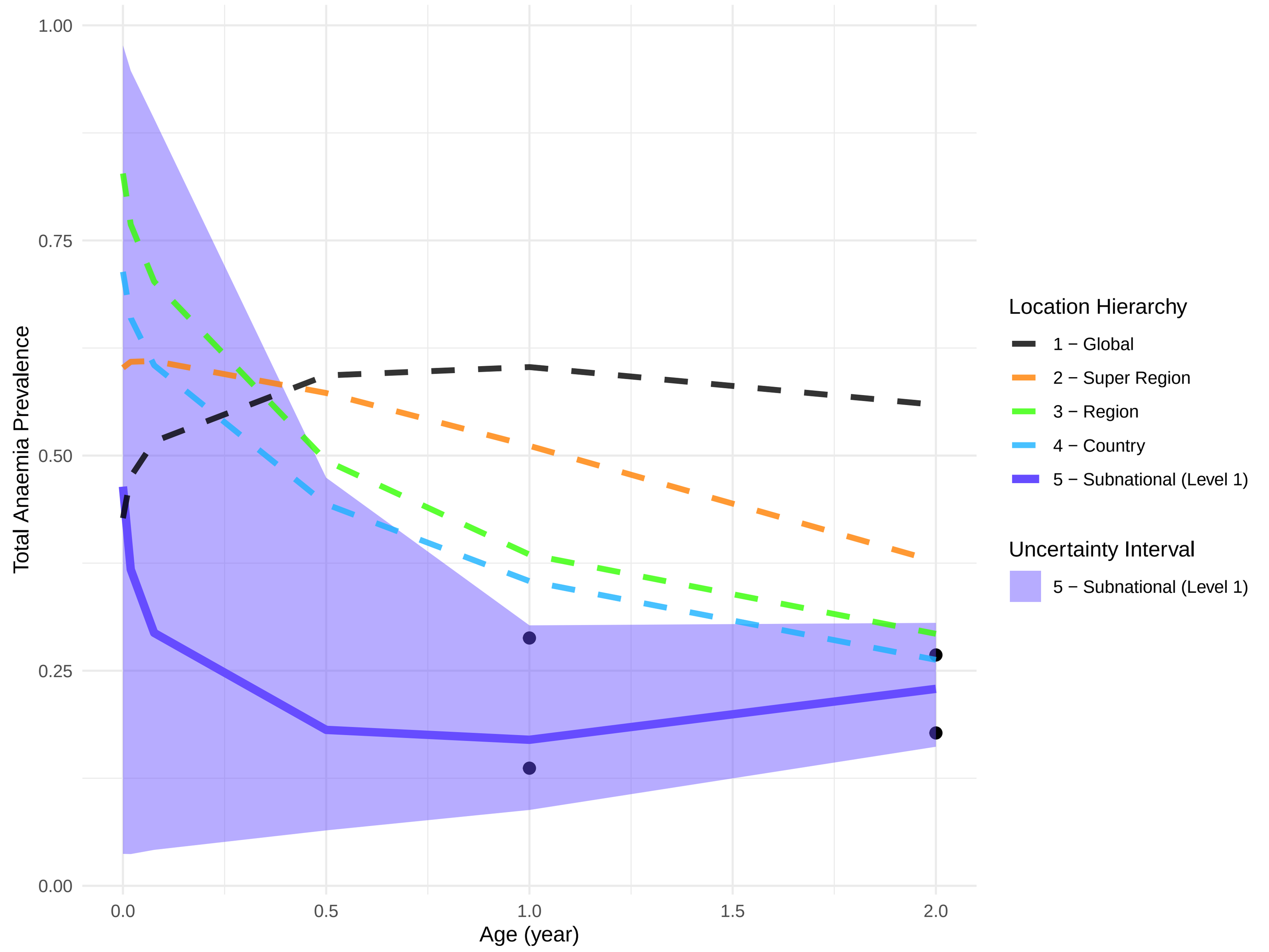

MR−BRT Age/Sex Fits − Non−pregnant Females <2 Years Old − Total Anaemia Prevalence − Baja California Sur
WHO−2024 Adjustment Method

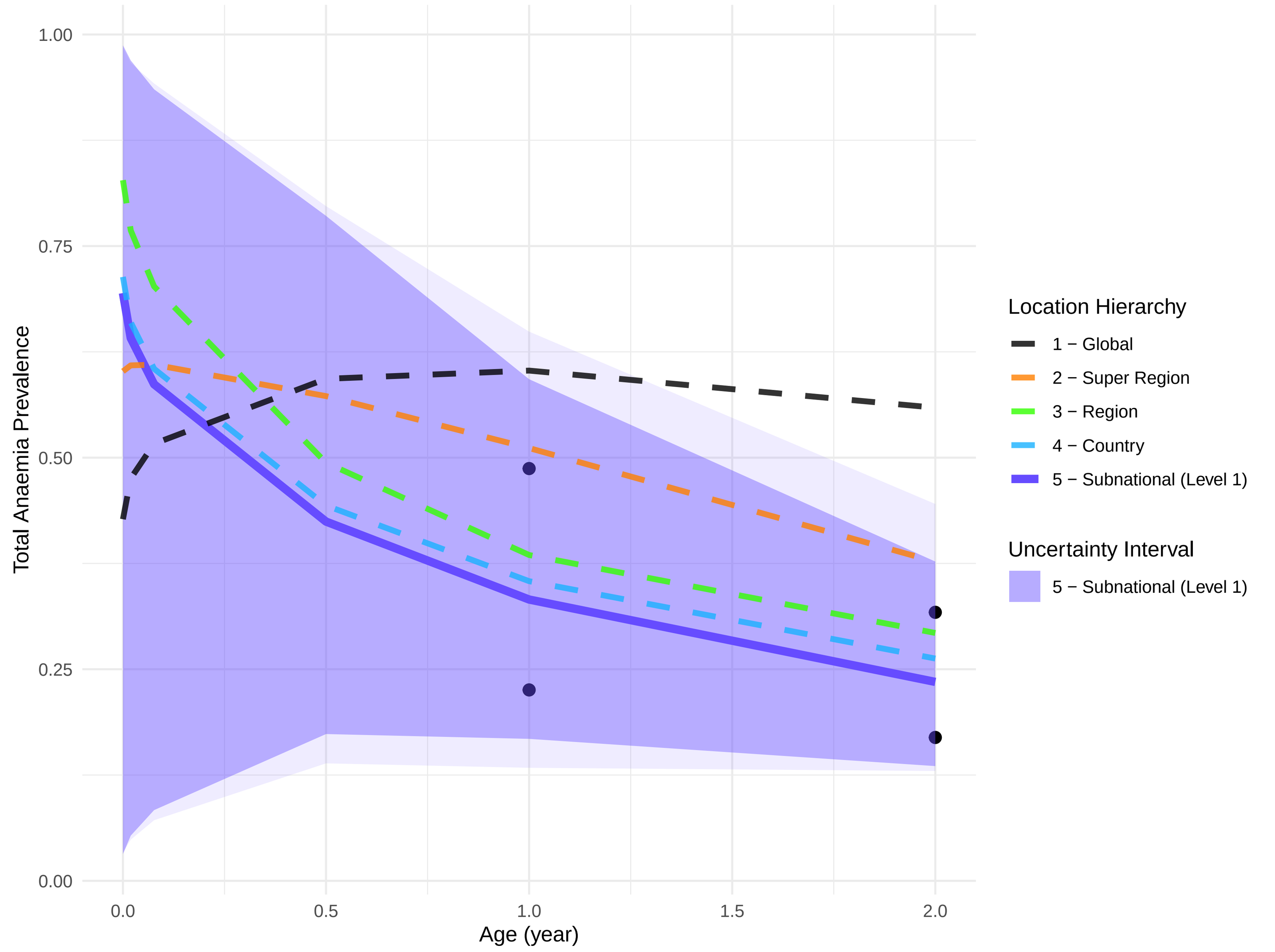

MR−BRT Age/Sex Fits − Non−pregnant Females <2 Years Old − Total Anaemia Prevalence − Campeche
WHO−2024 Adjustment Method

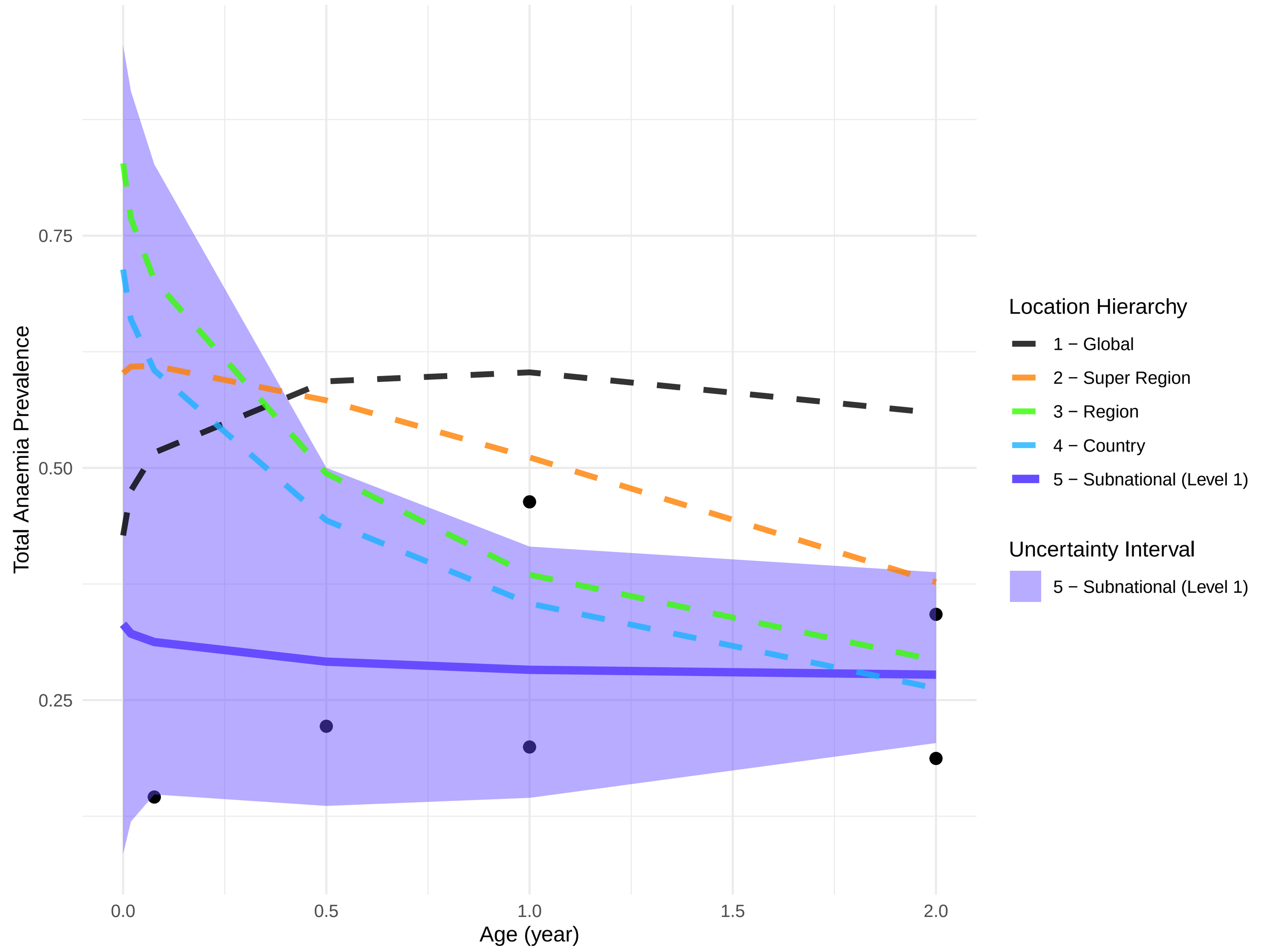

MR−BRT Age/Sex Fits − Non−pregnant Females <2 Years Old − Total Anaemia Prevalence − Chiapas
WHO−2024 Adjustment Method

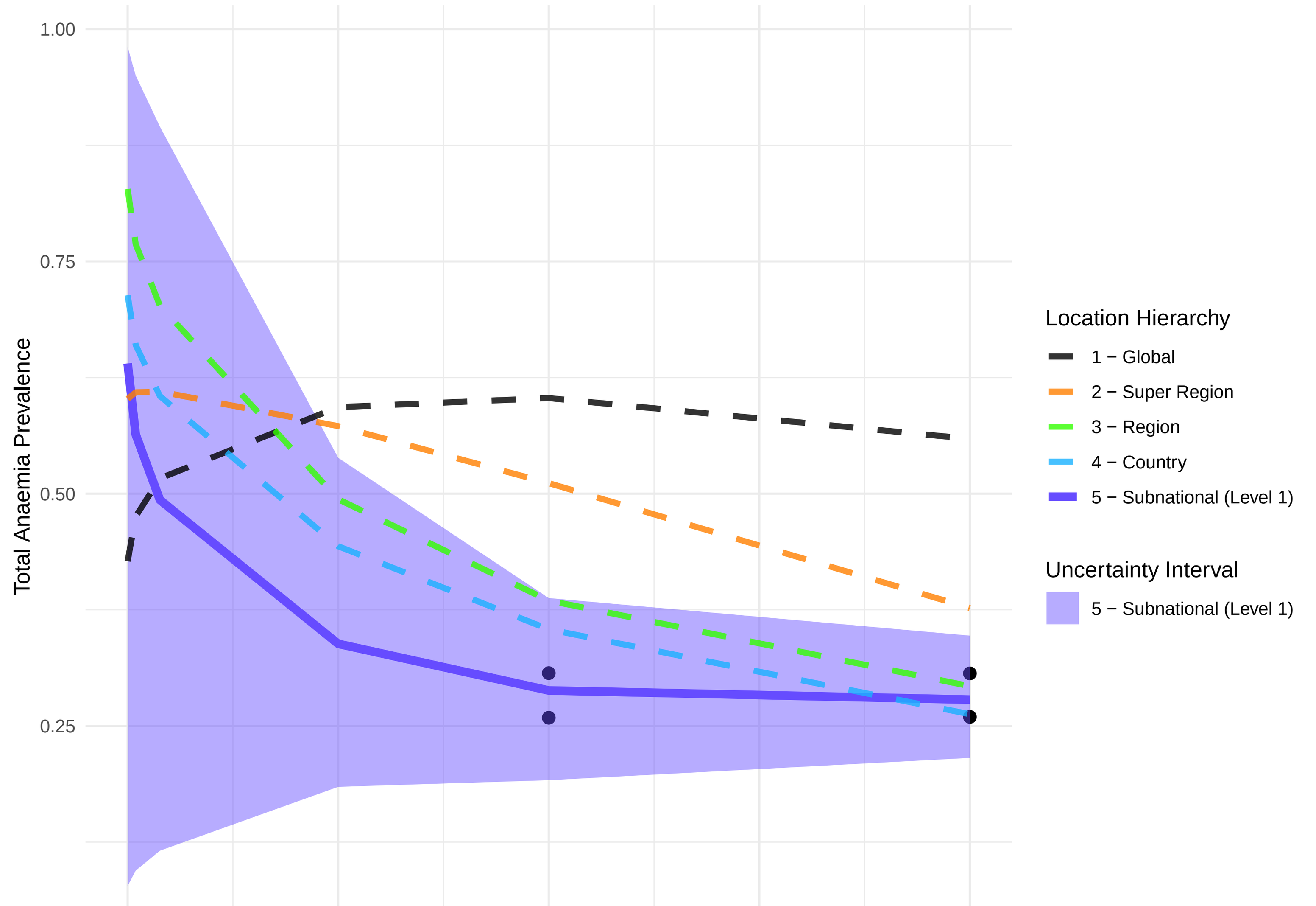

MR−BRT Age/Sex Fits − Non−pregnant Females <2 Years Old − Total Anaemia Prevalence − Chihuahua

WHO−2024 Adjustment Method

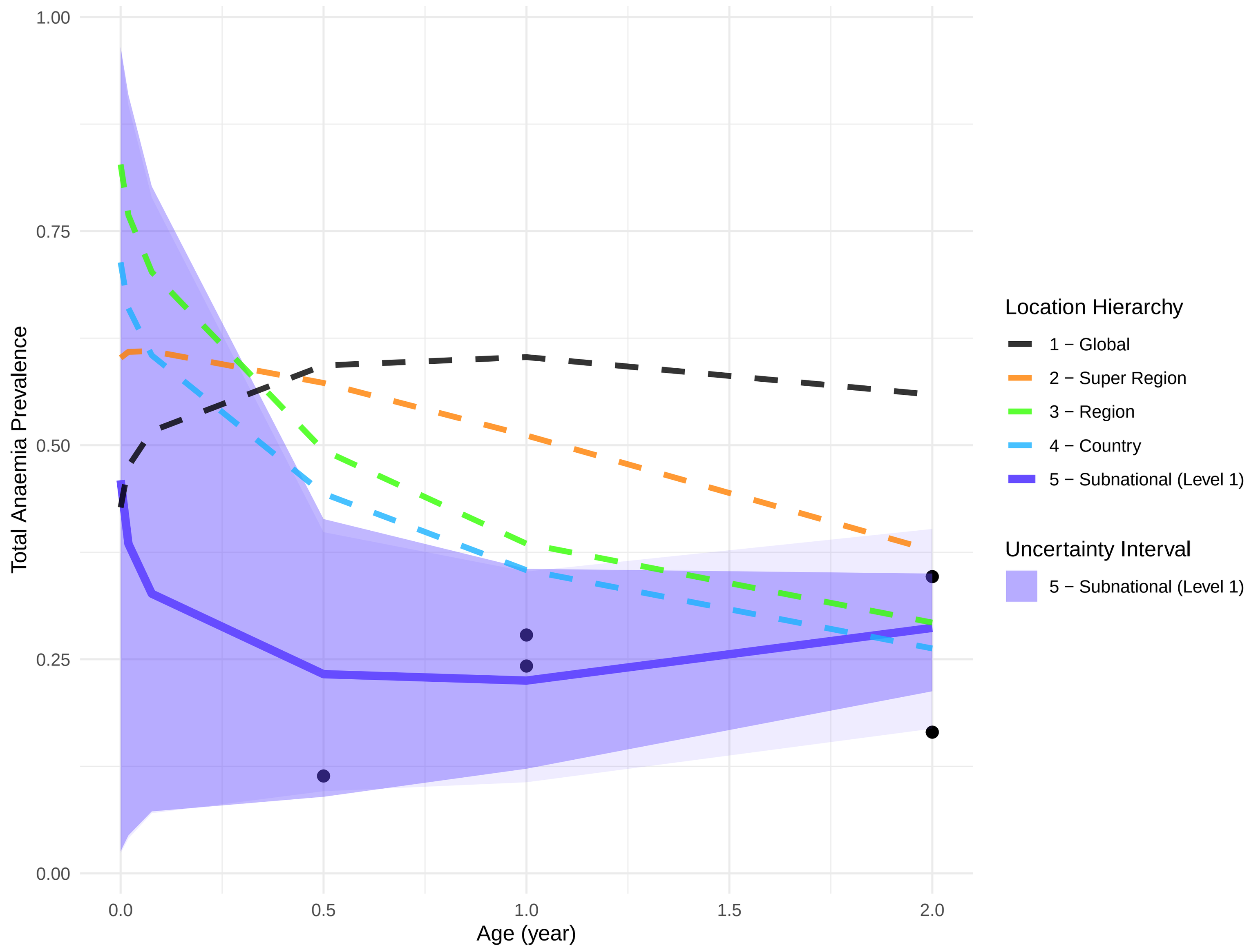

MR−BRT Age/Sex Fits − Non−pregnant Females <2 Years Old − Total Anaemia Prevalence − Coahuila

WHO−2024 Adjustment Method

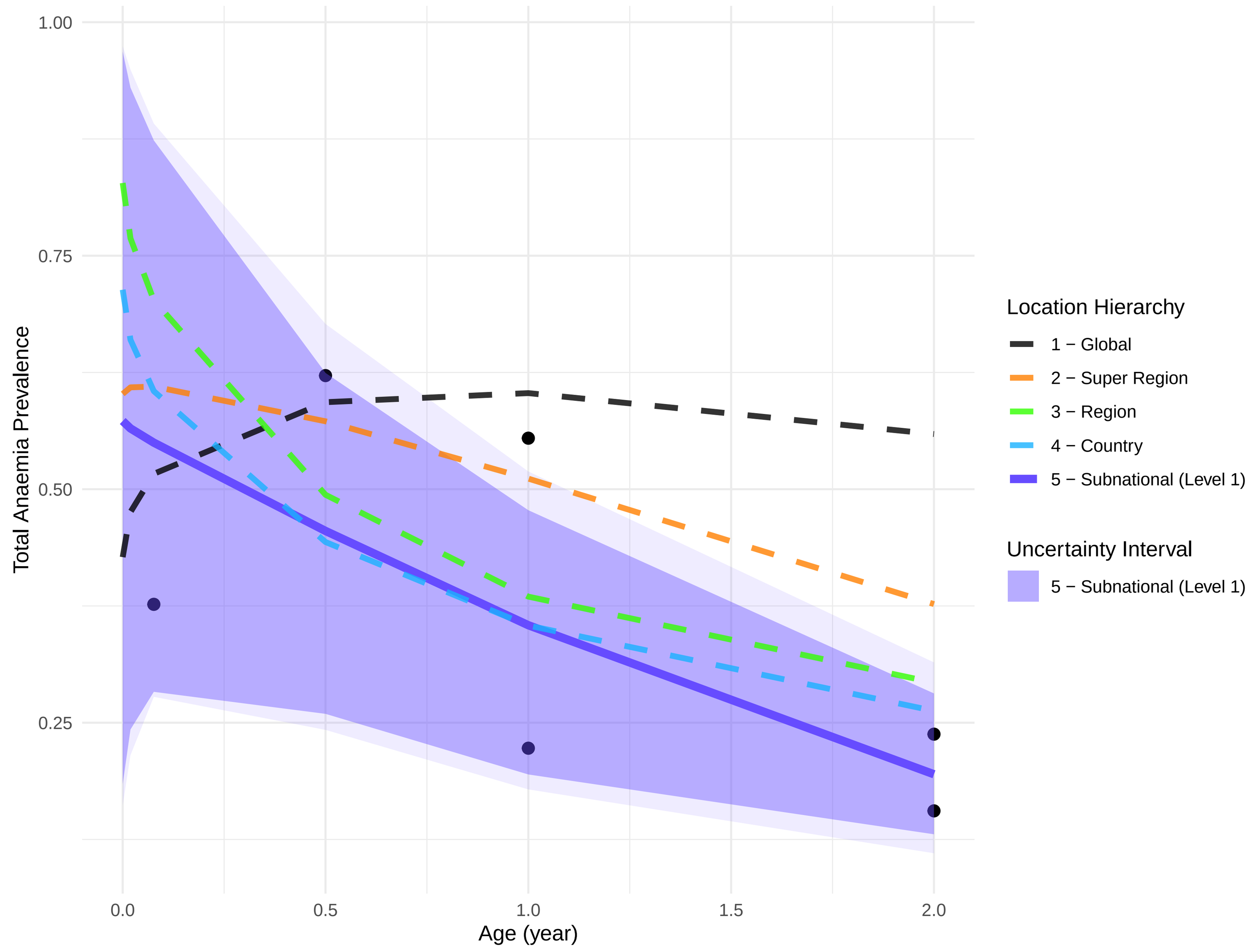

MR−BRT Age/Sex Fits − Non−pregnant Females <2 Years Old − Total Anaemia Prevalence − Colima

WHO−2024 Adjustment Method

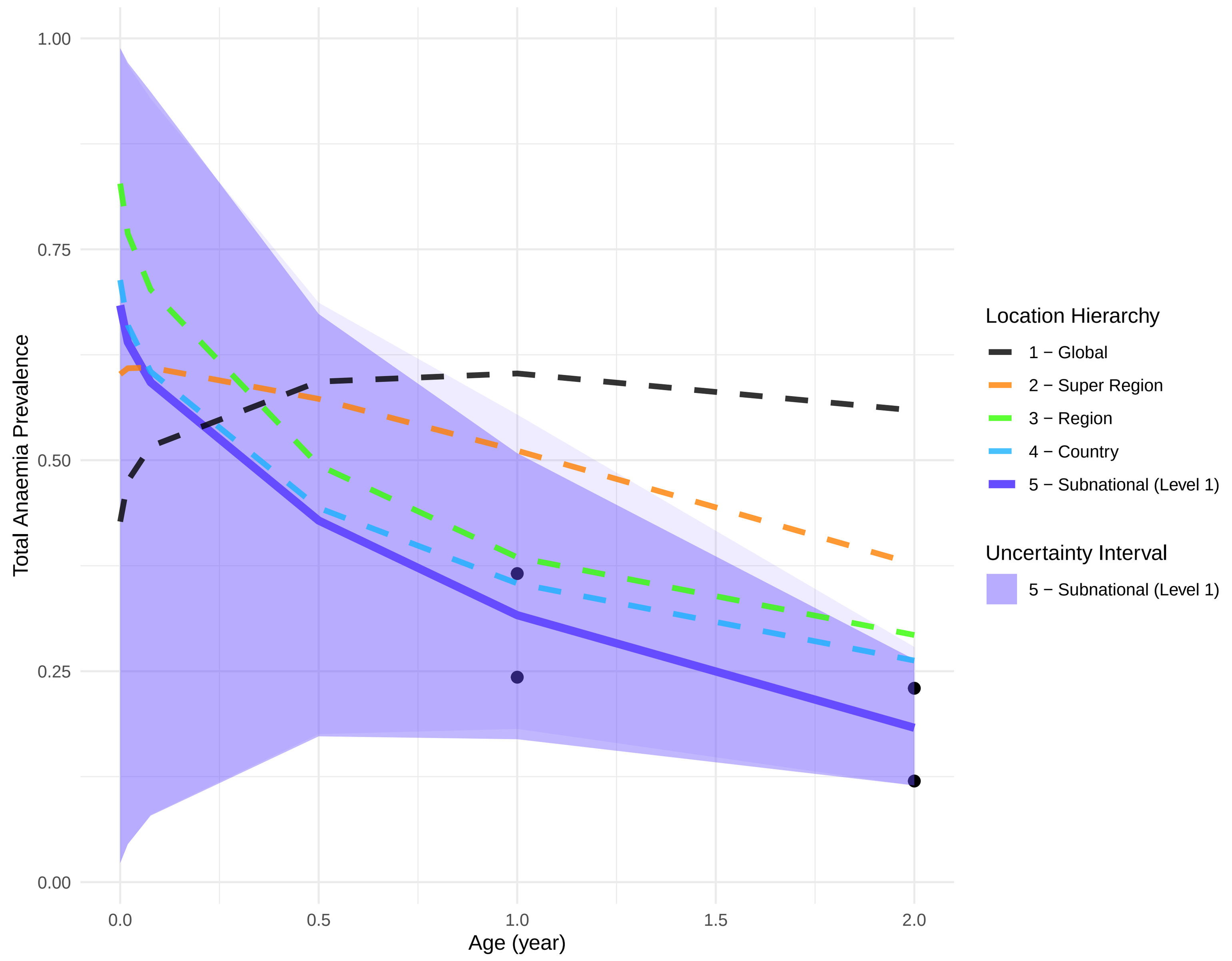

MR−BRT Age/Sex Fits − Non−pregnant Females <2 Years Old − Total Anaemia Prevalence − Durango

WHO−2024 Adjustment Method

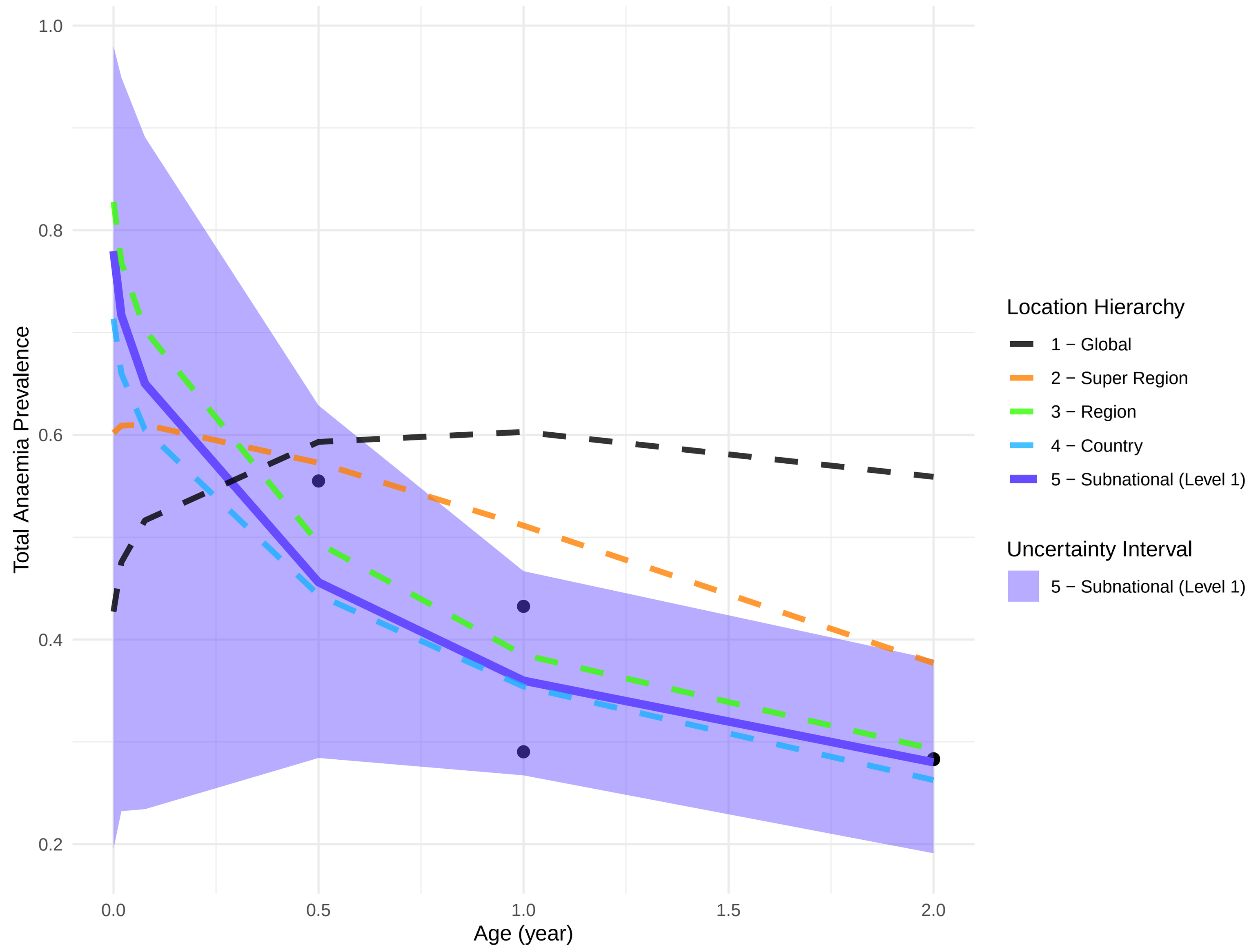

MR−BRT Age/Sex Fits − Non−pregnant Females <2 Years Old − Total Anaemia Prevalence − Guanajuato

WHO−2024 Adjustment Method

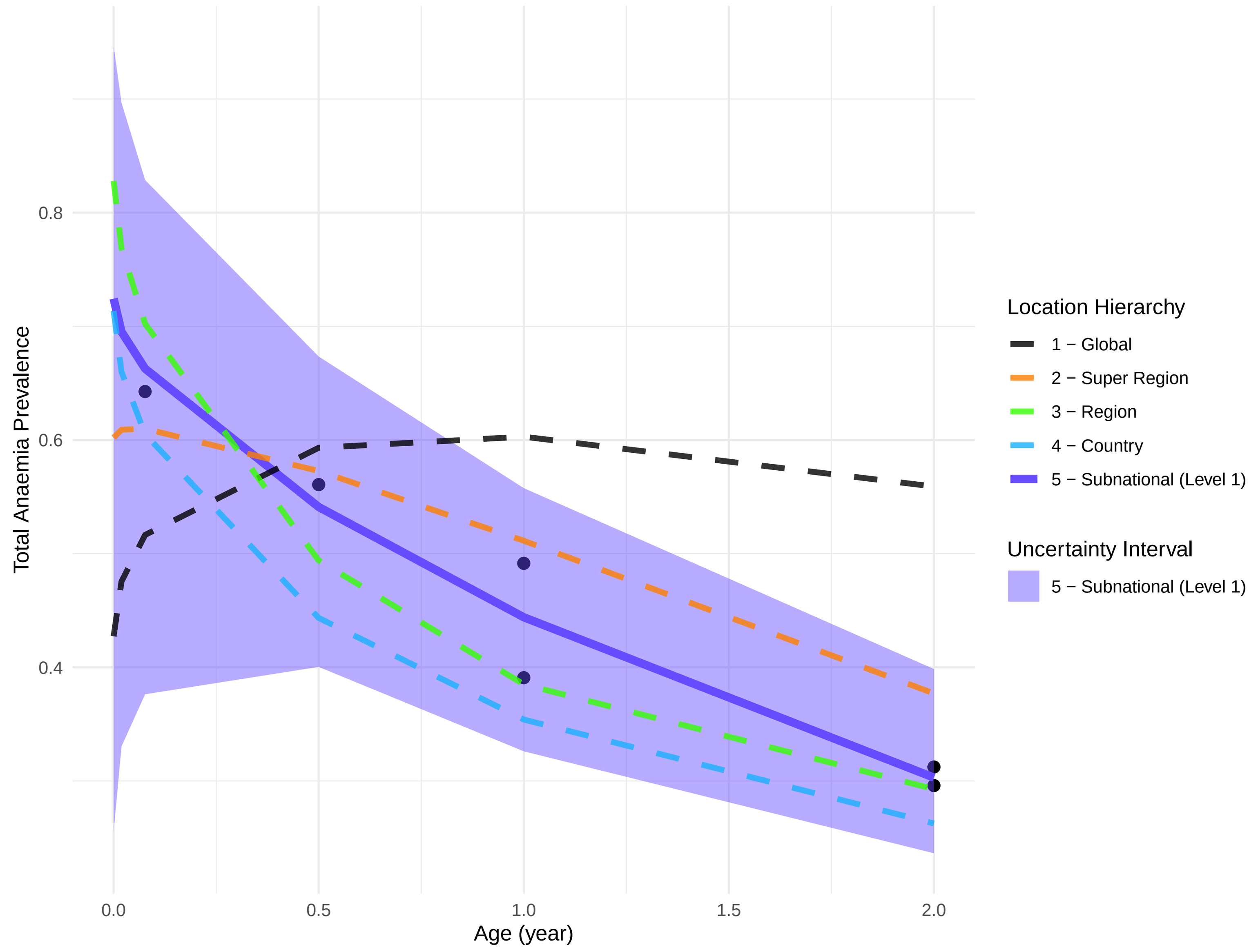

MR−BRT Age/Sex Fits − Non−pregnant Females <2 Years Old − Total Anaemia Prevalence − Guerrero

WHO−2024 Adjustment Method

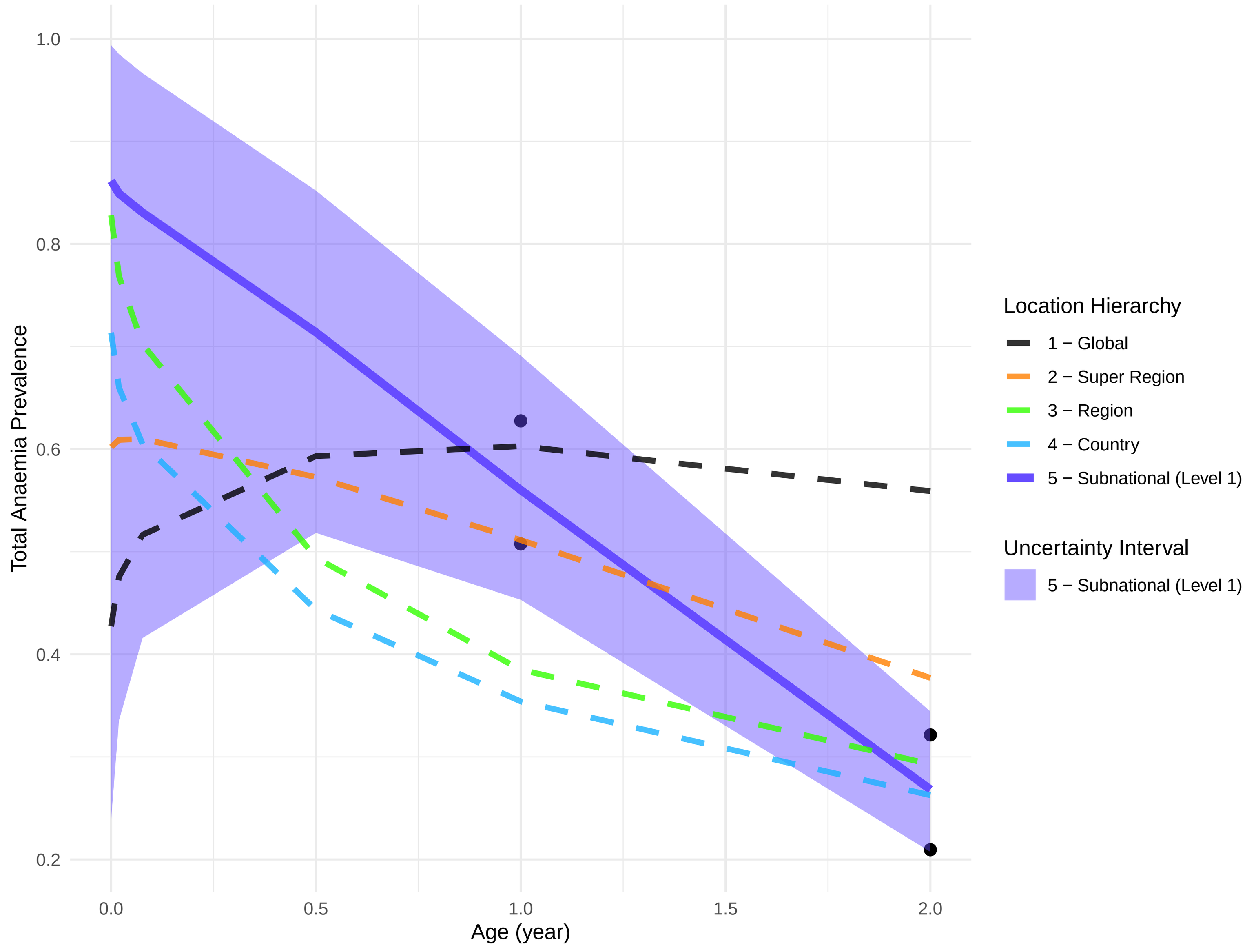

MR−BRT Age/Sex Fits − Non−pregnant Females <2 Years Old − Total Anaemia Prevalence − Hidalgo

WHO−2024 Adjustment Method

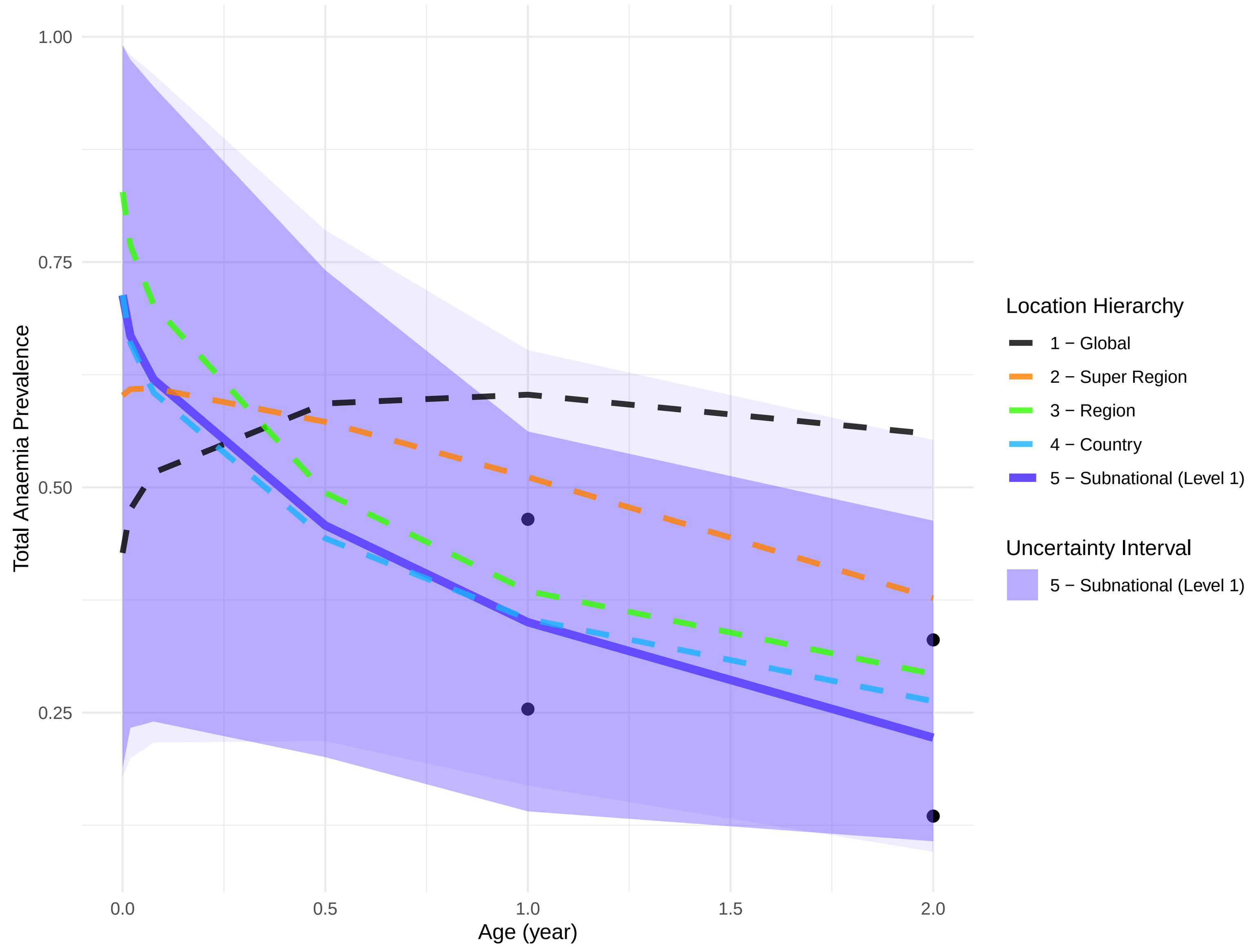

MR−BRT Age/Sex Fits − Non−pregnant Females <2 Years Old − Total Anaemia Prevalence − Jalisco

WHO−2024 Adjustment Method

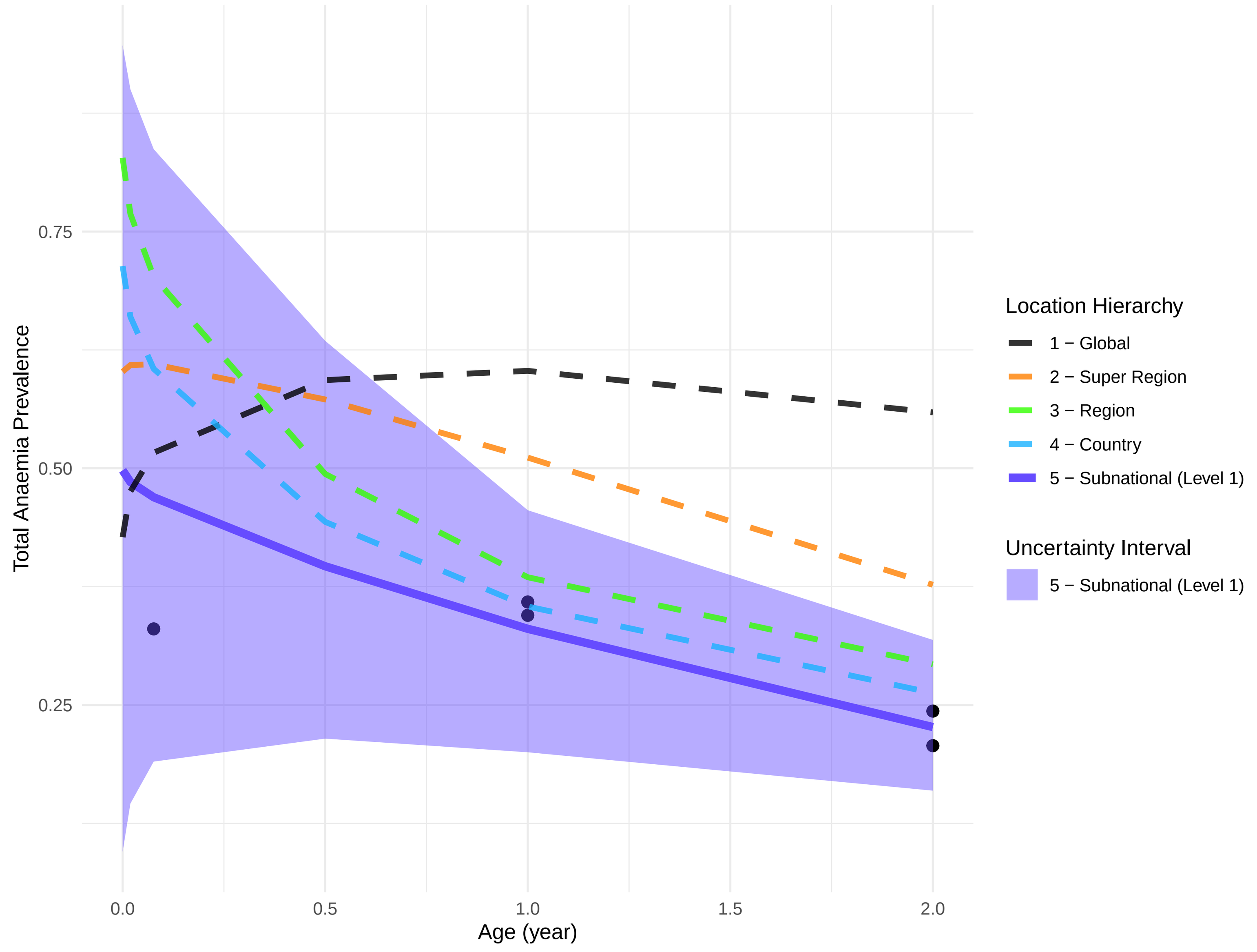

MR−BRT Age/Sex Fits − Non−pregnant Females <2 Years Old − Total Anaemia Prevalence − México

WHO−2024 Adjustment Method

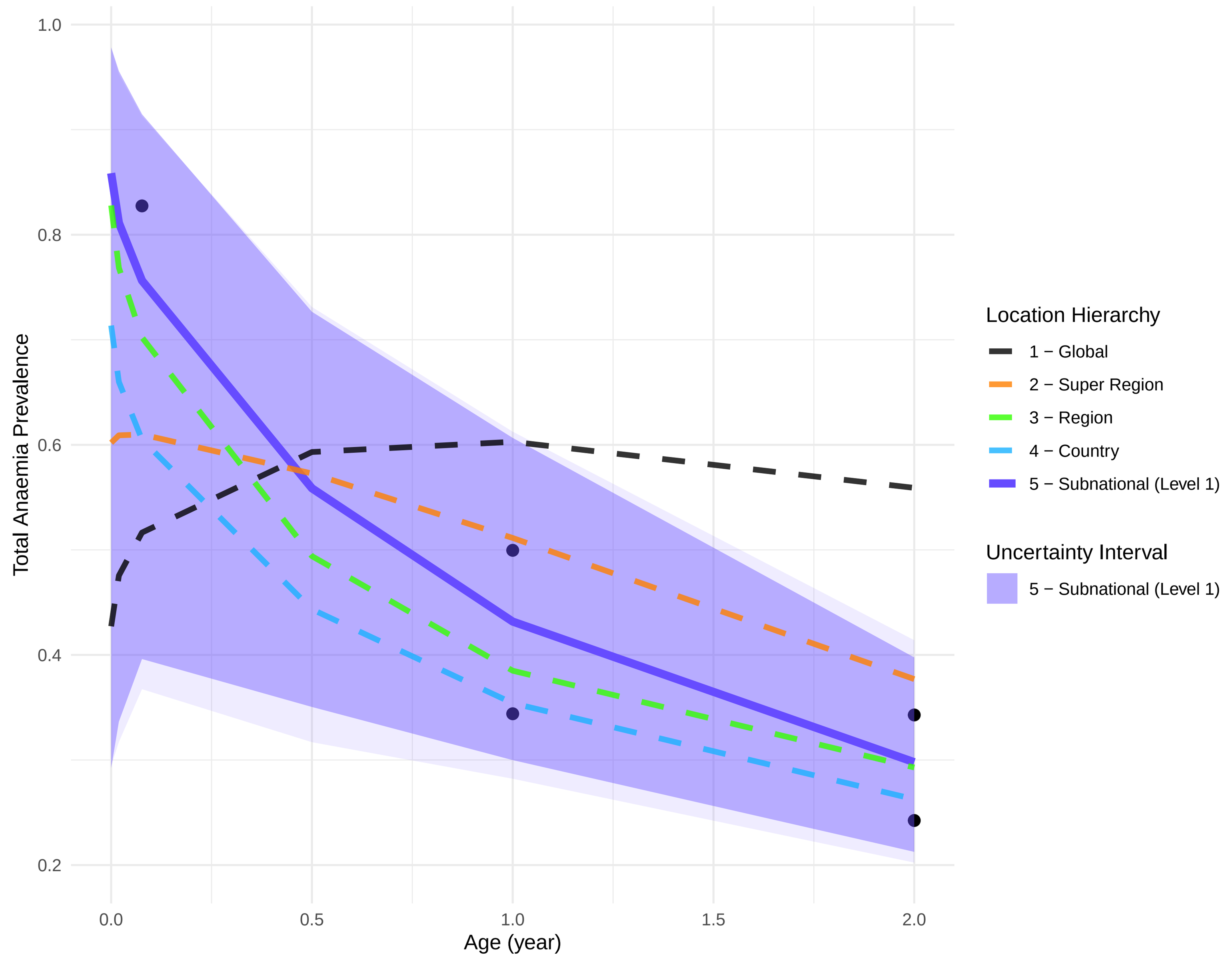

MR−BRT Age/Sex Fits − Non−pregnant Females <2 Years Old − Total Anaemia Prevalence − Mexico City

WHO−2024 Adjustment Method

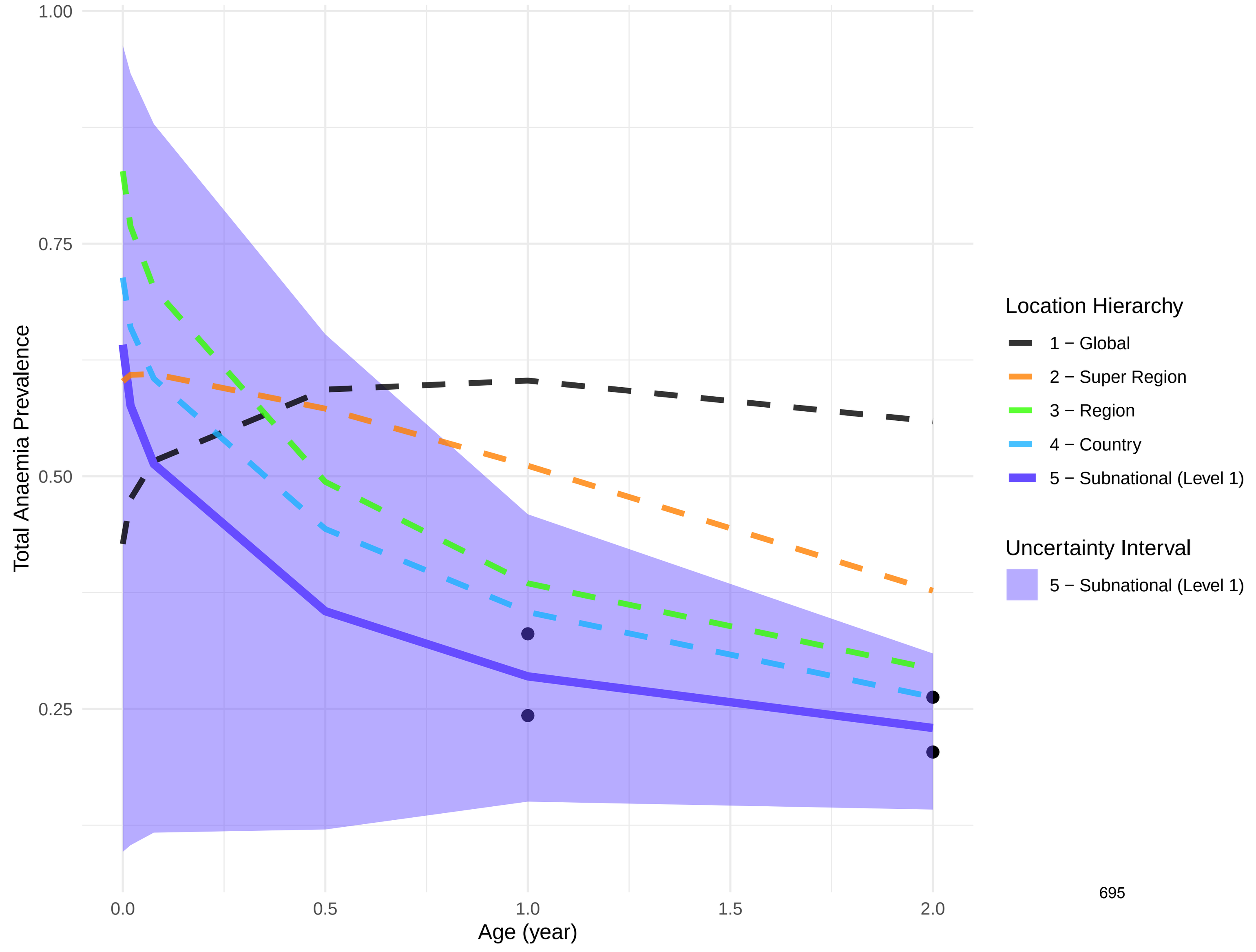

MR−BRT Age/Sex Fits − Non−pregnant Females <2 Years Old − Total Anaemia Prevalence − Michoacán de Ocampo
WHO−2024 Adjustment Method

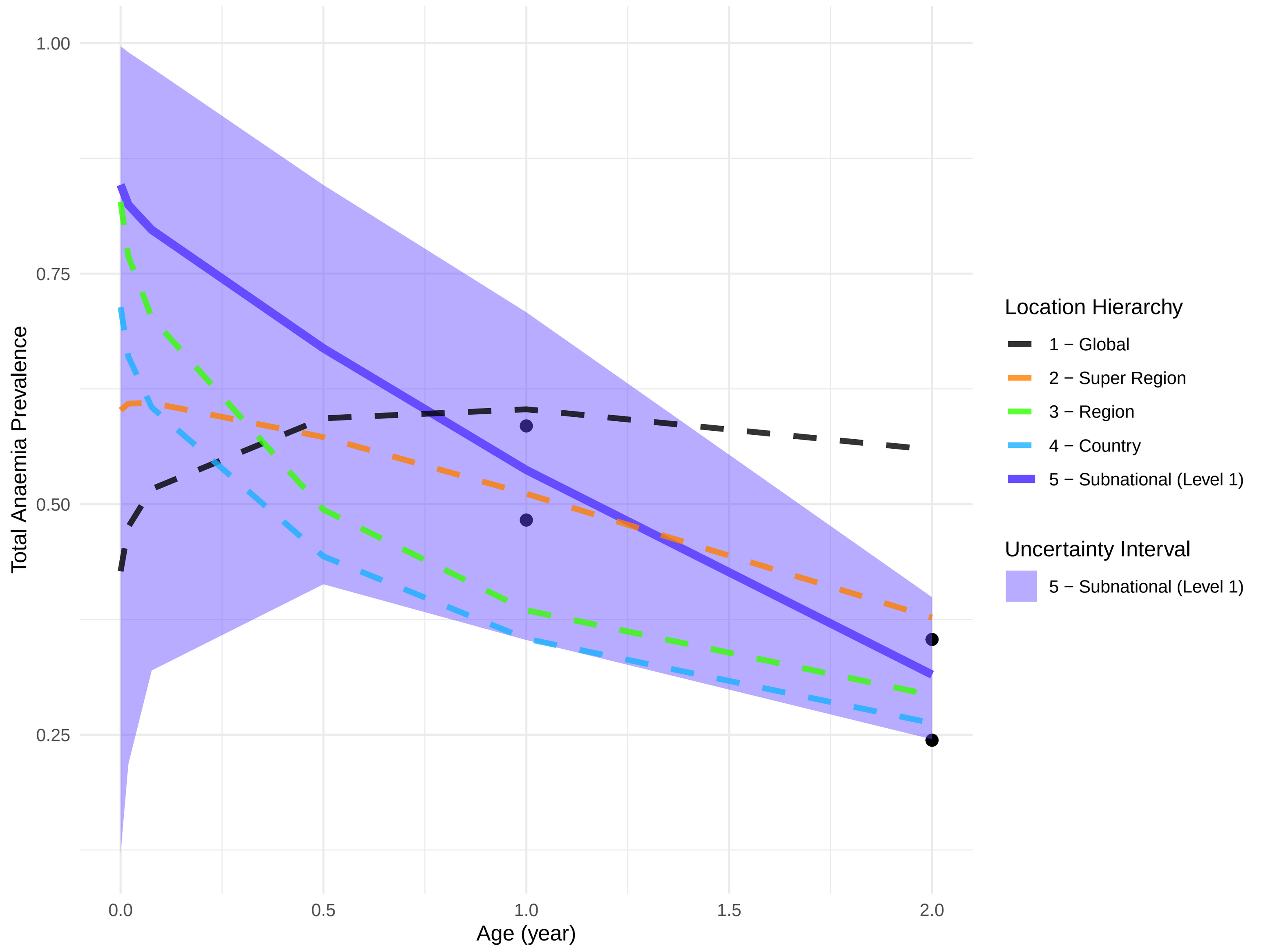

MR−BRT Age/Sex Fits − Non−pregnant Females <2 Years Old − Total Anaemia Prevalence − Morelos
WHO−2024 Adjustment Method

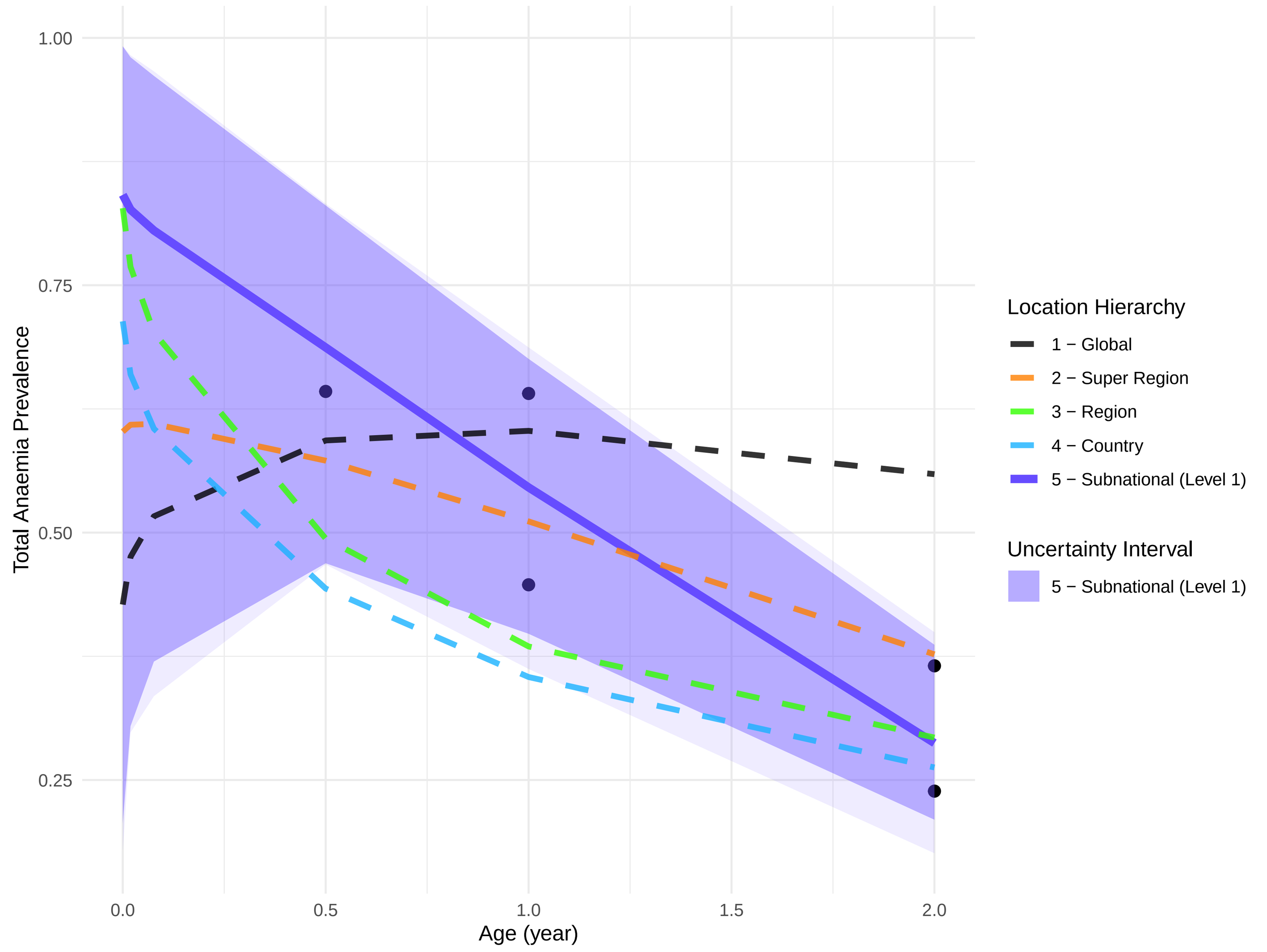

MR−BRT Age/Sex Fits − Non−pregnant Females <2 Years Old − Total Anaemia Prevalence − Nayarit
WHO−2024 Adjustment Method

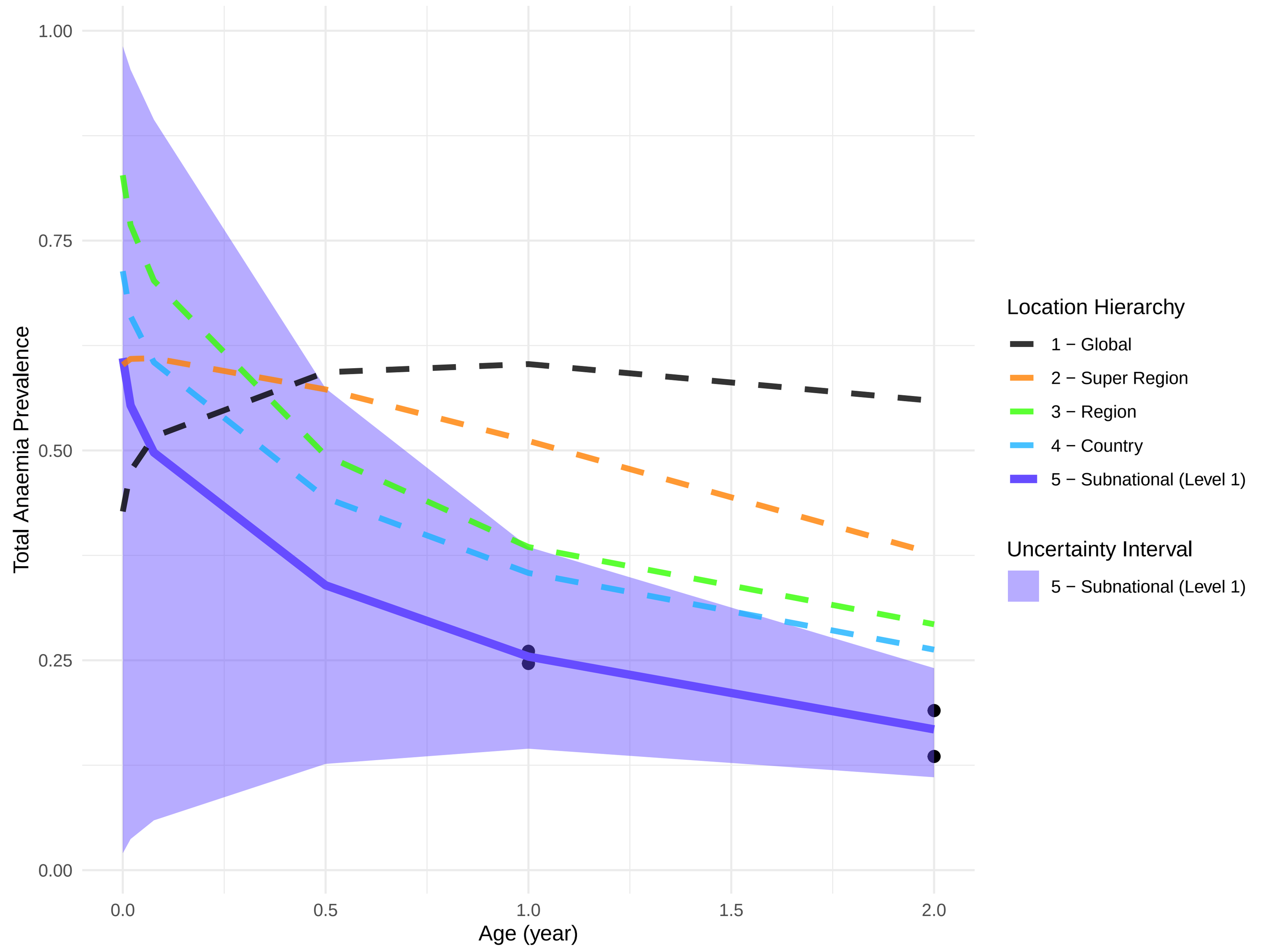

MR−BRT Age/Sex Fits − Non−pregnant Females <2 Years Old − Total Anaemia Prevalence − Nuevo León
WHO−2024 Adjustment Method

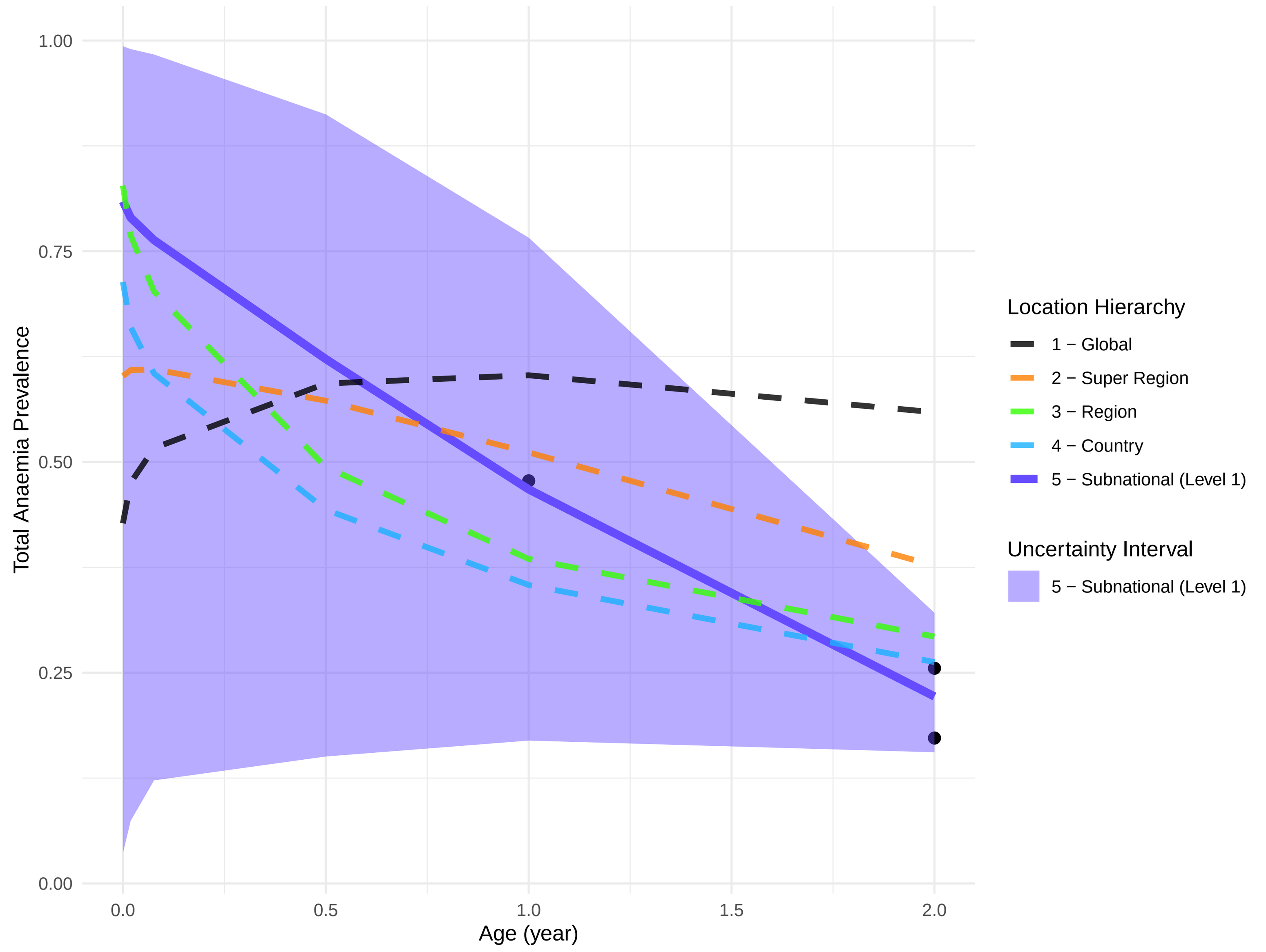

MR−BRT Age/Sex Fits − Non−pregnant Females <2 Years Old − Total Anaemia Prevalence − Oaxaca
WHO−2024 Adjustment Method

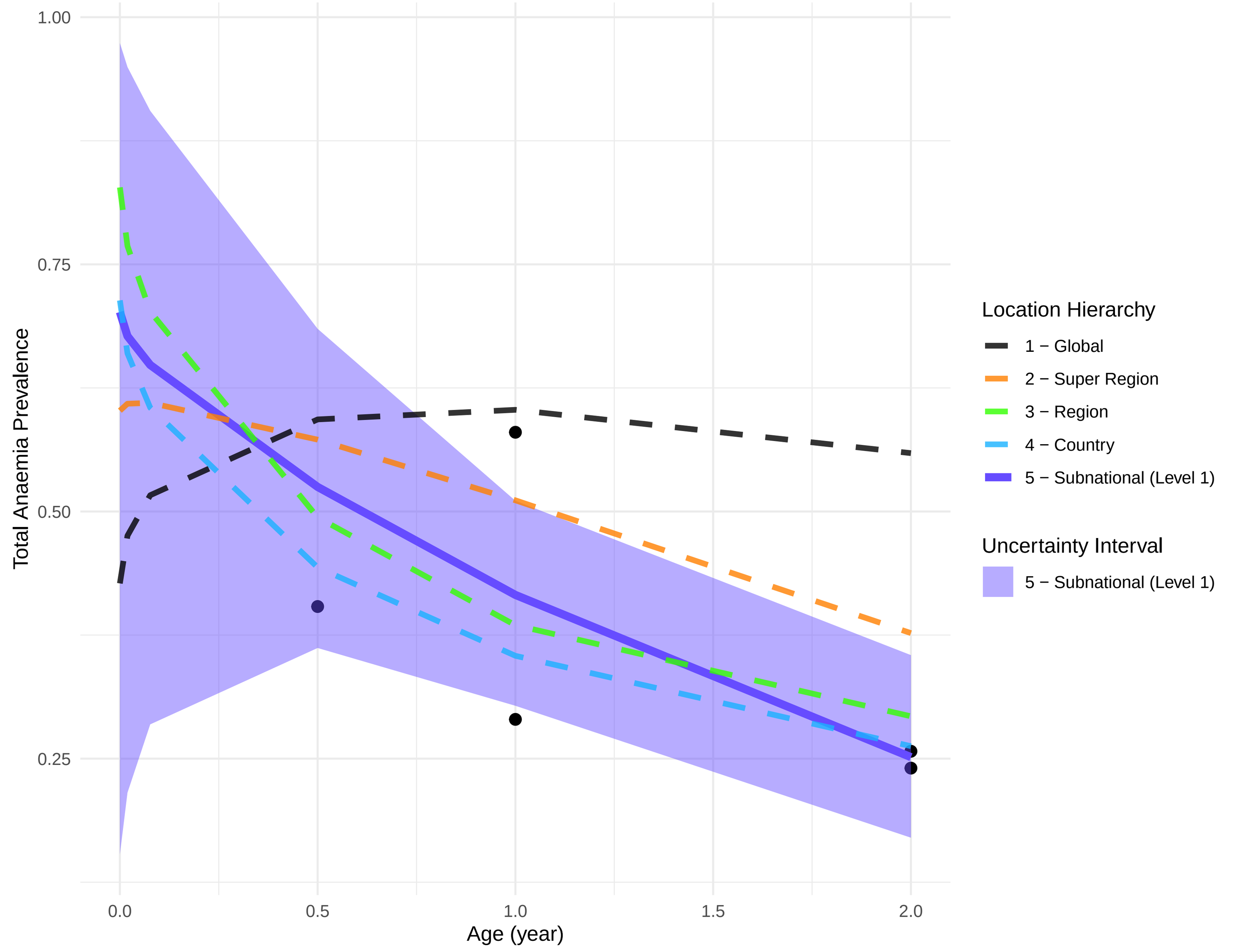

MR−BRT Age/Sex Fits − Non−pregnant Females <2 Years Old − Total Anaemia Prevalence − Puebla
WHO−2024 Adjustment Method

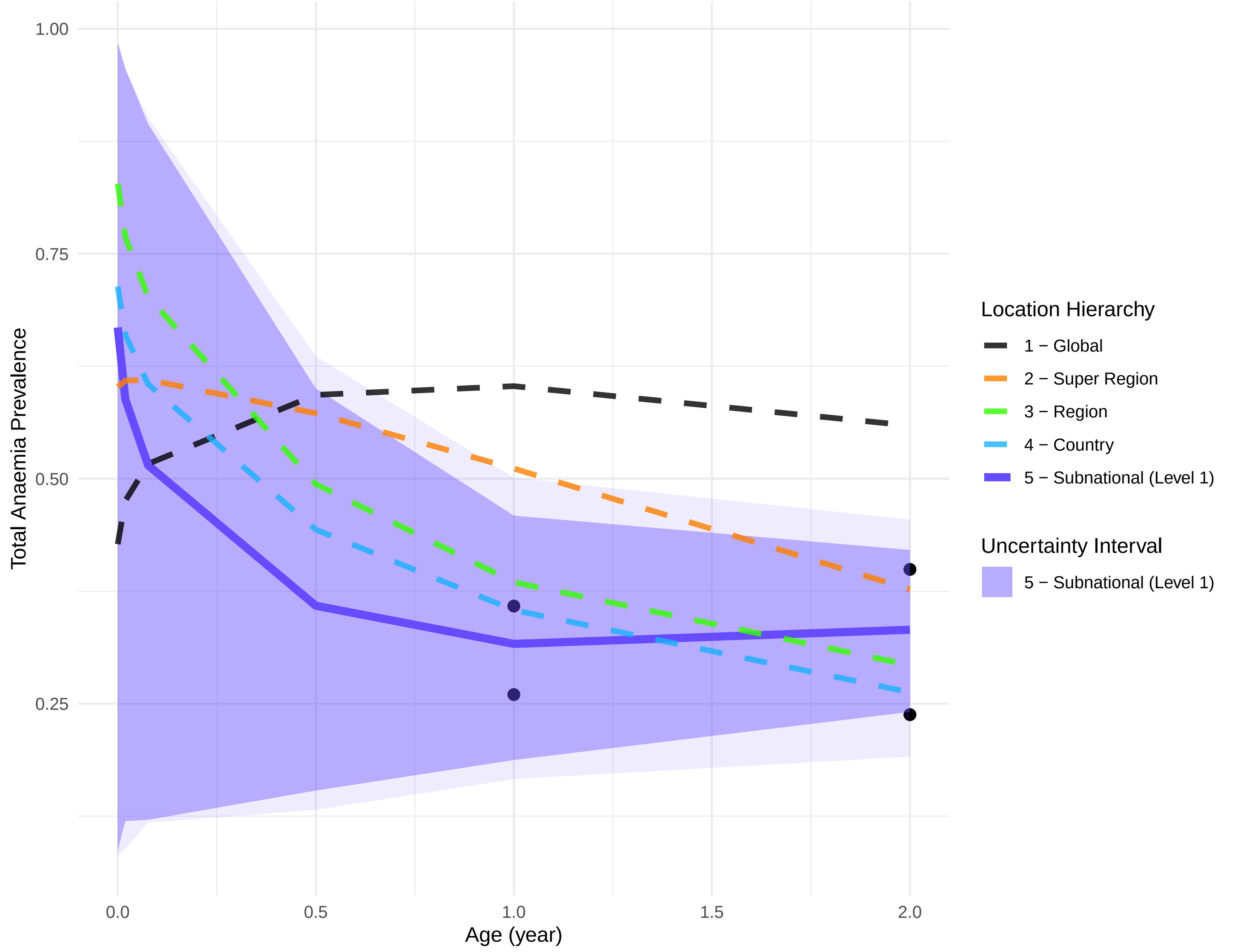

MR−BRT Age/Sex Fits − Non−pregnant Females <2 Years Old − Total Anaemia Prevalence − Querétaro
WHO−2024 Adjustment Method

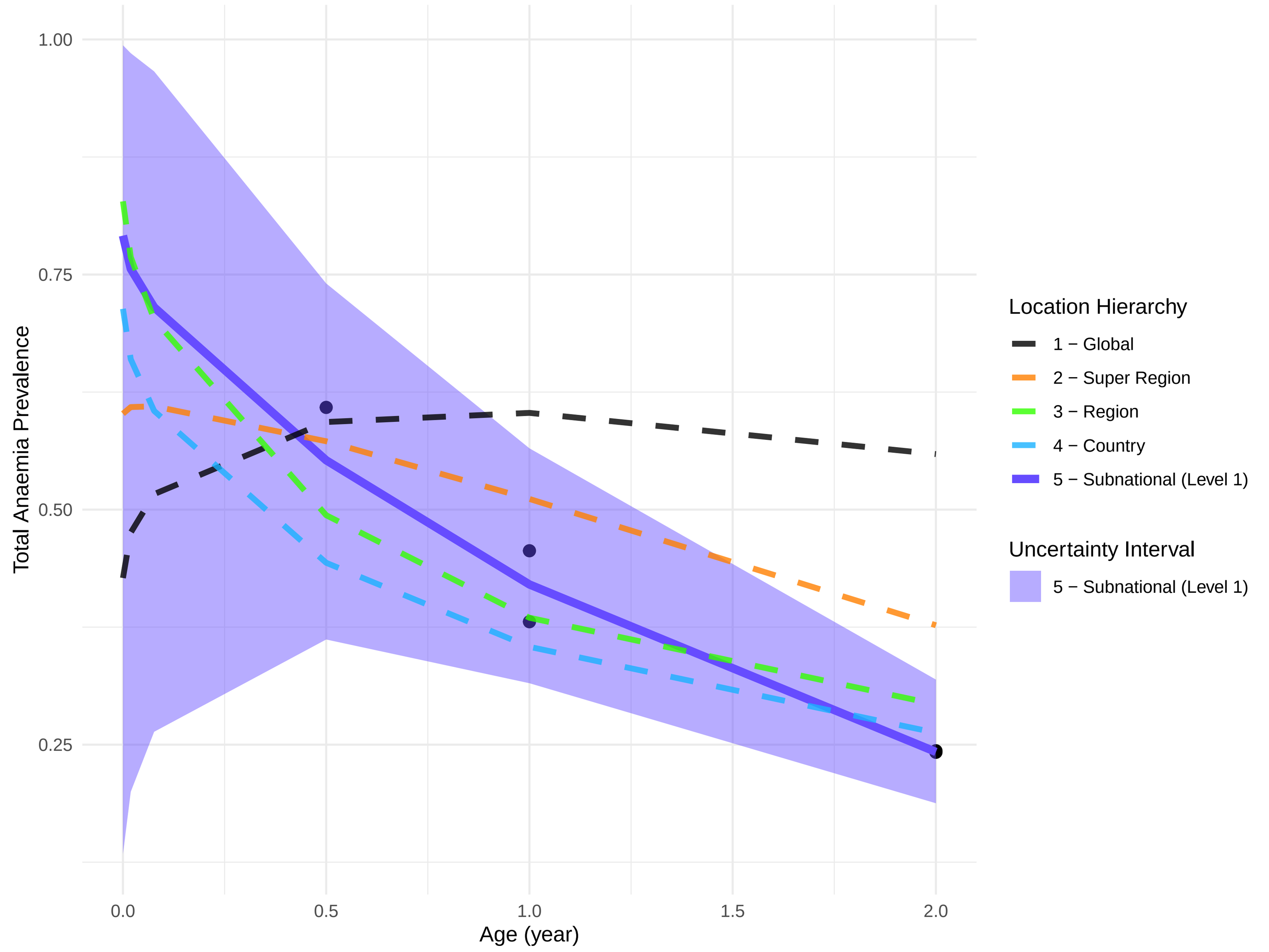

MR−BRT Age/Sex Fits − Non−pregnant Females <2 Years Old − Total Anaemia Prevalence − Quintana Roo
WHO−2024 Adjustment Method

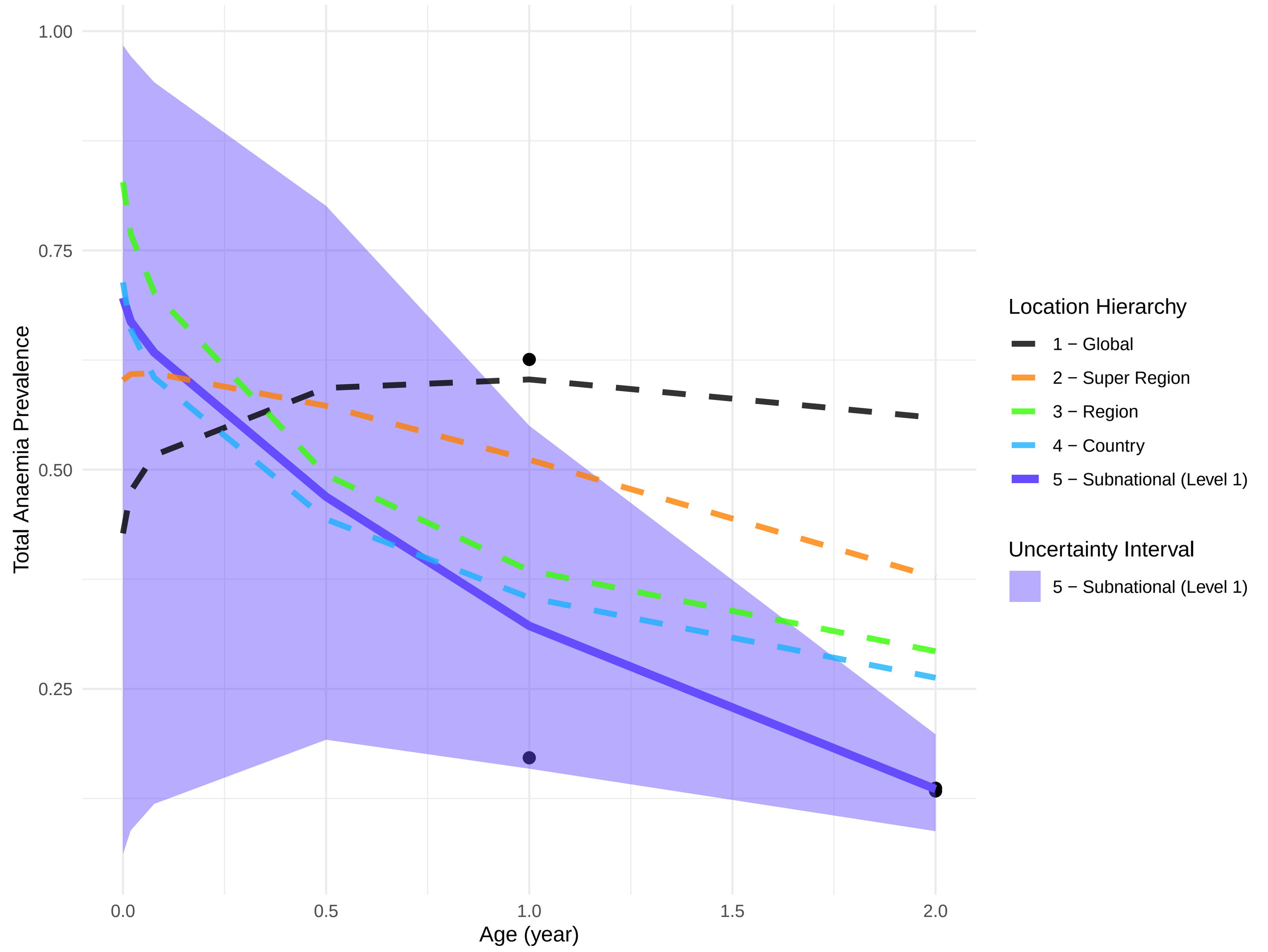

MR−BRT Age/Sex Fits − Non−pregnant Females <2 Years Old − Total Anaemia Prevalence − San Luis Potosí
WHO−2024 Adjustment Method

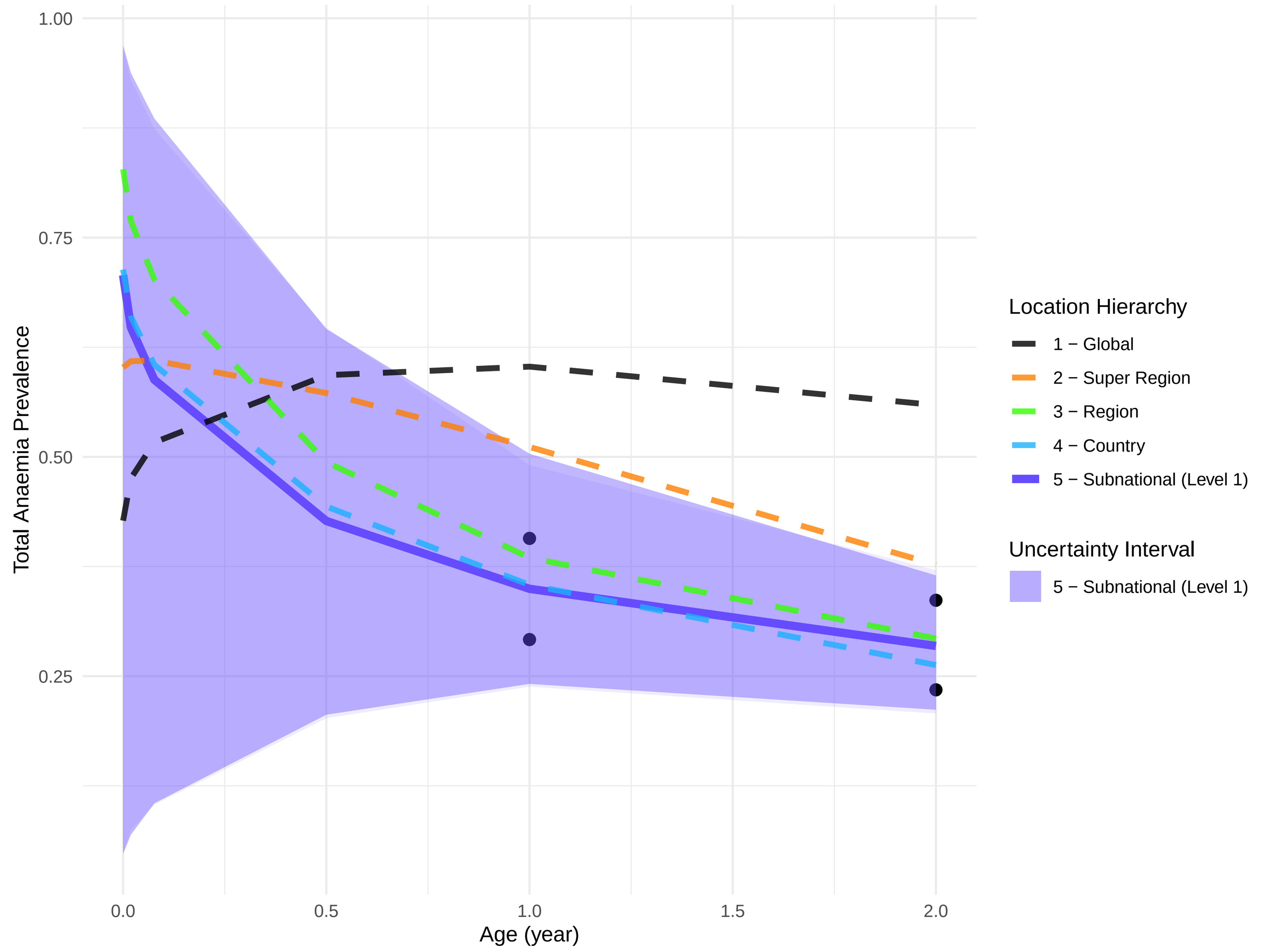

MR−BRT Age/Sex Fits − Non−pregnant Females <2 Years Old − Total Anaemia Prevalence − Sinaloa
WHO−2024 Adjustment Method

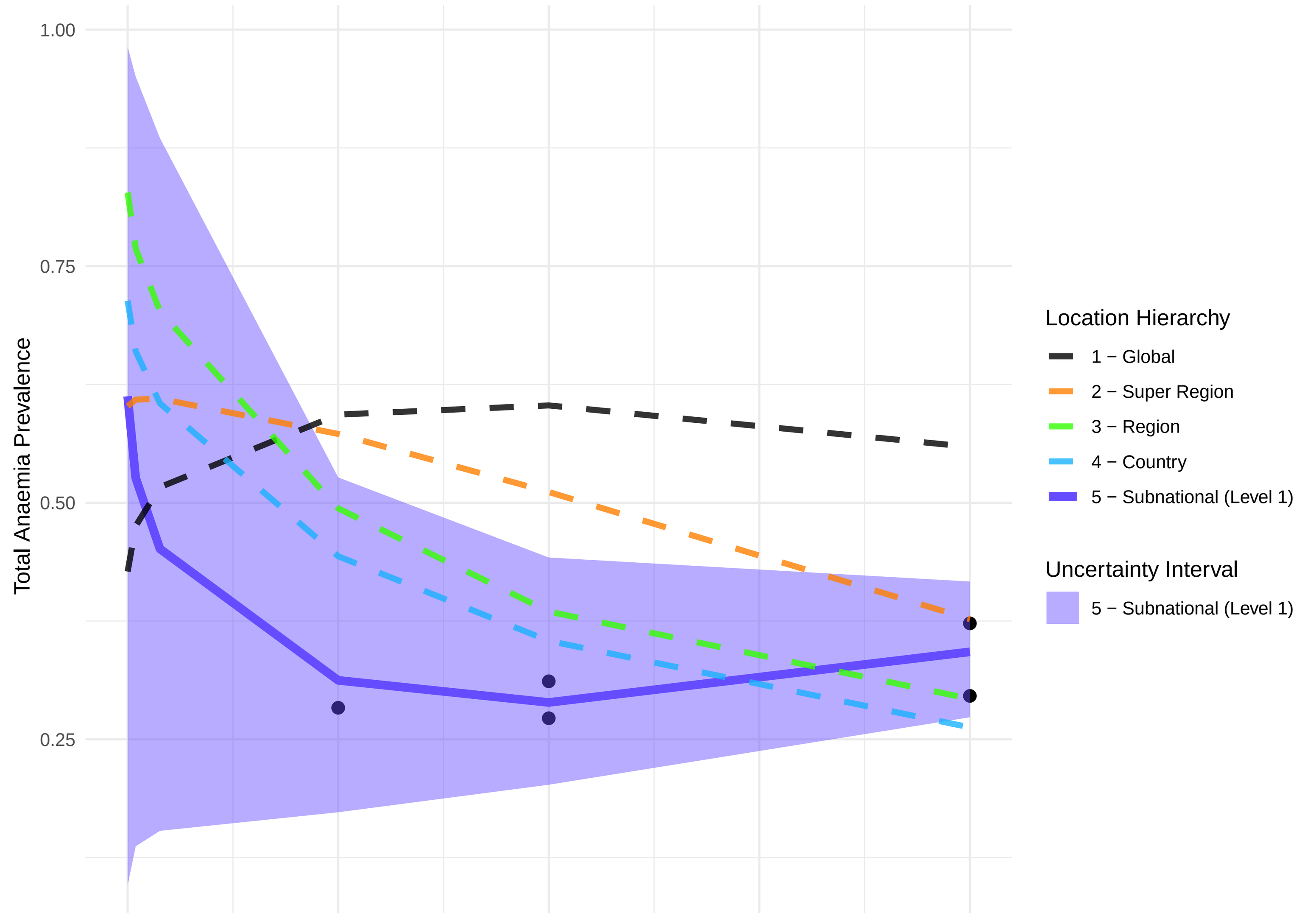

MR−BRT Age/Sex Fits − Non−pregnant Females <2 Years Old − Total Anaemia Prevalence − Sonora
WHO−2024 Adjustment Method

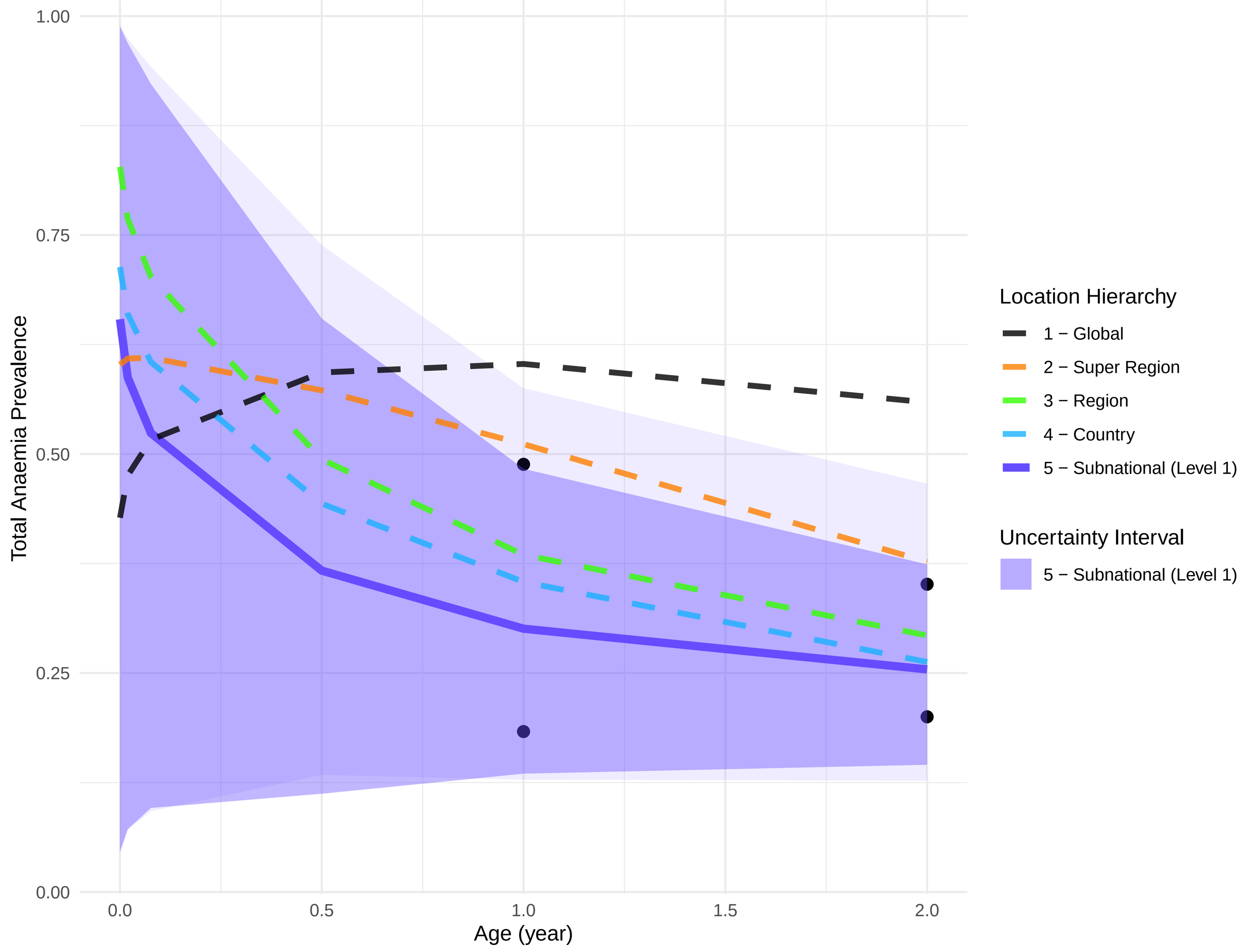

MR−BRT Age/Sex Fits − Non−pregnant Females <2 Years Old − Total Anaemia Prevalence − Tabasco
WHO−2024 Adjustment Method

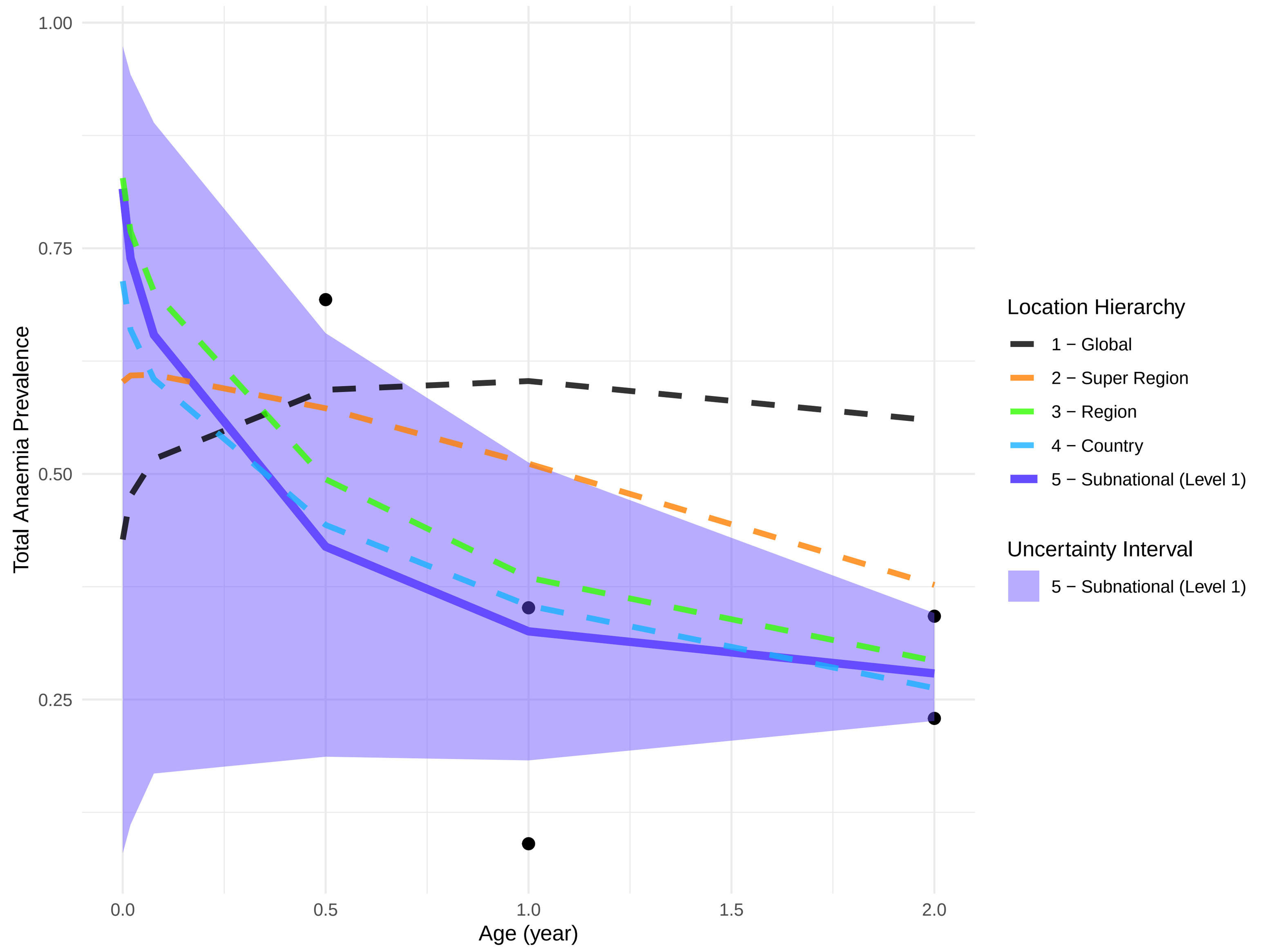

MR−BRT Age/Sex Fits − Non−pregnant Females <2 Years Old − Total Anaemia Prevalence − Tamaulipas
WHO−2024 Adjustment Method

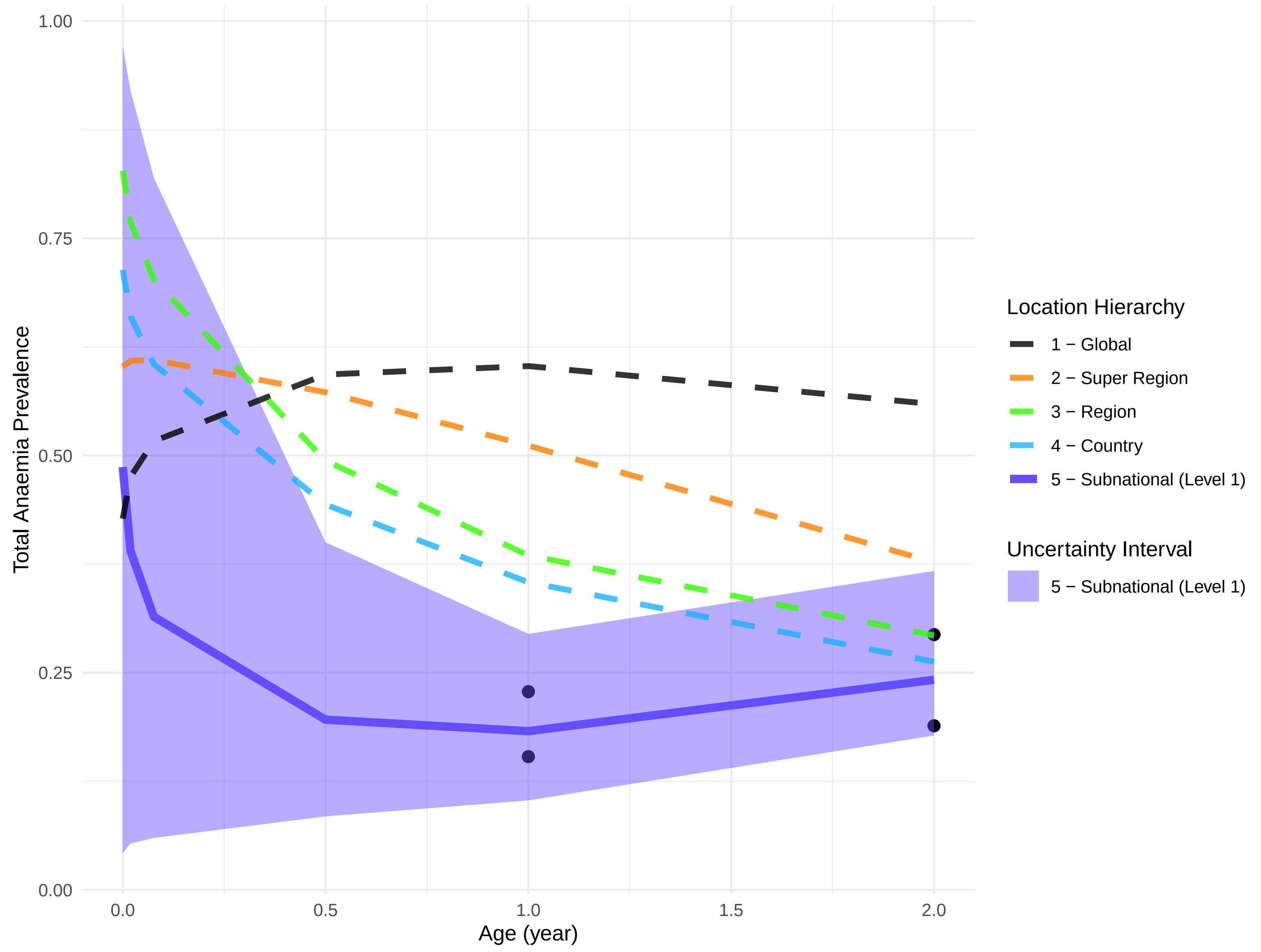

MR−BRT Age/Sex Fits − Non−pregnant Females <2 Years Old − Total Anaemia Prevalence − Tlaxcala
WHO−2024 Adjustment Method

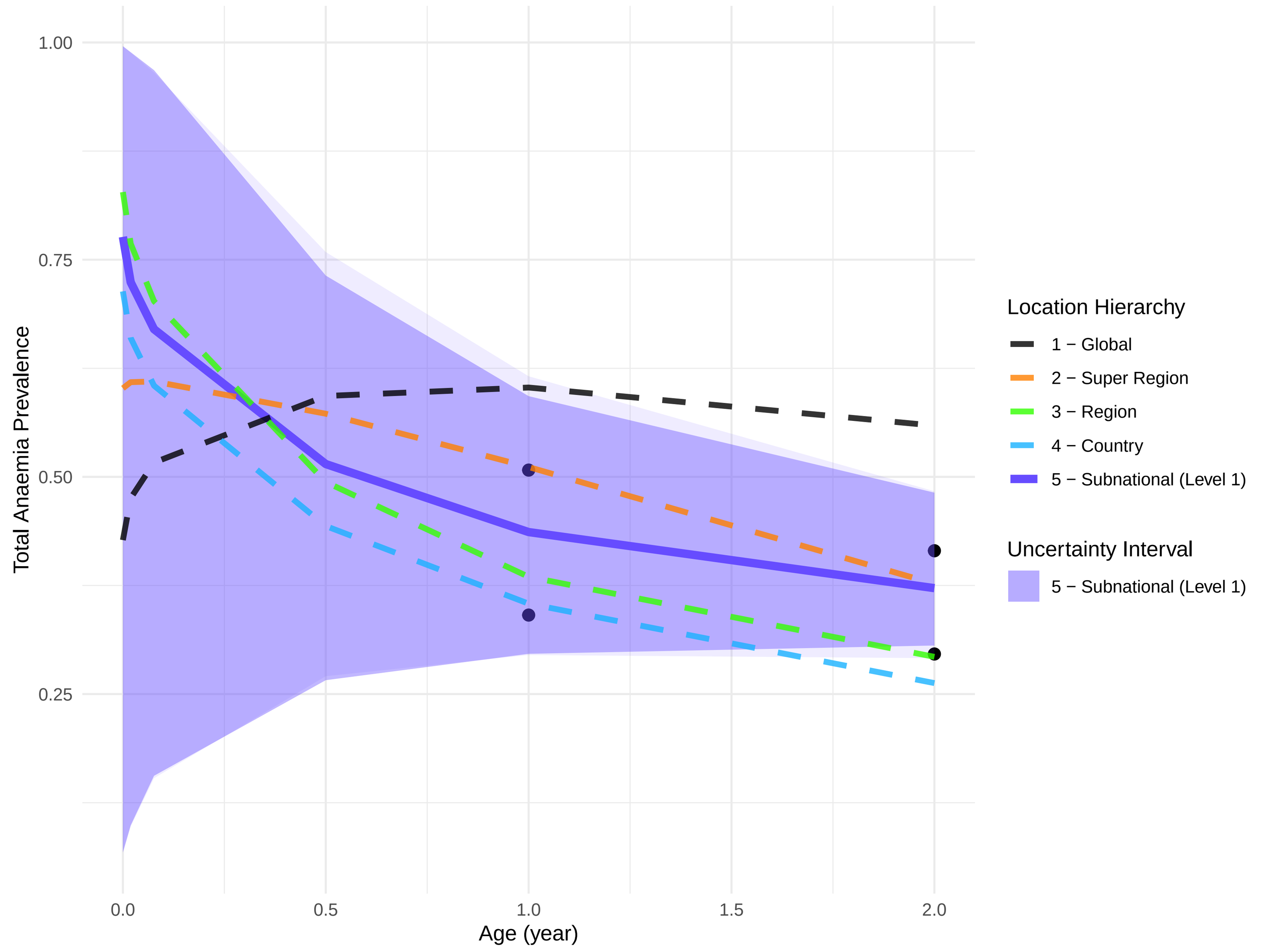

MR−BRT Age/Sex Fits − Non−pregnant Females <2 Years Old − Total Anaemia Prevalence − Veracruz de Ignacio de la Llave
WHO−2024 Adjustment Method

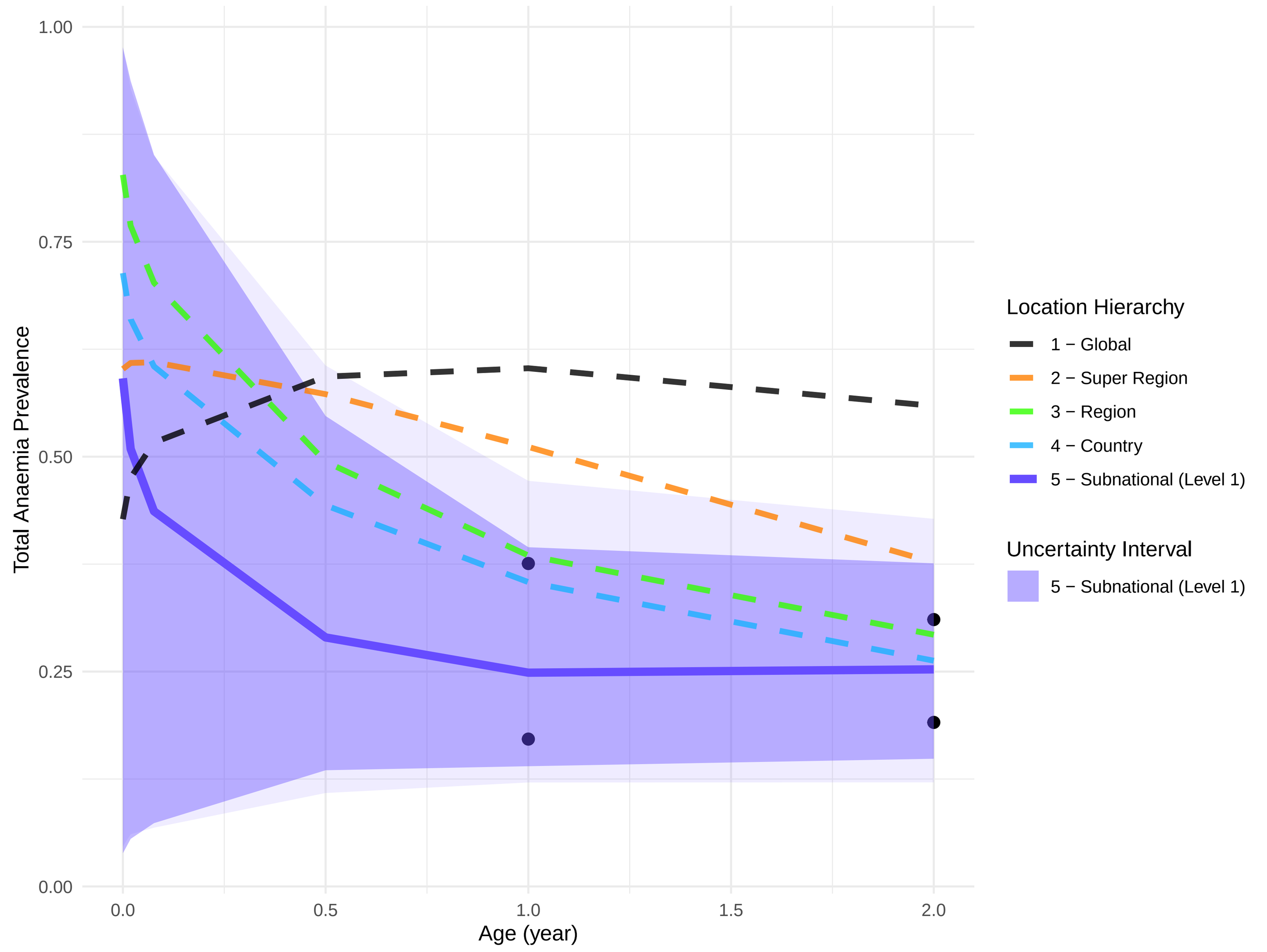

MR−BRT Age/Sex Fits − Non−pregnant Females <2 Years Old − Total Anaemia Prevalence − Yucatán
WHO−2024 Adjustment Method

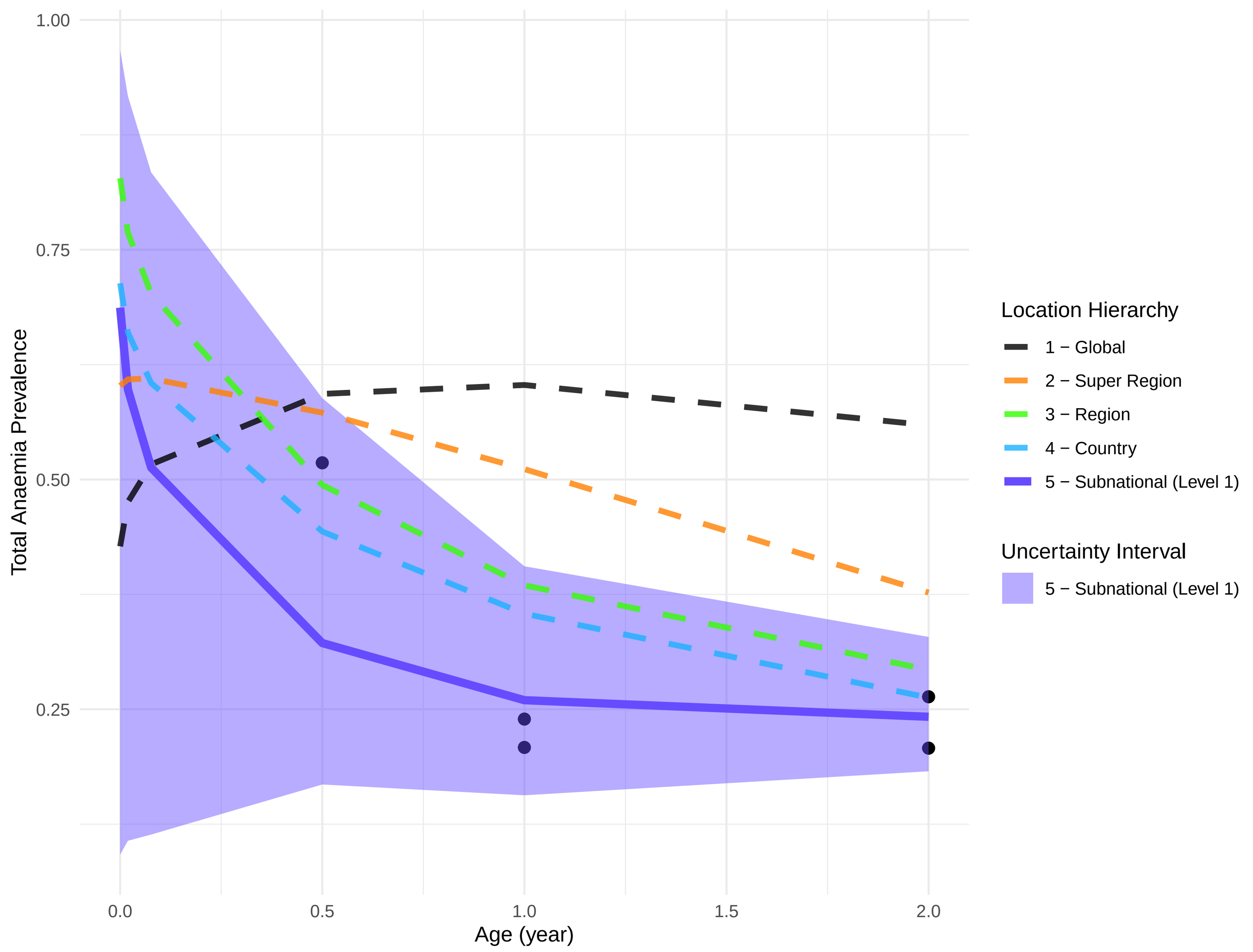

MR−BRT Age/Sex Fits − Non−pregnant Females <2 Years Old − Total Anaemia Prevalence − Zacatecas
WHO−2024 Adjustment Method

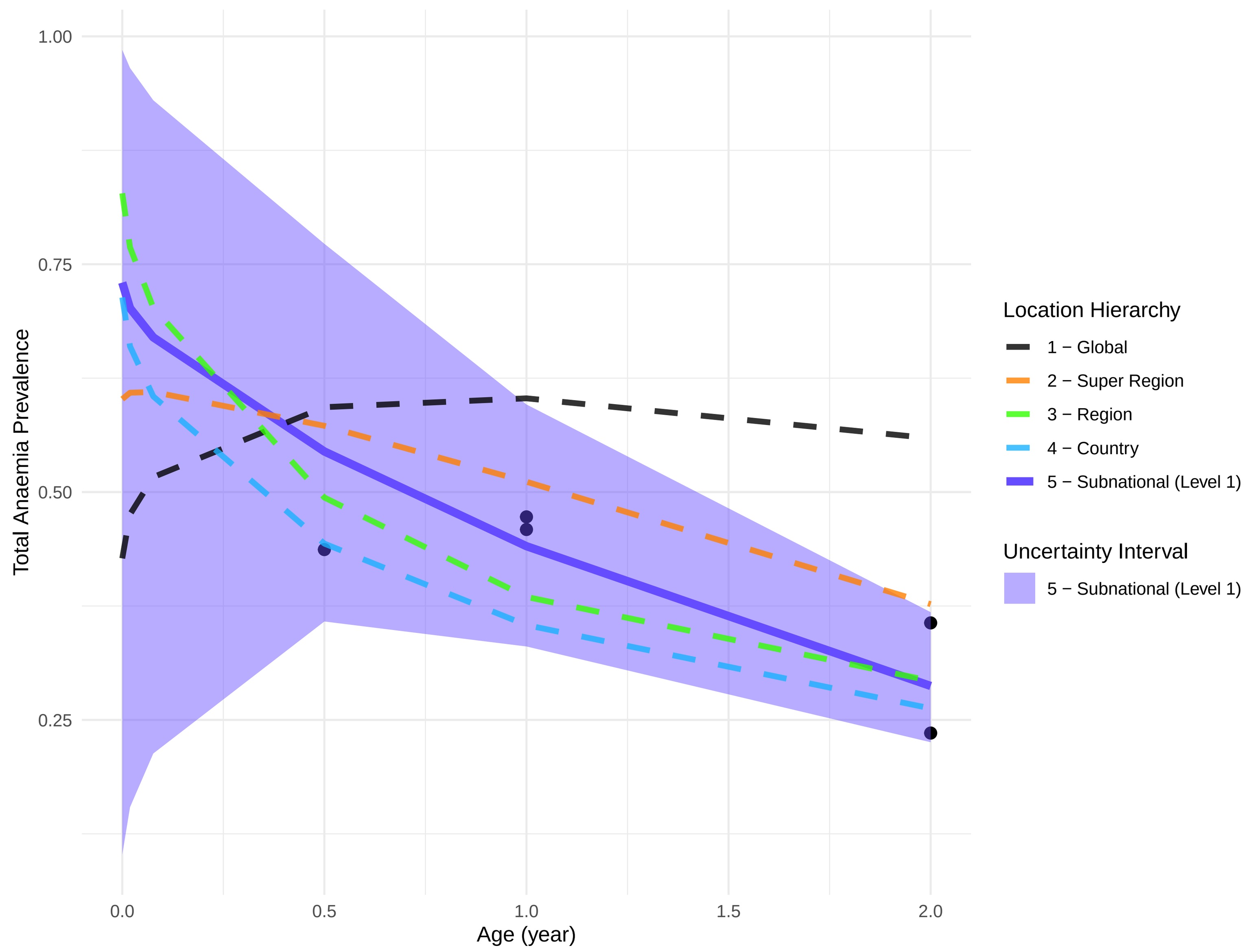

MR−BRT Age/Sex Fits − Non−pregnant Females <2 Years Old − Total Anaemia Prevalence − North Africa and Middle East
WHO−2024 Adjustment Method

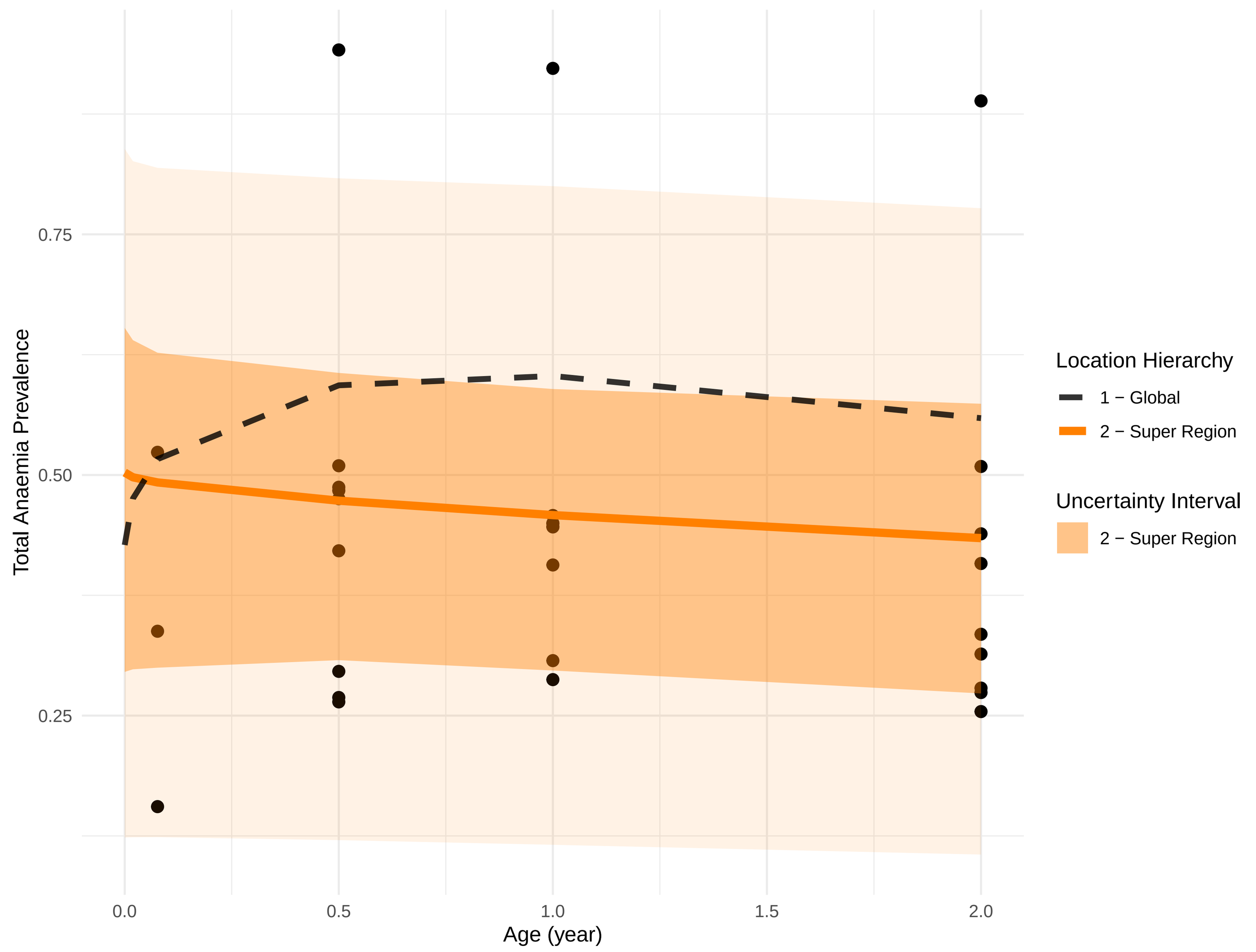

MR−BRT Age/Sex Fits − Non−pregnant Females <2 Years Old − Total Anaemia Prevalence − North Africa and Middle East
WHO−2024 Adjustment Method

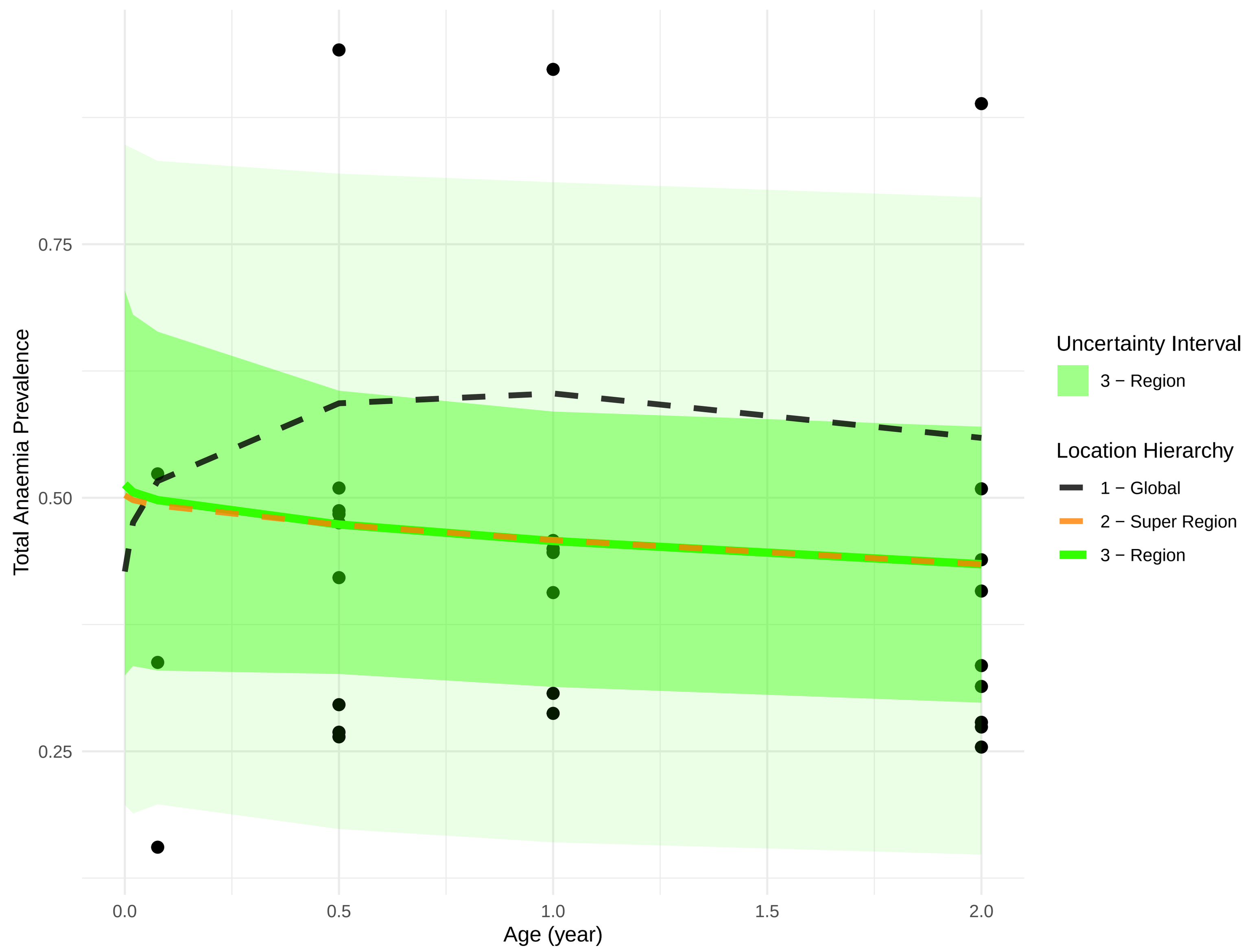

MR−BRT Age/Sex Fits − Non−pregnant Females <2 Years Old − Total Anaemia Prevalence − Egypt
WHO−2024 Adjustment Method

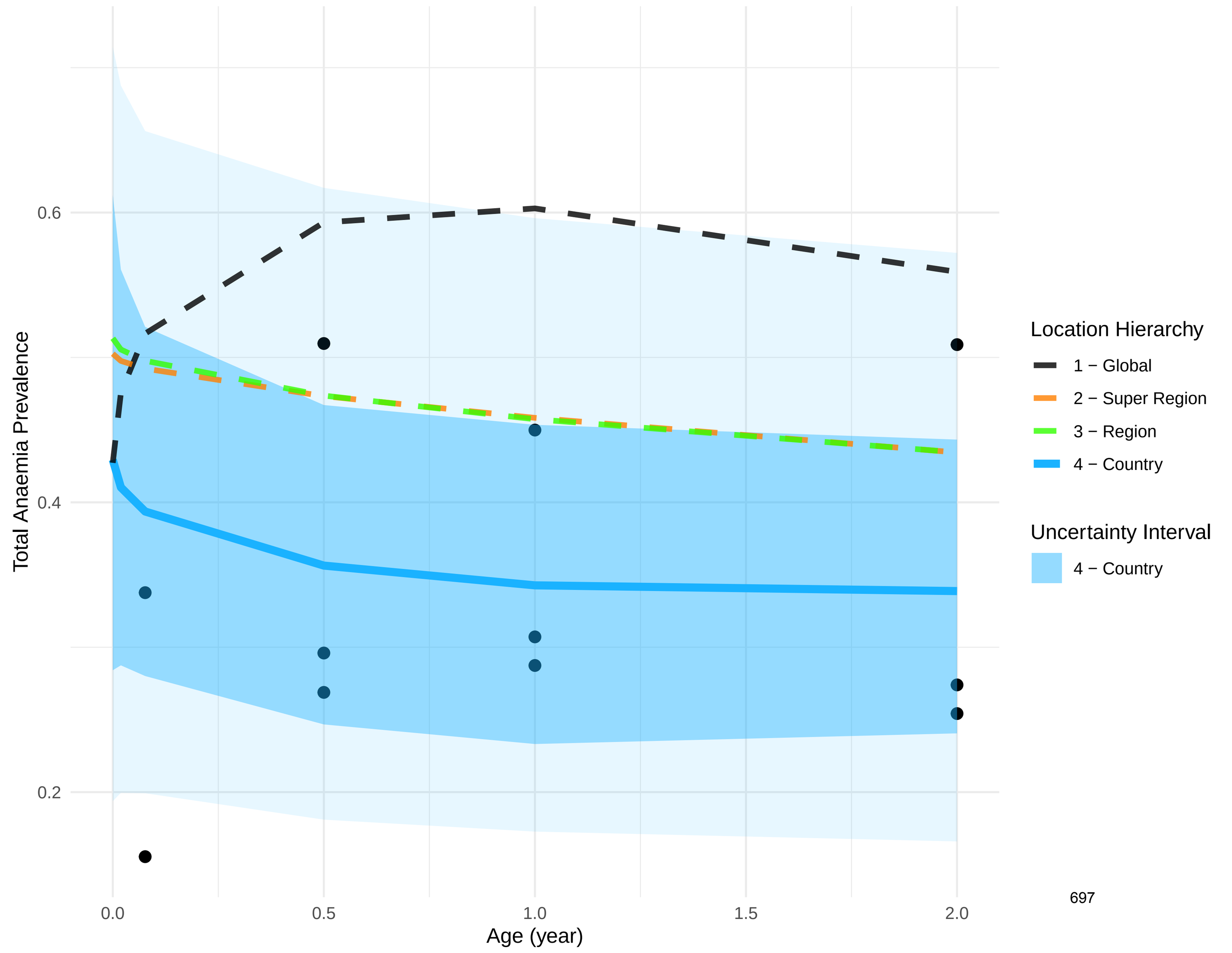

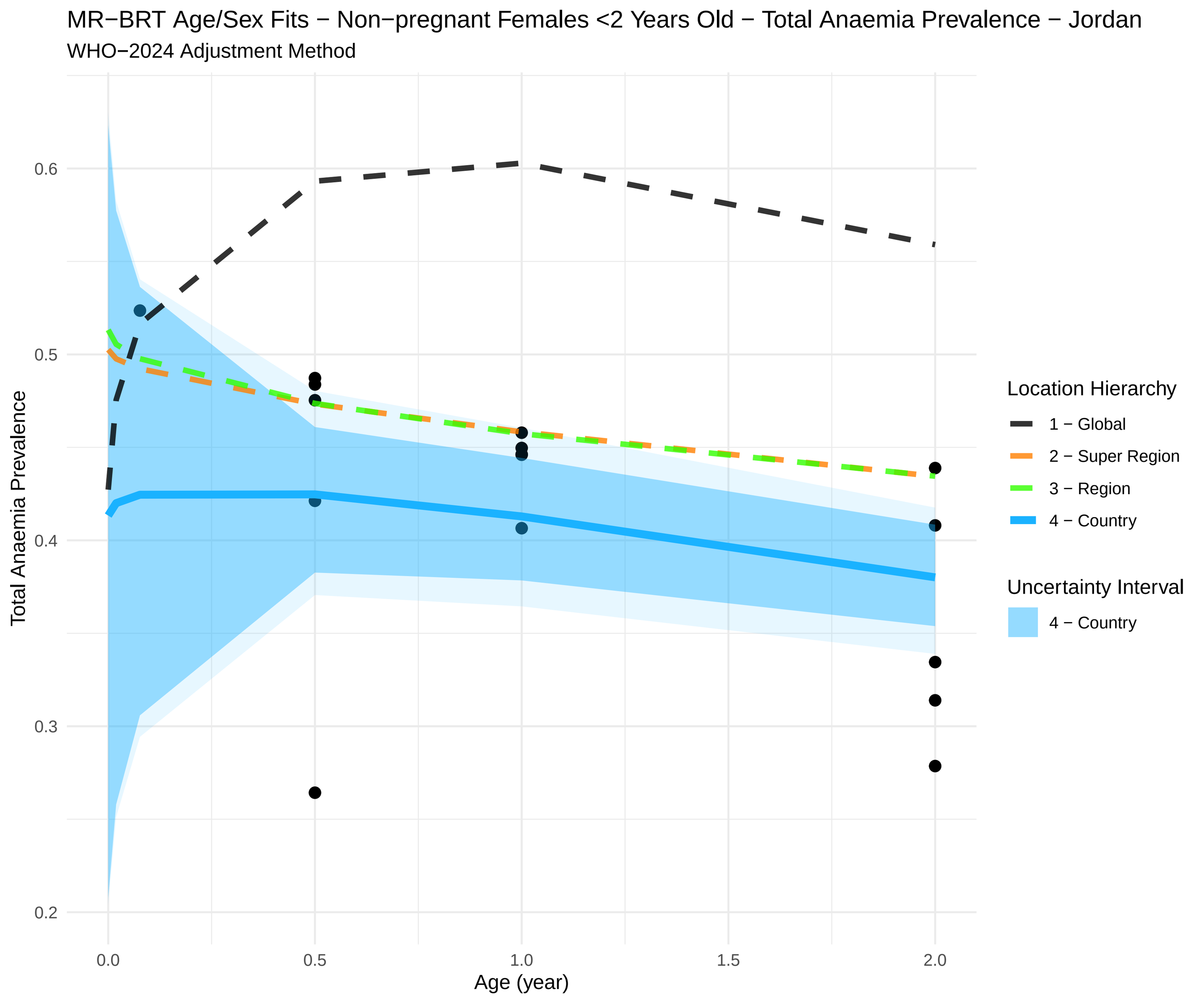
MR−BRT Age/Sex Fits − Non−pregnant Females <2 Years Old − Total Anaemia Prevalence − Jordan
WHO−2024 Adjustment Method
Total Anaemia Prevalence
Age (year)
Location Hierarchy
1 − Global
2 − Super Region
3 − Region
4 − Country
Uncertainty Interval
4 − Country

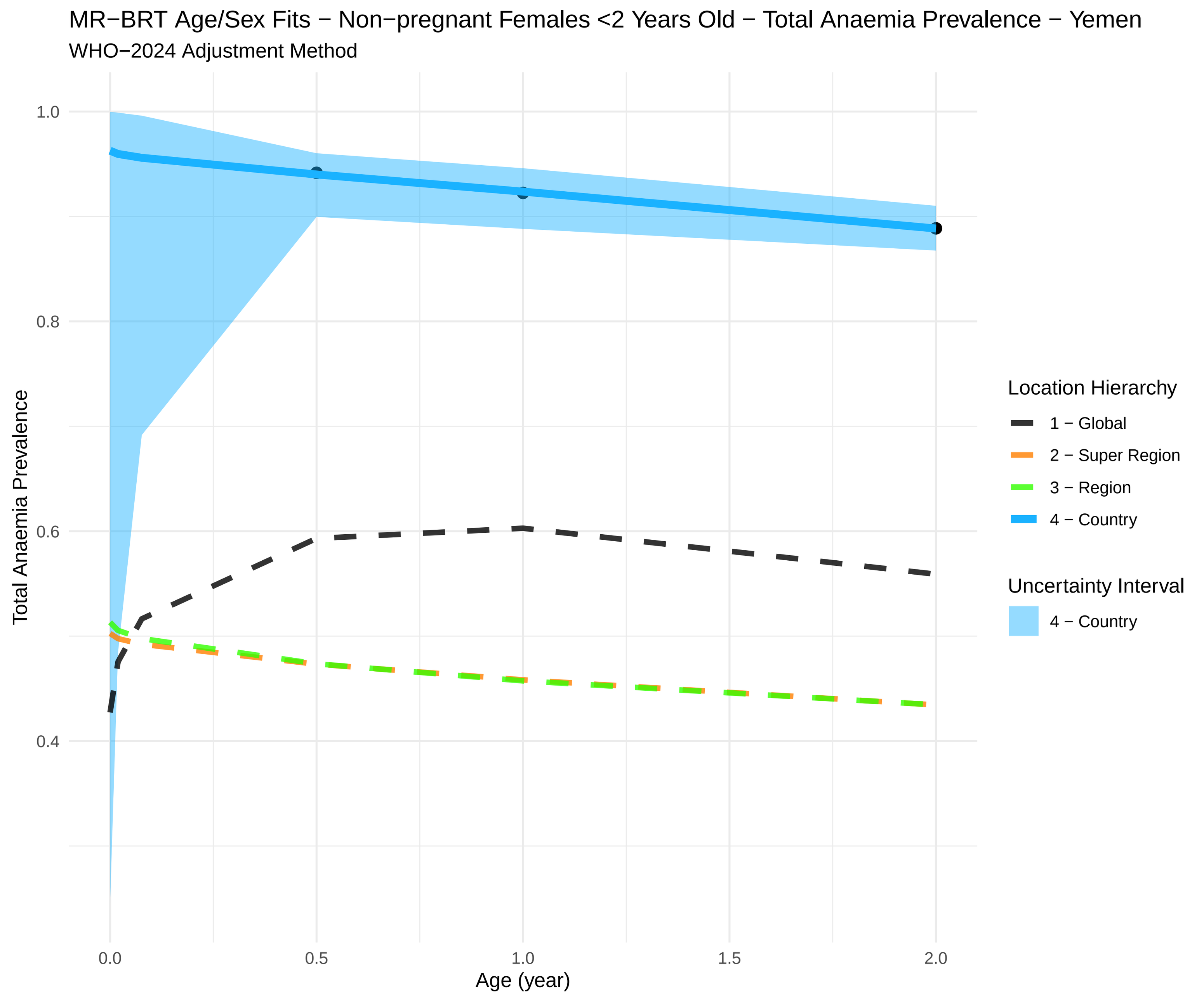
MR−BRT Age/Sex Fits − Non−pregnant Females <2 Years Old − Total Anaemia Prevalence − Yemen
WHO−2024 Adjustment Method
Total Anaemia Prevalence
Age (year)
Location Hierarchy
1 − Global
2 − Super Region
3 − Region
4 − Country
Uncertainty Interval
4 − Country

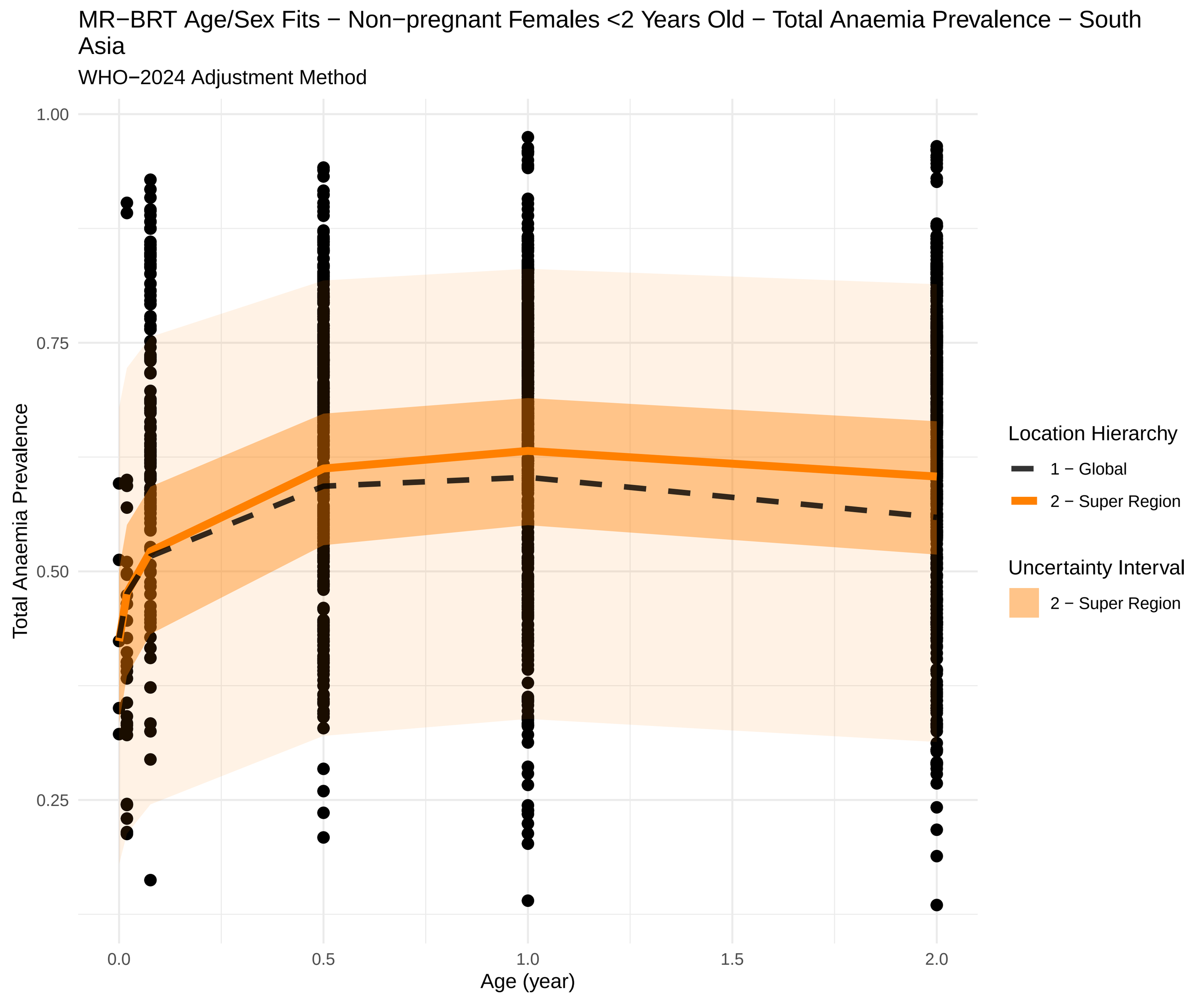
MR−BRT Age/Sex Fits − Non−pregnant Females <2 Years Old − Total Anaemia Prevalence − South Asia
WHO−2024 Adjustment Method
Total Anaemia Prevalence
Age (year)
Location Hierarchy
1 − Global
2 − Super Region
Uncertainty Interval
2 − Super Region

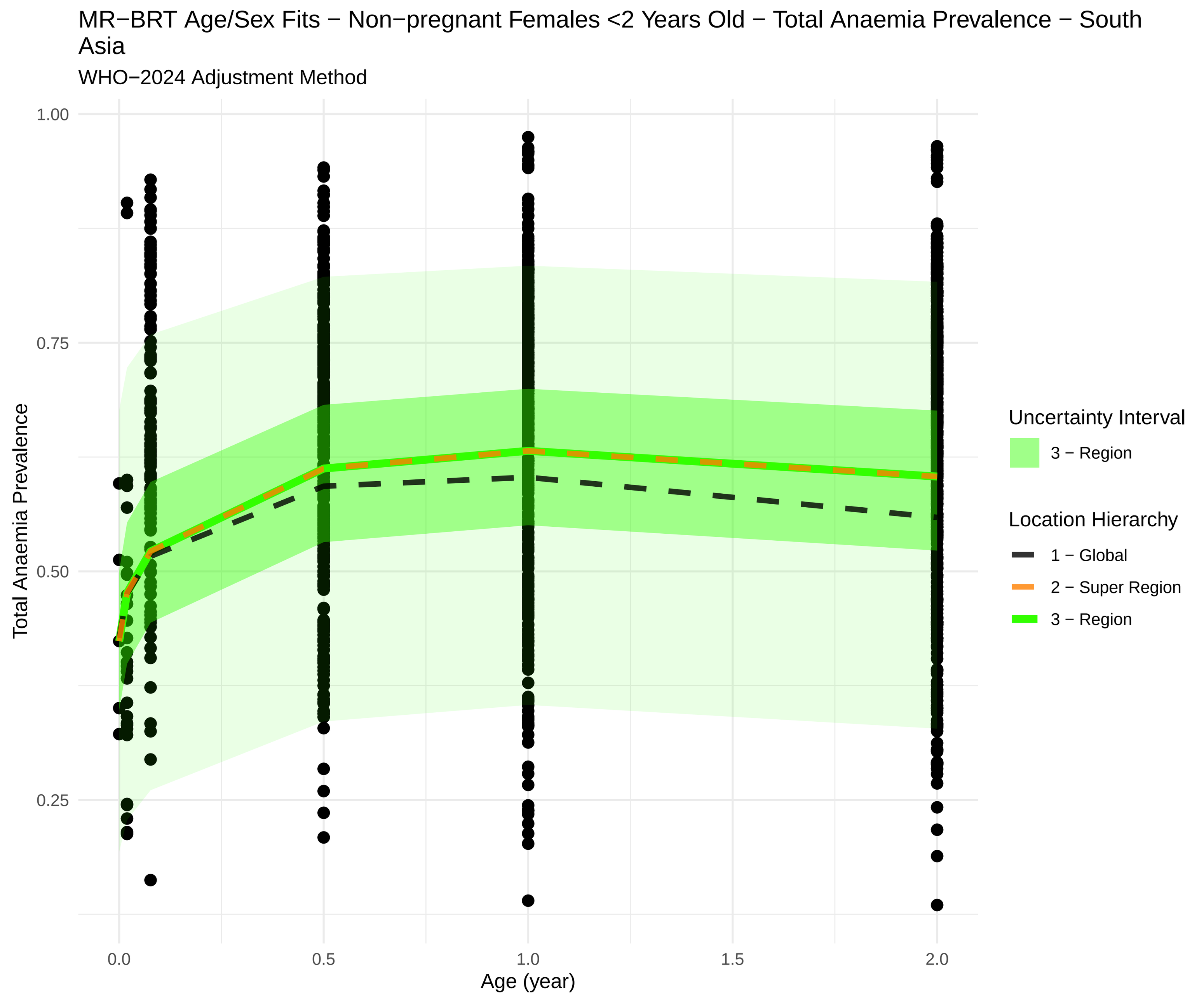
MR−BRT Age/Sex Fits − Non−pregnant Females <2 Years Old − Total Anaemia Prevalence − South Asia
WHO−2024 Adjustment Method
Total Anaemia Prevalence
Age (year)
Uncertainty Interval
3 − Region
Location Hierarchy
1 − Global
2 − Super Region
3 − Region

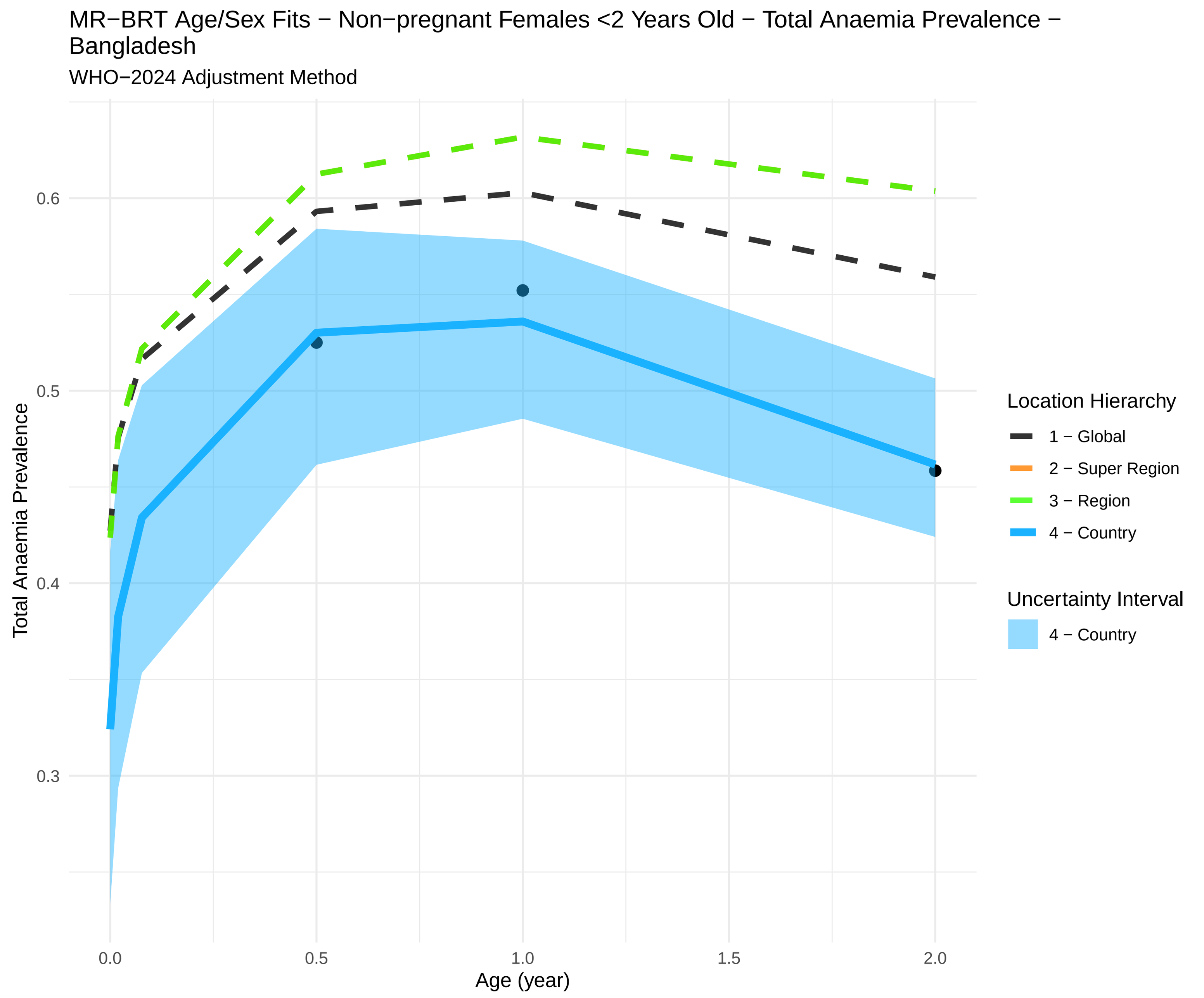
MR−BRT Age/Sex Fits − Non−pregnant Females <2 Years Old − Total Anaemia Prevalence − Bangladesh
WHO−2024 Adjustment Method
Total Anaemia Prevalence
Age (year)
Location Hierarchy
1 − Global
2 − Super Region
3 − Region
4 − Country
Uncertainty Interval
4 − Country

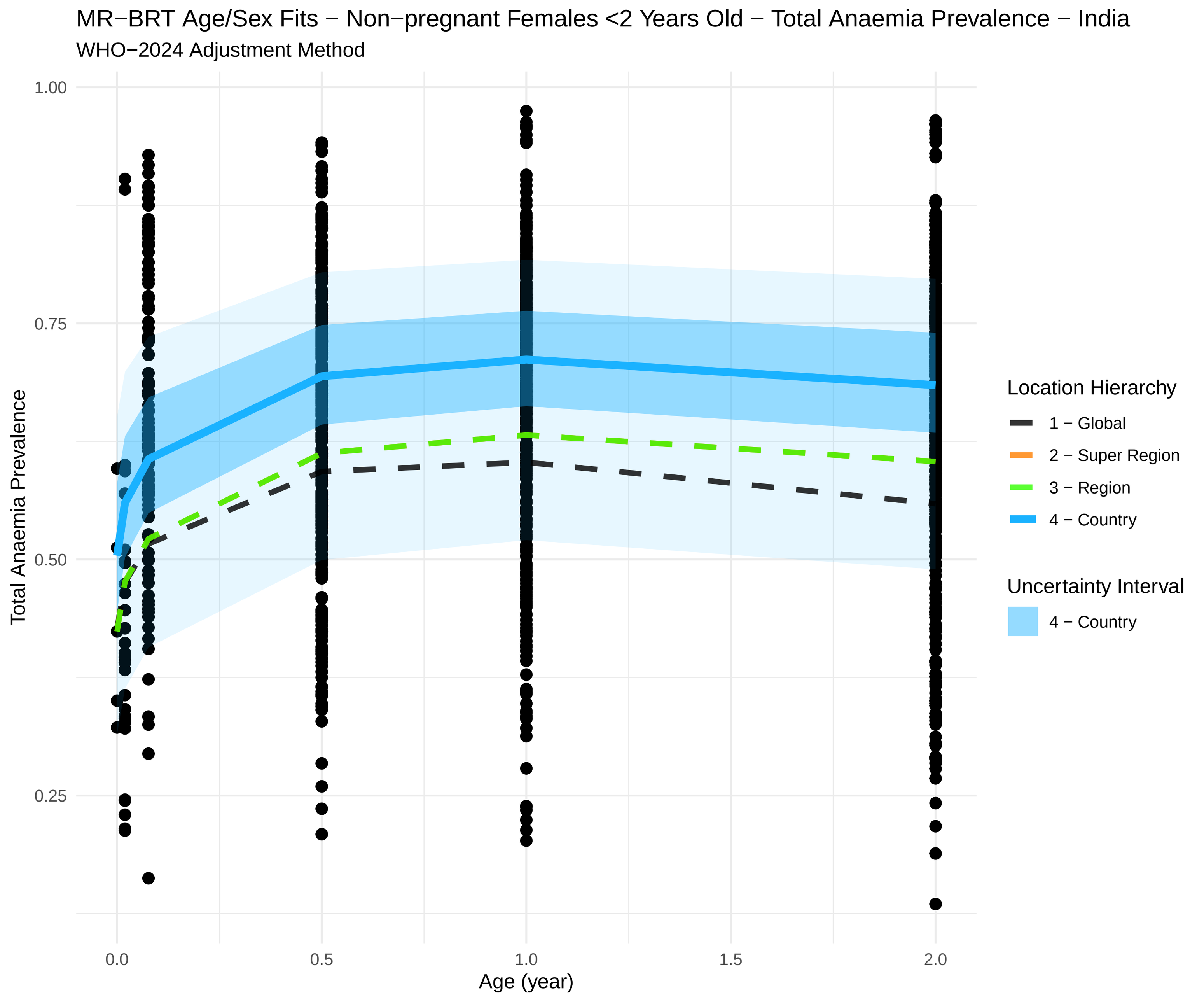
MR−BRT Age/Sex Fits − Non−pregnant Females <2 Years Old − Total Anaemia Prevalence − India
WHO−2024 Adjustment Method
Total Anaemia Prevalence
Age (year)
Location Hierarchy
1 − Global
2 − Super Region
3 − Region
4 − Country
Uncertainty Interval
4 − Country

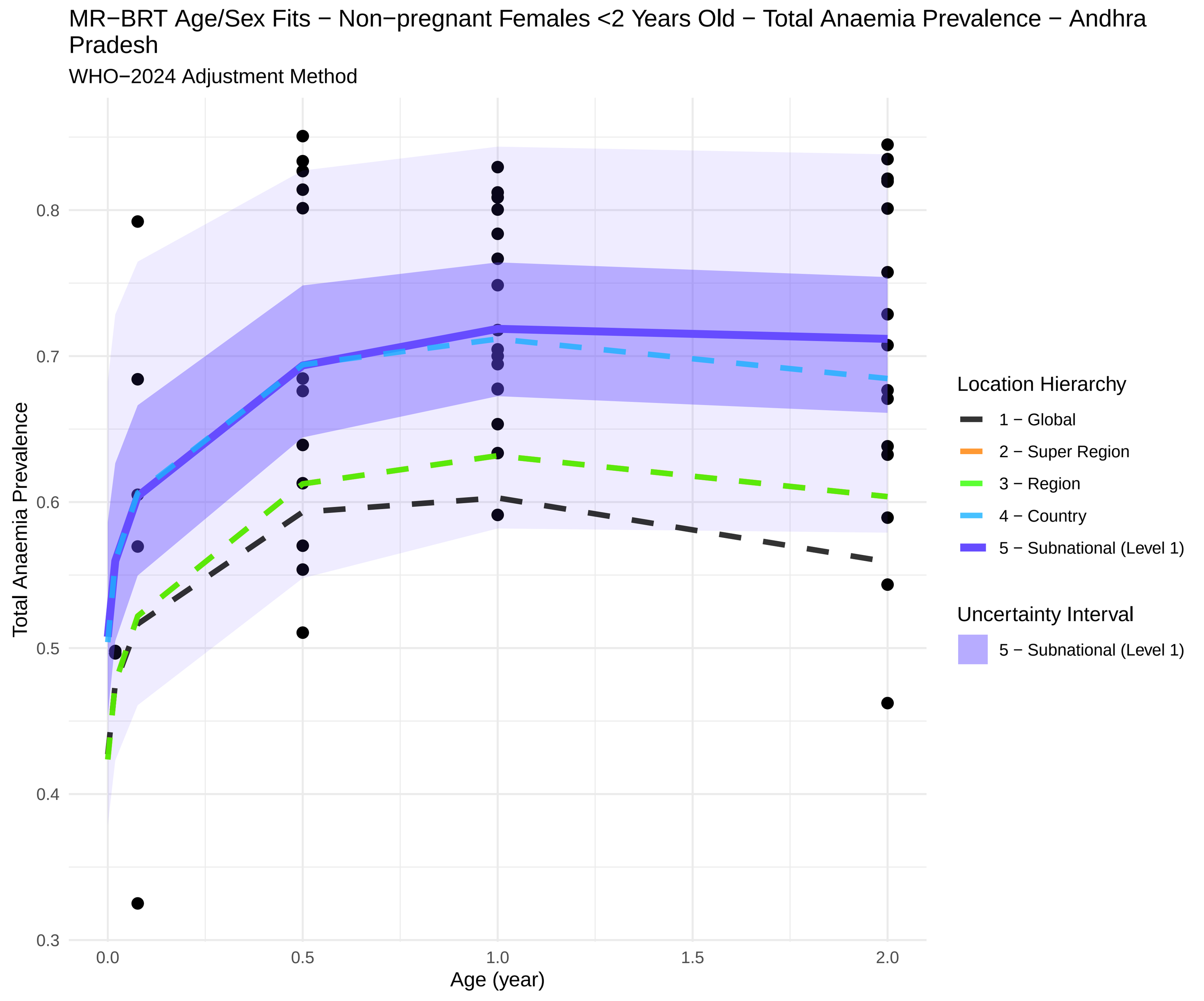
MR−BRT Age/Sex Fits − Non−pregnant Females <2 Years Old − Total Anaemia Prevalence − Andhra Pradesh
WHO−2024 Adjustment Method
Total Anaemia Prevalence
Age (year)
Location Hierarchy
1 − Global
2 − Super Region
3 − Region
4 − Country
5 − Subnational (Level 1)
Uncertainty Interval
5 − Subnational (Level 1)

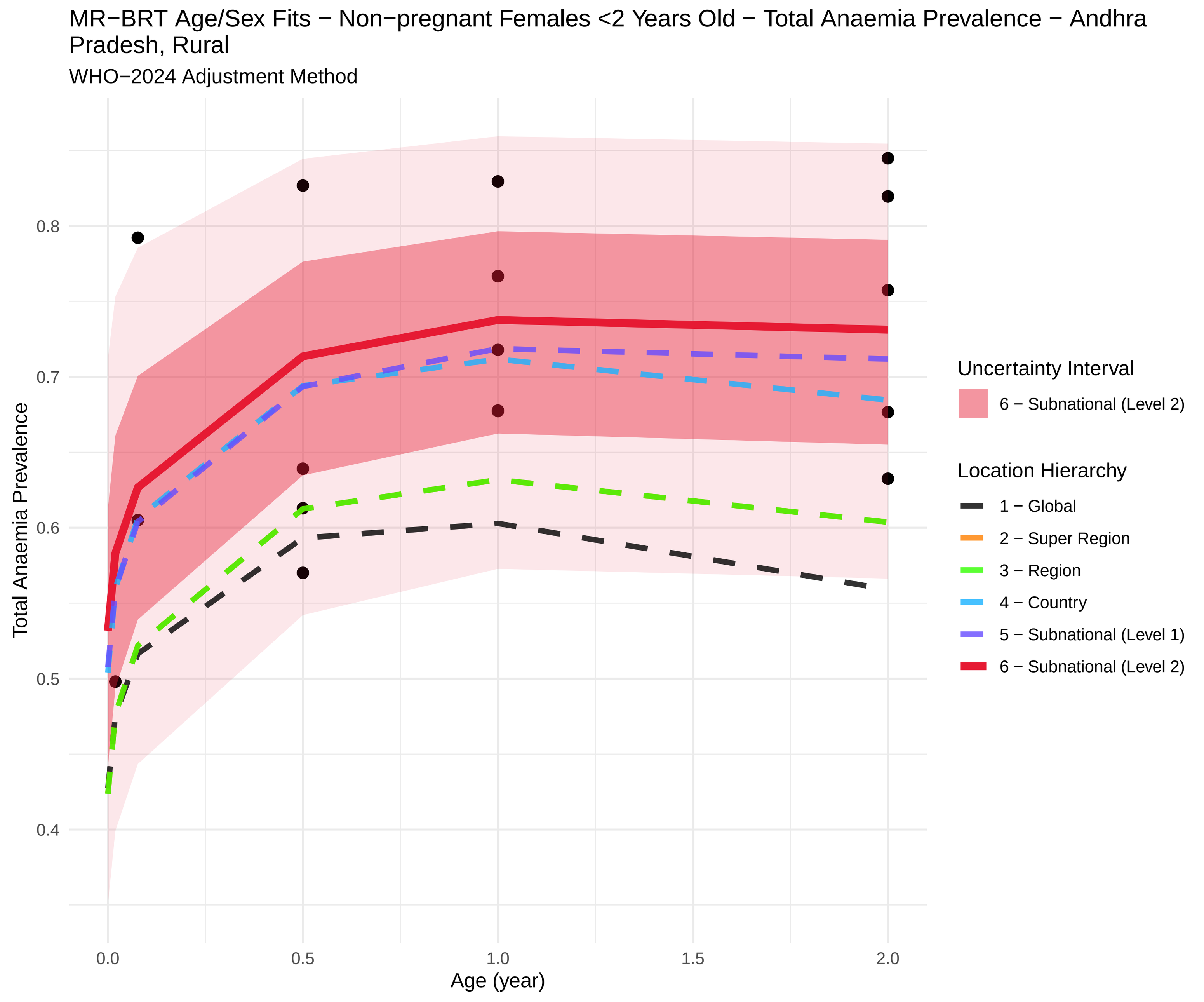
MR−BRT Age/Sex Fits − Non−pregnant Females <2 Years Old − Total Anaemia Prevalence − Andhra Pradesh, Rural
WHO−2024 Adjustment Method
Total Anaemia Prevalence
Age (year)
Uncertainty Interval
6 − Subnational (Level 2)
Location Hierarchy
1 − Global
2 − Super Region
3 − Region
4 − Country
5 − Subnational (Level 1)
6 − Subnational (Level 2)

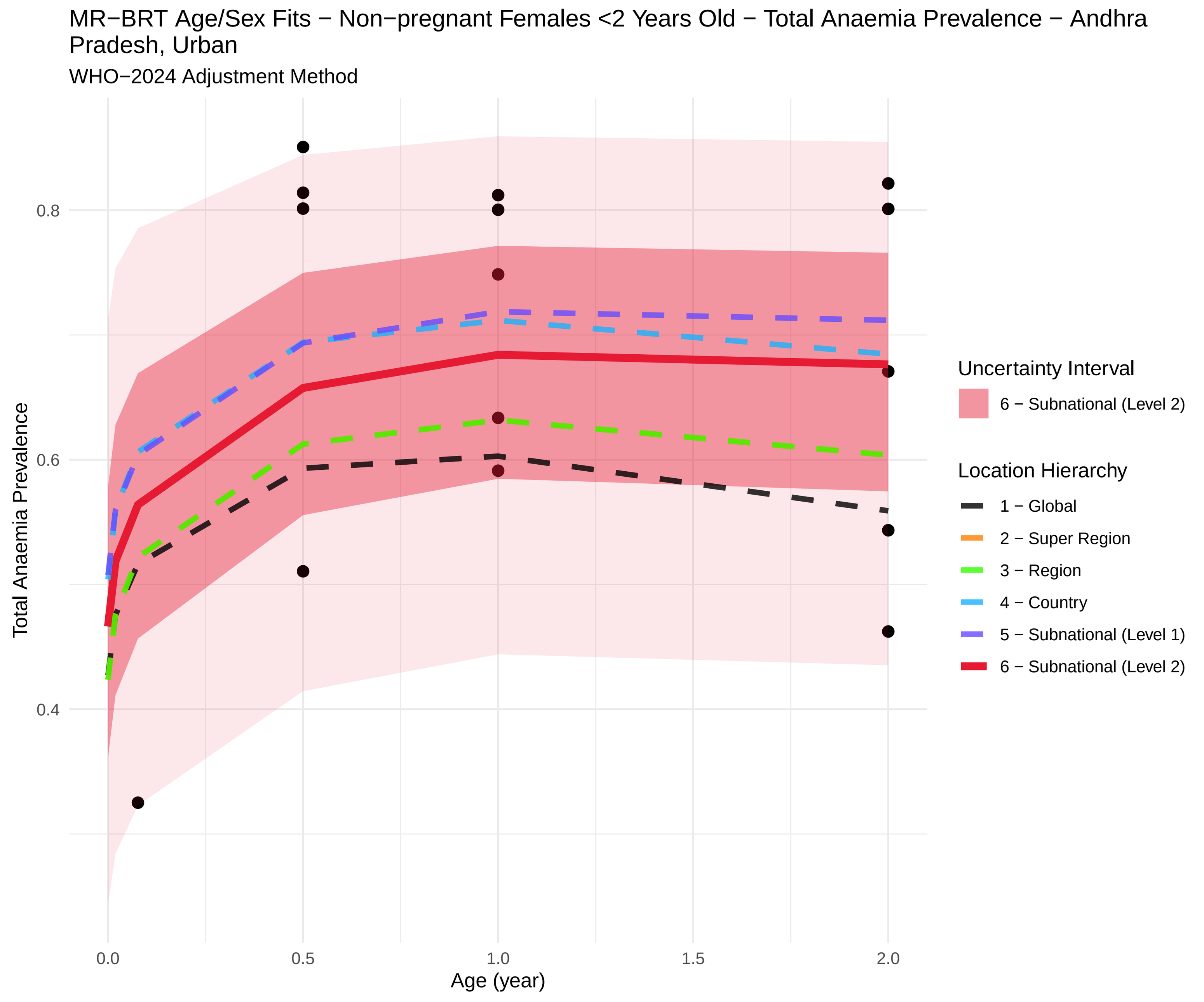
MR−BRT Age/Sex Fits − Non−pregnant Females <2 Years Old − Total Anaemia Prevalence − Andhra Pradesh, Urban
WHO−2024 Adjustment Method
Total Anaemia Prevalence
Age (year)
Uncertainty Interval
6 − Subnational (Level 2)
Location Hierarchy
1 − Global
2 − Super Region
3 − Region
4 − Country
5 − Subnational (Level 1)
6 − Subnational (Level 2)

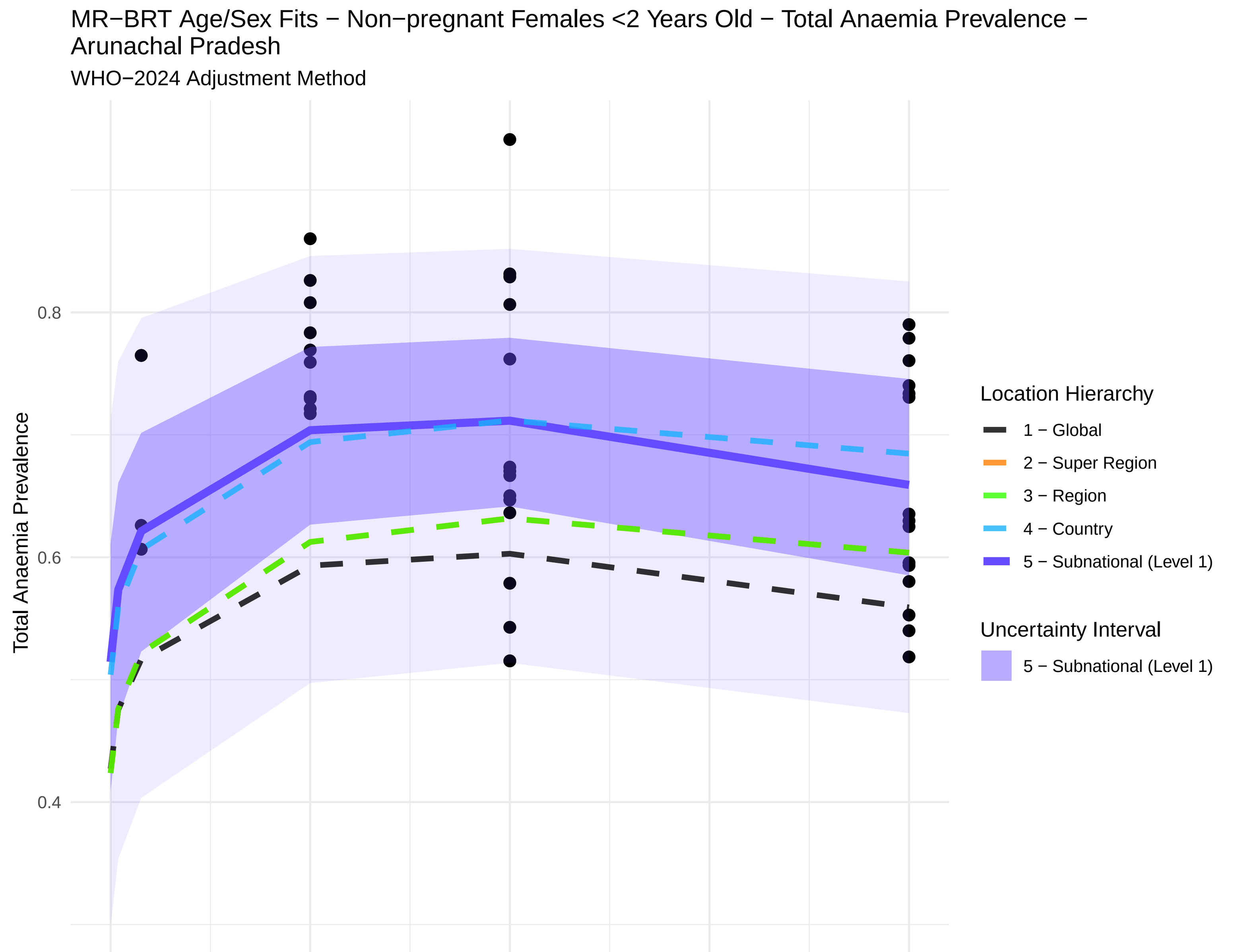
MR−BRT Age/Sex Fits − Non−pregnant Females <2 Years Old − Total Anaemia Prevalence − Arunachal Pradesh
WHO−2024 Adjustment Method
Total Anaemia Prevalence
Age (year)
Location Hierarchy
1 − Global
2 − Super Region
3 − Region
4 − Country
5 − Subnational (Level 1)
Uncertainty Interval
5 − Subnational (Level 1)

MR−BRT Age/Sex Fits − Non−pregnant Females <2 Years Old − Total Anaemia Prevalence − Arunachal Pradesh, Rural
WHO−2024 Adjustment Method

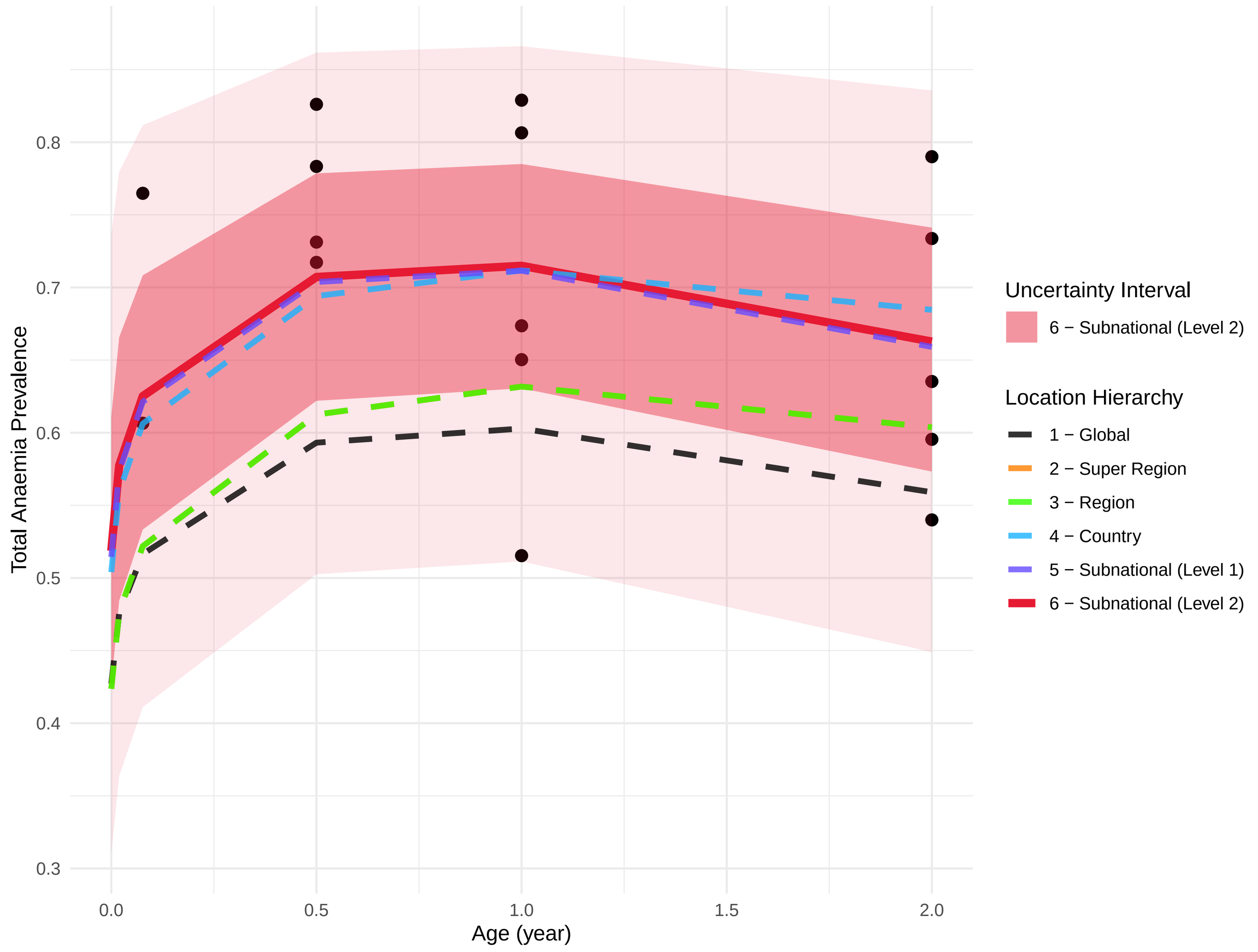

MR−BRT Age/Sex Fits − Non−pregnant Females <2 Years Old − Total Anaemia Prevalence − Arunachal Pradesh, Urban
WHO−2024 Adjustment Method

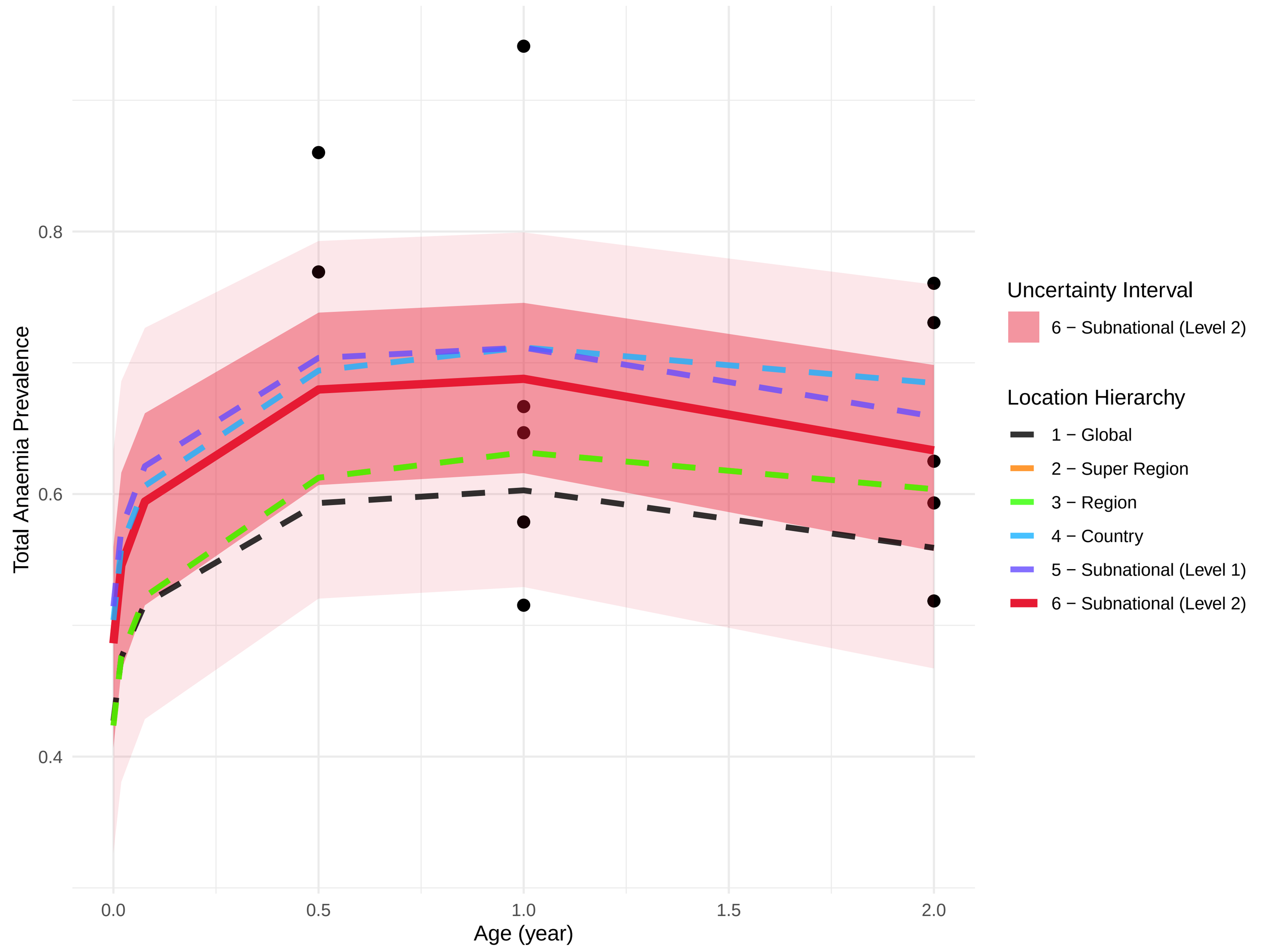

MR−BRT Age/Sex Fits − Non−pregnant Females <2 Years Old − Total Anaemia Prevalence − Assam
WHO−2024 Adjustment Method

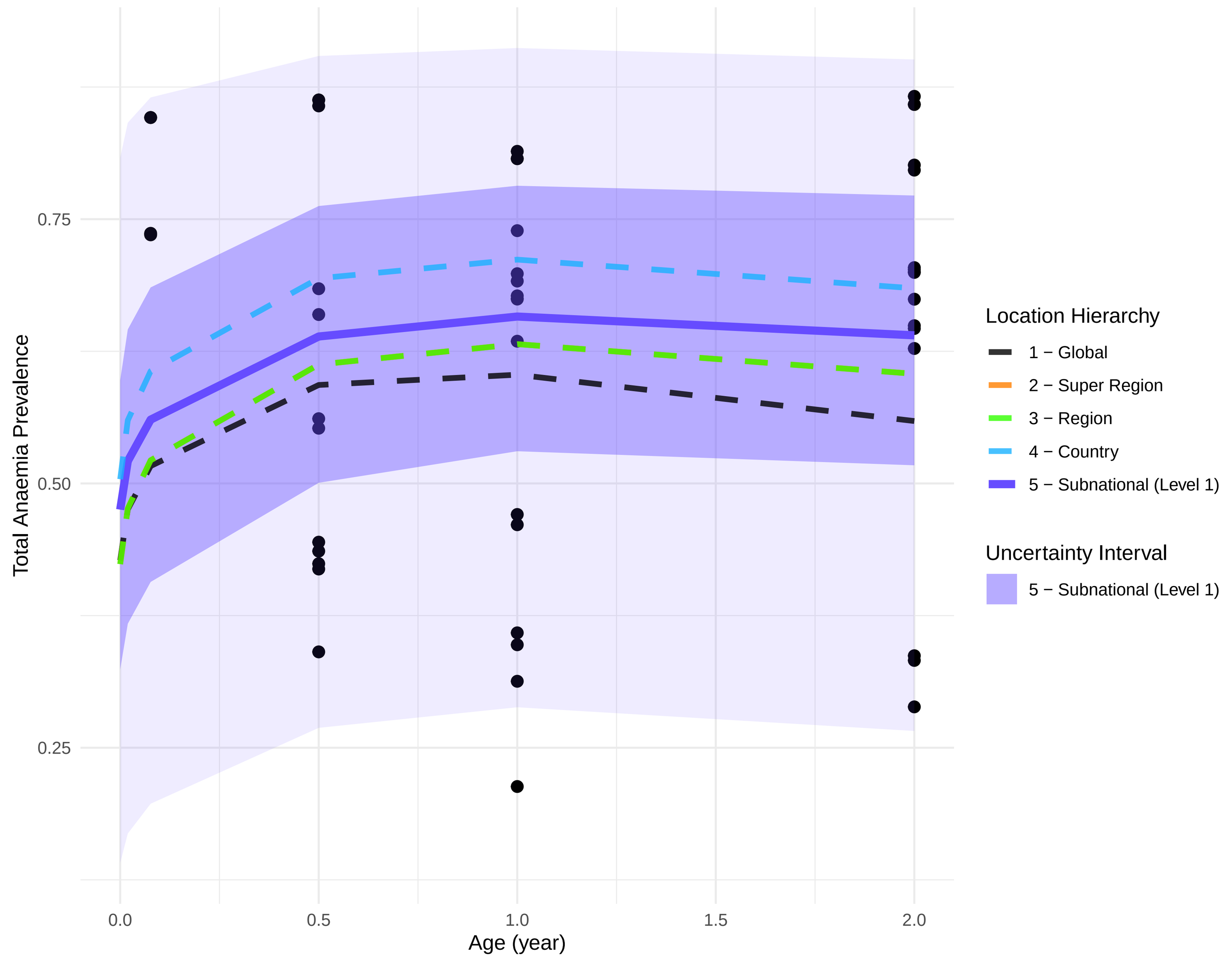

MR−BRT Age/Sex Fits − Non−pregnant Females <2 Years Old − Total Anaemia Prevalence − Assam, Rural
WHO−2024 Adjustment Method

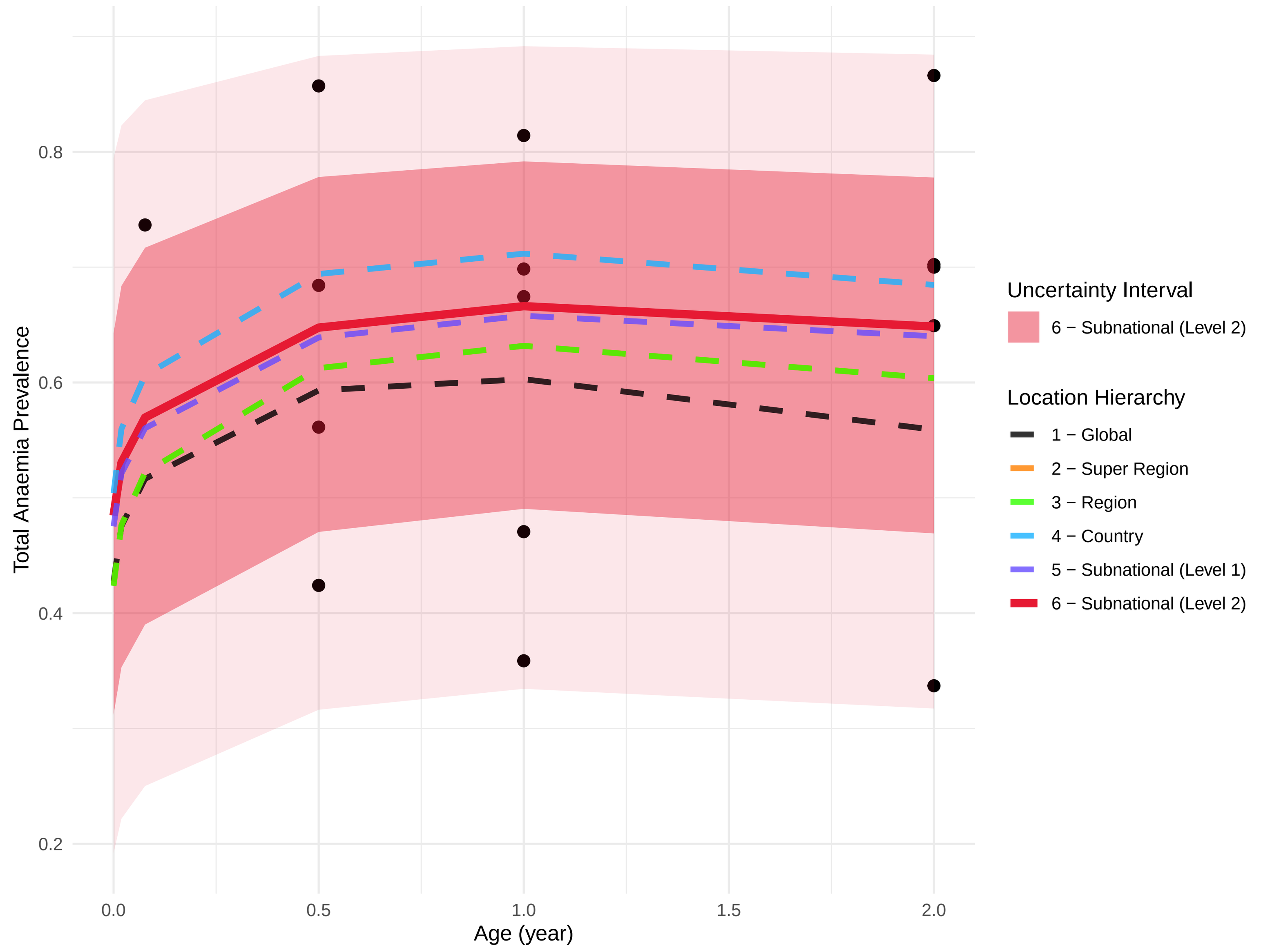

MR−BRT Age/Sex Fits − Non−pregnant Females <2 Years Old − Total Anaemia Prevalence − Assam, Urban
WHO−2024 Adjustment Method

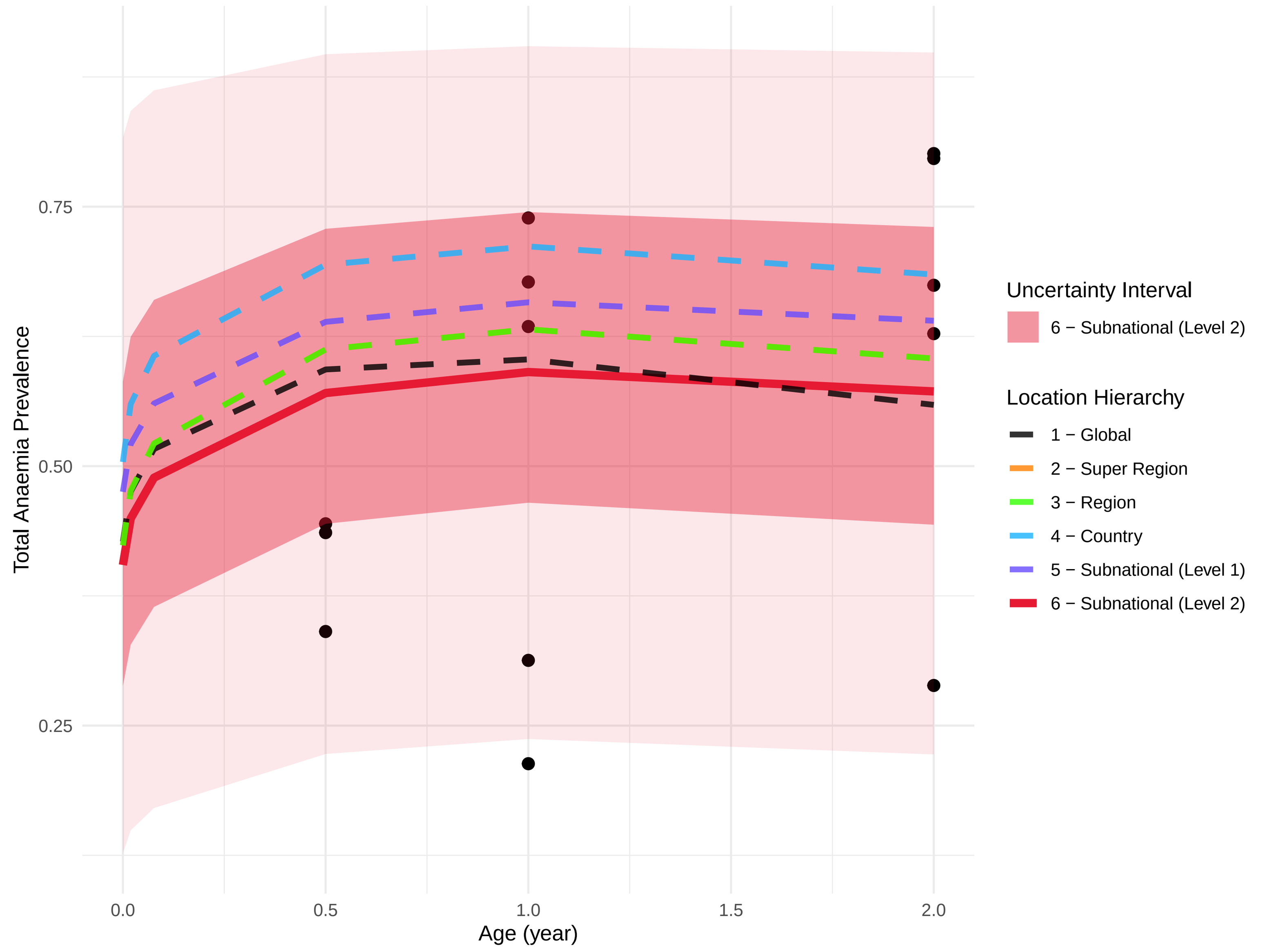

MR−BRT Age/Sex Fits − Non−pregnant Females <2 Years Old − Total Anaemia Prevalence − Bihar
WHO−2024 Adjustment Method

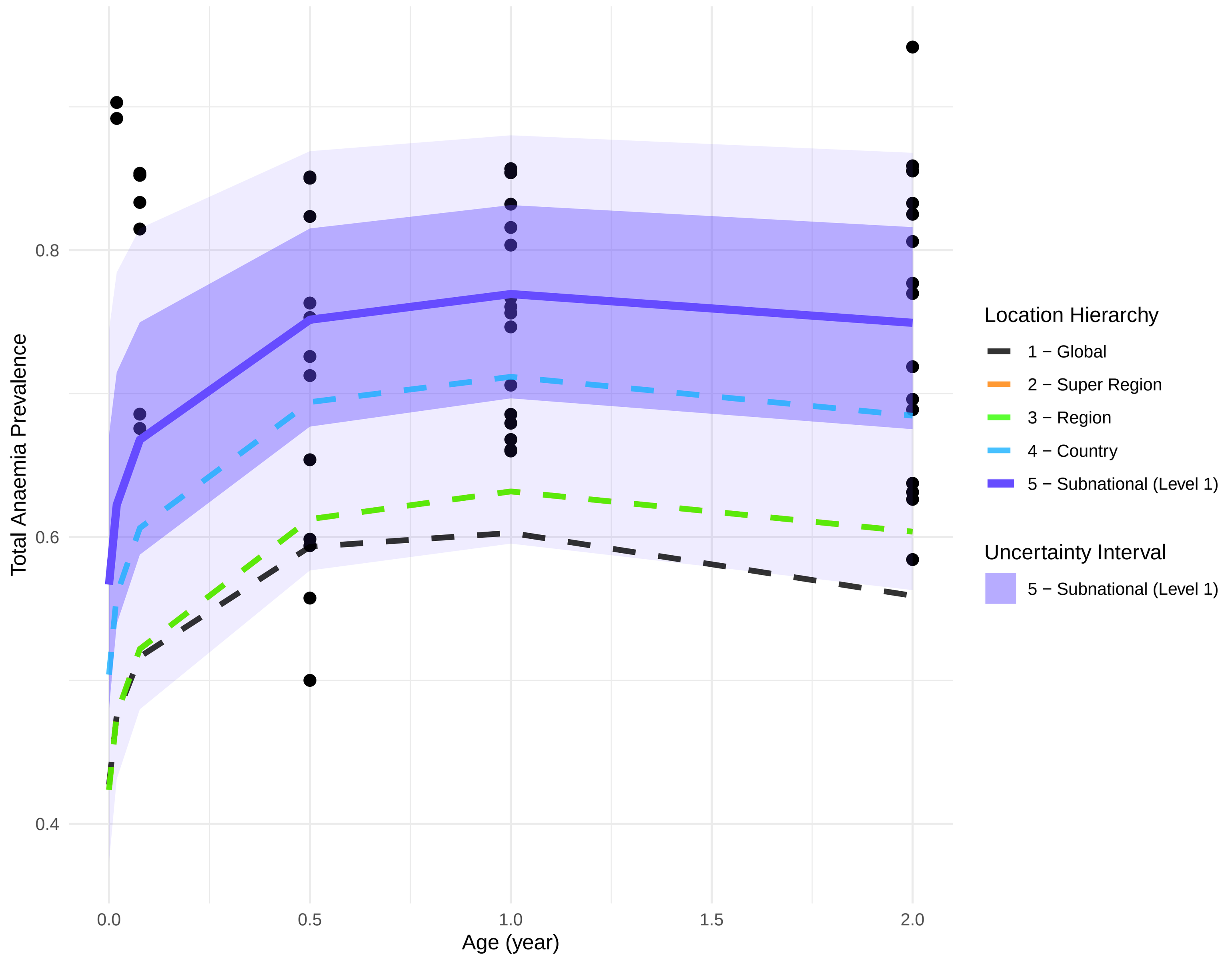

MR−BRT Age/Sex Fits − Non−pregnant Females <2 Years Old − Total Anaemia Prevalence − Bihar, Rural
WHO−2024 Adjustment Method

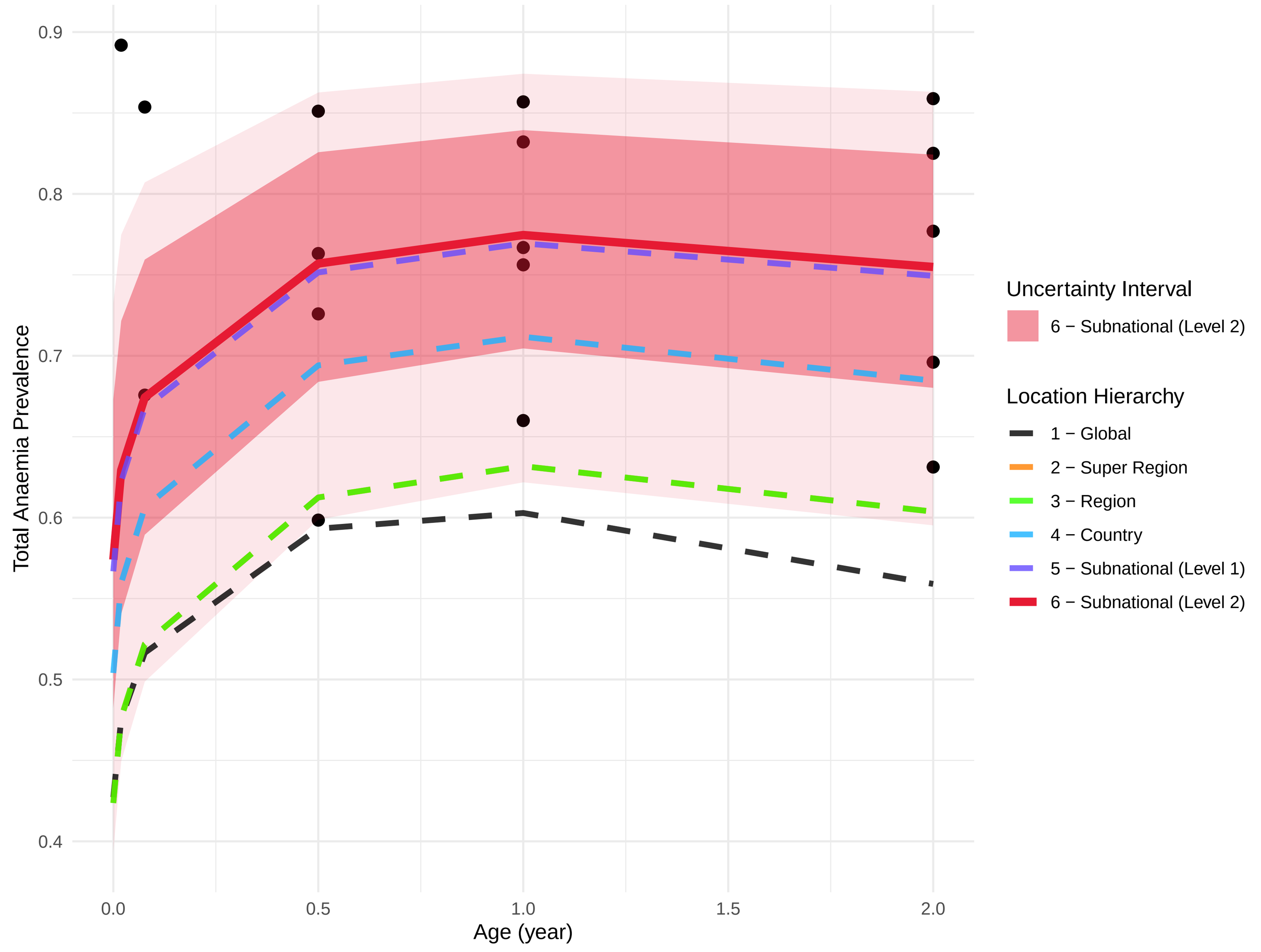

MR−BRT Age/Sex Fits − Non−pregnant Females <2 Years Old − Total Anaemia Prevalence − Bihar, Urban
WHO−2024 Adjustment Method

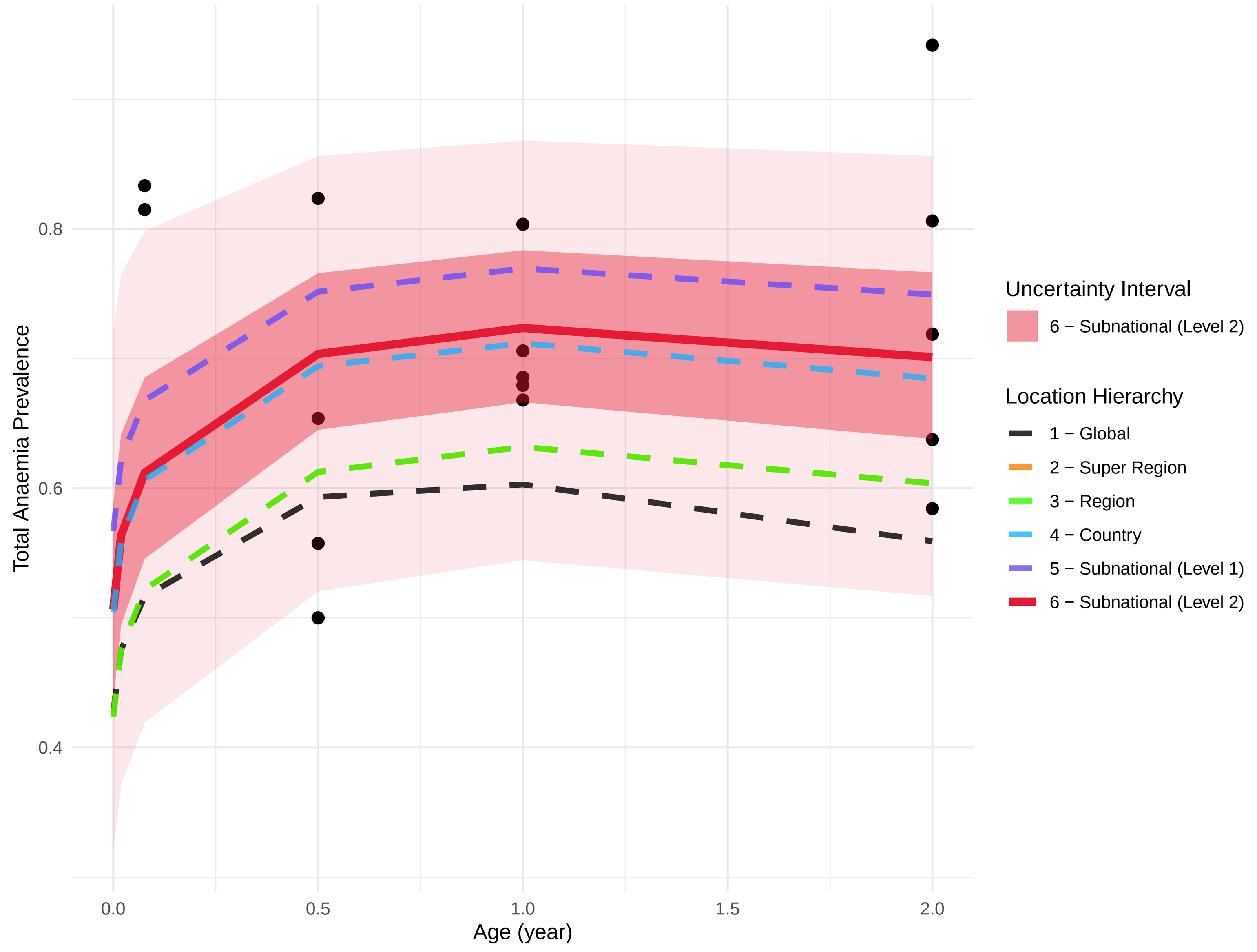

MR−BRT Age/Sex Fits − Non−pregnant Females <2 Years Old − Total Anaemia Prevalence − Chhattisgarh
WHO−2024 Adjustment Method

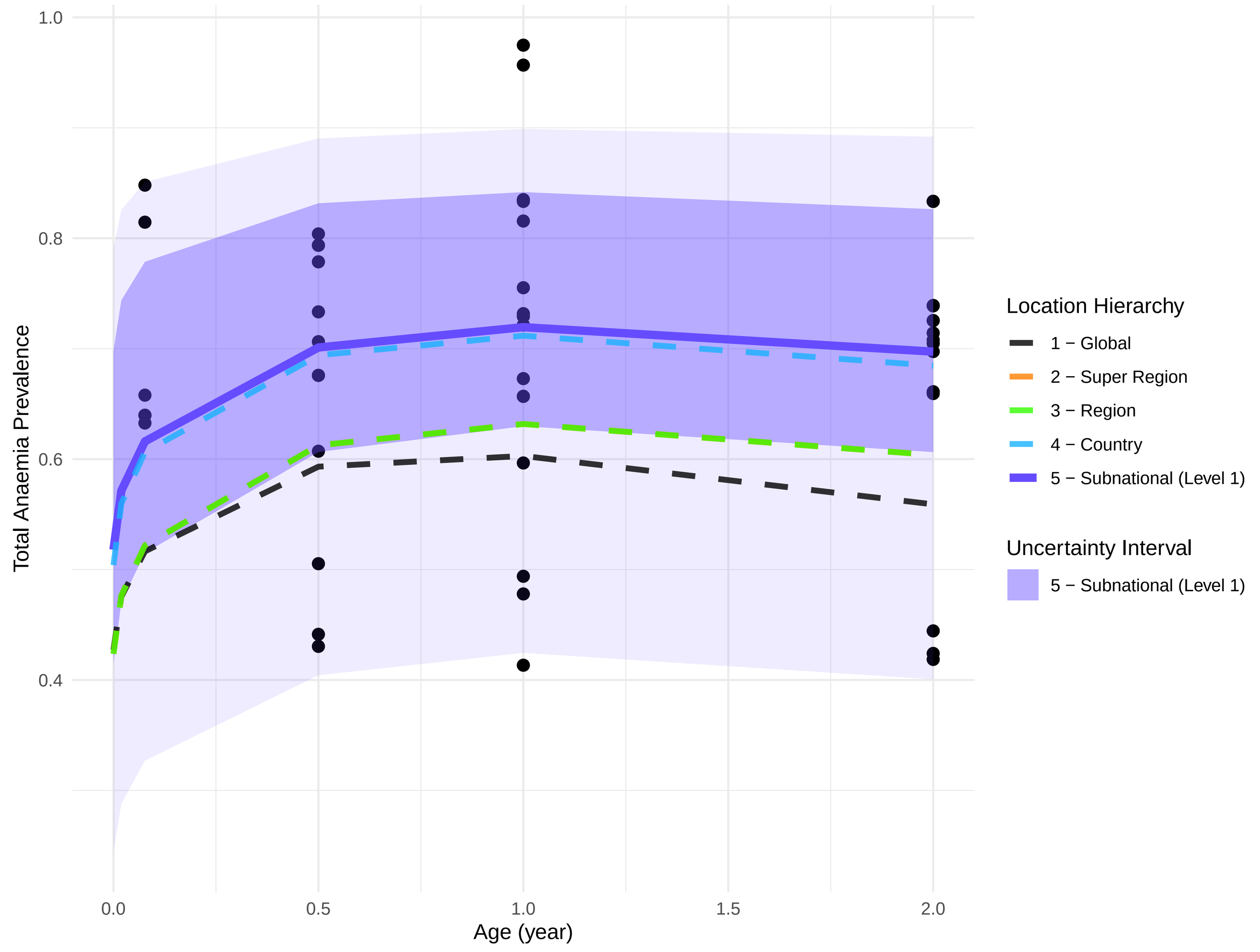

MR−BRT Age/Sex Fits − Non−pregnant Females <2 Years Old − Total Anaemia Prevalence − Chhattisgarh, Rural
WHO−2024 Adjustment Method

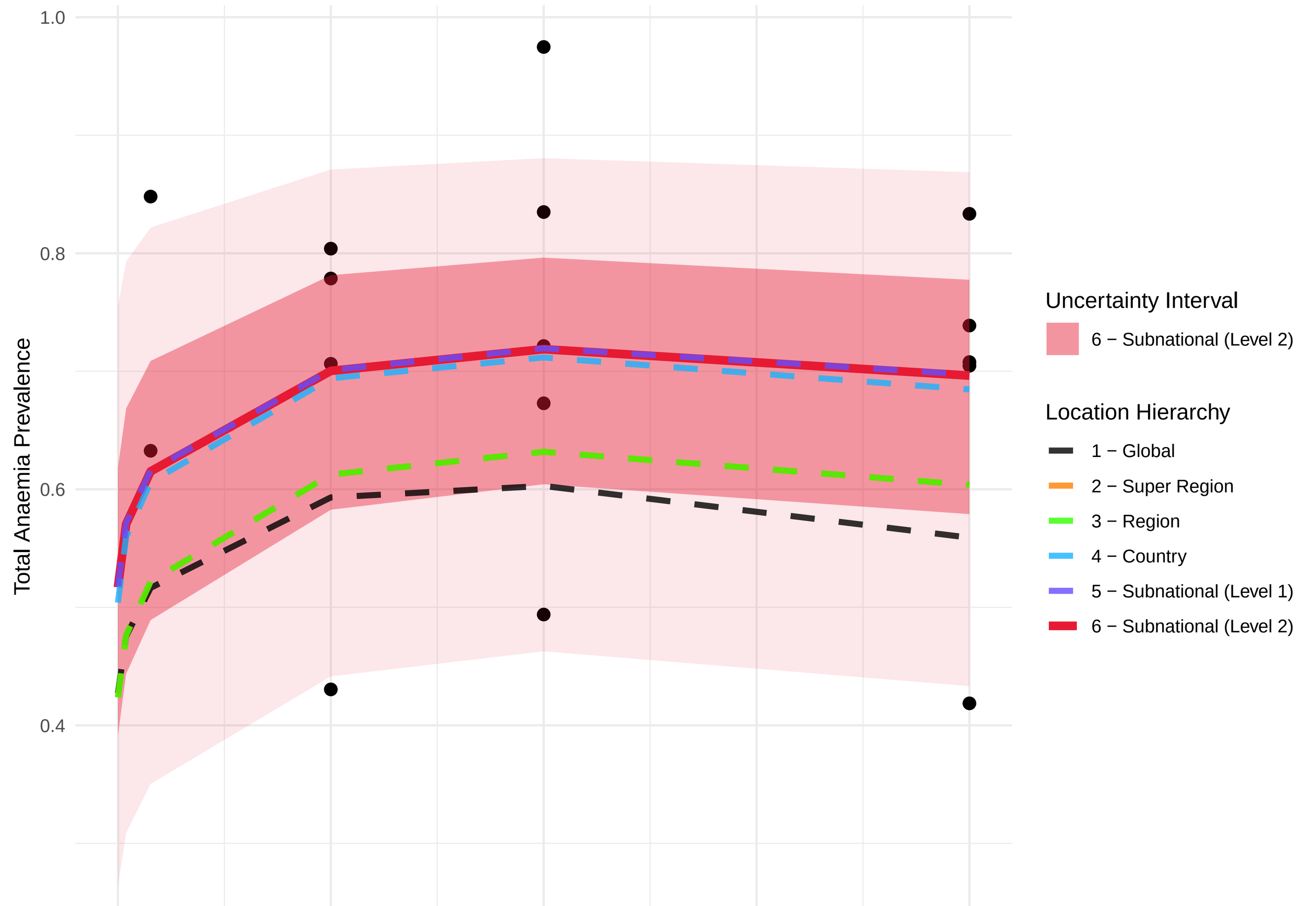

MR−BRT Age/Sex Fits − Non−pregnant Females <2 Years Old − Total Anaemia Prevalence − Chhattisgarh, Urban
WHO−2024 Adjustment Method

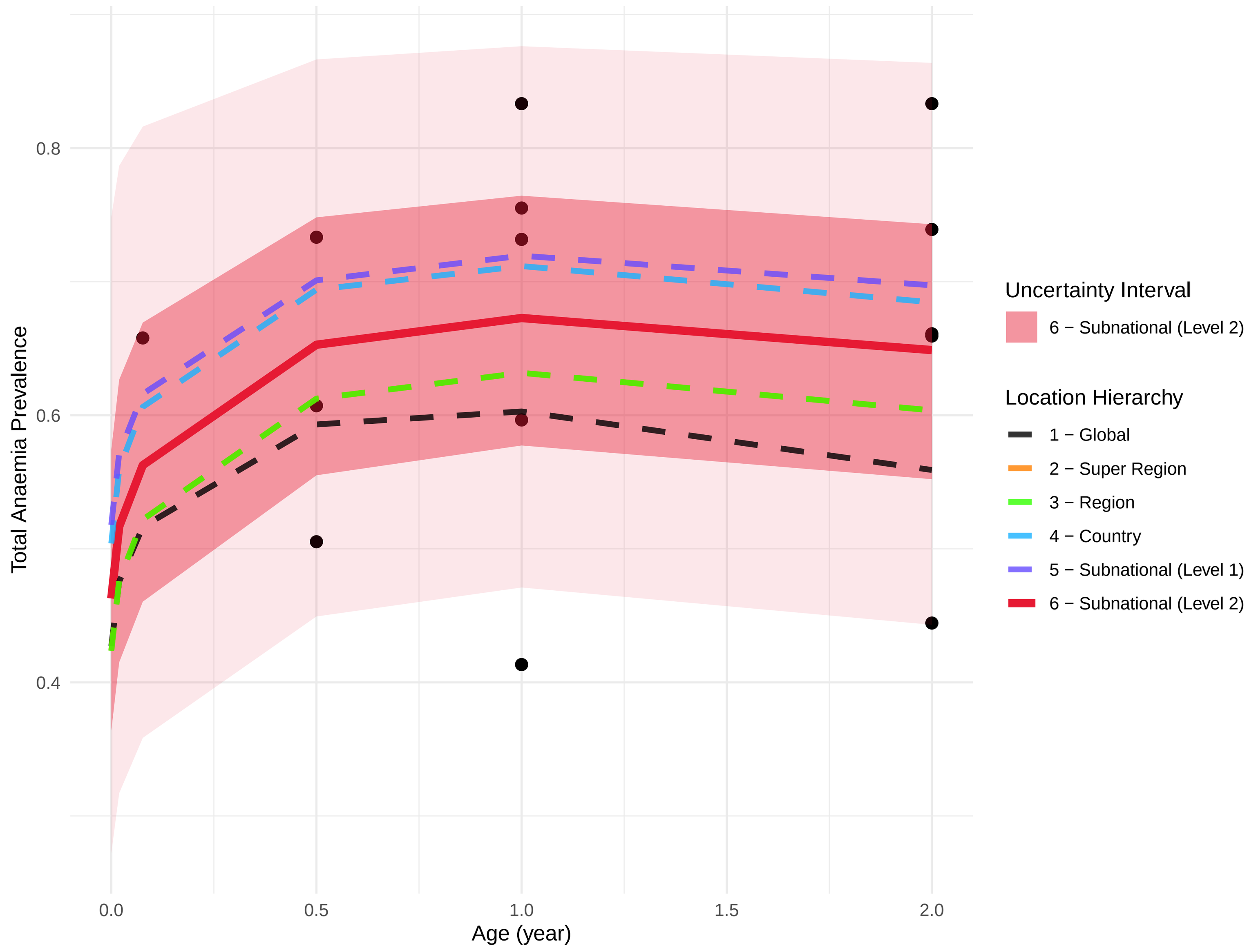

MR−BRT Age/Sex Fits − Non−pregnant Females <2 Years Old − Total Anaemia Prevalence − Delhi
WHO−2024 Adjustment Method

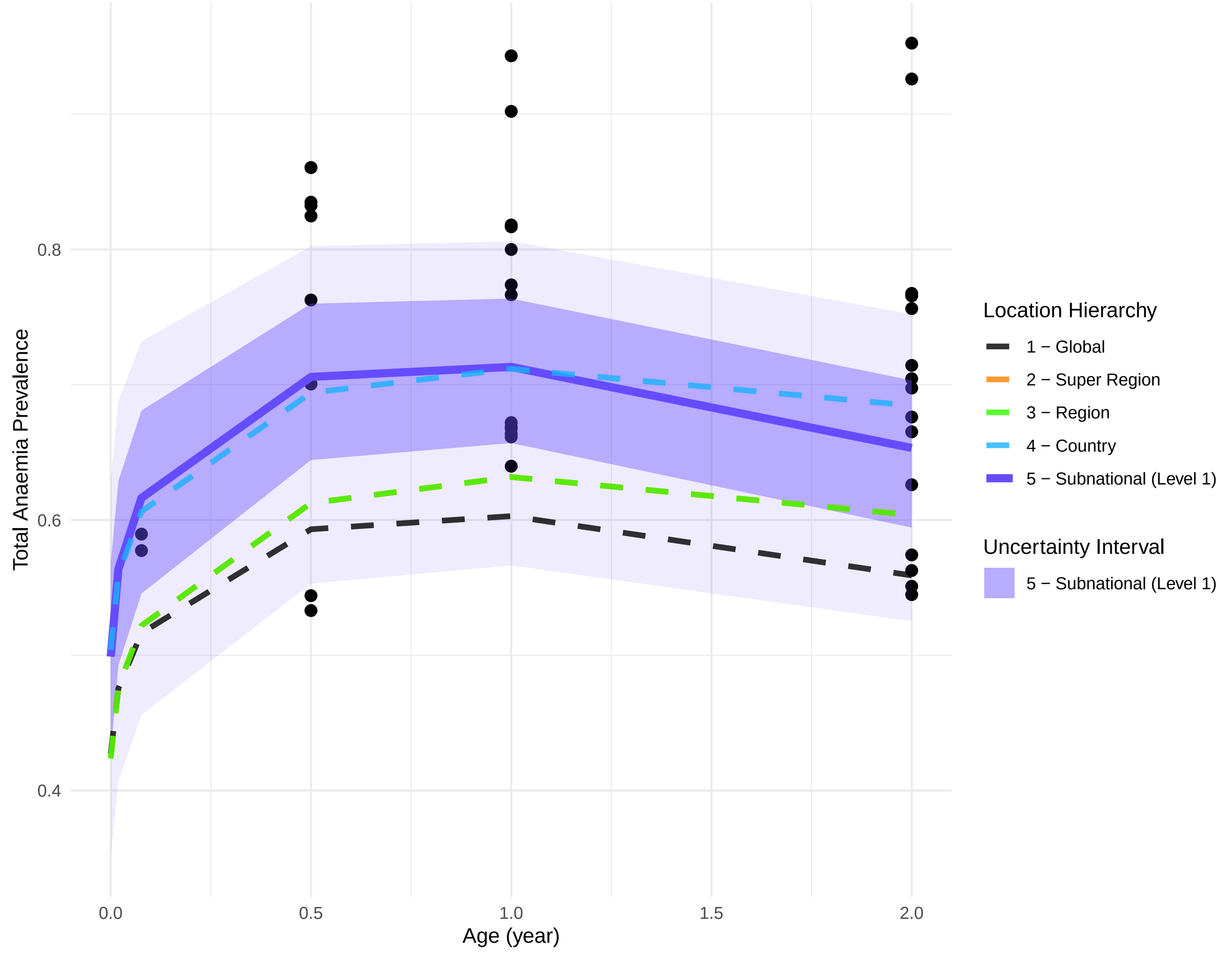

MR−BRT Age/Sex Fits − Non−pregnant Females <2 Years Old − Total Anaemia Prevalence − Delhi, Rural
WHO−2024 Adjustment Method

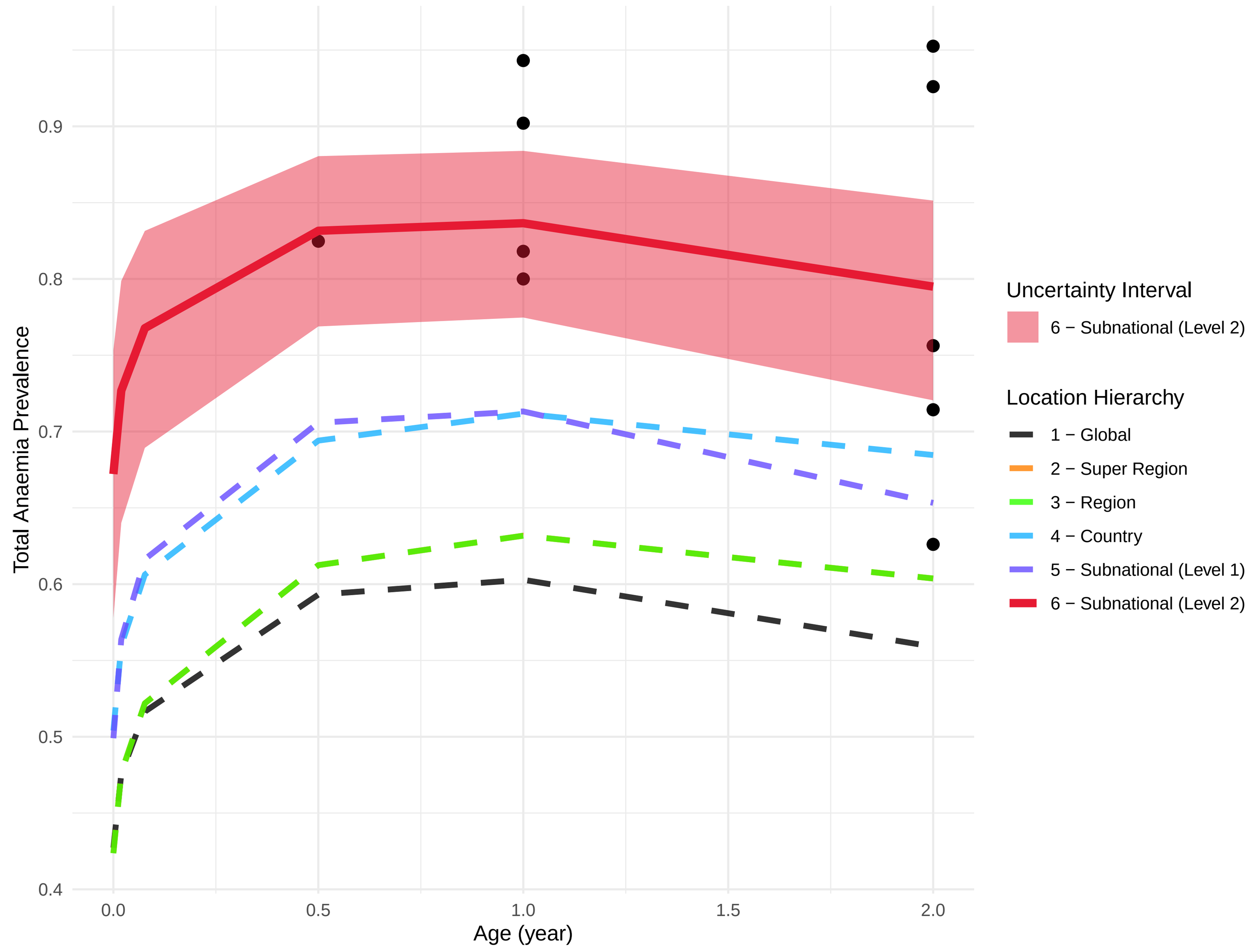

MR−BRT Age/Sex Fits − Non−pregnant Females <2 Years Old − Total Anaemia Prevalence − Delhi, Urban
WHO−2024 Adjustment Method

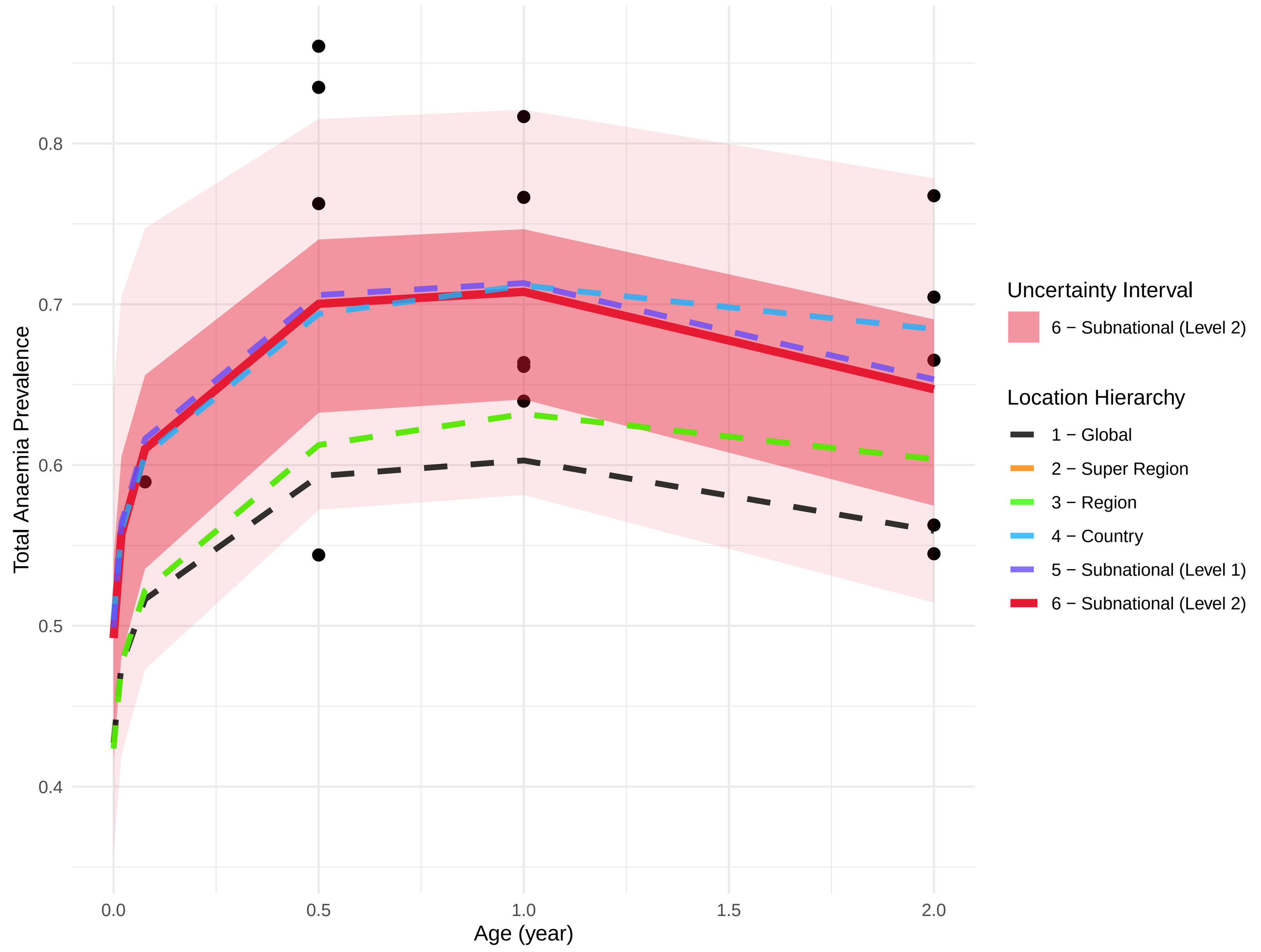

MR−BRT Age/Sex Fits − Non−pregnant Females <2 Years Old − Total Anaemia Prevalence − Goa
WHO−2024 Adjustment Method

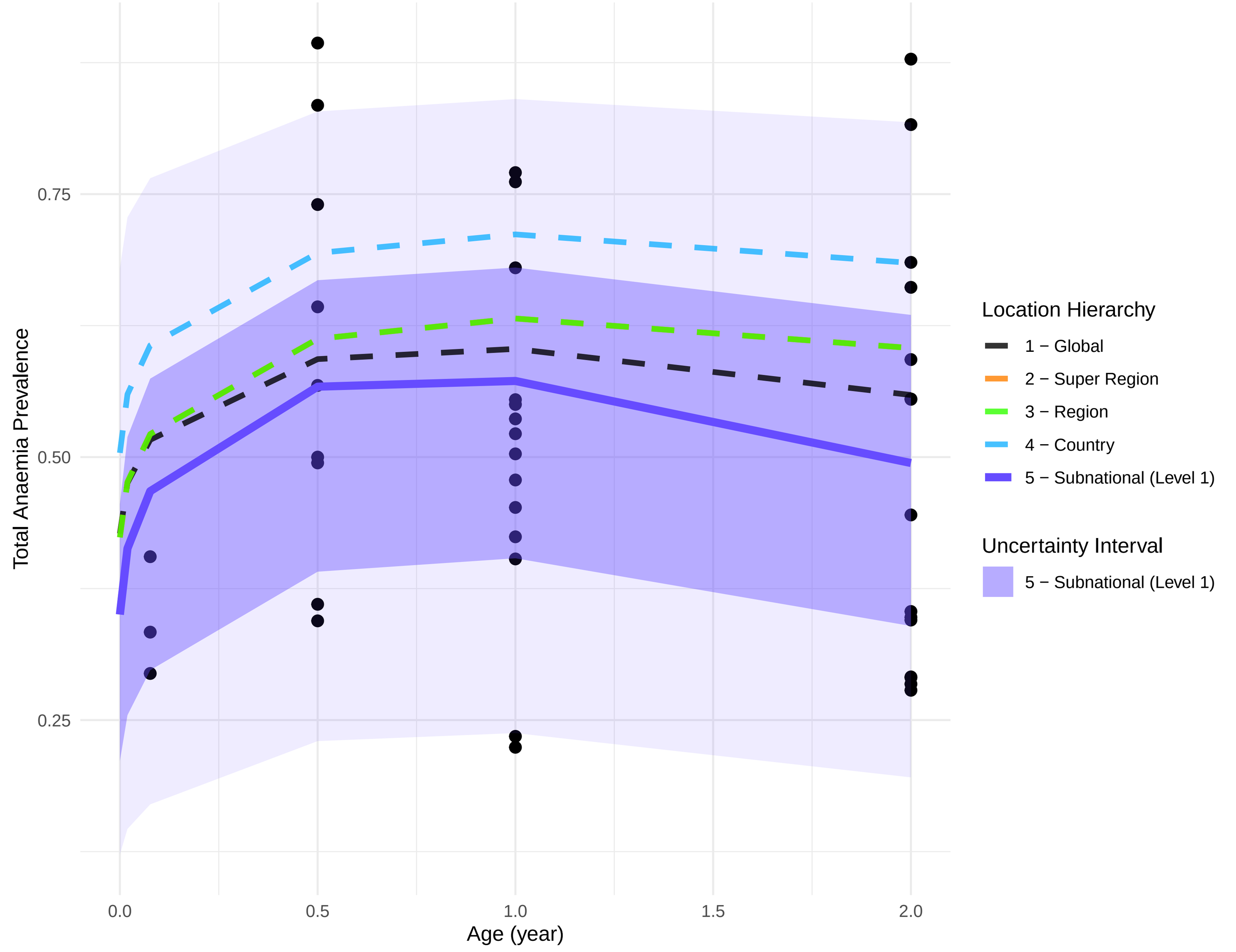

MR−BRT Age/Sex Fits − Non−pregnant Females <2 Years Old − Total Anaemia Prevalence − Goa, Rural
WHO−2024 Adjustment Method

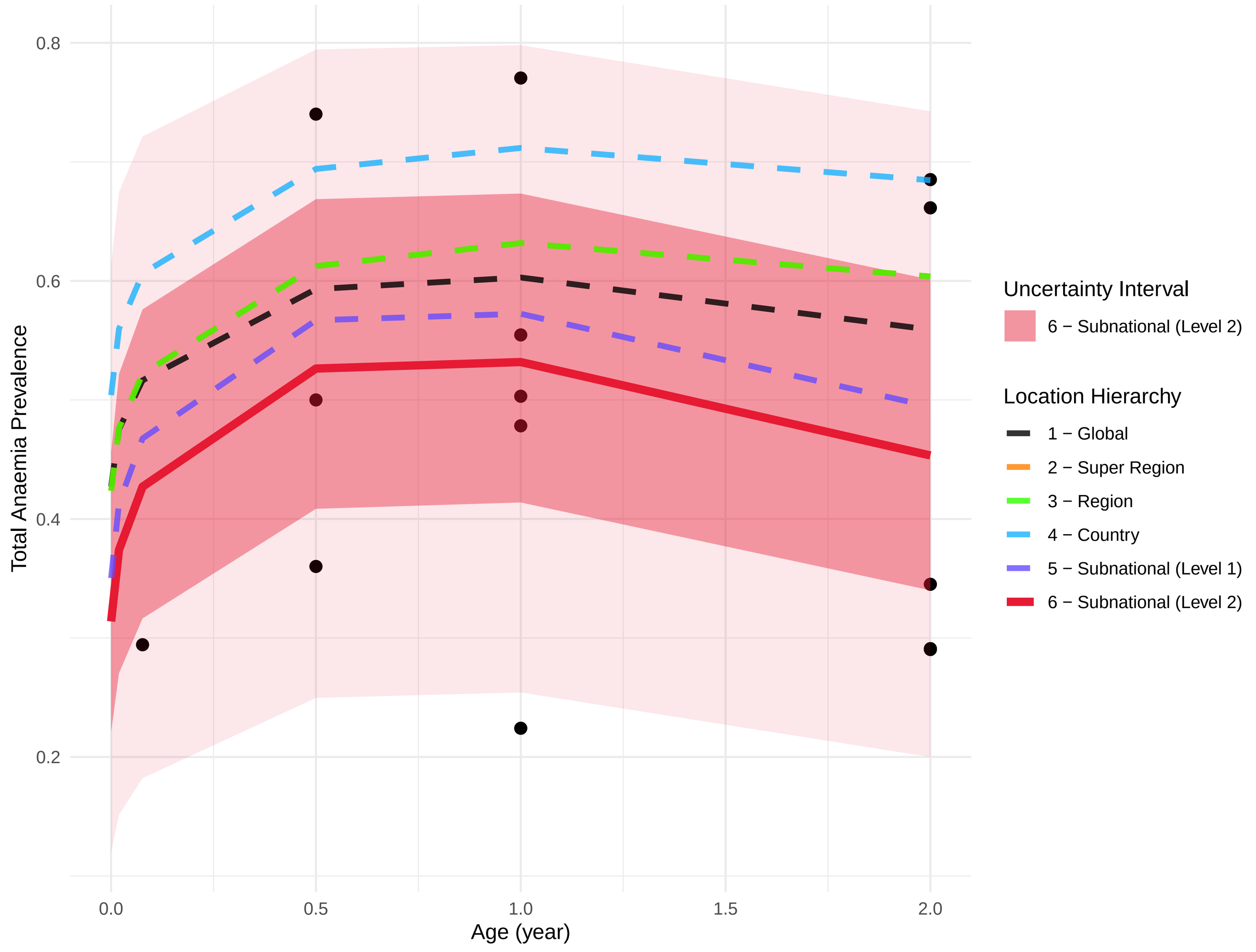

MR−BRT Age/Sex Fits − Non−pregnant Females <2 Years Old − Total Anaemia Prevalence − Goa, Urban
WHO−2024 Adjustment Method

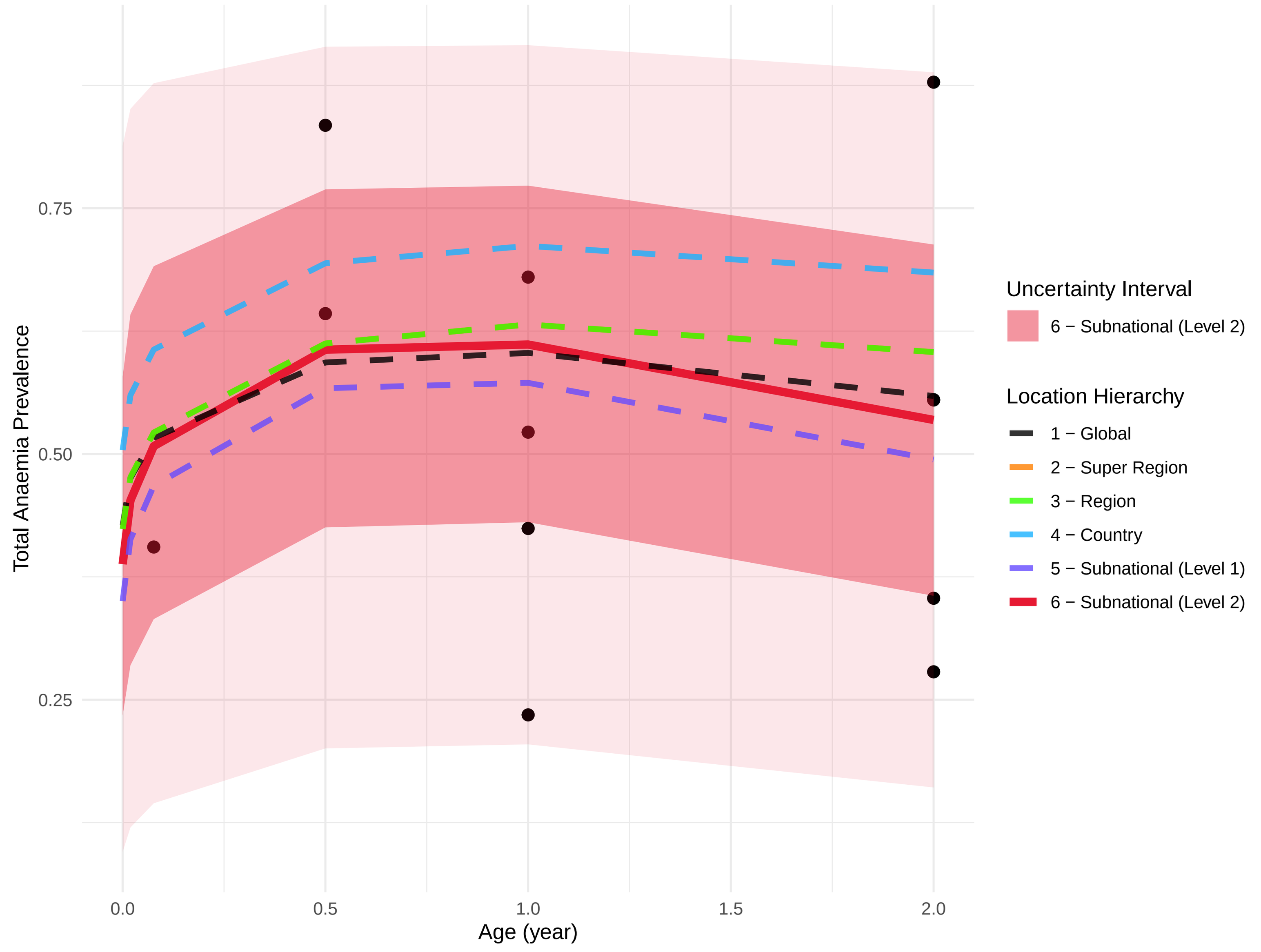

MR−BRT Age/Sex Fits − Non−pregnant Females <2 Years Old − Total Anaemia Prevalence − Gujarat
WHO−2024 Adjustment Method

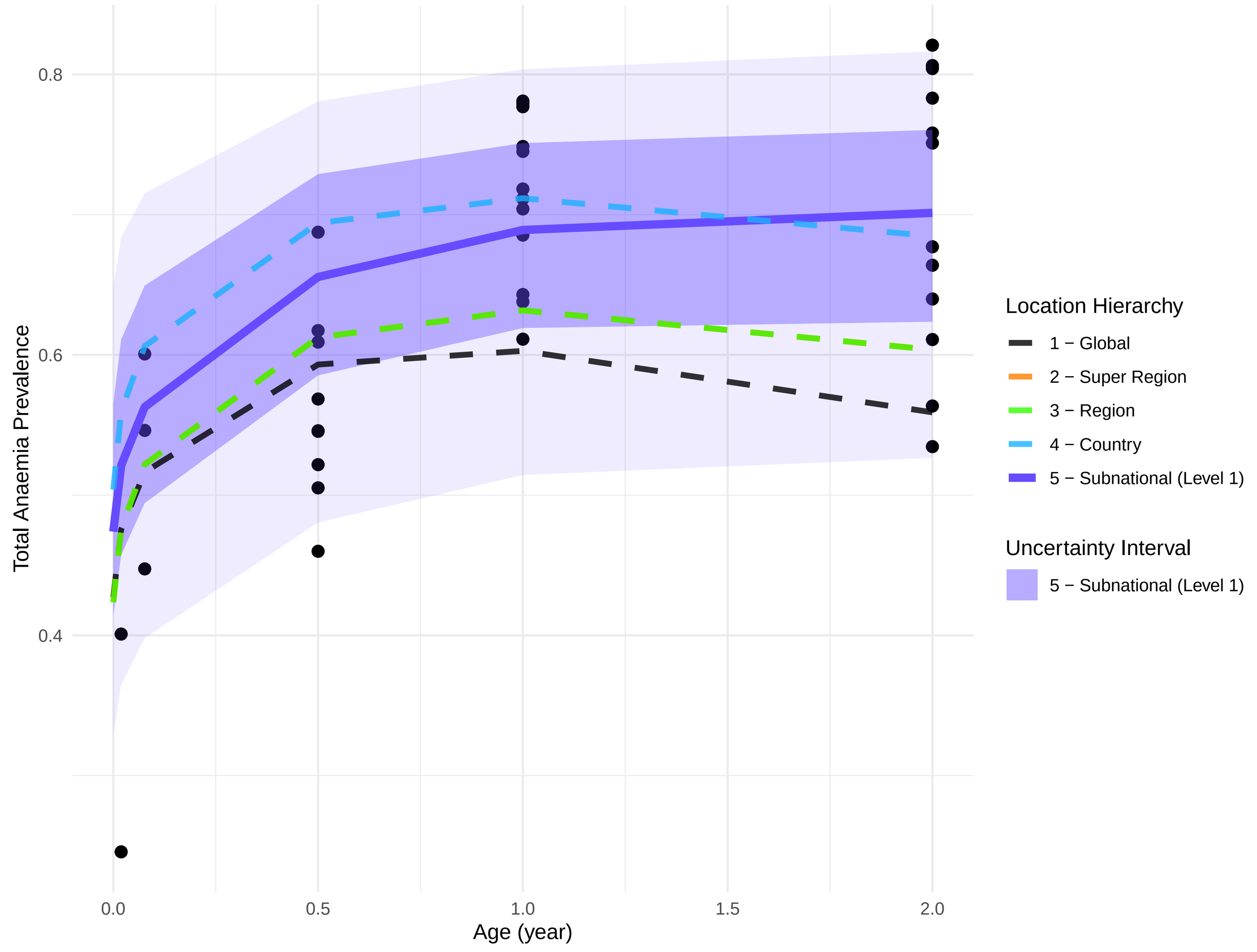

MR−BRT Age/Sex Fits − Non−pregnant Females <2 Years Old − Total Anaemia Prevalence − Gujarat, Rural
WHO−2024 Adjustment Method

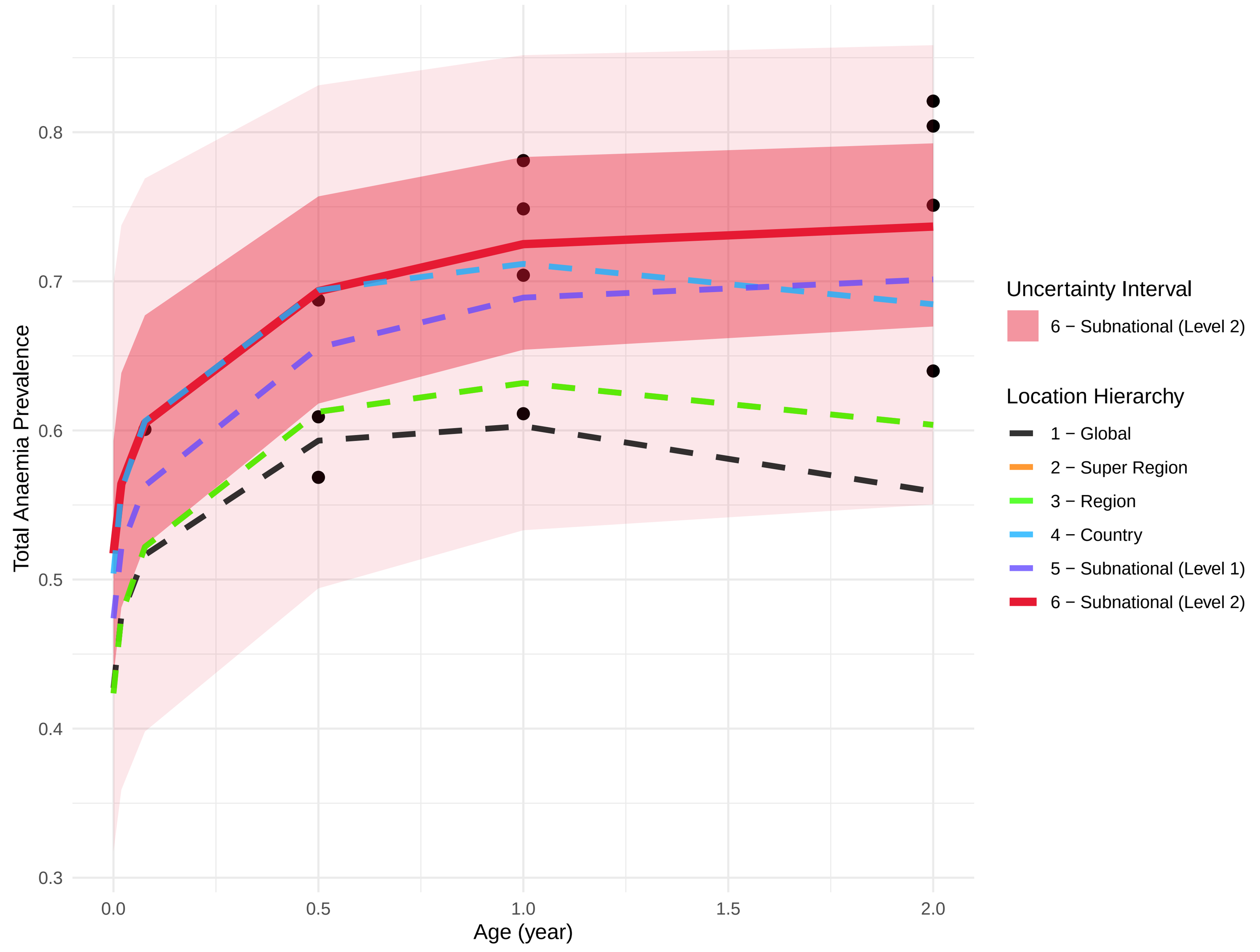

MR−BRT Age/Sex Fits − Non−pregnant Females <2 Years Old − Total Anaemia Prevalence − Gujarat, Urban
WHO−2024 Adjustment Method

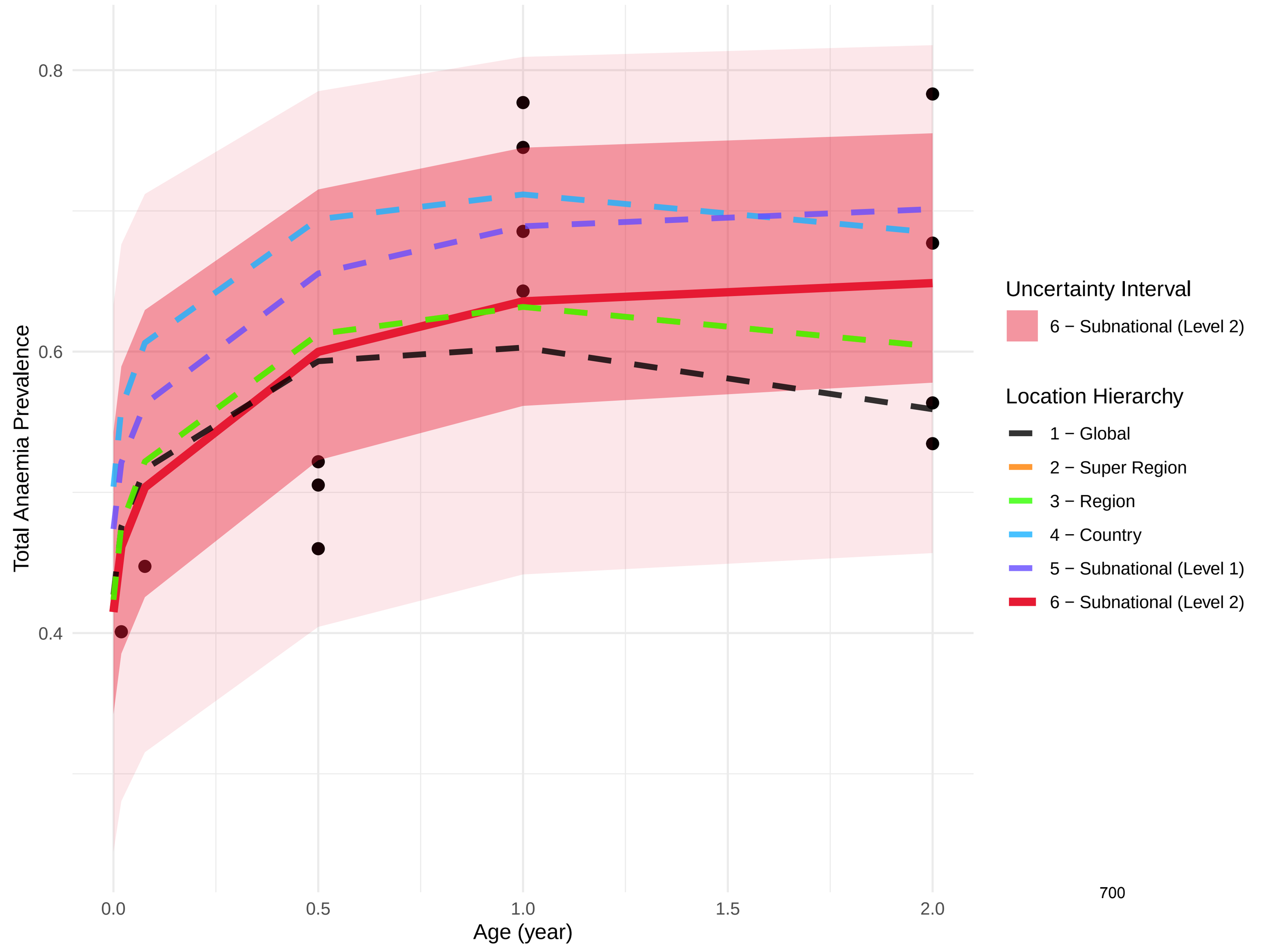

MR−BRT Age/Sex Fits − Non−pregnant Females <2 Years Old − Total Anaemia Prevalence − Haryana
WHO−2024 Adjustment Method

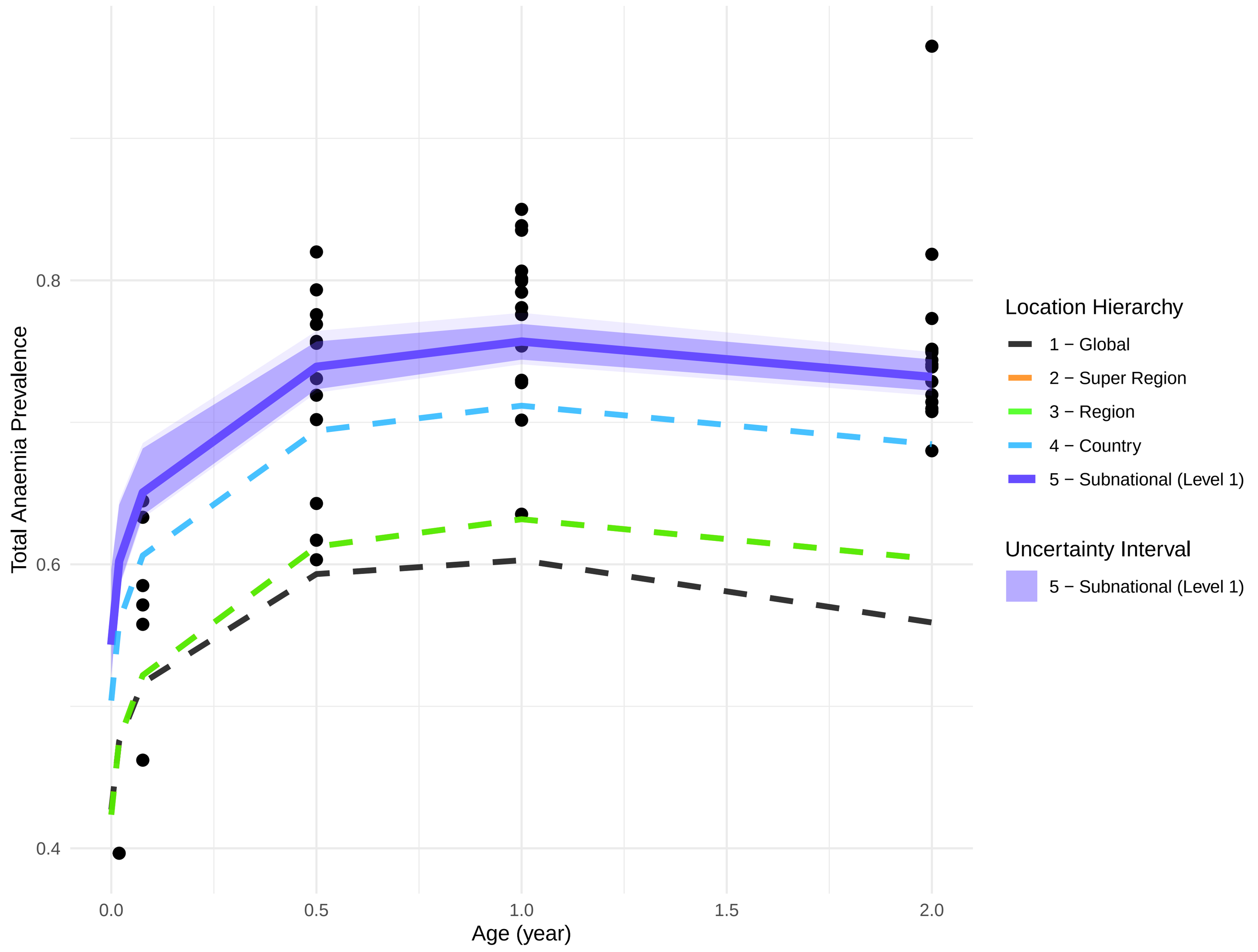

MR−BRT Age/Sex Fits − Non−pregnant Females <2 Years Old − Total Anaemia Prevalence − Haryana, Rural
WHO−2024 Adjustment Method

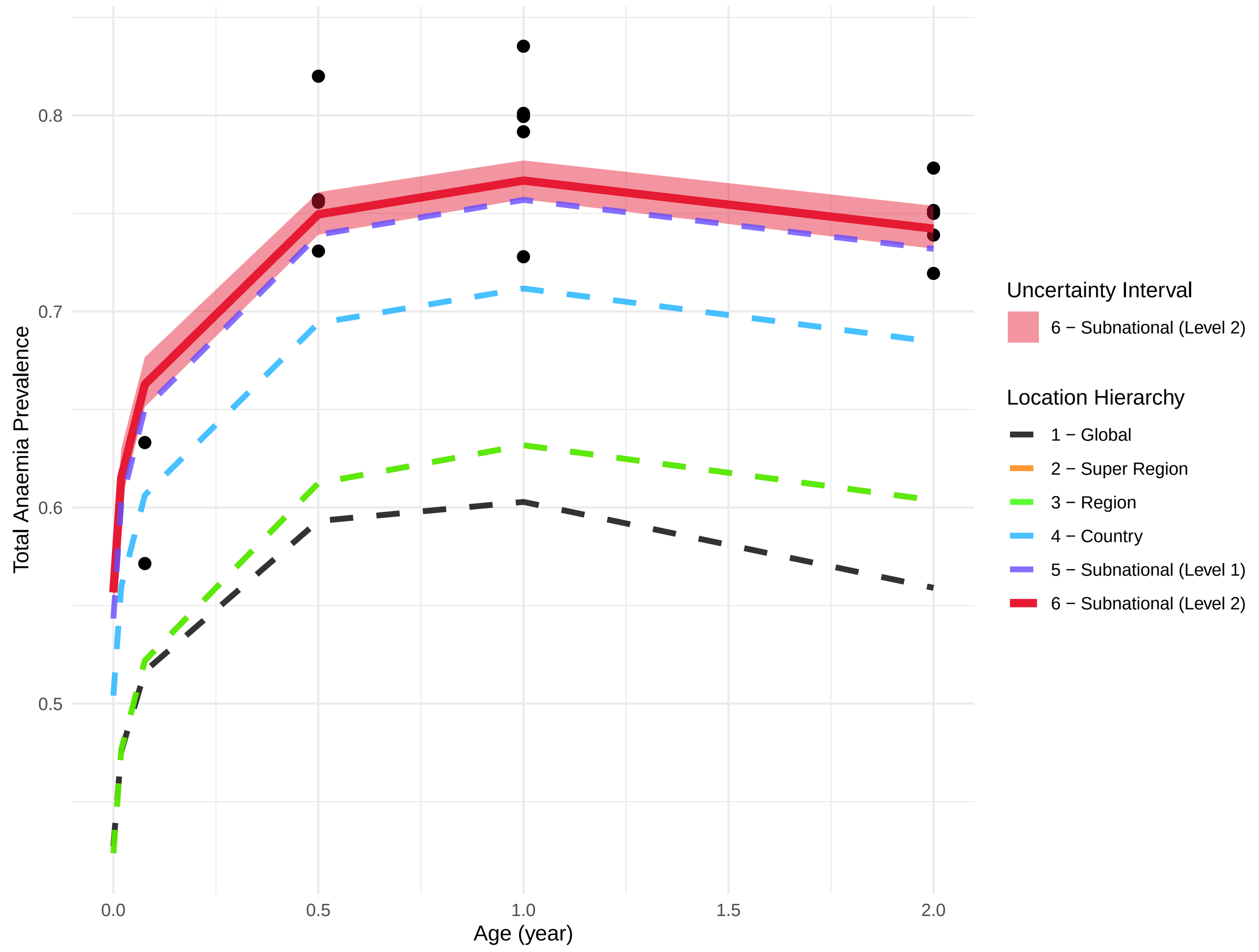

MR−BRT Age/Sex Fits − Non−pregnant Females <2 Years Old − Total Anaemia Prevalence − Haryana, Urban
WHO−2024 Adjustment Method

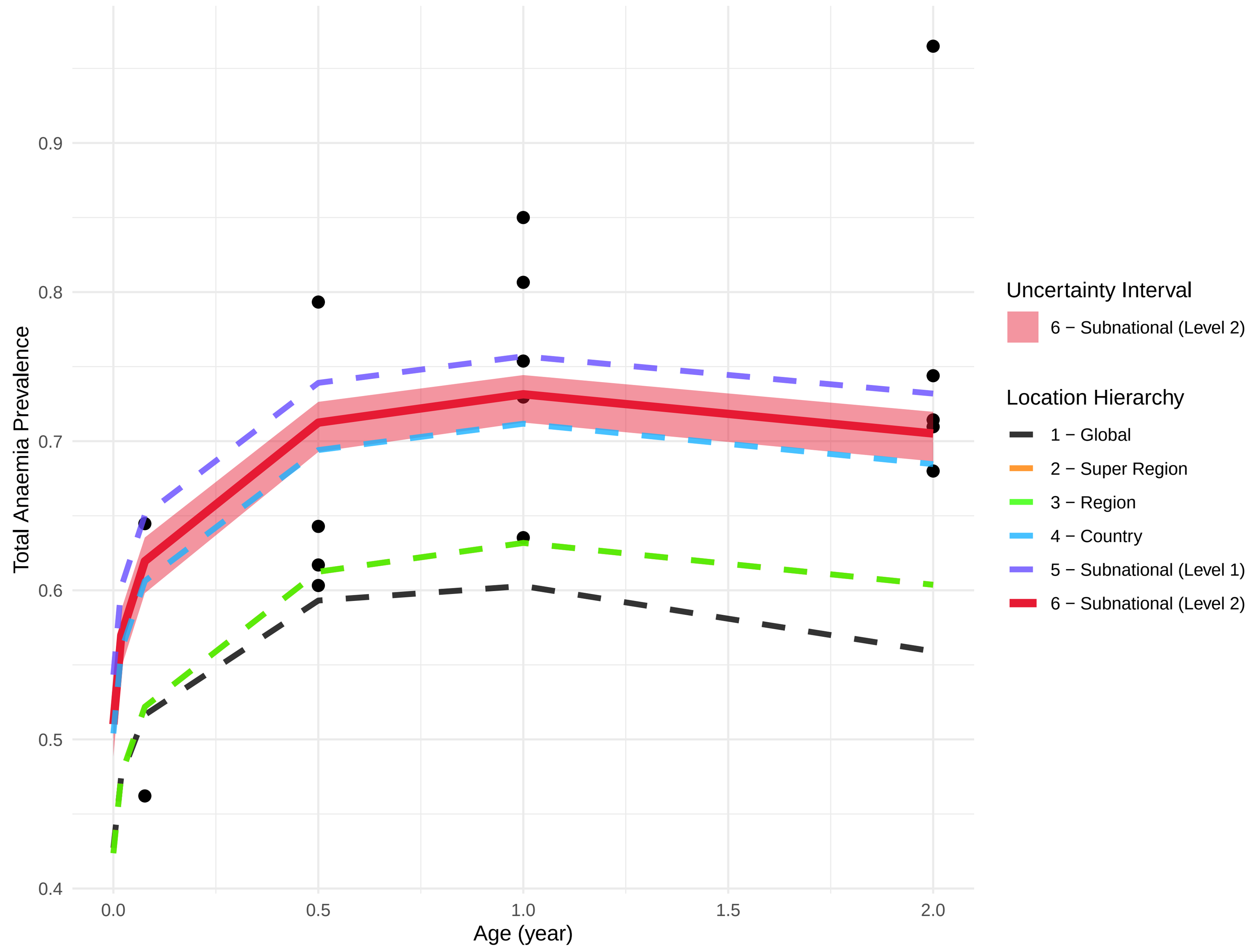

MR−BRT Age/Sex Fits − Non−pregnant Females <2 Years Old − Total Anaemia Prevalence − Himachal Pradesh
WHO−2024 Adjustment Method

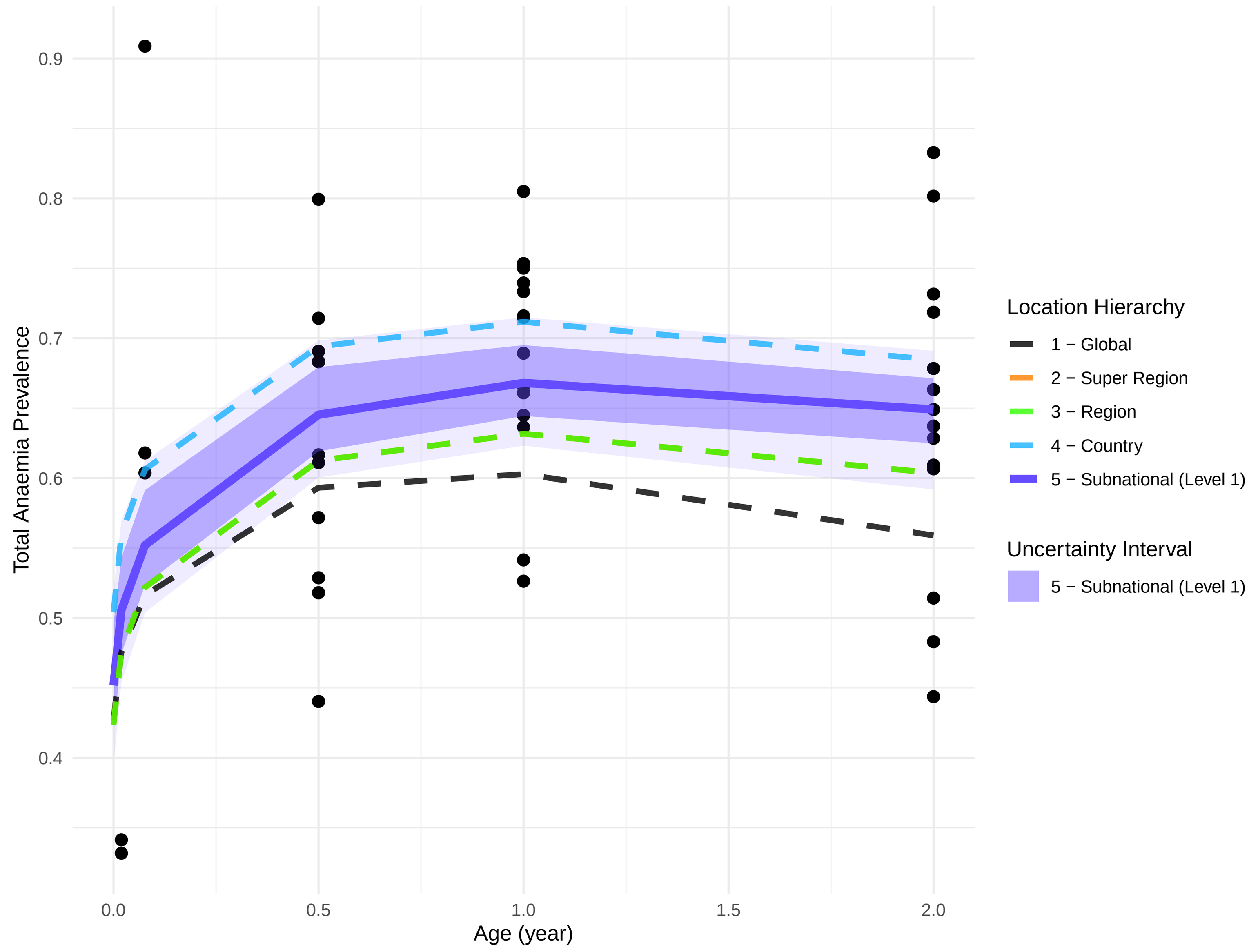

MR−BRT Age/Sex Fits − Non−pregnant Females <2 Years Old − Total Anaemia Prevalence − Himachal Pradesh, Rural
WHO−2024 Adjustment Method

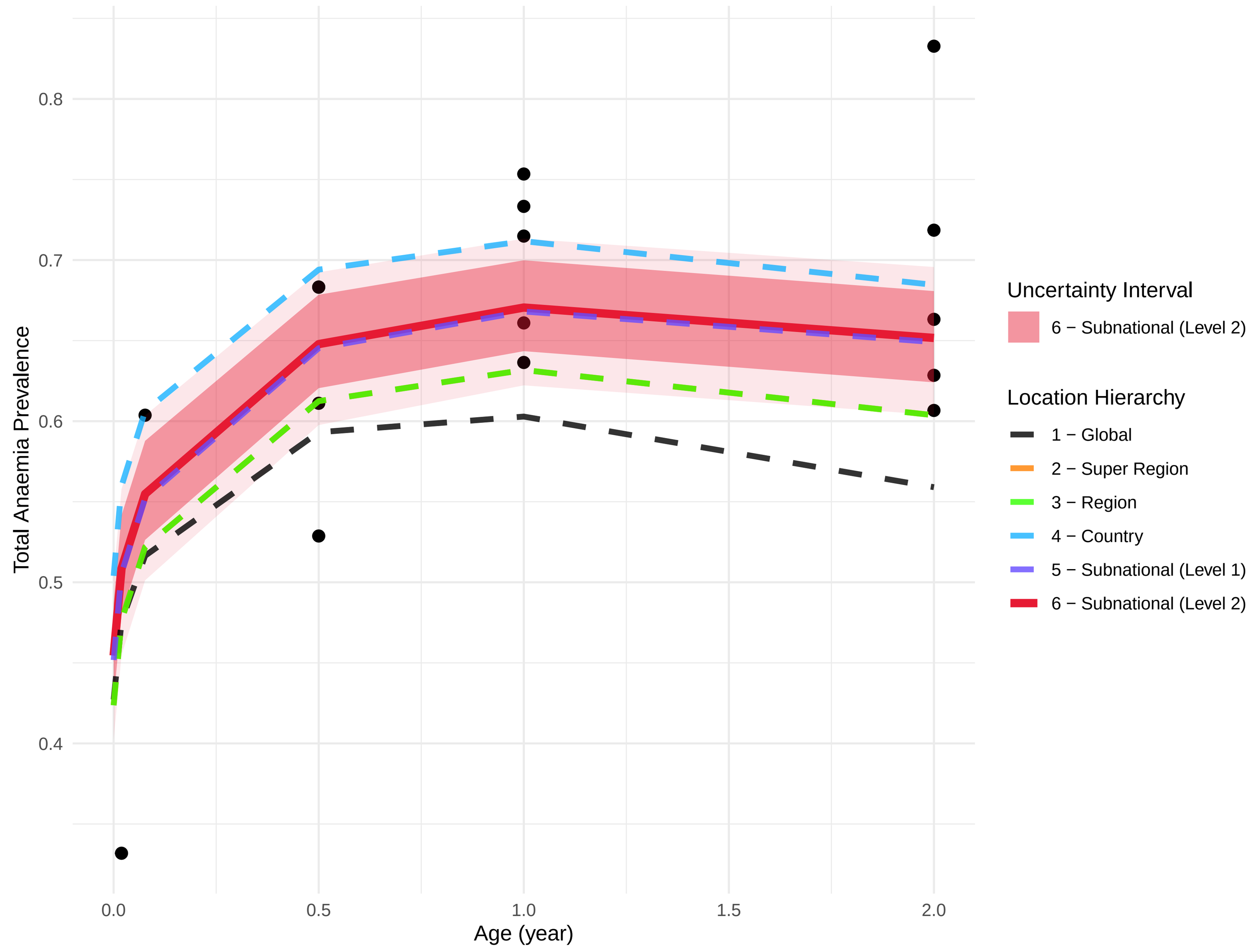

MR−BRT Age/Sex Fits − Non−pregnant Females <2 Years Old − Total Anaemia Prevalence − Himachal Pradesh, Urban
WHO−2024 Adjustment Method

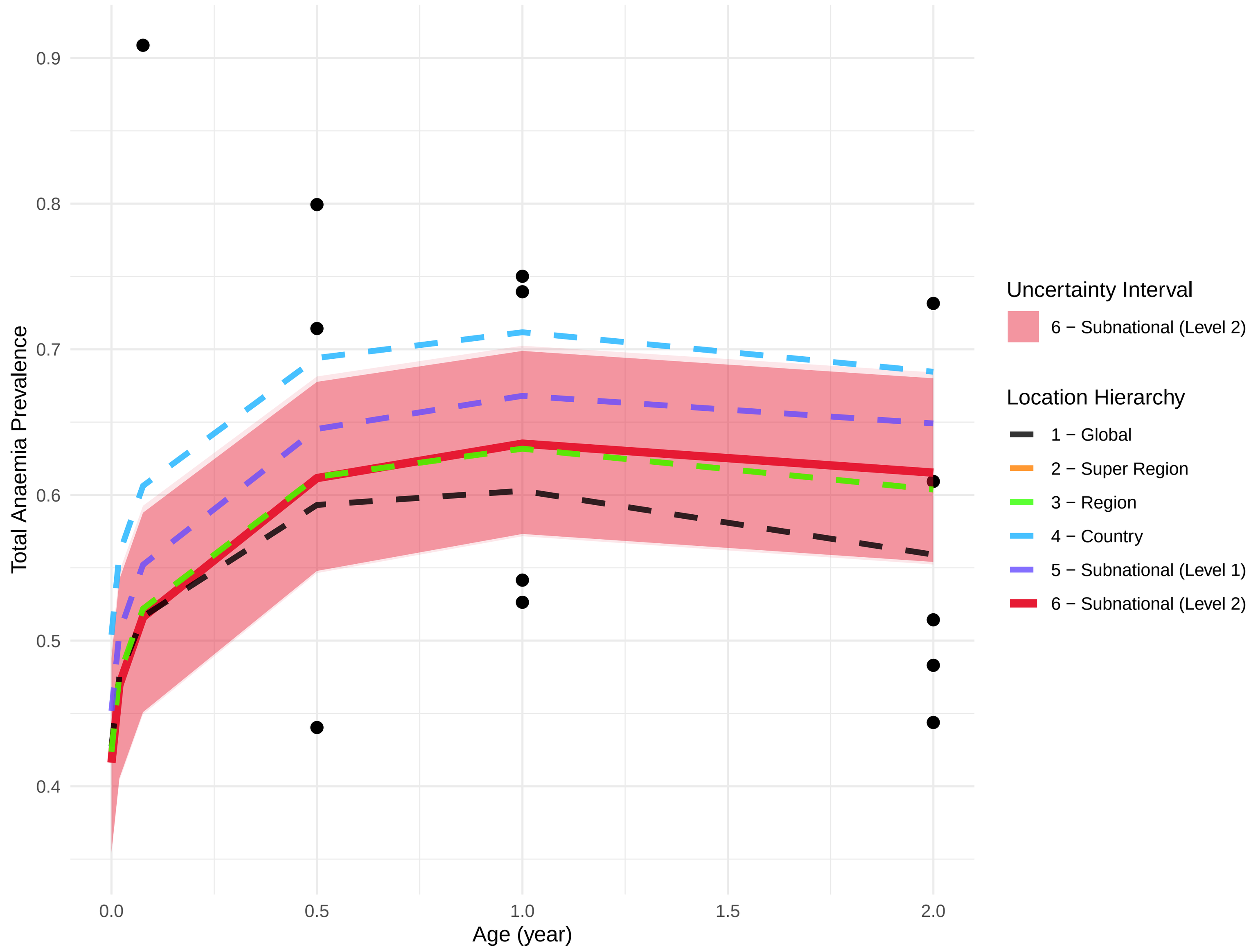

MR−BRT Age/Sex Fits − Non−pregnant Females <2 Years Old − Total Anaemia Prevalence − Jammu & Kashmir and Ladakh
WHO−2024 Adjustment Method

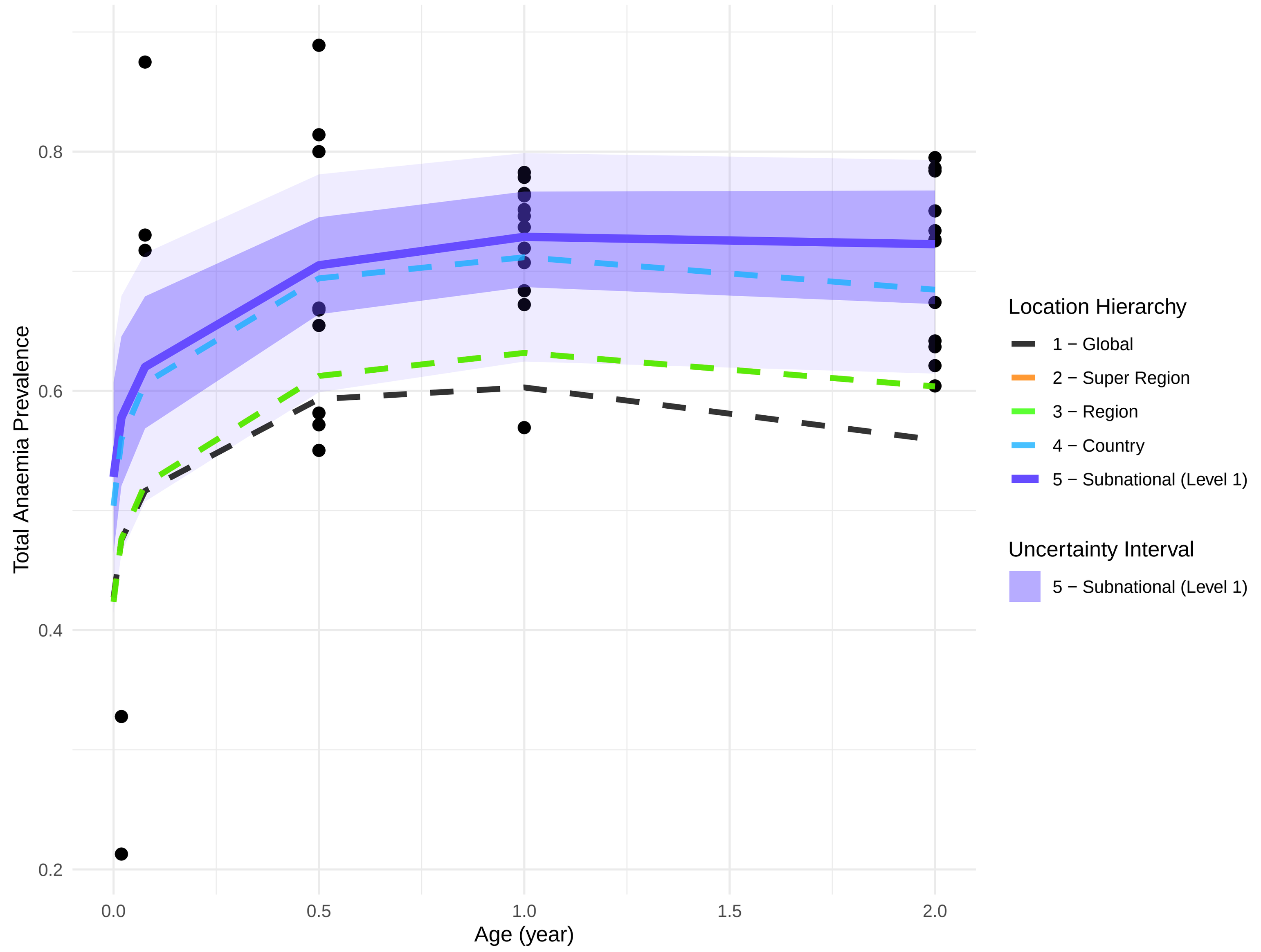

MR−BRT Age/Sex Fits − Non−pregnant Females <2 Years Old − Total Anaemia Prevalence − Jammu & Kashmir and Ladakh, Rural
WHO−2024 Adjustment Method

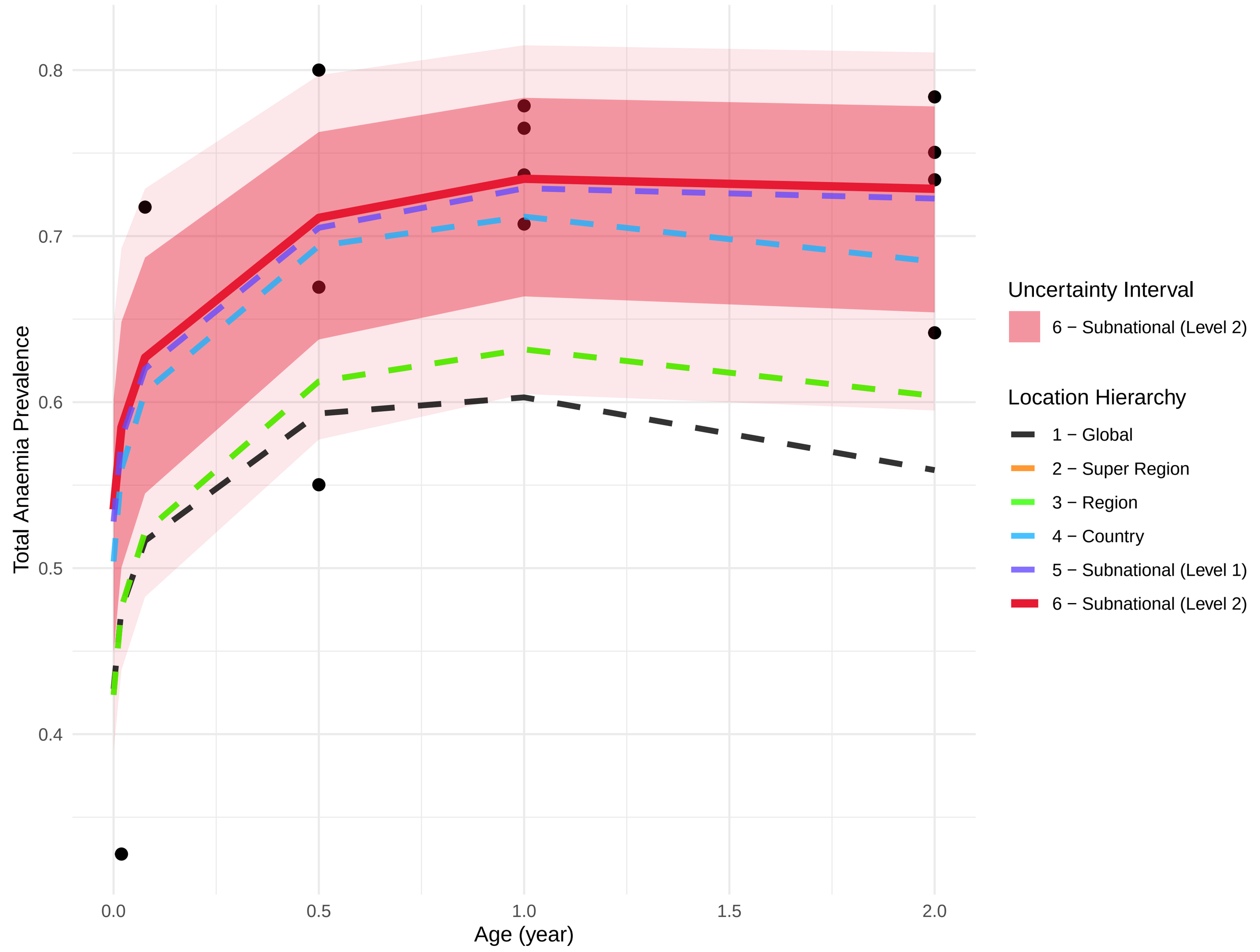

MR−BRT Age/Sex Fits − Non−pregnant Females <2 Years Old − Total Anaemia Prevalence − Jammu & Kashmir and Ladakh, Urban
WHO−2024 Adjustment Method

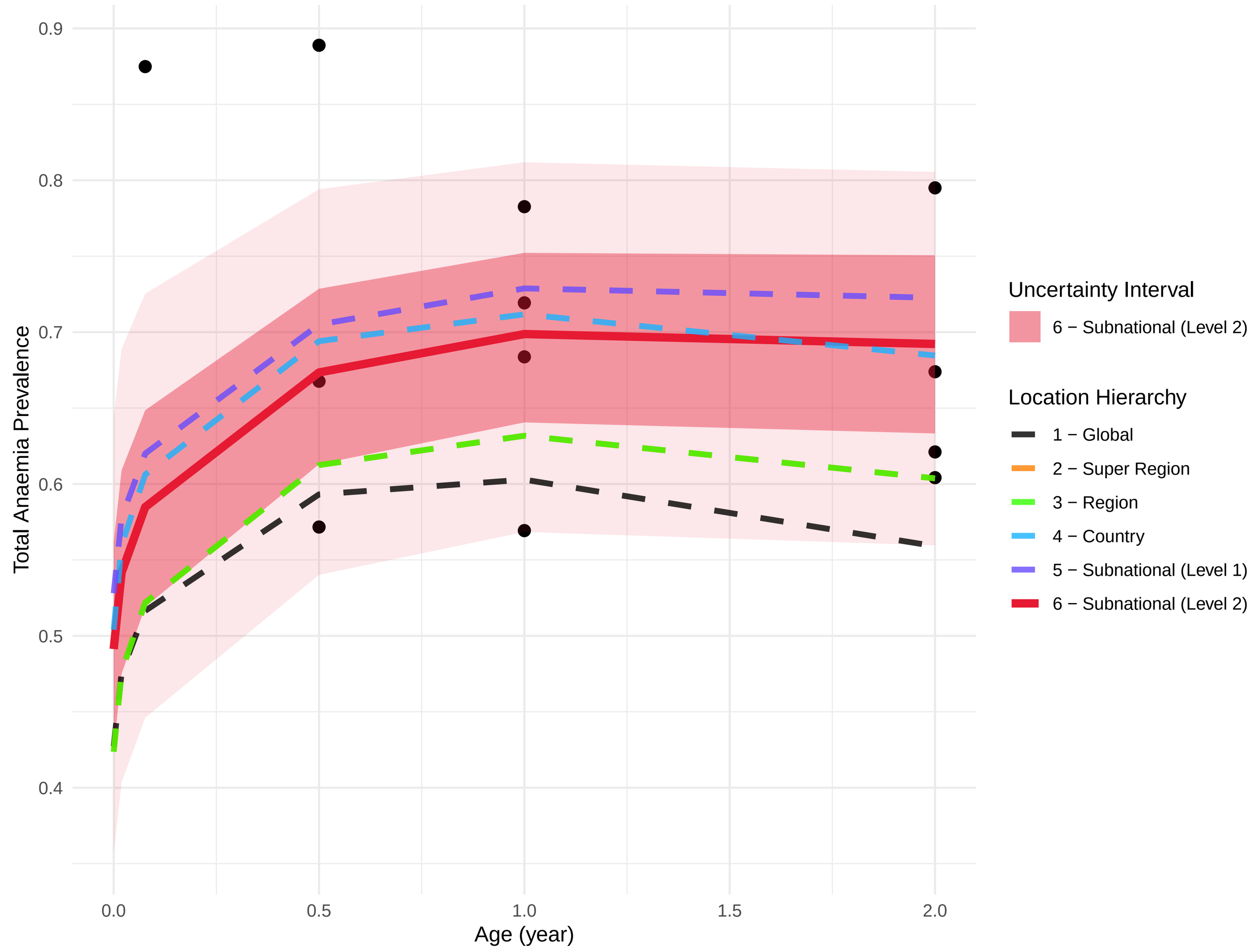

MR−BRT Age/Sex Fits − Non−pregnant Females <2 Years Old − Total Anaemia Prevalence − Jharkhand
WHO−2024 Adjustment Method

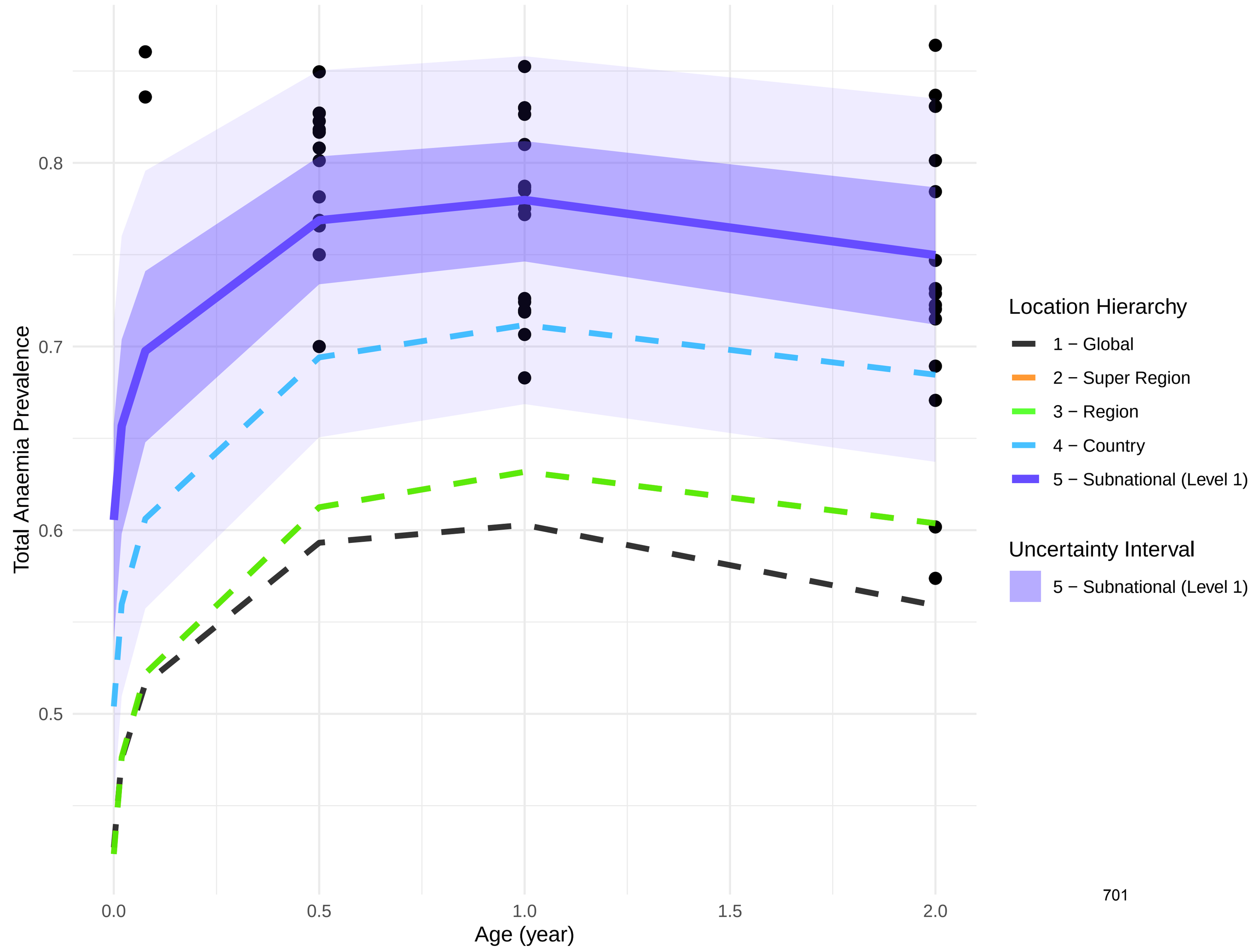

MR−BRT Age/Sex Fits − Non−pregnant Females <2 Years Old − Total Anaemia Prevalence − Jharkhand, Rural
WHO−2024 Adjustment Method

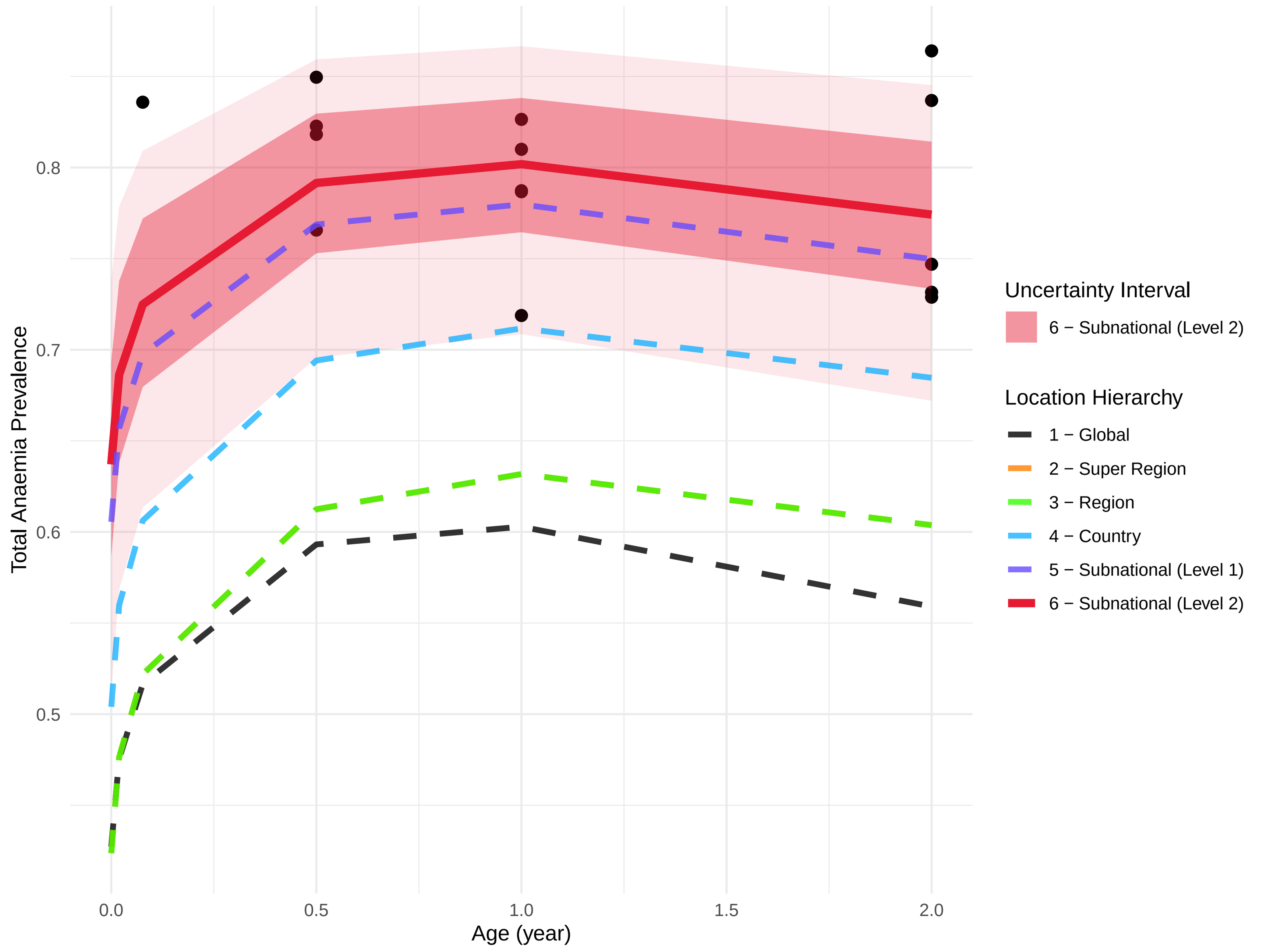

MR−BRT Age/Sex Fits − Non−pregnant Females <2 Years Old − Total Anaemia Prevalence − Jharkhand, Urban
WHO−2024 Adjustment Method

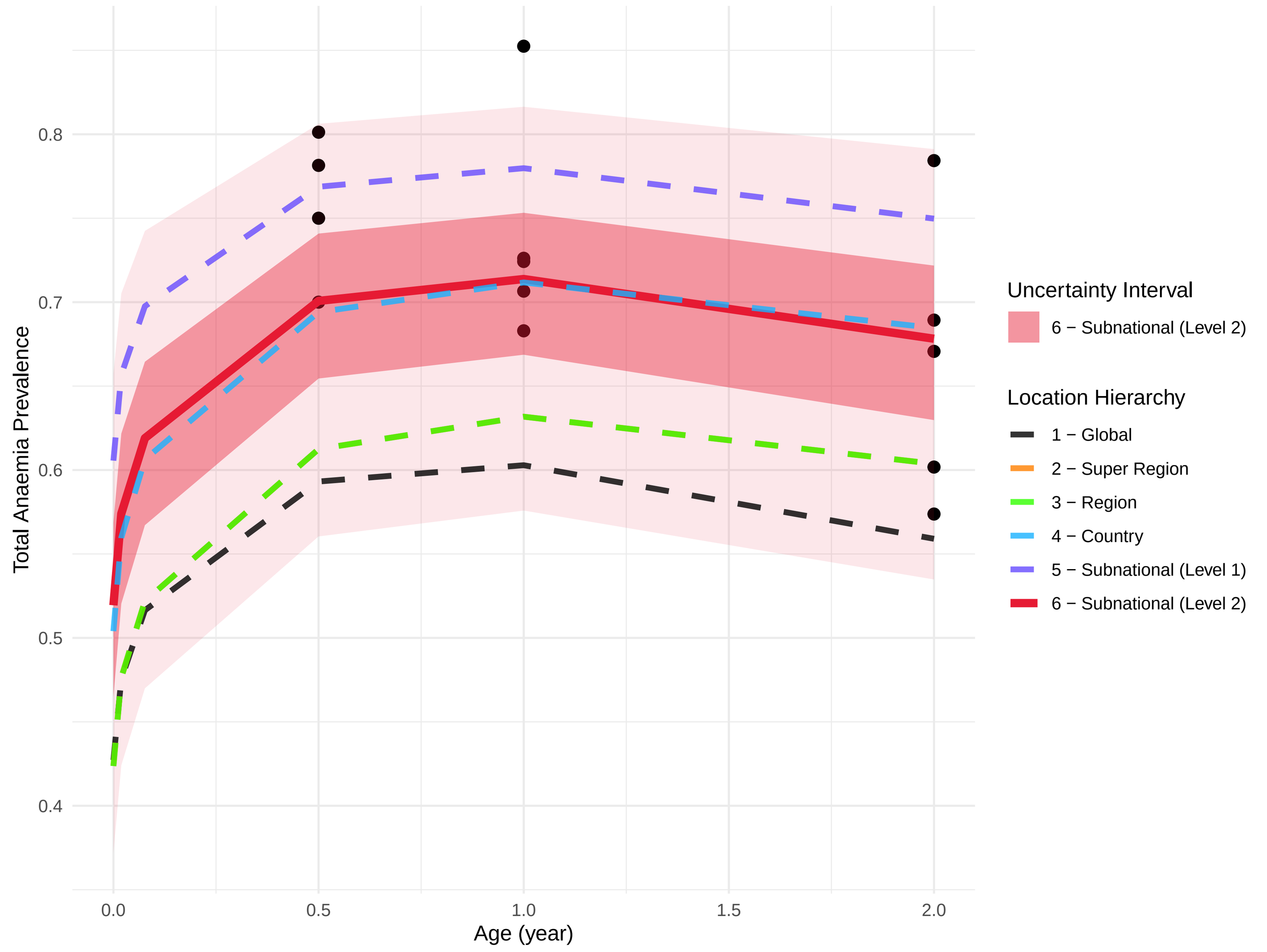

MR−BRT Age/Sex Fits − Non−pregnant Females <2 Years Old − Total Anaemia Prevalence − Karnataka
WHO−2024 Adjustment Method

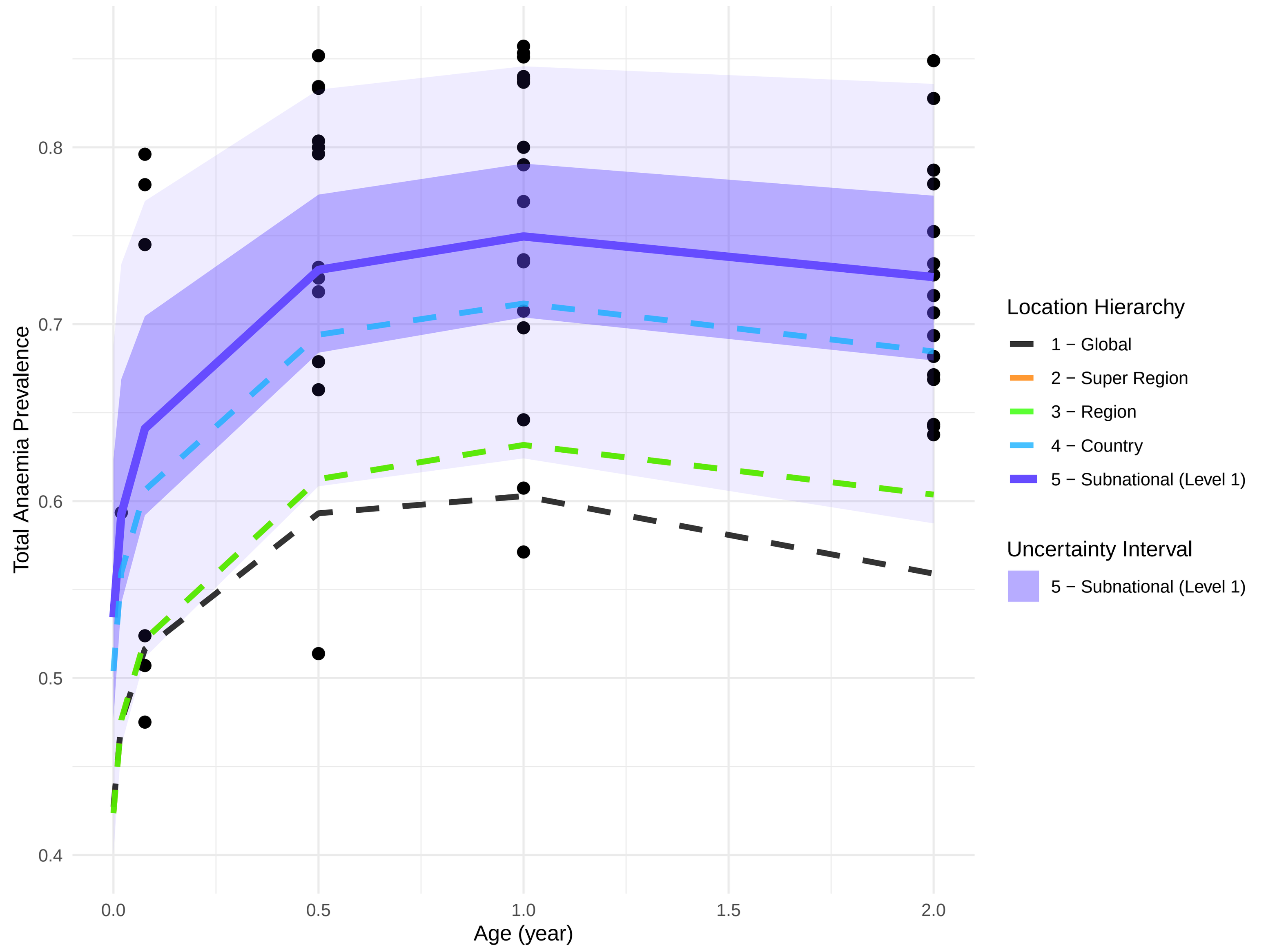

MR−BRT Age/Sex Fits − Non−pregnant Females <2 Years Old − Total Anaemia Prevalence − Karnataka, Rural
WHO−2024 Adjustment Method

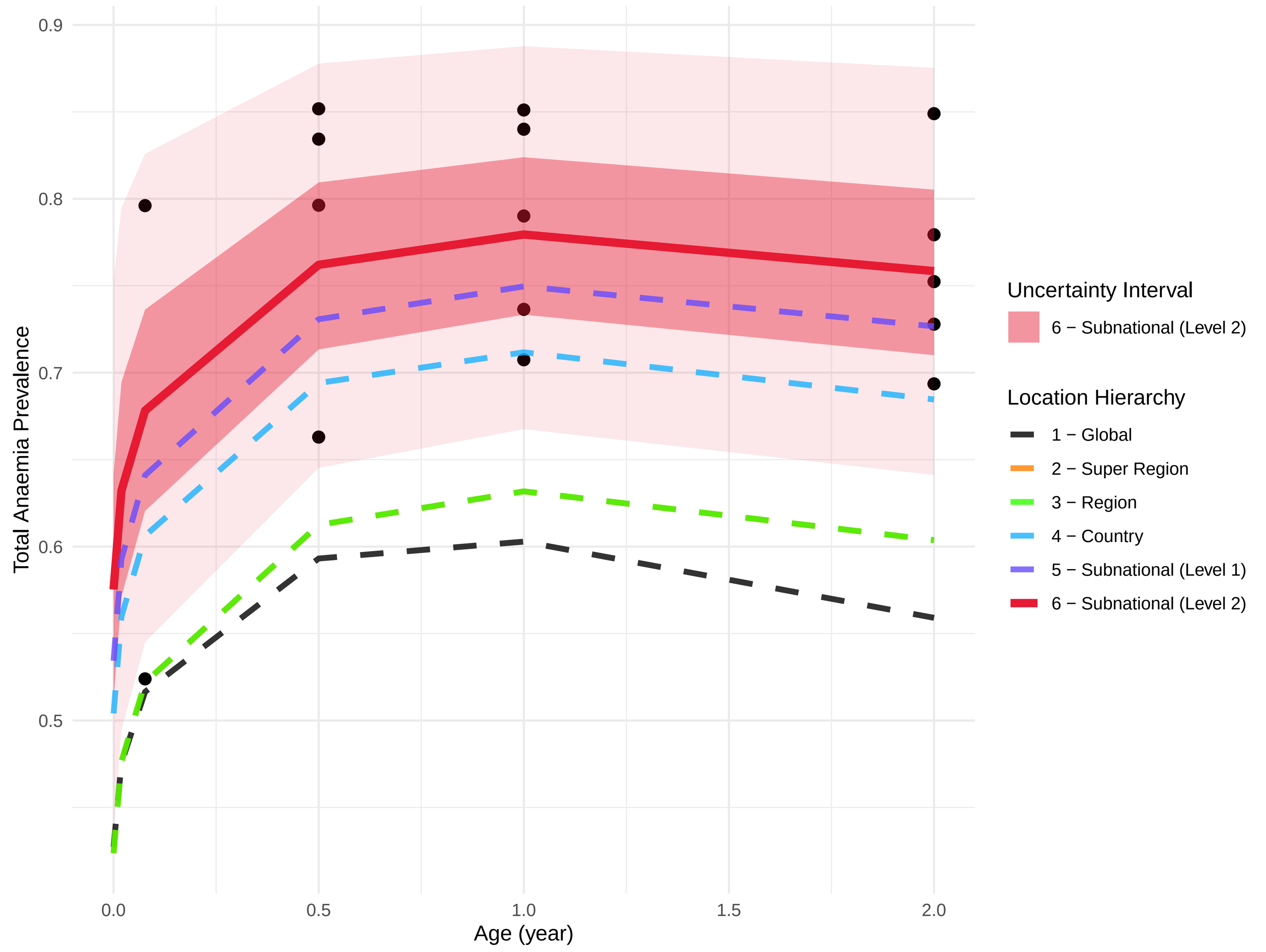

MR−BRT Age/Sex Fits − Non−pregnant Females <2 Years Old − Total Anaemia Prevalence − Karnataka, Urban
WHO−2024 Adjustment Method

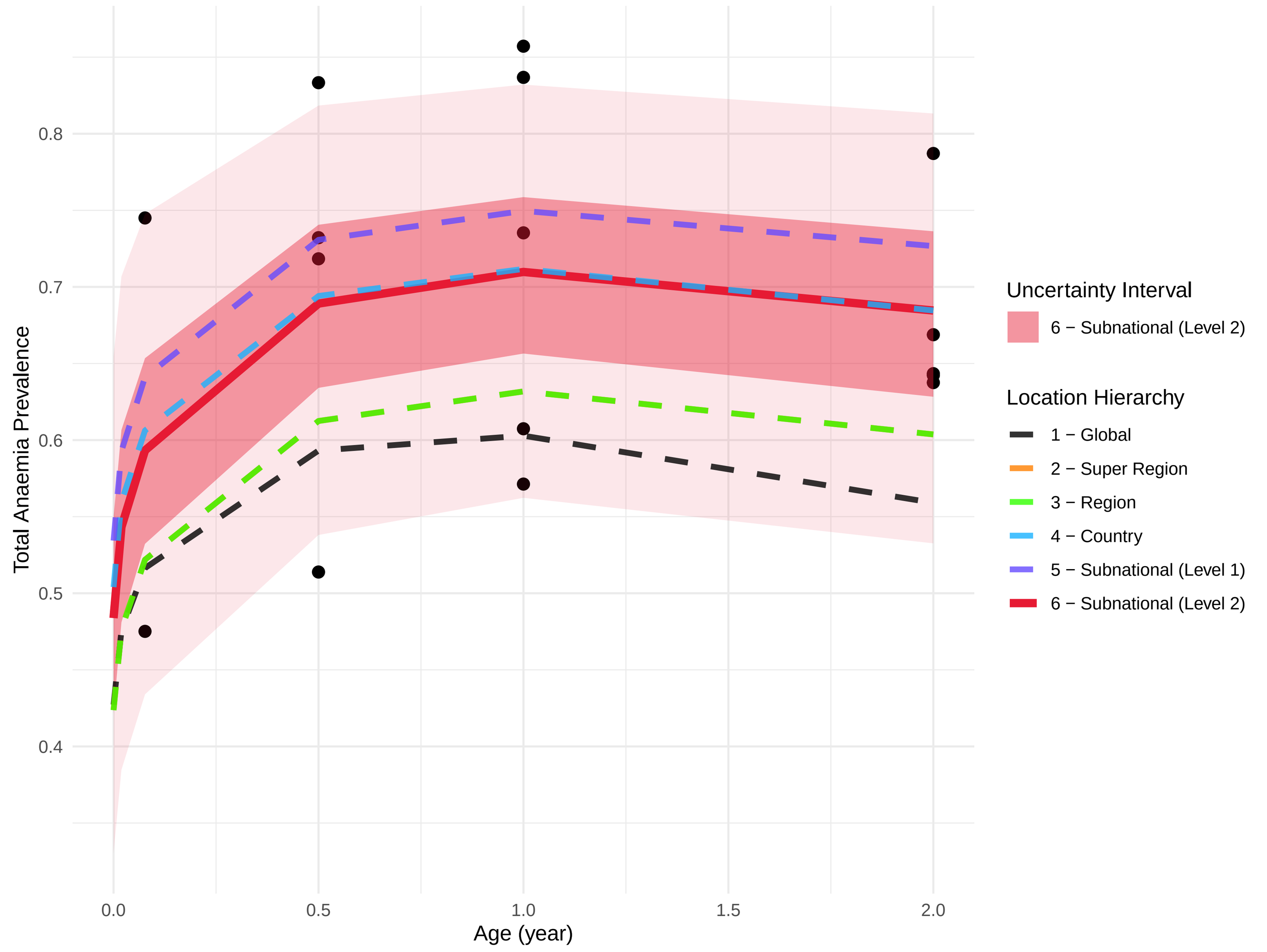

MR−BRT Age/Sex Fits − Non−pregnant Females <2 Years Old − Total Anaemia Prevalence − Kerala
WHO−2024 Adjustment Method

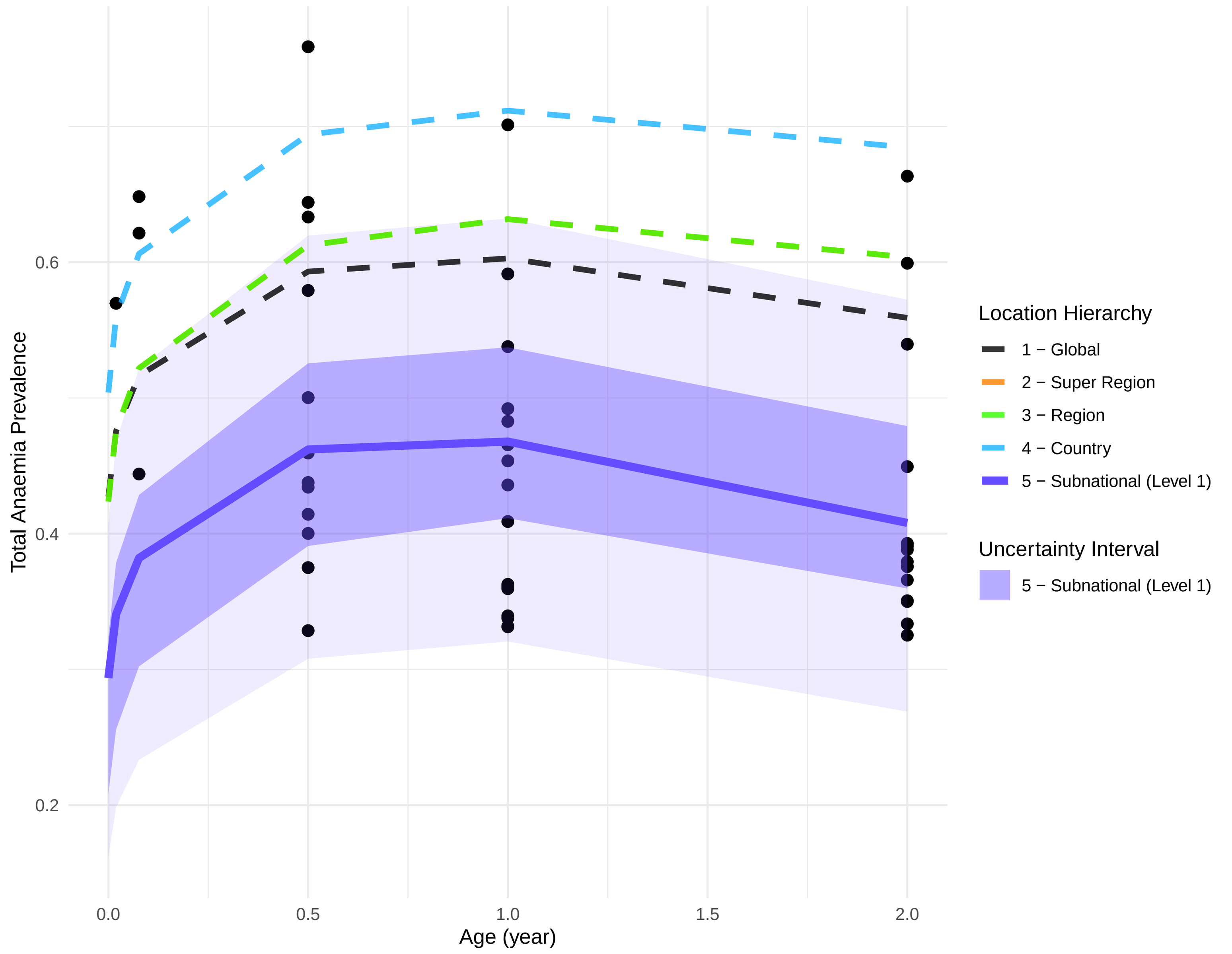

MR−BRT Age/Sex Fits − Non−pregnant Females <2 Years Old − Total Anaemia Prevalence − Kerala, Rural
WHO−2024 Adjustment Method

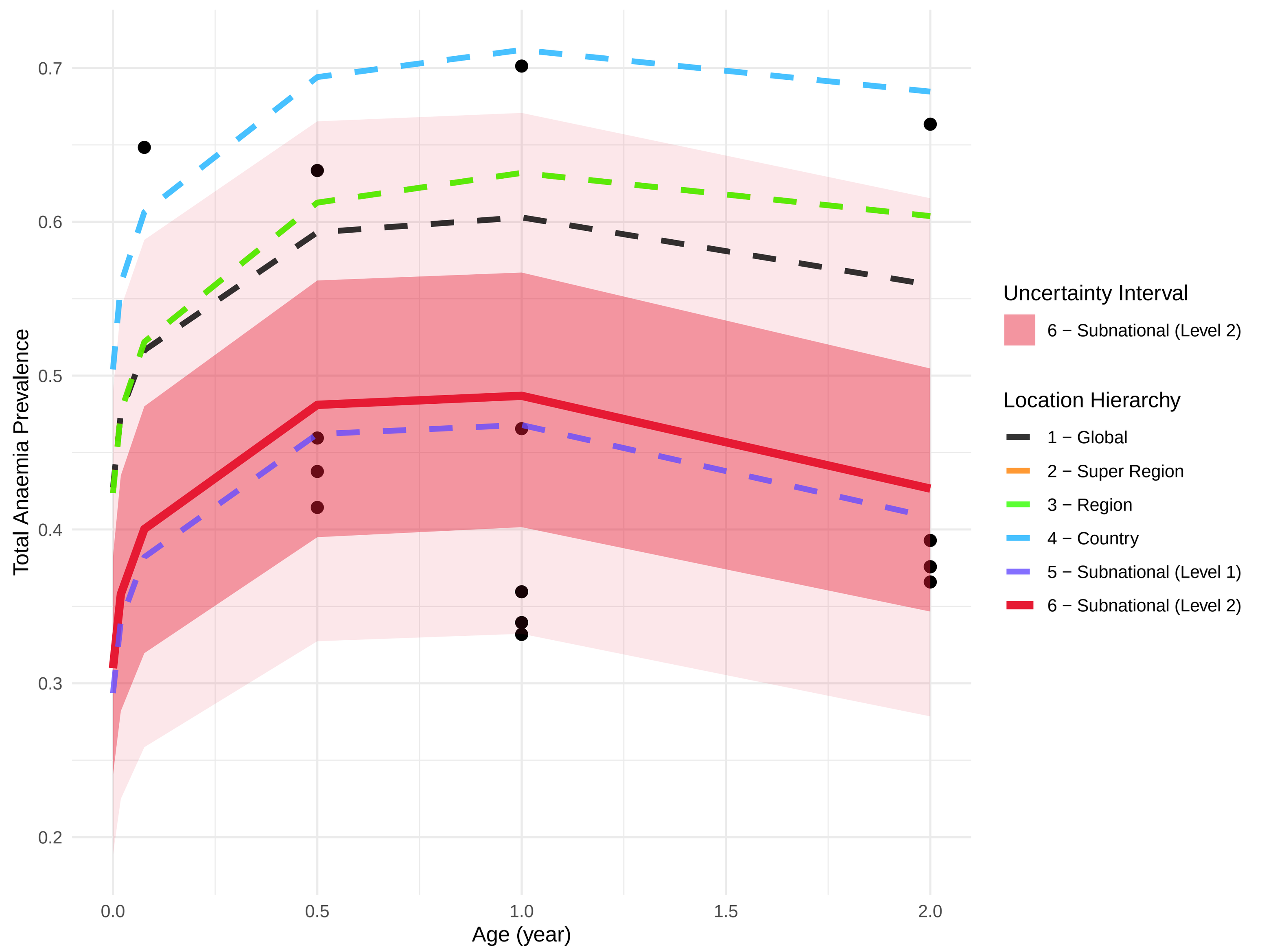

MR−BRT Age/Sex Fits − Non−pregnant Females <2 Years Old − Total Anaemia Prevalence − Kerala, Urban
WHO−2024 Adjustment Method

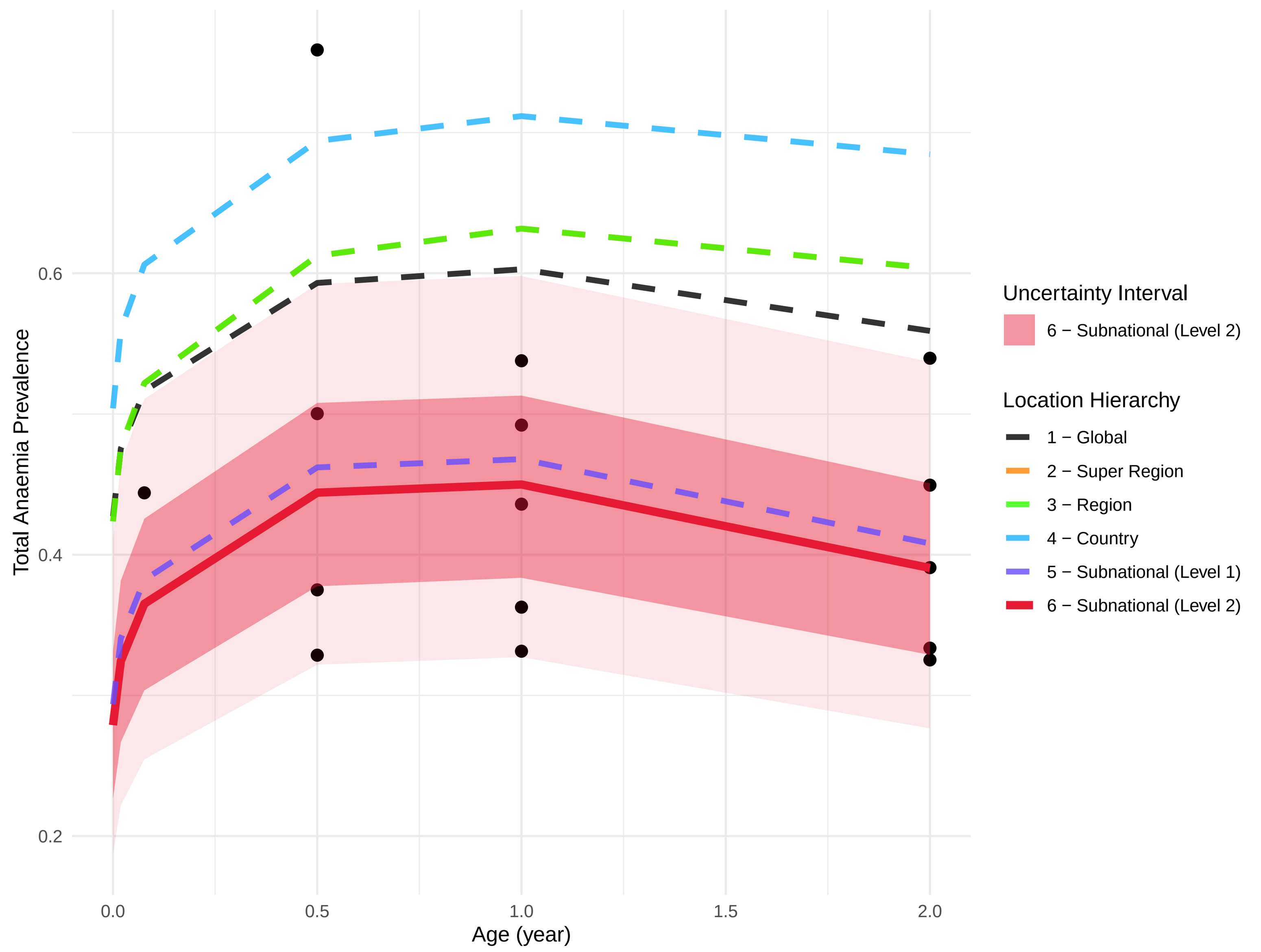

MR−BRT Age/Sex Fits − Non−pregnant Females <2 Years Old − Total Anaemia Prevalence − Madhya Pradesh
WHO−2024 Adjustment Method

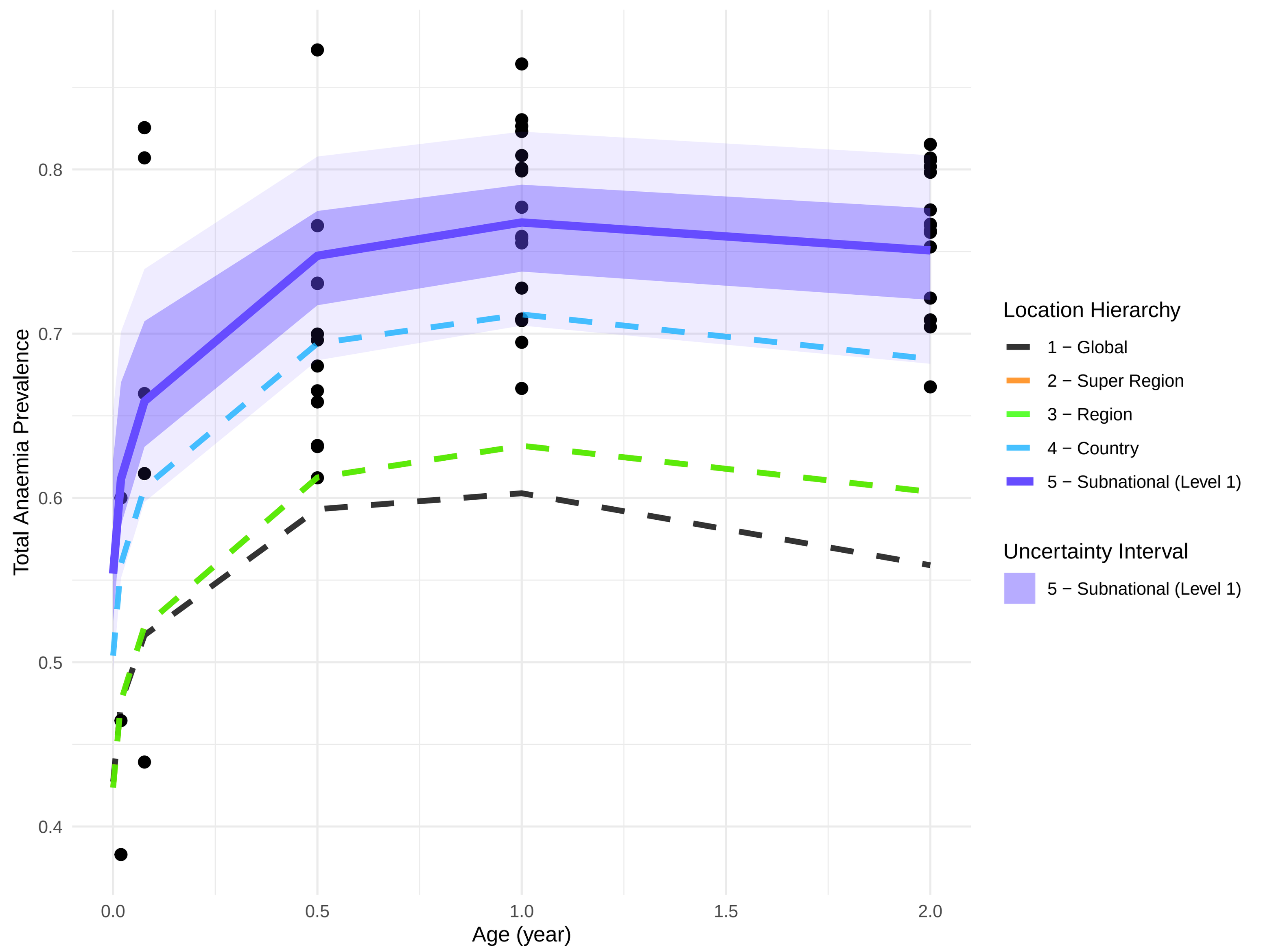

MR−BRT Age/Sex Fits − Non−pregnant Females <2 Years Old − Total Anaemia Prevalence − Madhya Pradesh, Rural
WHO−2024 Adjustment Method

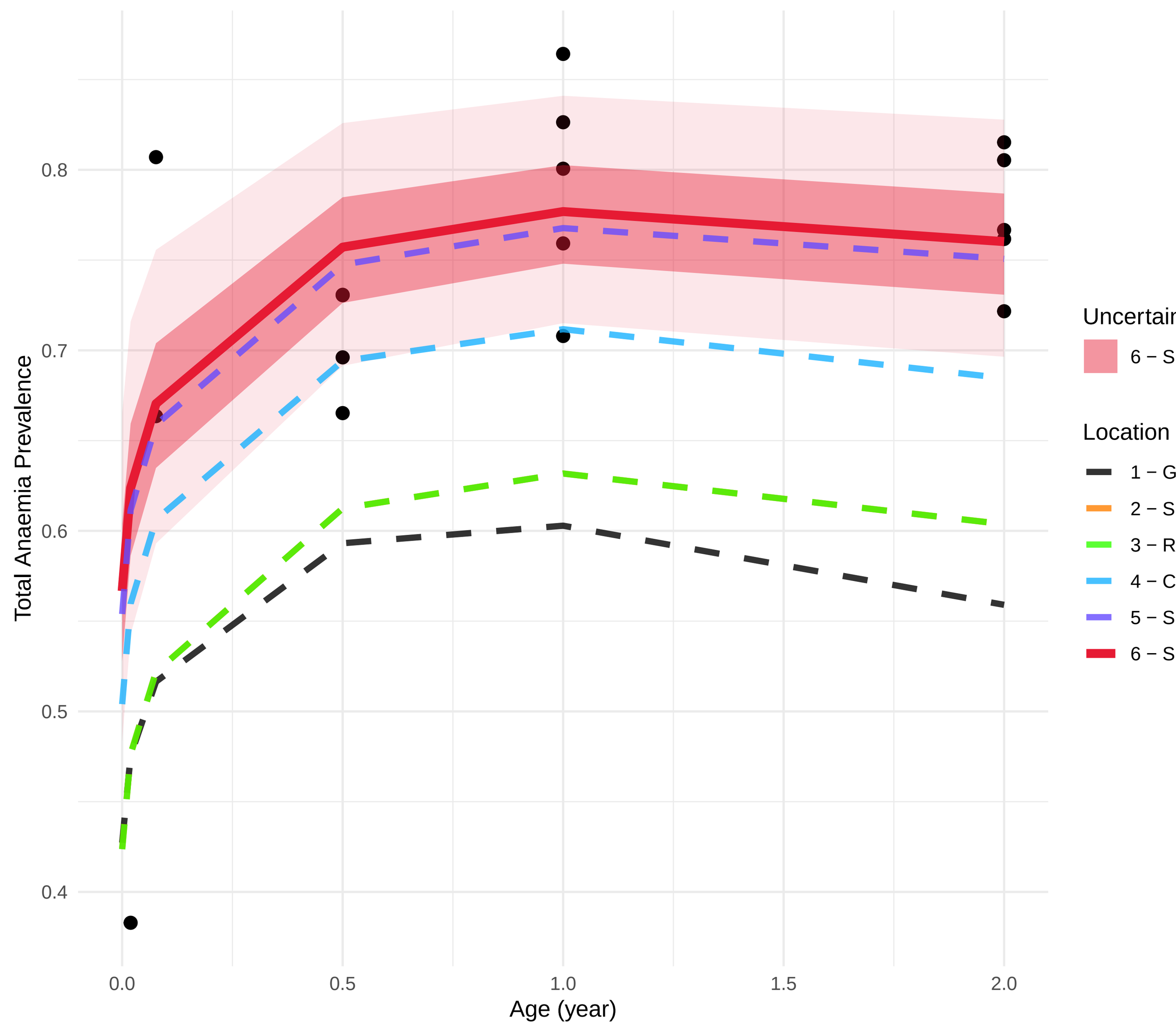

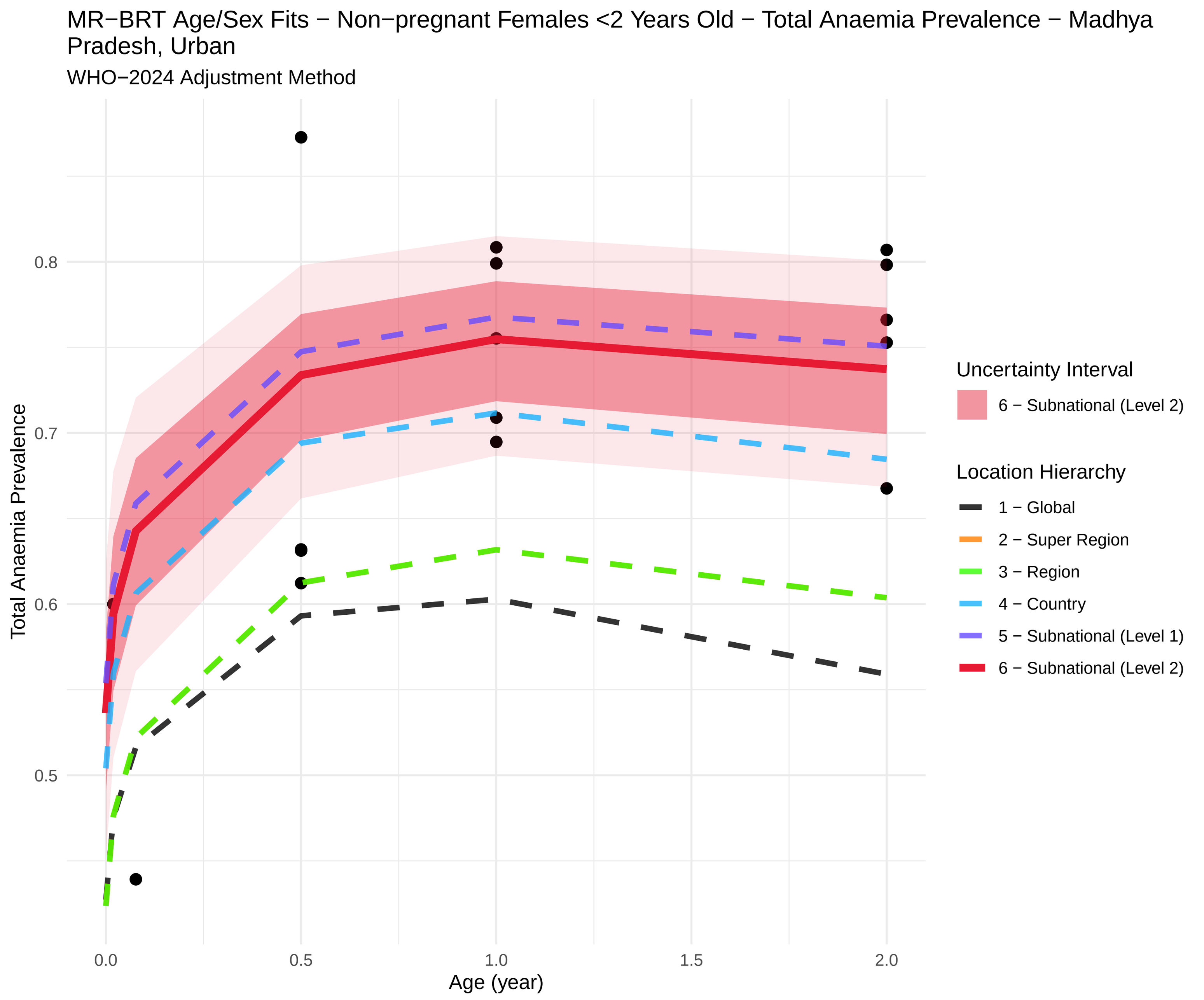
MR−BRT Age/Sex Fits − Non−pregnant Females <2 Years Old − Total Anaemia Prevalence − Madhya Pradesh, Urban
WHO−2024 Adjustment Method
Total Anaemia Prevalence
Age (year)
Uncertainty Interval
6 − Subnational (Level 2)
Location Hierarchy
1 − Global
2 − Super Region
3 − Region
4 − Country
5 − Subnational (Level 1)
6 − Subnational (Level 2)

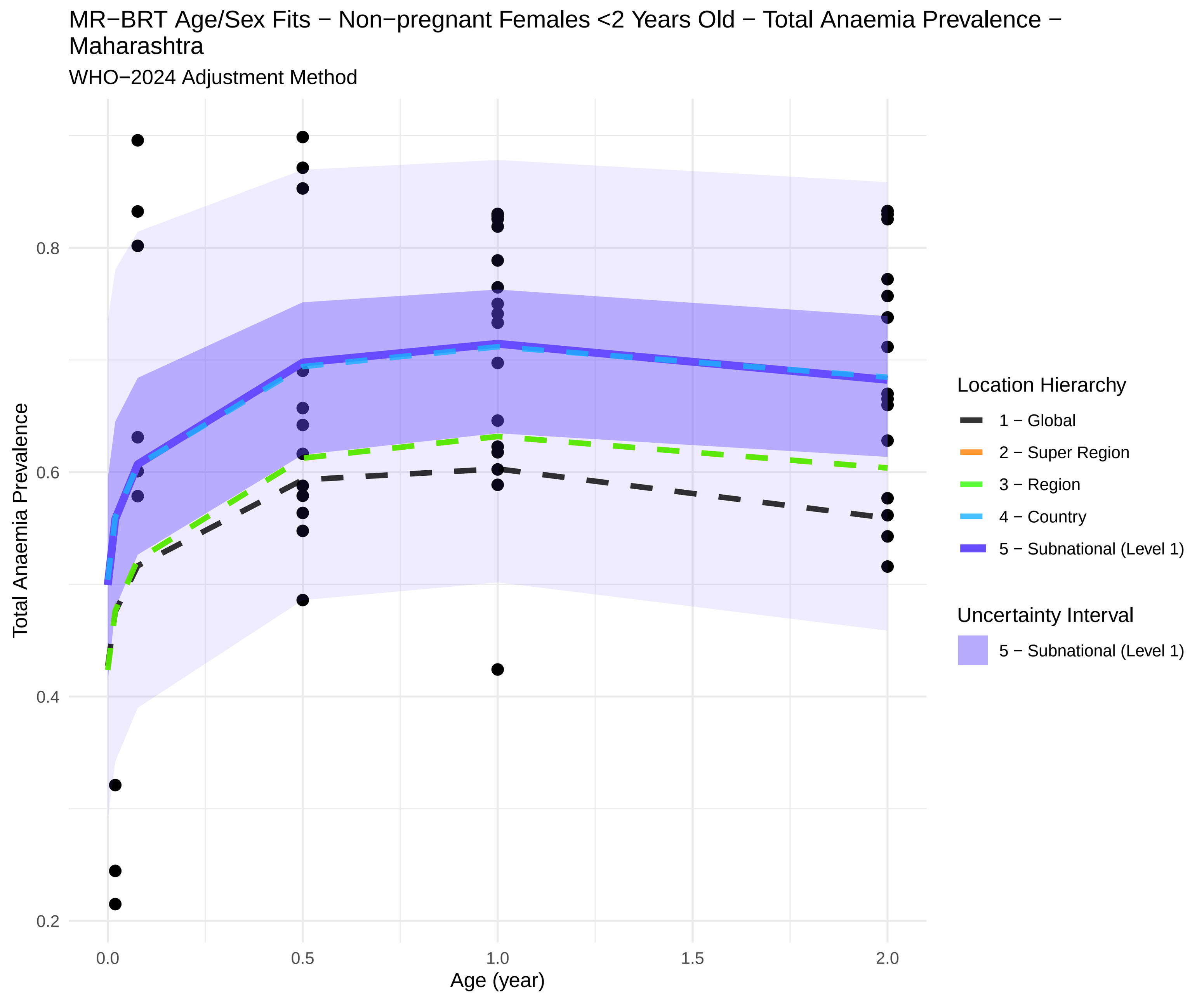
MR−BRT Age/Sex Fits − Non−pregnant Females <2 Years Old − Total Anaemia Prevalence − Maharashtra
WHO−2024 Adjustment Method
Total Anaemia Prevalence
Age (year)
Location Hierarchy
1 − Global
2 − Super Region
3 − Region
4 − Country
5 − Subnational (Level 1)
Uncertainty Interval
5 − Subnational (Level 1)

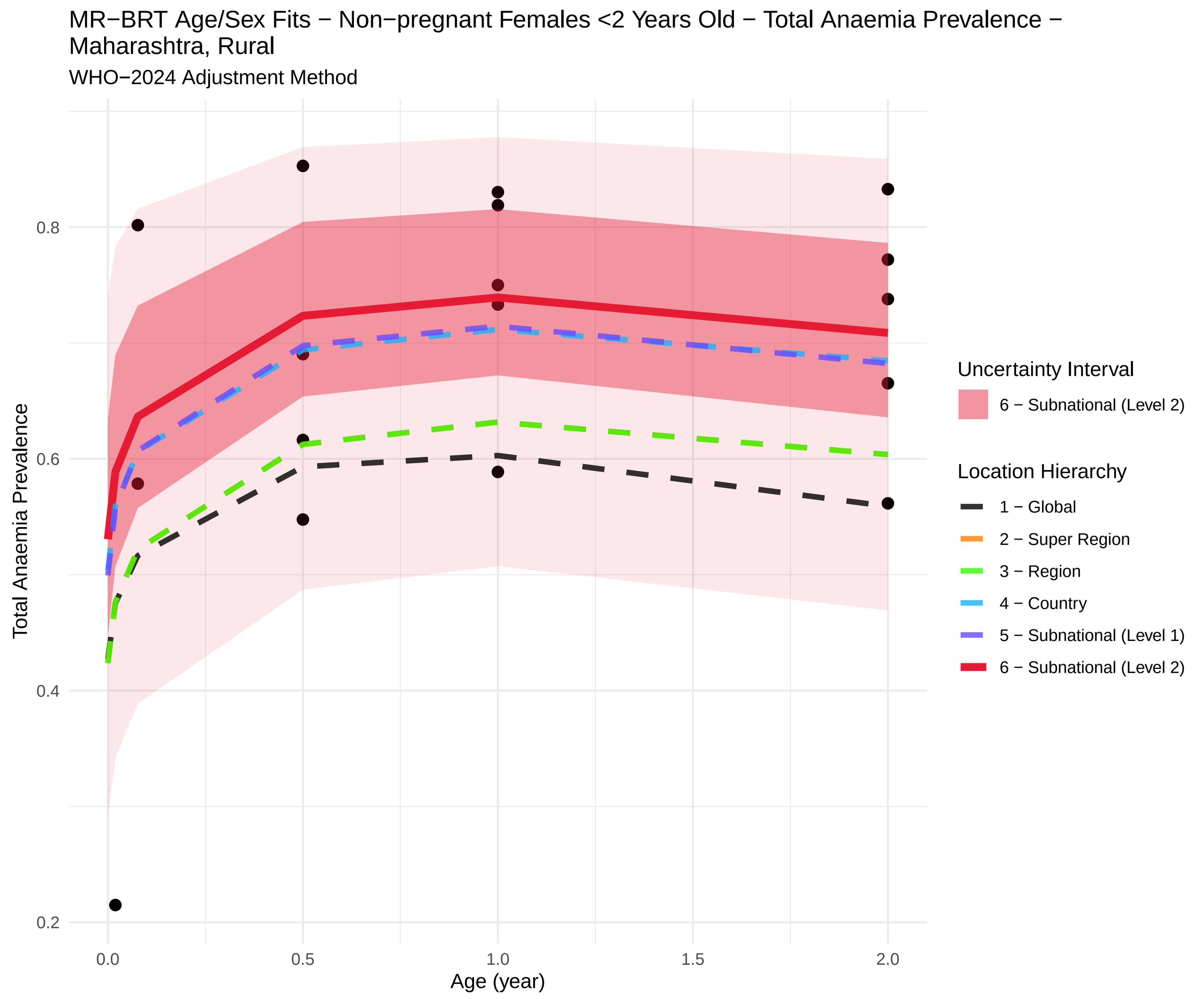
MR−BRT Age/Sex Fits − Non−pregnant Females <2 Years Old − Total Anaemia Prevalence − Maharashtra, Rural
WHO−2024 Adjustment Method
Total Anaemia Prevalence
Age (year)
Uncertainty Interval
6 − Subnational (Level 2)
Location Hierarchy
1 − Global
2 − Super Region
3 − Region
4 − Country
5 − Subnational (Level 1)
6 − Subnational (Level 2)

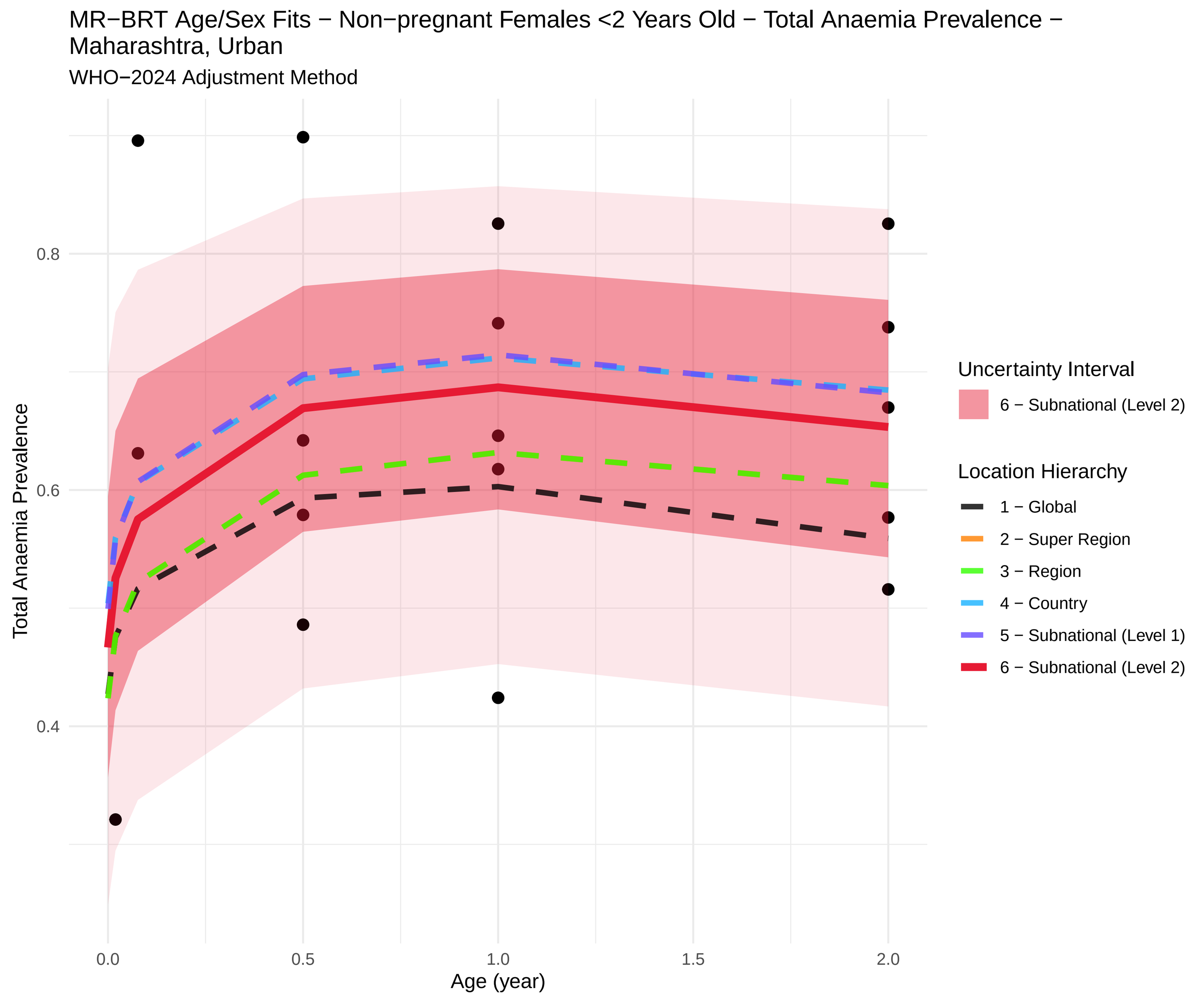
MR−BRT Age/Sex Fits − Non−pregnant Females <2 Years Old − Total Anaemia Prevalence − Maharashtra, Urban
WHO−2024 Adjustment Method
Total Anaemia Prevalence
Age (year)
Uncertainty Interval
6 − Subnational (Level 2)
Location Hierarchy
1 − Global
2 − Super Region
3 − Region
4 − Country
5 − Subnational (Level 1)
6 − Subnational (Level 2)

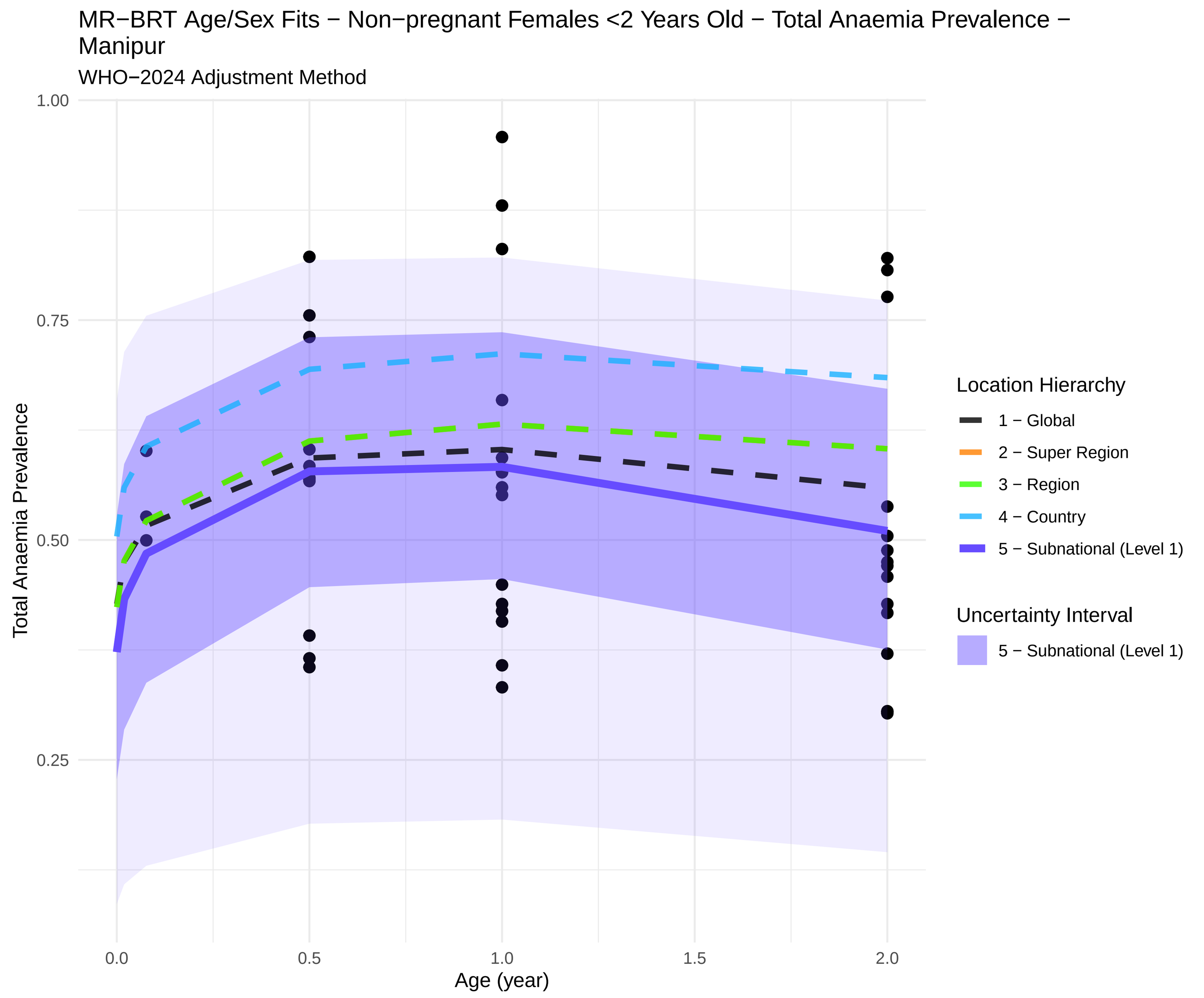
MR−BRT Age/Sex Fits − Non−pregnant Females <2 Years Old − Total Anaemia Prevalence − Manipur
WHO−2024 Adjustment Method
Total Anaemia Prevalence
Age (year)
Location Hierarchy
1 − Global
2 − Super Region
3 − Region
4 − Country
5 − Subnational (Level 1)
Uncertainty Interval
5 − Subnational (Level 1)

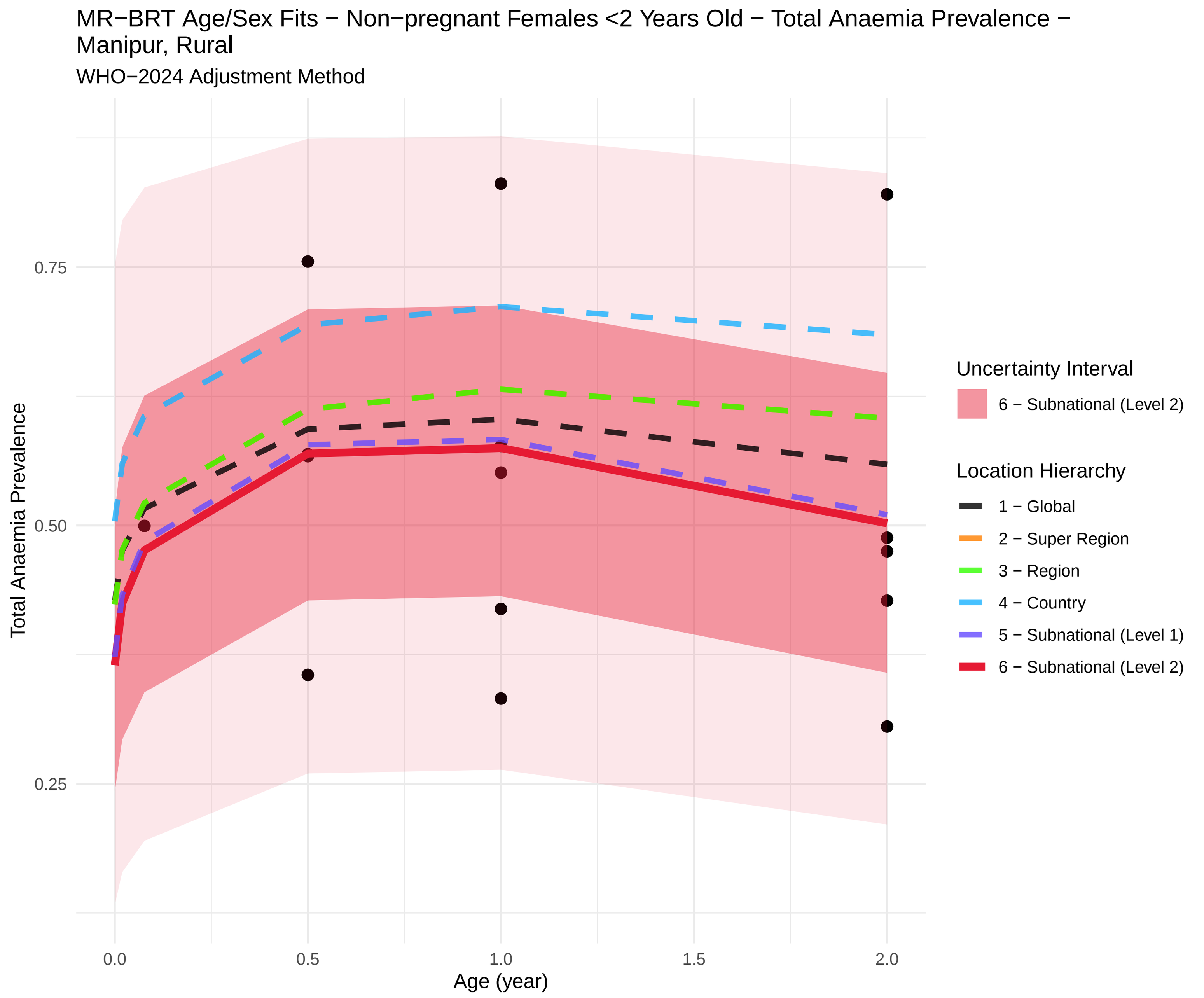
MR−BRT Age/Sex Fits − Non−pregnant Females <2 Years Old − Total Anaemia Prevalence − Manipur, Rural
WHO−2024 Adjustment Method
Total Anaemia Prevalence
Age (year)
Uncertainty Interval
6 − Subnational (Level 2)
Location Hierarchy
1 − Global
2 − Super Region
3 − Region
4 − Country
5 − Subnational (Level 1)
6 − Subnational (Level 2)

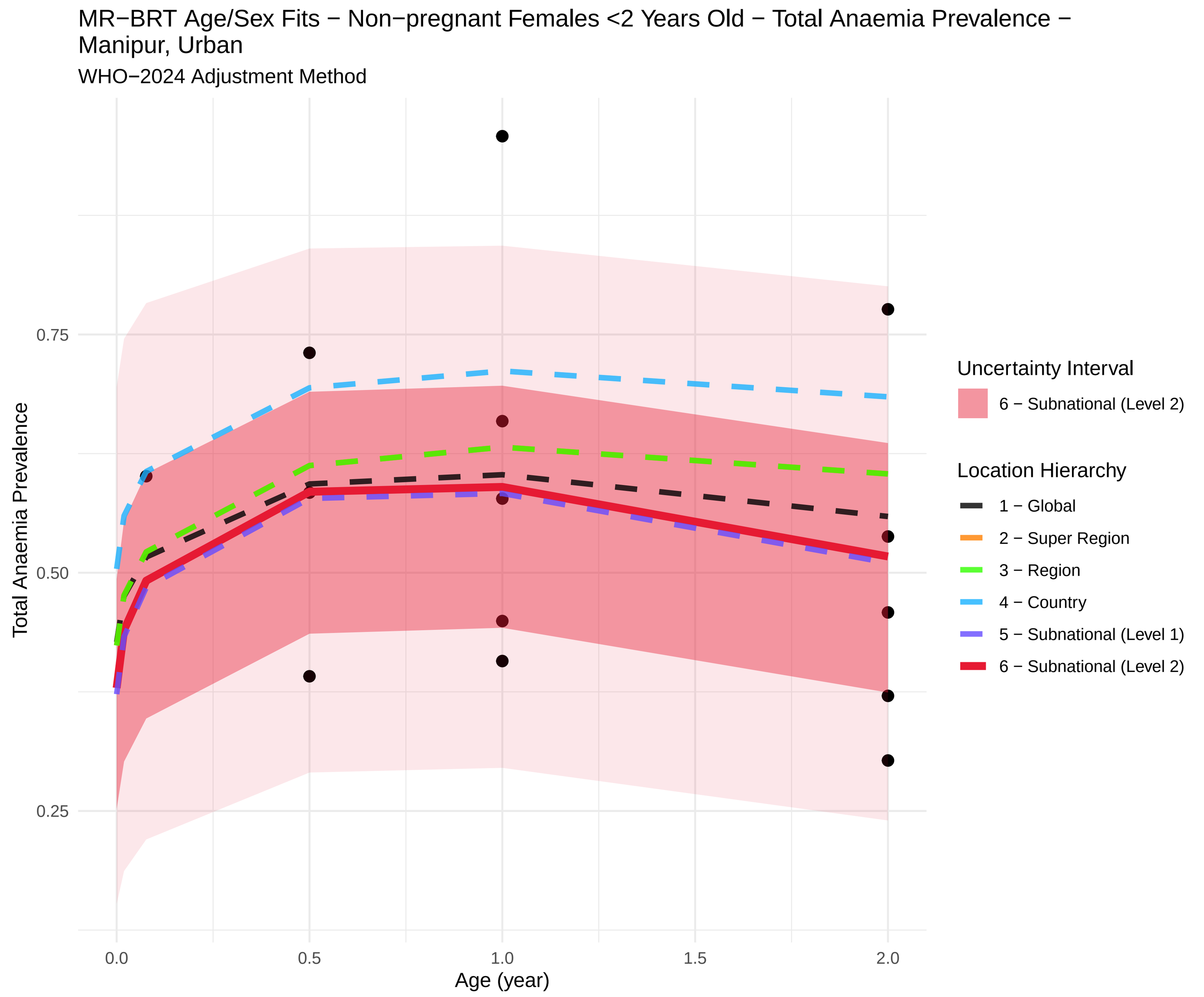
MR−BRT Age/Sex Fits − Non−pregnant Females <2 Years Old − Total Anaemia Prevalence − Manipur, Urban
WHO−2024 Adjustment Method
Total Anaemia Prevalence
Age (year)
Uncertainty Interval
6 − Subnational (Level 2)
Location Hierarchy
1 − Global
2 − Super Region
3 − Region
4 − Country
5 − Subnational (Level 1)
6 − Subnational (Level 2)

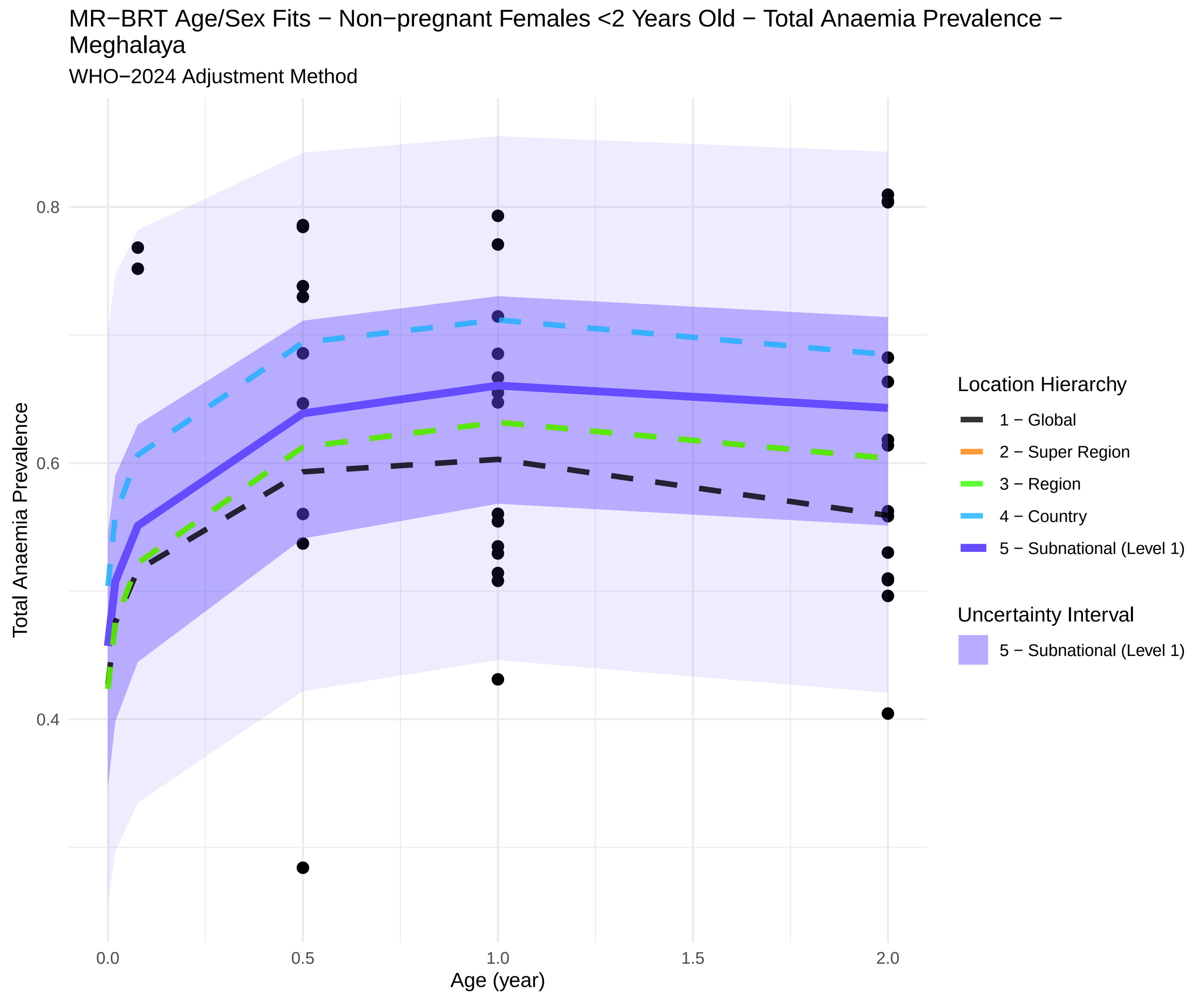
MR−BRT Age/Sex Fits − Non−pregnant Females <2 Years Old − Total Anaemia Prevalence − Meghalaya
WHO−2024 Adjustment Method
Total Anaemia Prevalence
Age (year)
Location Hierarchy
1 − Global
2 − Super Region
3 − Region
4 − Country
5 − Subnational (Level 1)
Uncertainty Interval
5 − Subnational (Level 1)

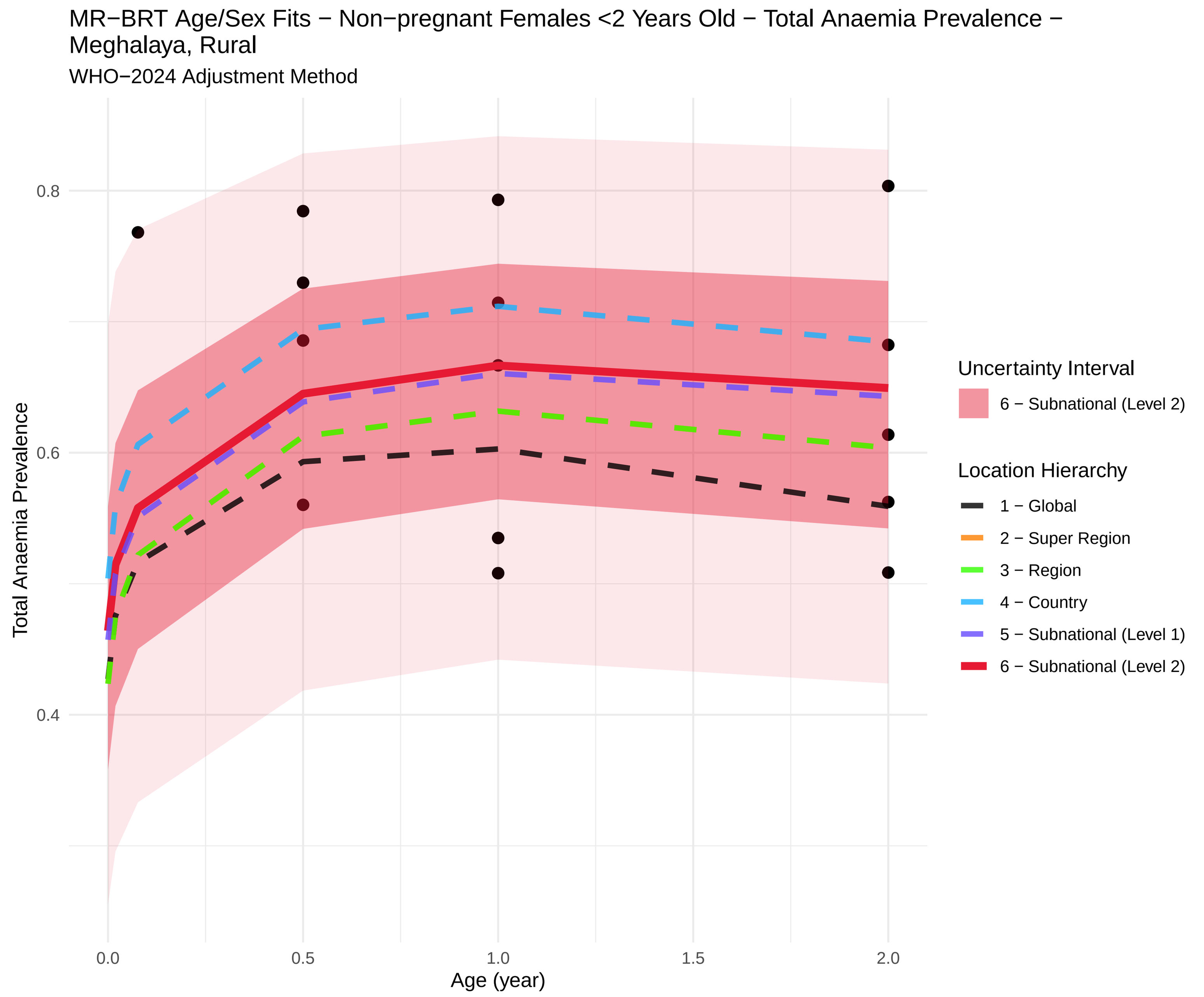
MR−BRT Age/Sex Fits − Non−pregnant Females <2 Years Old − Total Anaemia Prevalence − Meghalaya, Rural
WHO−2024 Adjustment Method
Total Anaemia Prevalence
Age (year)
Uncertainty Interval
6 − Subnational (Level 2)
Location Hierarchy
1 − Global
2 − Super Region
3 − Region
4 − Country
5 − Subnational (Level 1)
6 − Subnational (Level 2)

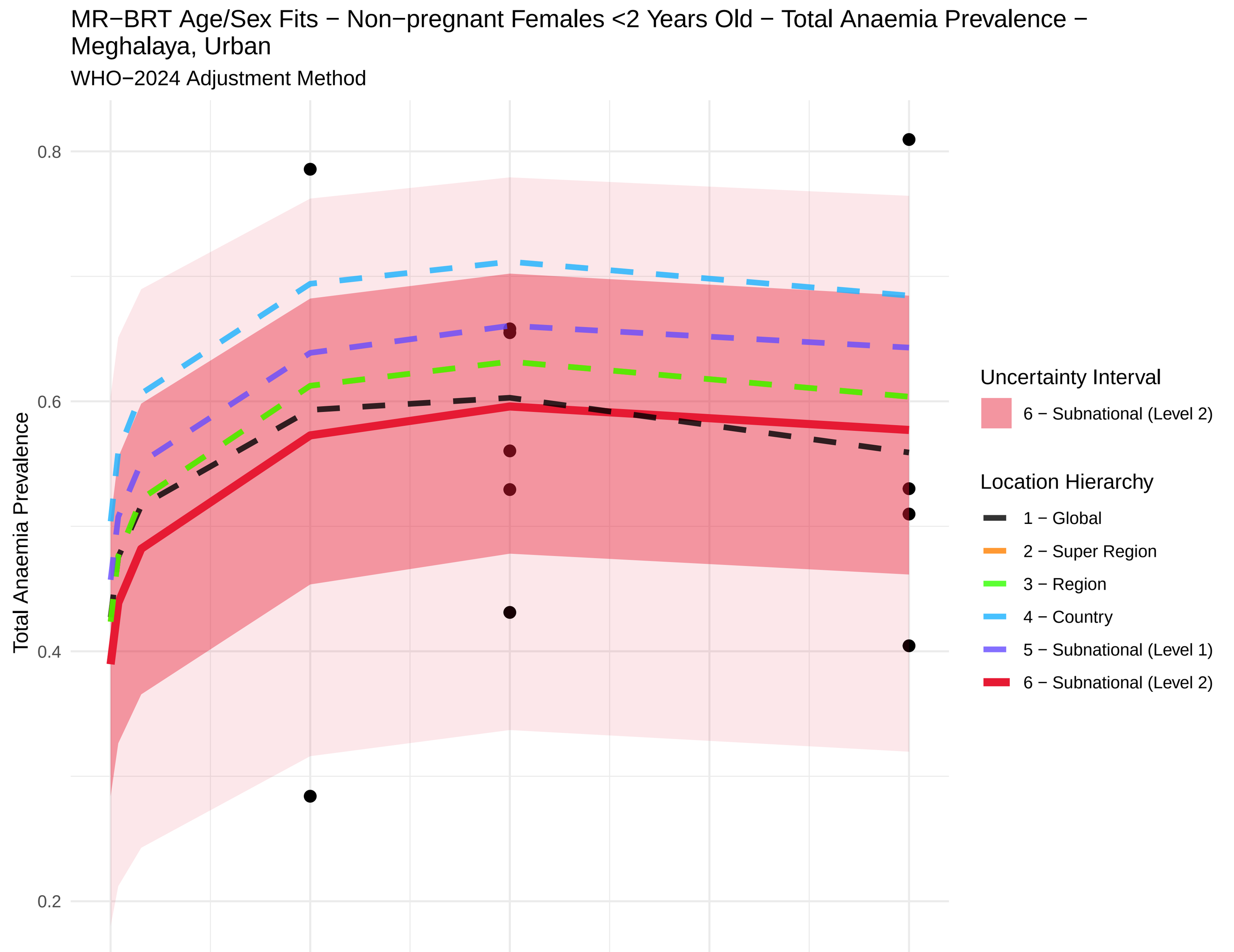
MR−BRT Age/Sex Fits − Non−pregnant Females <2 Years Old − Total Anaemia Prevalence − Meghalaya, Urban
WHO−2024 Adjustment Method
Total Anaemia Prevalence
Age (year)
Uncertainty Interval
6 − Subnational (Level 2)
Location Hierarchy
1 − Global
2 − Super Region
3 − Region
4 − Country
5 − Subnational (Level 1)
6 − Subnational (Level 2)

MR−BRT Age/Sex Fits − Non−pregnant Females <2 Years Old − Total Anaemia Prevalence − Mizoram
WHO−2024 Adjustment Method

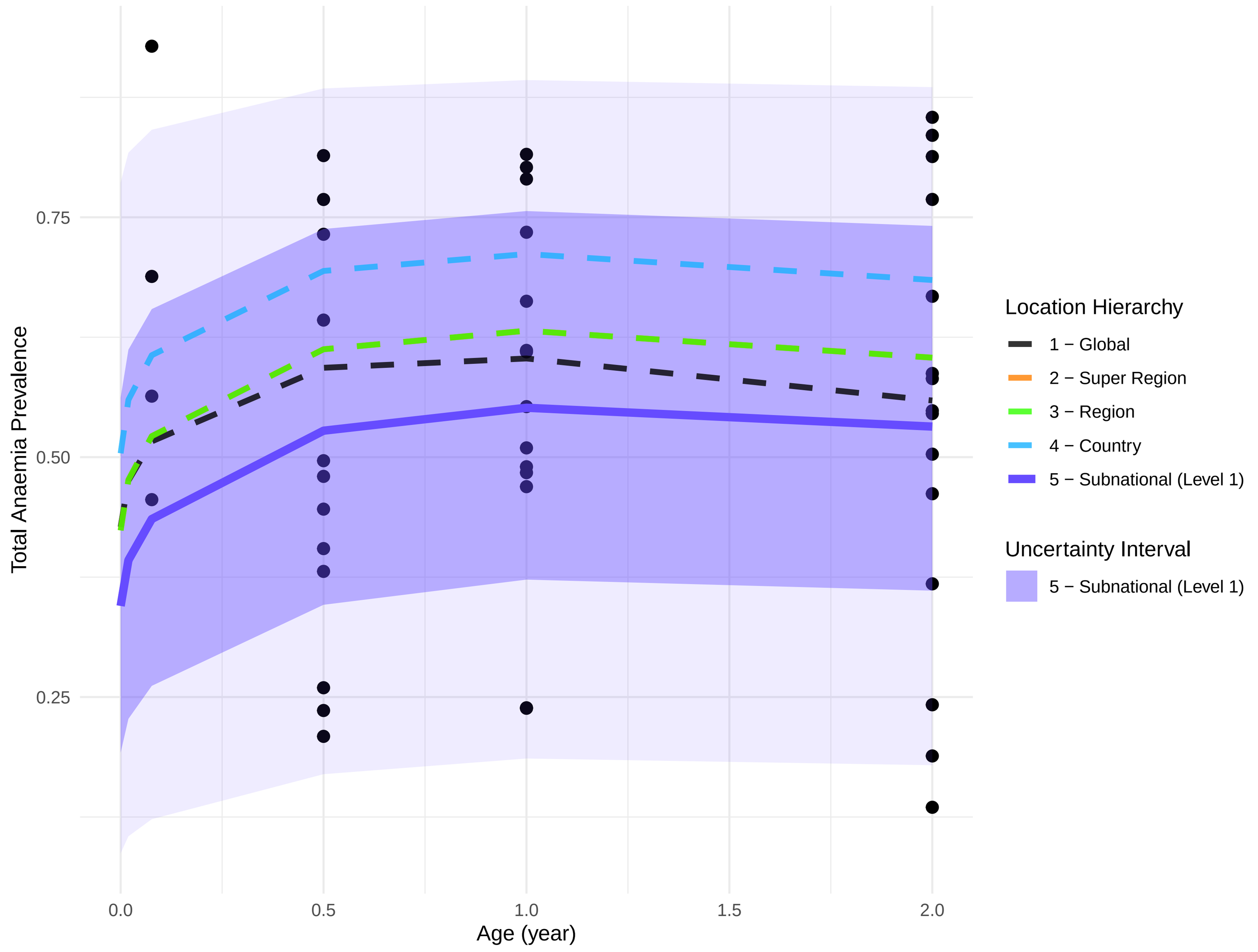

MR−BRT Age/Sex Fits − Non−pregnant Females <2 Years Old − Total Anaemia Prevalence − Mizoram, Rural
WHO−2024 Adjustment Method

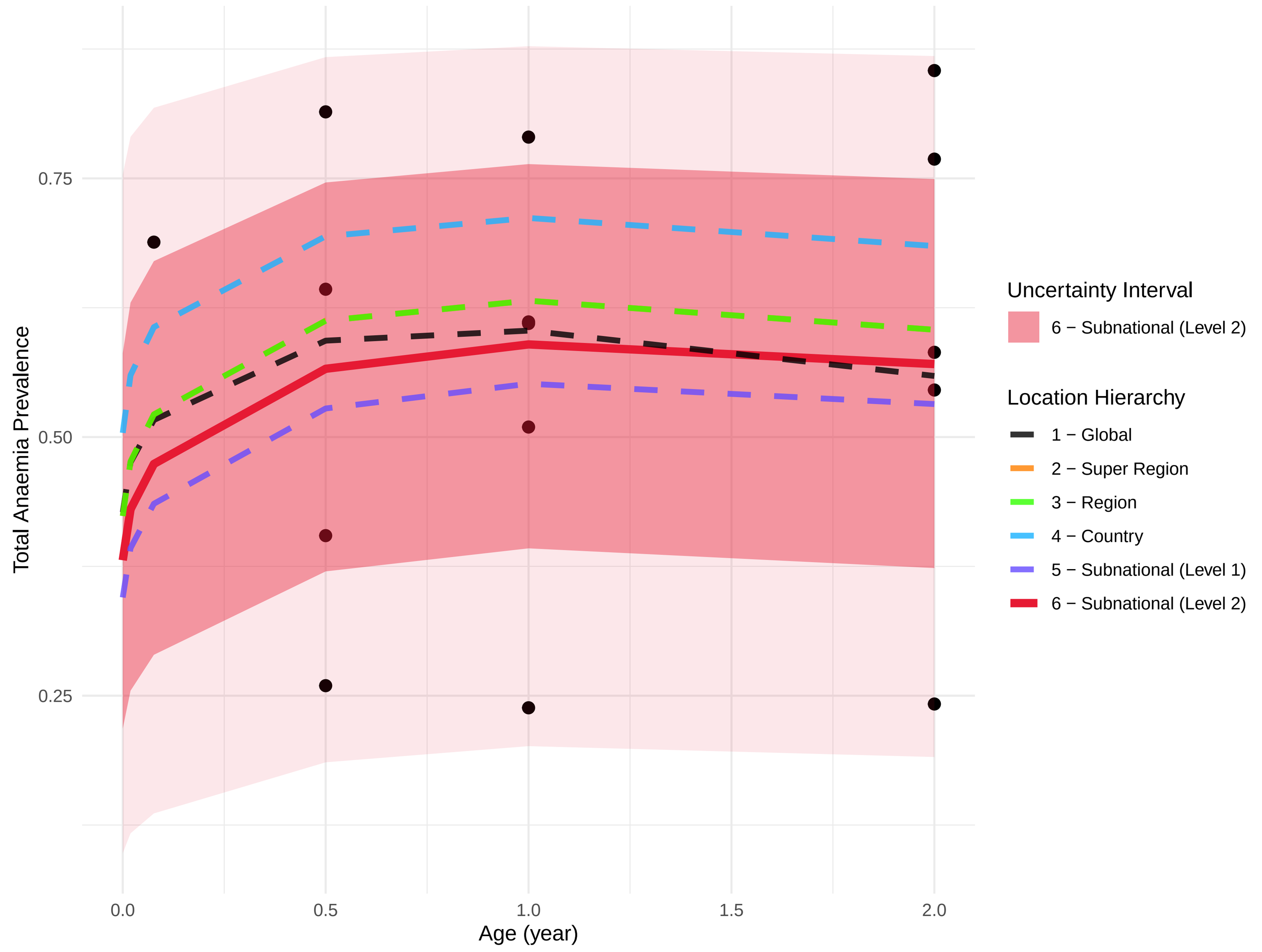

MR−BRT Age/Sex Fits − Non−pregnant Females <2 Years Old − Total Anaemia Prevalence − Mizoram, Urban
WHO−2024 Adjustment Method

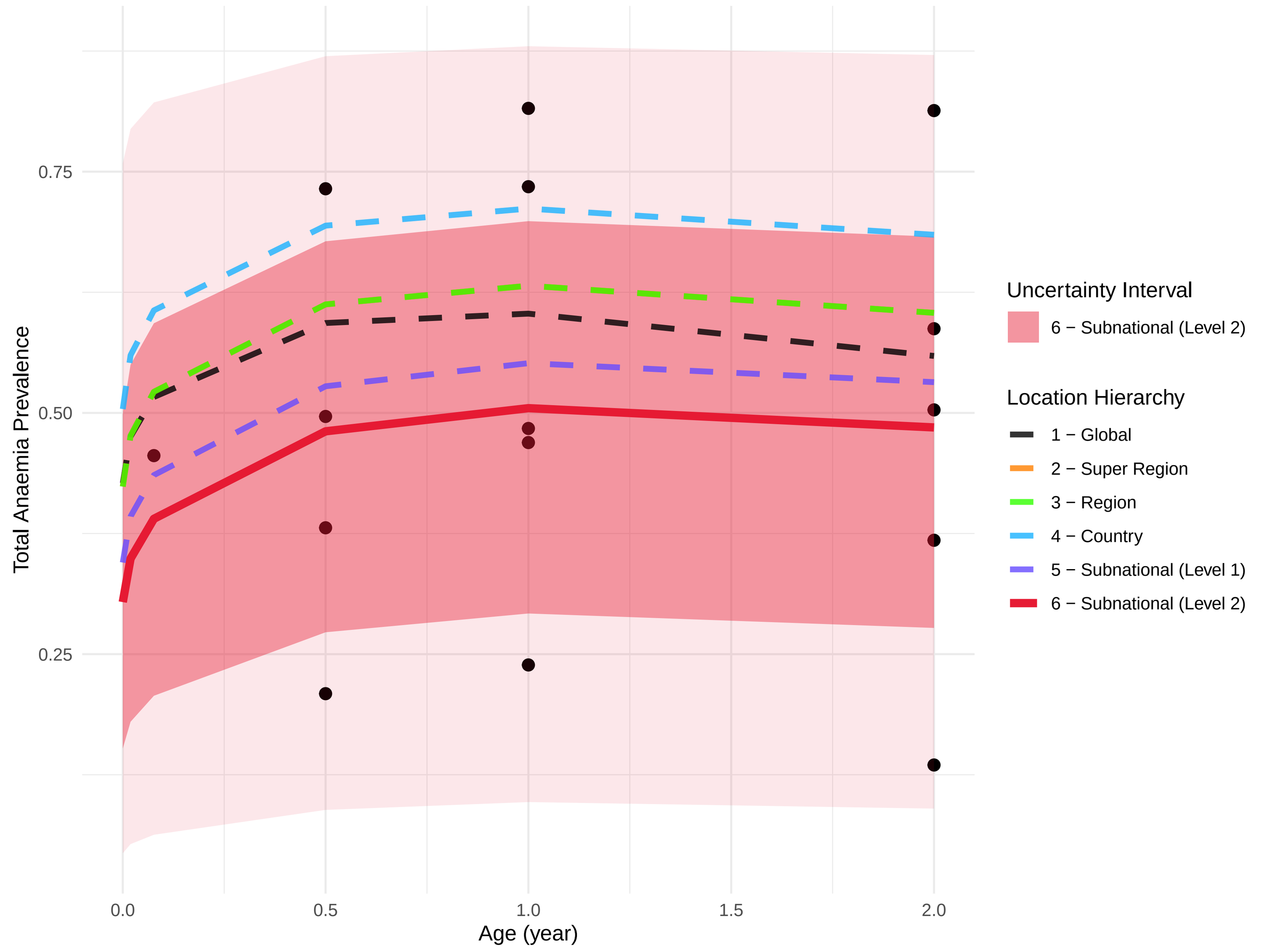

MR−BRT Age/Sex Fits − Non−pregnant Females <2 Years Old − Total Anaemia Prevalence − Nagaland
WHO−2024 Adjustment Method

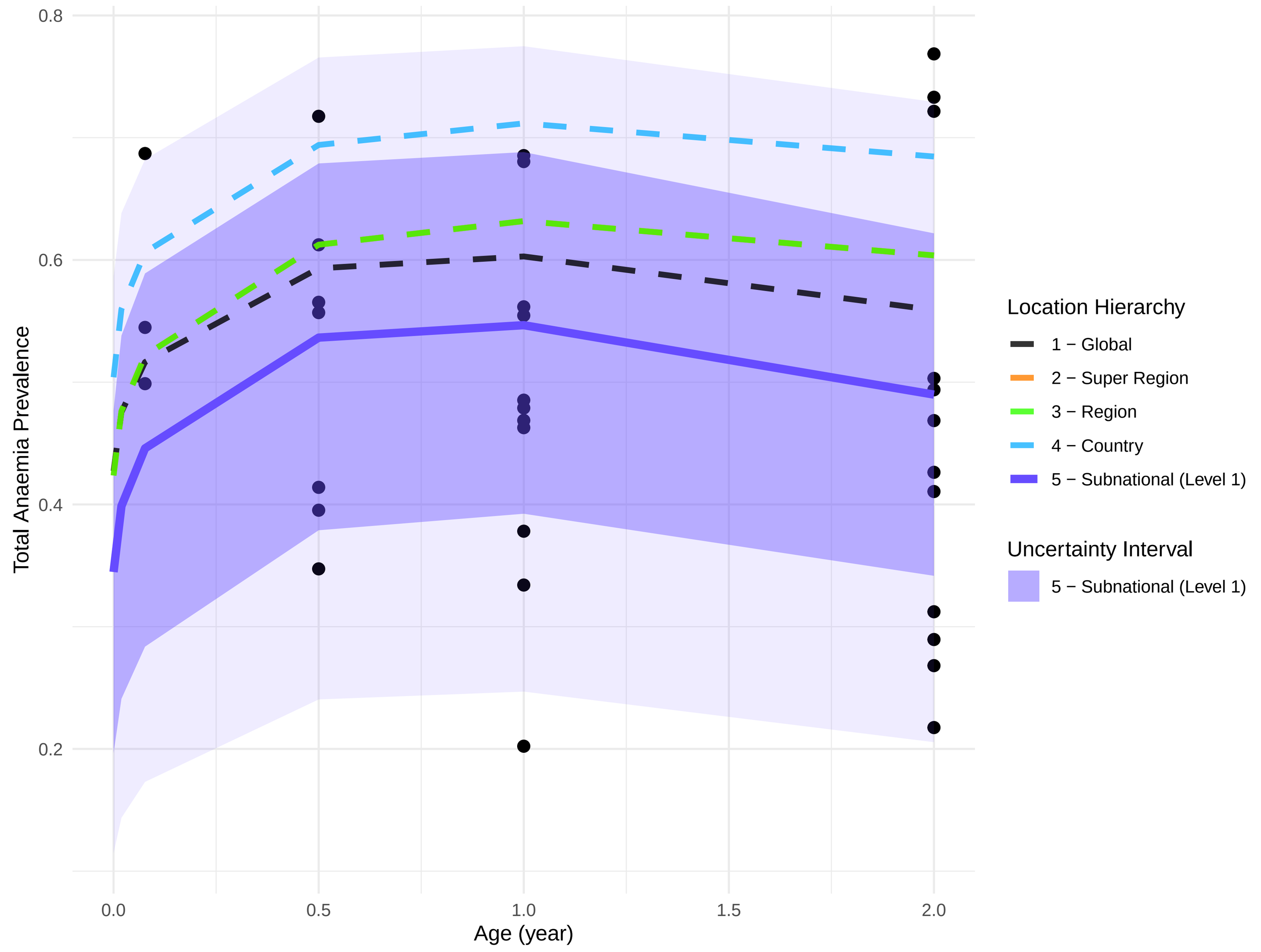

MR−BRT Age/Sex Fits − Non−pregnant Females <2 Years Old − Total Anaemia Prevalence − Nagaland, Rural
WHO−2024 Adjustment Method

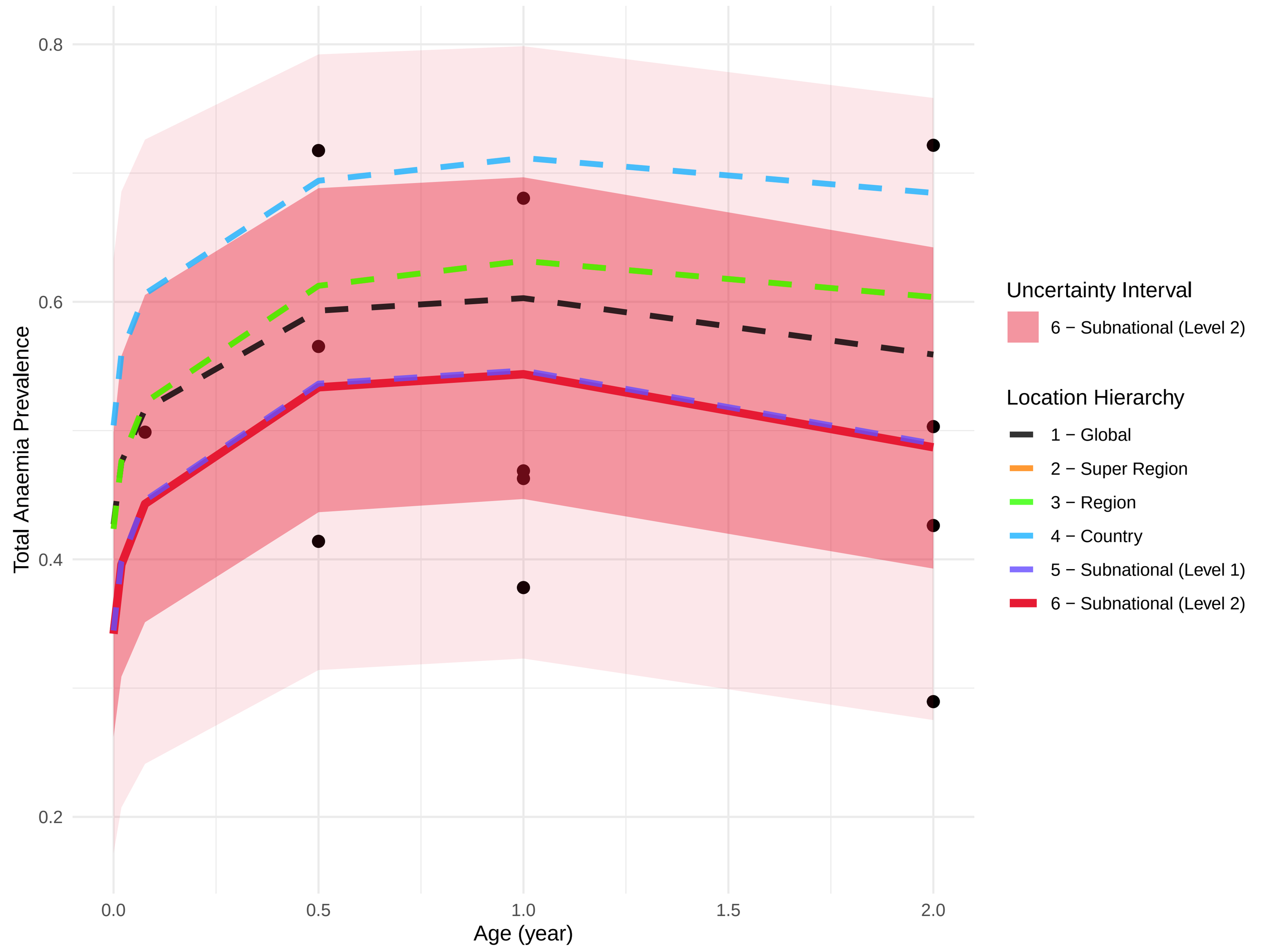

MR−BRT Age/Sex Fits − Non−pregnant Females <2 Years Old − Total Anaemia Prevalence − Nagaland, Urban
WHO−2024 Adjustment Method

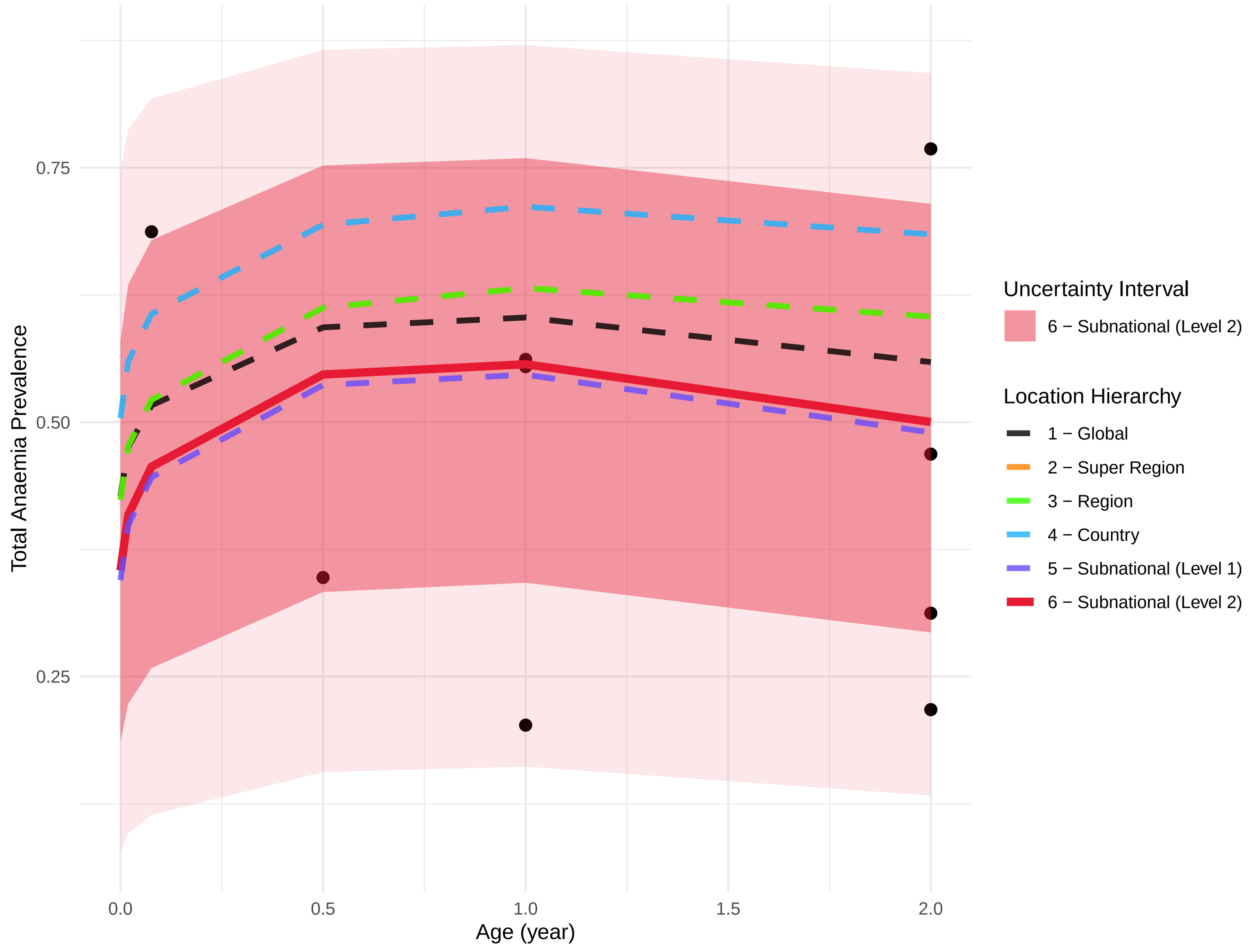

MR−BRT Age/Sex Fits − Non−pregnant Females <2 Years Old − Total Anaemia Prevalence − Odisha
WHO−2024 Adjustment Method

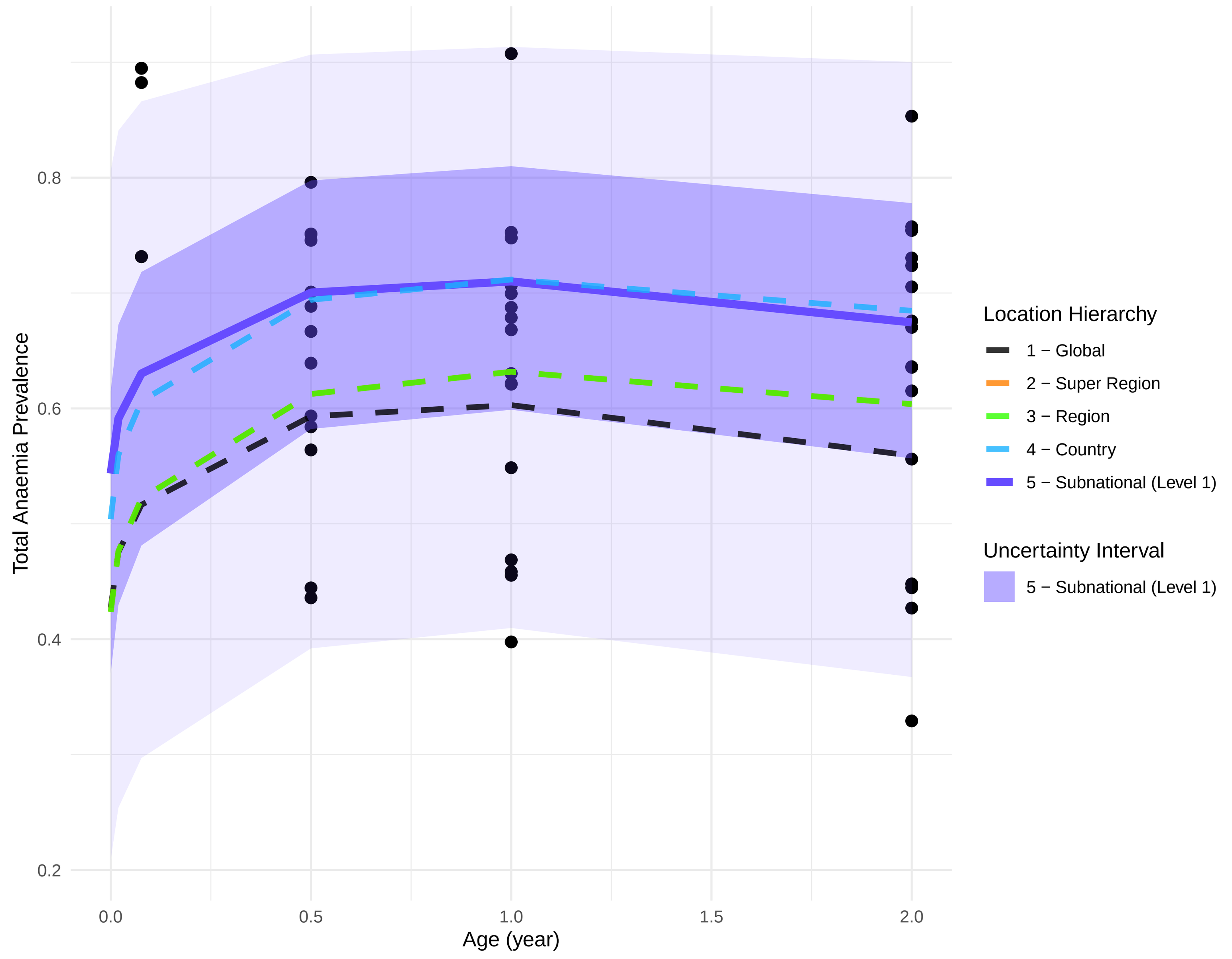

MR−BRT Age/Sex Fits − Non−pregnant Females <2 Years Old − Total Anaemia Prevalence − Odisha, Rural
WHO−2024 Adjustment Method

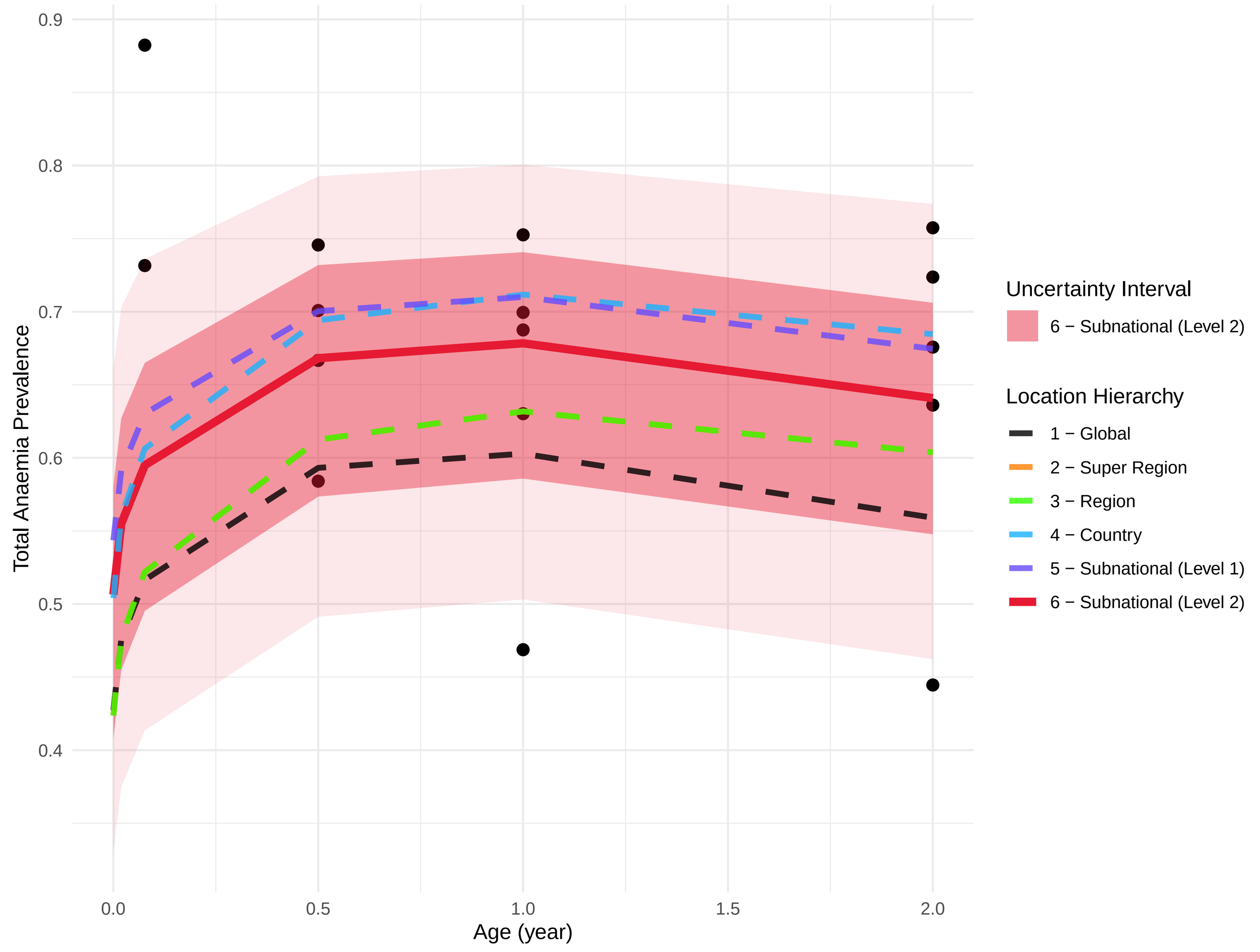

MR−BRT Age/Sex Fits − Non−pregnant Females <2 Years Old − Total Anaemia Prevalence − Odisha, Urban
WHO−2024 Adjustment Method

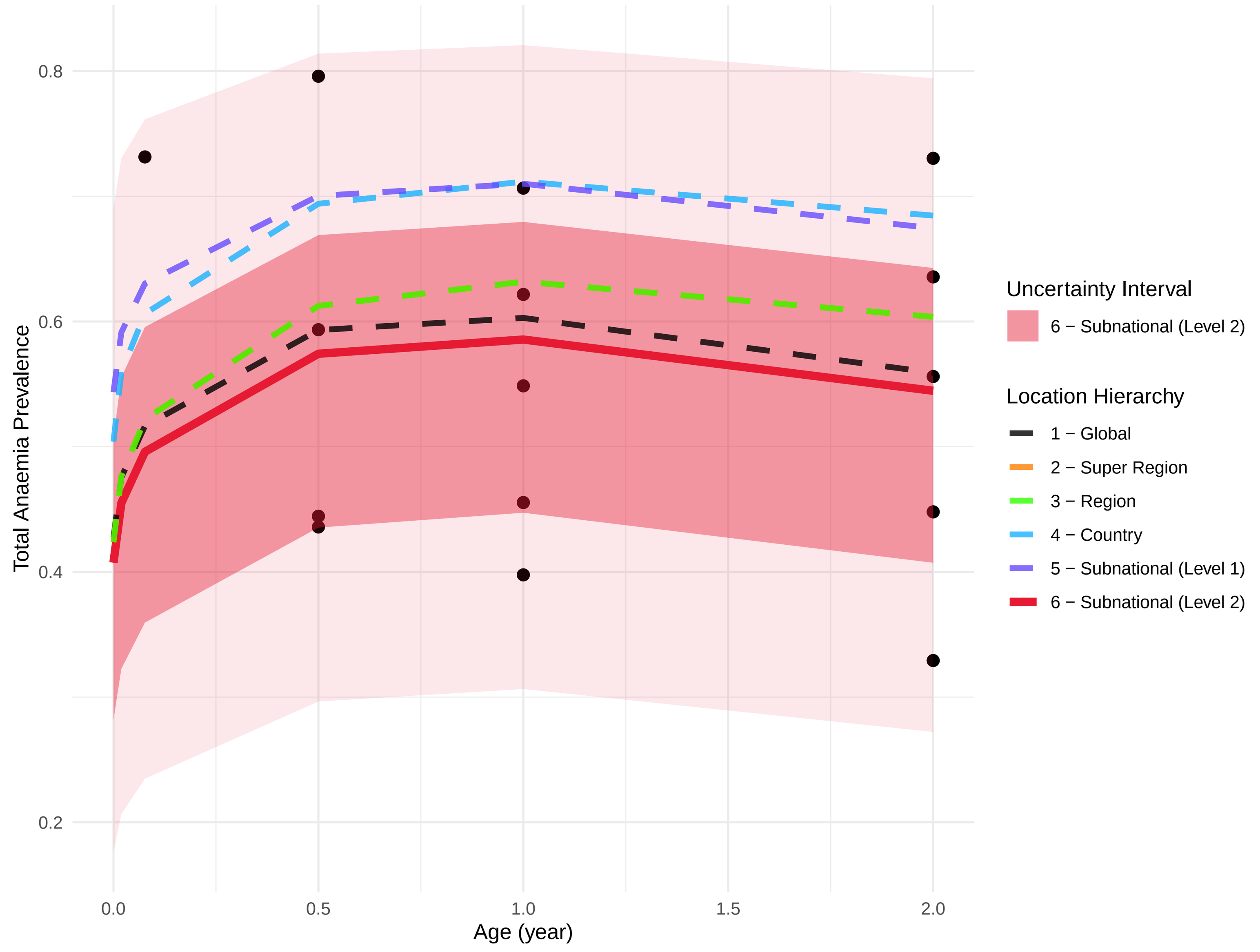

MR−BRT Age/Sex Fits − Non−pregnant Females <2 Years Old − Total Anaemia Prevalence − Other Union Territories
WHO−2024 Adjustment Method

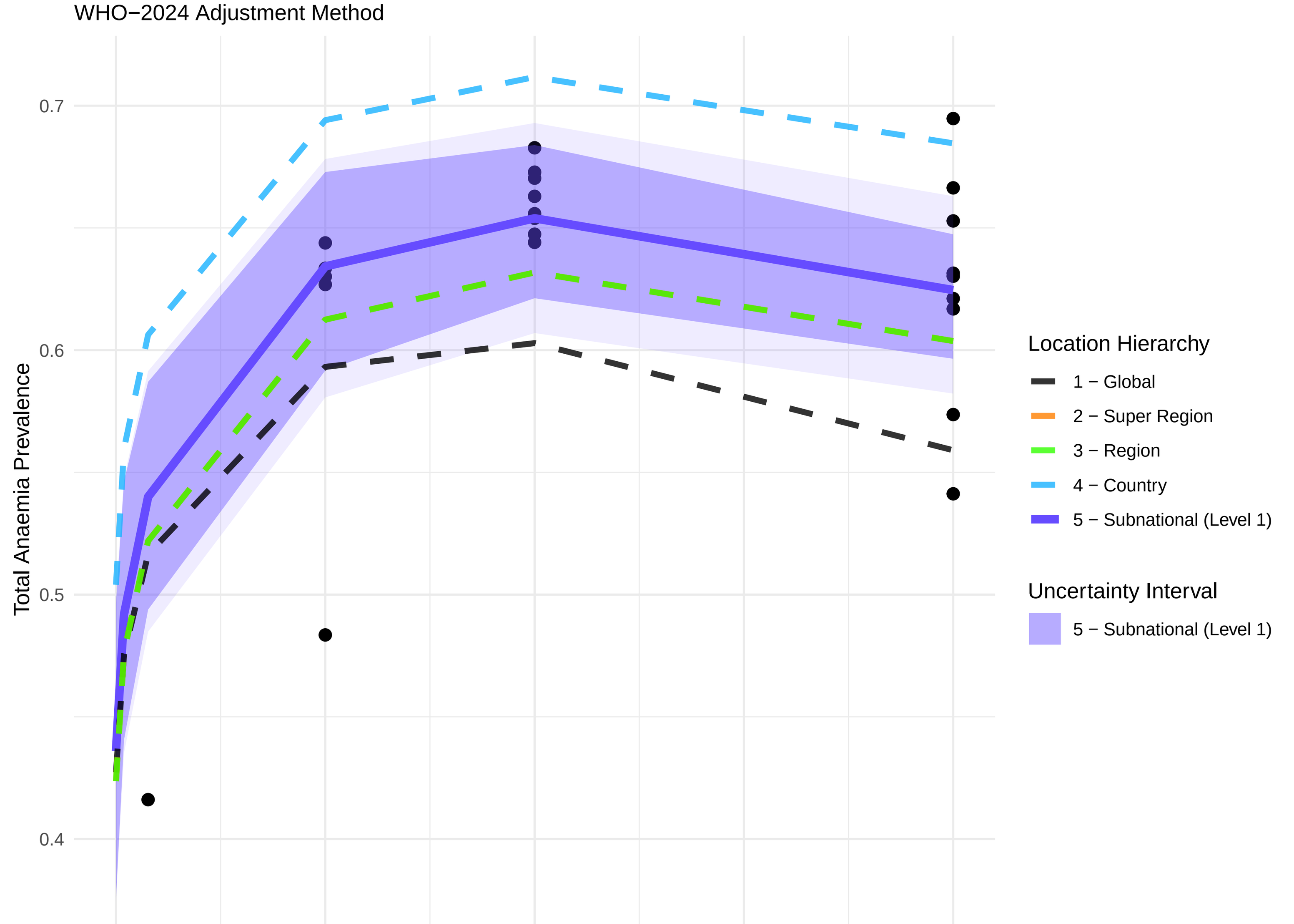

MR−BRT Age/Sex Fits − Non−pregnant Females <2 Years Old − Total Anaemia Prevalence − Other Union Territories, Rural
WHO−2024 Adjustment Method

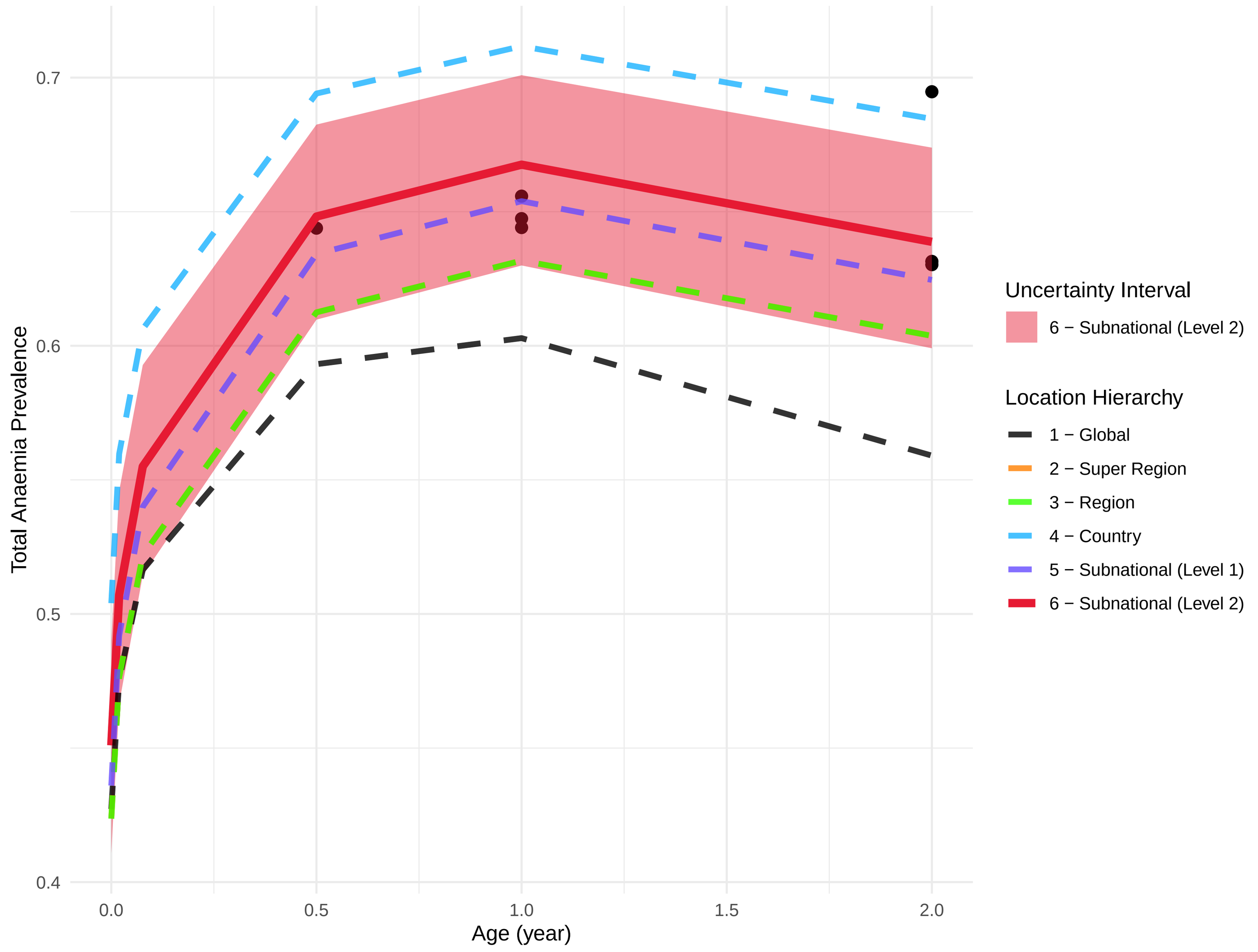

MR−BRT Age/Sex Fits − Non−pregnant Females <2 Years Old − Total Anaemia Prevalence − Other Union Territories, Urban
WHO−2024 Adjustment Method

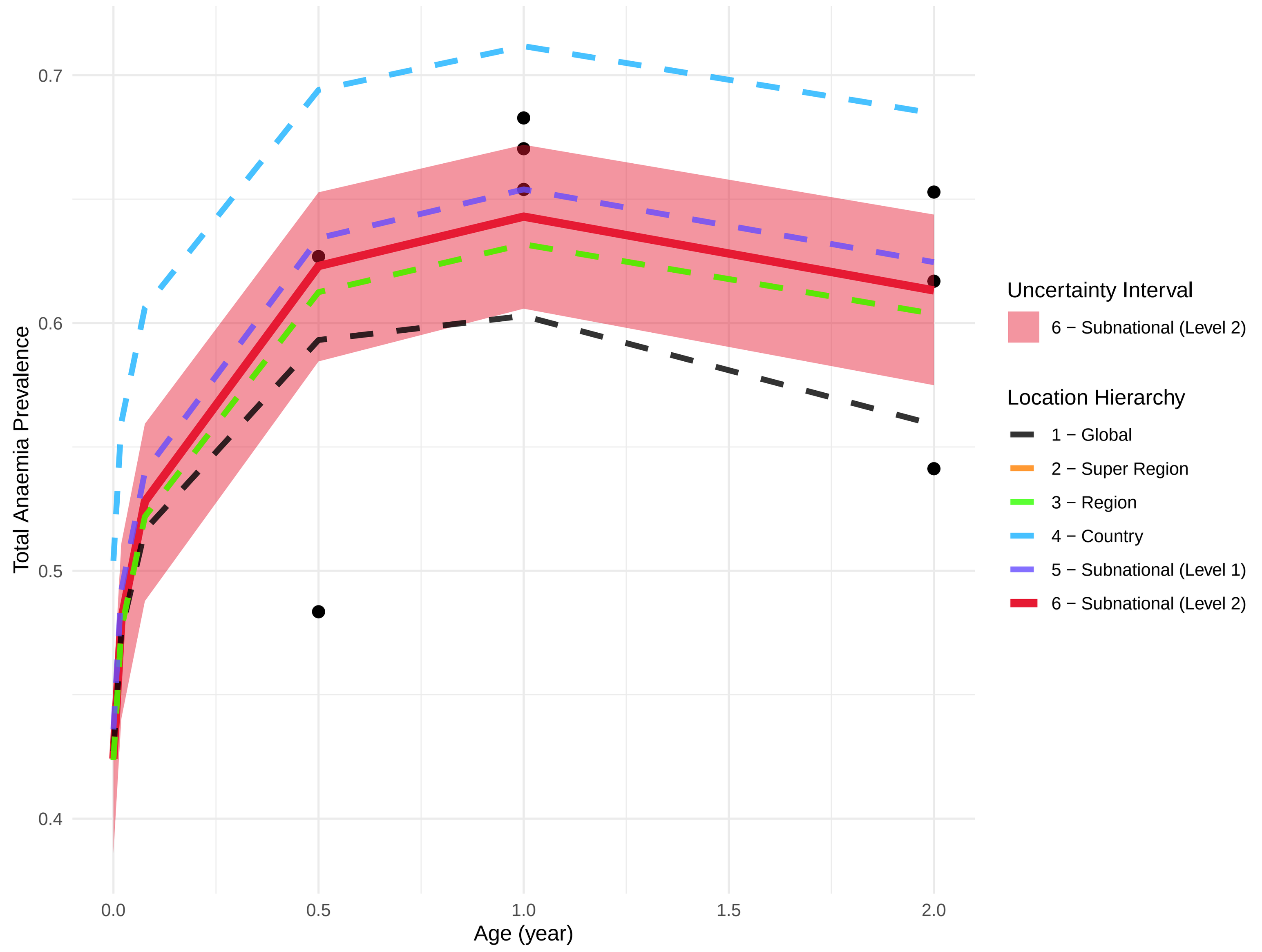

MR−BRT Age/Sex Fits − Non−pregnant Females <2 Years Old − Total Anaemia Prevalence − Punjab
WHO−2024 Adjustment Method

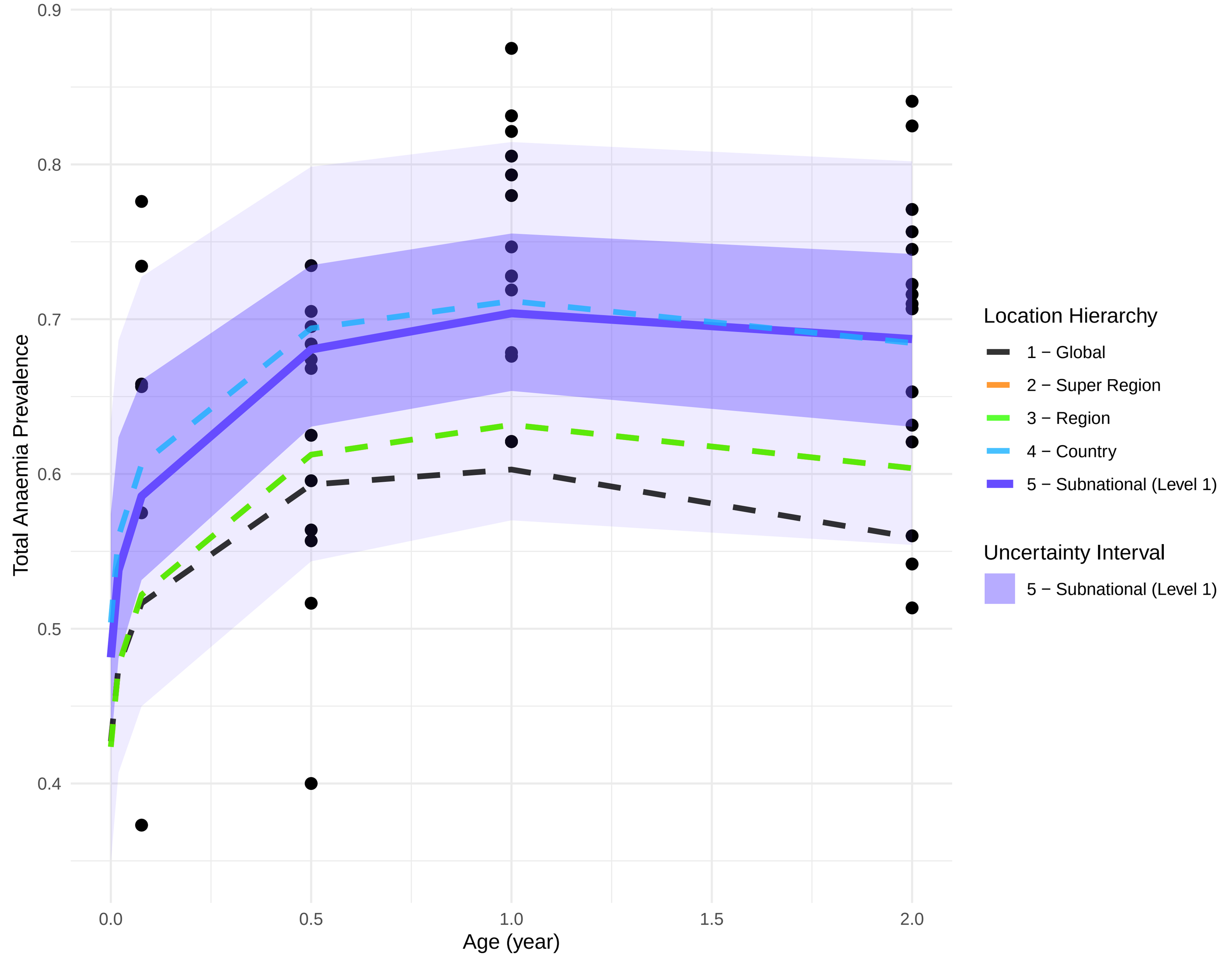

MR−BRT Age/Sex Fits − Non−pregnant Females <2 Years Old − Total Anaemia Prevalence − Punjab, Rural
WHO−2024 Adjustment Method

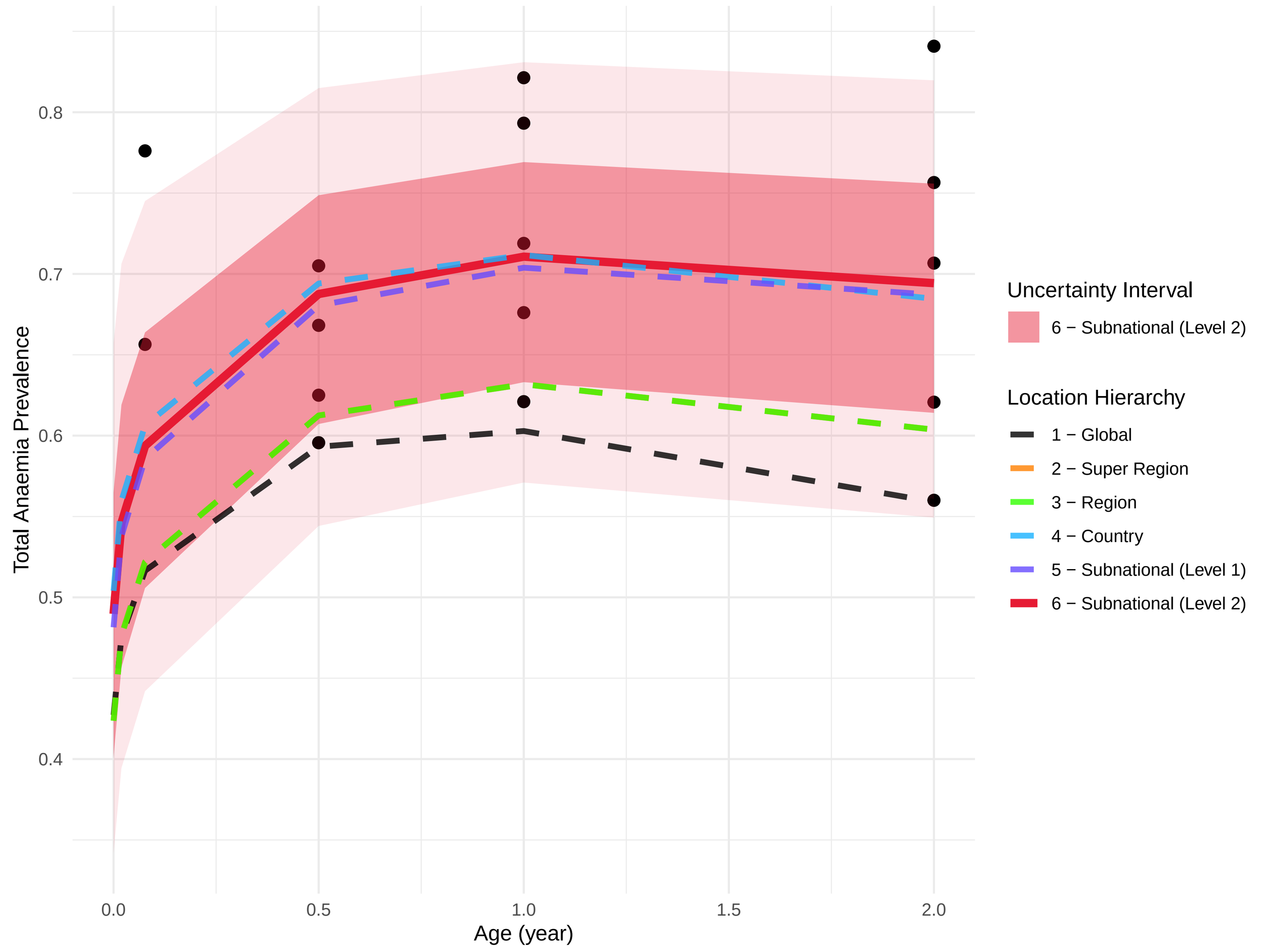

MR−BRT Age/Sex Fits − Non−pregnant Females <2 Years Old − Total Anaemia Prevalence − Punjab, Urban
WHO−2024 Adjustment Method

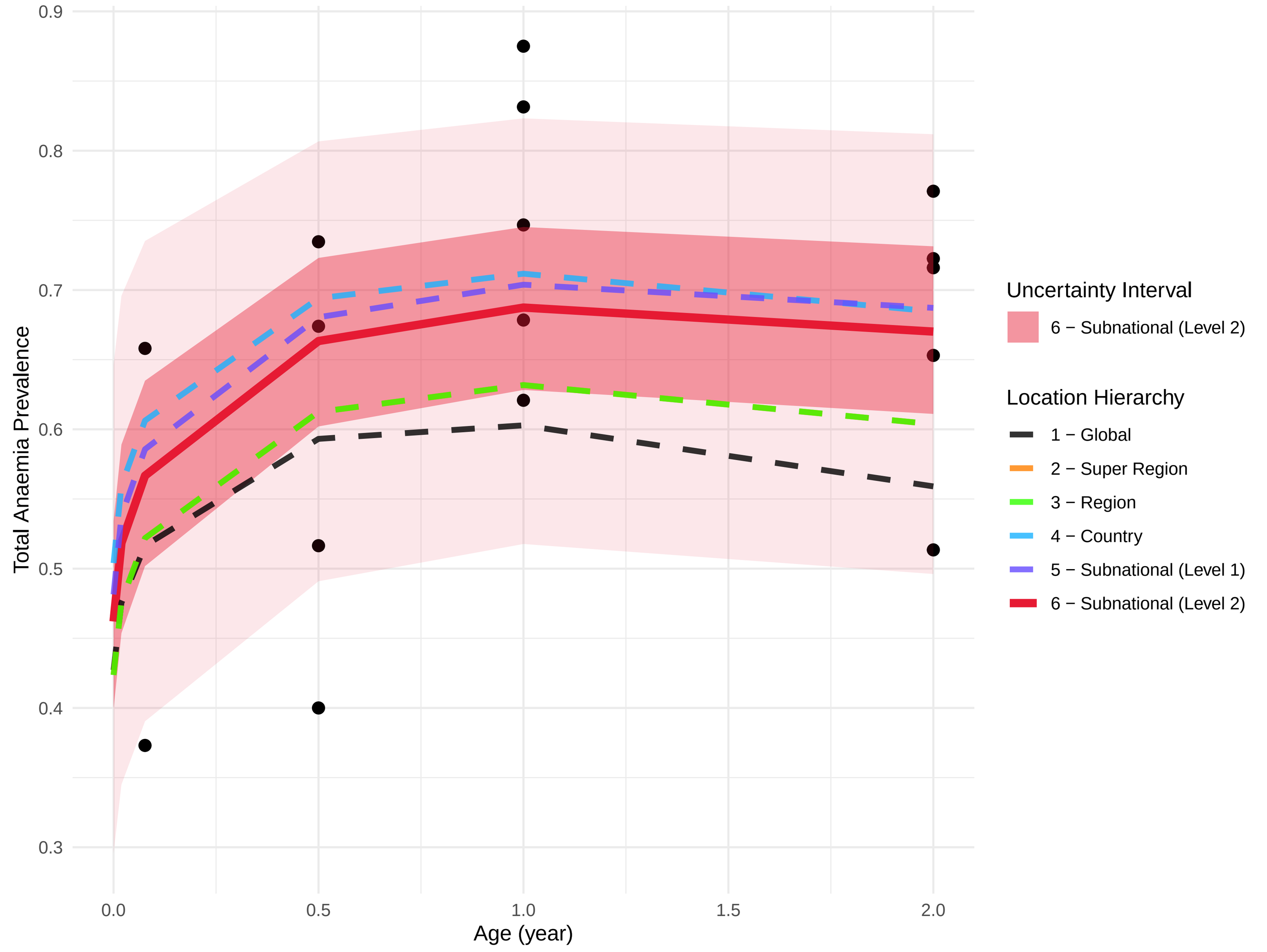

MR−BRT Age/Sex Fits − Non−pregnant Females <2 Years Old − Total Anaemia Prevalence − Rajasthan
WHO−2024 Adjustment Method

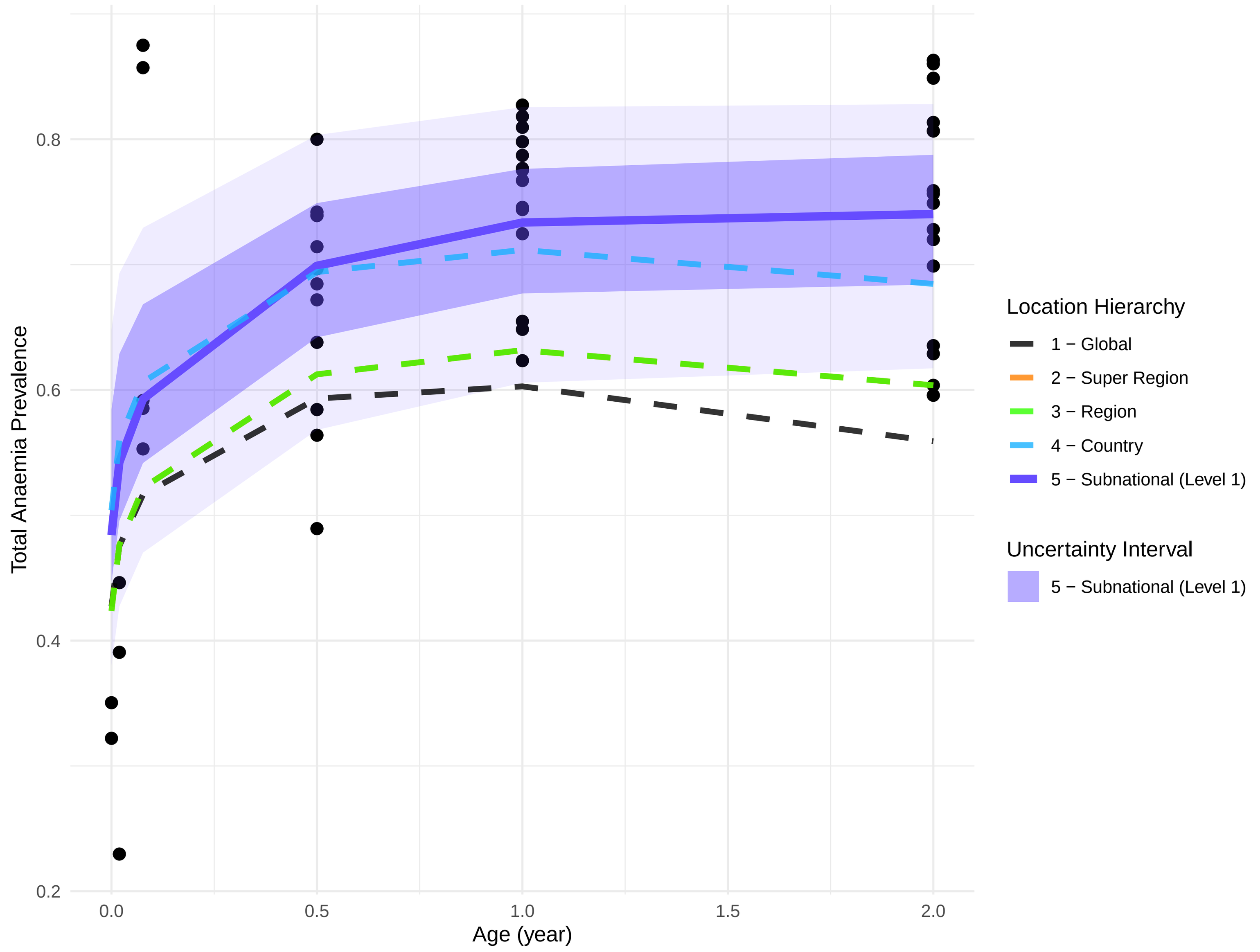

MR−BRT Age/Sex Fits − Non−pregnant Females <2 Years Old − Total Anaemia Prevalence − Rajasthan, Rural
WHO−2024 Adjustment Method

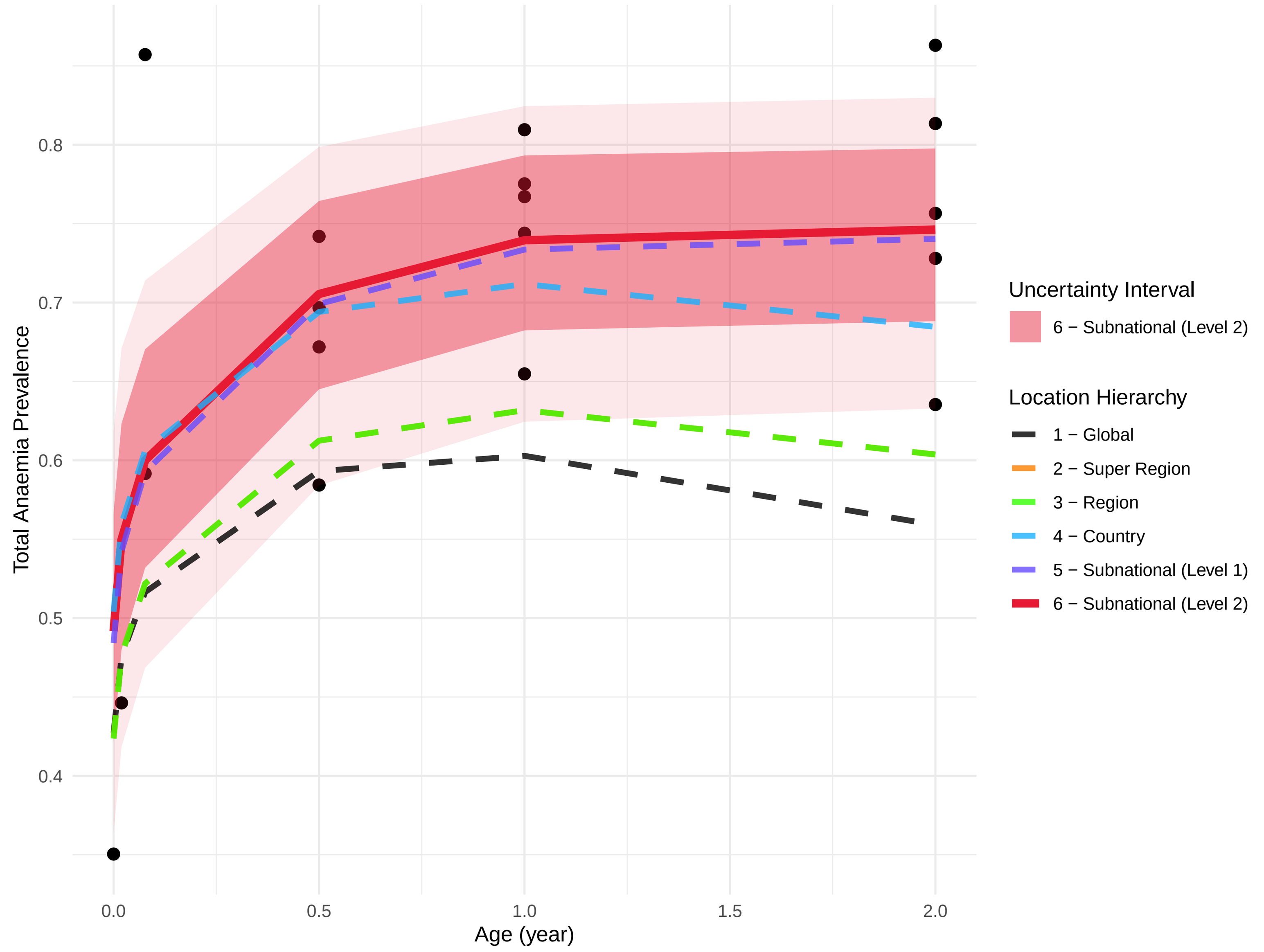

MR−BRT Age/Sex Fits − Non−pregnant Females <2 Years Old − Total Anaemia Prevalence − Rajasthan, Urban
WHO−2024 Adjustment Method

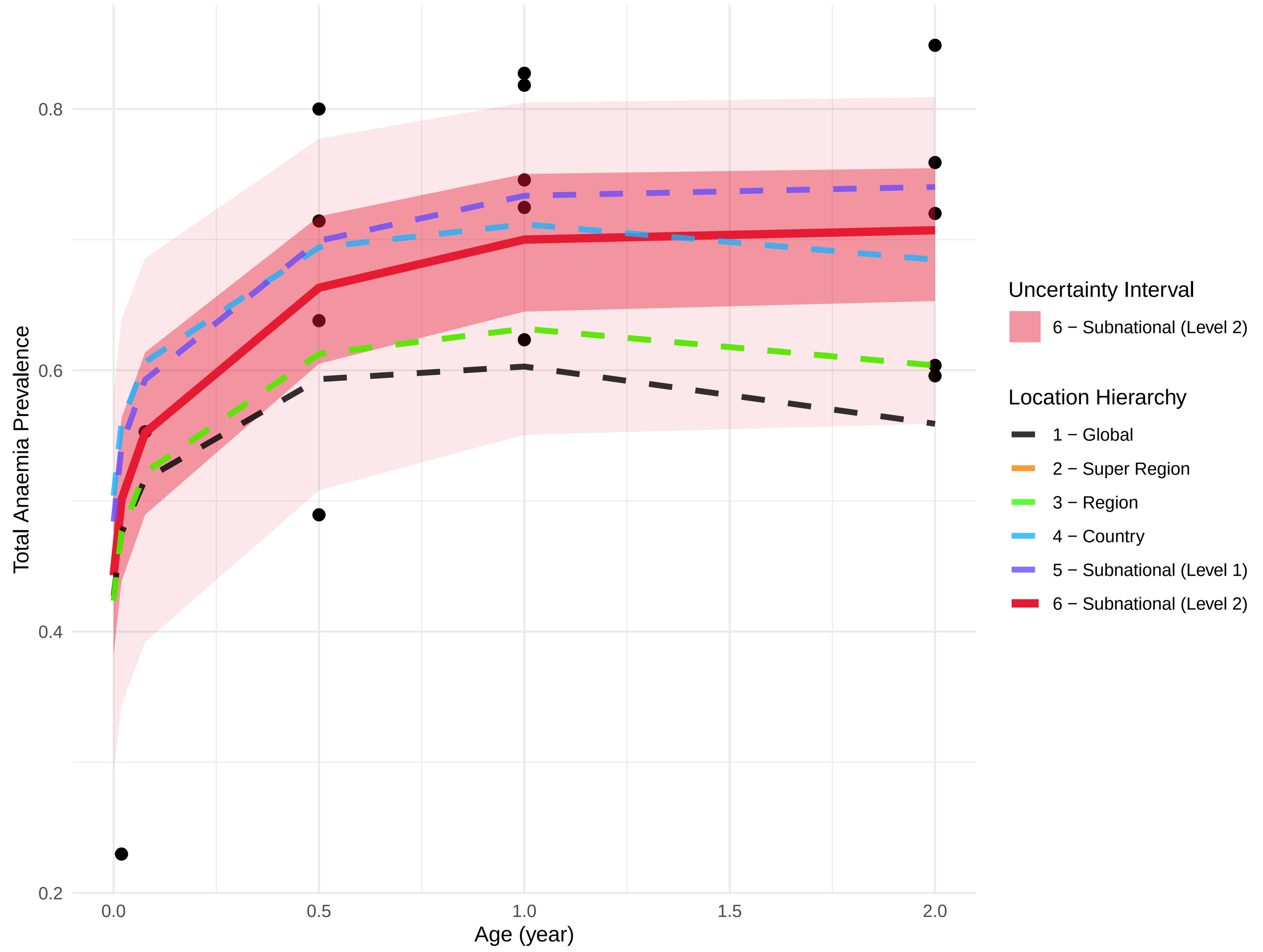

MR−BRT Age/Sex Fits − Non−pregnant Females <2 Years Old − Total Anaemia Prevalence − Sikkim
WHO−2024 Adjustment Method

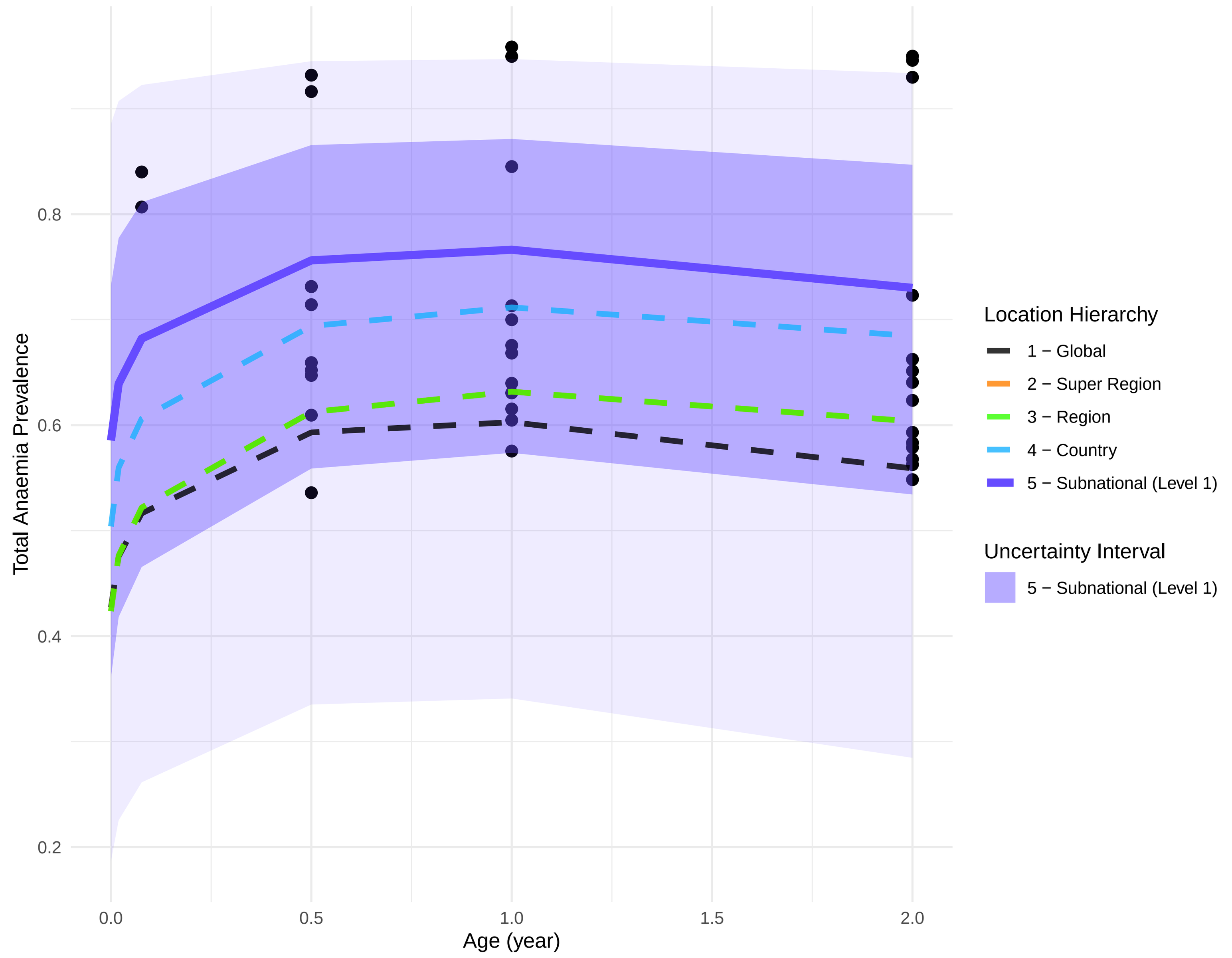

MR−BRT Age/Sex Fits − Non−pregnant Females <2 Years Old − Total Anaemia Prevalence − Sikkim, Rural
WHO−2024 Adjustment Method

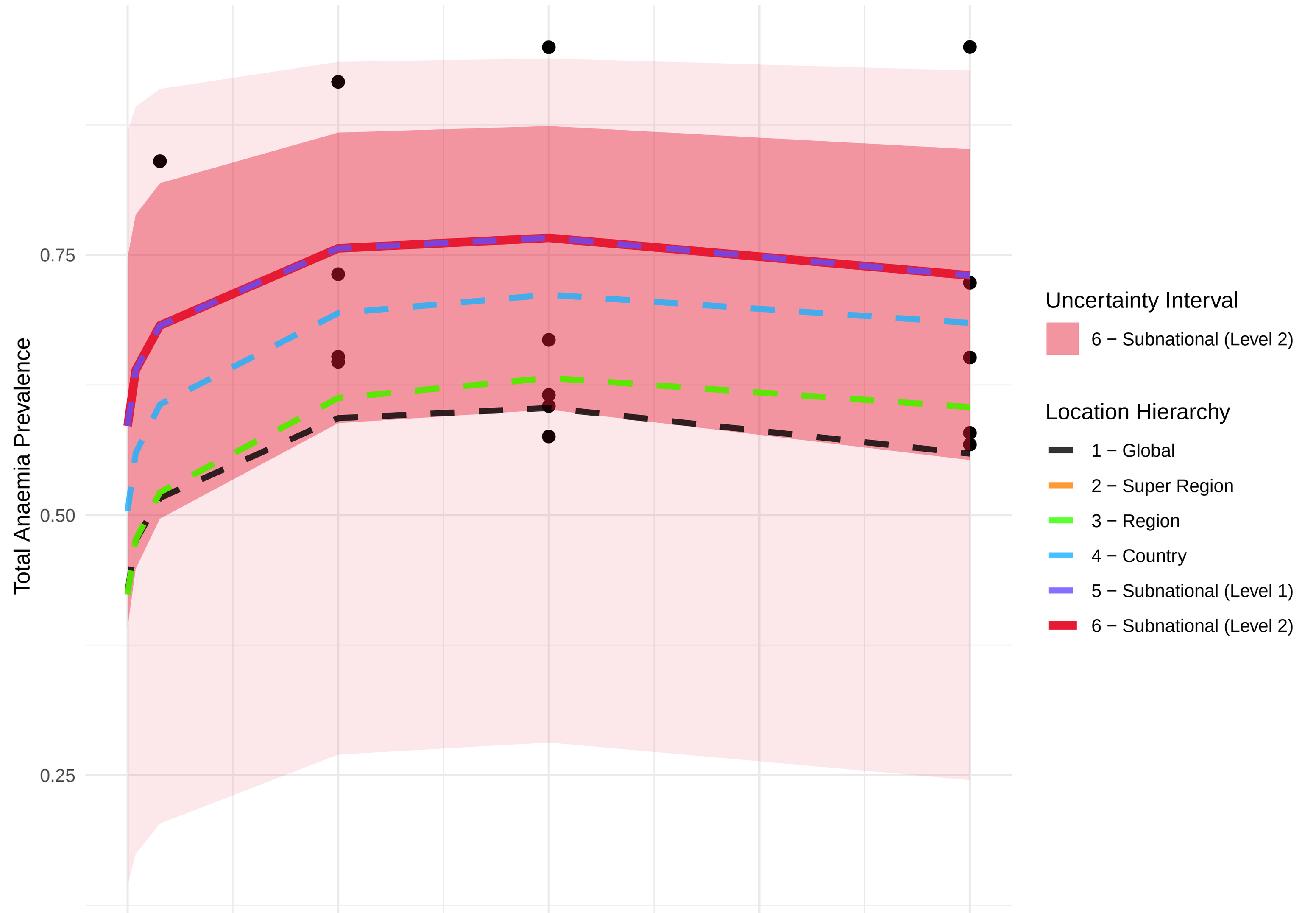

MR−BRT Age/Sex Fits − Non−pregnant Females <2 Years Old − Total Anaemia Prevalence − Sikkim, Urban
WHO−2024 Adjustment Method

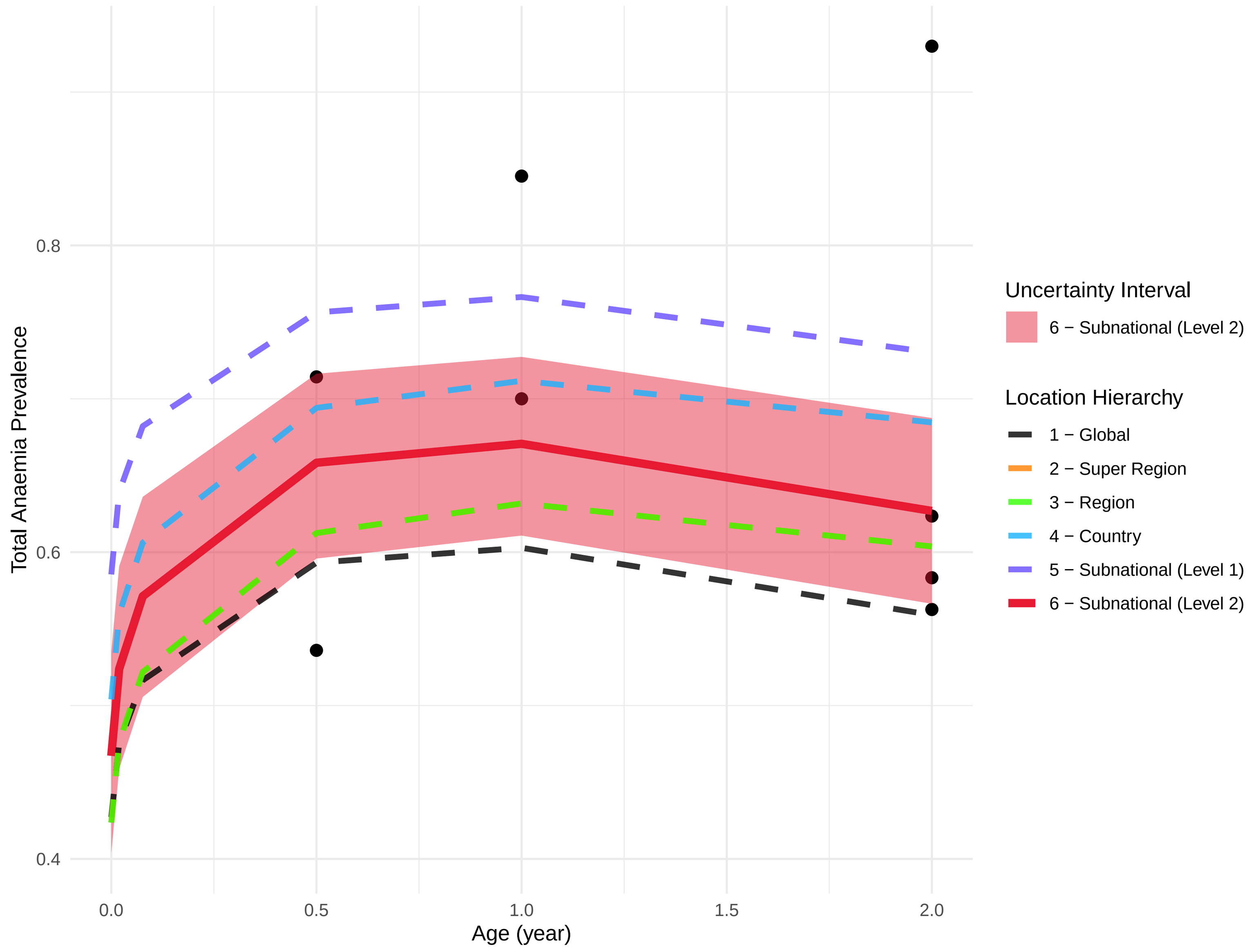

MR−BRT Age/Sex Fits − Non−pregnant Females <2 Years Old − Total Anaemia Prevalence − Tamil Nadu
WHO−2024 Adjustment Method

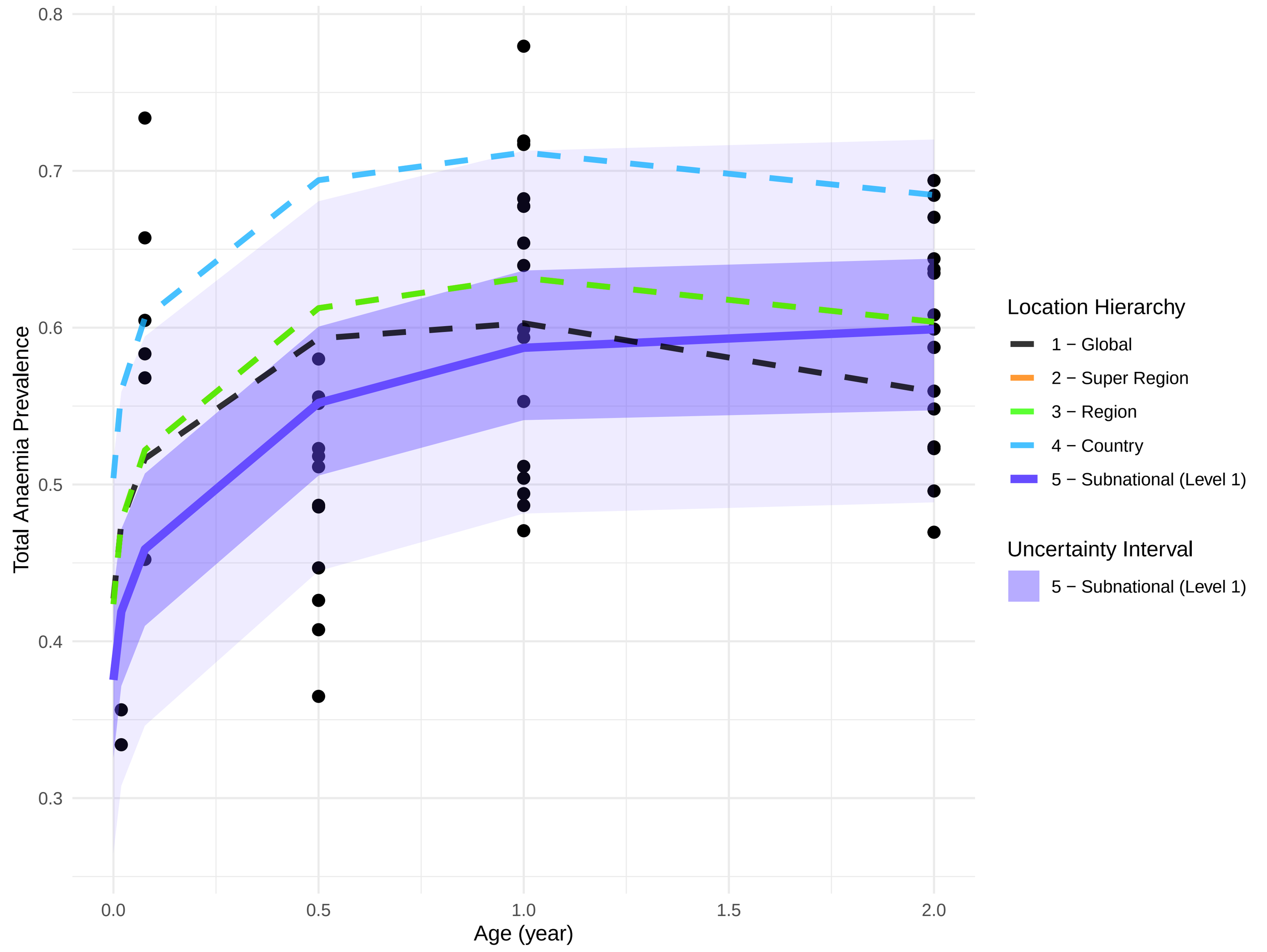

MR−BRT Age/Sex Fits − Non−pregnant Females <2 Years Old − Total Anaemia Prevalence − Tamil Nadu, Rural
WHO−2024 Adjustment Method

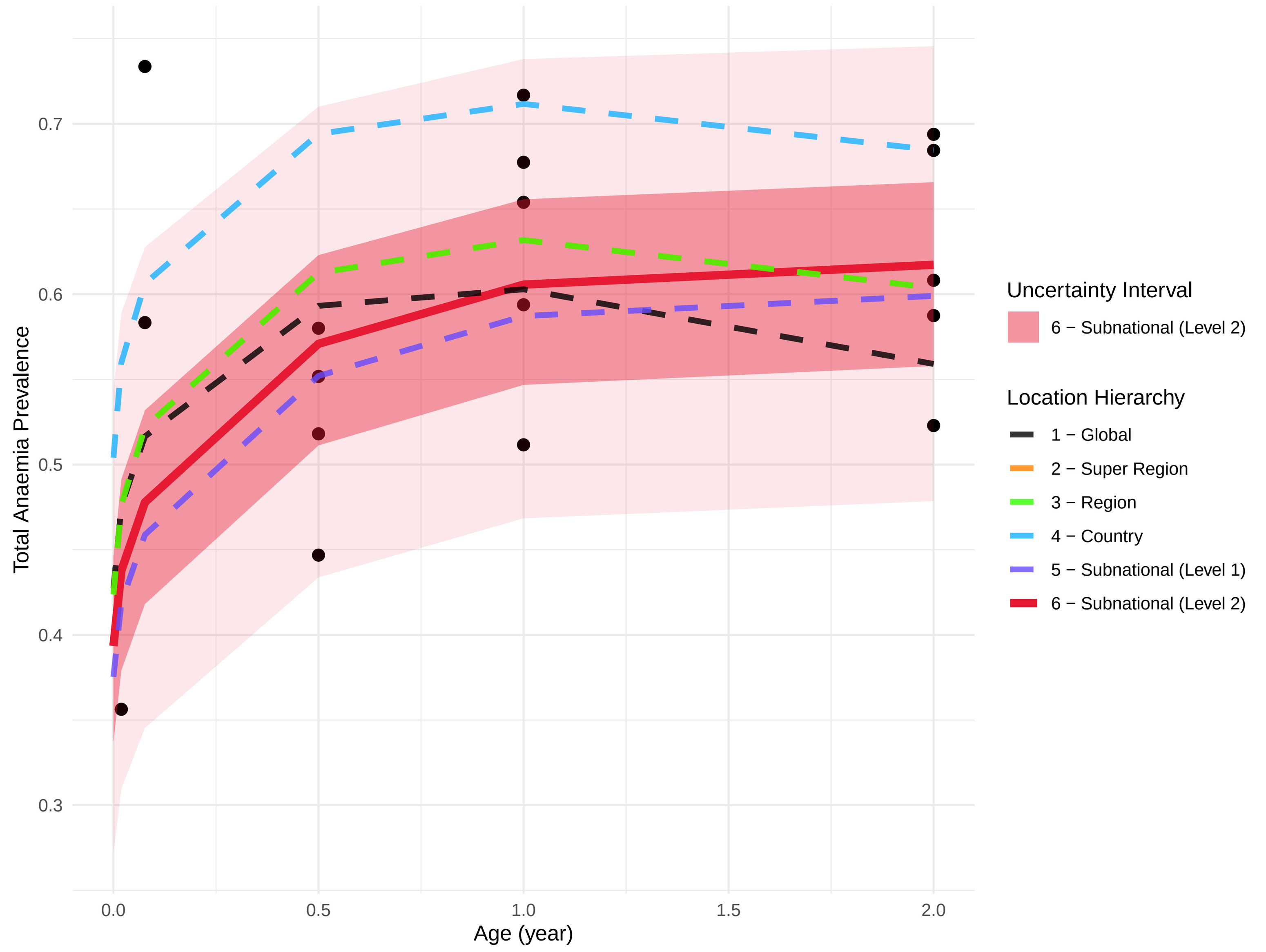

MR−BRT Age/Sex Fits − Non−pregnant Females <2 Years Old − Total Anaemia Prevalence − Tamil Nadu, Urban
WHO−2024 Adjustment Method

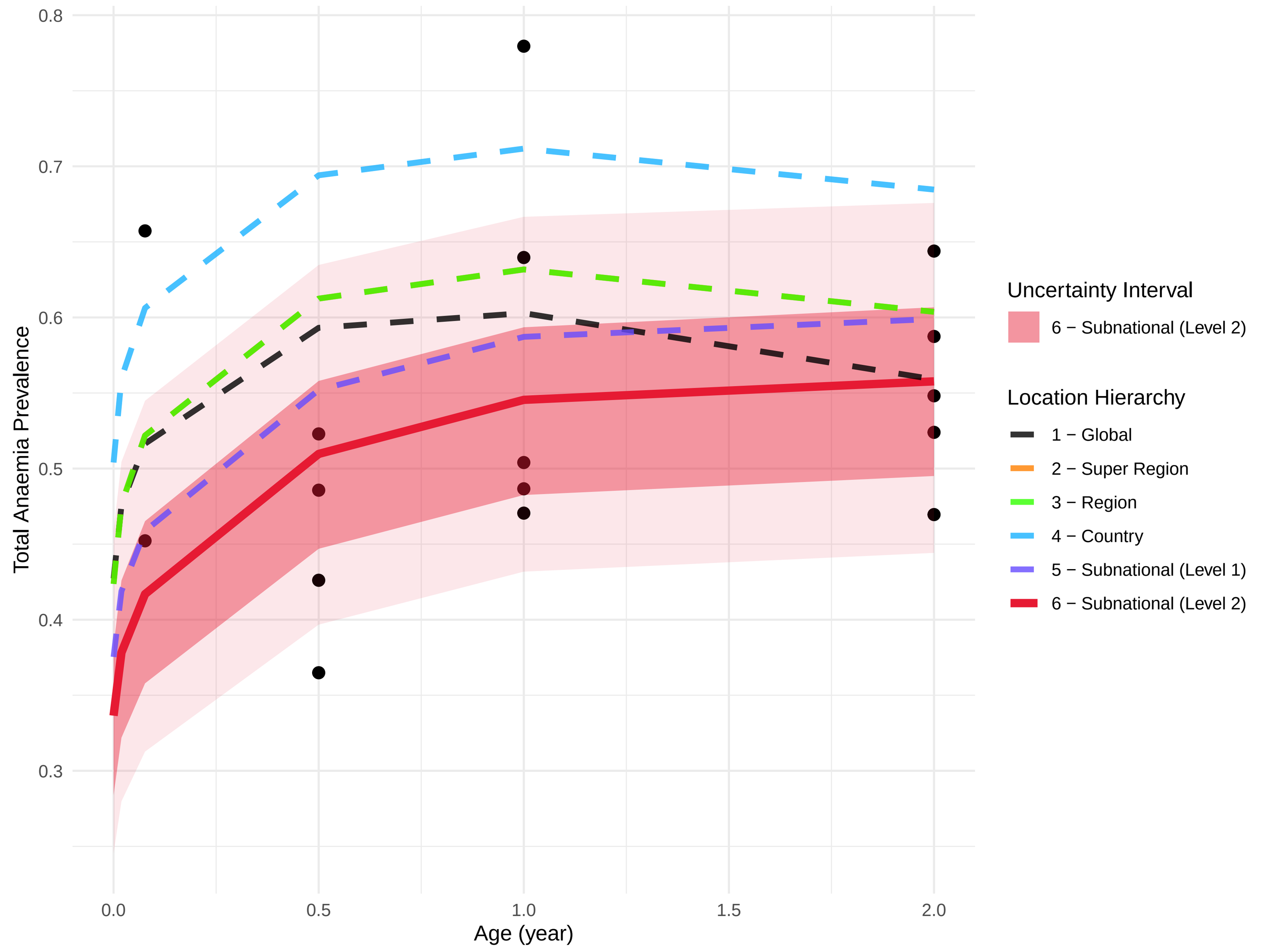

MR−BRT Age/Sex Fits − Non−pregnant Females <2 Years Old − Total Anaemia Prevalence − Telangana
WHO−2024 Adjustment Method

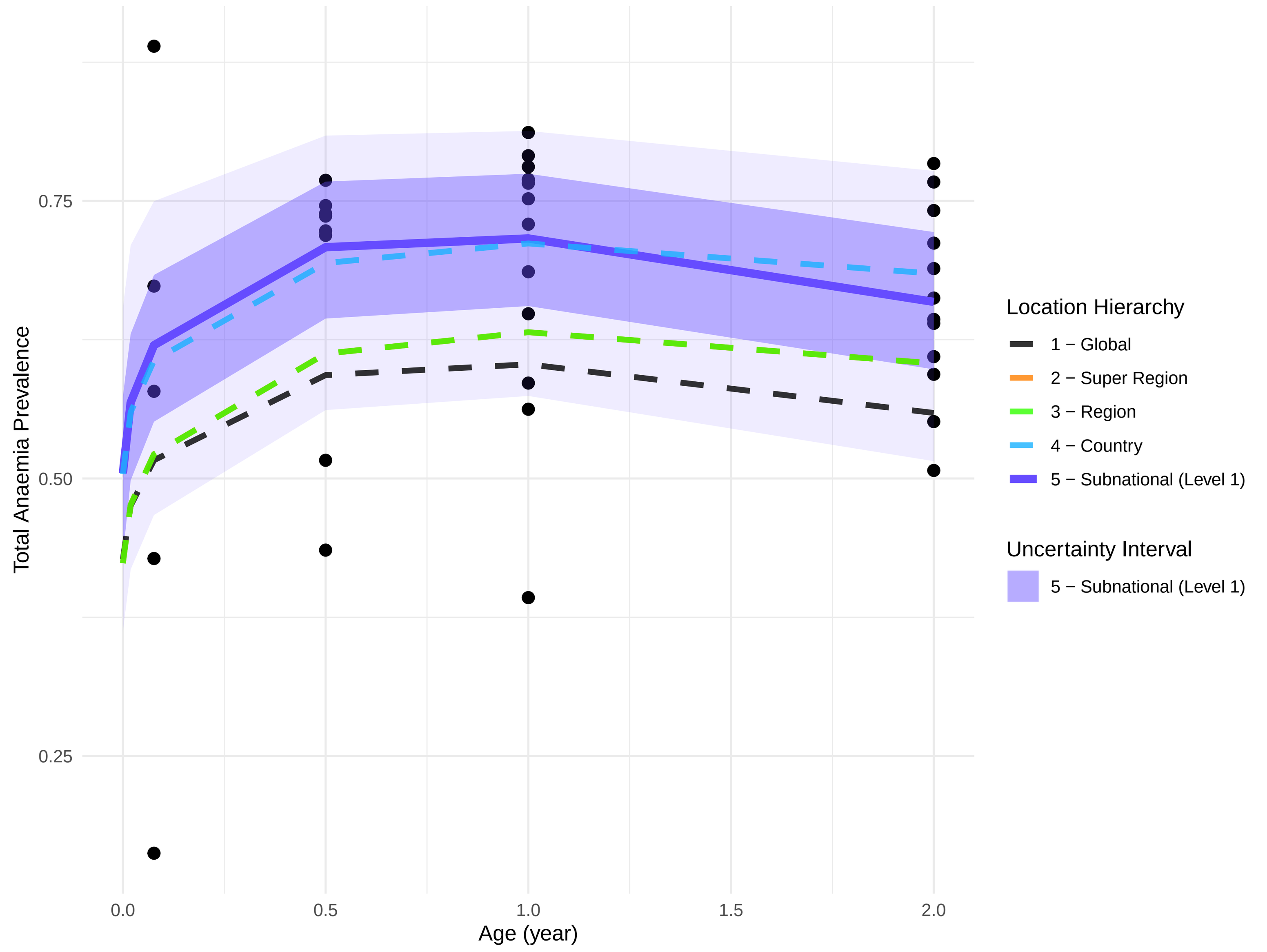

MR−BRT Age/Sex Fits − Non−pregnant Females <2 Years Old − Total Anaemia Prevalence − Telangana, Rural
WHO−2024 Adjustment Method

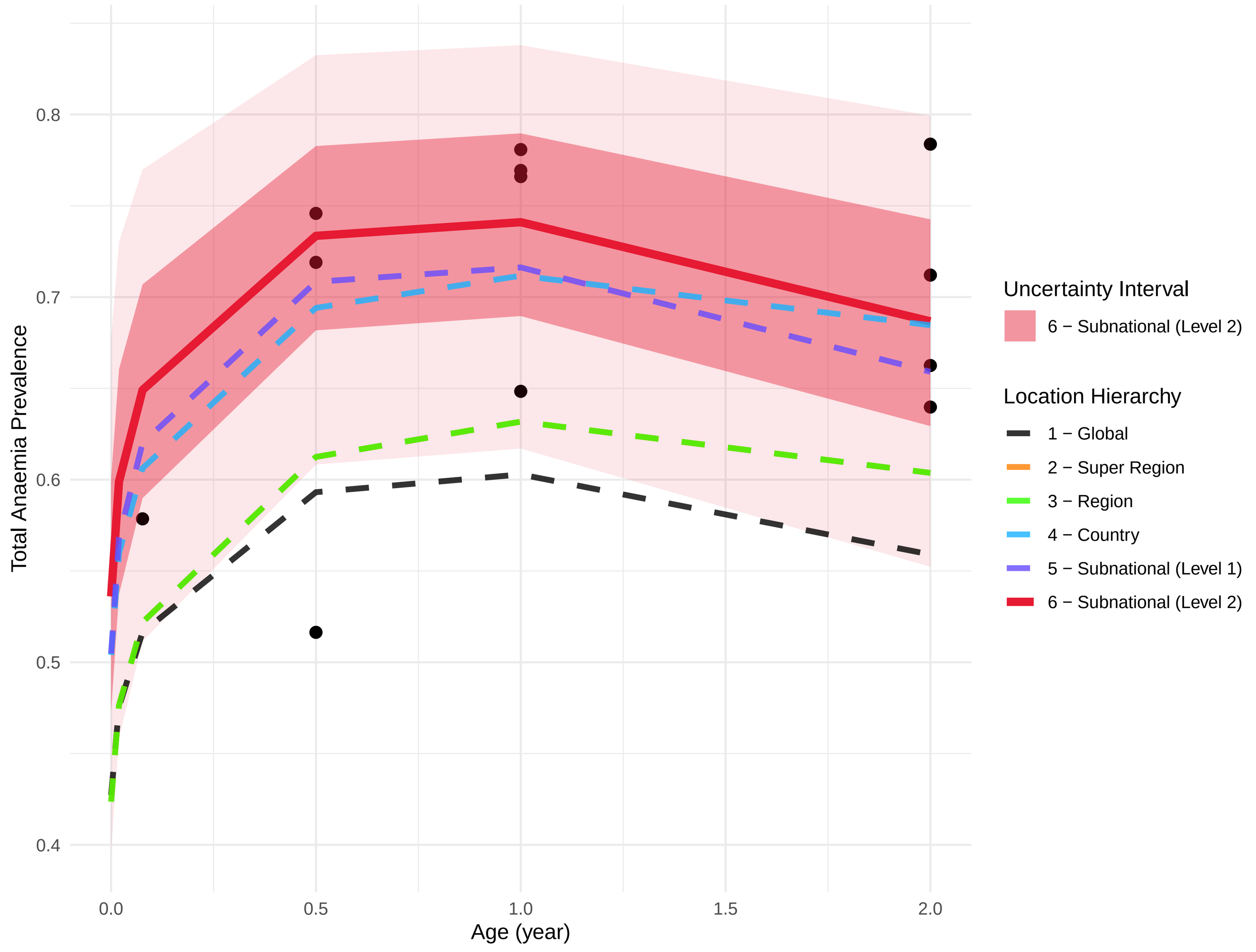

MR−BRT Age/Sex Fits − Non−pregnant Females <2 Years Old − Total Anaemia Prevalence − Telangana, Urban
WHO−2024 Adjustment Method

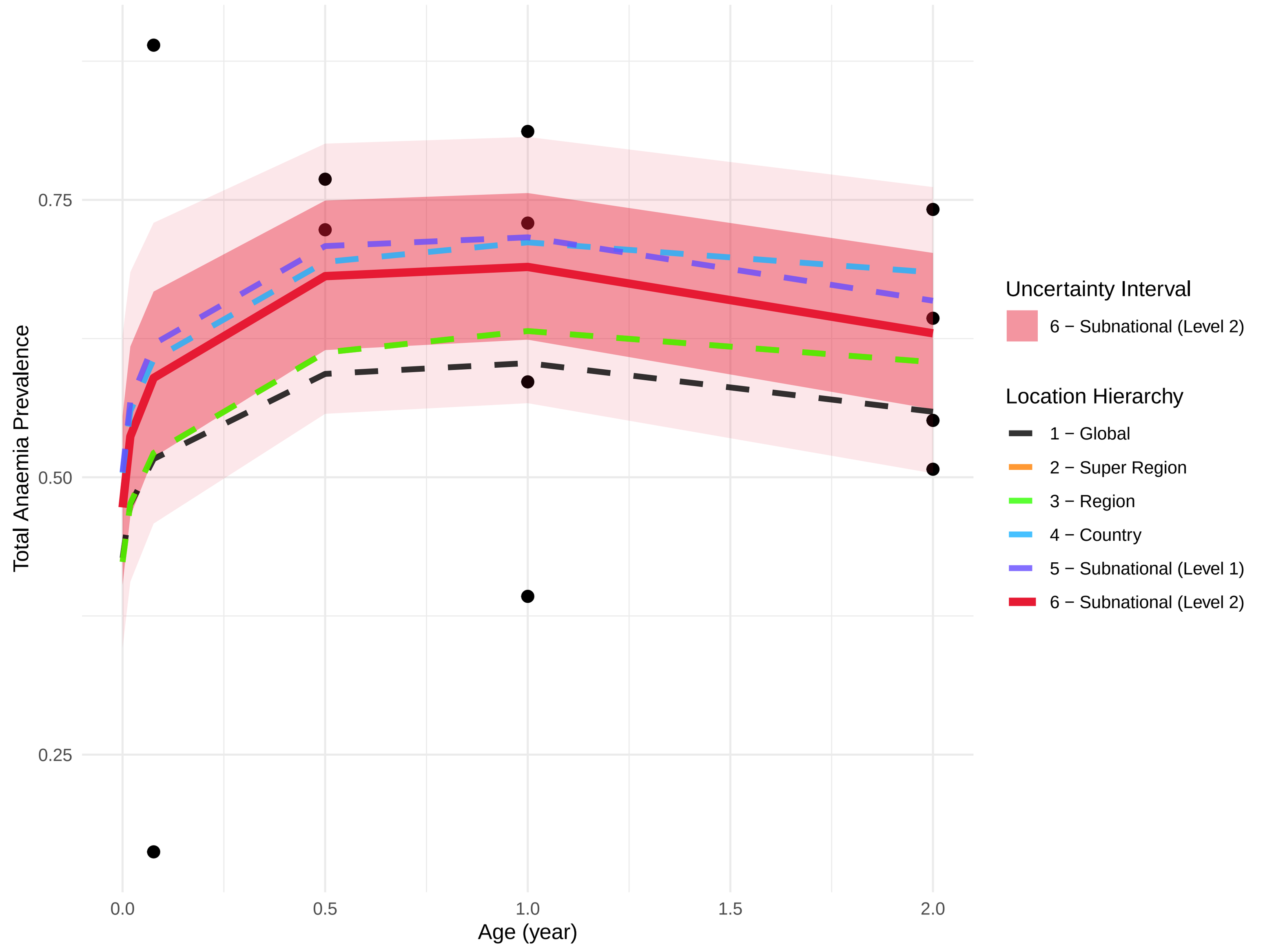

MR−BRT Age/Sex Fits − Non−pregnant Females <2 Years Old − Total Anaemia Prevalence − Tripura
WHO−2024 Adjustment Method

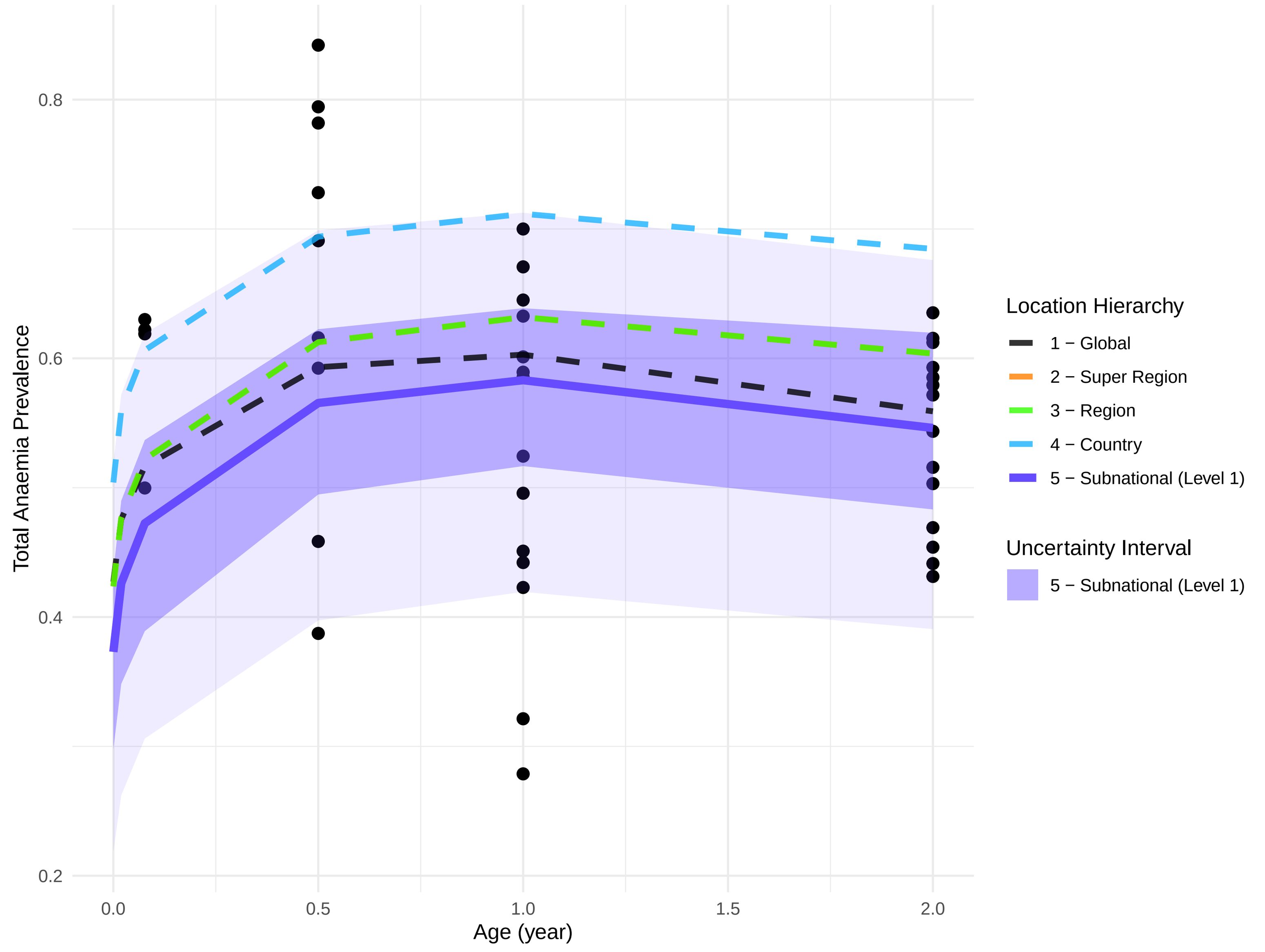

MR−BRT Age/Sex Fits − Non−pregnant Females <2 Years Old − Total Anaemia Prevalence − Tripura, Rural
WHO−2024 Adjustment Method

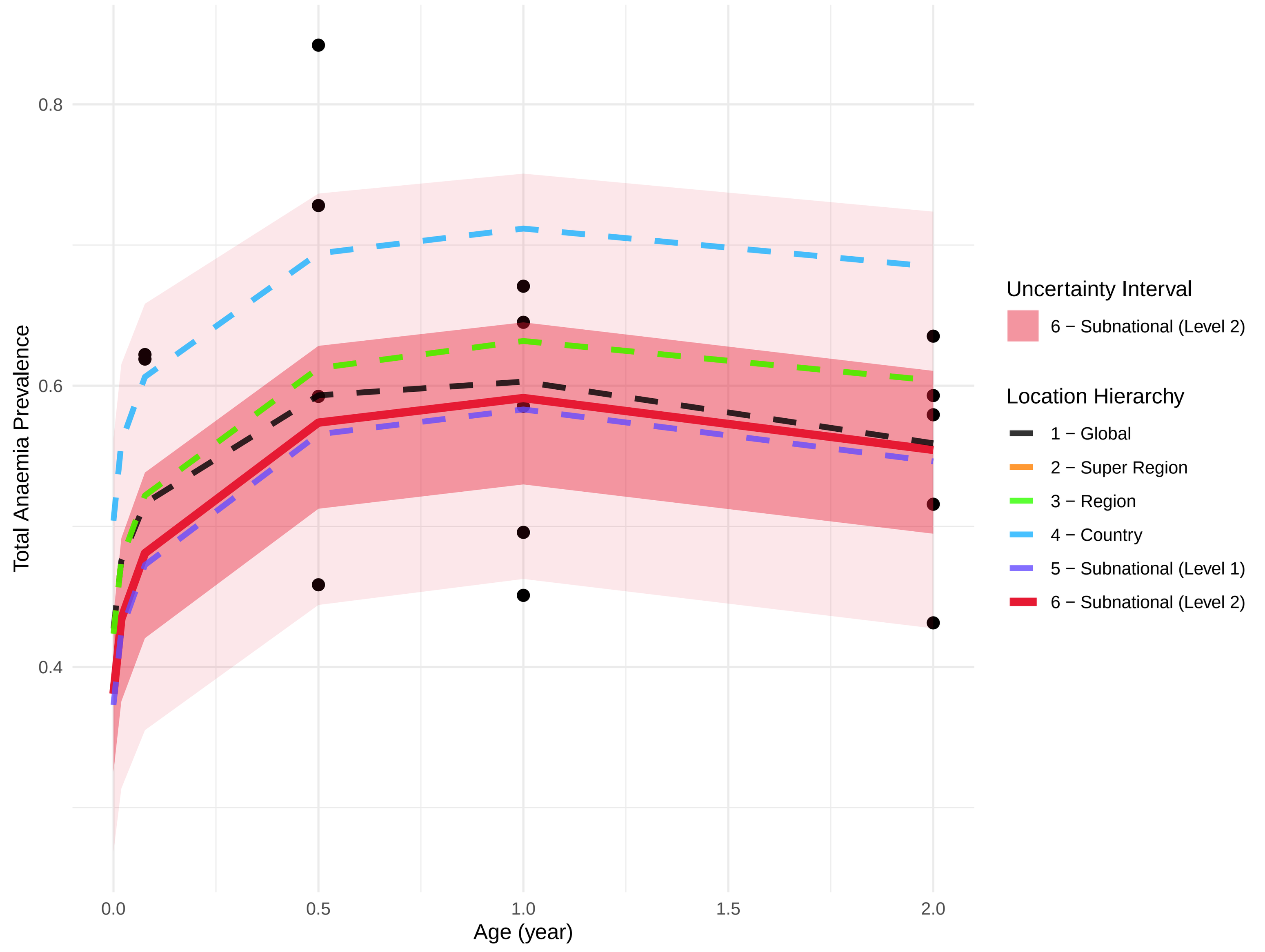

MR−BRT Age/Sex Fits − Non−pregnant Females <2 Years Old − Total Anaemia Prevalence − Tripura, Urban
WHO−2024 Adjustment Method

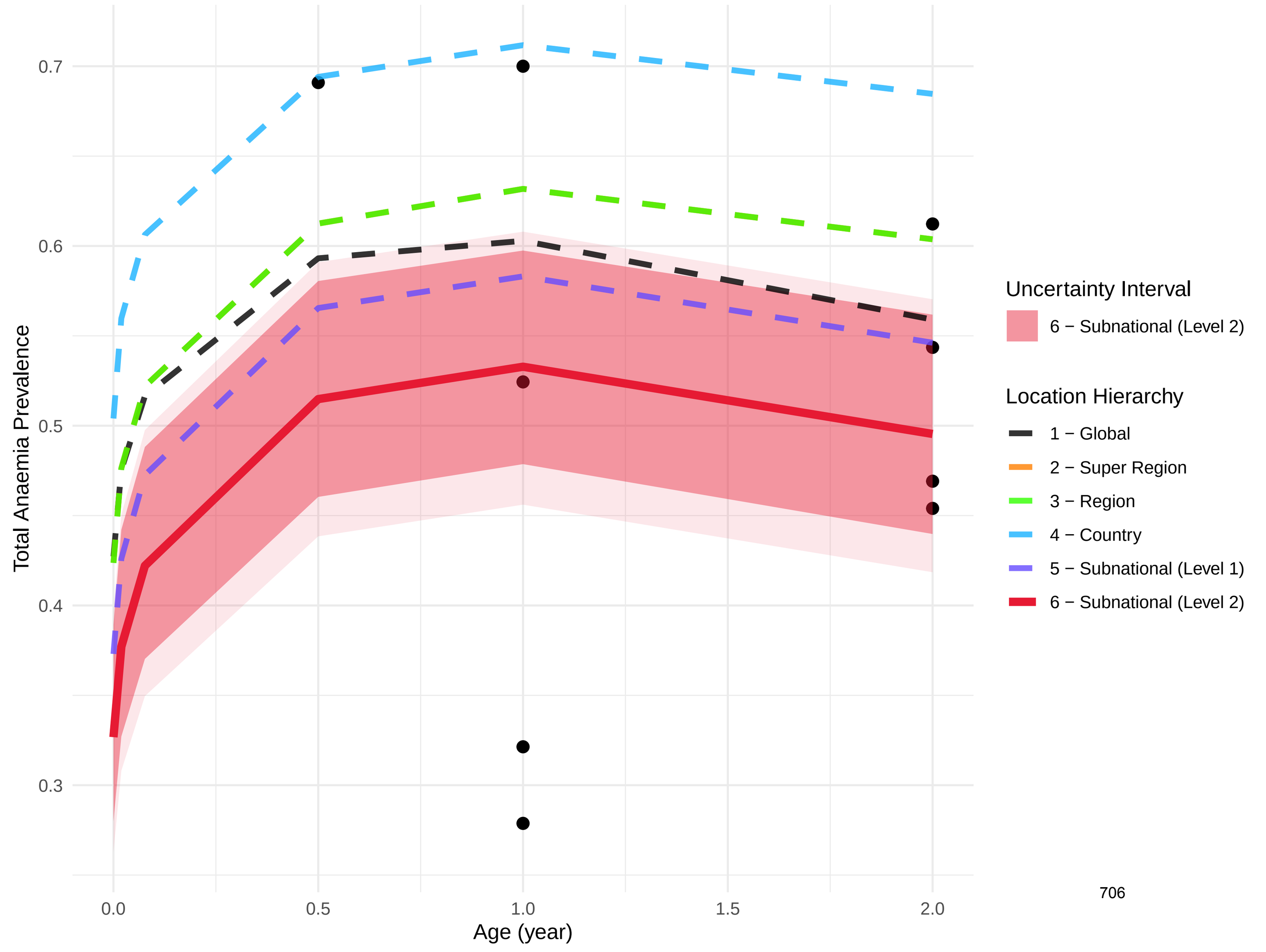

MR−BRT Age/Sex Fits − Non−pregnant Females <2 Years Old − Total Anaemia Prevalence − Uttar Pradesh
WHO−2024 Adjustment Method

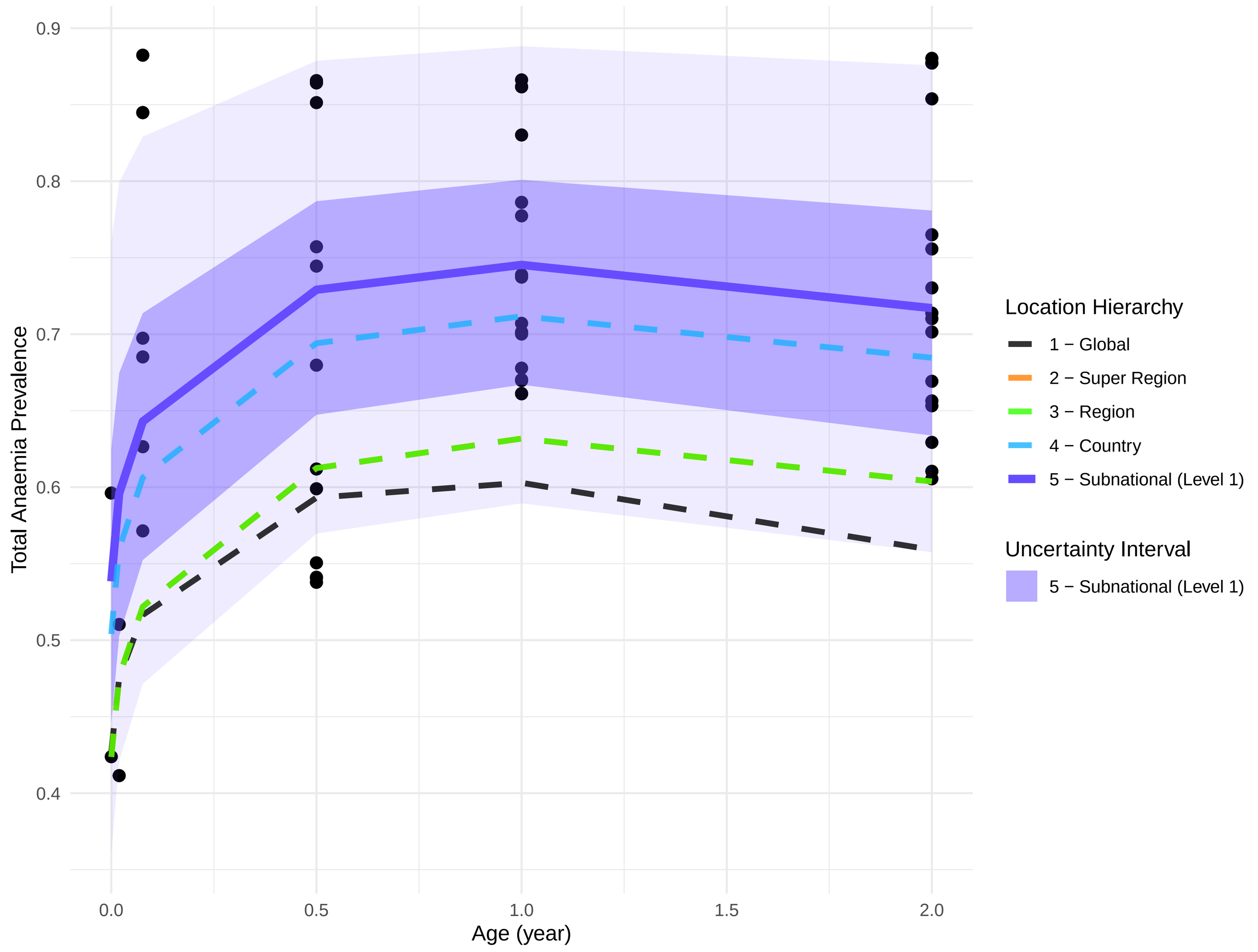

MR−BRT Age/Sex Fits − Non−pregnant Females <2 Years Old − Total Anaemia Prevalence − Uttar Pradesh, Rural
WHO−2024 Adjustment Method

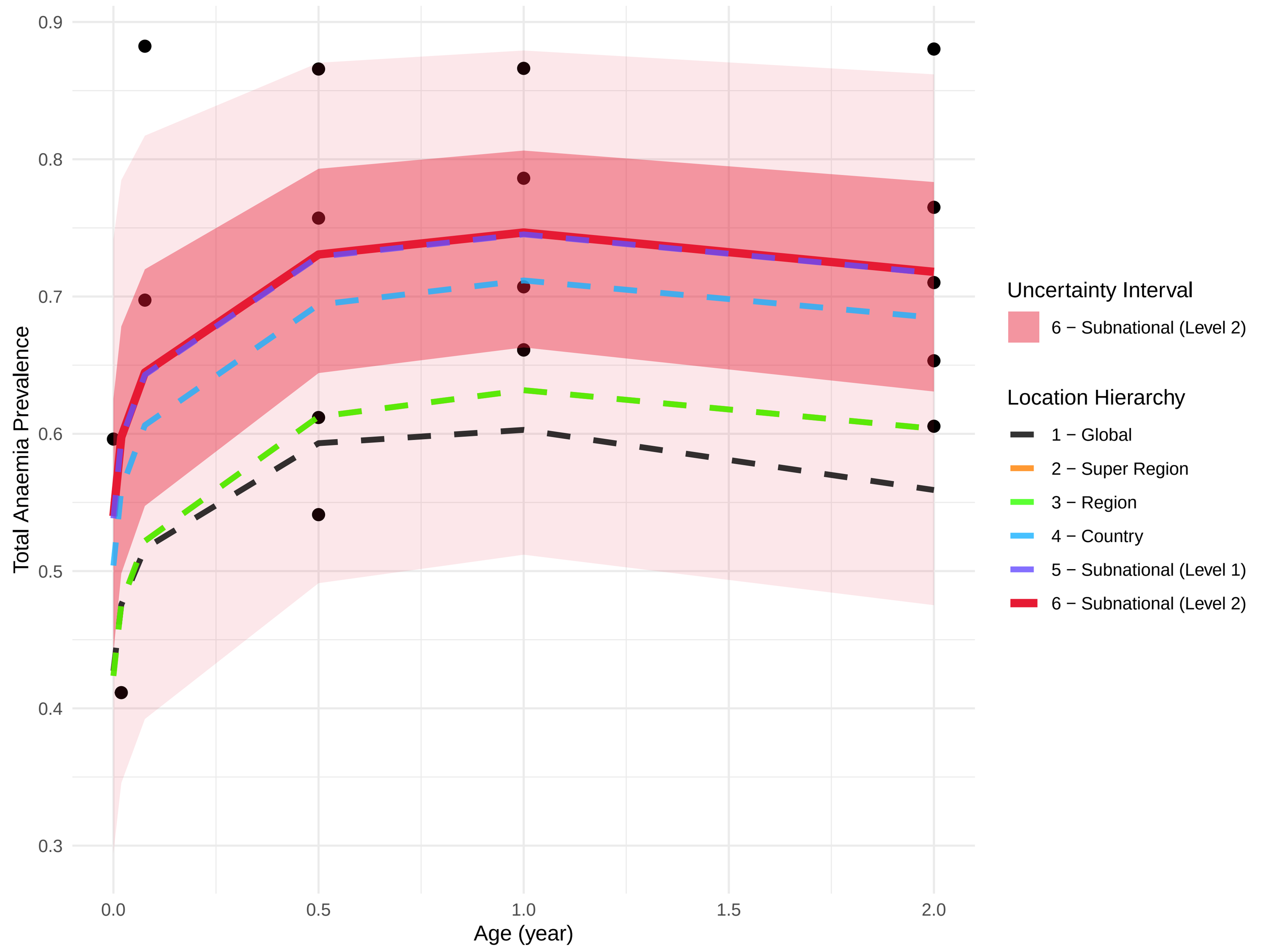

MR−BRT Age/Sex Fits − Non−pregnant Females <2 Years Old − Total Anaemia Prevalence − Uttar Pradesh, Urban
WHO−2024 Adjustment Method

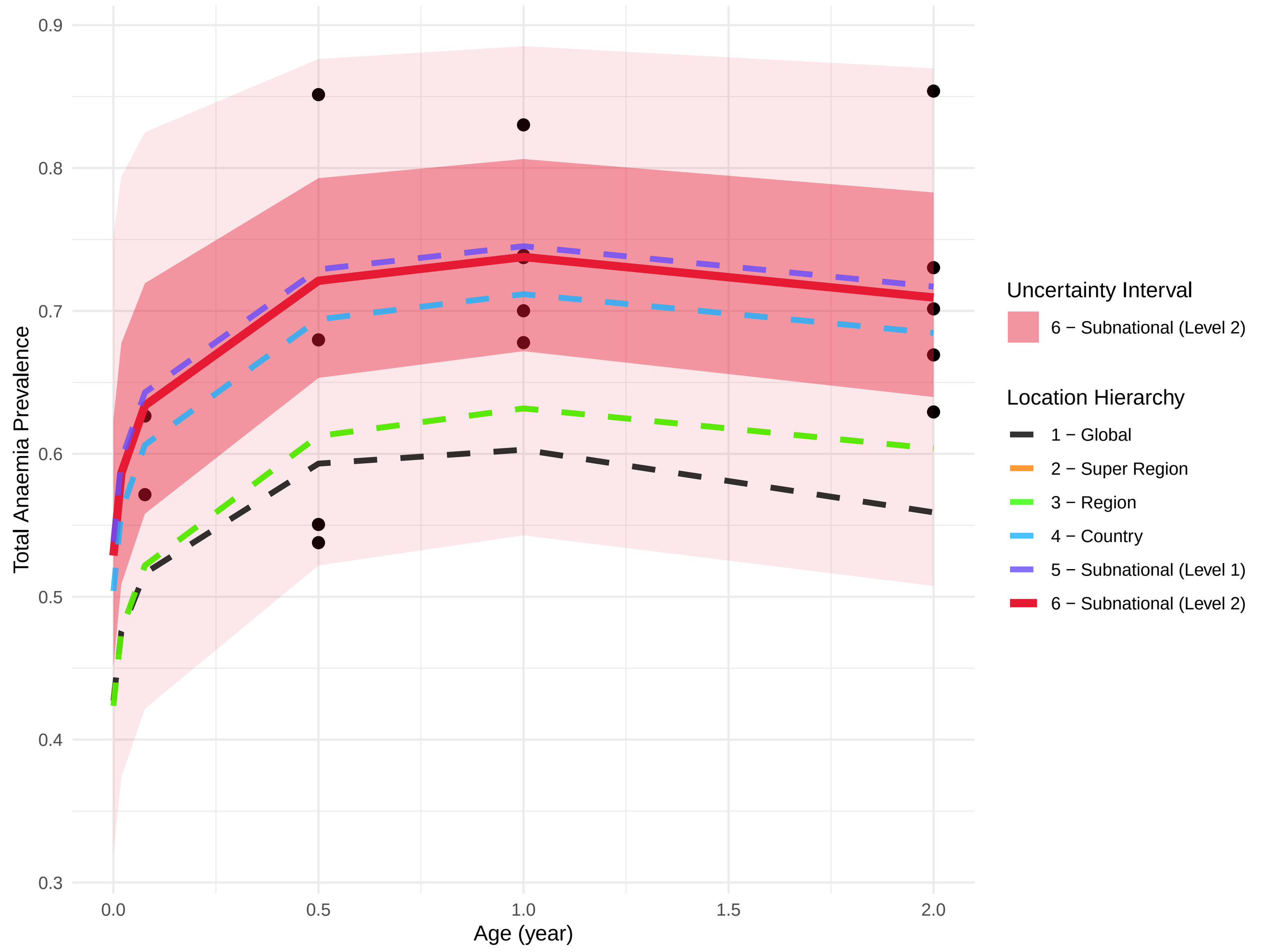

MR−BRT Age/Sex Fits − Non−pregnant Females <2 Years Old − Total Anaemia Prevalence − Uttarakhand
WHO−2024 Adjustment Method

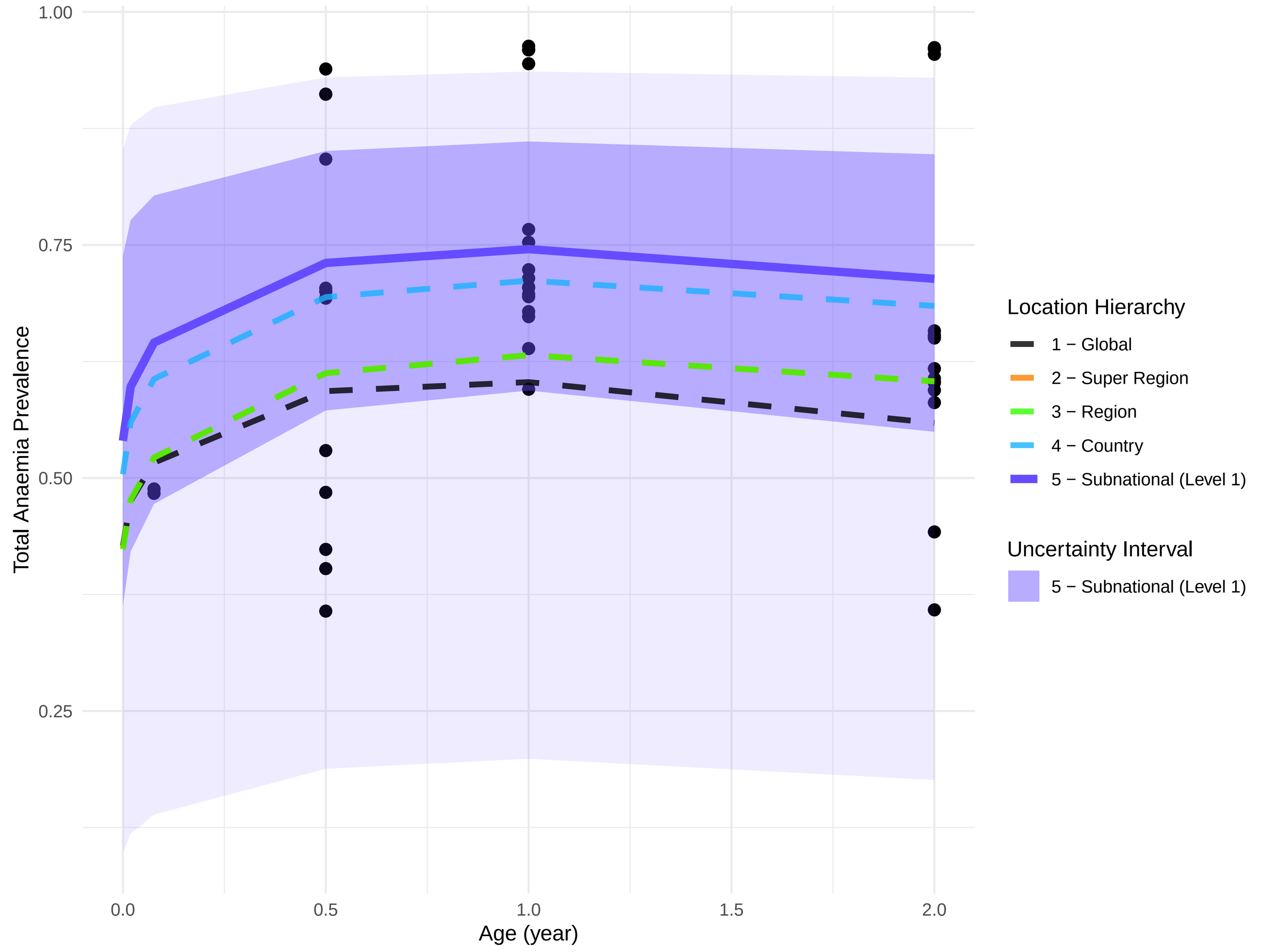

MR−BRT Age/Sex Fits − Non−pregnant Females <2 Years Old − Total Anaemia Prevalence − Uttarakhand, Rural
WHO−2024 Adjustment Method

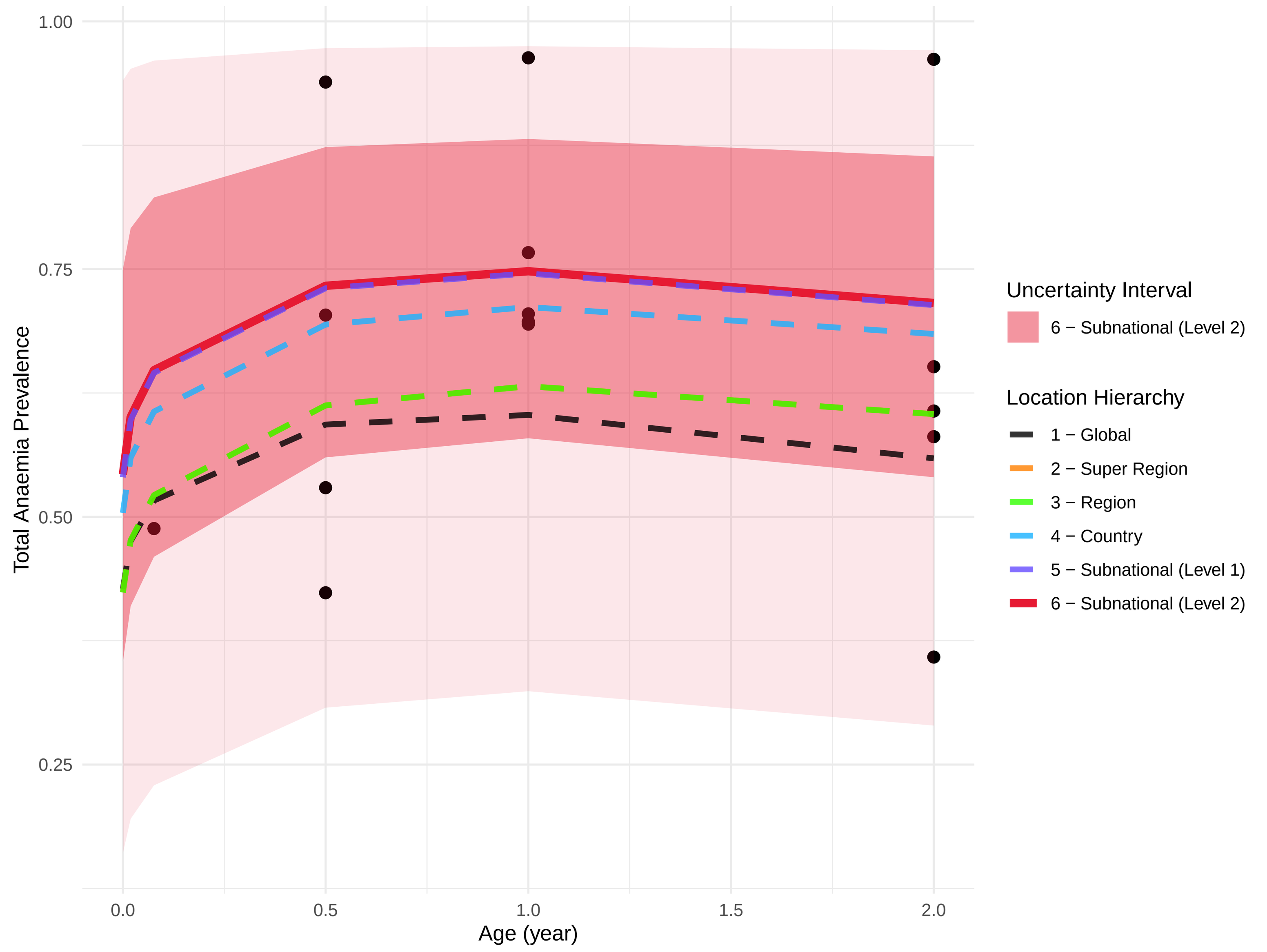

MR−BRT Age/Sex Fits − Non−pregnant Females <2 Years Old − Total Anaemia Prevalence − Uttarakhand, Urban
WHO−2024 Adjustment Method

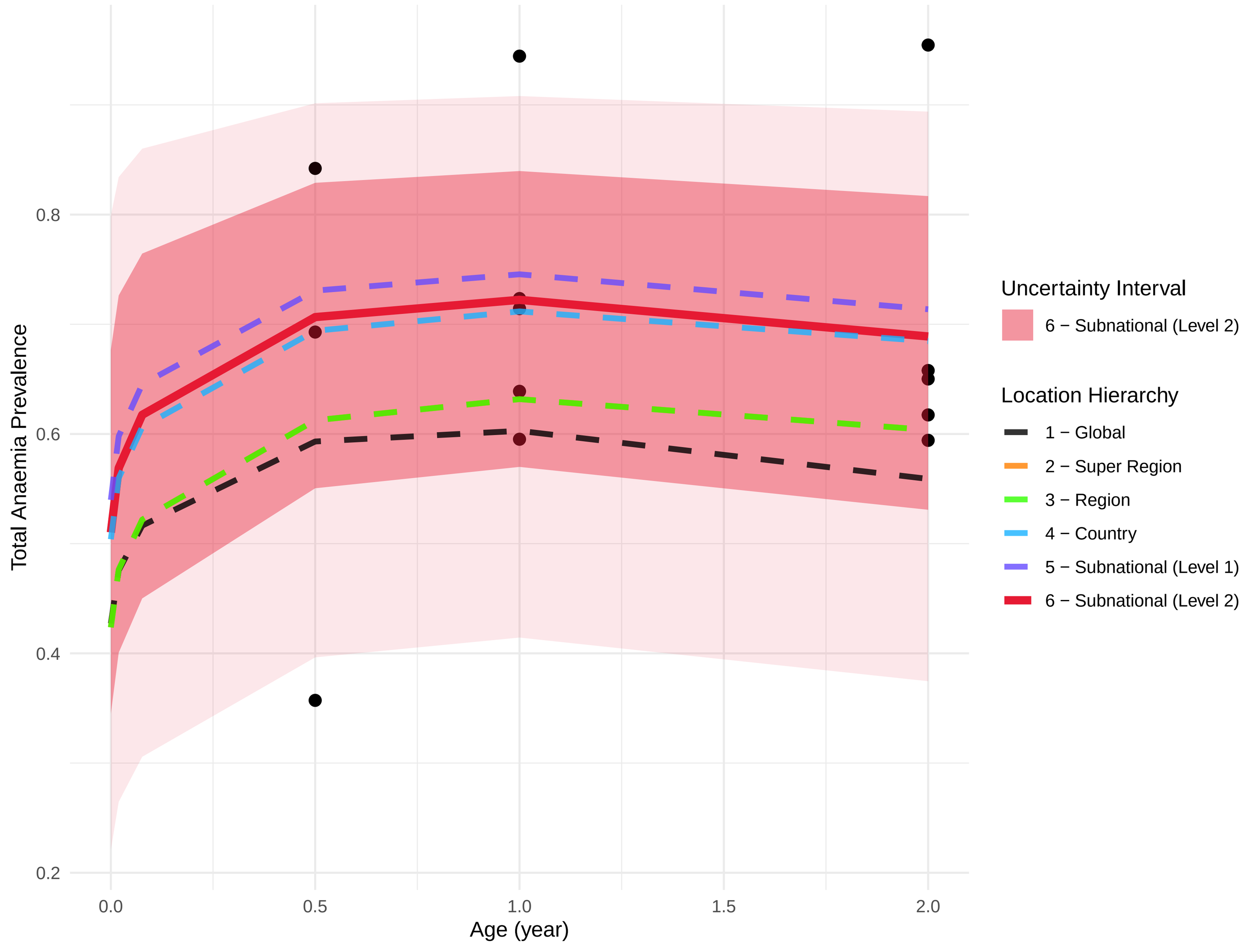

MR−BRT Age/Sex Fits − Non−pregnant Females <2 Years Old − Total Anaemia Prevalence − West Bengal
WHO−2024 Adjustment Method

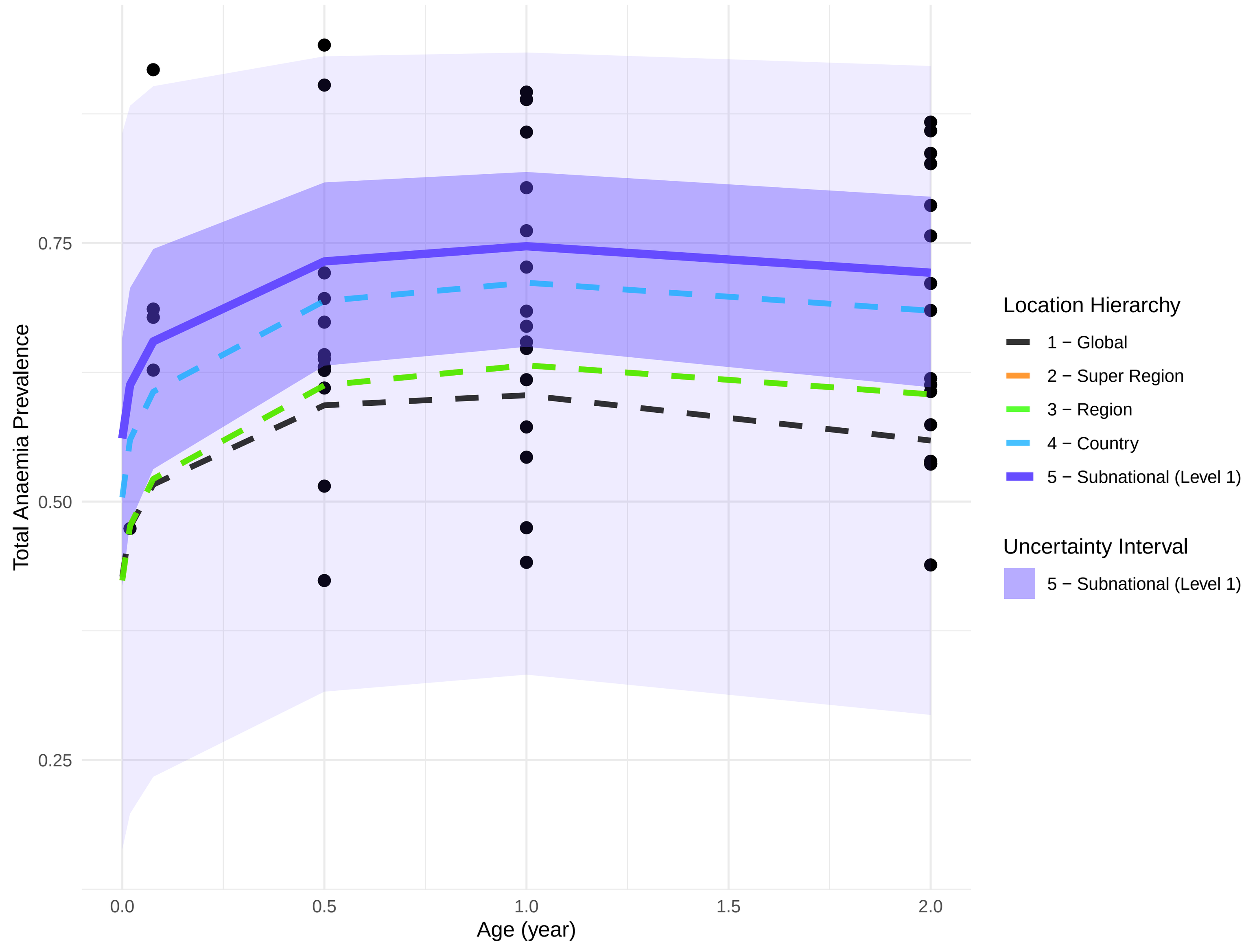

MR−BRT Age/Sex Fits − Non−pregnant Females <2 Years Old − Total Anaemia Prevalence − West Bengal, Rural
WHO−2024 Adjustment Method

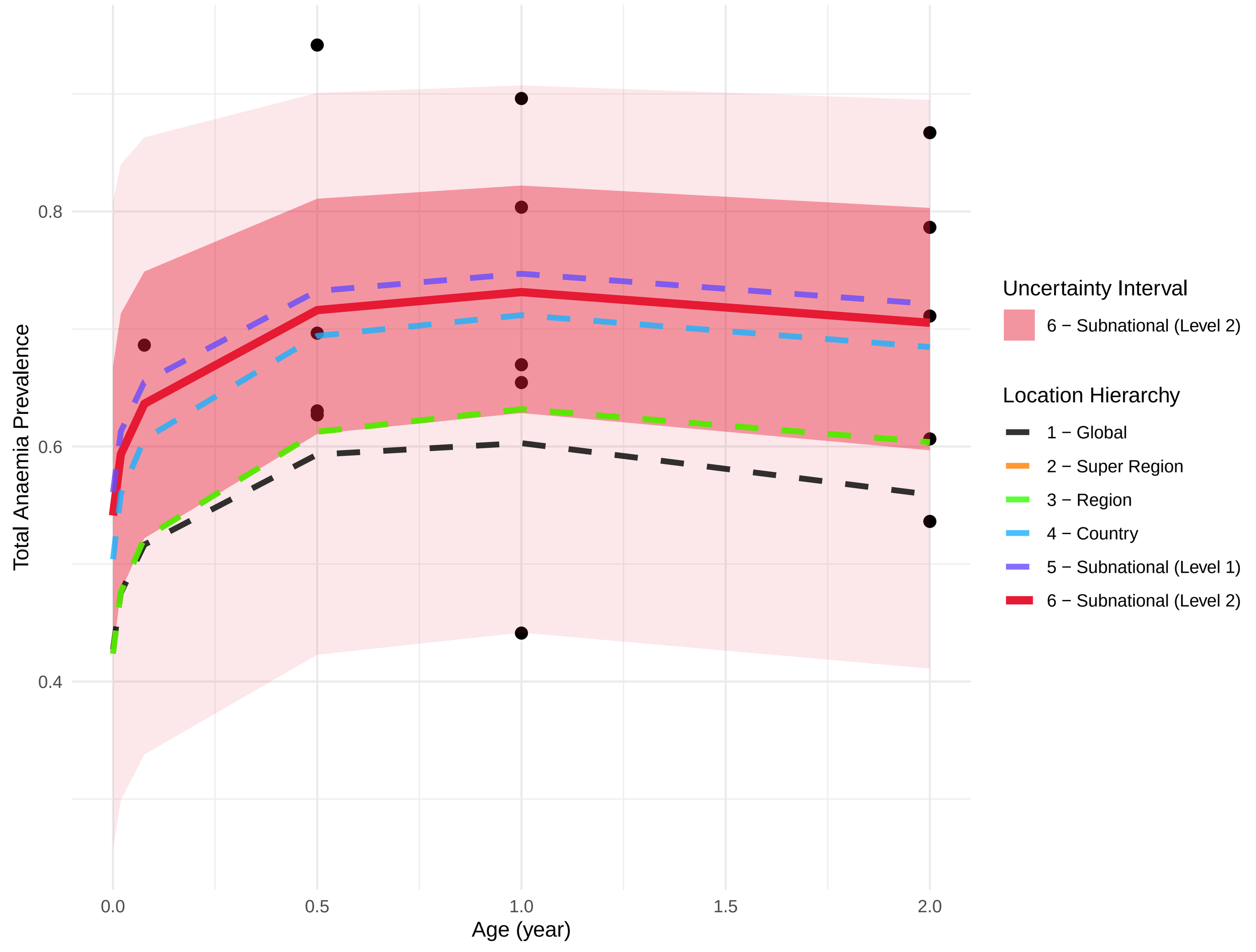

MR−BRT Age/Sex Fits − Non−pregnant Females <2 Years Old − Total Anaemia Prevalence − West Bengal, Urban
WHO−2024 Adjustment Method

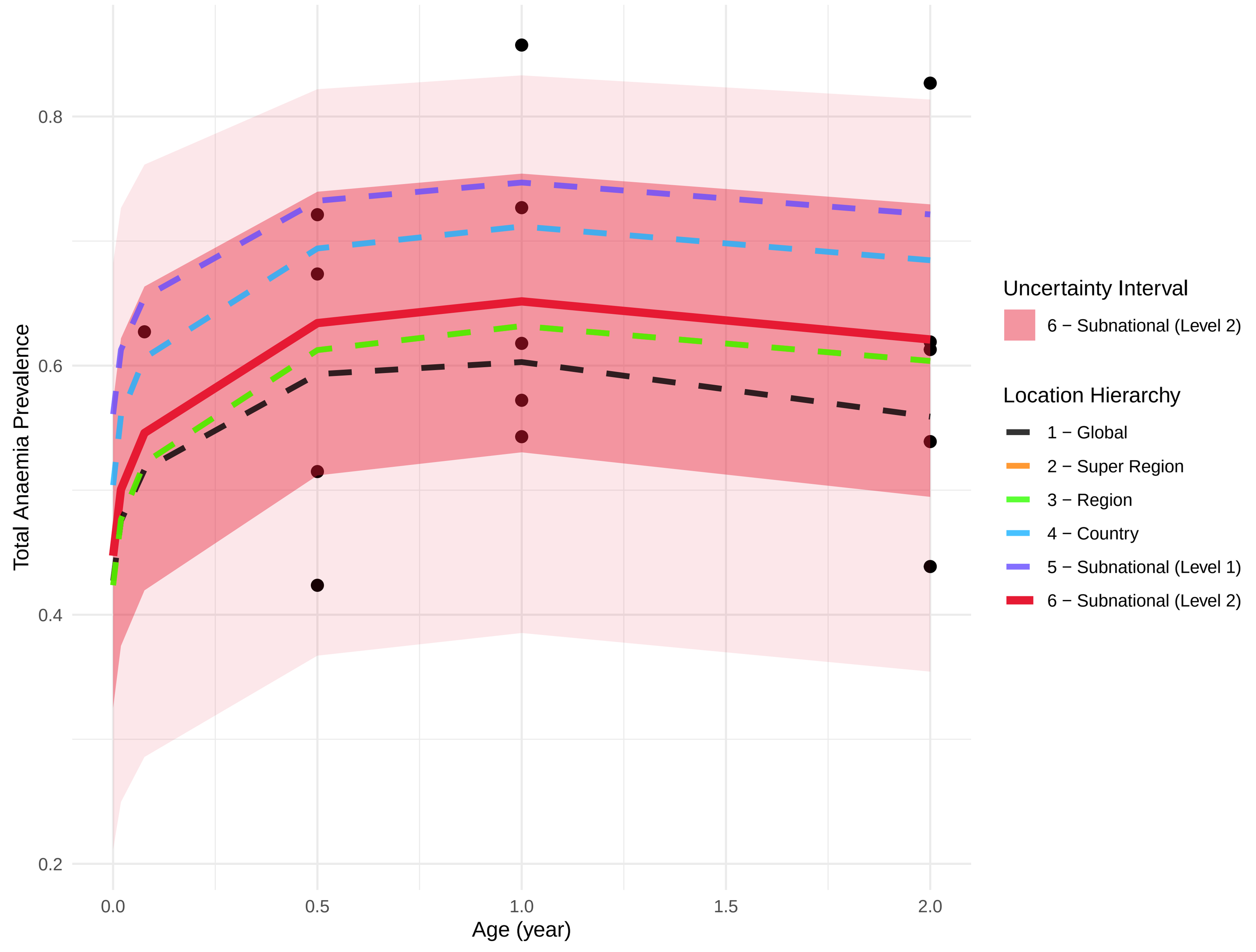

MR−BRT Age/Sex Fits − Non−pregnant Females <2 Years Old − Total Anaemia Prevalence − Nepal
WHO−2024 Adjustment Method

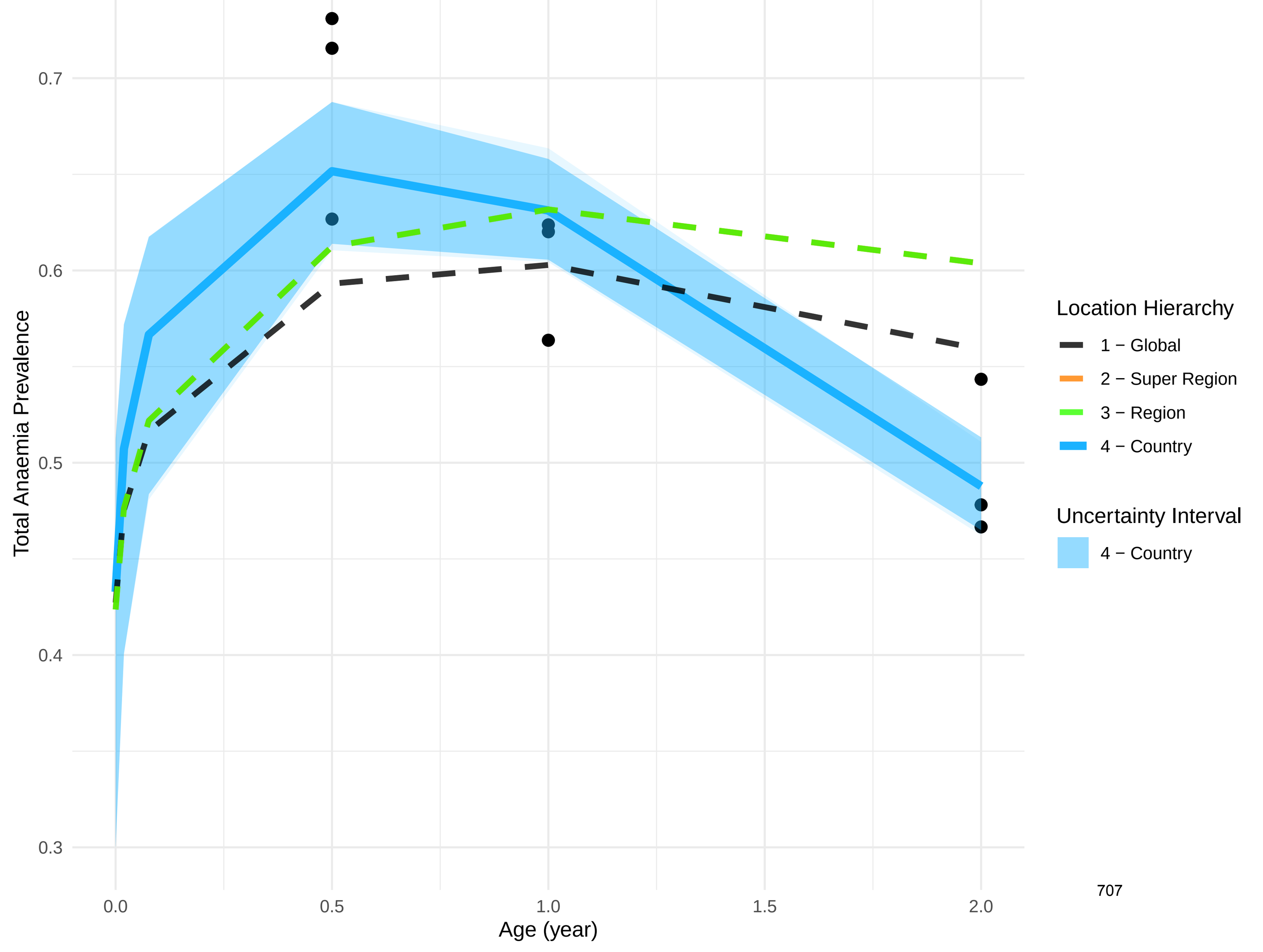

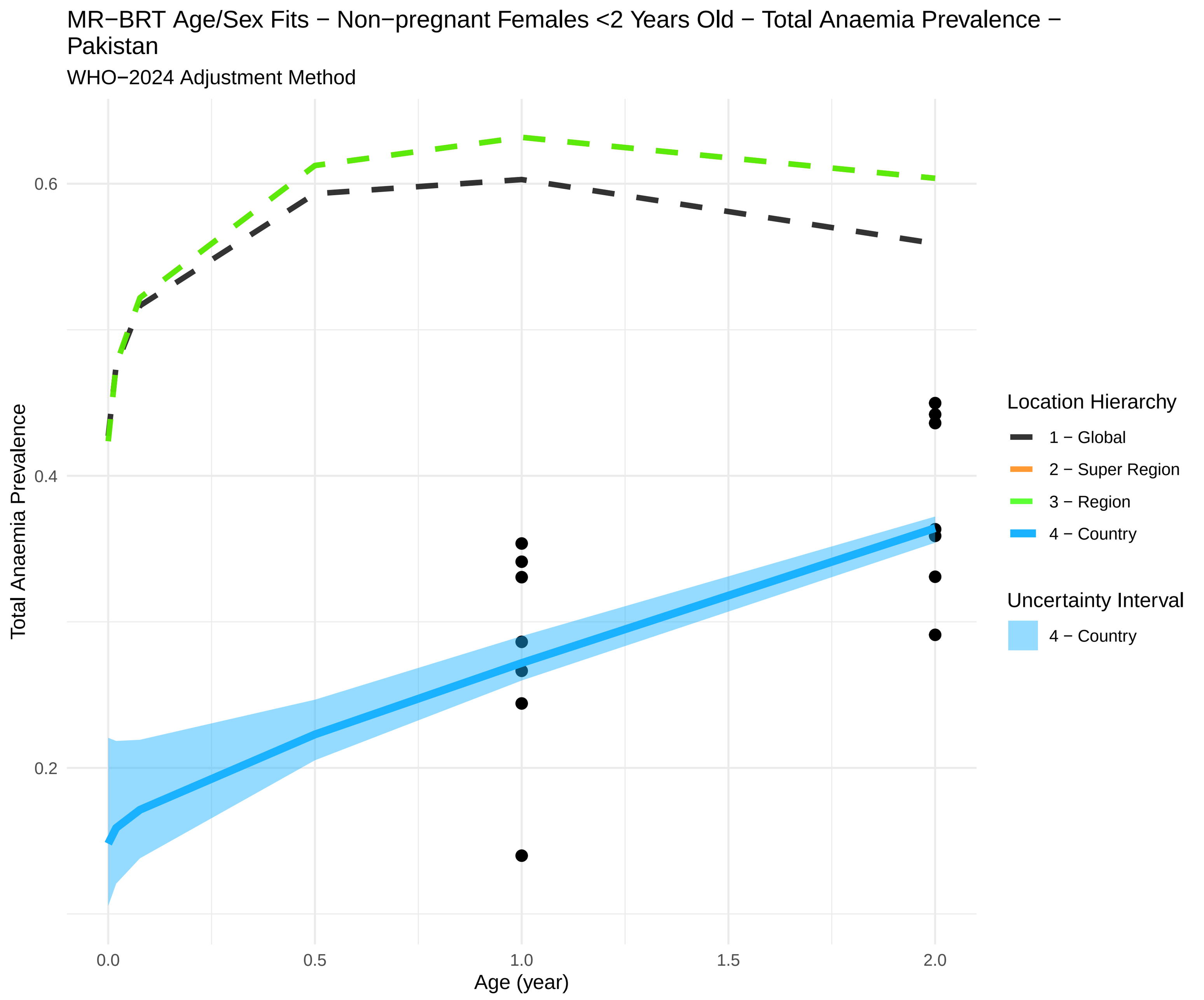
MR−BRT Age/Sex Fits − Non−pregnant Females <2 Years Old − Total Anaemia Prevalence − Pakistan
WHO−2024 Adjustment Method
Total Anaemia Prevalence
Age (year)
Location Hierarchy
1 − Global
2 − Super Region
3 − Region
4 − Country
Uncertainty Interval
4 − Country

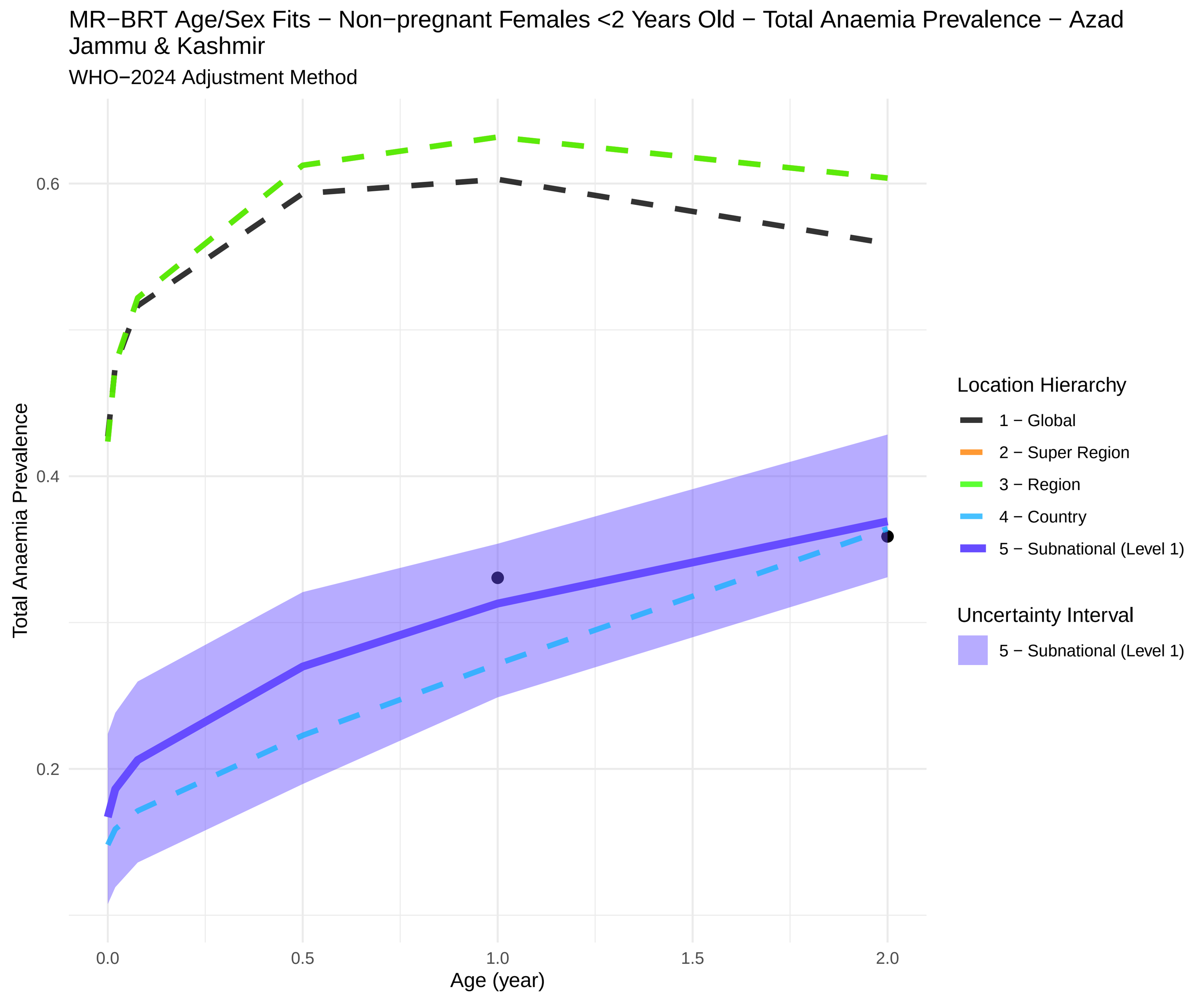
MR−BRT Age/Sex Fits − Non−pregnant Females <2 Years Old − Total Anaemia Prevalence − Azad Jammu & Kashmir
WHO−2024 Adjustment Method
Total Anaemia Prevalence
Age (year)
Location Hierarchy
1 − Global
2 − Super Region
3 − Region
4 − Country
5 − Subnational (Level 1)
Uncertainty Interval
5 − Subnational (Level 1)

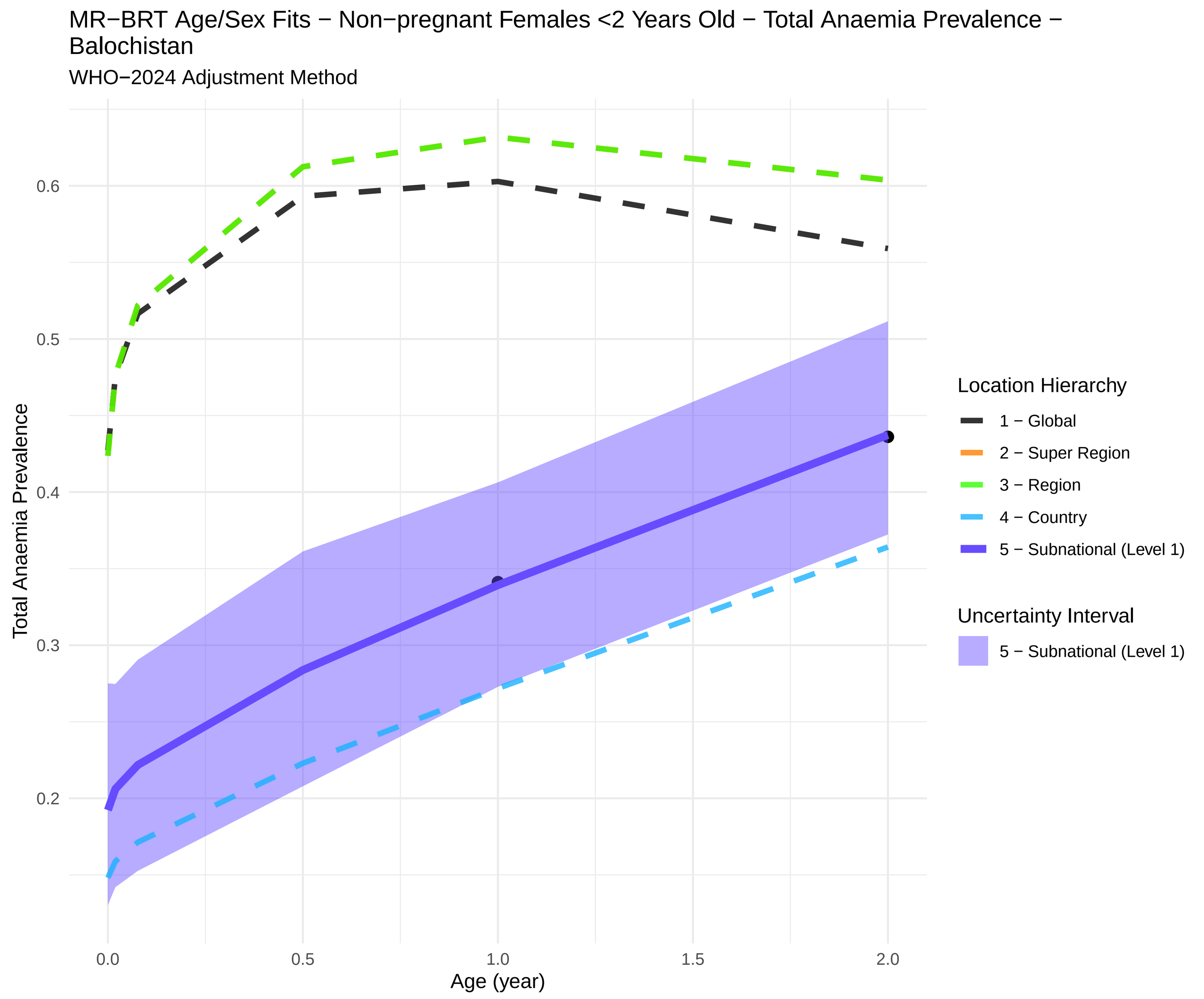
MR−BRT Age/Sex Fits − Non−pregnant Females <2 Years Old − Total Anaemia Prevalence − Balochistan
WHO−2024 Adjustment Method
Total Anaemia Prevalence
Age (year)
Location Hierarchy
1 − Global
2 − Super Region
3 − Region
4 − Country
5 − Subnational (Level 1)
Uncertainty Interval
5 − Subnational (Level 1)

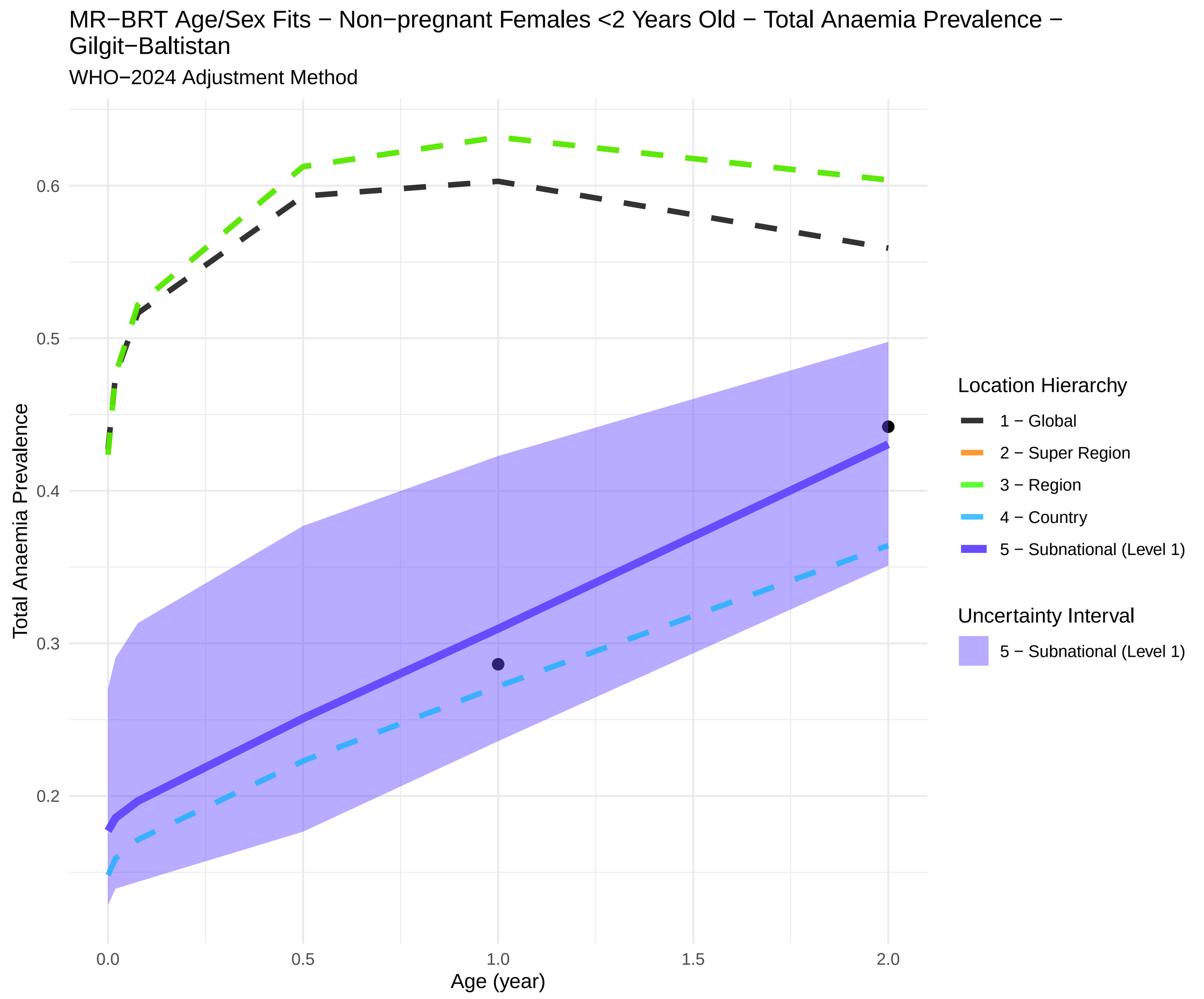
MR−BRT Age/Sex Fits − Non−pregnant Females <2 Years Old − Total Anaemia Prevalence − Gilgit−Baltistan
WHO−2024 Adjustment Method
Total Anaemia Prevalence
Age (year)
Location Hierarchy
1 − Global
2 − Super Region
3 − Region
4 − Country
5 − Subnational (Level 1)
Uncertainty Interval
5 − Subnational (Level 1)

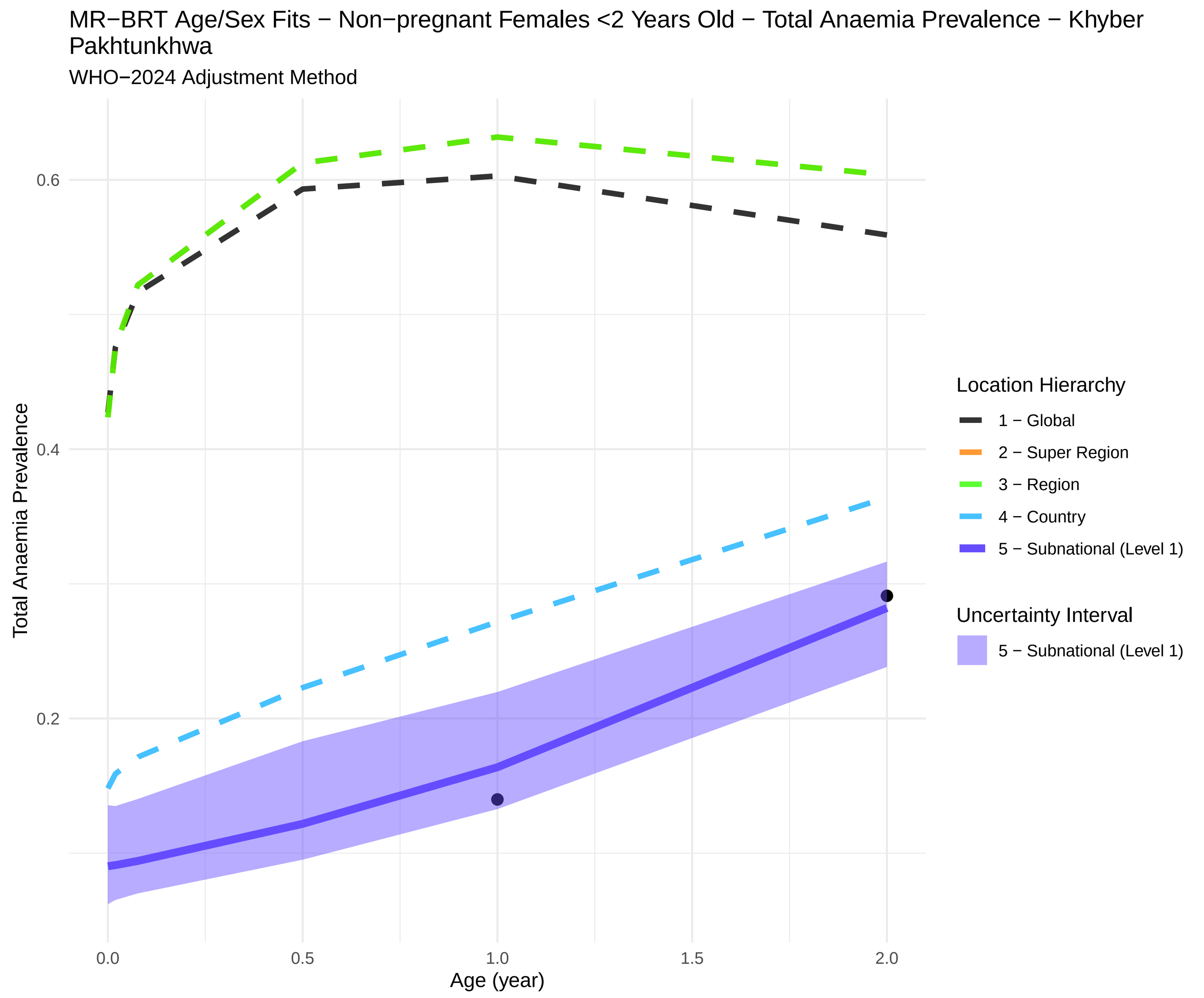
MR−BRT Age/Sex Fits − Non−pregnant Females <2 Years Old − Total Anaemia Prevalence − Khyber Pakhtunkhwa
WHO−2024 Adjustment Method
Total Anaemia Prevalence
Age (year)
Location Hierarchy
1 − Global
2 − Super Region
3 − Region
4 − Country
5 − Subnational (Level 1)
Uncertainty Interval
5 − Subnational (Level 1)

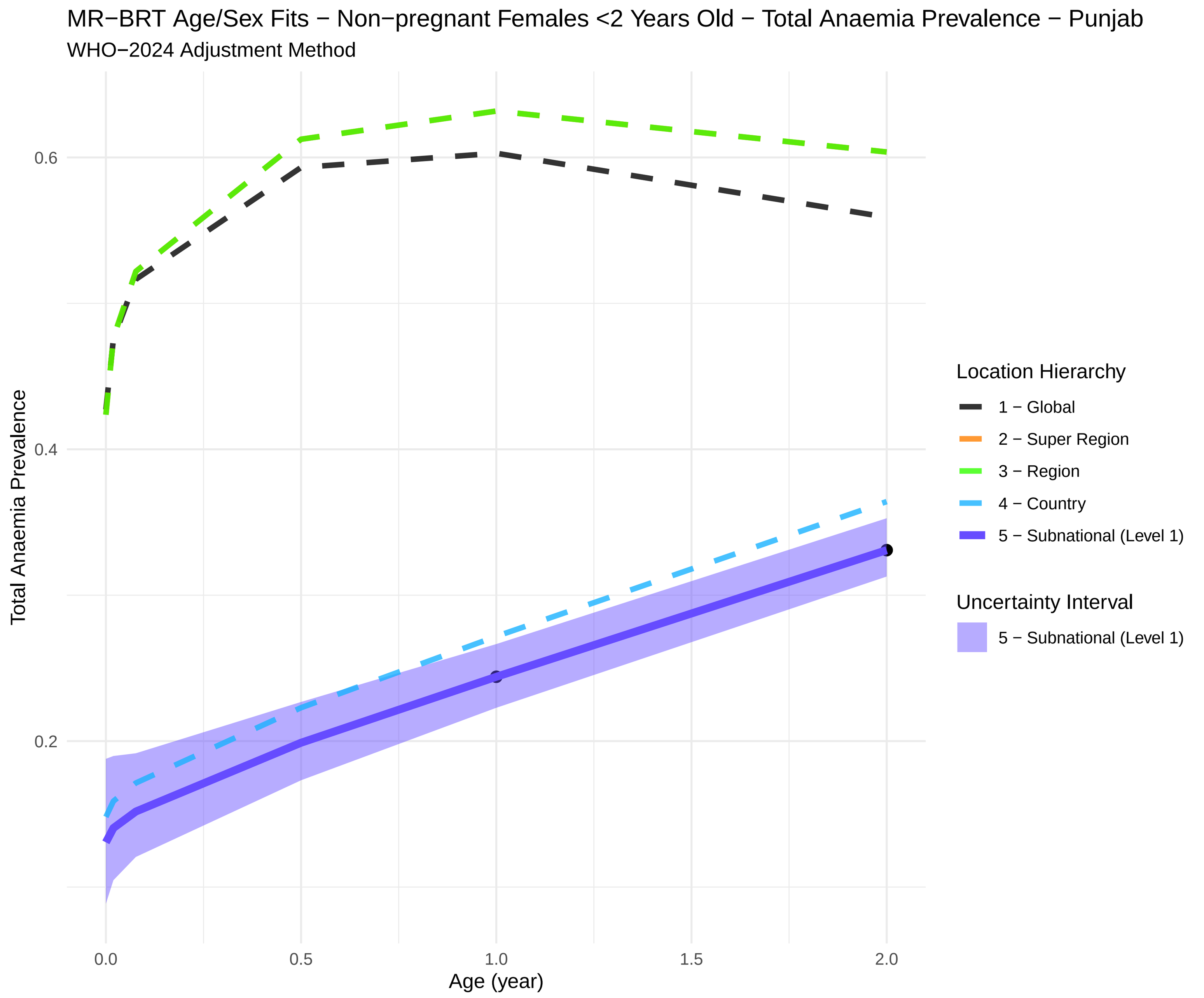
MR−BRT Age/Sex Fits − Non−pregnant Females <2 Years Old − Total Anaemia Prevalence − Punjab
WHO−2024 Adjustment Method
Total Anaemia Prevalence
Age (year)
Location Hierarchy
1 − Global
2 − Super Region
3 − Region
4 − Country
5 − Subnational (Level 1)
Uncertainty Interval
5 − Subnational (Level 1)

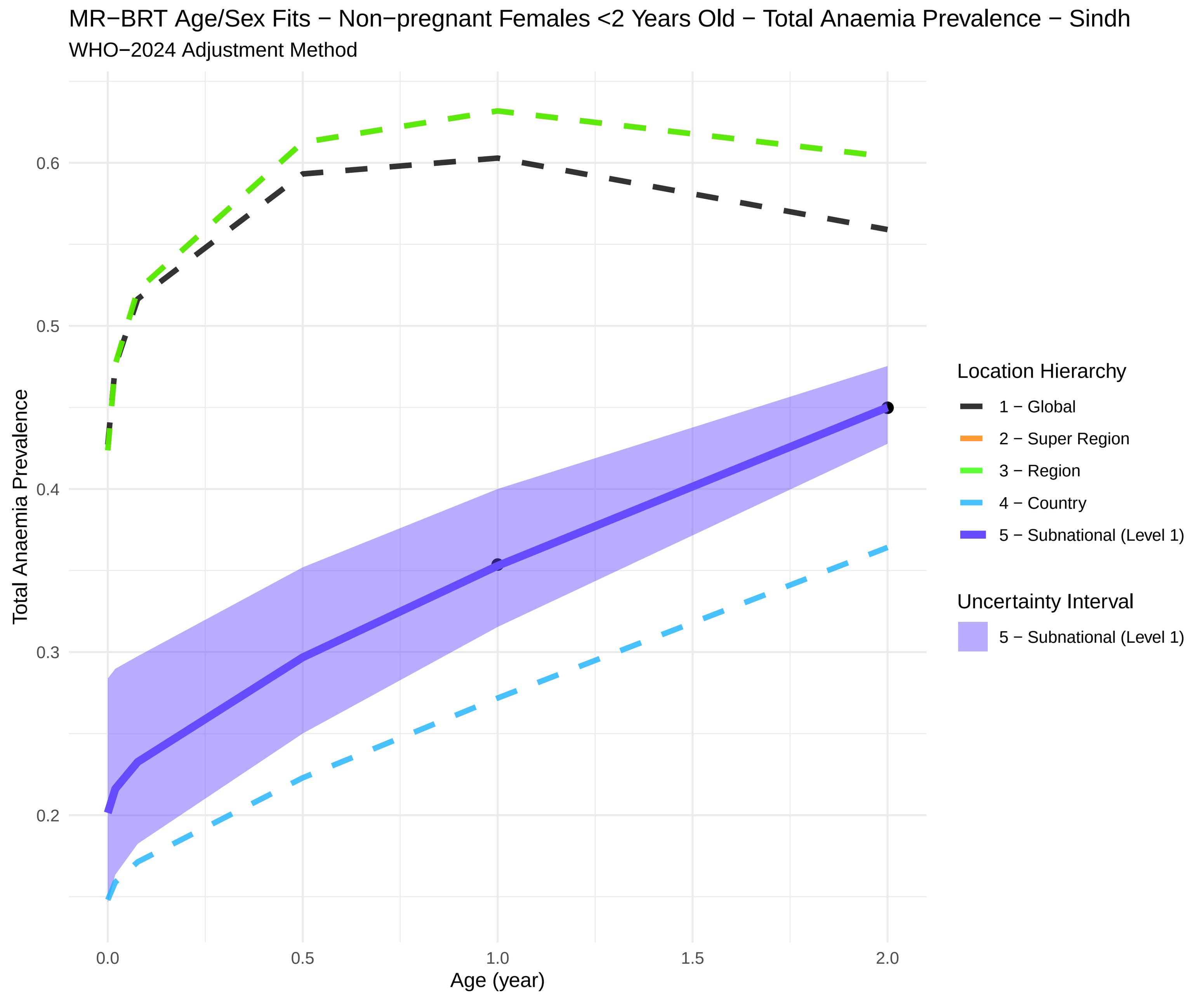
MR−BRT Age/Sex Fits − Non−pregnant Females <2 Years Old − Total Anaemia Prevalence − Sindh
WHO−2024 Adjustment Method
Total Anaemia Prevalence
Age (year)
Location Hierarchy
1 − Global
2 − Super Region
3 − Region
4 − Country
5 − Subnational (Level 1)
Uncertainty Interval
5 − Subnational (Level 1)

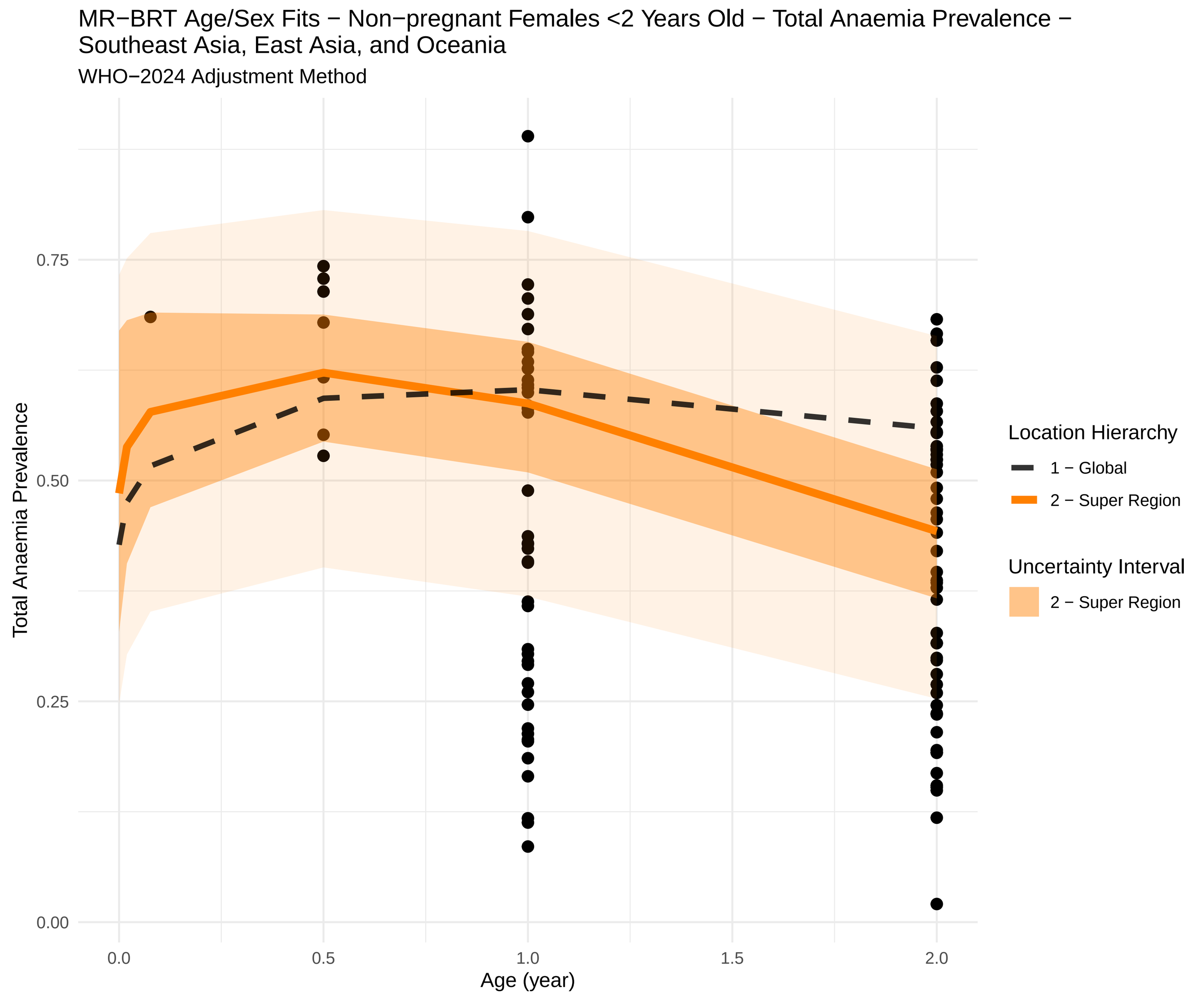
MR−BRT Age/Sex Fits − Non−pregnant Females <2 Years Old − Total Anaemia Prevalence − Southeast Asia, East Asia, and Oceania
WHO−2024 Adjustment Method
Total Anaemia Prevalence
Age (year)
Location Hierarchy
1 − Global
2 − Super Region
Uncertainty Interval
2 − Super Region

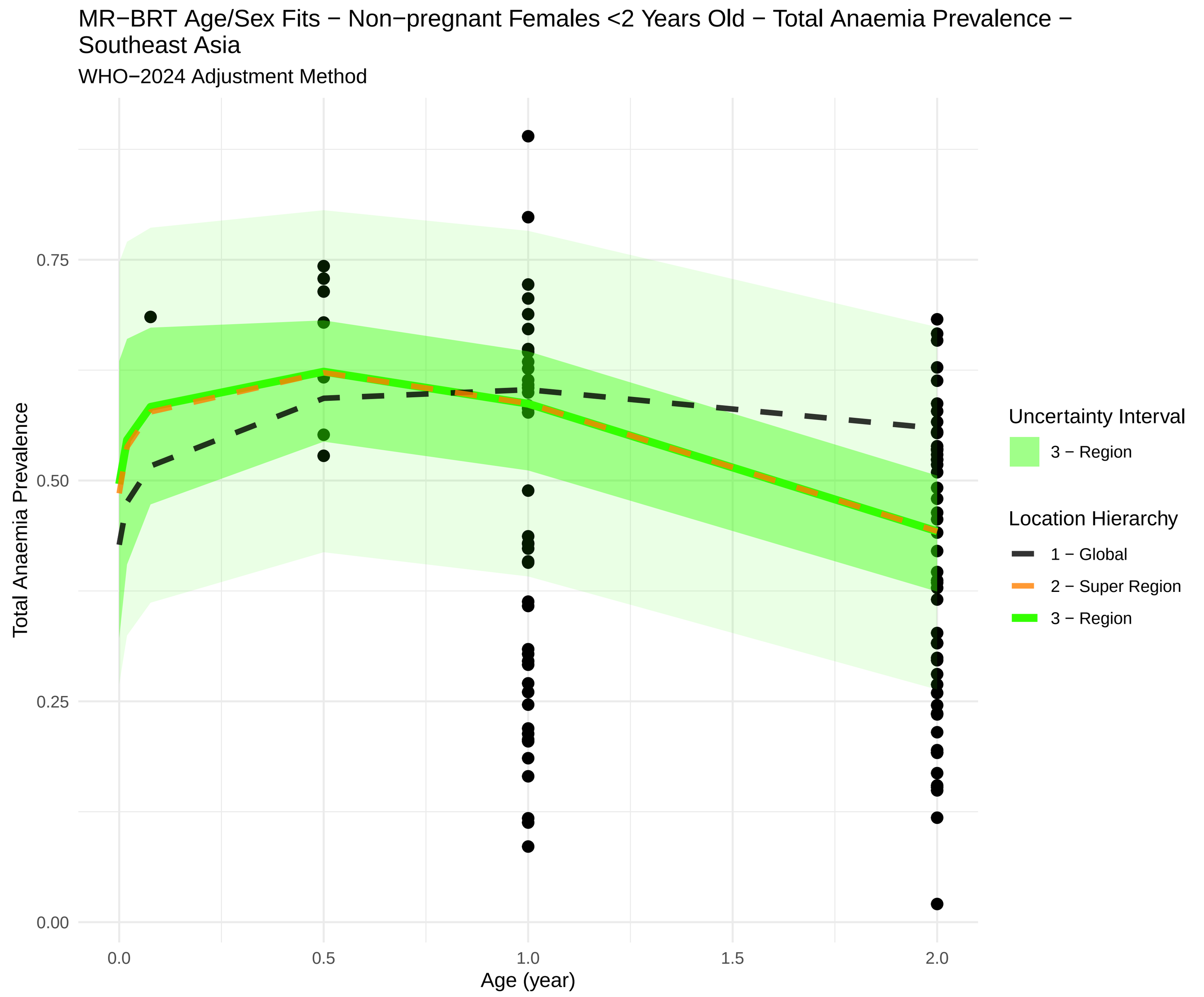
MR−BRT Age/Sex Fits − Non−pregnant Females <2 Years Old − Total Anaemia Prevalence − Southeast Asia
WHO−2024 Adjustment Method
Total Anaemia Prevalence
Age (year)
Uncertainty Interval
3 − Region
Location Hierarchy
1 − Global
2 − Super Region
3 − Region

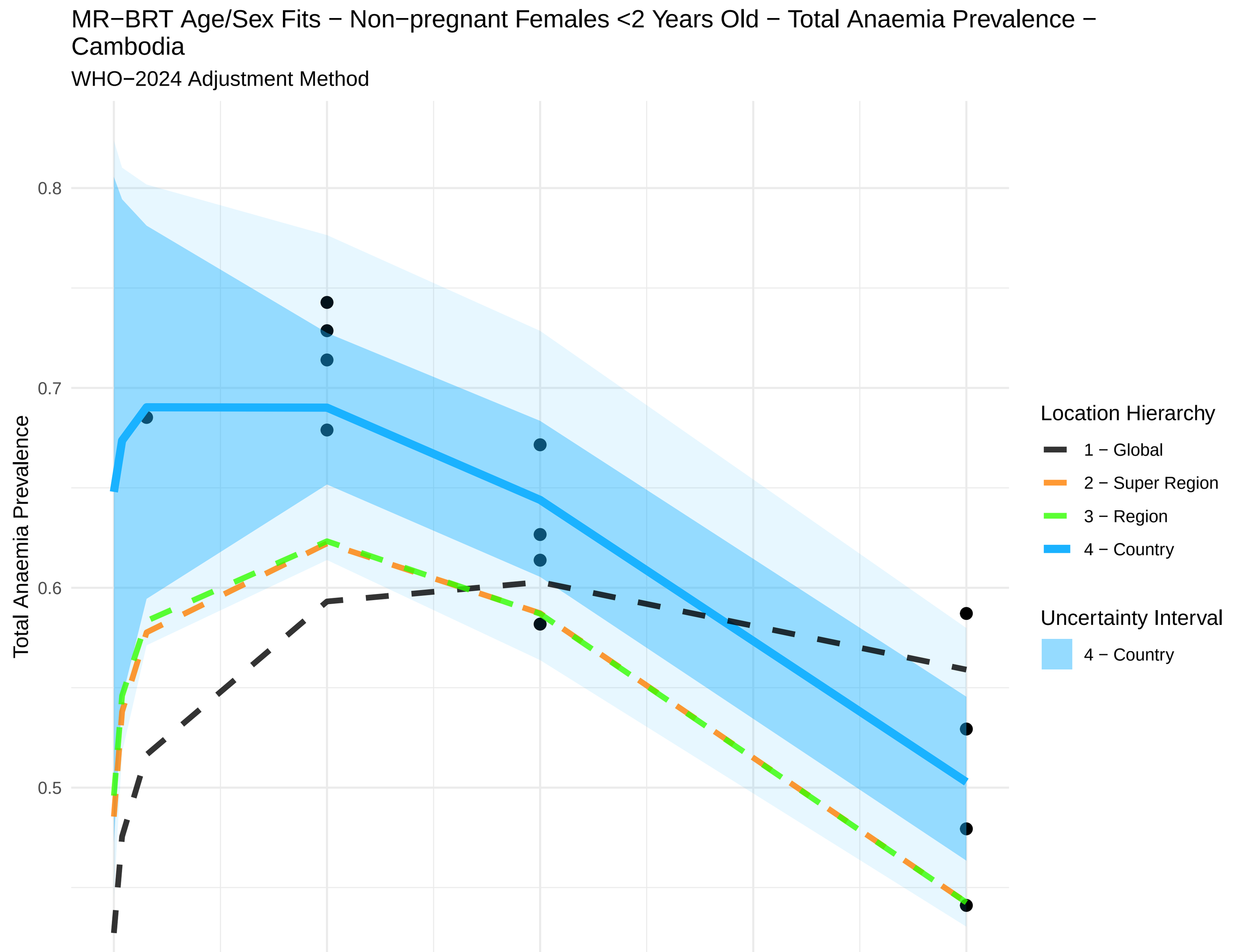
MR−BRT Age/Sex Fits − Non−pregnant Females <2 Years Old − Total Anaemia Prevalence − Cambodia
WHO−2024 Adjustment Method
Total Anaemia Prevalence
Age (year)
Location Hierarchy
1 − Global
2 − Super Region
3 − Region
4 − Country
Uncertainty Interval
4 − Country

MR−BRT Age/Sex Fits − Non−pregnant Females <2 Years Old − Total Anaemia Prevalence − Indonesia

WHO−2024 Adjustment Method

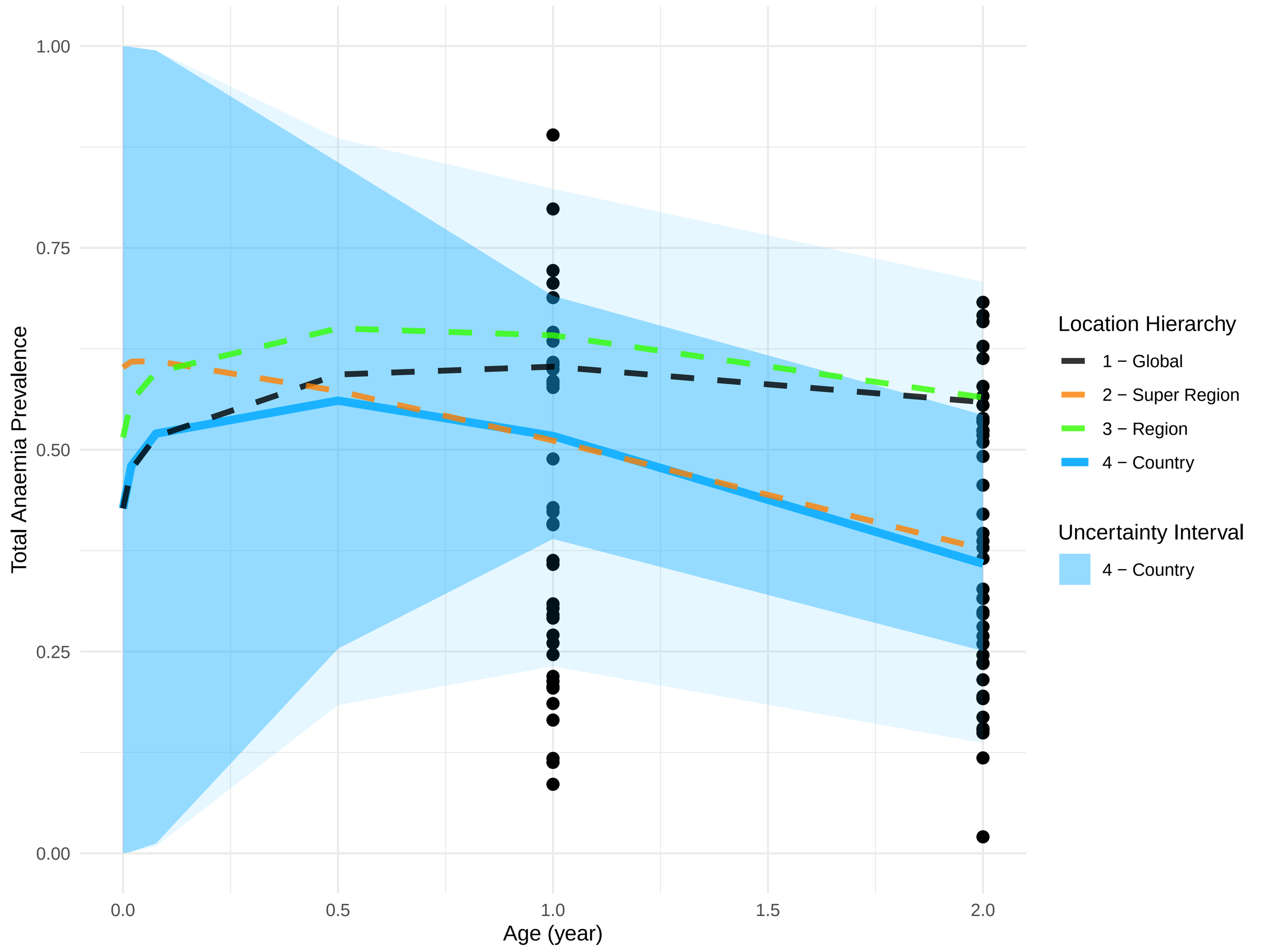

MR−BRT Age/Sex Fits − Non−pregnant Females <2 Years Old − Total Anaemia Prevalence − Bali

WHO−2024 Adjustment Method

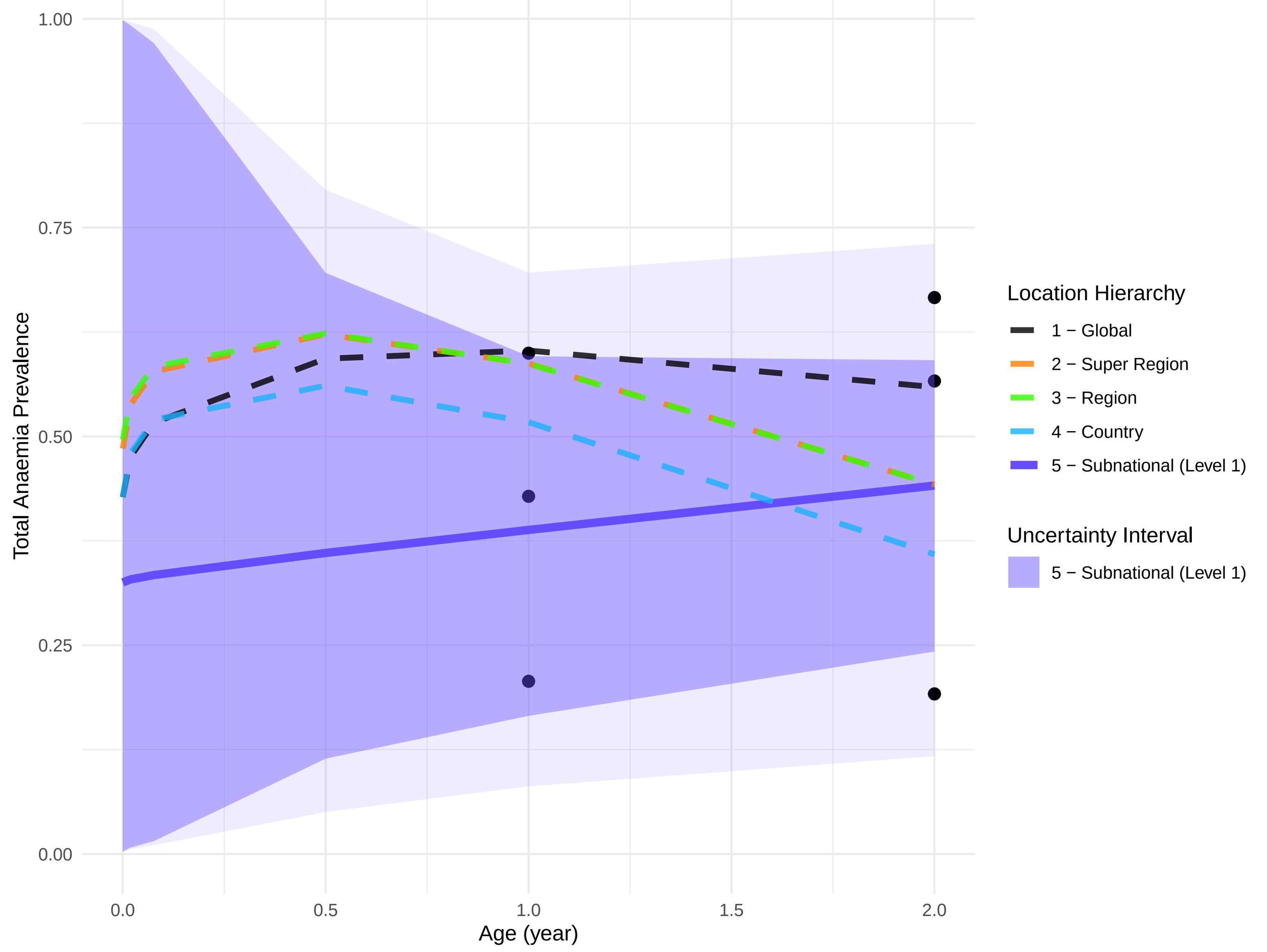

MR−BRT Age/Sex Fits − Non−pregnant Females <2 Years Old − Total Anaemia Prevalence − Bangka−Belitung Islands

WHO−2024 Adjustment Method

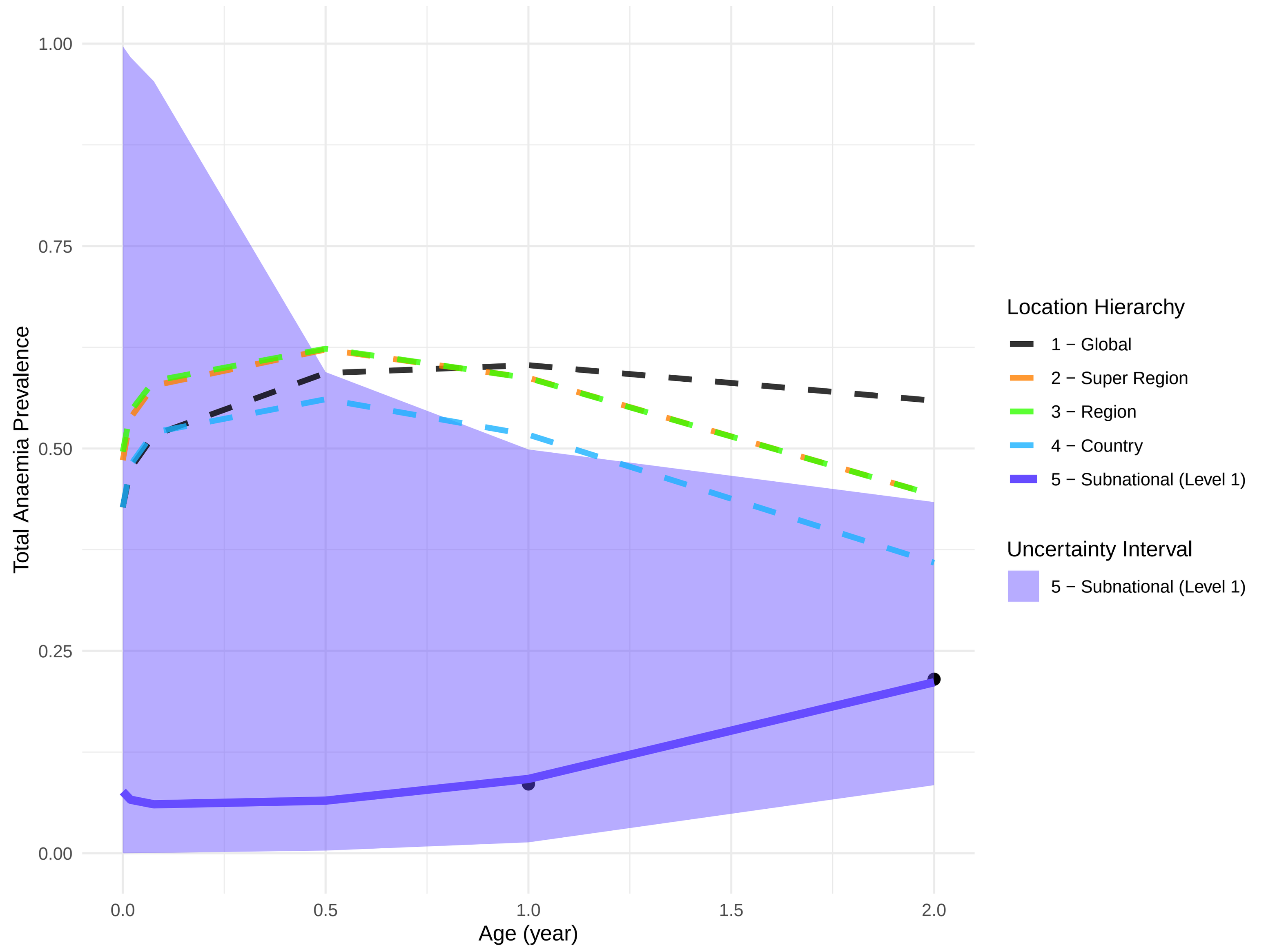

MR−BRT Age/Sex Fits − Non−pregnant Females <2 Years Old − Total Anaemia Prevalence − Banten

WHO−2024 Adjustment Method

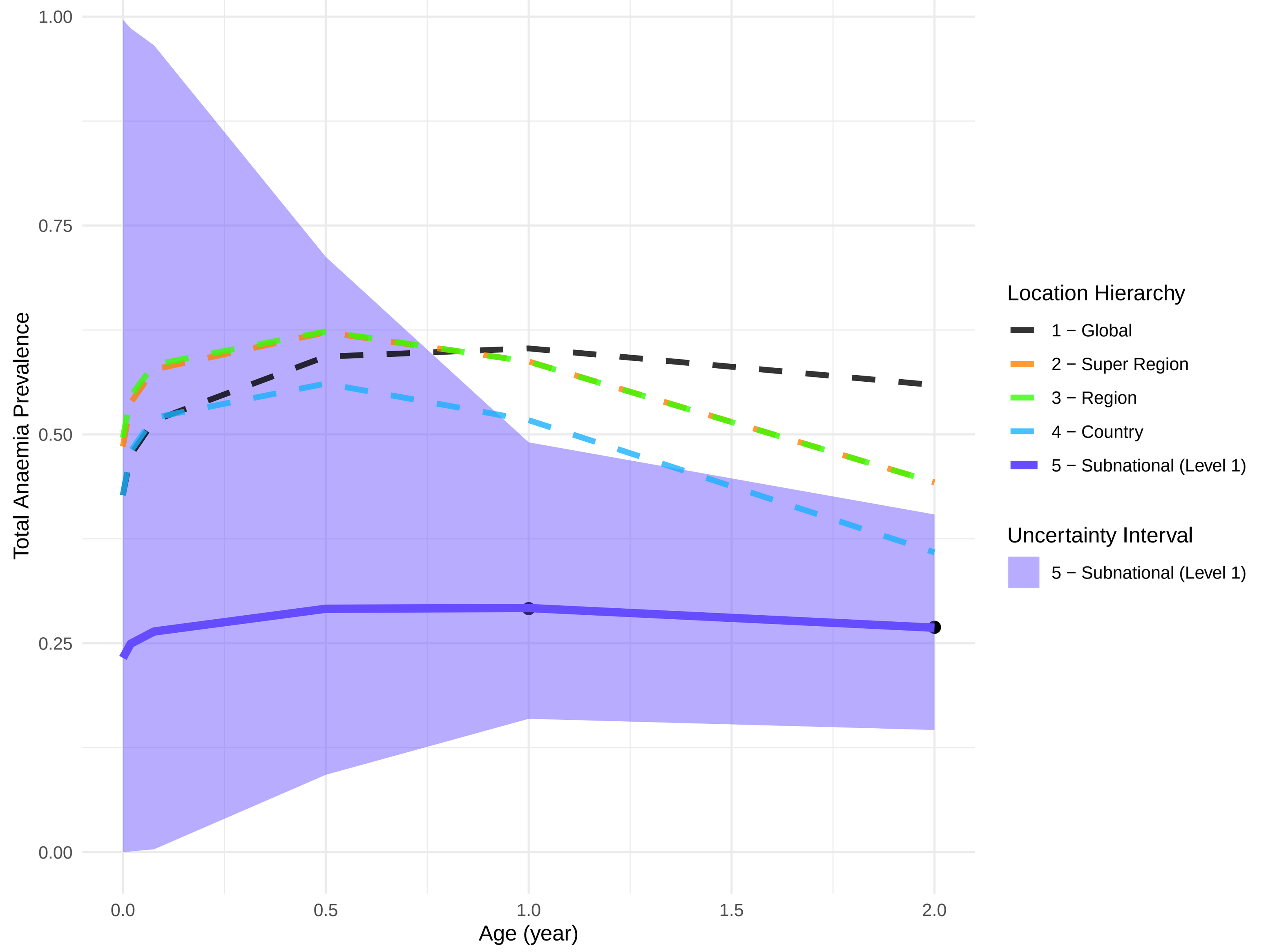

MR−BRT Age/Sex Fits − Non−pregnant Females <2 Years Old − Total Anaemia Prevalence − Jakarta

WHO−2024 Adjustment Method

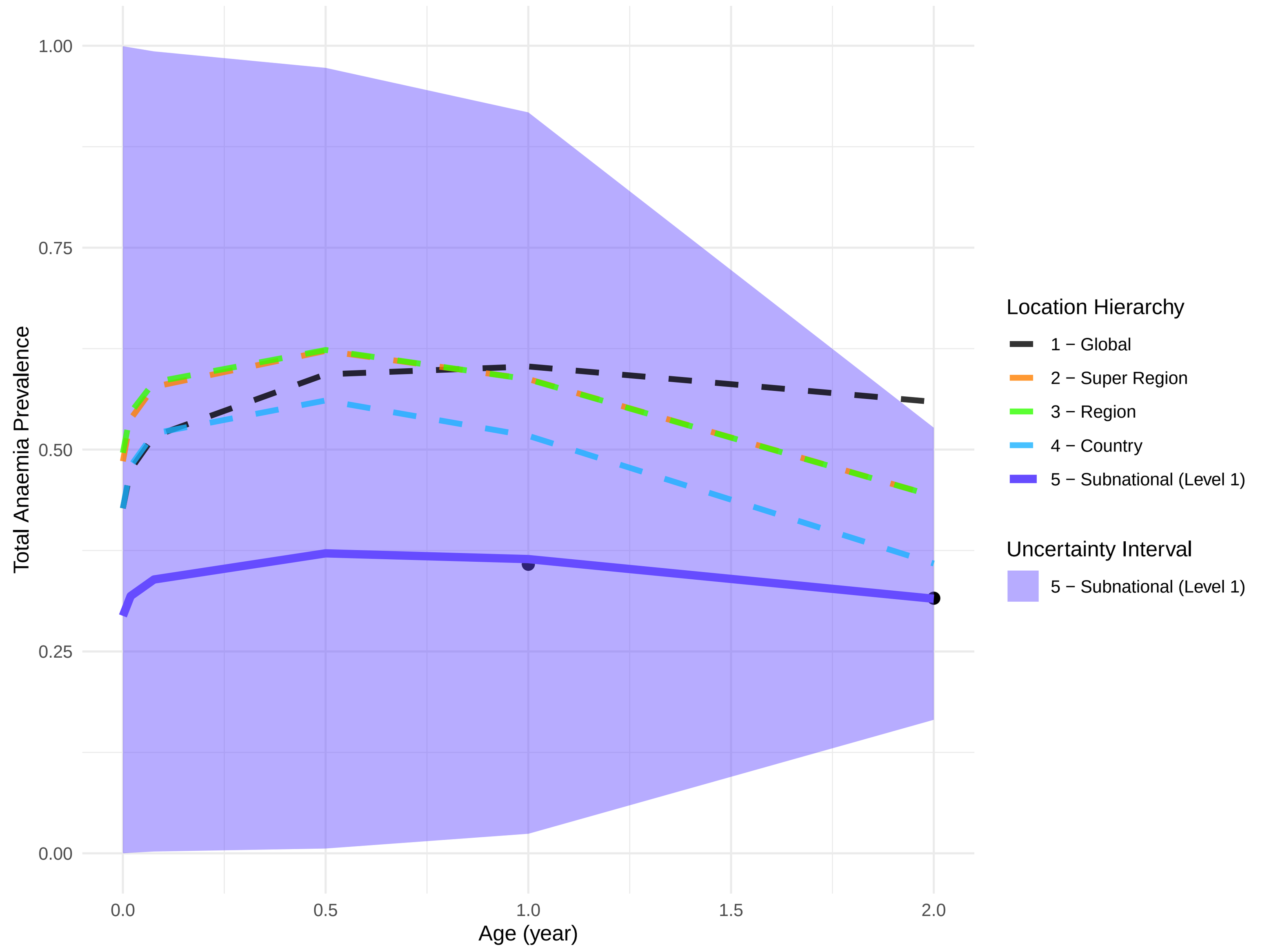

MR−BRT Age/Sex Fits − Non−pregnant Females <2 Years Old − Total Anaemia Prevalence − West Java

WHO−2024 Adjustment Method

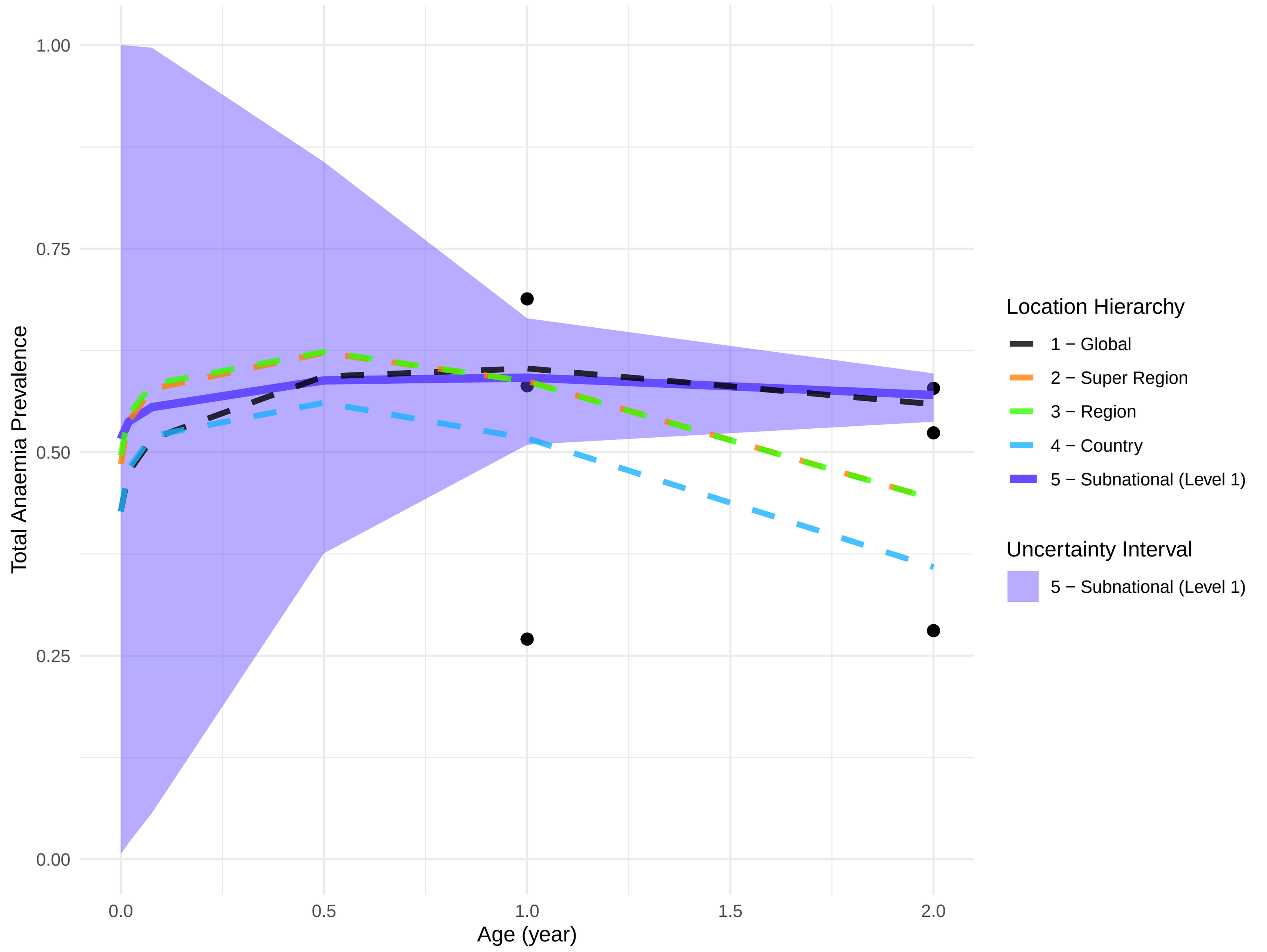

MR−BRT Age/Sex Fits − Non−pregnant Females <2 Years Old − Total Anaemia Prevalence − Central Java

WHO−2024 Adjustment Method

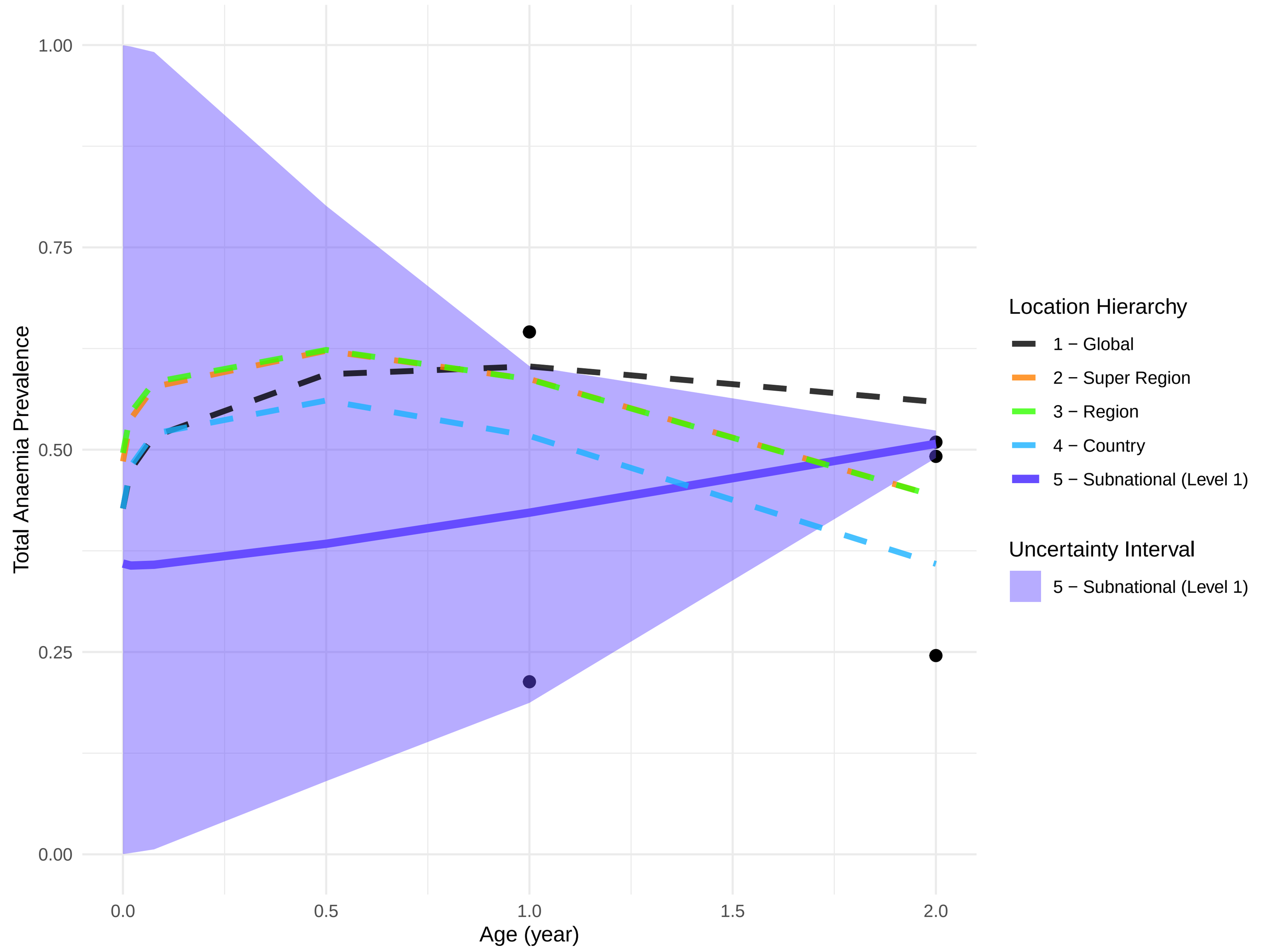

MR−BRT Age/Sex Fits − Non−pregnant Females <2 Years Old − Total Anaemia Prevalence − East Java

WHO−2024 Adjustment Method

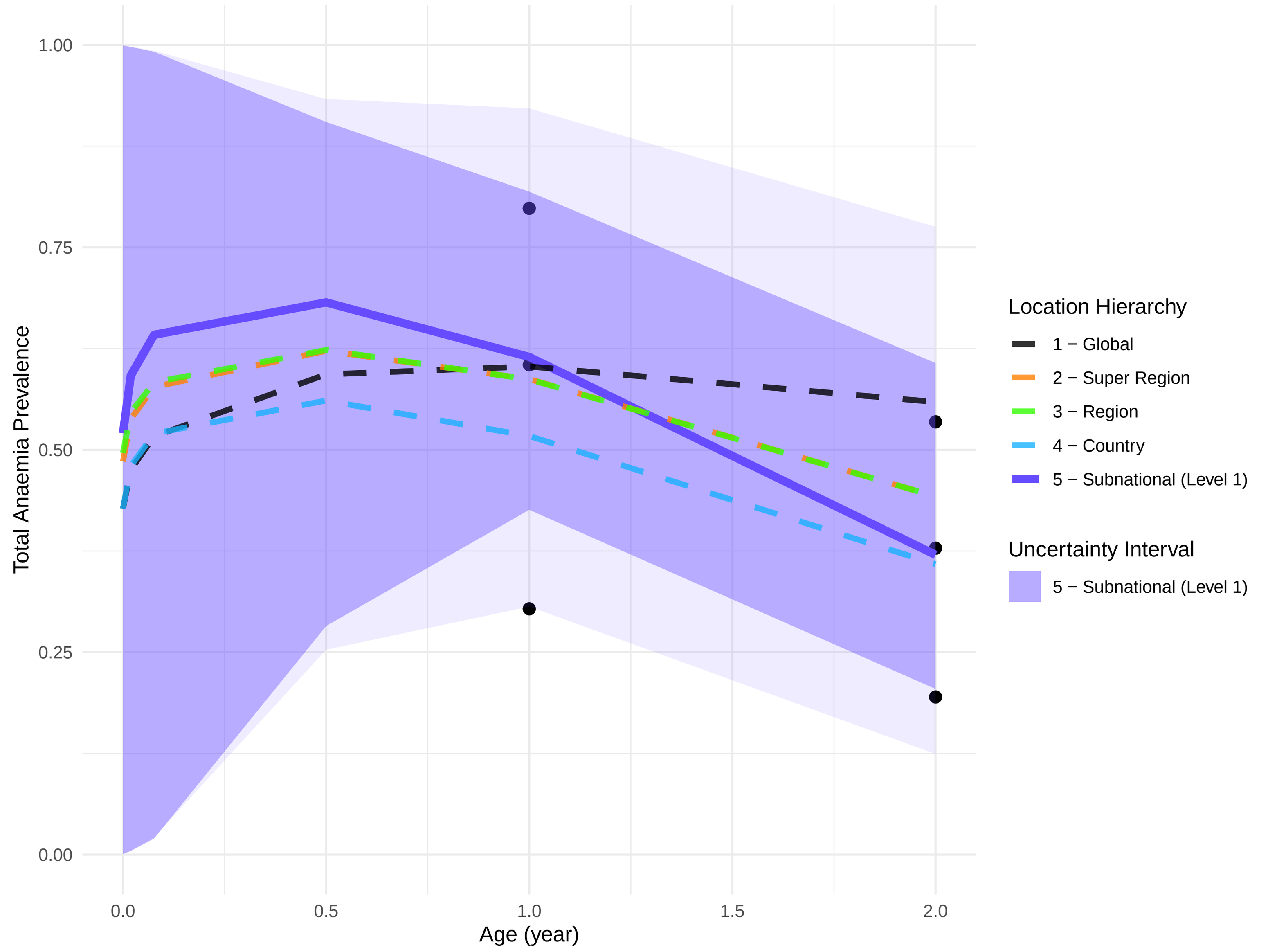

MR−BRT Age/Sex Fits − Non−pregnant Females <2 Years Old − Total Anaemia Prevalence − South Kalimantan

WHO−2024 Adjustment Method

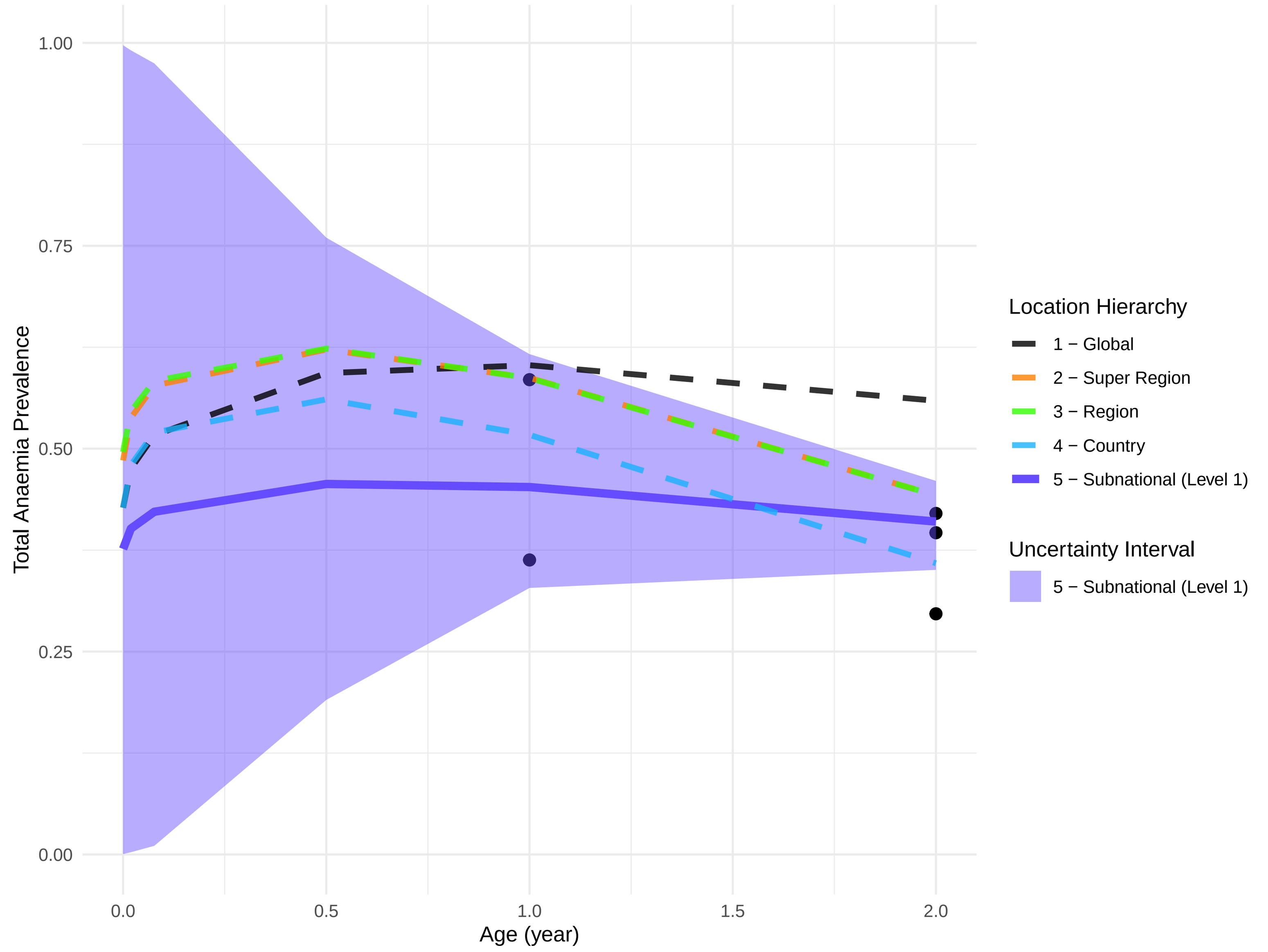

MR−BRT Age/Sex Fits − Non−pregnant Females <2 Years Old − Total Anaemia Prevalence − Lampung

WHO−2024 Adjustment Method

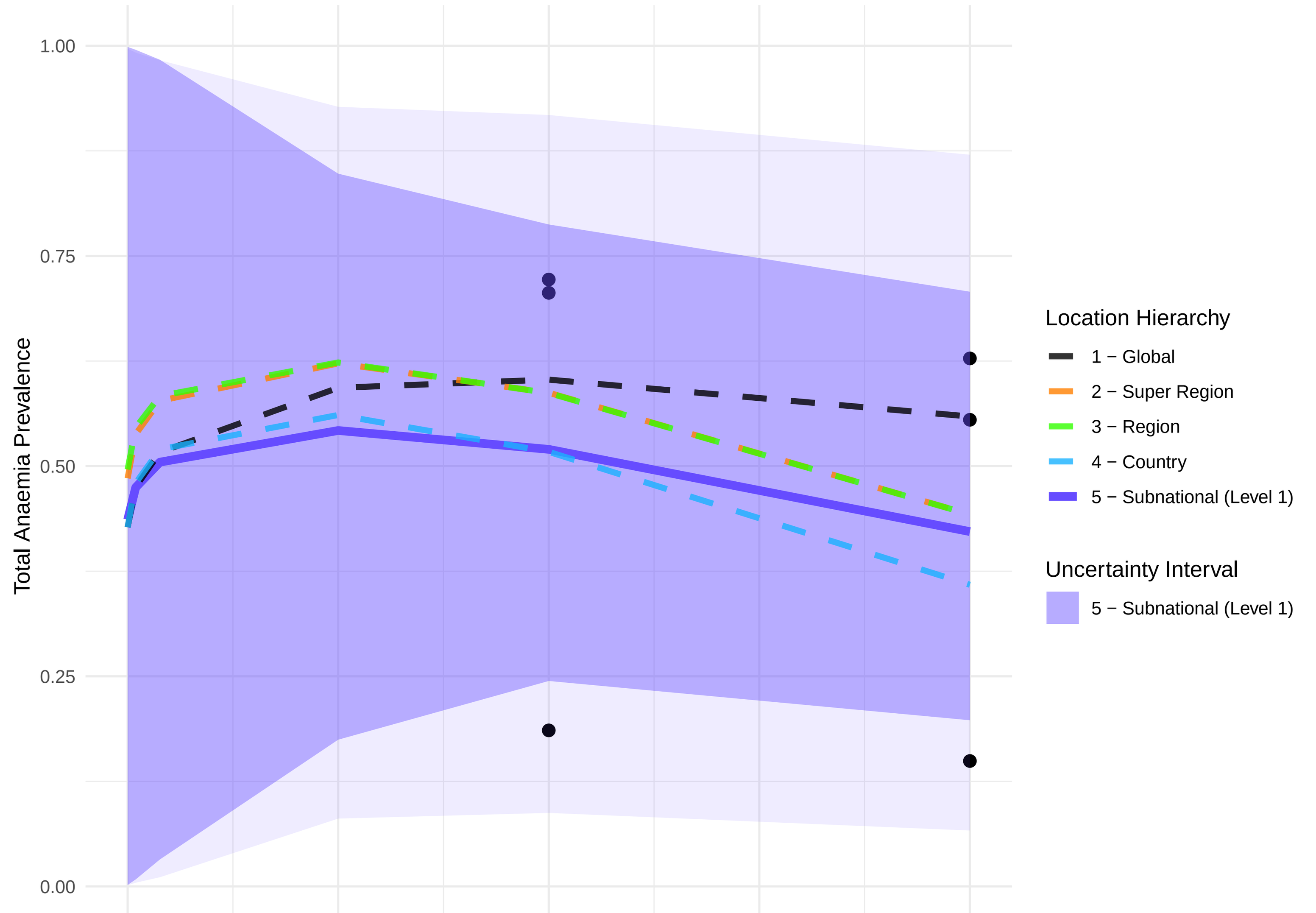

MR−BRT Age/Sex Fits − Non−pregnant Females <2 Years Old − Total Anaemia Prevalence − West Nusa Tenggara
WHO−2024 Adjustment Method

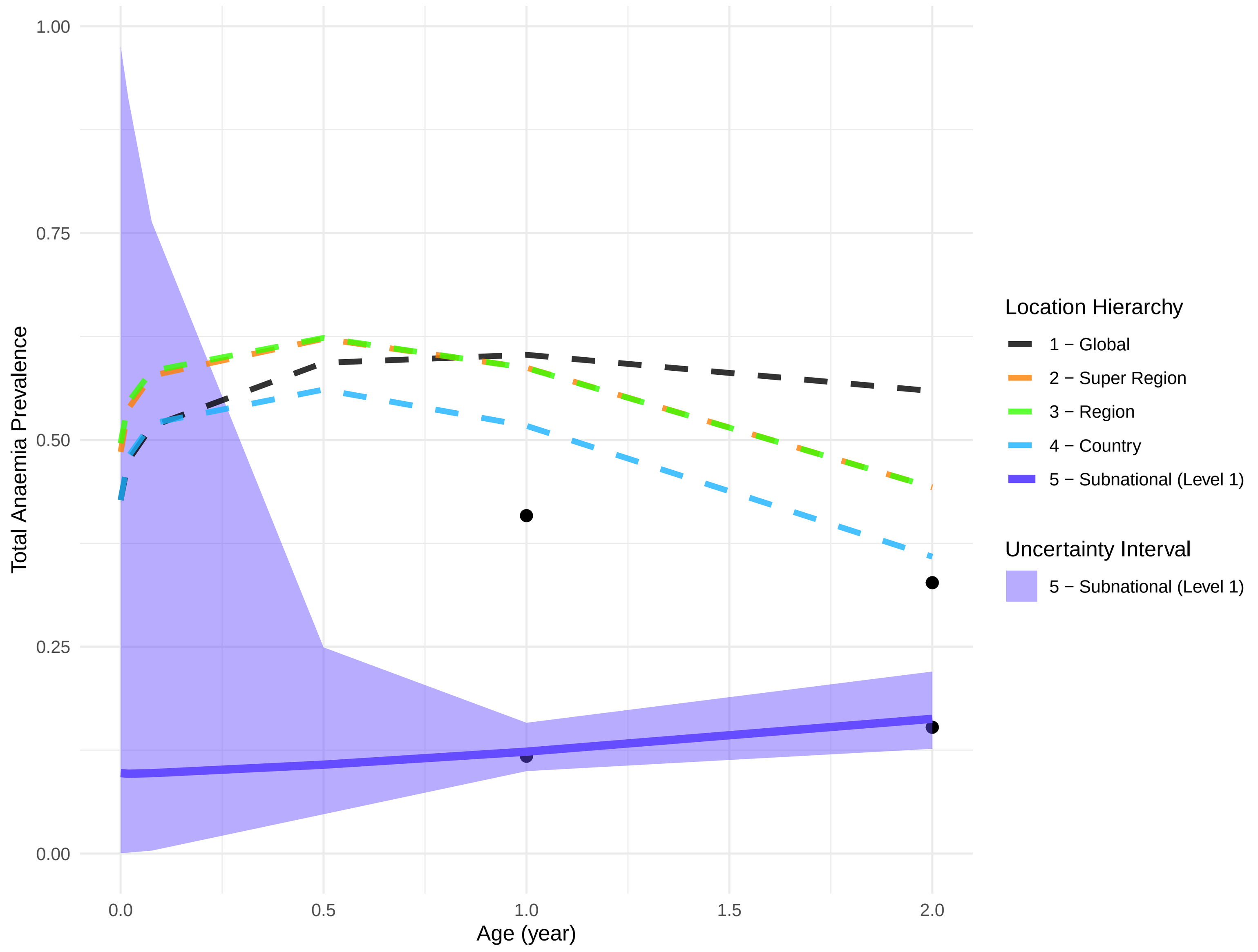

MR−BRT Age/Sex Fits − Non−pregnant Females <2 Years Old − Total Anaemia Prevalence − Riau
WHO−2024 Adjustment Method

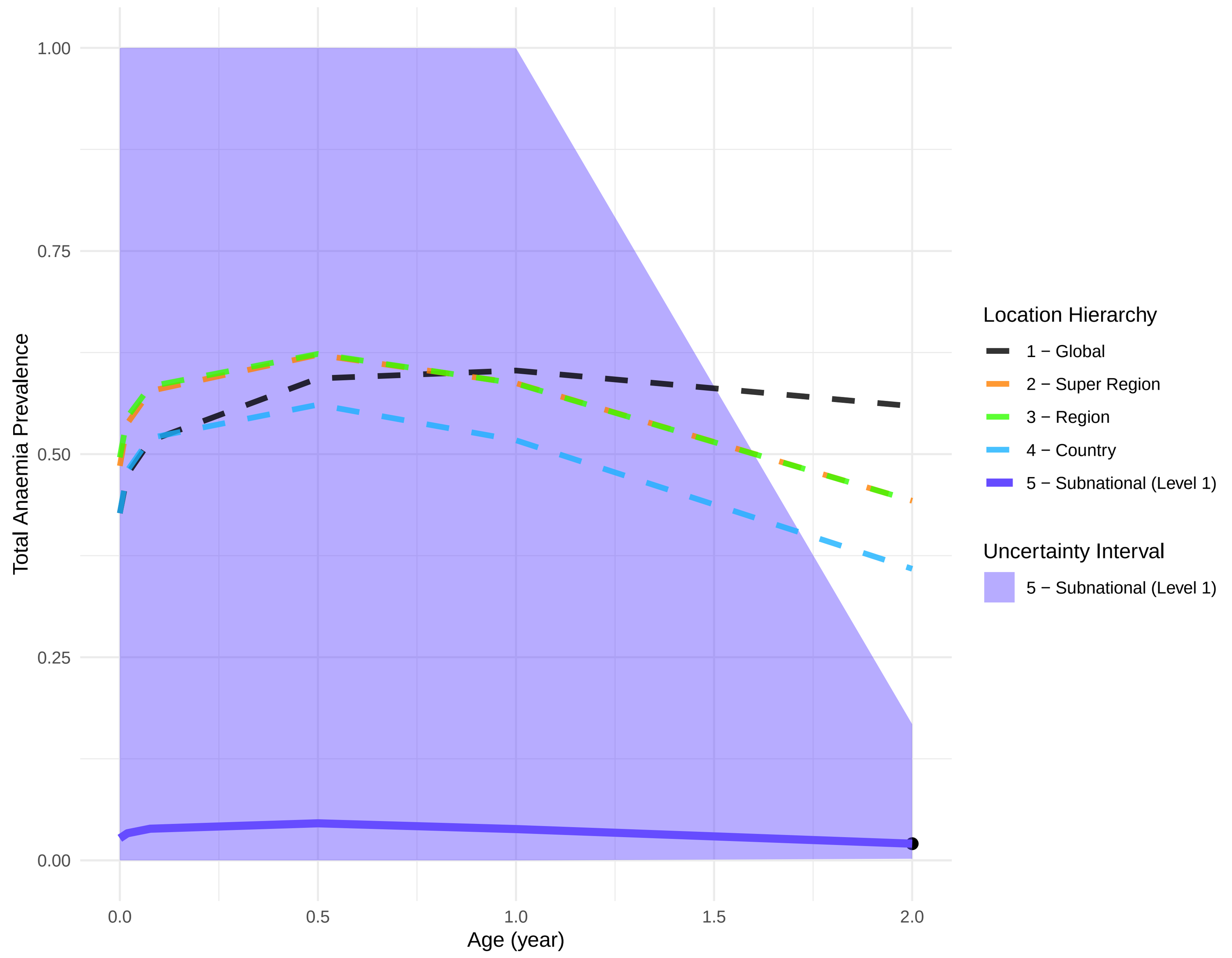

MR−BRT Age/Sex Fits − Non−pregnant Females <2 Years Old − Total Anaemia Prevalence − West Sulawesi
WHO−2024 Adjustment Method

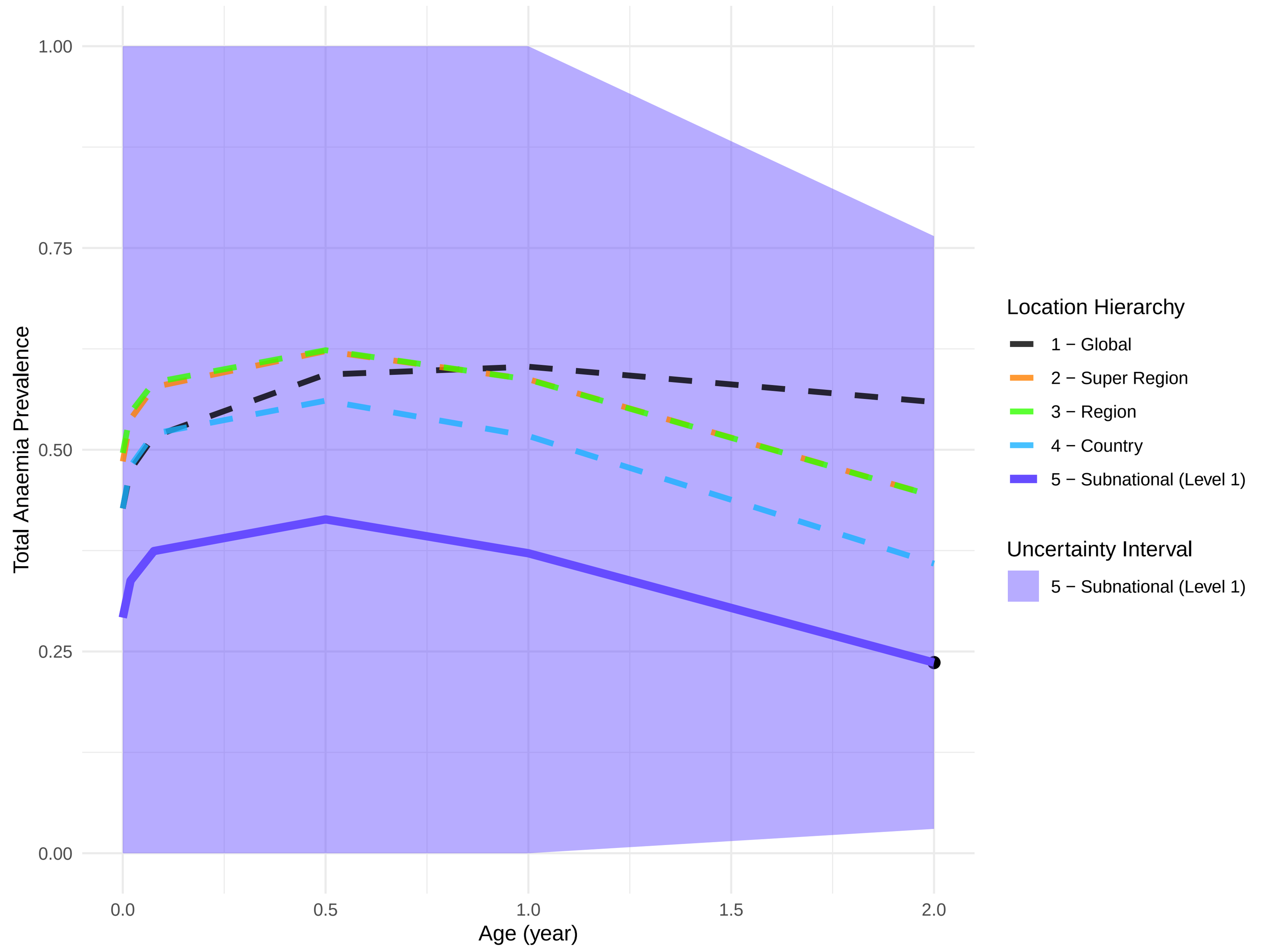

MR−BRT Age/Sex Fits − Non−pregnant Females <2 Years Old − Total Anaemia Prevalence − South Sulawesi
WHO−2024 Adjustment Method

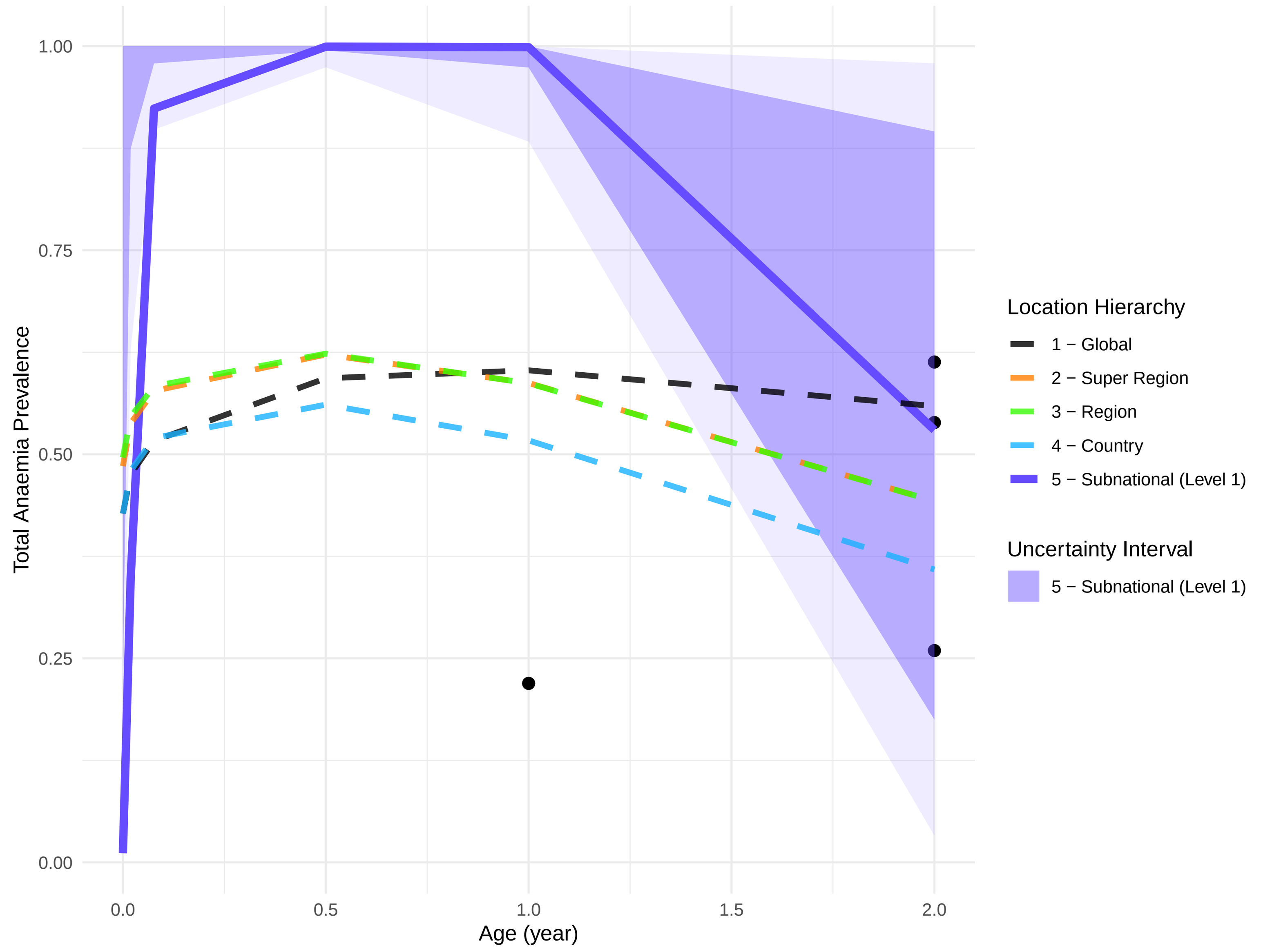

MR−BRT Age/Sex Fits − Non−pregnant Females <2 Years Old − Total Anaemia Prevalence − West Sumatra
WHO−2024 Adjustment Method

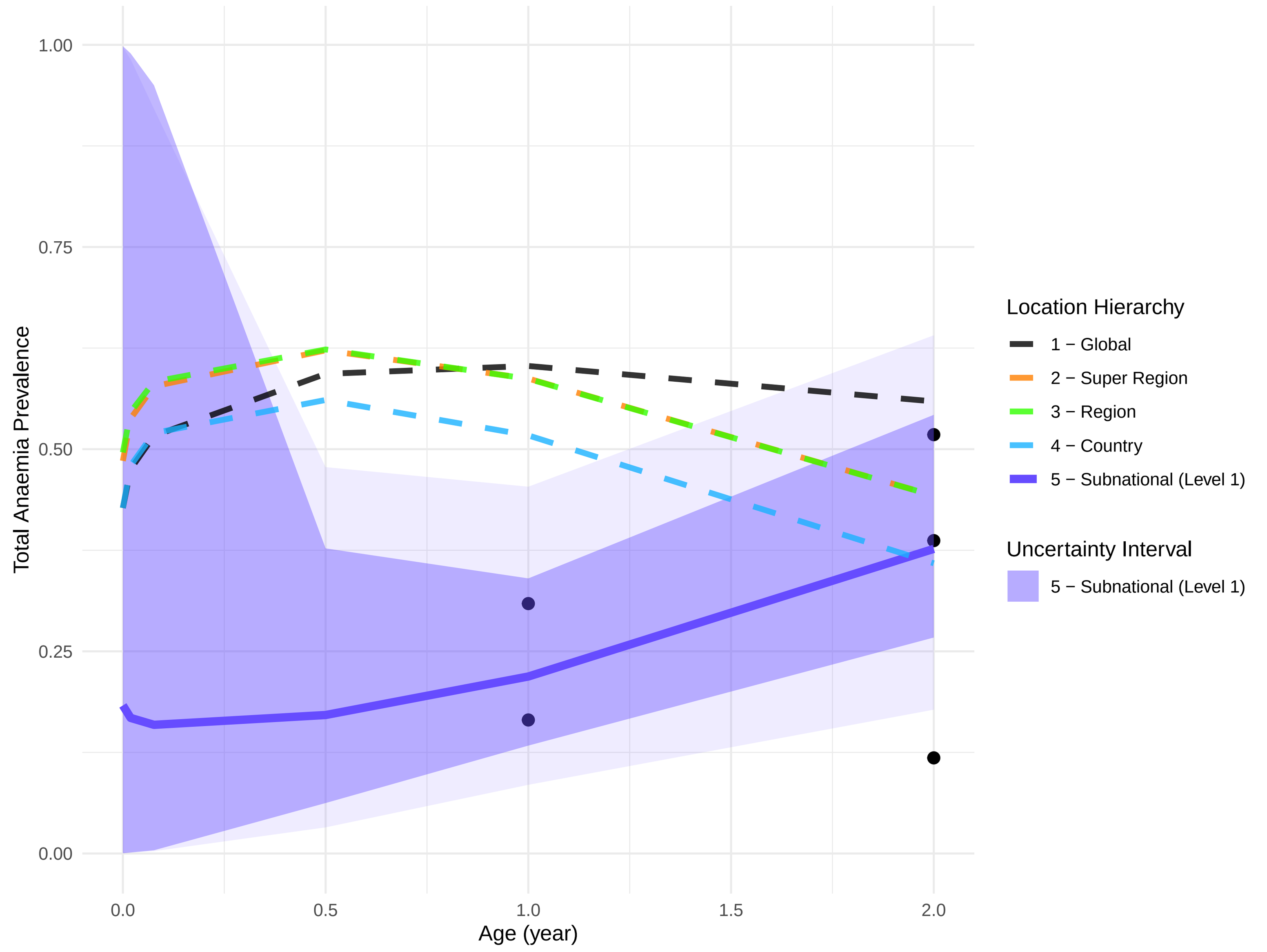

MR−BRT Age/Sex Fits − Non−pregnant Females <2 Years Old − Total Anaemia Prevalence − South Sumatra
WHO−2024 Adjustment Method

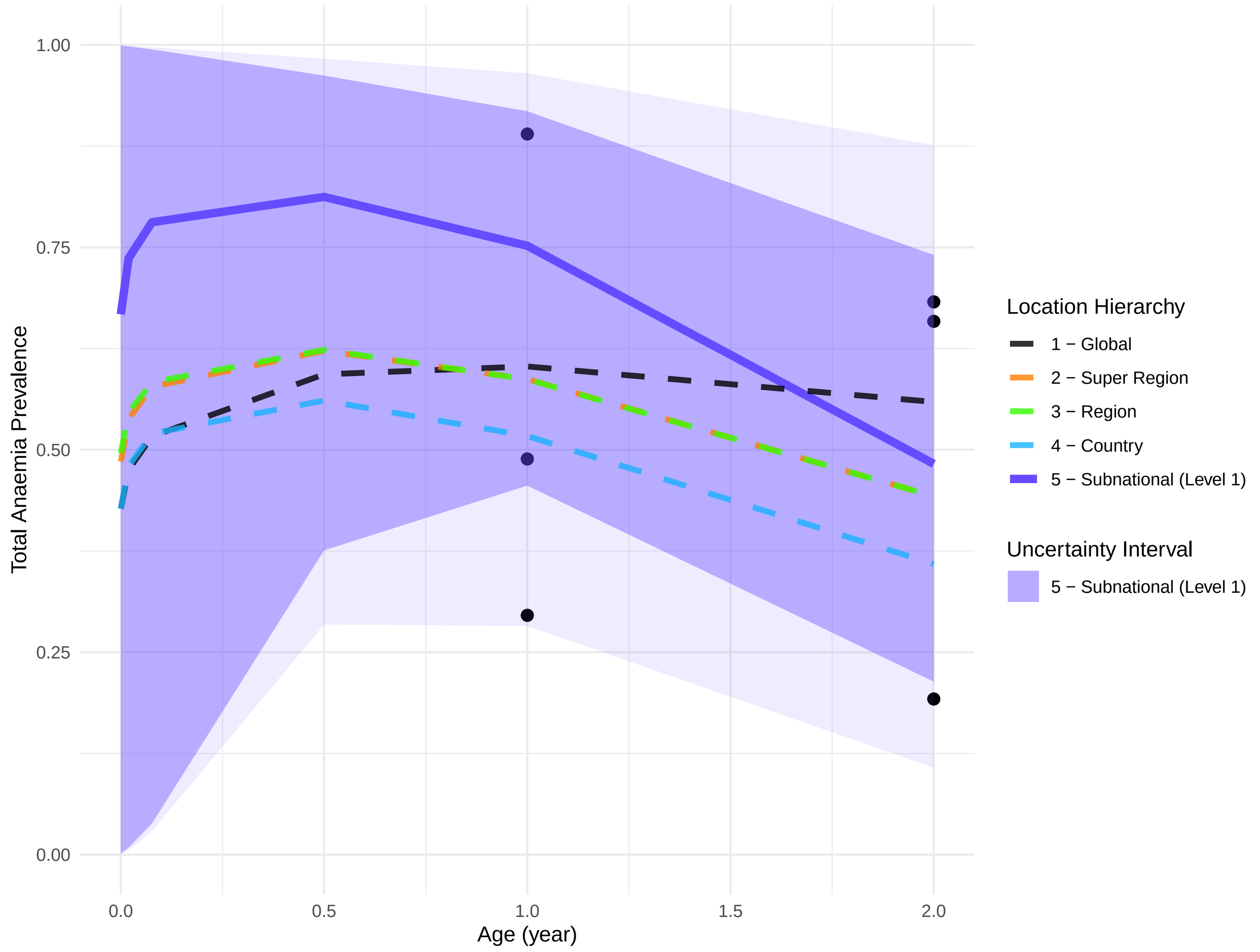

MR−BRT Age/Sex Fits − Non−pregnant Females <2 Years Old − Total Anaemia Prevalence − North Sumatra
WHO−2024 Adjustment Method

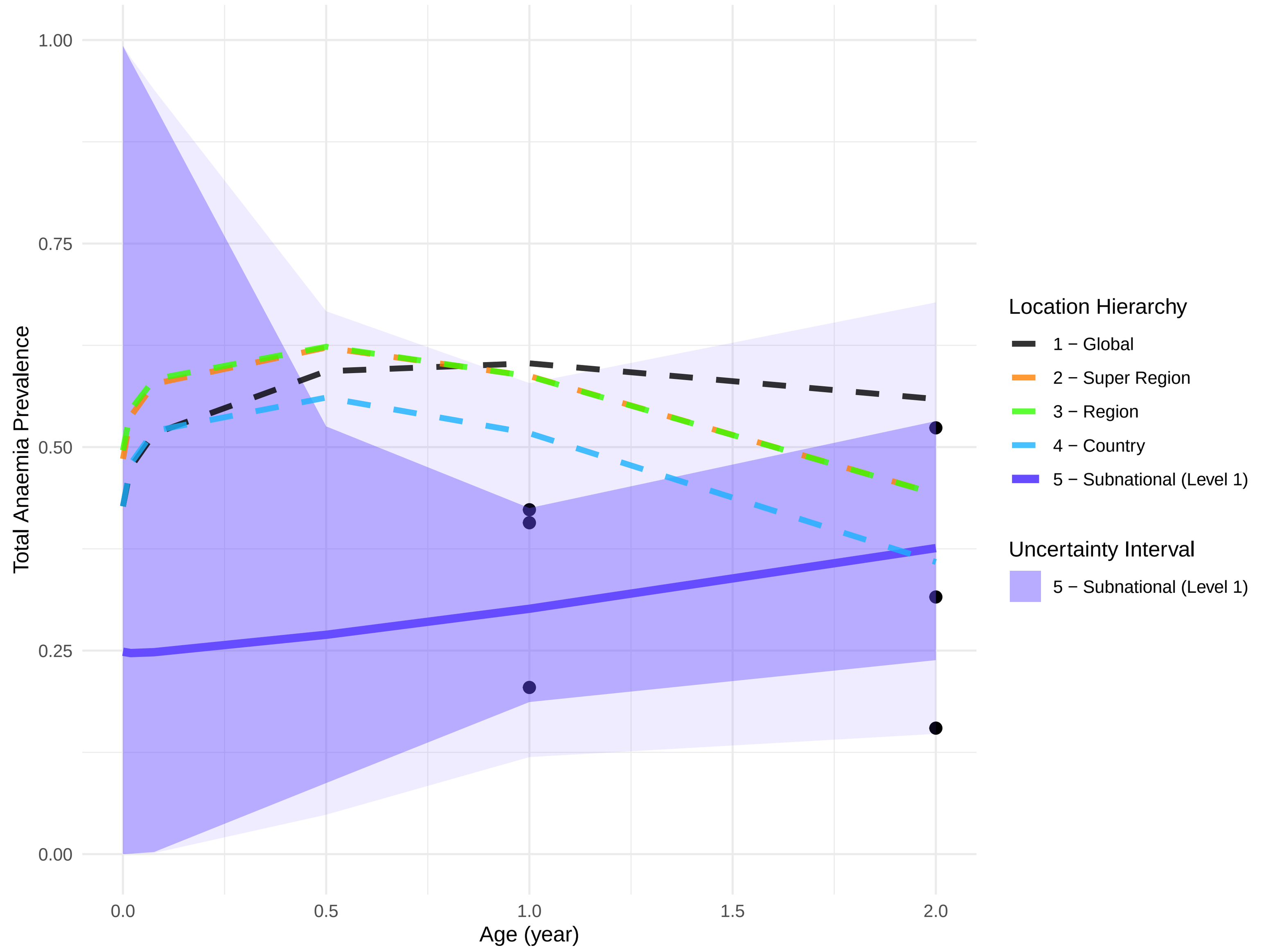

MR−BRT Age/Sex Fits − Non−pregnant Females <2 Years Old − Total Anaemia Prevalence − Yogyakarta
WHO−2024 Adjustment Method

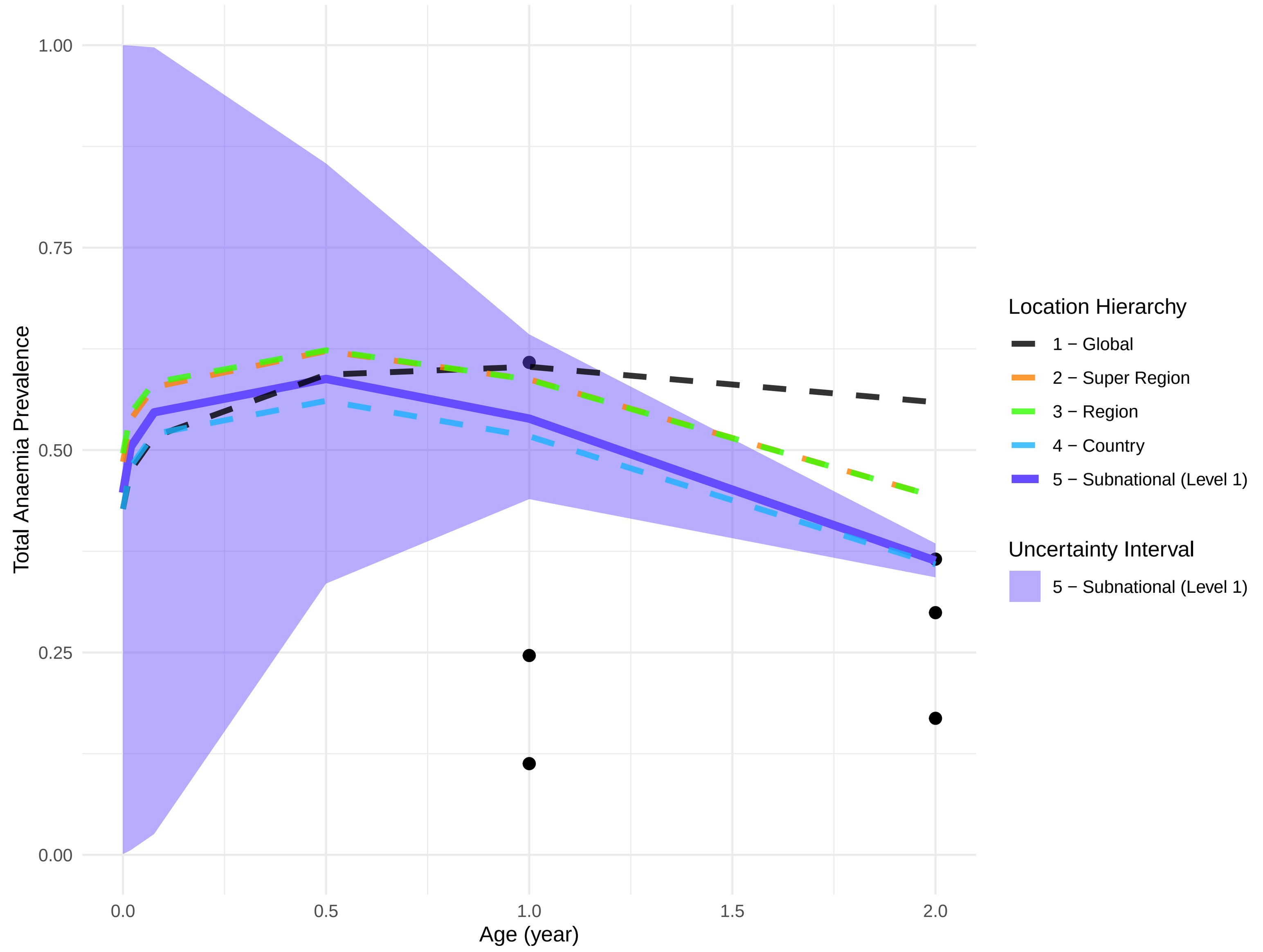

MR−BRT Age/Sex Fits − Non−pregnant Females <2 Years Old − Total Anaemia Prevalence − Myanmar
WHO−2024 Adjustment Method

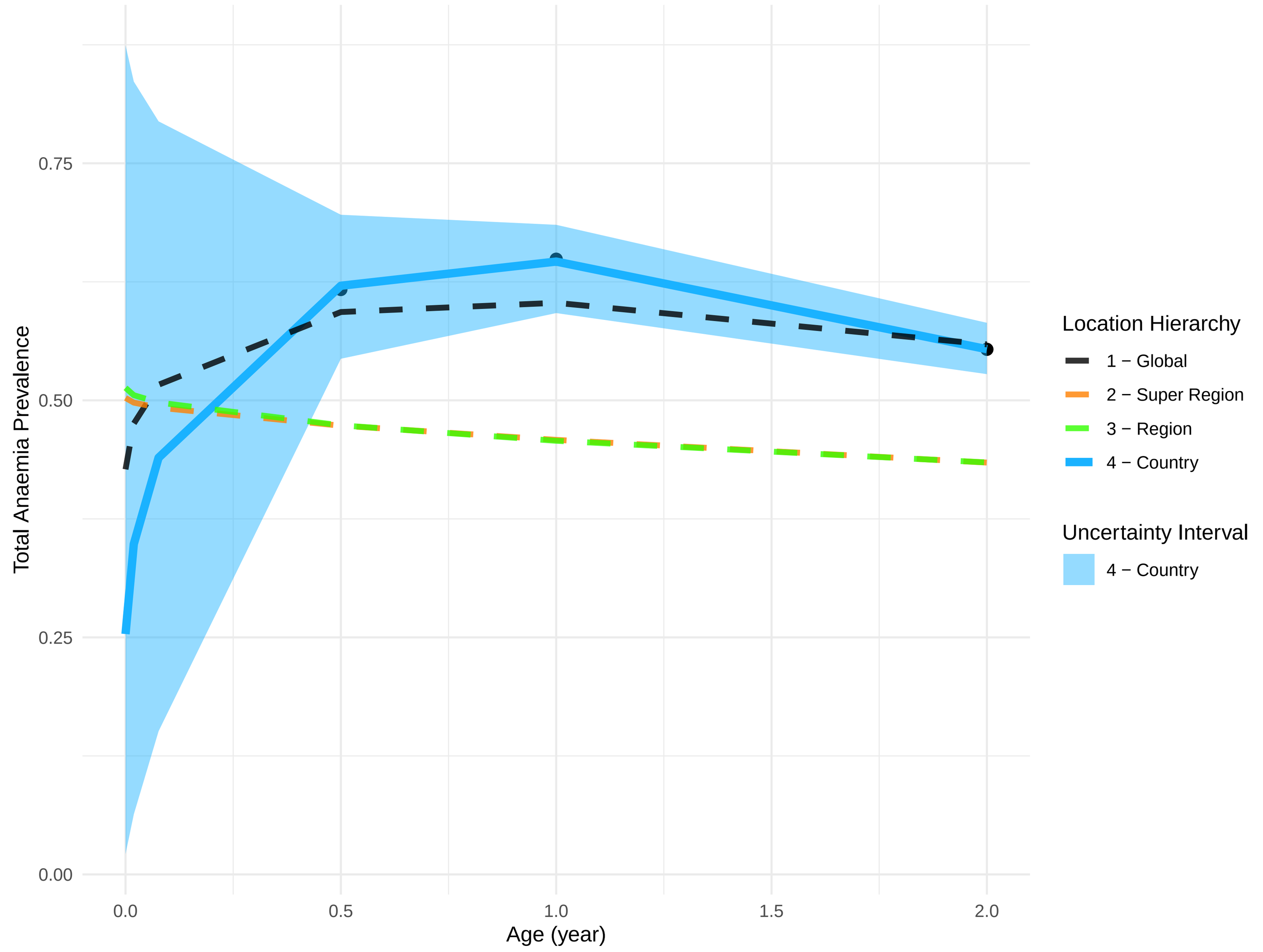

MR−BRT Age/Sex Fits − Non−pregnant Females <2 Years Old − Total Anaemia Prevalence − Timor−Leste
WHO−2024 Adjustment Method

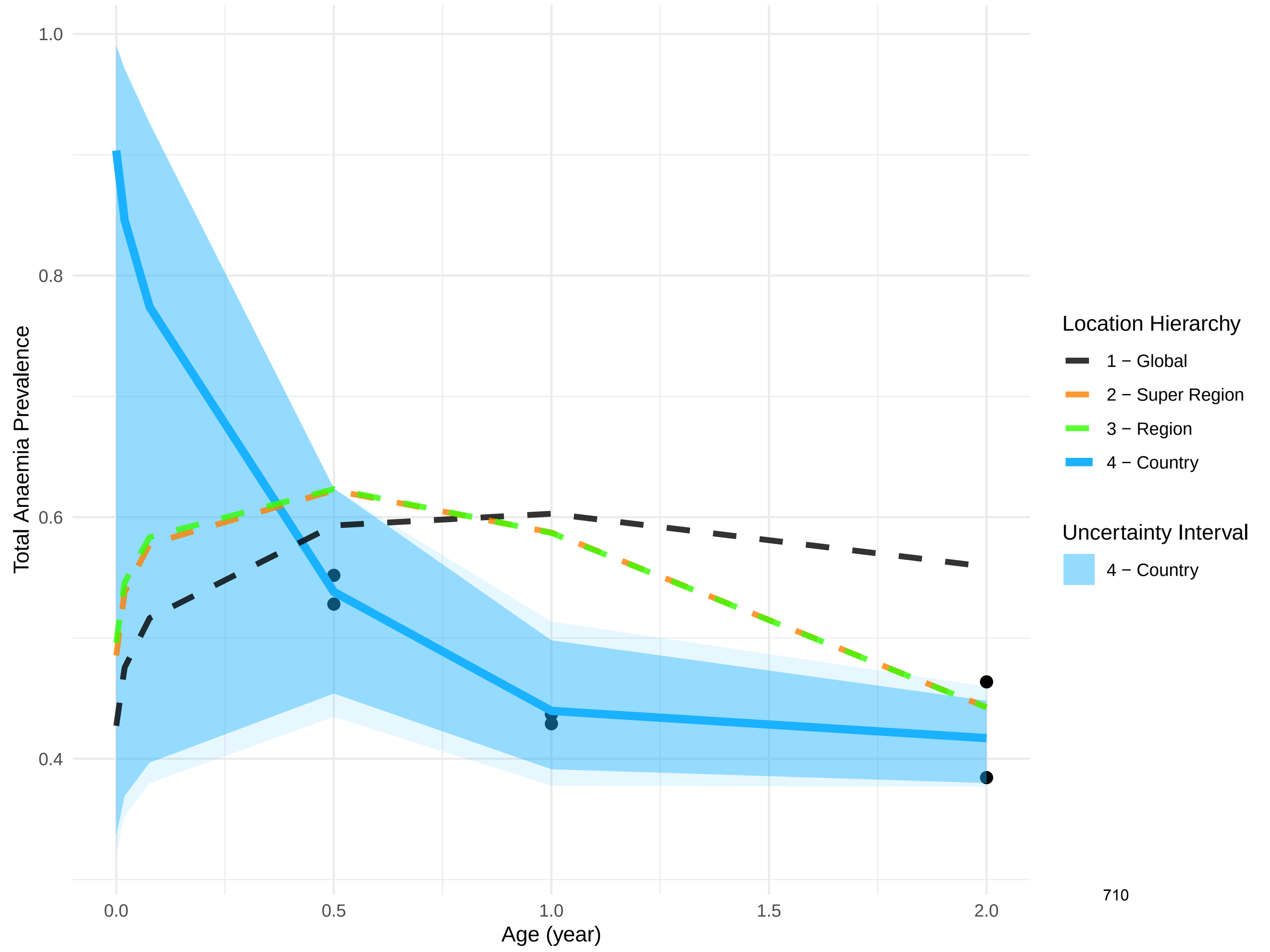

MR−BRT Age/Sex Fits − Non−pregnant Females <2 Years Old − Total Anaemia Prevalence − Sub−Saharan Africa
WHO−2024 Adjustment Method

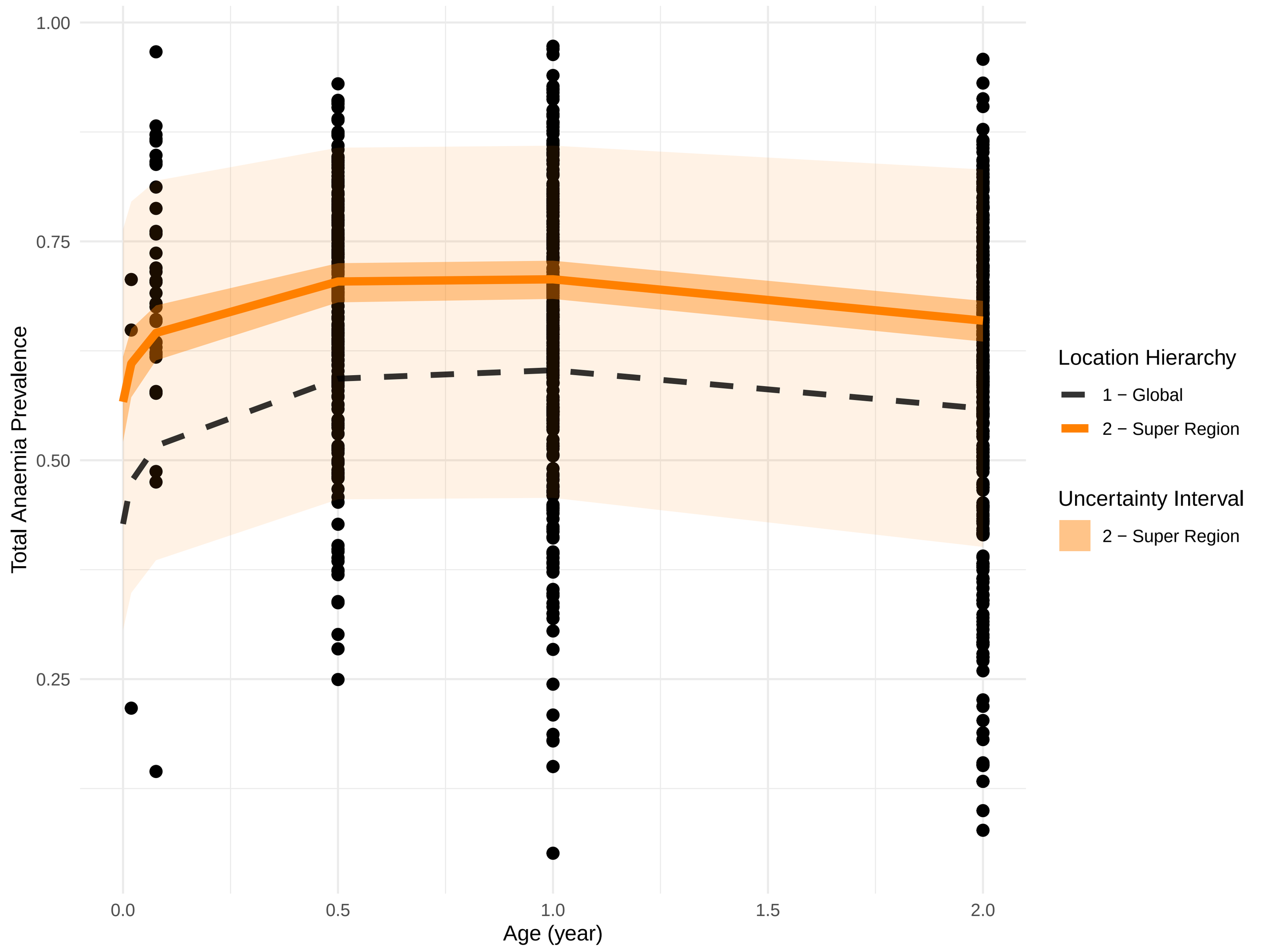

MR−BRT Age/Sex Fits − Non−pregnant Females <2 Years Old − Total Anaemia Prevalence − Central Sub−Saharan Africa
WHO−2024 Adjustment Method

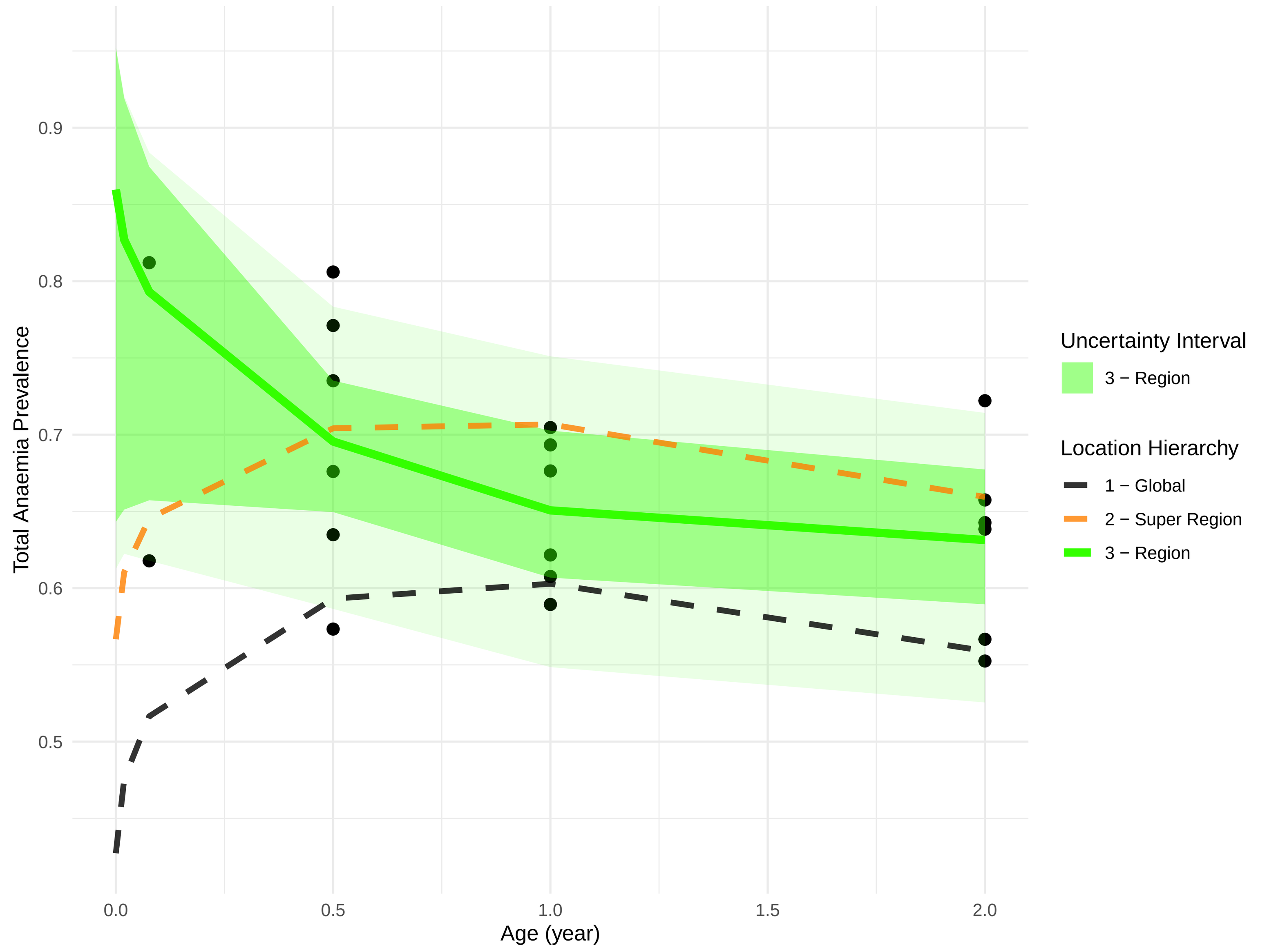

MR−BRT Age/Sex Fits − Non−pregnant Females <2 Years Old − Total Anaemia Prevalence − Angola
WHO−2024 Adjustment Method

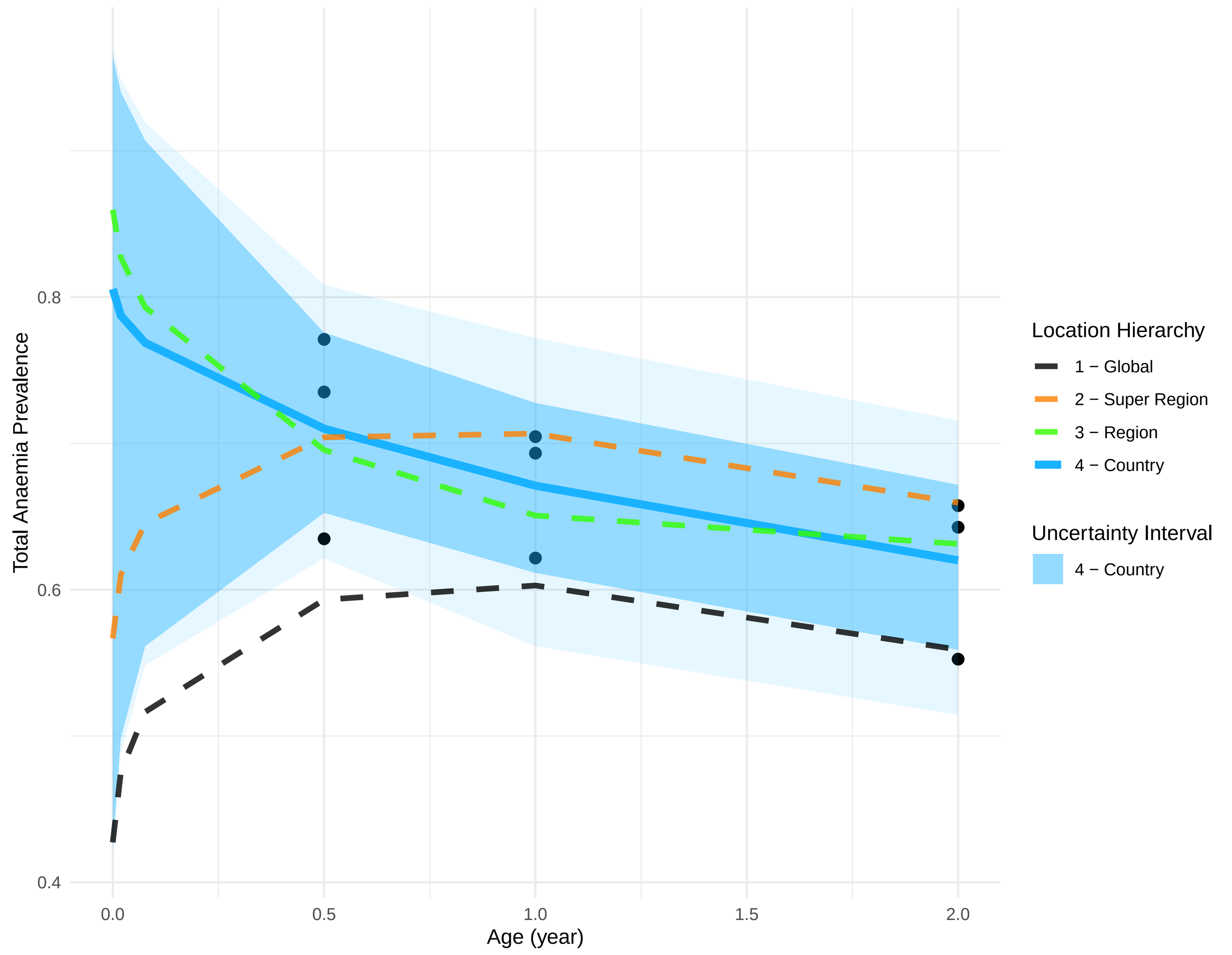

MR−BRT Age/Sex Fits − Non−pregnant Females <2 Years Old − Total Anaemia Prevalence − Democratic Republic of the Congo
WHO−2024 Adjustment Method

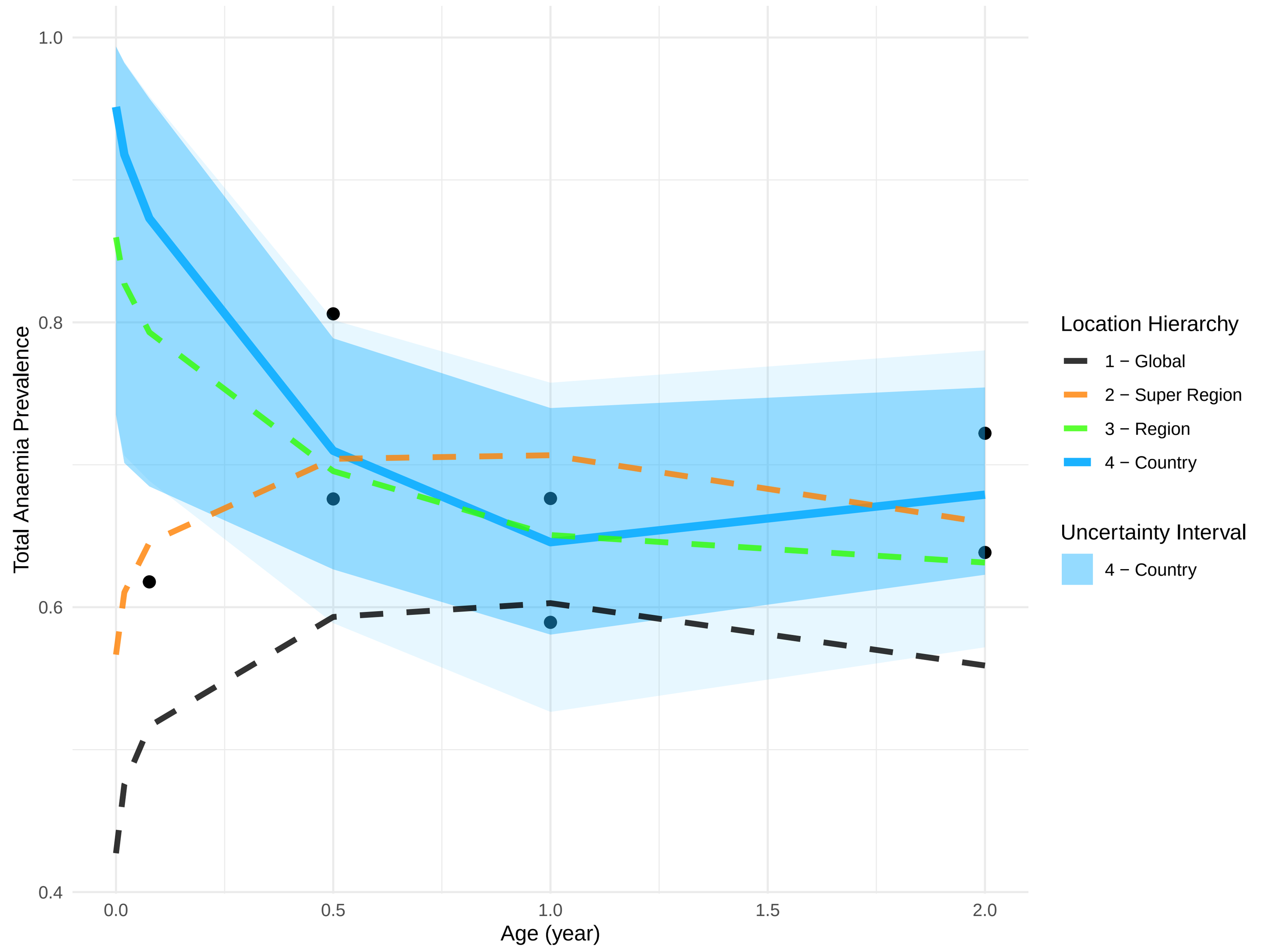

MR−BRT Age/Sex Fits − Non−pregnant Females <2 Years Old − Total Anaemia Prevalence − Gabon
WHO−2024 Adjustment Method

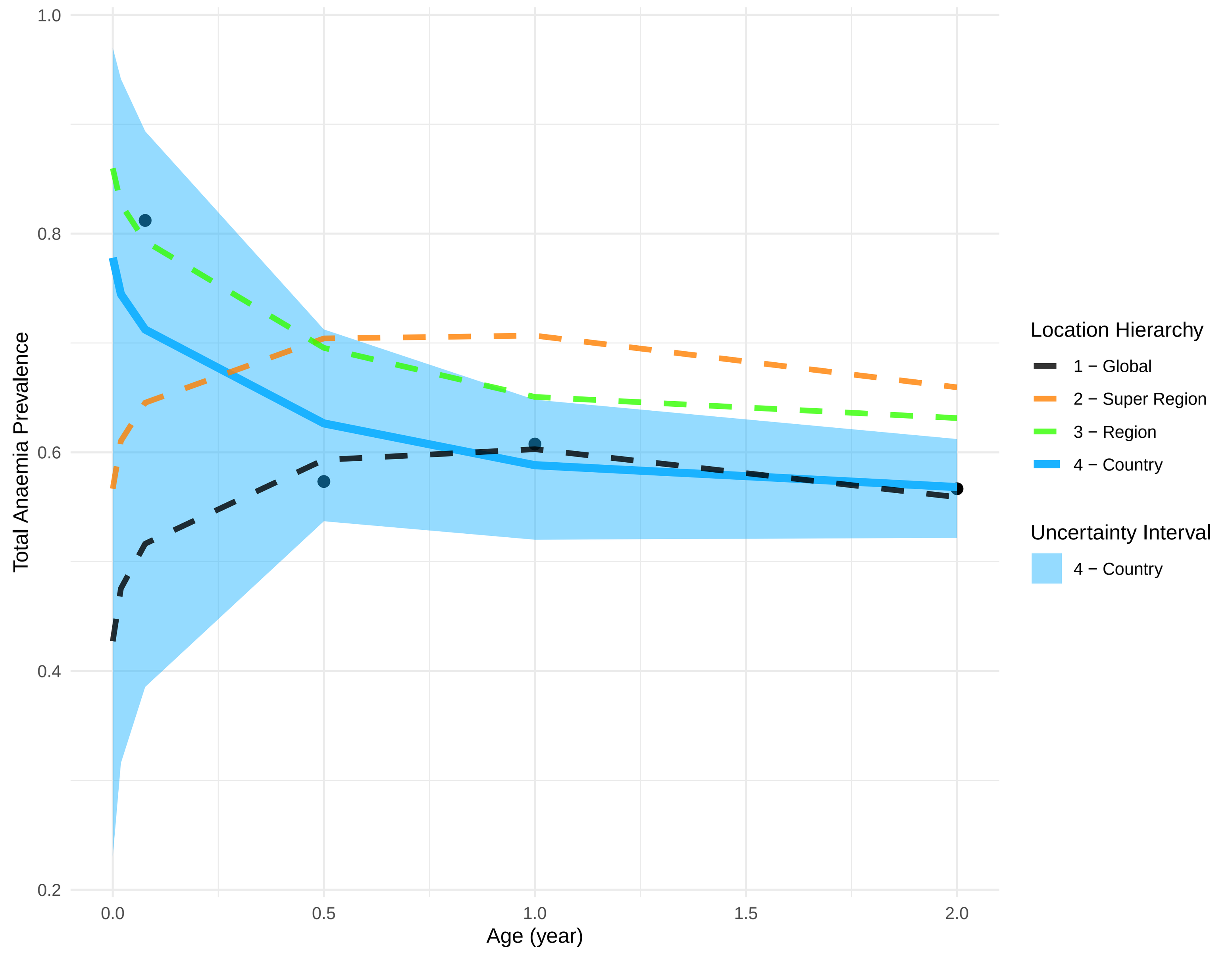

MR−BRT Age/Sex Fits − Non−pregnant Females <2 Years Old − Total Anaemia Prevalence − Eastern Sub−Saharan Africa
WHO−2024 Adjustment Method

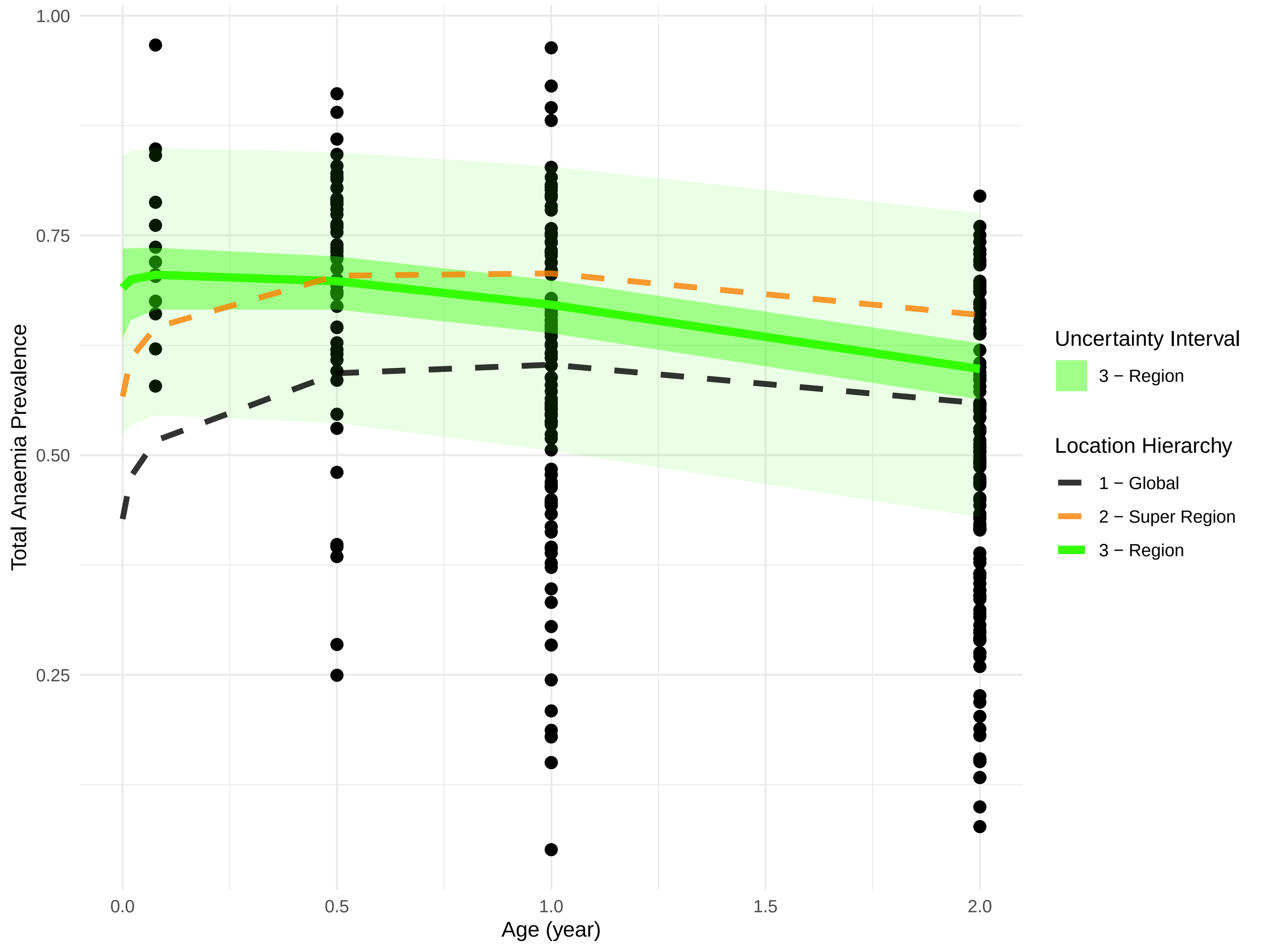

MR−BRT Age/Sex Fits − Non−pregnant Females <2 Years Old − Total Anaemia Prevalence − Burundi
WHO−2024 Adjustment Method

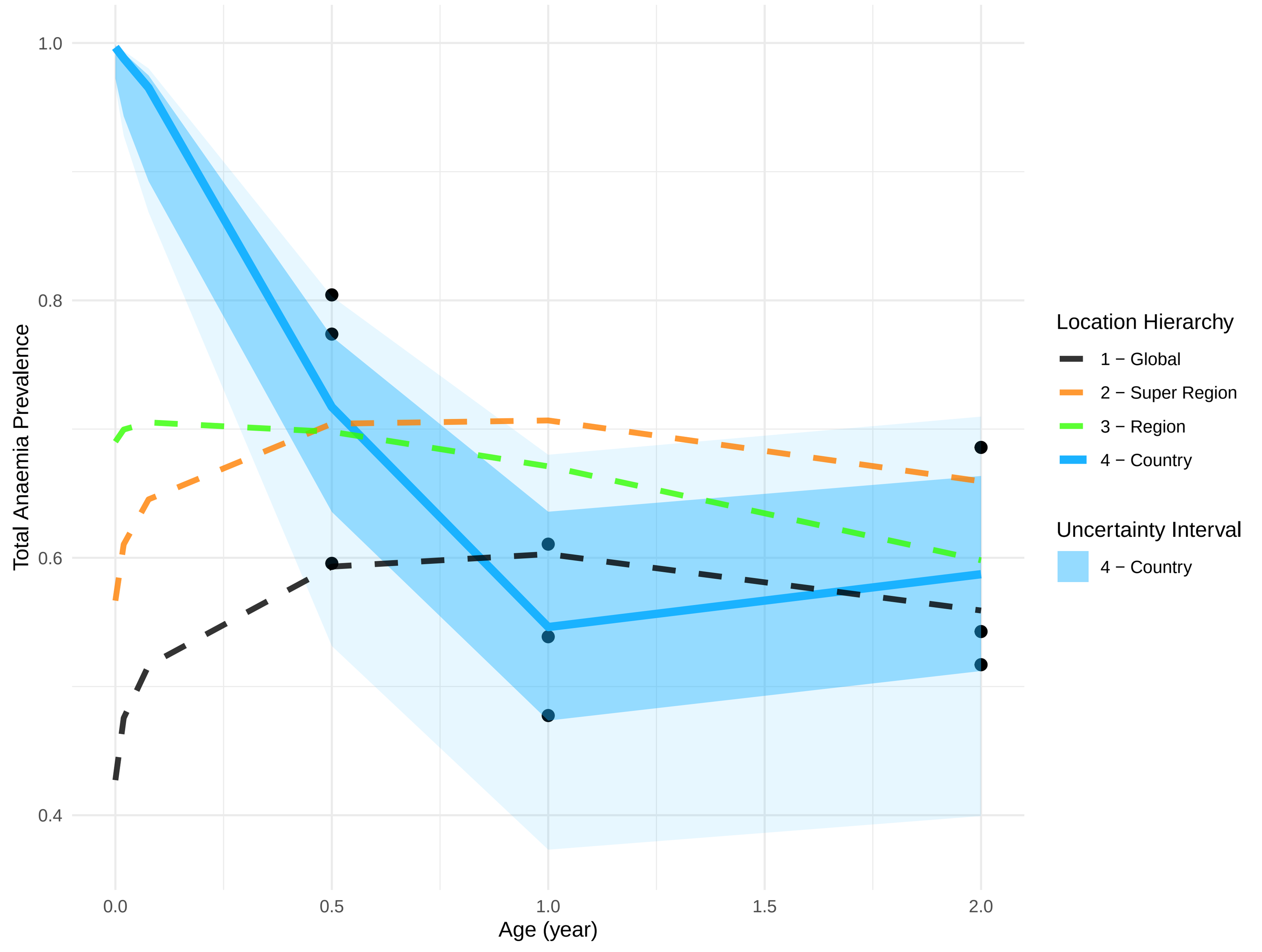

MR−BRT Age/Sex Fits − Non−pregnant Females <2 Years Old − Total Anaemia Prevalence − Ethiopia
WHO−2024 Adjustment Method

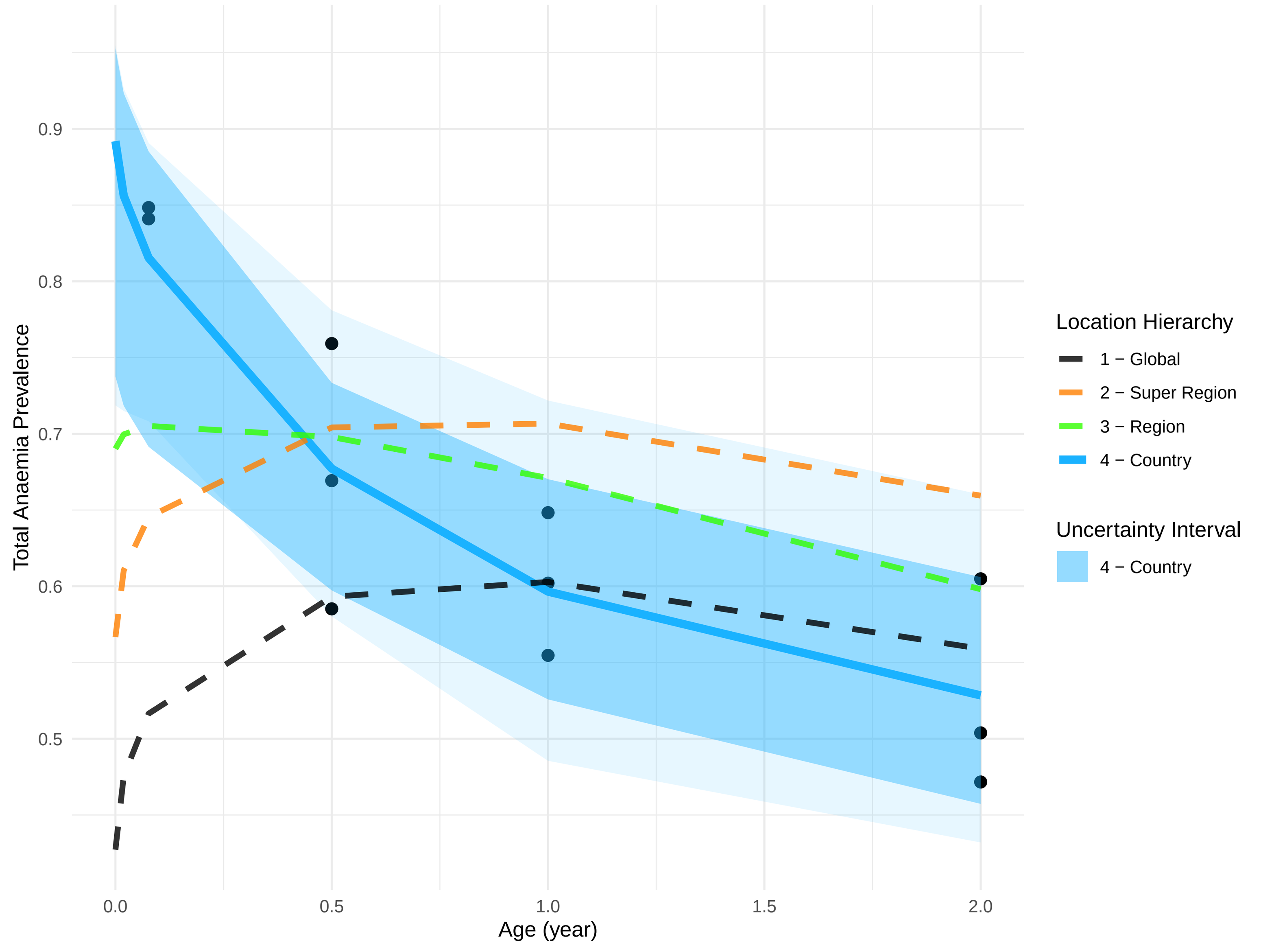

MR−BRT Age/Sex Fits − Non−pregnant Females <2 Years Old − Total Anaemia Prevalence − Kenya
WHO−2024 Adjustment Method

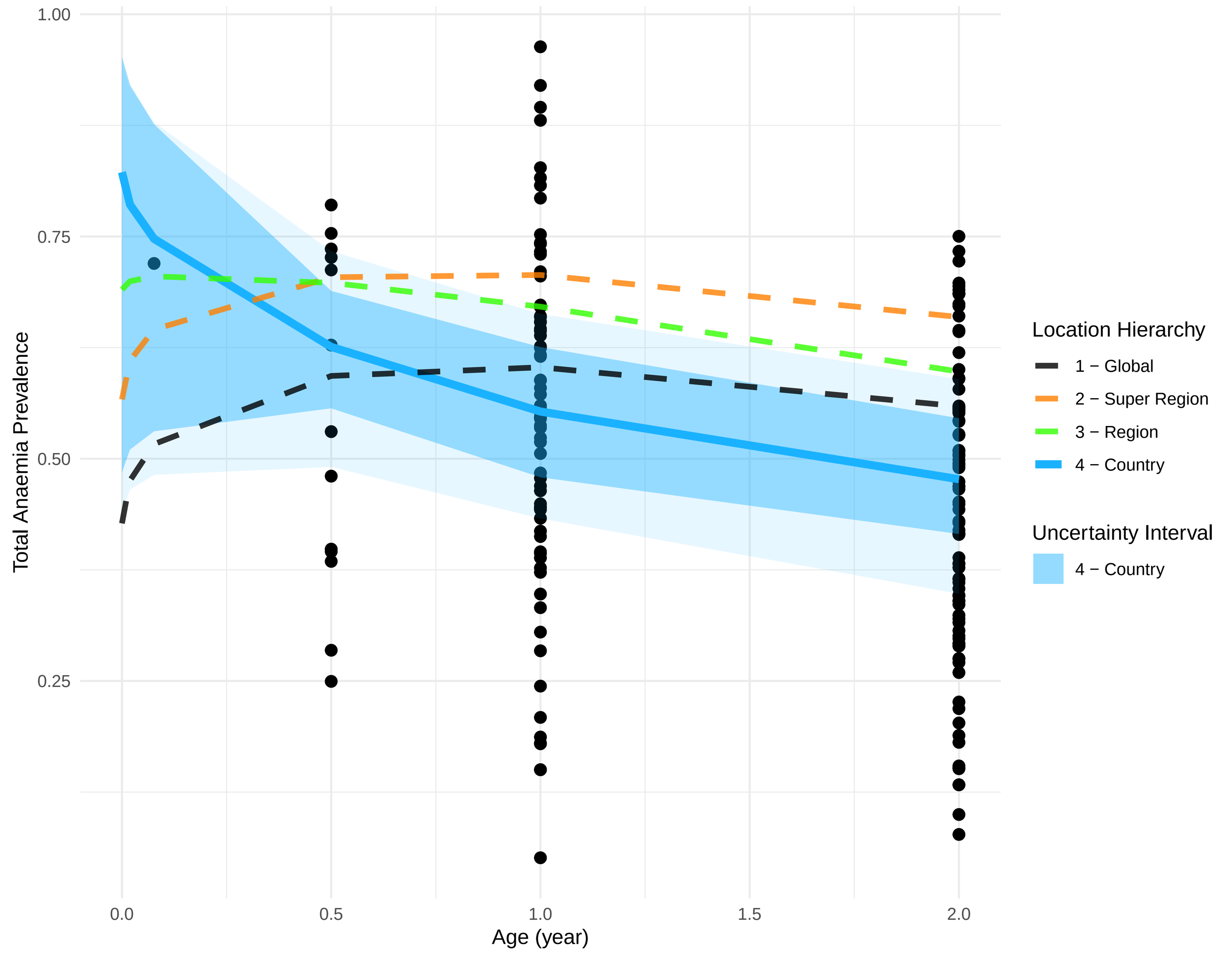

MR−BRT Age/Sex Fits − Non−pregnant Females <2 Years Old − Total Anaemia Prevalence − Baringo
WHO−2024 Adjustment Method

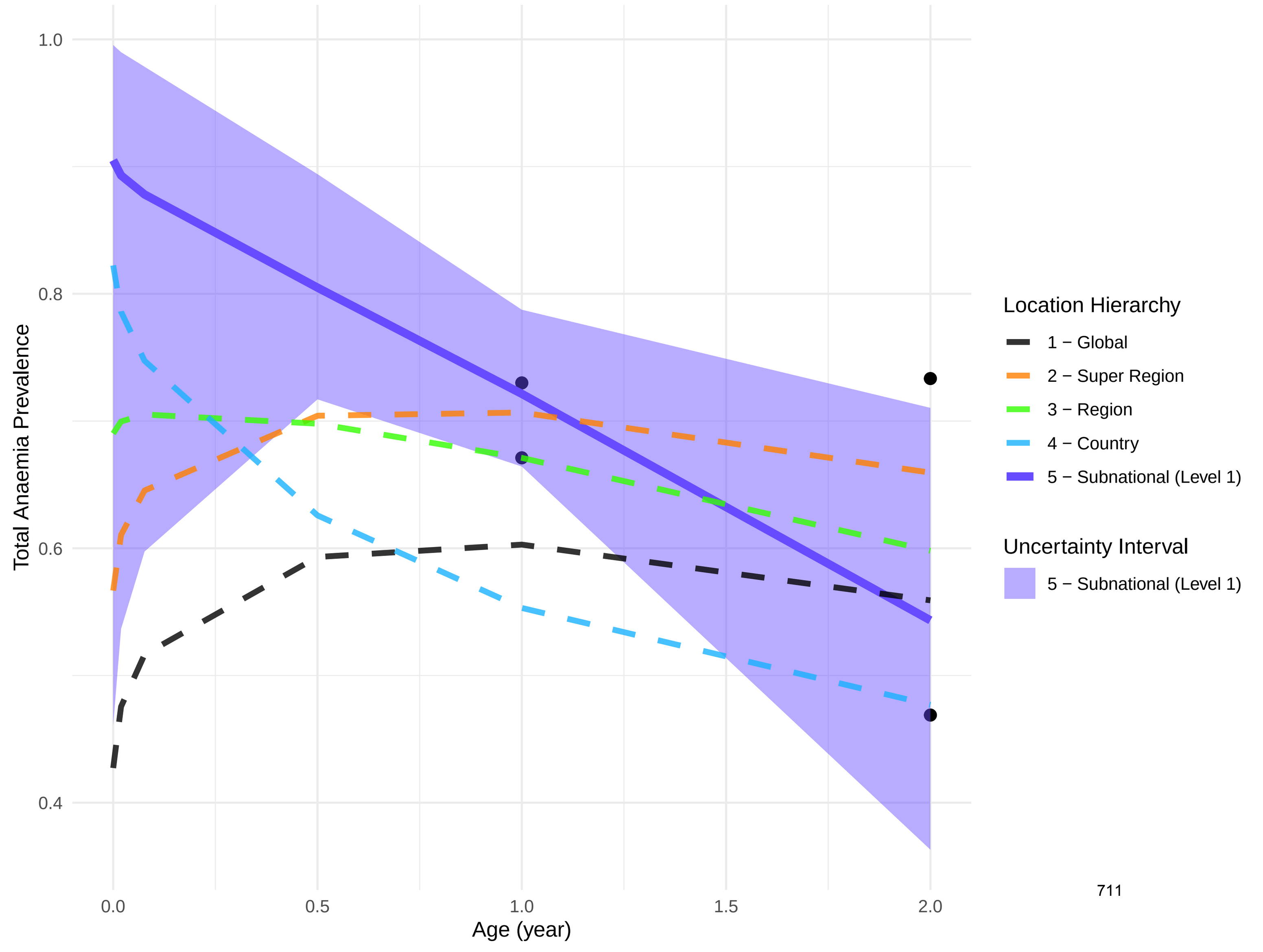

MR−BRT Age/Sex Fits − Non−pregnant Females <2 Years Old − Total Anaemia Prevalence − Bomet
WHO−2024 Adjustment Method

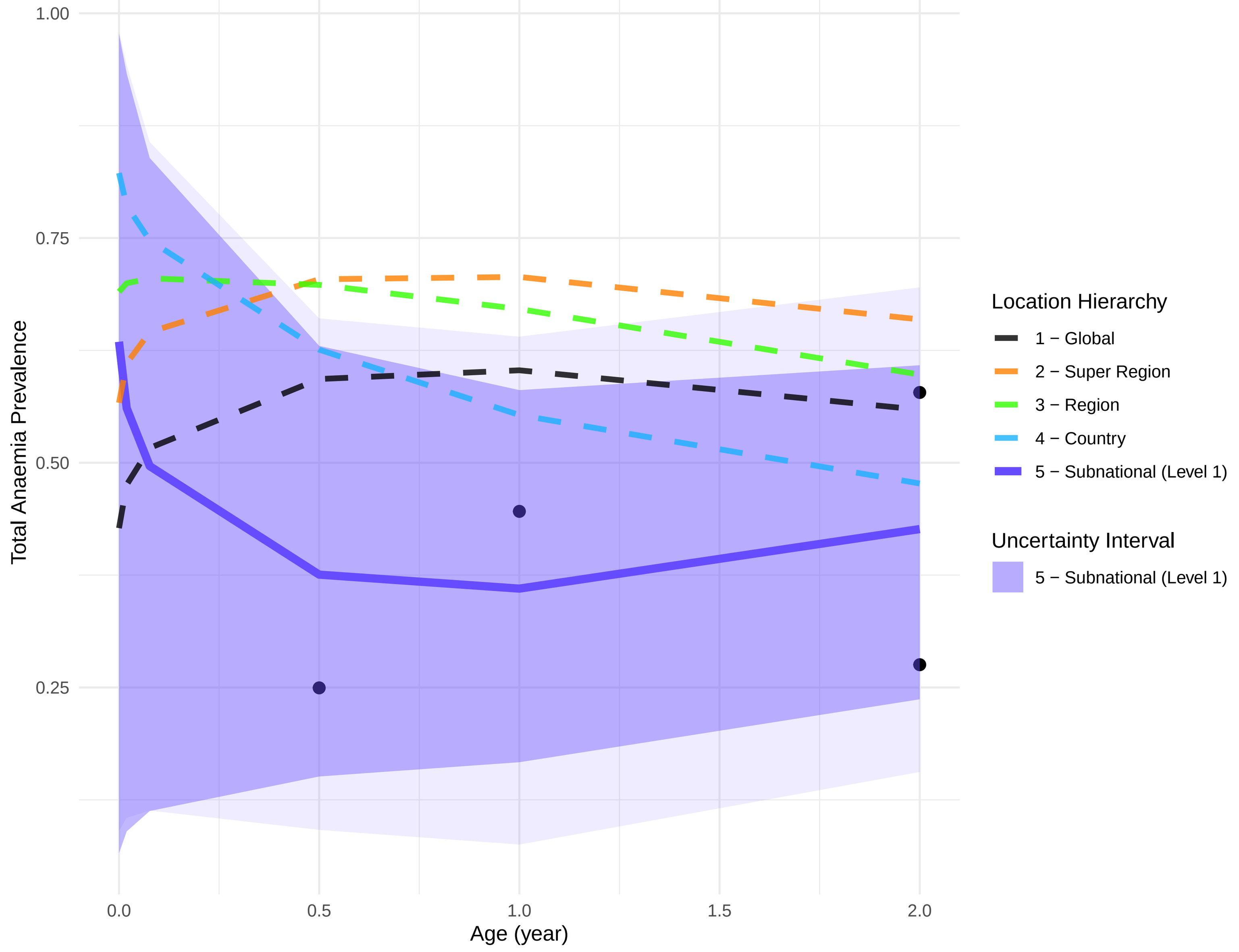

MR−BRT Age/Sex Fits − Non−pregnant Females <2 Years Old − Total Anaemia Prevalence − Bungoma
WHO−2024 Adjustment Method

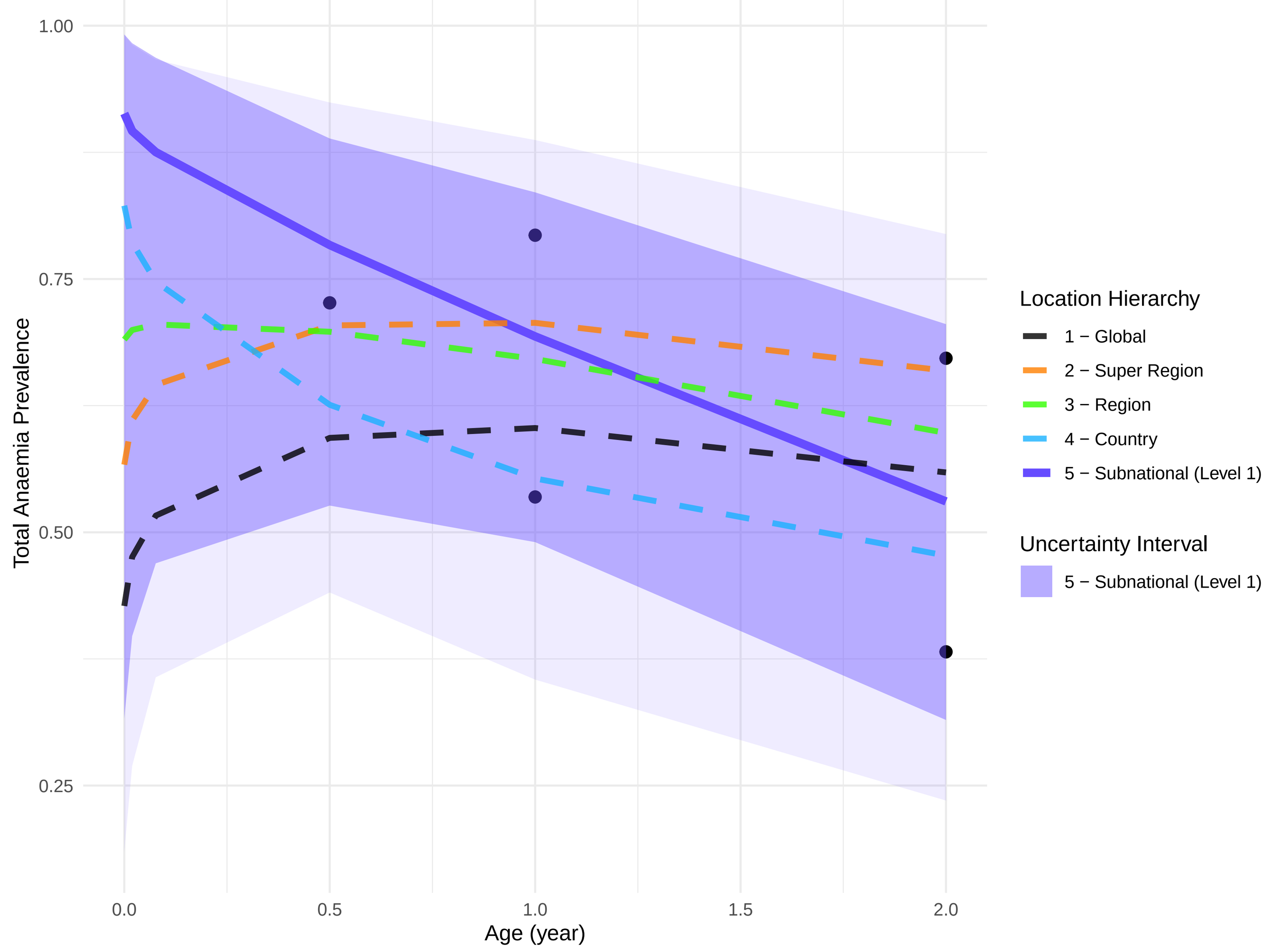

MR−BRT Age/Sex Fits − Non−pregnant Females <2 Years Old − Total Anaemia Prevalence − Busia
WHO−2024 Adjustment Method

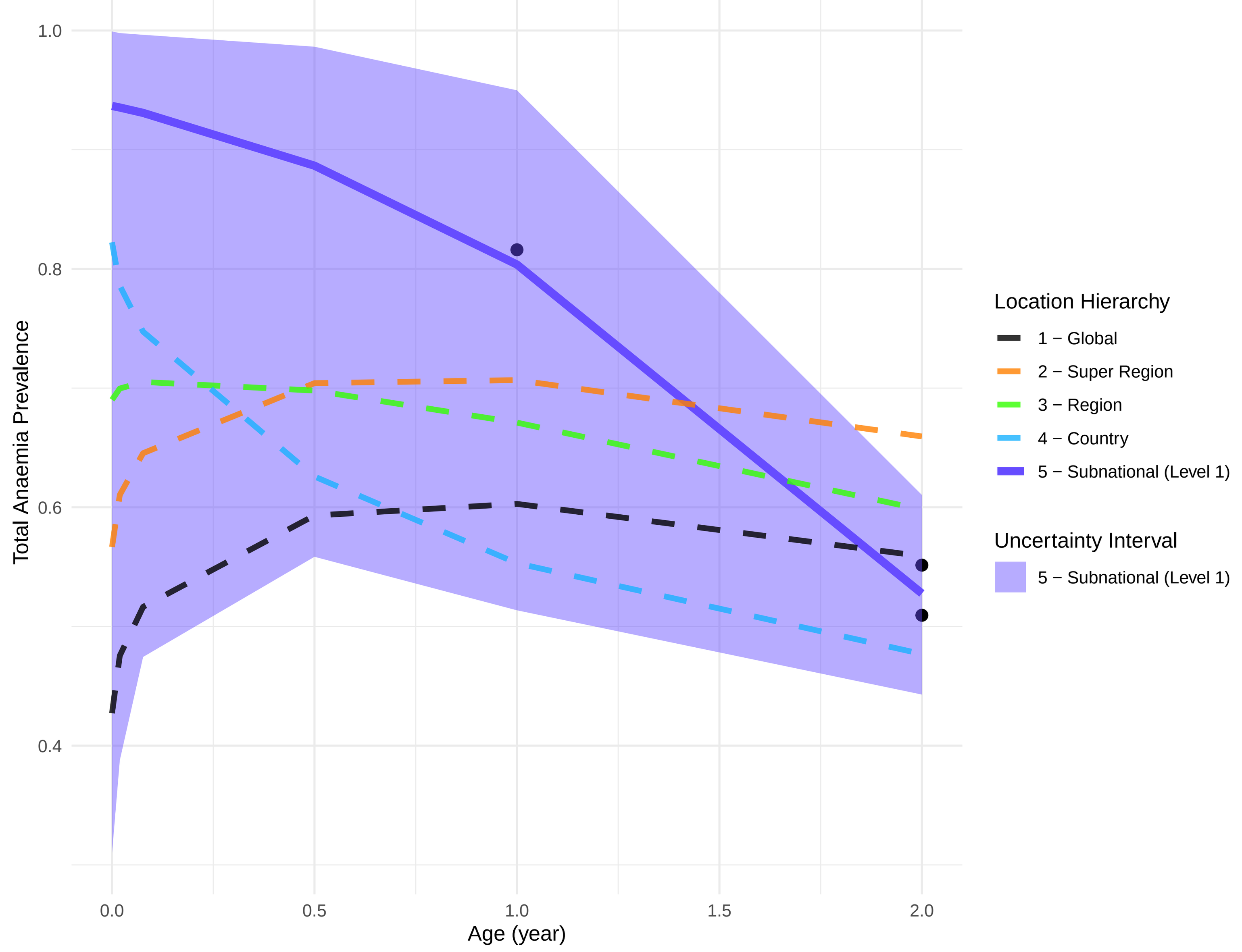

MR−BRT Age/Sex Fits − Non−pregnant Females <2 Years Old − Total Anaemia Prevalence − Elgeyo−Marakwet
WHO−2024 Adjustment Method

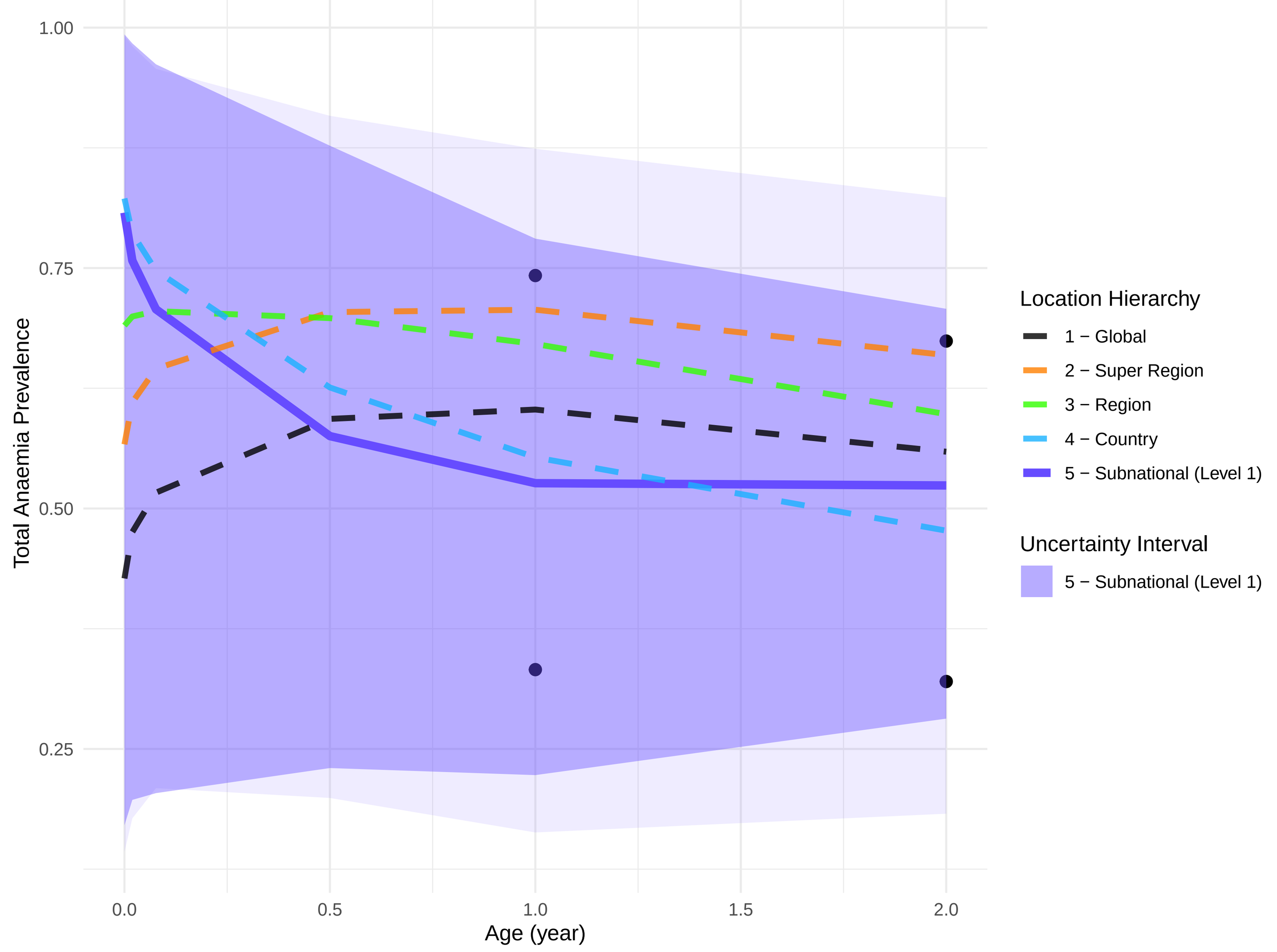

MR−BRT Age/Sex Fits − Non−pregnant Females <2 Years Old − Total Anaemia Prevalence − Embu
WHO−2024 Adjustment Method

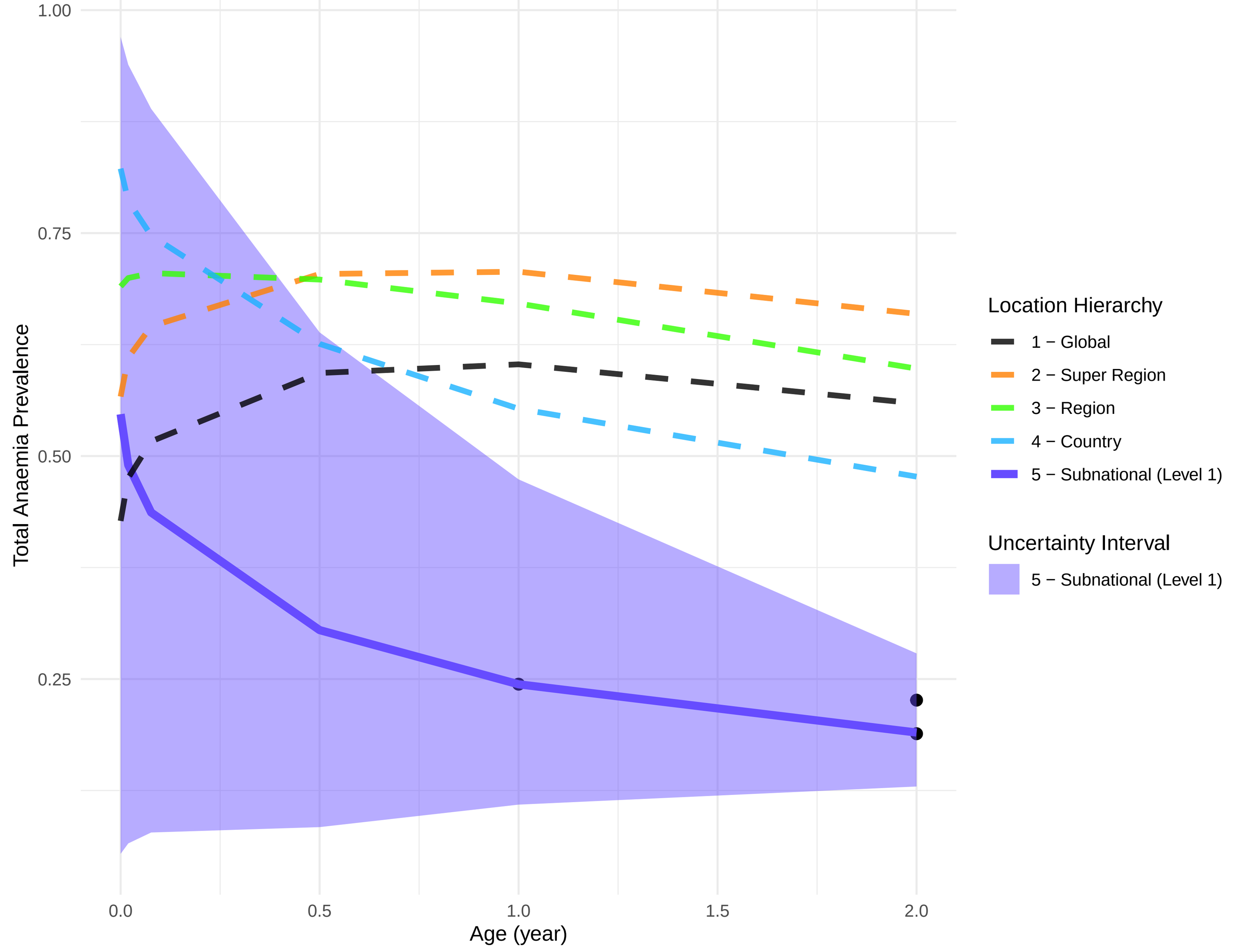

MR−BRT Age/Sex Fits − Non−pregnant Females <2 Years Old − Total Anaemia Prevalence − Garissa
WHO−2024 Adjustment Method

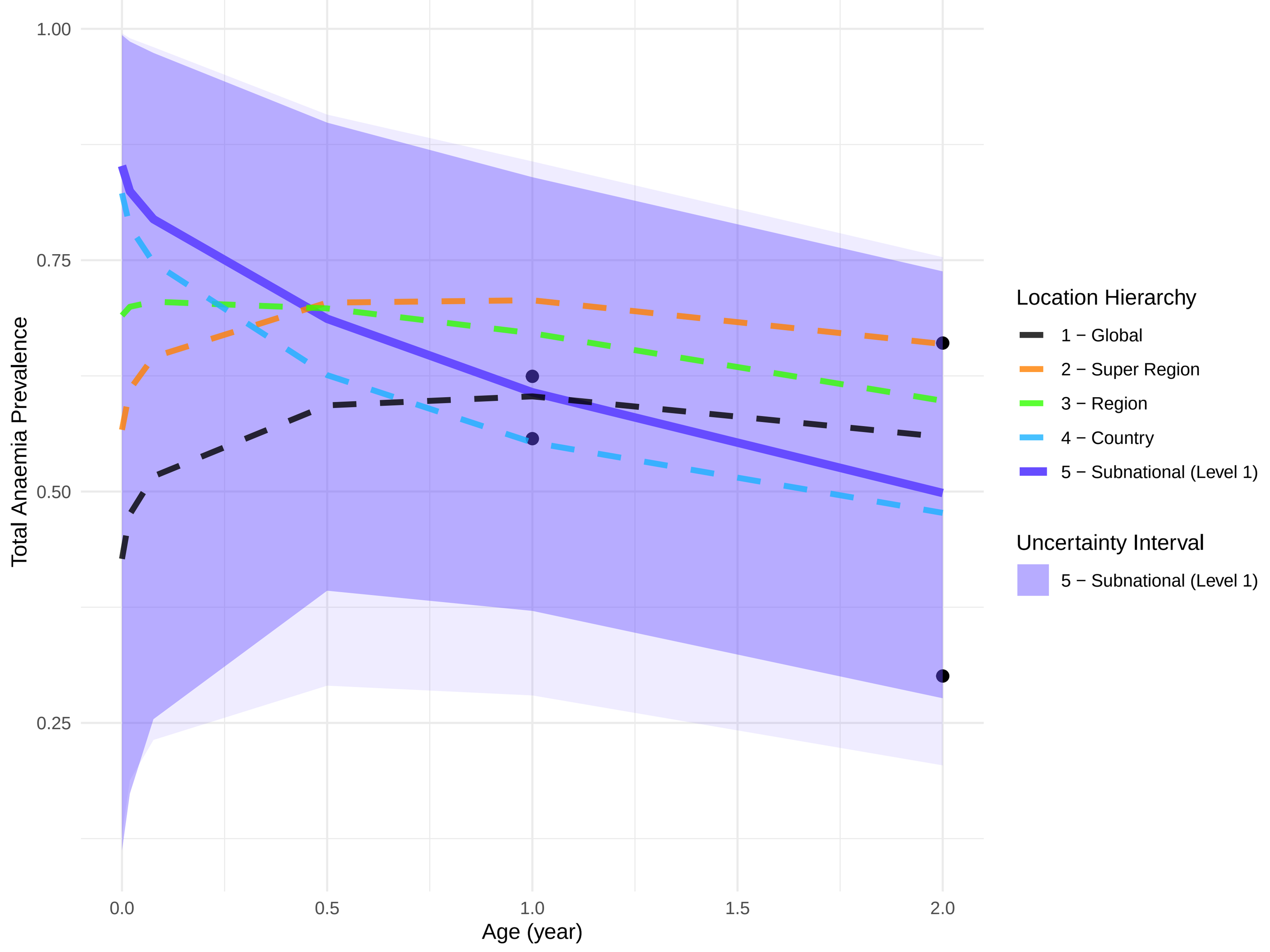

MR−BRT Age/Sex Fits − Non−pregnant Females <2 Years Old − Total Anaemia Prevalence − Homa Bay
WHO−2024 Adjustment Method

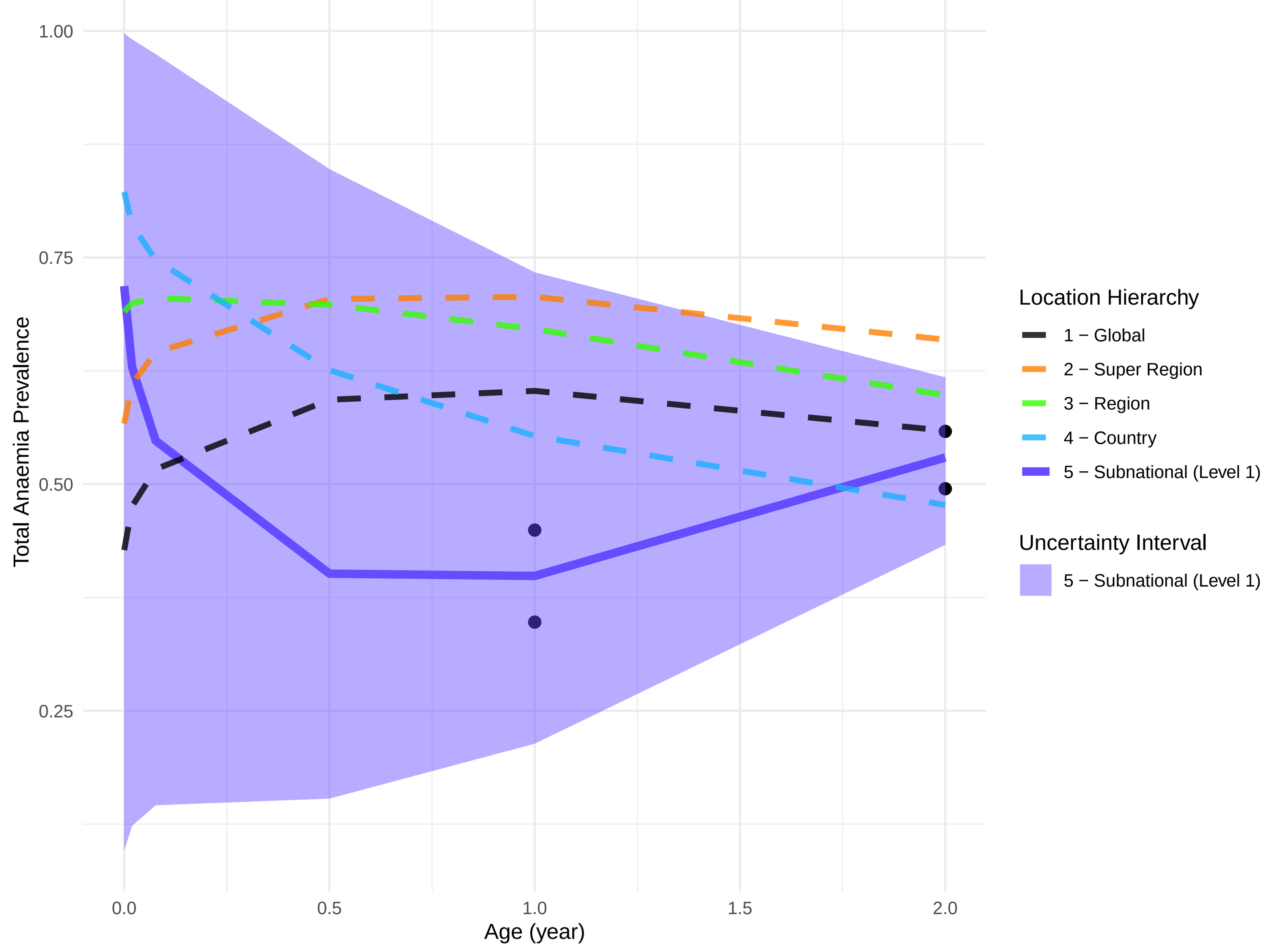

MR−BRT Age/Sex Fits − Non−pregnant Females <2 Years Old − Total Anaemia Prevalence − Isiolo
WHO−2024 Adjustment Method

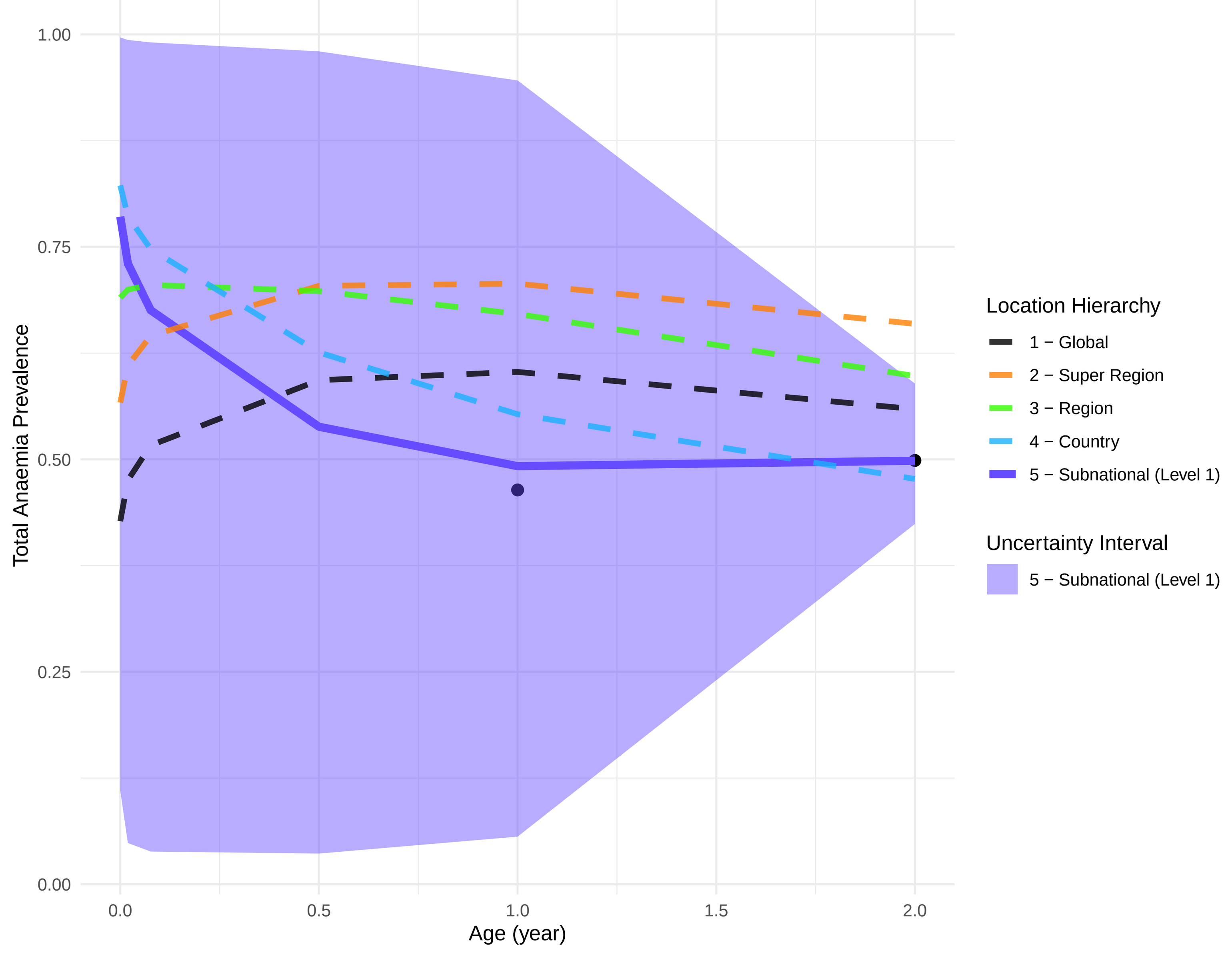

MR−BRT Age/Sex Fits − Non−pregnant Females <2 Years Old − Total Anaemia Prevalence − Kajiado
WHO−2024 Adjustment Method

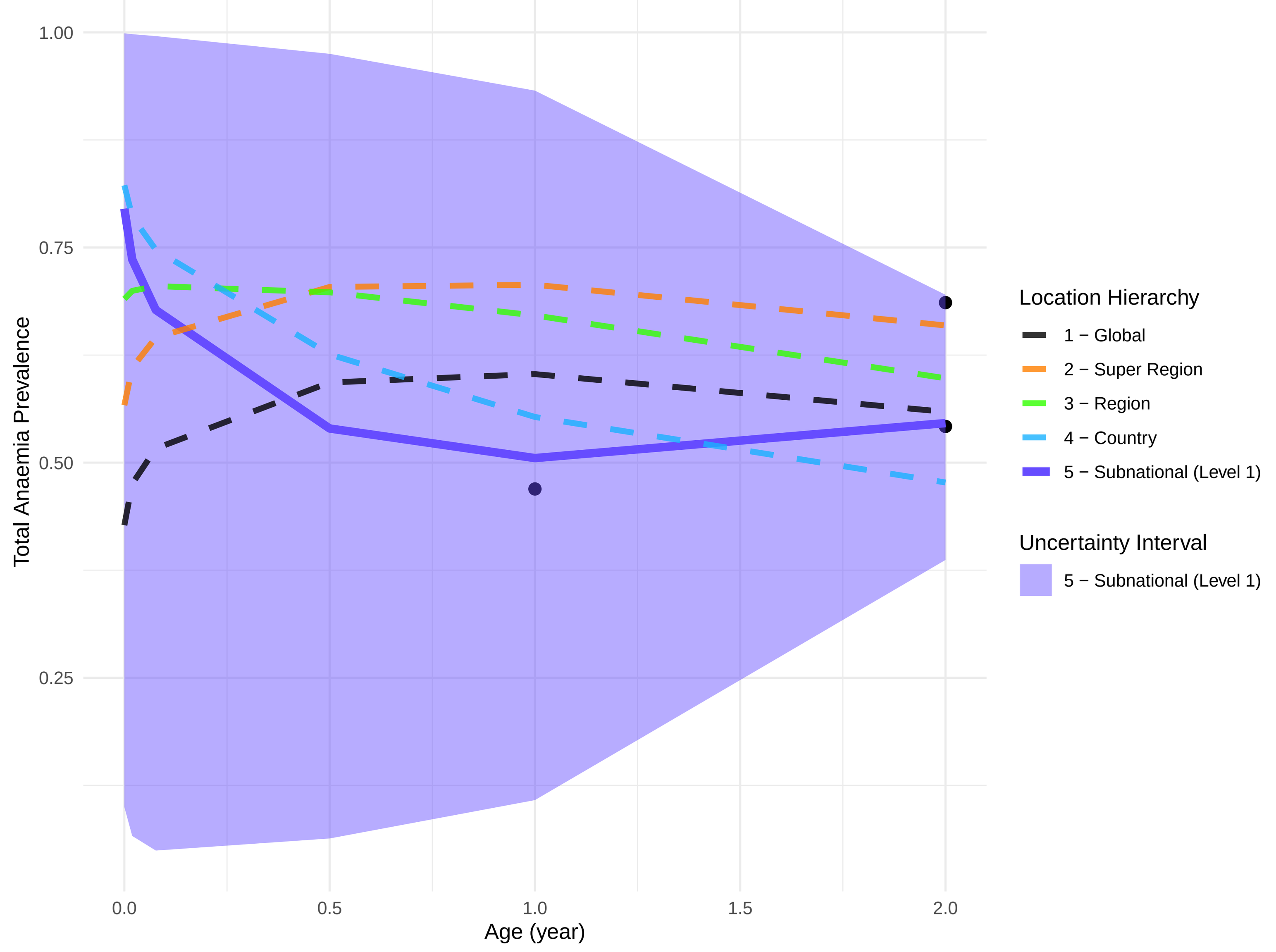

MR−BRT Age/Sex Fits − Non−pregnant Females <2 Years Old − Total Anaemia Prevalence − Kakamega
WHO−2024 Adjustment Method

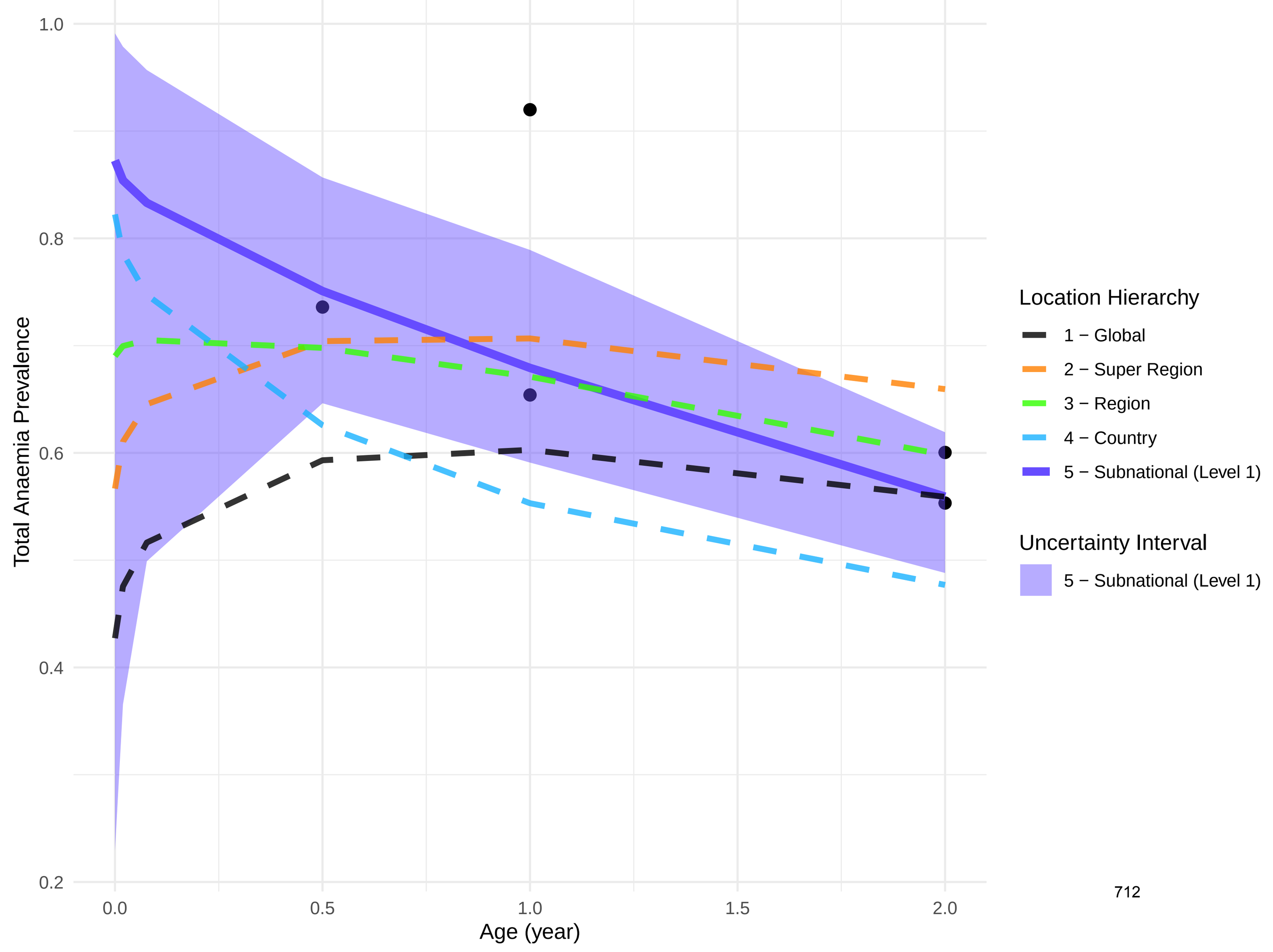

MR−BRT Age/Sex Fits − Non−pregnant Females <2 Years Old − Total Anaemia Prevalence − Kericho
WHO−2024 Adjustment Method

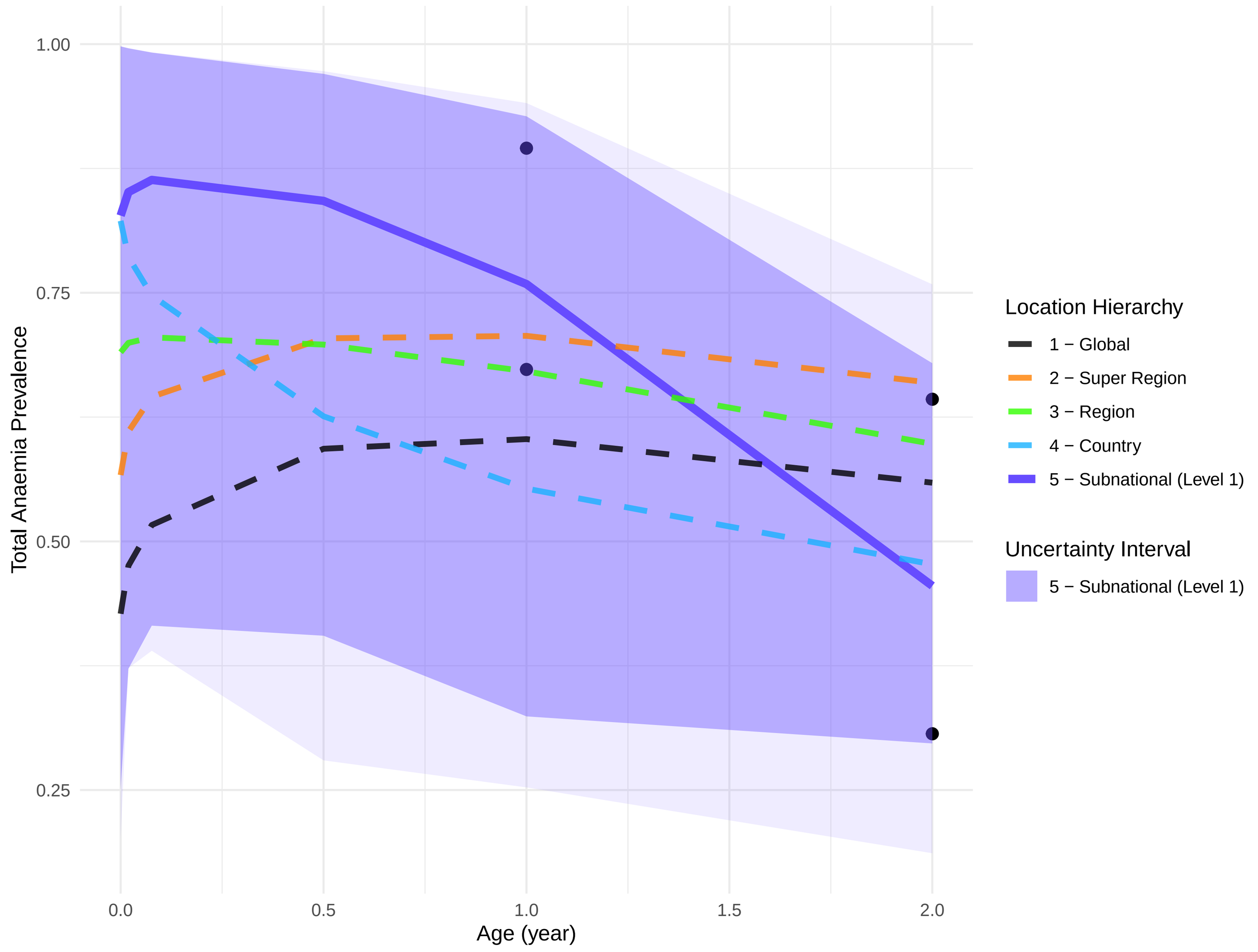

MR−BRT Age/Sex Fits − Non−pregnant Females <2 Years Old − Total Anaemia Prevalence − Kiambu
WHO−2024 Adjustment Method

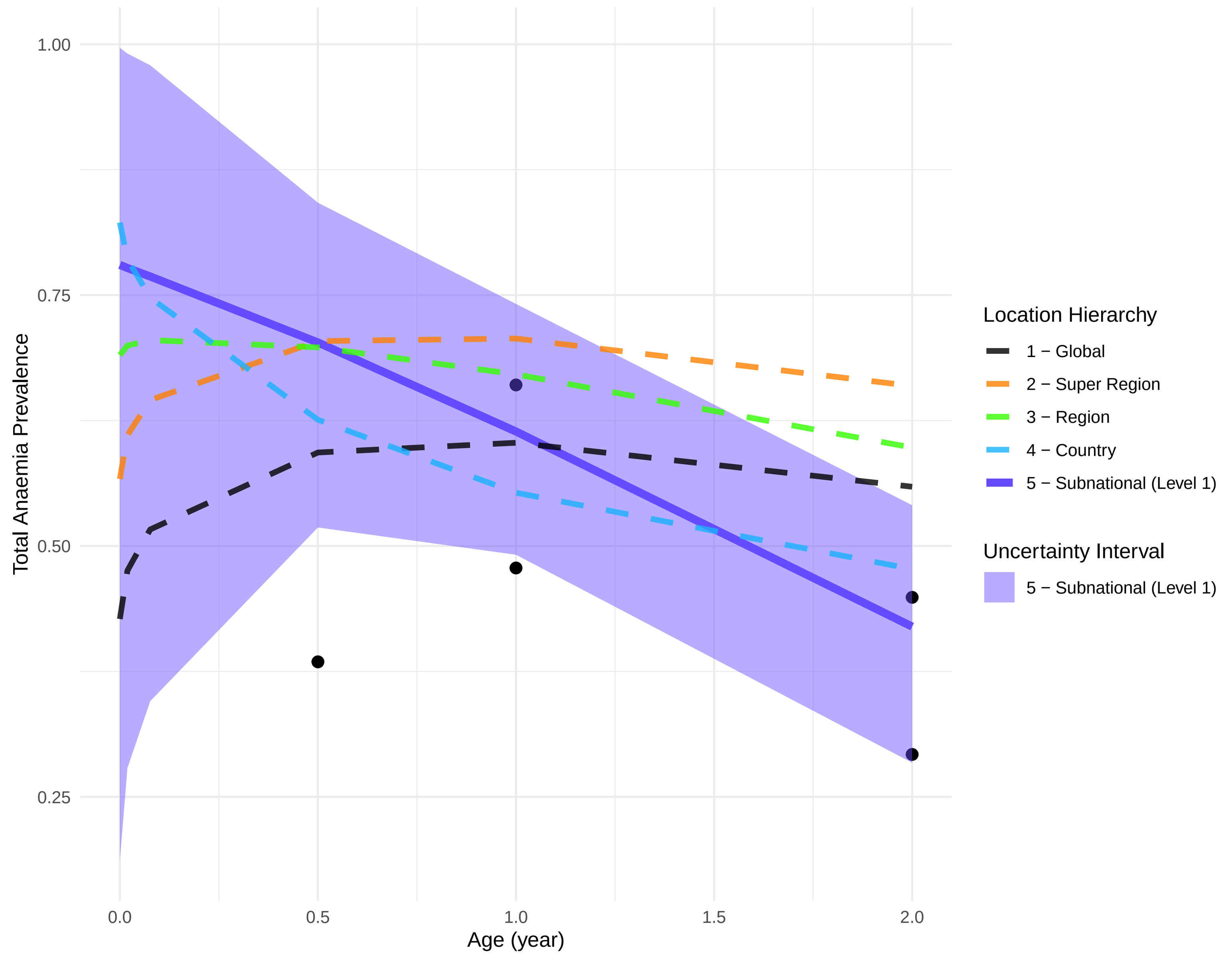

MR−BRT Age/Sex Fits − Non−pregnant Females <2 Years Old − Total Anaemia Prevalence − Kilifi
WHO−2024 Adjustment Method

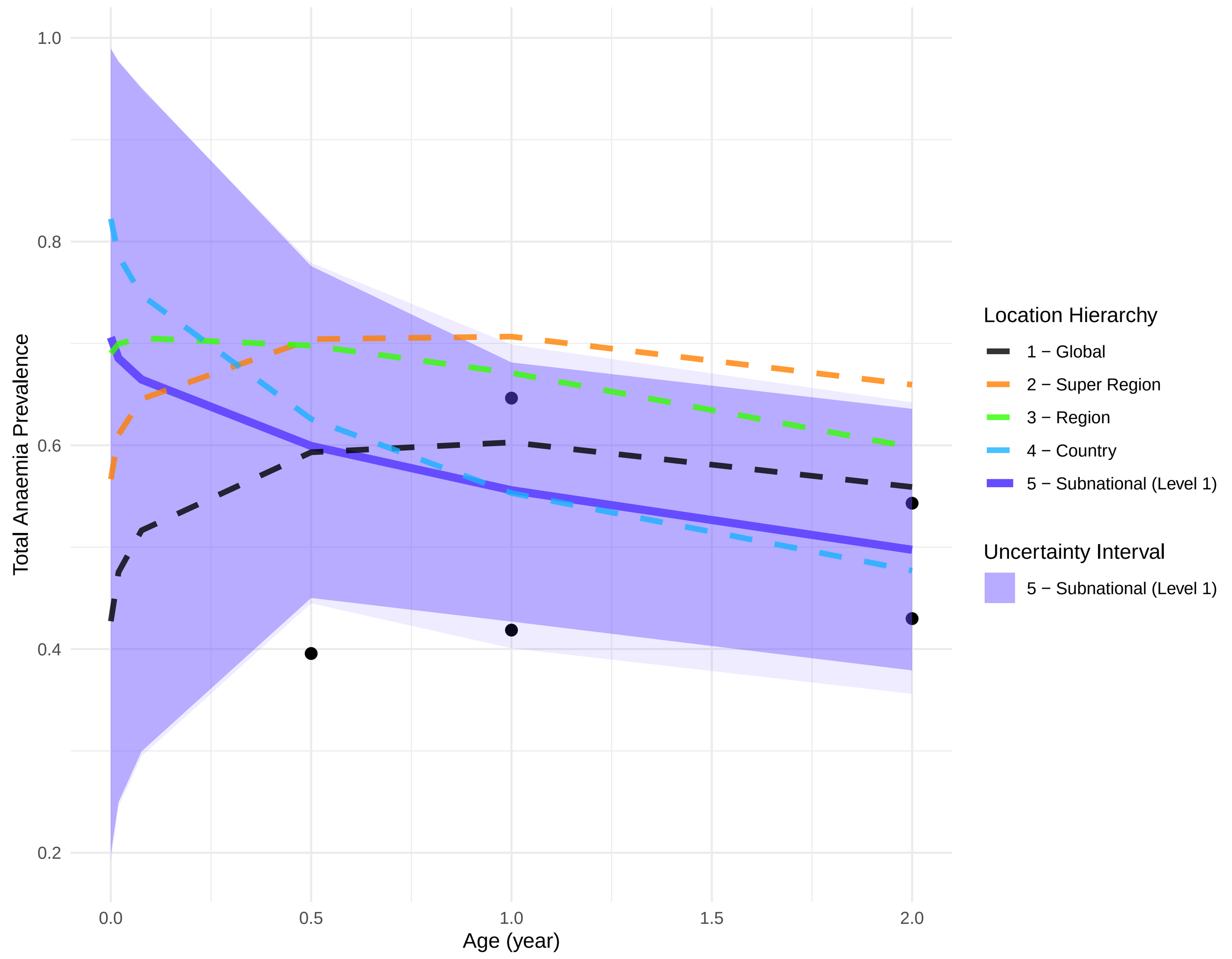

MR−BRT Age/Sex Fits − Non−pregnant Females <2 Years Old − Total Anaemia Prevalence − Kirinyaga
WHO−2024 Adjustment Method

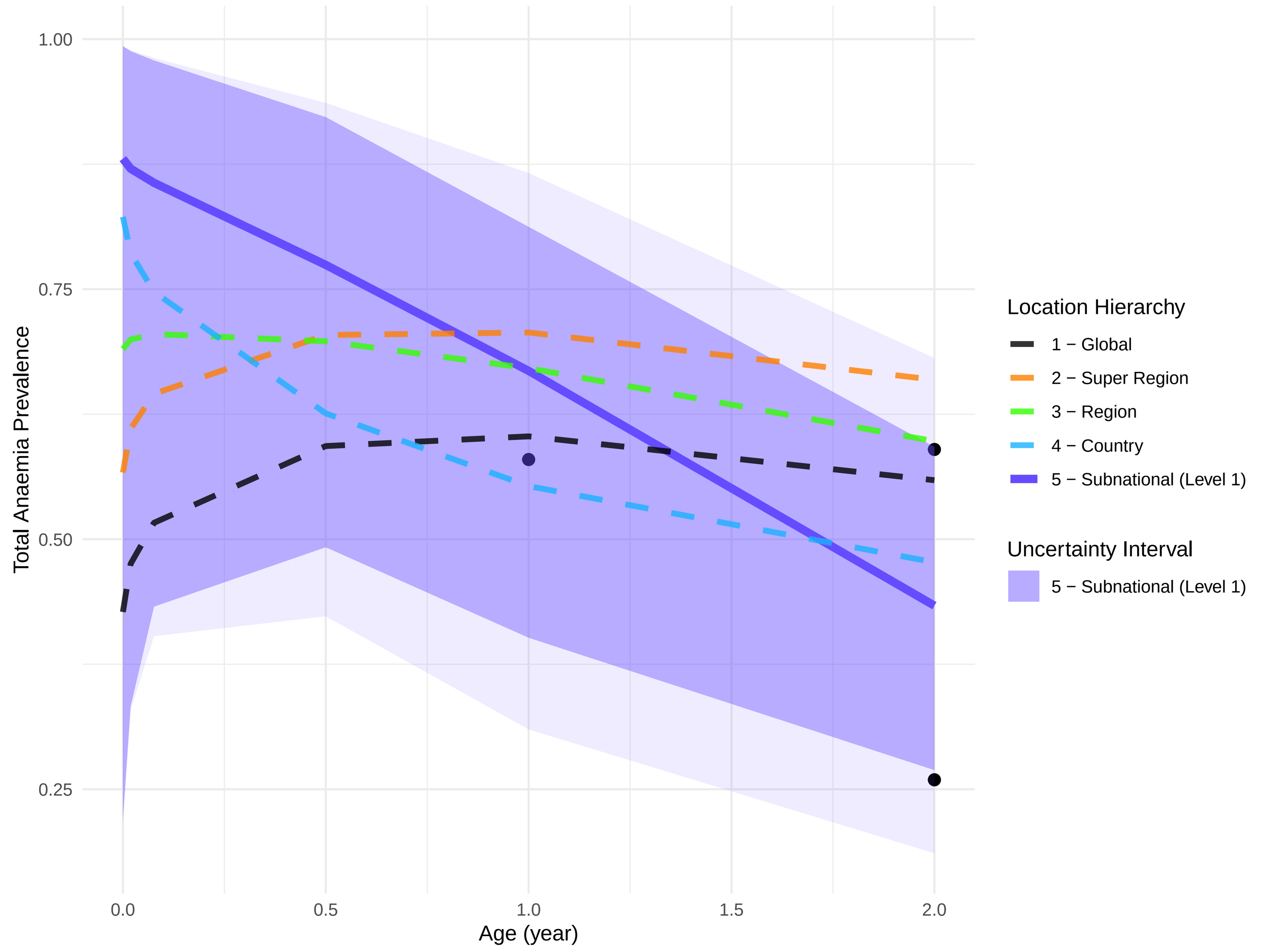

MR−BRT Age/Sex Fits − Non−pregnant Females <2 Years Old − Total Anaemia Prevalence − Kisii
WHO−2024 Adjustment Method

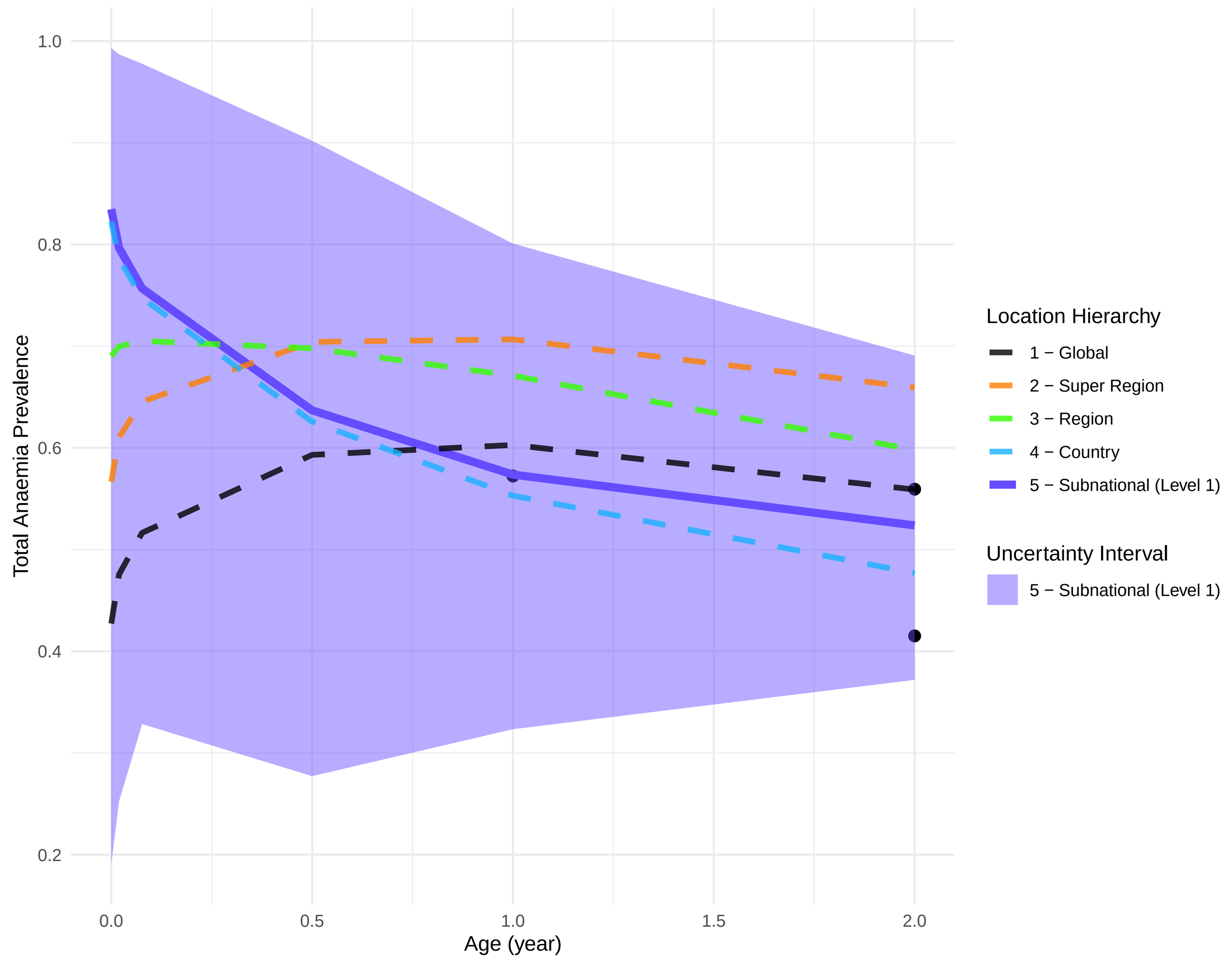

MR−BRT Age/Sex Fits − Non−pregnant Females <2 Years Old − Total Anaemia Prevalence − Kisumu
WHO−2024 Adjustment Method

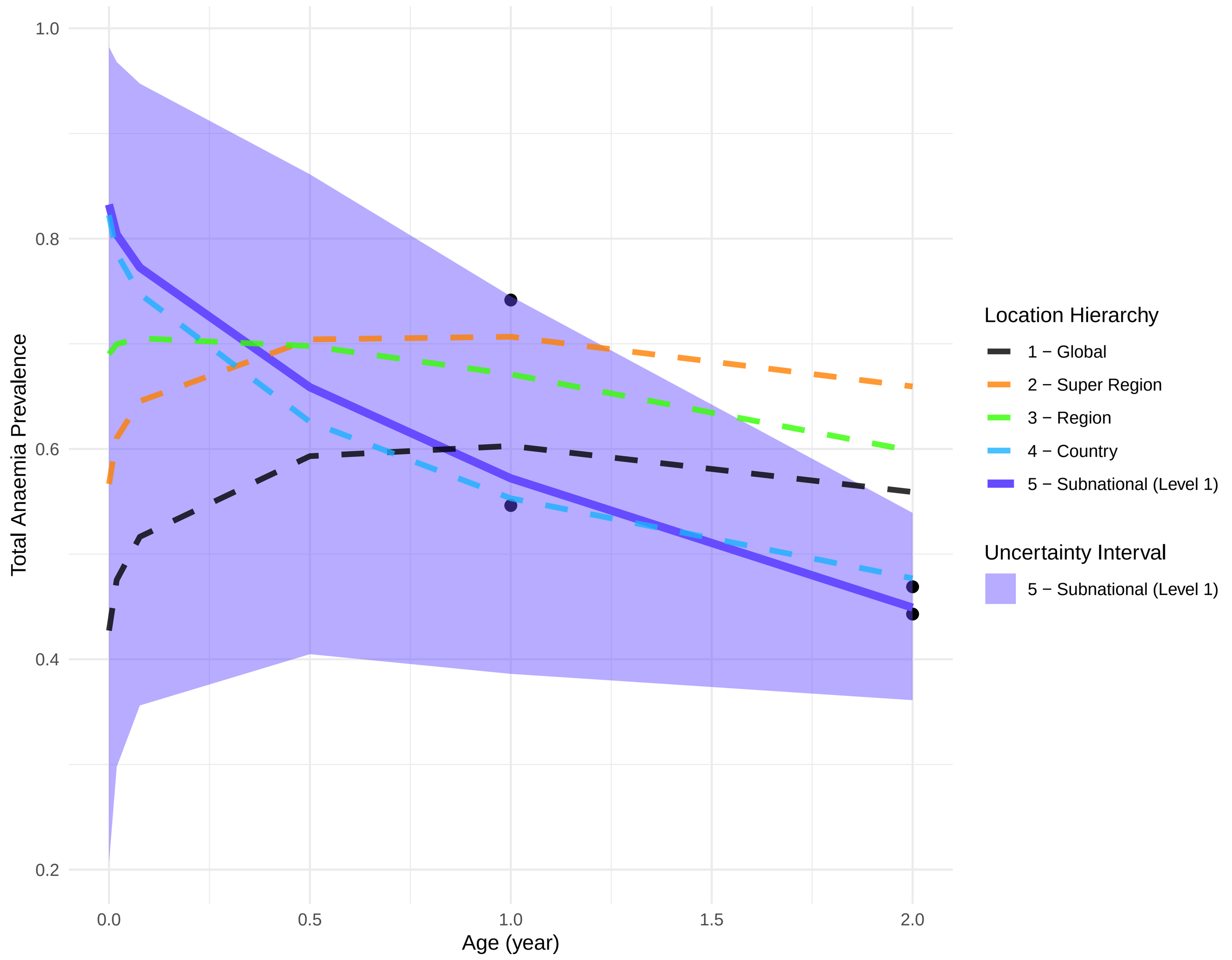

MR−BRT Age/Sex Fits − Non−pregnant Females <2 Years Old − Total Anaemia Prevalence − Kitui
WHO−2024 Adjustment Method

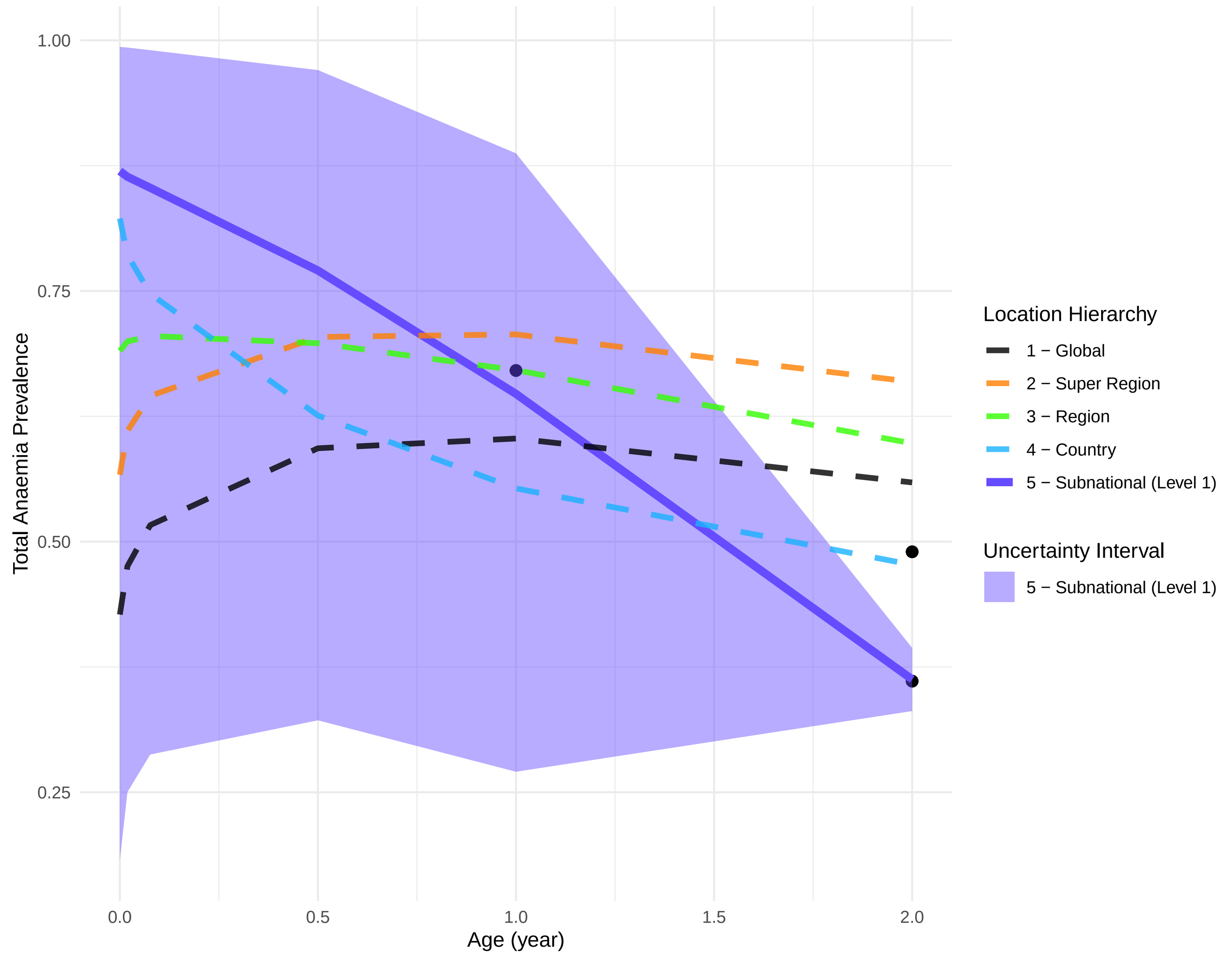

MR−BRT Age/Sex Fits − Non−pregnant Females <2 Years Old − Total Anaemia Prevalence − Kwale
WHO−2024 Adjustment Method

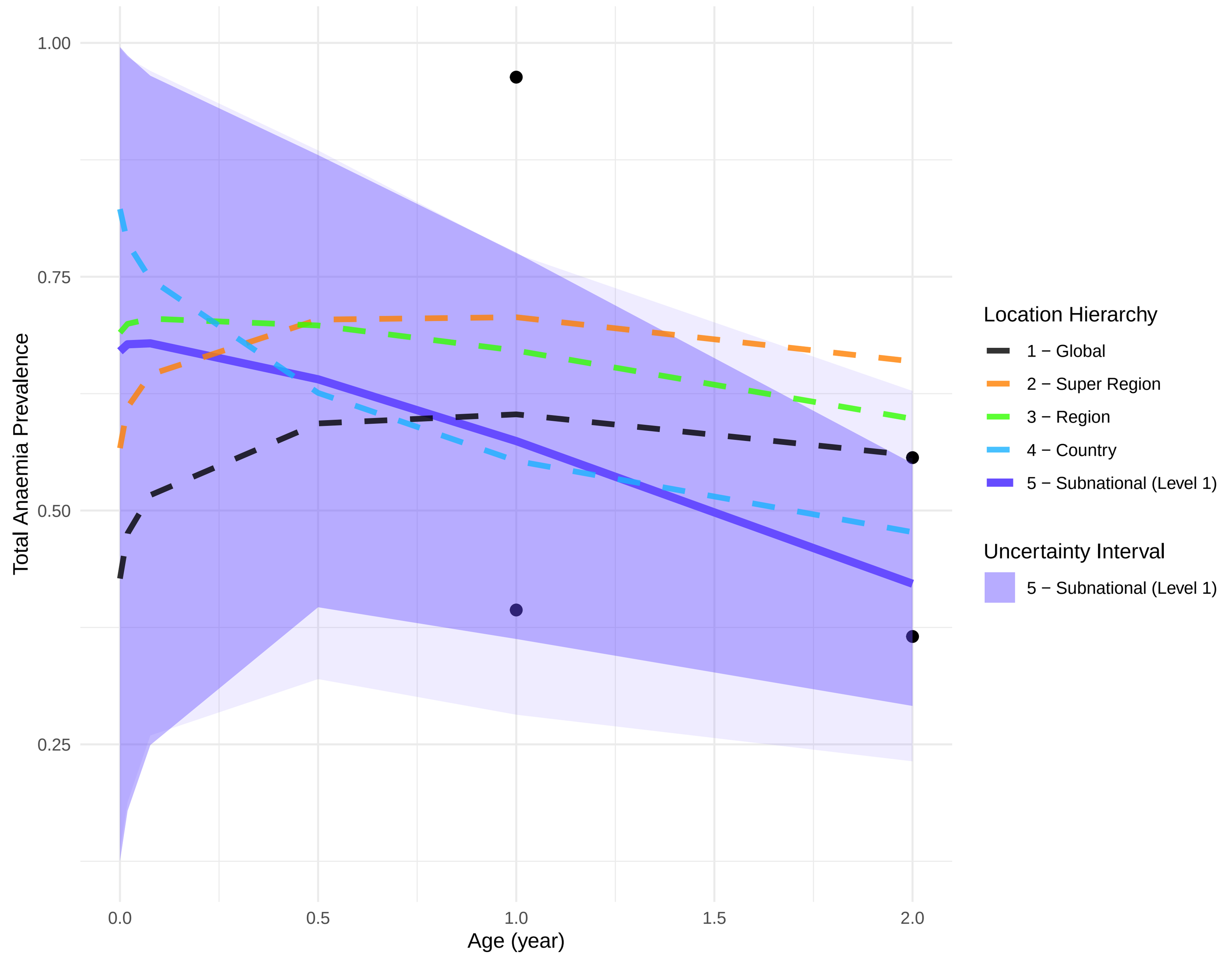

MR−BRT Age/Sex Fits − Non−pregnant Females <2 Years Old − Total Anaemia Prevalence − Laikipia
WHO−2024 Adjustment Method

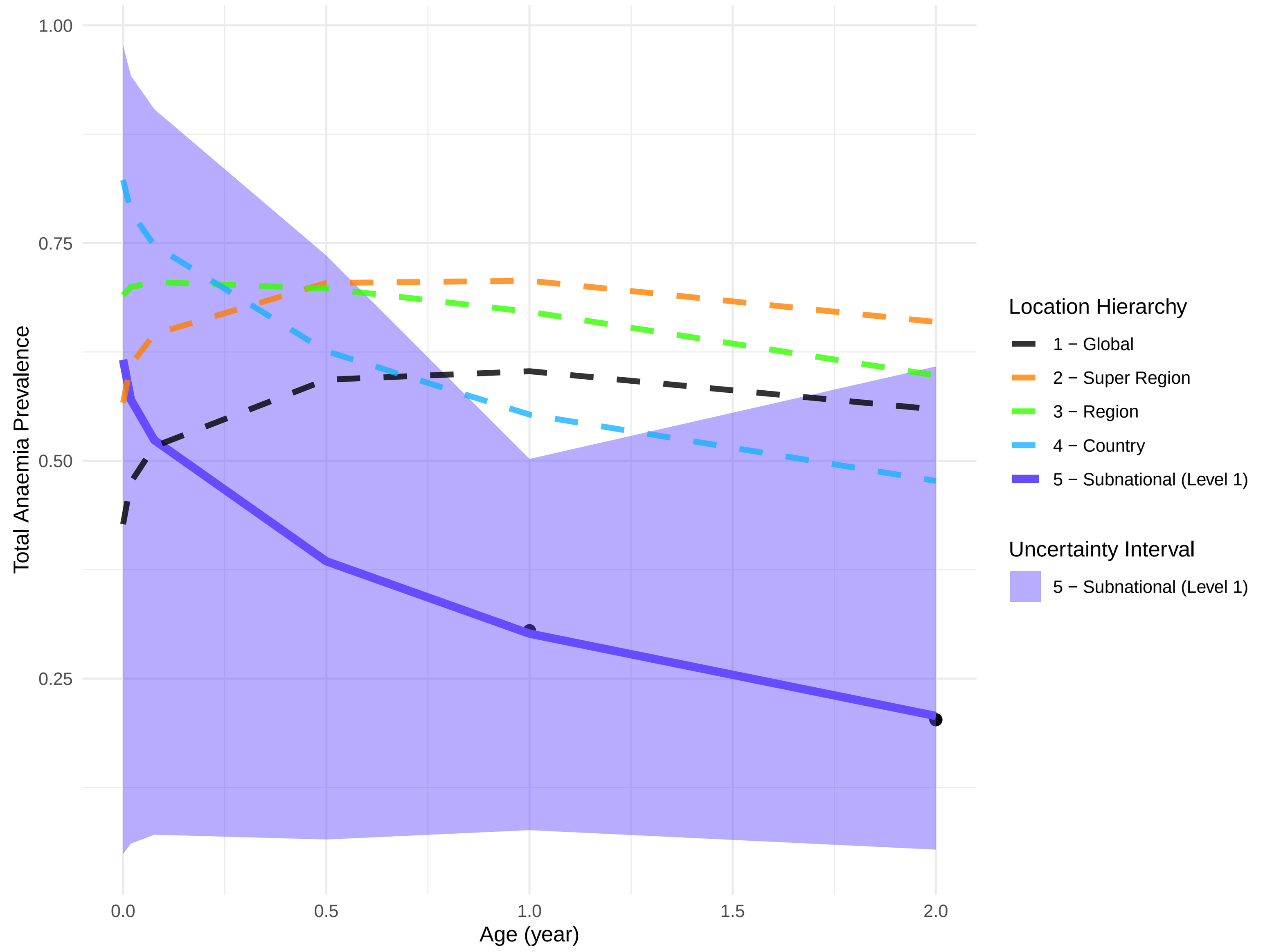

MR−BRT Age/Sex Fits − Non−pregnant Females <2 Years Old − Total Anaemia Prevalence − Lamu
WHO−2024 Adjustment Method

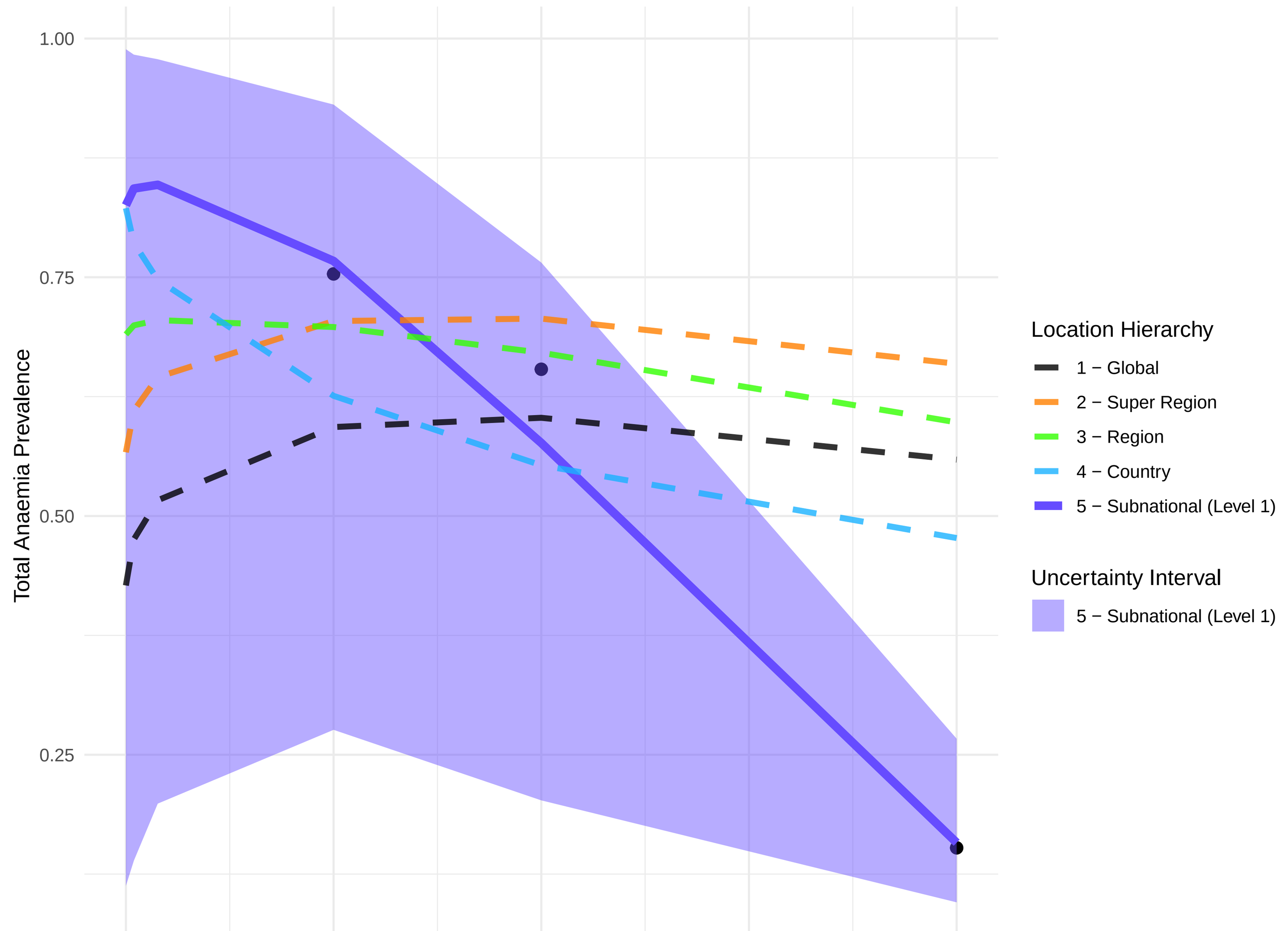

MR−BRT Age/Sex Fits − Non−pregnant Females <2 Years Old − Total Anaemia Prevalence − Machakos
WHO−2024 Adjustment Method

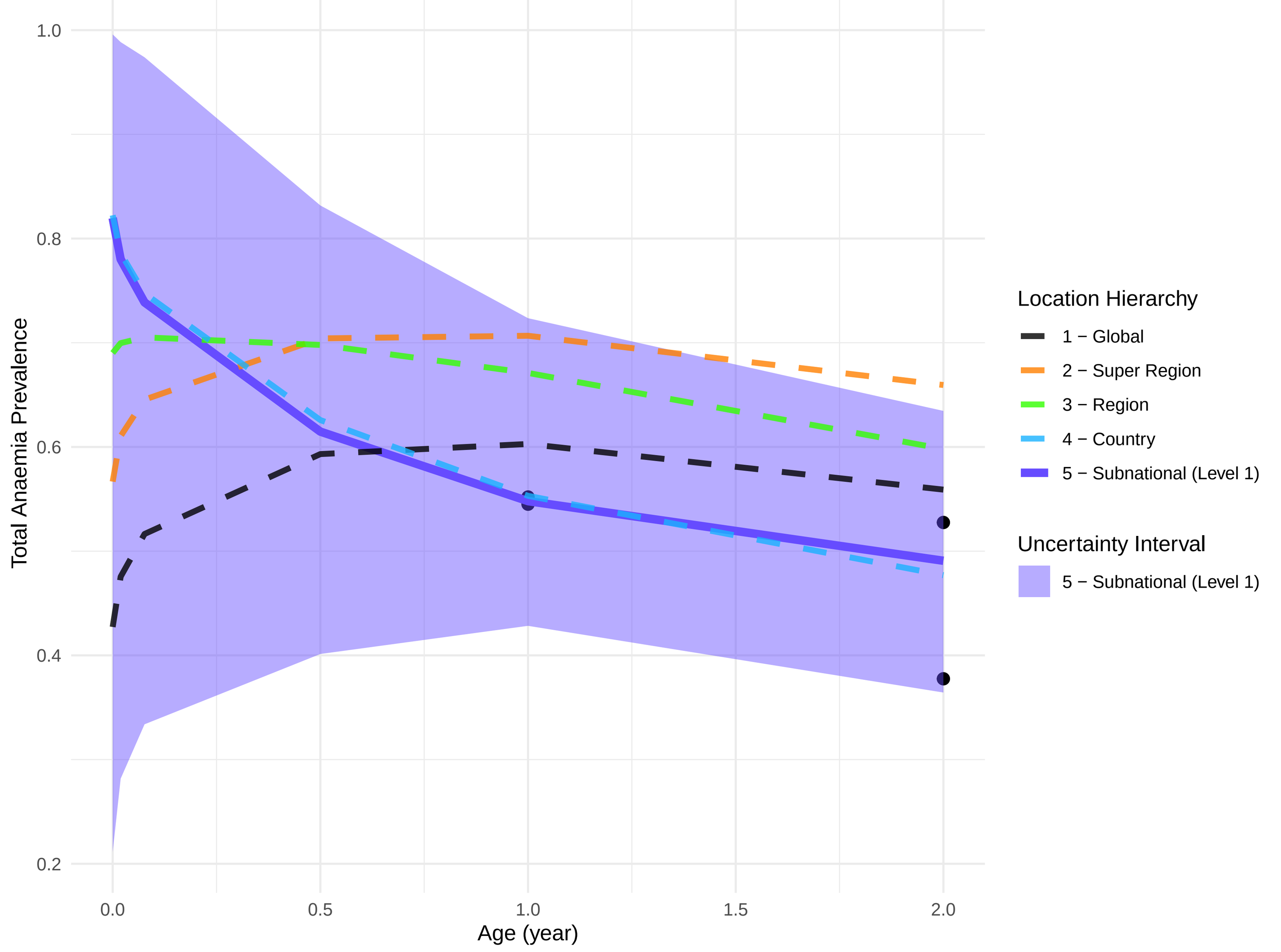

MR−BRT Age/Sex Fits − Non−pregnant Females <2 Years Old − Total Anaemia Prevalence − Makueni
WHO−2024 Adjustment Method

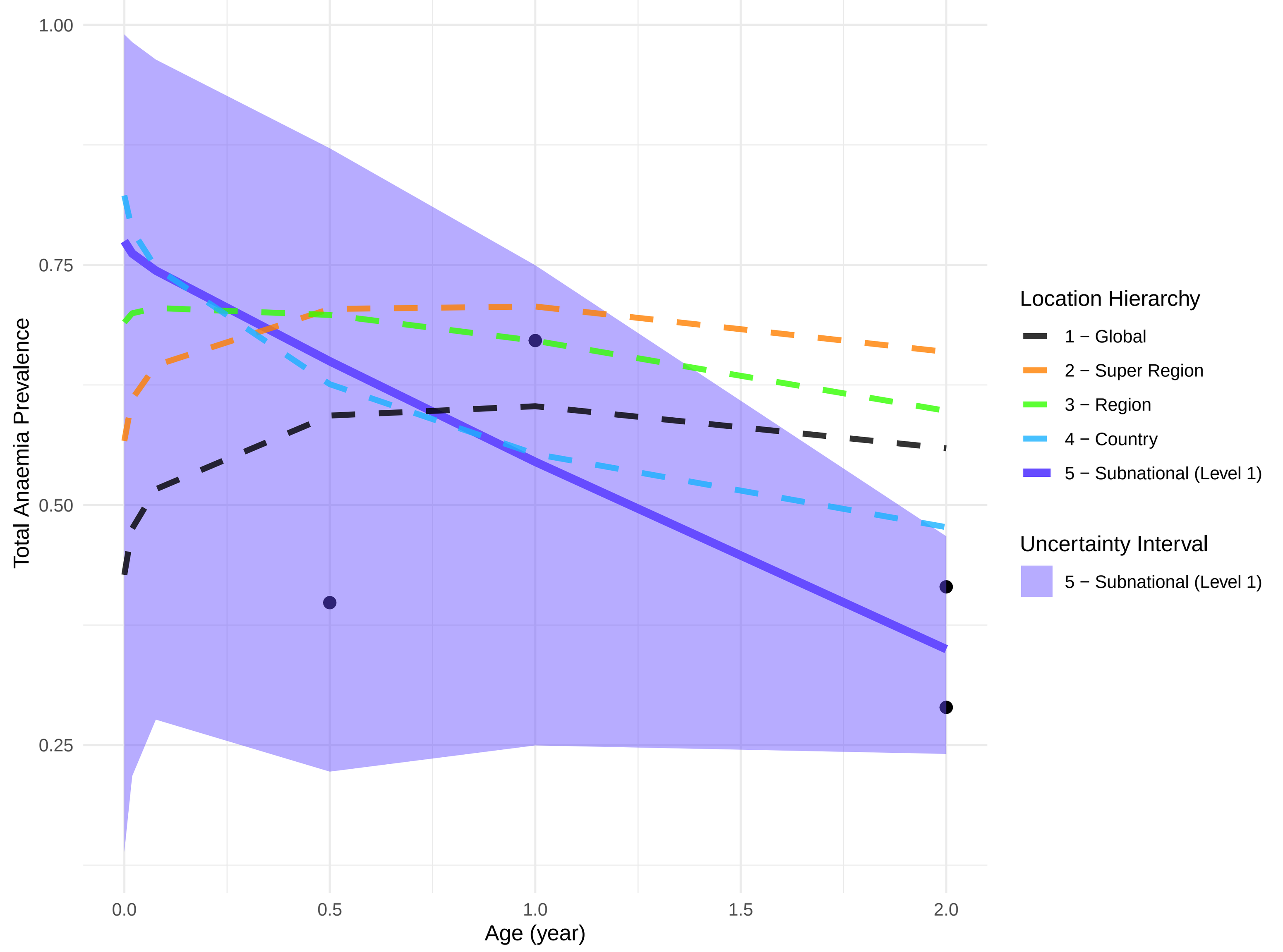

MR−BRT Age/Sex Fits − Non−pregnant Females <2 Years Old − Total Anaemia Prevalence − Mandera
WHO−2024 Adjustment Method

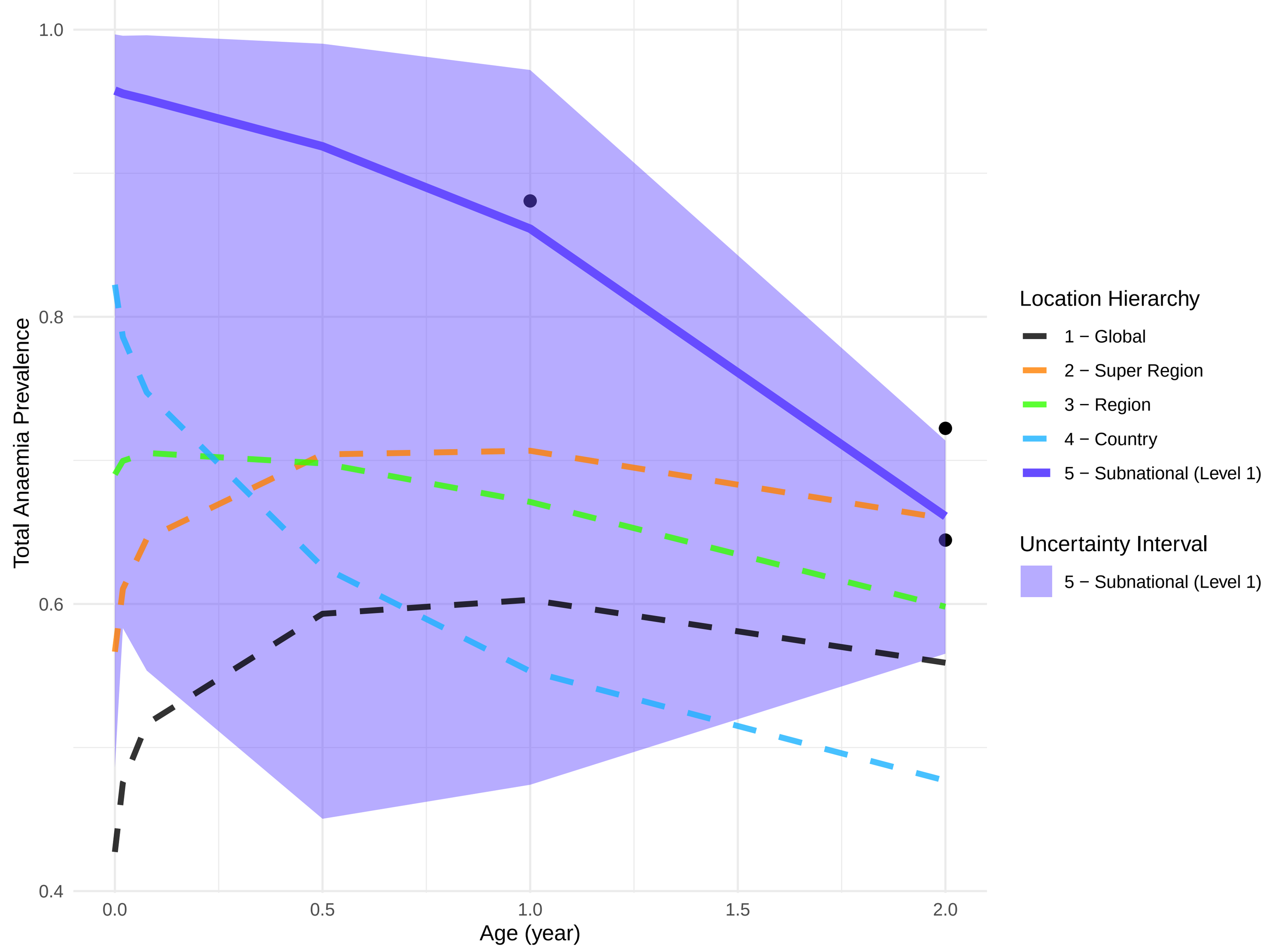

MR−BRT Age/Sex Fits − Non−pregnant Females <2 Years Old − Total Anaemia Prevalence − Marsabit
WHO−2024 Adjustment Method

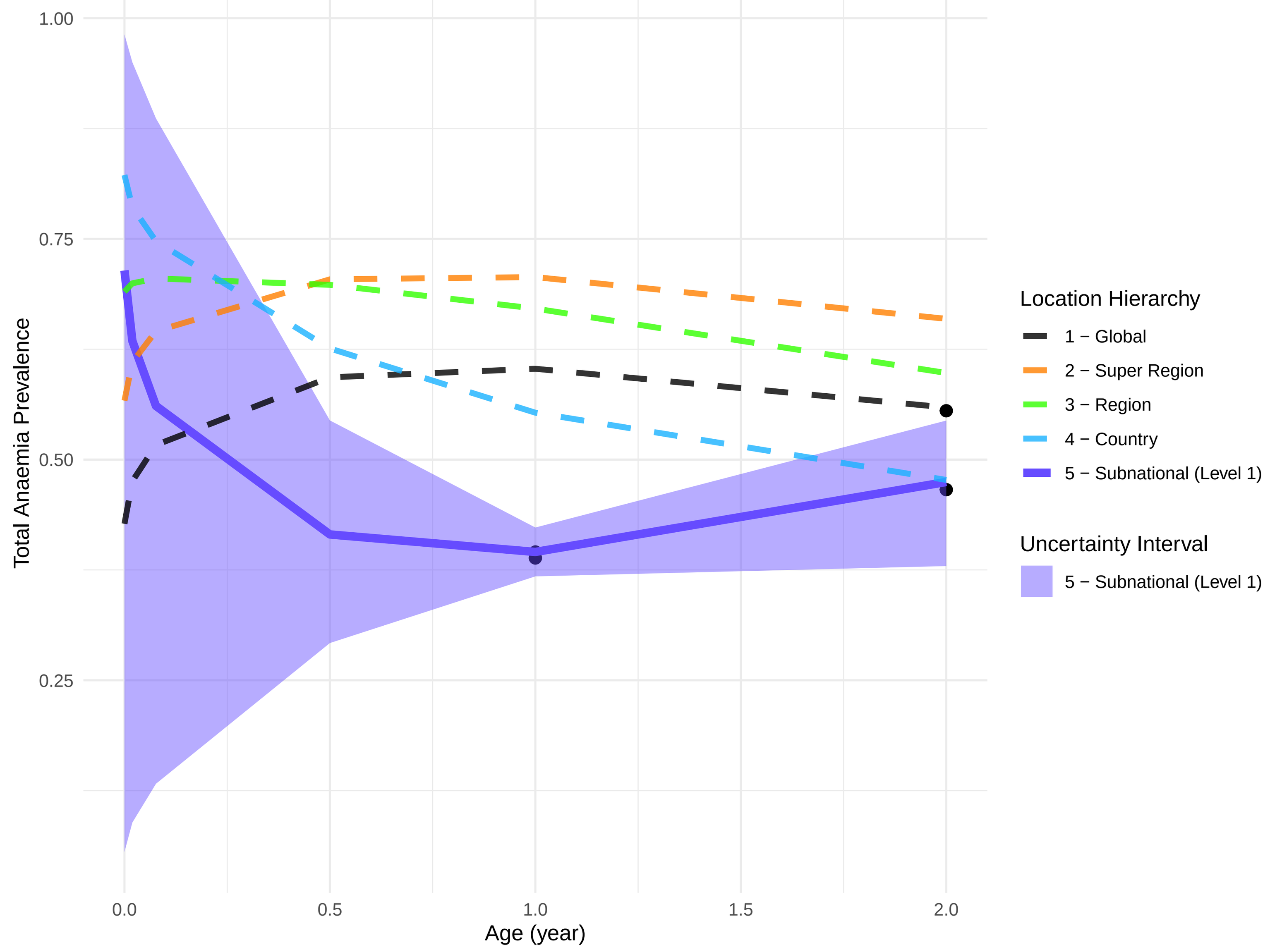

MR−BRT Age/Sex Fits − Non−pregnant Females <2 Years Old − Total Anaemia Prevalence − Meru
WHO−2024 Adjustment Method

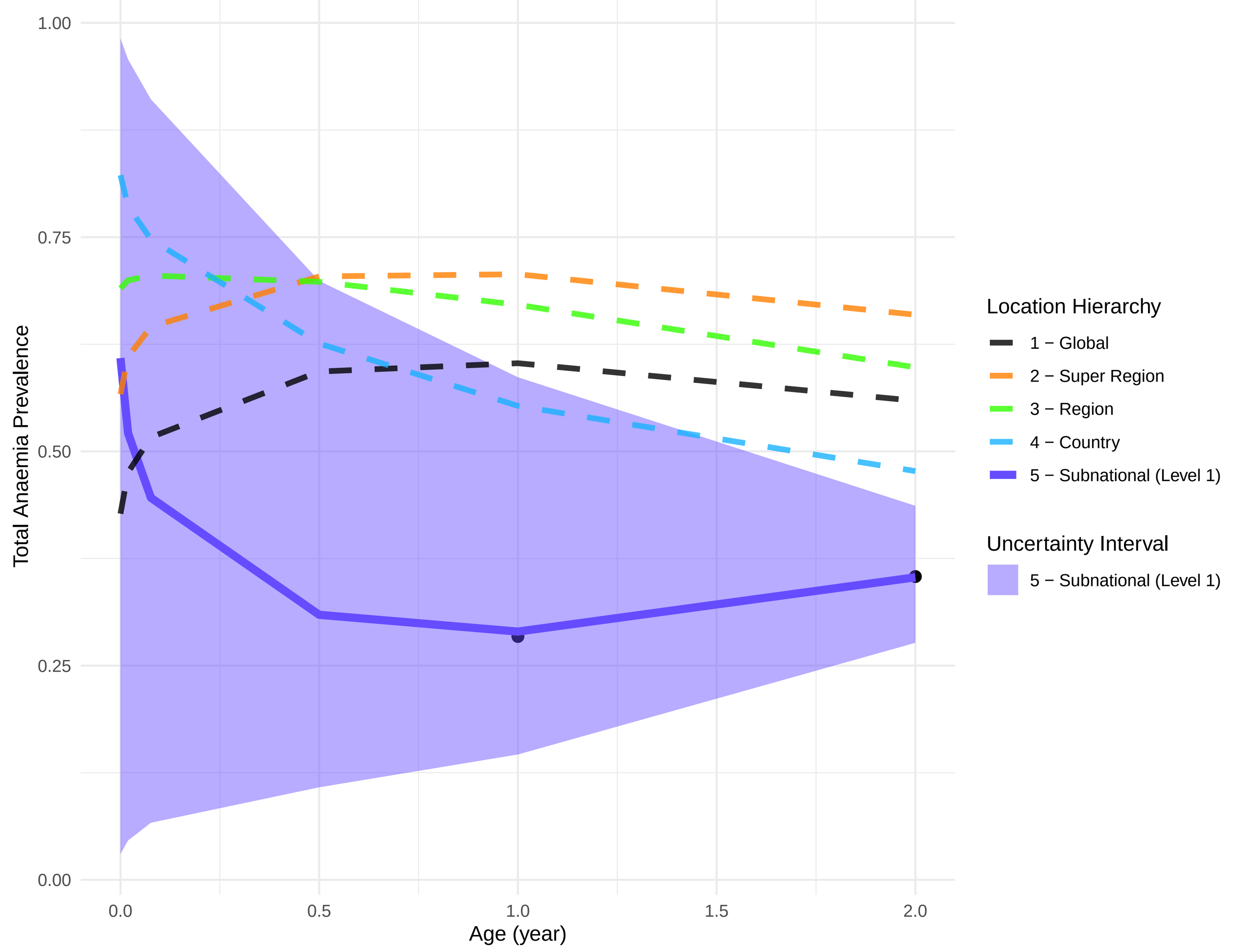

MR−BRT Age/Sex Fits − Non−pregnant Females <2 Years Old − Total Anaemia Prevalence − Migori
WHO−2024 Adjustment Method

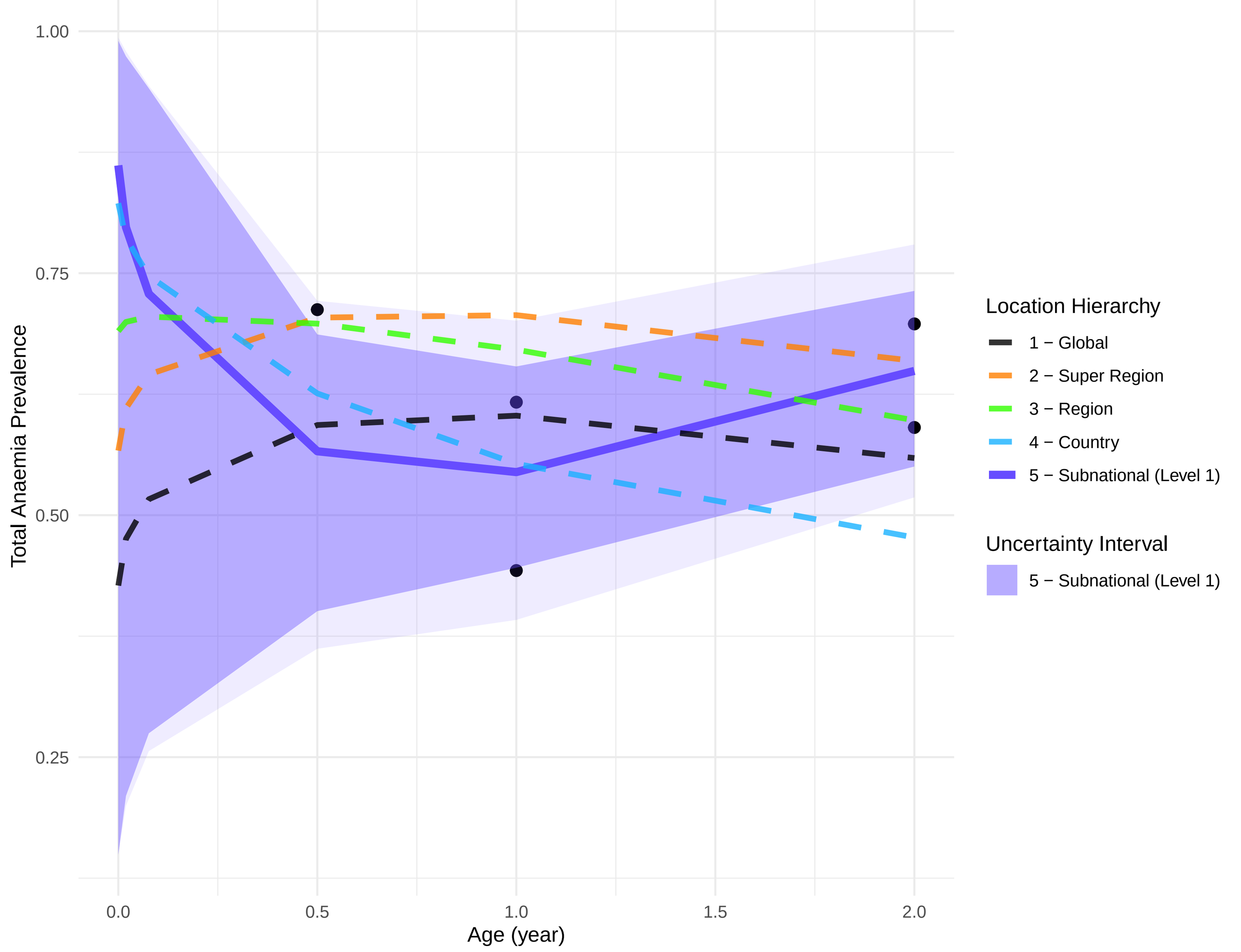

MR−BRT Age/Sex Fits − Non−pregnant Females <2 Years Old − Total Anaemia Prevalence − Mombasa
WHO−2024 Adjustment Method

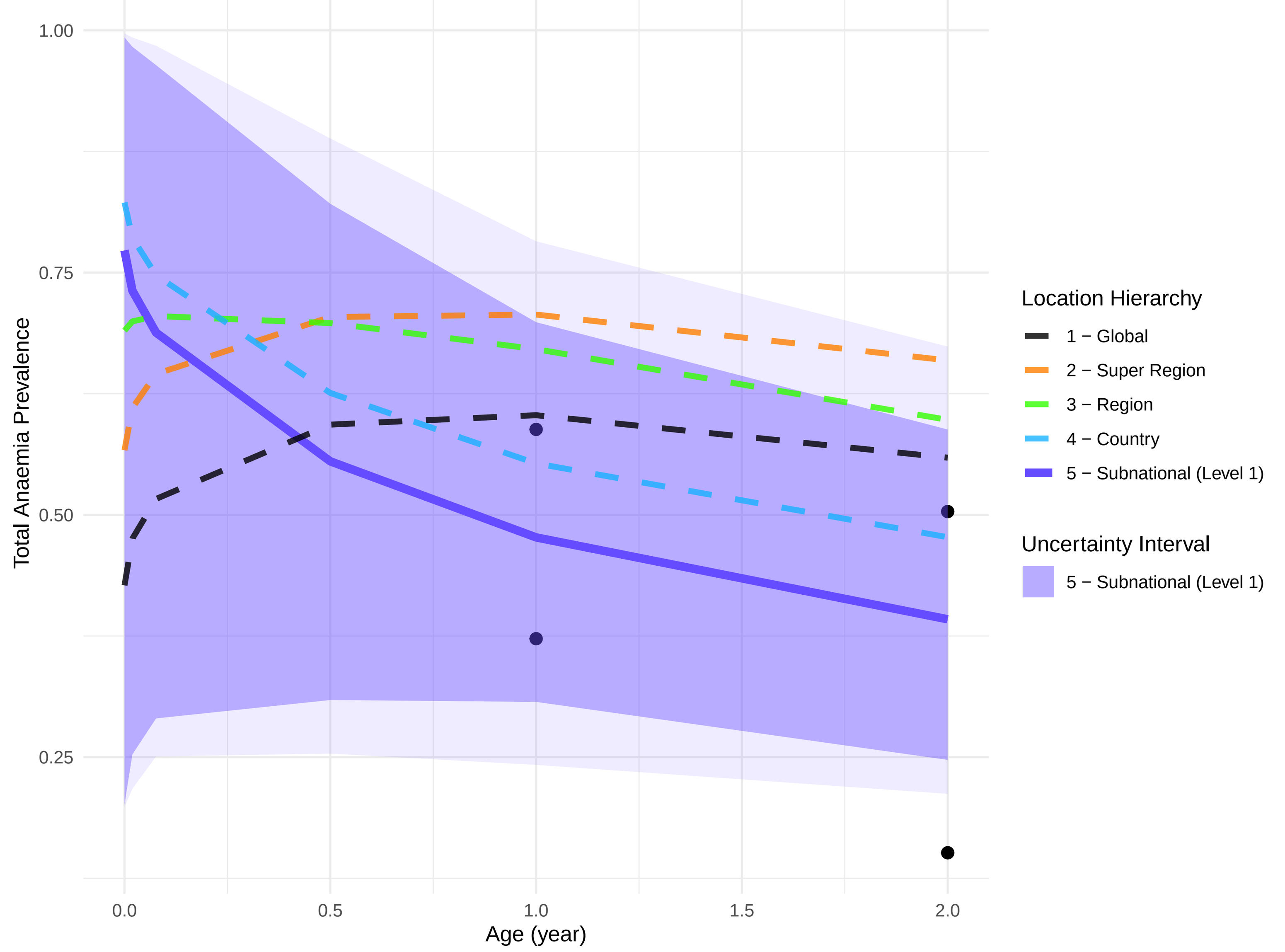

MR−BRT Age/Sex Fits − Non−pregnant Females <2 Years Old − Total Anaemia Prevalence − Murang'a
WHO−2024 Adjustment Method

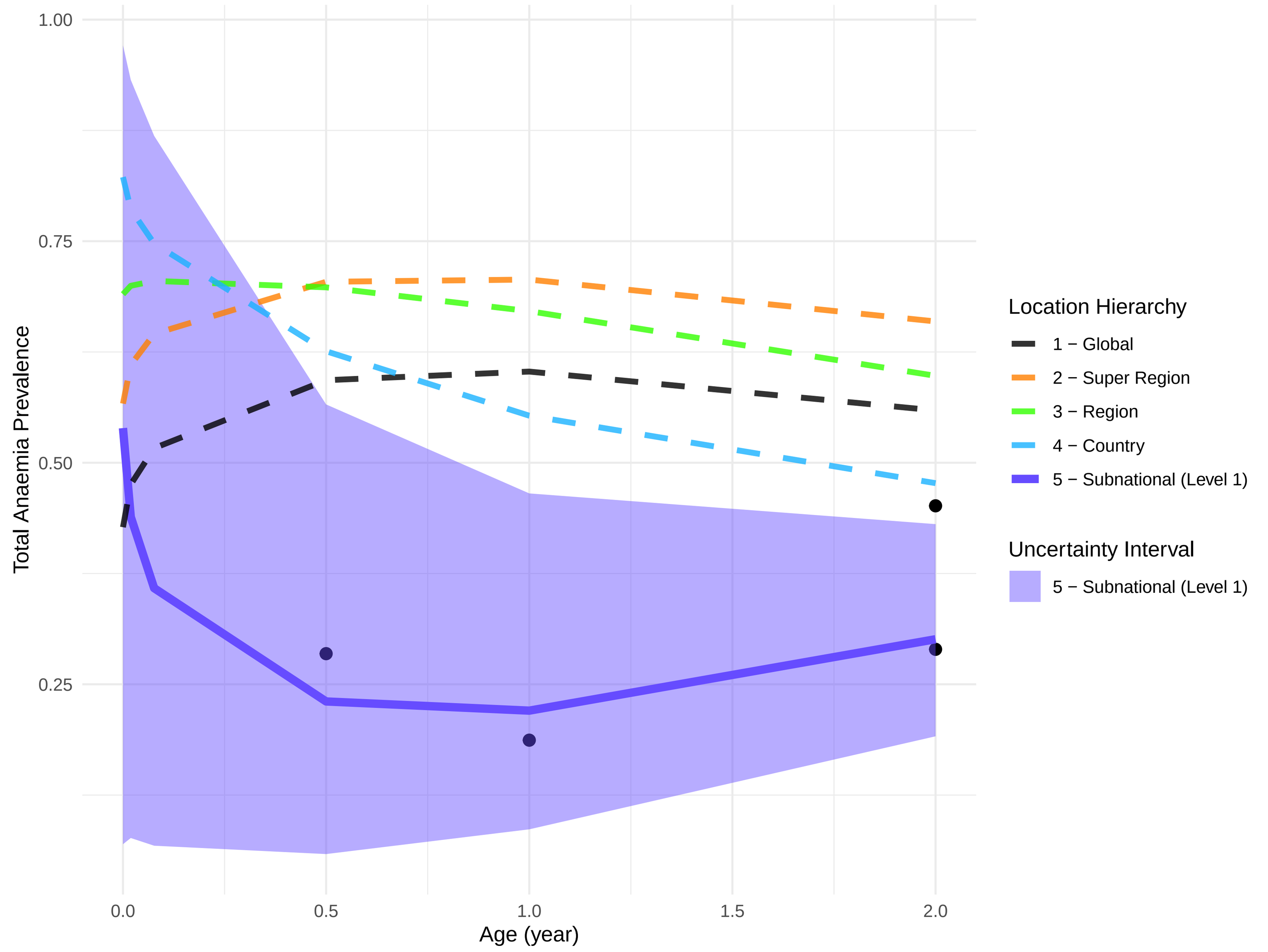

MR−BRT Age/Sex Fits − Non−pregnant Females <2 Years Old − Total Anaemia Prevalence − Nairobi
WHO−2024 Adjustment Method

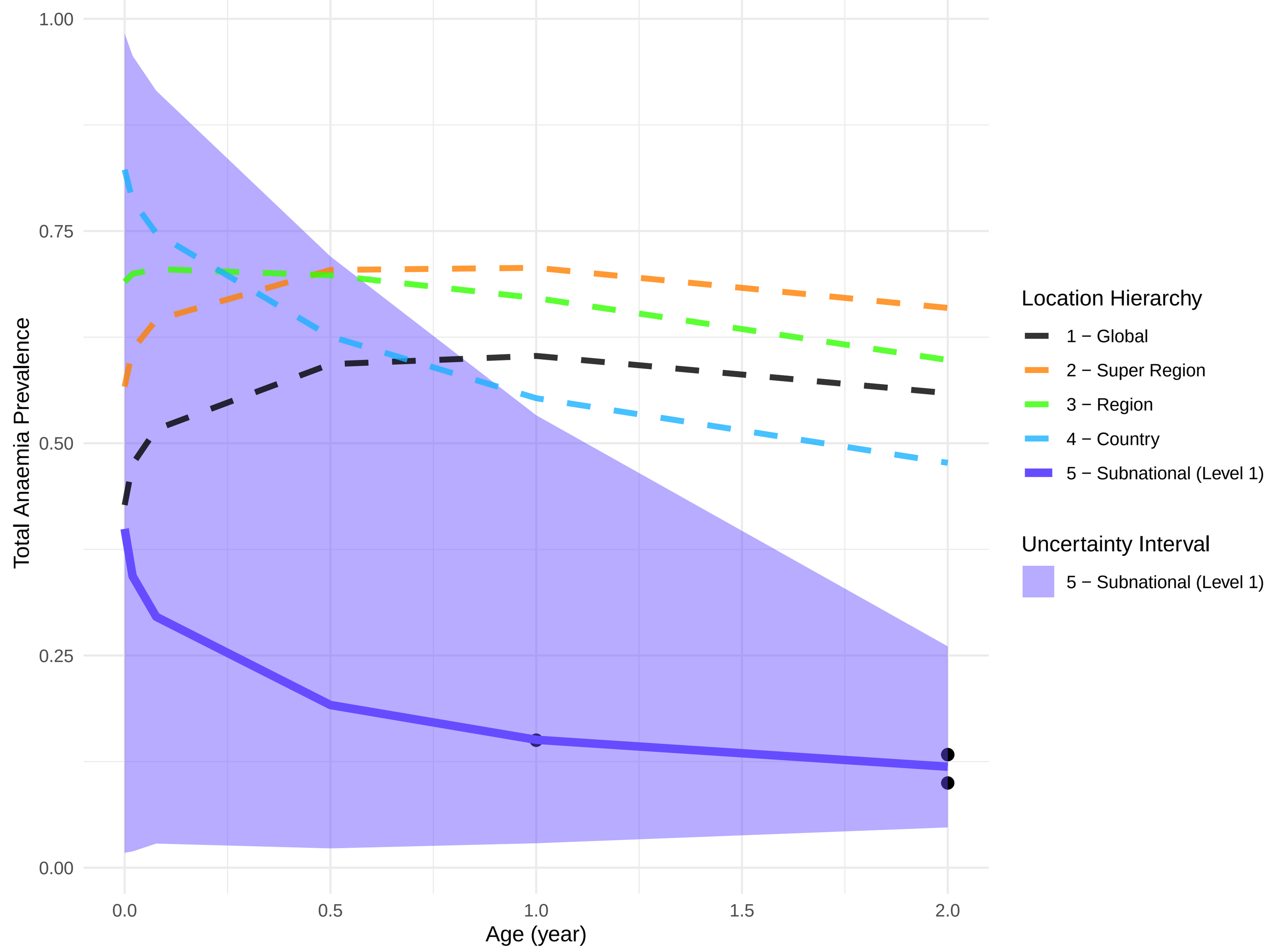

MR−BRT Age/Sex Fits − Non−pregnant Females <2 Years Old − Total Anaemia Prevalence − Nakuru
WHO−2024 Adjustment Method

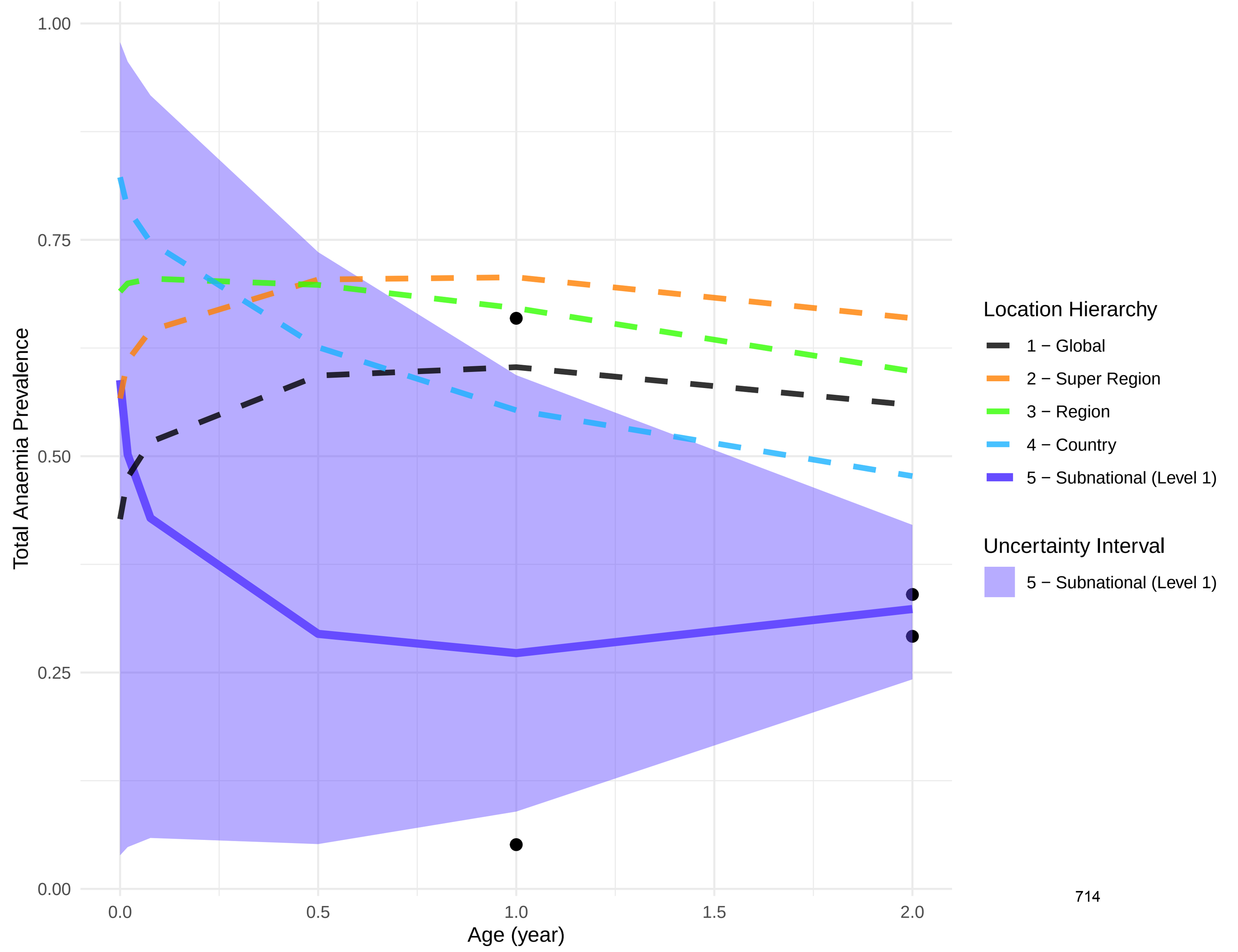

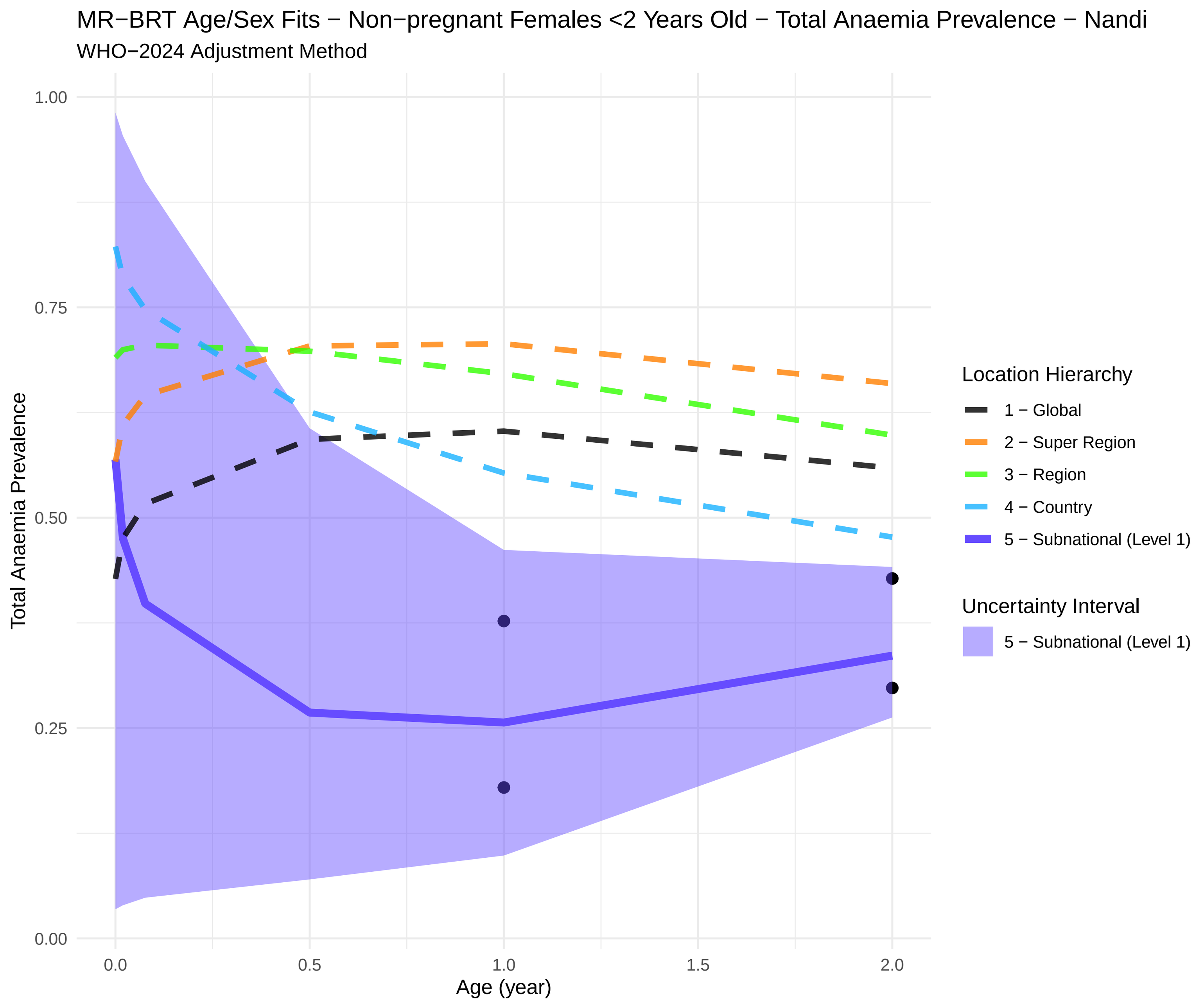
MR−BRT Age/Sex Fits − Non−pregnant Females <2 Years Old − Total Anaemia Prevalence − Nandi
WHO−2024 Adjustment Method
Total Anaemia Prevalence
Age (year)
Location Hierarchy
1 − Global
2 − Super Region
3 − Region
4 − Country
5 − Subnational (Level 1)
Uncertainty Interval
5 − Subnational (Level 1)

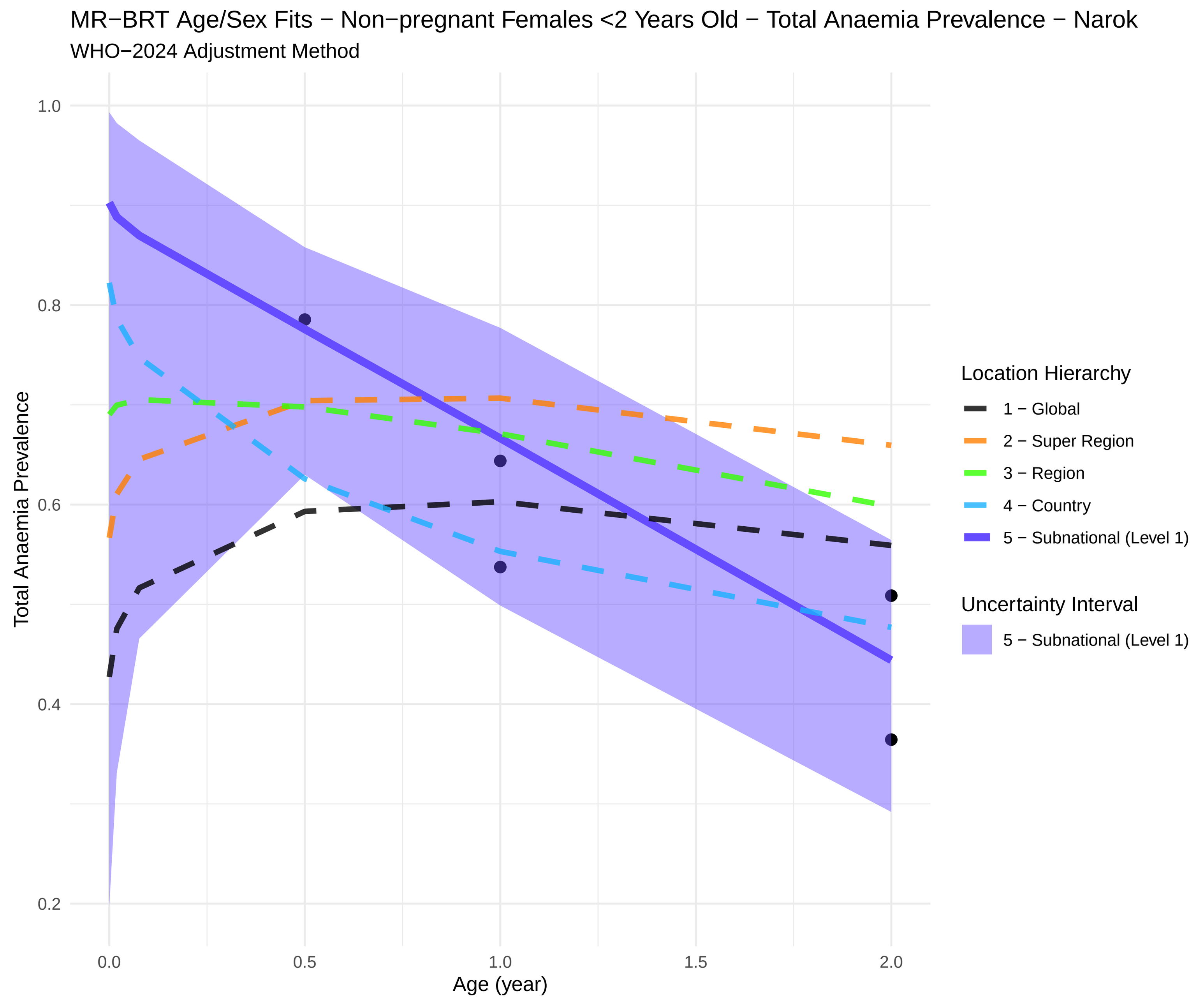
MR−BRT Age/Sex Fits − Non−pregnant Females <2 Years Old − Total Anaemia Prevalence − Narok
WHO−2024 Adjustment Method
Total Anaemia Prevalence
Age (year)
Location Hierarchy
1 − Global
2 − Super Region
3 − Region
4 − Country
5 − Subnational (Level 1)
Uncertainty Interval
5 − Subnational (Level 1)

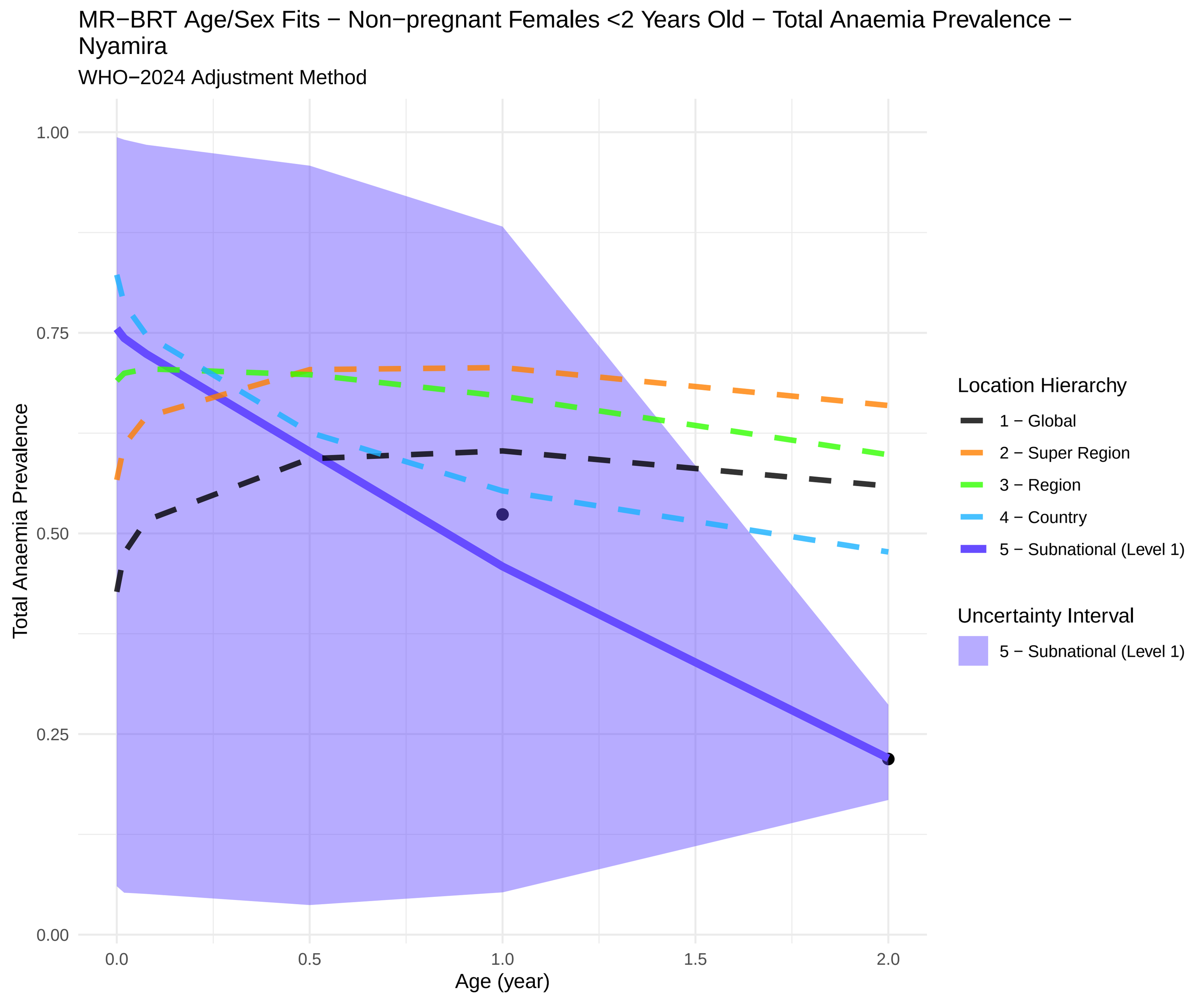
MR−BRT Age/Sex Fits − Non−pregnant Females <2 Years Old − Total Anaemia Prevalence − Nyamira
WHO−2024 Adjustment Method
Total Anaemia Prevalence
Age (year)
Location Hierarchy
1 − Global
2 − Super Region
3 − Region
4 − Country
5 − Subnational (Level 1)
Uncertainty Interval
5 − Subnational (Level 1)

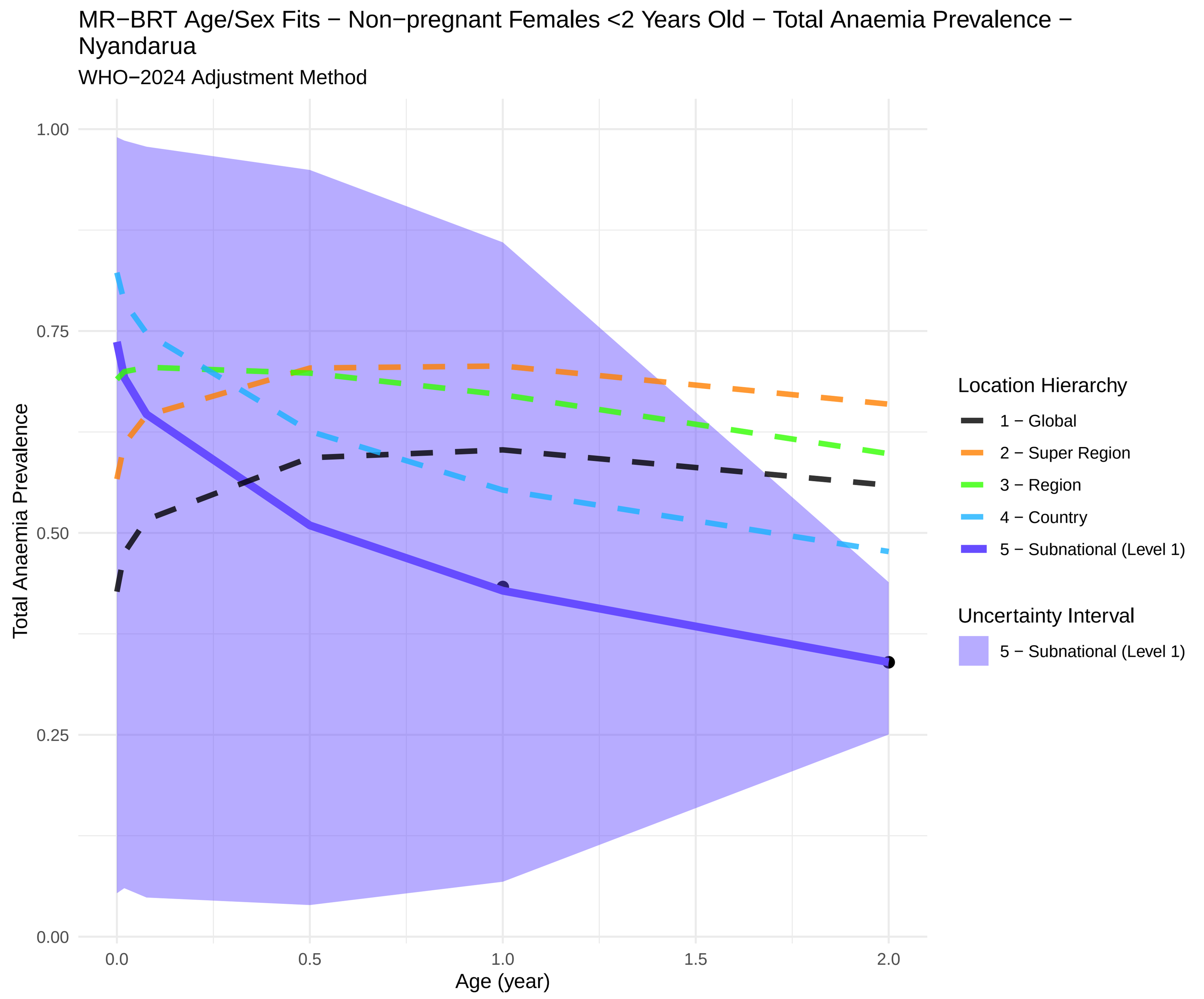
MR−BRT Age/Sex Fits − Non−pregnant Females <2 Years Old − Total Anaemia Prevalence − Nyandarua
WHO−2024 Adjustment Method
Total Anaemia Prevalence
Age (year)
Location Hierarchy
1 − Global
2 − Super Region
3 − Region
4 − Country
5 − Subnational (Level 1)
Uncertainty Interval
5 − Subnational (Level 1)

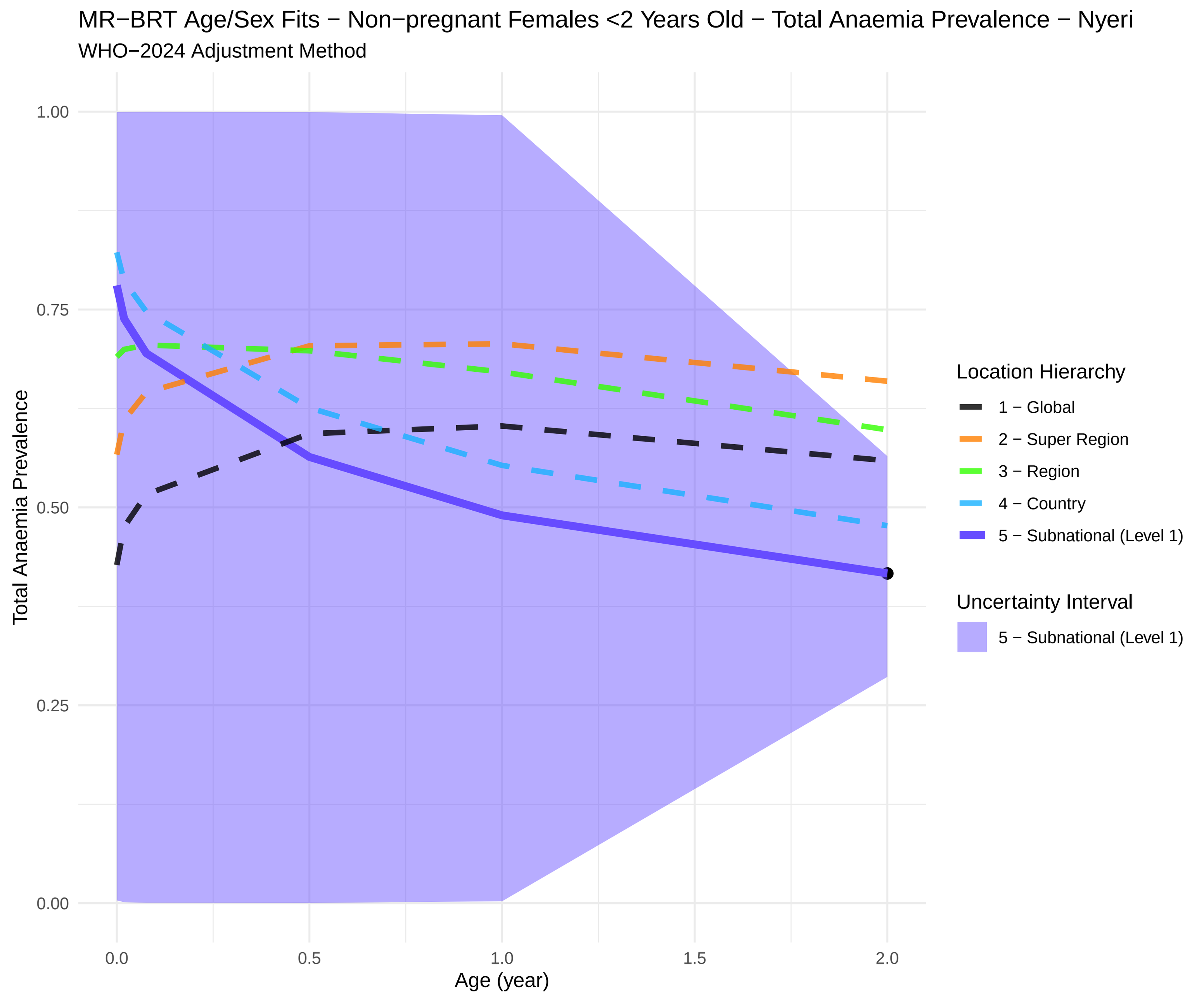
MR−BRT Age/Sex Fits − Non−pregnant Females <2 Years Old − Total Anaemia Prevalence − Nyeri
WHO−2024 Adjustment Method
Total Anaemia Prevalence
Age (year)
Location Hierarchy
1 − Global
2 − Super Region
3 − Region
4 − Country
5 − Subnational (Level 1)
Uncertainty Interval
5 − Subnational (Level 1)

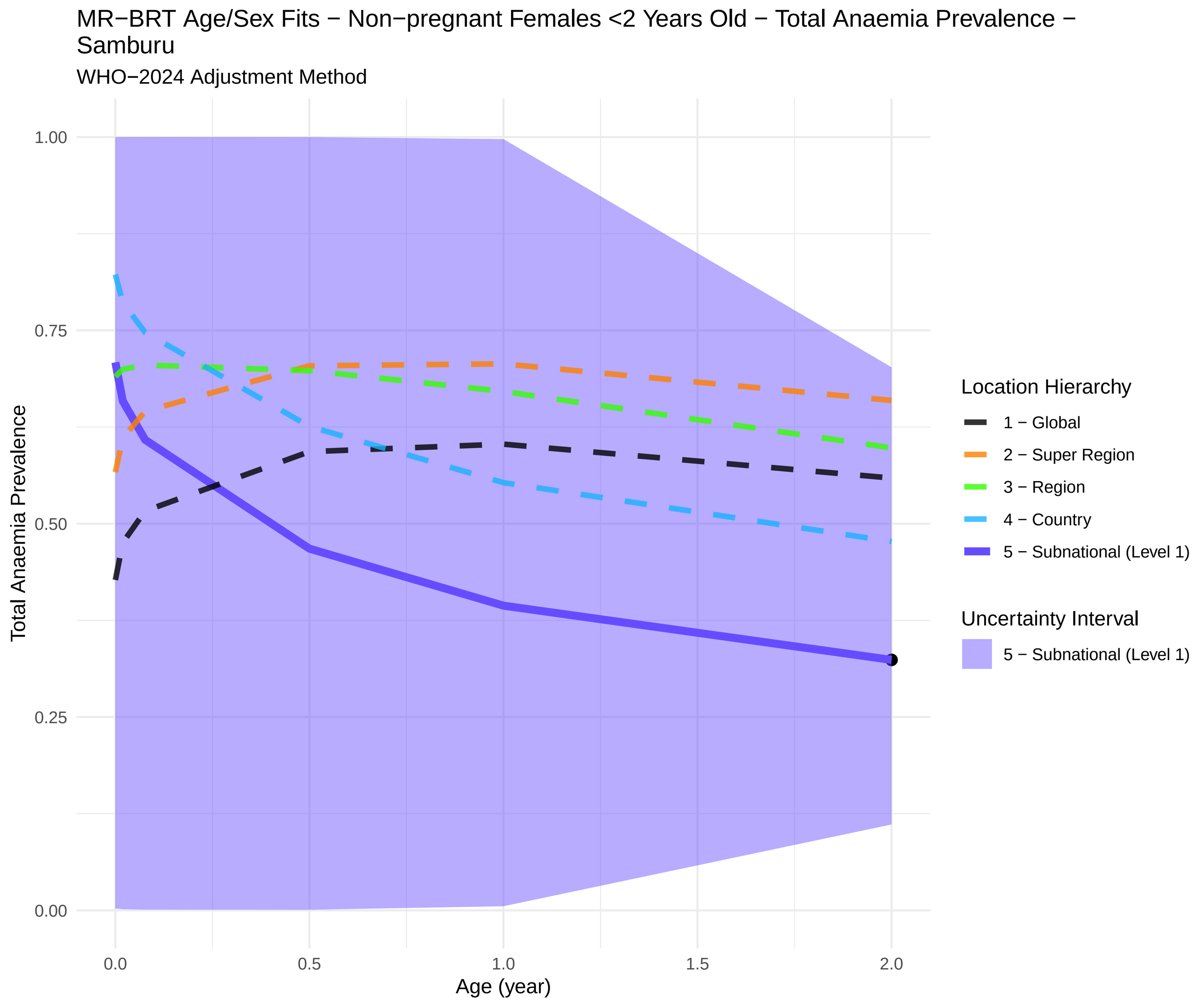
MR−BRT Age/Sex Fits − Non−pregnant Females <2 Years Old − Total Anaemia Prevalence − Samburu
WHO−2024 Adjustment Method
Total Anaemia Prevalence
Age (year)
Location Hierarchy
1 − Global
2 − Super Region
3 − Region
4 − Country
5 − Subnational (Level 1)
Uncertainty Interval
5 − Subnational (Level 1)

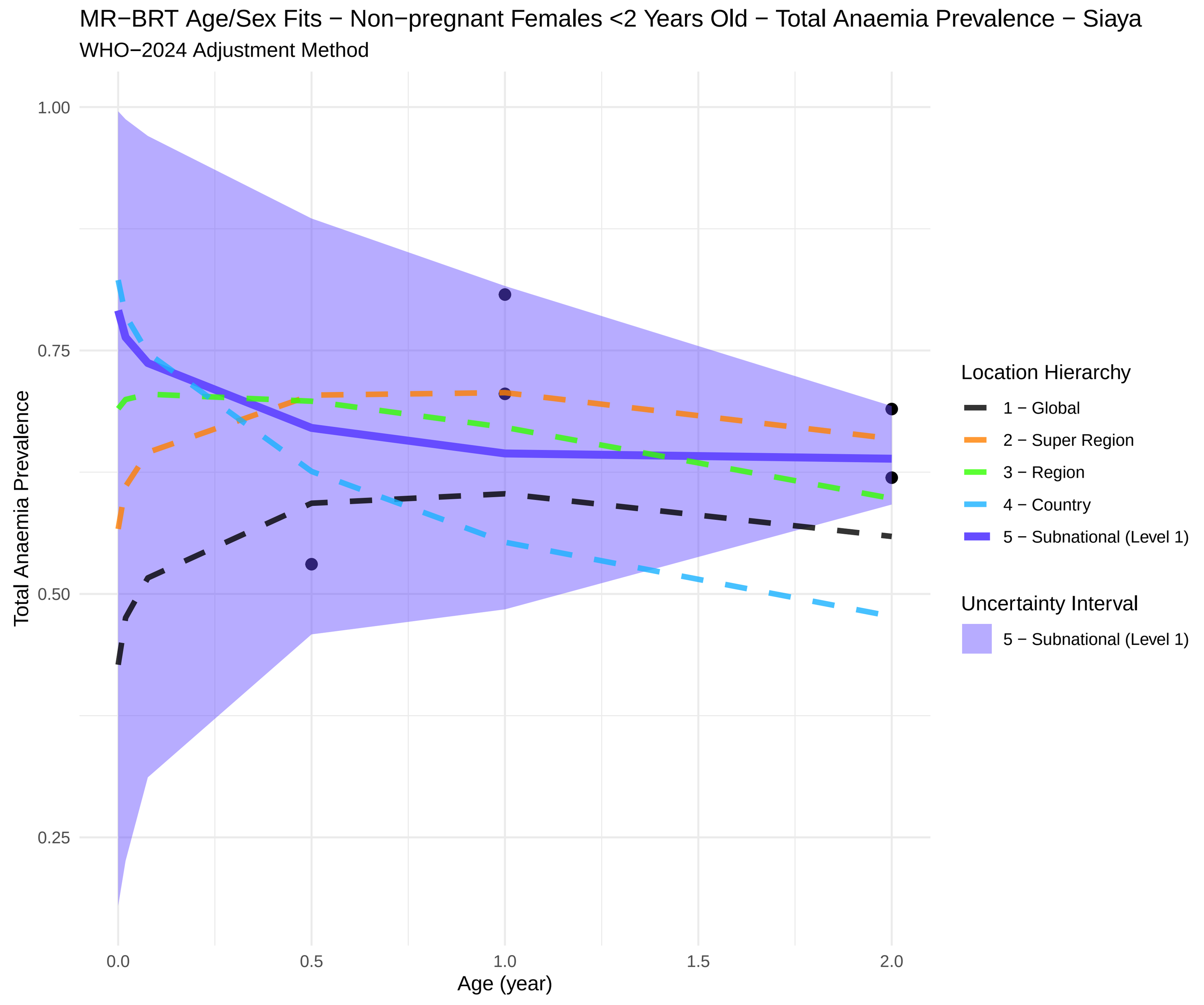
MR−BRT Age/Sex Fits − Non−pregnant Females <2 Years Old − Total Anaemia Prevalence − Siaya
WHO−2024 Adjustment Method
Total Anaemia Prevalence
Age (year)
Location Hierarchy
1 − Global
2 − Super Region
3 − Region
4 − Country
5 − Subnational (Level 1)
Uncertainty Interval
5 − Subnational (Level 1)

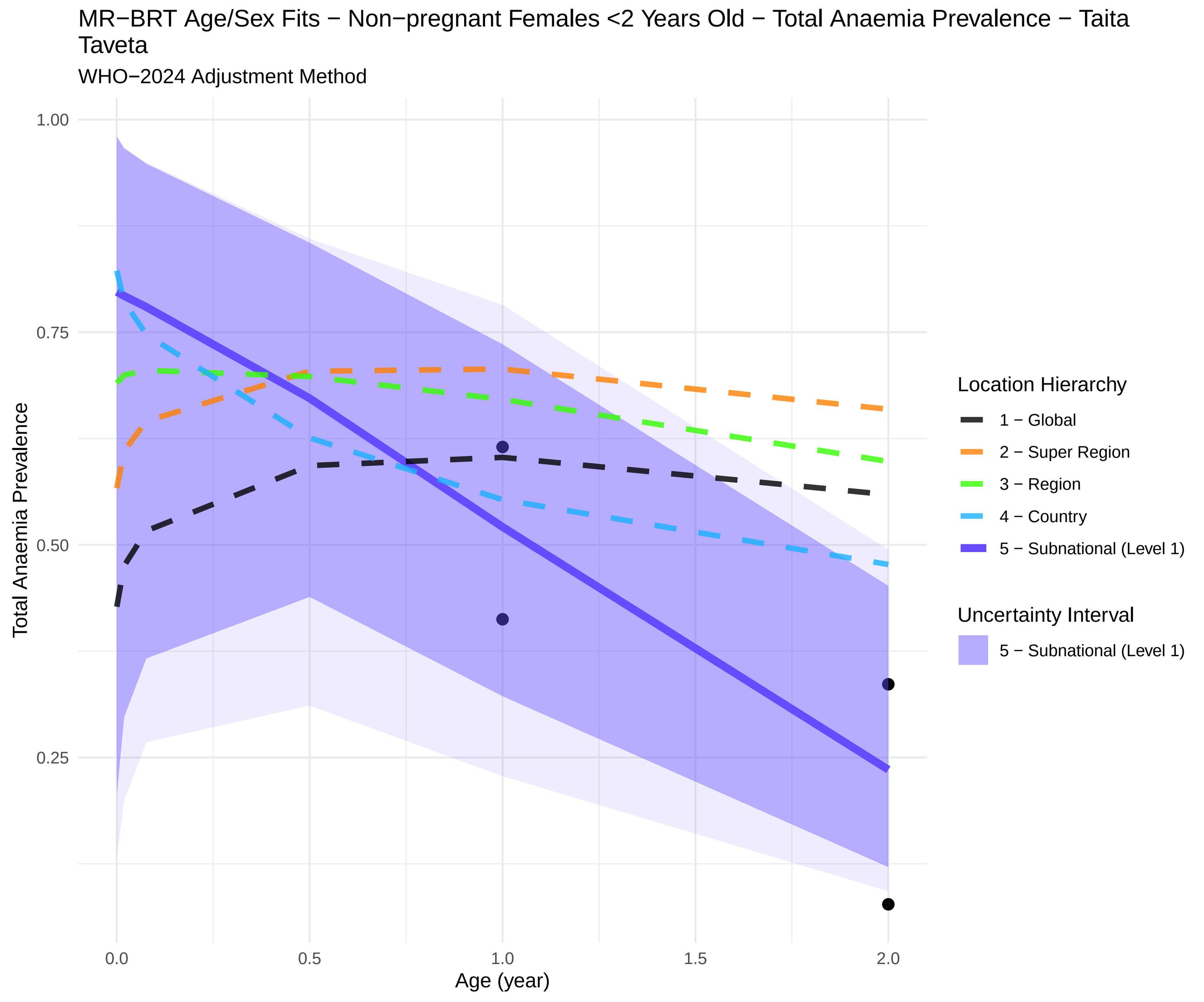
MR−BRT Age/Sex Fits − Non−pregnant Females <2 Years Old − Total Anaemia Prevalence − Taita Taveta
WHO−2024 Adjustment Method
Total Anaemia Prevalence
Age (year)
Location Hierarchy
1 − Global
2 − Super Region
3 − Region
4 − Country
5 − Subnational (Level 1)
Uncertainty Interval
5 − Subnational (Level 1)

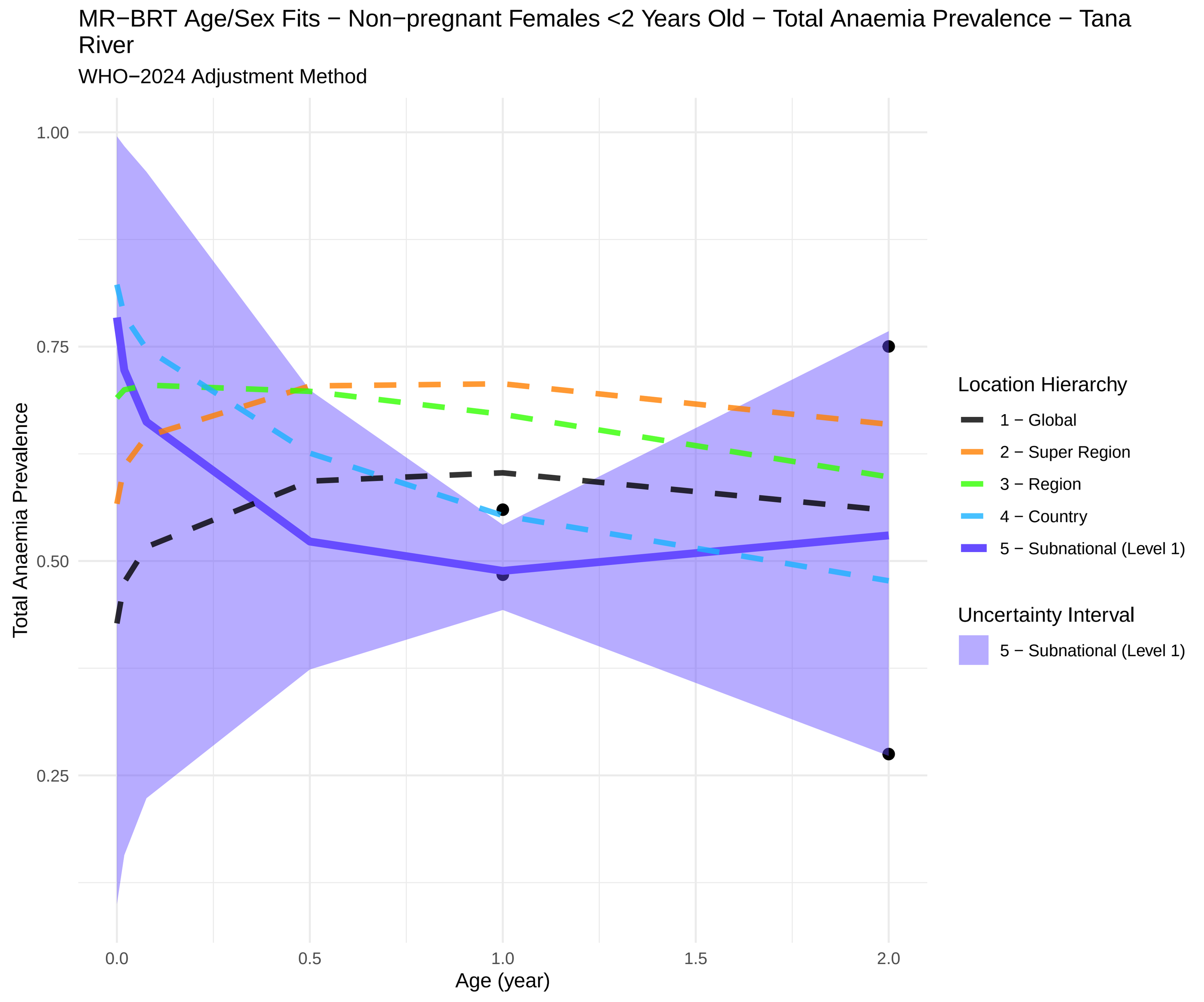
MR−BRT Age/Sex Fits − Non−pregnant Females <2 Years Old − Total Anaemia Prevalence − Tana River
WHO−2024 Adjustment Method
Total Anaemia Prevalence
Age (year)
Location Hierarchy
1 − Global
2 − Super Region
3 − Region
4 − Country
5 − Subnational (Level 1)
Uncertainty Interval
5 − Subnational (Level 1)

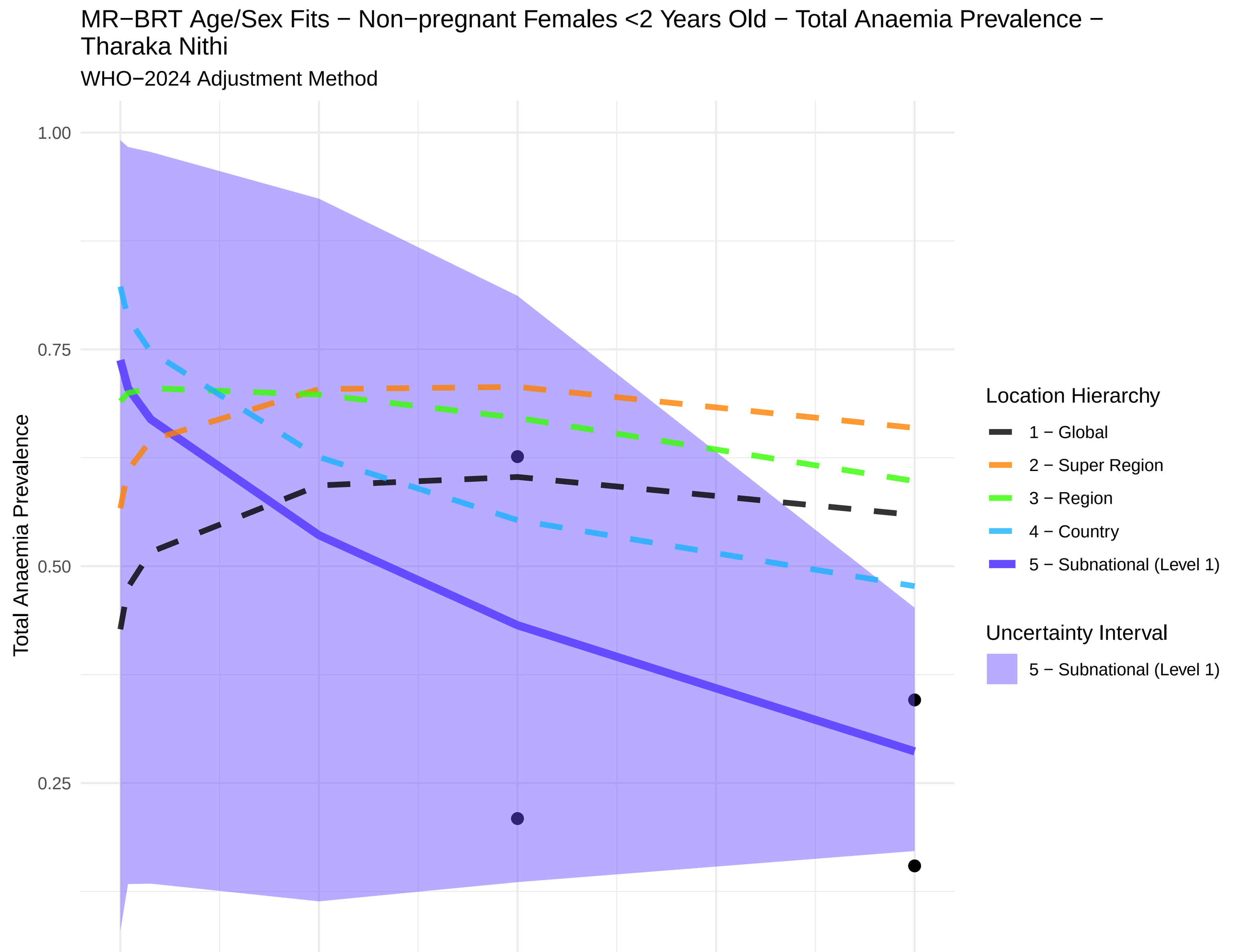
MR−BRT Age/Sex Fits − Non−pregnant Females <2 Years Old − Total Anaemia Prevalence − Tharaka Nithi
WHO−2024 Adjustment Method
Total Anaemia Prevalence
Age (year)
Location Hierarchy
1 − Global
2 − Super Region
3 − Region
4 − Country
5 − Subnational (Level 1)
Uncertainty Interval
5 − Subnational (Level 1)

MR−BRT Age/Sex Fits − Non−pregnant Females <2 Years Old − Total Anaemia Prevalence − Trans Nzoia
WHO−2024 Adjustment Method

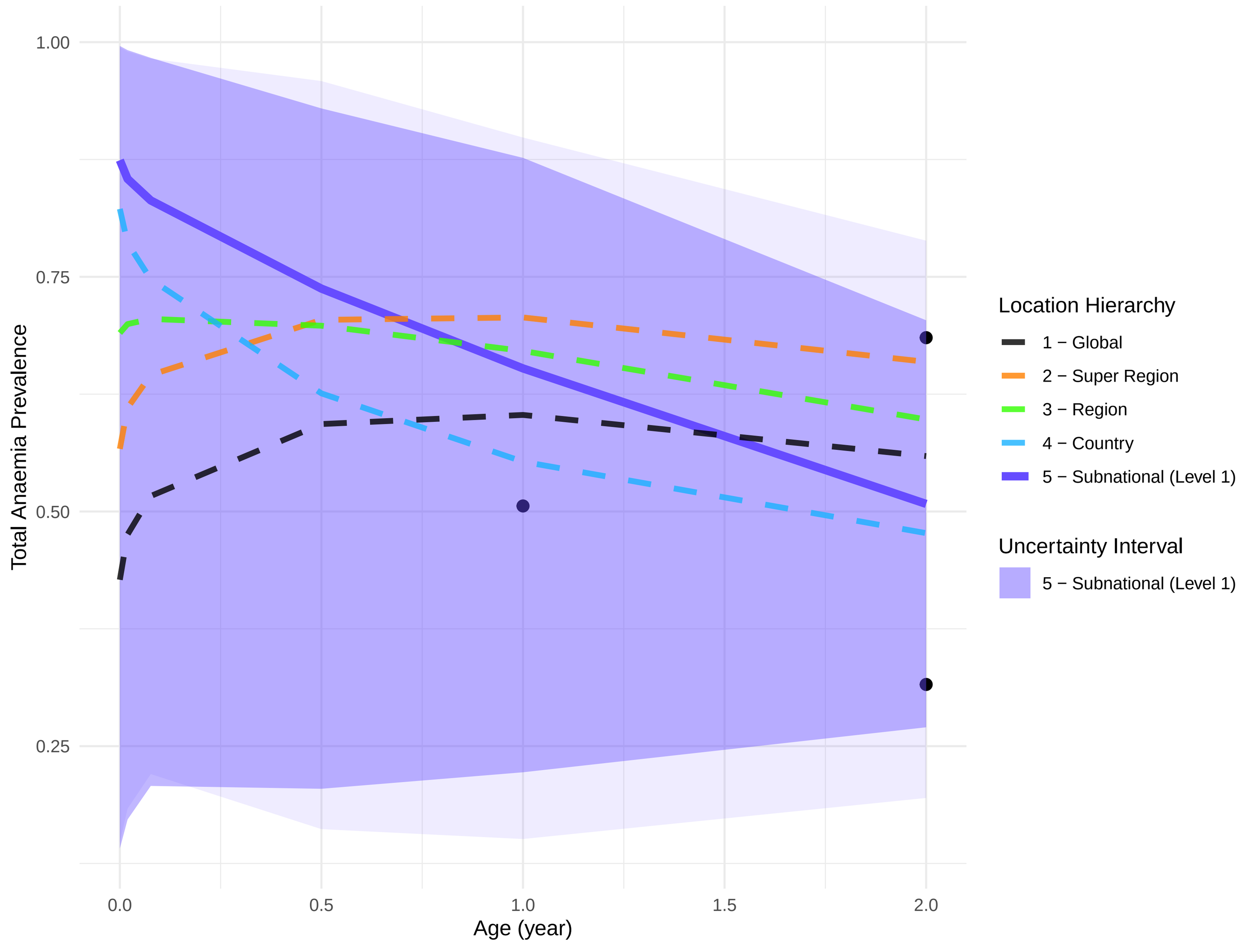

MR−BRT Age/Sex Fits − Non−pregnant Females <2 Years Old − Total Anaemia Prevalence − Turkana
WHO−2024 Adjustment Method

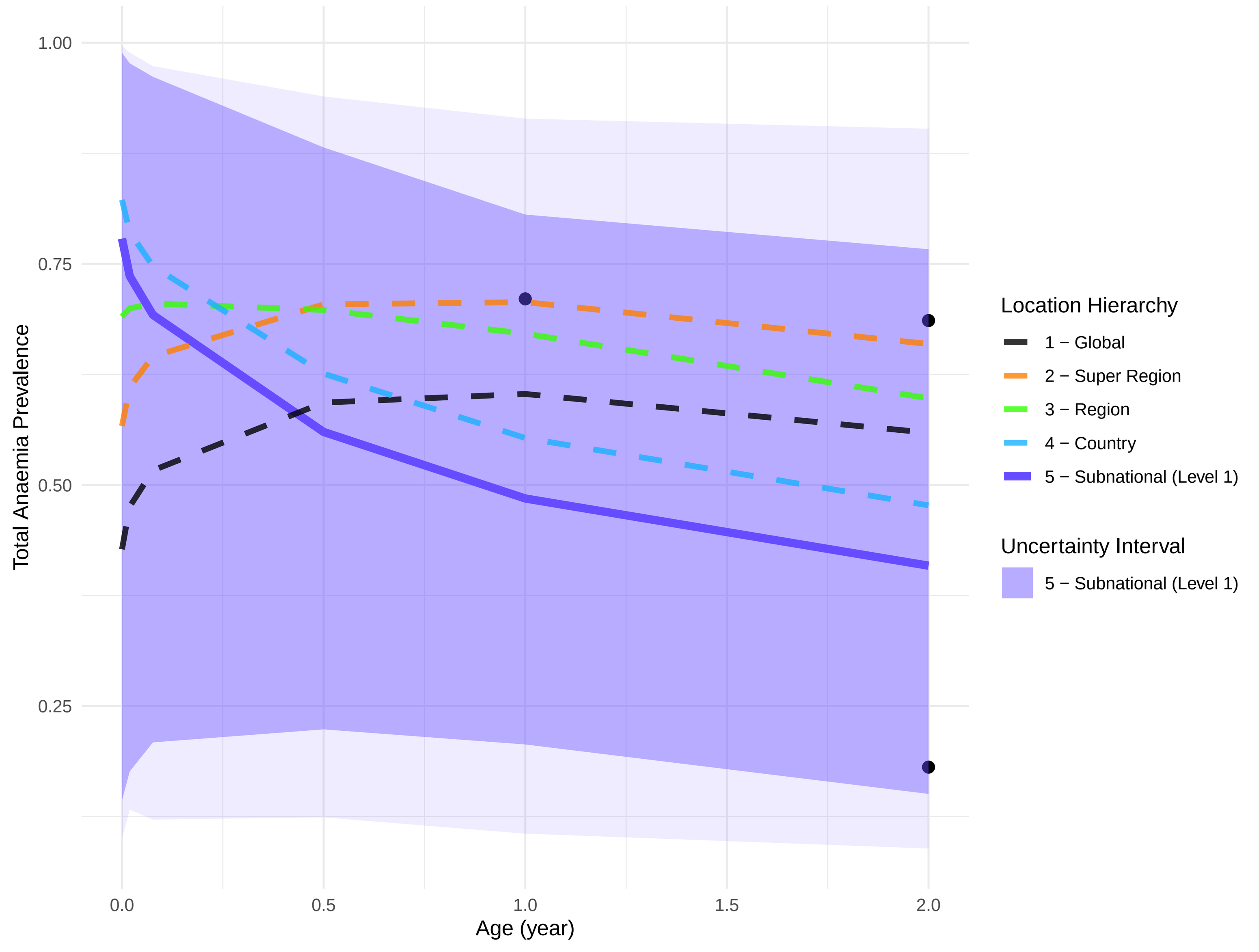

MR−BRT Age/Sex Fits − Non−pregnant Females <2 Years Old − Total Anaemia Prevalence − Uasin Gishu
WHO−2024 Adjustment Method

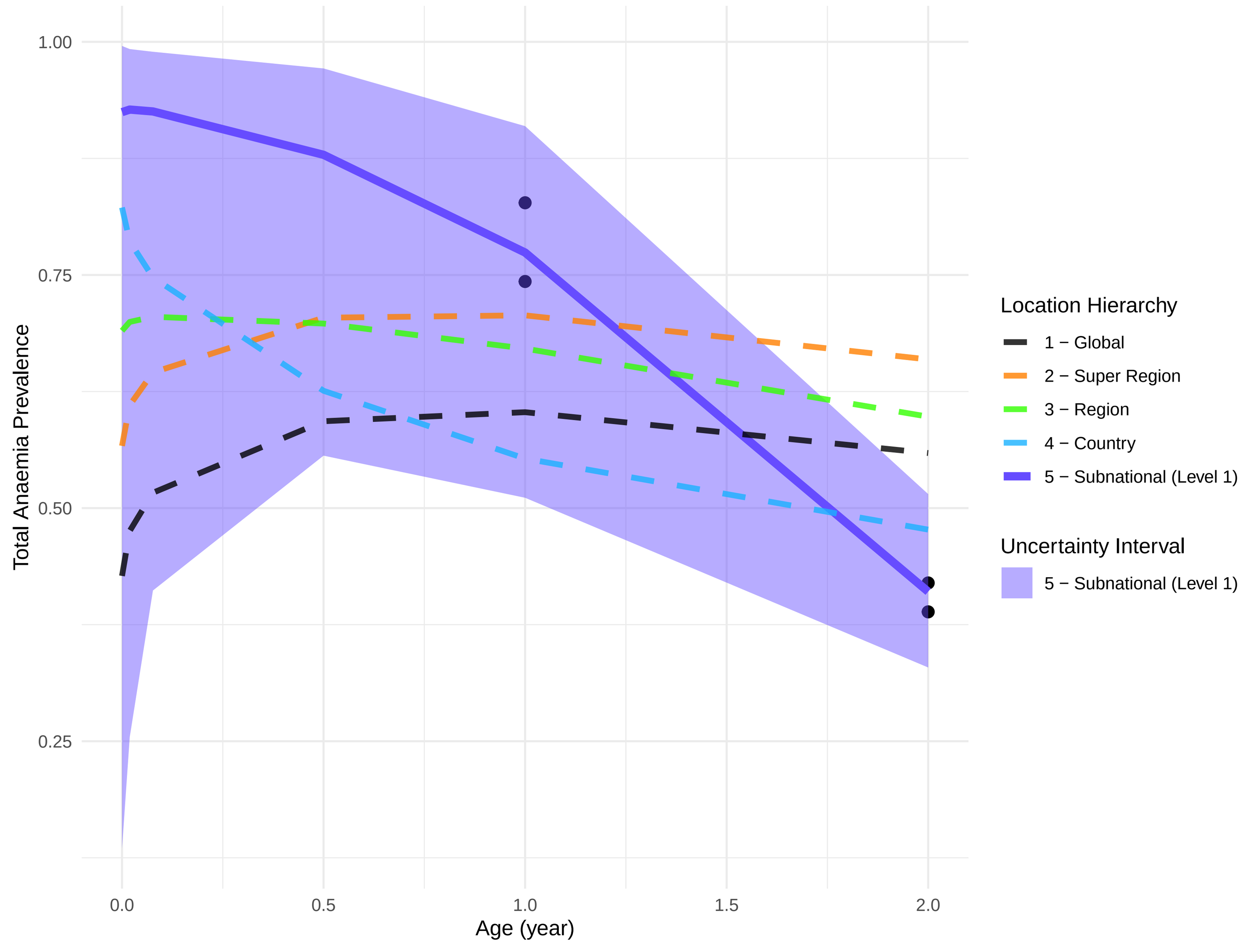

MR−BRT Age/Sex Fits − Non−pregnant Females <2 Years Old − Total Anaemia Prevalence − Vihiga
WHO−2024 Adjustment Method

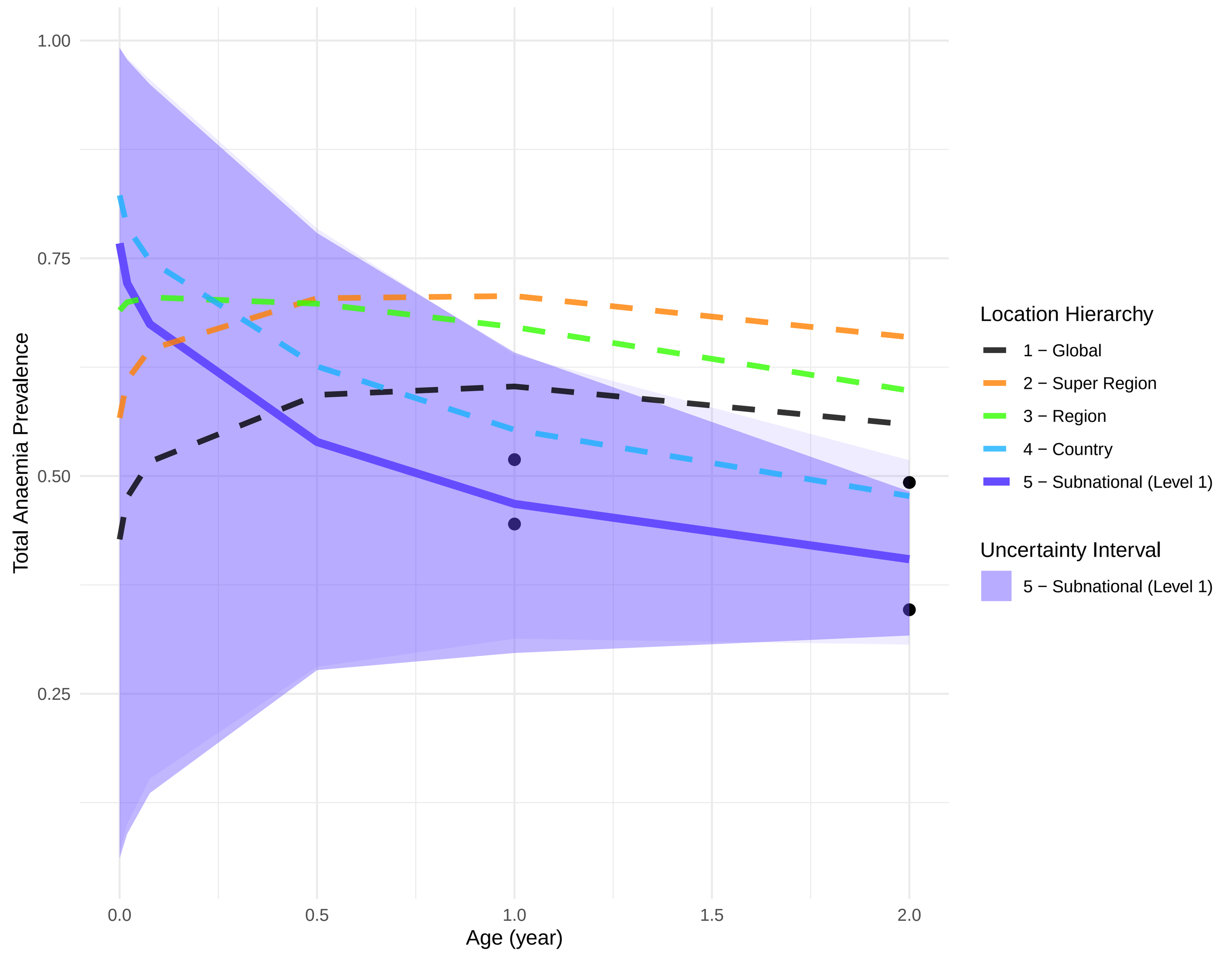

MR−BRT Age/Sex Fits − Non−pregnant Females <2 Years Old − Total Anaemia Prevalence − Wajir
WHO−2024 Adjustment Method

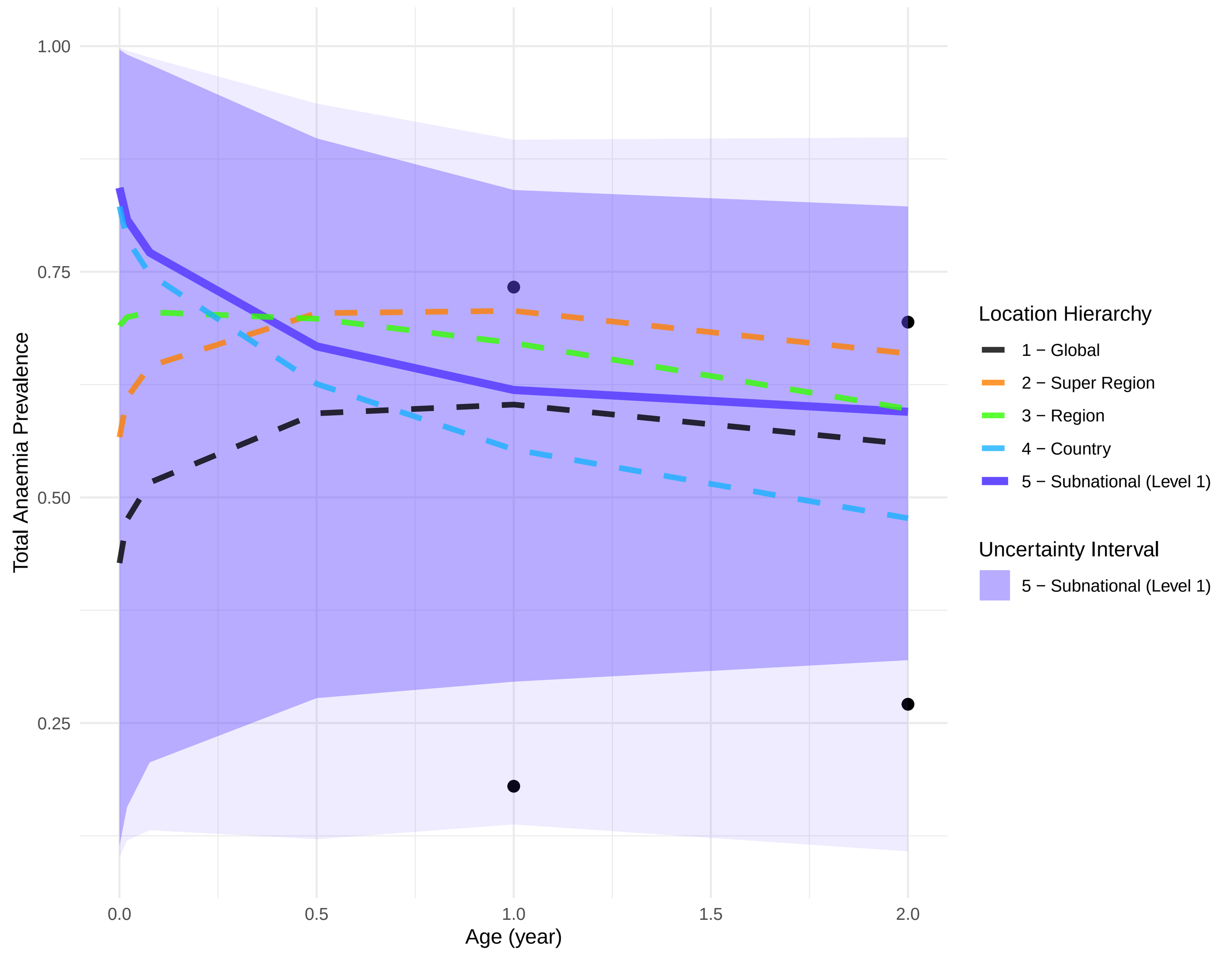

MR−BRT Age/Sex Fits − Non−pregnant Females <2 Years Old − Total Anaemia Prevalence − West Pokot
WHO−2024 Adjustment Method

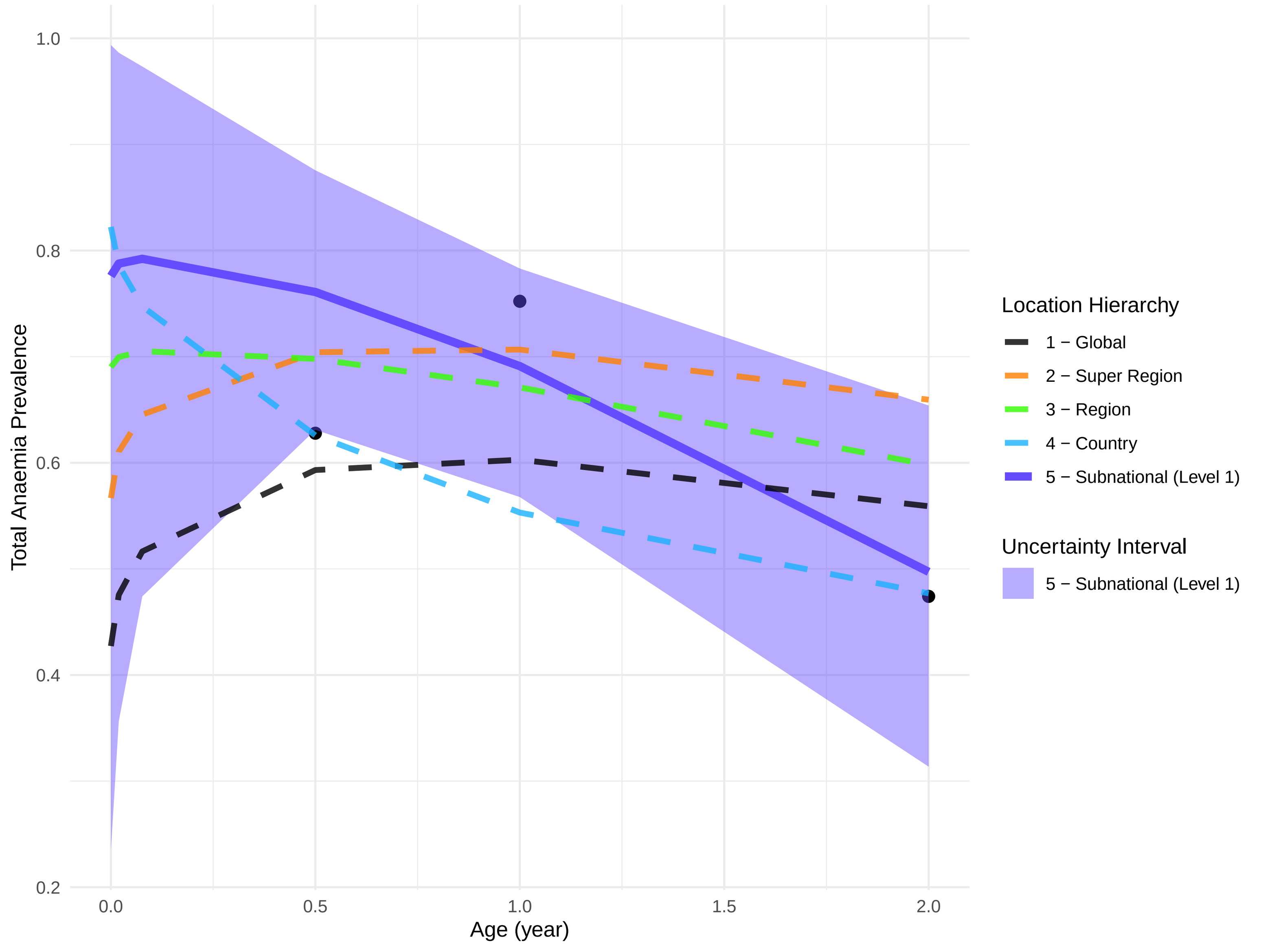

MR−BRT Age/Sex Fits − Non−pregnant Females <2 Years Old − Total Anaemia Prevalence − Madagascar
WHO−2024 Adjustment Method

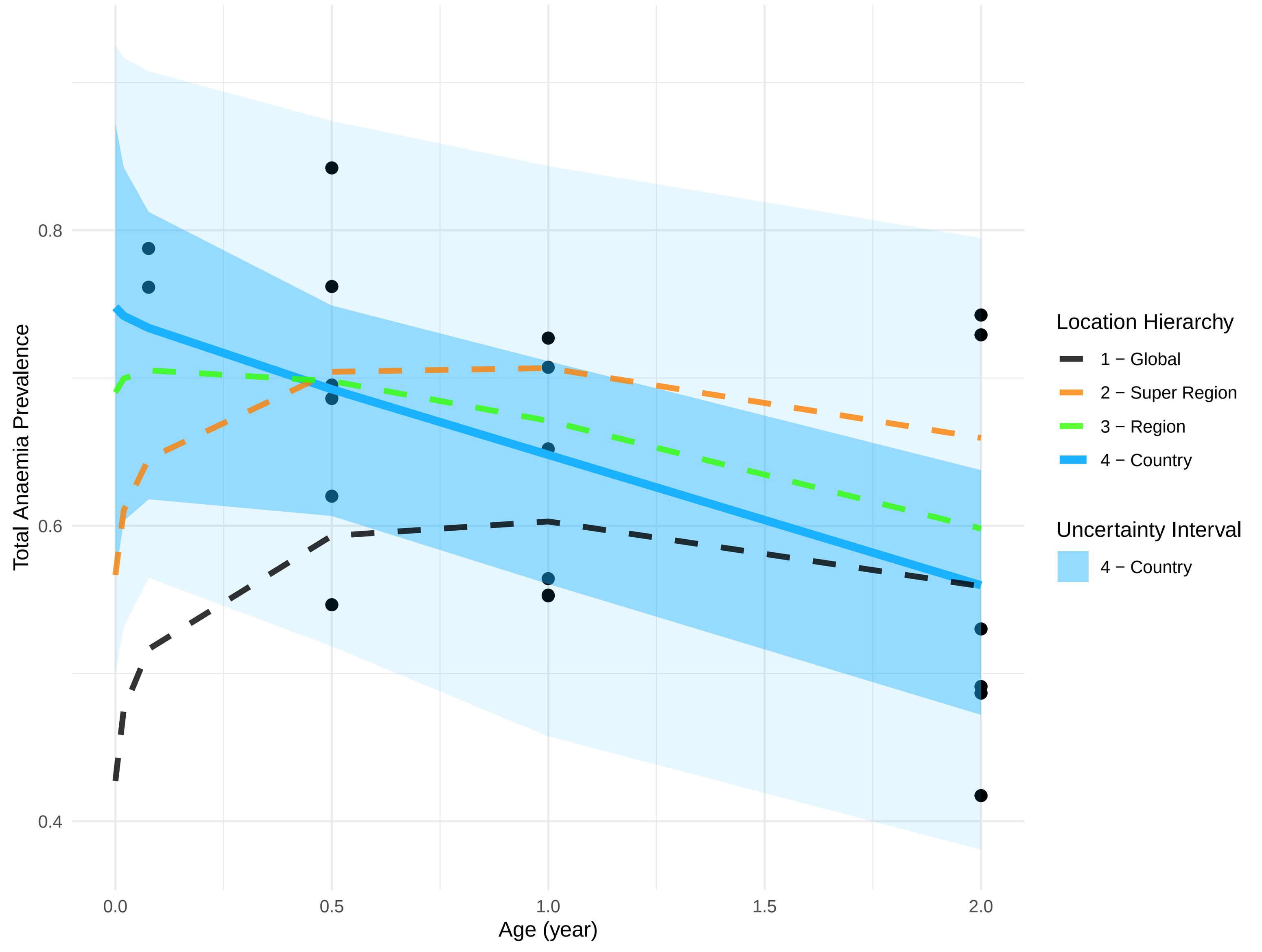

MR−BRT Age/Sex Fits − Non−pregnant Females <2 Years Old − Total Anaemia Prevalence − Malawi
WHO−2024 Adjustment Method

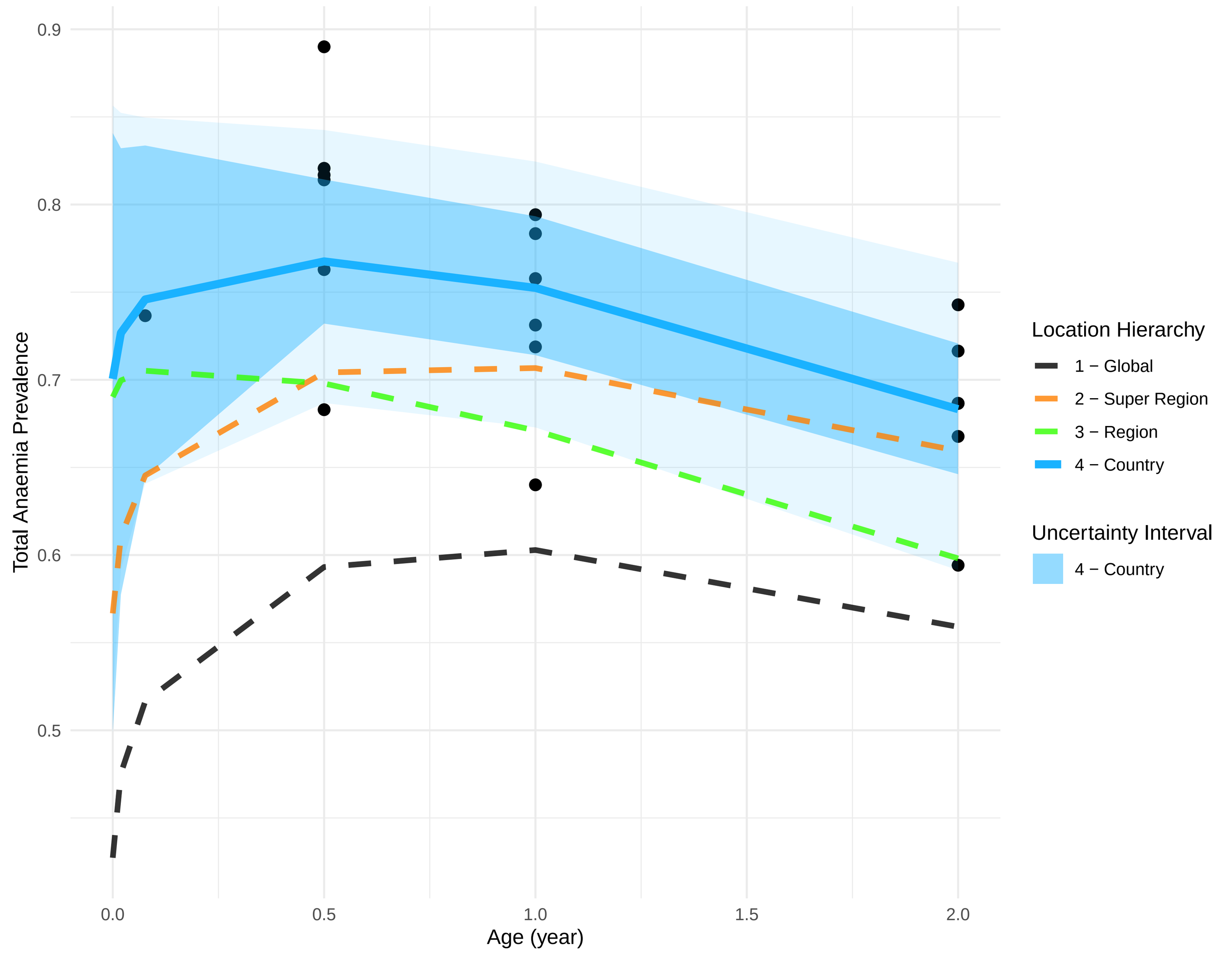

MR−BRT Age/Sex Fits − Non−pregnant Females <2 Years Old − Total Anaemia Prevalence − Mozambique
WHO−2024 Adjustment Method

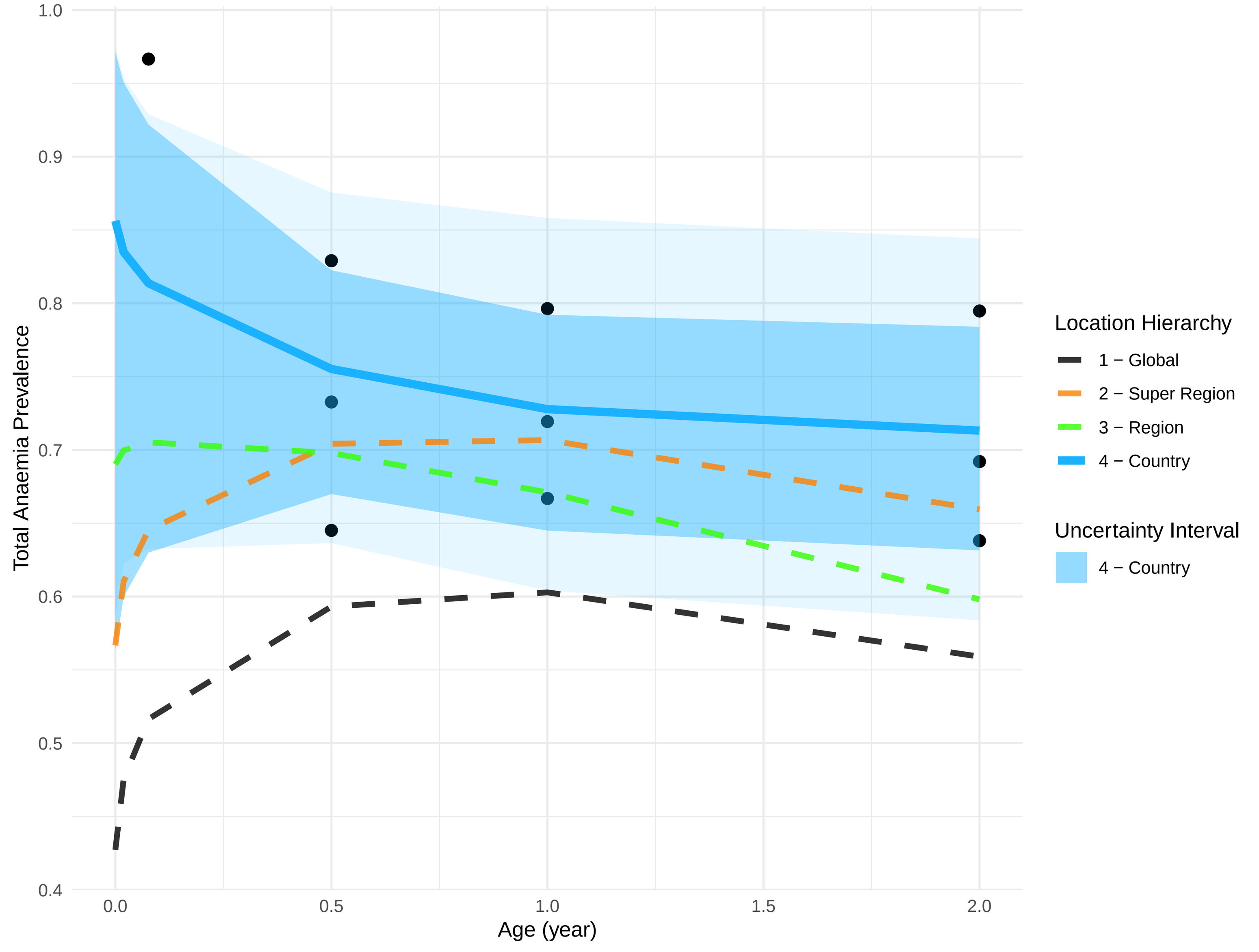

MR−BRT Age/Sex Fits − Non−pregnant Females <2 Years Old − Total Anaemia Prevalence − Rwanda
WHO−2024 Adjustment Method

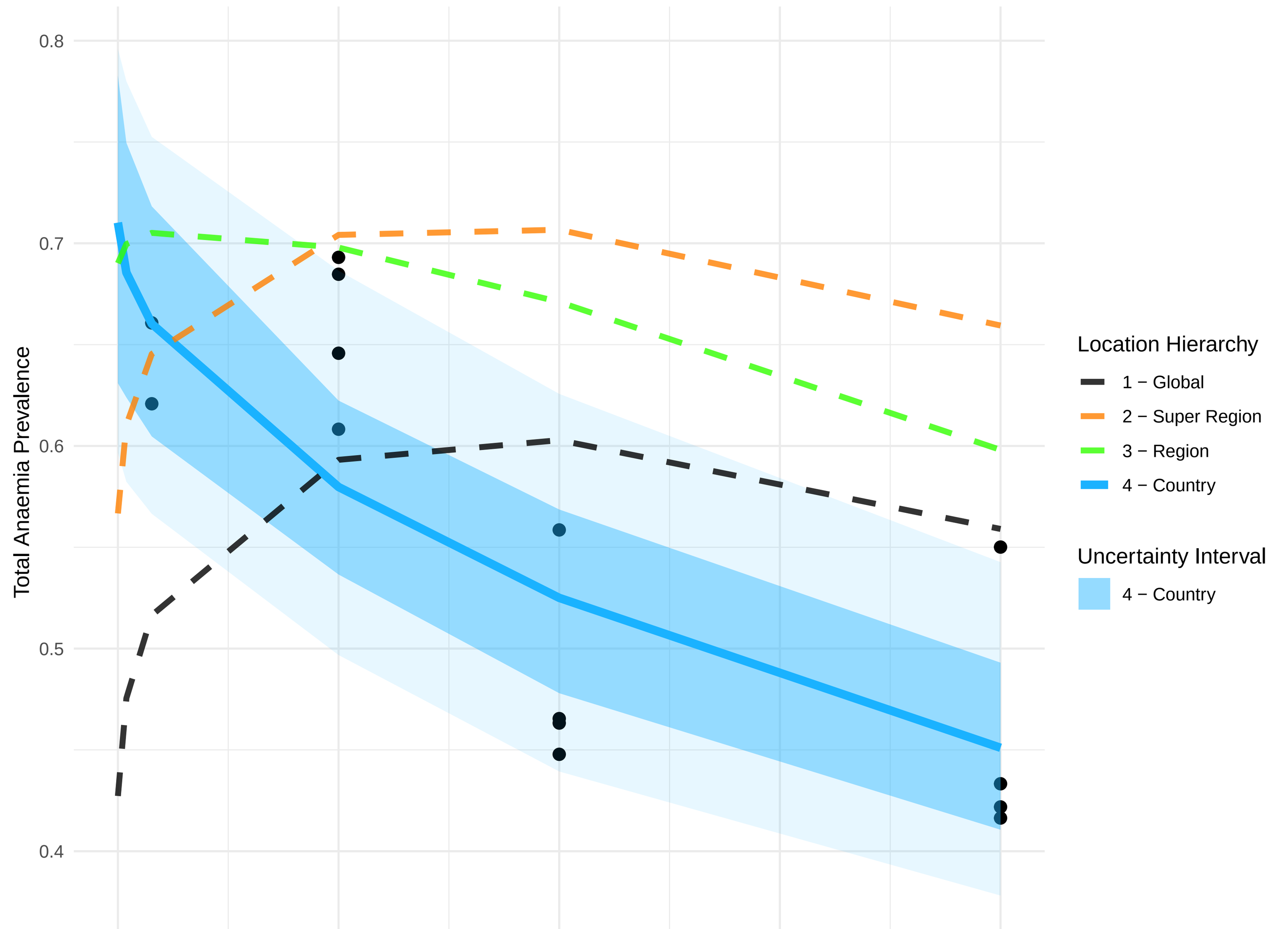

MR−BRT Age/Sex Fits − Non−pregnant Females <2 Years Old − Total Anaemia Prevalence − Uganda
WHO−2024 Adjustment Method

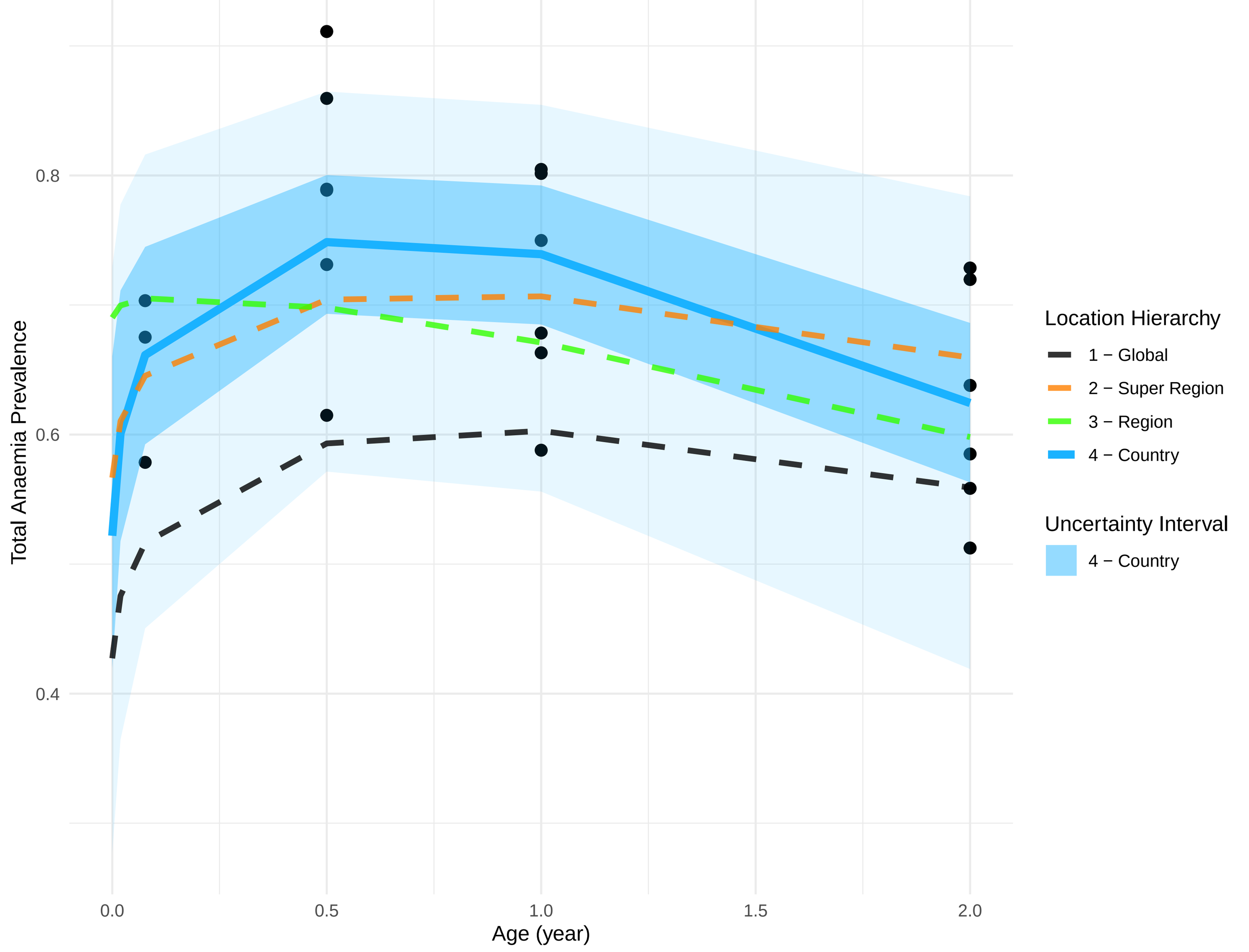

MR−BRT Age/Sex Fits − Non−pregnant Females <2 Years Old − Total Anaemia Prevalence − United Republic of Tanzania
WHO−2024 Adjustment Method

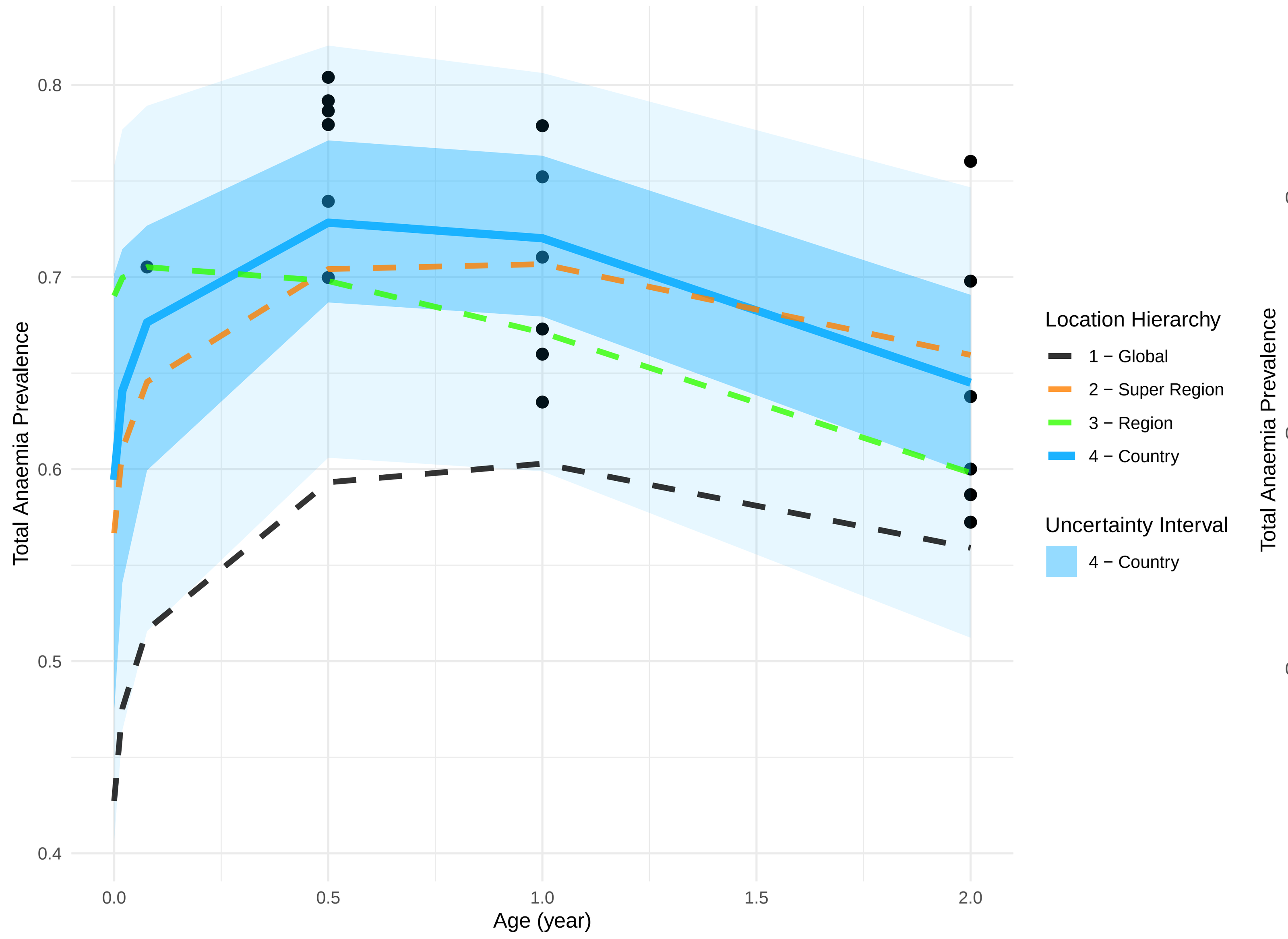

MR−BRT Age/Sex Fits − Non−pregnant Females <2 Years Old − Total Anaemia Prevalence − Zambia
WHO−2024 Adjustment Method

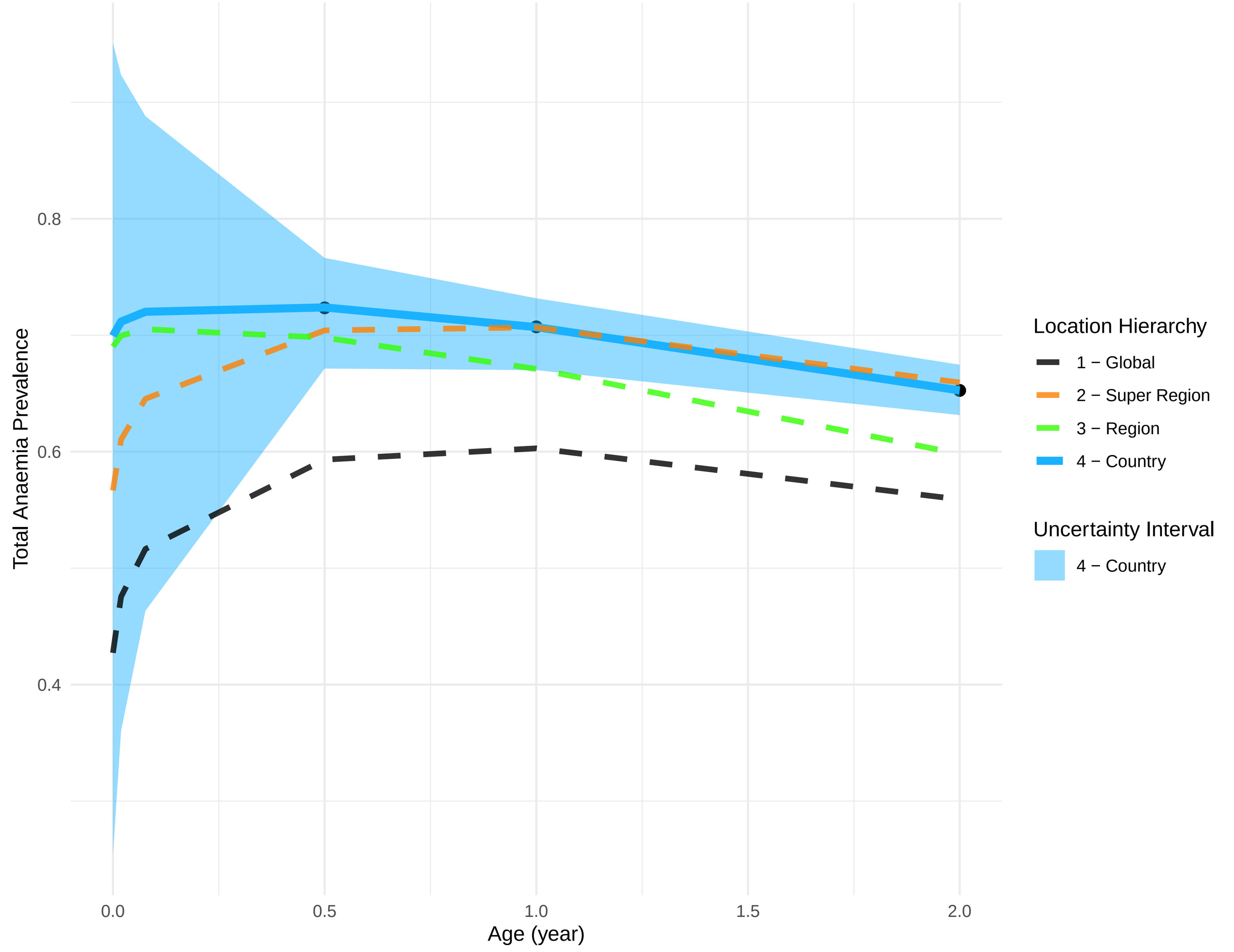

MR−BRT Age/Sex Fits − Non−pregnant Females <2 Years Old − Total Anaemia Prevalence − Southern Sub−Saharan Africa
WHO−2024 Adjustment Method

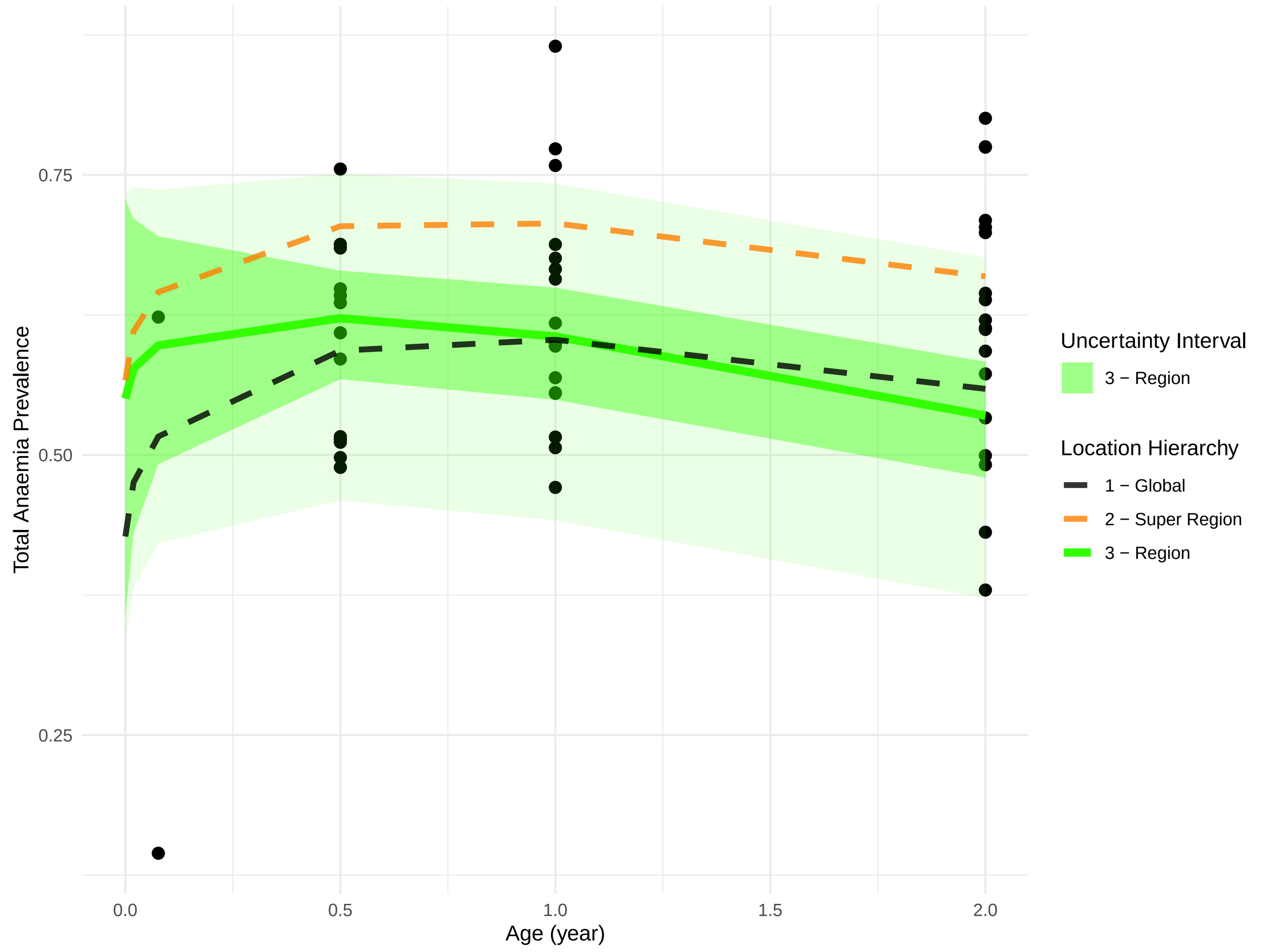

MR−BRT Age/Sex Fits − Non−pregnant Females <2 Years Old − Total Anaemia Prevalence − Eswatini
WHO−2024 Adjustment Method

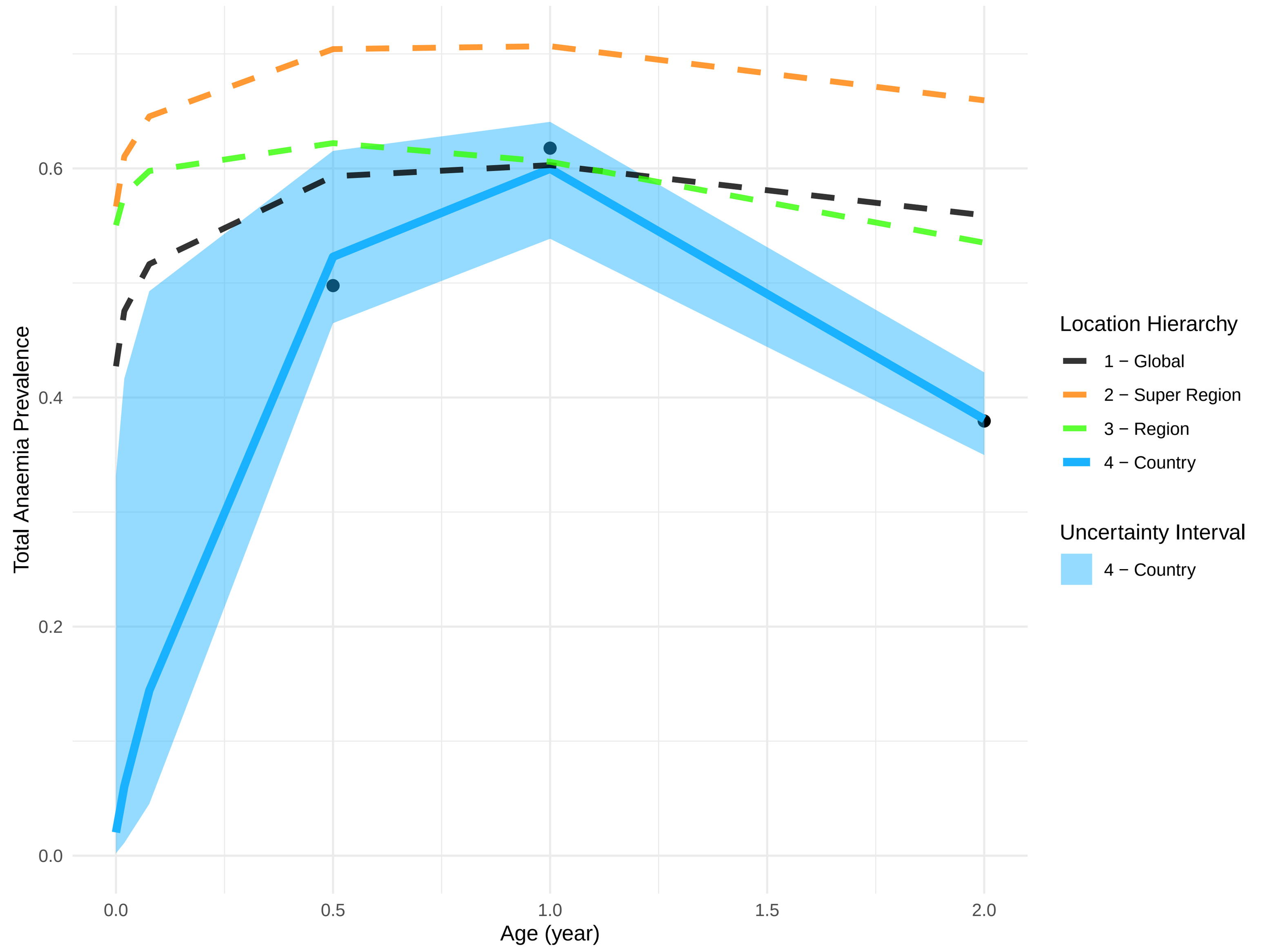

MR−BRT Age/Sex Fits − Non−pregnant Females <2 Years Old − Total Anaemia Prevalence − Lesotho
WHO−2024 Adjustment Method

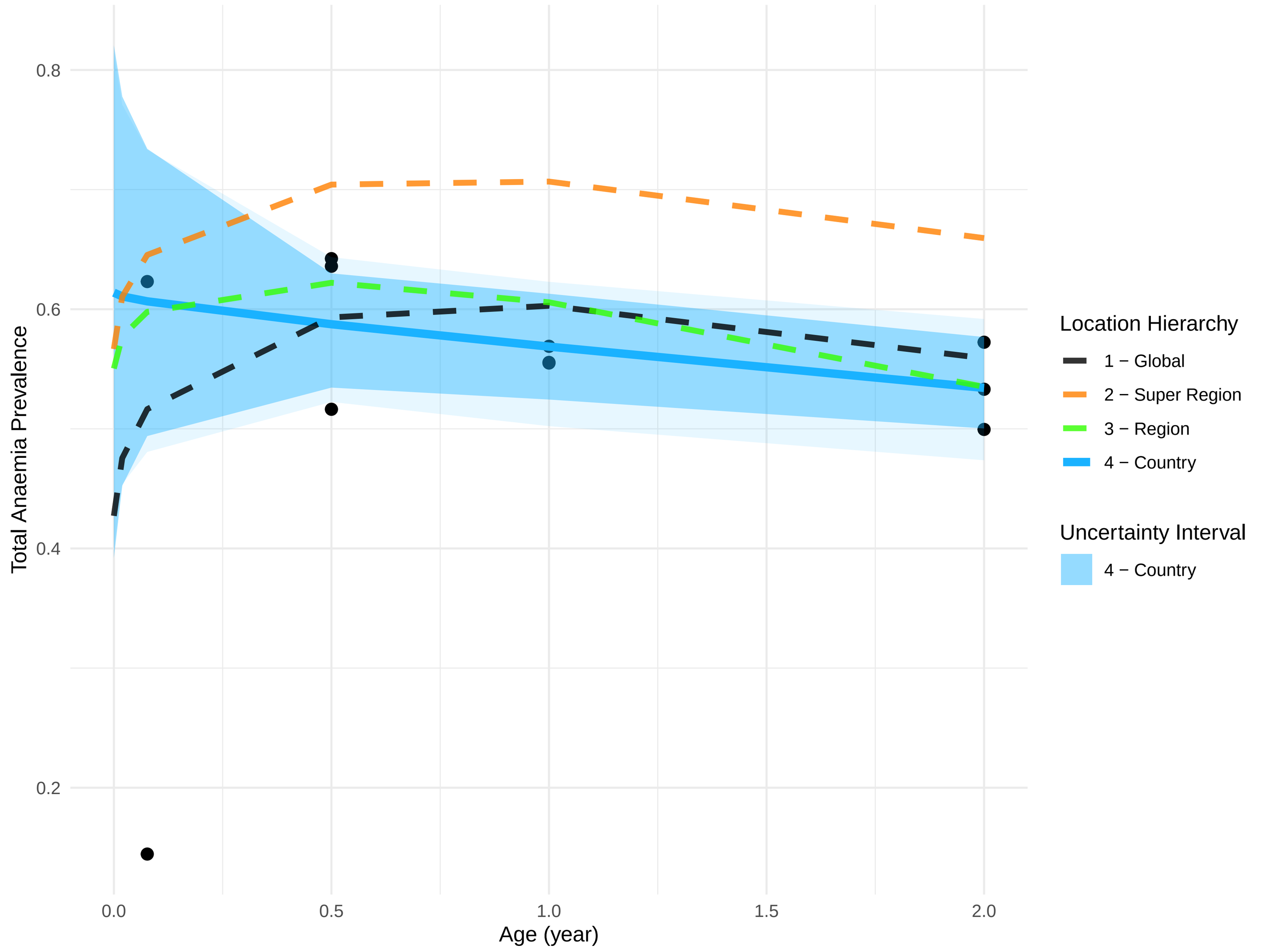

MR−BRT Age/Sex Fits − Non−pregnant Females <2 Years Old − Total Anaemia Prevalence − Namibia
WHO−2024 Adjustment Method

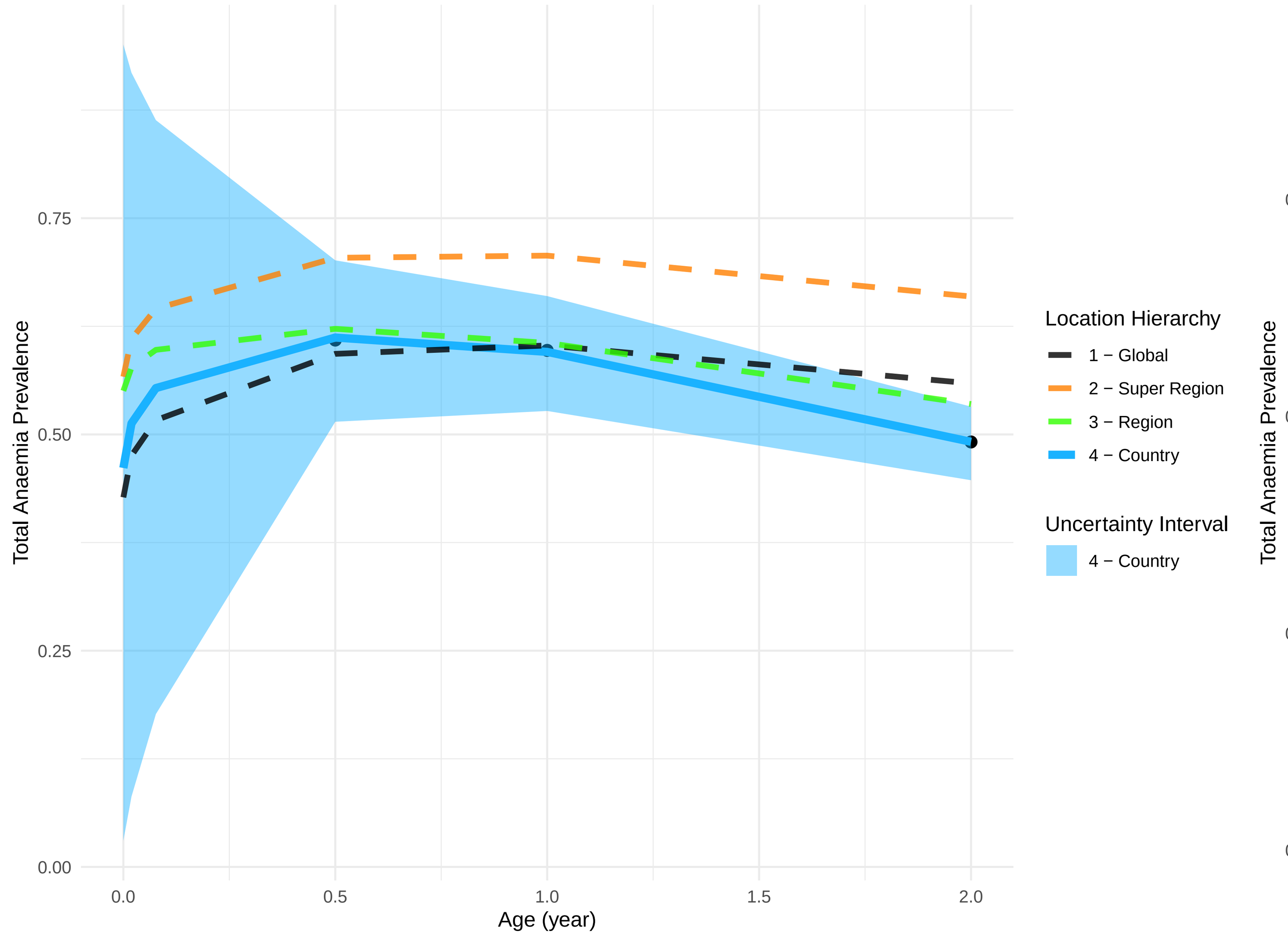

MR−BRT Age/Sex Fits − Non−pregnant Females <2 Years Old − Total Anaemia Prevalence − South Africa
WHO−2024 Adjustment Method

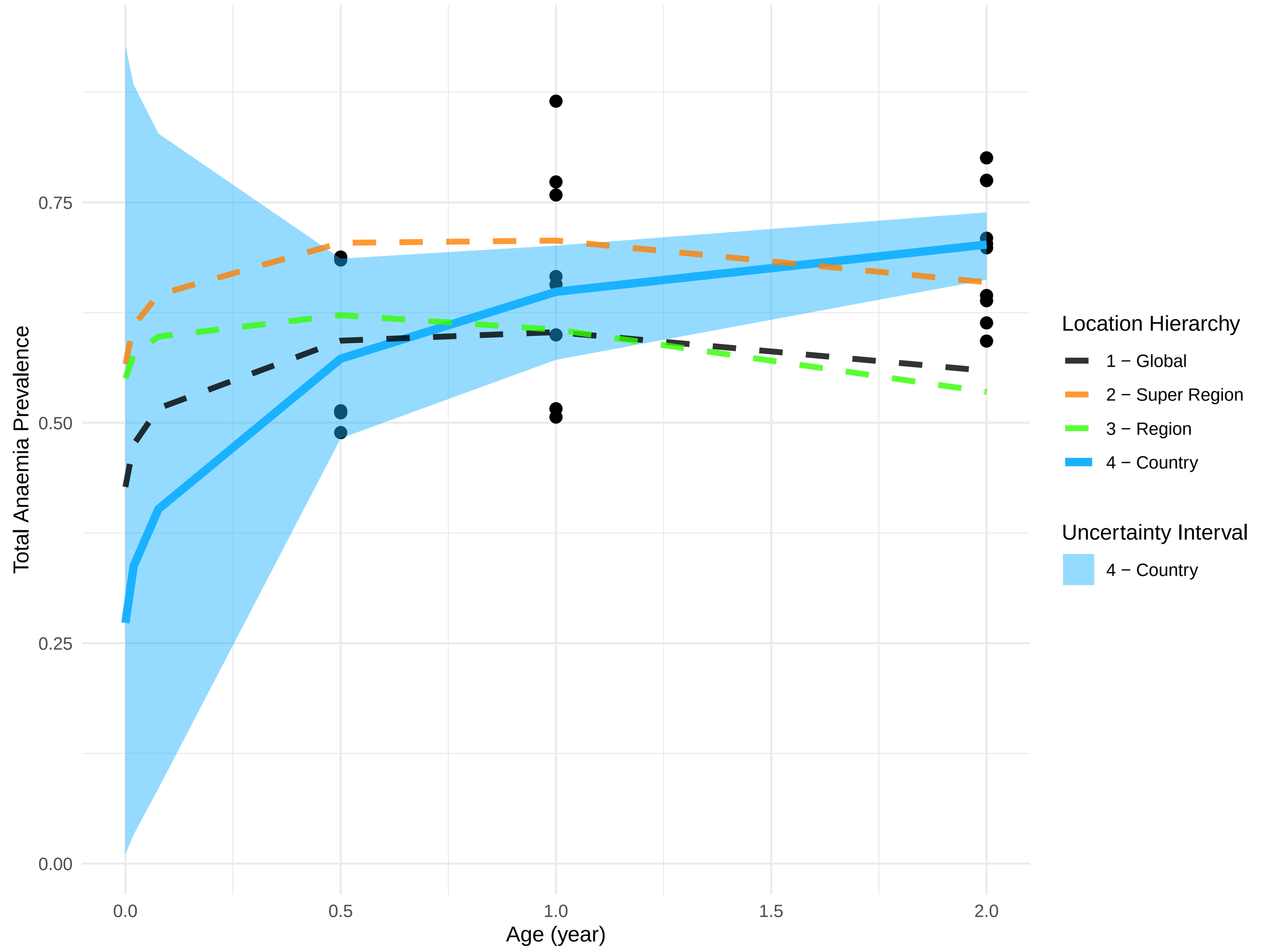

MR−BRT Age/Sex Fits − Non−pregnant Females <2 Years Old − Total Anaemia Prevalence − Eastern Cape
WHO−2024 Adjustment Method

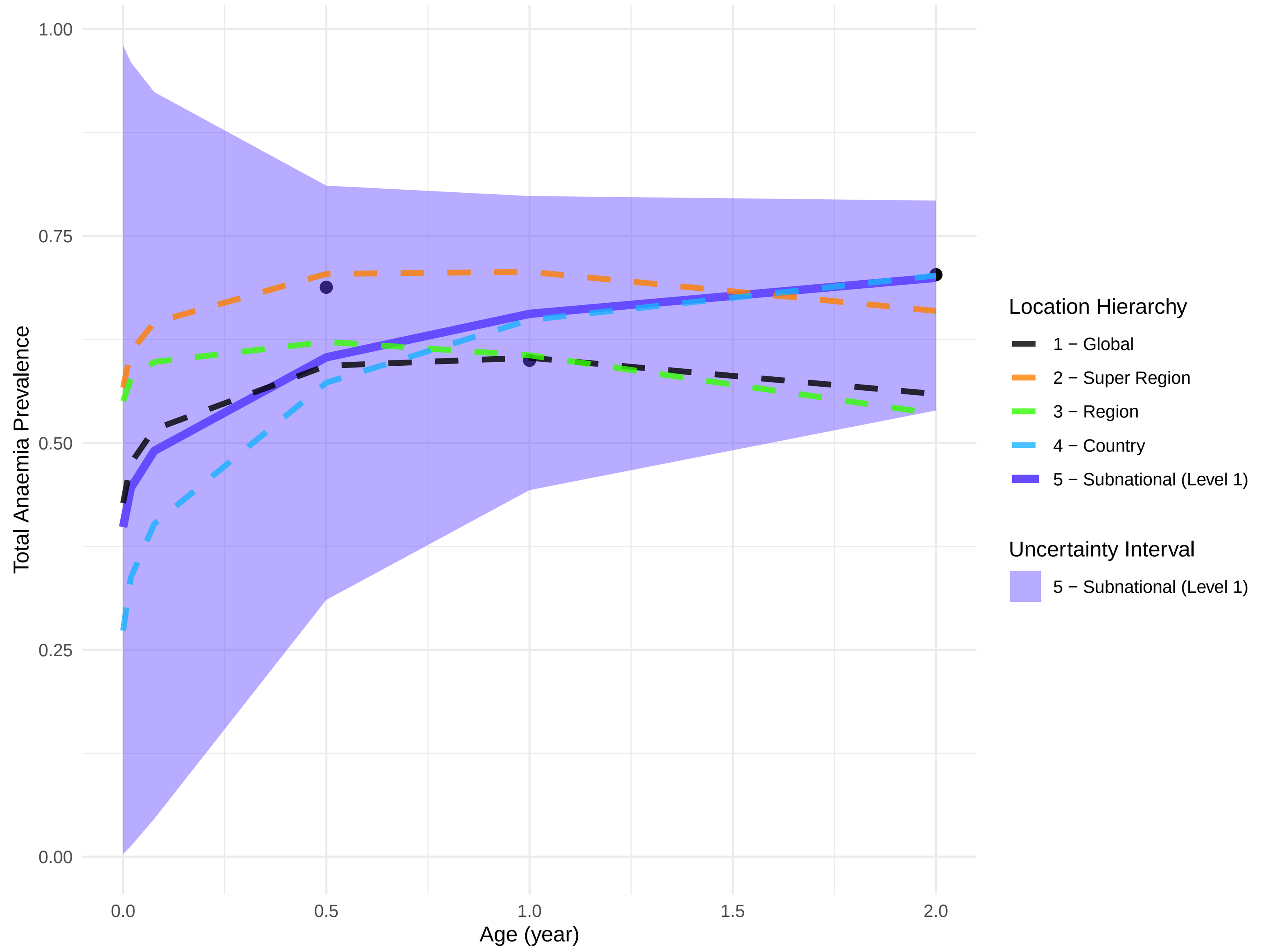

MR−BRT Age/Sex Fits − Non−pregnant Females <2 Years Old − Total Anaemia Prevalence − Free State
WHO−2024 Adjustment Method

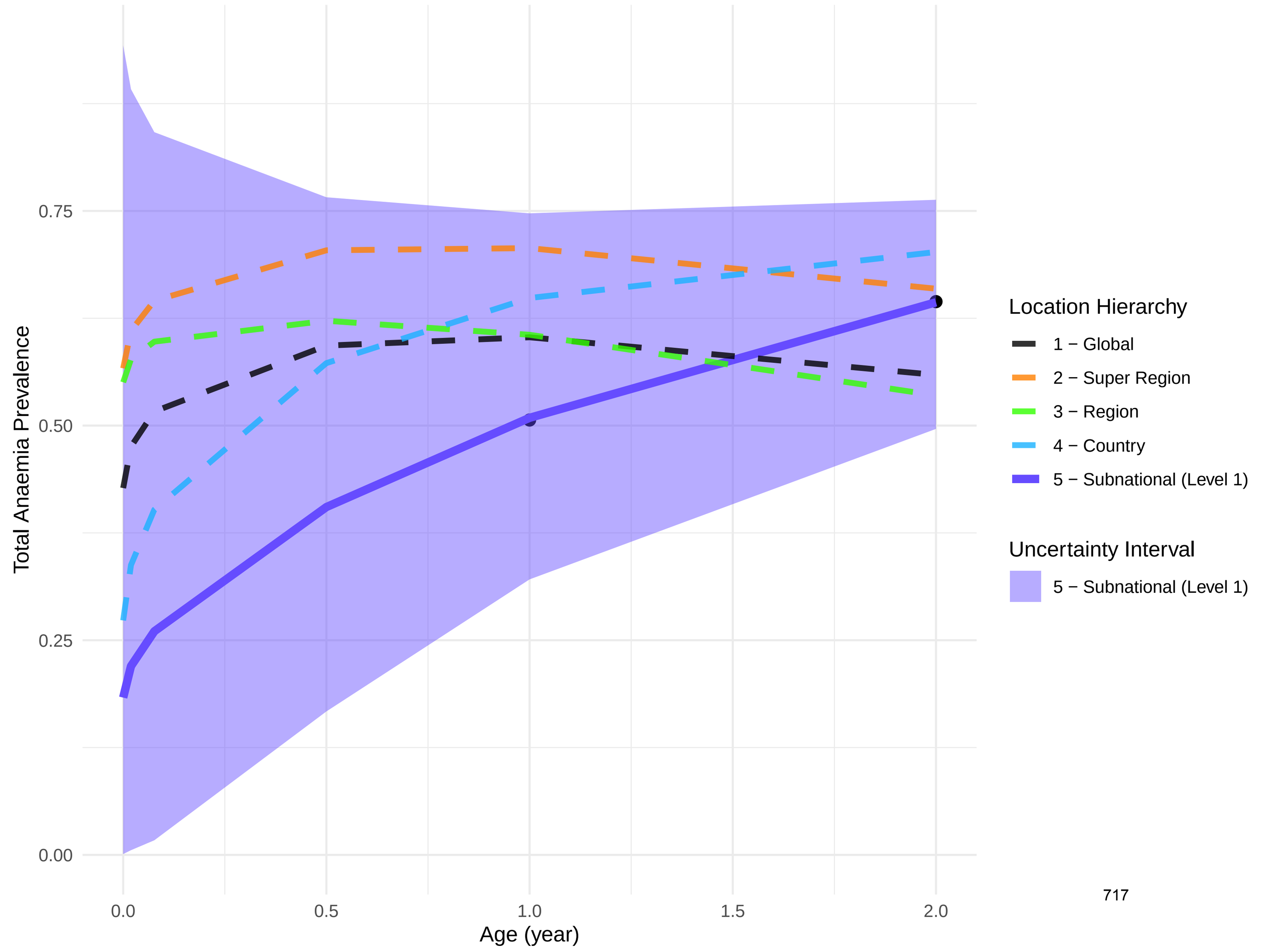

MR−BRT Age/Sex Fits − Non−pregnant Females <2 Years Old − Total Anaemia Prevalence − Gauteng
WHO−2024 Adjustment Method

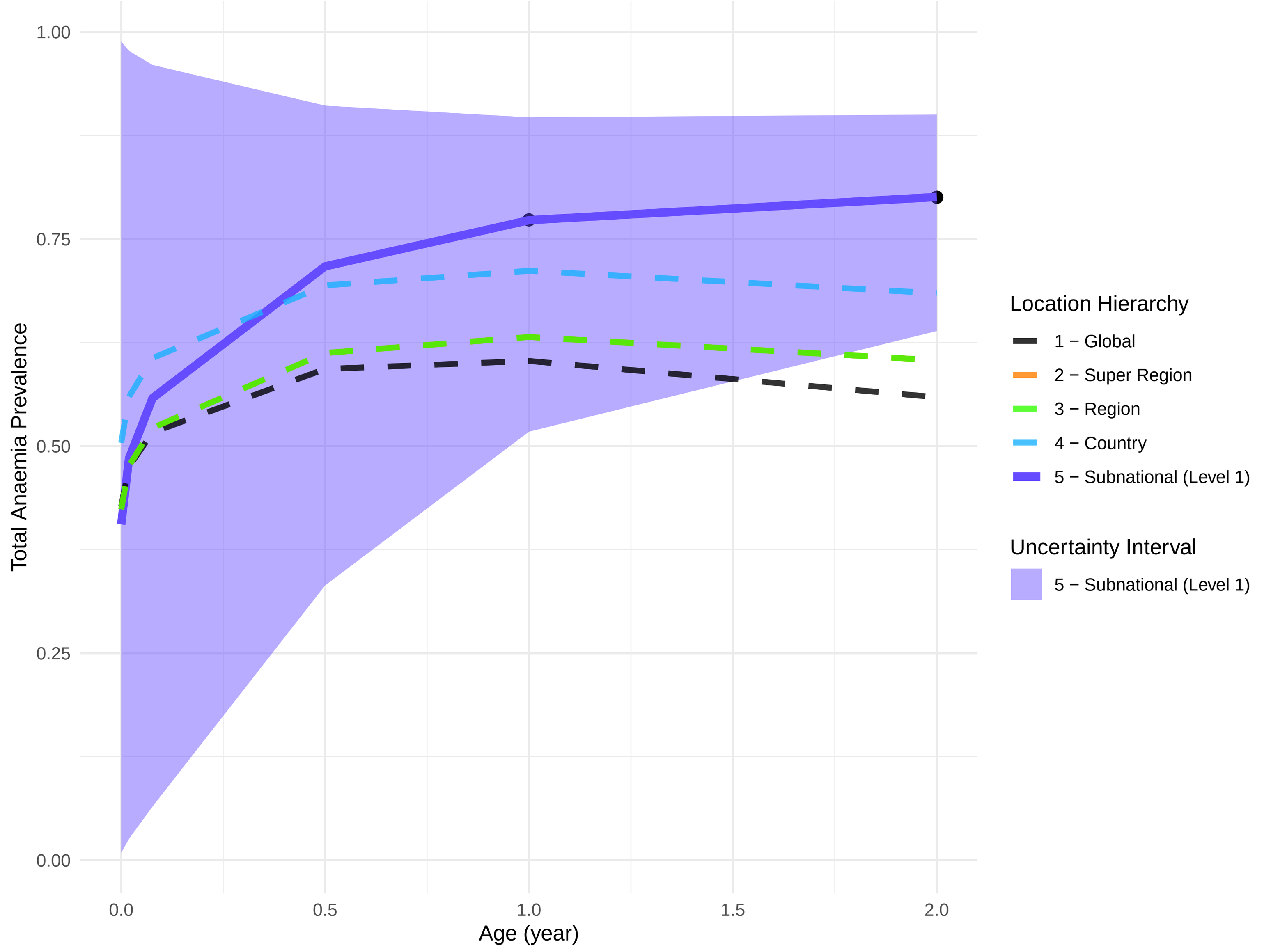

MR−BRT Age/Sex Fits − Non−pregnant Females <2 Years Old − Total Anaemia Prevalence − KwaZulu−Natal
WHO−2024 Adjustment Method

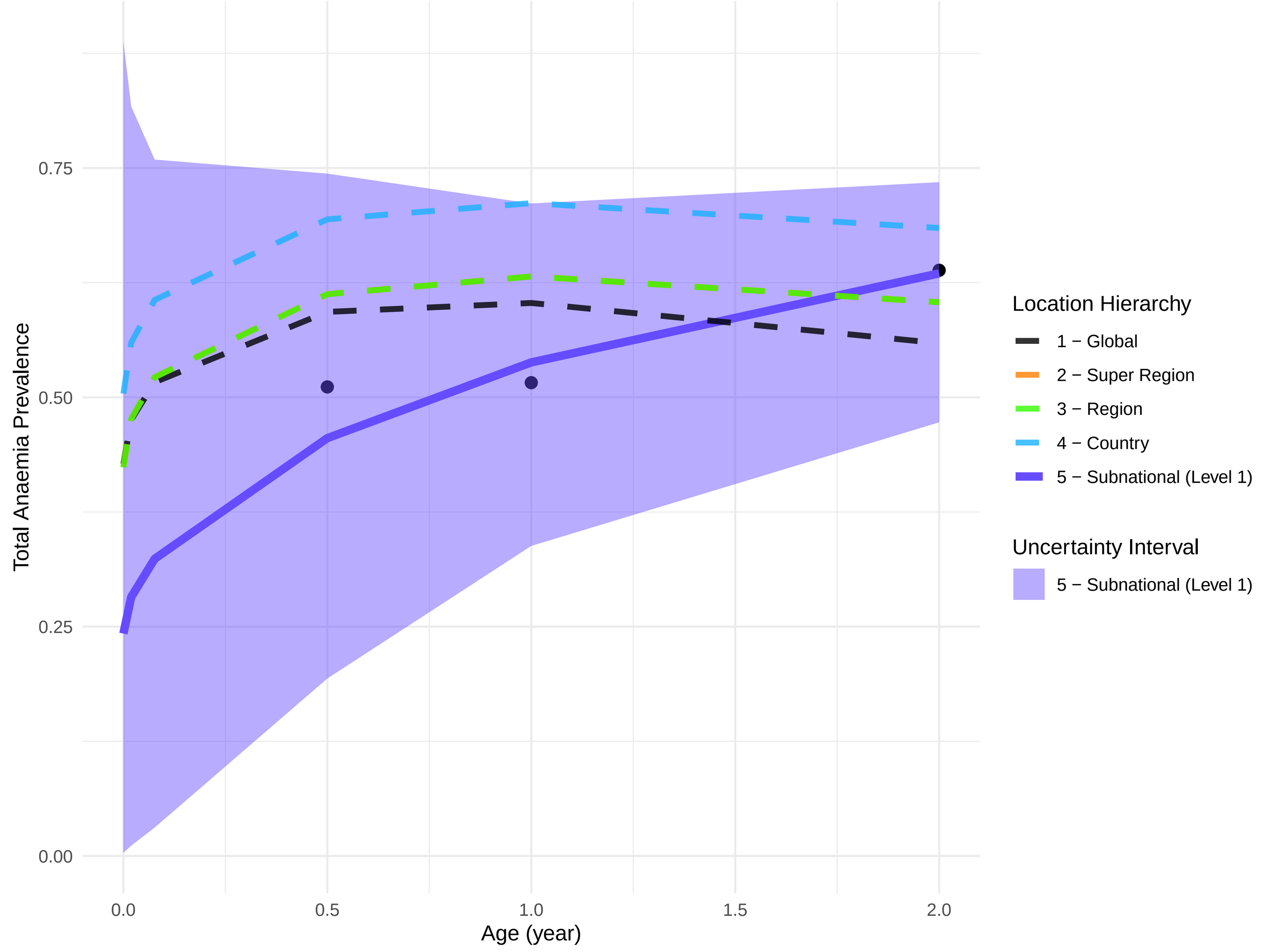

MR−BRT Age/Sex Fits − Non−pregnant Females <2 Years Old − Total Anaemia Prevalence − Limpopo
WHO−2024 Adjustment Method

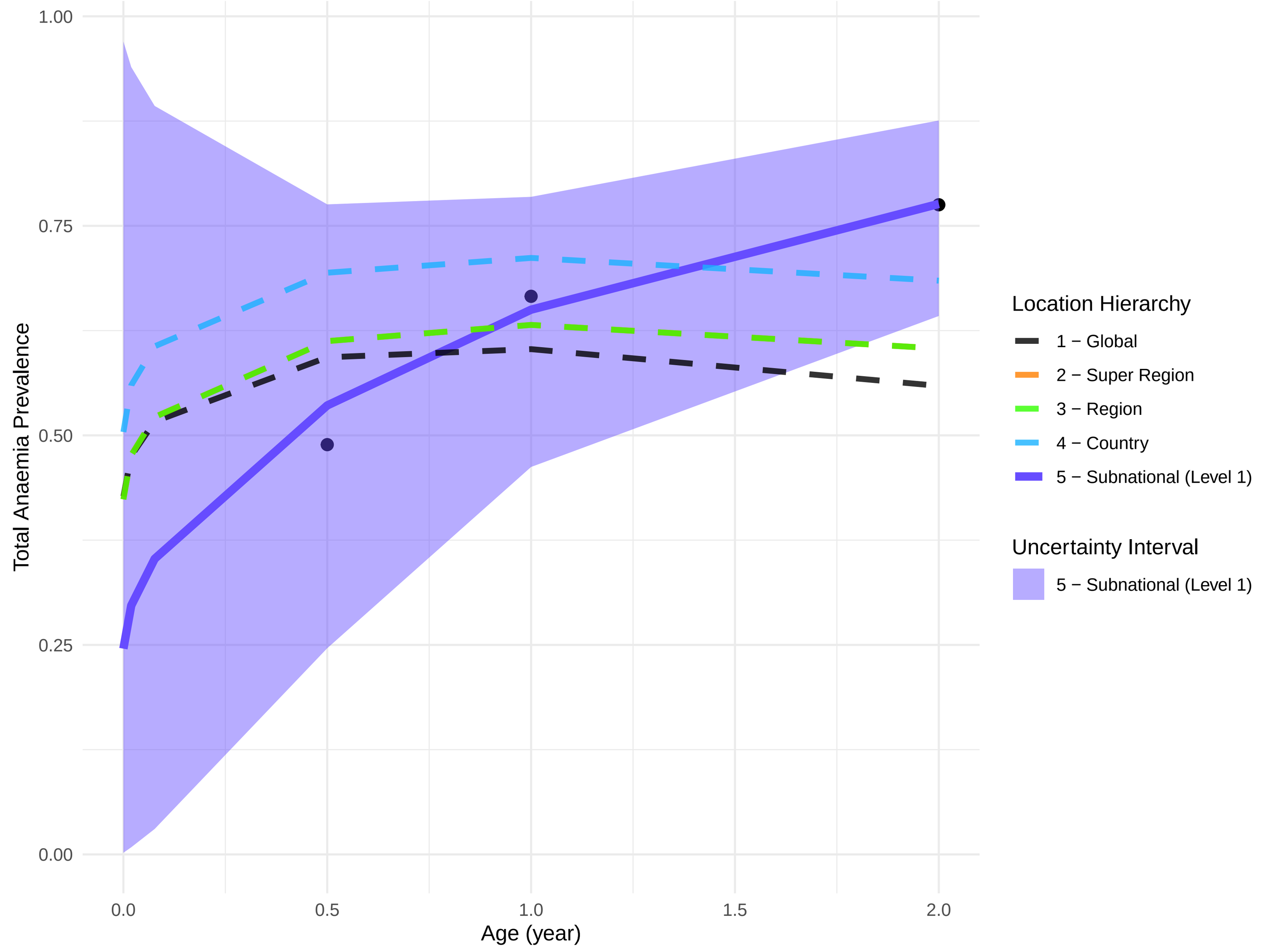

MR−BRT Age/Sex Fits − Non−pregnant Females <2 Years Old − Total Anaemia Prevalence − Mpumalanga
WHO−2024 Adjustment Method

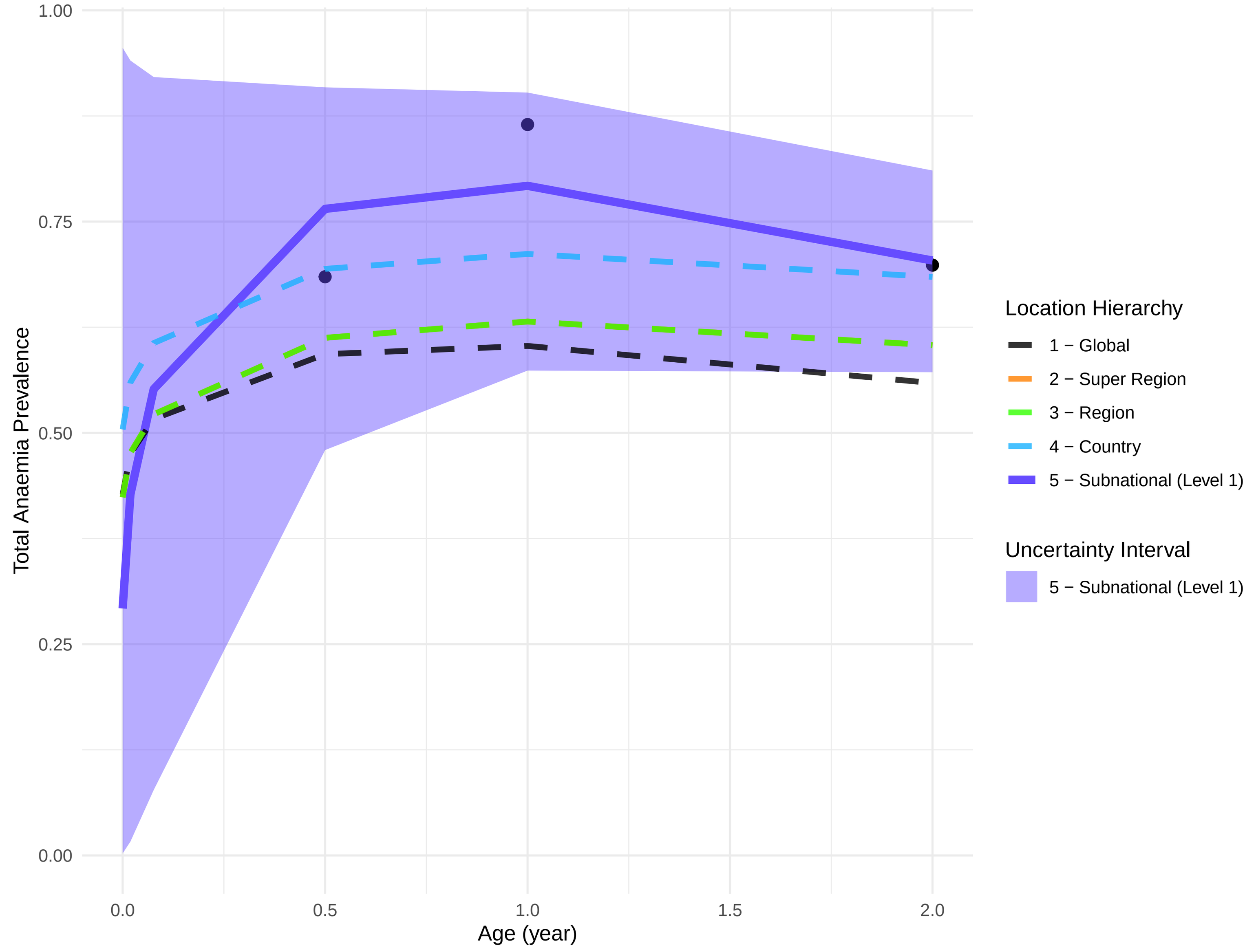

MR−BRT Age/Sex Fits − Non−pregnant Females <2 Years Old − Total Anaemia Prevalence − North−West
WHO−2024 Adjustment Method

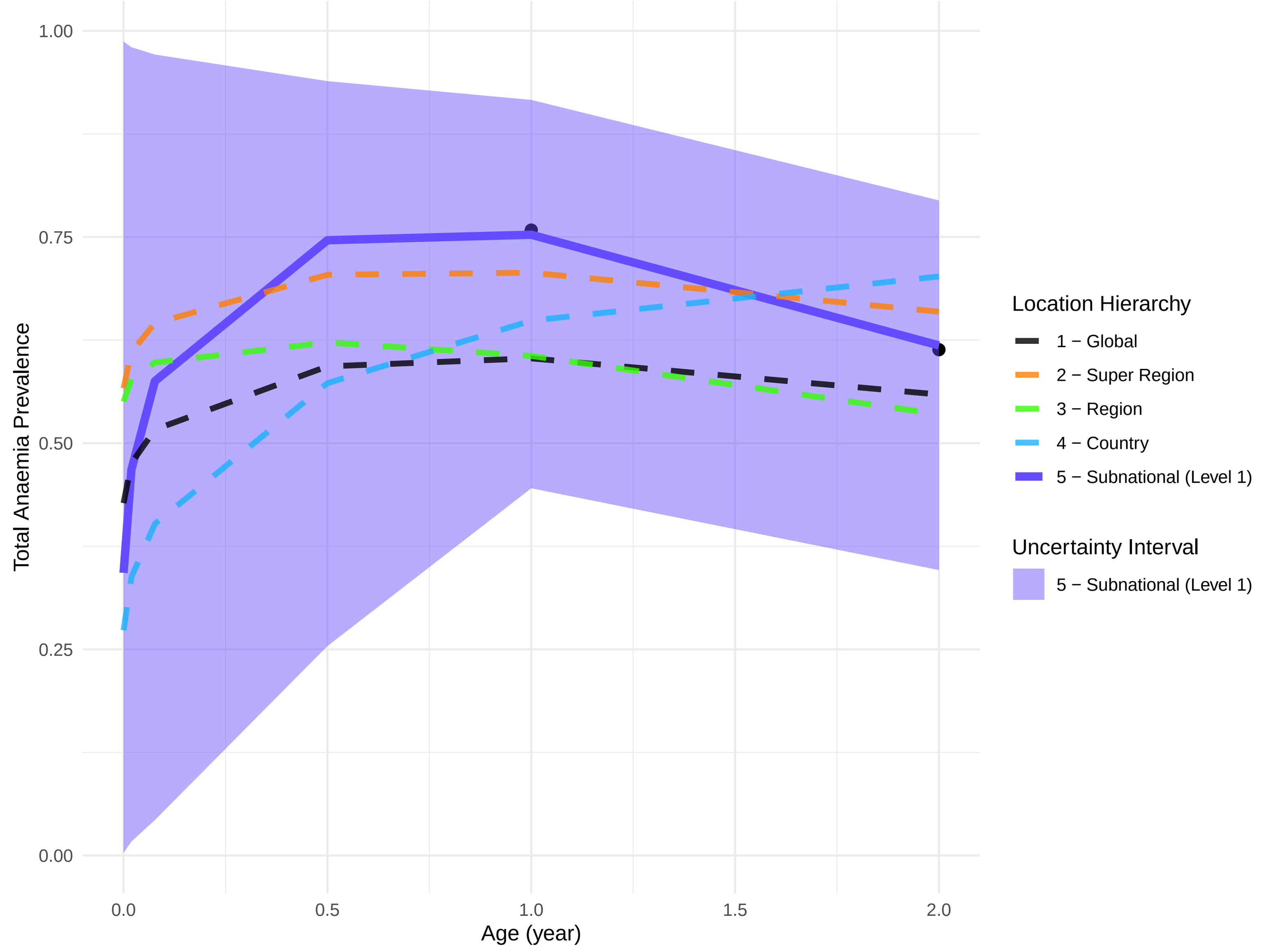

MR−BRT Age/Sex Fits − Non−pregnant Females <2 Years Old − Total Anaemia Prevalence − Northern Cape
WHO−2024 Adjustment Method

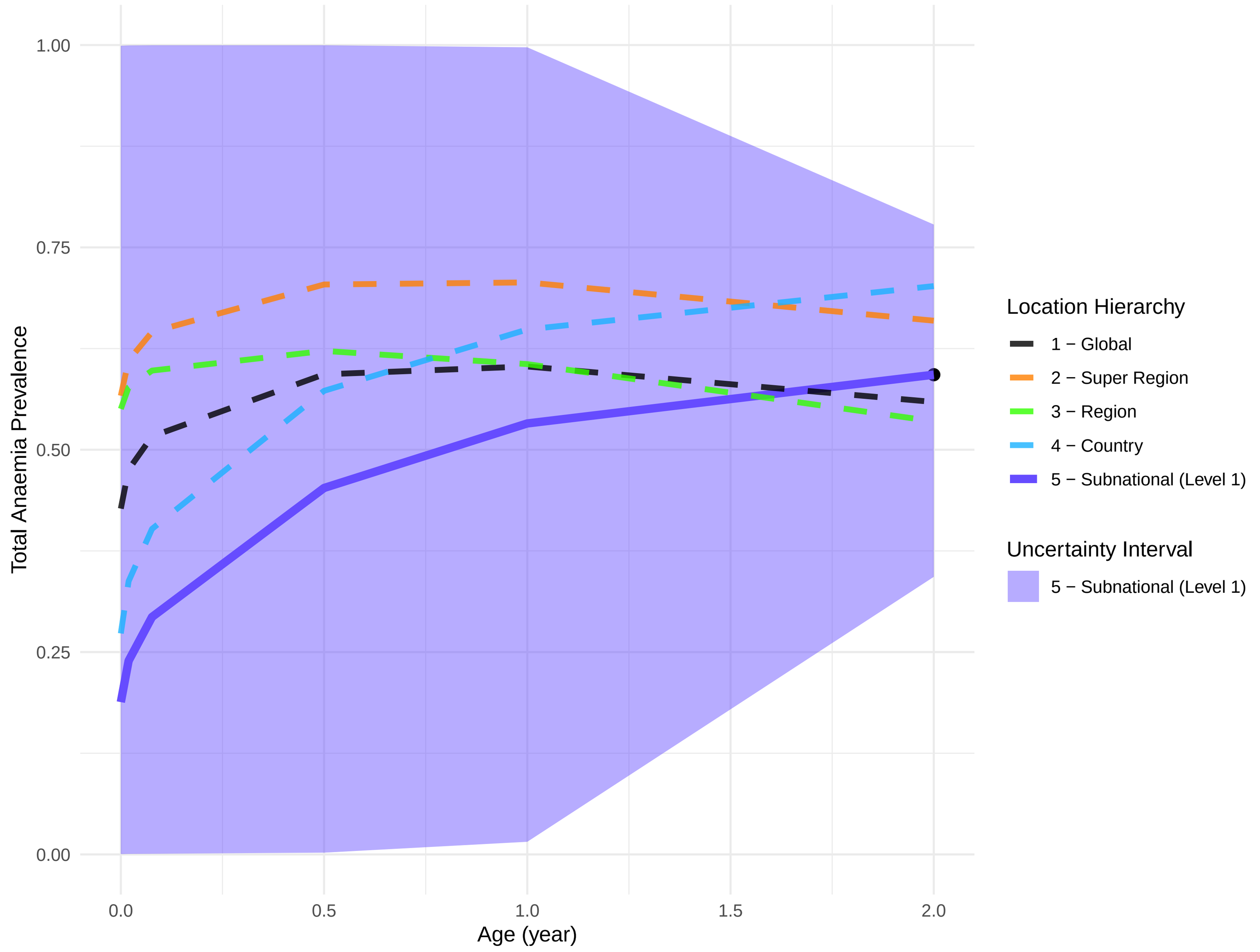

MR−BRT Age/Sex Fits − Non−pregnant Females <2 Years Old − Total Anaemia Prevalence − Western Cape
WHO−2024 Adjustment Method

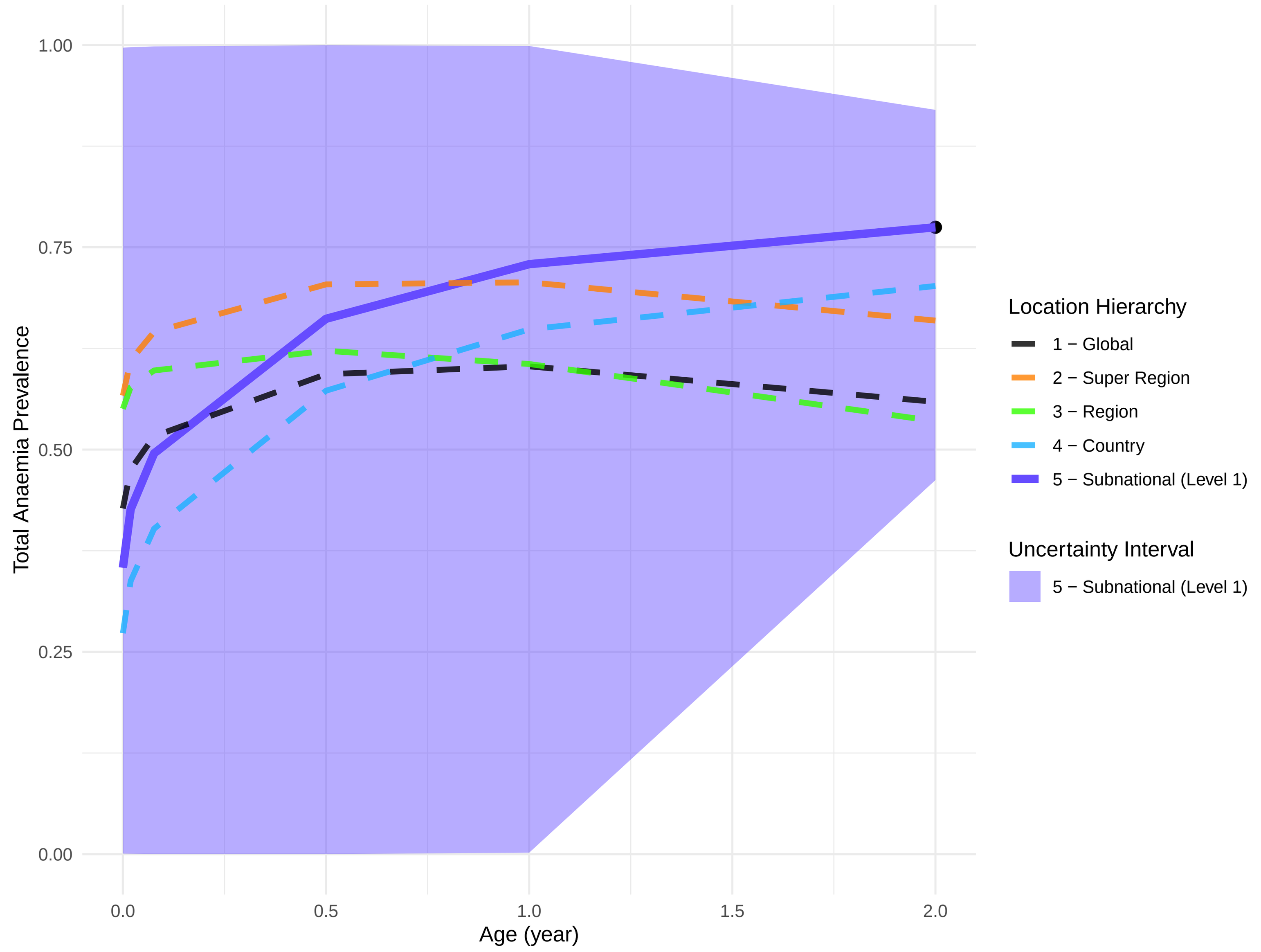

MR−BRT Age/Sex Fits − Non−pregnant Females <2 Years Old − Total Anaemia Prevalence − Zimbabwe
WHO−2024 Adjustment Method

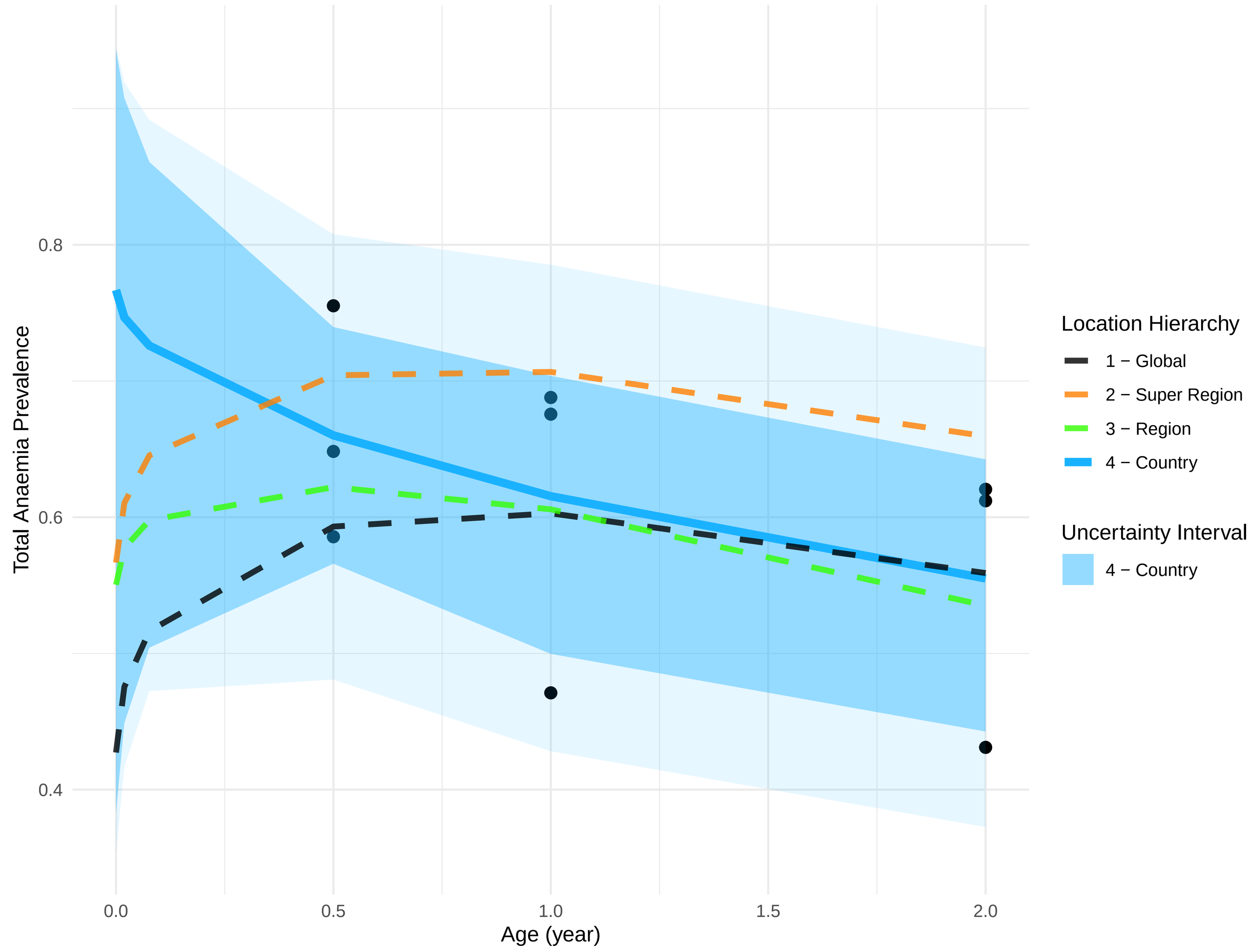

MR−BRT Age/Sex Fits − Non−pregnant Females <2 Years Old − Total Anaemia Prevalence − Western Sub−Saharan Africa
WHO−2024 Adjustment Method

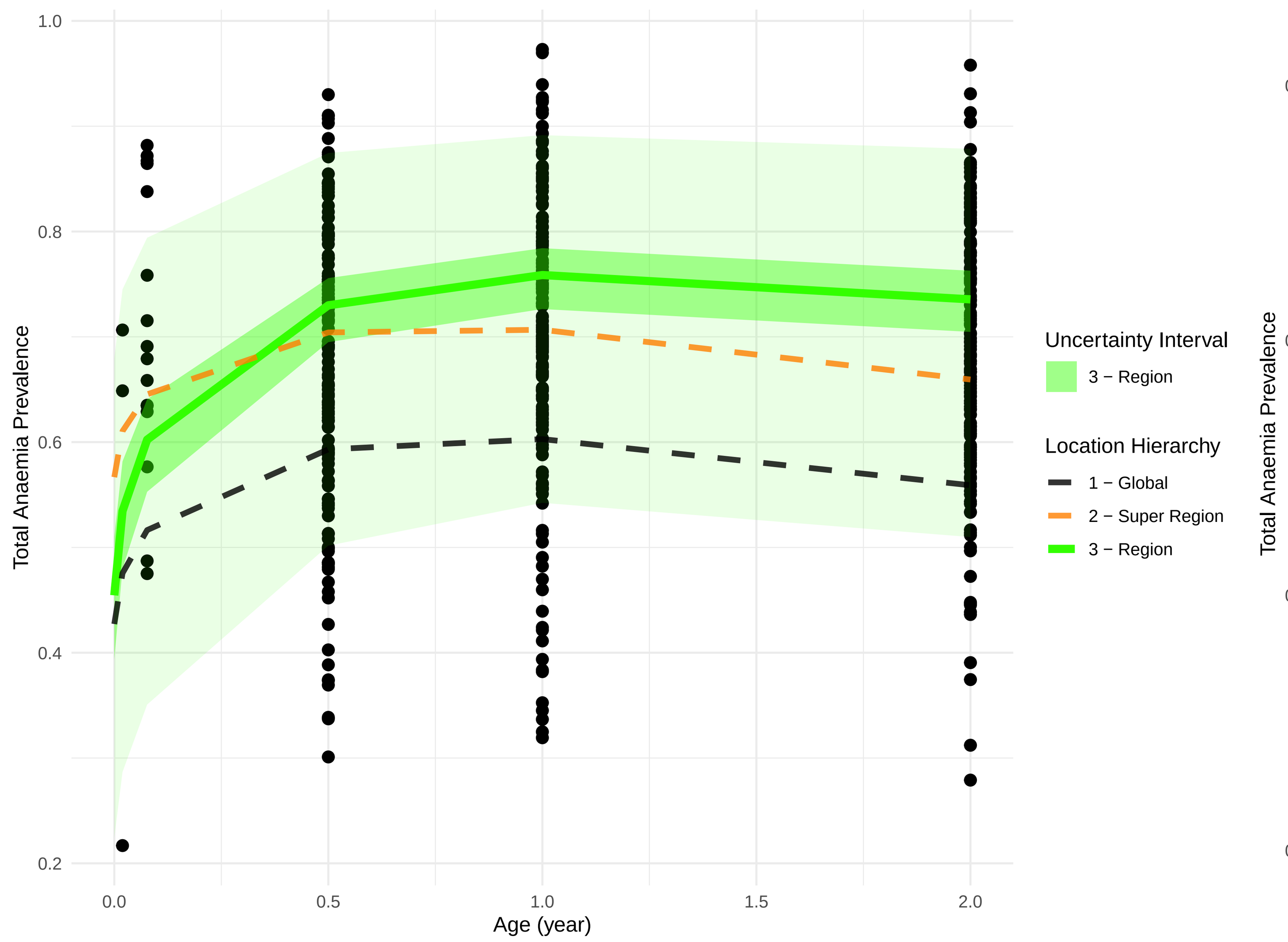

MR−BRT Age/Sex Fits − Non−pregnant Females <2 Years Old − Total Anaemia Prevalence − Benin
WHO−2024 Adjustment Method

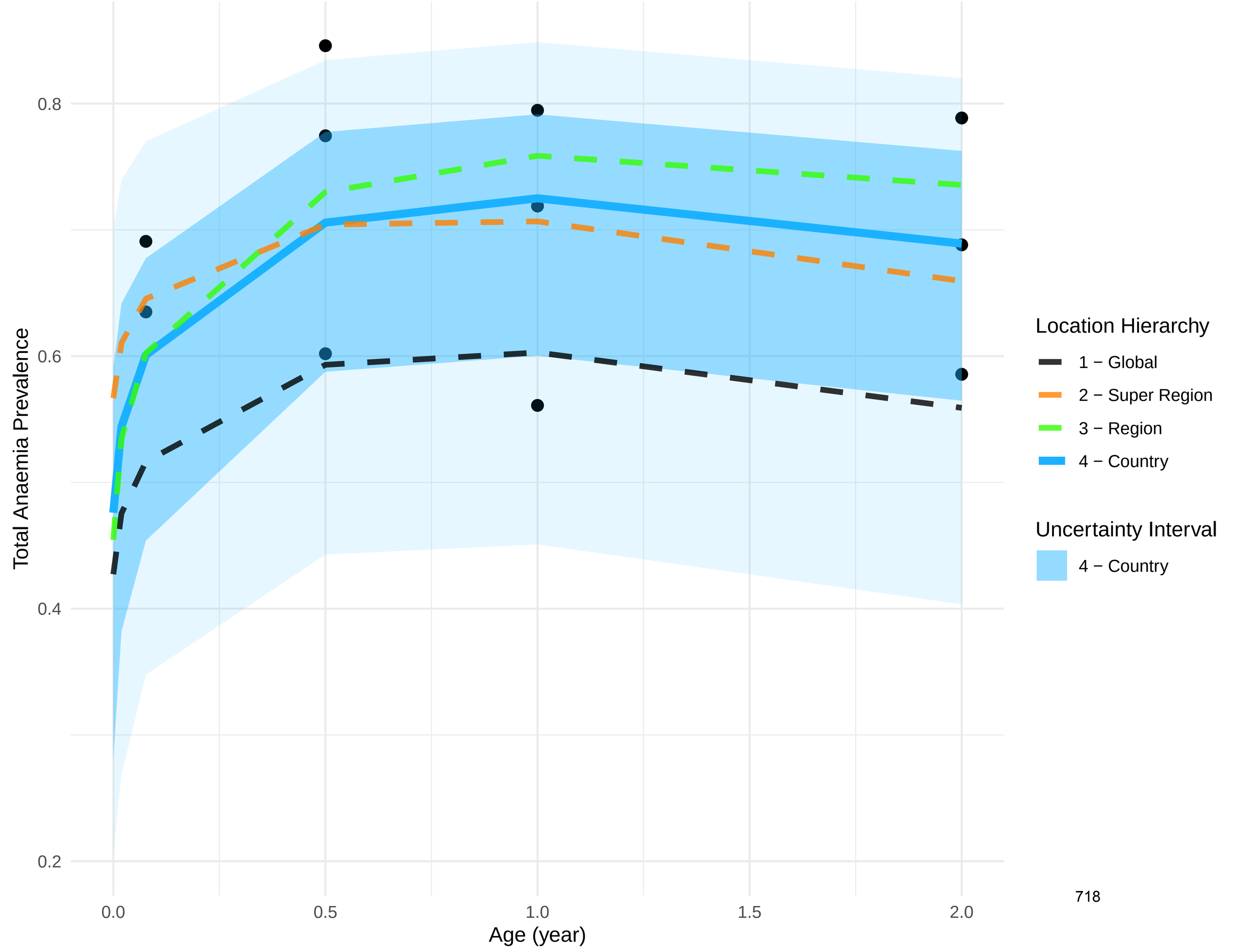

MR−BRT Age/Sex Fits − Non−pregnant Females <2 Years Old − Total Anaemia Prevalence − Burkina Faso
WHO−2024 Adjustment Method

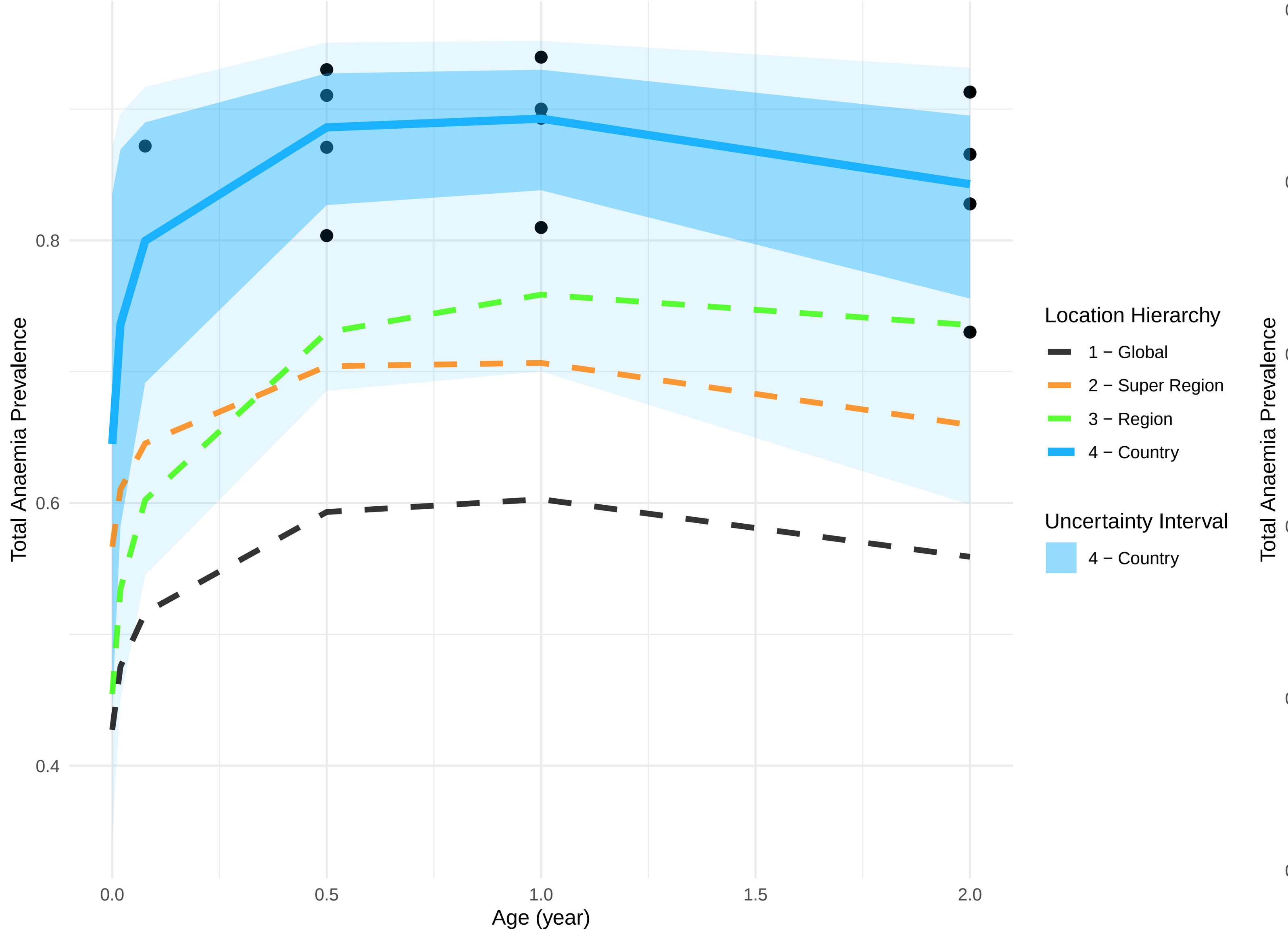

MR−BRT Age/Sex Fits − Non−pregnant Females <2 Years Old − Total Anaemia Prevalence − Cameroon
WHO−2024 Adjustment Method

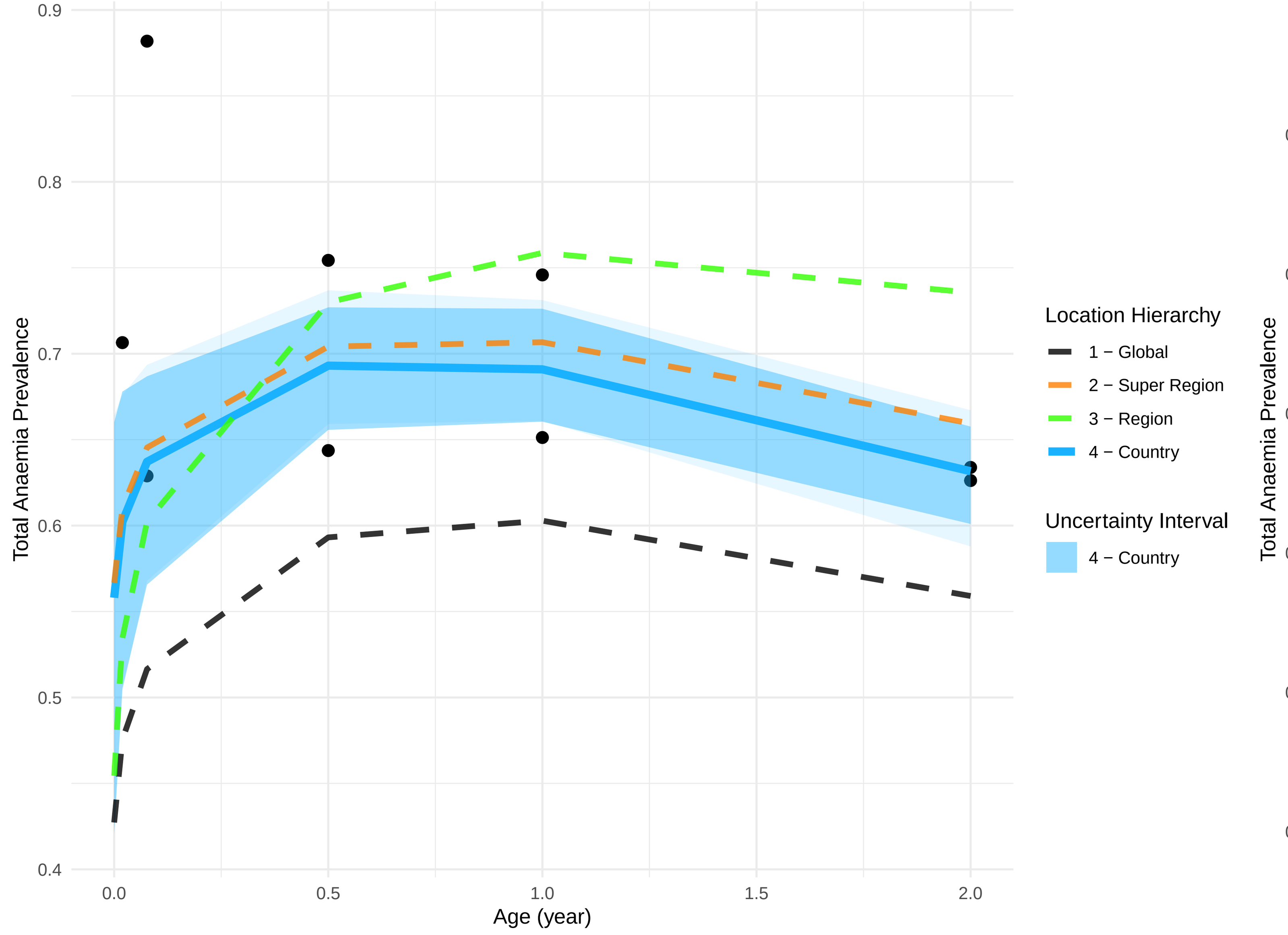

MR−BRT Age/Sex Fits − Non−pregnant Females <2 Years Old − Total Anaemia Prevalence − Côte d'Ivoire
WHO−2024 Adjustment Method

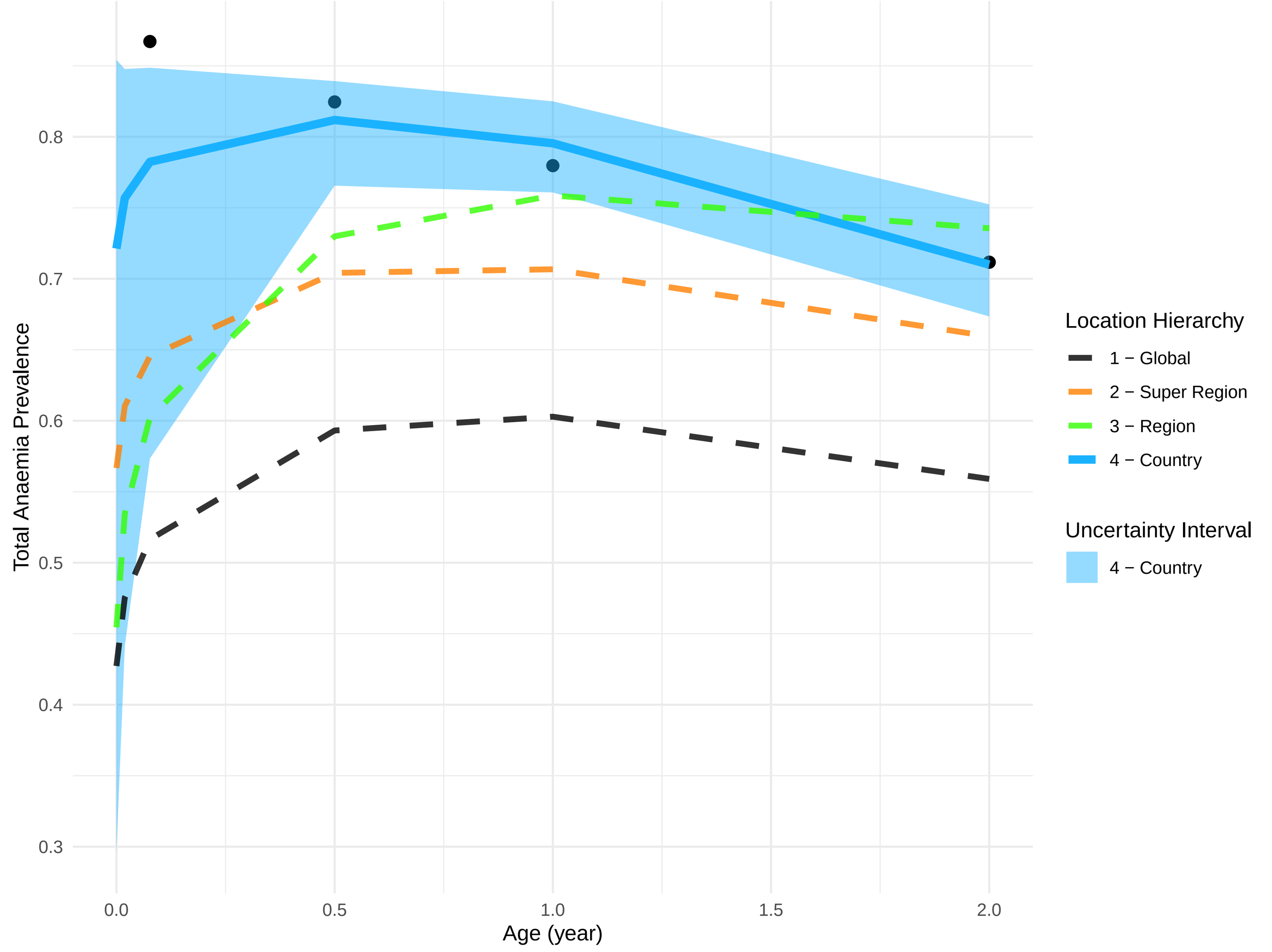

MR−BRT Age/Sex Fits − Non−pregnant Females <2 Years Old − Total Anaemia Prevalence − Gambia
WHO−2024 Adjustment Method

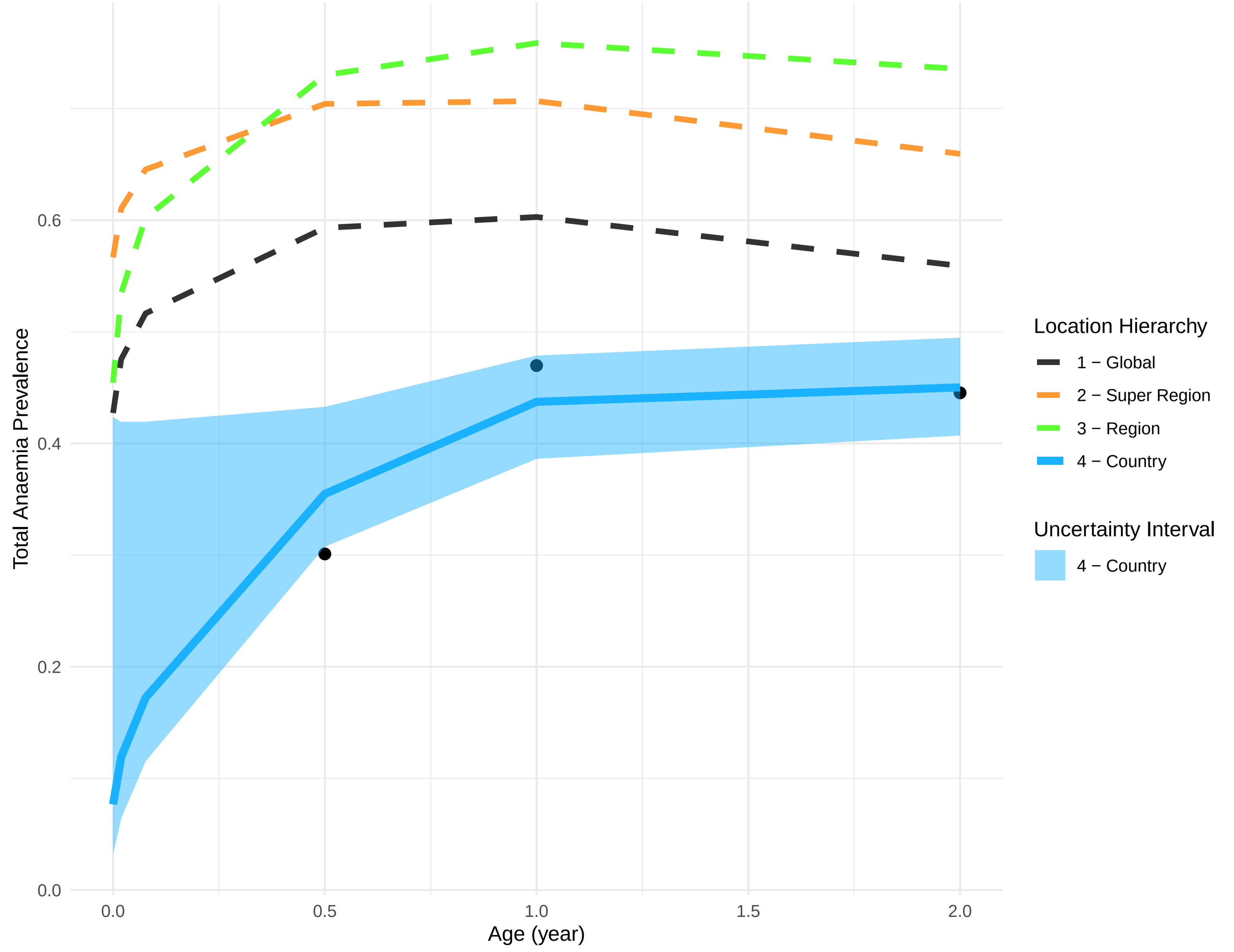

MR−BRT Age/Sex Fits − Non−pregnant Females <2 Years Old − Total Anaemia Prevalence − Ghana
WHO−2024 Adjustment Method

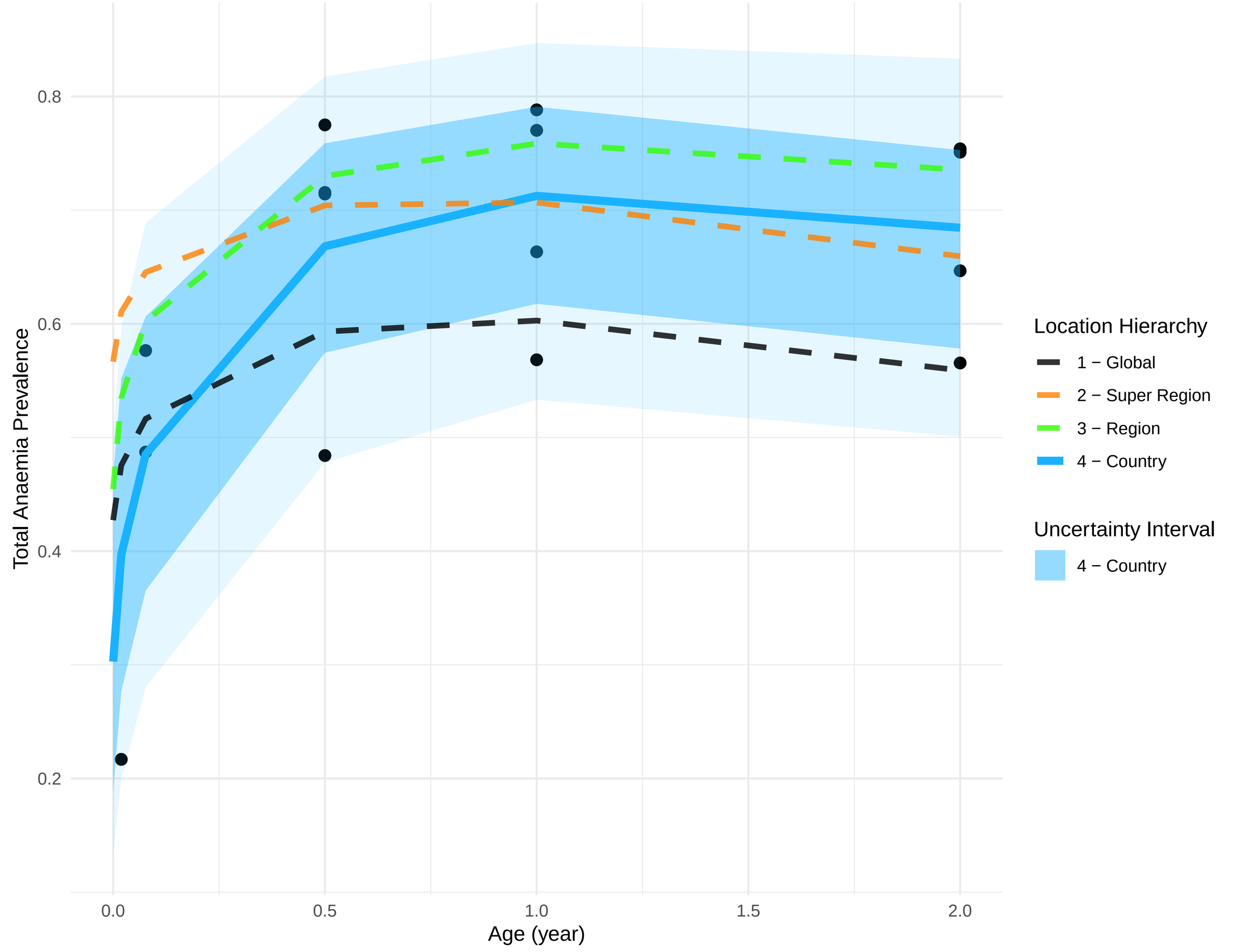

MR−BRT Age/Sex Fits − Non−pregnant Females <2 Years Old − Total Anaemia Prevalence − Guinea
WHO−2024 Adjustment Method

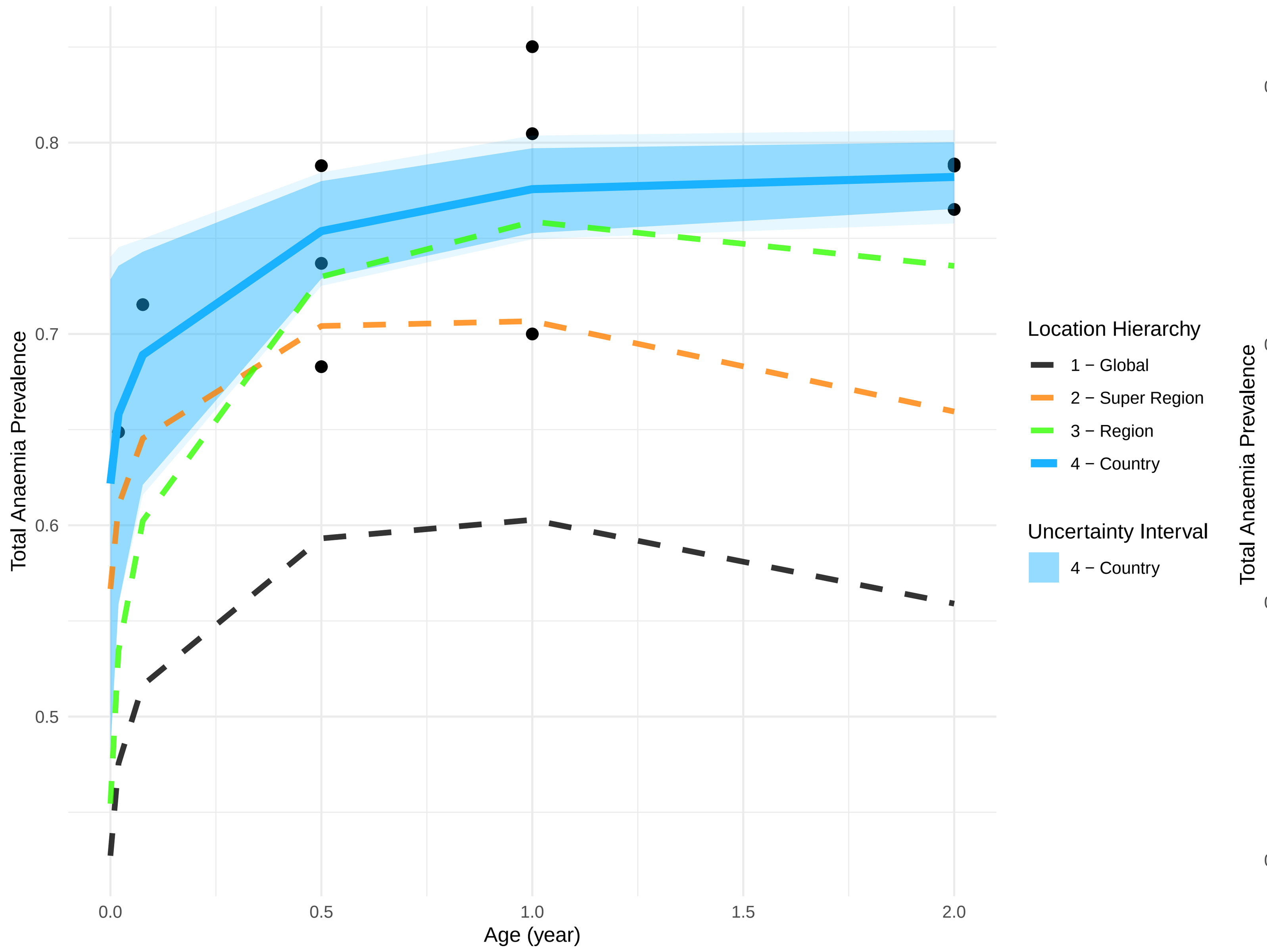

MR−BRT Age/Sex Fits − Non−pregnant Females <2 Years Old − Total Anaemia Prevalence − Liberia
WHO−2024 Adjustment Method

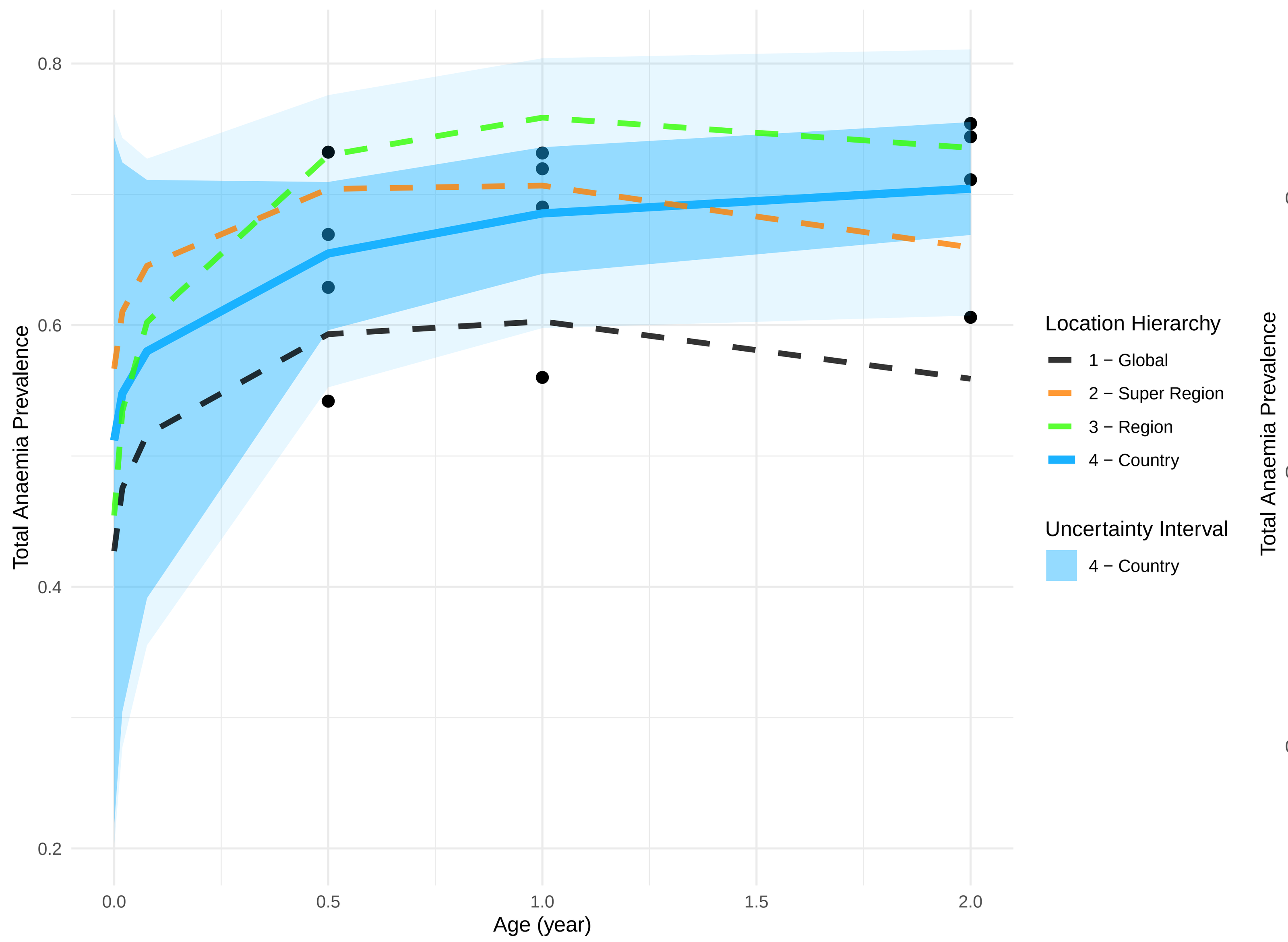

MR−BRT Age/Sex Fits − Non−pregnant Females <2 Years Old − Total Anaemia Prevalence − Mali
WHO−2024 Adjustment Method

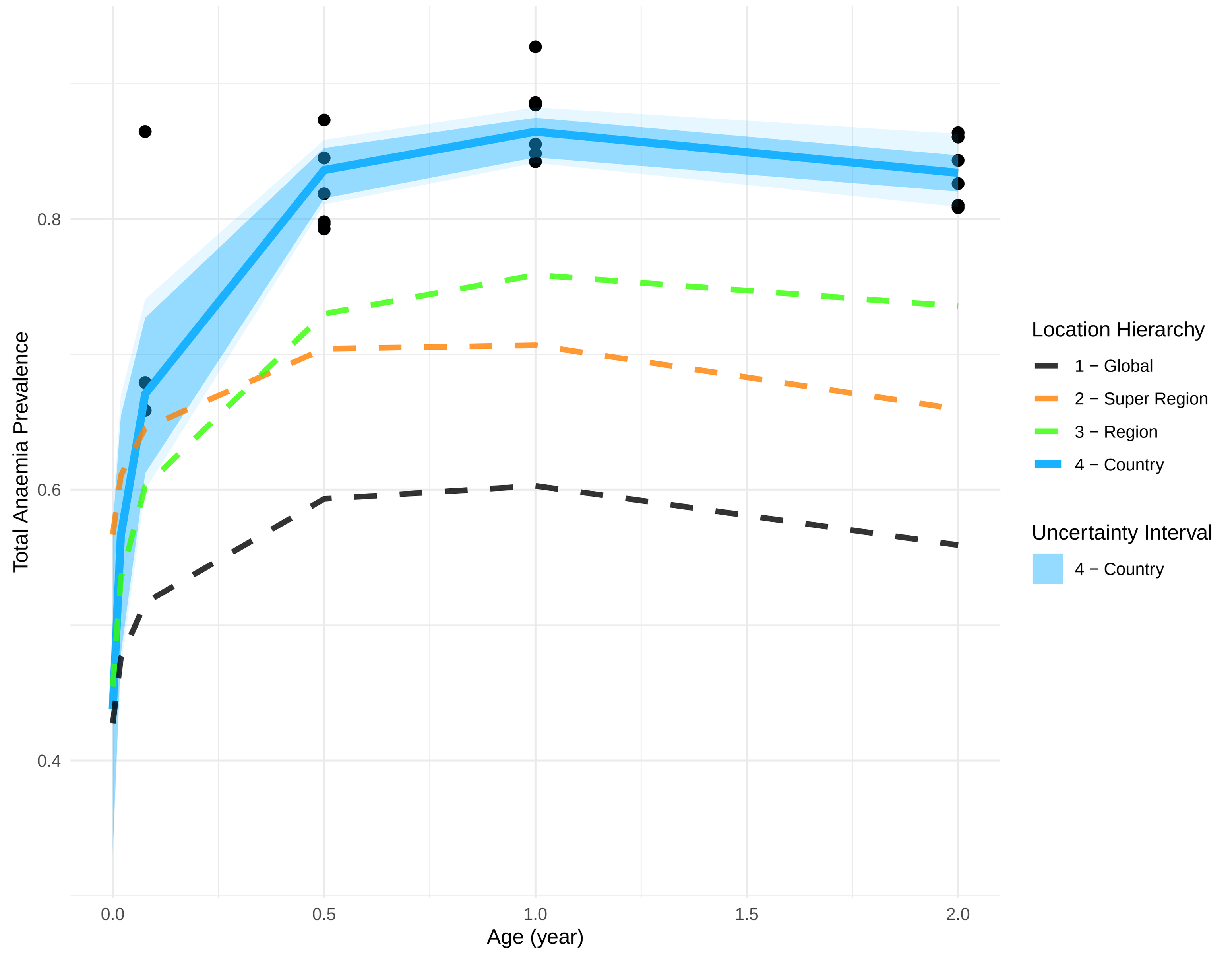

MR−BRT Age/Sex Fits − Non−pregnant Females <2 Years Old − Total Anaemia Prevalence − Niger
WHO−2024 Adjustment Method

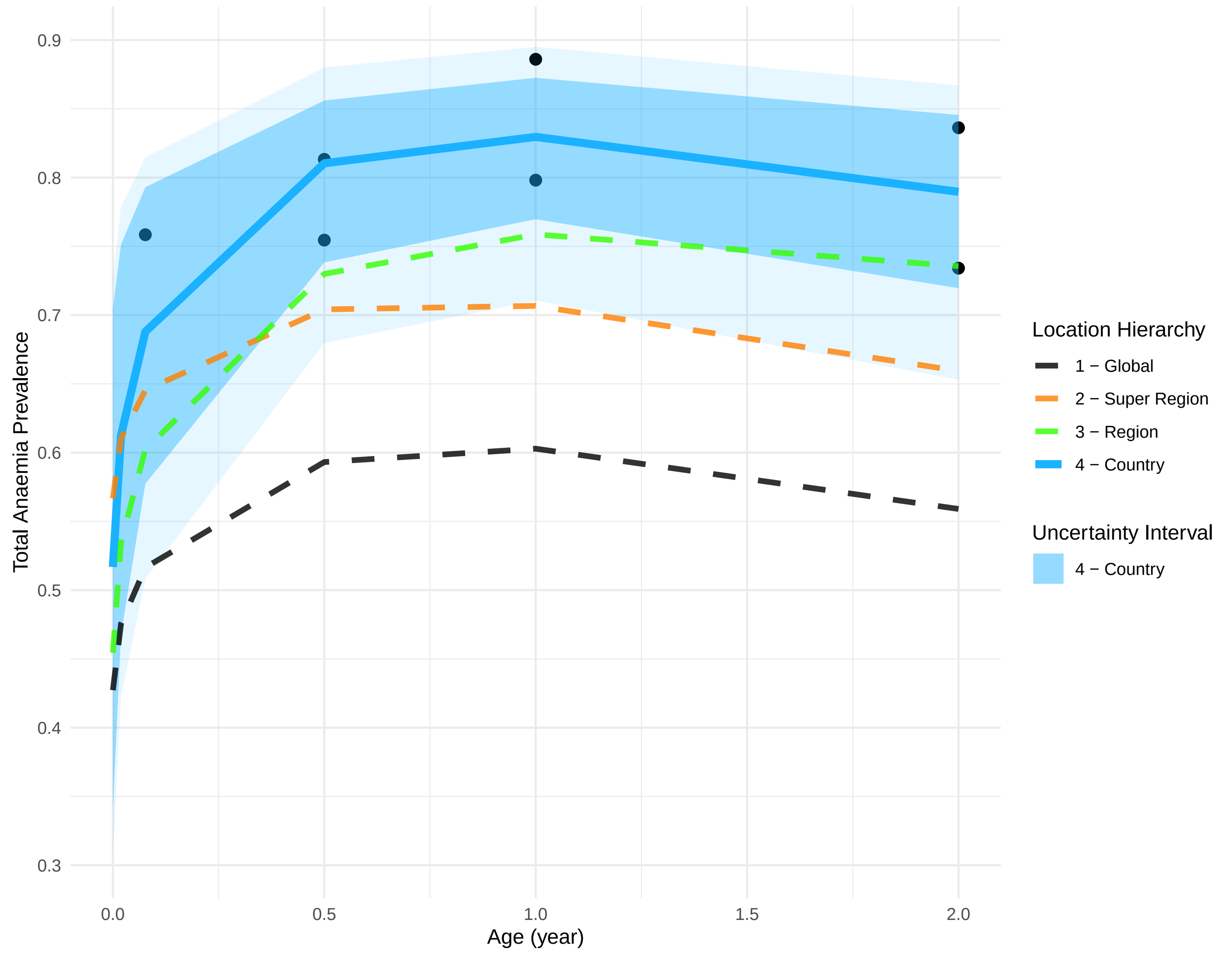

MR−BRT Age/Sex Fits − Non−pregnant Females <2 Years Old − Total Anaemia Prevalence − Nigeria
WHO−2024 Adjustment Method

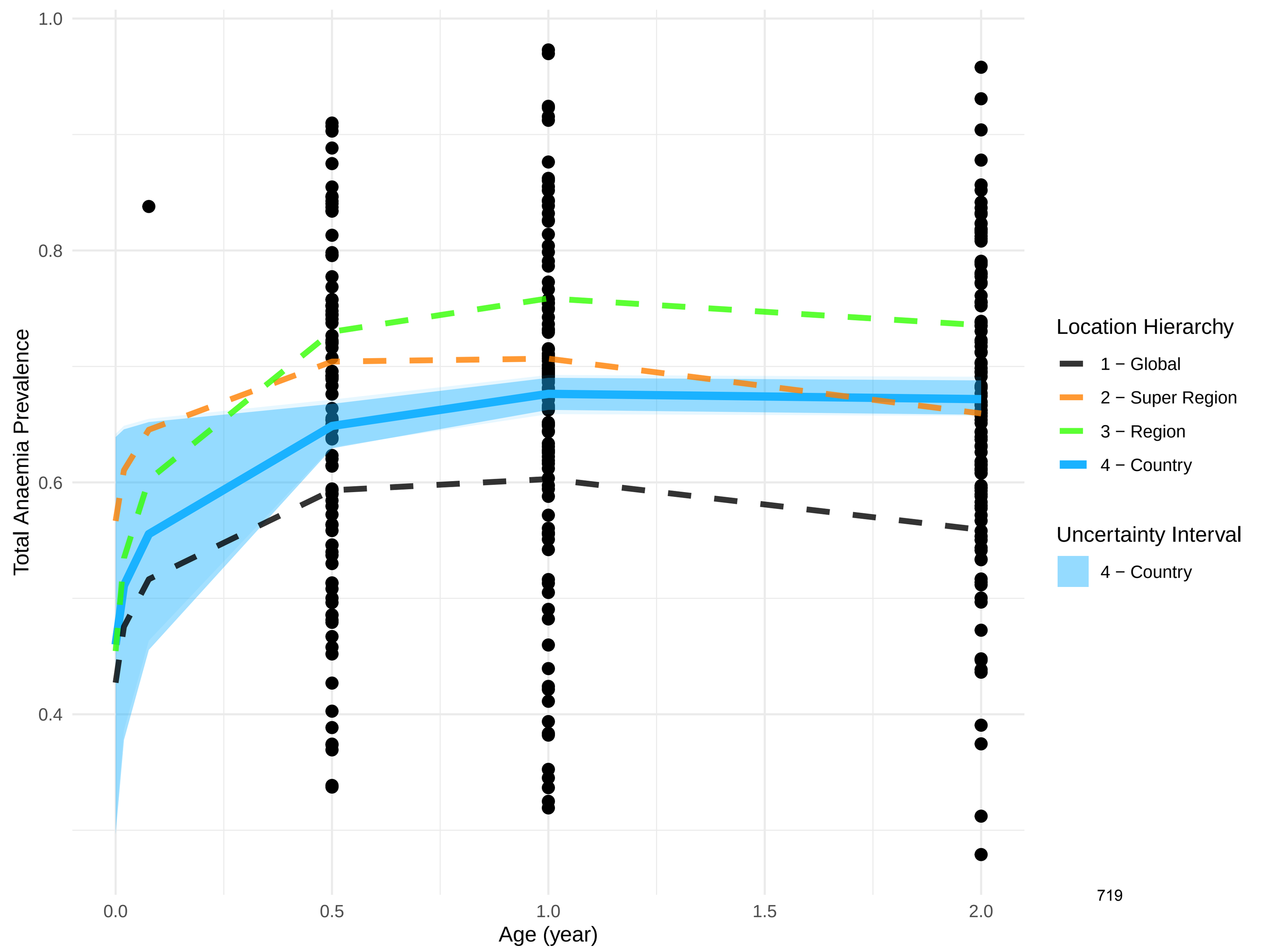

MR−BRT Age/Sex Fits − Non−pregnant Females <2 Years Old − Total Anaemia Prevalence − Abia
WHO−2024 Adjustment Method

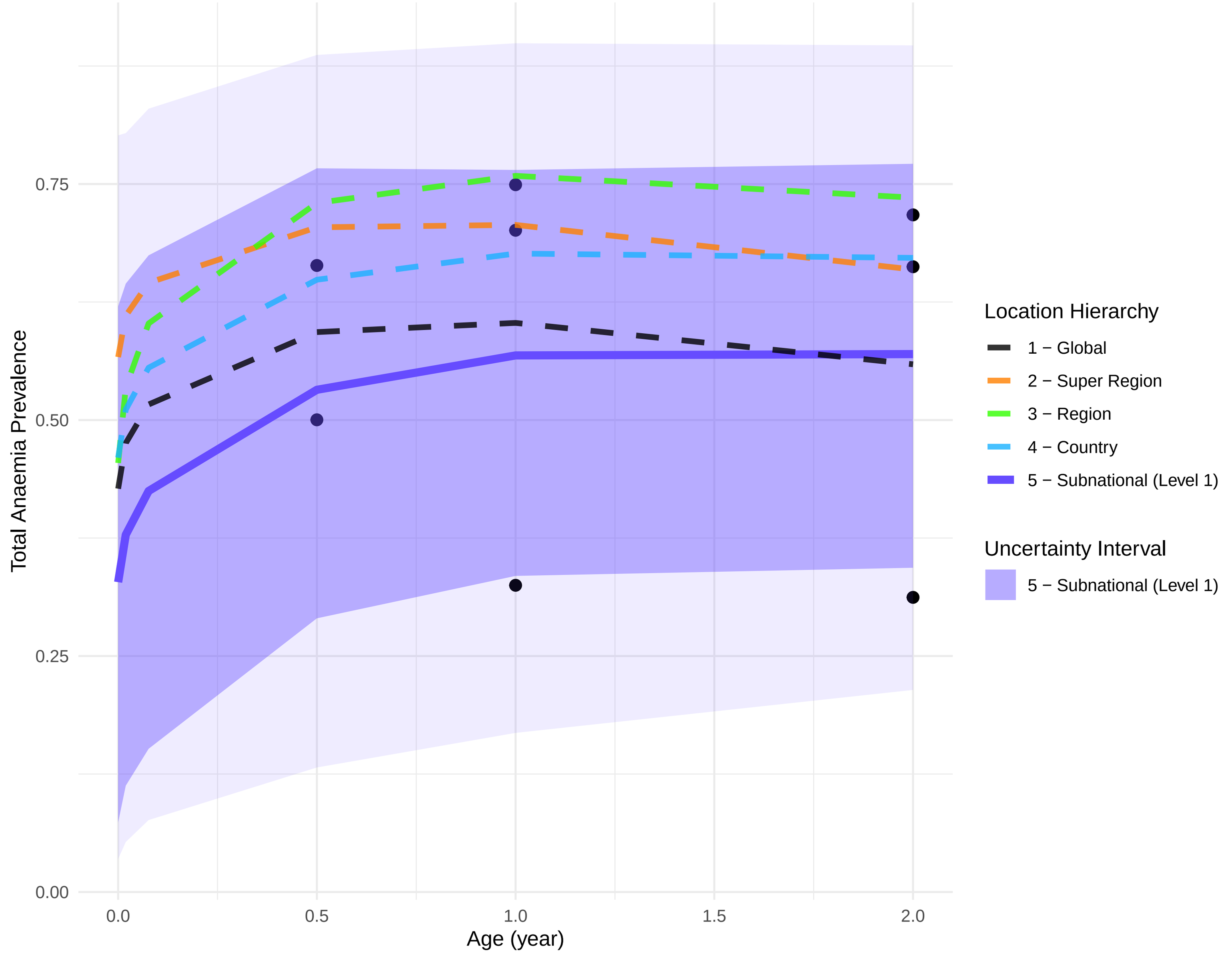

MR−BRT Age/Sex Fits − Non−pregnant Females <2 Years Old − Total Anaemia Prevalence − Adamawa
WHO−2024 Adjustment Method

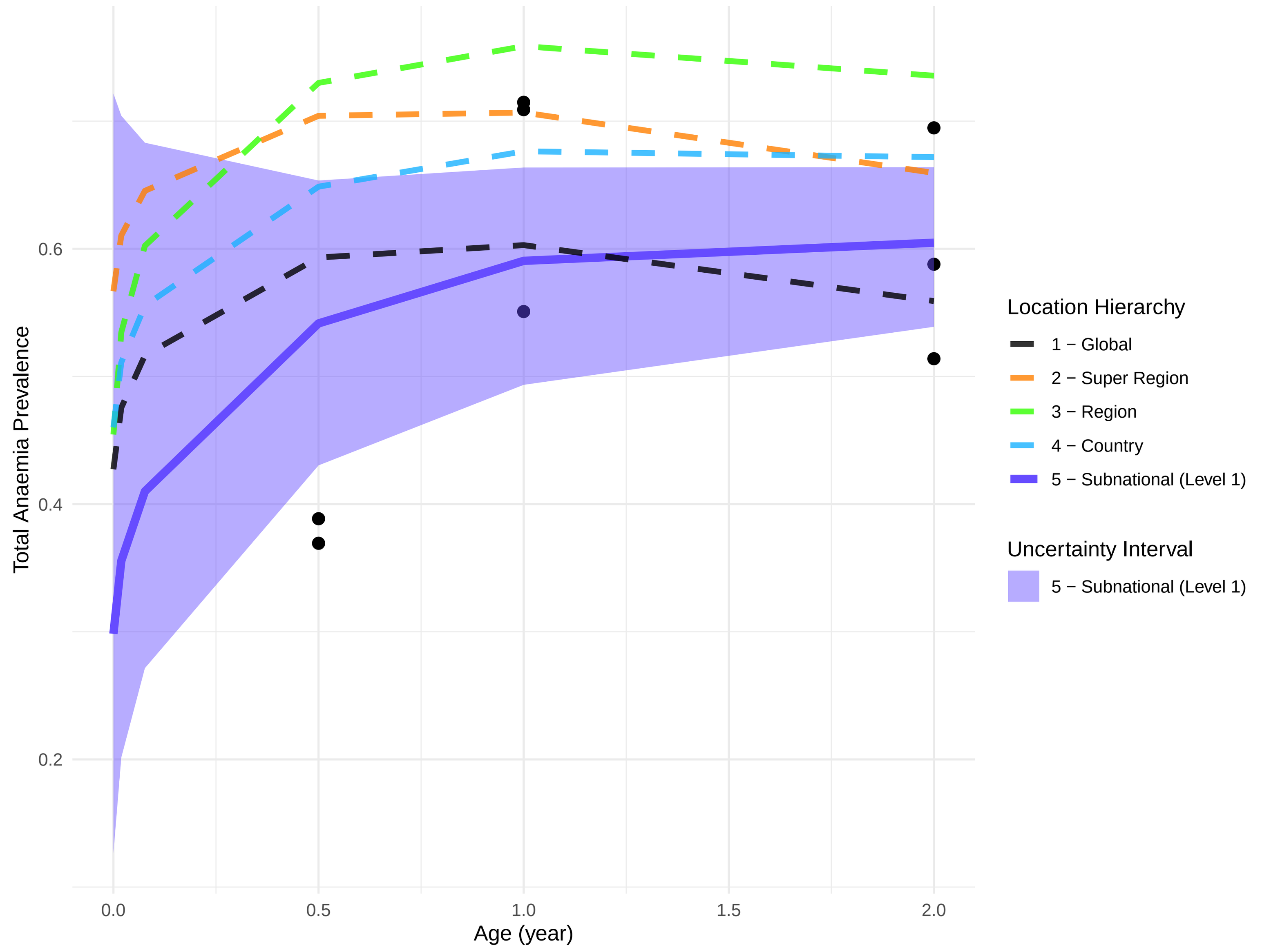

MR−BRT Age/Sex Fits − Non−pregnant Females <2 Years Old − Total Anaemia Prevalence − Akwa Ibom
WHO−2024 Adjustment Method

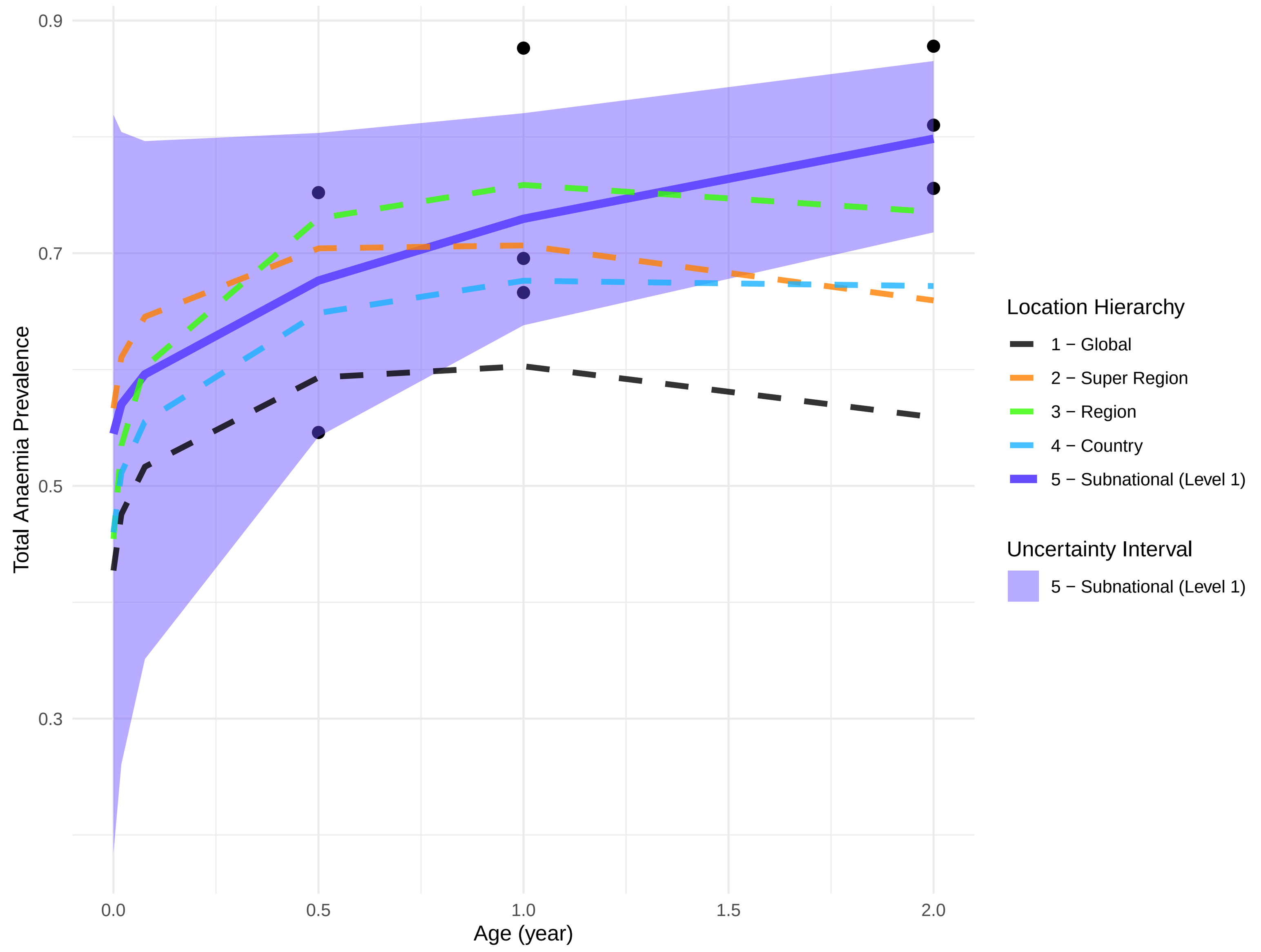

MR−BRT Age/Sex Fits − Non−pregnant Females <2 Years Old − Total Anaemia Prevalence − Anambra
WHO−2024 Adjustment Method

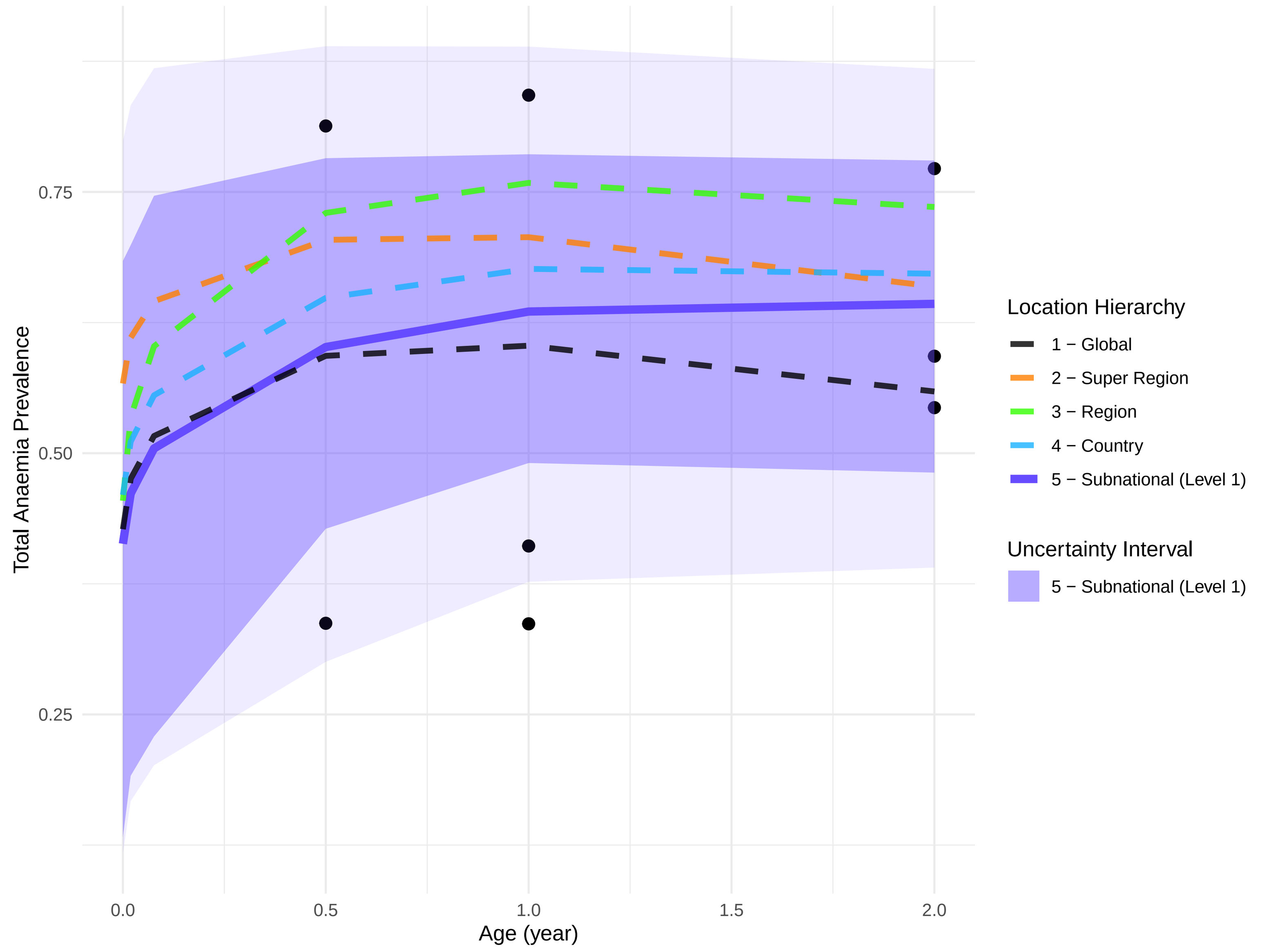

MR−BRT Age/Sex Fits − Non−pregnant Females <2 Years Old − Total Anaemia Prevalence − Bauchi
WHO−2024 Adjustment Method

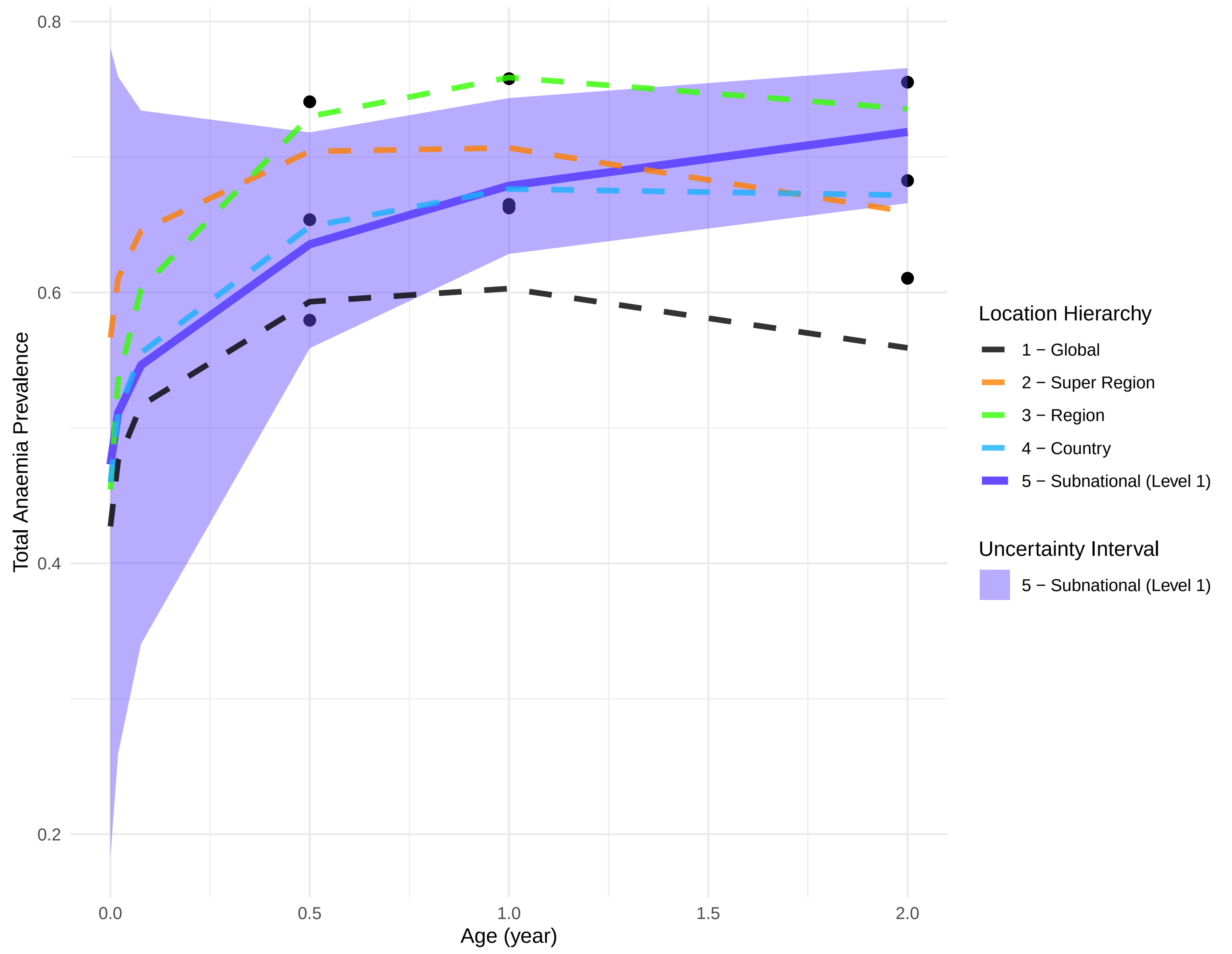

MR−BRT Age/Sex Fits − Non−pregnant Females <2 Years Old − Total Anaemia Prevalence − Bayelsa
WHO−2024 Adjustment Method

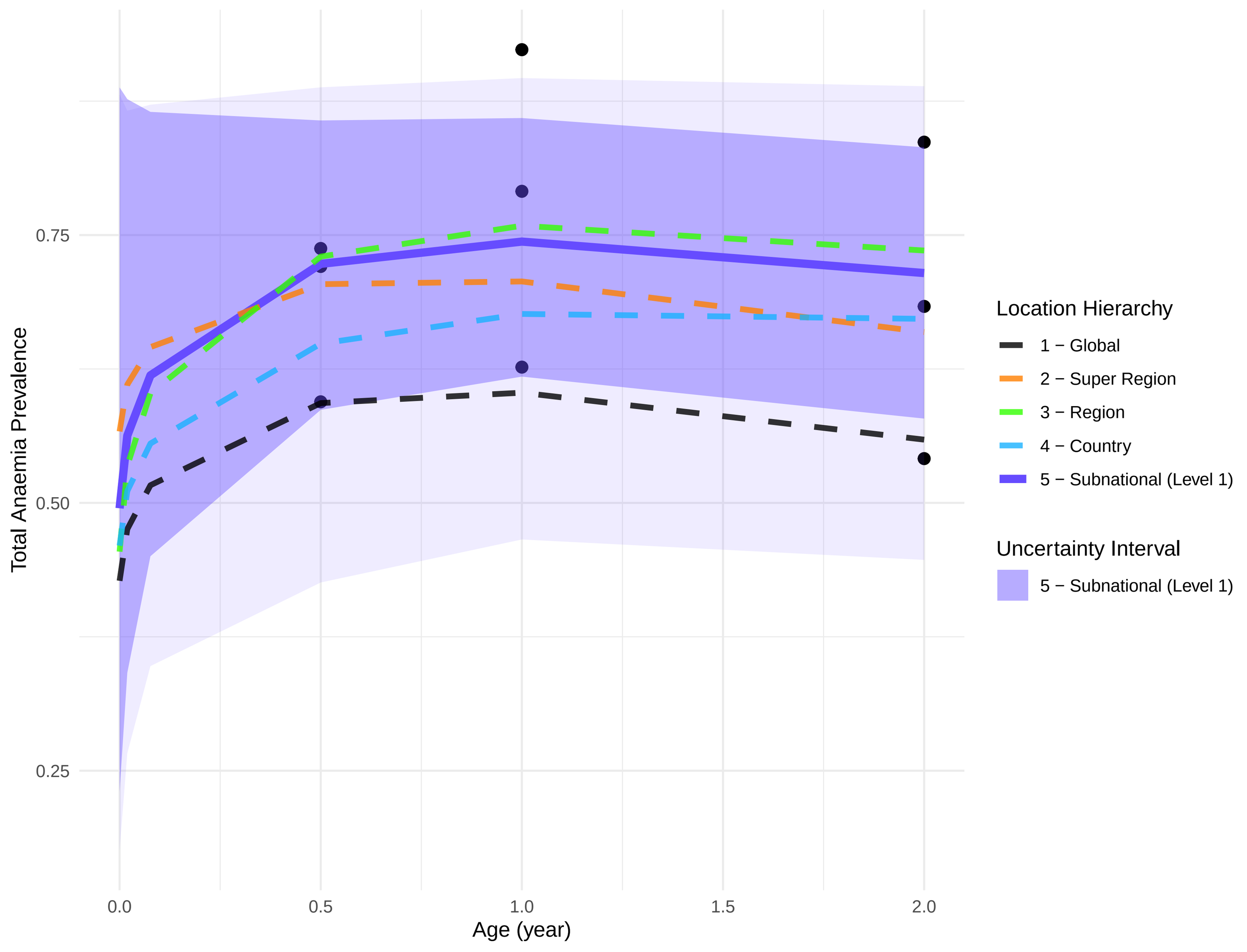

MR−BRT Age/Sex Fits − Non−pregnant Females <2 Years Old − Total Anaemia Prevalence − Benue
WHO−2024 Adjustment Method

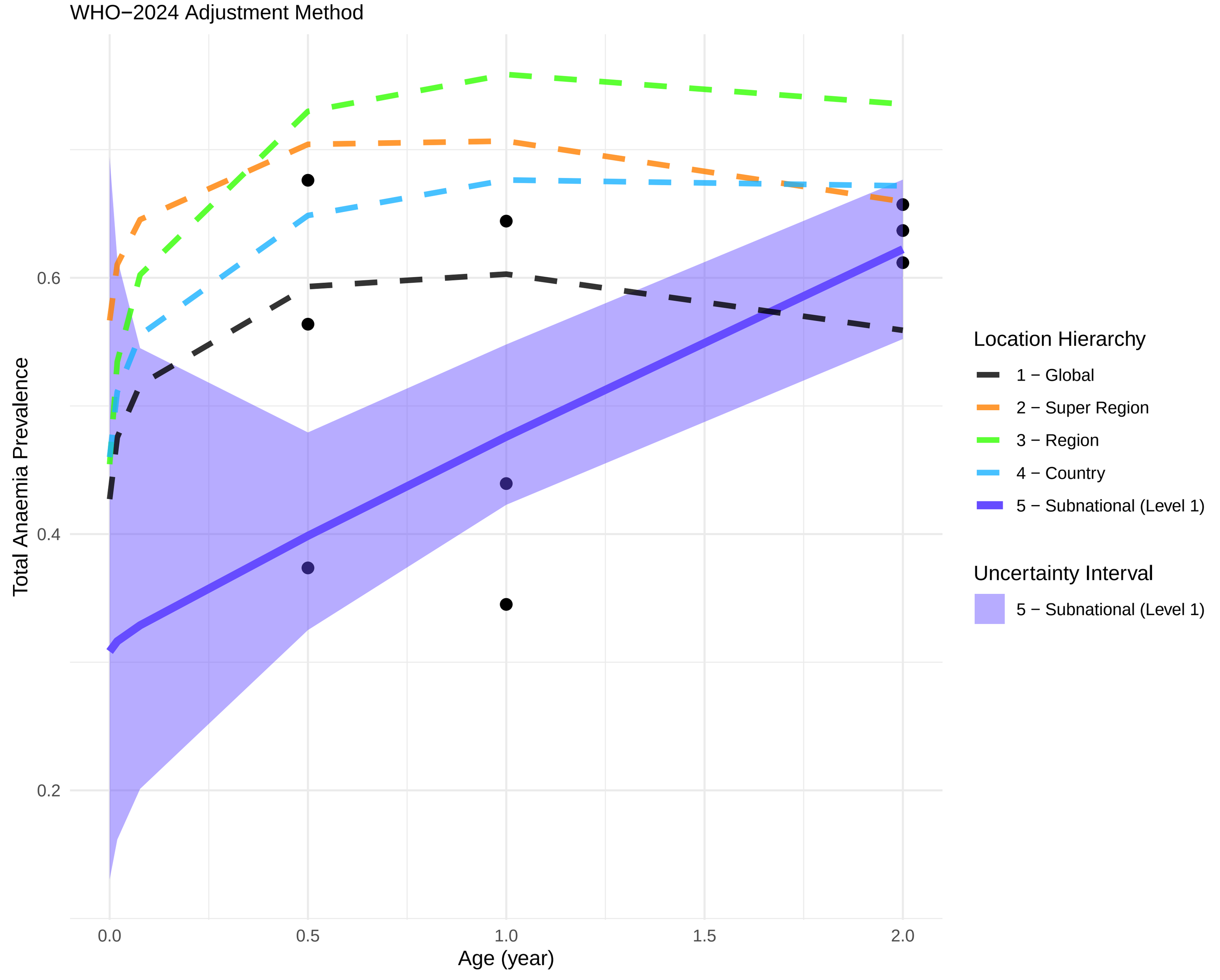

MR−BRT Age/Sex Fits − Non−pregnant Females <2 Years Old − Total Anaemia Prevalence − Borno
WHO−2024 Adjustment Method

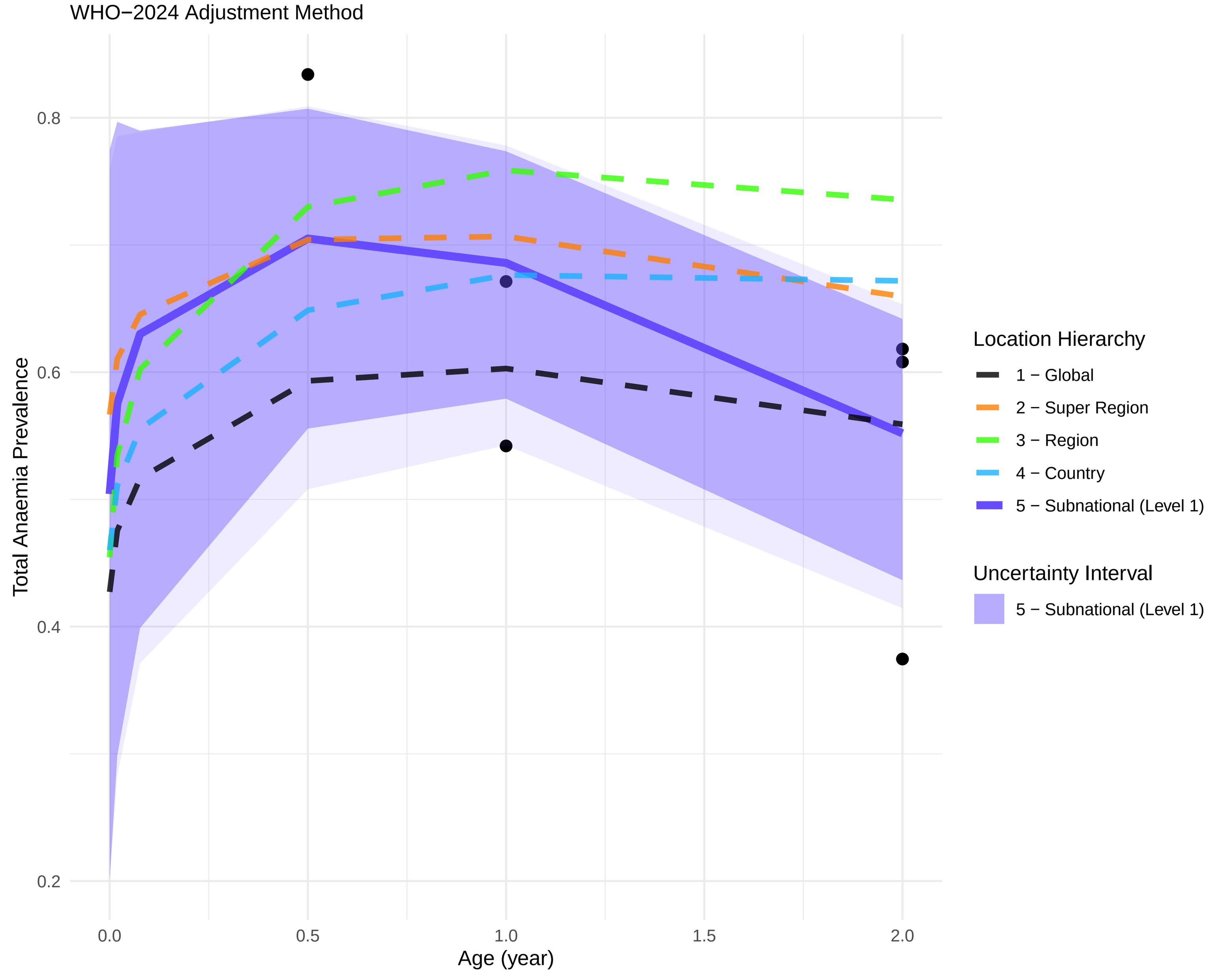

MR−BRT Age/Sex Fits − Non−pregnant Females <2 Years Old − Total Anaemia Prevalence − Cross River
WHO−2024 Adjustment Method

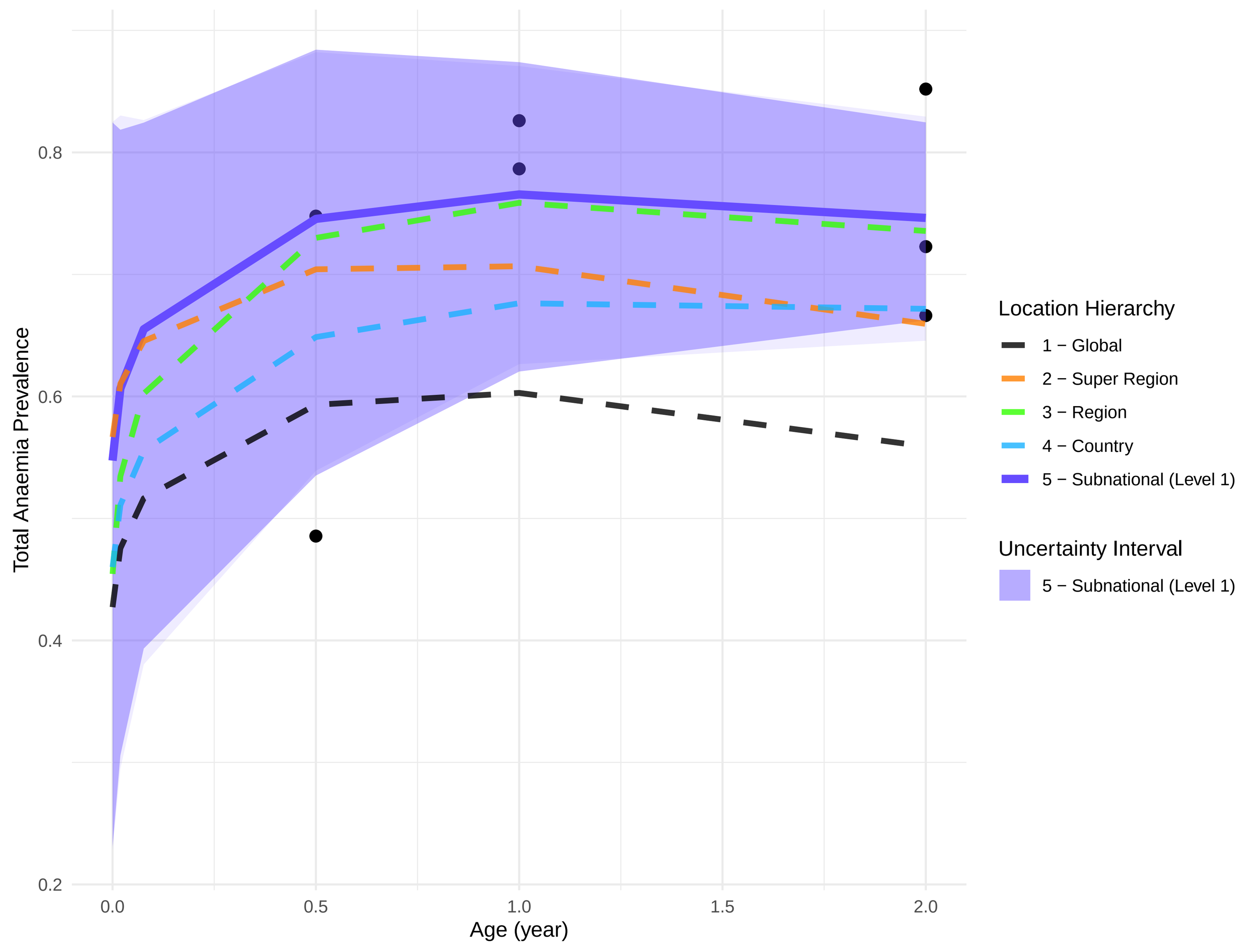

MR−BRT Age/Sex Fits − Non−pregnant Females <2 Years Old − Total Anaemia Prevalence − Delta
WHO−2024 Adjustment Method

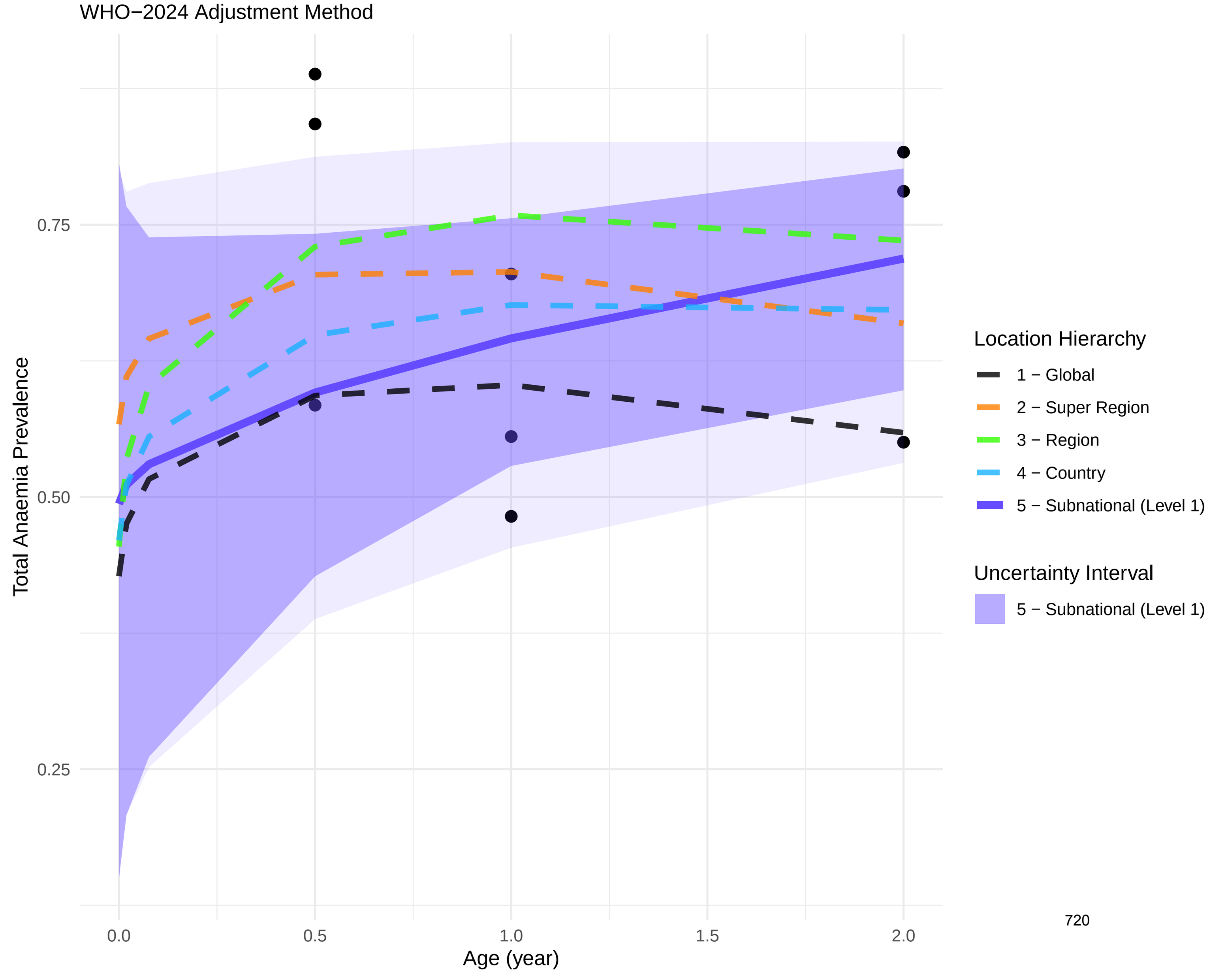

MR−BRT Age/Sex Fits − Non-pregnant Females <2 Years Old − Total Anaemia Prevalence − Ebonyi
WHO−2024 Adjustment Method

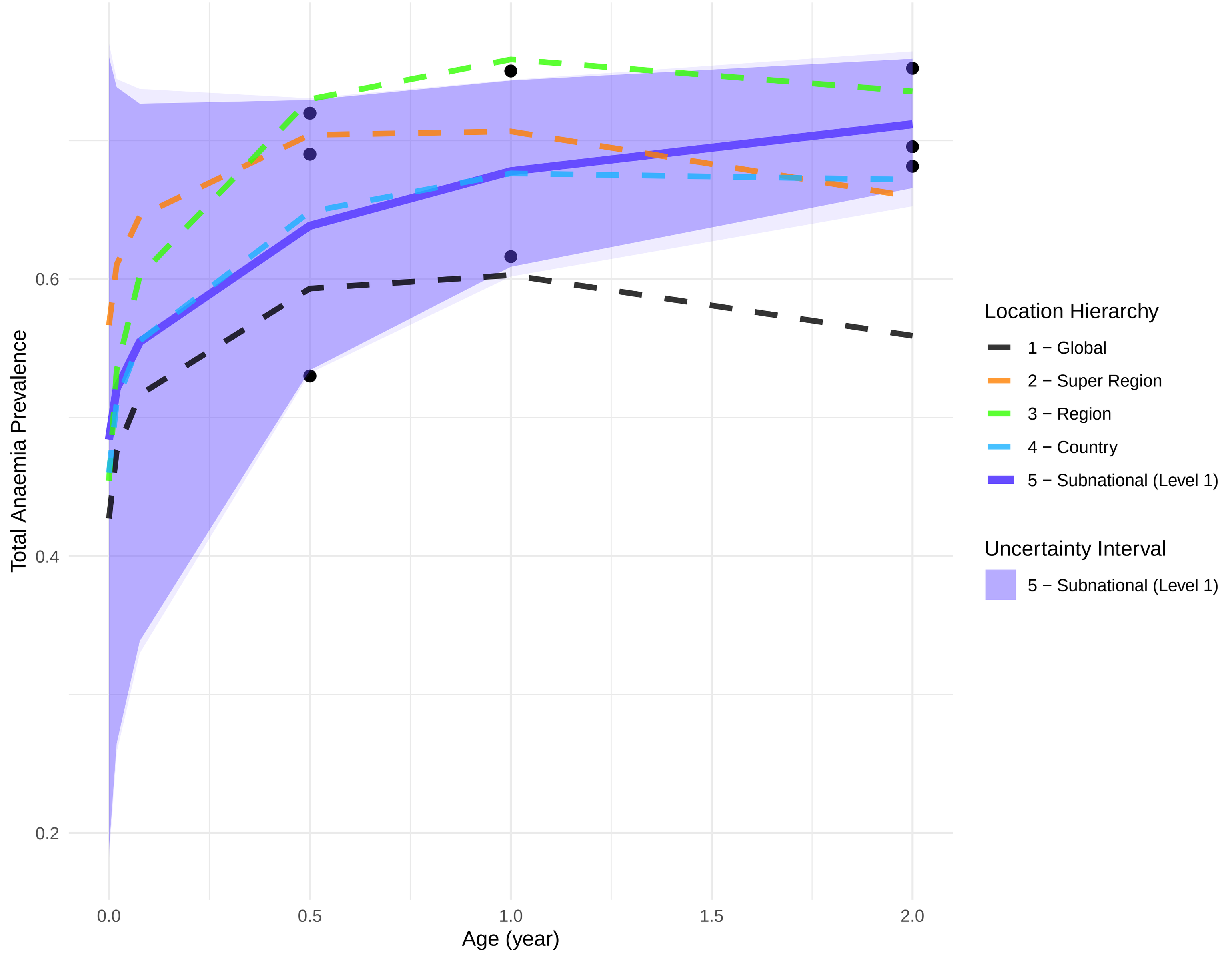

MR−BRT Age/Sex Fits − Non-pregnant Females <2 Years Old − Total Anaemia Prevalence − Edo
WHO−2024 Adjustment Method

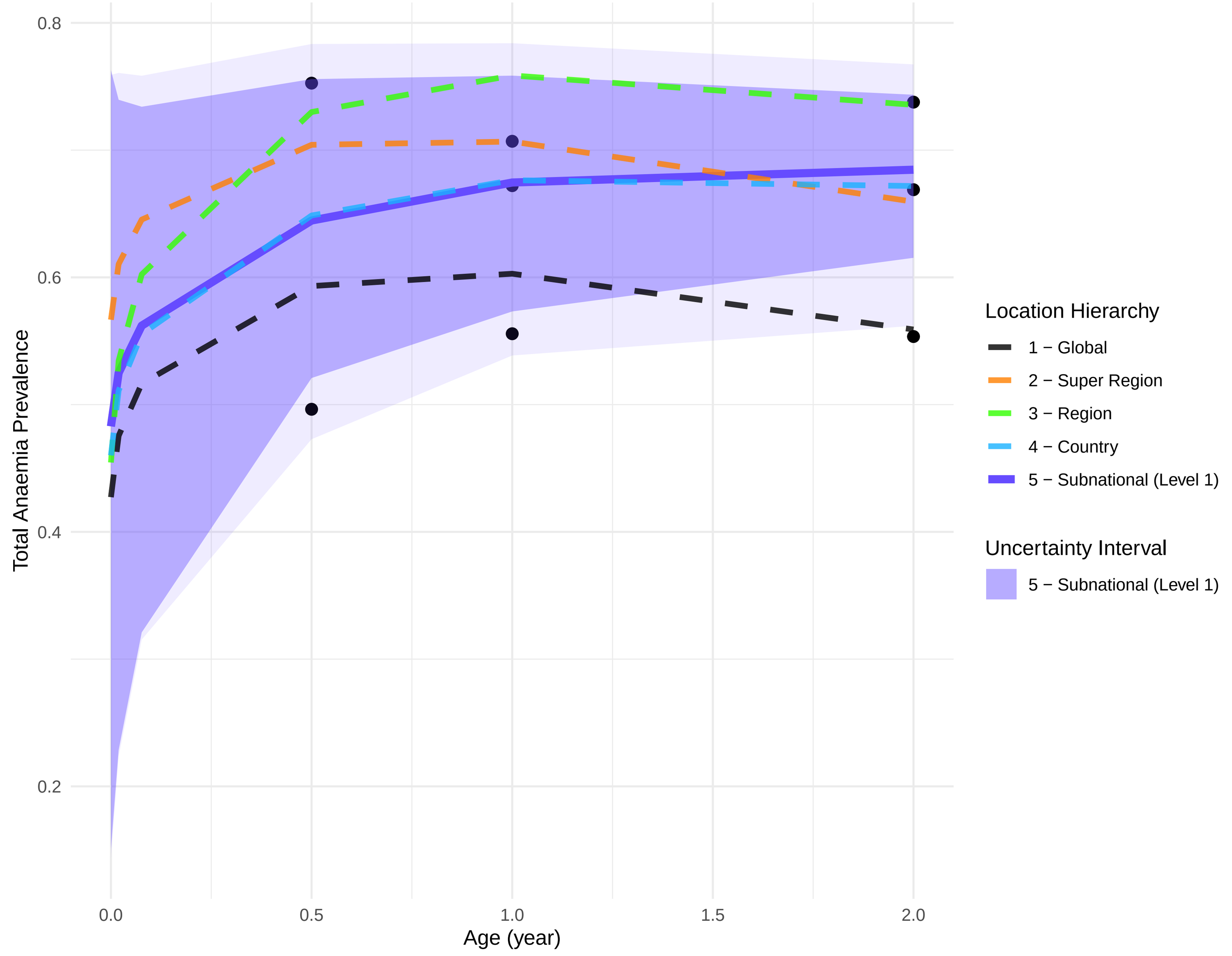

MR−BRT Age/Sex Fits − Non-pregnant Females <2 Years Old − Total Anaemia Prevalence − Ekiti
WHO−2024 Adjustment Method

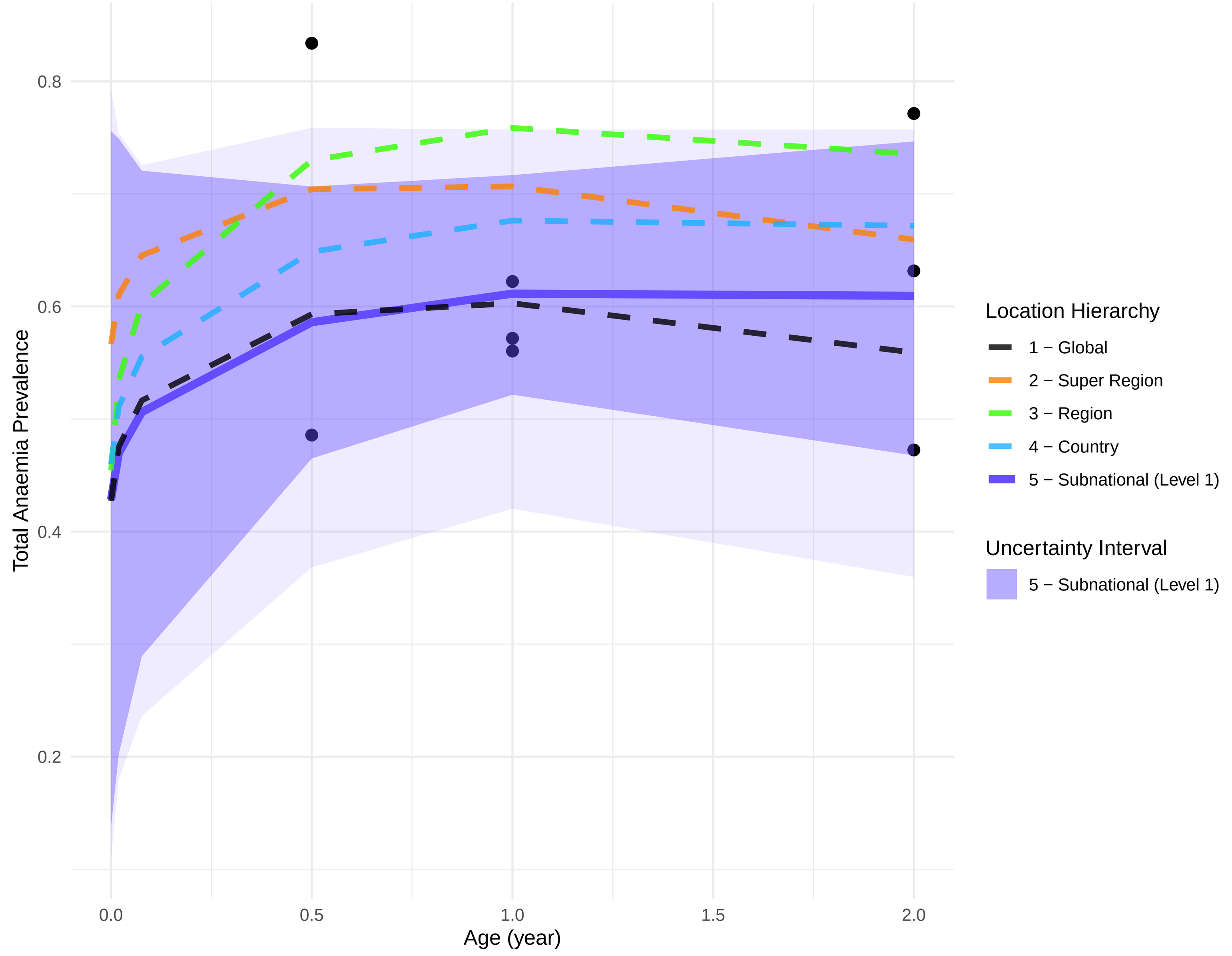

MR−BRT Age/Sex Fits − Non-pregnant Females <2 Years Old − Total Anaemia Prevalence − Enugu
WHO−2024 Adjustment Method

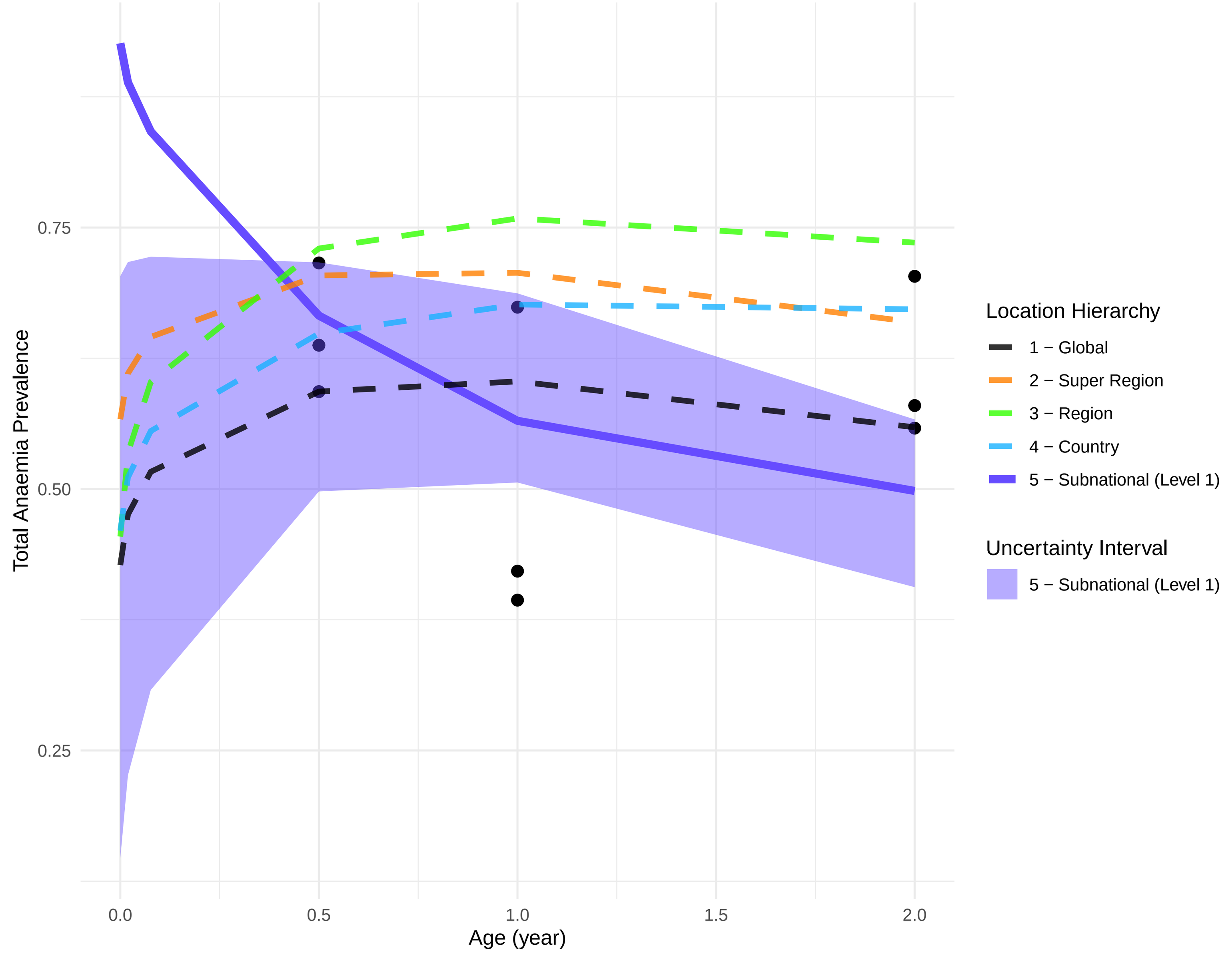

MR−BRT Age/Sex Fits − Non-pregnant Females <2 Years Old − Total Anaemia Prevalence − FCT (Abuja)
WHO−2024 Adjustment Method

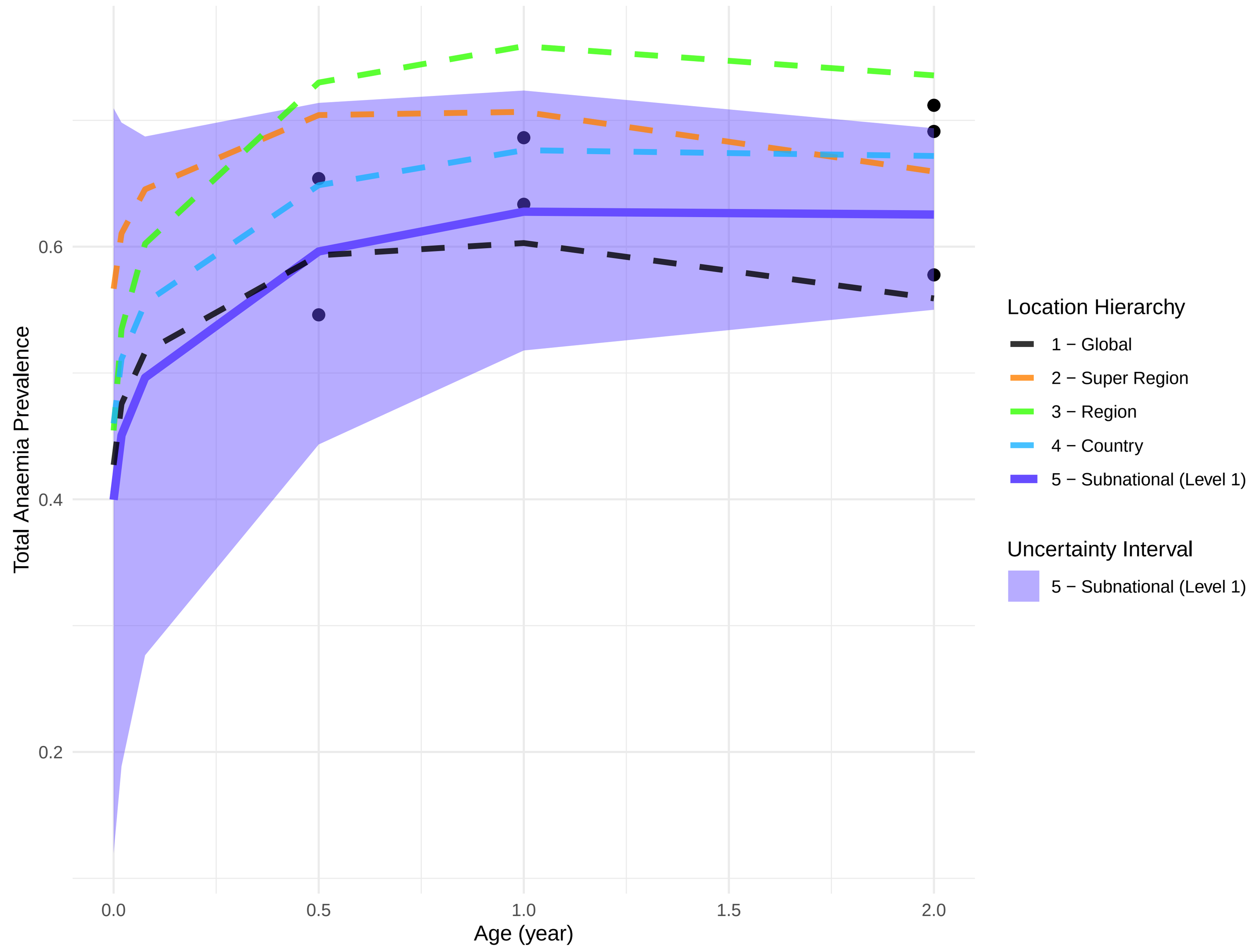

MR−BRT Age/Sex Fits − Non-pregnant Females <2 Years Old − Total Anaemia Prevalence − Gombe
WHO−2024 Adjustment Method

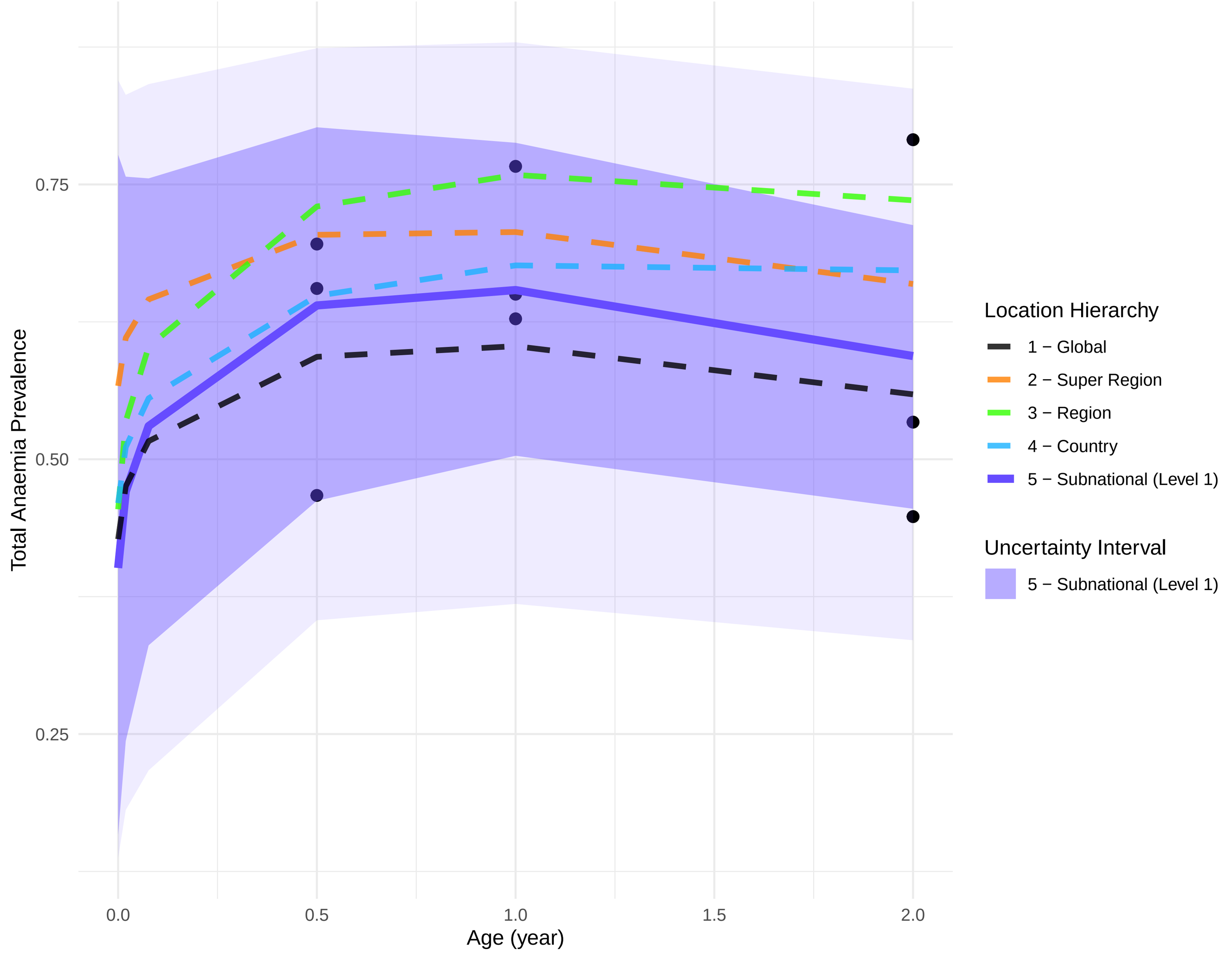

MR−BRT Age/Sex Fits − Non-pregnant Females <2 Years Old − Total Anaemia Prevalence − Imo
WHO−2024 Adjustment Method

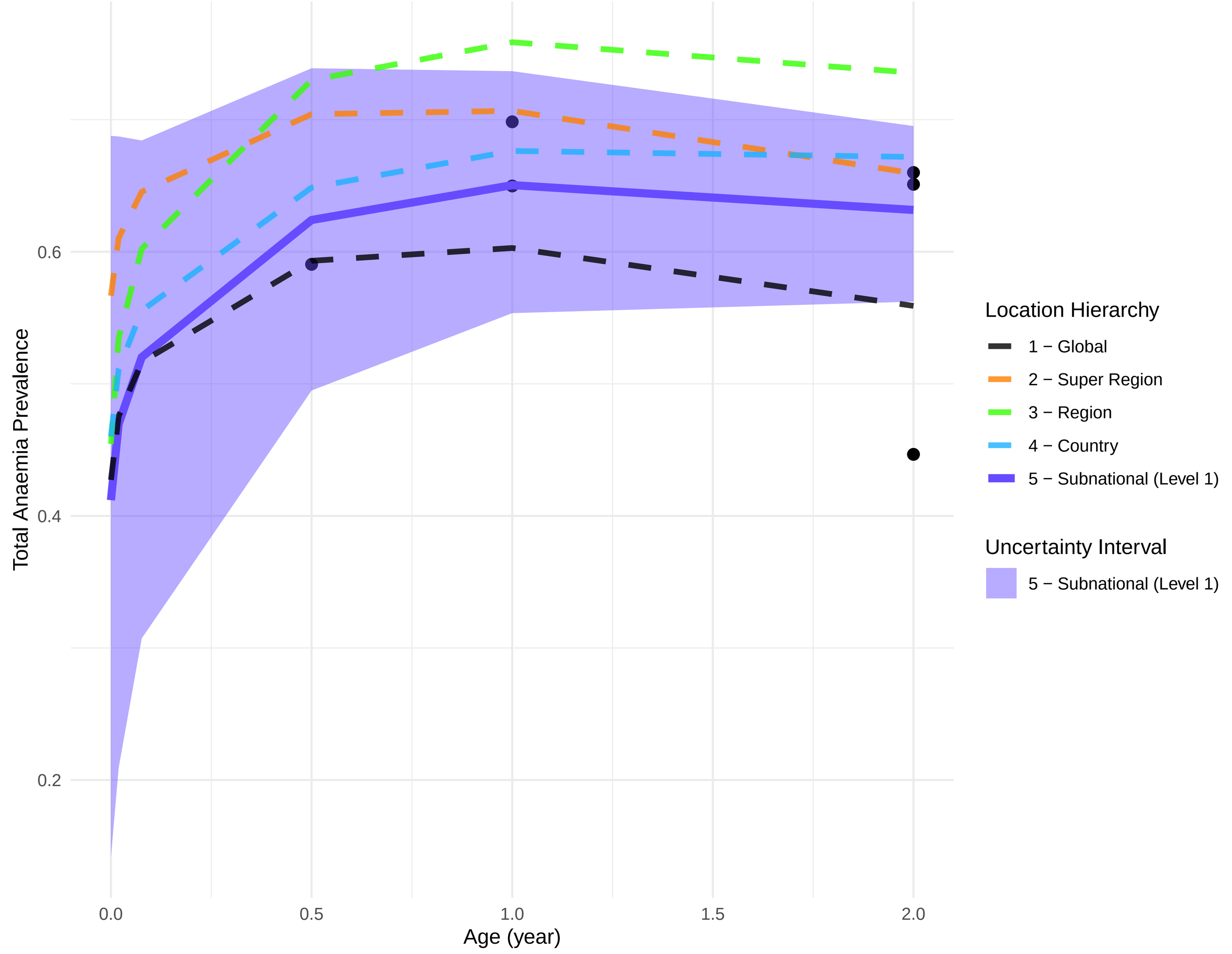

MR−BRT Age/Sex Fits − Non-pregnant Females <2 Years Old − Total Anaemia Prevalence − Jigawa
WHO−2024 Adjustment Method

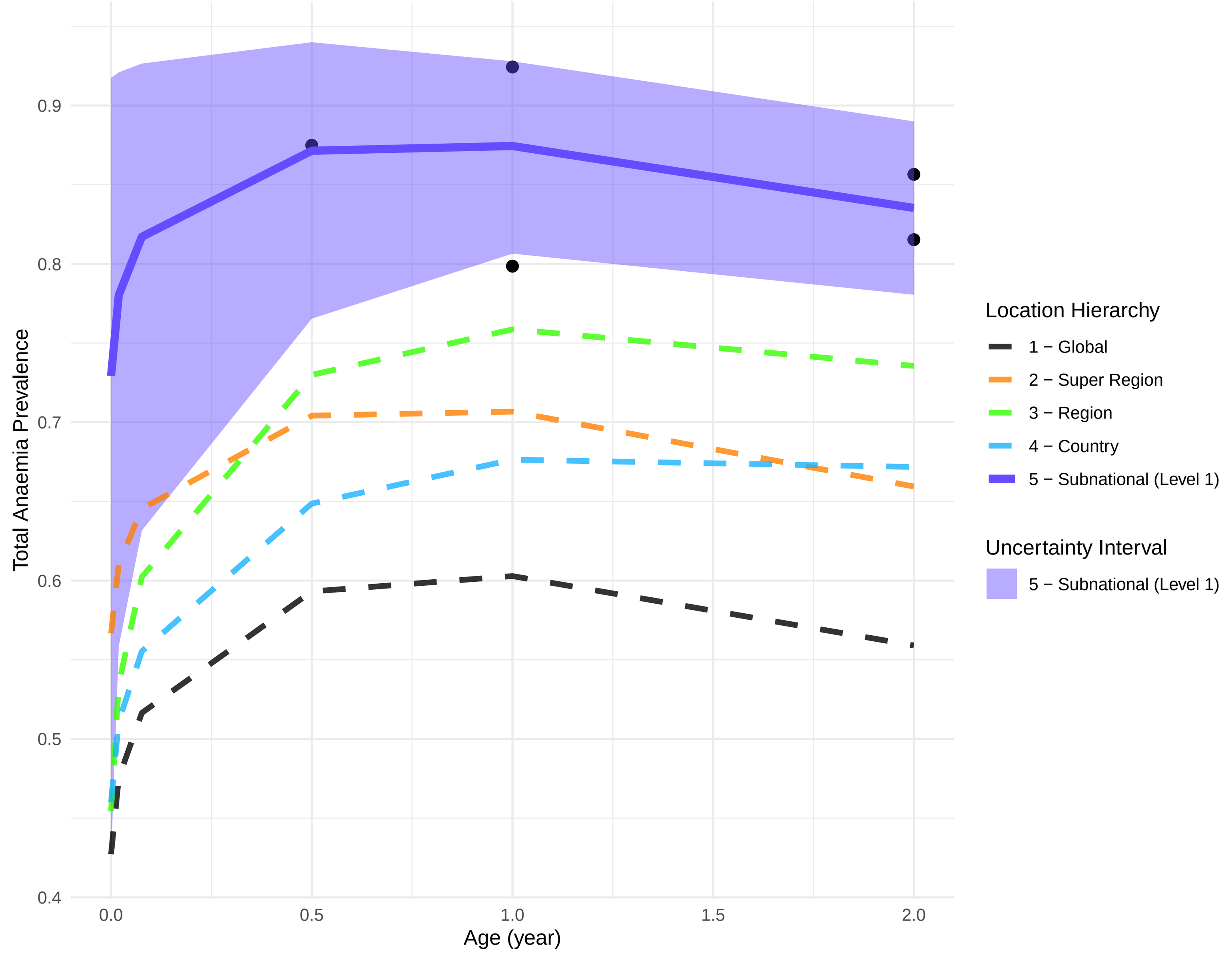

MR−BRT Age/Sex Fits − Non-pregnant Females <2 Years Old − Total Anaemia Prevalence − Kaduna
WHO−2024 Adjustment Method

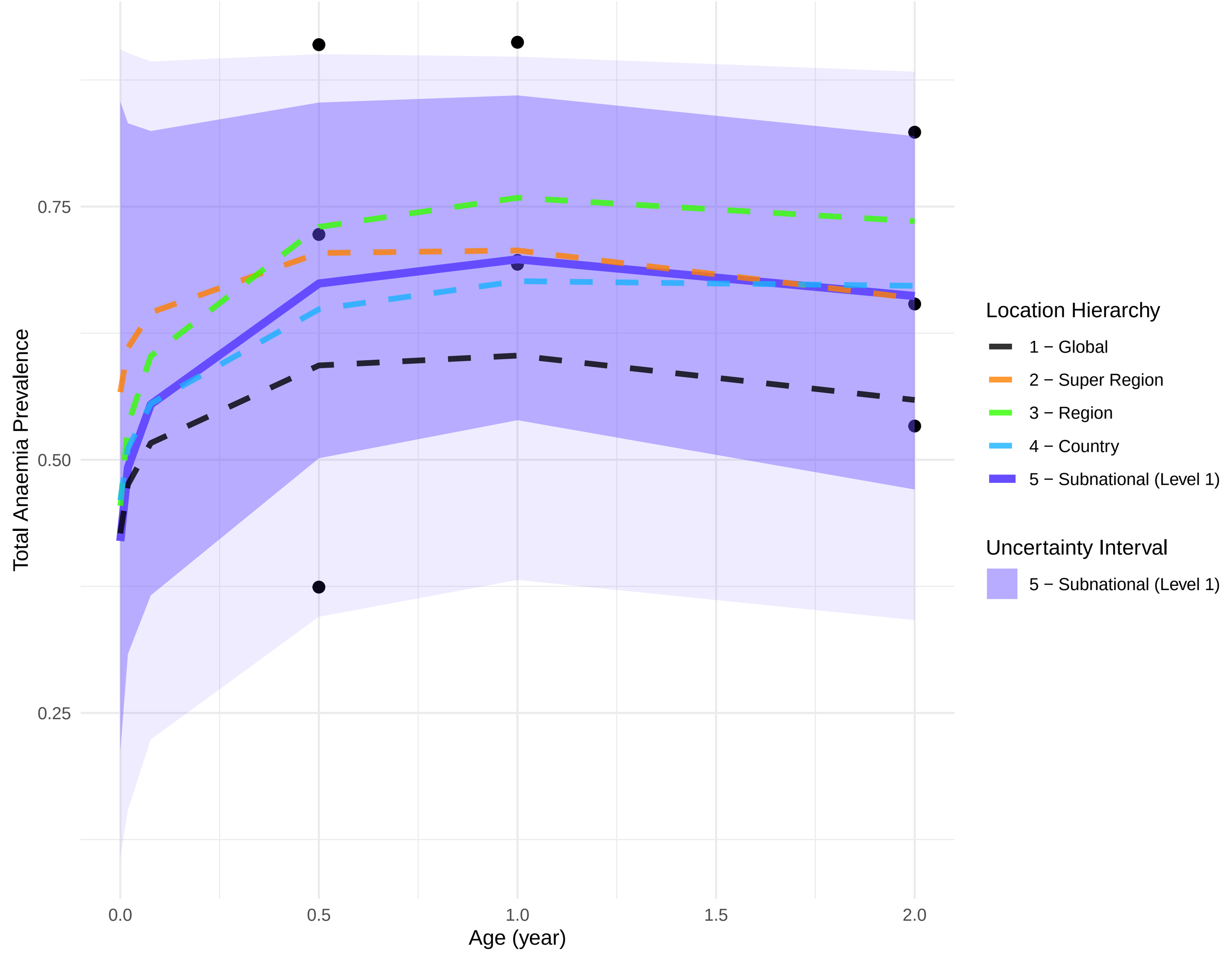

MR−BRT Age/Sex Fits − Non-pregnant Females <2 Years Old − Total Anaemia Prevalence − Kano
WHO−2024 Adjustment Method

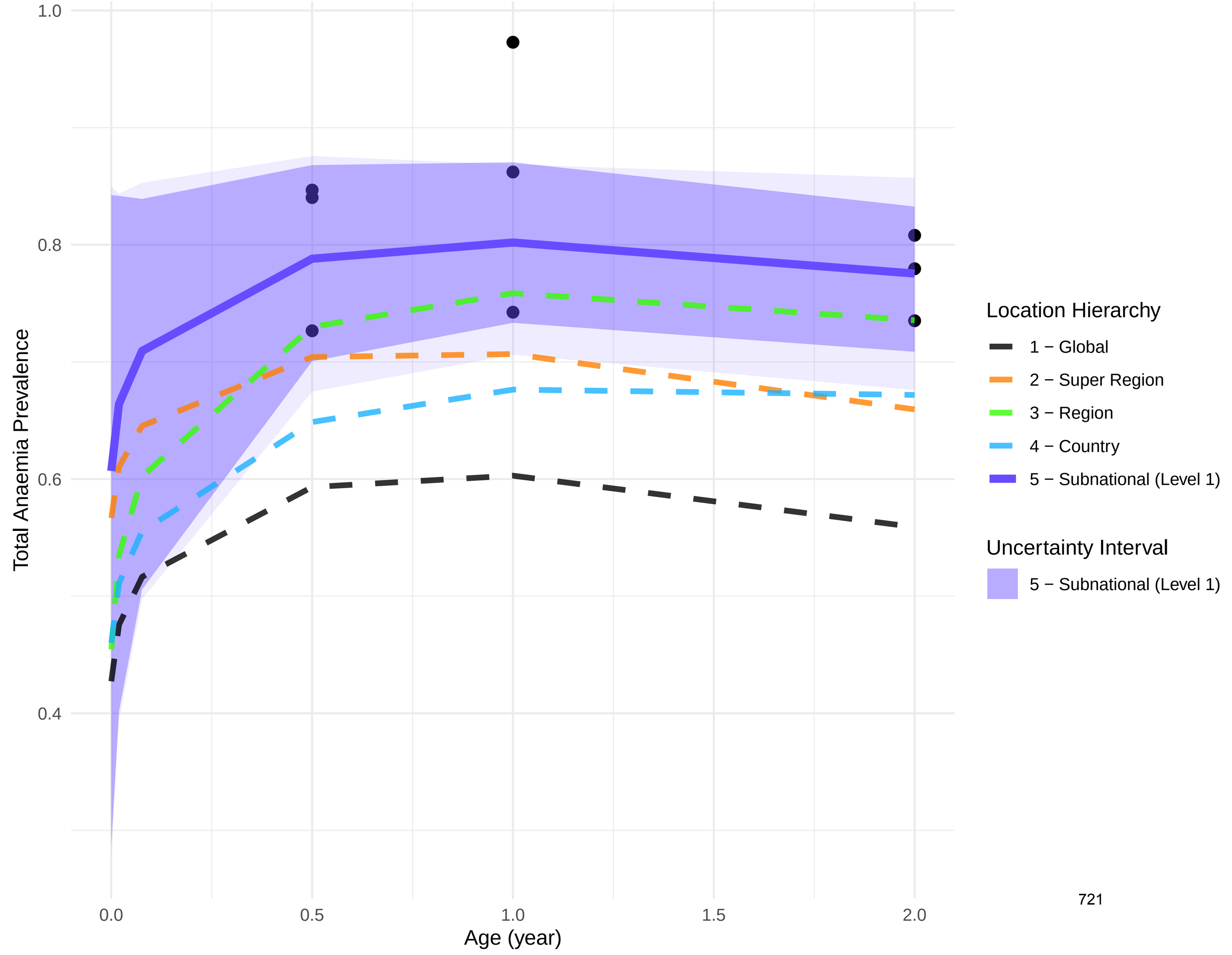

MR−BRT Age/Sex Fits − Non−pregnant Females <2 Years Old − Total Anaemia Prevalence − Katsina
WHO−2024 Adjustment Method

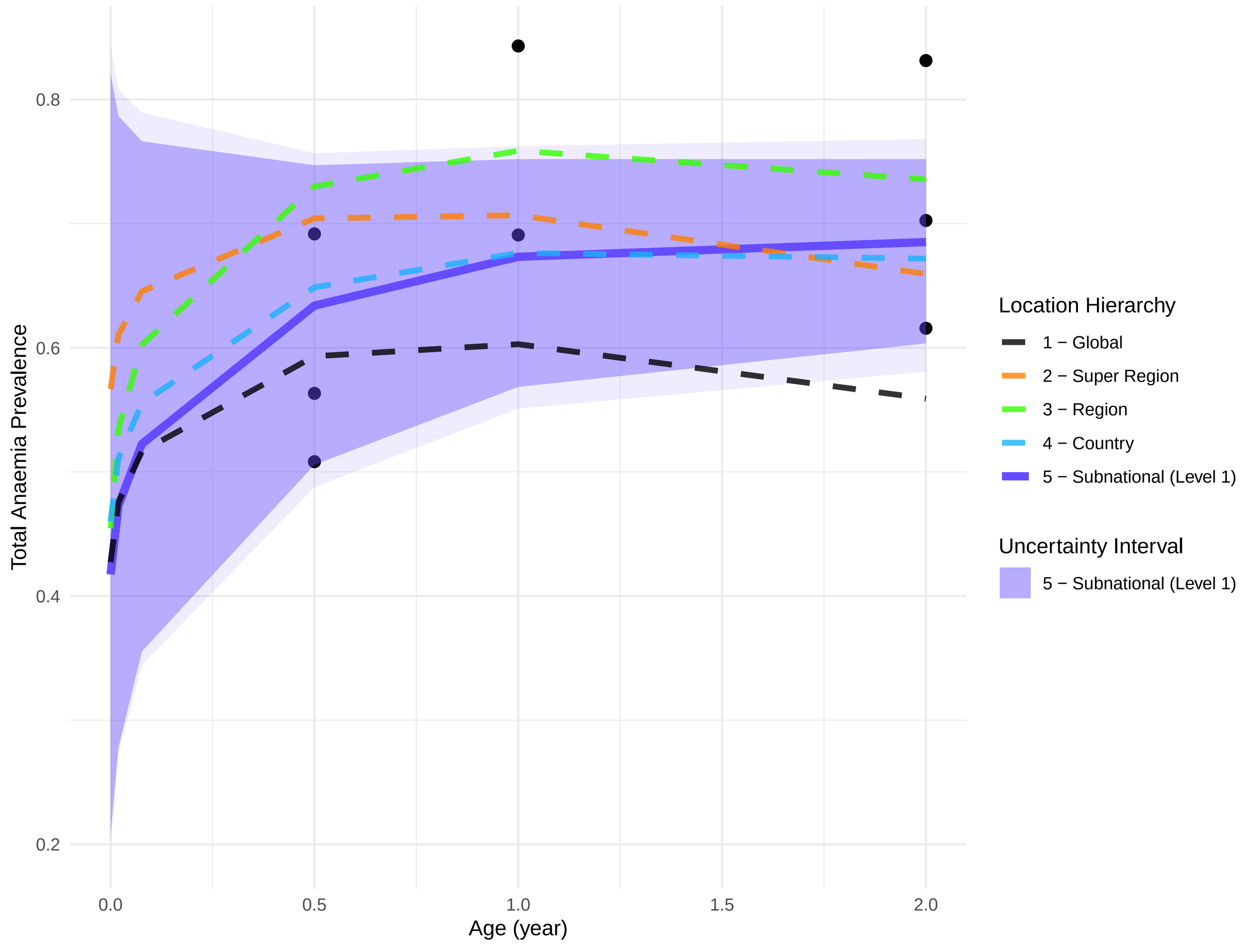

MR−BRT Age/Sex Fits − Non−pregnant Females <2 Years Old − Total Anaemia Prevalence − Kebbi
WHO−2024 Adjustment Method

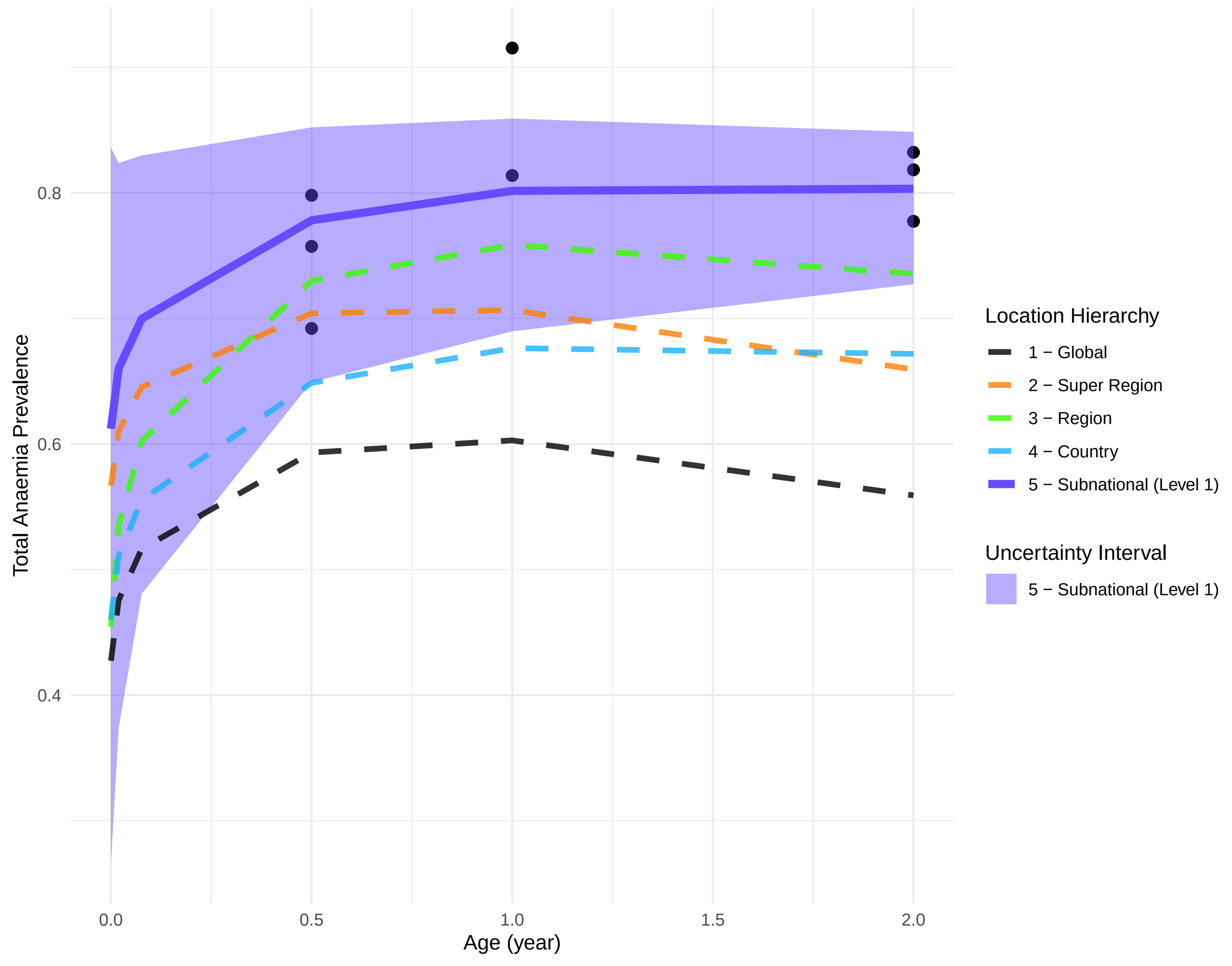

MR−BRT Age/Sex Fits − Non−pregnant Females <2 Years Old − Total Anaemia Prevalence − Kogi
WHO−2024 Adjustment Method

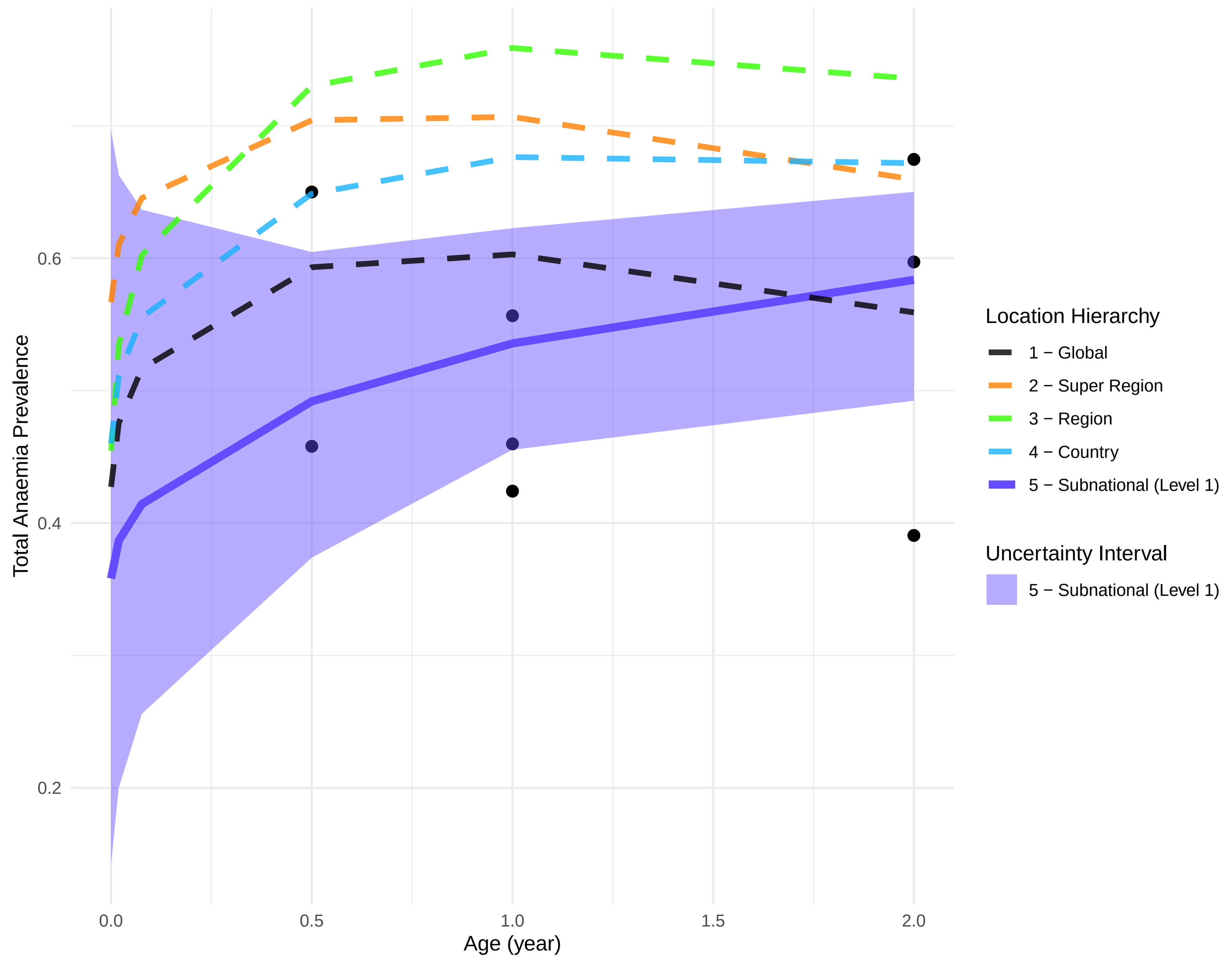

MR−BRT Age/Sex Fits − Non−pregnant Females <2 Years Old − Total Anaemia Prevalence − Kwara
WHO−2024 Adjustment Method

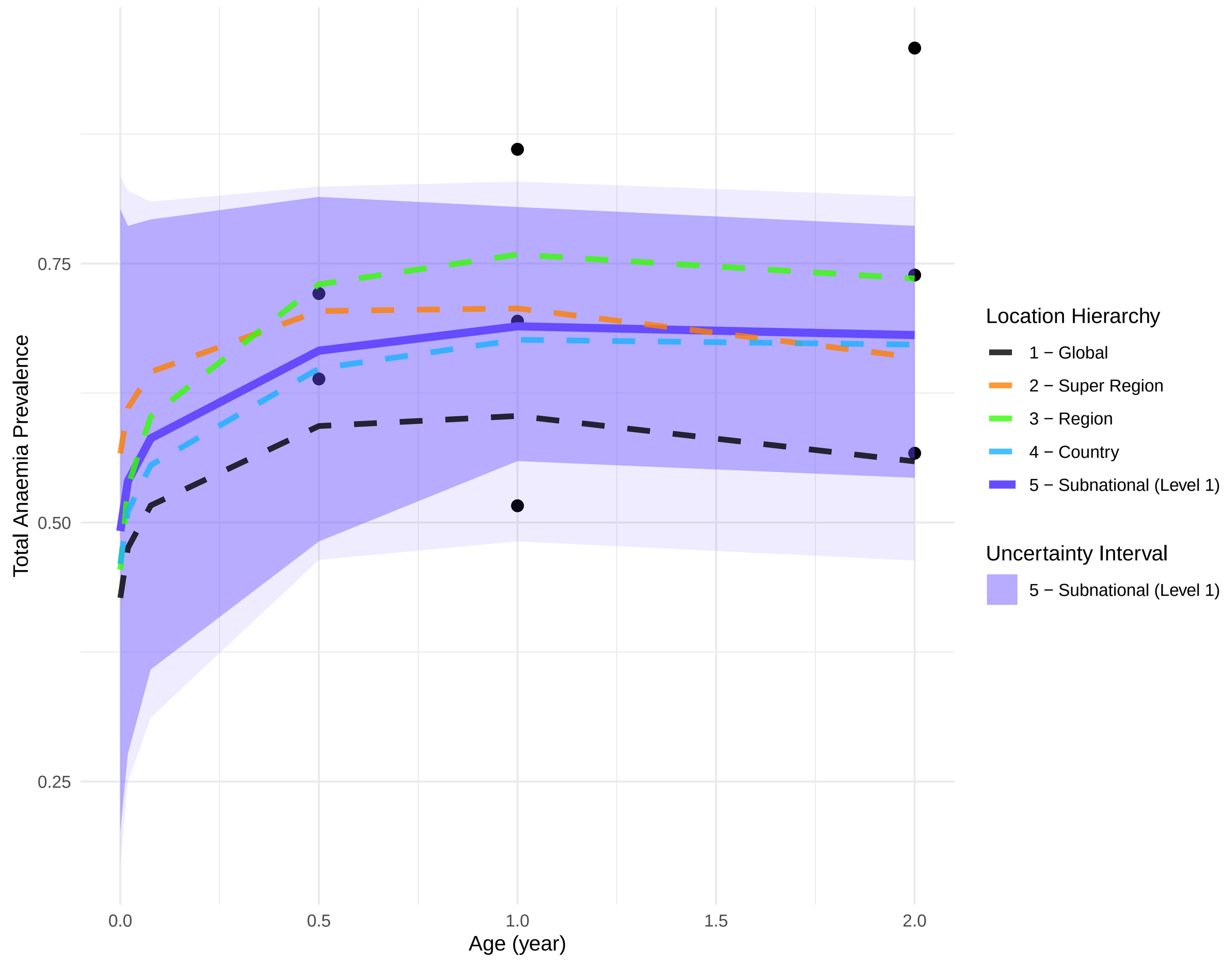

MR−BRT Age/Sex Fits − Non−pregnant Females <2 Years Old − Total Anaemia Prevalence − Lagos
WHO−2024 Adjustment Method

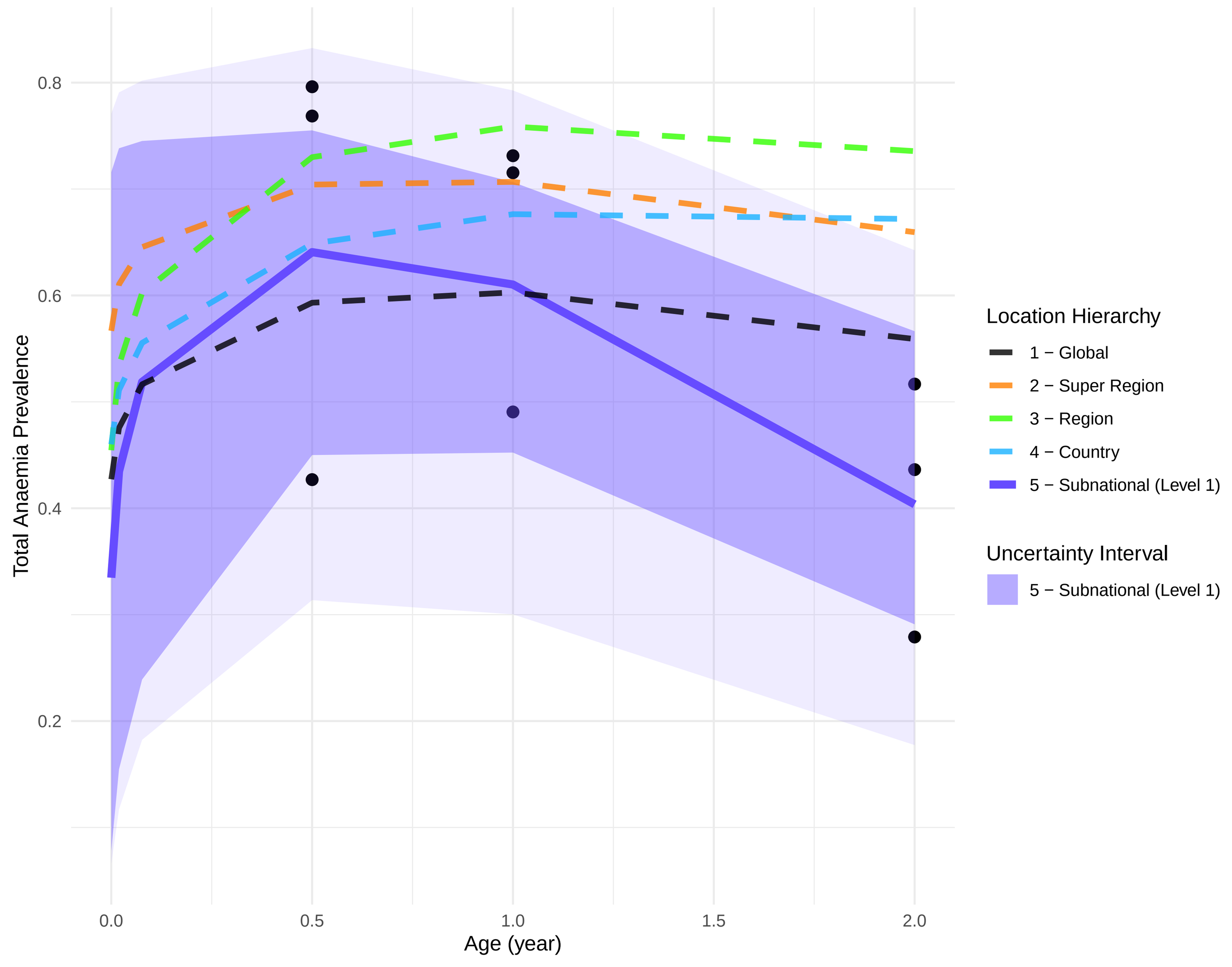

MR−BRT Age/Sex Fits − Non−pregnant Females <2 Years Old − Total Anaemia Prevalence − Nasarawa
WHO−2024 Adjustment Method

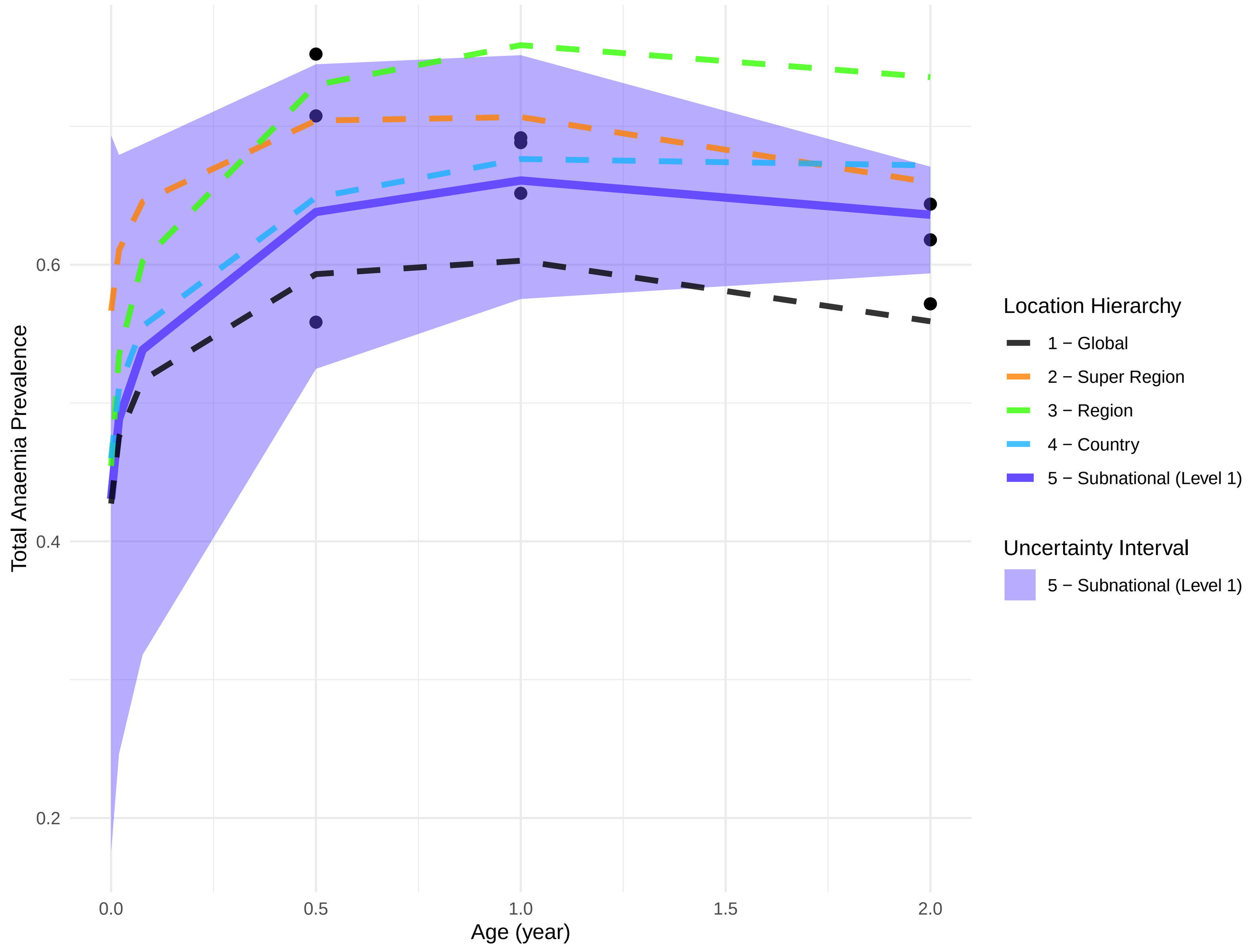

MR−BRT Age/Sex Fits − Non−pregnant Females <2 Years Old − Total Anaemia Prevalence − Niger
WHO−2024 Adjustment Method

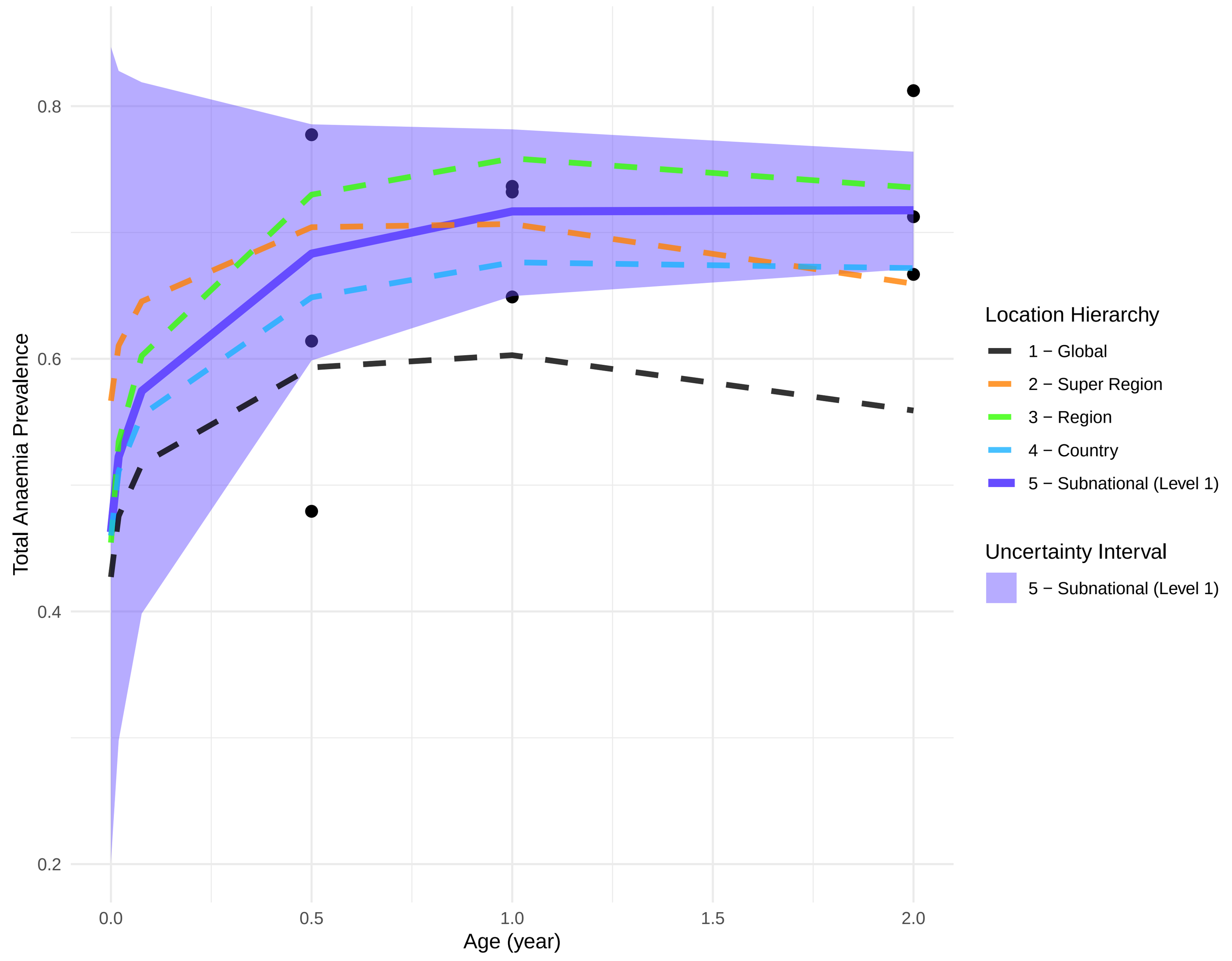

MR−BRT Age/Sex Fits − Non−pregnant Females <2 Years Old − Total Anaemia Prevalence − Ogun
WHO−2024 Adjustment Method

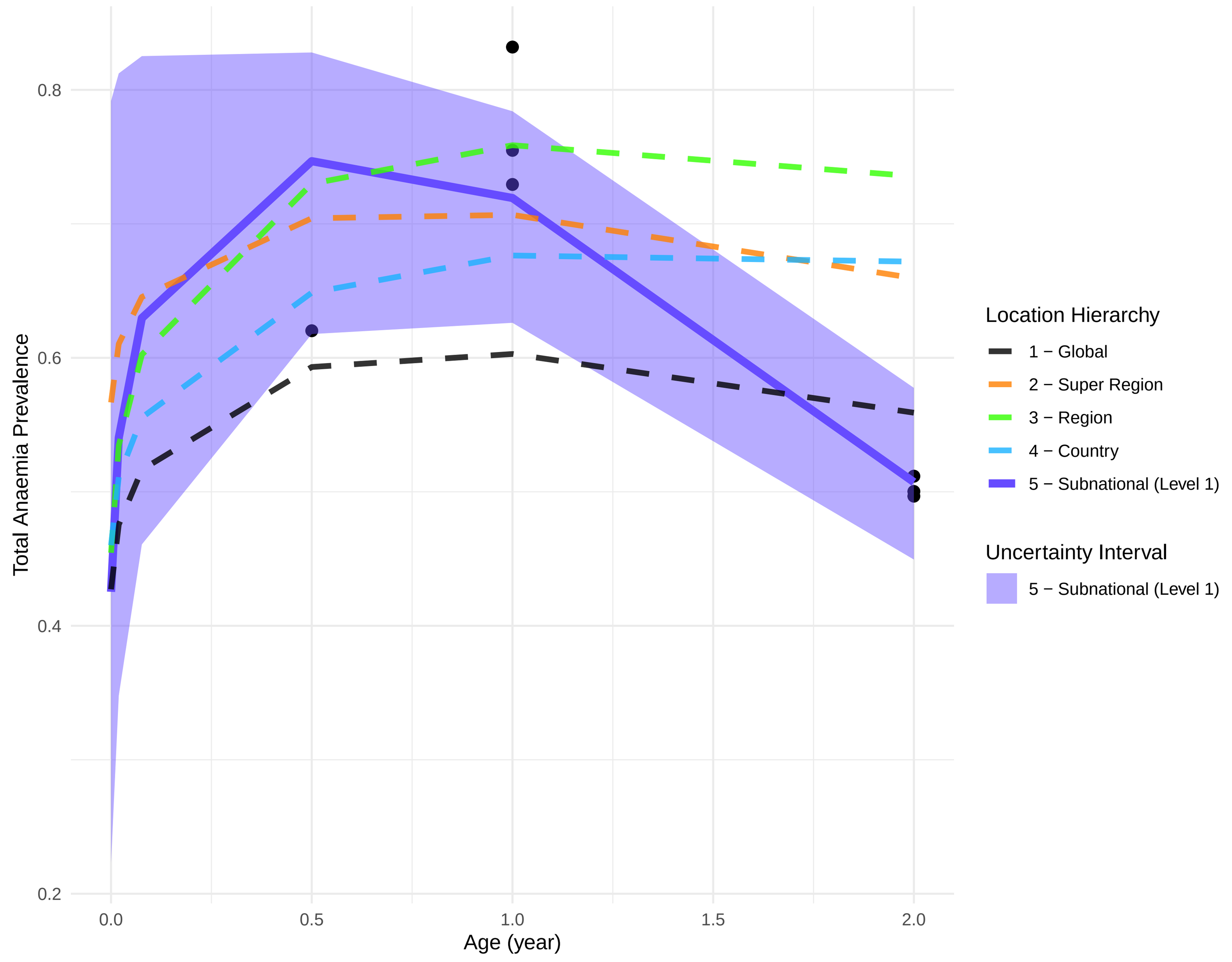

MR−BRT Age/Sex Fits − Non−pregnant Females <2 Years Old − Total Anaemia Prevalence − Ondo
WHO−2024 Adjustment Method

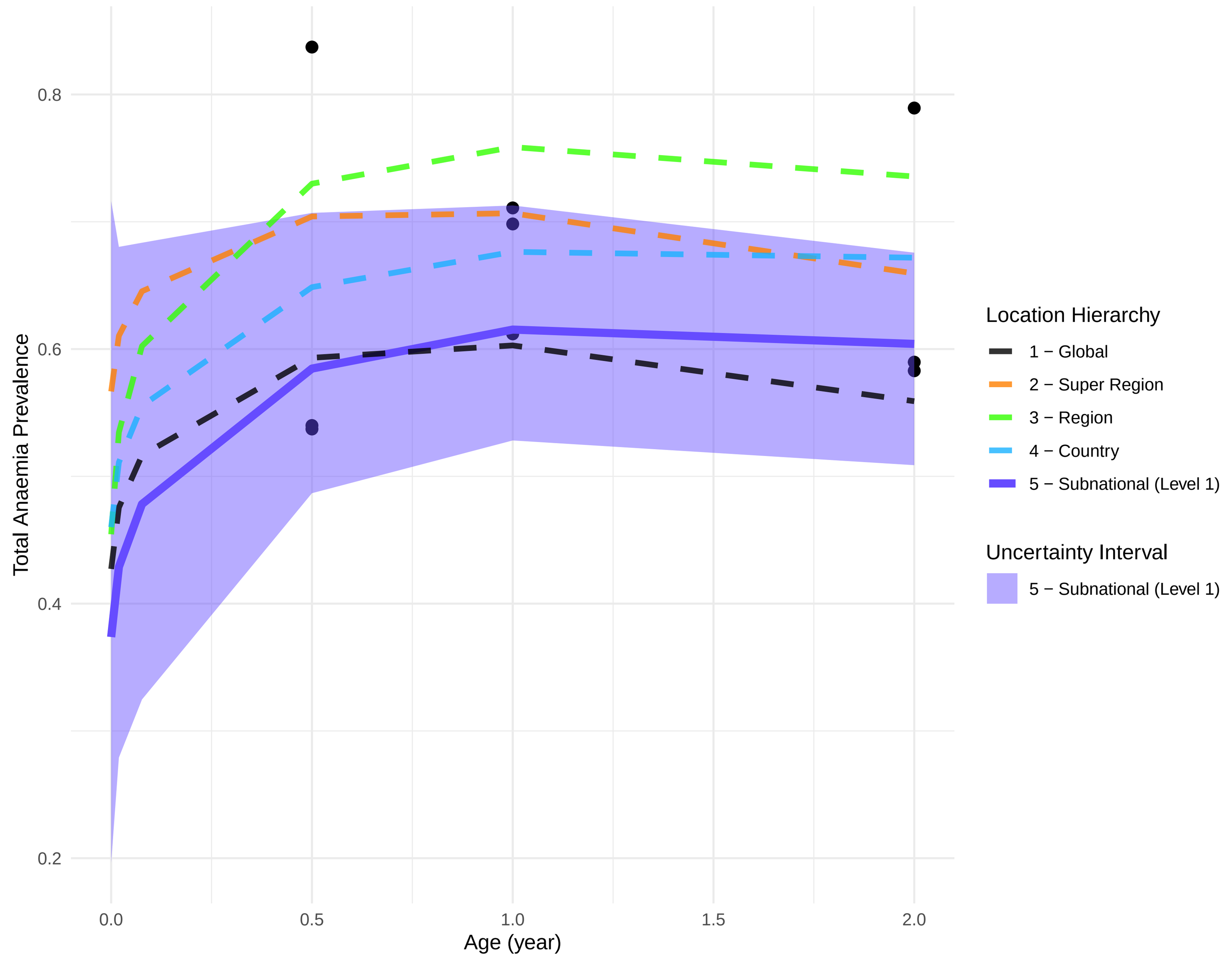

MR−BRT Age/Sex Fits − Non−pregnant Females <2 Years Old − Total Anaemia Prevalence − Osun
WHO−2024 Adjustment Method

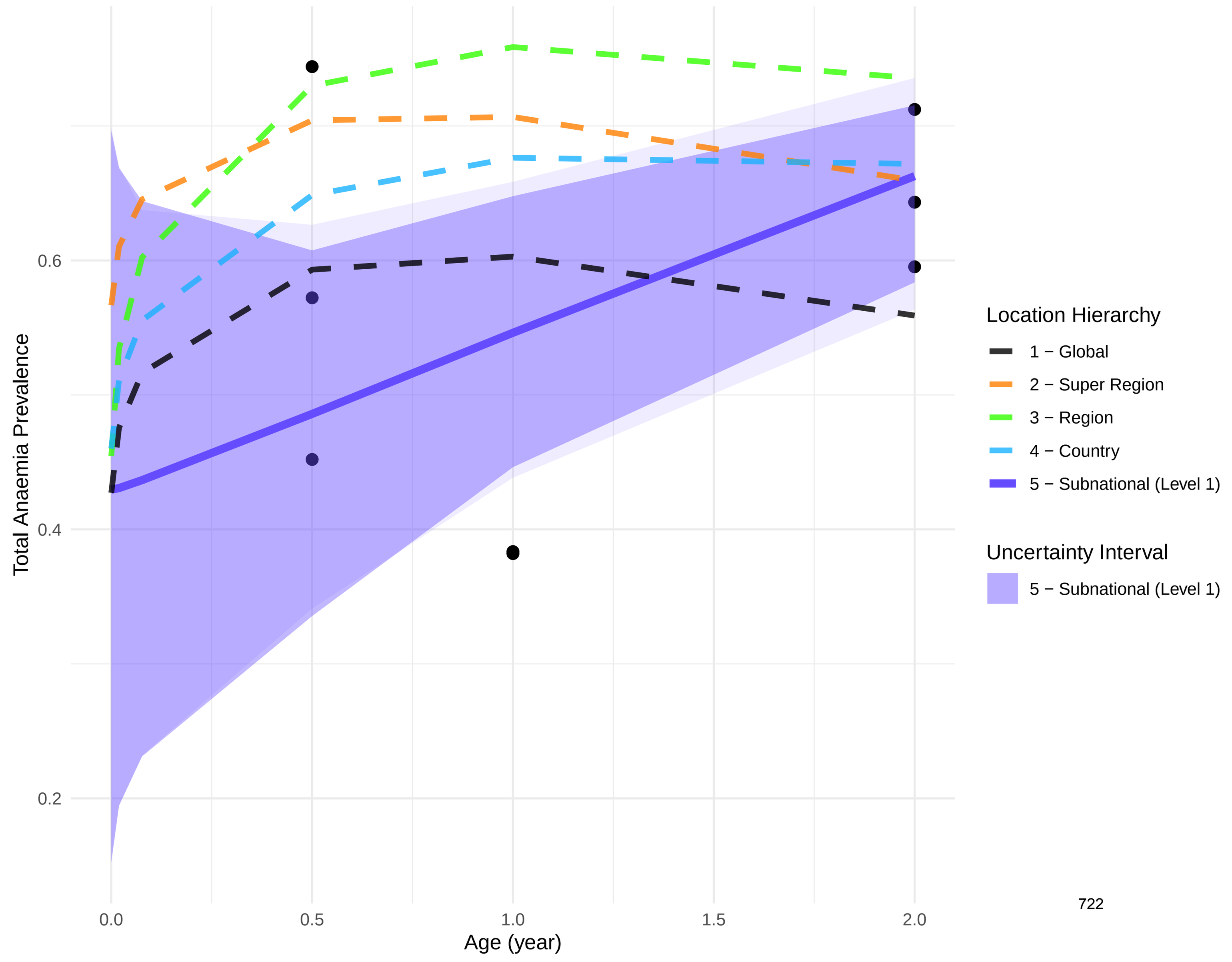

MR−BRT Age/Sex Fits − Non−pregnant Females <2 Years Old − Total Anaemia Prevalence − Oyo
WHO−2024 Adjustment Method

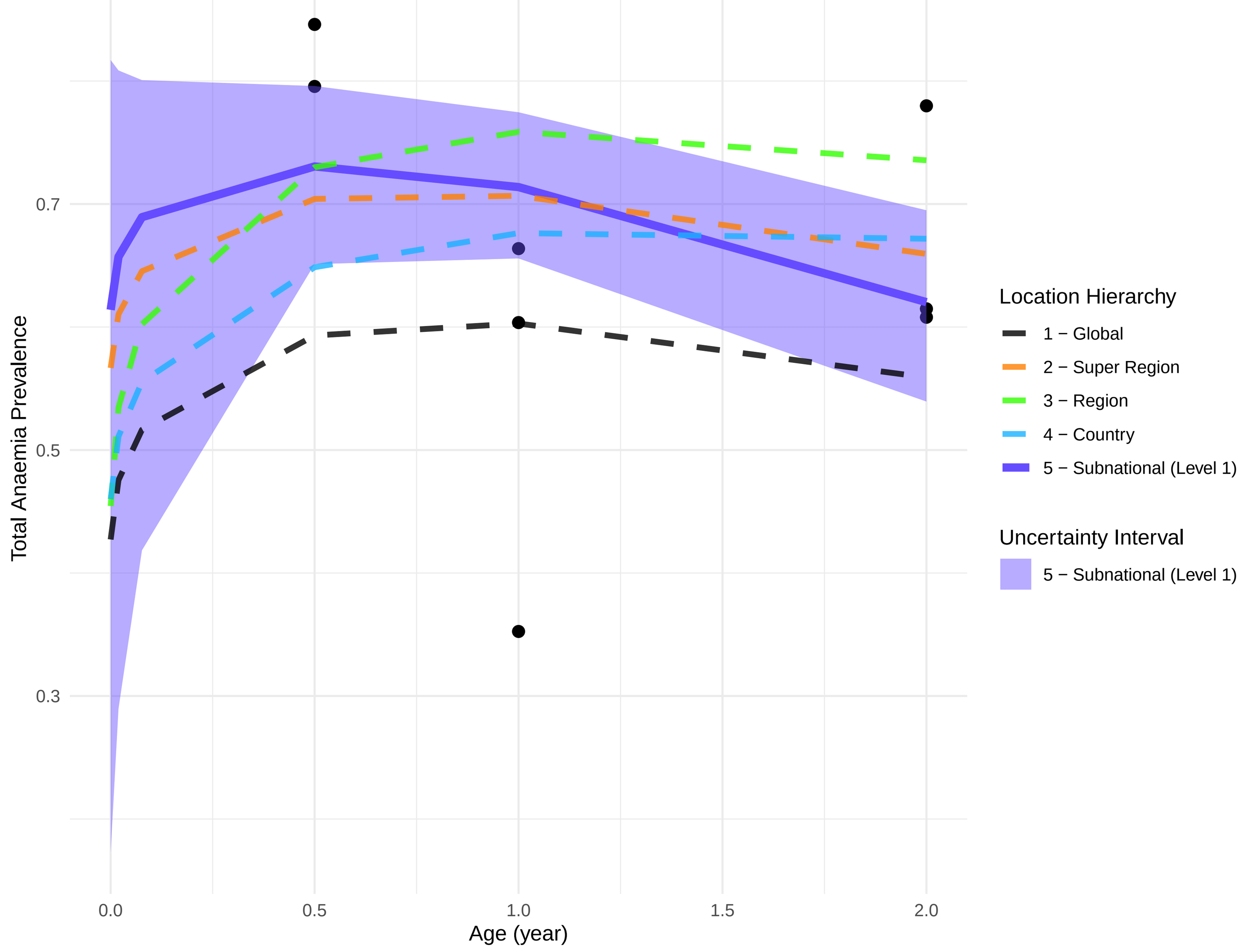

MR−BRT Age/Sex Fits − Non−pregnant Females <2 Years Old − Total Anaemia Prevalence − Plateau
WHO−2024 Adjustment Method

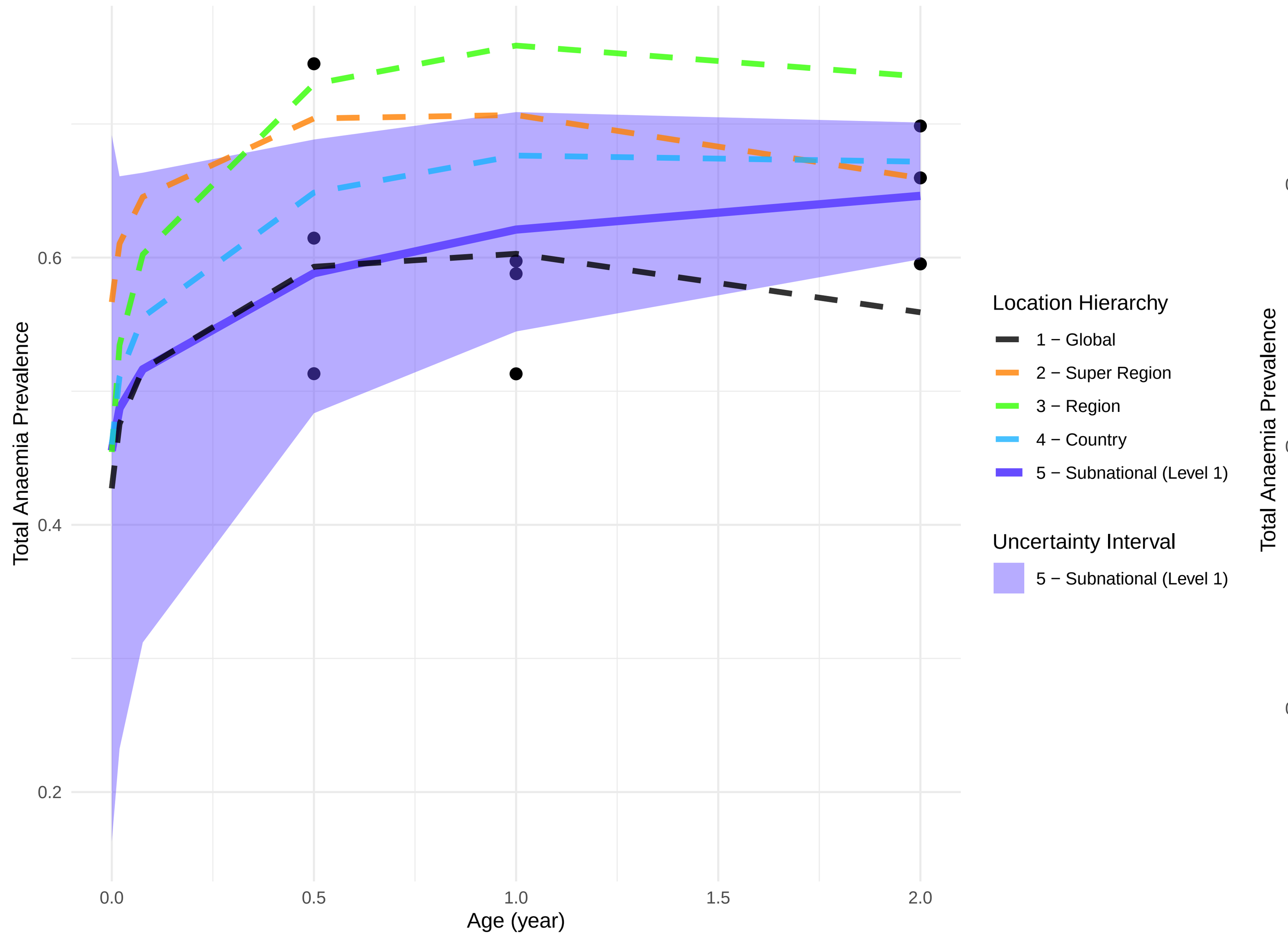

MR−BRT Age/Sex Fits − Non−pregnant Females <2 Years Old − Total Anaemia Prevalence − Rivers
WHO−2024 Adjustment Method

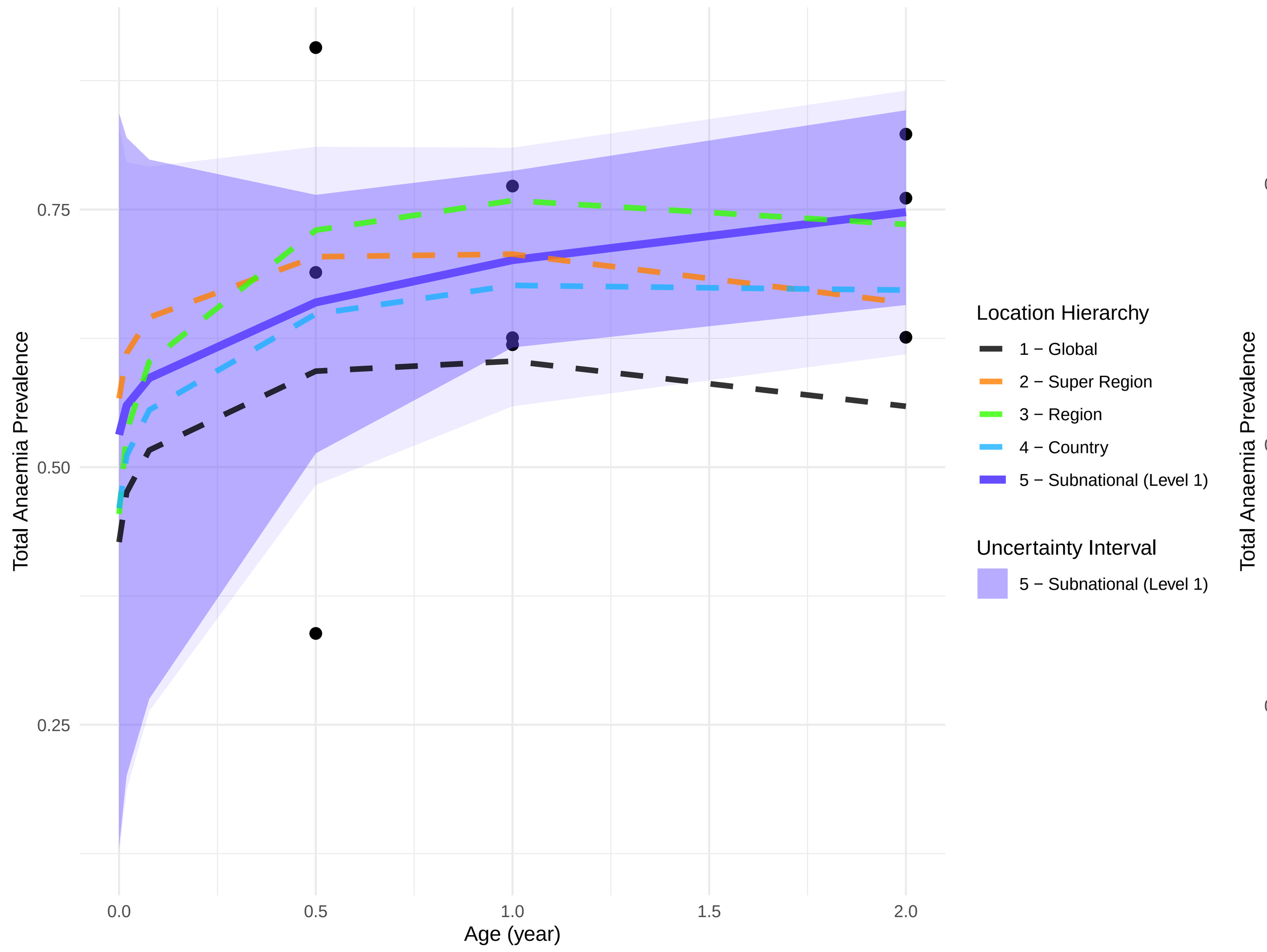

MR−BRT Age/Sex Fits − Non−pregnant Females <2 Years Old − Total Anaemia Prevalence − Sokoto
WHO−2024 Adjustment Method

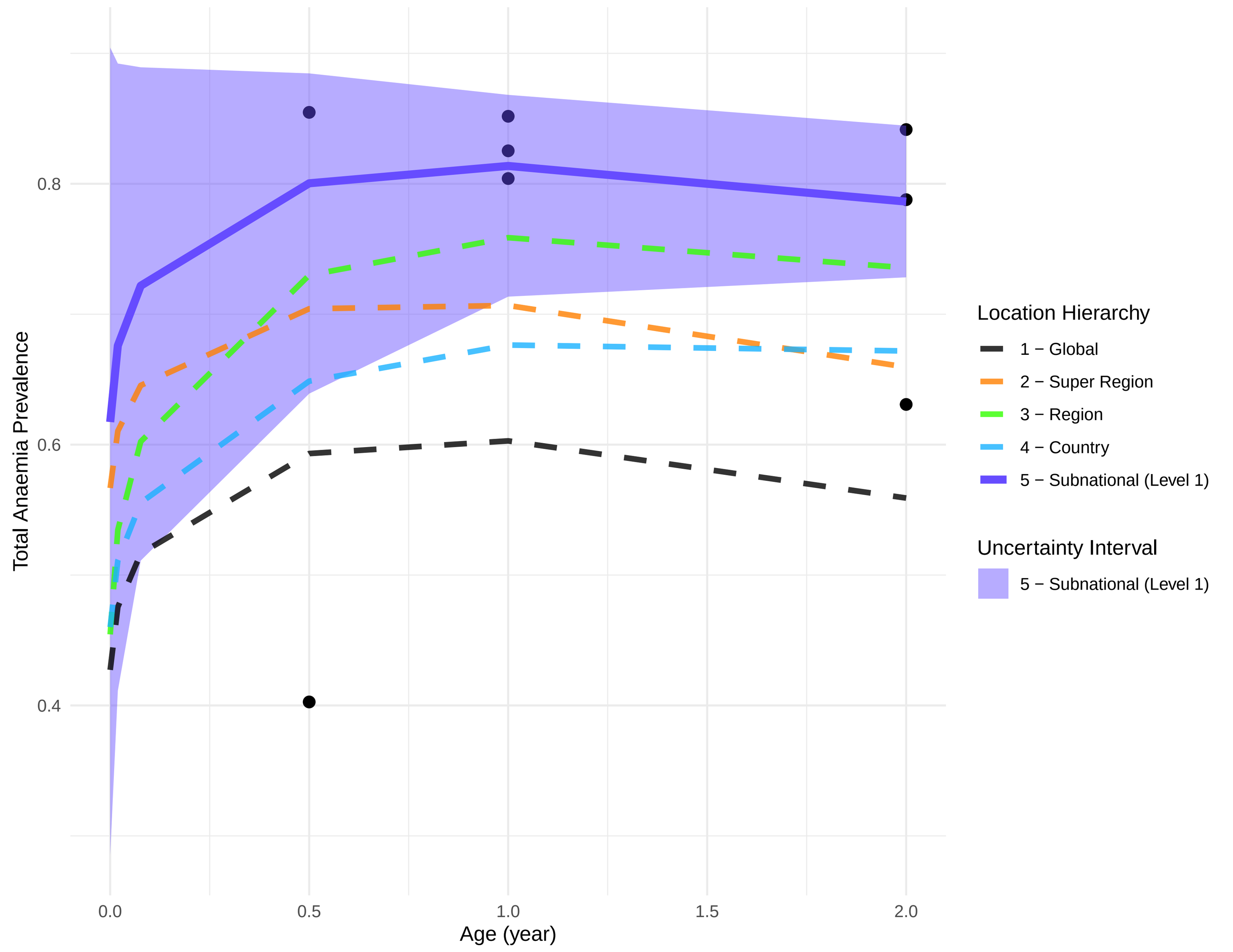

MR−BRT Age/Sex Fits − Non−pregnant Females <2 Years Old − Total Anaemia Prevalence − Taraba
WHO−2024 Adjustment Method

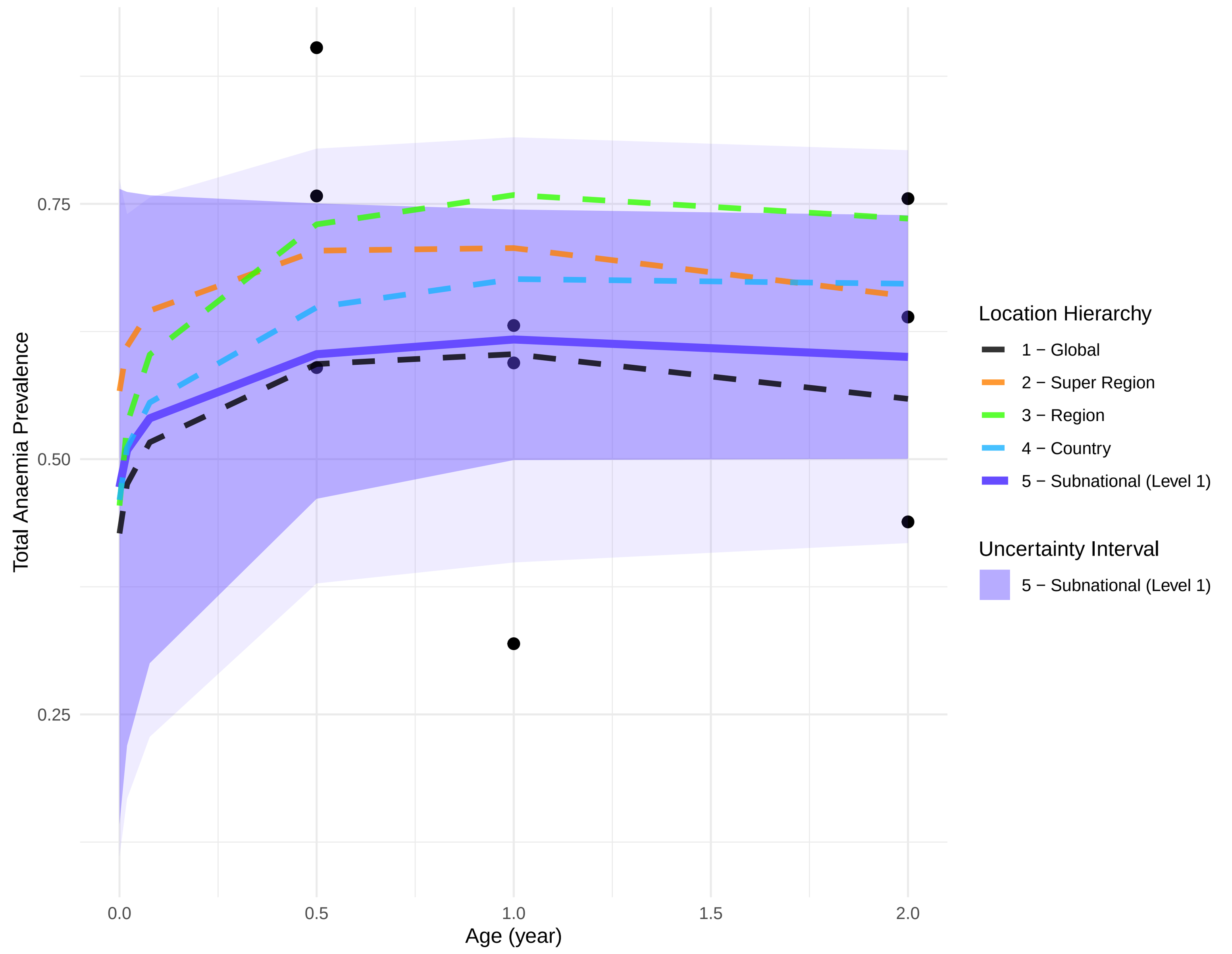

MR−BRT Age/Sex Fits − Non−pregnant Females <2 Years Old − Total Anaemia Prevalence − Yobe
WHO−2024 Adjustment Method

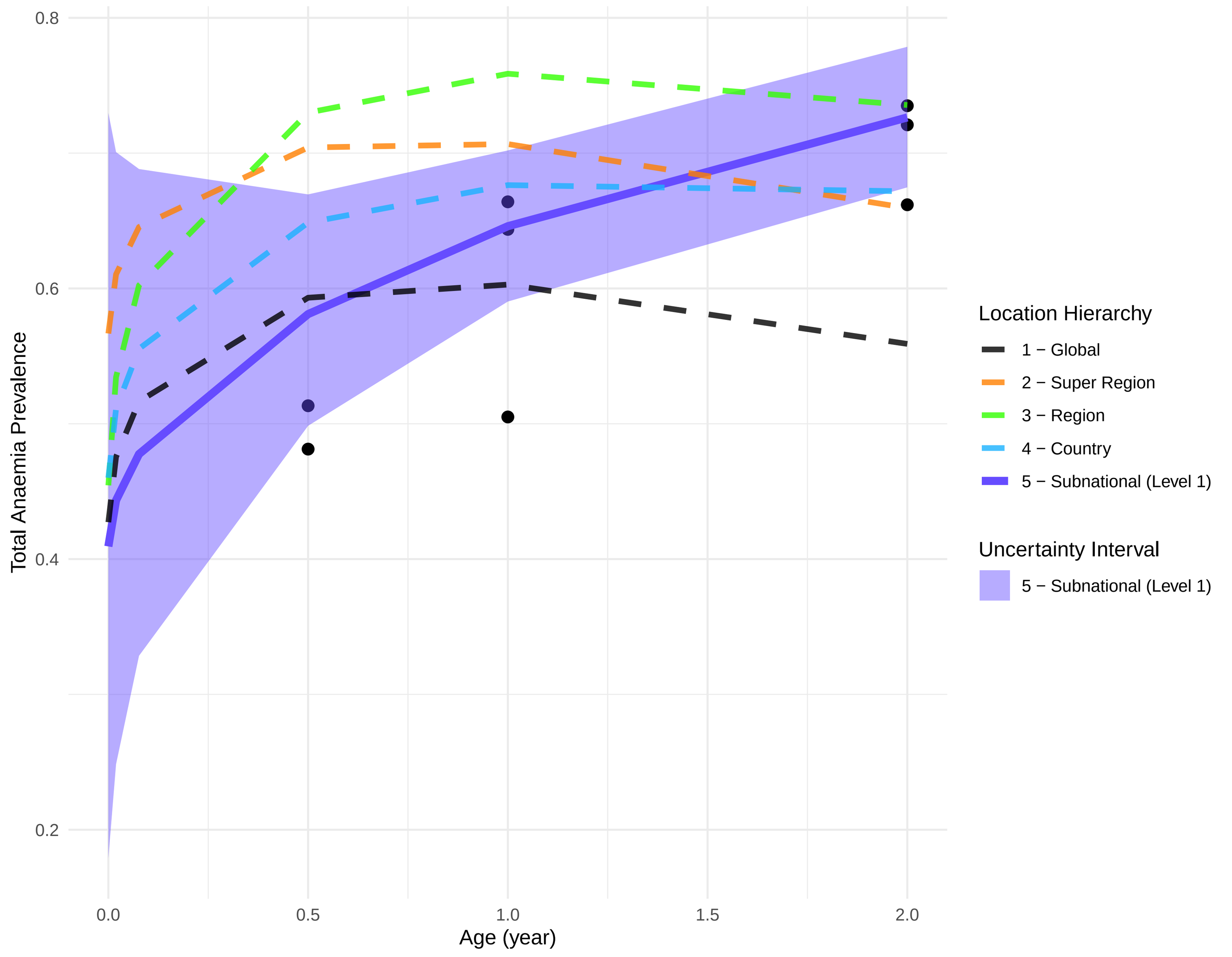

MR−BRT Age/Sex Fits − Non−pregnant Females <2 Years Old − Total Anaemia Prevalence − Zamfara
WHO−2024 Adjustment Method

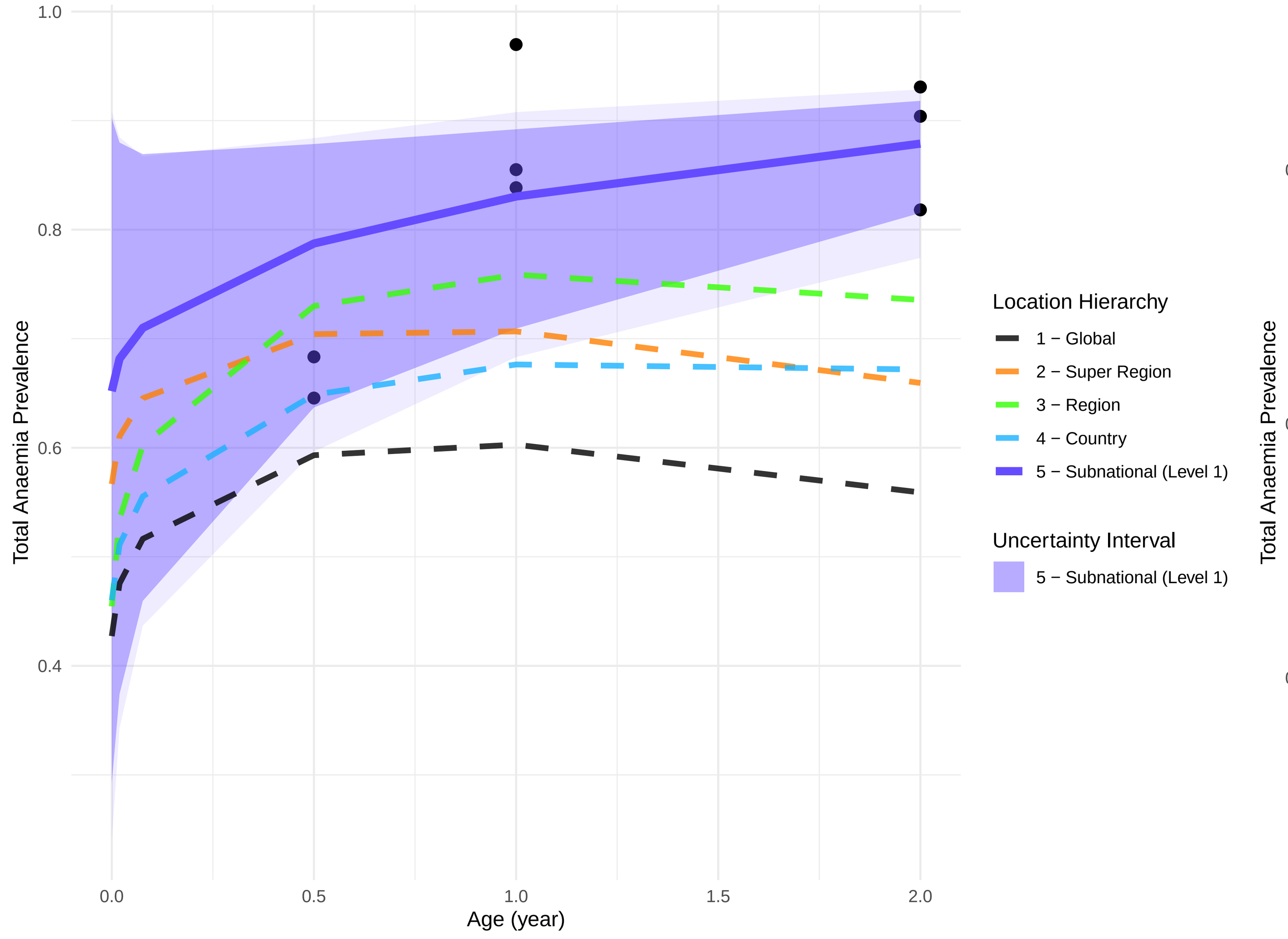

MR−BRT Age/Sex Fits − Non−pregnant Females <2 Years Old − Total Anaemia Prevalence − Senegal
WHO−2024 Adjustment Method

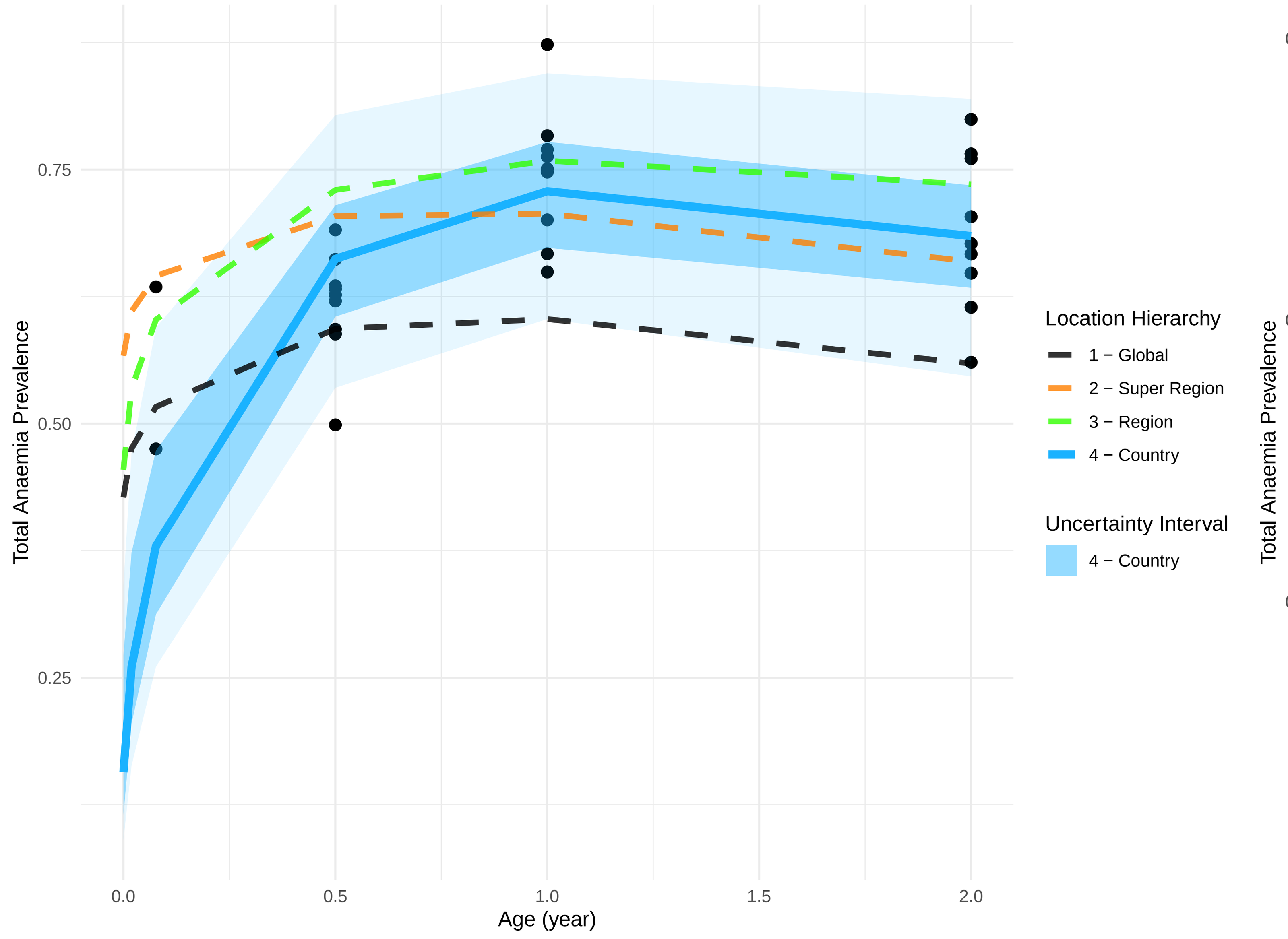

MR−BRT Age/Sex Fits − Non−pregnant Females <2 Years Old − Total Anaemia Prevalence − Sierra Leone
WHO−2024 Adjustment Method

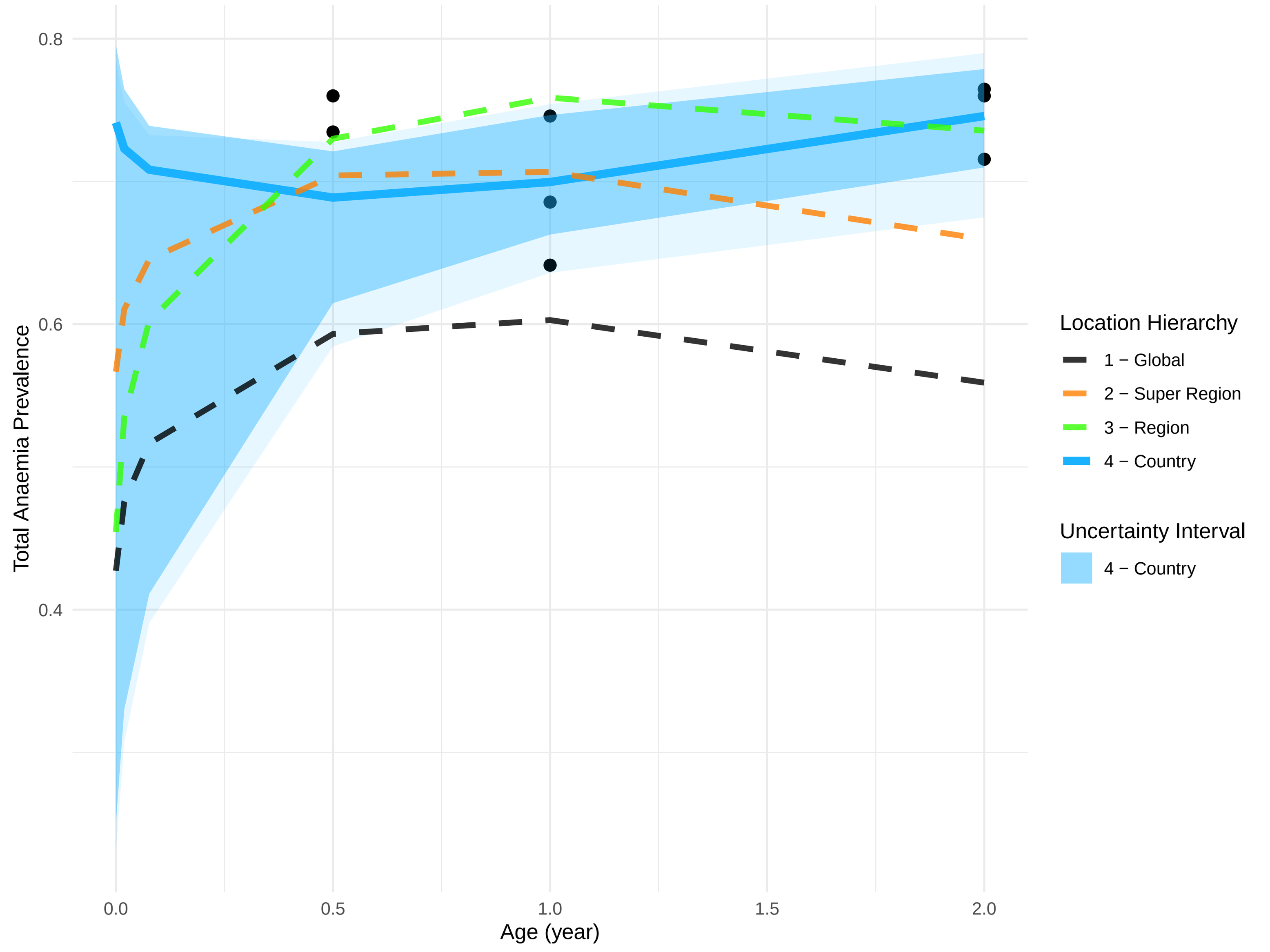

MR−BRT Age/Sex Fits − Non−pregnant Females <2 Years Old − Total Anaemia Prevalence − Togo
WHO−2024 Adjustment Method

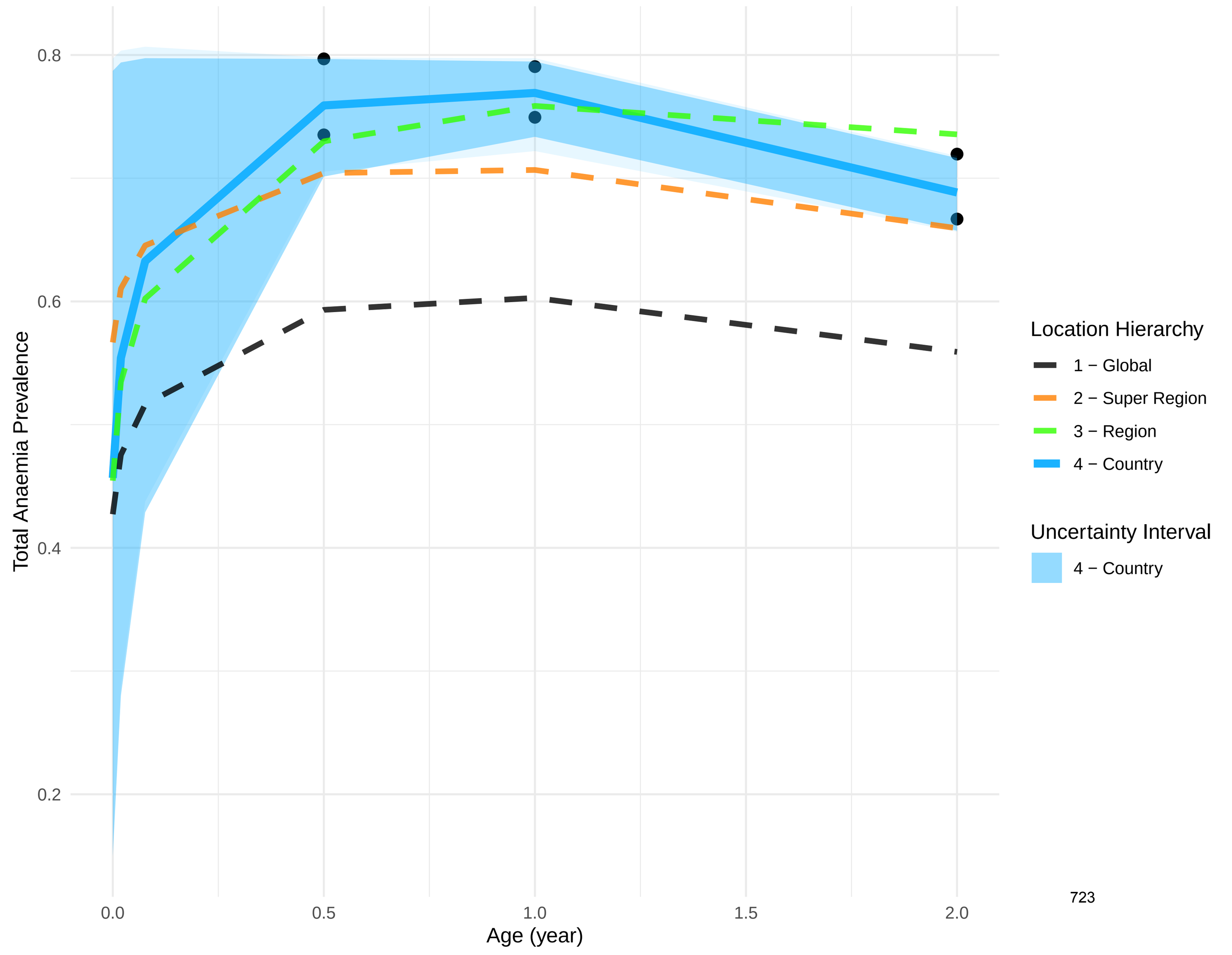

MR−BRT Age/Sex Fits − Males <2 Years Old − Total Anaemia Prevalence − Global
WHO−2001 Adjustment Method

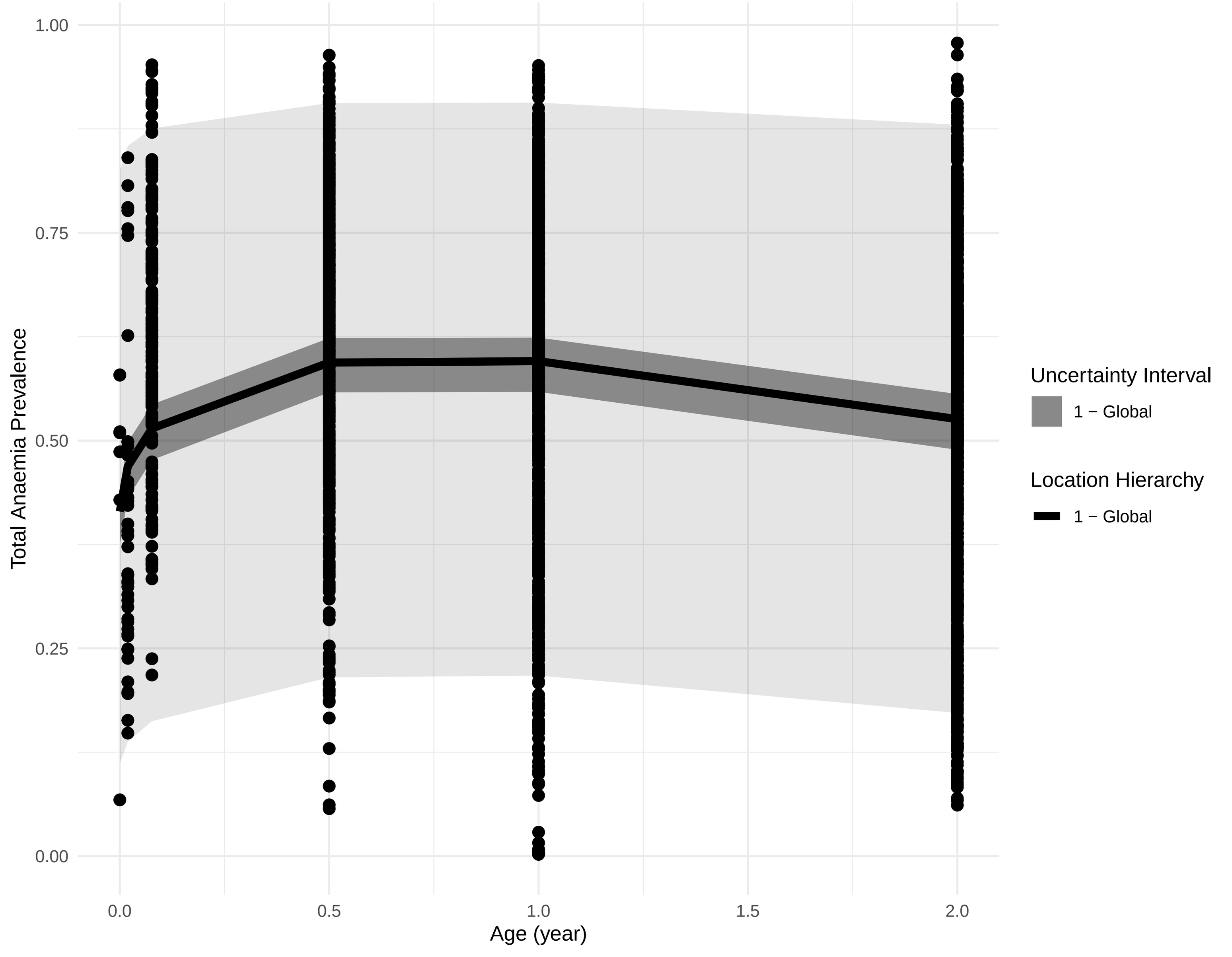

MR−BRT Age/Sex Fits − Males <2 Years Old − Total Anaemia Prevalence − Central Europe, Eastern Europe, and Central Asia
WHO−2001 Adjustment Method

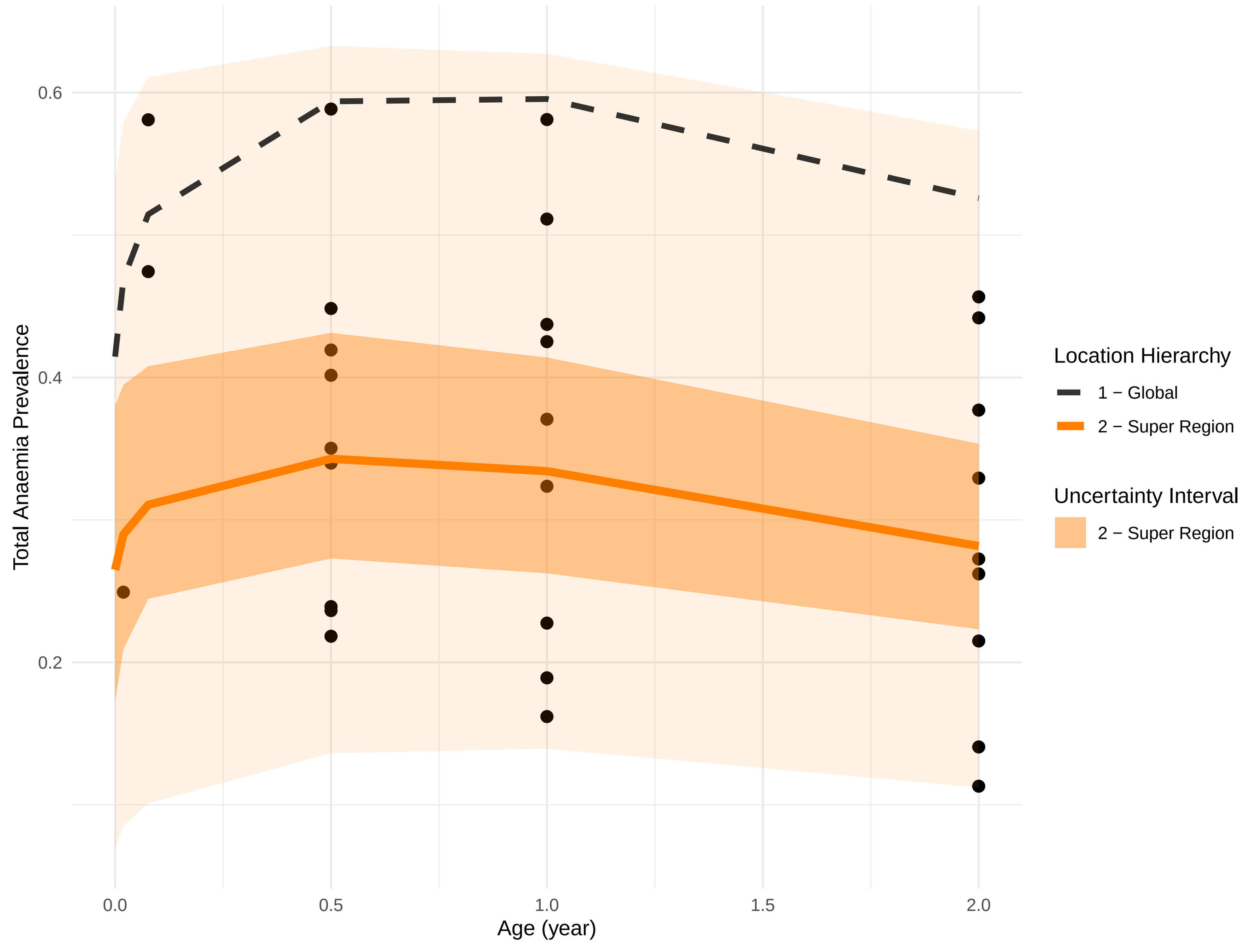

MR−BRT Age/Sex Fits − Males <2 Years Old − Total Anaemia Prevalence − Central Asia
WHO−2001 Adjustment Method

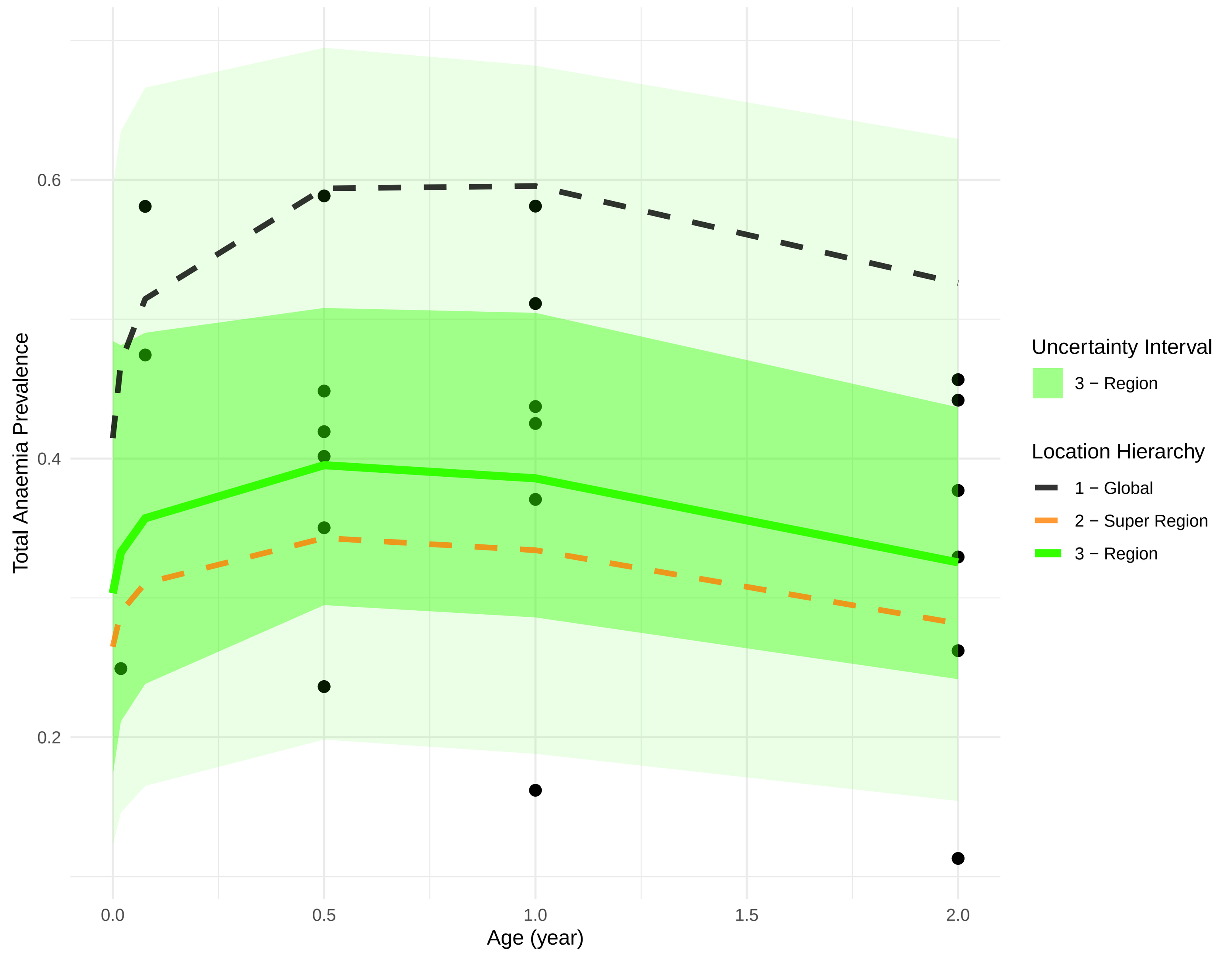

MR−BRT Age/Sex Fits − Males <2 Years Old − Total Anaemia Prevalence − Armenia
WHO−2001 Adjustment Method

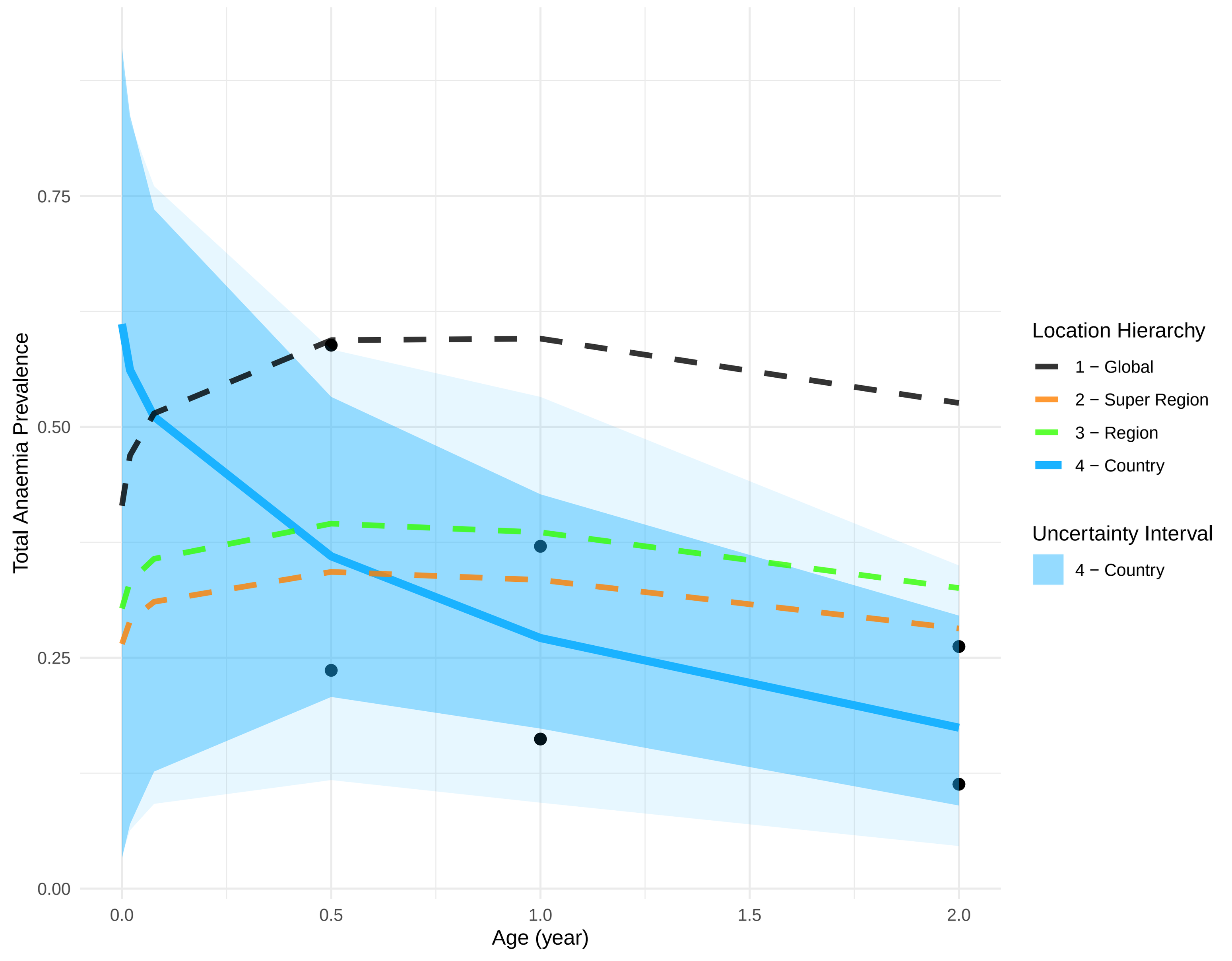

MR−BRT Age/Sex Fits − Males <2 Years Old − Total Anaemia Prevalence − Azerbaijan
WHO−2001 Adjustment Method

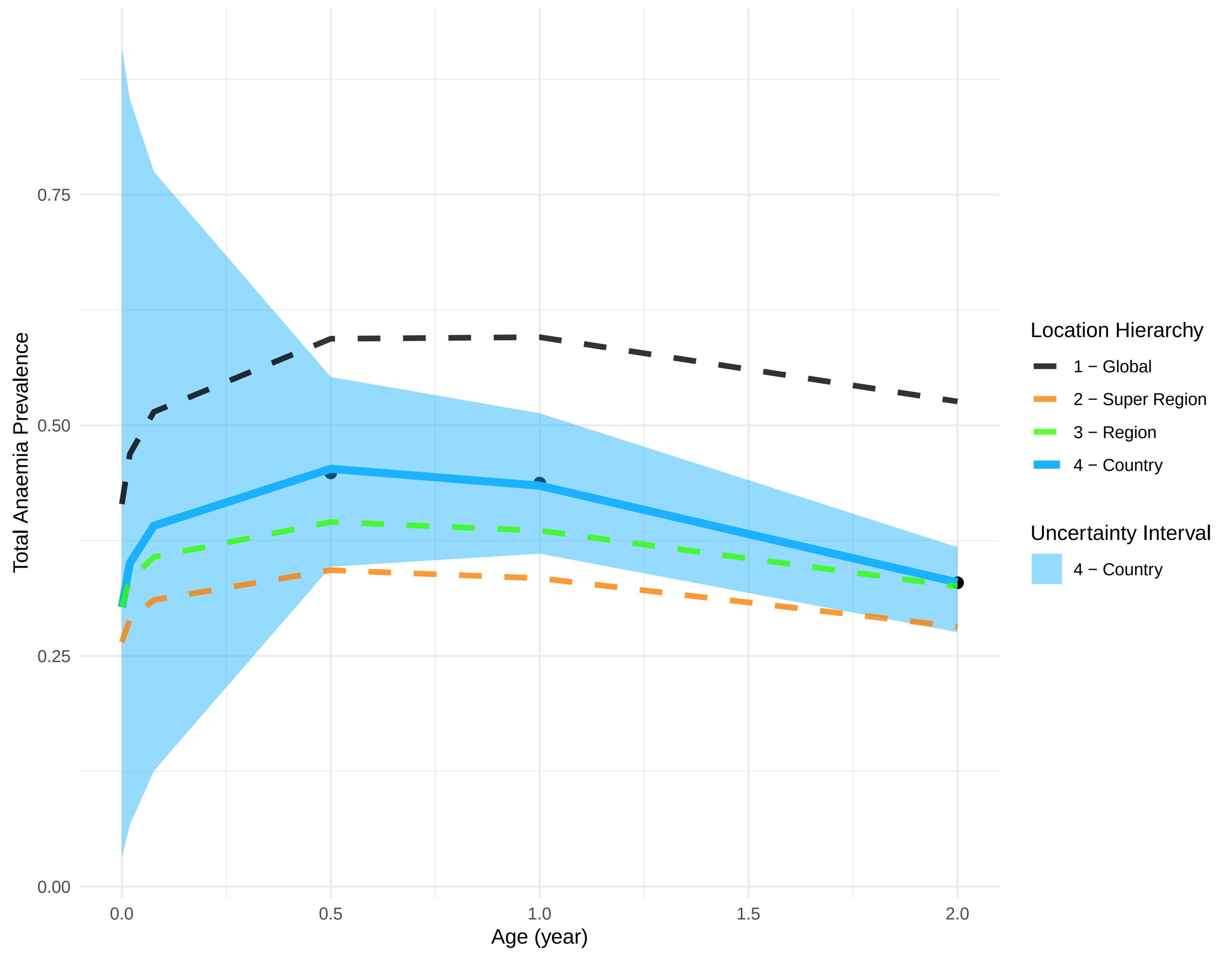

MR−BRT Age/Sex Fits − Males <2 Years Old − Total Anaemia Prevalence − Kyrgyzstan
WHO−2001 Adjustment Method

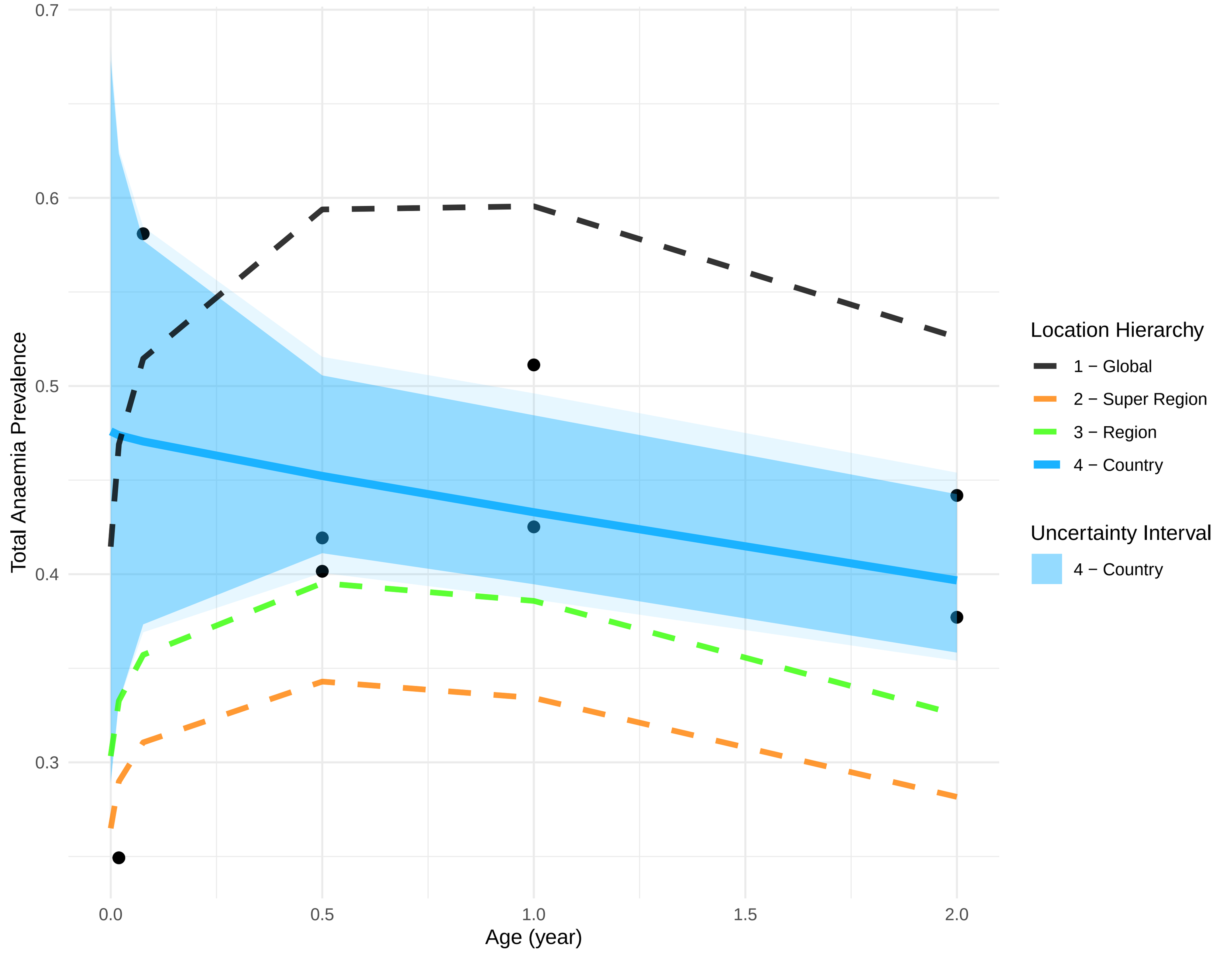

MR−BRT Age/Sex Fits − Males <2 Years Old − Total Anaemia Prevalence − Uzbekistan
WHO−2001 Adjustment Method

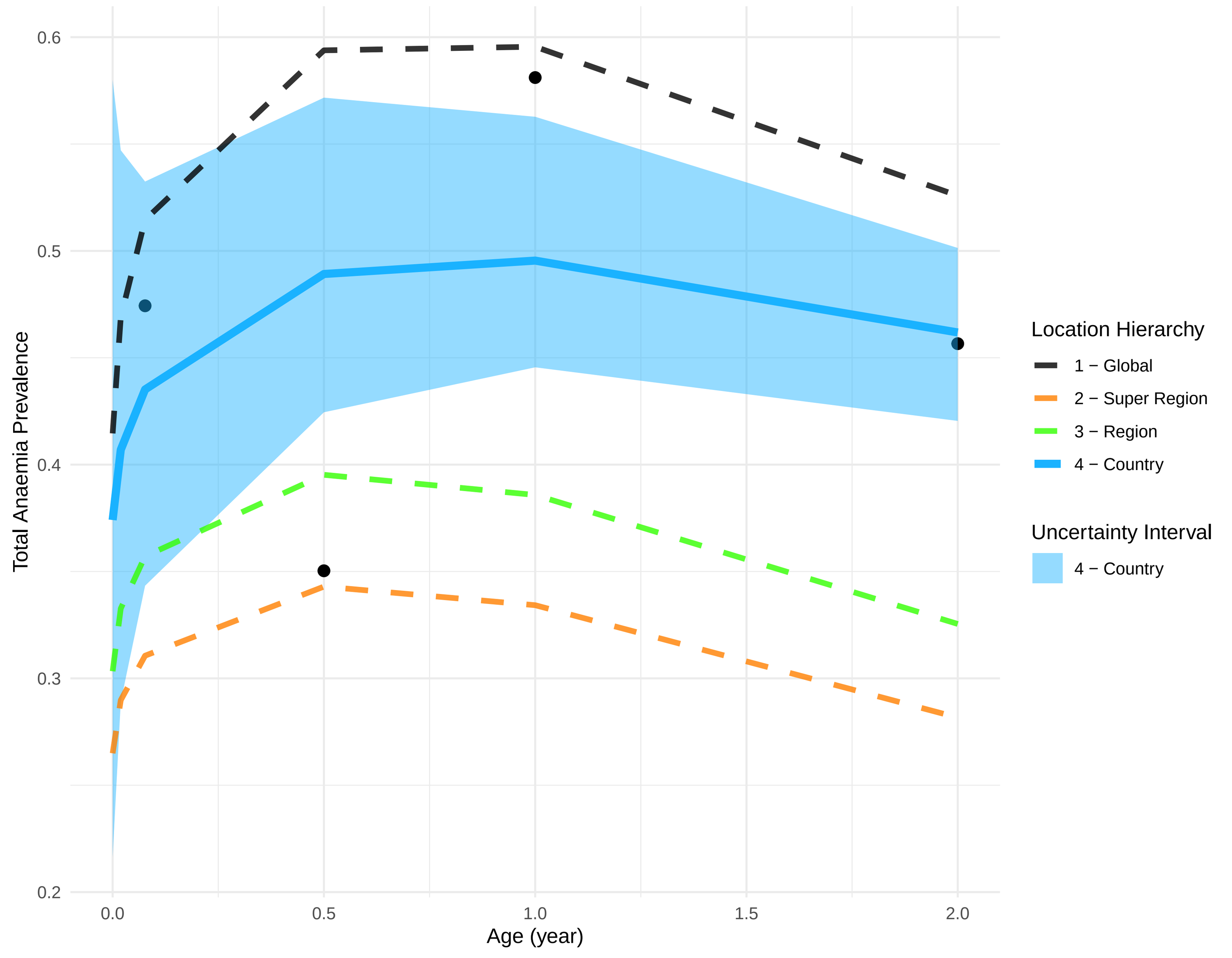

MR−BRT Age/Sex Fits − Males <2 Years Old − Total Anaemia Prevalence − Central Europe
WHO−2001 Adjustment Method

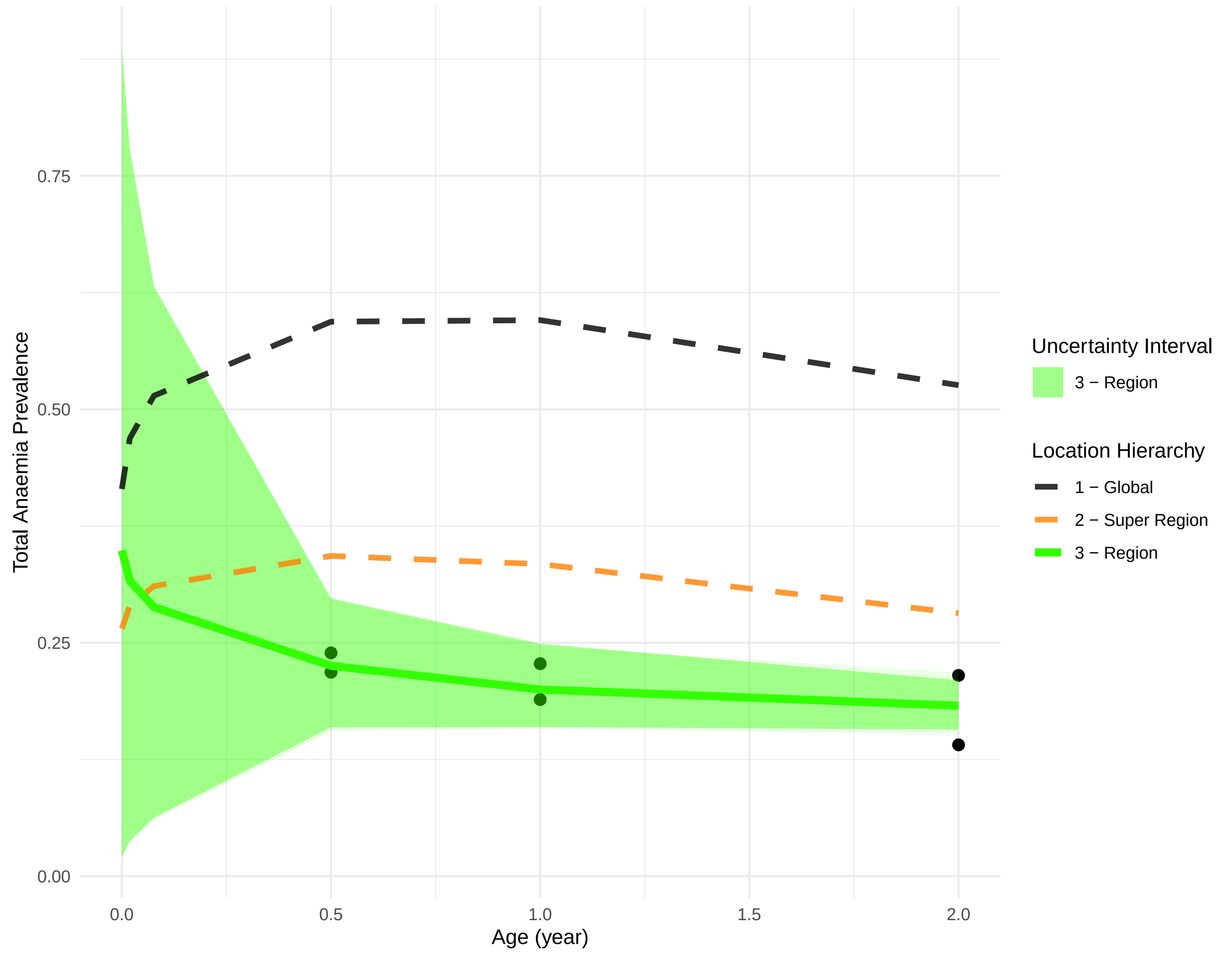

MR−BRT Age/Sex Fits − Males <2 Years Old − Total Anaemia Prevalence − Albania
WHO−2001 Adjustment Method

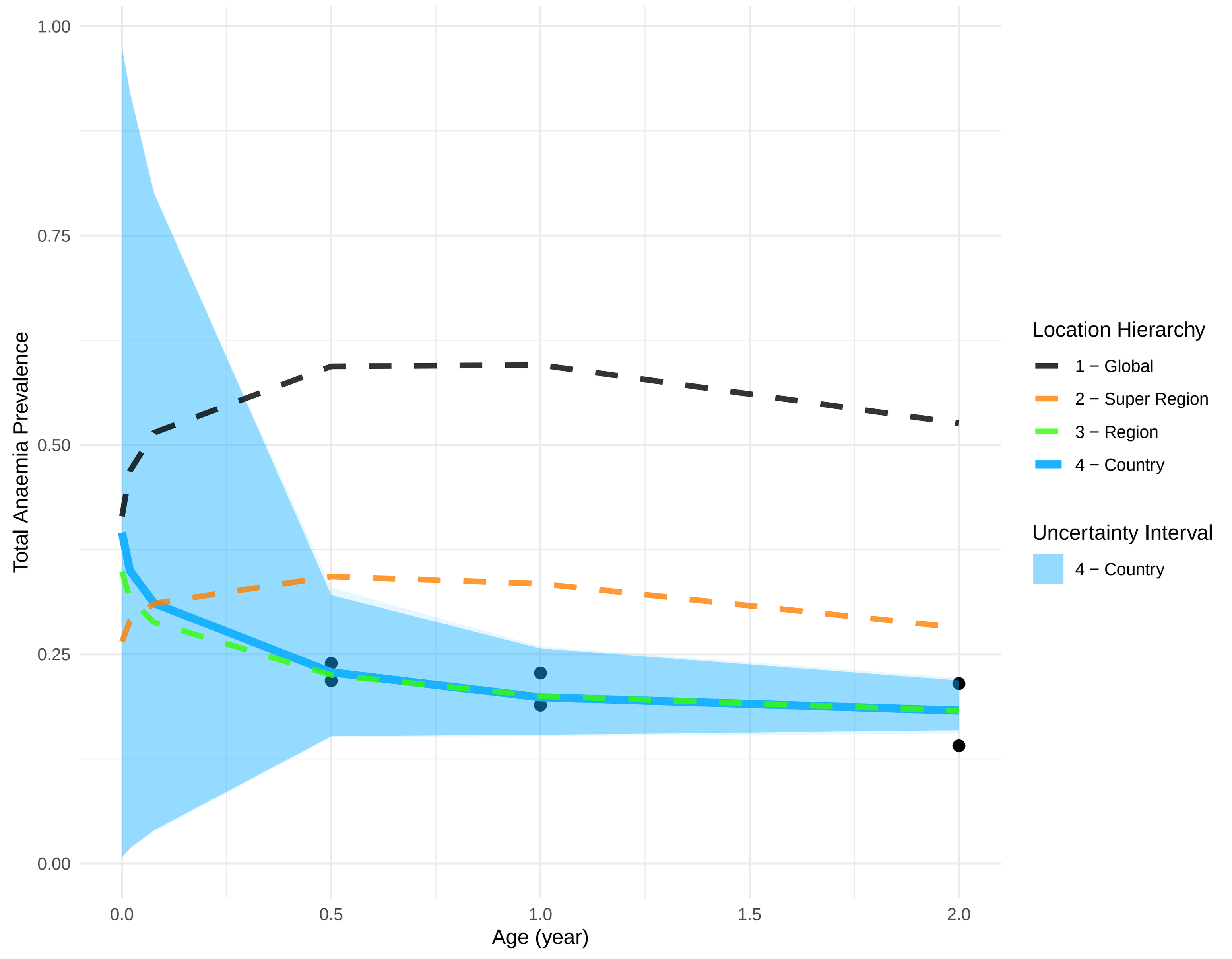

MR−BRT Age/Sex Fits − Males <2 Years Old − Total Anaemia Prevalence − Eastern Europe
WHO−2001 Adjustment Method

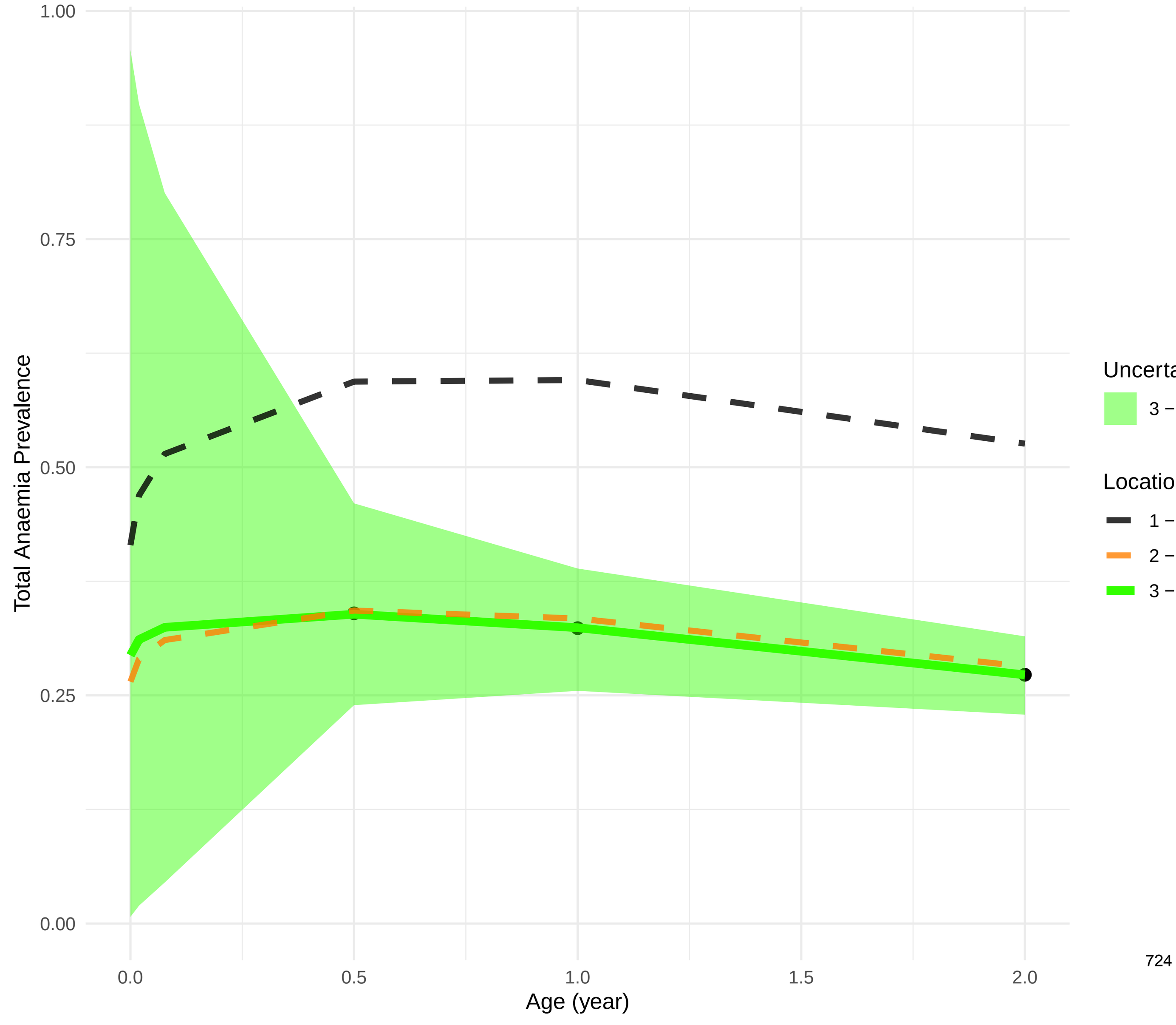

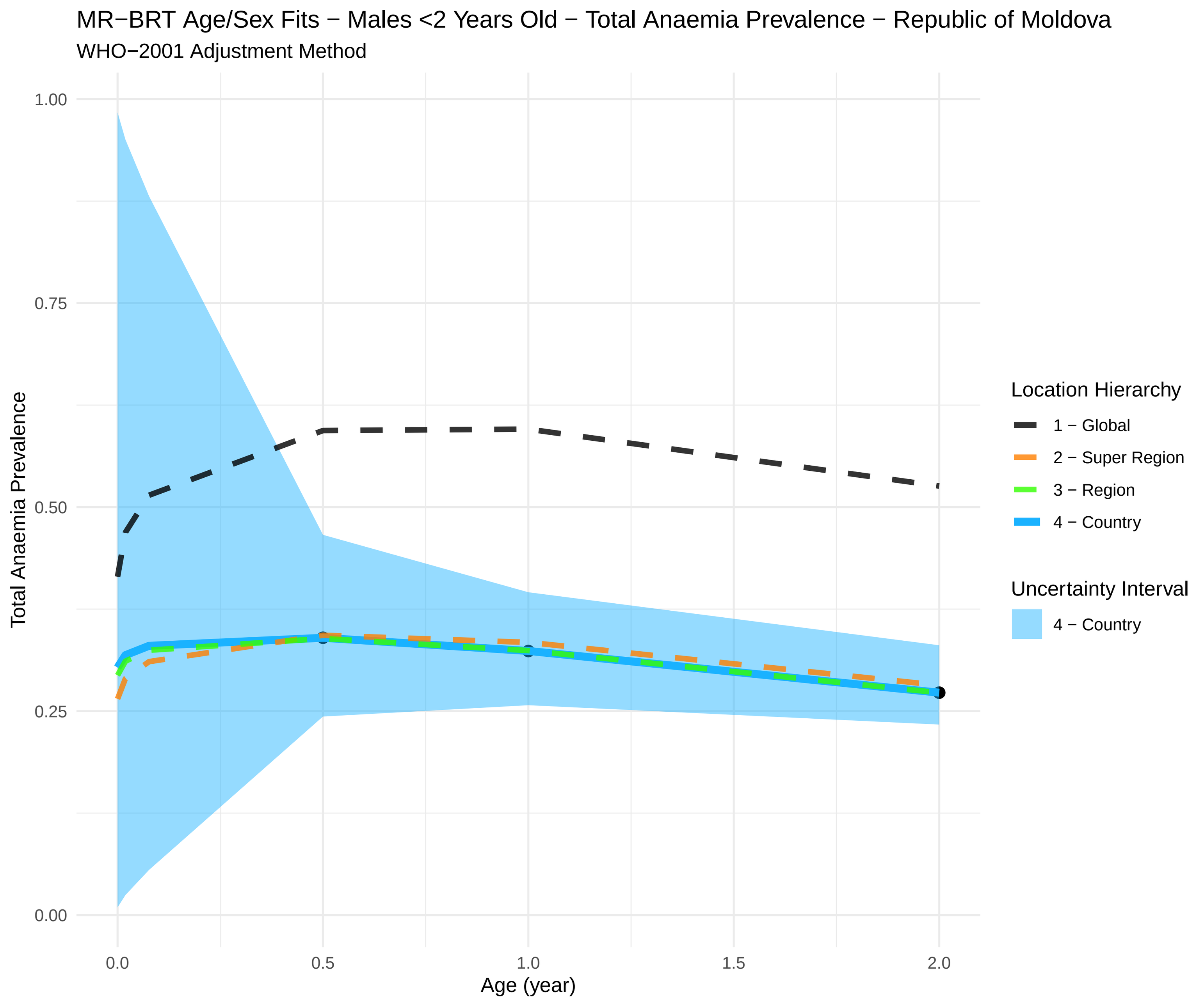
MR−BRT Age/Sex Fits − Males <2 Years Old − Total Anaemia Prevalence − Republic of Moldova
WHO−2001 Adjustment Method
Total Anaemia Prevalence
Age (year)
Location Hierarchy
1 − Global
2 − Super Region
3 − Region
4 − Country
Uncertainty Interval
4 − Country

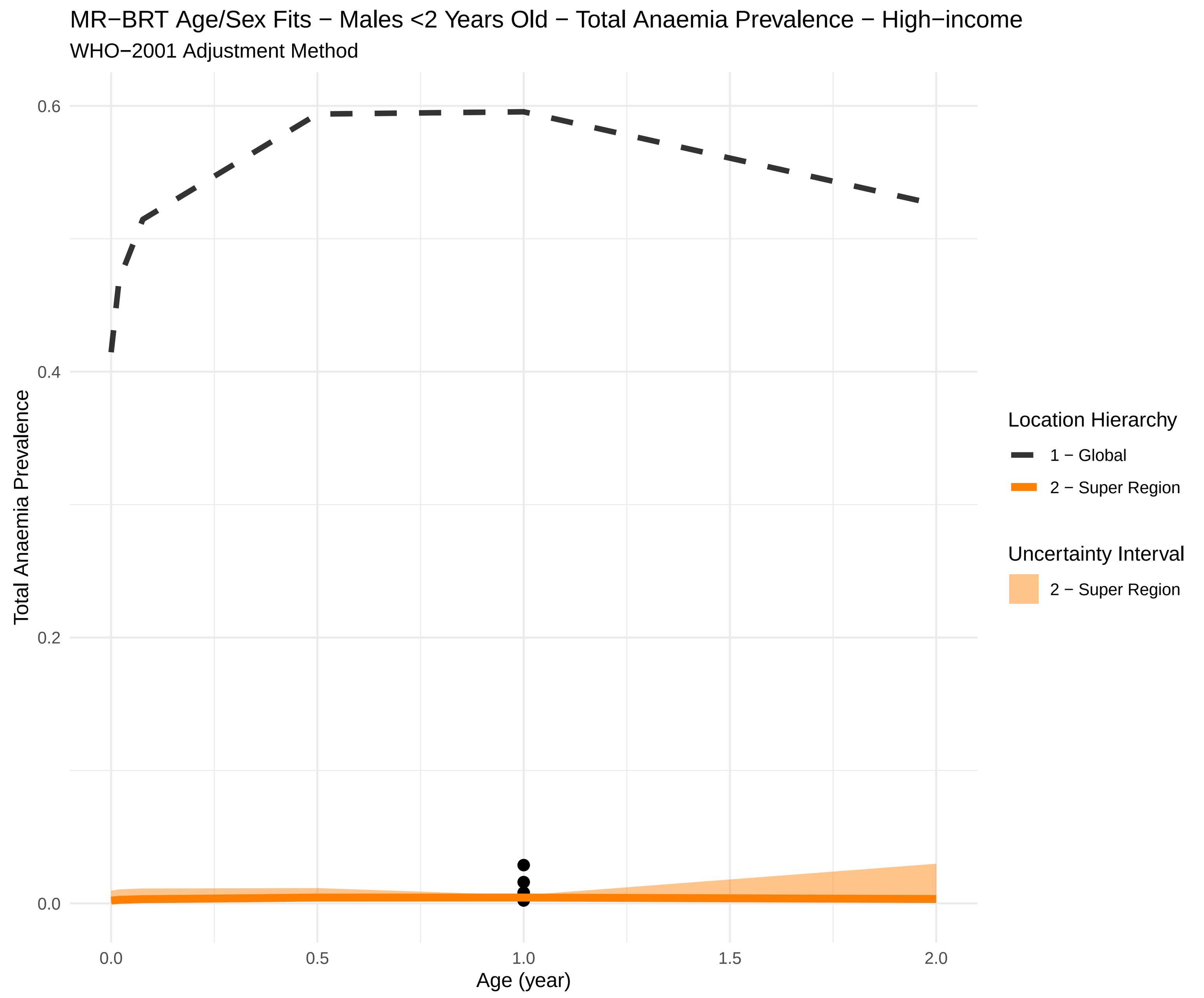
MR−BRT Age/Sex Fits − Males <2 Years Old − Total Anaemia Prevalence − High−income
WHO−2001 Adjustment Method
Total Anaemia Prevalence
Age (year)
Location Hierarchy
1 − Global
2 − Super Region
Uncertainty Interval
2 − Super Region

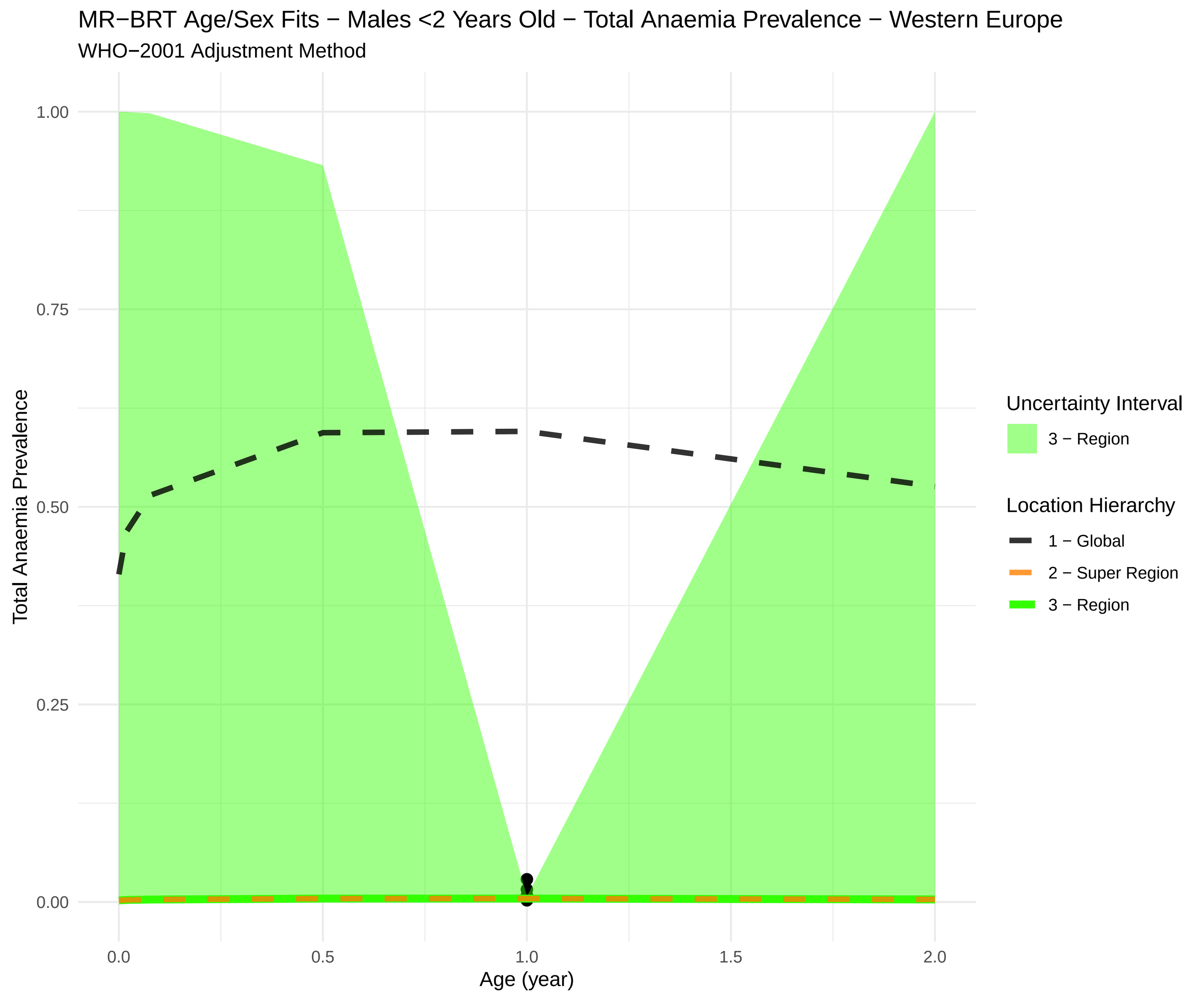
MR−BRT Age/Sex Fits − Males <2 Years Old − Total Anaemia Prevalence − Western Europe
WHO−2001 Adjustment Method
Total Anaemia Prevalence
Age (year)
Uncertainty Interval
3 − Region
Location Hierarchy
1 − Global
2 − Super Region
3 − Region

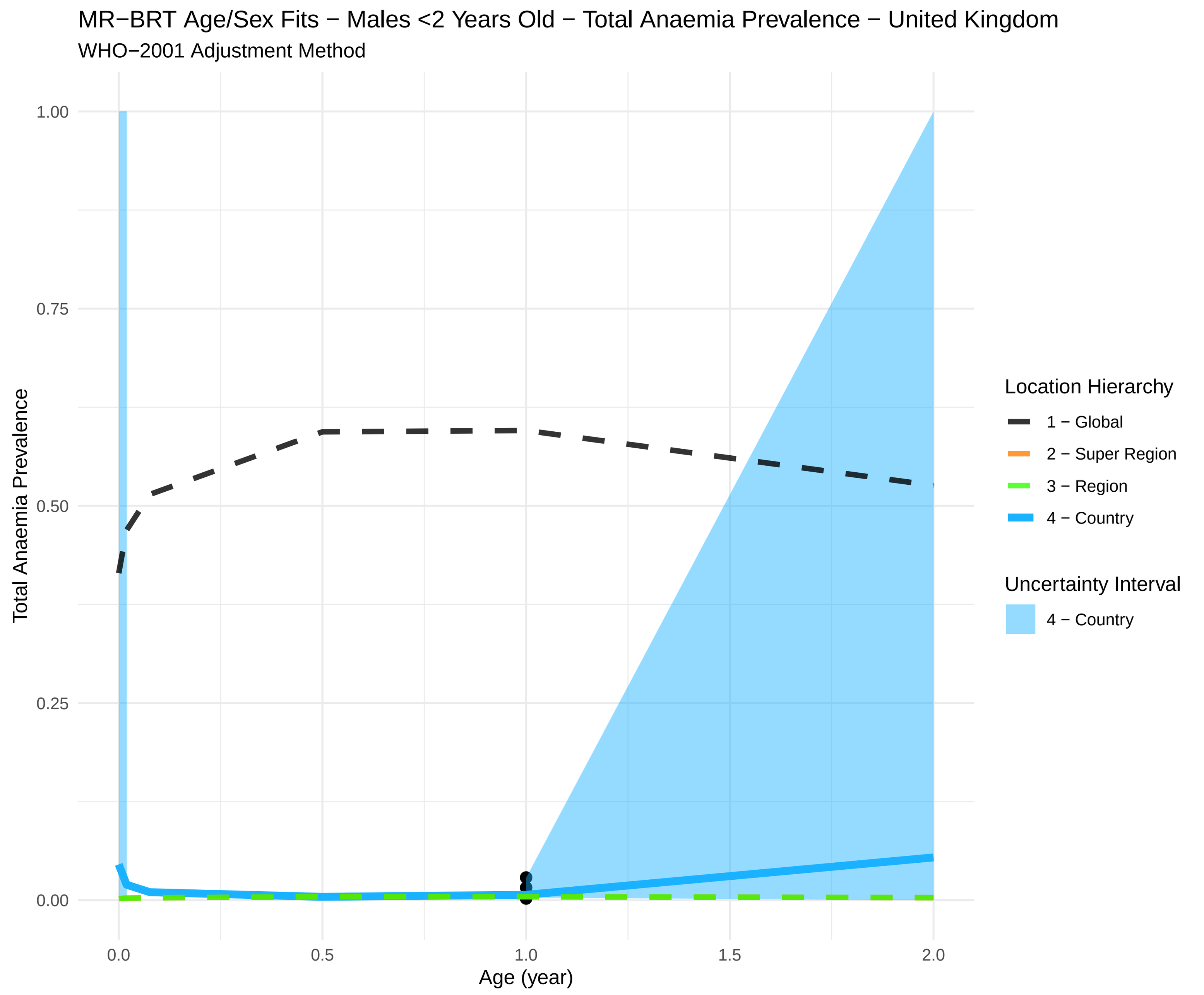
MR−BRT Age/Sex Fits − Males <2 Years Old − Total Anaemia Prevalence − United Kingdom
WHO−2001 Adjustment Method
Total Anaemia Prevalence
Age (year)
Location Hierarchy
1 − Global
2 − Super Region
3 − Region
4 − Country
Uncertainty Interval
4 − Country

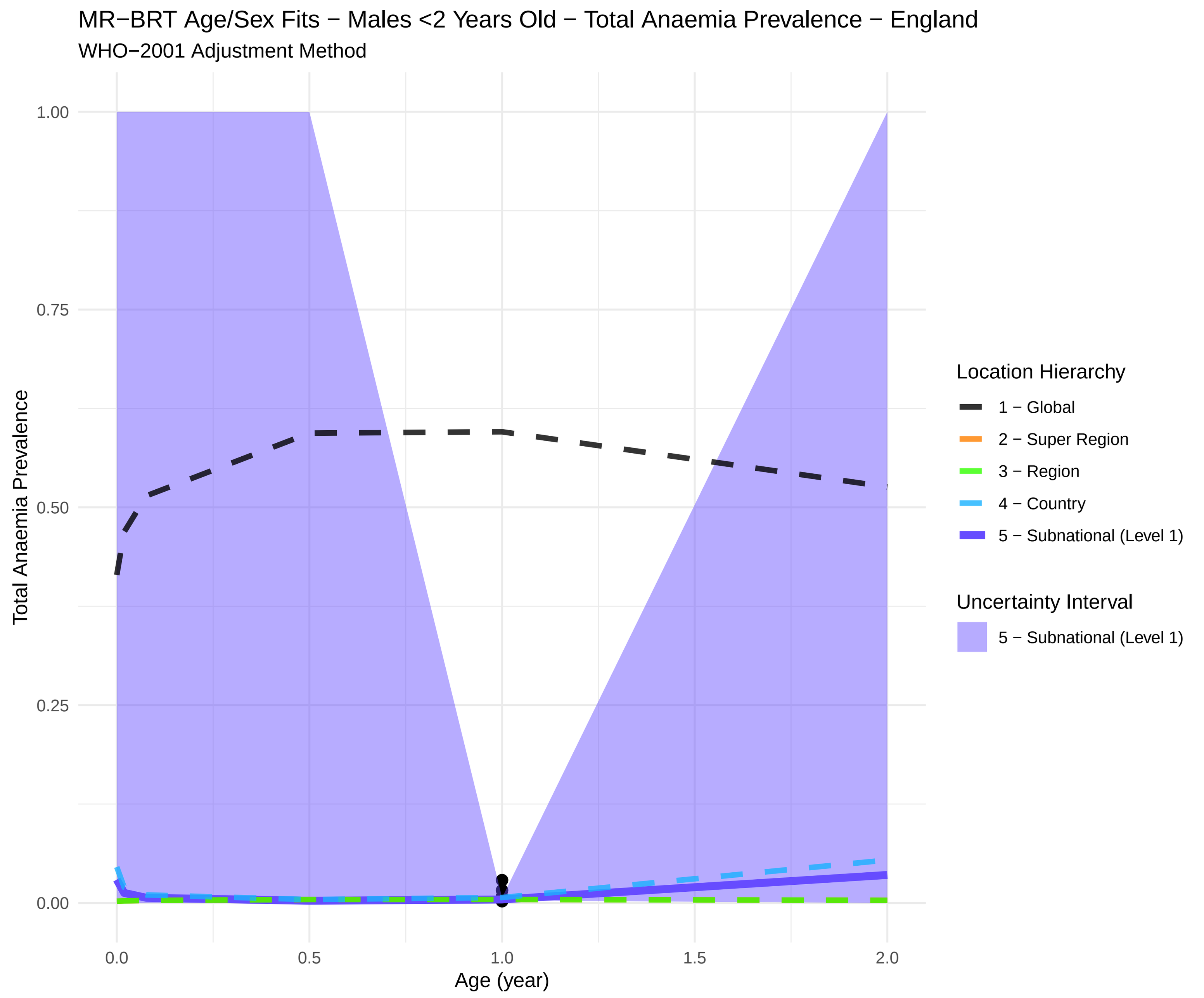
MR−BRT Age/Sex Fits − Males <2 Years Old − Total Anaemia Prevalence − England
WHO−2001 Adjustment Method
Total Anaemia Prevalence
Age (year)
Location Hierarchy
1 − Global
2 − Super Region
3 − Region
4 − Country
5 − Subnational (Level 1)
Uncertainty Interval
5 − Subnational (Level 1)

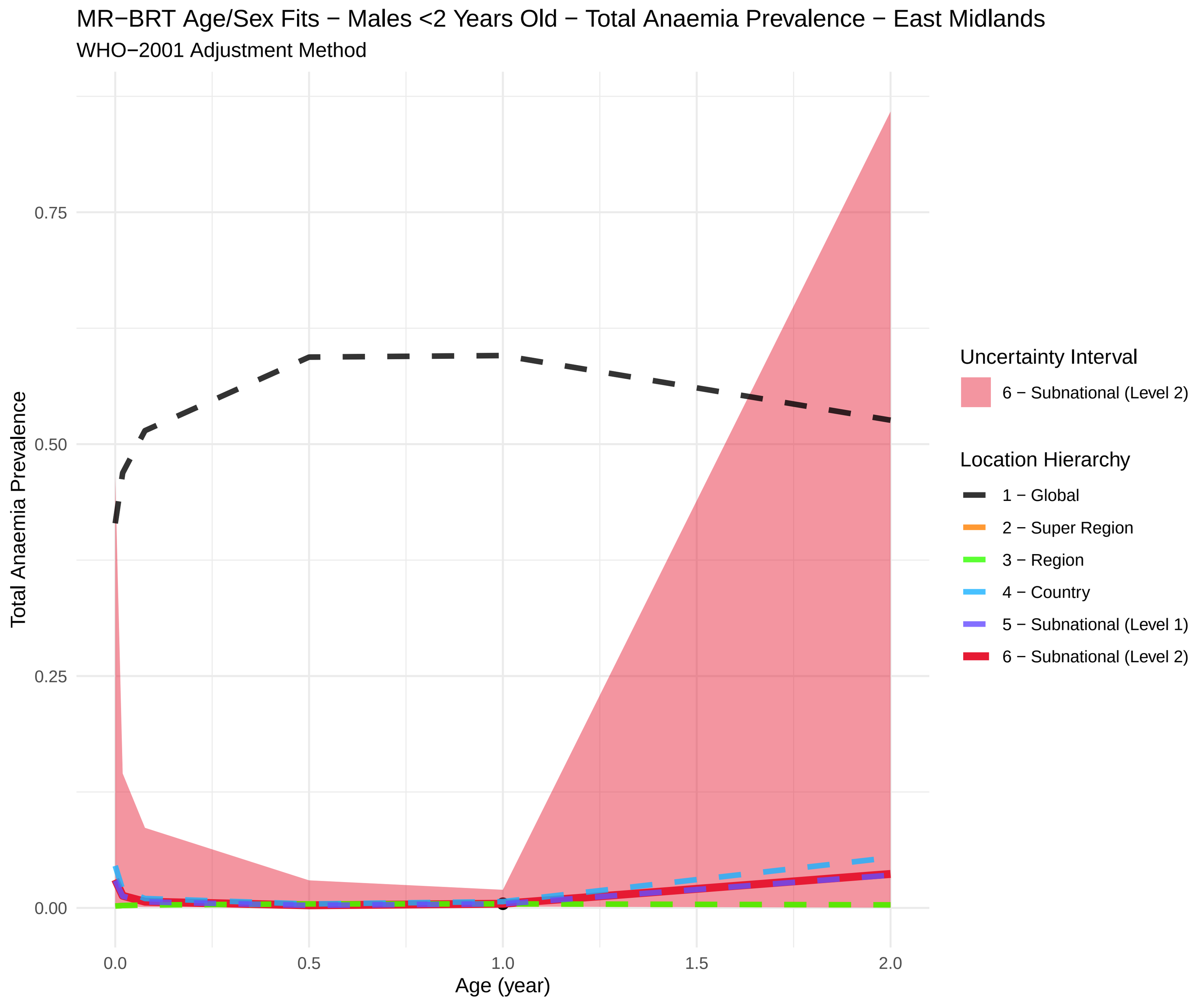
MR−BRT Age/Sex Fits − Males <2 Years Old − Total Anaemia Prevalence − East Midlands
WHO−2001 Adjustment Method
Total Anaemia Prevalence
Age (year)
Uncertainty Interval
6 − Subnational (Level 2)
Location Hierarchy
1 − Global
2 − Super Region
3 − Region
4 − Country
5 − Subnational (Level 1)
6 − Subnational (Level 2)

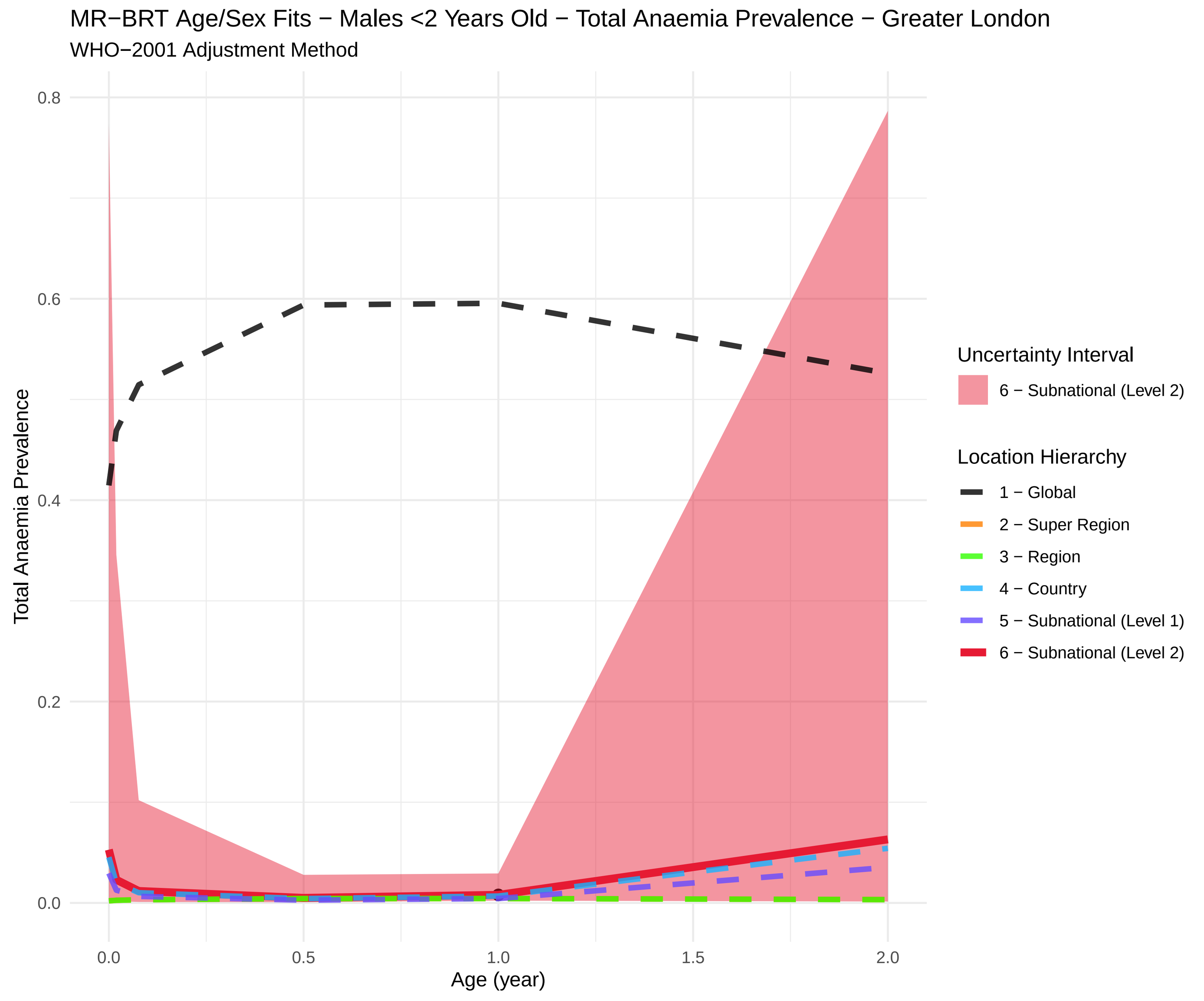
MR−BRT Age/Sex Fits − Males <2 Years Old − Total Anaemia Prevalence − Greater London
WHO−2001 Adjustment Method
Total Anaemia Prevalence
Age (year)
Uncertainty Interval
6 − Subnational (Level 2)
Location Hierarchy
1 − Global
2 − Super Region
3 − Region
4 − Country
5 − Subnational (Level 1)
6 − Subnational (Level 2)

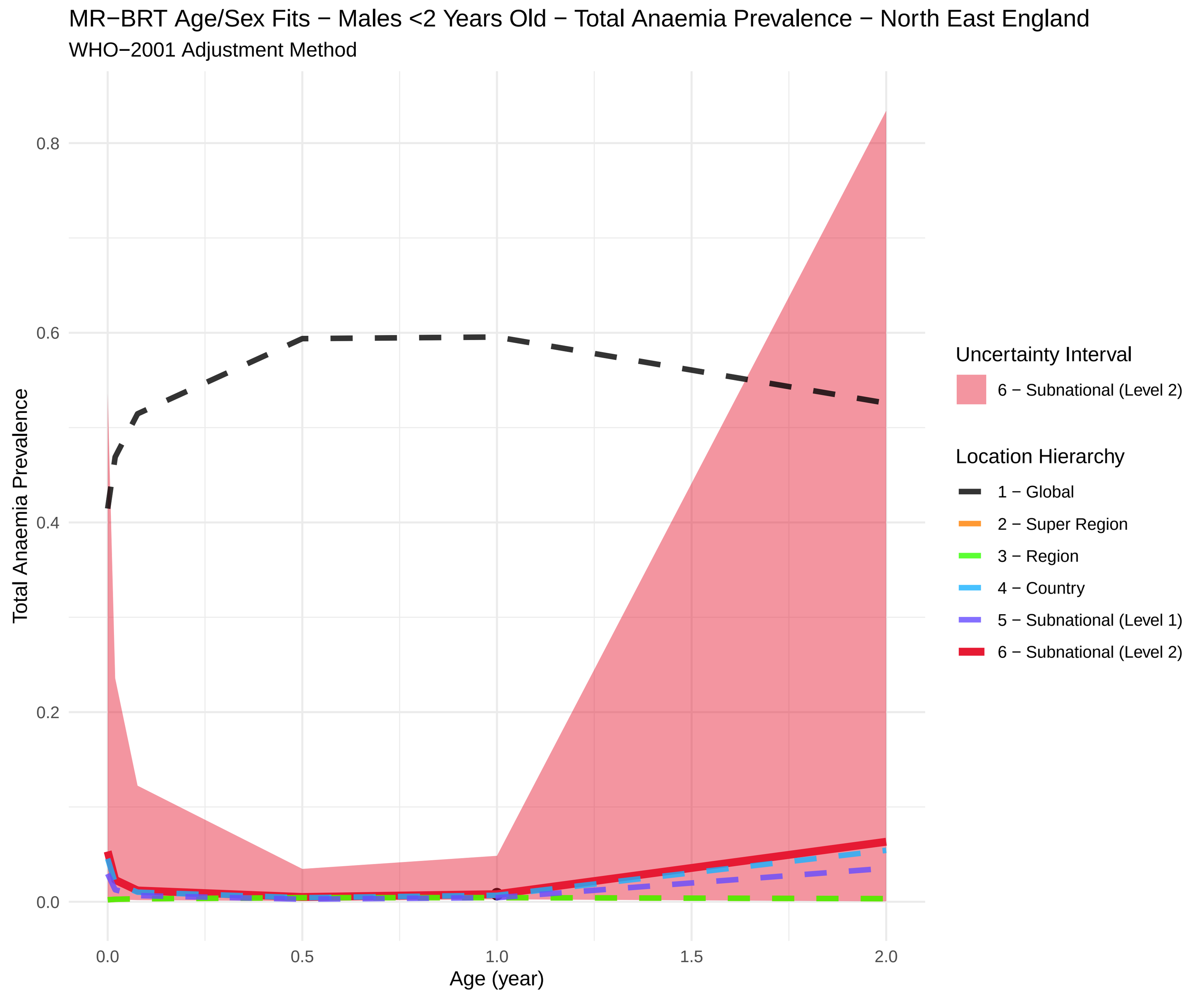
MR−BRT Age/Sex Fits − Males <2 Years Old − Total Anaemia Prevalence − North East England
WHO−2001 Adjustment Method
Total Anaemia Prevalence
Age (year)
Uncertainty Interval
6 − Subnational (Level 2)
Location Hierarchy
1 − Global
2 − Super Region
3 − Region
4 − Country
5 − Subnational (Level 1)
6 − Subnational (Level 2)

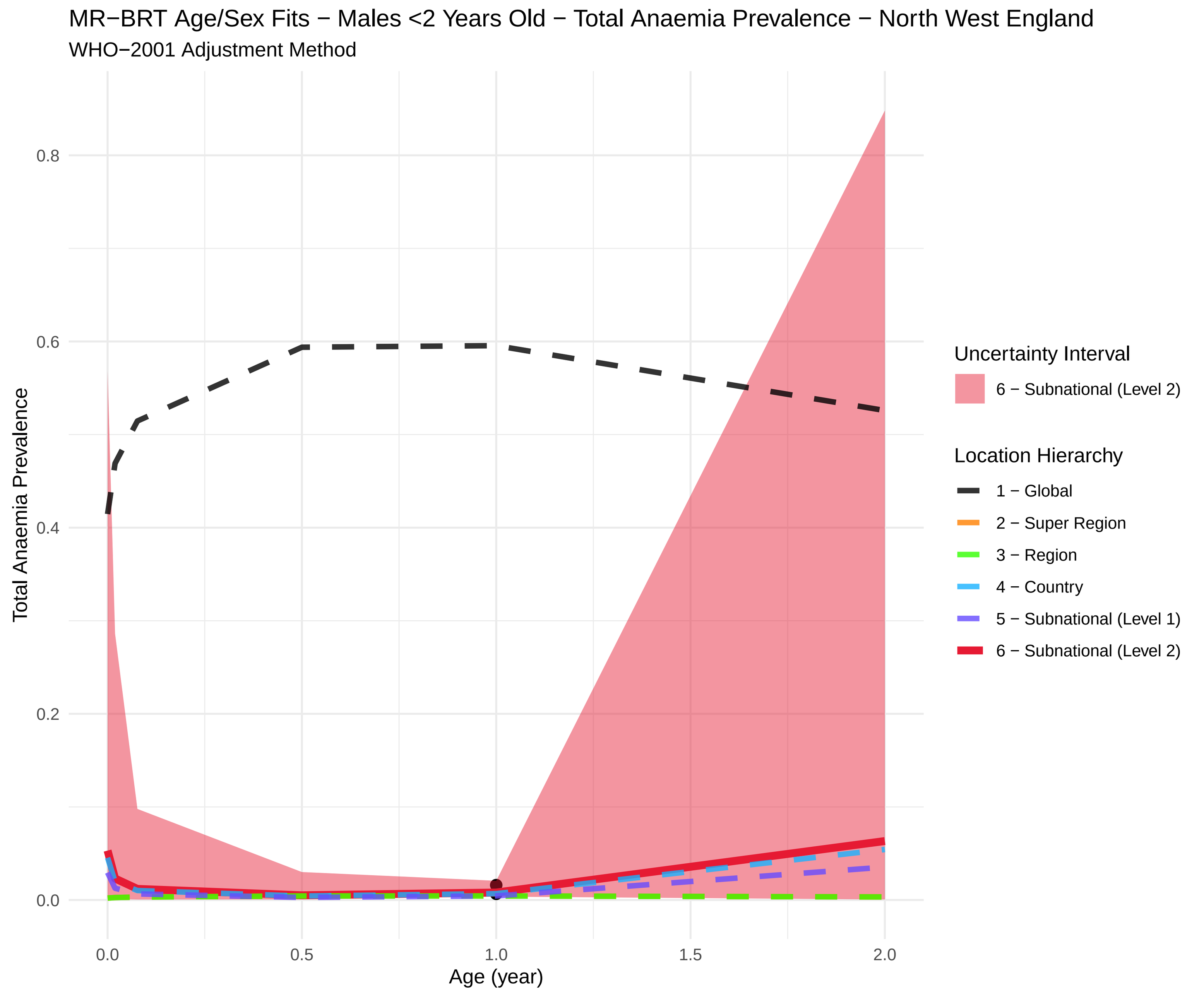
MR−BRT Age/Sex Fits − Males <2 Years Old − Total Anaemia Prevalence − North West England
WHO−2001 Adjustment Method
Total Anaemia Prevalence
Age (year)
Uncertainty Interval
6 − Subnational (Level 2)
Location Hierarchy
1 − Global
2 − Super Region
3 − Region
4 − Country
5 − Subnational (Level 1)
6 − Subnational (Level 2)

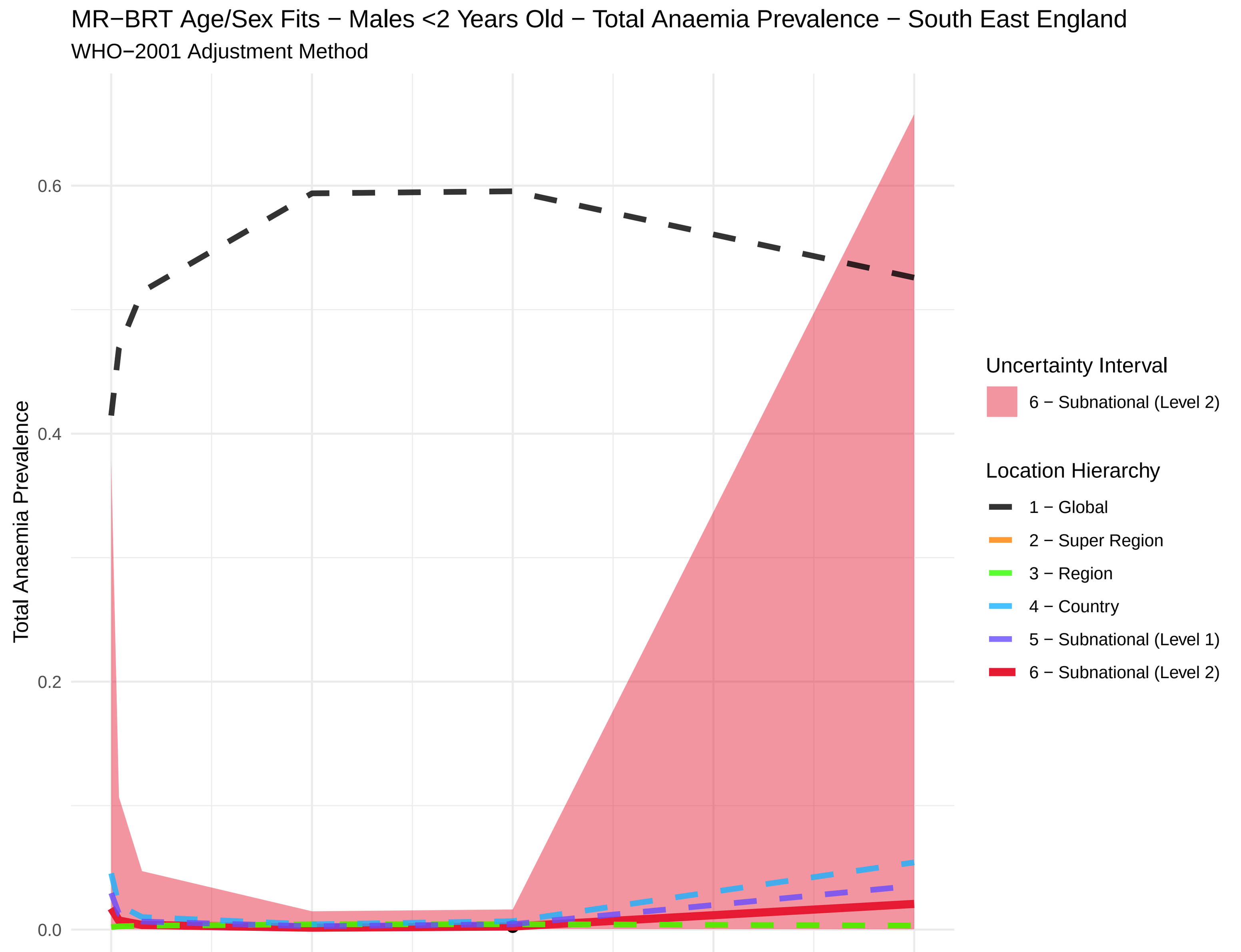
MR−BRT Age/Sex Fits − Males <2 Years Old − Total Anaemia Prevalence − South East England
WHO−2001 Adjustment Method
Total Anaemia Prevalence
Age (year)
Uncertainty Interval
6 − Subnational (Level 2)
Location Hierarchy
1 − Global
2 − Super Region
3 − Region
4 − Country
5 − Subnational (Level 1)
6 − Subnational (Level 2)

MR−BRT Age/Sex Fits − Males <2 Years Old − Total Anaemia Prevalence − South West England
WHO−2001 Adjustment Method

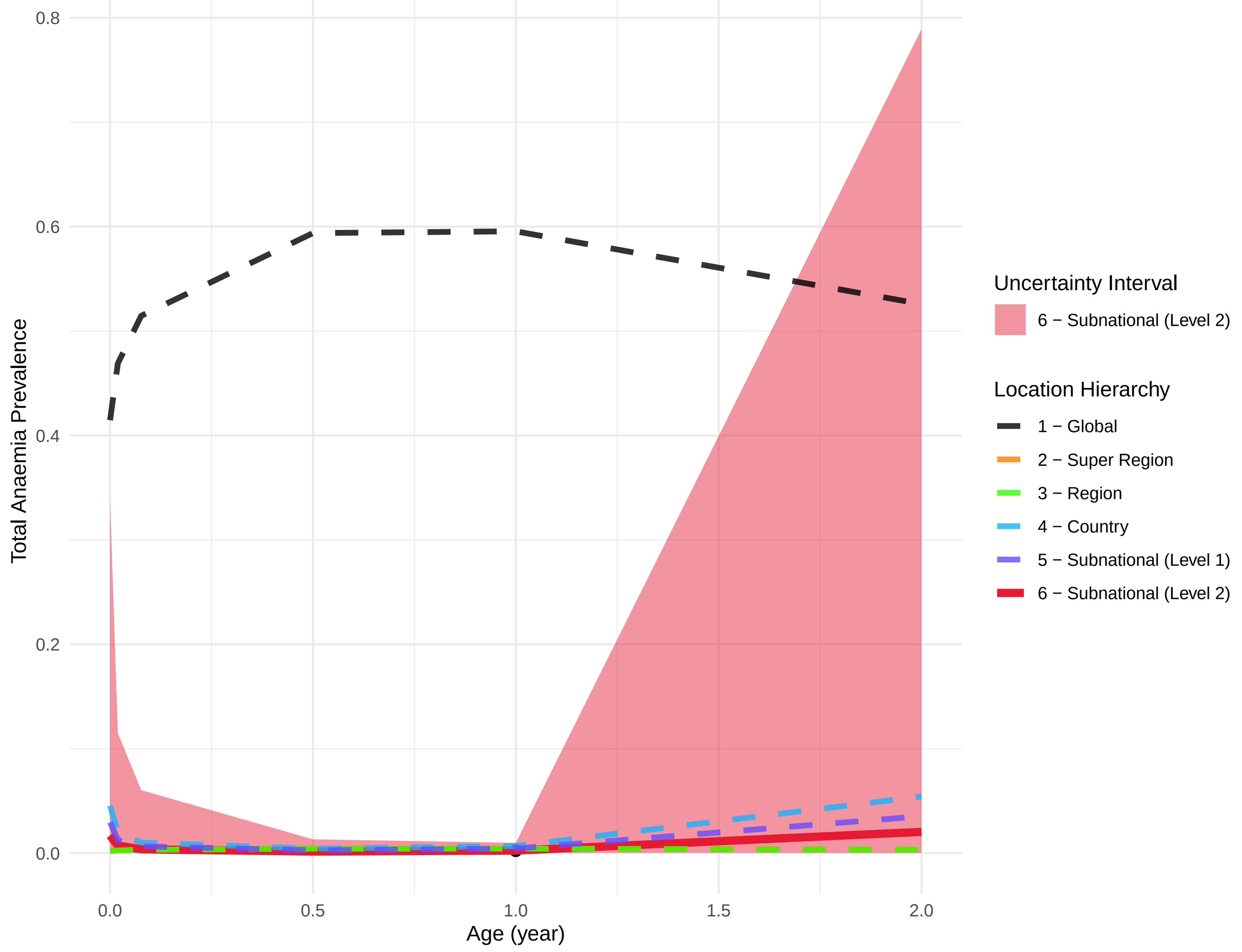

MR−BRT Age/Sex Fits − Males <2 Years Old − Total Anaemia Prevalence − West Midlands
WHO−2001 Adjustment Method

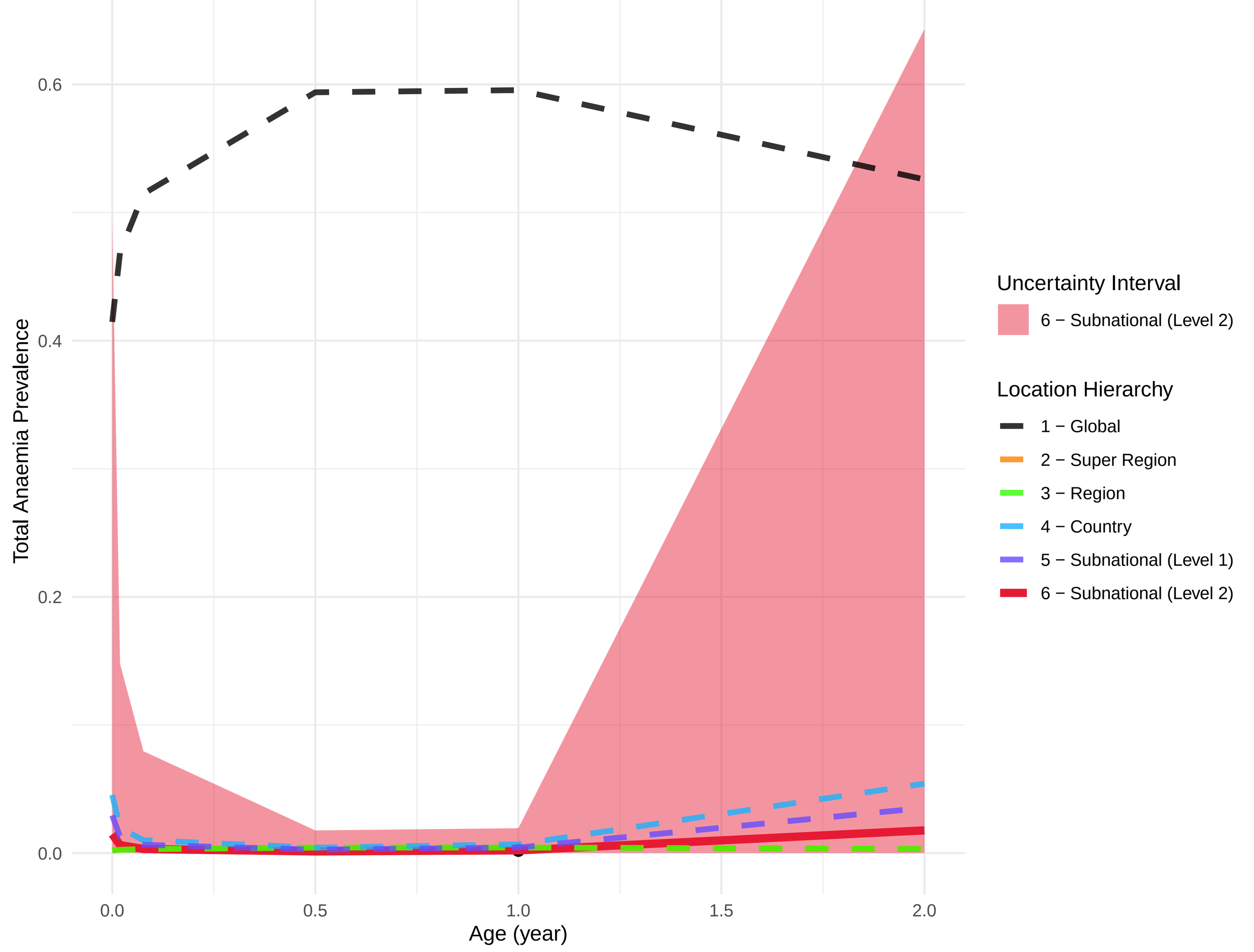

MR−BRT Age/Sex Fits − Males <2 Years Old − Total Anaemia Prevalence − Yorkshire and the Humber
WHO−2001 Adjustment Method

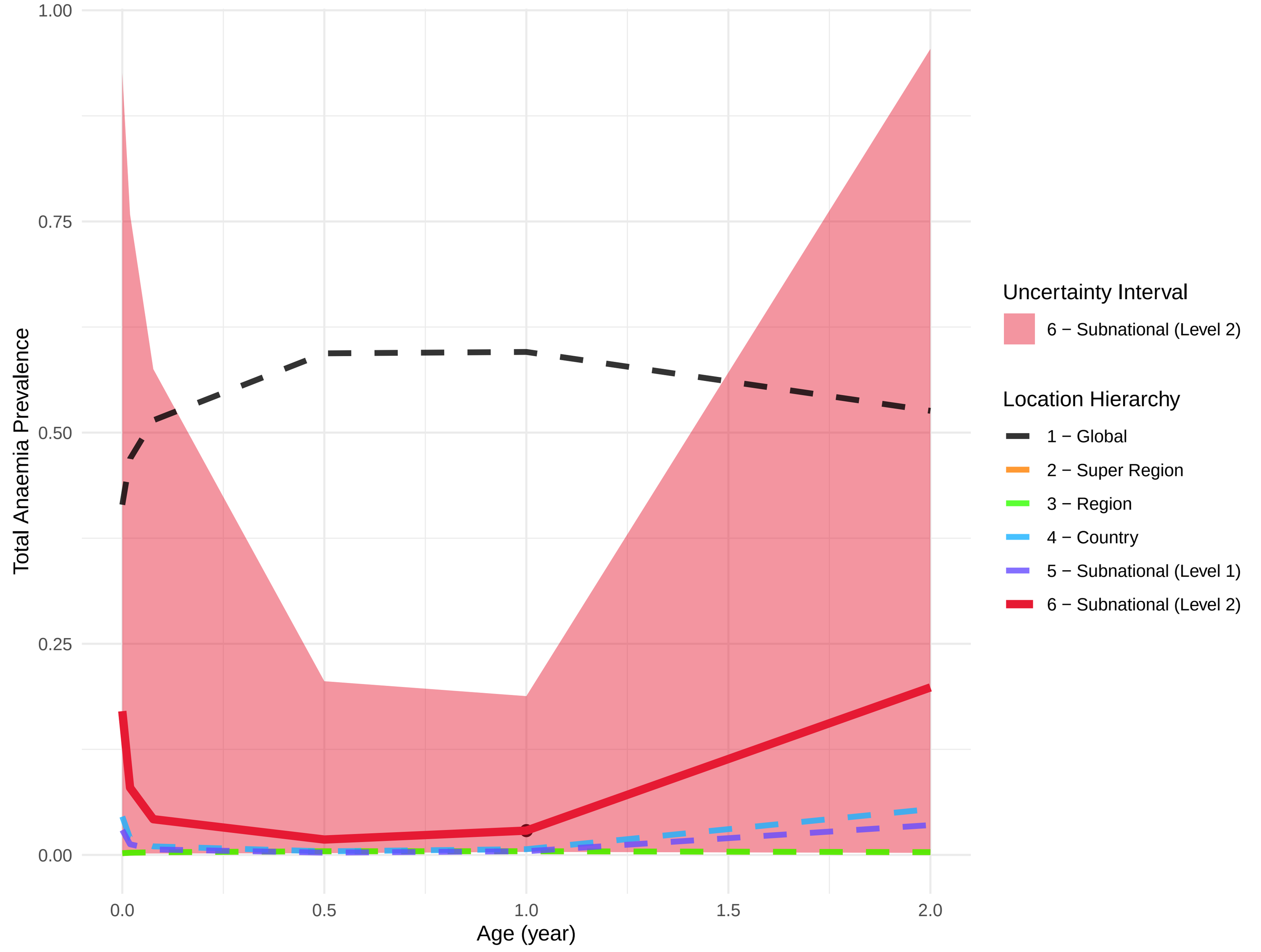

MR−BRT Age/Sex Fits − Males <2 Years Old − Total Anaemia Prevalence − Latin America and Caribbean
WHO−2001 Adjustment Method

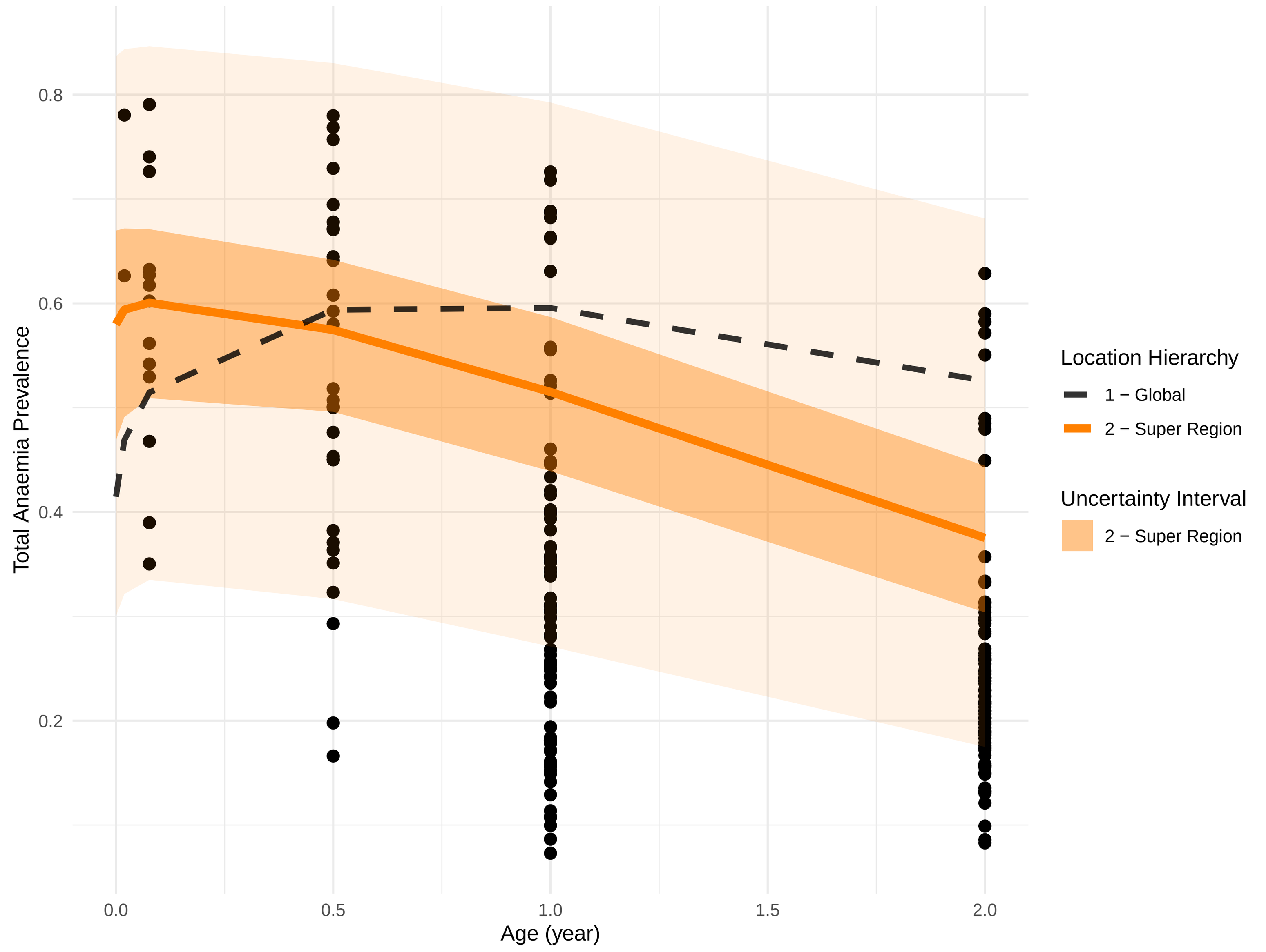

MR−BRT Age/Sex Fits − Males <2 Years Old − Total Anaemia Prevalence − Andean Latin America
WHO−2001 Adjustment Method

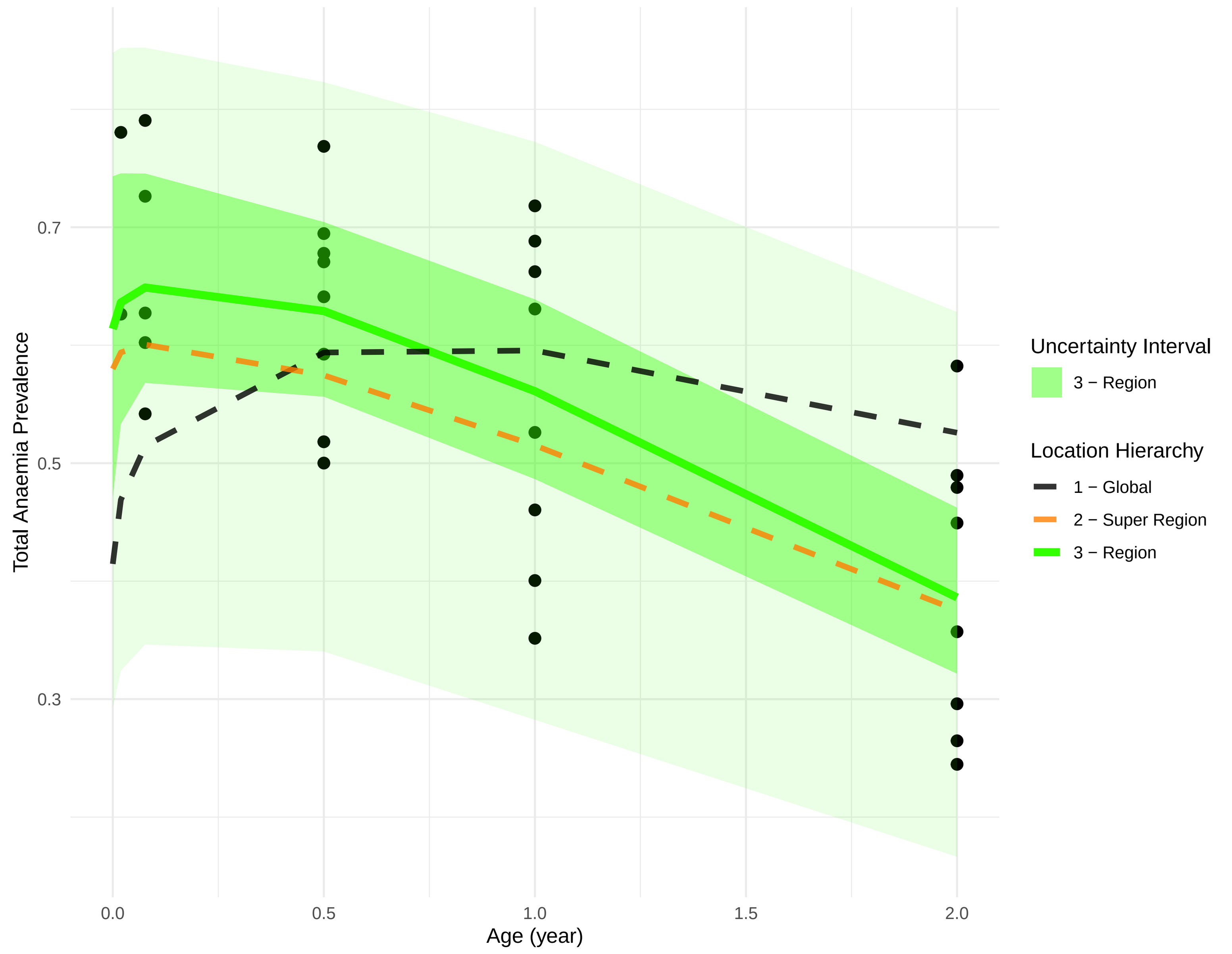

MR−BRT Age/Sex Fits − Males <2 Years Old − Total Anaemia Prevalence − Bolivia (Plurinational State of)
WHO−2001 Adjustment Method

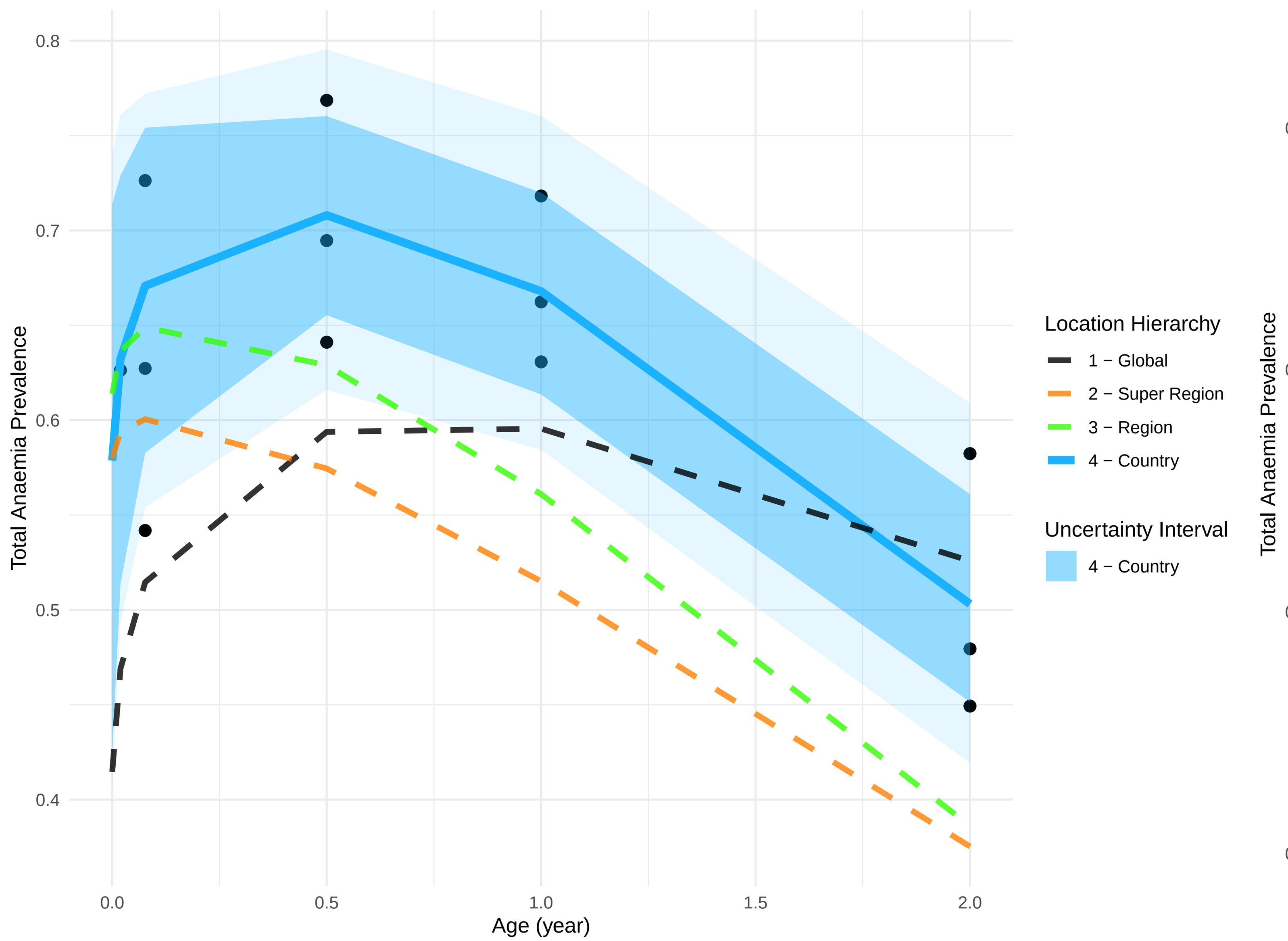

MR−BRT Age/Sex Fits − Males <2 Years Old − Total Anaemia Prevalence − Peru
WHO−2001 Adjustment Method

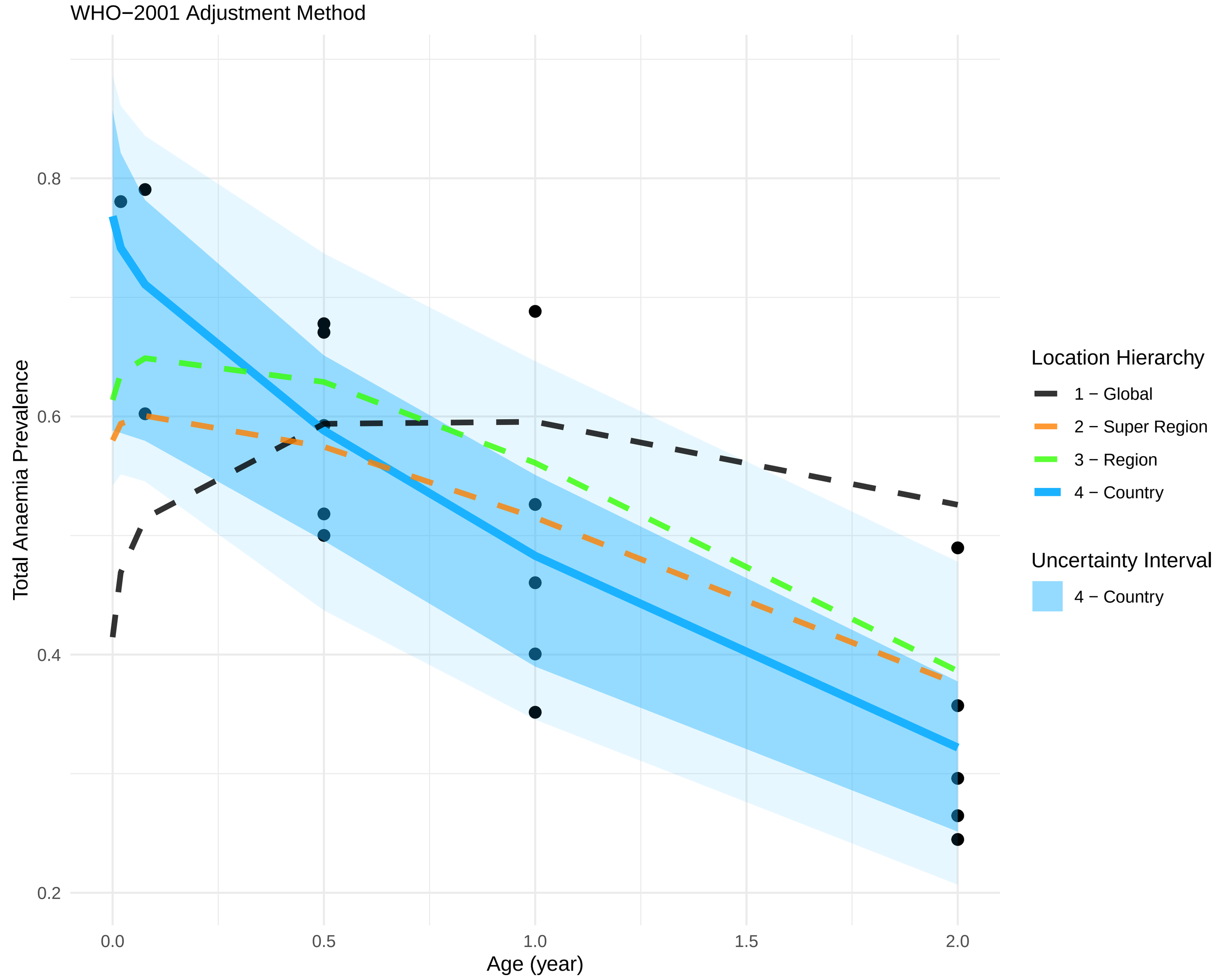

MR−BRT Age/Sex Fits − Males <2 Years Old − Total Anaemia Prevalence − Caribbean
WHO−2001 Adjustment Method

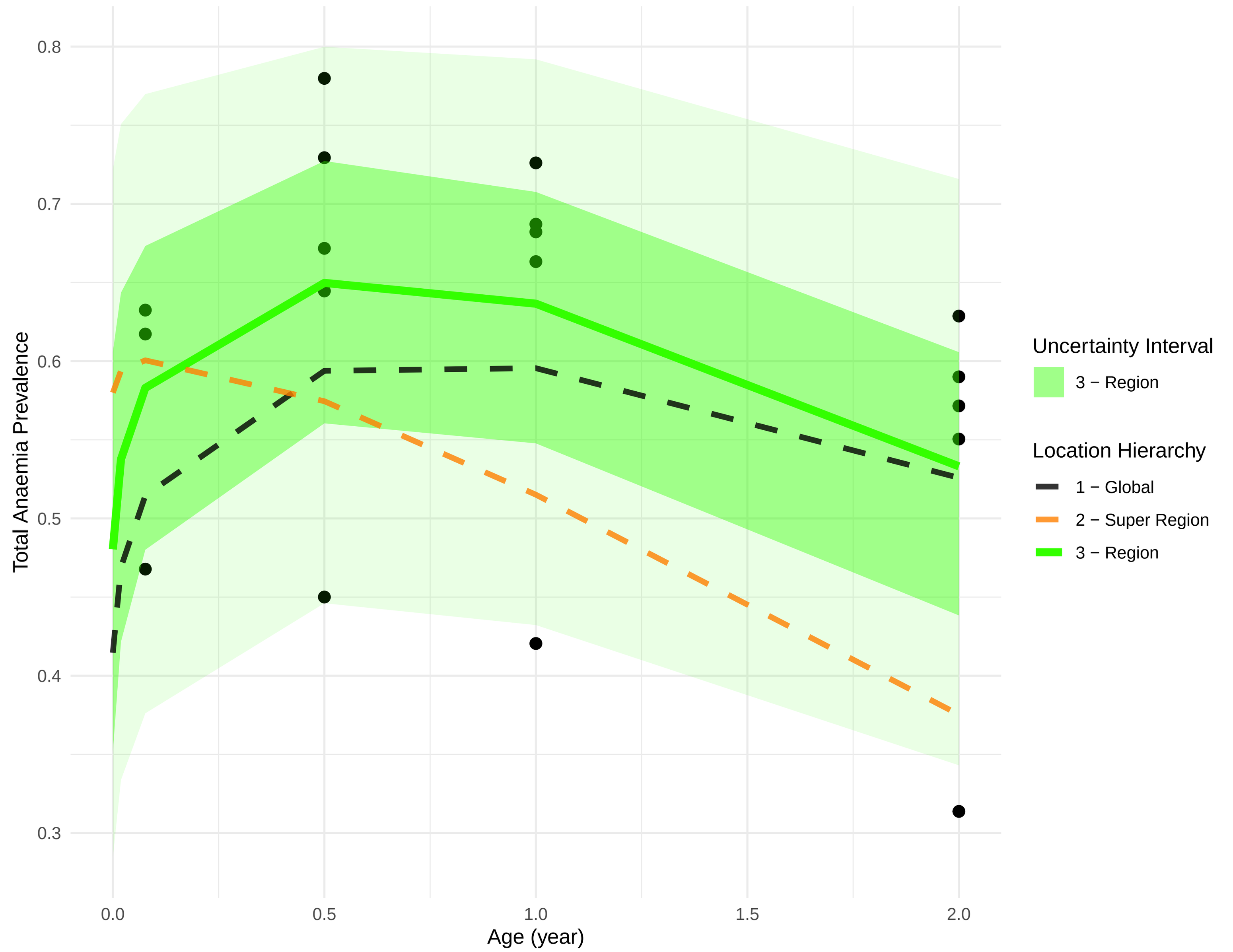

MR−BRT Age/Sex Fits − Males <2 Years Old − Total Anaemia Prevalence − Guyana
WHO−2001 Adjustment Method

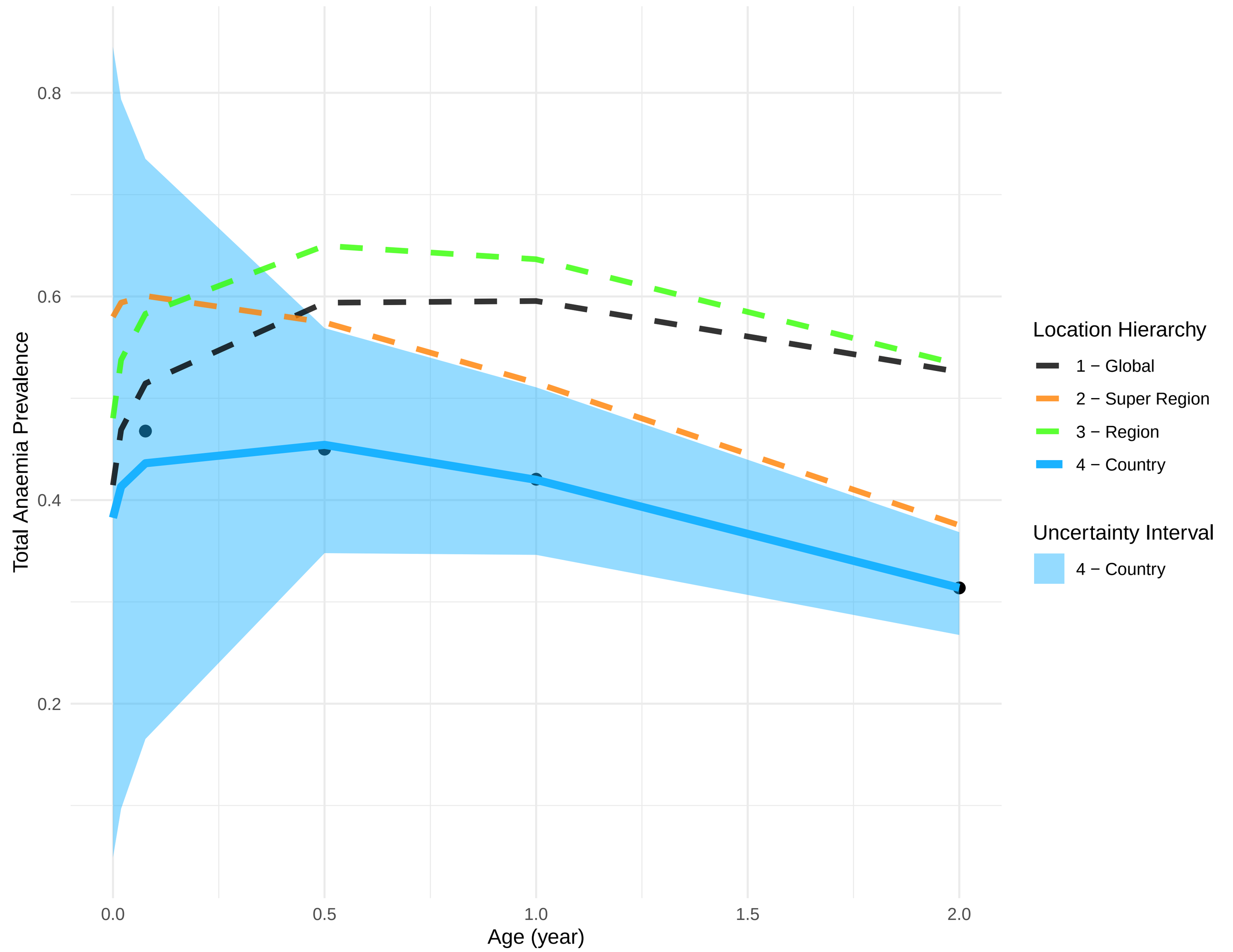

MR−BRT Age/Sex Fits − Males <2 Years Old − Total Anaemia Prevalence − Haiti
WHO−2001 Adjustment Method

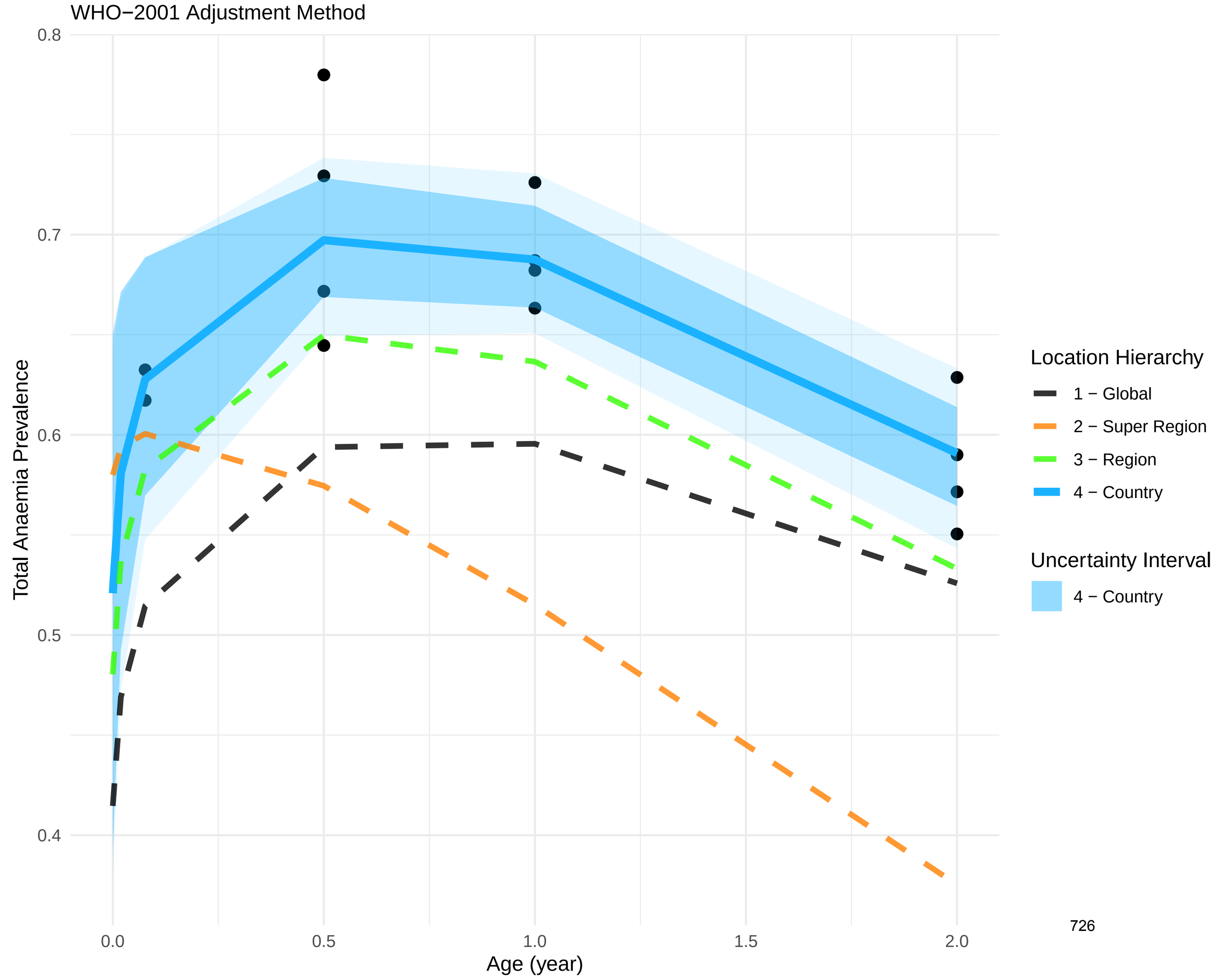

MR−BRT Age/Sex Fits − Males <2 Years Old − Total Anaemia Prevalence − Central Latin America
WHO−2001 Adjustment Method

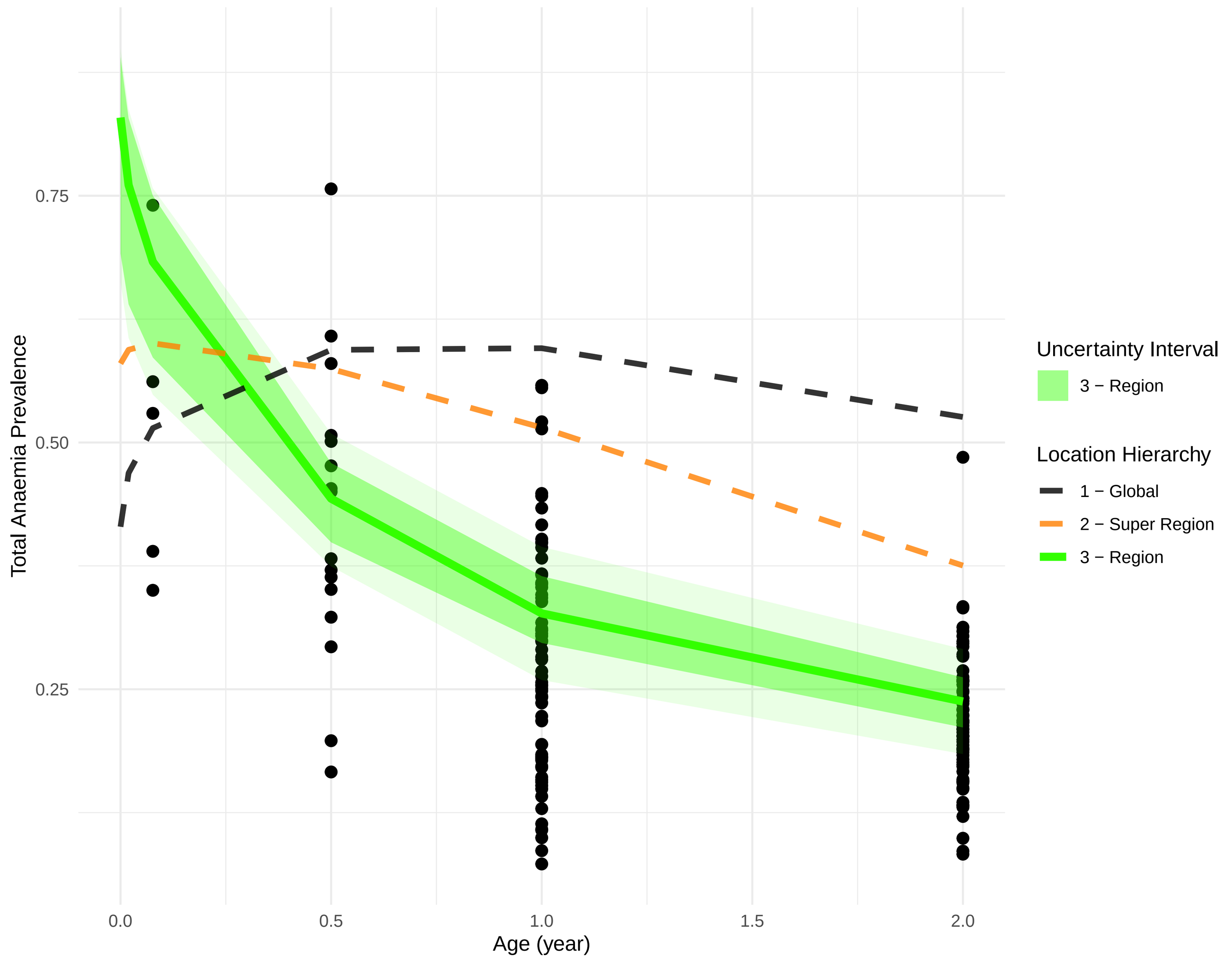

MR−BRT Age/Sex Fits − Males <2 Years Old − Total Anaemia Prevalence − Guatemala
WHO−2001 Adjustment Method

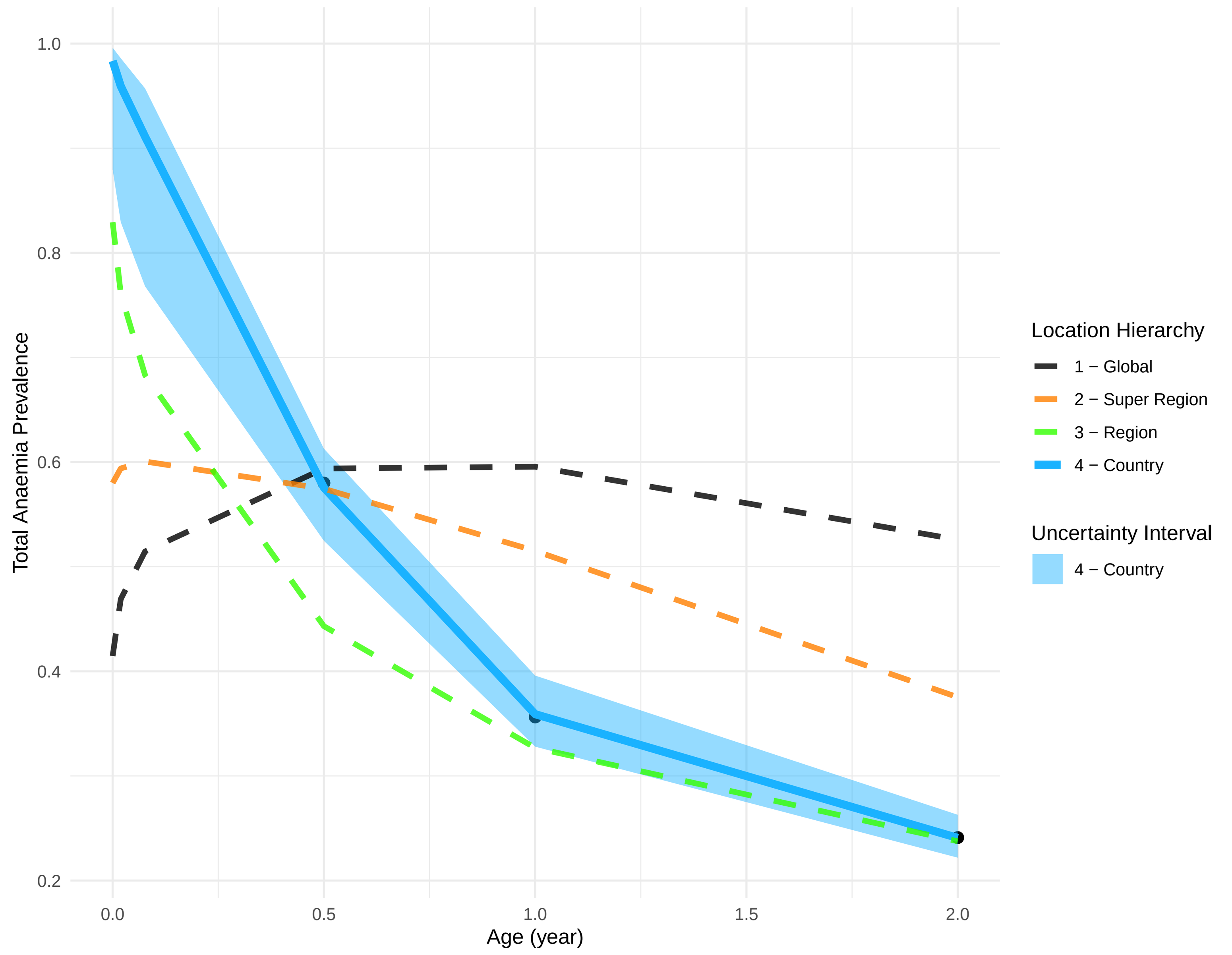

MR−BRT Age/Sex Fits − Males <2 Years Old − Total Anaemia Prevalence − Honduras
WHO−2001 Adjustment Method

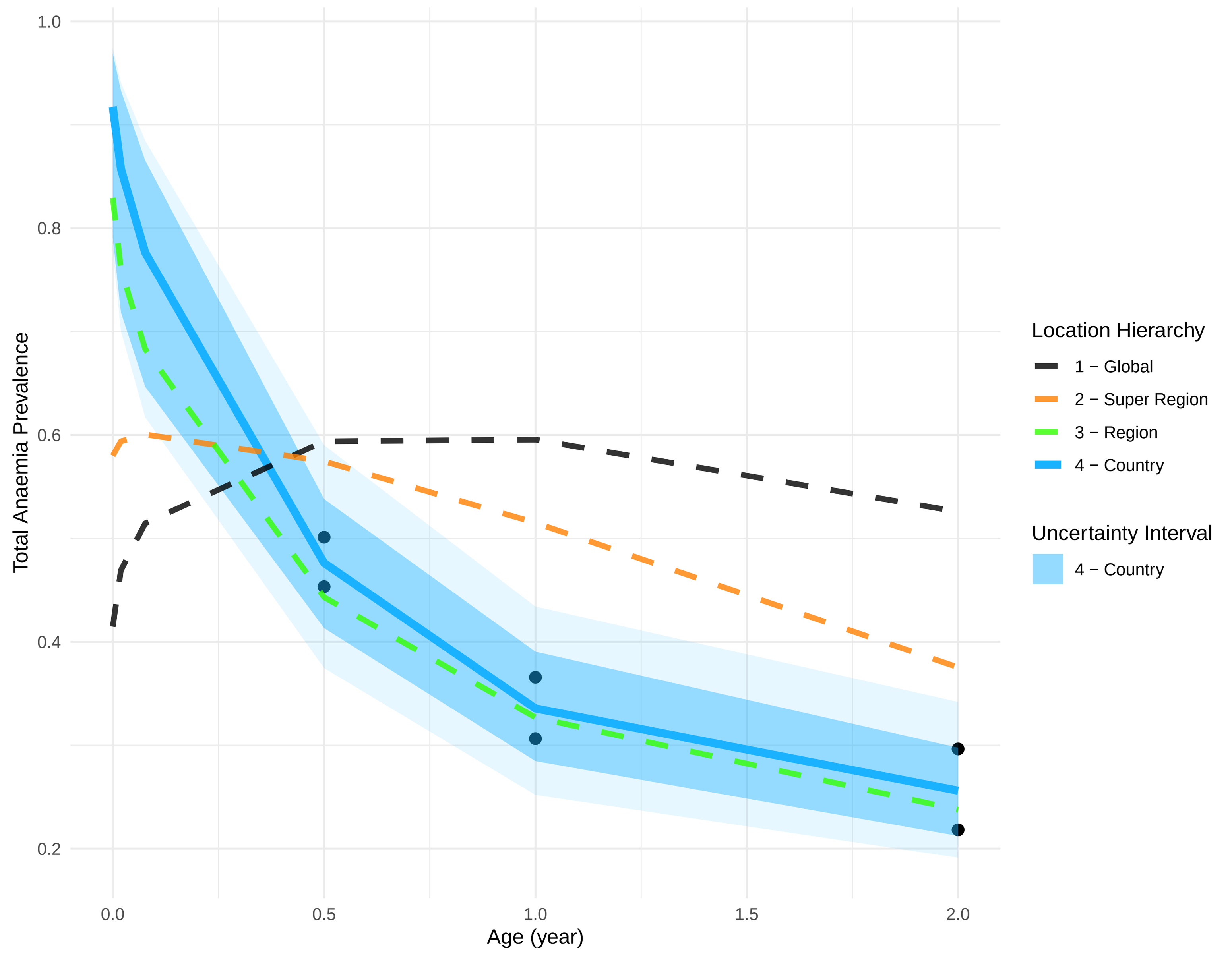

MR−BRT Age/Sex Fits − Males <2 Years Old − Total Anaemia Prevalence − Mexico
WHO−2001 Adjustment Method

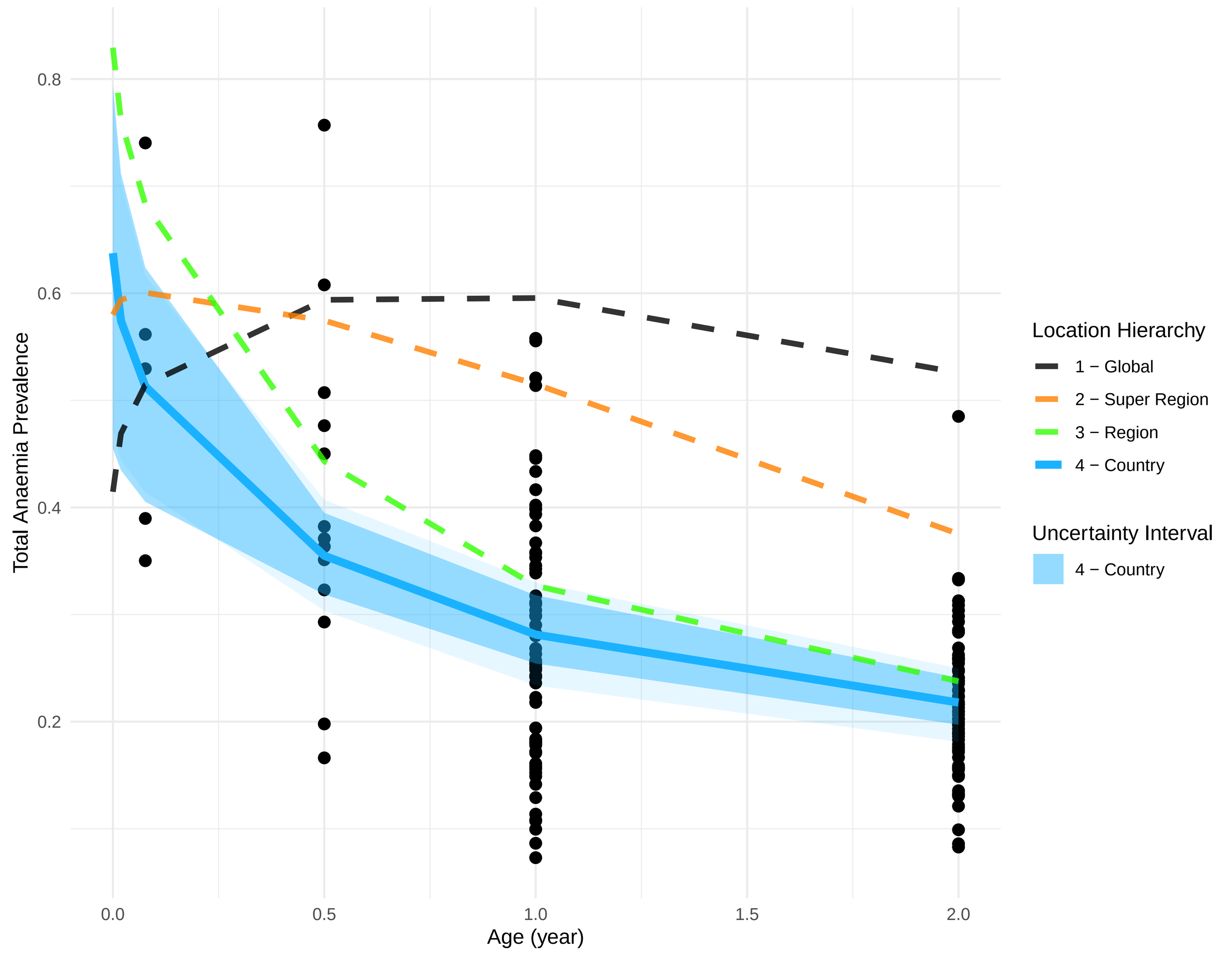

MR−BRT Age/Sex Fits − Males <2 Years Old − Total Anaemia Prevalence − Aguascalientes
WHO−2001 Adjustment Method

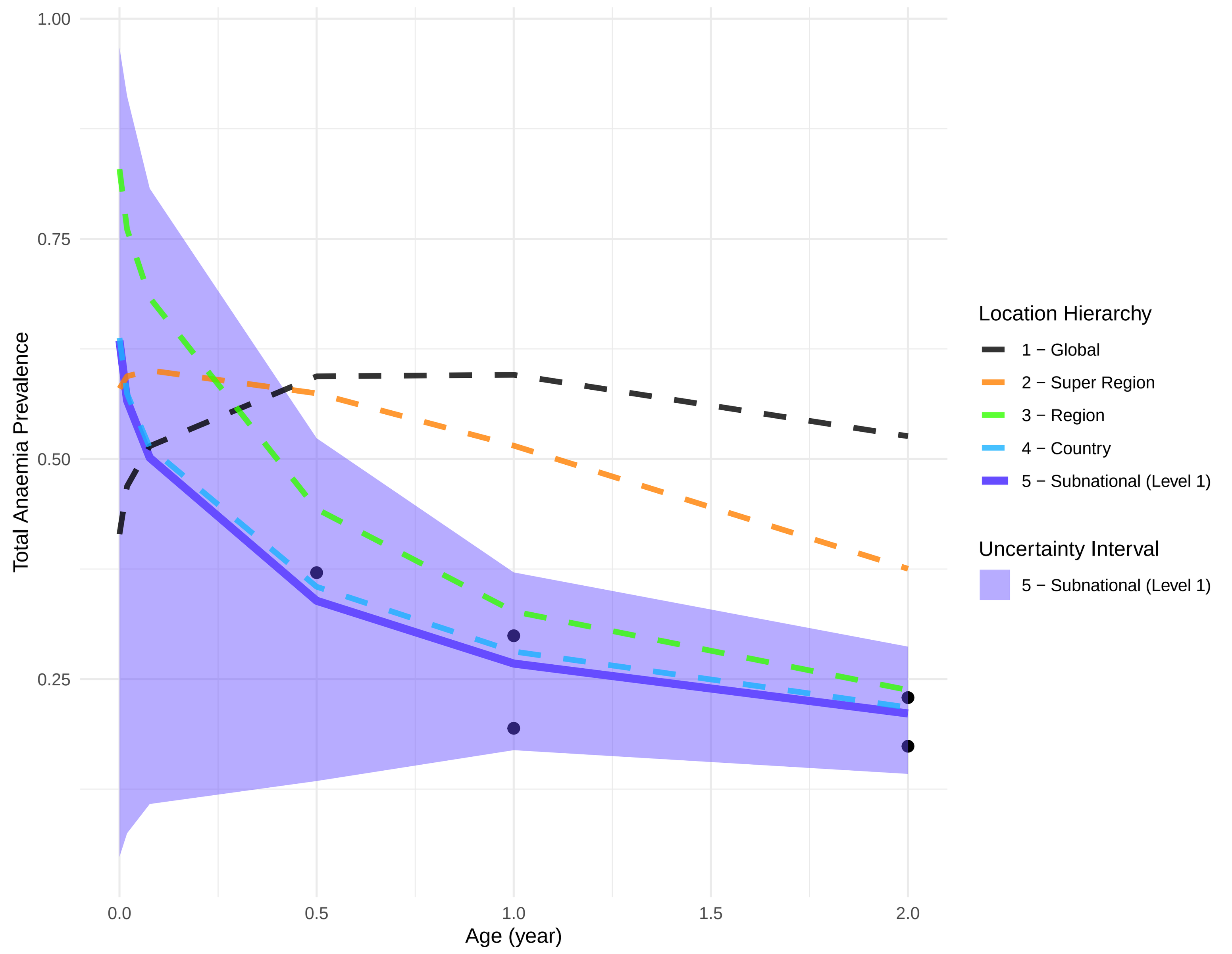

MR−BRT Age/Sex Fits − Males <2 Years Old − Total Anaemia Prevalence − Baja California
WHO−2001 Adjustment Method

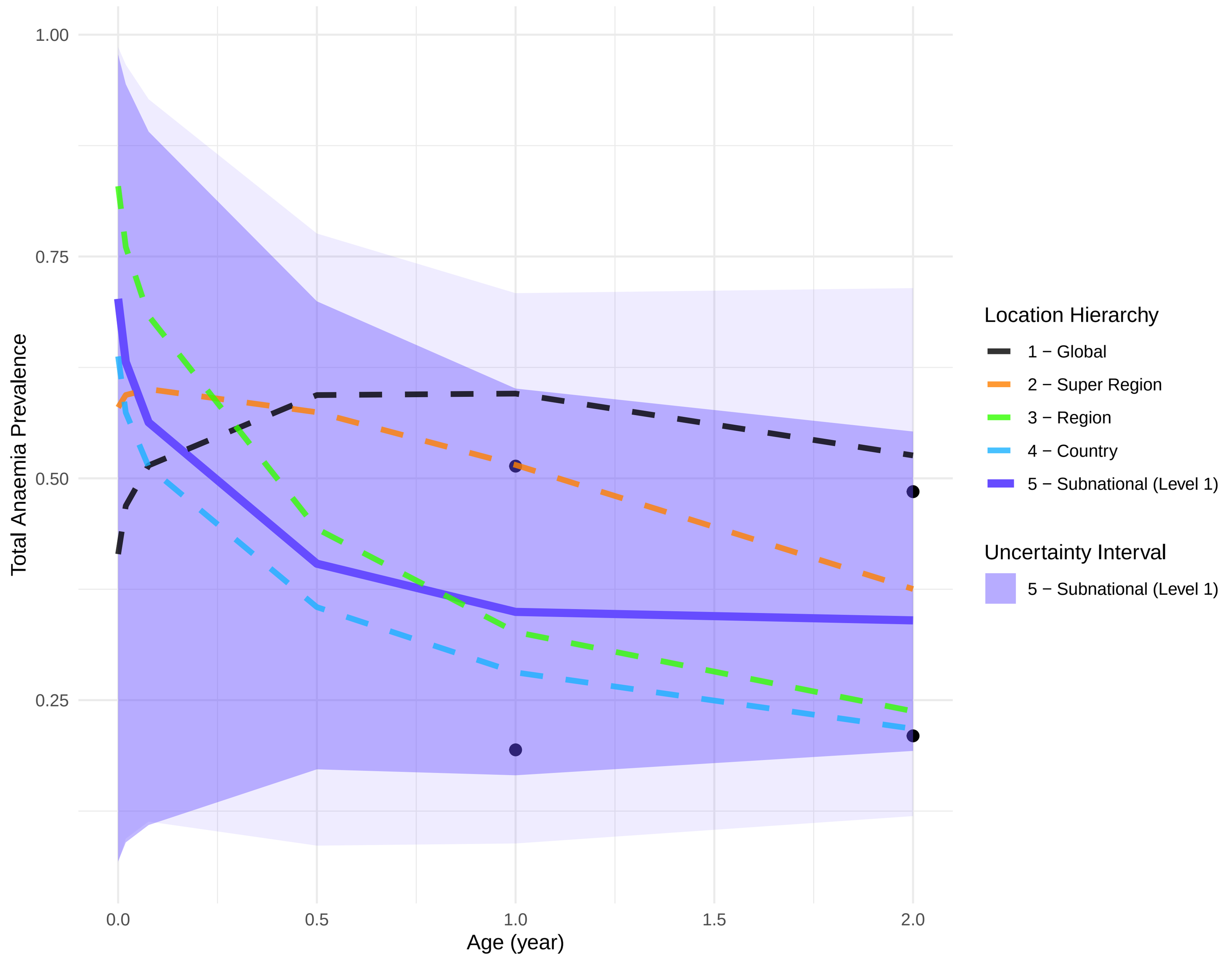

MR−BRT Age/Sex Fits − Males <2 Years Old − Total Anaemia Prevalence − Baja California Sur
WHO−2001 Adjustment Method

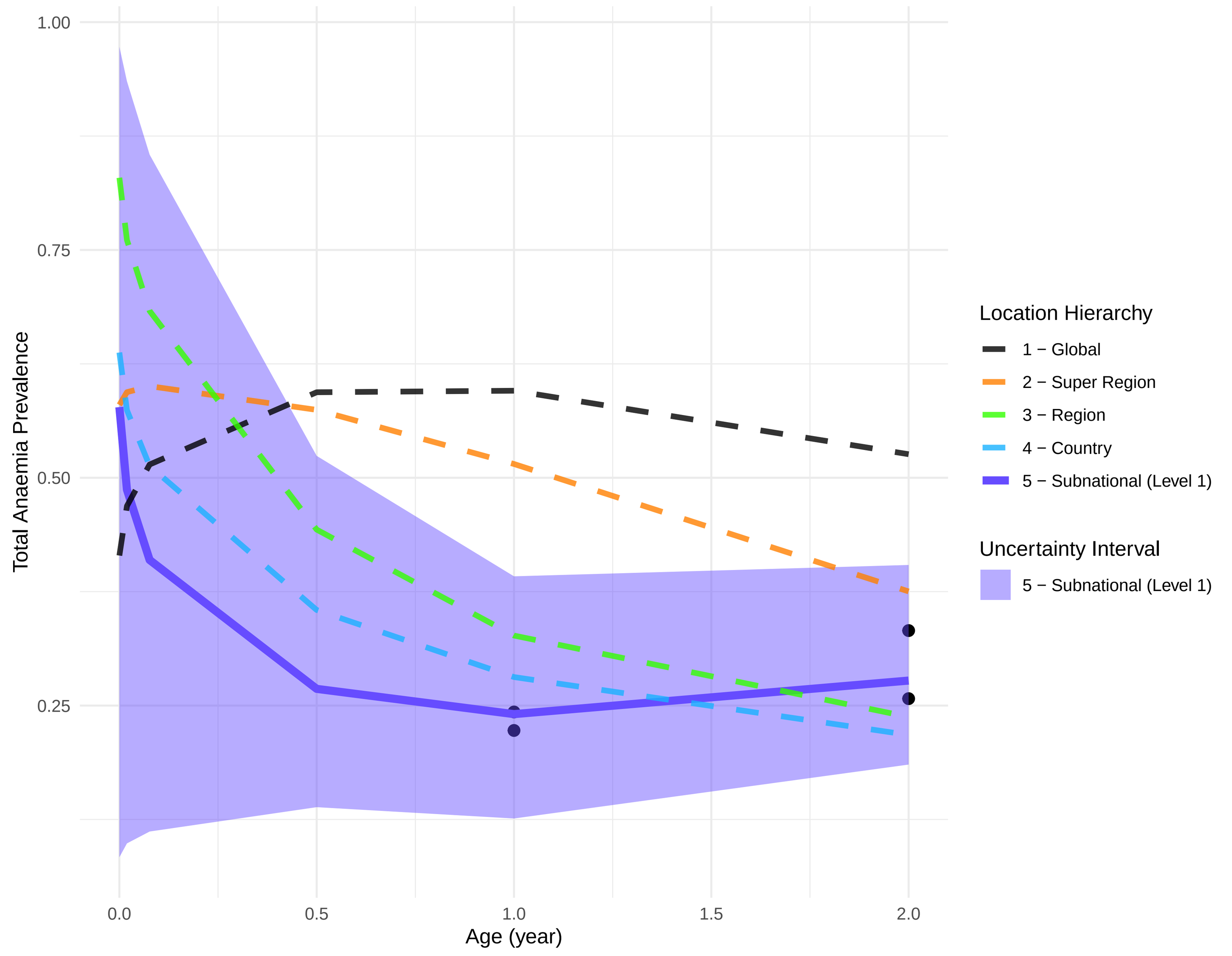

MR−BRT Age/Sex Fits − Males <2 Years Old − Total Anaemia Prevalence − Campeche
WHO−2001 Adjustment Method

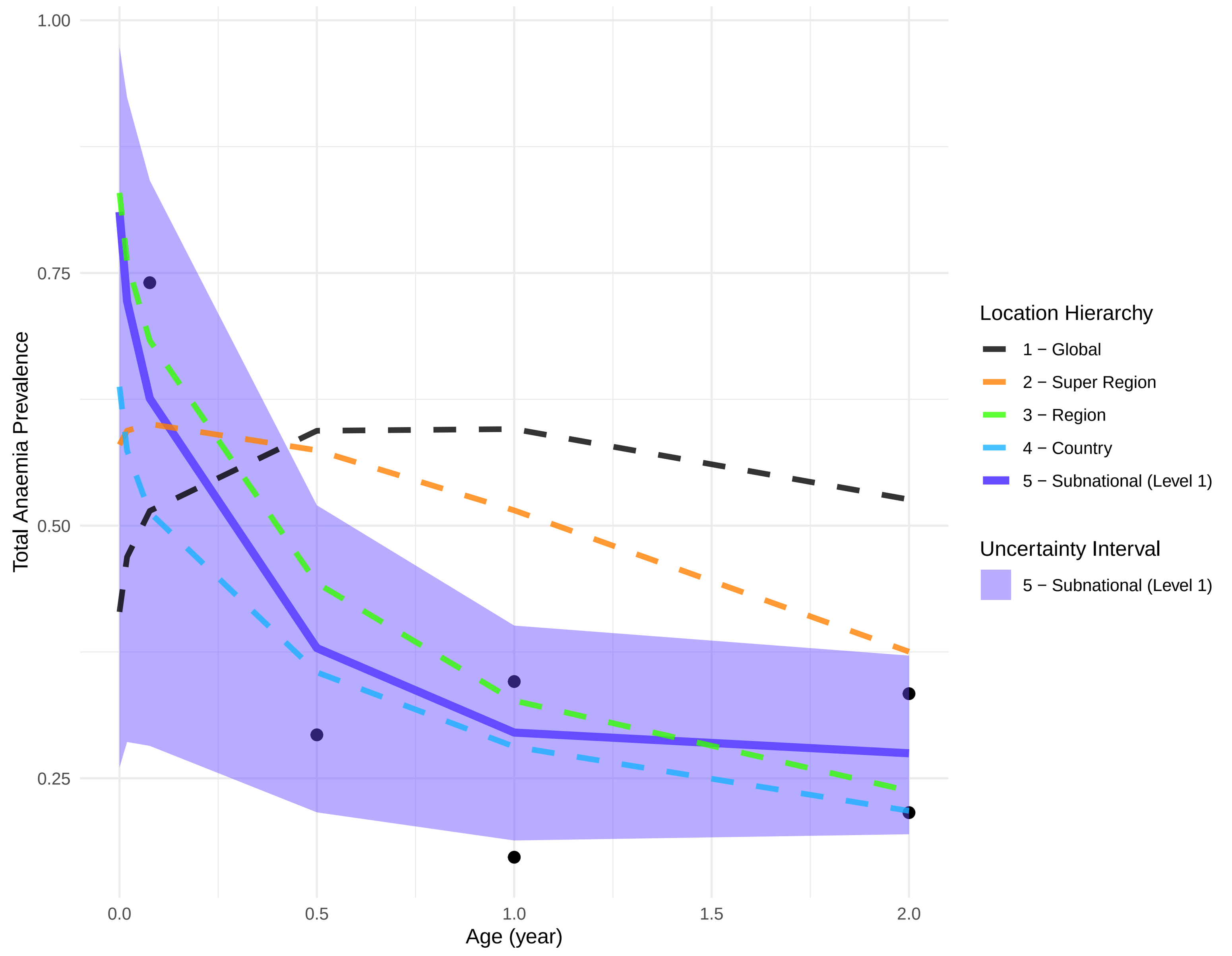

MR−BRT Age/Sex Fits − Males <2 Years Old − Total Anaemia Prevalence − Chiapas
WHO−2001 Adjustment Method

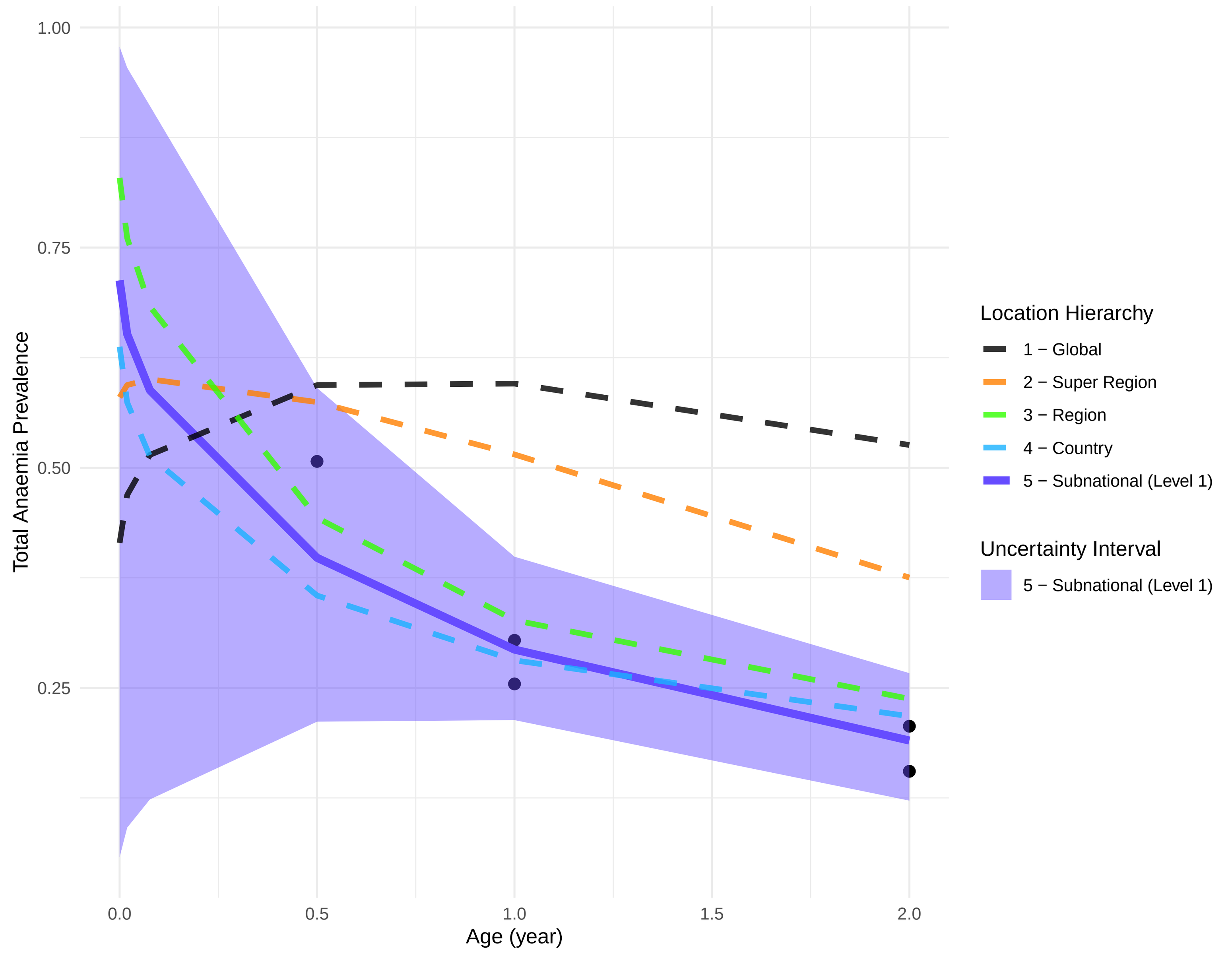

MR−BRT Age/Sex Fits − Males <2 Years Old − Total Anaemia Prevalence − Chihuahua
WHO−2001 Adjustment Method

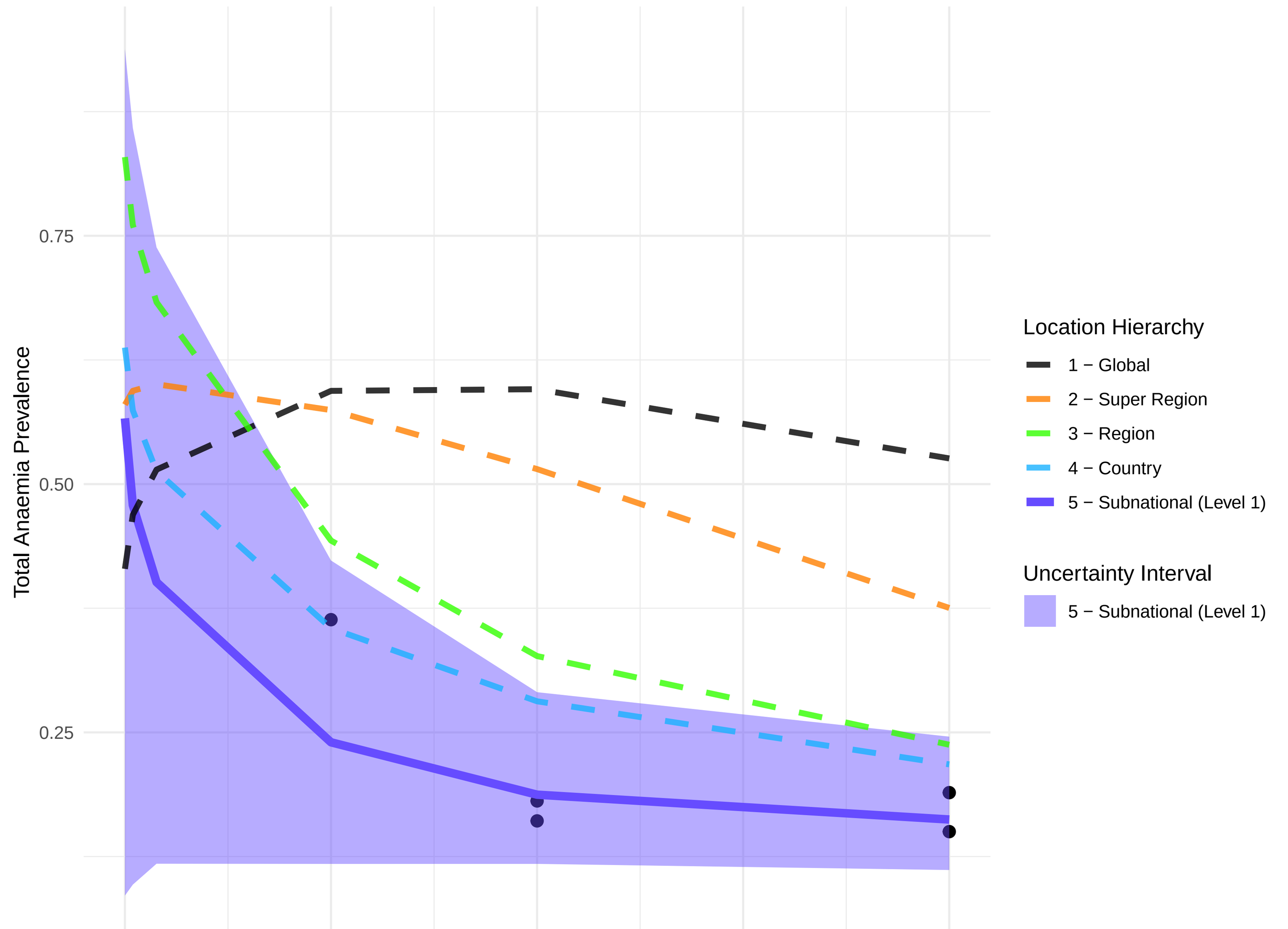

MR−BRT Age/Sex Fits − Males <2 Years Old − Total Anaemia Prevalence − Coahuila
WHO−2001 Adjustment Method

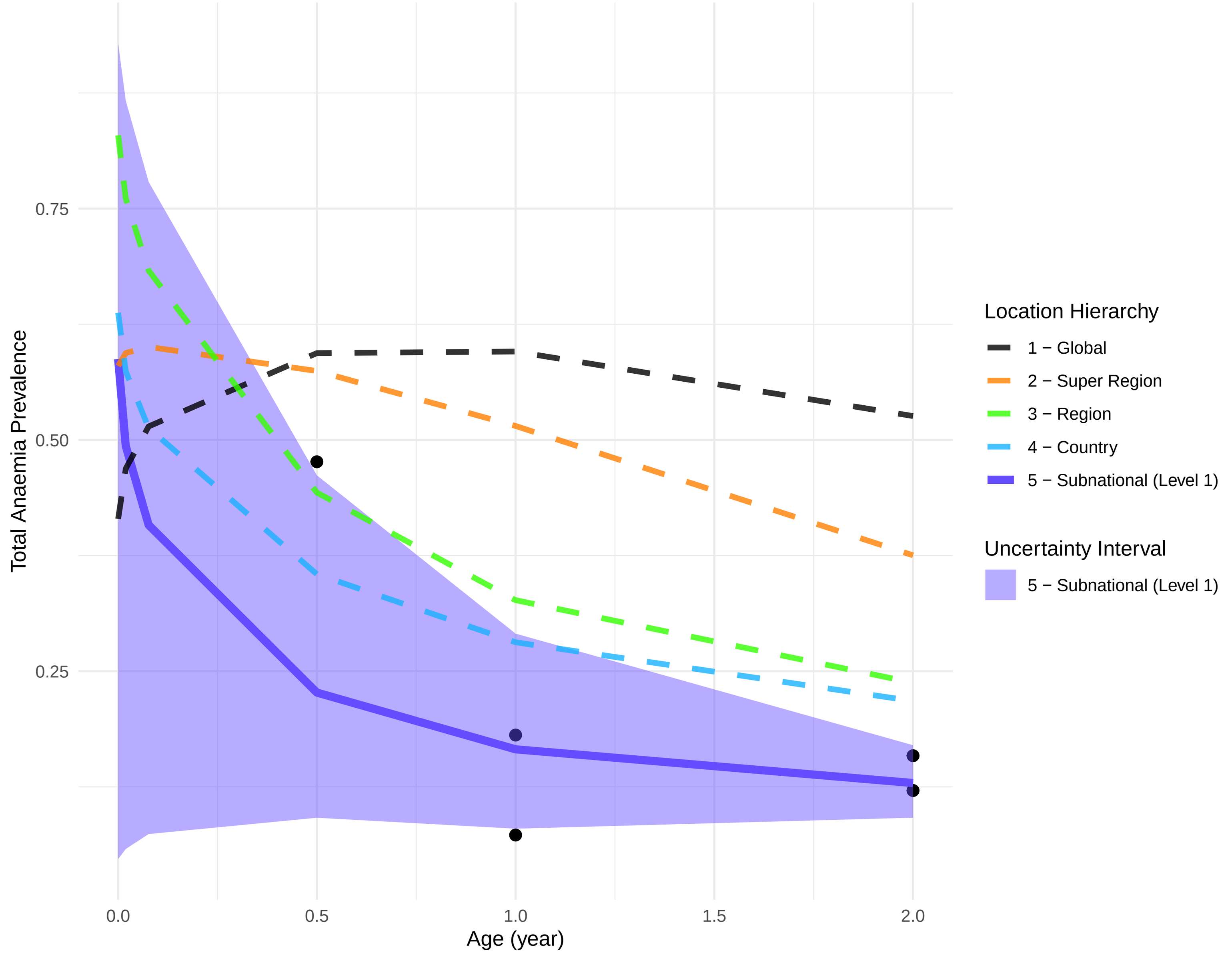

MR−BRT Age/Sex Fits − Males <2 Years Old − Total Anaemia Prevalence − Colima
WHO−2001 Adjustment Method

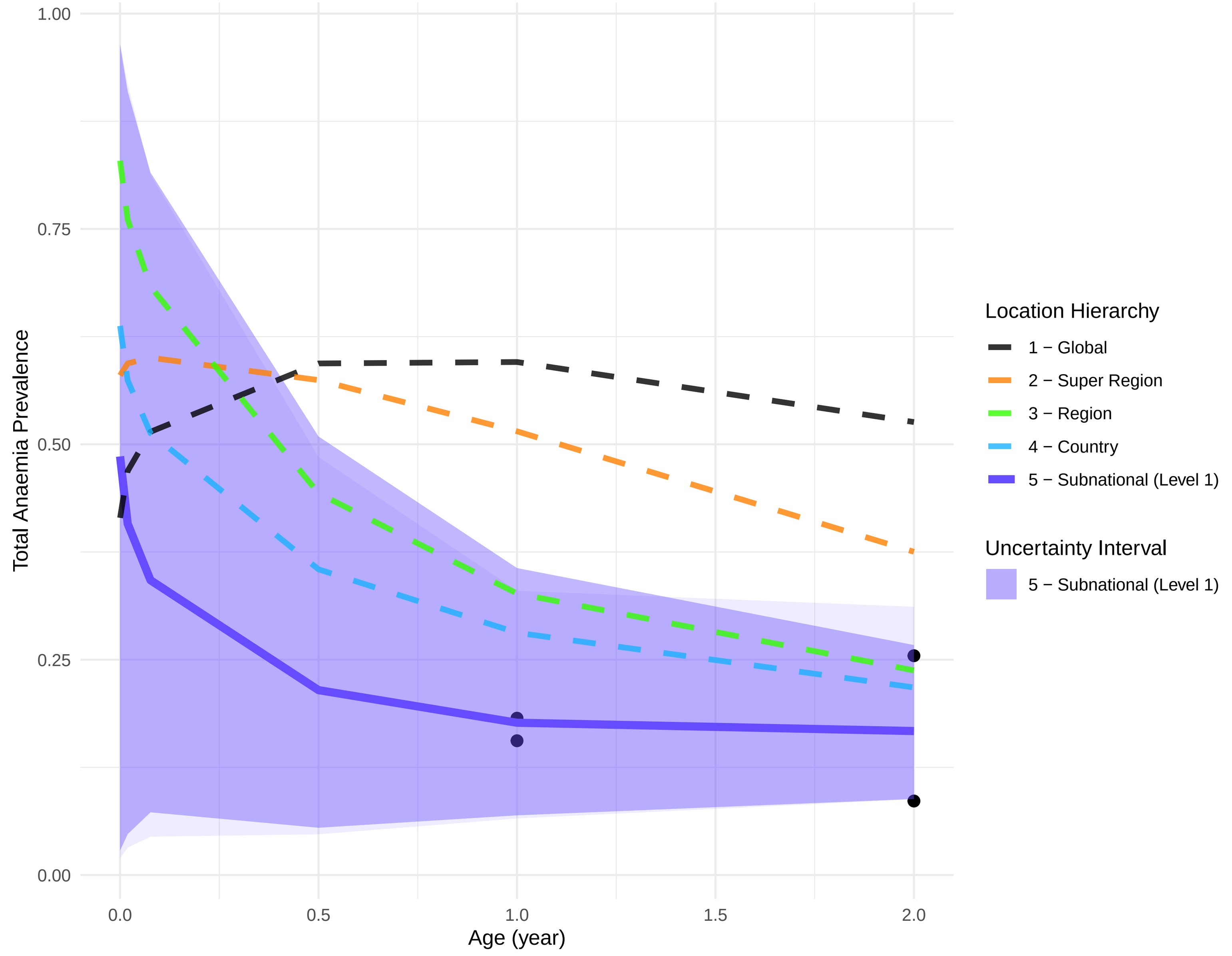

MR−BRT Age/Sex Fits − Males <2 Years Old − Total Anaemia Prevalence − Durango
WHO−2001 Adjustment Method

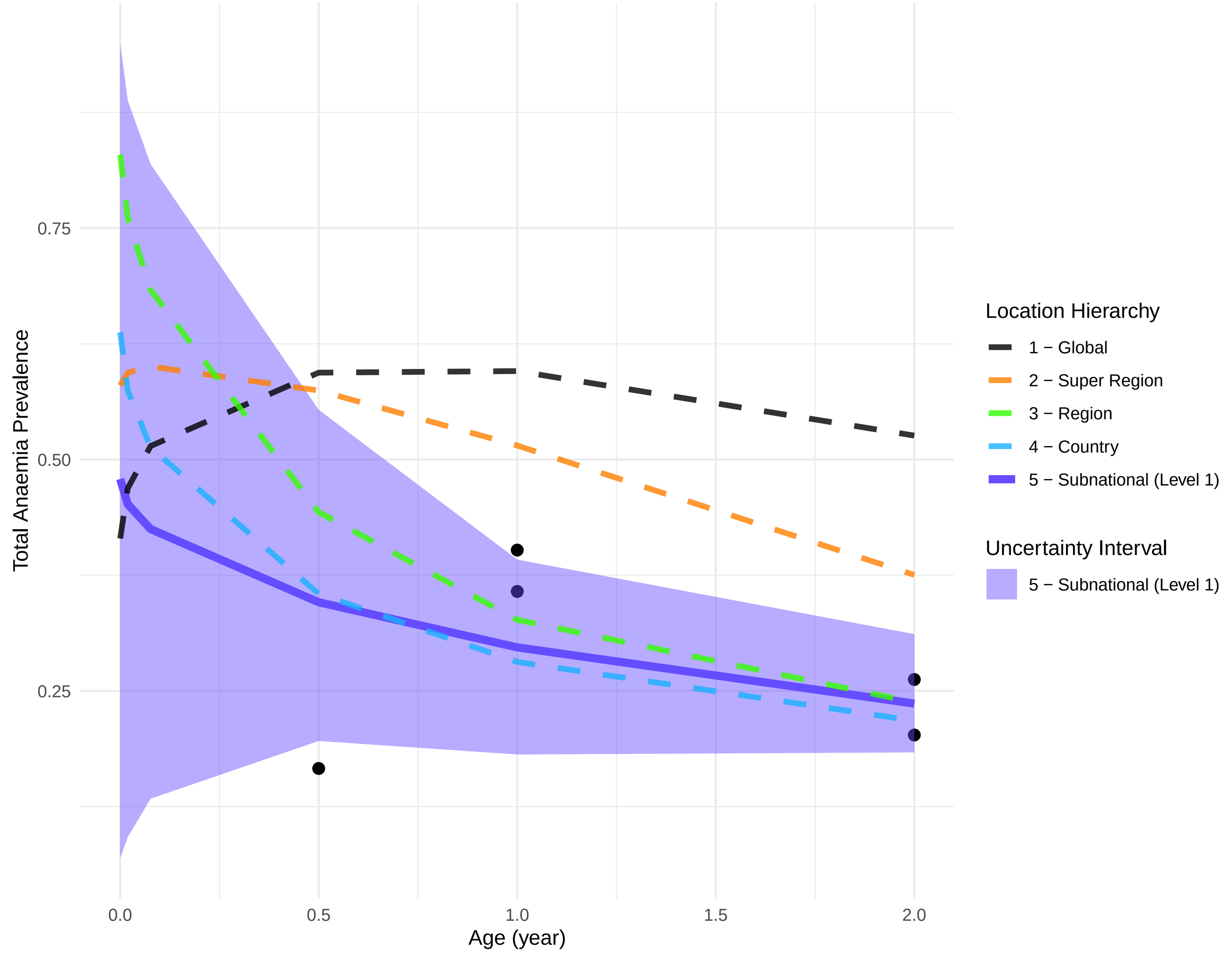

MR−BRT Age/Sex Fits − Males <2 Years Old − Total Anaemia Prevalence − Guanajuato
WHO−2001 Adjustment Method

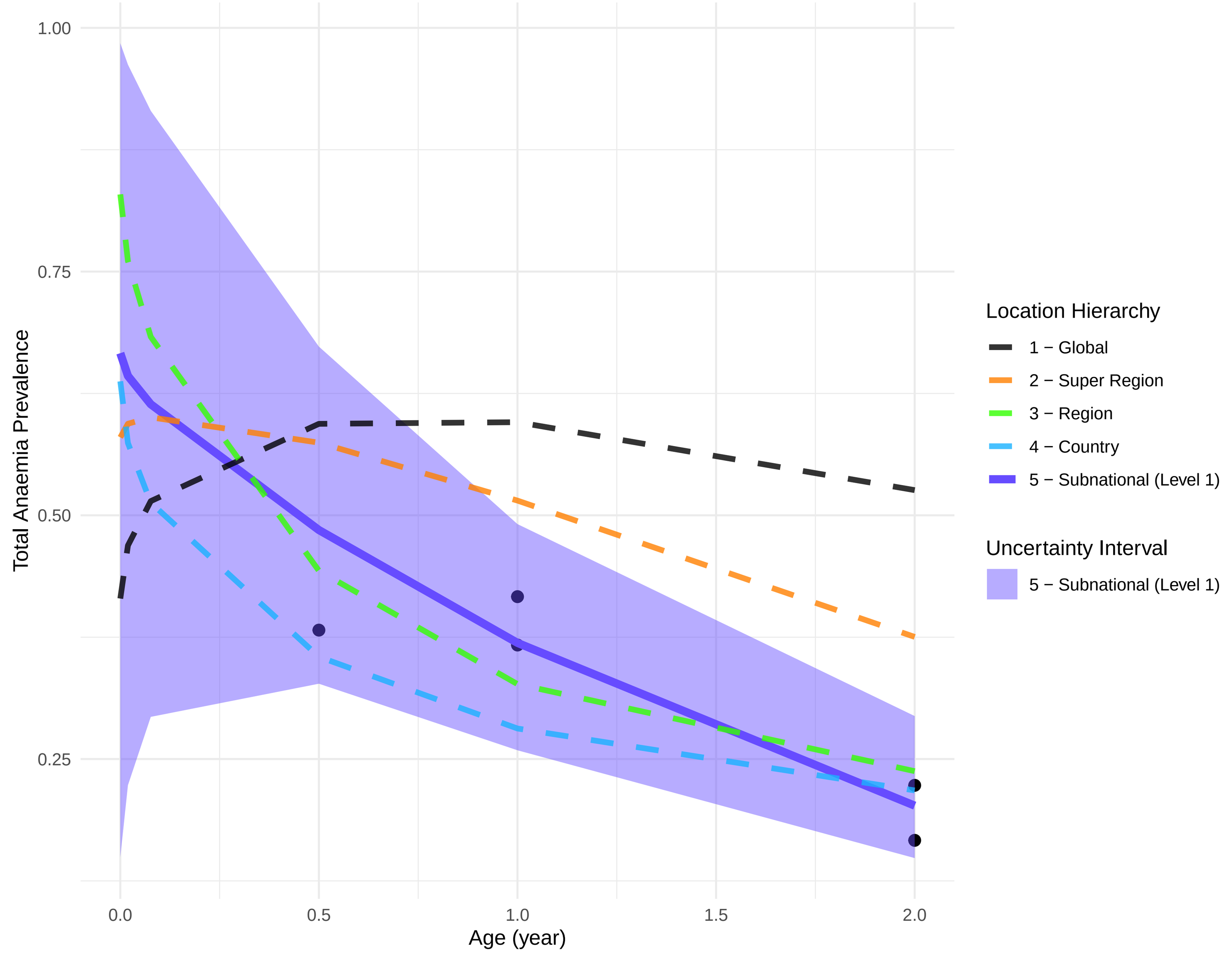

MR−BRT Age/Sex Fits − Males <2 Years Old − Total Anaemia Prevalence − Guerrero
WHO−2001 Adjustment Method

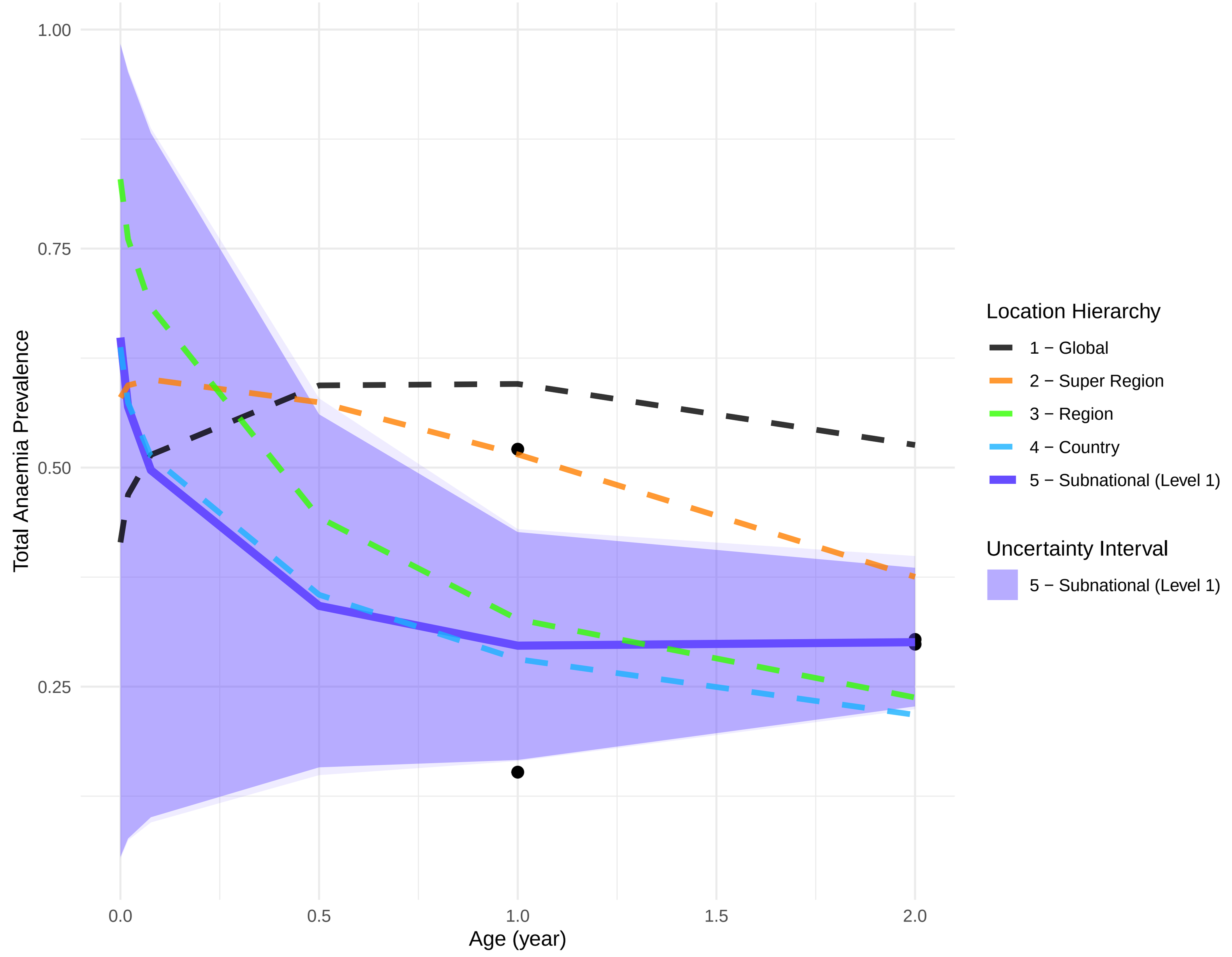

MR−BRT Age/Sex Fits − Males <2 Years Old − Total Anaemia Prevalence − Hidalgo
WHO−2001 Adjustment Method

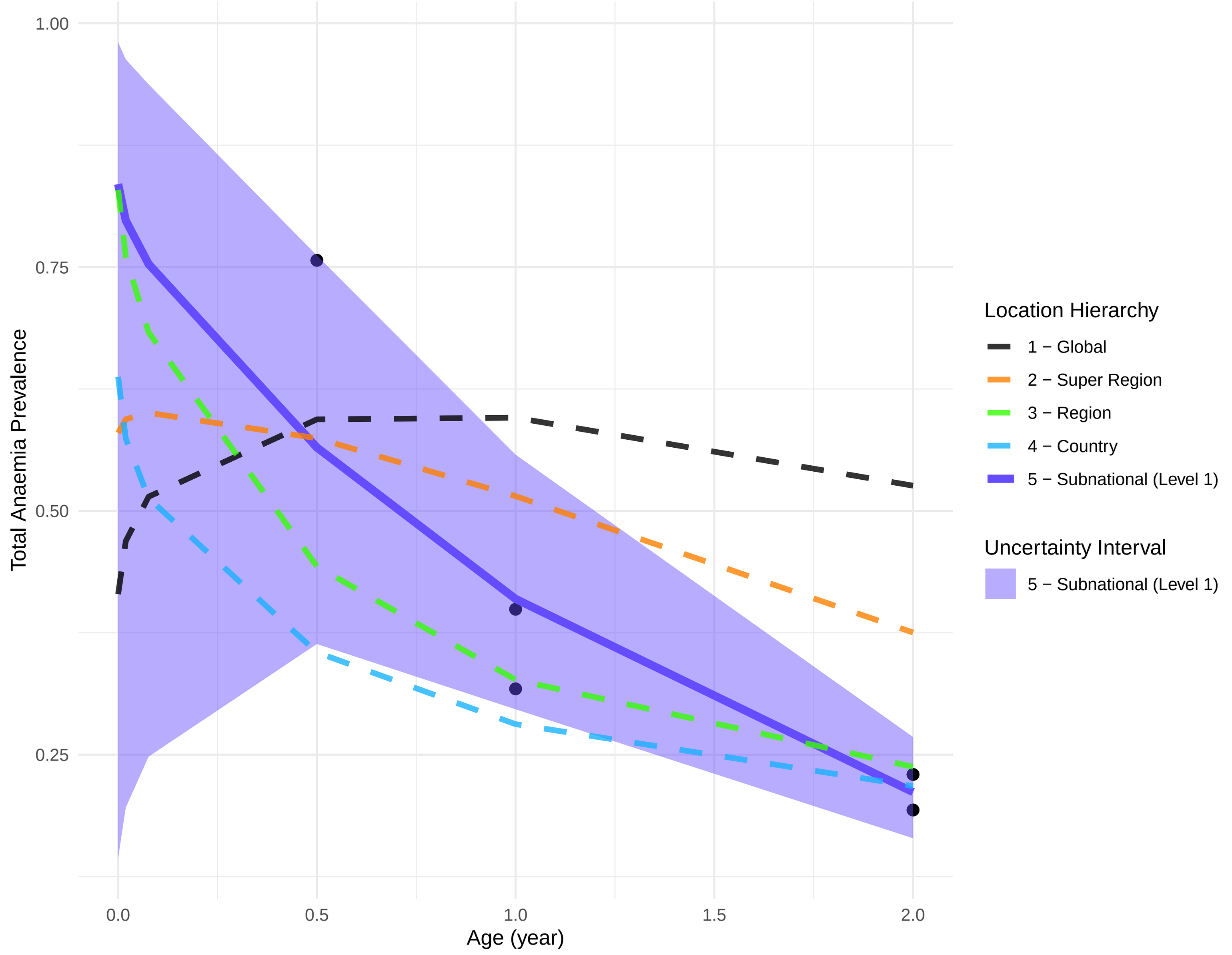

MR−BRT Age/Sex Fits − Males <2 Years Old − Total Anaemia Prevalence − Jalisco
WHO−2001 Adjustment Method

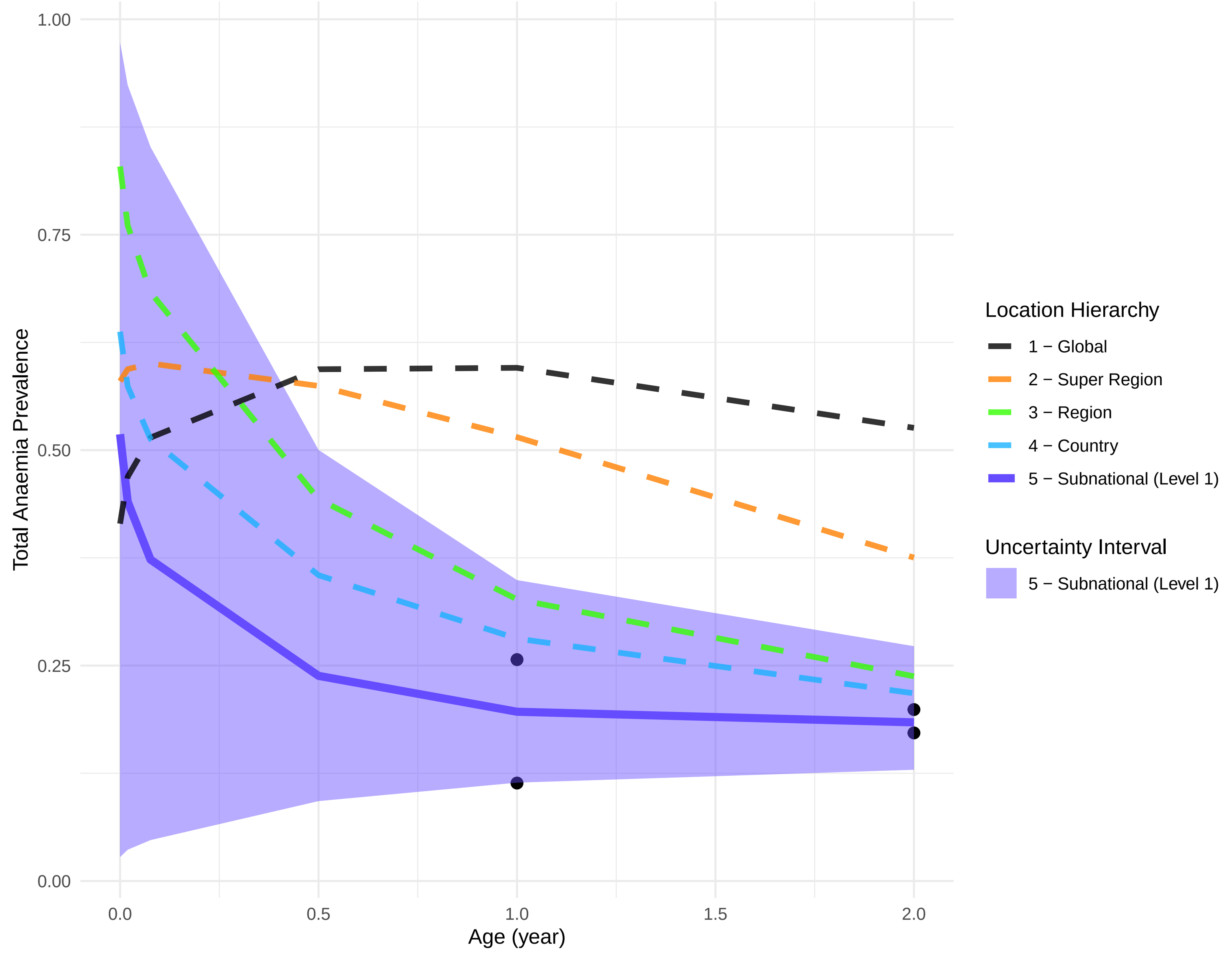

MR−BRT Age/Sex Fits − Males <2 Years Old − Total Anaemia Prevalence − México
WHO−2001 Adjustment Method

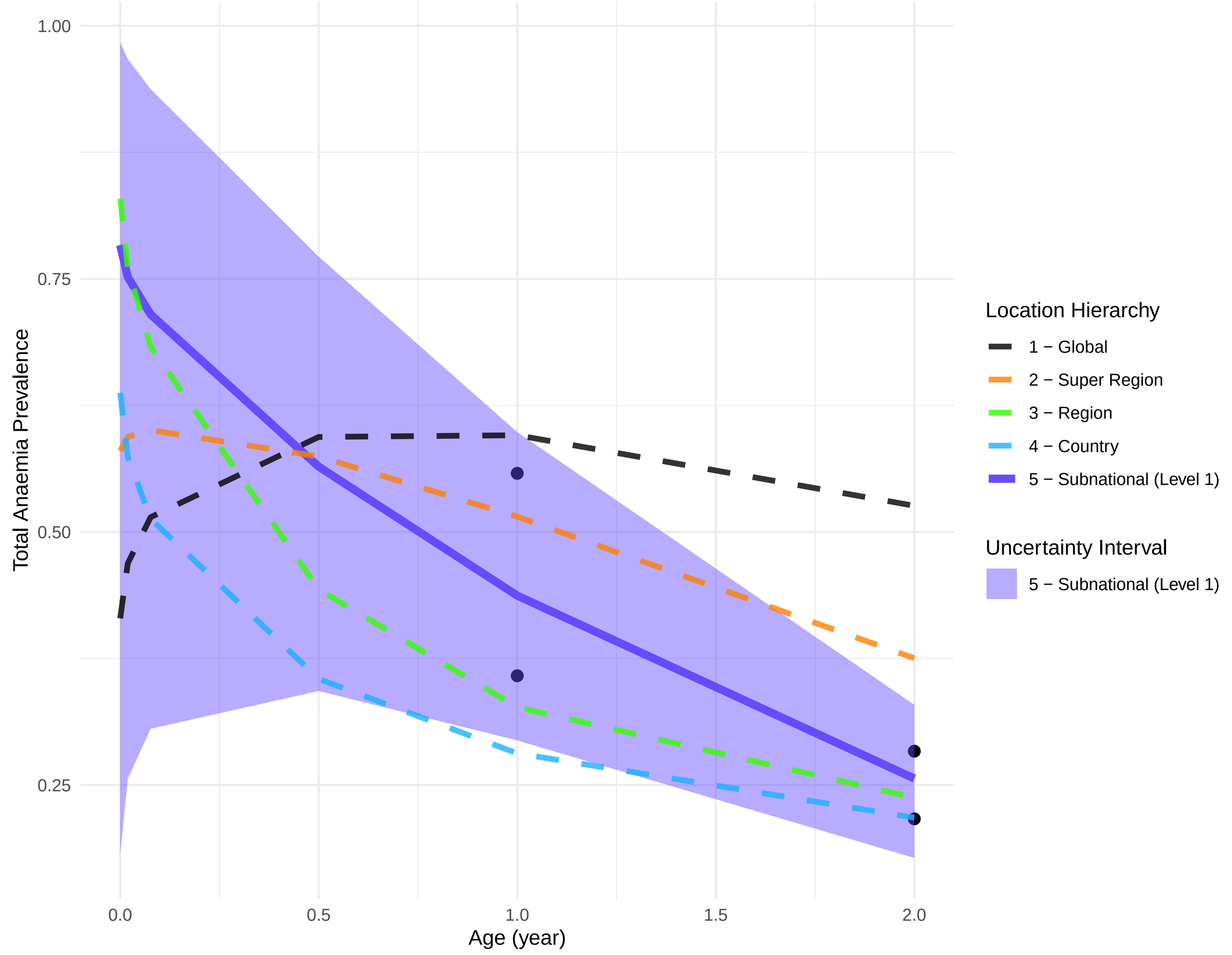

MR−BRT Age/Sex Fits − Males <2 Years Old − Total Anaemia Prevalence − Mexico City
WHO−2001 Adjustment Method

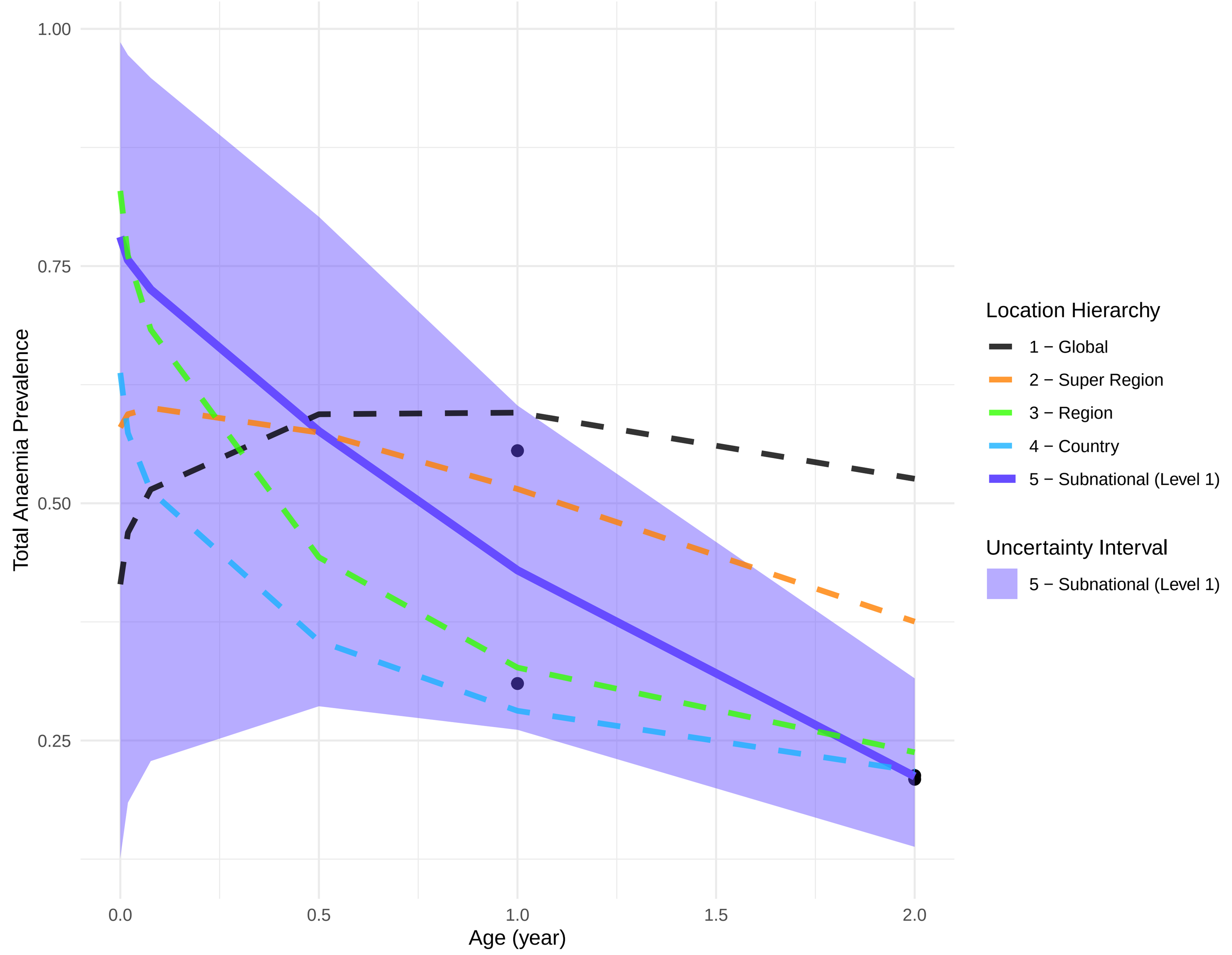

MR−BRT Age/Sex Fits − Males <2 Years Old − Total Anaemia Prevalence − Michoacán de Ocampo
WHO−2001 Adjustment Method

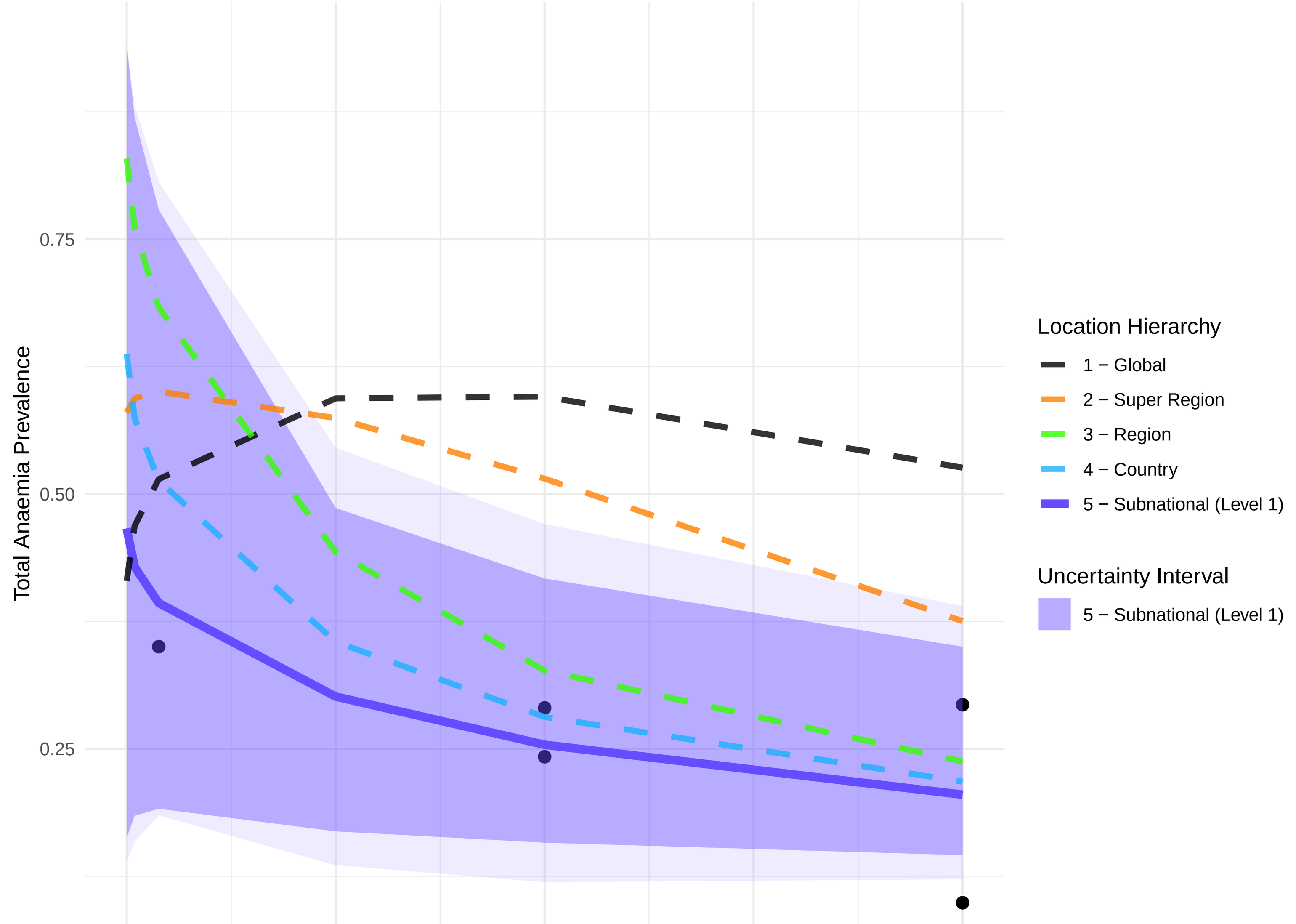

MR−BRT Age/Sex Fits − Males <2 Years Old − Total Anaemia Prevalence − Morelos
WHO−2001 Adjustment Method

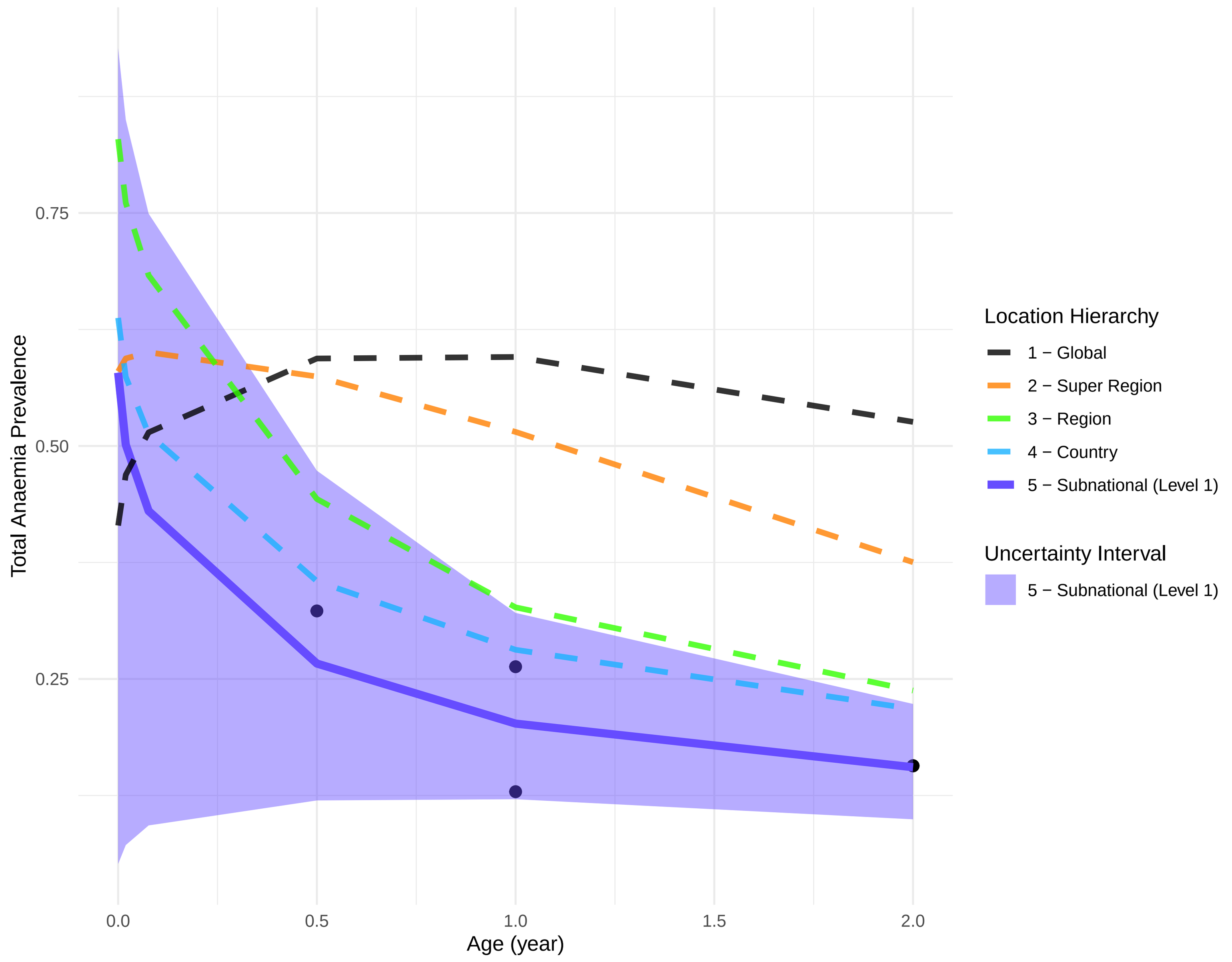

MR−BRT Age/Sex Fits − Males <2 Years Old − Total Anaemia Prevalence − Nayarit
WHO−2001 Adjustment Method

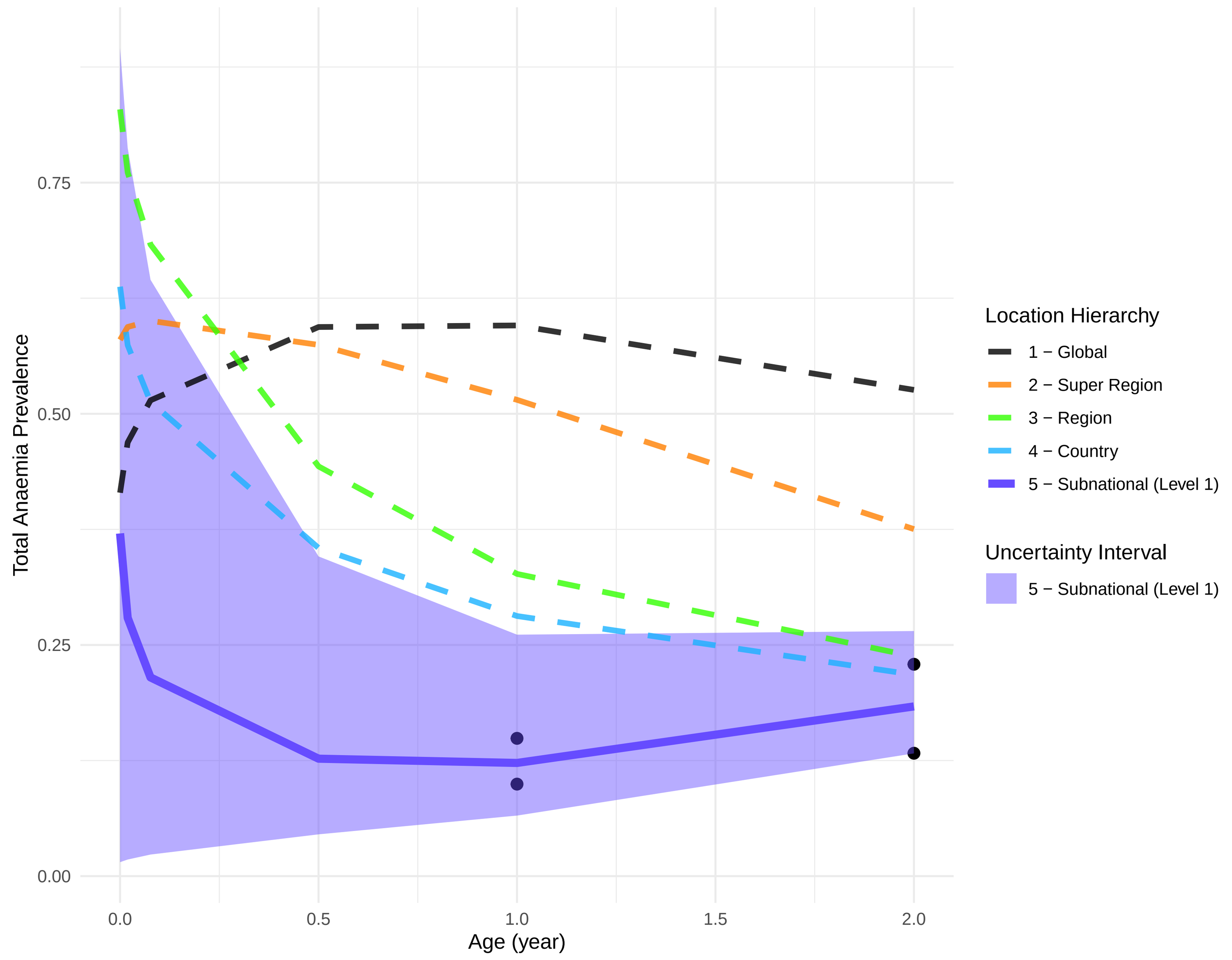

MR−BRT Age/Sex Fits − Males <2 Years Old − Total Anaemia Prevalence − Nuevo León
WHO−2001 Adjustment Method

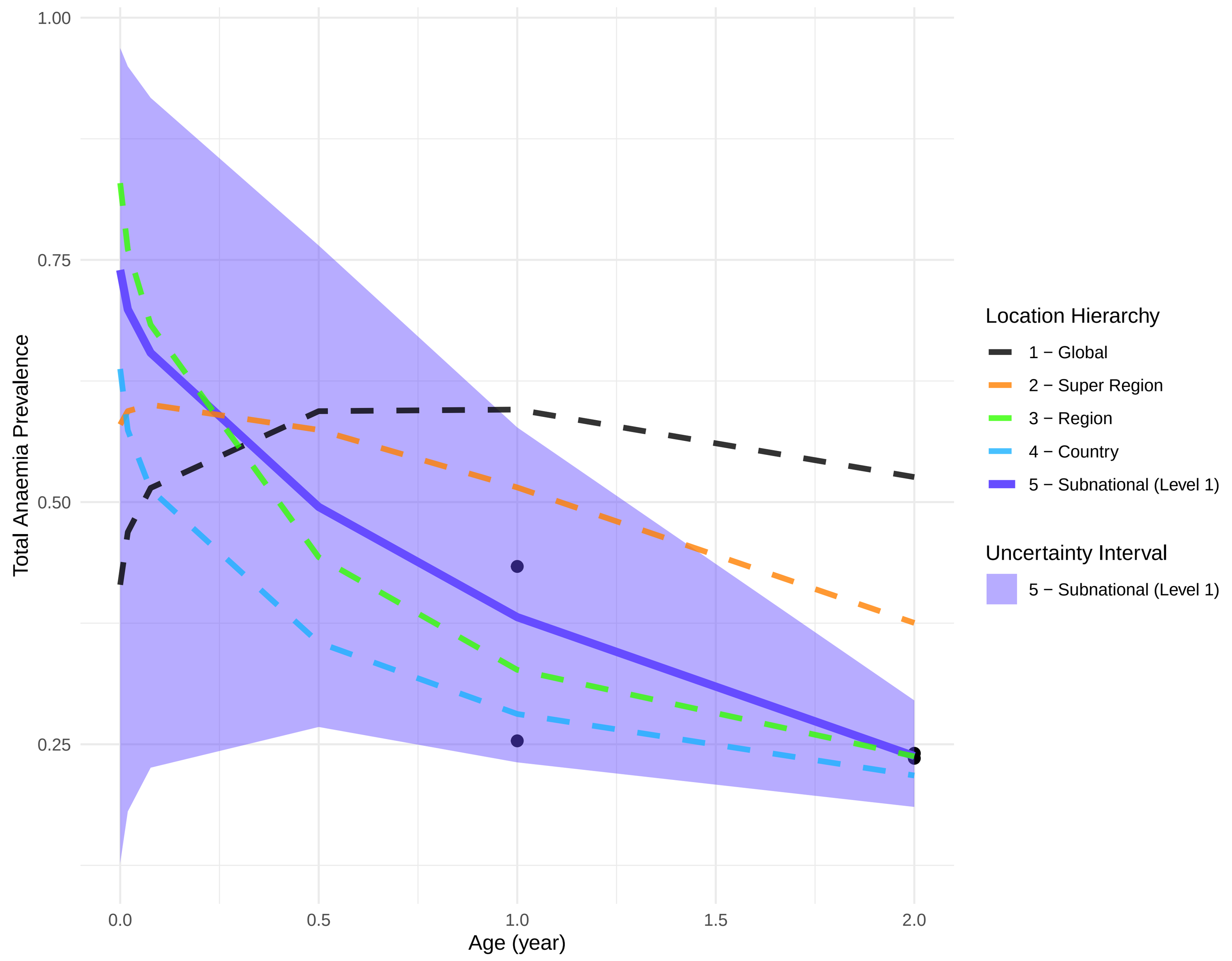

MR−BRT Age/Sex Fits − Males <2 Years Old − Total Anaemia Prevalence − Oaxaca
WHO−2001 Adjustment Method

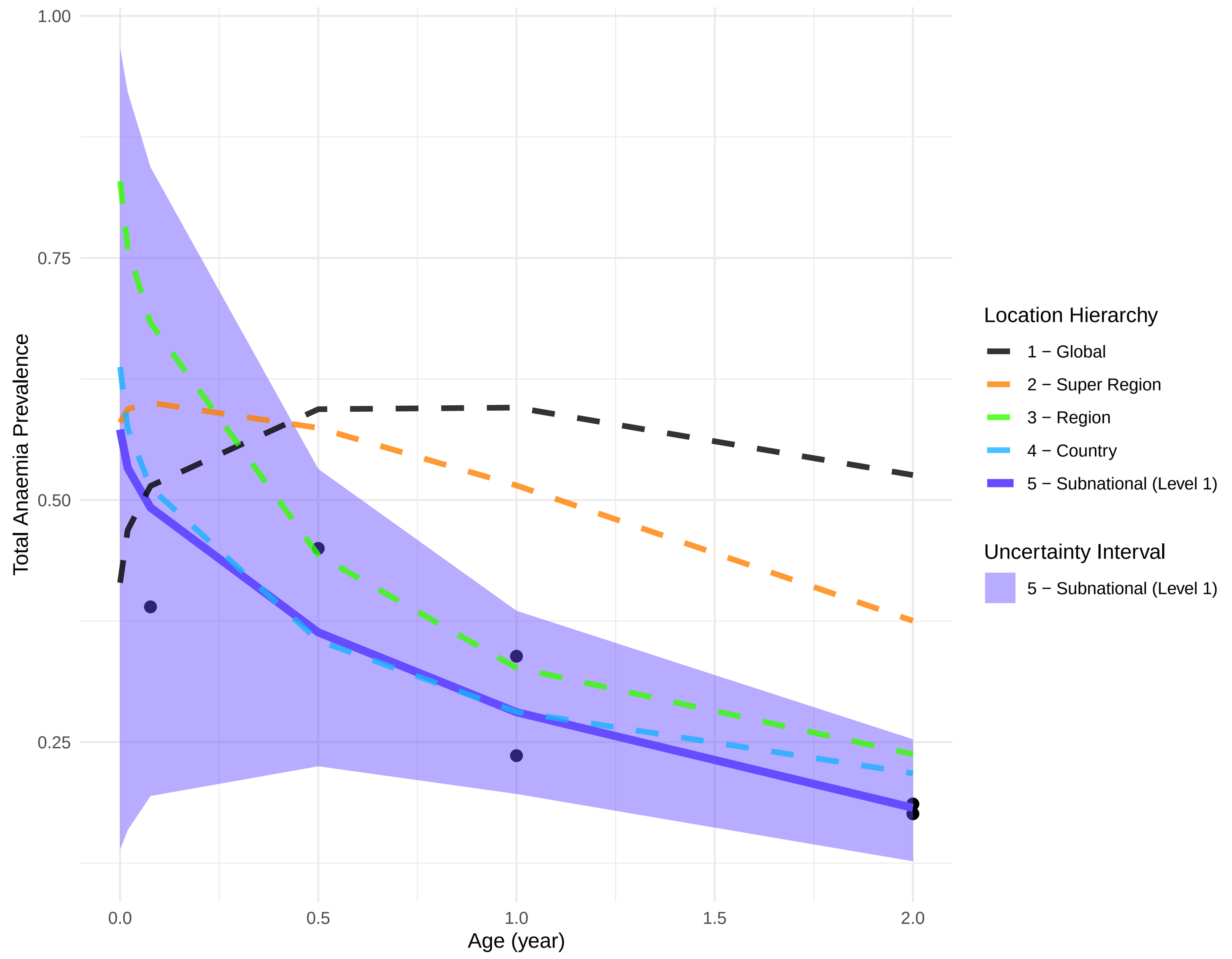

MR−BRT Age/Sex Fits − Males <2 Years Old − Total Anaemia Prevalence − Puebla
WHO−2001 Adjustment Method

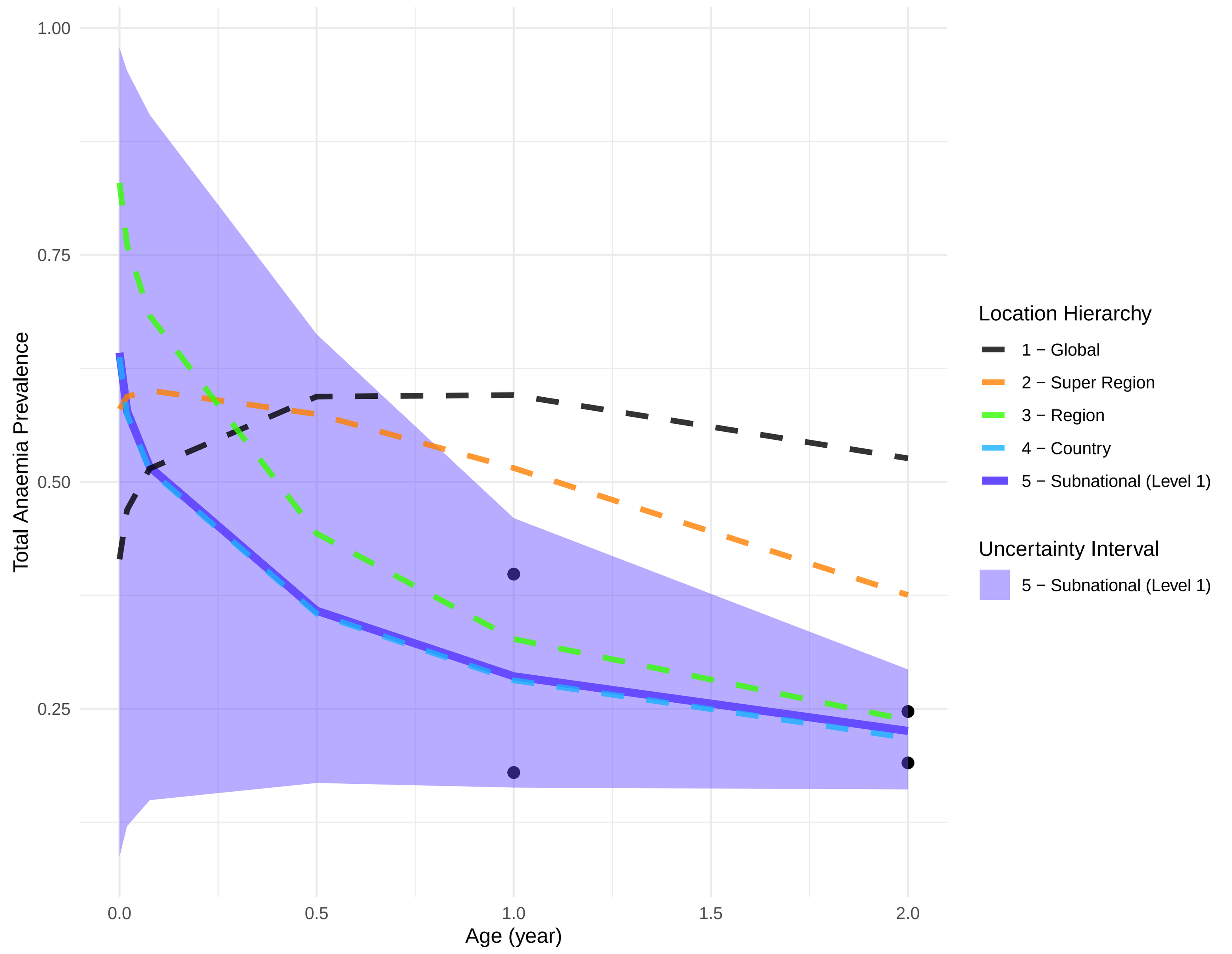

MR−BRT Age/Sex Fits − Males <2 Years Old − Total Anaemia Prevalence − Querétaro
WHO−2001 Adjustment Method

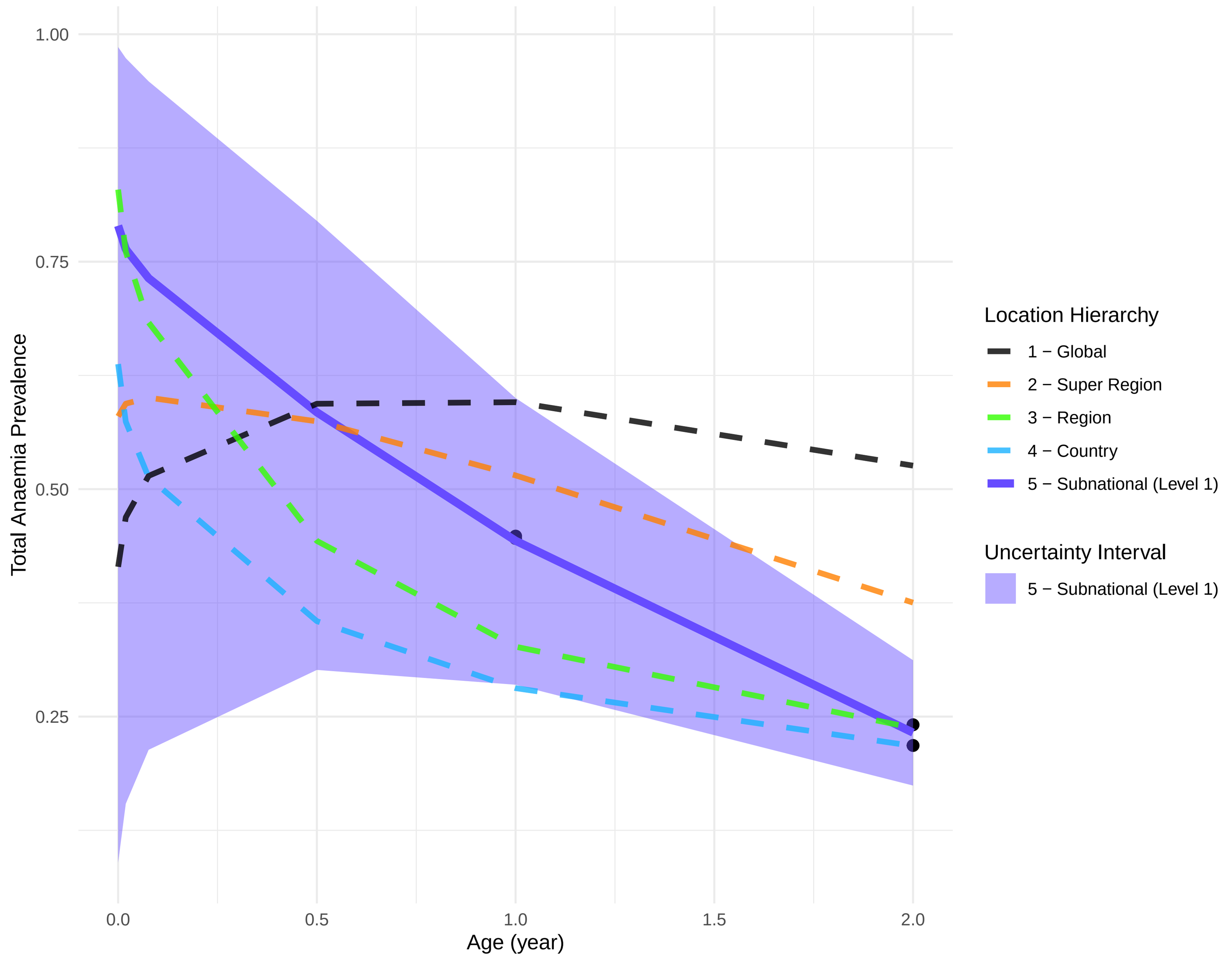

MR−BRT Age/Sex Fits − Males <2 Years Old − Total Anaemia Prevalence − Quintana Roo
WHO−2001 Adjustment Method

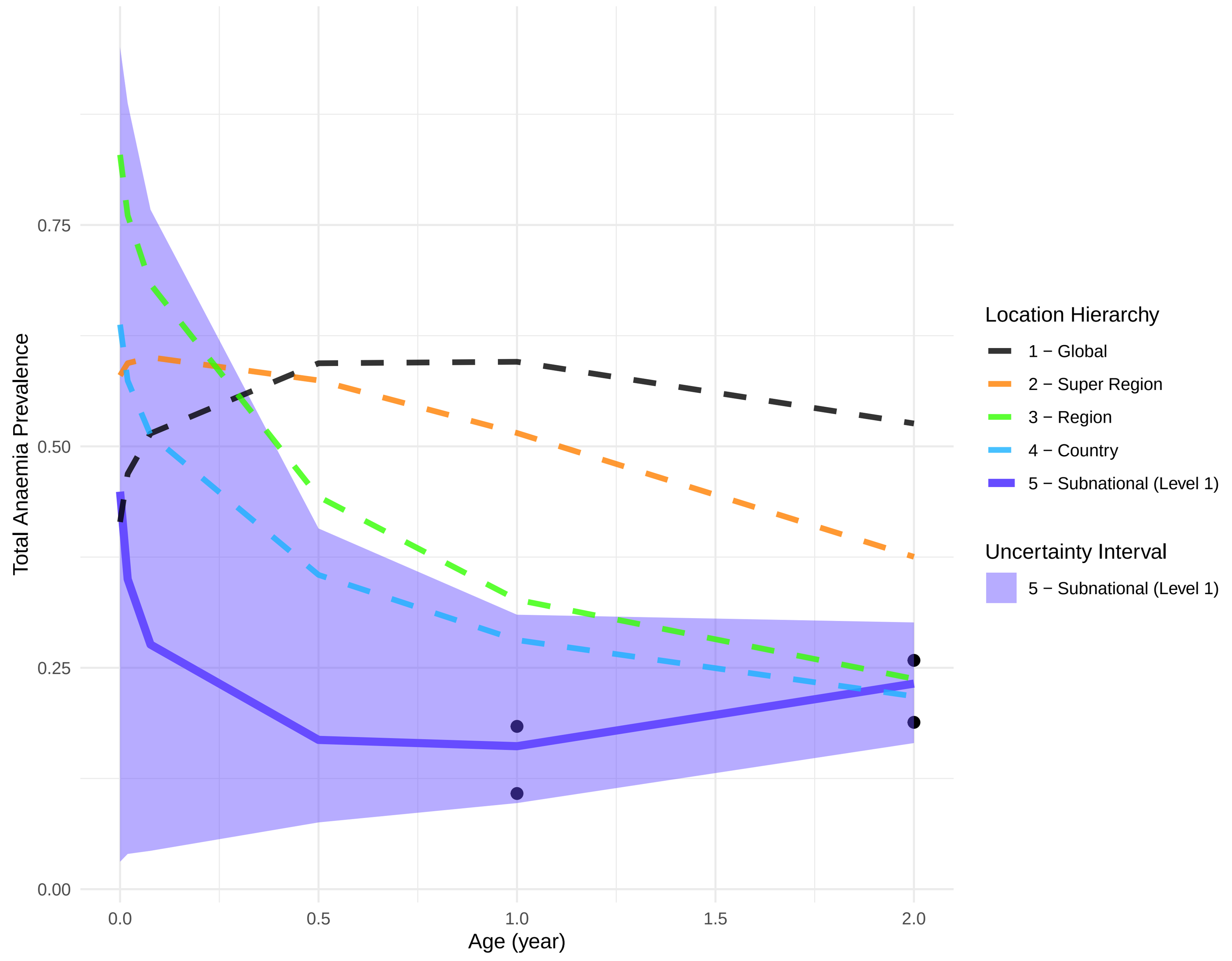

MR−BRT Age/Sex Fits − Males <2 Years Old − Total Anaemia Prevalence − San Luis Potosí
WHO−2001 Adjustment Method

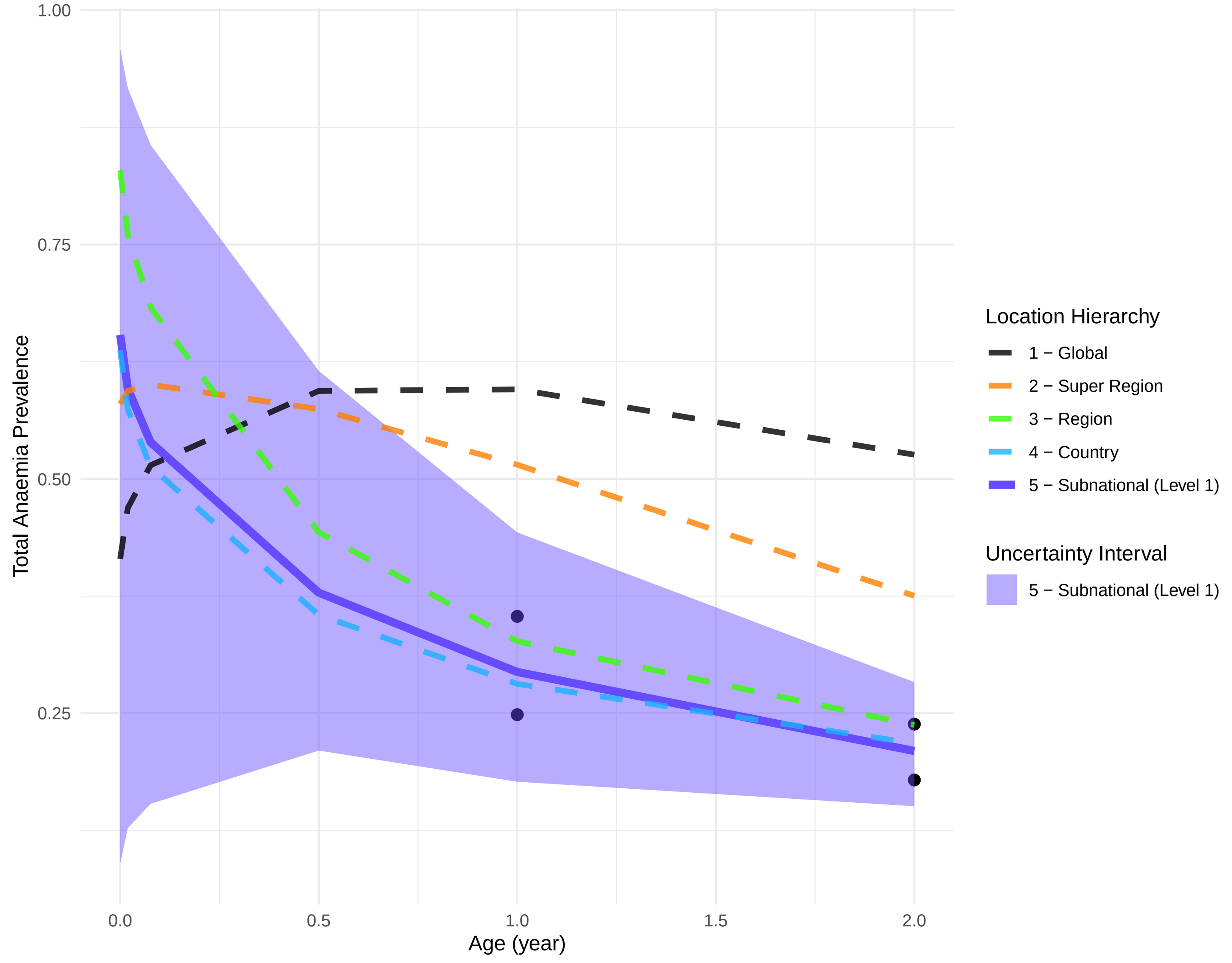

MR−BRT Age/Sex Fits − Males <2 Years Old − Total Anaemia Prevalence − Sinaloa
WHO−2001 Adjustment Method

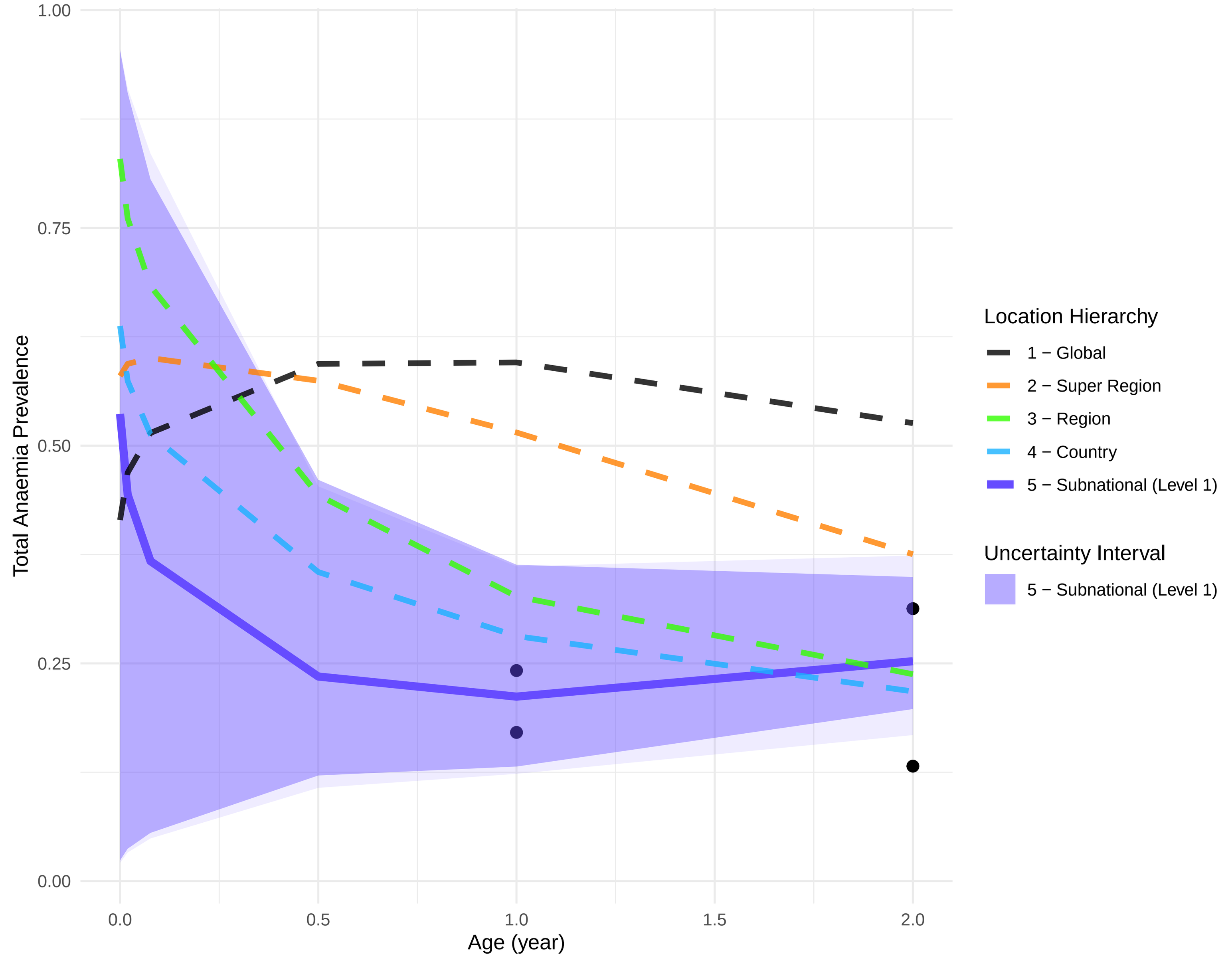

MR−BRT Age/Sex Fits − Males <2 Years Old − Total Anaemia Prevalence − Sonora
WHO−2001 Adjustment Method

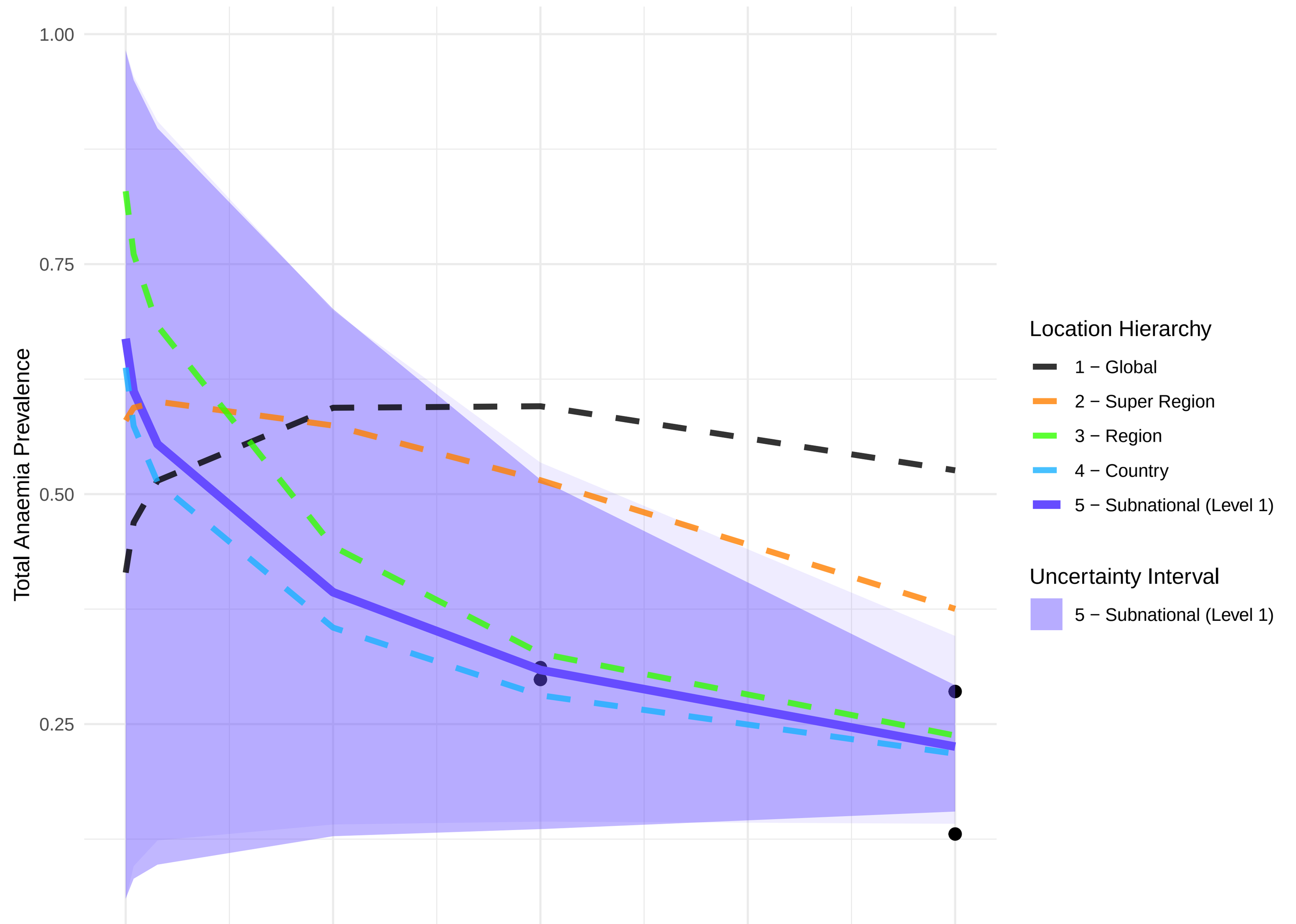

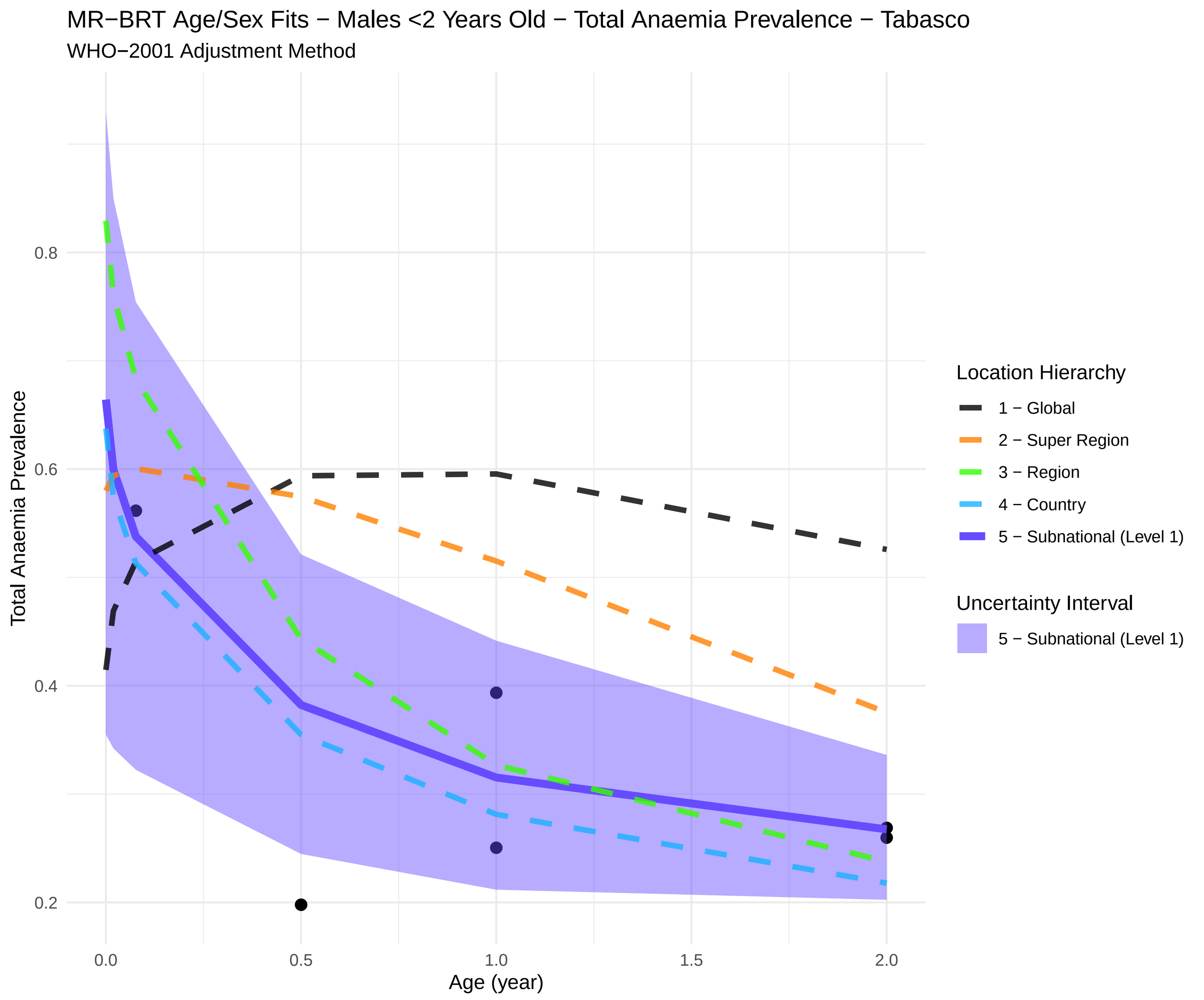
MR−BRT Age/Sex Fits − Males <2 Years Old − Total Anaemia Prevalence − Tabasco
WHO−2001 Adjustment Method
Total Anaemia Prevalence
Age (year)
Location Hierarchy
1 − Global
2 − Super Region
3 − Region
4 − Country
5 − Subnational (Level 1)
Uncertainty Interval
5 − Subnational (Level 1)

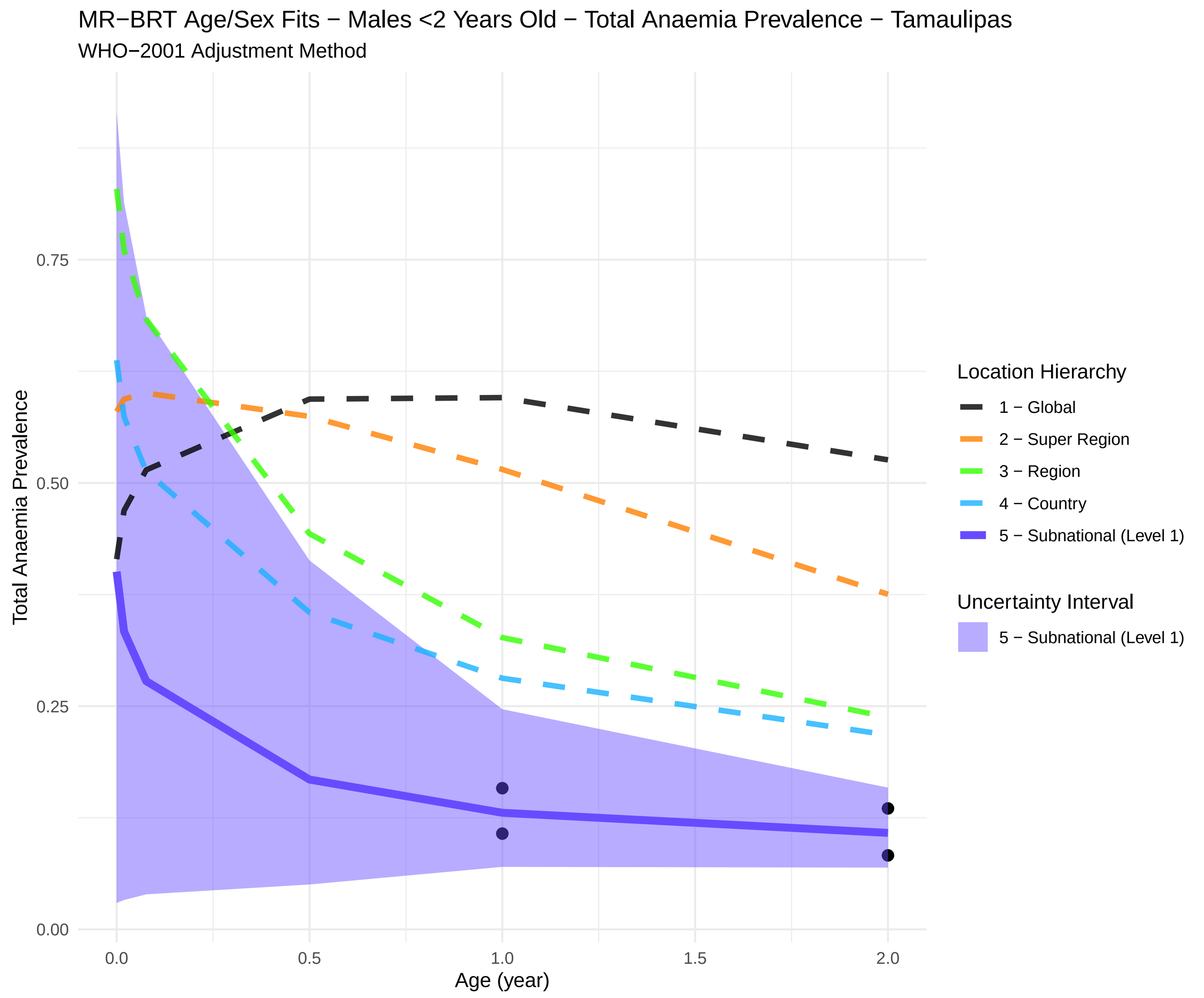
MR−BRT Age/Sex Fits − Males <2 Years Old − Total Anaemia Prevalence − Tamaulipas
WHO−2001 Adjustment Method
Total Anaemia Prevalence
Age (year)
Location Hierarchy
1 − Global
2 − Super Region
3 − Region
4 − Country
5 − Subnational (Level 1)
Uncertainty Interval
5 − Subnational (Level 1)

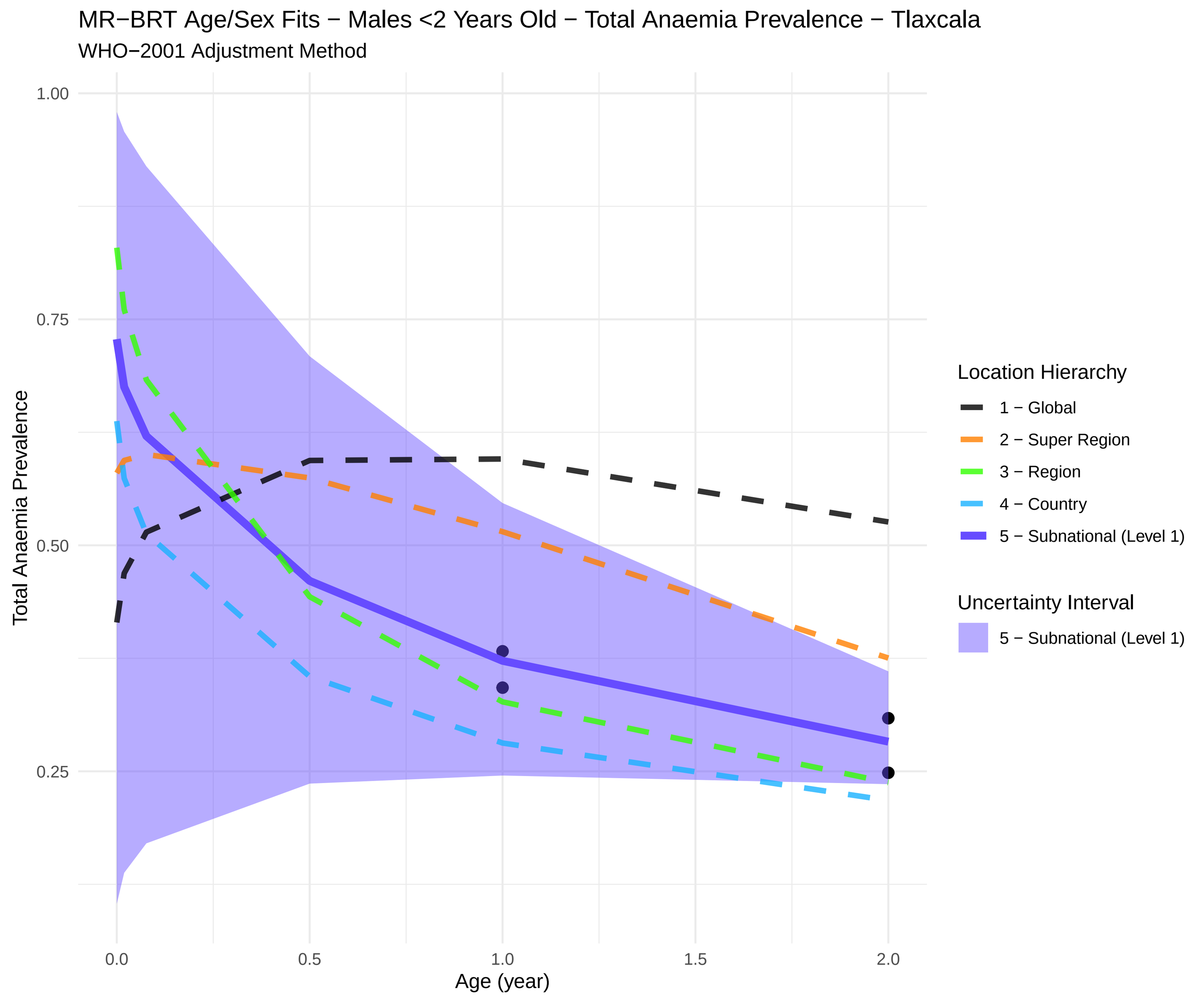
MR−BRT Age/Sex Fits − Males <2 Years Old − Total Anaemia Prevalence − Tlaxcala
WHO−2001 Adjustment Method
Total Anaemia Prevalence
Age (year)
Location Hierarchy
1 − Global
2 − Super Region
3 − Region
4 − Country
5 − Subnational (Level 1)
Uncertainty Interval
5 − Subnational (Level 1)

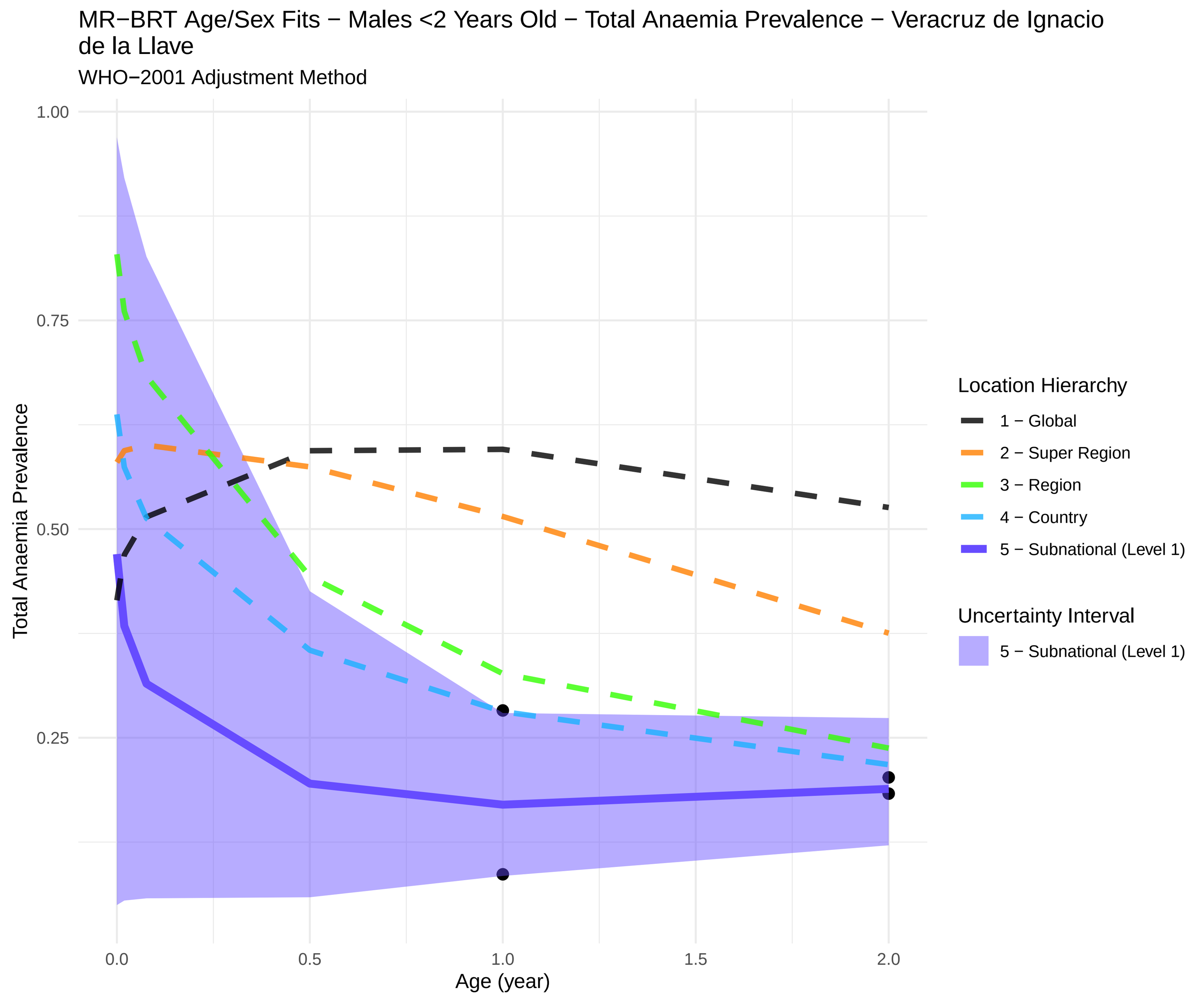
MR−BRT Age/Sex Fits − Males <2 Years Old − Total Anaemia Prevalence − Veracruz de Ignacio de la Llave
WHO−2001 Adjustment Method
Total Anaemia Prevalence
Age (year)
Location Hierarchy
1 − Global
2 − Super Region
3 − Region
4 − Country
5 − Subnational (Level 1)
Uncertainty Interval
5 − Subnational (Level 1)

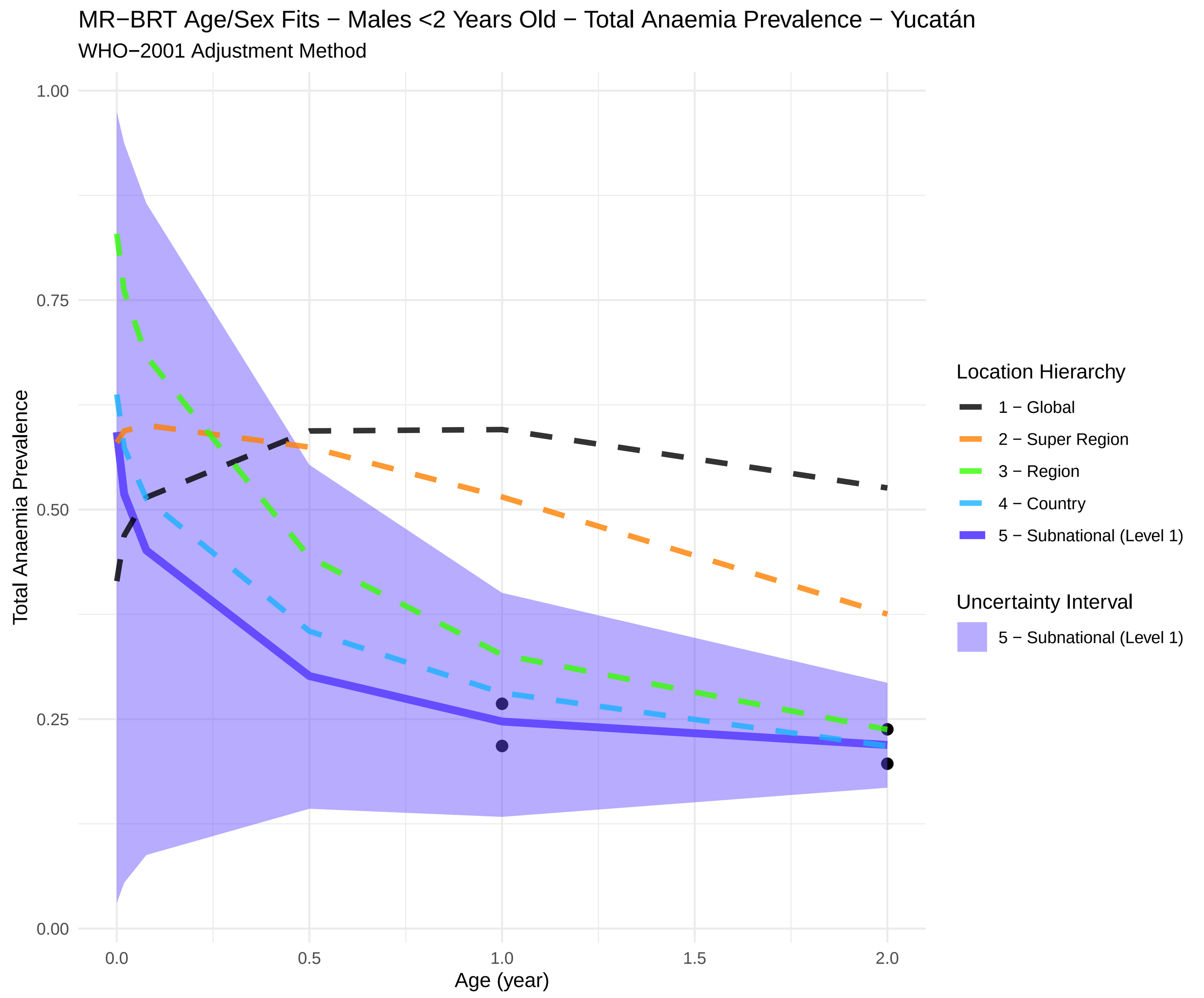
MR−BRT Age/Sex Fits − Males <2 Years Old − Total Anaemia Prevalence − Yucatán
WHO−2001 Adjustment Method
Total Anaemia Prevalence
Age (year)
Location Hierarchy
1 − Global
2 − Super Region
3 − Region
4 − Country
5 − Subnational (Level 1)
Uncertainty Interval
5 − Subnational (Level 1)

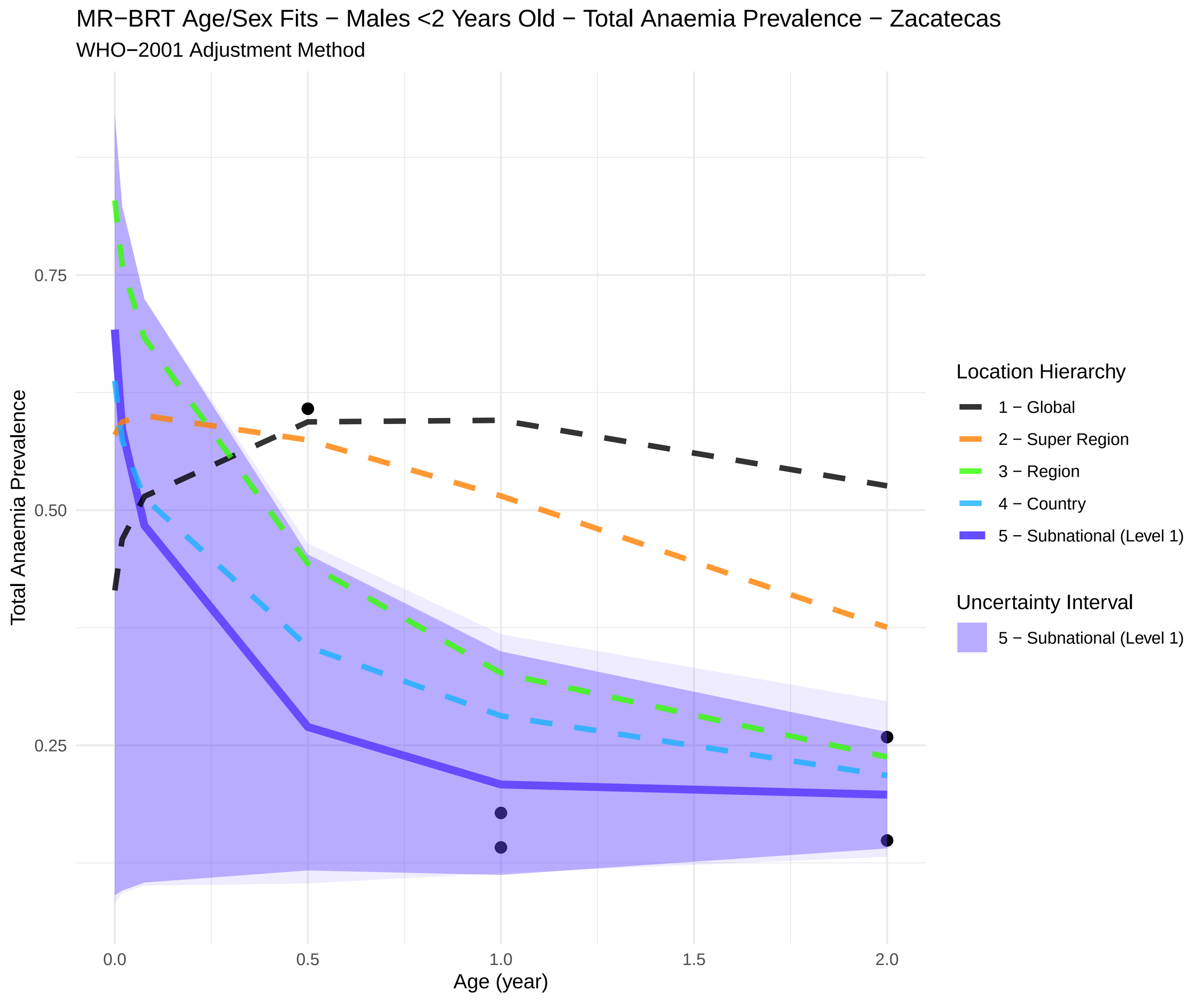
MR−BRT Age/Sex Fits − Males <2 Years Old − Total Anaemia Prevalence − Zacatecas
WHO−2001 Adjustment Method
Total Anaemia Prevalence
Age (year)
Location Hierarchy
1 − Global
2 − Super Region
3 − Region
4 − Country
5 − Subnational (Level 1)
Uncertainty Interval
5 − Subnational (Level 1)

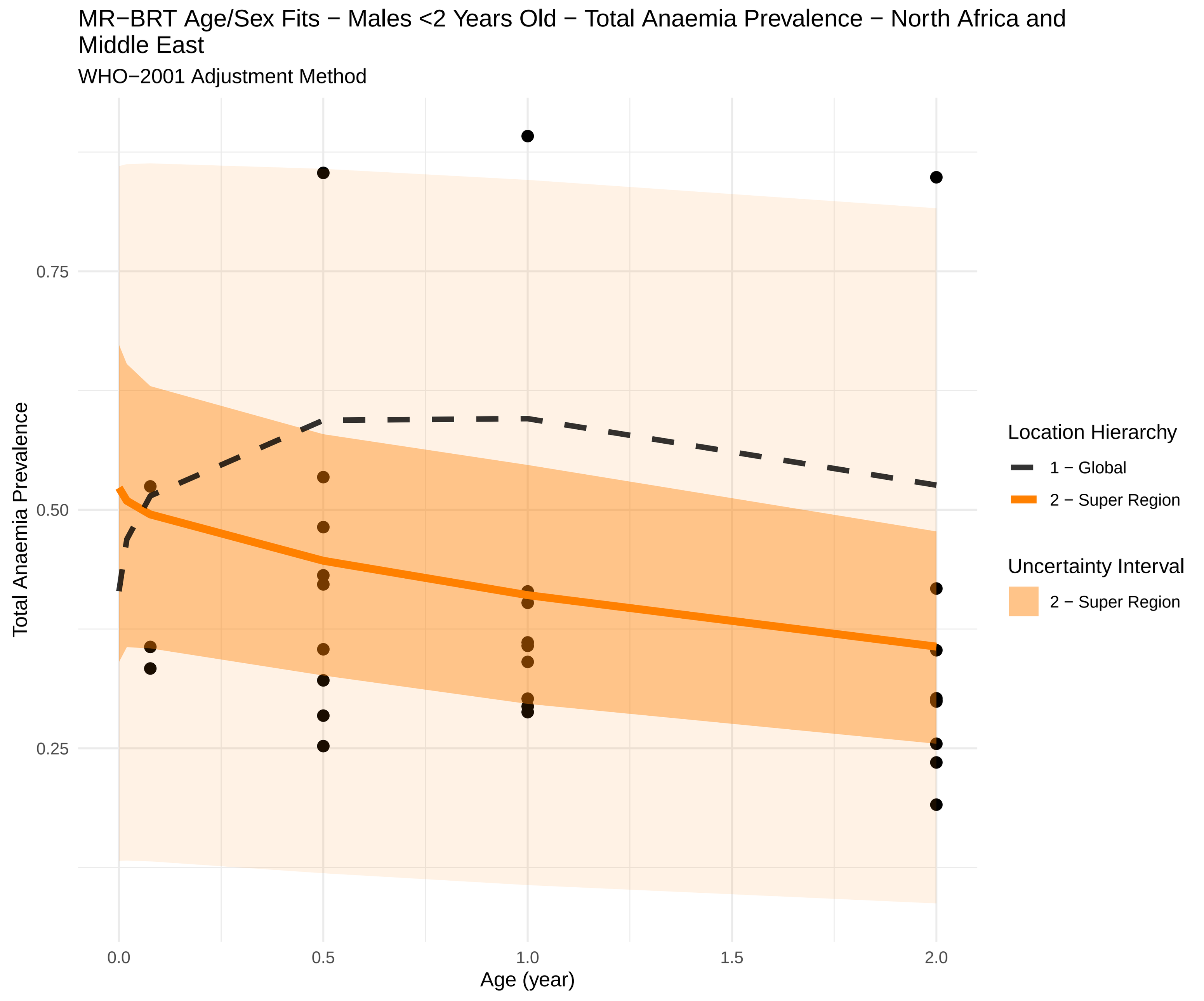
MR−BRT Age/Sex Fits − Males <2 Years Old − Total Anaemia Prevalence − North Africa and Middle East
WHO−2001 Adjustment Method
Total Anaemia Prevalence
Age (year)
Location Hierarchy
1 − Global
2 − Super Region
Uncertainty Interval
2 − Super Region

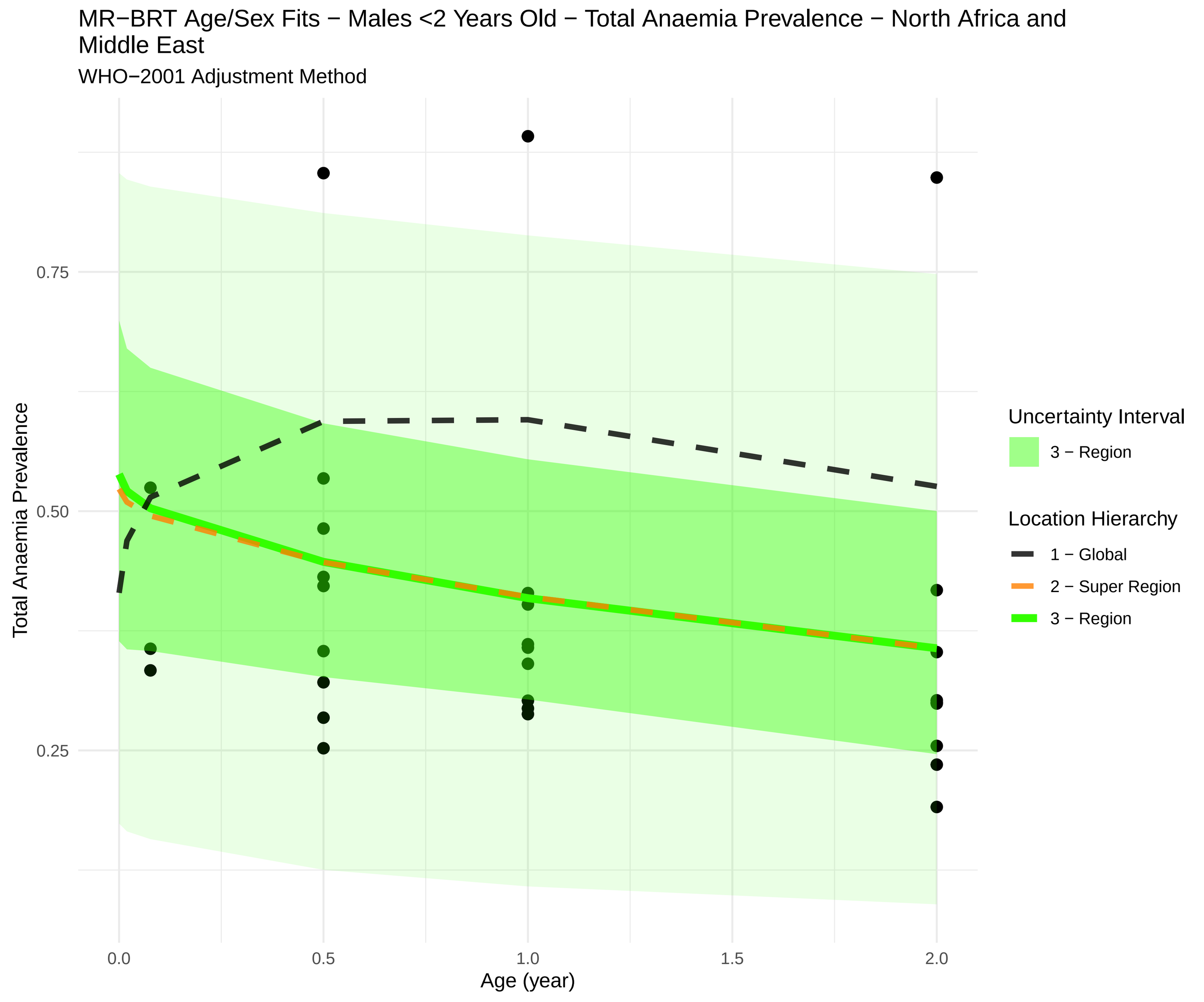
MR−BRT Age/Sex Fits − Males <2 Years Old − Total Anaemia Prevalence − North Africa and Middle East
WHO−2001 Adjustment Method
Total Anaemia Prevalence
Age (year)
Uncertainty Interval
3 − Region
Location Hierarchy
1 − Global
2 − Super Region
3 − Region

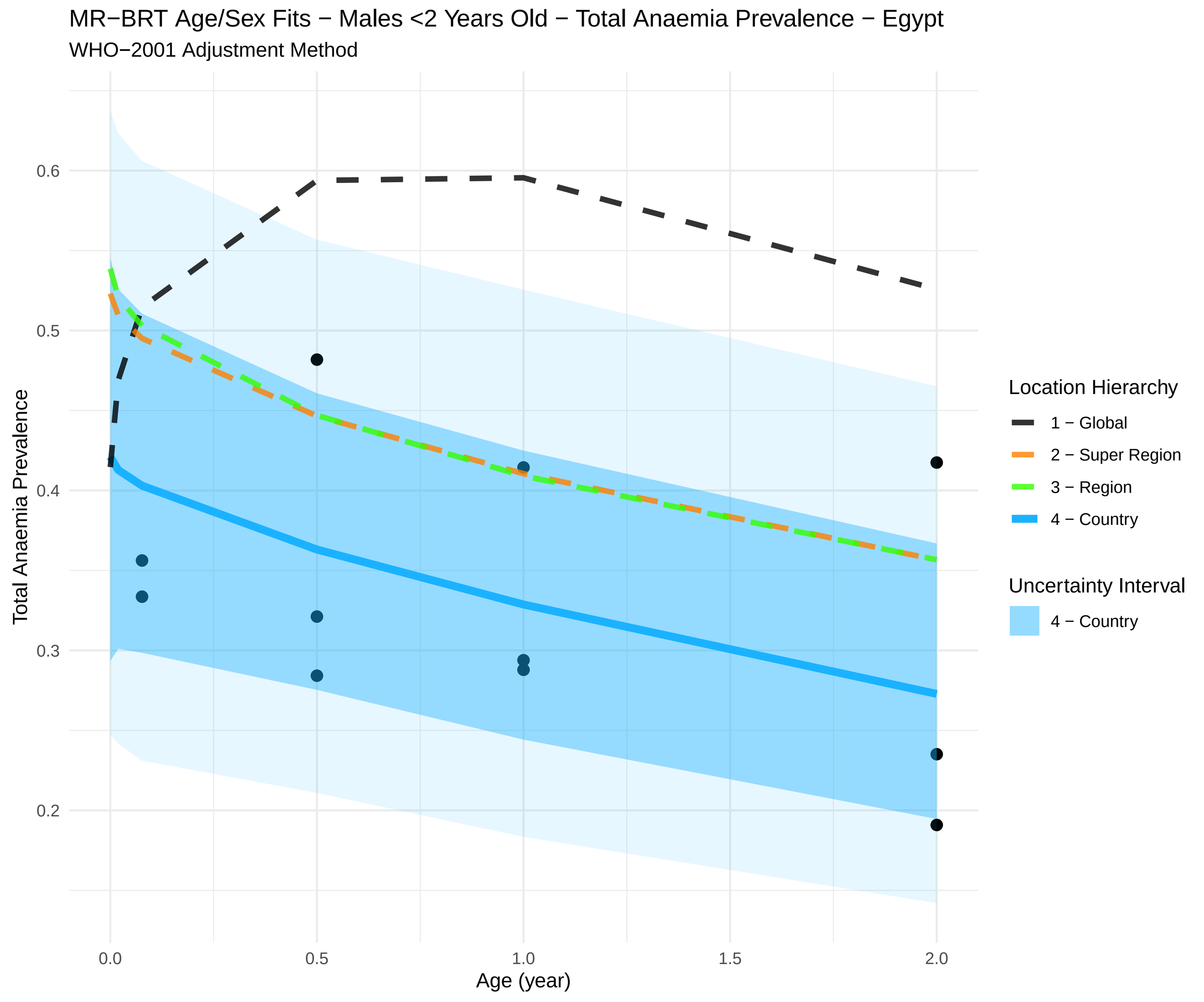
MR−BRT Age/Sex Fits − Males <2 Years Old − Total Anaemia Prevalence − Egypt
WHO−2001 Adjustment Method
Total Anaemia Prevalence
Age (year)
Location Hierarchy
1 − Global
2 − Super Region
3 − Region
4 − Country
Uncertainty Interval
4 − Country

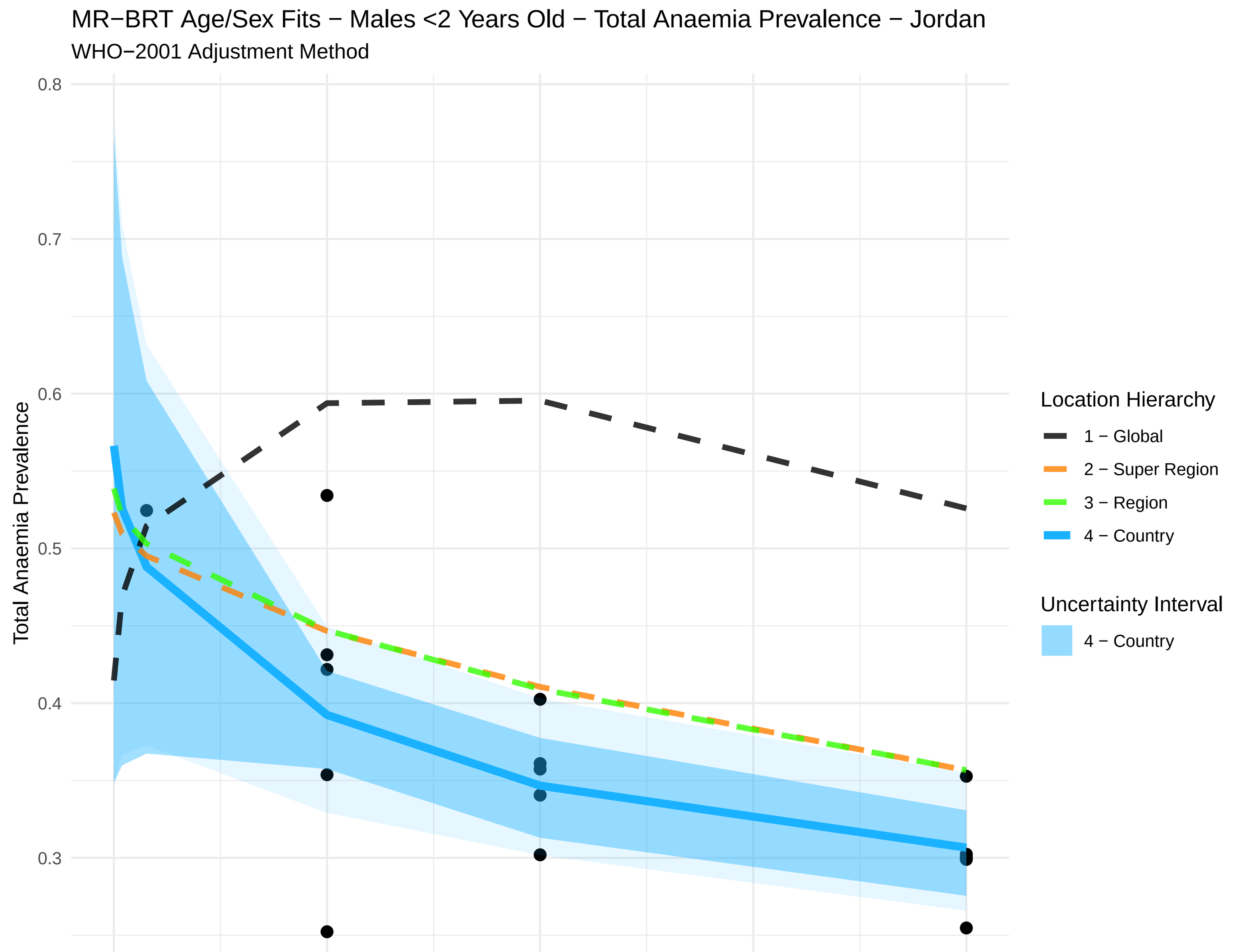
MR−BRT Age/Sex Fits − Males <2 Years Old − Total Anaemia Prevalence − Jordan
WHO−2001 Adjustment Method
Total Anaemia Prevalence
Age (year)
Location Hierarchy
1 − Global
2 − Super Region
3 − Region
4 − Country
Uncertainty Interval
4 − Country

MR−BRT Age/Sex Fits − Males <2 Years Old − Total Anaemia Prevalence − Yemen
WHO−2001 Adjustment Method

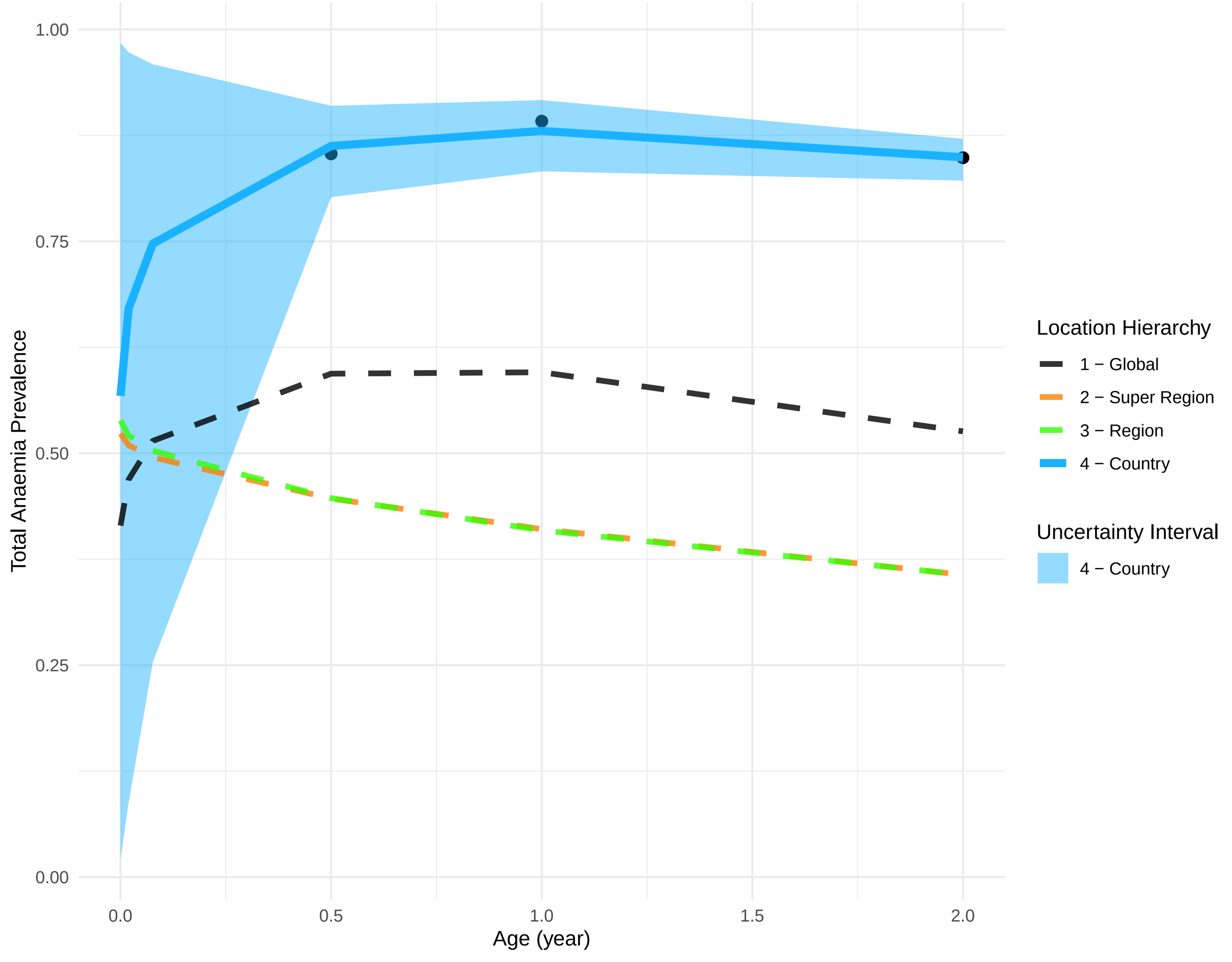

MR−BRT Age/Sex Fits − Males <2 Years Old − Total Anaemia Prevalence − South Asia
WHO−2001 Adjustment Method

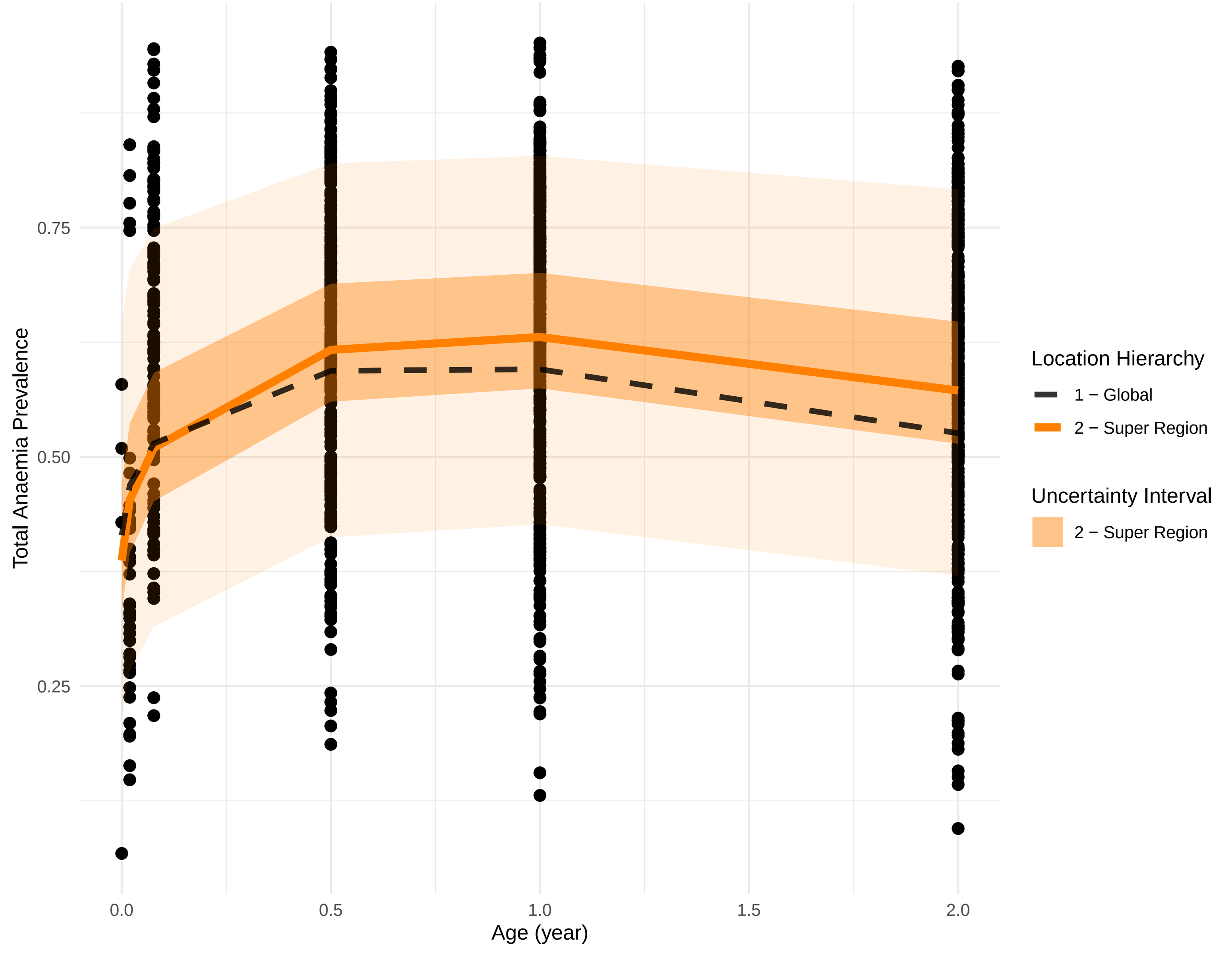

MR−BRT Age/Sex Fits − Males <2 Years Old − Total Anaemia Prevalence − South Asia
WHO−2001 Adjustment Method

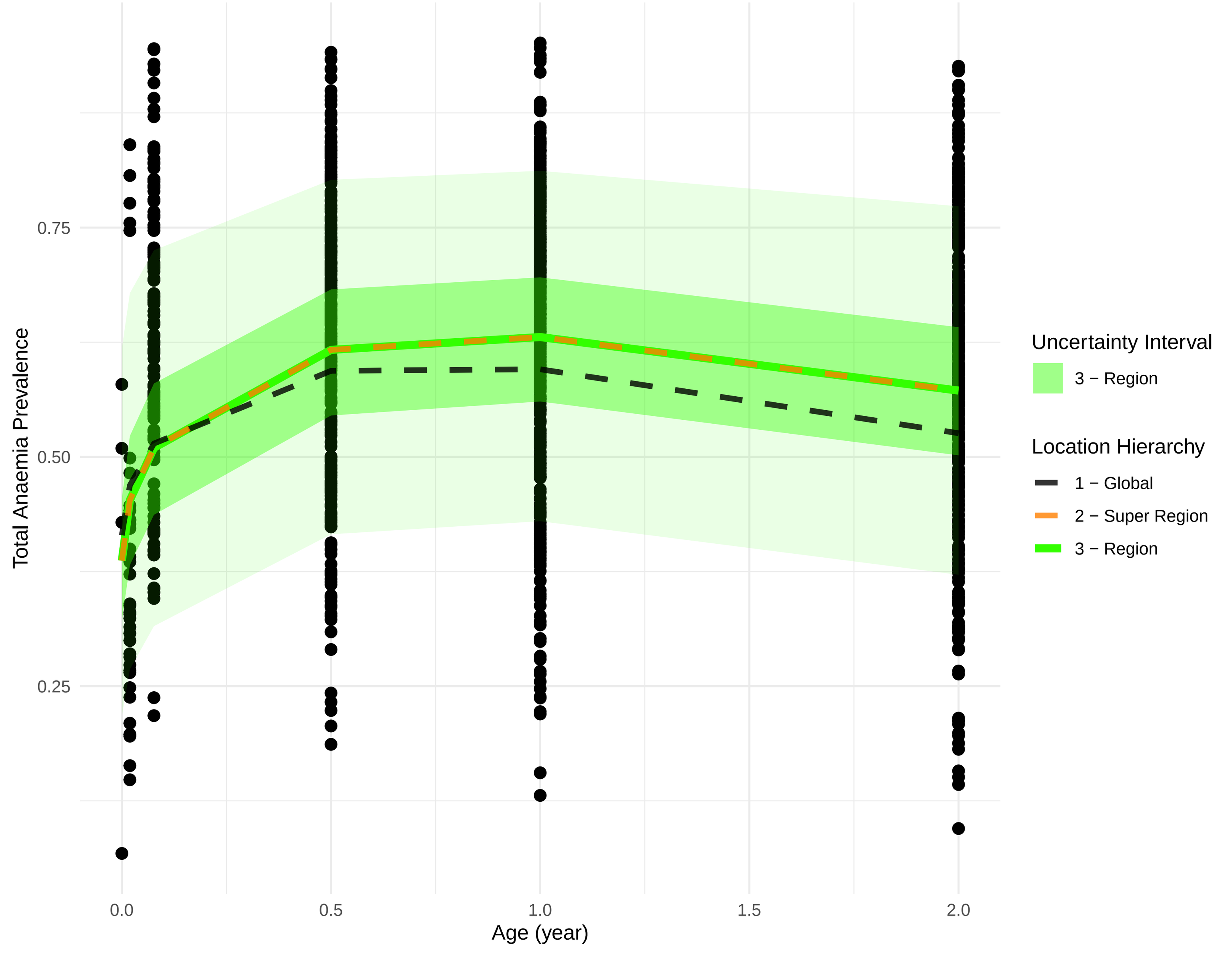

MR−BRT Age/Sex Fits − Males <2 Years Old − Total Anaemia Prevalence − Bangladesh
WHO−2001 Adjustment Method

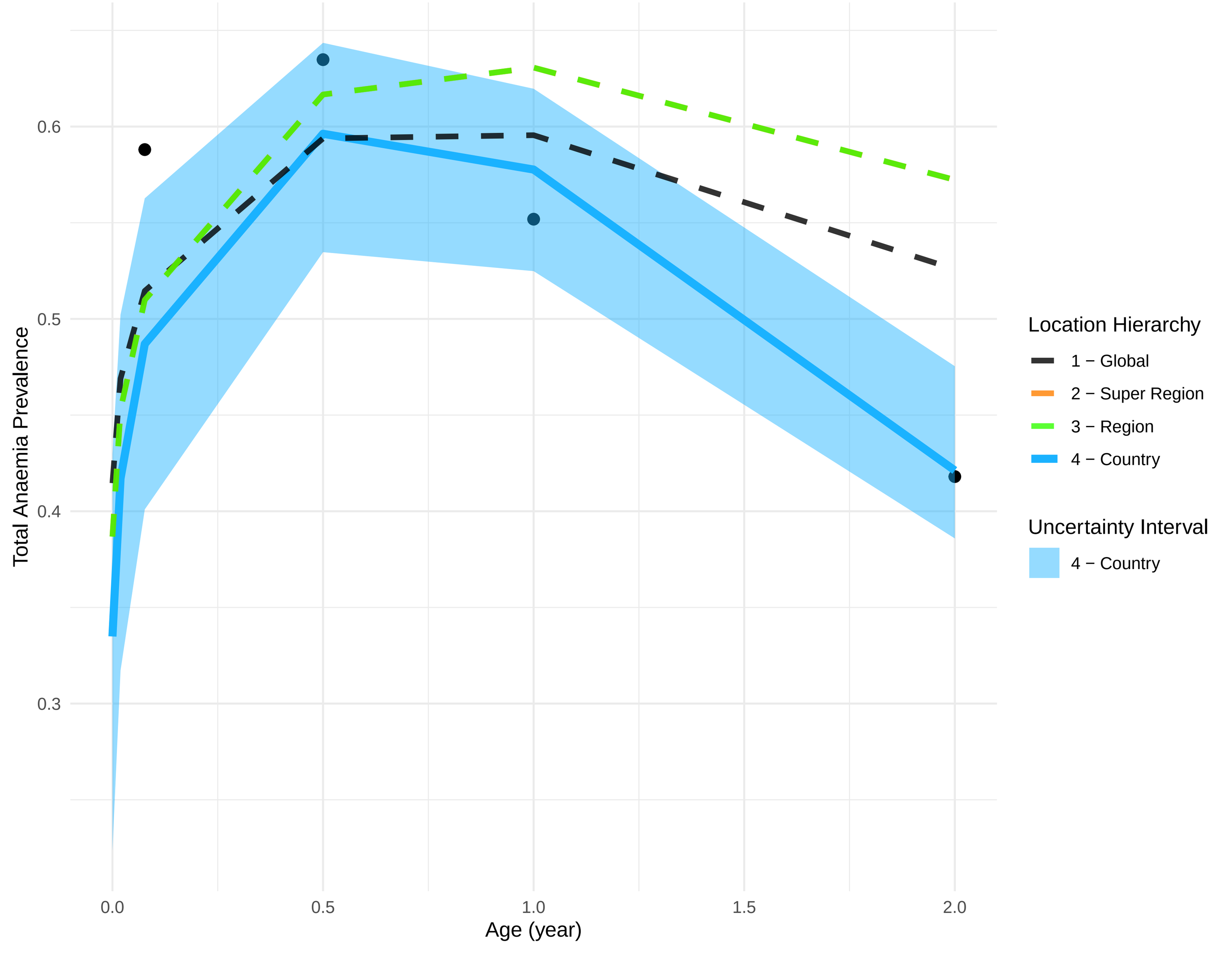

MR−BRT Age/Sex Fits − Males <2 Years Old − Total Anaemia Prevalence − India
WHO−2001 Adjustment Method

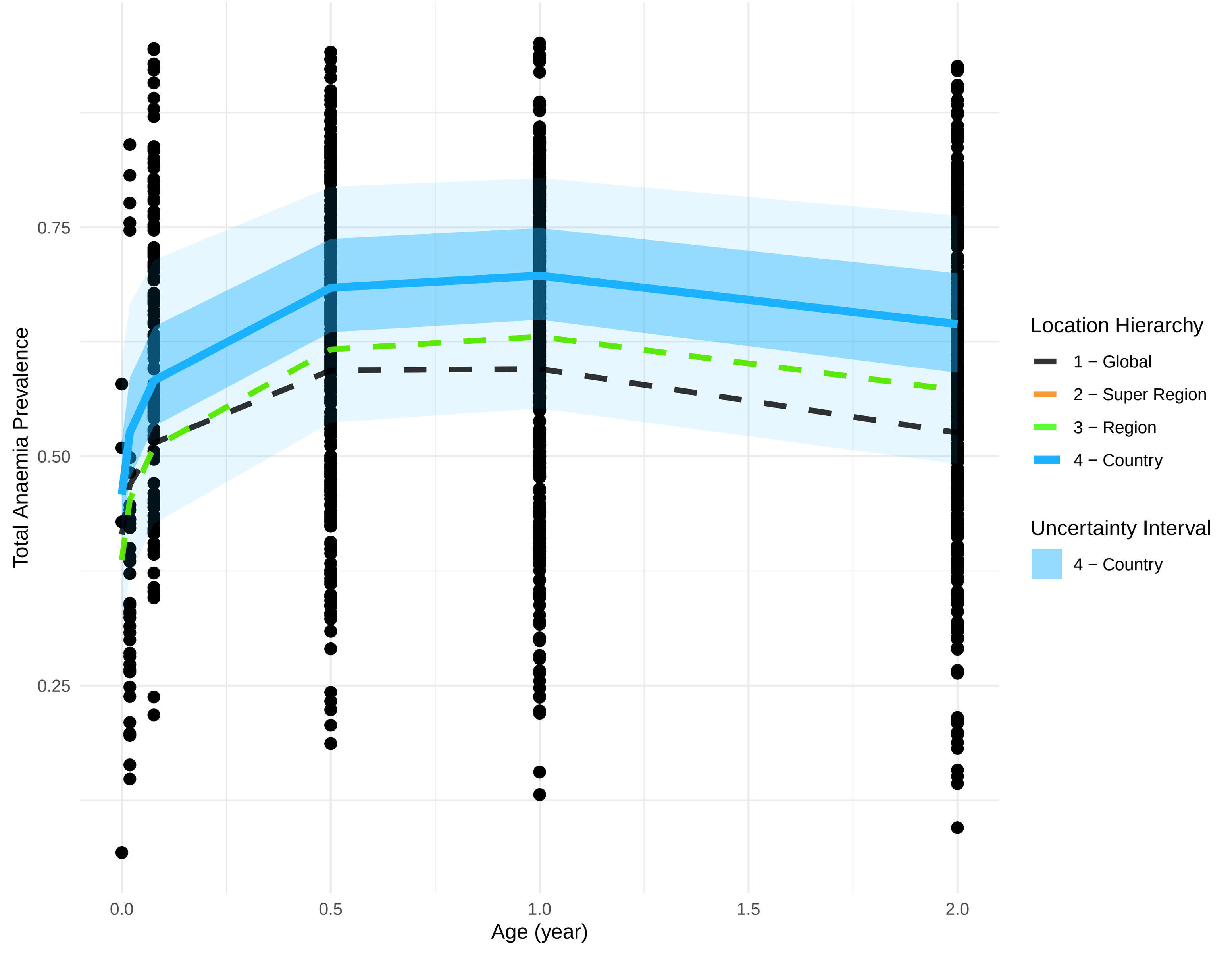

MR−BRT Age/Sex Fits − Males <2 Years Old − Total Anaemia Prevalence − Andhra Pradesh
WHO−2001 Adjustment Method

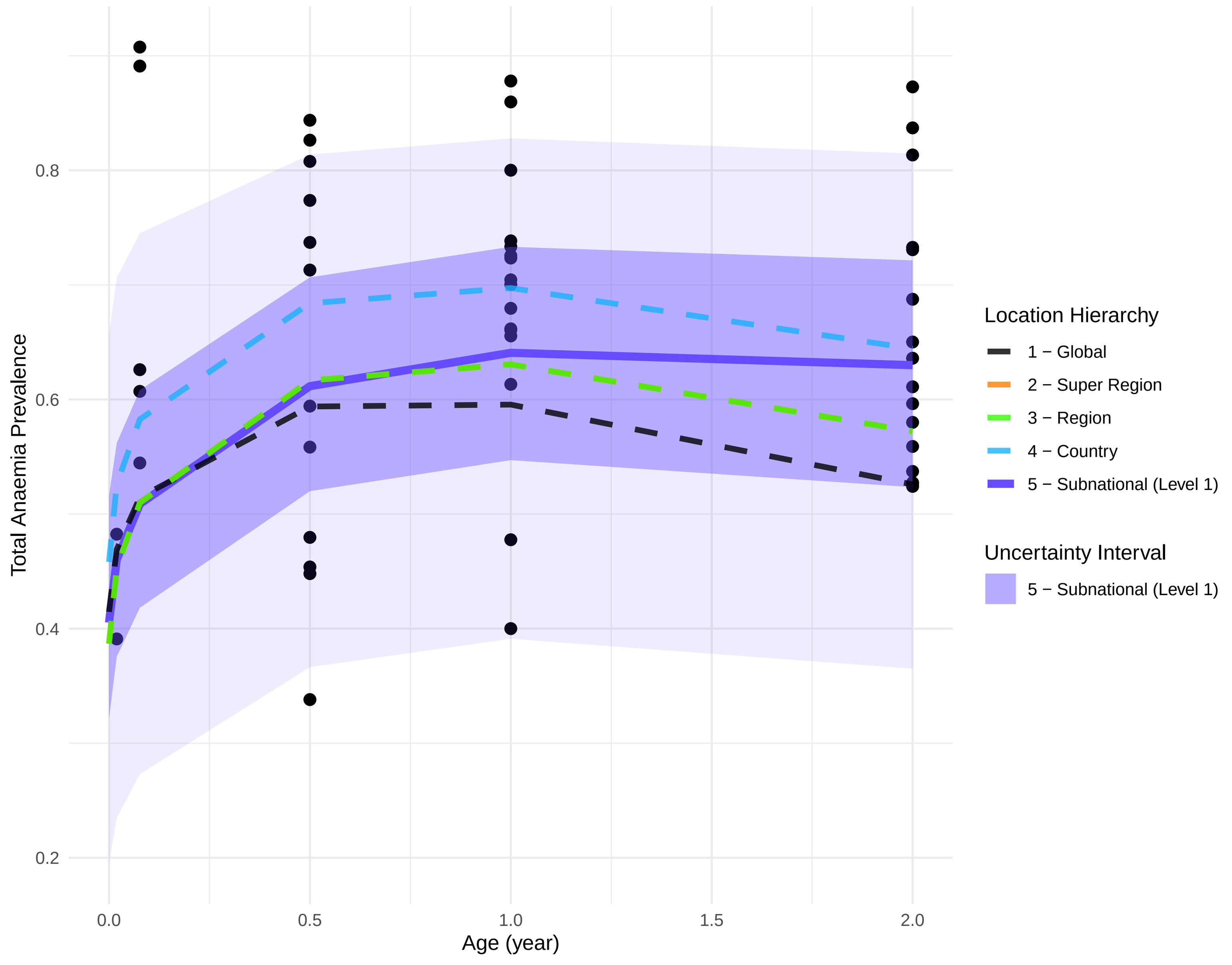

MR−BRT Age/Sex Fits − Males <2 Years Old − Total Anaemia Prevalence − Andhra Pradesh, Rural
WHO−2001 Adjustment Method

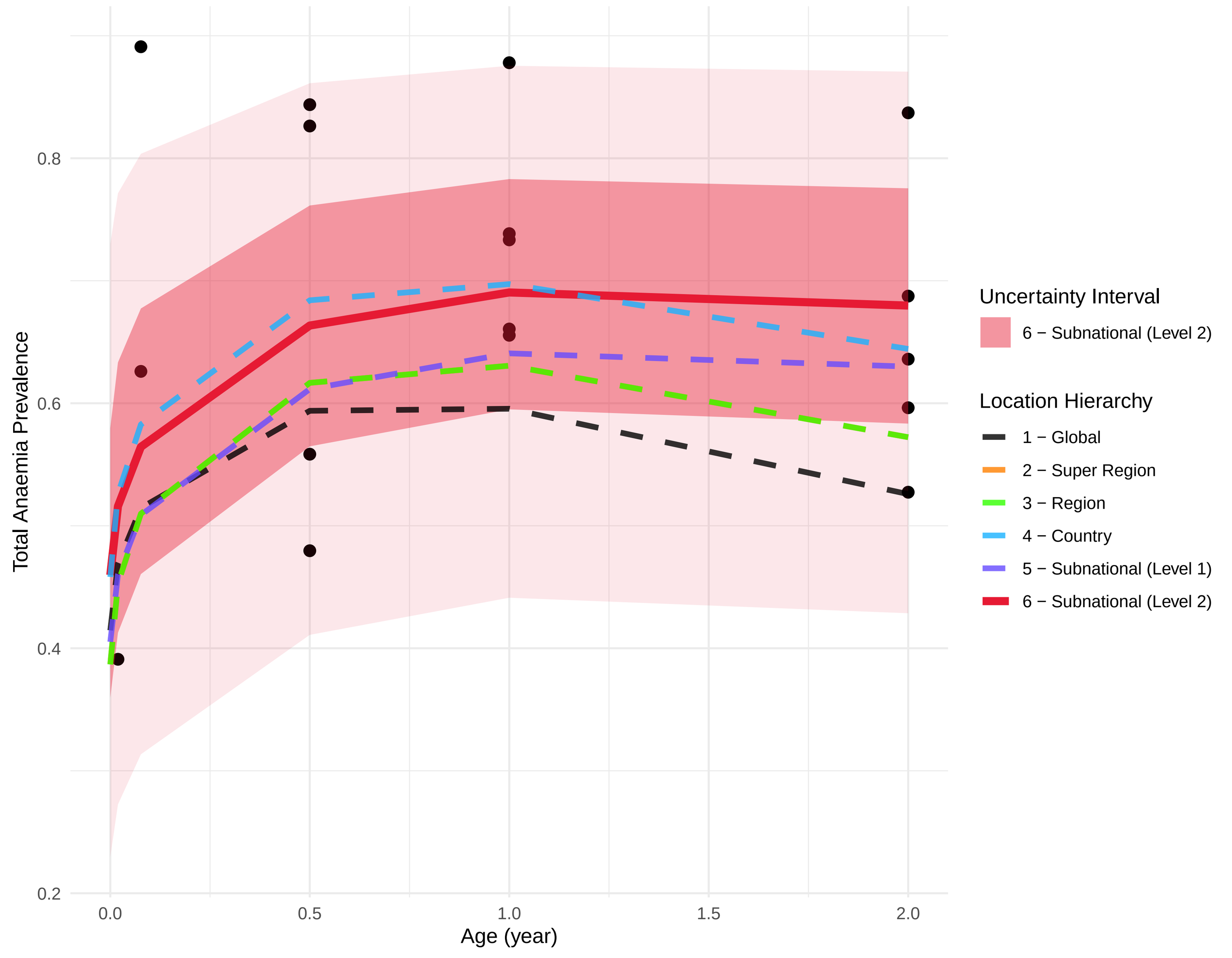

MR−BRT Age/Sex Fits − Males <2 Years Old − Total Anaemia Prevalence − Andhra Pradesh, Urban
WHO−2001 Adjustment Method

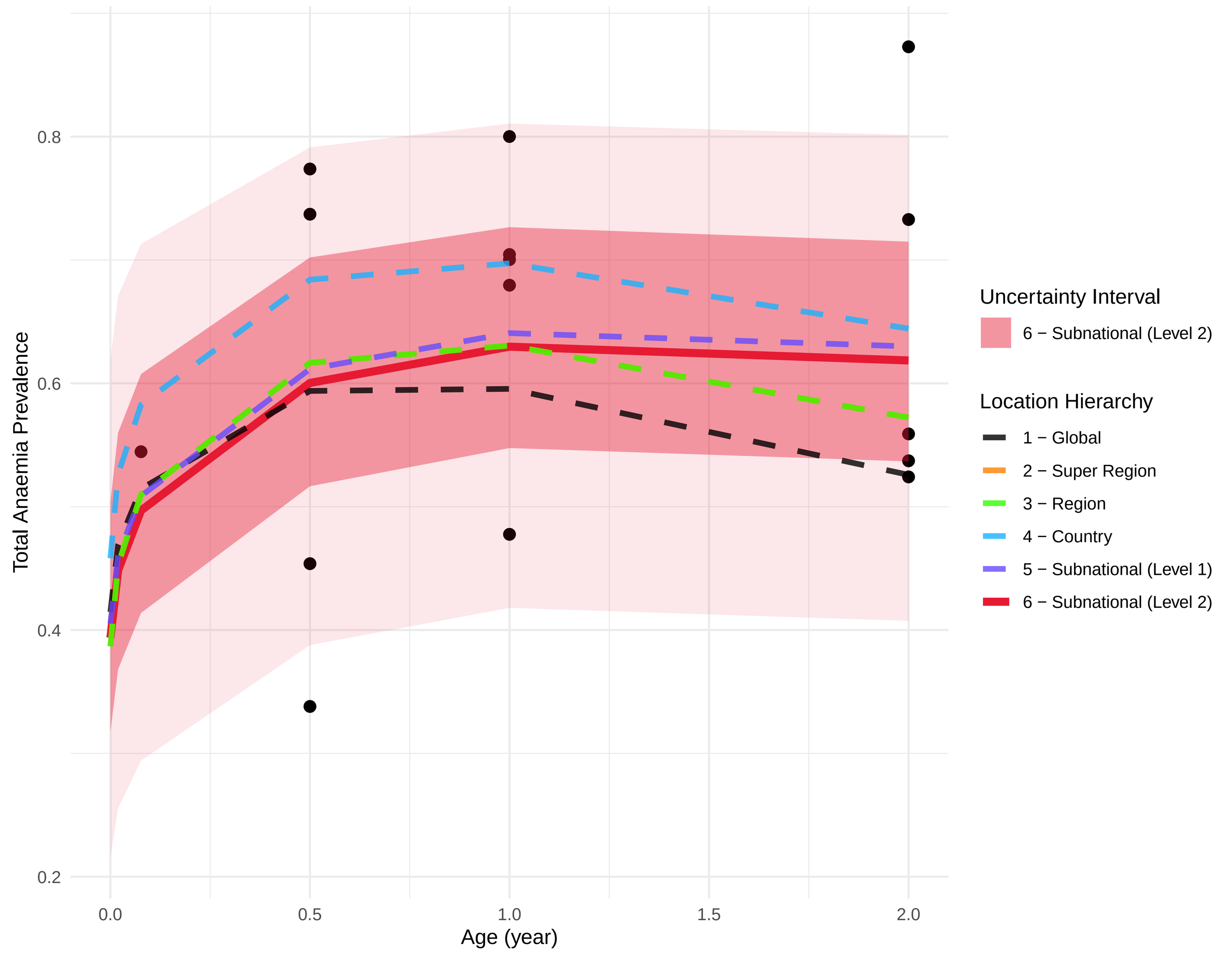

MR−BRT Age/Sex Fits − Males <2 Years Old − Total Anaemia Prevalence − Arunachal Pradesh
WHO−2001 Adjustment Method

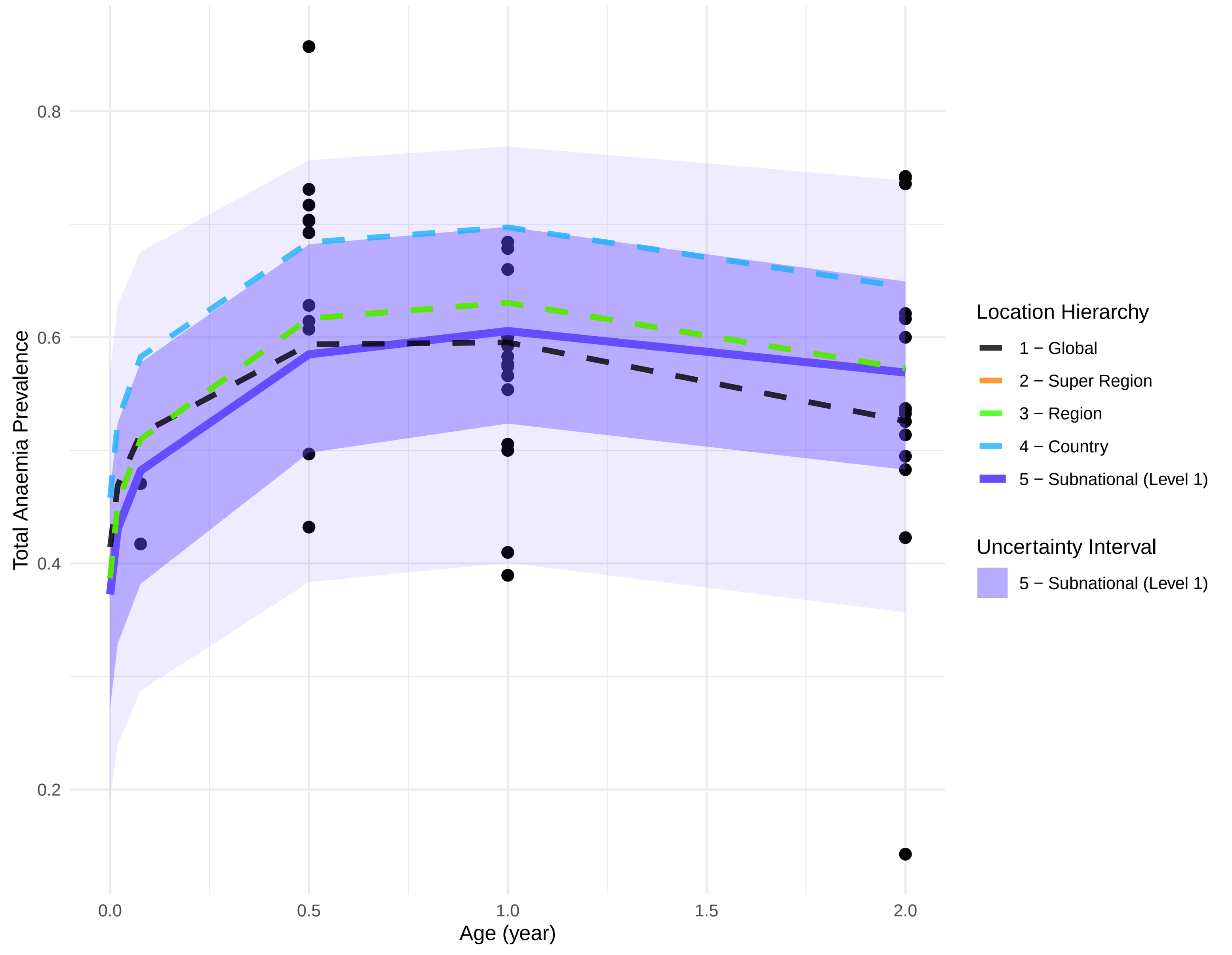

MR−BRT Age/Sex Fits − Males <2 Years Old − Total Anaemia Prevalence − Arunachal Pradesh, Rural
WHO−2001 Adjustment Method

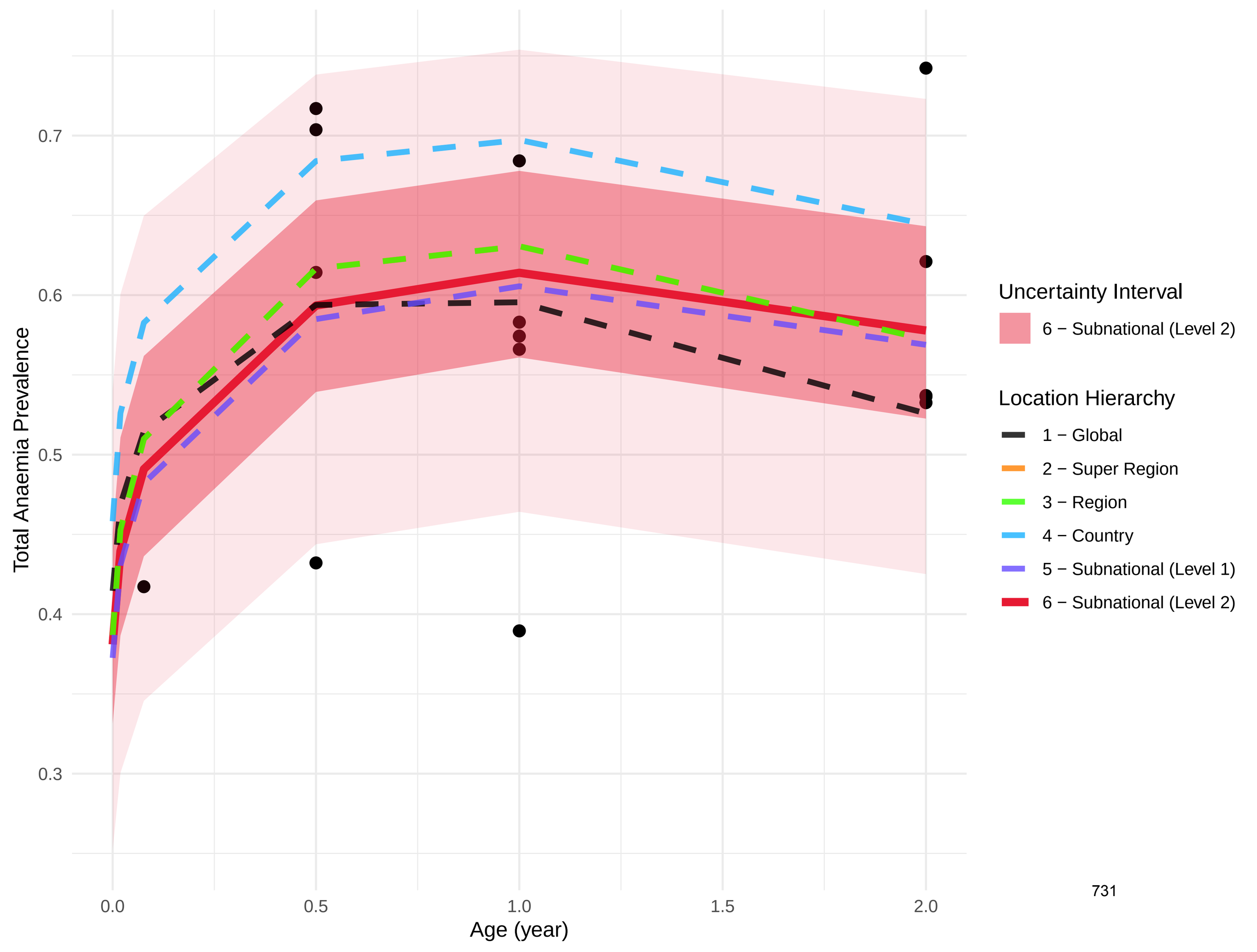

MR−BRT Age/Sex Fits − Males <2 Years Old − Total Anaemia Prevalence − Arunachal Pradesh, Urban
WHO−2001 Adjustment Method

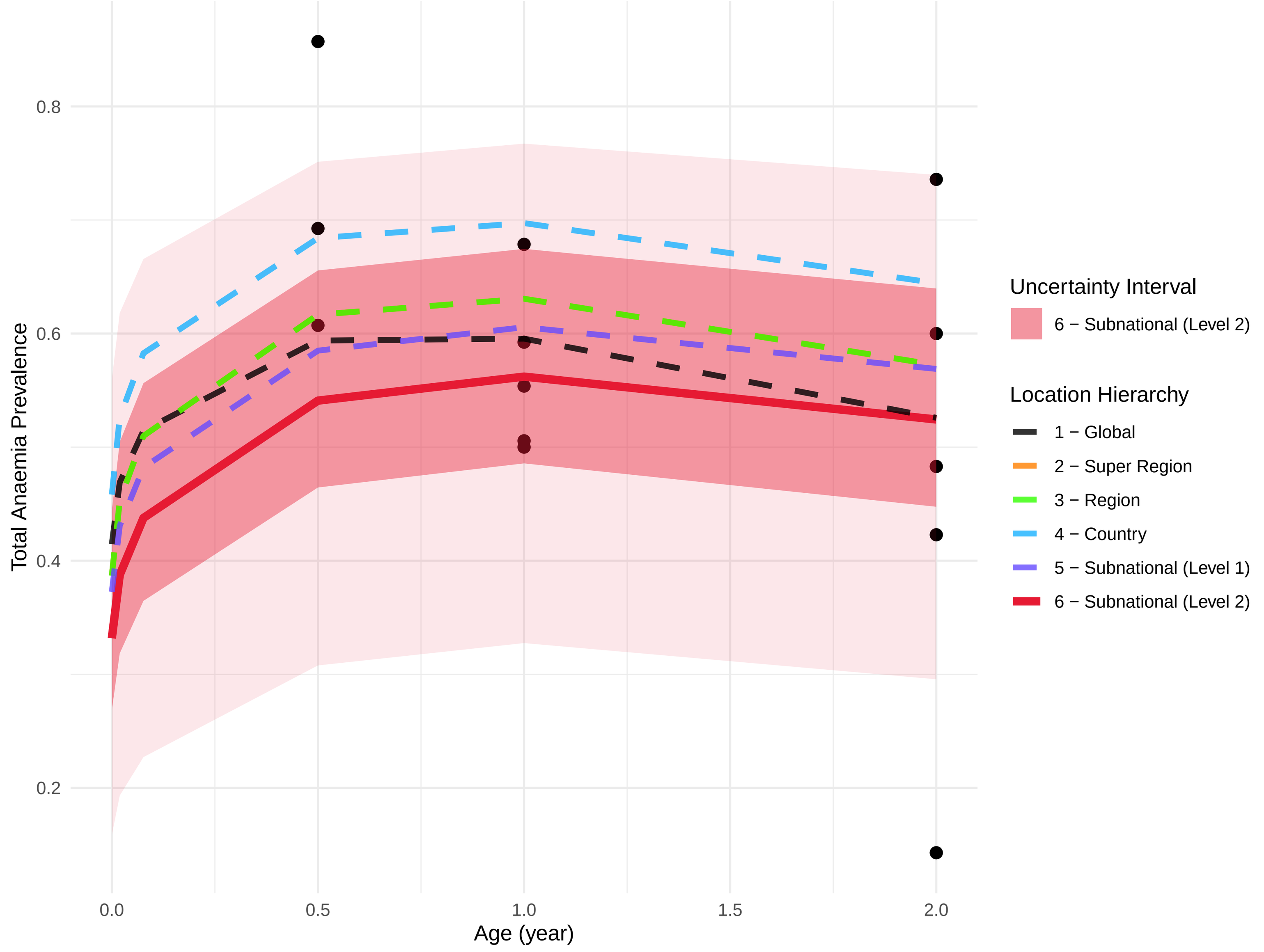

MR−BRT Age/Sex Fits − Males <2 Years Old − Total Anaemia Prevalence − Assam
WHO−2001 Adjustment Method

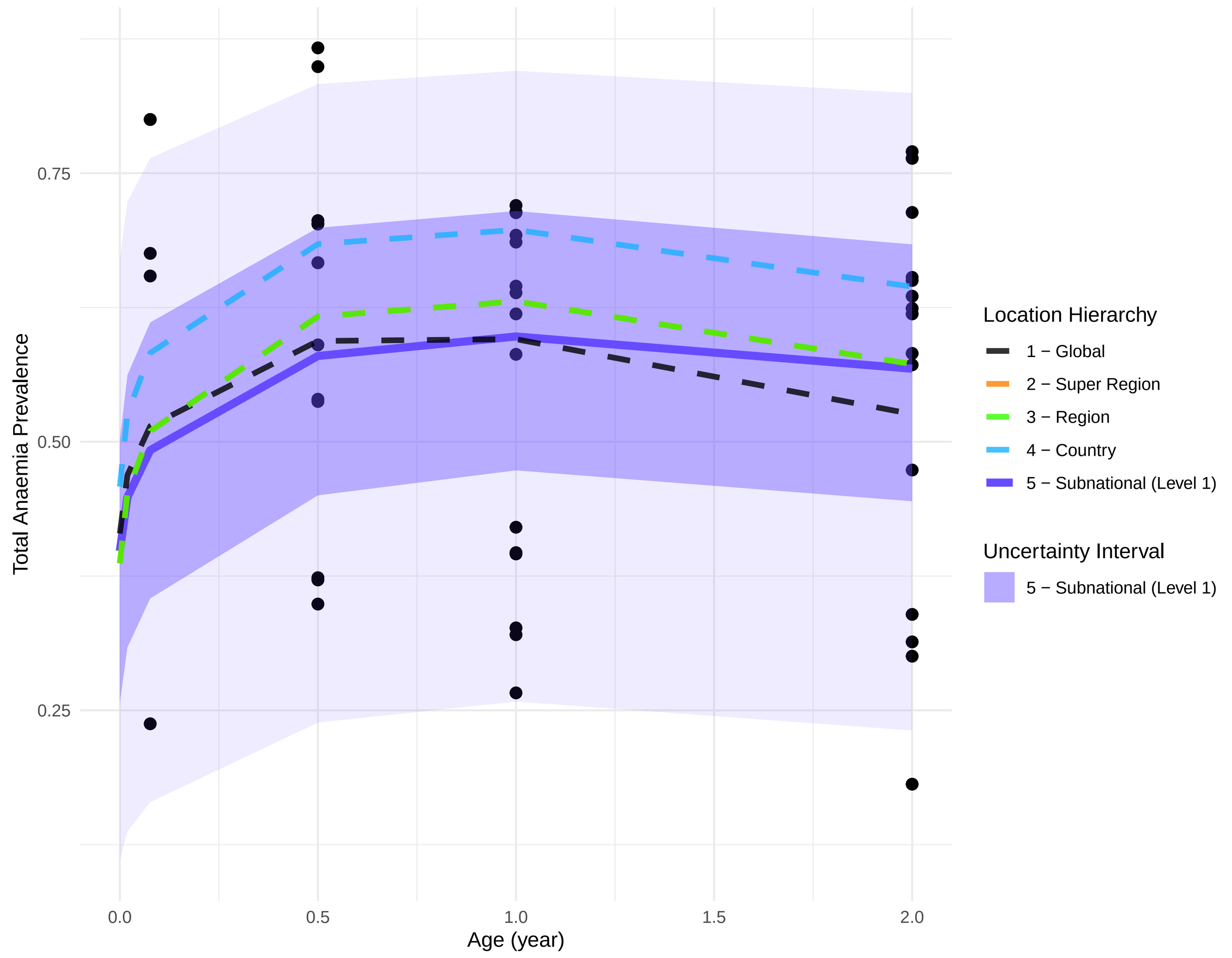

MR−BRT Age/Sex Fits − Males <2 Years Old − Total Anaemia Prevalence − Assam, Rural
WHO−2001 Adjustment Method

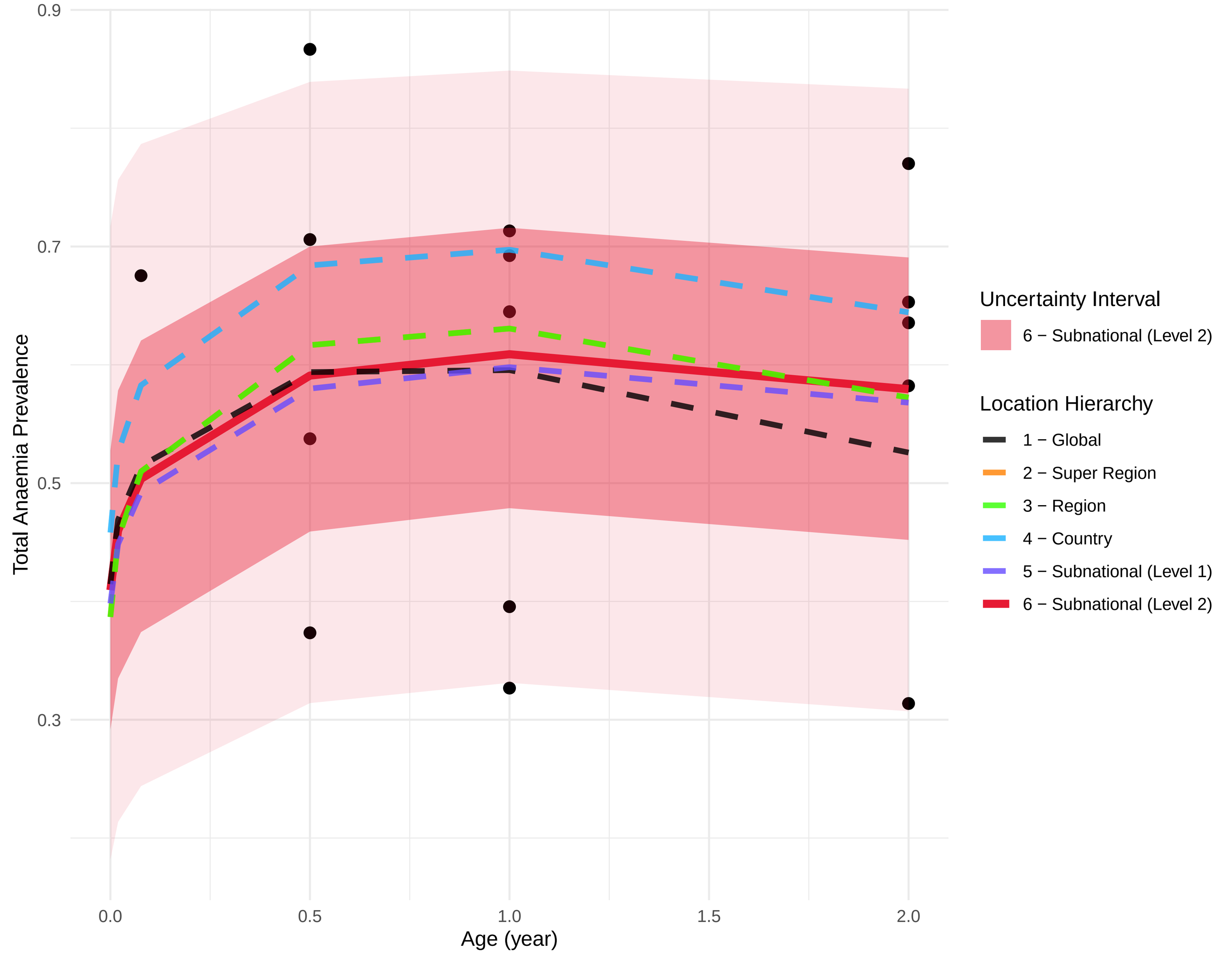

MR−BRT Age/Sex Fits − Males <2 Years Old − Total Anaemia Prevalence − Assam, Urban
WHO−2001 Adjustment Method

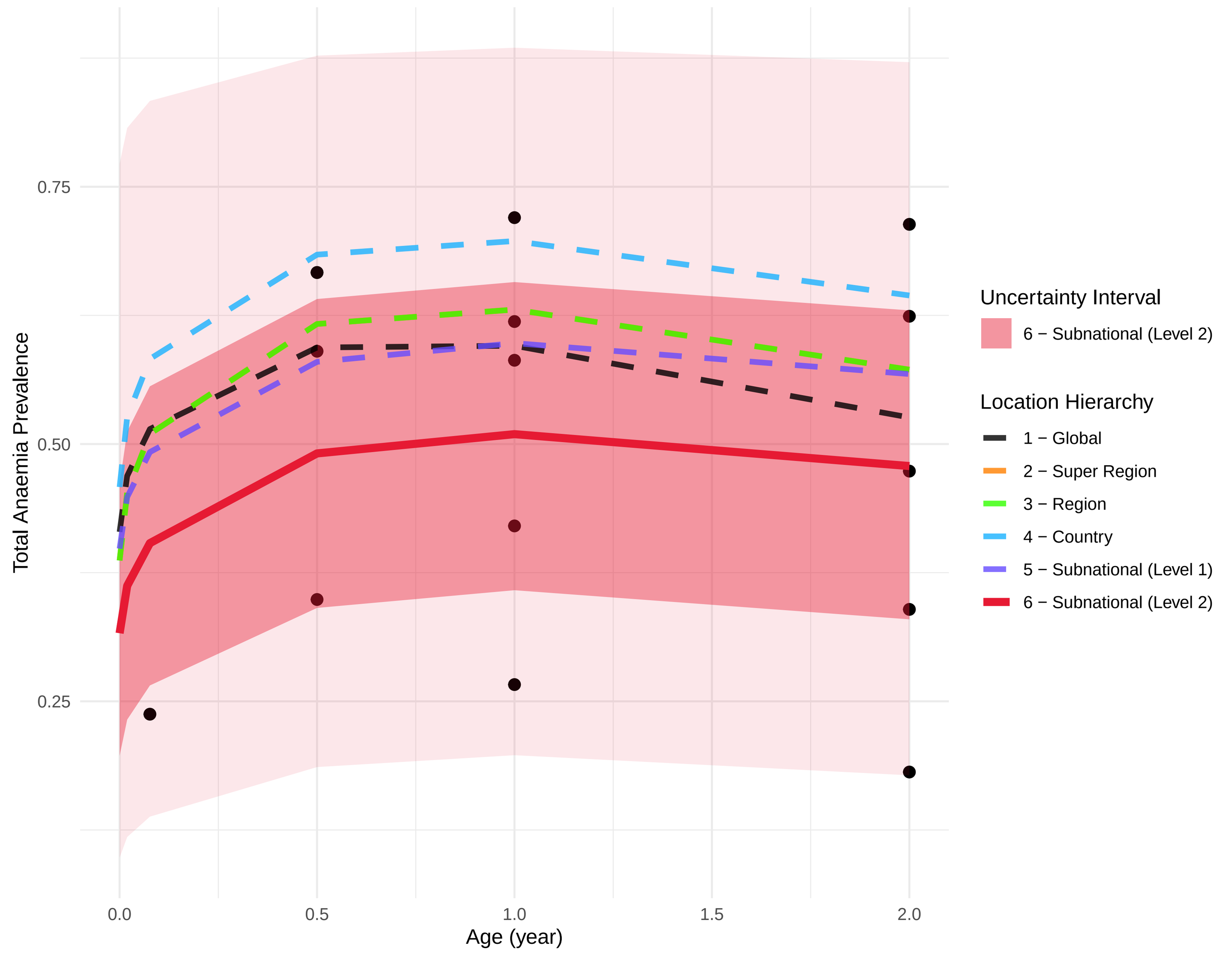

MR−BRT Age/Sex Fits − Males <2 Years Old − Total Anaemia Prevalence − Bihar
WHO−2001 Adjustment Method

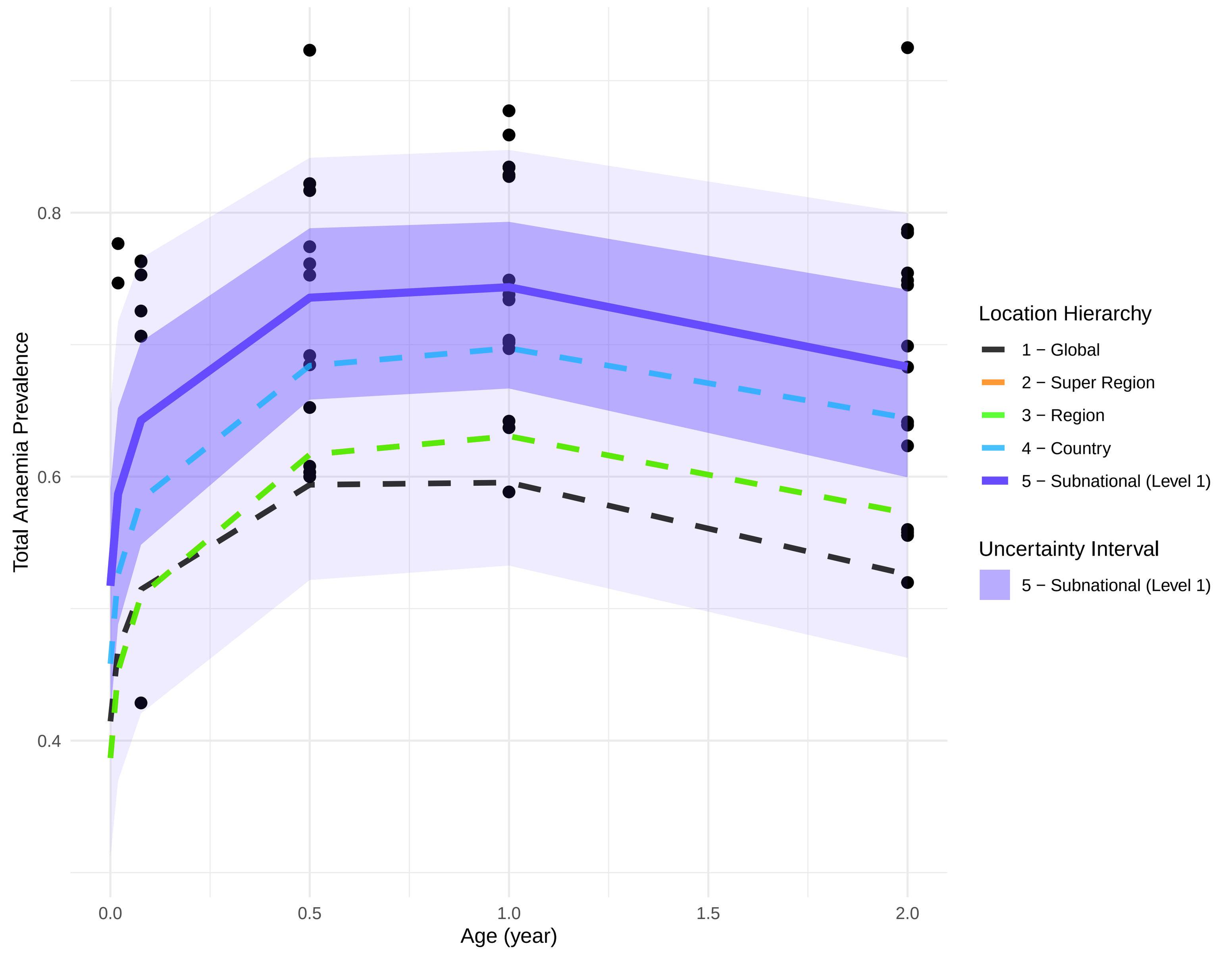

MR−BRT Age/Sex Fits − Males <2 Years Old − Total Anaemia Prevalence − Bihar, Rural
WHO−2001 Adjustment Method

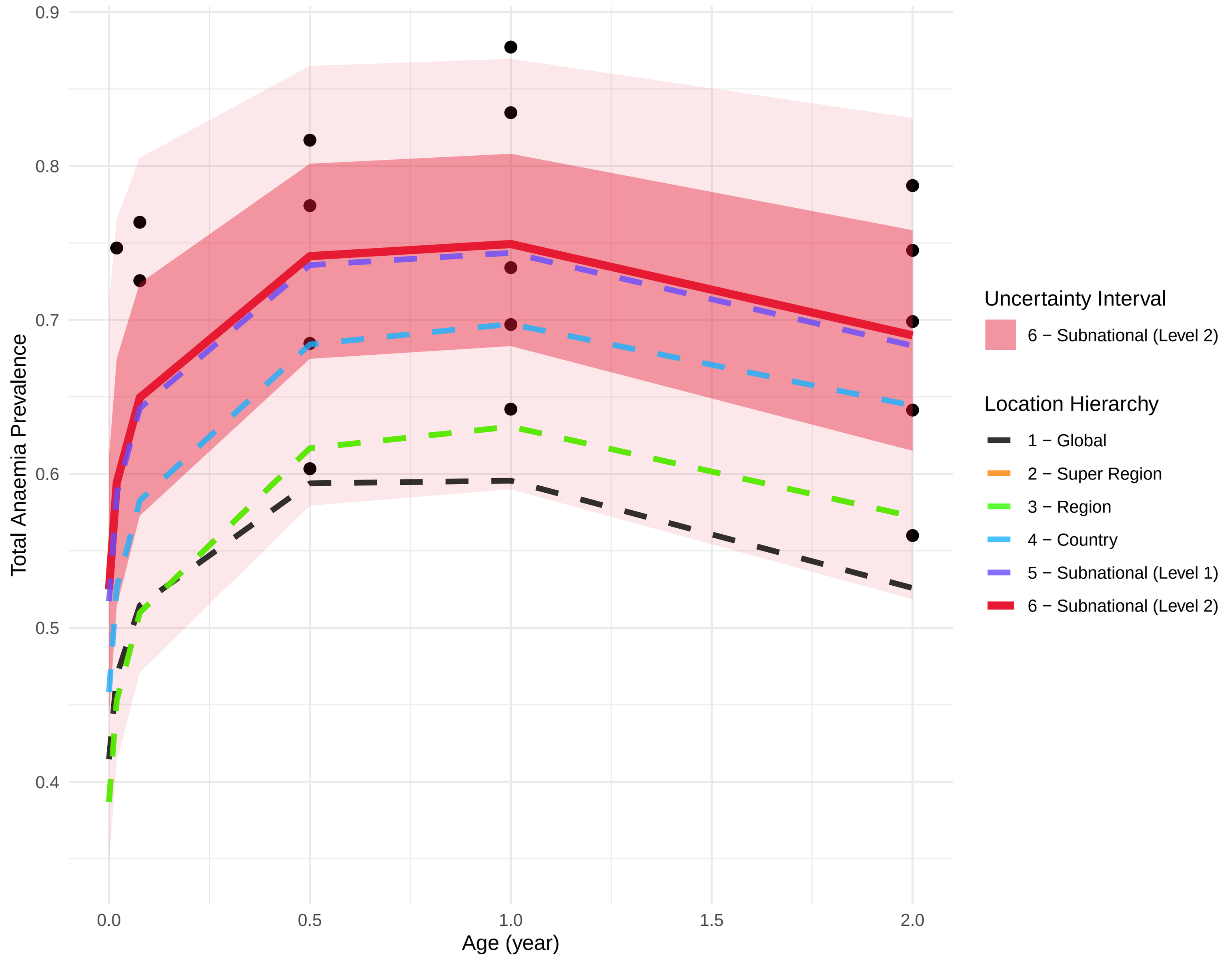

MR−BRT Age/Sex Fits − Males <2 Years Old − Total Anaemia Prevalence − Bihar, Urban
WHO−2001 Adjustment Method

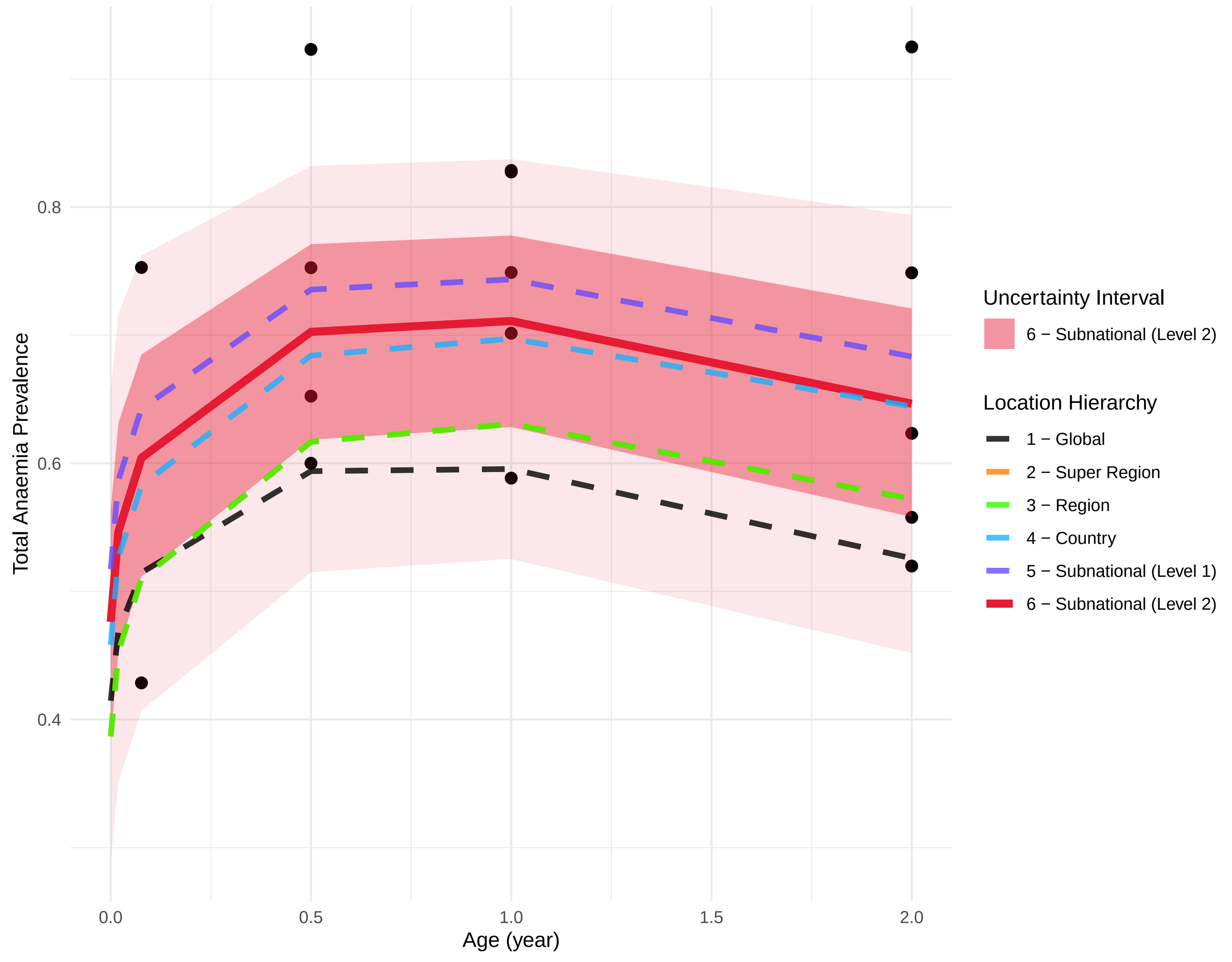

MR−BRT Age/Sex Fits − Males <2 Years Old − Total Anaemia Prevalence − Chhattisgarh
WHO−2001 Adjustment Method

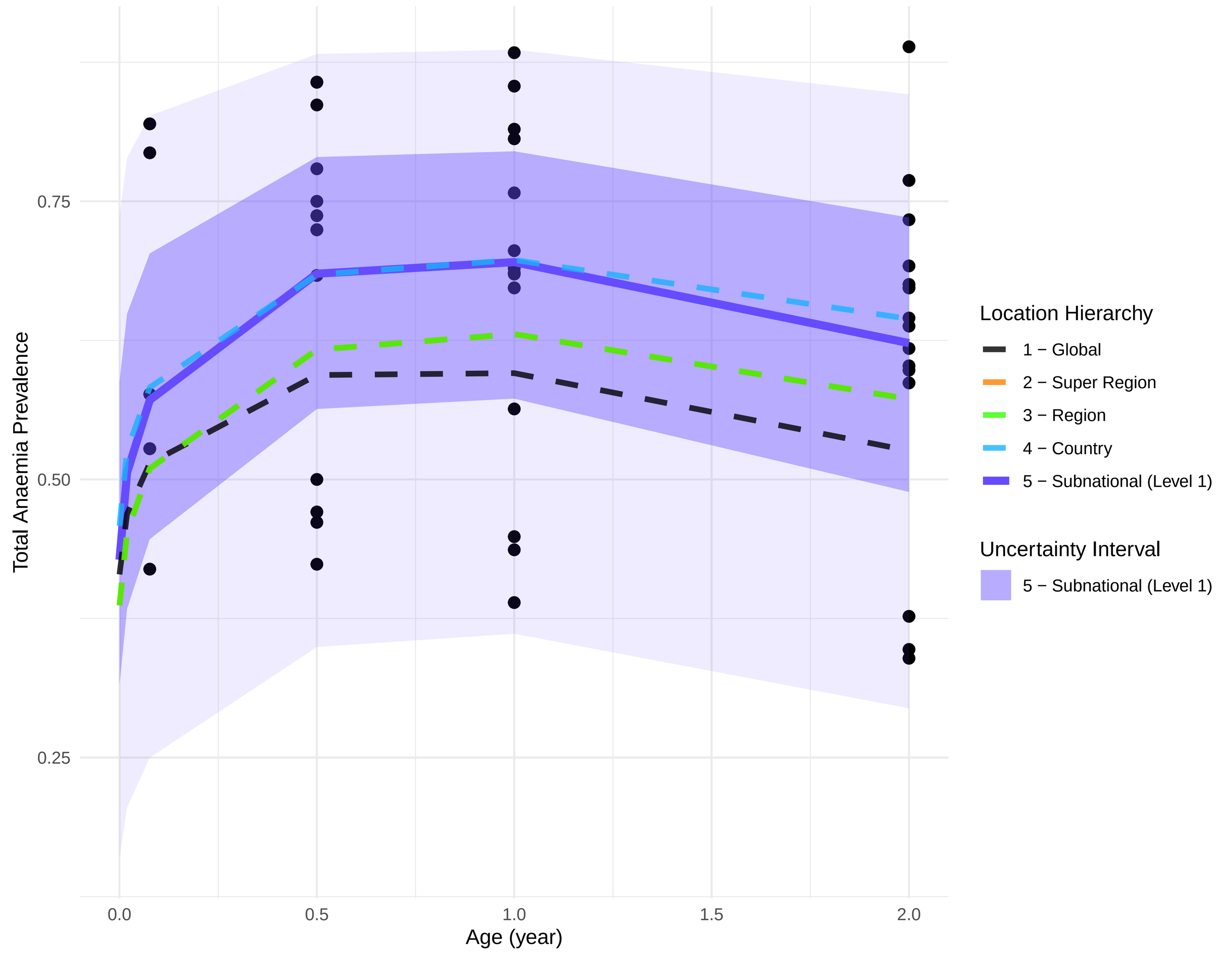

MR−BRT Age/Sex Fits − Males <2 Years Old − Total Anaemia Prevalence − Chhattisgarh, Rural
WHO−2001 Adjustment Method

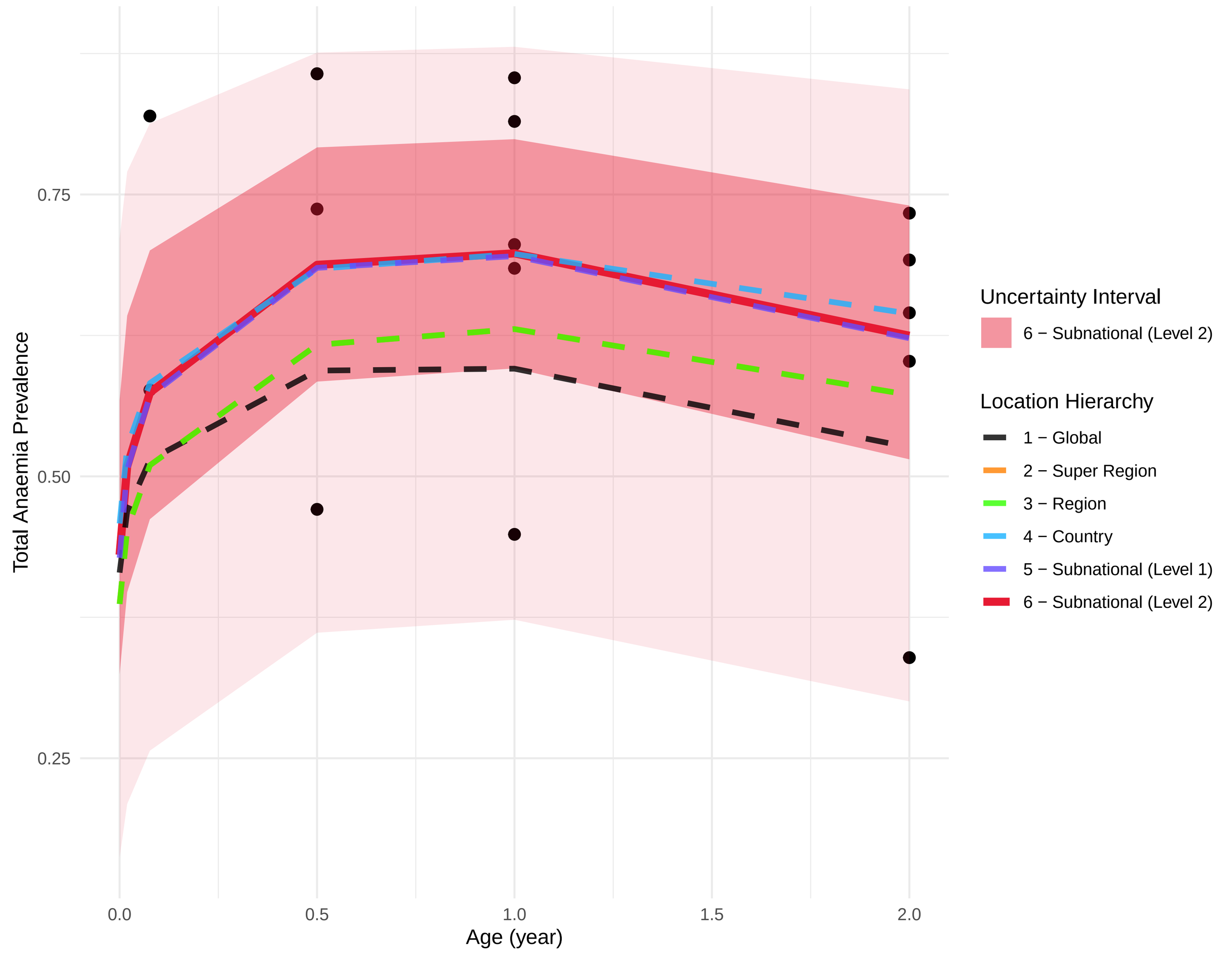

MR−BRT Age/Sex Fits − Males <2 Years Old − Total Anaemia Prevalence − Chhattisgarh, Urban
WHO−2001 Adjustment Method

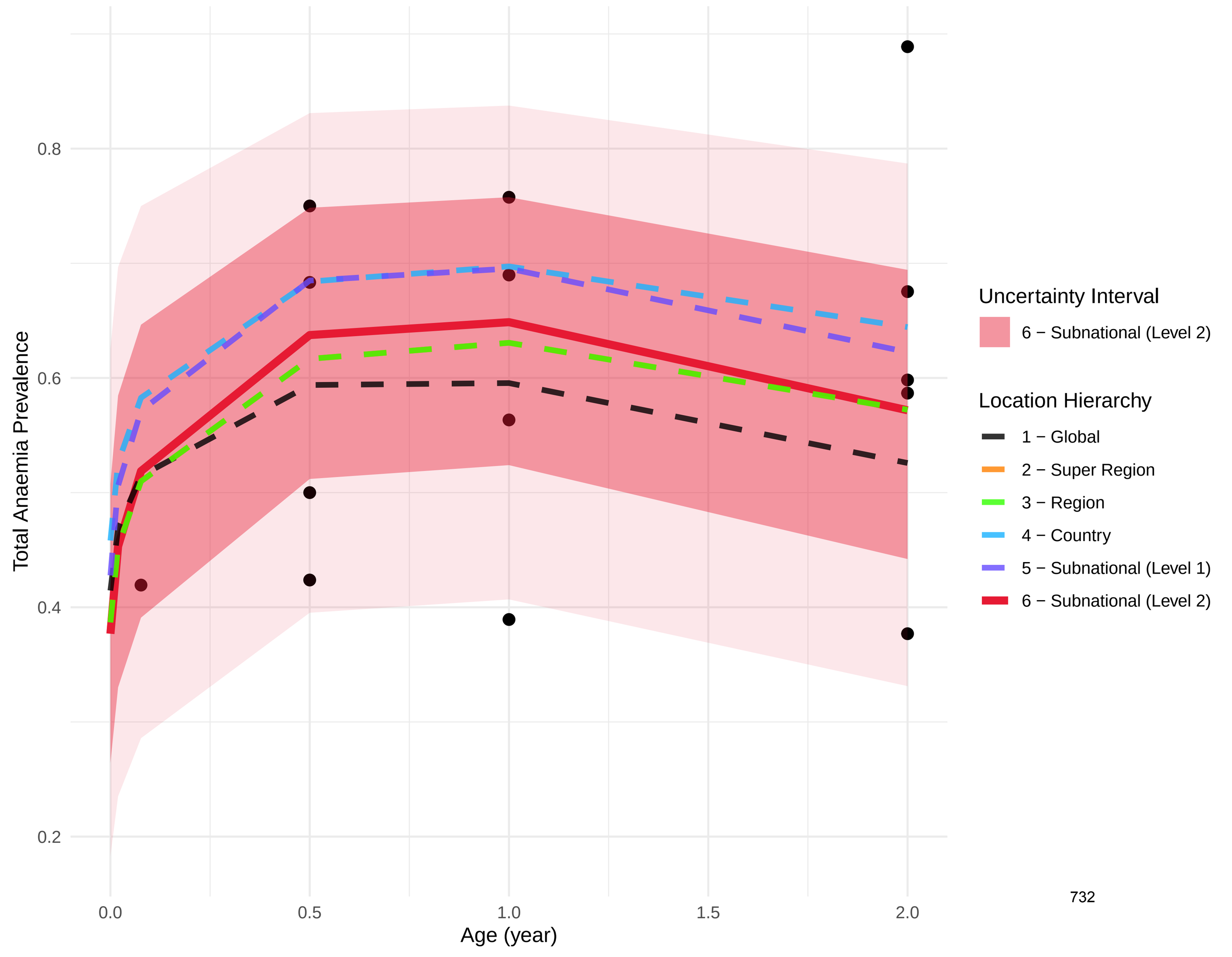

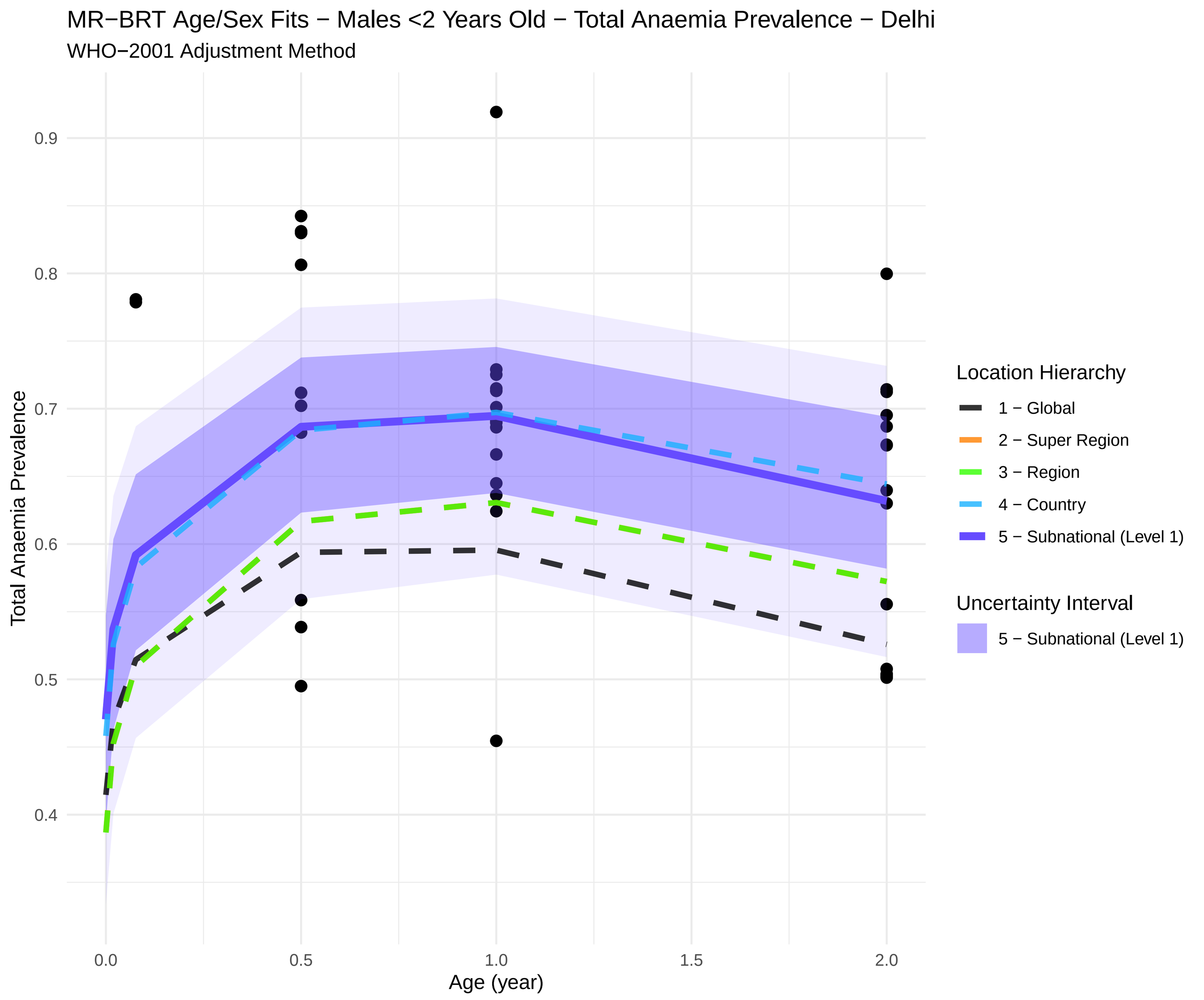
MR−BRT Age/Sex Fits − Males <2 Years Old − Total Anaemia Prevalence − Delhi
WHO−2001 Adjustment Method
Total Anaemia Prevalence
Age (year)
Location Hierarchy
1 − Global
2 − Super Region
3 − Region
4 − Country
5 − Subnational (Level 1)
Uncertainty Interval
5 − Subnational (Level 1)

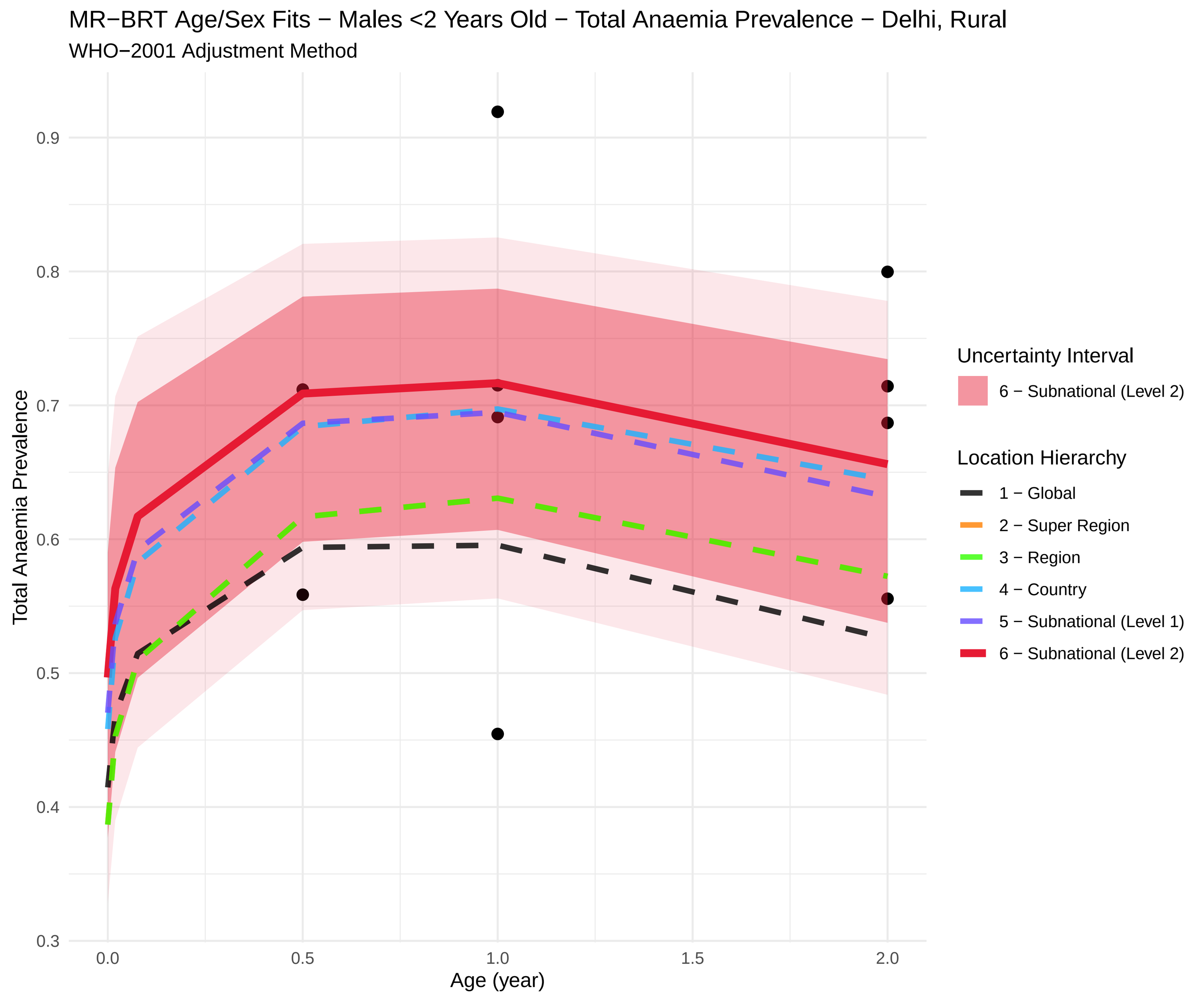
MR−BRT Age/Sex Fits − Males <2 Years Old − Total Anaemia Prevalence − Delhi, Rural
WHO−2001 Adjustment Method
Total Anaemia Prevalence
Age (year)
Uncertainty Interval
6 − Subnational (Level 2)
Location Hierarchy
1 − Global
2 − Super Region
3 − Region
4 − Country
5 − Subnational (Level 1)
6 − Subnational (Level 2)

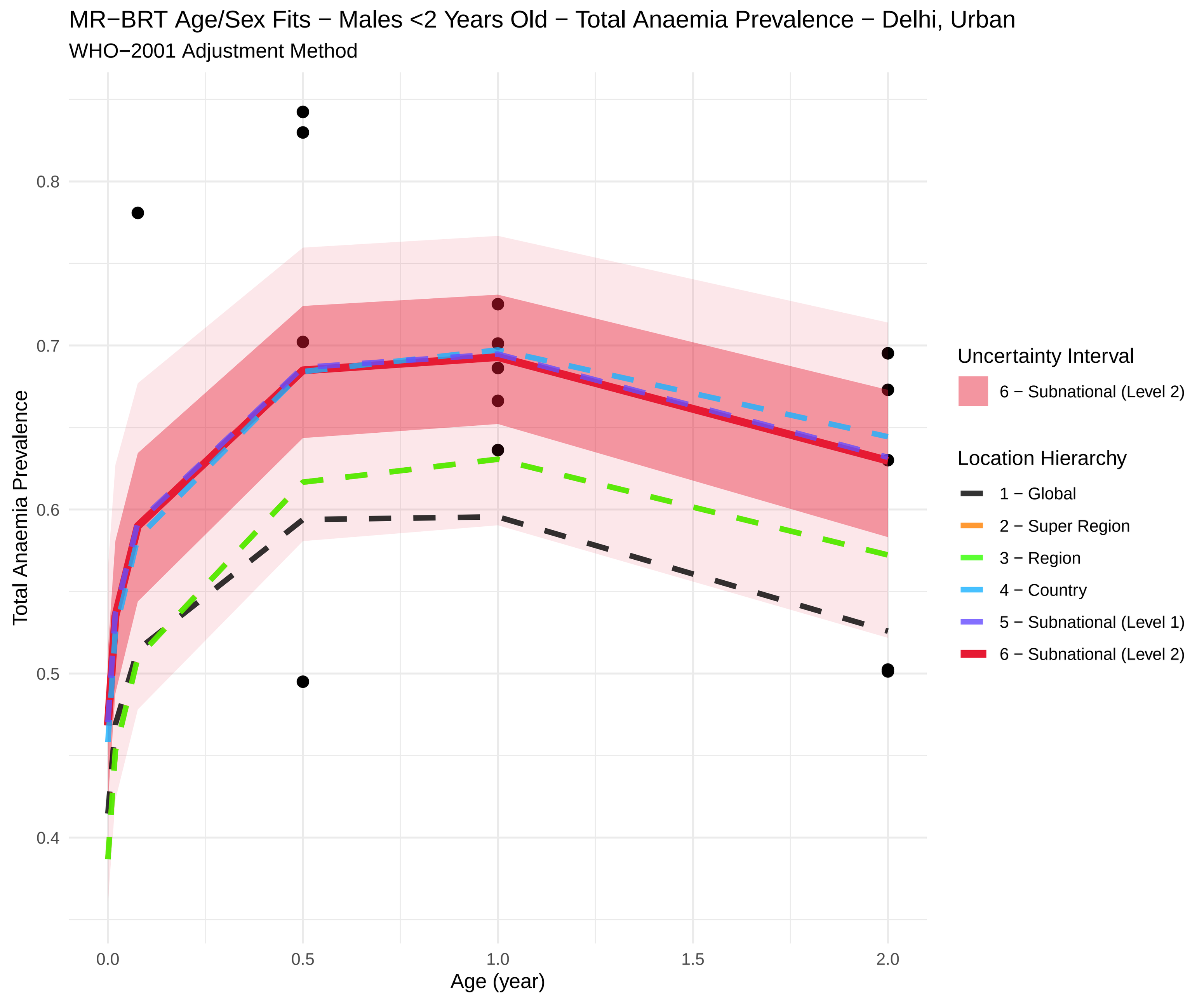
MR−BRT Age/Sex Fits − Males <2 Years Old − Total Anaemia Prevalence − Delhi, Urban
WHO−2001 Adjustment Method
Total Anaemia Prevalence
Age (year)
Uncertainty Interval
6 − Subnational (Level 2)
Location Hierarchy
1 − Global
2 − Super Region
3 − Region
4 − Country
5 − Subnational (Level 1)
6 − Subnational (Level 2)

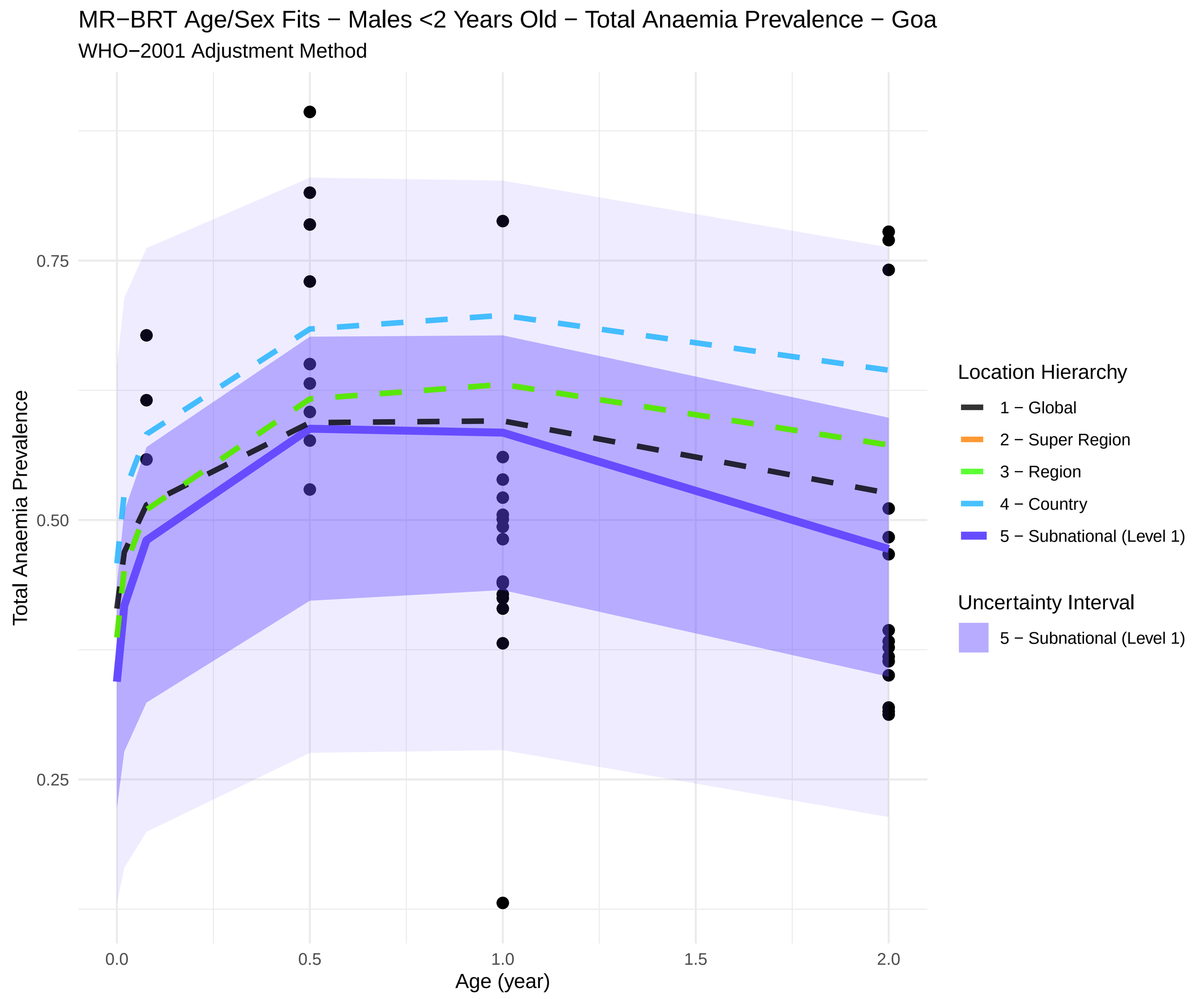
MR−BRT Age/Sex Fits − Males <2 Years Old − Total Anaemia Prevalence − Goa
WHO−2001 Adjustment Method
Total Anaemia Prevalence
Age (year)
Location Hierarchy
1 − Global
2 − Super Region
3 − Region
4 − Country
5 − Subnational (Level 1)
Uncertainty Interval
5 − Subnational (Level 1)

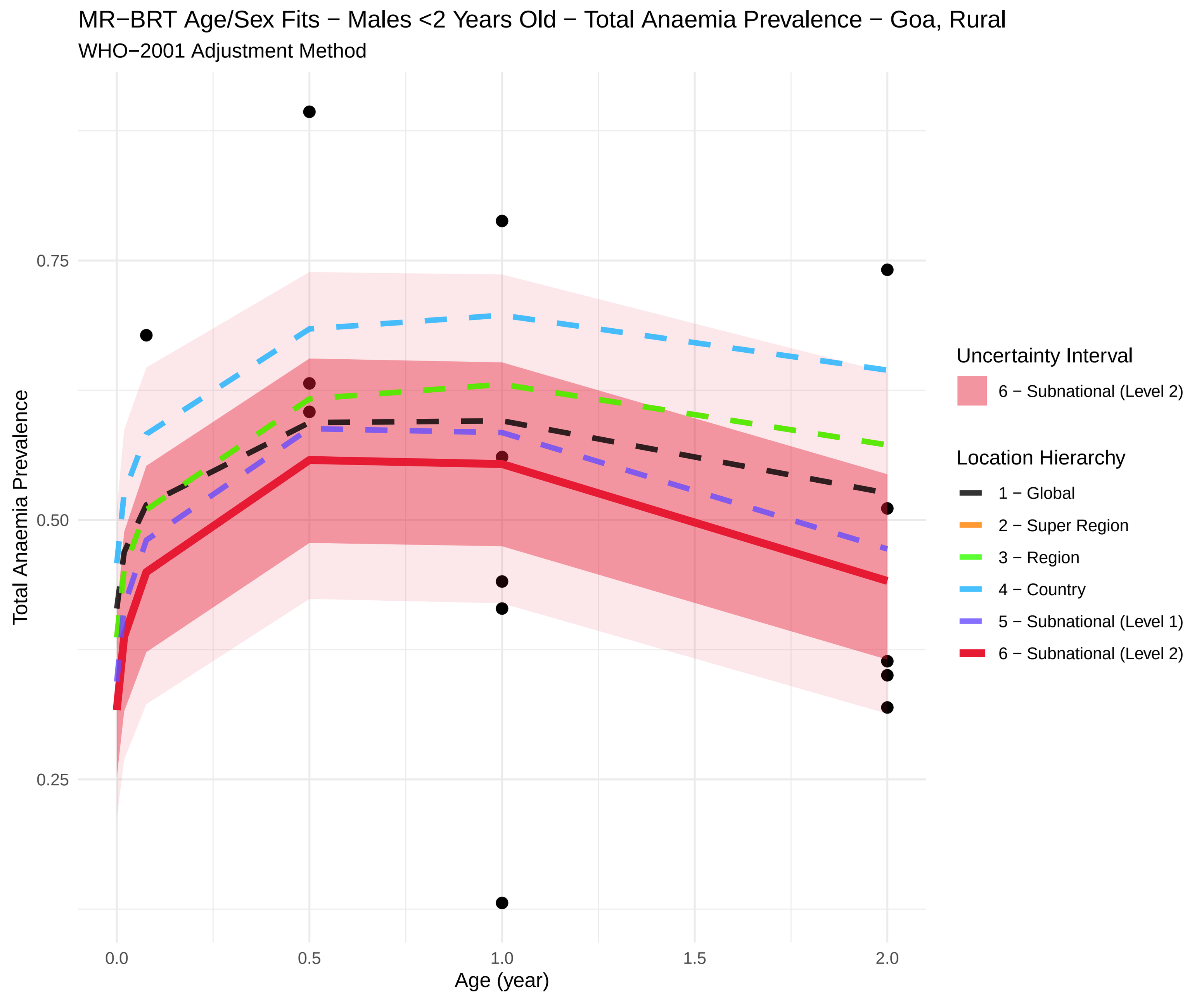
MR−BRT Age/Sex Fits − Males <2 Years Old − Total Anaemia Prevalence − Goa, Rural
WHO−2001 Adjustment Method
Total Anaemia Prevalence
Age (year)
Uncertainty Interval
6 − Subnational (Level 2)
Location Hierarchy
1 − Global
2 − Super Region
3 − Region
4 − Country
5 − Subnational (Level 1)
6 − Subnational (Level 2)

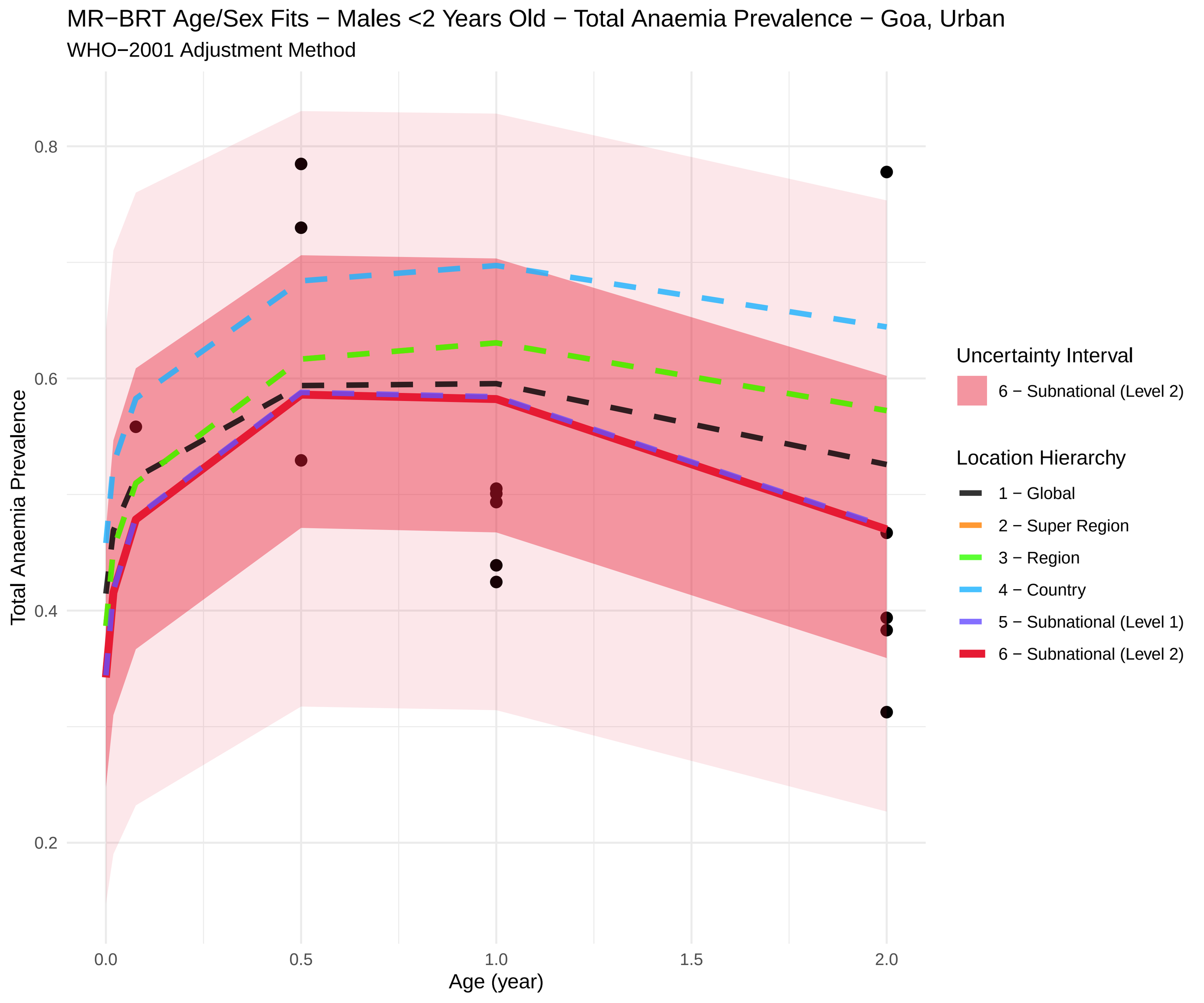
MR−BRT Age/Sex Fits − Males <2 Years Old − Total Anaemia Prevalence − Goa, Urban
WHO−2001 Adjustment Method
Total Anaemia Prevalence
Age (year)
Uncertainty Interval
6 − Subnational (Level 2)
Location Hierarchy
1 − Global
2 − Super Region
3 − Region
4 − Country
5 − Subnational (Level 1)
6 − Subnational (Level 2)

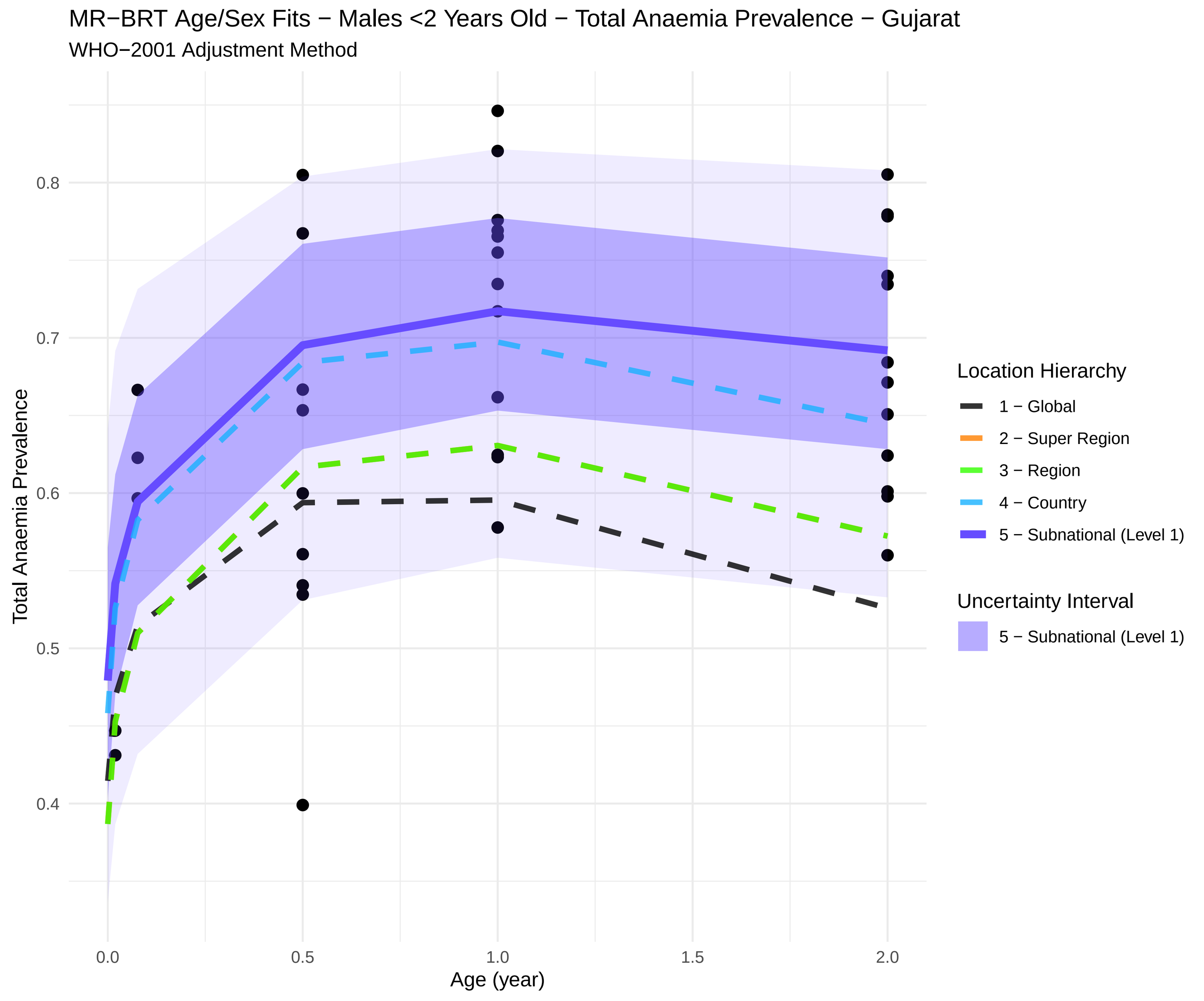
MR−BRT Age/Sex Fits − Males <2 Years Old − Total Anaemia Prevalence − Gujarat
WHO−2001 Adjustment Method
Total Anaemia Prevalence
Age (year)
Location Hierarchy
1 − Global
2 − Super Region
3 − Region
4 − Country
5 − Subnational (Level 1)
Uncertainty Interval
5 − Subnational (Level 1)

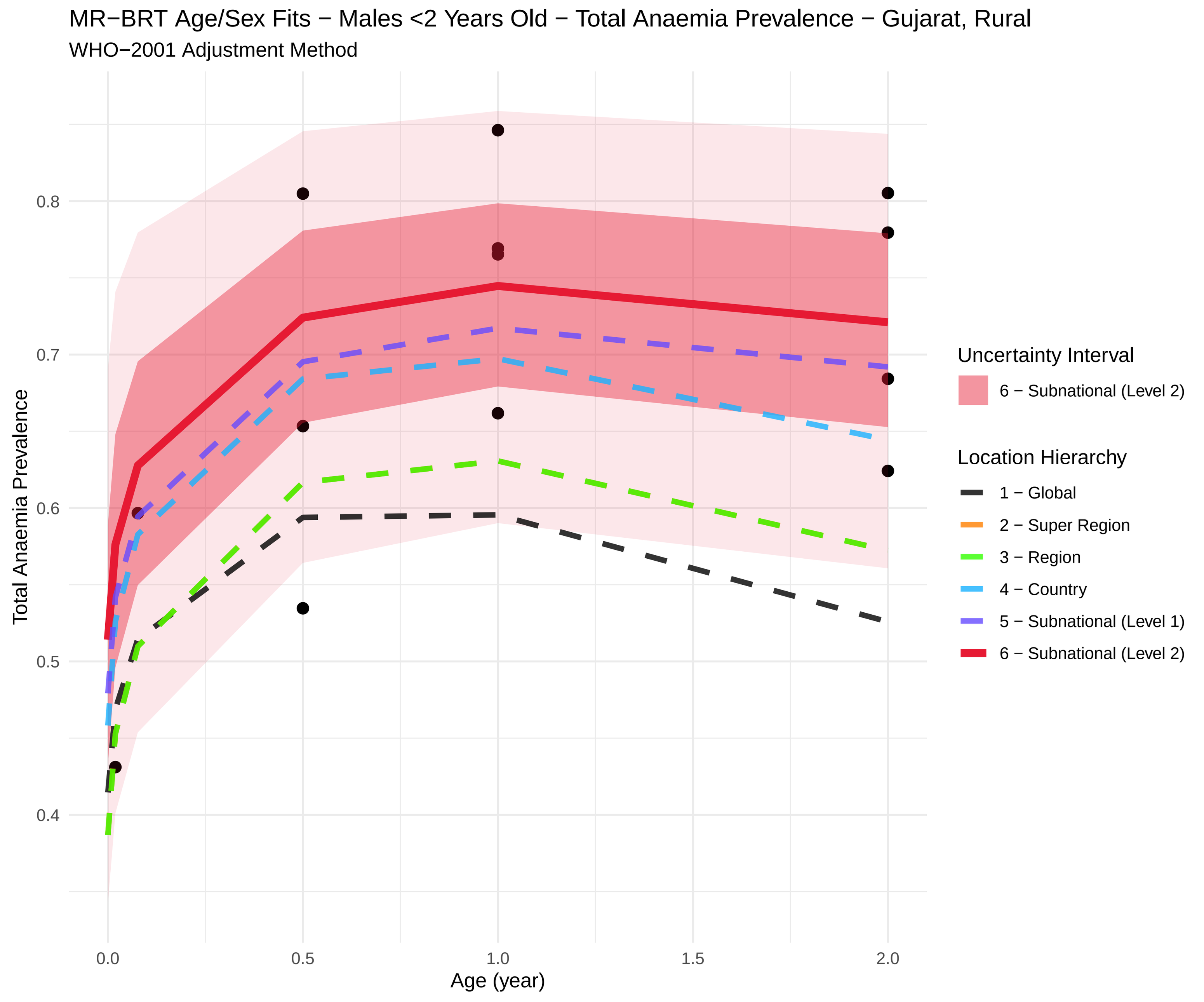
MR−BRT Age/Sex Fits − Males <2 Years Old − Total Anaemia Prevalence − Gujarat, Rural
WHO−2001 Adjustment Method
Total Anaemia Prevalence
Age (year)
Uncertainty Interval
6 − Subnational (Level 2)
Location Hierarchy
1 − Global
2 − Super Region
3 − Region
4 − Country
5 − Subnational (Level 1)
6 − Subnational (Level 2)

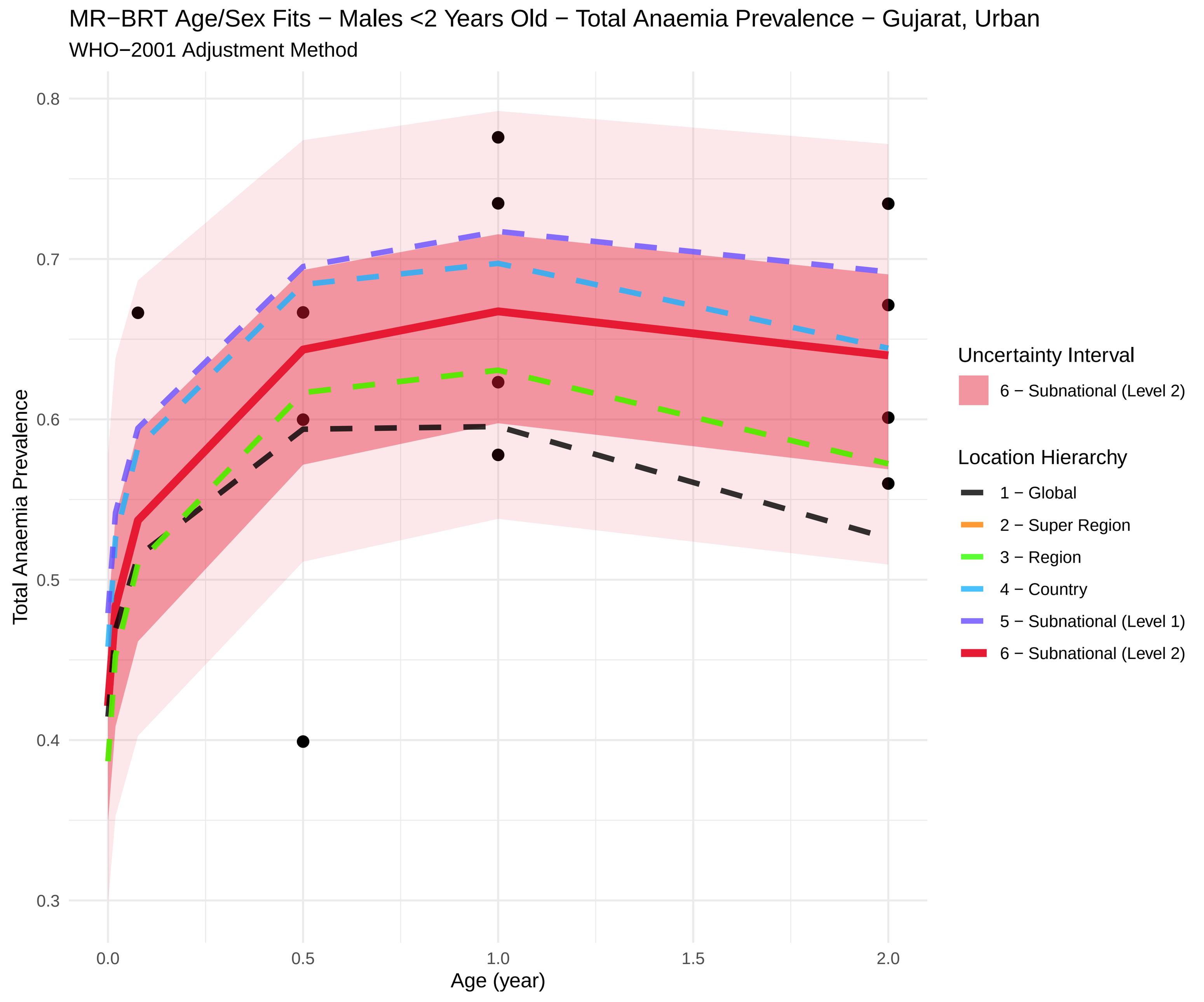
MR−BRT Age/Sex Fits − Males <2 Years Old − Total Anaemia Prevalence − Gujarat, Urban
WHO−2001 Adjustment Method
Total Anaemia Prevalence
Age (year)
Uncertainty Interval
6 − Subnational (Level 2)
Location Hierarchy
1 − Global
2 − Super Region
3 − Region
4 − Country
5 − Subnational (Level 1)
6 − Subnational (Level 2)

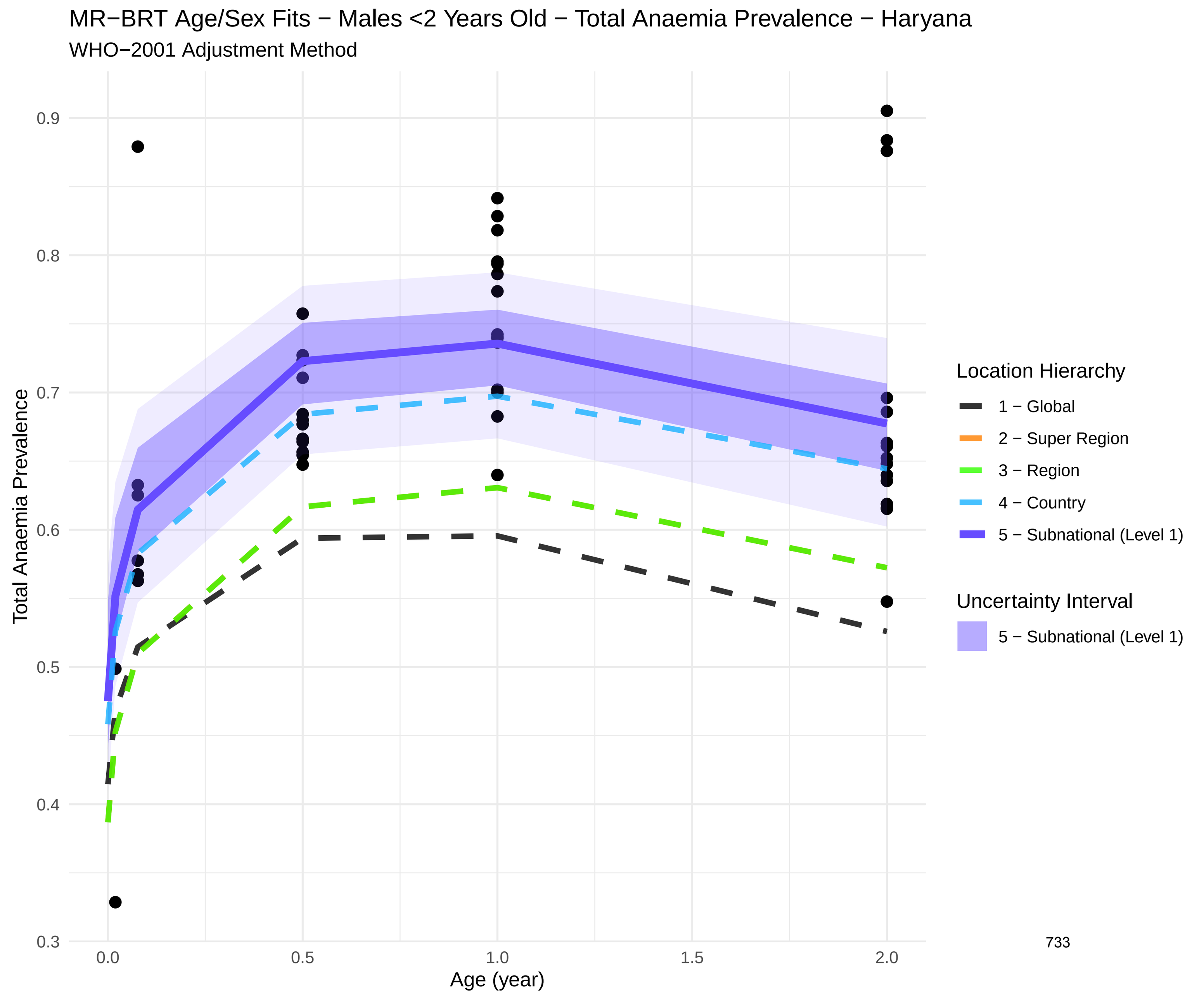
MR−BRT Age/Sex Fits − Males <2 Years Old − Total Anaemia Prevalence − Haryana
WHO−2001 Adjustment Method
Total Anaemia Prevalence
Age (year)
Location Hierarchy
1 − Global
2 − Super Region
3 − Region
4 − Country
5 − Subnational (Level 1)
Uncertainty Interval
5 − Subnational (Level 1)

MR−BRT Age/Sex Fits − Males <2 Years Old − Total Anaemia Prevalence − Haryana, Rural
WHO−2001 Adjustment Method

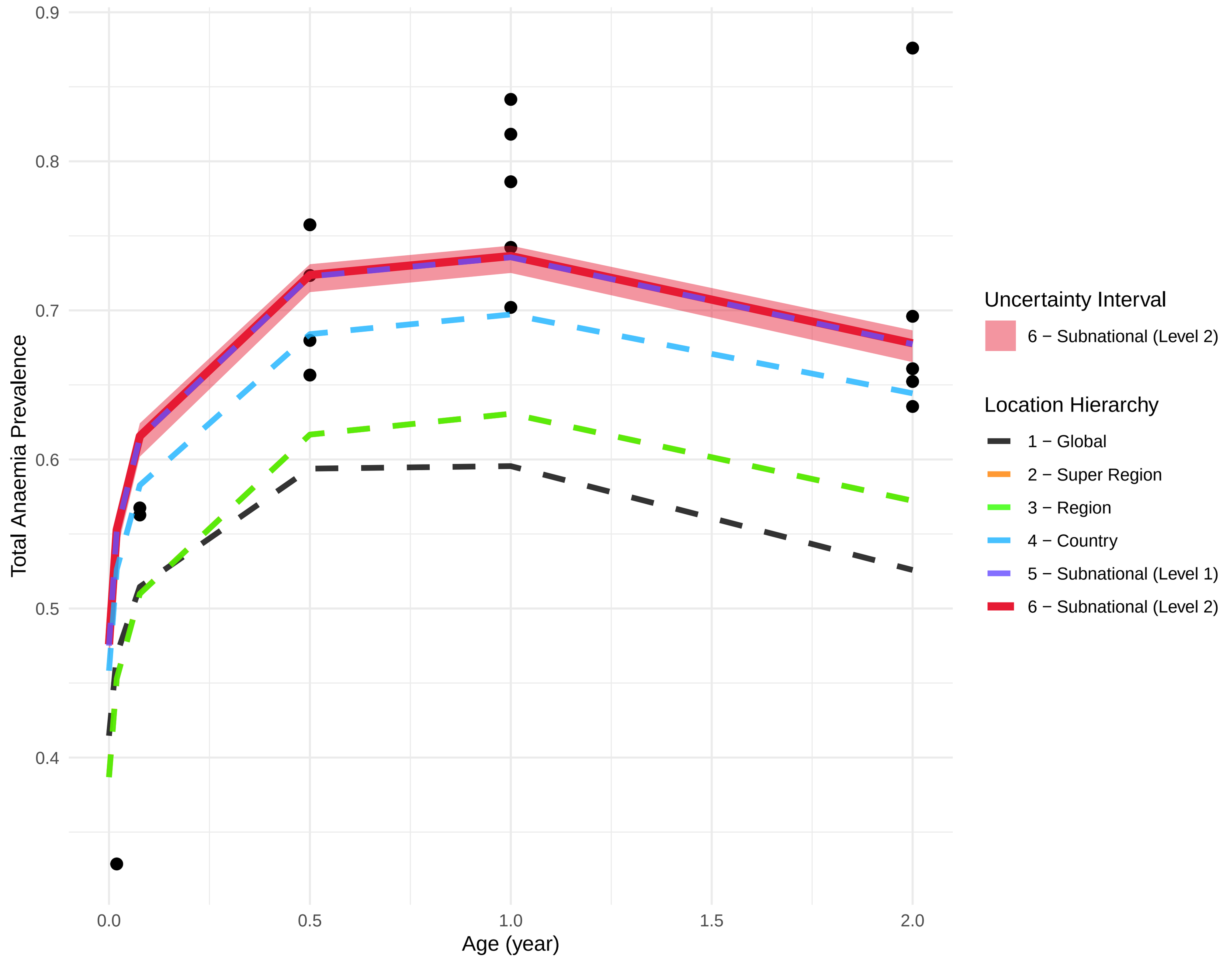

MR−BRT Age/Sex Fits − Males <2 Years Old − Total Anaemia Prevalence − Haryana, Urban
WHO−2001 Adjustment Method

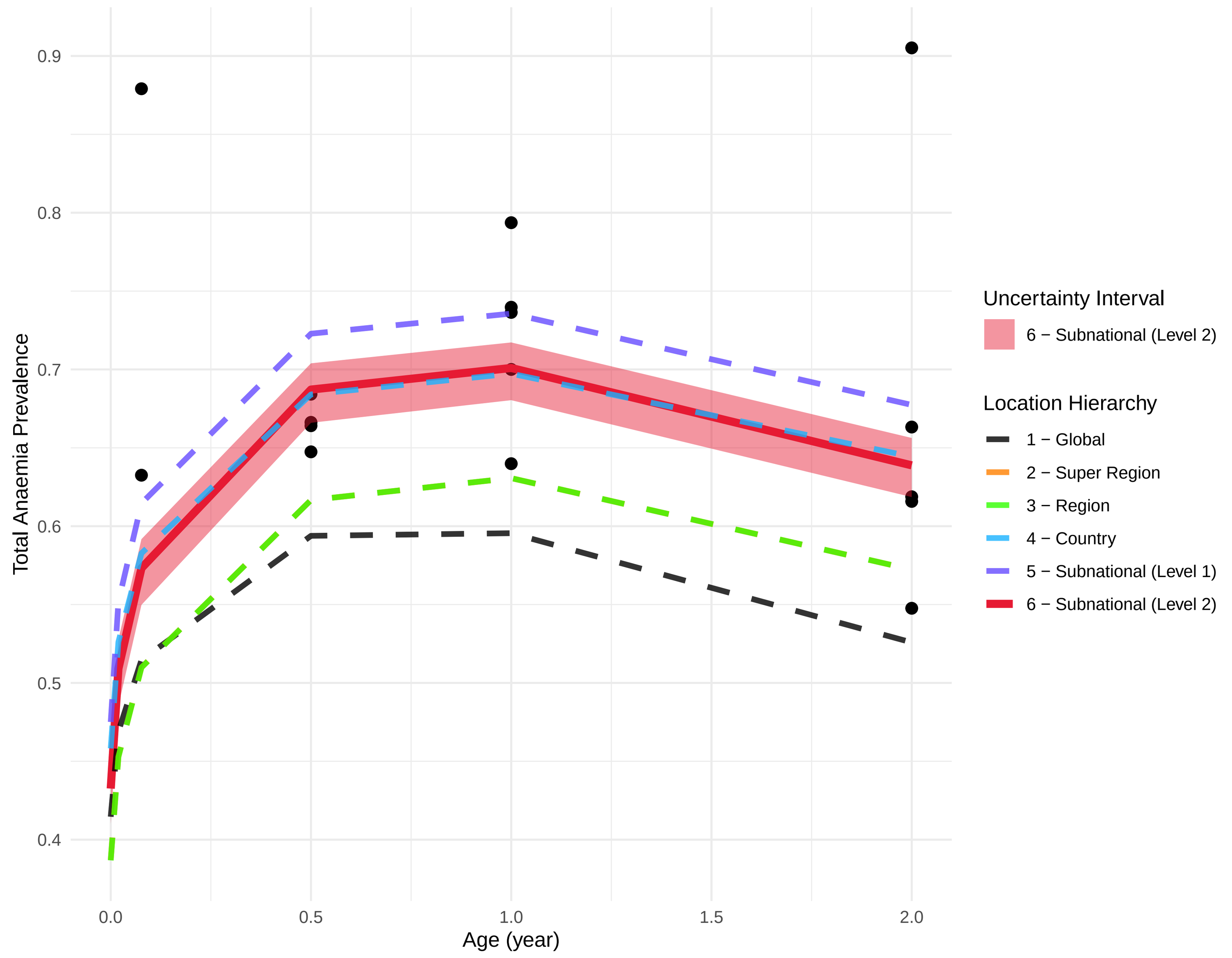

MR−BRT Age/Sex Fits − Males <2 Years Old − Total Anaemia Prevalence − Himachal Pradesh
WHO−2001 Adjustment Method

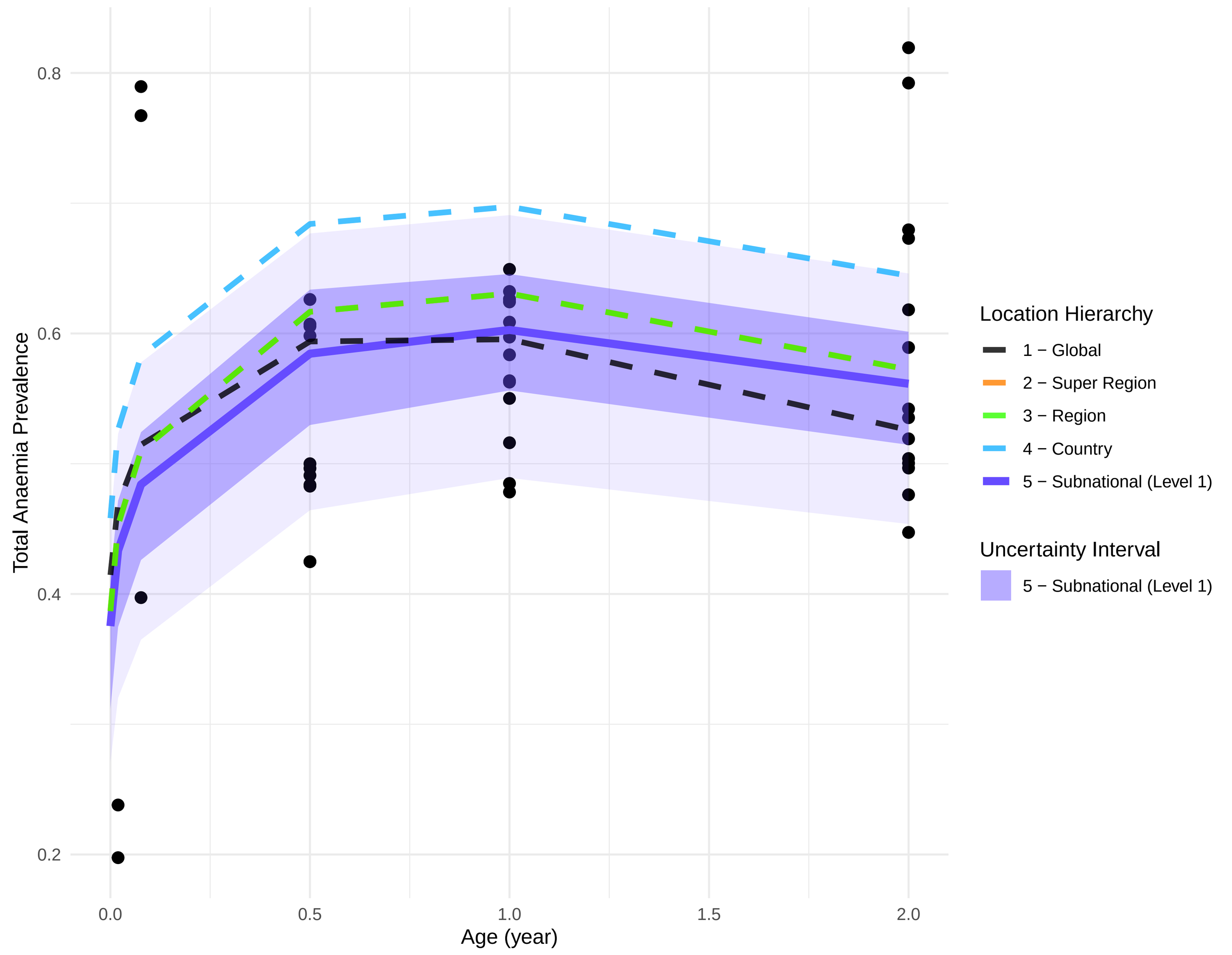

MR−BRT Age/Sex Fits − Males <2 Years Old − Total Anaemia Prevalence − Himachal Pradesh, Rural
WHO−2001 Adjustment Method

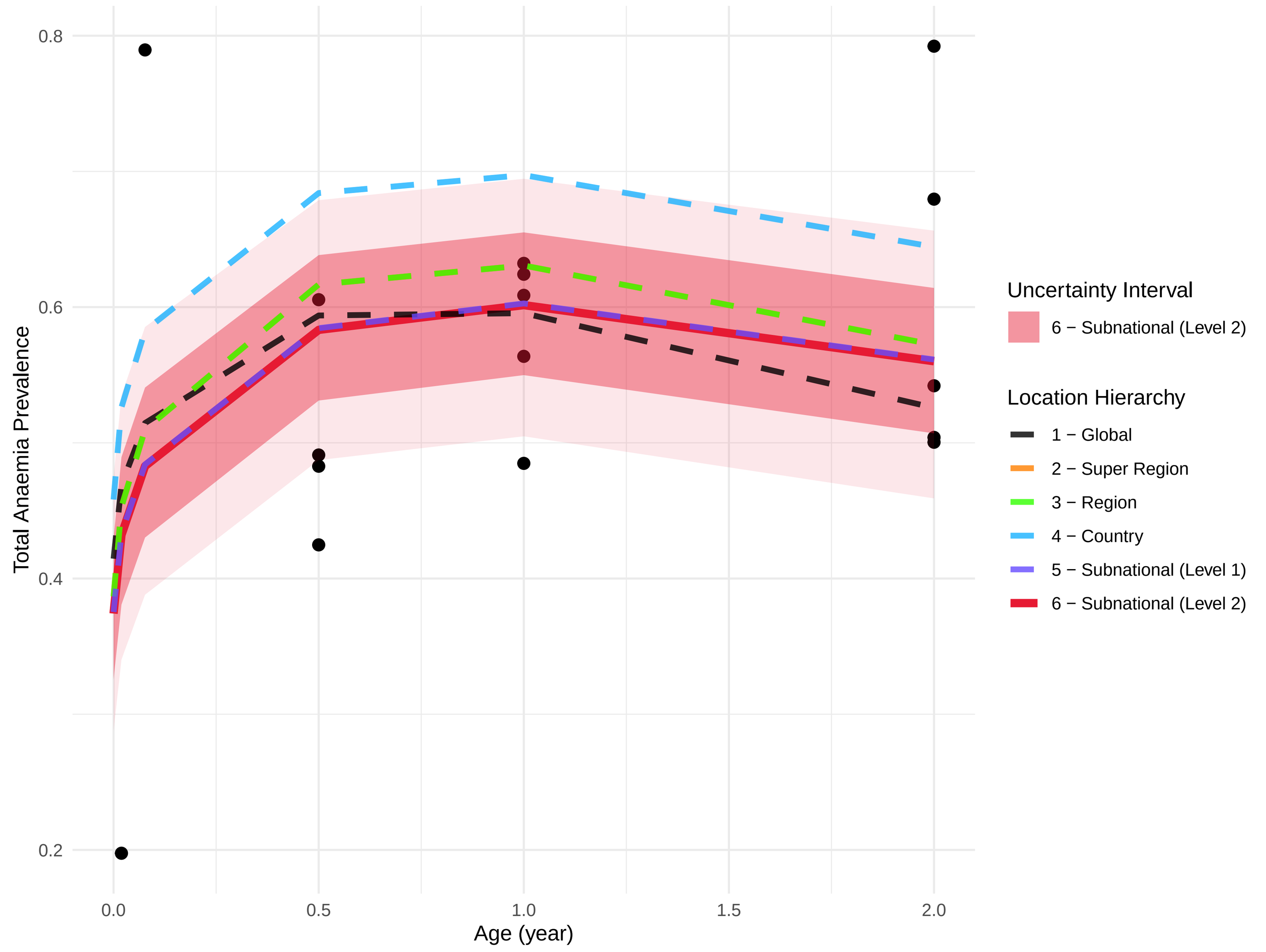

MR−BRT Age/Sex Fits − Males <2 Years Old − Total Anaemia Prevalence − Himachal Pradesh, Urban
WHO−2001 Adjustment Method

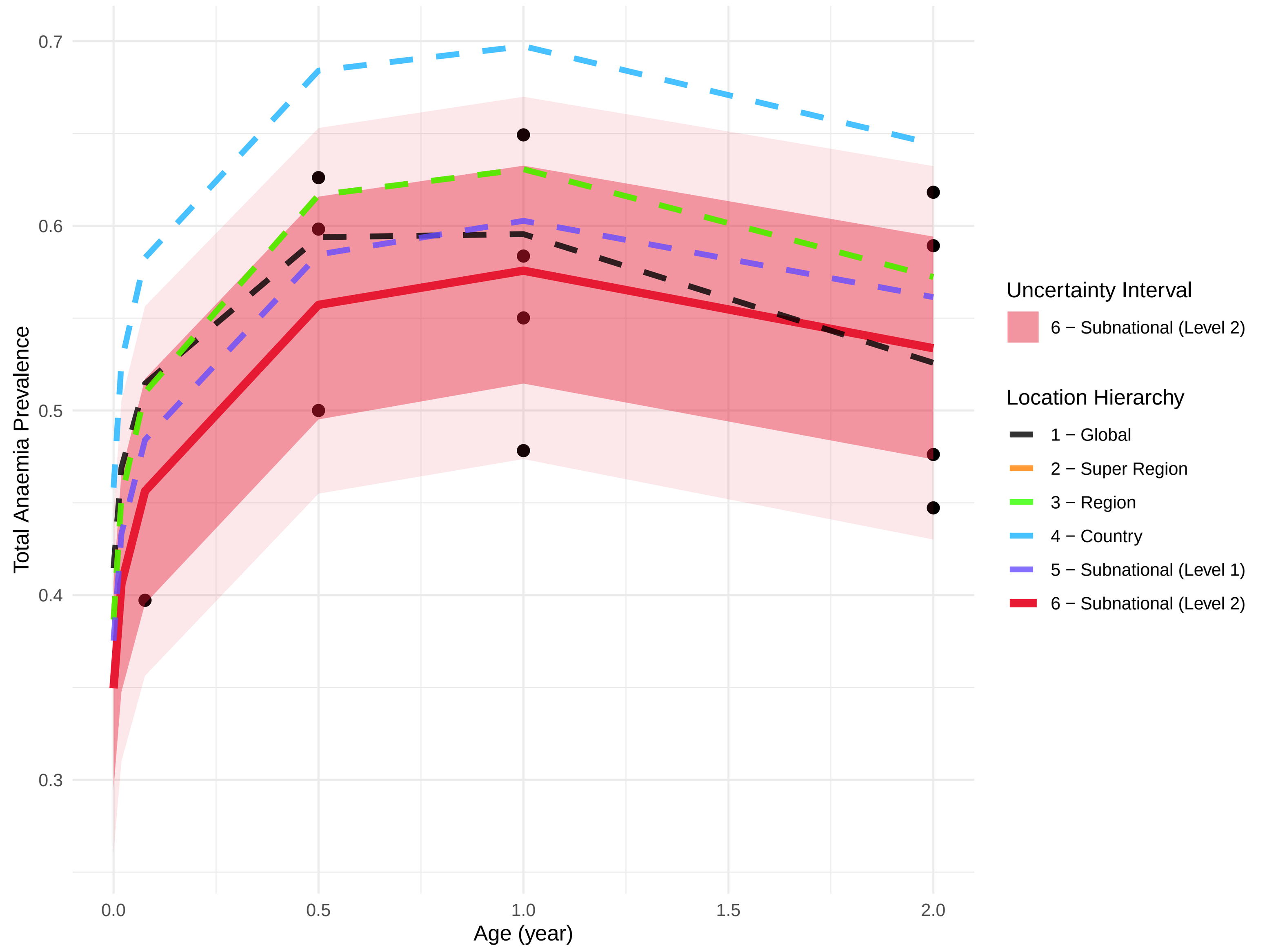

MR−BRT Age/Sex Fits − Males <2 Years Old − Total Anaemia Prevalence − Jammu & Kashmir and Ladakh
WHO−2001 Adjustment Method

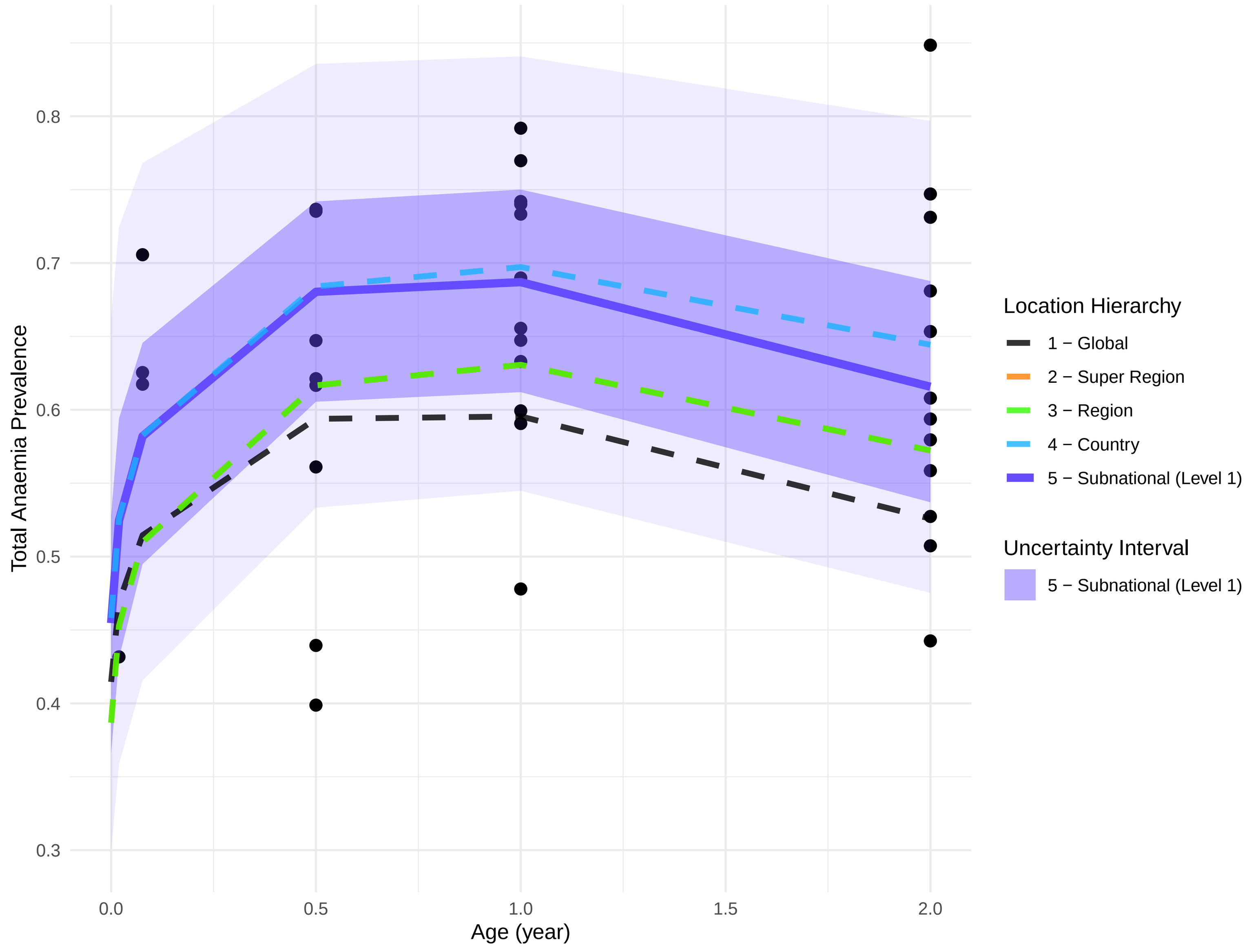

MR−BRT Age/Sex Fits − Males <2 Years Old − Total Anaemia Prevalence − Jammu & Kashmir and Ladakh, Rural
WHO−2001 Adjustment Method

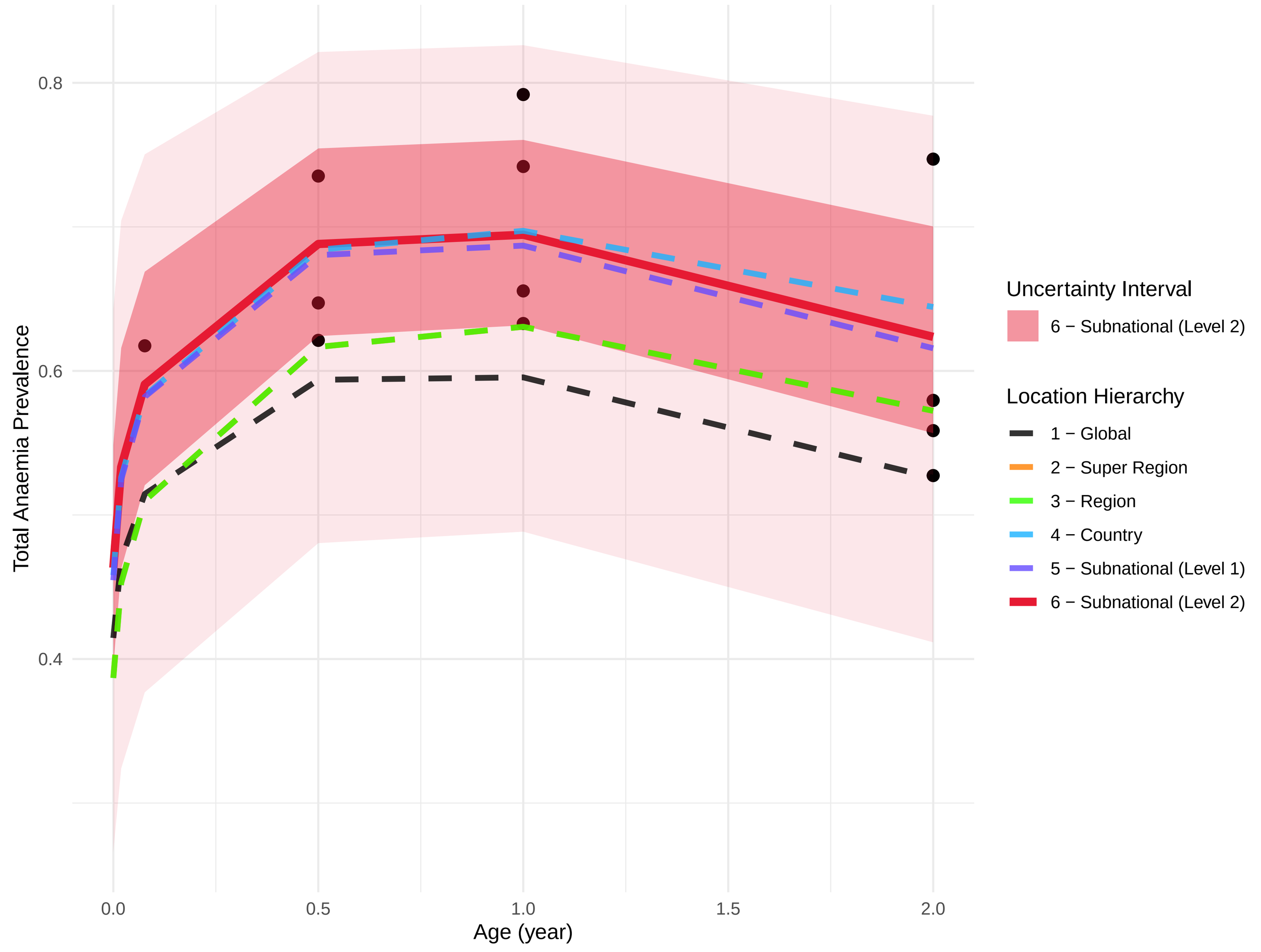

MR−BRT Age/Sex Fits − Males <2 Years Old − Total Anaemia Prevalence − Jammu & Kashmir and Ladakh, Urban
WHO−2001 Adjustment Method

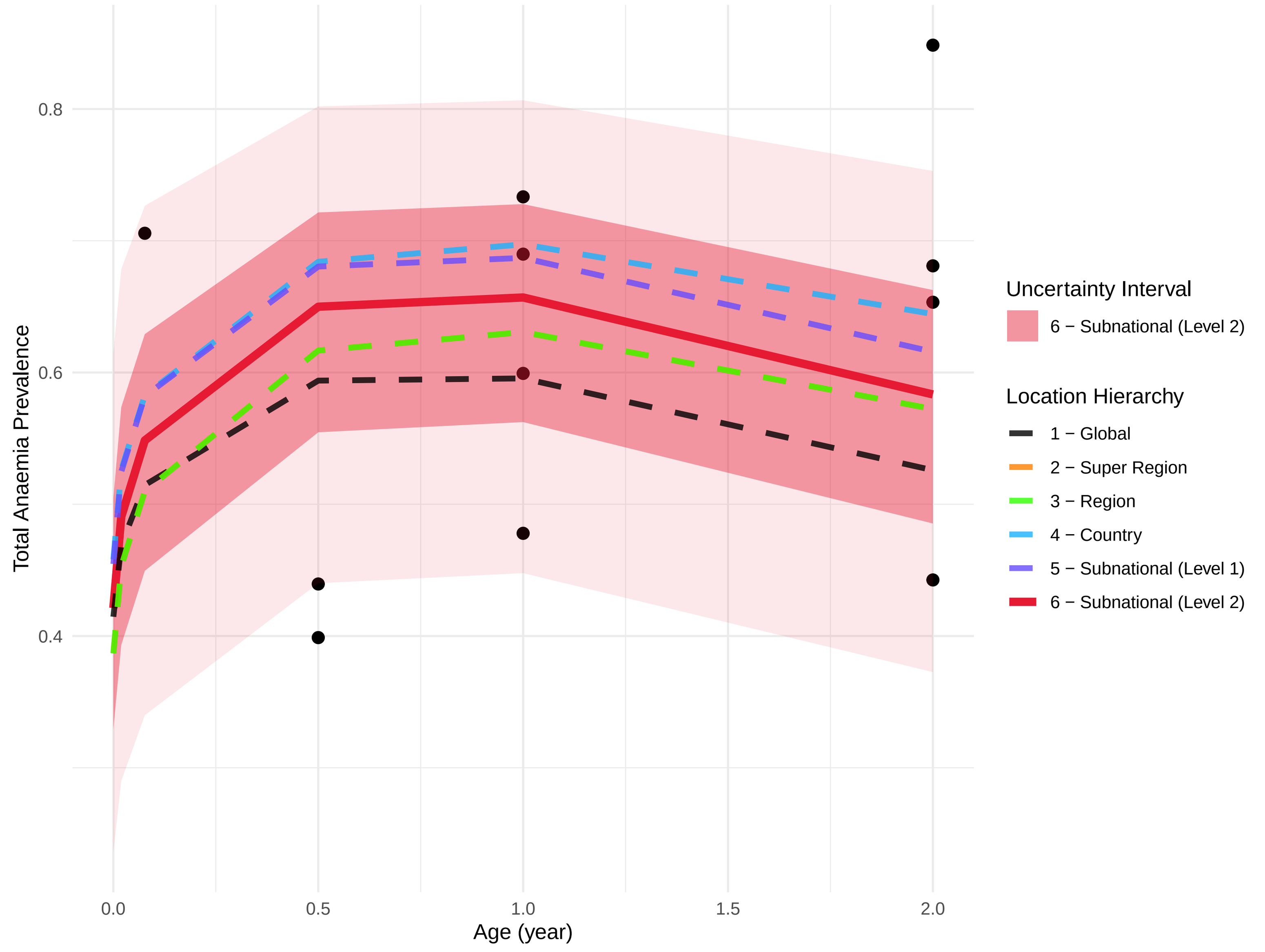

MR−BRT Age/Sex Fits − Males <2 Years Old − Total Anaemia Prevalence − Jharkhand
WHO−2001 Adjustment Method

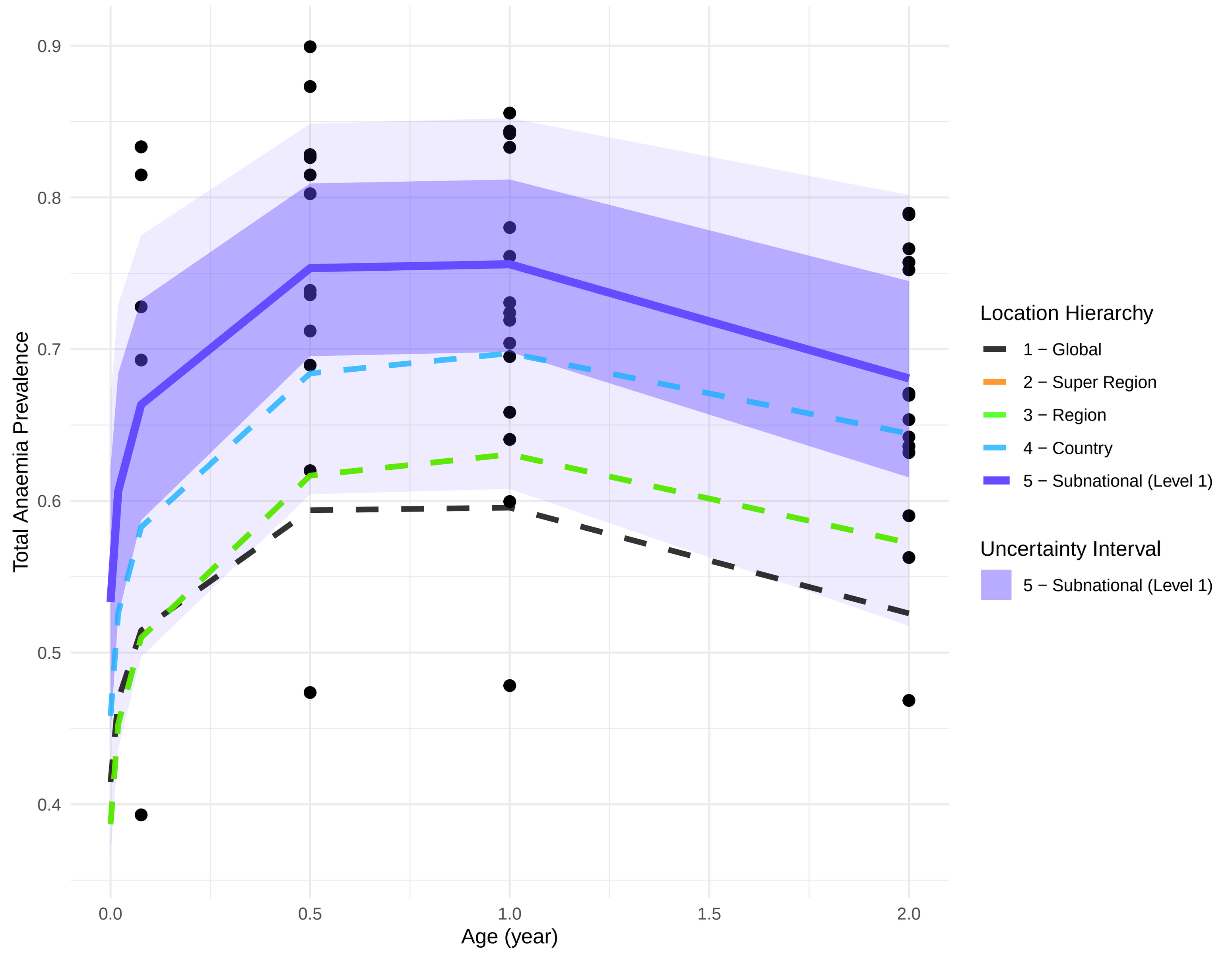

MR−BRT Age/Sex Fits − Males <2 Years Old − Total Anaemia Prevalence − Jharkhand, Rural
WHO−2001 Adjustment Method

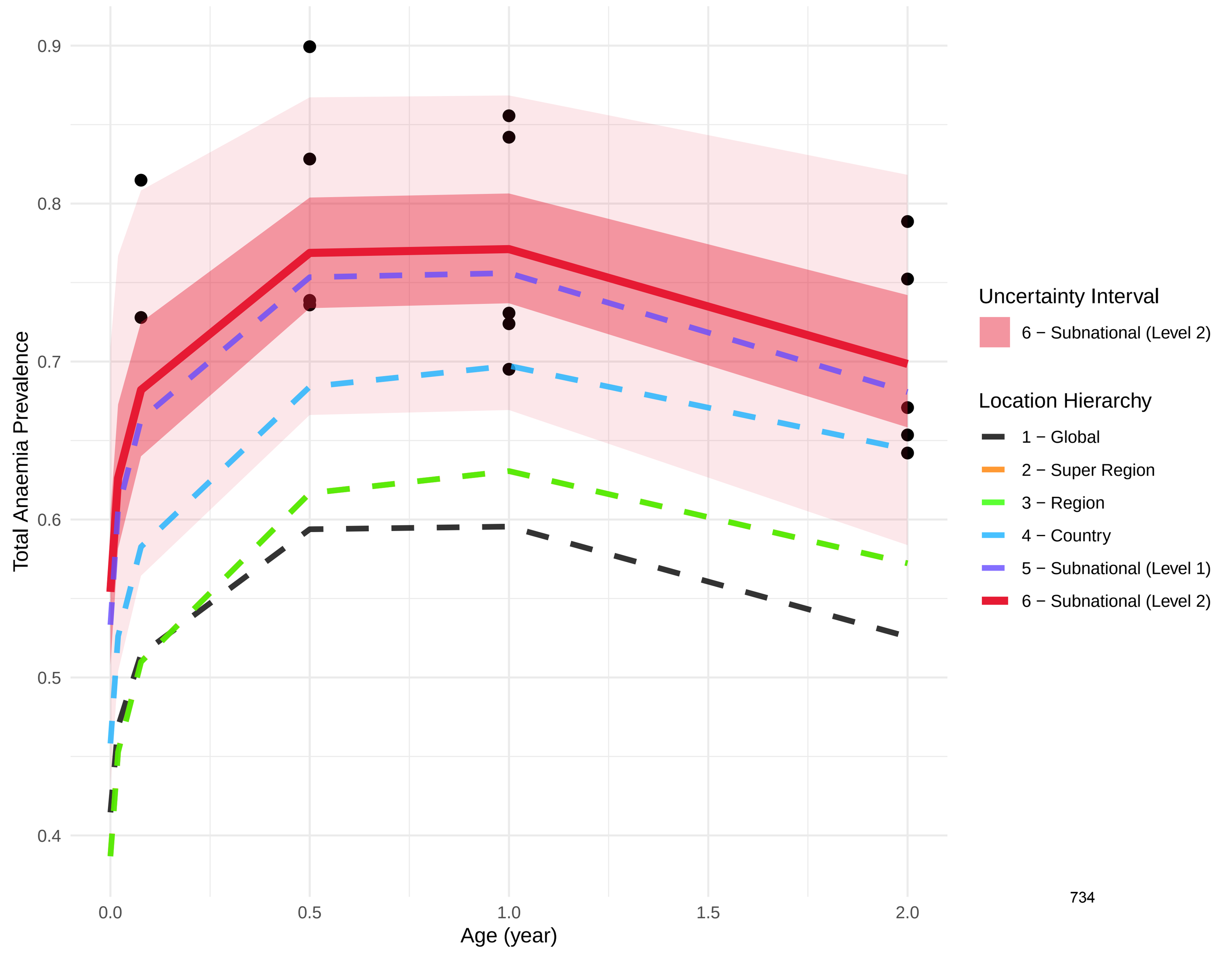

MR−BRT Age/Sex Fits − Males <2 Years Old − Total Anaemia Prevalence − Jharkhand, Urban

WHO−2001 Adjustment Method

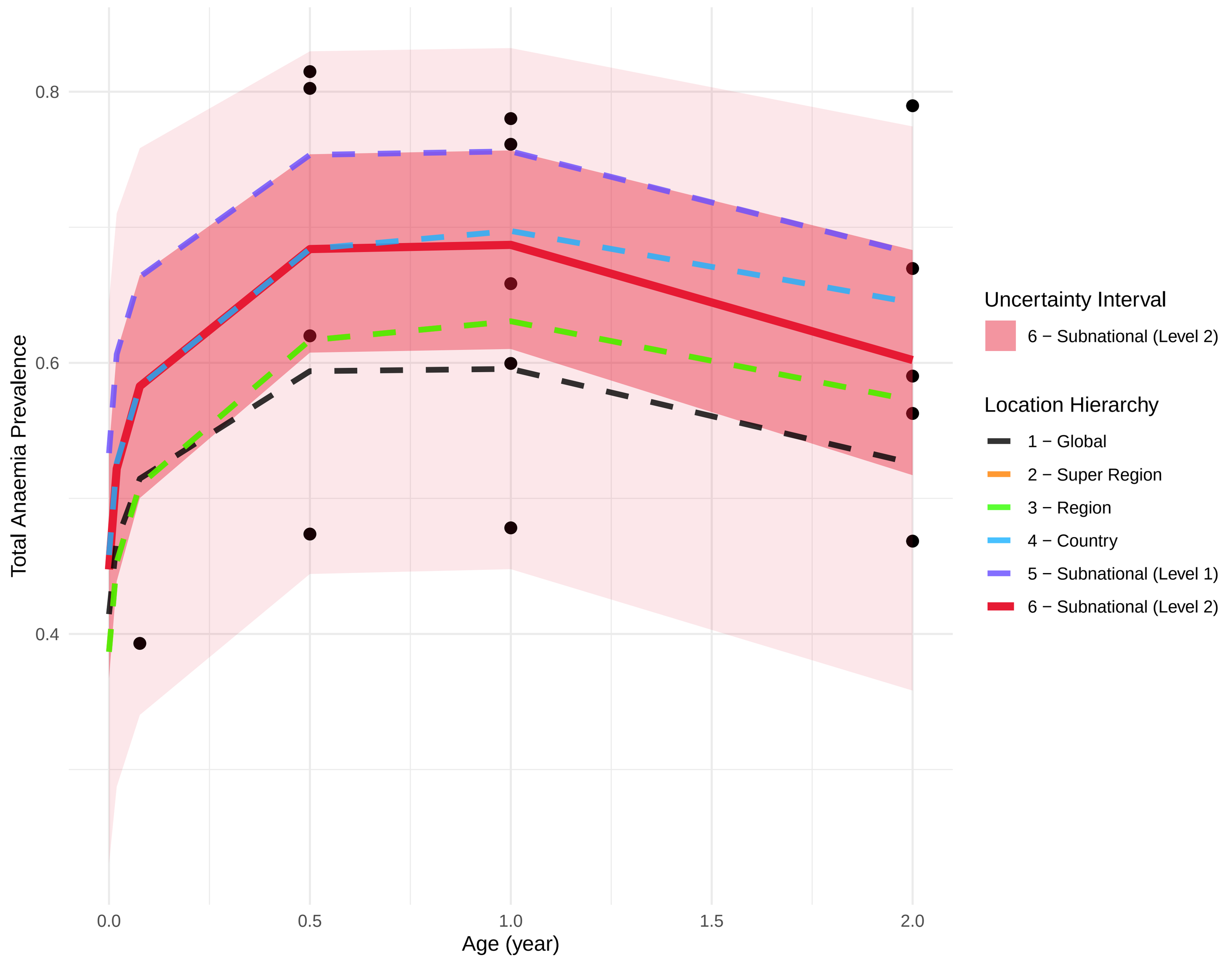

MR−BRT Age/Sex Fits − Males <2 Years Old − Total Anaemia Prevalence − Karnataka

WHO−2001 Adjustment Method

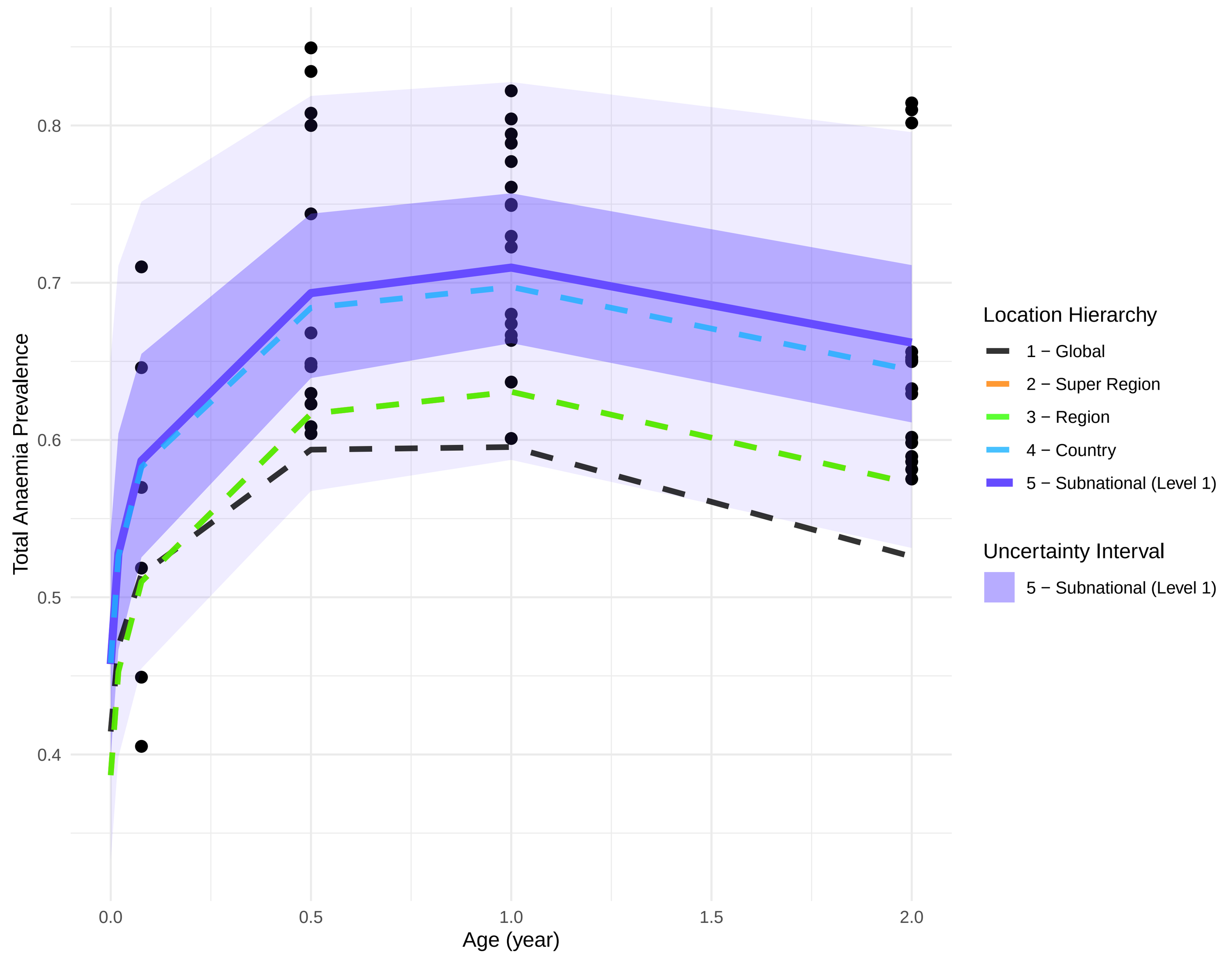

MR−BRT Age/Sex Fits − Males <2 Years Old − Total Anaemia Prevalence − Karnataka, Rural

WHO−2001 Adjustment Method

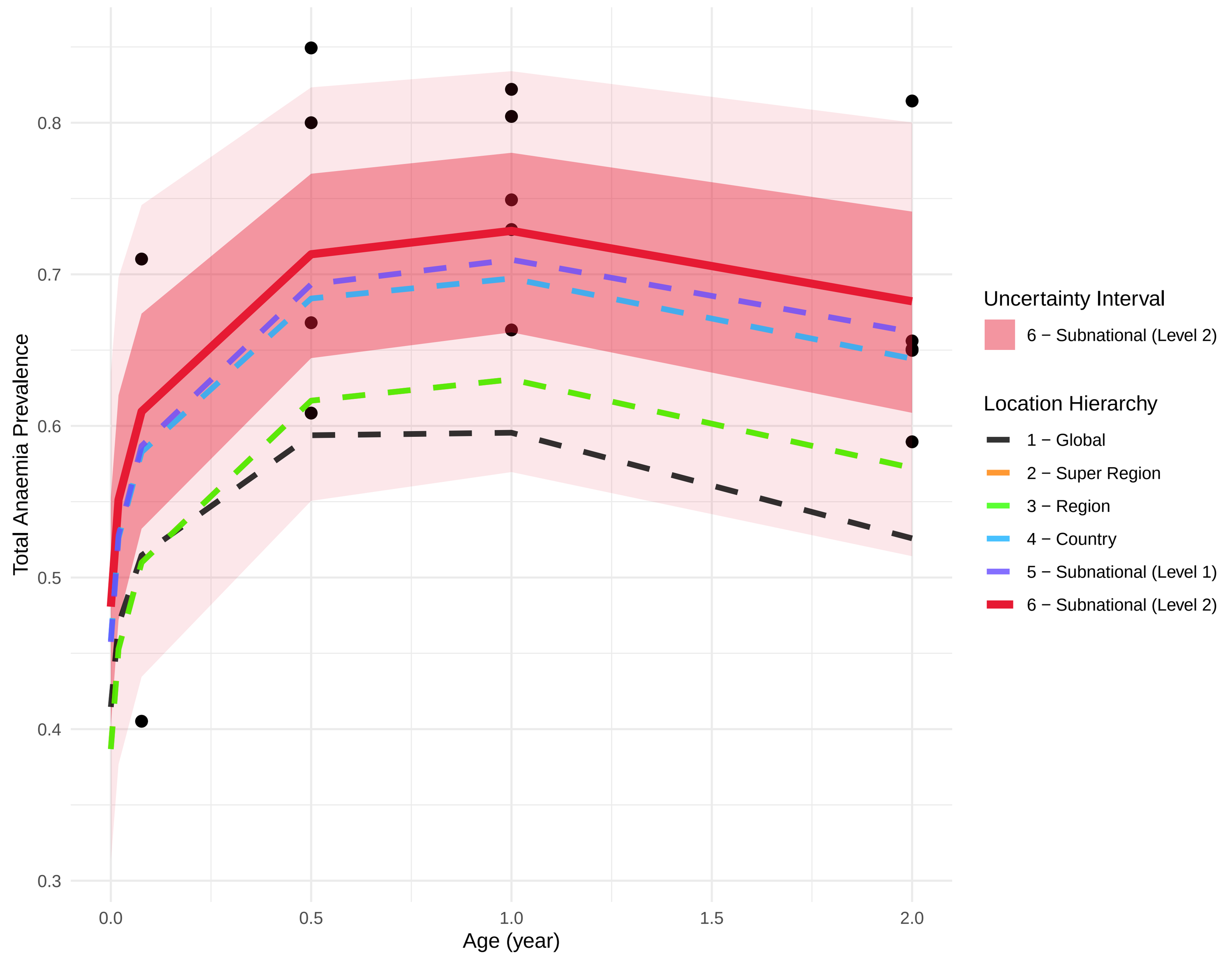

MR−BRT Age/Sex Fits − Males <2 Years Old − Total Anaemia Prevalence − Karnataka, Urban

WHO−2001 Adjustment Method

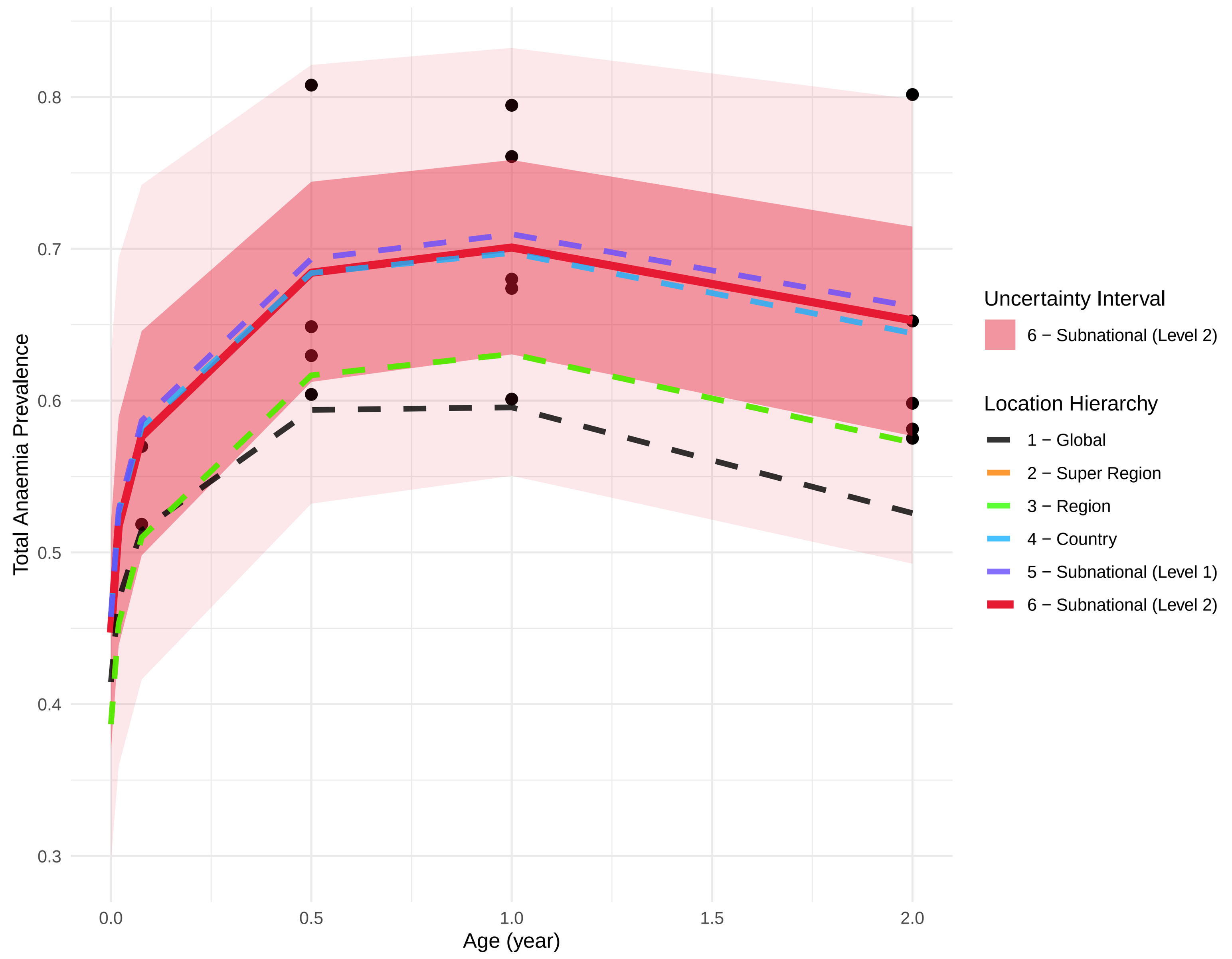

MR−BRT Age/Sex Fits − Males <2 Years Old − Total Anaemia Prevalence − Kerala

WHO−2001 Adjustment Method

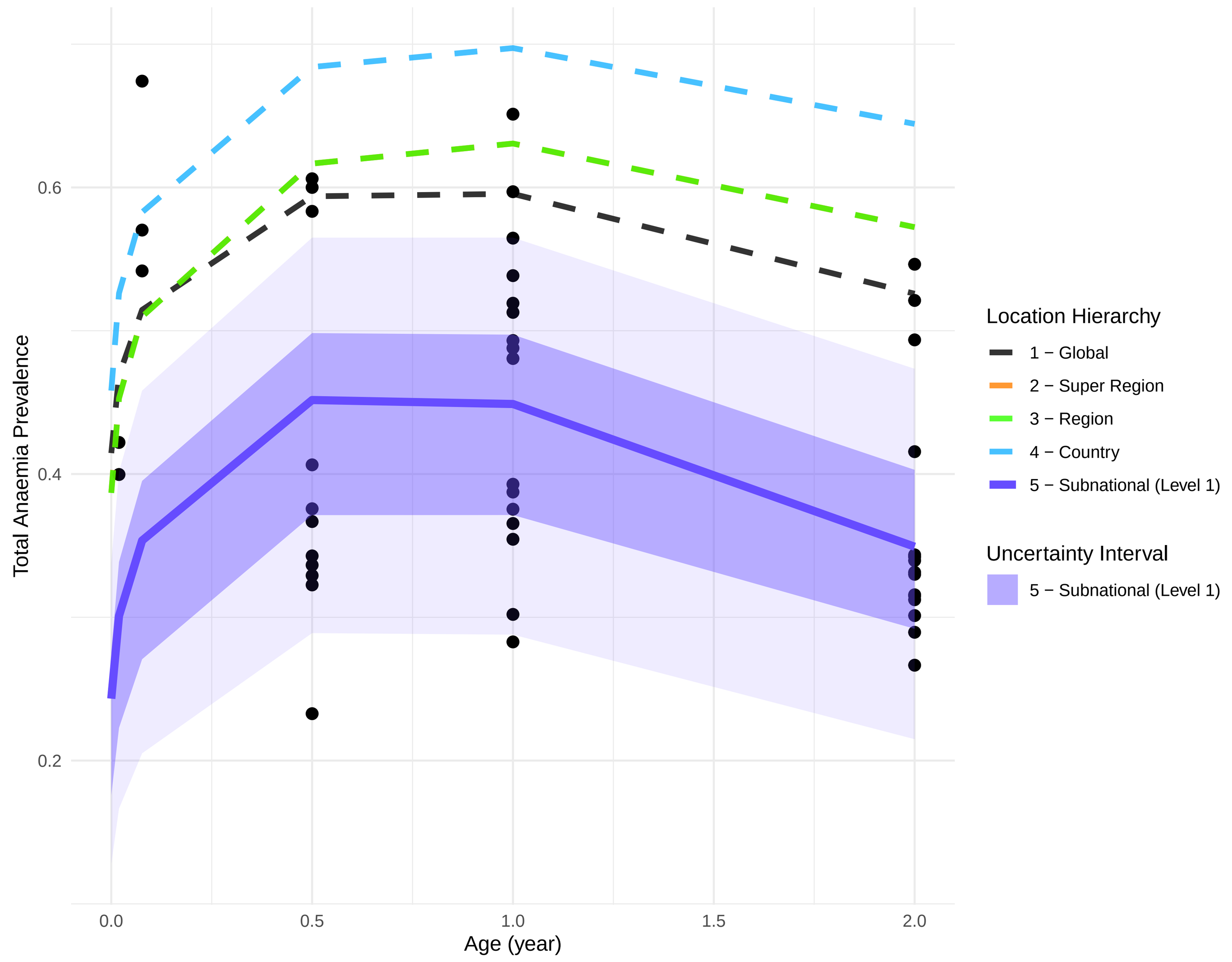

MR−BRT Age/Sex Fits − Males <2 Years Old − Total Anaemia Prevalence − Kerala, Rural

WHO−2001 Adjustment Method

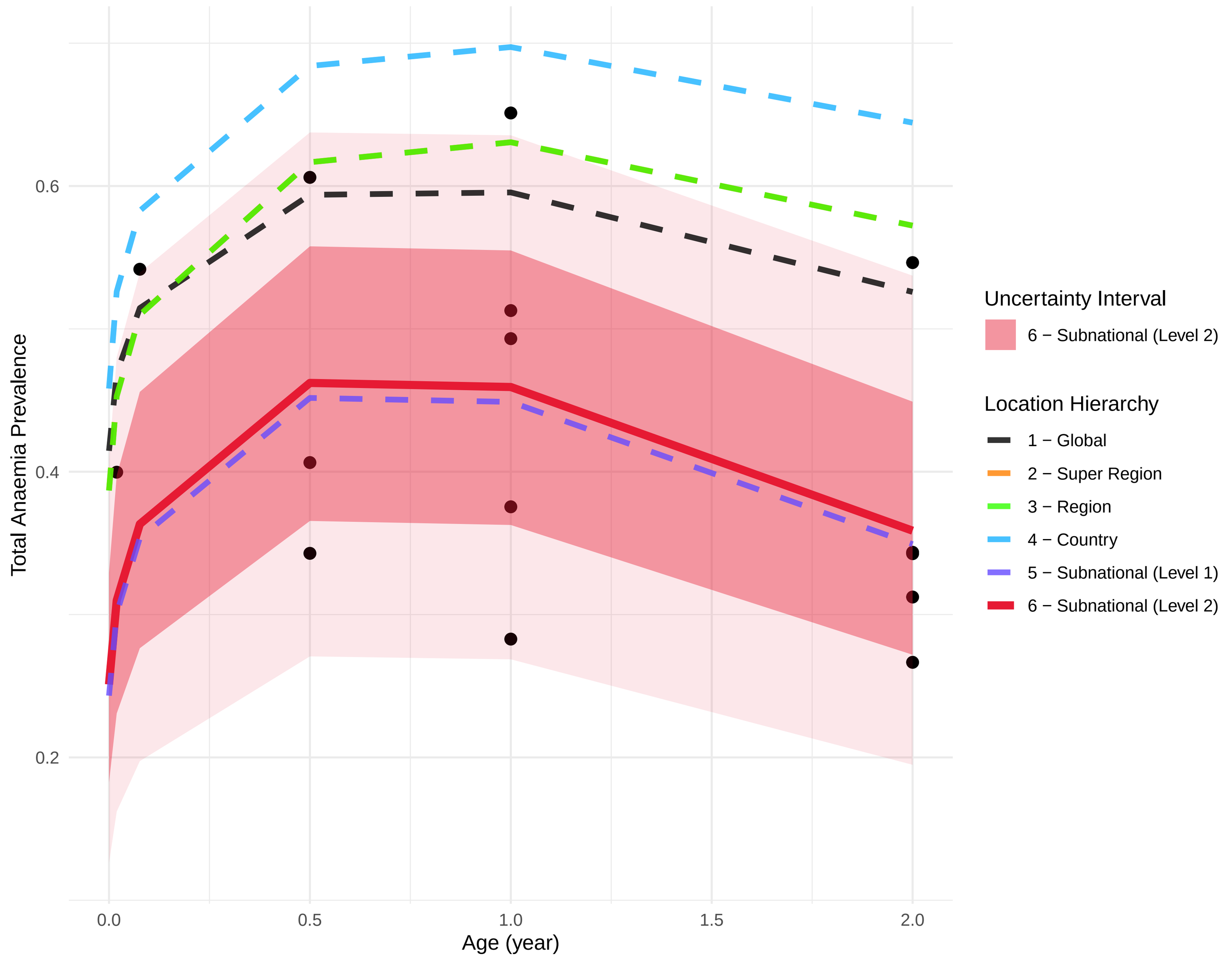

MR−BRT Age/Sex Fits − Males <2 Years Old − Total Anaemia Prevalence − Kerala, Urban

WHO−2001 Adjustment Method

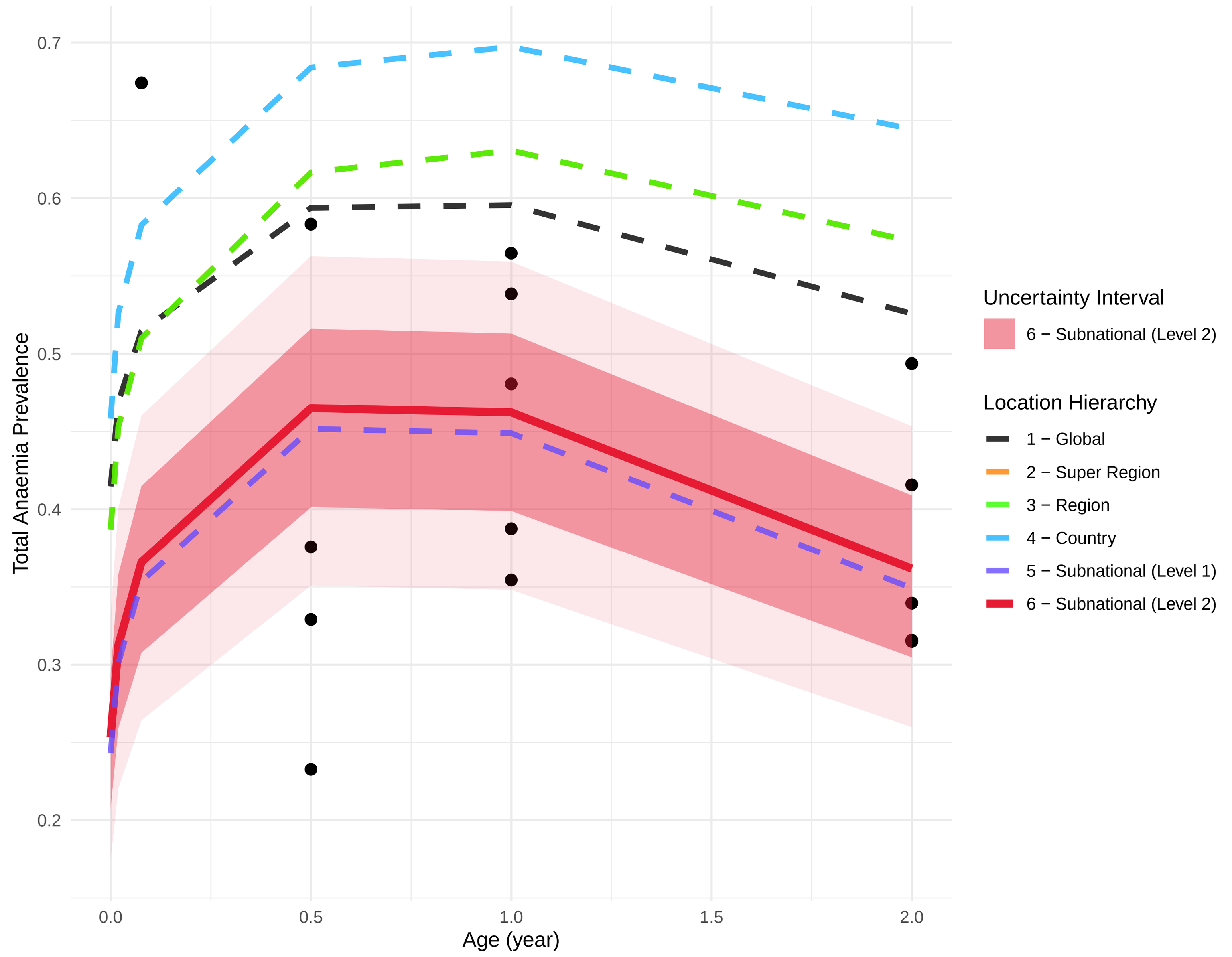

MR−BRT Age/Sex Fits − Males <2 Years Old − Total Anaemia Prevalence − Madhya Pradesh

WHO−2001 Adjustment Method

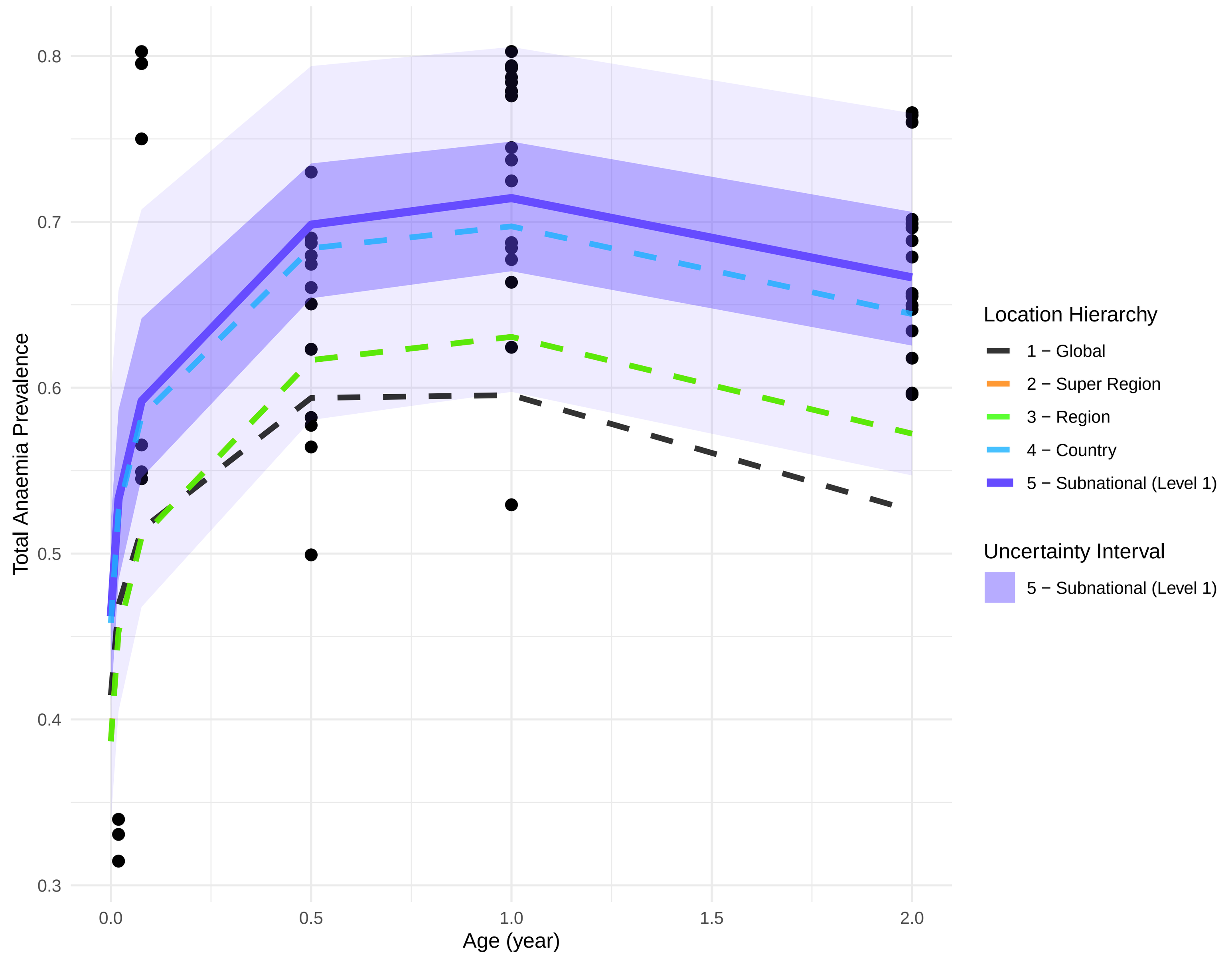

MR−BRT Age/Sex Fits − Males <2 Years Old − Total Anaemia Prevalence − Madhya Pradesh, Rural

WHO−2001 Adjustment Method

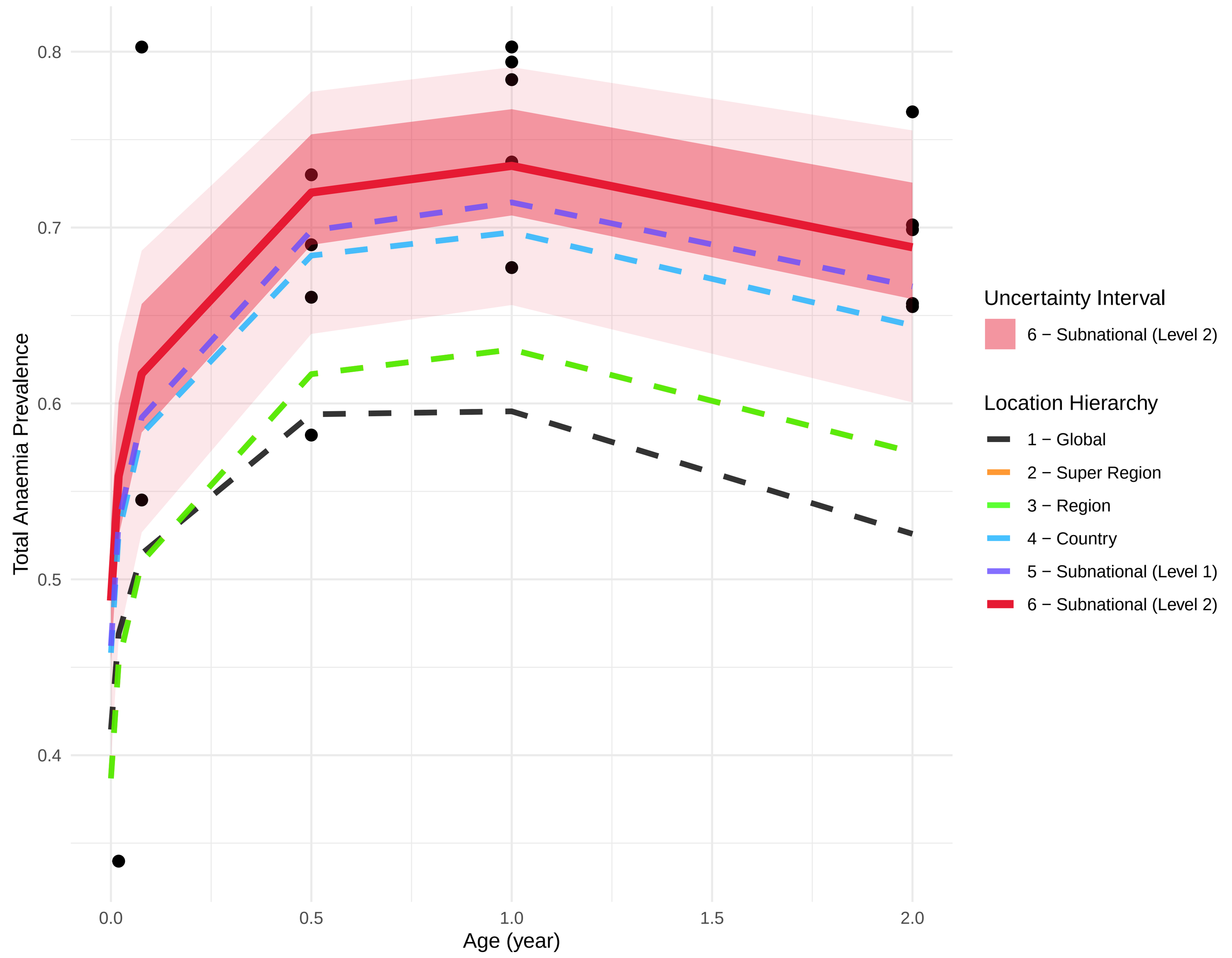

MR−BRT Age/Sex Fits − Males <2 Years Old − Total Anaemia Prevalence − Madhya Pradesh, Urban

WHO−2001 Adjustment Method

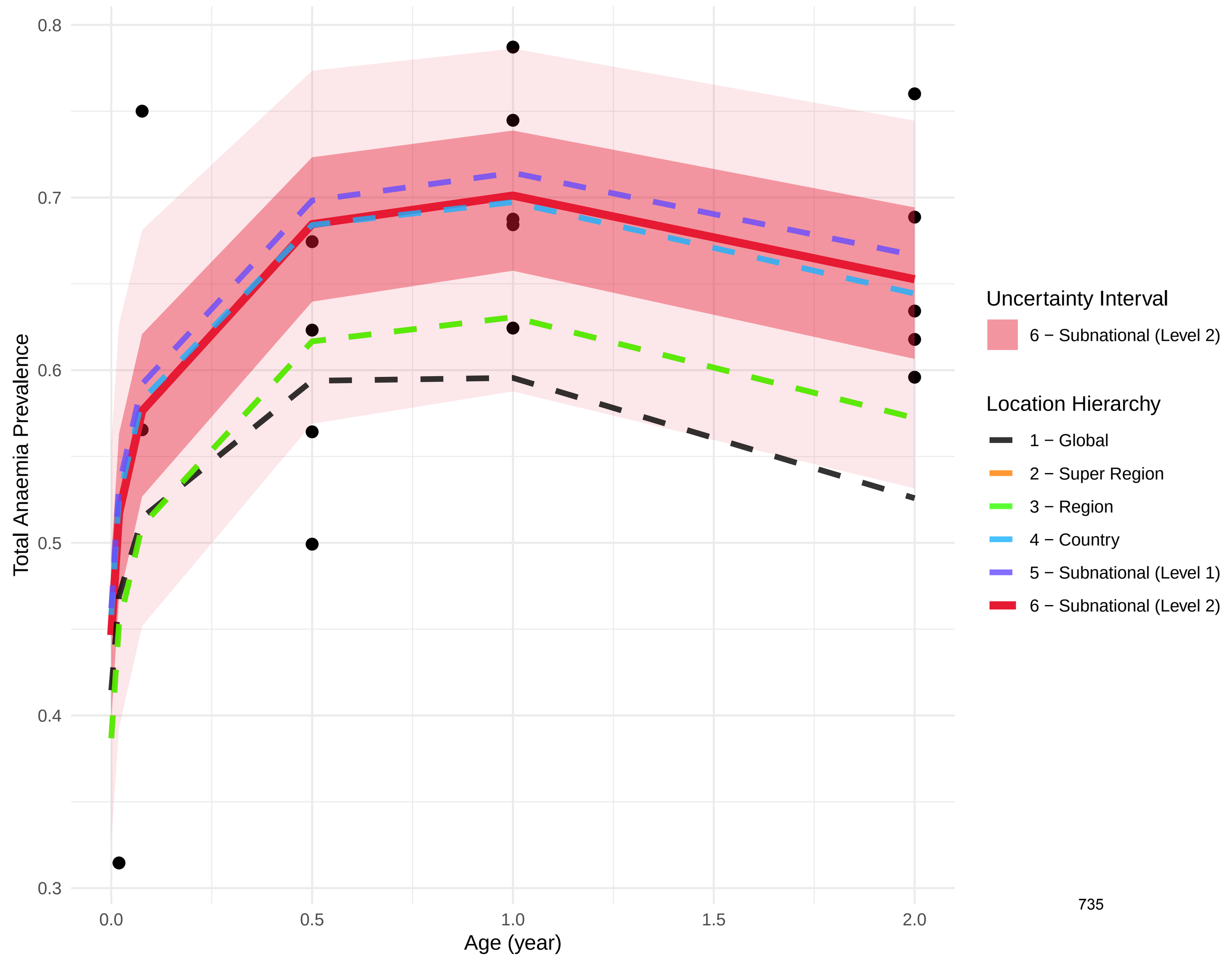

MR−BRT Age/Sex Fits − Males <2 Years Old − Total Anaemia Prevalence − Maharashtra
WHO−2001 Adjustment Method

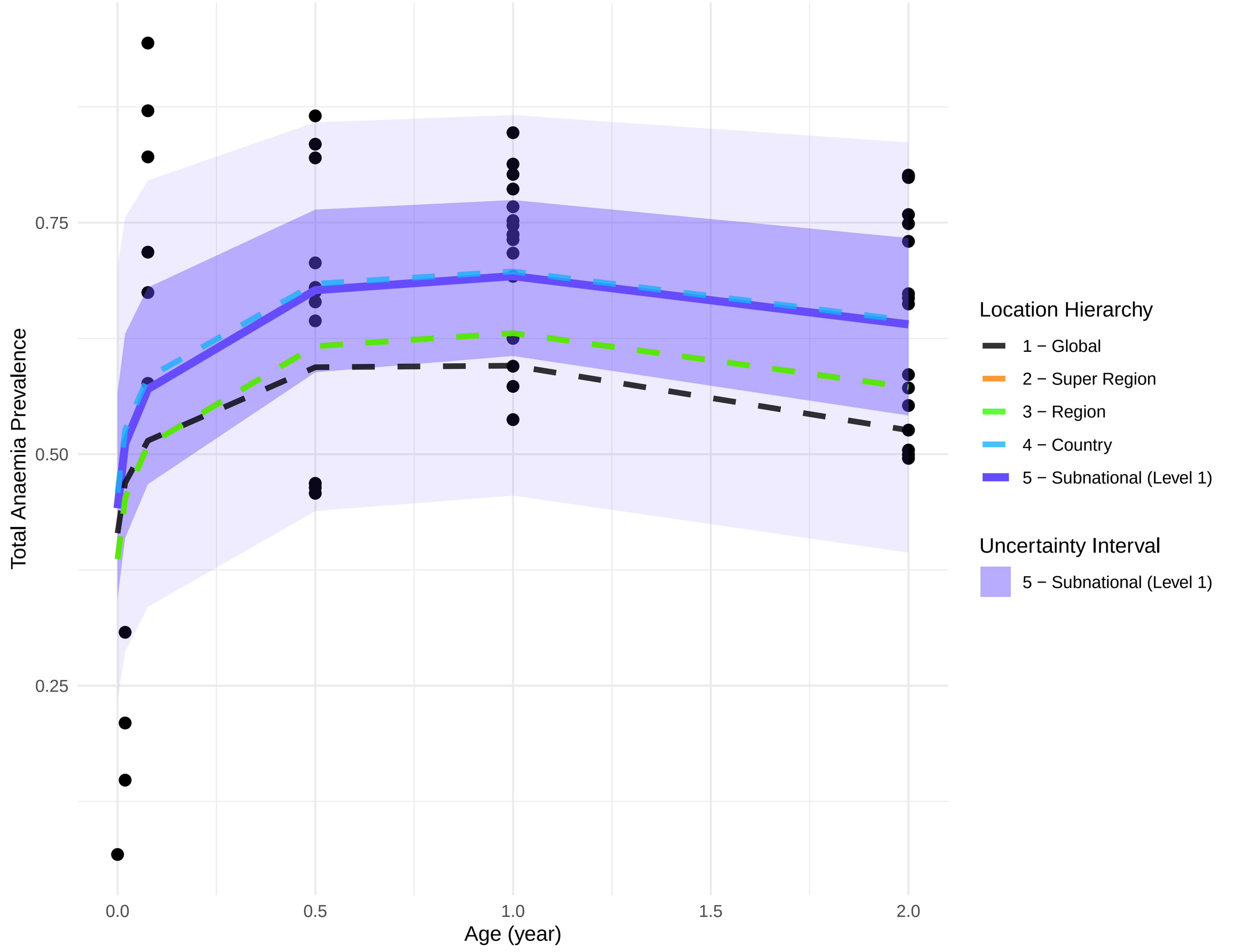

MR−BRT Age/Sex Fits − Males <2 Years Old − Total Anaemia Prevalence − Maharashtra, Rural
WHO−2001 Adjustment Method

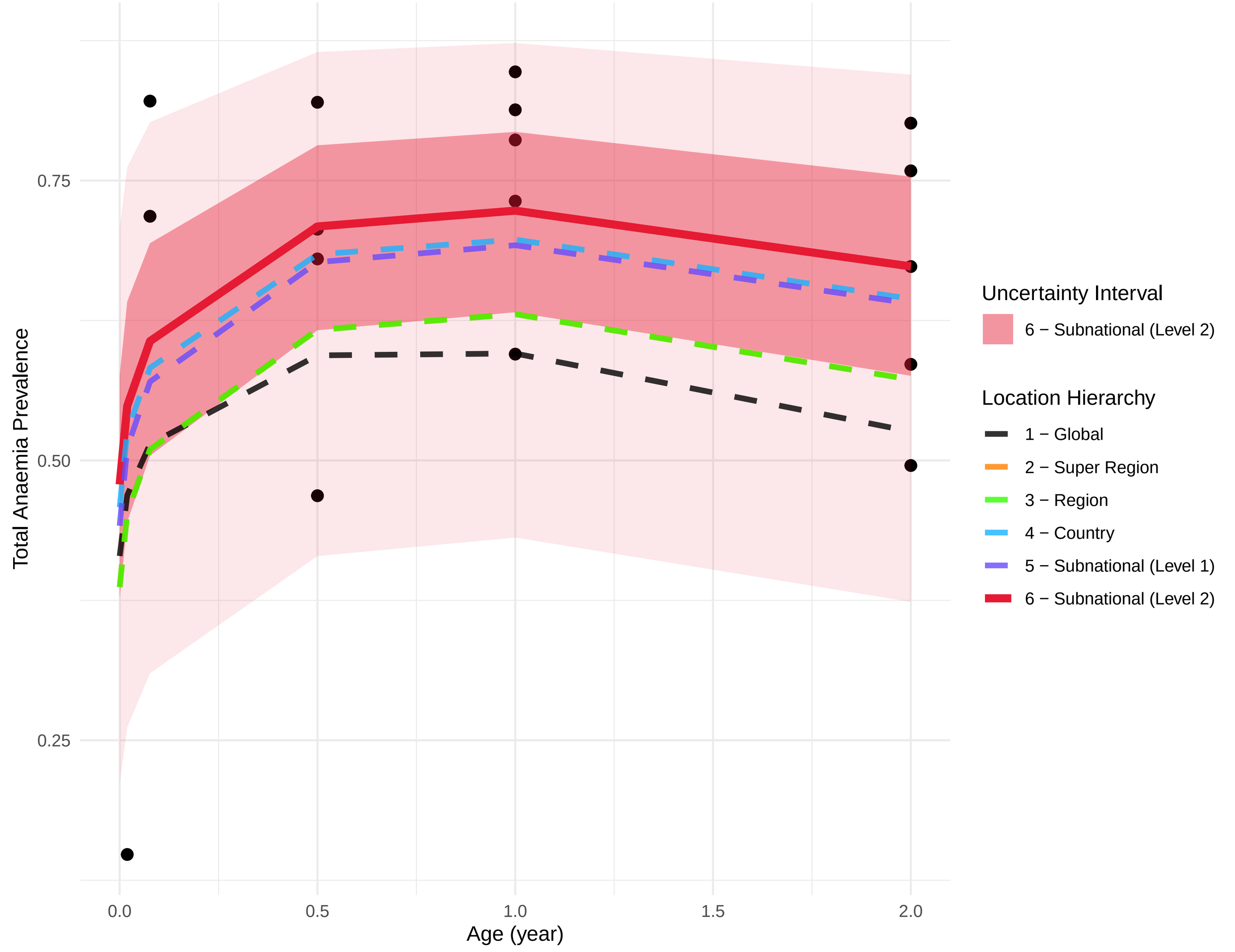

MR−BRT Age/Sex Fits − Males <2 Years Old − Total Anaemia Prevalence − Maharashtra, Urban
WHO−2001 Adjustment Method

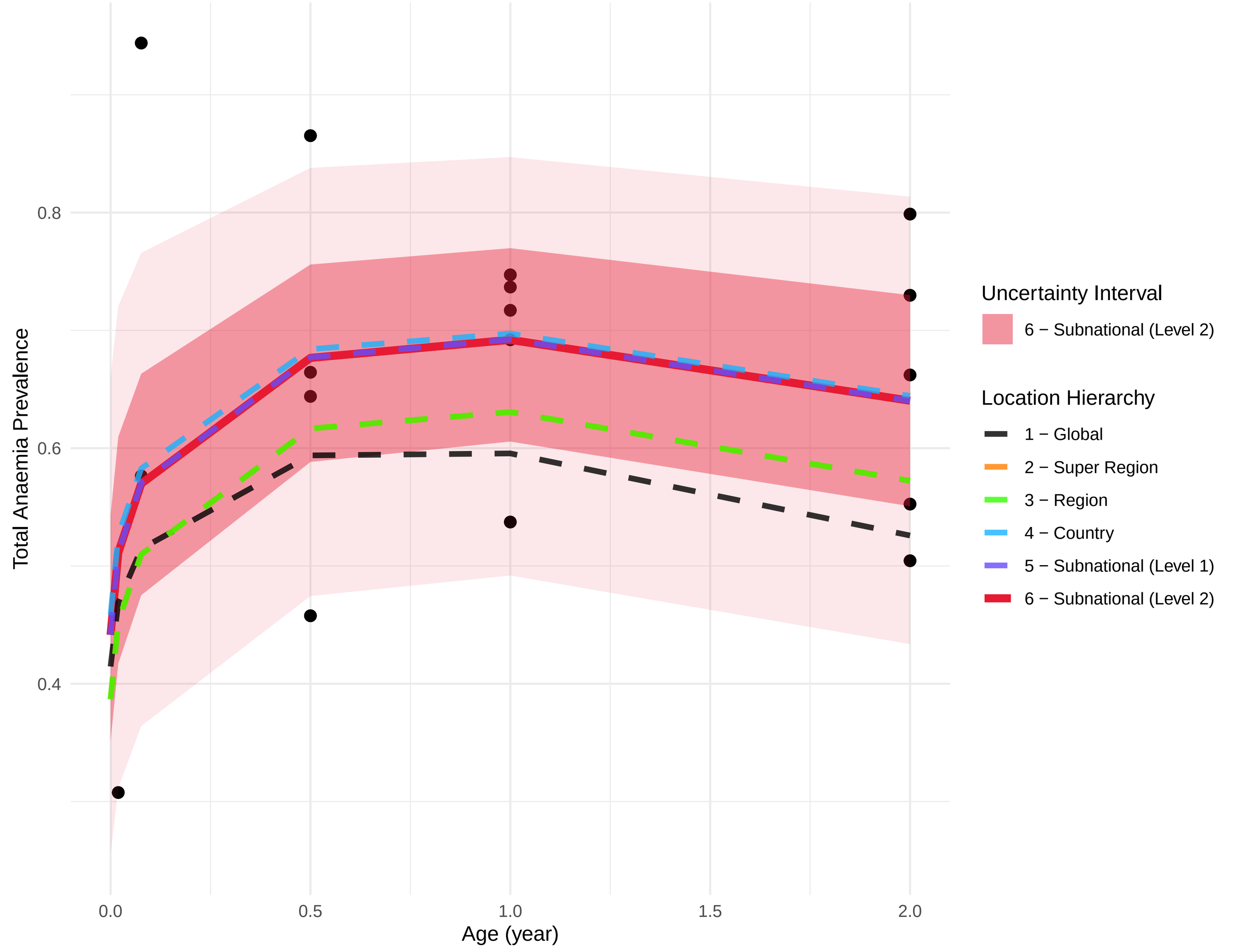

MR−BRT Age/Sex Fits − Males <2 Years Old − Total Anaemia Prevalence − Manipur
WHO−2001 Adjustment Method

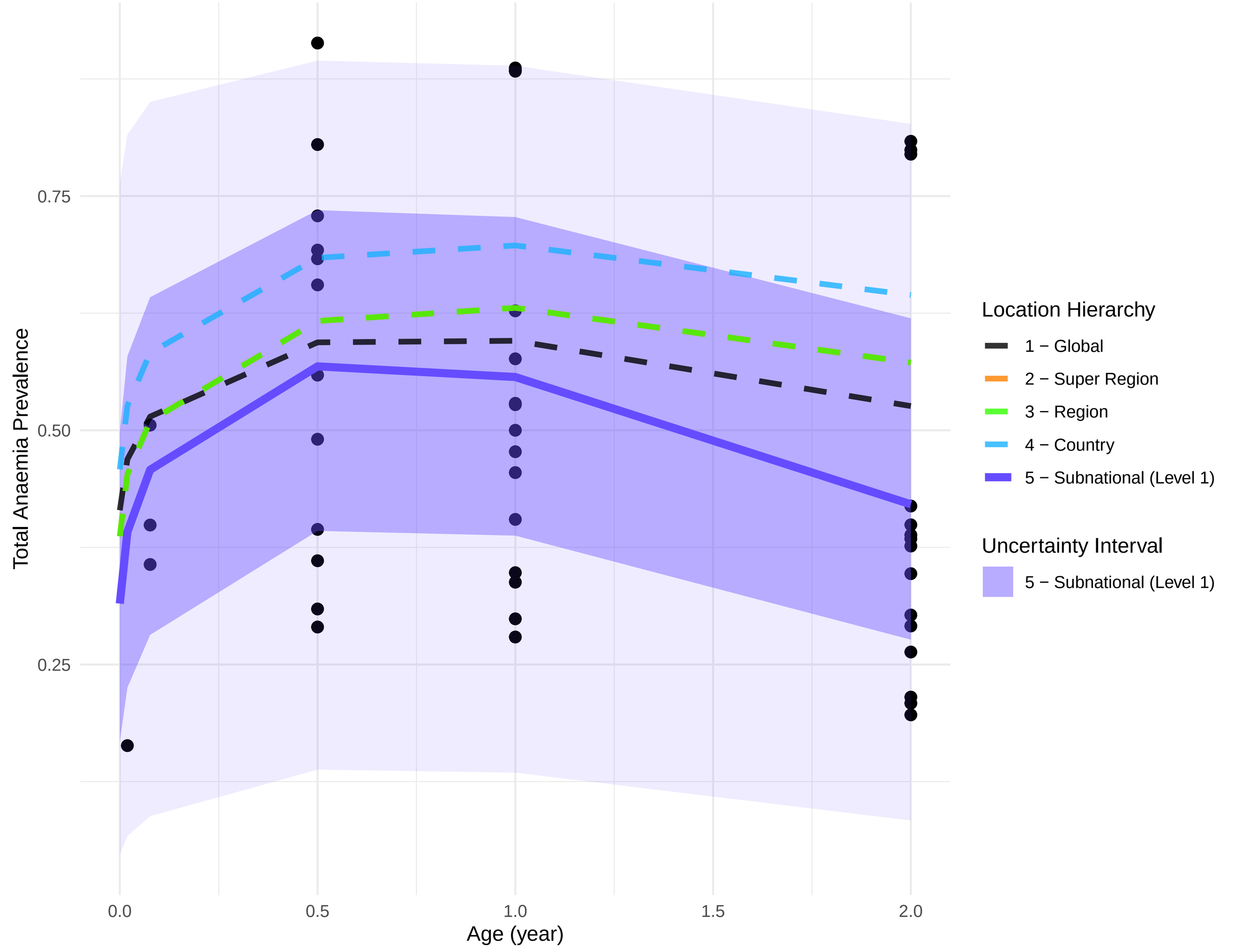

MR−BRT Age/Sex Fits − Males <2 Years Old − Total Anaemia Prevalence − Manipur, Rural
WHO−2001 Adjustment Method

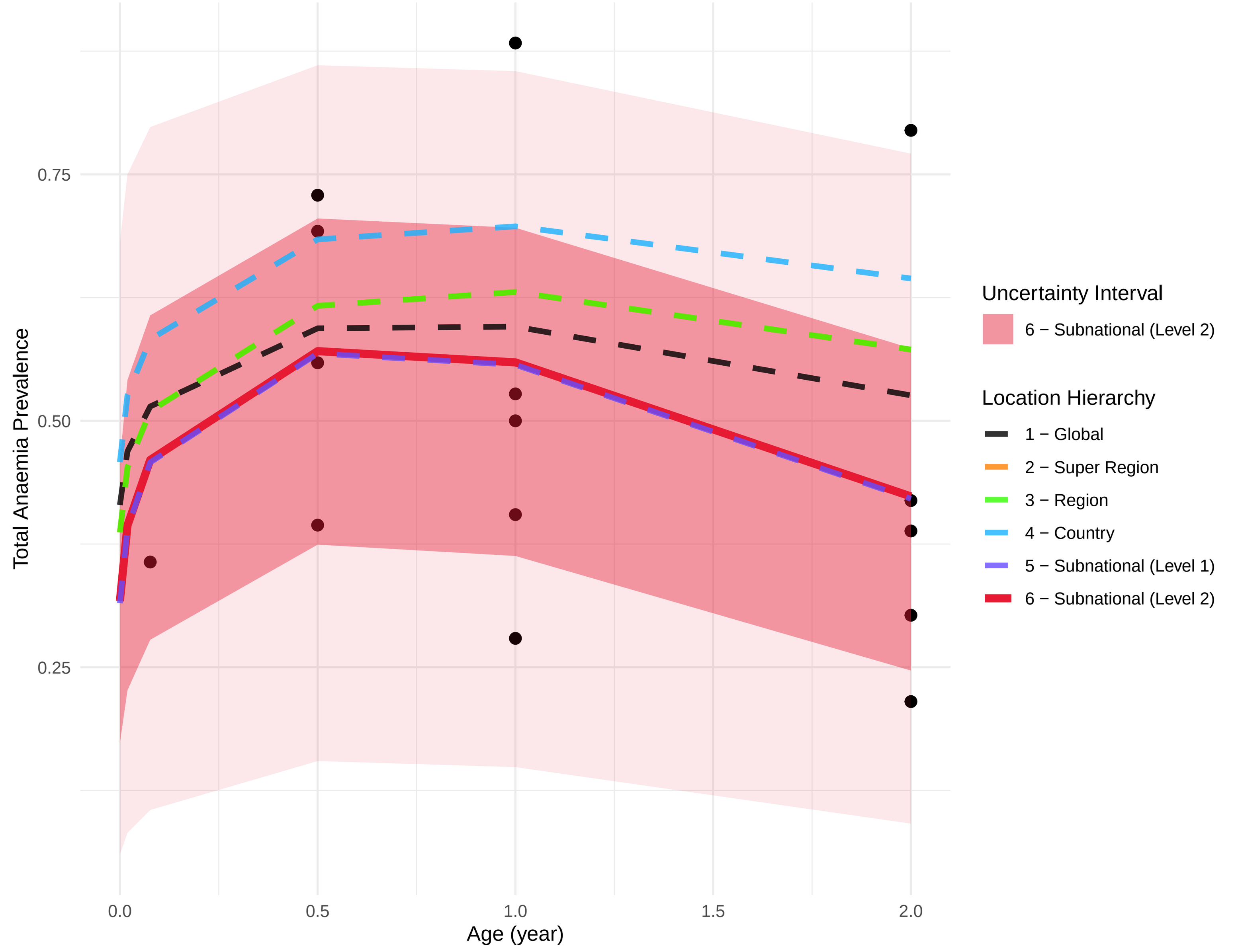

MR−BRT Age/Sex Fits − Males <2 Years Old − Total Anaemia Prevalence − Manipur, Urban
WHO−2001 Adjustment Method

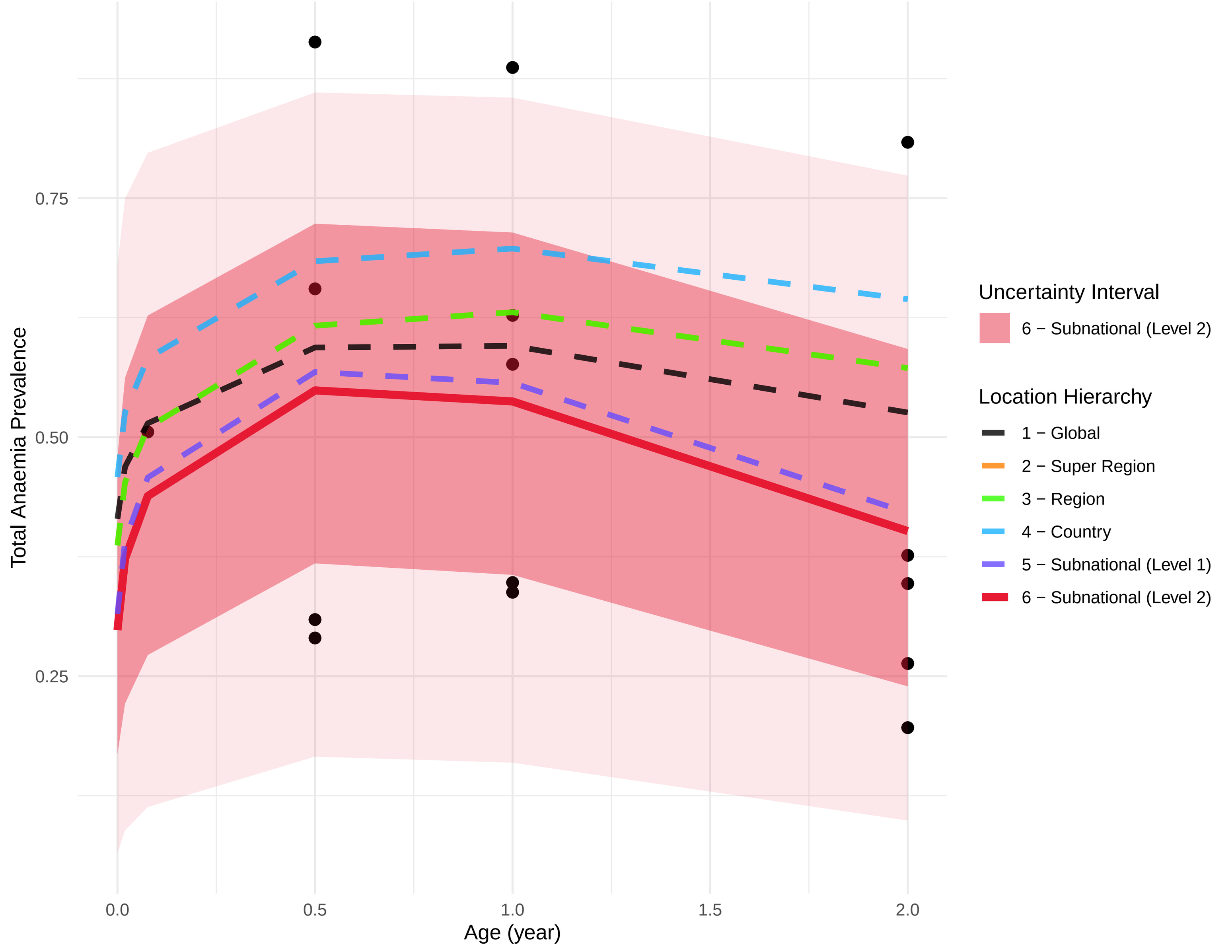

MR−BRT Age/Sex Fits − Males <2 Years Old − Total Anaemia Prevalence − Meghalaya
WHO−2001 Adjustment Method

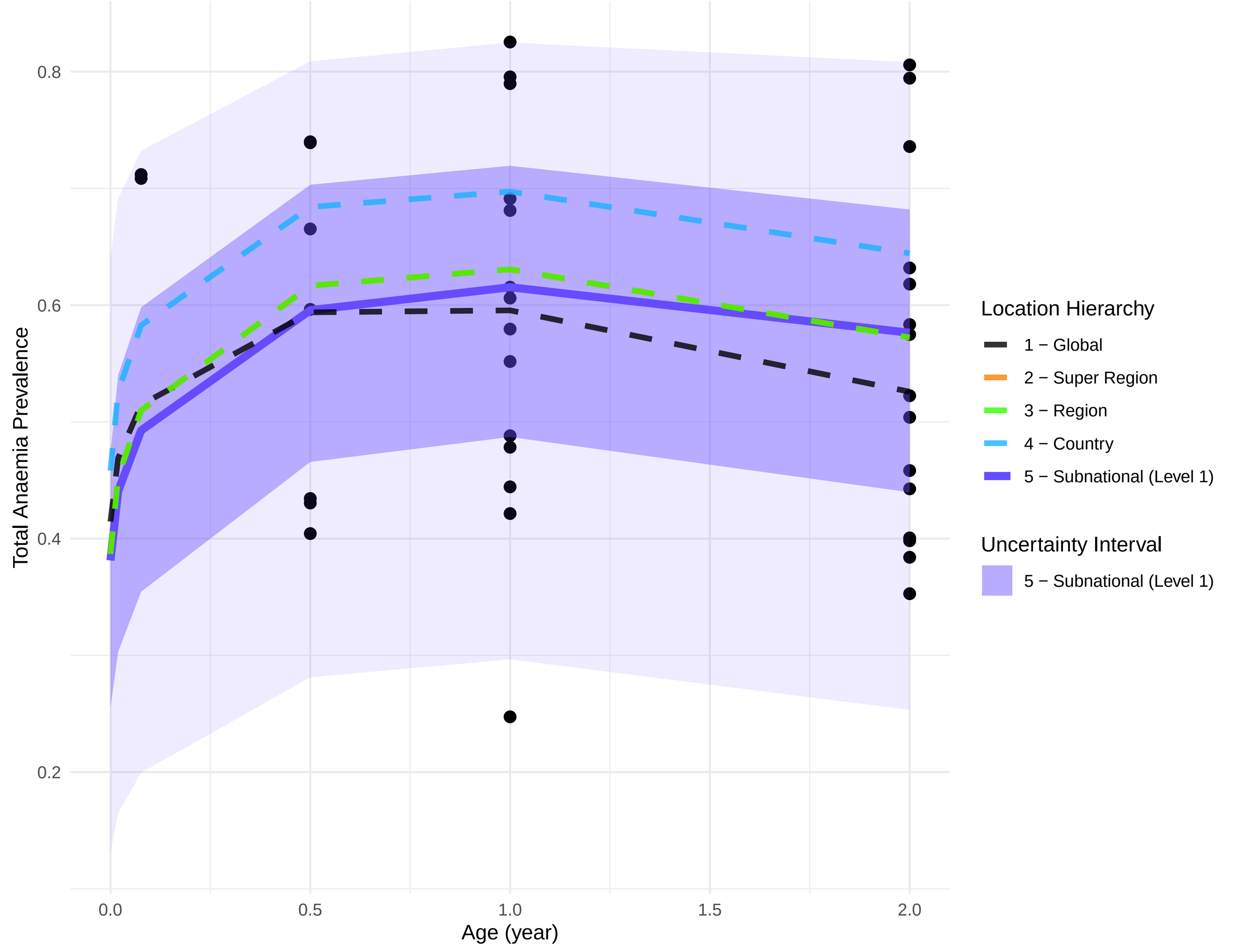

MR−BRT Age/Sex Fits − Males <2 Years Old − Total Anaemia Prevalence − Meghalaya, Rural
WHO−2001 Adjustment Method

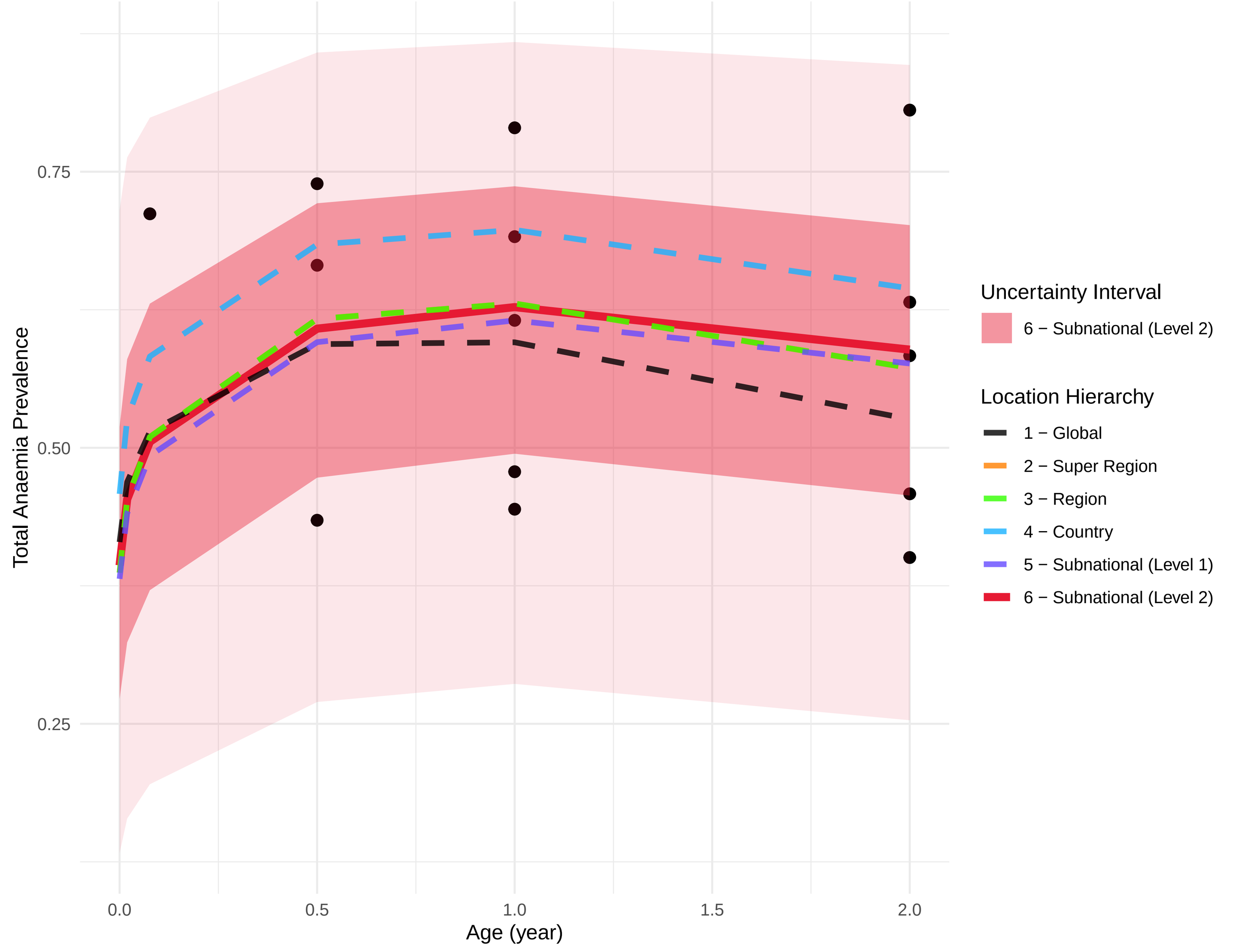

MR−BRT Age/Sex Fits − Males <2 Years Old − Total Anaemia Prevalence − Meghalaya, Urban
WHO−2001 Adjustment Method

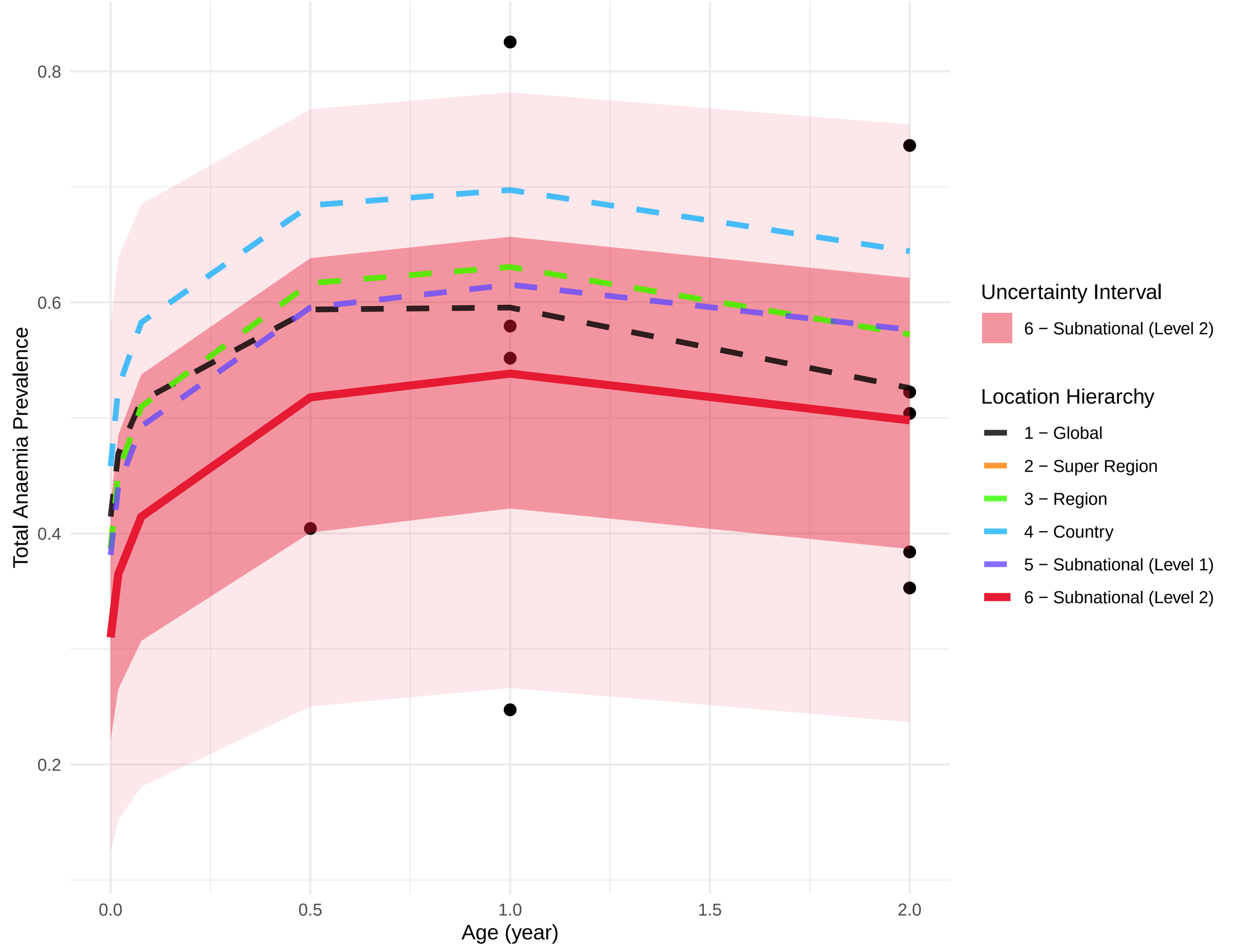

MR−BRT Age/Sex Fits − Males <2 Years Old − Total Anaemia Prevalence − Mizoram
WHO−2001 Adjustment Method

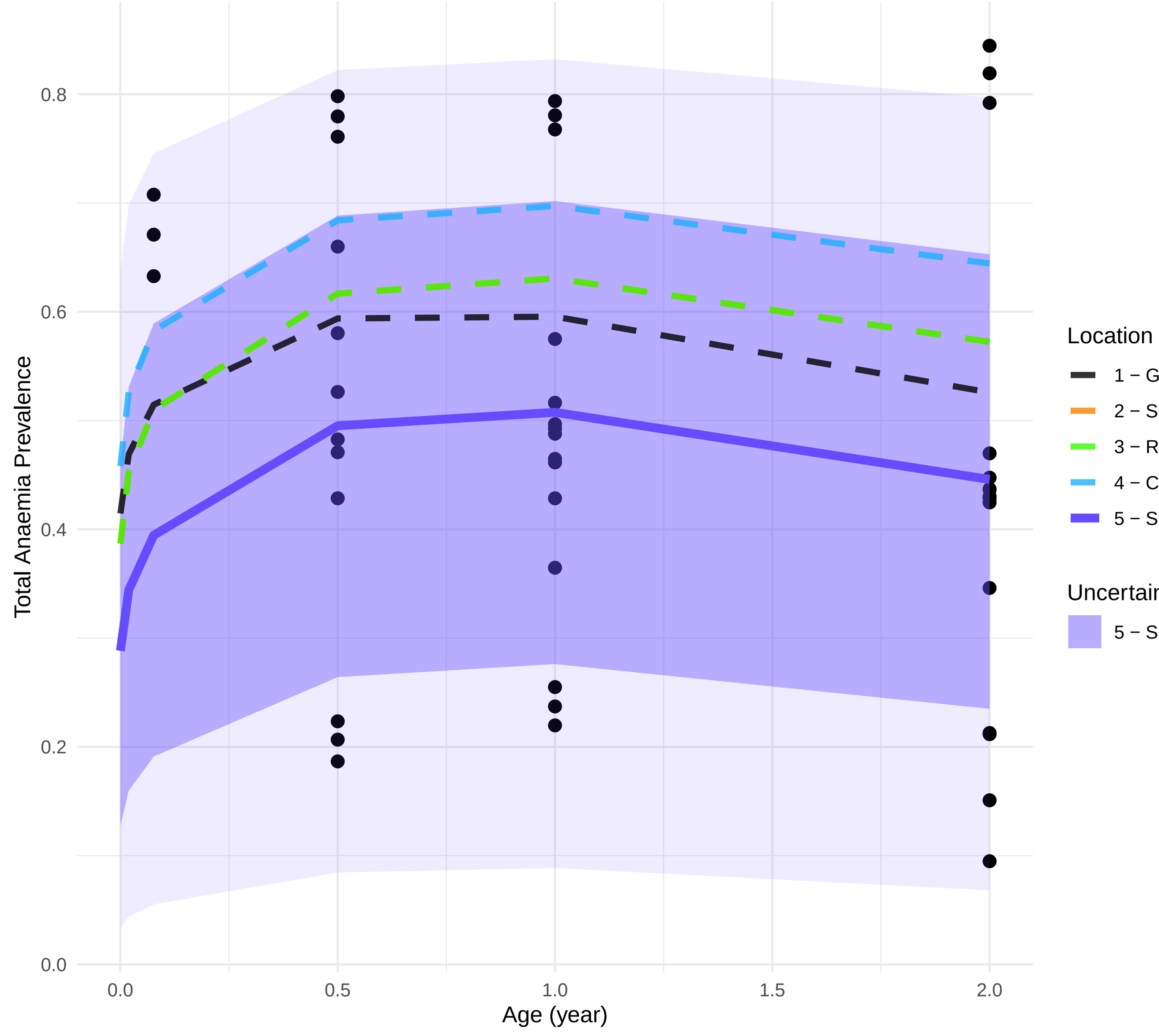

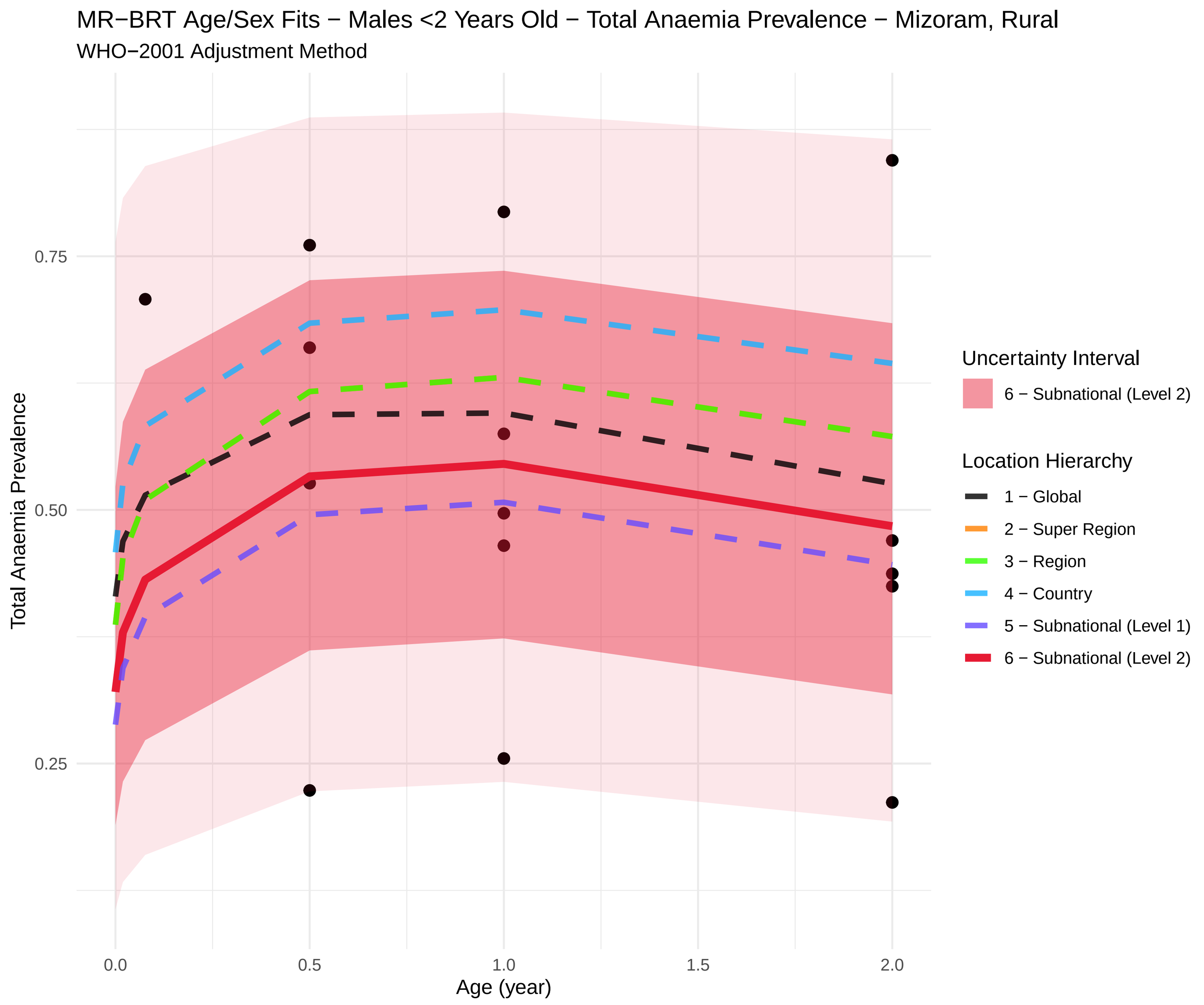
MR−BRT Age/Sex Fits − Males <2 Years Old − Total Anaemia Prevalence − Mizoram, Rural
WHO−2001 Adjustment Method
Total Anaemia Prevalence
Age (year)
Uncertainty Interval
6 − Subnational (Level 2)
Location Hierarchy
1 − Global
2 − Super Region
3 − Region
4 − Country
5 − Subnational (Level 1)
6 − Subnational (Level 2)

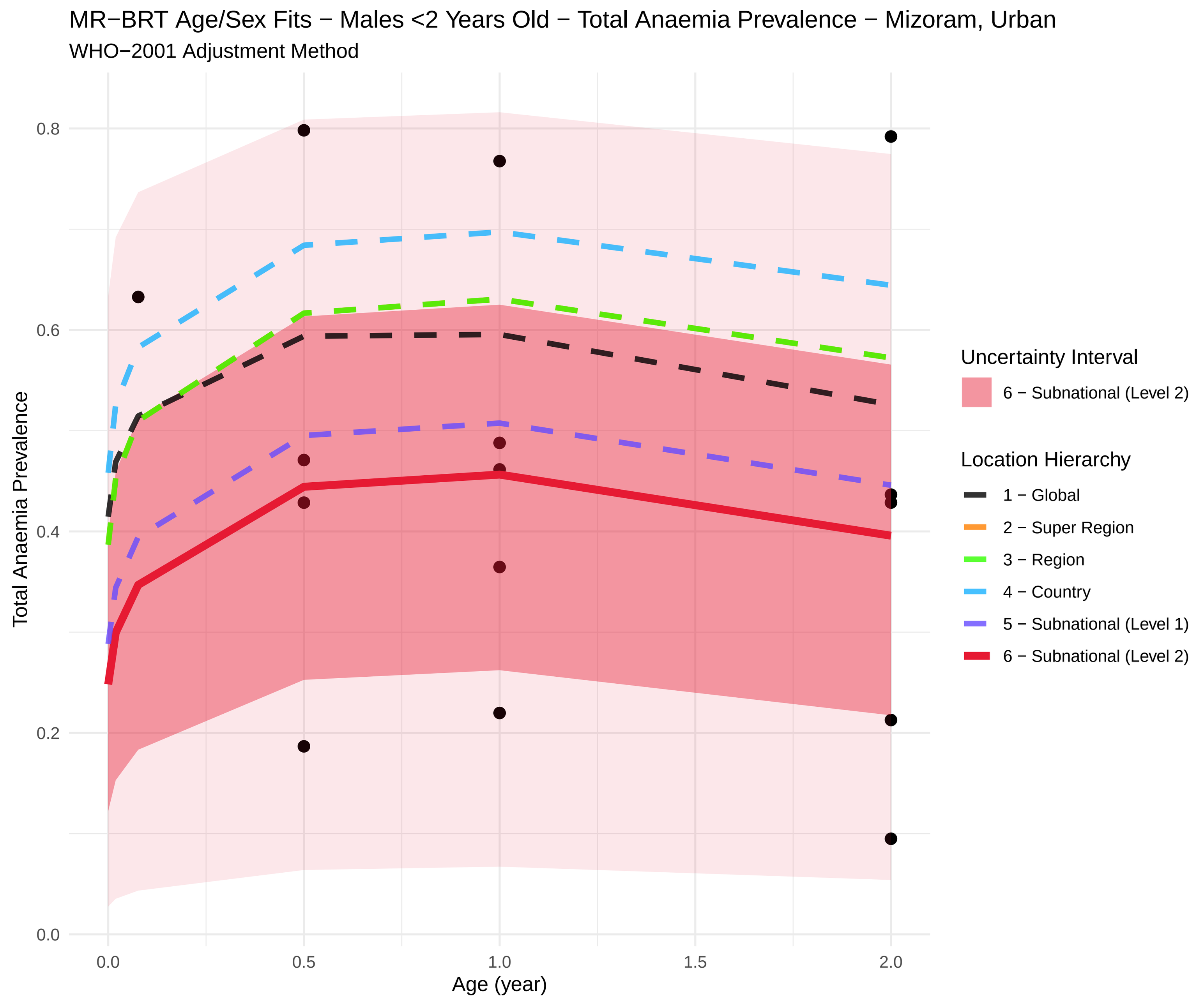
MR−BRT Age/Sex Fits − Males <2 Years Old − Total Anaemia Prevalence − Mizoram, Urban
WHO−2001 Adjustment Method
Total Anaemia Prevalence
Age (year)
Uncertainty Interval
6 − Subnational (Level 2)
Location Hierarchy
1 − Global
2 − Super Region
3 − Region
4 − Country
5 − Subnational (Level 1)
6 − Subnational (Level 2)

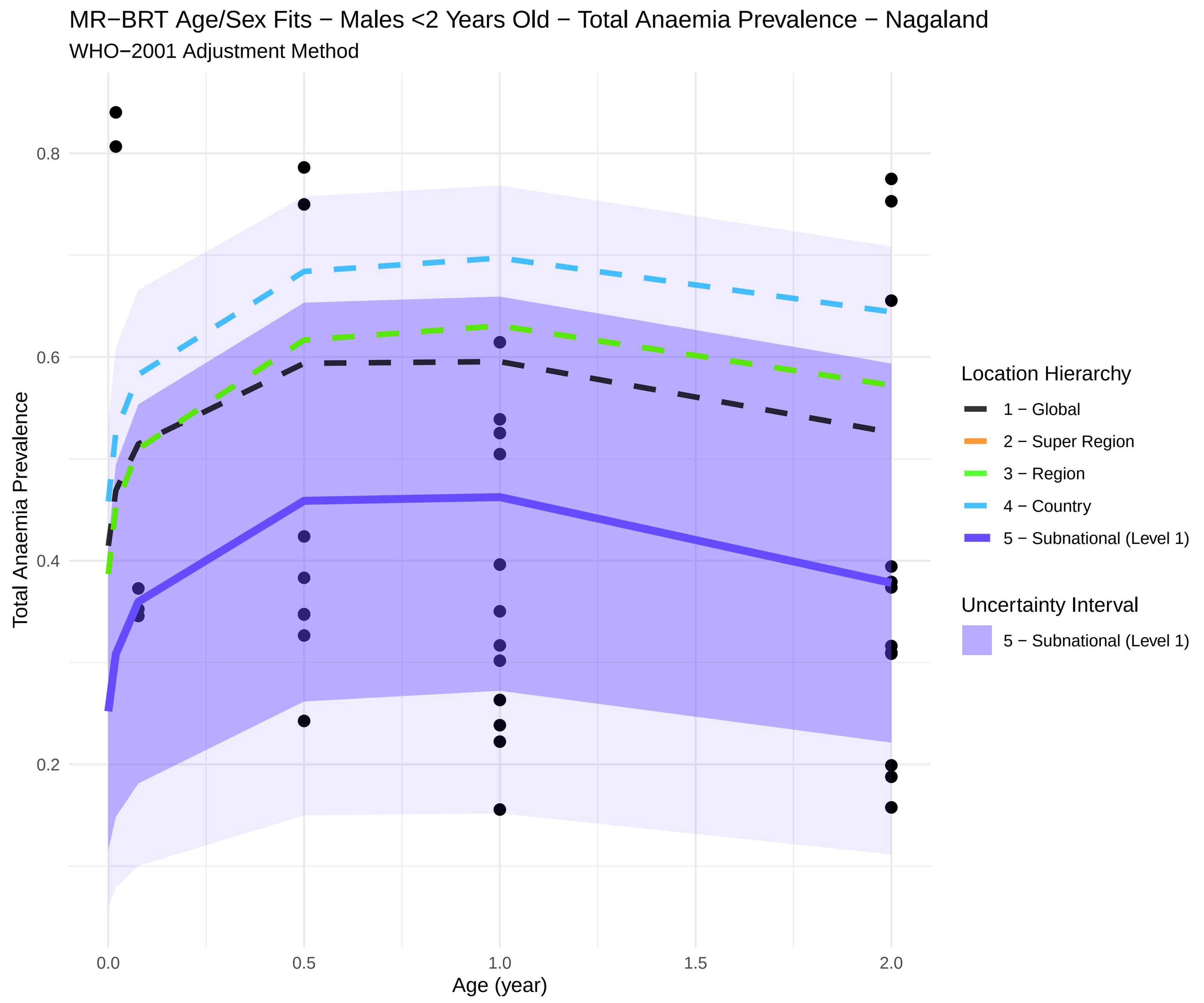
MR−BRT Age/Sex Fits − Males <2 Years Old − Total Anaemia Prevalence − Nagaland
WHO−2001 Adjustment Method
Total Anaemia Prevalence
Age (year)
Location Hierarchy
1 − Global
2 − Super Region
3 − Region
4 − Country
5 − Subnational (Level 1)
Uncertainty Interval
5 − Subnational (Level 1)

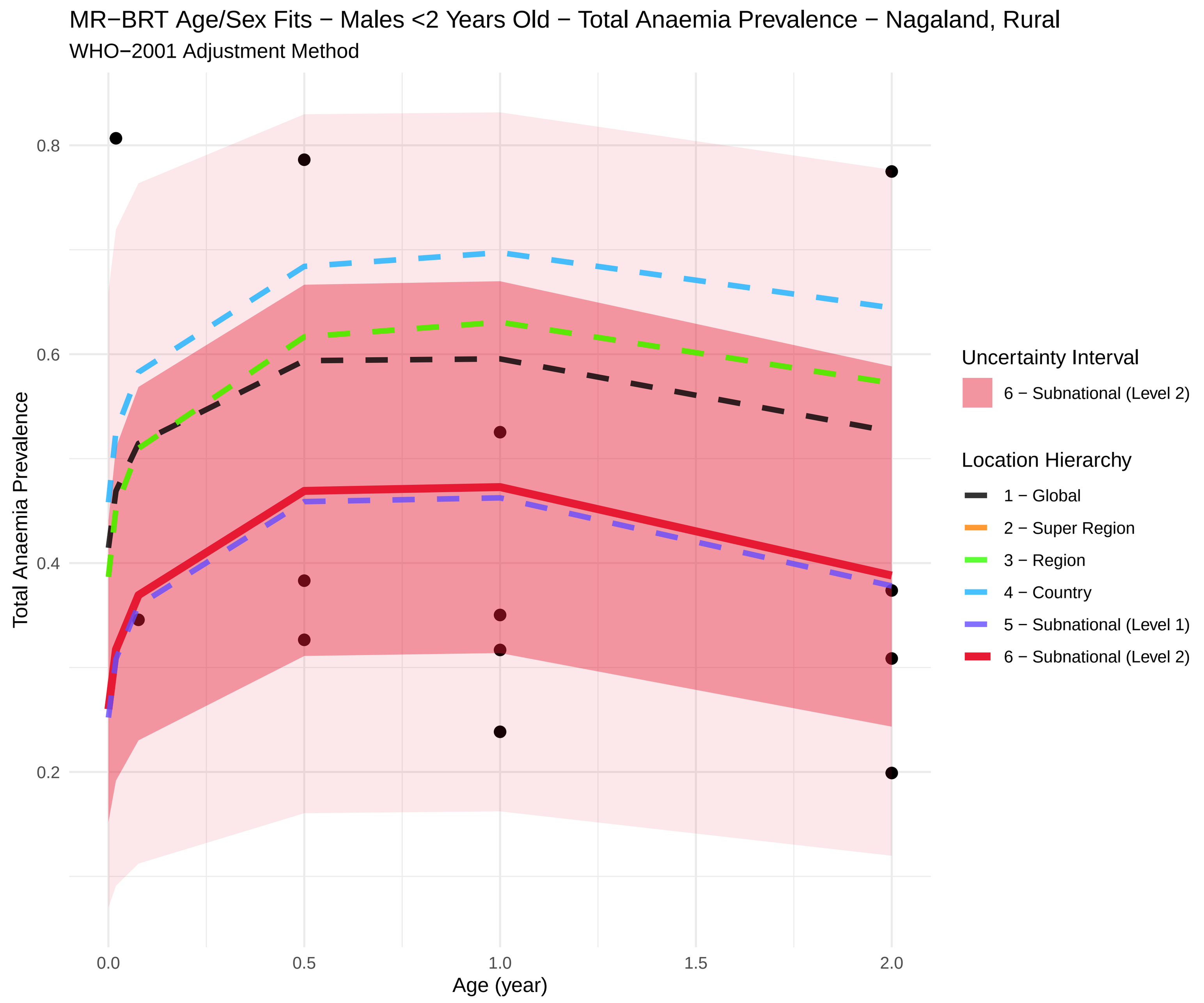
MR−BRT Age/Sex Fits − Males <2 Years Old − Total Anaemia Prevalence − Nagaland, Rural
WHO−2001 Adjustment Method
Total Anaemia Prevalence
Age (year)
Uncertainty Interval
6 − Subnational (Level 2)
Location Hierarchy
1 − Global
2 − Super Region
3 − Region
4 − Country
5 − Subnational (Level 1)
6 − Subnational (Level 2)

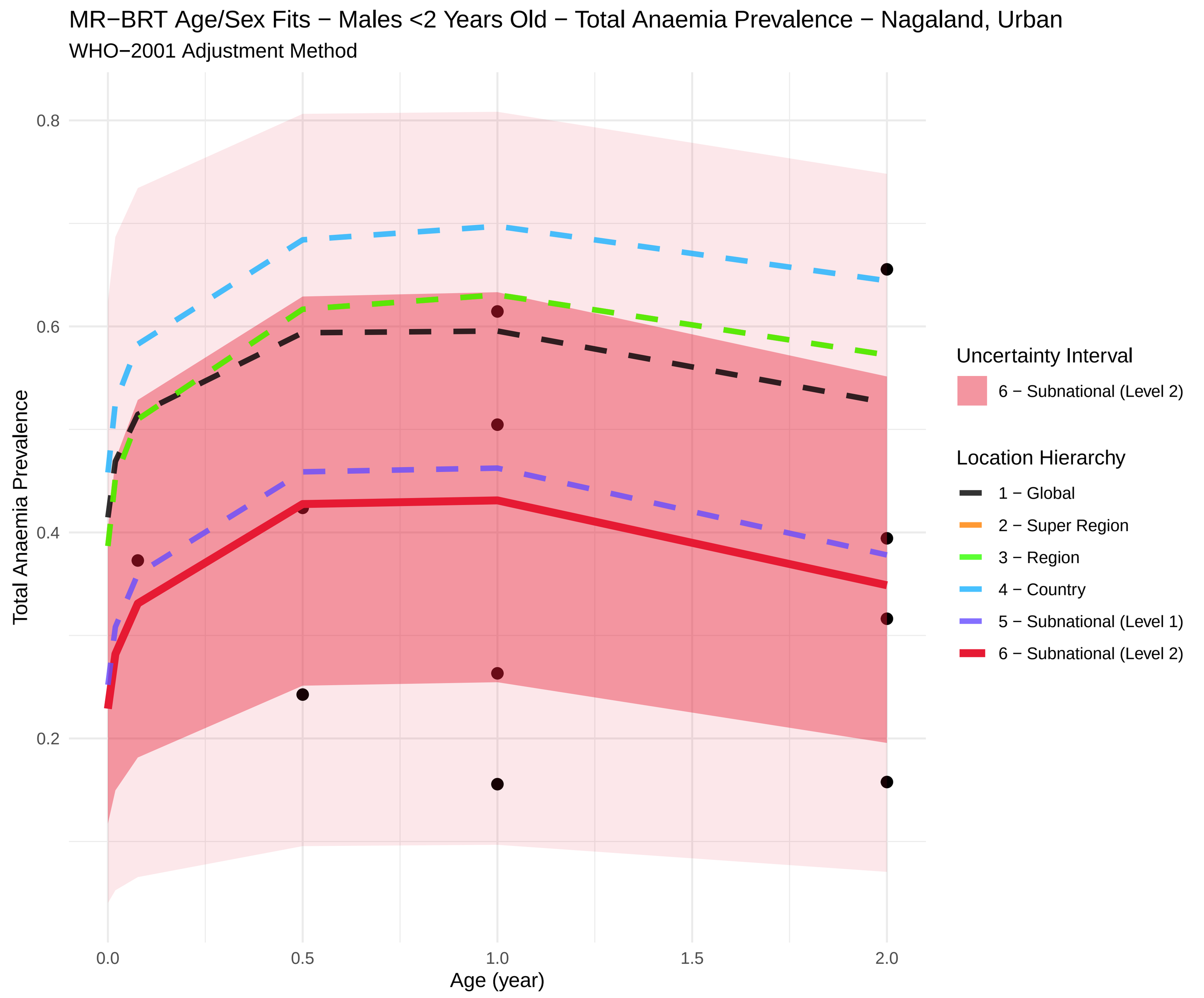
MR−BRT Age/Sex Fits − Males <2 Years Old − Total Anaemia Prevalence − Nagaland, Urban
WHO−2001 Adjustment Method
Total Anaemia Prevalence
Age (year)
Uncertainty Interval
6 − Subnational (Level 2)
Location Hierarchy
1 − Global
2 − Super Region
3 − Region
4 − Country
5 − Subnational (Level 1)
6 − Subnational (Level 2)

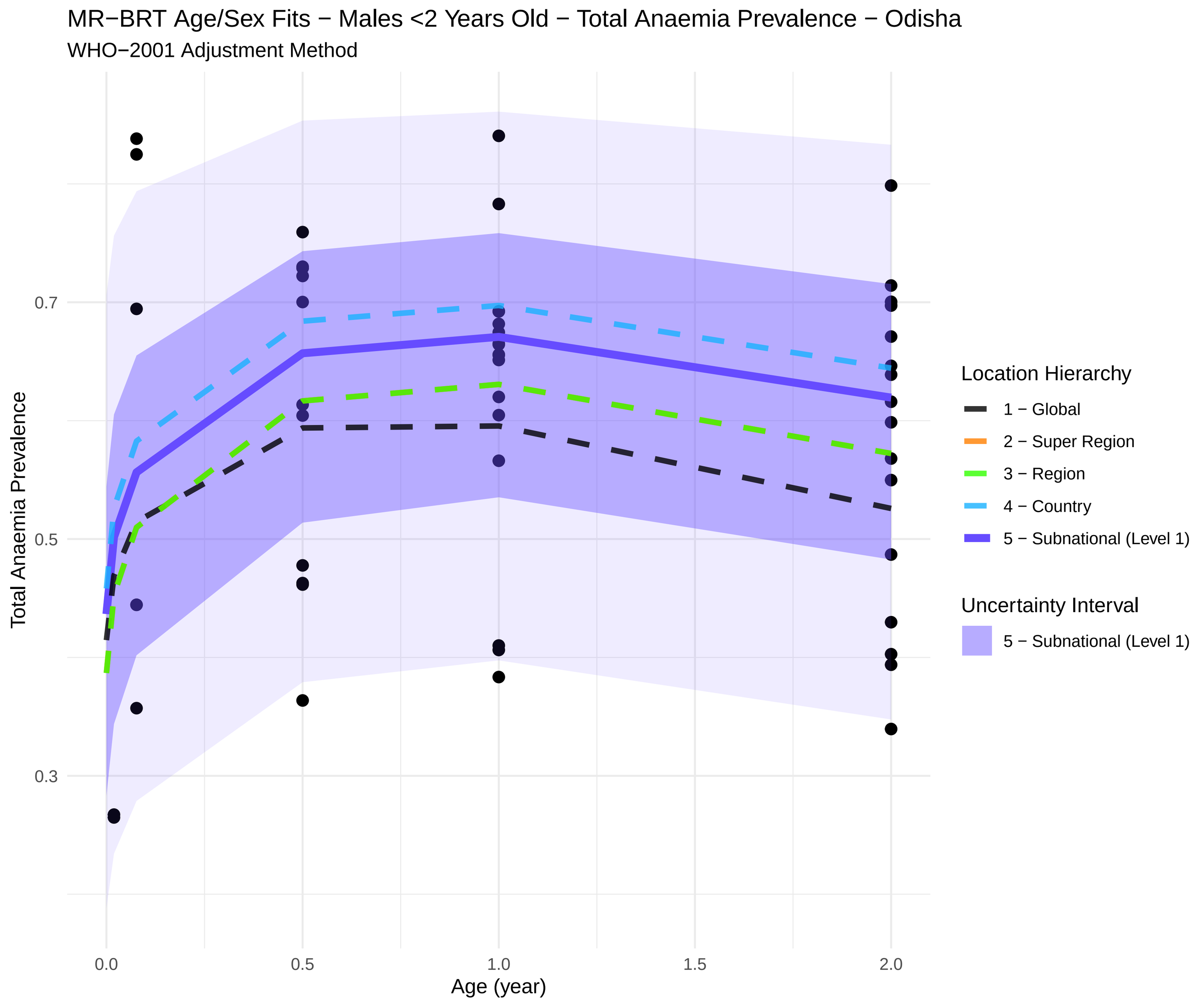
MR−BRT Age/Sex Fits − Males <2 Years Old − Total Anaemia Prevalence − Odisha
WHO−2001 Adjustment Method
Total Anaemia Prevalence
Age (year)
Location Hierarchy
1 − Global
2 − Super Region
3 − Region
4 − Country
5 − Subnational (Level 1)
Uncertainty Interval
5 − Subnational (Level 1)

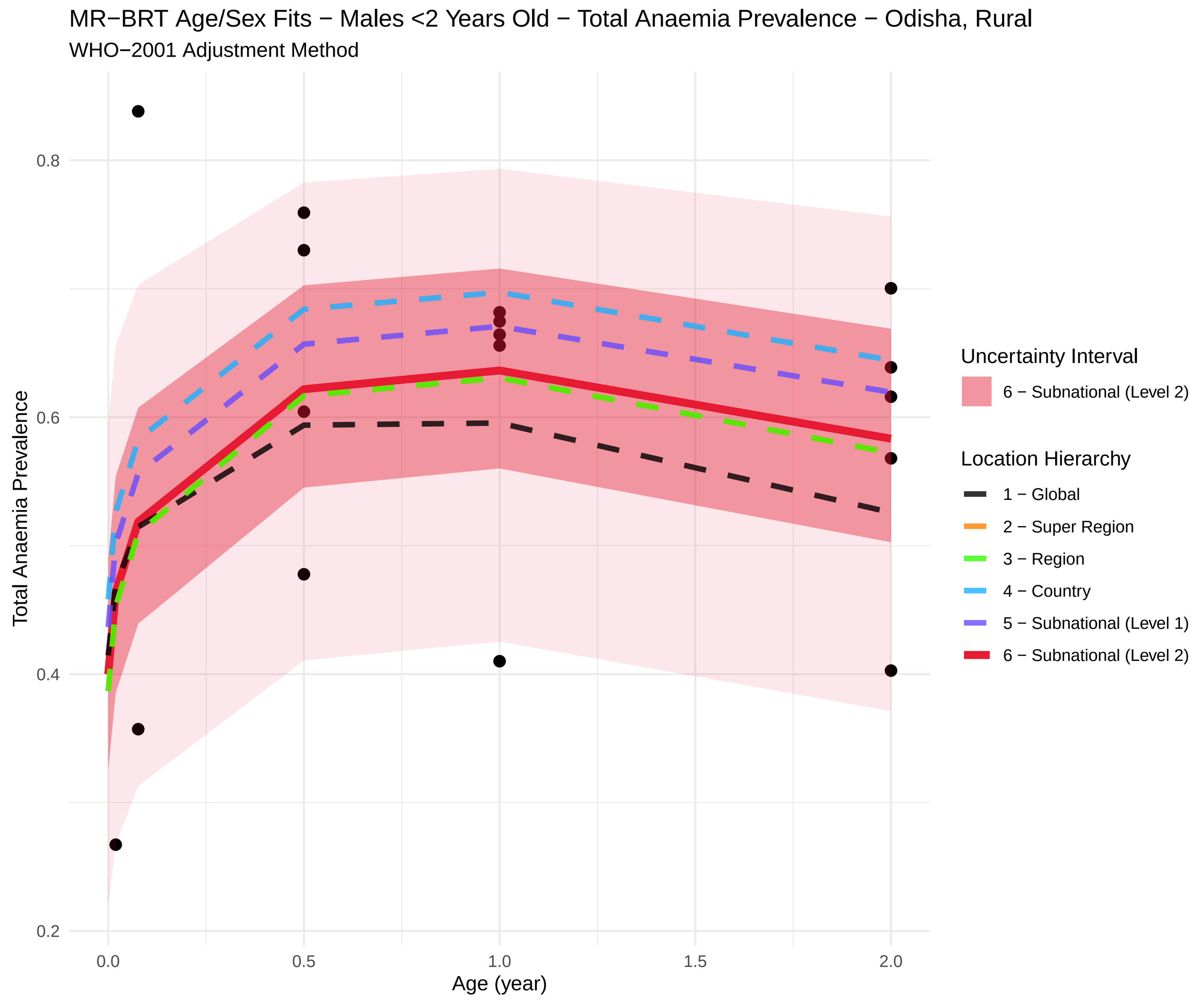
MR−BRT Age/Sex Fits − Males <2 Years Old − Total Anaemia Prevalence − Odisha, Rural
WHO−2001 Adjustment Method
Total Anaemia Prevalence
Age (year)
Uncertainty Interval
6 − Subnational (Level 2)
Location Hierarchy
1 − Global
2 − Super Region
3 − Region
4 − Country
5 − Subnational (Level 1)
6 − Subnational (Level 2)

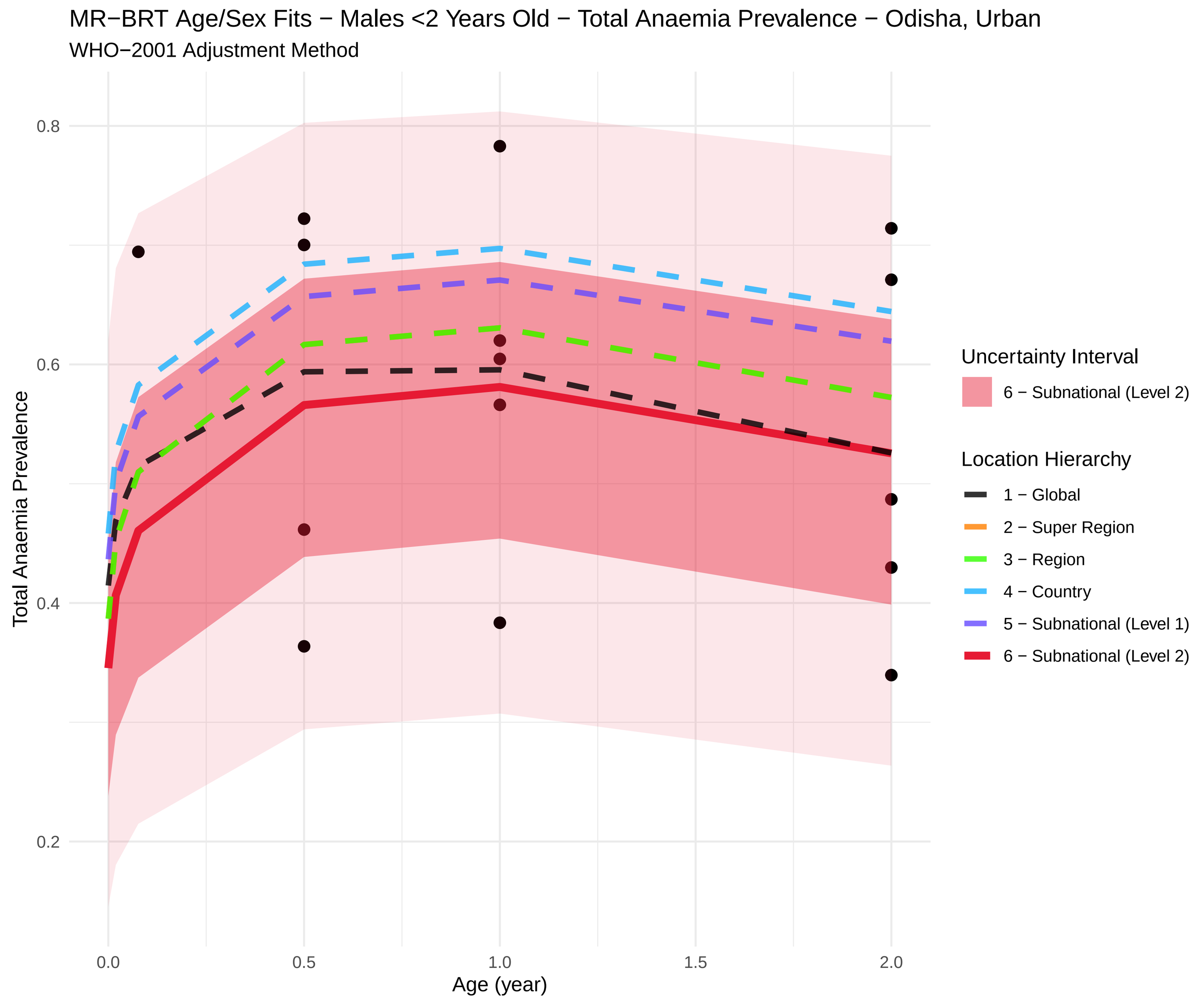
MR−BRT Age/Sex Fits − Males <2 Years Old − Total Anaemia Prevalence − Odisha, Urban
WHO−2001 Adjustment Method
Total Anaemia Prevalence
Age (year)
Uncertainty Interval
6 − Subnational (Level 2)
Location Hierarchy
1 − Global
2 − Super Region
3 − Region
4 − Country
5 − Subnational (Level 1)
6 − Subnational (Level 2)

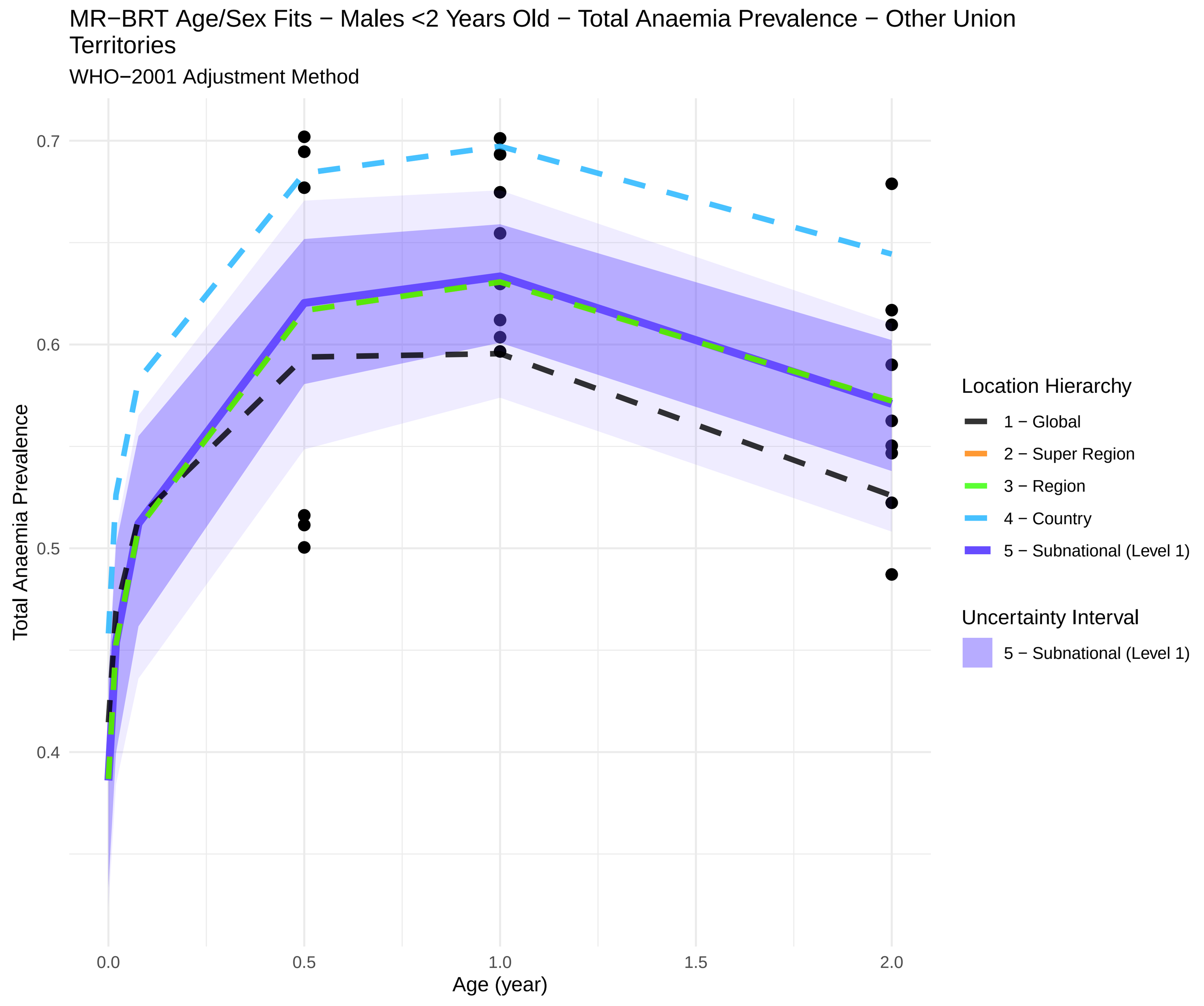
MR−BRT Age/Sex Fits − Males <2 Years Old − Total Anaemia Prevalence − Other Union Territories
WHO−2001 Adjustment Method
Total Anaemia Prevalence
Age (year)
Location Hierarchy
1 − Global
2 − Super Region
3 − Region
4 − Country
5 − Subnational (Level 1)
Uncertainty Interval
5 − Subnational (Level 1)

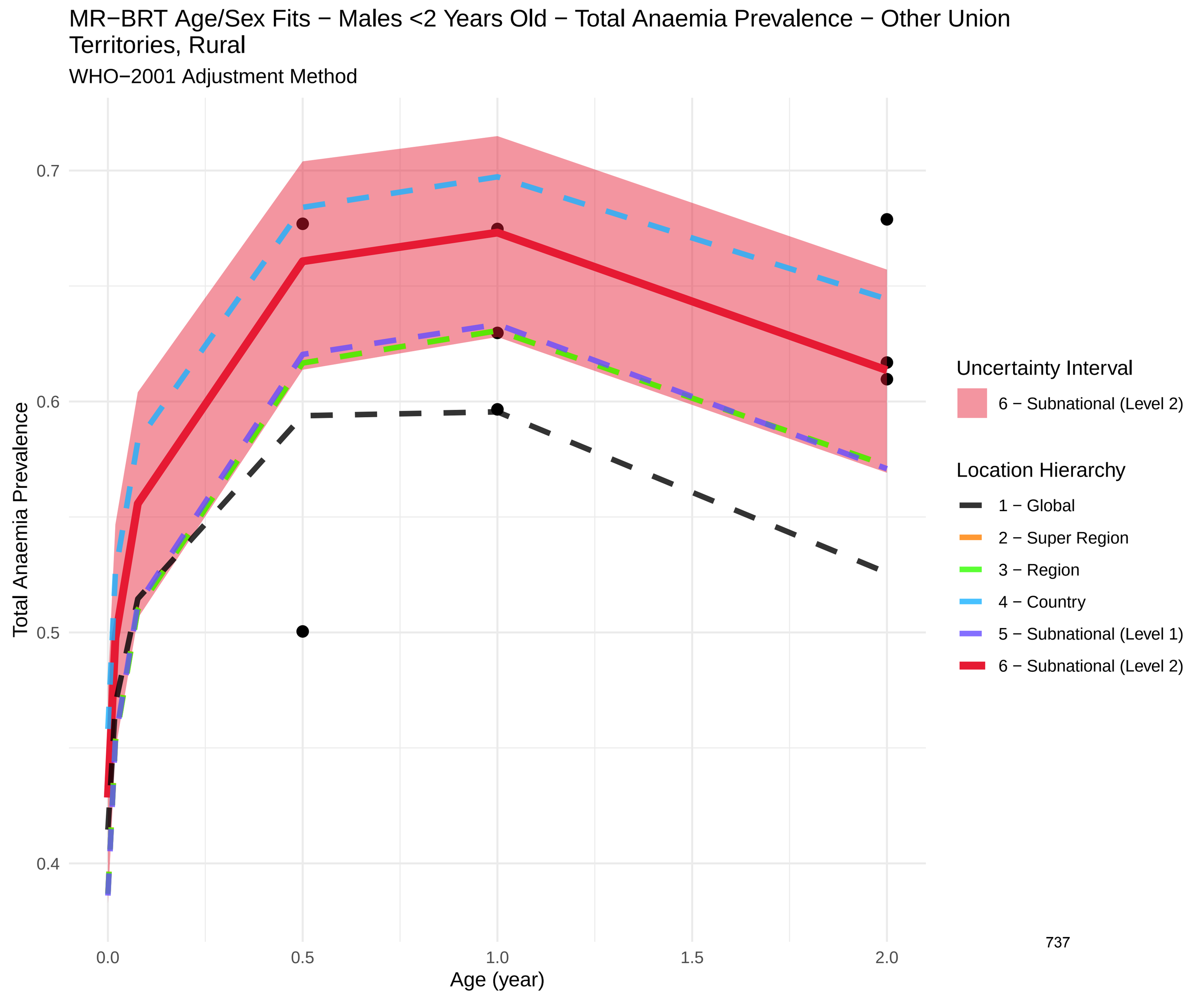
MR−BRT Age/Sex Fits − Males <2 Years Old − Total Anaemia Prevalence − Other Union Territories, Rural
WHO−2001 Adjustment Method
Total Anaemia Prevalence
Age (year)
Uncertainty Interval
6 − Subnational (Level 2)
Location Hierarchy
1 − Global
2 − Super Region
3 − Region
4 − Country
5 − Subnational (Level 1)
6 − Subnational (Level 2)

MR−BRT Age/Sex Fits − Males <2 Years Old − Total Anaemia Prevalence − Other Union Territories, Urban
WHO−2001 Adjustment Method

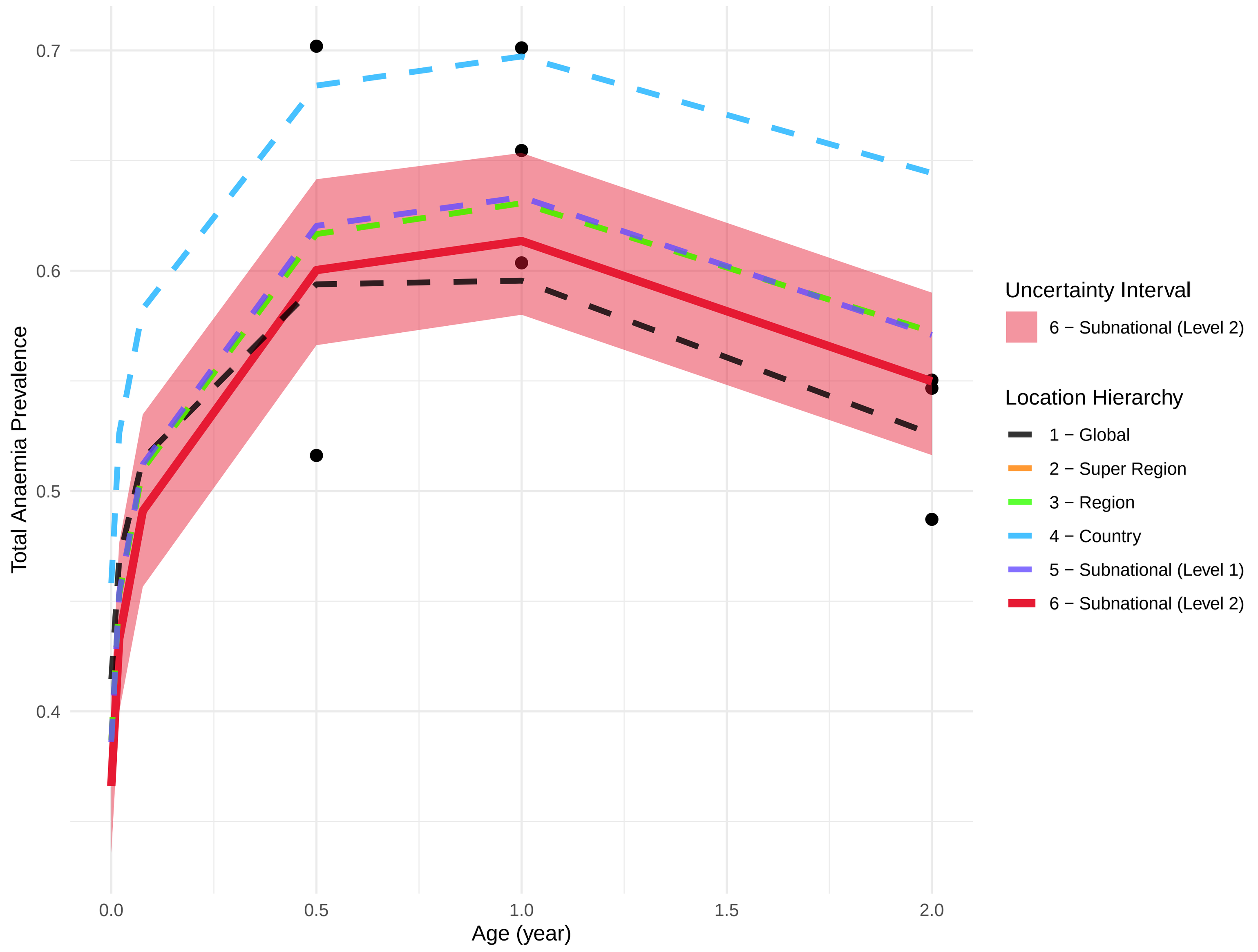

MR−BRT Age/Sex Fits − Males <2 Years Old − Total Anaemia Prevalence − Punjab
WHO−2001 Adjustment Method

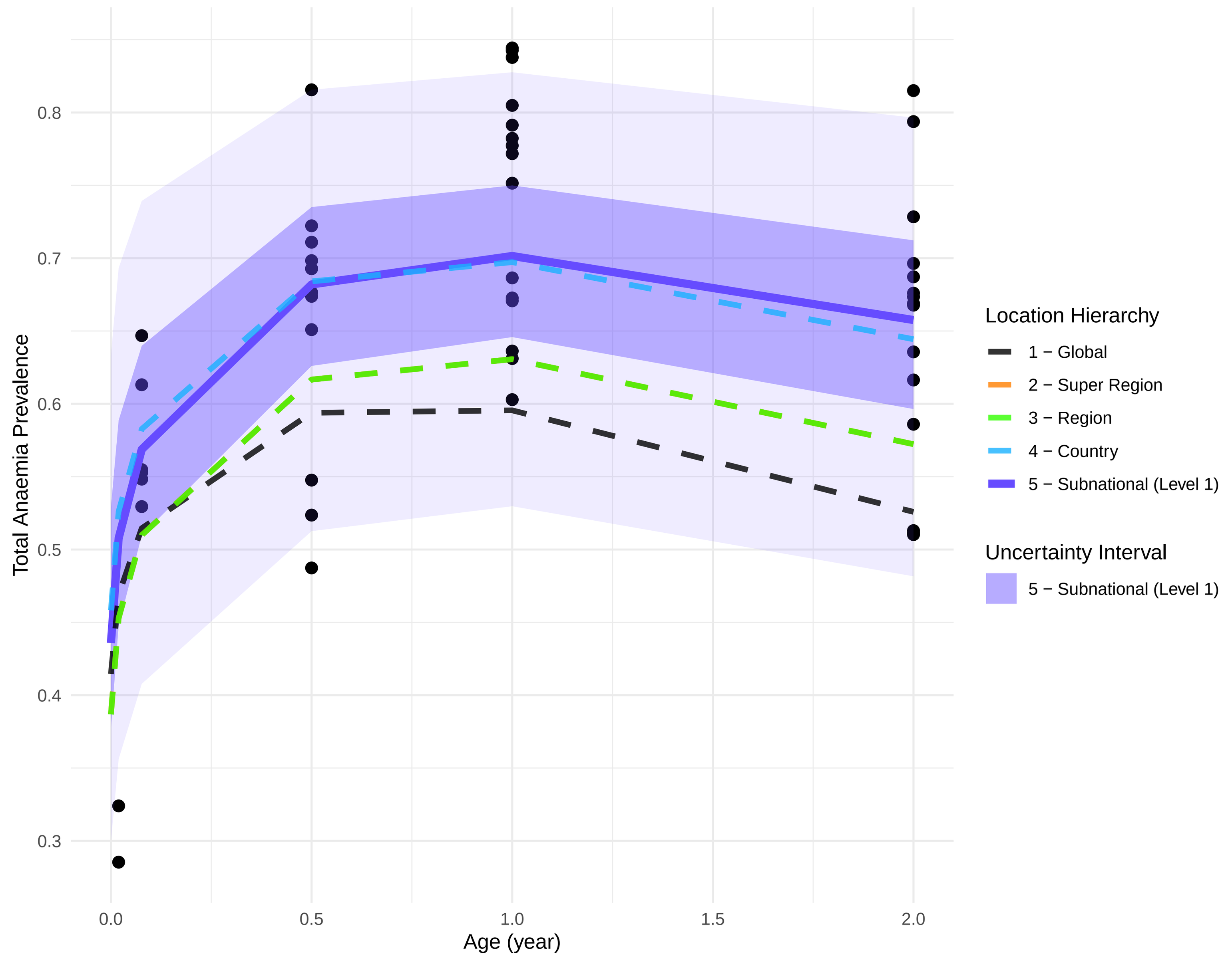

MR−BRT Age/Sex Fits − Males <2 Years Old − Total Anaemia Prevalence − Punjab, Rural
WHO−2001 Adjustment Method

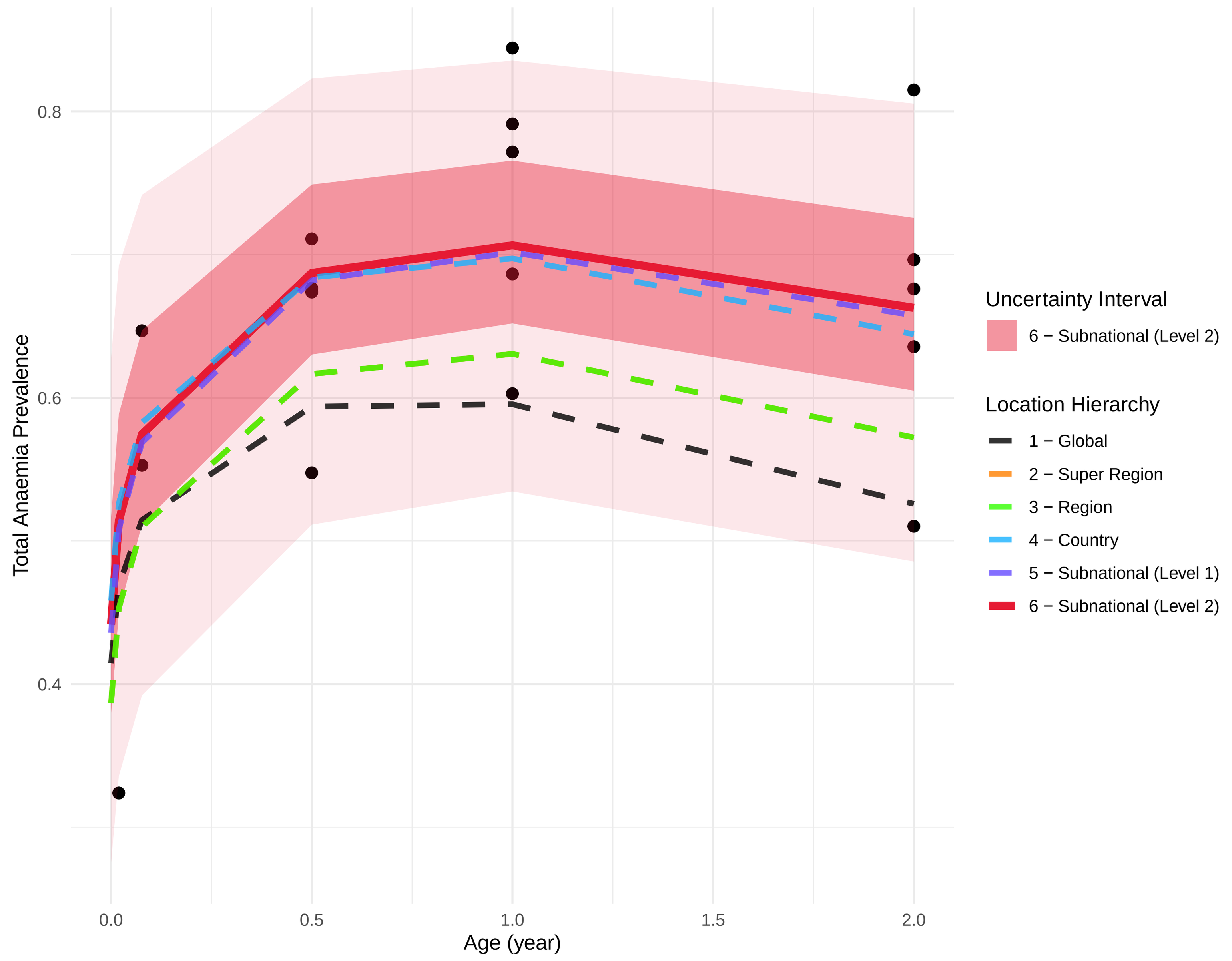

MR−BRT Age/Sex Fits − Males <2 Years Old − Total Anaemia Prevalence − Punjab, Urban
WHO−2001 Adjustment Method

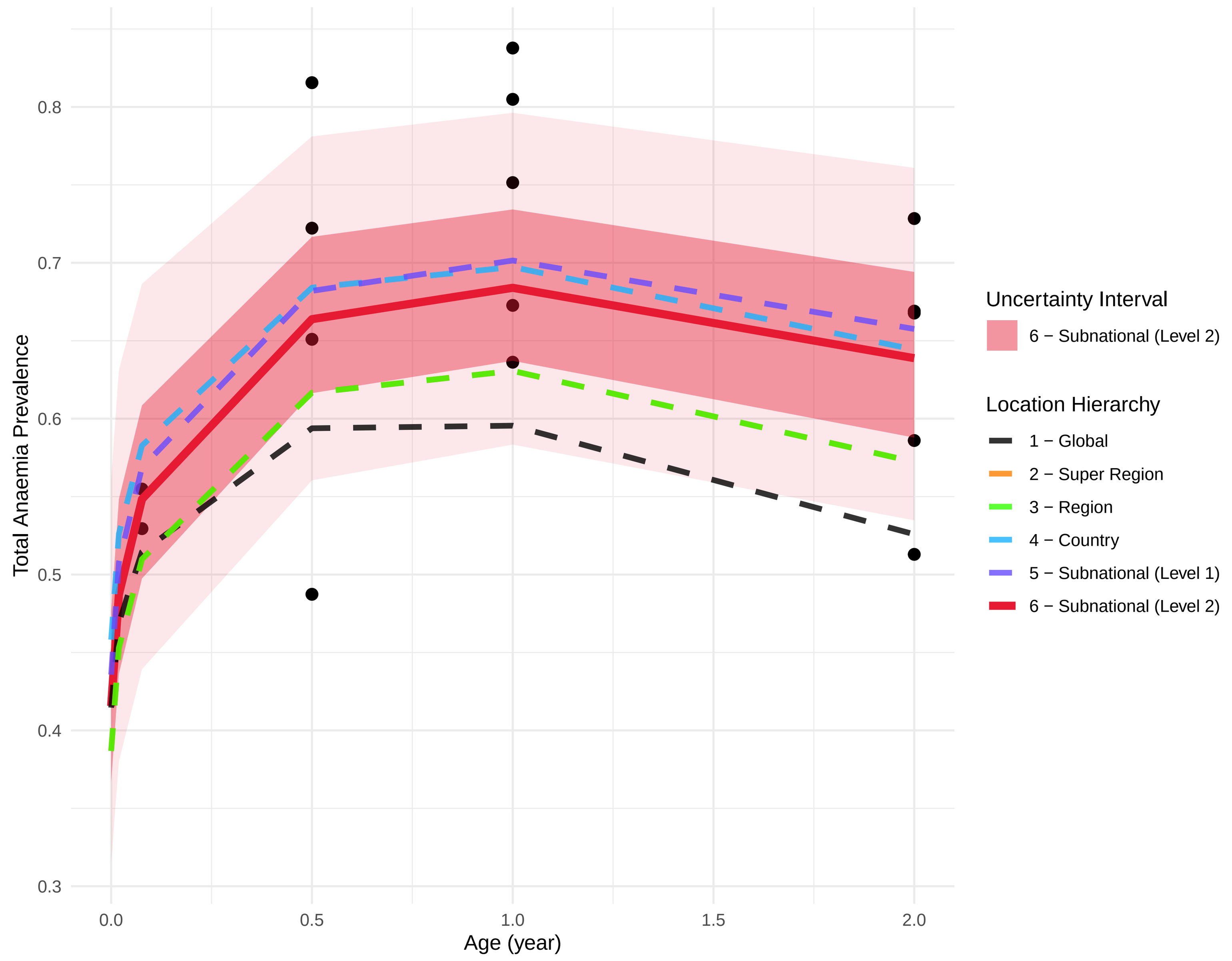

MR−BRT Age/Sex Fits − Males <2 Years Old − Total Anaemia Prevalence − Rajasthan
WHO−2001 Adjustment Method

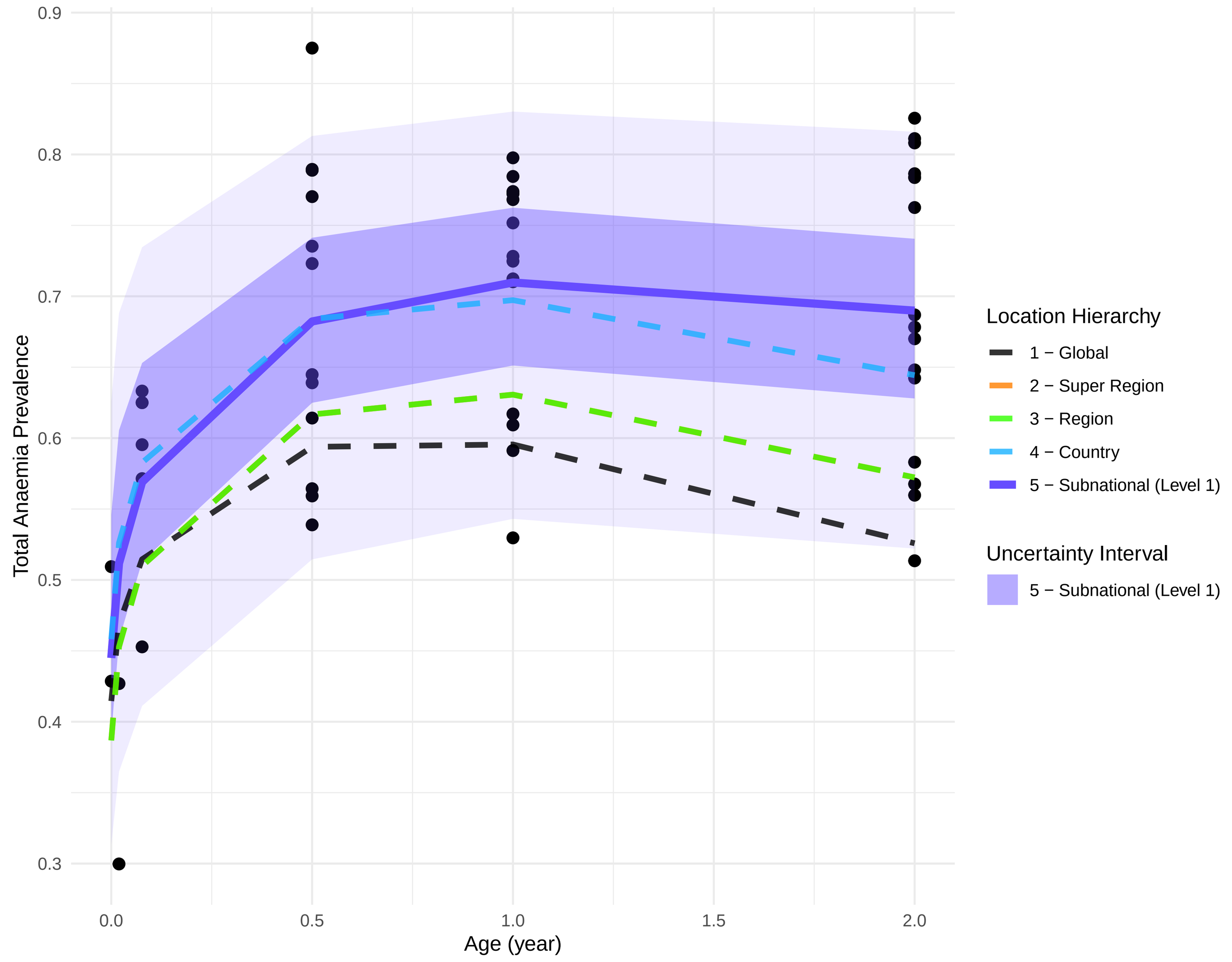

MR−BRT Age/Sex Fits − Males <2 Years Old − Total Anaemia Prevalence − Rajasthan, Rural
WHO−2001 Adjustment Method

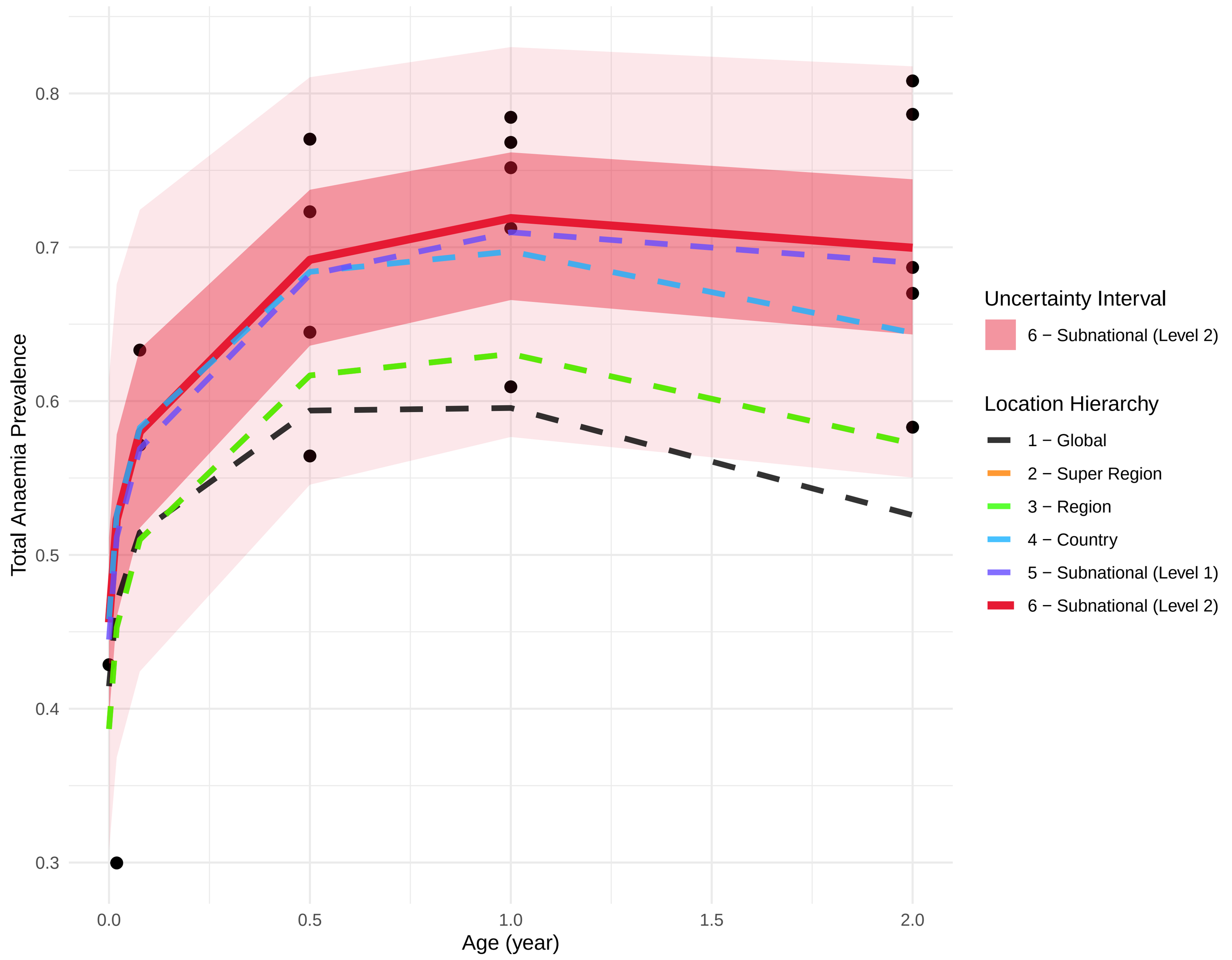

MR−BRT Age/Sex Fits − Males <2 Years Old − Total Anaemia Prevalence − Rajasthan, Urban
WHO−2001 Adjustment Method

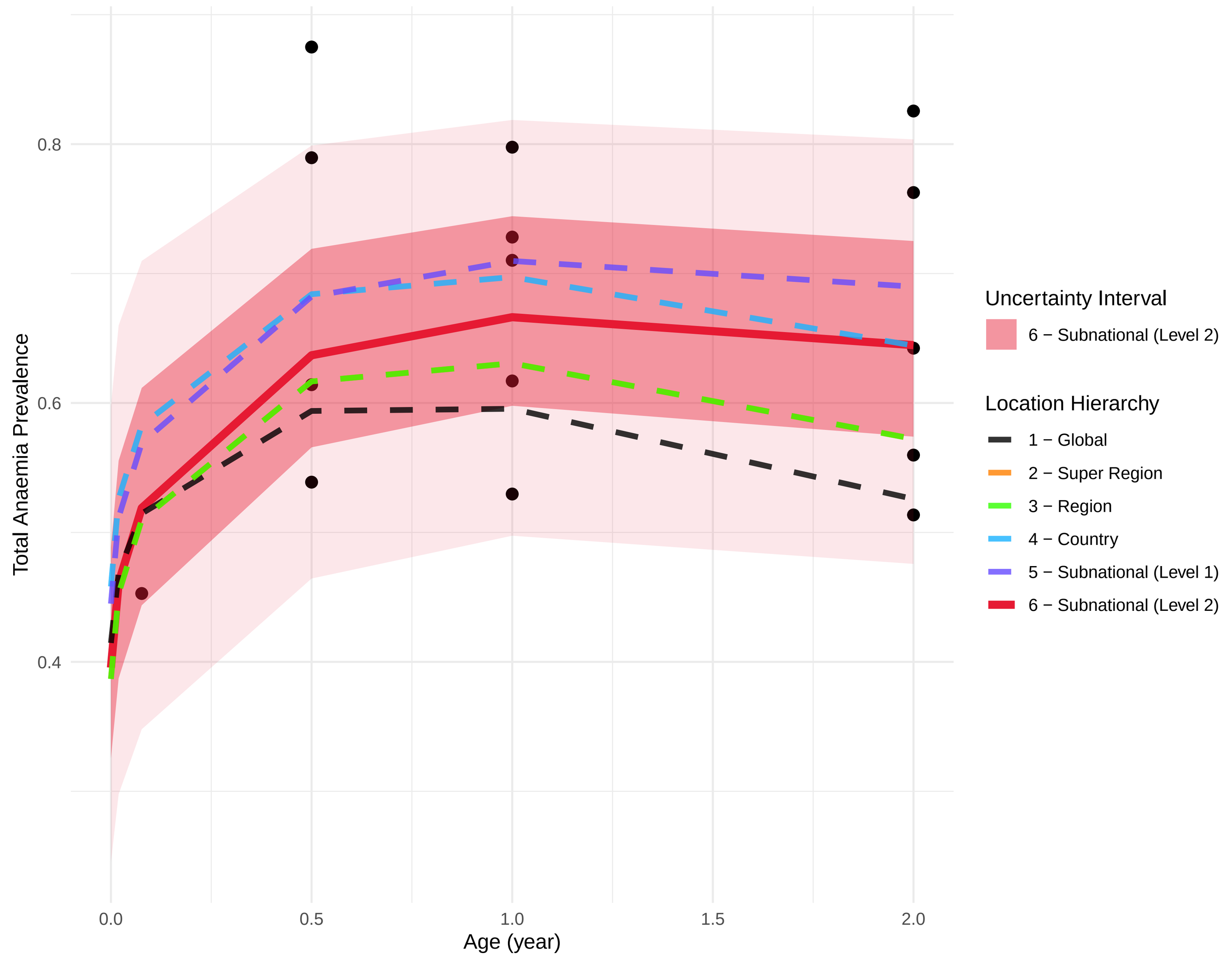

MR−BRT Age/Sex Fits − Males <2 Years Old − Total Anaemia Prevalence − Sikkim
WHO−2001 Adjustment Method

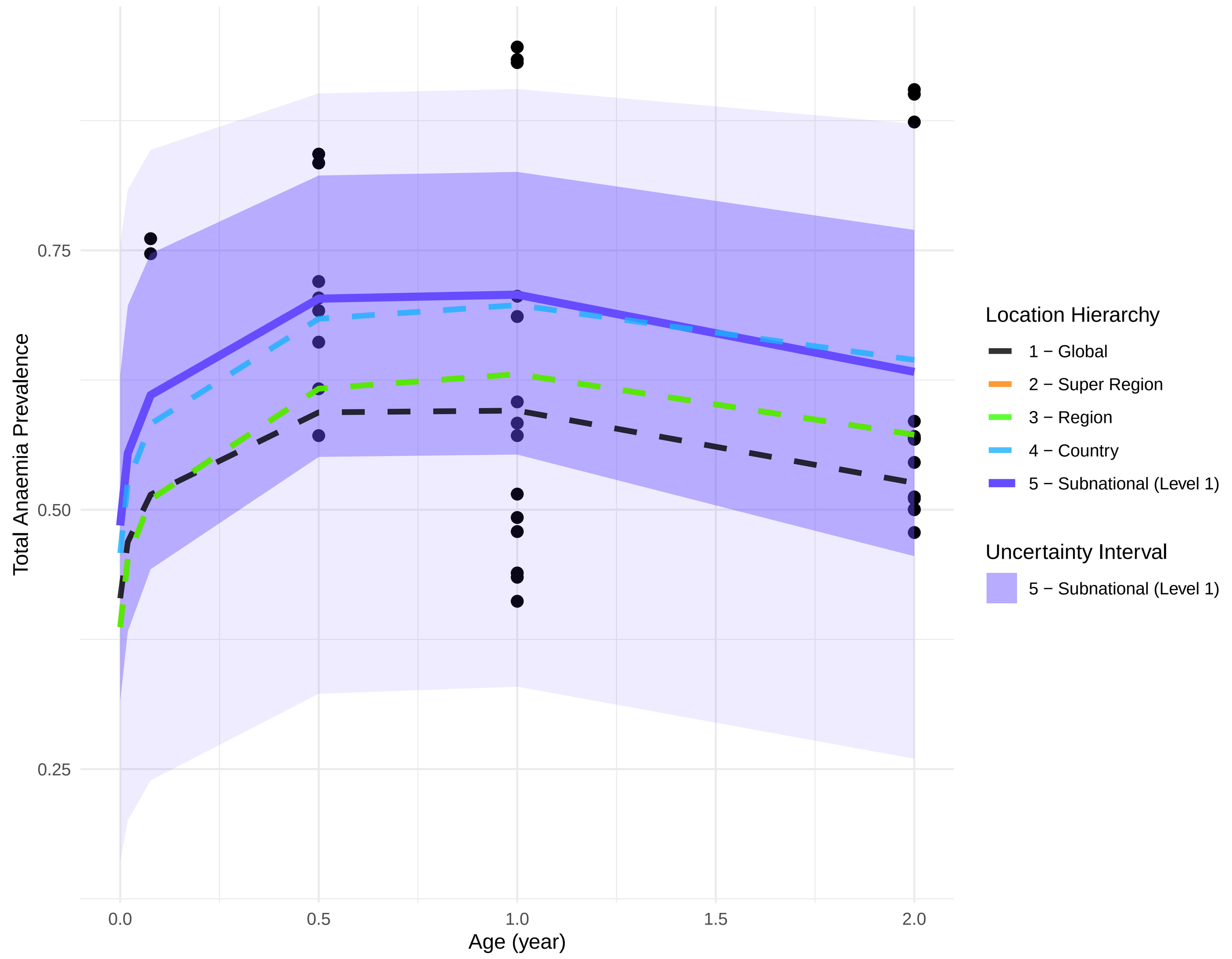

MR−BRT Age/Sex Fits − Males <2 Years Old − Total Anaemia Prevalence − Sikkim, Rural
WHO−2001 Adjustment Method

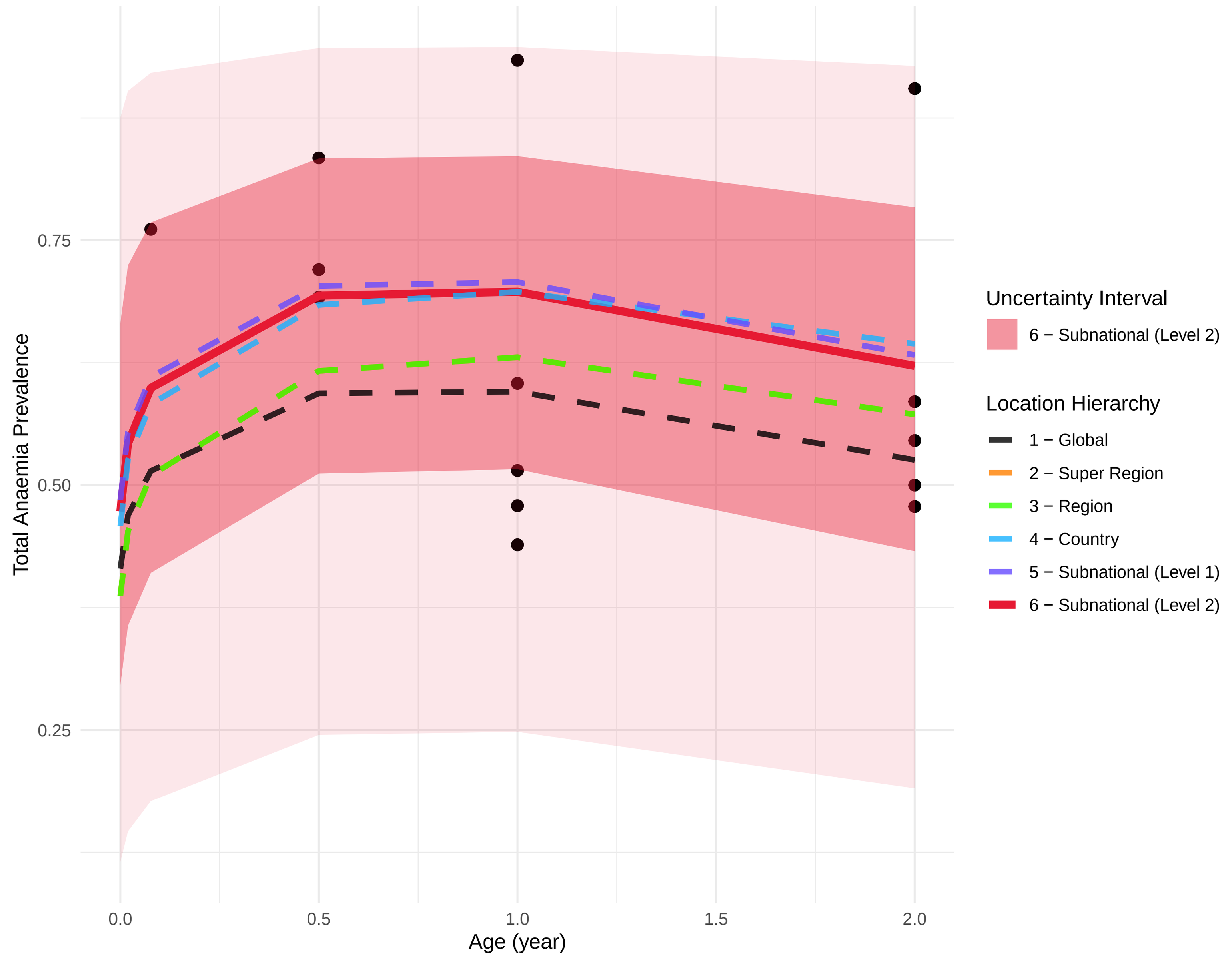

MR−BRT Age/Sex Fits − Males <2 Years Old − Total Anaemia Prevalence − Sikkim, Urban
WHO−2001 Adjustment Method

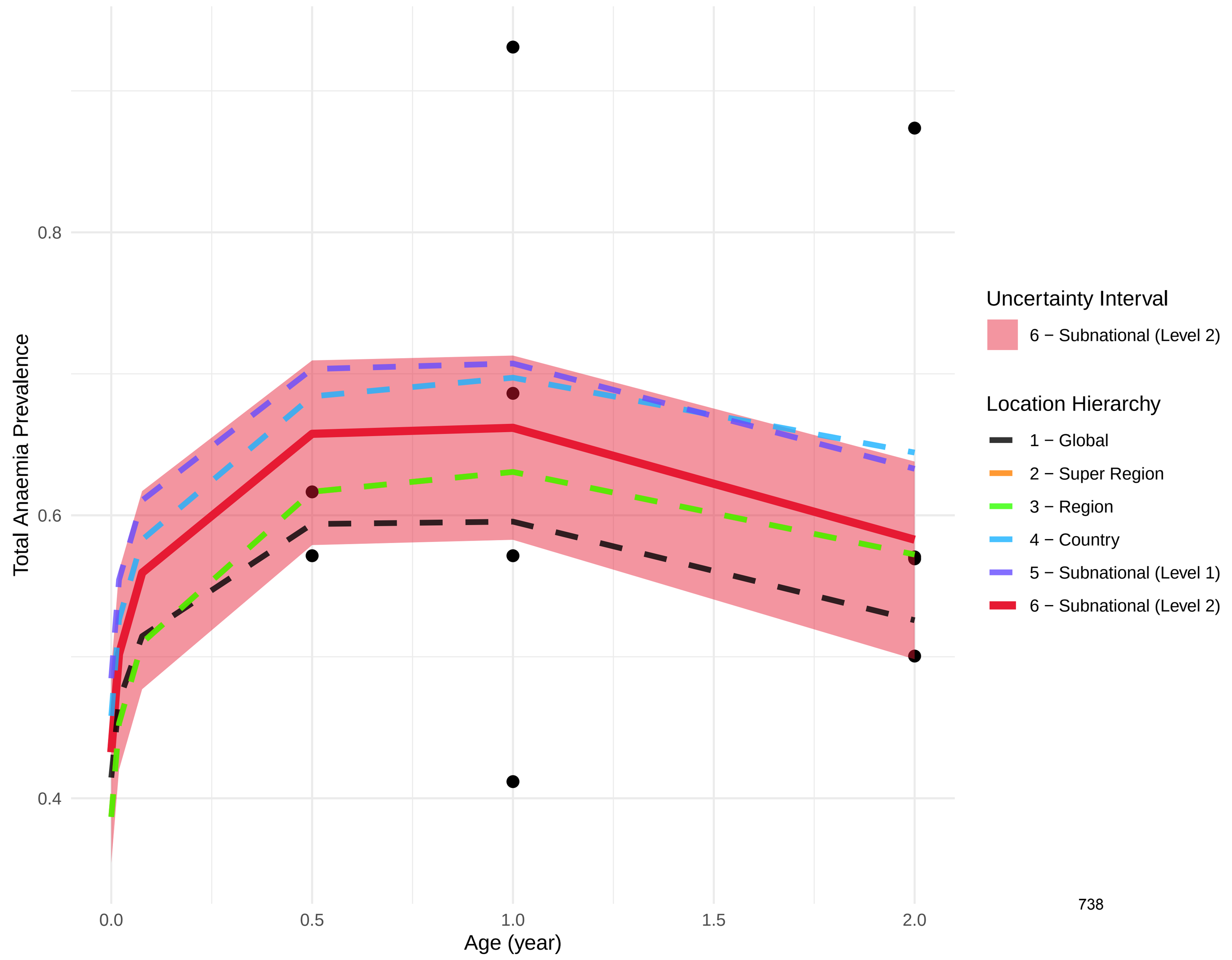

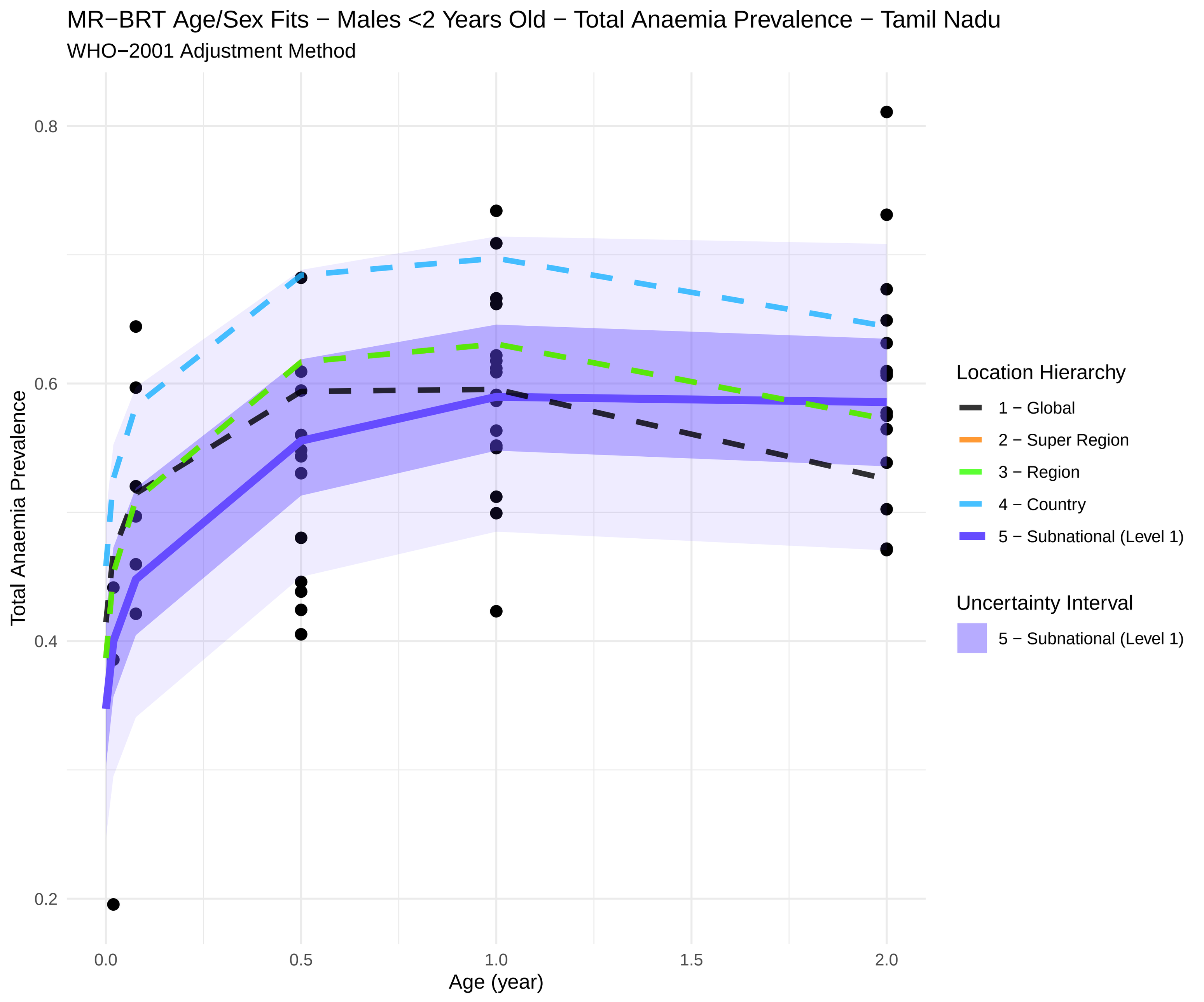
MR−BRT Age/Sex Fits − Males <2 Years Old − Total Anaemia Prevalence − Tamil Nadu
WHO−2001 Adjustment Method
Total Anaemia Prevalence
Age (year)
Location Hierarchy
1 − Global
2 − Super Region
3 − Region
4 − Country
5 − Subnational (Level 1)
Uncertainty Interval
5 − Subnational (Level 1)

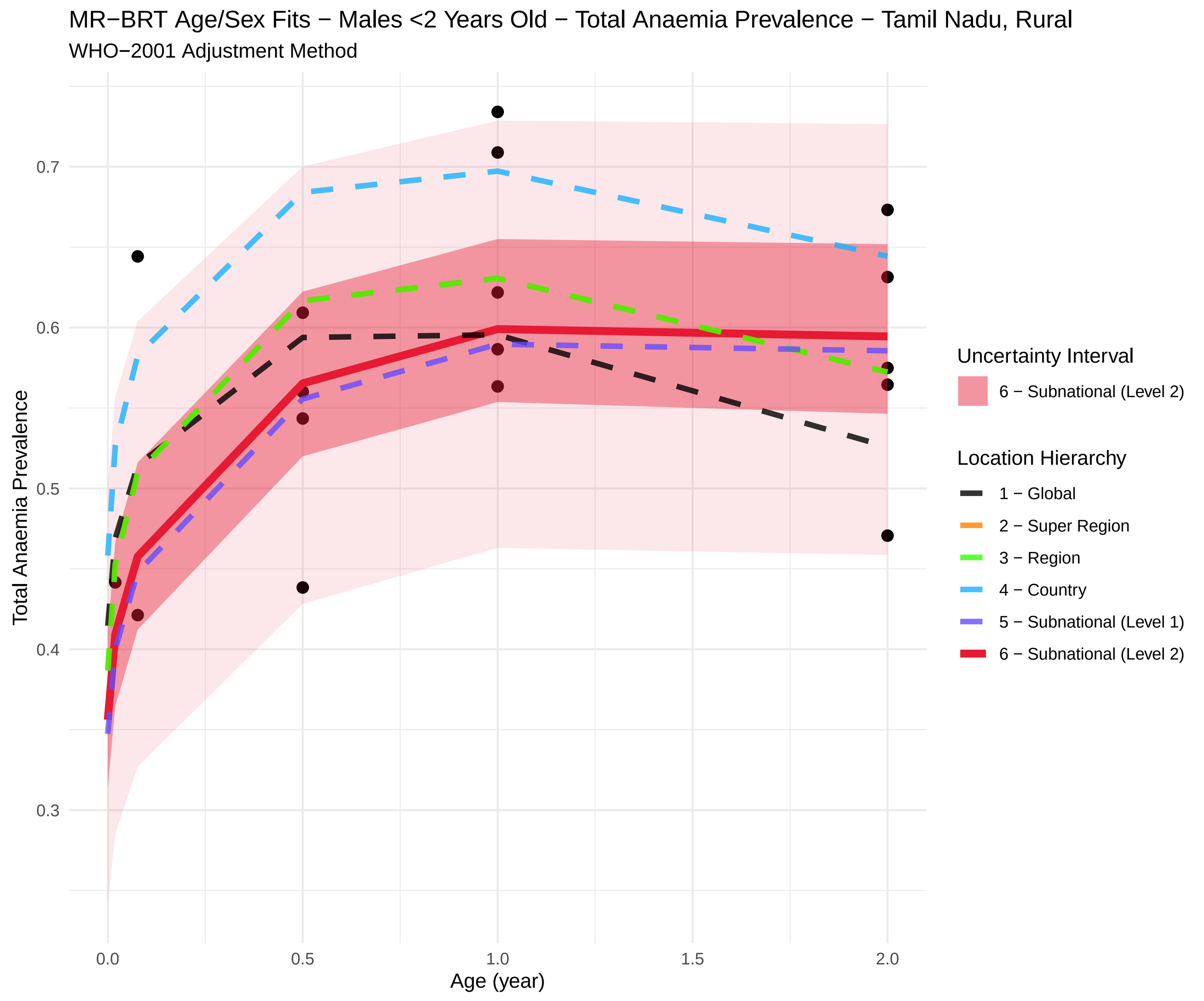
MR−BRT Age/Sex Fits − Males <2 Years Old − Total Anaemia Prevalence − Tamil Nadu, Rural
WHO−2001 Adjustment Method
Total Anaemia Prevalence
Age (year)
Uncertainty Interval
6 − Subnational (Level 2)
Location Hierarchy
1 − Global
2 − Super Region
3 − Region
4 − Country
5 − Subnational (Level 1)
6 − Subnational (Level 2)

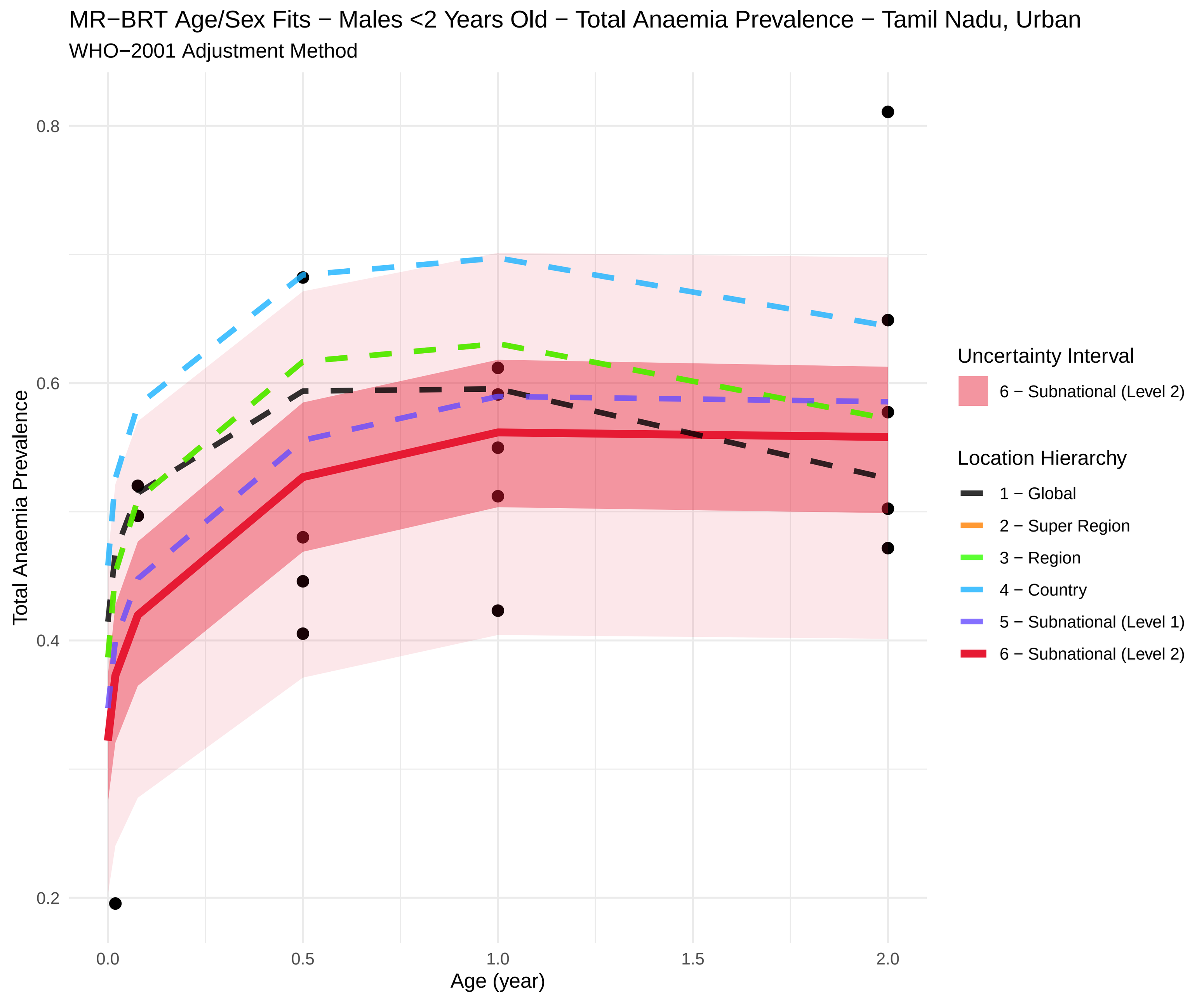
MR−BRT Age/Sex Fits − Males <2 Years Old − Total Anaemia Prevalence − Tamil Nadu, Urban
WHO−2001 Adjustment Method
Total Anaemia Prevalence
Age (year)
Uncertainty Interval
6 − Subnational (Level 2)
Location Hierarchy
1 − Global
2 − Super Region
3 − Region
4 − Country
5 − Subnational (Level 1)
6 − Subnational (Level 2)

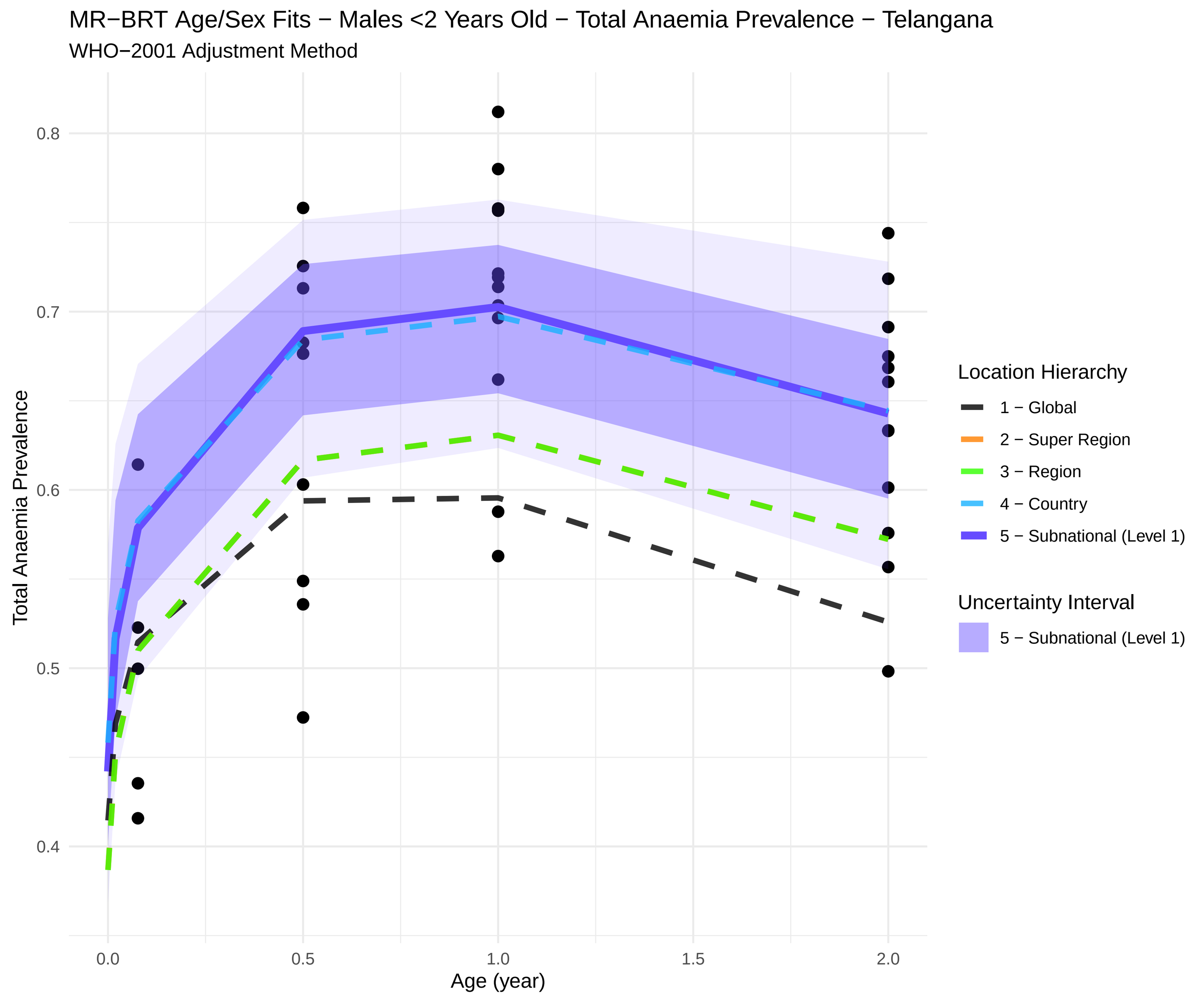
MR−BRT Age/Sex Fits − Males <2 Years Old − Total Anaemia Prevalence − Telangana
WHO−2001 Adjustment Method
Total Anaemia Prevalence
Age (year)
Location Hierarchy
1 − Global
2 − Super Region
3 − Region
4 − Country
5 − Subnational (Level 1)
Uncertainty Interval
5 − Subnational (Level 1)

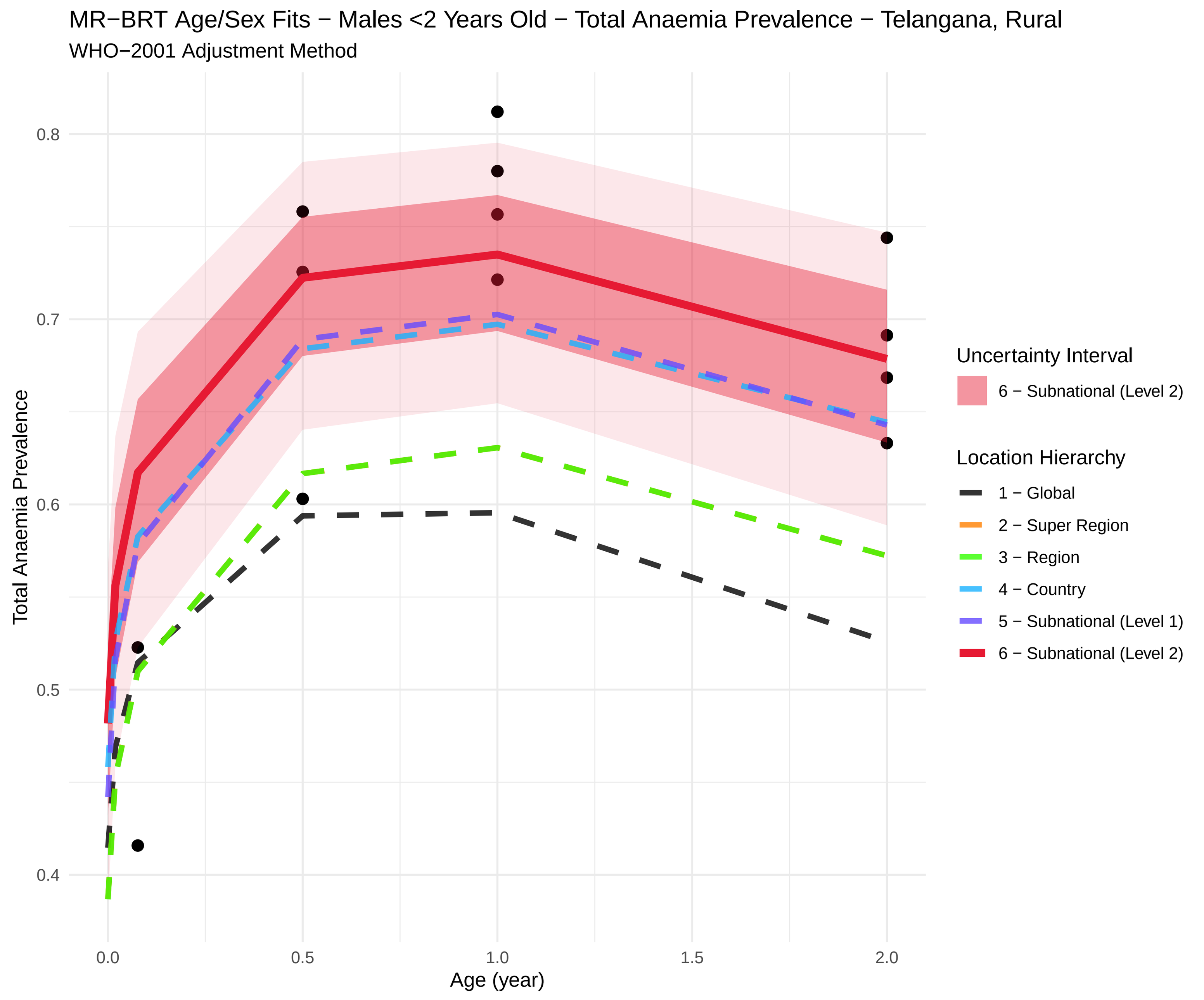
MR−BRT Age/Sex Fits − Males <2 Years Old − Total Anaemia Prevalence − Telangana, Rural
WHO−2001 Adjustment Method
Total Anaemia Prevalence
Age (year)
Uncertainty Interval
6 − Subnational (Level 2)
Location Hierarchy
1 − Global
2 − Super Region
3 − Region
4 − Country
5 − Subnational (Level 1)
6 − Subnational (Level 2)

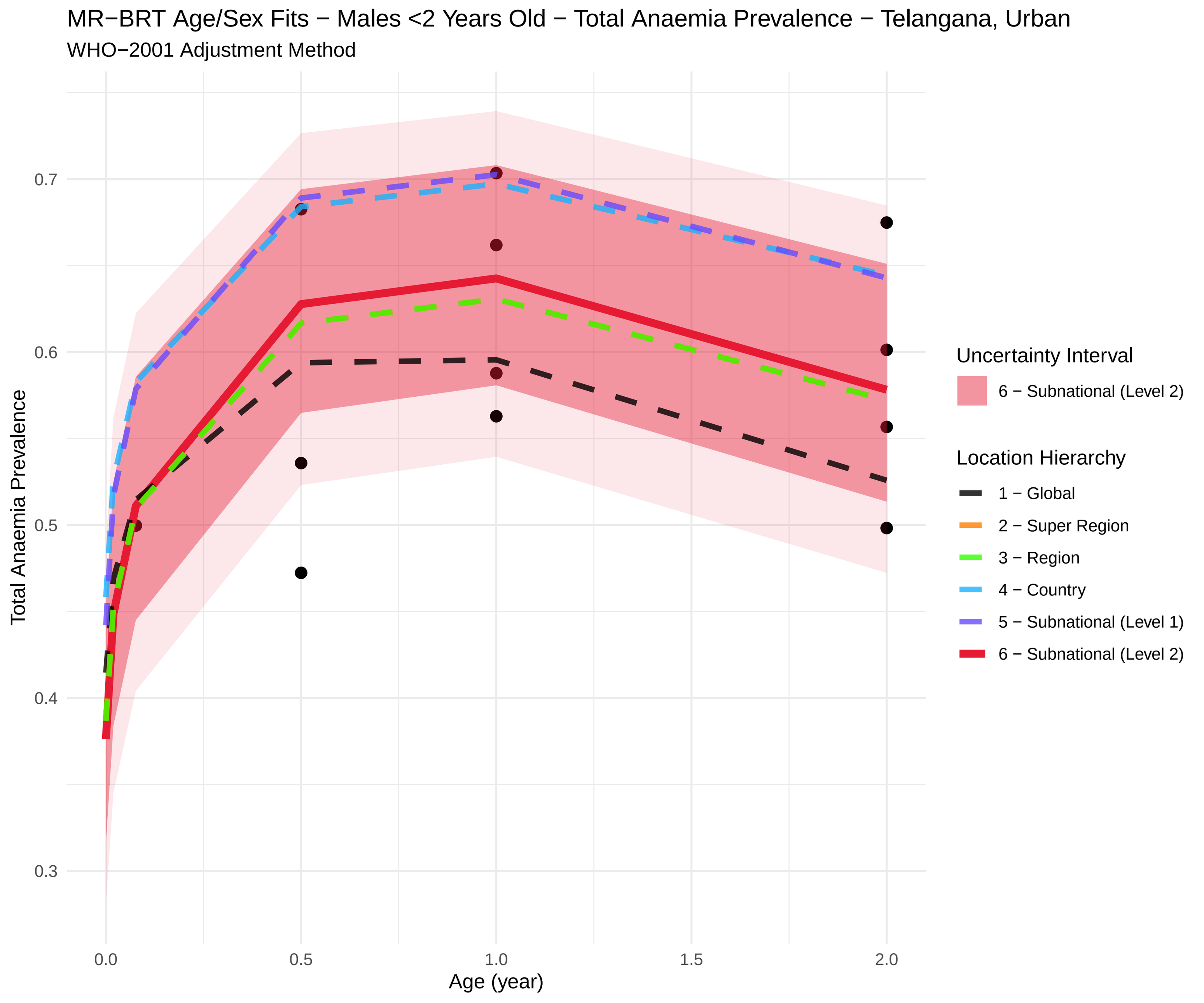
MR−BRT Age/Sex Fits − Males <2 Years Old − Total Anaemia Prevalence − Telangana, Urban
WHO−2001 Adjustment Method
Total Anaemia Prevalence
Age (year)
Uncertainty Interval
6 − Subnational (Level 2)
Location Hierarchy
1 − Global
2 − Super Region
3 − Region
4 − Country
5 − Subnational (Level 1)
6 − Subnational (Level 2)

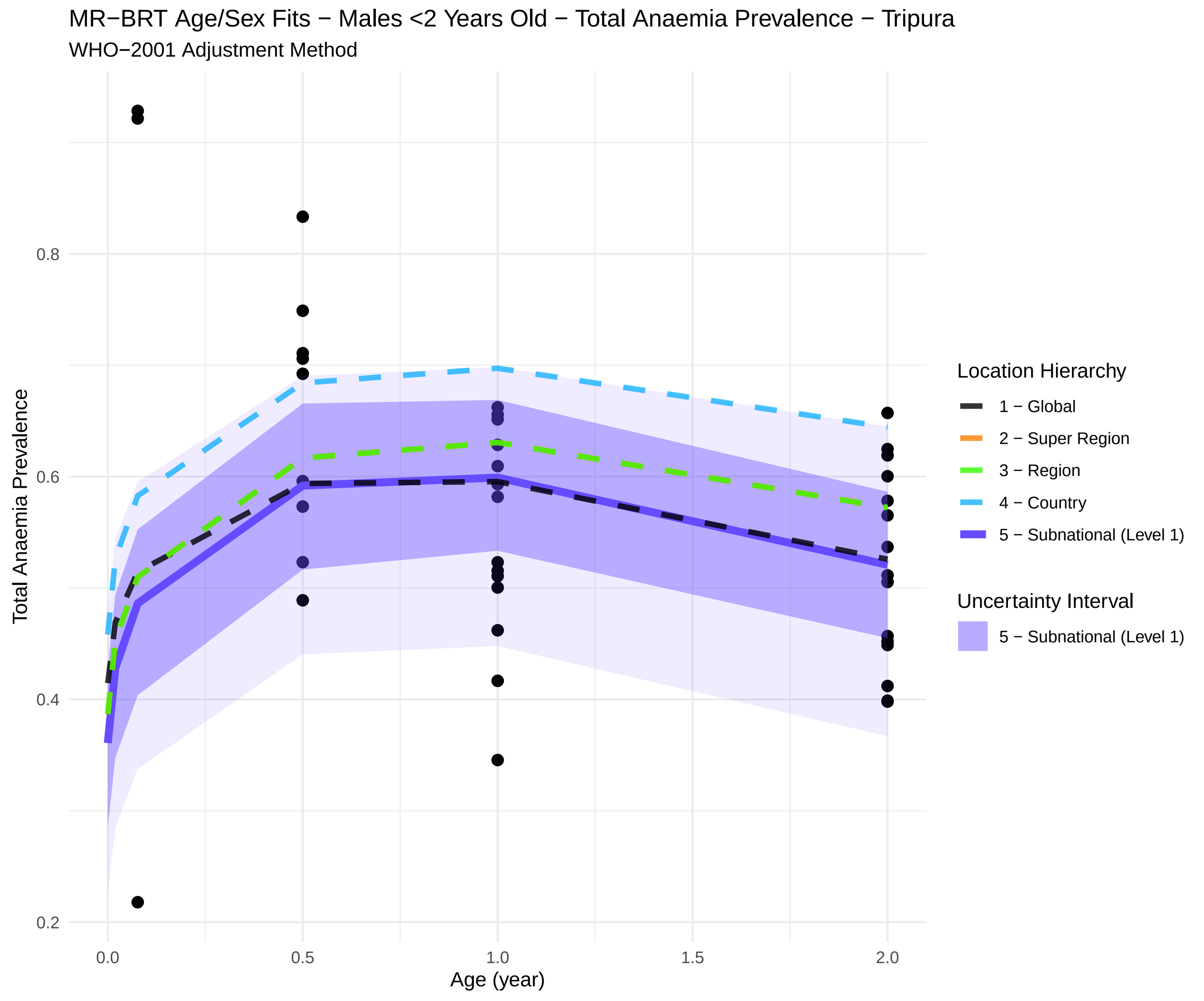
MR−BRT Age/Sex Fits − Males <2 Years Old − Total Anaemia Prevalence − Tripura
WHO−2001 Adjustment Method
Total Anaemia Prevalence
Age (year)
Location Hierarchy
1 − Global
2 − Super Region
3 − Region
4 − Country
5 − Subnational (Level 1)
Uncertainty Interval
5 − Subnational (Level 1)

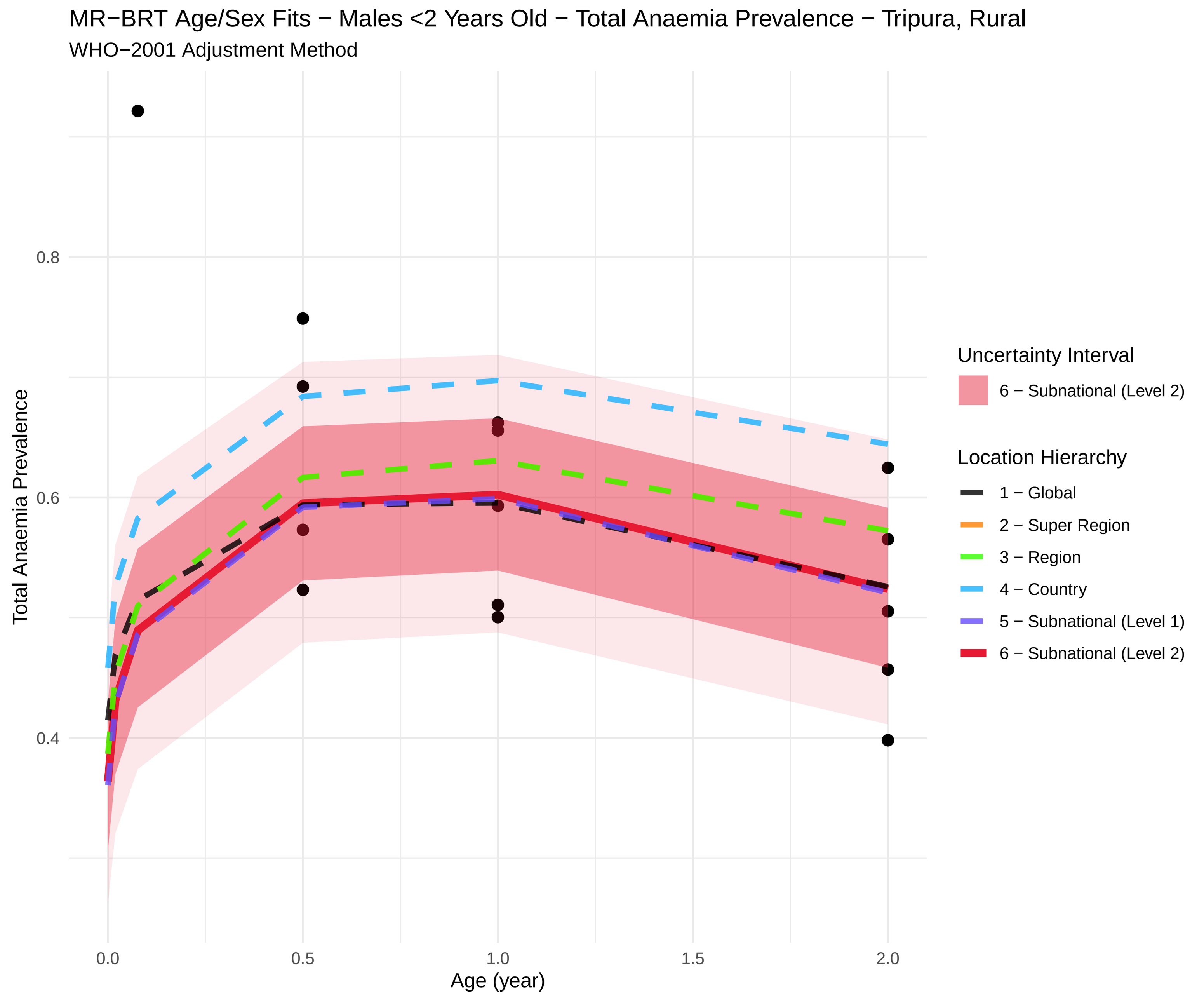
MR−BRT Age/Sex Fits − Males <2 Years Old − Total Anaemia Prevalence − Tripura, Rural
WHO−2001 Adjustment Method
Total Anaemia Prevalence
Age (year)
Uncertainty Interval
6 − Subnational (Level 2)
Location Hierarchy
1 − Global
2 − Super Region
3 − Region
4 − Country
5 − Subnational (Level 1)
6 − Subnational (Level 2)

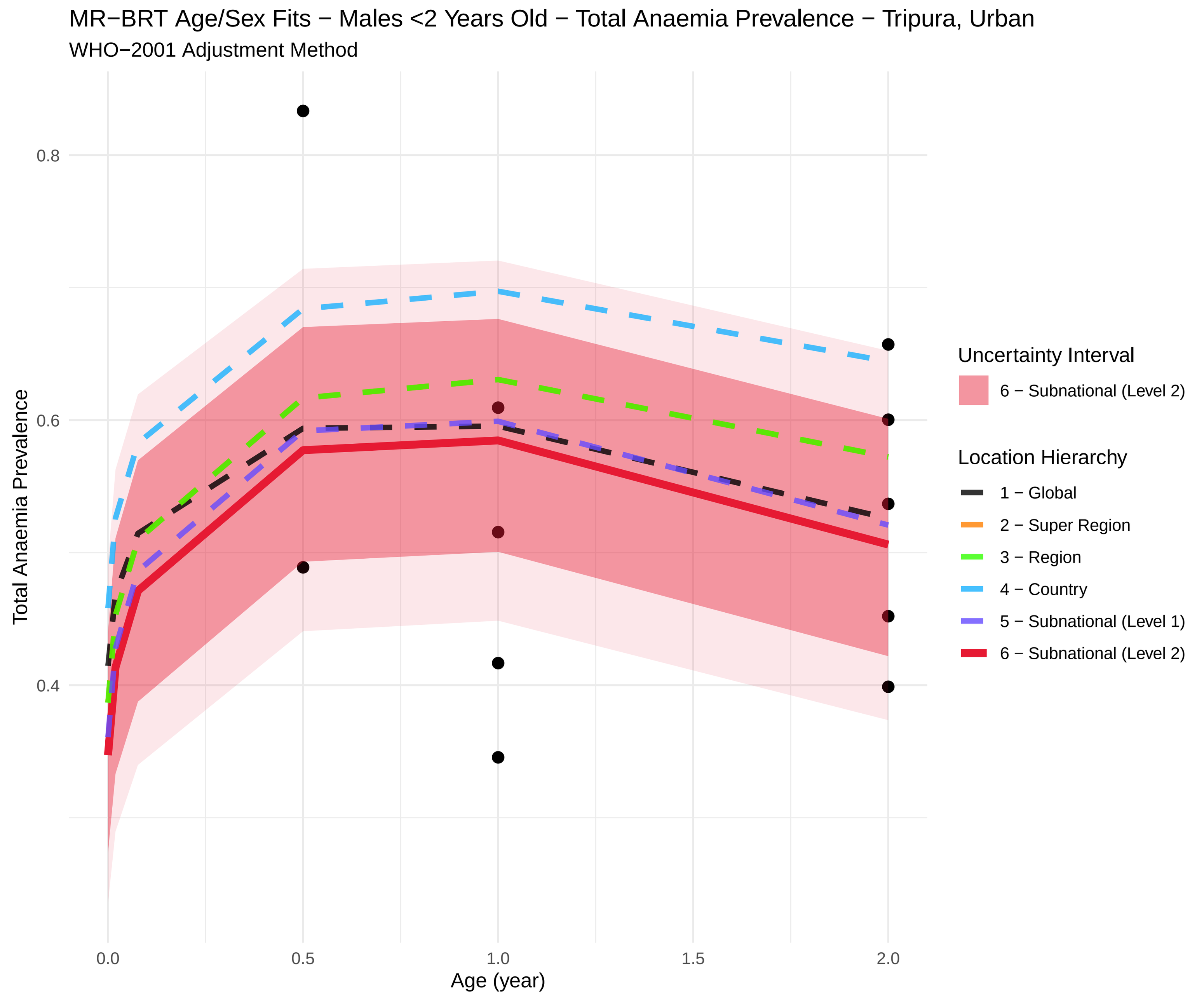
MR−BRT Age/Sex Fits − Males <2 Years Old − Total Anaemia Prevalence − Tripura, Urban
WHO−2001 Adjustment Method
Total Anaemia Prevalence
Age (year)
Uncertainty Interval
6 − Subnational (Level 2)
Location Hierarchy
1 − Global
2 − Super Region
3 − Region
4 − Country
5 − Subnational (Level 1)
6 − Subnational (Level 2)

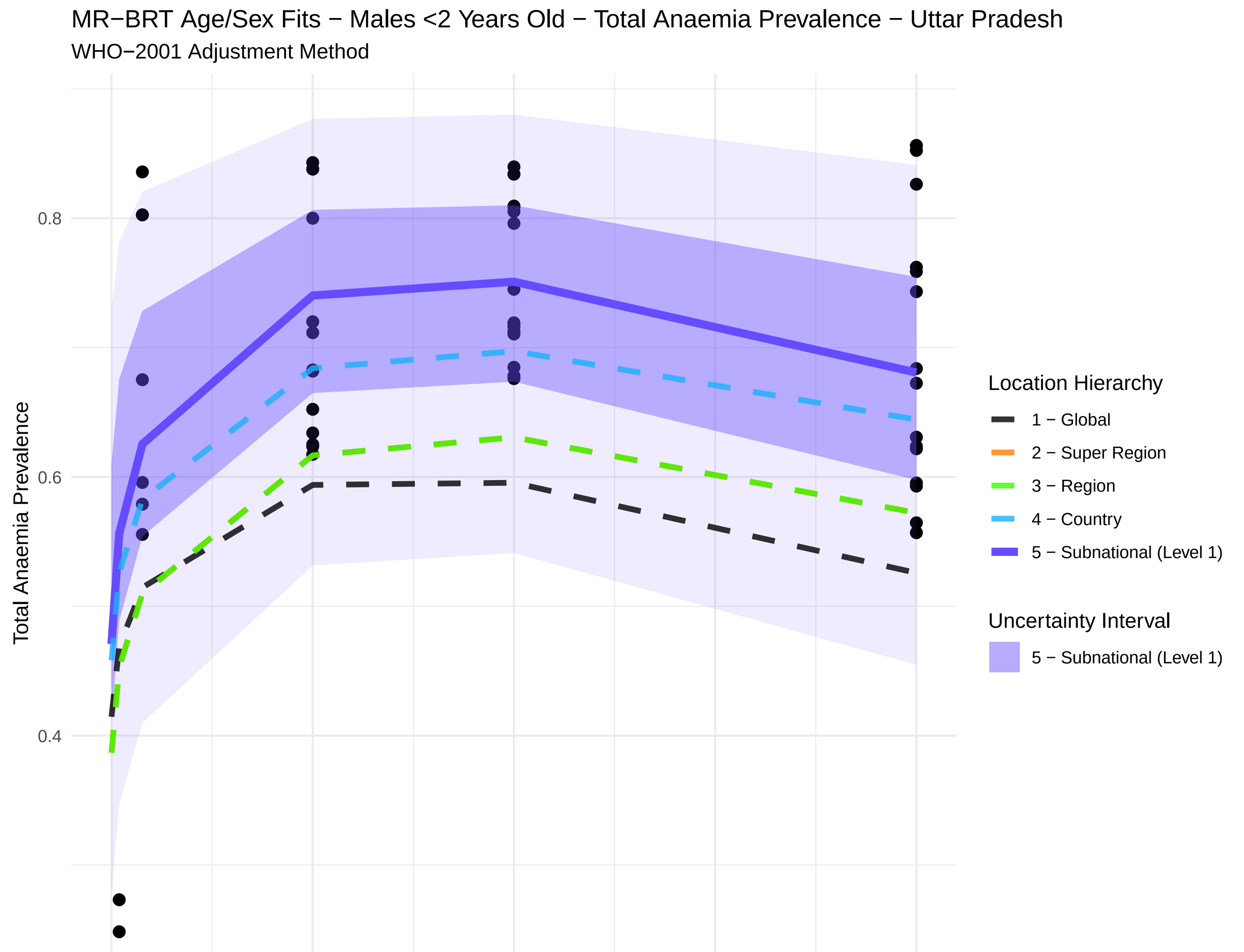
MR−BRT Age/Sex Fits − Males <2 Years Old − Total Anaemia Prevalence − Uttar Pradesh
WHO−2001 Adjustment Method
Total Anaemia Prevalence
Age (year)
Location Hierarchy
1 − Global
2 − Super Region
3 − Region
4 − Country
5 − Subnational (Level 1)
Uncertainty Interval
5 − Subnational (Level 1)

MR−BRT Age/Sex Fits − Males <2 Years Old − Total Anaemia Prevalence − Uttar Pradesh, Rural
WHO−2001 Adjustment Method

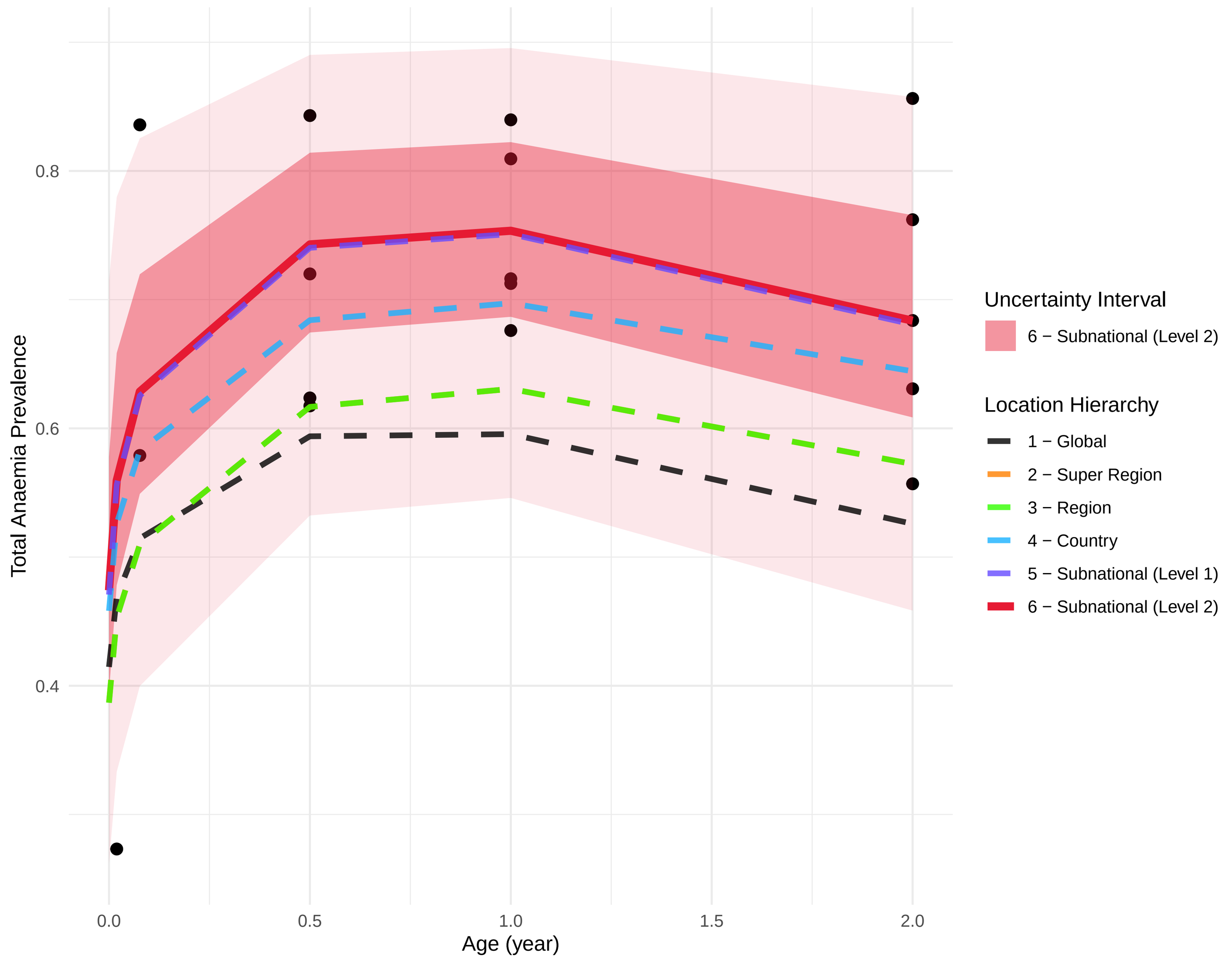

MR−BRT Age/Sex Fits − Males <2 Years Old − Total Anaemia Prevalence − Uttar Pradesh, Urban
WHO−2001 Adjustment Method

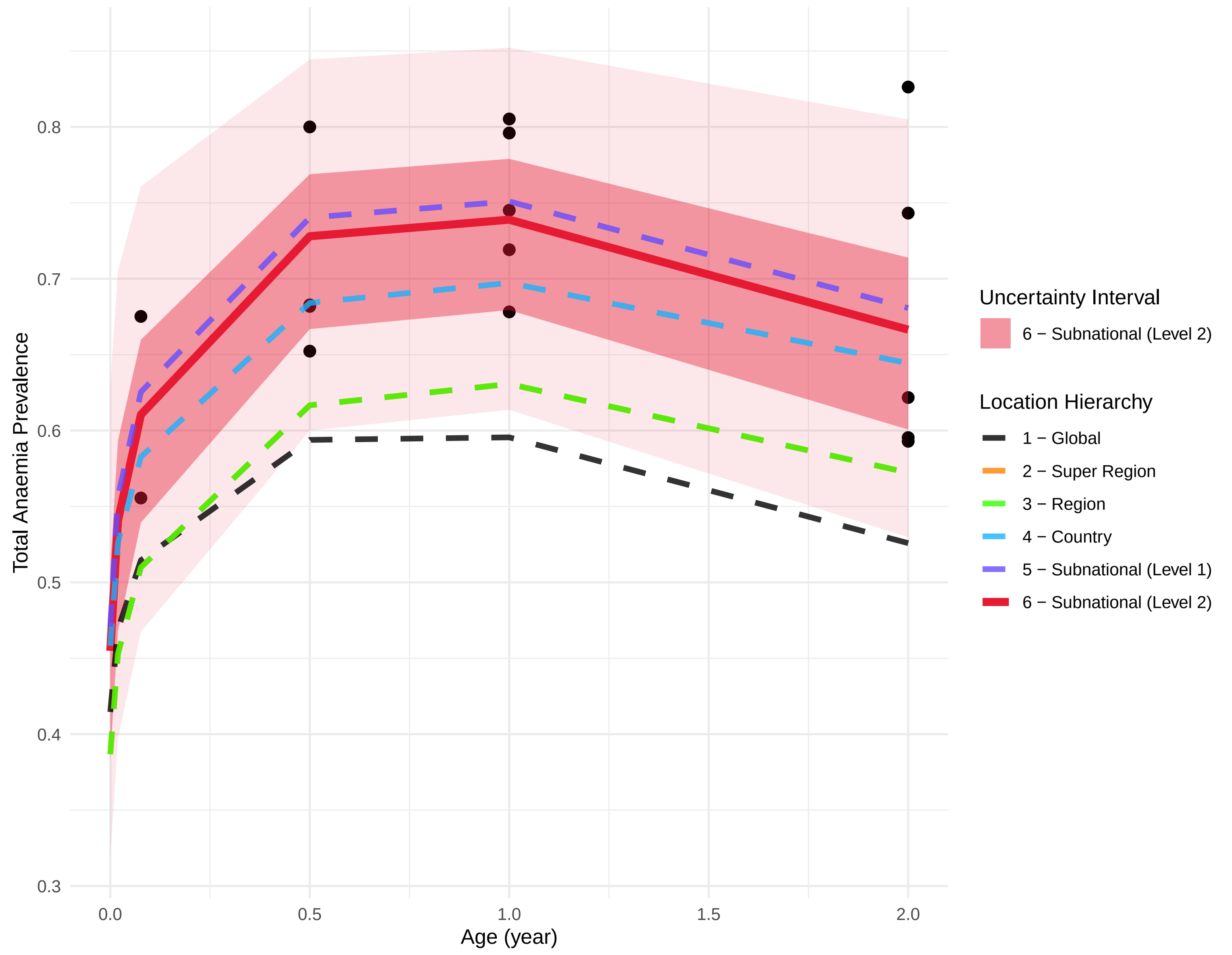

MR−BRT Age/Sex Fits − Males <2 Years Old − Total Anaemia Prevalence − Uttarakhand
WHO−2001 Adjustment Method

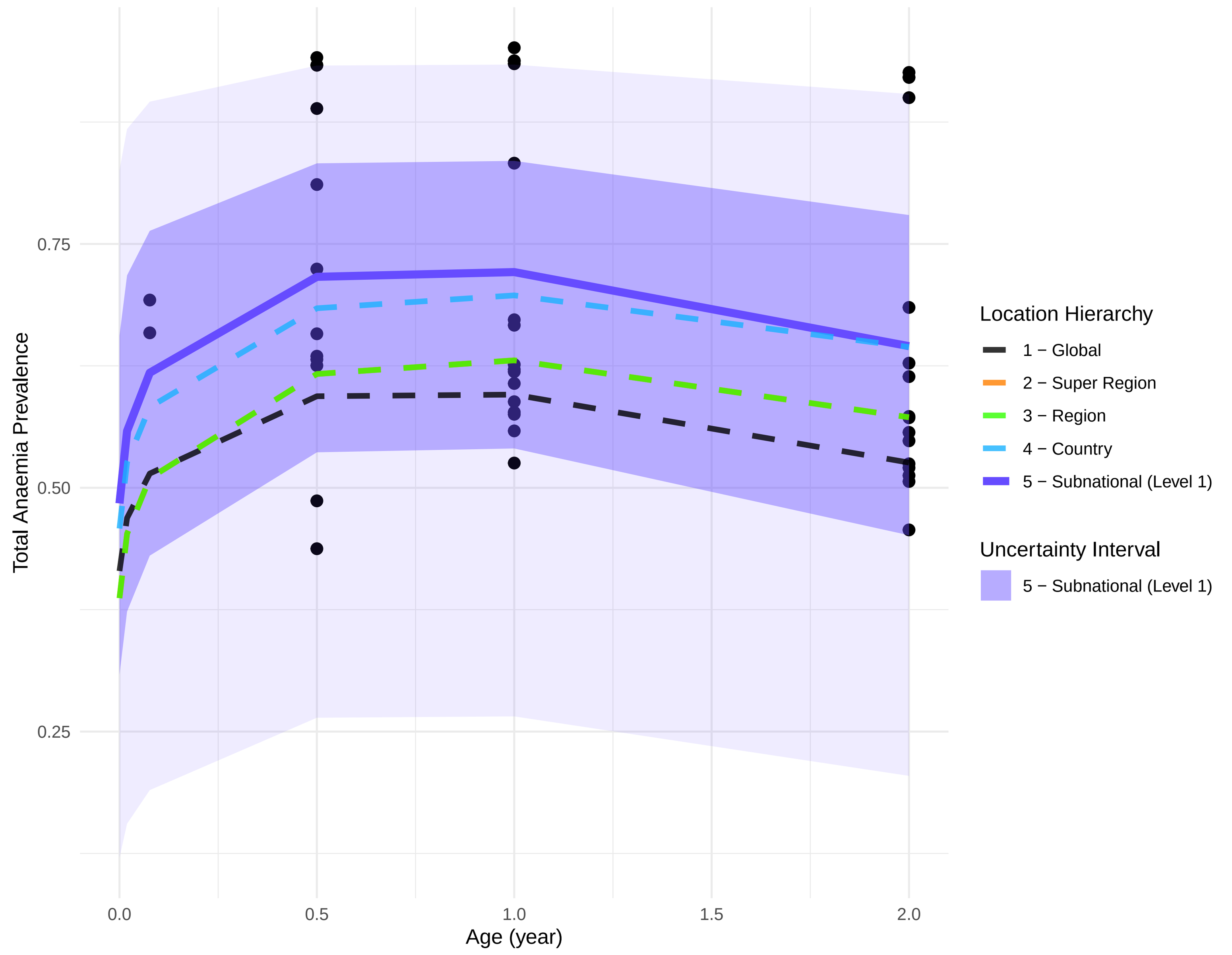

MR−BRT Age/Sex Fits − Males <2 Years Old − Total Anaemia Prevalence − Uttarakhand, Rural
WHO−2001 Adjustment Method

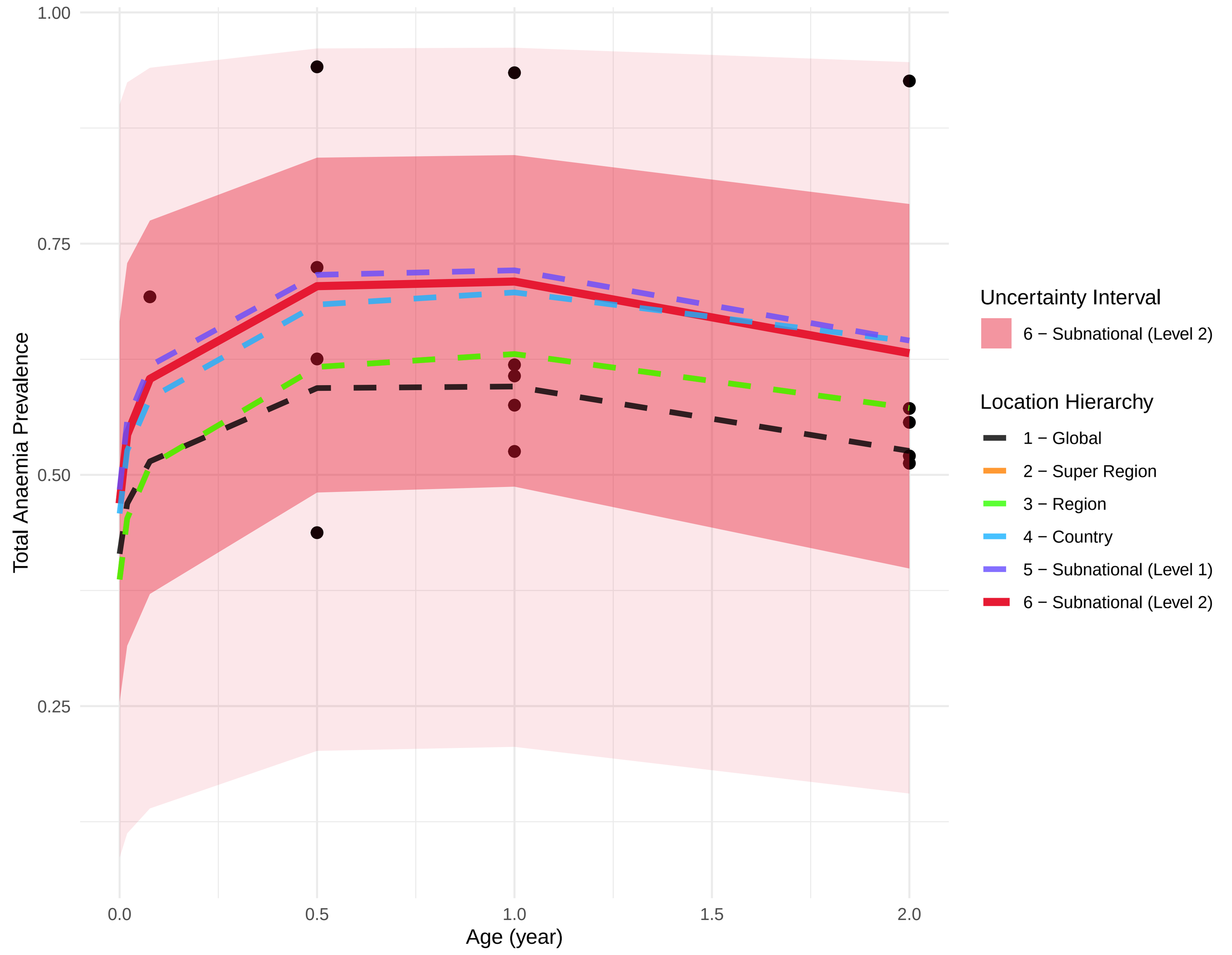

MR−BRT Age/Sex Fits − Males <2 Years Old − Total Anaemia Prevalence − Uttarakhand, Urban
WHO−2001 Adjustment Method

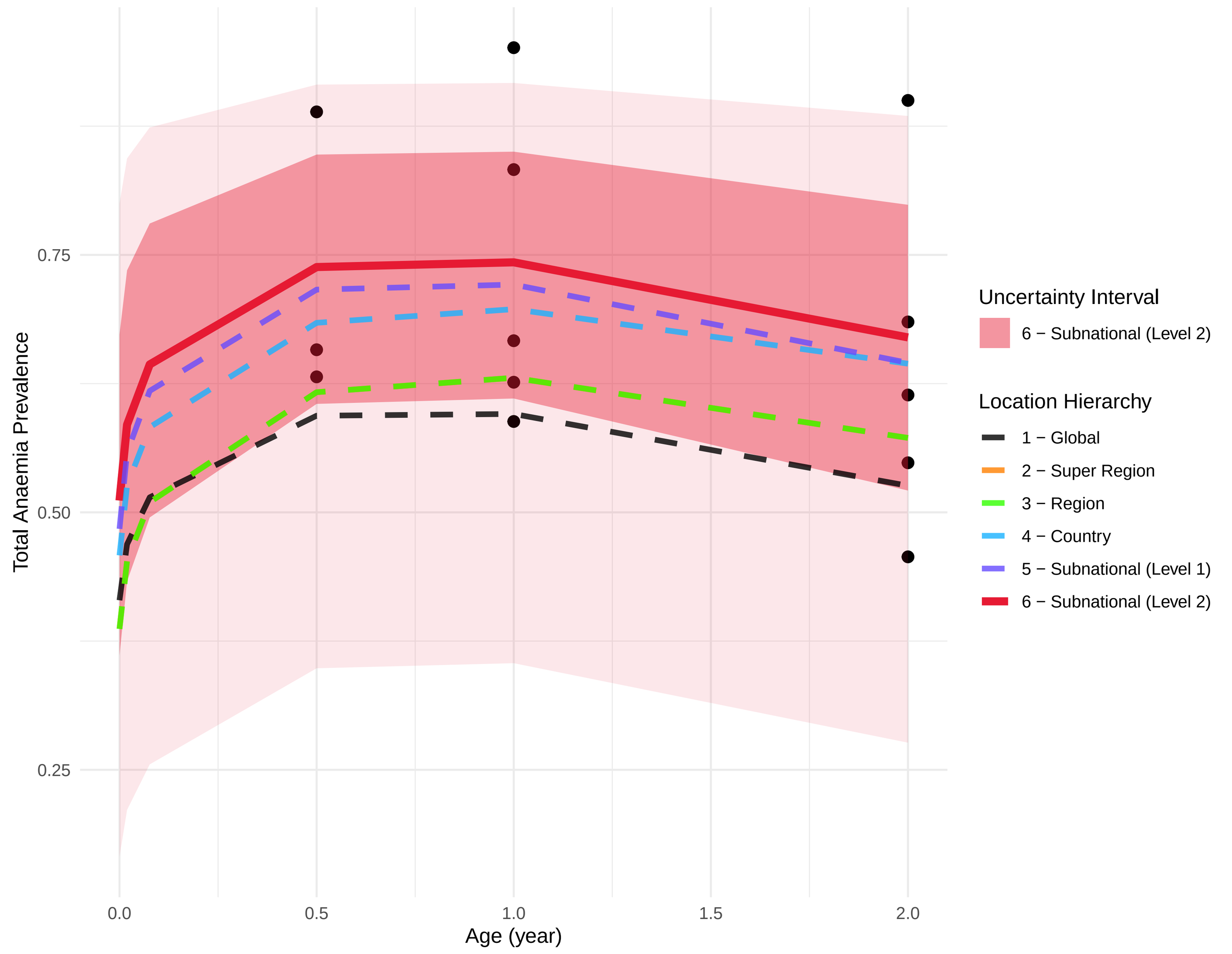

MR−BRT Age/Sex Fits − Males <2 Years Old − Total Anaemia Prevalence − West Bengal
WHO−2001 Adjustment Method

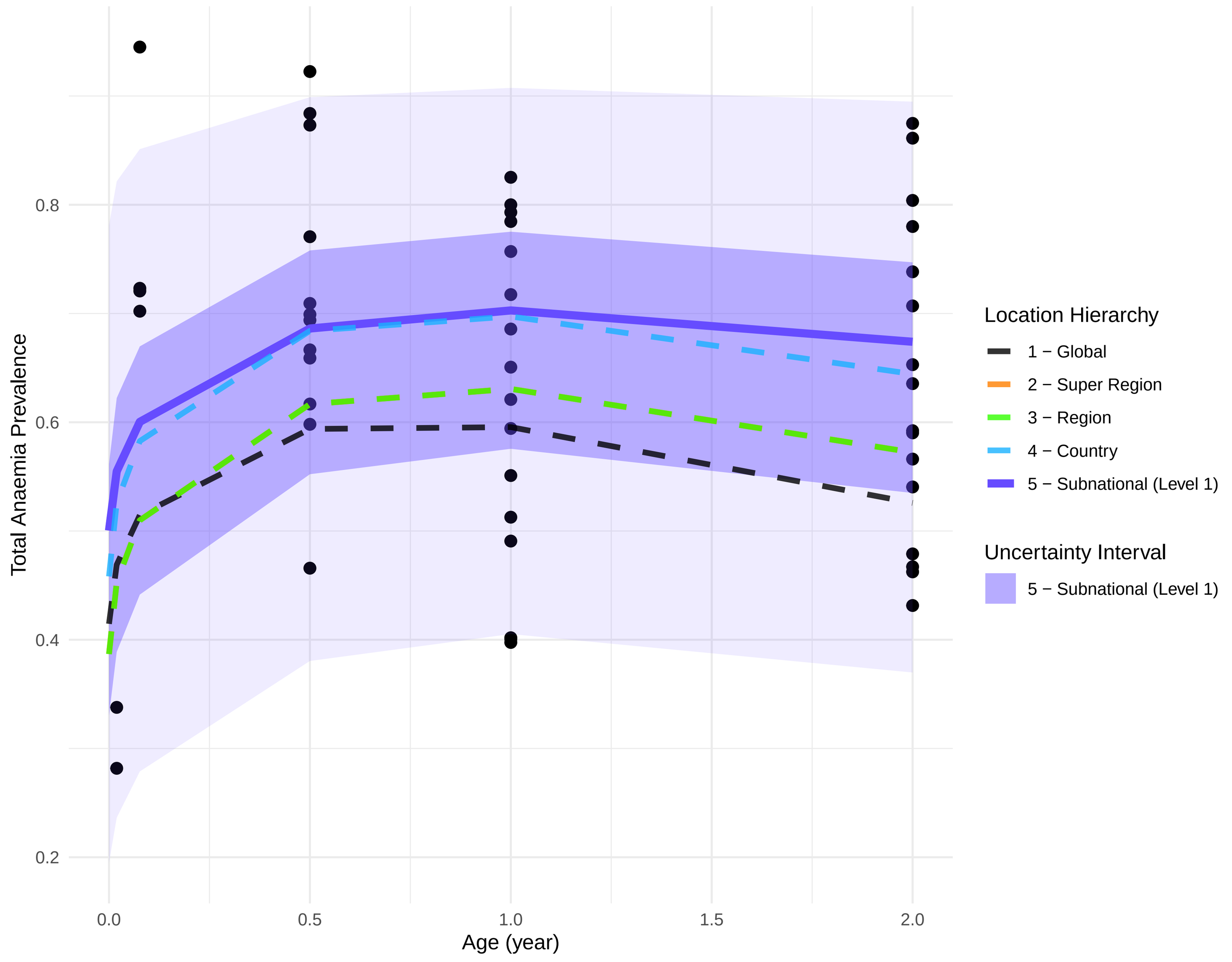

MR−BRT Age/Sex Fits − Males <2 Years Old − Total Anaemia Prevalence − West Bengal, Rural
WHO−2001 Adjustment Method

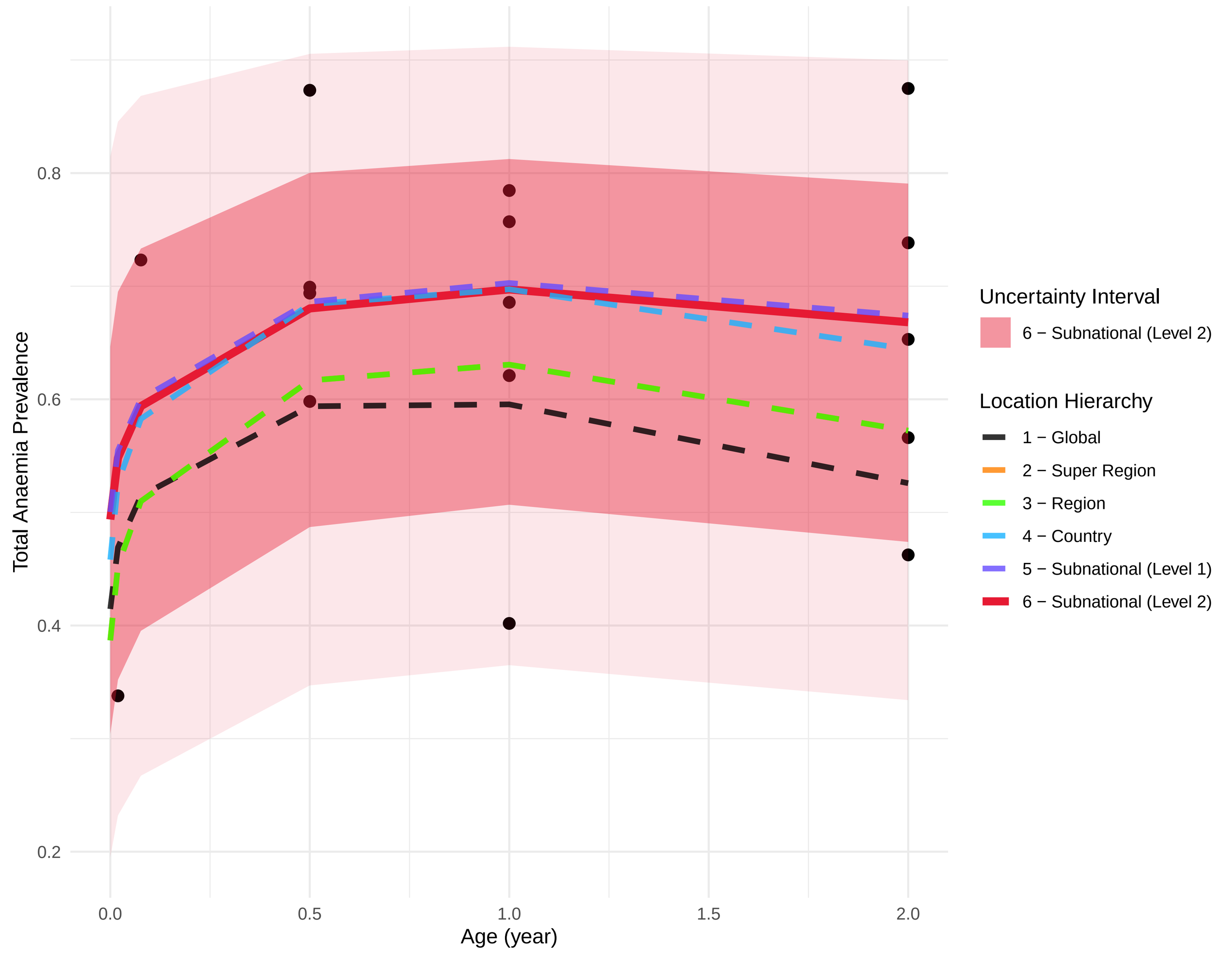

MR−BRT Age/Sex Fits − Males <2 Years Old − Total Anaemia Prevalence − West Bengal, Urban
WHO−2001 Adjustment Method

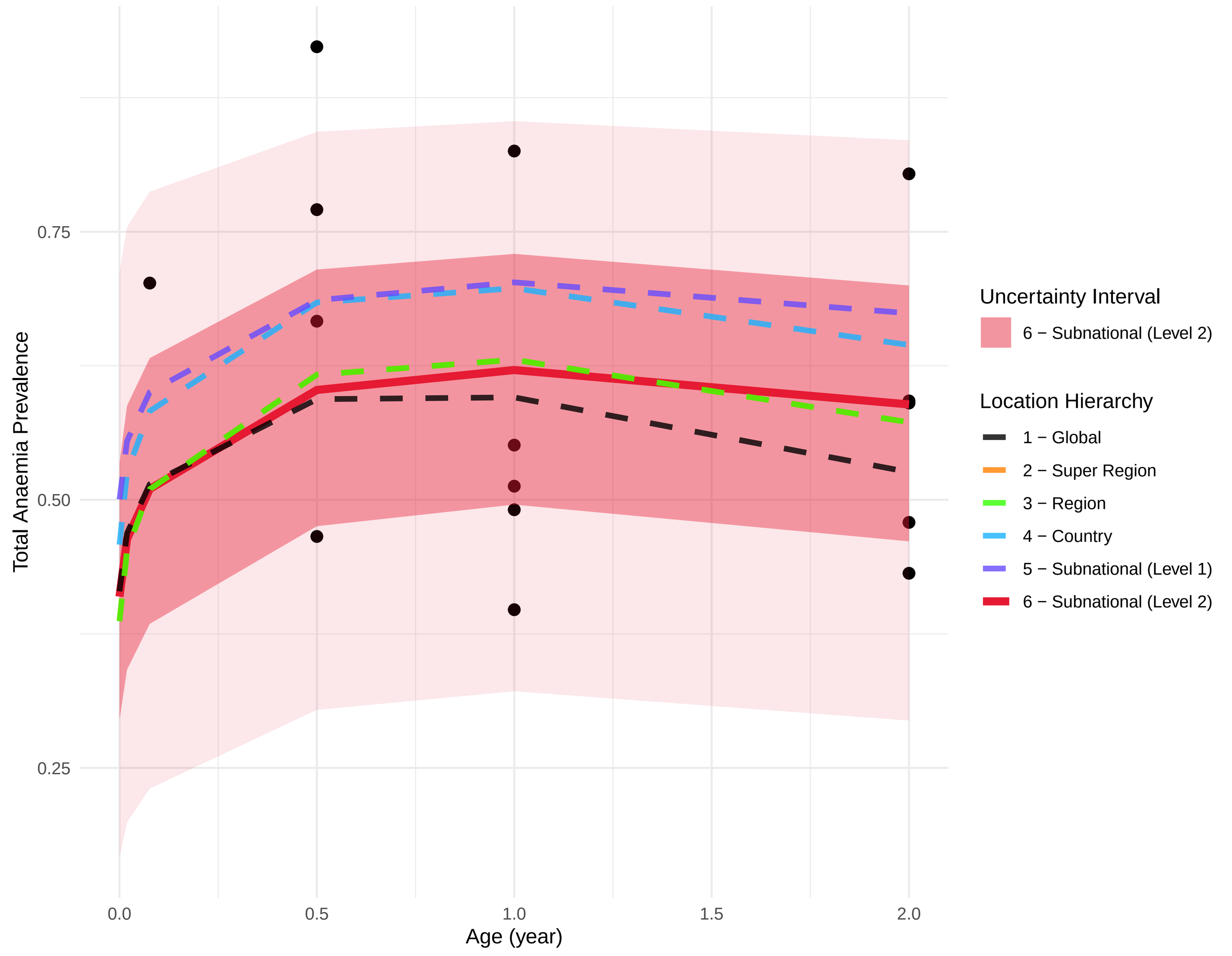

MR−BRT Age/Sex Fits − Males <2 Years Old − Total Anaemia Prevalence − Nepal
WHO−2001 Adjustment Method

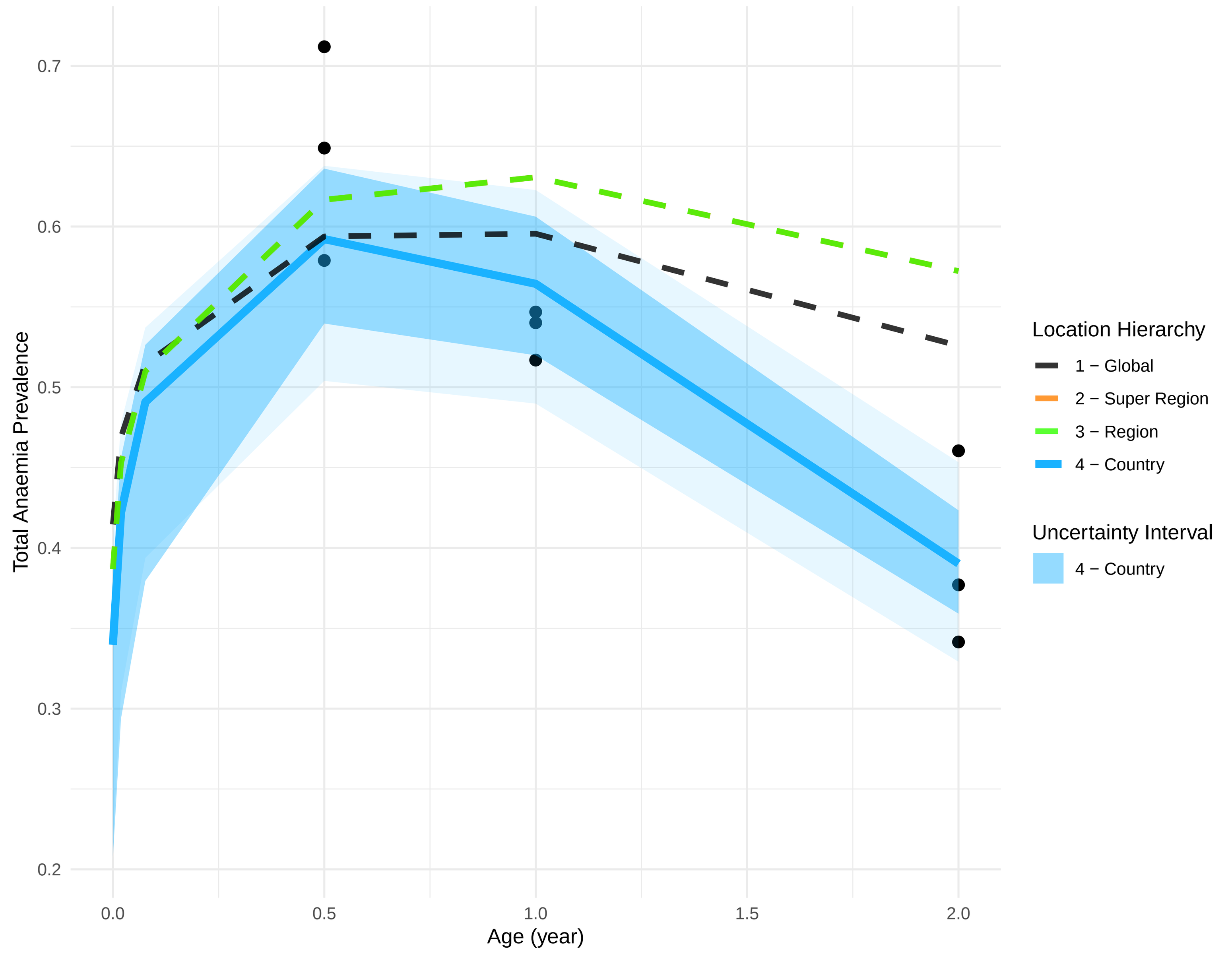

MR−BRT Age/Sex Fits − Males <2 Years Old − Total Anaemia Prevalence − Southeast Asia, East Asia, and Oceania
WHO−2001 Adjustment Method

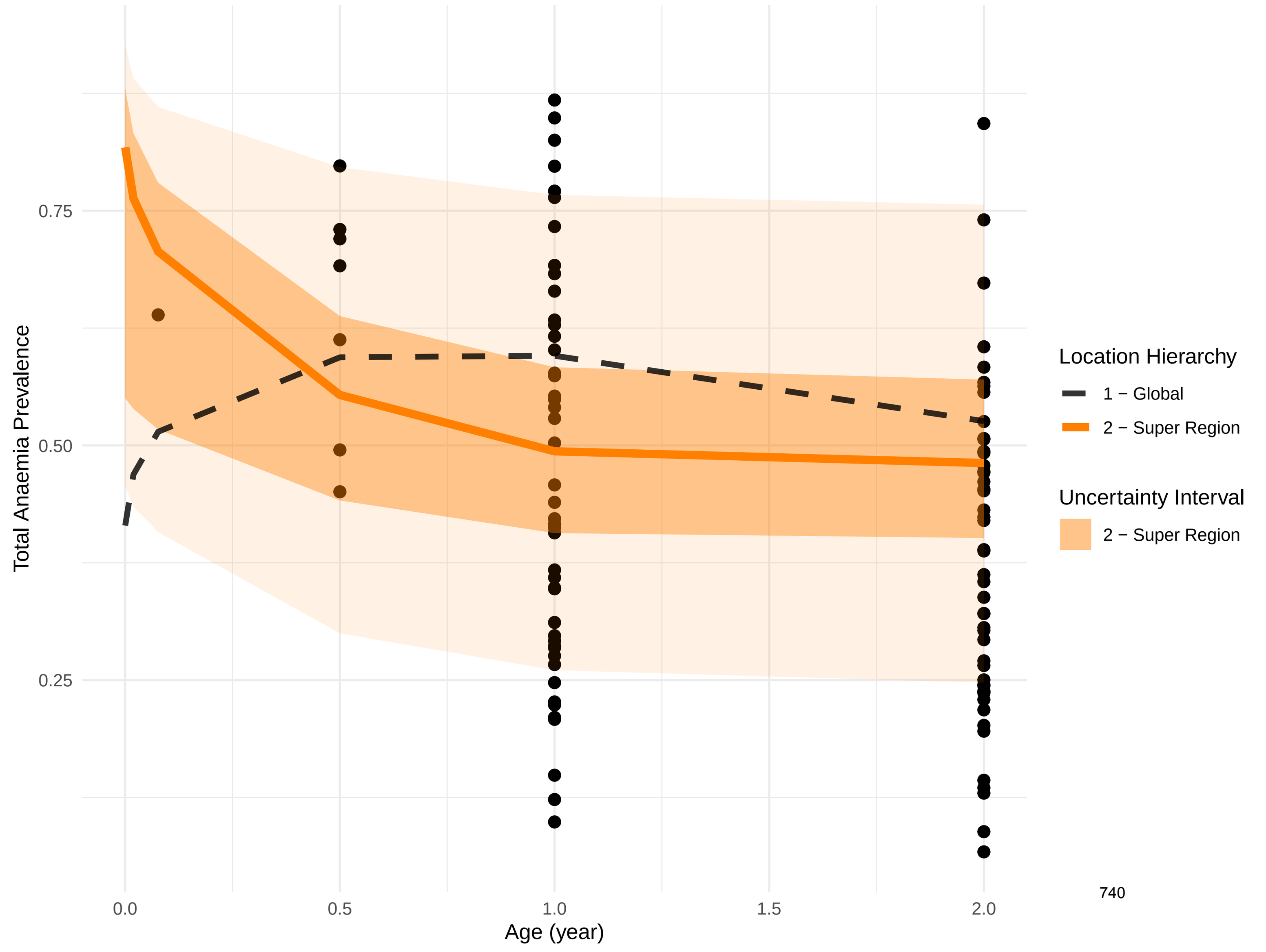

MR−BRT Age/Sex Fits − Males <2 Years Old − Total Anaemia Prevalence − Southeast Asia
WHO−2001 Adjustment Method

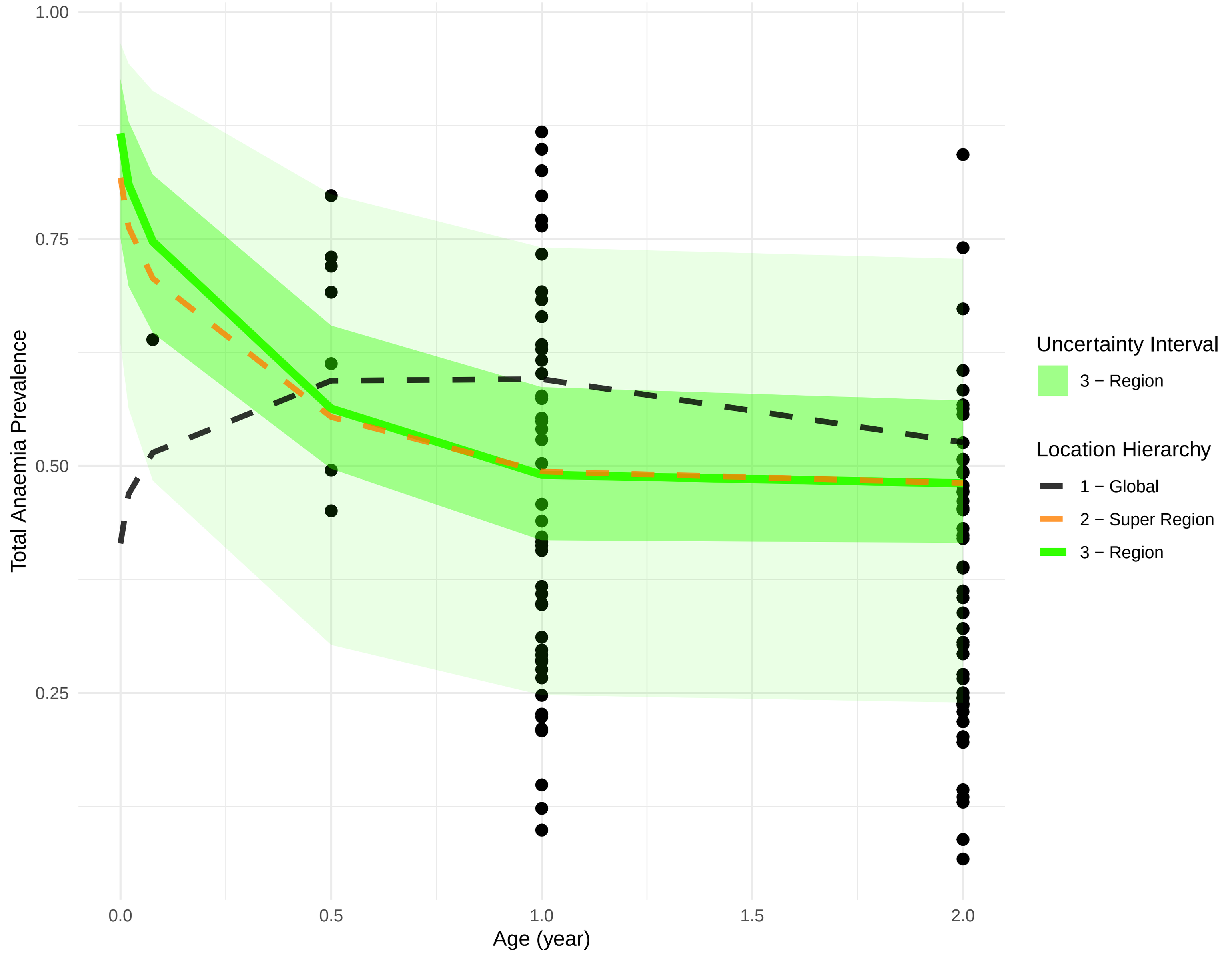

MR−BRT Age/Sex Fits − Males <2 Years Old − Total Anaemia Prevalence − Cambodia
WHO−2001 Adjustment Method

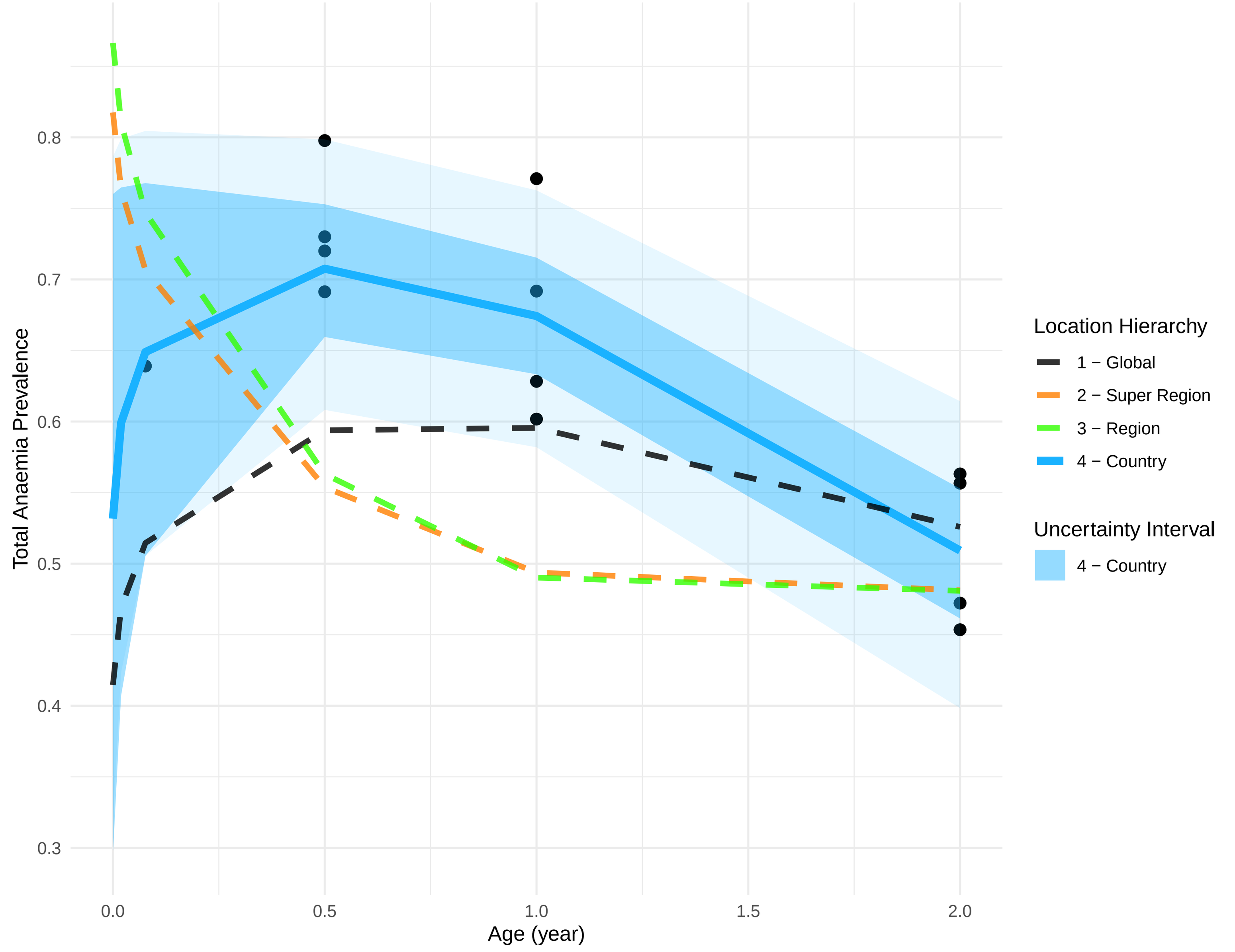

MR−BRT Age/Sex Fits − Males <2 Years Old − Total Anaemia Prevalence − Indonesia
WHO−2001 Adjustment Method

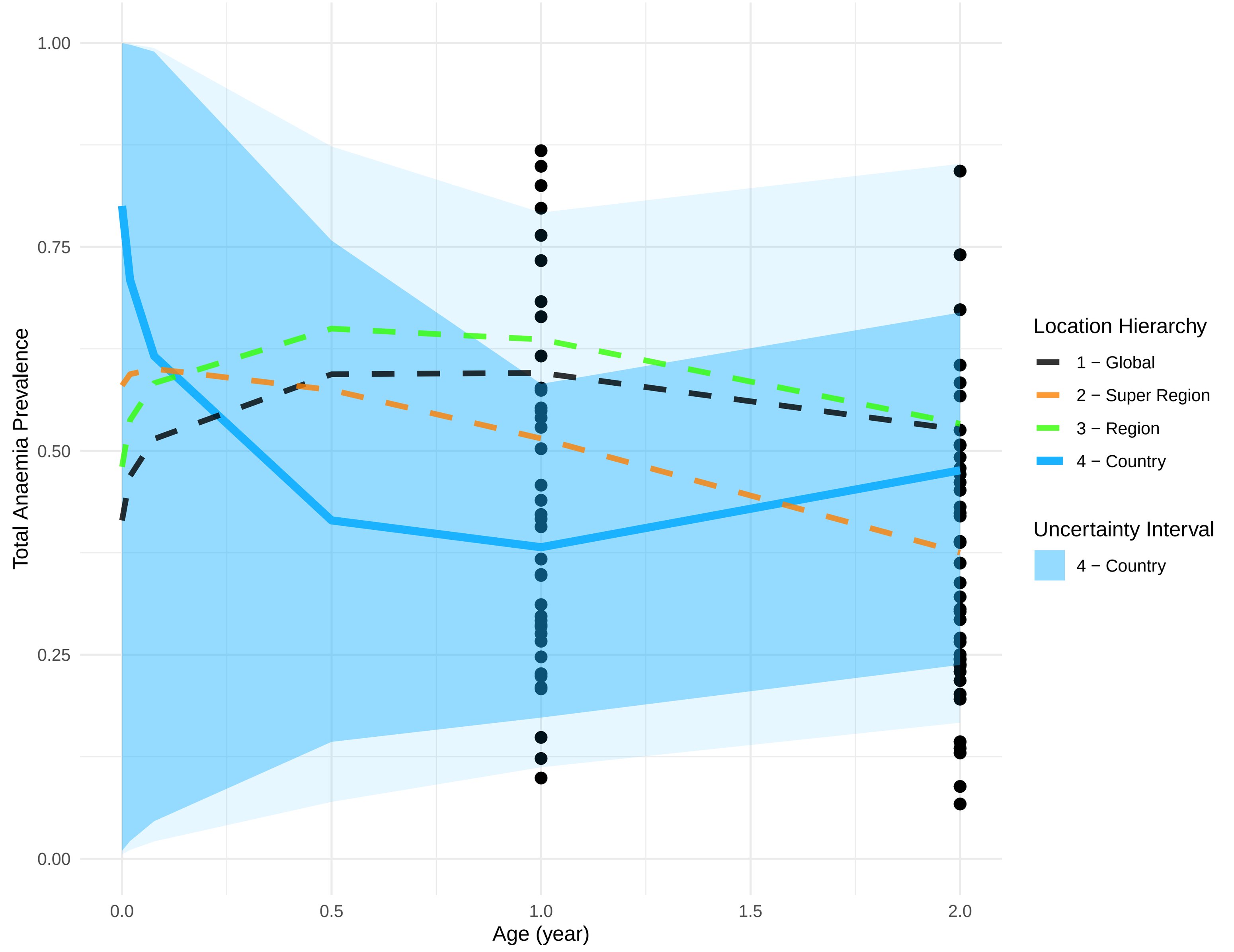

MR−BRT Age/Sex Fits − Males <2 Years Old − Total Anaemia Prevalence − Bali
WHO−2001 Adjustment Method

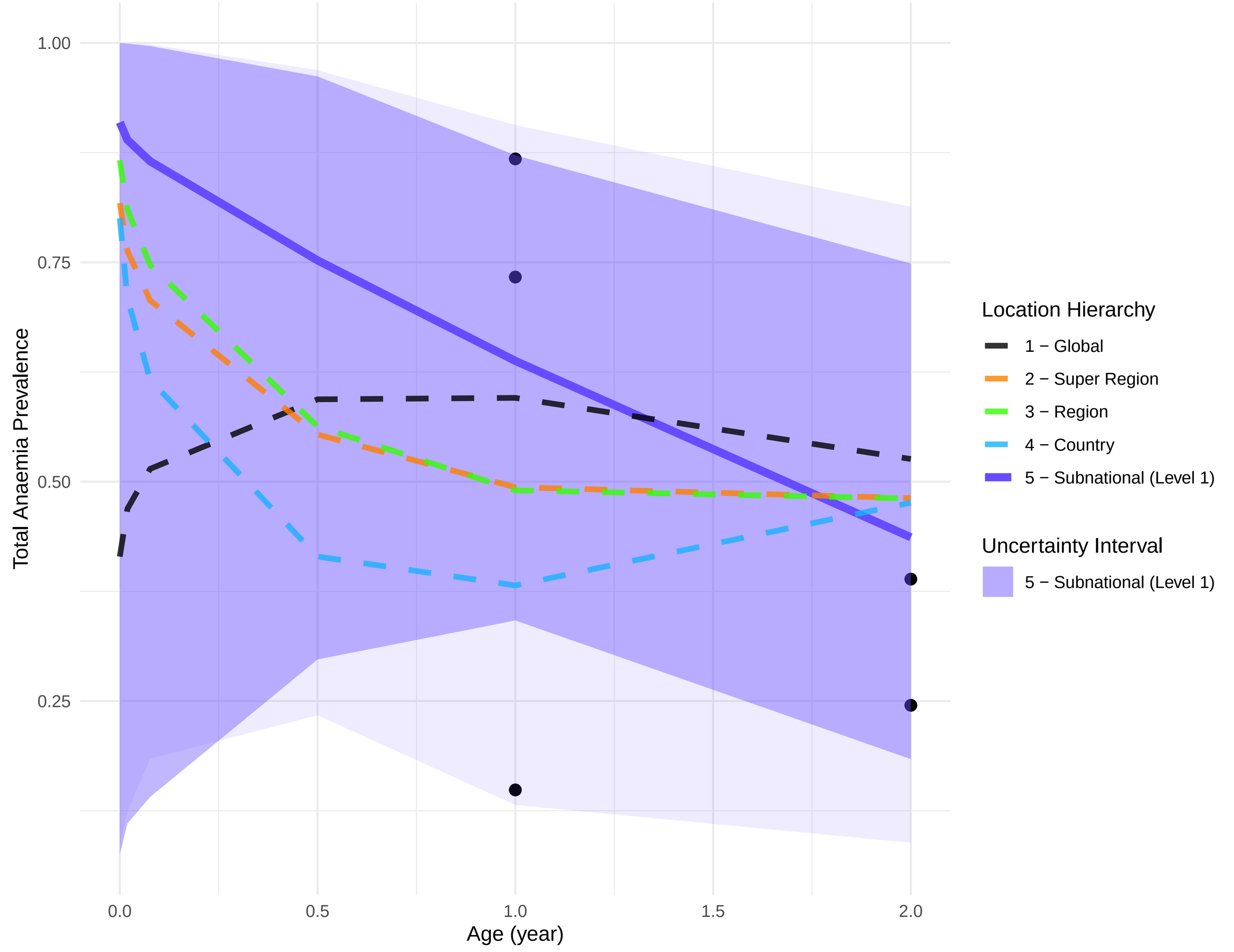

MR−BRT Age/Sex Fits − Males <2 Years Old − Total Anaemia Prevalence − Bangka−Belitung Islands
WHO−2001 Adjustment Method

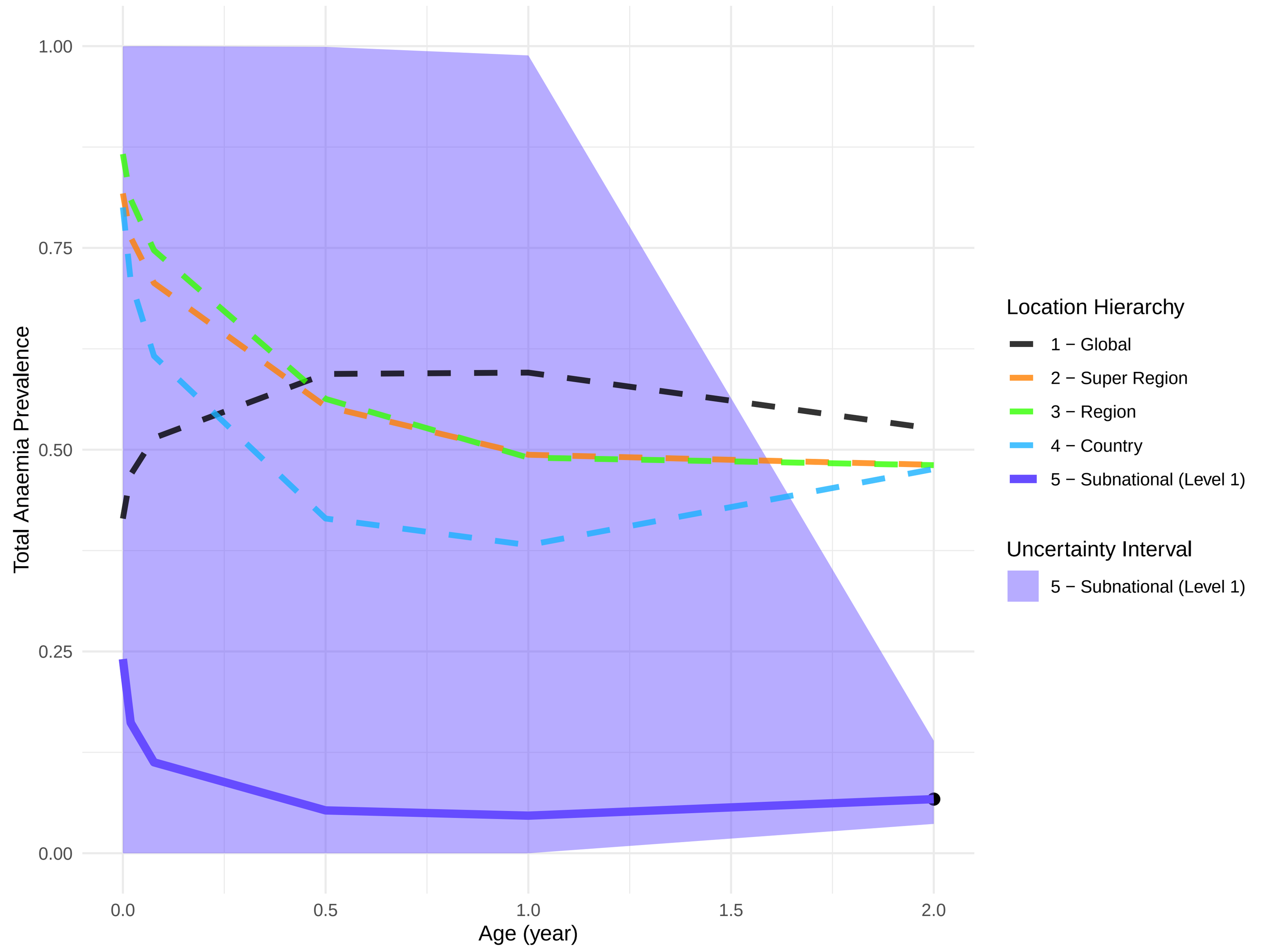

MR−BRT Age/Sex Fits − Males <2 Years Old − Total Anaemia Prevalence − Banten
WHO−2001 Adjustment Method

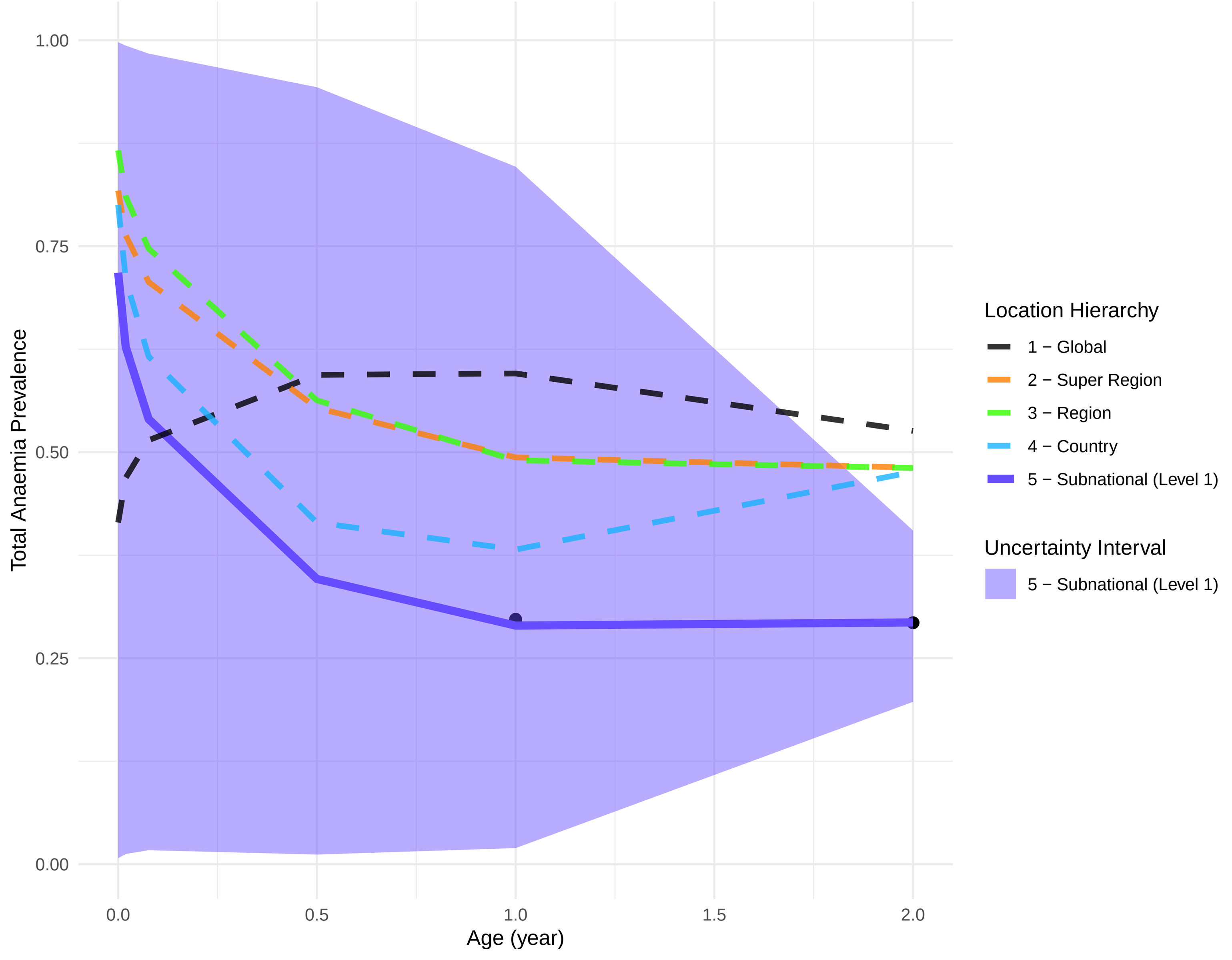

MR−BRT Age/Sex Fits − Males <2 Years Old − Total Anaemia Prevalence − Jakarta
WHO−2001 Adjustment Method

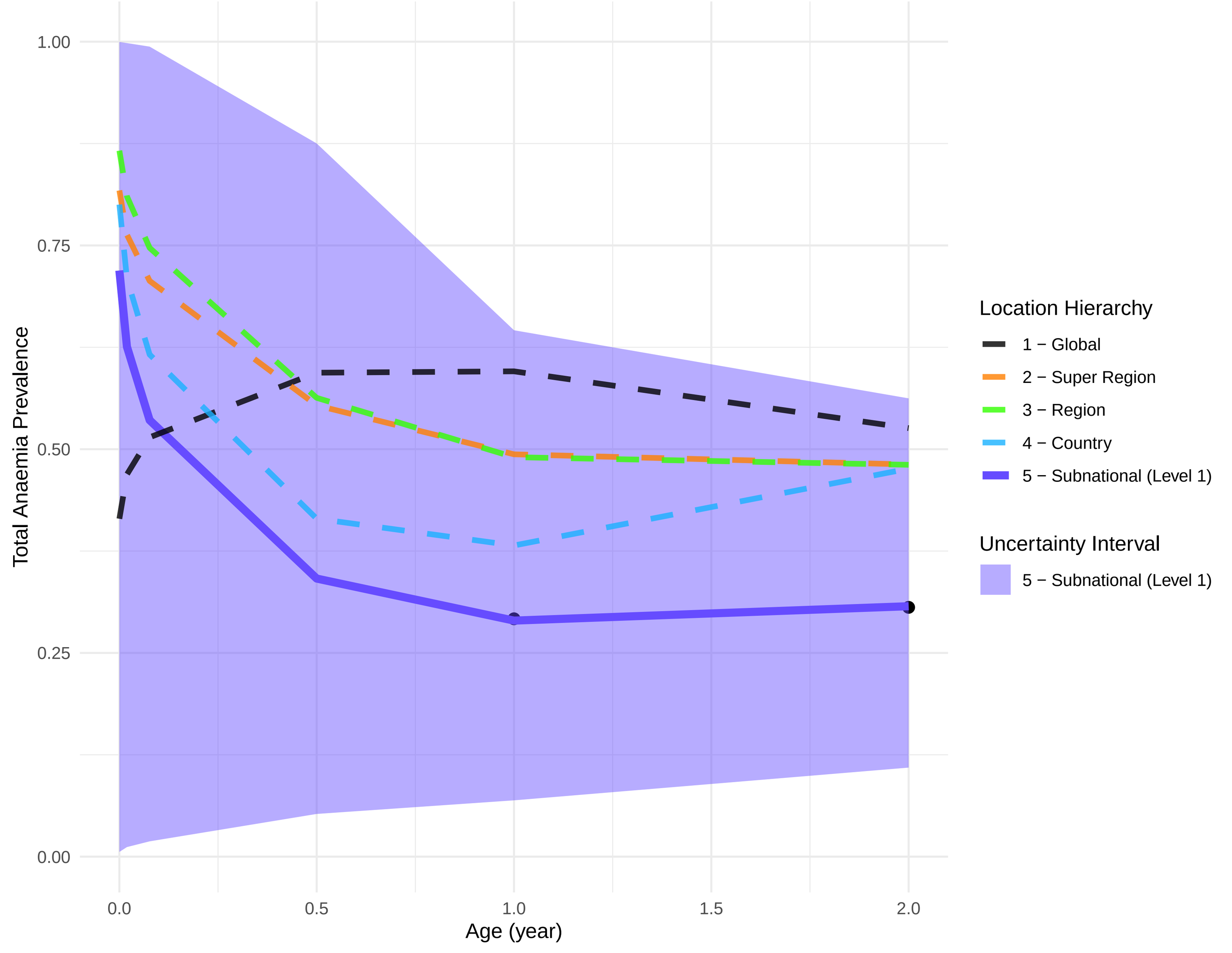

MR−BRT Age/Sex Fits − Males <2 Years Old − Total Anaemia Prevalence − West Java
WHO−2001 Adjustment Method

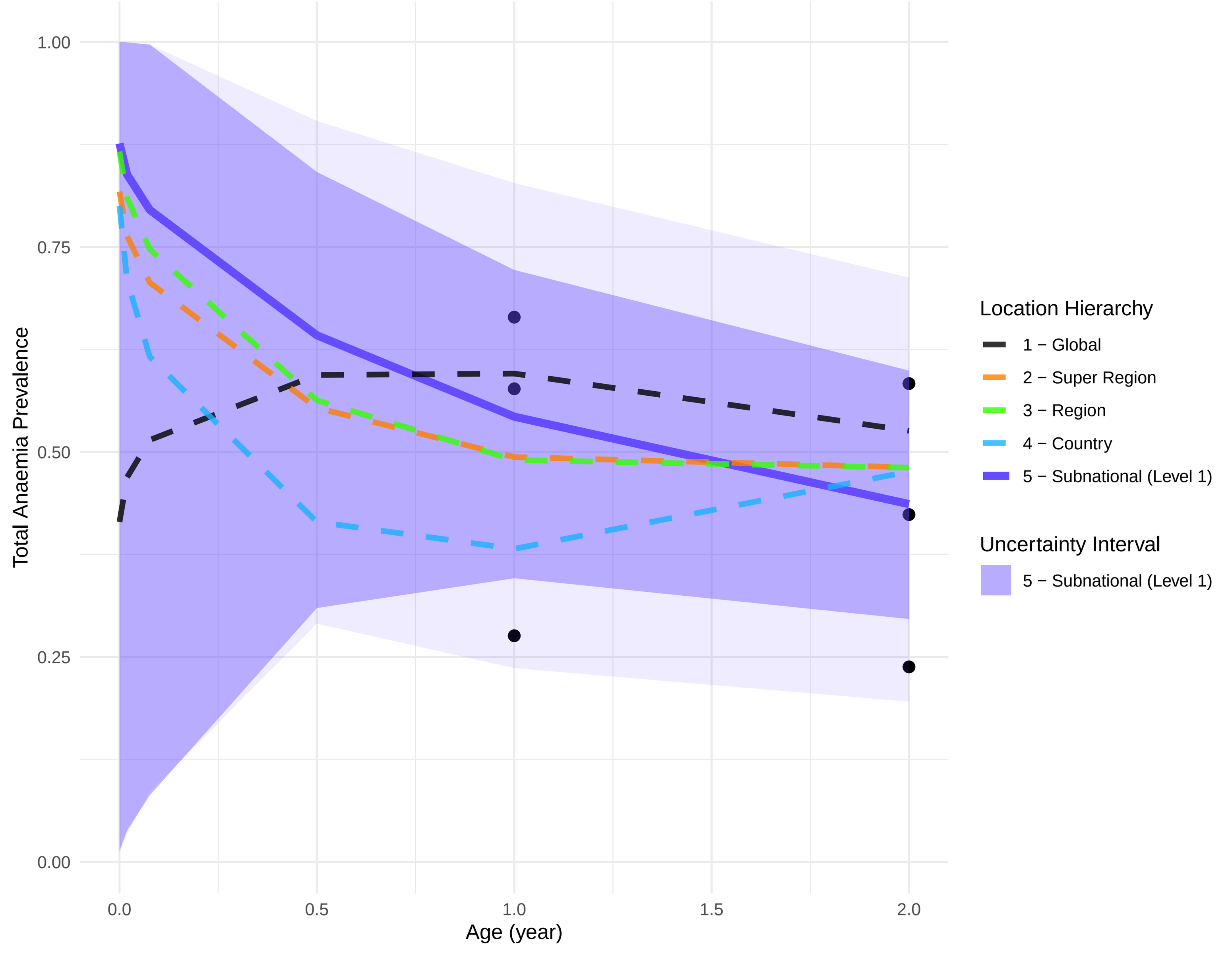

MR−BRT Age/Sex Fits − Males <2 Years Old − Total Anaemia Prevalence − Central Java
WHO−2001 Adjustment Method

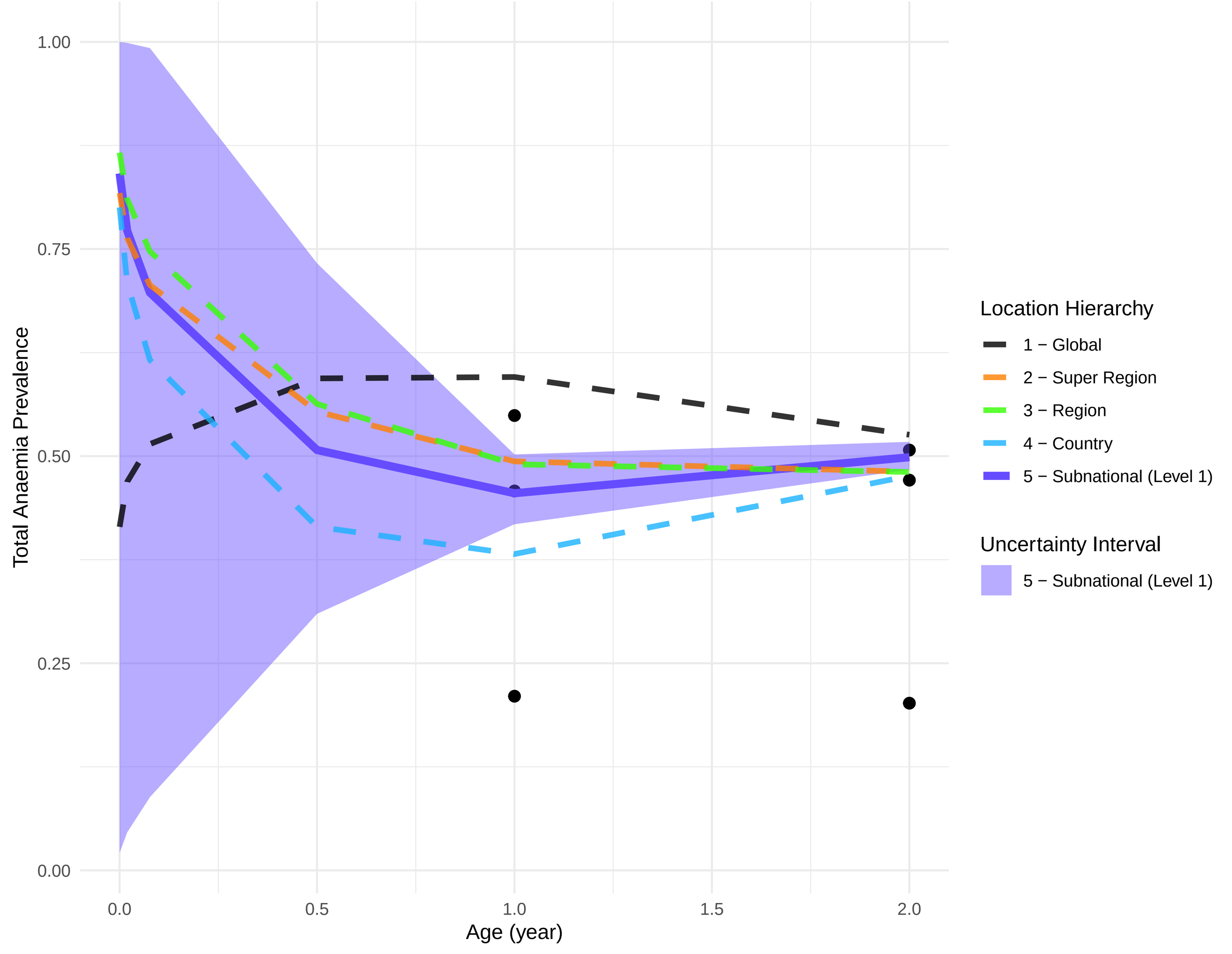

MR−BRT Age/Sex Fits − Males <2 Years Old − Total Anaemia Prevalence − East Java
WHO−2001 Adjustment Method

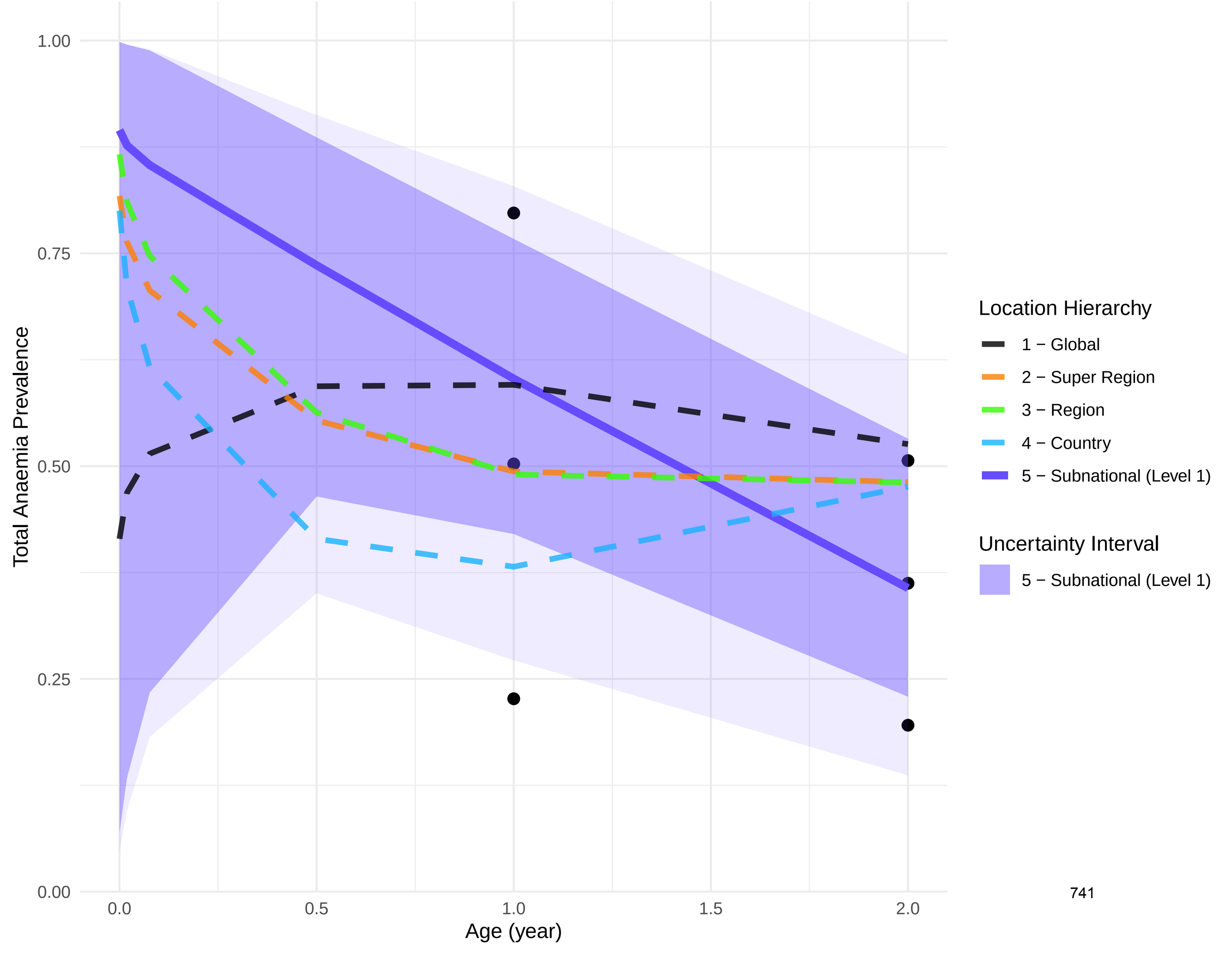

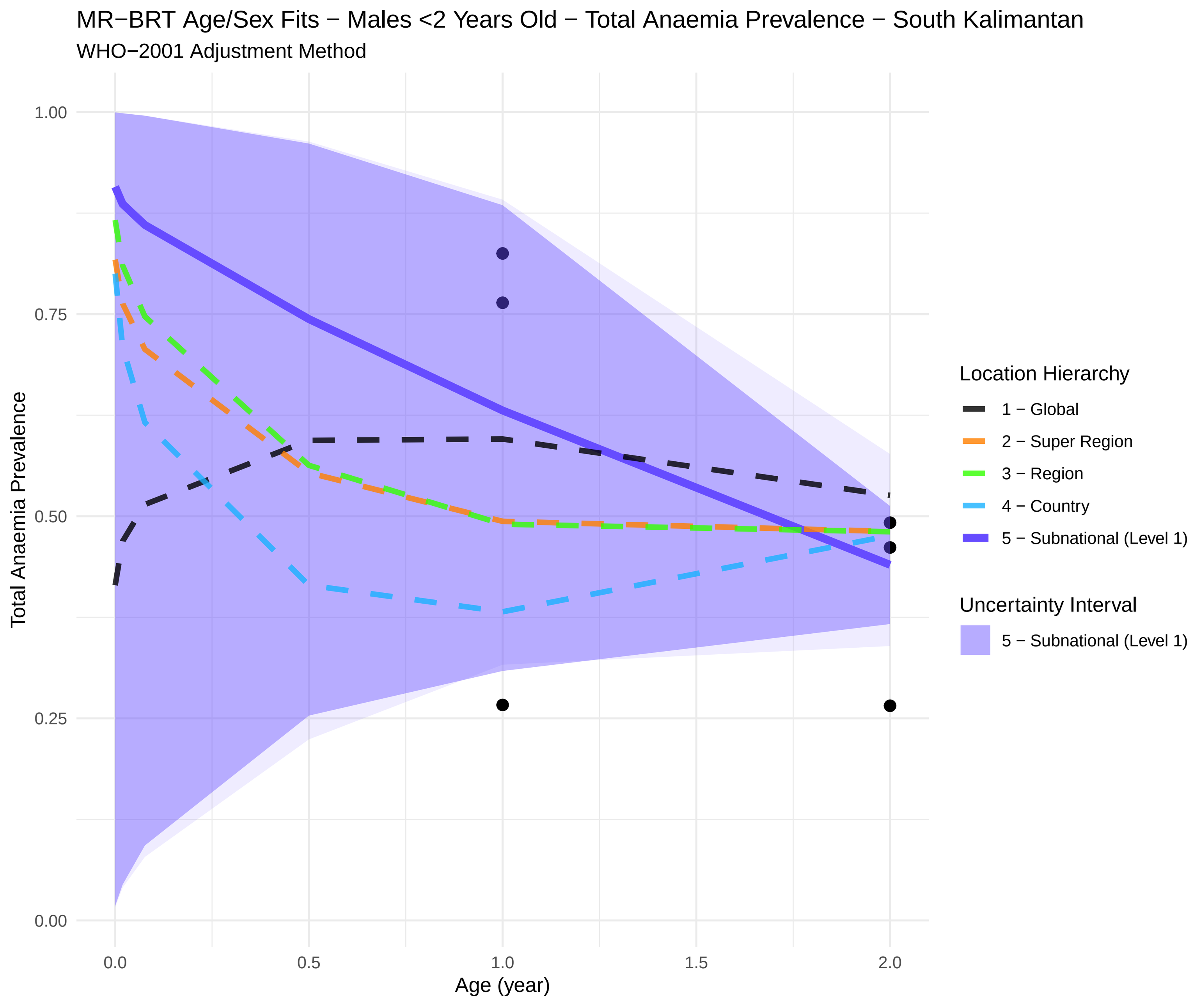
MR−BRT Age/Sex Fits − Males <2 Years Old − Total Anaemia Prevalence − South Kalimantan
WHO−2001 Adjustment Method
Total Anaemia Prevalence
Age (year)
Location Hierarchy
1 − Global
2 − Super Region
3 − Region
4 − Country
5 − Subnational (Level 1)
Uncertainty Interval
5 − Subnational (Level 1)

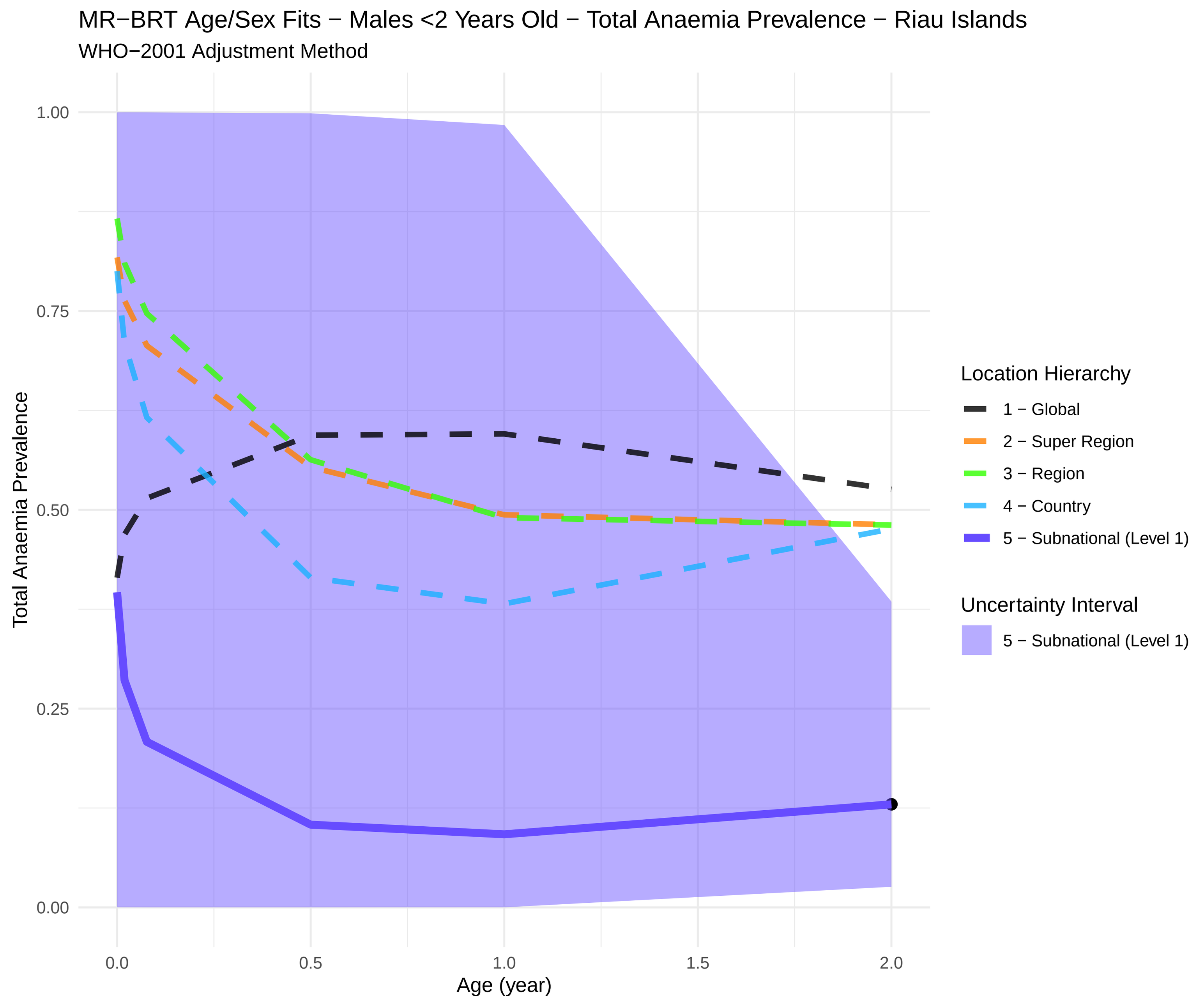
MR−BRT Age/Sex Fits − Males <2 Years Old − Total Anaemia Prevalence − Riau Islands
WHO−2001 Adjustment Method
Total Anaemia Prevalence
Age (year)
Location Hierarchy
1 − Global
2 − Super Region
3 − Region
4 − Country
5 − Subnational (Level 1)
Uncertainty Interval
5 − Subnational (Level 1)

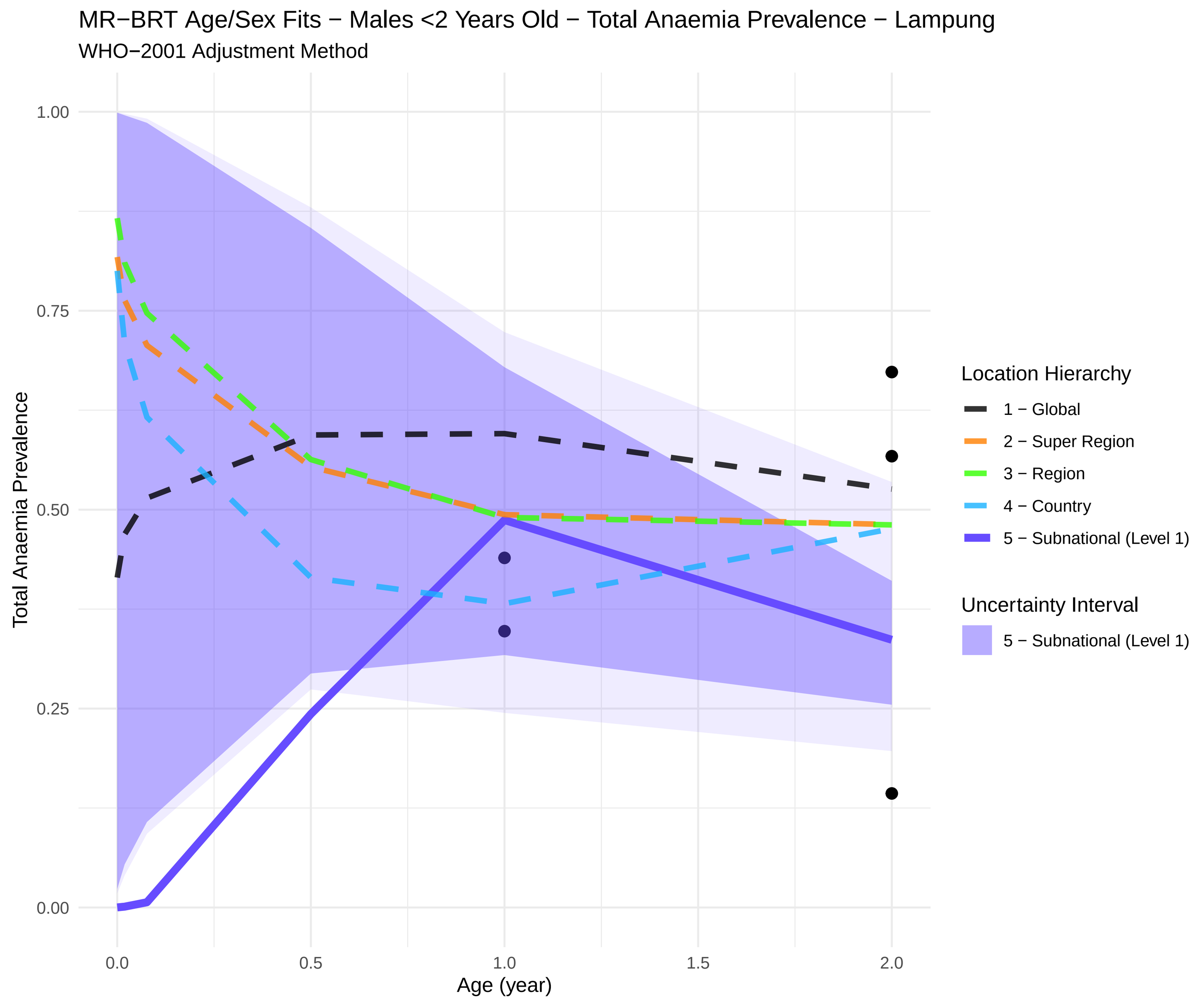
MR−BRT Age/Sex Fits − Males <2 Years Old − Total Anaemia Prevalence − Lampung
WHO−2001 Adjustment Method
Total Anaemia Prevalence
Age (year)
Location Hierarchy
1 − Global
2 − Super Region
3 − Region
4 − Country
5 − Subnational (Level 1)
Uncertainty Interval
5 − Subnational (Level 1)

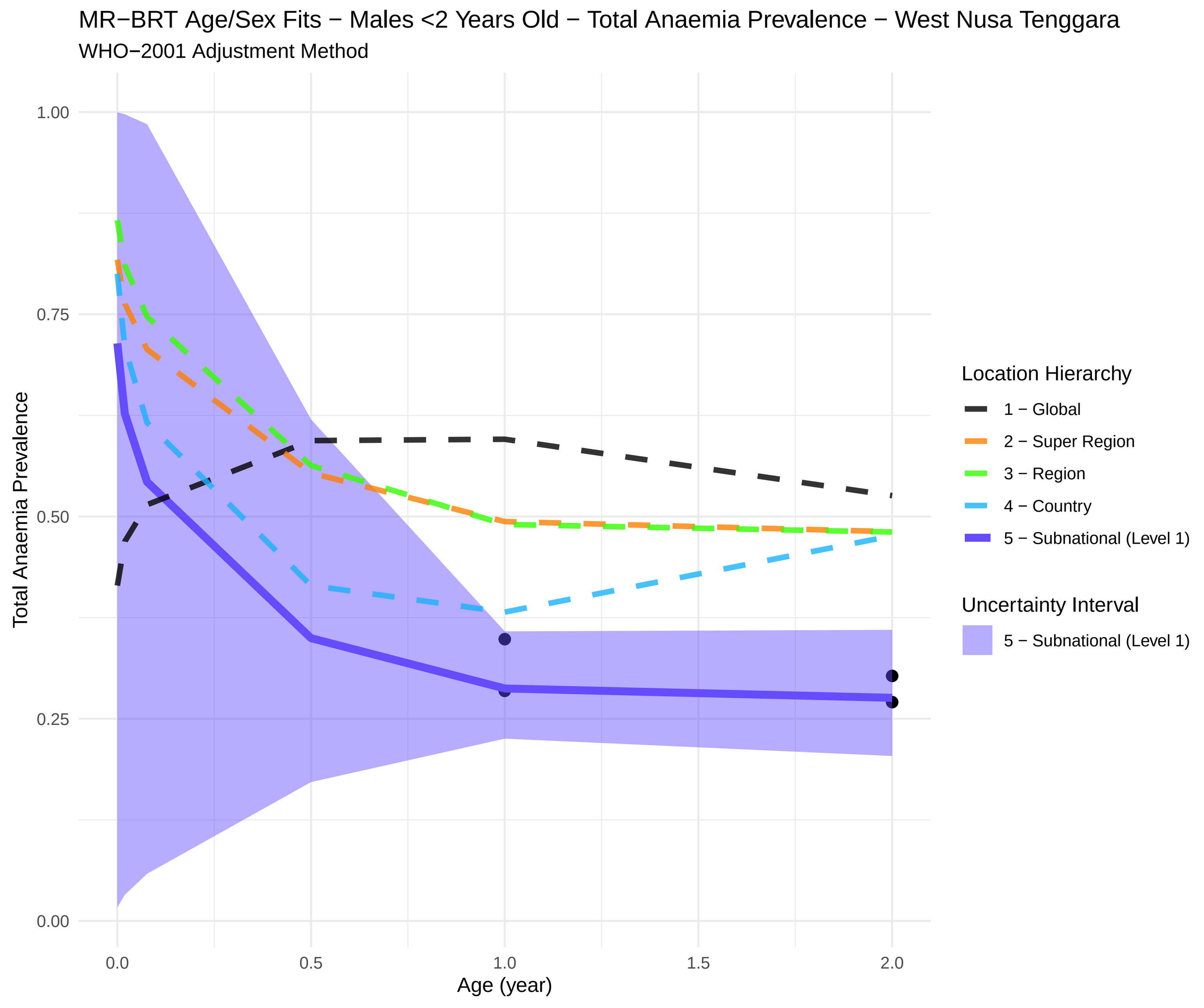
MR−BRT Age/Sex Fits − Males <2 Years Old − Total Anaemia Prevalence − West Nusa Tenggara
WHO−2001 Adjustment Method
Total Anaemia Prevalence
Age (year)
Location Hierarchy
1 − Global
2 − Super Region
3 − Region
4 − Country
5 − Subnational (Level 1)
Uncertainty Interval
5 − Subnational (Level 1)

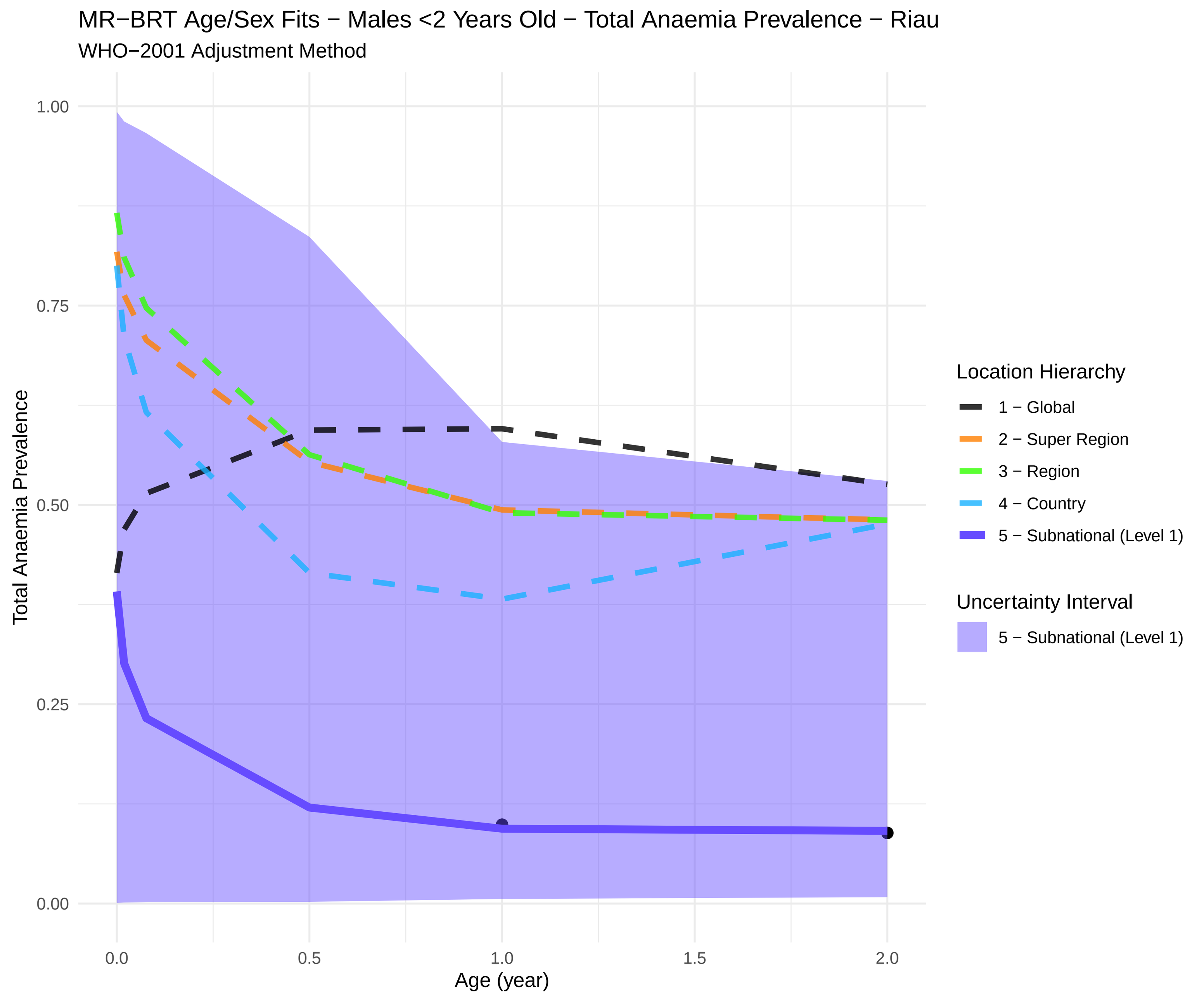
MR−BRT Age/Sex Fits − Males <2 Years Old − Total Anaemia Prevalence − Riau
WHO−2001 Adjustment Method
Total Anaemia Prevalence
Age (year)
Location Hierarchy
1 − Global
2 − Super Region
3 − Region
4 − Country
5 − Subnational (Level 1)
Uncertainty Interval
5 − Subnational (Level 1)

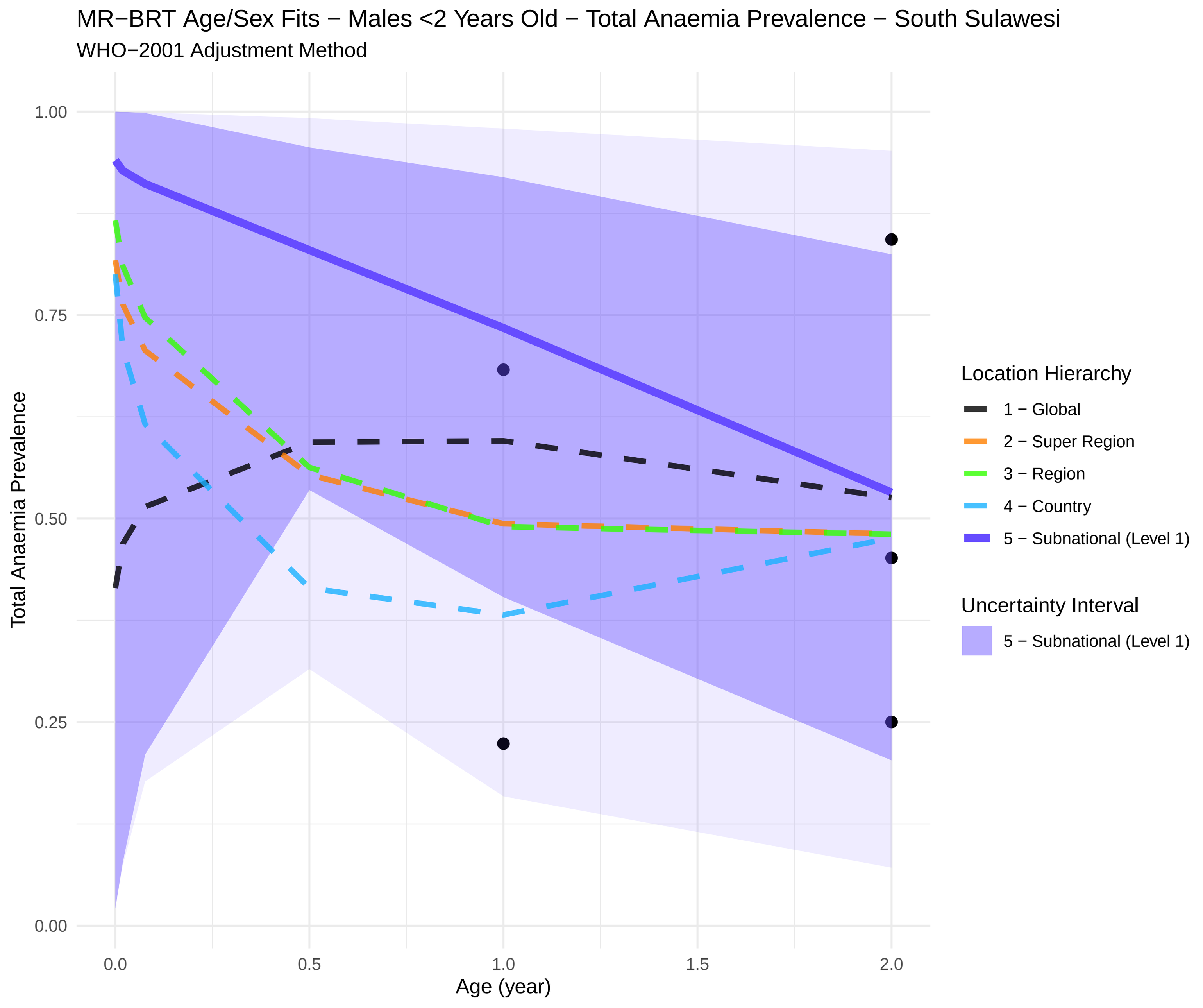
MR−BRT Age/Sex Fits − Males <2 Years Old − Total Anaemia Prevalence − South Sulawesi
WHO−2001 Adjustment Method
Total Anaemia Prevalence
Age (year)
Location Hierarchy
1 − Global
2 − Super Region
3 − Region
4 − Country
5 − Subnational (Level 1)
Uncertainty Interval
5 − Subnational (Level 1)

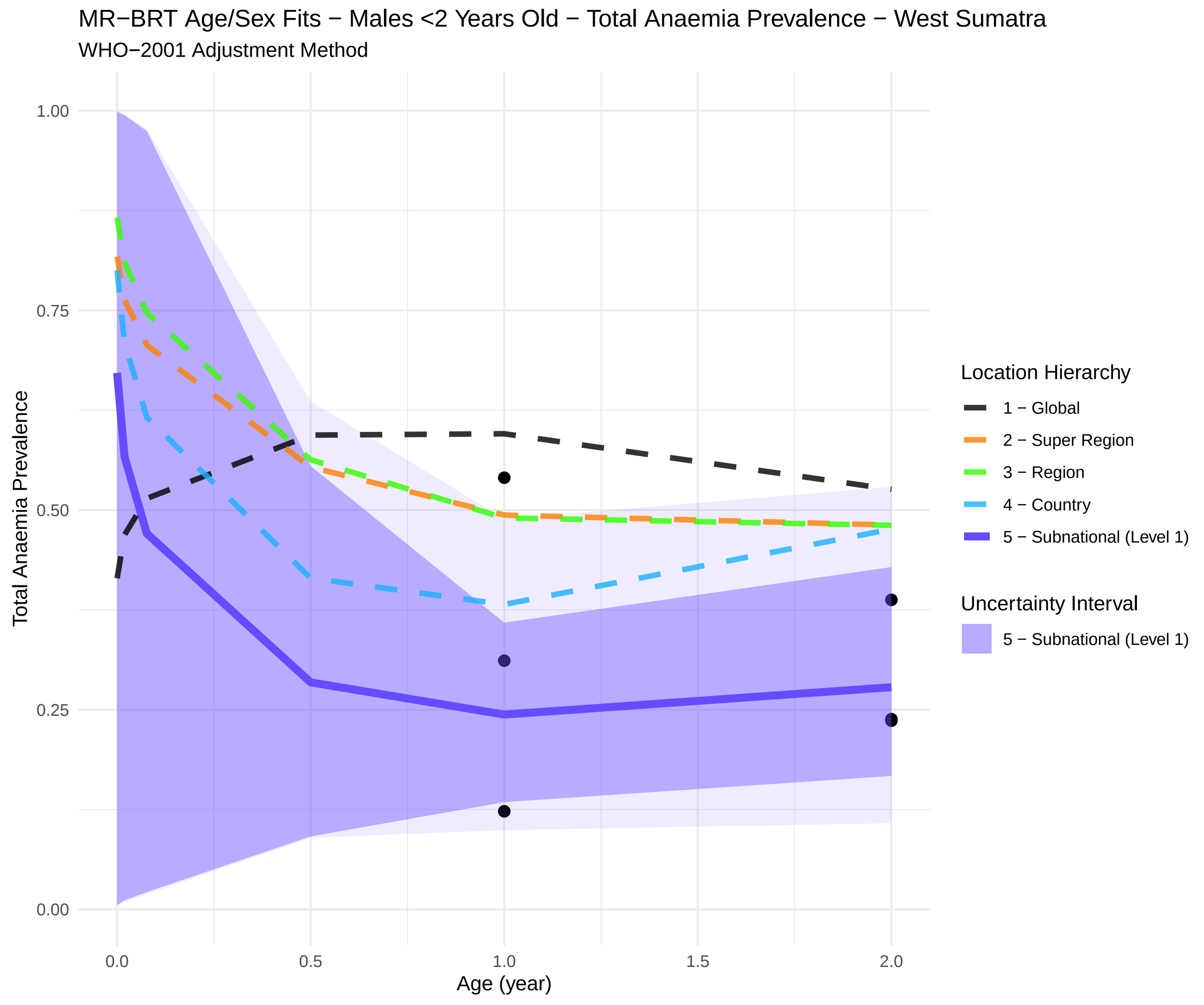
MR−BRT Age/Sex Fits − Males <2 Years Old − Total Anaemia Prevalence − West Sumatra
WHO−2001 Adjustment Method
Total Anaemia Prevalence
Age (year)
Location Hierarchy
1 − Global
2 − Super Region
3 − Region
4 − Country
5 − Subnational (Level 1)
Uncertainty Interval
5 − Subnational (Level 1)

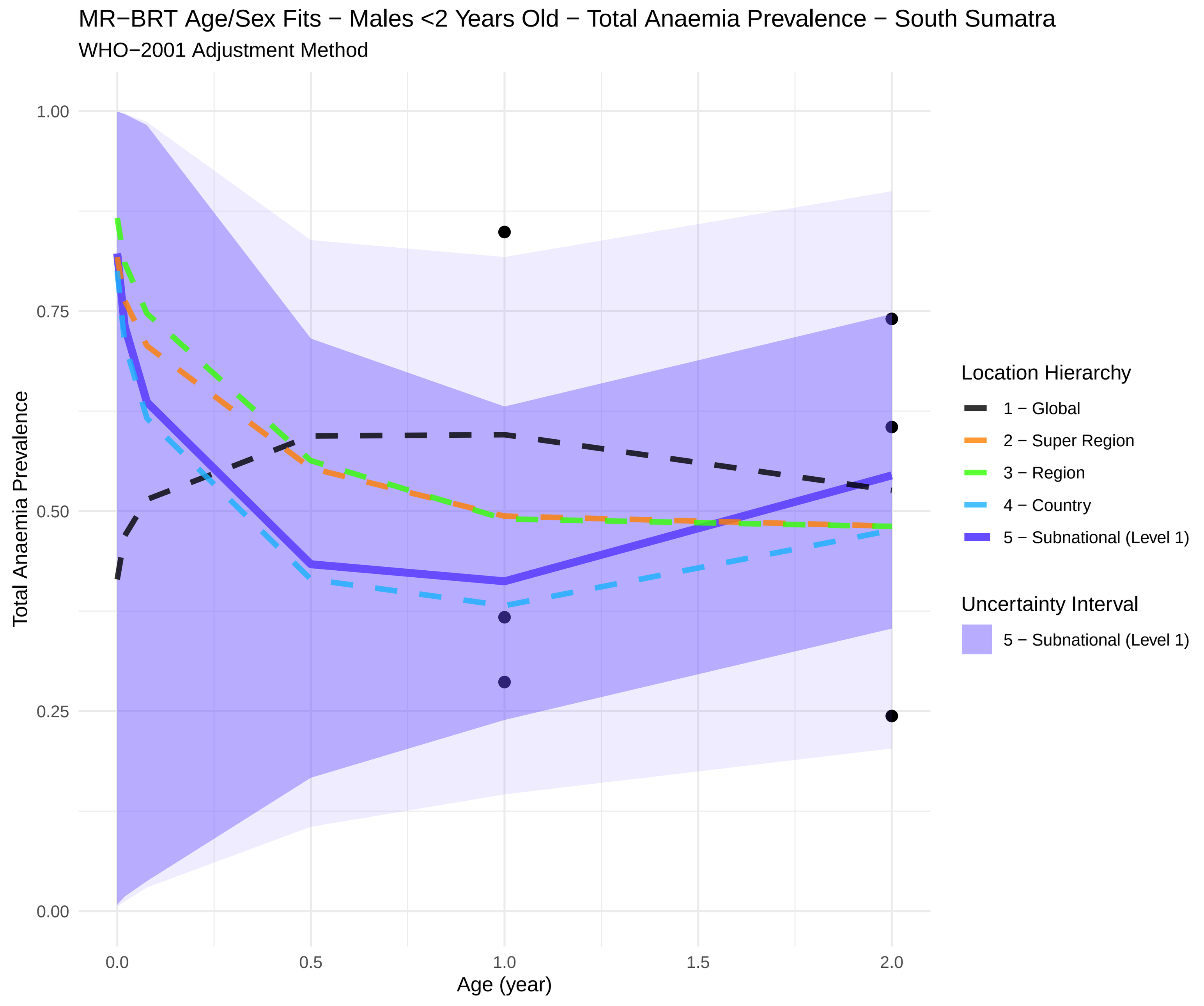
MR−BRT Age/Sex Fits − Males <2 Years Old − Total Anaemia Prevalence − South Sumatra
WHO−2001 Adjustment Method
Total Anaemia Prevalence
Age (year)
Location Hierarchy
1 − Global
2 − Super Region
3 − Region
4 − Country
5 − Subnational (Level 1)
Uncertainty Interval
5 − Subnational (Level 1)

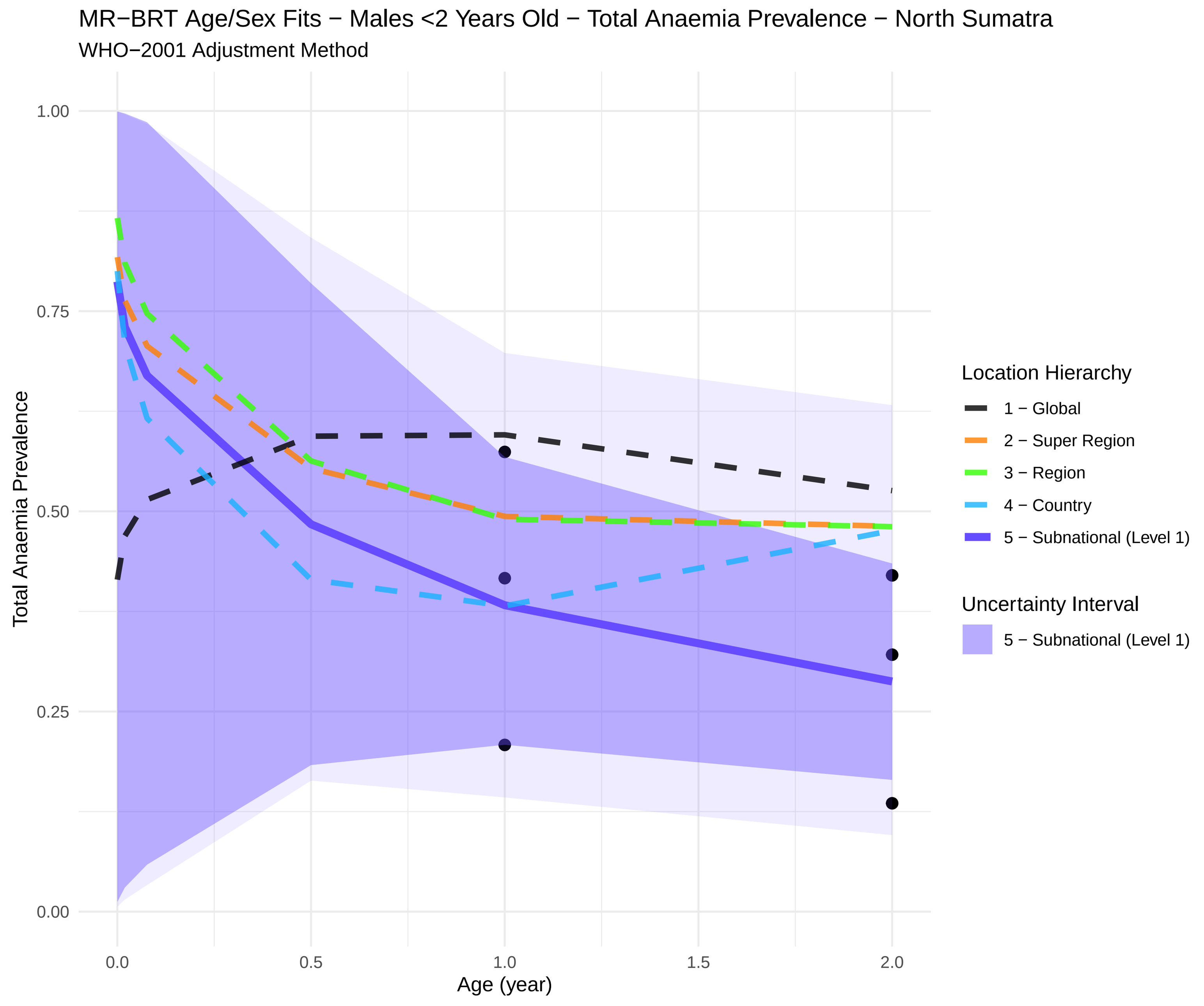
MR−BRT Age/Sex Fits − Males <2 Years Old − Total Anaemia Prevalence − North Sumatra
WHO−2001 Adjustment Method
Total Anaemia Prevalence
Age (year)
Location Hierarchy
1 − Global
2 − Super Region
3 − Region
4 − Country
5 − Subnational (Level 1)
Uncertainty Interval
5 − Subnational (Level 1)

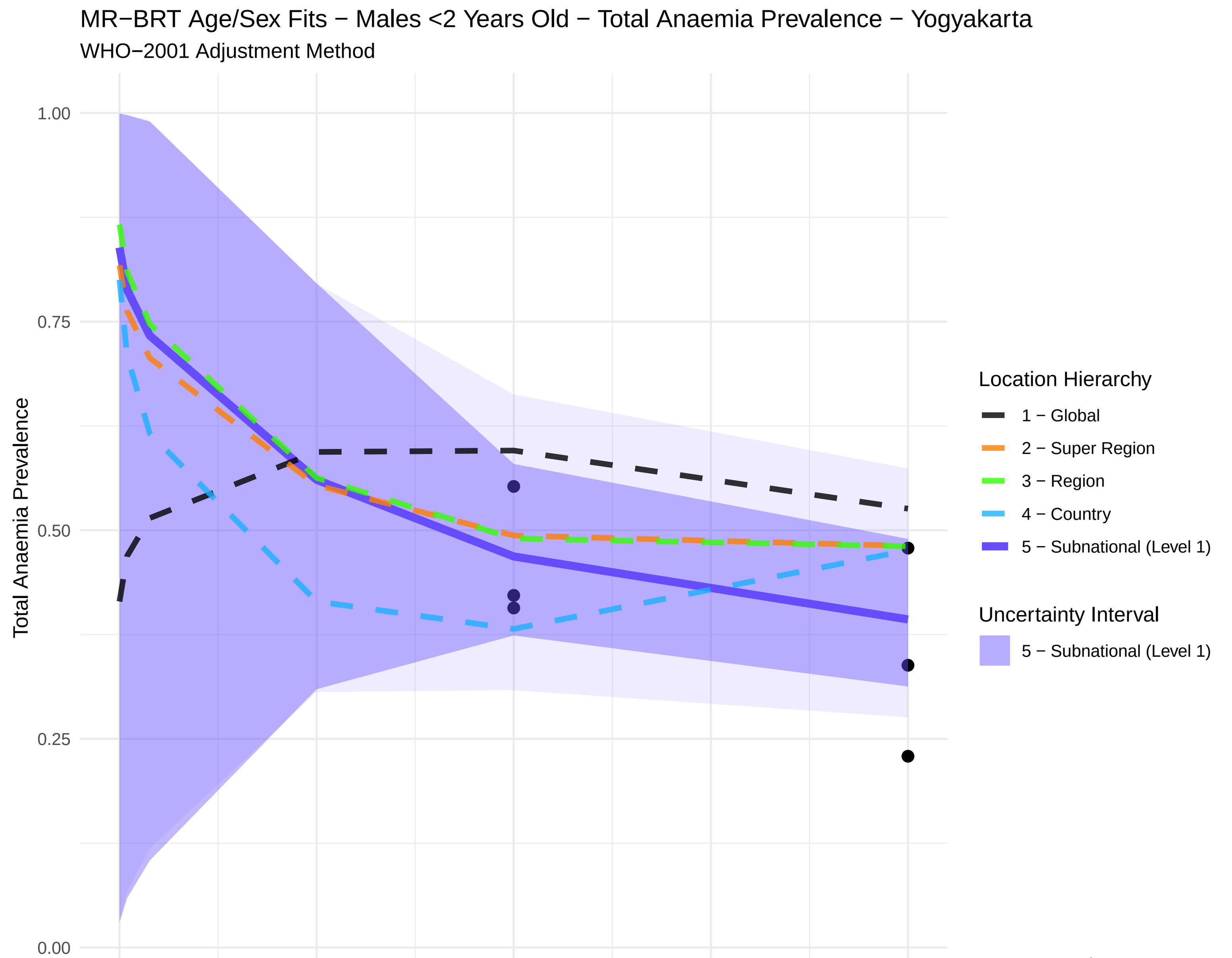
MR−BRT Age/Sex Fits − Males <2 Years Old − Total Anaemia Prevalence − Yogyakarta
WHO−2001 Adjustment Method
Total Anaemia Prevalence
Age (year)
Location Hierarchy
1 − Global
2 − Super Region
3 − Region
4 − Country
5 − Subnational (Level 1)
Uncertainty Interval
5 − Subnational (Level 1)

MR−BRT Age/Sex Fits − Males <2 Years Old − Total Anaemia Prevalence − Myanmar
WHO−2001 Adjustment Method

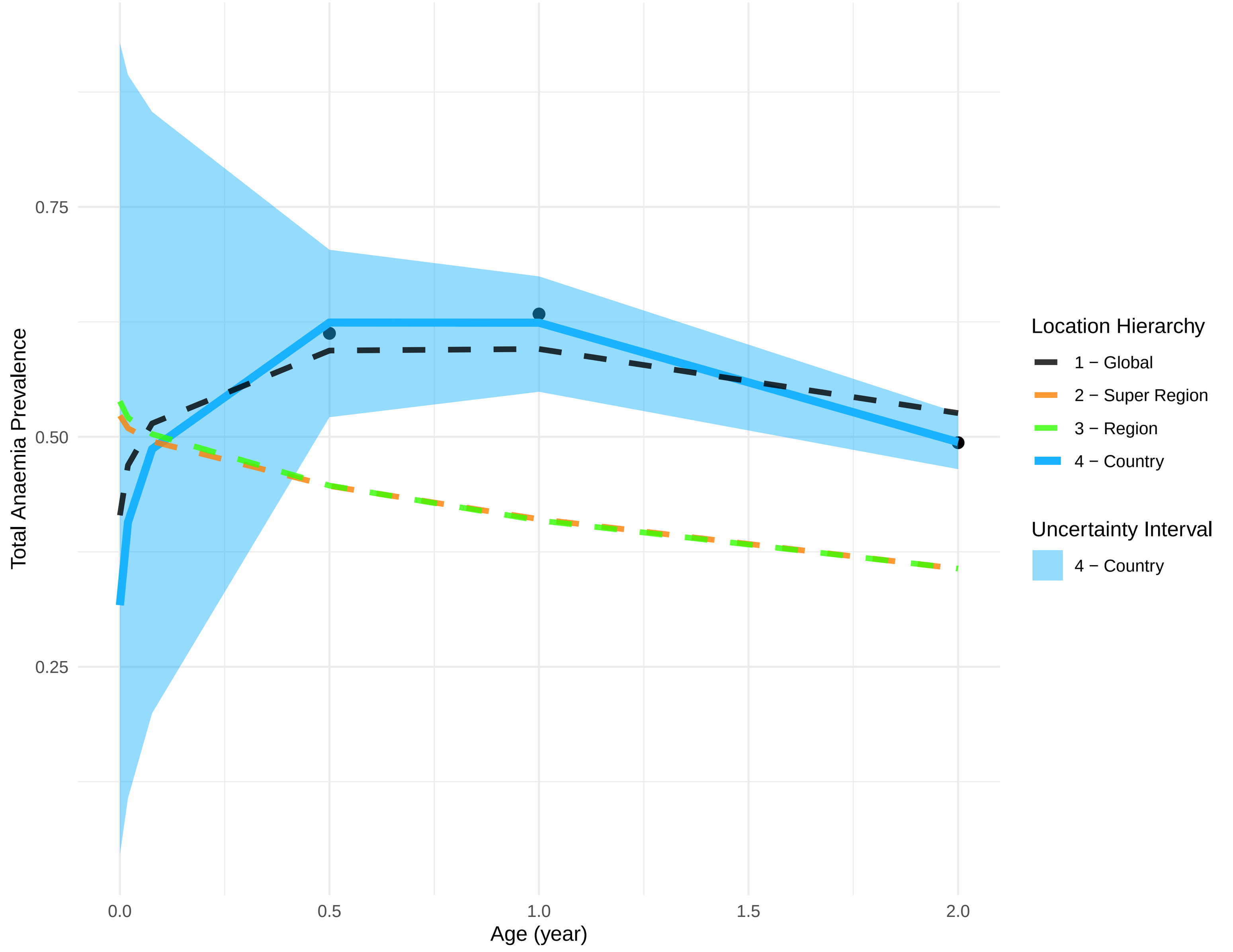

MR−BRT Age/Sex Fits − Males <2 Years Old − Total Anaemia Prevalence − Timor−Leste
WHO−2001 Adjustment Method

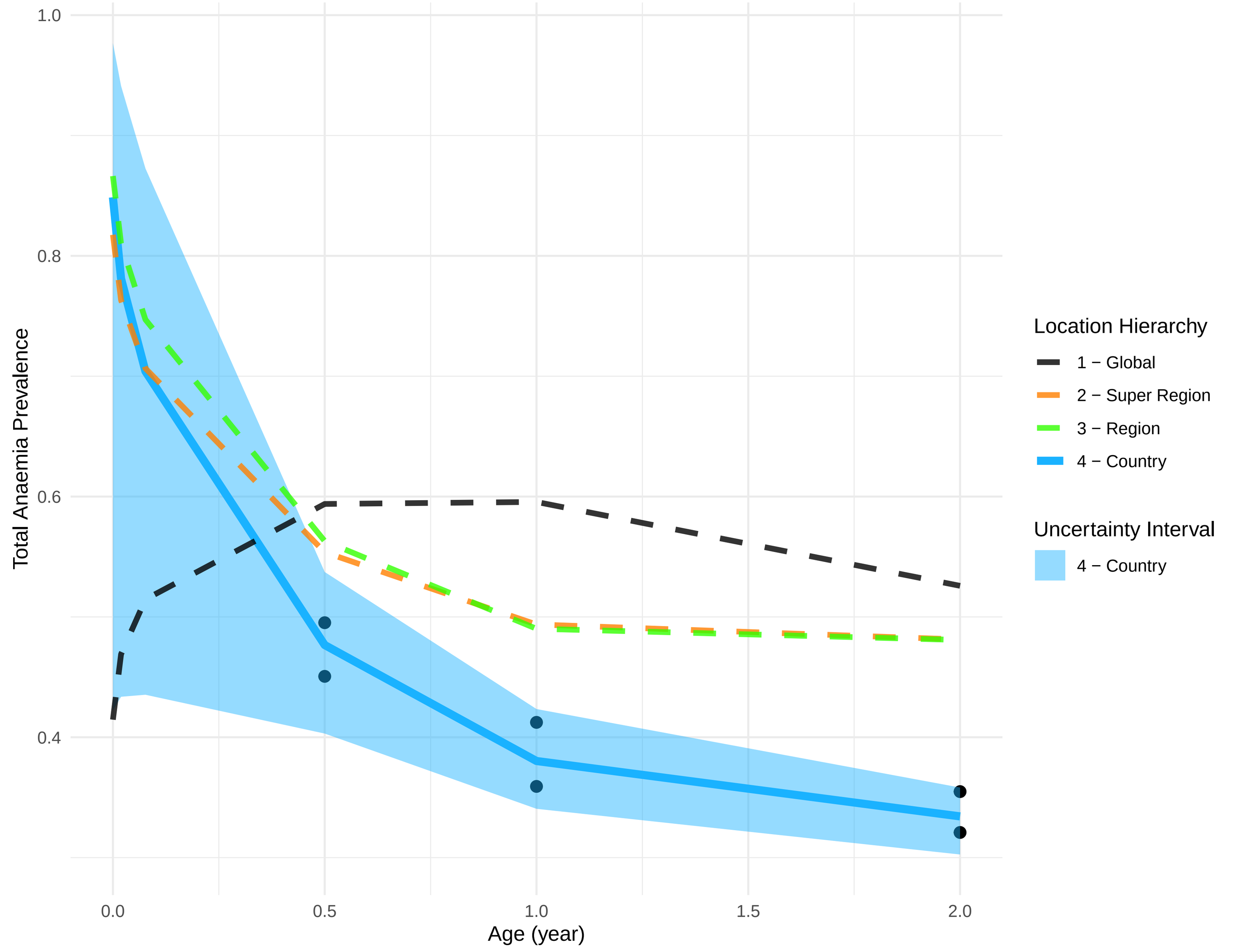

MR−BRT Age/Sex Fits − Males <2 Years Old − Total Anaemia Prevalence − Sub−Saharan Africa
WHO−2001 Adjustment Method

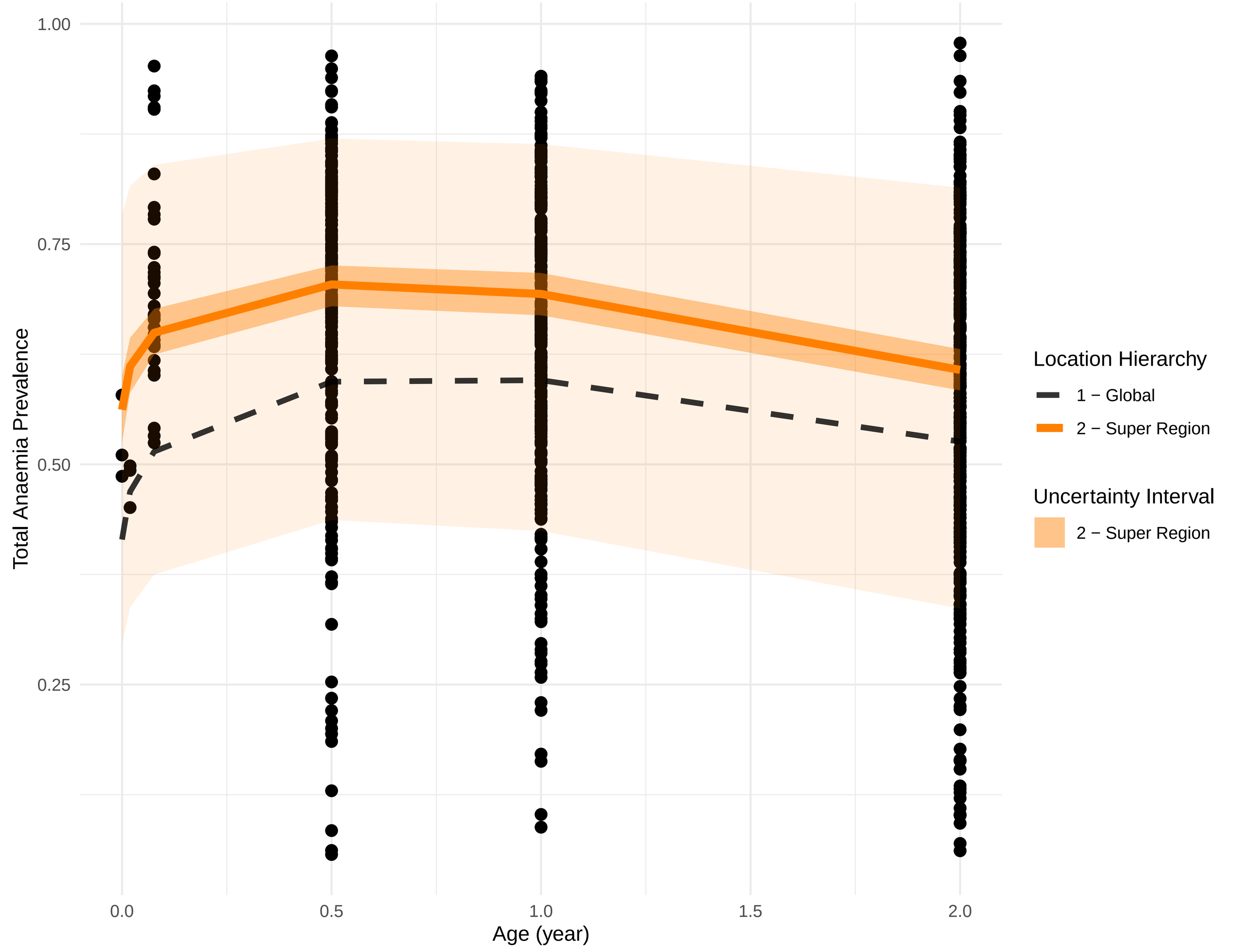

MR−BRT Age/Sex Fits − Males <2 Years Old − Total Anaemia Prevalence − Central Sub−Saharan Africa
WHO−2001 Adjustment Method

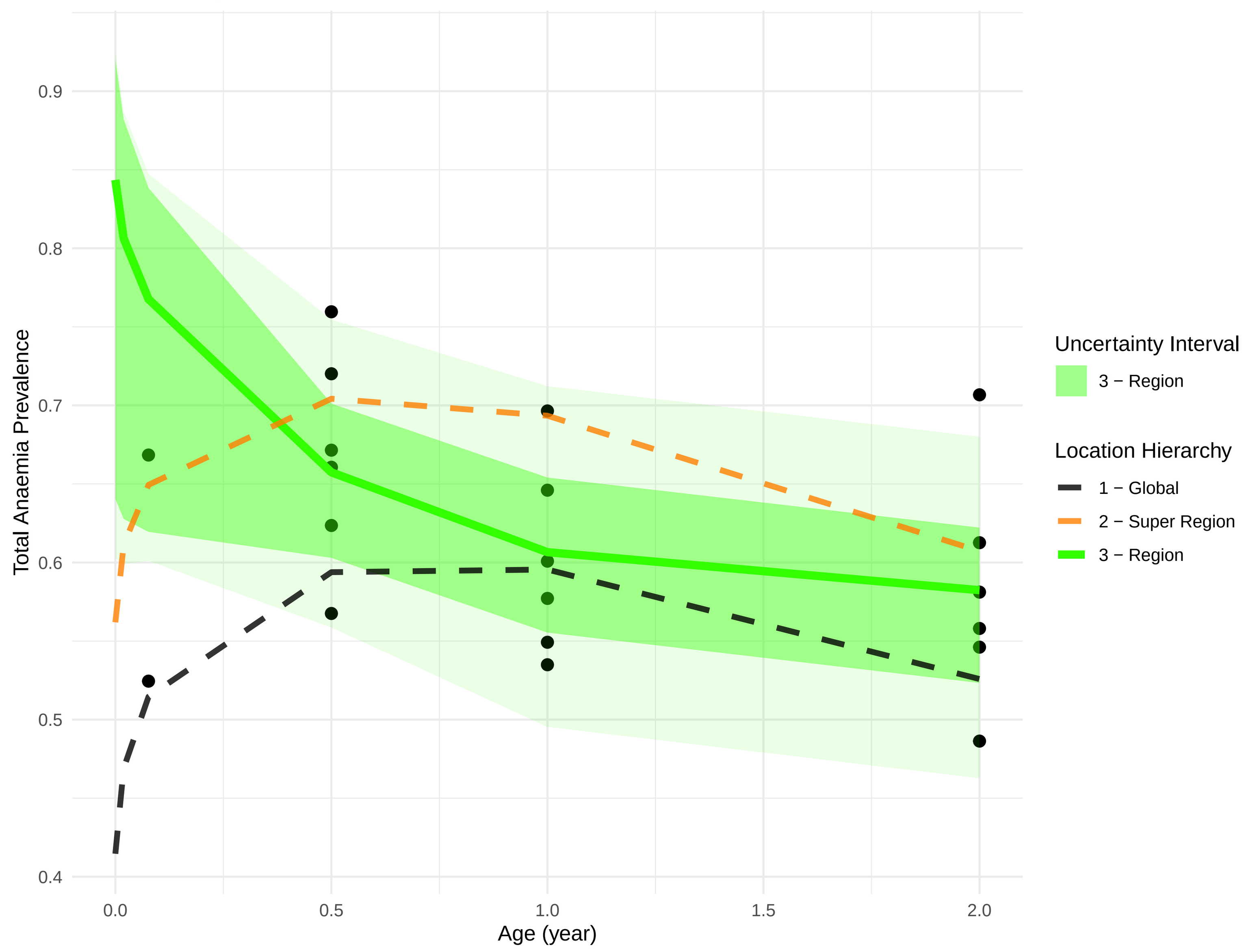

MR−BRT Age/Sex Fits − Males <2 Years Old − Total Anaemia Prevalence − Angola
WHO−2001 Adjustment Method

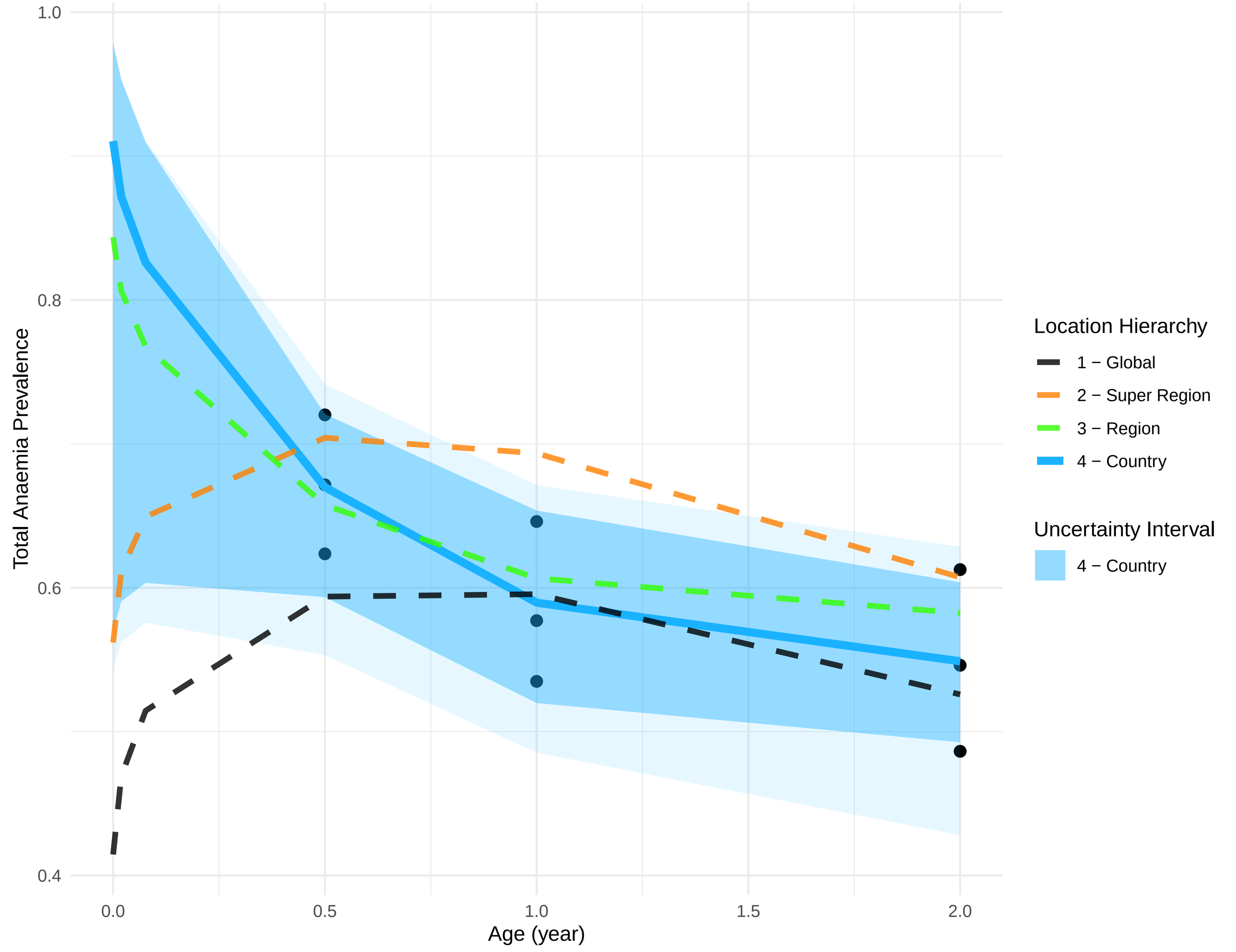

MR−BRT Age/Sex Fits − Males <2 Years Old − Total Anaemia Prevalence − Democratic Republic of the Congo
WHO−2001 Adjustment Method

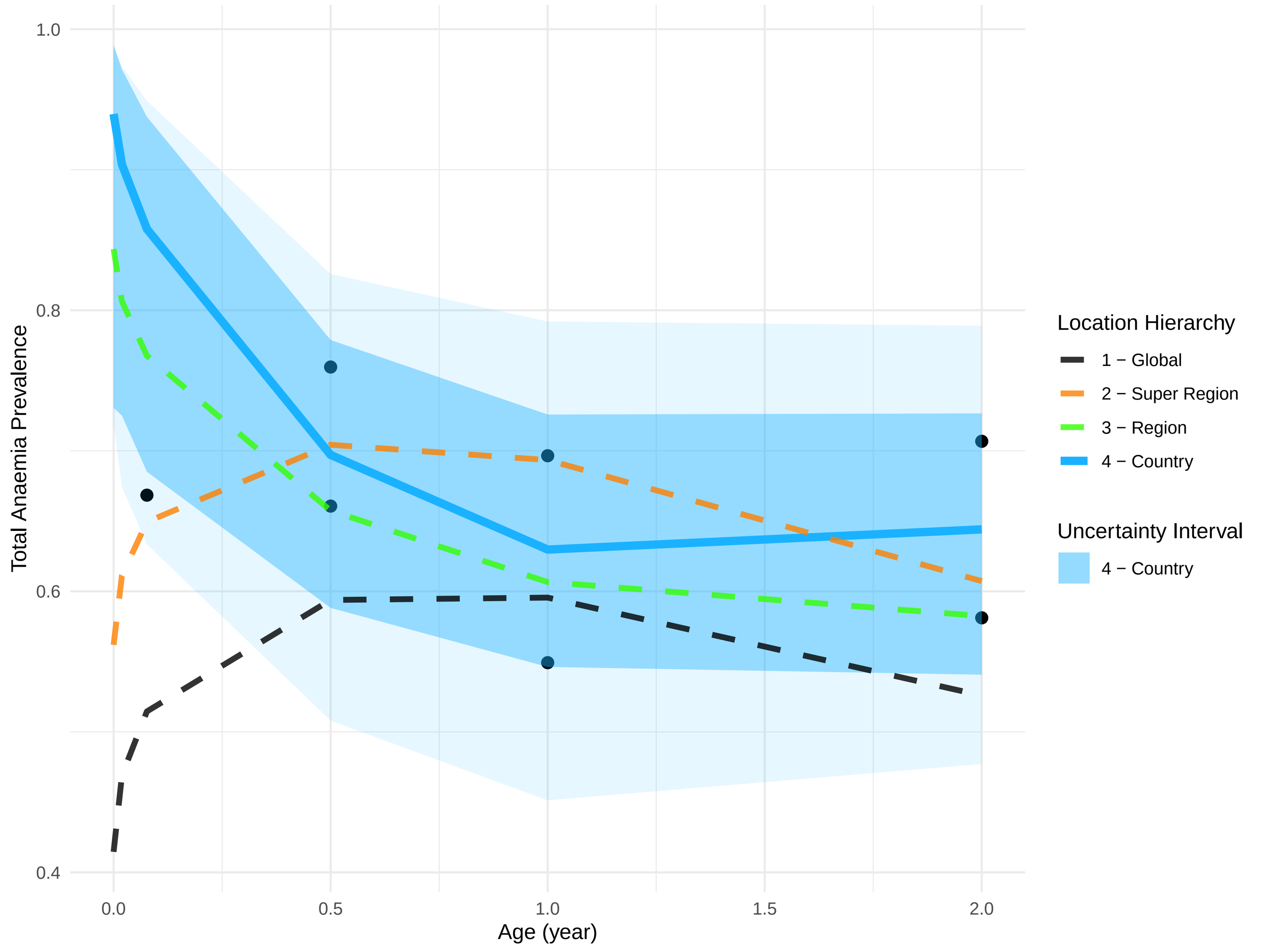

MR−BRT Age/Sex Fits − Males <2 Years Old − Total Anaemia Prevalence − Gabon
WHO−2001 Adjustment Method

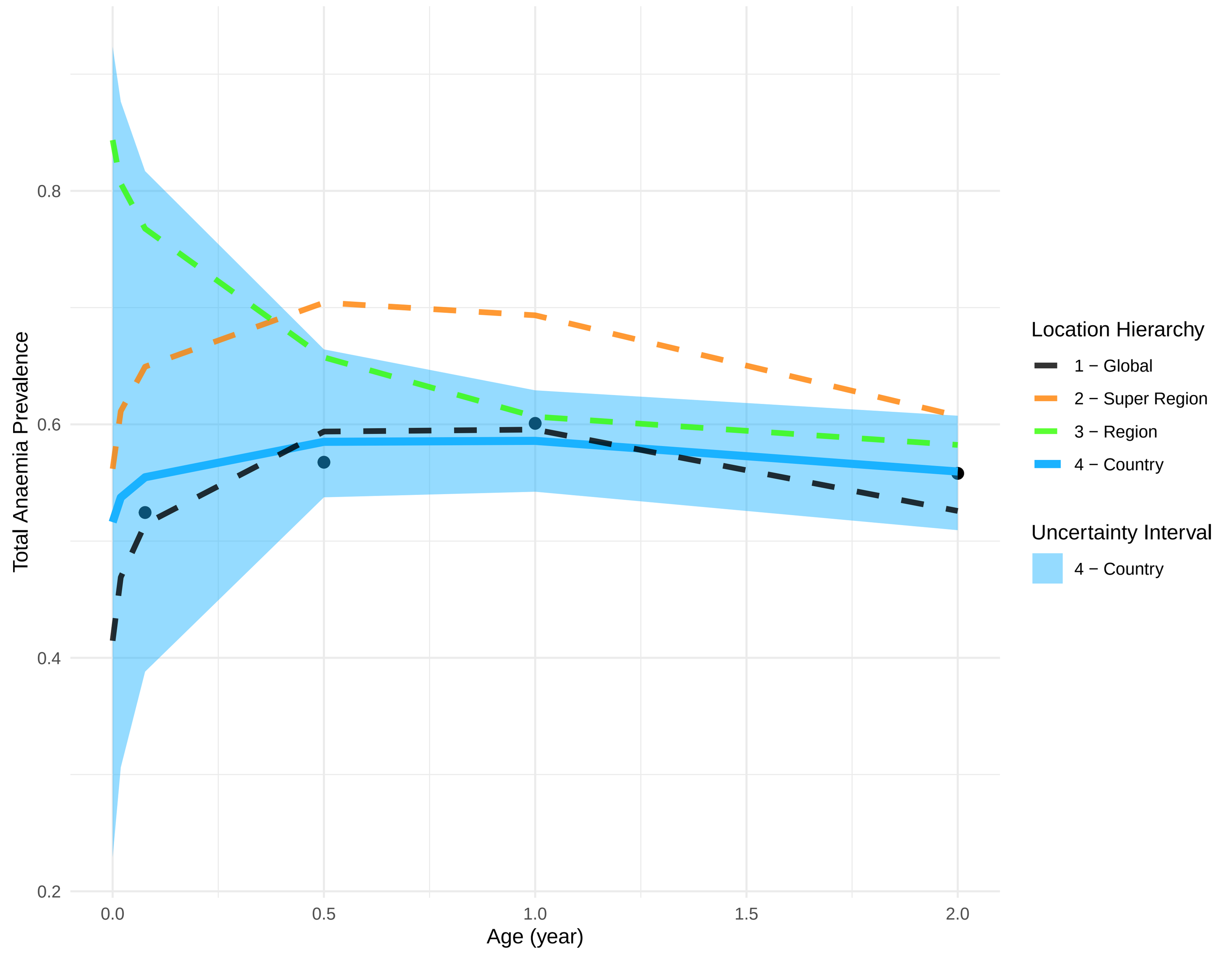

MR−BRT Age/Sex Fits − Males <2 Years Old − Total Anaemia Prevalence − Eastern Sub−Saharan Africa
WHO−2001 Adjustment Method

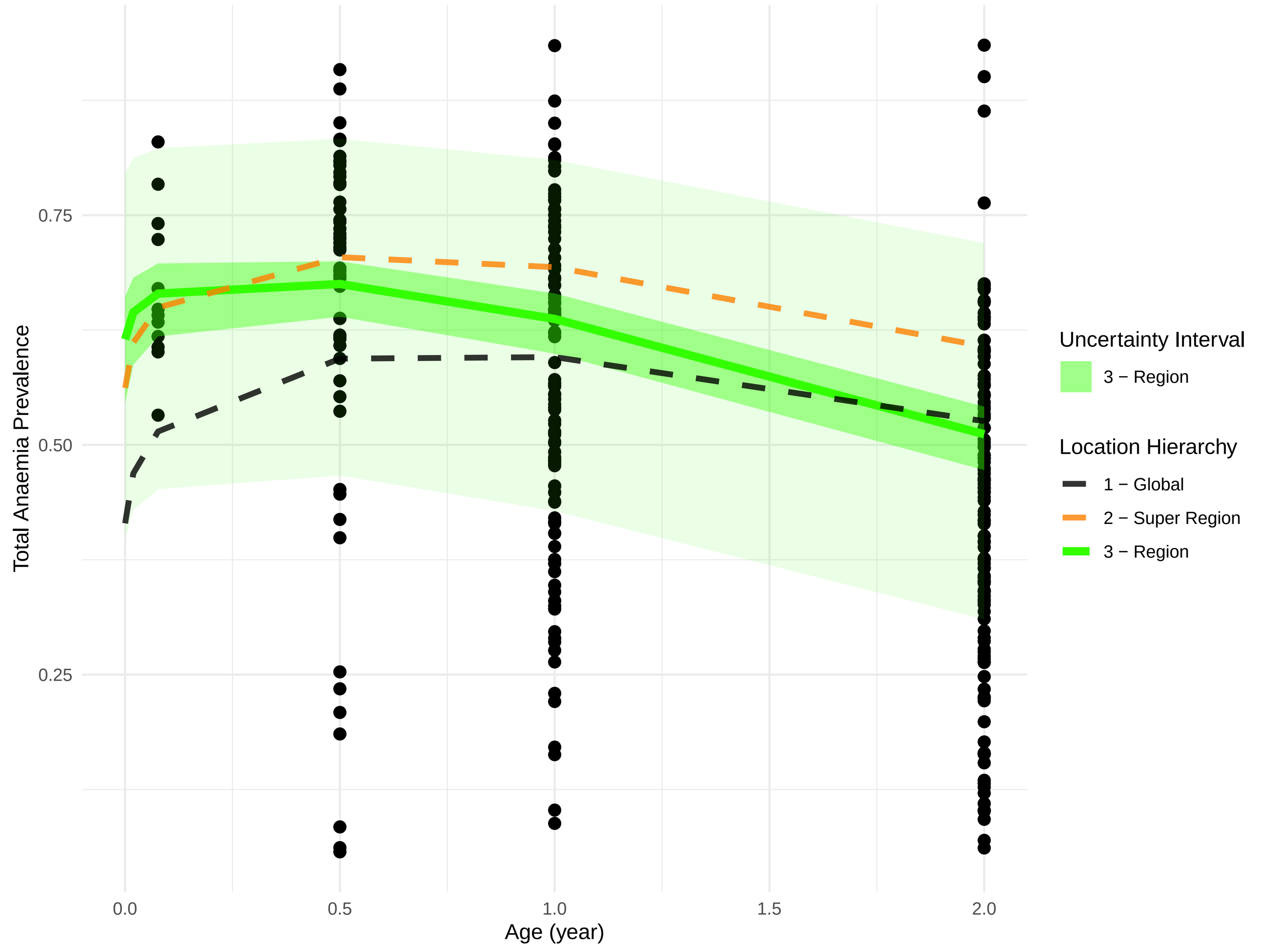

MR−BRT Age/Sex Fits − Males <2 Years Old − Total Anaemia Prevalence − Burundi
WHO−2001 Adjustment Method

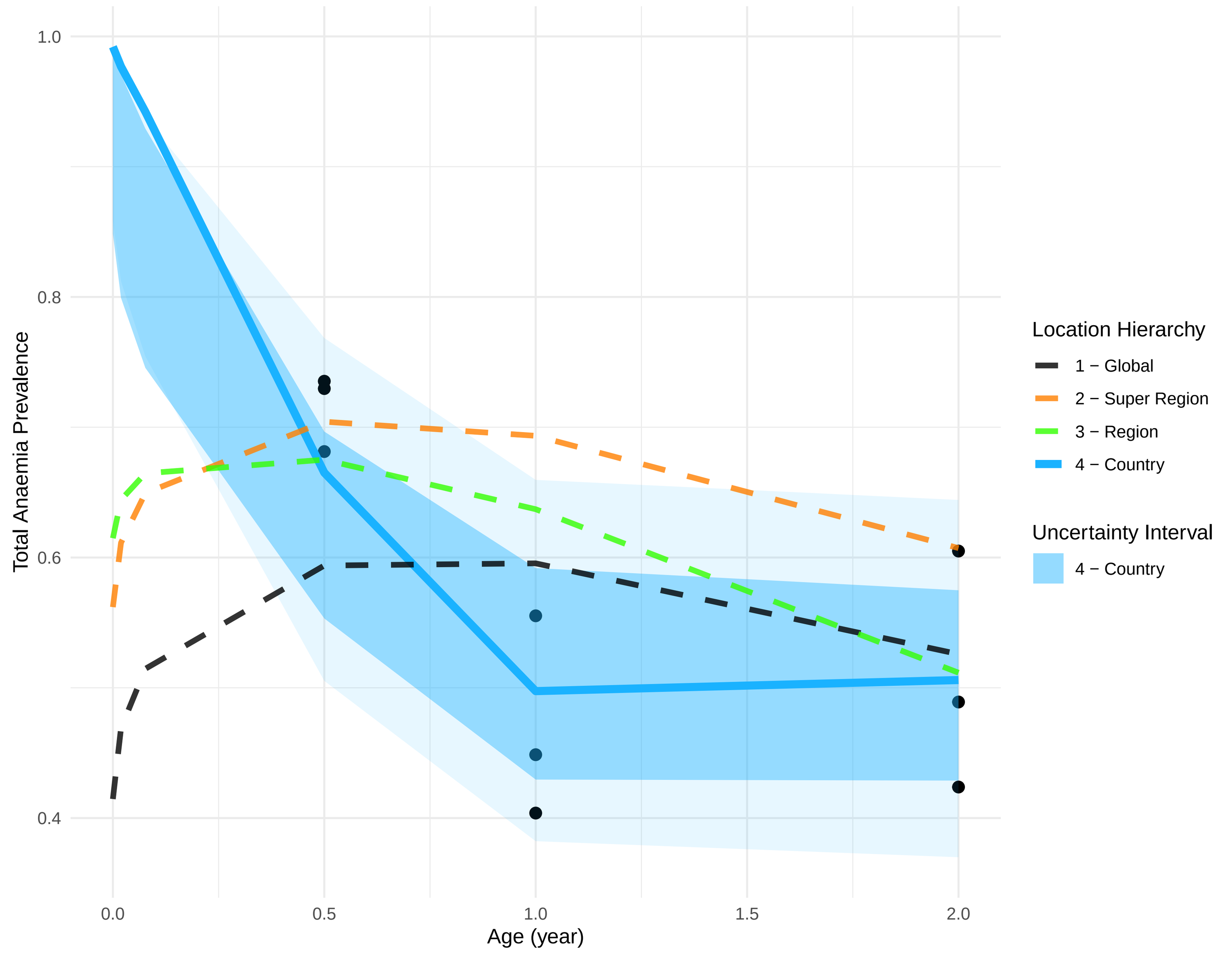

MR−BRT Age/Sex Fits − Males <2 Years Old − Total Anaemia Prevalence − Ethiopia
WHO−2001 Adjustment Method

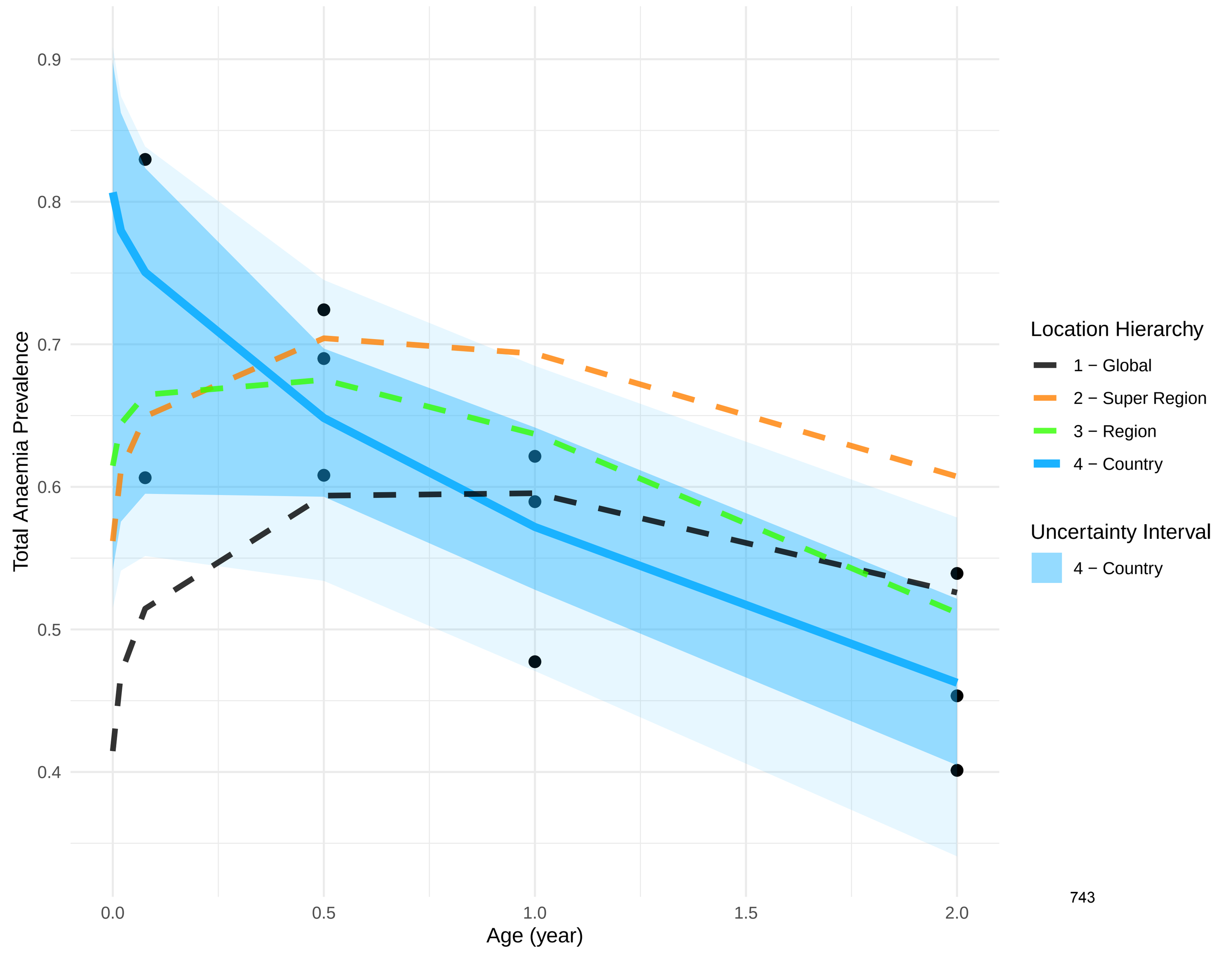

MR−BRT Age/Sex Fits − Males <2 Years Old − Total Anaemia Prevalence − Kenya
WHO−2001 Adjustment Method

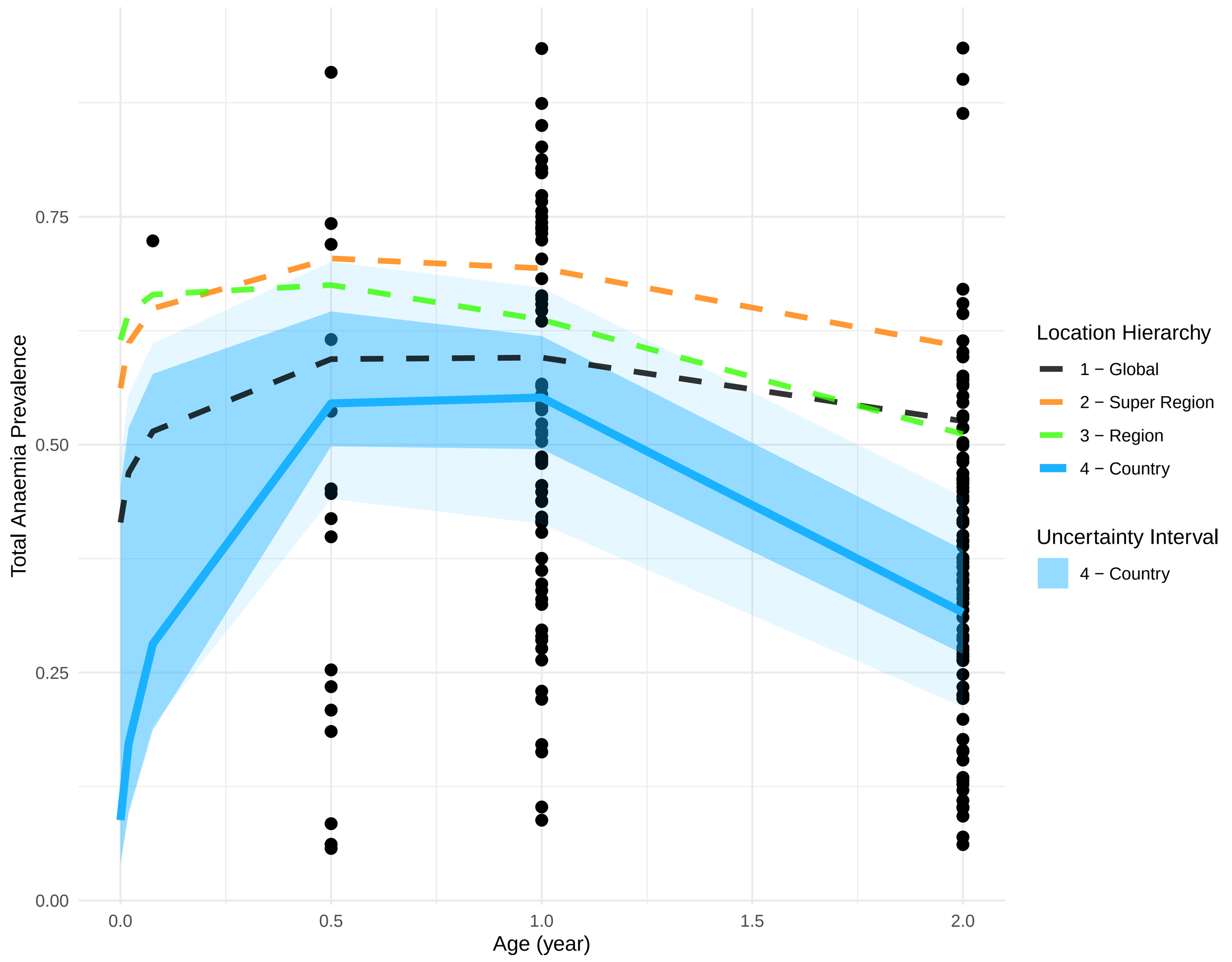

MR−BRT Age/Sex Fits − Males <2 Years Old − Total Anaemia Prevalence − Baringo
WHO−2001 Adjustment Method

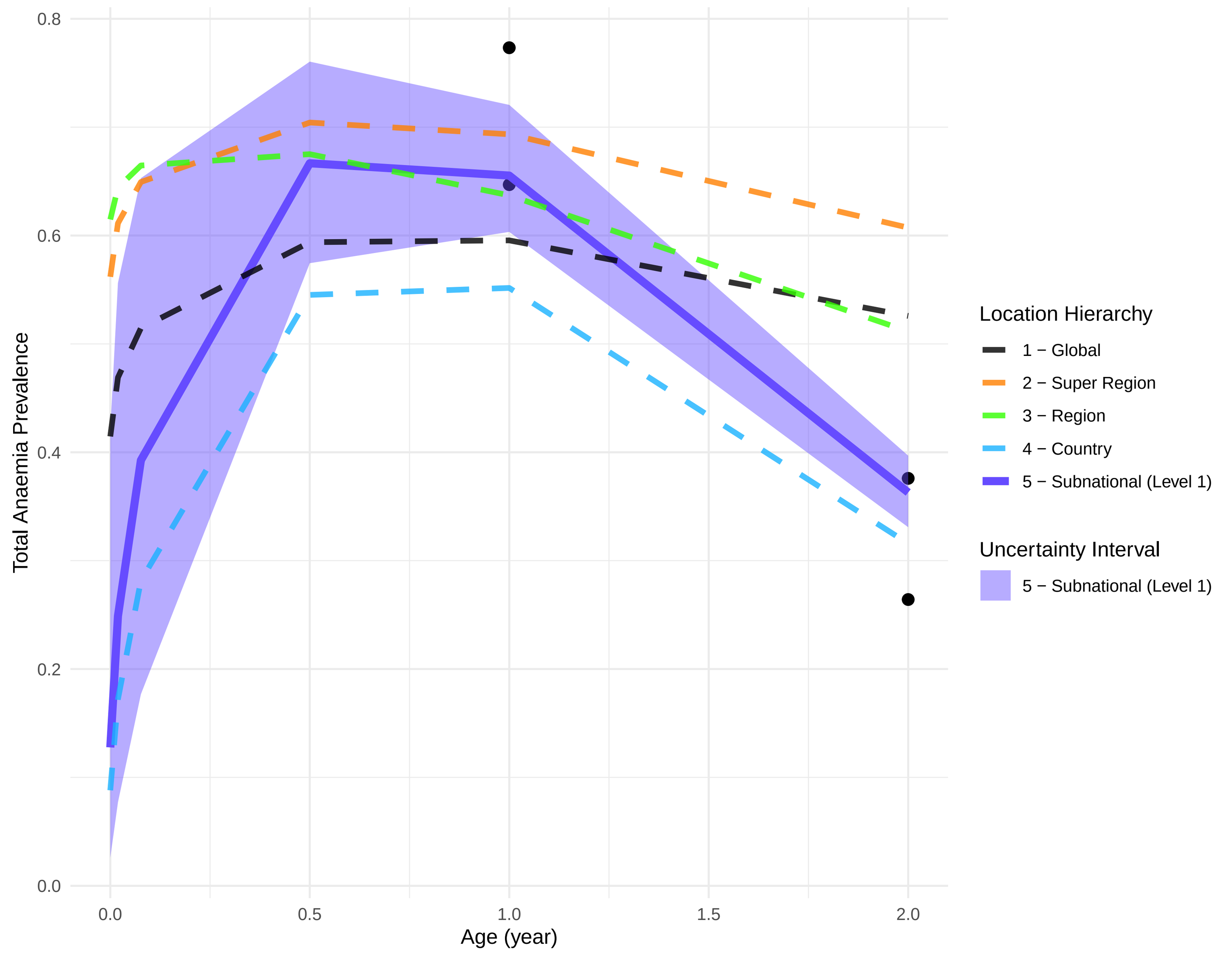

MR−BRT Age/Sex Fits − Males <2 Years Old − Total Anaemia Prevalence − Bomet
WHO−2001 Adjustment Method

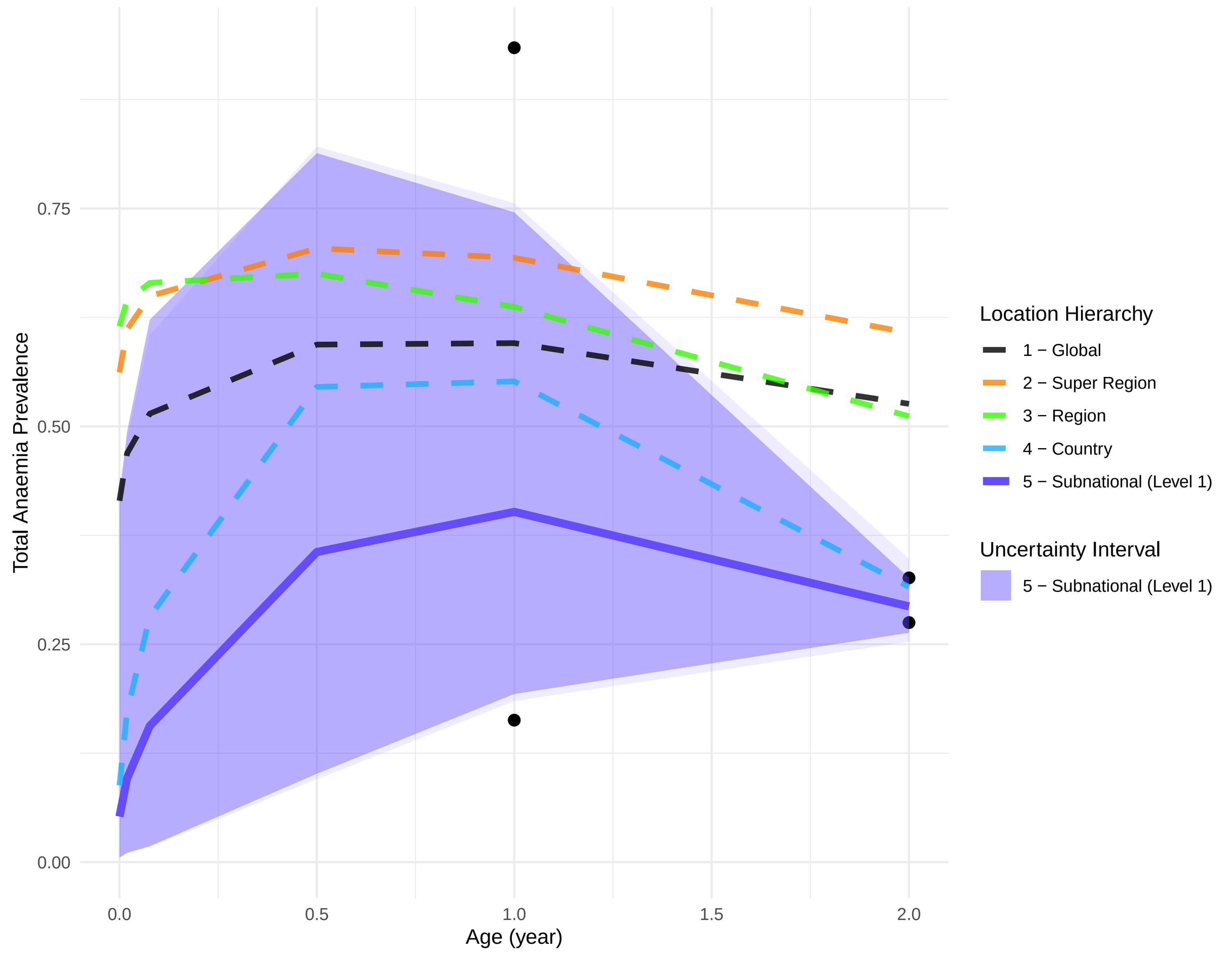

MR−BRT Age/Sex Fits − Males <2 Years Old − Total Anaemia Prevalence − Bungoma
WHO−2001 Adjustment Method

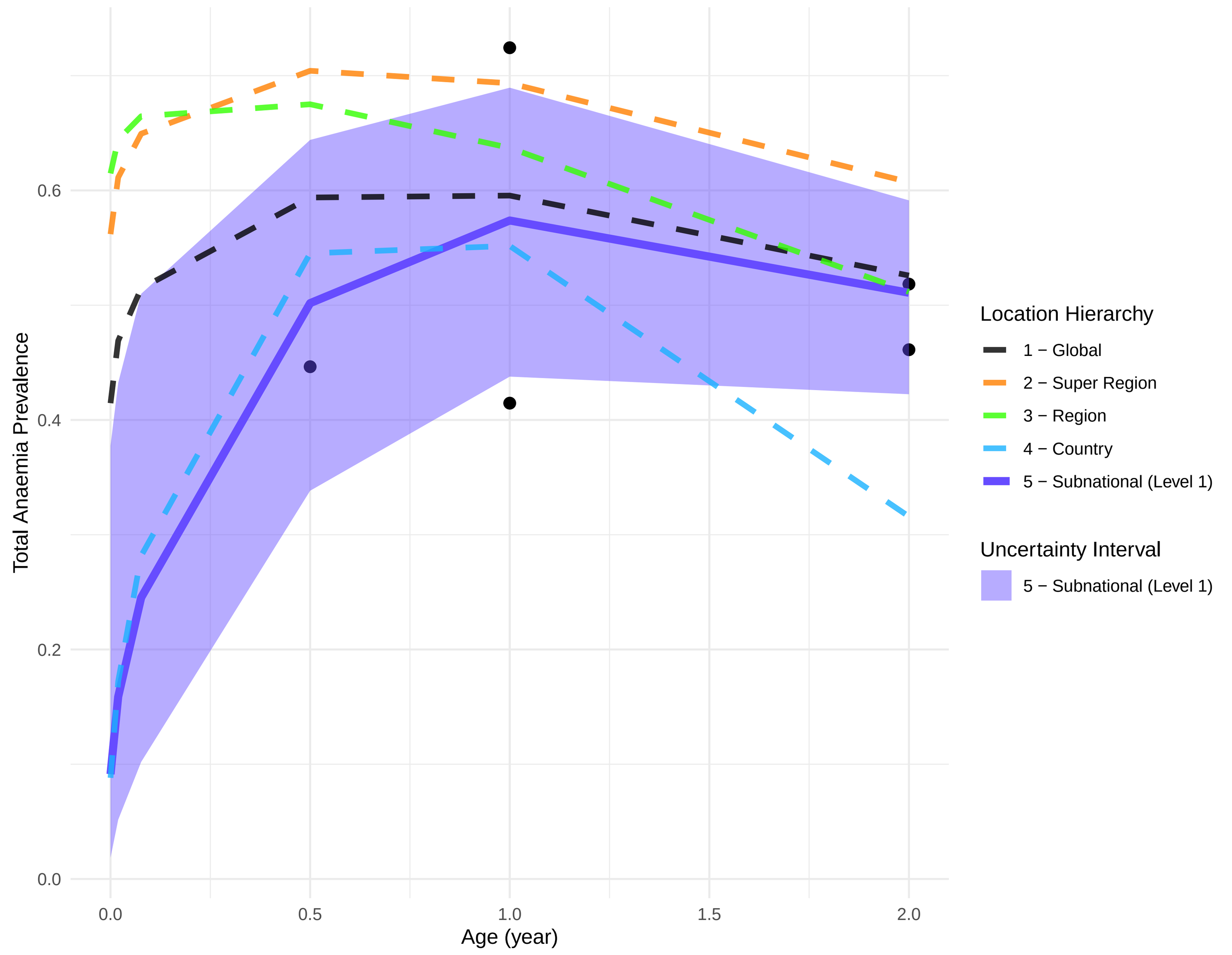

MR−BRT Age/Sex Fits − Males <2 Years Old − Total Anaemia Prevalence − Busia
WHO−2001 Adjustment Method

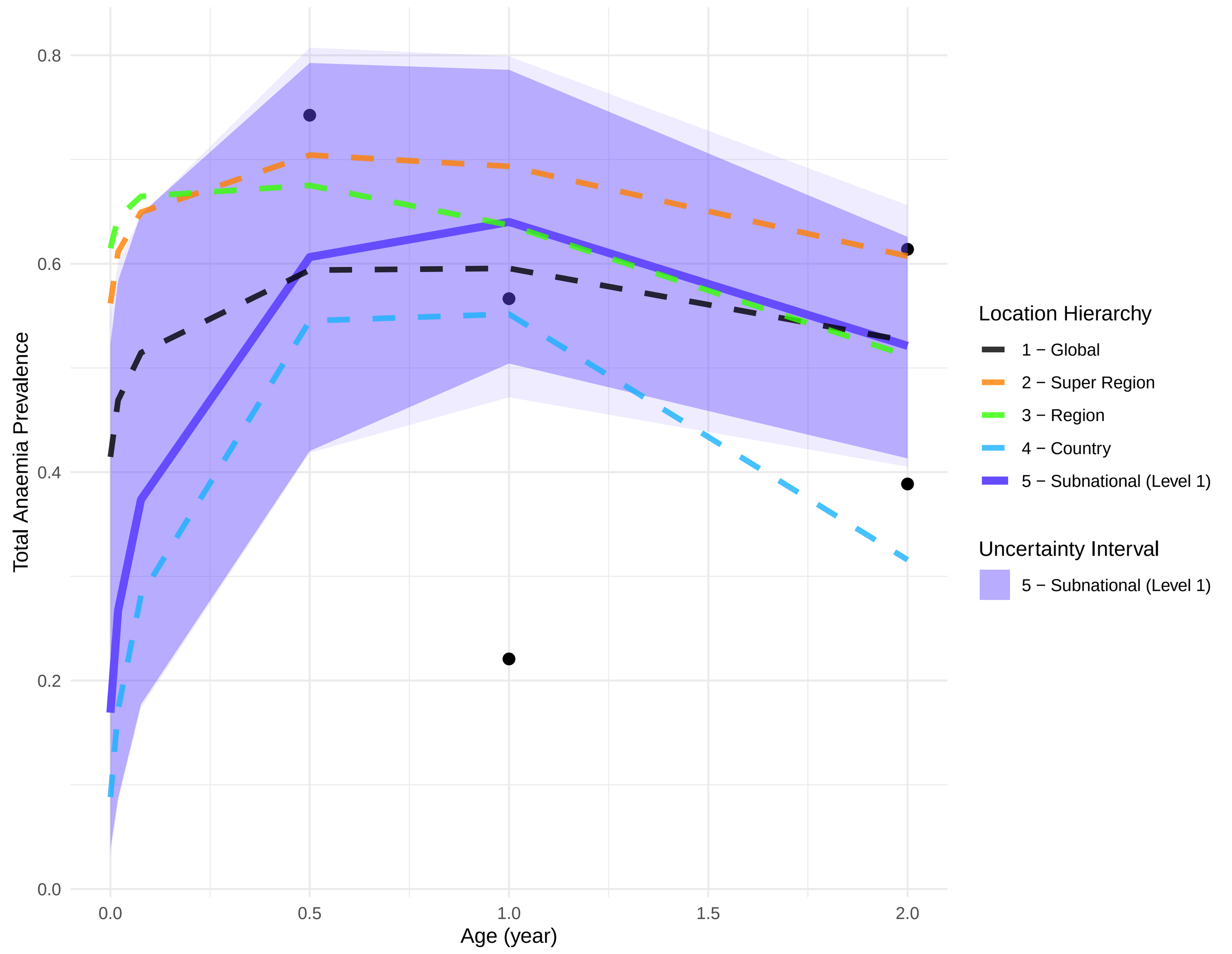

MR−BRT Age/Sex Fits − Males <2 Years Old − Total Anaemia Prevalence − Elgeyo−Marakwet
WHO−2001 Adjustment Method

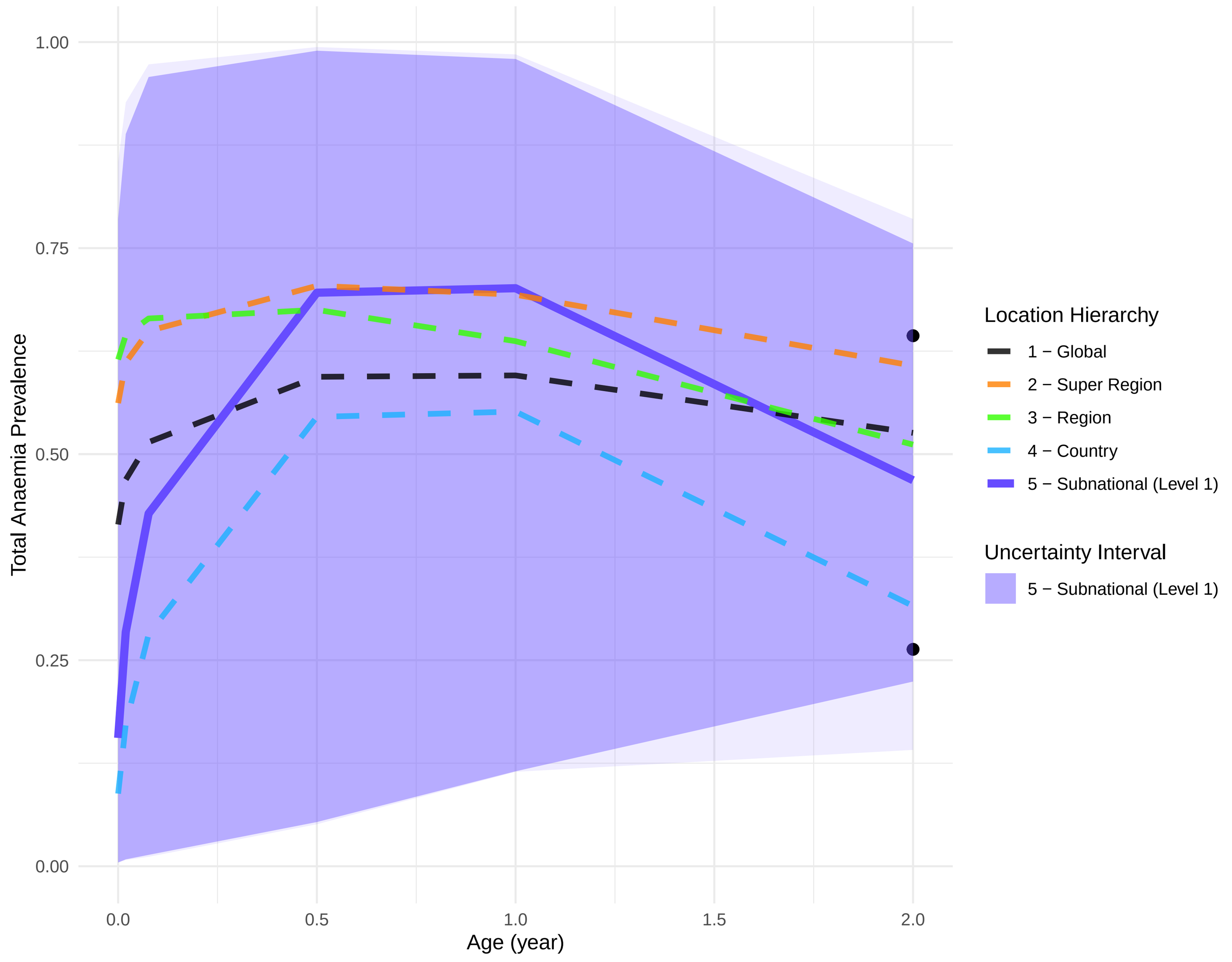

MR−BRT Age/Sex Fits − Males <2 Years Old − Total Anaemia Prevalence − Embu
WHO−2001 Adjustment Method

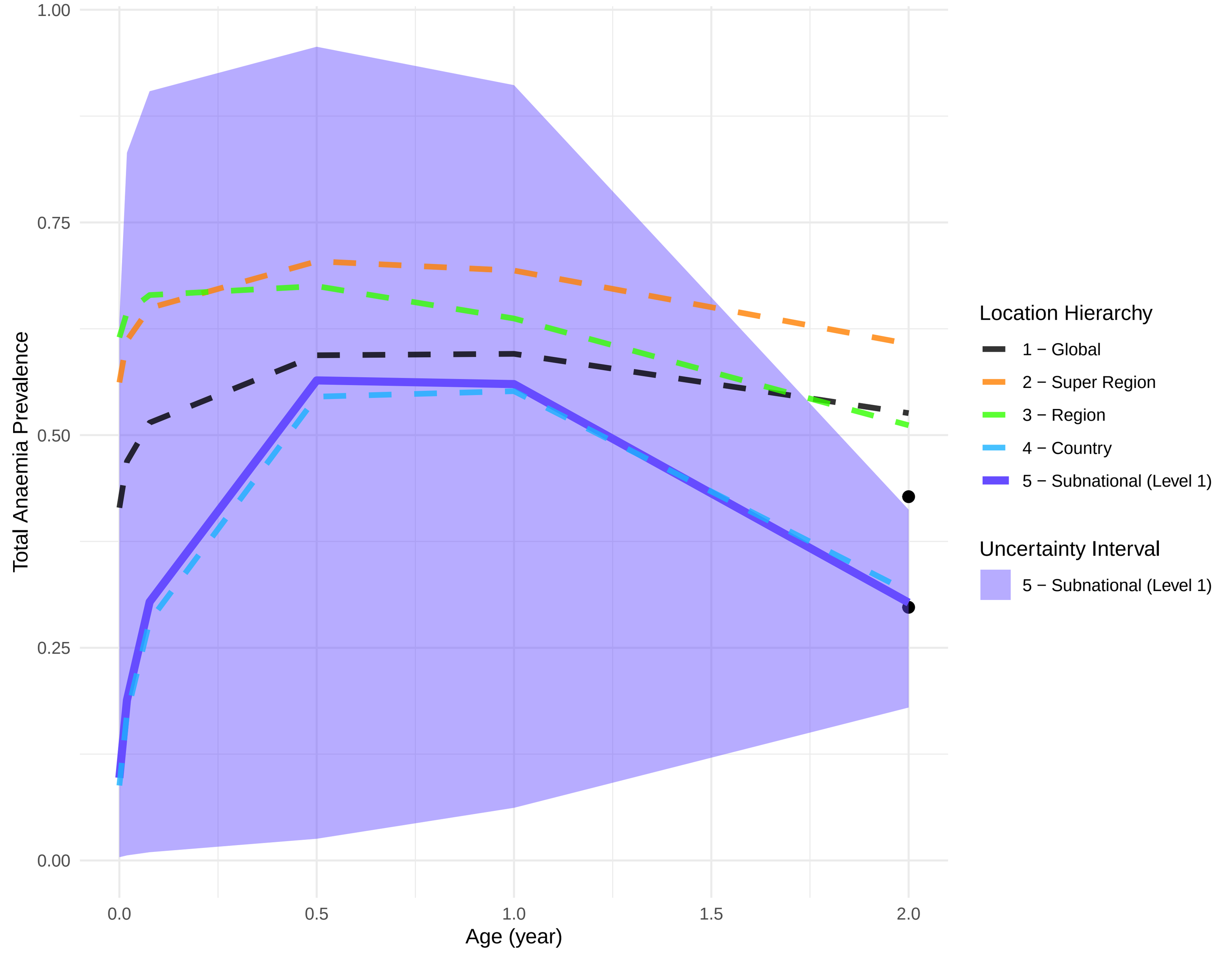

MR−BRT Age/Sex Fits − Males <2 Years Old − Total Anaemia Prevalence − Garissa
WHO−2001 Adjustment Method

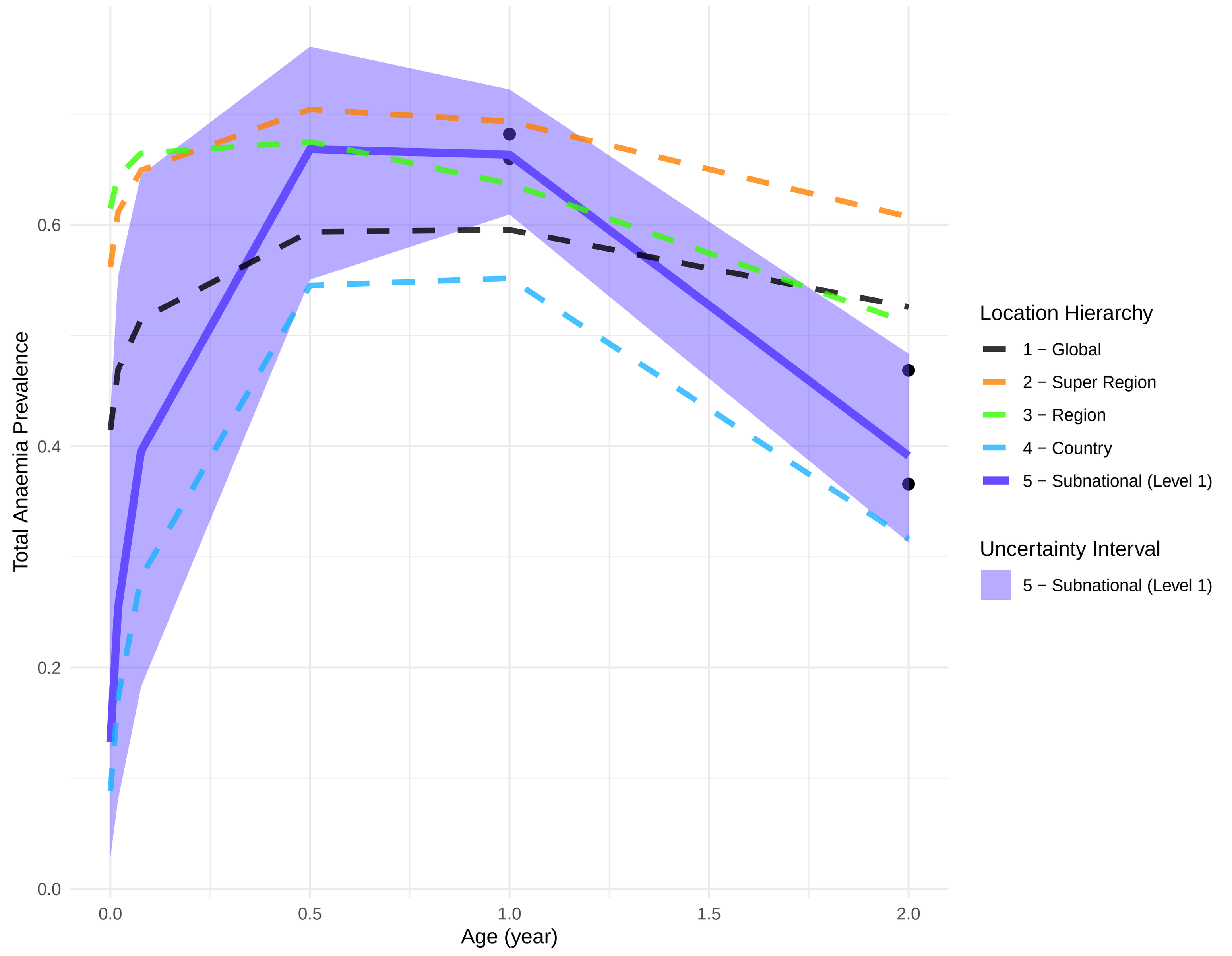

MR−BRT Age/Sex Fits − Males <2 Years Old − Total Anaemia Prevalence − Homa Bay
WHO−2001 Adjustment Method

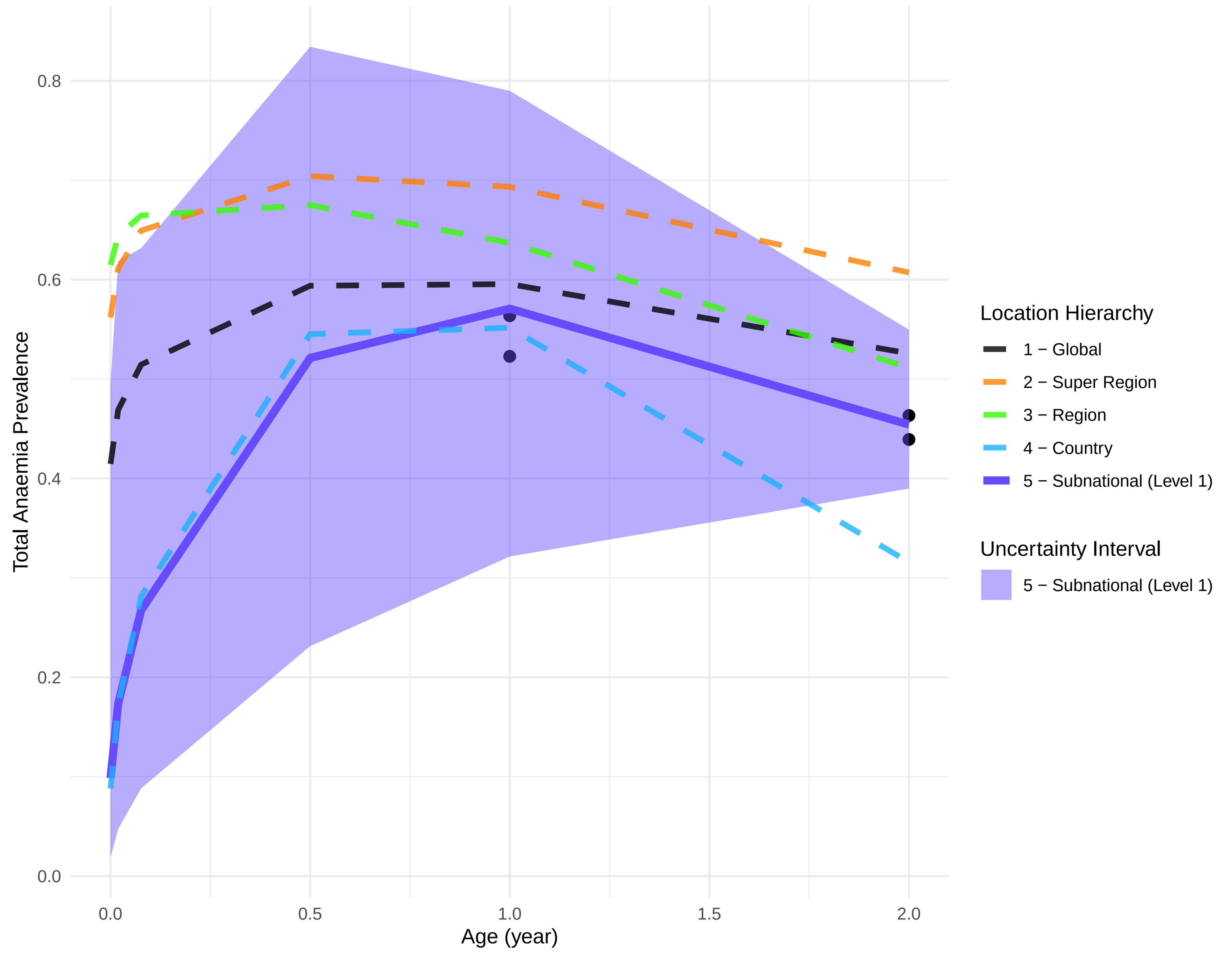

MR−BRT Age/Sex Fits − Males <2 Years Old − Total Anaemia Prevalence − Isiolo
WHO−2001 Adjustment Method

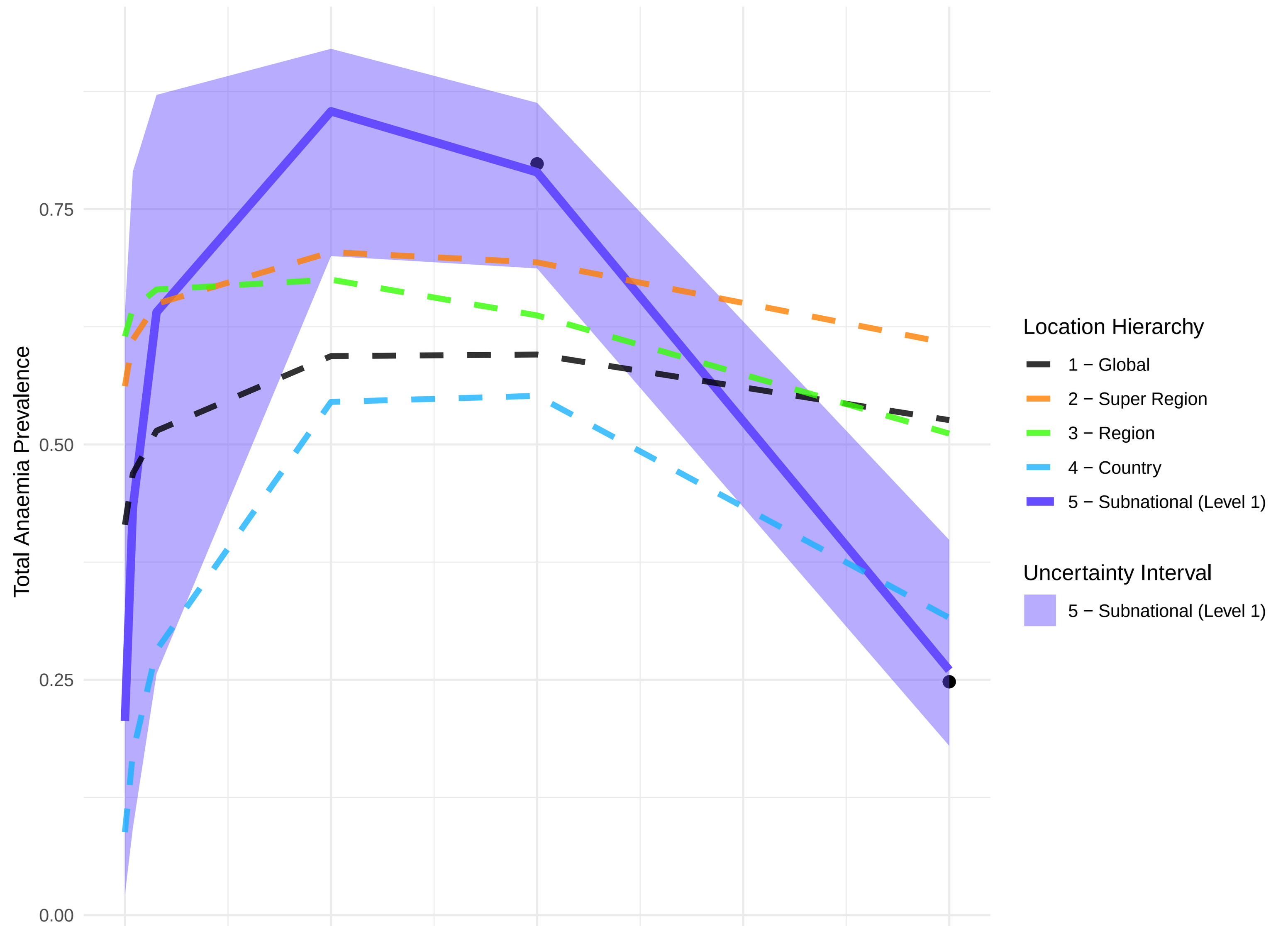

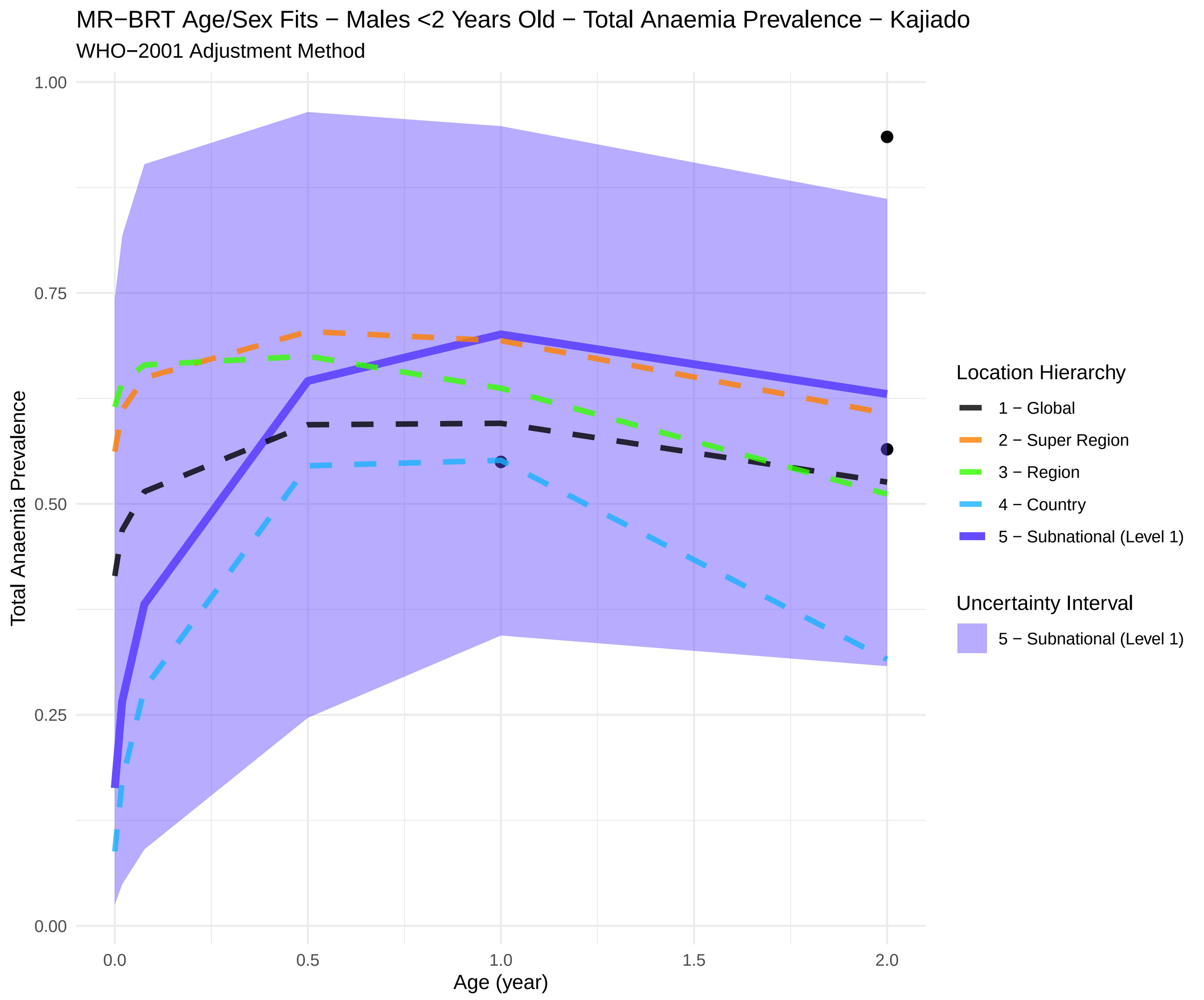
MR−BRT Age/Sex Fits − Males <2 Years Old − Total Anaemia Prevalence − Kajiado
WHO−2001 Adjustment Method
Total Anaemia Prevalence
Age (year)
Location Hierarchy
1 − Global
2 − Super Region
3 − Region
4 − Country
5 − Subnational (Level 1)
Uncertainty Interval
5 − Subnational (Level 1)

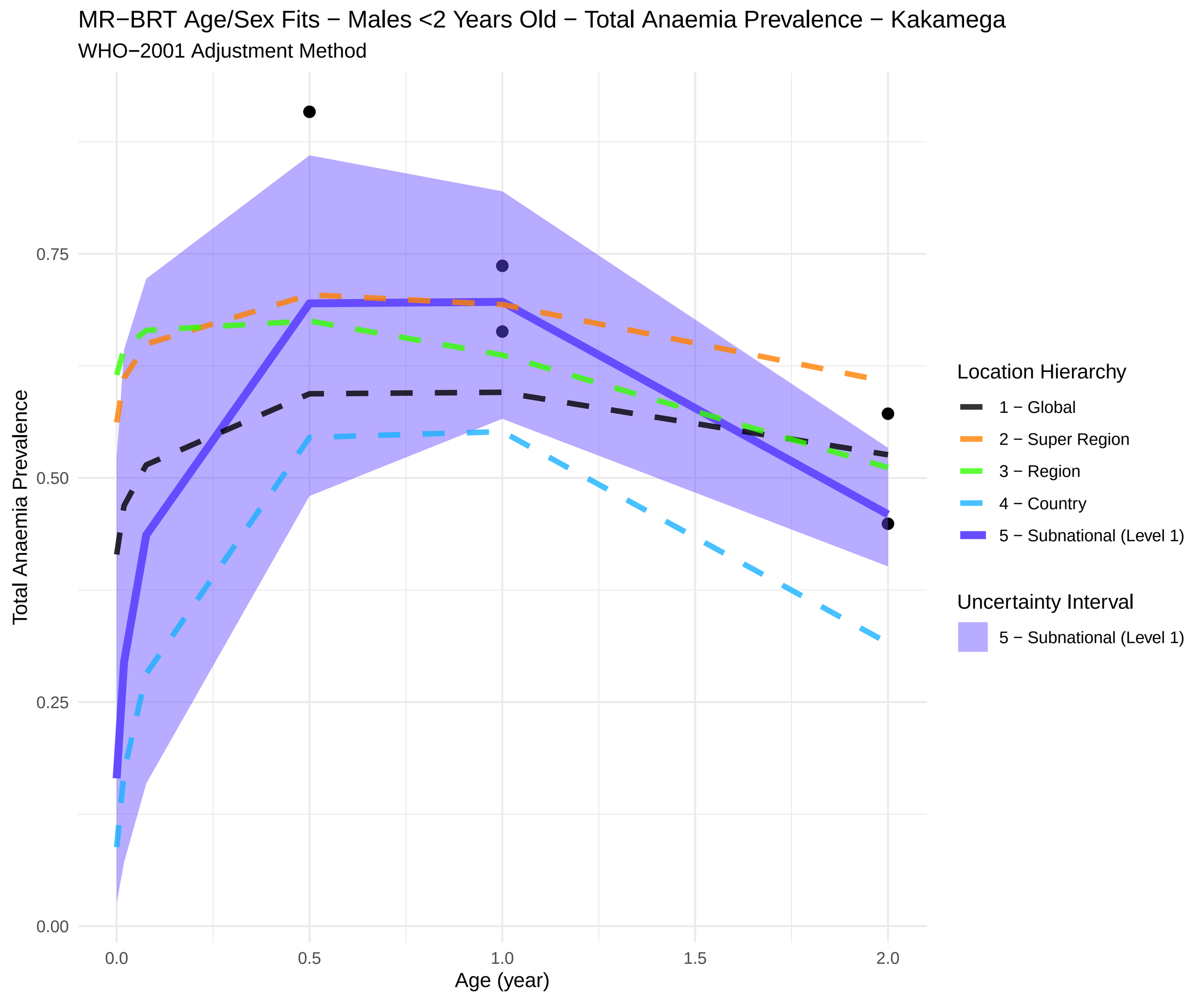
MR−BRT Age/Sex Fits − Males <2 Years Old − Total Anaemia Prevalence − Kakamega
WHO−2001 Adjustment Method
Total Anaemia Prevalence
Age (year)
Location Hierarchy
1 − Global
2 − Super Region
3 − Region
4 − Country
5 − Subnational (Level 1)
Uncertainty Interval
5 − Subnational (Level 1)

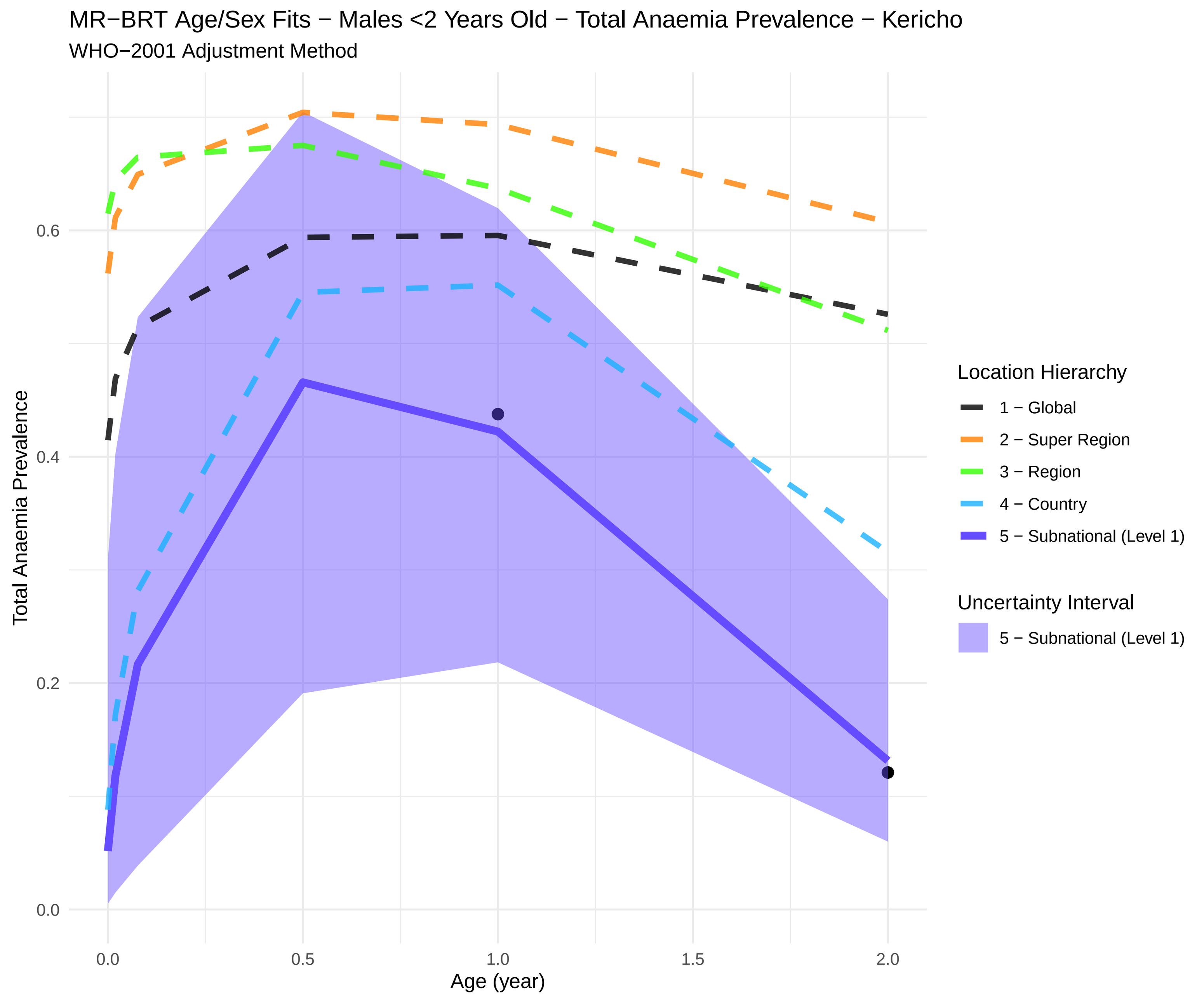
MR−BRT Age/Sex Fits − Males <2 Years Old − Total Anaemia Prevalence − Kericho
WHO−2001 Adjustment Method
Total Anaemia Prevalence
Age (year)
Location Hierarchy
1 − Global
2 − Super Region
3 − Region
4 − Country
5 − Subnational (Level 1)
Uncertainty Interval
5 − Subnational (Level 1)

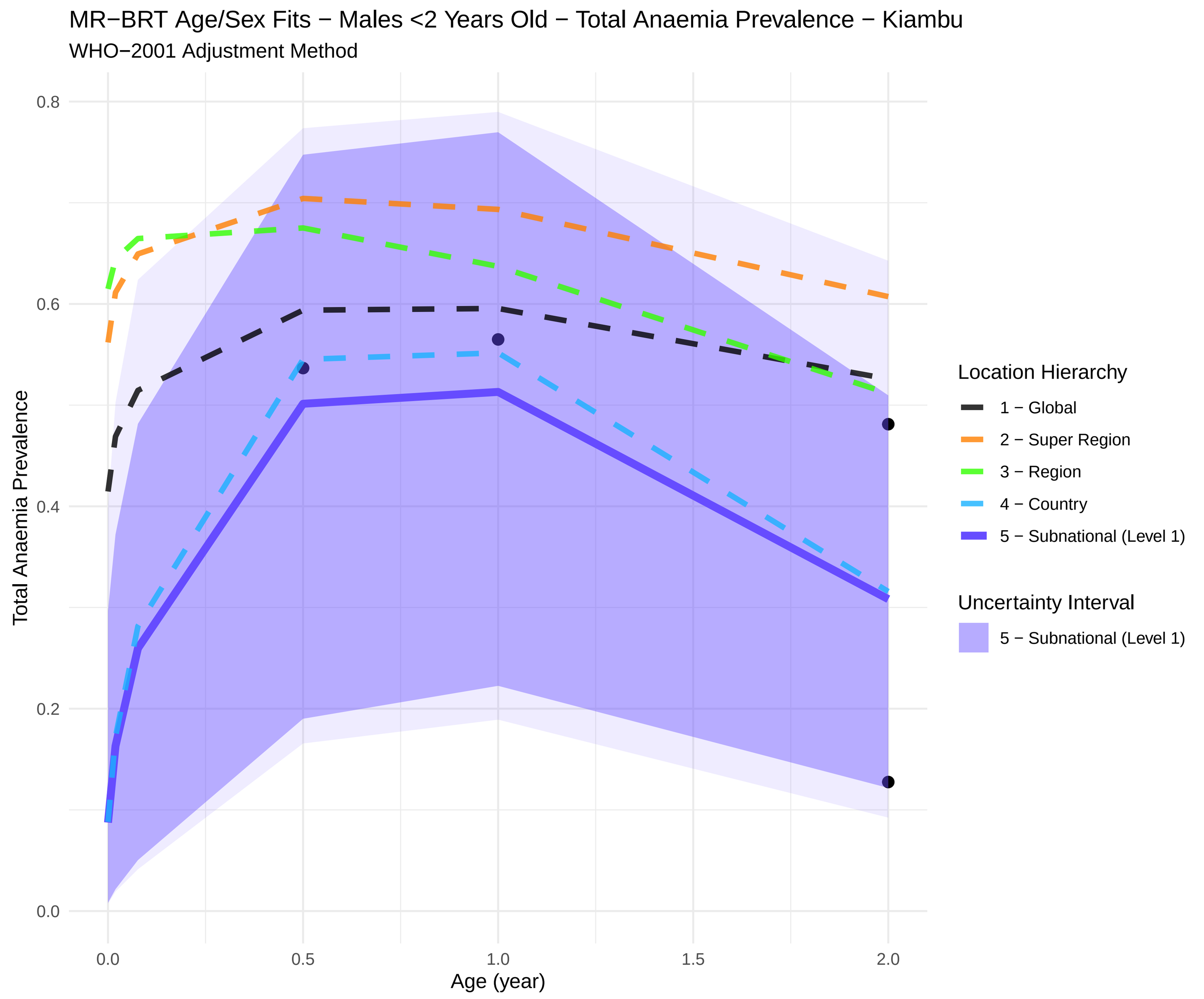
MR−BRT Age/Sex Fits − Males <2 Years Old − Total Anaemia Prevalence − Kiambu
WHO−2001 Adjustment Method
Total Anaemia Prevalence
Age (year)
Location Hierarchy
1 − Global
2 − Super Region
3 − Region
4 − Country
5 − Subnational (Level 1)
Uncertainty Interval
5 − Subnational (Level 1)

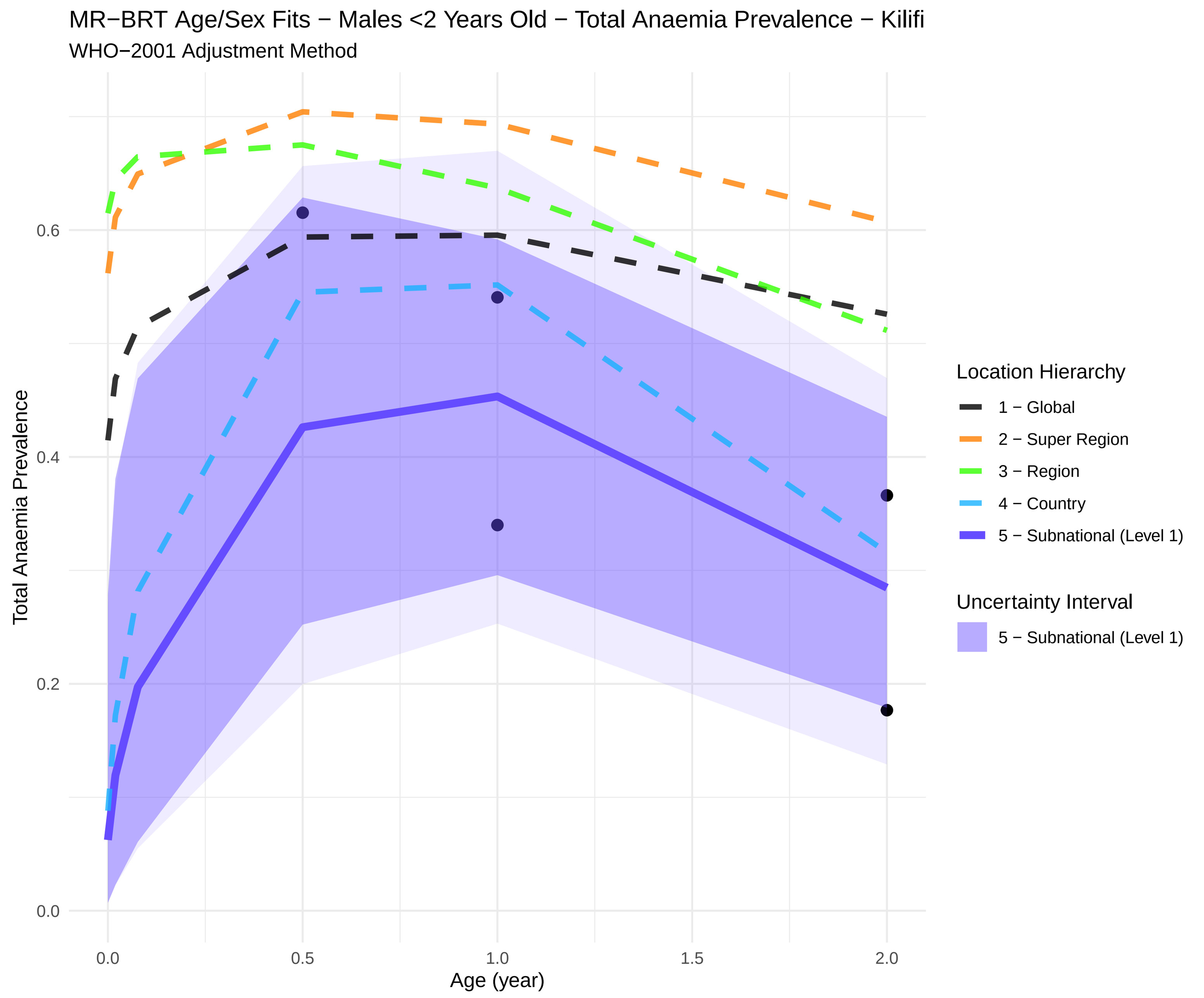
MR−BRT Age/Sex Fits − Males <2 Years Old − Total Anaemia Prevalence − Kilifi
WHO−2001 Adjustment Method
Total Anaemia Prevalence
Age (year)
Location Hierarchy
1 − Global
2 − Super Region
3 − Region
4 − Country
5 − Subnational (Level 1)
Uncertainty Interval
5 − Subnational (Level 1)

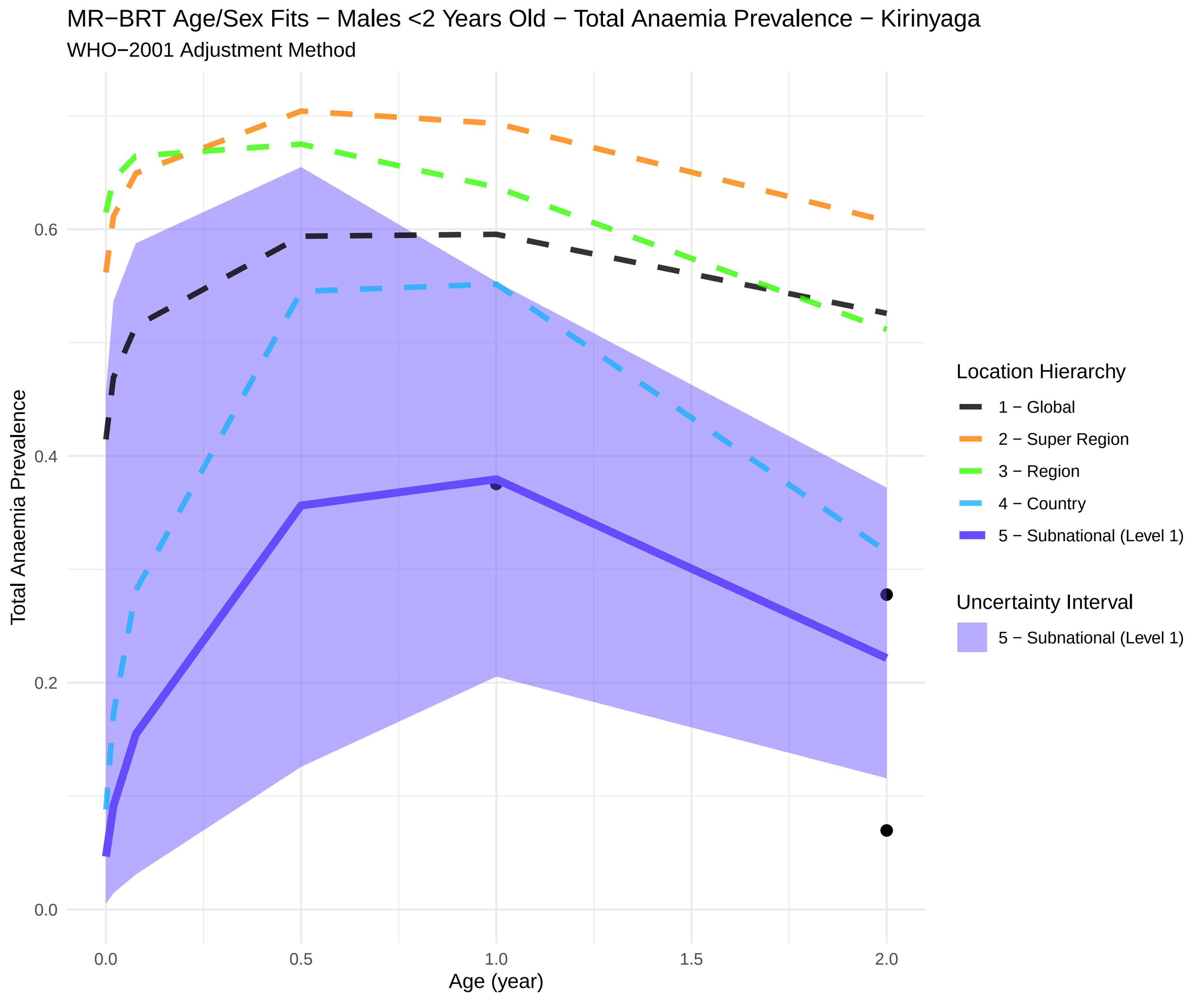
MR−BRT Age/Sex Fits − Males <2 Years Old − Total Anaemia Prevalence − Kirinyaga
WHO−2001 Adjustment Method
Total Anaemia Prevalence
Age (year)
Location Hierarchy
1 − Global
2 − Super Region
3 − Region
4 − Country
5 − Subnational (Level 1)
Uncertainty Interval
5 − Subnational (Level 1)

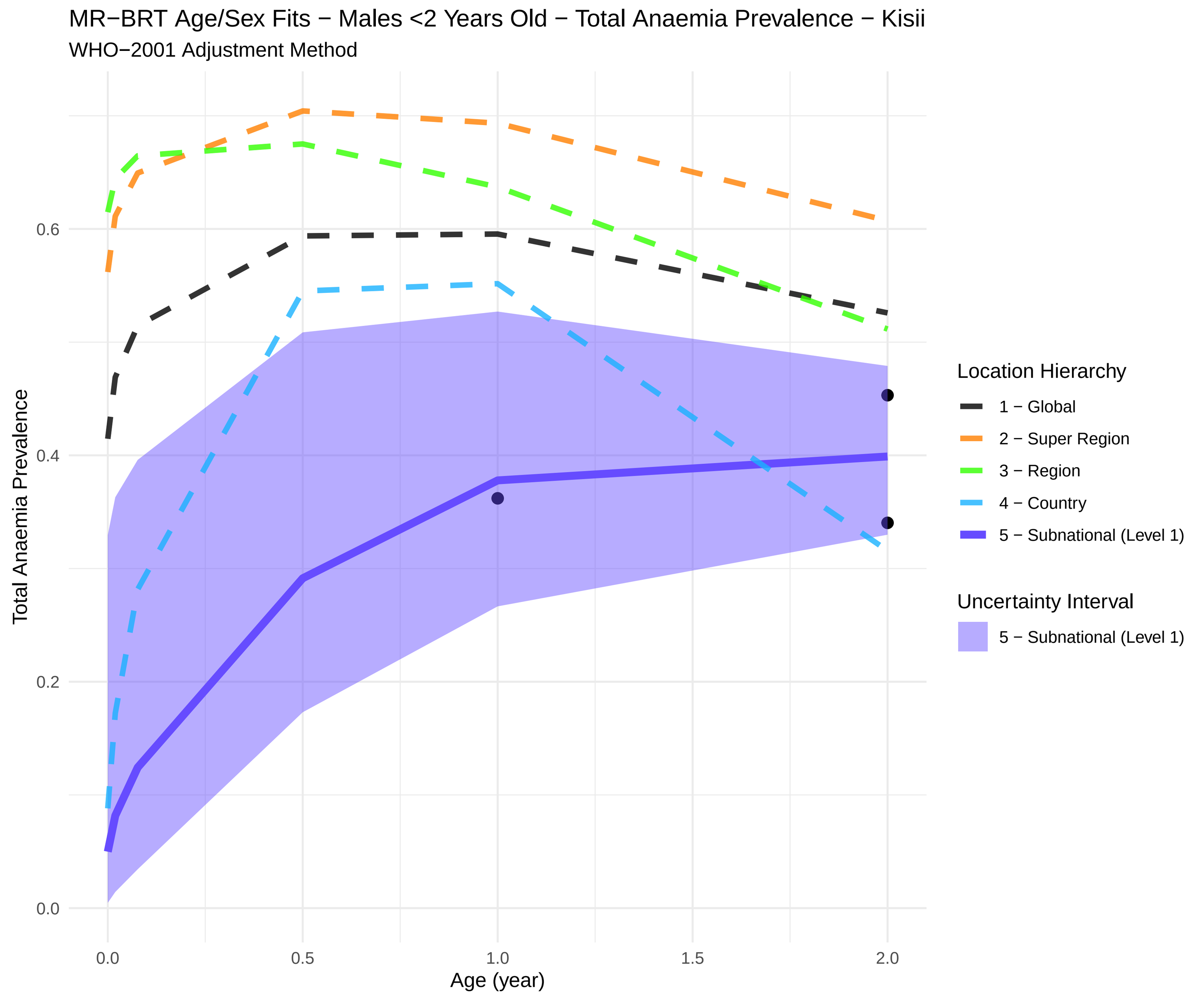
MR−BRT Age/Sex Fits − Males <2 Years Old − Total Anaemia Prevalence − Kisii
WHO−2001 Adjustment Method
Total Anaemia Prevalence
Age (year)
Location Hierarchy
1 − Global
2 − Super Region
3 − Region
4 − Country
5 − Subnational (Level 1)
Uncertainty Interval
5 − Subnational (Level 1)

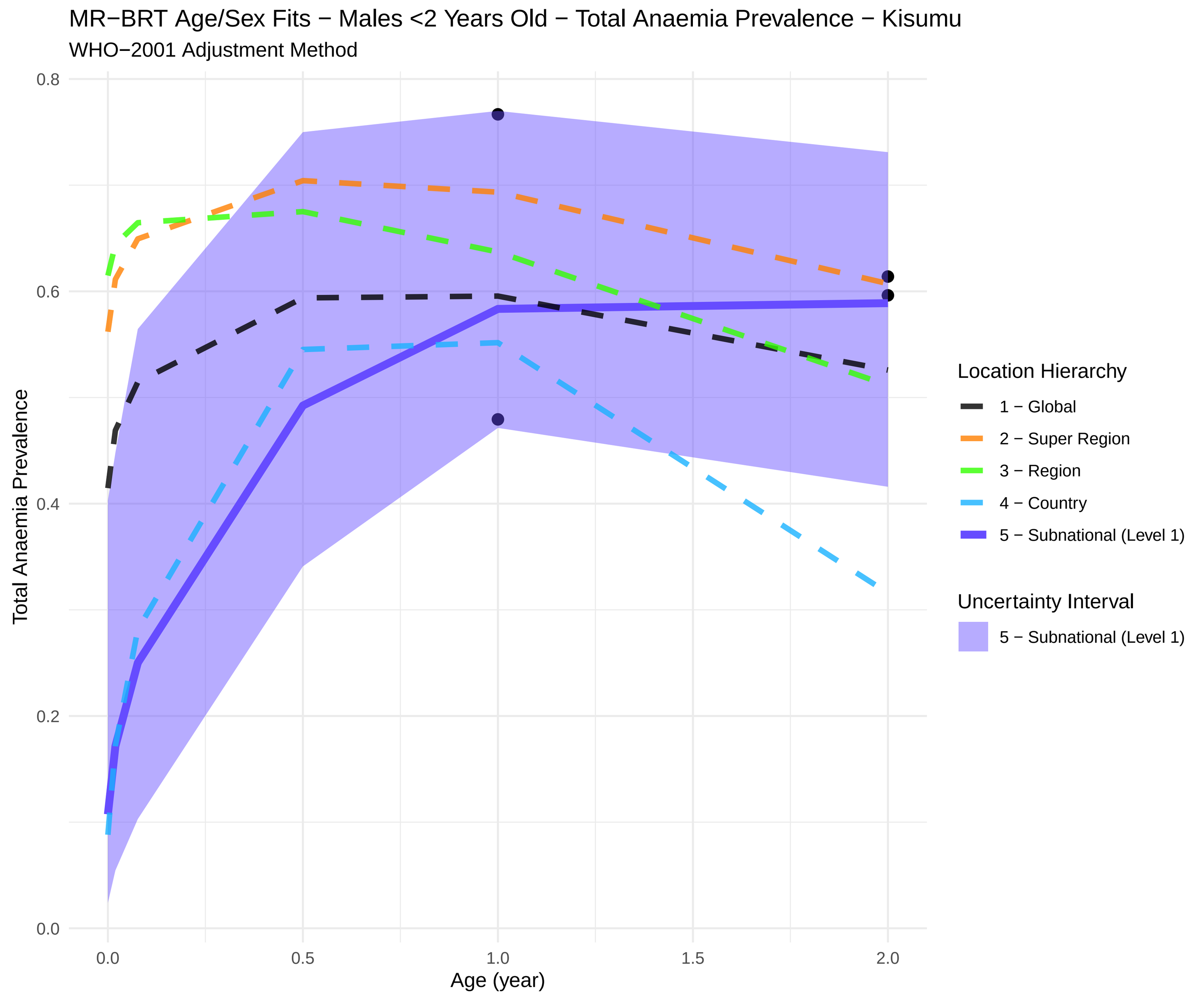
MR−BRT Age/Sex Fits − Males <2 Years Old − Total Anaemia Prevalence − Kisumu
WHO−2001 Adjustment Method
Total Anaemia Prevalence
Age (year)
Location Hierarchy
1 − Global
2 − Super Region
3 − Region
4 − Country
5 − Subnational (Level 1)
Uncertainty Interval
5 − Subnational (Level 1)

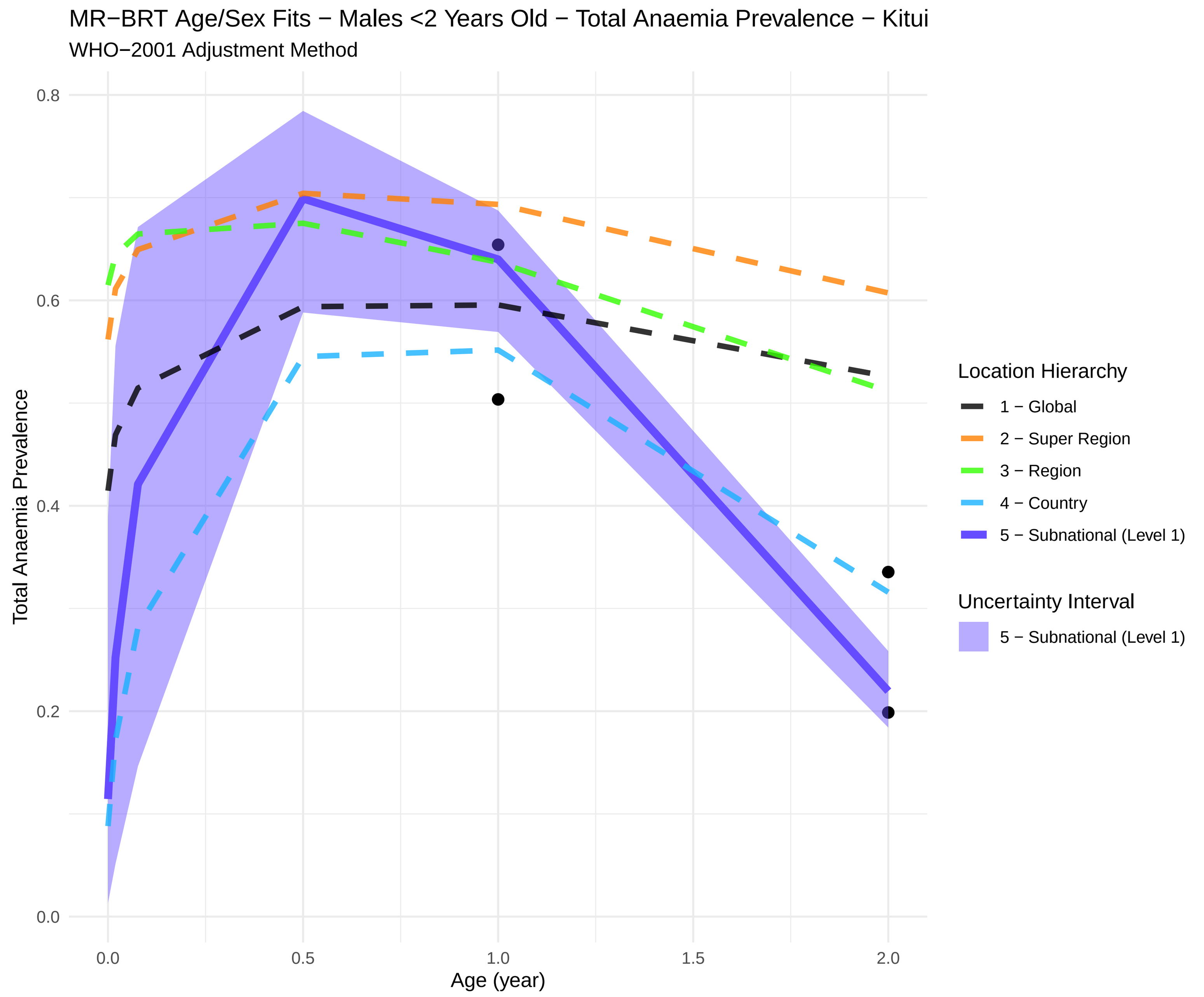
MR−BRT Age/Sex Fits − Males <2 Years Old − Total Anaemia Prevalence − Kitui
WHO−2001 Adjustment Method
Total Anaemia Prevalence
Age (year)
Location Hierarchy
1 − Global
2 − Super Region
3 − Region
4 − Country
5 − Subnational (Level 1)
Uncertainty Interval
5 − Subnational (Level 1)

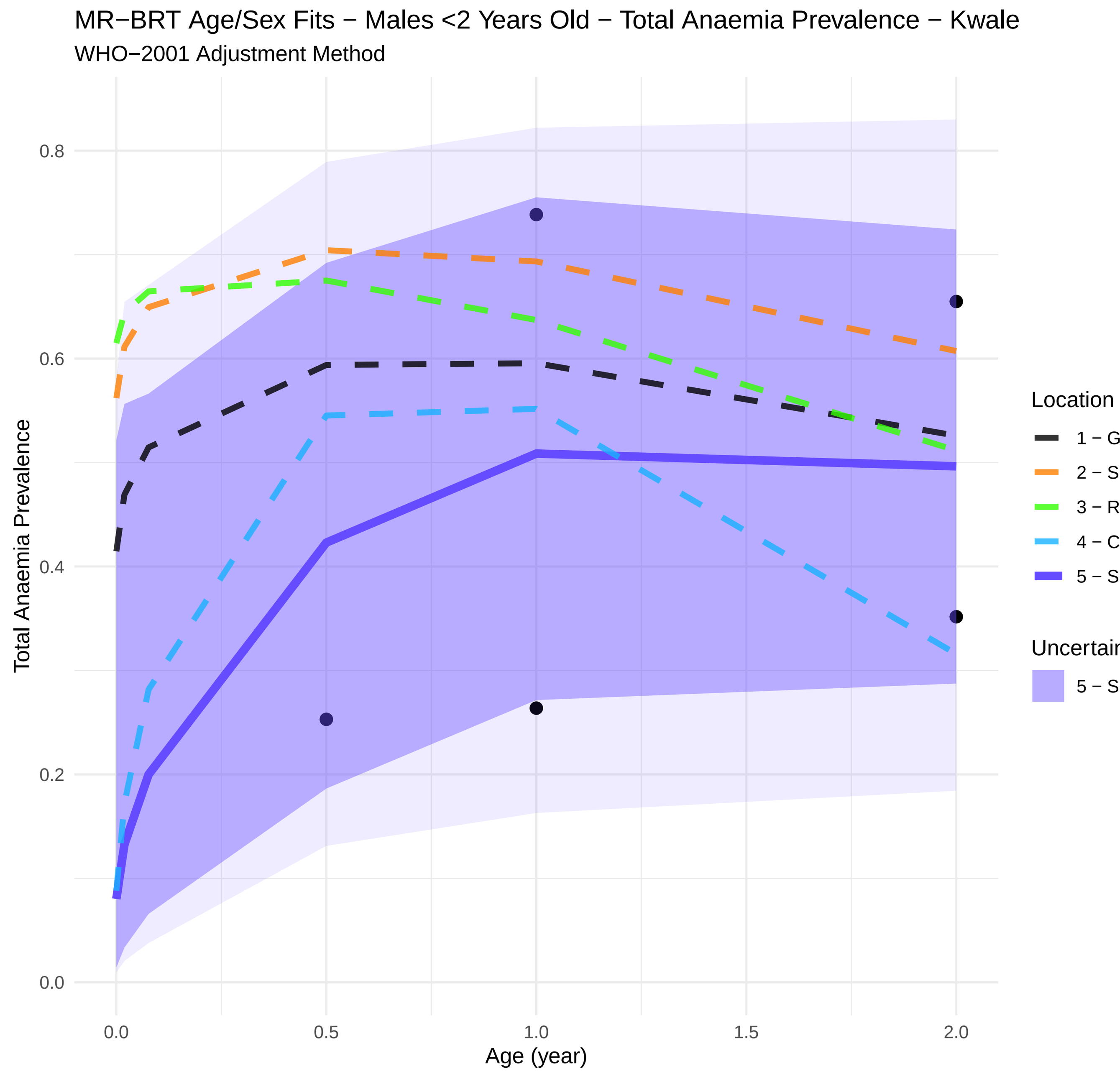
MR−BRT Age/Sex Fits − Males <2 Years Old − Total Anaemia Prevalence − Kwale
WHO−2001 Adjustment Method
Total Anaemia Prevalence
Age (year)
Location Hierarchy
1 − Global
2 − Super Region
3 − Region
4 − Country
5 − Subnational (Level 1)
Uncertainty Interval
5 − Subnational (Level 1)

MR−BRT Age/Sex Fits − Males <2 Years Old − Total Anaemia Prevalence − Laikipia
WHO−2001 Adjustment Method

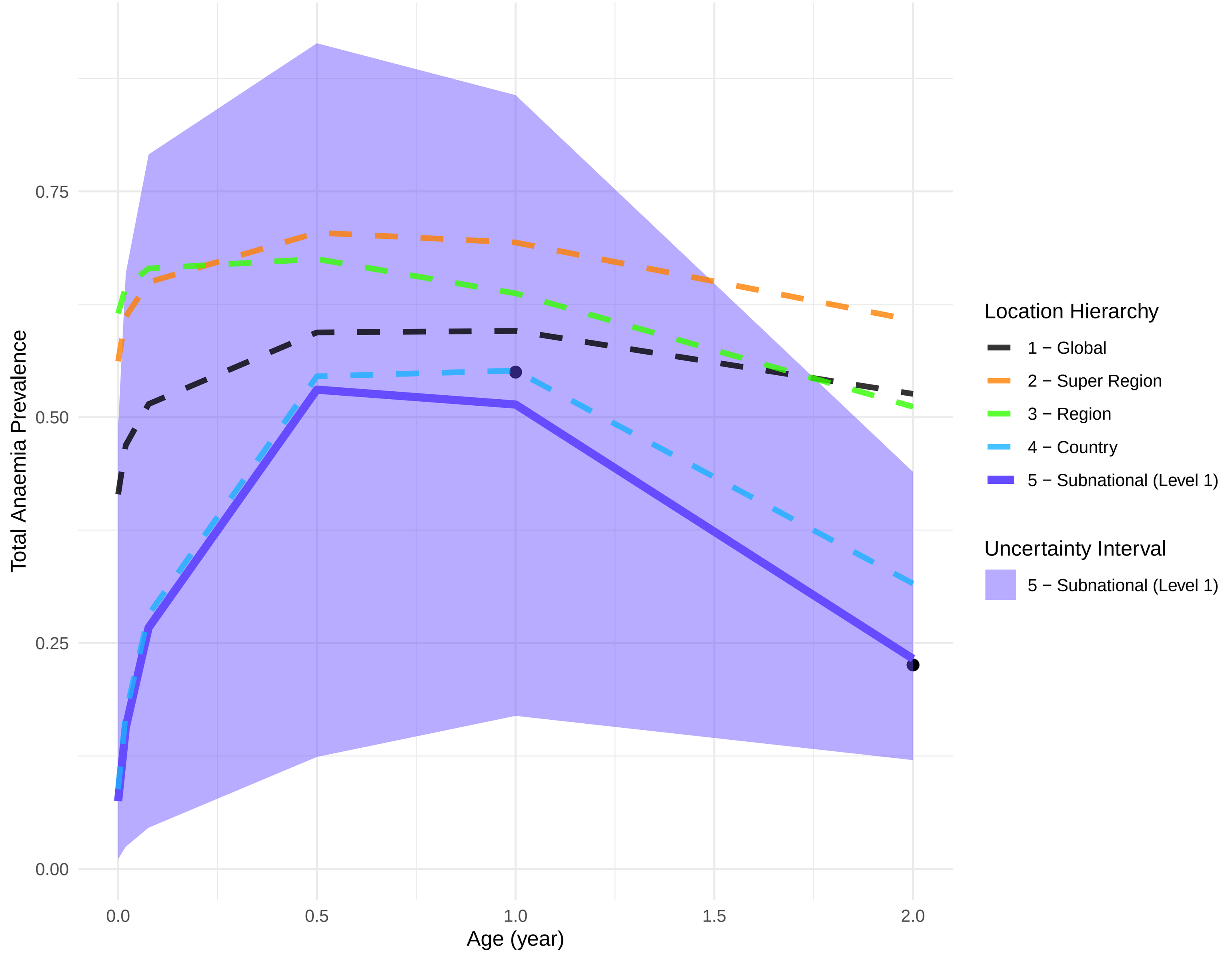

MR−BRT Age/Sex Fits − Males <2 Years Old − Total Anaemia Prevalence − Lamu
WHO−2001 Adjustment Method

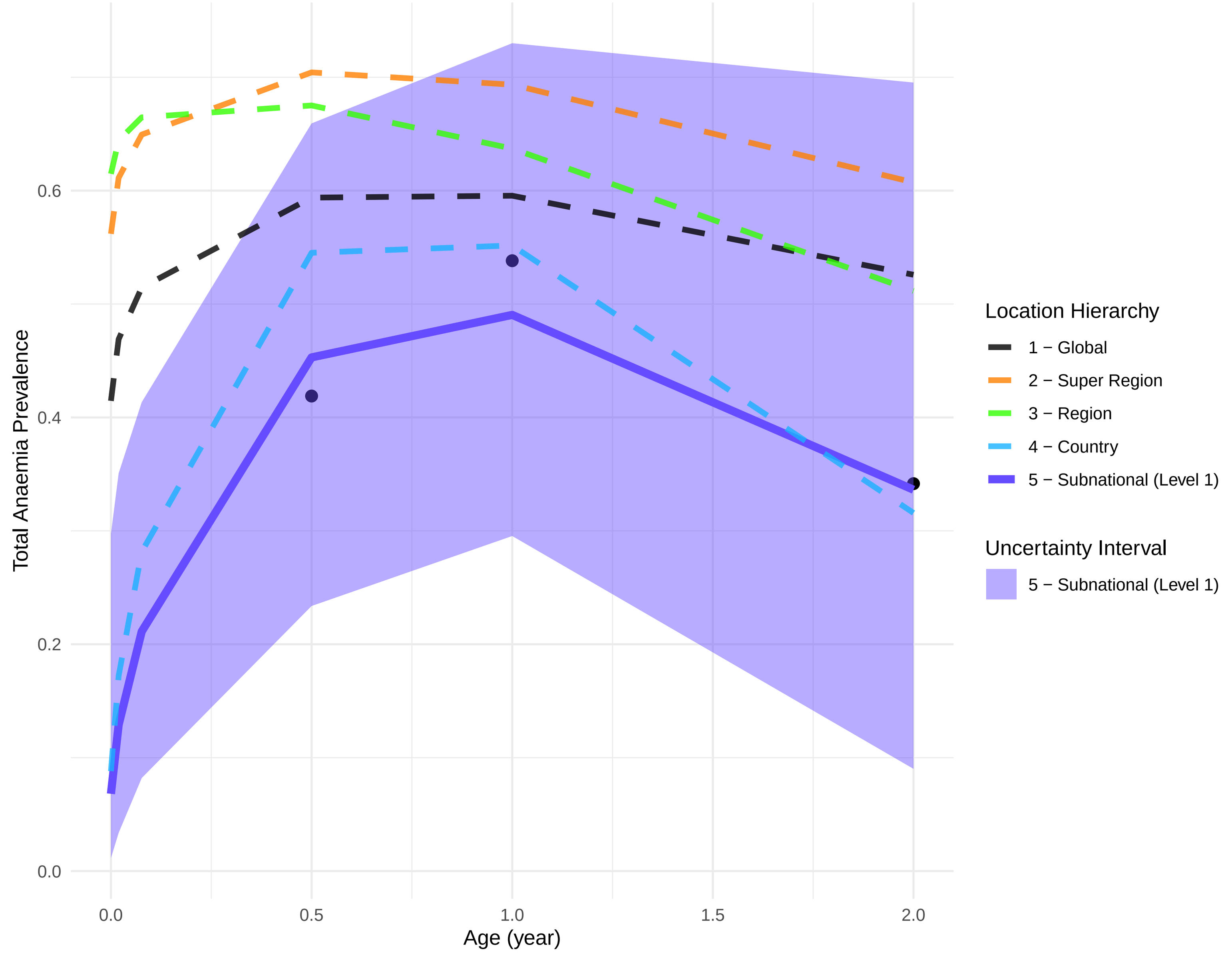

MR−BRT Age/Sex Fits − Males <2 Years Old − Total Anaemia Prevalence − Machakos
WHO−2001 Adjustment Method

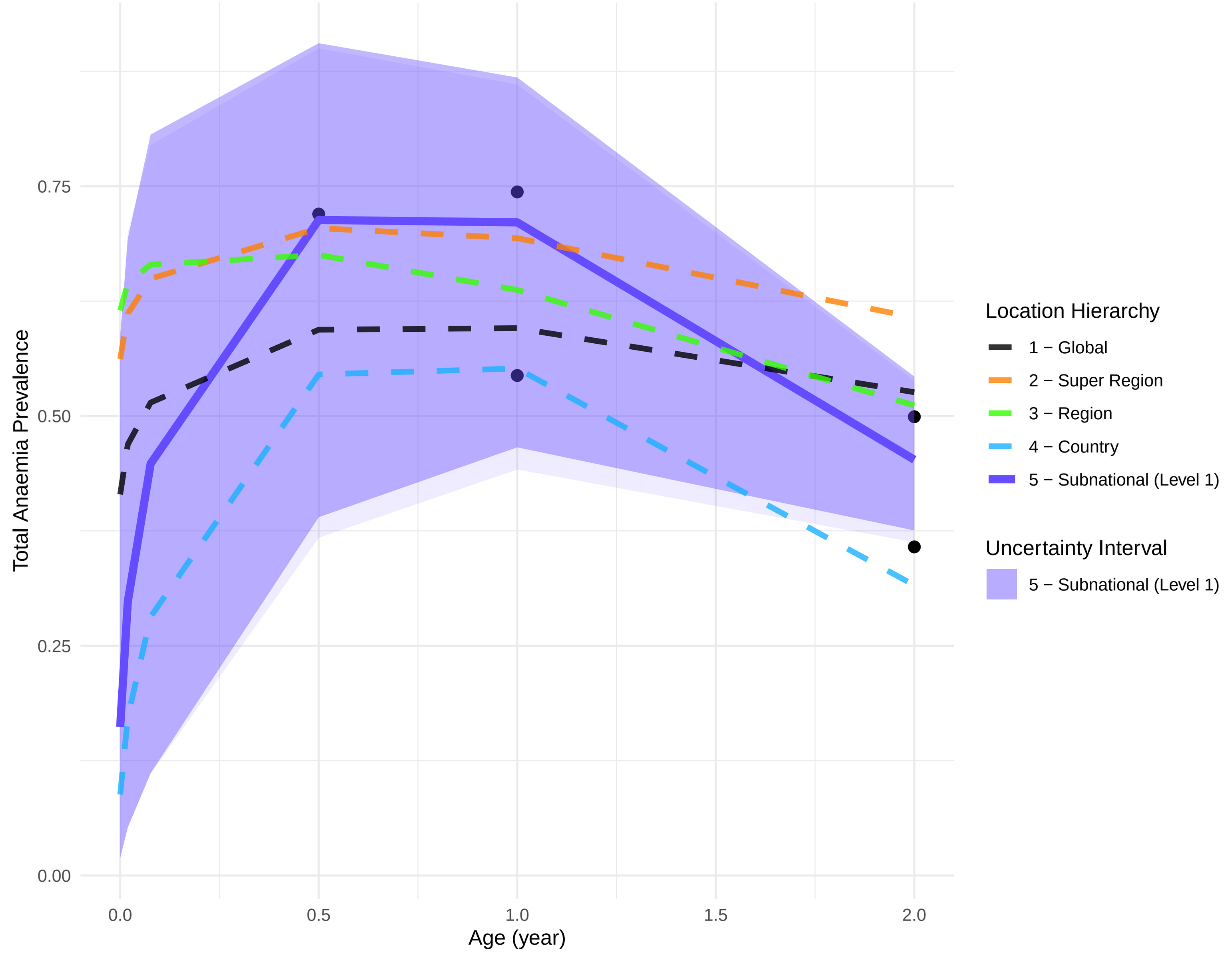

MR−BRT Age/Sex Fits − Males <2 Years Old − Total Anaemia Prevalence − Makueni
WHO−2001 Adjustment Method

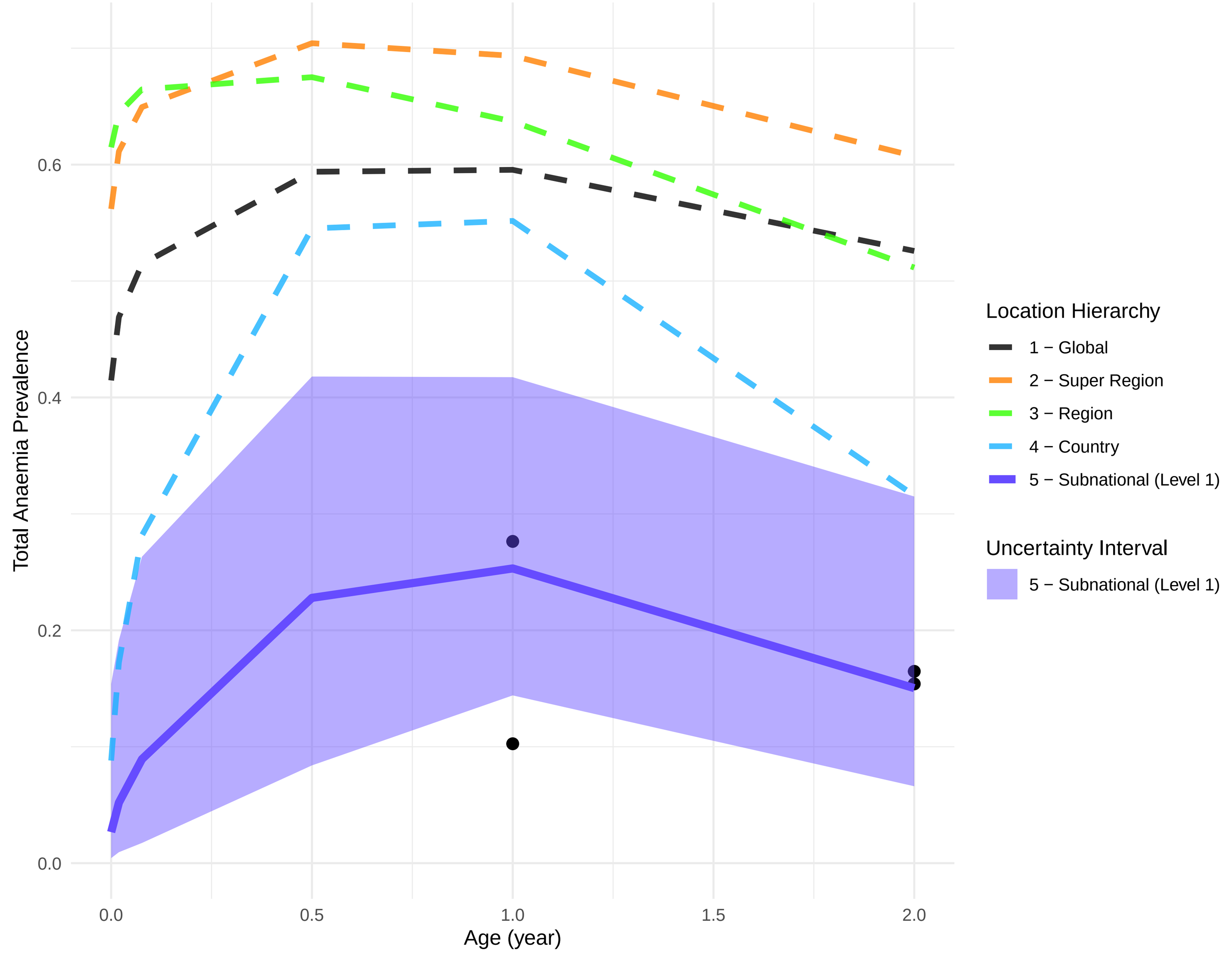

MR−BRT Age/Sex Fits − Males <2 Years Old − Total Anaemia Prevalence − Mandera
WHO−2001 Adjustment Method

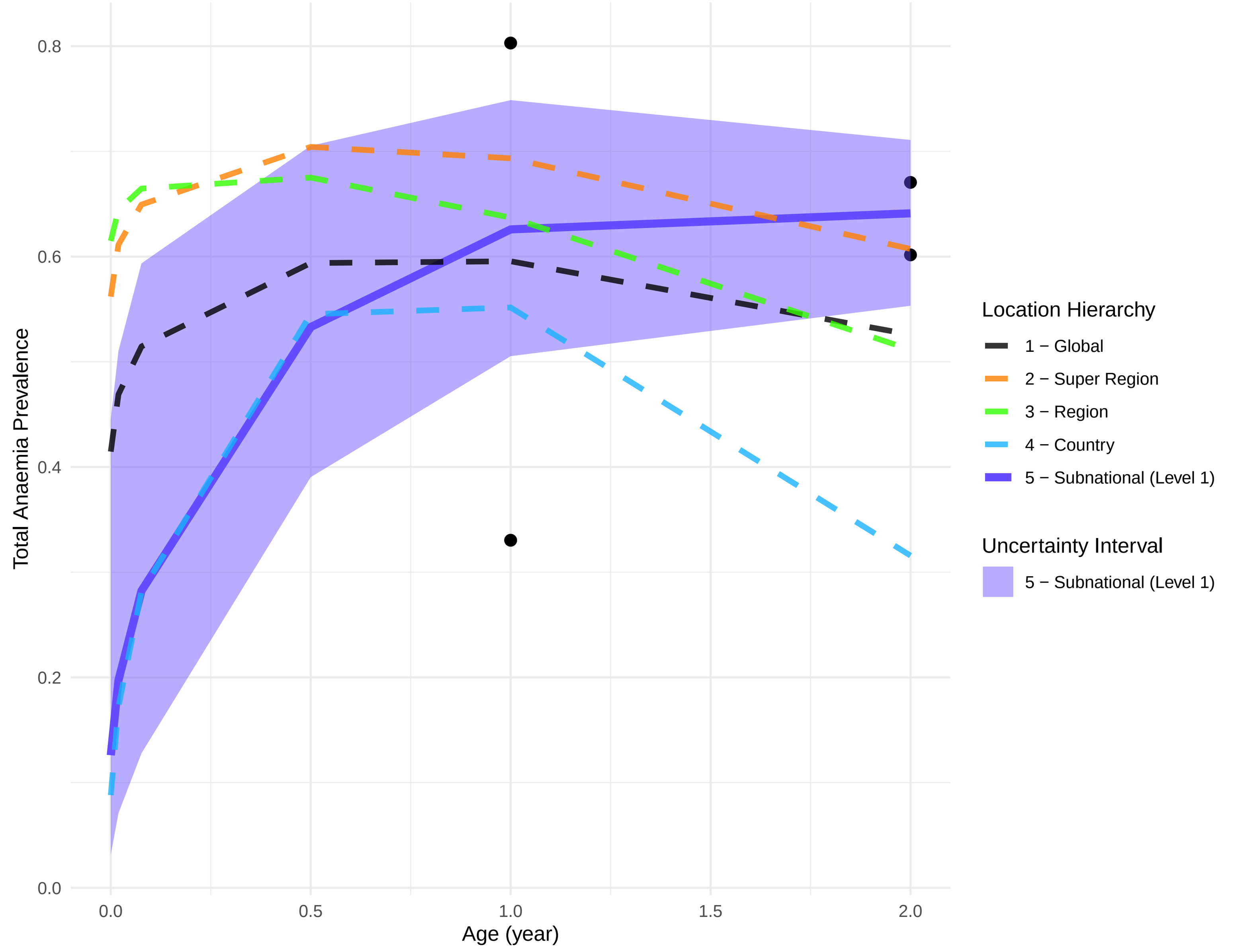

MR−BRT Age/Sex Fits − Males <2 Years Old − Total Anaemia Prevalence − Marsabit
WHO−2001 Adjustment Method

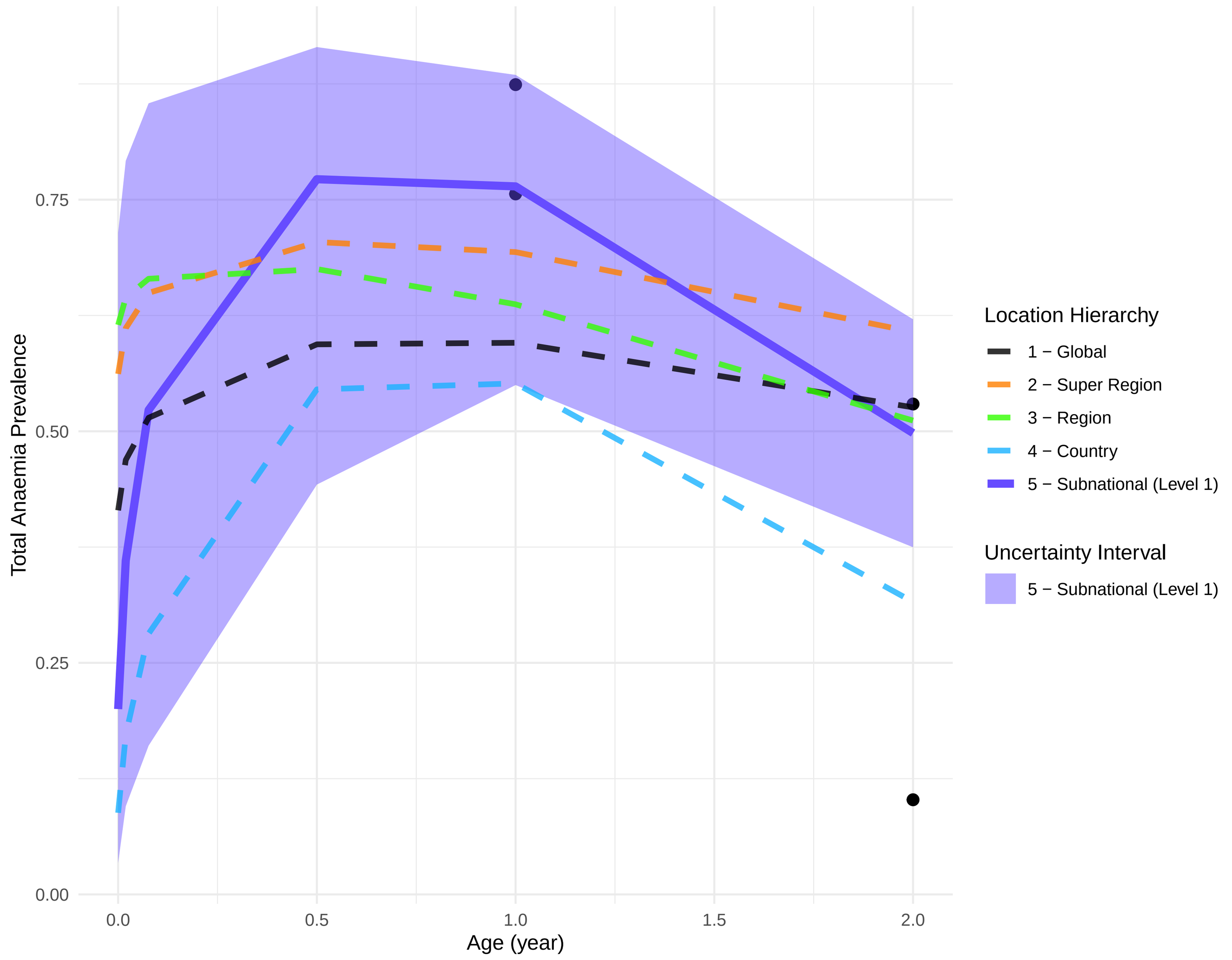

MR−BRT Age/Sex Fits − Males <2 Years Old − Total Anaemia Prevalence − Meru
WHO−2001 Adjustment Method

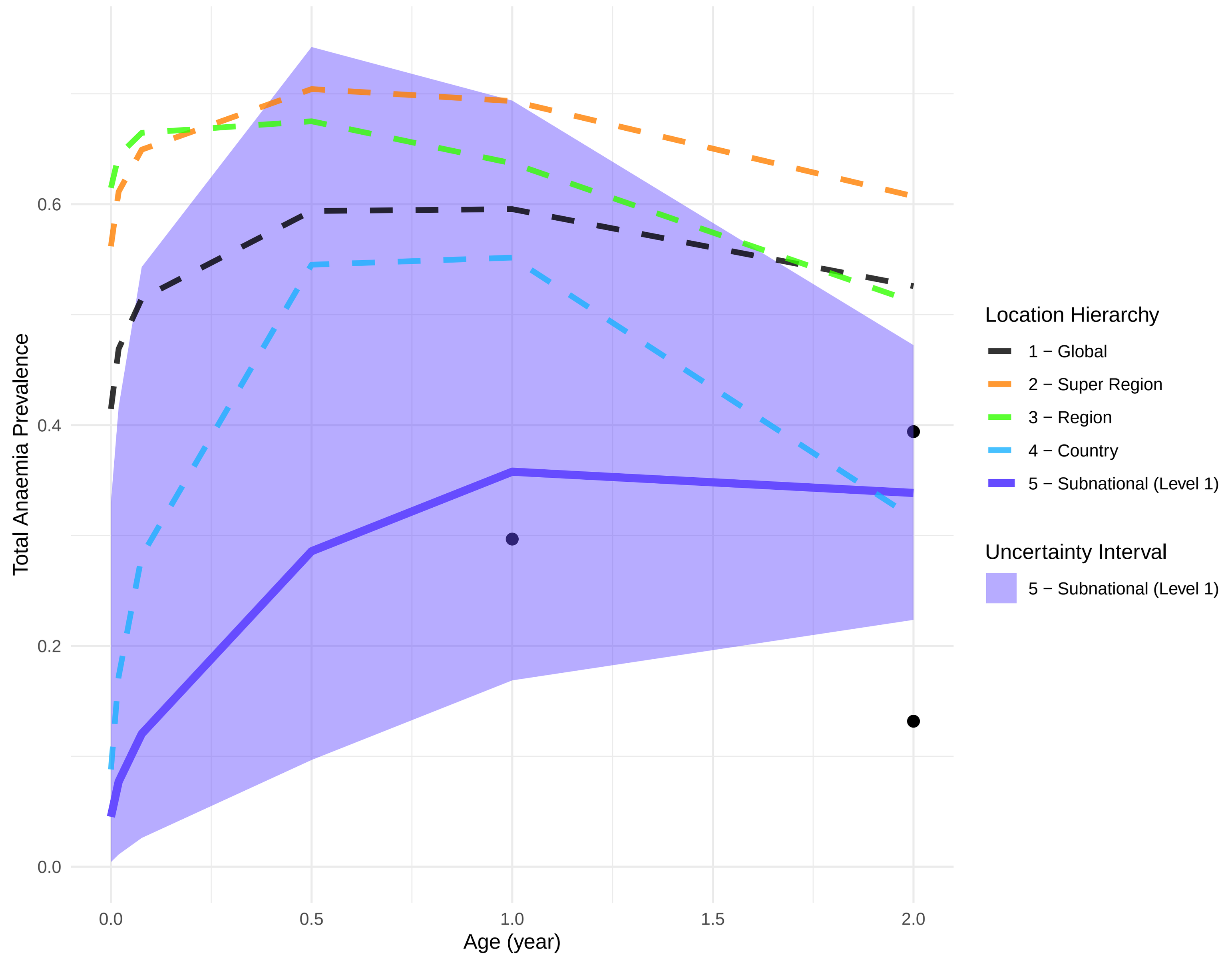

MR−BRT Age/Sex Fits − Males <2 Years Old − Total Anaemia Prevalence − Migori
WHO−2001 Adjustment Method

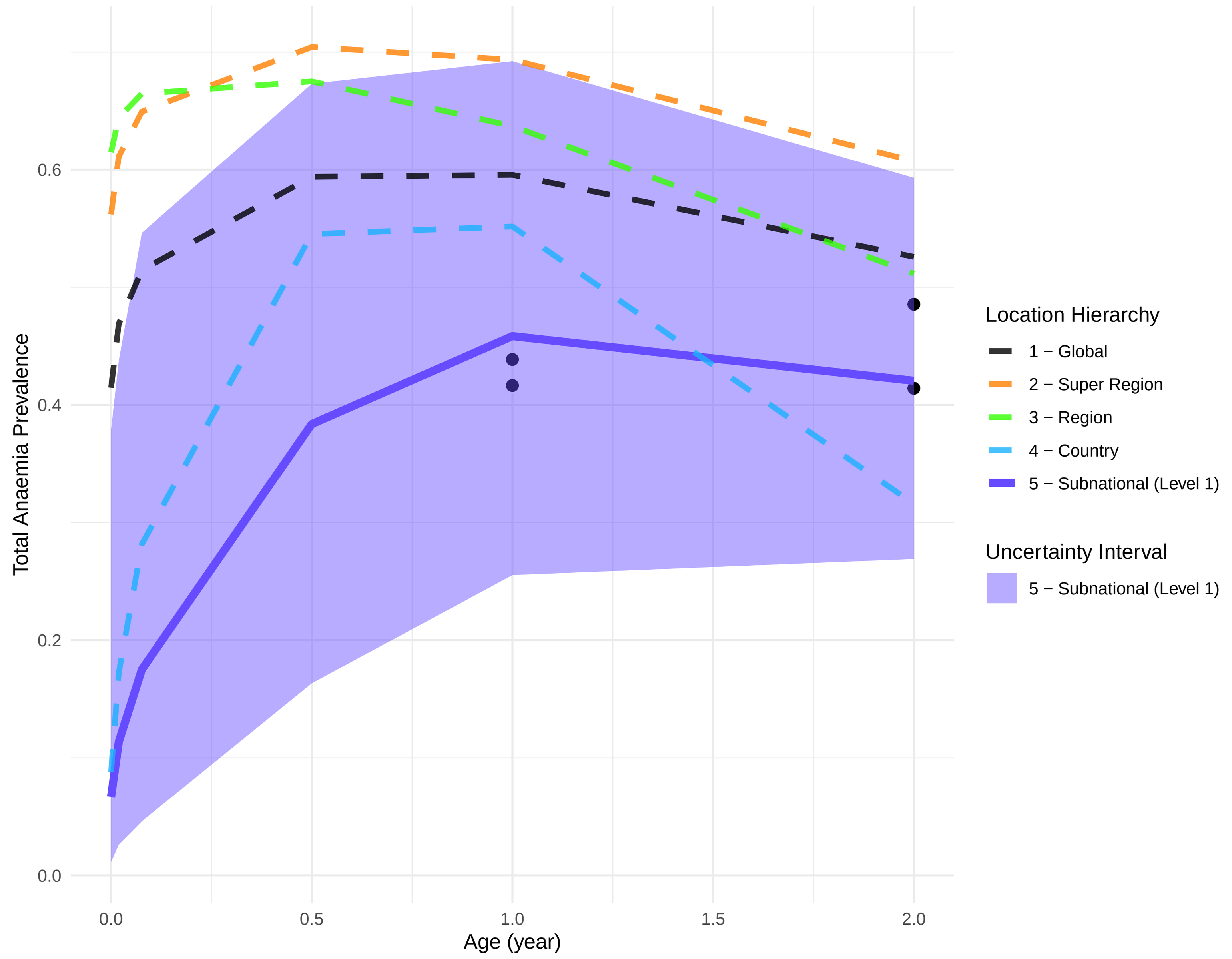

MR−BRT Age/Sex Fits − Males <2 Years Old − Total Anaemia Prevalence − Mombasa
WHO−2001 Adjustment Method

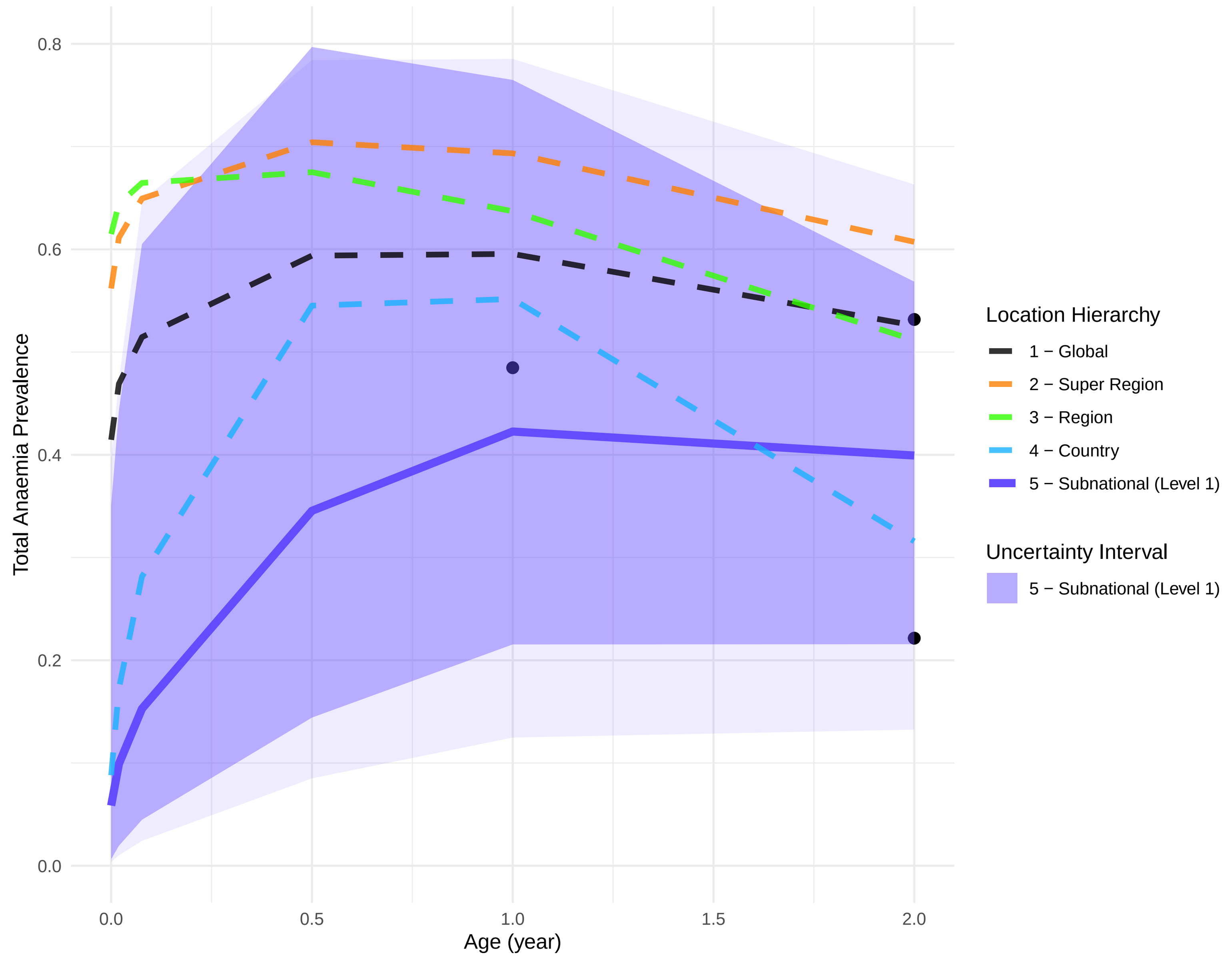

MR−BRT Age/Sex Fits − Males <2 Years Old − Total Anaemia Prevalence − Murang'a
WHO−2001 Adjustment Method

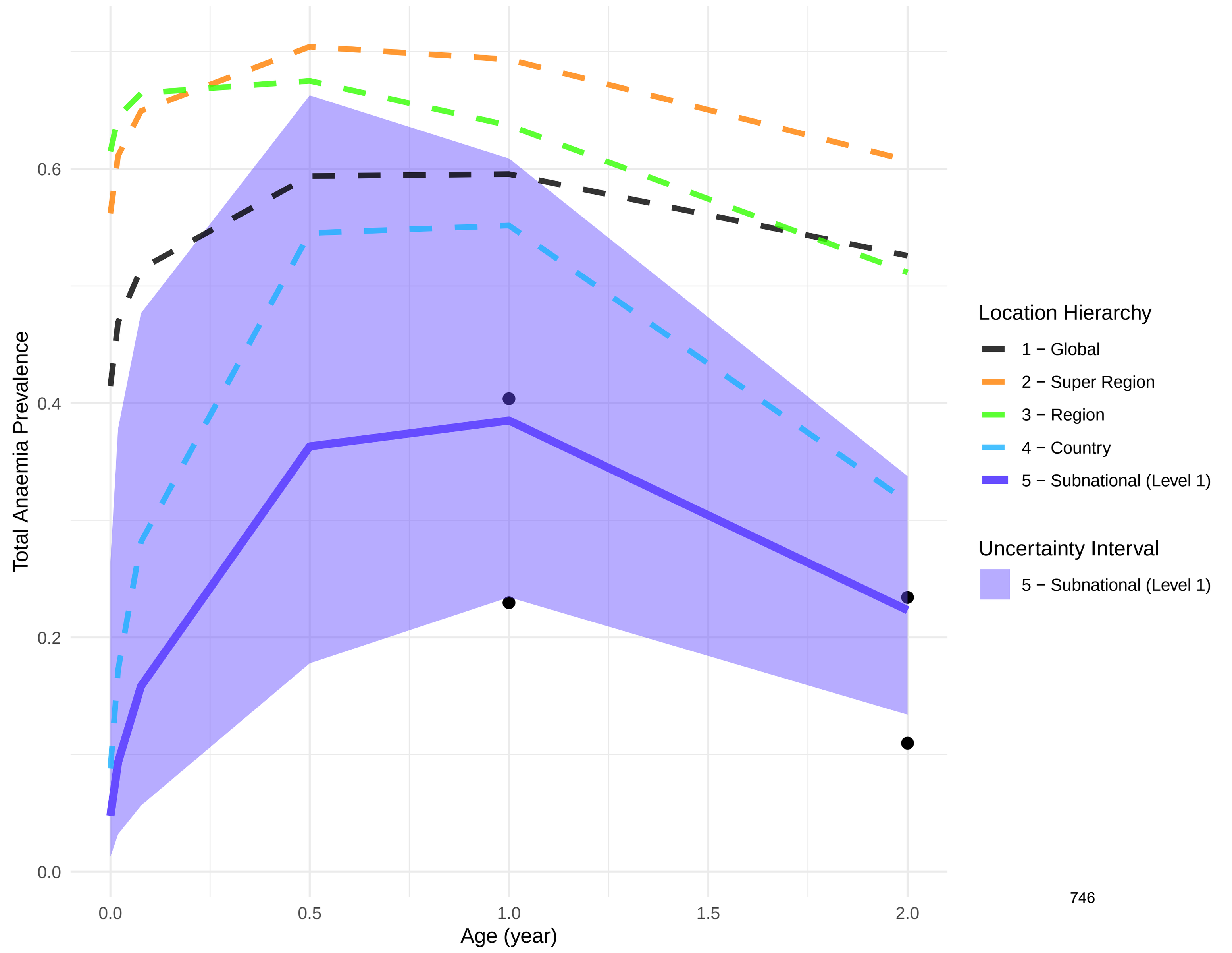

MR−BRT Age/Sex Fits − Males <2 Years Old − Total Anaemia Prevalence − Nairobi
WHO−2001 Adjustment Method

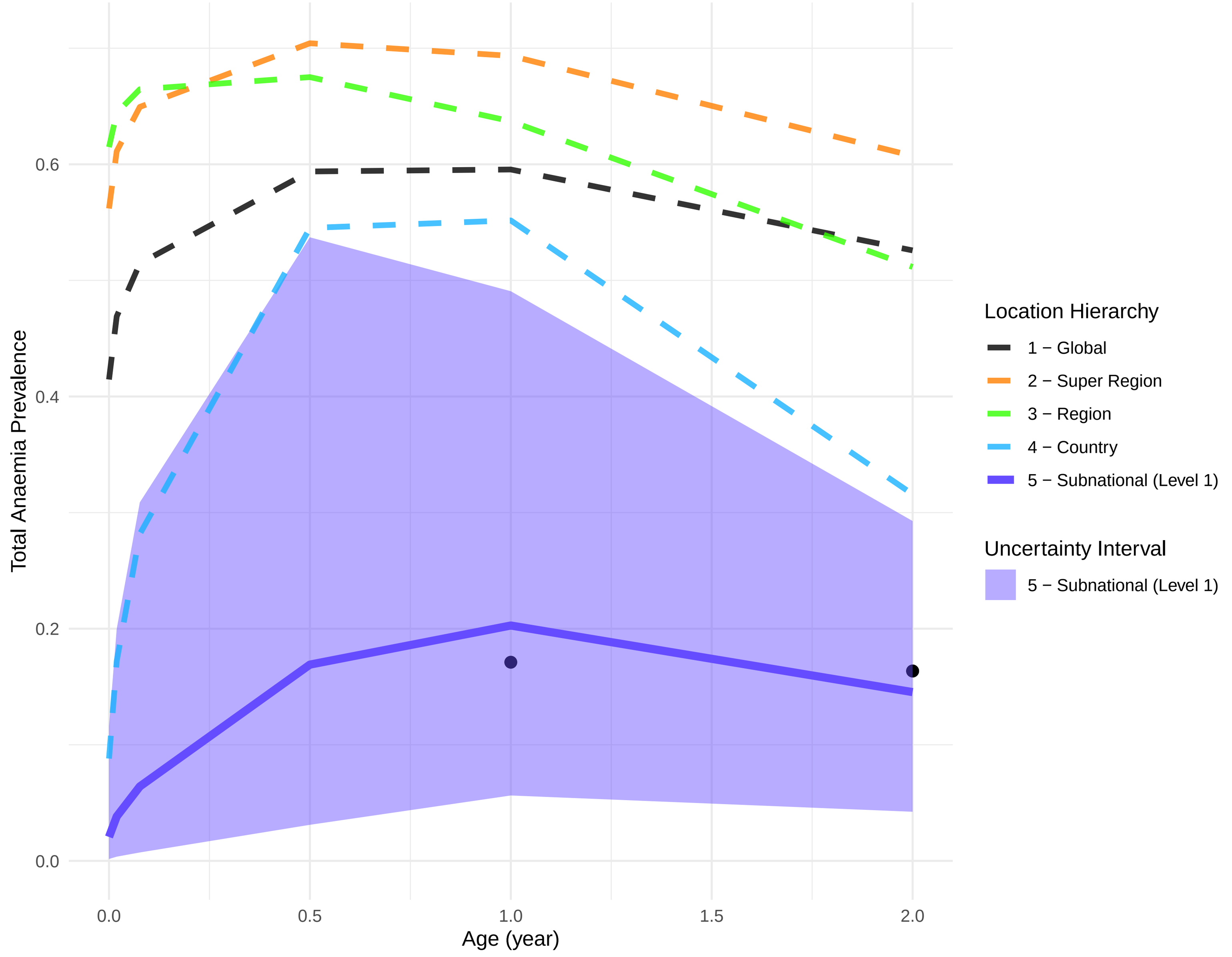

MR−BRT Age/Sex Fits − Males <2 Years Old − Total Anaemia Prevalence − Nakuru
WHO−2001 Adjustment Method

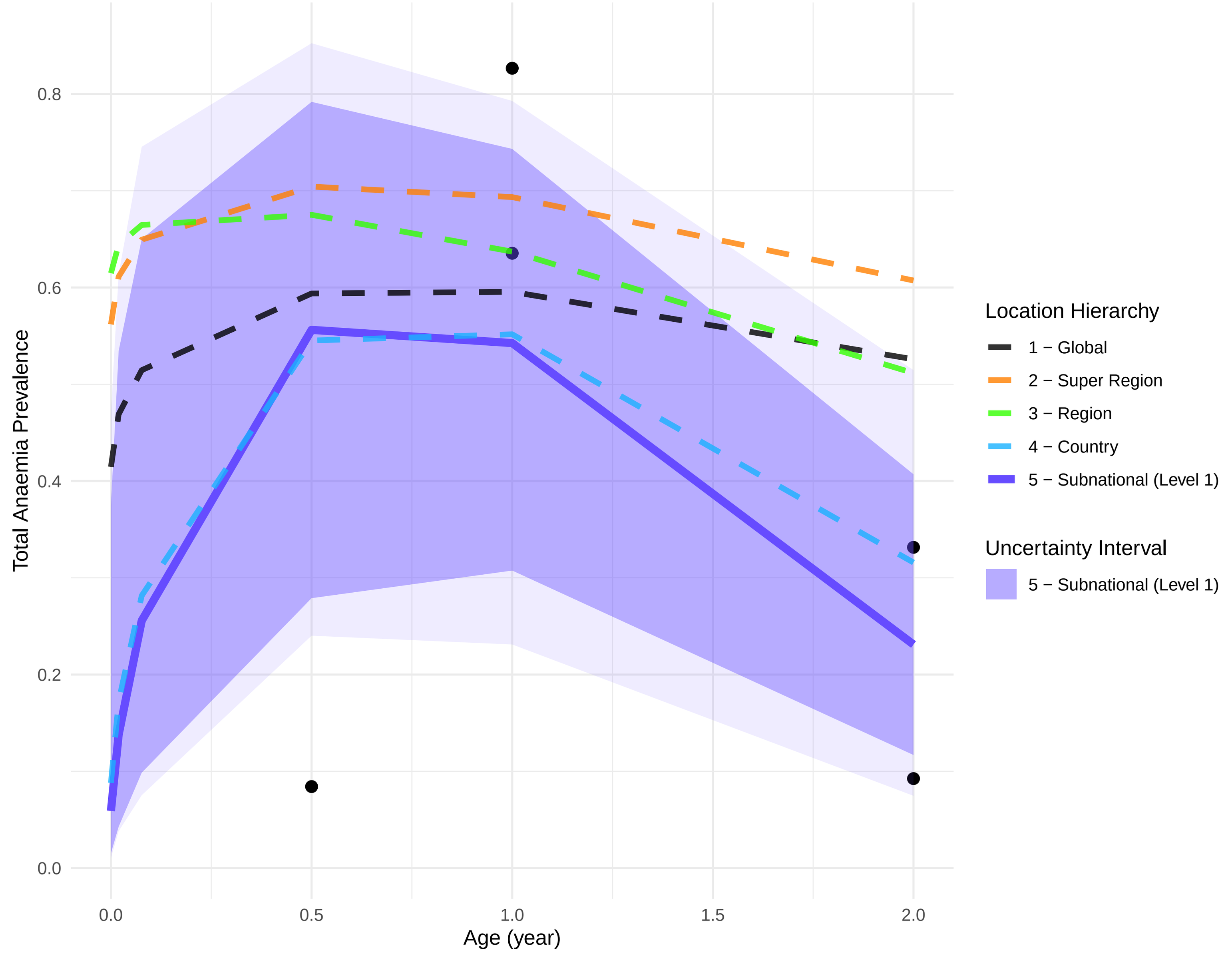

MR−BRT Age/Sex Fits − Males <2 Years Old − Total Anaemia Prevalence − Nandi
WHO−2001 Adjustment Method

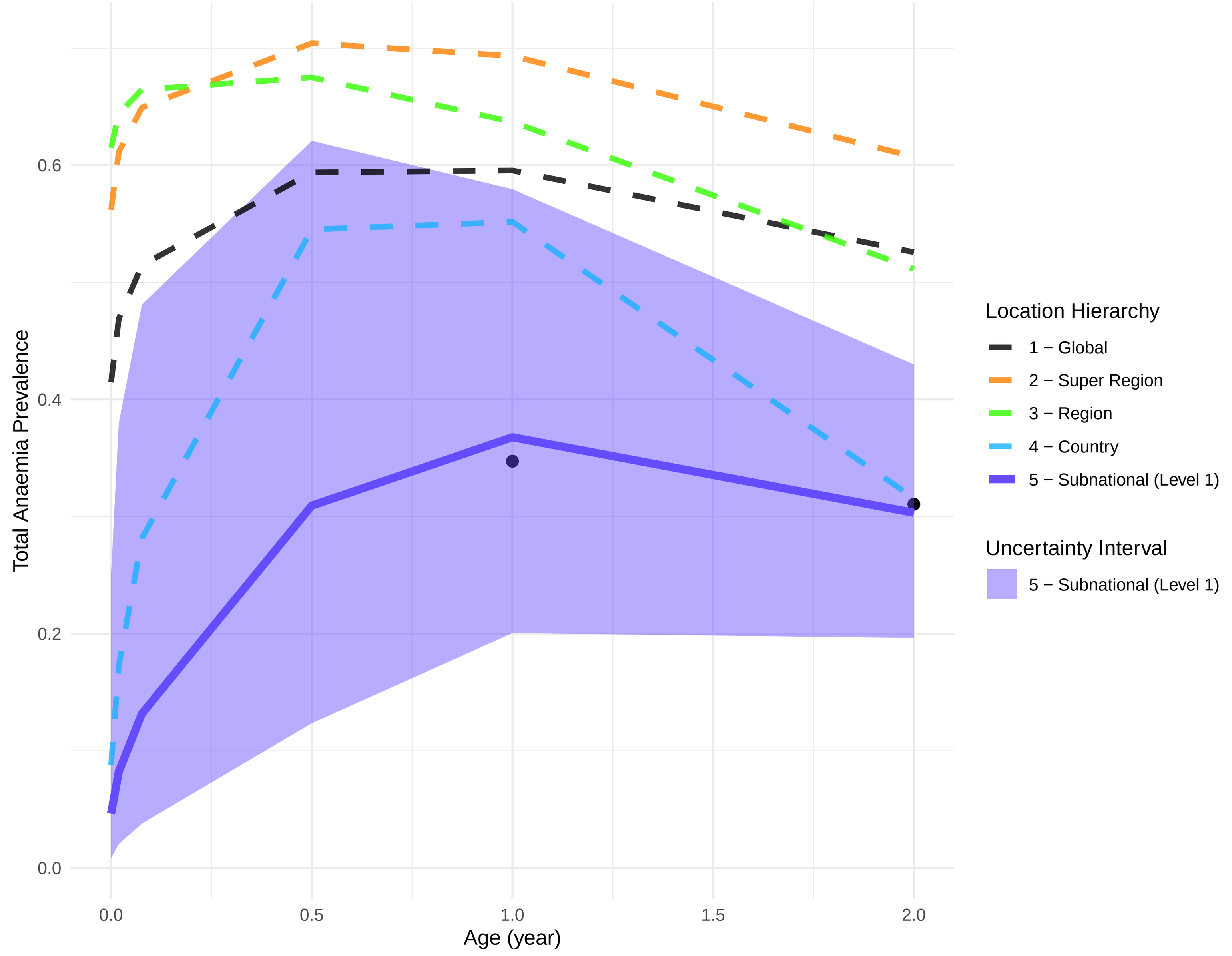

MR−BRT Age/Sex Fits − Males <2 Years Old − Total Anaemia Prevalence − Narok
WHO−2001 Adjustment Method

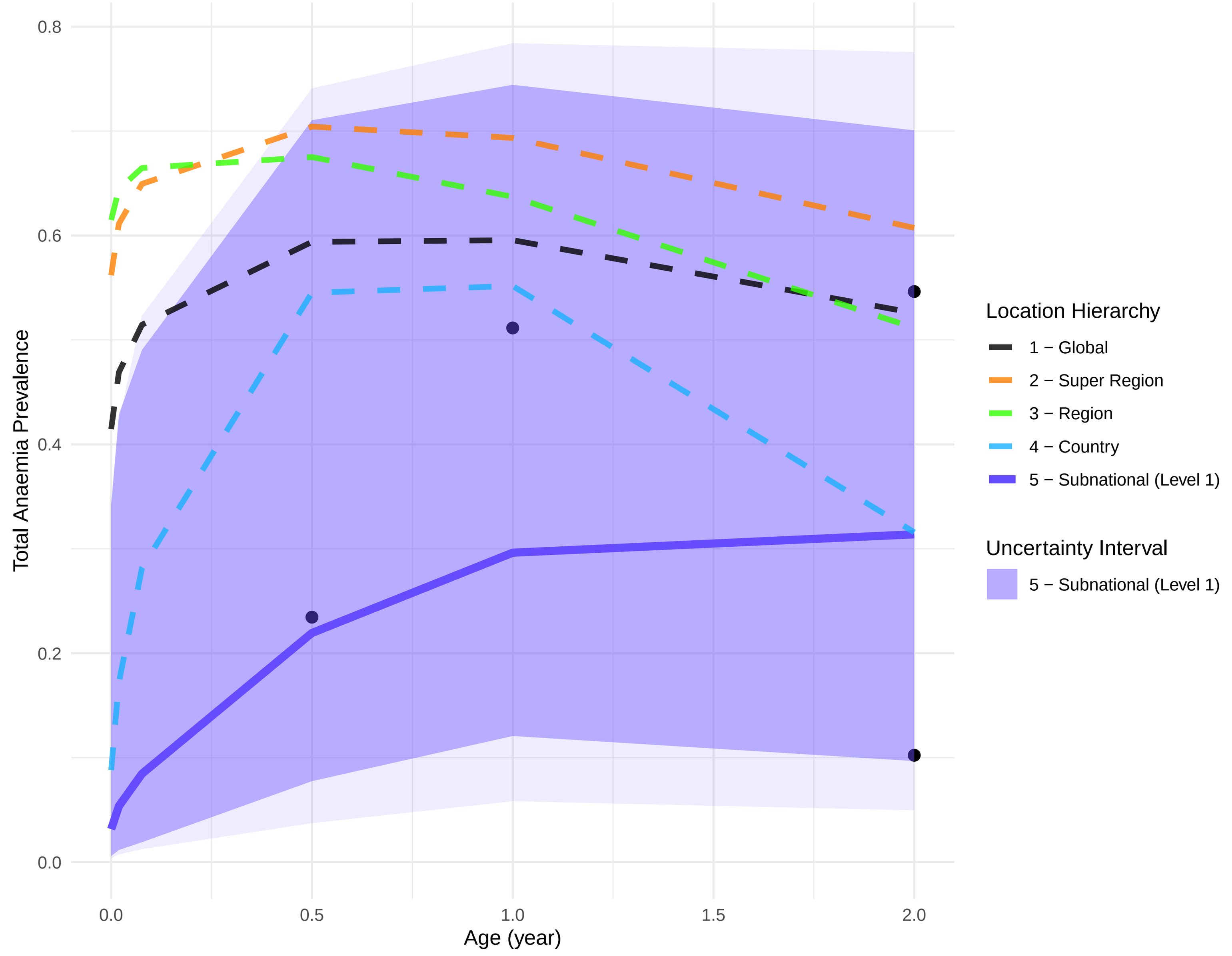

MR−BRT Age/Sex Fits − Males <2 Years Old − Total Anaemia Prevalence − Nyamira
WHO−2001 Adjustment Method

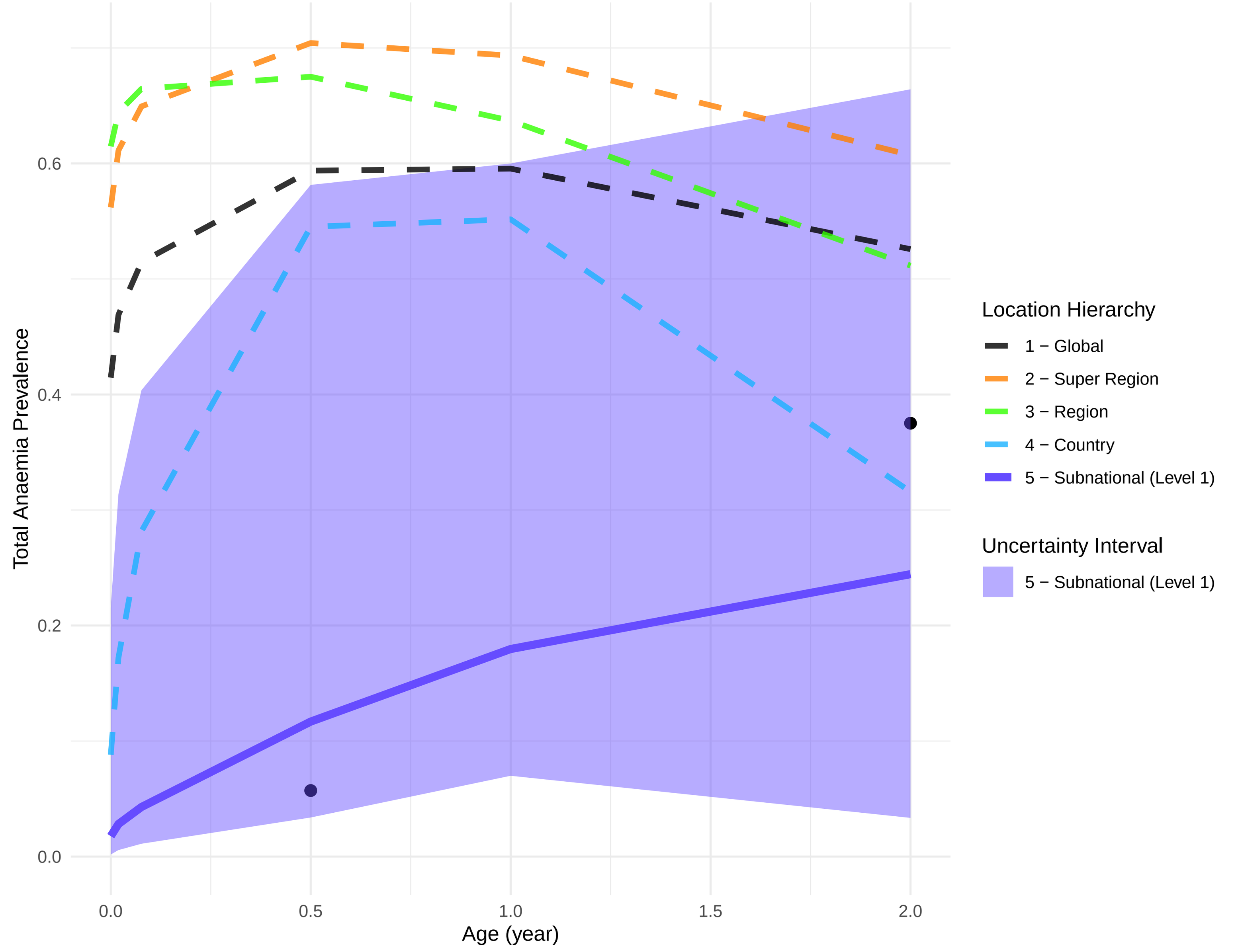

MR−BRT Age/Sex Fits − Males <2 Years Old − Total Anaemia Prevalence − Nyandarua
WHO−2001 Adjustment Method

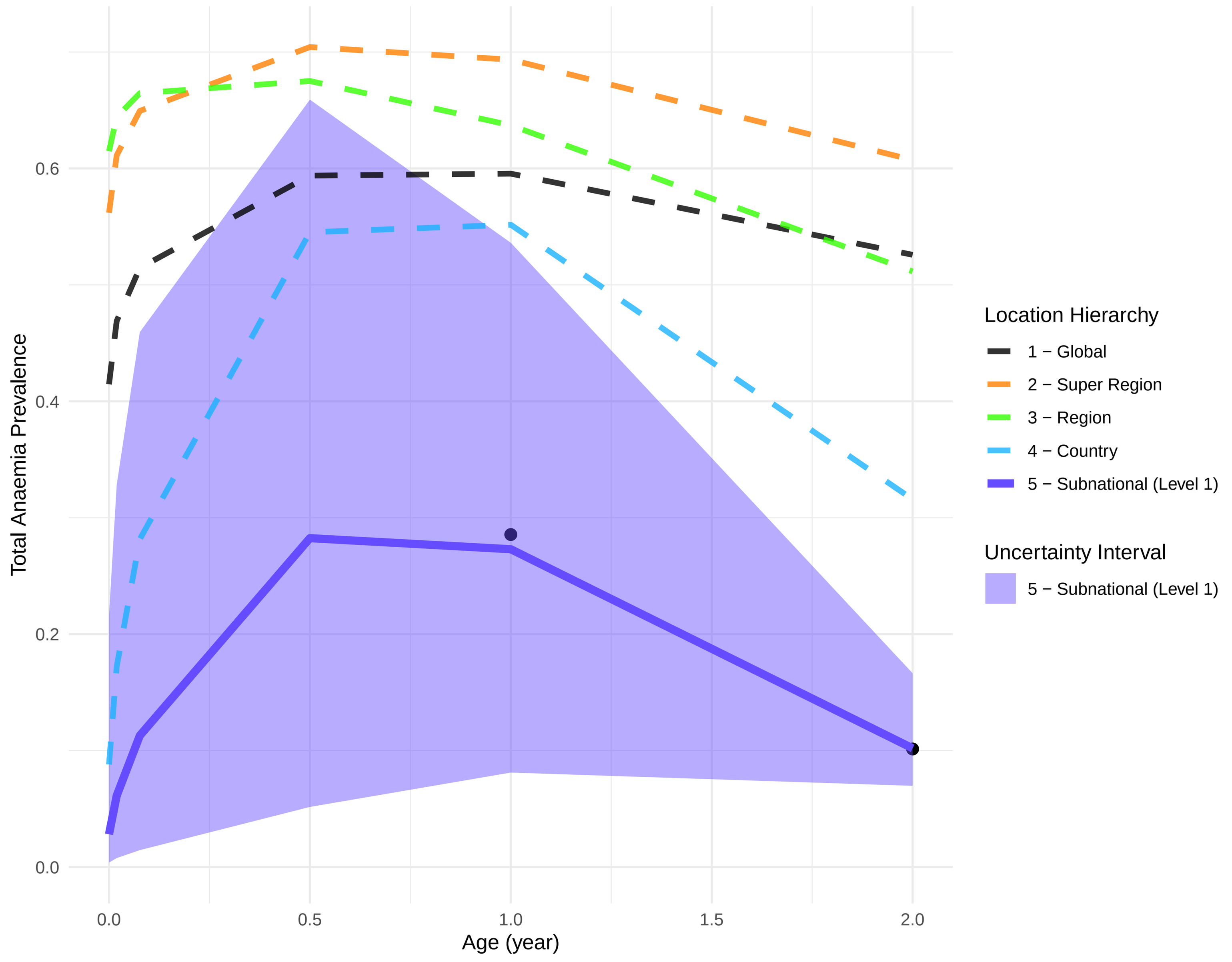

MR−BRT Age/Sex Fits − Males <2 Years Old − Total Anaemia Prevalence − Nyeri
WHO−2001 Adjustment Method

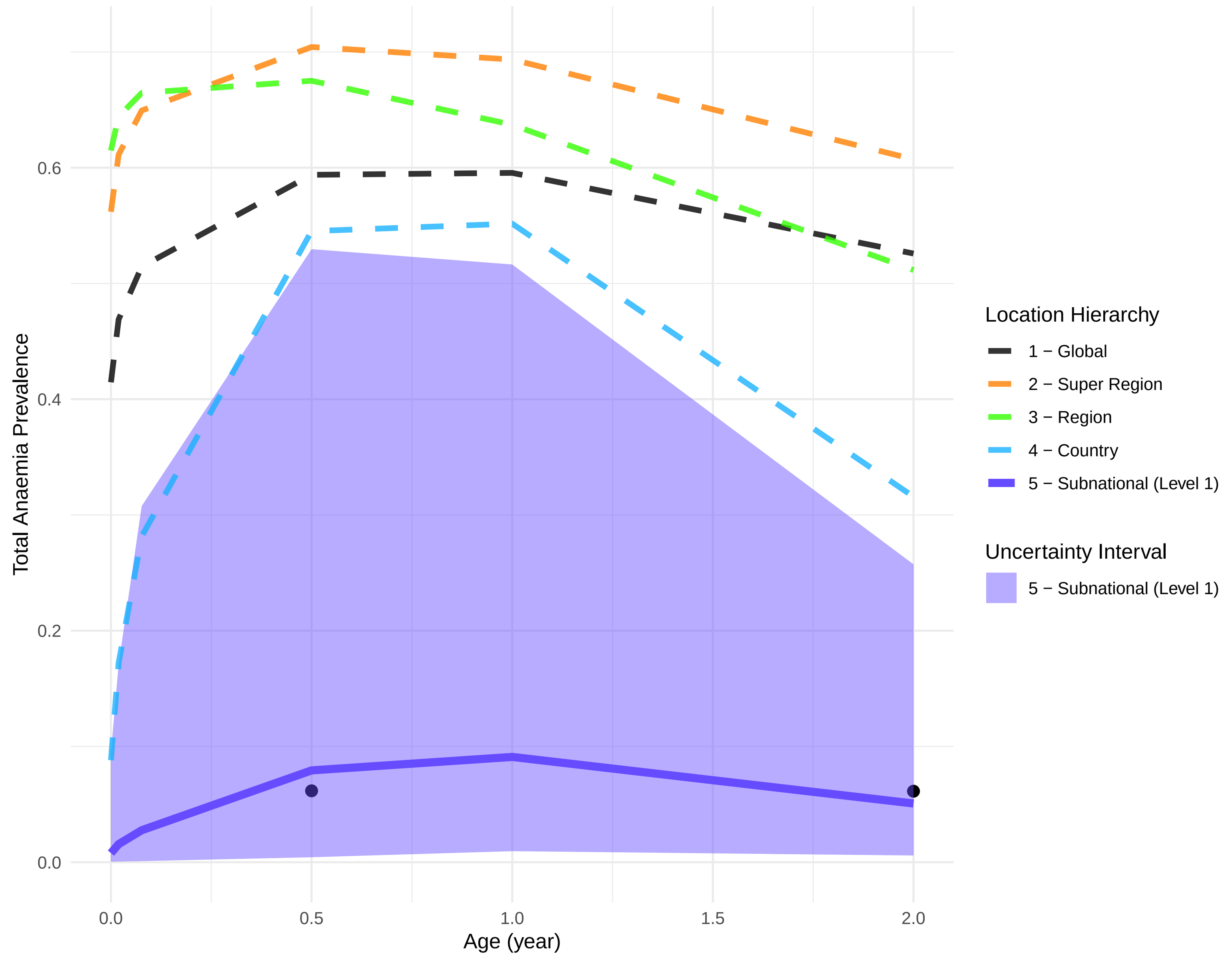

MR−BRT Age/Sex Fits − Males <2 Years Old − Total Anaemia Prevalence − Samburu
WHO−2001 Adjustment Method

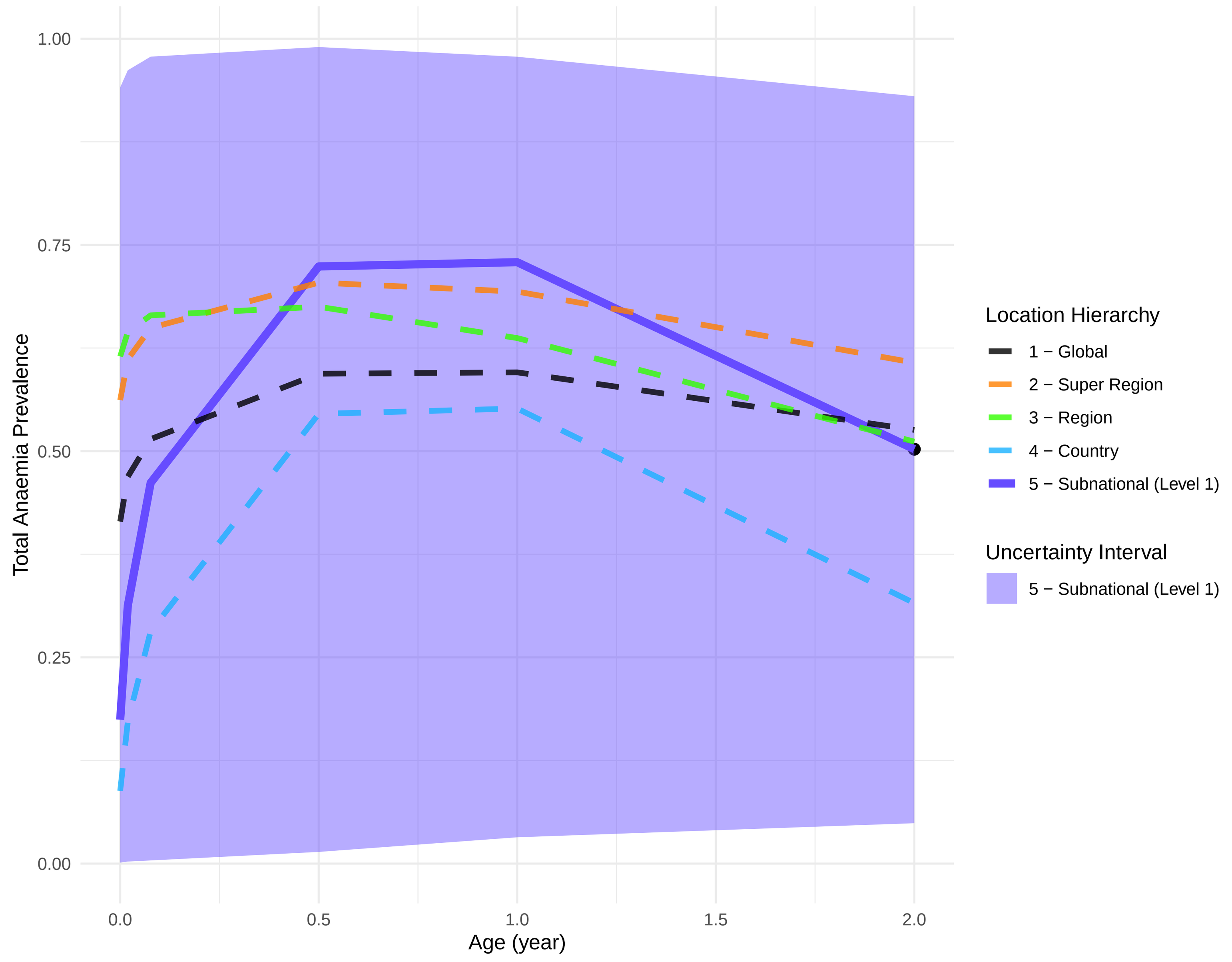

MR−BRT Age/Sex Fits − Males <2 Years Old − Total Anaemia Prevalence − Siaya
WHO−2001 Adjustment Method

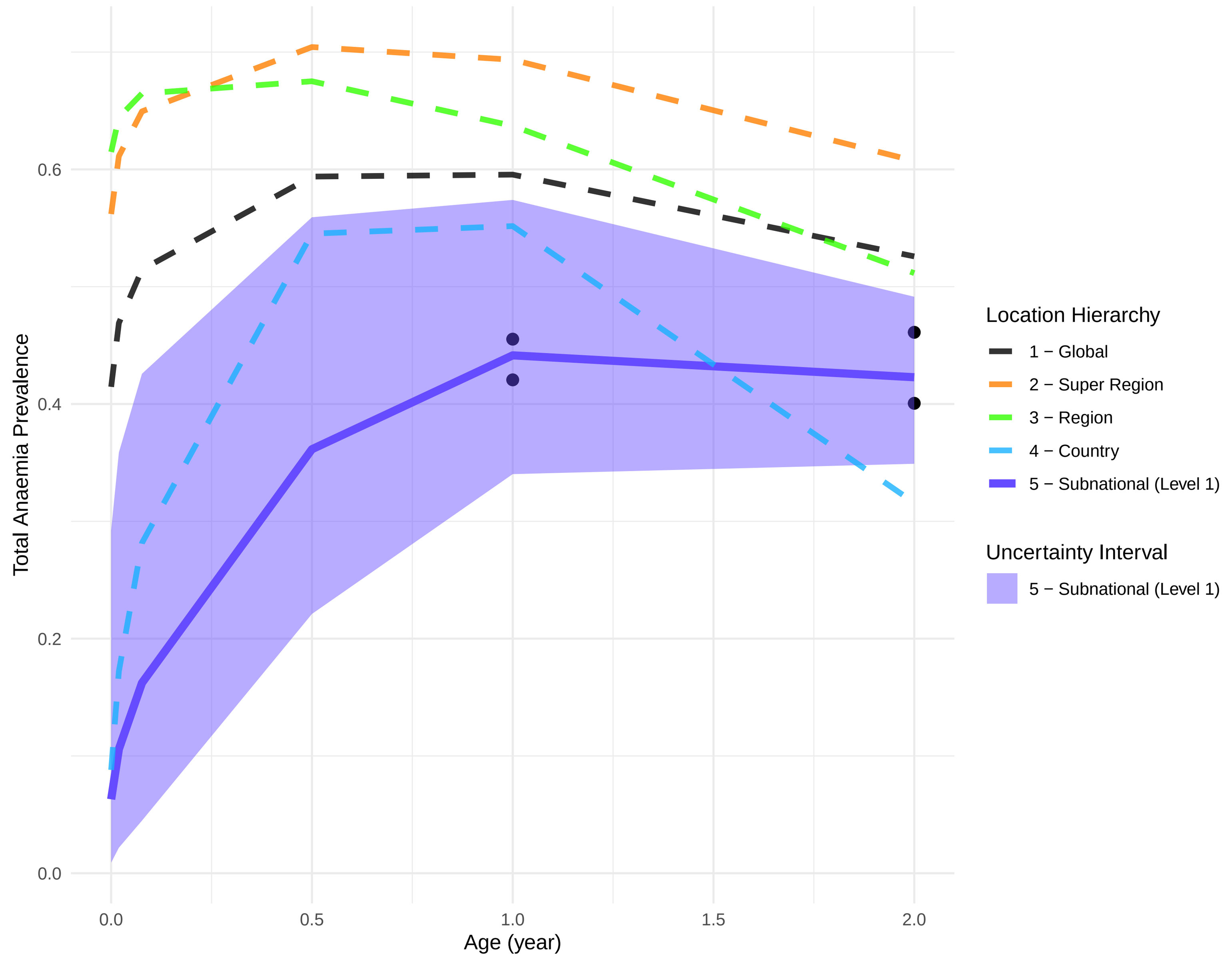

MR−BRT Age/Sex Fits − Males <2 Years Old − Total Anaemia Prevalence − Taita Taveta
WHO−2001 Adjustment Method

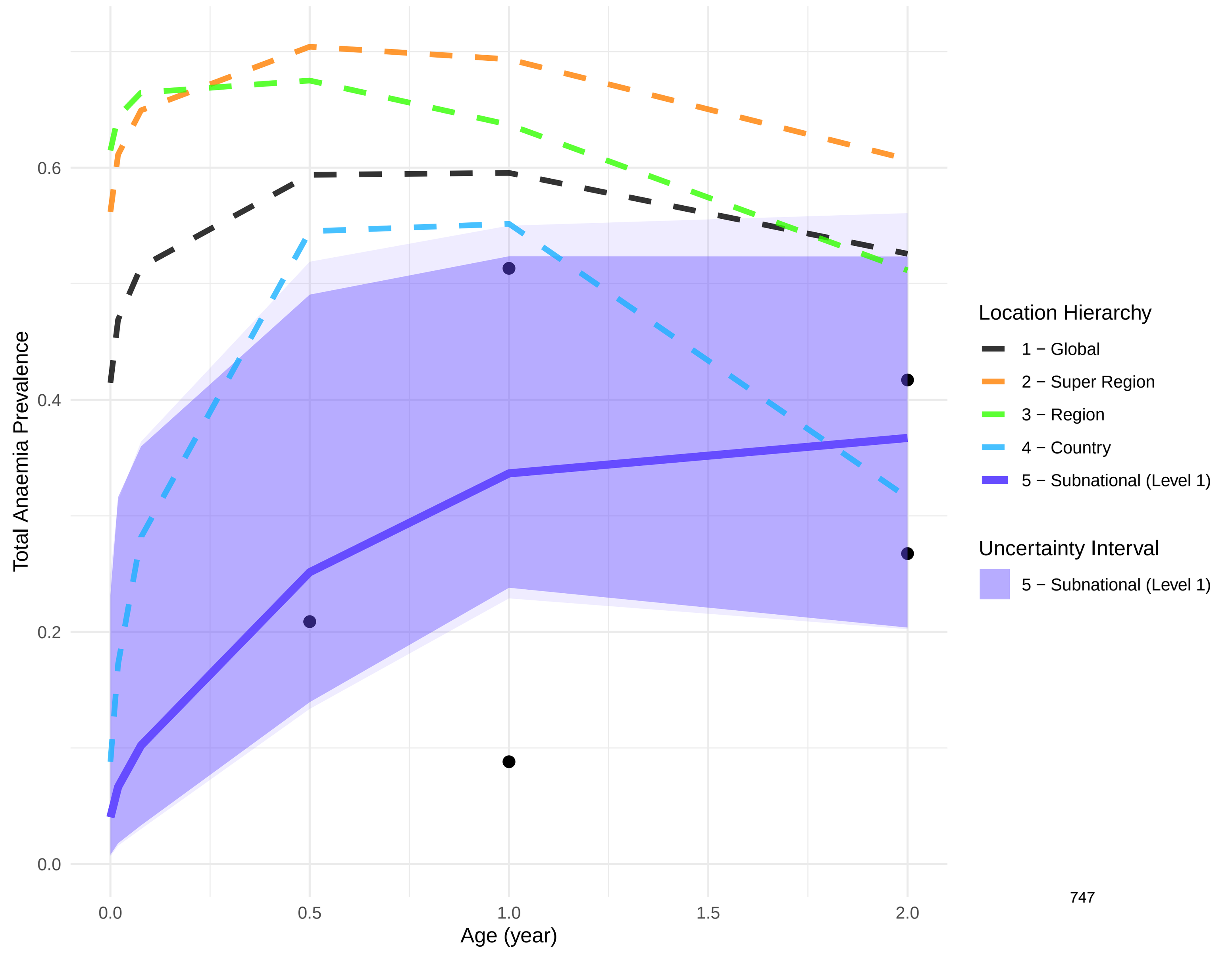

MR−BRT Age/Sex Fits − Males <2 Years Old − Total Anaemia Prevalence − Tana River
WHO−2001 Adjustment Method

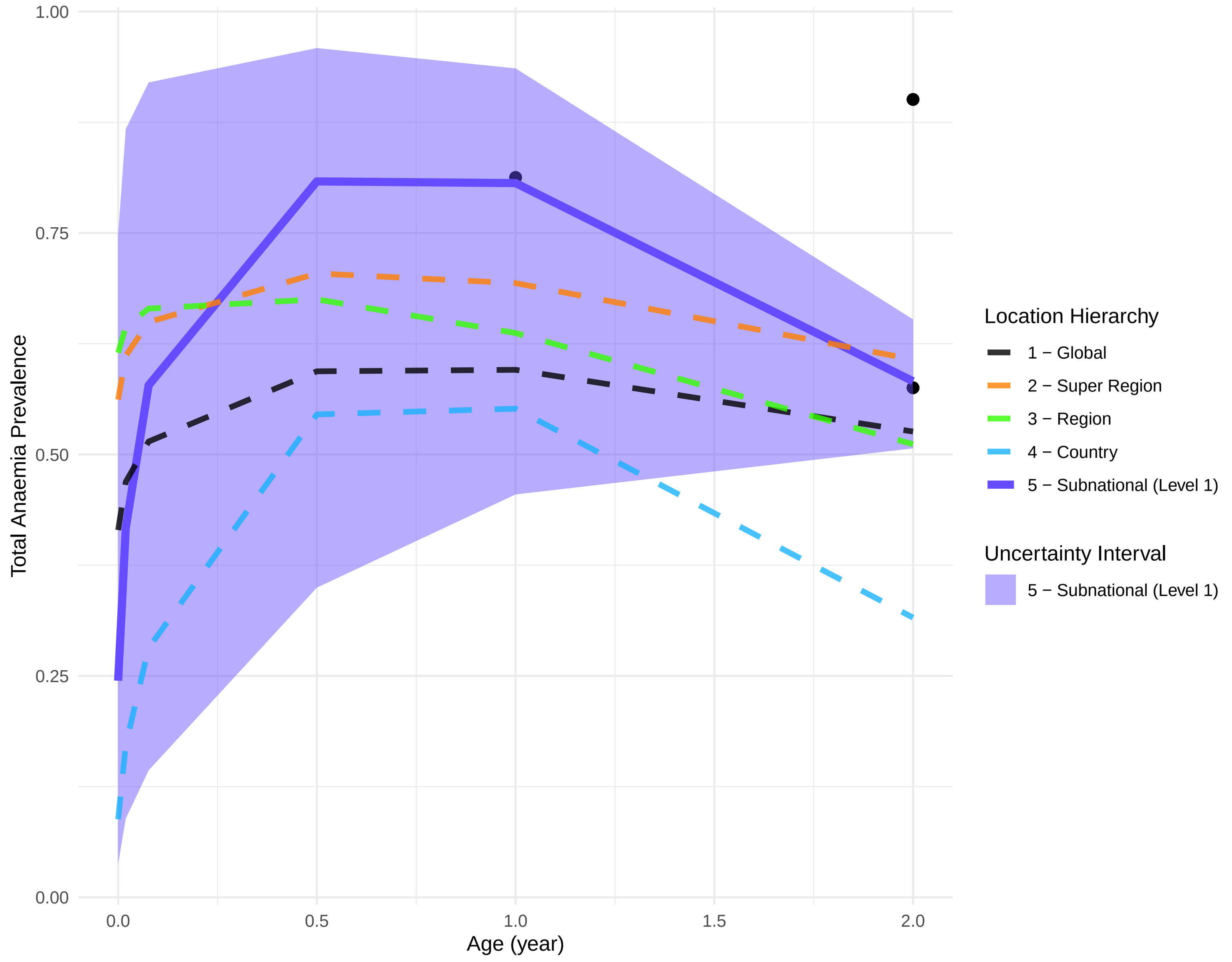

MR−BRT Age/Sex Fits − Males <2 Years Old − Total Anaemia Prevalence − Tharaka Nithi
WHO−2001 Adjustment Method

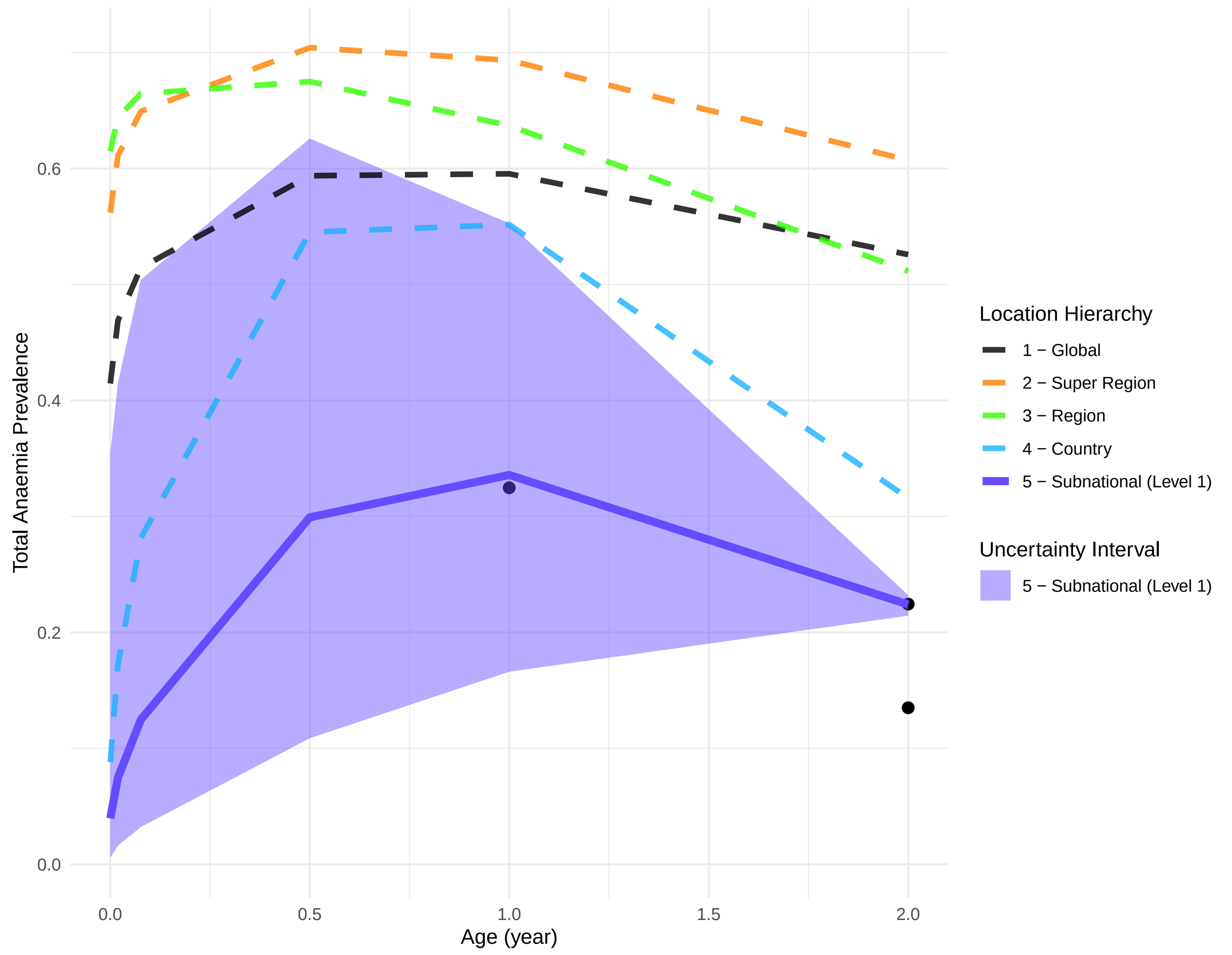

MR−BRT Age/Sex Fits − Males <2 Years Old − Total Anaemia Prevalence − Trans Nzoia
WHO−2001 Adjustment Method

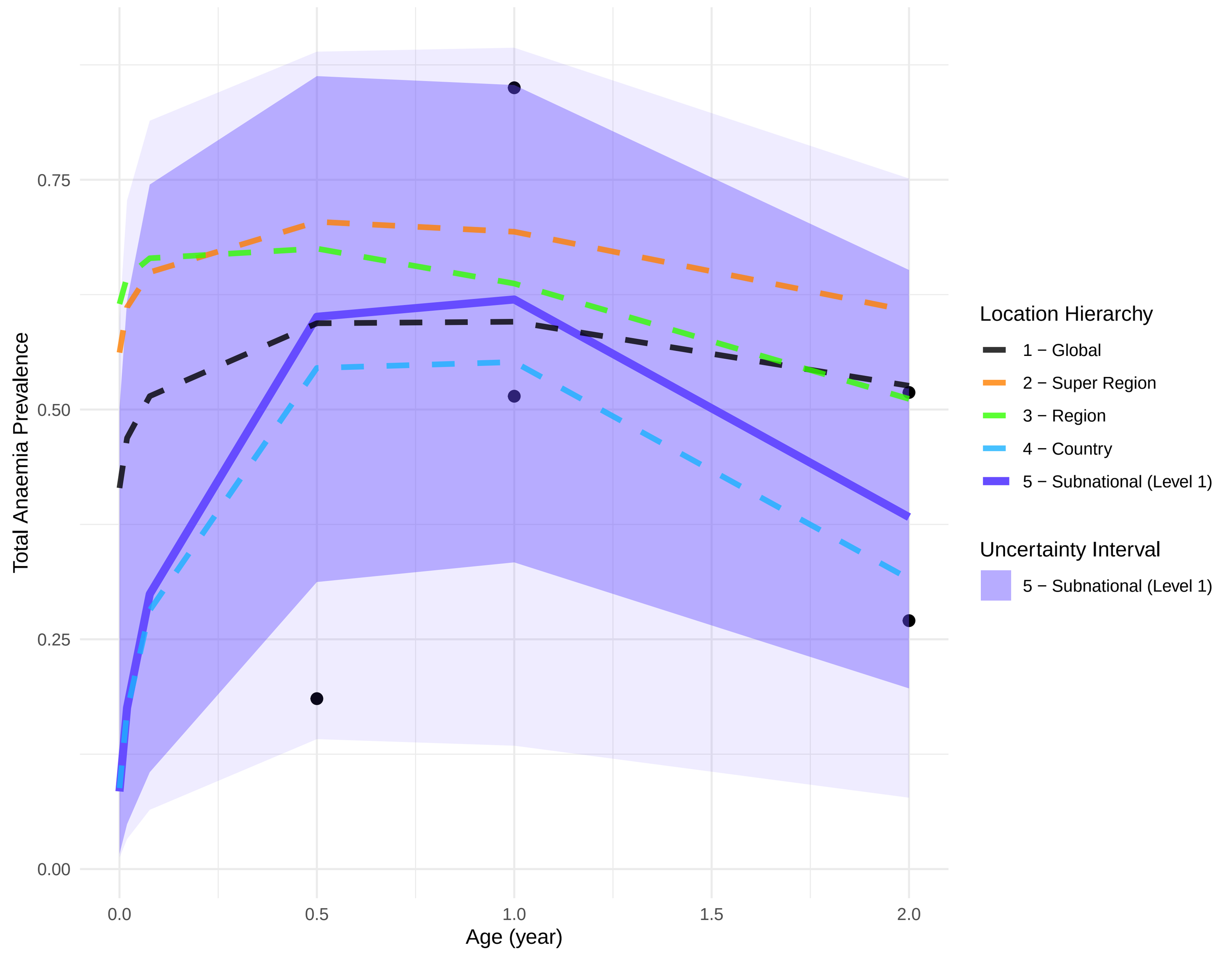

MR−BRT Age/Sex Fits − Males <2 Years Old − Total Anaemia Prevalence − Turkana
WHO−2001 Adjustment Method

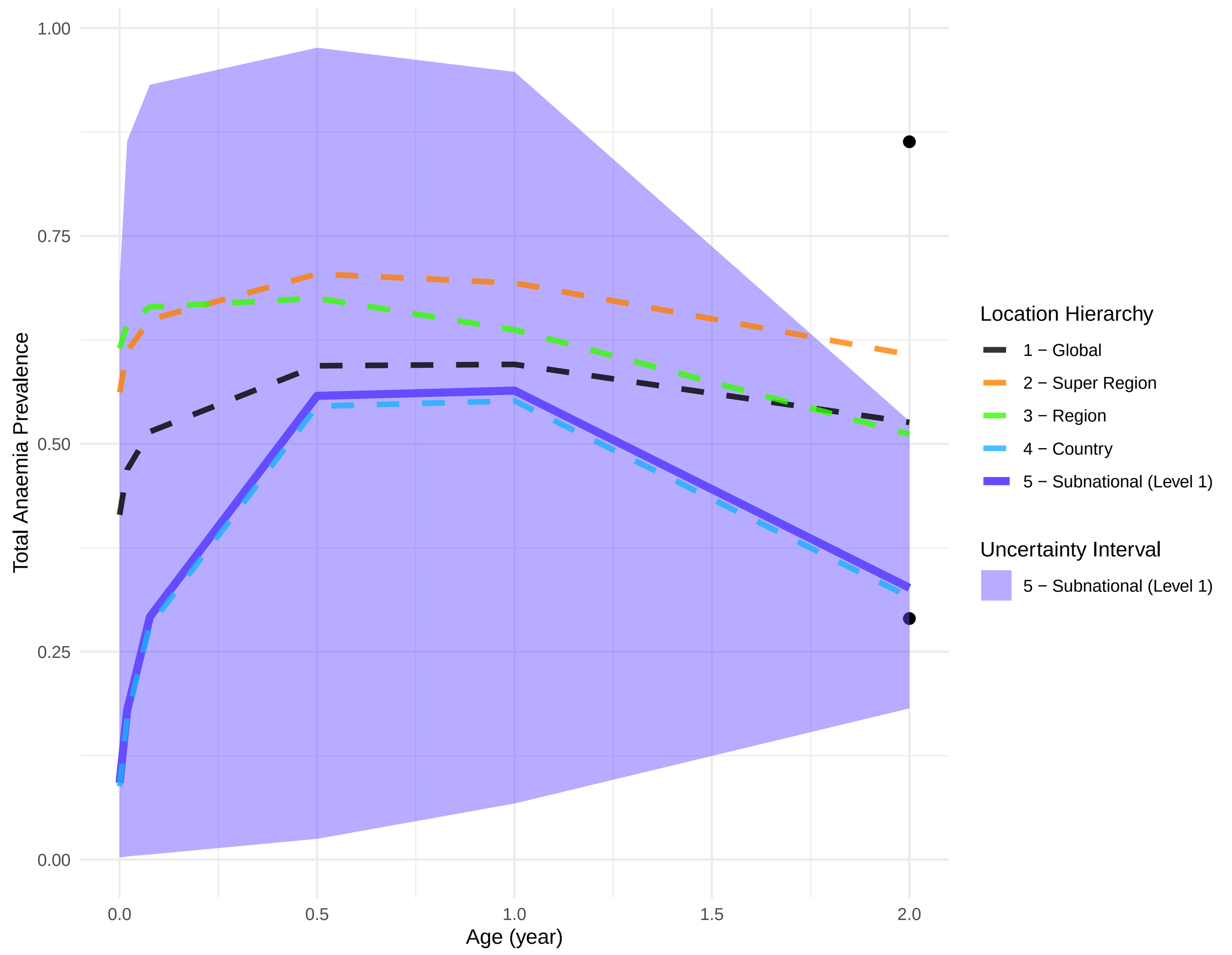

MR−BRT Age/Sex Fits − Males <2 Years Old − Total Anaemia Prevalence − Uasin Gishu
WHO−2001 Adjustment Method

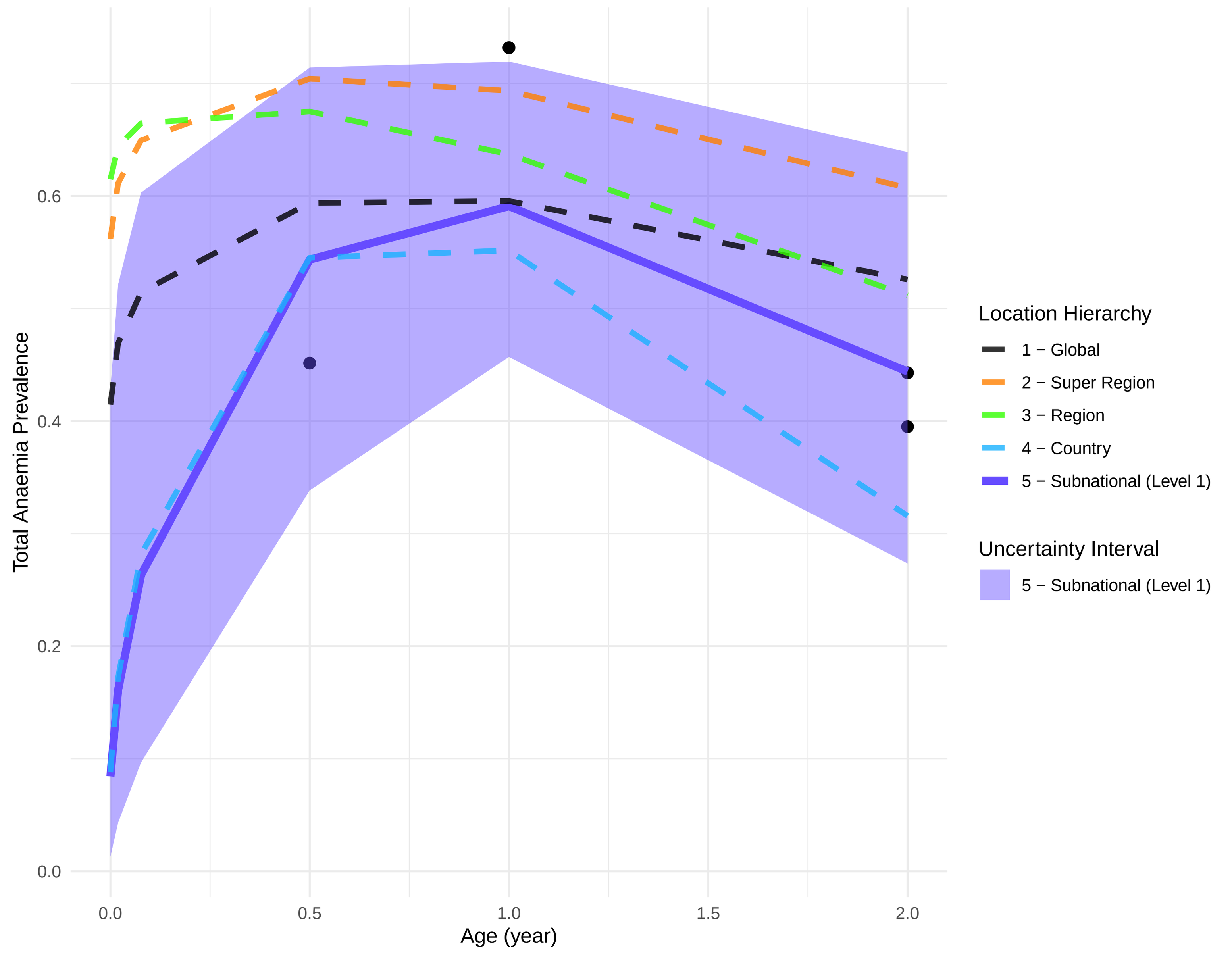

MR−BRT Age/Sex Fits − Males <2 Years Old − Total Anaemia Prevalence − Vihiga
WHO−2001 Adjustment Method

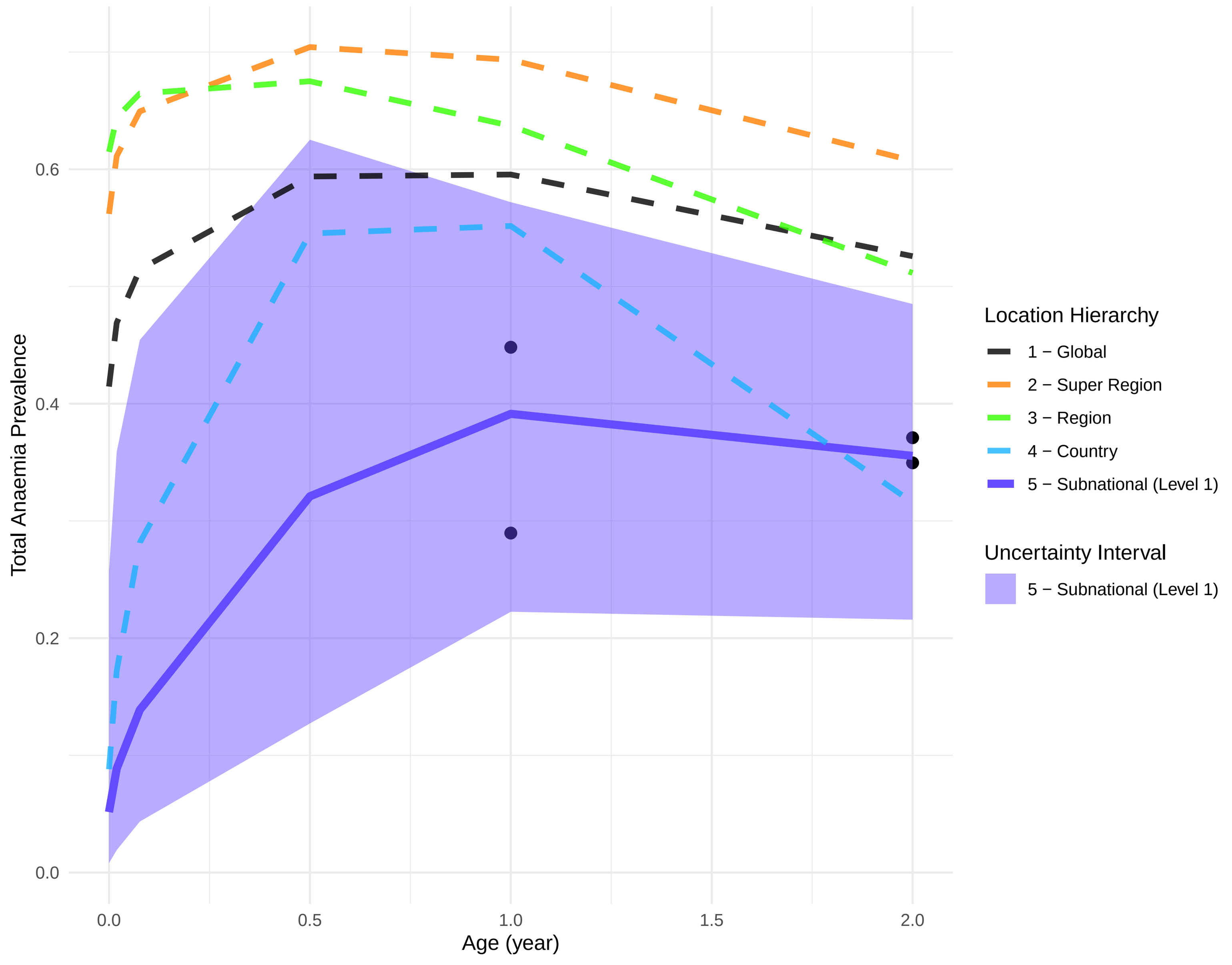

MR−BRT Age/Sex Fits − Males <2 Years Old − Total Anaemia Prevalence − Wajir
WHO−2001 Adjustment Method

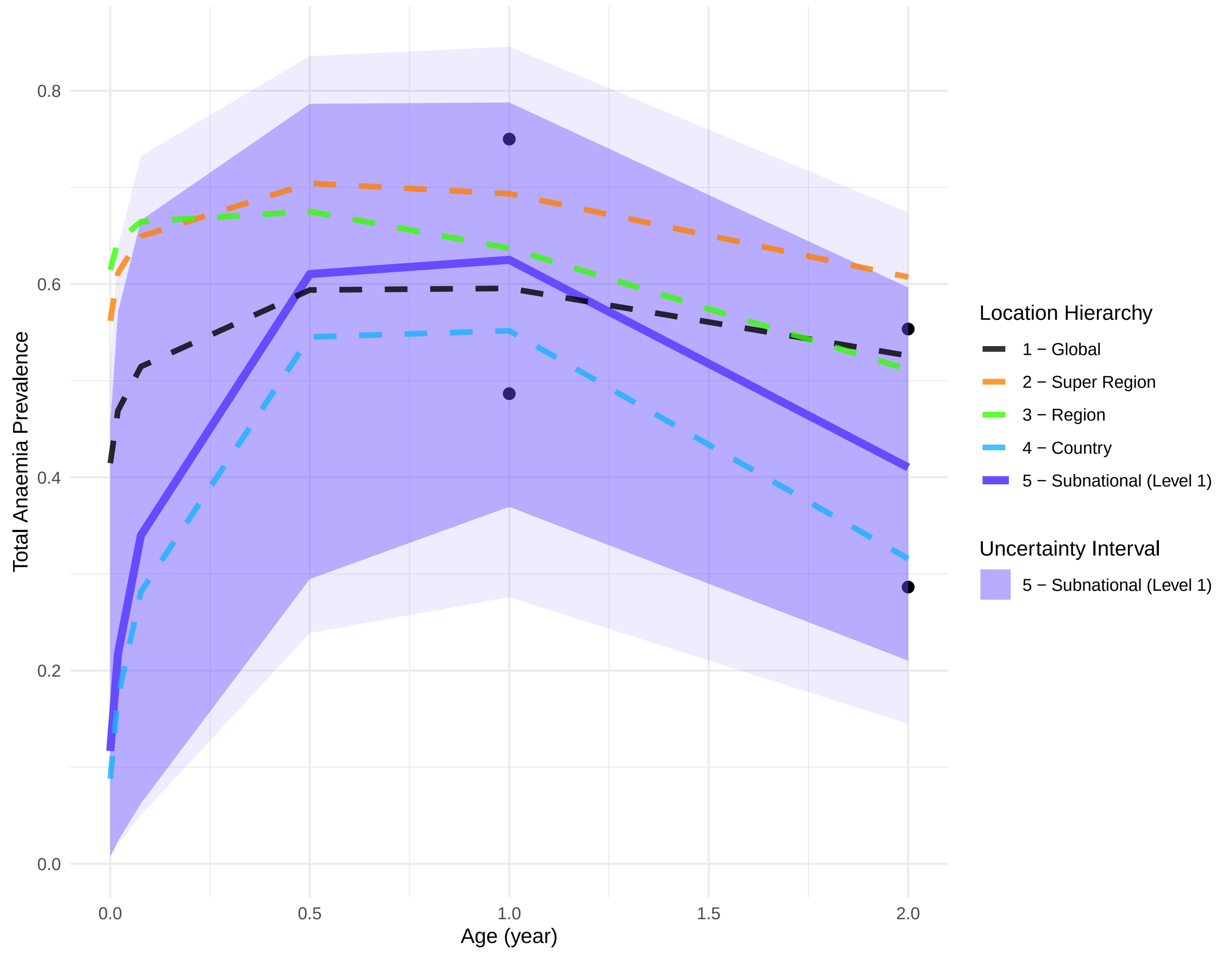

MR−BRT Age/Sex Fits − Males <2 Years Old − Total Anaemia Prevalence − West Pokot
WHO−2001 Adjustment Method

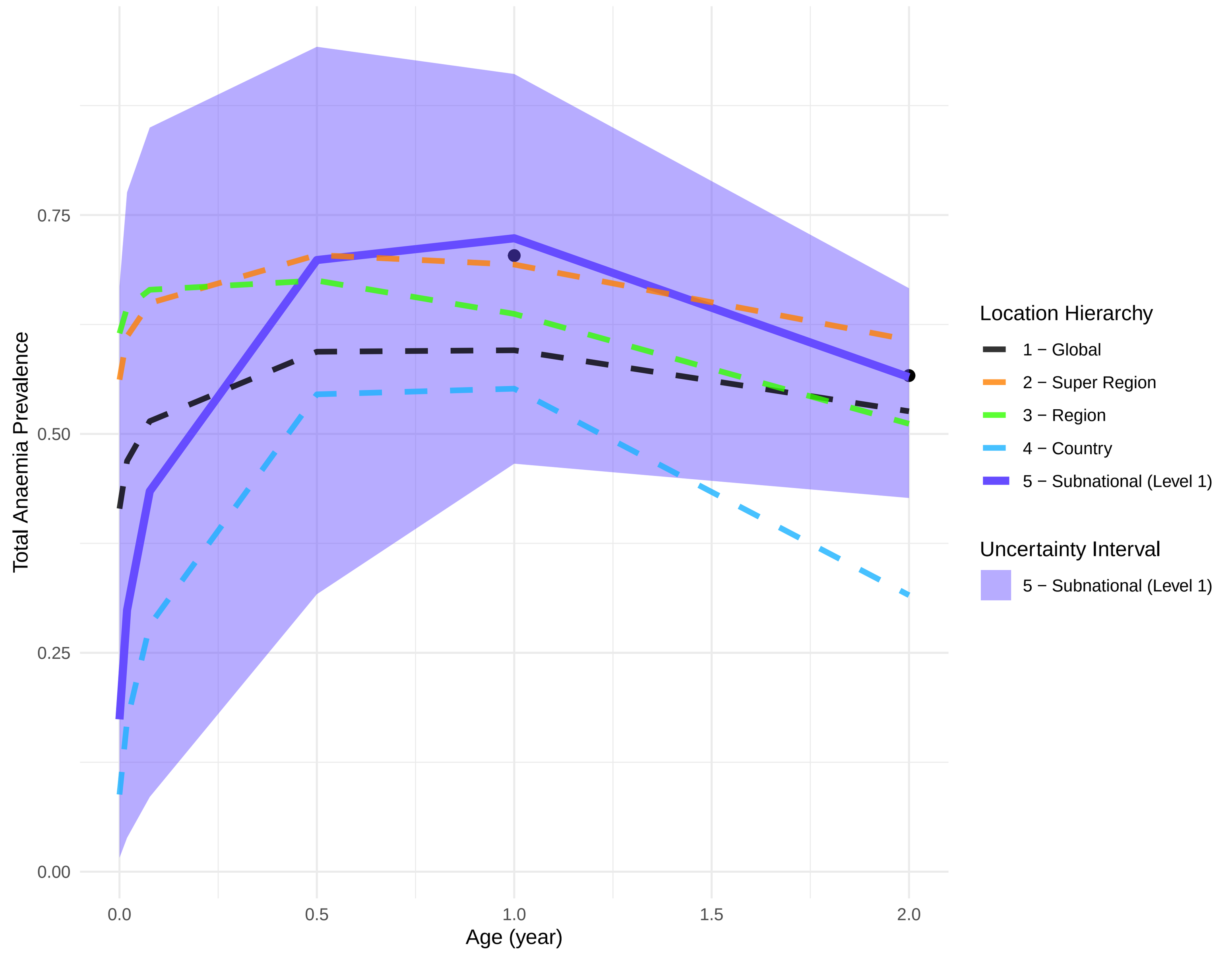

MR−BRT Age/Sex Fits − Males <2 Years Old − Total Anaemia Prevalence − Madagascar
WHO−2001 Adjustment Method

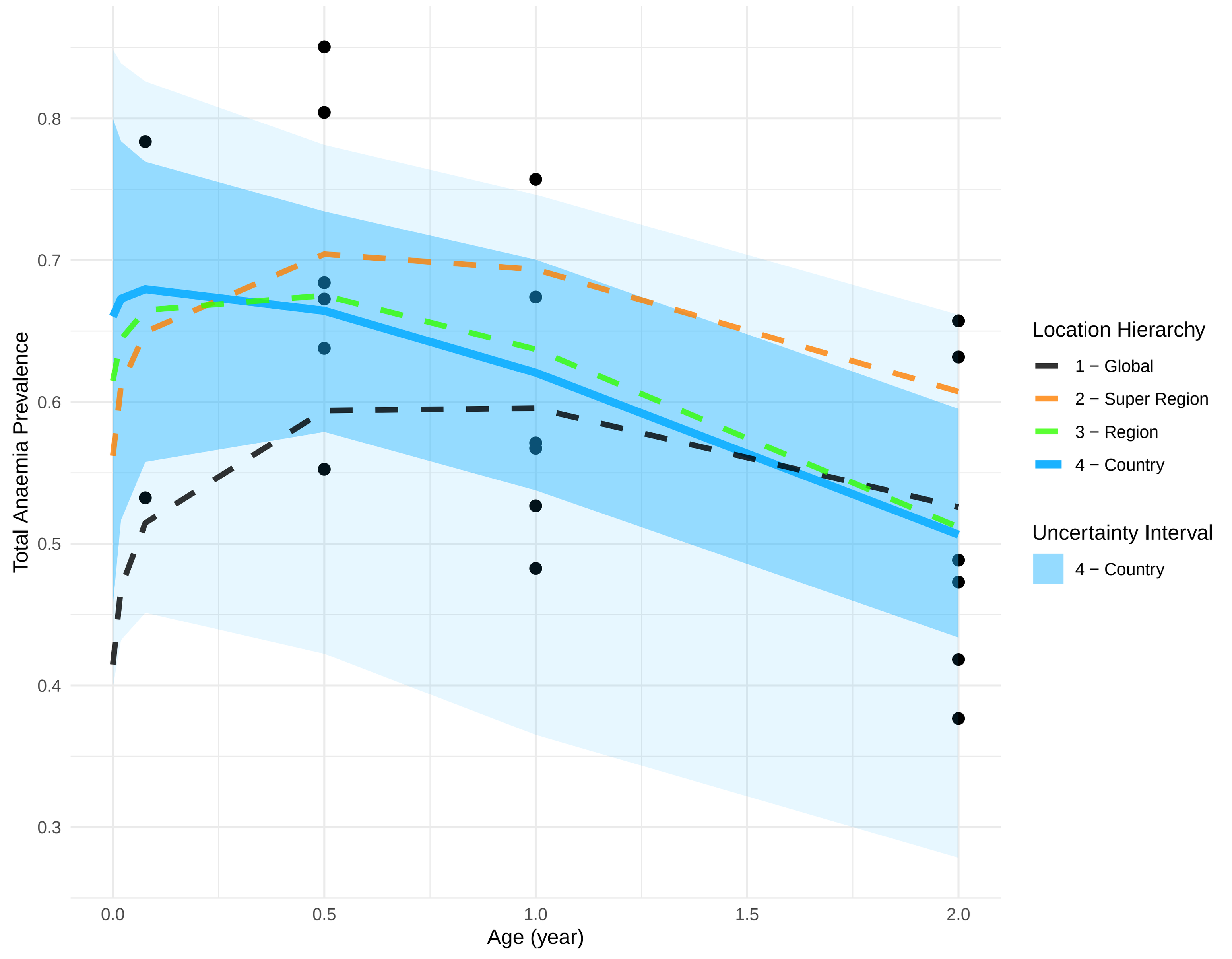

MR−BRT Age/Sex Fits − Males <2 Years Old − Total Anaemia Prevalence − Malawi
WHO−2001 Adjustment Method

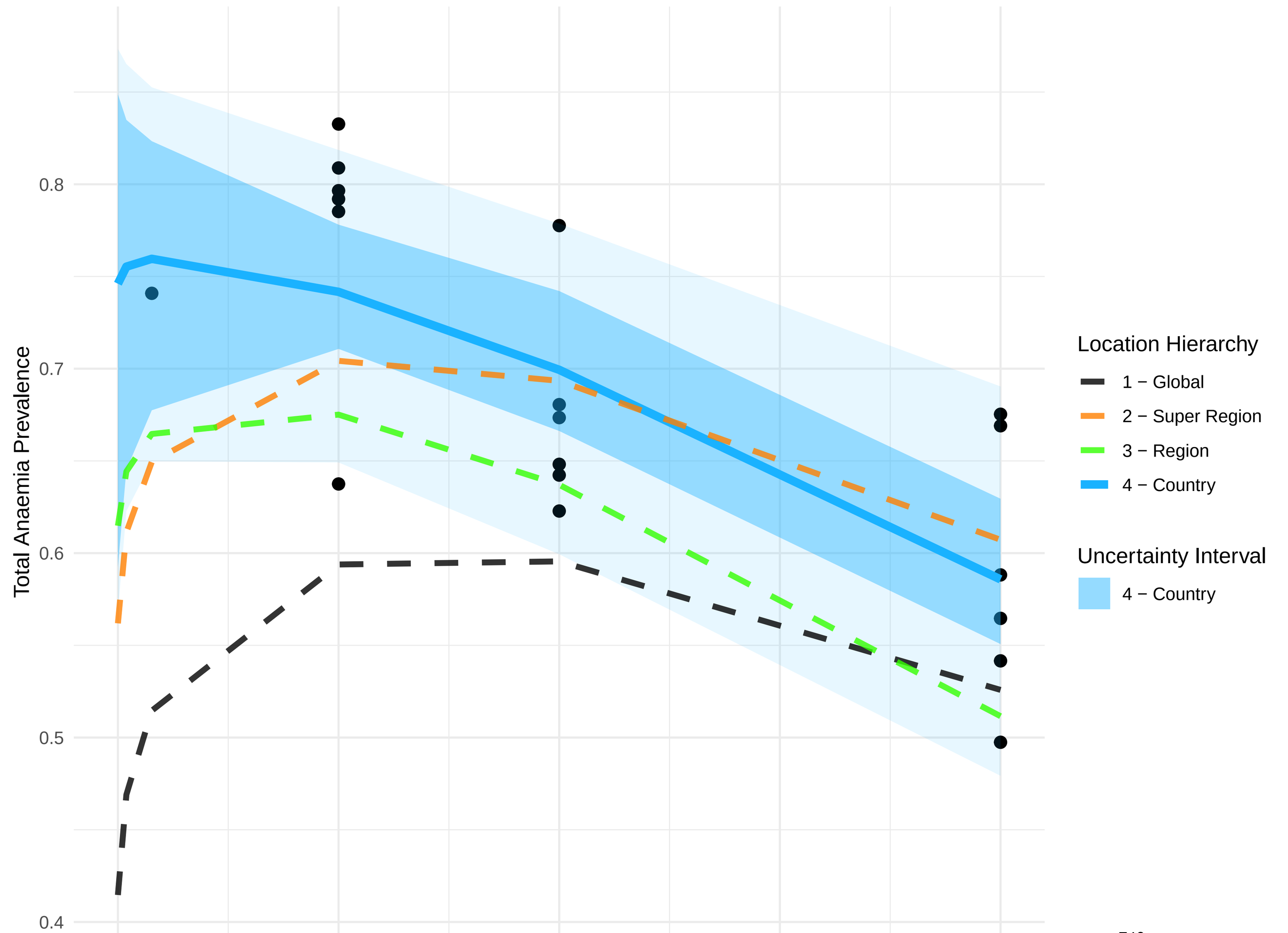

MR−BRT Age/Sex Fits − Males <2 Years Old − Total Anaemia Prevalence − Mozambique
WHO−2001 Adjustment Method

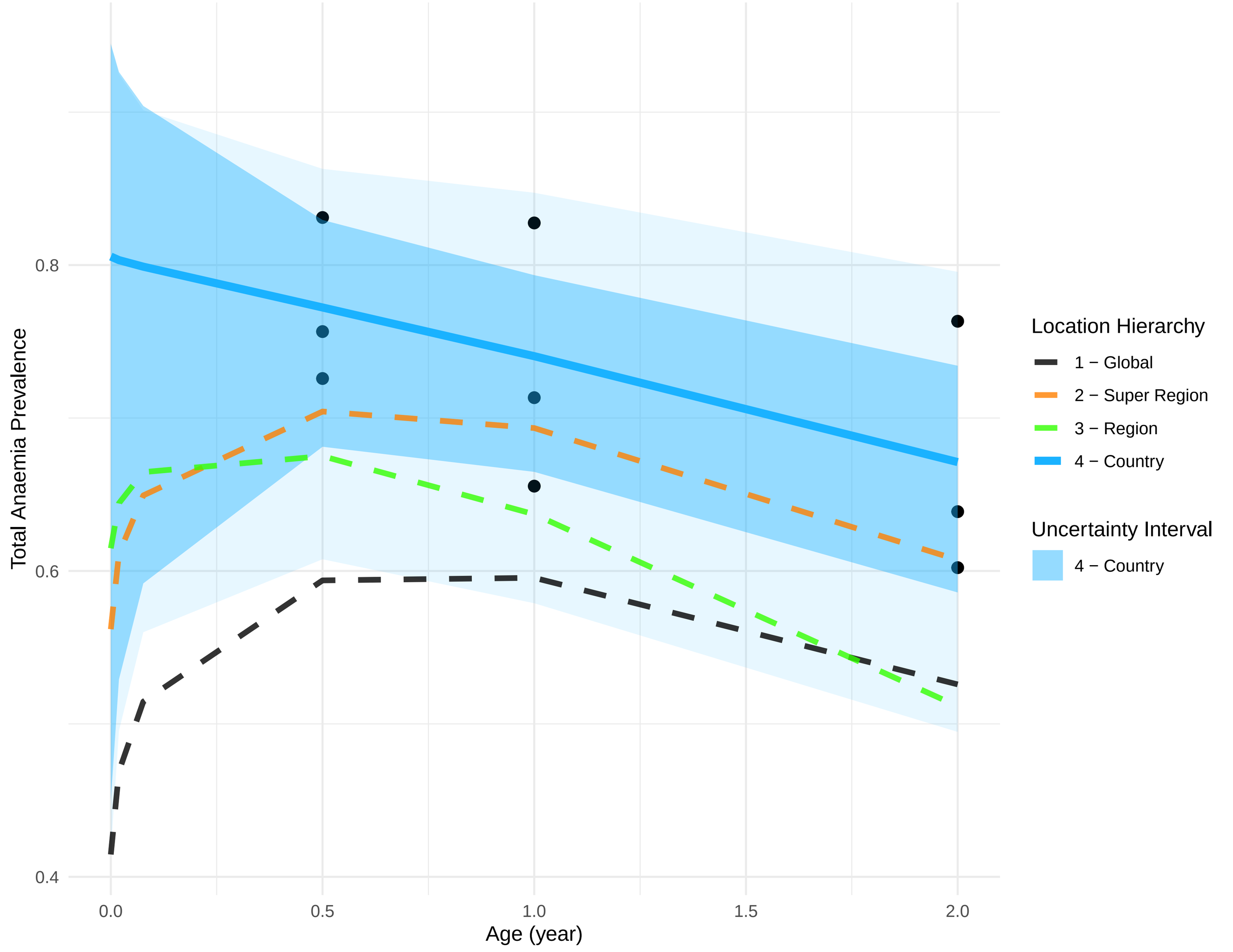

MR−BRT Age/Sex Fits − Males <2 Years Old − Total Anaemia Prevalence − Rwanda
WHO−2001 Adjustment Method

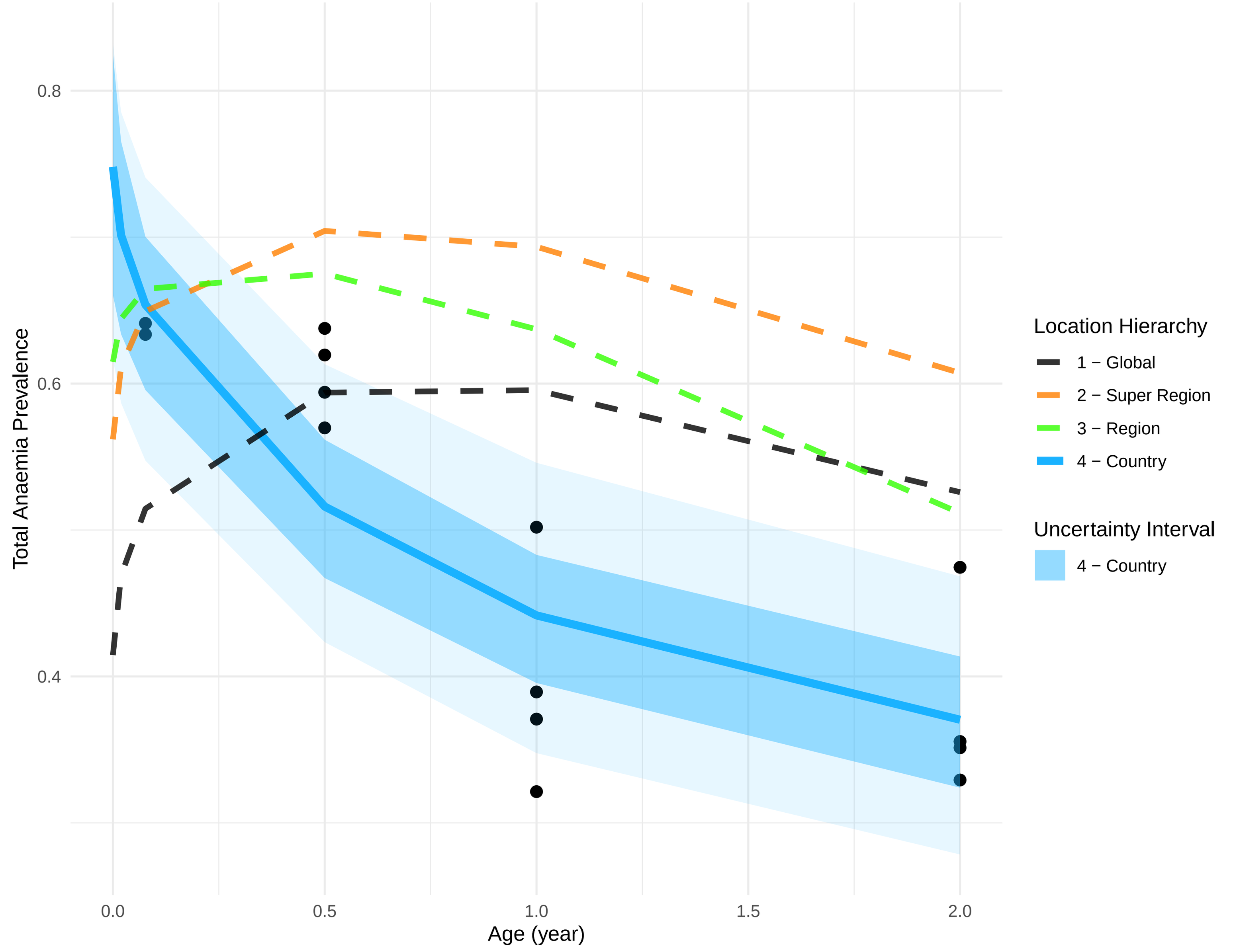

MR−BRT Age/Sex Fits − Males <2 Years Old − Total Anaemia Prevalence − Uganda
WHO−2001 Adjustment Method

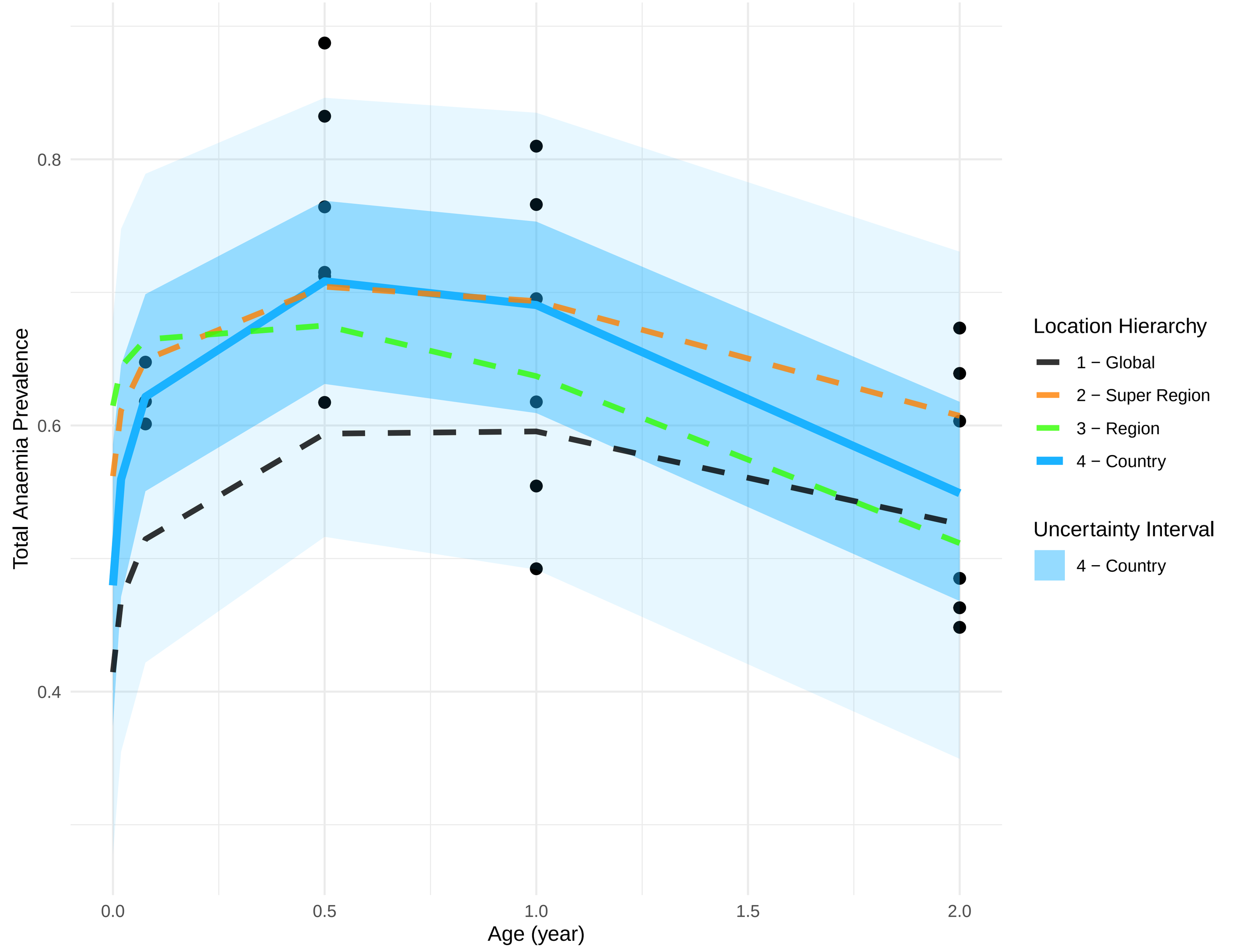

MR−BRT Age/Sex Fits − Males <2 Years Old − Total Anaemia Prevalence − United Republic of Tanzania
WHO−2001 Adjustment Method

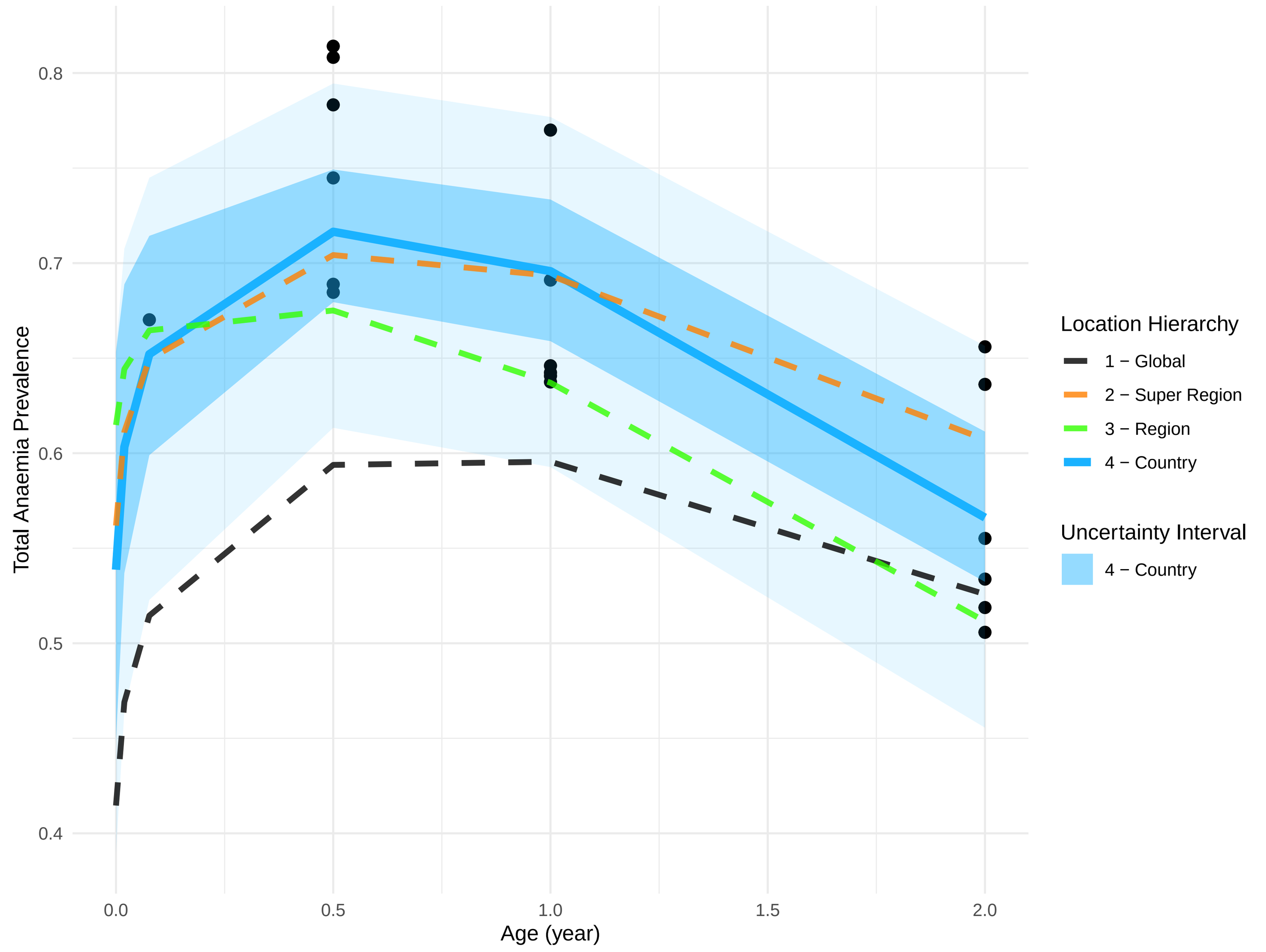

MR−BRT Age/Sex Fits − Males <2 Years Old − Total Anaemia Prevalence − Zambia
WHO−2001 Adjustment Method

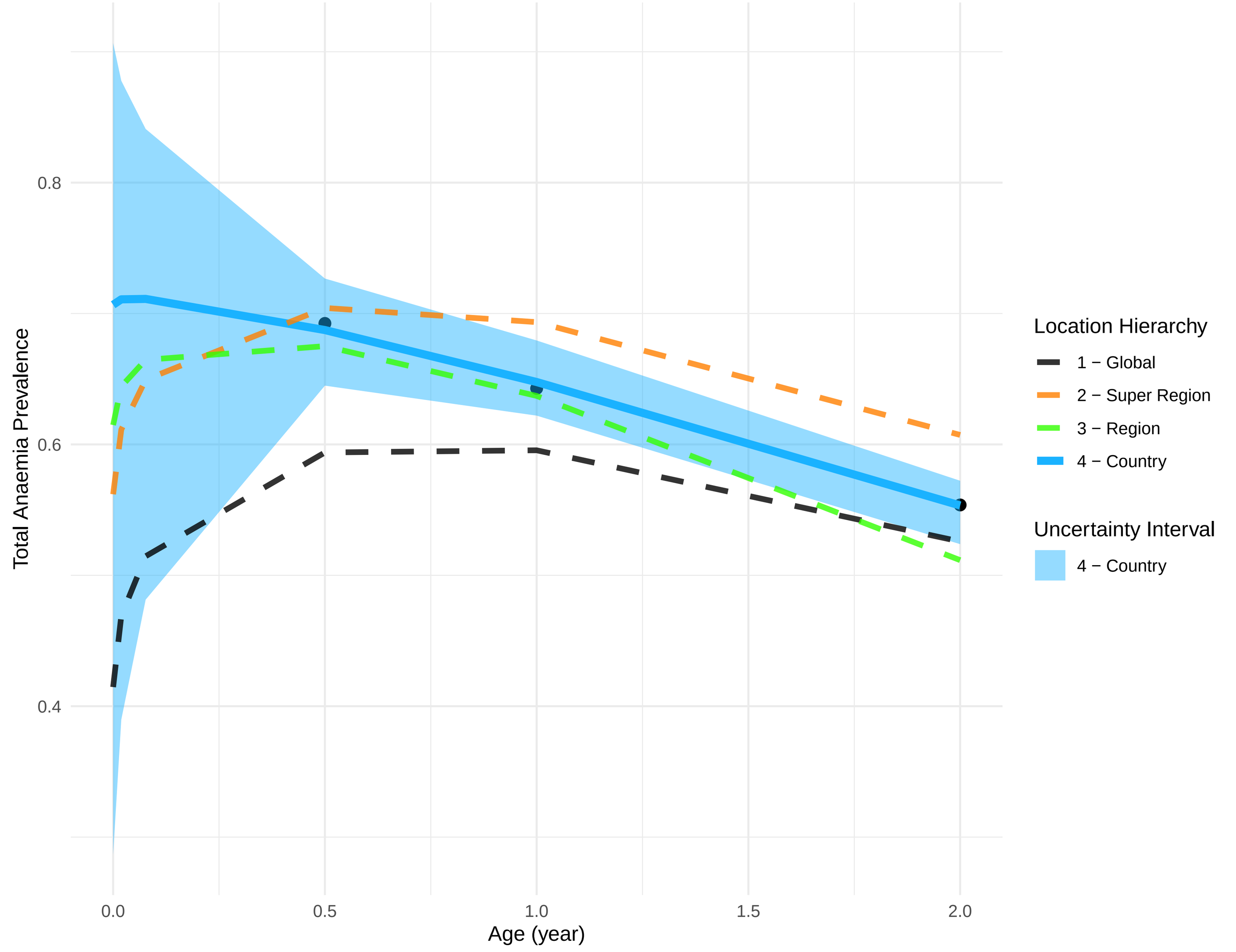

MR−BRT Age/Sex Fits − Males <2 Years Old − Total Anaemia Prevalence − Southern Sub-Saharan Africa
WHO−2001 Adjustment Method

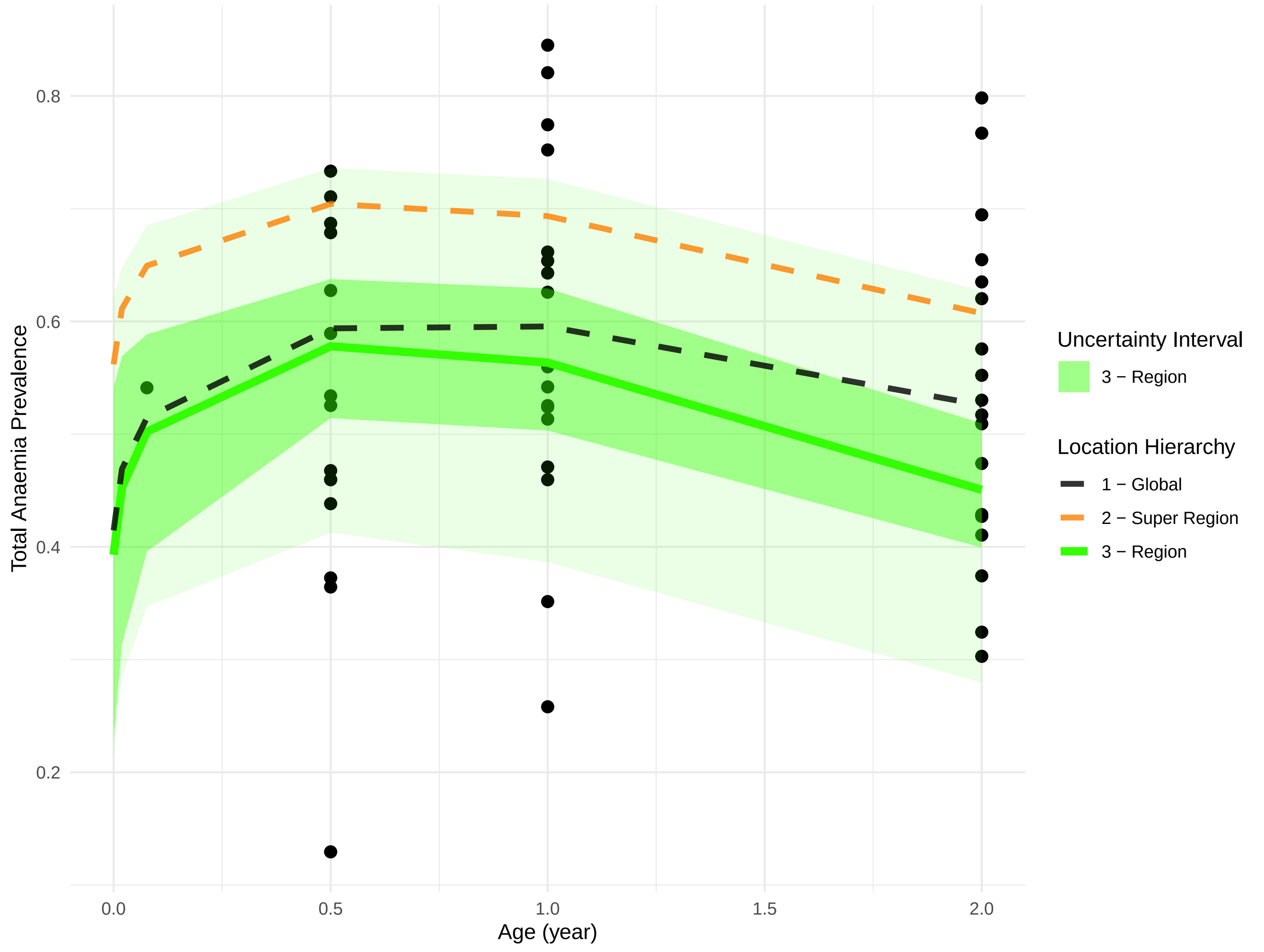

MR−BRT Age/Sex Fits − Males <2 Years Old − Total Anaemia Prevalence − Eswatini
WHO−2001 Adjustment Method

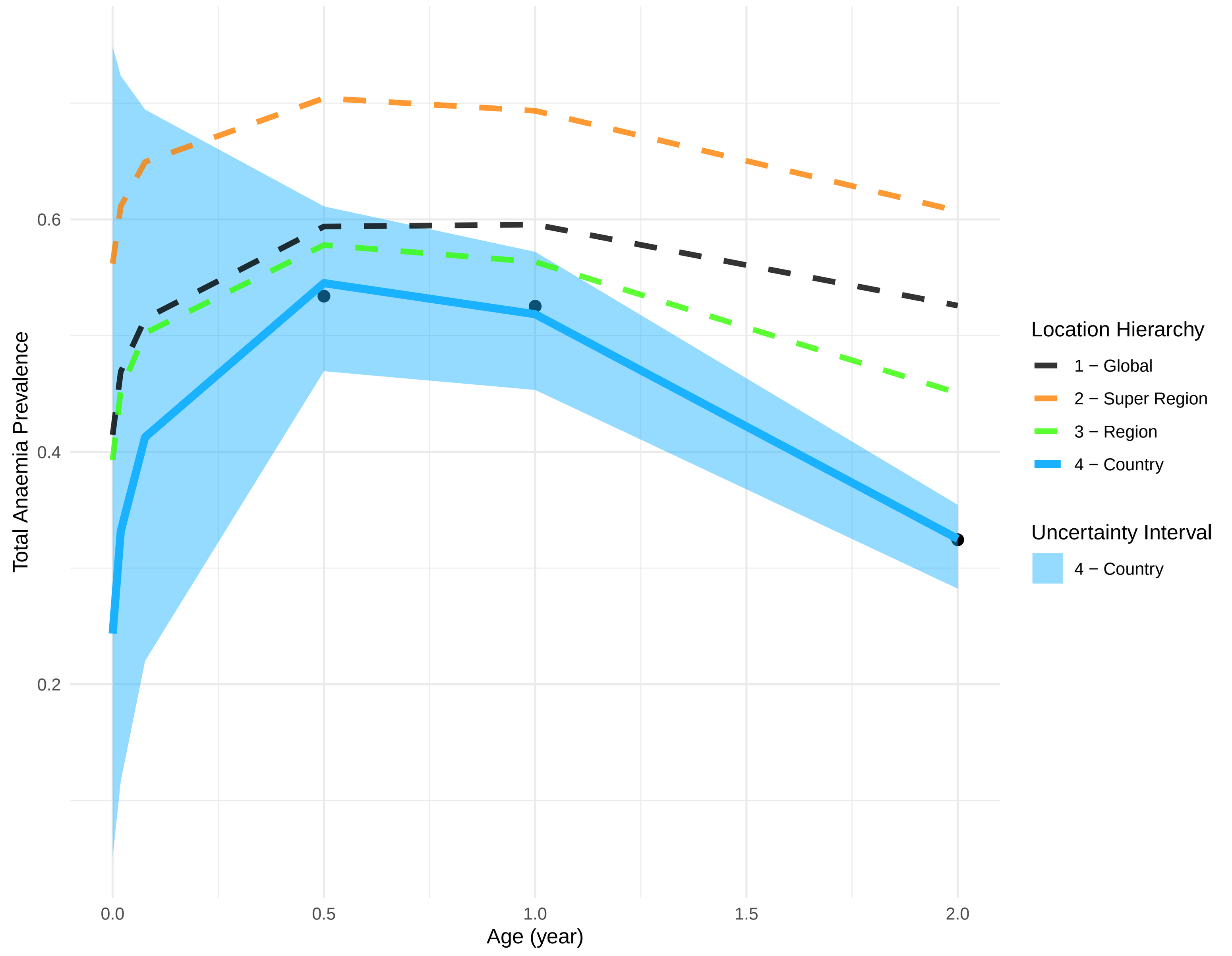

MR−BRT Age/Sex Fits − Males <2 Years Old − Total Anaemia Prevalence − Lesotho
WHO−2001 Adjustment Method

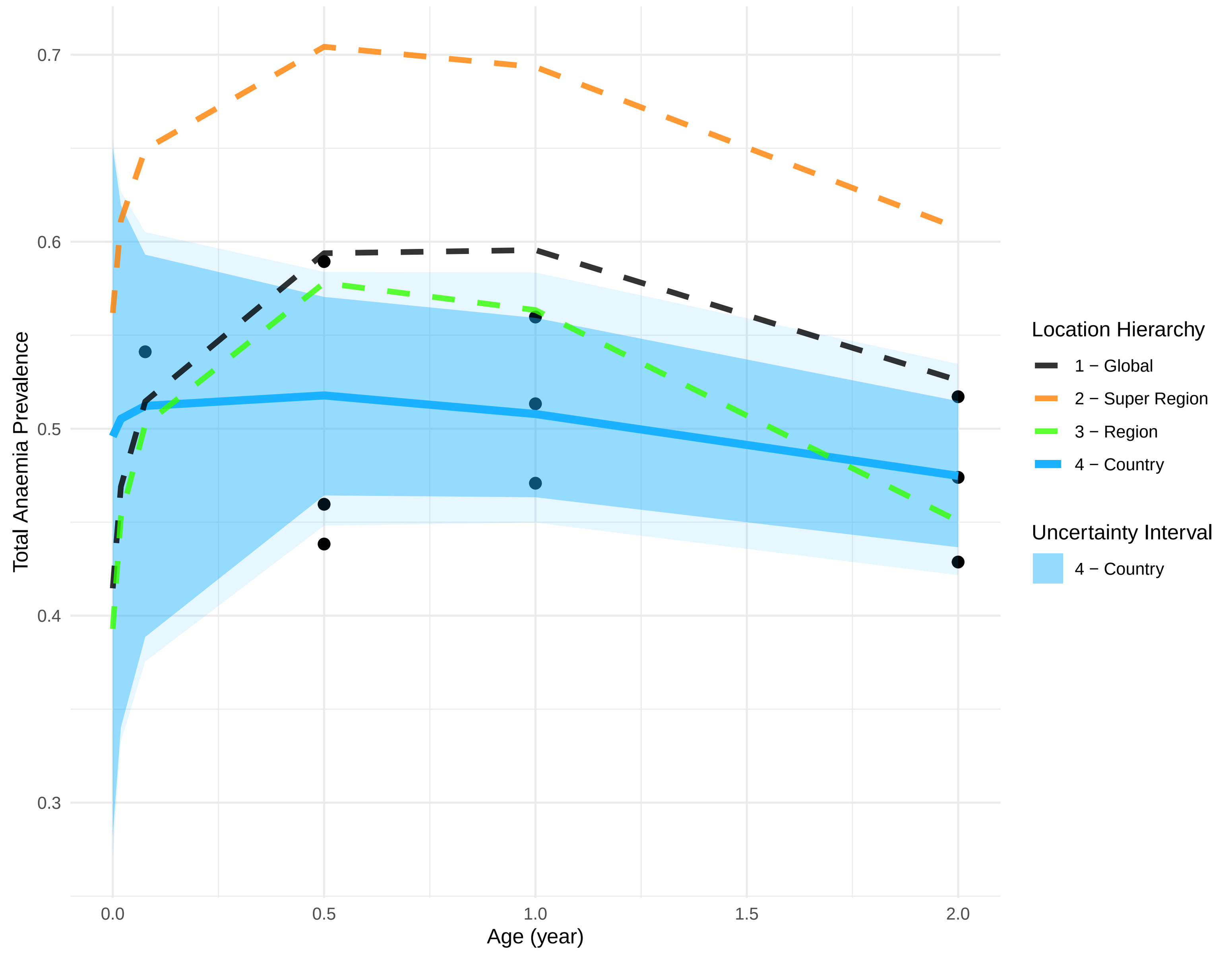

MR−BRT Age/Sex Fits − Males <2 Years Old − Total Anaemia Prevalence − Namibia
WHO−2001 Adjustment Method

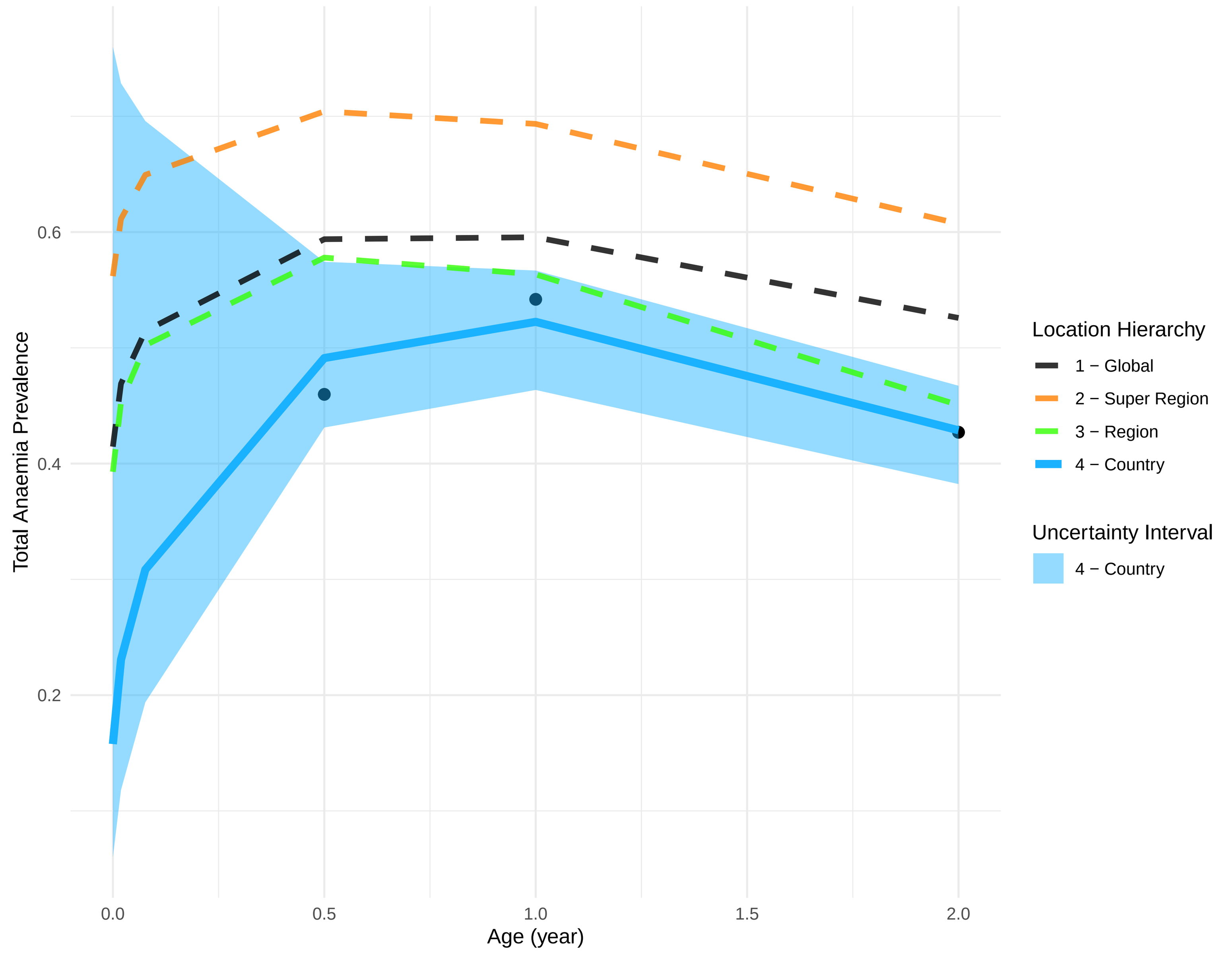

MR−BRT Age/Sex Fits − Males <2 Years Old − Total Anaemia Prevalence − South Africa
WHO−2001 Adjustment Method

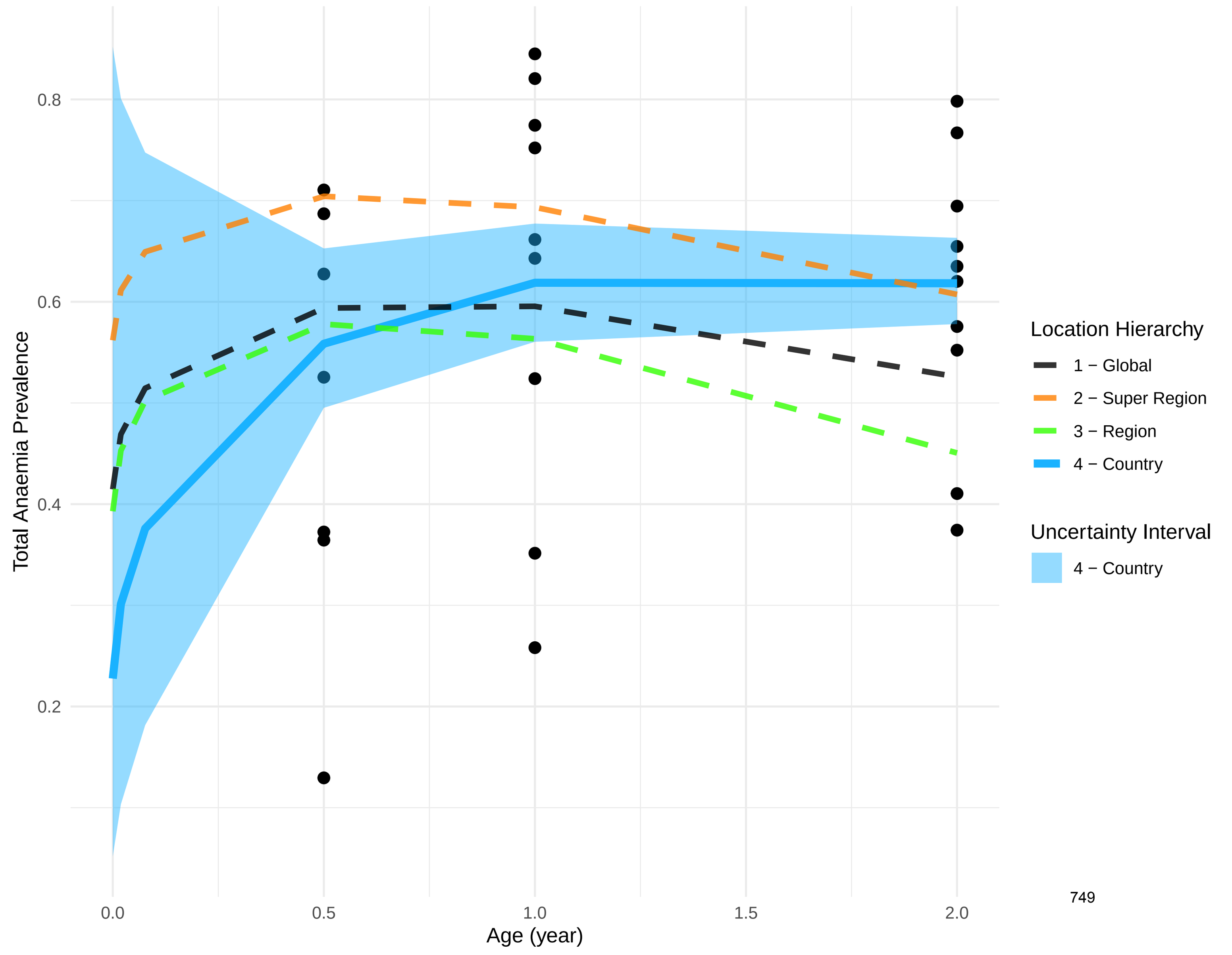

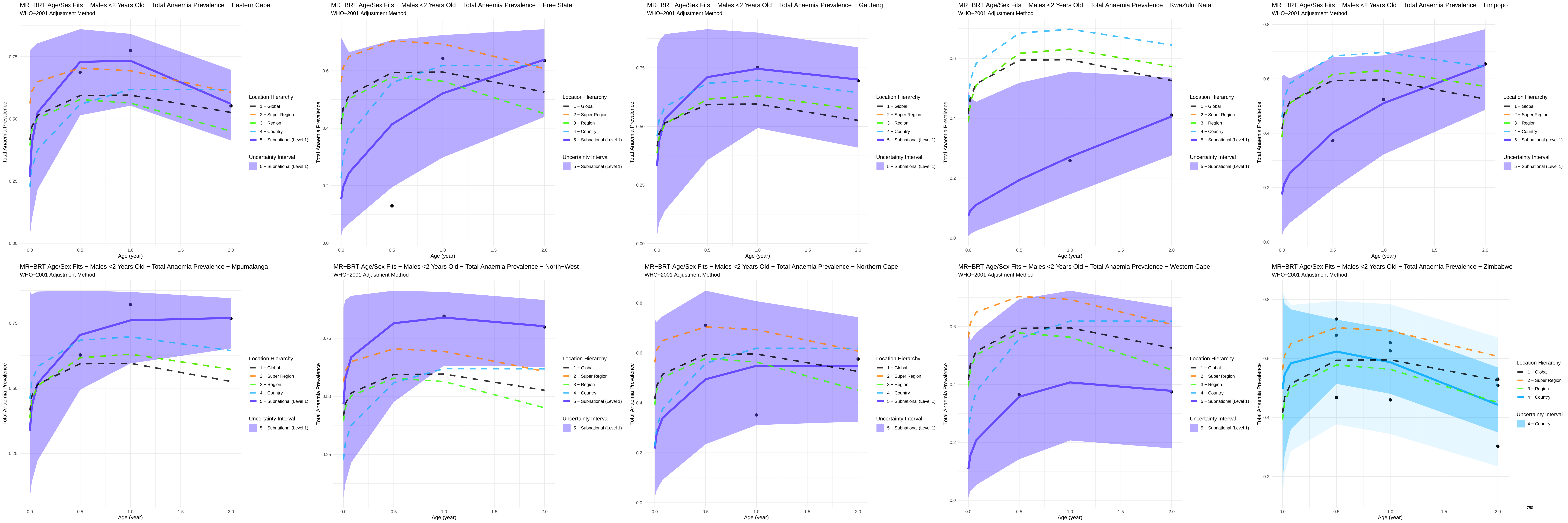

MR−BRT Age/Sex Fits − Males <2 Years Old − Total Anaemia Prevalence − Eastern Cape
WHO−2001 Adjustment Method
MR−BRT Age/Sex Fits − Males <2 Years Old − Total Anaemia Prevalence − Free State
WHO−2001 Adjustment Method
MR−BRT Age/Sex Fits − Males <2 Years Old − Total Anaemia Prevalence − Gauteng
WHO−2001 Adjustment Method
MR−BRT Age/Sex Fits − Males <2 Years Old − Total Anaemia Prevalence − KwaZulu−Natal
WHO−2001 Adjustment Method
MR−BRT Age/Sex Fits − Males <2 Years Old − Total Anaemia Prevalence − Limpopo
WHO−2001 Adjustment Method
MR−BRT Age/Sex Fits − Males <2 Years Old − Total Anaemia Prevalence − Mpumalanga
WHO−2001 Adjustment Method
MR−BRT Age/Sex Fits − Males <2 Years Old − Total Anaemia Prevalence − North−West
WHO−2001 Adjustment Method
MR−BRT Age/Sex Fits − Males <2 Years Old − Total Anaemia Prevalence − Northern Cape
WHO−2001 Adjustment Method
MR−BRT Age/Sex Fits − Males <2 Years Old − Total Anaemia Prevalence − Western Cape
WHO−2001 Adjustment Method
MR−BRT Age/Sex Fits − Males <2 Years Old − Total Anaemia Prevalence − Zimbabwe
WHO−2001 Adjustment Method
Total Anaemia Prevalence
Age (year)
Location Hierarchy
1 − Global
2 − Super Region
3 − Region
4 − Country
5 − Subnational (Level 1)
Uncertainty Interval
5 − Subnational (Level 1)
4 − Country

MR−BRT Age/Sex Fits − Males <2 Years Old − Total Anaemia Prevalence − Western Sub-Saharan Africa
WHO−2001 Adjustment Method

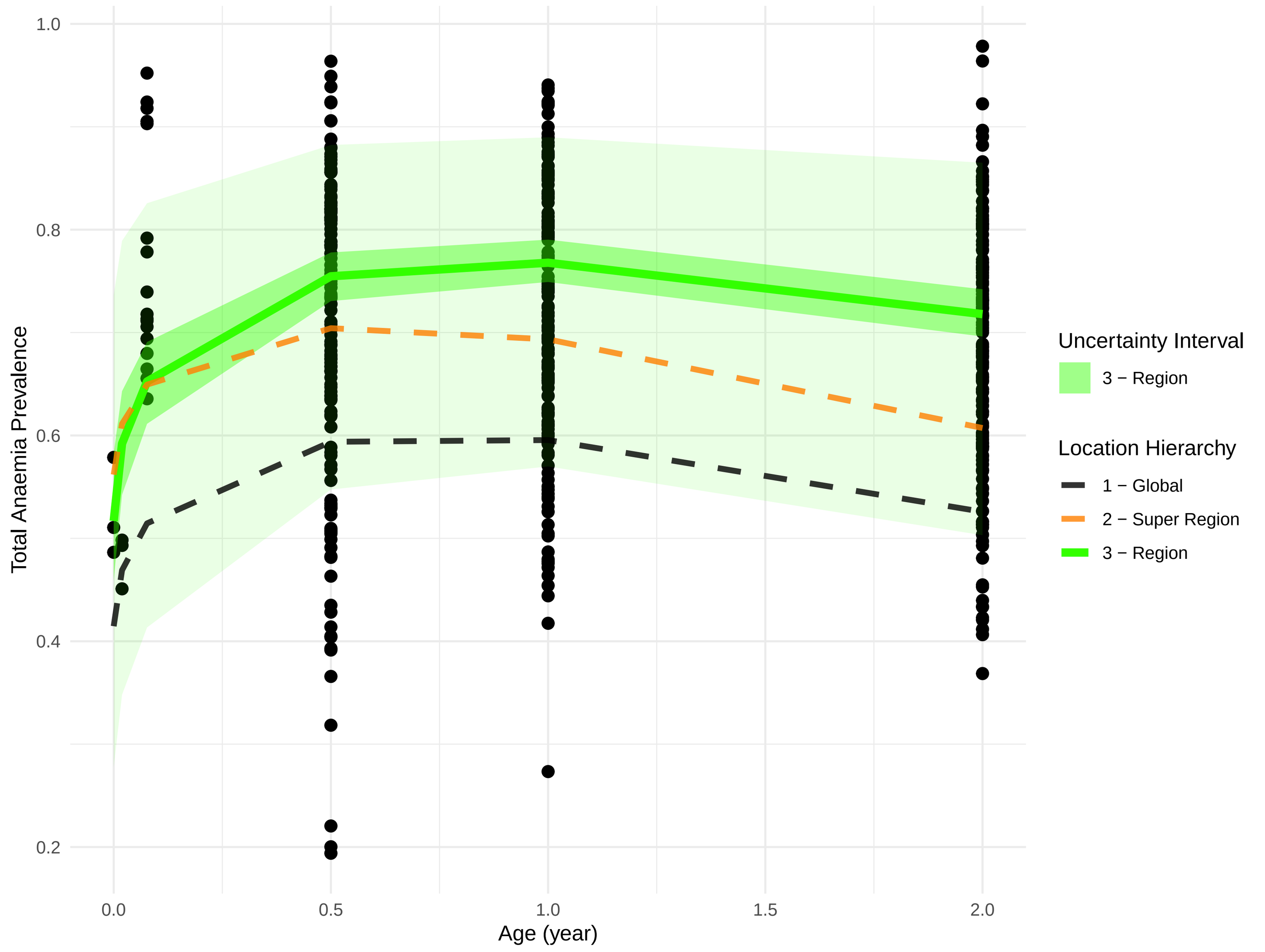

MR−BRT Age/Sex Fits − Males <2 Years Old − Total Anaemia Prevalence − Benin
WHO−2001 Adjustment Method

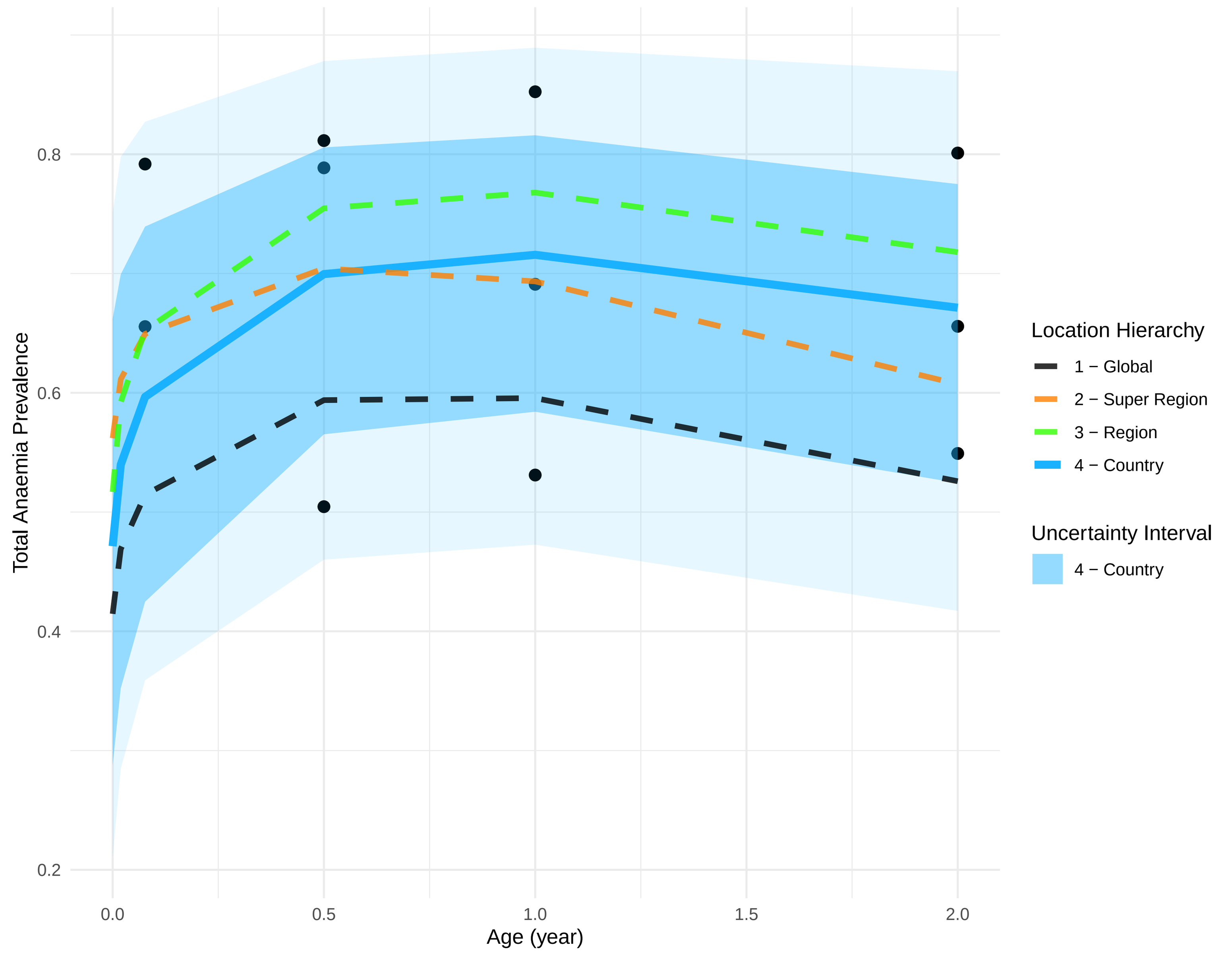

MR−BRT Age/Sex Fits − Males <2 Years Old − Total Anaemia Prevalence − Burkina Faso
WHO−2001 Adjustment Method

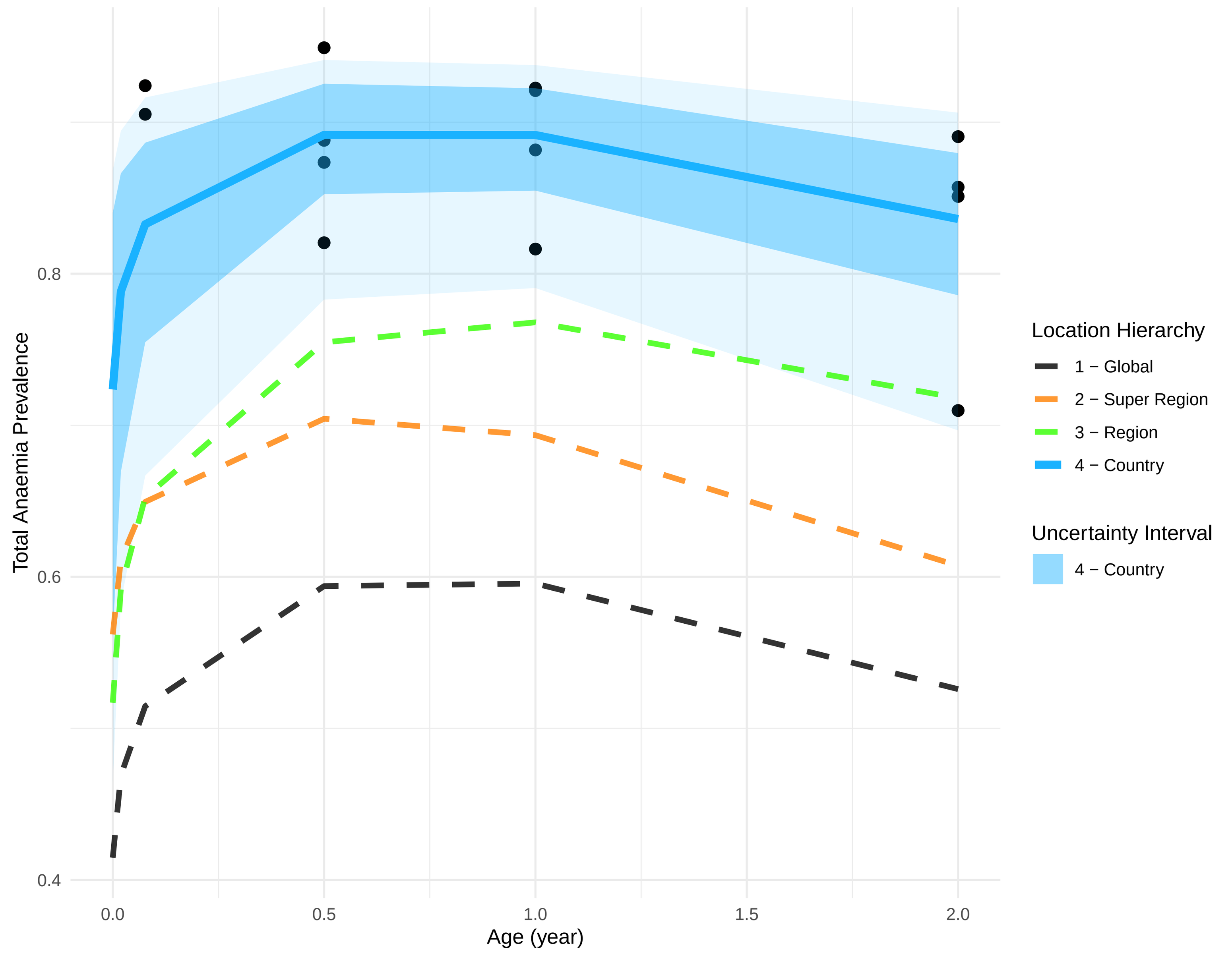

MR−BRT Age/Sex Fits − Males <2 Years Old − Total Anaemia Prevalence − Cameroon
WHO−2001 Adjustment Method

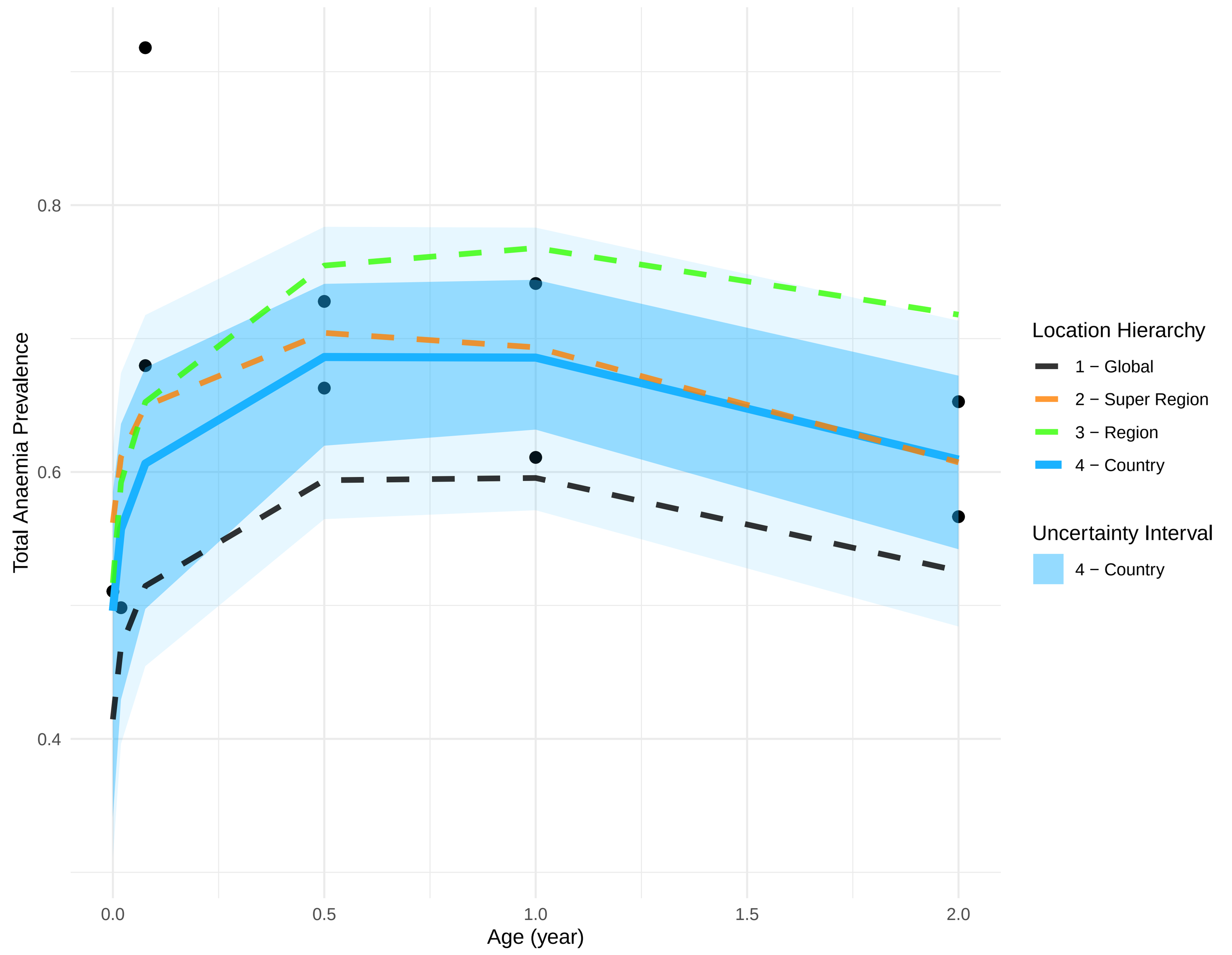

MR−BRT Age/Sex Fits − Males <2 Years Old − Total Anaemia Prevalence − Côte d'Ivoire
WHO−2001 Adjustment Method

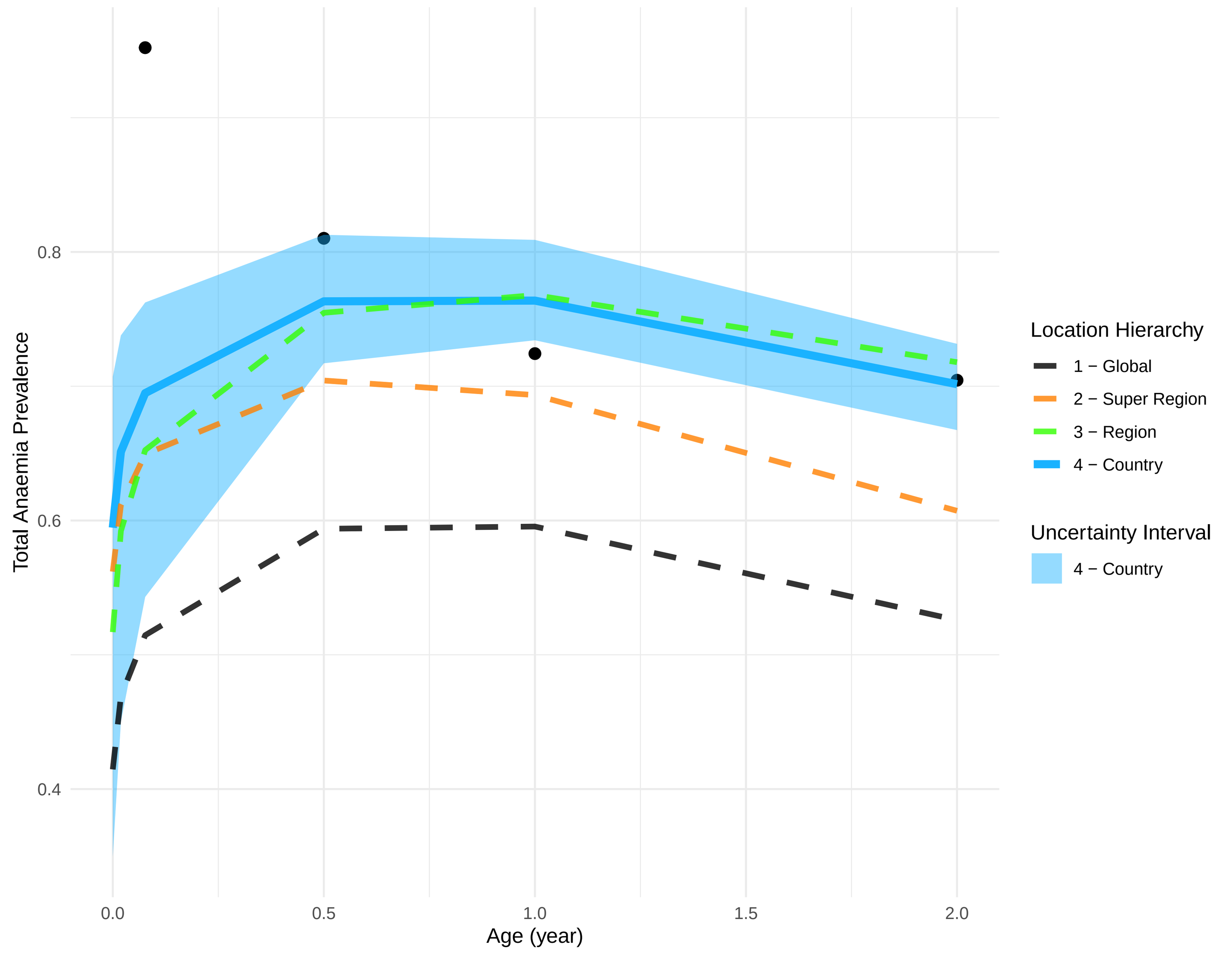

MR−BRT Age/Sex Fits − Males <2 Years Old − Total Anaemia Prevalence − Gambia
WHO−2001 Adjustment Method

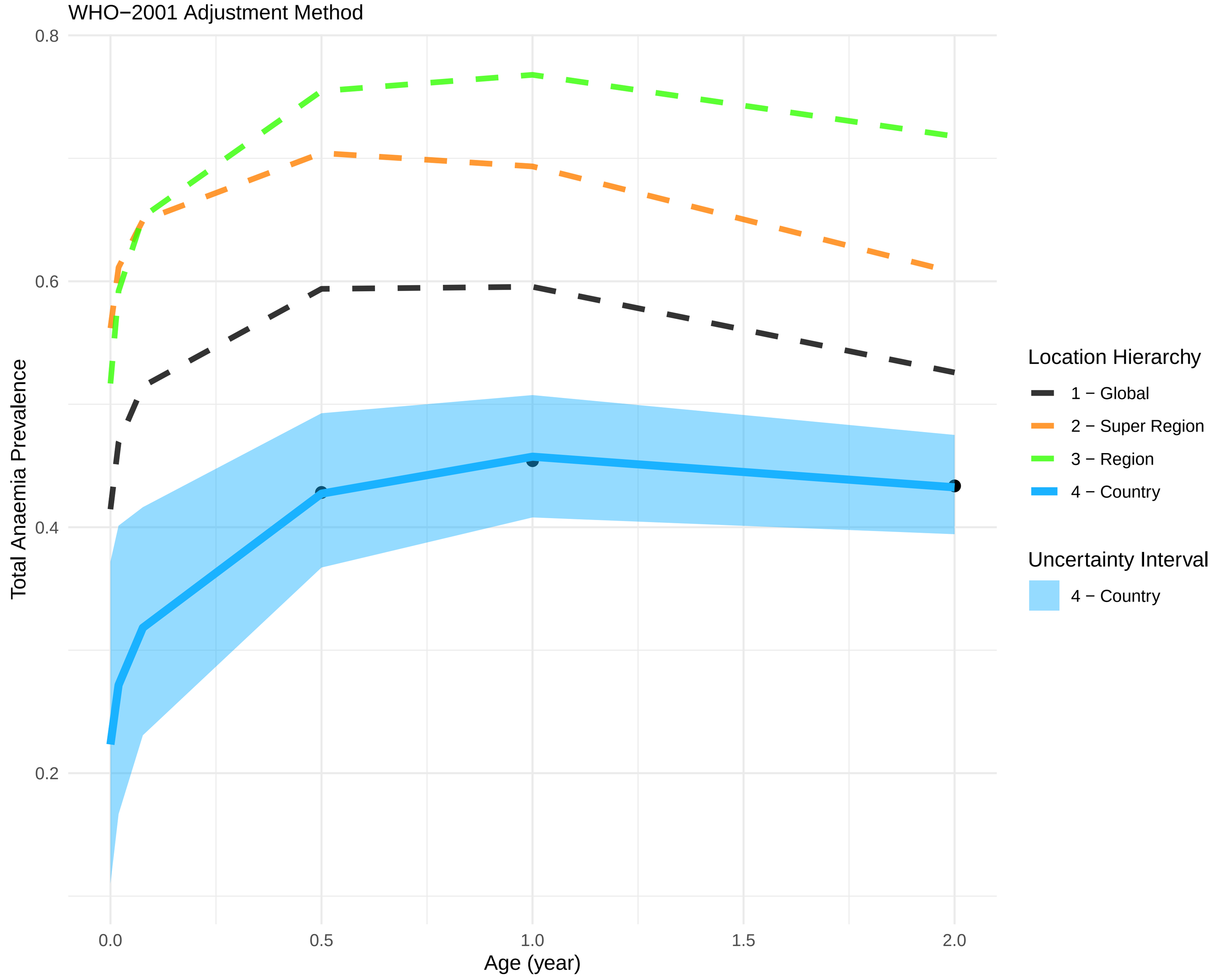

MR−BRT Age/Sex Fits − Males <2 Years Old − Total Anaemia Prevalence − Ghana
WHO−2001 Adjustment Method

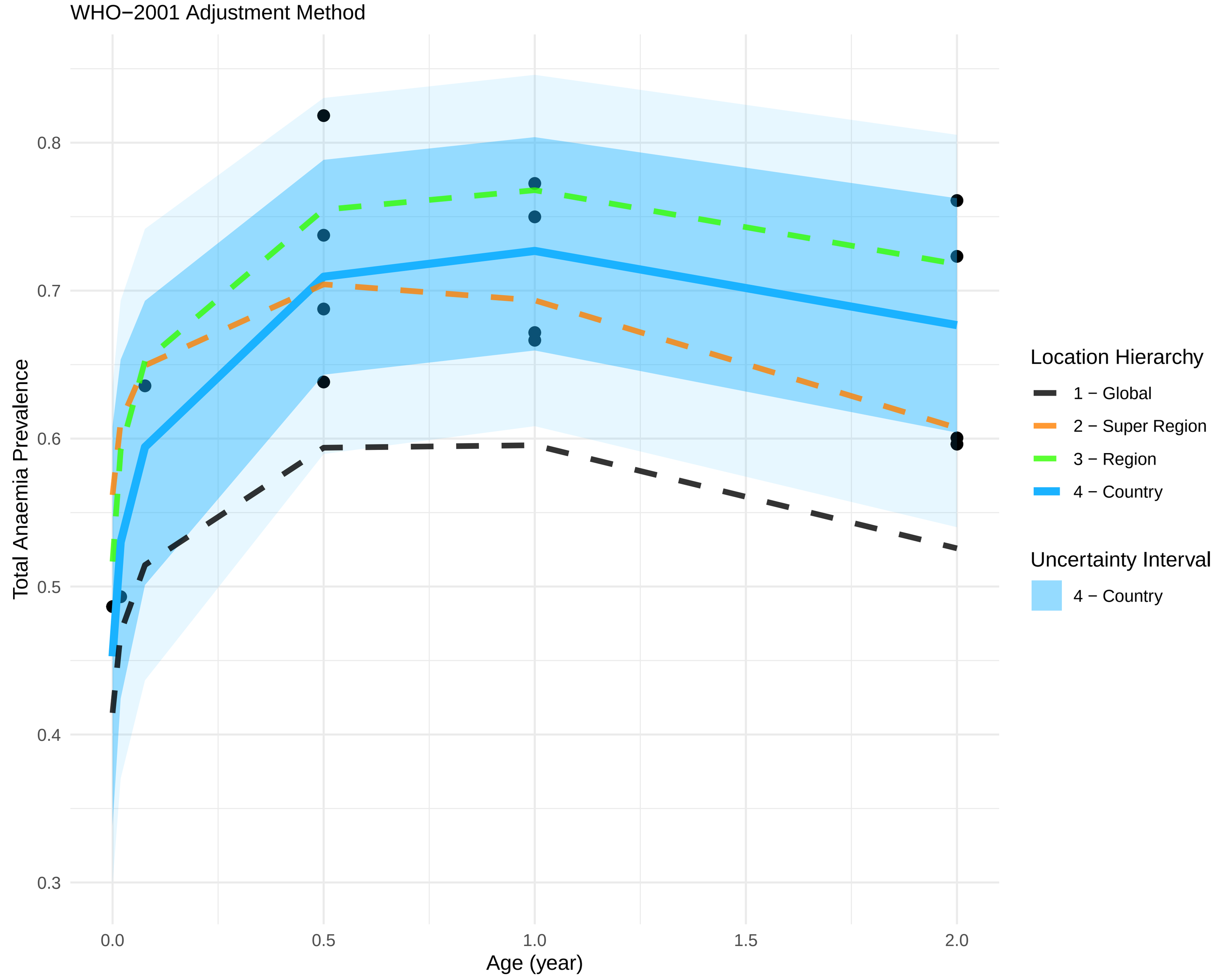

MR−BRT Age/Sex Fits − Males <2 Years Old − Total Anaemia Prevalence − Guinea
WHO−2001 Adjustment Method

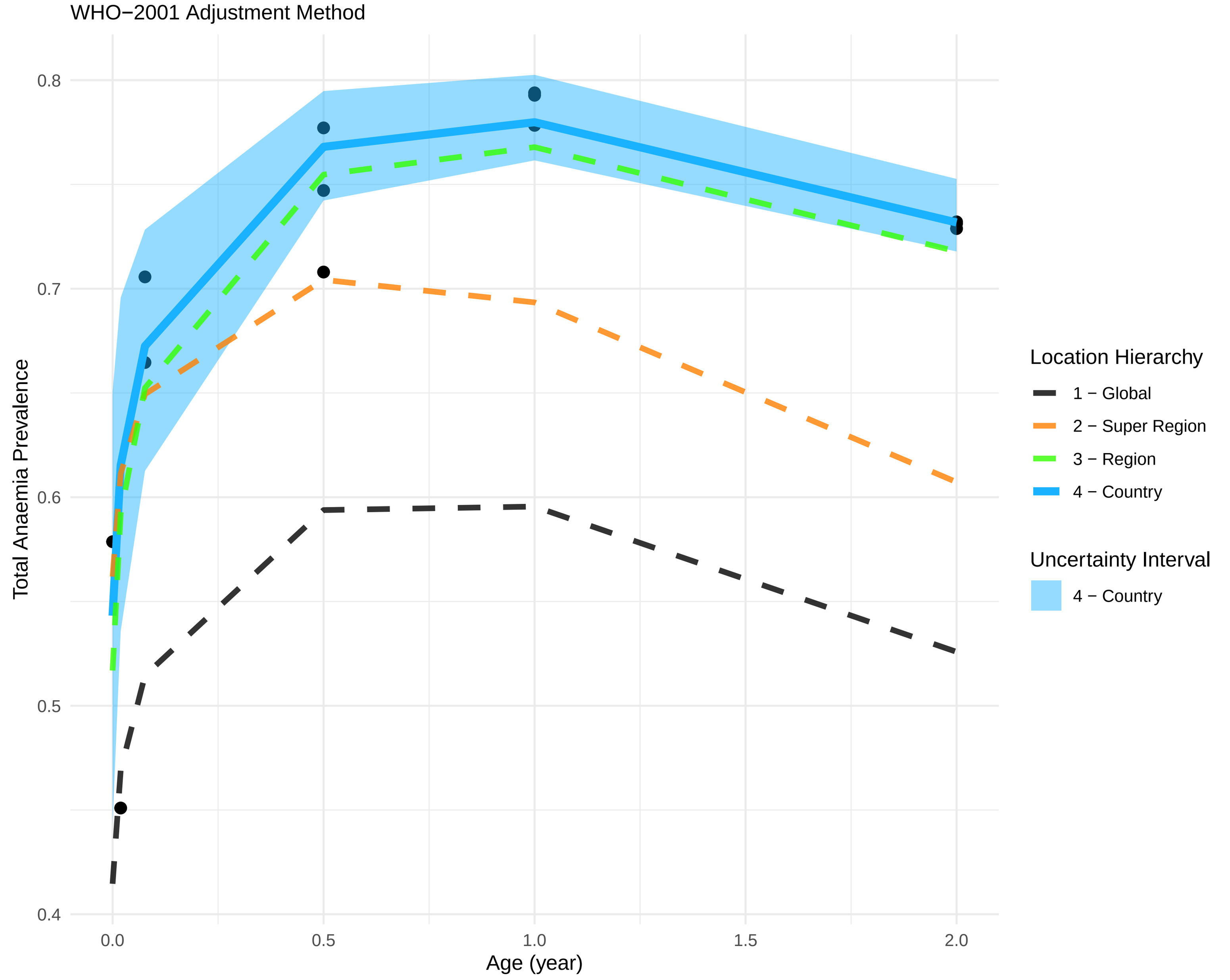

MR−BRT Age/Sex Fits − Males <2 Years Old − Total Anaemia Prevalence − Liberia
WHO−2001 Adjustment Method

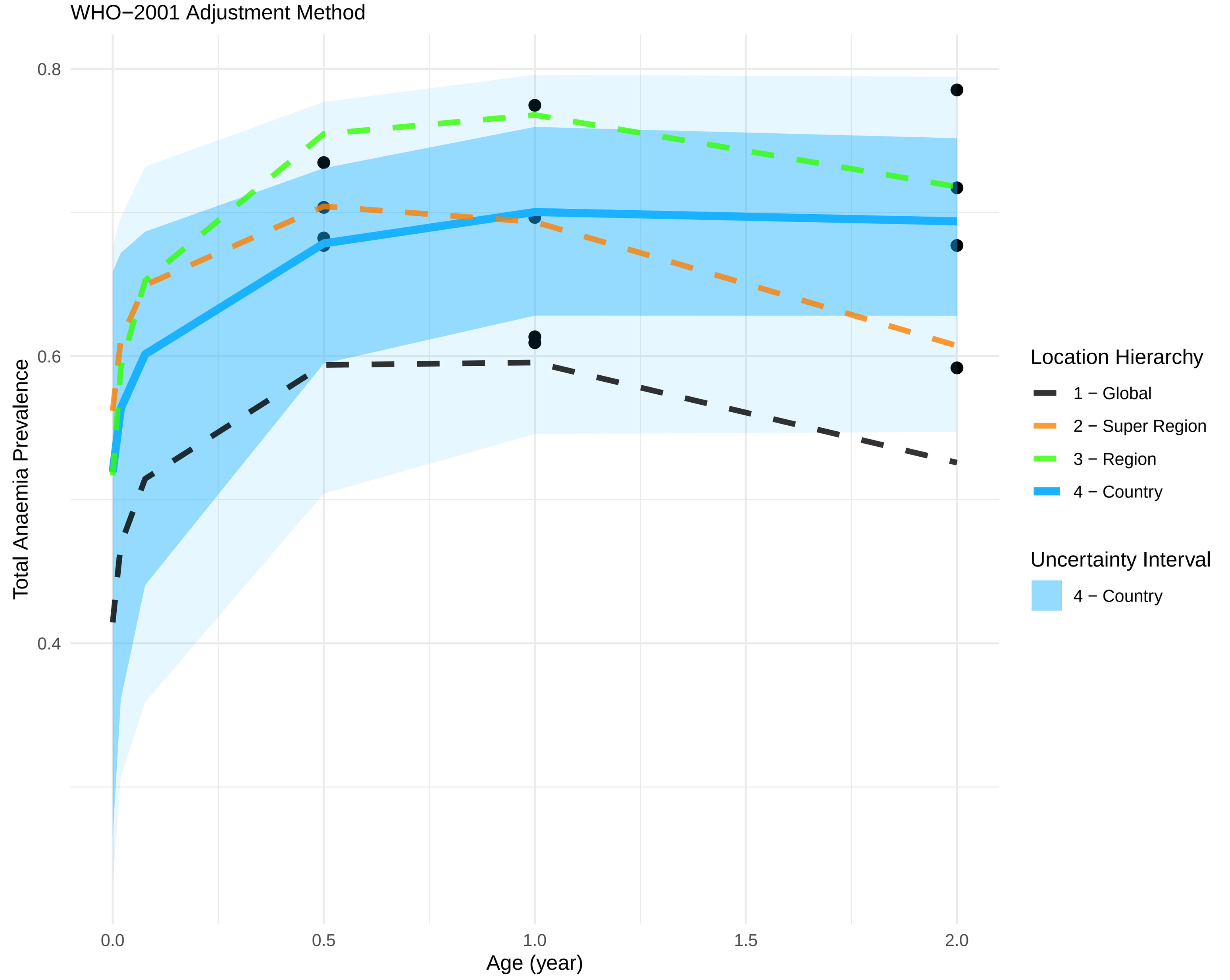

MR−BRT Age/Sex Fits − Males <2 Years Old − Total Anaemia Prevalence − Mali
WHO−2001 Adjustment Method

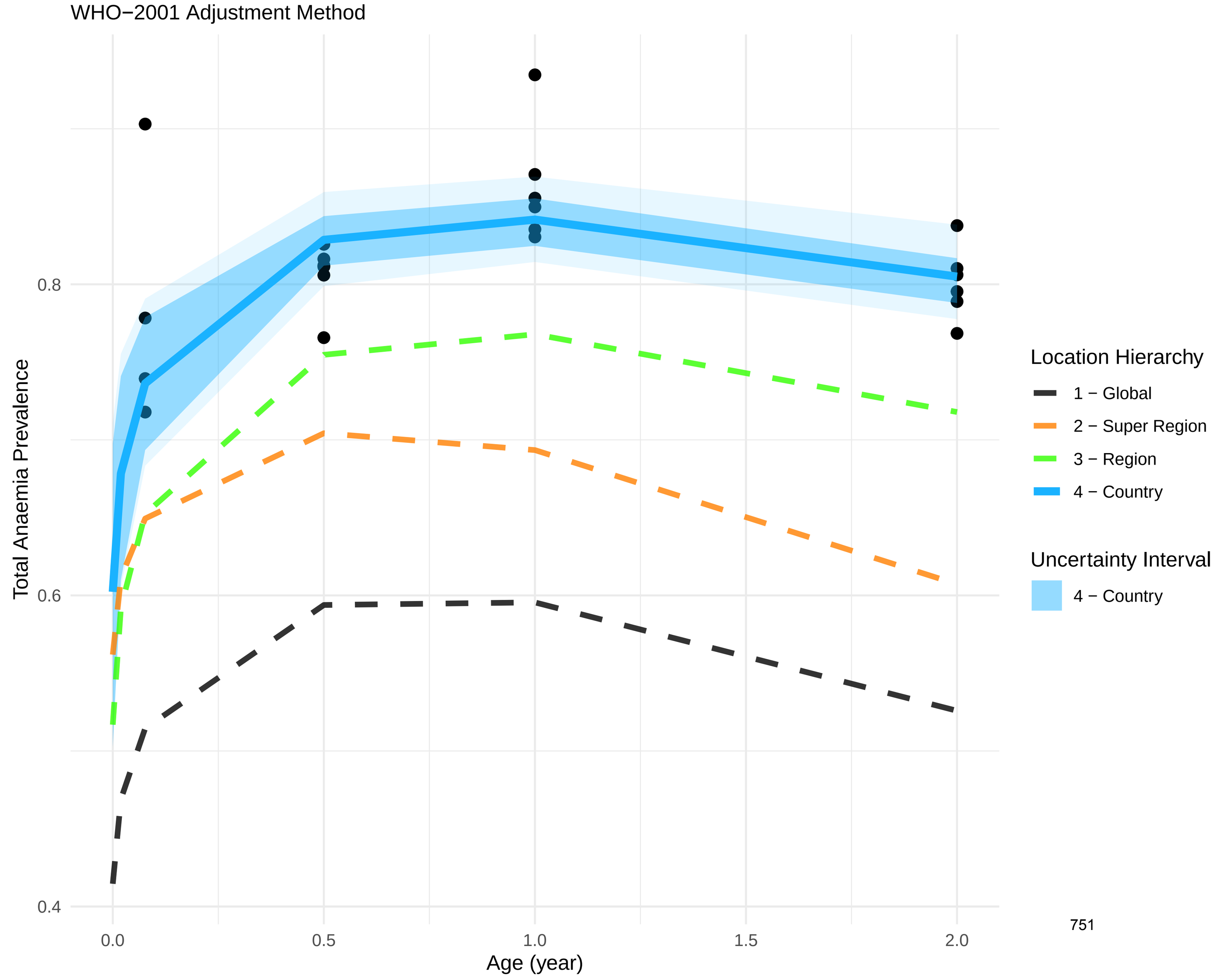

MR−BRT Age/Sex Fits − Males <2 Years Old − Total Anaemia Prevalence − Niger
WHO−2001 Adjustment Method

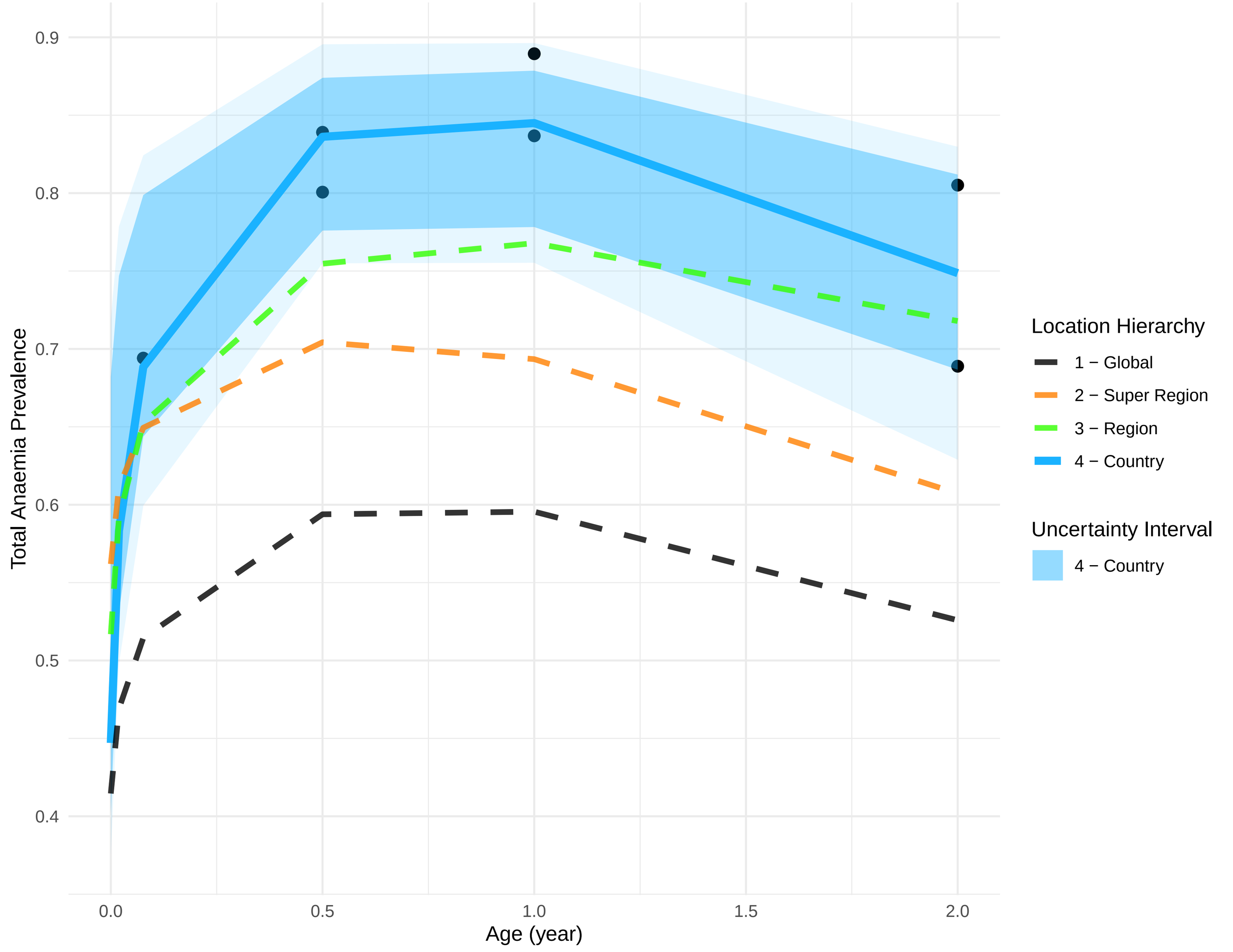

MR−BRT Age/Sex Fits − Males <2 Years Old − Total Anaemia Prevalence − Nigeria
WHO−2001 Adjustment Method

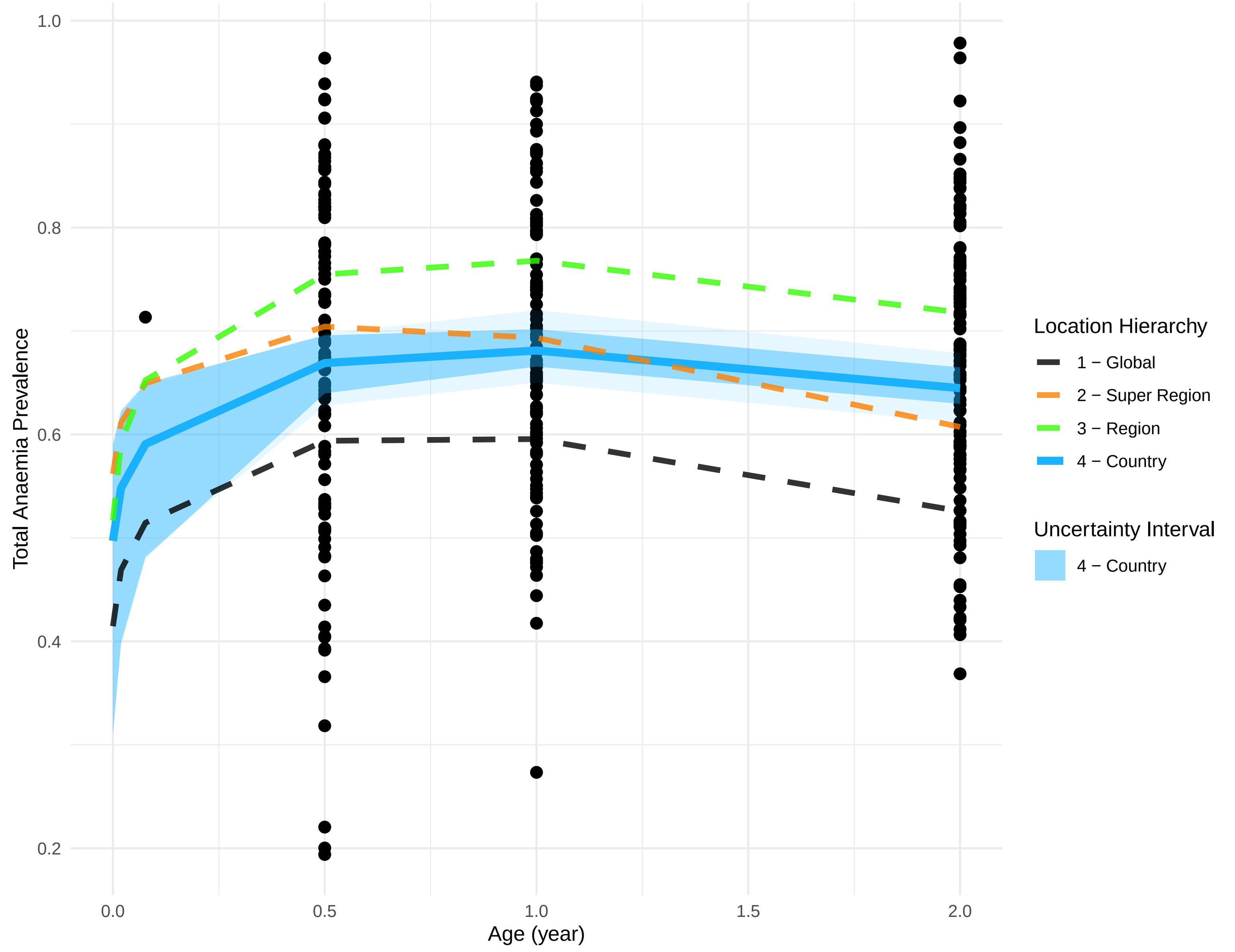

MR−BRT Age/Sex Fits − Males <2 Years Old − Total Anaemia Prevalence − Abia
WHO−2001 Adjustment Method

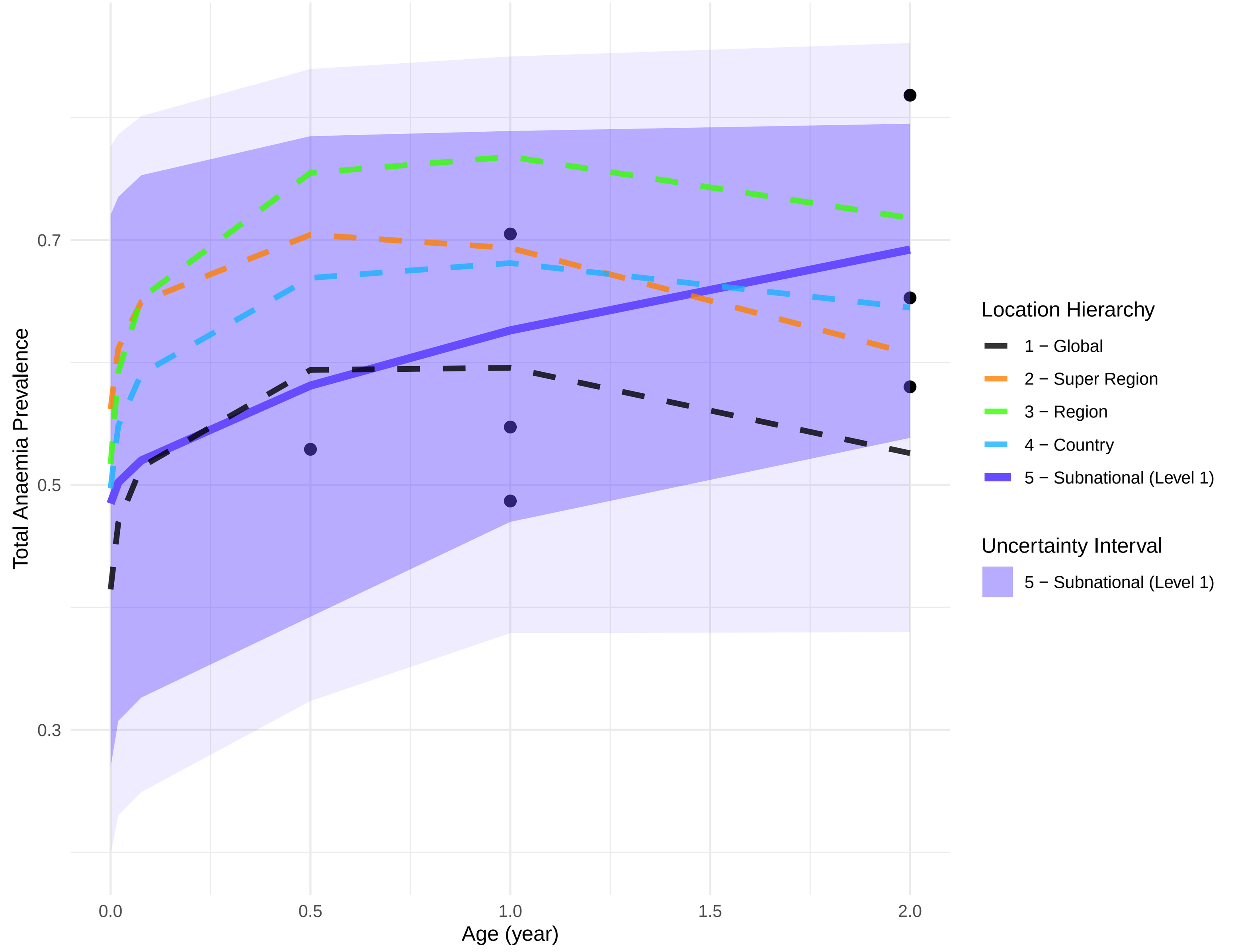

MR−BRT Age/Sex Fits − Males <2 Years Old − Total Anaemia Prevalence − Adamawa
WHO−2001 Adjustment Method

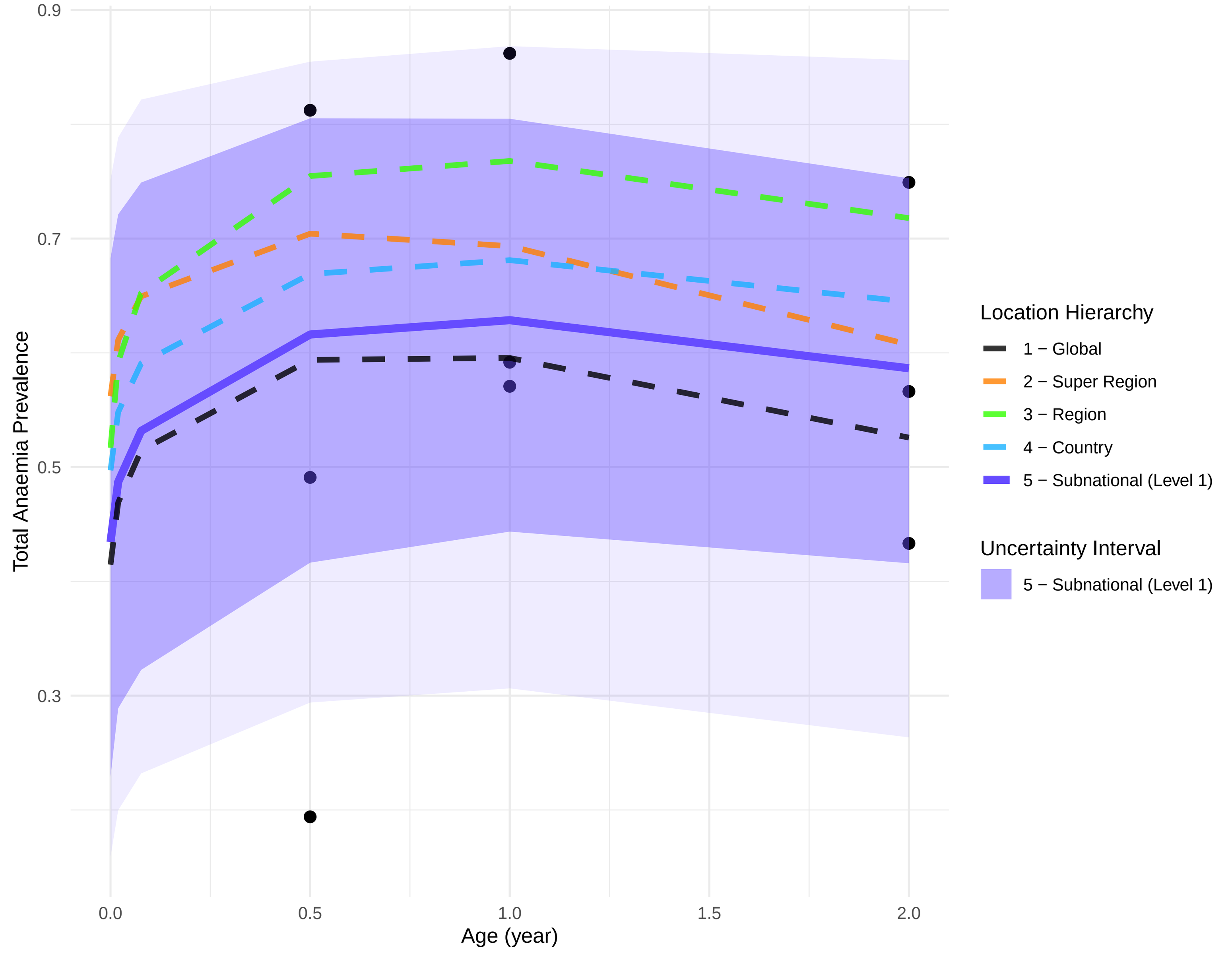

MR−BRT Age/Sex Fits − Males <2 Years Old − Total Anaemia Prevalence − Akwa Ibom
WHO−2001 Adjustment Method

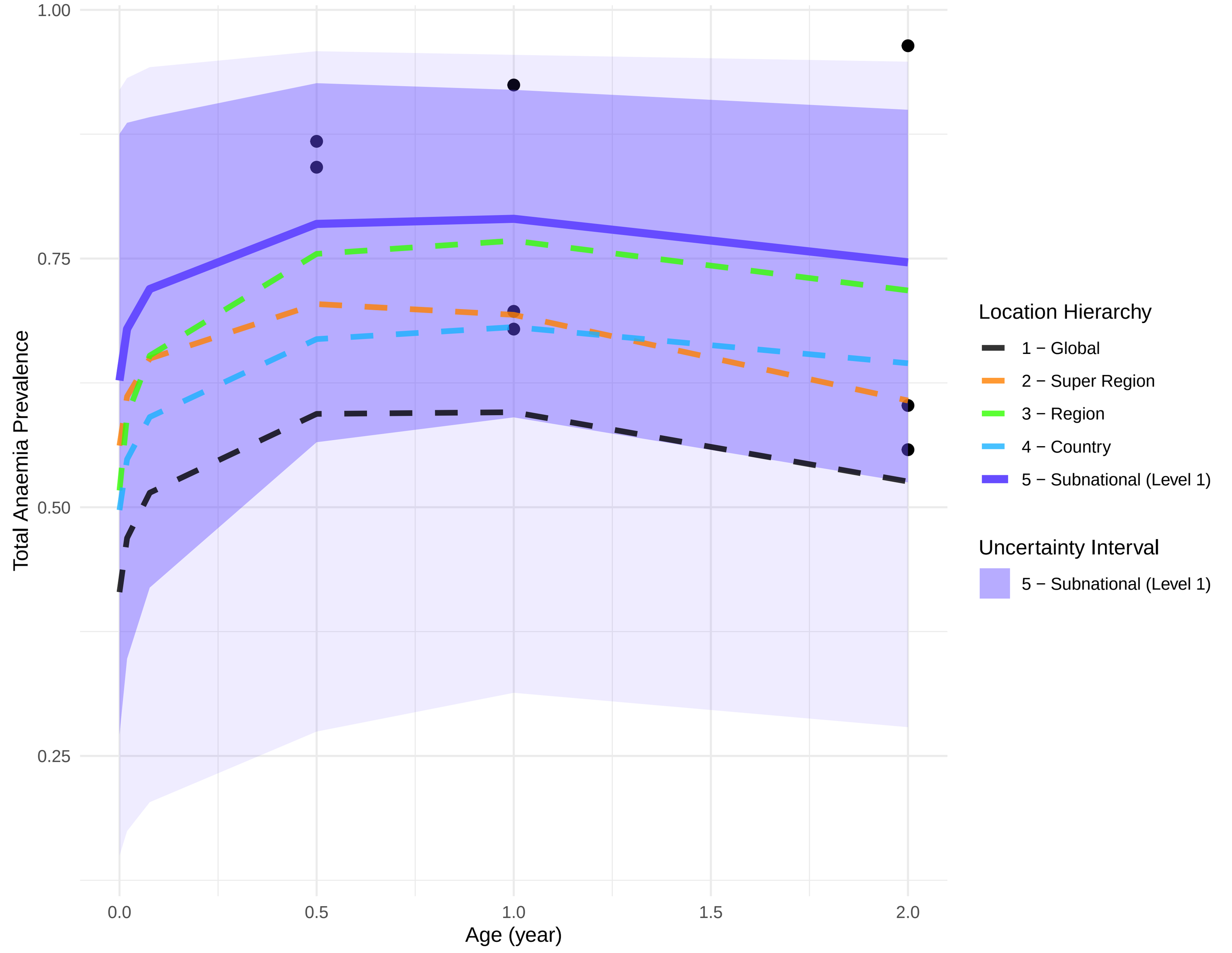

MR−BRT Age/Sex Fits − Males <2 Years Old − Total Anaemia Prevalence − Anambra
WHO−2001 Adjustment Method

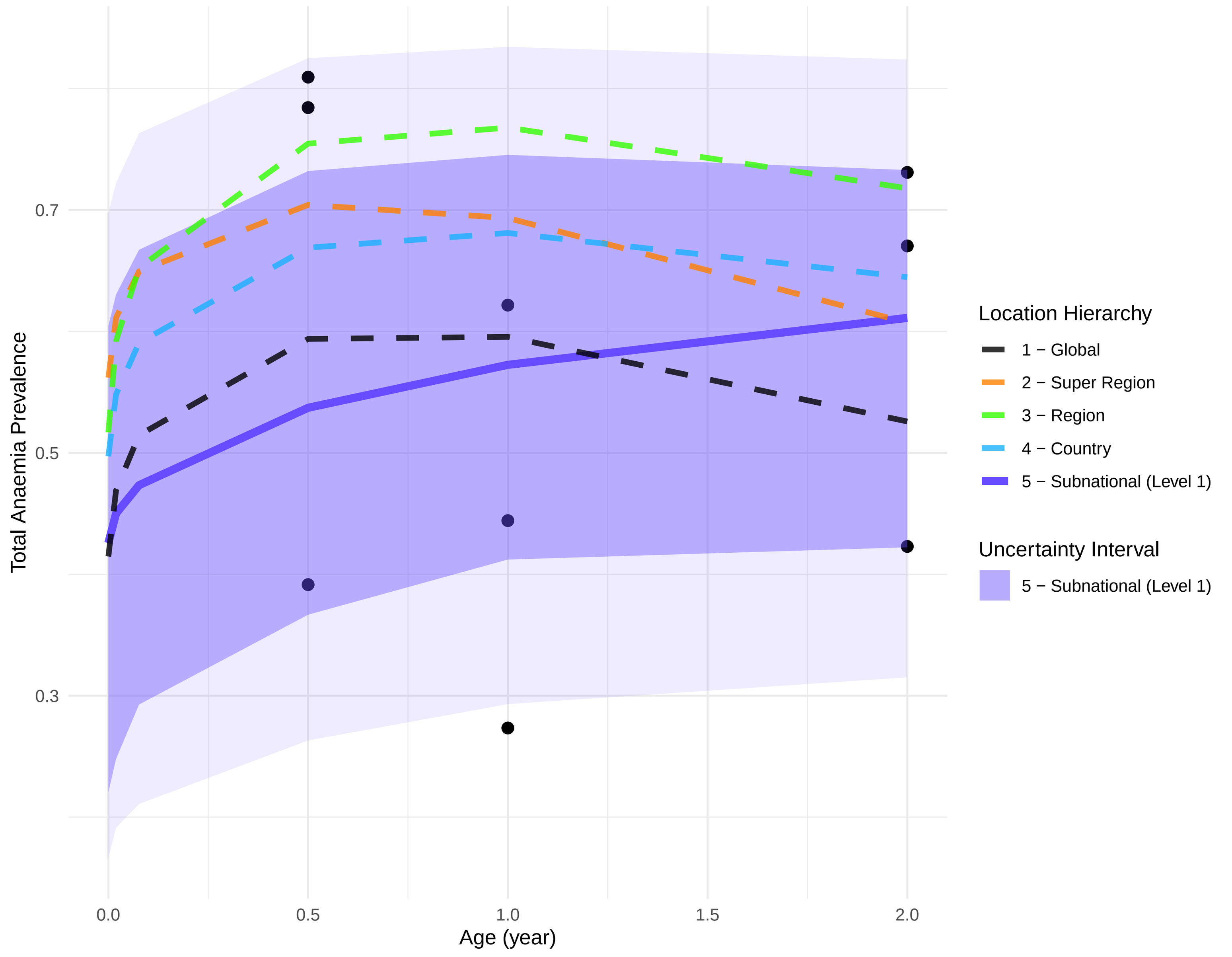

MR−BRT Age/Sex Fits − Males <2 Years Old − Total Anaemia Prevalence − Bauchi
WHO−2001 Adjustment Method

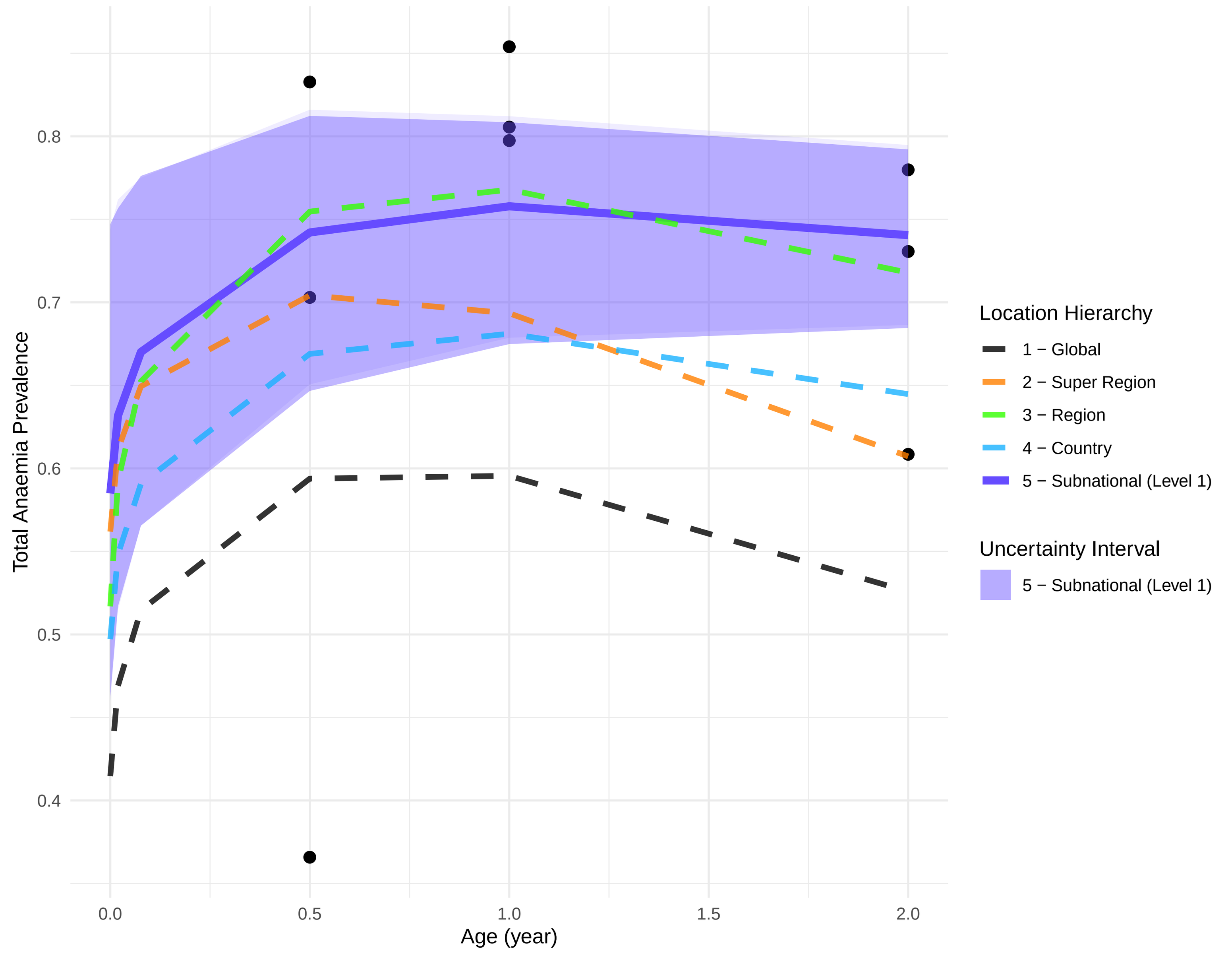

MR−BRT Age/Sex Fits − Males <2 Years Old − Total Anaemia Prevalence − Bayelsa
WHO−2001 Adjustment Method

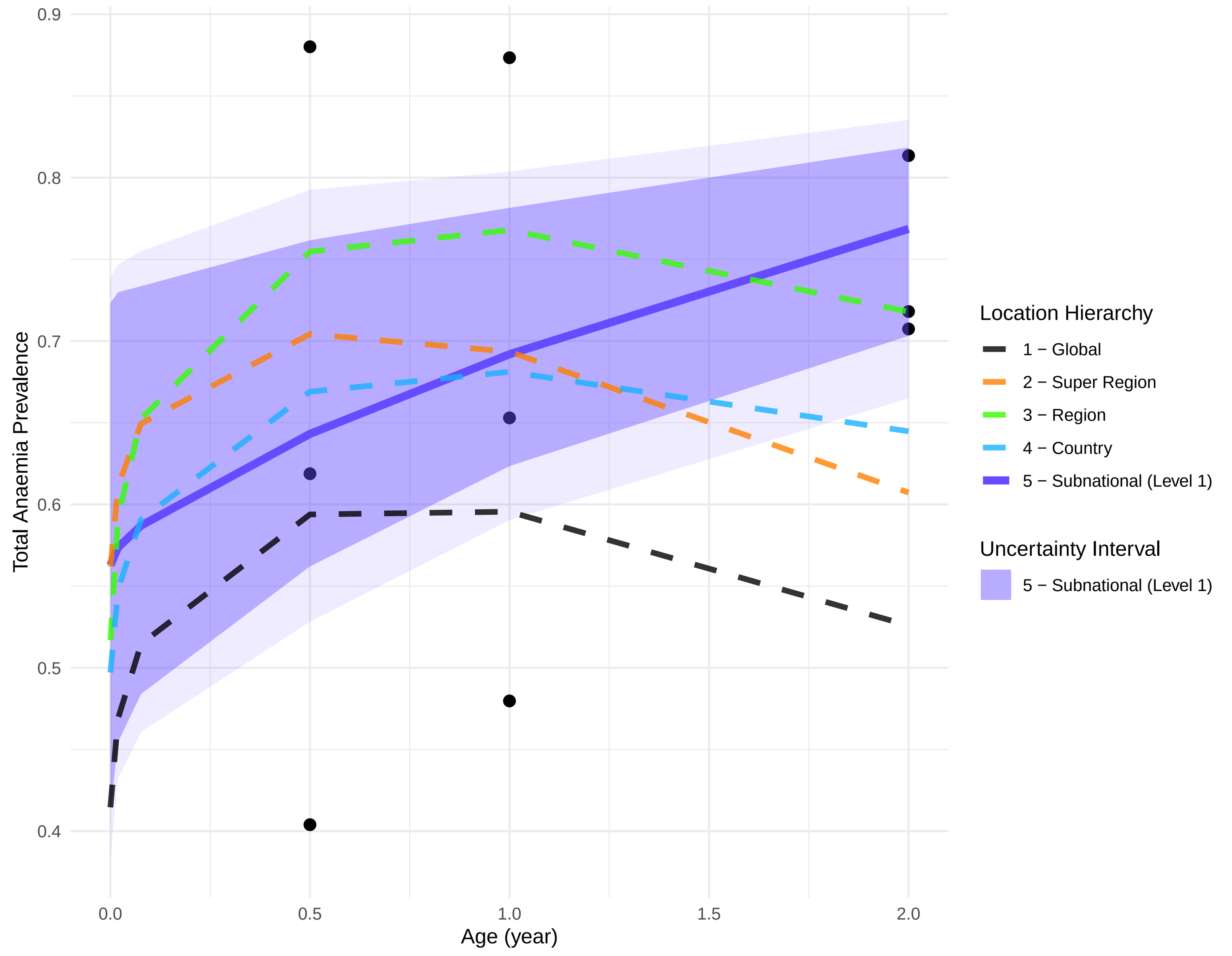

MR−BRT Age/Sex Fits − Males <2 Years Old − Total Anaemia Prevalence − Benue
WHO−2001 Adjustment Method

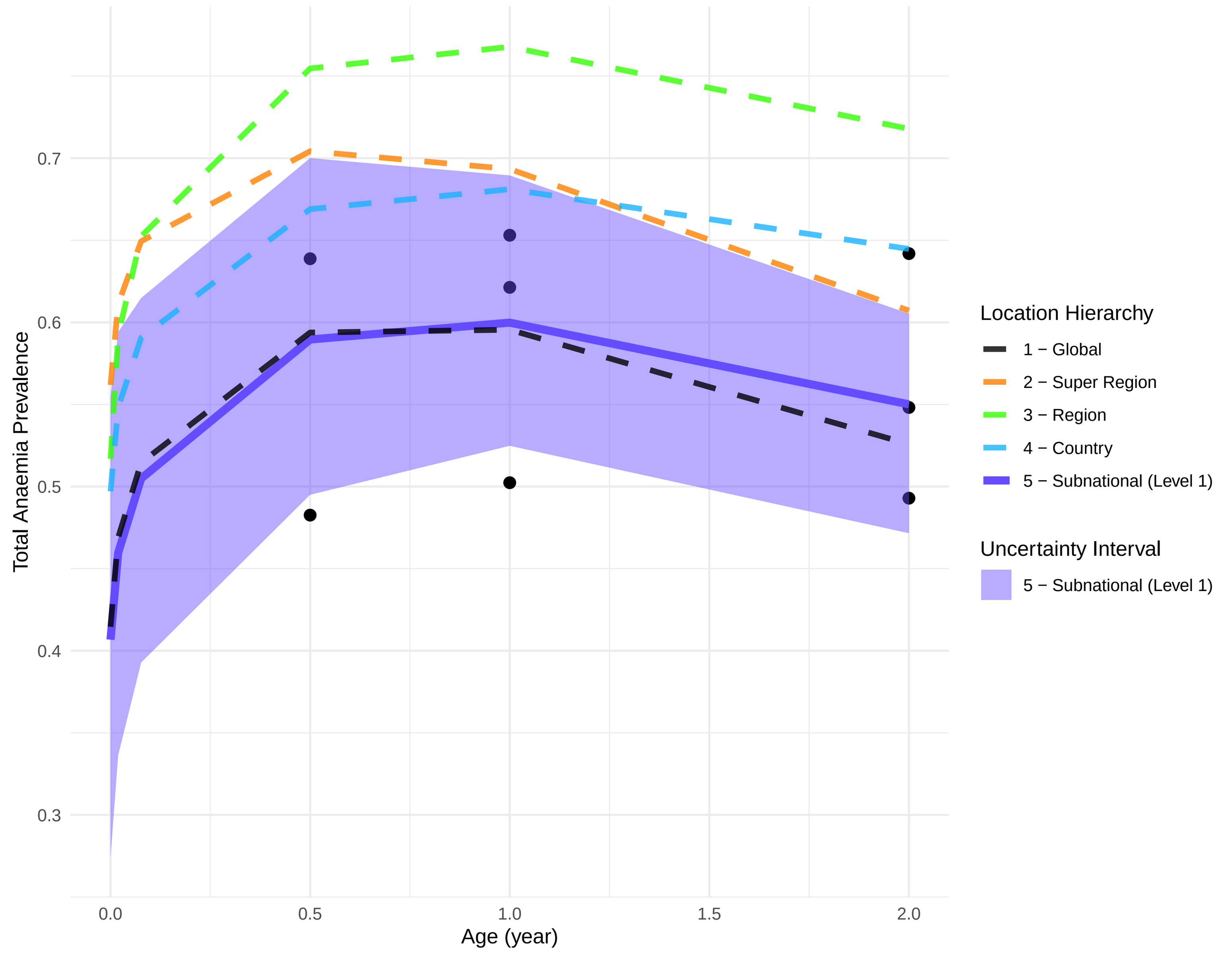

MR−BRT Age/Sex Fits − Males <2 Years Old − Total Anaemia Prevalence − Borno
WHO−2001 Adjustment Method

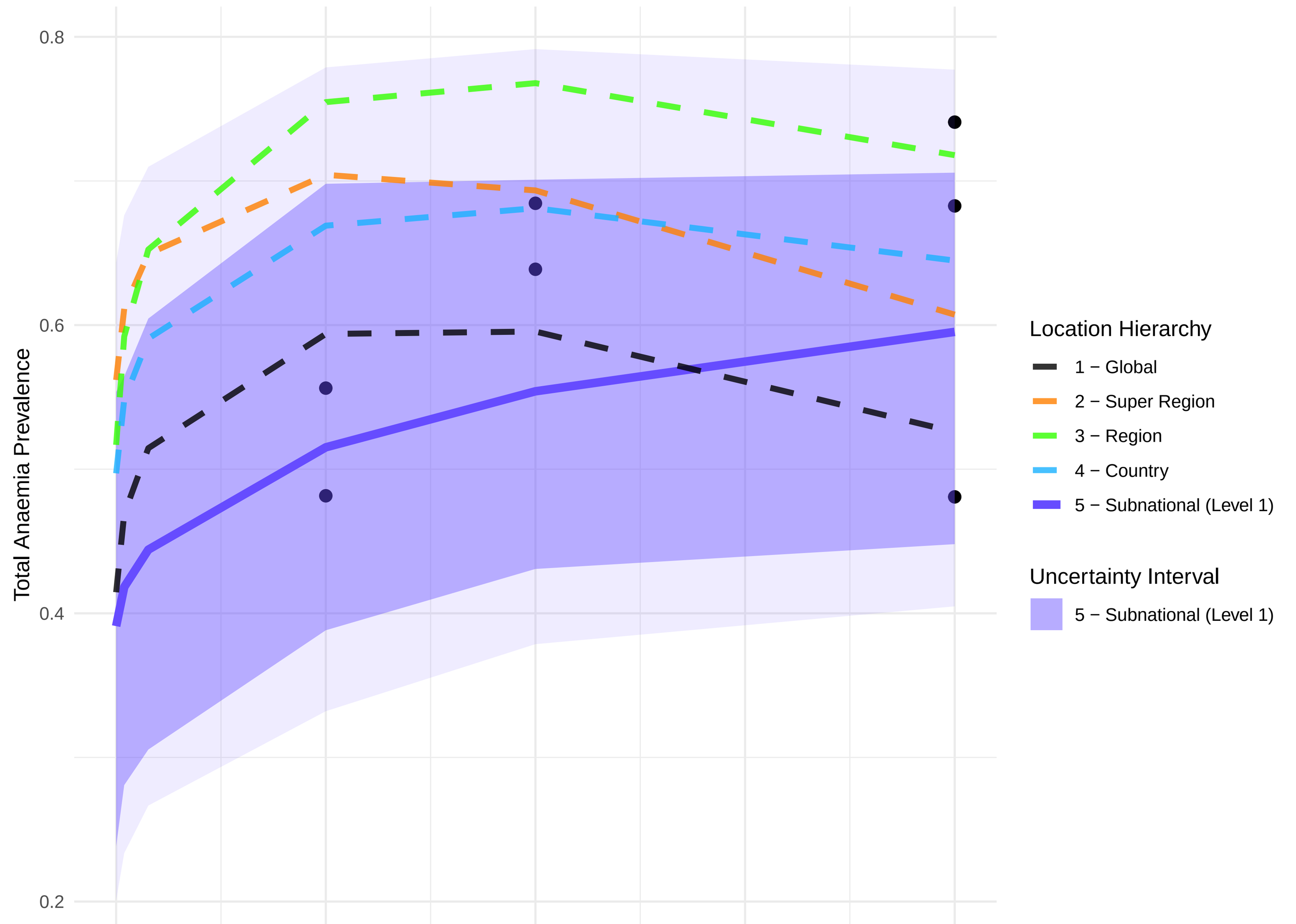

MR−BRT Age/Sex Fits − Males <2 Years Old − Total Anaemia Prevalence − Cross River
WHO−2001 Adjustment Method

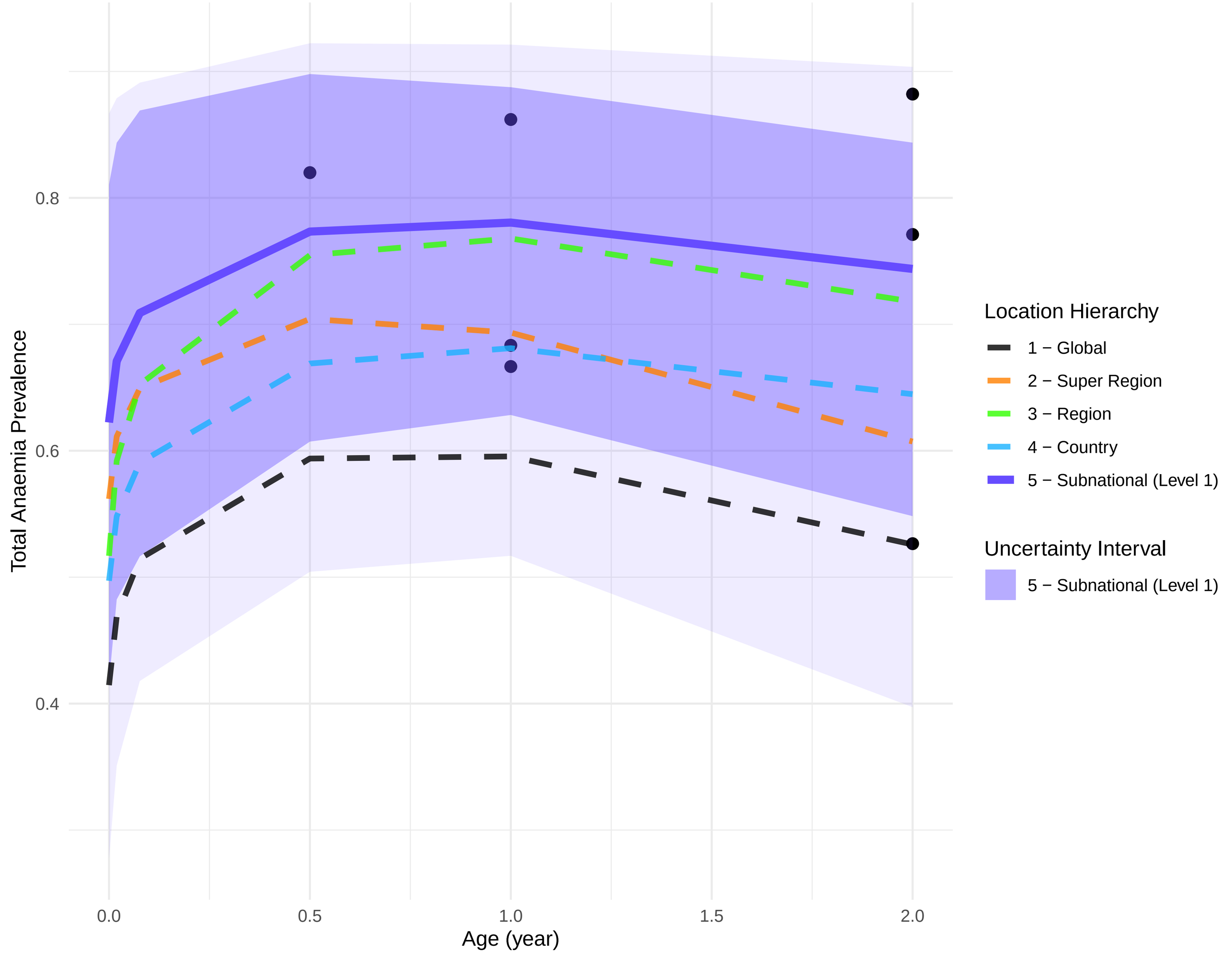

MR−BRT Age/Sex Fits − Males <2 Years Old − Total Anaemia Prevalence − Delta
WHO−2001 Adjustment Method

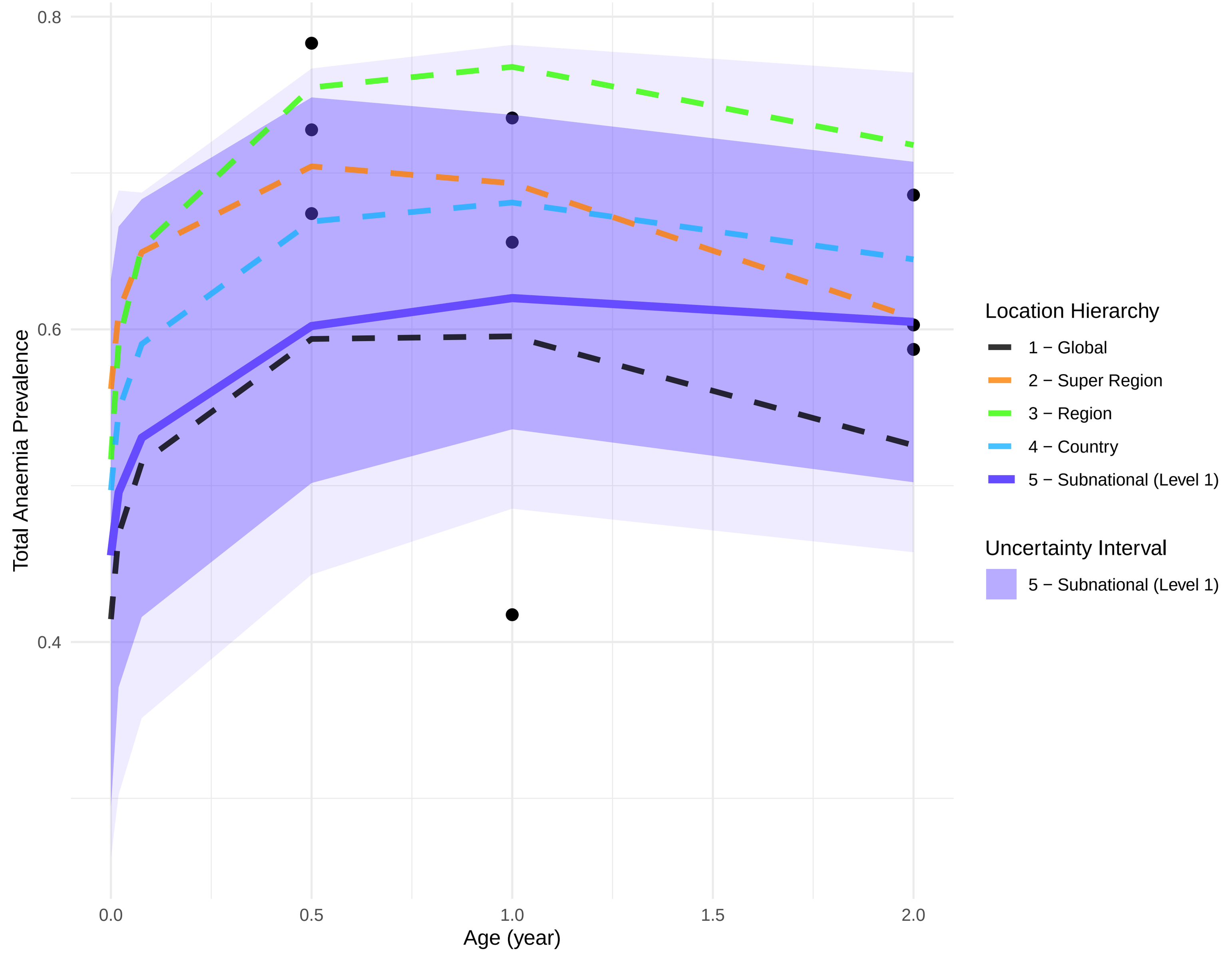

MR−BRT Age/Sex Fits − Males <2 Years Old − Total Anaemia Prevalence − Ebonyi
WHO−2001 Adjustment Method

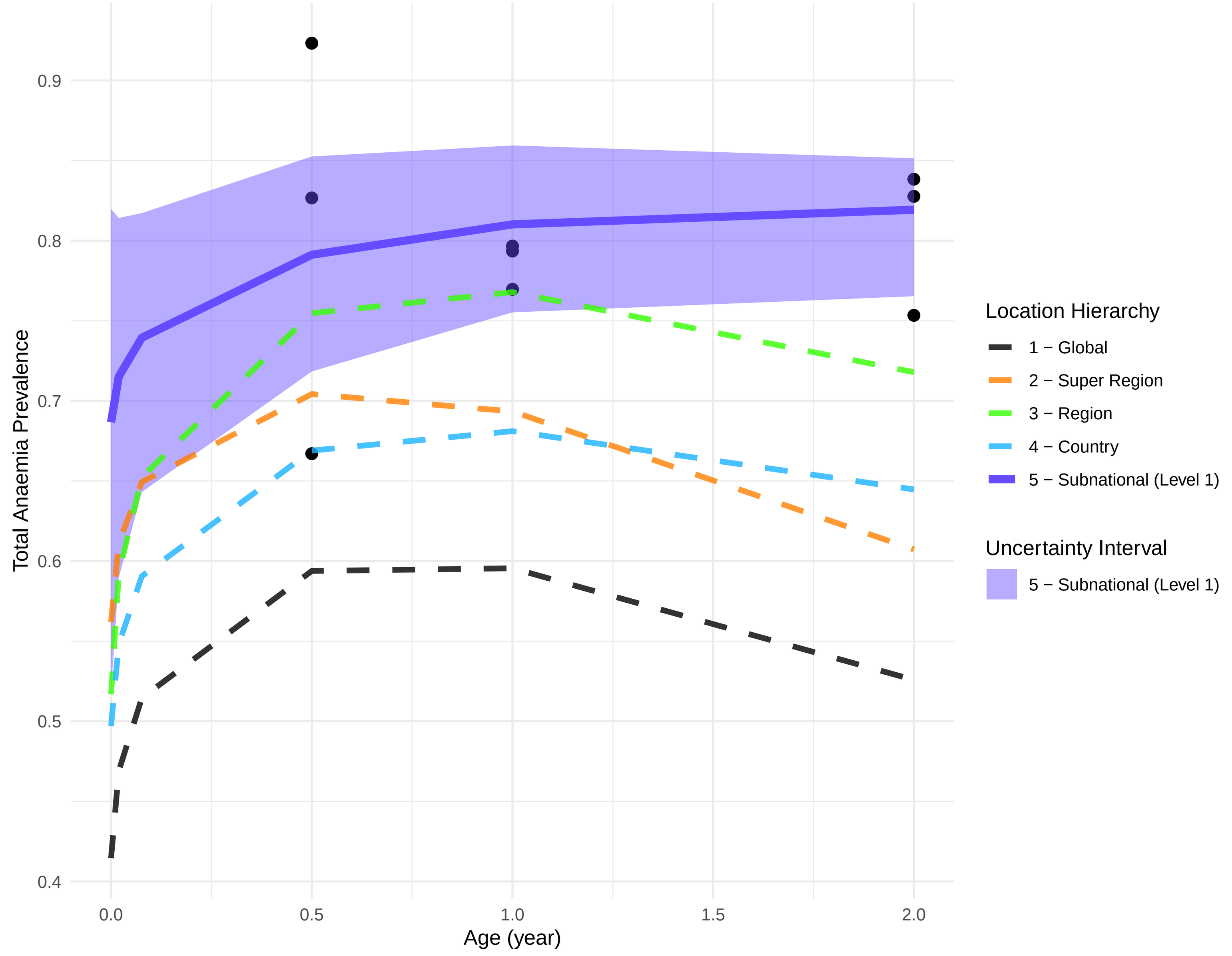

MR−BRT Age/Sex Fits − Males <2 Years Old − Total Anaemia Prevalence − Edo
WHO−2001 Adjustment Method

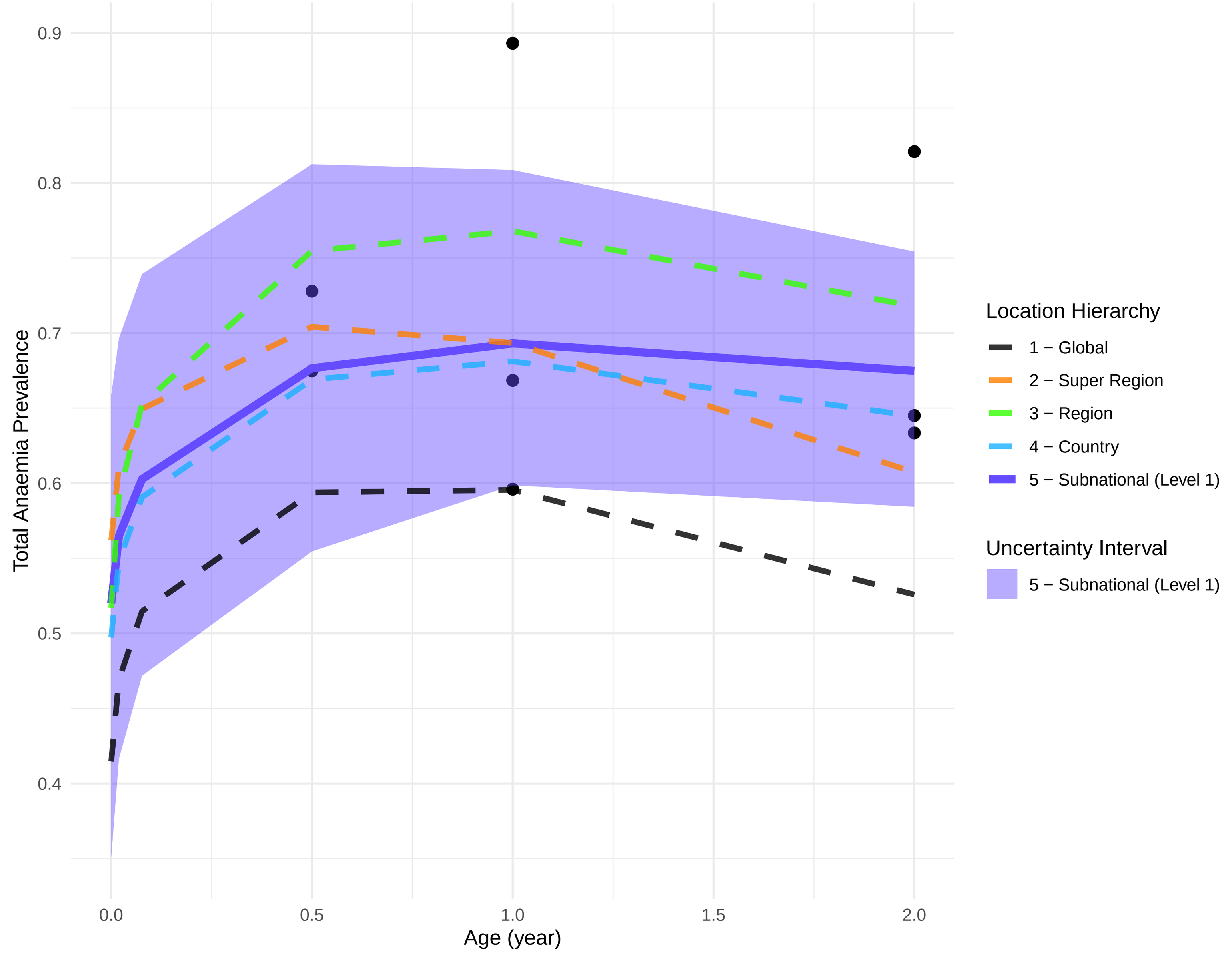

MR−BRT Age/Sex Fits − Males <2 Years Old − Total Anaemia Prevalence − Ekiti
WHO−2001 Adjustment Method

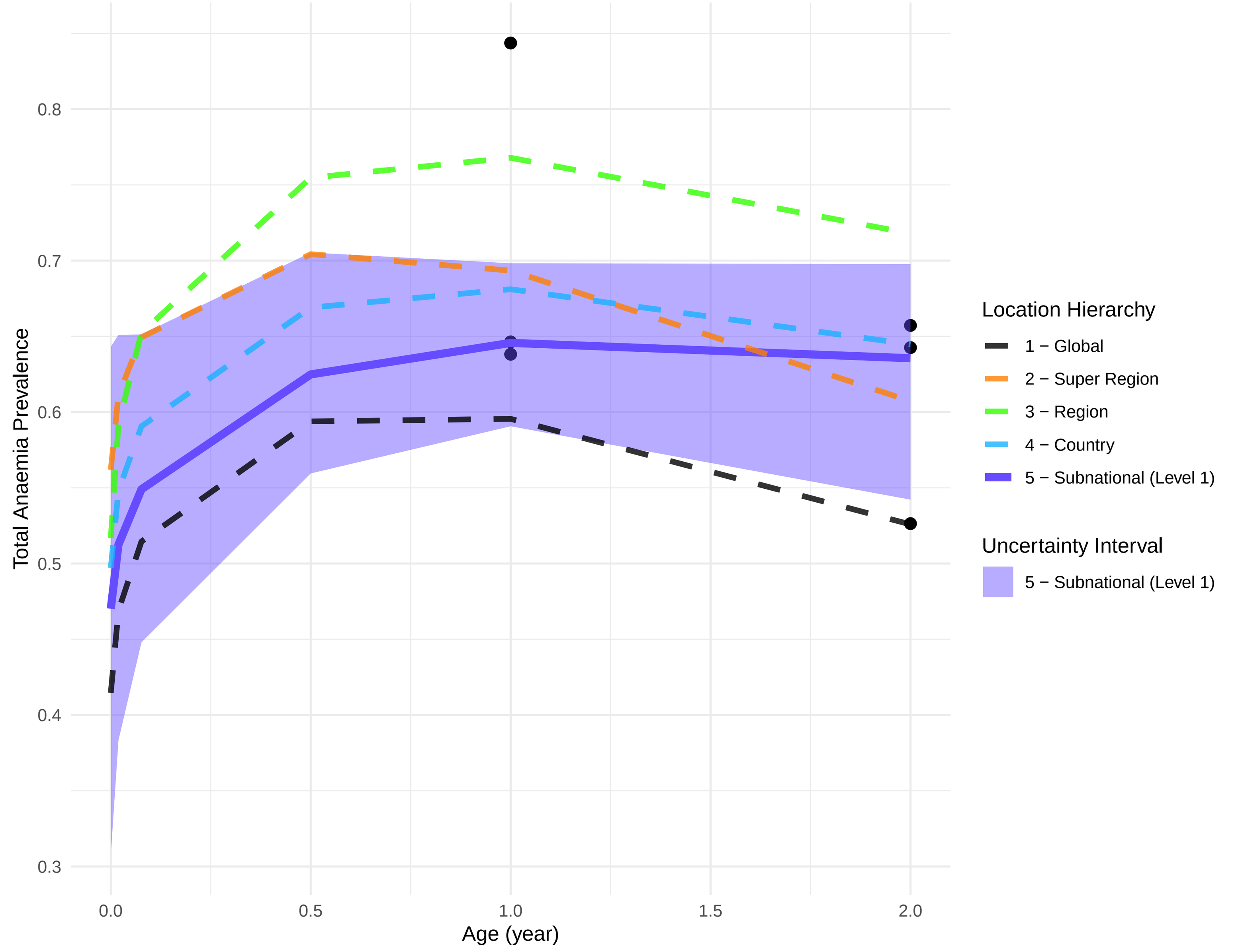

MR−BRT Age/Sex Fits − Males <2 Years Old − Total Anaemia Prevalence − Enugu
WHO−2001 Adjustment Method

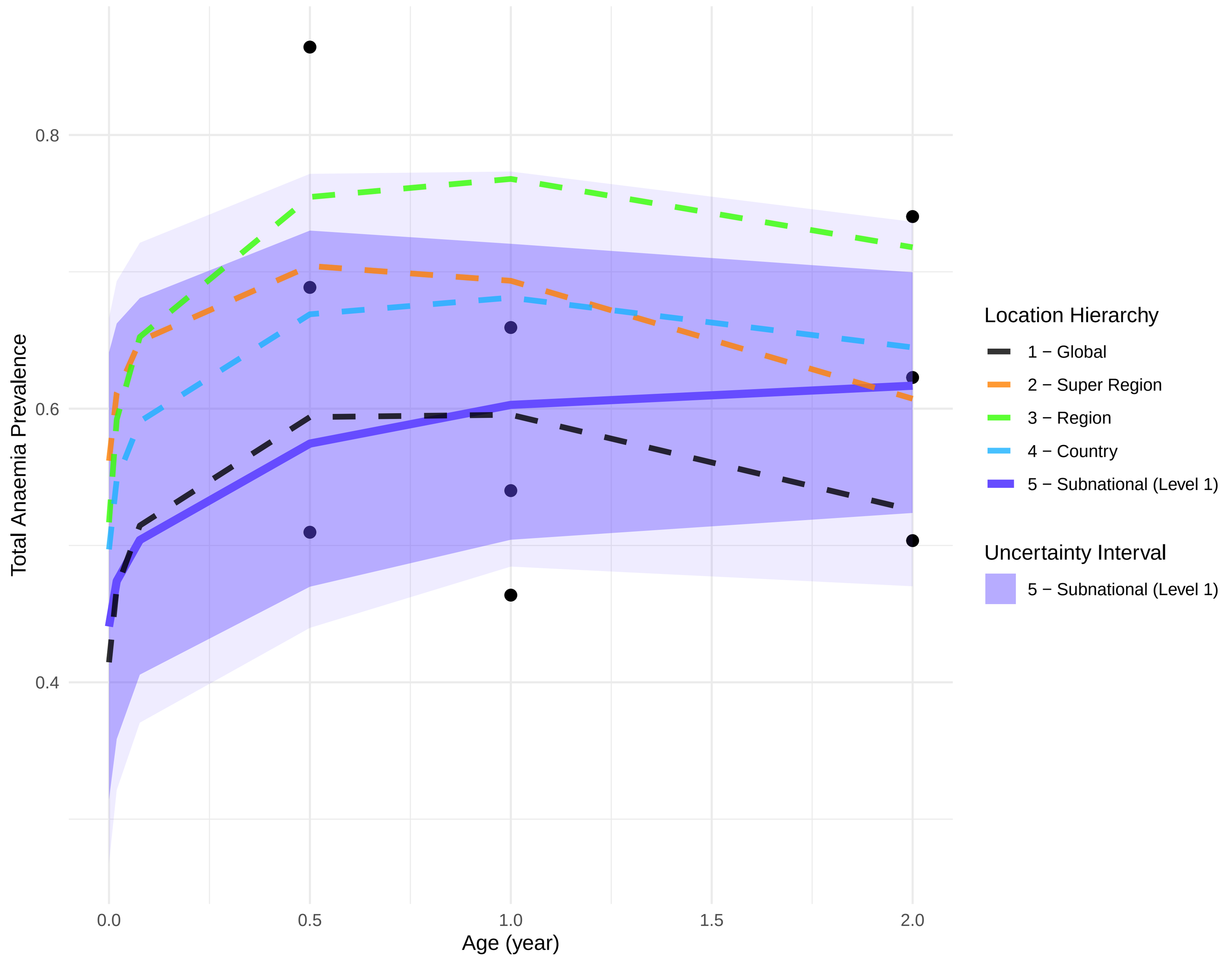

MR−BRT Age/Sex Fits − Males <2 Years Old − Total Anaemia Prevalence − FCT (Abuja)
WHO−2001 Adjustment Method

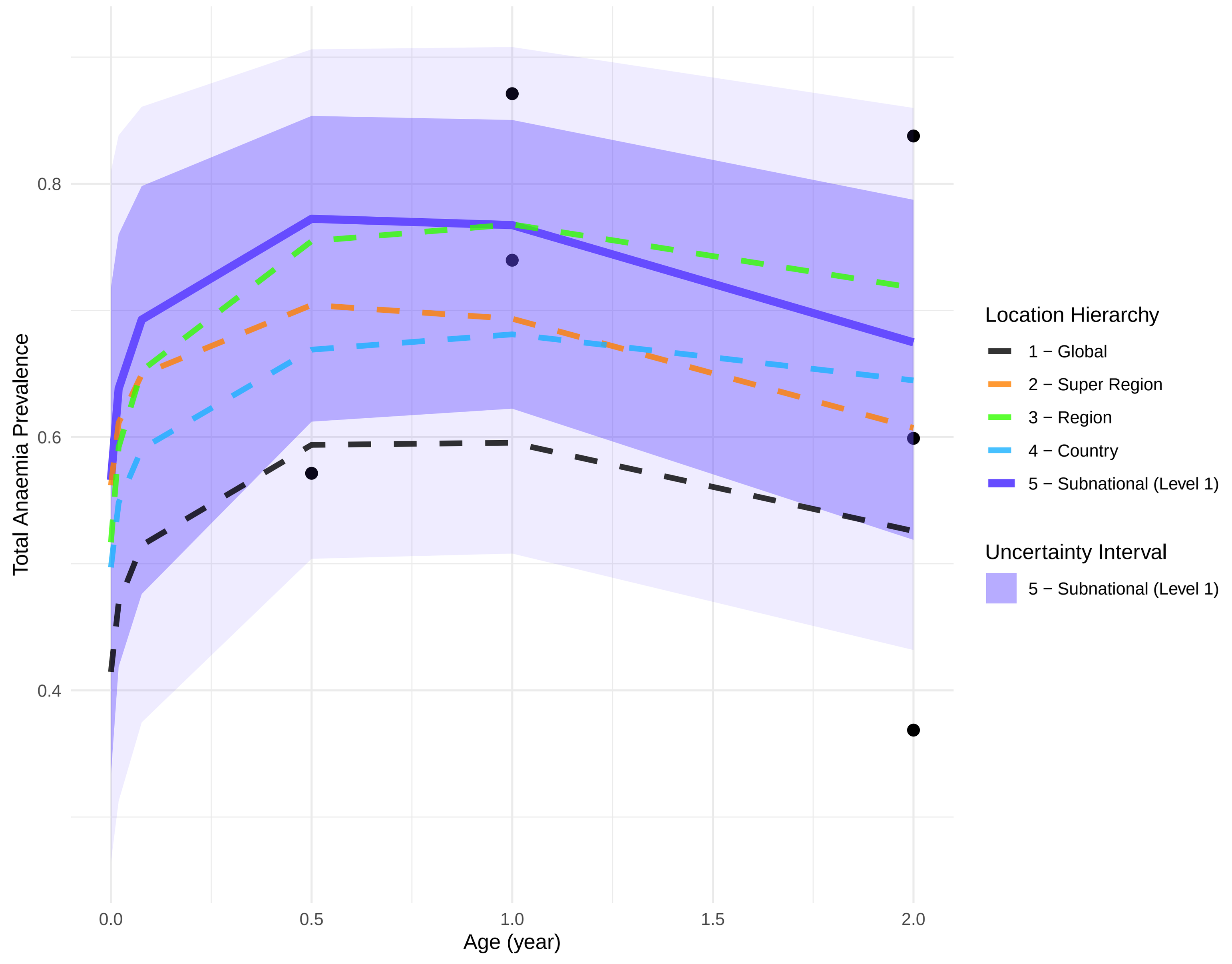

MR−BRT Age/Sex Fits − Males <2 Years Old − Total Anaemia Prevalence − Gombe
WHO−2001 Adjustment Method

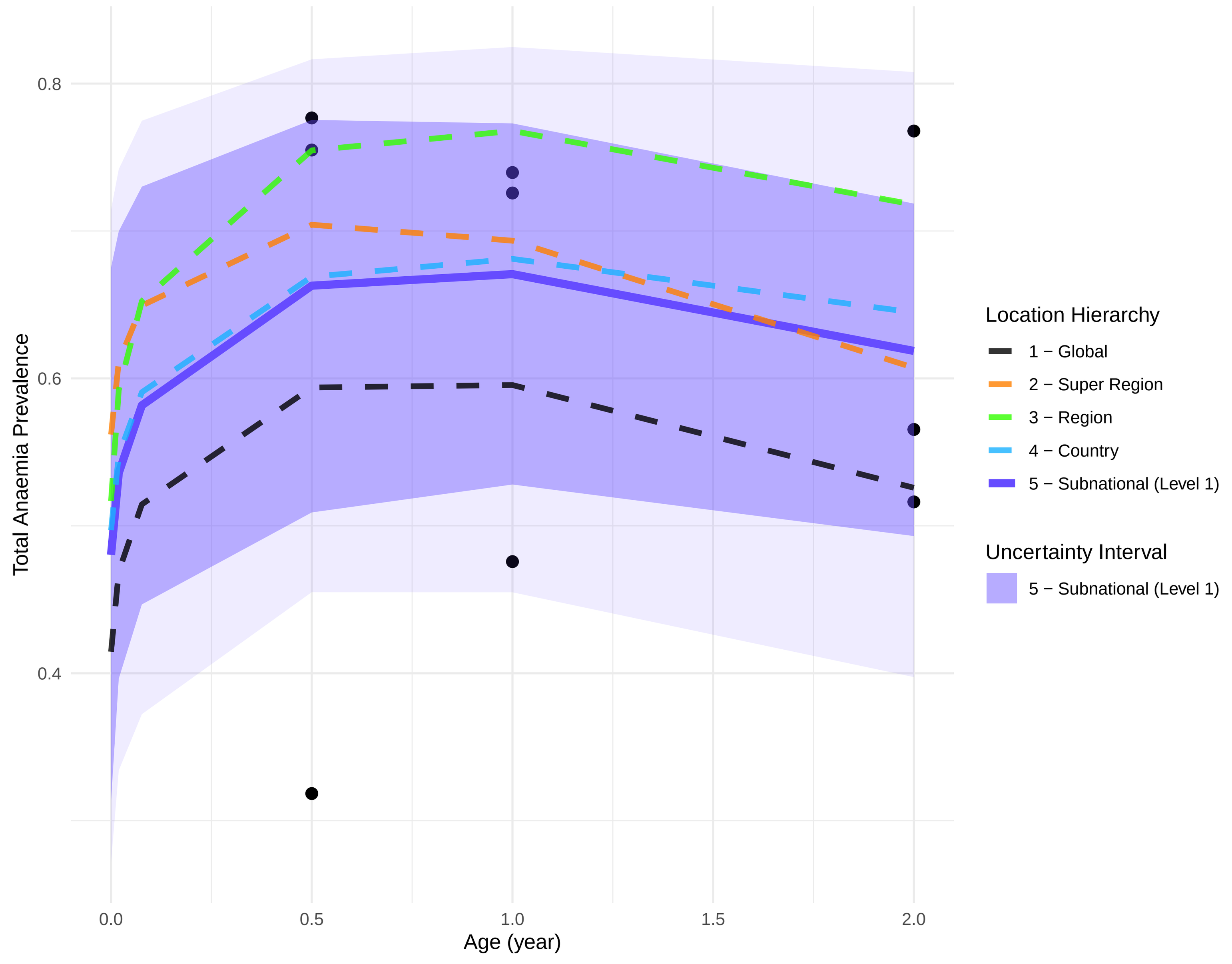

MR−BRT Age/Sex Fits − Males <2 Years Old − Total Anaemia Prevalence − Imo
WHO−2001 Adjustment Method

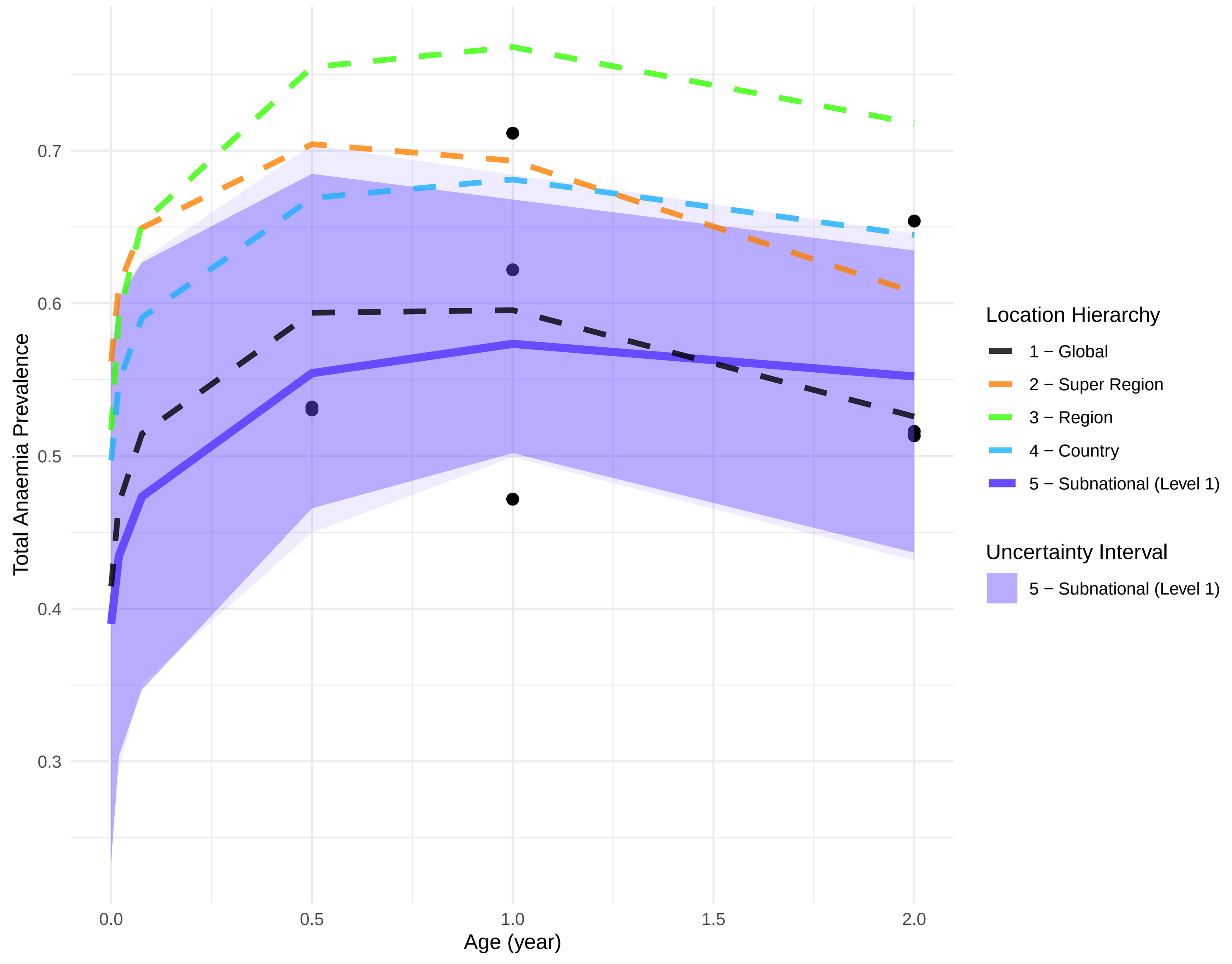

MR−BRT Age/Sex Fits − Males <2 Years Old − Total Anaemia Prevalence − Jigawa
WHO−2001 Adjustment Method

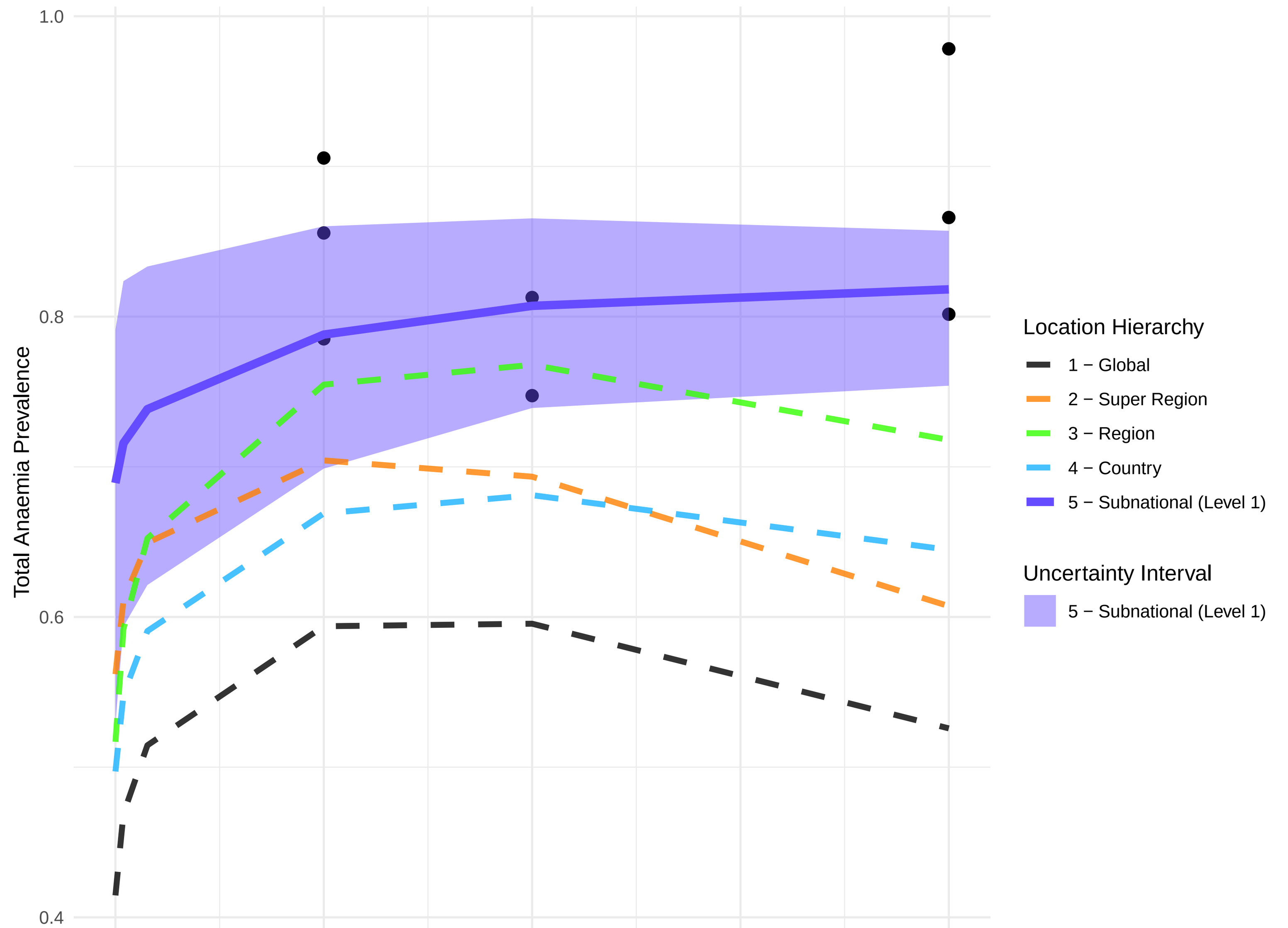

MR−BRT Age/Sex Fits − Males <2 Years Old − Total Anaemia Prevalence − Kaduna
WHO−2001 Adjustment Method

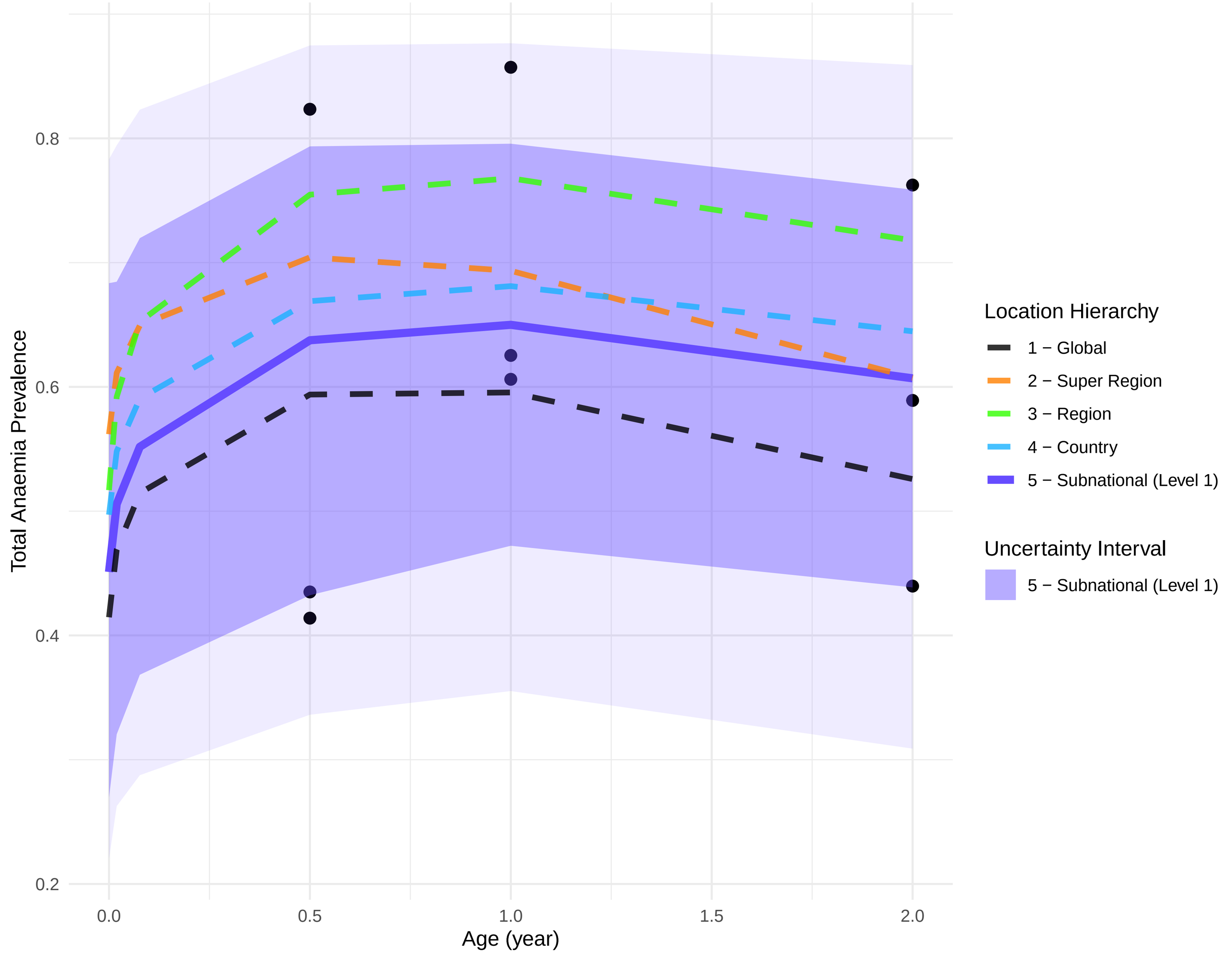

MR−BRT Age/Sex Fits − Males <2 Years Old − Total Anaemia Prevalence − Kano
WHO−2001 Adjustment Method

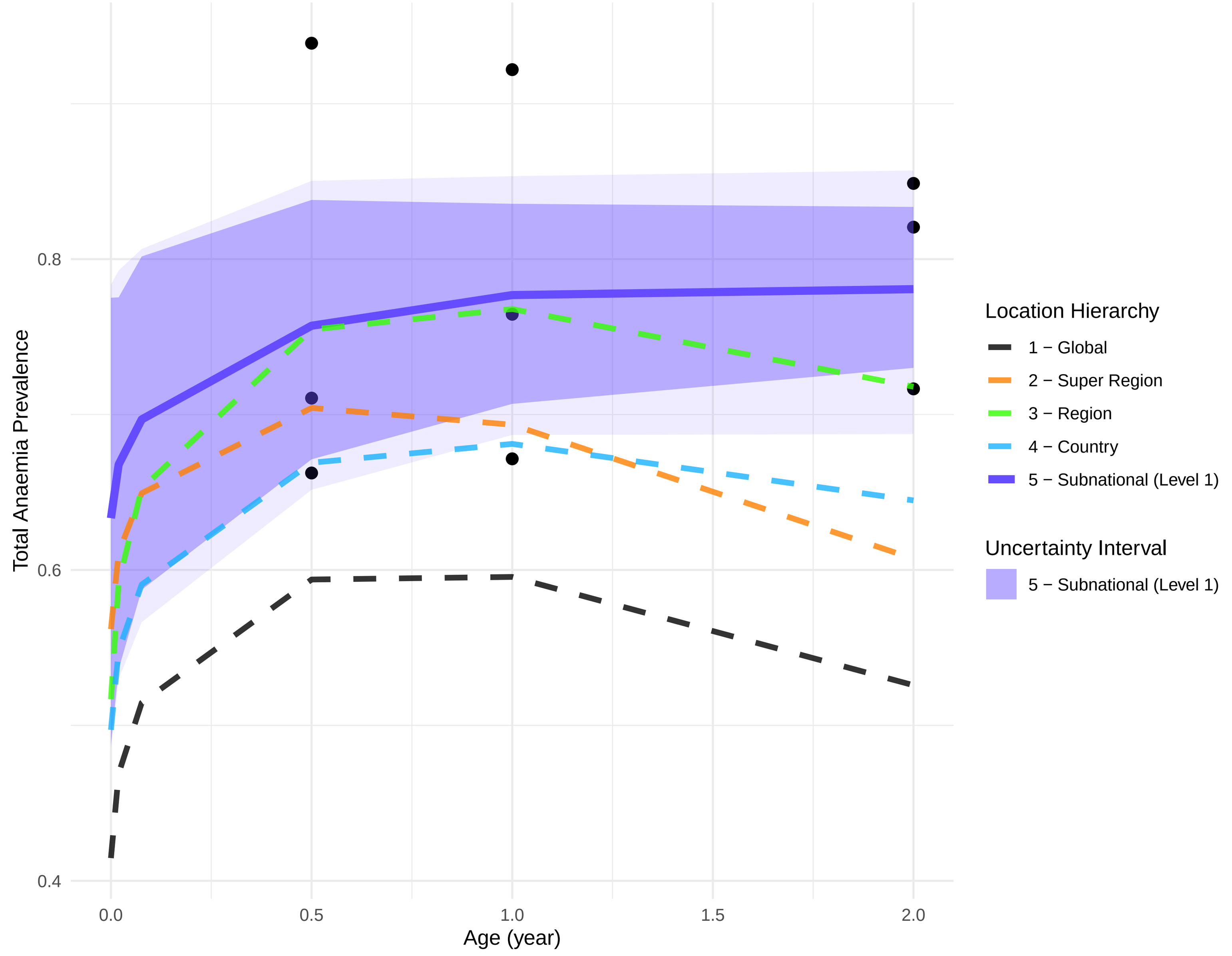

MR−BRT Age/Sex Fits − Males <2 Years Old − Total Anaemia Prevalence − Katsina
WHO−2001 Adjustment Method

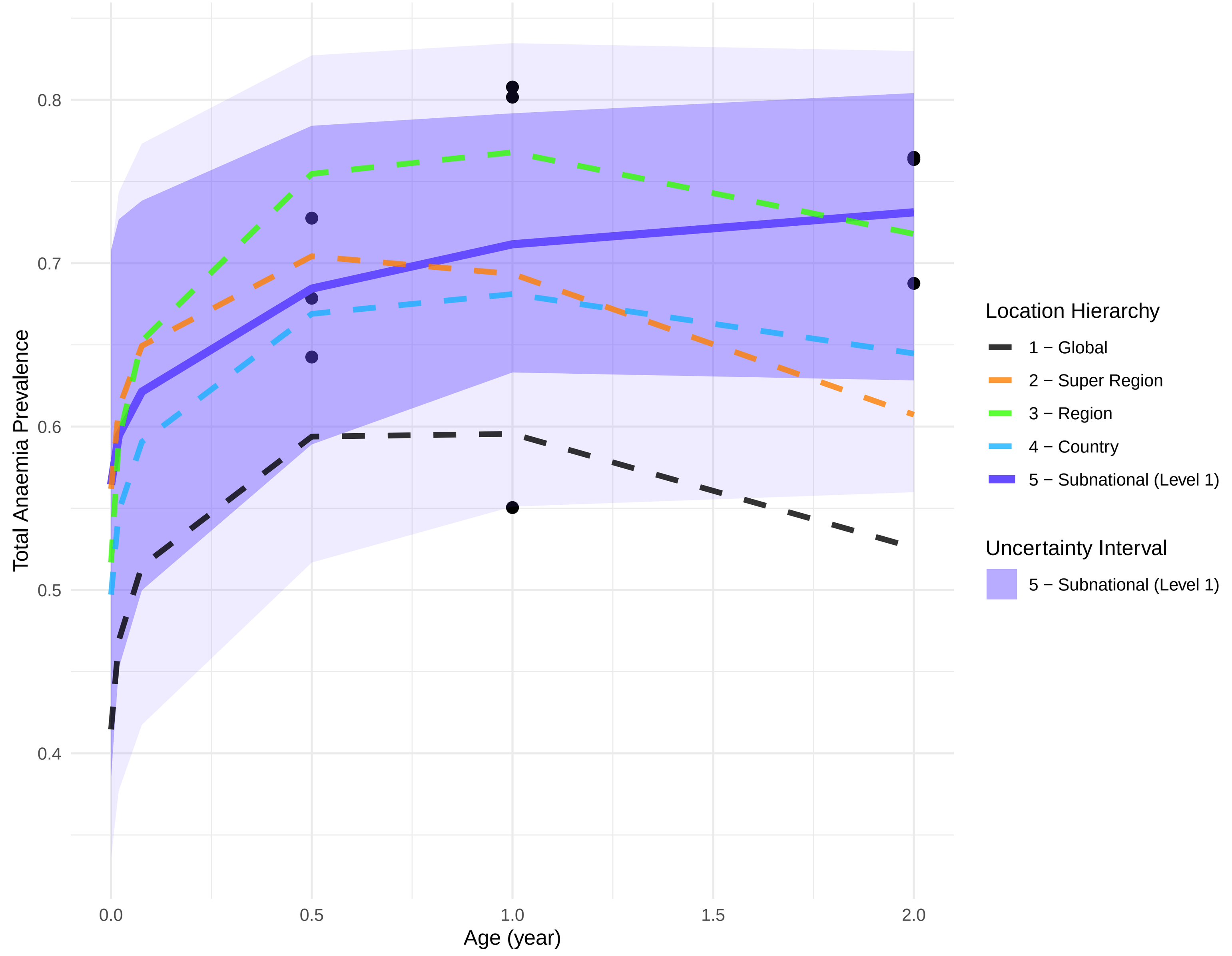

MR−BRT Age/Sex Fits − Males <2 Years Old − Total Anaemia Prevalence − Kebbi
WHO−2001 Adjustment Method

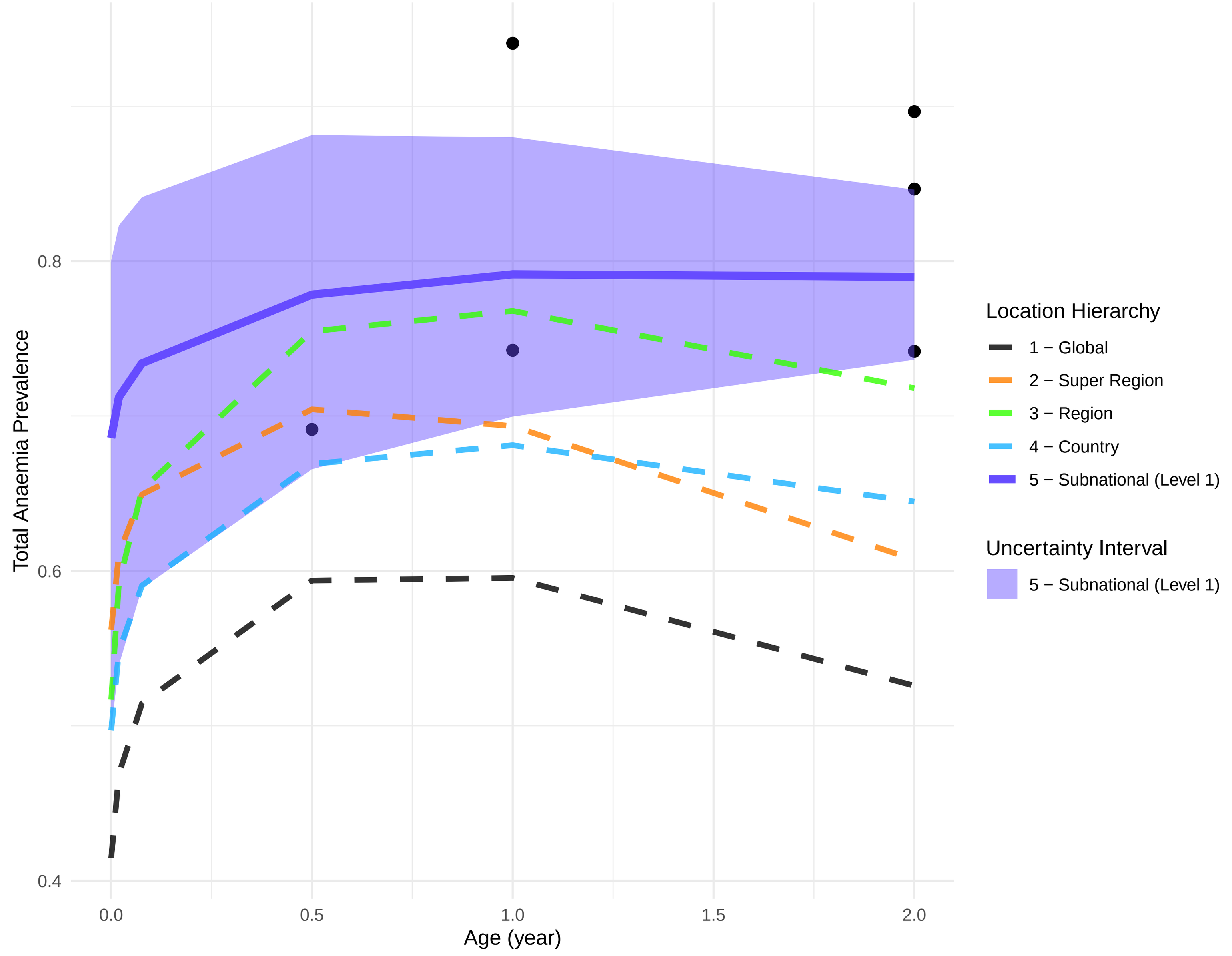

MR−BRT Age/Sex Fits − Males <2 Years Old − Total Anaemia Prevalence − Kogi
WHO−2001 Adjustment Method

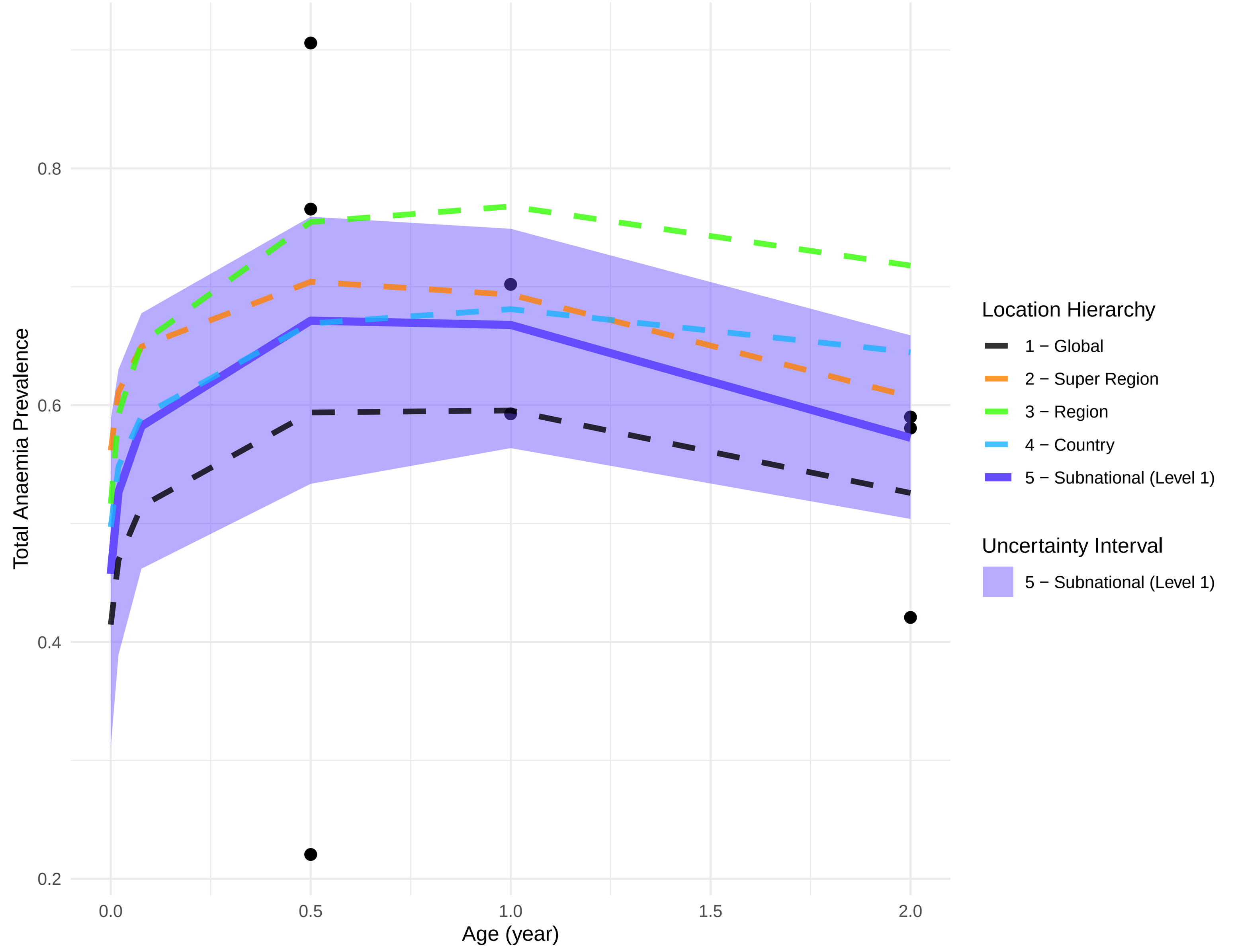

MR−BRT Age/Sex Fits − Males <2 Years Old − Total Anaemia Prevalence − Kwara
WHO−2001 Adjustment Method

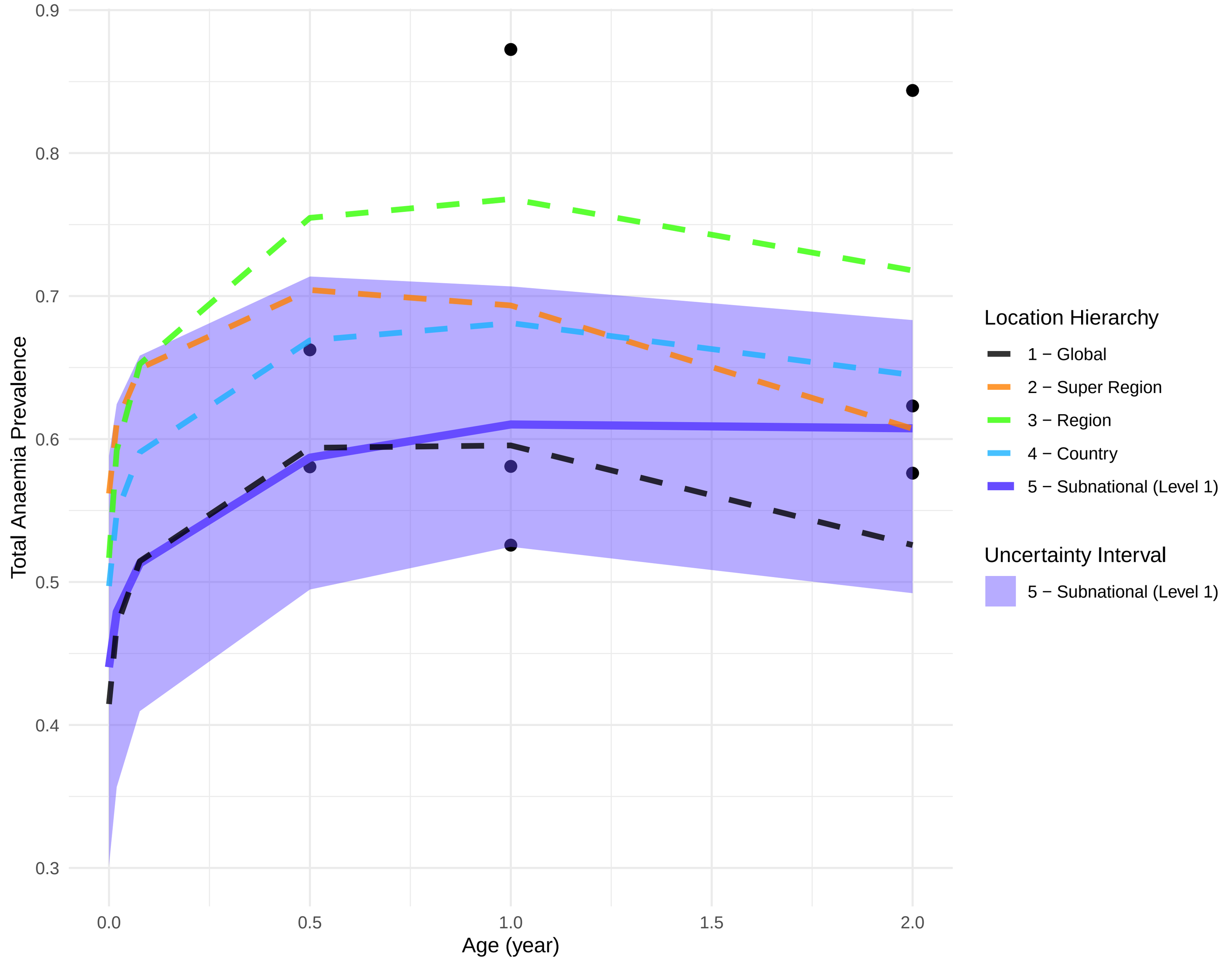

MR−BRT Age/Sex Fits − Males <2 Years Old − Total Anaemia Prevalence − Lagos
WHO−2001 Adjustment Method

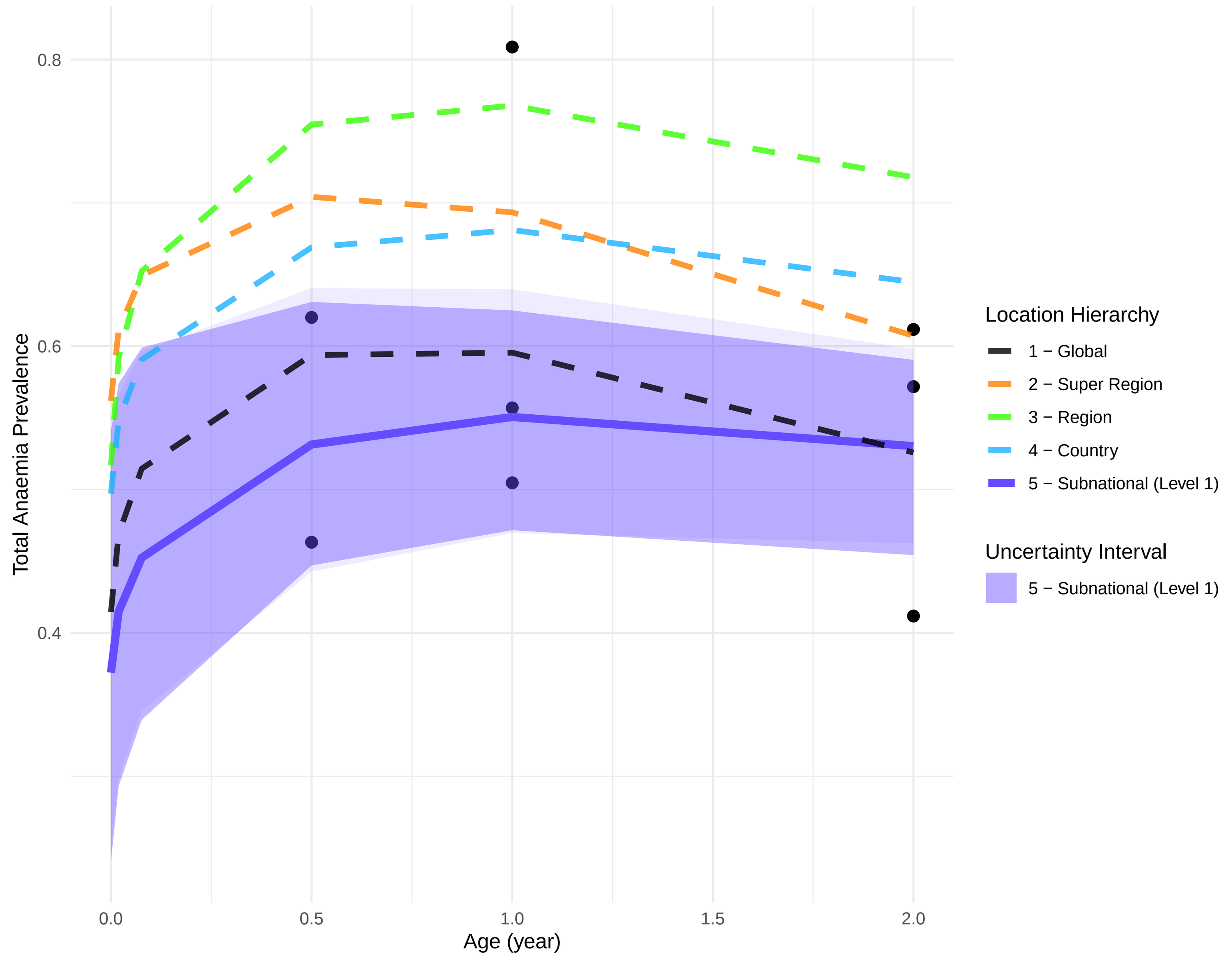

MR−BRT Age/Sex Fits − Males <2 Years Old − Total Anaemia Prevalence − Nasarawa
WHO−2001 Adjustment Method

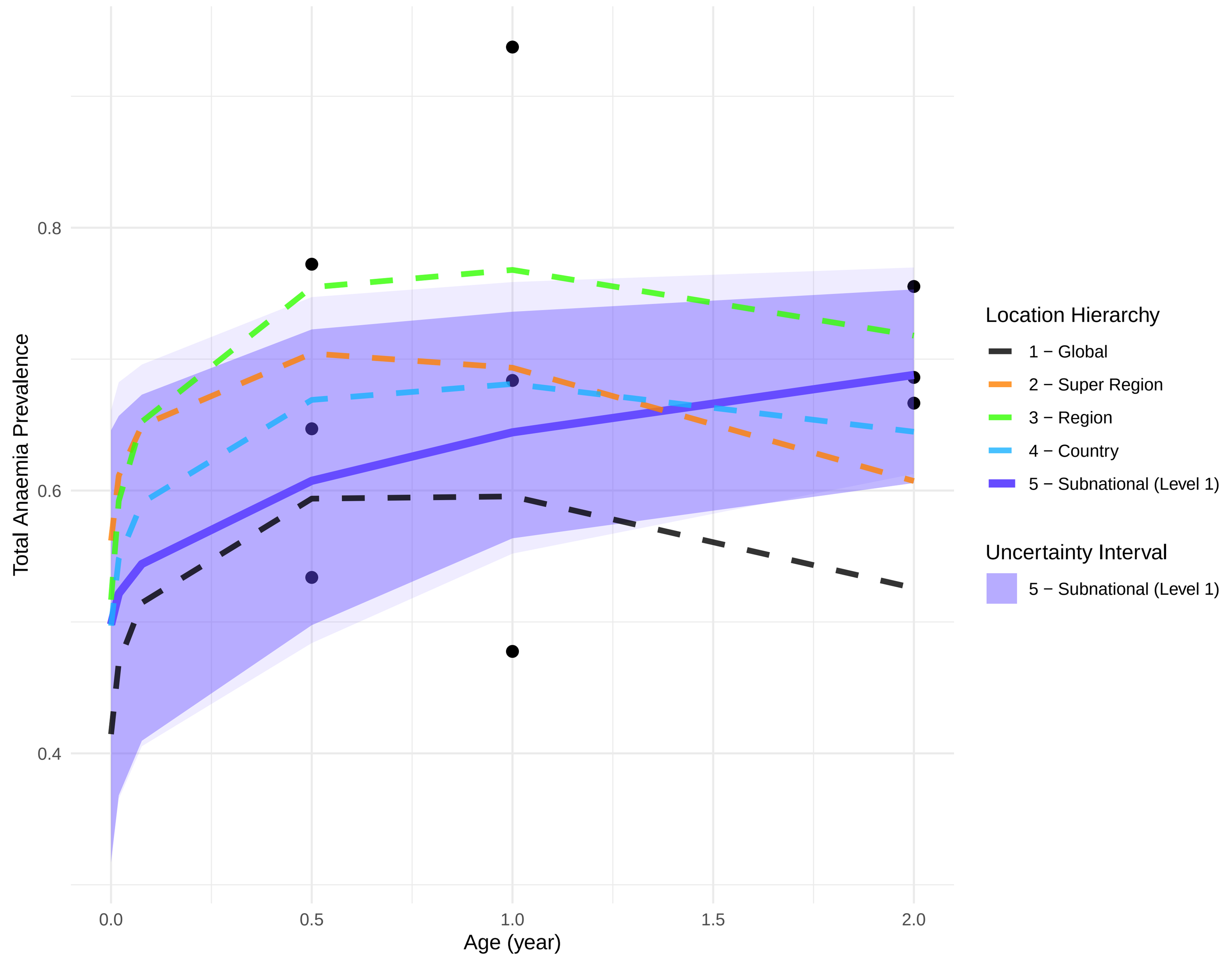

MR−BRT Age/Sex Fits − Males <2 Years Old − Total Anaemia Prevalence − Niger
WHO−2001 Adjustment Method

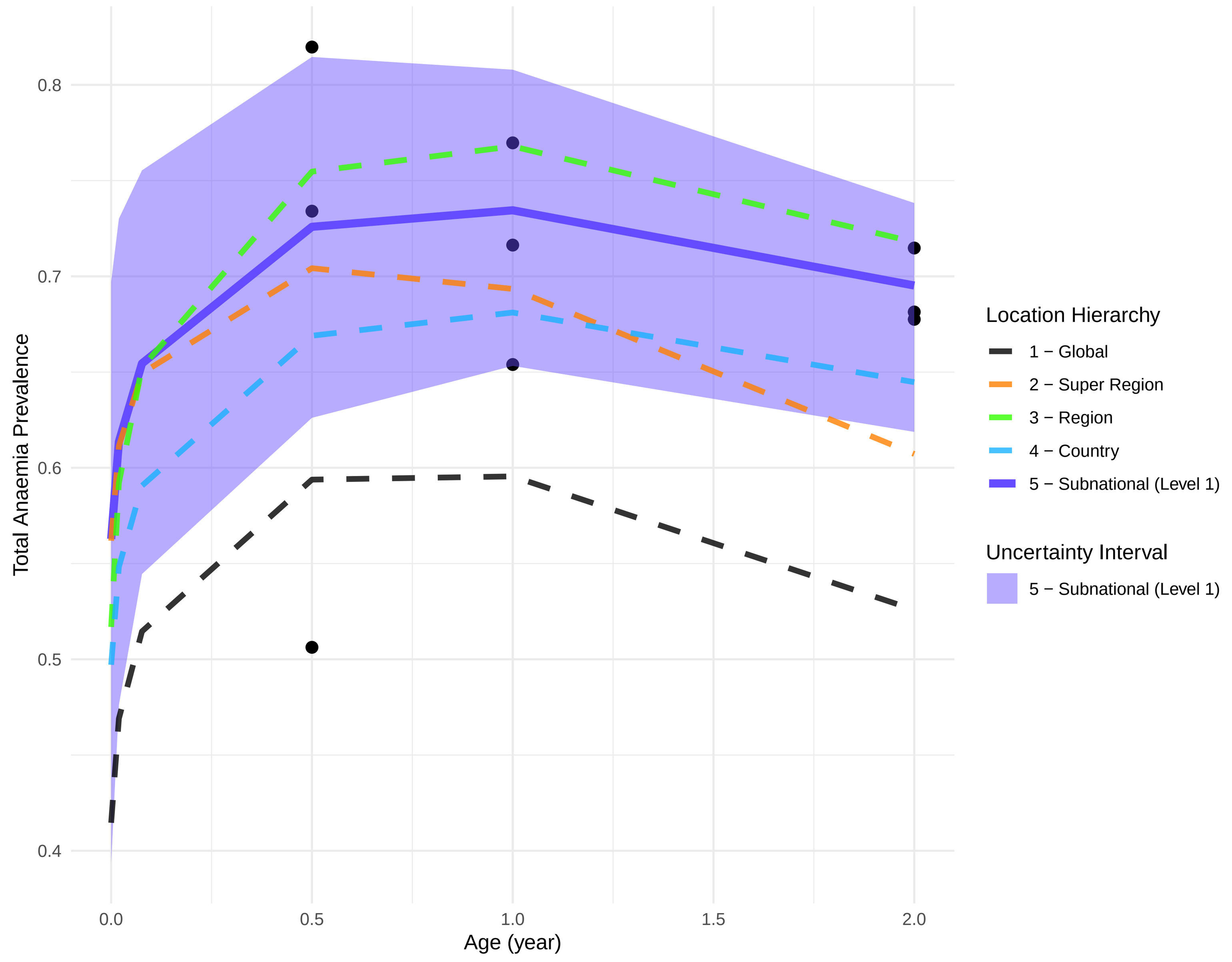

MR−BRT Age/Sex Fits − Males <2 Years Old − Total Anaemia Prevalence − Ogun
WHO−2001 Adjustment Method

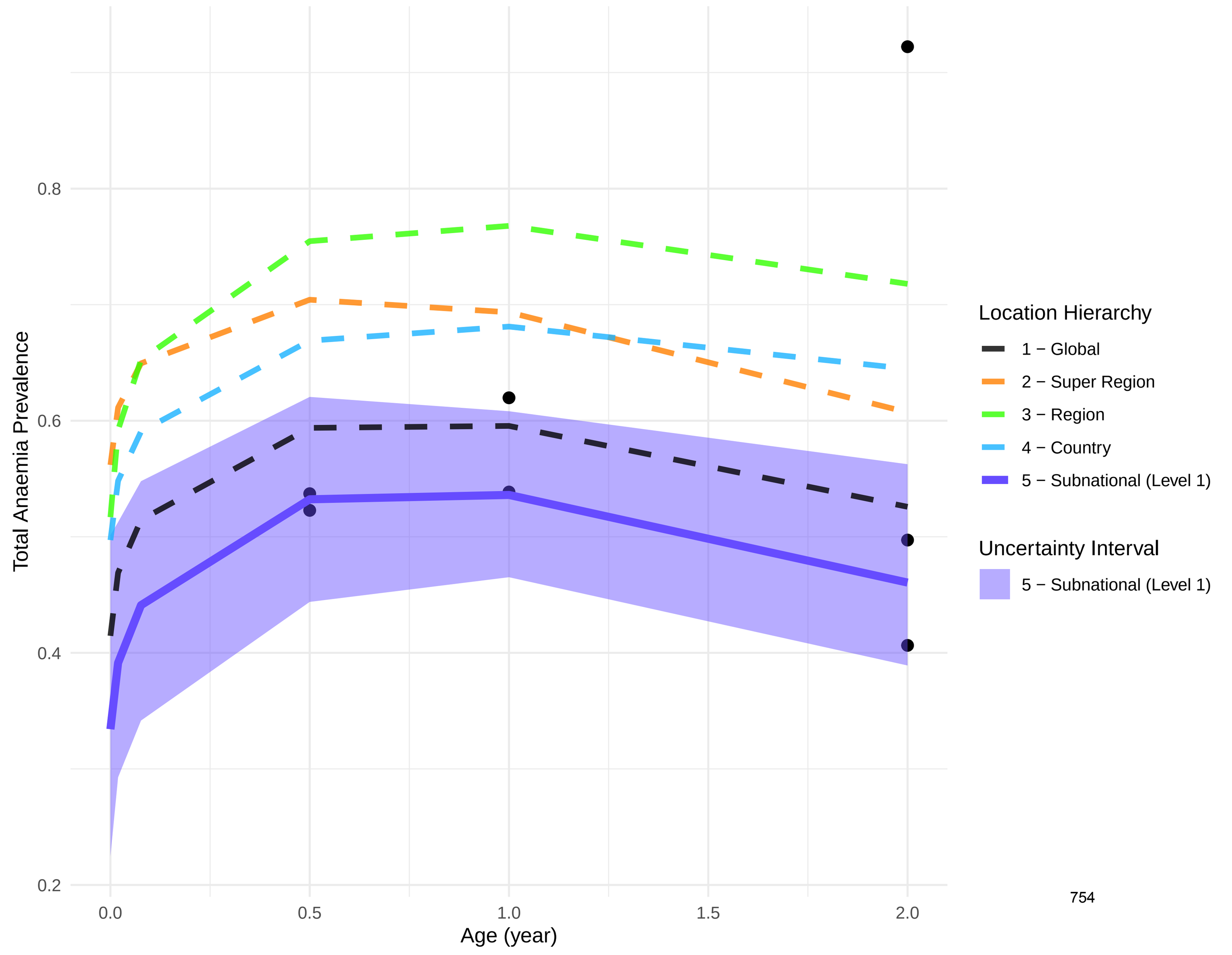

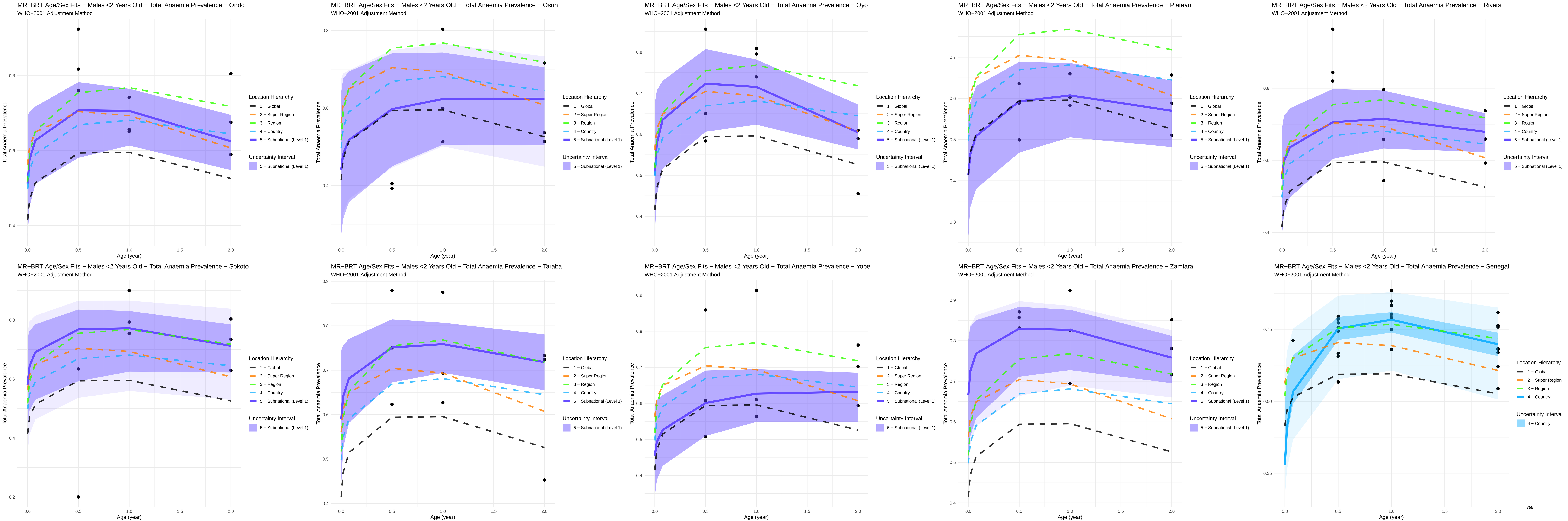
MR−BRT Age/Sex Fits − Males <2 Years Old − Total Anaemia Prevalence − Ondo
WHO−2001 Adjustment Method
MR−BRT Age/Sex Fits − Males <2 Years Old − Total Anaemia Prevalence − Osun
WHO−2001 Adjustment Method
MR−BRT Age/Sex Fits − Males <2 Years Old − Total Anaemia Prevalence − Oyo
WHO−2001 Adjustment Method
MR−BRT Age/Sex Fits − Males <2 Years Old − Total Anaemia Prevalence − Plateau
WHO−2001 Adjustment Method
MR−BRT Age/Sex Fits − Males <2 Years Old − Total Anaemia Prevalence − Rivers
WHO−2001 Adjustment Method
MR−BRT Age/Sex Fits − Males <2 Years Old − Total Anaemia Prevalence − Sokoto
WHO−2001 Adjustment Method
MR−BRT Age/Sex Fits − Males <2 Years Old − Total Anaemia Prevalence − Taraba
WHO−2001 Adjustment Method
MR−BRT Age/Sex Fits − Males <2 Years Old − Total Anaemia Prevalence − Yobe
WHO−2001 Adjustment Method
MR−BRT Age/Sex Fits − Males <2 Years Old − Total Anaemia Prevalence − Zamfara
WHO−2001 Adjustment Method
MR−BRT Age/Sex Fits − Males <2 Years Old − Total Anaemia Prevalence − Senegal
WHO−2001 Adjustment Method
Total Anaemia Prevalence
Age (year)
Location Hierarchy
1 − Global
2 − Super Region
3 − Region
4 − Country
5 − Subnational (Level 1)
Uncertainty Interval
5 − Subnational (Level 1)
4 − Country

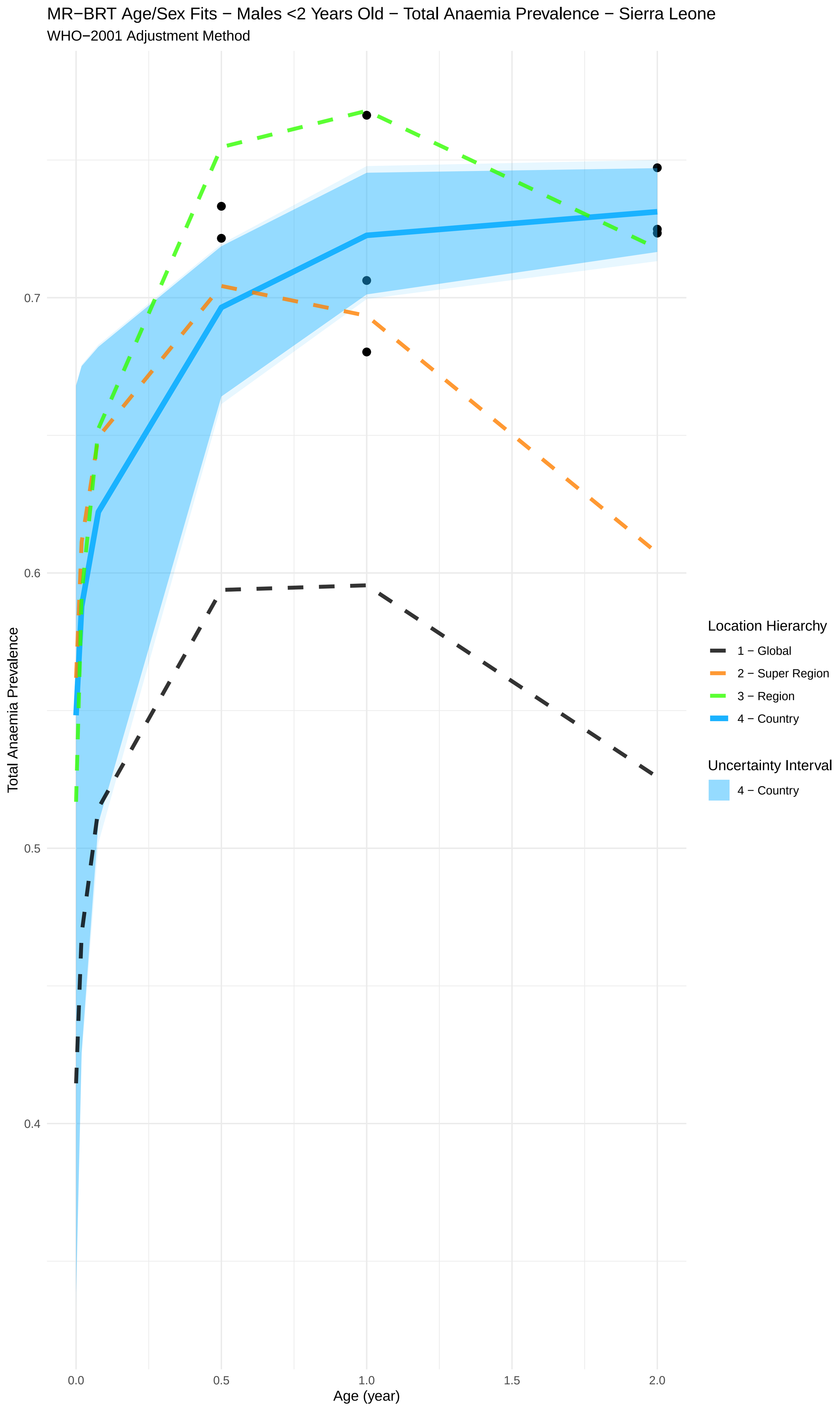
MR−BRT Age/Sex Fits − Males <2 Years Old − Total Anaemia Prevalence − Sierra Leone
WHO−2001 Adjustment Method
Total Anaemia Prevalence
Age (year)
Location Hierarchy
1 − Global
2 − Super Region
3 − Region
4 − Country
Uncertainty Interval
4 − Country

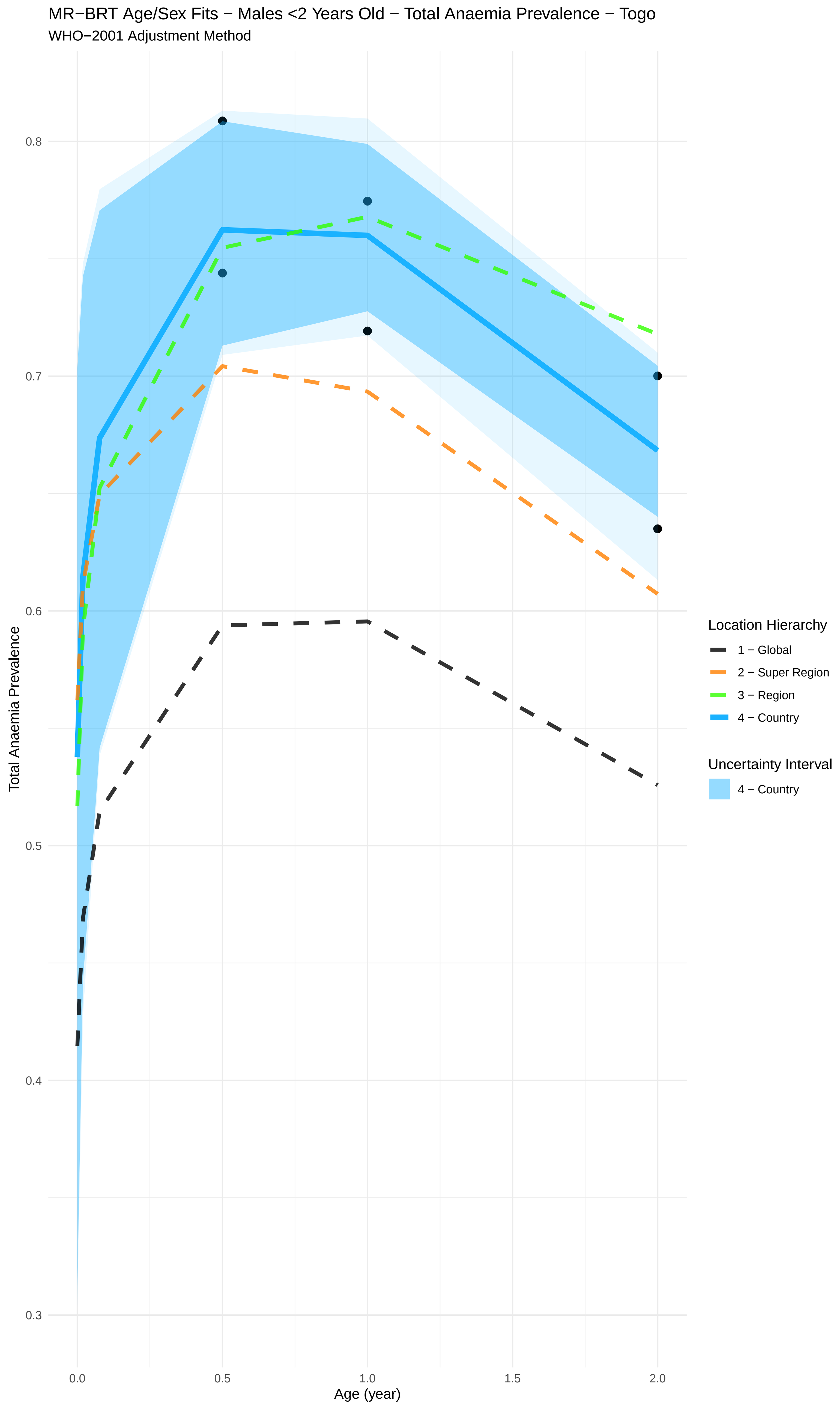
MR−BRT Age/Sex Fits − Males <2 Years Old − Total Anaemia Prevalence − Togo
WHO−2001 Adjustment Method
Total Anaemia Prevalence
Age (year)
Location Hierarchy
1 − Global
2 − Super Region
3 − Region
4 − Country
Uncertainty Interval
4 − Country

MR−BRT Age/Sex Fits − Non−pregnant Females <2 Years Old − Total Anaemia Prevalence − Global
WHO−2001 Adjustment Method

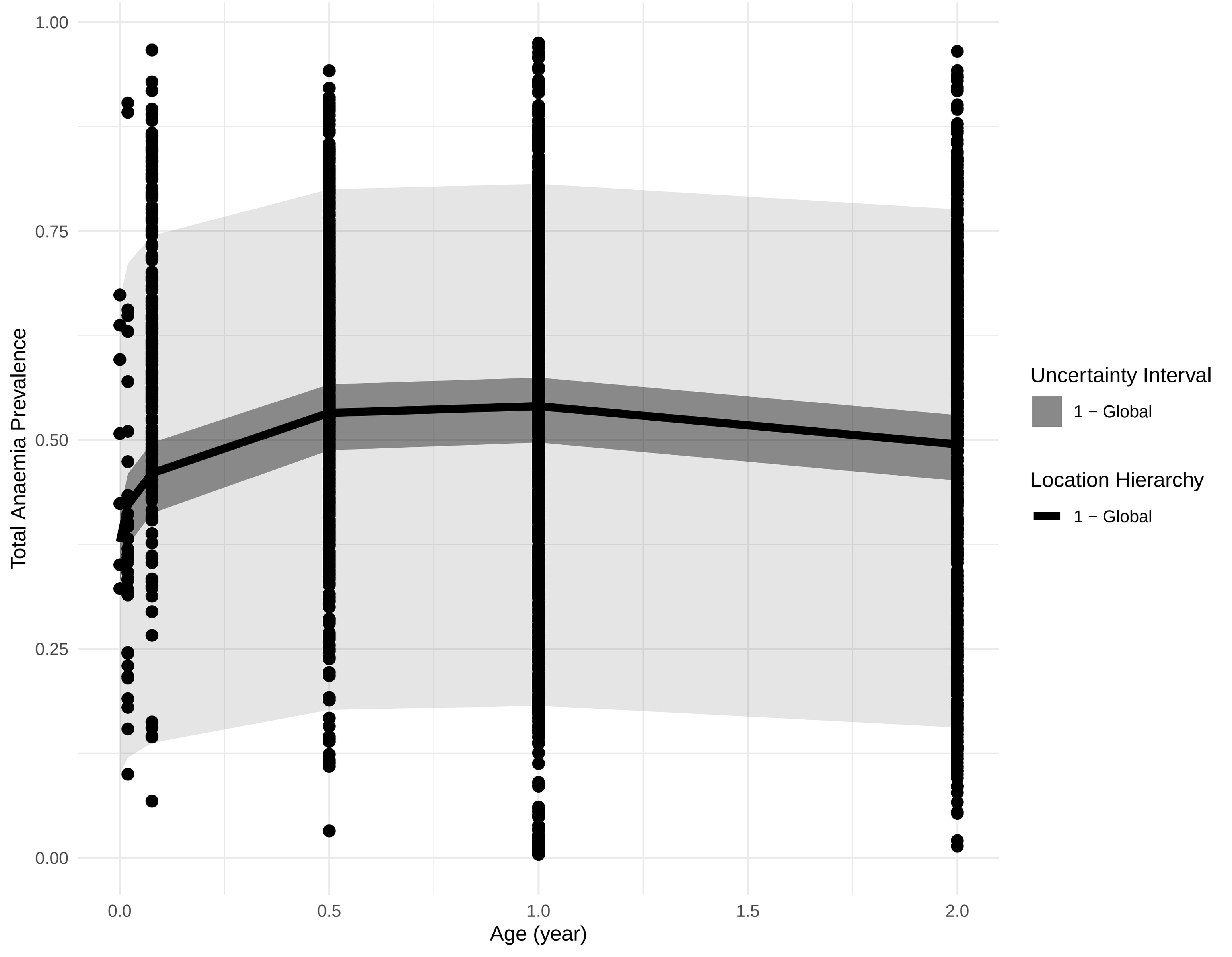

MR−BRT Age/Sex Fits − Non−pregnant Females <2 Years Old − Total Anaemia Prevalence − Central Europe, Eastern Europe, and Central Asia
WHO−2001 Adjustment Method

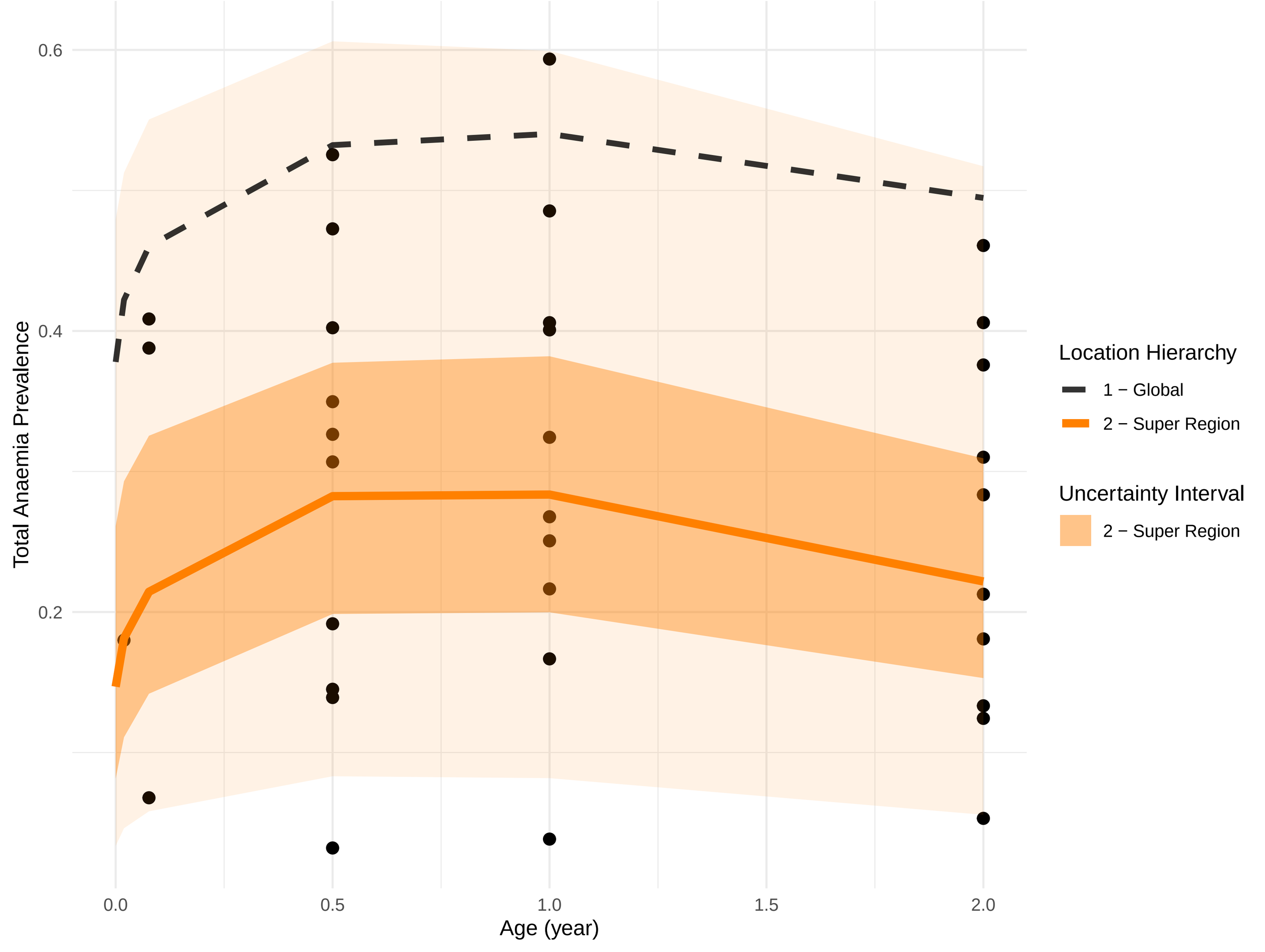

MR−BRT Age/Sex Fits − Non−pregnant Females <2 Years Old − Total Anaemia Prevalence − Central Asia
WHO−2001 Adjustment Method

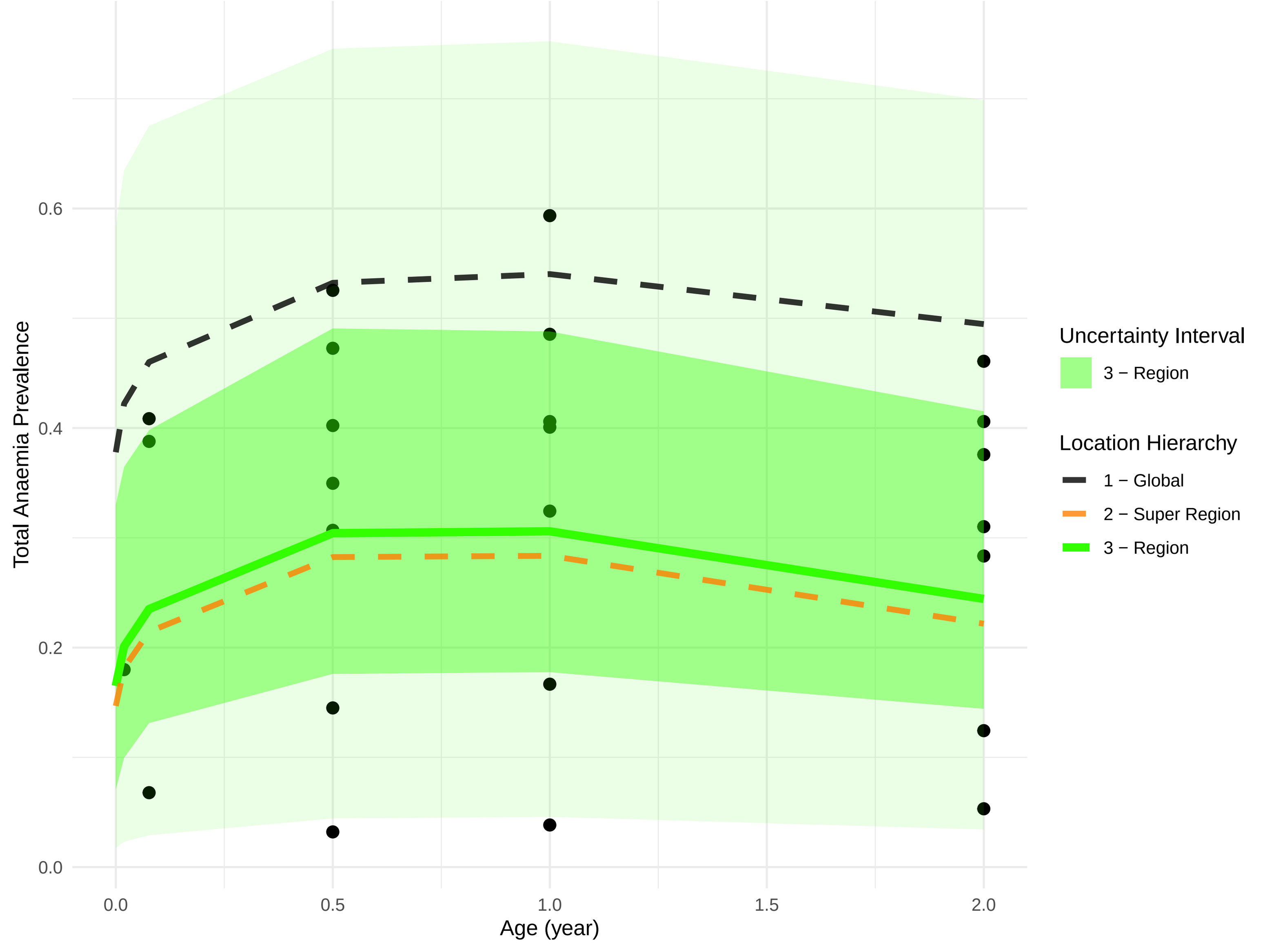

MR−BRT Age/Sex Fits − Non−pregnant Females <2 Years Old − Total Anaemia Prevalence − Armenia
WHO−2001 Adjustment Method

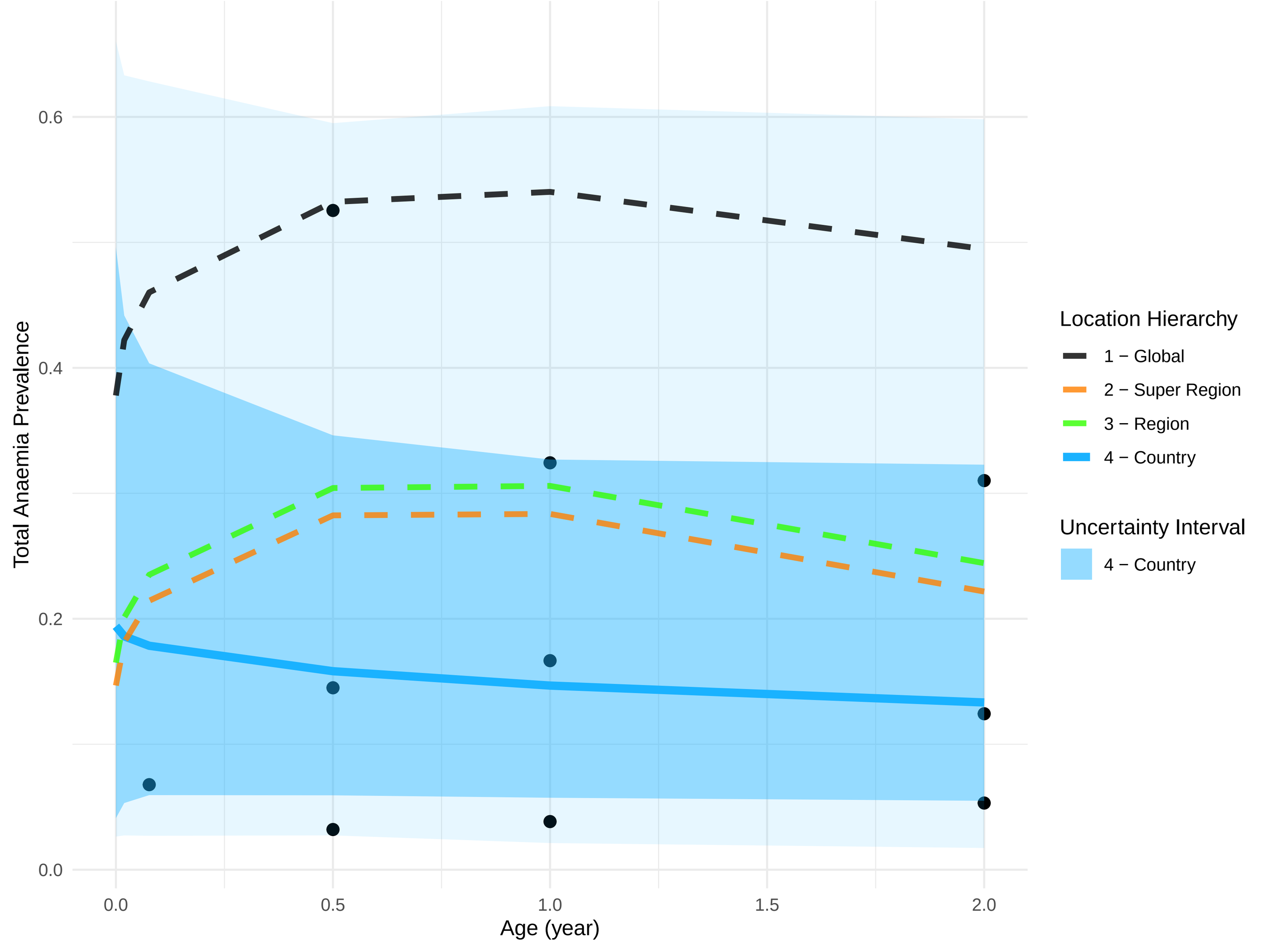

MR−BRT Age/Sex Fits − Non−pregnant Females <2 Years Old − Total Anaemia Prevalence − Azerbaijan
WHO−2001 Adjustment Method

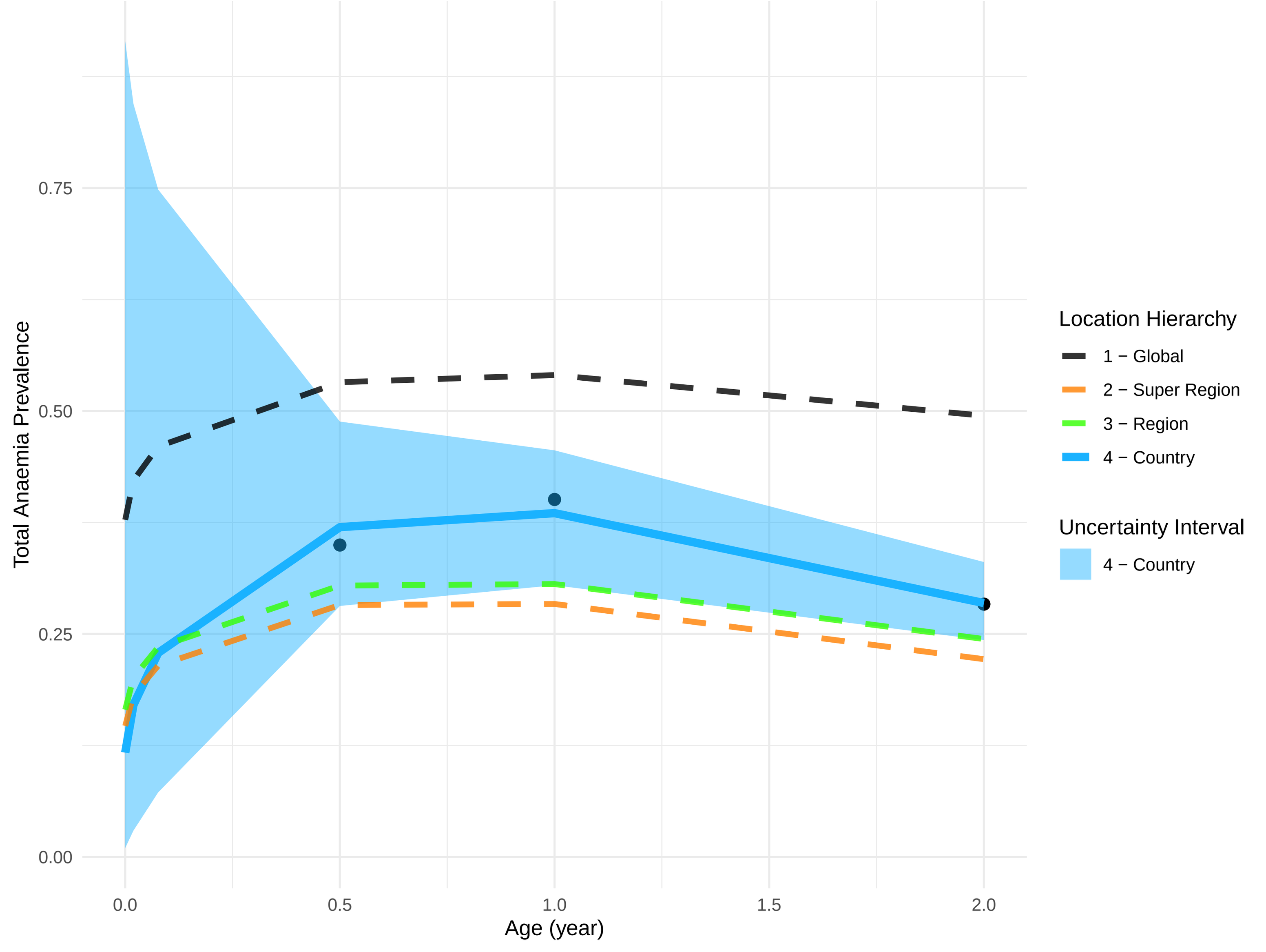

MR−BRT Age/Sex Fits − Non−pregnant Females <2 Years Old − Total Anaemia Prevalence − Kyrgyzstan
WHO−2001 Adjustment Method

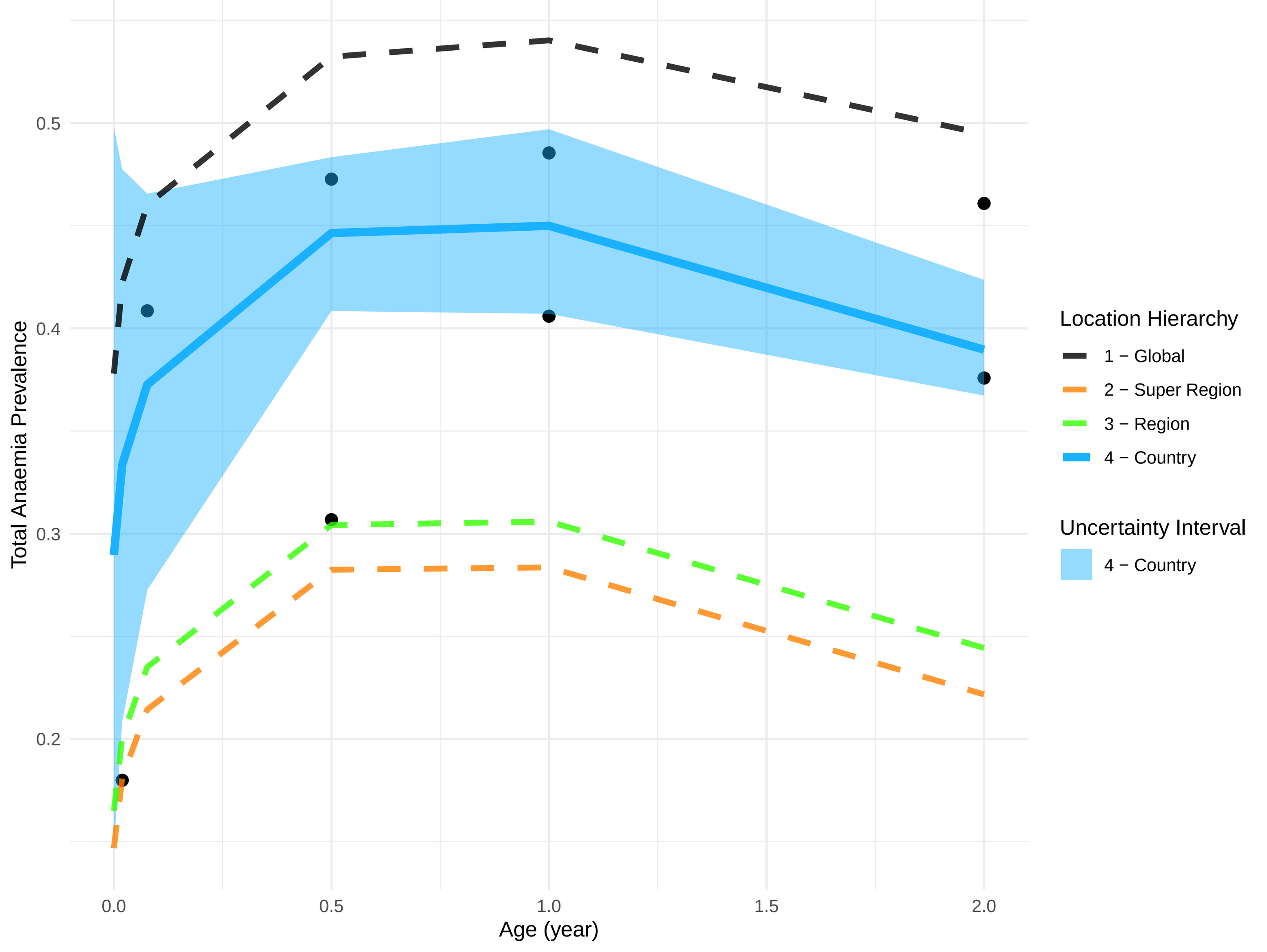

MR−BRT Age/Sex Fits − Non−pregnant Females <2 Years Old − Total Anaemia Prevalence − Uzbekistan
WHO−2001 Adjustment Method

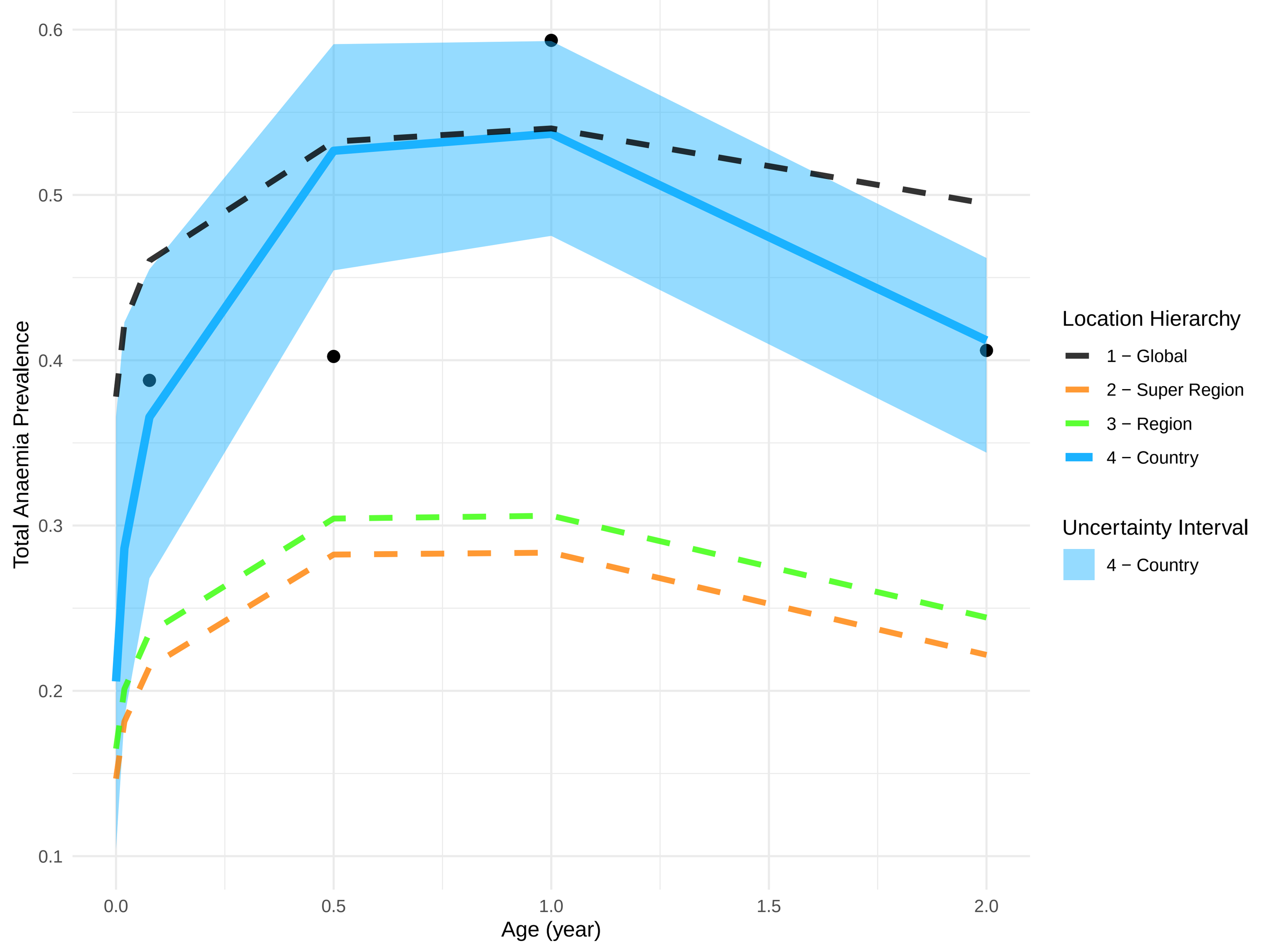

MR−BRT Age/Sex Fits − Non−pregnant Females <2 Years Old − Total Anaemia Prevalence − Central Europe
WHO−2001 Adjustment Method

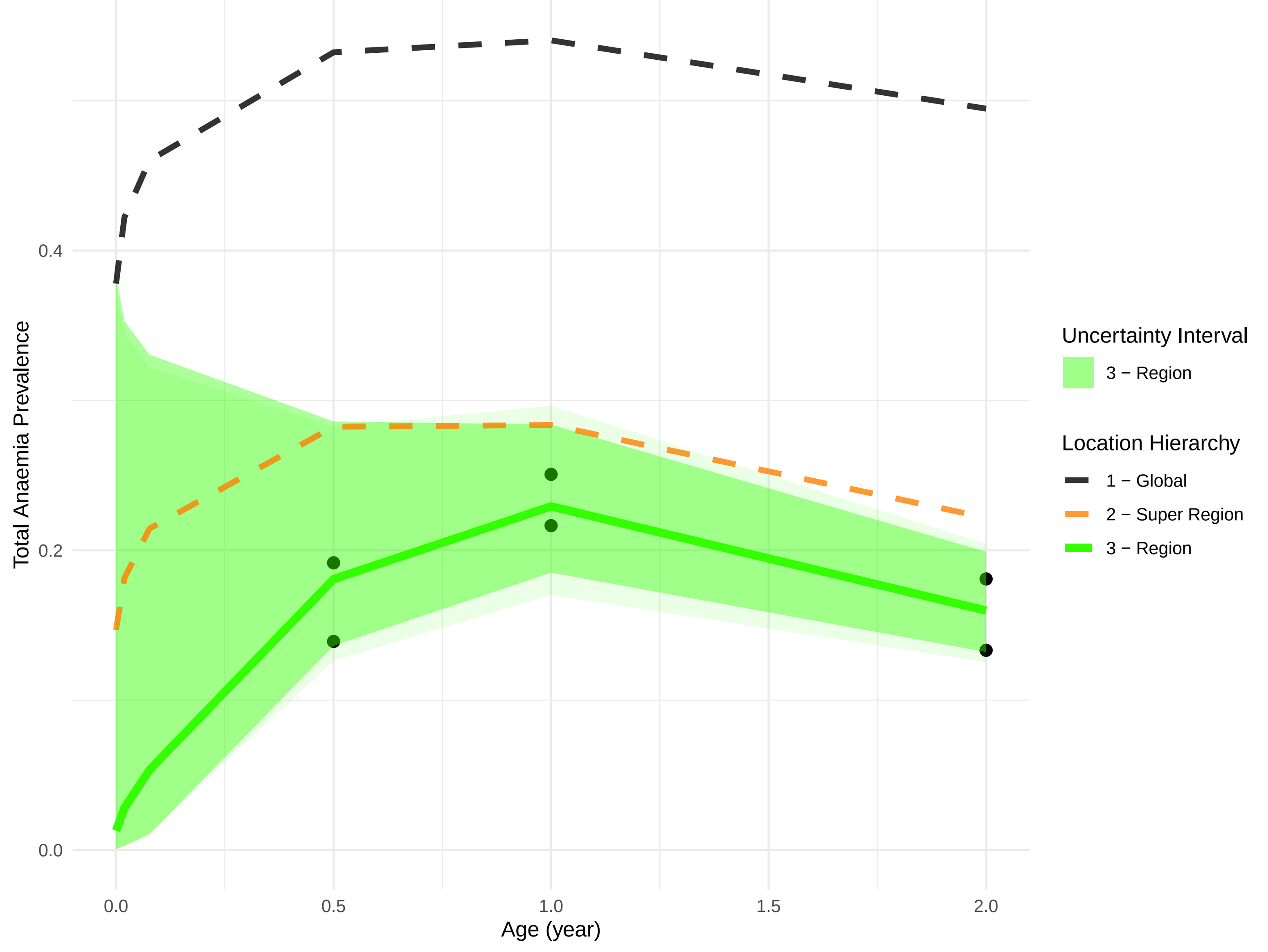

MR−BRT Age/Sex Fits − Non−pregnant Females <2 Years Old − Total Anaemia Prevalence − Albania
WHO−2001 Adjustment Method

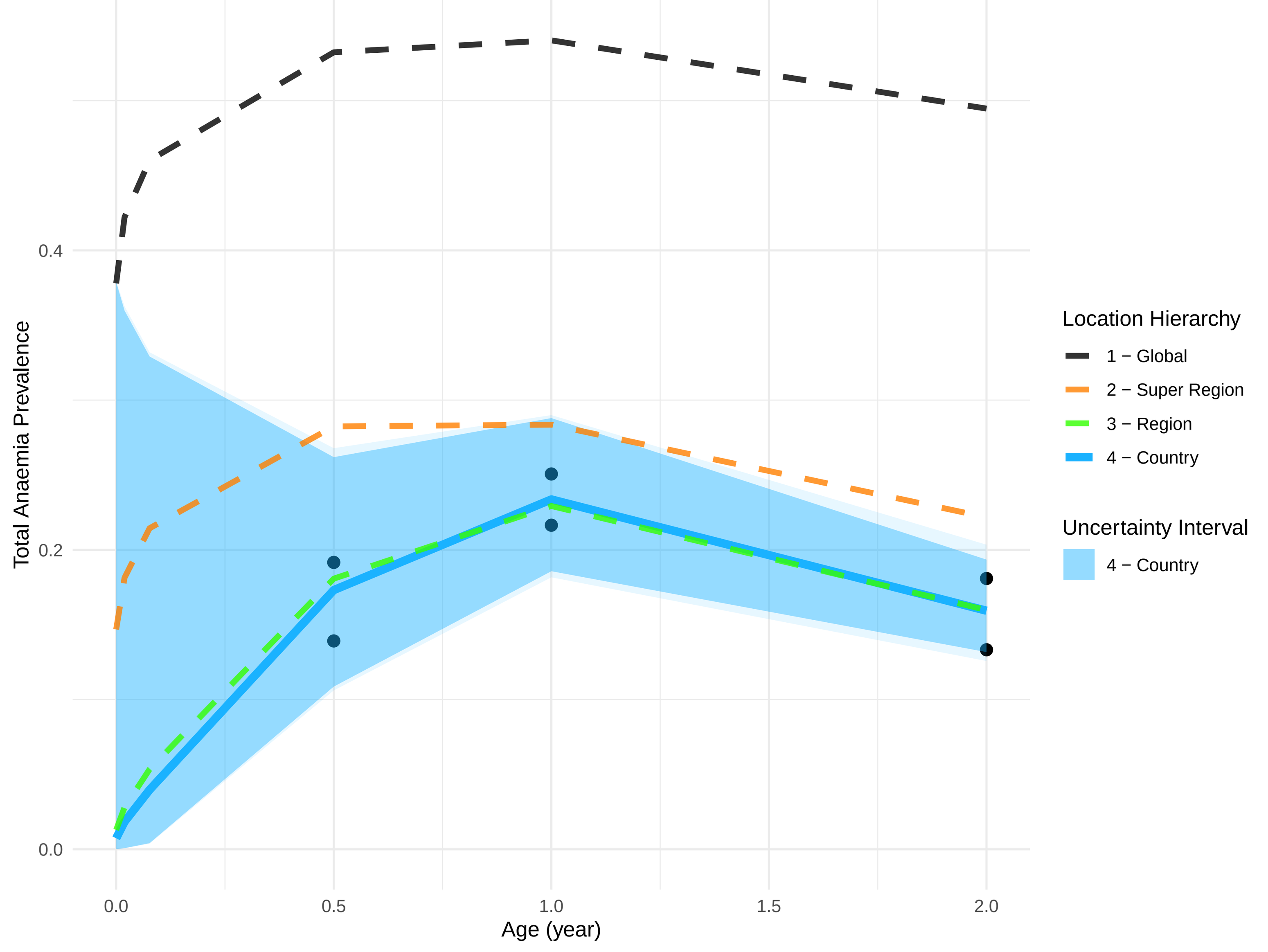

MR−BRT Age/Sex Fits − Non−pregnant Females <2 Years Old − Total Anaemia Prevalence − Eastern Europe
WHO−2001 Adjustment Method

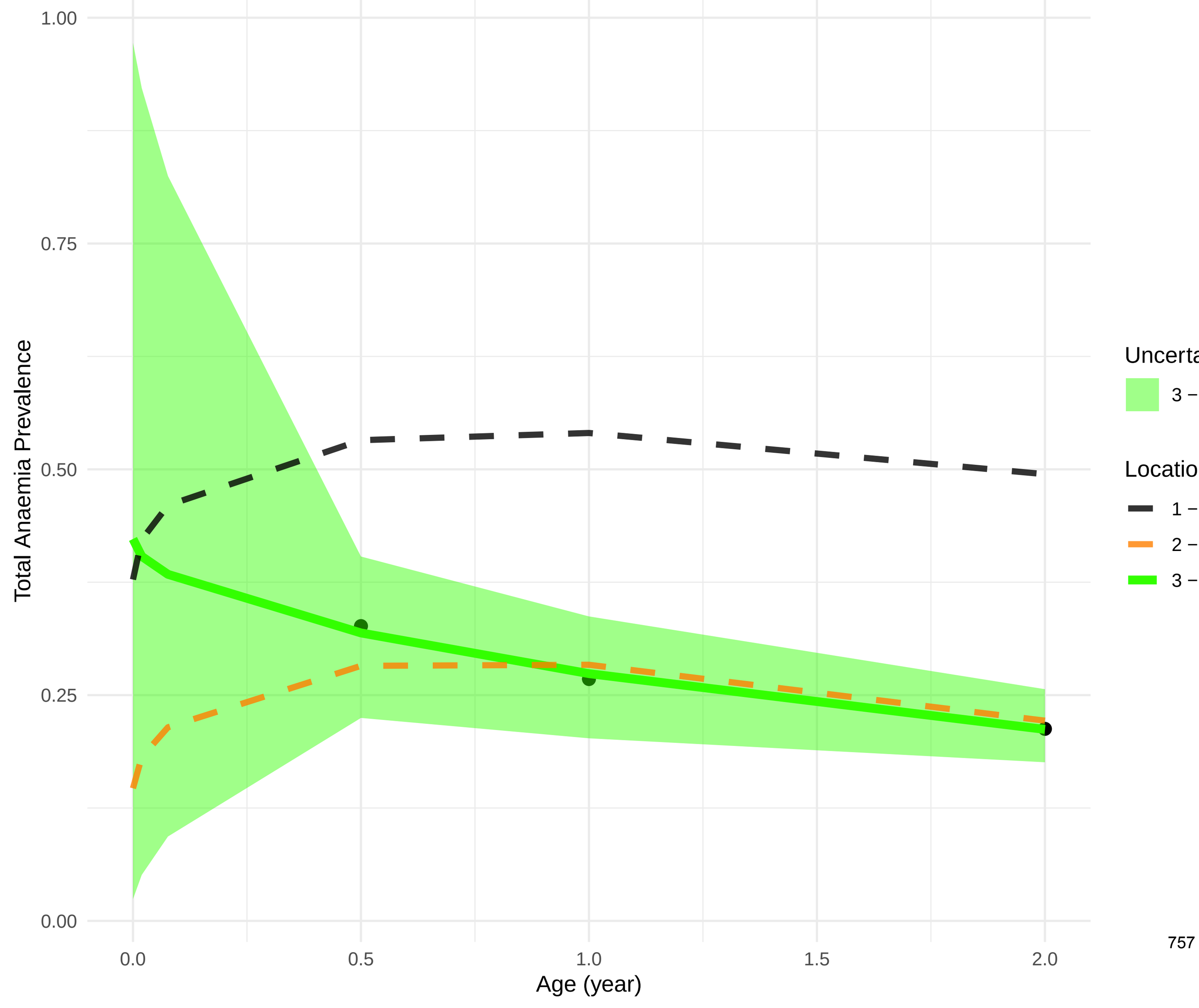

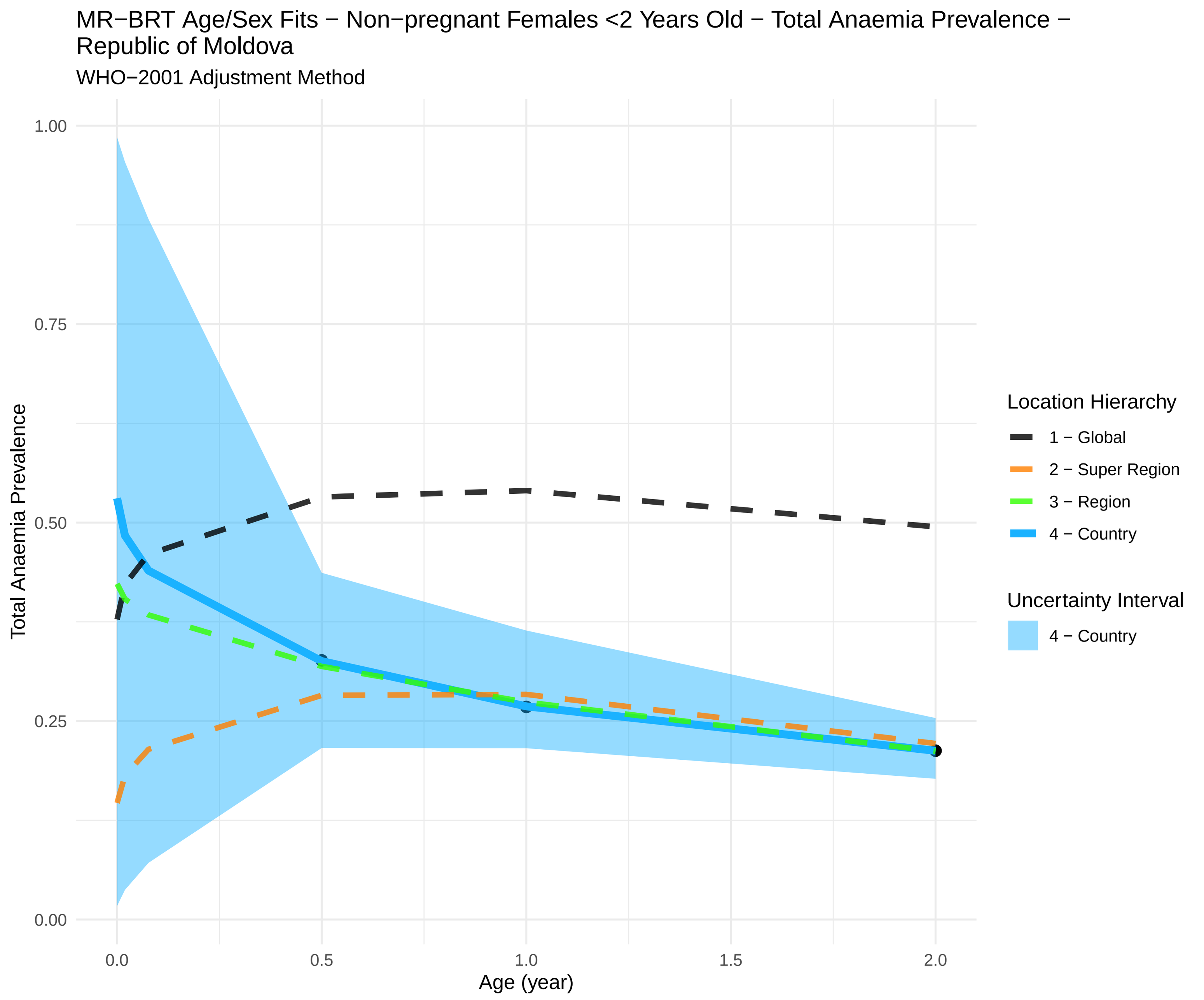
MR−BRT Age/Sex Fits − Non−pregnant Females <2 Years Old − Total Anaemia Prevalence − Republic of Moldova
WHO−2001 Adjustment Method
Total Anaemia Prevalence
Age (year)
Location Hierarchy
1 − Global
2 − Super Region
3 − Region
4 − Country
Uncertainty Interval
4 − Country

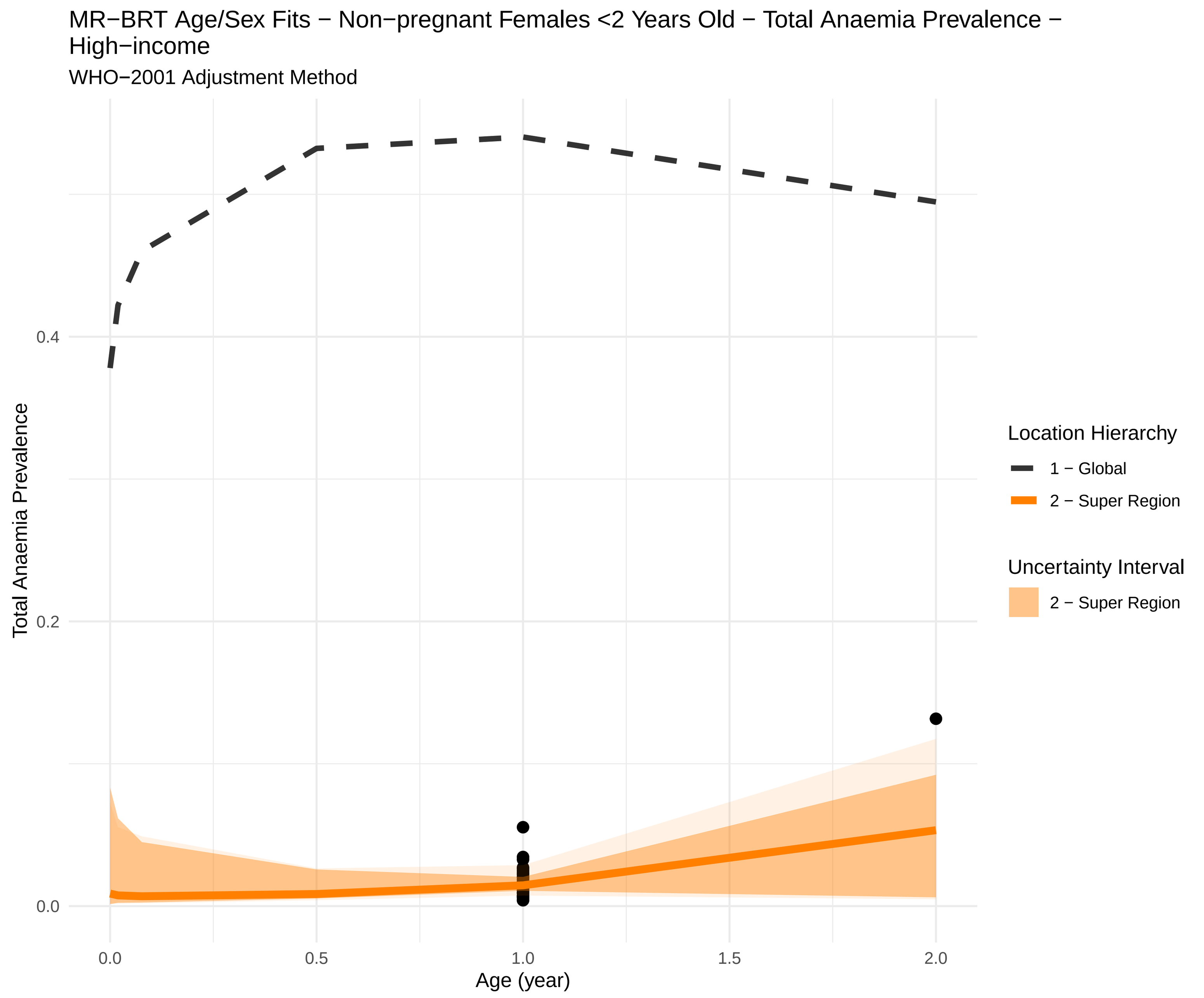
MR−BRT Age/Sex Fits − Non−pregnant Females <2 Years Old − Total Anaemia Prevalence − High−income
WHO−2001 Adjustment Method
Total Anaemia Prevalence
Age (year)
Location Hierarchy
1 − Global
2 − Super Region
Uncertainty Interval
2 − Super Region

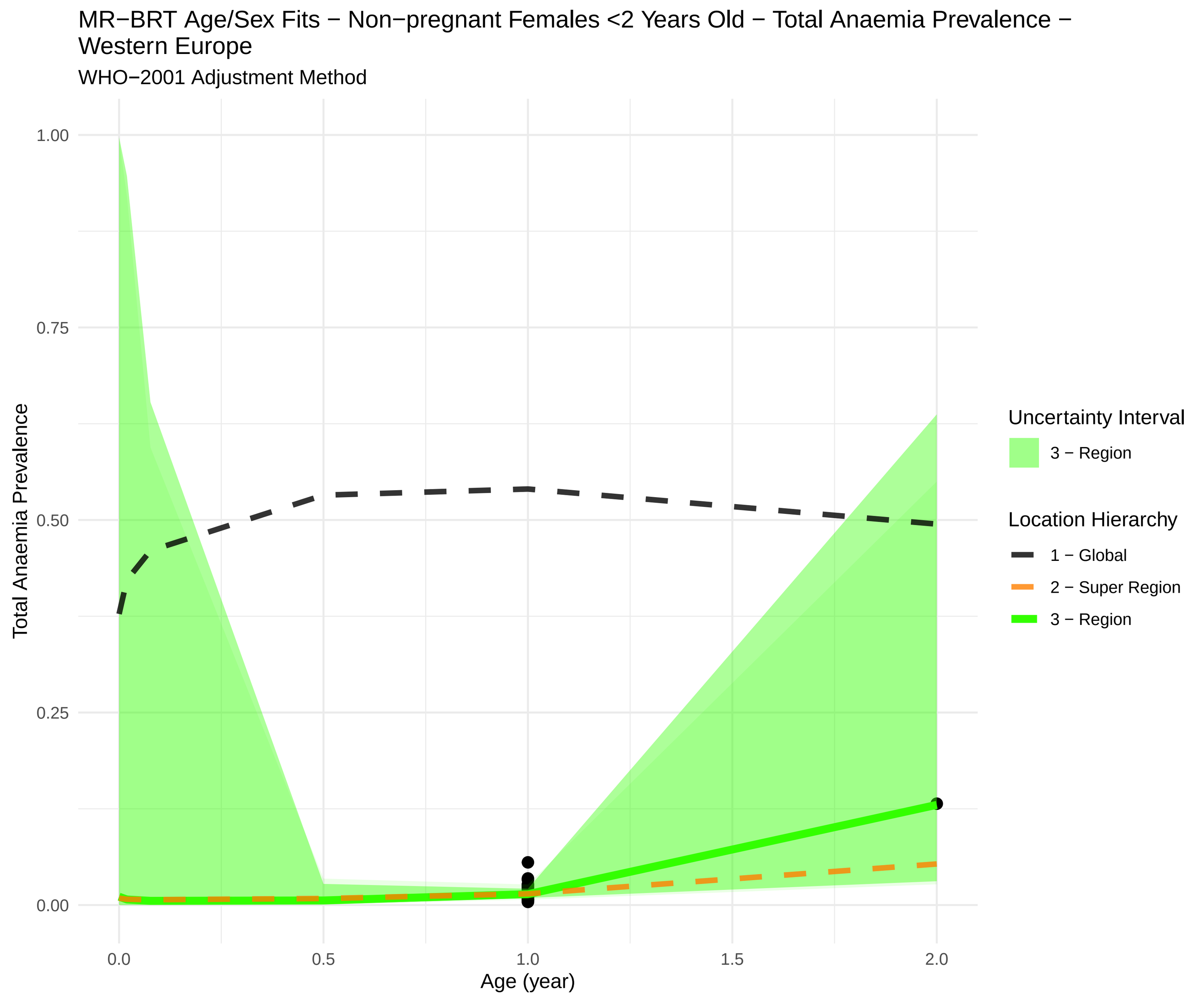
MR−BRT Age/Sex Fits − Non−pregnant Females <2 Years Old − Total Anaemia Prevalence − Western Europe
WHO−2001 Adjustment Method
Total Anaemia Prevalence
Age (year)
Uncertainty Interval
3 − Region
Location Hierarchy
1 − Global
2 − Super Region
3 − Region

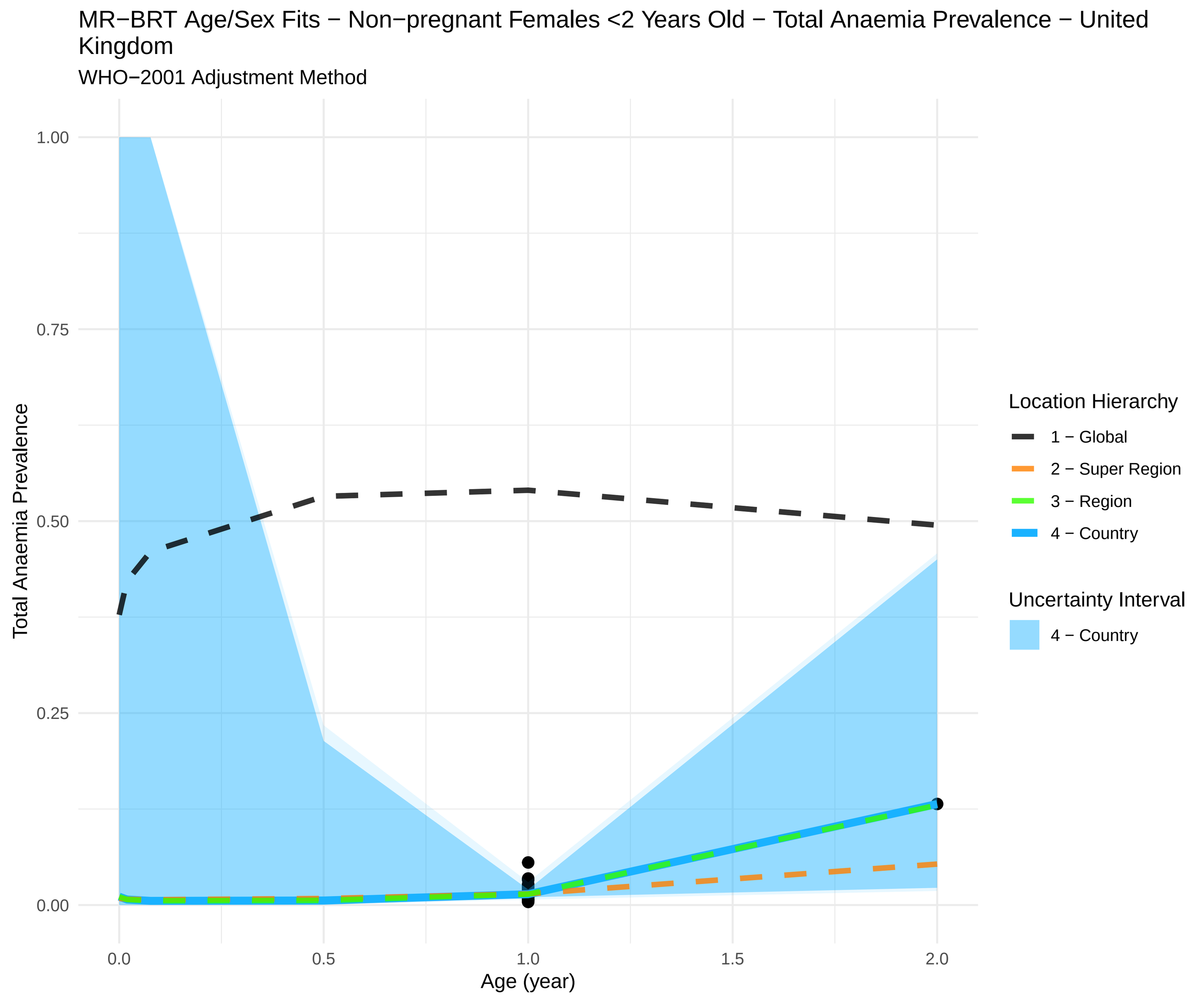
MR−BRT Age/Sex Fits − Non−pregnant Females <2 Years Old − Total Anaemia Prevalence − United Kingdom
WHO−2001 Adjustment Method
Total Anaemia Prevalence
Age (year)
Location Hierarchy
1 − Global
2 − Super Region
3 − Region
4 − Country
Uncertainty Interval
4 − Country

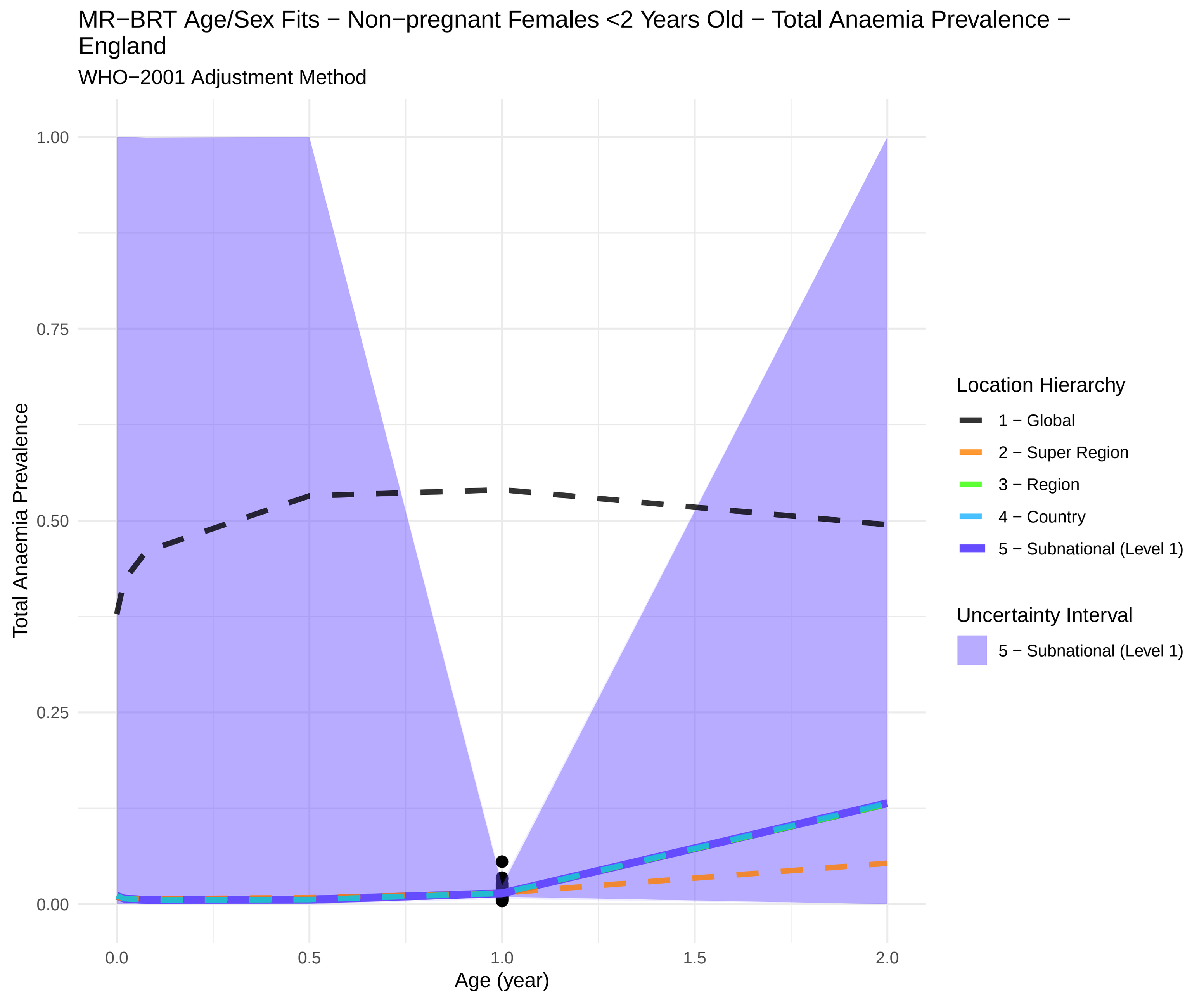
MR−BRT Age/Sex Fits − Non−pregnant Females <2 Years Old − Total Anaemia Prevalence − England
WHO−2001 Adjustment Method
Total Anaemia Prevalence
Age (year)
Location Hierarchy
1 − Global
2 − Super Region
3 − Region
4 − Country
5 − Subnational (Level 1)
Uncertainty Interval
5 − Subnational (Level 1)

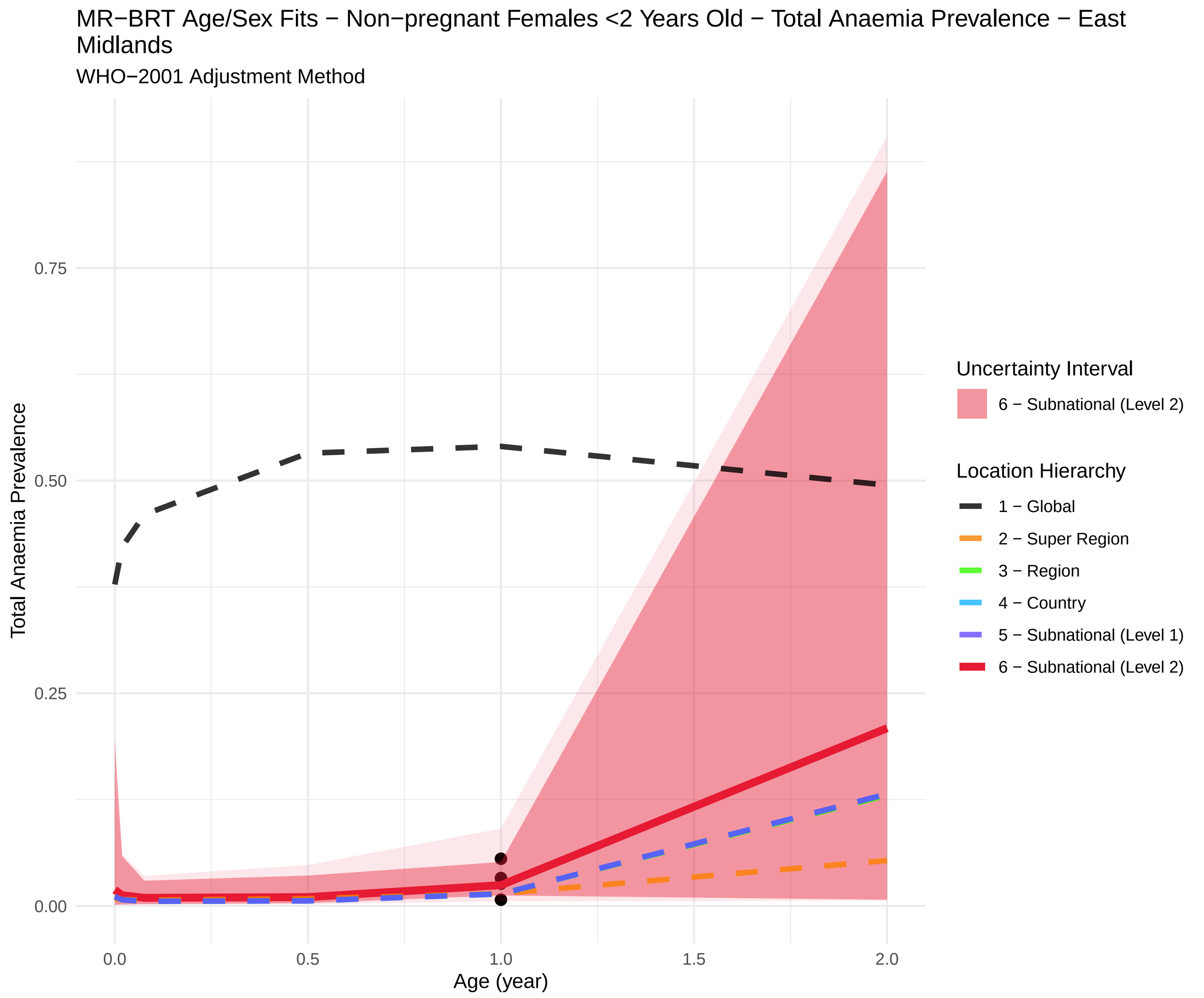
MR−BRT Age/Sex Fits − Non−pregnant Females <2 Years Old − Total Anaemia Prevalence − East Midlands
WHO−2001 Adjustment Method
Total Anaemia Prevalence
Age (year)
Uncertainty Interval
6 − Subnational (Level 2)
Location Hierarchy
1 − Global
2 − Super Region
3 − Region
4 − Country
5 − Subnational (Level 1)
6 − Subnational (Level 2)

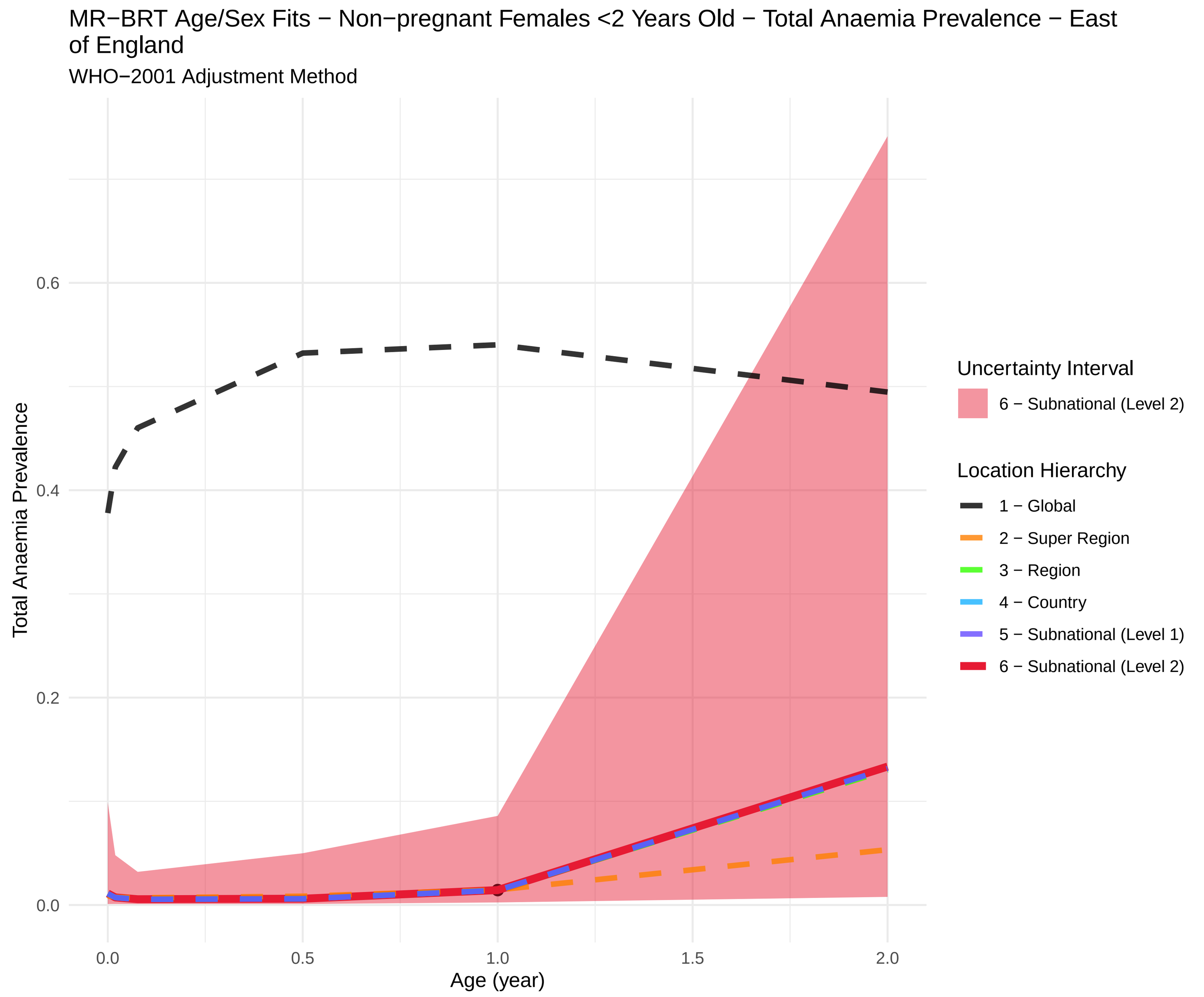
MR−BRT Age/Sex Fits − Non−pregnant Females <2 Years Old − Total Anaemia Prevalence − East of England
WHO−2001 Adjustment Method
Total Anaemia Prevalence
Age (year)
Uncertainty Interval
6 − Subnational (Level 2)
Location Hierarchy
1 − Global
2 − Super Region
3 − Region
4 − Country
5 − Subnational (Level 1)
6 − Subnational (Level 2)

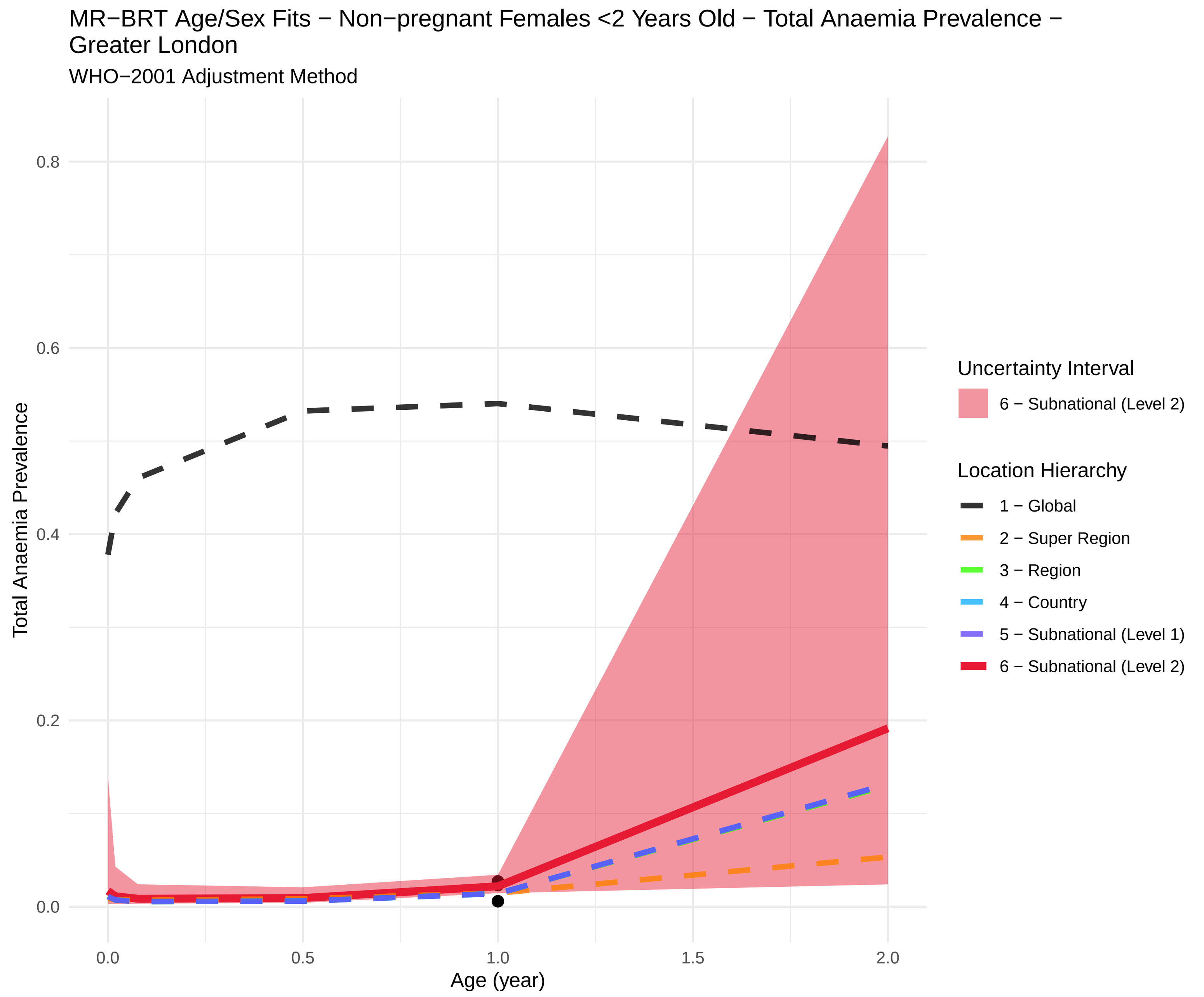
MR−BRT Age/Sex Fits − Non−pregnant Females <2 Years Old − Total Anaemia Prevalence − Greater London
WHO−2001 Adjustment Method
Total Anaemia Prevalence
Age (year)
Uncertainty Interval
6 − Subnational (Level 2)
Location Hierarchy
1 − Global
2 − Super Region
3 − Region
4 − Country
5 − Subnational (Level 1)
6 − Subnational (Level 2)

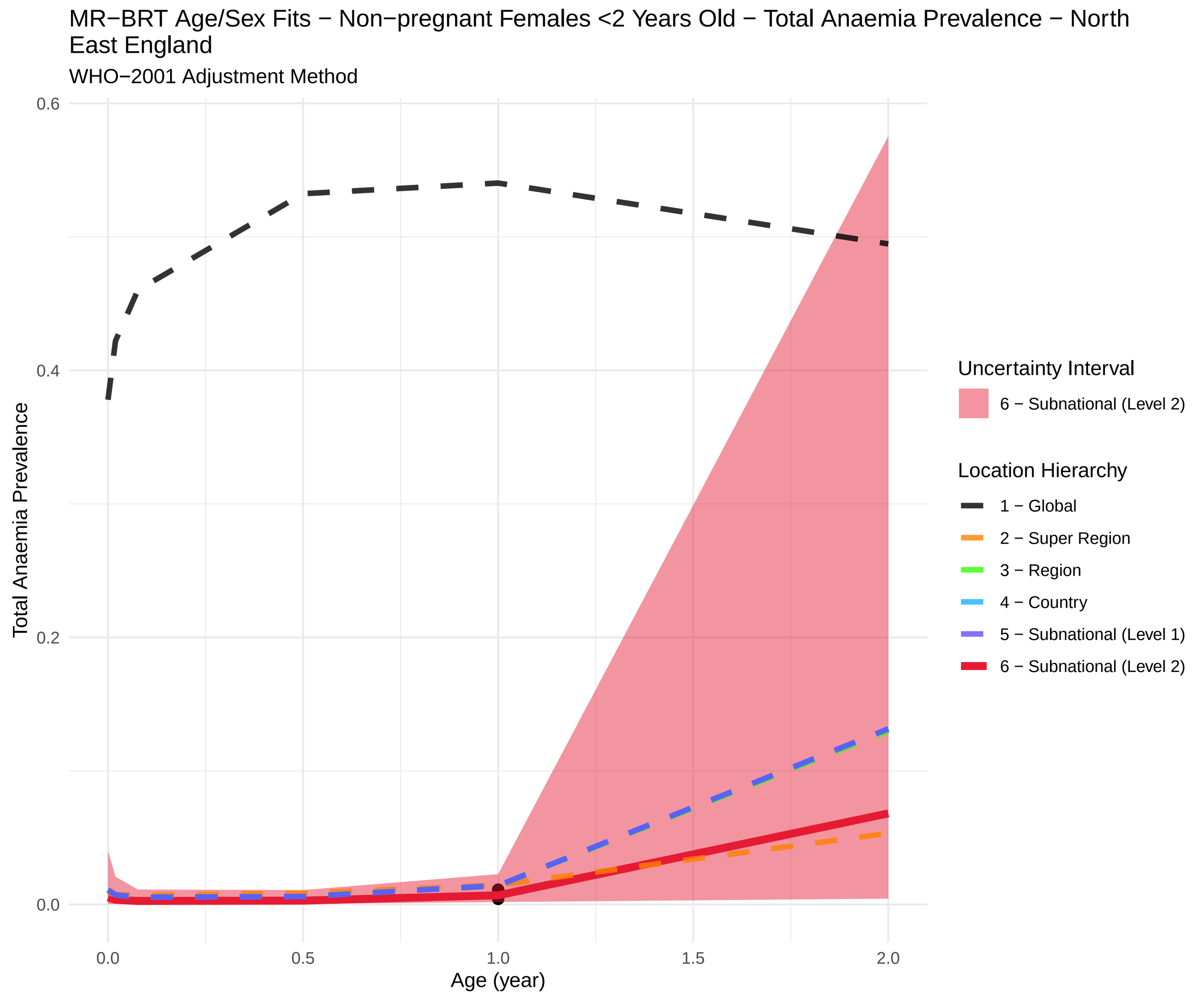
MR−BRT Age/Sex Fits − Non−pregnant Females <2 Years Old − Total Anaemia Prevalence − North East England
WHO−2001 Adjustment Method
Total Anaemia Prevalence
Age (year)
Uncertainty Interval
6 − Subnational (Level 2)
Location Hierarchy
1 − Global
2 − Super Region
3 − Region
4 − Country
5 − Subnational (Level 1)
6 − Subnational (Level 2)

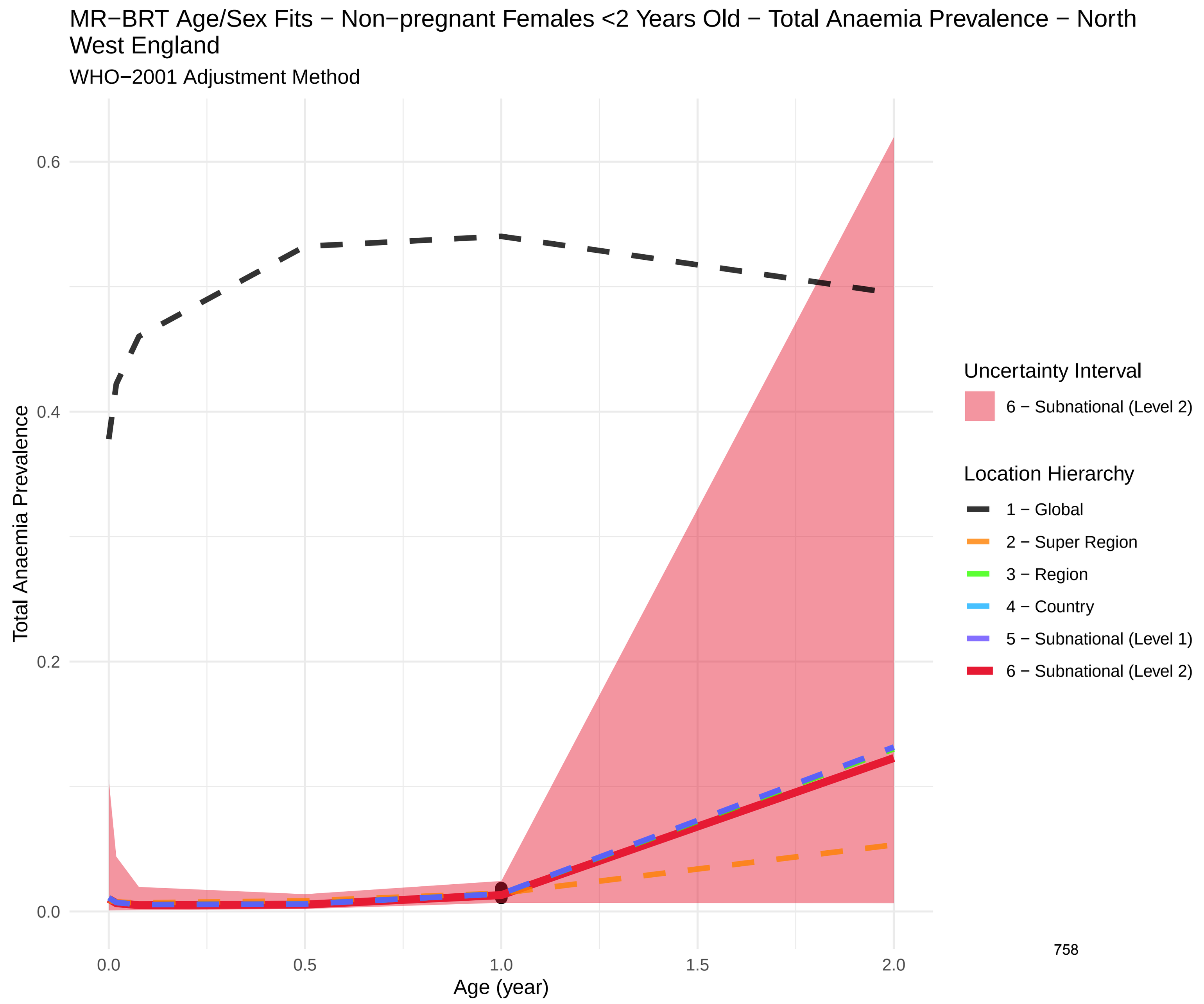
MR−BRT Age/Sex Fits − Non−pregnant Females <2 Years Old − Total Anaemia Prevalence − North West England
WHO−2001 Adjustment Method
Total Anaemia Prevalence
Age (year)
Uncertainty Interval
6 − Subnational (Level 2)
Location Hierarchy
1 − Global
2 − Super Region
3 − Region
4 − Country
5 − Subnational (Level 1)
6 − Subnational (Level 2)

MR−BRT Age/Sex Fits − Non−pregnant Females <2 Years Old − Total Anaemia Prevalence − South East England
WHO−2001 Adjustment Method

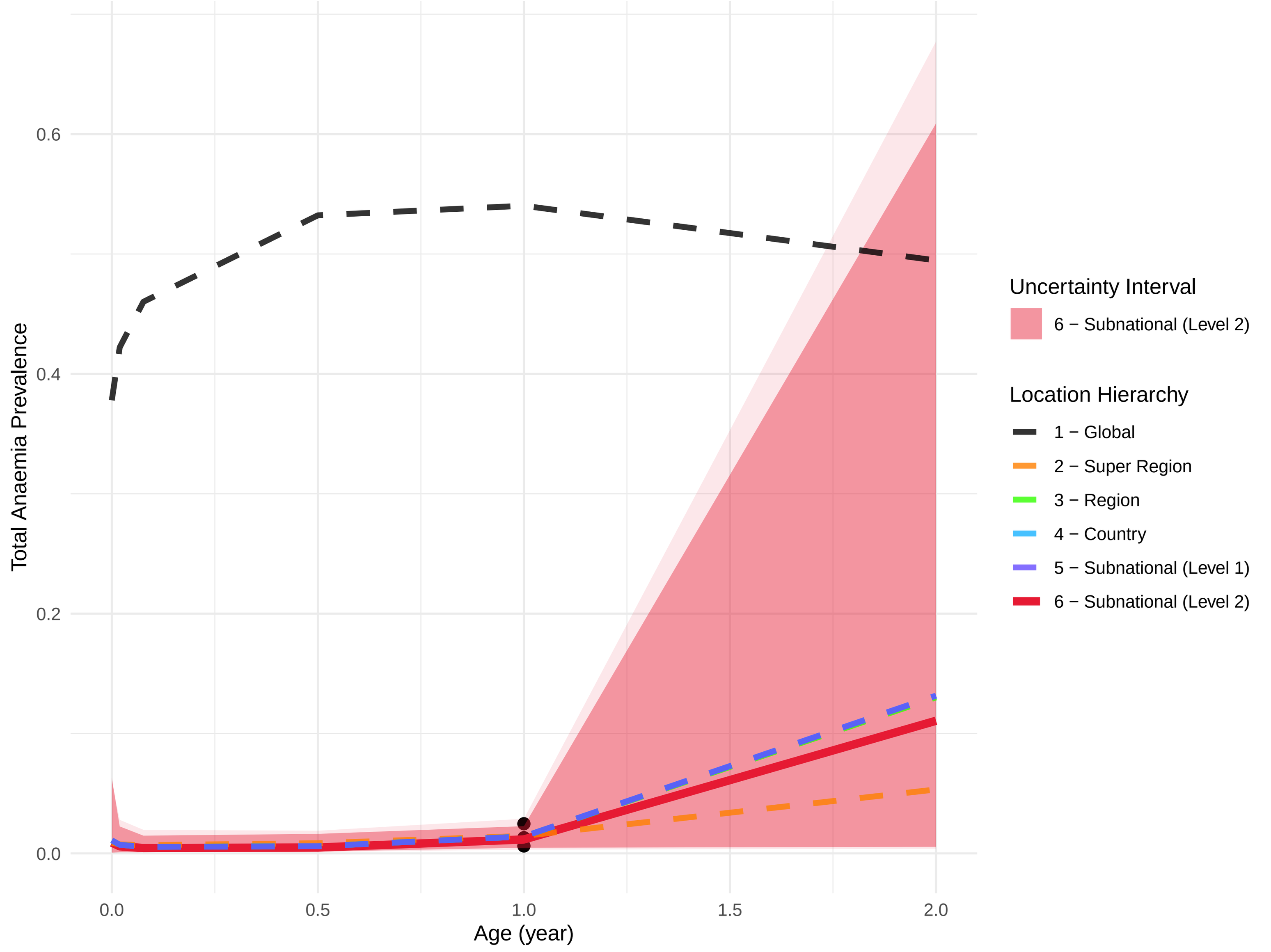

MR−BRT Age/Sex Fits − Non−pregnant Females <2 Years Old − Total Anaemia Prevalence − South West England
WHO−2001 Adjustment Method

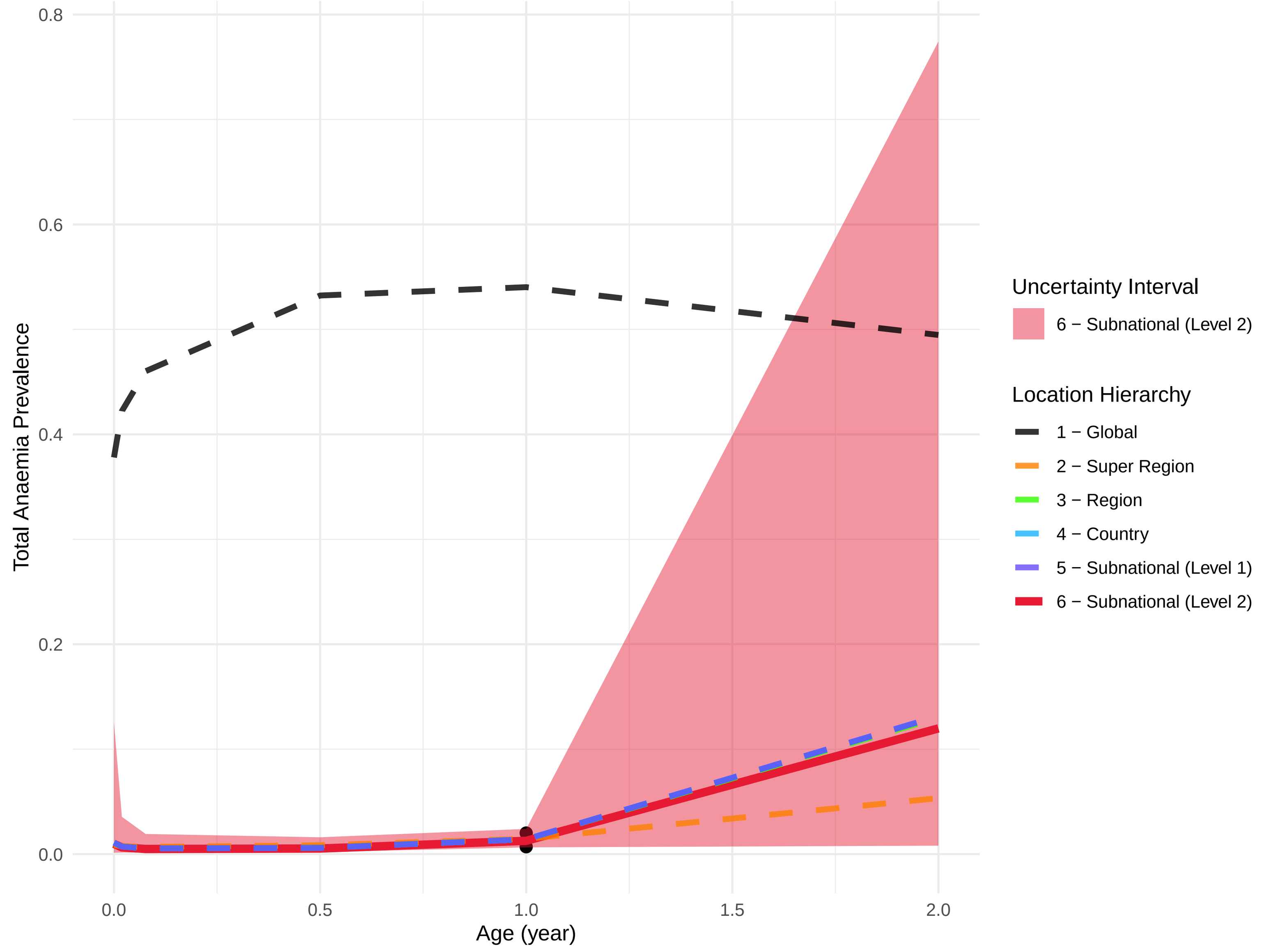

MR−BRT Age/Sex Fits − Non−pregnant Females <2 Years Old − Total Anaemia Prevalence − West Midlands
WHO−2001 Adjustment Method

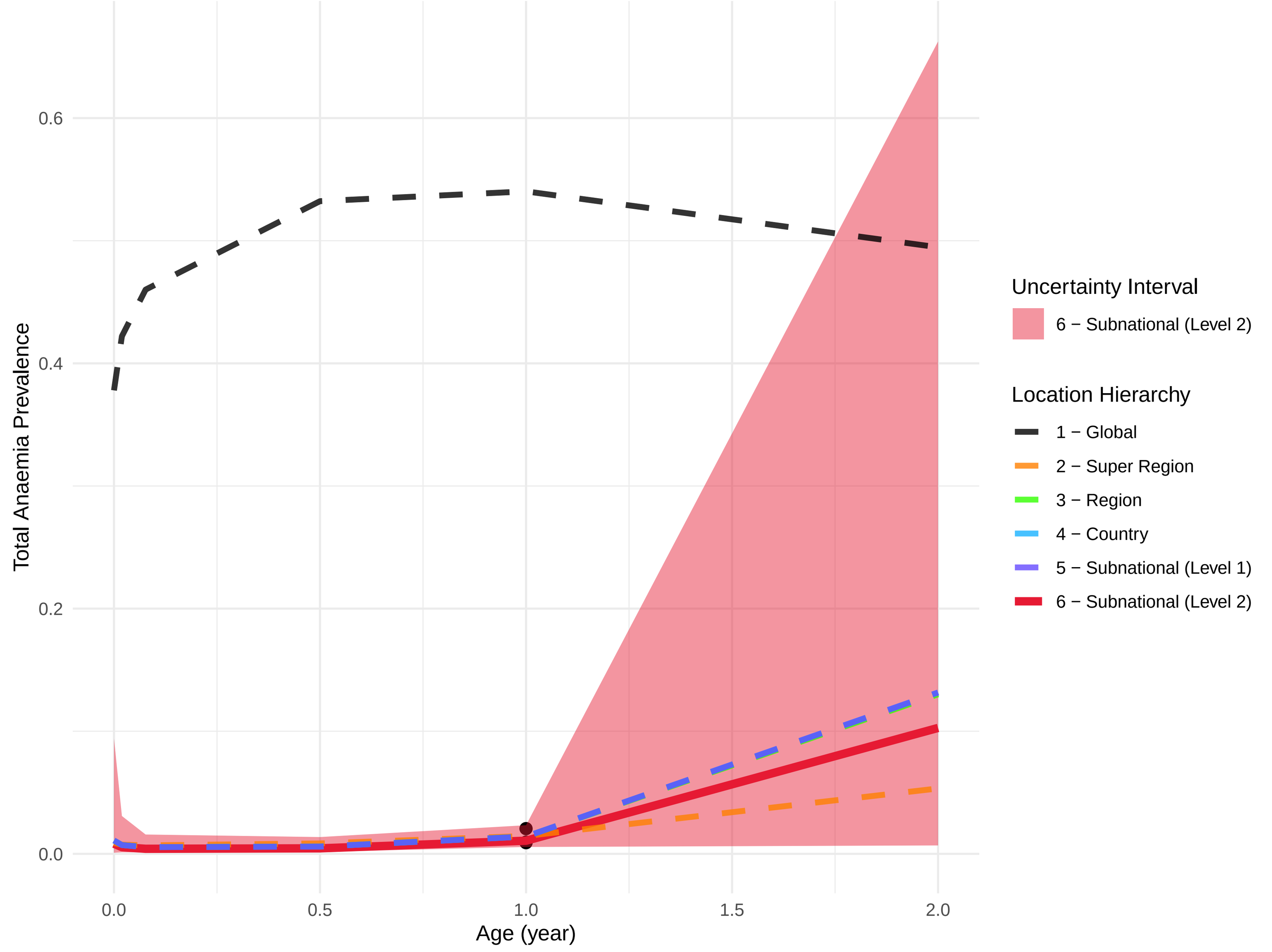

MR−BRT Age/Sex Fits − Non−pregnant Females <2 Years Old − Total Anaemia Prevalence − Yorkshire and the Humber
WHO−2001 Adjustment Method

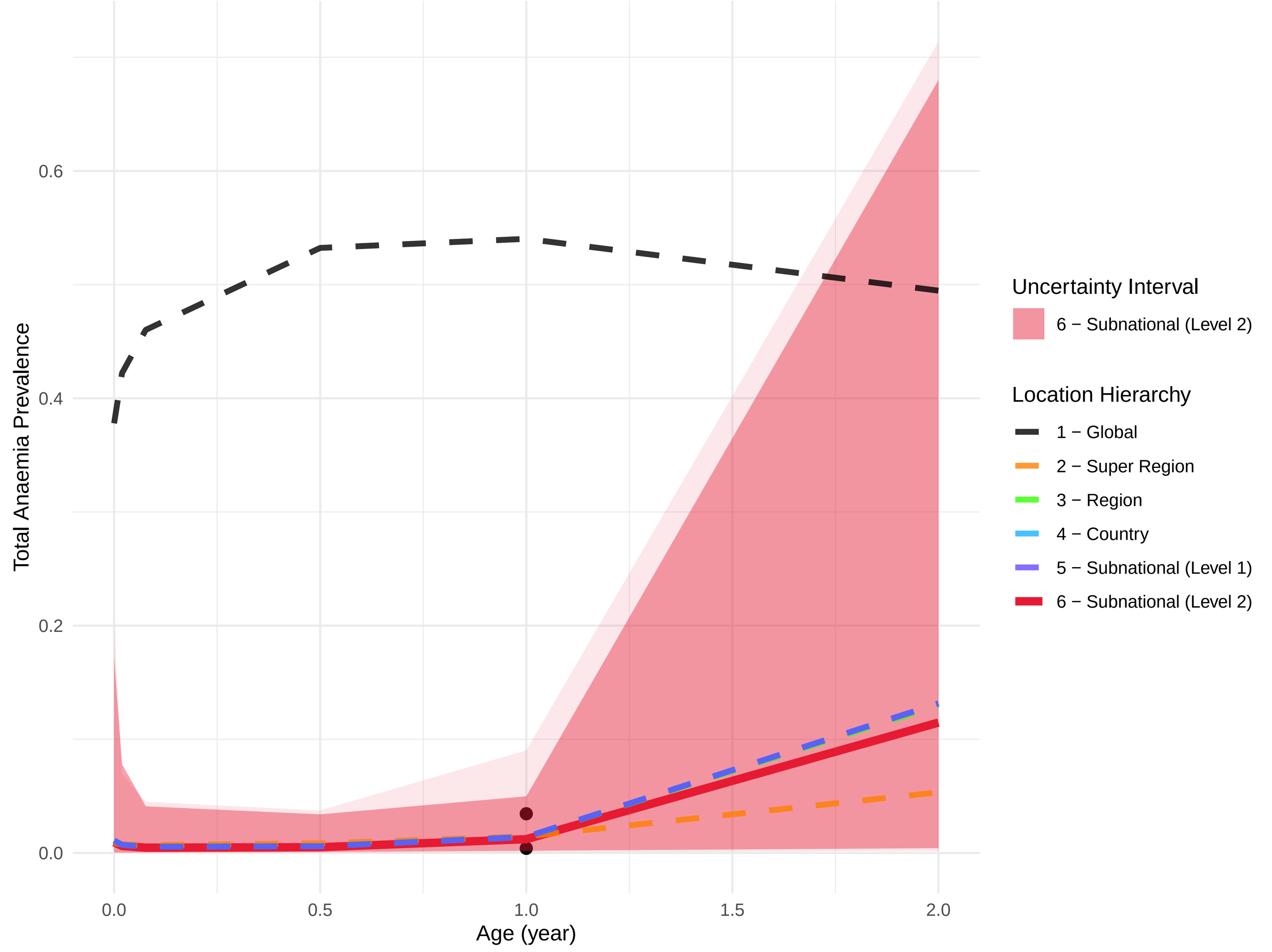

MR−BRT Age/Sex Fits − Non−pregnant Females <2 Years Old − Total Anaemia Prevalence − Latin America and Caribbean
WHO−2001 Adjustment Method

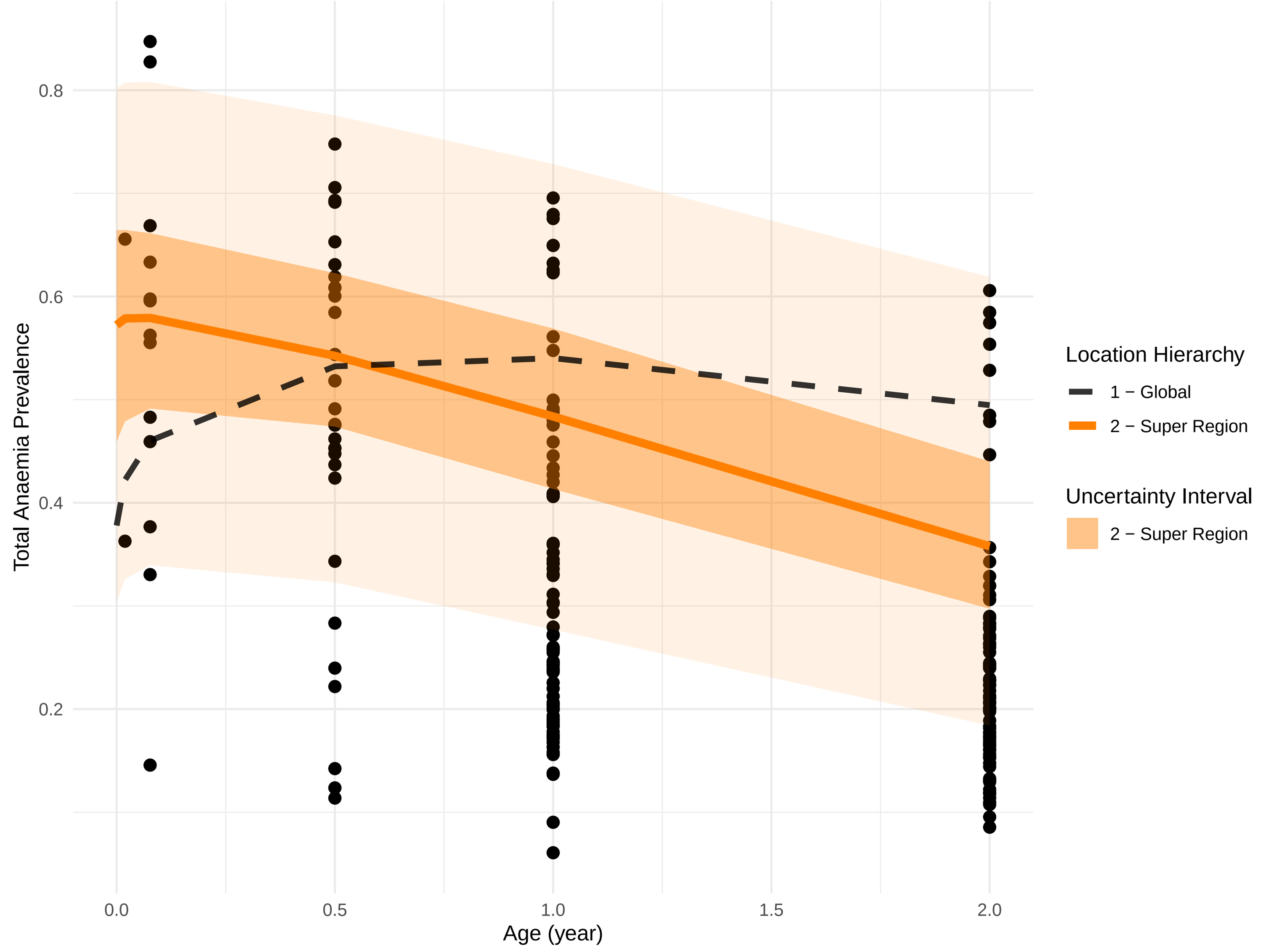

MR−BRT Age/Sex Fits − Non−pregnant Females <2 Years Old − Total Anaemia Prevalence − Andean Latin America
WHO−2001 Adjustment Method

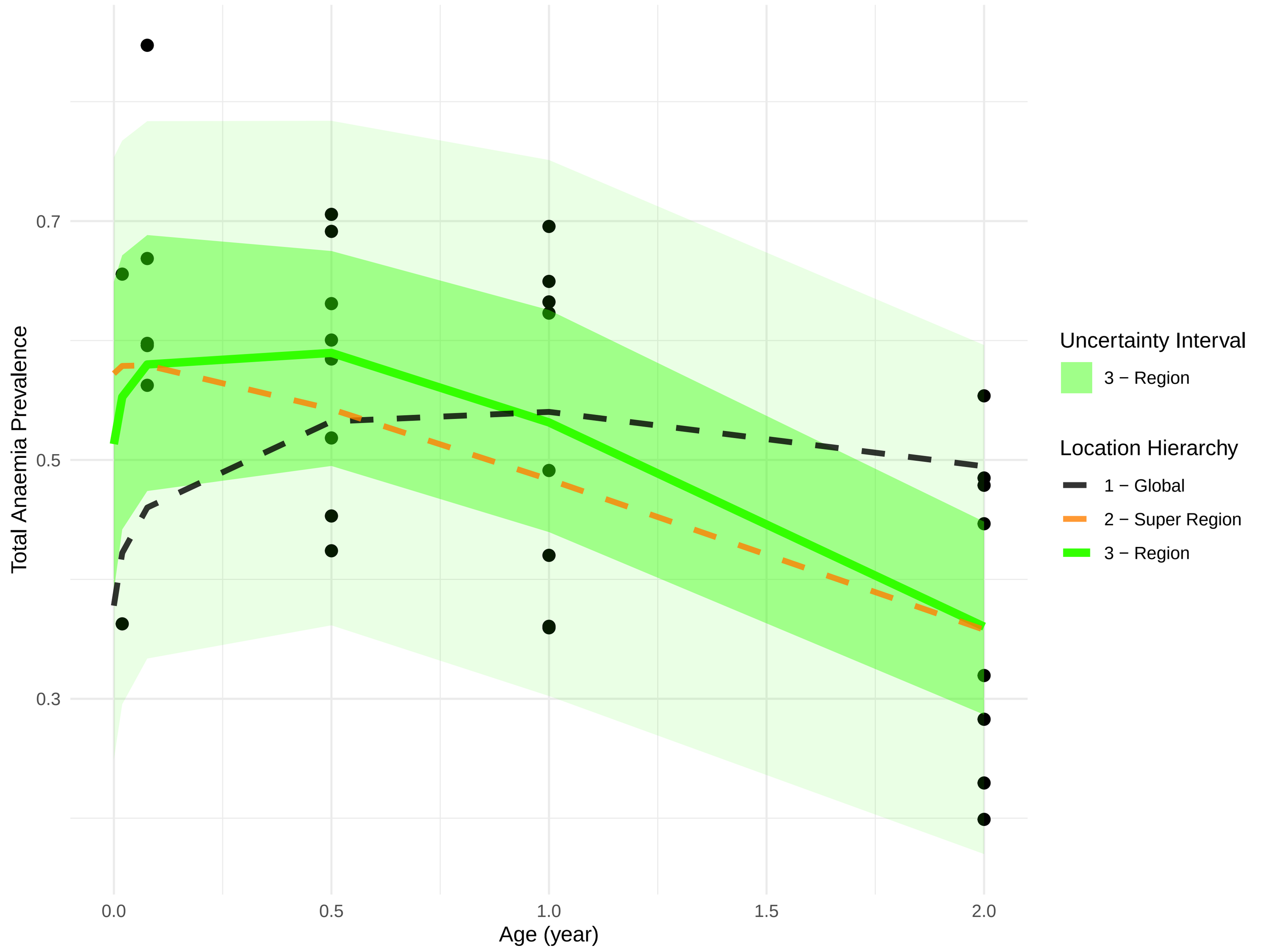

MR−BRT Age/Sex Fits − Non−pregnant Females <2 Years Old − Total Anaemia Prevalence − Bolivia (Plurinational State of)
WHO−2001 Adjustment Method

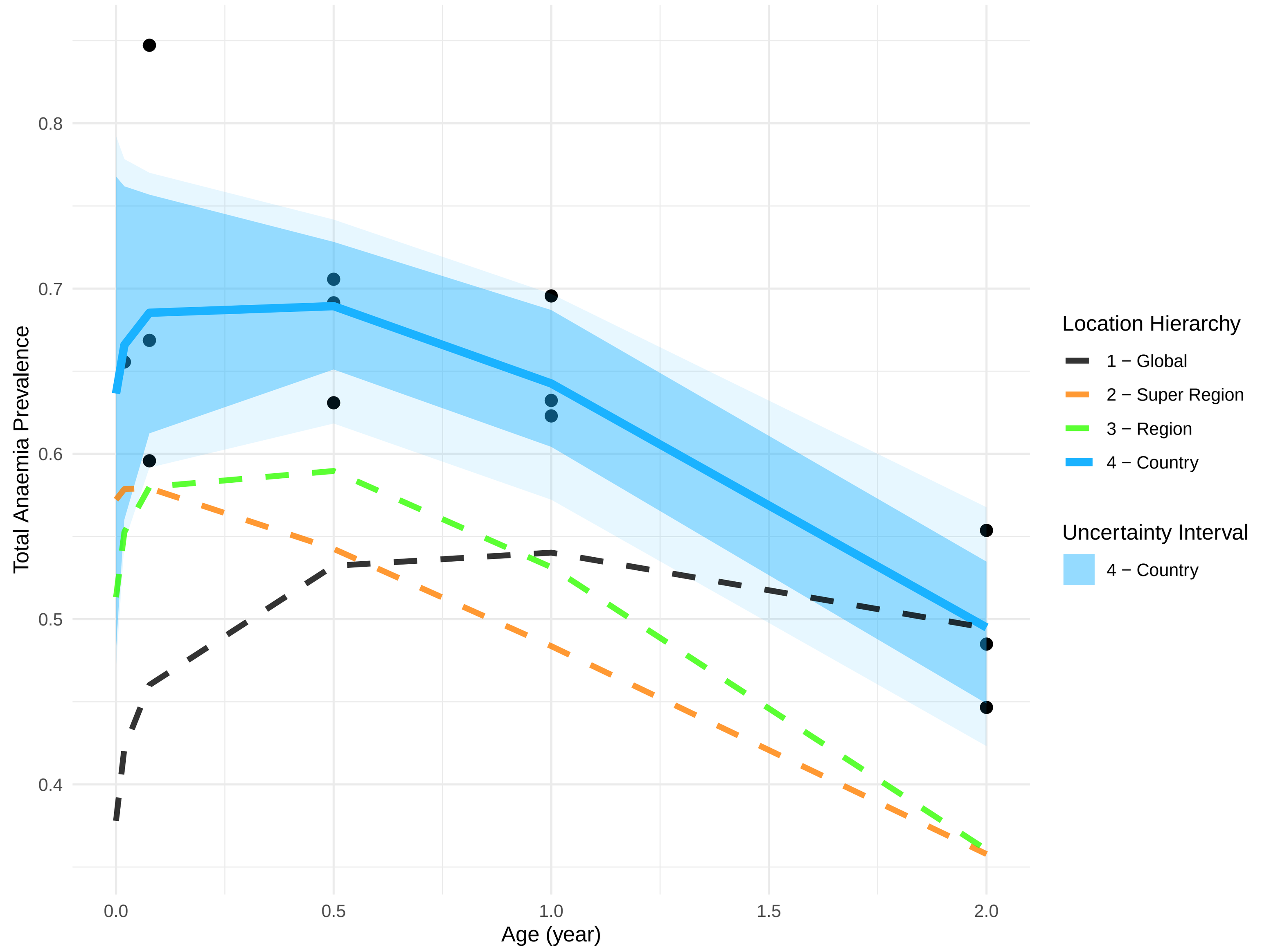

MR−BRT Age/Sex Fits − Non−pregnant Females <2 Years Old − Total Anaemia Prevalence − Peru
WHO−2001 Adjustment Method

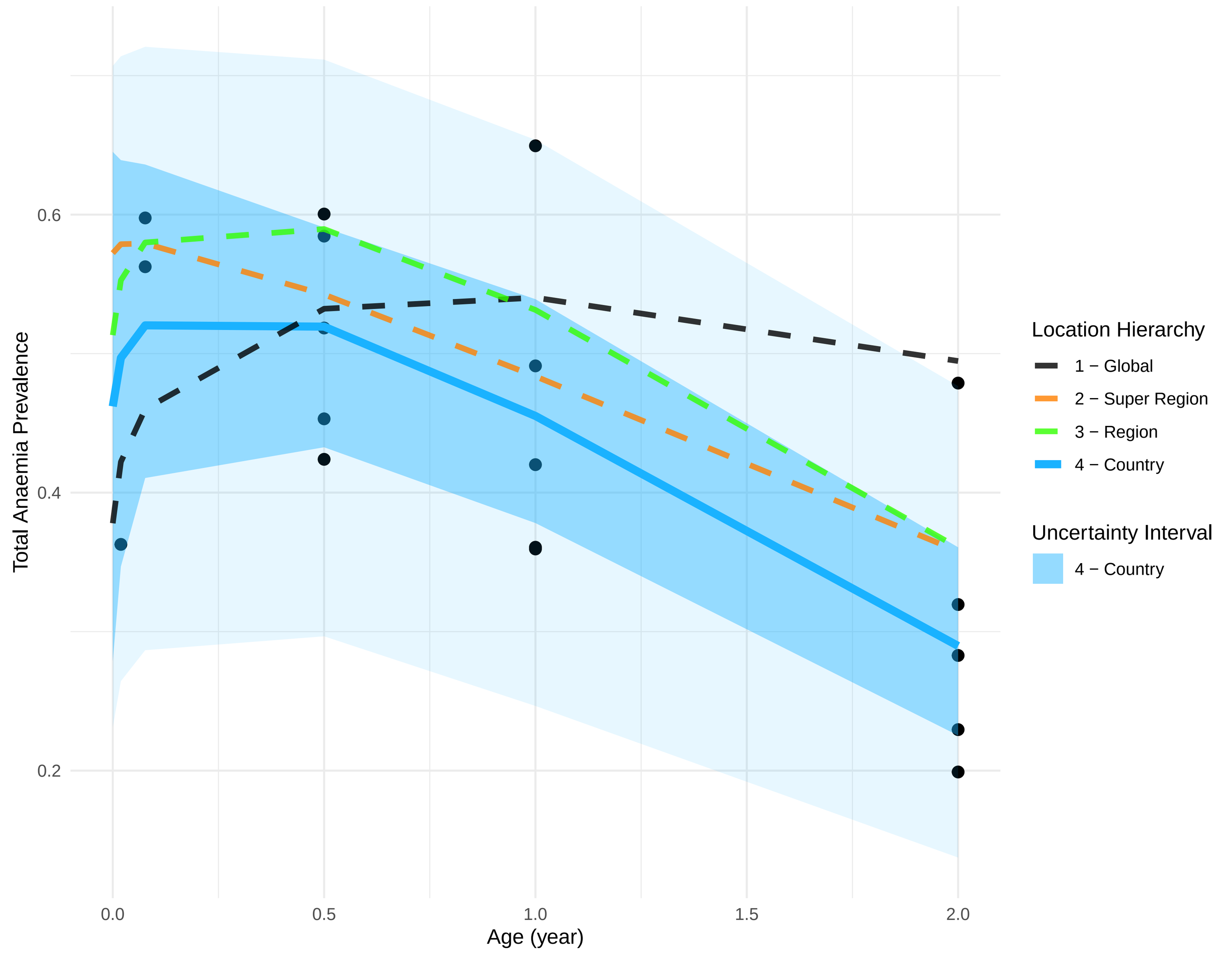

MR−BRT Age/Sex Fits − Non−pregnant Females <2 Years Old − Total Anaemia Prevalence − Caribbean
WHO−2001 Adjustment Method

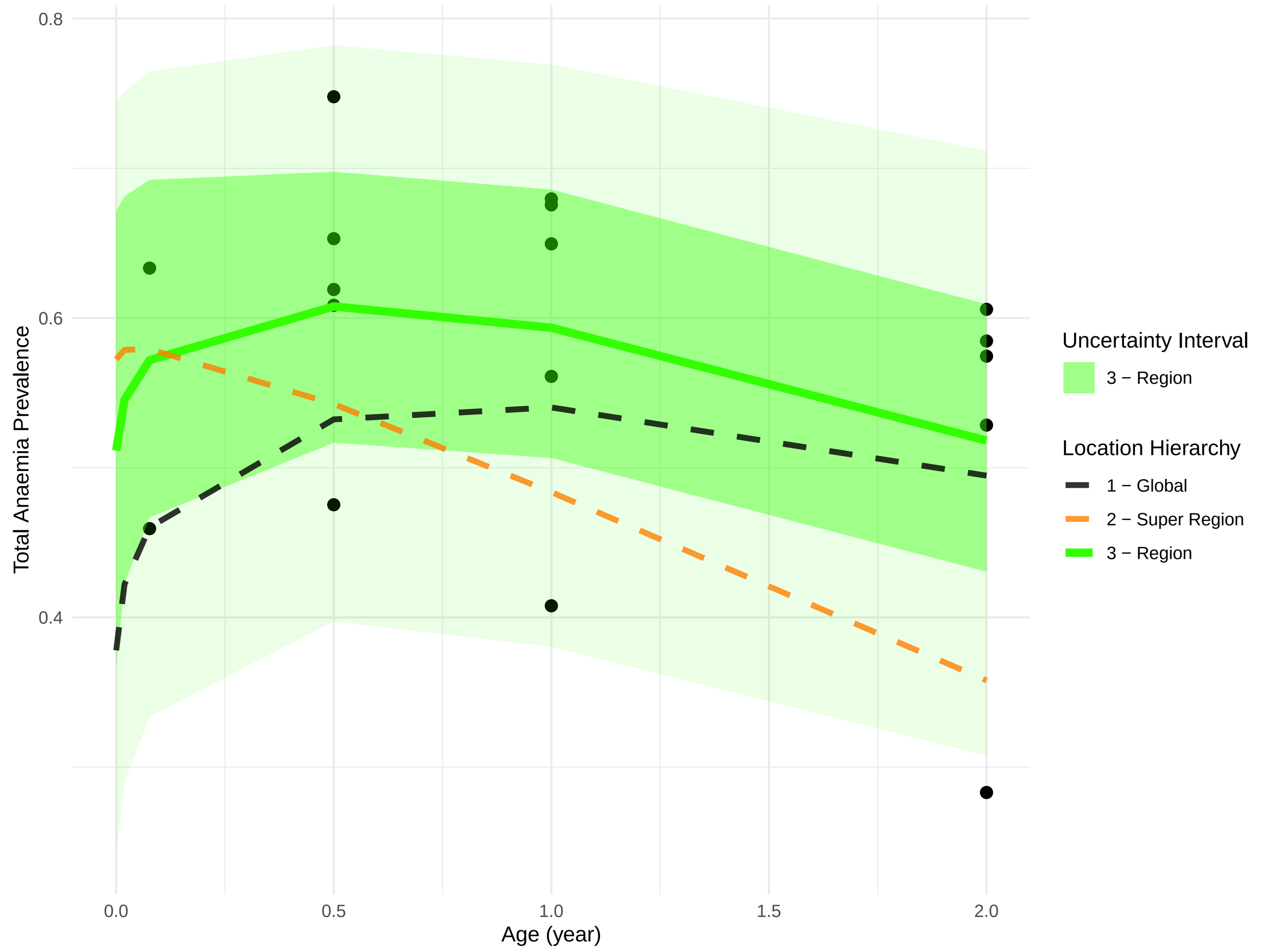

MR−BRT Age/Sex Fits − Non−pregnant Females <2 Years Old − Total Anaemia Prevalence − Guyana
WHO−2001 Adjustment Method

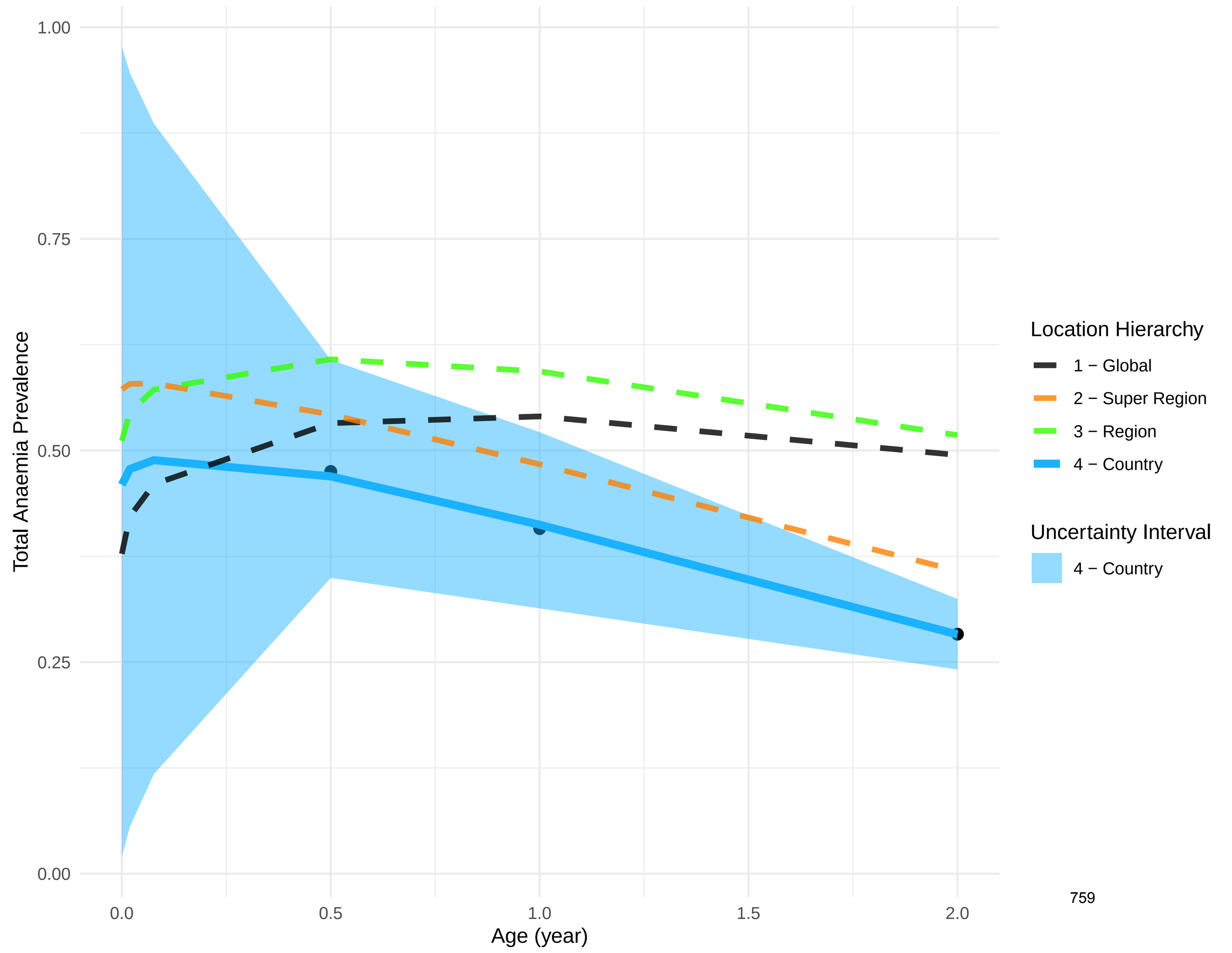

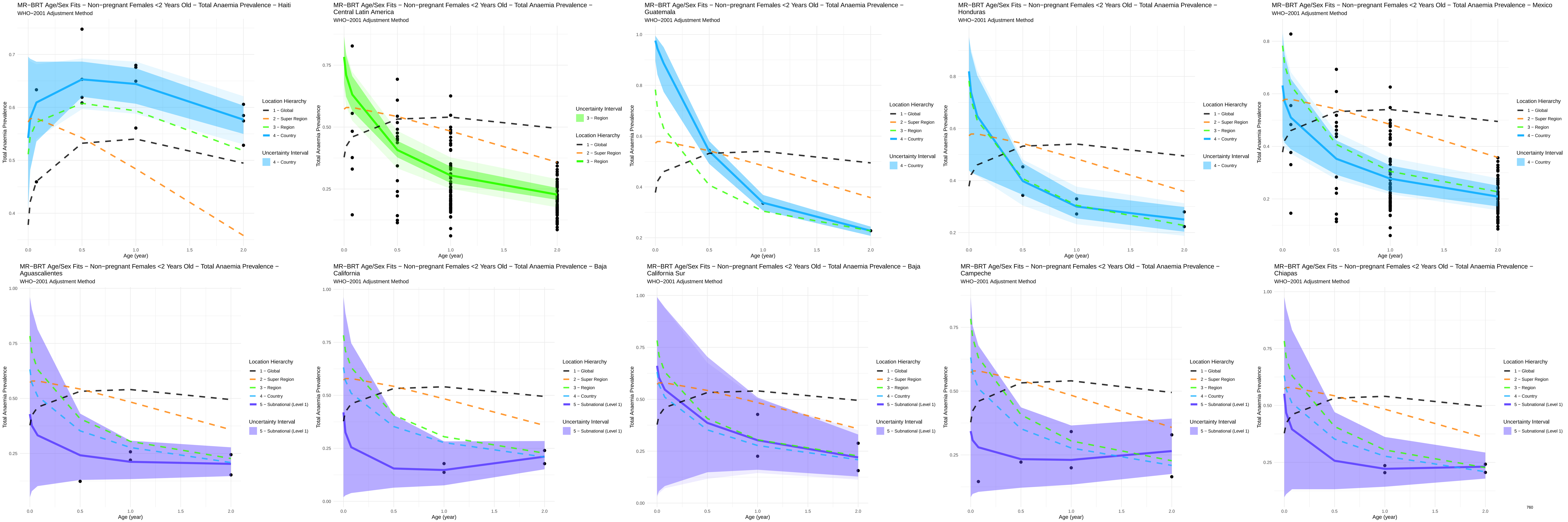
MR−BRT Age/Sex Fits − Non−pregnant Females <2 Years Old − Total Anaemia Prevalence − Haiti
WHO−2001 Adjustment Method
MR−BRT Age/Sex Fits − Non−pregnant Females <2 Years Old − Total Anaemia Prevalence − Central Latin America
WHO−2001 Adjustment Method
MR−BRT Age/Sex Fits − Non−pregnant Females <2 Years Old − Total Anaemia Prevalence − Guatemala
WHO−2001 Adjustment Method
MR−BRT Age/Sex Fits − Non−pregnant Females <2 Years Old − Total Anaemia Prevalence − Honduras
WHO−2001 Adjustment Method
MR−BRT Age/Sex Fits − Non−pregnant Females <2 Years Old − Total Anaemia Prevalence − Mexico
WHO−2001 Adjustment Method
MR−BRT Age/Sex Fits − Non−pregnant Females <2 Years Old − Total Anaemia Prevalence − Aguascalientes
WHO−2001 Adjustment Method
MR−BRT Age/Sex Fits − Non−pregnant Females <2 Years Old − Total Anaemia Prevalence − Baja California
WHO−2001 Adjustment Method
MR−BRT Age/Sex Fits − Non−pregnant Females <2 Years Old − Total Anaemia Prevalence − Baja California Sur
WHO−2001 Adjustment Method
MR−BRT Age/Sex Fits − Non−pregnant Females <2 Years Old − Total Anaemia Prevalence − Campeche
WHO−2001 Adjustment Method
MR−BRT Age/Sex Fits − Non−pregnant Females <2 Years Old − Total Anaemia Prevalence − Chiapas
WHO−2001 Adjustment Method
Total Anaemia Prevalence
Age (year)
Location Hierarchy
1 − Global
2 − Super Region
3 − Region
4 − Country
5 − Subnational (Level 1)
Uncertainty Interval
3 − Region
4 − Country
5 − Subnational (Level 1)

MR−BRT Age/Sex Fits − Non−pregnant Females <2 Years Old − Total Anaemia Prevalence − Chihuahua

WHO−2001 Adjustment Method

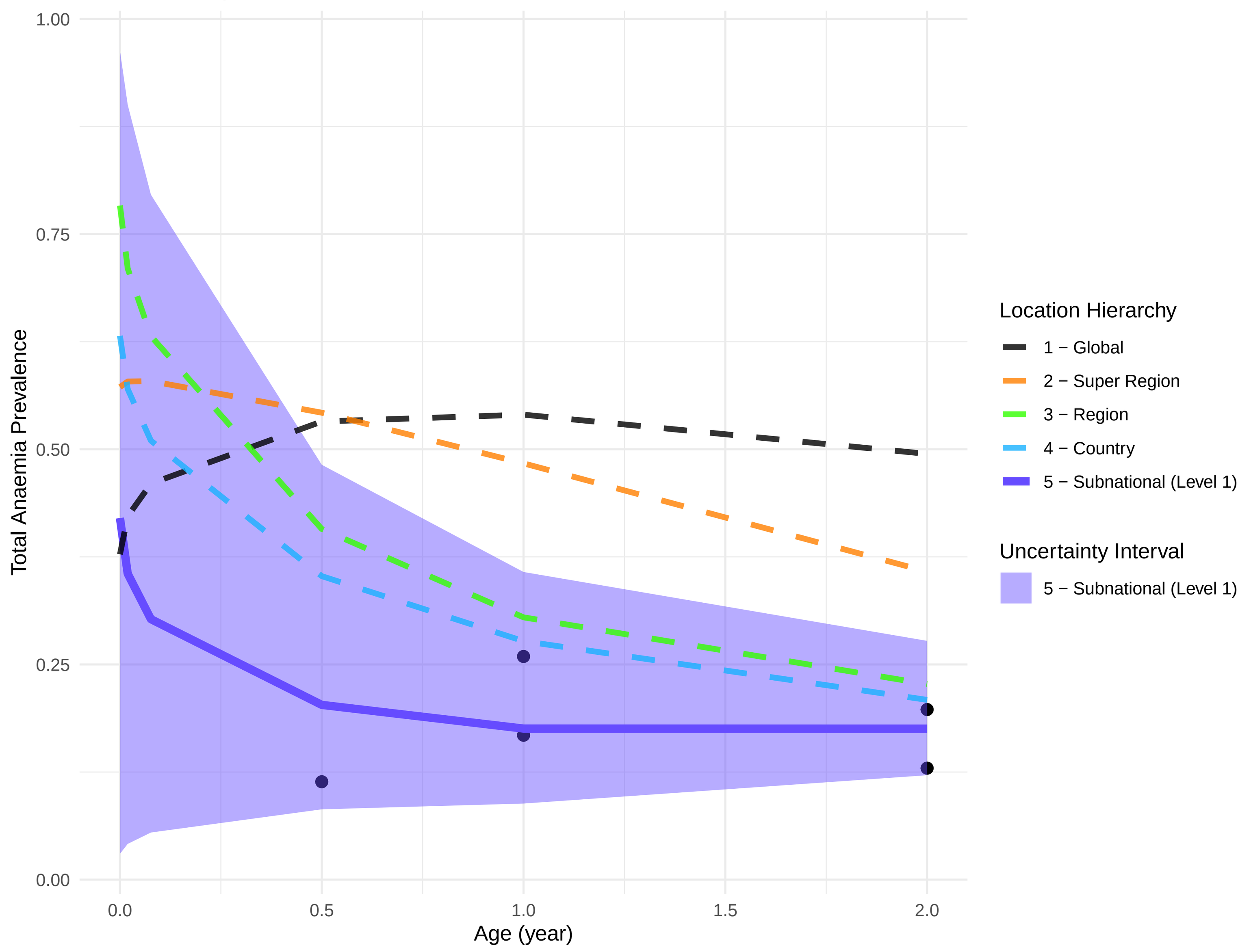

MR−BRT Age/Sex Fits − Non−pregnant Females <2 Years Old − Total Anaemia Prevalence − Coahuila

WHO−2001 Adjustment Method

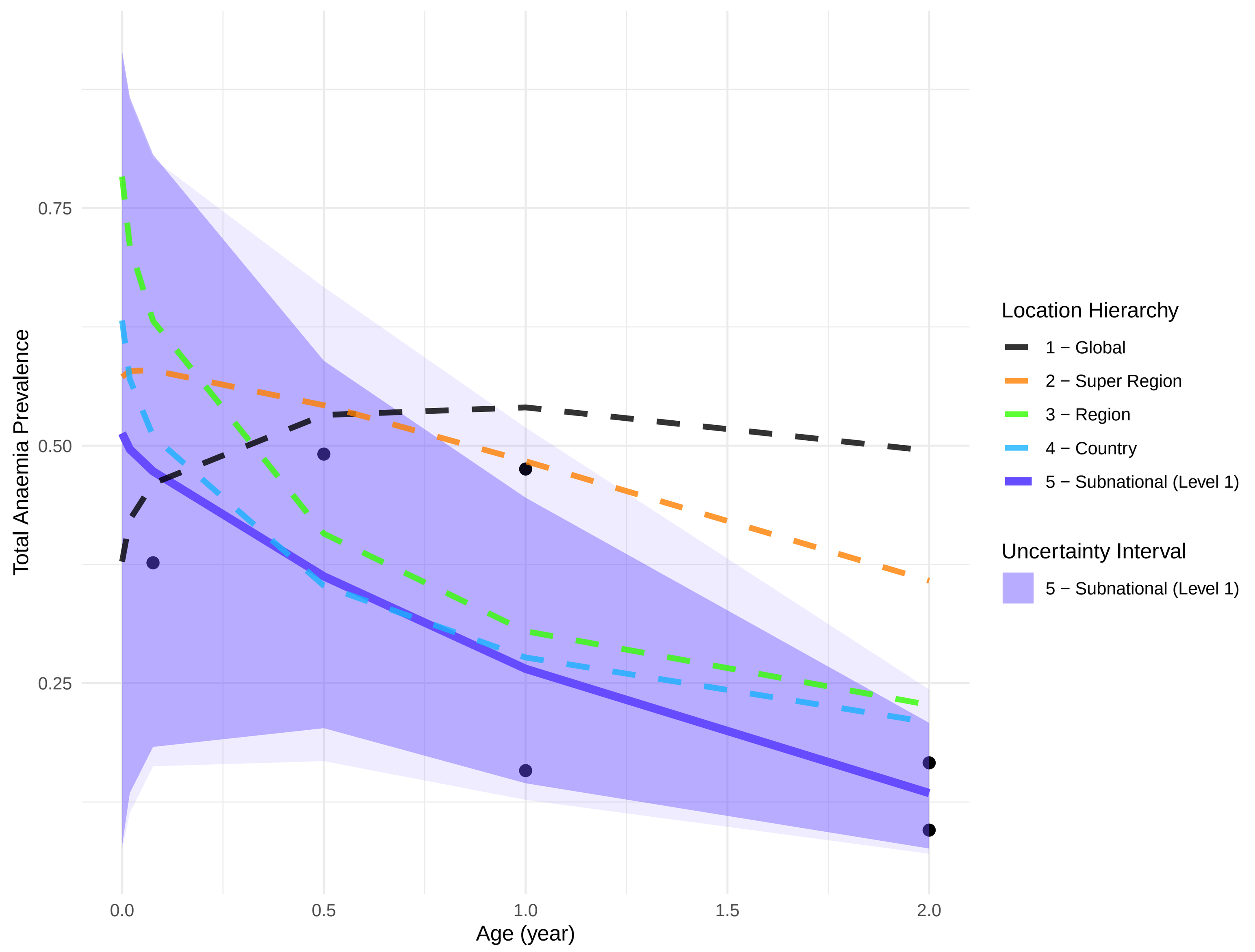

MR−BRT Age/Sex Fits − Non−pregnant Females <2 Years Old − Total Anaemia Prevalence − Colima

WHO−2001 Adjustment Method

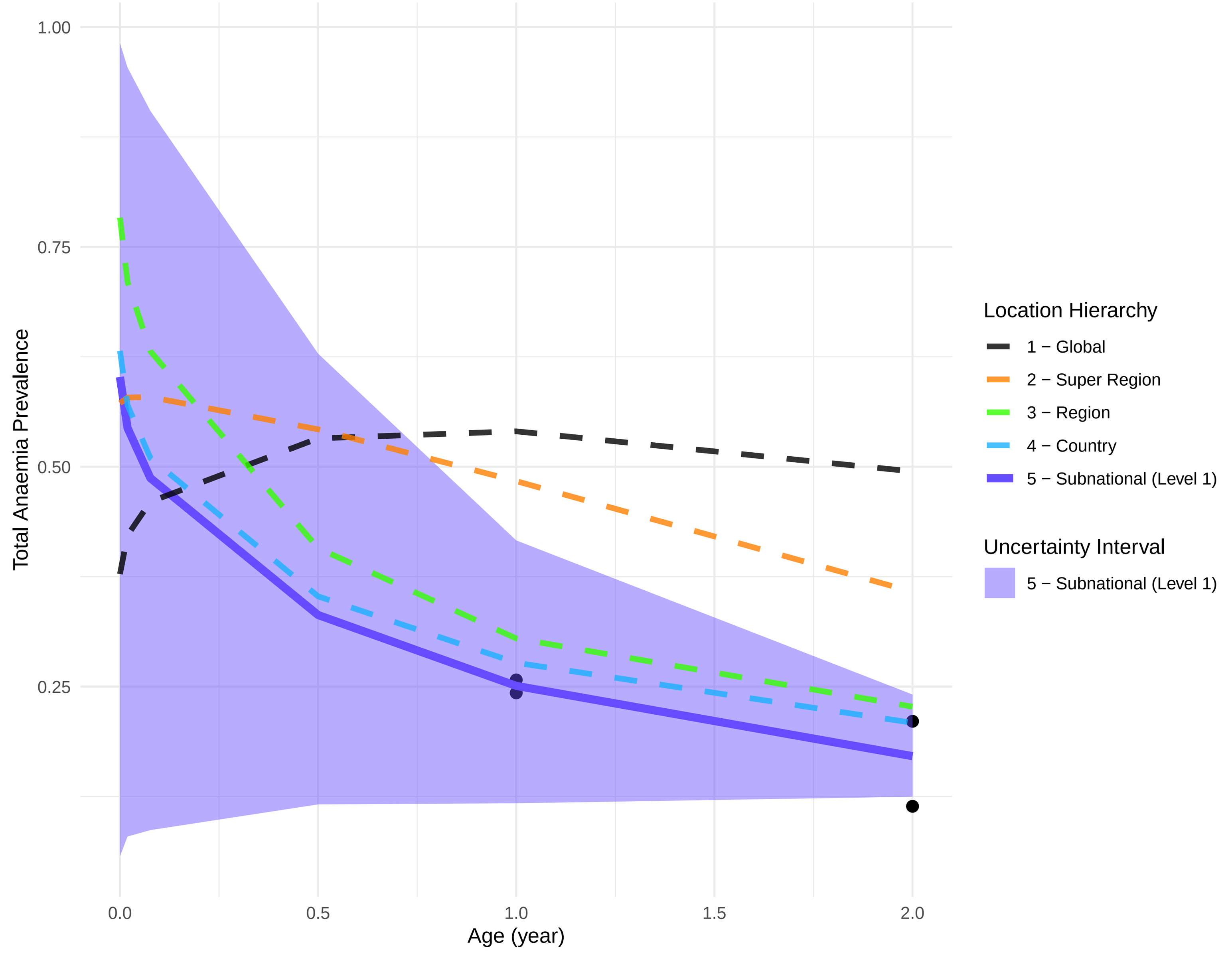

MR−BRT Age/Sex Fits − Non−pregnant Females <2 Years Old − Total Anaemia Prevalence − Durango

WHO−2001 Adjustment Method

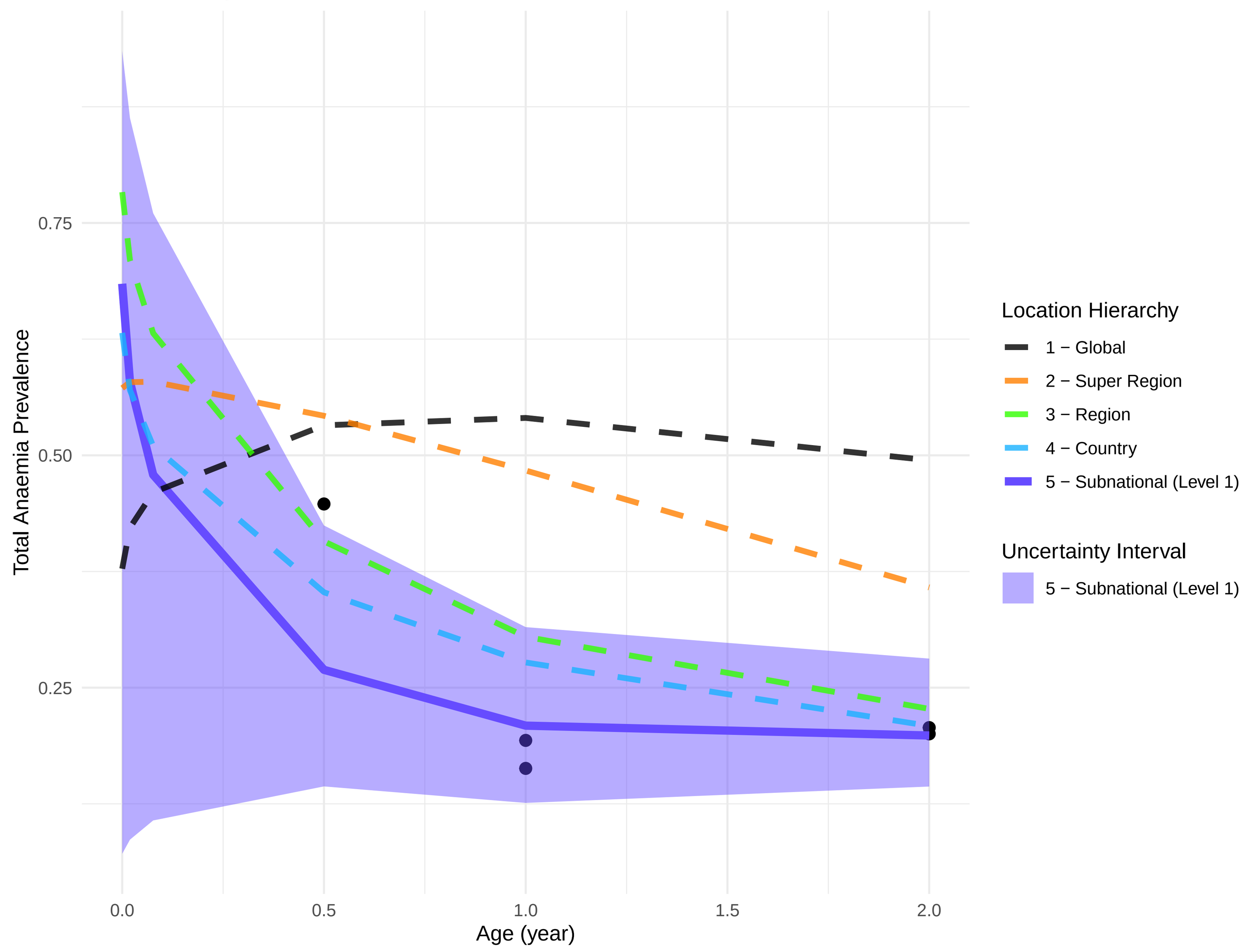

MR−BRT Age/Sex Fits − Non−pregnant Females <2 Years Old − Total Anaemia Prevalence − Guanajuato

WHO−2001 Adjustment Method

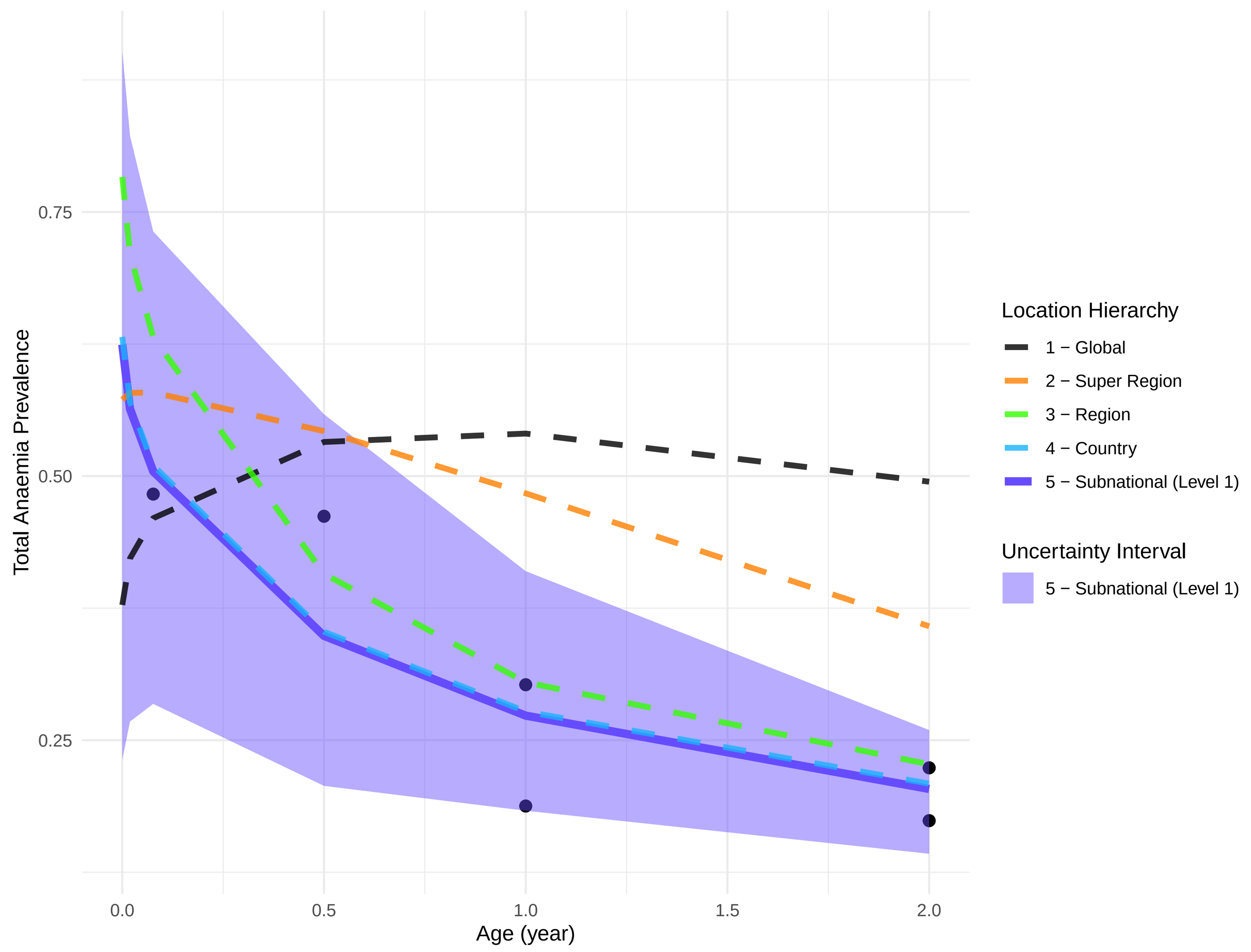

MR−BRT Age/Sex Fits − Non−pregnant Females <2 Years Old − Total Anaemia Prevalence − Guerrero

WHO−2001 Adjustment Method

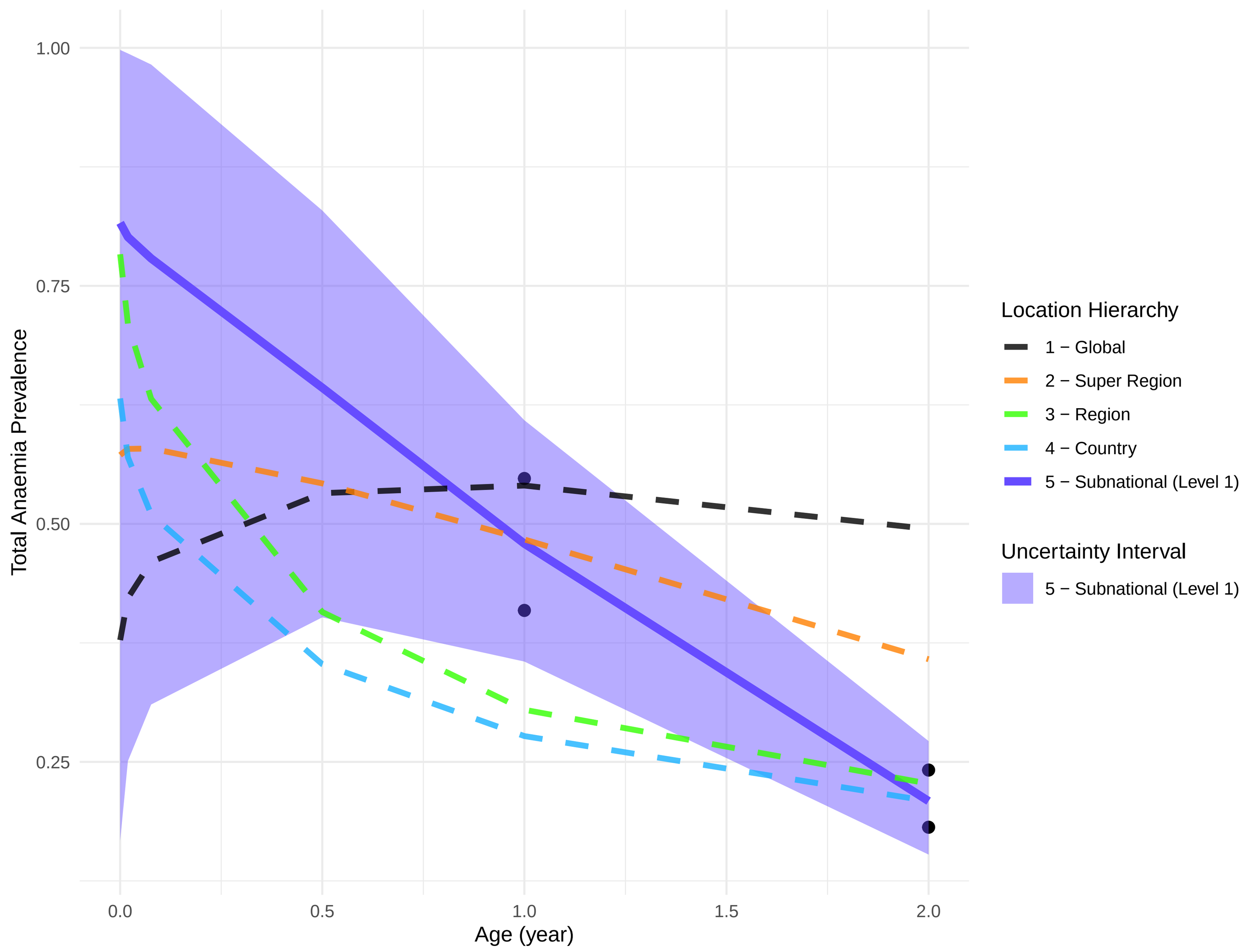

MR−BRT Age/Sex Fits − Non−pregnant Females <2 Years Old − Total Anaemia Prevalence − Hidalgo

WHO−2001 Adjustment Method

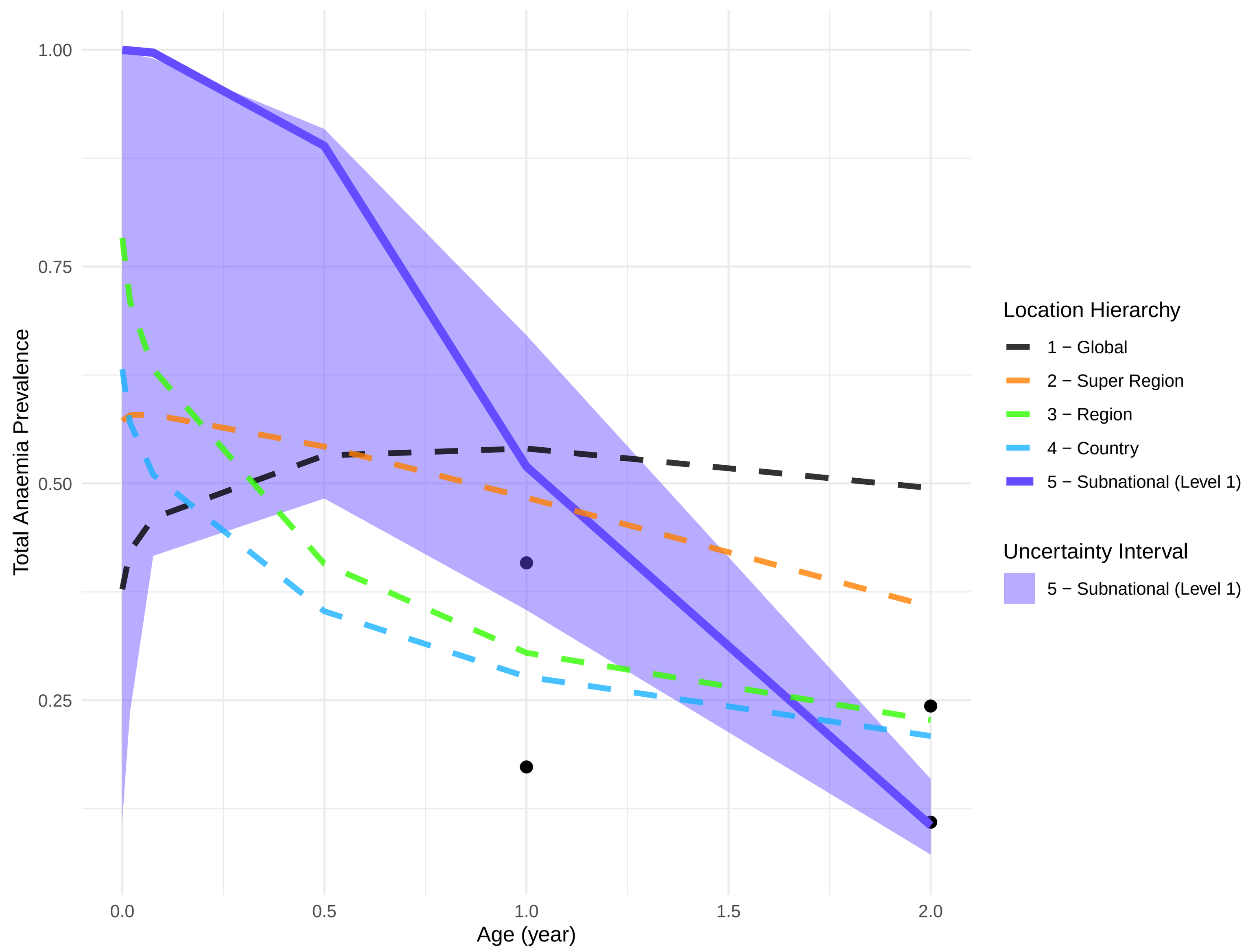

MR−BRT Age/Sex Fits − Non−pregnant Females <2 Years Old − Total Anaemia Prevalence − Jalisco

WHO−2001 Adjustment Method

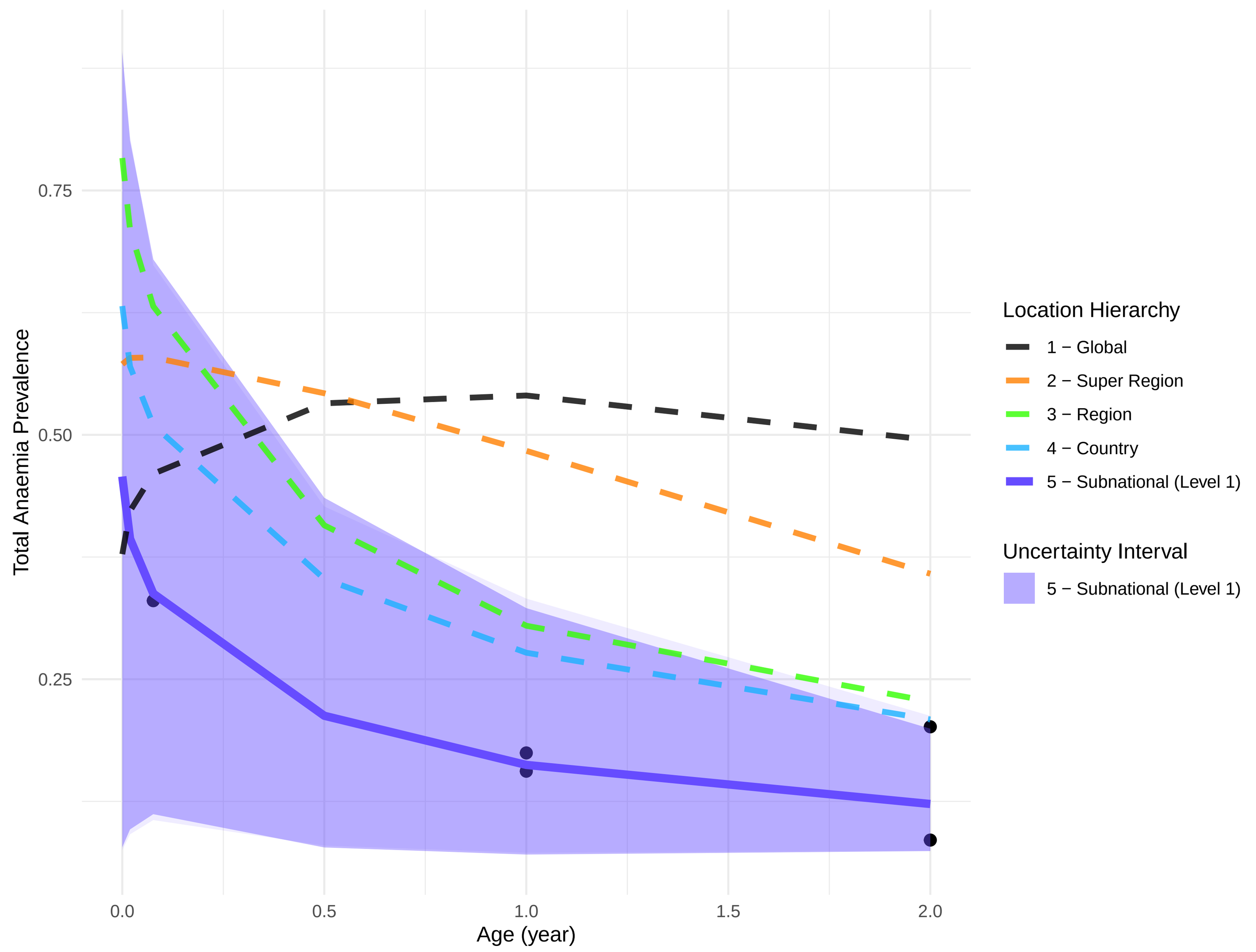

MR−BRT Age/Sex Fits − Non−pregnant Females <2 Years Old − Total Anaemia Prevalence − México

WHO−2001 Adjustment Method

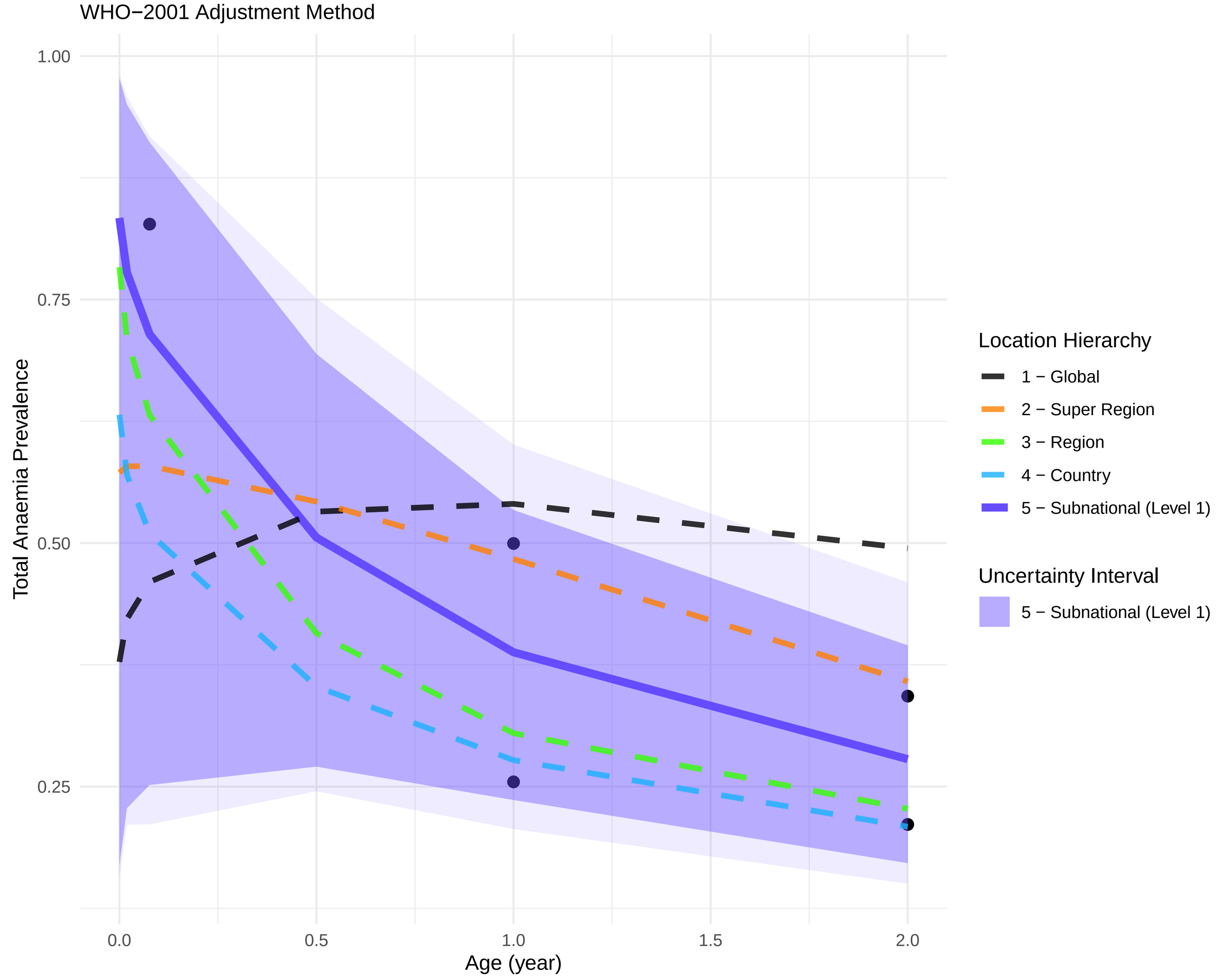

MR−BRT Age/Sex Fits − Non−pregnant Females <2 Years Old − Total Anaemia Prevalence − Mexico City

WHO−2001 Adjustment Method

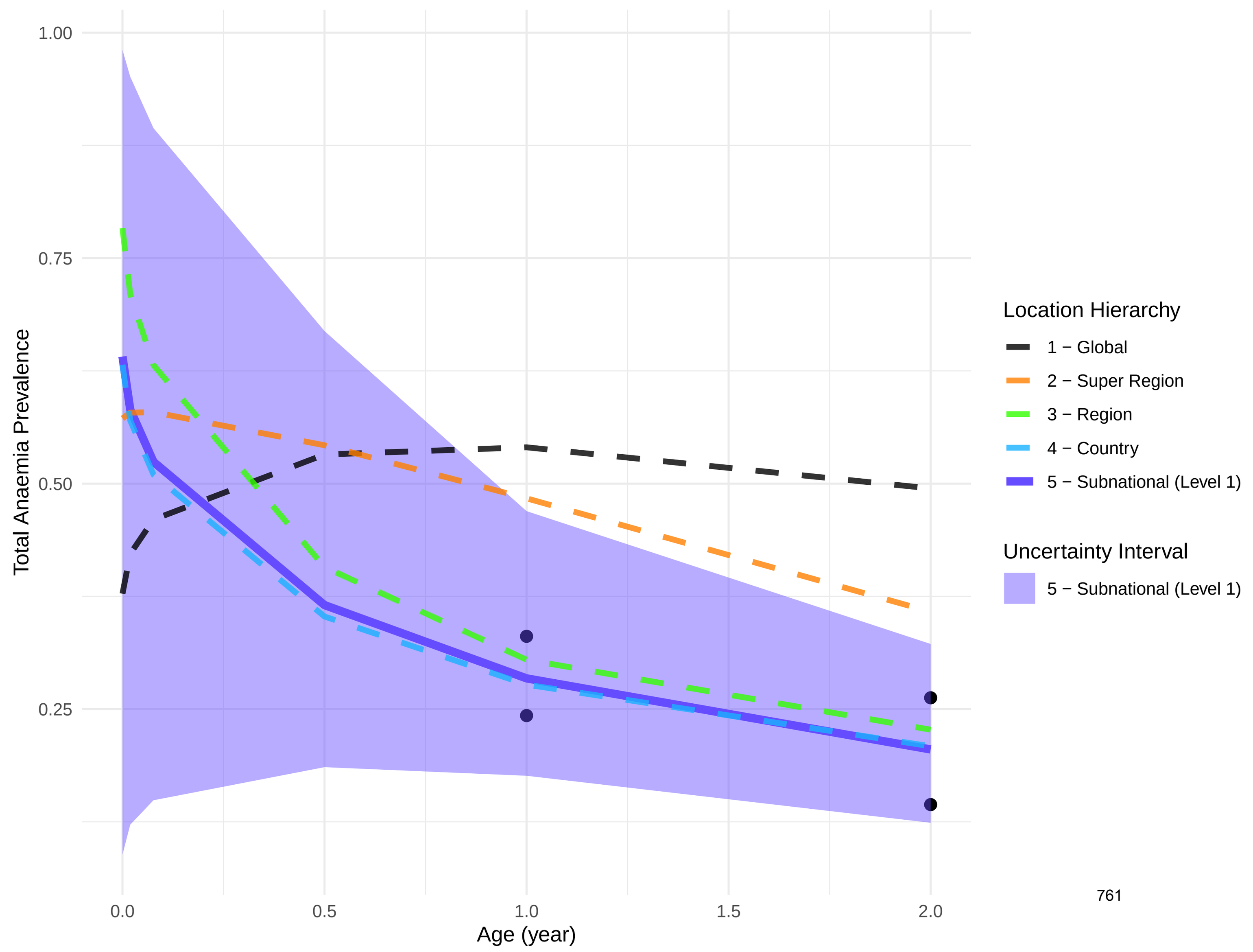

MR−BRT Age/Sex Fits − Non−pregnant Females <2 Years Old − Total Anaemia Prevalence − Michoacán de Ocampo
WHO−2001 Adjustment Method

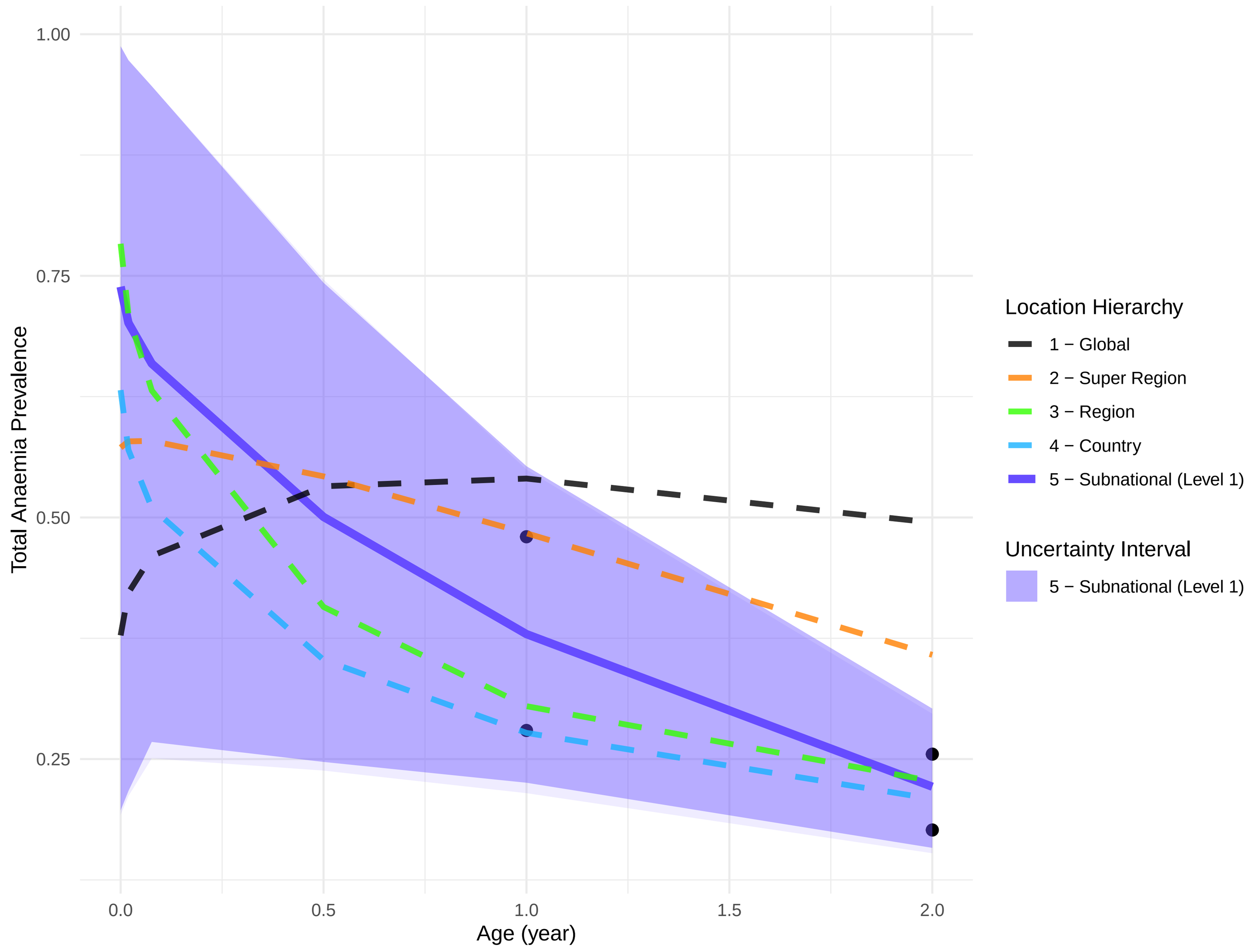

MR−BRT Age/Sex Fits − Non−pregnant Females <2 Years Old − Total Anaemia Prevalence − Morelos
WHO−2001 Adjustment Method

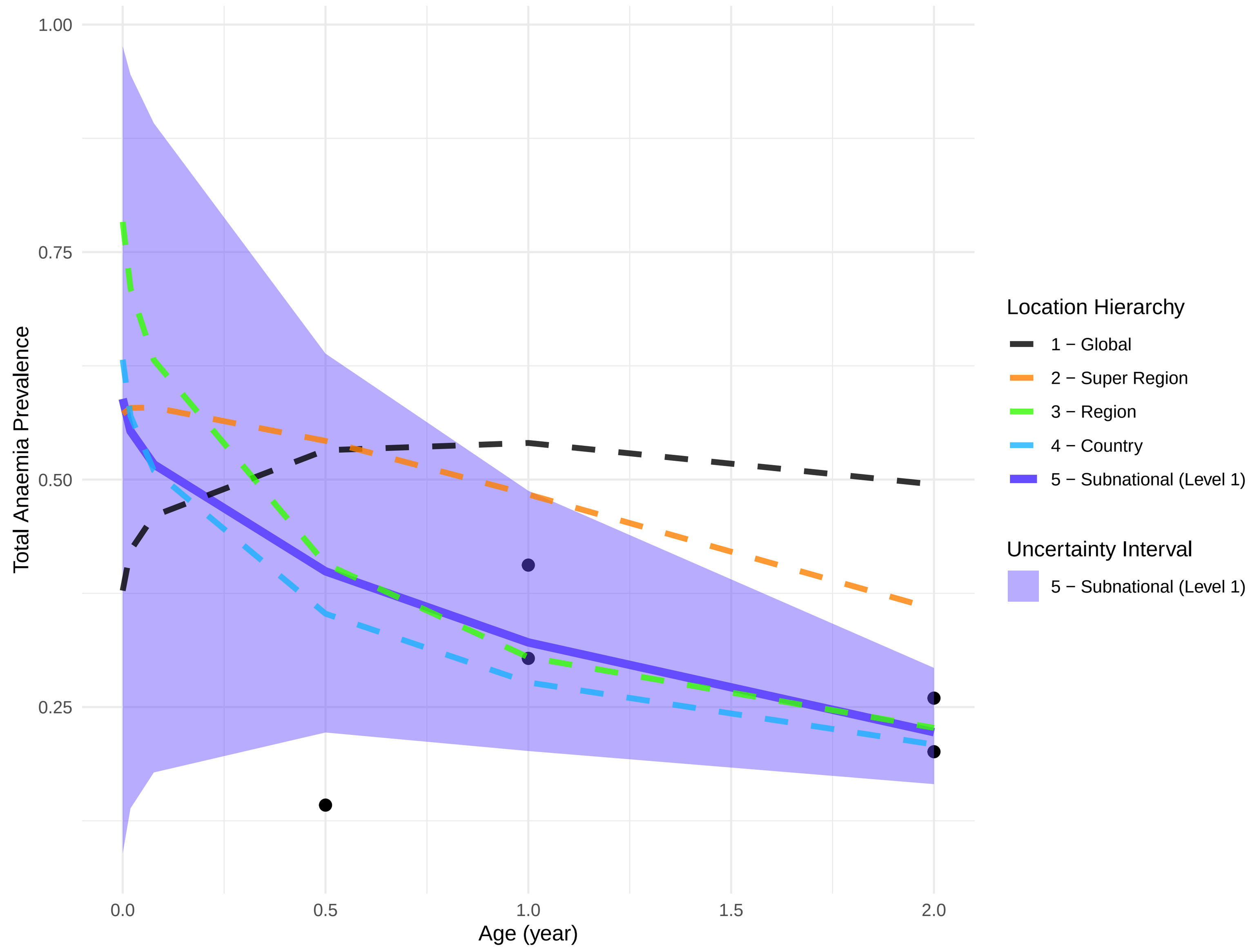

MR−BRT Age/Sex Fits − Non−pregnant Females <2 Years Old − Total Anaemia Prevalence − Nayarit
WHO−2001 Adjustment Method

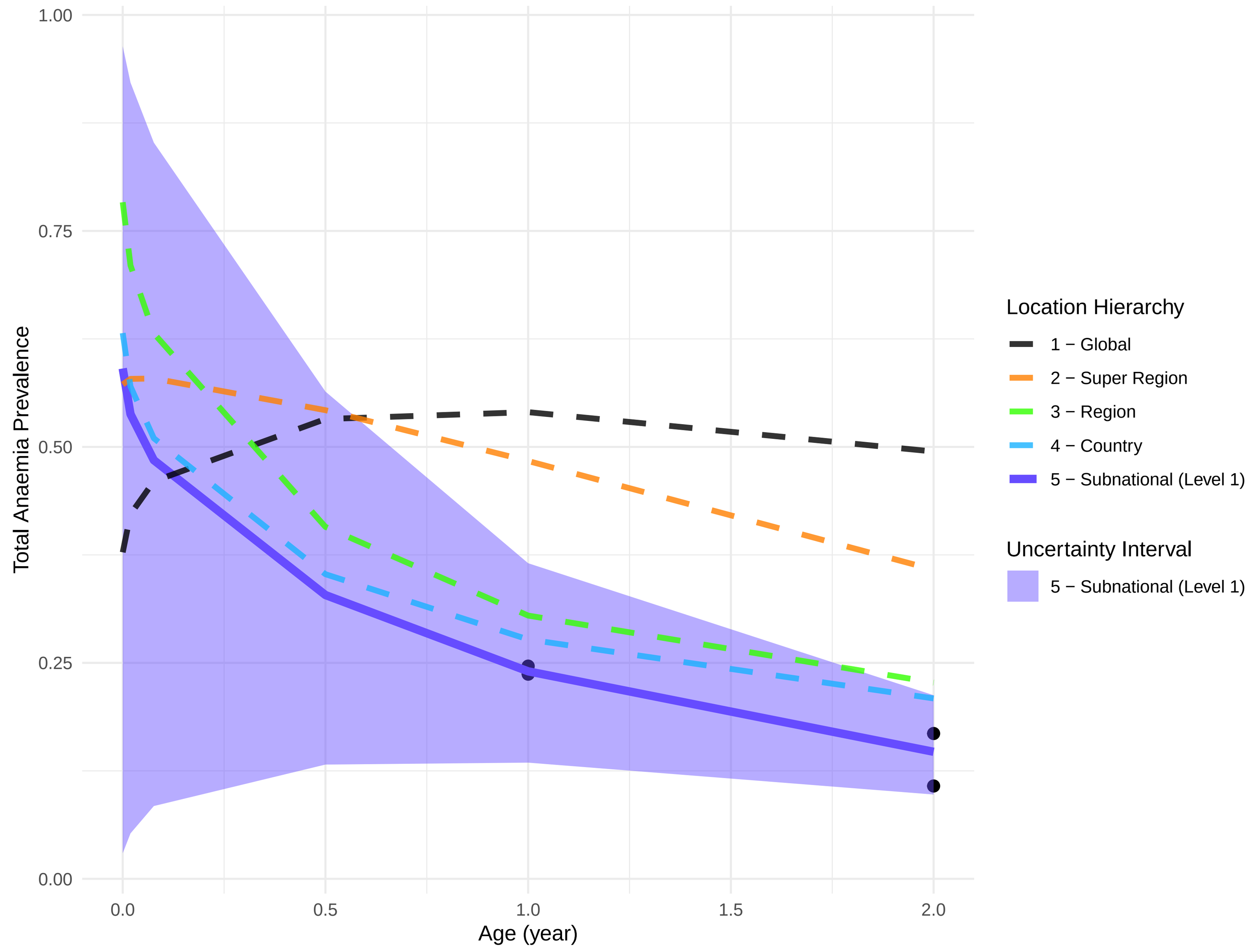

MR−BRT Age/Sex Fits − Non−pregnant Females <2 Years Old − Total Anaemia Prevalence − Nuevo León
WHO−2001 Adjustment Method

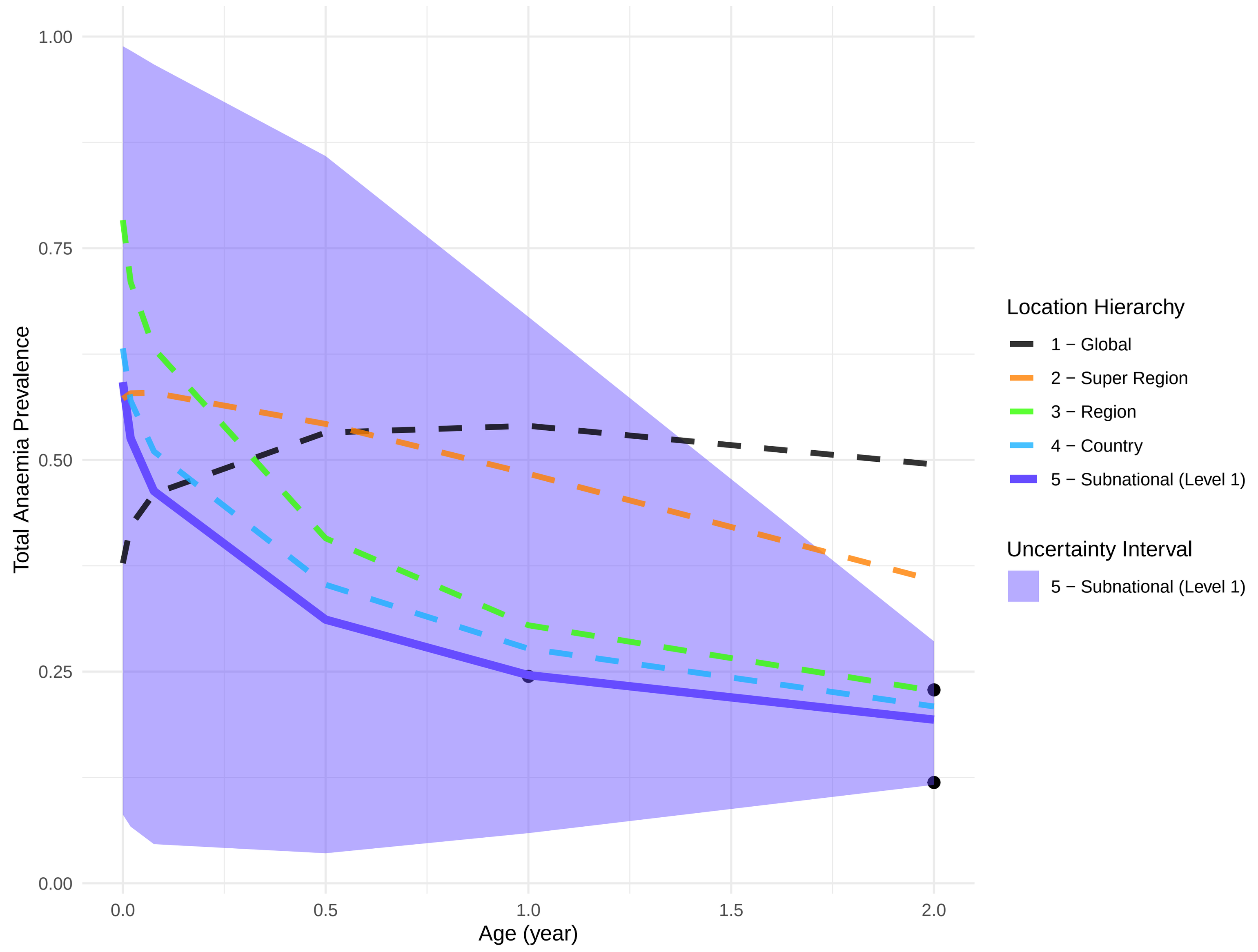

MR−BRT Age/Sex Fits − Non−pregnant Females <2 Years Old − Total Anaemia Prevalence − Oaxaca
WHO−2001 Adjustment Method

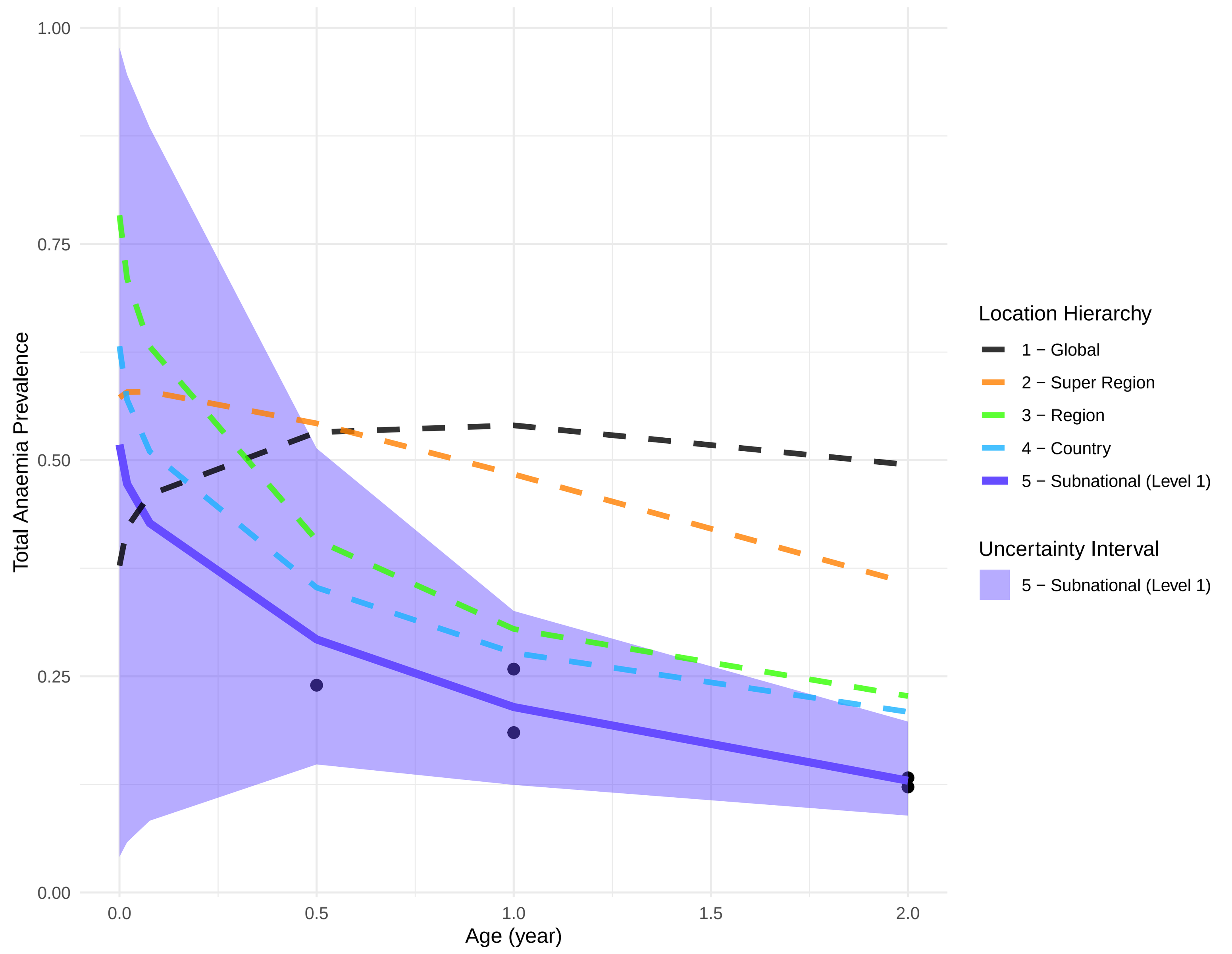

MR−BRT Age/Sex Fits − Non−pregnant Females <2 Years Old − Total Anaemia Prevalence − Puebla
WHO−2001 Adjustment Method

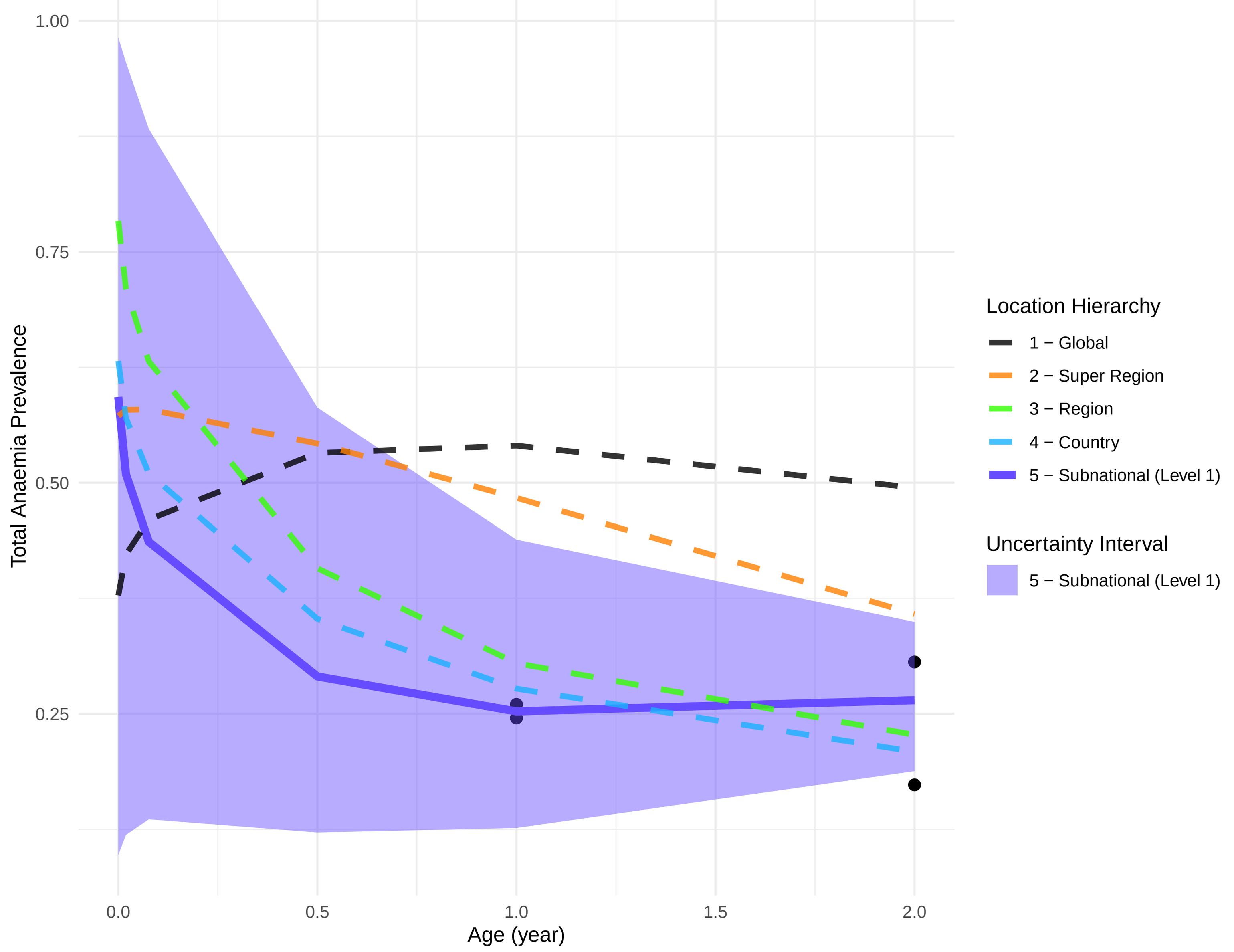

MR−BRT Age/Sex Fits − Non−pregnant Females <2 Years Old − Total Anaemia Prevalence − Querétaro
WHO−2001 Adjustment Method

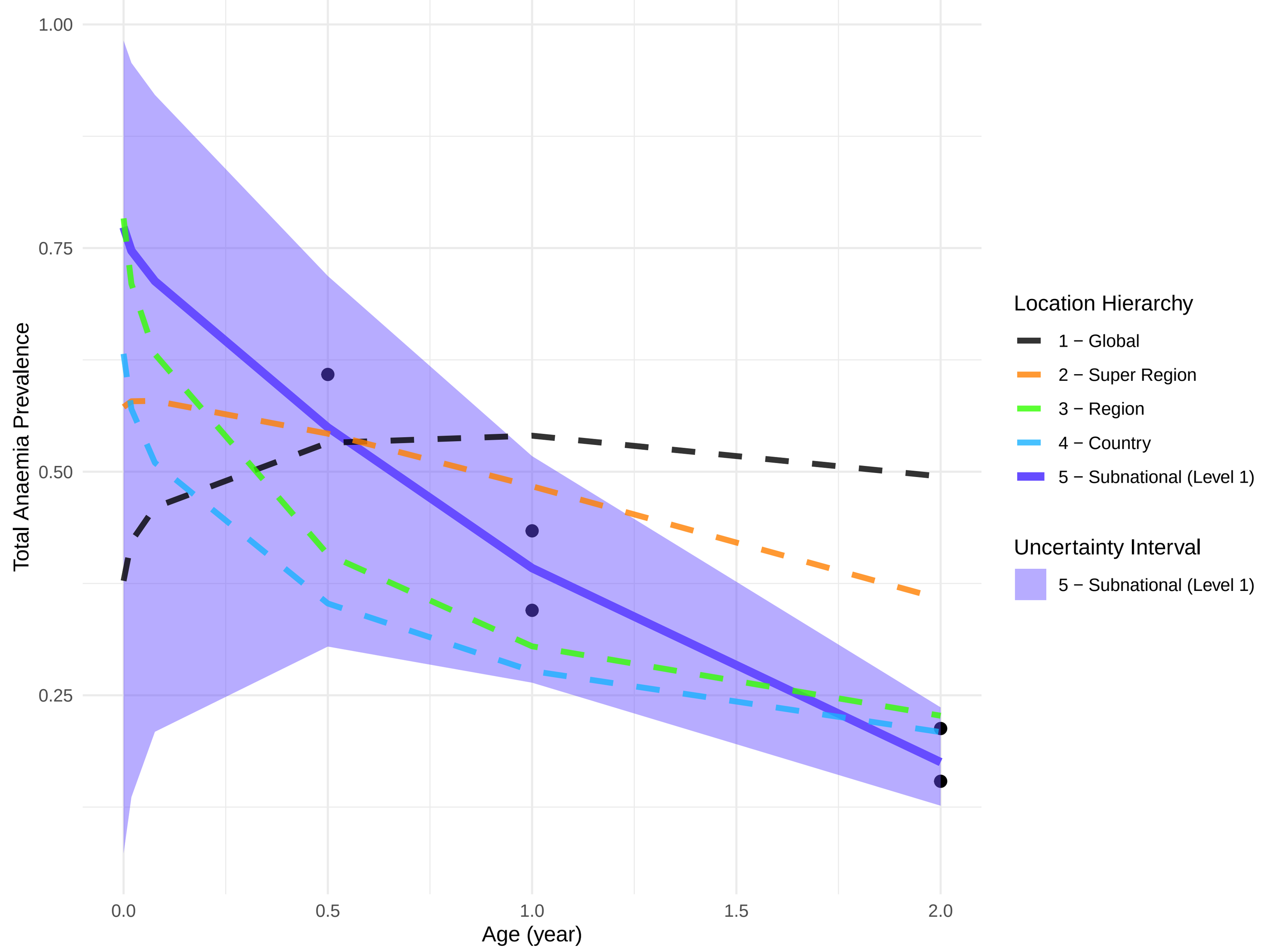

MR−BRT Age/Sex Fits − Non−pregnant Females <2 Years Old − Total Anaemia Prevalence − Quintana Roo
WHO−2001 Adjustment Method

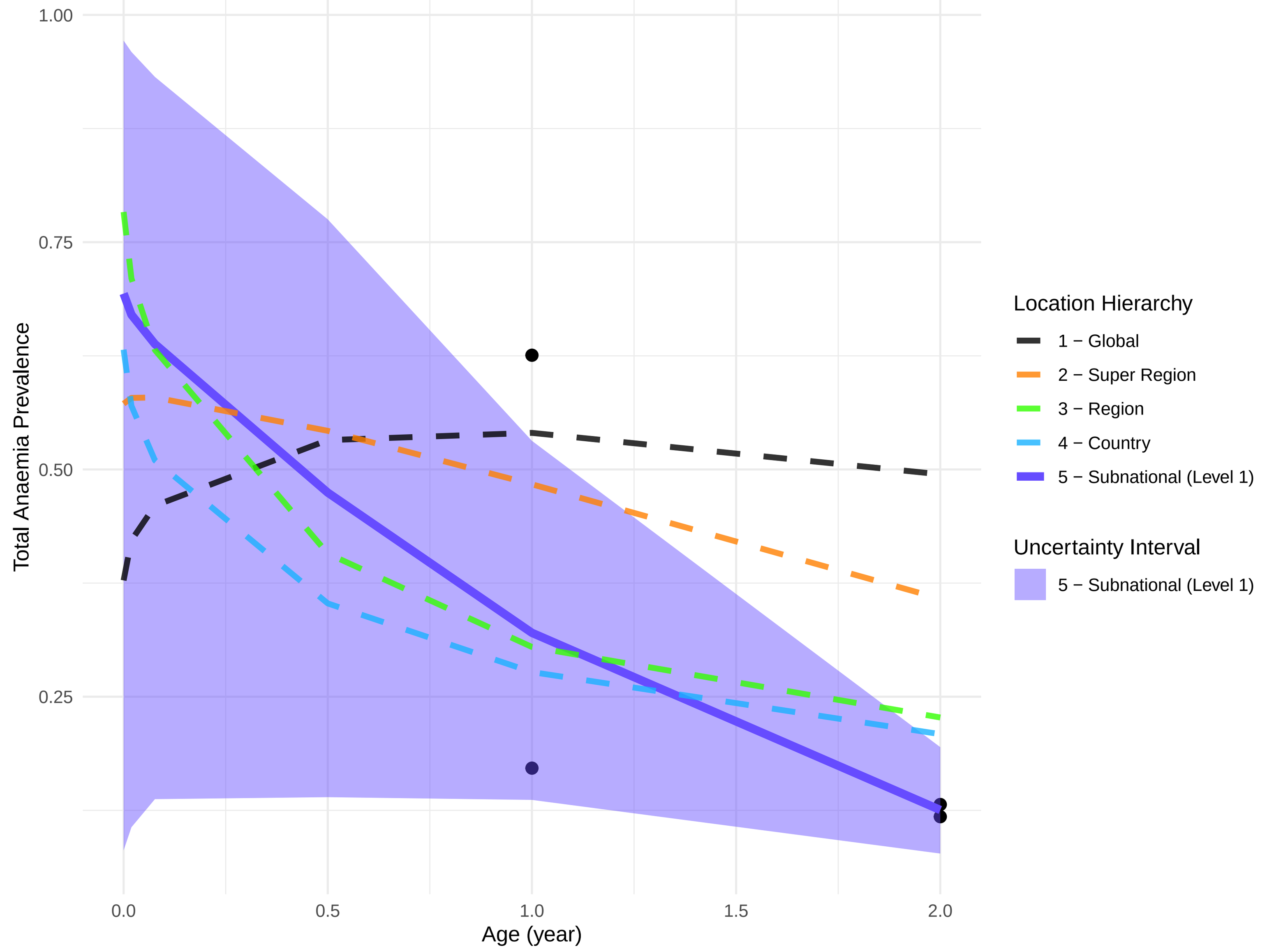

MR−BRT Age/Sex Fits − Non−pregnant Females <2 Years Old − Total Anaemia Prevalence − San Luis Potosí
WHO−2001 Adjustment Method

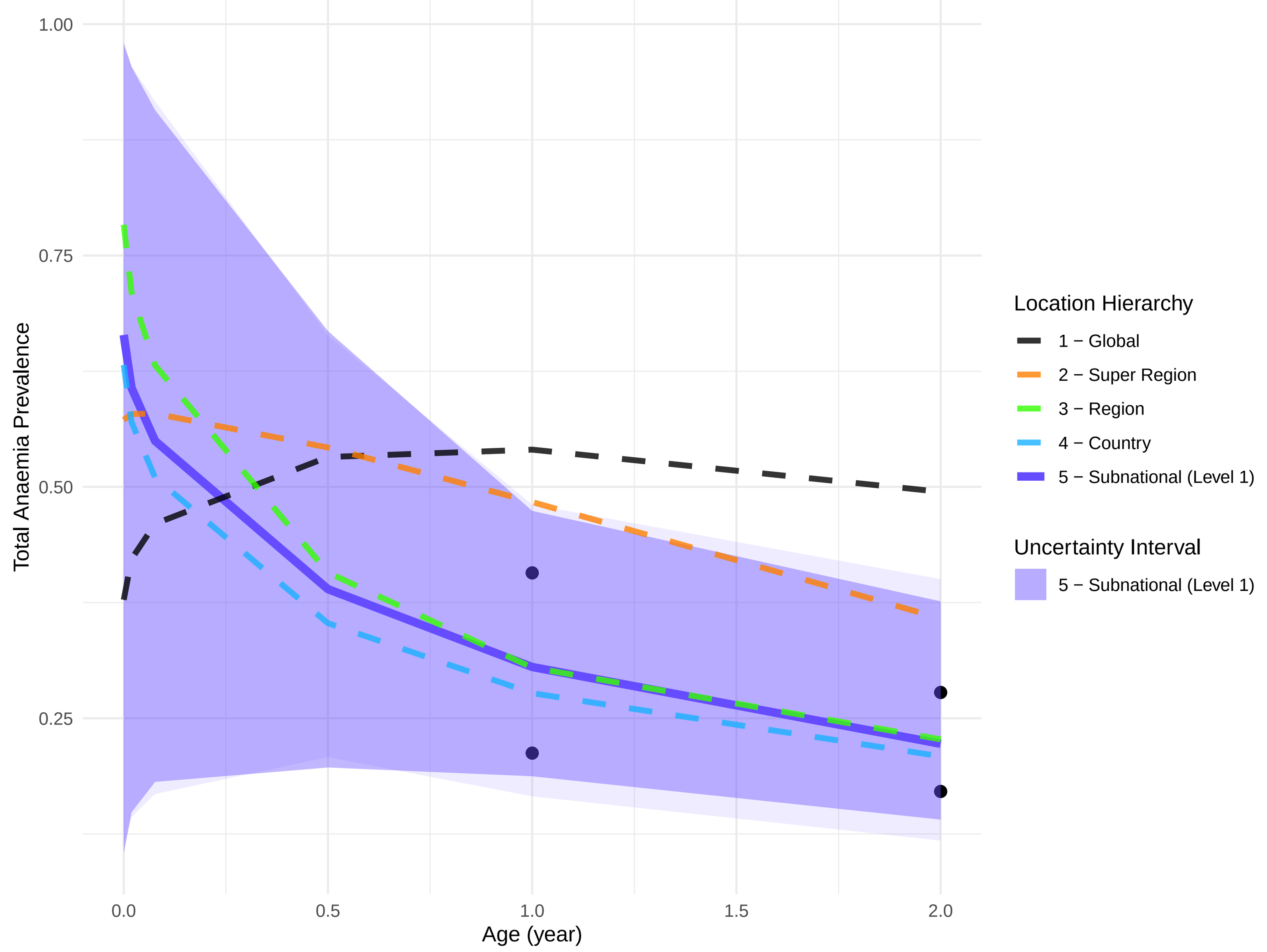

MR−BRT Age/Sex Fits − Non−pregnant Females <2 Years Old − Total Anaemia Prevalence − Sinaloa
WHO−2001 Adjustment Method

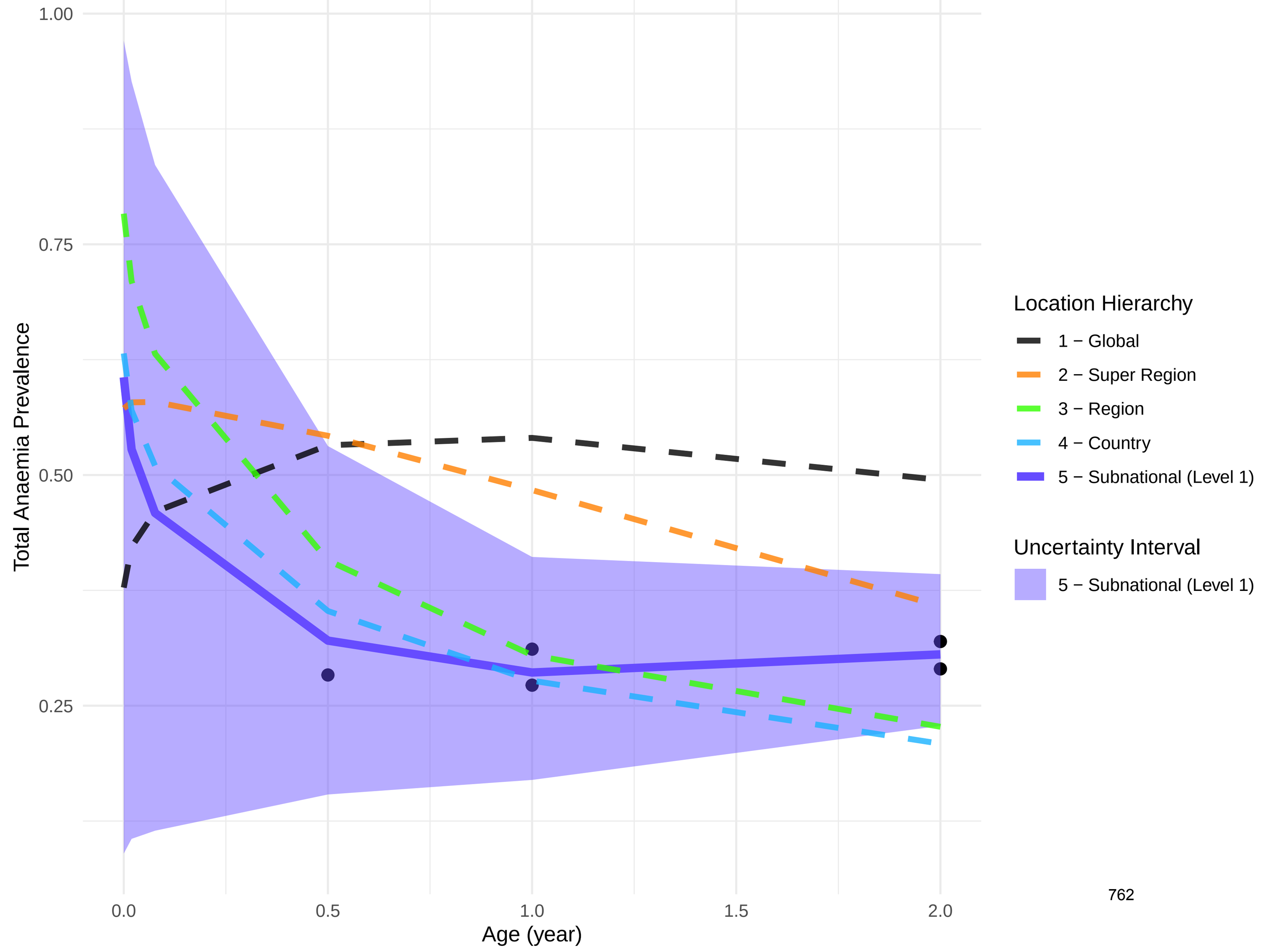

MR−BRT Age/Sex Fits − Non−pregnant Females <2 Years Old − Total Anaemia Prevalence − Sonora
WHO−2001 Adjustment Method

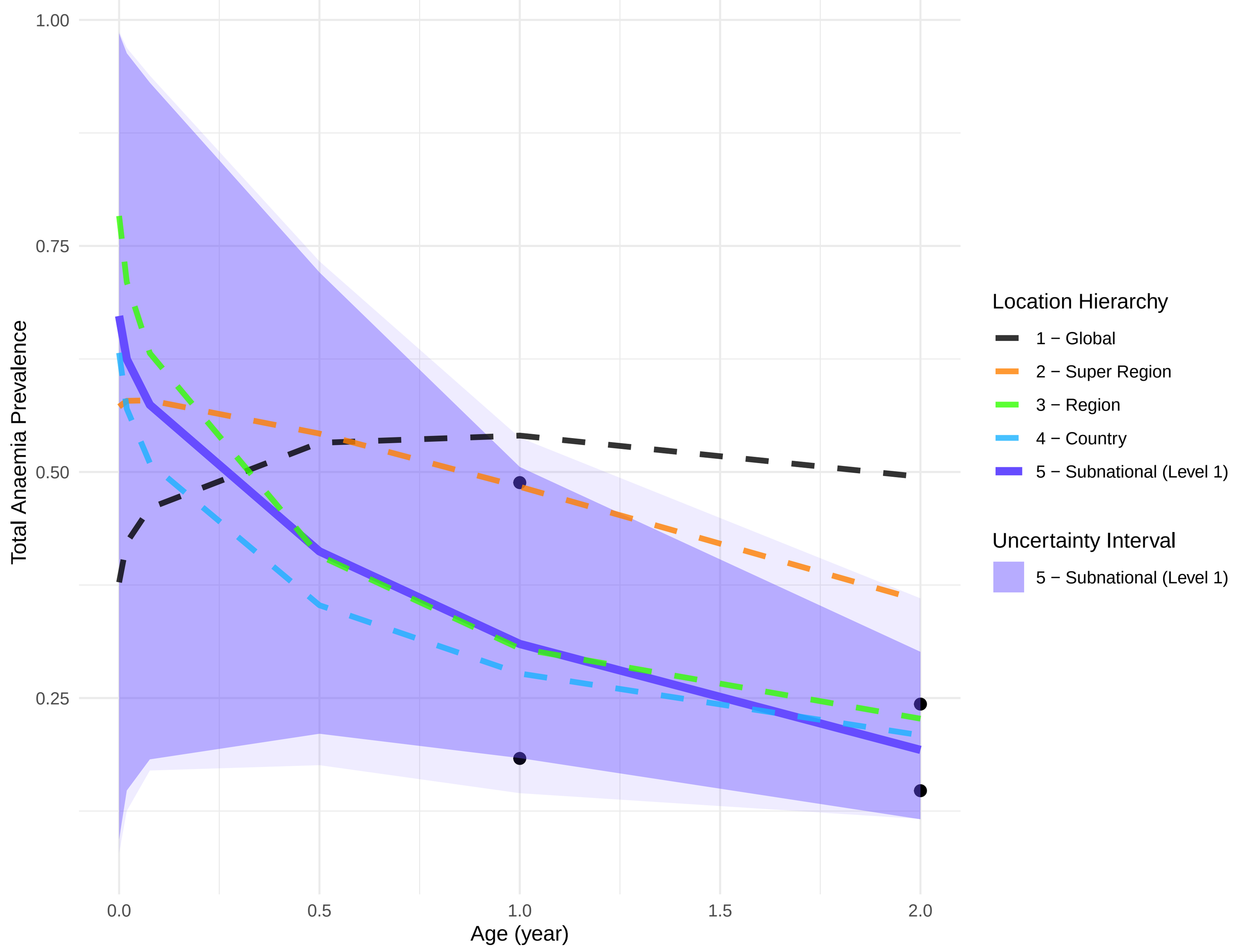

MR−BRT Age/Sex Fits − Non−pregnant Females <2 Years Old − Total Anaemia Prevalence − Tabasco
WHO−2001 Adjustment Method

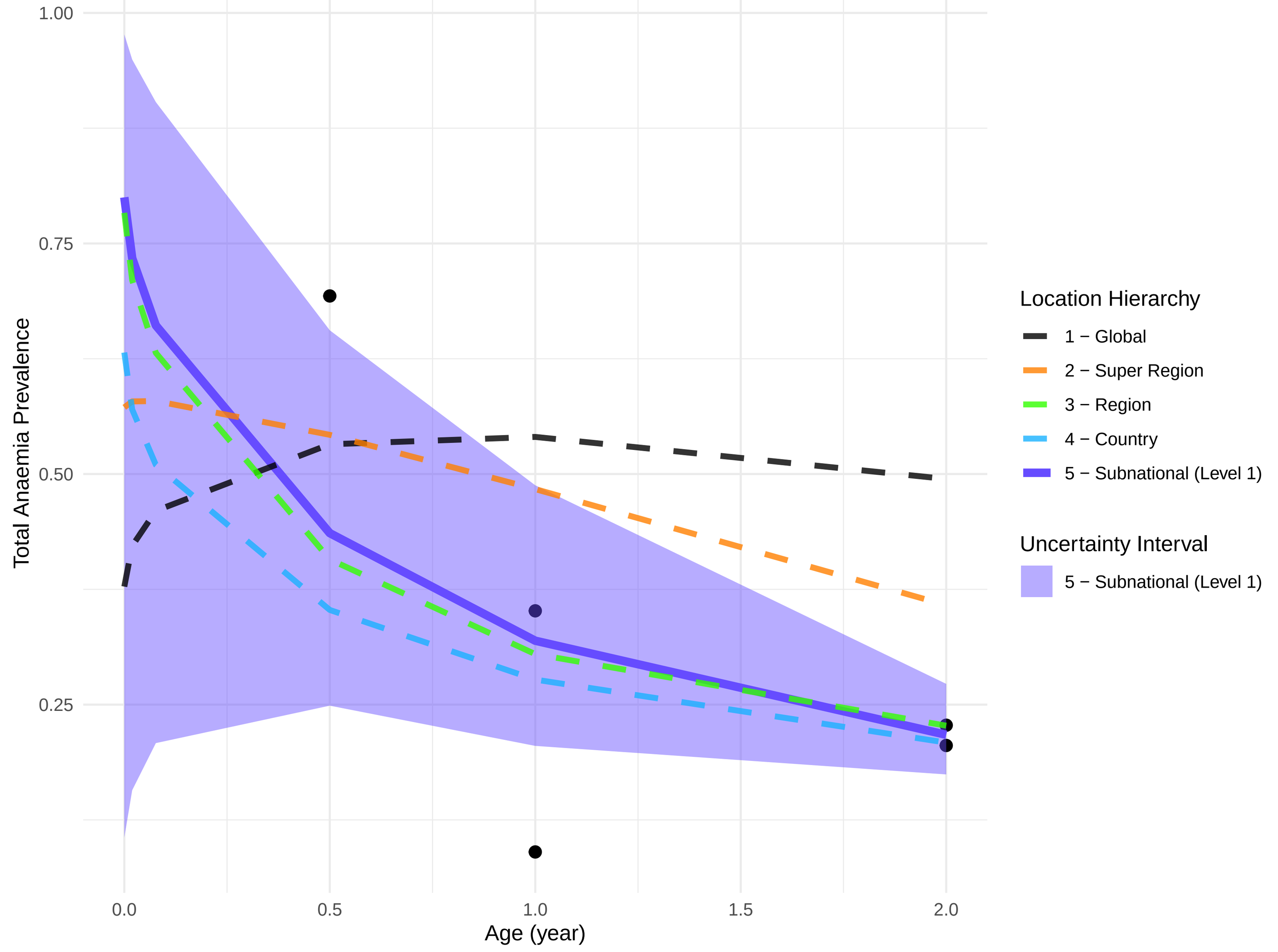

MR−BRT Age/Sex Fits − Non−pregnant Females <2 Years Old − Total Anaemia Prevalence − Tamaulipas
WHO−2001 Adjustment Method

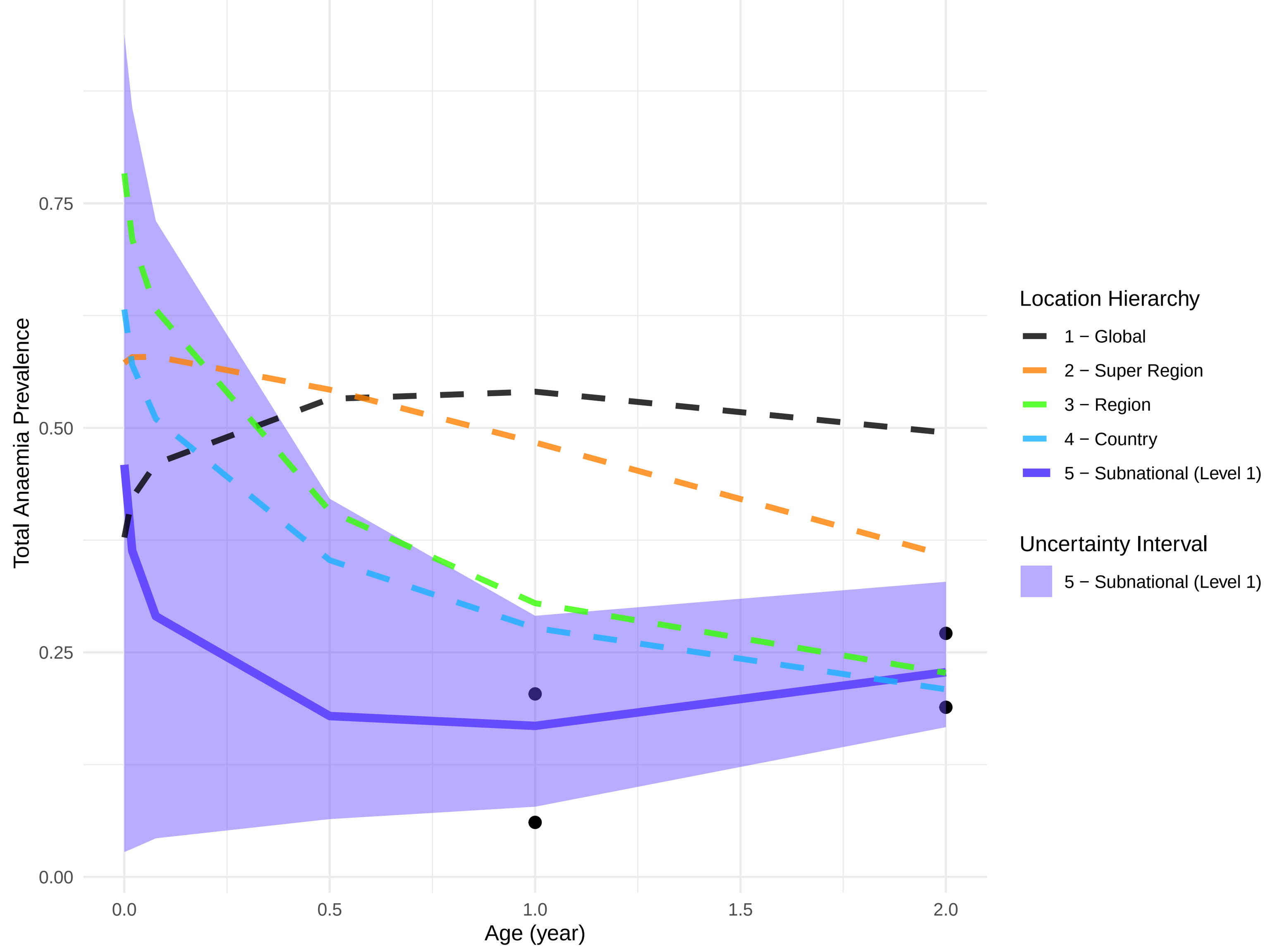

MR−BRT Age/Sex Fits − Non−pregnant Females <2 Years Old − Total Anaemia Prevalence − Tlaxcala
WHO−2001 Adjustment Method

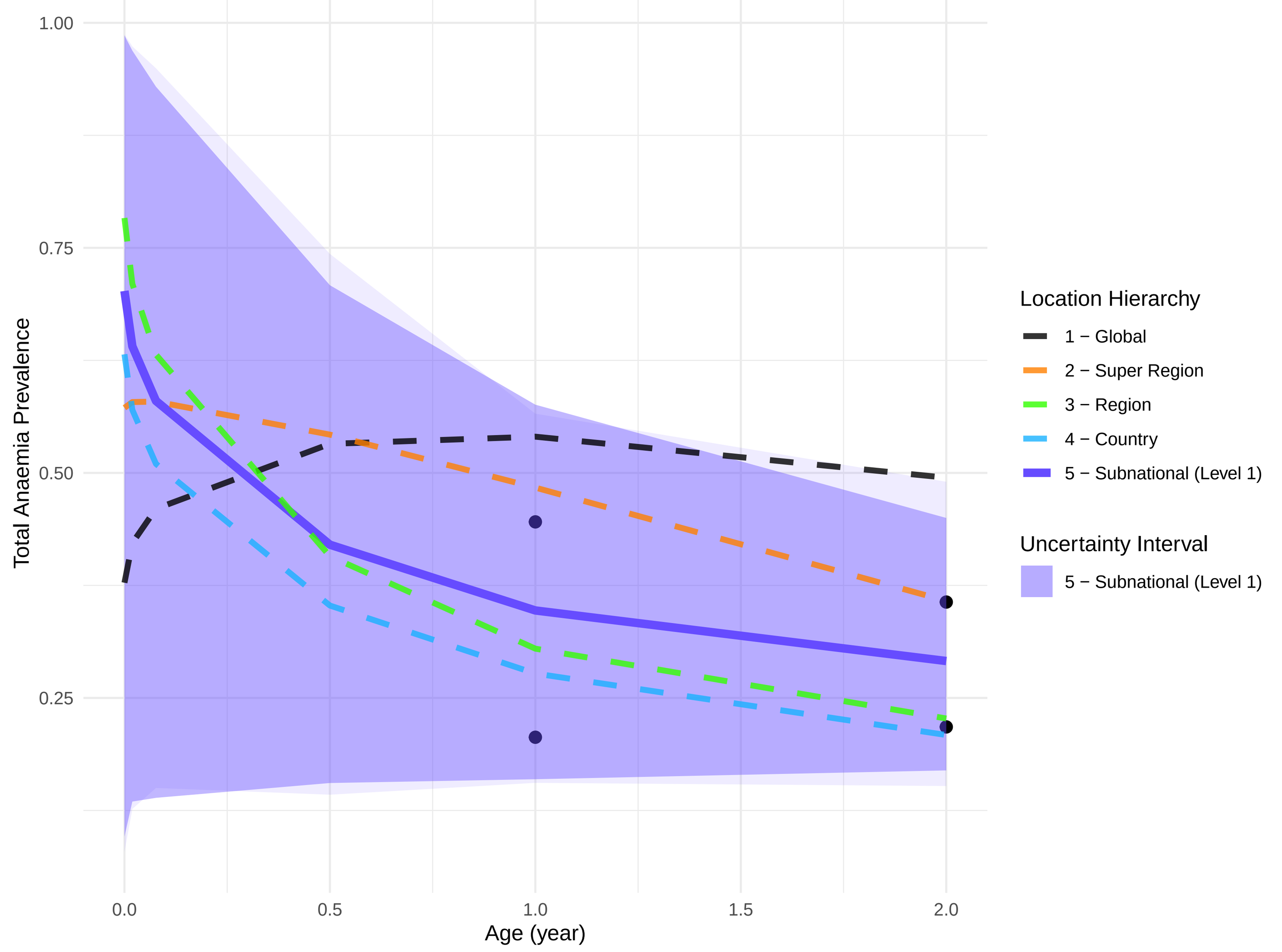

MR−BRT Age/Sex Fits − Non−pregnant Females <2 Years Old − Total Anaemia Prevalence − Veracruz de Ignacio de la Llave
WHO−2001 Adjustment Method

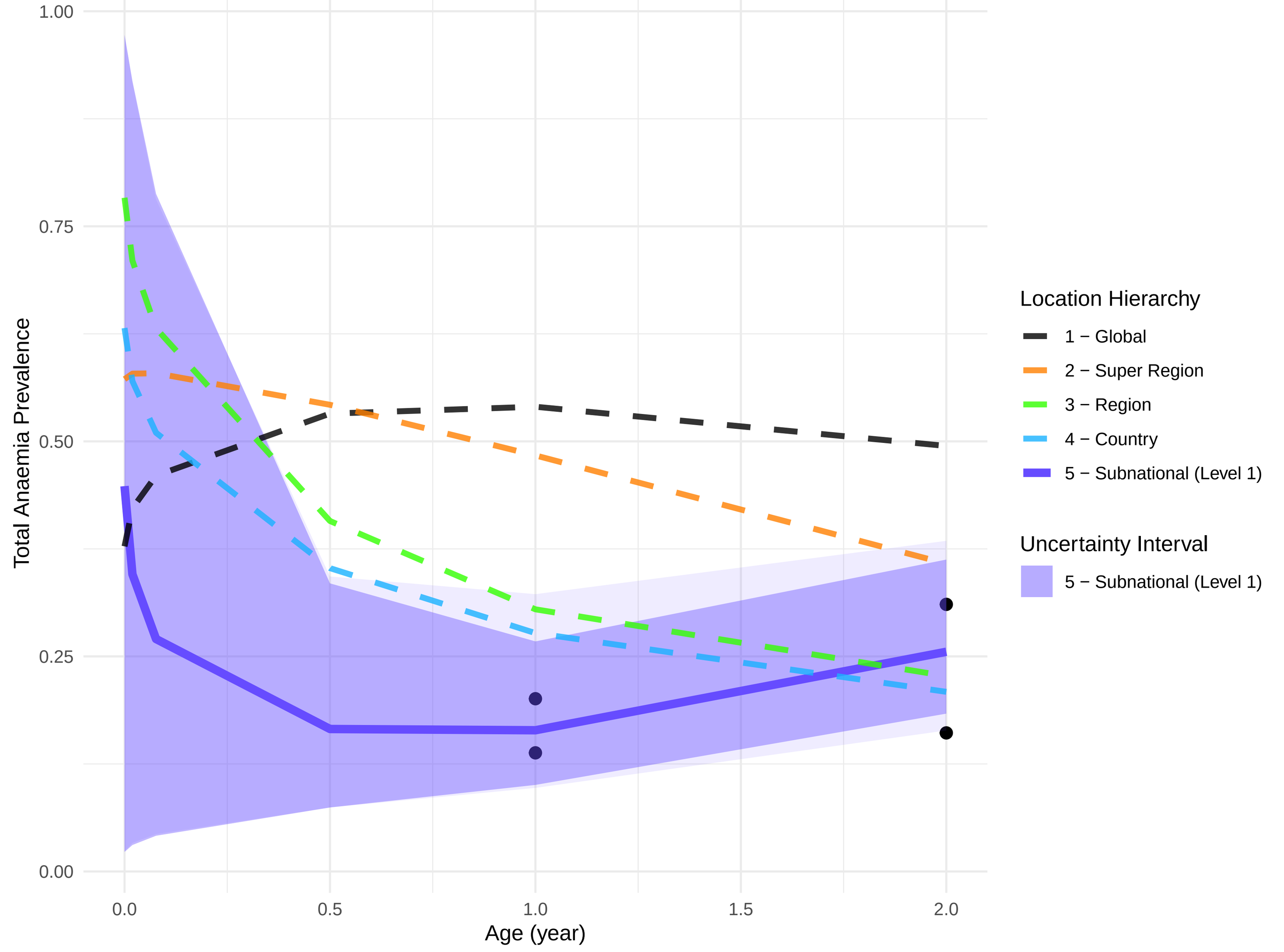

MR−BRT Age/Sex Fits − Non−pregnant Females <2 Years Old − Total Anaemia Prevalence − Yucatán
WHO−2001 Adjustment Method

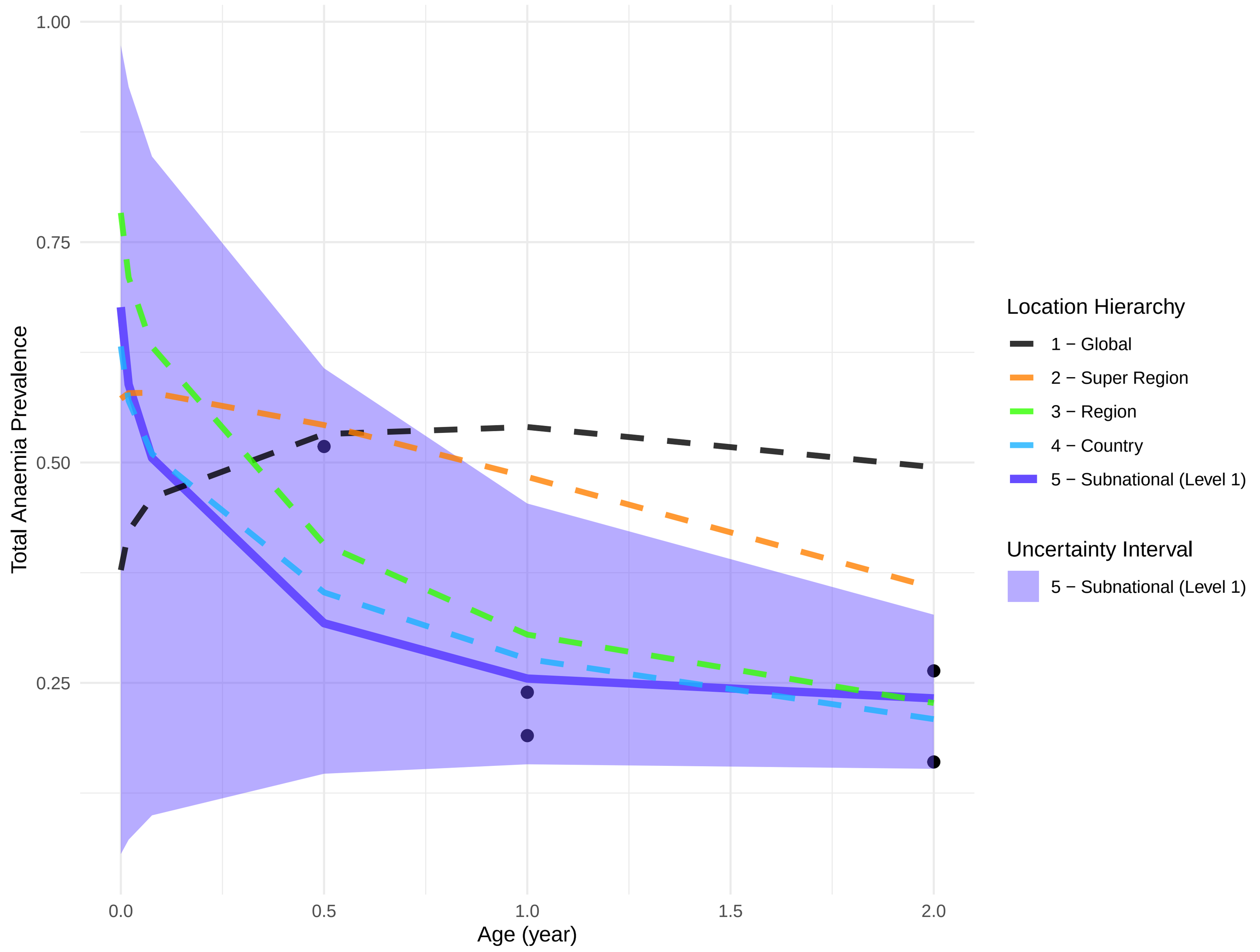

MR−BRT Age/Sex Fits − Non−pregnant Females <2 Years Old − Total Anaemia Prevalence − Zacatecas
WHO−2001 Adjustment Method

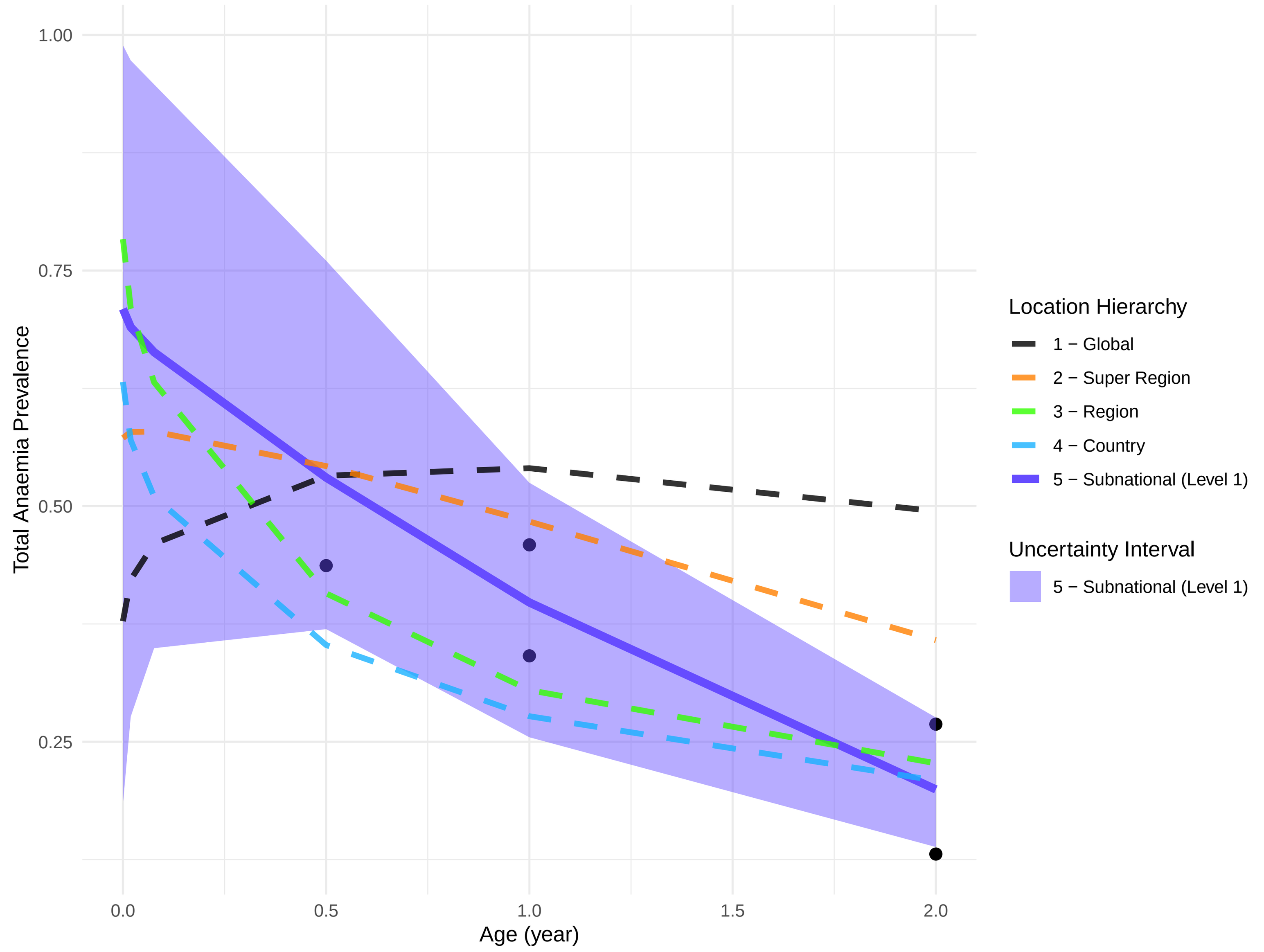

MR−BRT Age/Sex Fits − Non−pregnant Females <2 Years Old − Total Anaemia Prevalence − North Africa and Middle East
WHO−2001 Adjustment Method

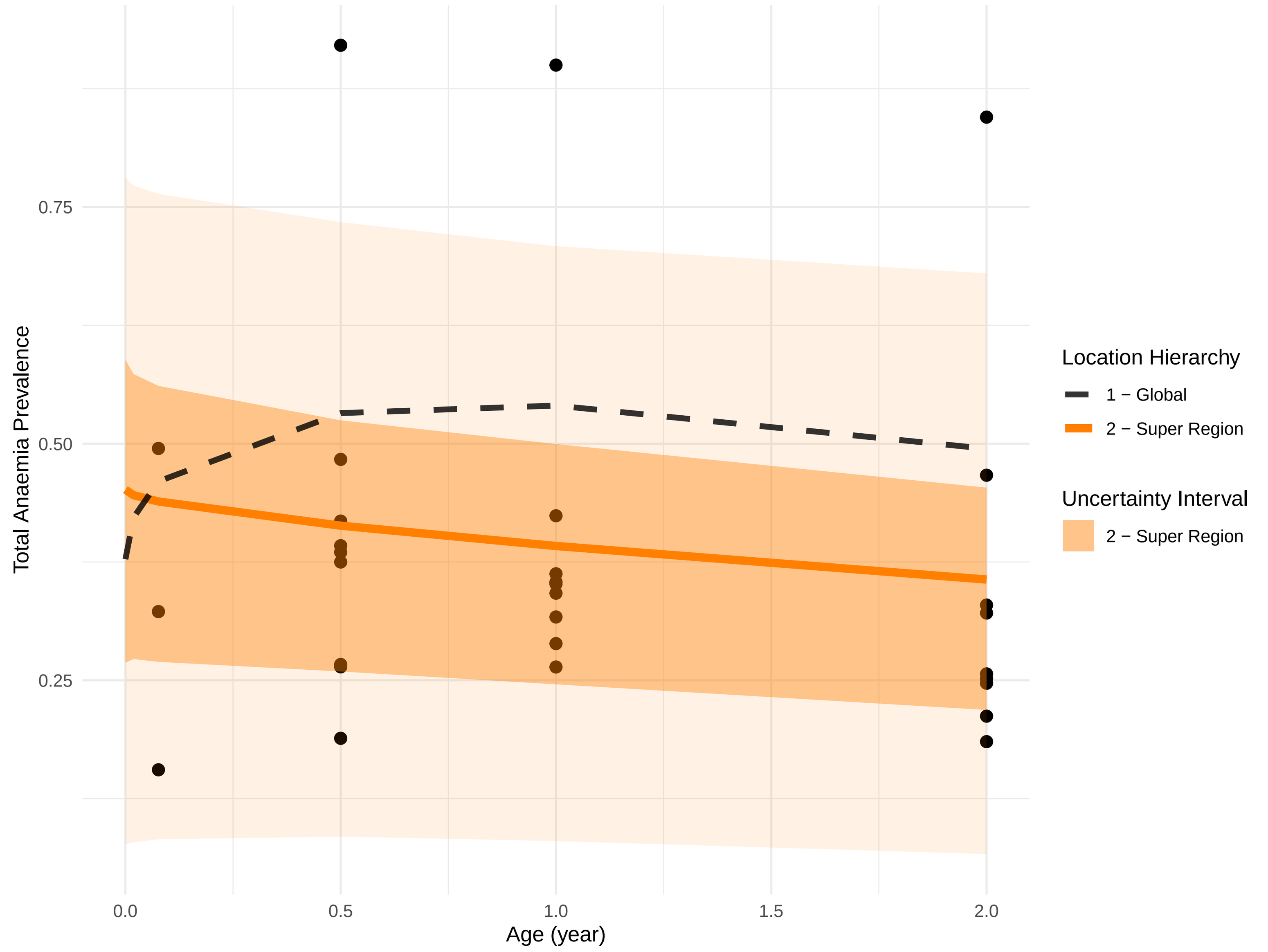

MR−BRT Age/Sex Fits − Non−pregnant Females <2 Years Old − Total Anaemia Prevalence − North Africa and Middle East
WHO−2001 Adjustment Method

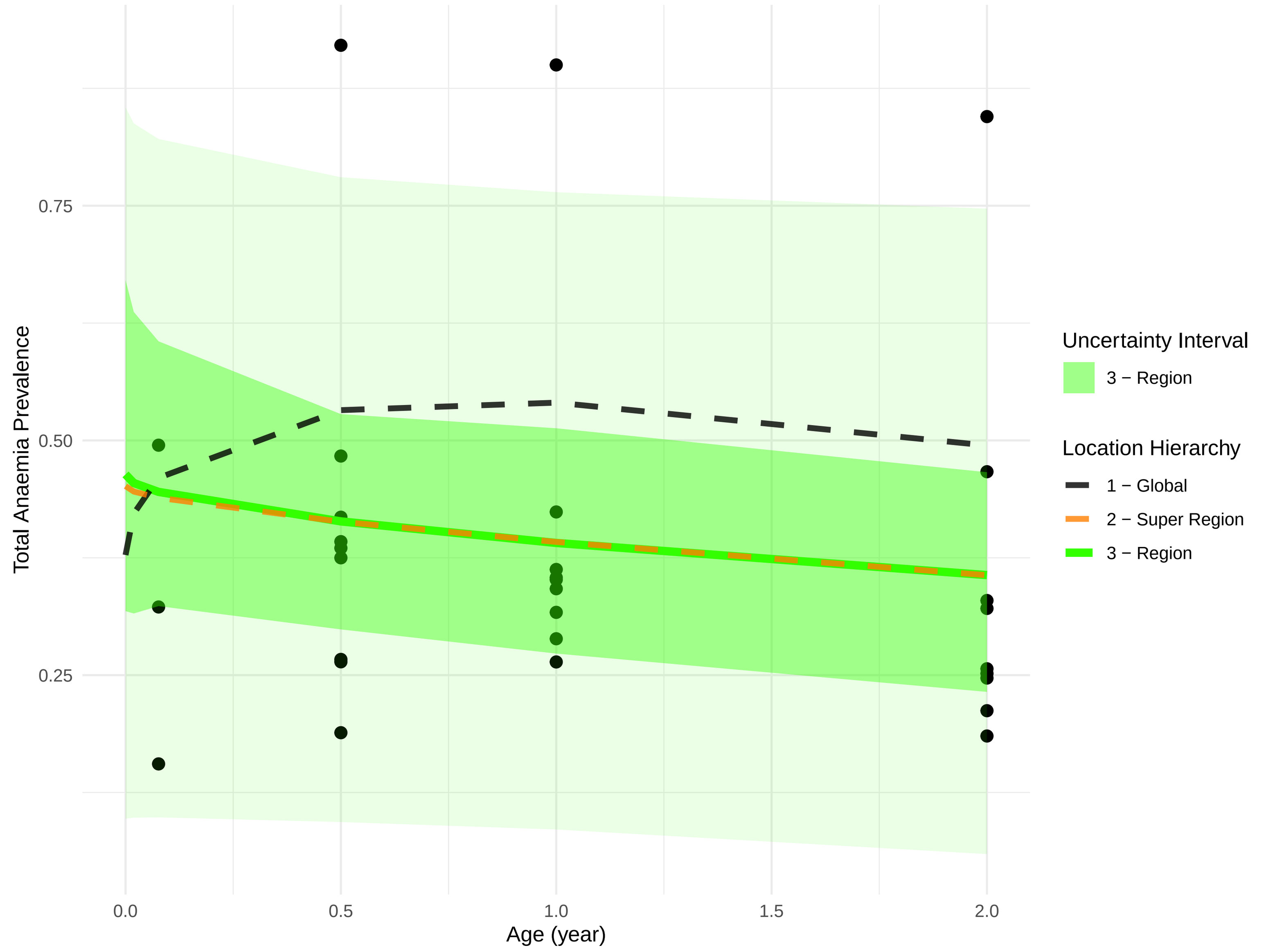

MR−BRT Age/Sex Fits − Non−pregnant Females <2 Years Old − Total Anaemia Prevalence − Egypt
WHO−2001 Adjustment Method

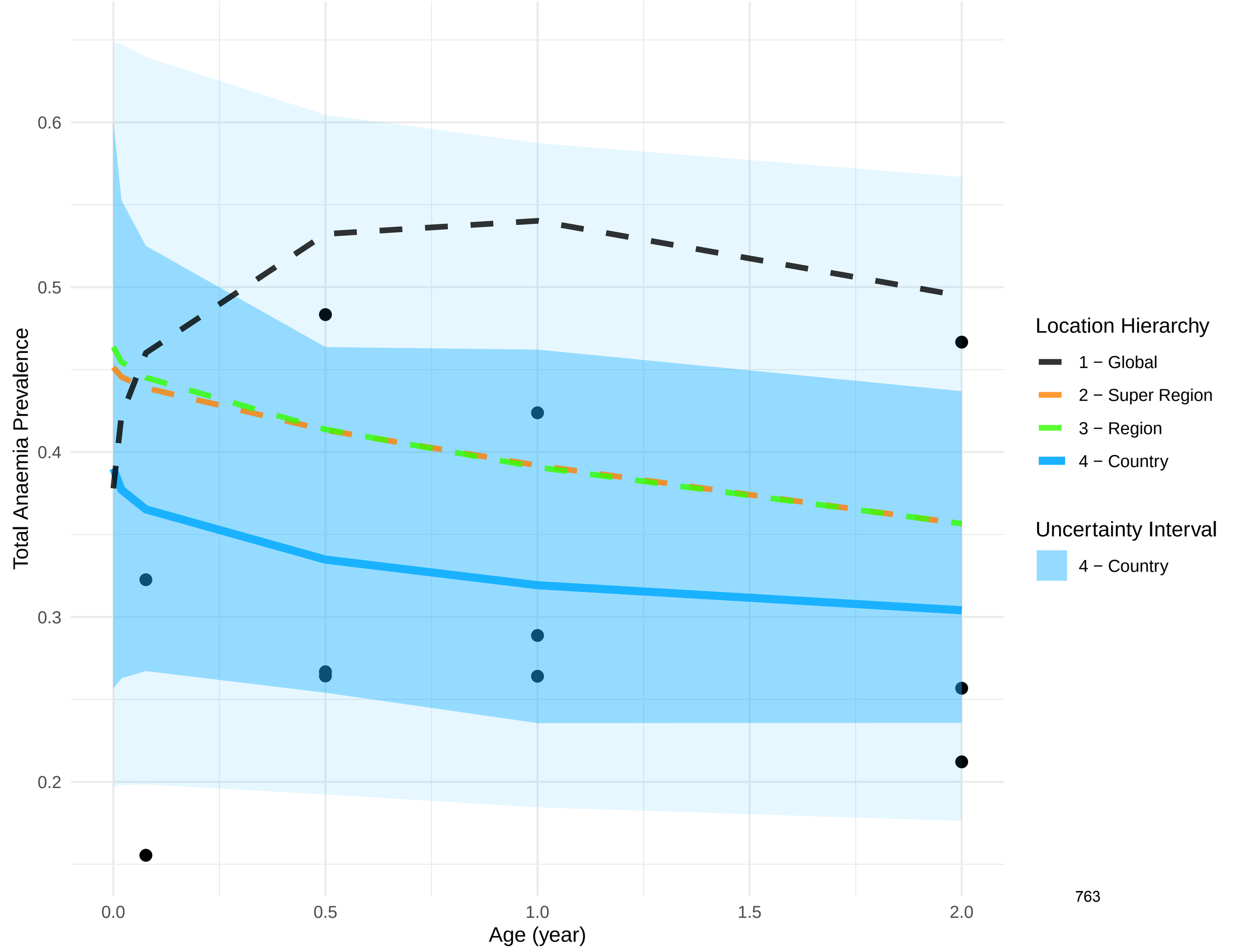

MR−BRT Age/Sex Fits − Non−pregnant Females <2 Years Old − Total Anaemia Prevalence − Jordan
WHO−2001 Adjustment Method

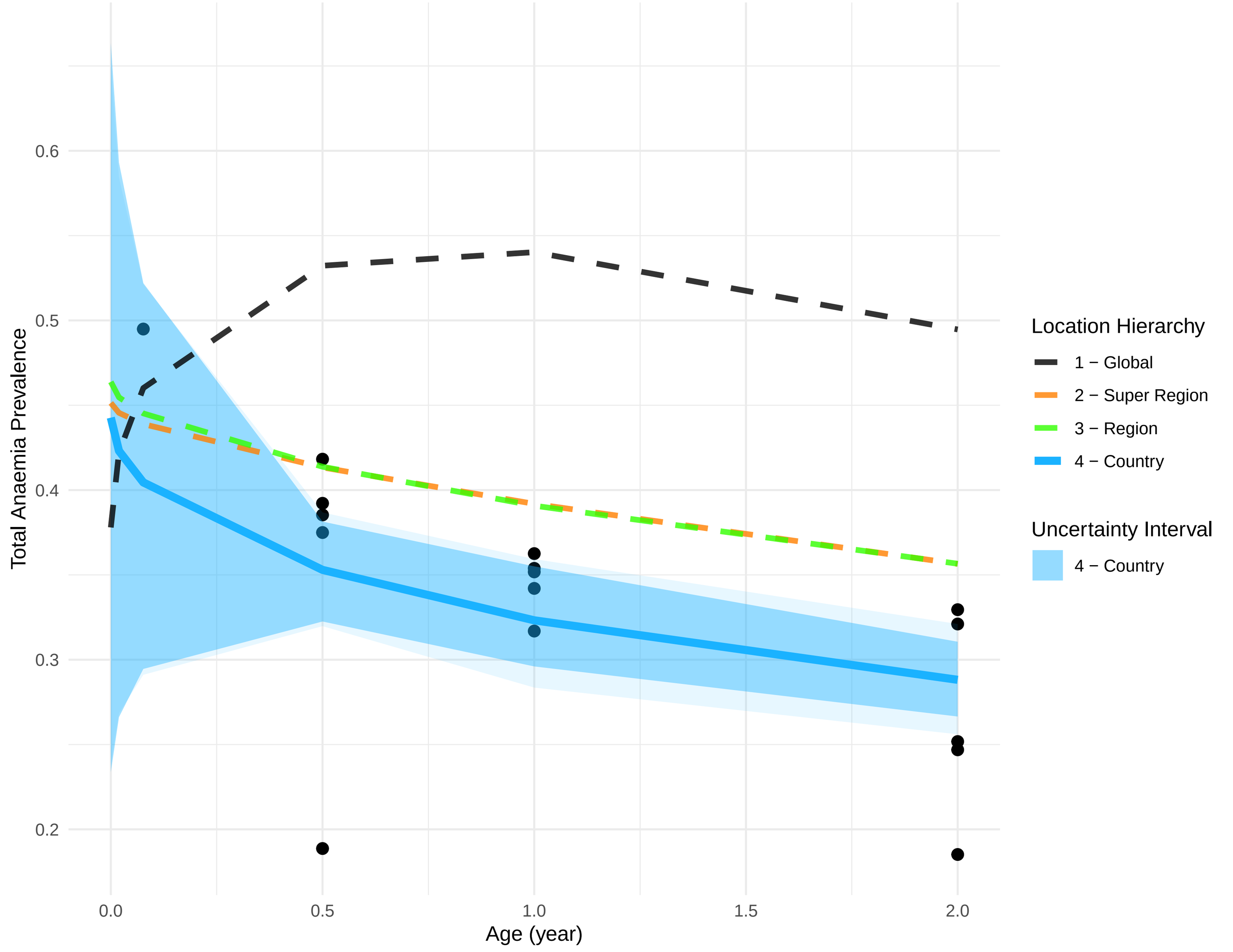

MR−BRT Age/Sex Fits − Non−pregnant Females <2 Years Old − Total Anaemia Prevalence − Yemen
WHO−2001 Adjustment Method

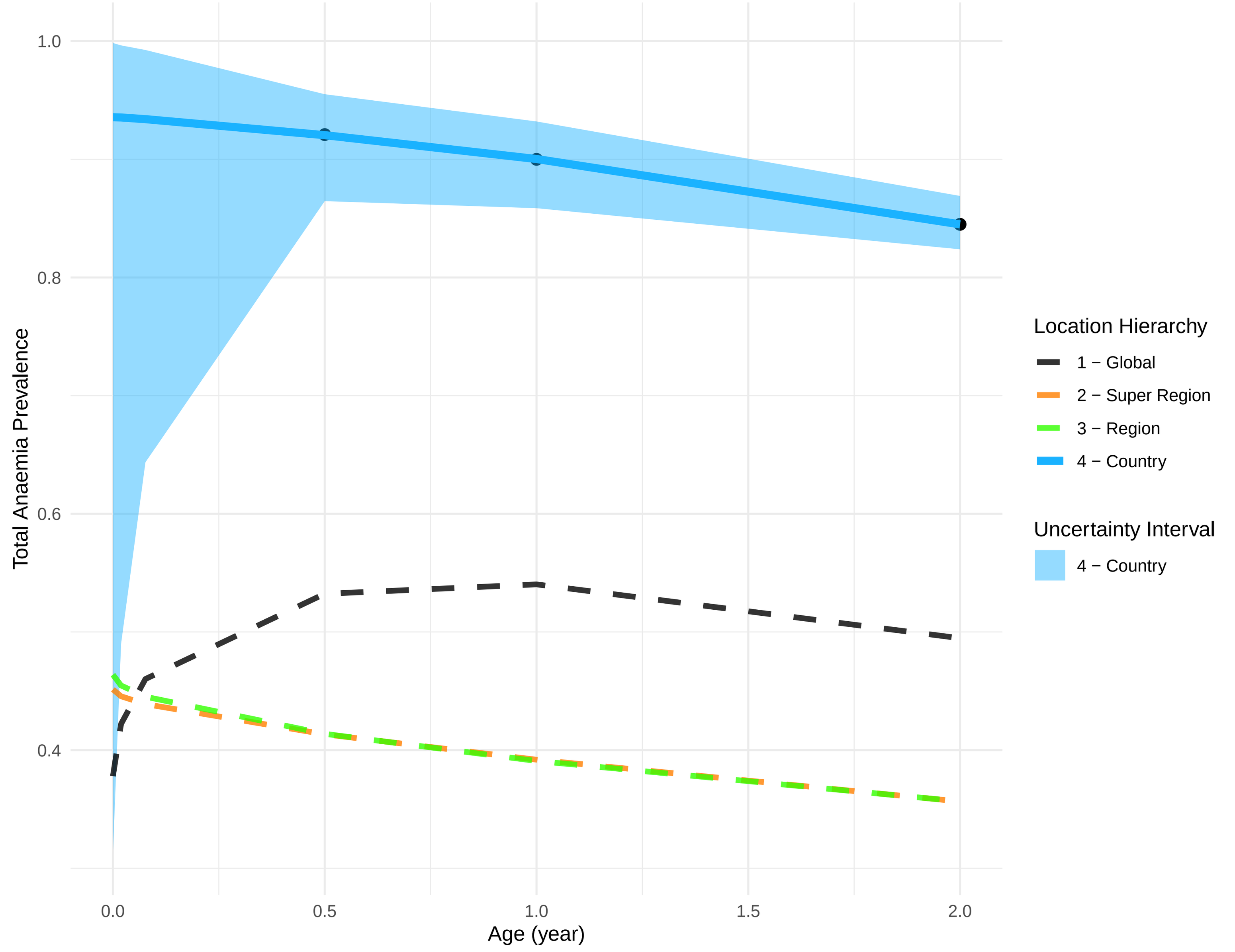

MR−BRT Age/Sex Fits − Non−pregnant Females <2 Years Old − Total Anaemia Prevalence − South Asia
WHO−2001 Adjustment Method

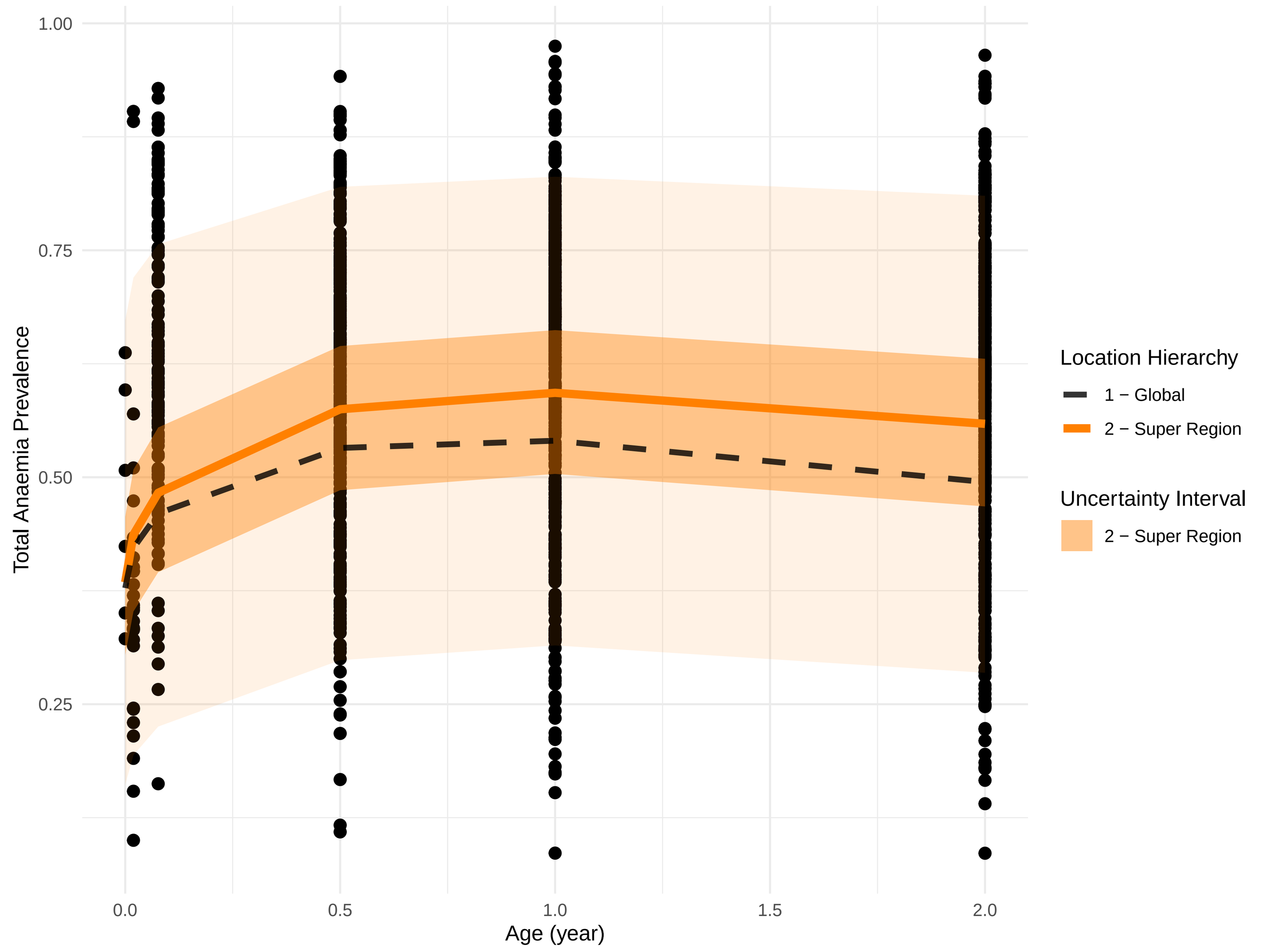

MR−BRT Age/Sex Fits − Non−pregnant Females <2 Years Old − Total Anaemia Prevalence − South Asia
WHO−2001 Adjustment Method

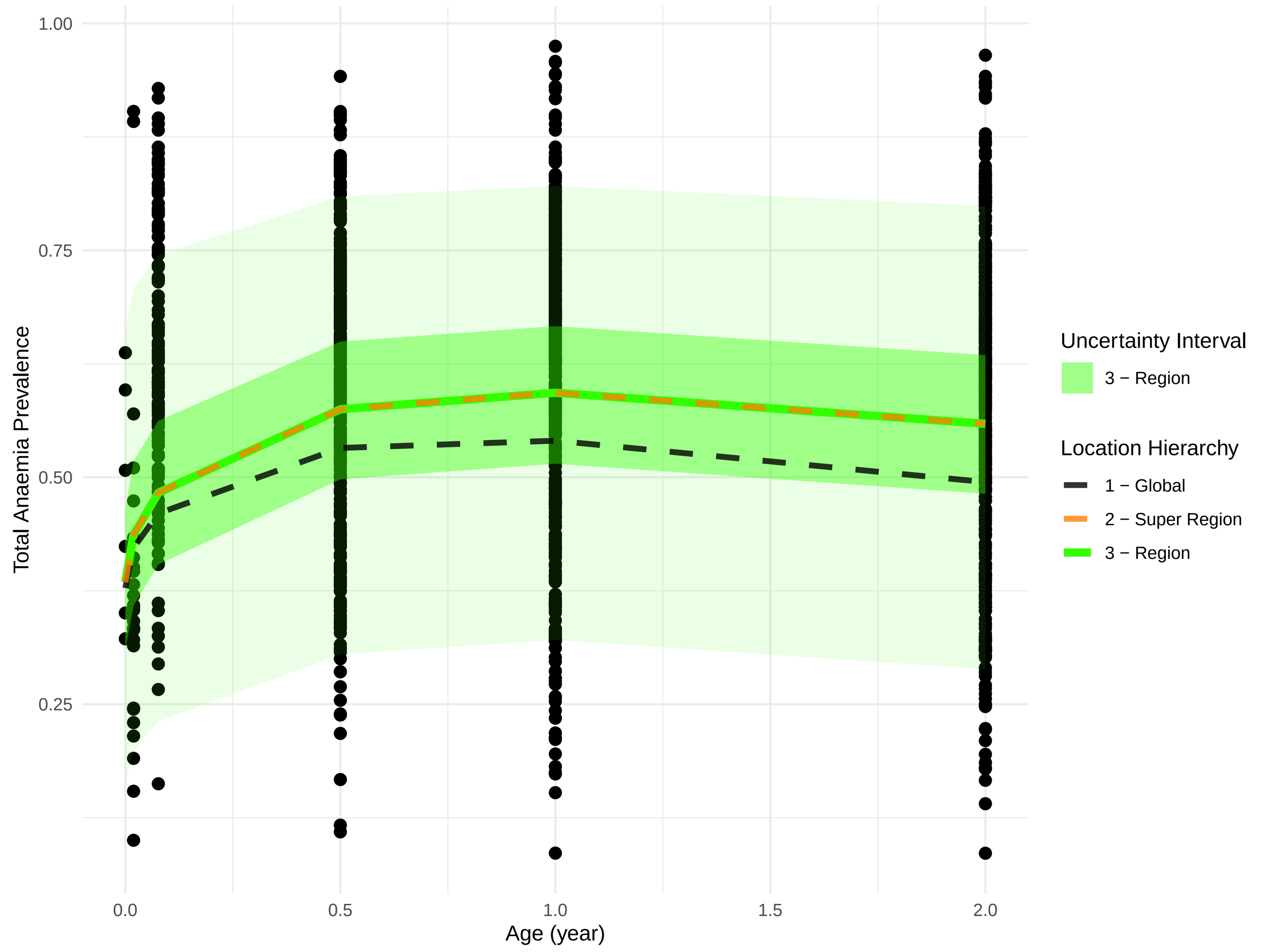

MR−BRT Age/Sex Fits − Non−pregnant Females <2 Years Old − Total Anaemia Prevalence − Bangladesh
WHO−2001 Adjustment Method

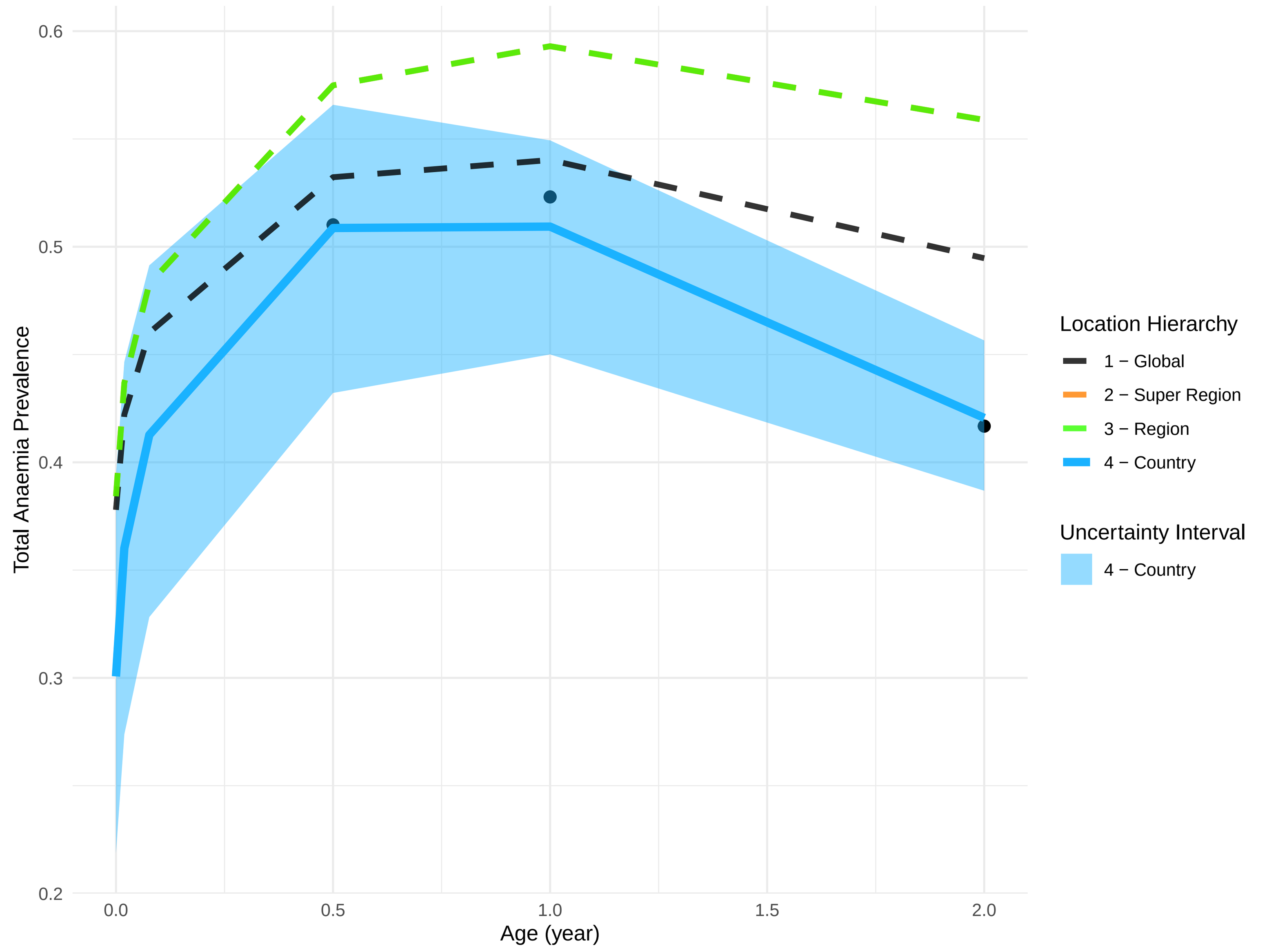

MR−BRT Age/Sex Fits − Non−pregnant Females <2 Years Old − Total Anaemia Prevalence − India
WHO−2001 Adjustment Method

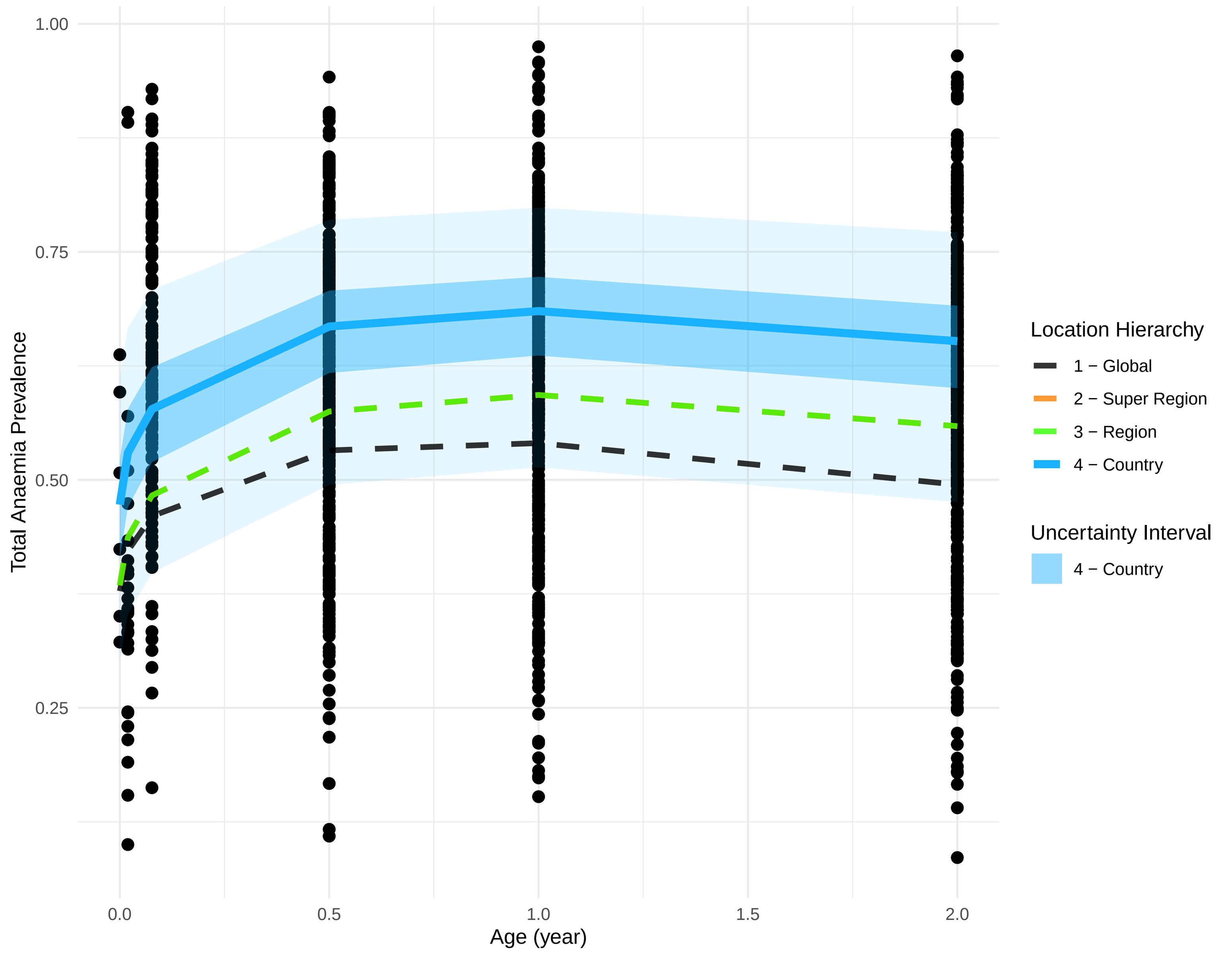

MR−BRT Age/Sex Fits − Non−pregnant Females <2 Years Old − Total Anaemia Prevalence − Andhra Pradesh
WHO−2001 Adjustment Method

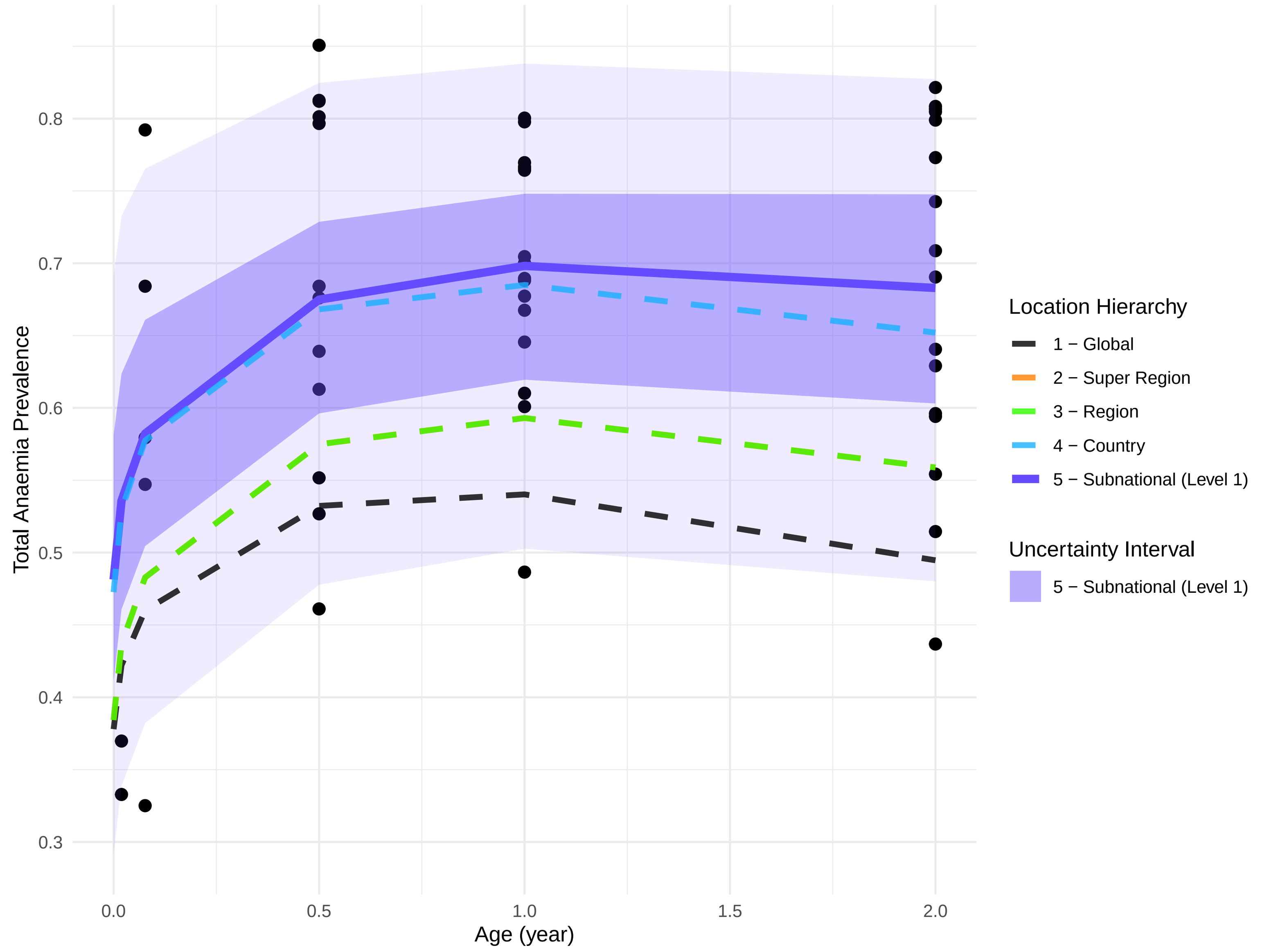

MR−BRT Age/Sex Fits − Non−pregnant Females <2 Years Old − Total Anaemia Prevalence − Andhra Pradesh, Rural
WHO−2001 Adjustment Method

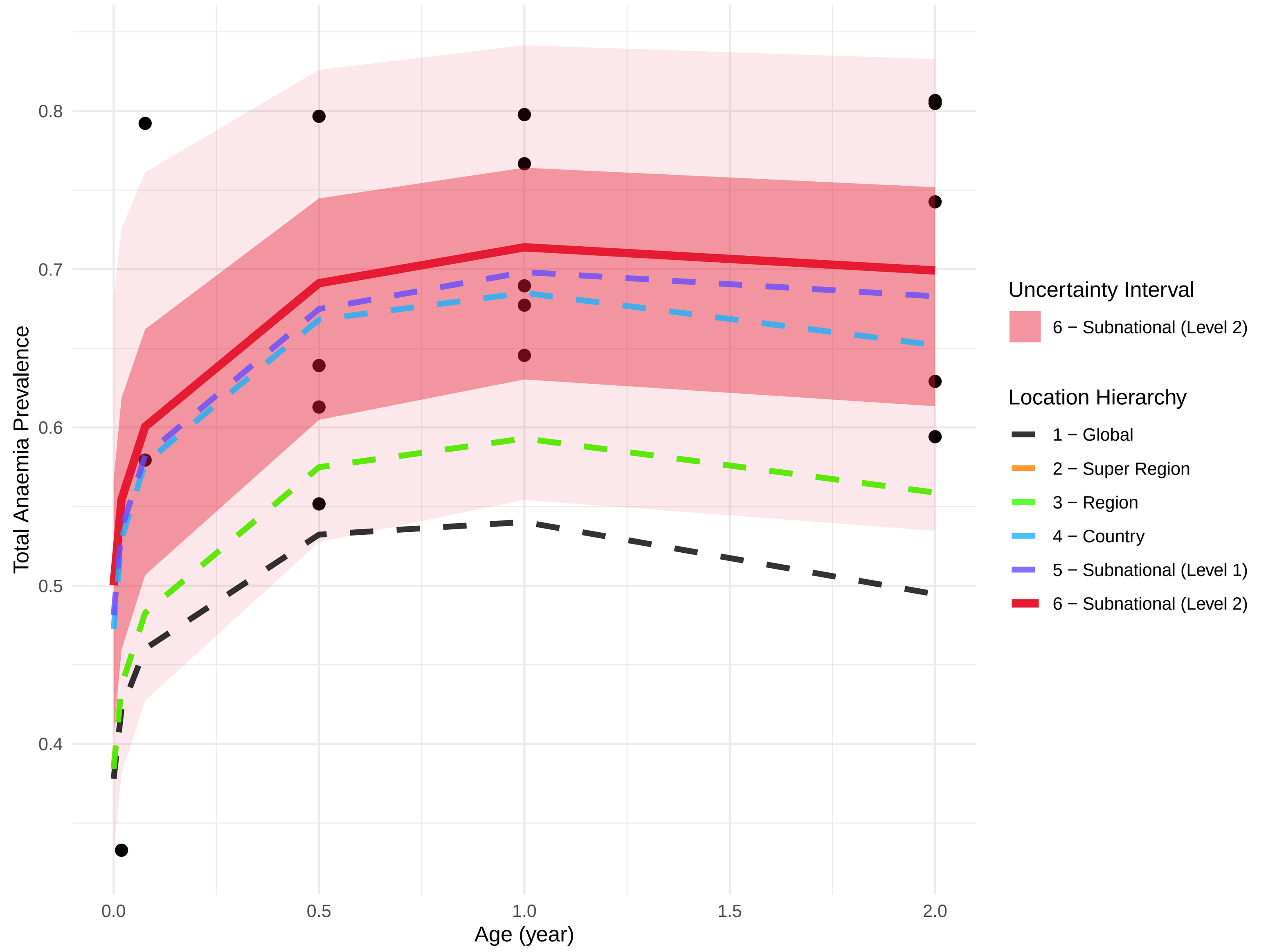

MR−BRT Age/Sex Fits − Non−pregnant Females <2 Years Old − Total Anaemia Prevalence − Andhra Pradesh, Urban
WHO−2001 Adjustment Method

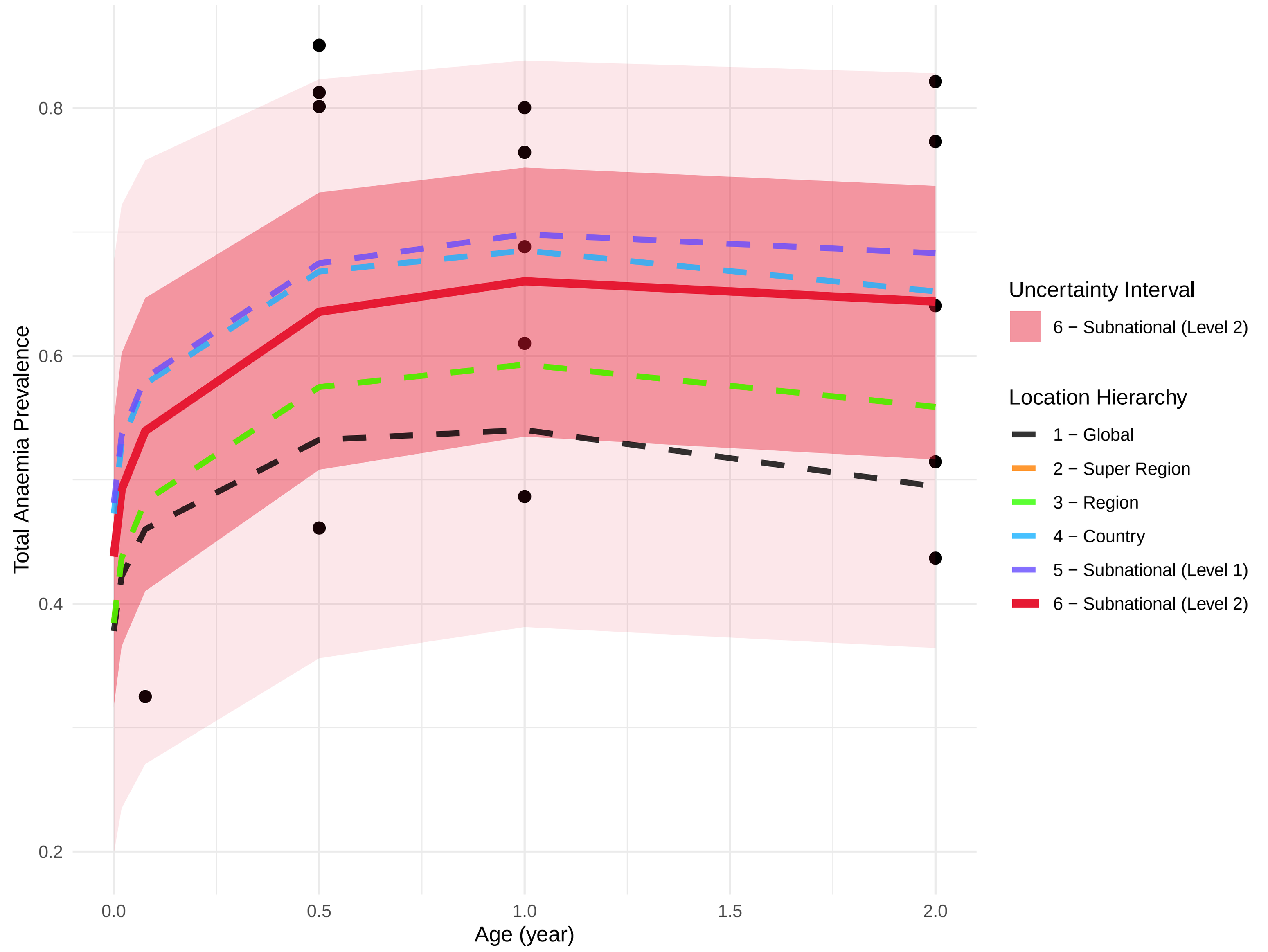

MR−BRT Age/Sex Fits − Non−pregnant Females <2 Years Old − Total Anaemia Prevalence − Arunachal Pradesh
WHO−2001 Adjustment Method

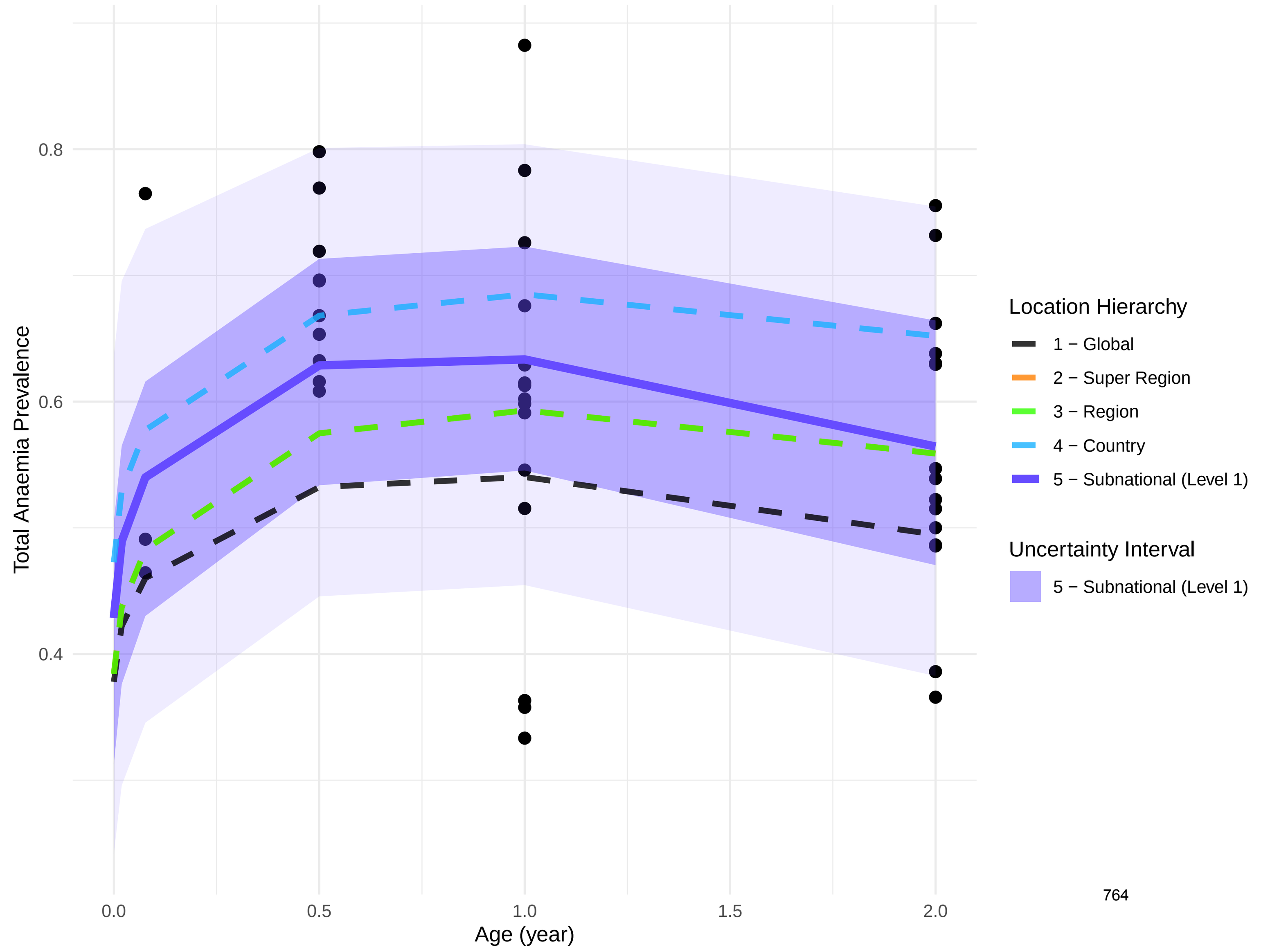

MR−BRT Age/Sex Fits − Non−pregnant Females <2 Years Old − Total Anaemia Prevalence − Arunachal Pradesh, Rural
WHO−2001 Adjustment Method

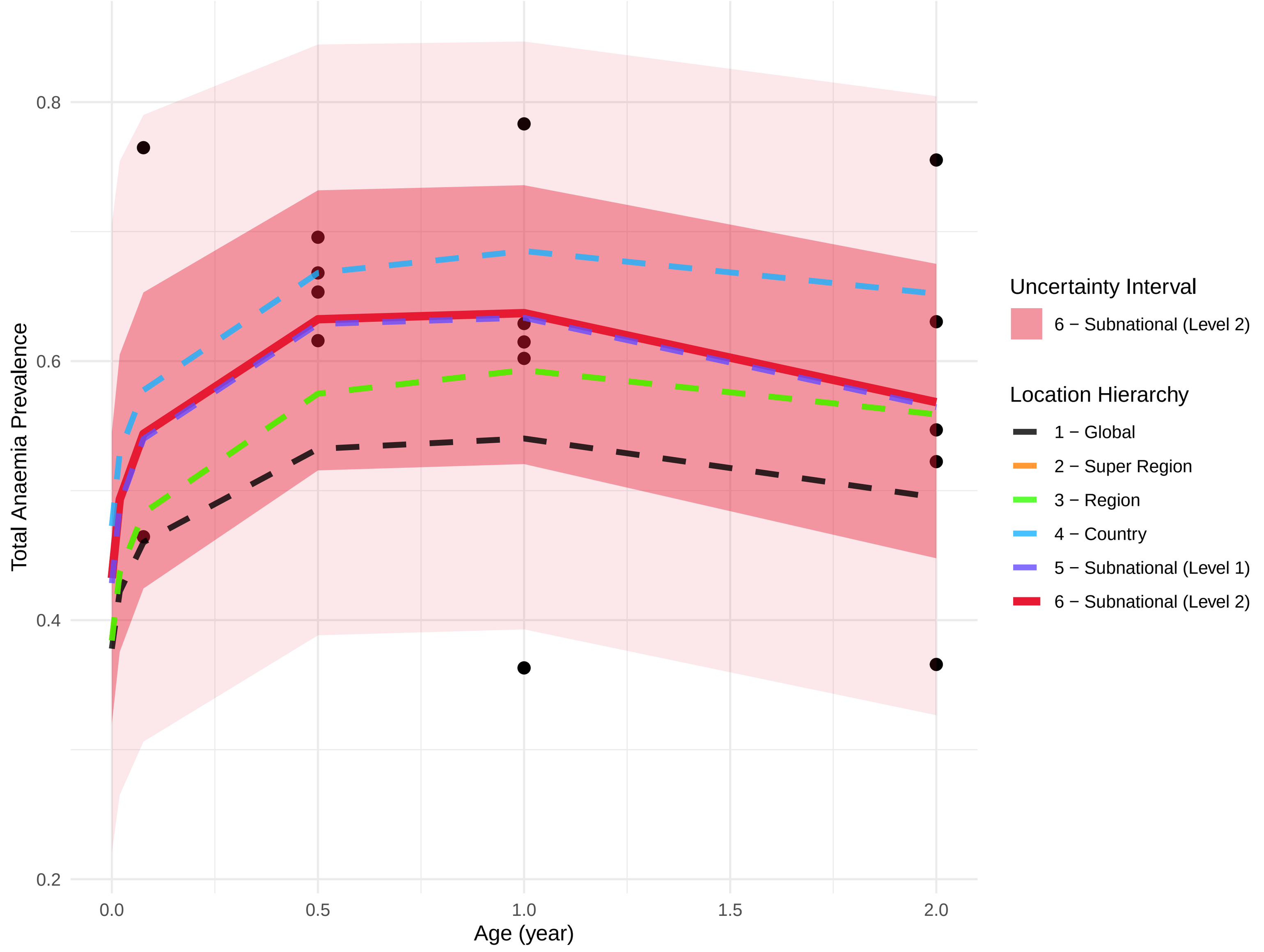

MR−BRT Age/Sex Fits − Non−pregnant Females <2 Years Old − Total Anaemia Prevalence − Arunachal Pradesh, Urban
WHO−2001 Adjustment Method

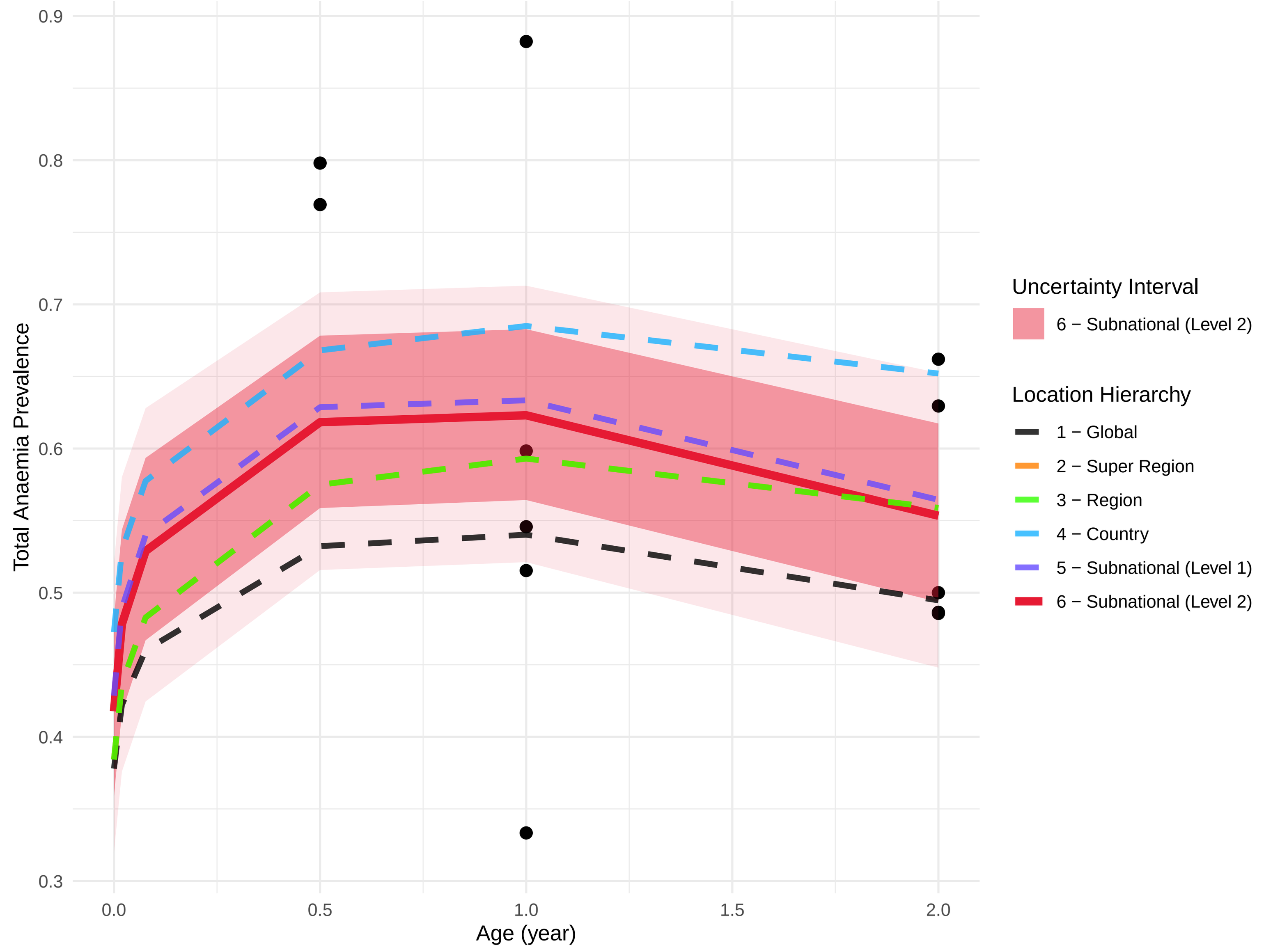

MR−BRT Age/Sex Fits − Non−pregnant Females <2 Years Old − Total Anaemia Prevalence − Assam
WHO−2001 Adjustment Method

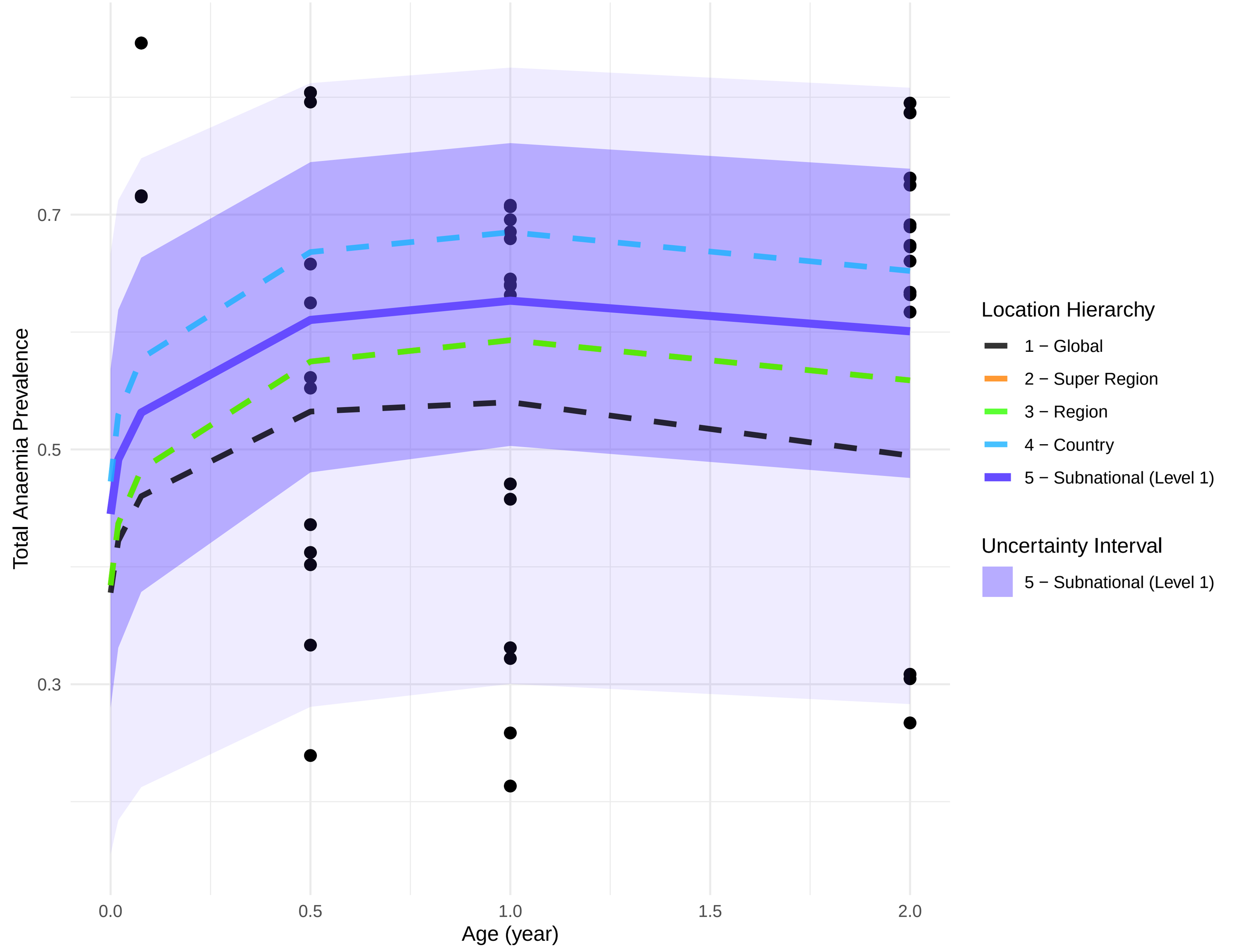

MR−BRT Age/Sex Fits − Non−pregnant Females <2 Years Old − Total Anaemia Prevalence − Assam, Rural
WHO−2001 Adjustment Method

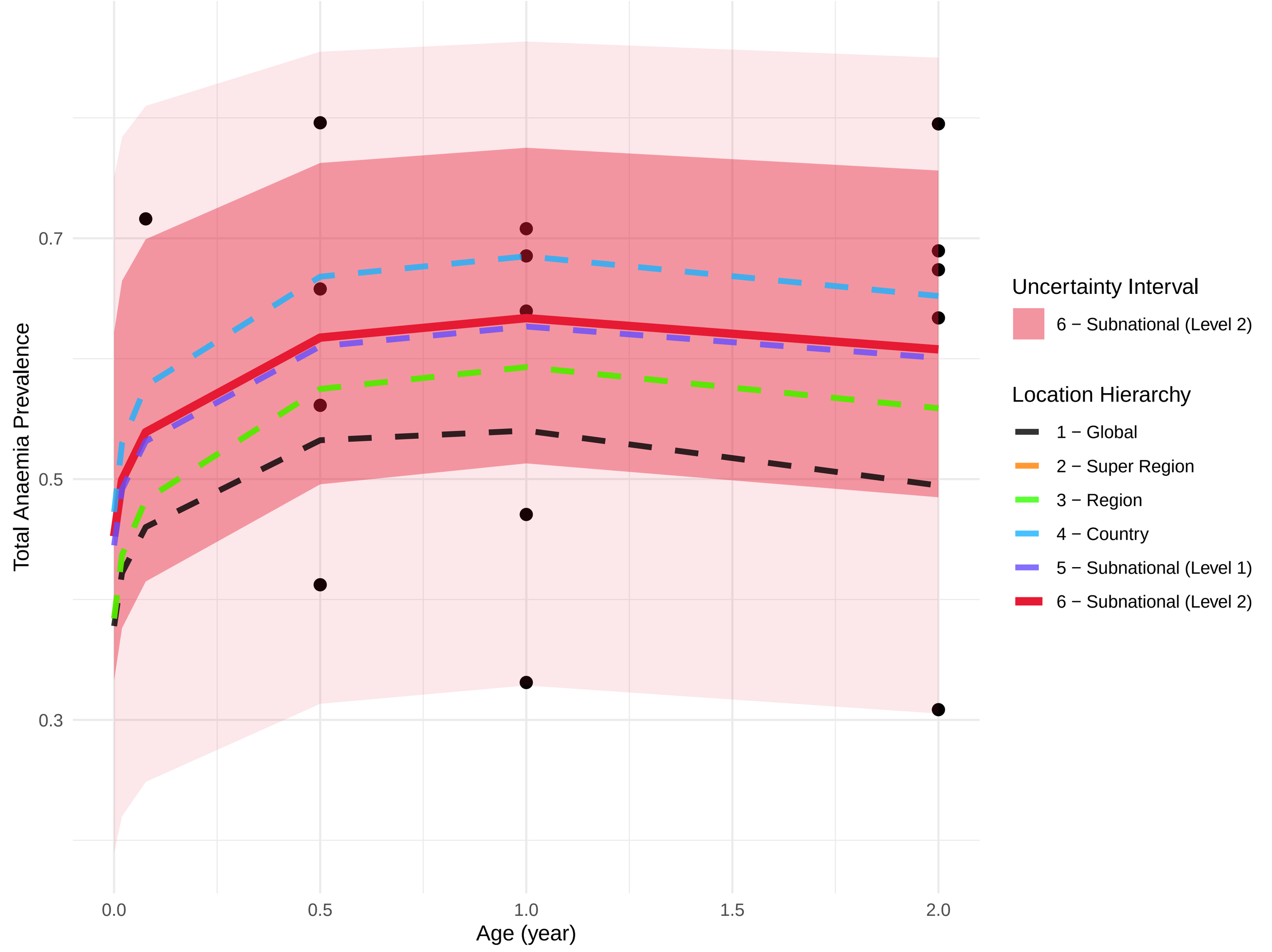

MR−BRT Age/Sex Fits − Non−pregnant Females <2 Years Old − Total Anaemia Prevalence − Assam, Urban
WHO−2001 Adjustment Method

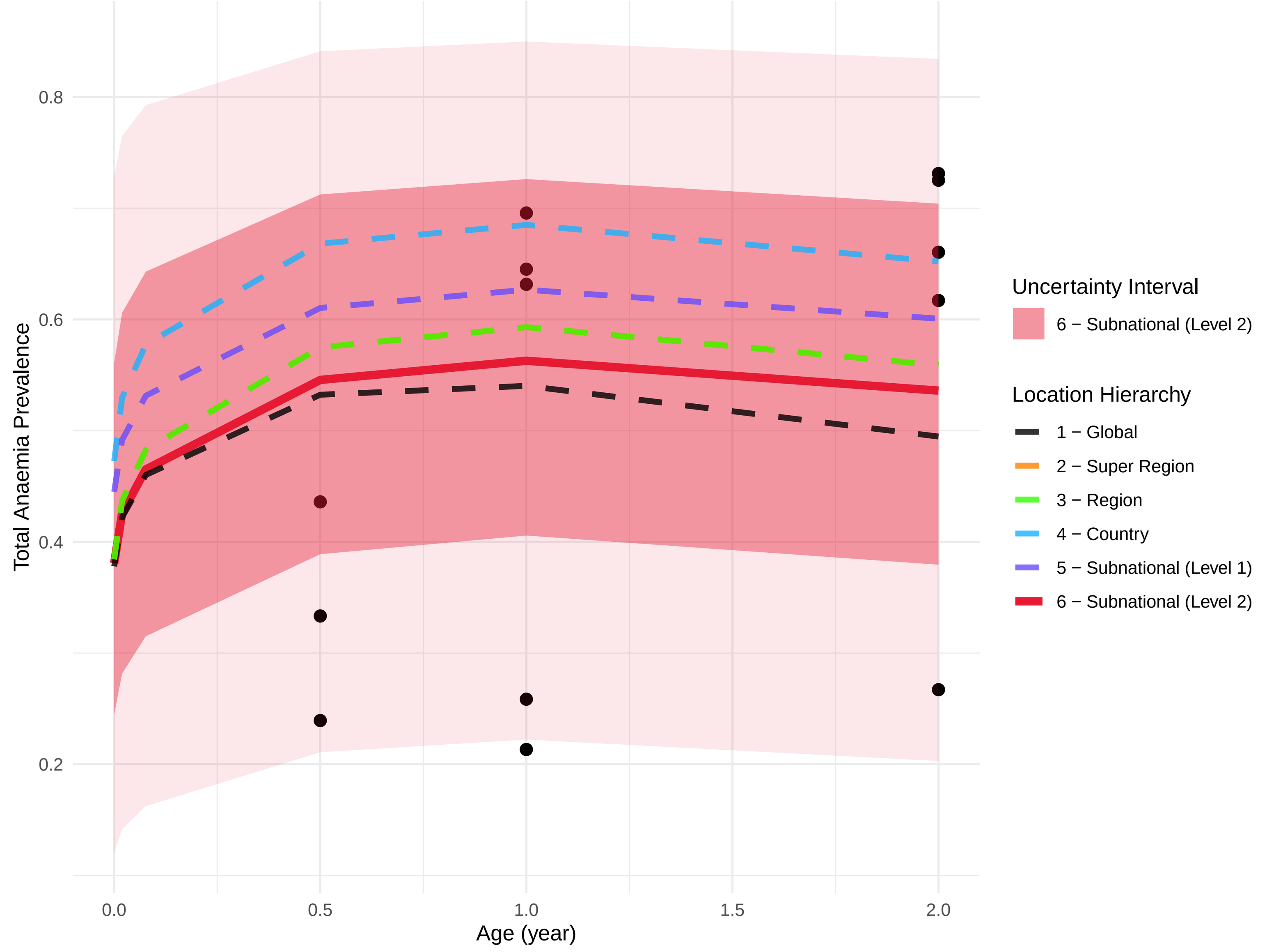

MR−BRT Age/Sex Fits − Non−pregnant Females <2 Years Old − Total Anaemia Prevalence − Bihar
WHO−2001 Adjustment Method

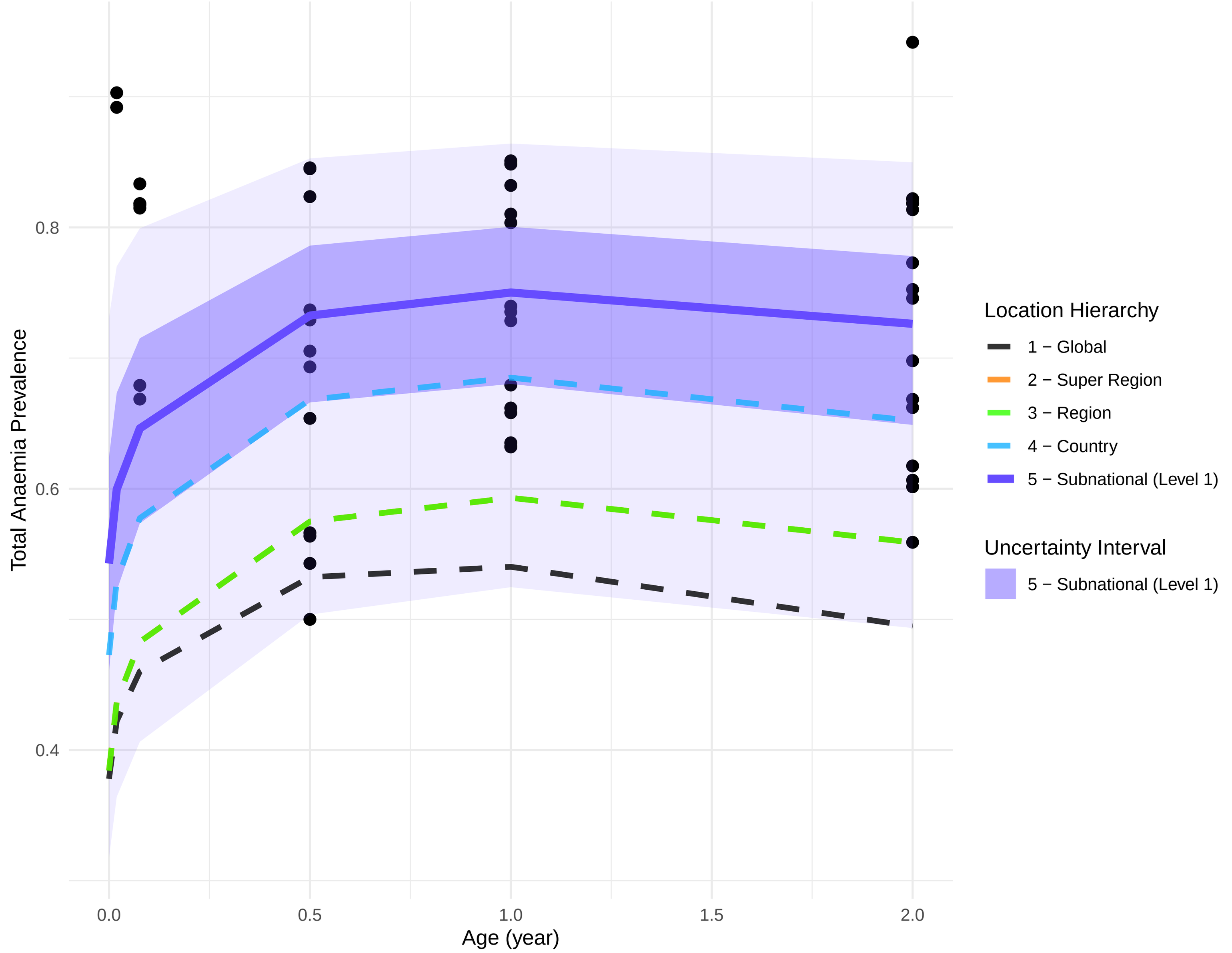

MR−BRT Age/Sex Fits − Non−pregnant Females <2 Years Old − Total Anaemia Prevalence − Bihar, Rural
WHO−2001 Adjustment Method

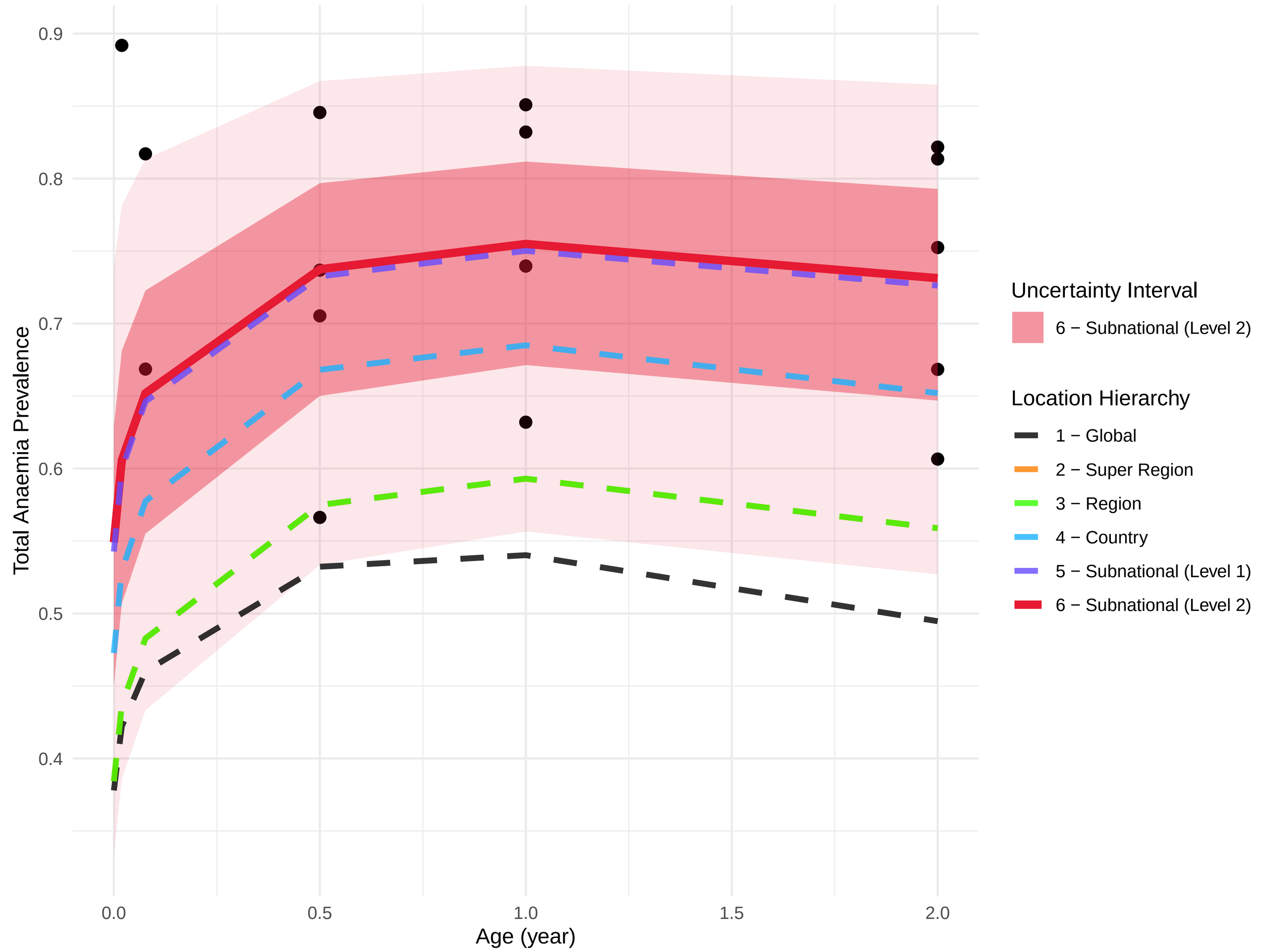

MR−BRT Age/Sex Fits − Non−pregnant Females <2 Years Old − Total Anaemia Prevalence − Bihar, Urban
WHO−2001 Adjustment Method

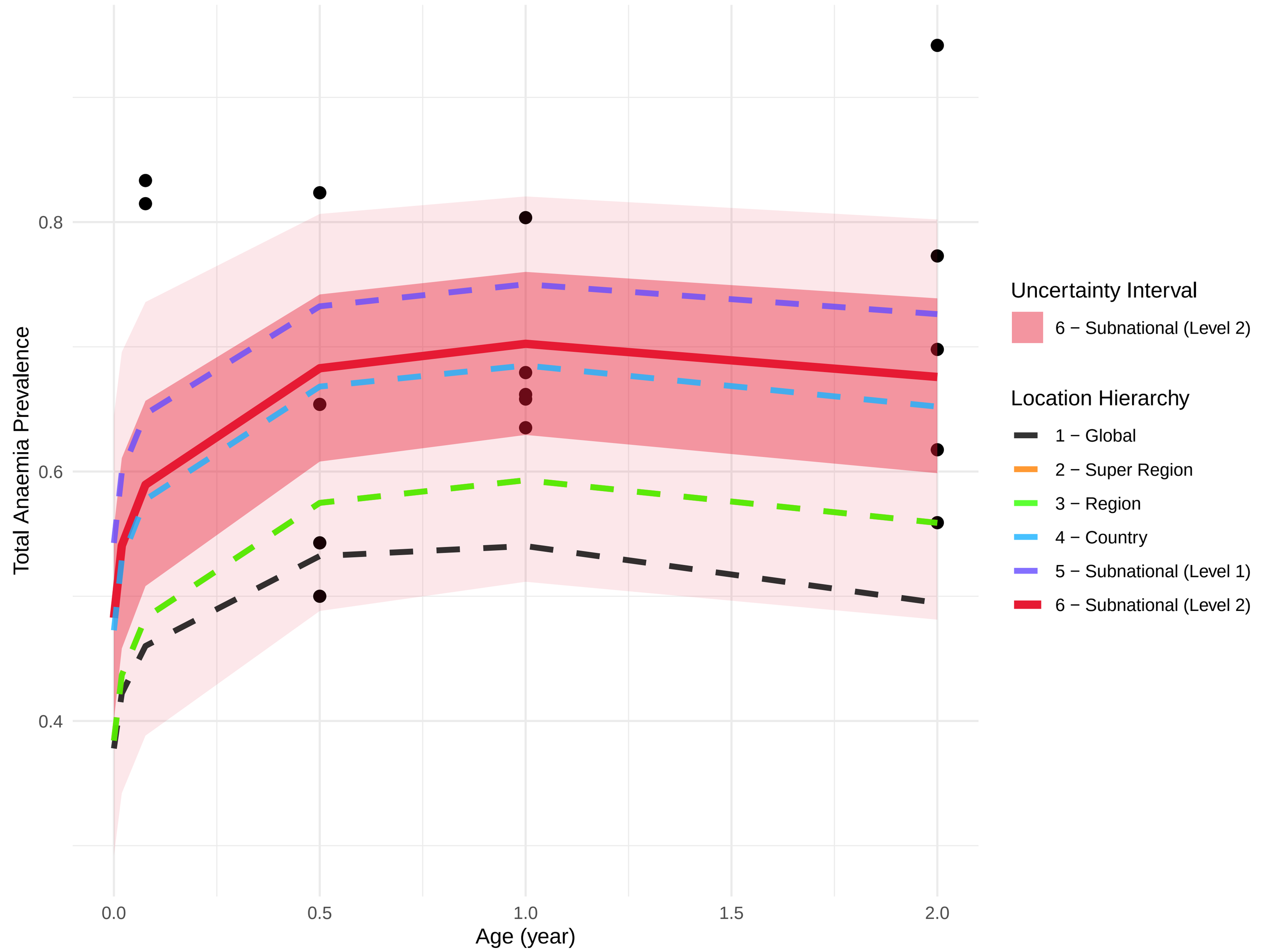

MR−BRT Age/Sex Fits − Non−pregnant Females <2 Years Old − Total Anaemia Prevalence − Chhattisgarh
WHO−2001 Adjustment Method

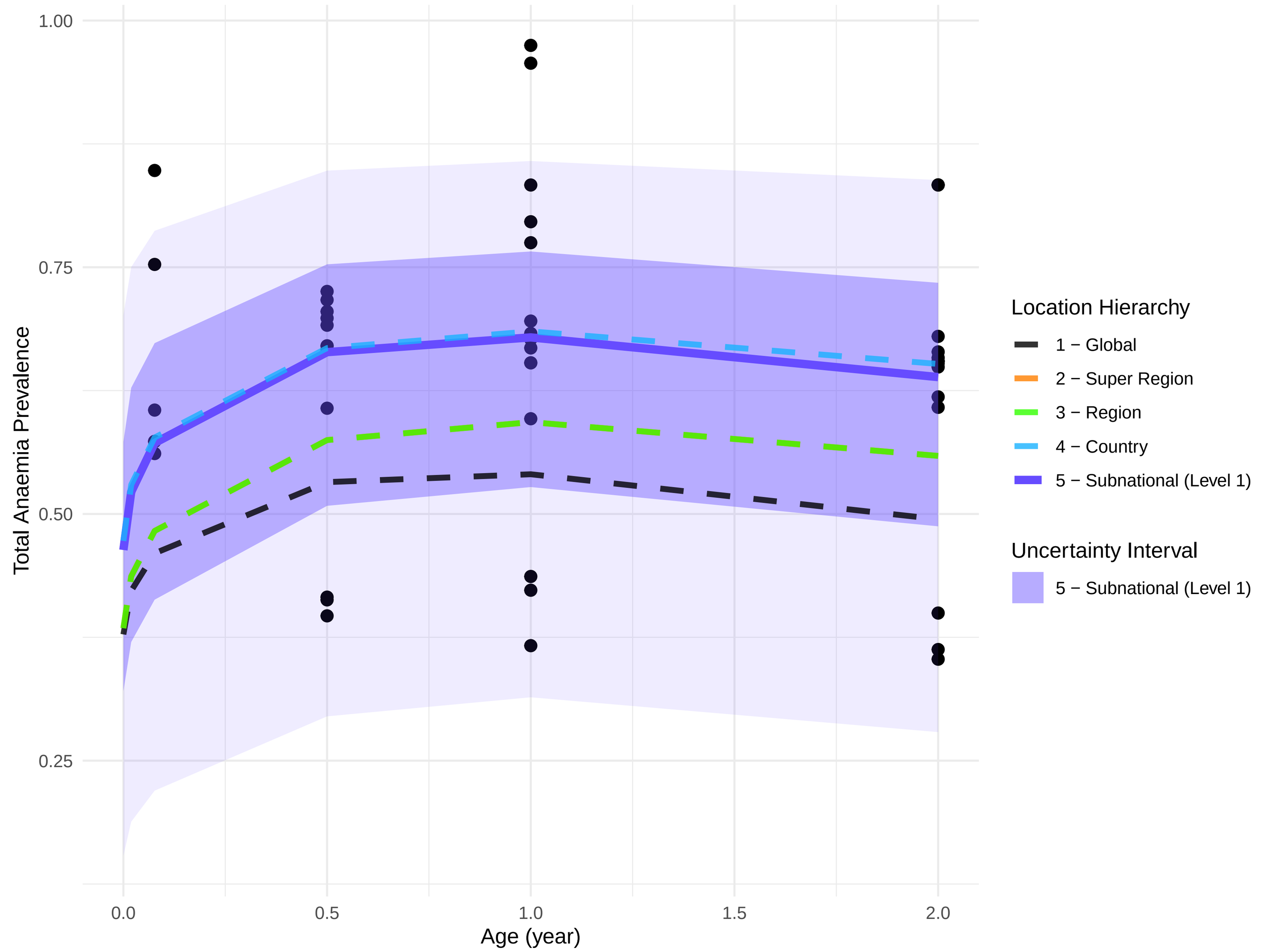

MR−BRT Age/Sex Fits − Non−pregnant Females <2 Years Old − Total Anaemia Prevalence − Chhattisgarh, Rural
WHO−2001 Adjustment Method

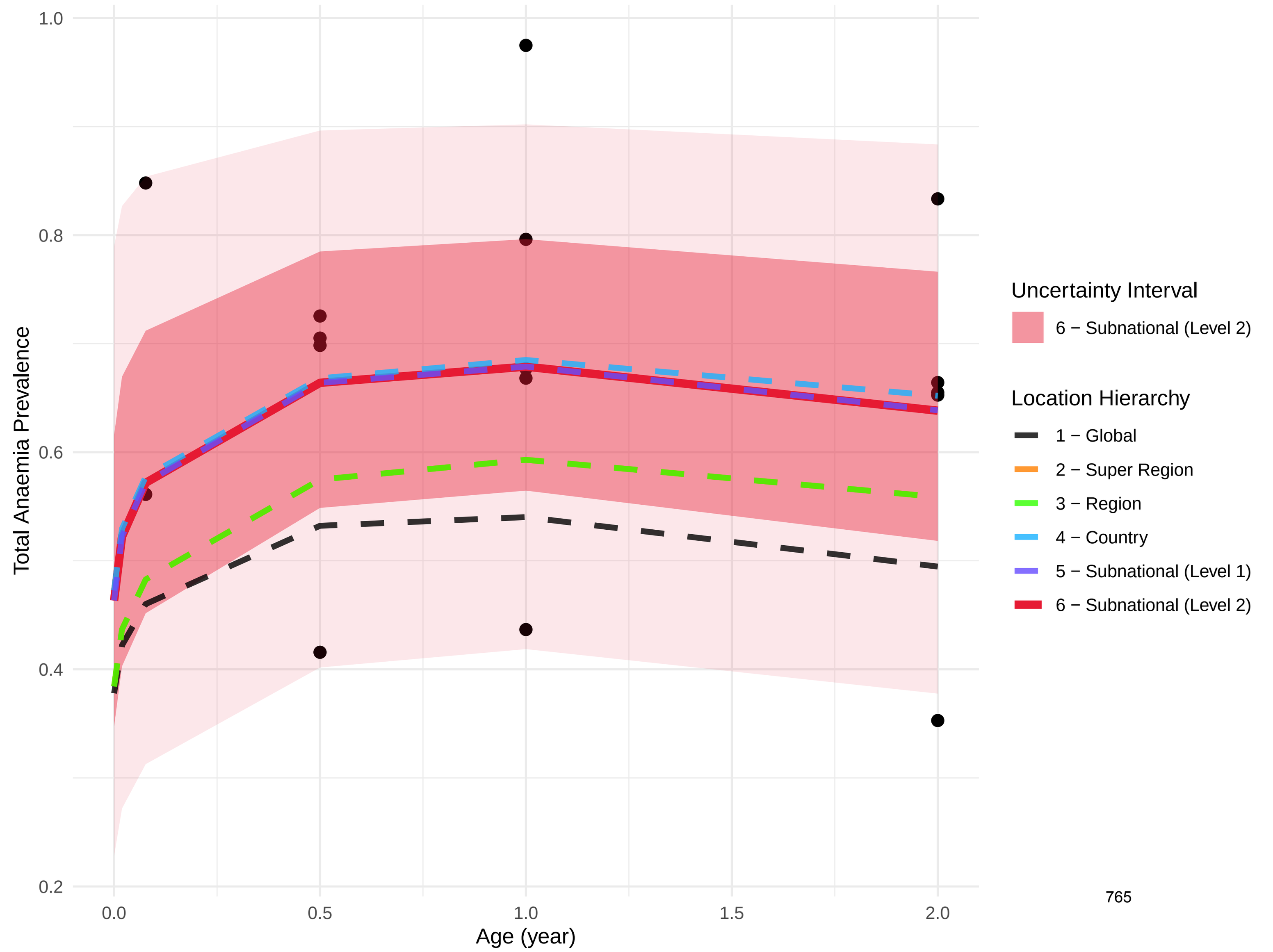

MR−BRT Age/Sex Fits − Non−pregnant Females <2 Years Old − Total Anaemia Prevalence − Chhattisgarh, Urban
WHO−2001 Adjustment Method

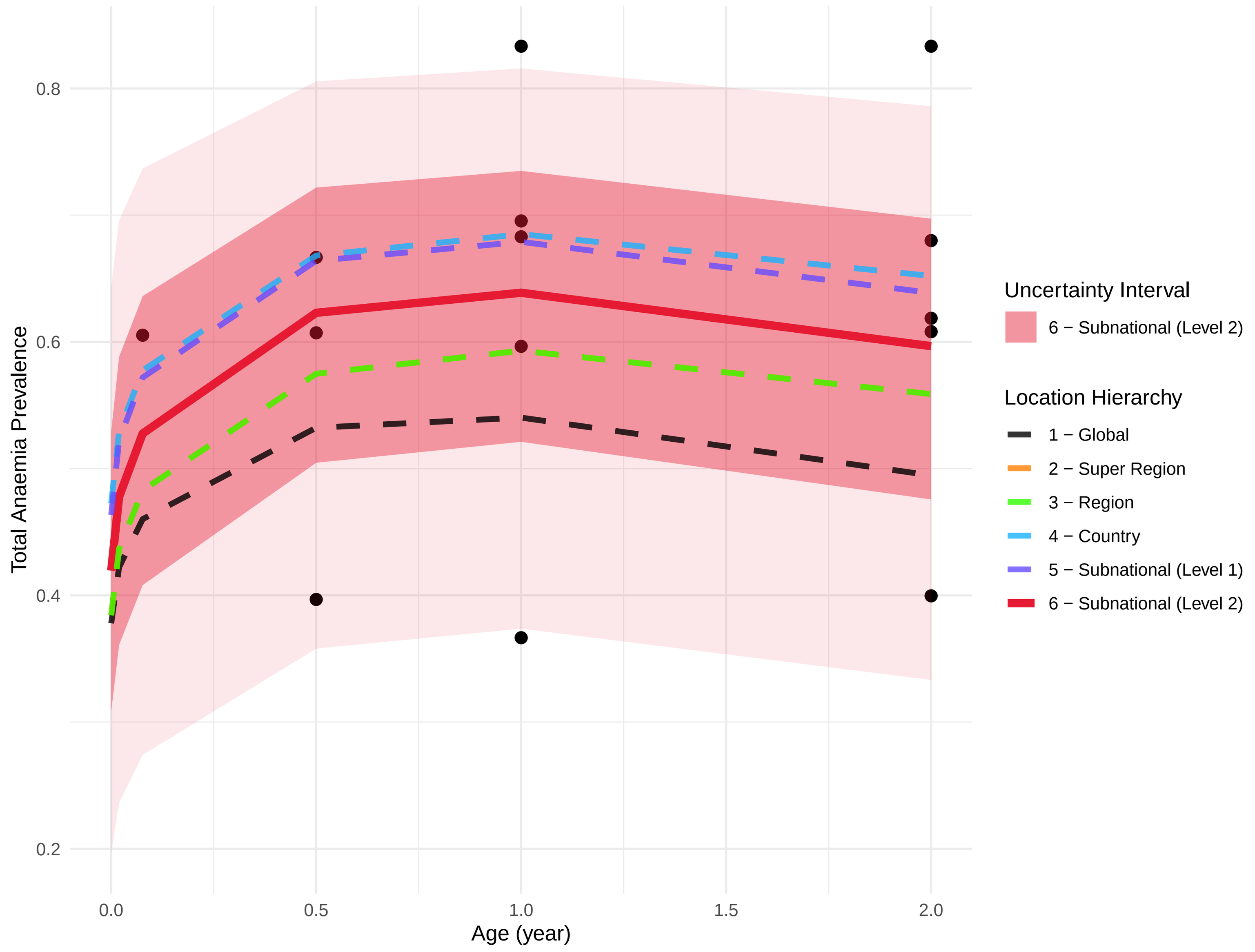

MR−BRT Age/Sex Fits − Non−pregnant Females <2 Years Old − Total Anaemia Prevalence − Delhi
WHO−2001 Adjustment Method

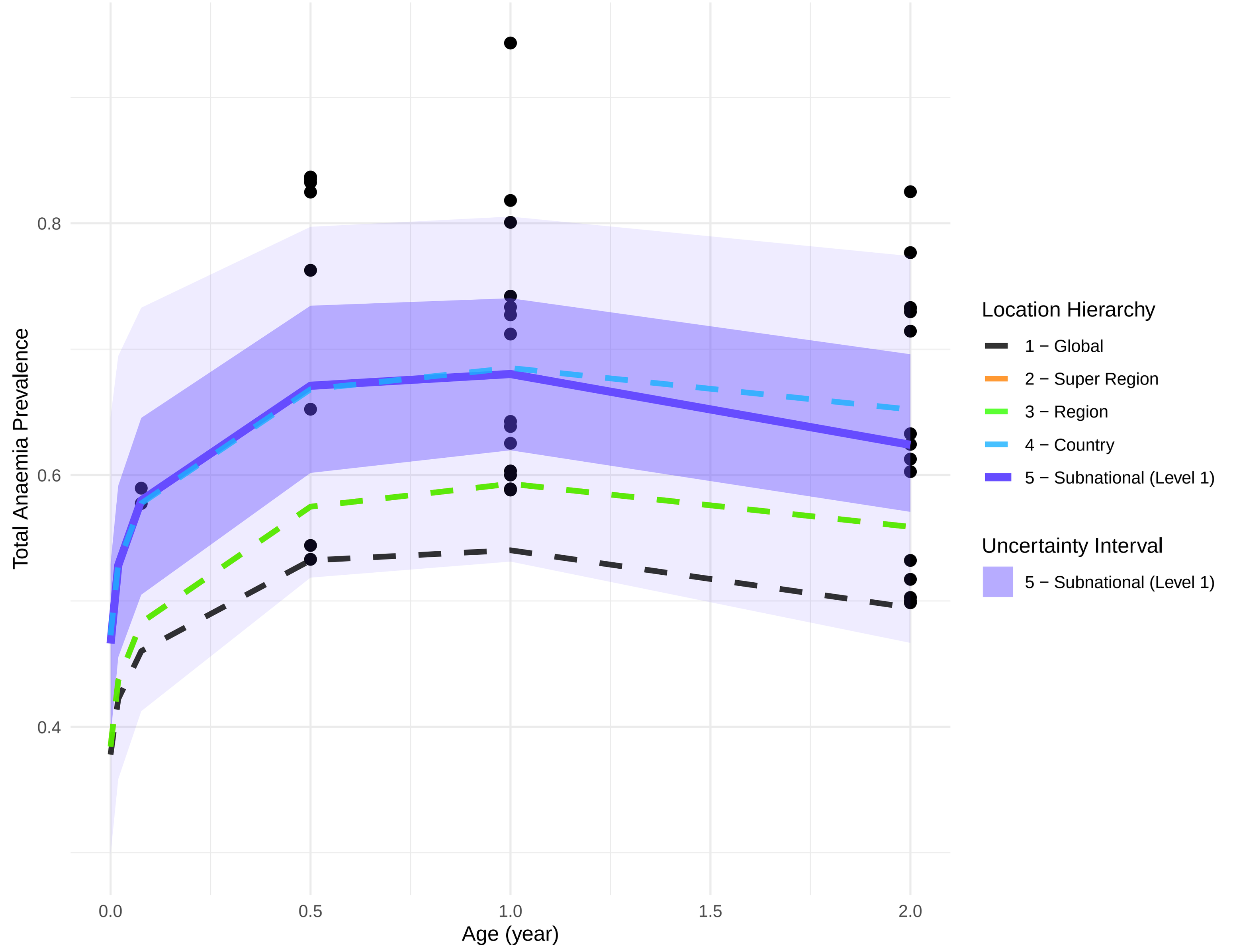

MR−BRT Age/Sex Fits − Non−pregnant Females <2 Years Old − Total Anaemia Prevalence − Delhi, Rural
WHO−2001 Adjustment Method

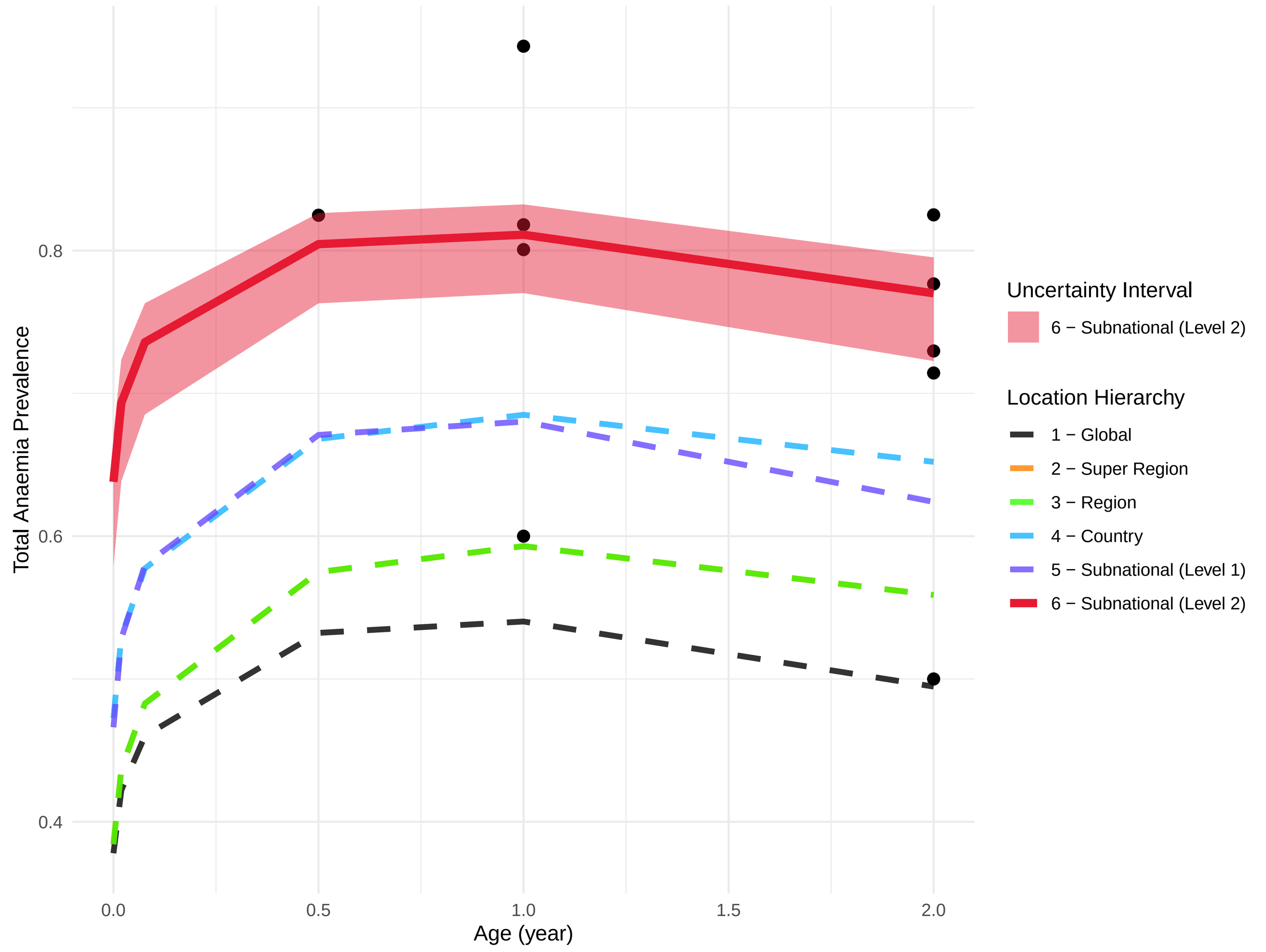

MR−BRT Age/Sex Fits − Non−pregnant Females <2 Years Old − Total Anaemia Prevalence − Delhi, Urban
WHO−2001 Adjustment Method

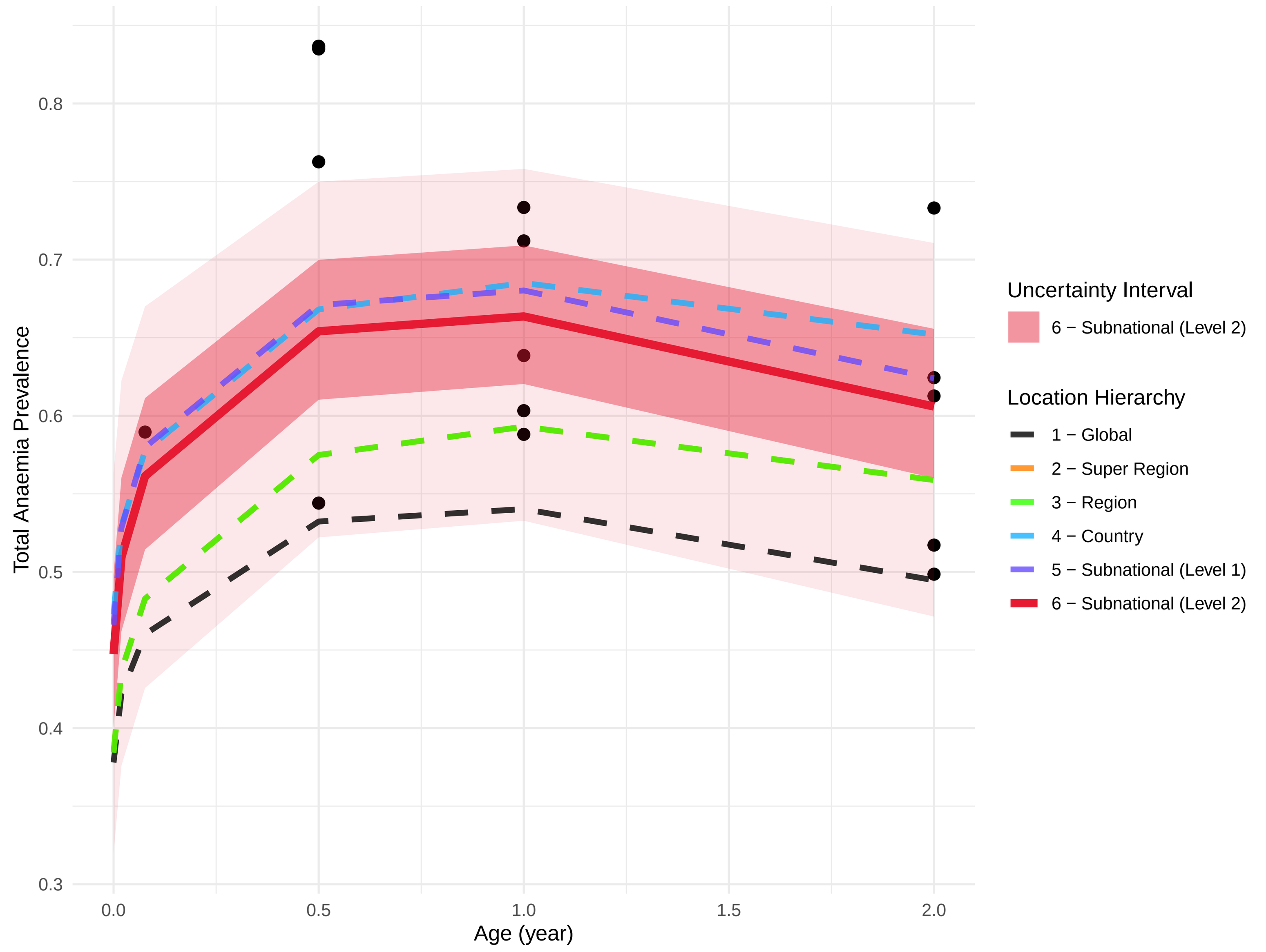

MR−BRT Age/Sex Fits − Non−pregnant Females <2 Years Old − Total Anaemia Prevalence − Goa
WHO−2001 Adjustment Method

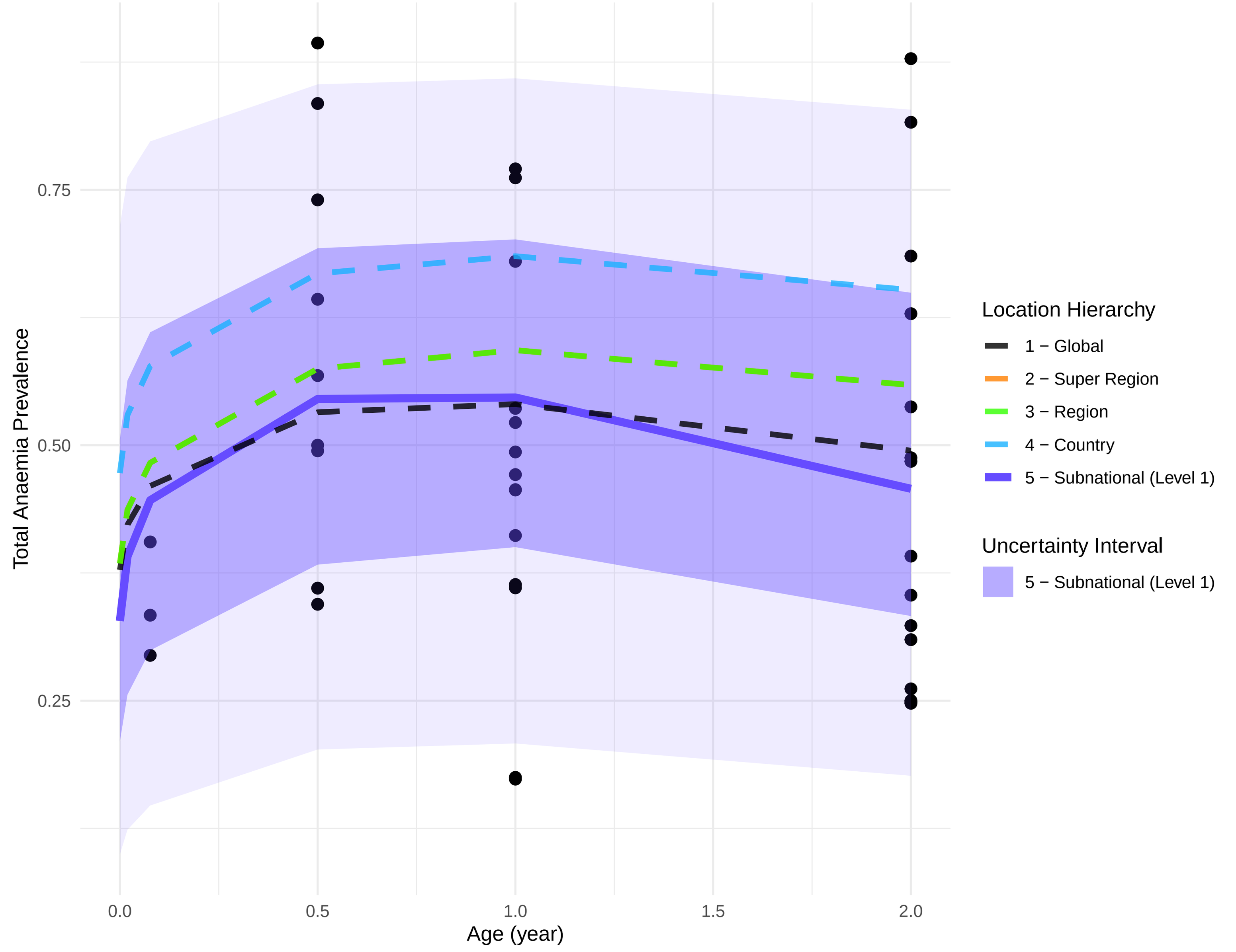

MR−BRT Age/Sex Fits − Non−pregnant Females <2 Years Old − Total Anaemia Prevalence − Goa, Rural
WHO−2001 Adjustment Method

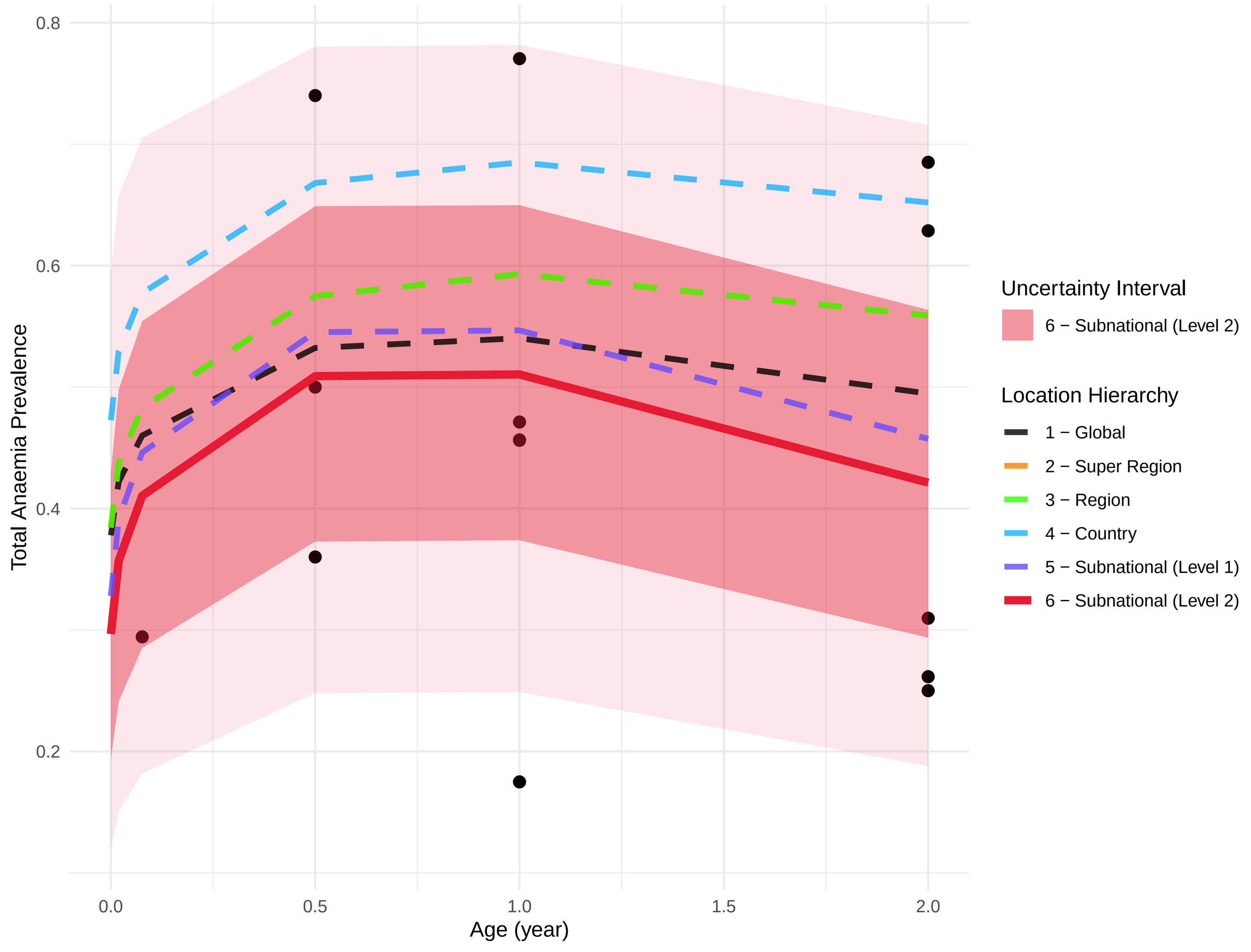

MR−BRT Age/Sex Fits − Non−pregnant Females <2 Years Old − Total Anaemia Prevalence − Goa, Urban
WHO−2001 Adjustment Method

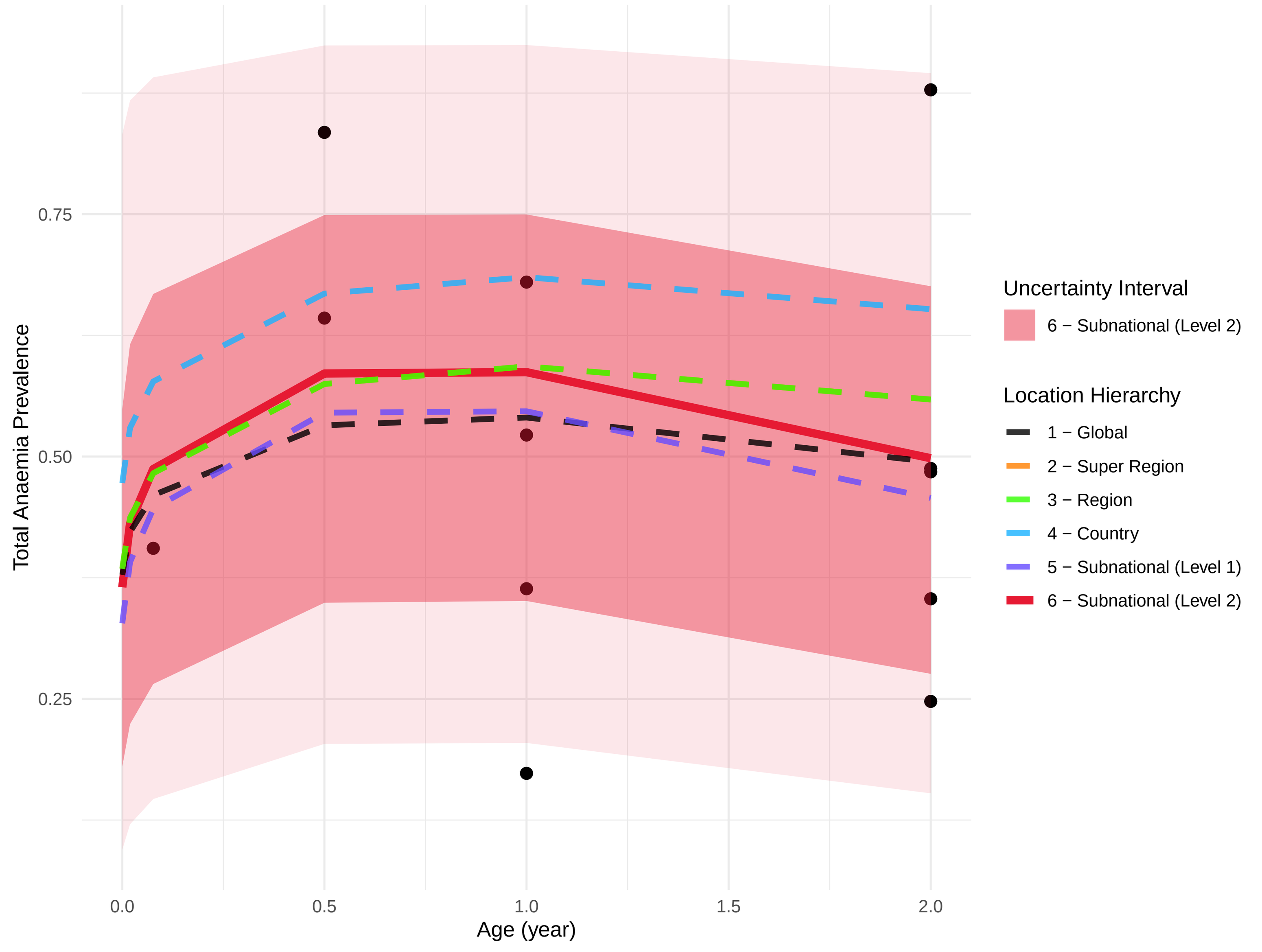

MR−BRT Age/Sex Fits − Non−pregnant Females <2 Years Old − Total Anaemia Prevalence − Gujarat
WHO−2001 Adjustment Method

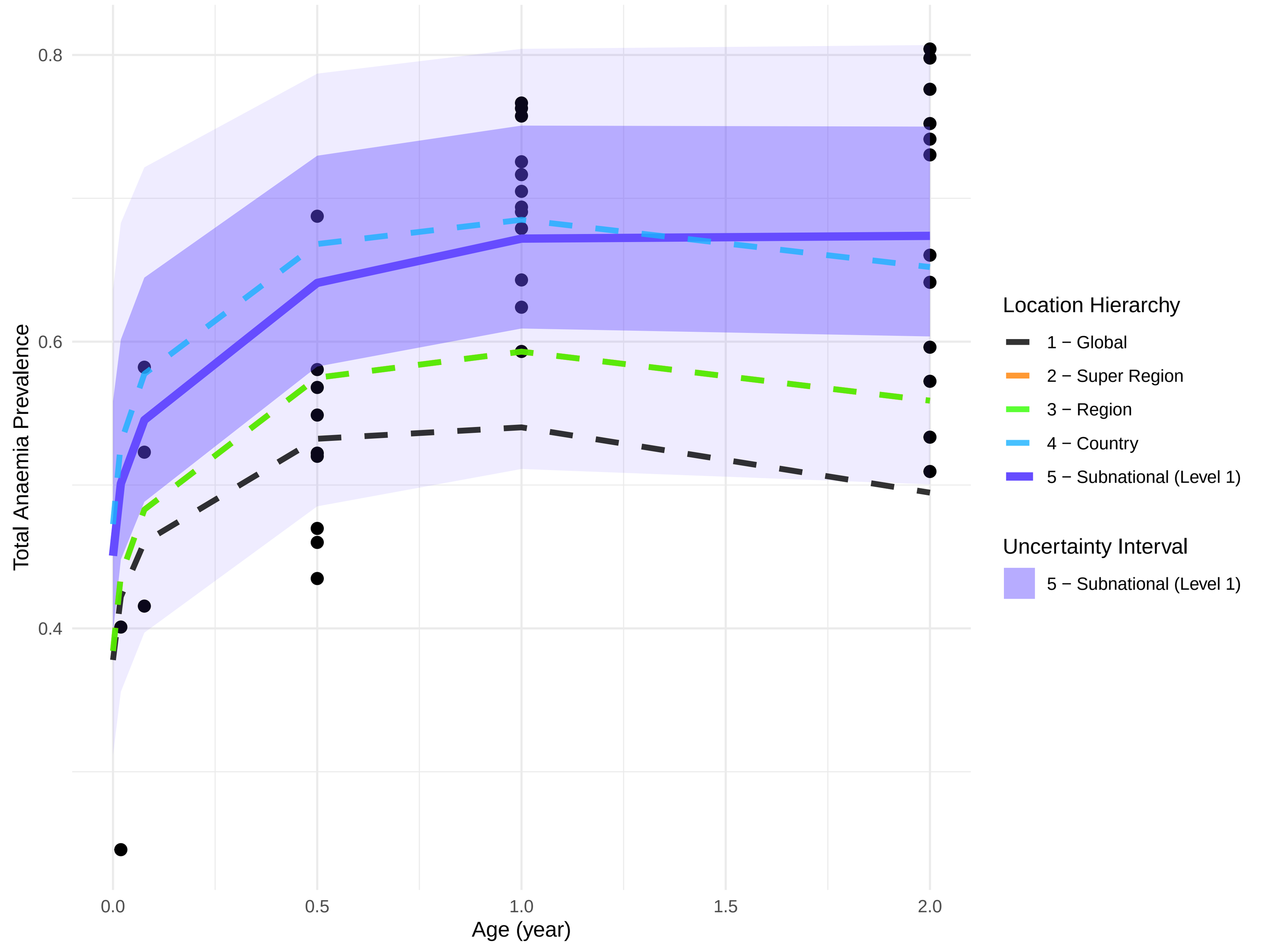

MR−BRT Age/Sex Fits − Non−pregnant Females <2 Years Old − Total Anaemia Prevalence − Gujarat, Rural
WHO−2001 Adjustment Method

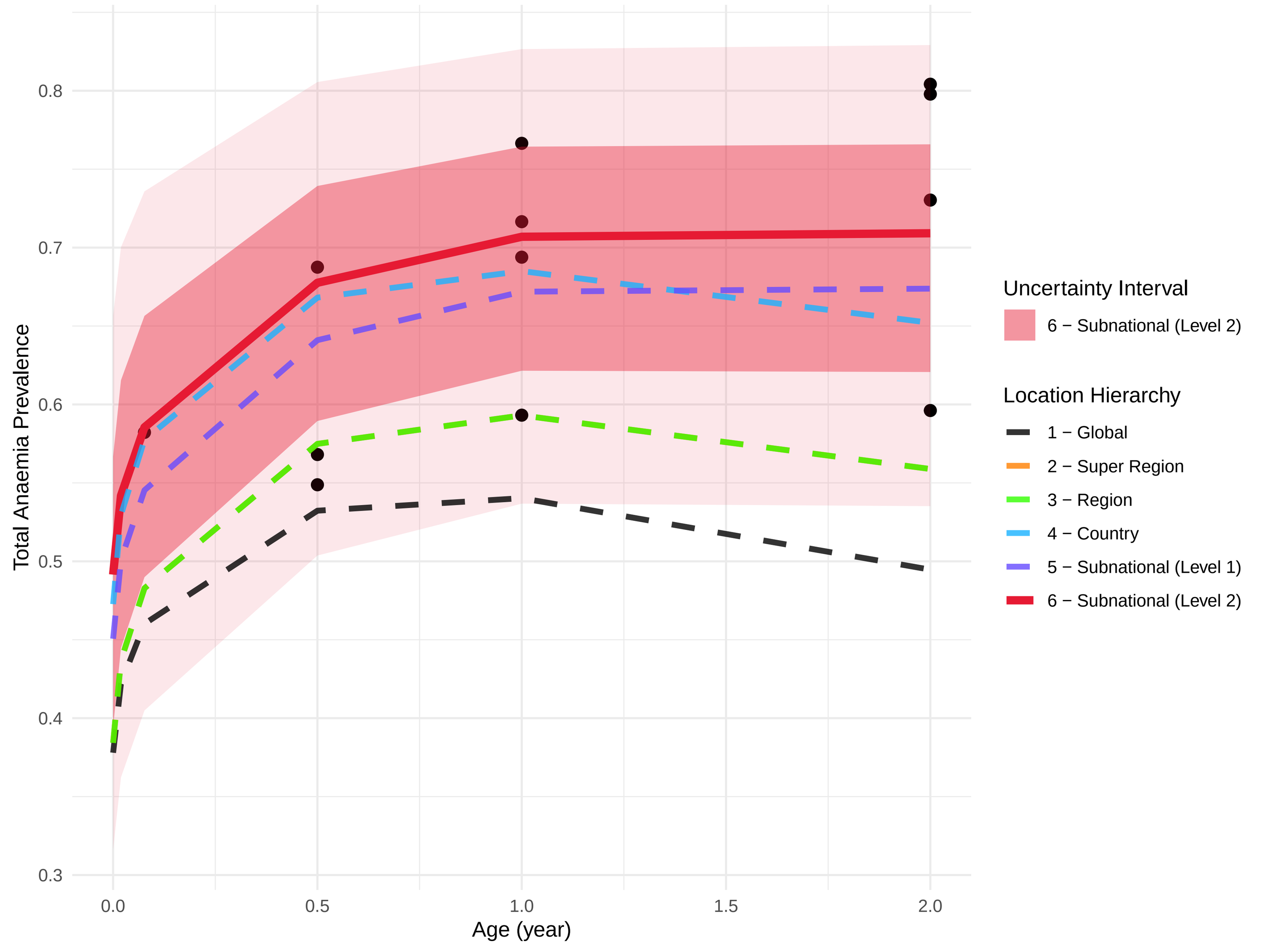

MR−BRT Age/Sex Fits − Non−pregnant Females <2 Years Old − Total Anaemia Prevalence − Gujarat, Urban
WHO−2001 Adjustment Method

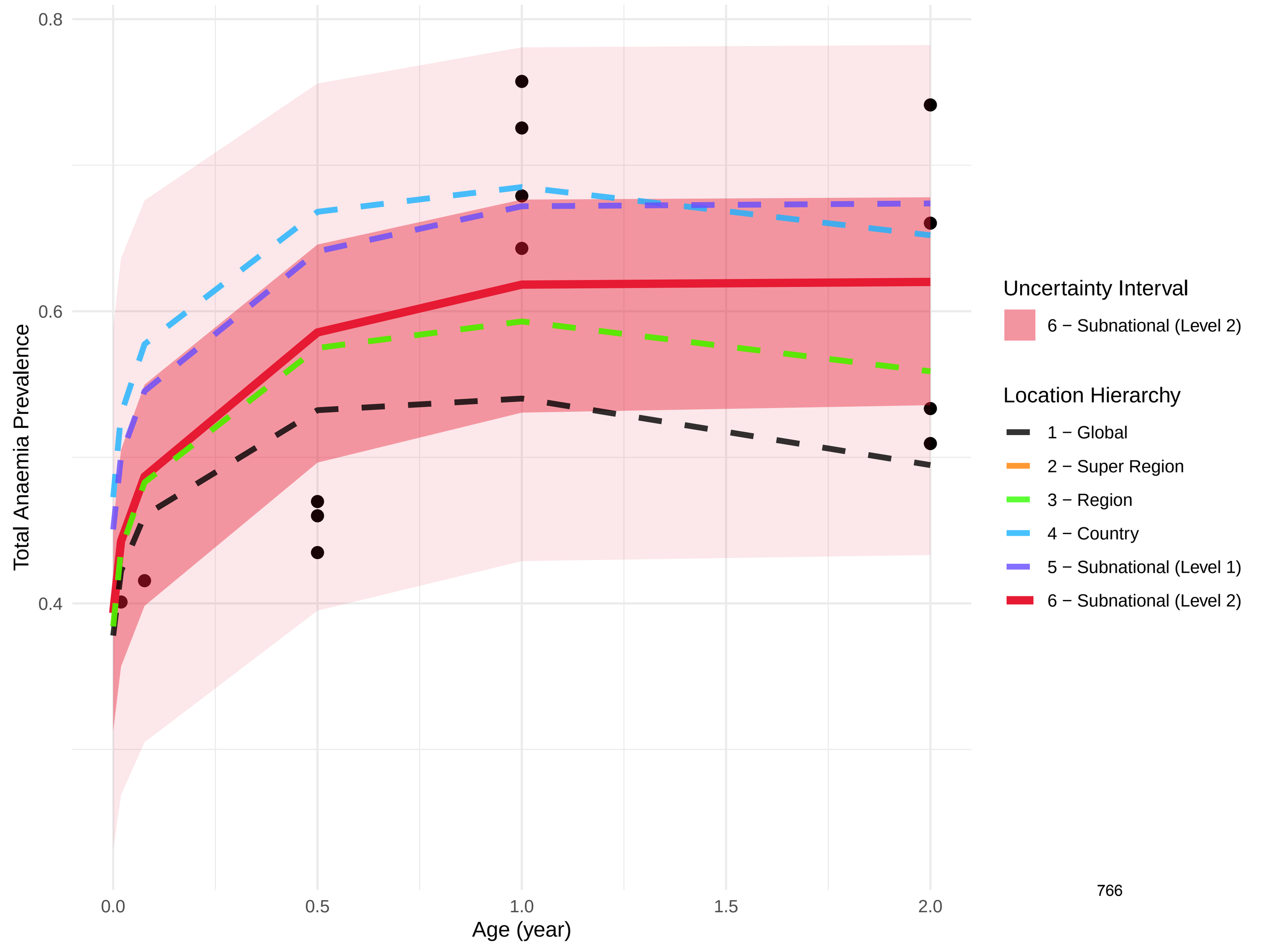

MR−BRT Age/Sex Fits − Non−pregnant Females <2 Years Old − Total Anaemia Prevalence − Haryana
WHO−2001 Adjustment Method

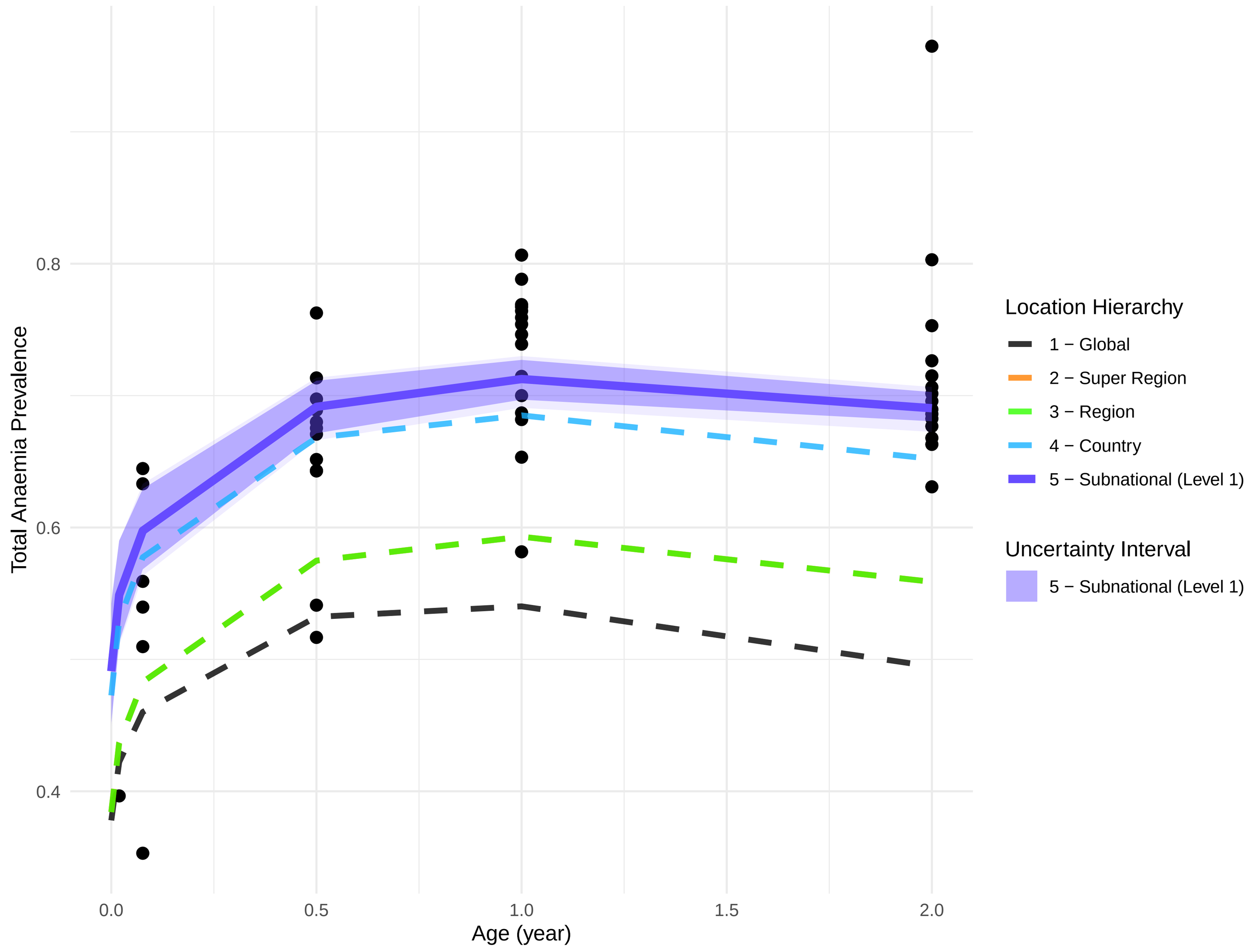

MR−BRT Age/Sex Fits − Non−pregnant Females <2 Years Old − Total Anaemia Prevalence − Haryana, Rural
WHO−2001 Adjustment Method

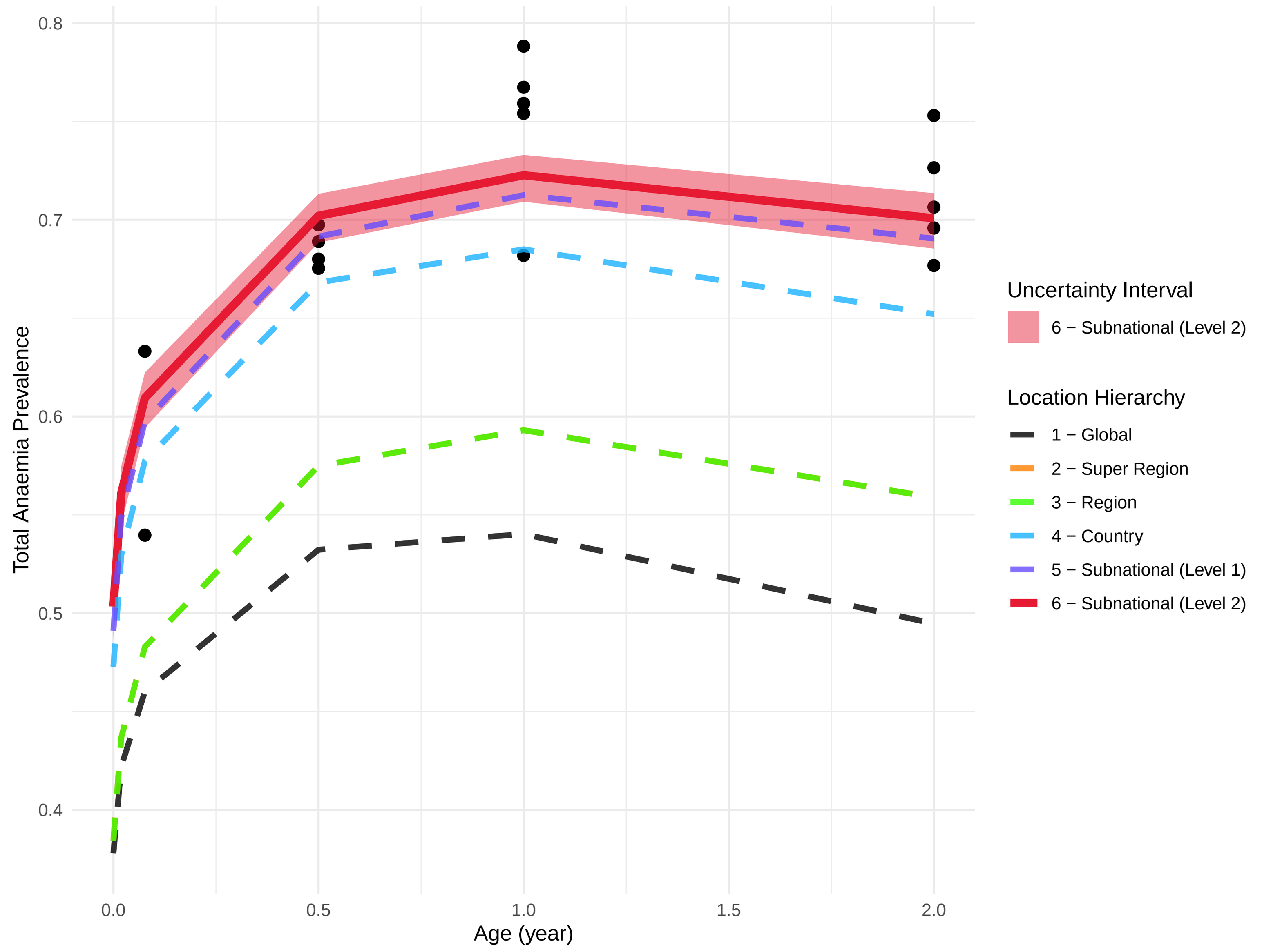

MR−BRT Age/Sex Fits − Non−pregnant Females <2 Years Old − Total Anaemia Prevalence − Haryana, Urban
WHO−2001 Adjustment Method

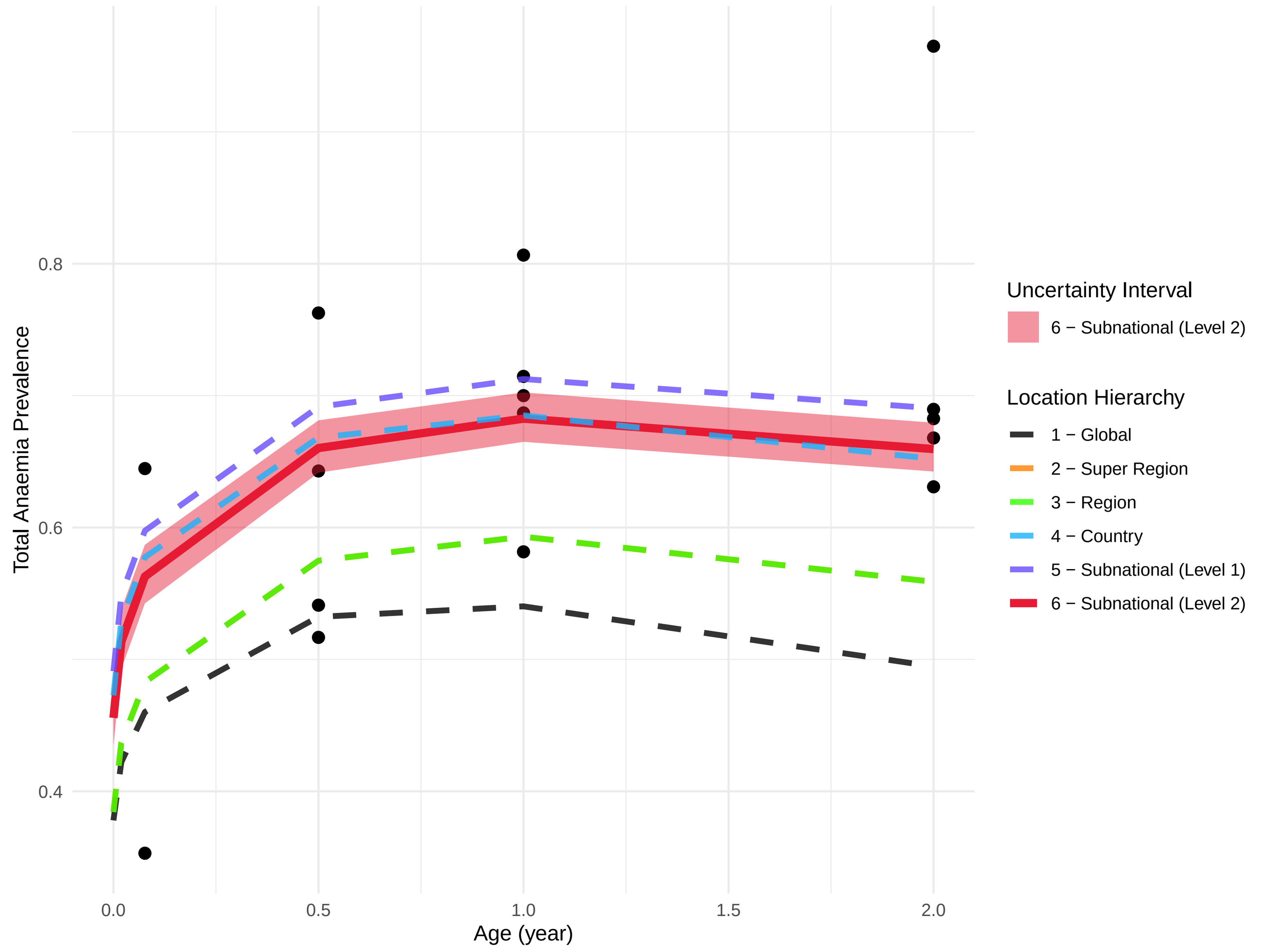

MR−BRT Age/Sex Fits − Non−pregnant Females <2 Years Old − Total Anaemia Prevalence − Himachal Pradesh
WHO−2001 Adjustment Method

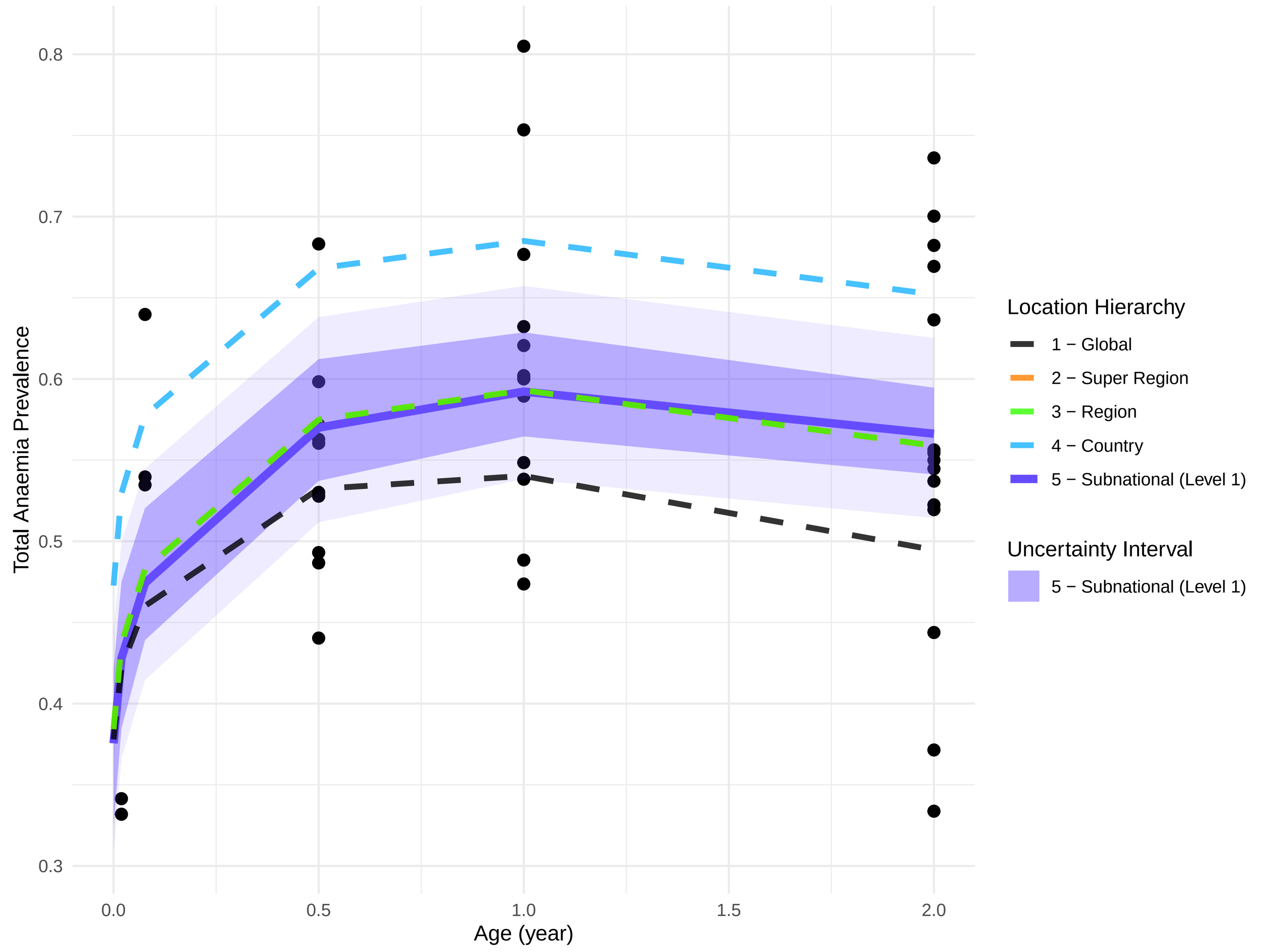

MR−BRT Age/Sex Fits − Non−pregnant Females <2 Years Old − Total Anaemia Prevalence − Himachal Pradesh, Rural
WHO−2001 Adjustment Method

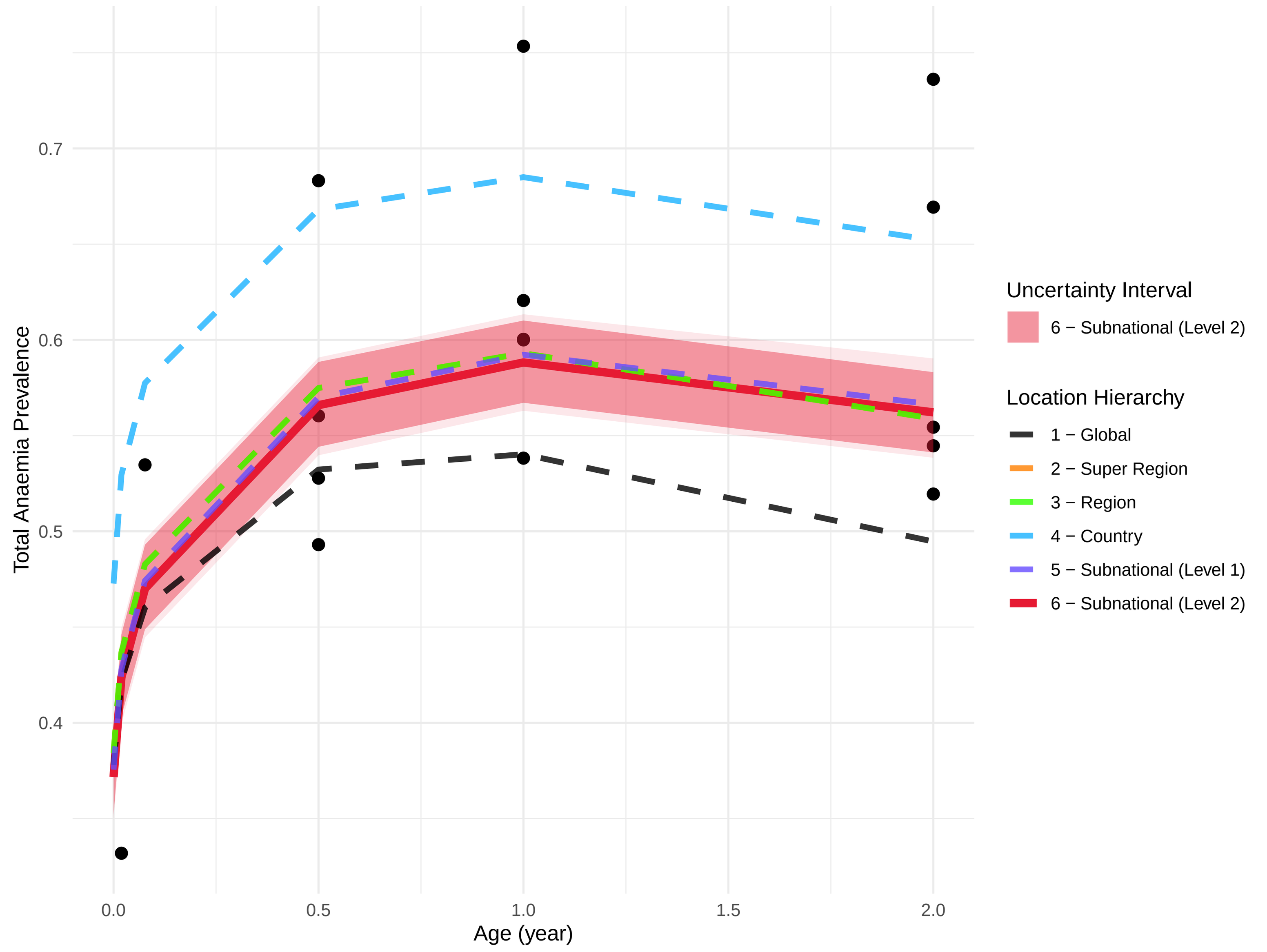

MR−BRT Age/Sex Fits − Non−pregnant Females <2 Years Old − Total Anaemia Prevalence − Himachal Pradesh, Urban
WHO−2001 Adjustment Method

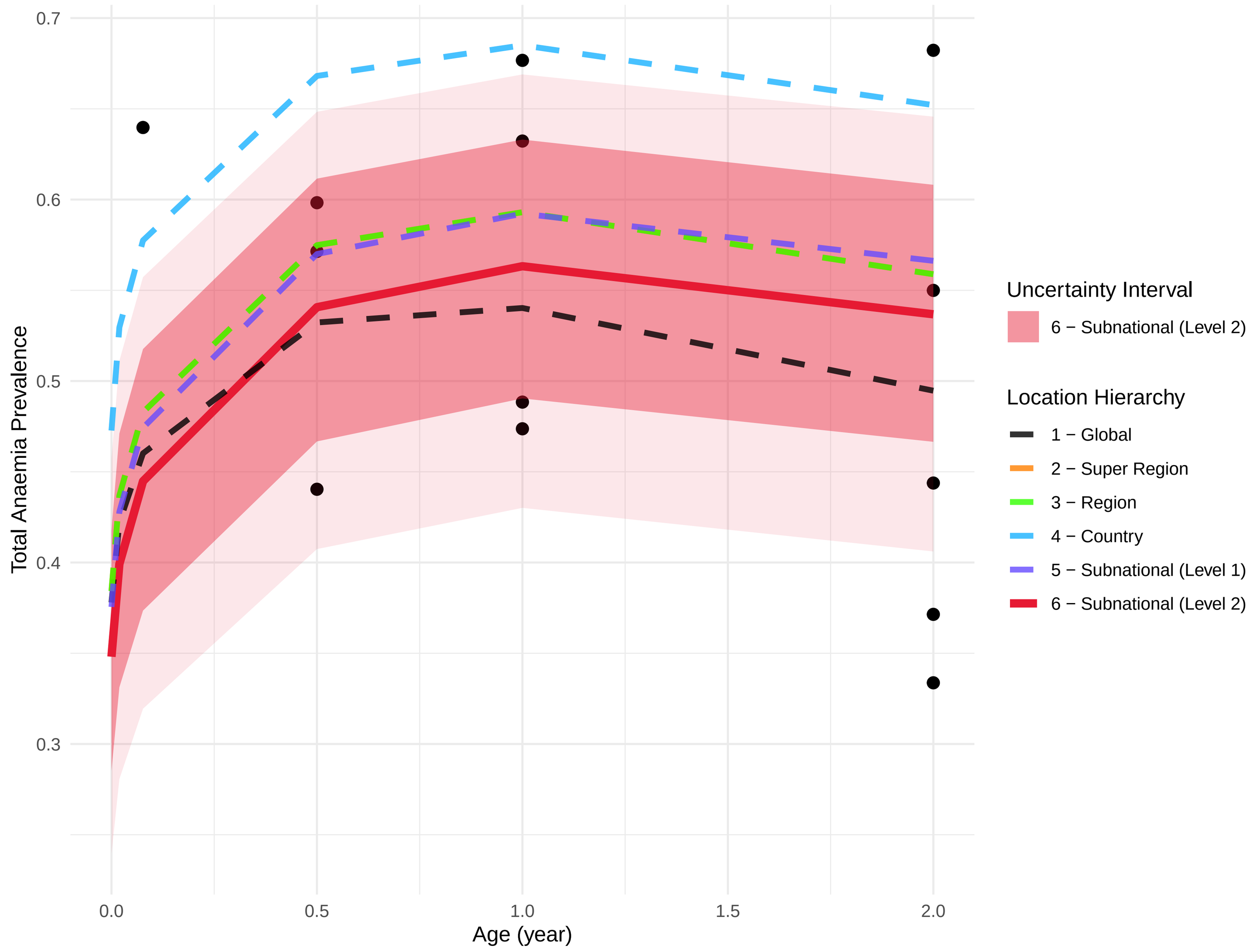

MR−BRT Age/Sex Fits − Non−pregnant Females <2 Years Old − Total Anaemia Prevalence − Jammu & Kashmir and Ladakh
WHO−2001 Adjustment Method

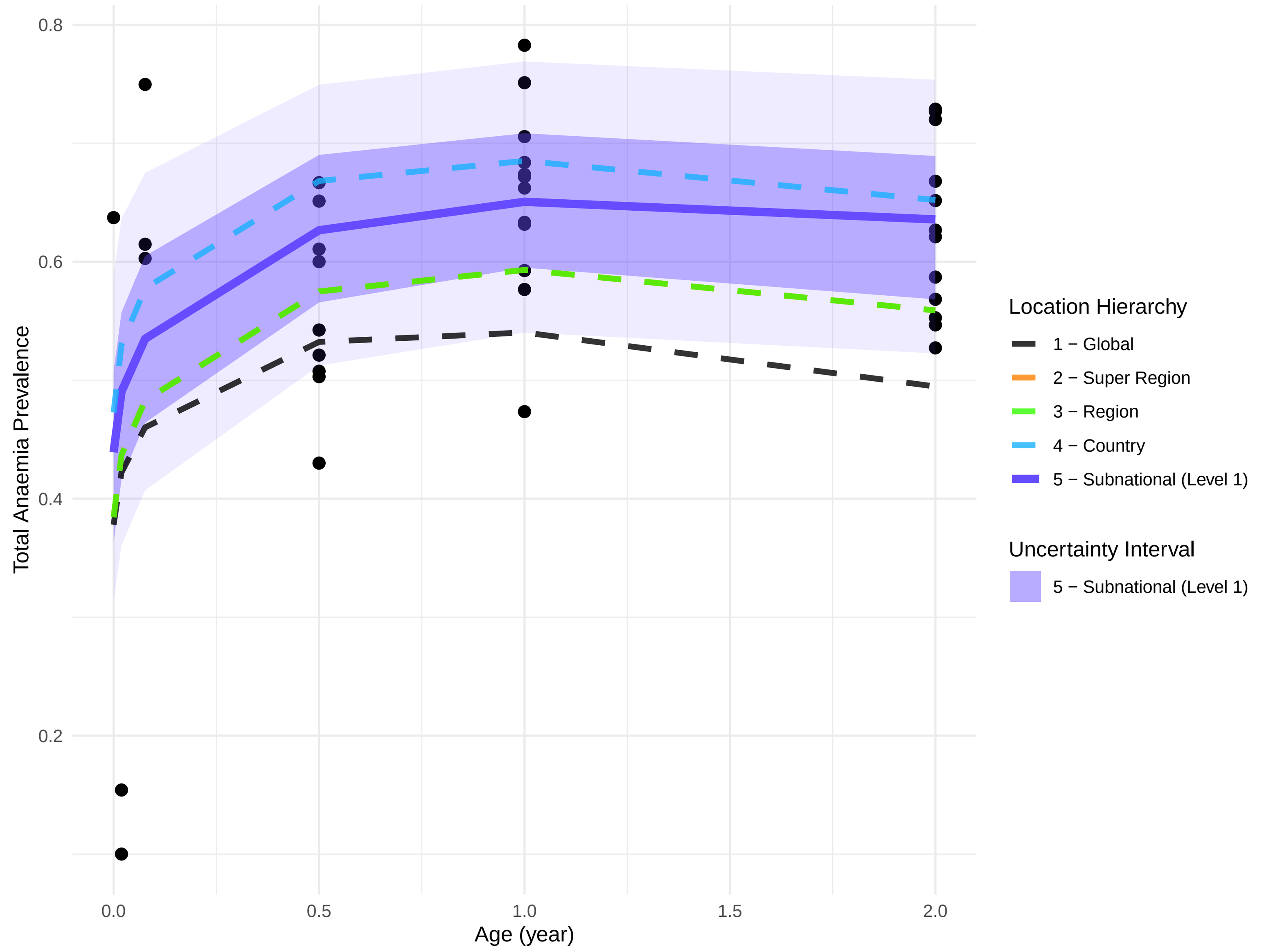

MR−BRT Age/Sex Fits − Non−pregnant Females <2 Years Old − Total Anaemia Prevalence − Jammu & Kashmir and Ladakh, Rural
WHO−2001 Adjustment Method

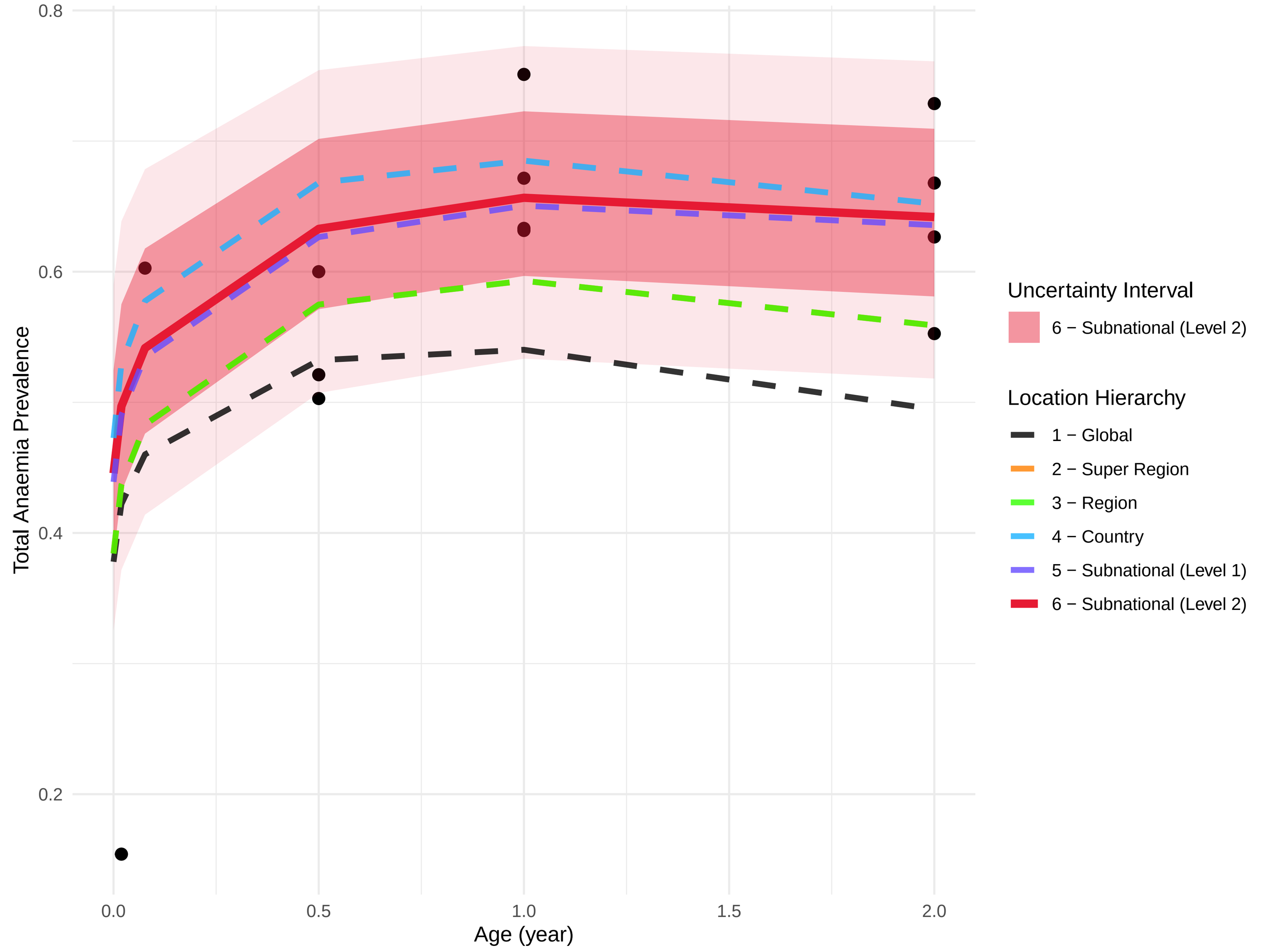

MR−BRT Age/Sex Fits − Non−pregnant Females <2 Years Old − Total Anaemia Prevalence − Jammu & Kashmir and Ladakh, Urban
WHO−2001 Adjustment Method

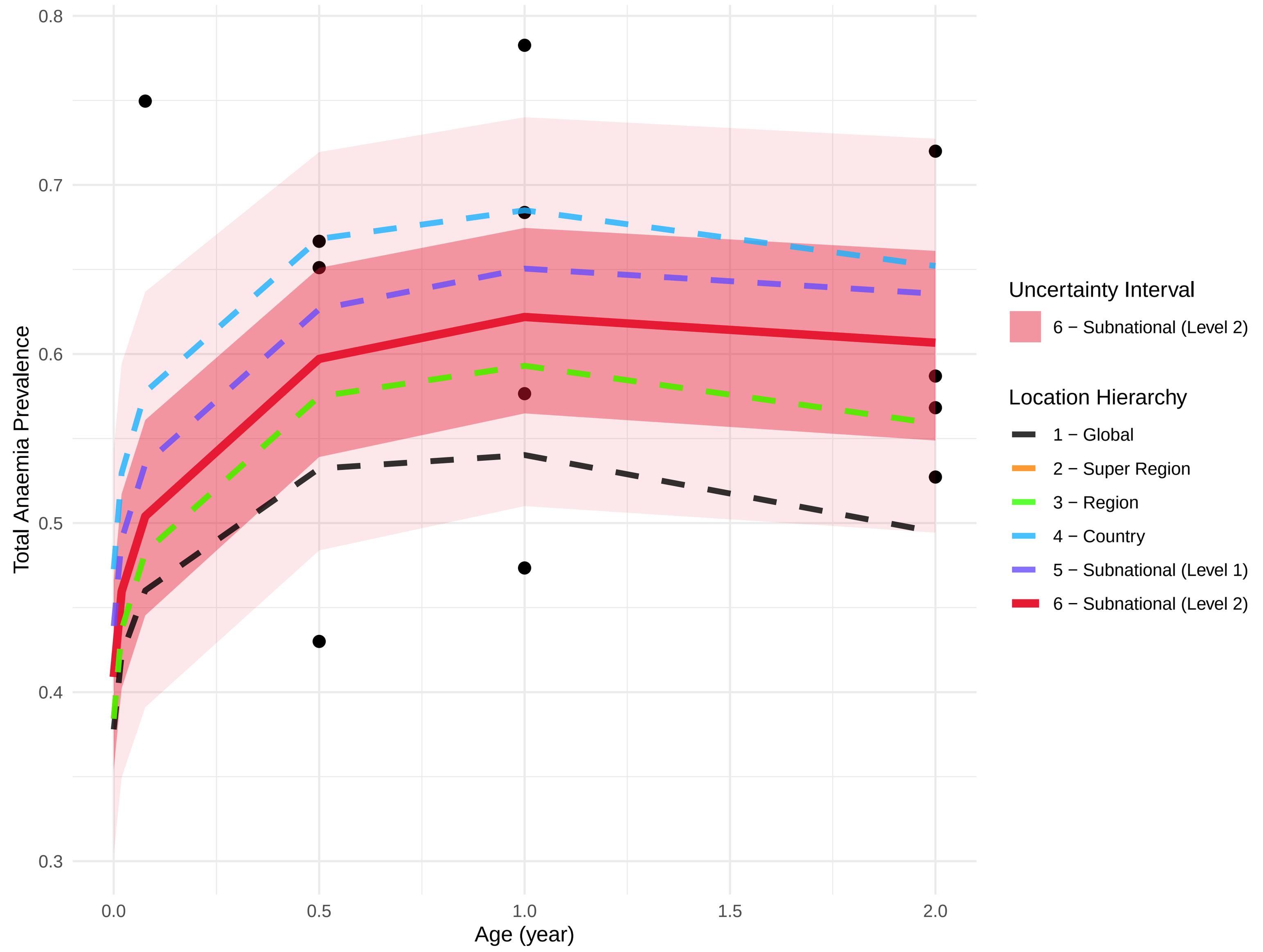

MR−BRT Age/Sex Fits − Non−pregnant Females <2 Years Old − Total Anaemia Prevalence − Jharkhand
WHO−2001 Adjustment Method

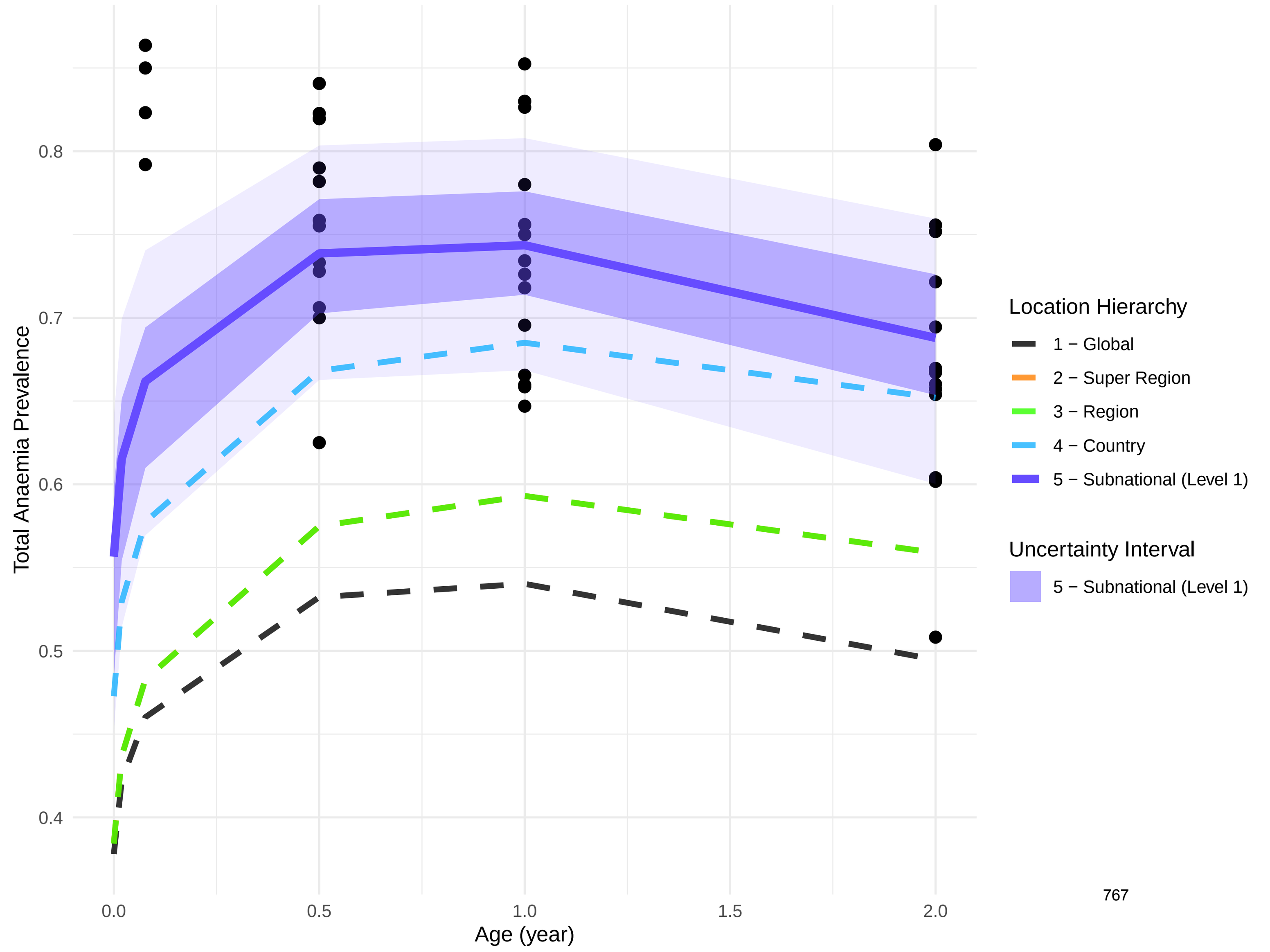

MR−BRT Age/Sex Fits − Non−pregnant Females <2 Years Old − Total Anaemia Prevalence − Jharkhand, Rural
WHO−2001 Adjustment Method

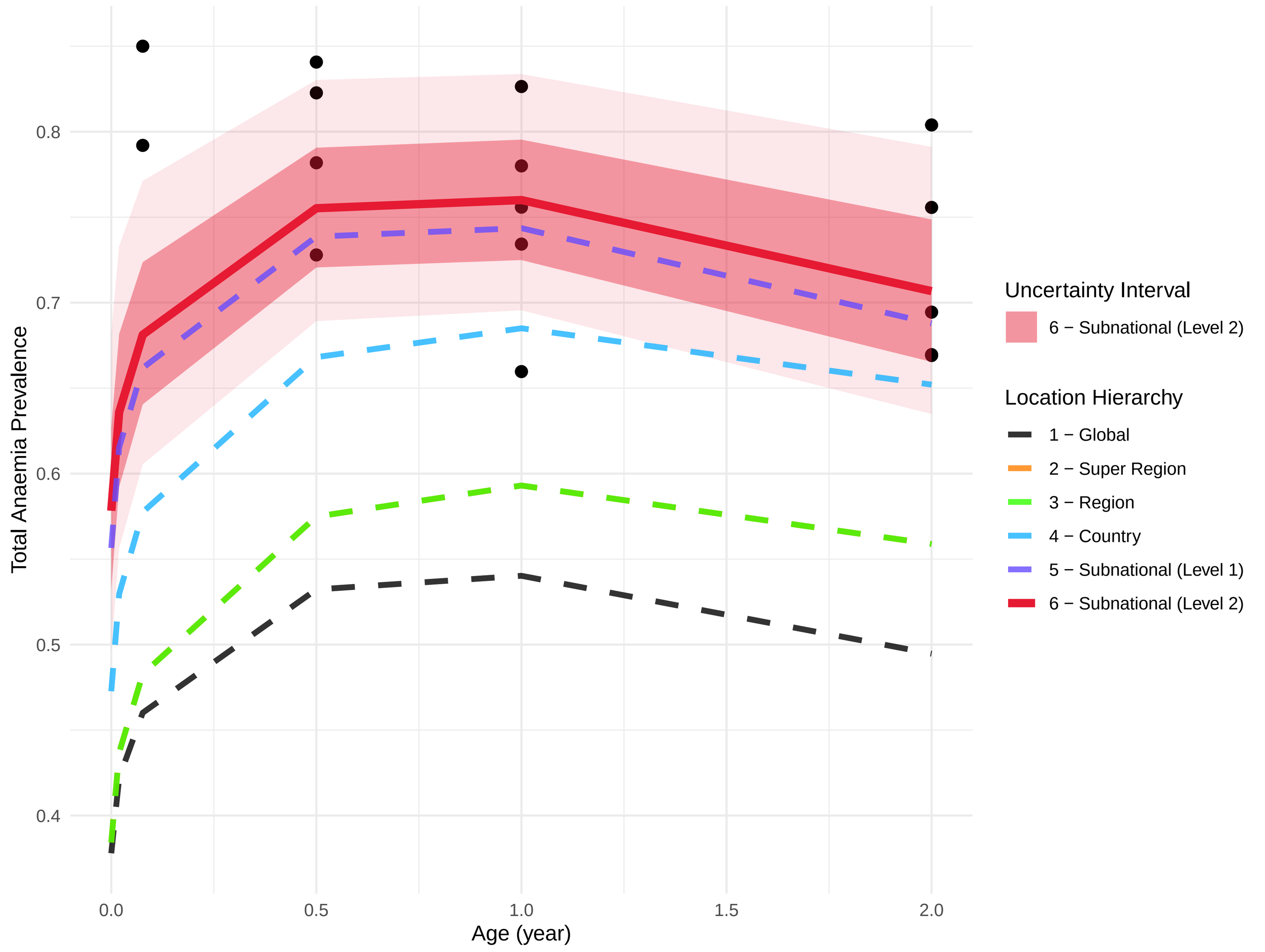

MR−BRT Age/Sex Fits − Non−pregnant Females <2 Years Old − Total Anaemia Prevalence − Jharkhand, Urban
WHO−2001 Adjustment Method

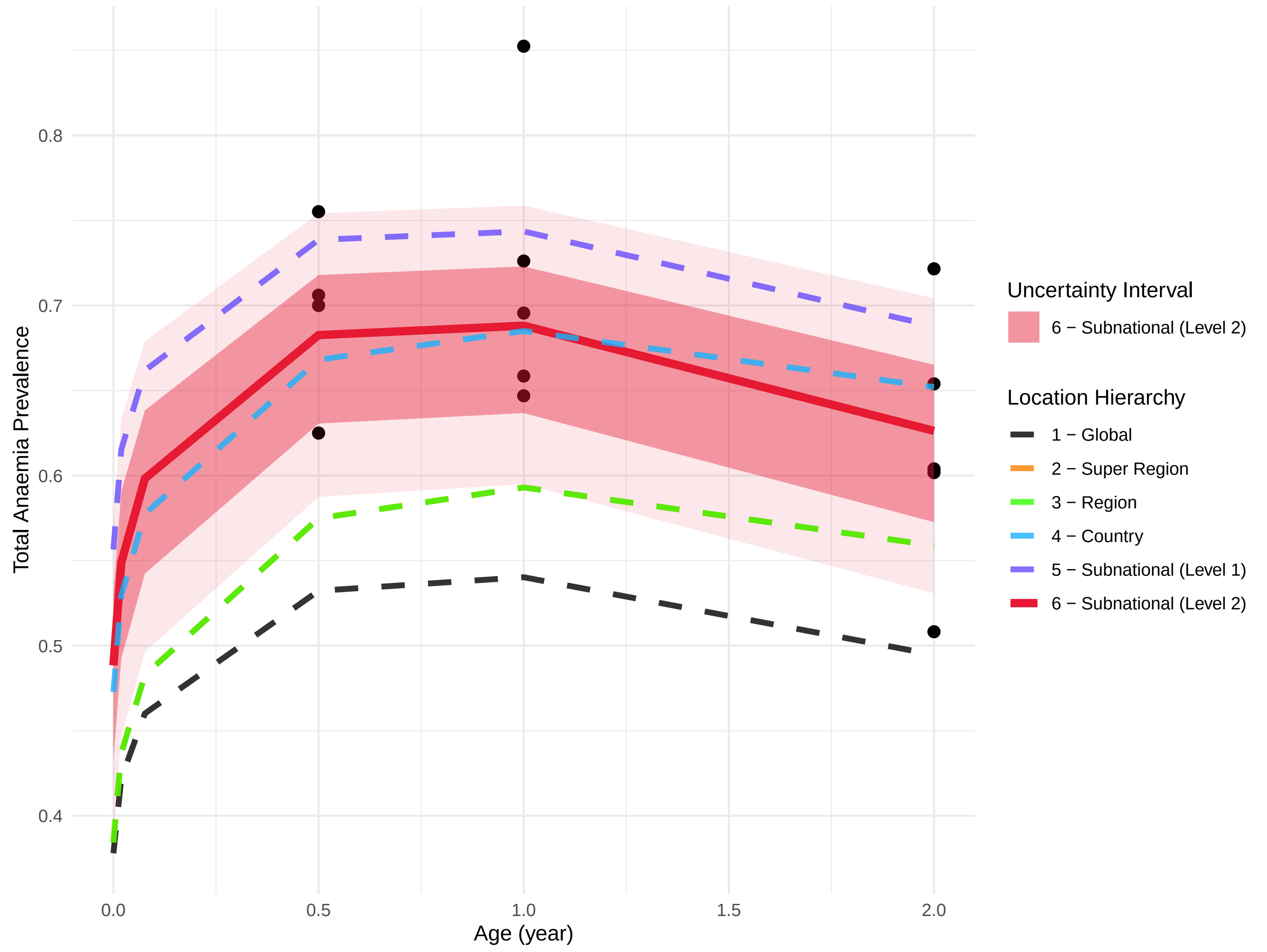

MR−BRT Age/Sex Fits − Non−pregnant Females <2 Years Old − Total Anaemia Prevalence − Karnataka
WHO−2001 Adjustment Method

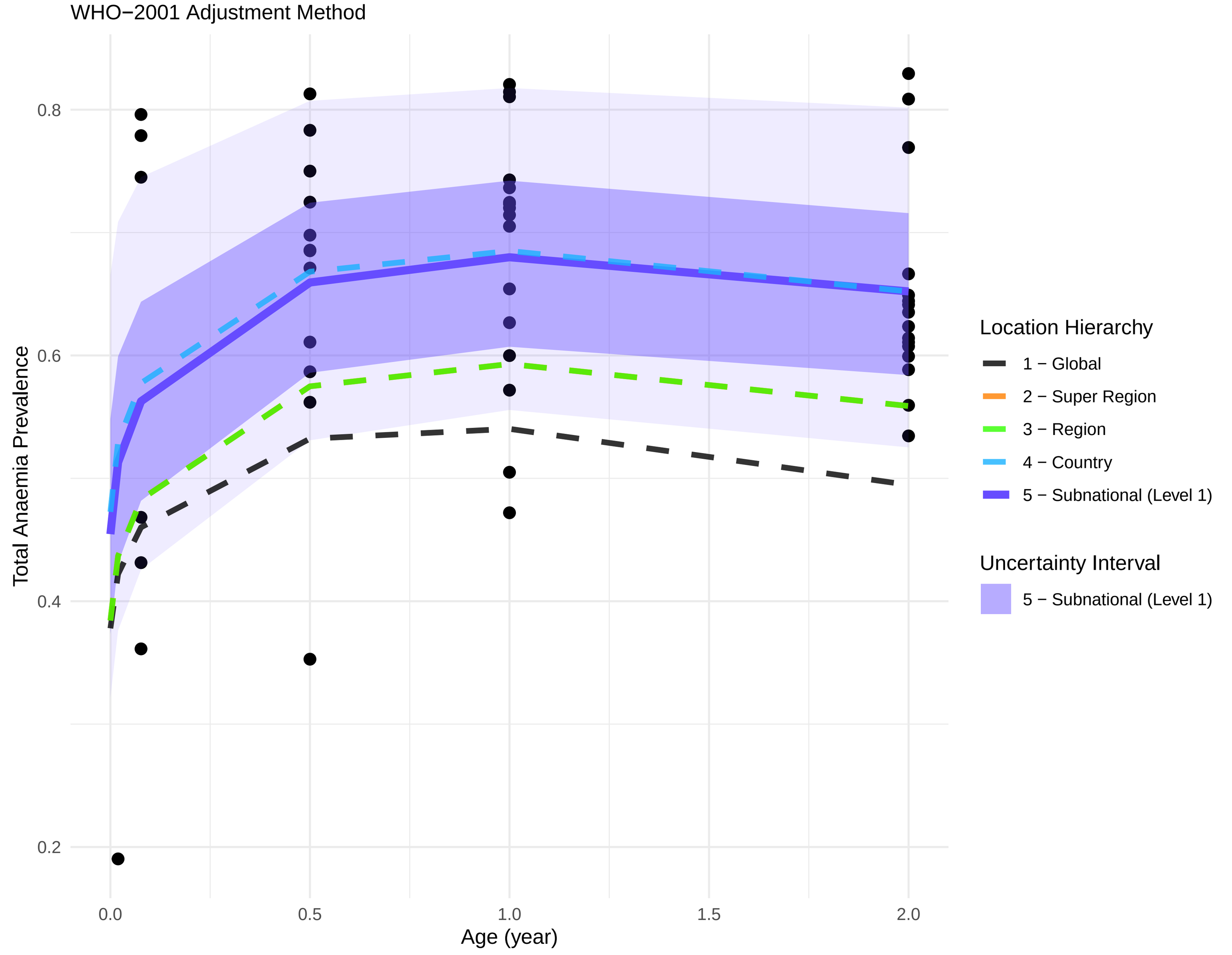

MR−BRT Age/Sex Fits − Non−pregnant Females <2 Years Old − Total Anaemia Prevalence − Karnataka, Rural
WHO−2001 Adjustment Method

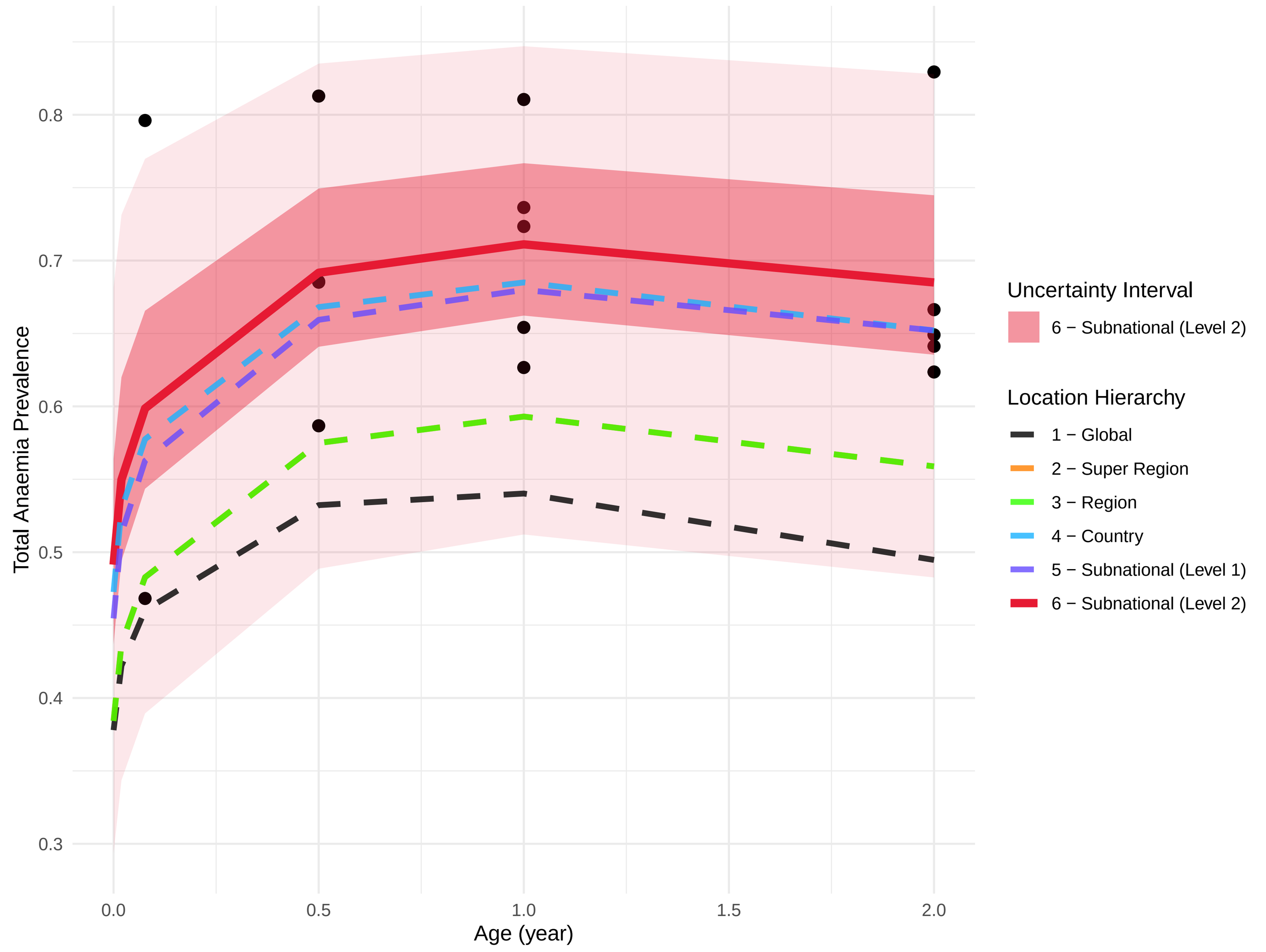

MR−BRT Age/Sex Fits − Non−pregnant Females <2 Years Old − Total Anaemia Prevalence − Karnataka, Urban
WHO−2001 Adjustment Method

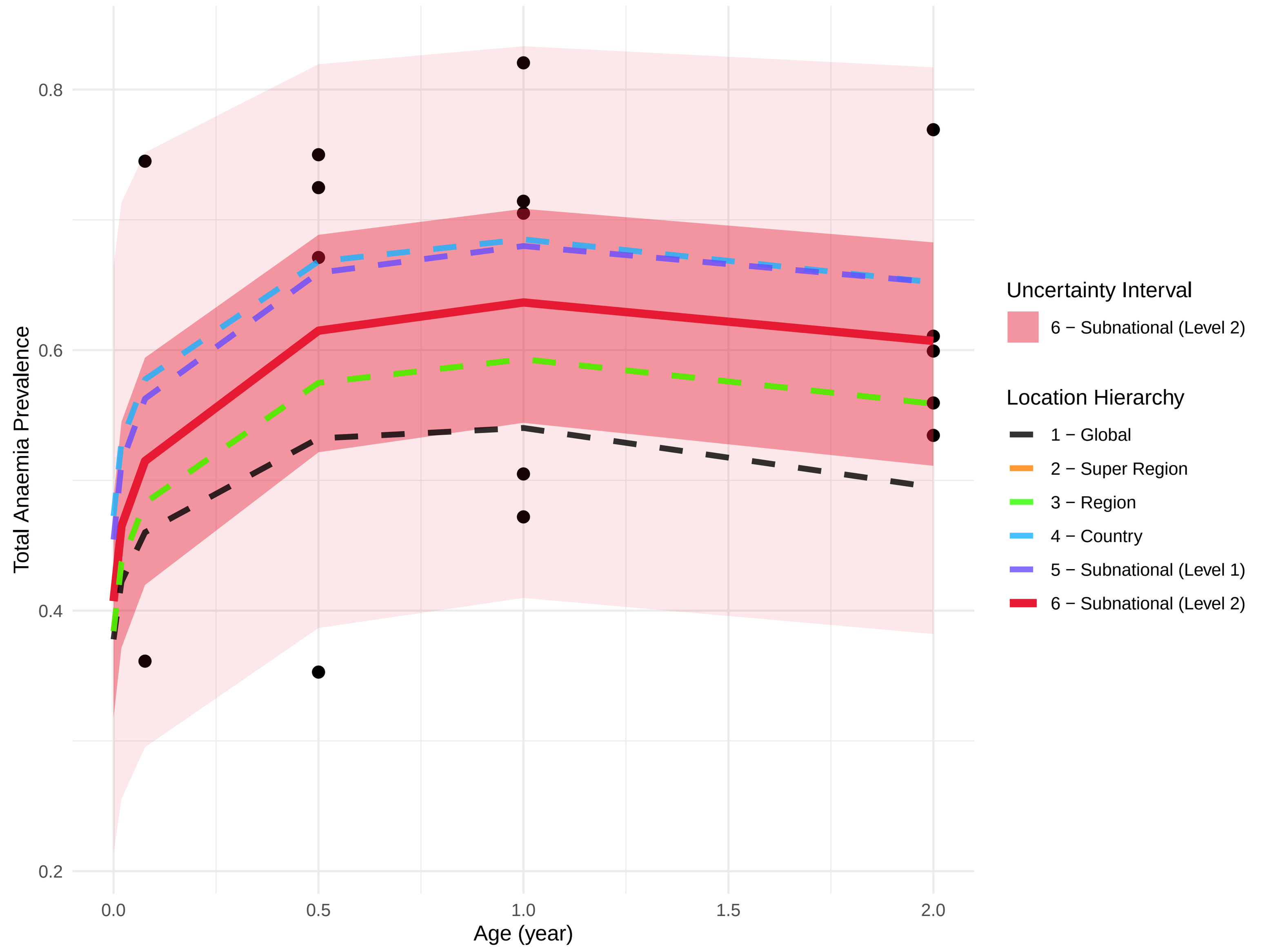

MR−BRT Age/Sex Fits − Non−pregnant Females <2 Years Old − Total Anaemia Prevalence − Kerala
WHO−2001 Adjustment Method

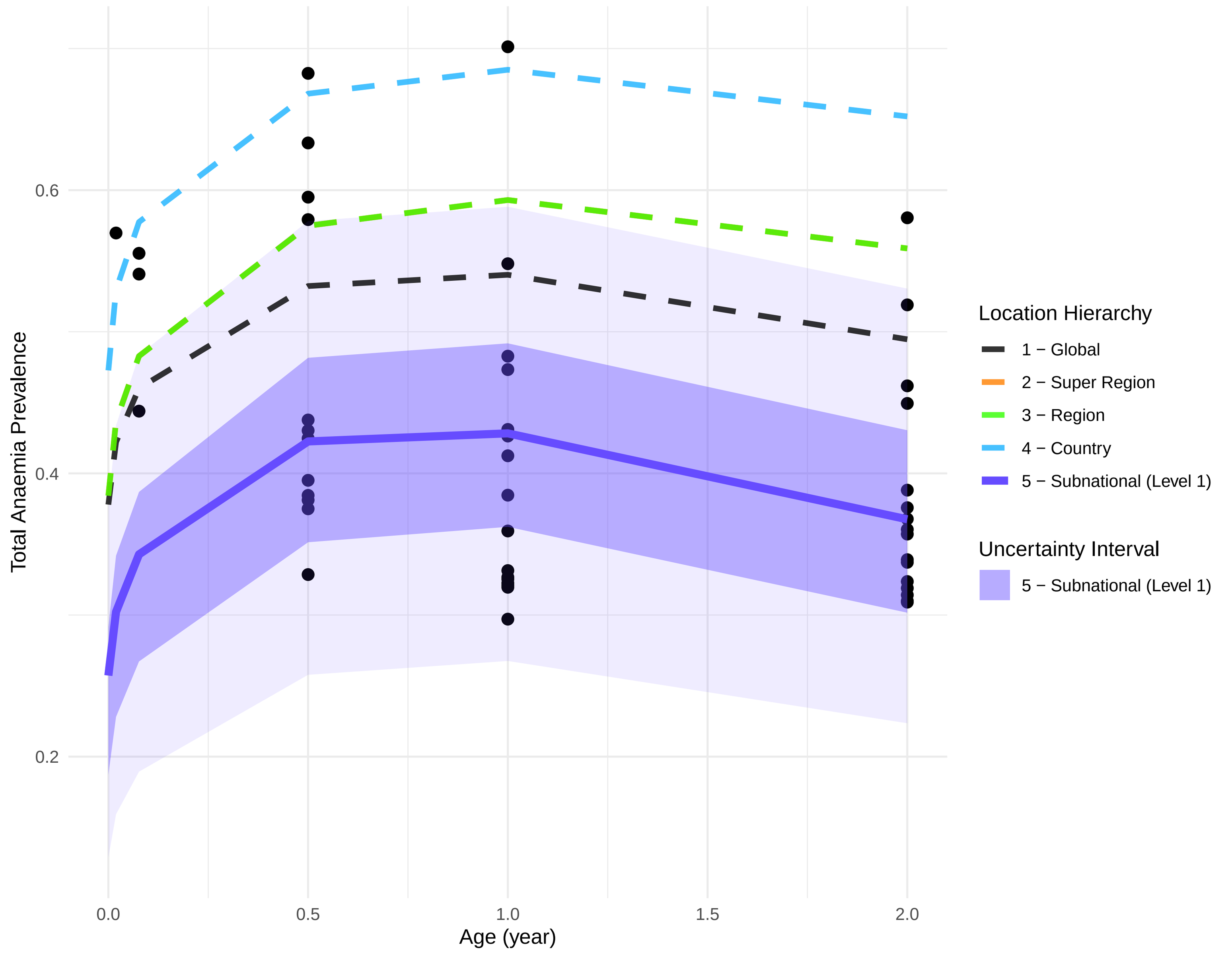

MR−BRT Age/Sex Fits − Non−pregnant Females <2 Years Old − Total Anaemia Prevalence − Kerala, Rural
WHO−2001 Adjustment Method

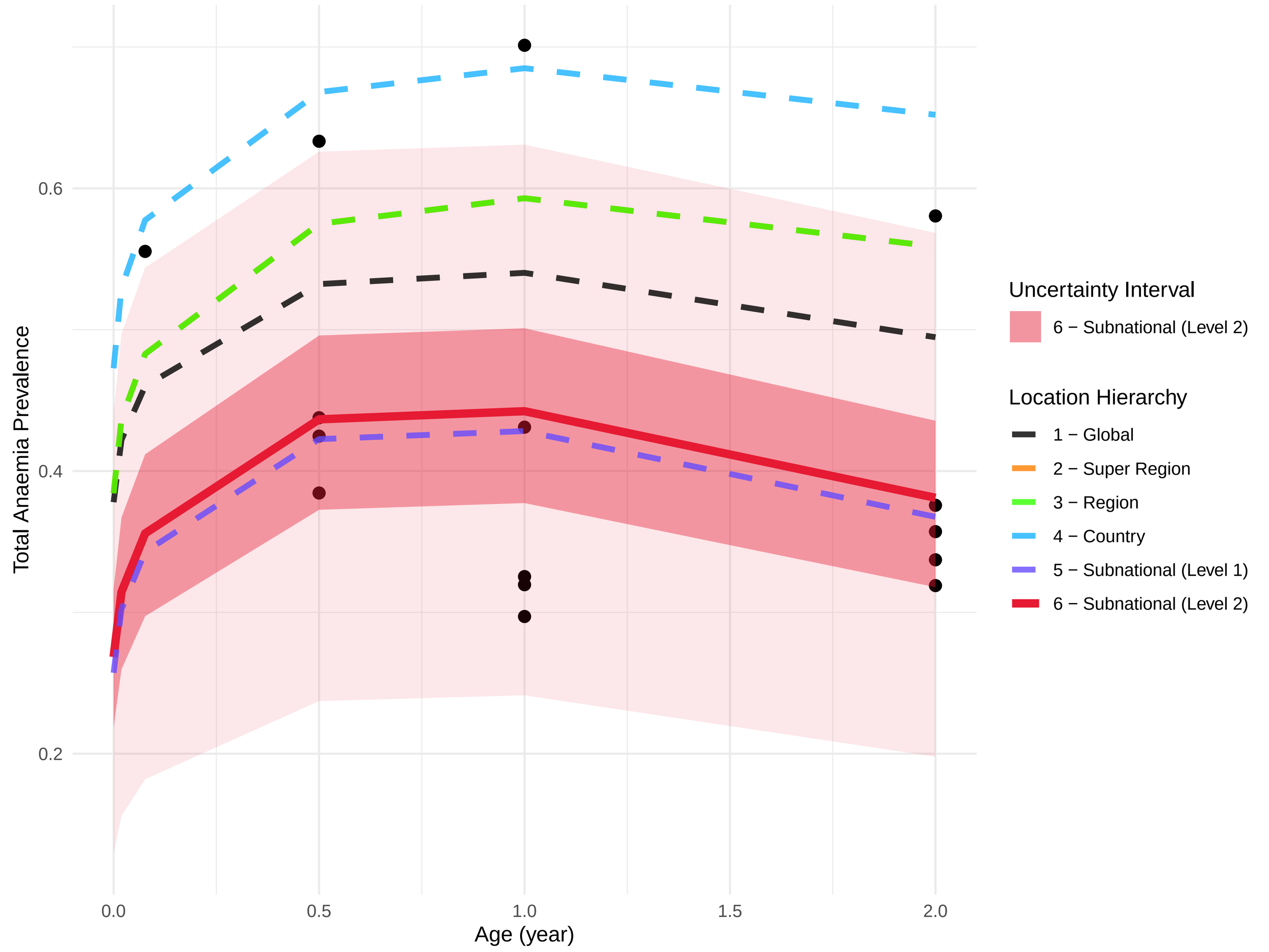

MR−BRT Age/Sex Fits − Non−pregnant Females <2 Years Old − Total Anaemia Prevalence − Kerala, Urban
WHO−2001 Adjustment Method

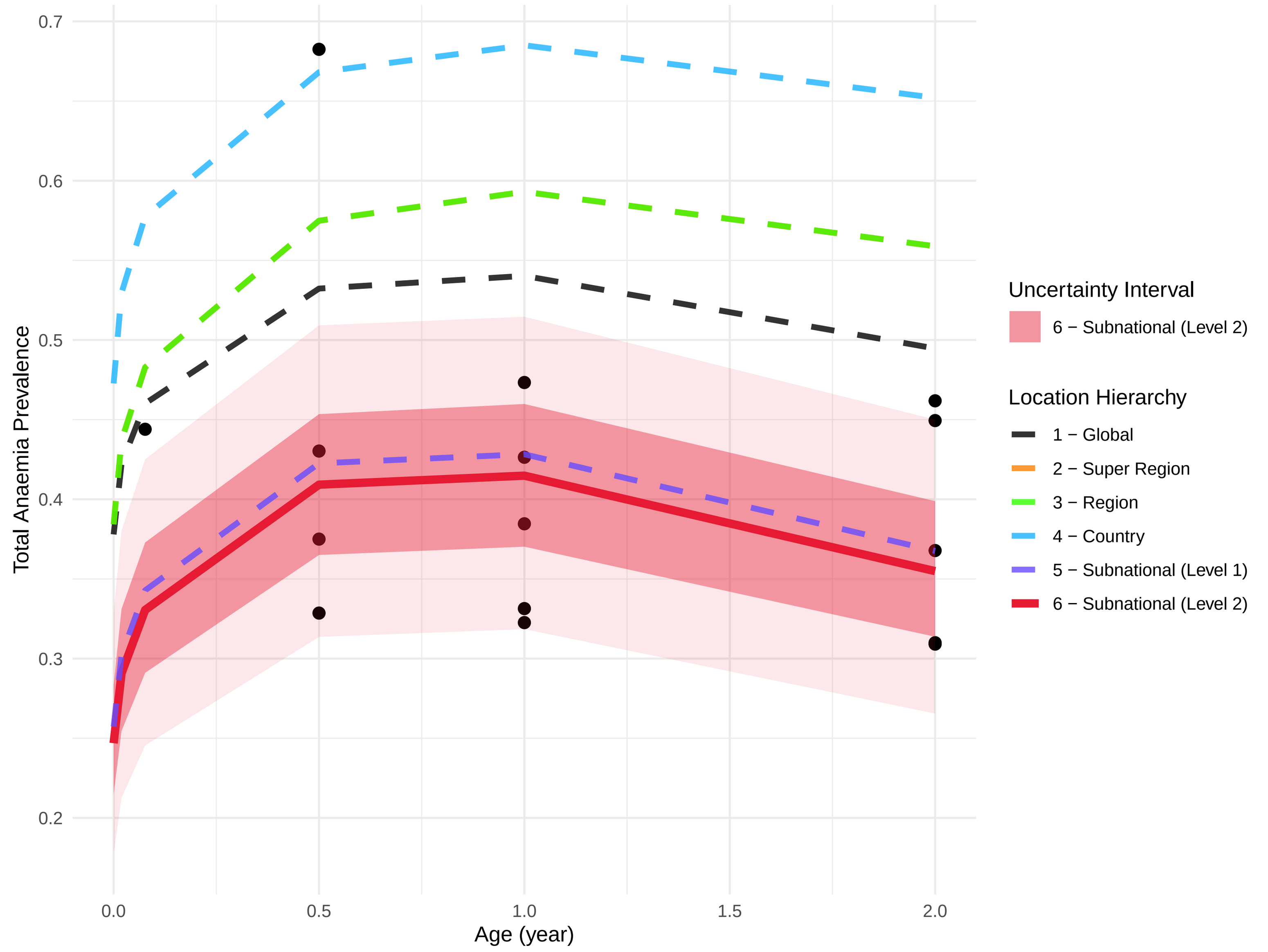

MR−BRT Age/Sex Fits − Non−pregnant Females <2 Years Old − Total Anaemia Prevalence − Madhya Pradesh
WHO−2001 Adjustment Method

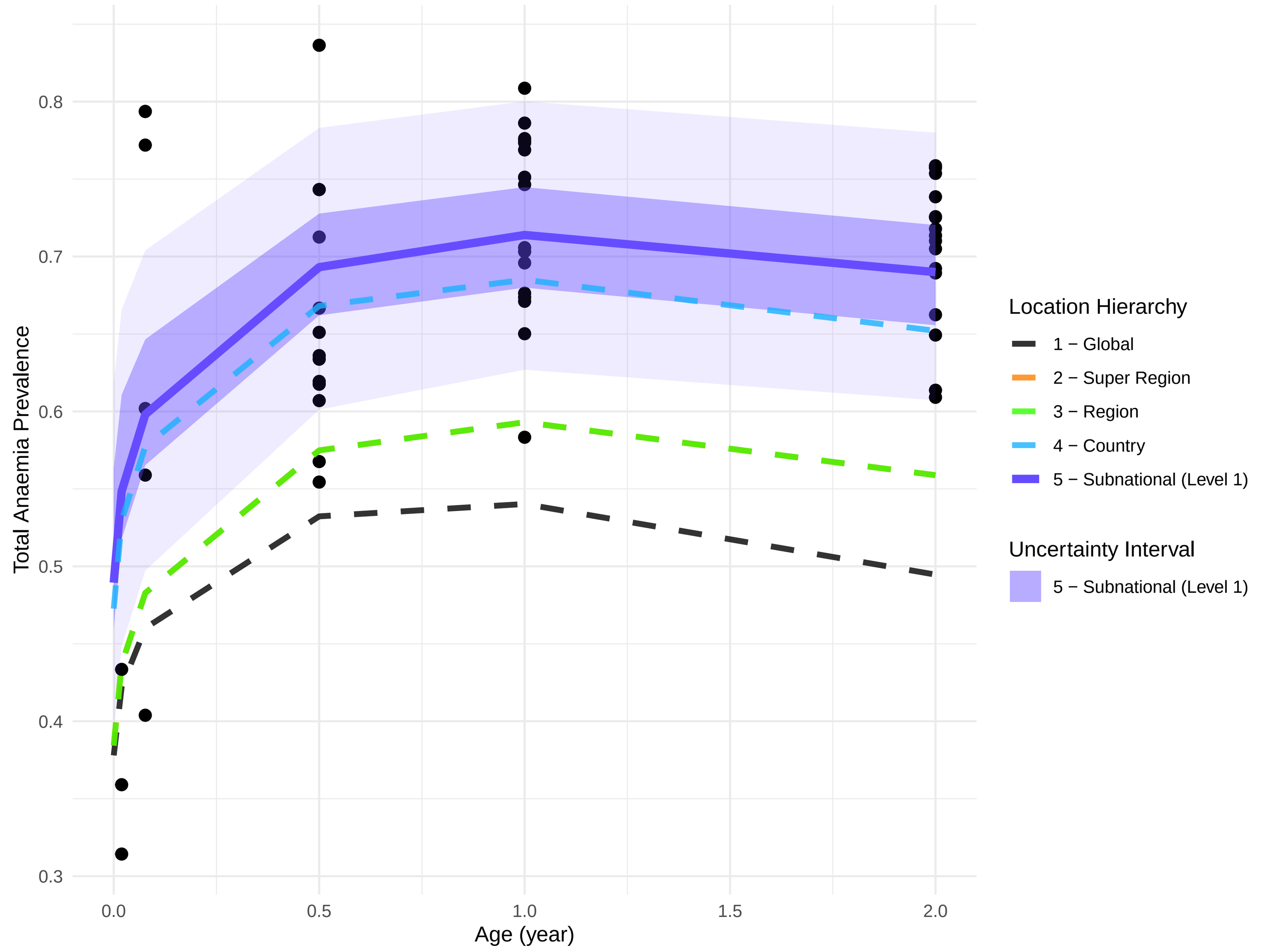

MR−BRT Age/Sex Fits − Non−pregnant Females <2 Years Old − Total Anaemia Prevalence − Madhya Pradesh, Rural
WHO−2001 Adjustment Method

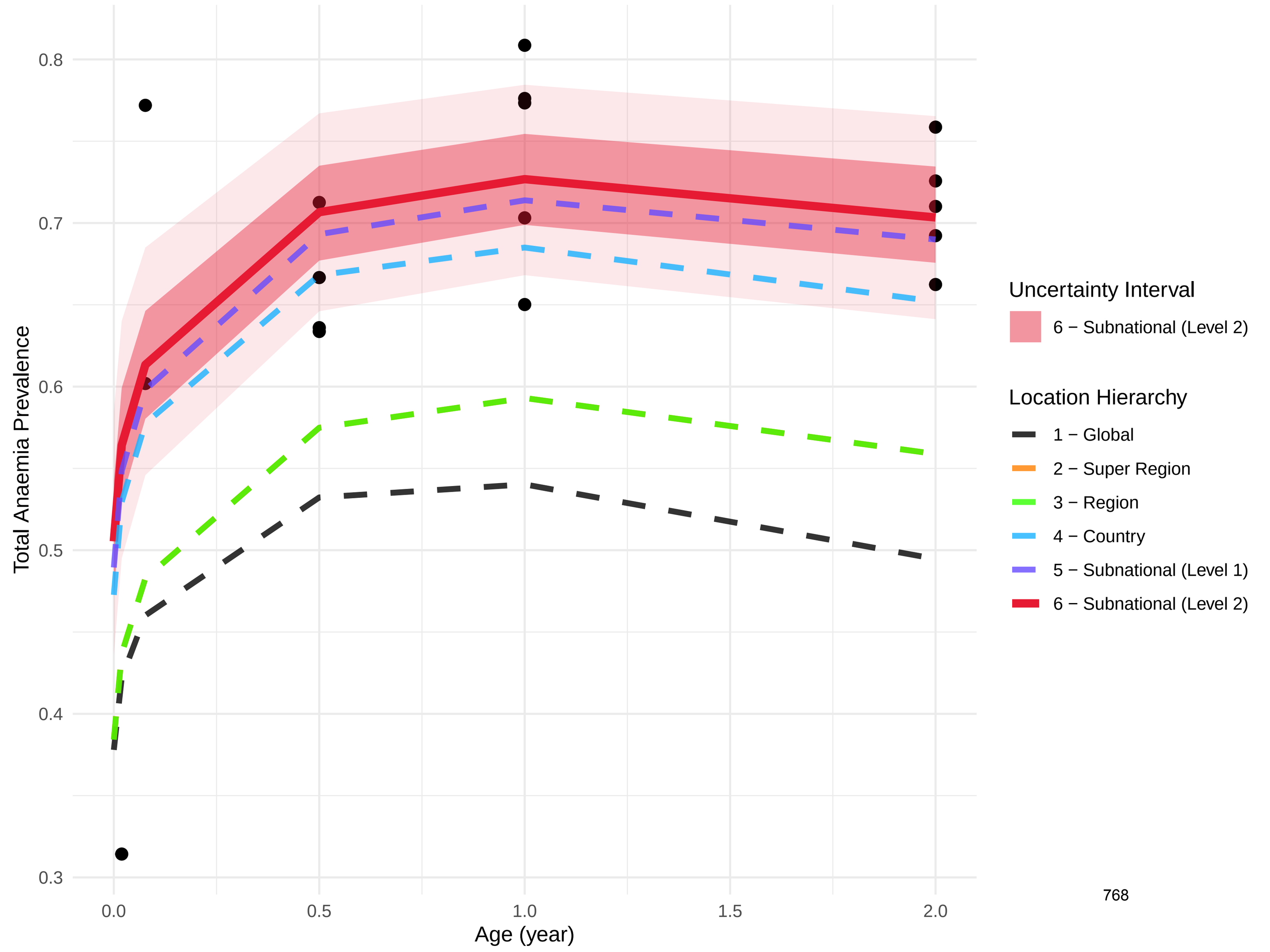

MR−BRT Age/Sex Fits − Non−pregnant Females <2 Years Old − Total Anaemia Prevalence − Madhya Pradesh, Urban
WHO−2001 Adjustment Method

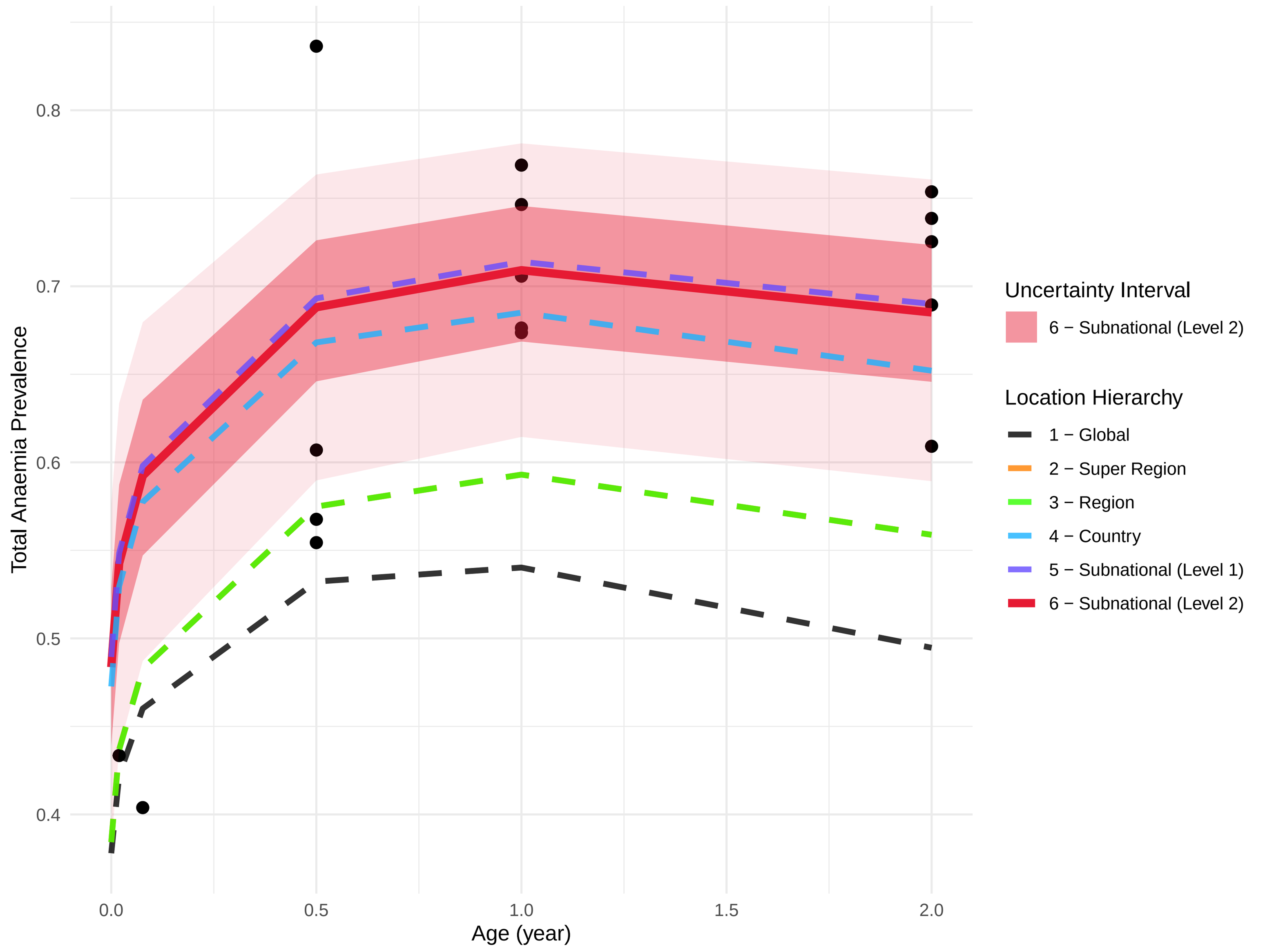

MR−BRT Age/Sex Fits − Non−pregnant Females <2 Years Old − Total Anaemia Prevalence − Maharashtra
WHO−2001 Adjustment Method

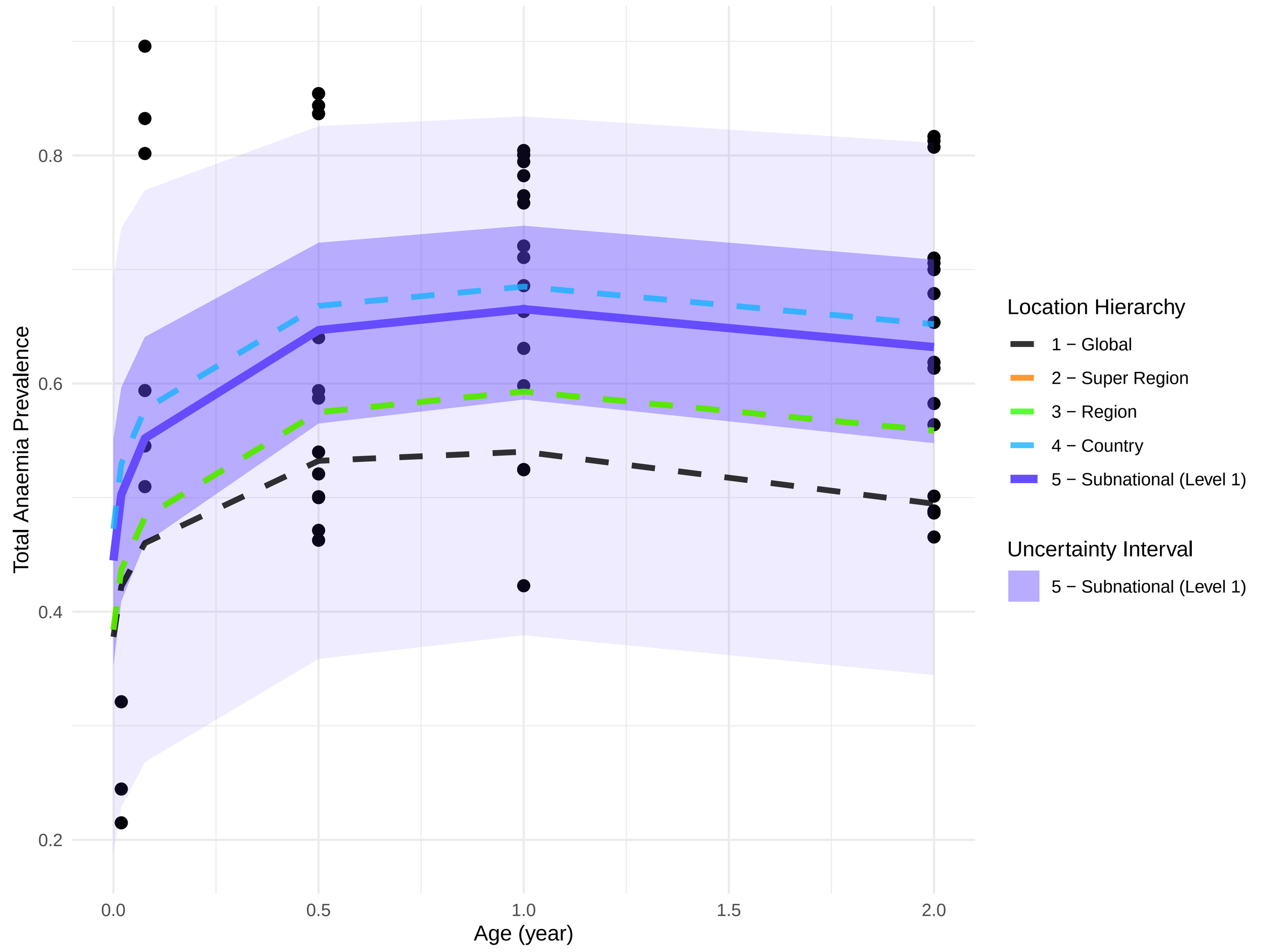

MR−BRT Age/Sex Fits − Non−pregnant Females <2 Years Old − Total Anaemia Prevalence − Maharashtra, Rural
WHO−2001 Adjustment Method

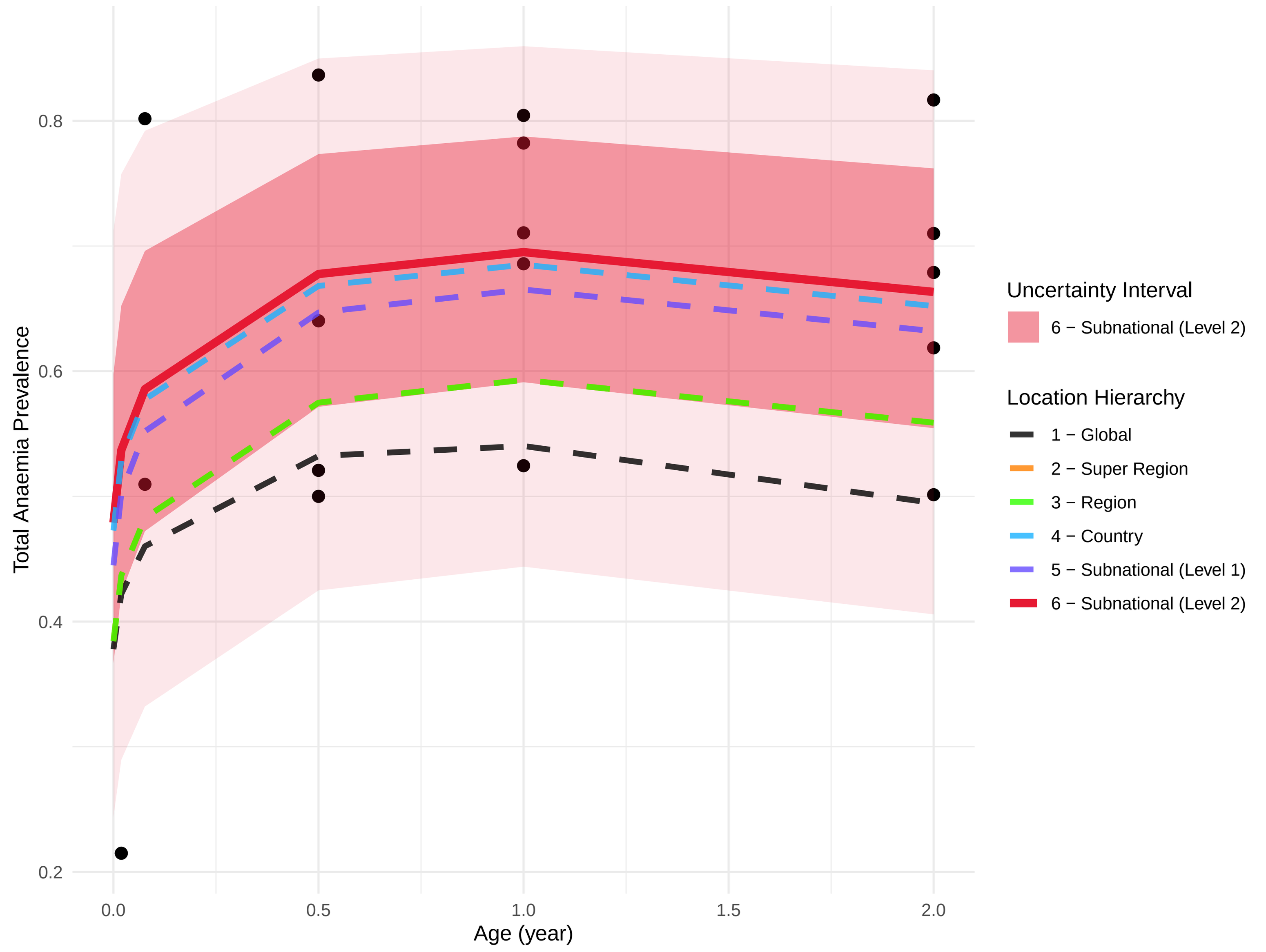

MR−BRT Age/Sex Fits − Non−pregnant Females <2 Years Old − Total Anaemia Prevalence − Maharashtra, Urban
WHO−2001 Adjustment Method

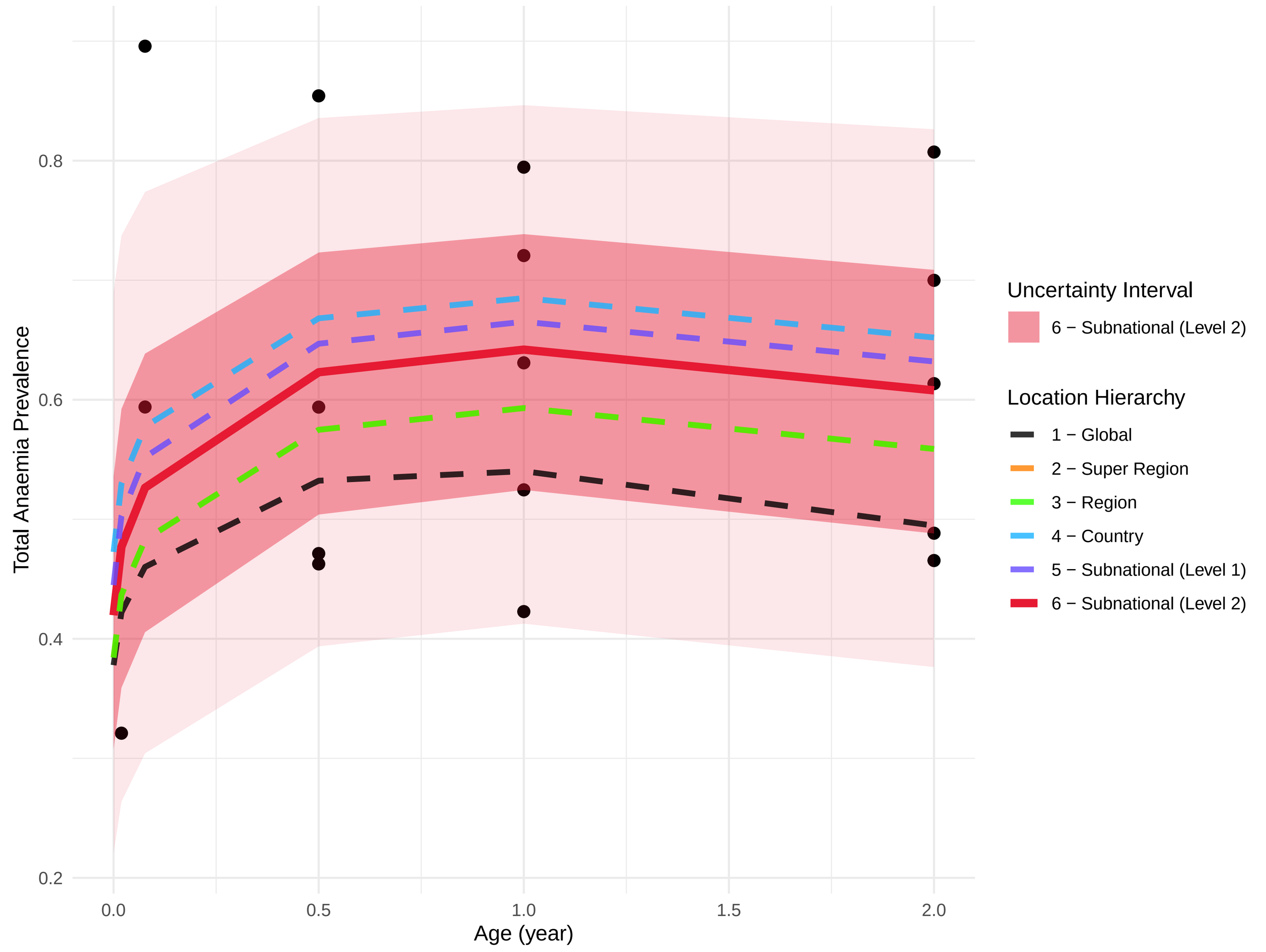

MR−BRT Age/Sex Fits − Non−pregnant Females <2 Years Old − Total Anaemia Prevalence − Manipur
WHO−2001 Adjustment Method

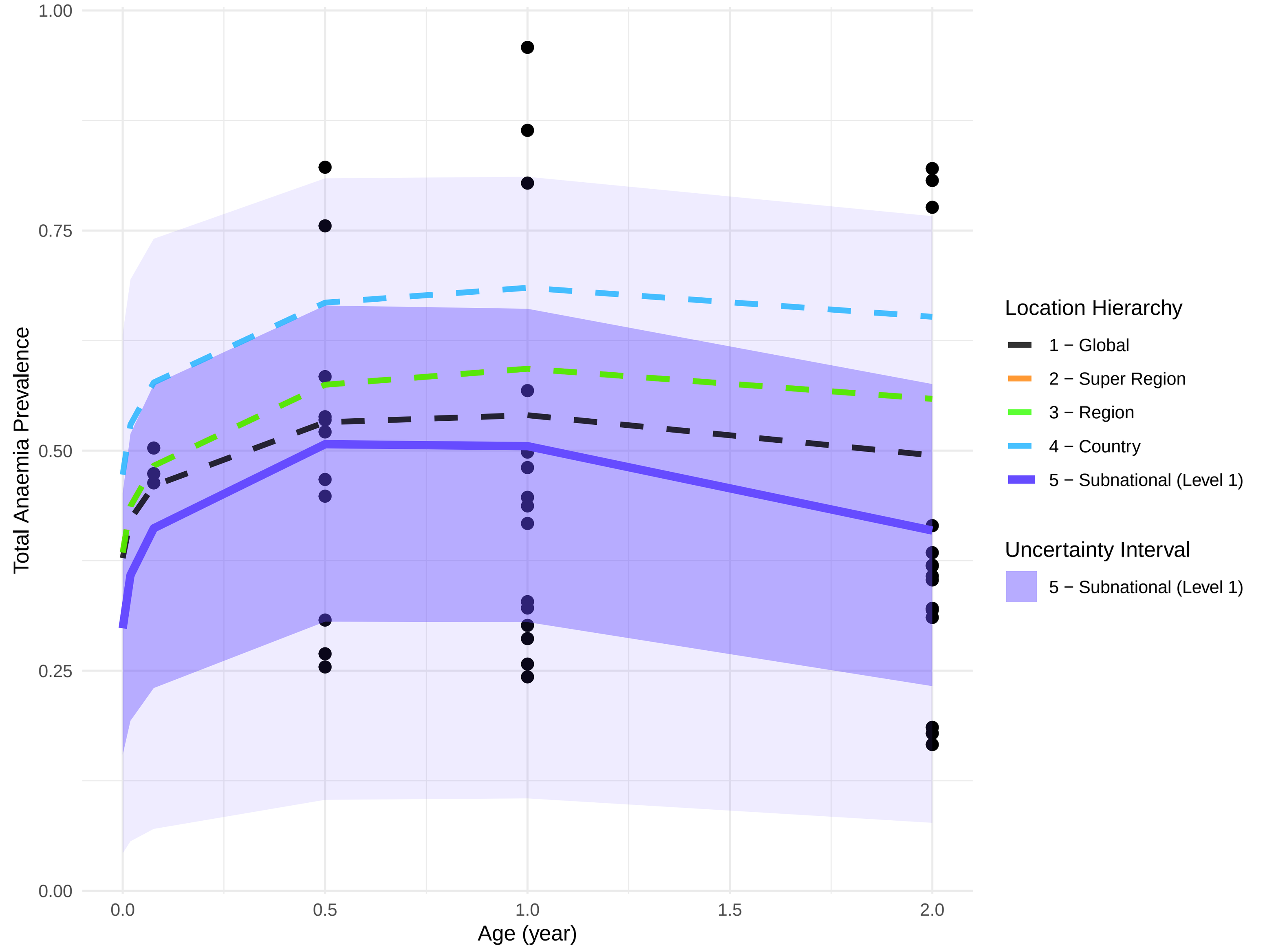

MR−BRT Age/Sex Fits − Non−pregnant Females <2 Years Old − Total Anaemia Prevalence − Manipur, Rural
WHO−2001 Adjustment Method

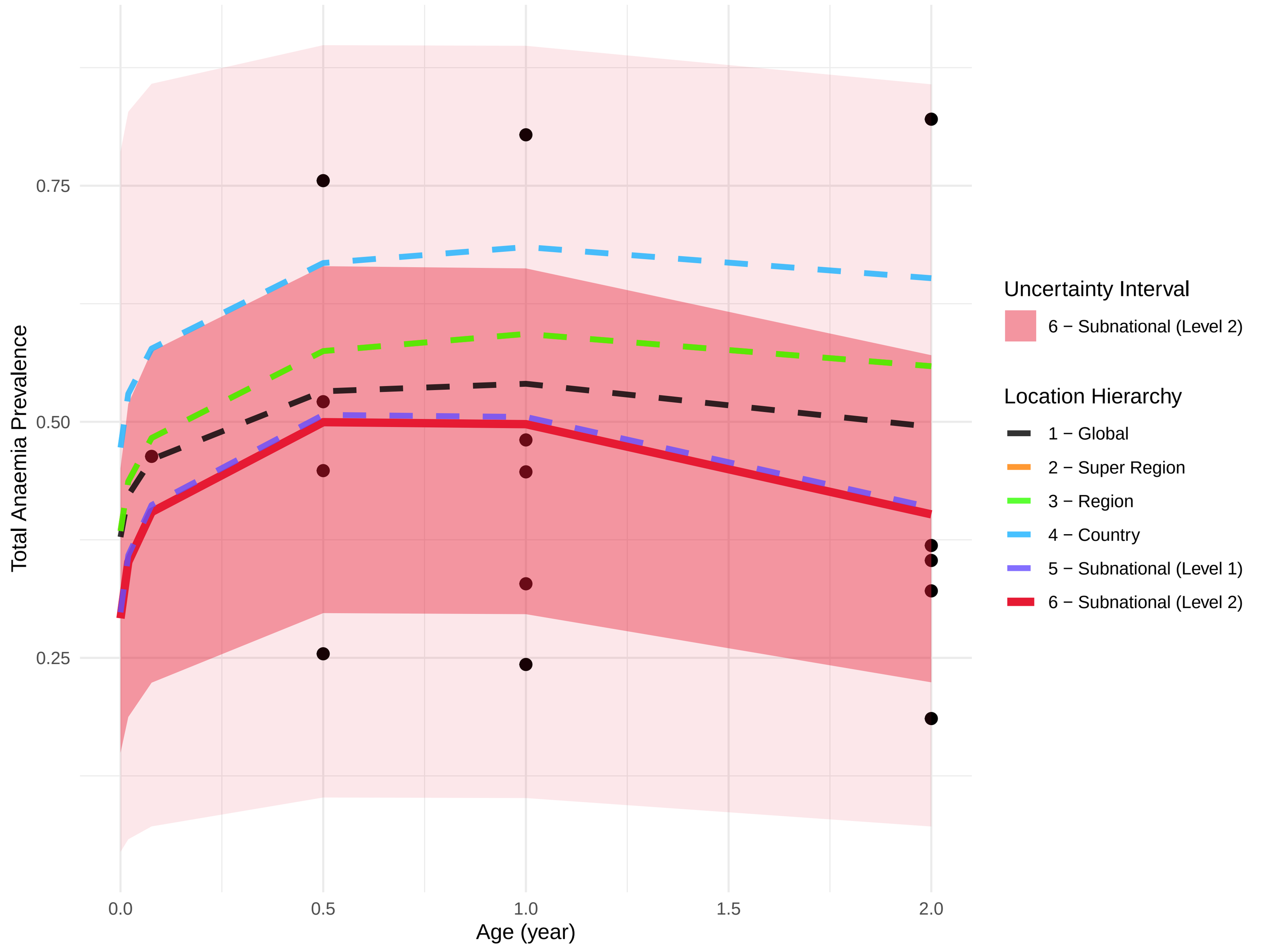

MR−BRT Age/Sex Fits − Non−pregnant Females <2 Years Old − Total Anaemia Prevalence − Manipur, Urban
WHO−2001 Adjustment Method

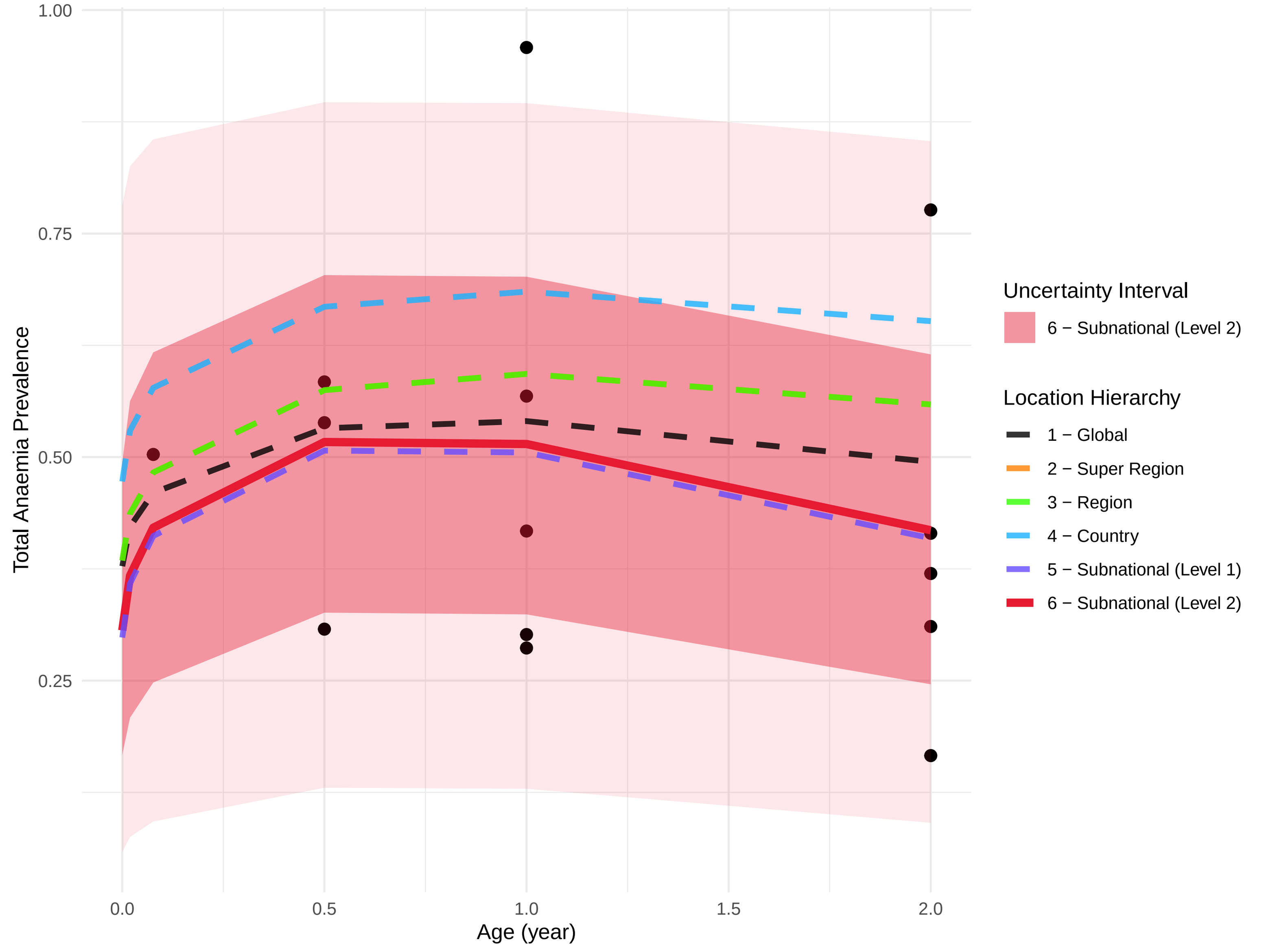

MR−BRT Age/Sex Fits − Non−pregnant Females <2 Years Old − Total Anaemia Prevalence − Meghalaya
WHO−2001 Adjustment Method

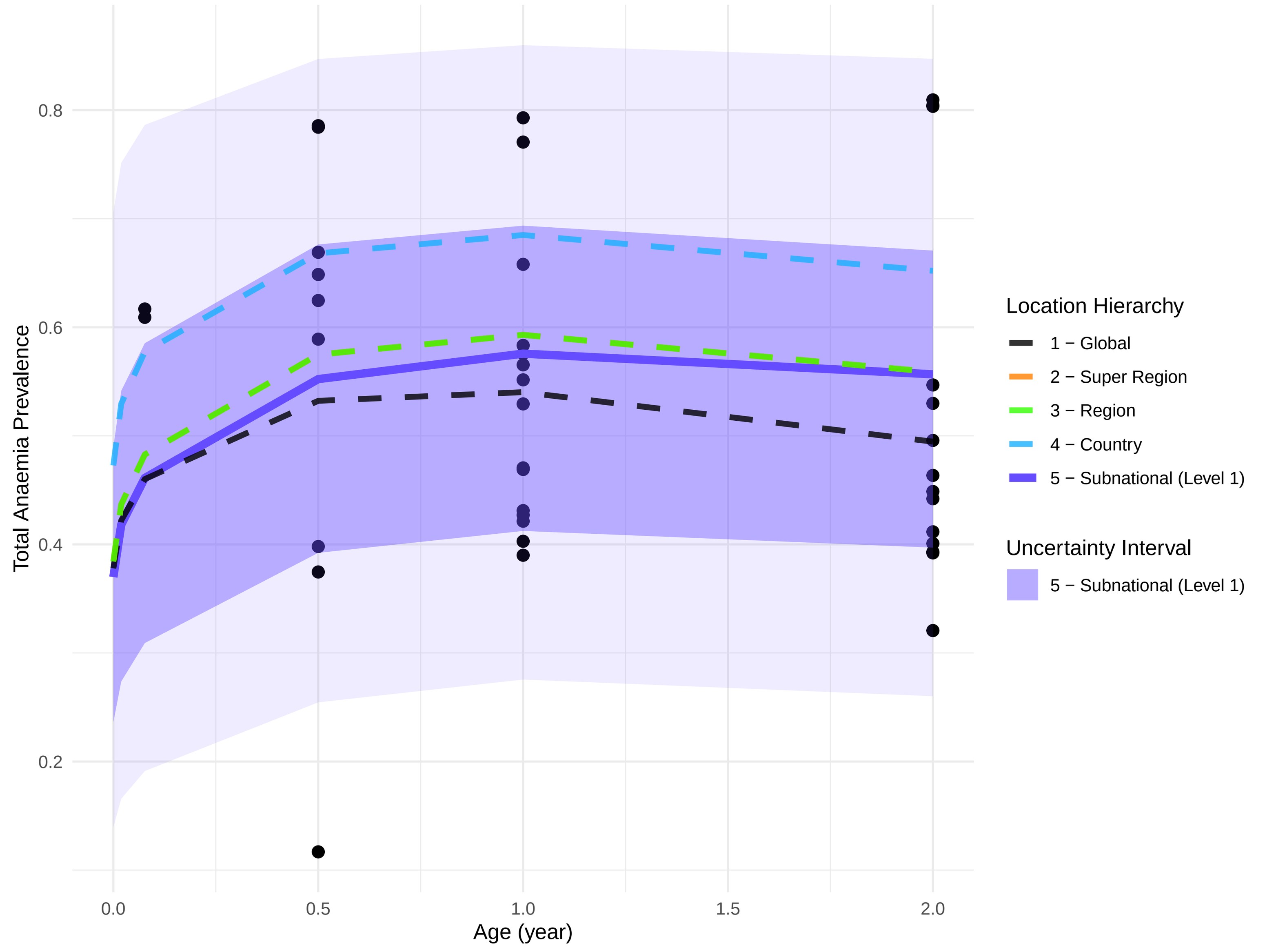

MR−BRT Age/Sex Fits − Non−pregnant Females <2 Years Old − Total Anaemia Prevalence − Meghalaya, Rural
WHO−2001 Adjustment Method

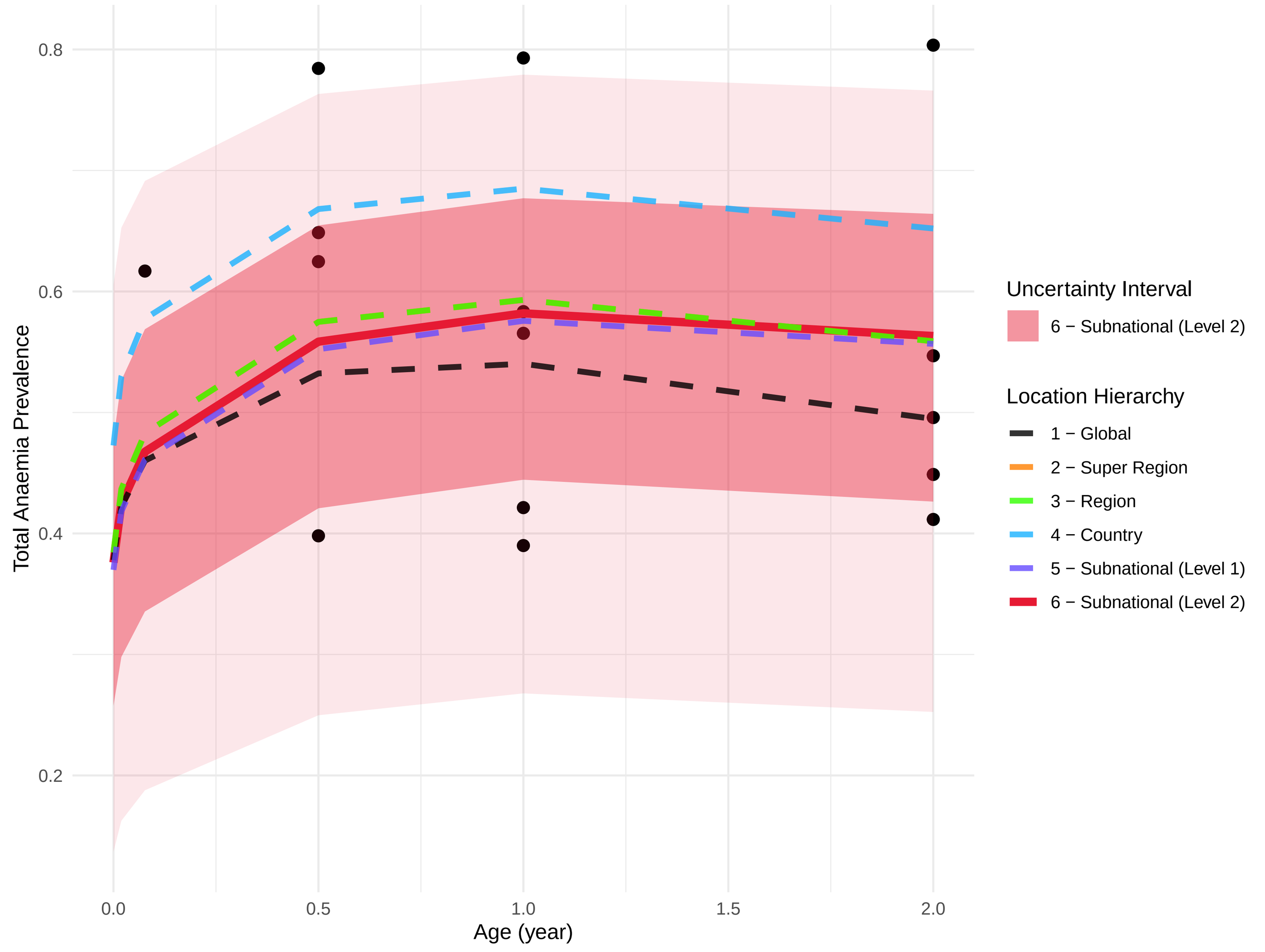

MR−BRT Age/Sex Fits − Non−pregnant Females <2 Years Old − Total Anaemia Prevalence − Meghalaya, Urban
WHO−2001 Adjustment Method

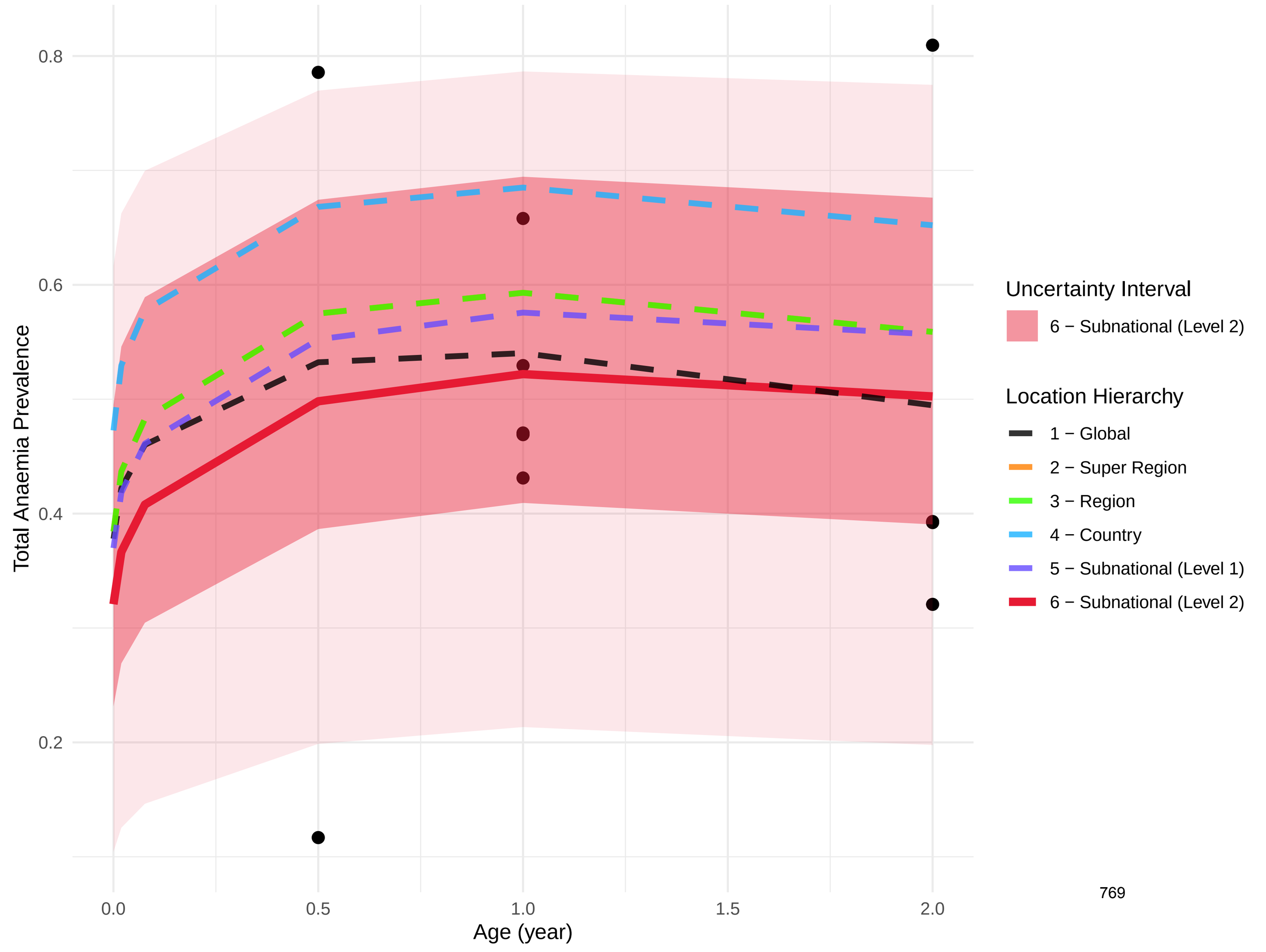

MR−BRT Age/Sex Fits − Non−pregnant Females <2 Years Old − Total Anaemia Prevalence − Mizoram
WHO−2001 Adjustment Method

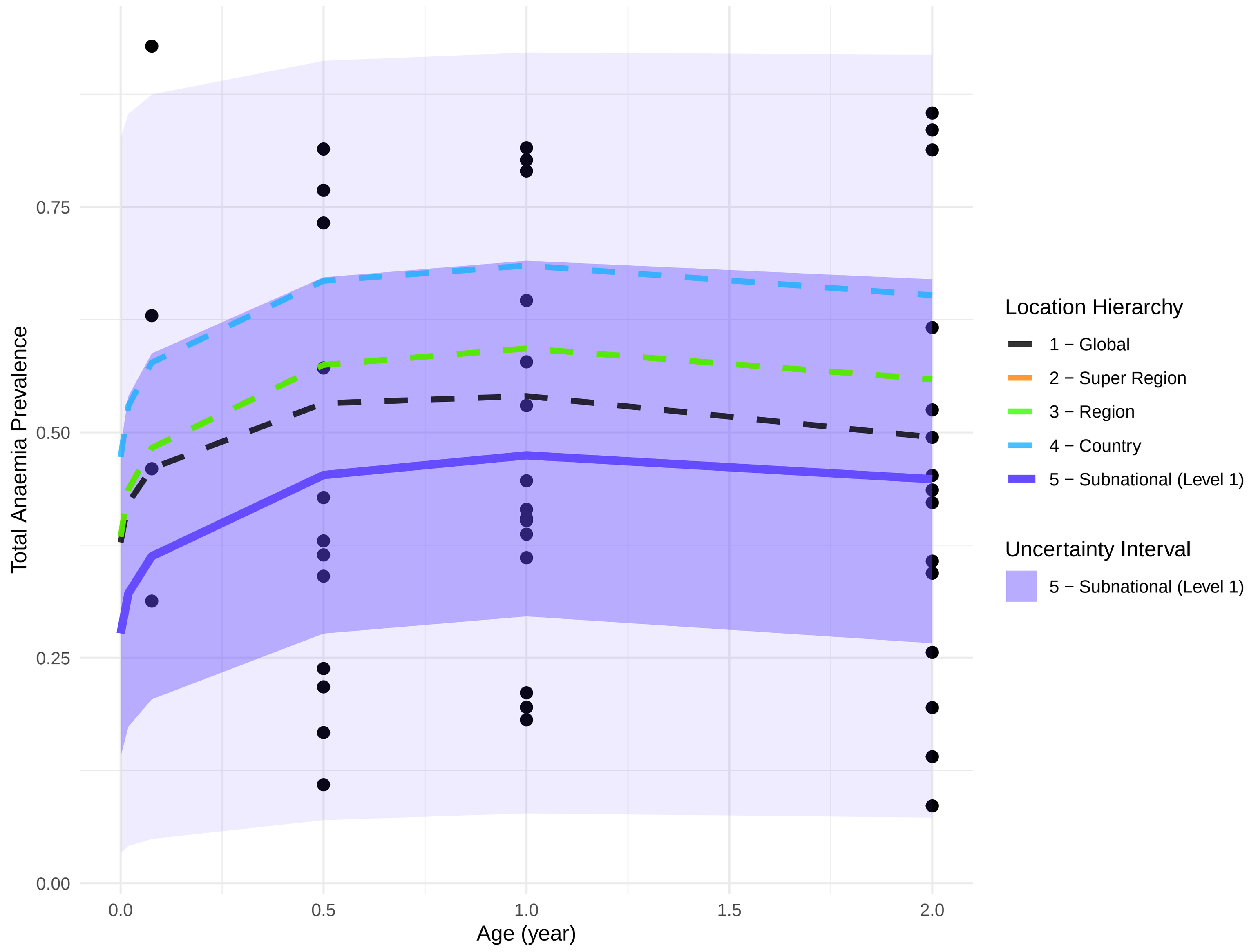

MR−BRT Age/Sex Fits − Non−pregnant Females <2 Years Old − Total Anaemia Prevalence − Mizoram, Rural
WHO−2001 Adjustment Method

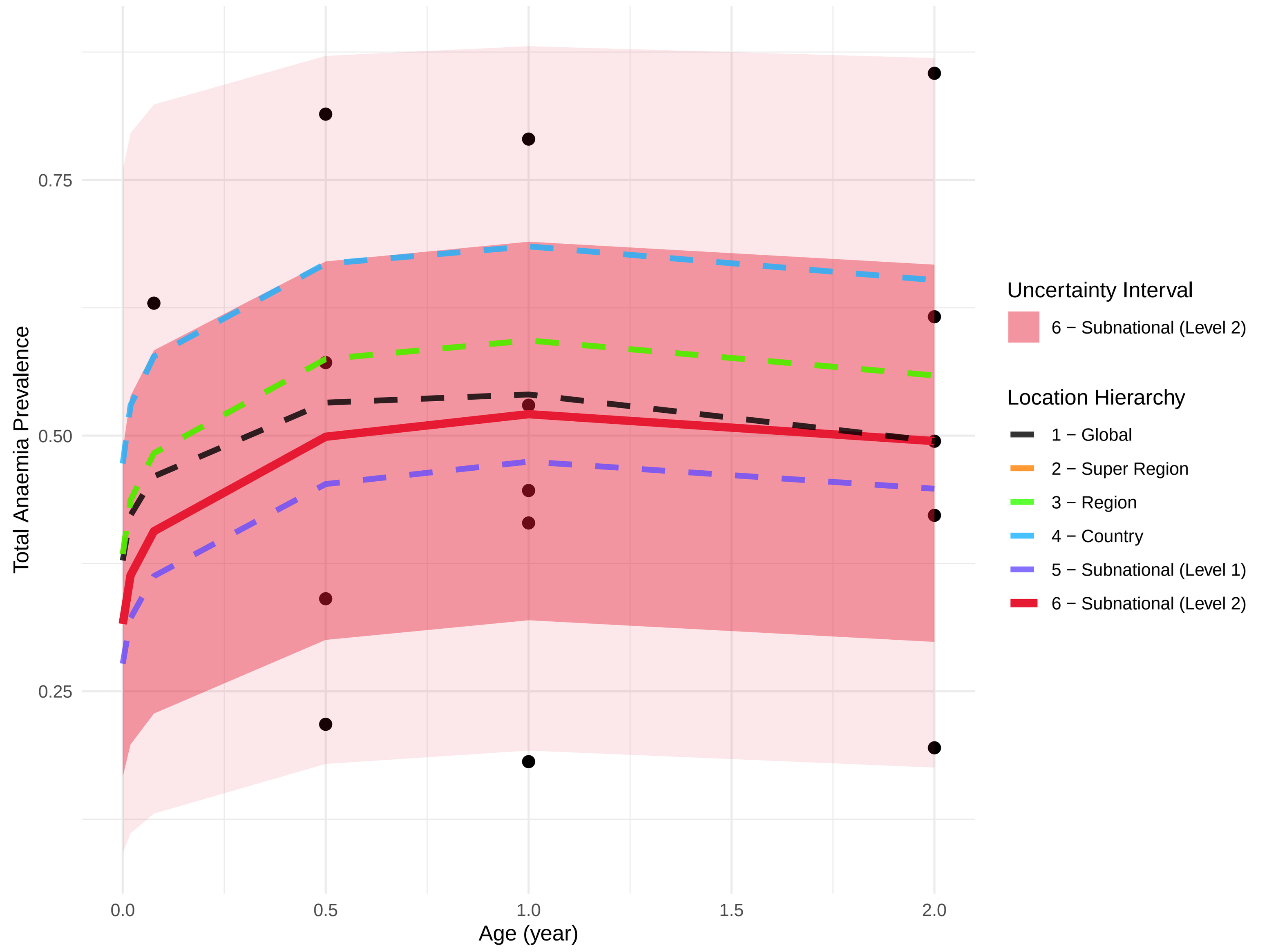

MR−BRT Age/Sex Fits − Non−pregnant Females <2 Years Old − Total Anaemia Prevalence − Mizoram, Urban
WHO−2001 Adjustment Method

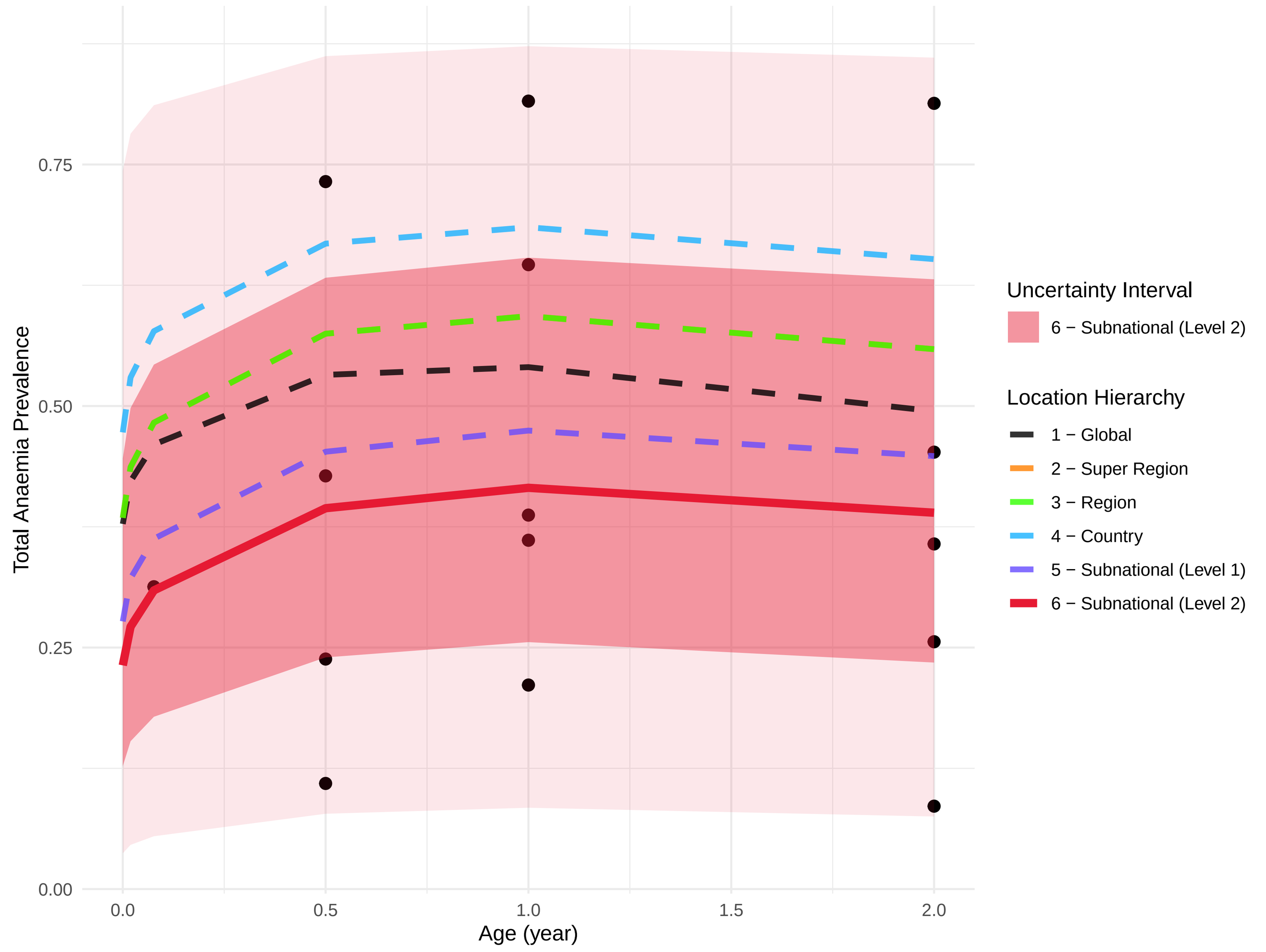

MR−BRT Age/Sex Fits − Non−pregnant Females <2 Years Old − Total Anaemia Prevalence − Nagaland
WHO−2001 Adjustment Method

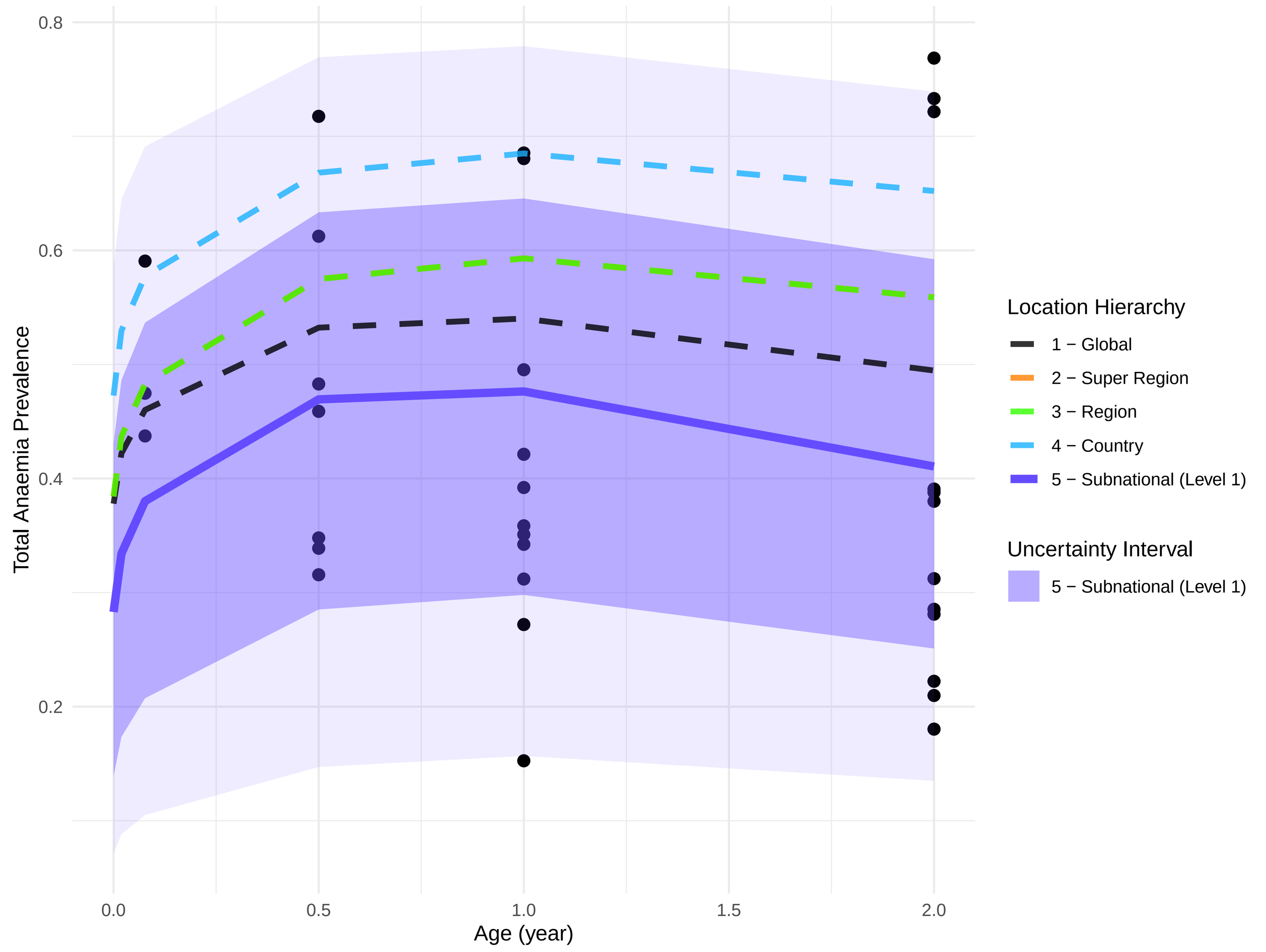

MR−BRT Age/Sex Fits − Non−pregnant Females <2 Years Old − Total Anaemia Prevalence − Nagaland, Rural
WHO−2001 Adjustment Method

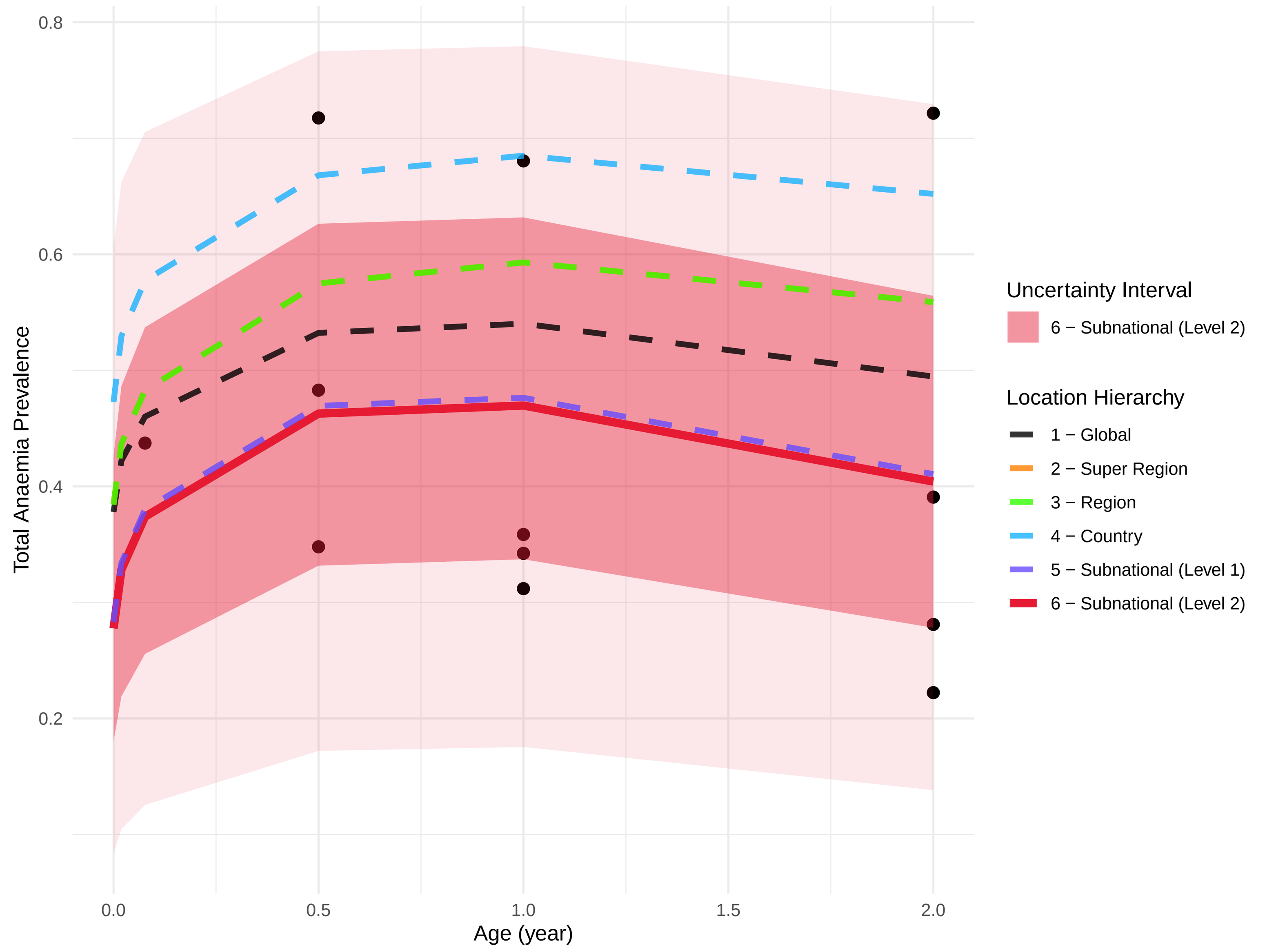

MR−BRT Age/Sex Fits − Non−pregnant Females <2 Years Old − Total Anaemia Prevalence − Nagaland, Urban
WHO−2001 Adjustment Method

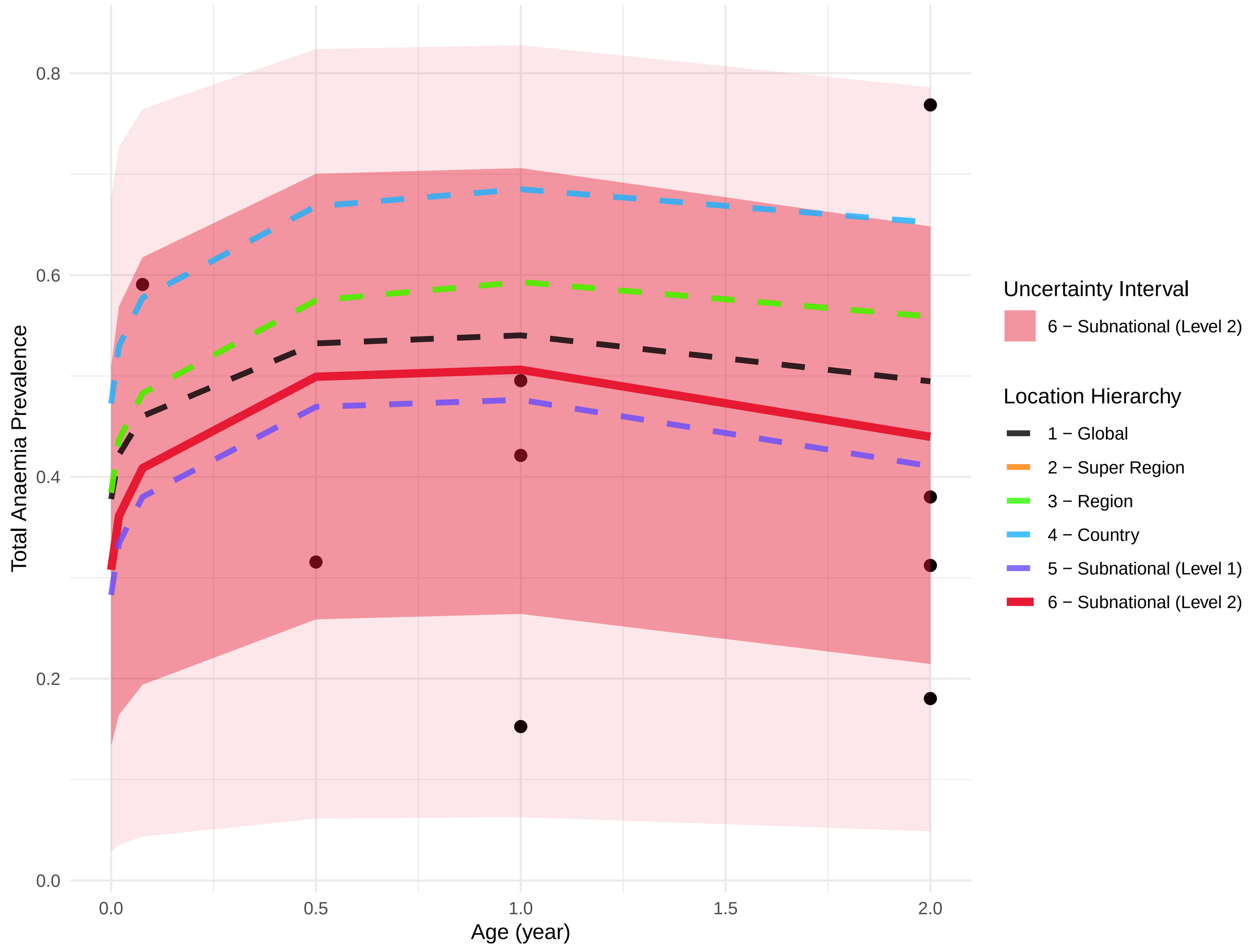

MR−BRT Age/Sex Fits − Non−pregnant Females <2 Years Old − Total Anaemia Prevalence − Odisha
WHO−2001 Adjustment Method

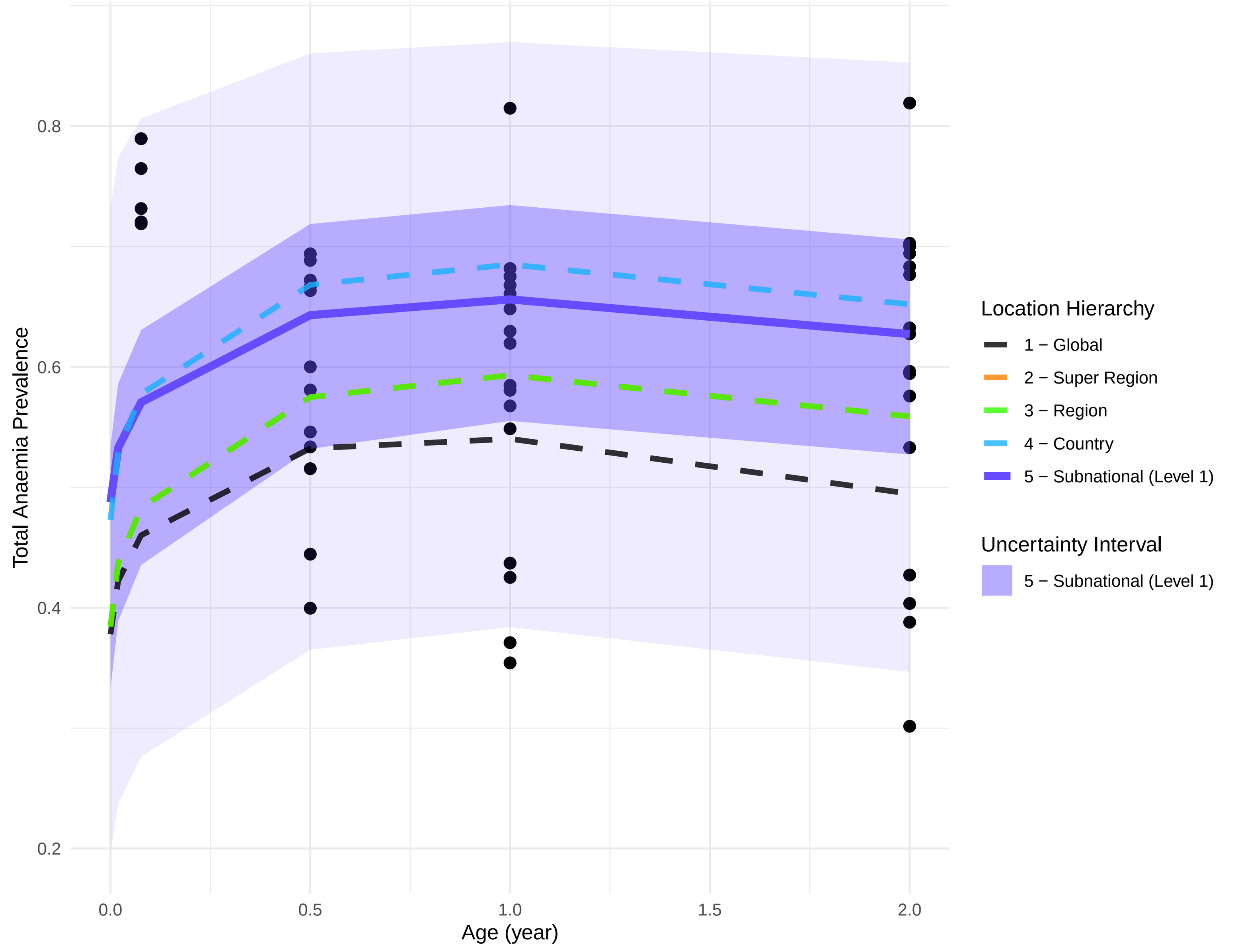

MR−BRT Age/Sex Fits − Non−pregnant Females <2 Years Old − Total Anaemia Prevalence − Odisha, Rural
WHO−2001 Adjustment Method

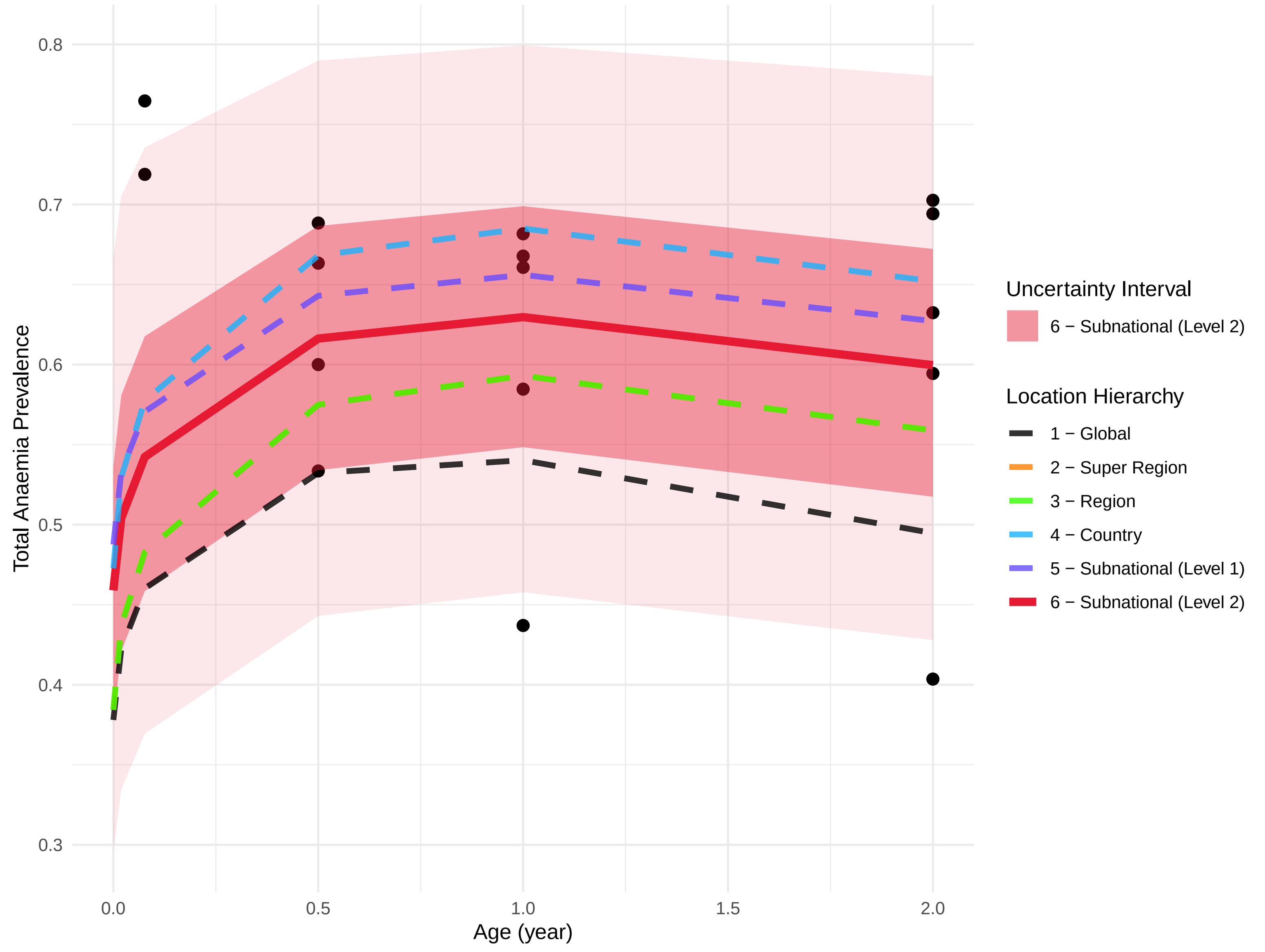

MR−BRT Age/Sex Fits − Non−pregnant Females <2 Years Old − Total Anaemia Prevalence − Odisha, Urban
WHO−2001 Adjustment Method

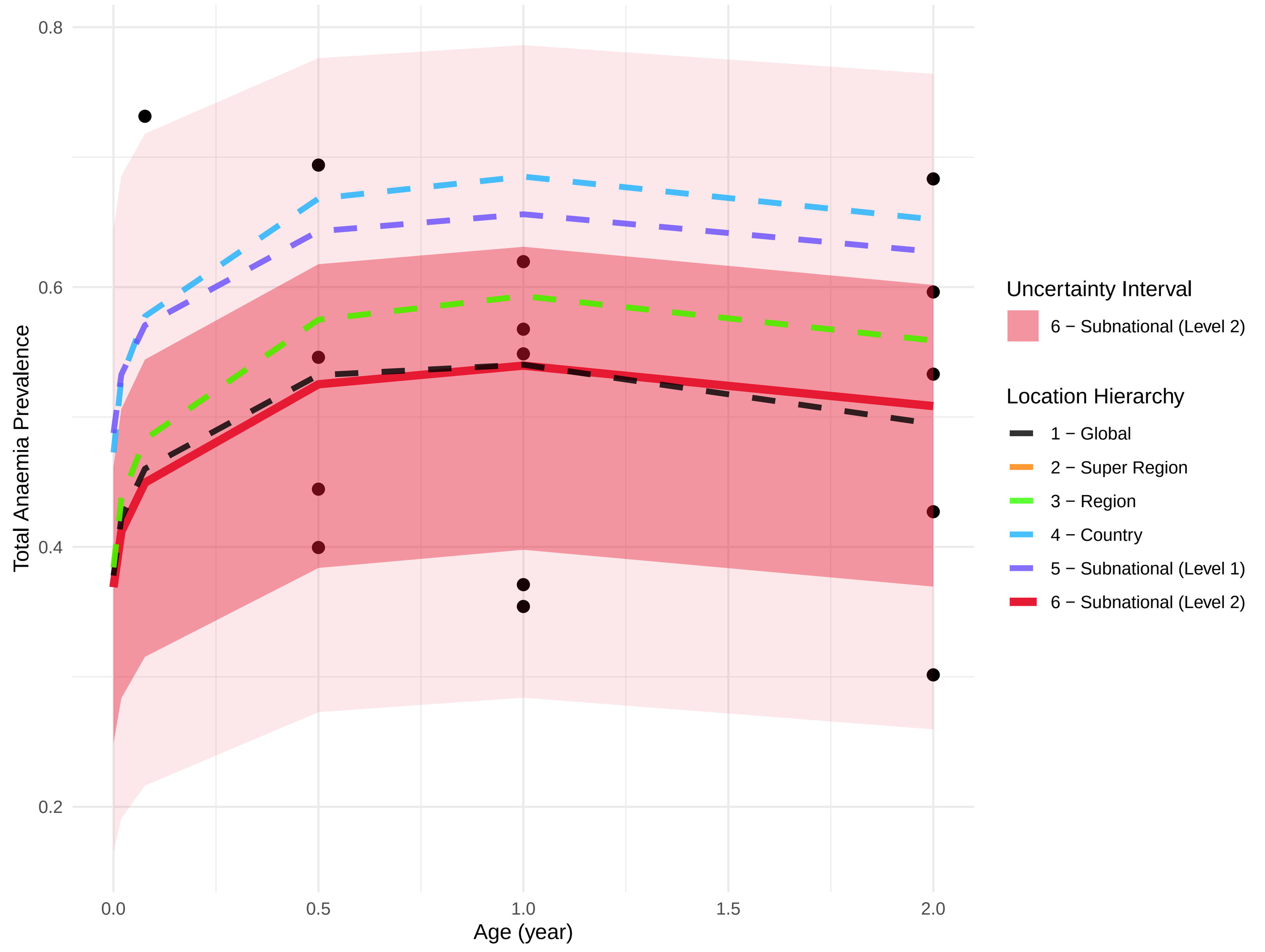

MR−BRT Age/Sex Fits − Non−pregnant Females <2 Years Old − Total Anaemia Prevalence − Other Union Territories
WHO−2001 Adjustment Method

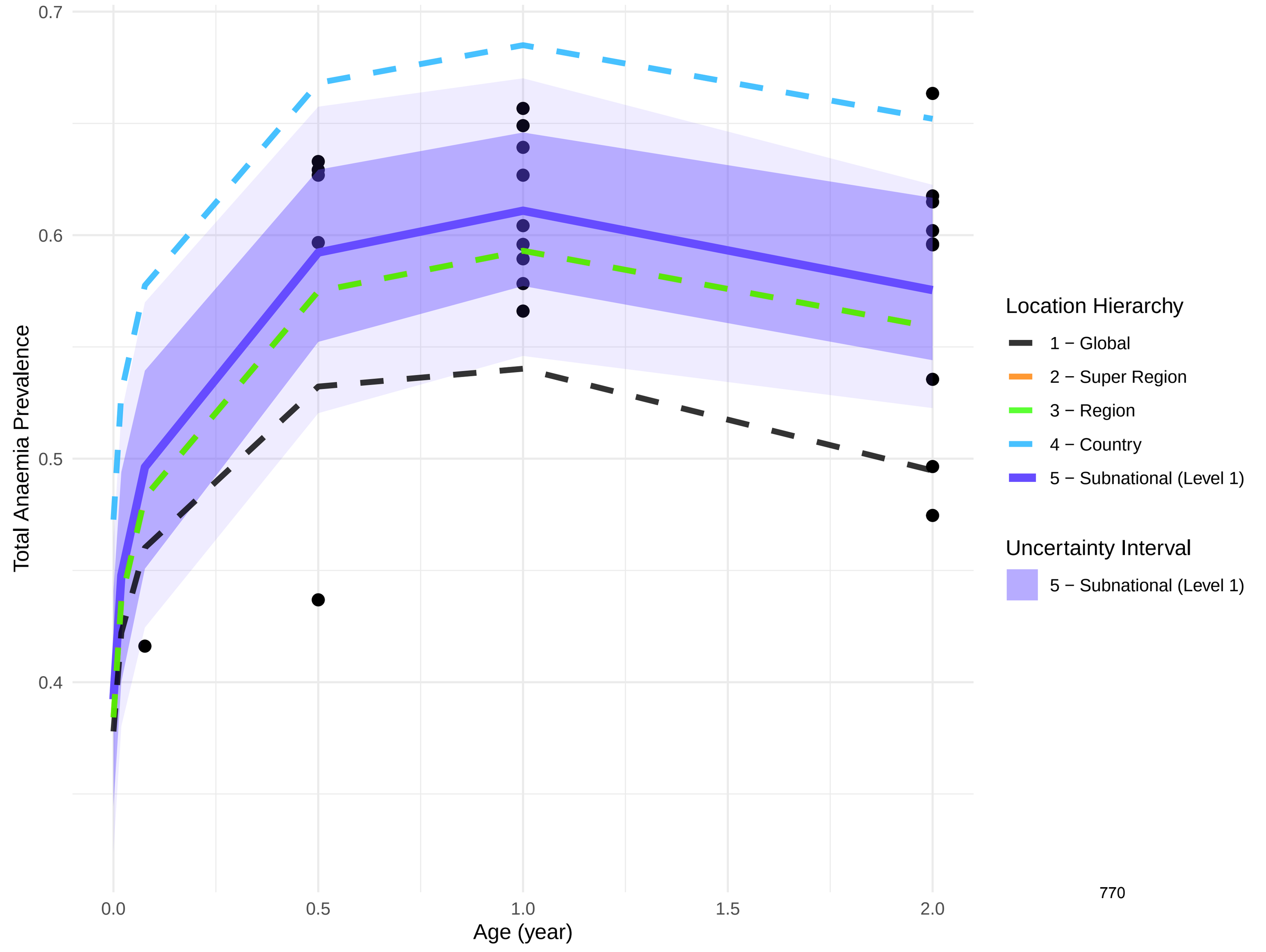

MR−BRT Age/Sex Fits − Non−pregnant Females <2 Years Old − Total Anaemia Prevalence − Other Union Territories, Rural
WHO−2001 Adjustment Method

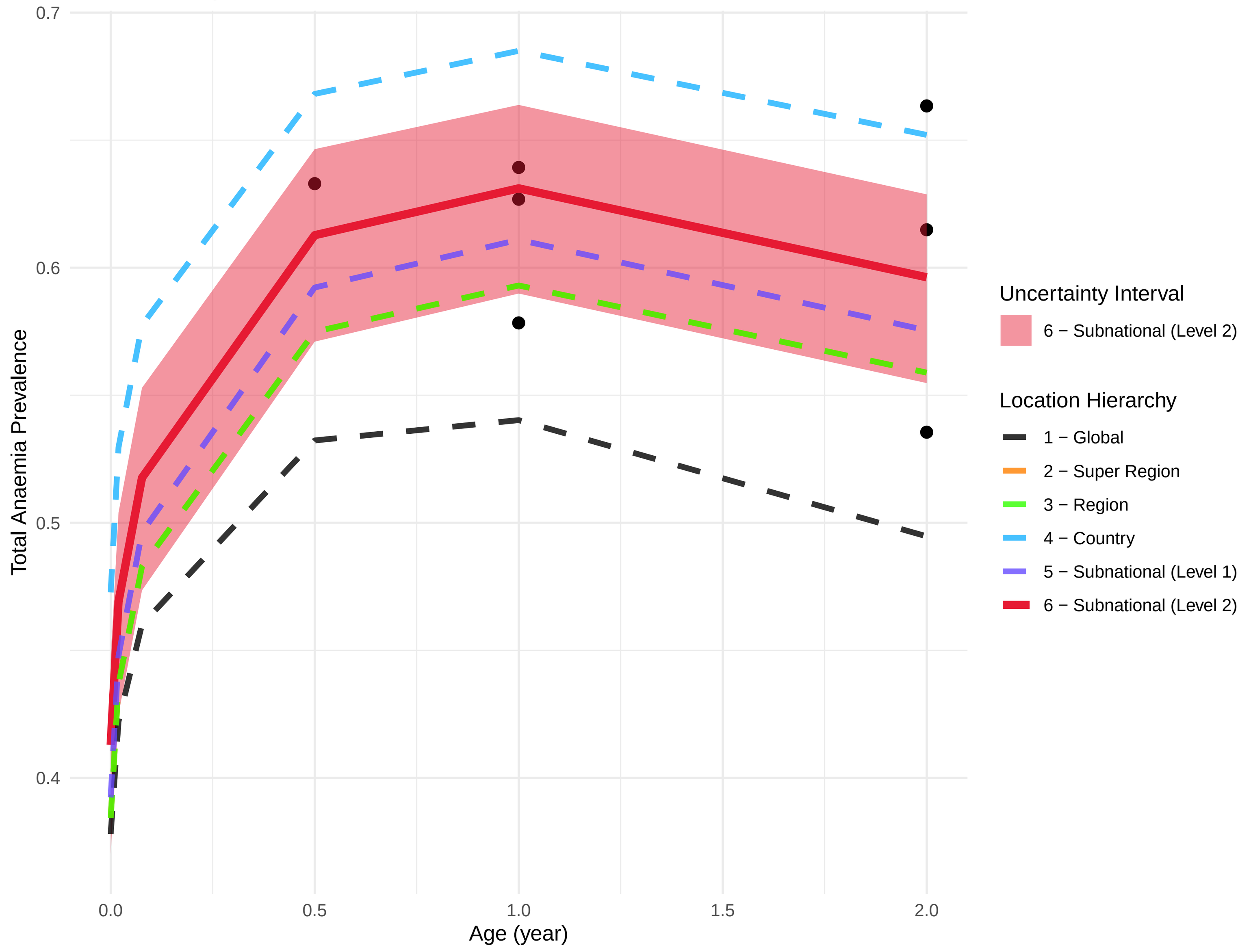

MR−BRT Age/Sex Fits − Non−pregnant Females <2 Years Old − Total Anaemia Prevalence − Other Union Territories, Urban
WHO−2001 Adjustment Method

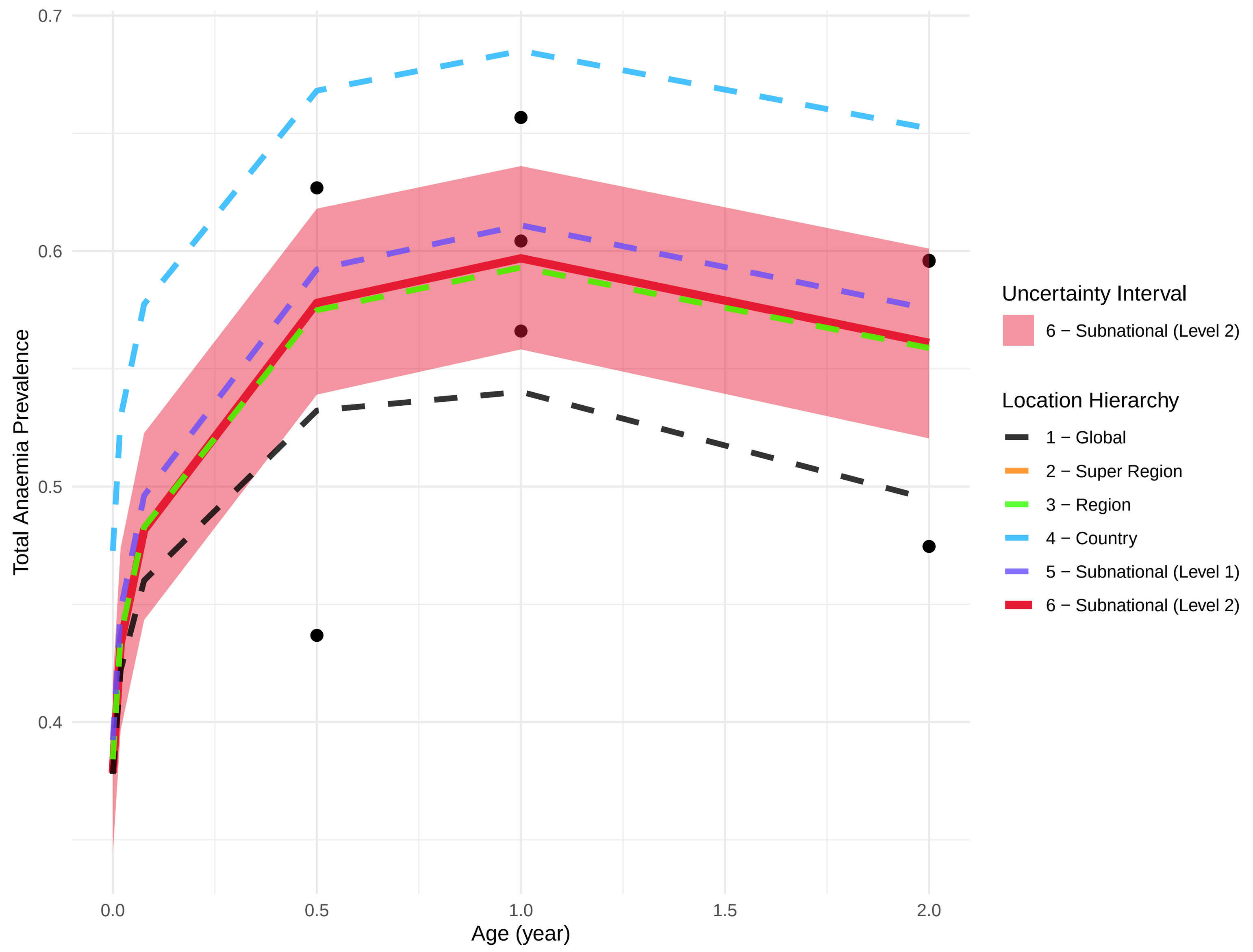

MR−BRT Age/Sex Fits − Non−pregnant Females <2 Years Old − Total Anaemia Prevalence − Punjab
WHO−2001 Adjustment Method

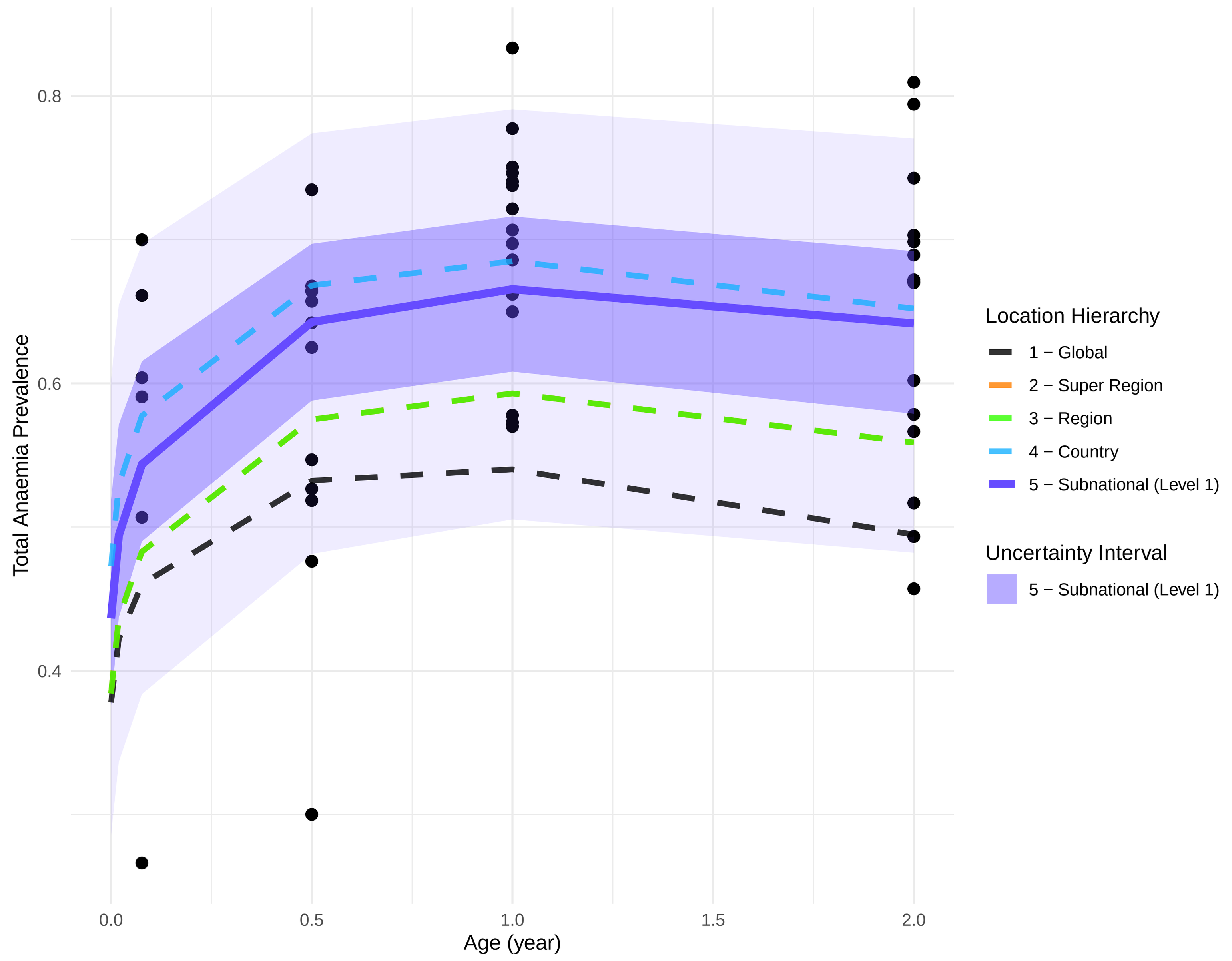

MR−BRT Age/Sex Fits − Non−pregnant Females <2 Years Old − Total Anaemia Prevalence − Punjab, Rural
WHO−2001 Adjustment Method

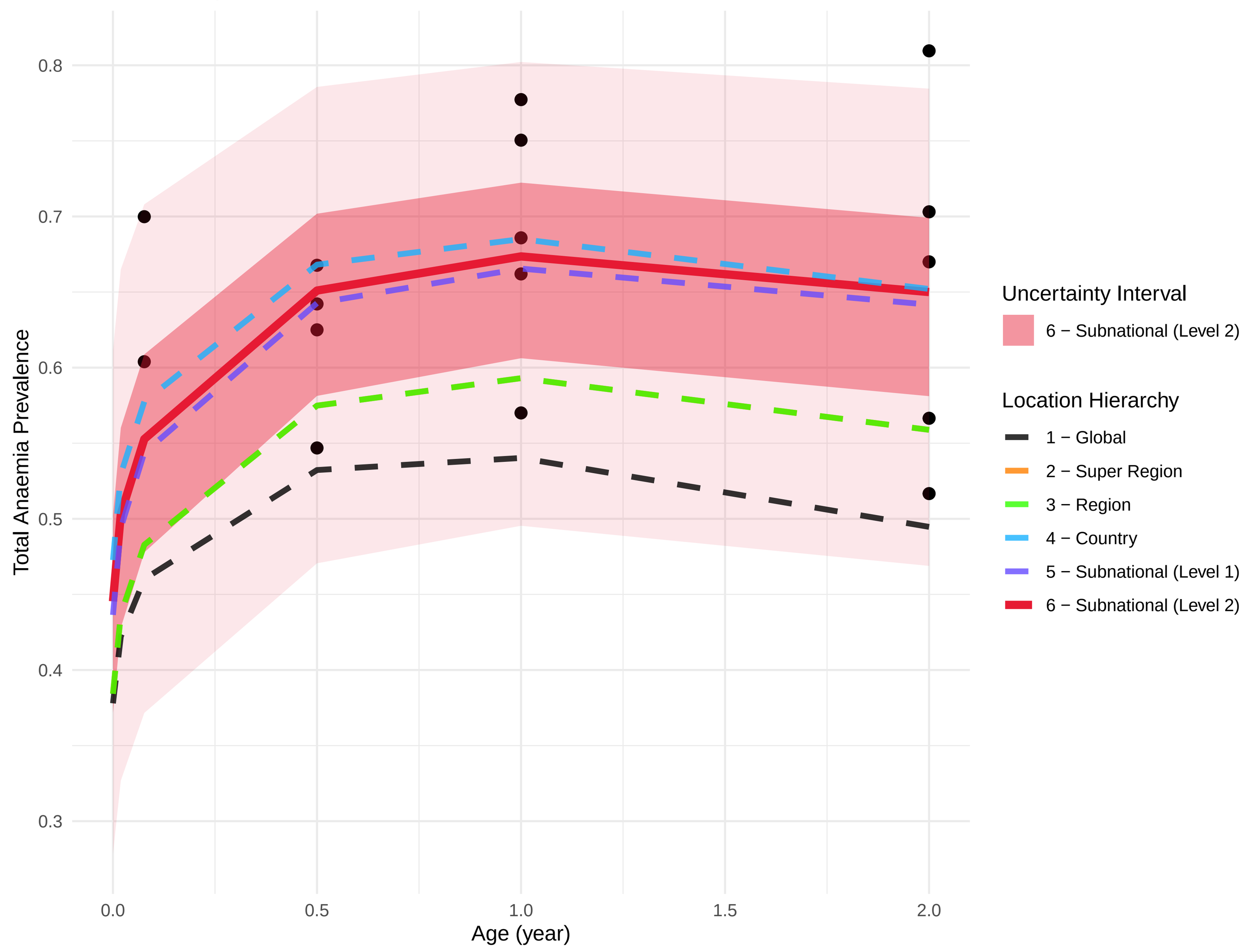

MR−BRT Age/Sex Fits − Non−pregnant Females <2 Years Old − Total Anaemia Prevalence − Punjab, Urban
WHO−2001 Adjustment Method

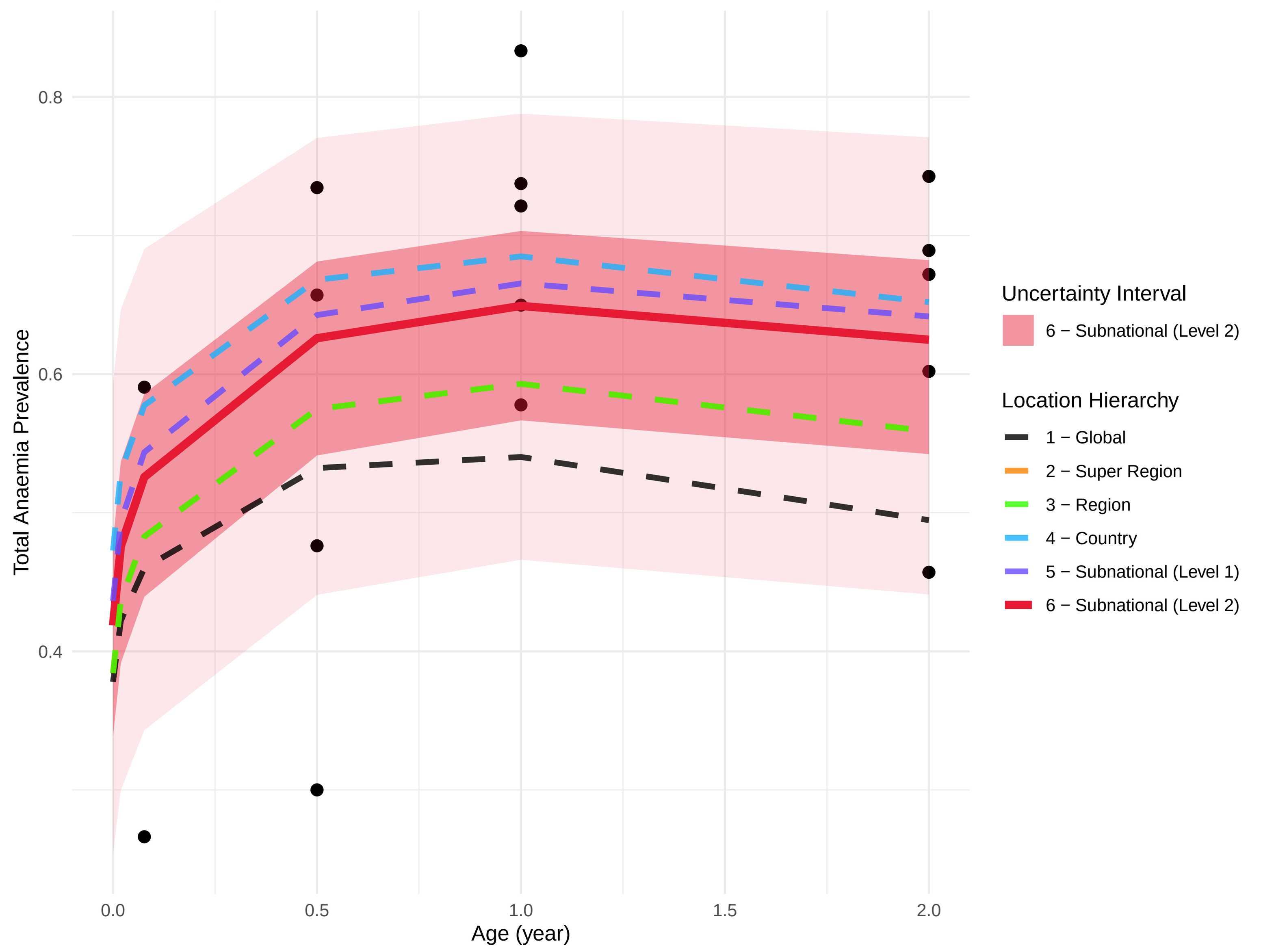

MR−BRT Age/Sex Fits − Non−pregnant Females <2 Years Old − Total Anaemia Prevalence − Rajasthan
WHO−2001 Adjustment Method

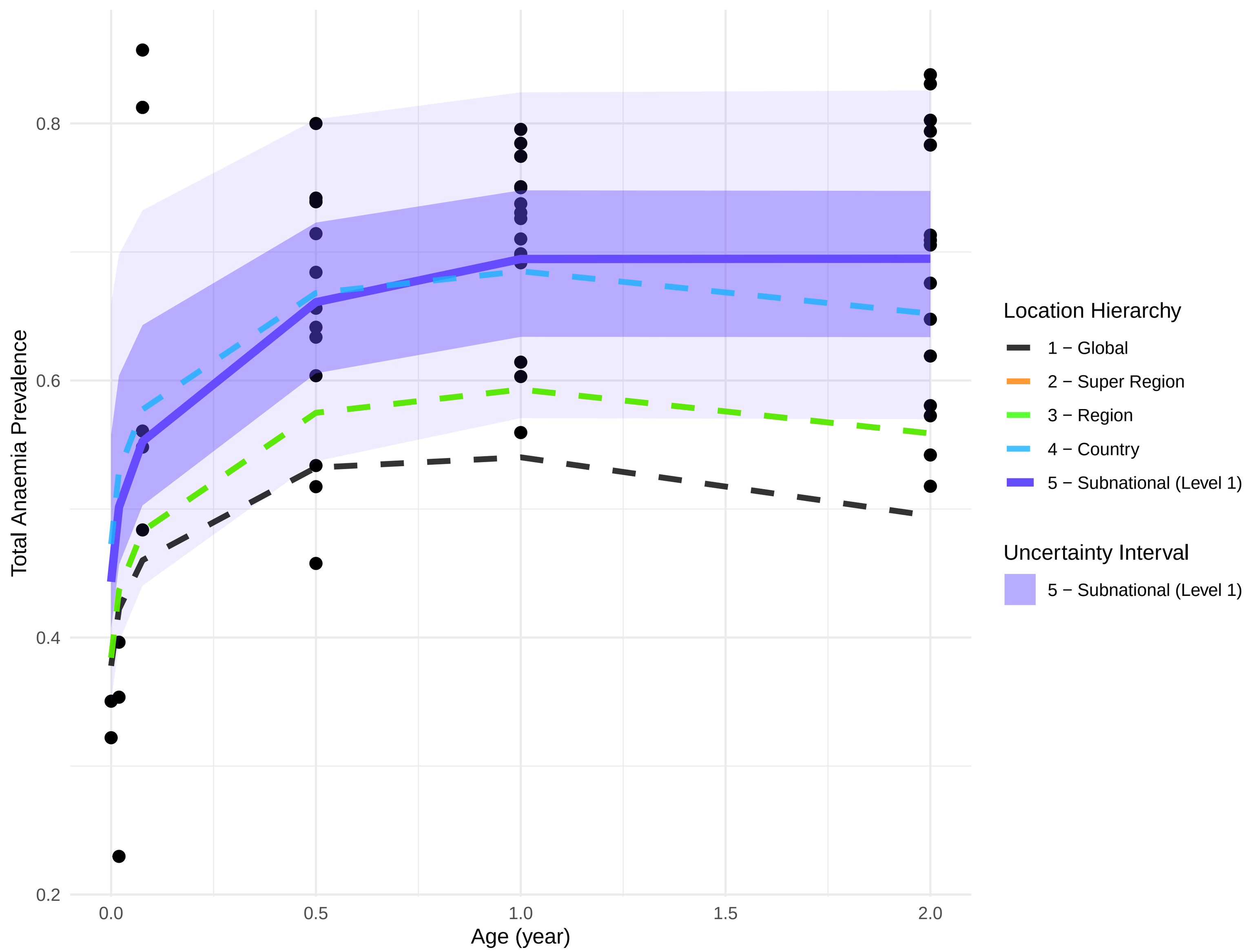

MR−BRT Age/Sex Fits − Non−pregnant Females <2 Years Old − Total Anaemia Prevalence − Rajasthan, Rural
WHO−2001 Adjustment Method

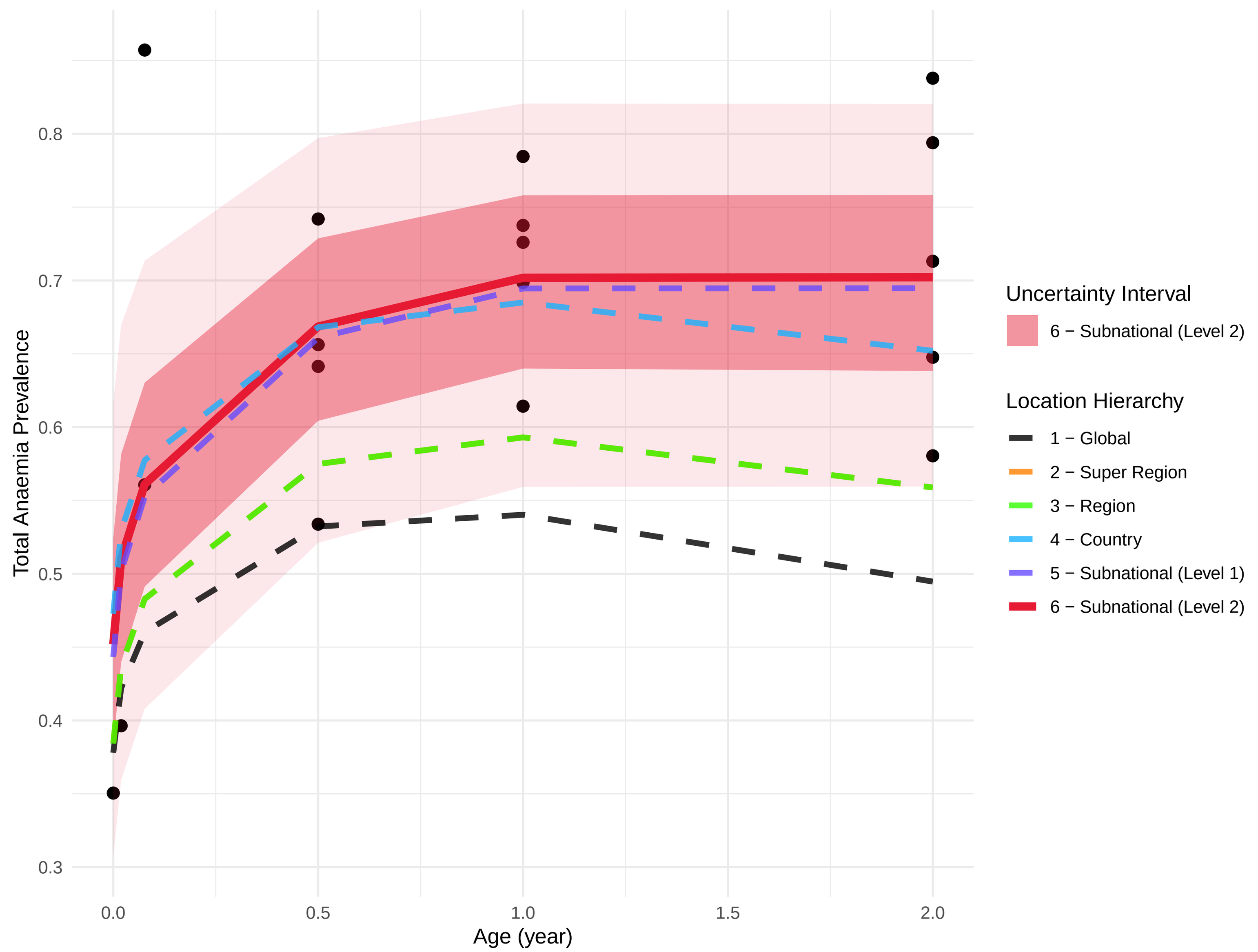

MR−BRT Age/Sex Fits − Non−pregnant Females <2 Years Old − Total Anaemia Prevalence − Rajasthan, Urban
WHO−2001 Adjustment Method

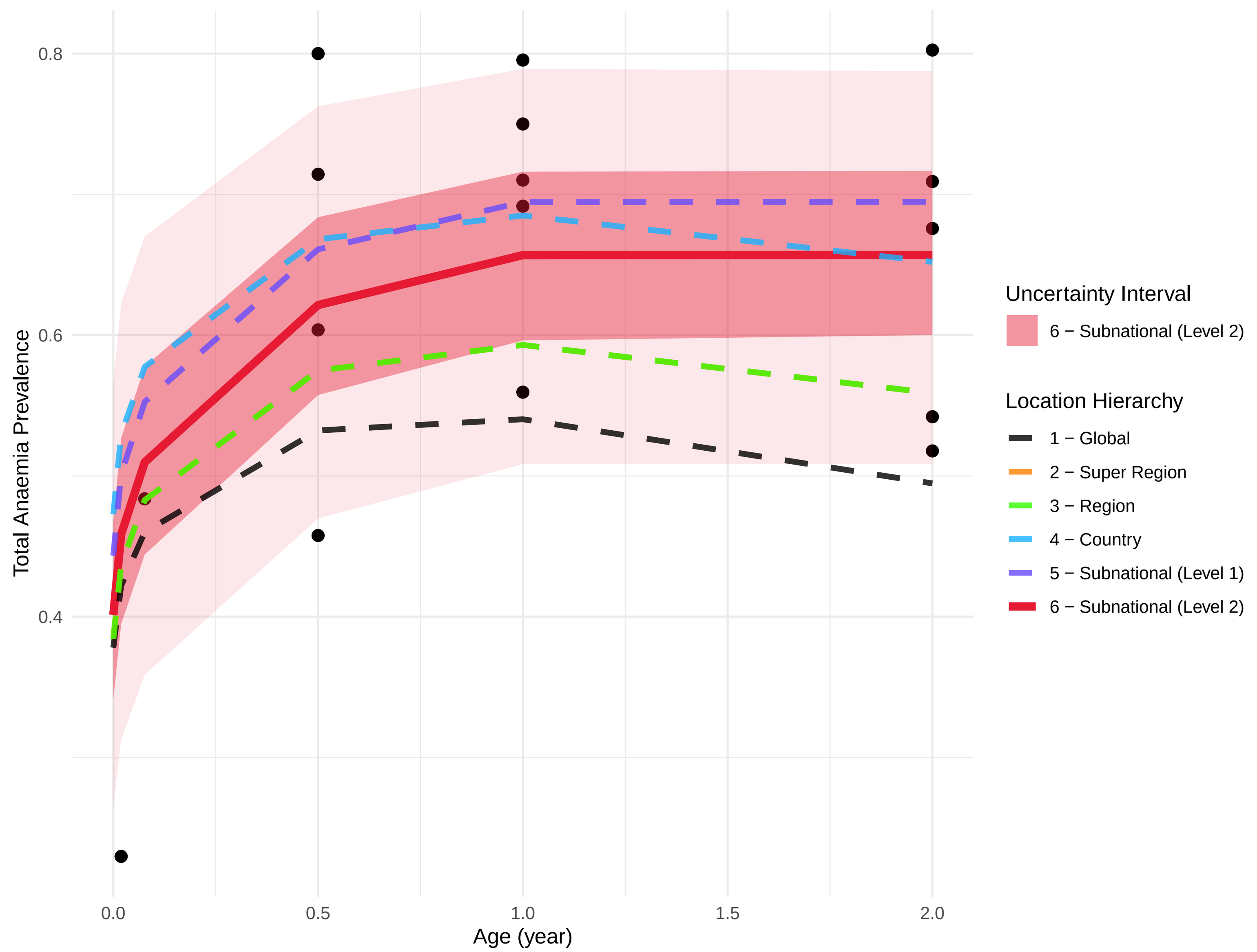

MR−BRT Age/Sex Fits − Non−pregnant Females <2 Years Old − Total Anaemia Prevalence − Sikkim
WHO−2001 Adjustment Method

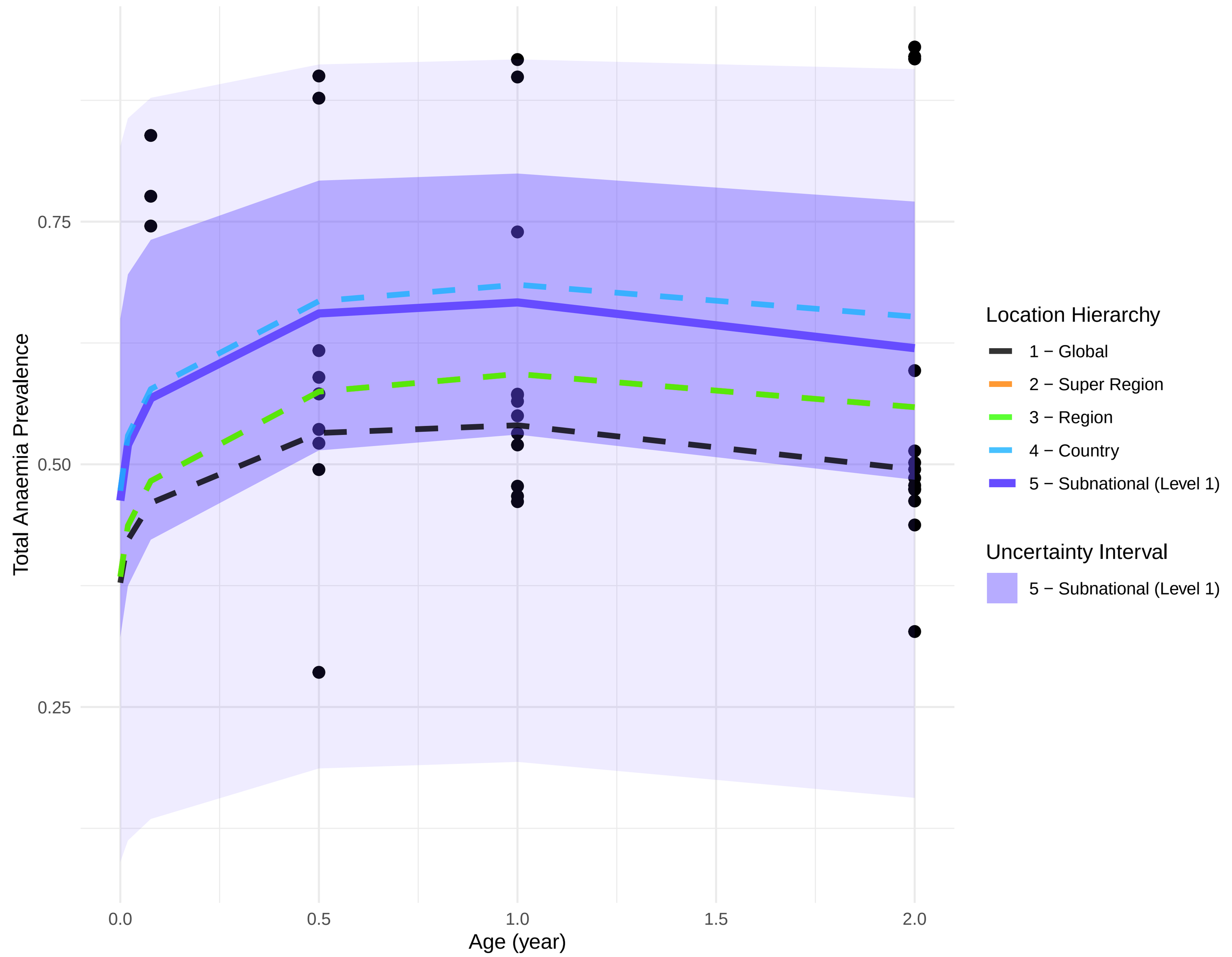

MR−BRT Age/Sex Fits − Non−pregnant Females <2 Years Old − Total Anaemia Prevalence − Sikkim, Rural
WHO−2001 Adjustment Method

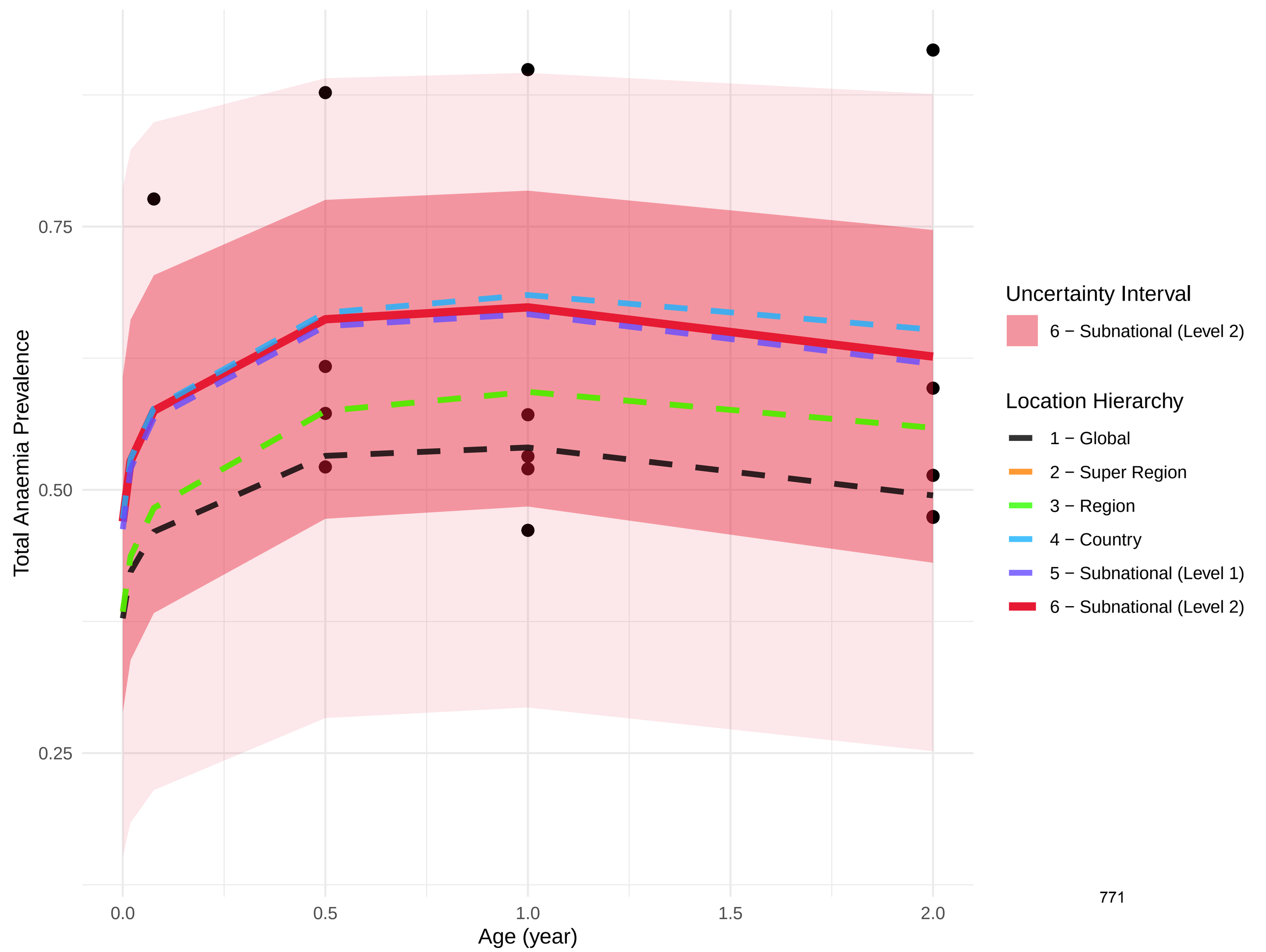

MR−BRT Age/Sex Fits − Non−pregnant Females <2 Years Old − Total Anaemia Prevalence − Sikkim, Urban
WHO−2001 Adjustment Method

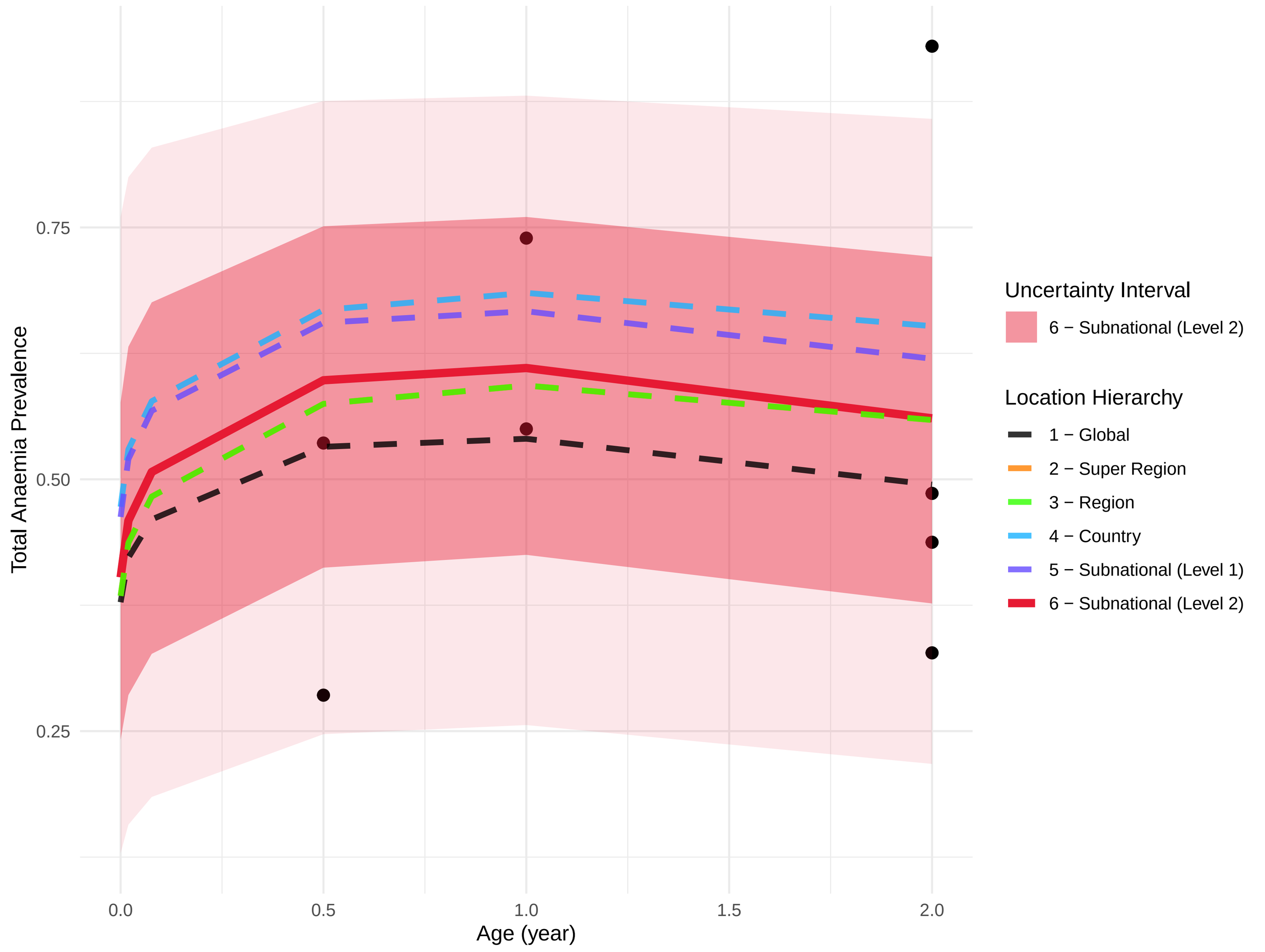

MR−BRT Age/Sex Fits − Non−pregnant Females <2 Years Old − Total Anaemia Prevalence − Tamil Nadu
WHO−2001 Adjustment Method

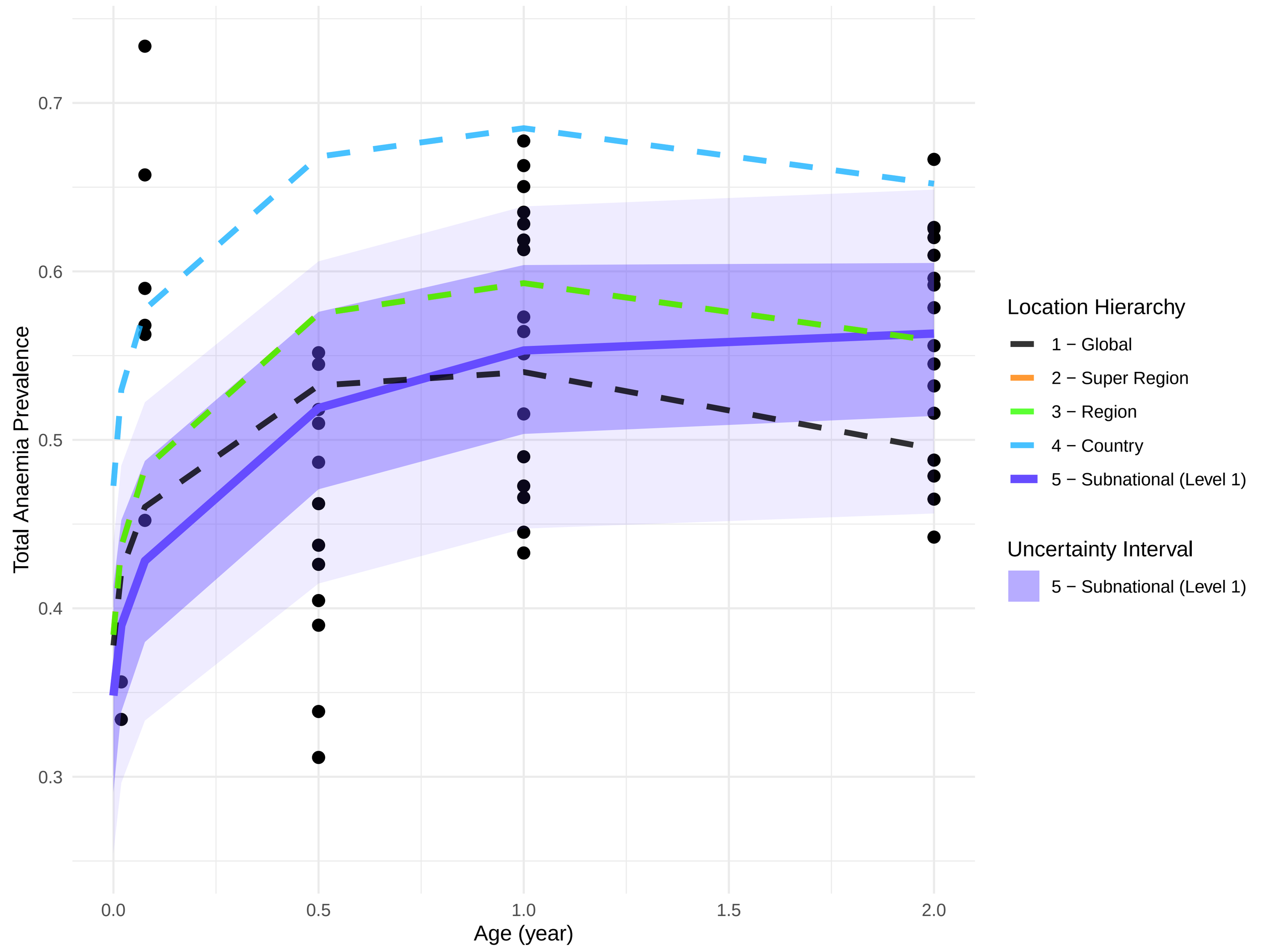

MR−BRT Age/Sex Fits − Non−pregnant Females <2 Years Old − Total Anaemia Prevalence − Tamil Nadu, Rural
WHO−2001 Adjustment Method

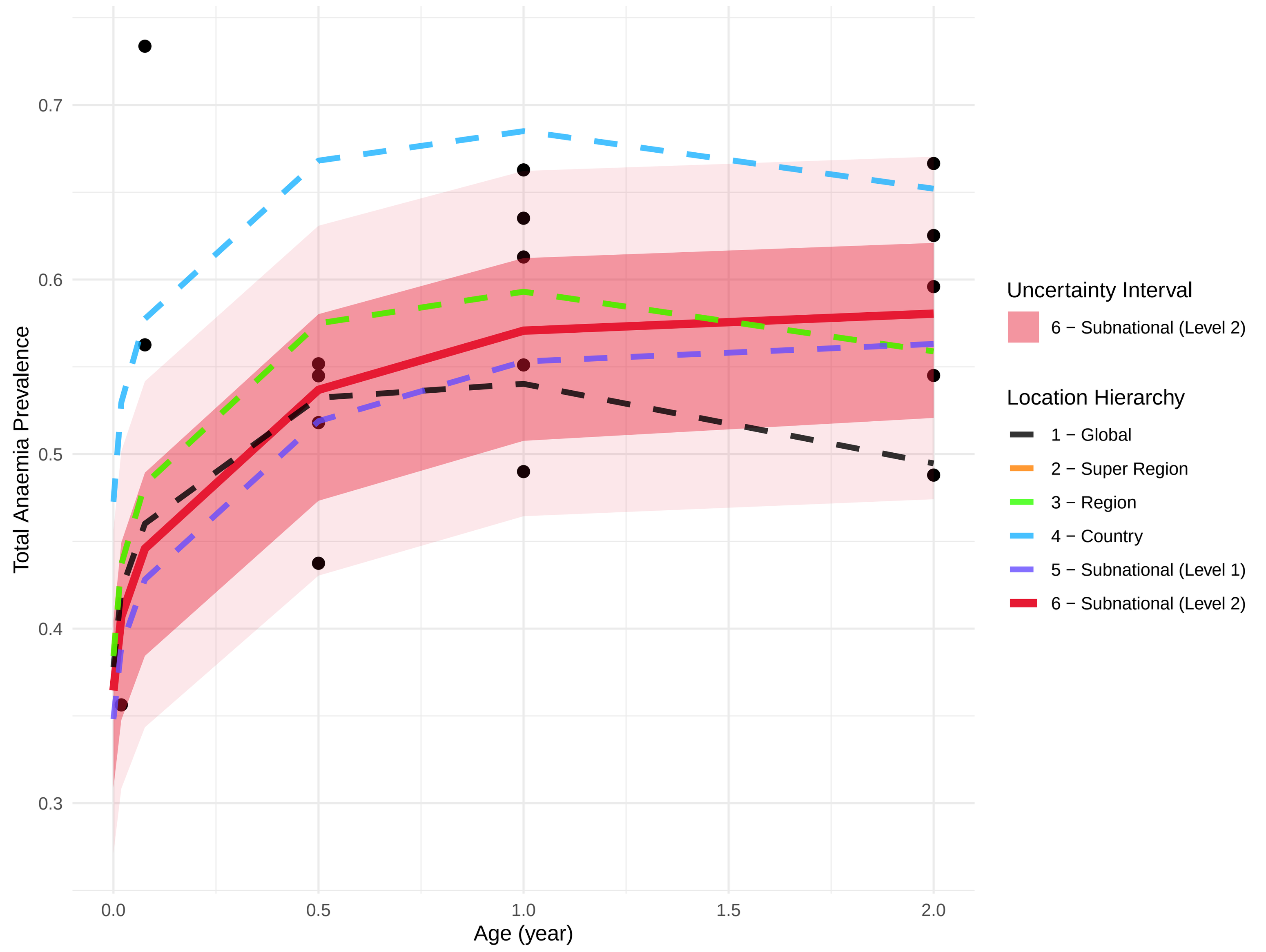

MR−BRT Age/Sex Fits − Non−pregnant Females <2 Years Old − Total Anaemia Prevalence − Tamil Nadu, Urban
WHO−2001 Adjustment Method

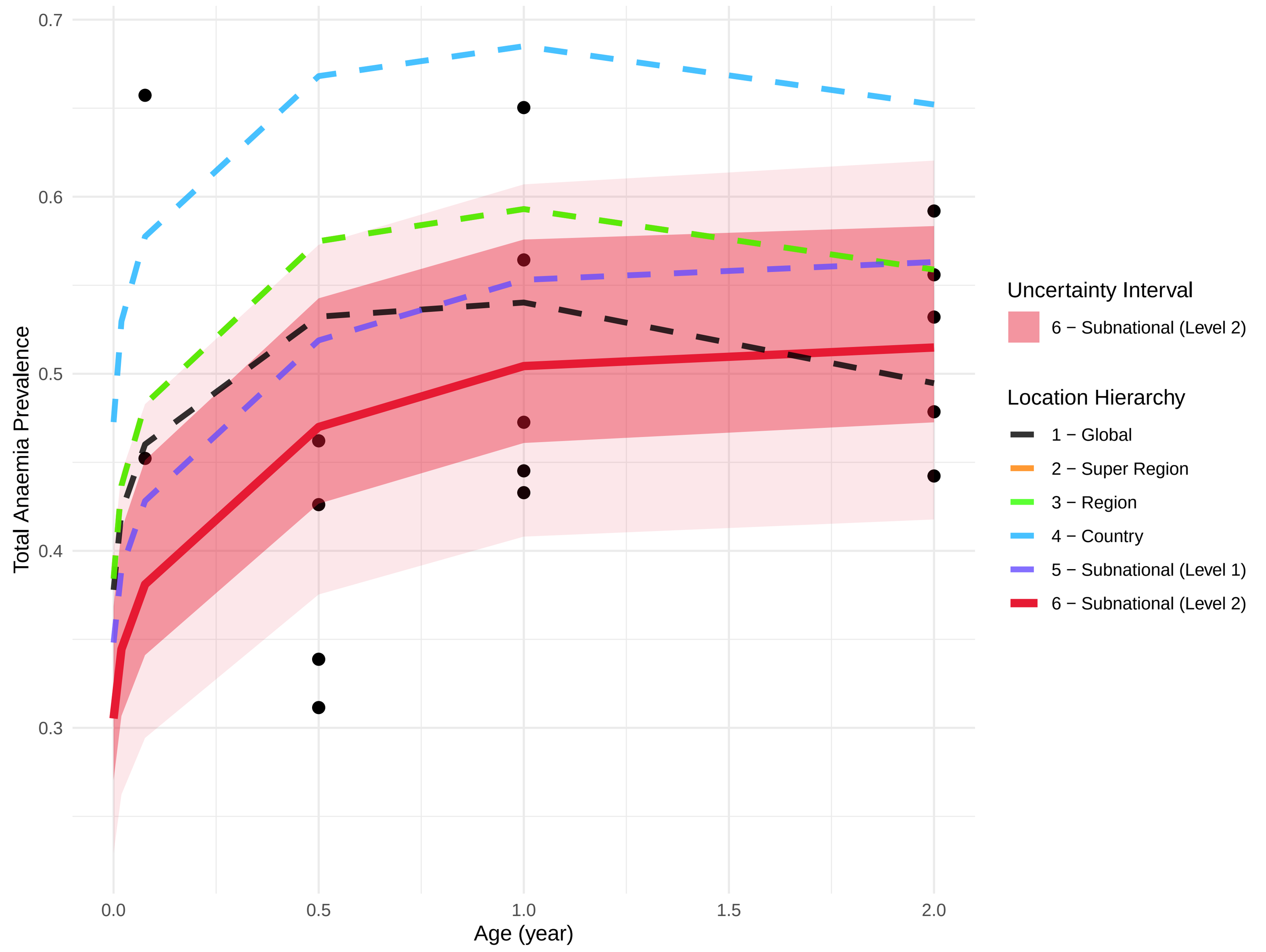

MR−BRT Age/Sex Fits − Non−pregnant Females <2 Years Old − Total Anaemia Prevalence − Telangana
WHO−2001 Adjustment Method

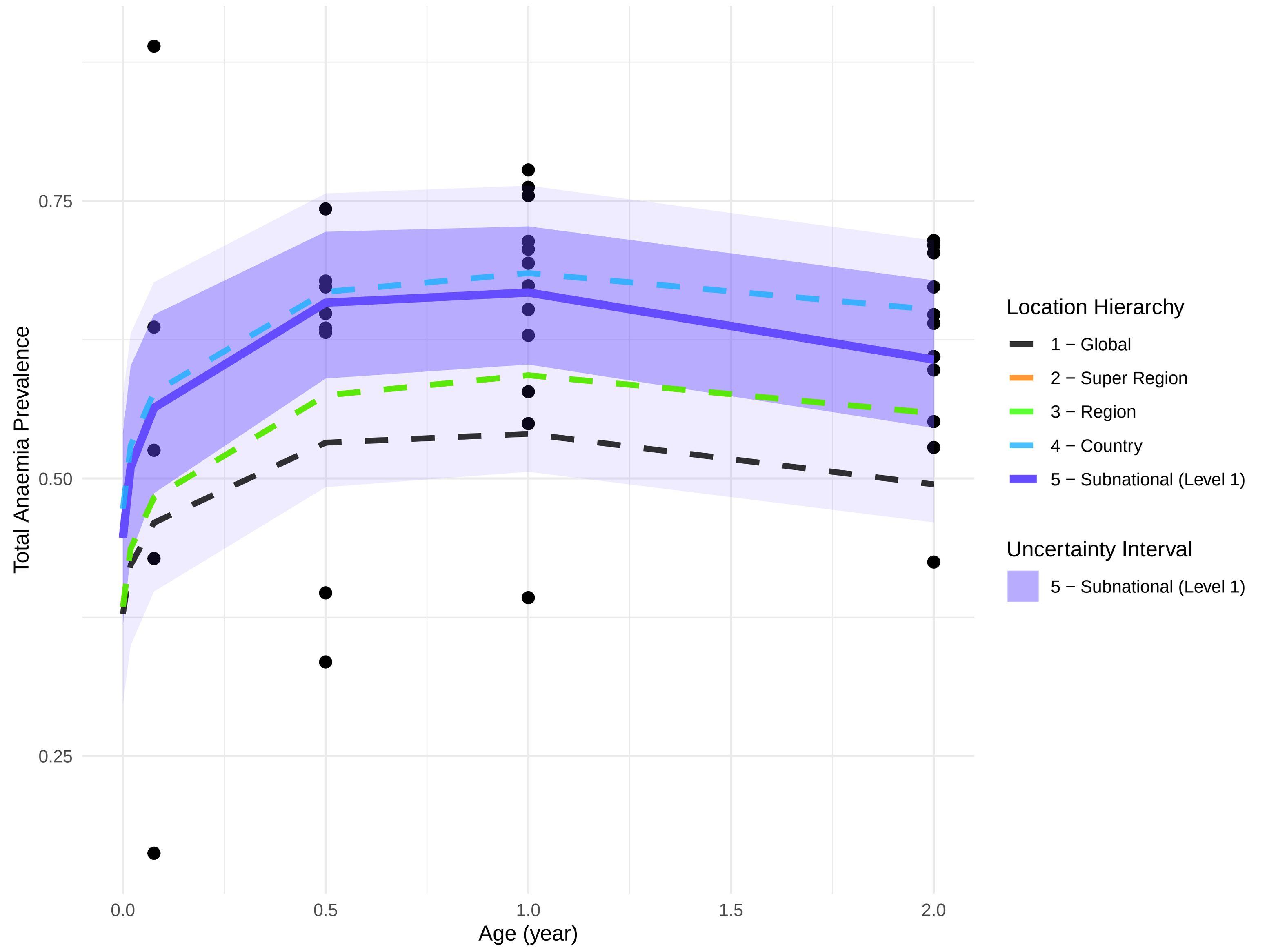

MR−BRT Age/Sex Fits − Non−pregnant Females <2 Years Old − Total Anaemia Prevalence − Telangana, Rural
WHO−2001 Adjustment Method

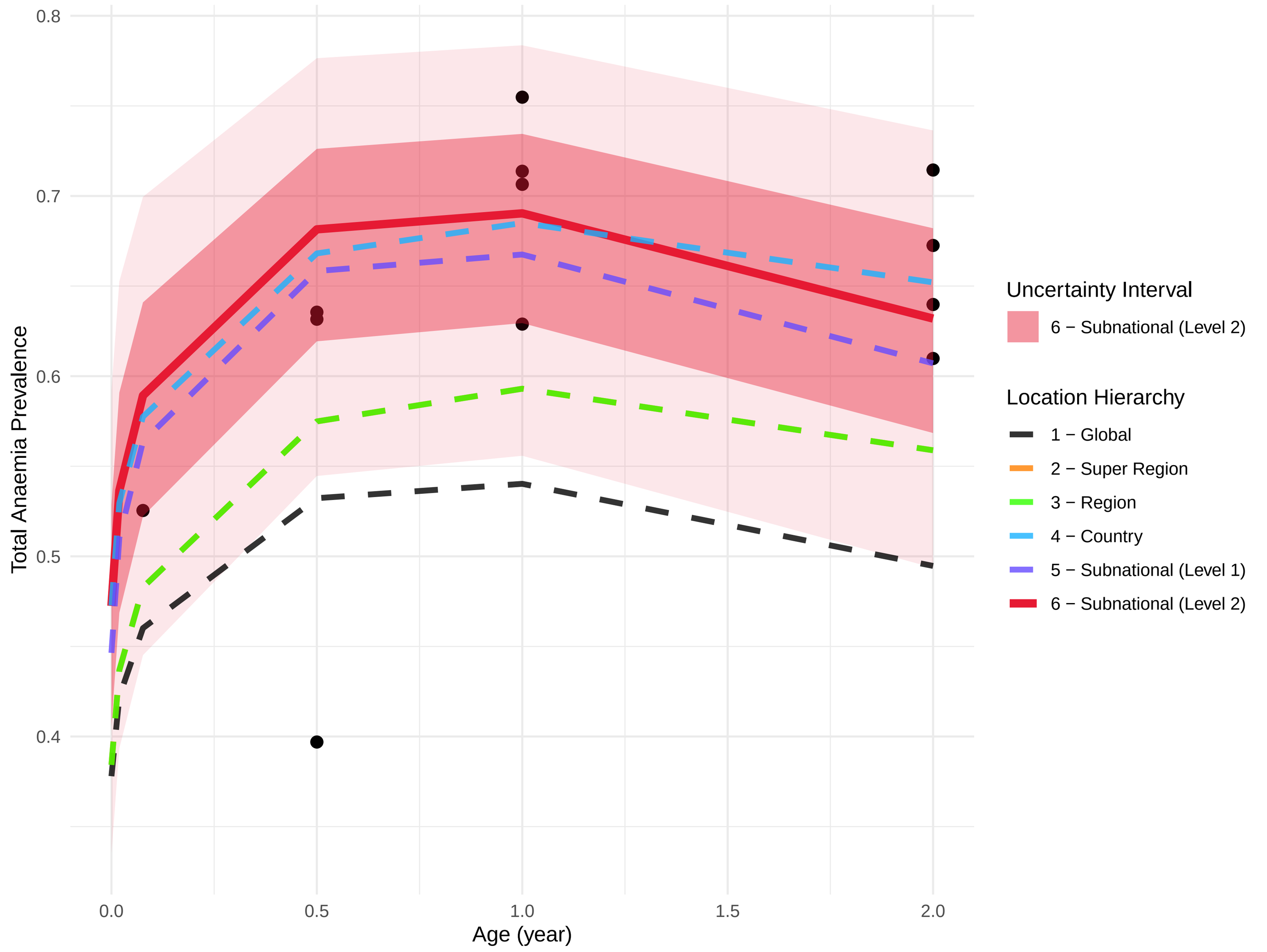

MR−BRT Age/Sex Fits − Non−pregnant Females <2 Years Old − Total Anaemia Prevalence − Telangana, Urban
WHO−2001 Adjustment Method

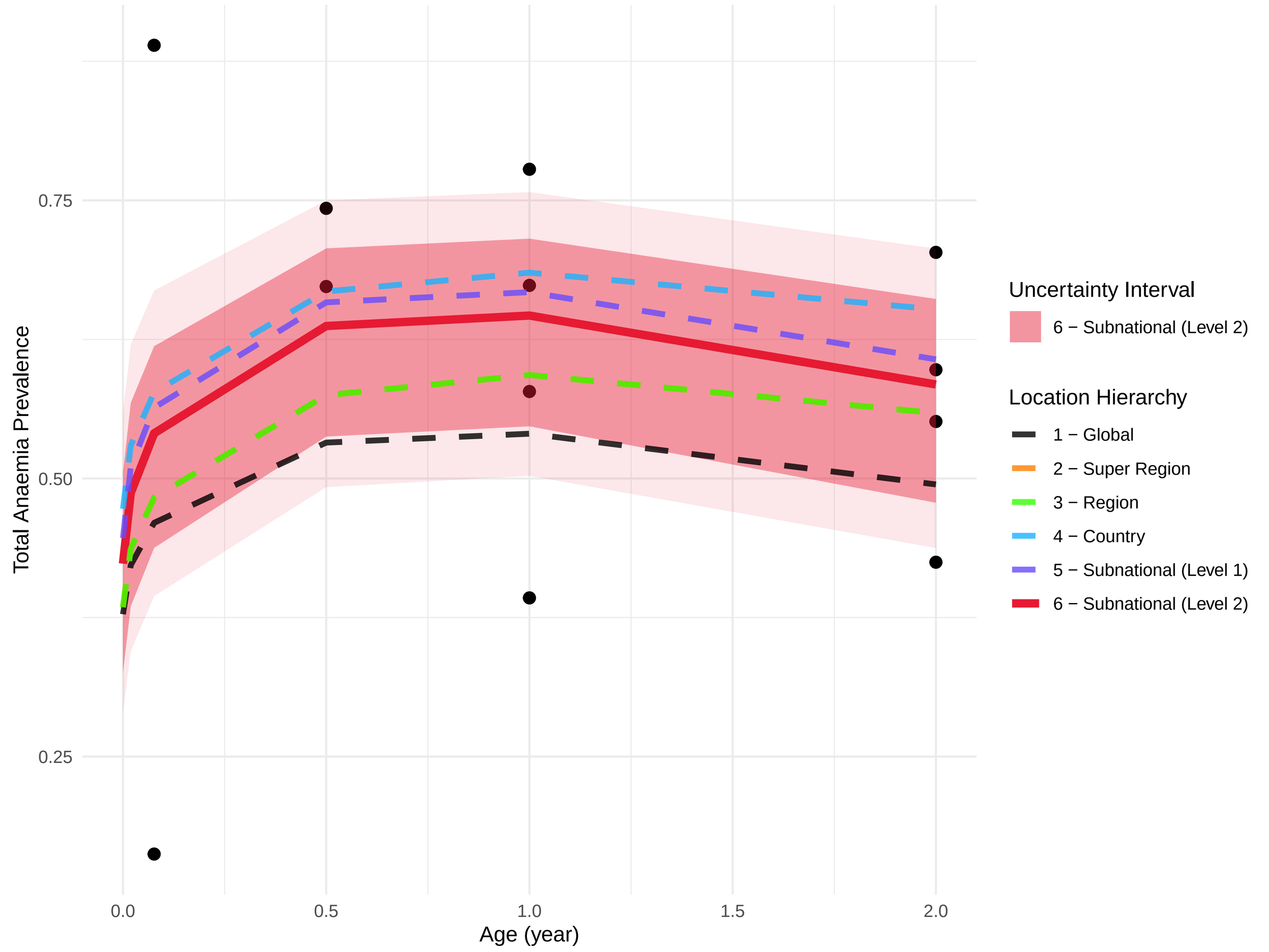

MR−BRT Age/Sex Fits − Non−pregnant Females <2 Years Old − Total Anaemia Prevalence − Tripura
WHO−2001 Adjustment Method

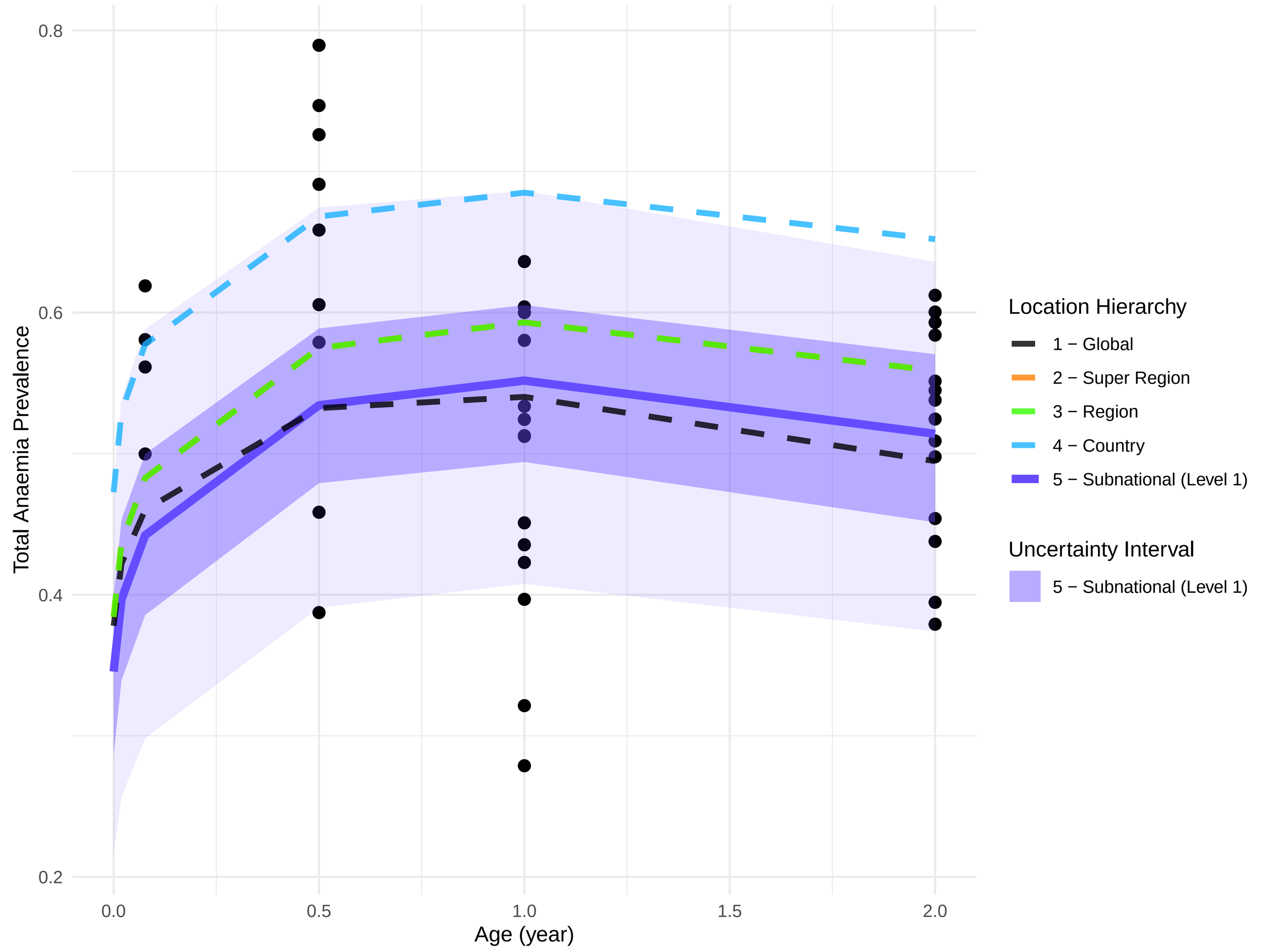

MR−BRT Age/Sex Fits − Non−pregnant Females <2 Years Old − Total Anaemia Prevalence − Tripura, Rural
WHO−2001 Adjustment Method

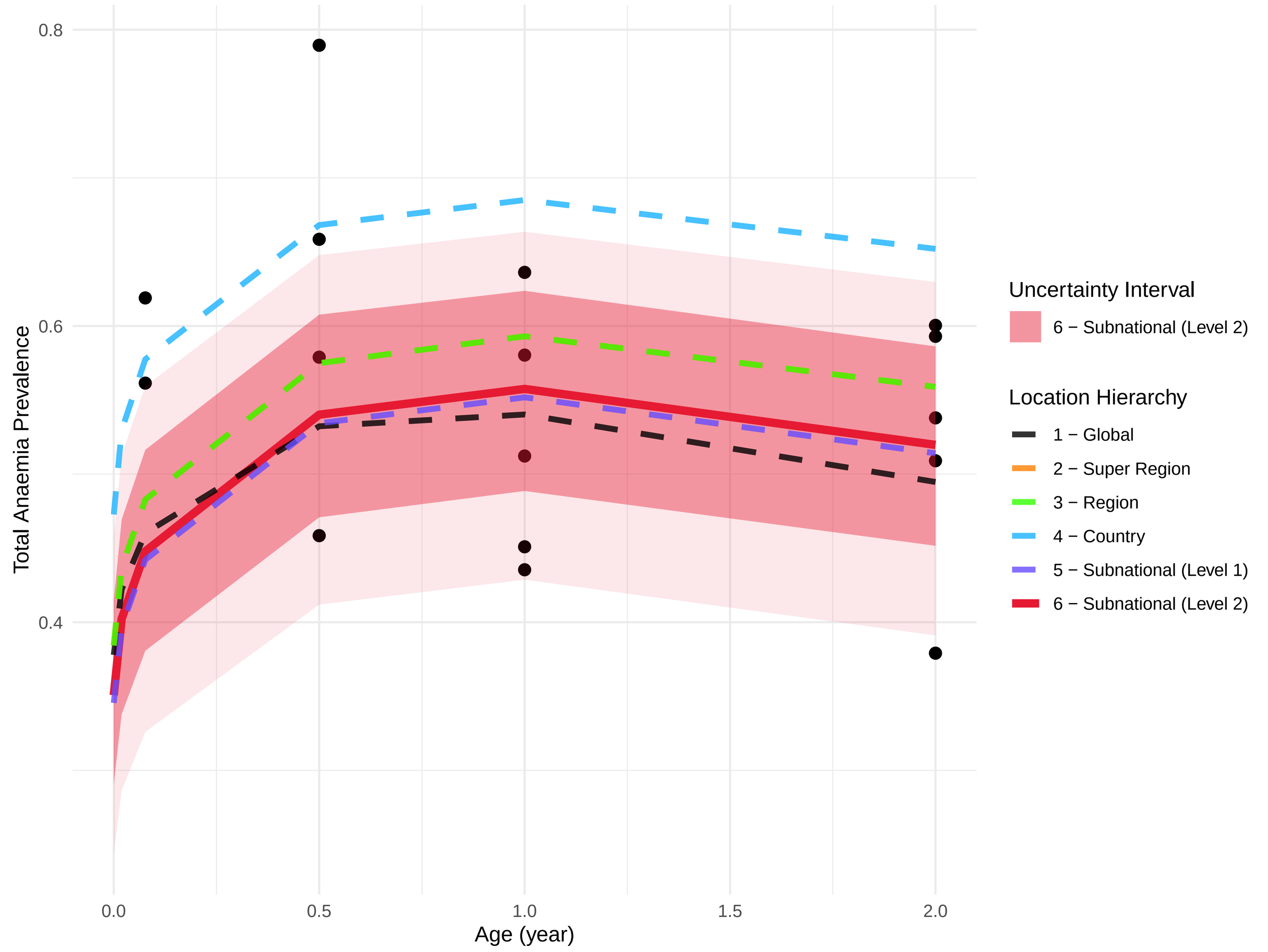

MR−BRT Age/Sex Fits − Non−pregnant Females <2 Years Old − Total Anaemia Prevalence − Tripura, Urban
WHO−2001 Adjustment Method

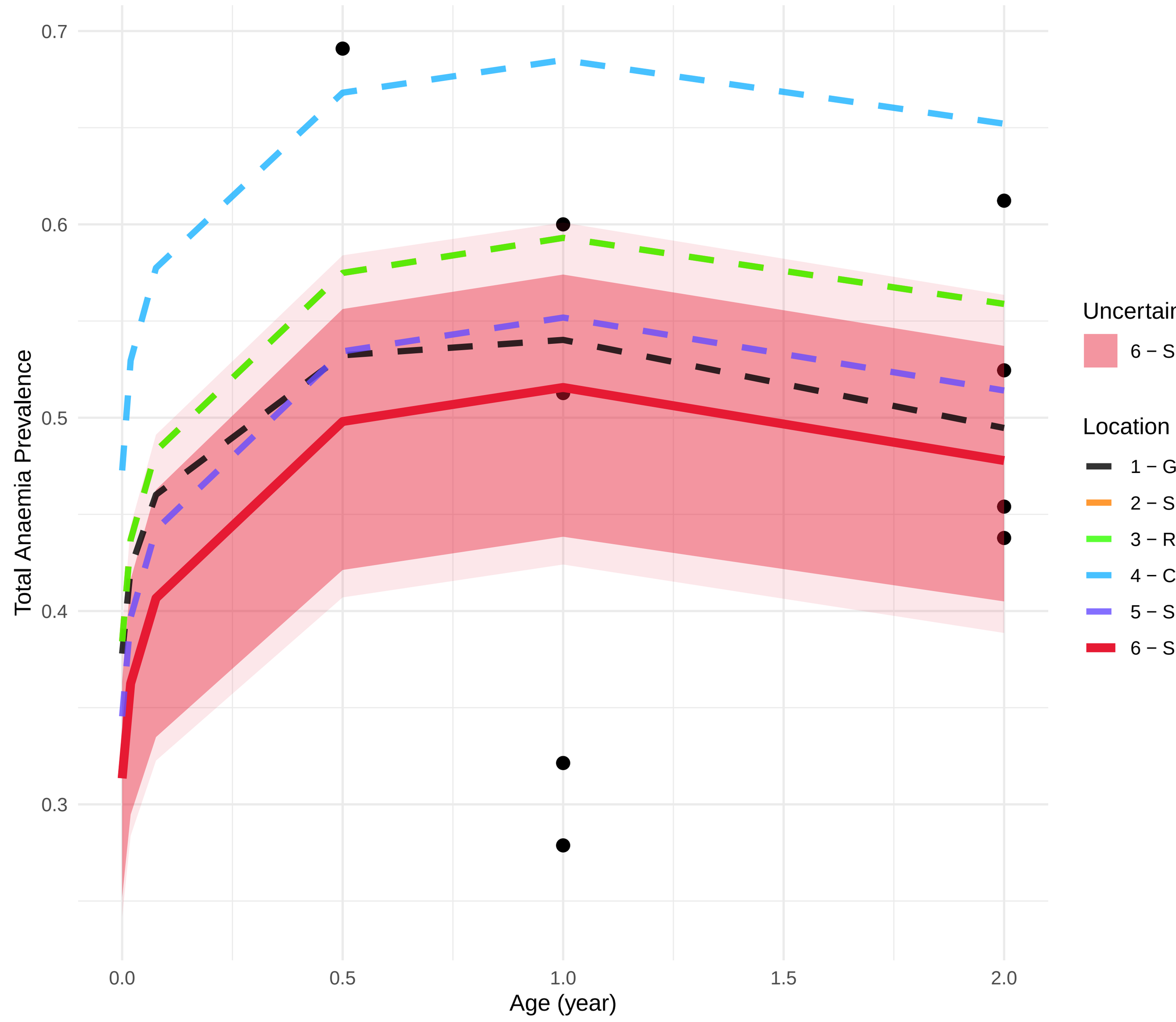

MR−BRT Age/Sex Fits − Non−pregnant Females <2 Years Old − Total Anaemia Prevalence − Uttar Pradesh
WHO−2001 Adjustment Method

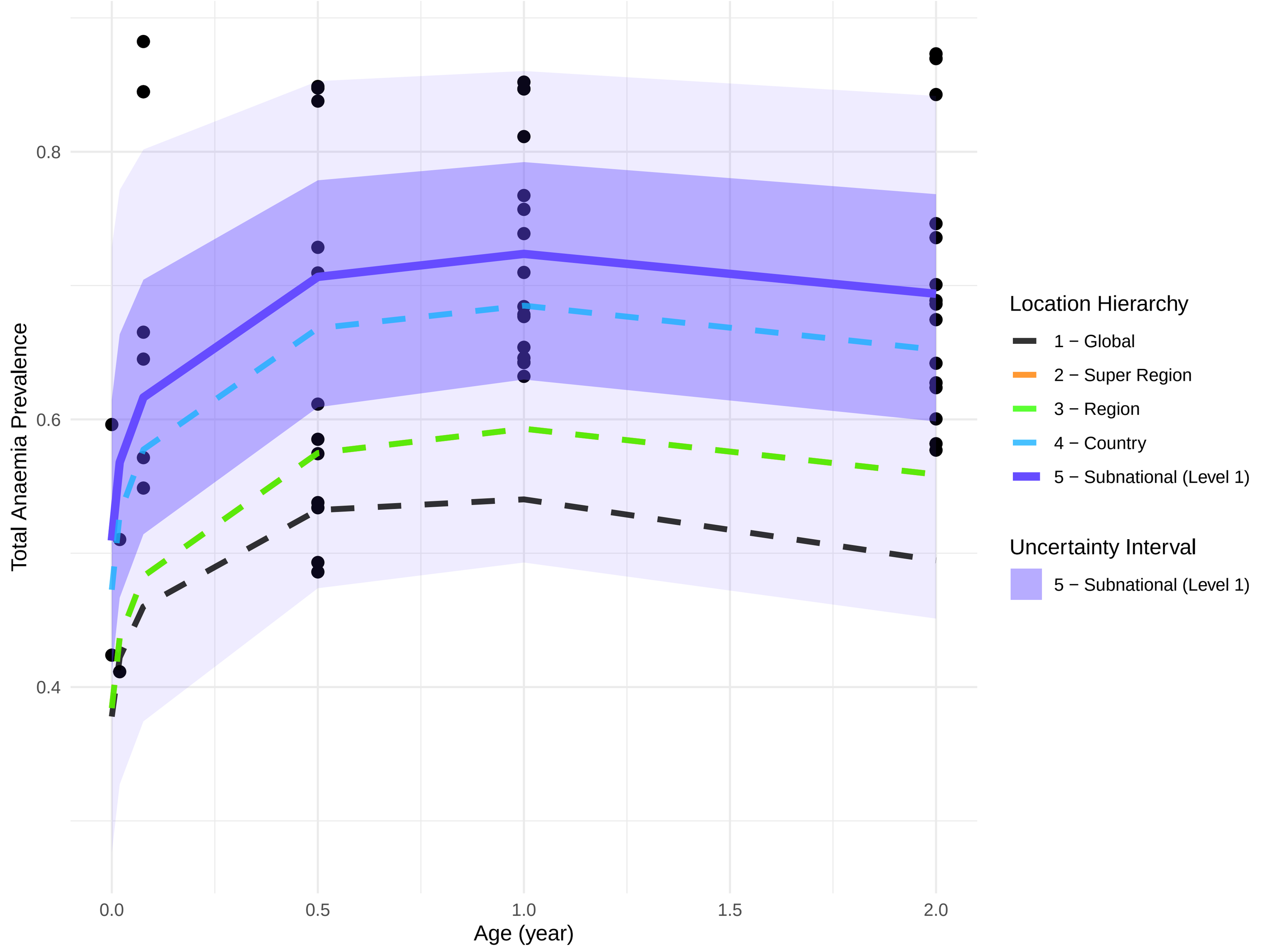

MR−BRT Age/Sex Fits − Non−pregnant Females <2 Years Old − Total Anaemia Prevalence − Uttar Pradesh, Rural
WHO−2001 Adjustment Method

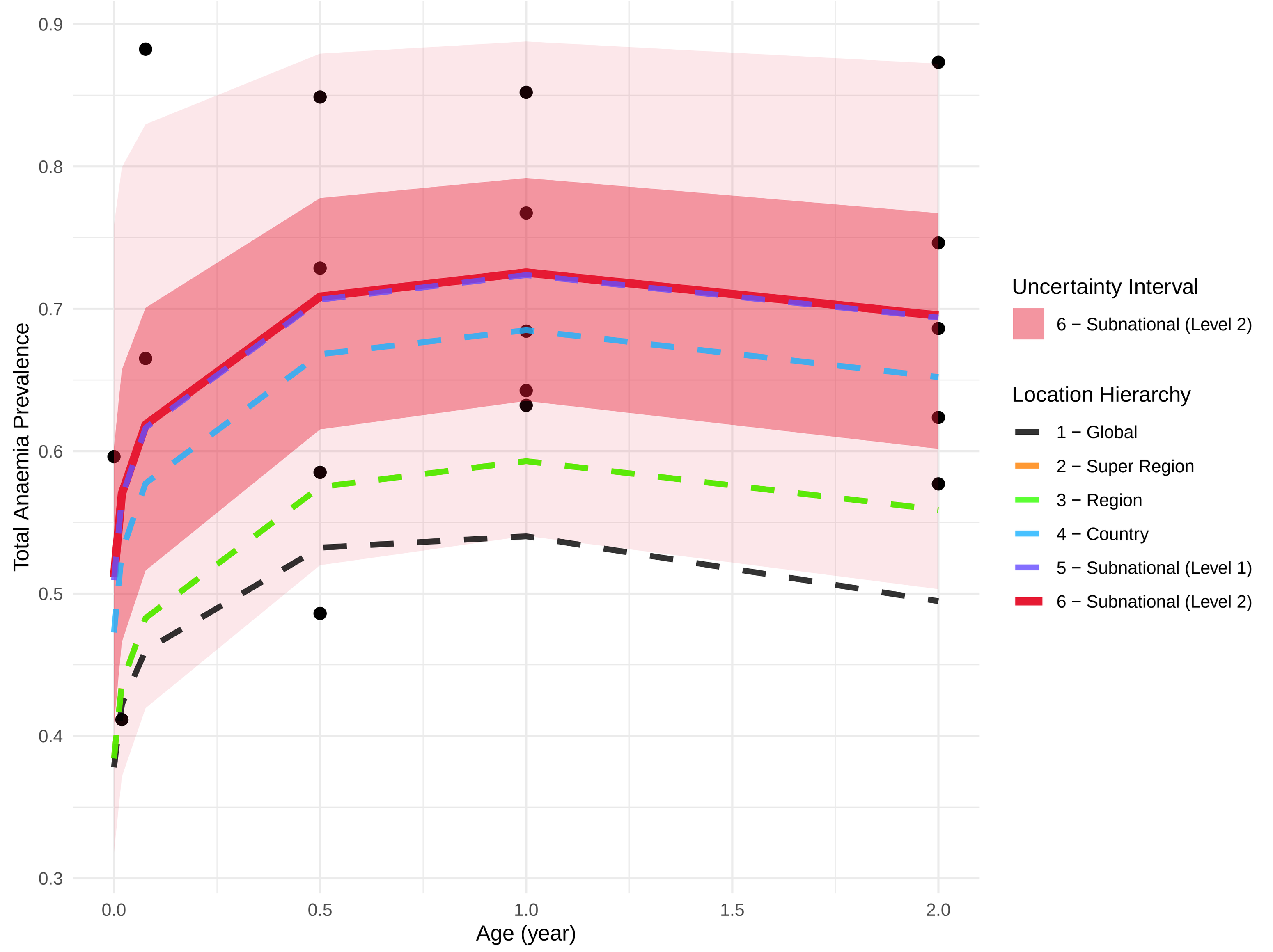

MR−BRT Age/Sex Fits − Non−pregnant Females <2 Years Old − Total Anaemia Prevalence − Uttar Pradesh, Urban
WHO−2001 Adjustment Method

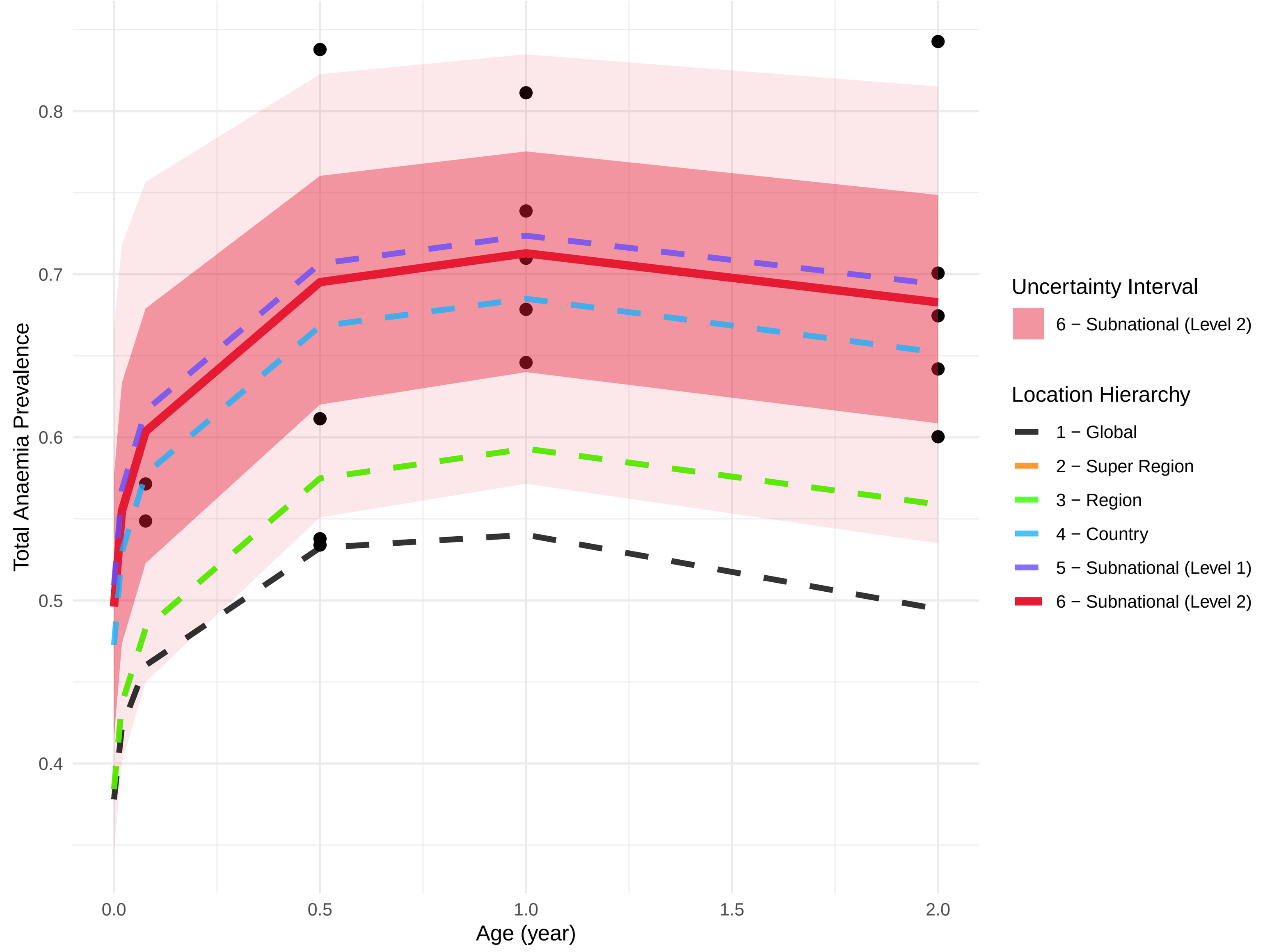

MR−BRT Age/Sex Fits − Non−pregnant Females <2 Years Old − Total Anaemia Prevalence − Uttarakhand
WHO−2001 Adjustment Method

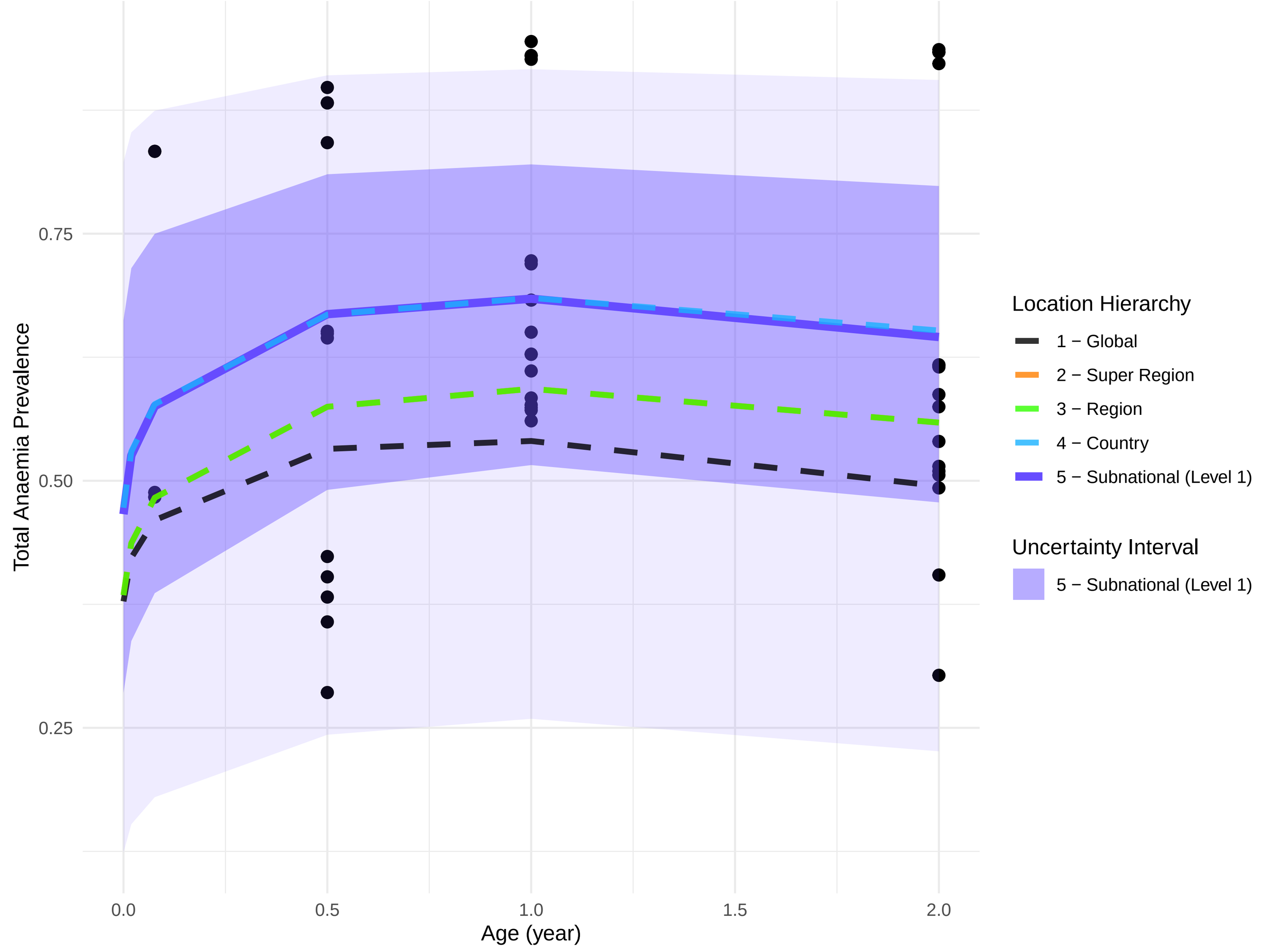

MR−BRT Age/Sex Fits − Non−pregnant Females <2 Years Old − Total Anaemia Prevalence − Uttarakhand, Rural
WHO−2001 Adjustment Method

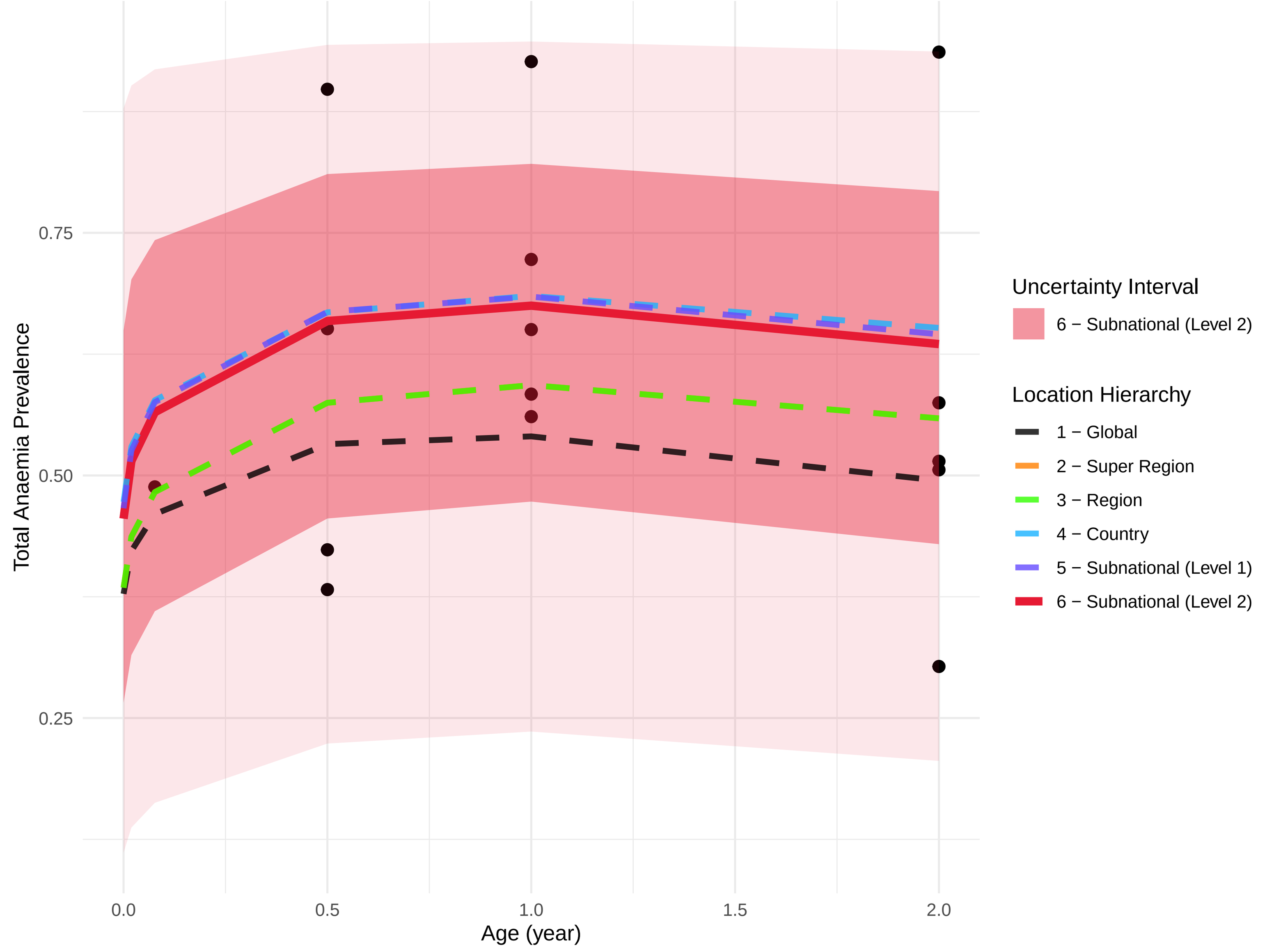

MR−BRT Age/Sex Fits − Non−pregnant Females <2 Years Old − Total Anaemia Prevalence − Uttarakhand, Urban
WHO−2001 Adjustment Method

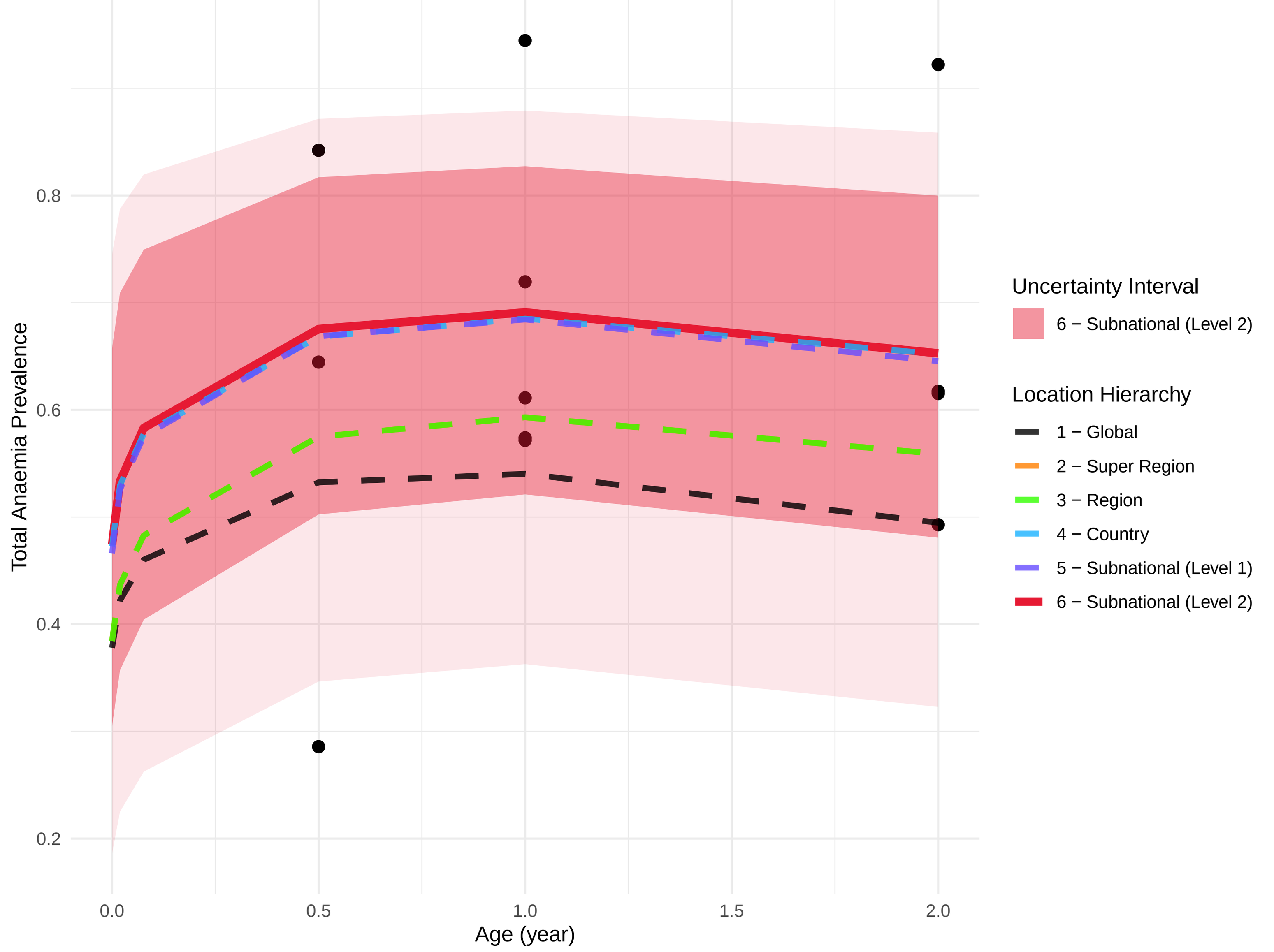

MR−BRT Age/Sex Fits − Non−pregnant Females <2 Years Old − Total Anaemia Prevalence − West Bengal
WHO−2001 Adjustment Method

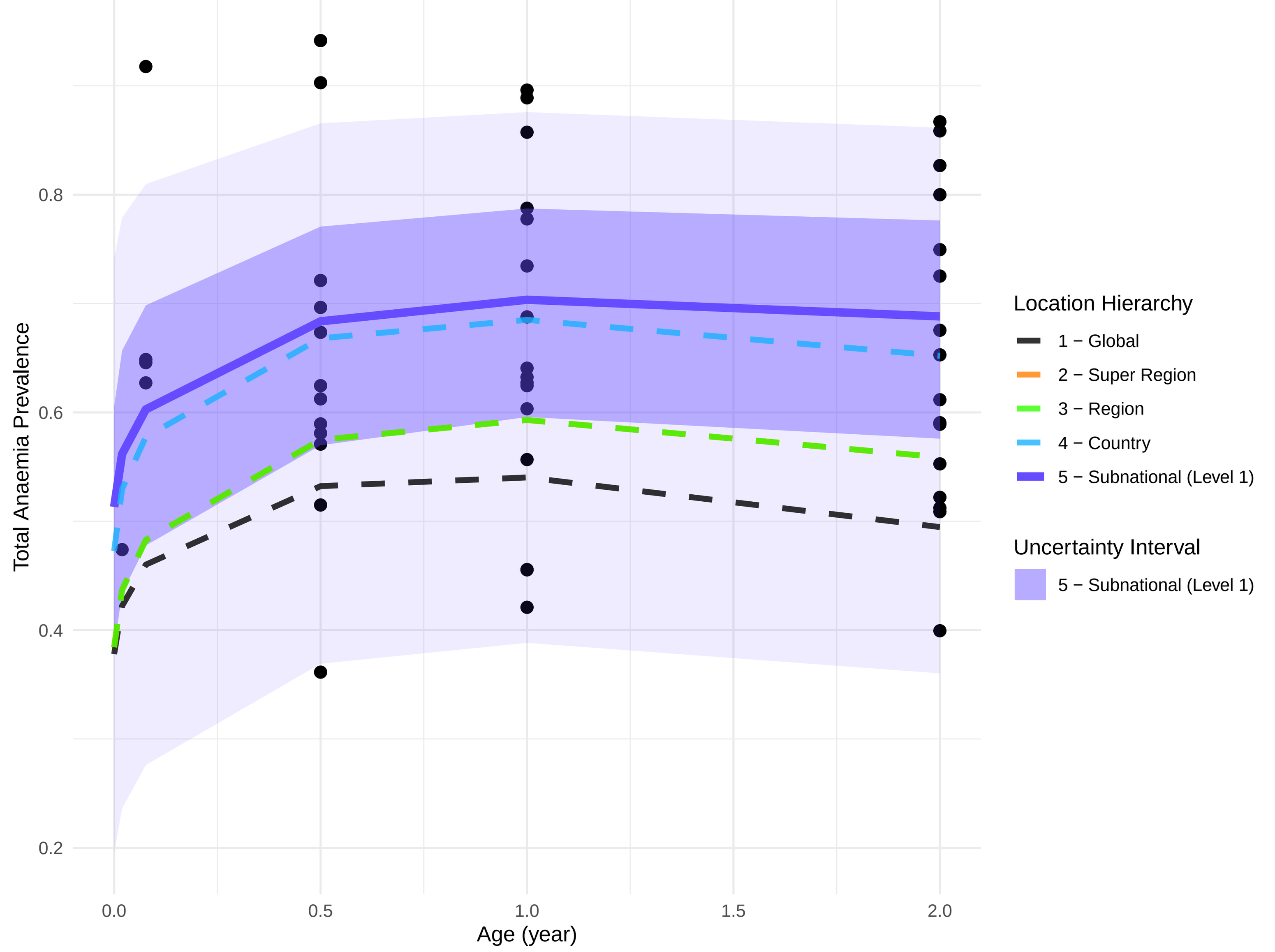

MR−BRT Age/Sex Fits − Non−pregnant Females <2 Years Old − Total Anaemia Prevalence − West Bengal, Rural
WHO−2001 Adjustment Method

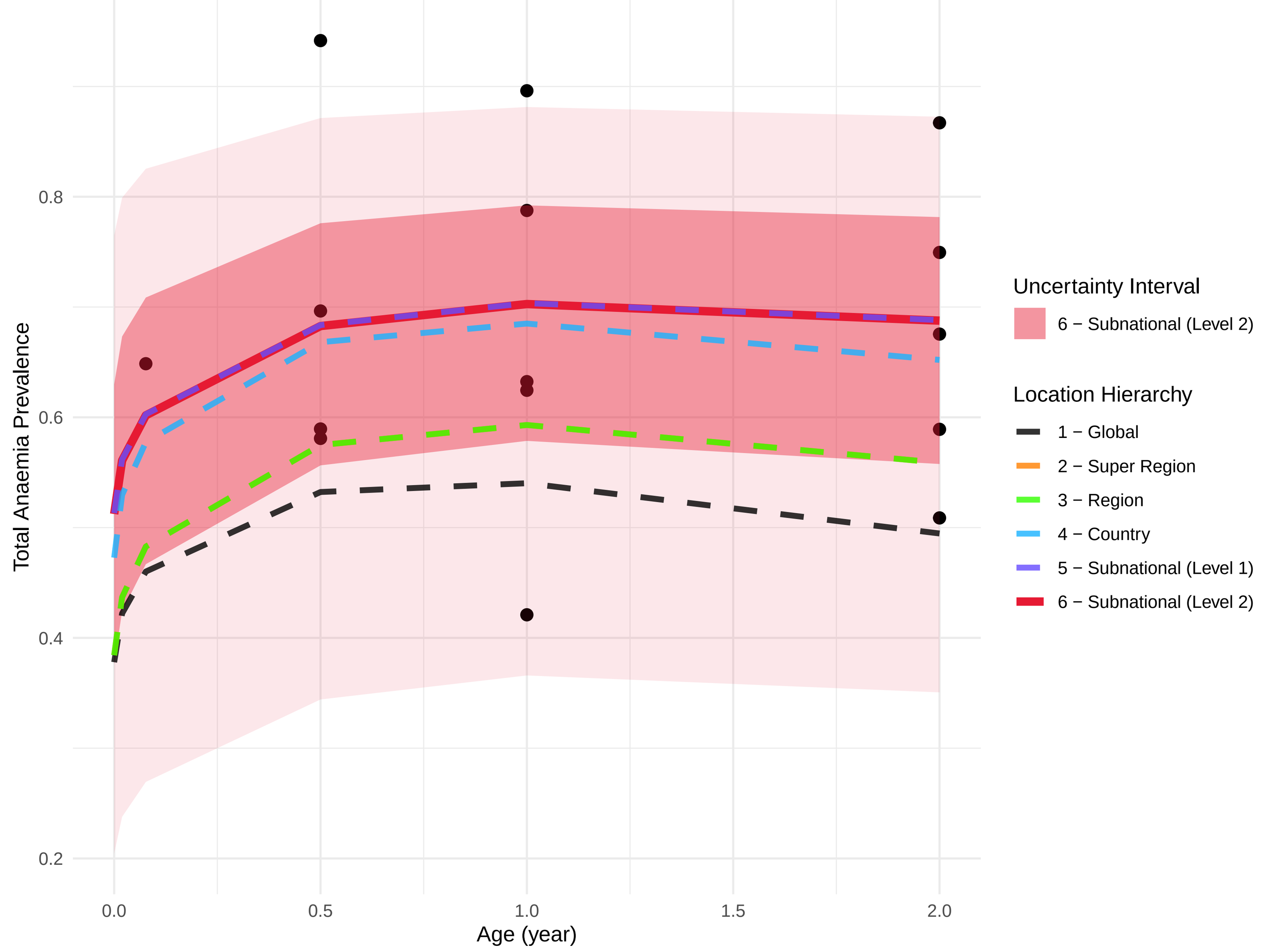

MR−BRT Age/Sex Fits − Non−pregnant Females <2 Years Old − Total Anaemia Prevalence − West Bengal, Urban
WHO−2001 Adjustment Method

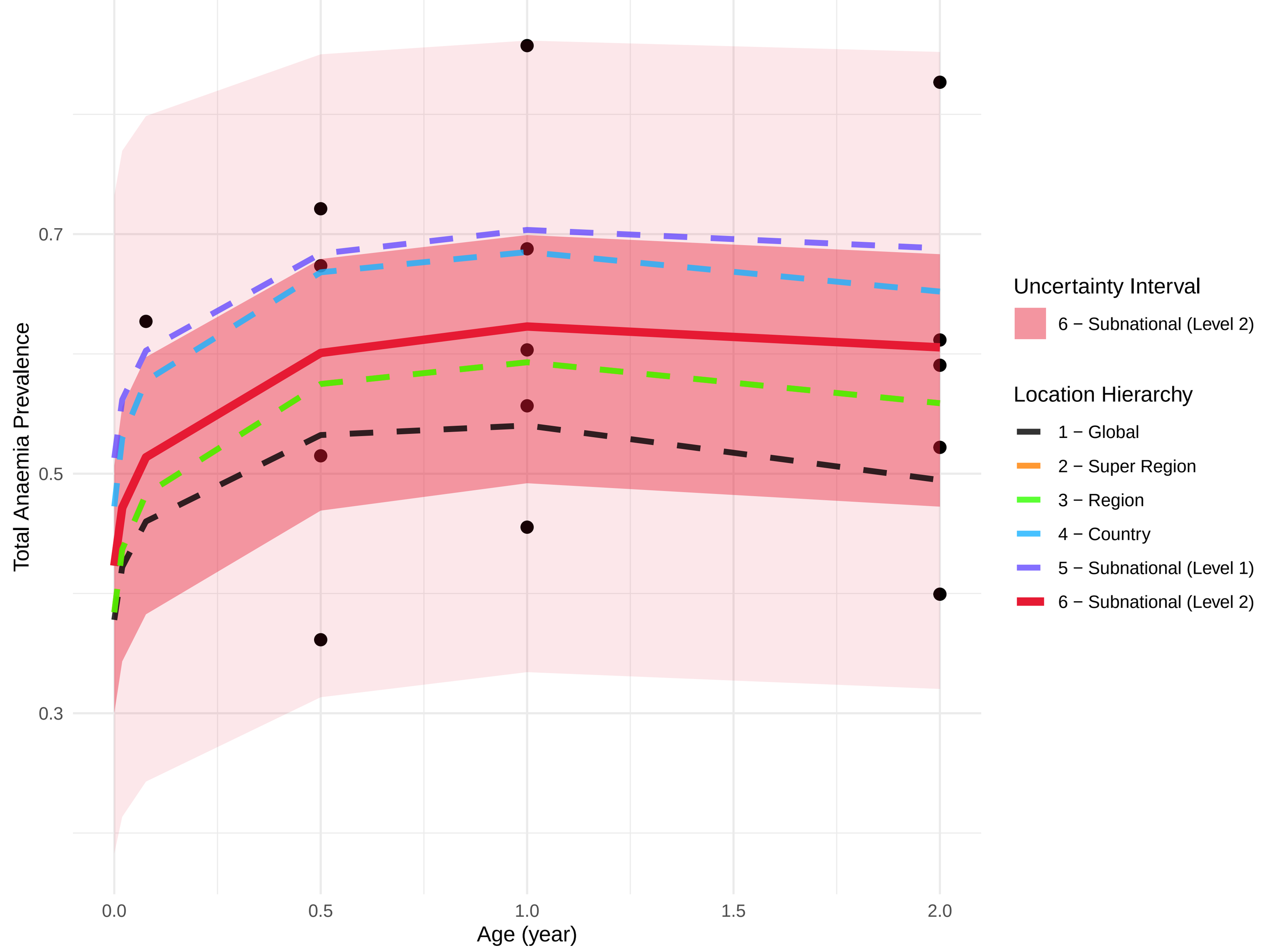

MR−BRT Age/Sex Fits − Non−pregnant Females <2 Years Old − Total Anaemia Prevalence − Nepal
WHO−2001 Adjustment Method

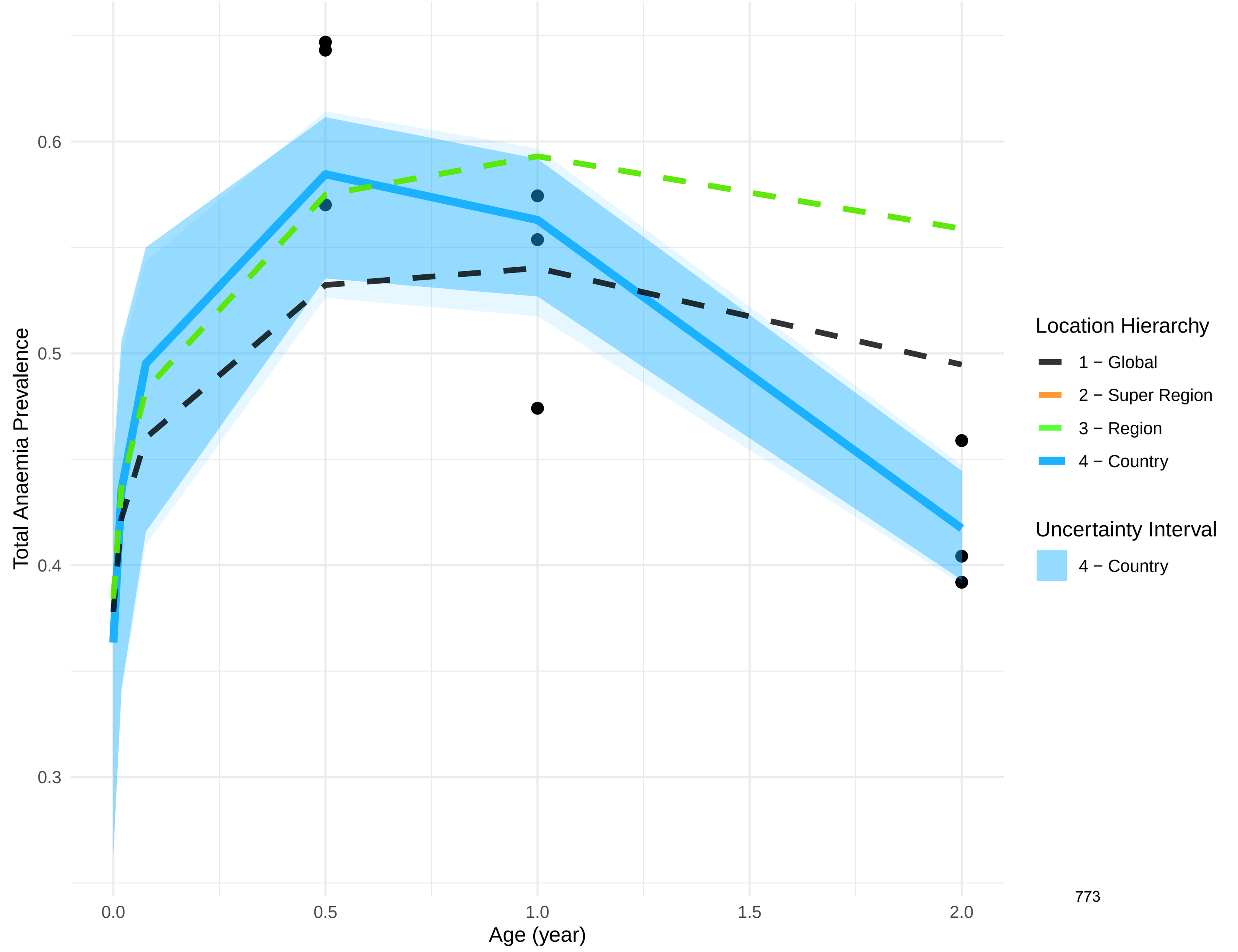

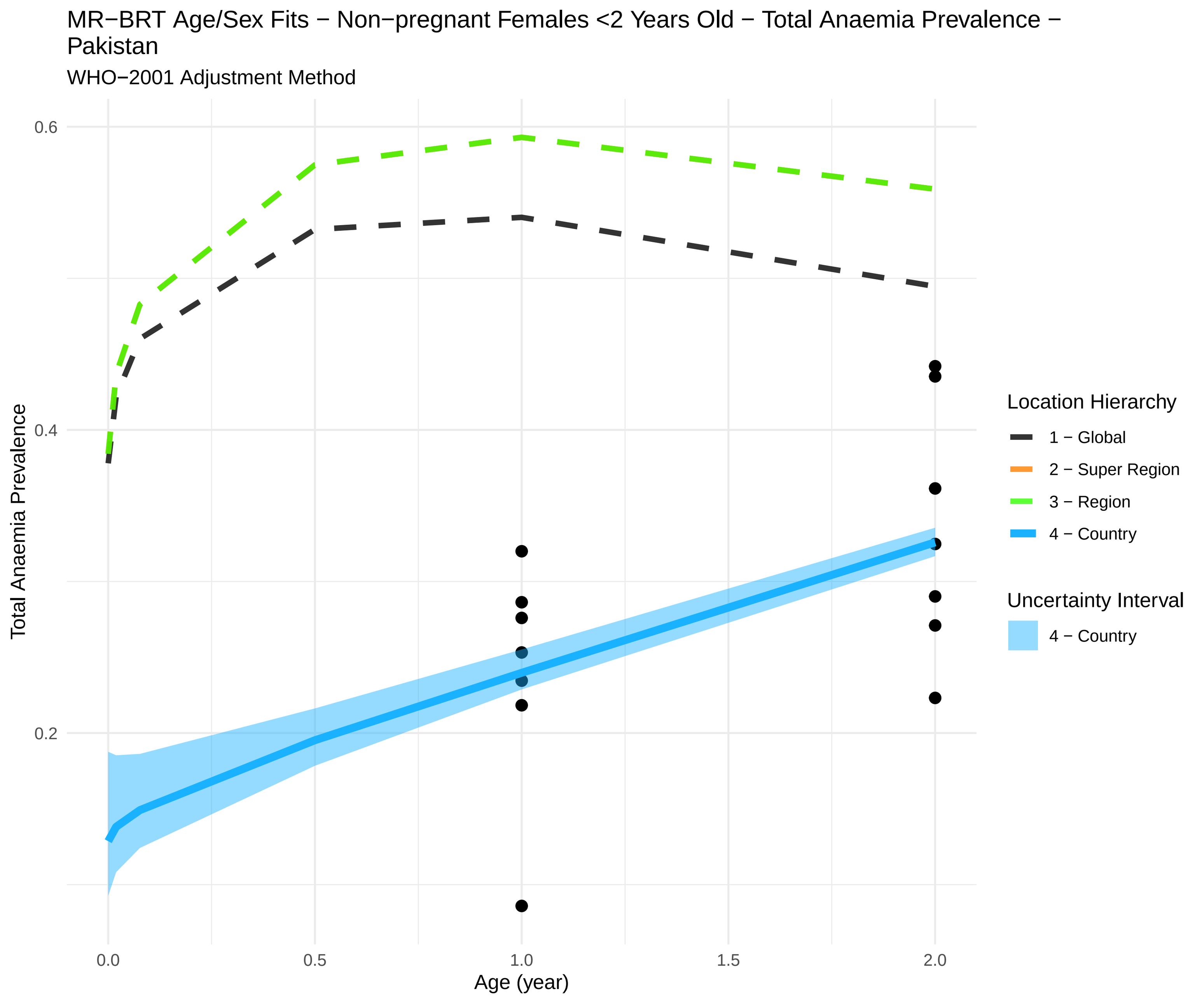
MR−BRT Age/Sex Fits − Non−pregnant Females <2 Years Old − Total Anaemia Prevalence − Pakistan
WHO−2001 Adjustment Method
Total Anaemia Prevalence
Age (year)
Location Hierarchy
1 − Global
2 − Super Region
3 − Region
4 − Country
Uncertainty Interval
4 − Country

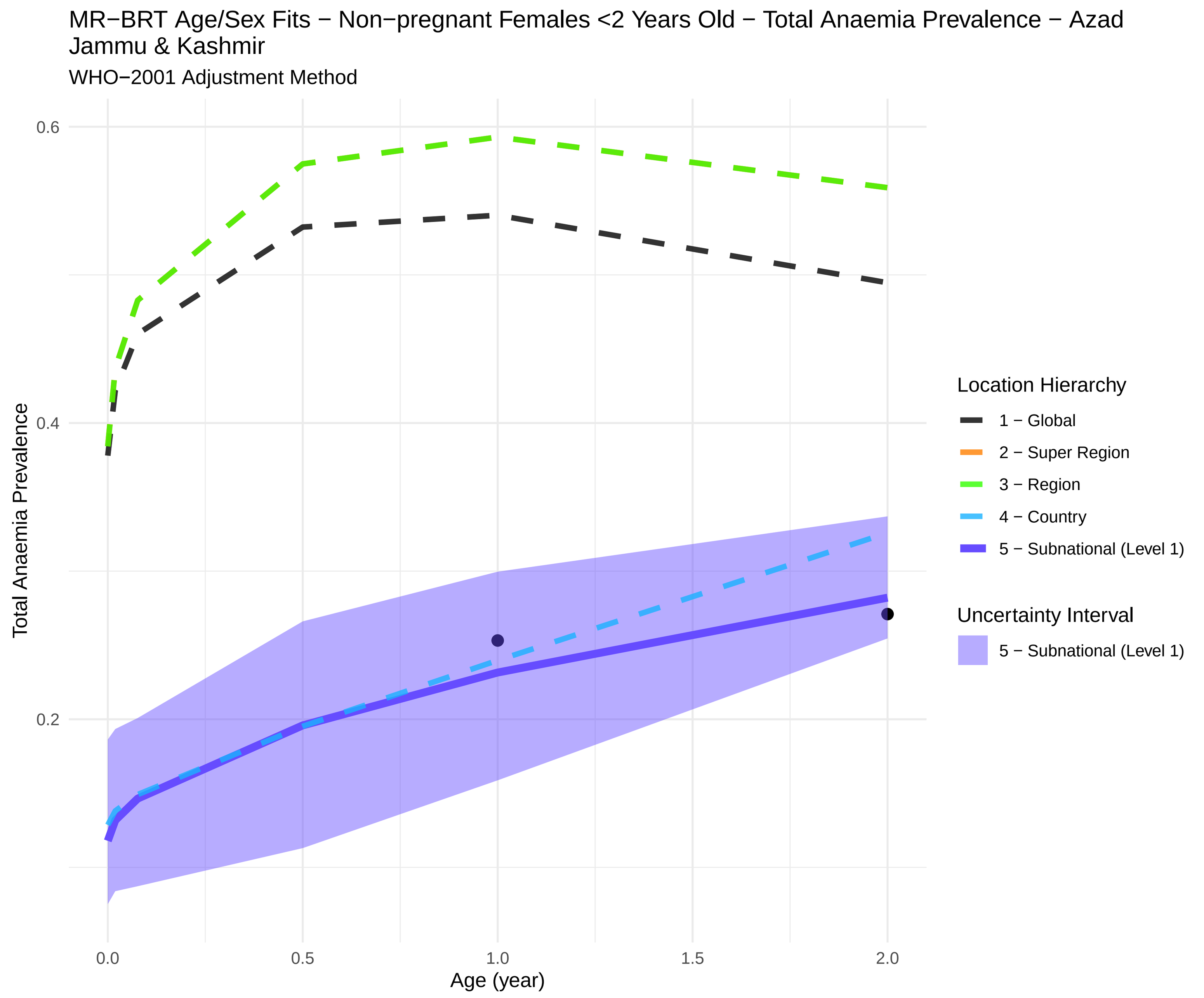
MR−BRT Age/Sex Fits − Non−pregnant Females <2 Years Old − Total Anaemia Prevalence − Azad Jammu & Kashmir
WHO−2001 Adjustment Method
Total Anaemia Prevalence
Age (year)
Location Hierarchy
1 − Global
2 − Super Region
3 − Region
4 − Country
5 − Subnational (Level 1)
Uncertainty Interval
5 − Subnational (Level 1)

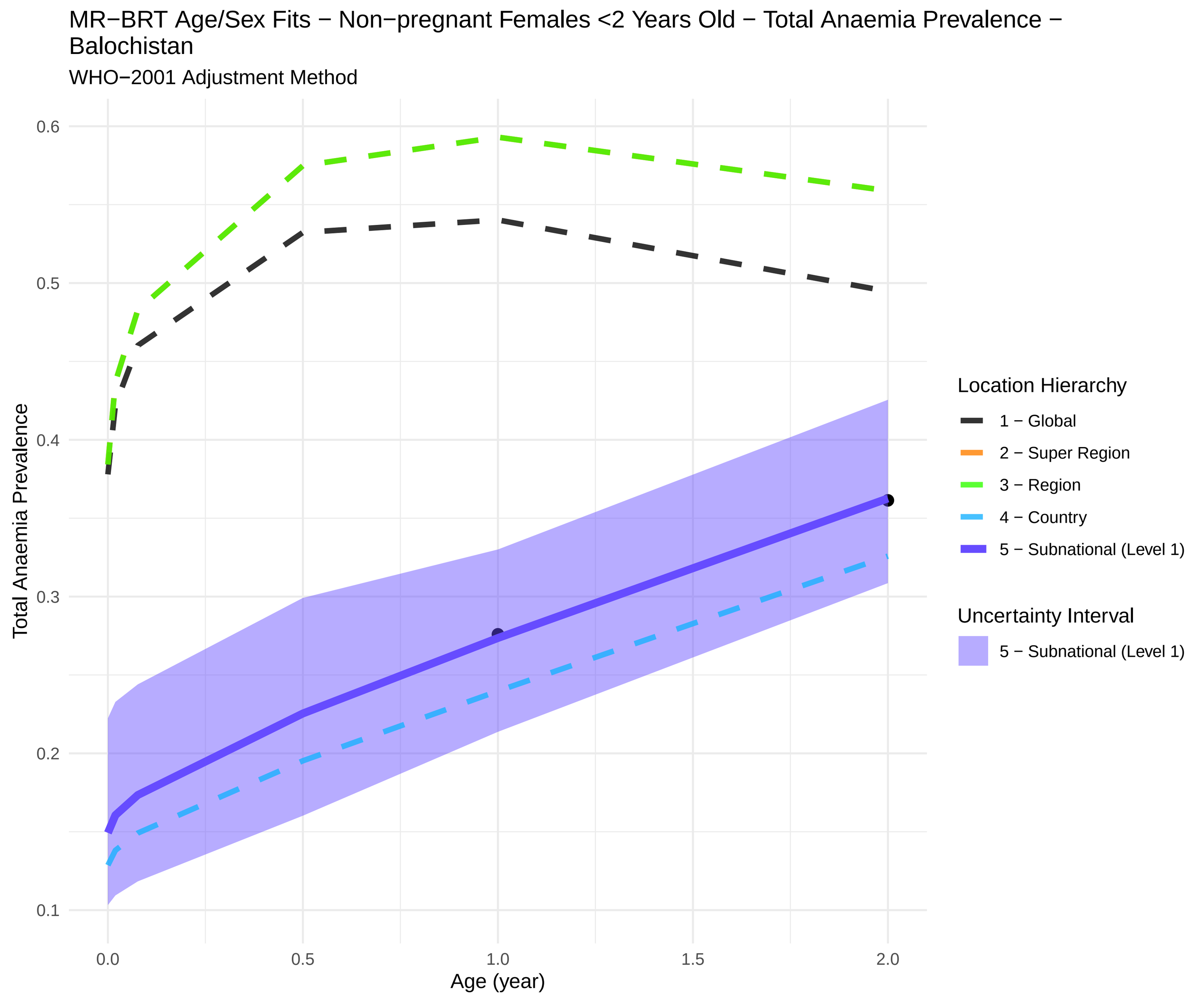
MR−BRT Age/Sex Fits − Non−pregnant Females <2 Years Old − Total Anaemia Prevalence − Balochistan
WHO−2001 Adjustment Method
Total Anaemia Prevalence
Age (year)
Location Hierarchy
1 − Global
2 − Super Region
3 − Region
4 − Country
5 − Subnational (Level 1)
Uncertainty Interval
5 − Subnational (Level 1)

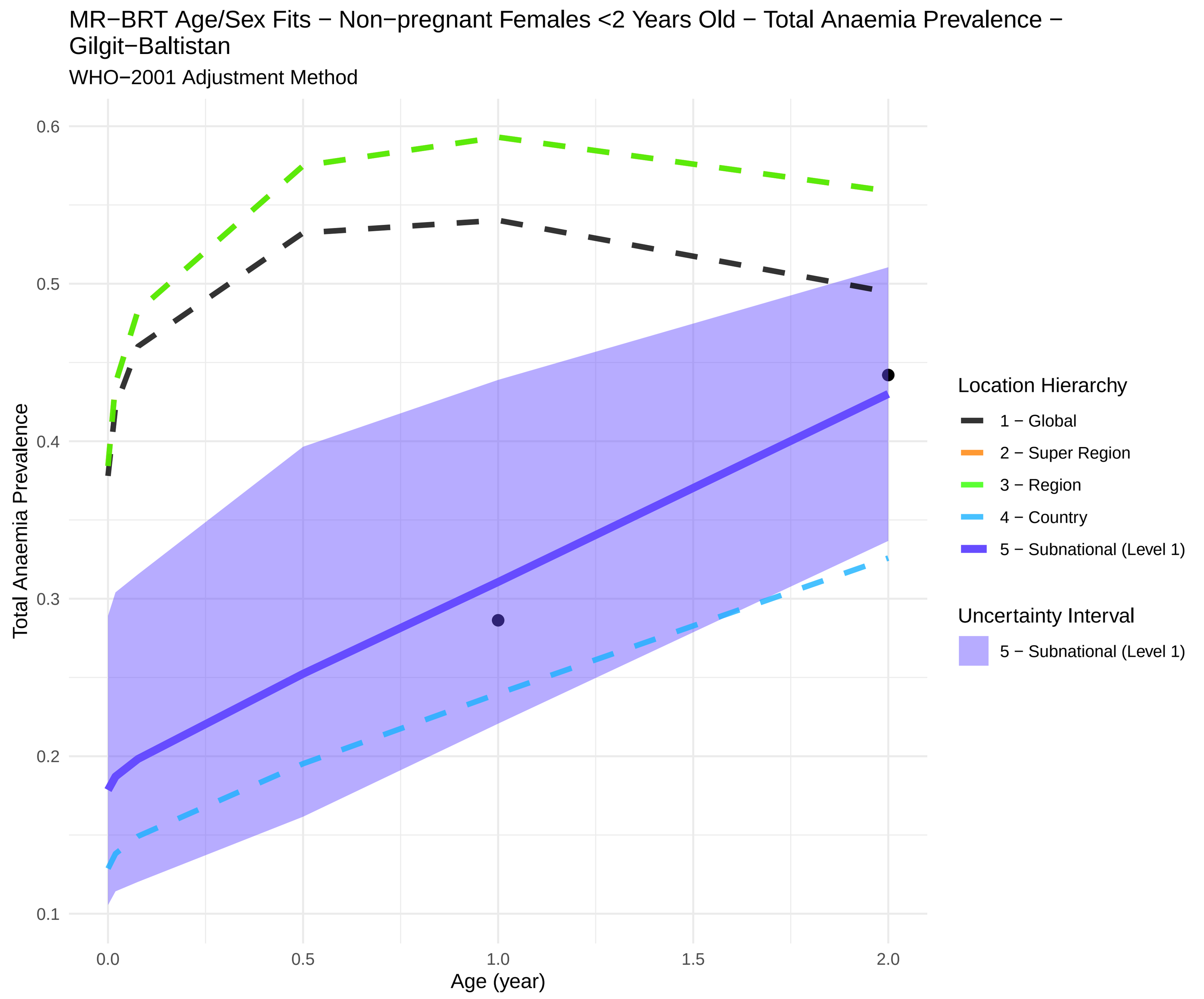
MR−BRT Age/Sex Fits − Non−pregnant Females <2 Years Old − Total Anaemia Prevalence − Gilgit−Baltistan
WHO−2001 Adjustment Method
Total Anaemia Prevalence
Age (year)
Location Hierarchy
1 − Global
2 − Super Region
3 − Region
4 − Country
5 − Subnational (Level 1)
Uncertainty Interval
5 − Subnational (Level 1)

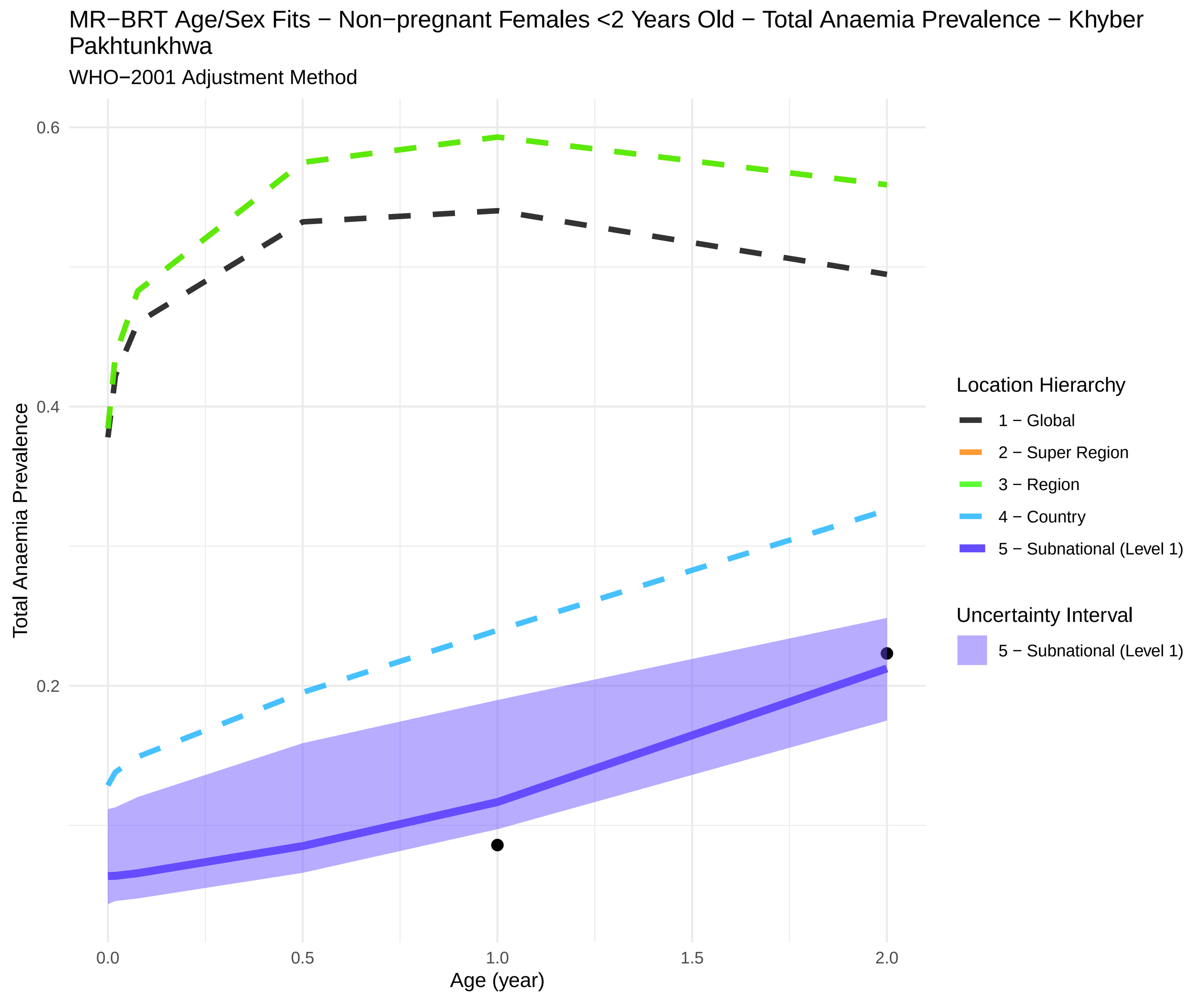
MR−BRT Age/Sex Fits − Non−pregnant Females <2 Years Old − Total Anaemia Prevalence − Khyber Pakhtunkhwa
WHO−2001 Adjustment Method
Total Anaemia Prevalence
Age (year)
Location Hierarchy
1 − Global
2 − Super Region
3 − Region
4 − Country
5 − Subnational (Level 1)
Uncertainty Interval
5 − Subnational (Level 1)

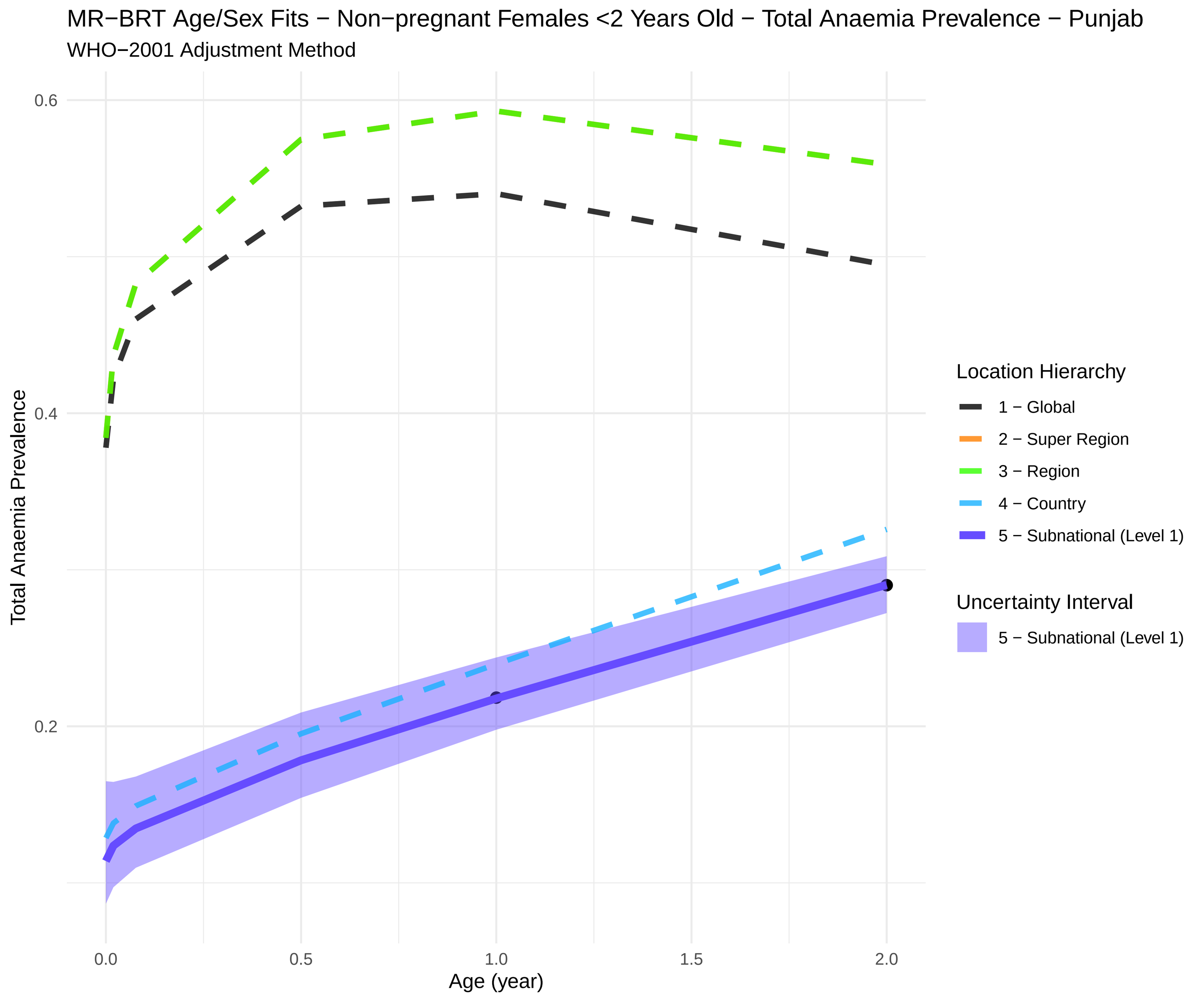
MR−BRT Age/Sex Fits − Non−pregnant Females <2 Years Old − Total Anaemia Prevalence − Punjab
WHO−2001 Adjustment Method
Total Anaemia Prevalence
Age (year)
Location Hierarchy
1 − Global
2 − Super Region
3 − Region
4 − Country
5 − Subnational (Level 1)
Uncertainty Interval
5 − Subnational (Level 1)

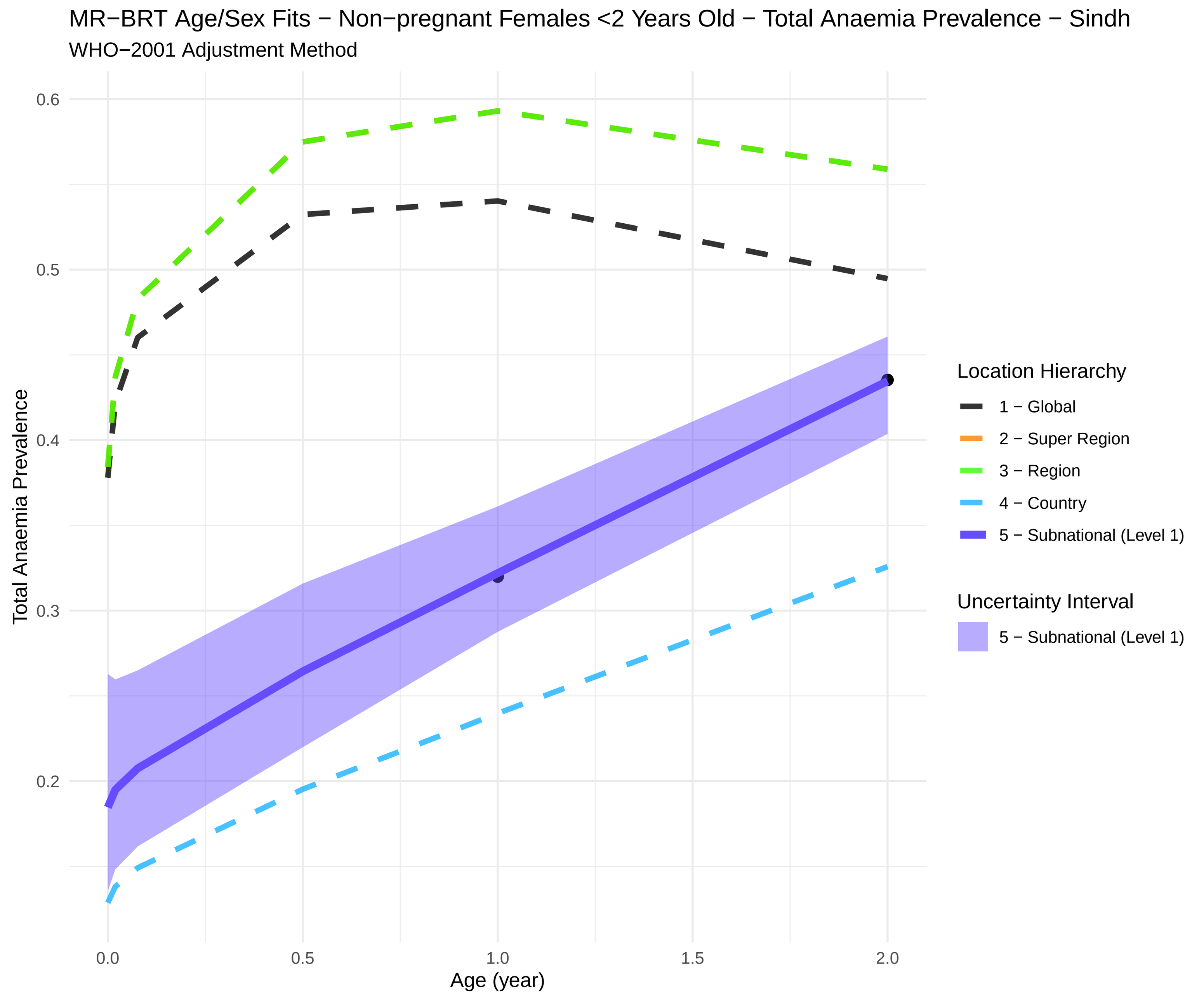
MR−BRT Age/Sex Fits − Non−pregnant Females <2 Years Old − Total Anaemia Prevalence − Sindh
WHO−2001 Adjustment Method
Total Anaemia Prevalence
Age (year)
Location Hierarchy
1 − Global
2 − Super Region
3 − Region
4 − Country
5 − Subnational (Level 1)
Uncertainty Interval
5 − Subnational (Level 1)

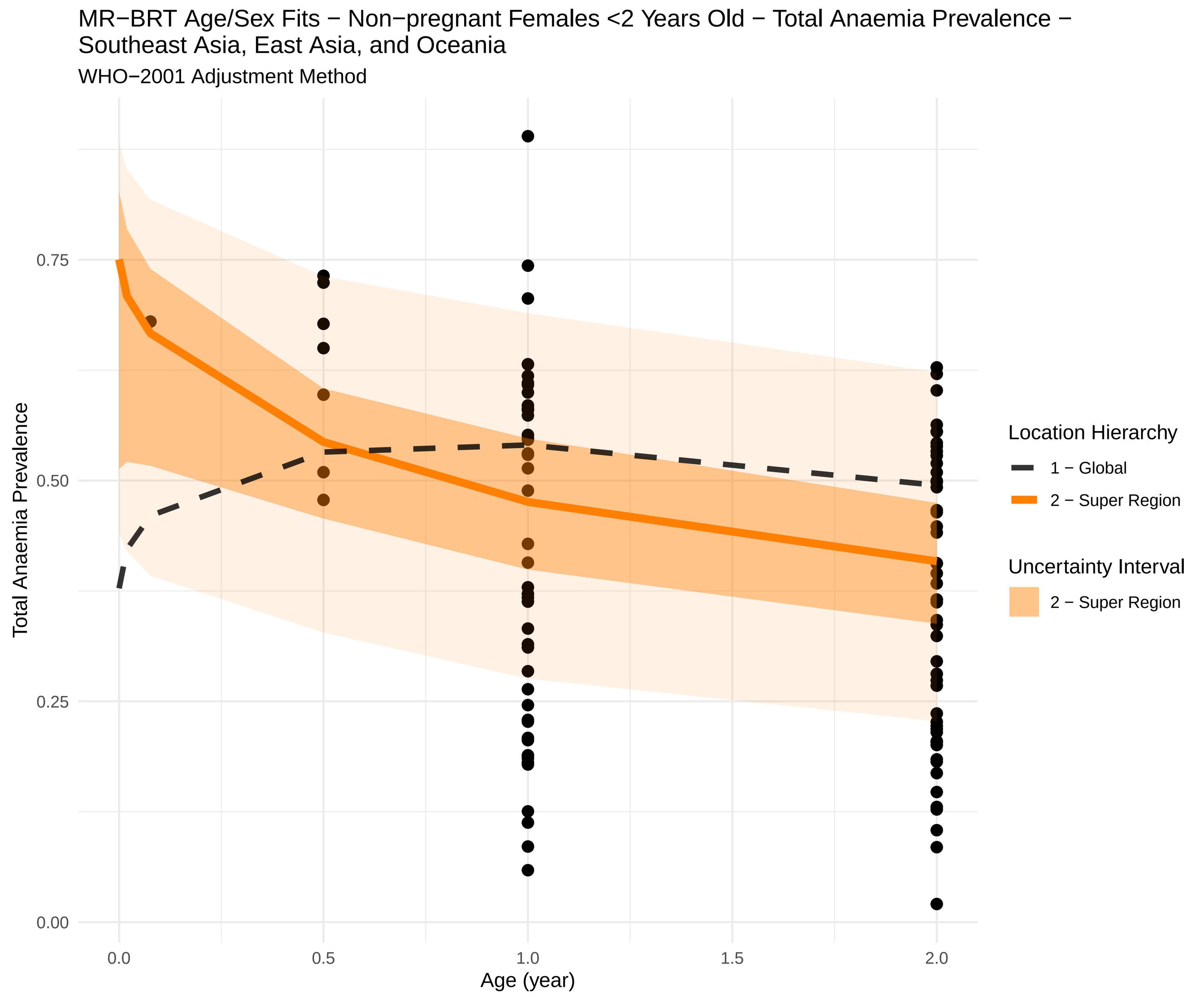
MR−BRT Age/Sex Fits − Non−pregnant Females <2 Years Old − Total Anaemia Prevalence − Southeast Asia, East Asia, and Oceania
WHO−2001 Adjustment Method
Total Anaemia Prevalence
Age (year)
Location Hierarchy
1 − Global
2 − Super Region
Uncertainty Interval
2 − Super Region

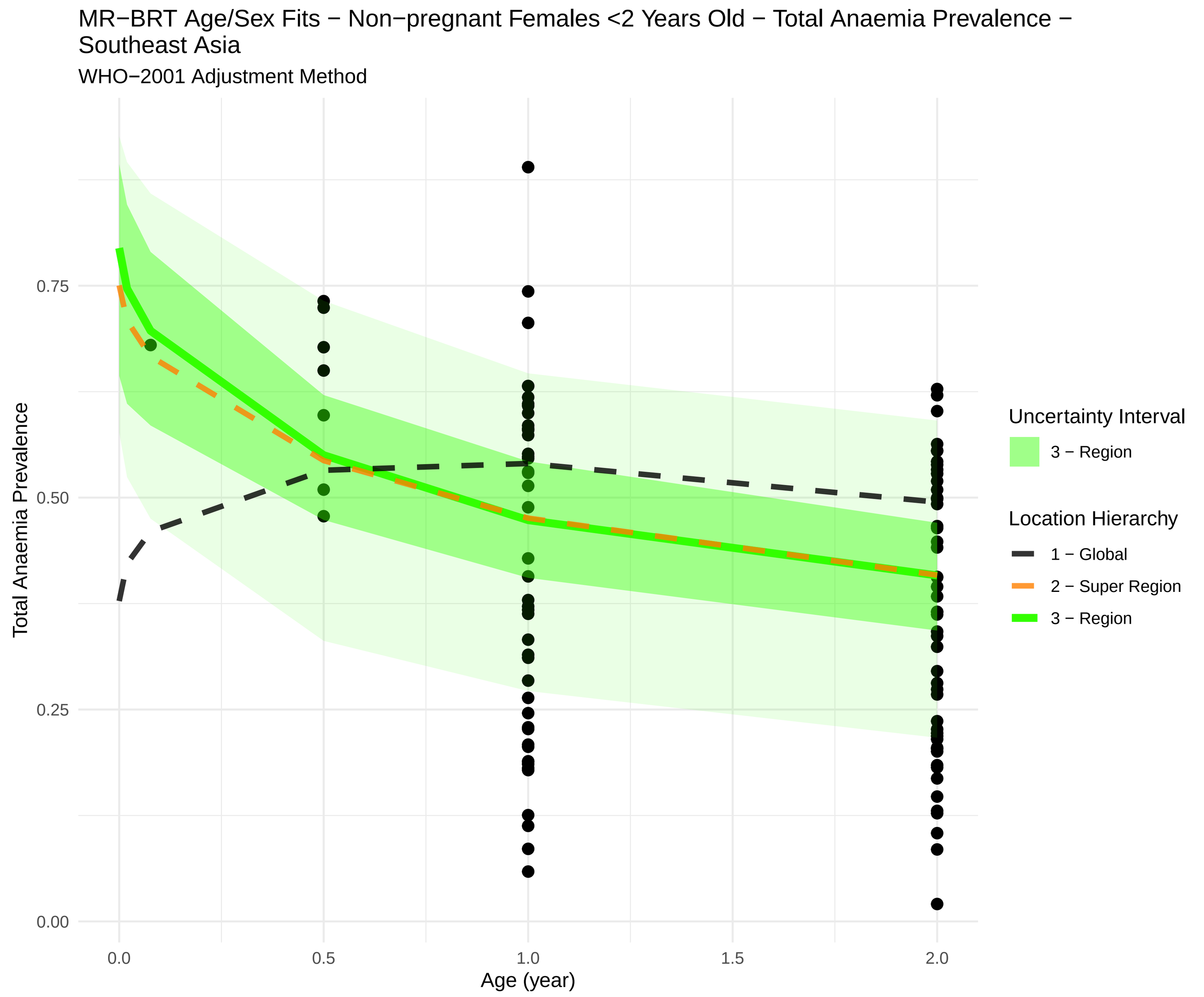
MR−BRT Age/Sex Fits − Non−pregnant Females <2 Years Old − Total Anaemia Prevalence − Southeast Asia
WHO−2001 Adjustment Method
Total Anaemia Prevalence
Age (year)
Uncertainty Interval
3 − Region
Location Hierarchy
1 − Global
2 − Super Region
3 − Region

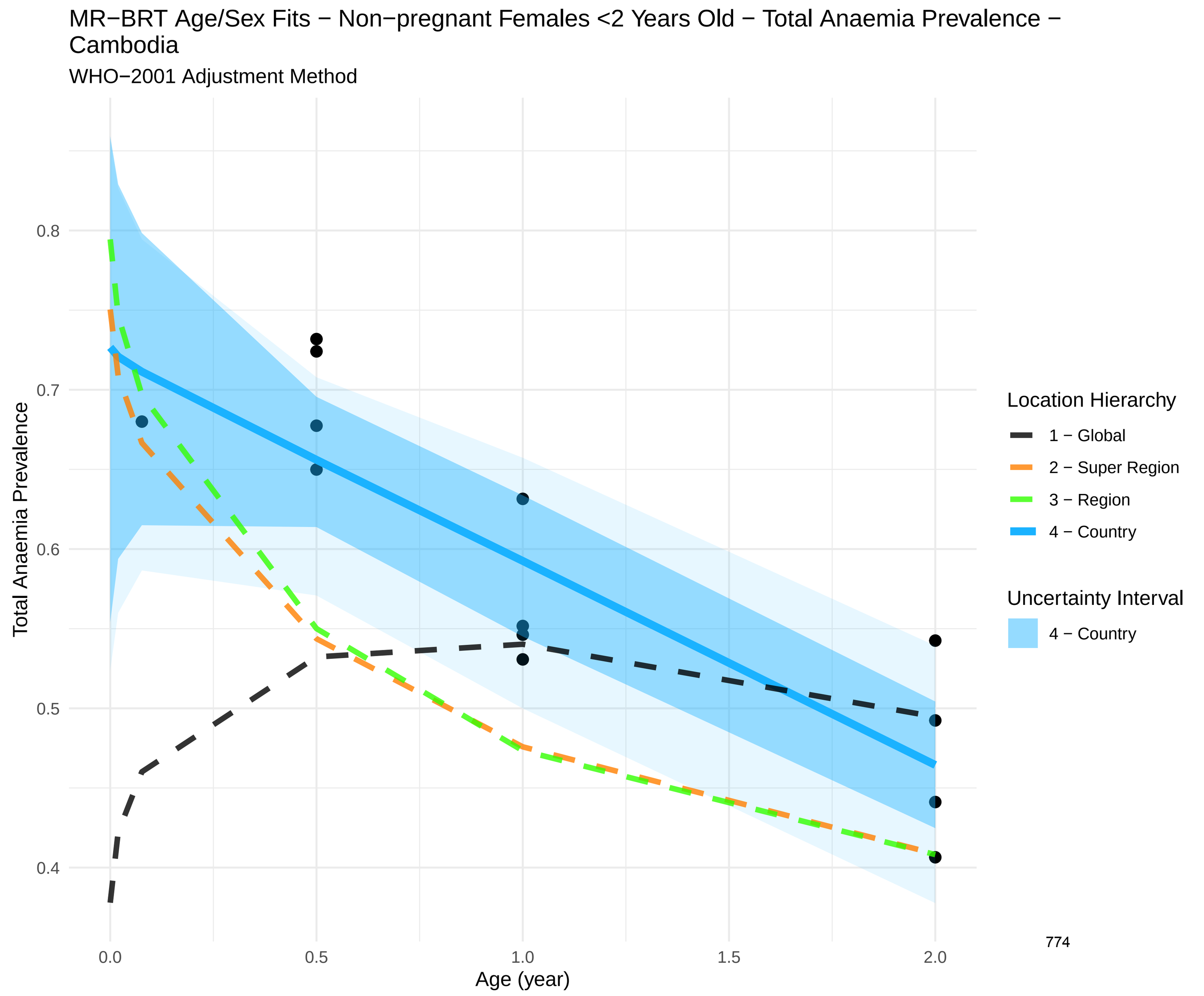
MR−BRT Age/Sex Fits − Non−pregnant Females <2 Years Old − Total Anaemia Prevalence − Cambodia
WHO−2001 Adjustment Method
Total Anaemia Prevalence
Age (year)
Location Hierarchy
1 − Global
2 − Super Region
3 − Region
4 − Country
Uncertainty Interval
4 − Country

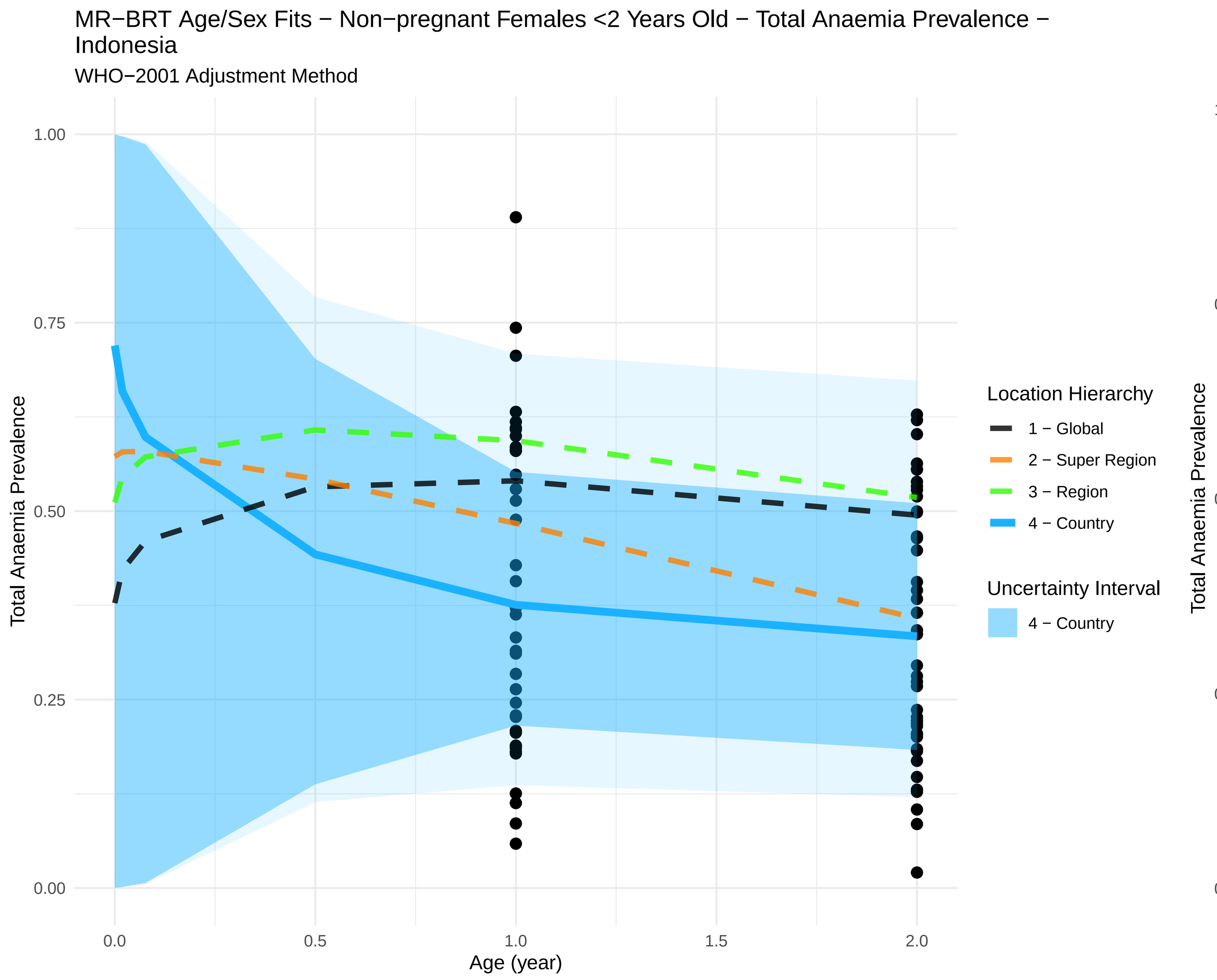
MR−BRT Age/Sex Fits − Non−pregnant Females <2 Years Old − Total Anaemia Prevalence − Indonesia
WHO−2001 Adjustment Method
Total Anaemia Prevalence
Age (year)
Location Hierarchy
1 − Global
2 − Super Region
3 − Region
4 − Country
Uncertainty Interval
4 − Country

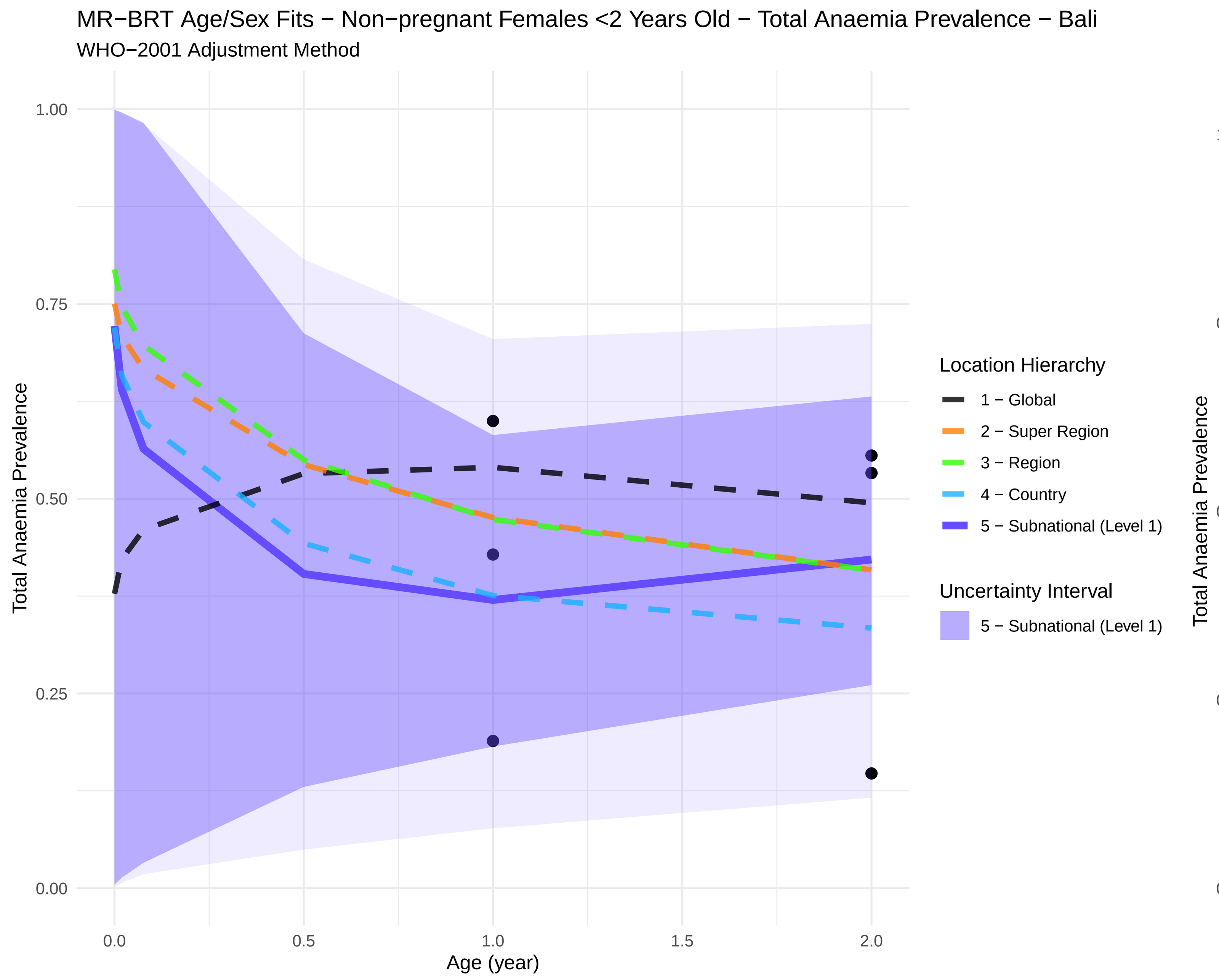
MR−BRT Age/Sex Fits − Non−pregnant Females <2 Years Old − Total Anaemia Prevalence − Bali
WHO−2001 Adjustment Method
Total Anaemia Prevalence
Age (year)
Location Hierarchy
1 − Global
2 − Super Region
3 − Region
4 − Country
5 − Subnational (Level 1)
Uncertainty Interval
5 − Subnational (Level 1)

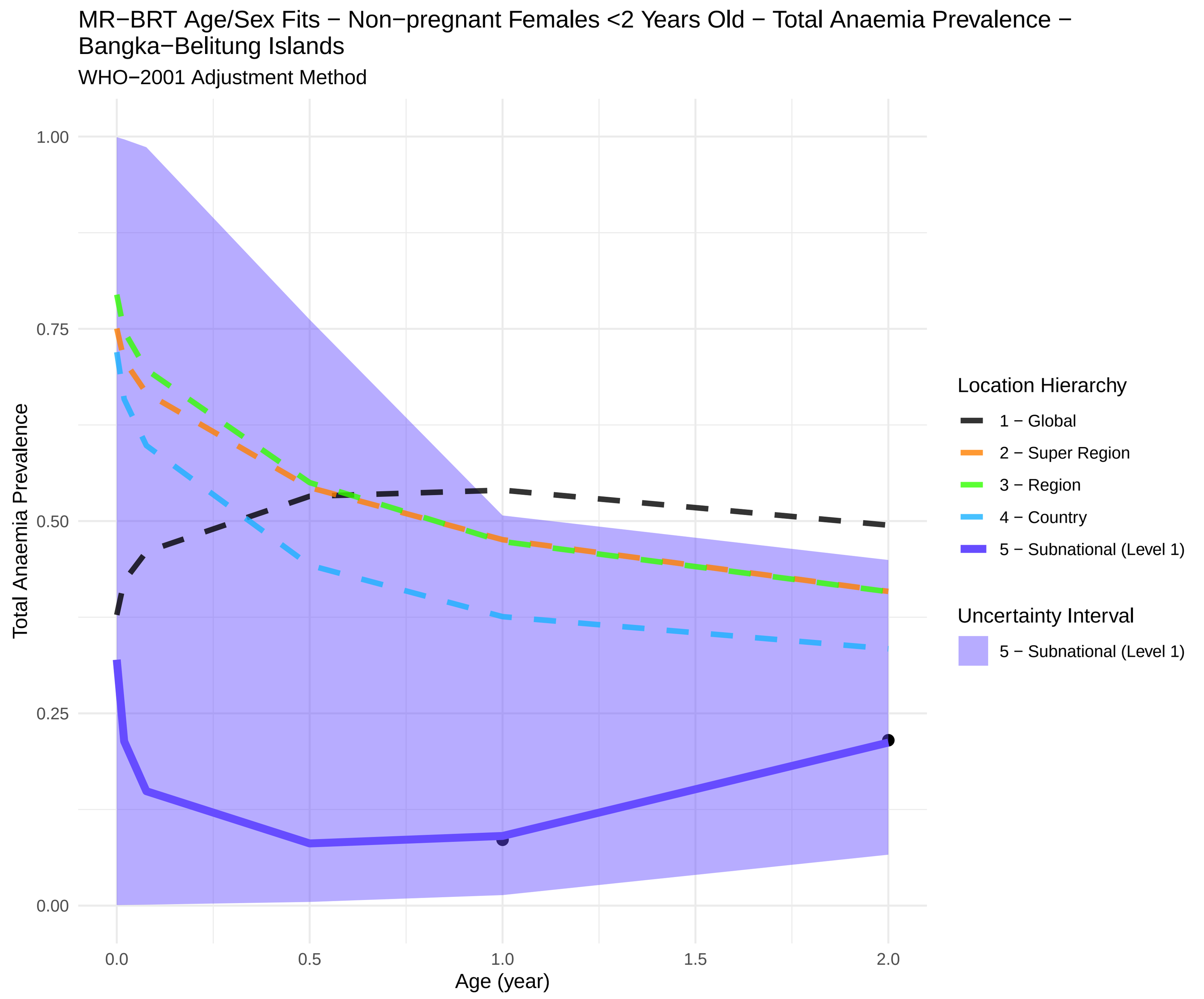
MR−BRT Age/Sex Fits − Non−pregnant Females <2 Years Old − Total Anaemia Prevalence − Bangka−Belitung Islands
WHO−2001 Adjustment Method
Total Anaemia Prevalence
Age (year)
Location Hierarchy
1 − Global
2 − Super Region
3 − Region
4 − Country
5 − Subnational (Level 1)
Uncertainty Interval
5 − Subnational (Level 1)

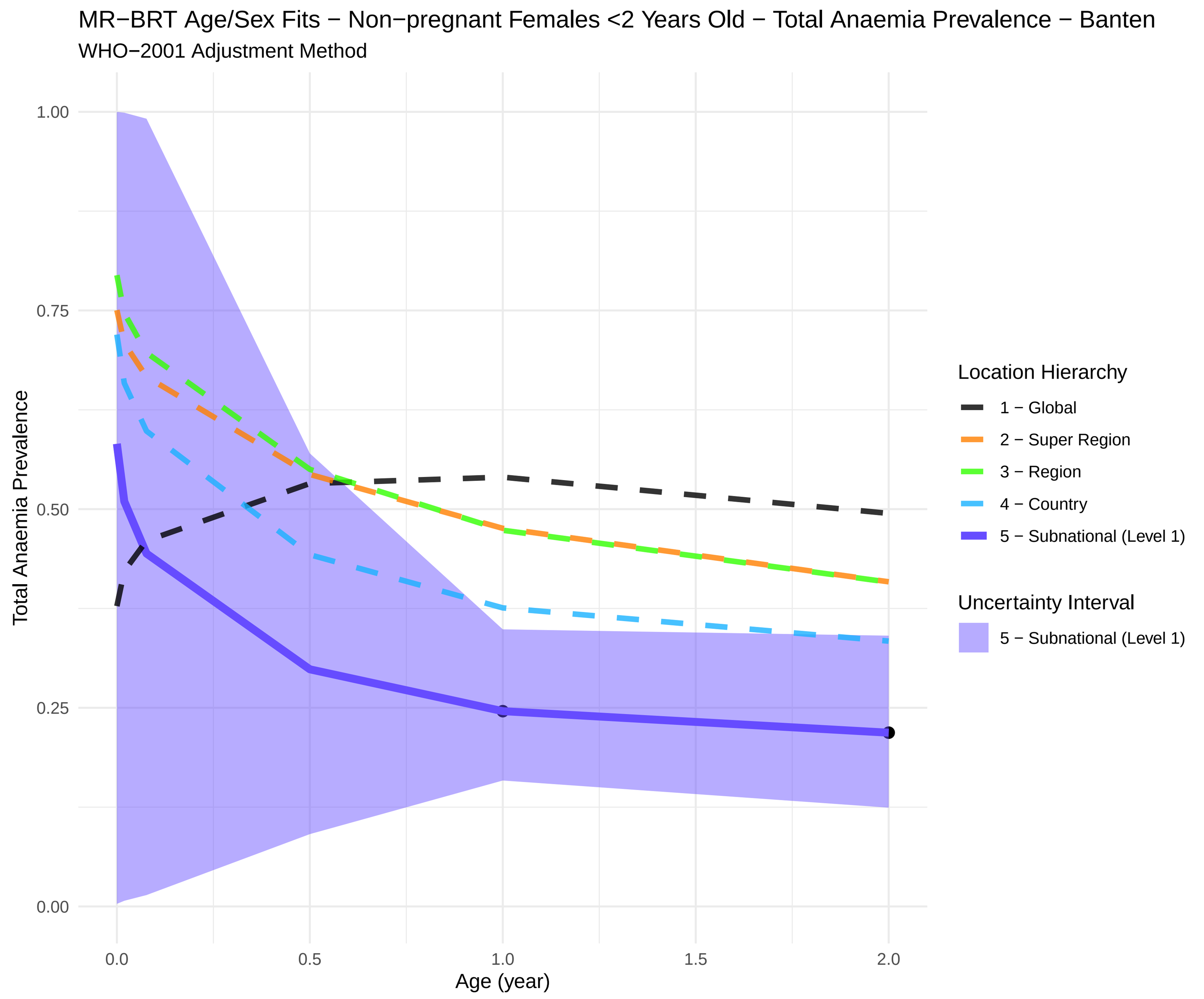
MR−BRT Age/Sex Fits − Non−pregnant Females <2 Years Old − Total Anaemia Prevalence − Banten
WHO−2001 Adjustment Method
Total Anaemia Prevalence
Age (year)
Location Hierarchy
1 − Global
2 − Super Region
3 − Region
4 − Country
5 − Subnational (Level 1)
Uncertainty Interval
5 − Subnational (Level 1)

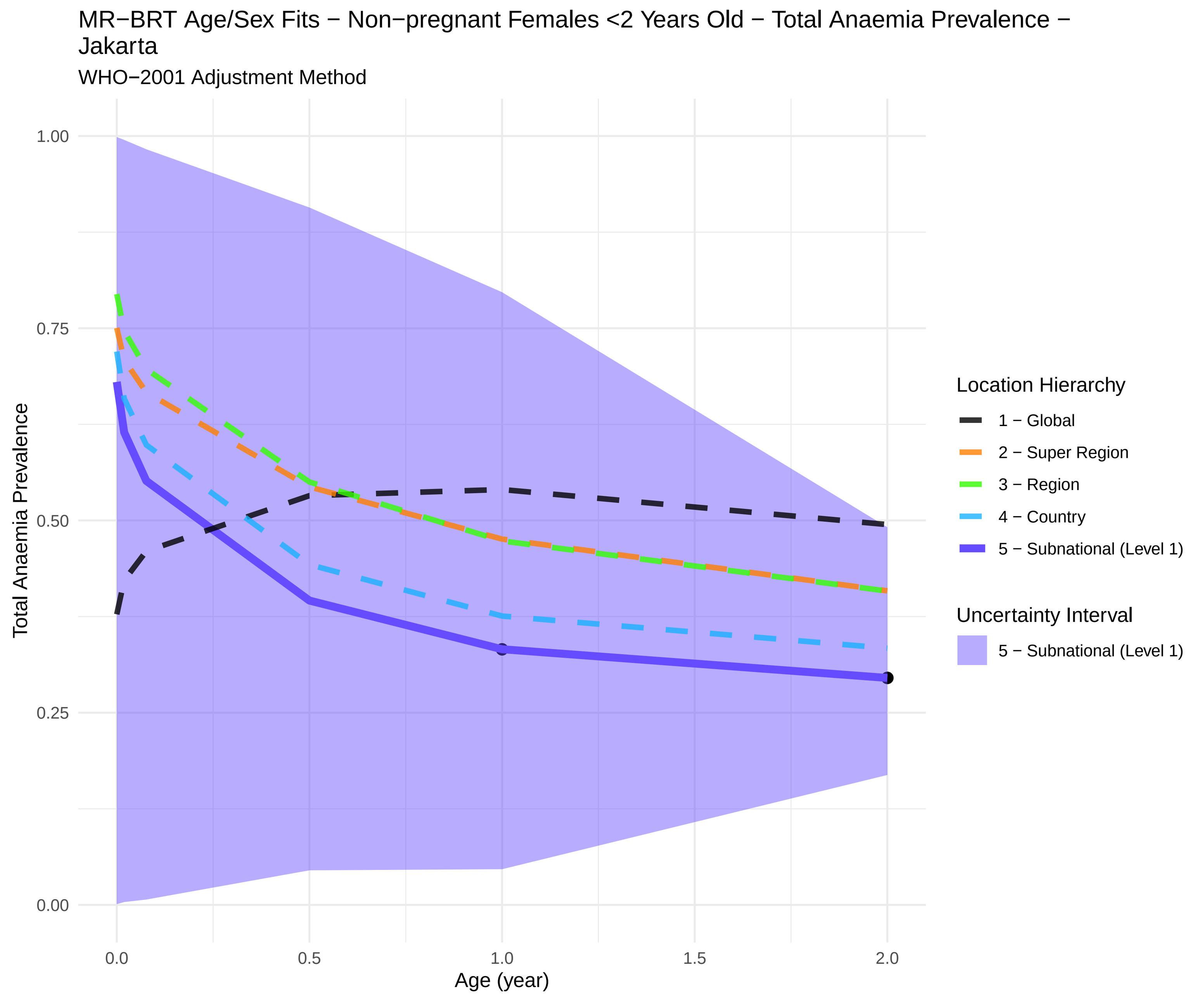
MR−BRT Age/Sex Fits − Non−pregnant Females <2 Years Old − Total Anaemia Prevalence − Jakarta
WHO−2001 Adjustment Method
Total Anaemia Prevalence
Age (year)
Location Hierarchy
1 − Global
2 − Super Region
3 − Region
4 − Country
5 − Subnational (Level 1)
Uncertainty Interval
5 − Subnational (Level 1)

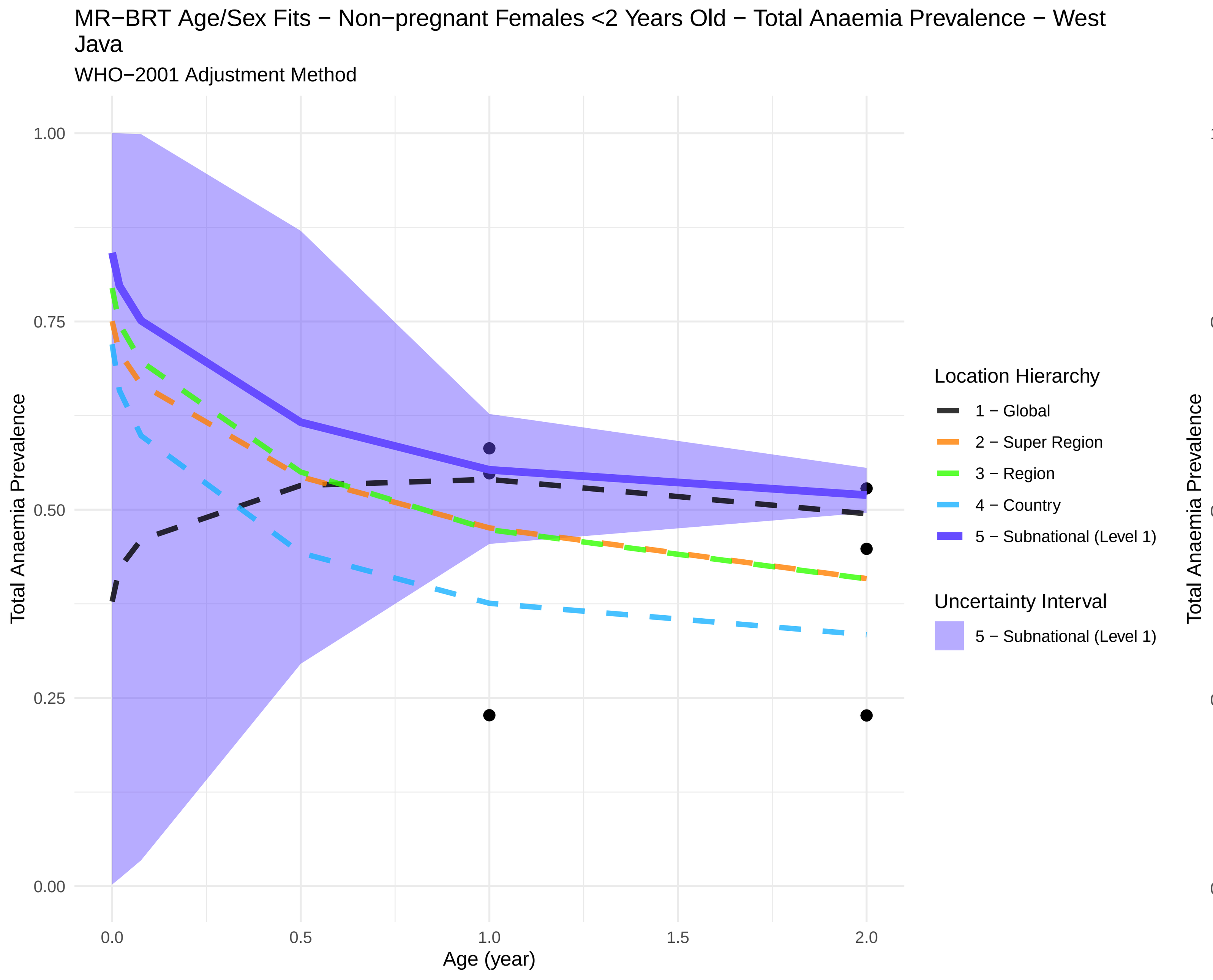
MR−BRT Age/Sex Fits − Non−pregnant Females <2 Years Old − Total Anaemia Prevalence − West Java
WHO−2001 Adjustment Method
Total Anaemia Prevalence
Age (year)
Location Hierarchy
1 − Global
2 − Super Region
3 − Region
4 − Country
5 − Subnational (Level 1)
Uncertainty Interval
5 − Subnational (Level 1)

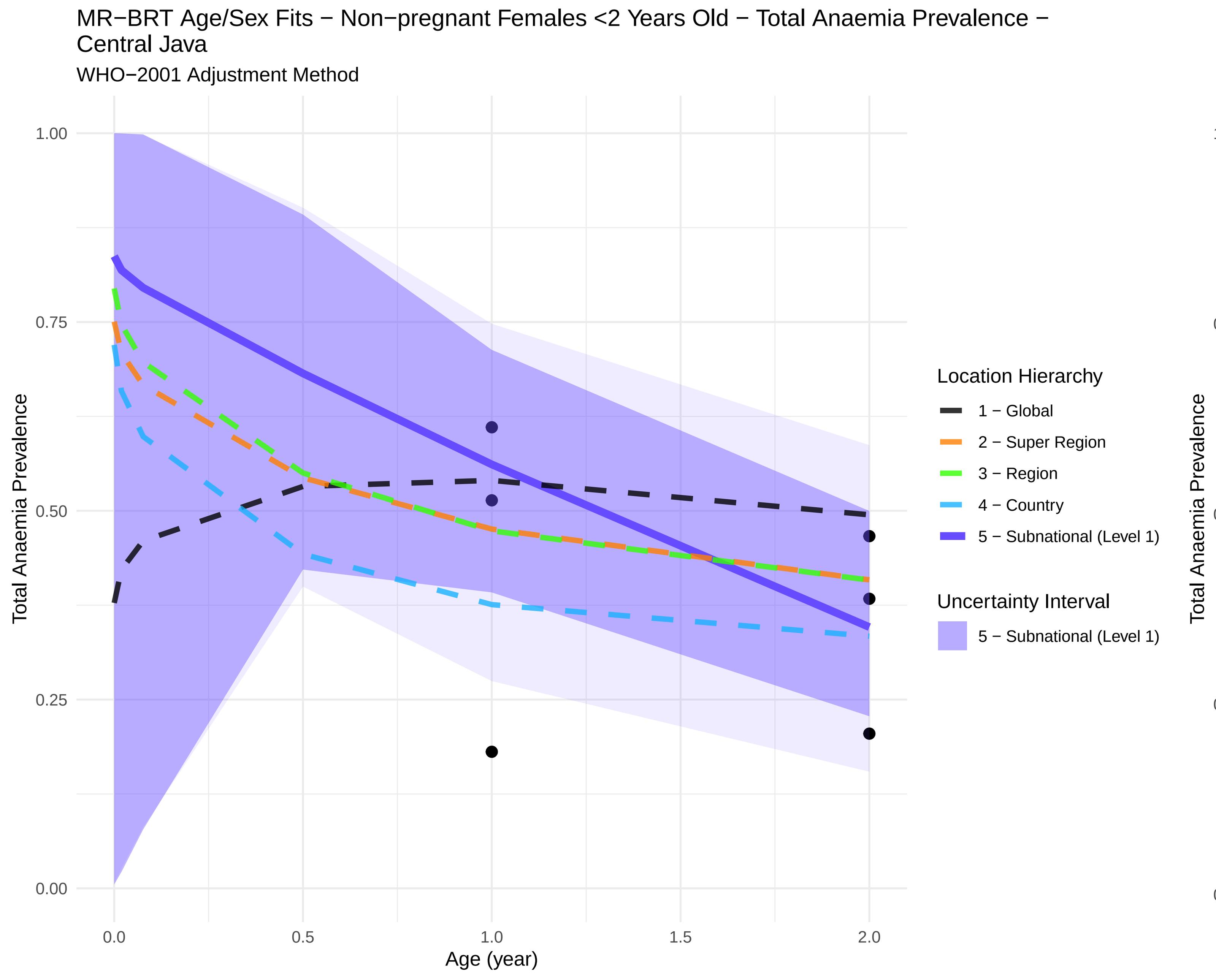
MR−BRT Age/Sex Fits − Non−pregnant Females <2 Years Old − Total Anaemia Prevalence − Central Java
WHO−2001 Adjustment Method
Total Anaemia Prevalence
Age (year)
Location Hierarchy
1 − Global
2 − Super Region
3 − Region
4 − Country
5 − Subnational (Level 1)
Uncertainty Interval
5 − Subnational (Level 1)

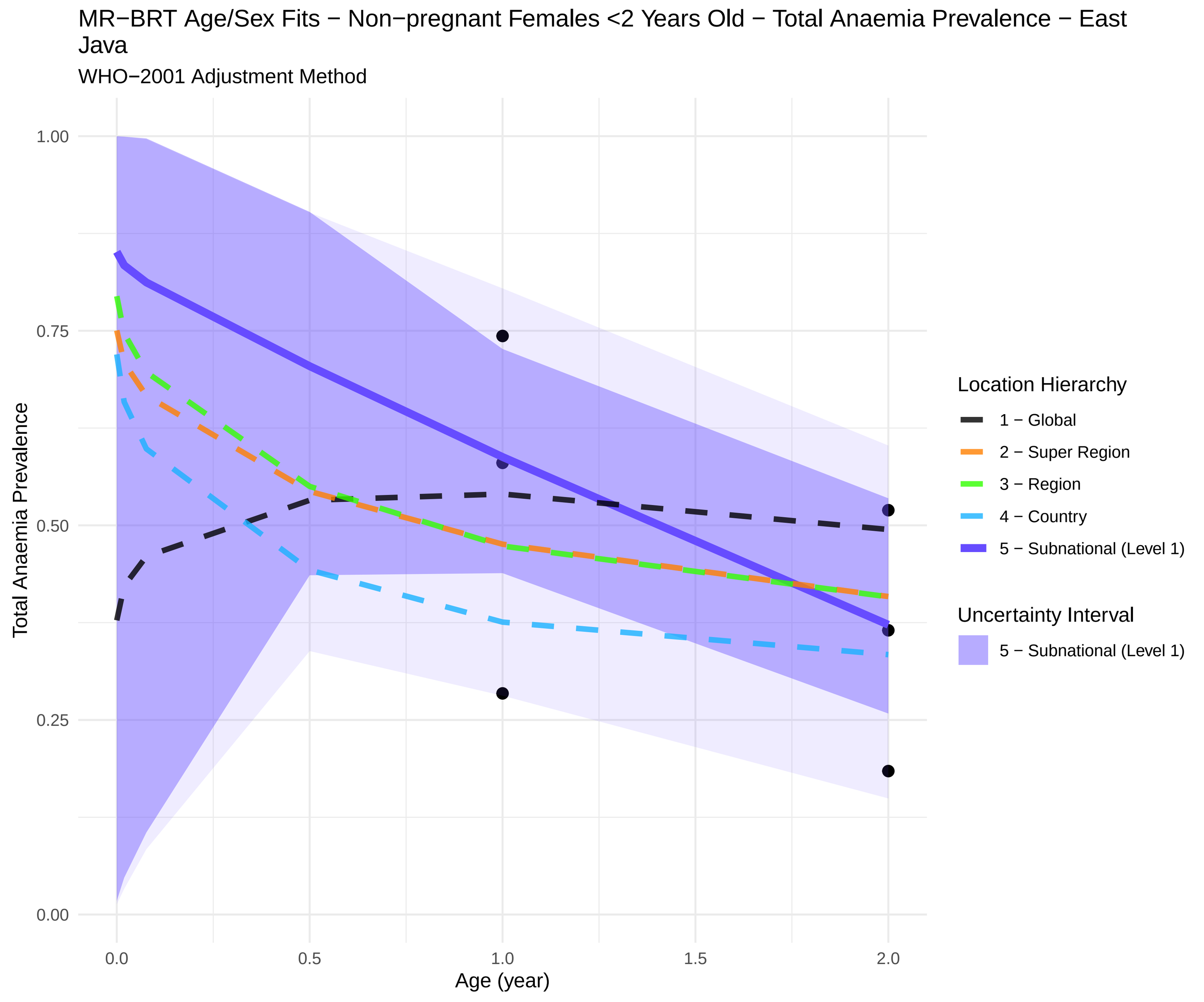
MR−BRT Age/Sex Fits − Non−pregnant Females <2 Years Old − Total Anaemia Prevalence − East Java
WHO−2001 Adjustment Method
Total Anaemia Prevalence
Age (year)
Location Hierarchy
1 − Global
2 − Super Region
3 − Region
4 − Country
5 − Subnational (Level 1)
Uncertainty Interval
5 − Subnational (Level 1)

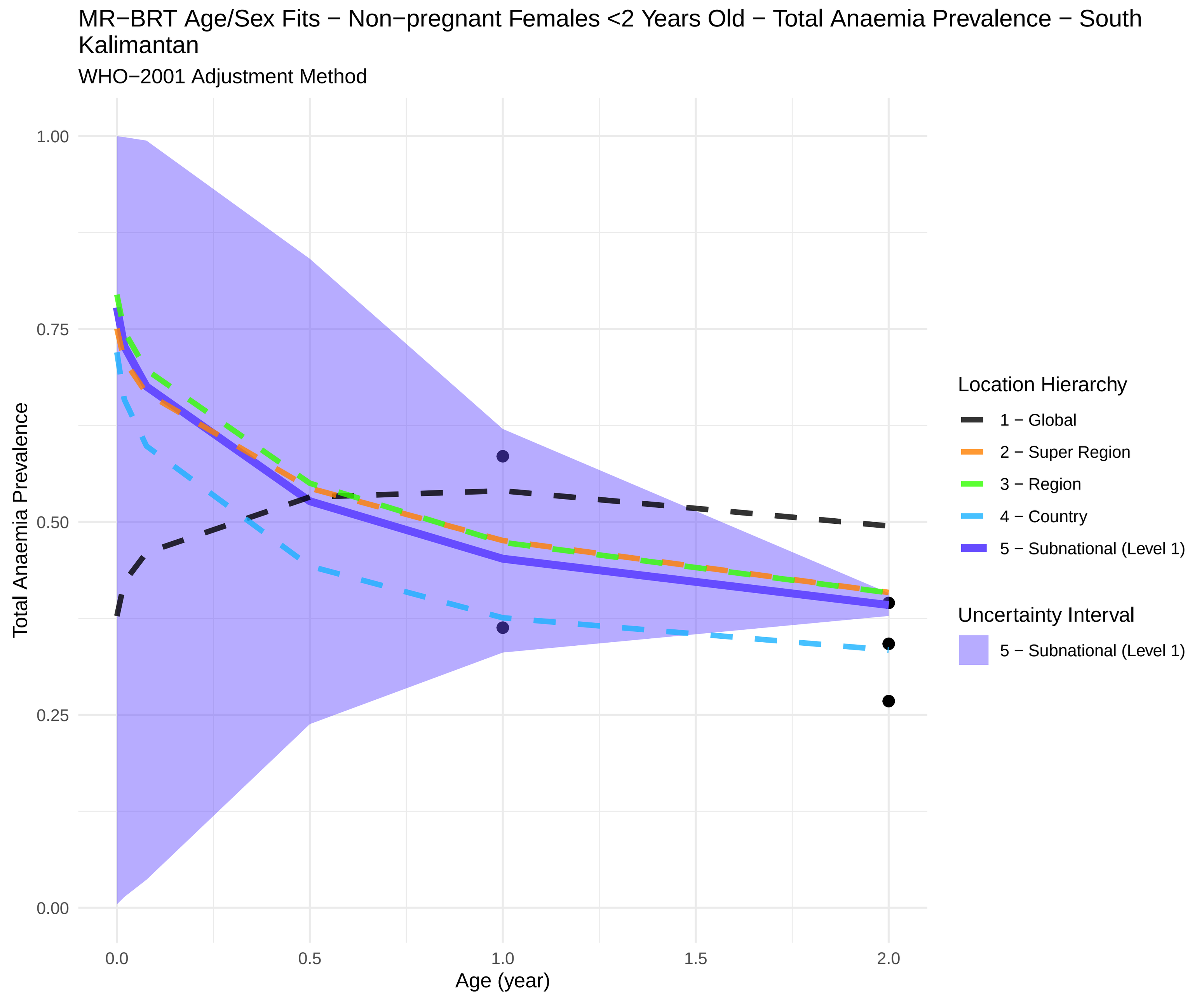
MR−BRT Age/Sex Fits − Non−pregnant Females <2 Years Old − Total Anaemia Prevalence − South Kalimantan
WHO−2001 Adjustment Method
Total Anaemia Prevalence
Age (year)
Location Hierarchy
1 − Global
2 − Super Region
3 − Region
4 − Country
5 − Subnational (Level 1)
Uncertainty Interval
5 − Subnational (Level 1)

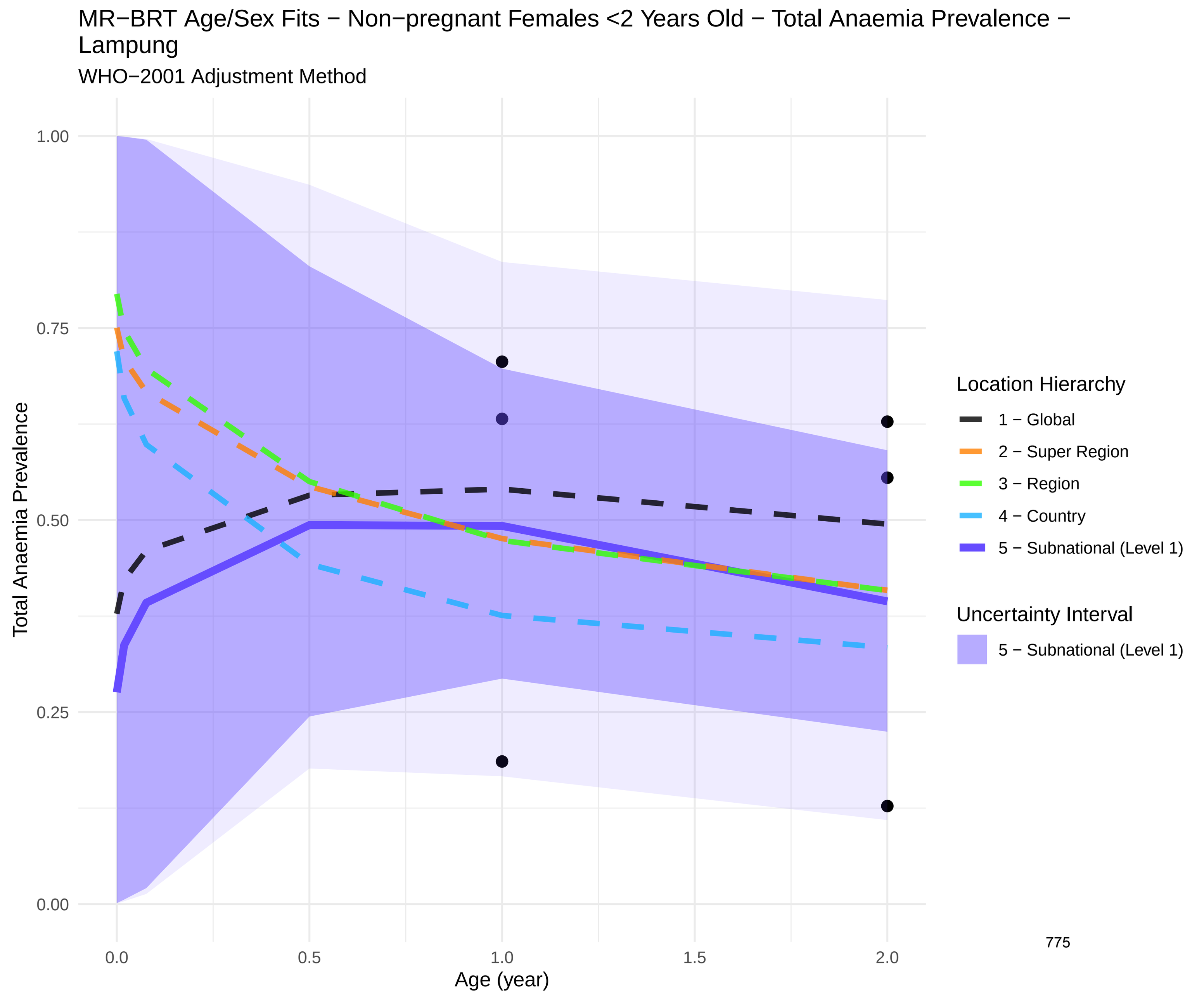
MR−BRT Age/Sex Fits − Non−pregnant Females <2 Years Old − Total Anaemia Prevalence − Lampung
WHO−2001 Adjustment Method
Total Anaemia Prevalence
Age (year)
Location Hierarchy
1 − Global
2 − Super Region
3 − Region
4 − Country
5 − Subnational (Level 1)
Uncertainty Interval
5 − Subnational (Level 1)

MR−BRT Age/Sex Fits − Non−pregnant Females <2 Years Old − Total Anaemia Prevalence − West Nusa Tenggara
WHO−2001 Adjustment Method

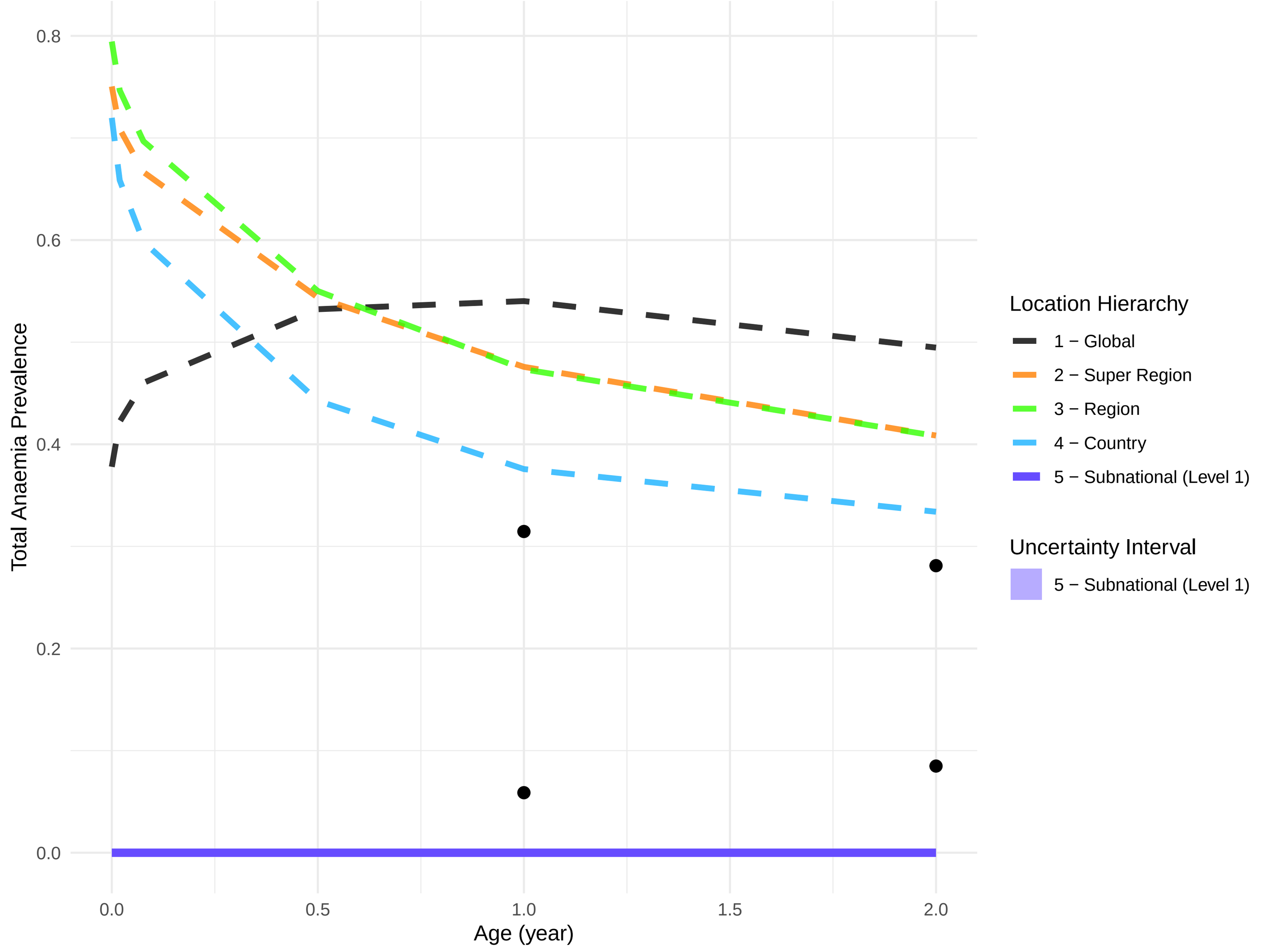

MR−BRT Age/Sex Fits − Non−pregnant Females <2 Years Old − Total Anaemia Prevalence − Riau
WHO−2001 Adjustment Method

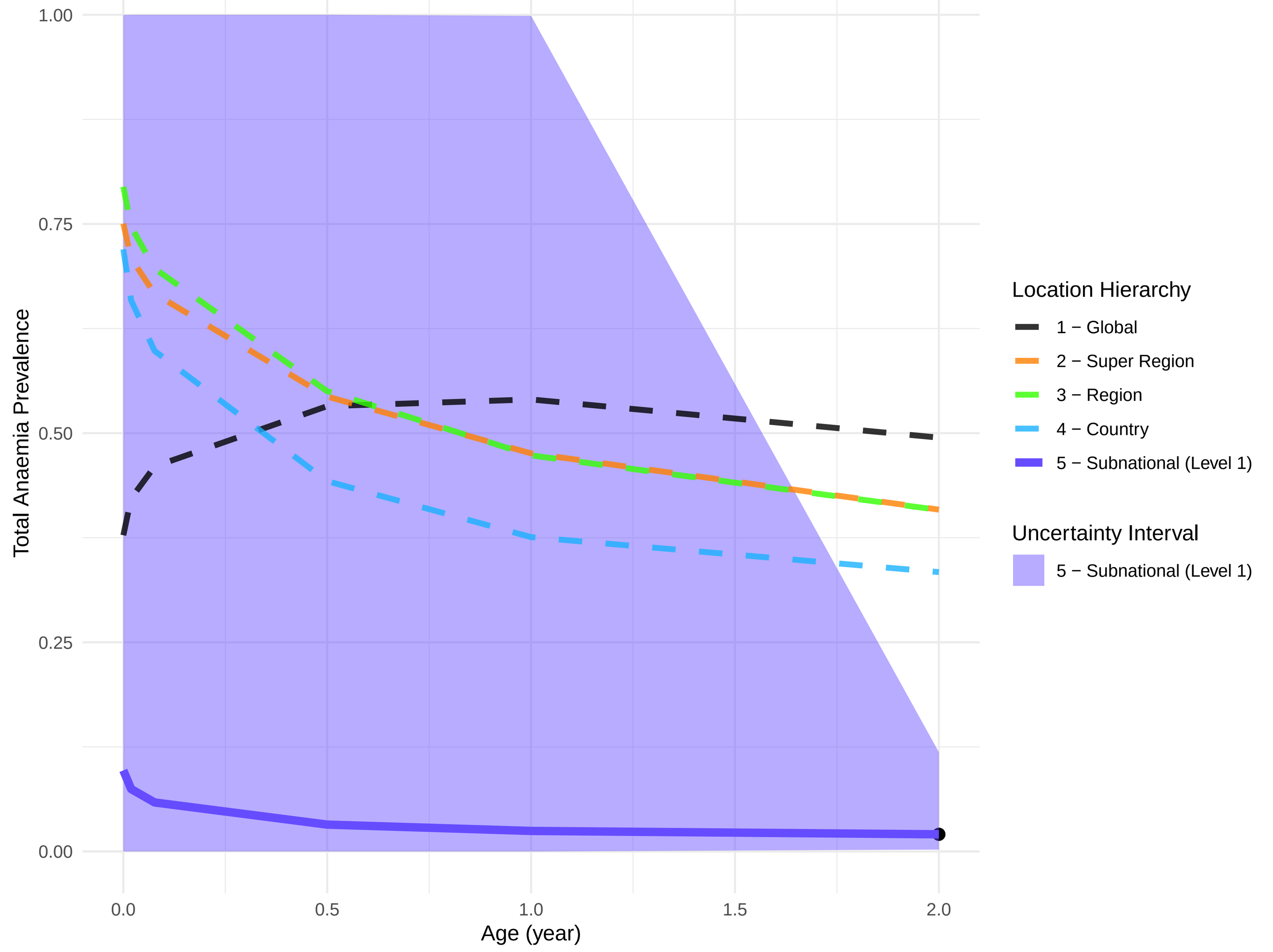

MR−BRT Age/Sex Fits − Non−pregnant Females <2 Years Old − Total Anaemia Prevalence − West Sulawesi
WHO−2001 Adjustment Method

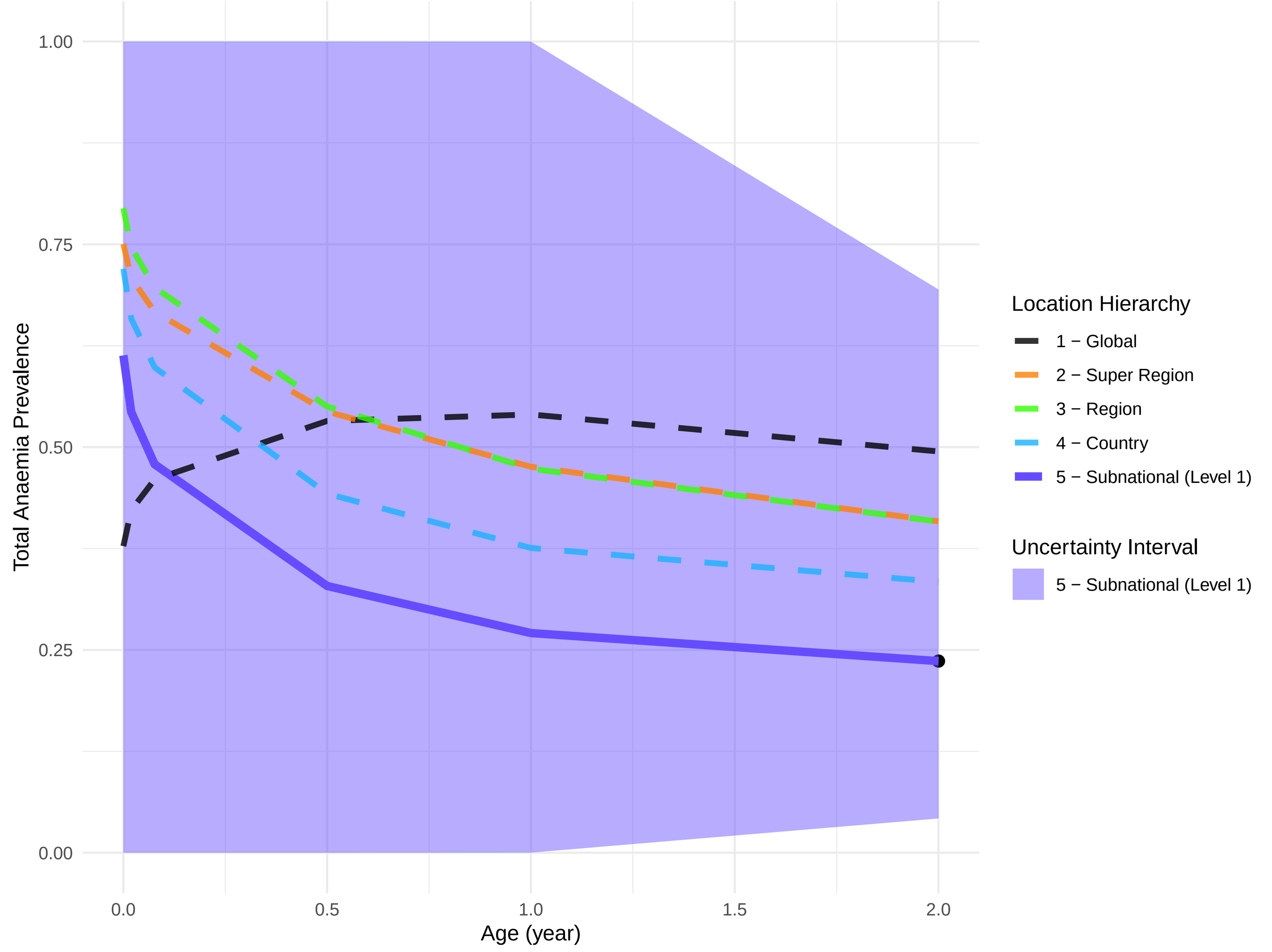

MR−BRT Age/Sex Fits − Non−pregnant Females <2 Years Old − Total Anaemia Prevalence − South Sulawesi
WHO−2001 Adjustment Method

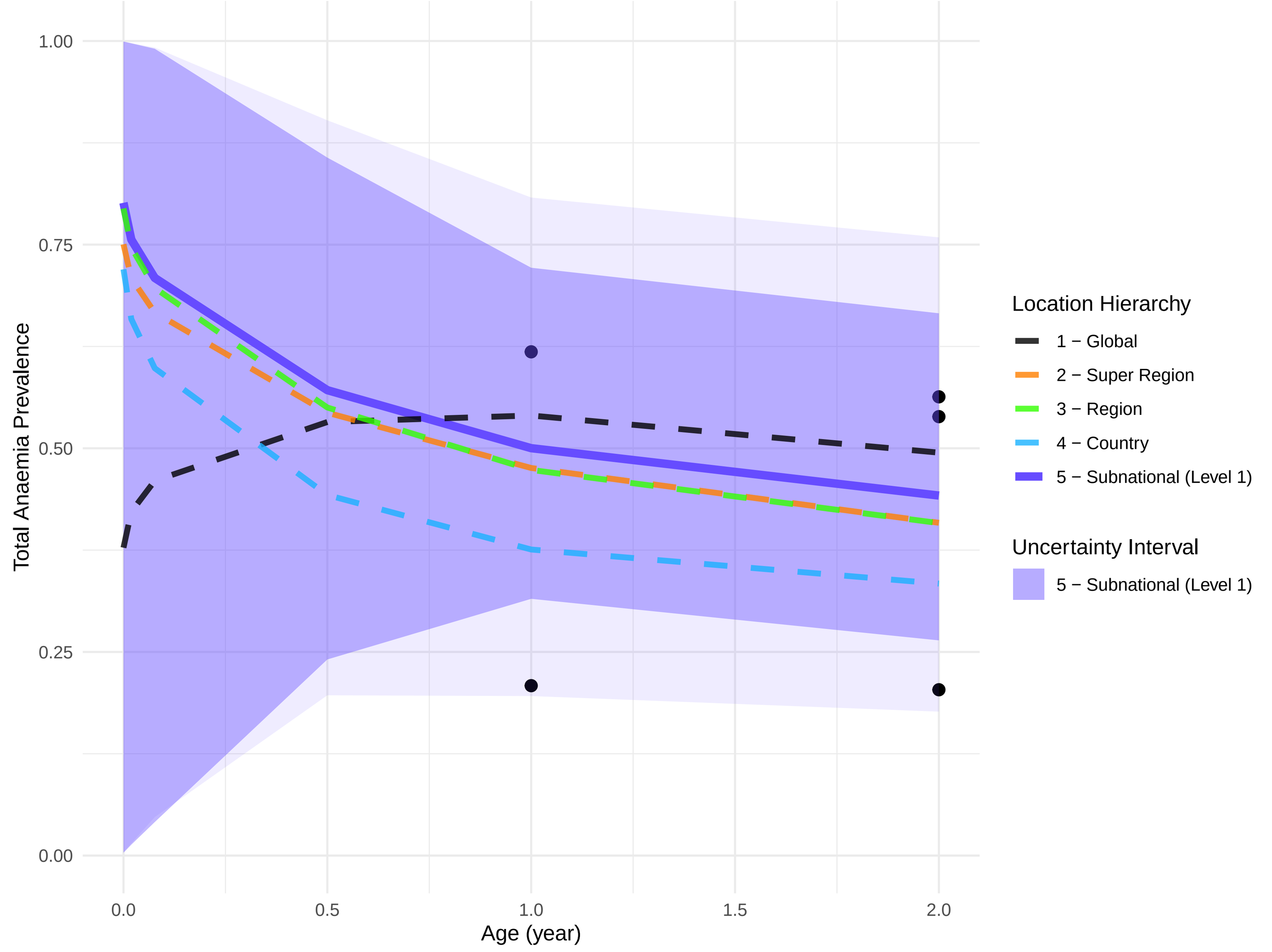

MR−BRT Age/Sex Fits − Non−pregnant Females <2 Years Old − Total Anaemia Prevalence − West Sumatra
WHO−2001 Adjustment Method

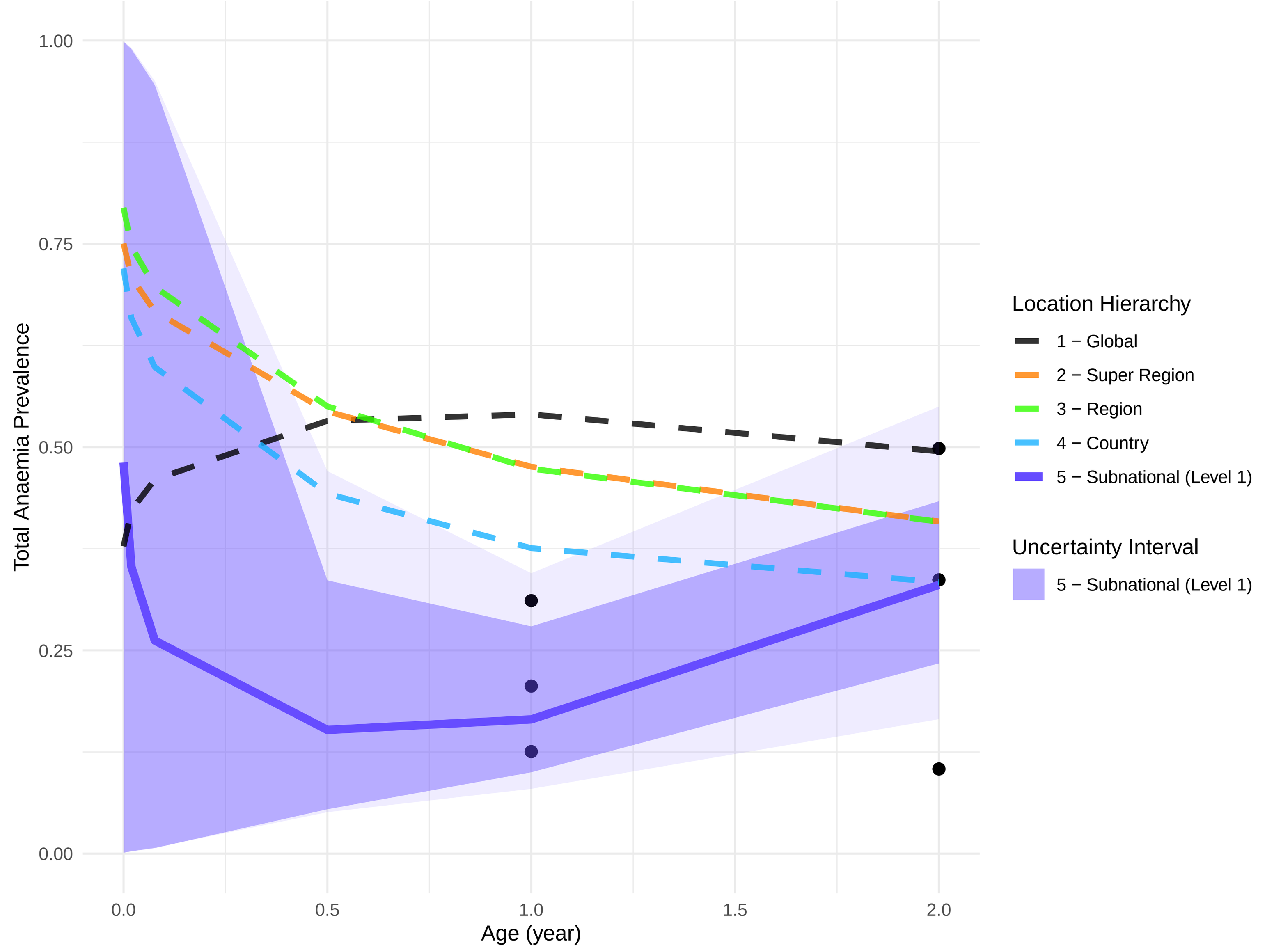

MR−BRT Age/Sex Fits − Non−pregnant Females <2 Years Old − Total Anaemia Prevalence − South Sumatra
WHO−2001 Adjustment Method

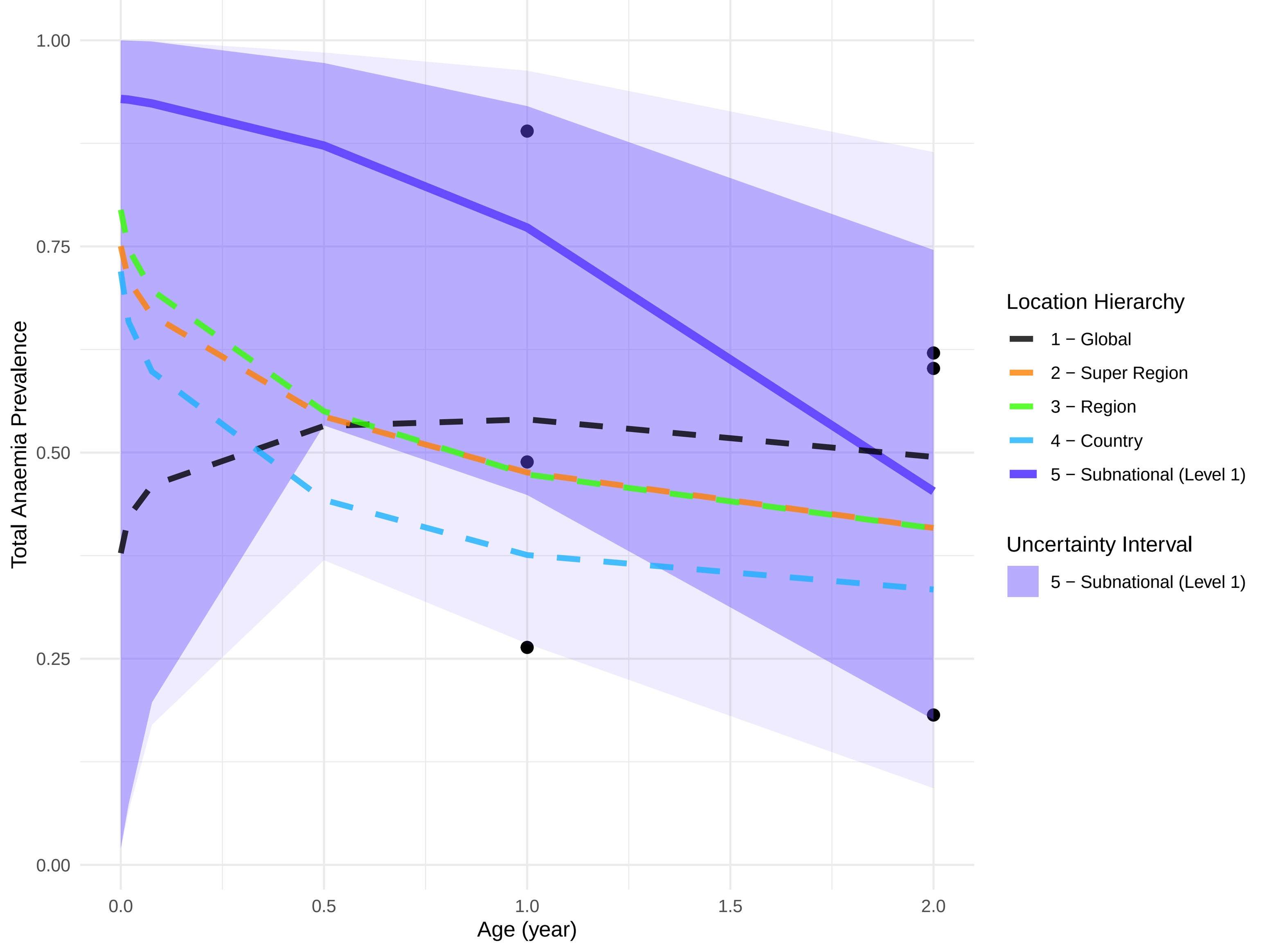

MR−BRT Age/Sex Fits − Non−pregnant Females <2 Years Old − Total Anaemia Prevalence − North Sumatra
WHO−2001 Adjustment Method

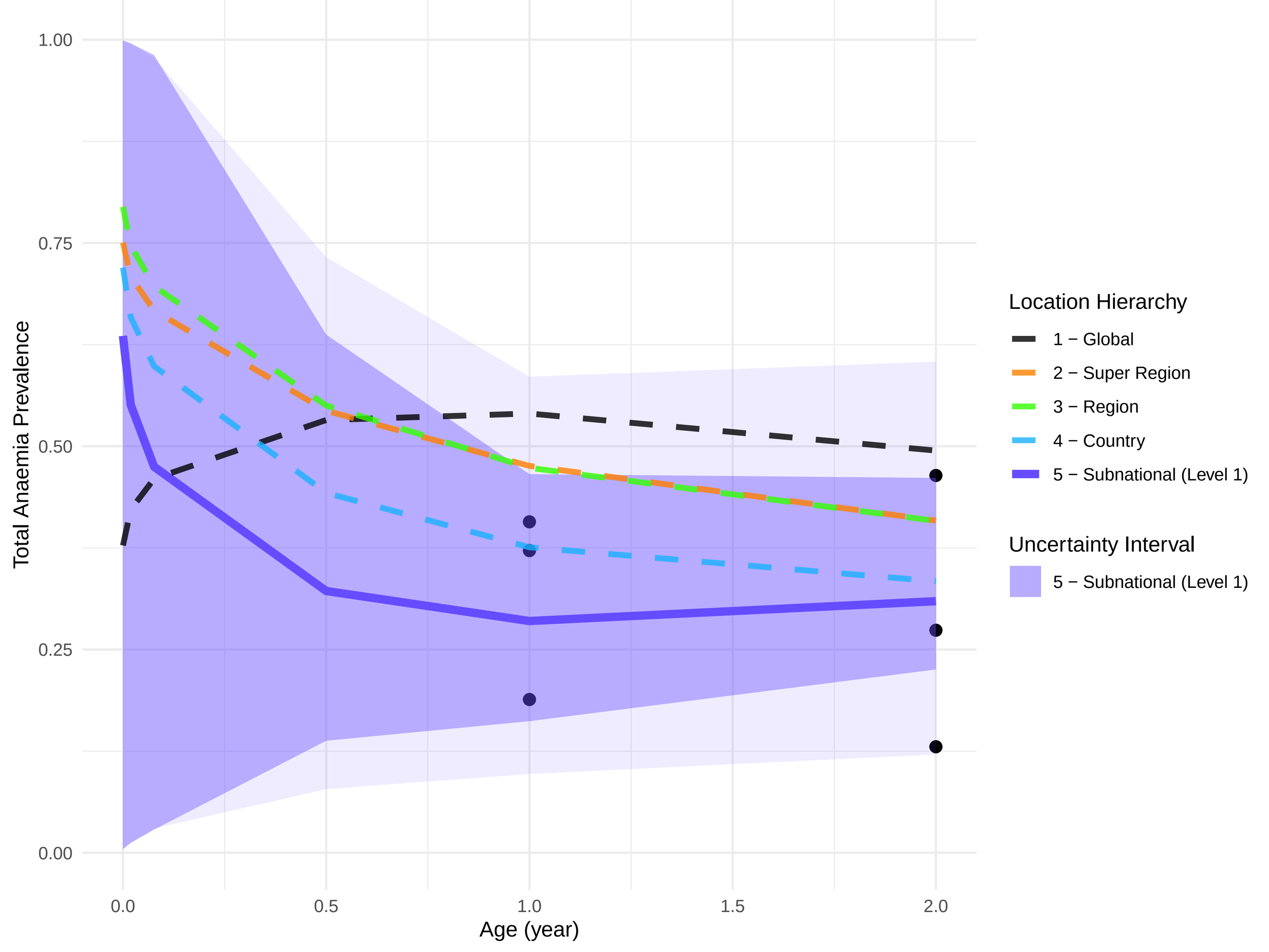

MR−BRT Age/Sex Fits − Non−pregnant Females <2 Years Old − Total Anaemia Prevalence − Yogyakarta
WHO−2001 Adjustment Method

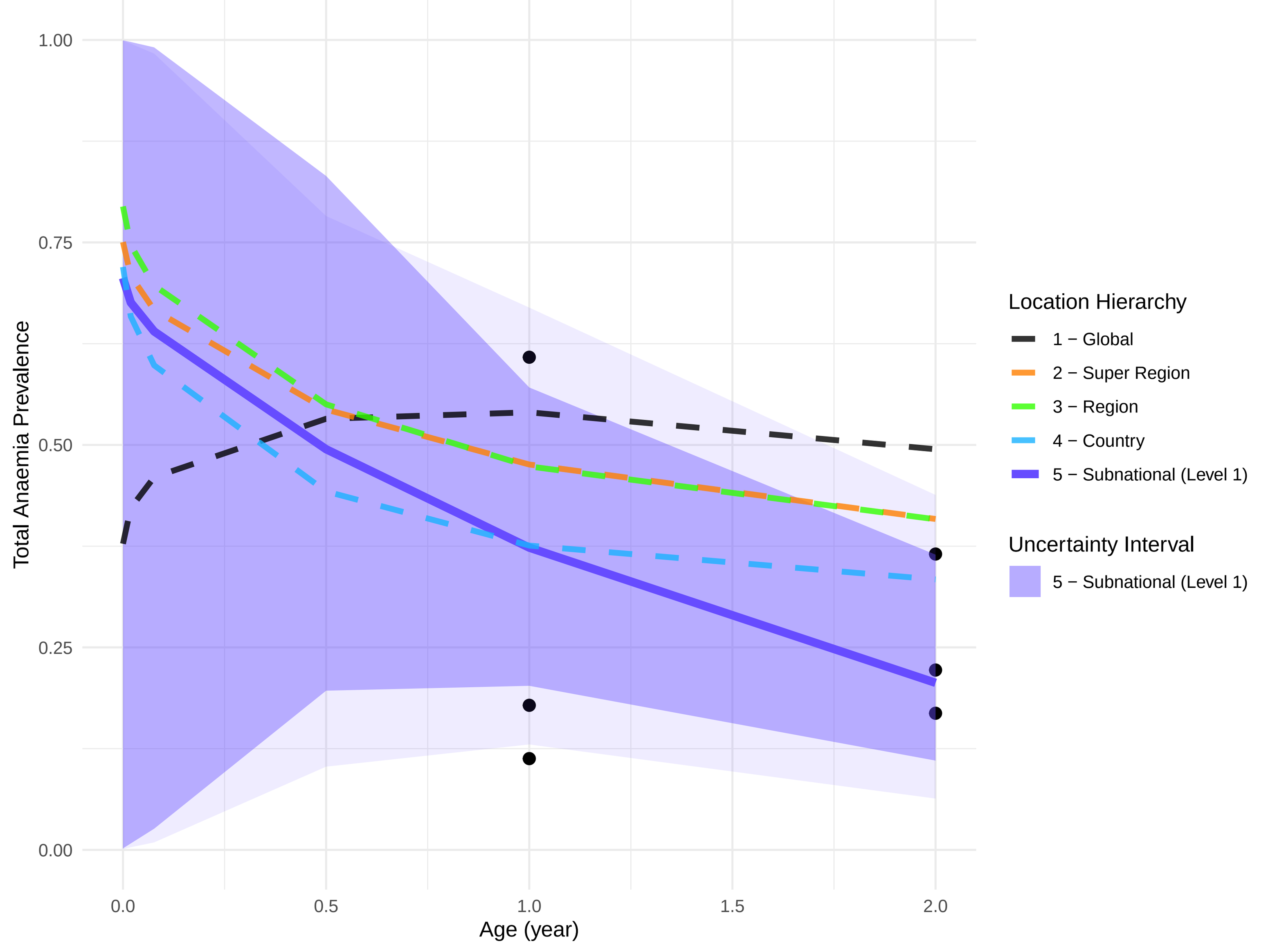

MR−BRT Age/Sex Fits − Non−pregnant Females <2 Years Old − Total Anaemia Prevalence − Myanmar
WHO−2001 Adjustment Method

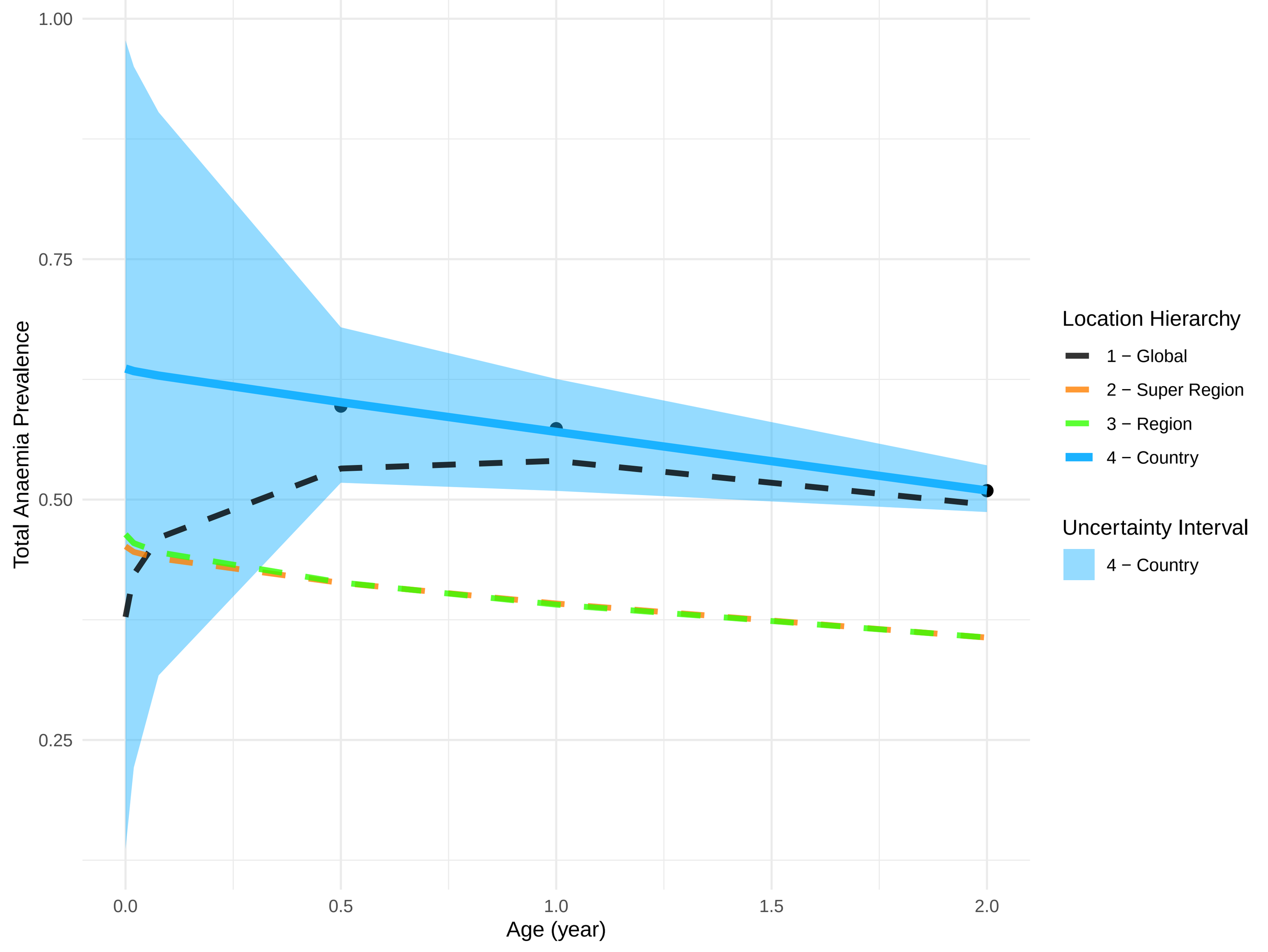

MR−BRT Age/Sex Fits − Non−pregnant Females <2 Years Old − Total Anaemia Prevalence − Timor−Leste
WHO−2001 Adjustment Method

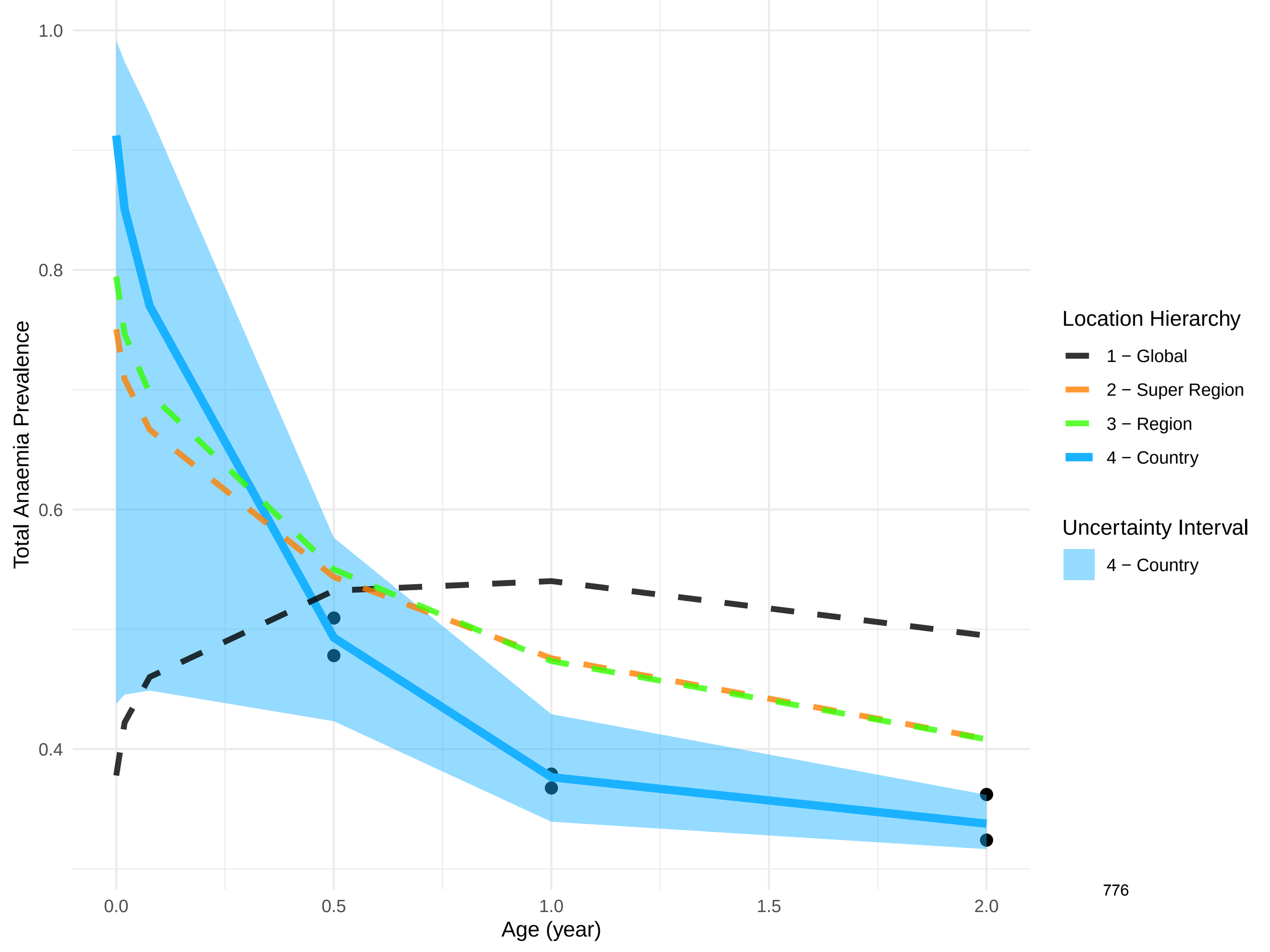

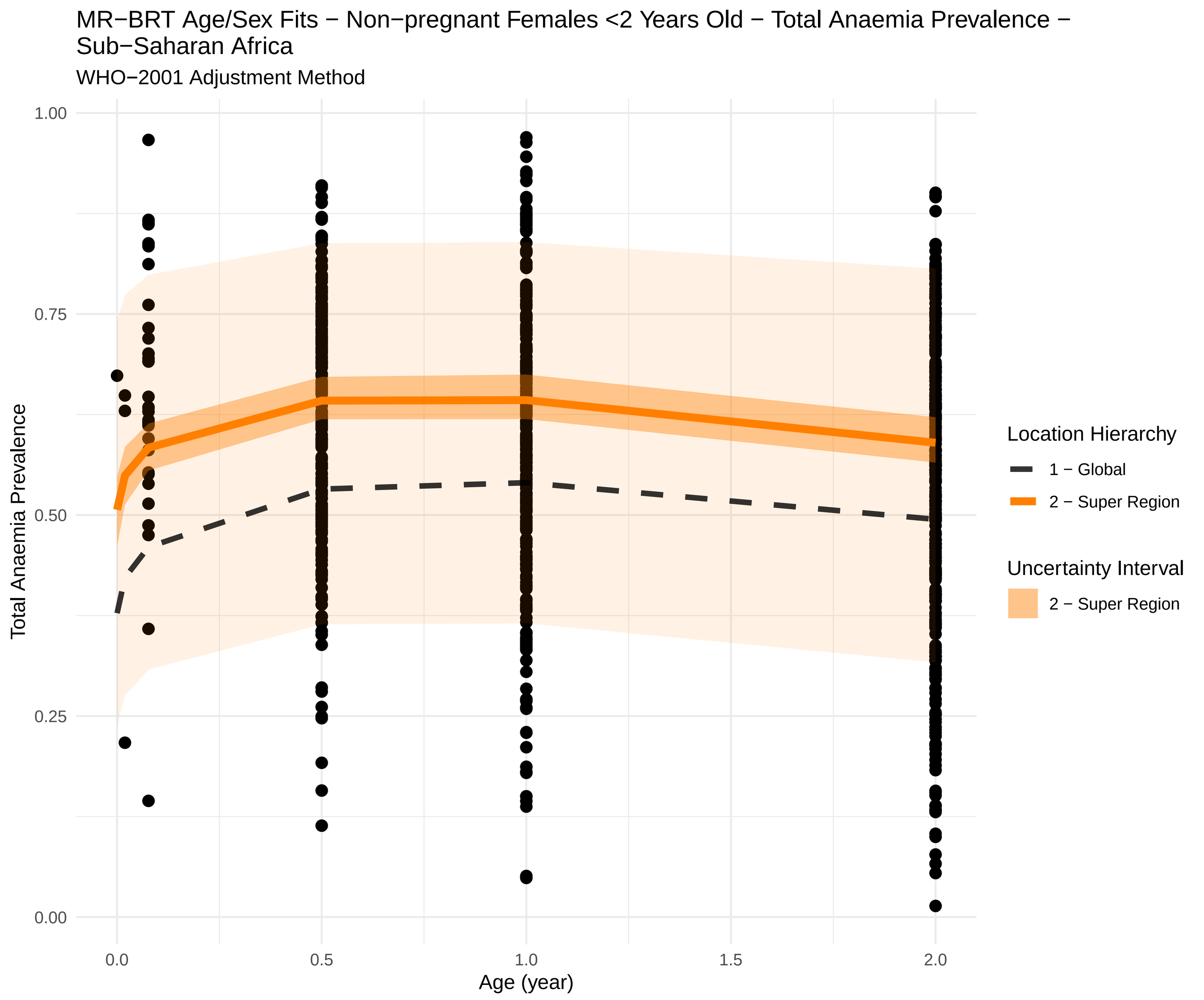
MR−BRT Age/Sex Fits − Non−pregnant Females <2 Years Old − Total Anaemia Prevalence − Sub−Saharan Africa
WHO−2001 Adjustment Method
Total Anaemia Prevalence
Age (year)
Location Hierarchy
1 − Global
2 − Super Region
Uncertainty Interval
2 − Super Region

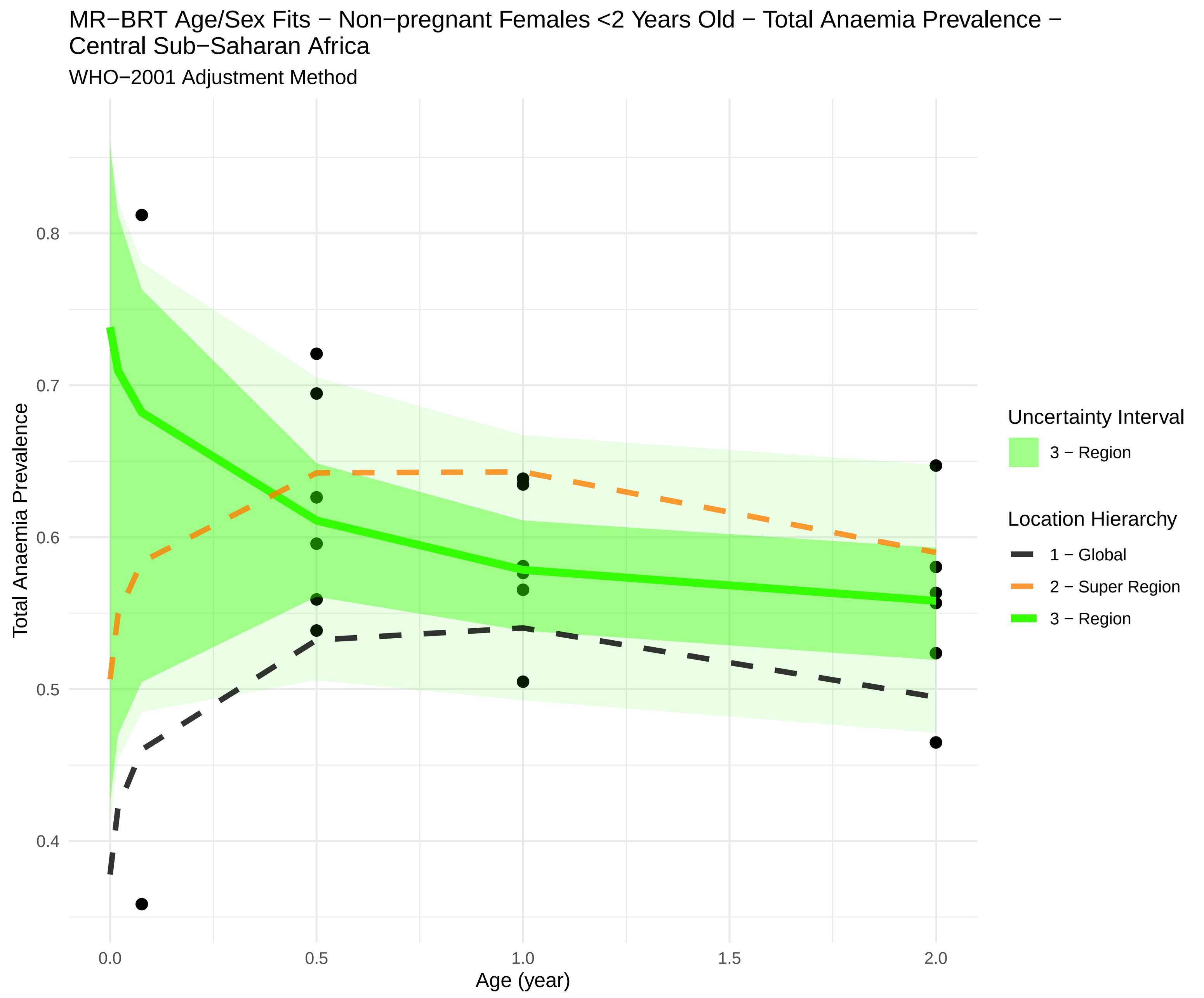
MR−BRT Age/Sex Fits − Non−pregnant Females <2 Years Old − Total Anaemia Prevalence − Central Sub−Saharan Africa
WHO−2001 Adjustment Method
Total Anaemia Prevalence
Age (year)
Uncertainty Interval
3 − Region
Location Hierarchy
1 − Global
2 − Super Region
3 − Region

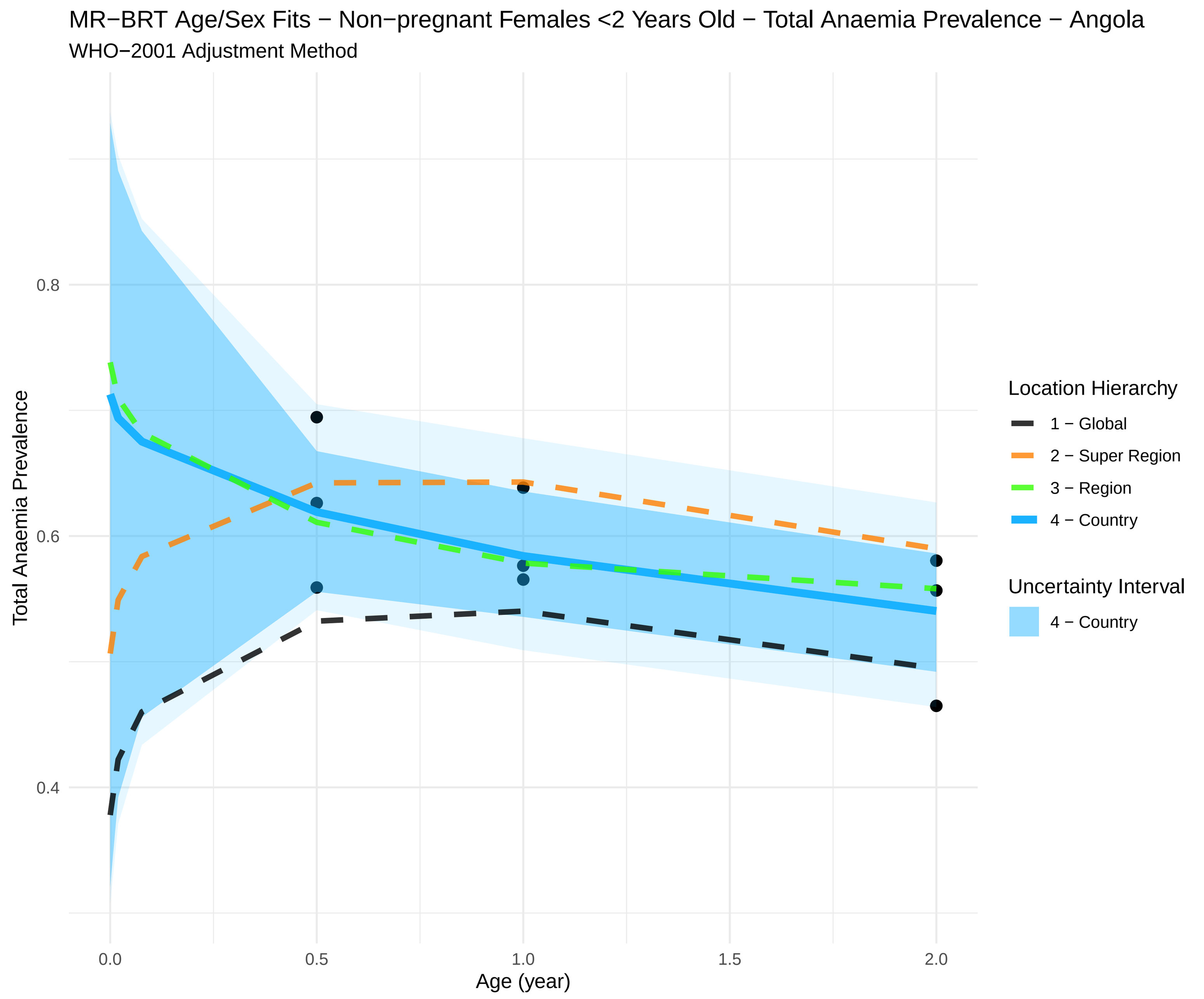
MR−BRT Age/Sex Fits − Non−pregnant Females <2 Years Old − Total Anaemia Prevalence − Angola
WHO−2001 Adjustment Method
Total Anaemia Prevalence
Age (year)
Location Hierarchy
1 − Global
2 − Super Region
3 − Region
4 − Country
Uncertainty Interval
4 − Country

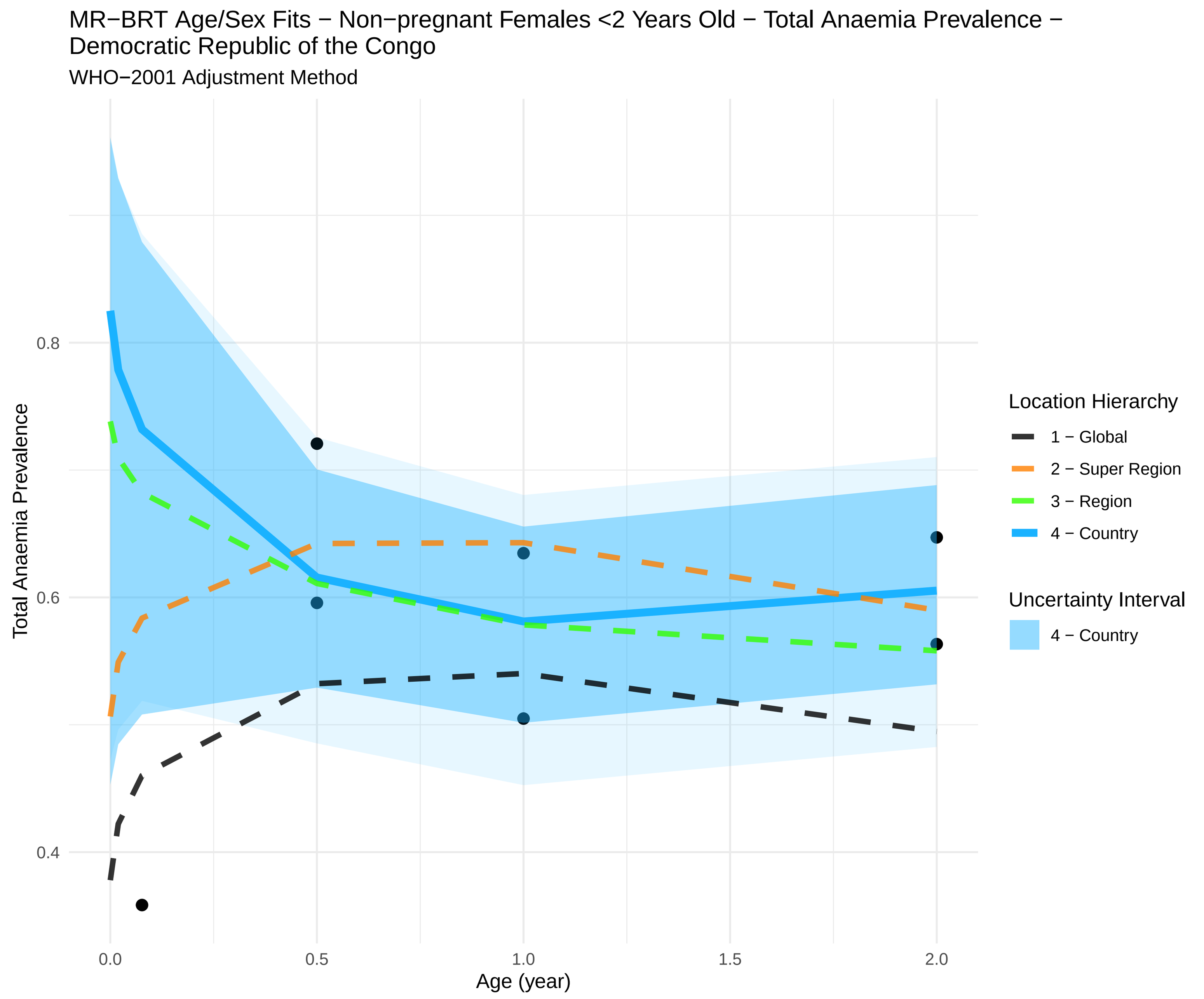
MR−BRT Age/Sex Fits − Non−pregnant Females <2 Years Old − Total Anaemia Prevalence − Democratic Republic of the Congo
WHO−2001 Adjustment Method
Total Anaemia Prevalence
Age (year)
Location Hierarchy
1 − Global
2 − Super Region
3 − Region
4 − Country
Uncertainty Interval
4 − Country

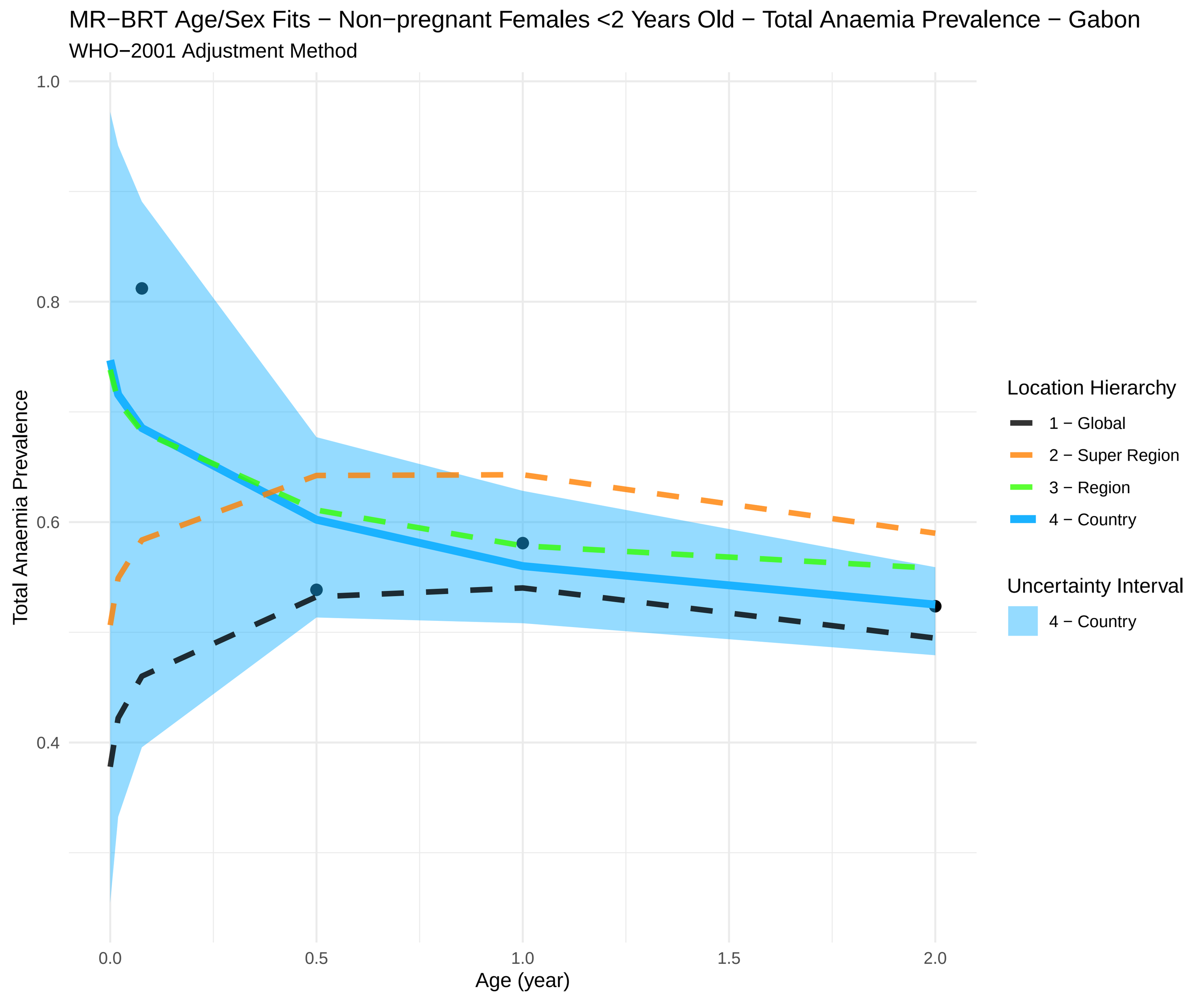
MR−BRT Age/Sex Fits − Non−pregnant Females <2 Years Old − Total Anaemia Prevalence − Gabon
WHO−2001 Adjustment Method
Total Anaemia Prevalence
Age (year)
Location Hierarchy
1 − Global
2 − Super Region
3 − Region
4 − Country
Uncertainty Interval
4 − Country

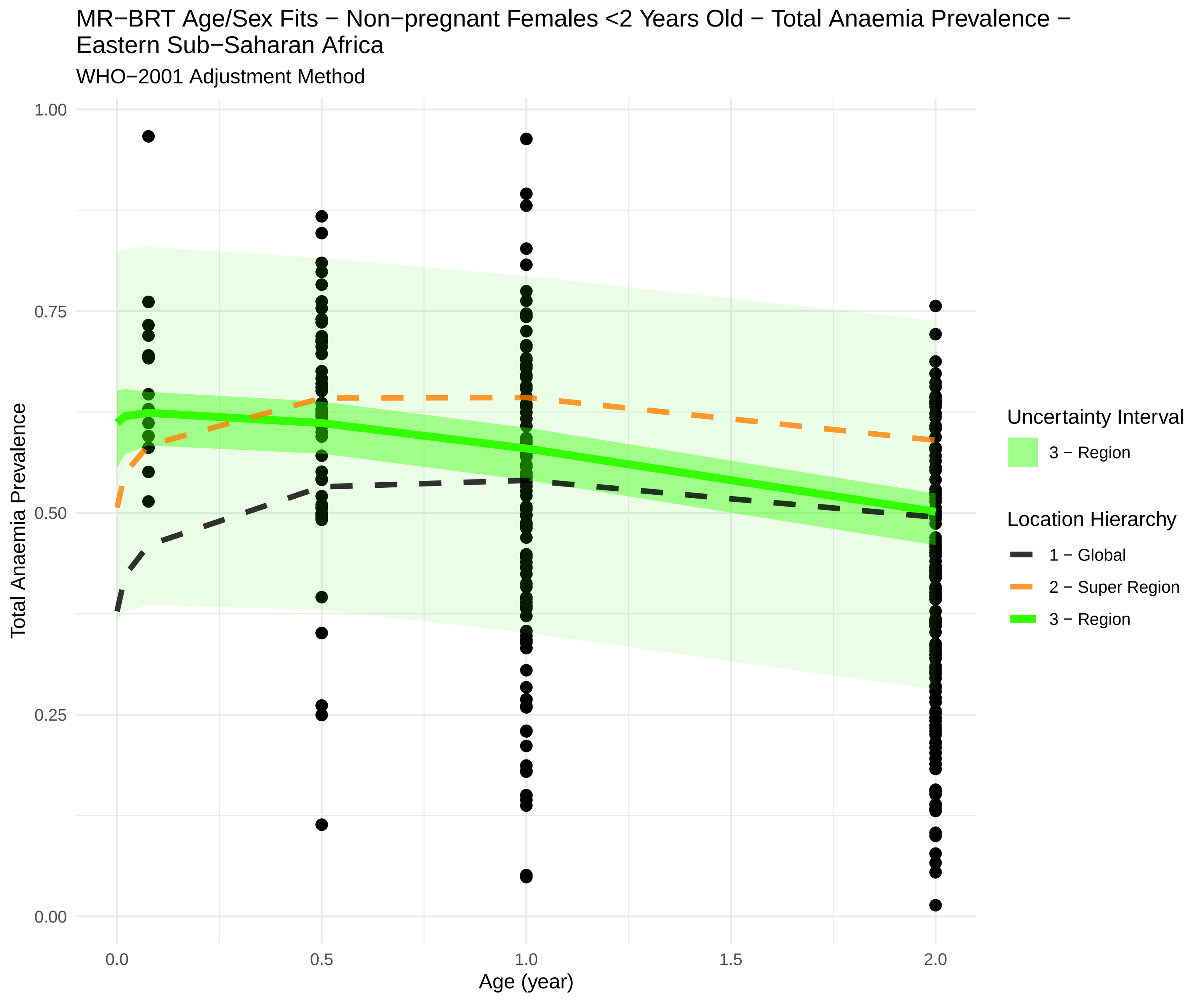
MR−BRT Age/Sex Fits − Non−pregnant Females <2 Years Old − Total Anaemia Prevalence − Eastern Sub−Saharan Africa
WHO−2001 Adjustment Method
Total Anaemia Prevalence
Age (year)
Uncertainty Interval
3 − Region
Location Hierarchy
1 − Global
2 − Super Region
3 − Region

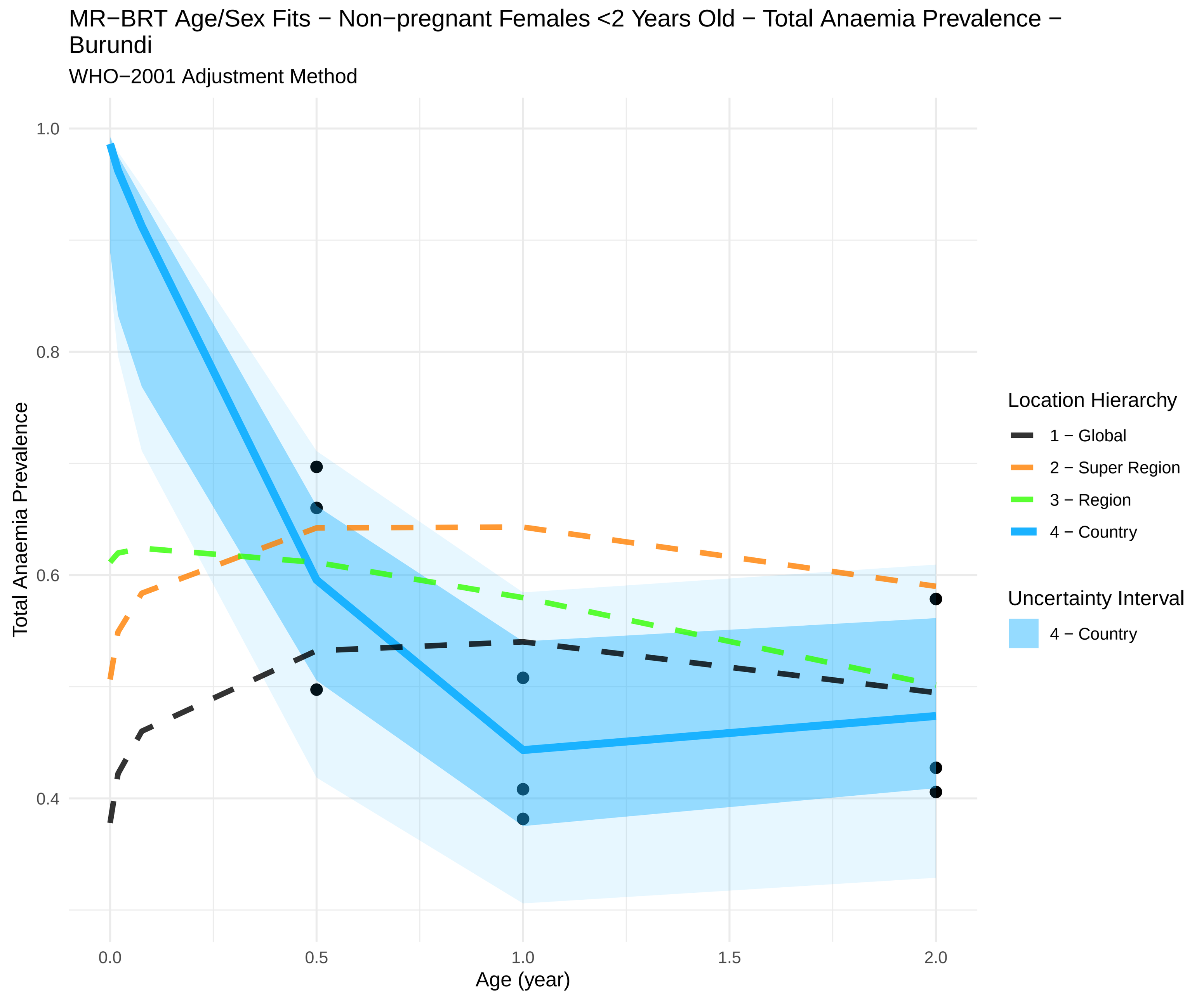
MR−BRT Age/Sex Fits − Non−pregnant Females <2 Years Old − Total Anaemia Prevalence − Burundi
WHO−2001 Adjustment Method
Total Anaemia Prevalence
Age (year)
Location Hierarchy
1 − Global
2 − Super Region
3 − Region
4 − Country
Uncertainty Interval
4 − Country

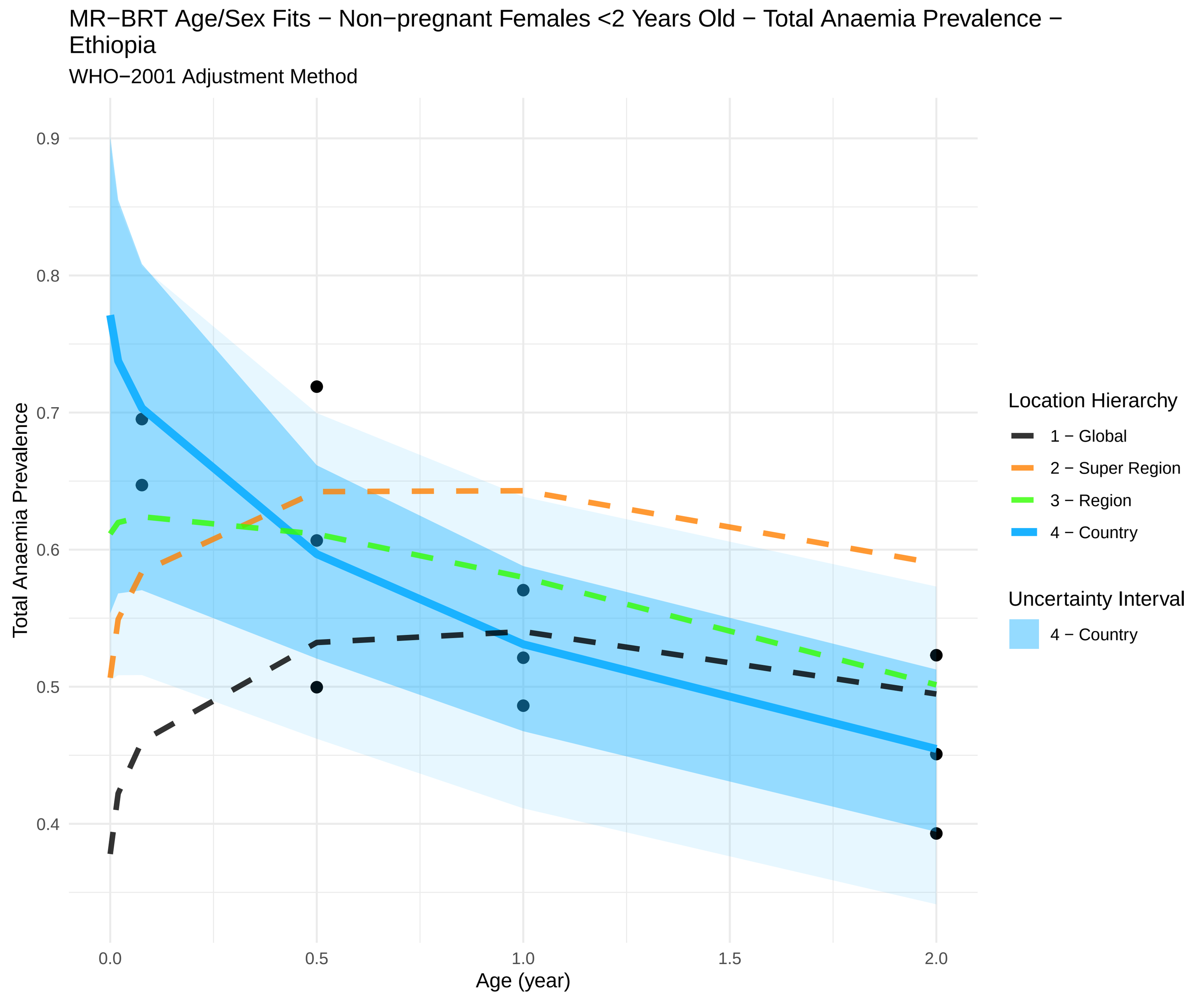
MR−BRT Age/Sex Fits − Non−pregnant Females <2 Years Old − Total Anaemia Prevalence − Ethiopia
WHO−2001 Adjustment Method
Total Anaemia Prevalence
Age (year)
Location Hierarchy
1 − Global
2 − Super Region
3 − Region
4 − Country
Uncertainty Interval
4 − Country

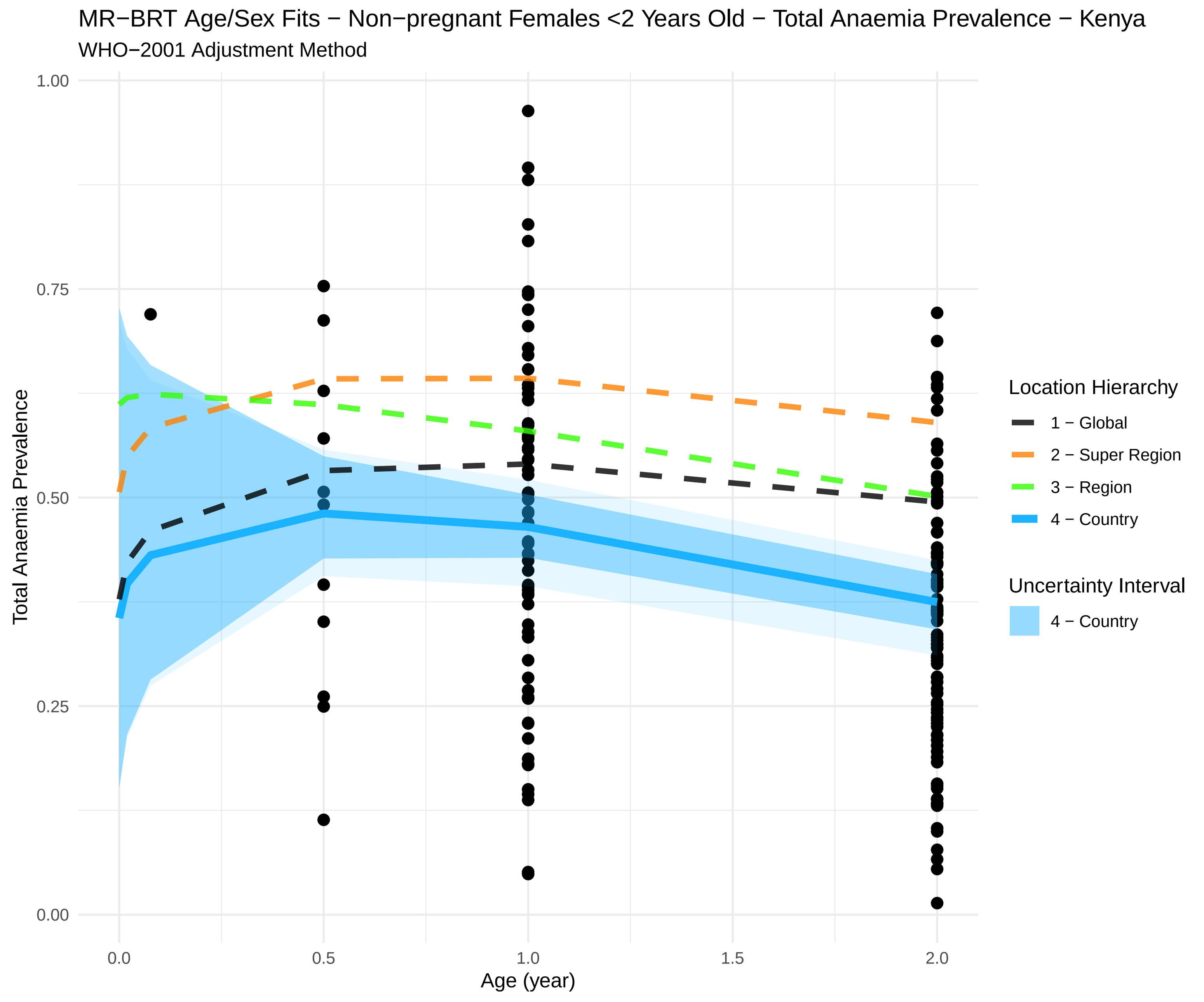
MR−BRT Age/Sex Fits − Non−pregnant Females <2 Years Old − Total Anaemia Prevalence − Kenya
WHO−2001 Adjustment Method
Total Anaemia Prevalence
Age (year)
Location Hierarchy
1 − Global
2 − Super Region
3 − Region
4 − Country
Uncertainty Interval
4 − Country

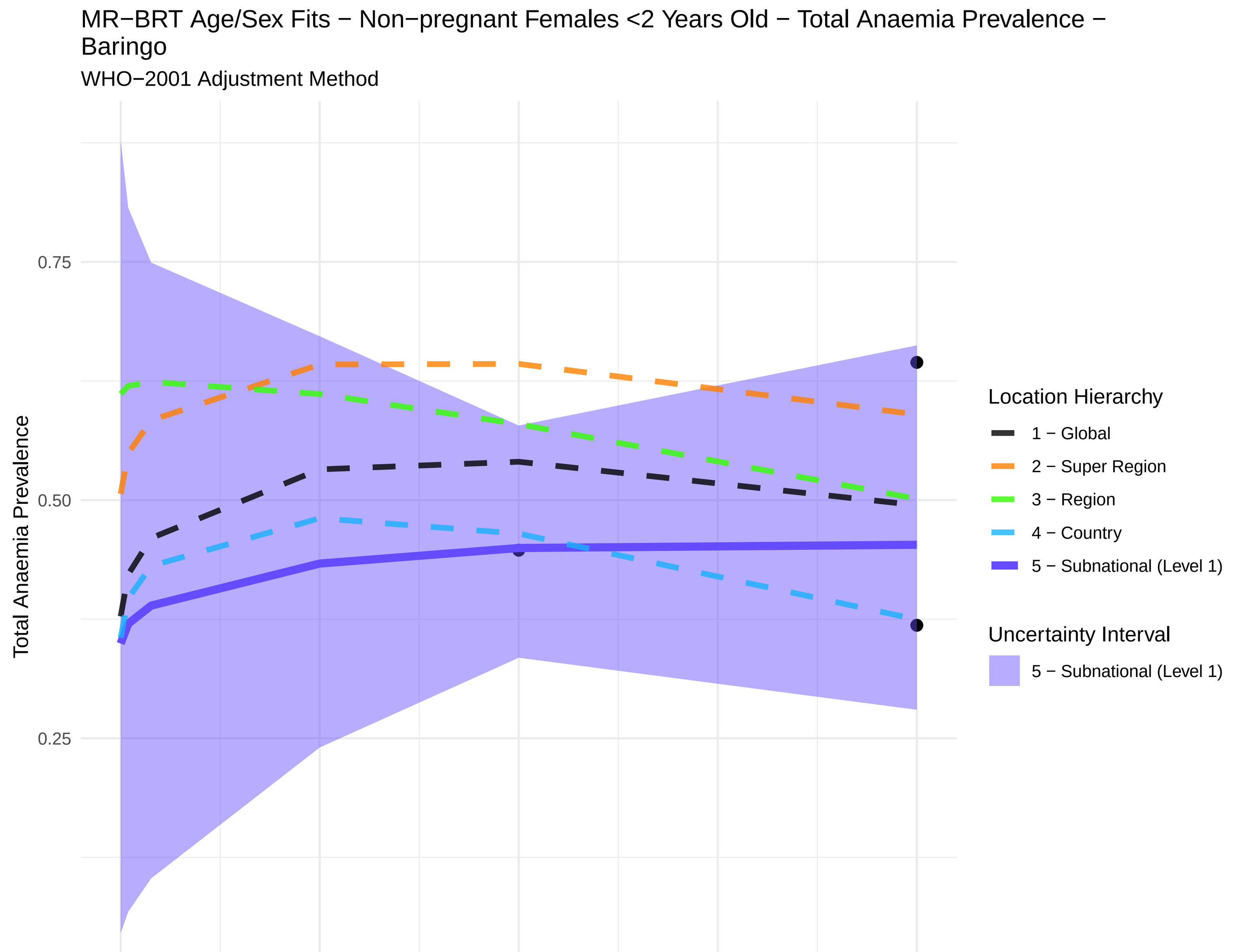
MR−BRT Age/Sex Fits − Non−pregnant Females <2 Years Old − Total Anaemia Prevalence − Baringo
WHO−2001 Adjustment Method
Total Anaemia Prevalence
Age (year)
Location Hierarchy
1 − Global
2 − Super Region
3 − Region
4 − Country
5 − Subnational (Level 1)
Uncertainty Interval
5 − Subnational (Level 1)

MR−BRT Age/Sex Fits − Non−pregnant Females <2 Years Old − Total Anaemia Prevalence − Bomet

WHO−2001 Adjustment Method

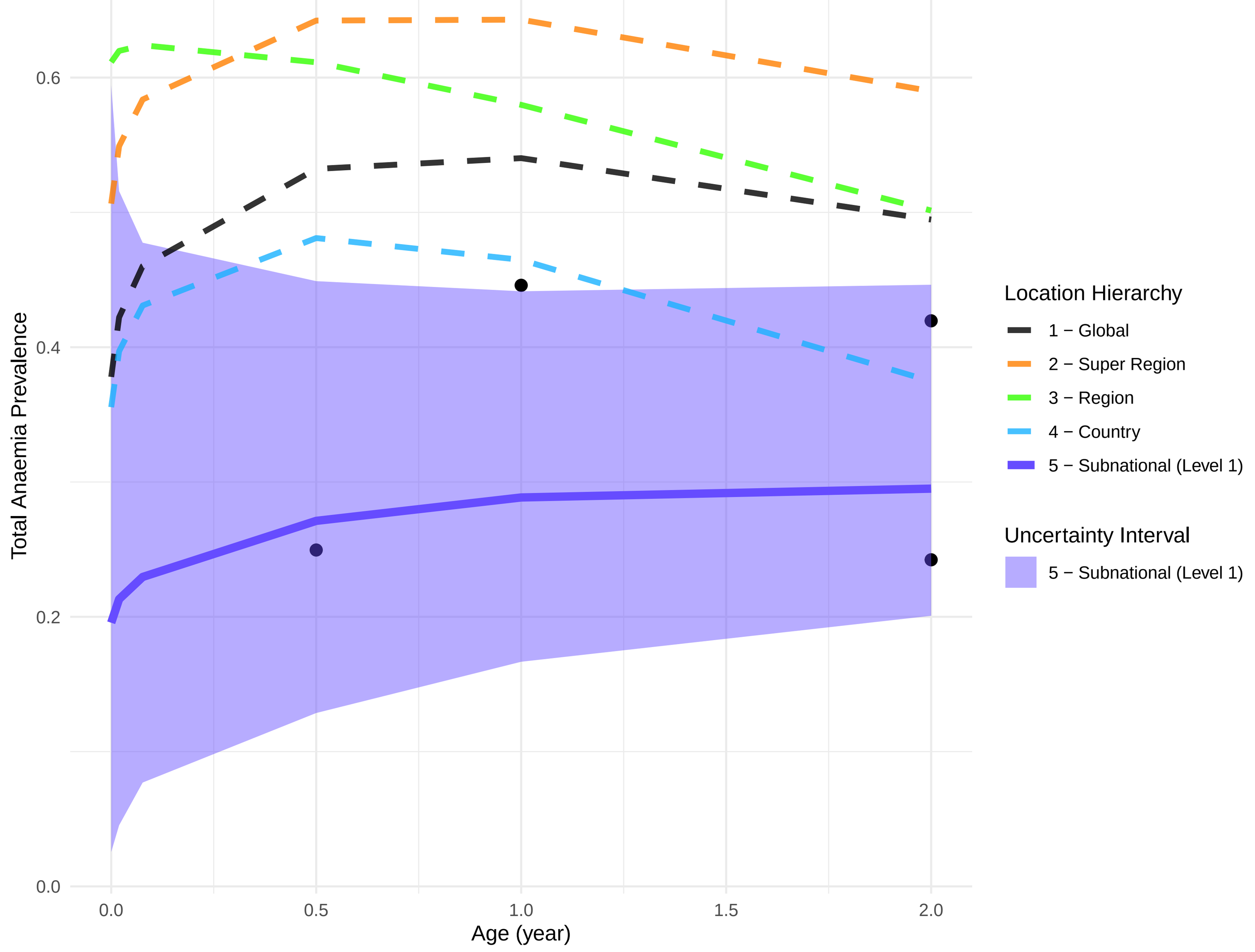

MR−BRT Age/Sex Fits − Non−pregnant Females <2 Years Old − Total Anaemia Prevalence − Bungoma

WHO−2001 Adjustment Method

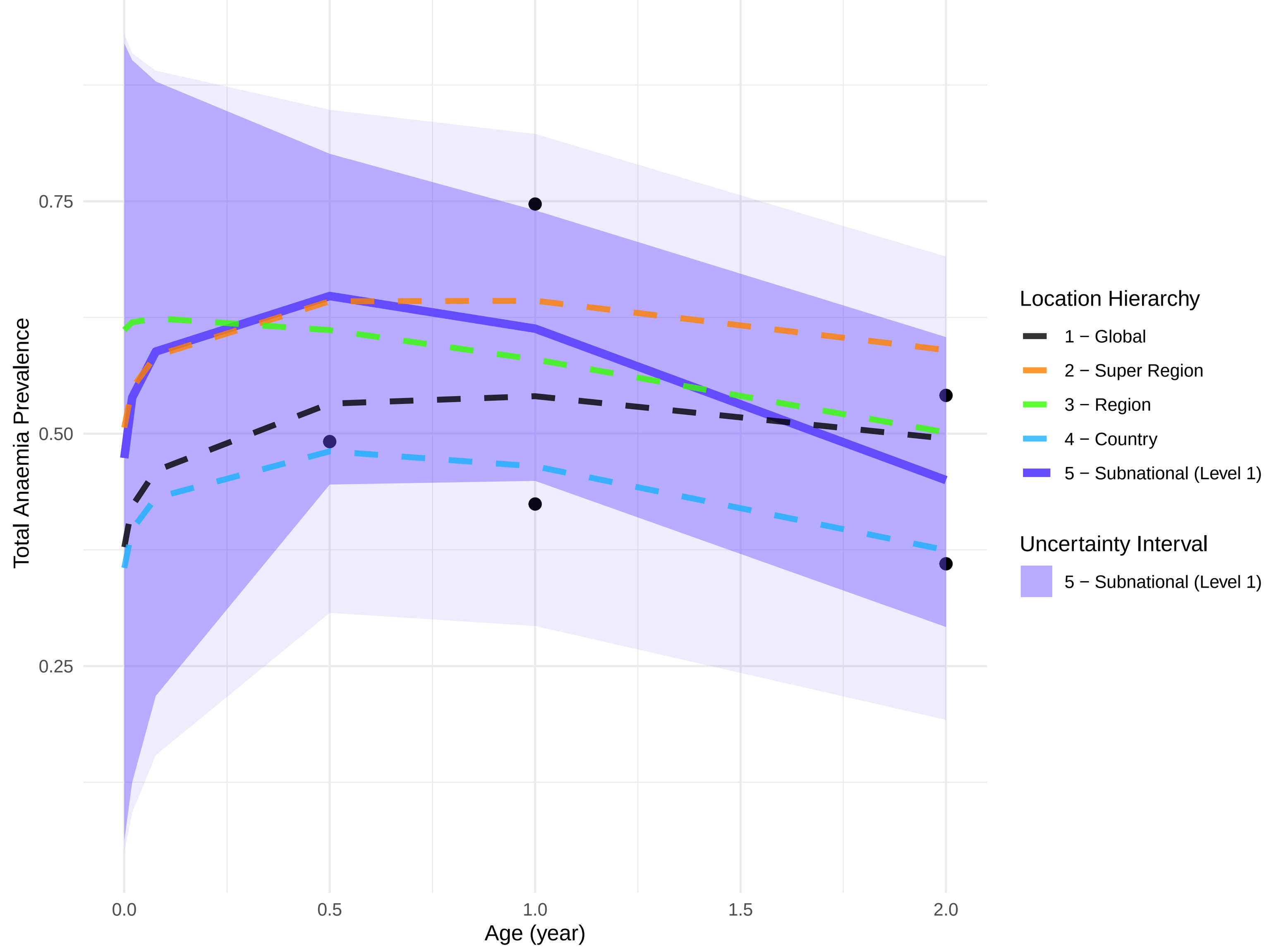

MR−BRT Age/Sex Fits − Non−pregnant Females <2 Years Old − Total Anaemia Prevalence − Busia

WHO−2001 Adjustment Method

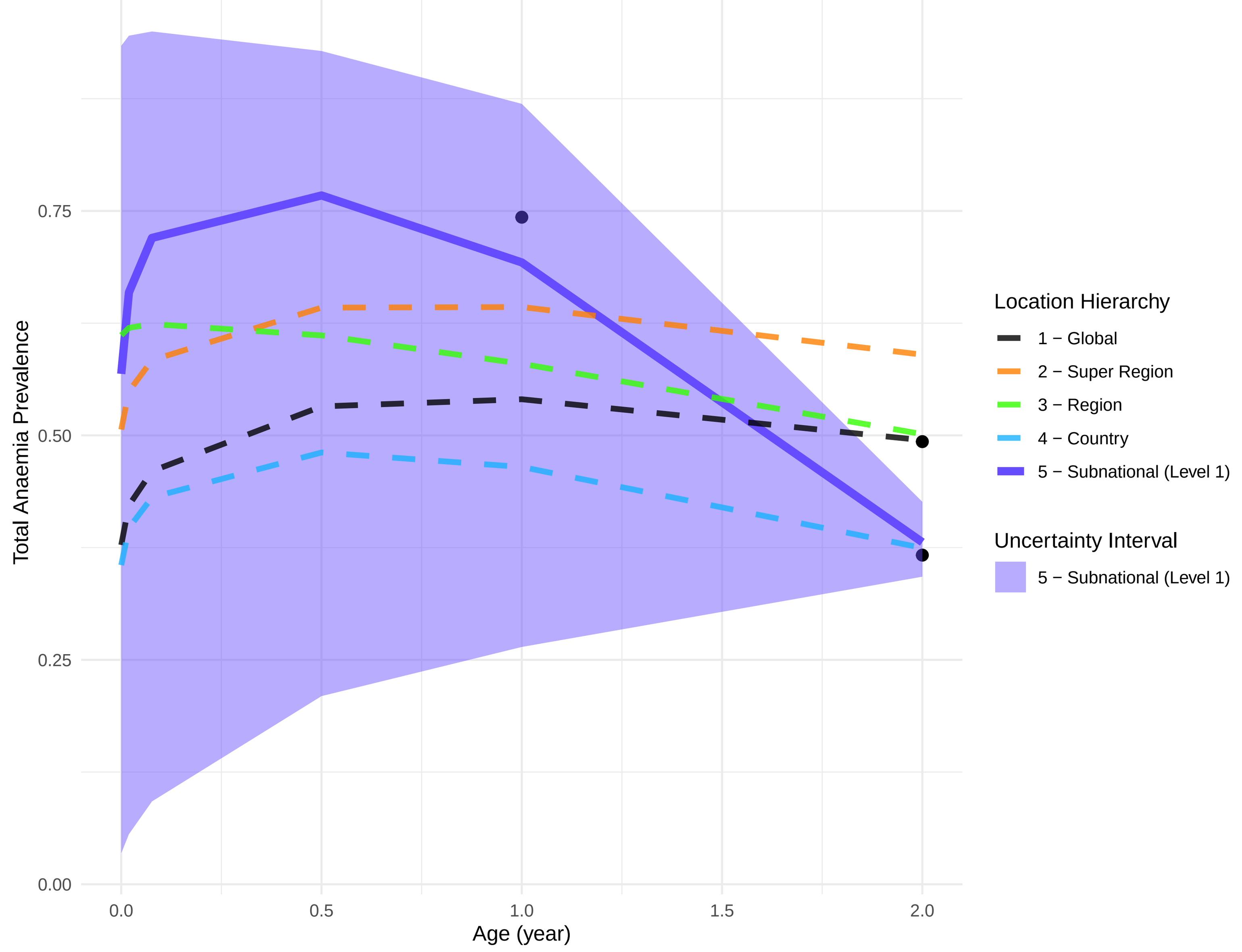

MR−BRT Age/Sex Fits − Non−pregnant Females <2 Years Old − Total Anaemia Prevalence − Elgeyo−Marakwet

WHO−2001 Adjustment Method

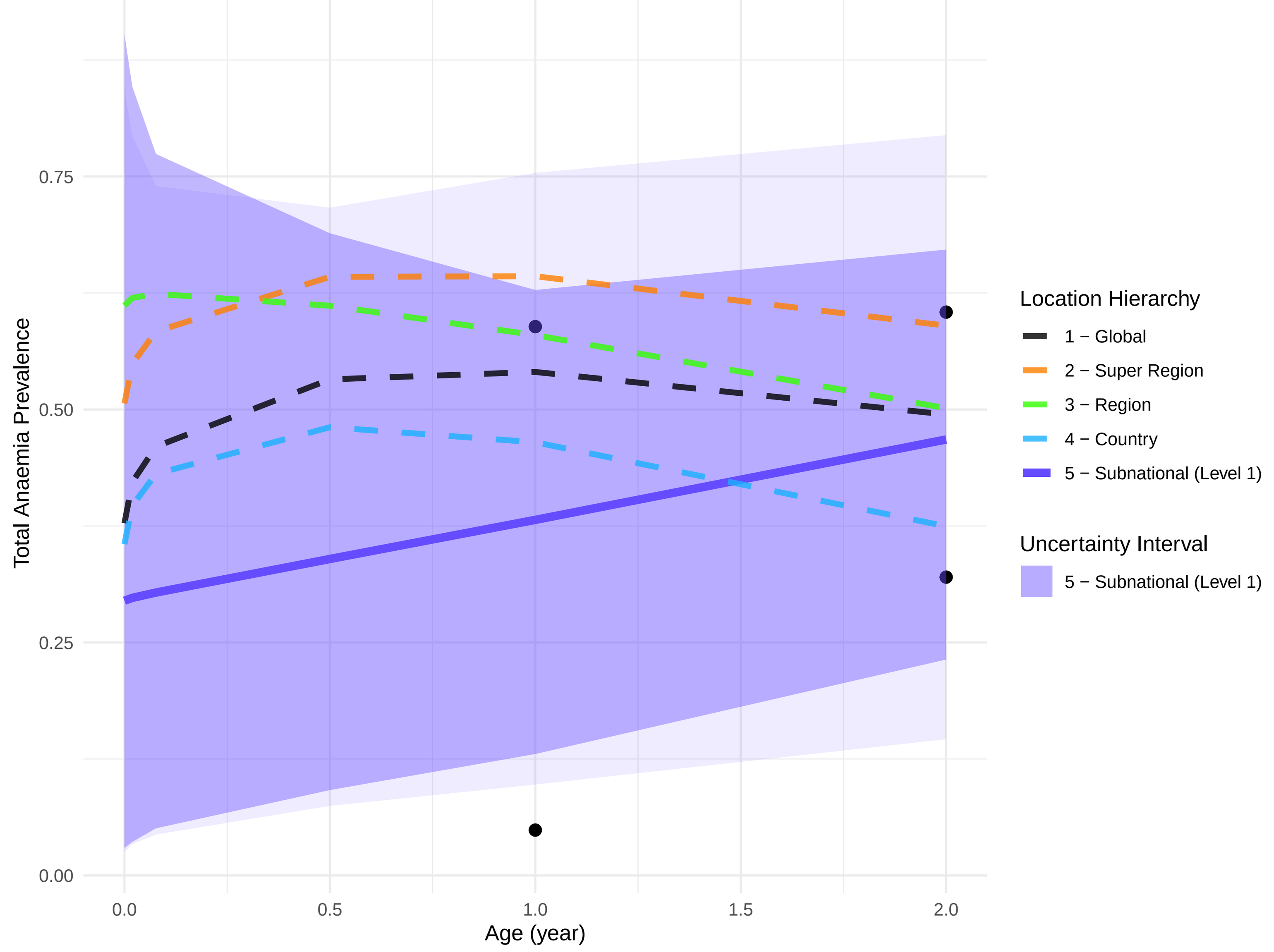

MR−BRT Age/Sex Fits − Non−pregnant Females <2 Years Old − Total Anaemia Prevalence − Embu

WHO−2001 Adjustment Method

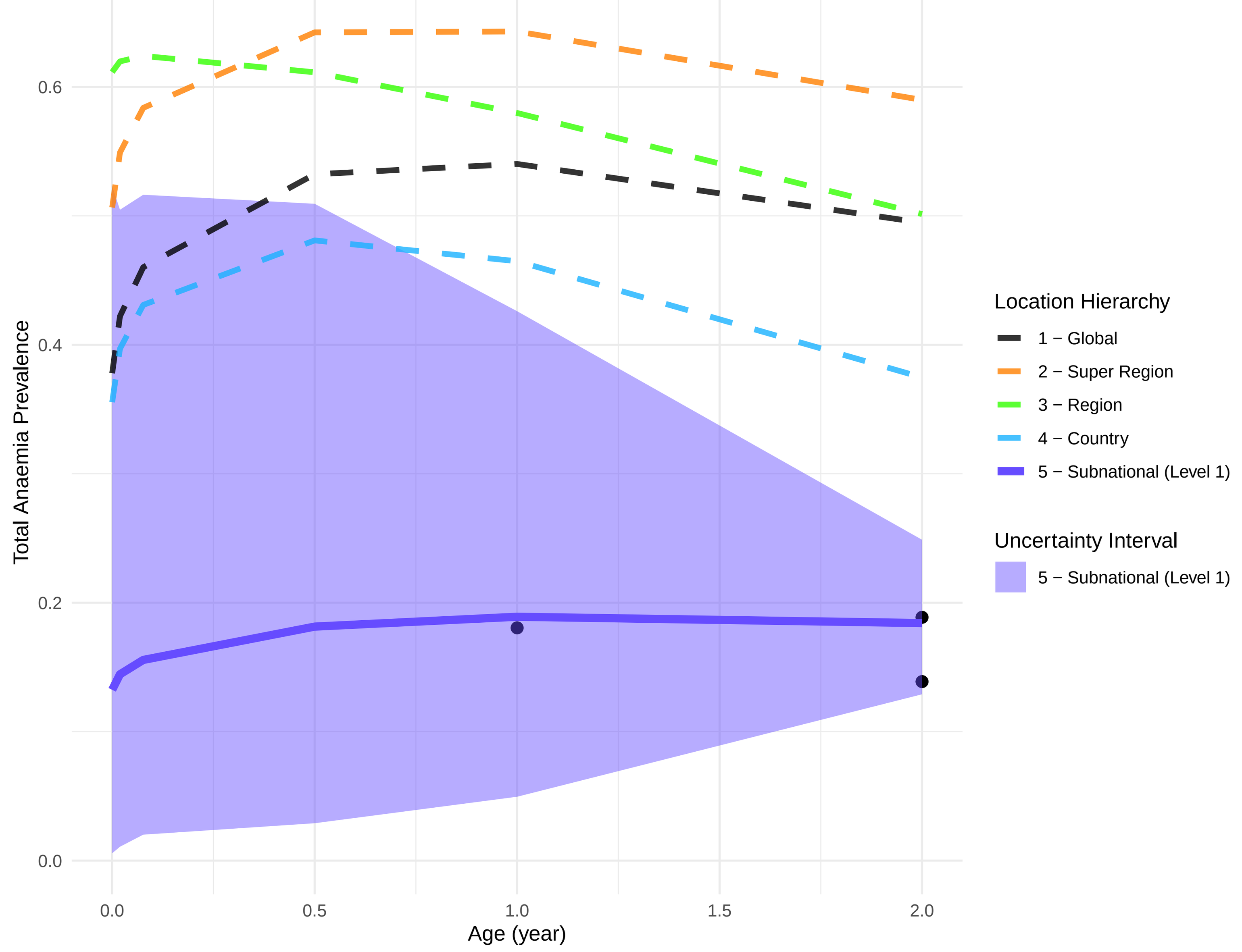

MR−BRT Age/Sex Fits − Non−pregnant Females <2 Years Old − Total Anaemia Prevalence − Garissa

WHO−2001 Adjustment Method

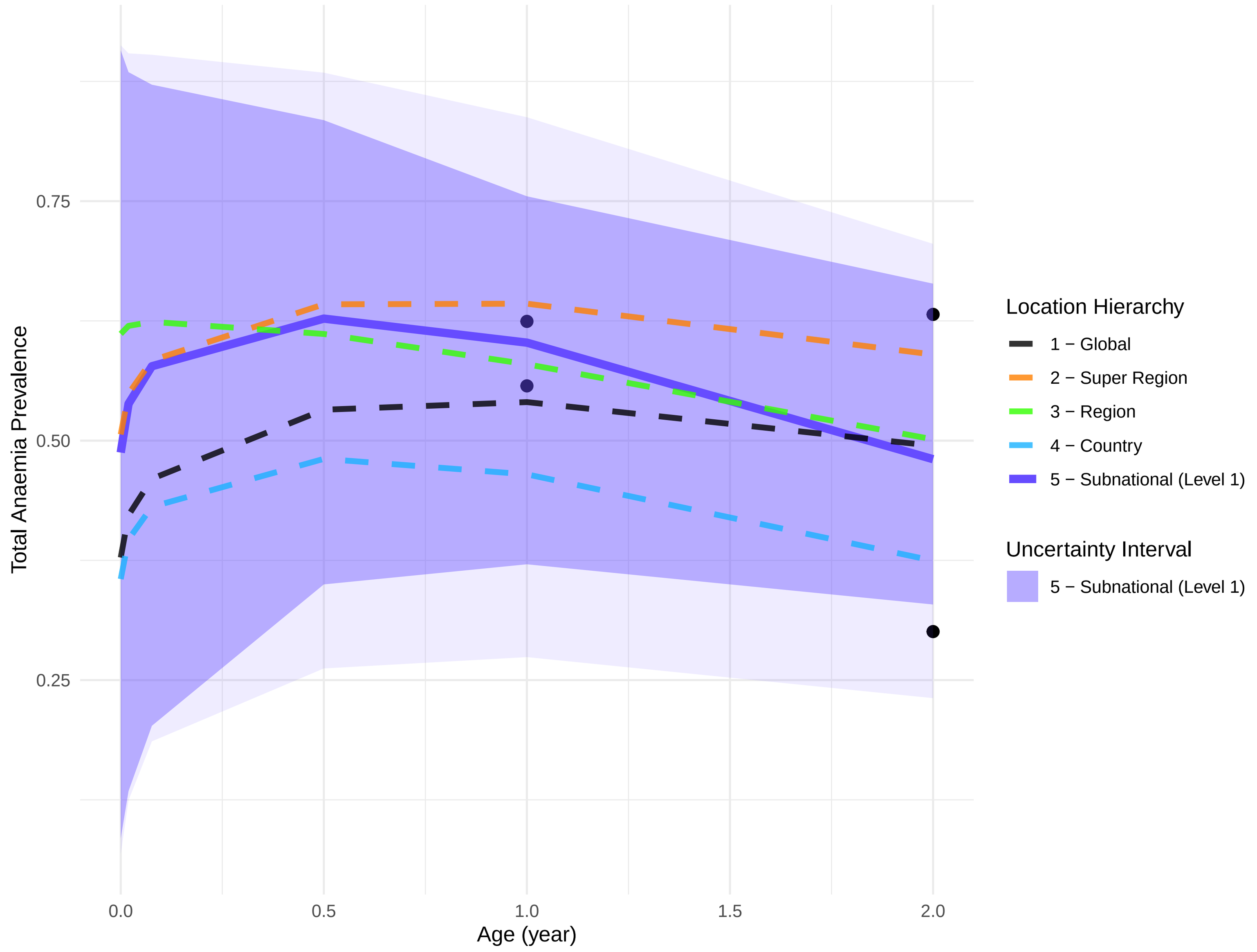

MR−BRT Age/Sex Fits − Non−pregnant Females <2 Years Old − Total Anaemia Prevalence − Homa Bay

WHO−2001 Adjustment Method

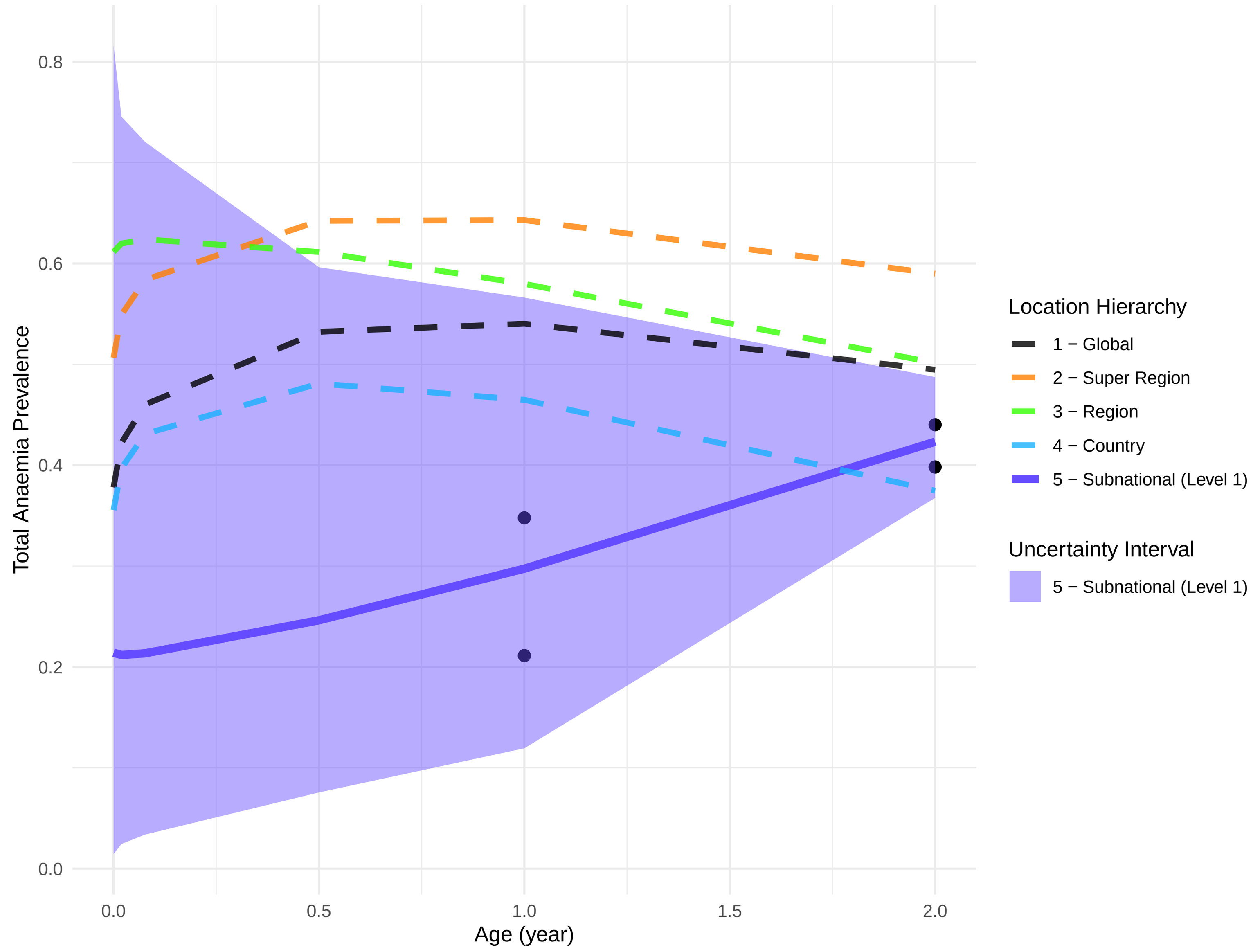

MR−BRT Age/Sex Fits − Non−pregnant Females <2 Years Old − Total Anaemia Prevalence − Isiolo

WHO−2001 Adjustment Method

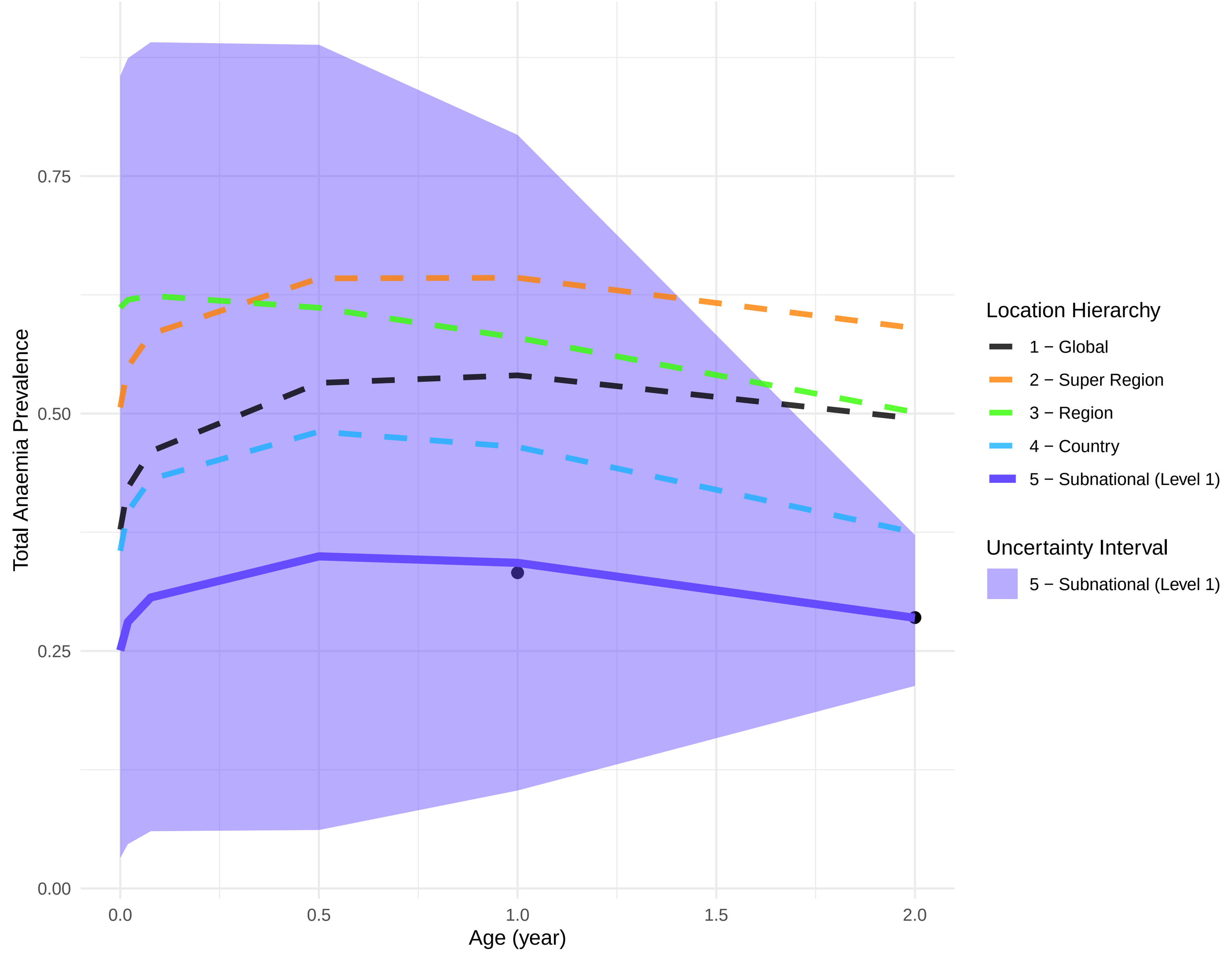

MR−BRT Age/Sex Fits − Non−pregnant Females <2 Years Old − Total Anaemia Prevalence − Kajiado

WHO−2001 Adjustment Method

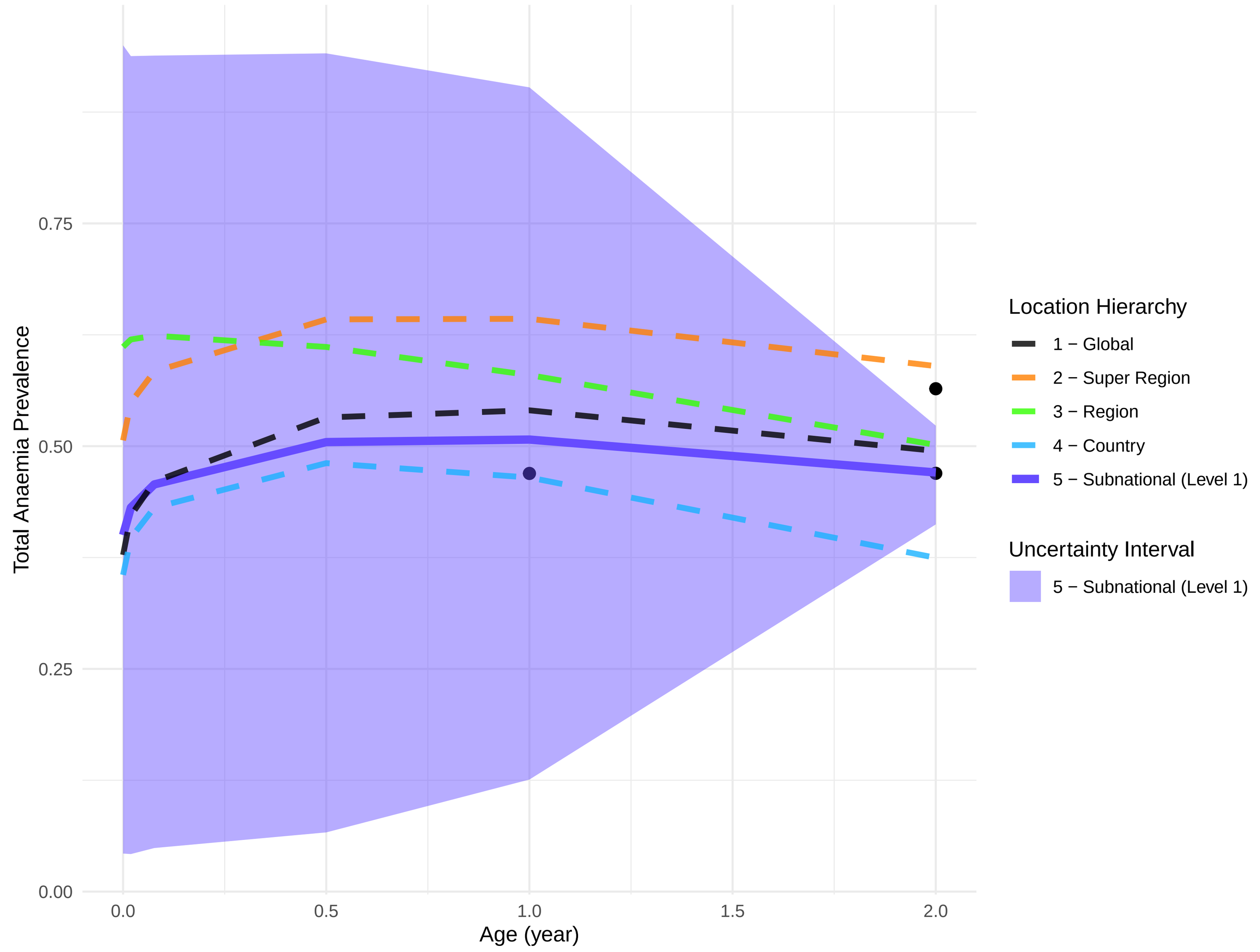

MR−BRT Age/Sex Fits − Non−pregnant Females <2 Years Old − Total Anaemia Prevalence − Kakamega

WHO−2001 Adjustment Method

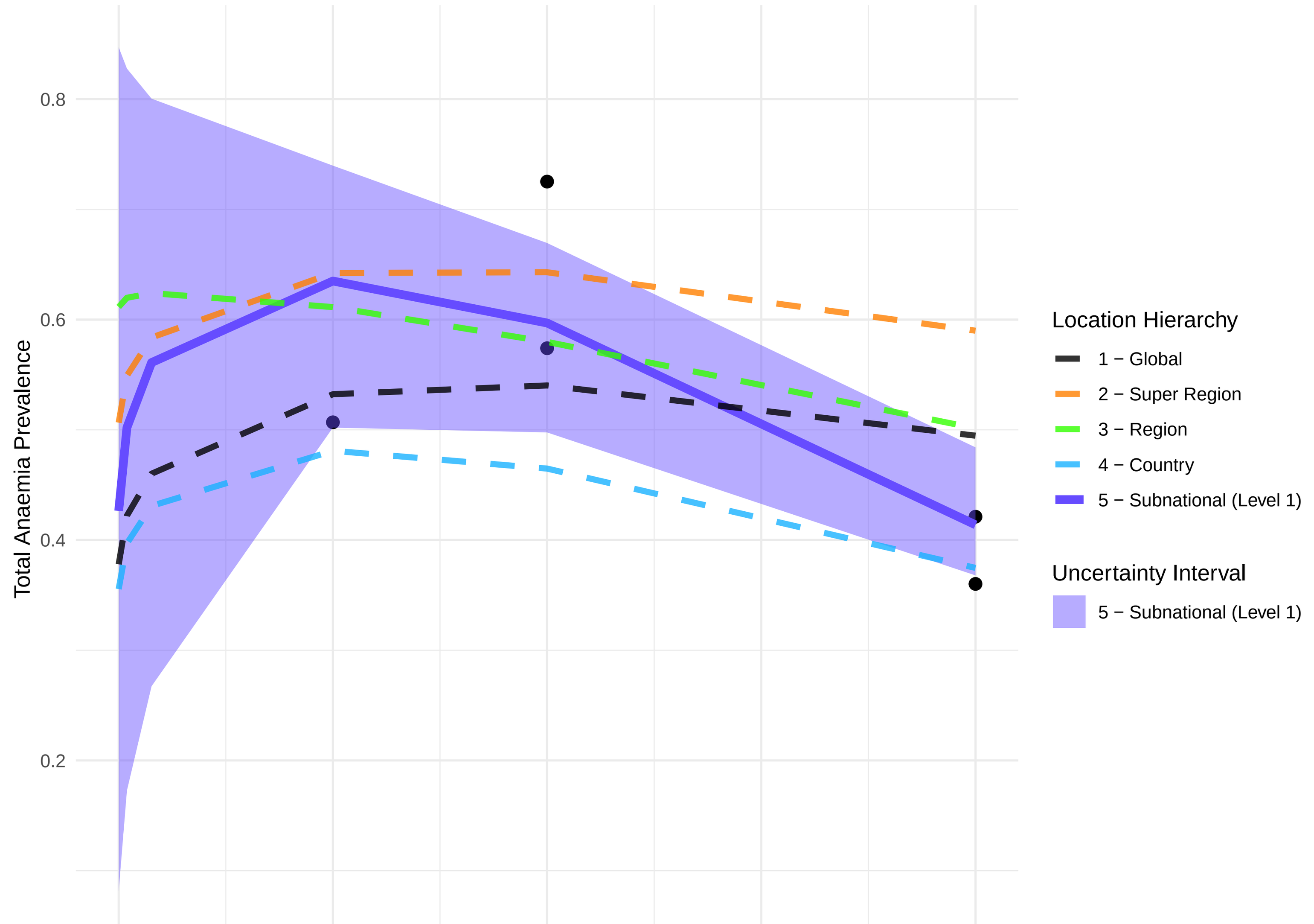

MR−BRT Age/Sex Fits − Non−pregnant Females <2 Years Old − Total Anaemia Prevalence − Kericho
WHO−2001 Adjustment Method

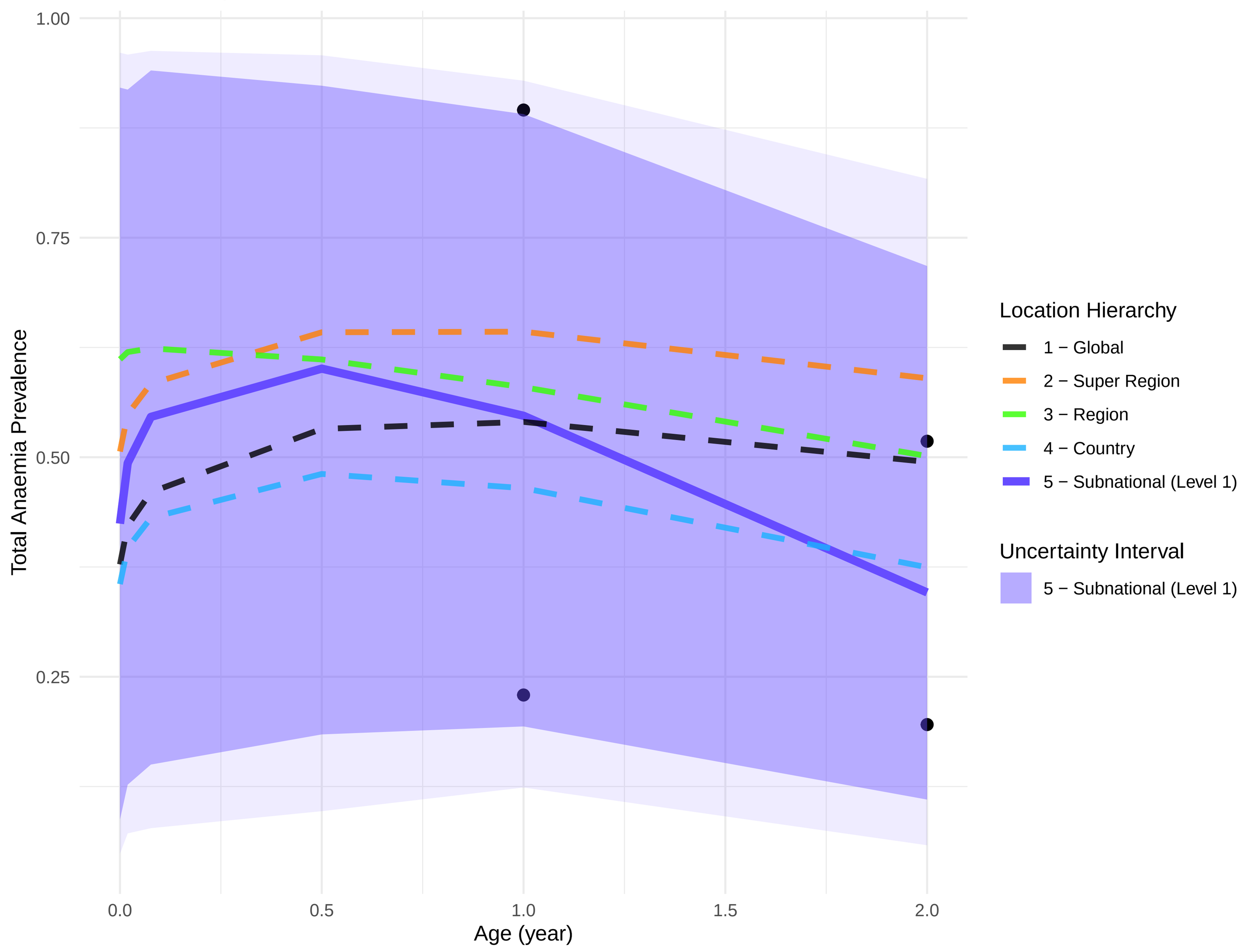

MR−BRT Age/Sex Fits − Non−pregnant Females <2 Years Old − Total Anaemia Prevalence − Kiambu
WHO−2001 Adjustment Method

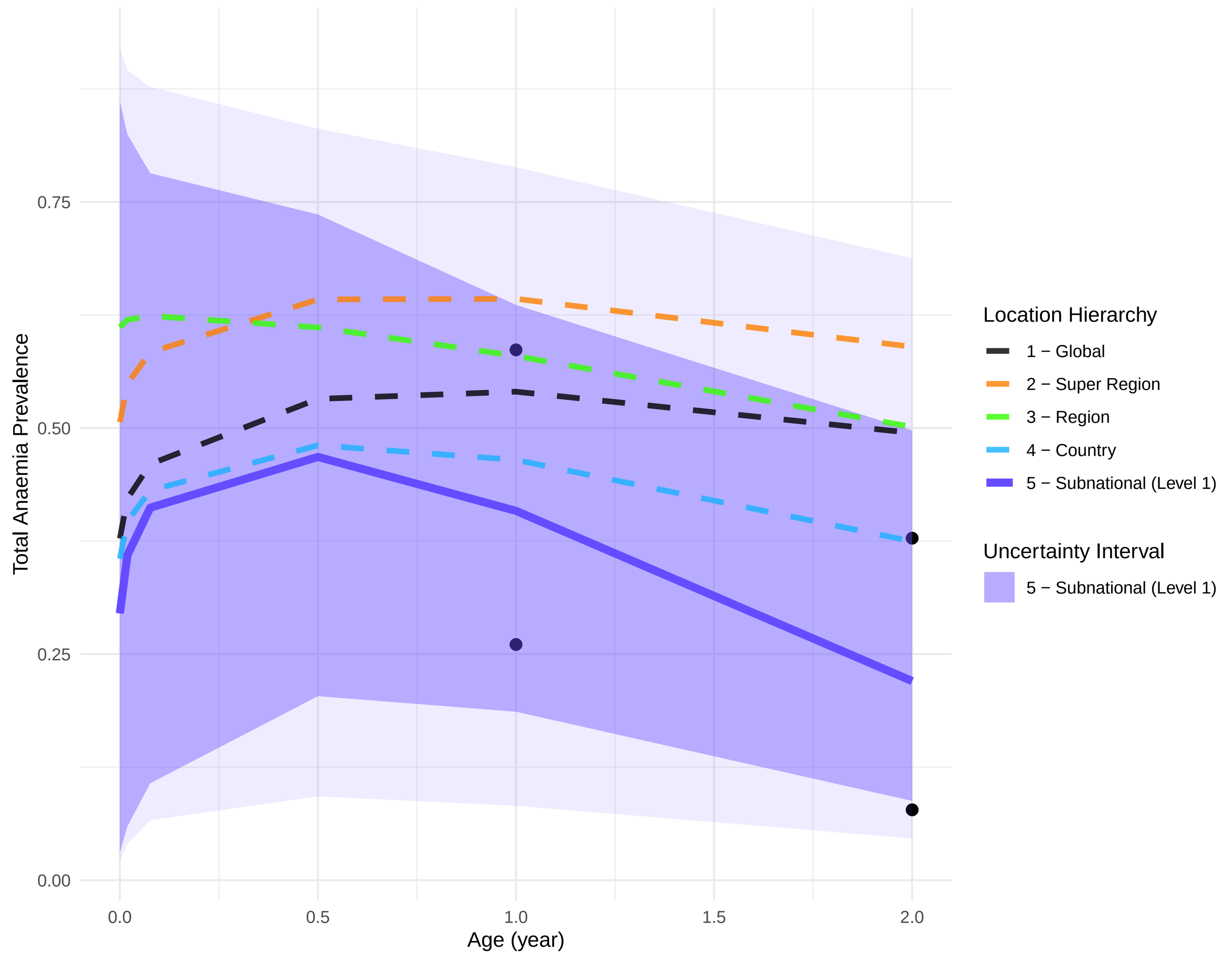

MR−BRT Age/Sex Fits − Non−pregnant Females <2 Years Old − Total Anaemia Prevalence − Kilifi
WHO−2001 Adjustment Method

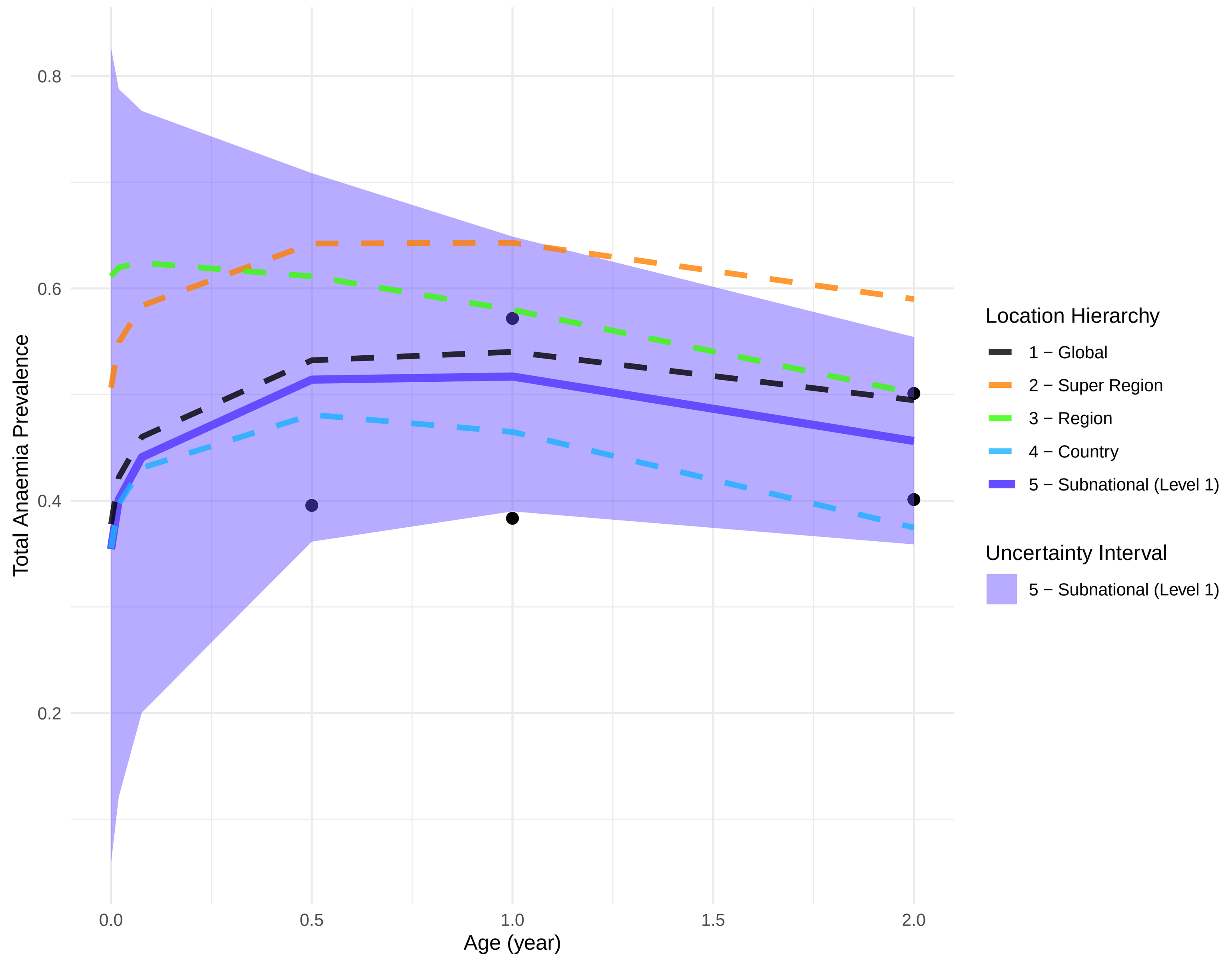

MR−BRT Age/Sex Fits − Non−pregnant Females <2 Years Old − Total Anaemia Prevalence − Kirinyaga
WHO−2001 Adjustment Method

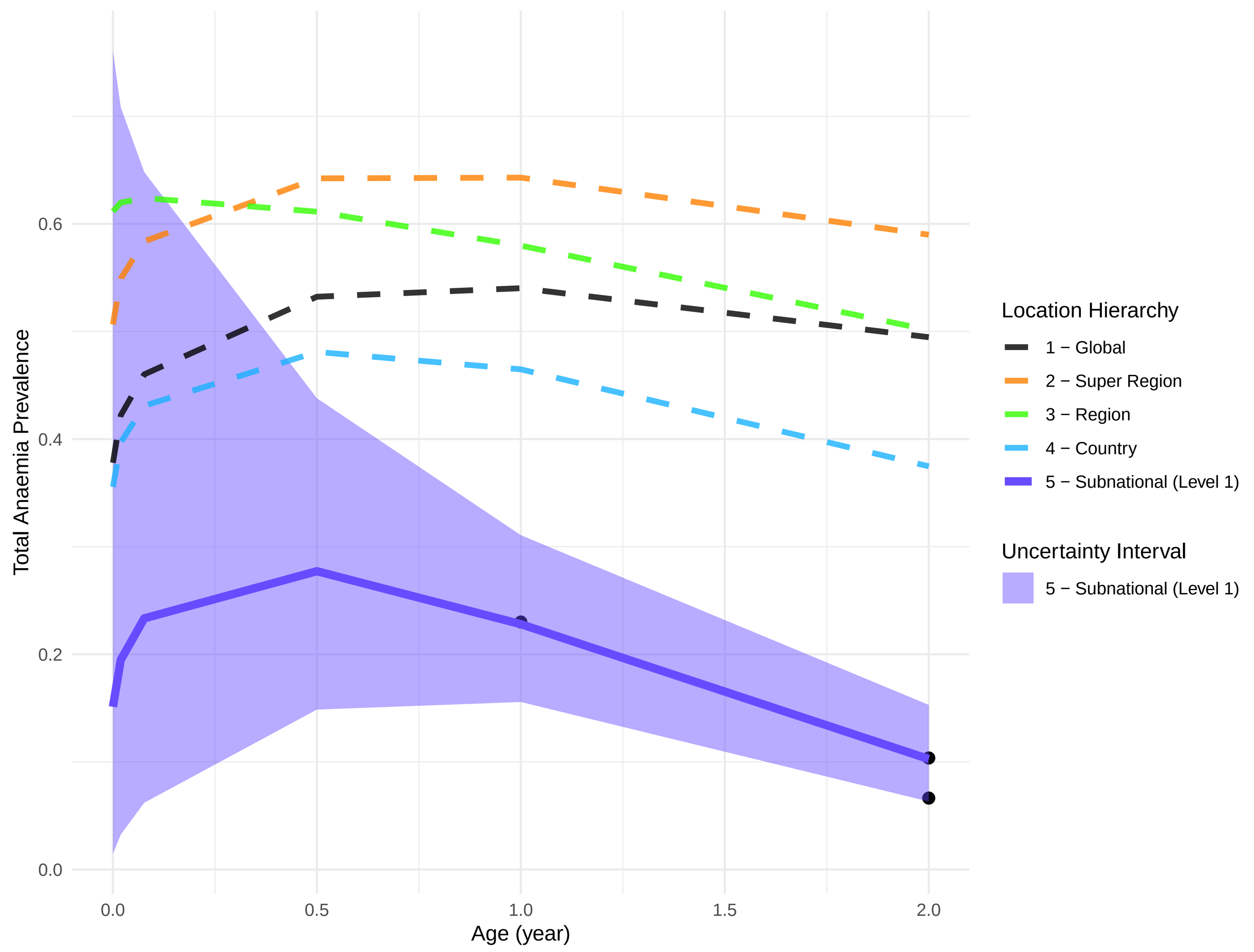

MR−BRT Age/Sex Fits − Non−pregnant Females <2 Years Old − Total Anaemia Prevalence − Kisii
WHO−2001 Adjustment Method

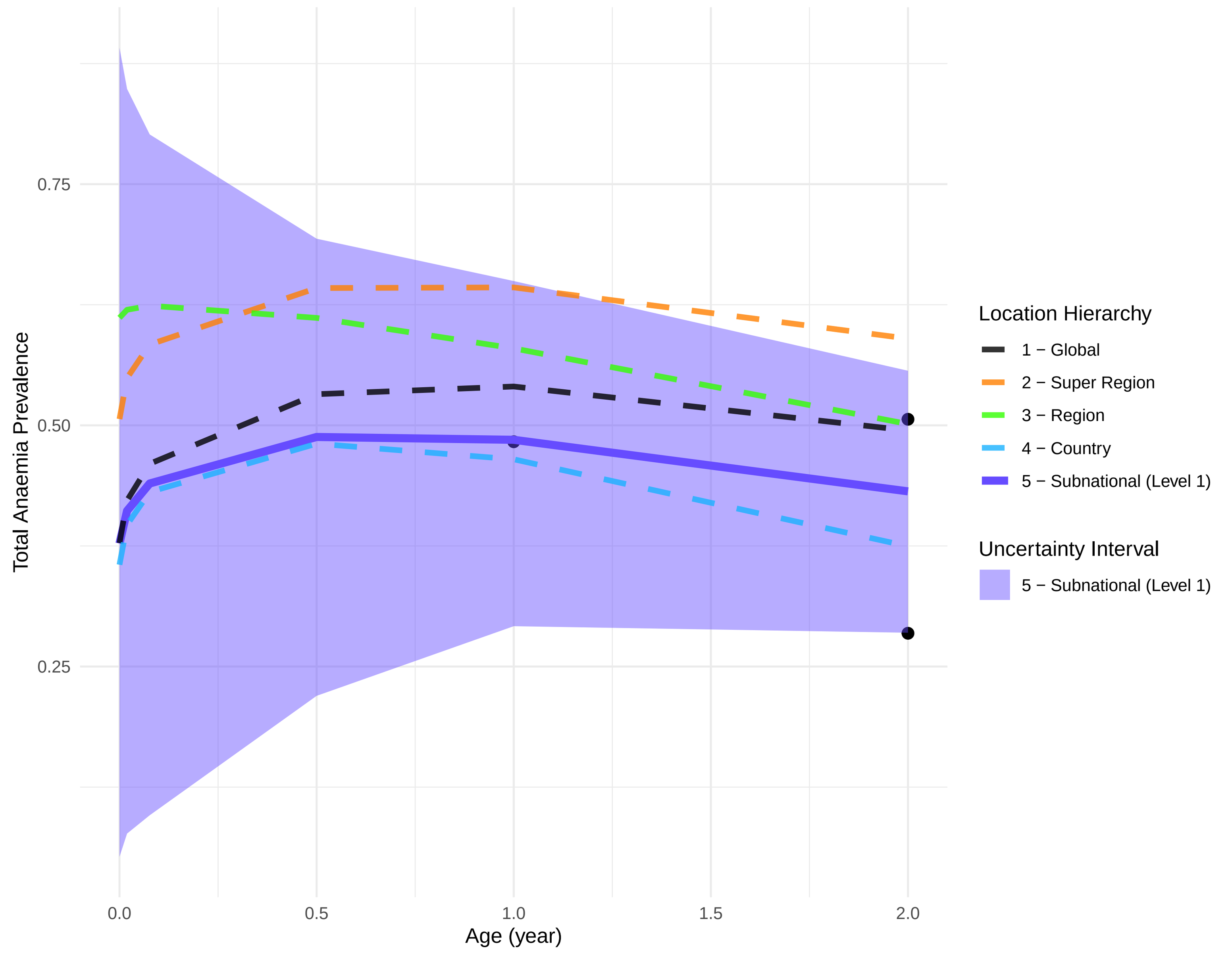

MR−BRT Age/Sex Fits − Non−pregnant Females <2 Years Old − Total Anaemia Prevalence − Kisumu
WHO−2001 Adjustment Method

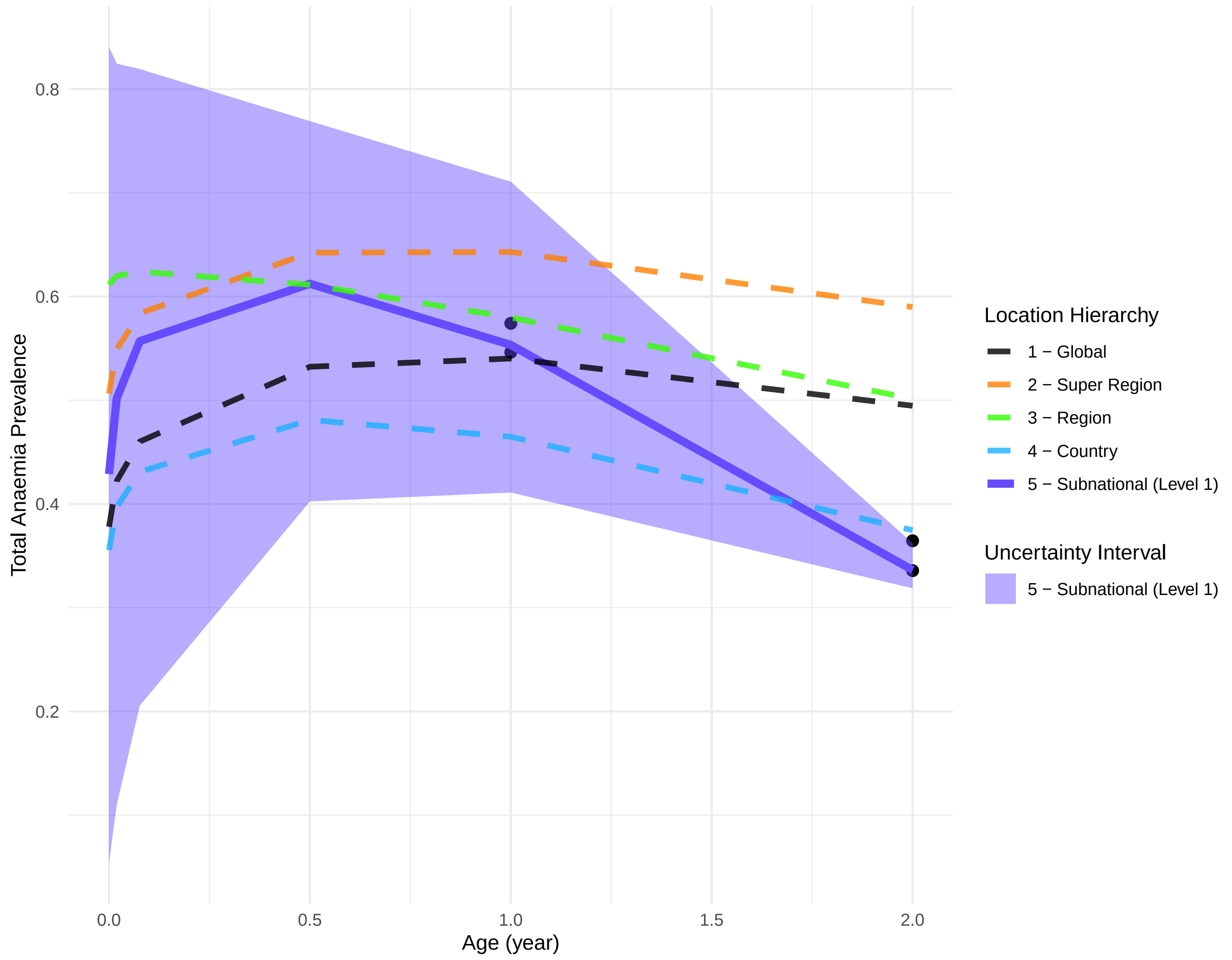

MR−BRT Age/Sex Fits − Non−pregnant Females <2 Years Old − Total Anaemia Prevalence − Kitui
WHO−2001 Adjustment Method

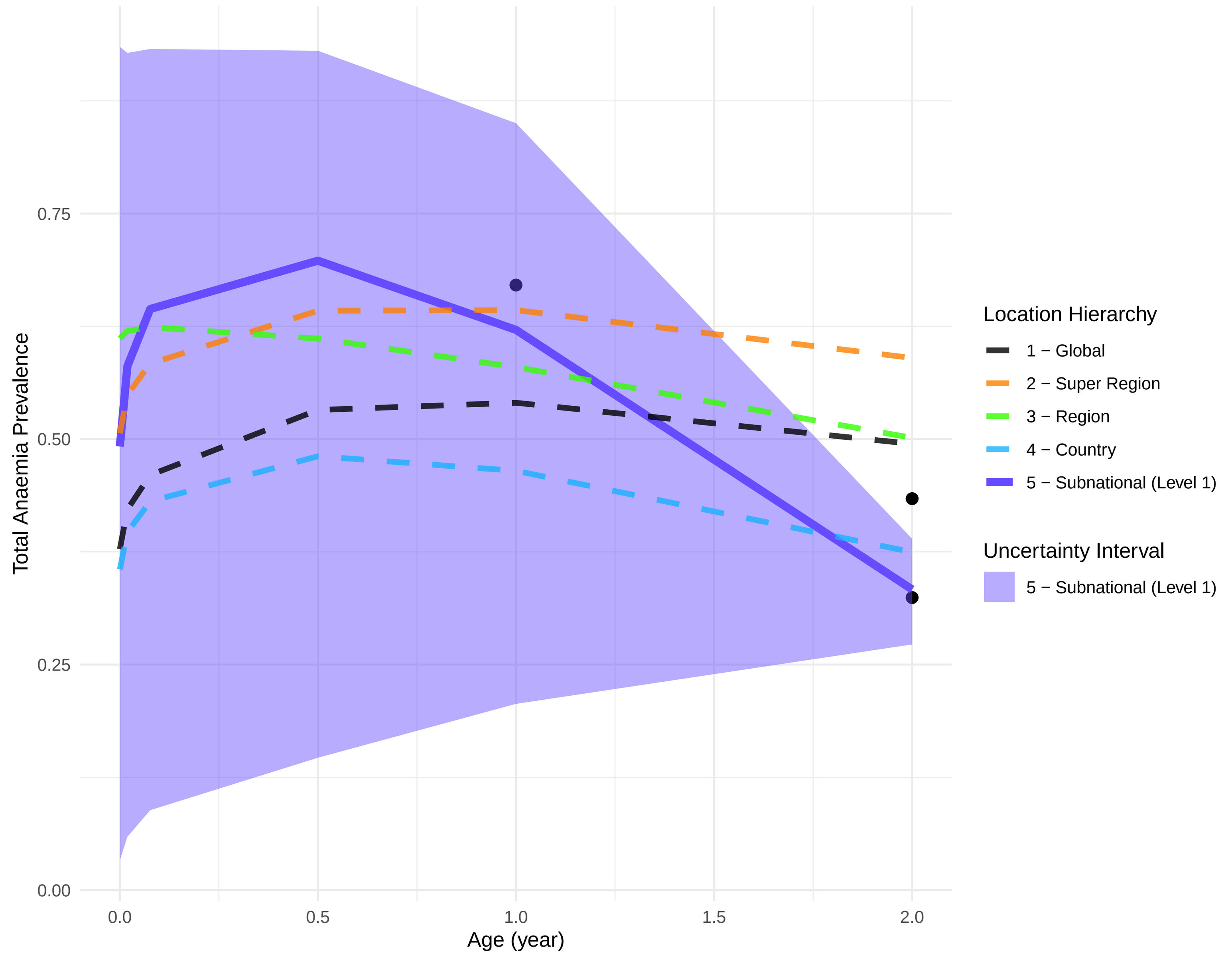

MR−BRT Age/Sex Fits − Non−pregnant Females <2 Years Old − Total Anaemia Prevalence − Kwale
WHO−2001 Adjustment Method

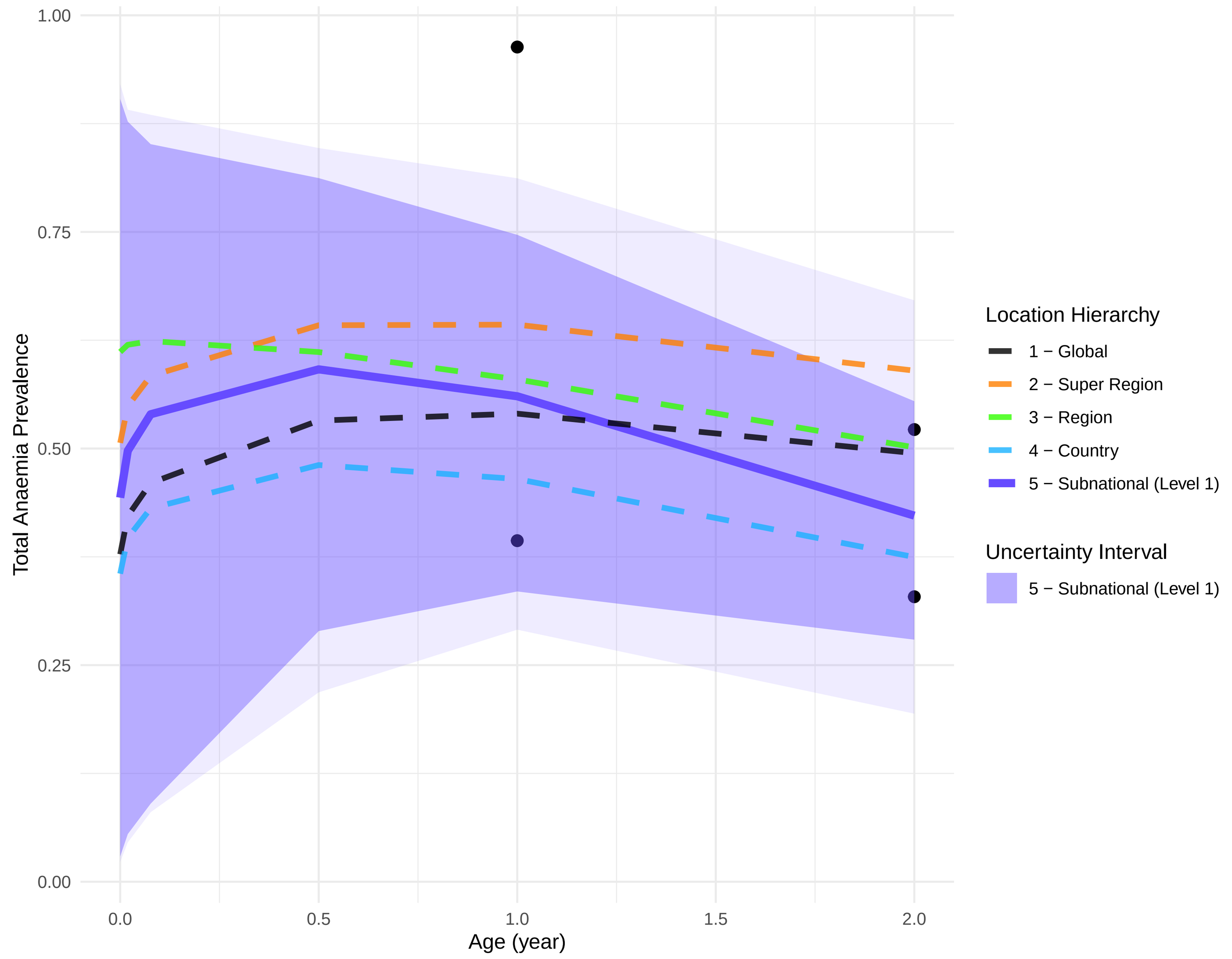

MR−BRT Age/Sex Fits − Non−pregnant Females <2 Years Old − Total Anaemia Prevalence − Laikipia
WHO−2001 Adjustment Method

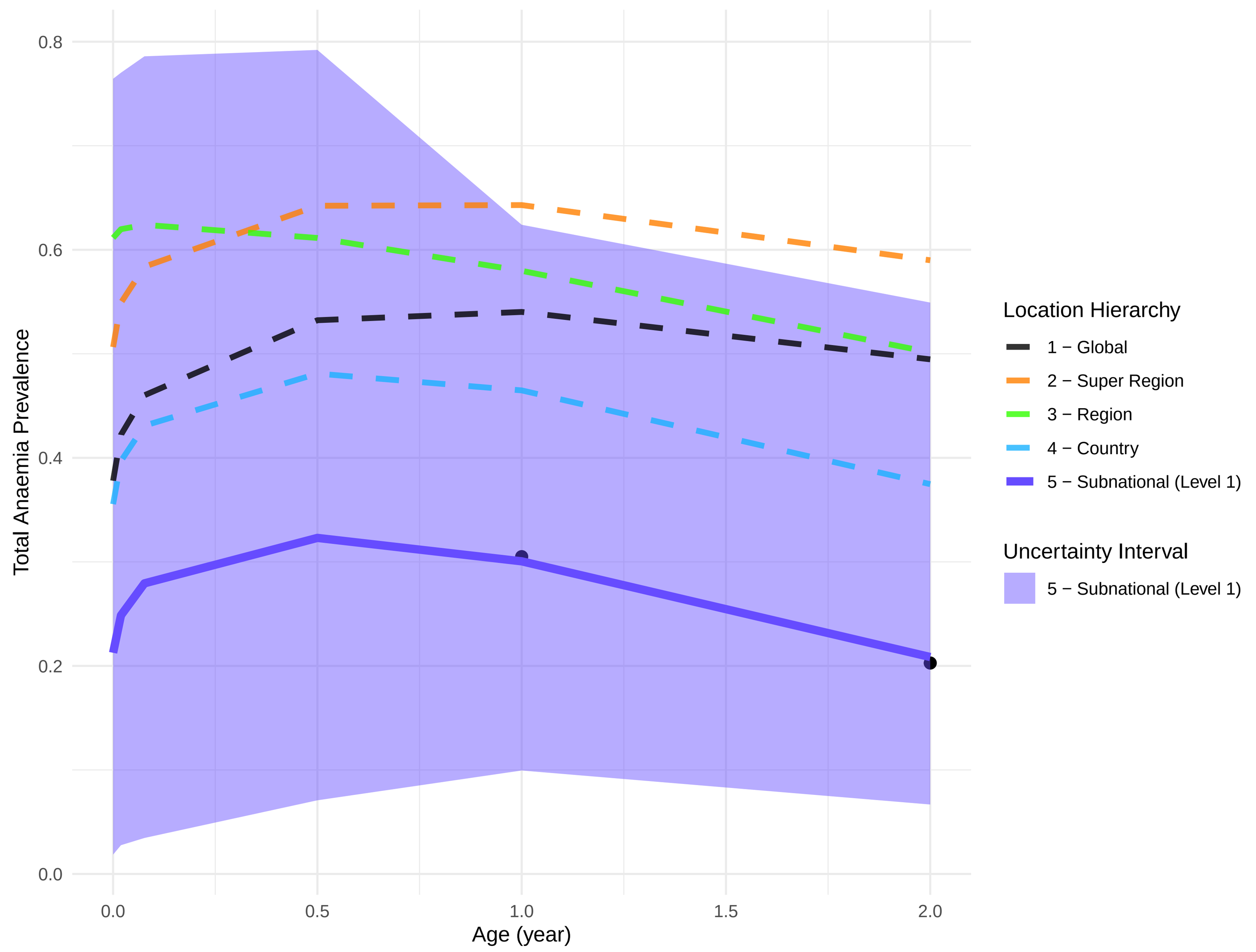

MR−BRT Age/Sex Fits − Non−pregnant Females <2 Years Old − Total Anaemia Prevalence − Lamu
WHO−2001 Adjustment Method

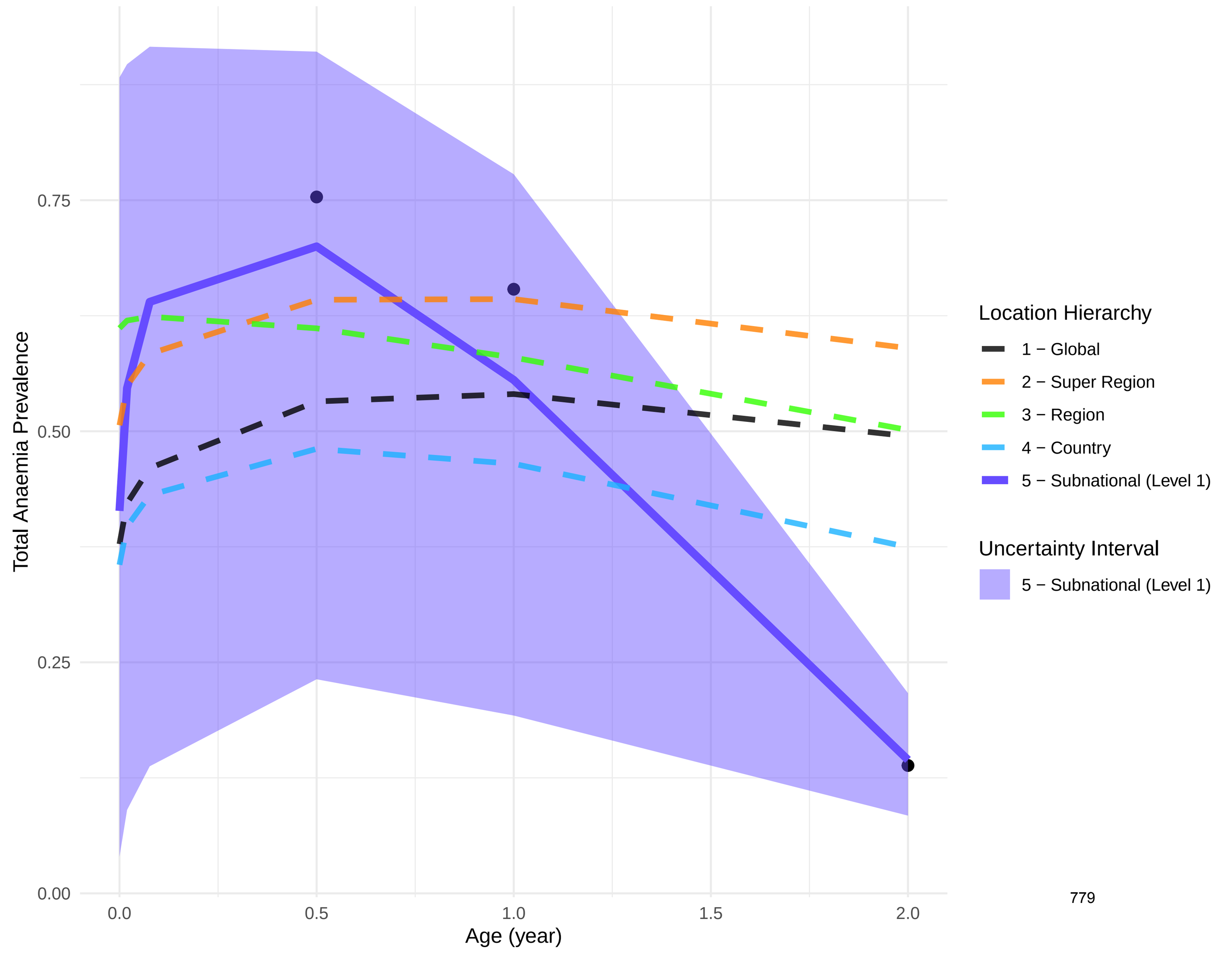

MR−BRT Age/Sex Fits − Non−pregnant Females <2 Years Old − Total Anaemia Prevalence − Machakos
WHO−2001 Adjustment Method

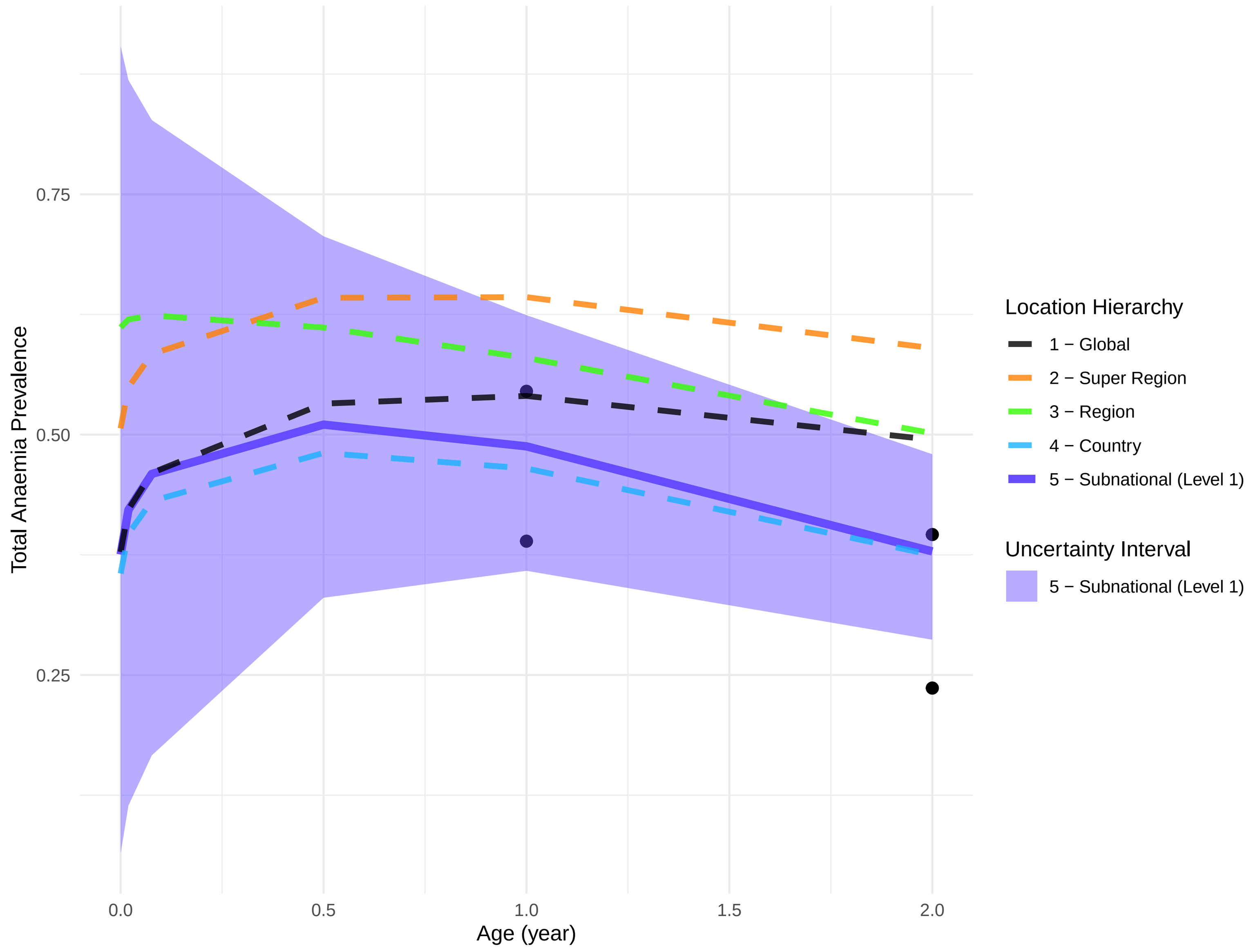

MR−BRT Age/Sex Fits − Non−pregnant Females <2 Years Old − Total Anaemia Prevalence − Makueni
WHO−2001 Adjustment Method

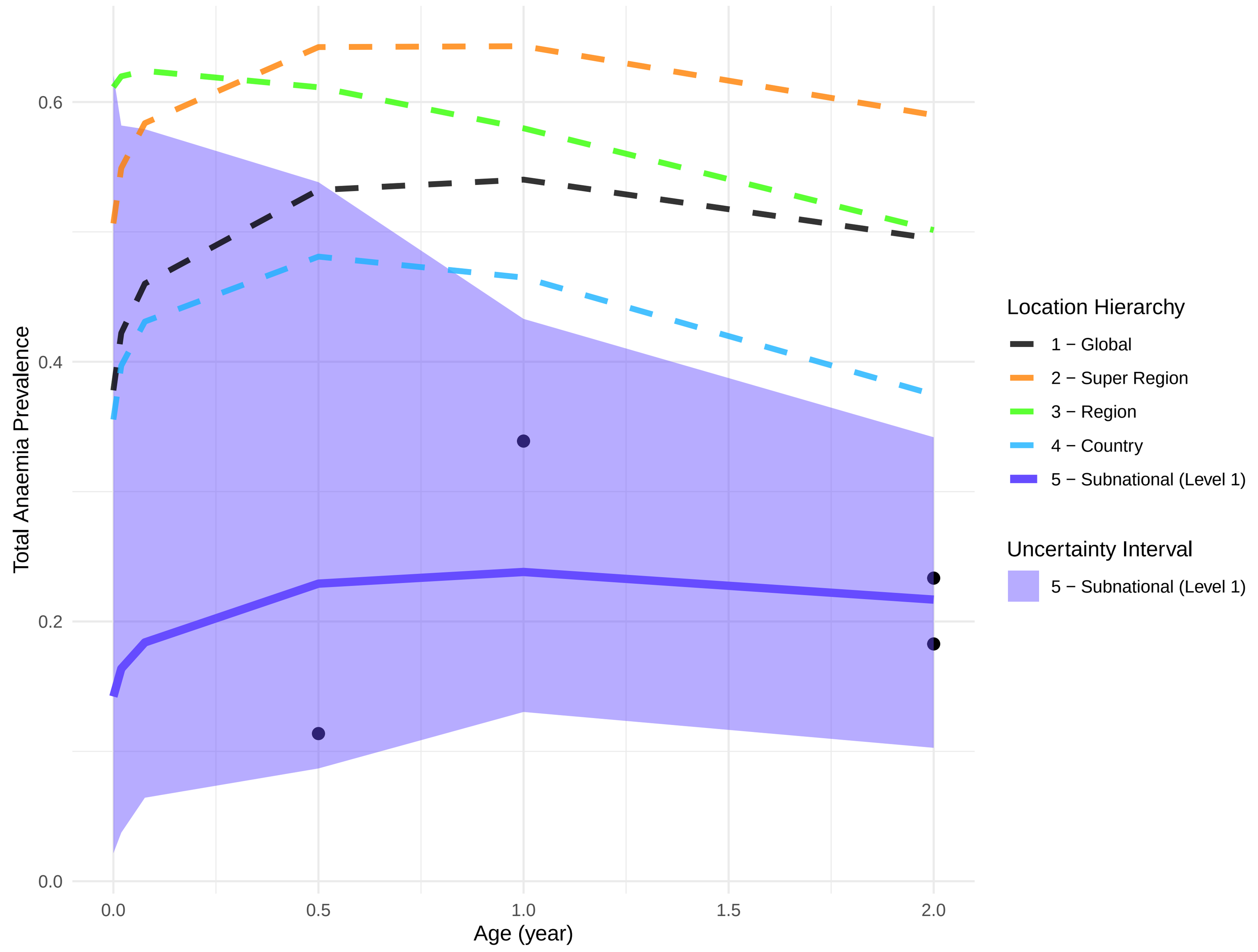

MR−BRT Age/Sex Fits − Non−pregnant Females <2 Years Old − Total Anaemia Prevalence − Mandera
WHO−2001 Adjustment Method

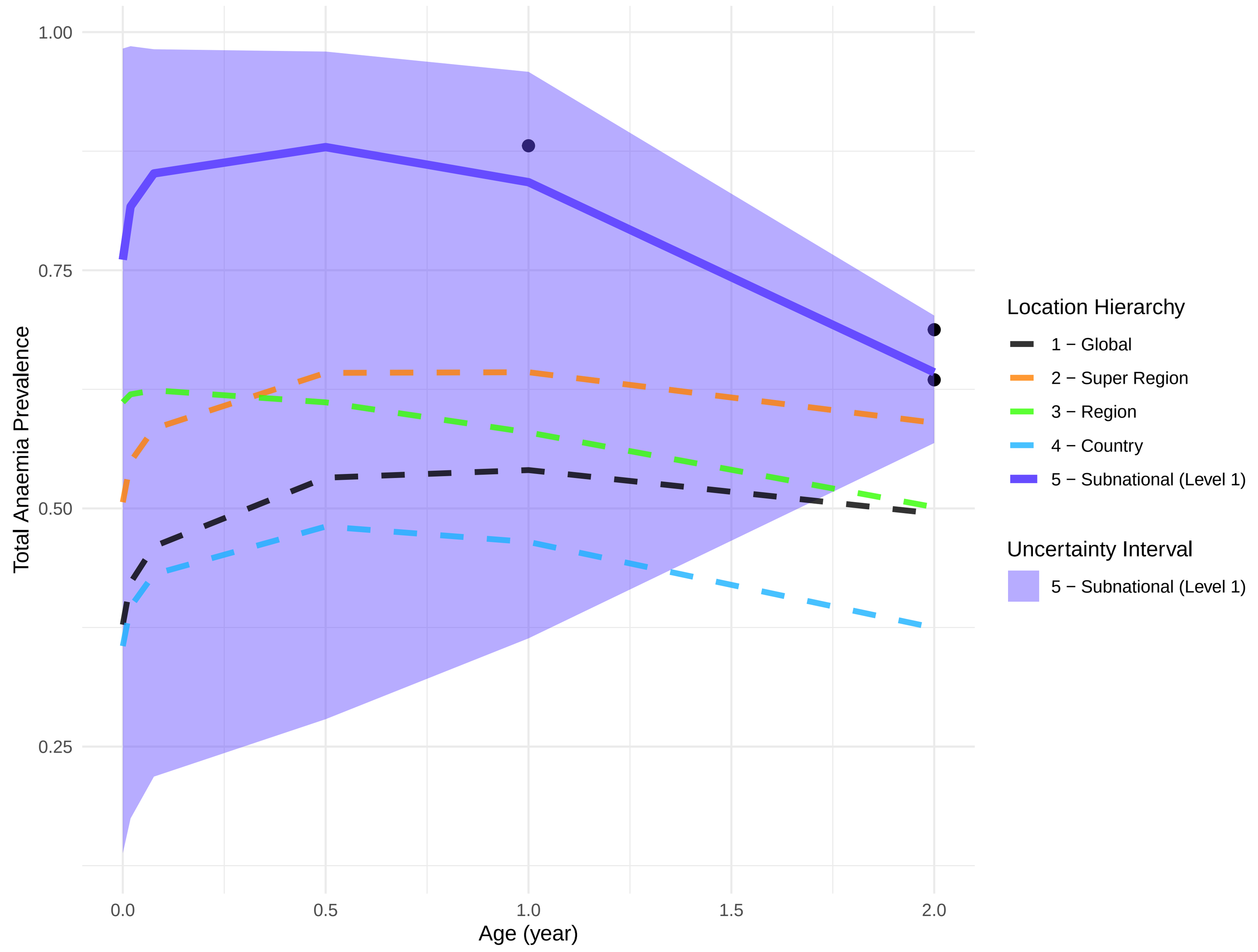

MR−BRT Age/Sex Fits − Non−pregnant Females <2 Years Old − Total Anaemia Prevalence − Marsabit
WHO−2001 Adjustment Method

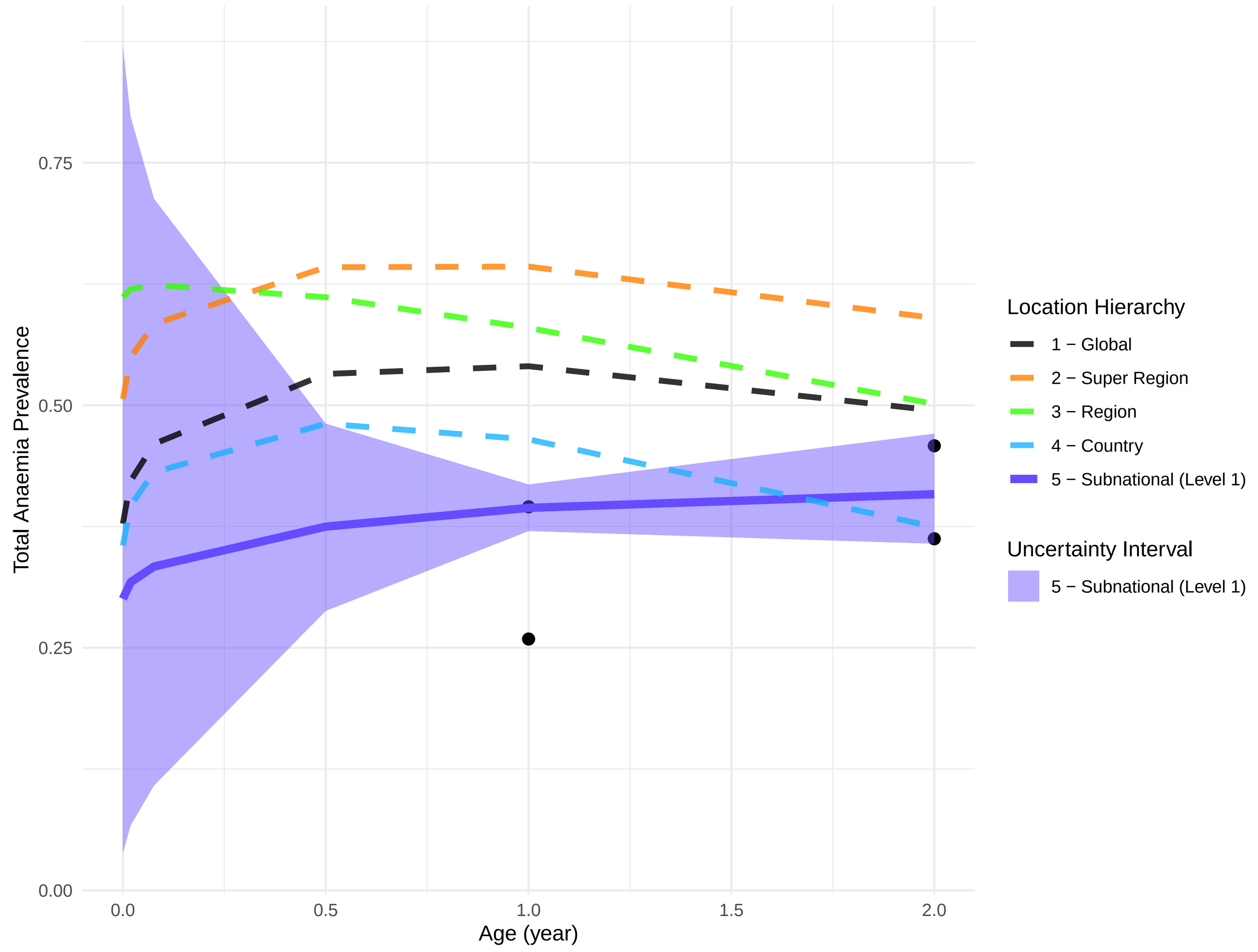

MR−BRT Age/Sex Fits − Non−pregnant Females <2 Years Old − Total Anaemia Prevalence − Meru
WHO−2001 Adjustment Method

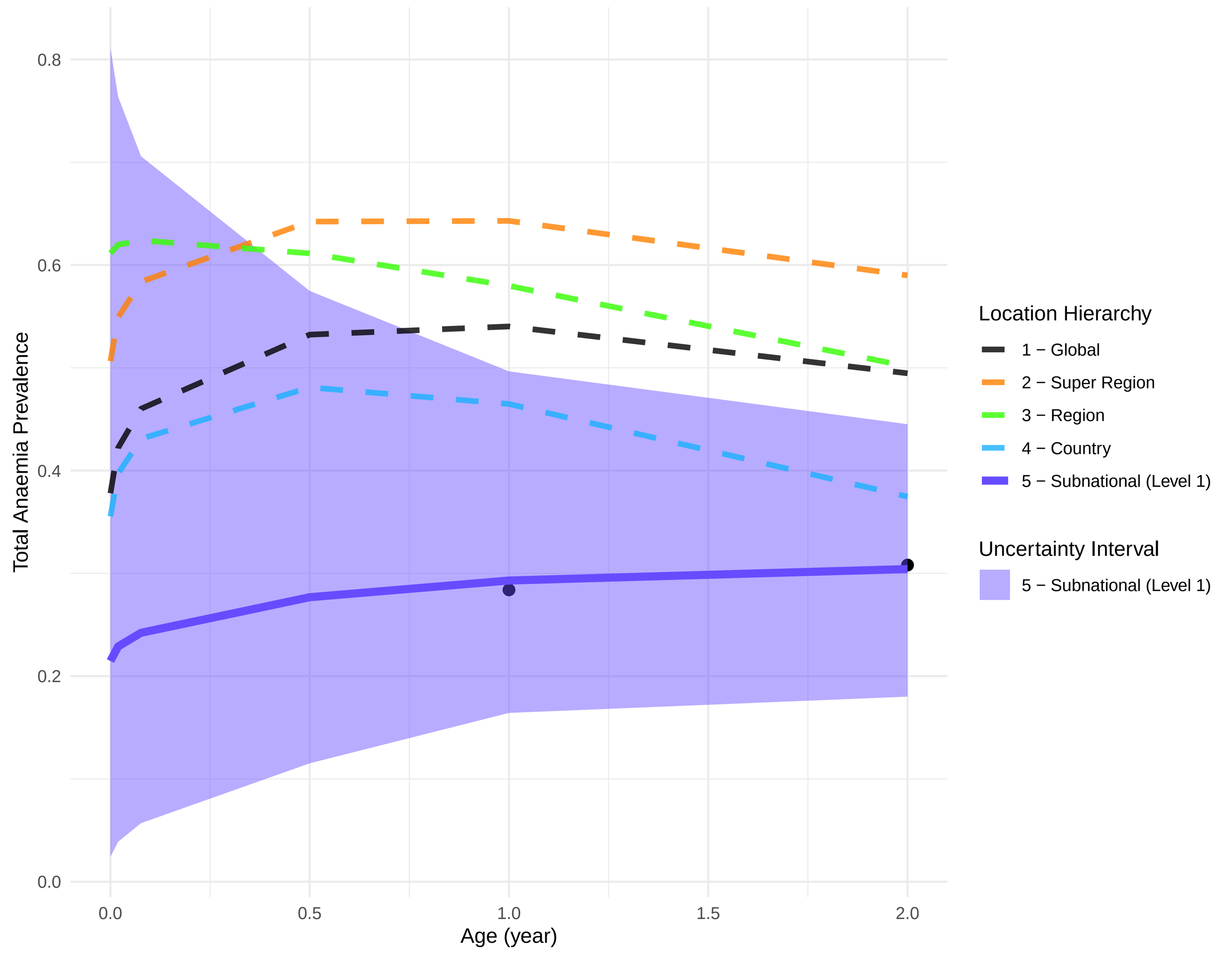

MR−BRT Age/Sex Fits − Non−pregnant Females <2 Years Old − Total Anaemia Prevalence − Migori
WHO−2001 Adjustment Method

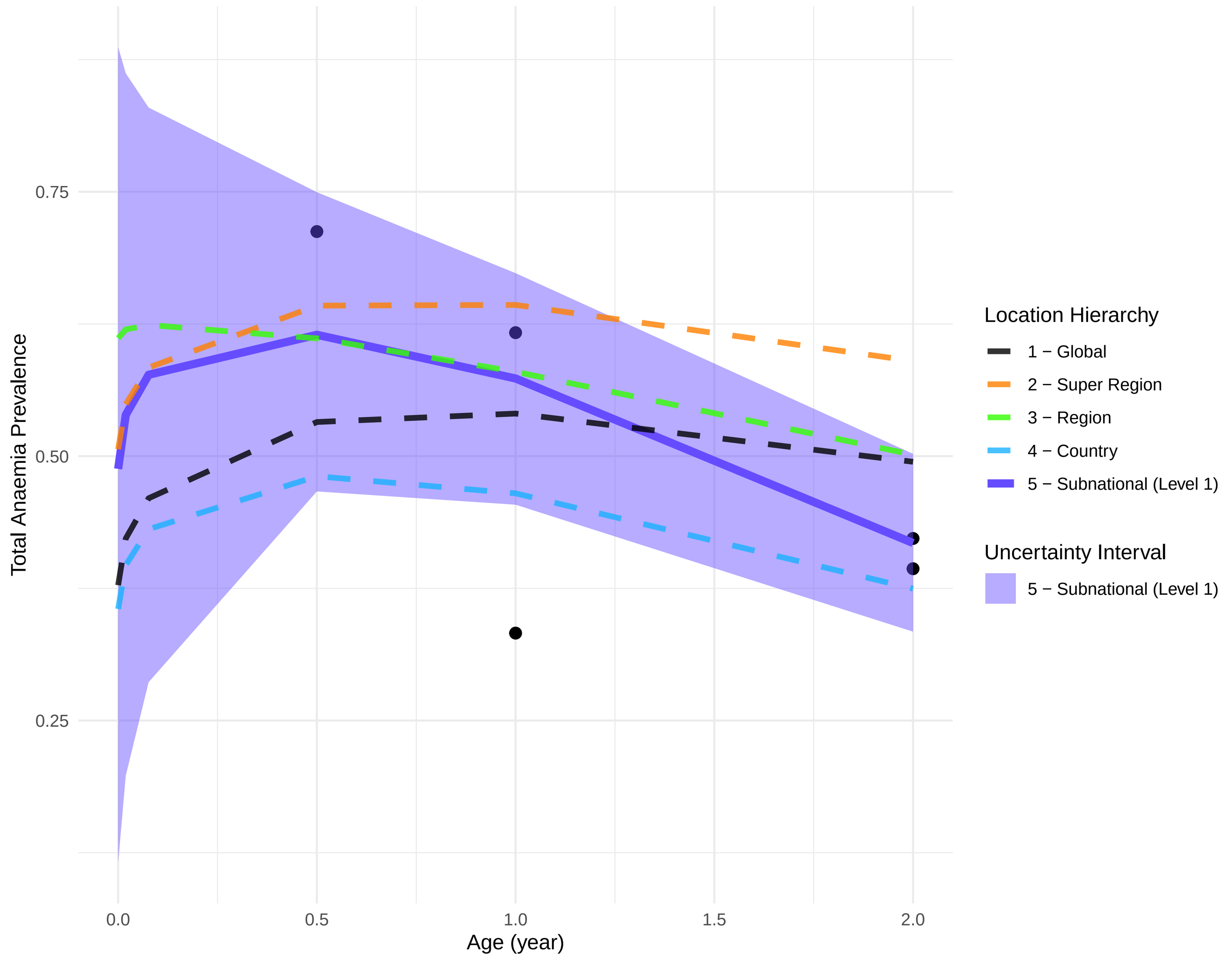

MR−BRT Age/Sex Fits − Non−pregnant Females <2 Years Old − Total Anaemia Prevalence − Mombasa
WHO−2001 Adjustment Method

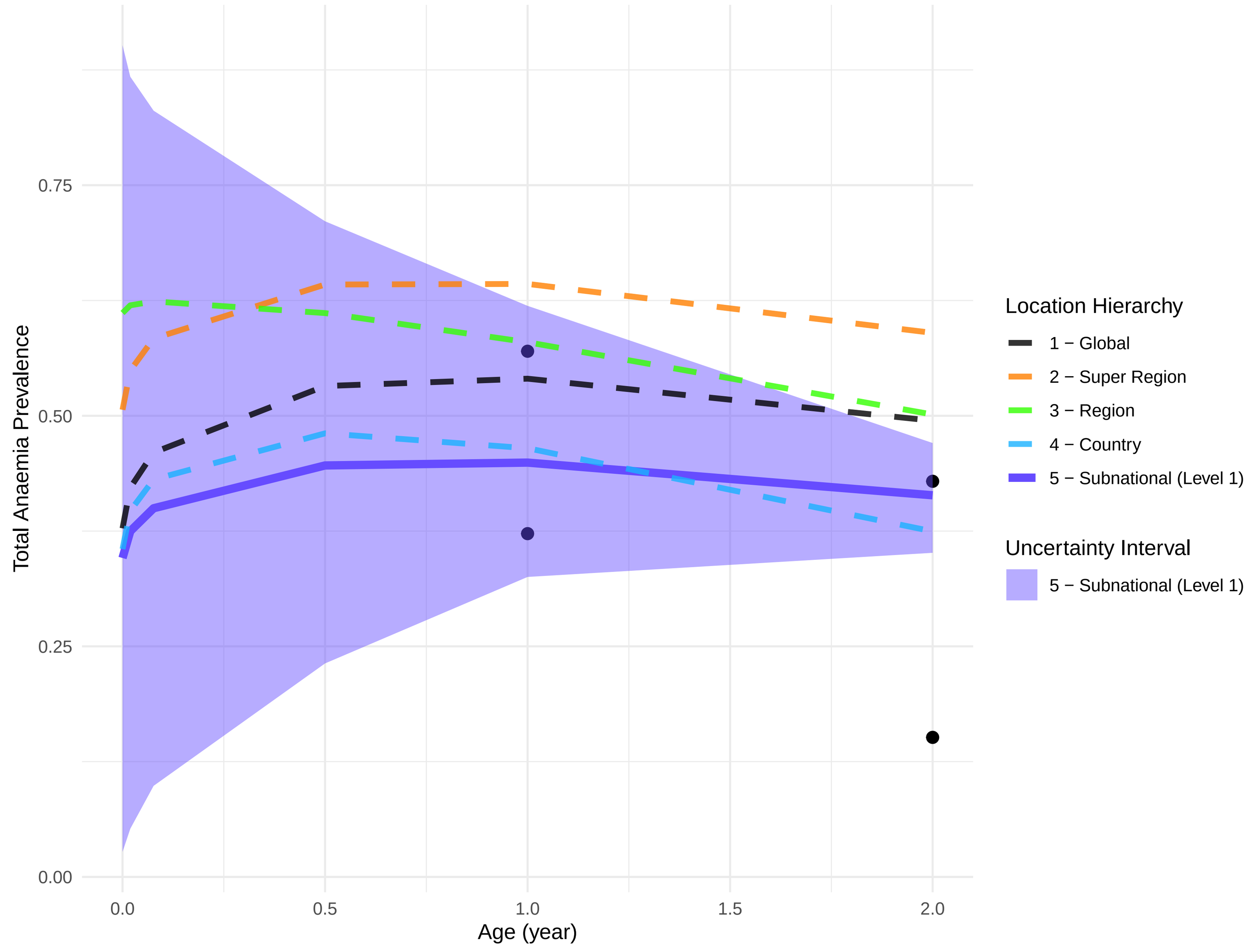

MR−BRT Age/Sex Fits − Non−pregnant Females <2 Years Old − Total Anaemia Prevalence − Murang'a
WHO−2001 Adjustment Method

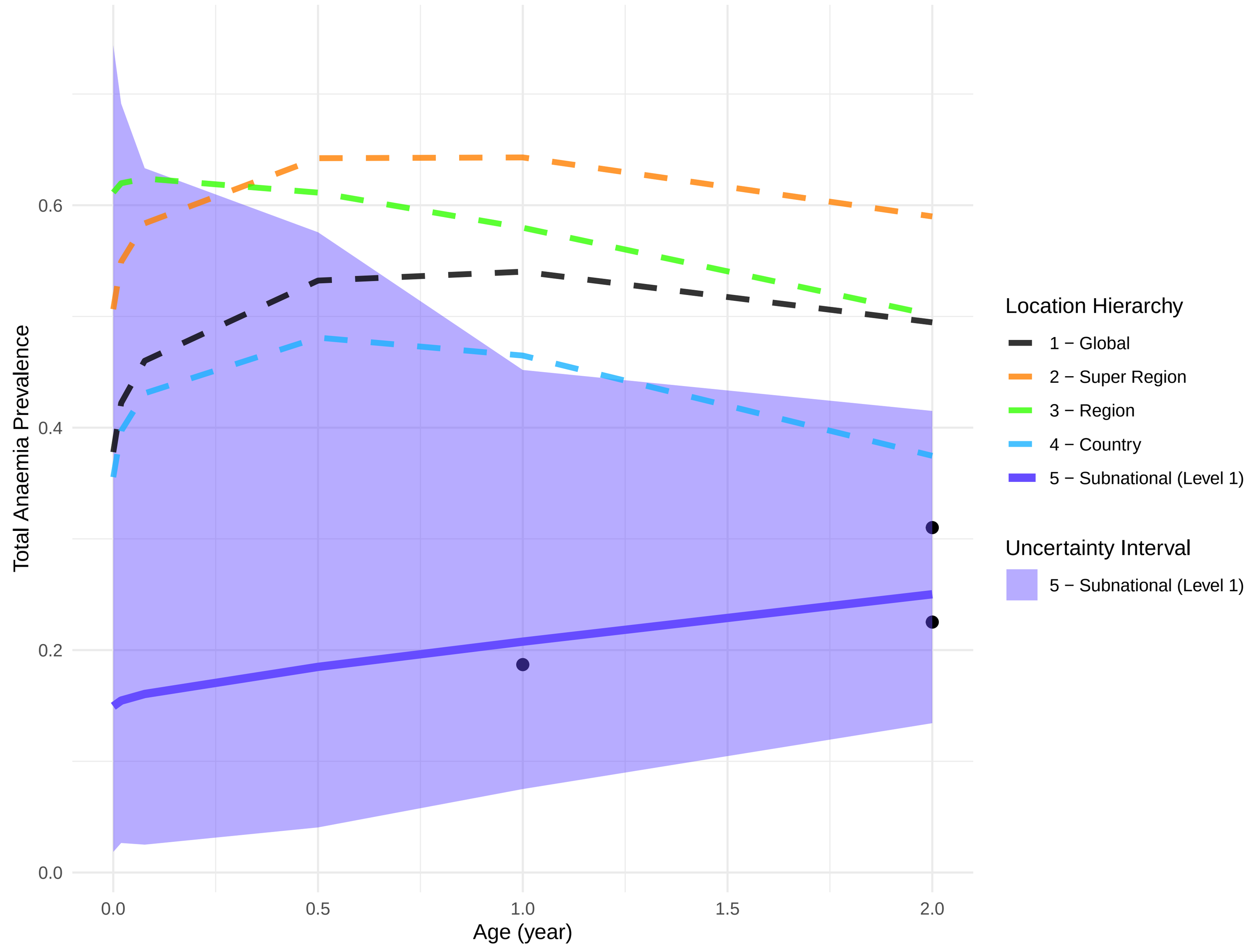

MR−BRT Age/Sex Fits − Non−pregnant Females <2 Years Old − Total Anaemia Prevalence − Nairobi
WHO−2001 Adjustment Method

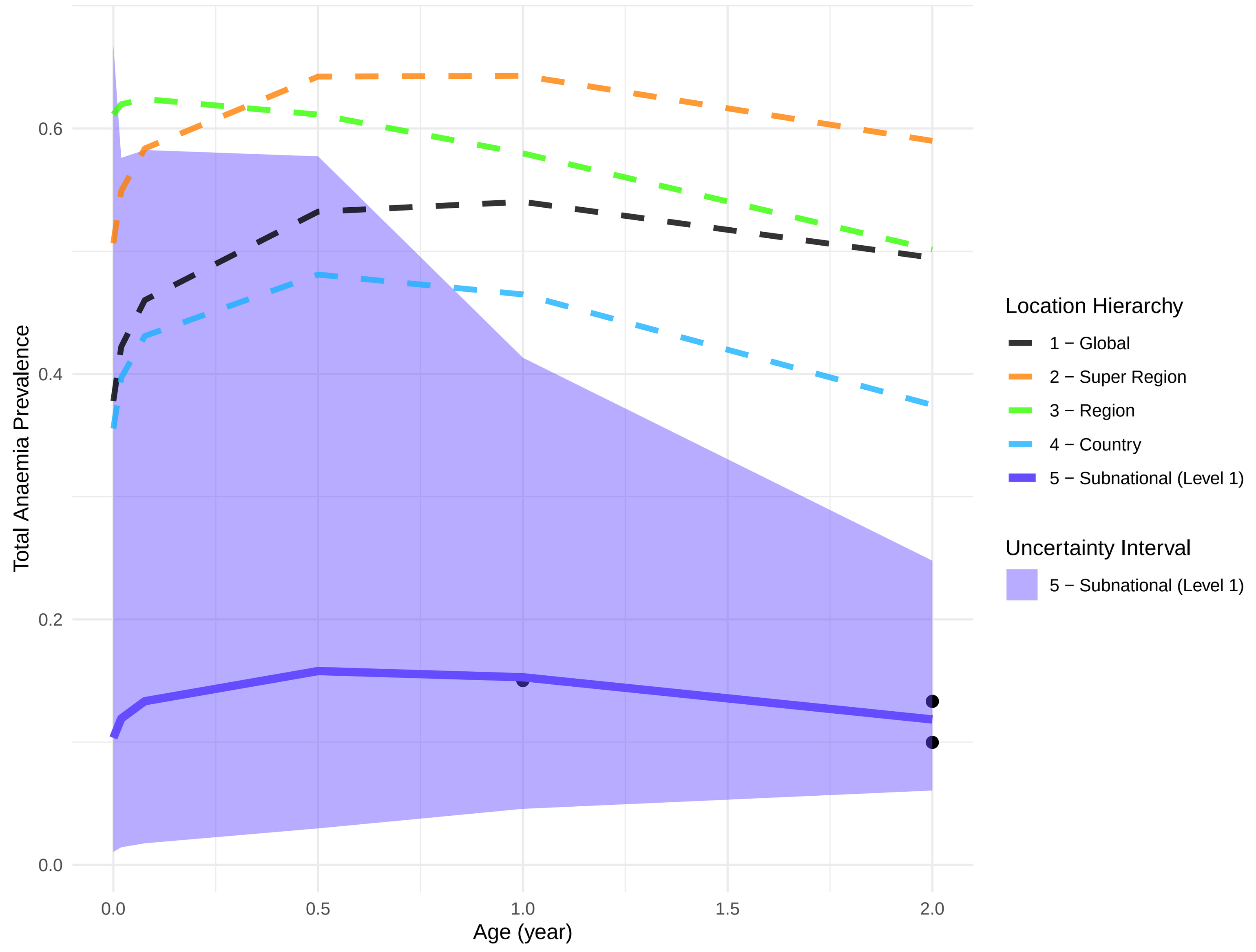

MR−BRT Age/Sex Fits − Non−pregnant Females <2 Years Old − Total Anaemia Prevalence − Nakuru
WHO−2001 Adjustment Method

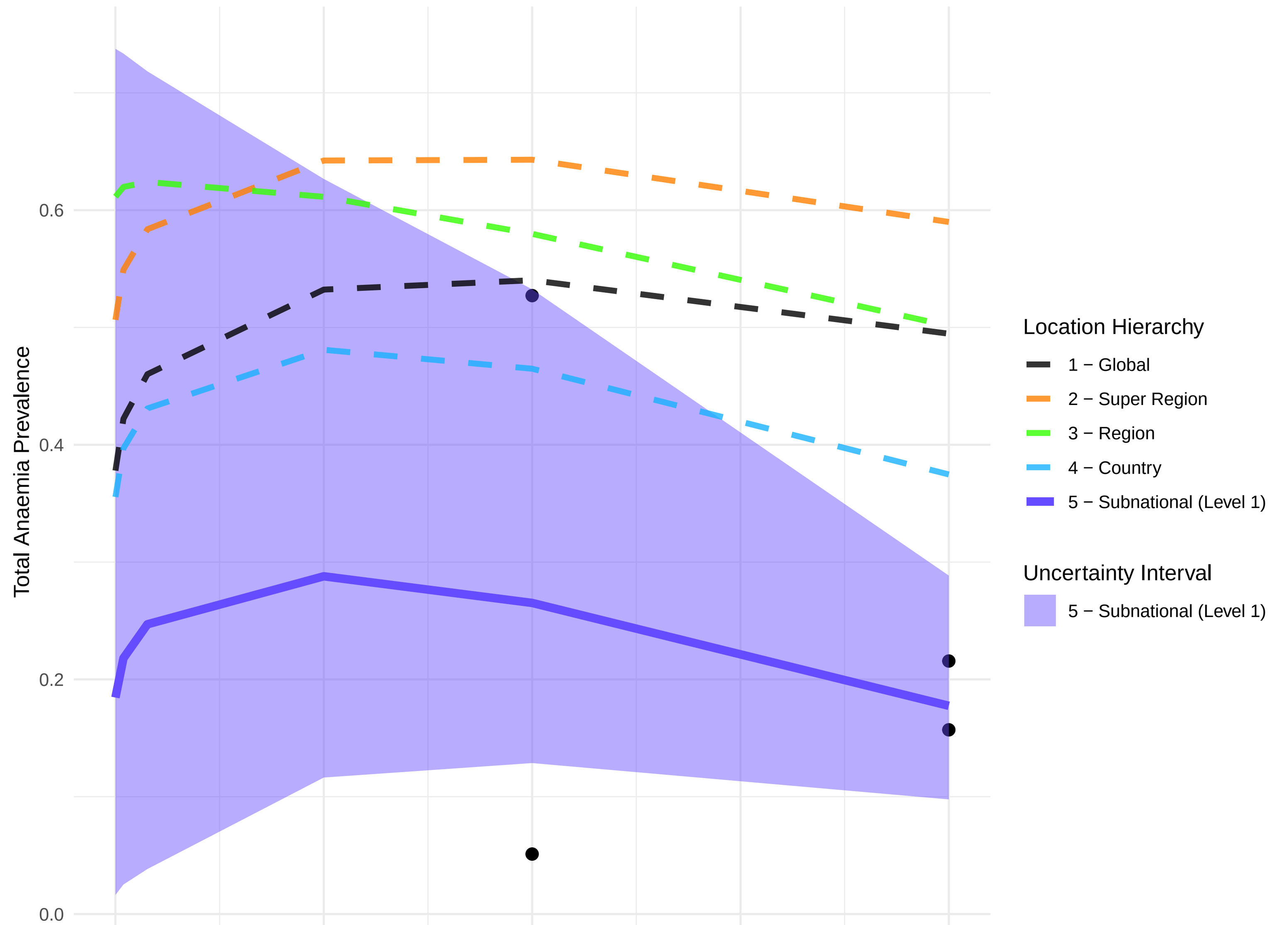

MR−BRT Age/Sex Fits − Non−pregnant Females <2 Years Old − Total Anaemia Prevalence − Nandi
WHO−2001 Adjustment Method

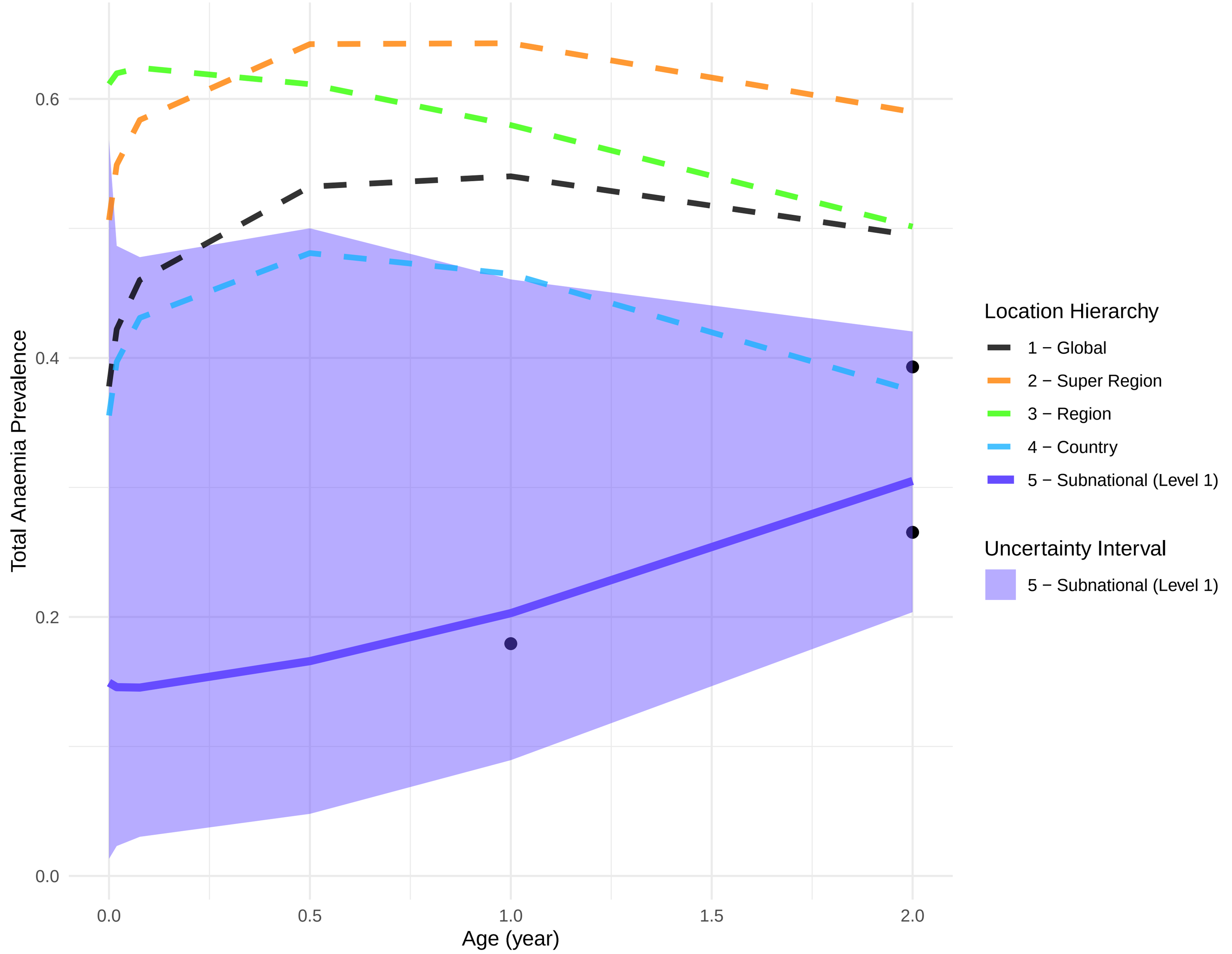

MR−BRT Age/Sex Fits − Non−pregnant Females <2 Years Old − Total Anaemia Prevalence − Narok
WHO−2001 Adjustment Method

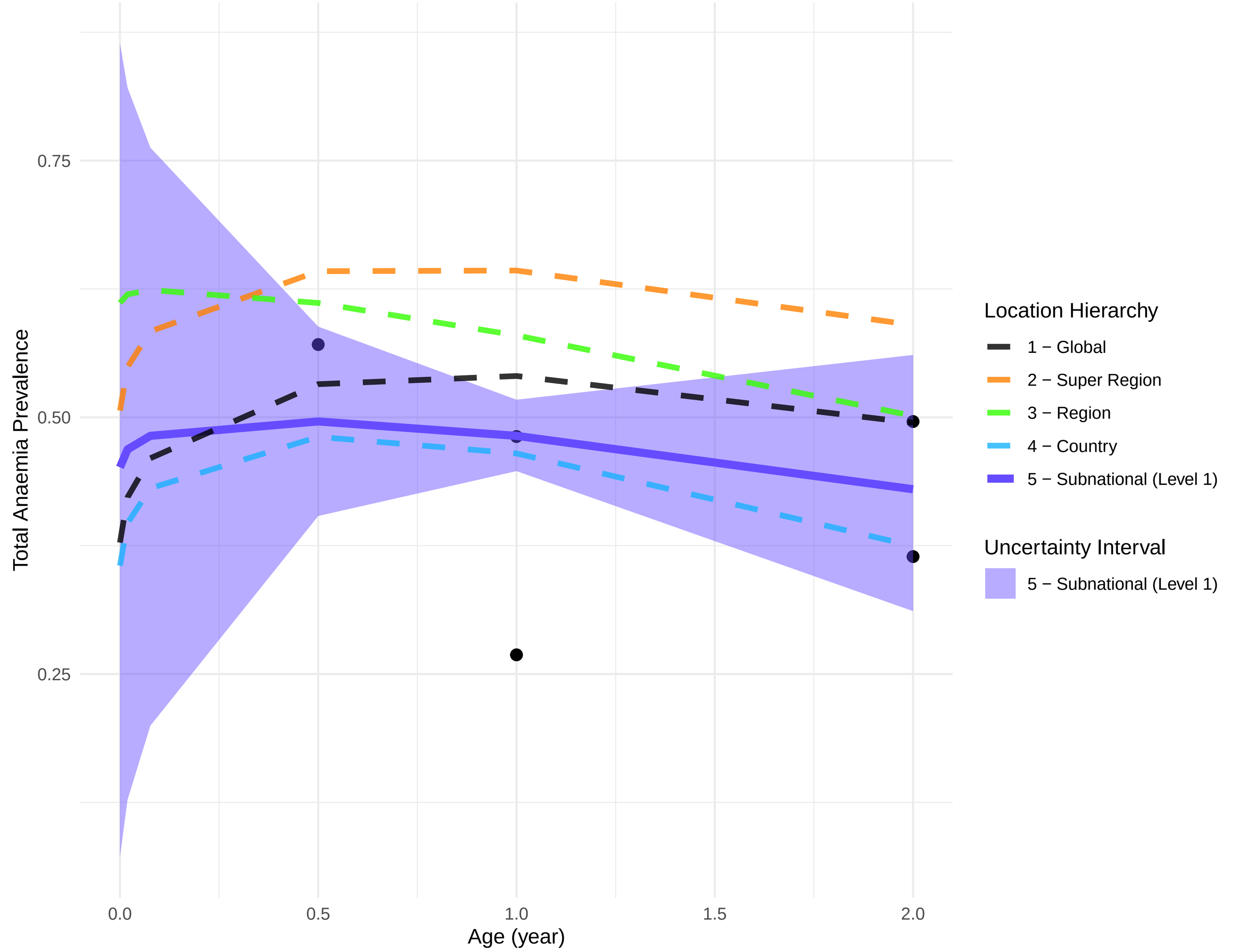

MR−BRT Age/Sex Fits − Non−pregnant Females <2 Years Old − Total Anaemia Prevalence − Nyamira
WHO−2001 Adjustment Method

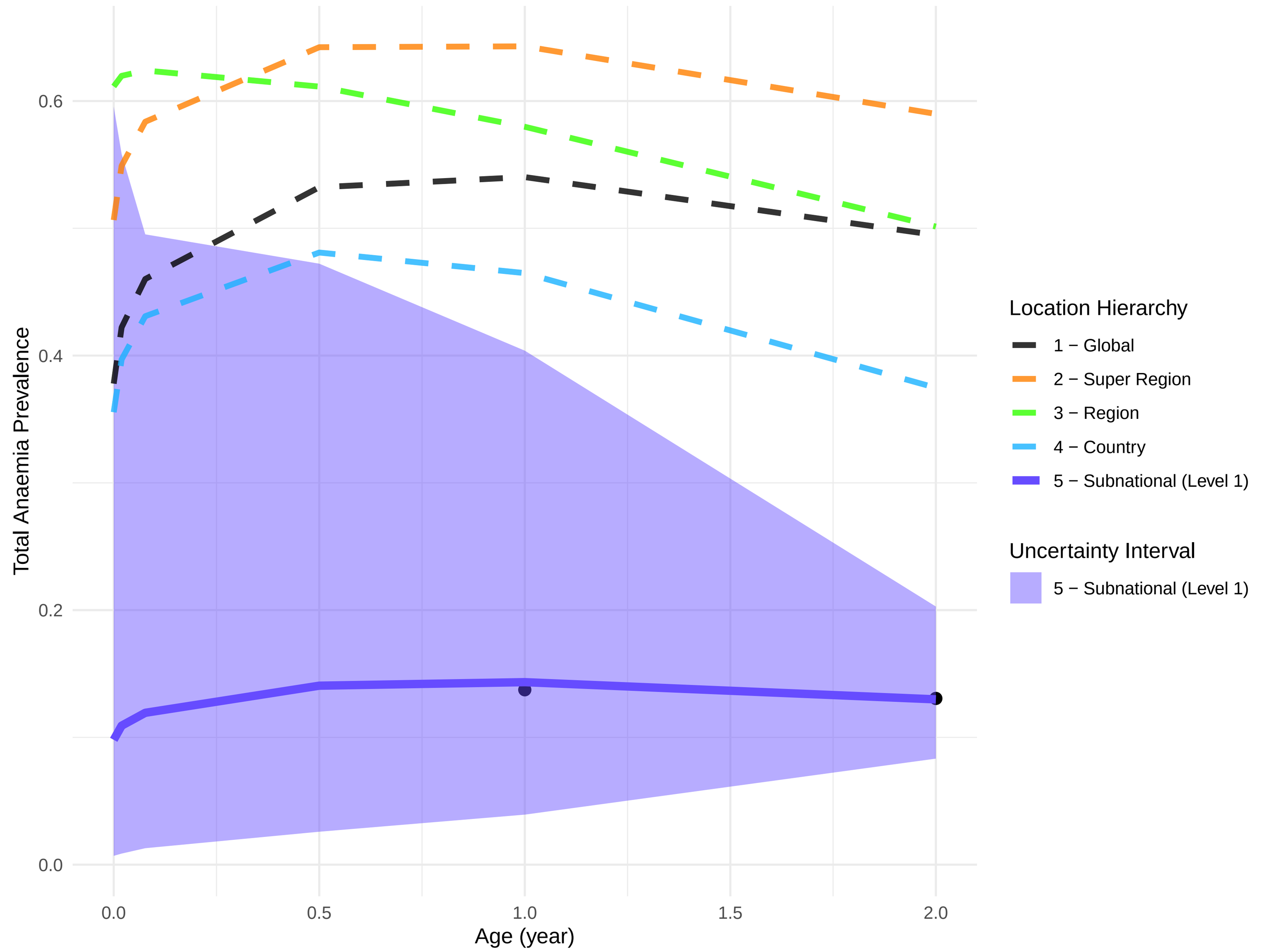

MR−BRT Age/Sex Fits − Non−pregnant Females <2 Years Old − Total Anaemia Prevalence − Nyandarua
WHO−2001 Adjustment Method

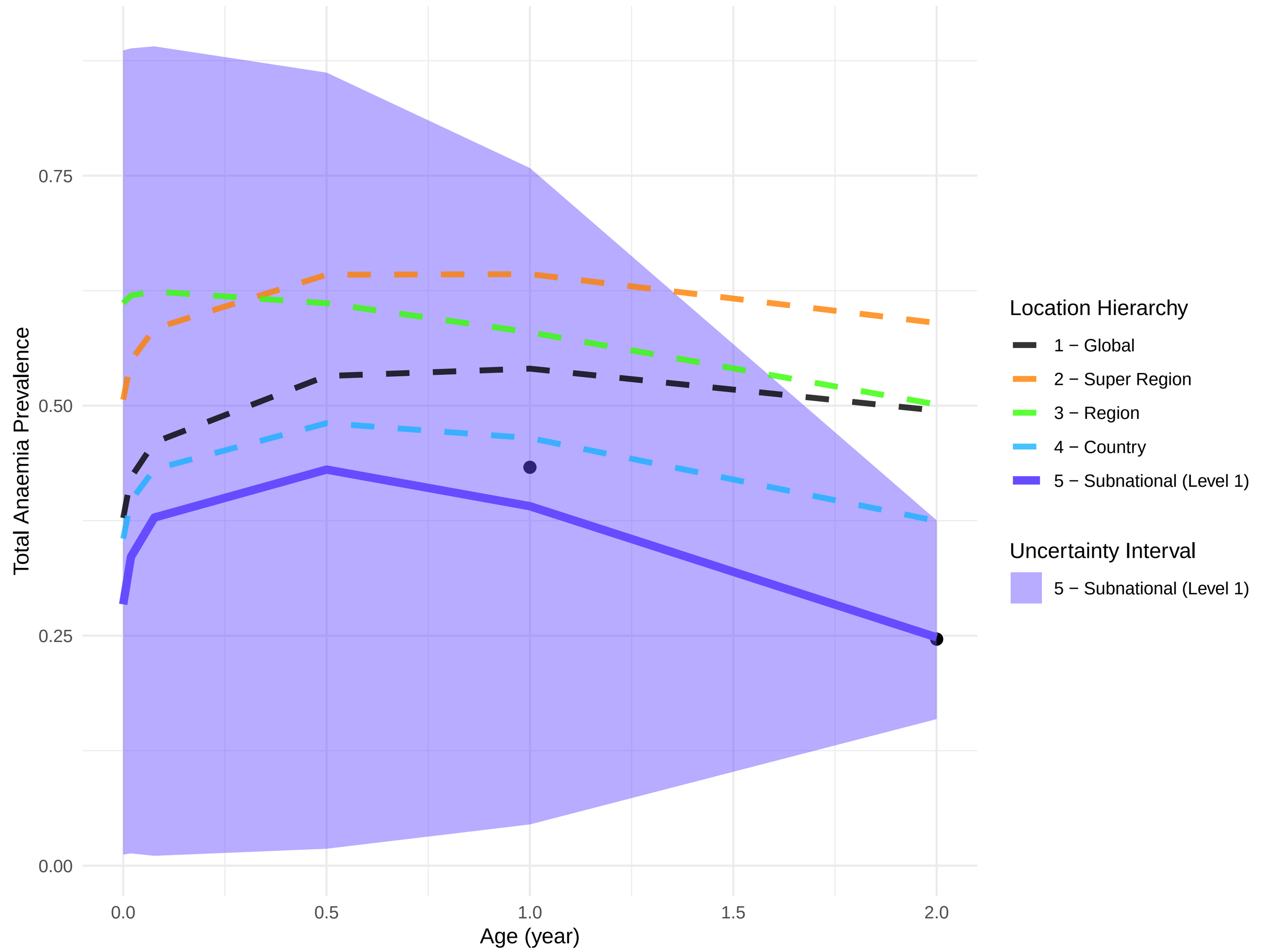

MR−BRT Age/Sex Fits − Non−pregnant Females <2 Years Old − Total Anaemia Prevalence − Nyeri
WHO−2001 Adjustment Method

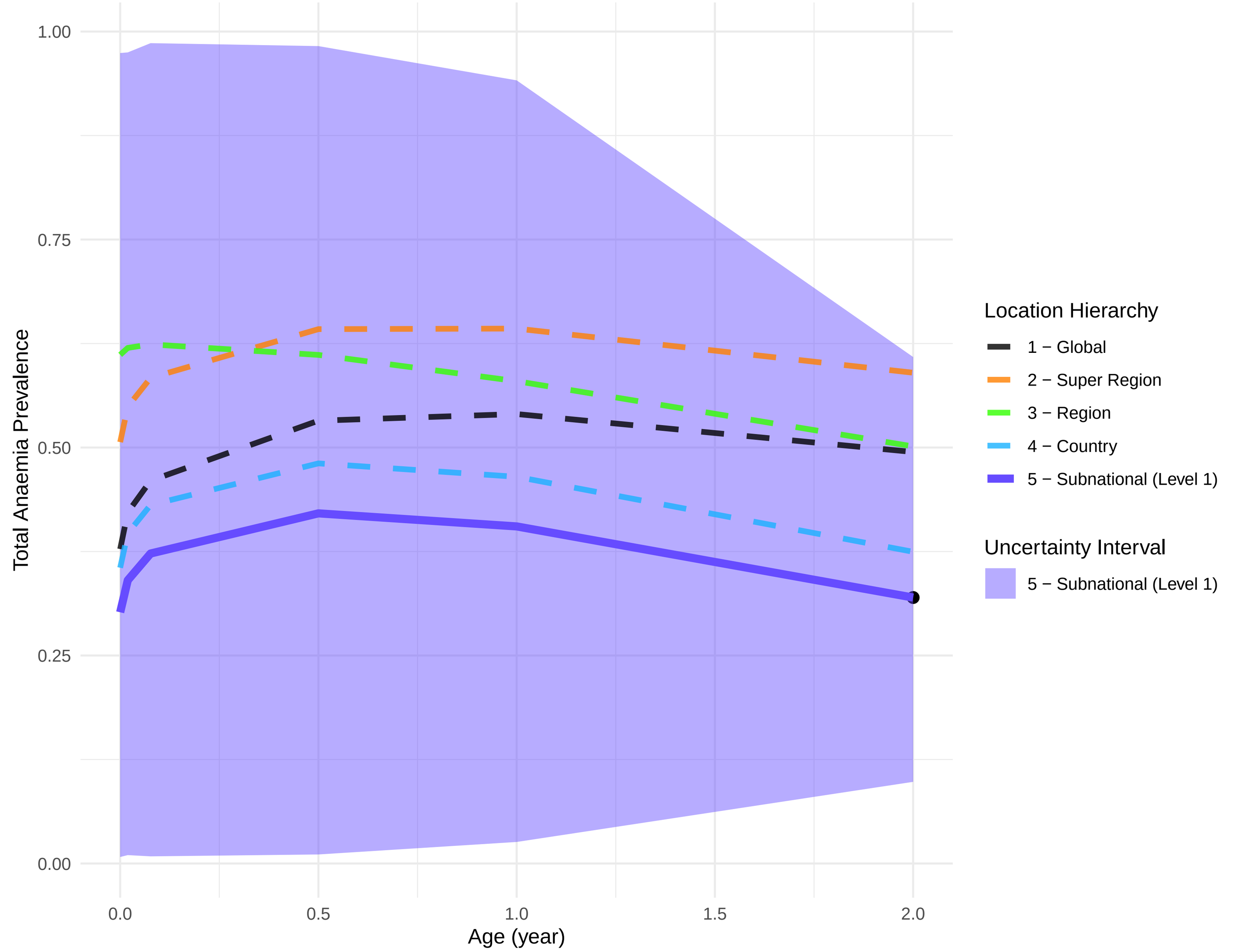

MR−BRT Age/Sex Fits − Non−pregnant Females <2 Years Old − Total Anaemia Prevalence − Samburu
WHO−2001 Adjustment Method

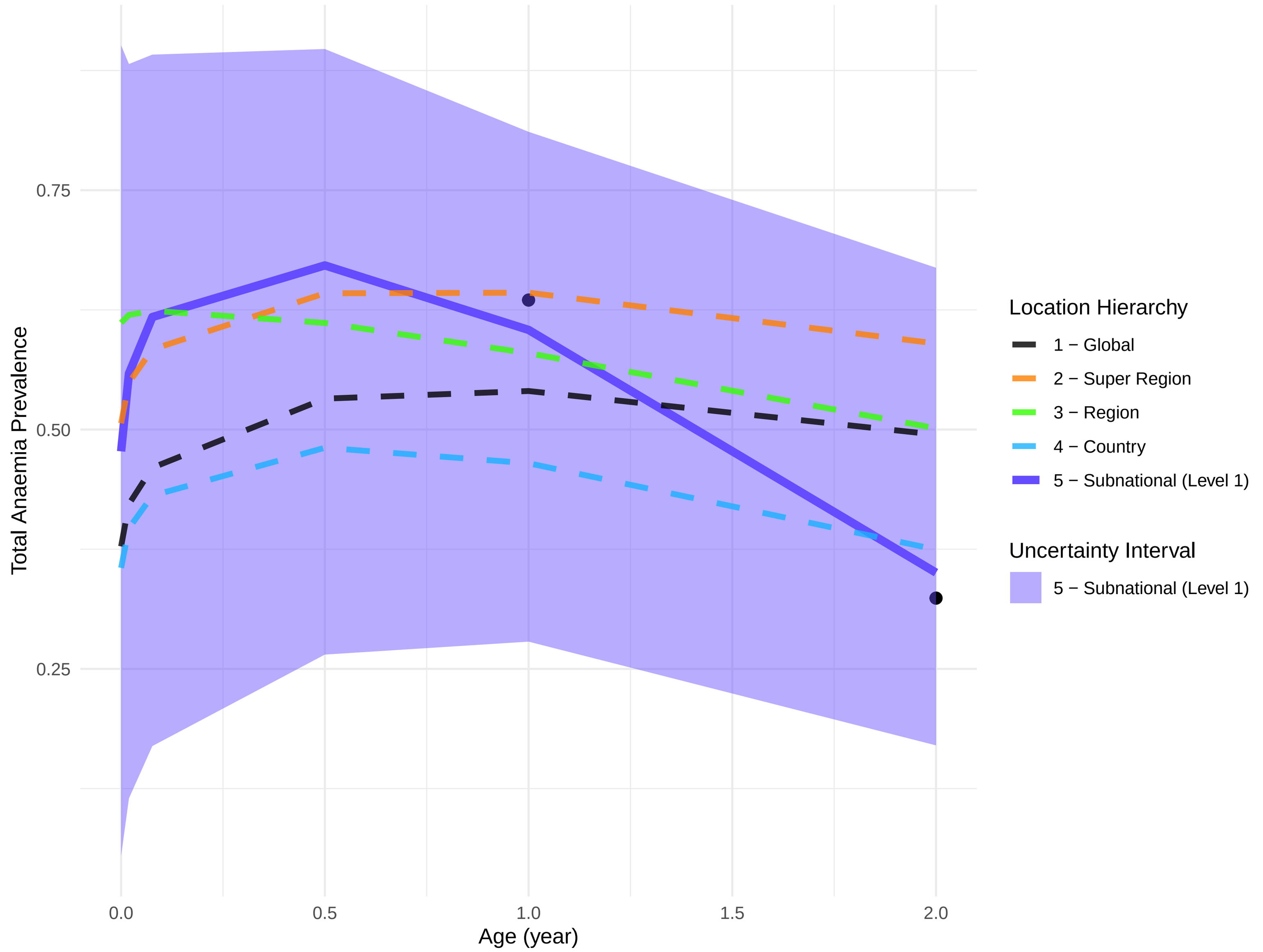

MR−BRT Age/Sex Fits − Non−pregnant Females <2 Years Old − Total Anaemia Prevalence − Siaya
WHO−2001 Adjustment Method

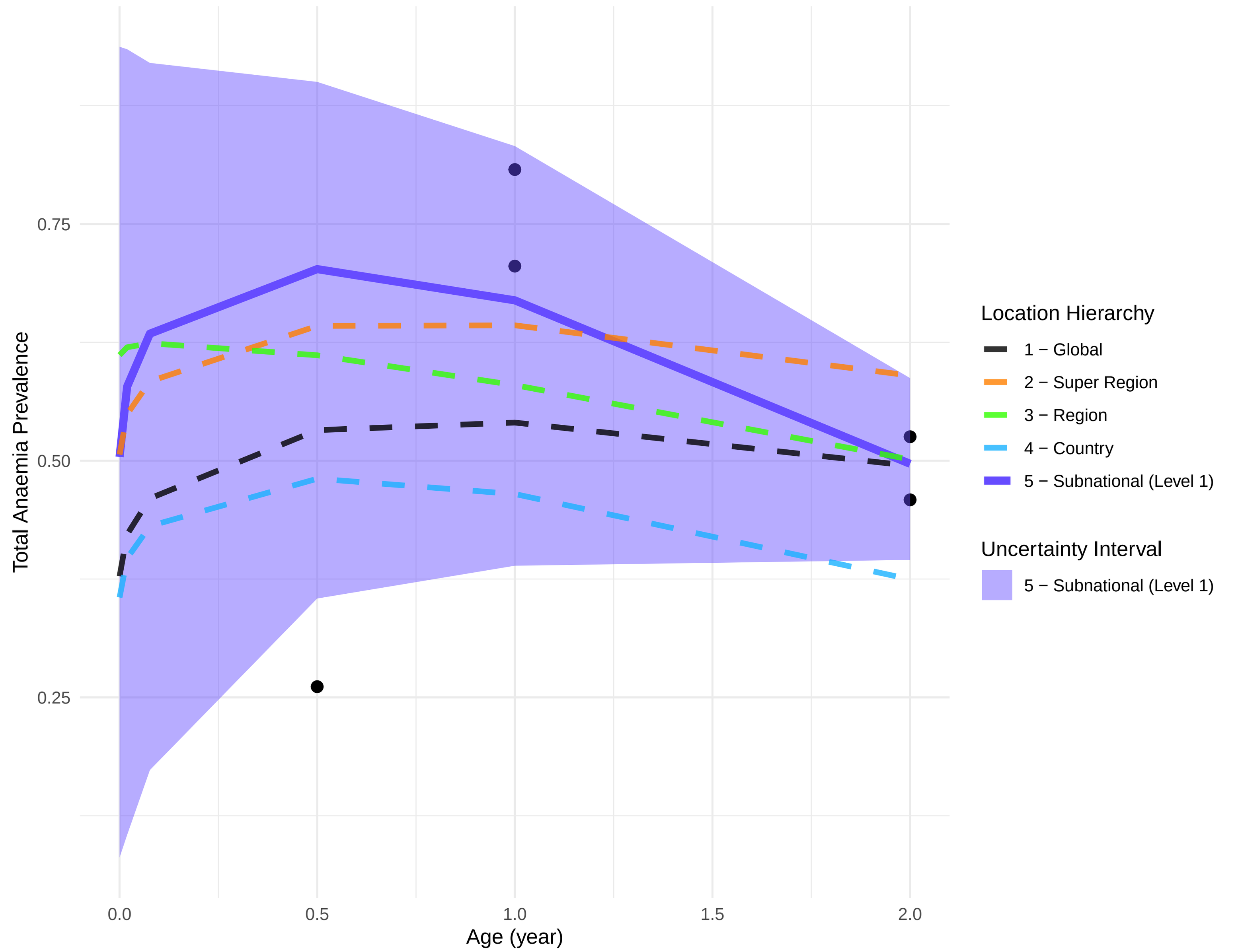

MR−BRT Age/Sex Fits − Non−pregnant Females <2 Years Old − Total Anaemia Prevalence − Taita Taveta
WHO−2001 Adjustment Method

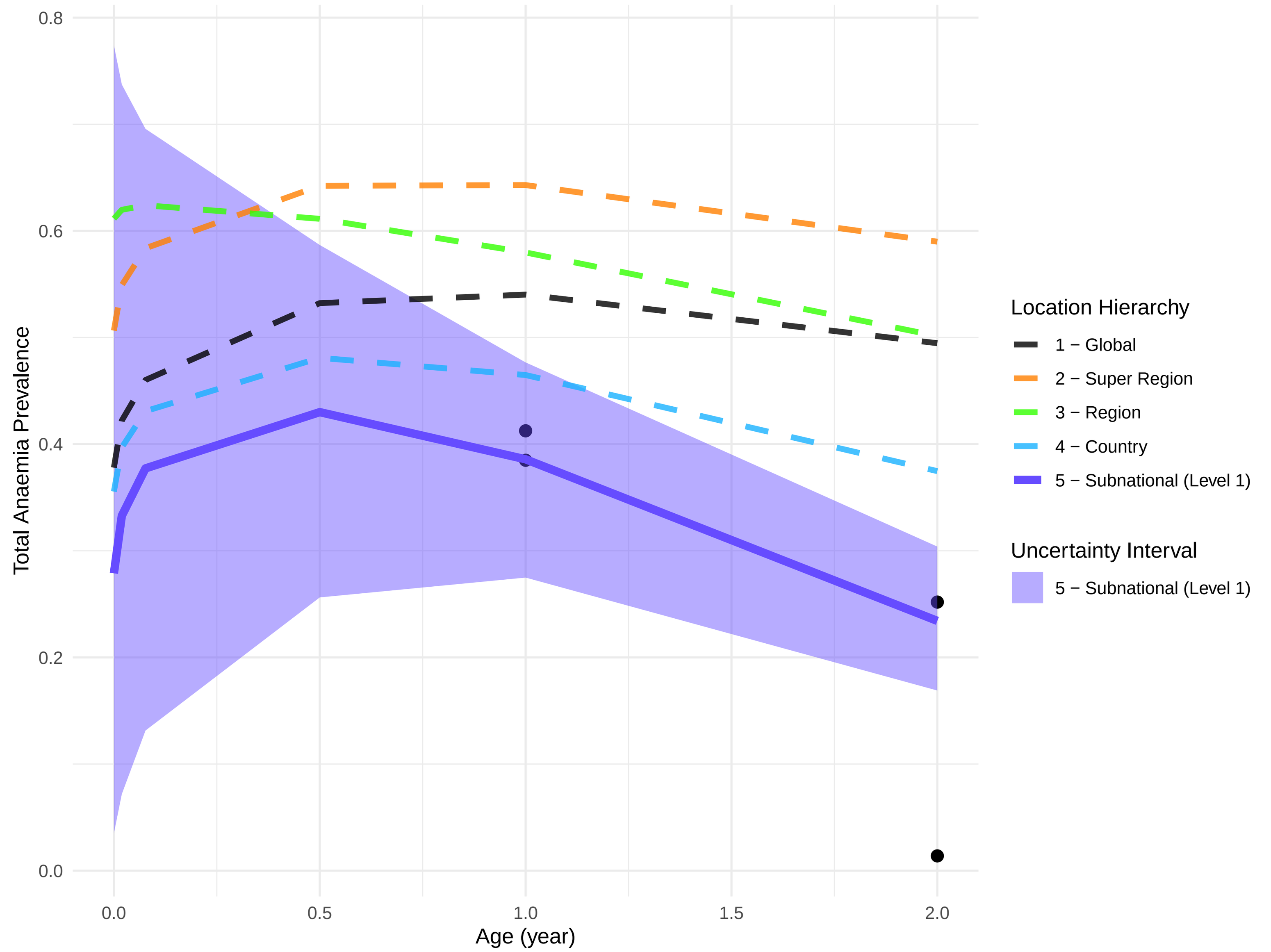

MR−BRT Age/Sex Fits − Non−pregnant Females <2 Years Old − Total Anaemia Prevalence − Tana River
WHO−2001 Adjustment Method

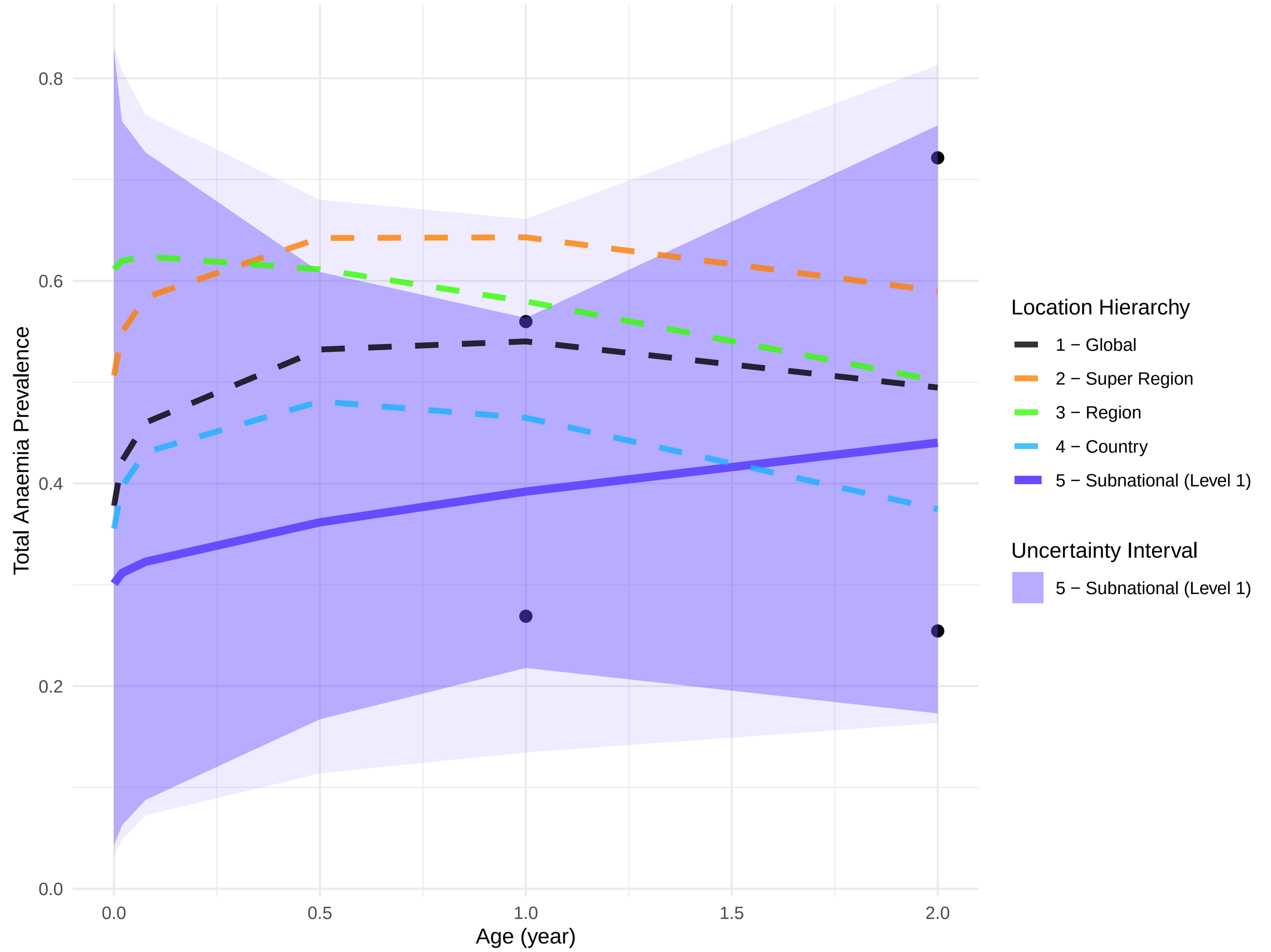

MR−BRT Age/Sex Fits − Non−pregnant Females <2 Years Old − Total Anaemia Prevalence − Tharaka Nithi
WHO−2001 Adjustment Method

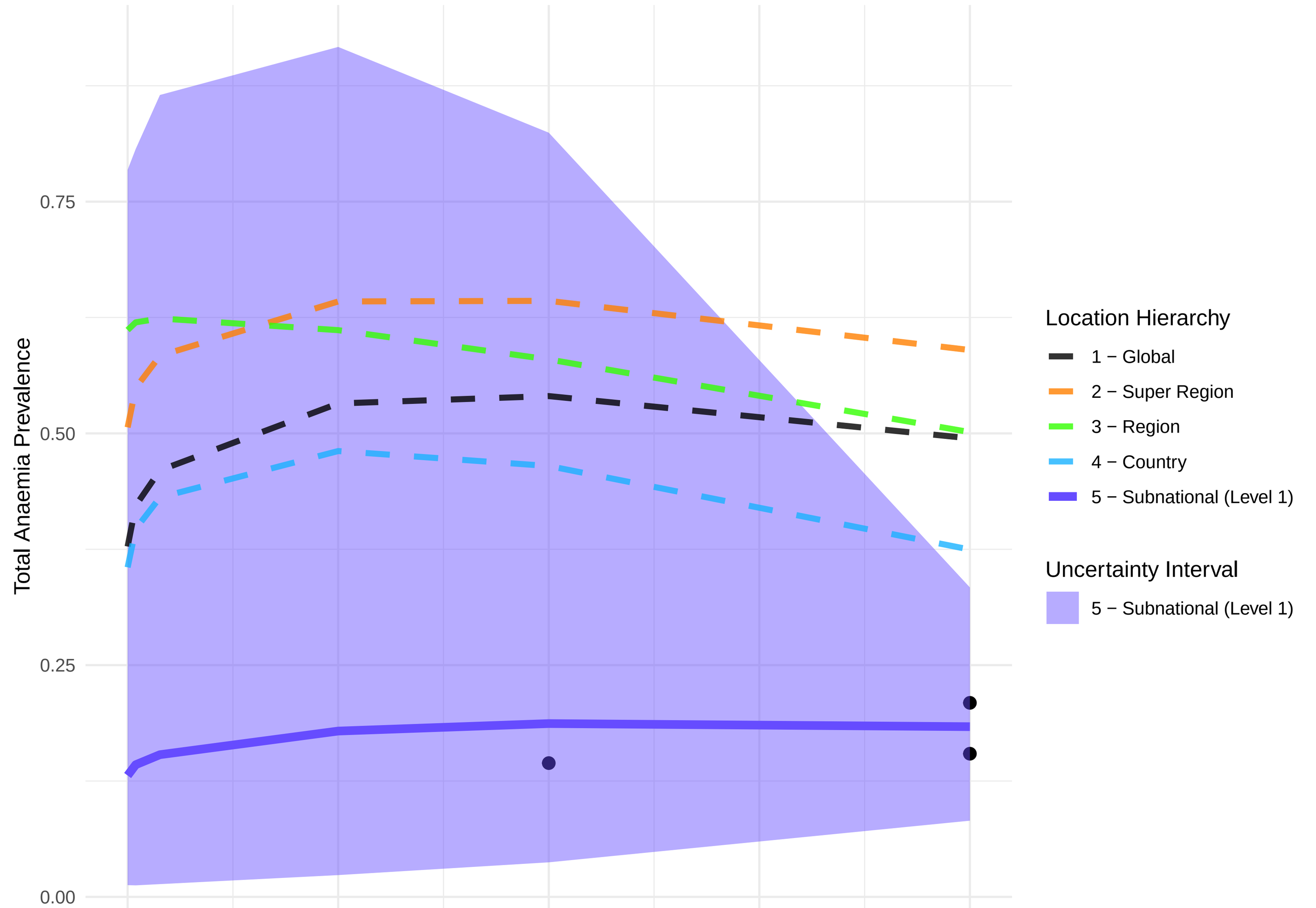

MR−BRT Age/Sex Fits − Non−pregnant Females <2 Years Old − Total Anaemia Prevalence − Trans Nzoia
WHO−2001 Adjustment Method

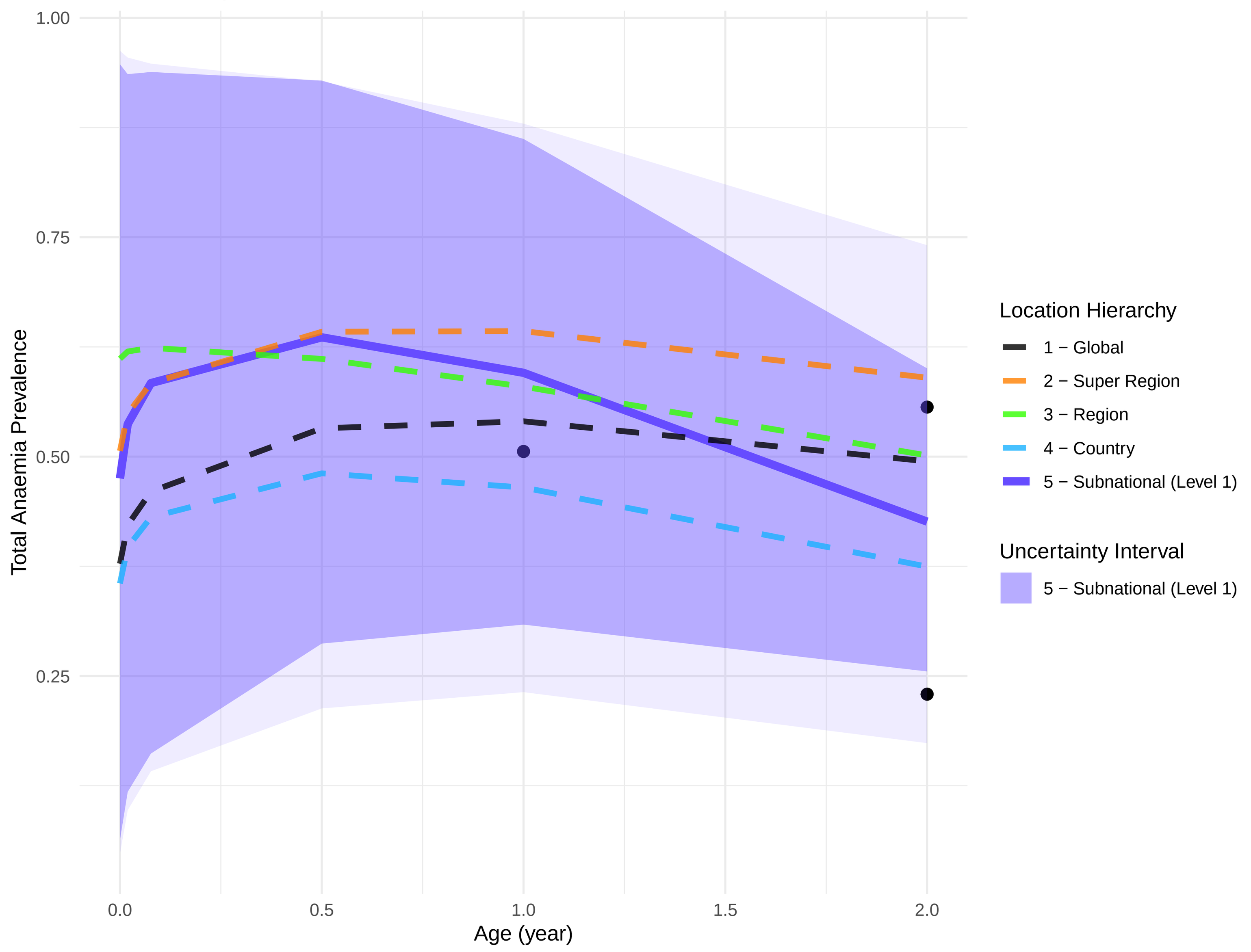

MR−BRT Age/Sex Fits − Non−pregnant Females <2 Years Old − Total Anaemia Prevalence − Turkana
WHO−2001 Adjustment Method

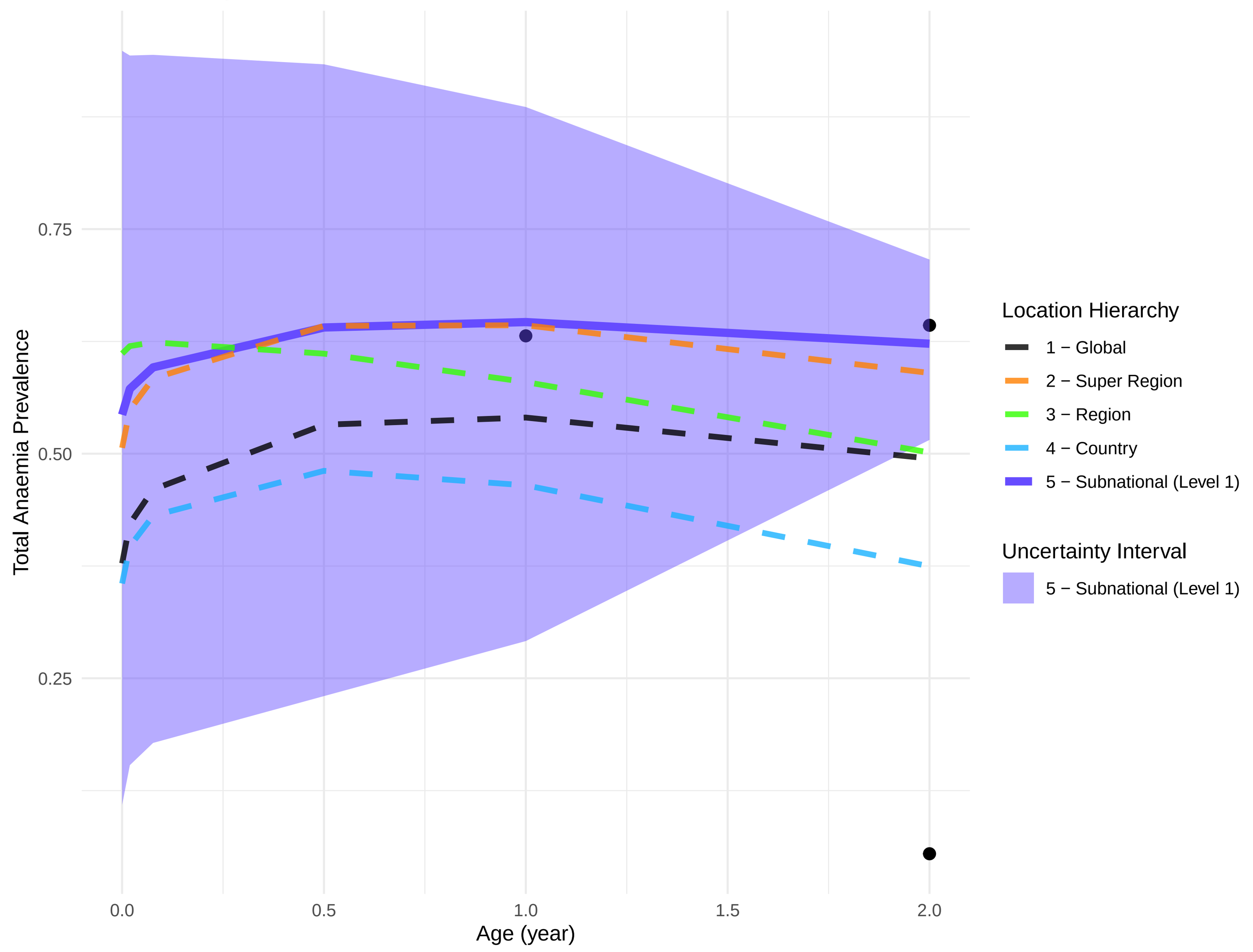

MR−BRT Age/Sex Fits − Non−pregnant Females <2 Years Old − Total Anaemia Prevalence − Uasin Gishu
WHO−2001 Adjustment Method

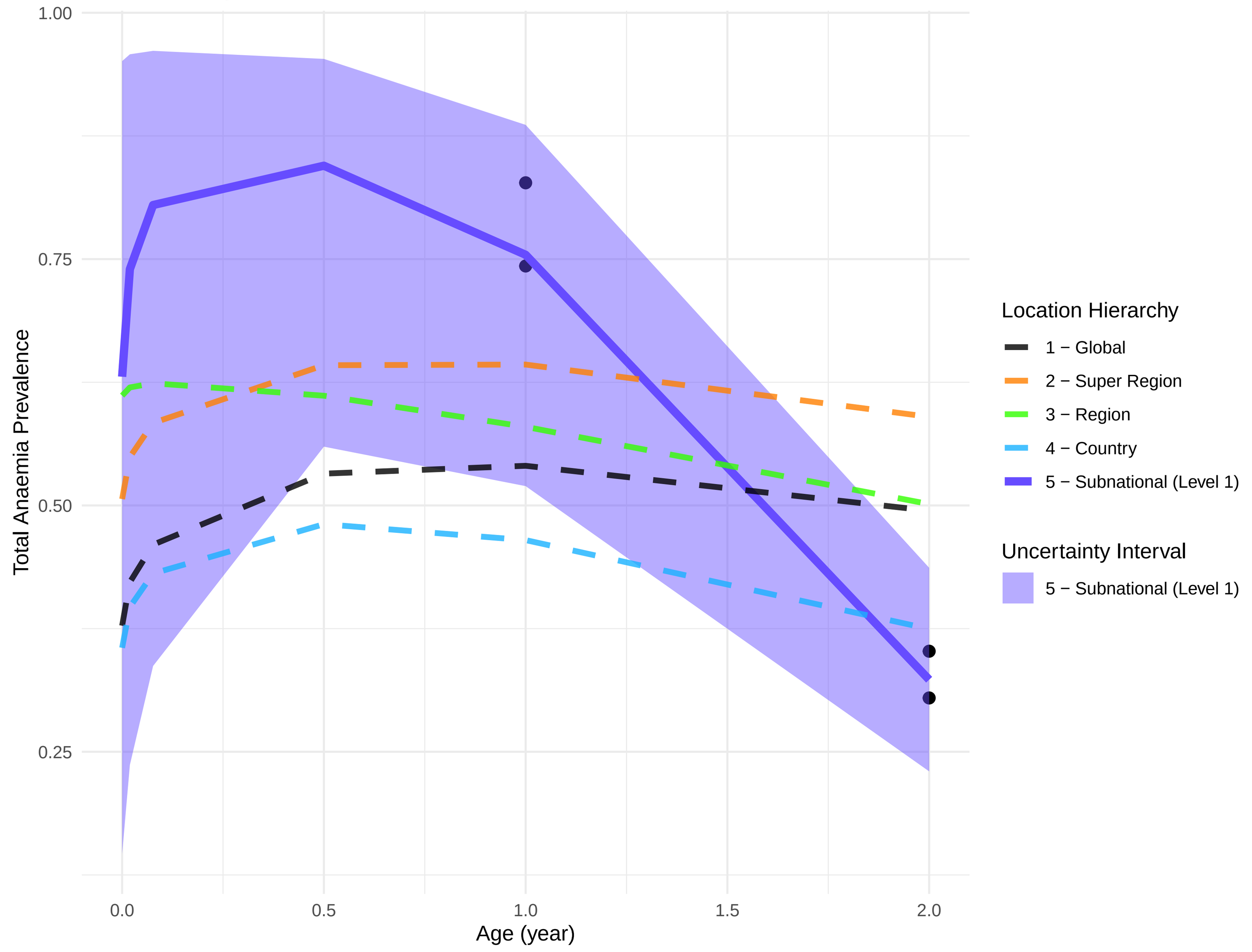

MR−BRT Age/Sex Fits − Non−pregnant Females <2 Years Old − Total Anaemia Prevalence − Vihiga
WHO−2001 Adjustment Method

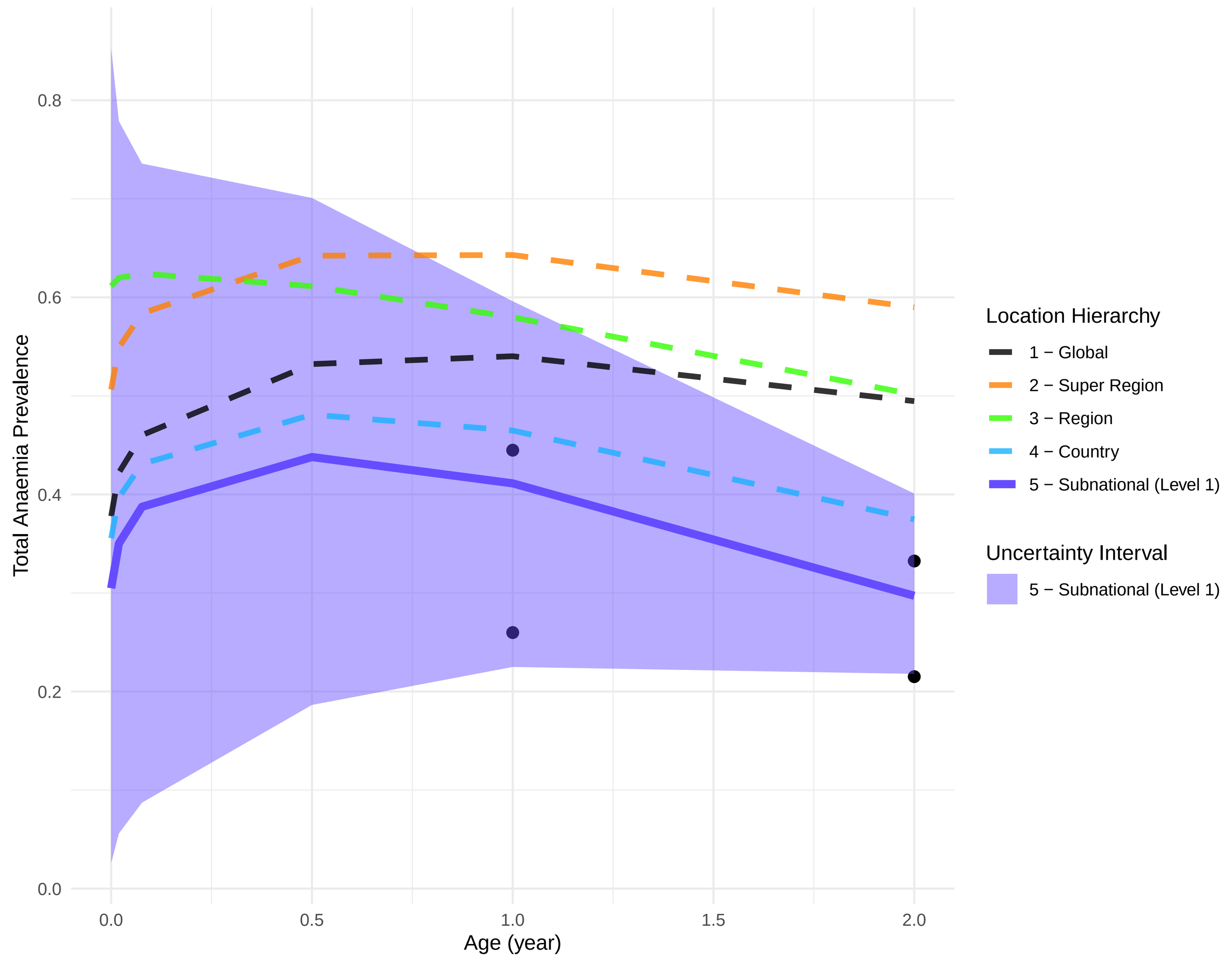

MR−BRT Age/Sex Fits − Non−pregnant Females <2 Years Old − Total Anaemia Prevalence − Wajir
WHO−2001 Adjustment Method

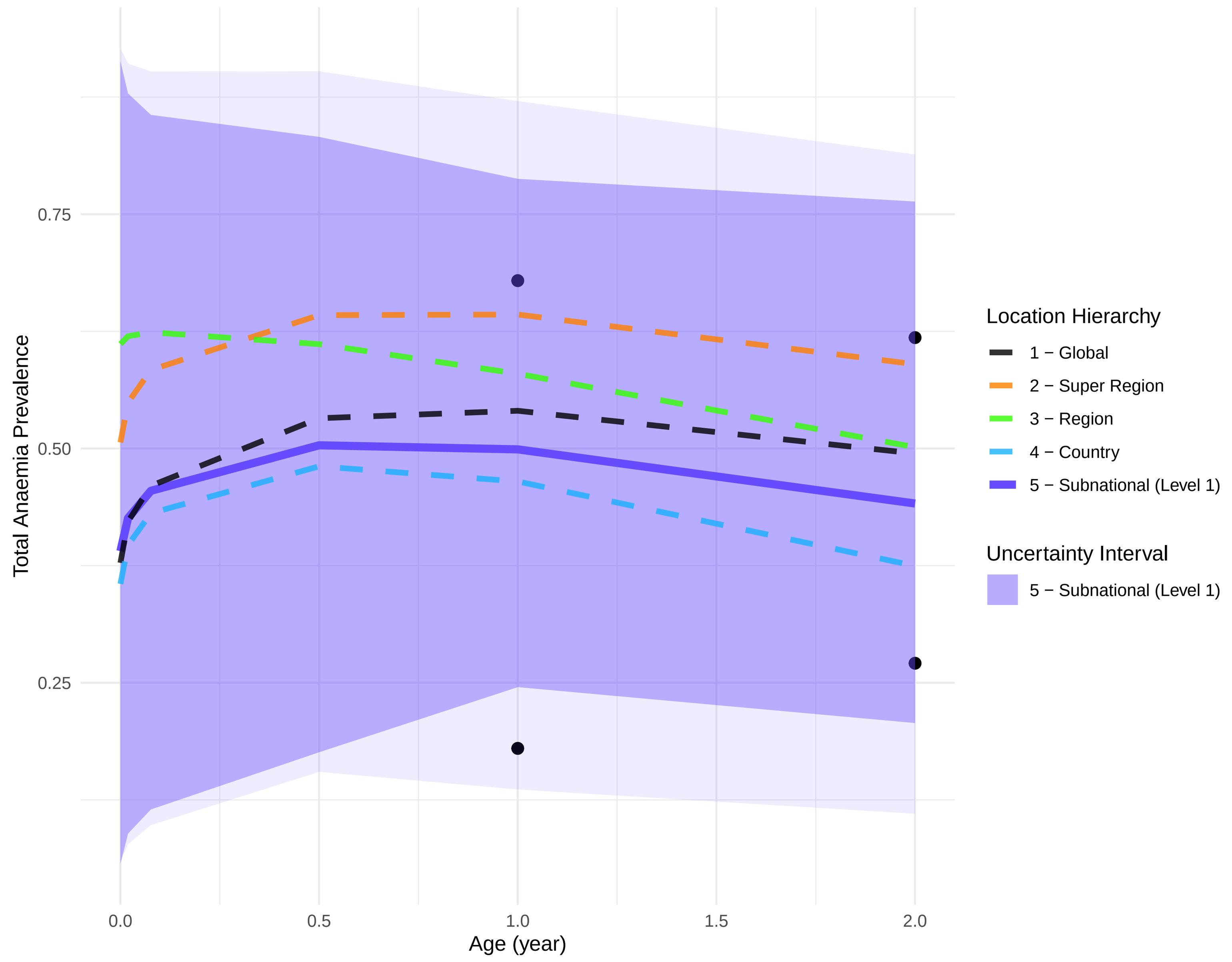

MR−BRT Age/Sex Fits − Non−pregnant Females <2 Years Old − Total Anaemia Prevalence − West Pokot
WHO−2001 Adjustment Method

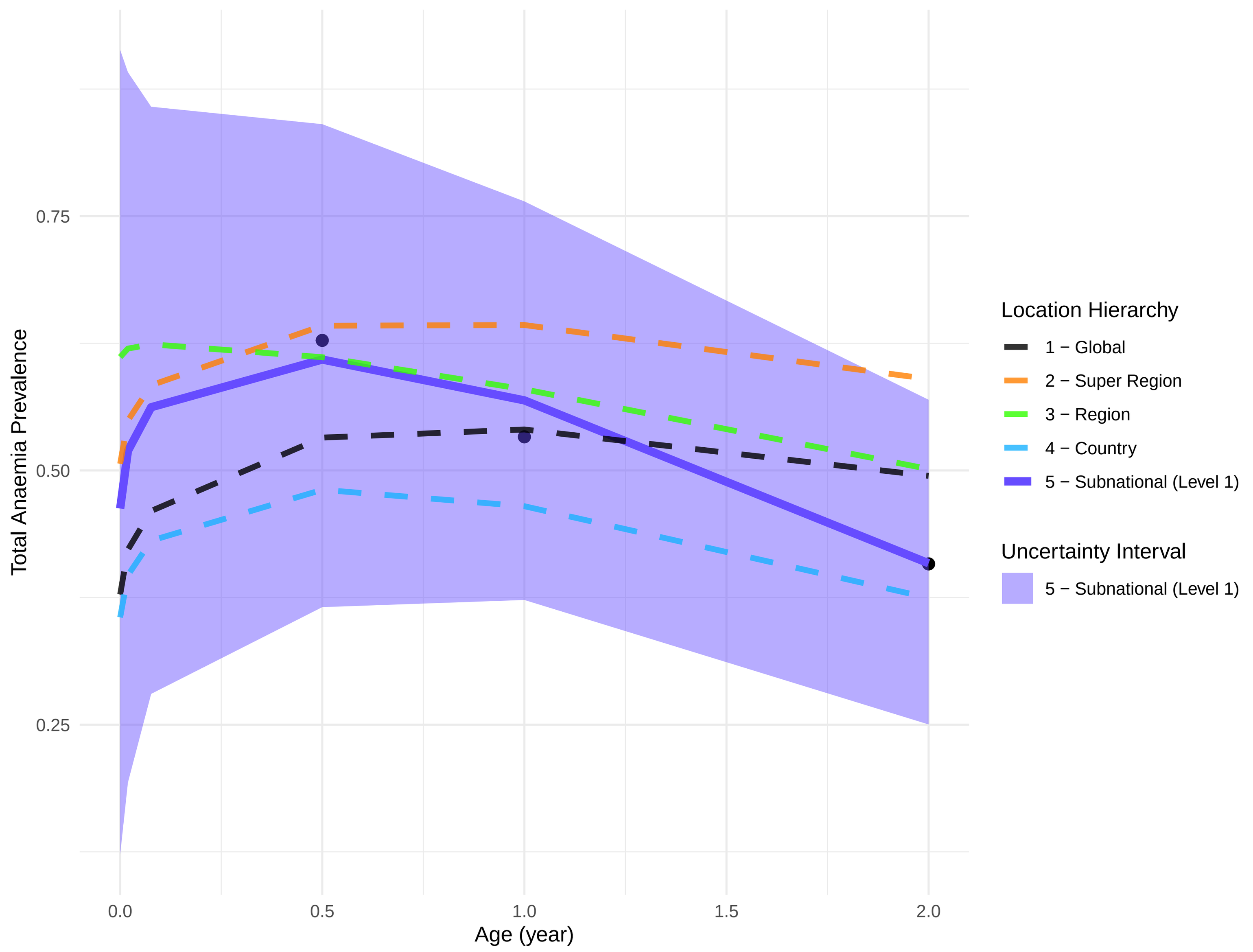

MR−BRT Age/Sex Fits − Non−pregnant Females <2 Years Old − Total Anaemia Prevalence − Madagascar
WHO−2001 Adjustment Method

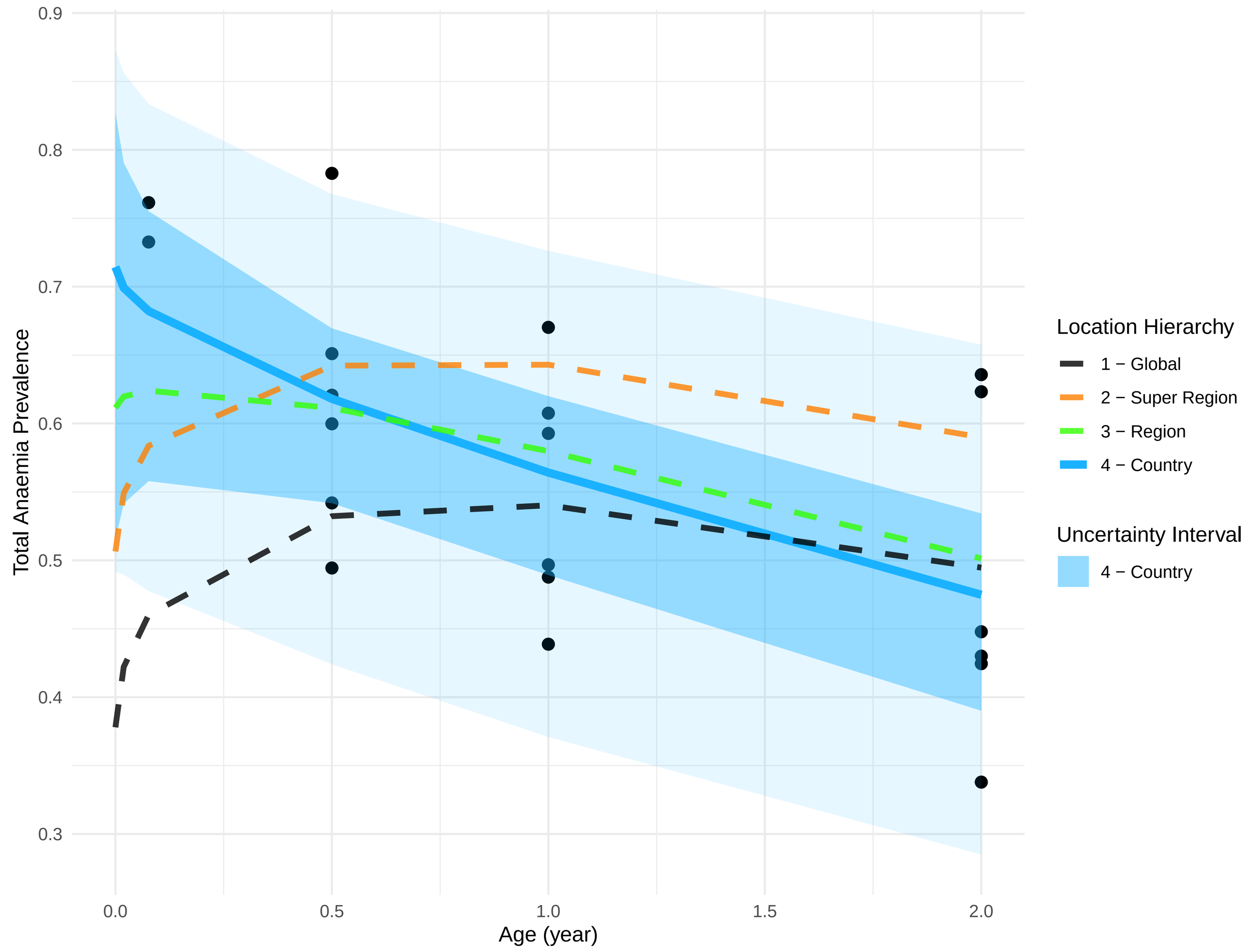

MR−BRT Age/Sex Fits − Non−pregnant Females <2 Years Old − Total Anaemia Prevalence − Malawi
WHO−2001 Adjustment Method

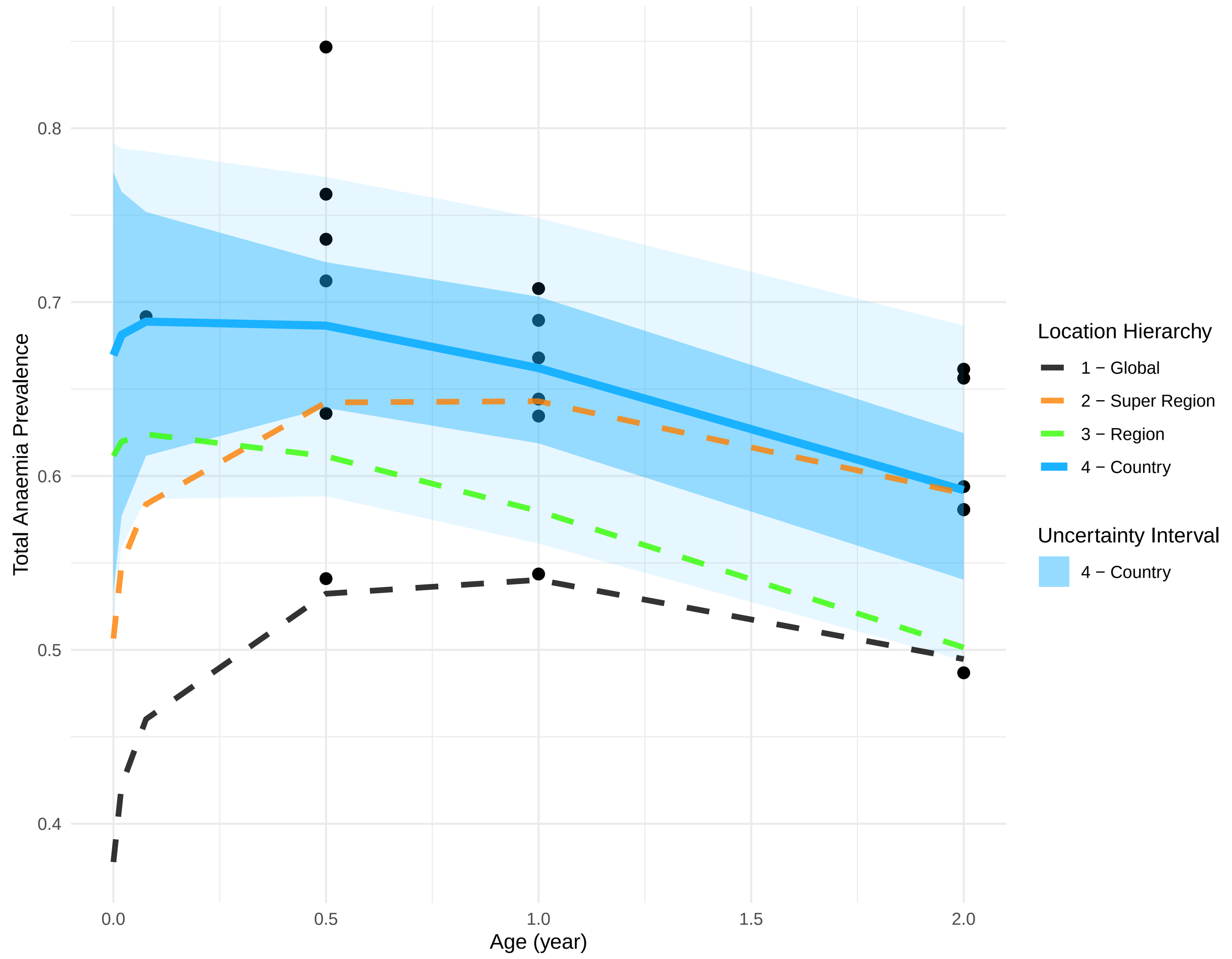

MR−BRT Age/Sex Fits − Non−pregnant Females <2 Years Old − Total Anaemia Prevalence − Mozambique
WHO−2001 Adjustment Method

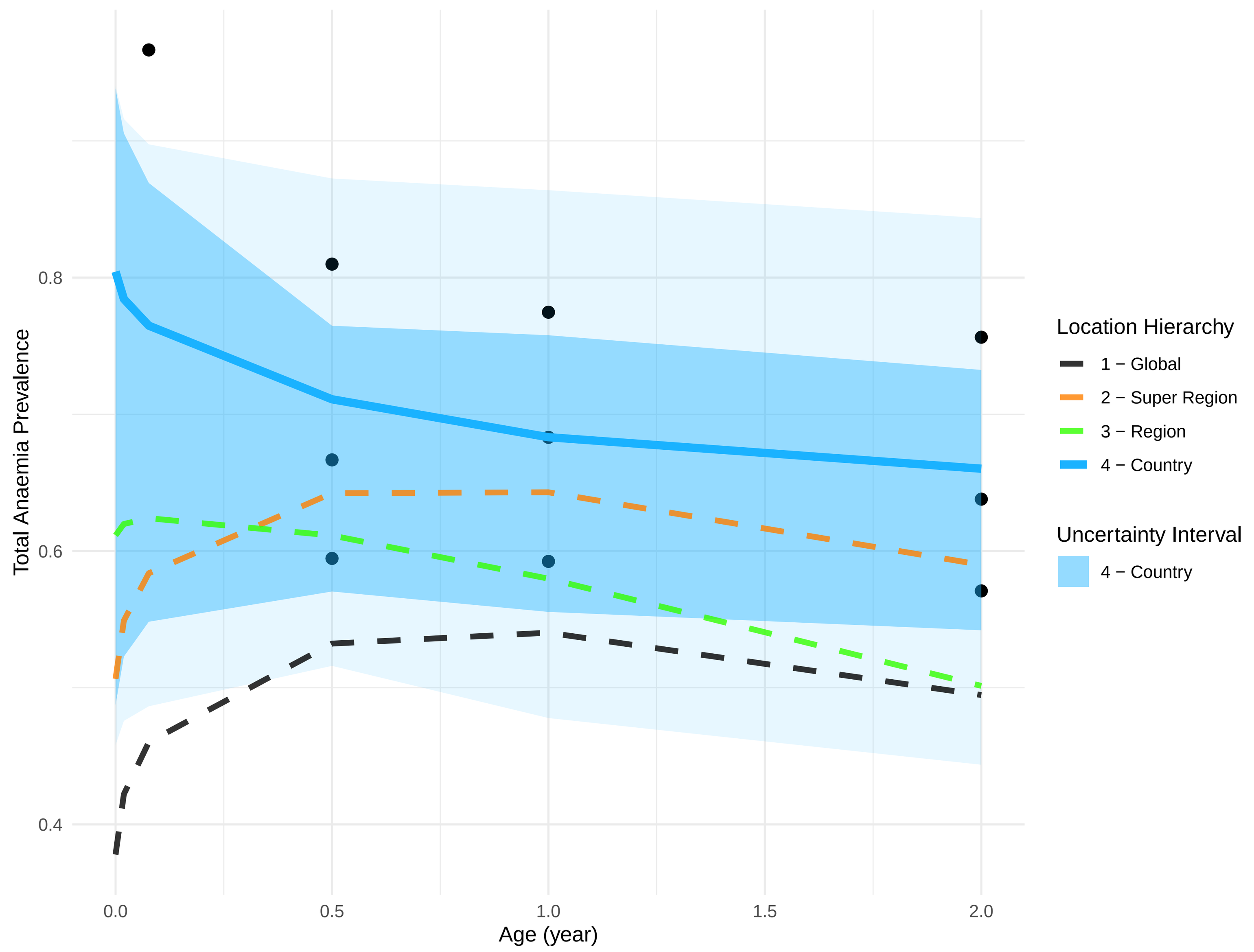

MR−BRT Age/Sex Fits − Non−pregnant Females <2 Years Old − Total Anaemia Prevalence − Rwanda
WHO−2001 Adjustment Method

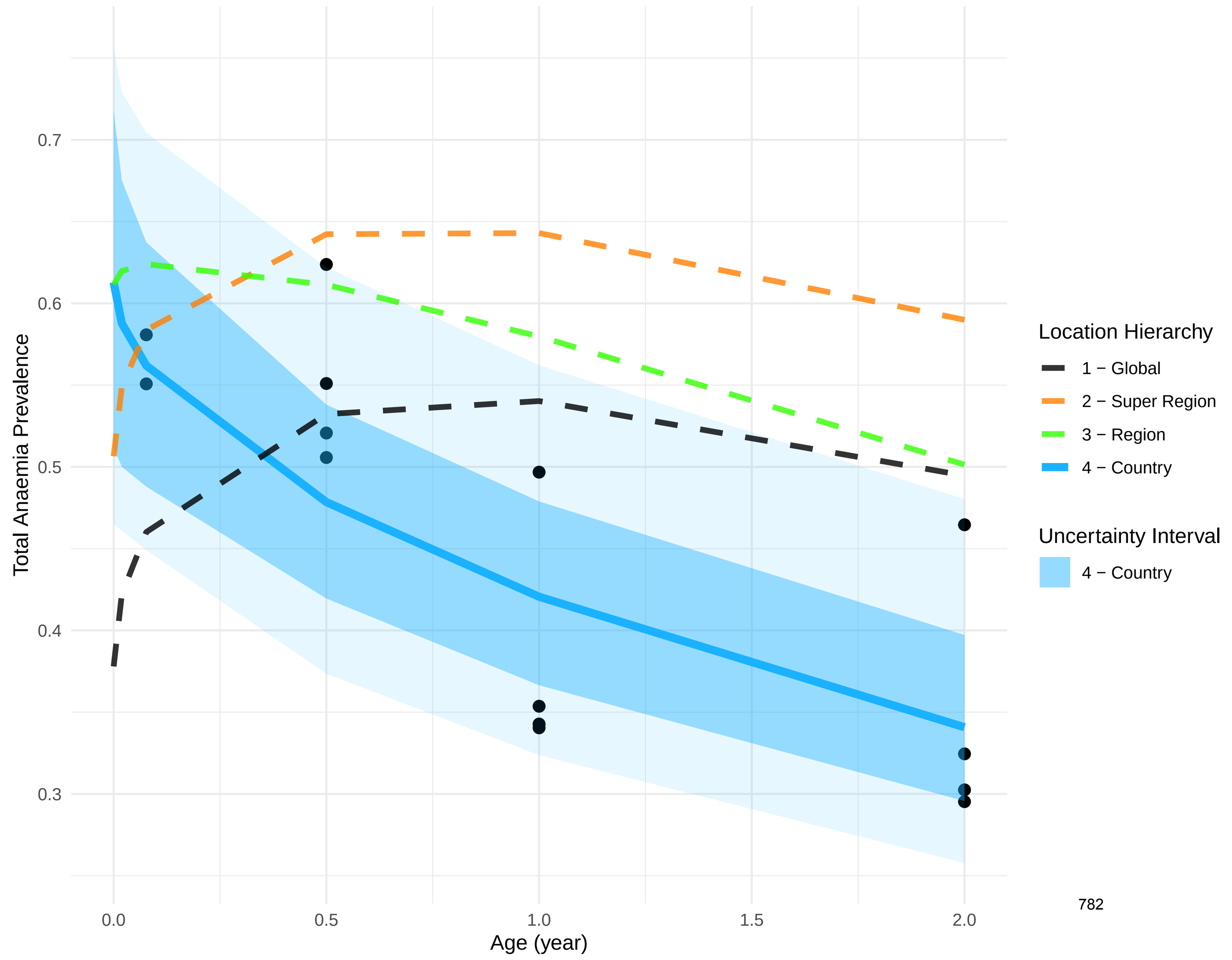

MR−BRT Age/Sex Fits − Non−pregnant Females <2 Years Old − Total Anaemia Prevalence − Uganda
WHO−2001 Adjustment Method

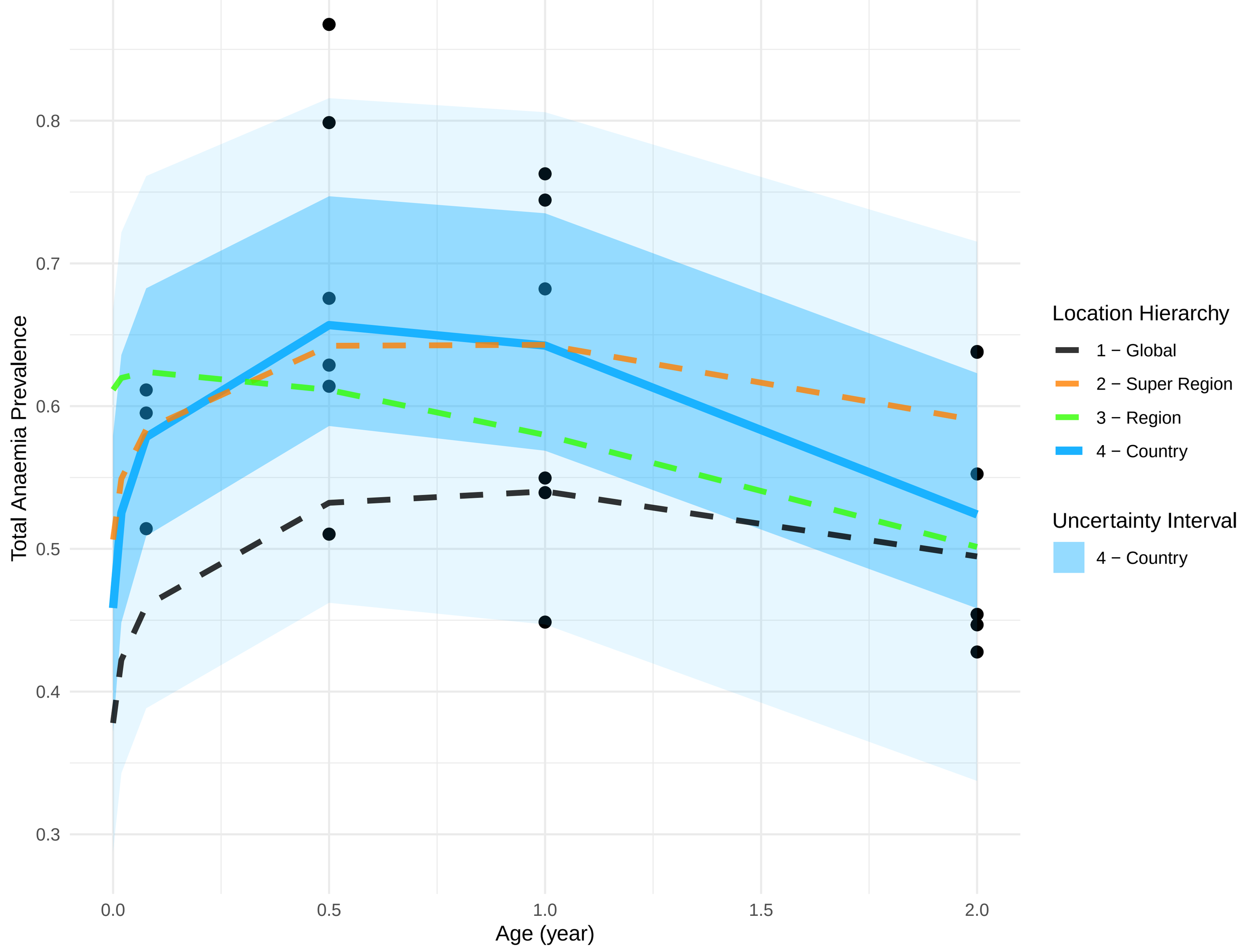

MR−BRT Age/Sex Fits − Non−pregnant Females <2 Years Old − Total Anaemia Prevalence − United Republic of Tanzania
WHO−2001 Adjustment Method

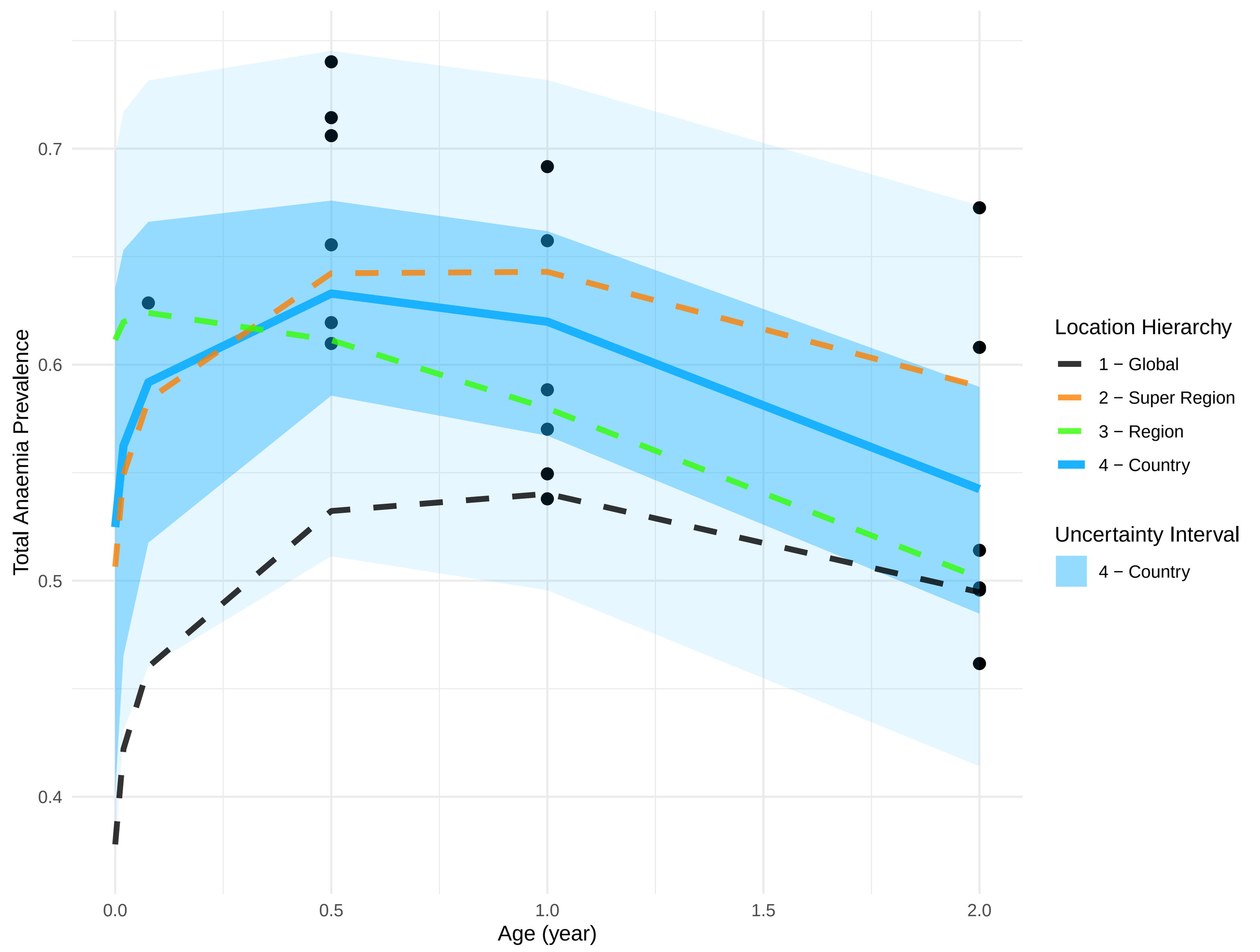

MR−BRT Age/Sex Fits − Non−pregnant Females <2 Years Old − Total Anaemia Prevalence − Zambia
WHO−2001 Adjustment Method

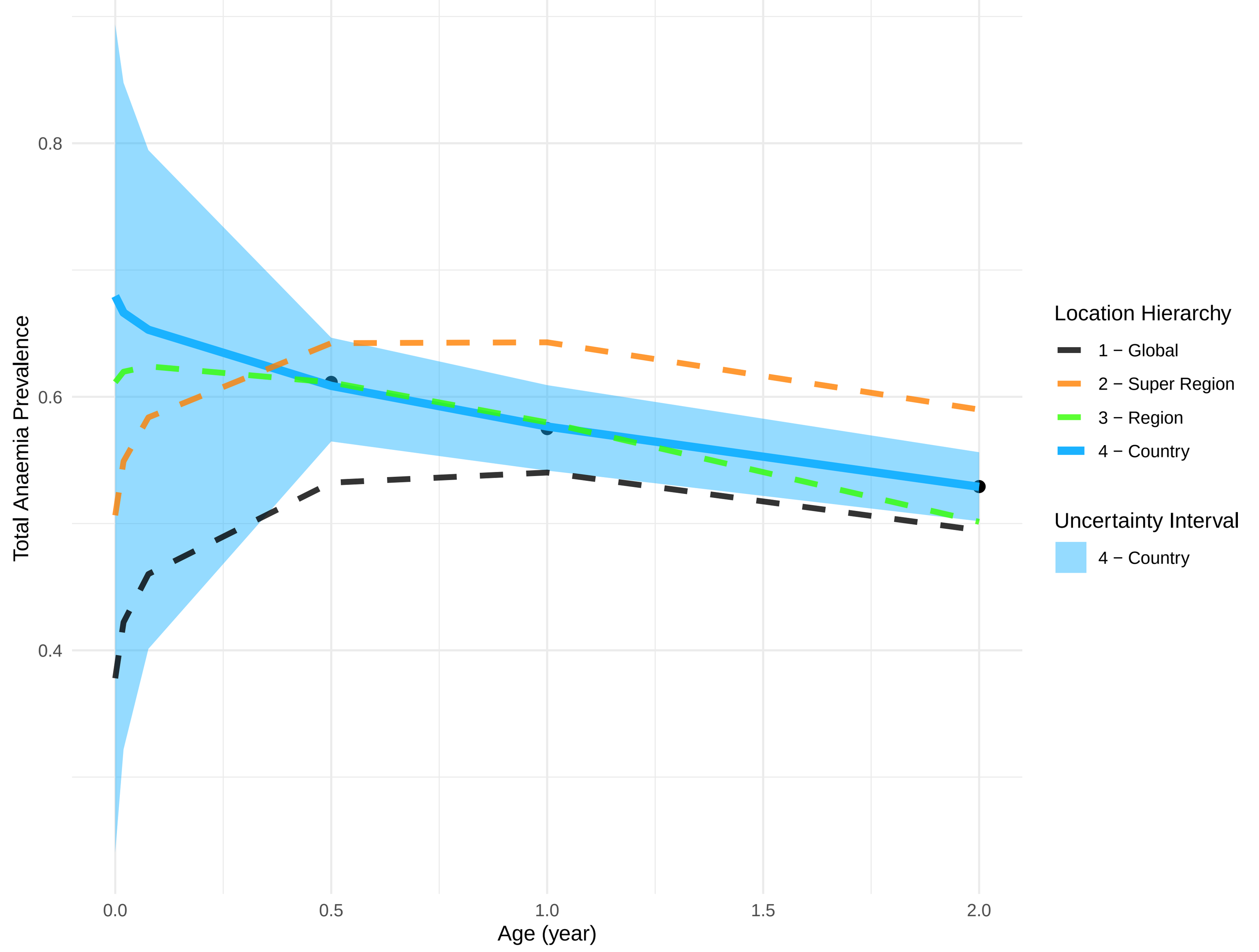

MR−BRT Age/Sex Fits − Non−pregnant Females <2 Years Old − Total Anaemia Prevalence − Southern Sub−Saharan Africa
WHO−2001 Adjustment Method

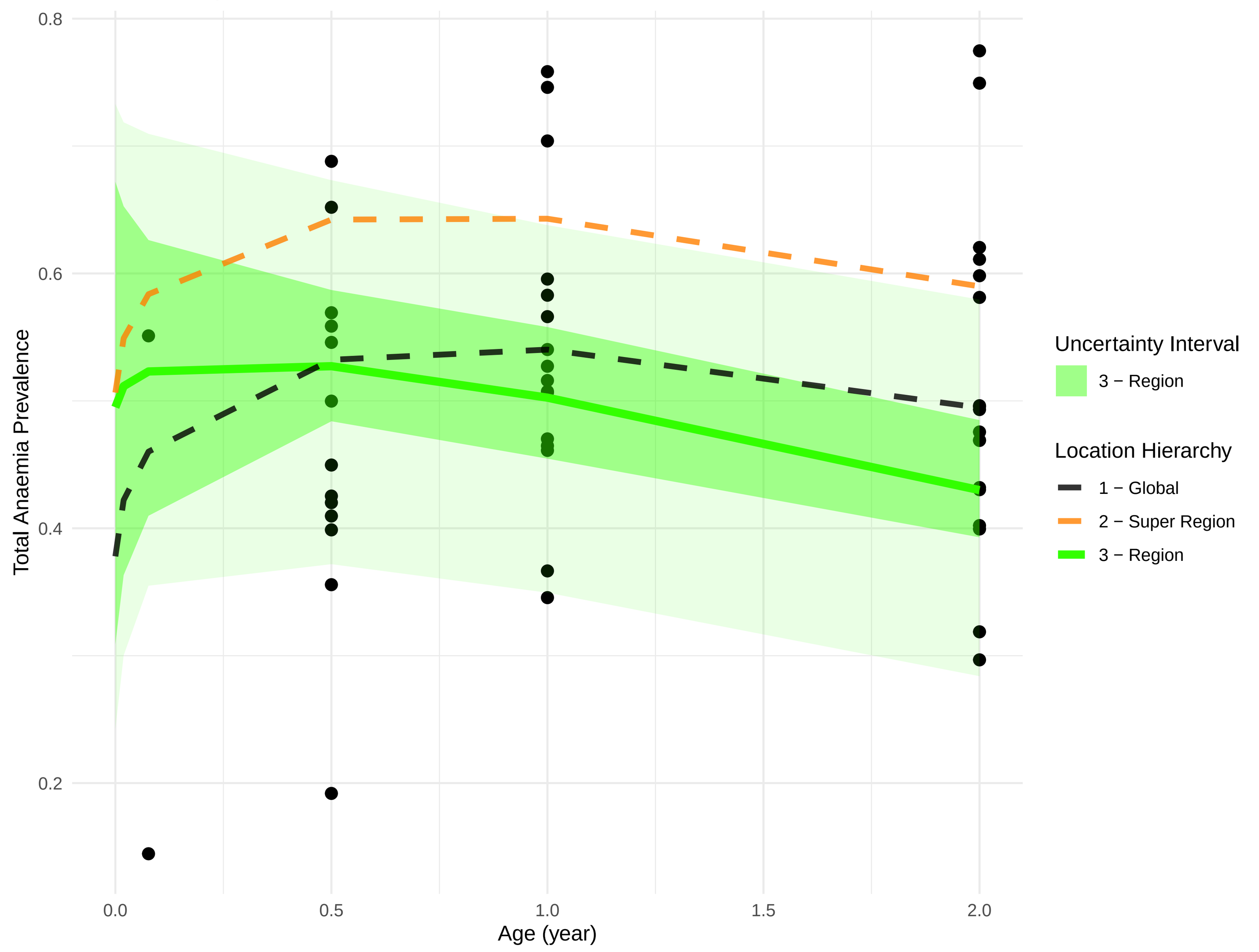

MR−BRT Age/Sex Fits − Non−pregnant Females <2 Years Old − Total Anaemia Prevalence − Eswatini
WHO−2001 Adjustment Method

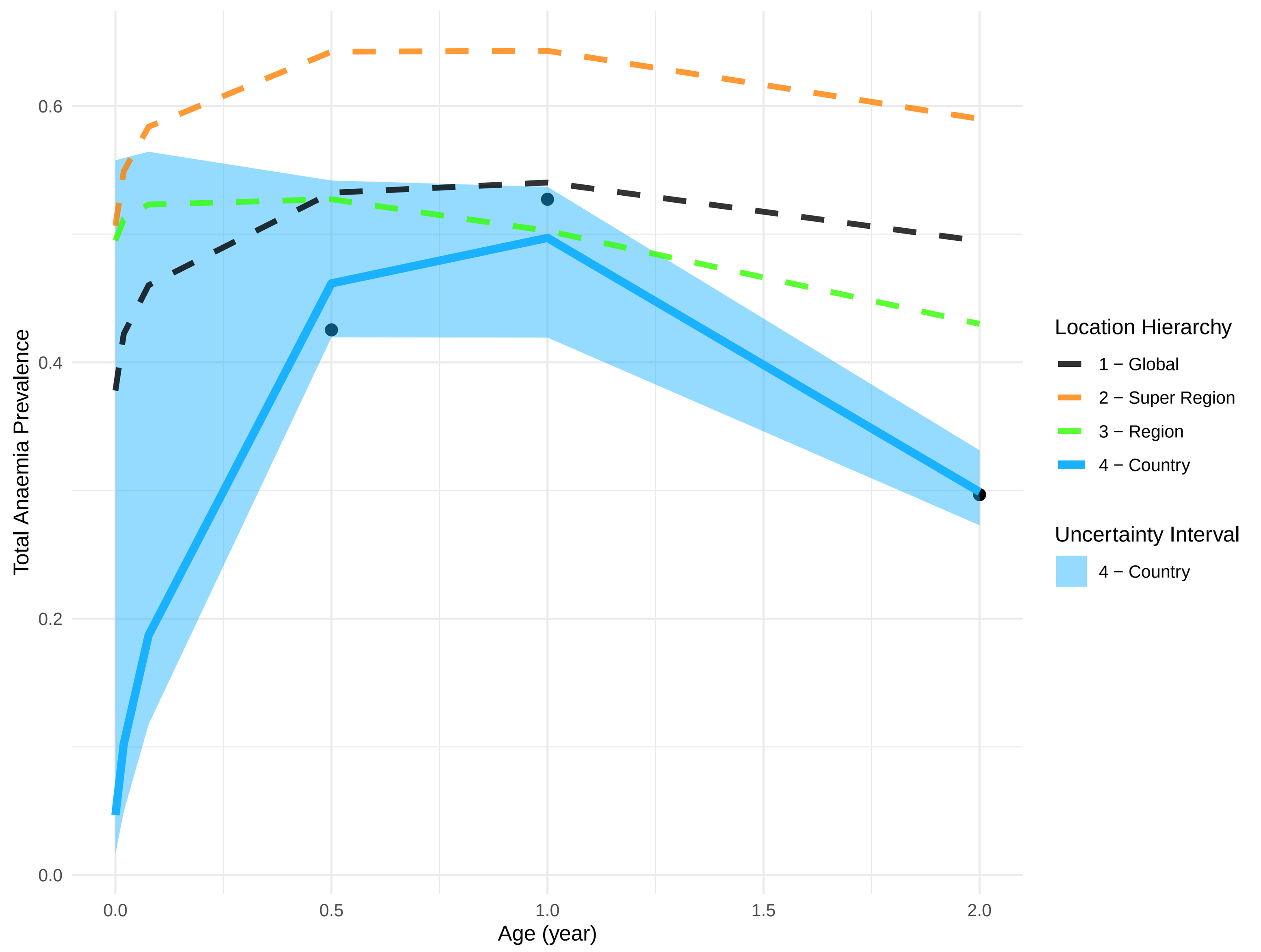

MR−BRT Age/Sex Fits − Non−pregnant Females <2 Years Old − Total Anaemia Prevalence − Lesotho
WHO−2001 Adjustment Method

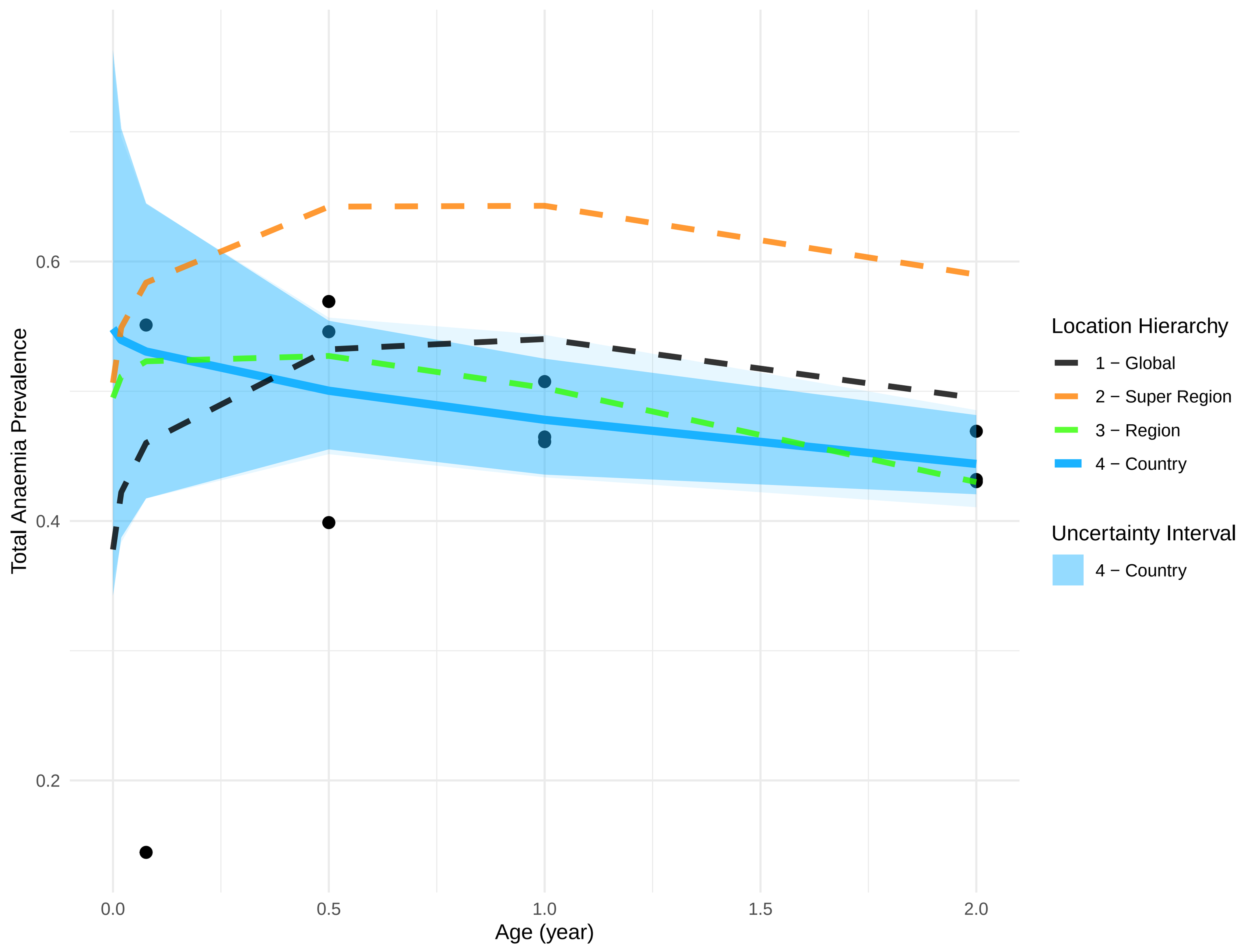

MR−BRT Age/Sex Fits − Non−pregnant Females <2 Years Old − Total Anaemia Prevalence − Namibia
WHO−2001 Adjustment Method

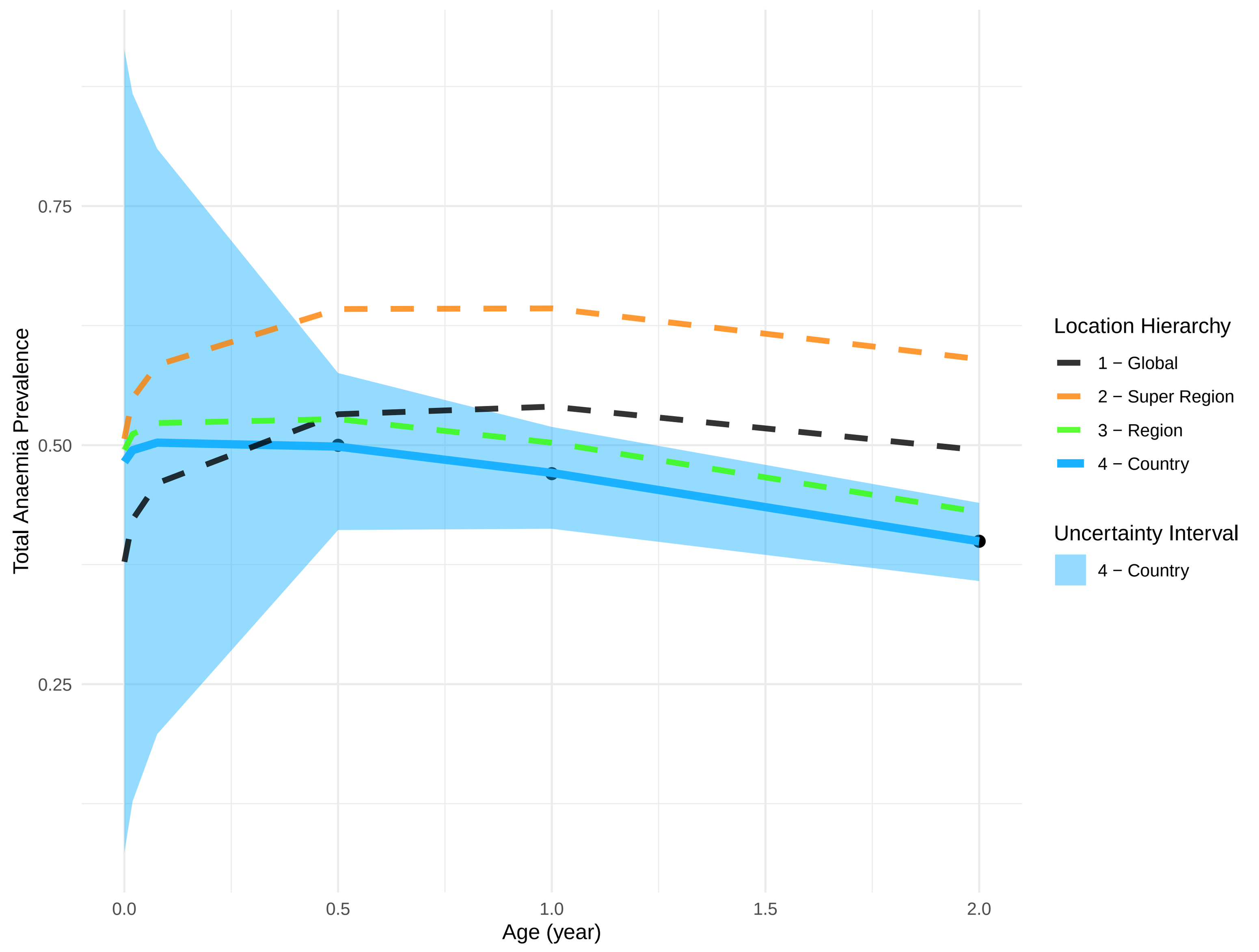

MR−BRT Age/Sex Fits − Non−pregnant Females <2 Years Old − Total Anaemia Prevalence − South Africa
WHO−2001 Adjustment Method

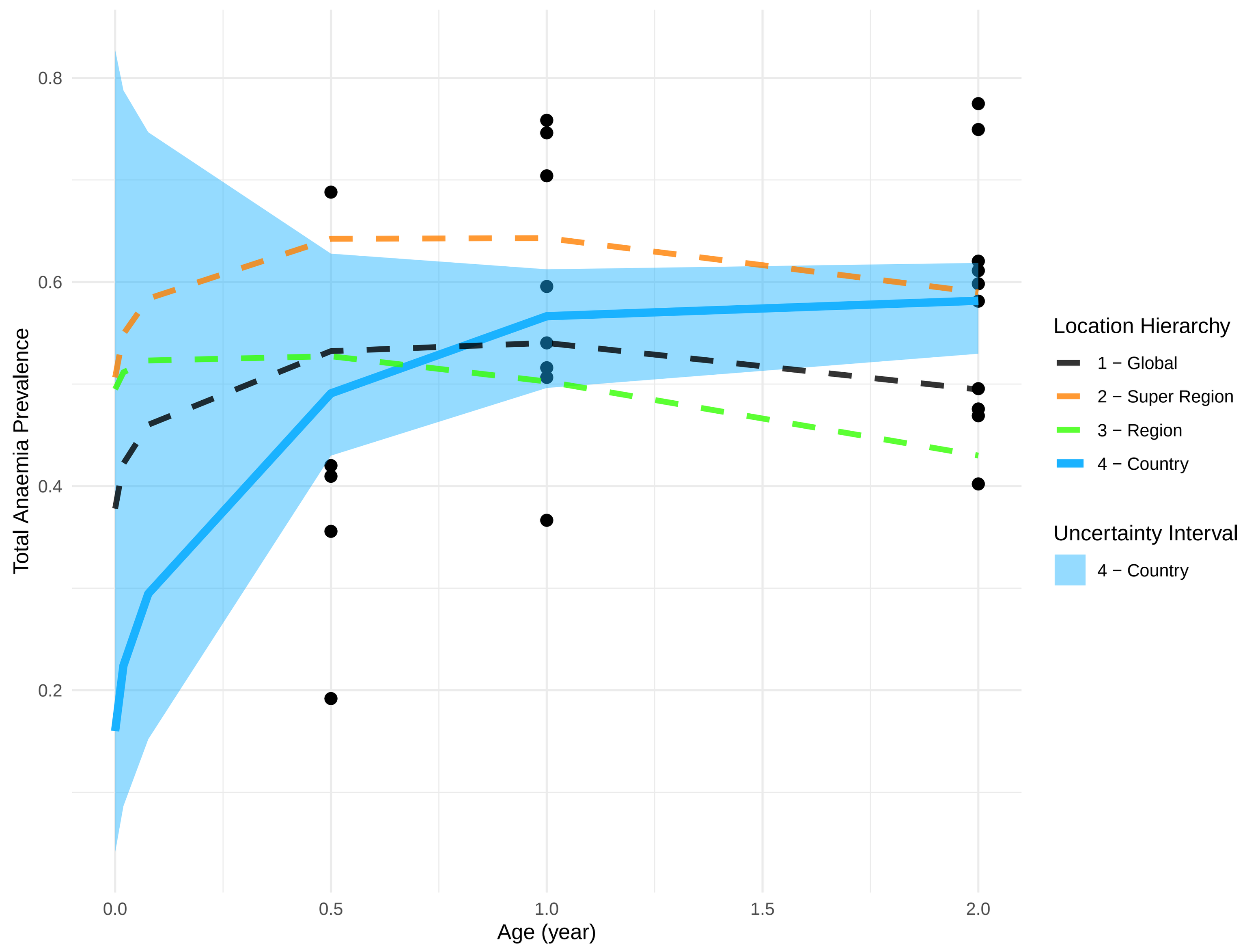

MR−BRT Age/Sex Fits − Non−pregnant Females <2 Years Old − Total Anaemia Prevalence − Eastern Cape
WHO−2001 Adjustment Method

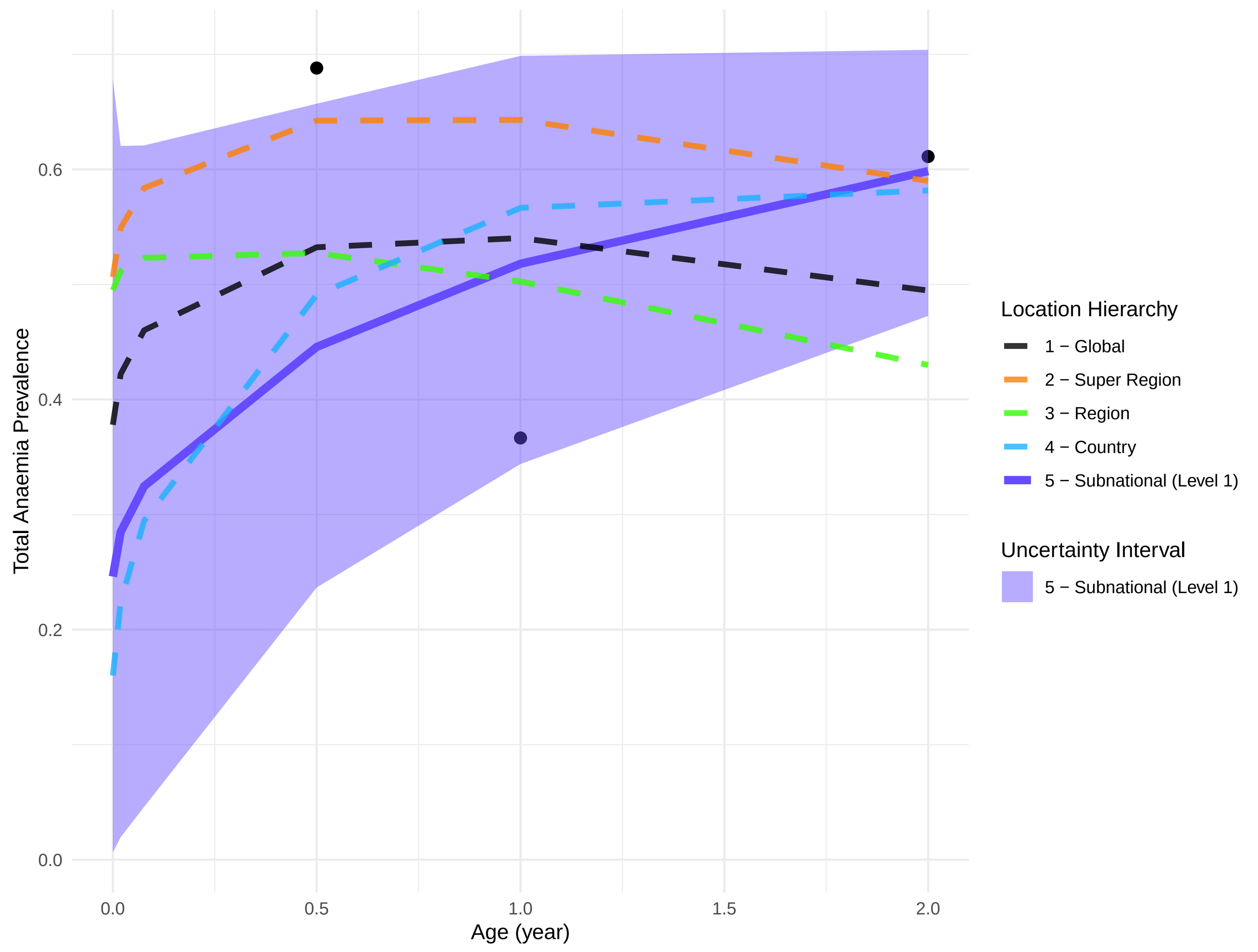

MR−BRT Age/Sex Fits − Non−pregnant Females <2 Years Old − Total Anaemia Prevalence − Free State
WHO−2001 Adjustment Method

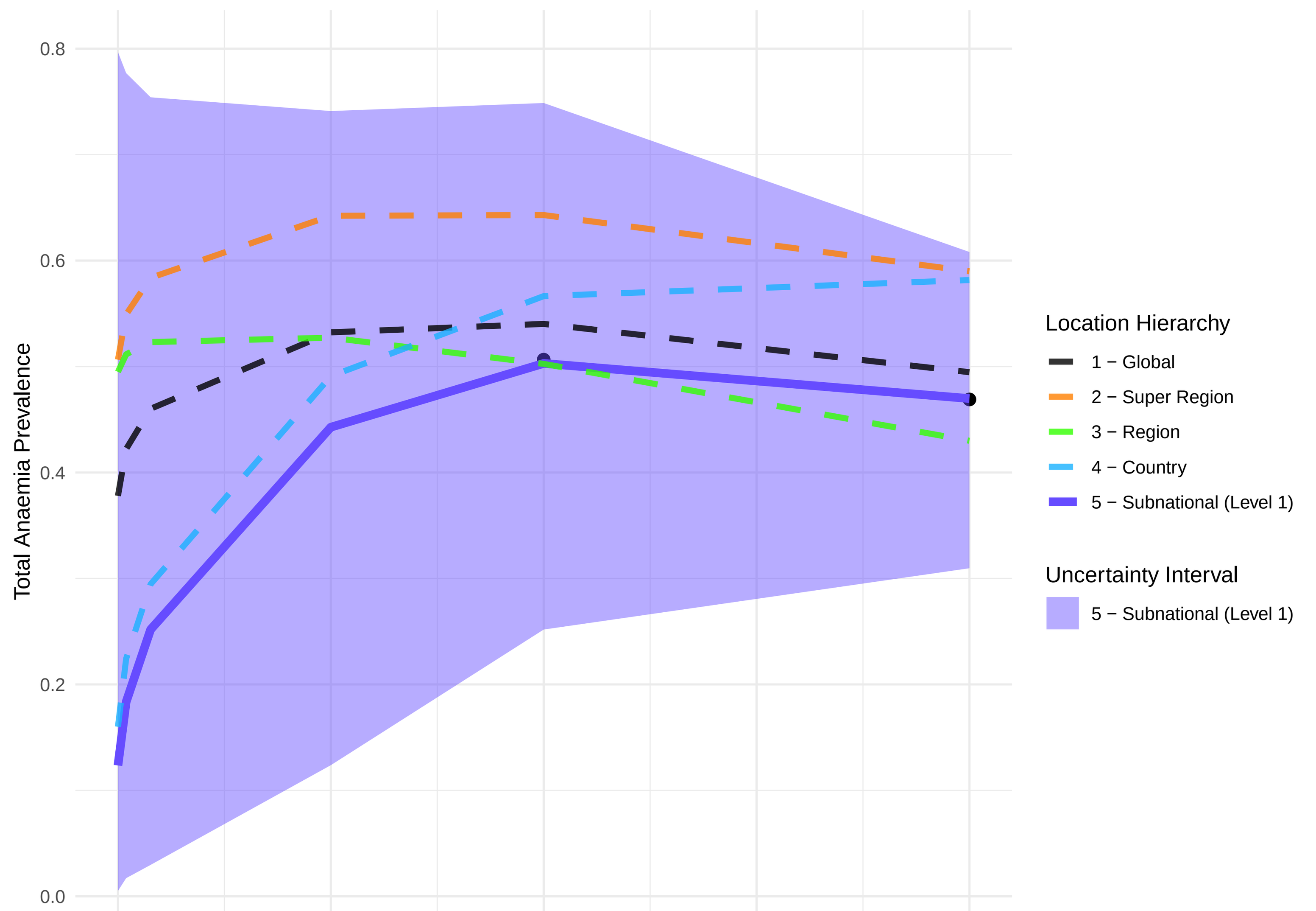

MR−BRT Age/Sex Fits − Non−pregnant Females <2 Years Old − Total Anaemia Prevalence − Gauteng
WHO−2001 Adjustment Method

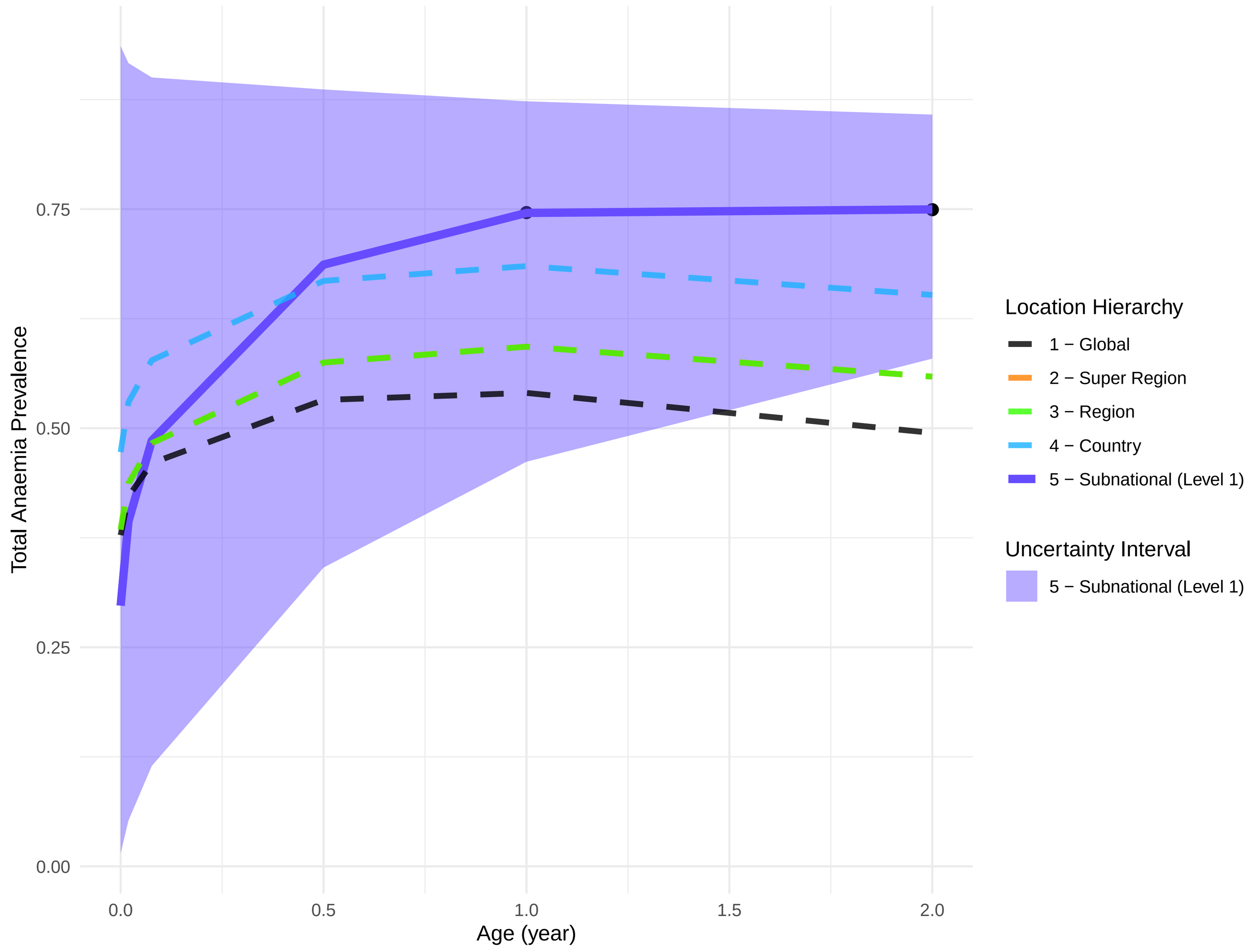

MR−BRT Age/Sex Fits − Non−pregnant Females <2 Years Old − Total Anaemia Prevalence − KwaZulu−Natal
WHO−2001 Adjustment Method

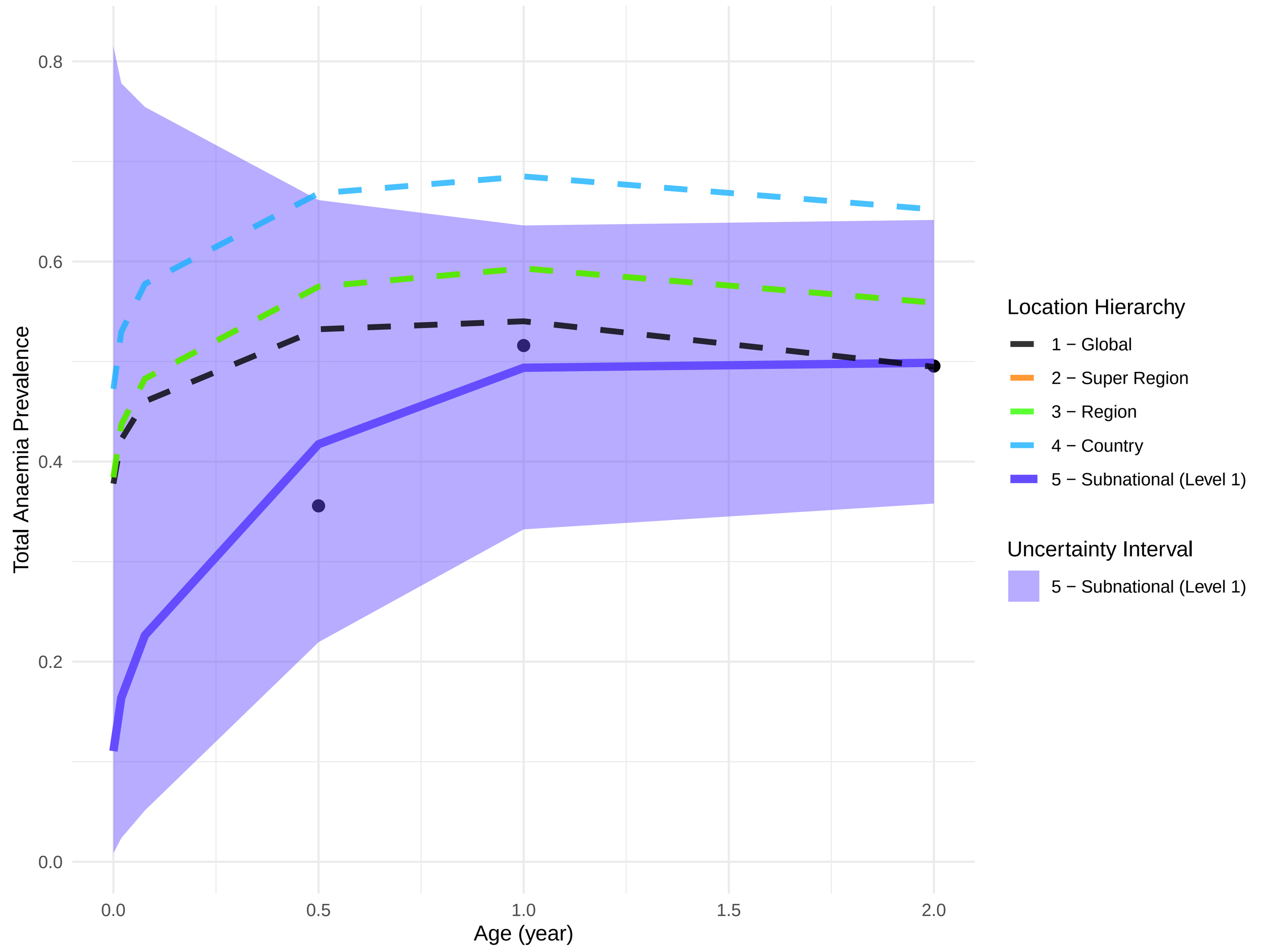

MR−BRT Age/Sex Fits − Non−pregnant Females <2 Years Old − Total Anaemia Prevalence − Limpopo
WHO−2001 Adjustment Method

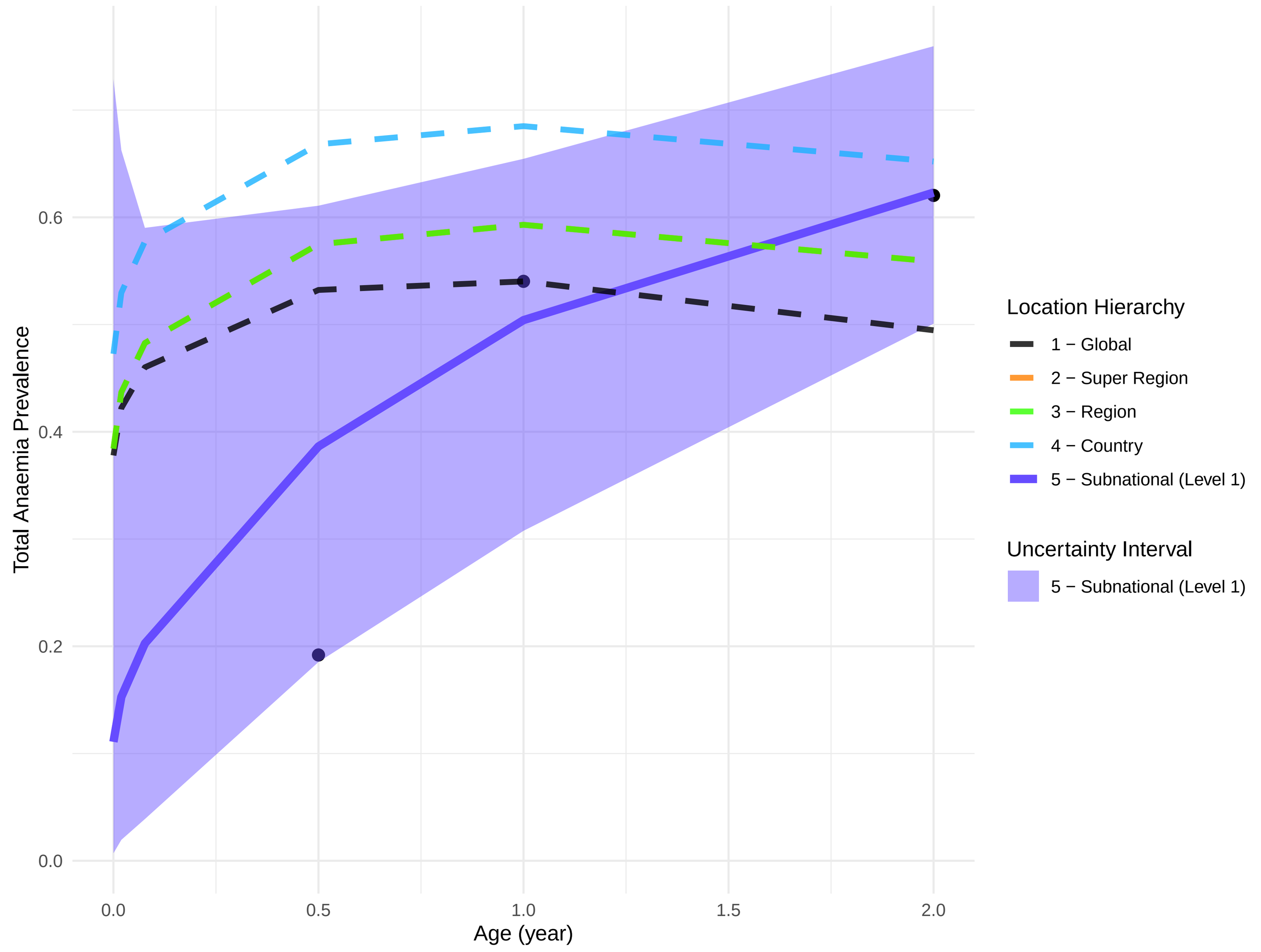

MR−BRT Age/Sex Fits − Non−pregnant Females <2 Years Old − Total Anaemia Prevalence − Mpumalanga
WHO−2001 Adjustment Method

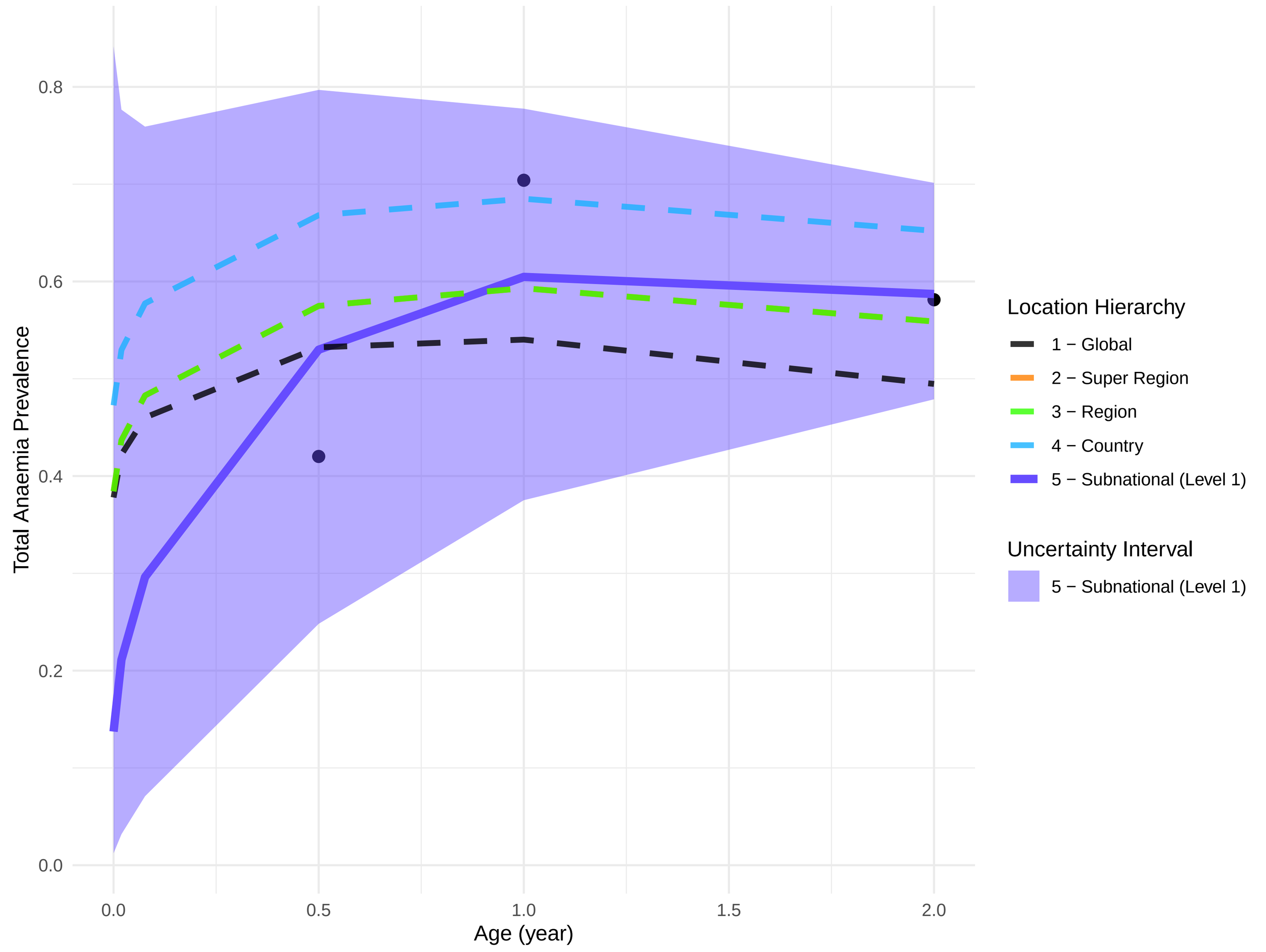

MR−BRT Age/Sex Fits − Non−pregnant Females <2 Years Old − Total Anaemia Prevalence − North−West
WHO−2001 Adjustment Method

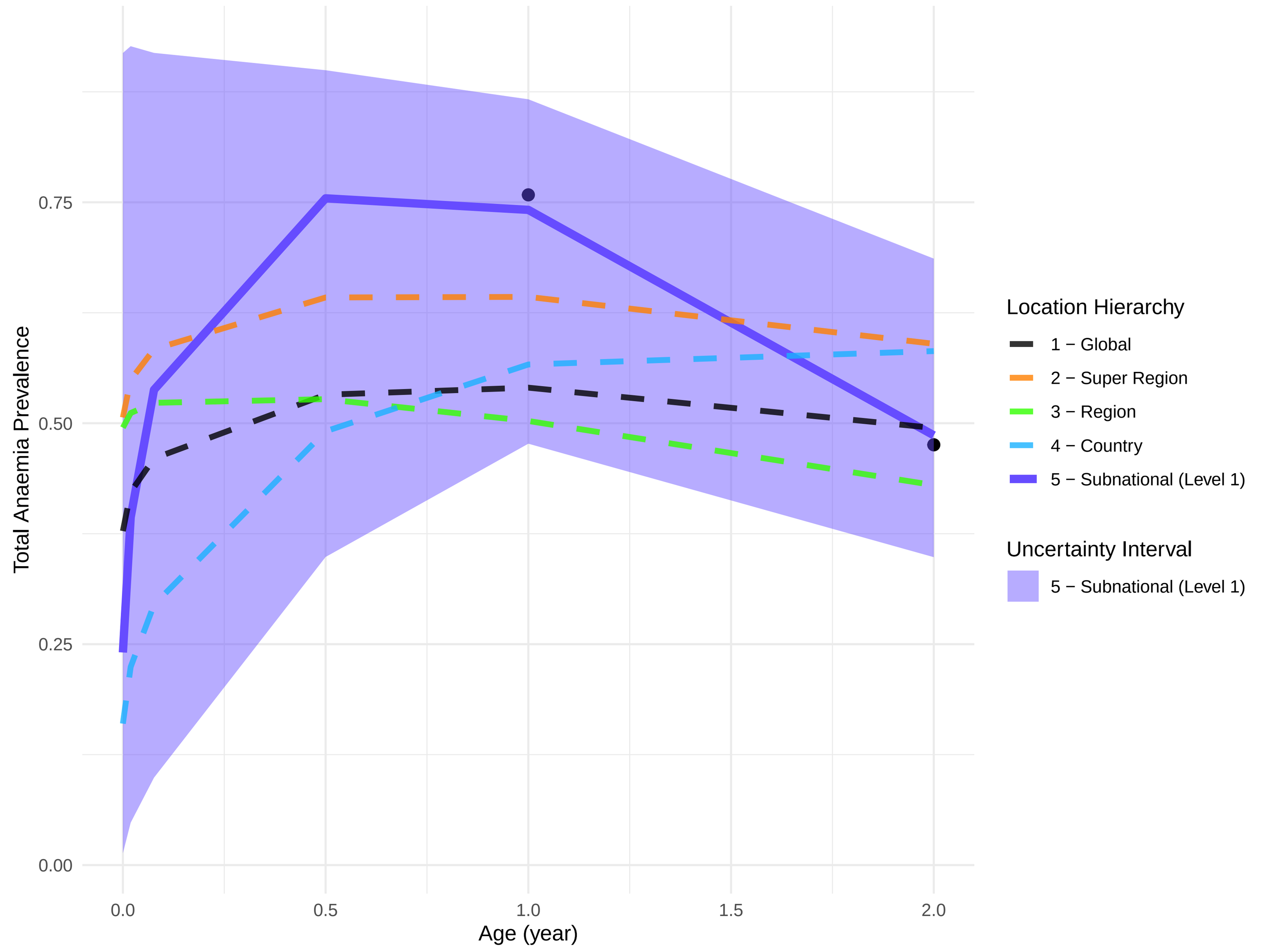

MR−BRT Age/Sex Fits − Non−pregnant Females <2 Years Old − Total Anaemia Prevalence − Northern Cape
WHO−2001 Adjustment Method

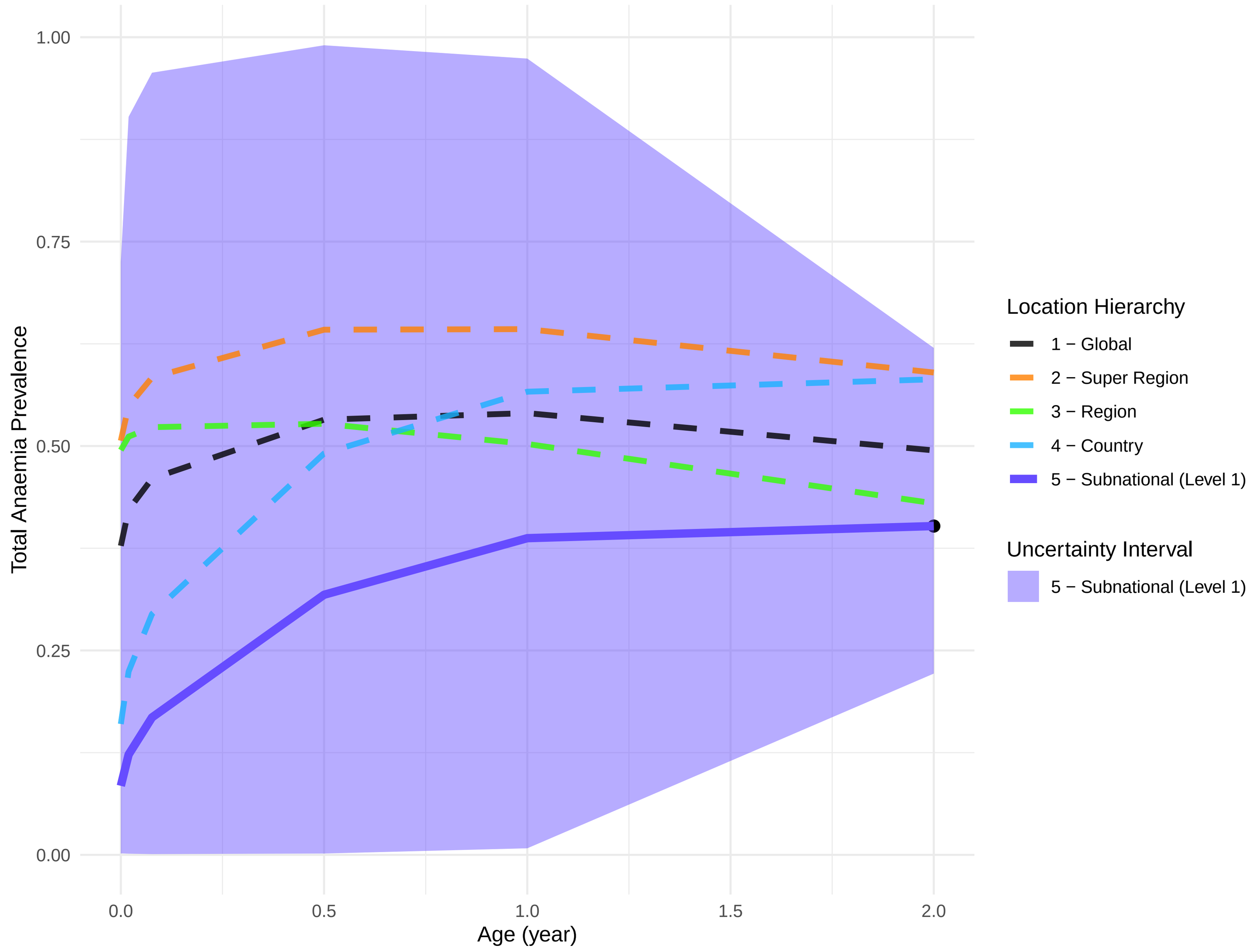

MR−BRT Age/Sex Fits − Non−pregnant Females <2 Years Old − Total Anaemia Prevalence − Western Cape
WHO−2001 Adjustment Method

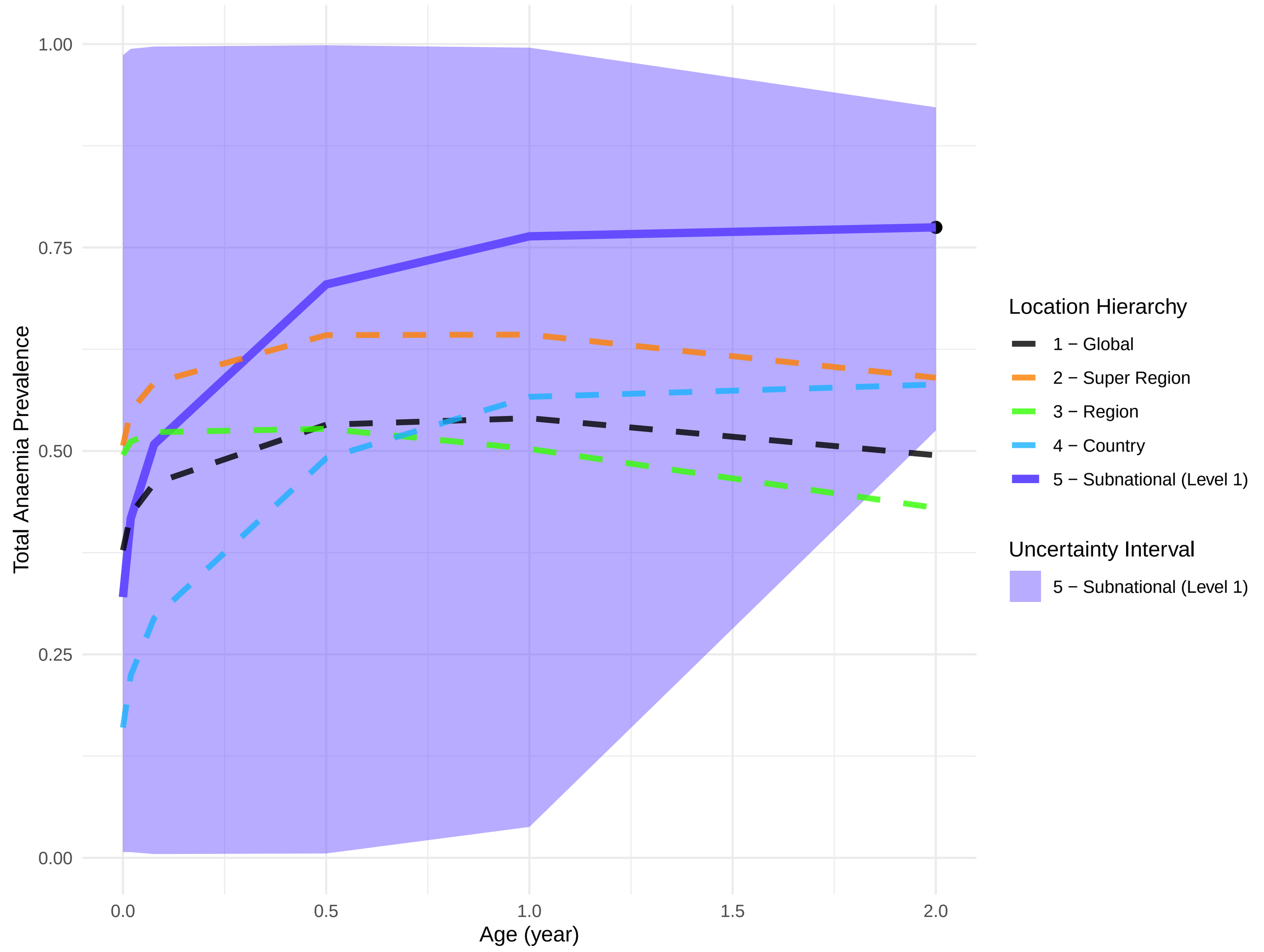

MR−BRT Age/Sex Fits − Non−pregnant Females <2 Years Old − Total Anaemia Prevalence − Zimbabwe
WHO−2001 Adjustment Method

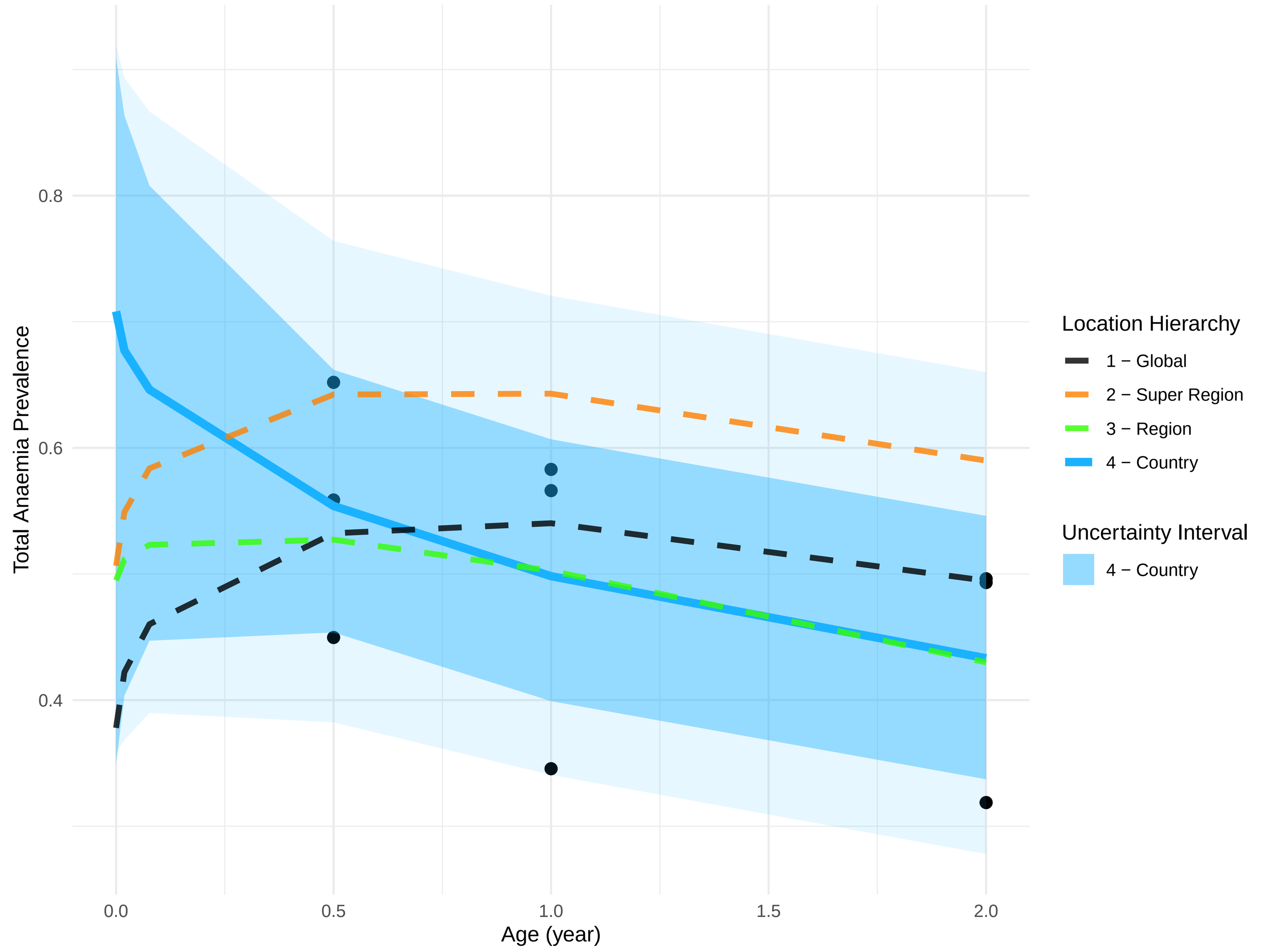

MR−BRT Age/Sex Fits − Non−pregnant Females <2 Years Old − Total Anaemia Prevalence − Western Sub−Saharan Africa
WHO−2001 Adjustment Method

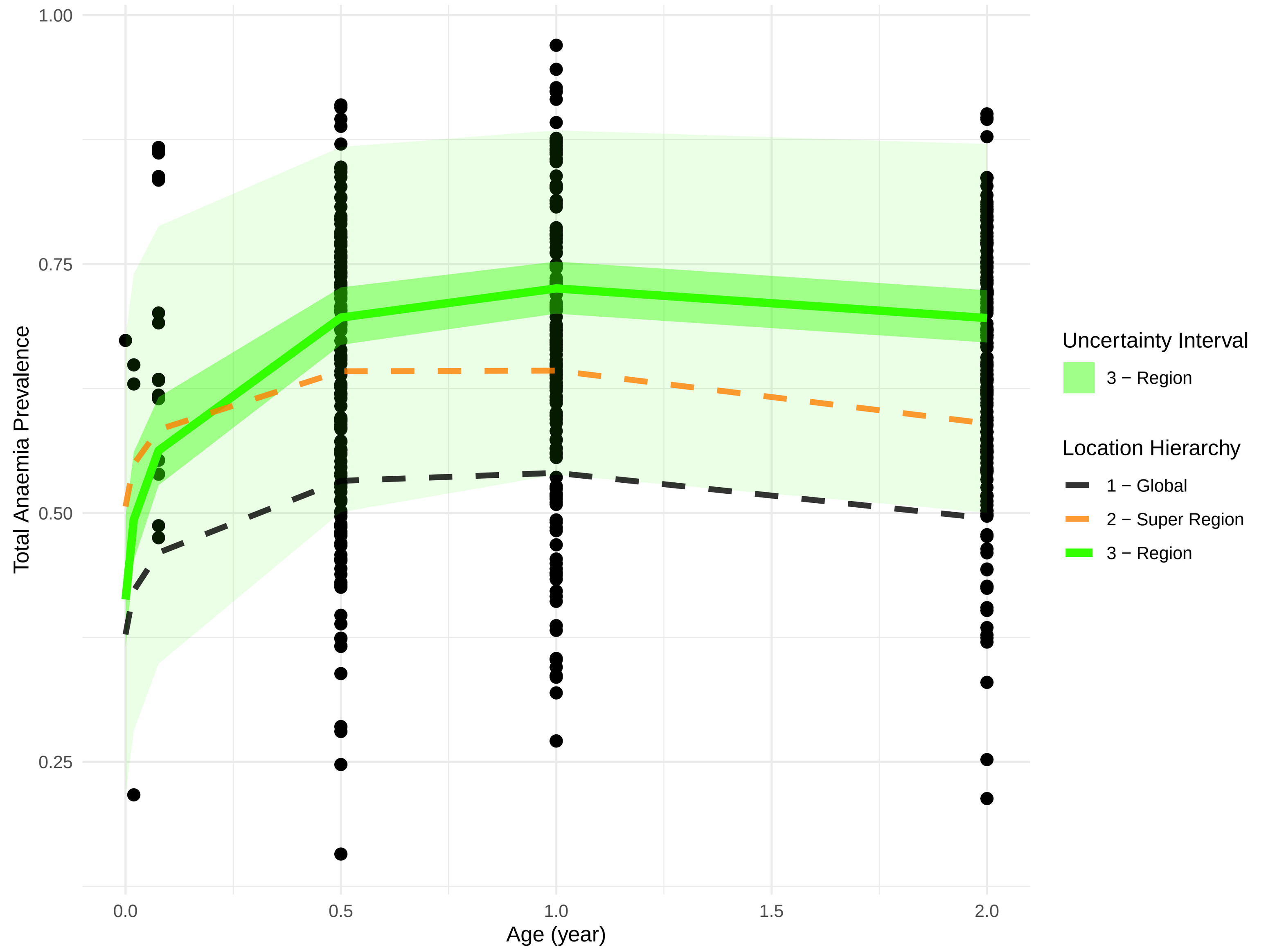

MR−BRT Age/Sex Fits − Non−pregnant Females <2 Years Old − Total Anaemia Prevalence − Benin
WHO−2001 Adjustment Method

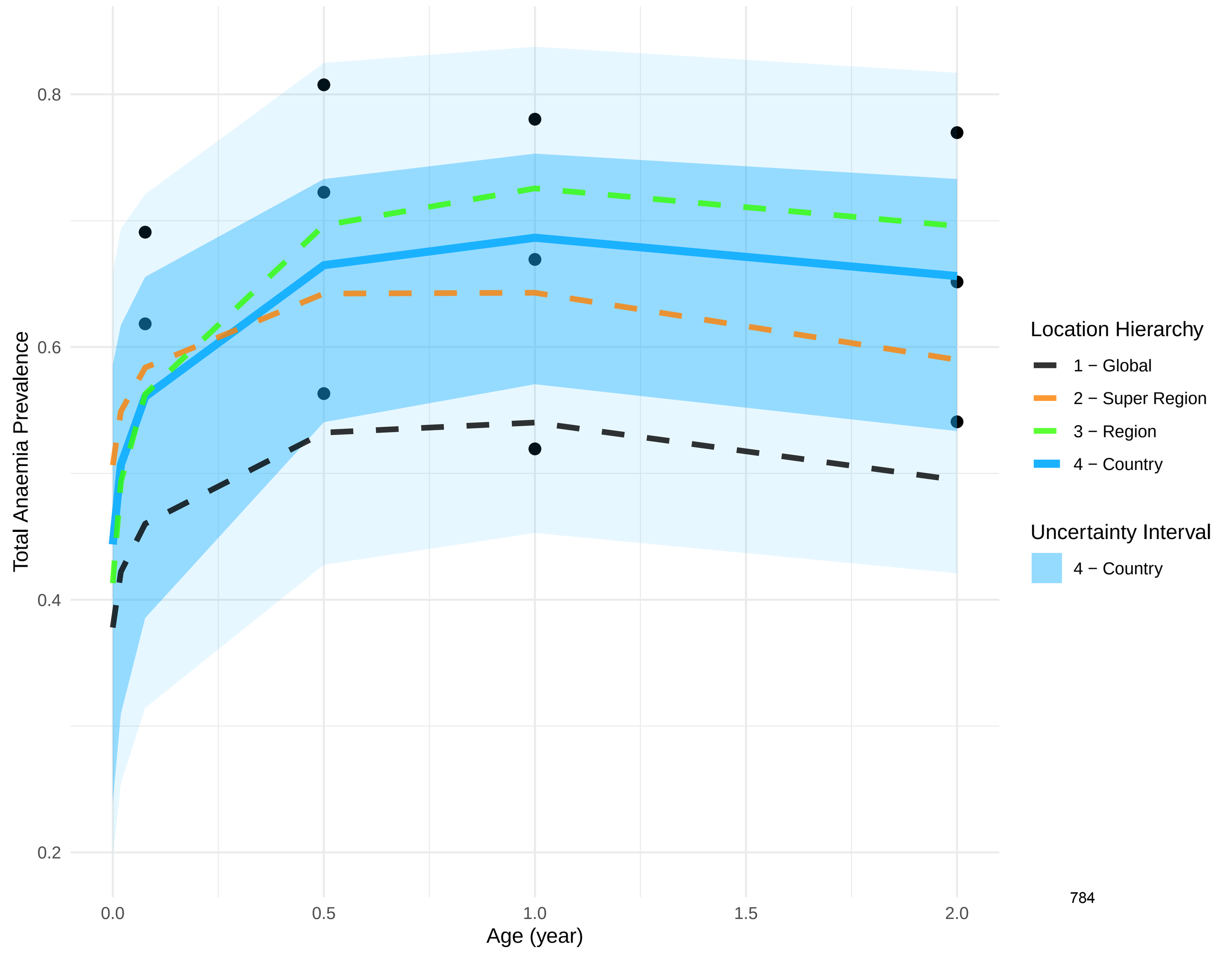

MR−BRT Age/Sex Fits − Non-pregnant Females <2 Years Old − Total Anaemia Prevalence − Burkina Faso
WHO−2001 Adjustment Method

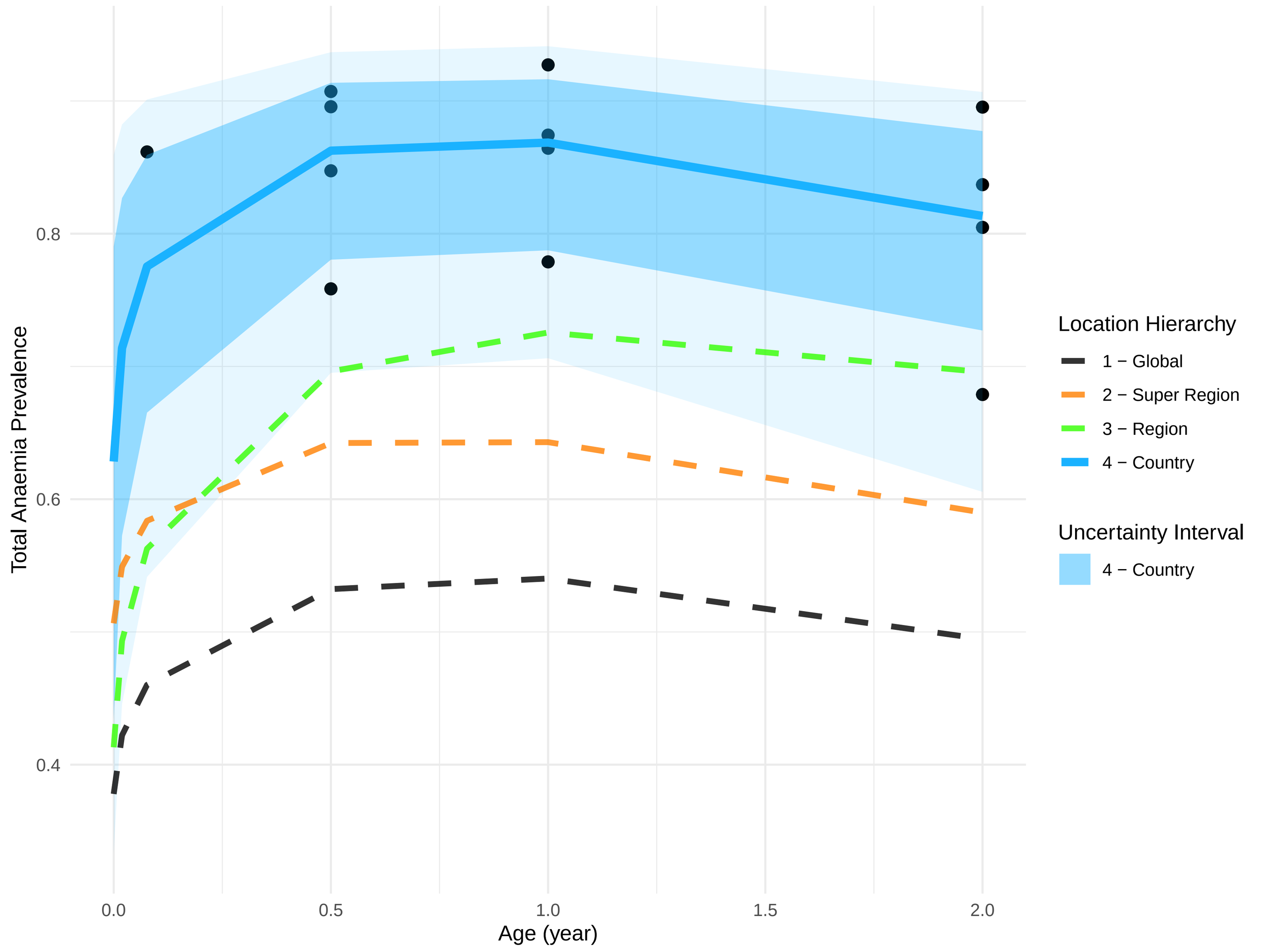

MR−BRT Age/Sex Fits − Non-pregnant Females <2 Years Old − Total Anaemia Prevalence − Cameroon
WHO−2001 Adjustment Method

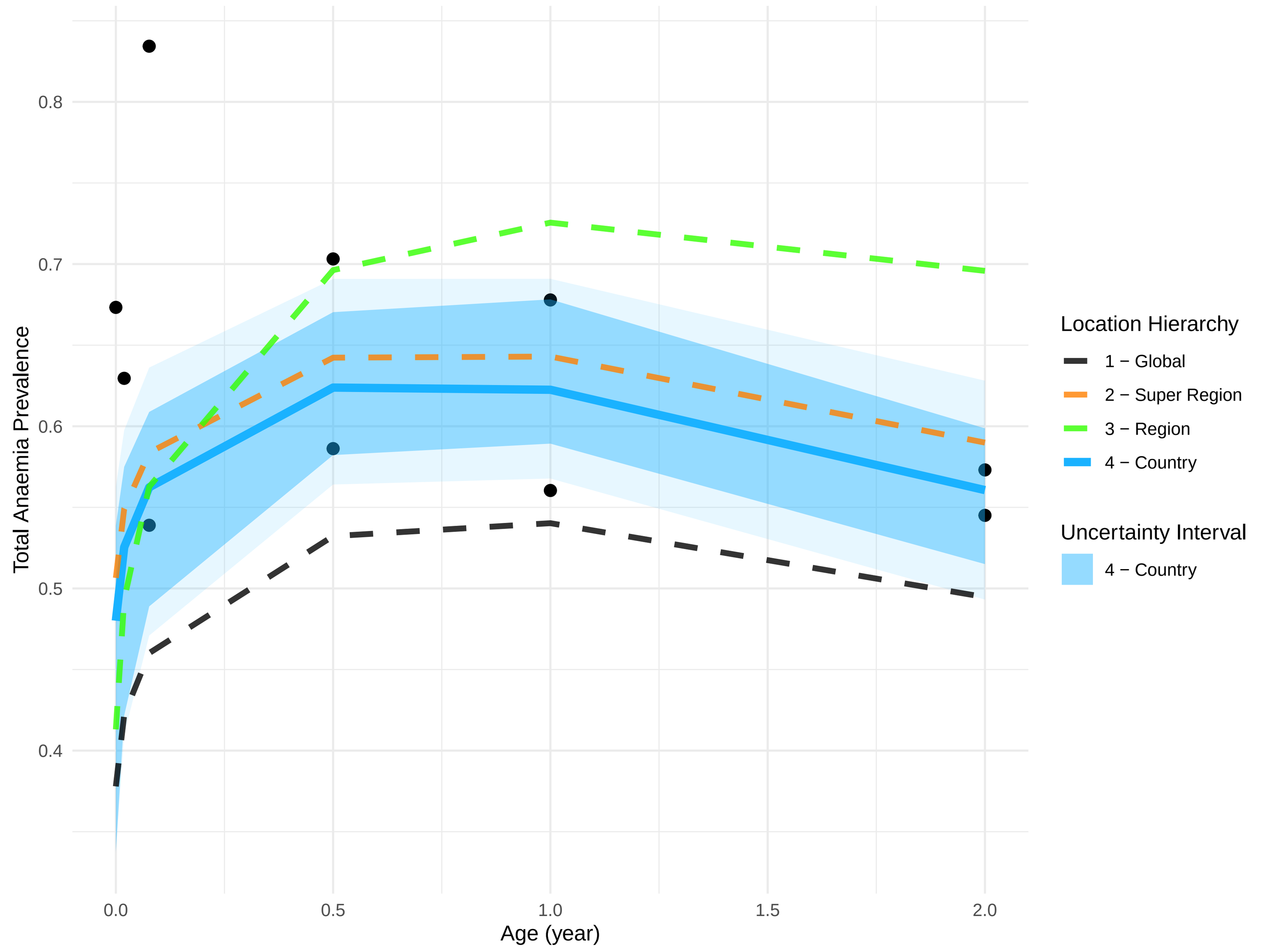

MR−BRT Age/Sex Fits − Non-pregnant Females <2 Years Old − Total Anaemia Prevalence − Côte d'Ivoire
WHO−2001 Adjustment Method

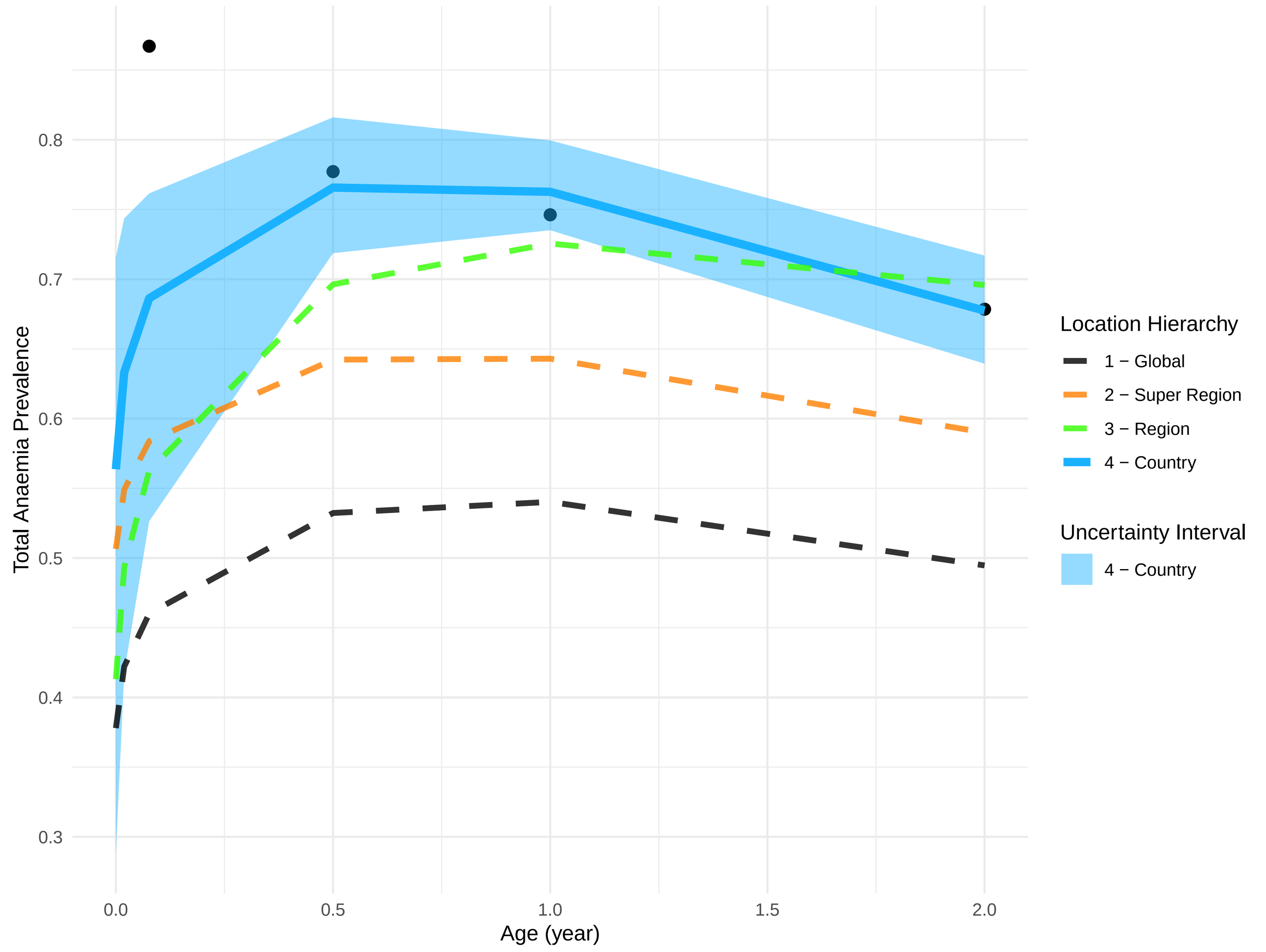

MR−BRT Age/Sex Fits − Non-pregnant Females <2 Years Old − Total Anaemia Prevalence − Gambia
WHO−2001 Adjustment Method

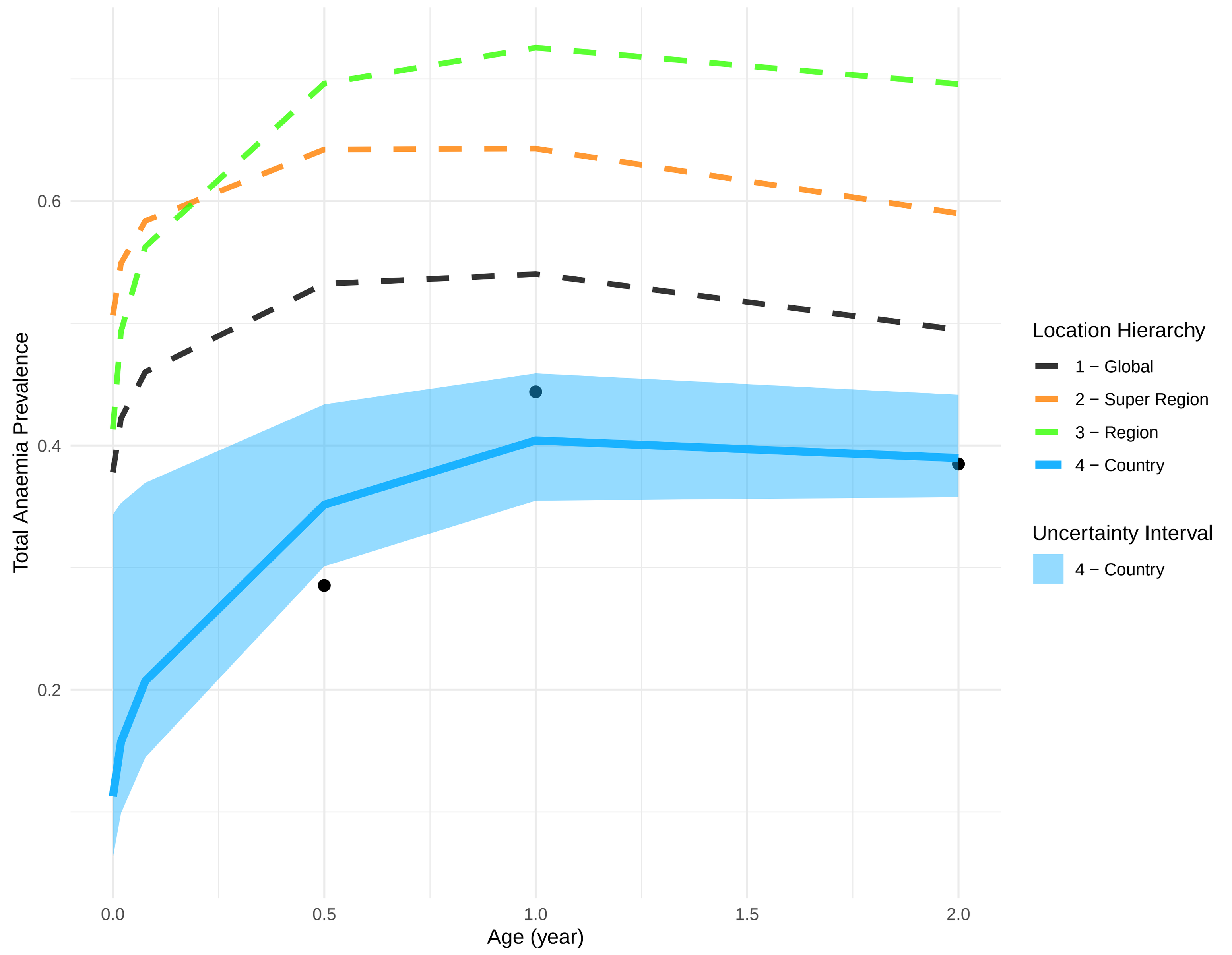

MR−BRT Age/Sex Fits − Non-pregnant Females <2 Years Old − Total Anaemia Prevalence − Ghana
WHO−2001 Adjustment Method

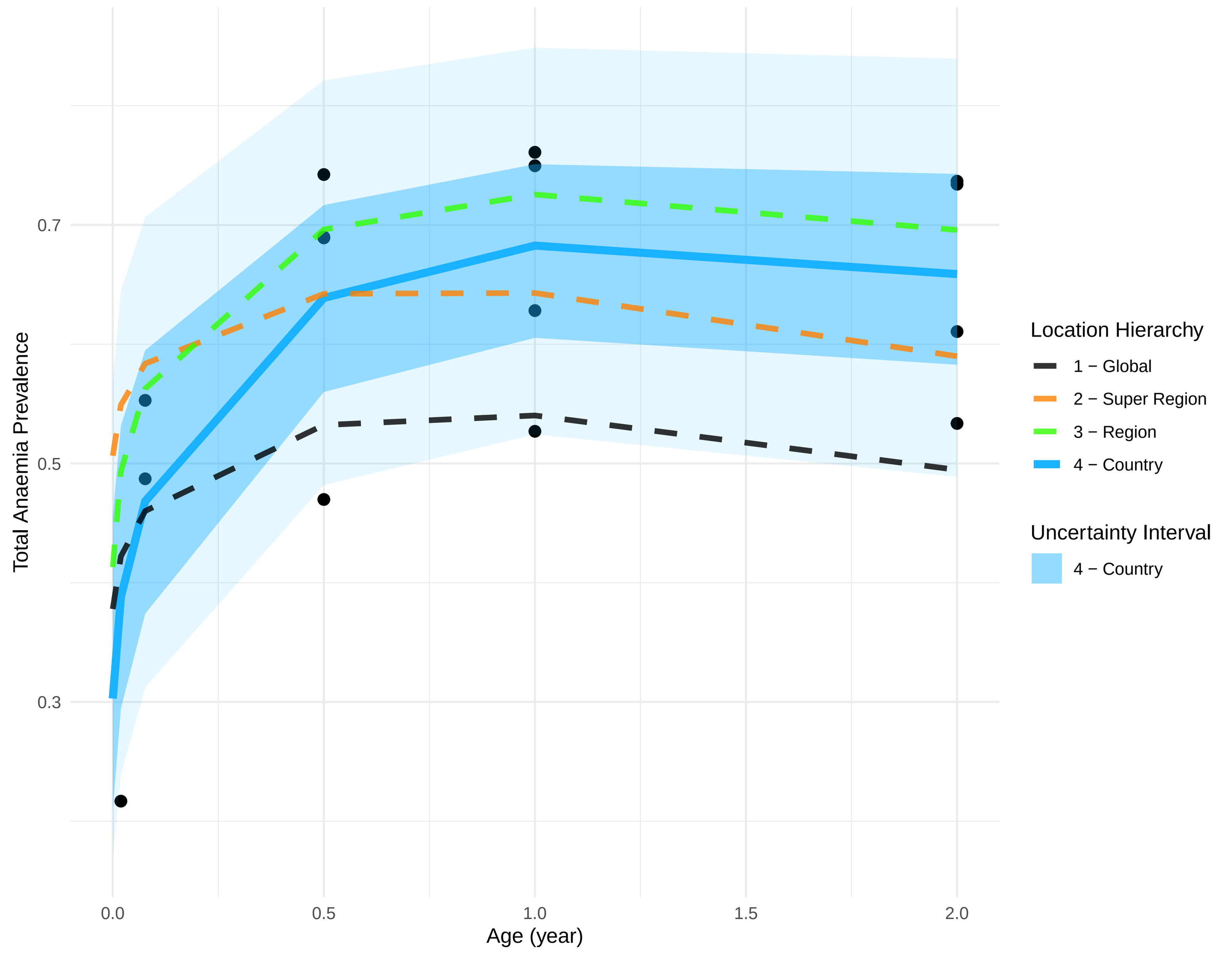

MR−BRT Age/Sex Fits − Non-pregnant Females <2 Years Old − Total Anaemia Prevalence − Guinea
WHO−2001 Adjustment Method

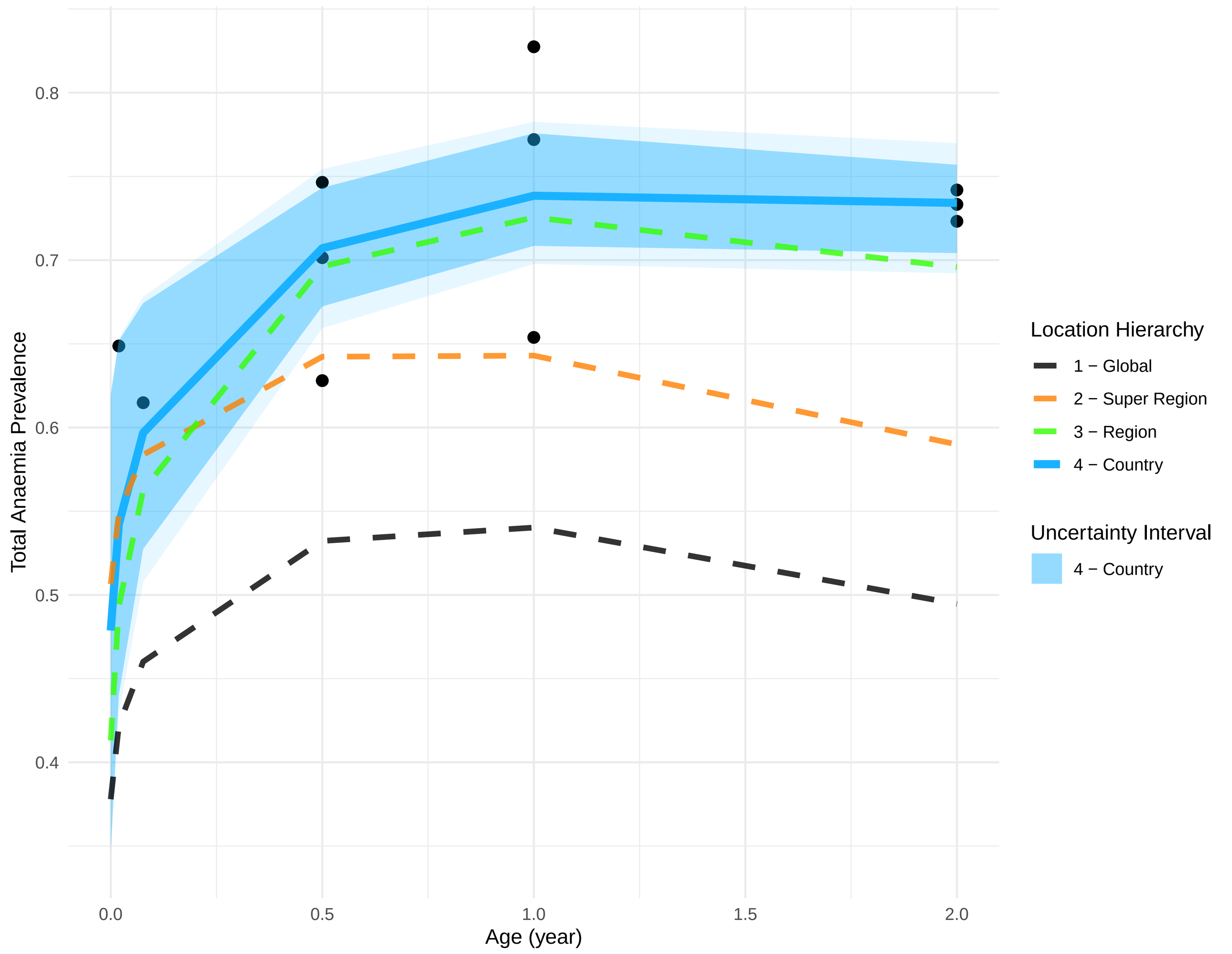

MR−BRT Age/Sex Fits − Non-pregnant Females <2 Years Old − Total Anaemia Prevalence − Liberia
WHO−2001 Adjustment Method

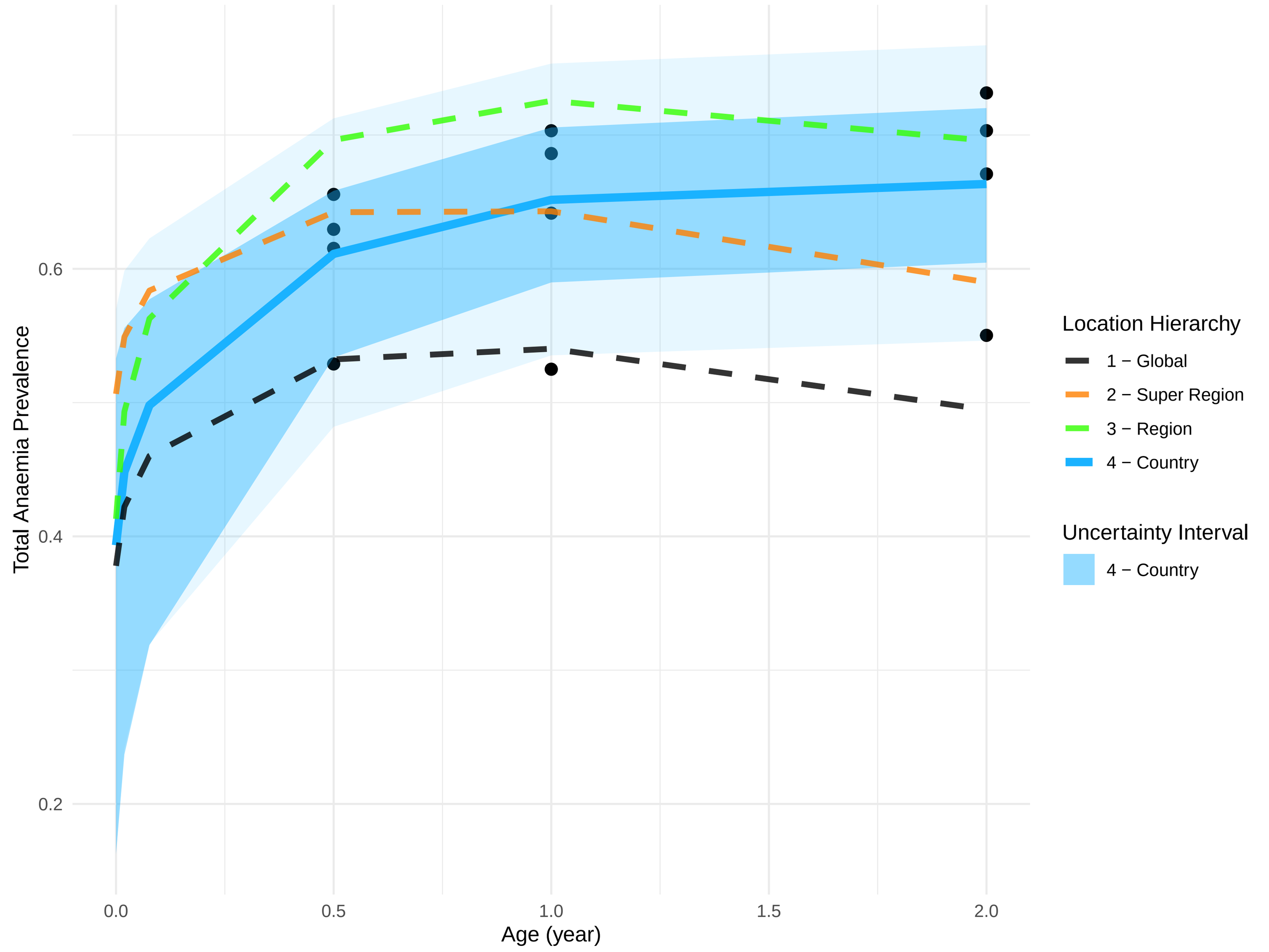

MR−BRT Age/Sex Fits − Non-pregnant Females <2 Years Old − Total Anaemia Prevalence − Mali
WHO−2001 Adjustment Method

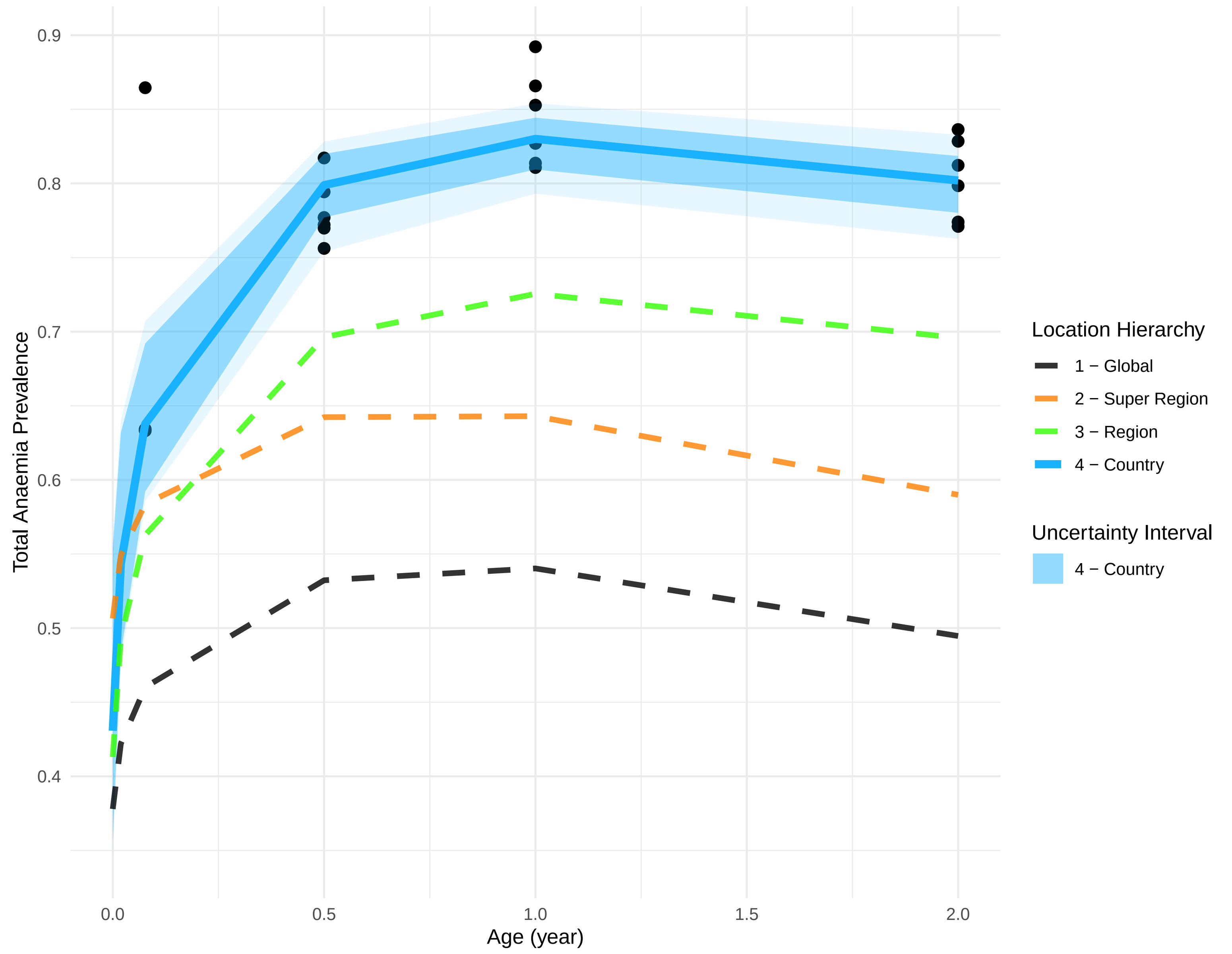

MR−BRT Age/Sex Fits − Non-pregnant Females <2 Years Old − Total Anaemia Prevalence − Niger
WHO−2001 Adjustment Method

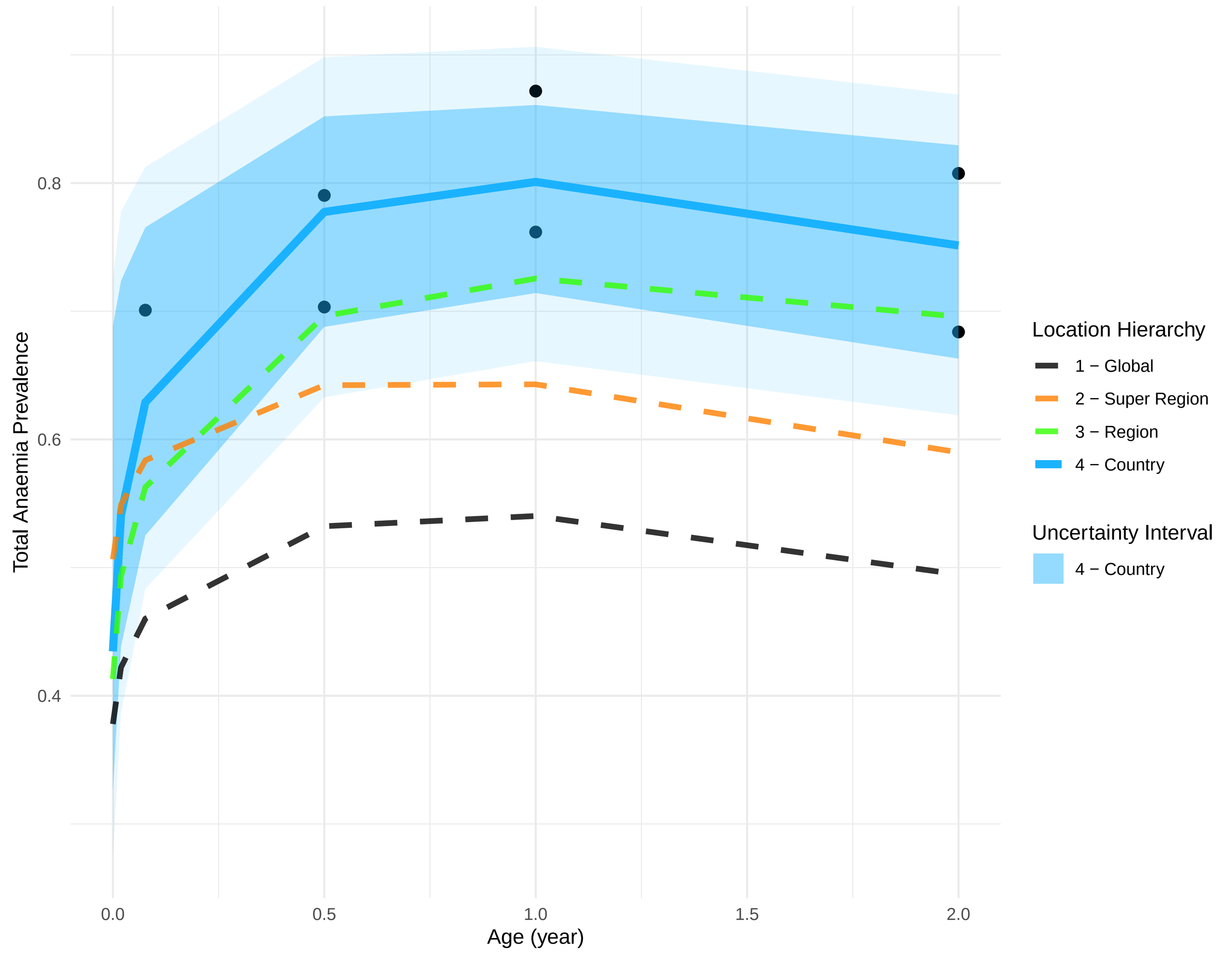

MR−BRT Age/Sex Fits − Non-pregnant Females <2 Years Old − Total Anaemia Prevalence − Nigeria
WHO−2001 Adjustment Method

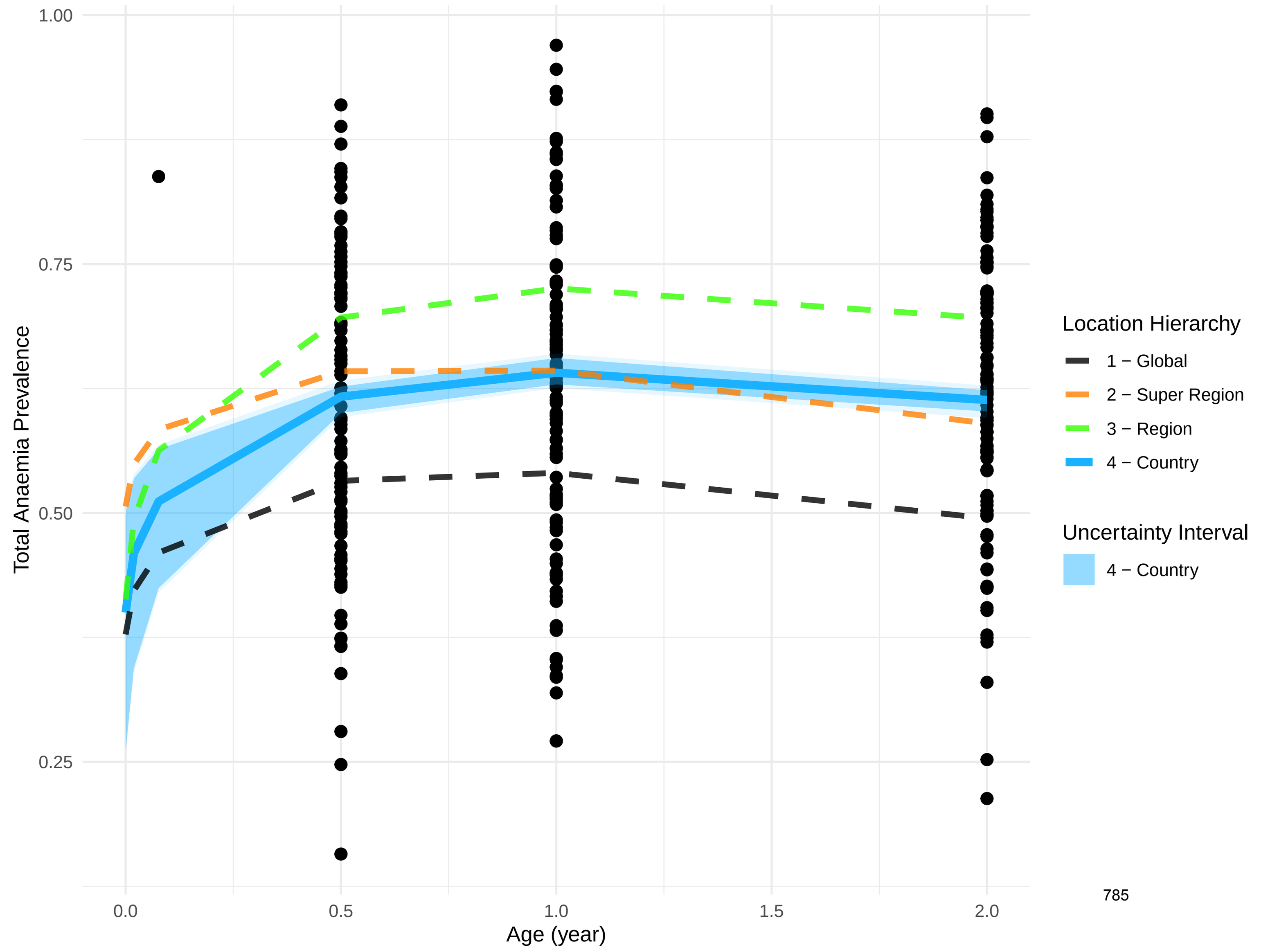

MR−BRT Age/Sex Fits − Non−pregnant Females <2 Years Old − Total Anaemia Prevalence − Abia
WHO−2001 Adjustment Method

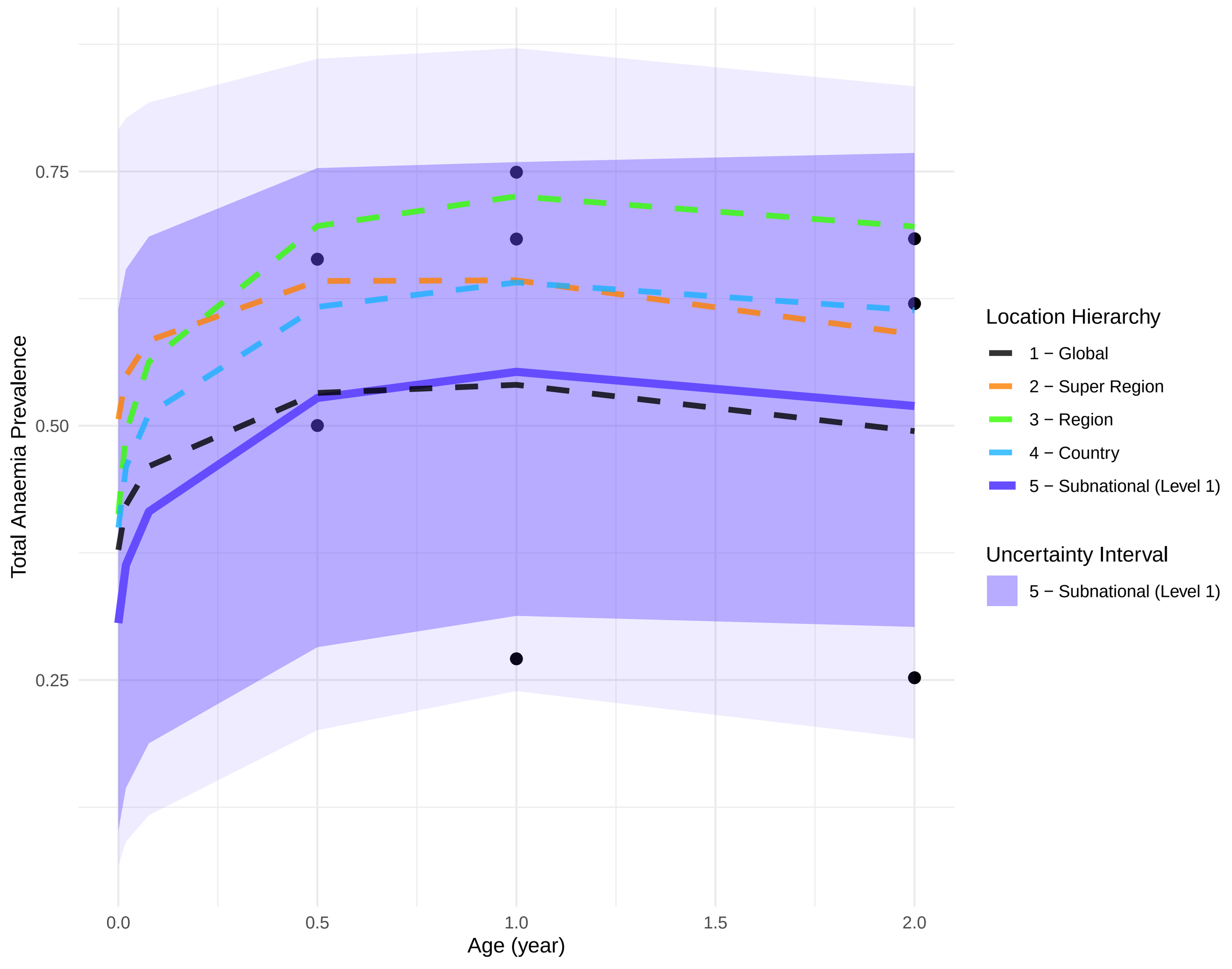

MR−BRT Age/Sex Fits − Non−pregnant Females <2 Years Old − Total Anaemia Prevalence − Adamawa
WHO−2001 Adjustment Method

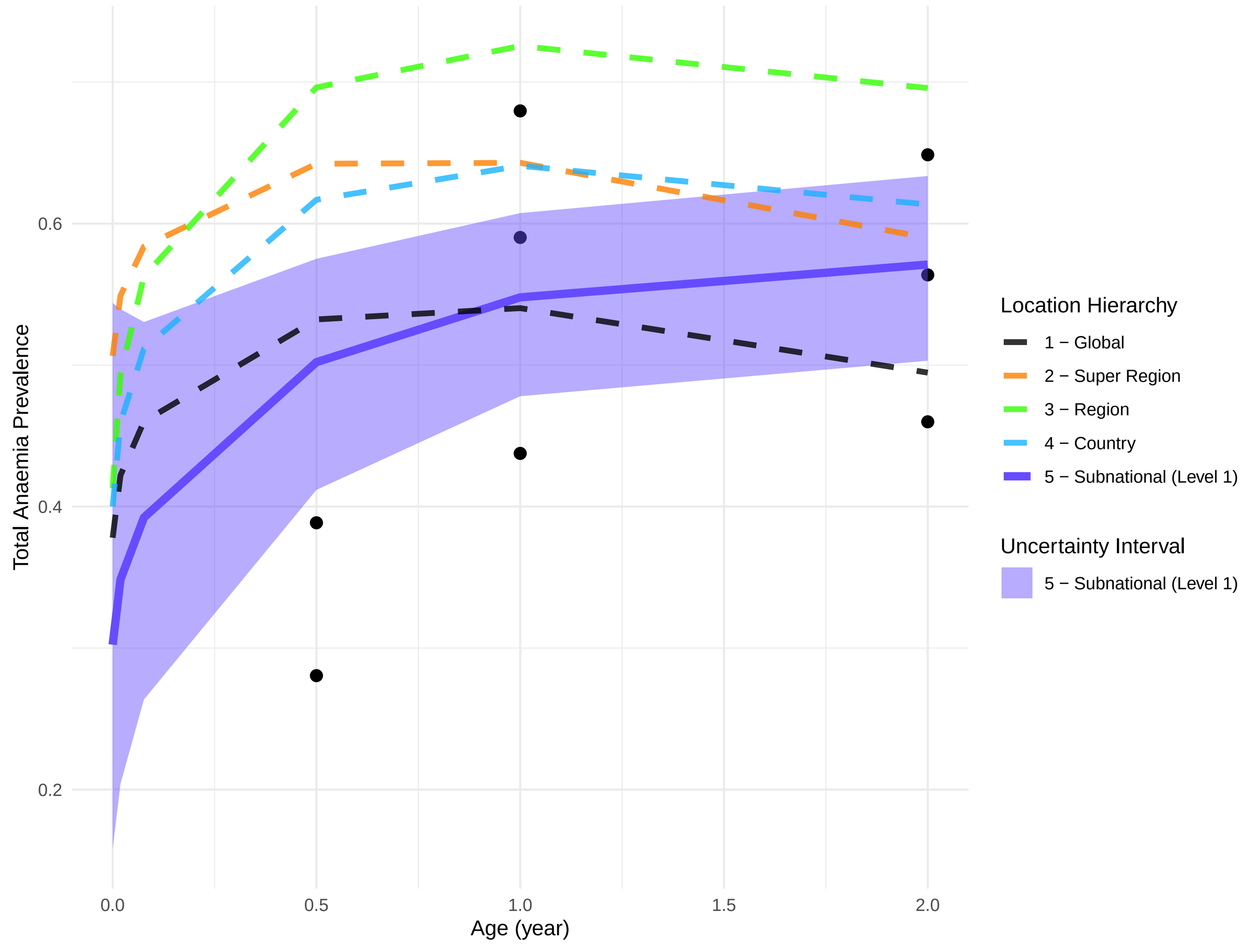

MR−BRT Age/Sex Fits − Non−pregnant Females <2 Years Old − Total Anaemia Prevalence − Akwa Ibom
WHO−2001 Adjustment Method

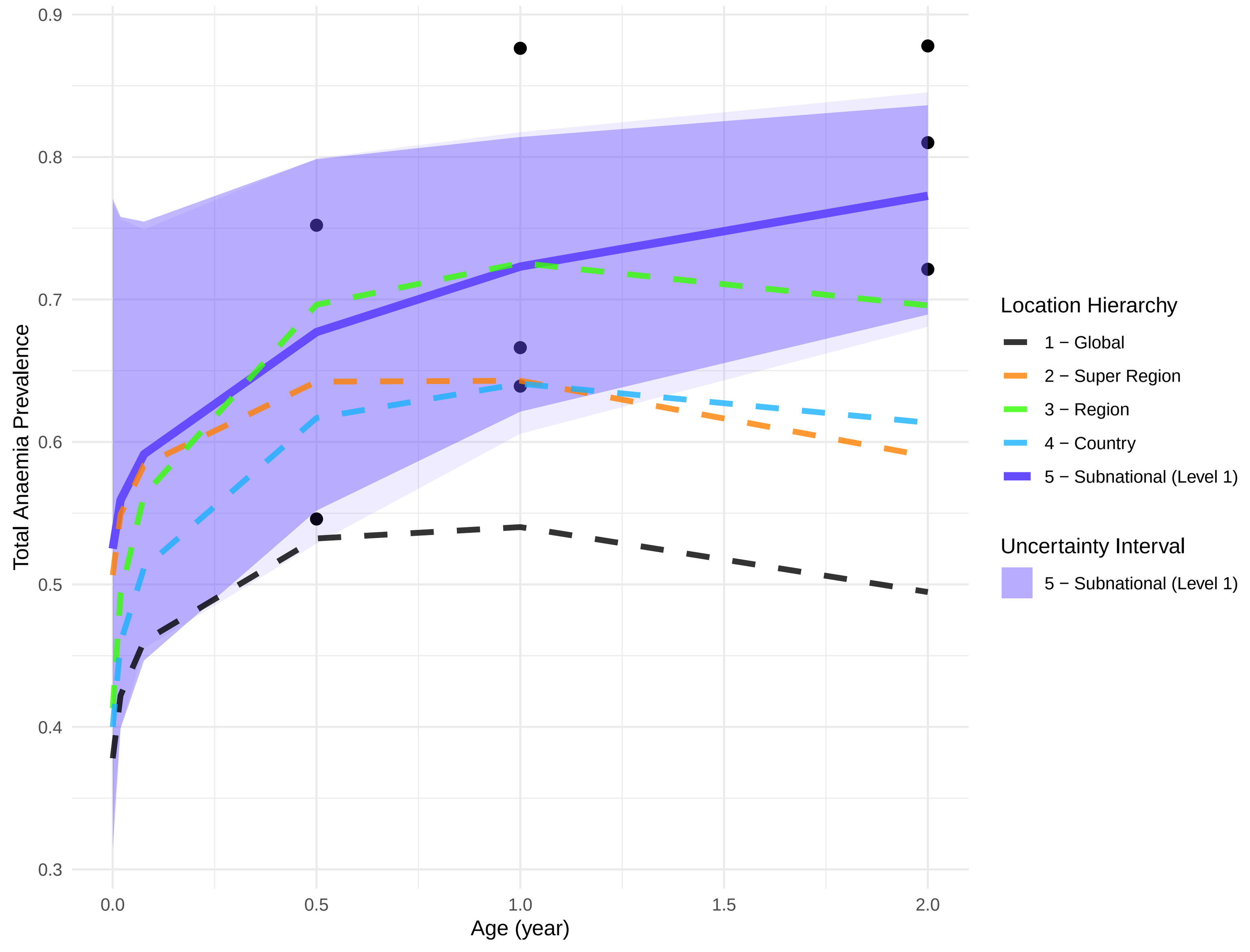

MR−BRT Age/Sex Fits − Non−pregnant Females <2 Years Old − Total Anaemia Prevalence − Anambra
WHO−2001 Adjustment Method

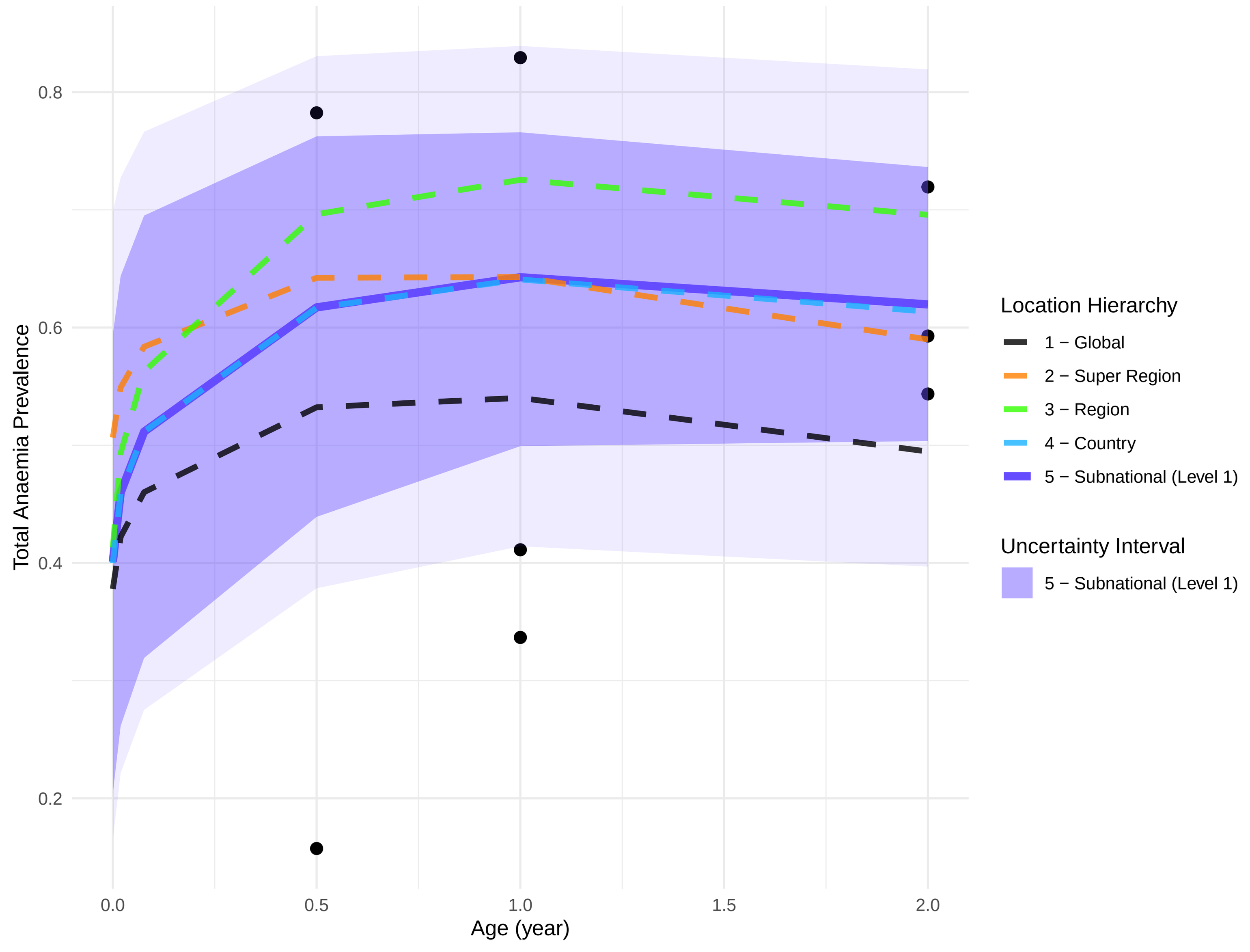

MR−BRT Age/Sex Fits − Non−pregnant Females <2 Years Old − Total Anaemia Prevalence − Bauchi
WHO−2001 Adjustment Method

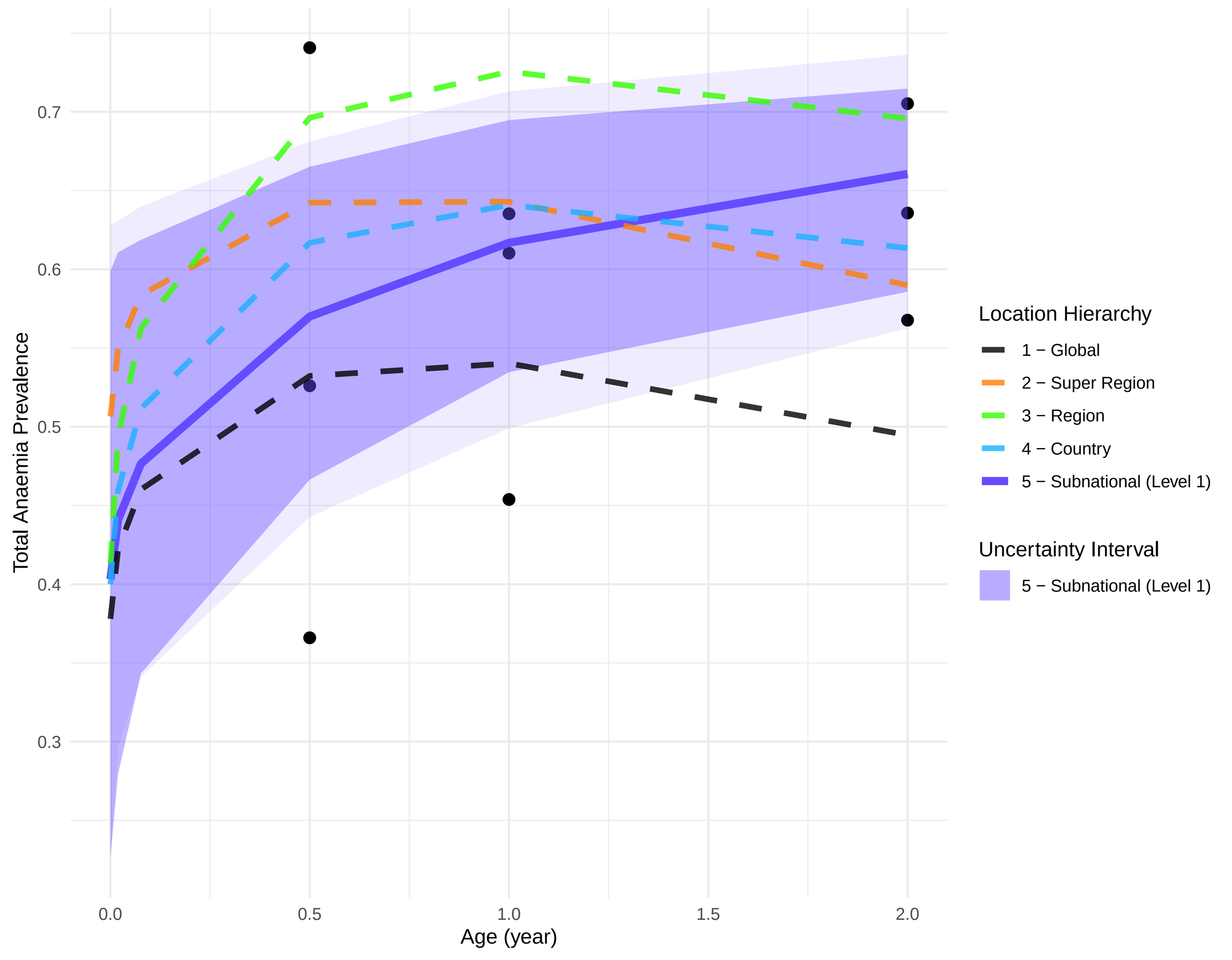

MR−BRT Age/Sex Fits − Non−pregnant Females <2 Years Old − Total Anaemia Prevalence − Bayelsa
WHO−2001 Adjustment Method

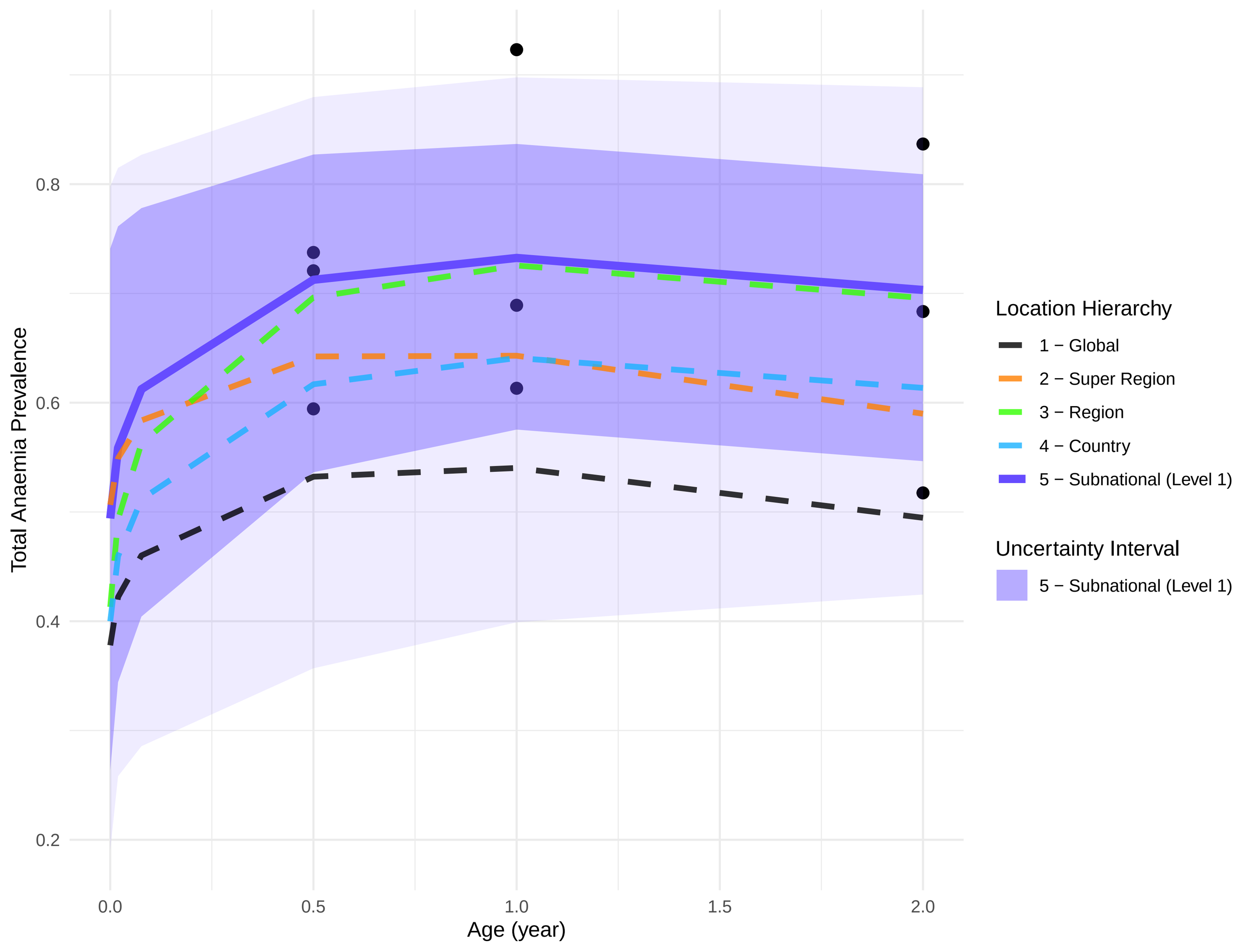

MR−BRT Age/Sex Fits − Non−pregnant Females <2 Years Old − Total Anaemia Prevalence − Benue
WHO−2001 Adjustment Method

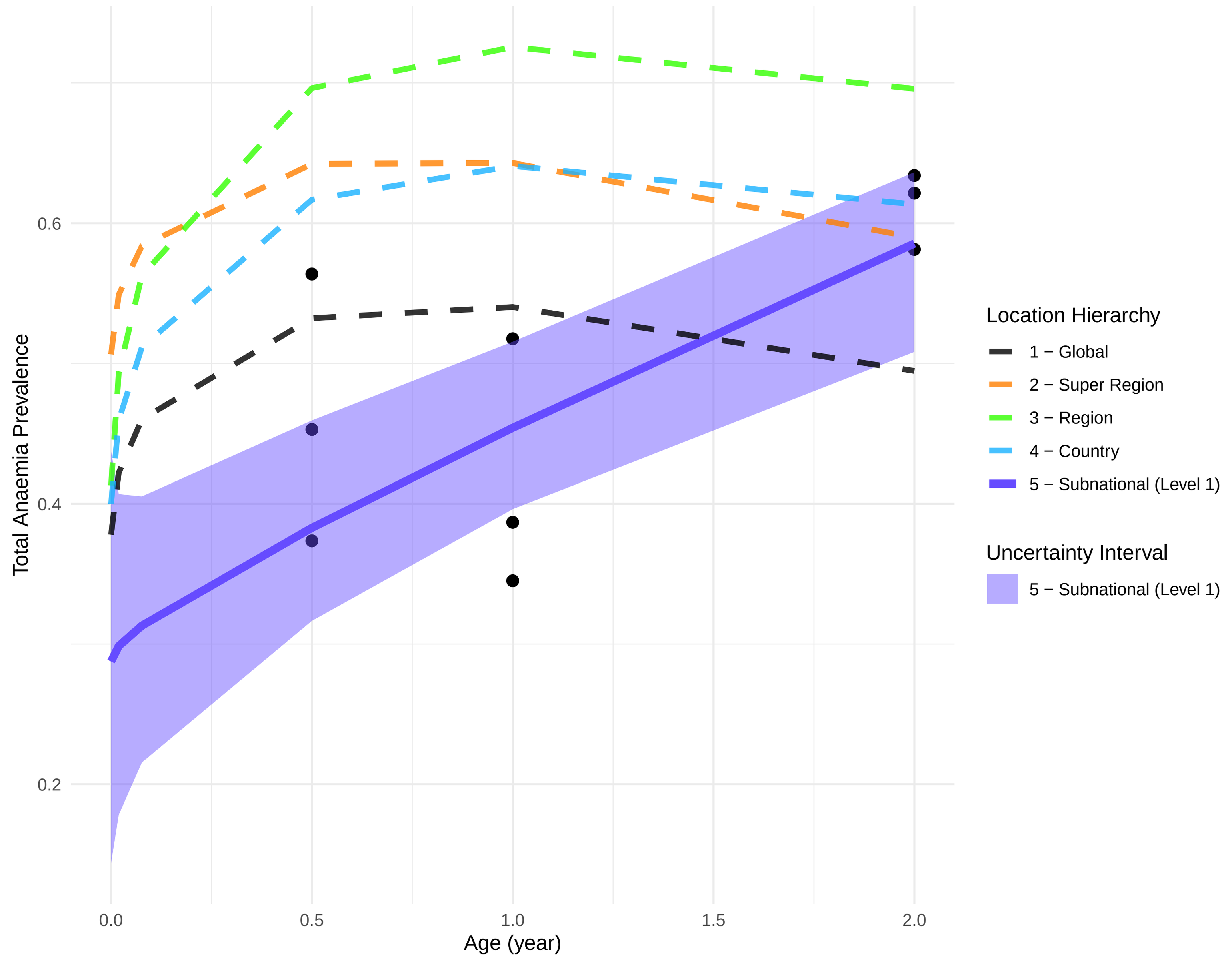

MR−BRT Age/Sex Fits − Non−pregnant Females <2 Years Old − Total Anaemia Prevalence − Borno
WHO−2001 Adjustment Method

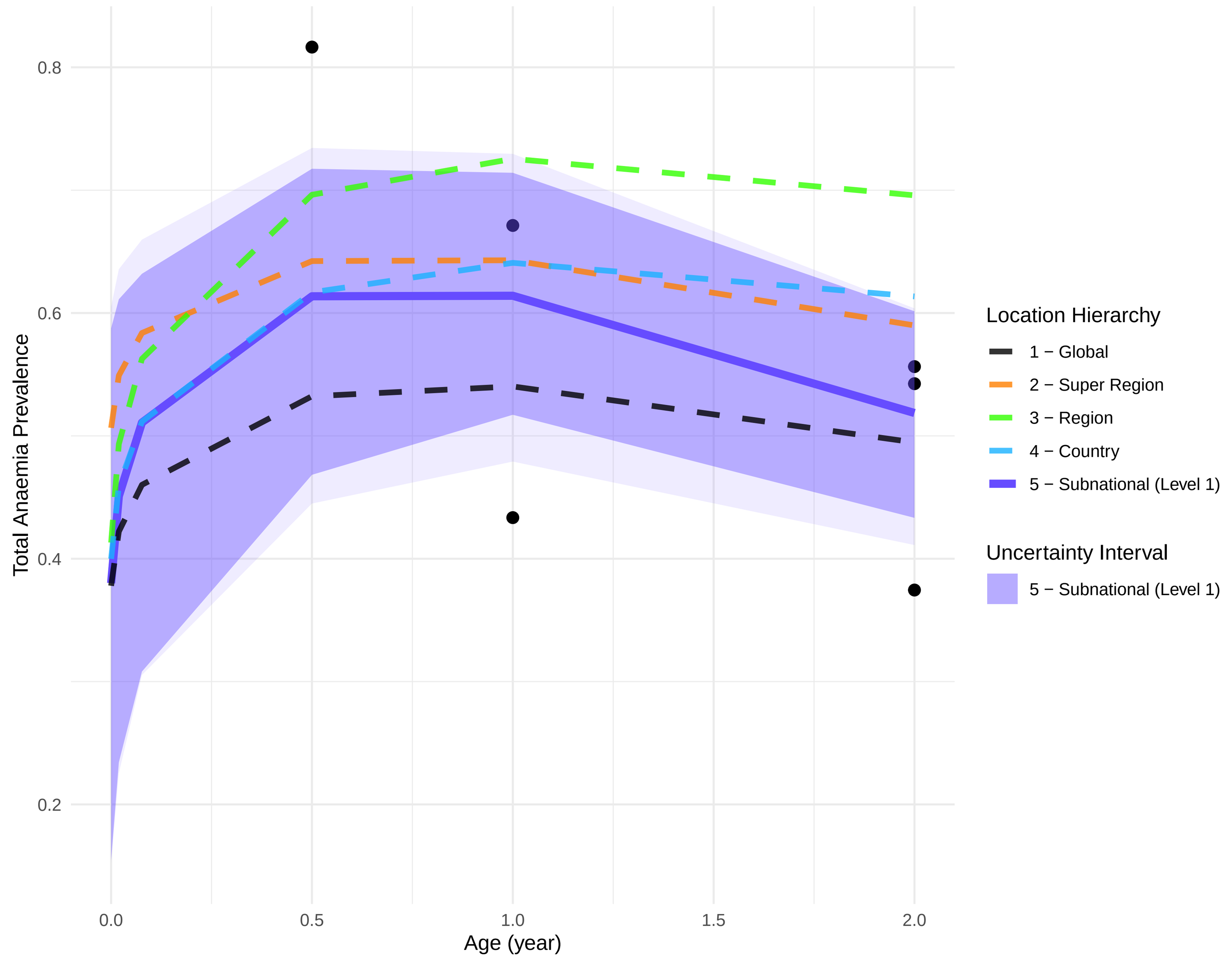

MR−BRT Age/Sex Fits − Non−pregnant Females <2 Years Old − Total Anaemia Prevalence − Cross River
WHO−2001 Adjustment Method

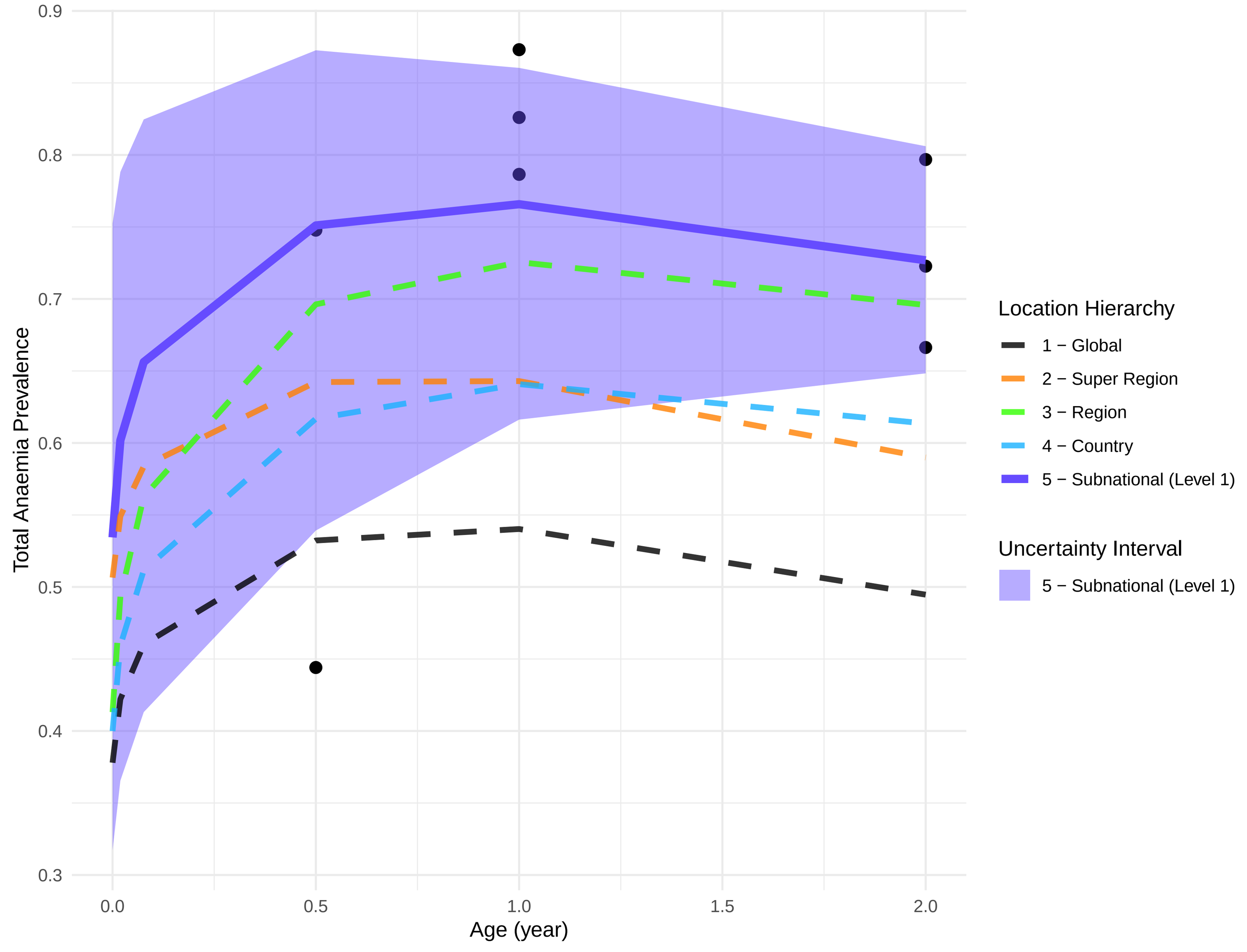

MR−BRT Age/Sex Fits − Non−pregnant Females <2 Years Old − Total Anaemia Prevalence − Delta
WHO−2001 Adjustment Method

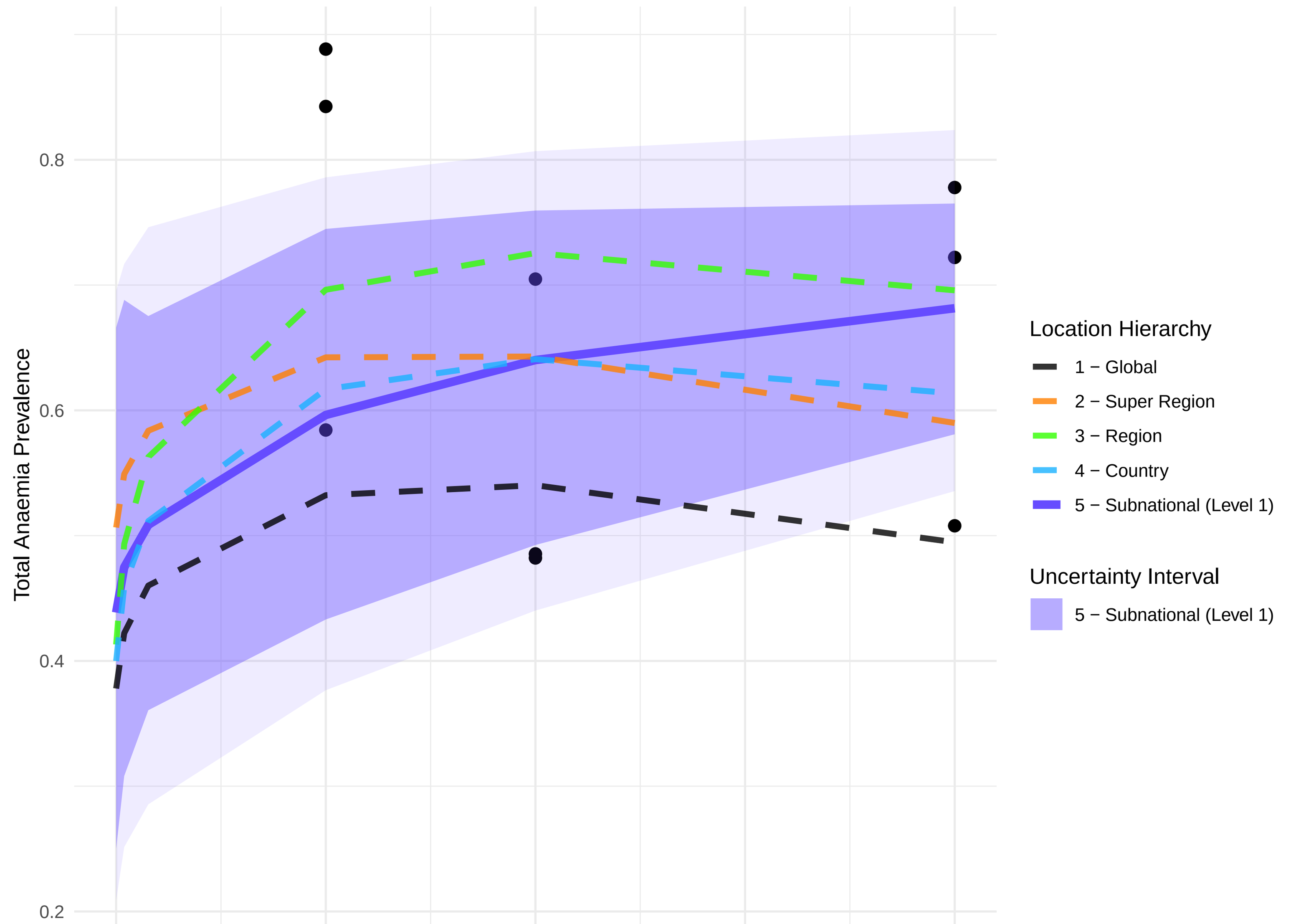

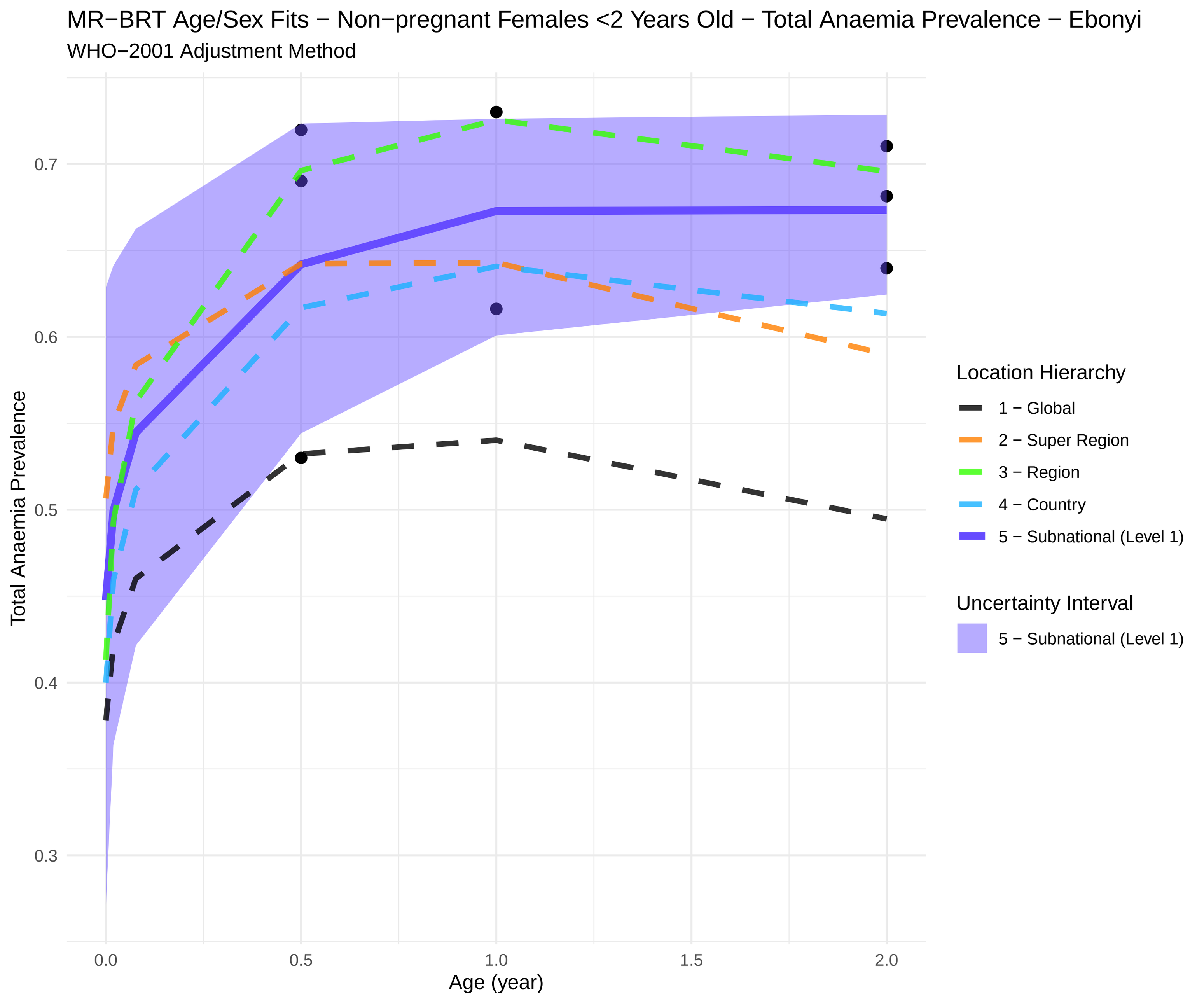
MR−BRT Age/Sex Fits − Non−pregnant Females <2 Years Old − Total Anaemia Prevalence − Ebonyi
WHO−2001 Adjustment Method
Total Anaemia Prevalence
Age (year)
Location Hierarchy
1 − Global
2 − Super Region
3 − Region
4 − Country
5 − Subnational (Level 1)
Uncertainty Interval
5 − Subnational (Level 1)

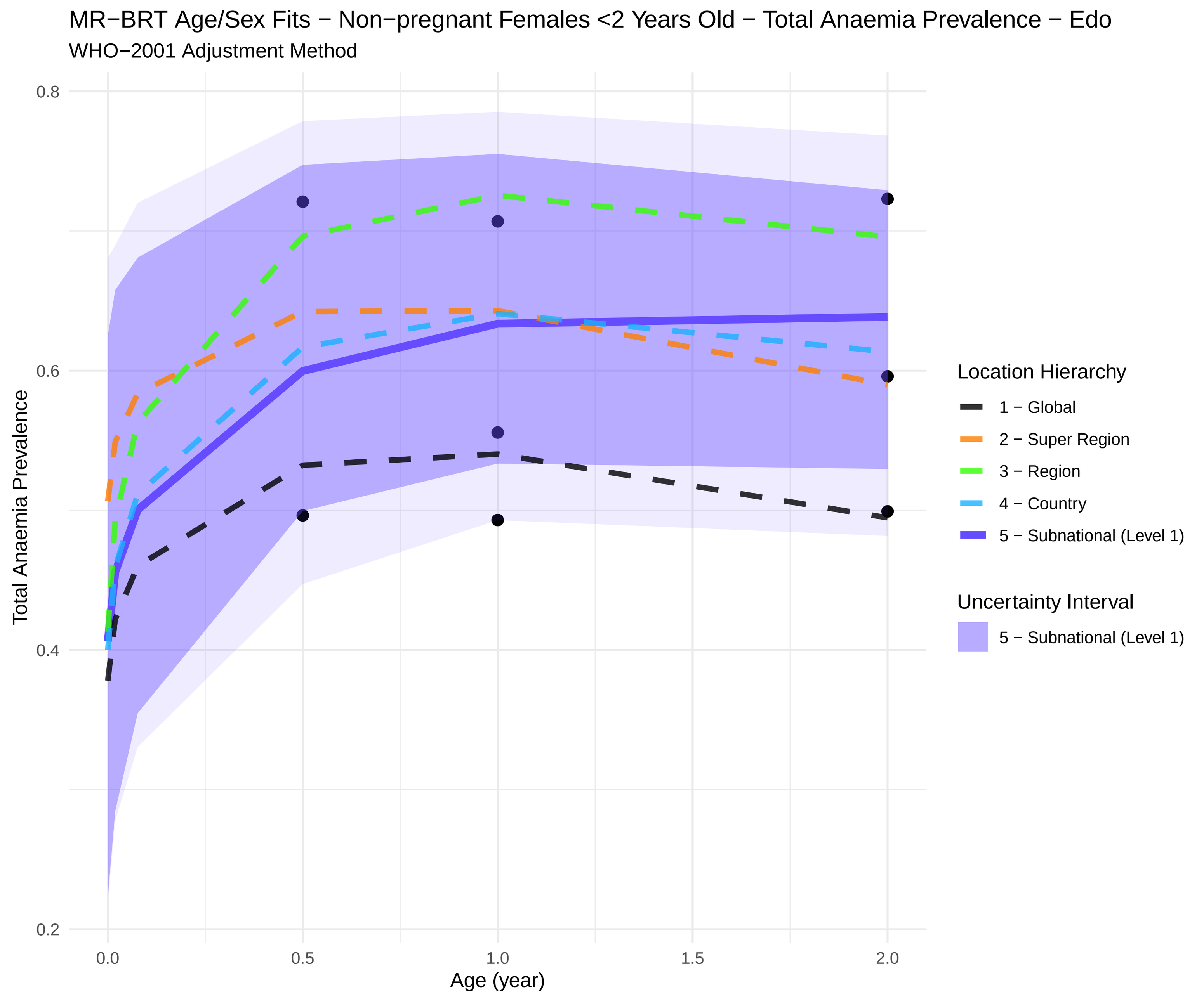
MR−BRT Age/Sex Fits − Non−pregnant Females <2 Years Old − Total Anaemia Prevalence − Edo
WHO−2001 Adjustment Method
Total Anaemia Prevalence
Age (year)
Location Hierarchy
1 − Global
2 − Super Region
3 − Region
4 − Country
5 − Subnational (Level 1)
Uncertainty Interval
5 − Subnational (Level 1)

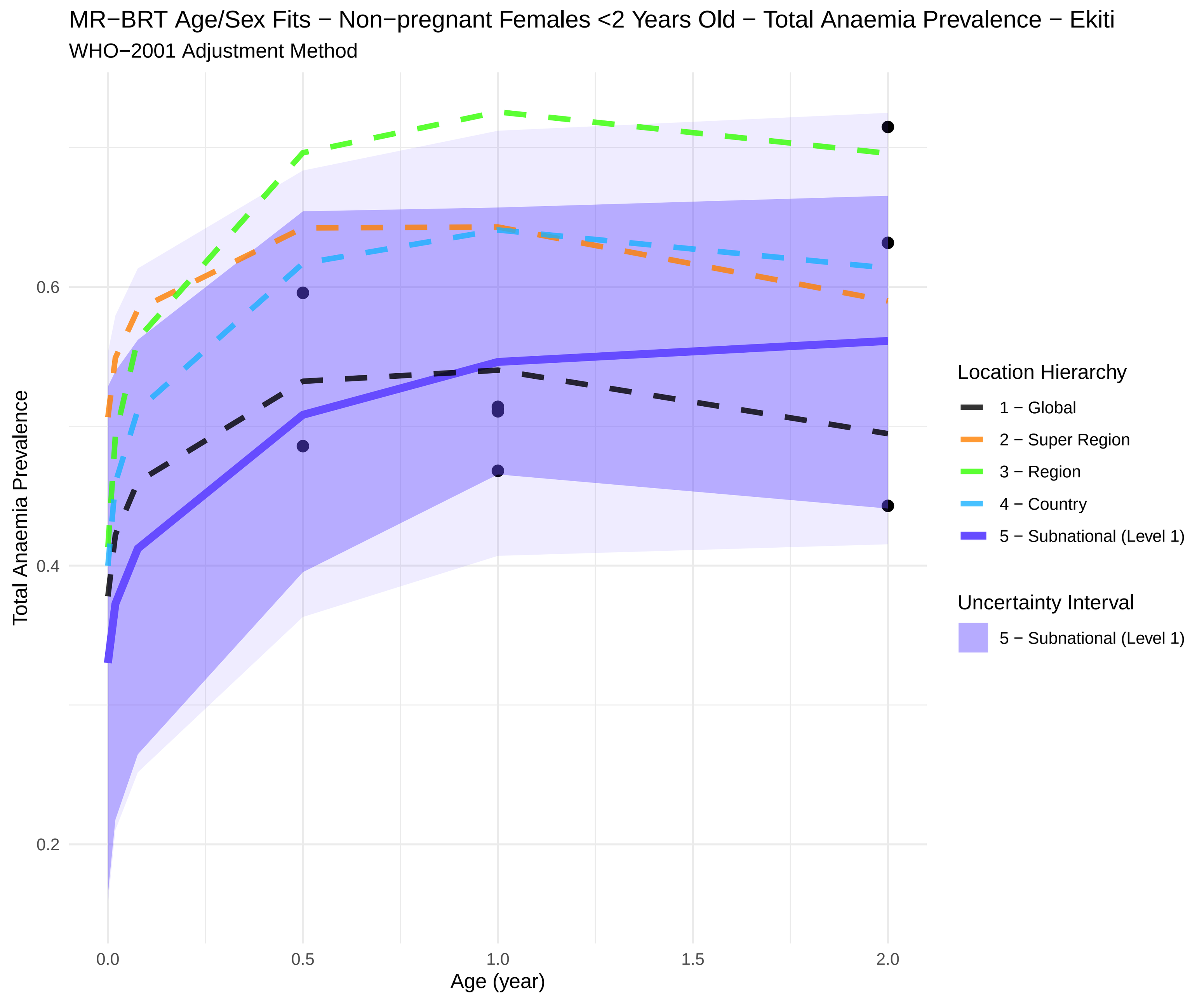
MR−BRT Age/Sex Fits − Non−pregnant Females <2 Years Old − Total Anaemia Prevalence − Ekiti
WHO−2001 Adjustment Method
Total Anaemia Prevalence
Age (year)
Location Hierarchy
1 − Global
2 − Super Region
3 − Region
4 − Country
5 − Subnational (Level 1)
Uncertainty Interval
5 − Subnational (Level 1)

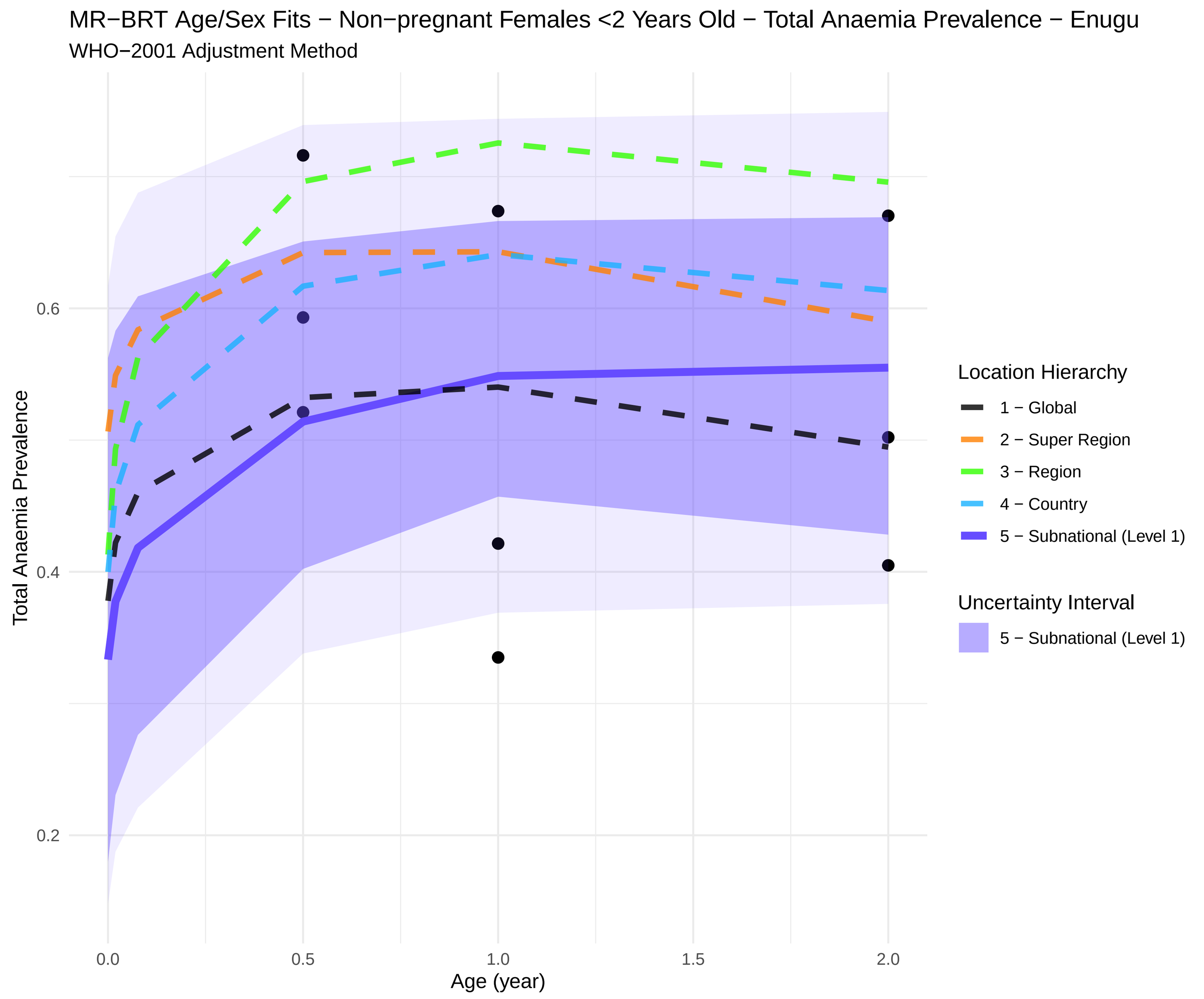
MR−BRT Age/Sex Fits − Non−pregnant Females <2 Years Old − Total Anaemia Prevalence − Enugu
WHO−2001 Adjustment Method
Total Anaemia Prevalence
Age (year)
Location Hierarchy
1 − Global
2 − Super Region
3 − Region
4 − Country
5 − Subnational (Level 1)
Uncertainty Interval
5 − Subnational (Level 1)

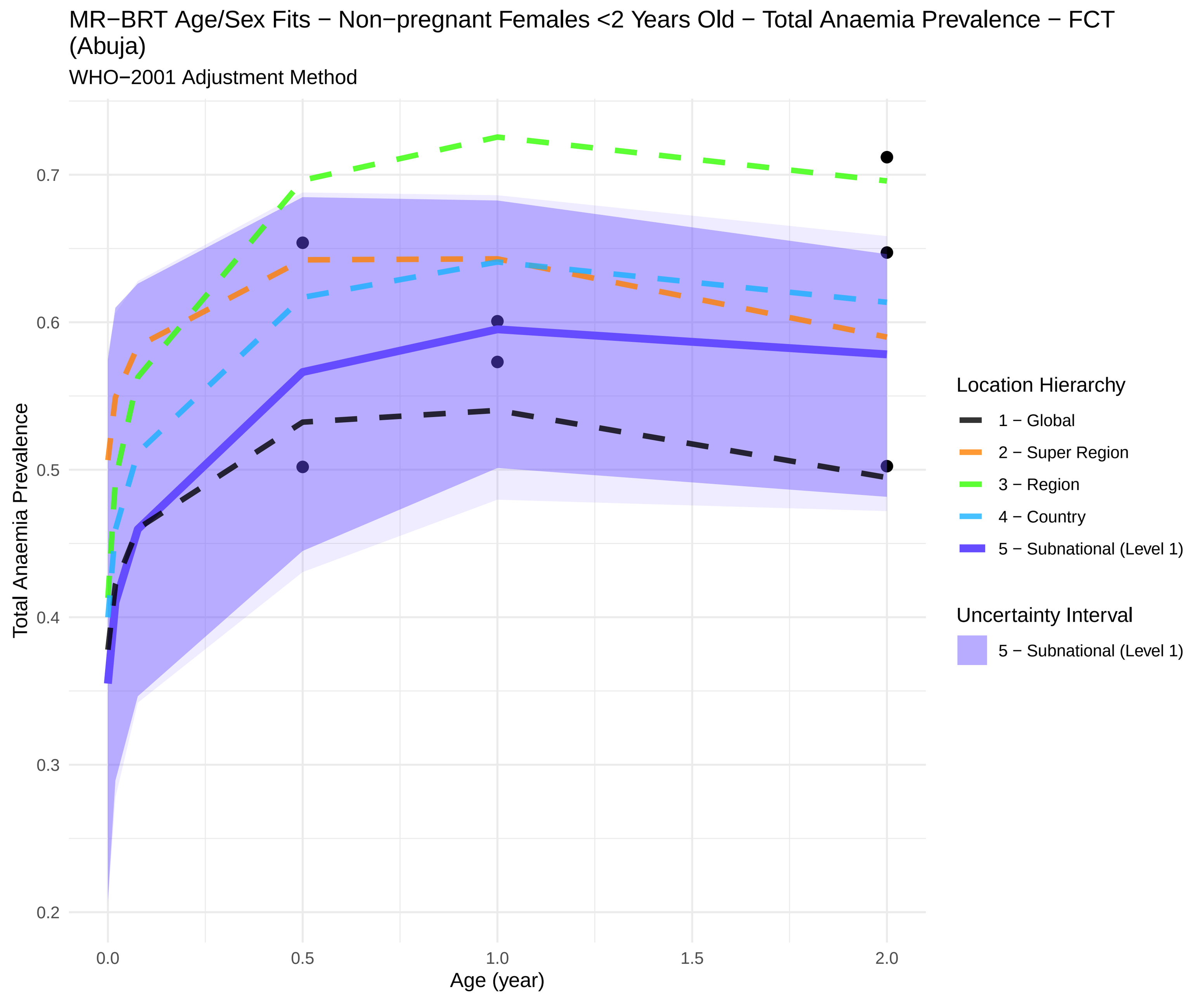
MR−BRT Age/Sex Fits − Non−pregnant Females <2 Years Old − Total Anaemia Prevalence − FCT (Abuja)
WHO−2001 Adjustment Method
Total Anaemia Prevalence
Age (year)
Location Hierarchy
1 − Global
2 − Super Region
3 − Region
4 − Country
5 − Subnational (Level 1)
Uncertainty Interval
5 − Subnational (Level 1)

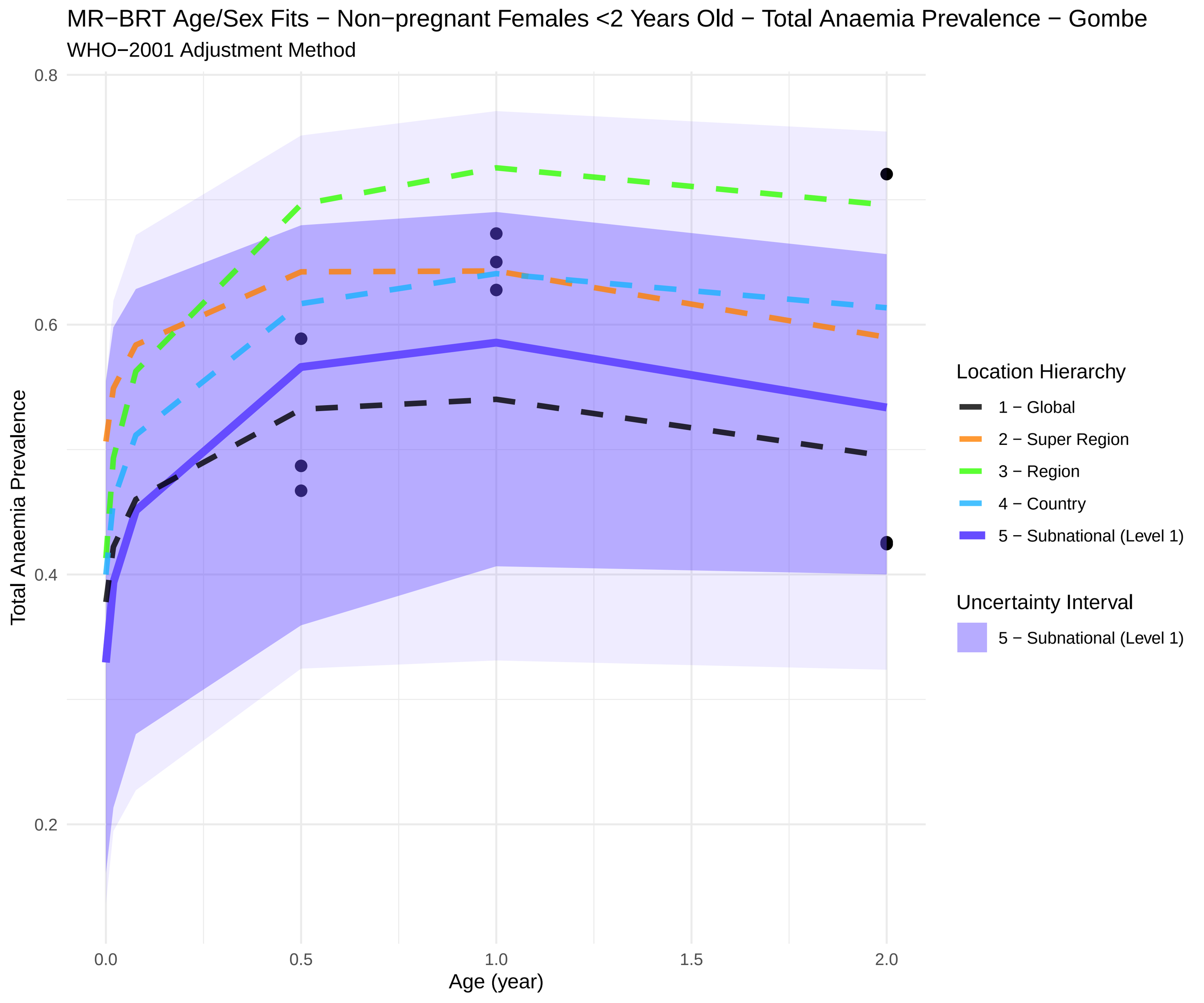
MR−BRT Age/Sex Fits − Non−pregnant Females <2 Years Old − Total Anaemia Prevalence − Gombe
WHO−2001 Adjustment Method
Total Anaemia Prevalence
Age (year)
Location Hierarchy
1 − Global
2 − Super Region
3 − Region
4 − Country
5 − Subnational (Level 1)
Uncertainty Interval
5 − Subnational (Level 1)

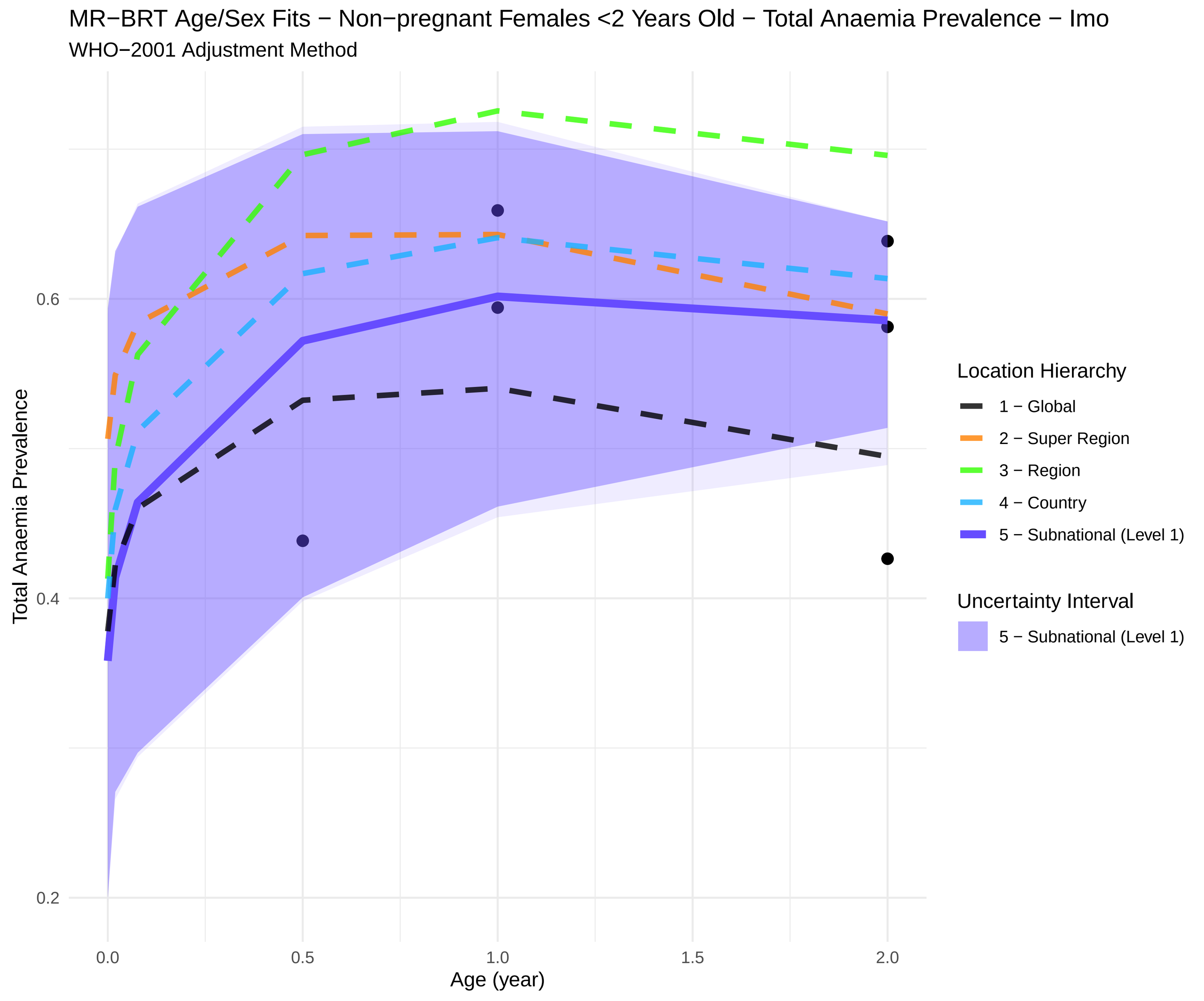
MR−BRT Age/Sex Fits − Non−pregnant Females <2 Years Old − Total Anaemia Prevalence − Imo
WHO−2001 Adjustment Method
Total Anaemia Prevalence
Age (year)
Location Hierarchy
1 − Global
2 − Super Region
3 − Region
4 − Country
5 − Subnational (Level 1)
Uncertainty Interval
5 − Subnational (Level 1)

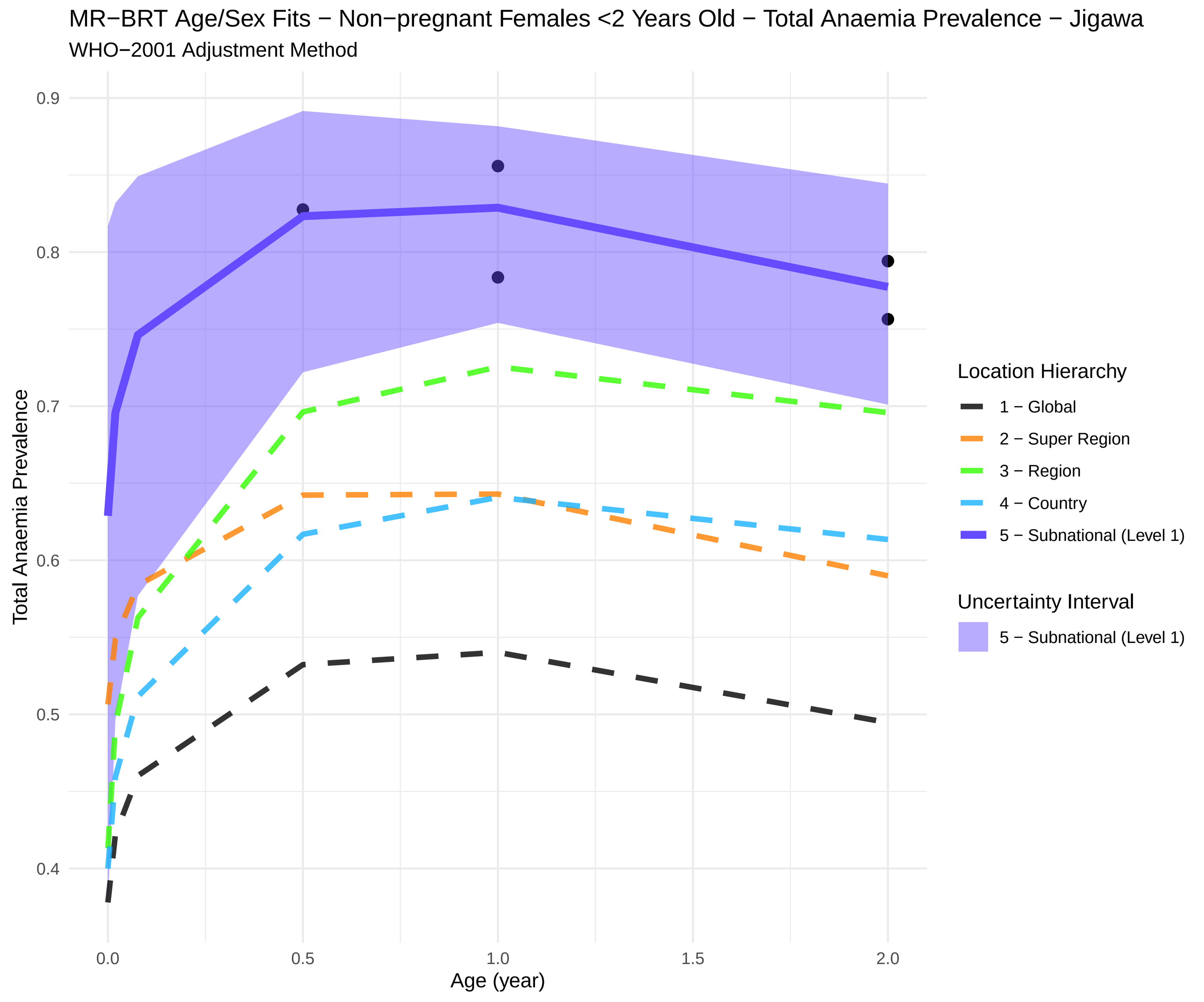
MR−BRT Age/Sex Fits − Non−pregnant Females <2 Years Old − Total Anaemia Prevalence − Jigawa
WHO−2001 Adjustment Method
Total Anaemia Prevalence
Age (year)
Location Hierarchy
1 − Global
2 − Super Region
3 − Region
4 − Country
5 − Subnational (Level 1)
Uncertainty Interval
5 − Subnational (Level 1)

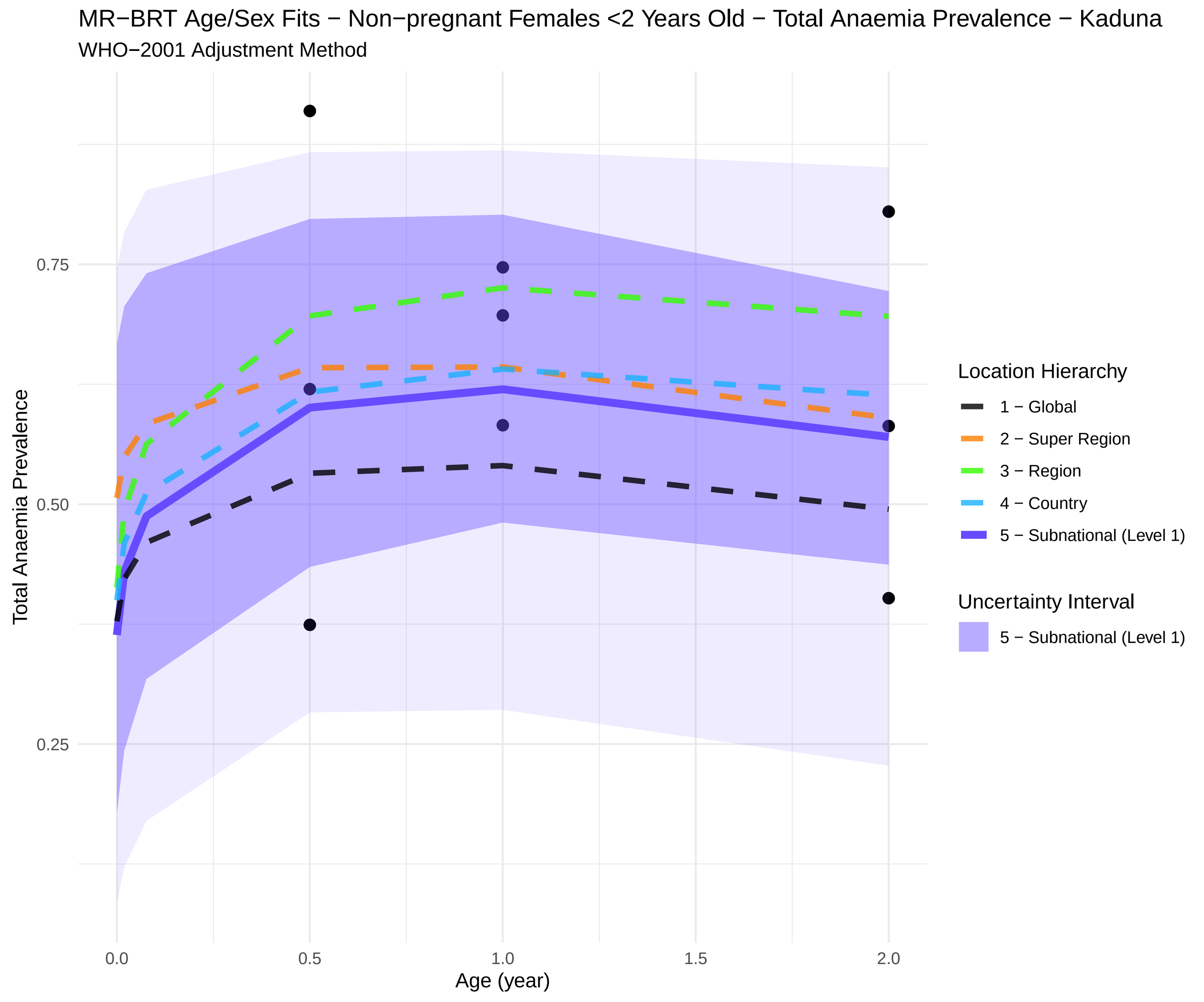
MR−BRT Age/Sex Fits − Non−pregnant Females <2 Years Old − Total Anaemia Prevalence − Kaduna
WHO−2001 Adjustment Method
Total Anaemia Prevalence
Age (year)
Location Hierarchy
1 − Global
2 − Super Region
3 − Region
4 − Country
5 − Subnational (Level 1)
Uncertainty Interval
5 − Subnational (Level 1)

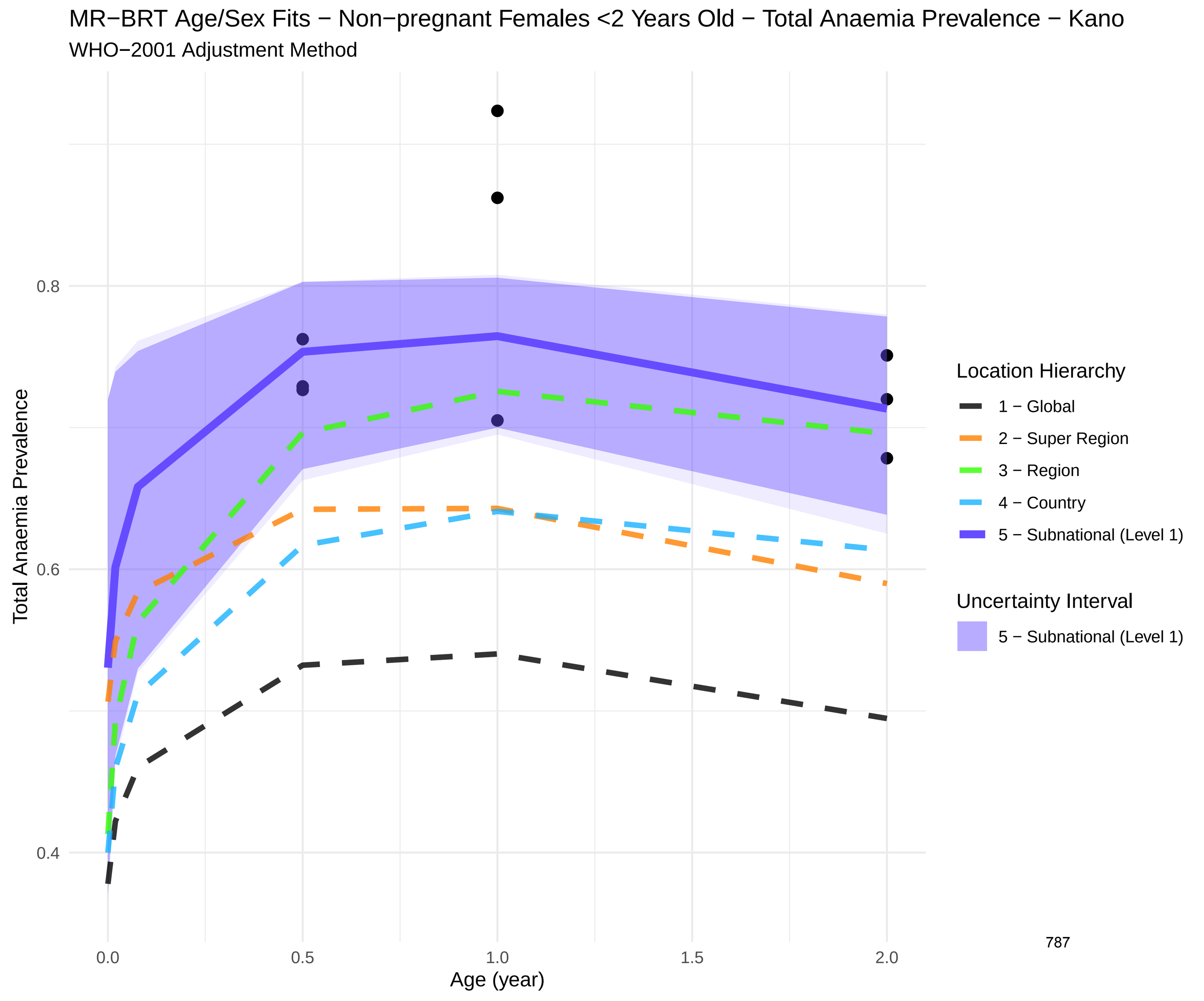
MR−BRT Age/Sex Fits − Non−pregnant Females <2 Years Old − Total Anaemia Prevalence − Kano
WHO−2001 Adjustment Method
Total Anaemia Prevalence
Age (year)
Location Hierarchy
1 − Global
2 − Super Region
3 − Region
4 − Country
5 − Subnational (Level 1)
Uncertainty Interval
5 − Subnational (Level 1)

MR−BRT Age/Sex Fits − Non−pregnant Females <2 Years Old − Total Anaemia Prevalence − Katsina
WHO−2001 Adjustment Method

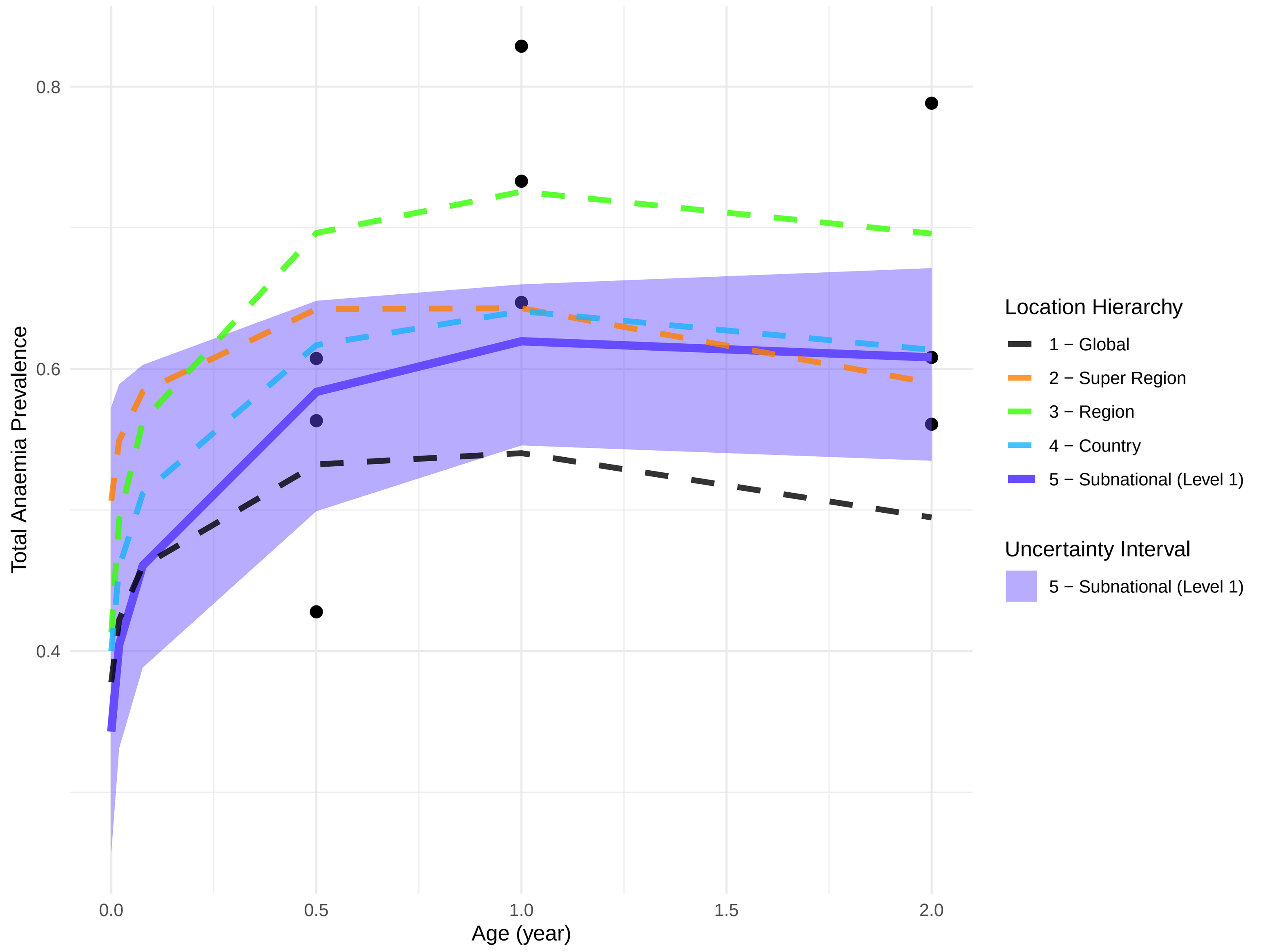

MR−BRT Age/Sex Fits − Non−pregnant Females <2 Years Old − Total Anaemia Prevalence − Kebbi
WHO−2001 Adjustment Method

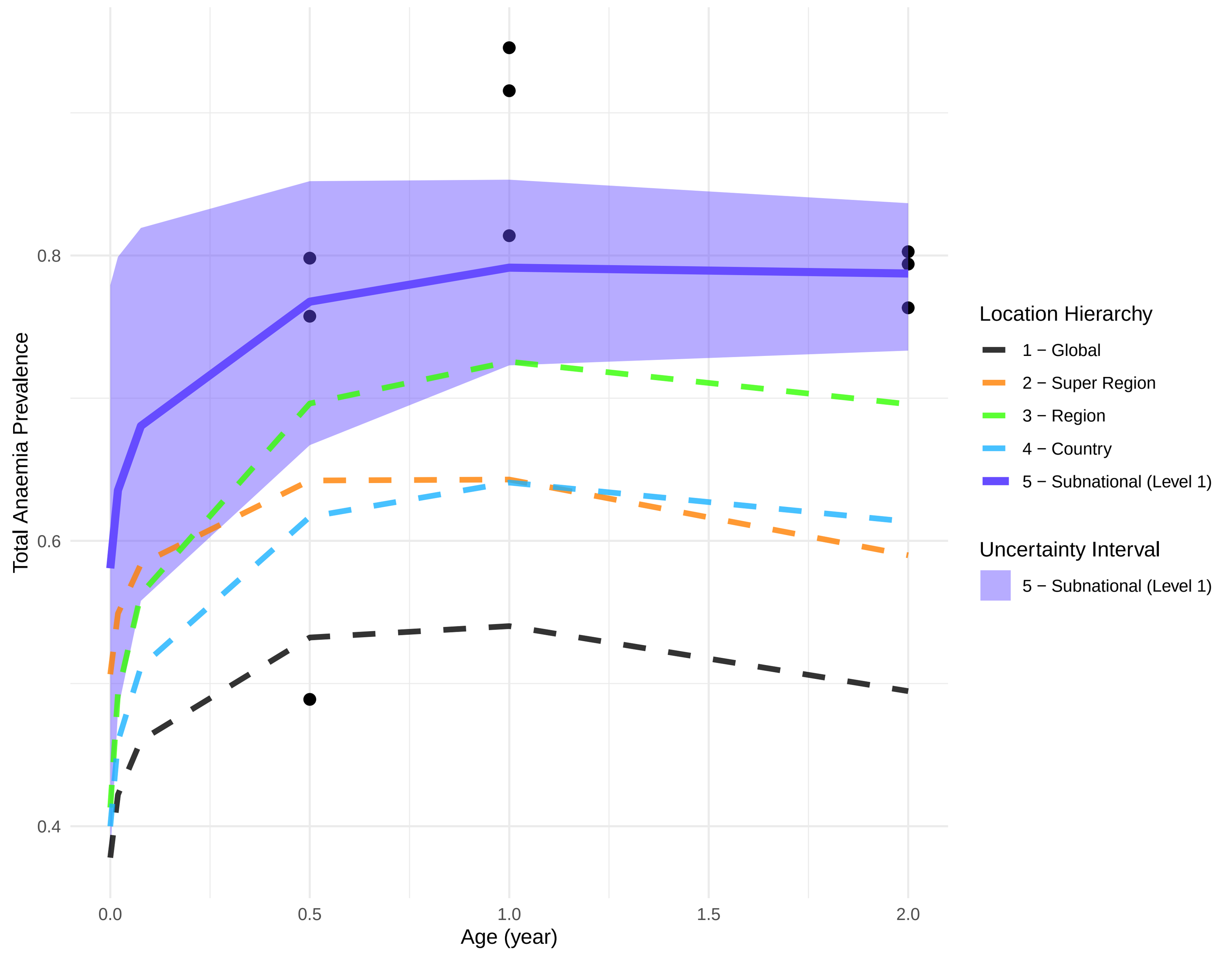

MR−BRT Age/Sex Fits − Non−pregnant Females <2 Years Old − Total Anaemia Prevalence − Kogi
WHO−2001 Adjustment Method

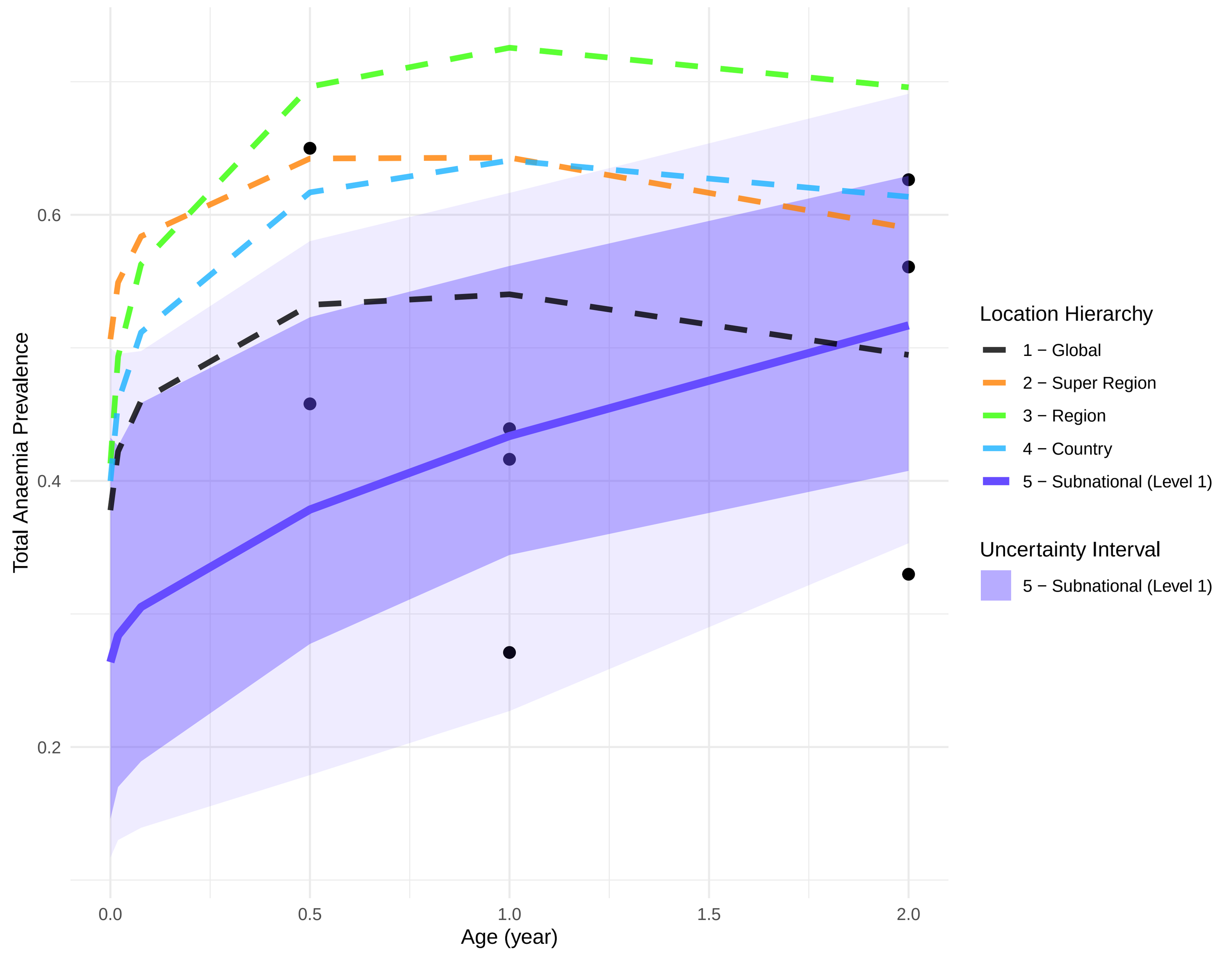

MR−BRT Age/Sex Fits − Non−pregnant Females <2 Years Old − Total Anaemia Prevalence − Kwara
WHO−2001 Adjustment Method

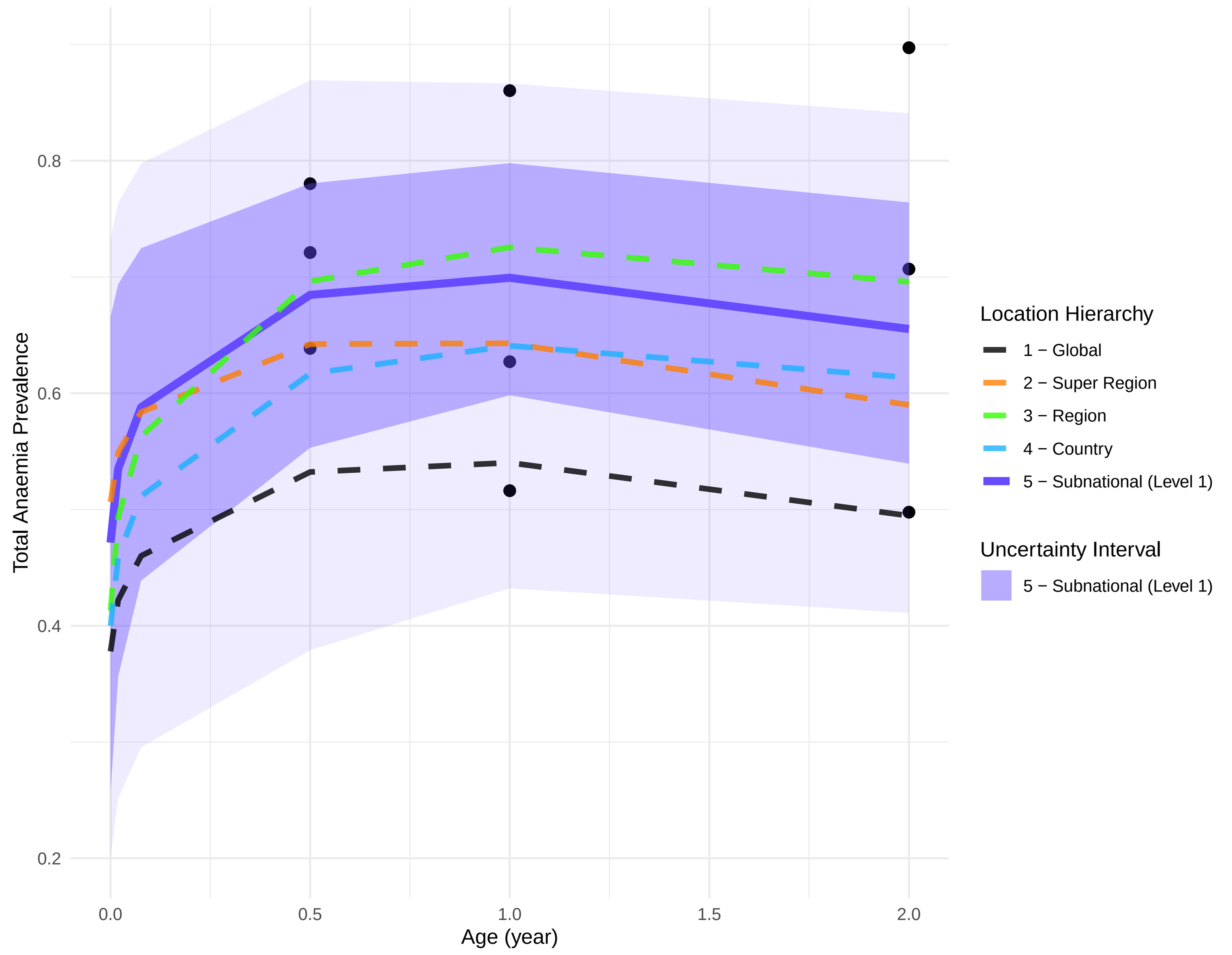

MR−BRT Age/Sex Fits − Non−pregnant Females <2 Years Old − Total Anaemia Prevalence − Lagos
WHO−2001 Adjustment Method

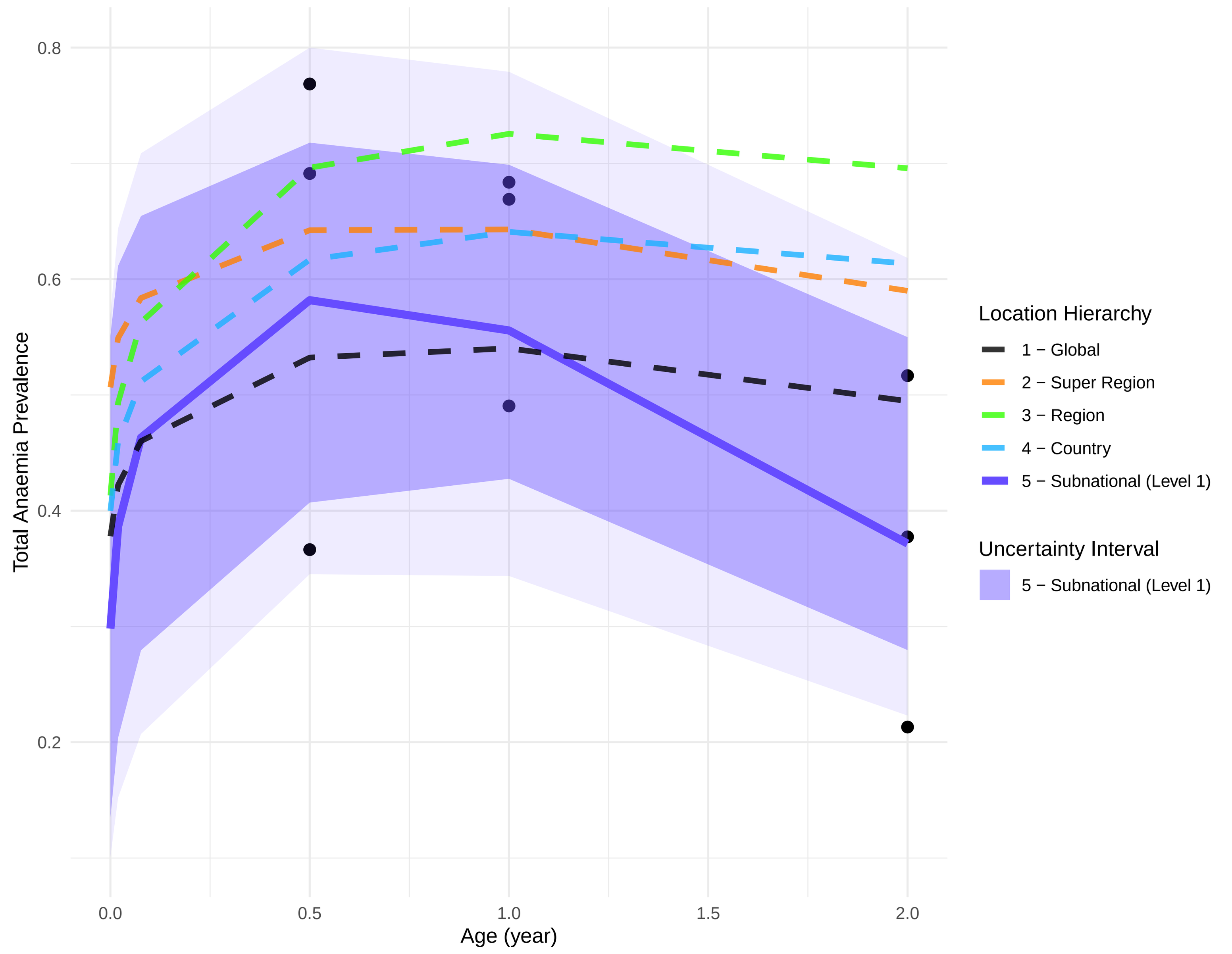

MR−BRT Age/Sex Fits − Non−pregnant Females <2 Years Old − Total Anaemia Prevalence − Nasarawa
WHO−2001 Adjustment Method

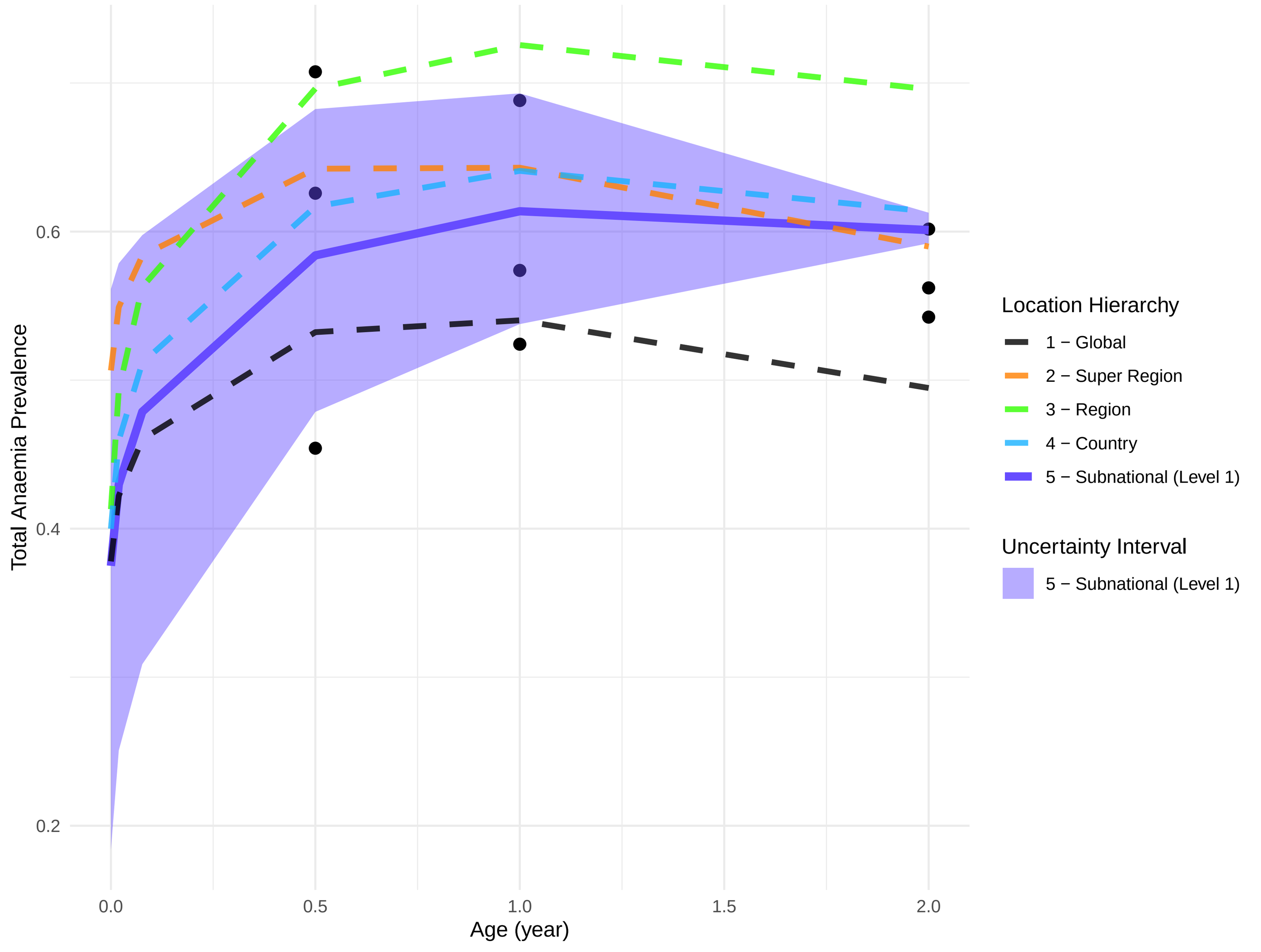

MR−BRT Age/Sex Fits − Non−pregnant Females <2 Years Old − Total Anaemia Prevalence − Niger
WHO−2001 Adjustment Method

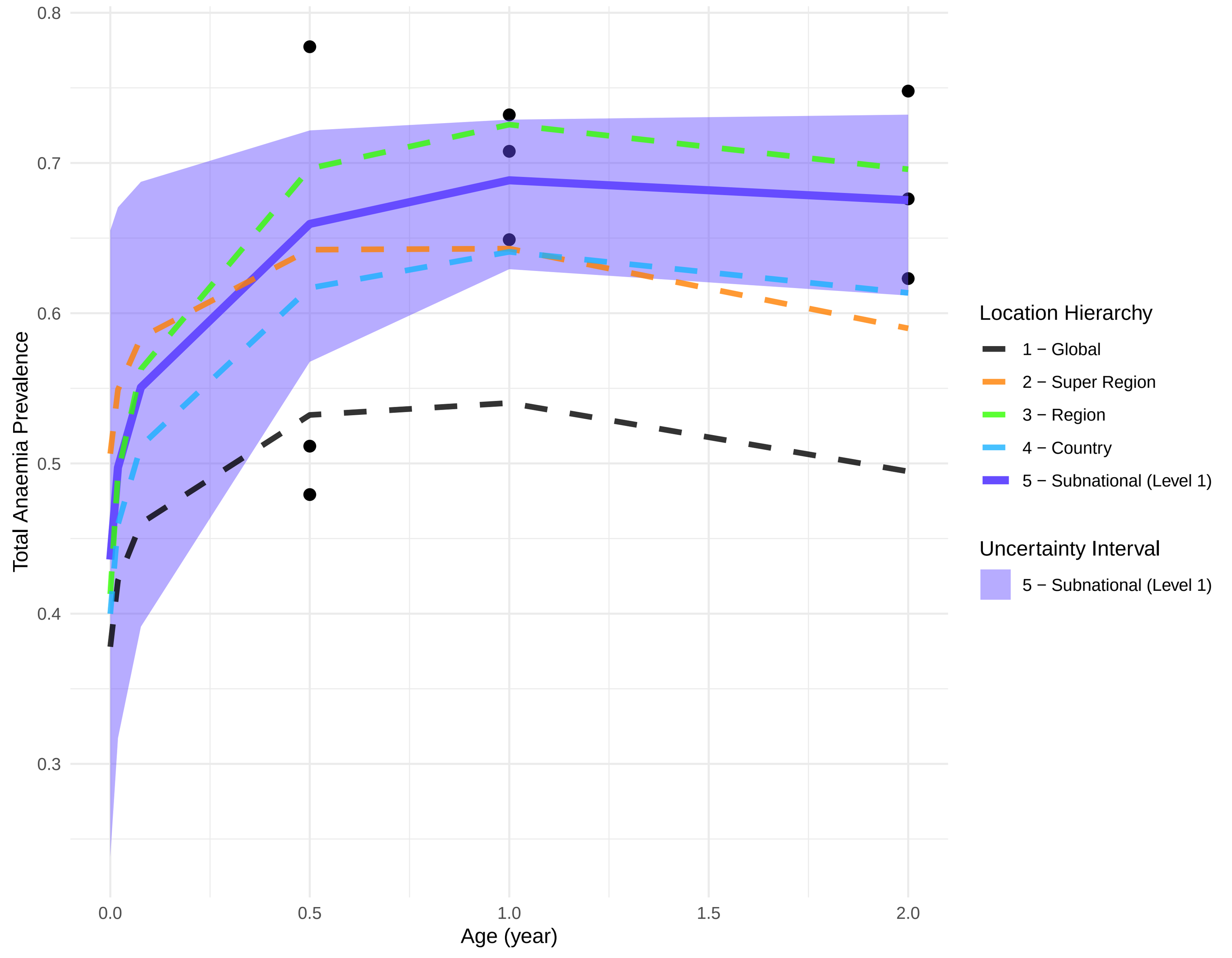

MR−BRT Age/Sex Fits − Non−pregnant Females <2 Years Old − Total Anaemia Prevalence − Ogun
WHO−2001 Adjustment Method

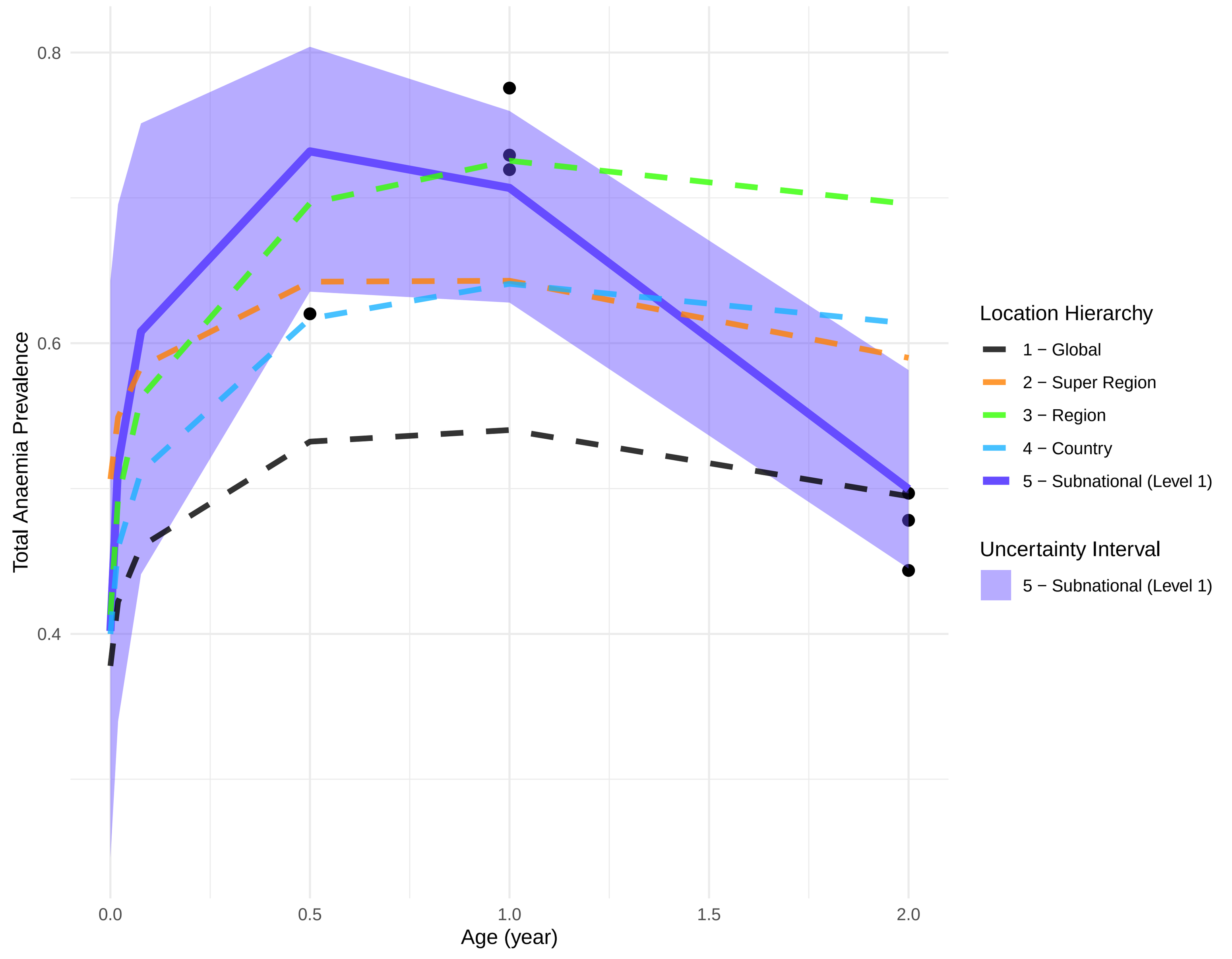

MR−BRT Age/Sex Fits − Non−pregnant Females <2 Years Old − Total Anaemia Prevalence − Ondo
WHO−2001 Adjustment Method

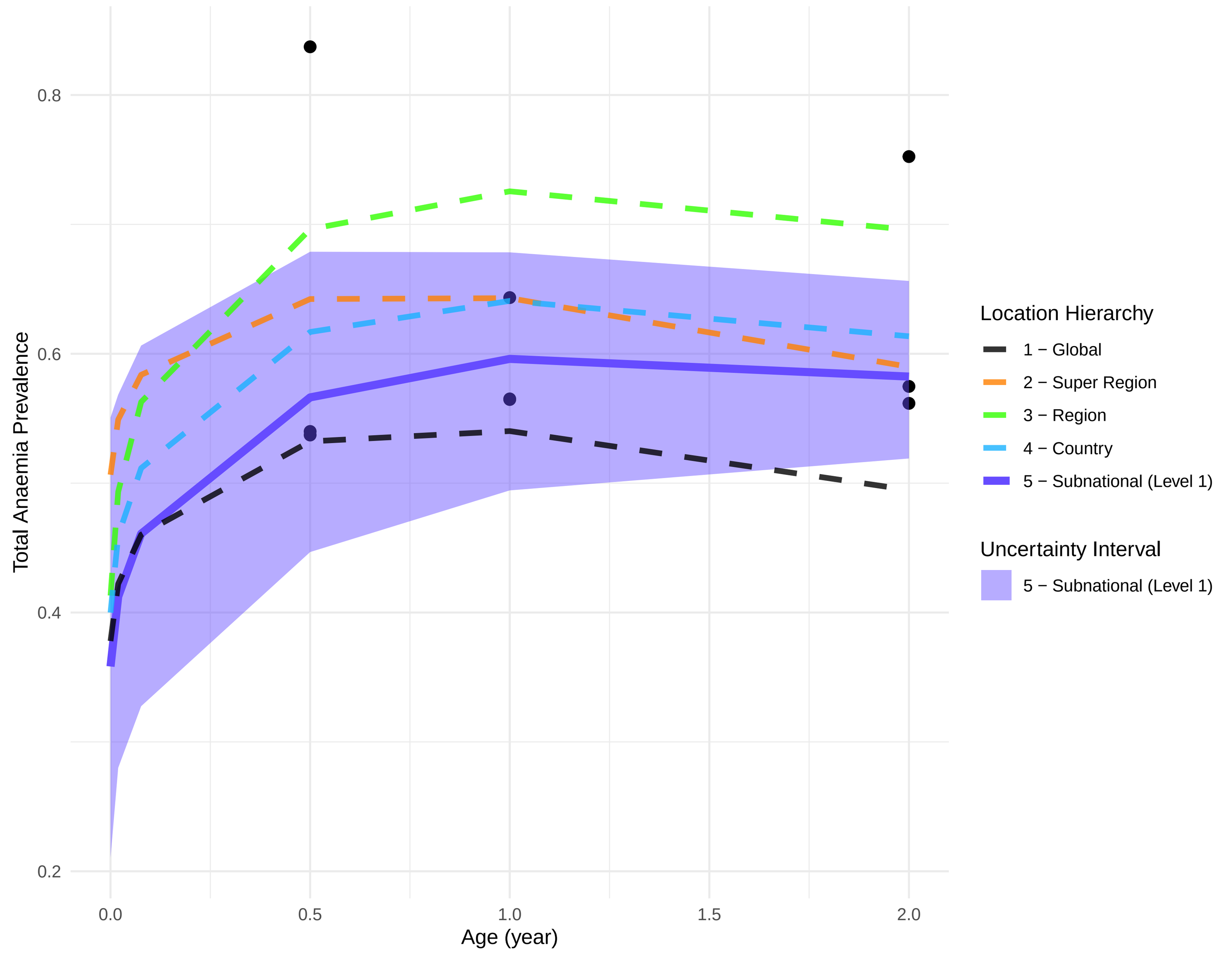

MR−BRT Age/Sex Fits − Non−pregnant Females <2 Years Old − Total Anaemia Prevalence − Osun
WHO−2001 Adjustment Method

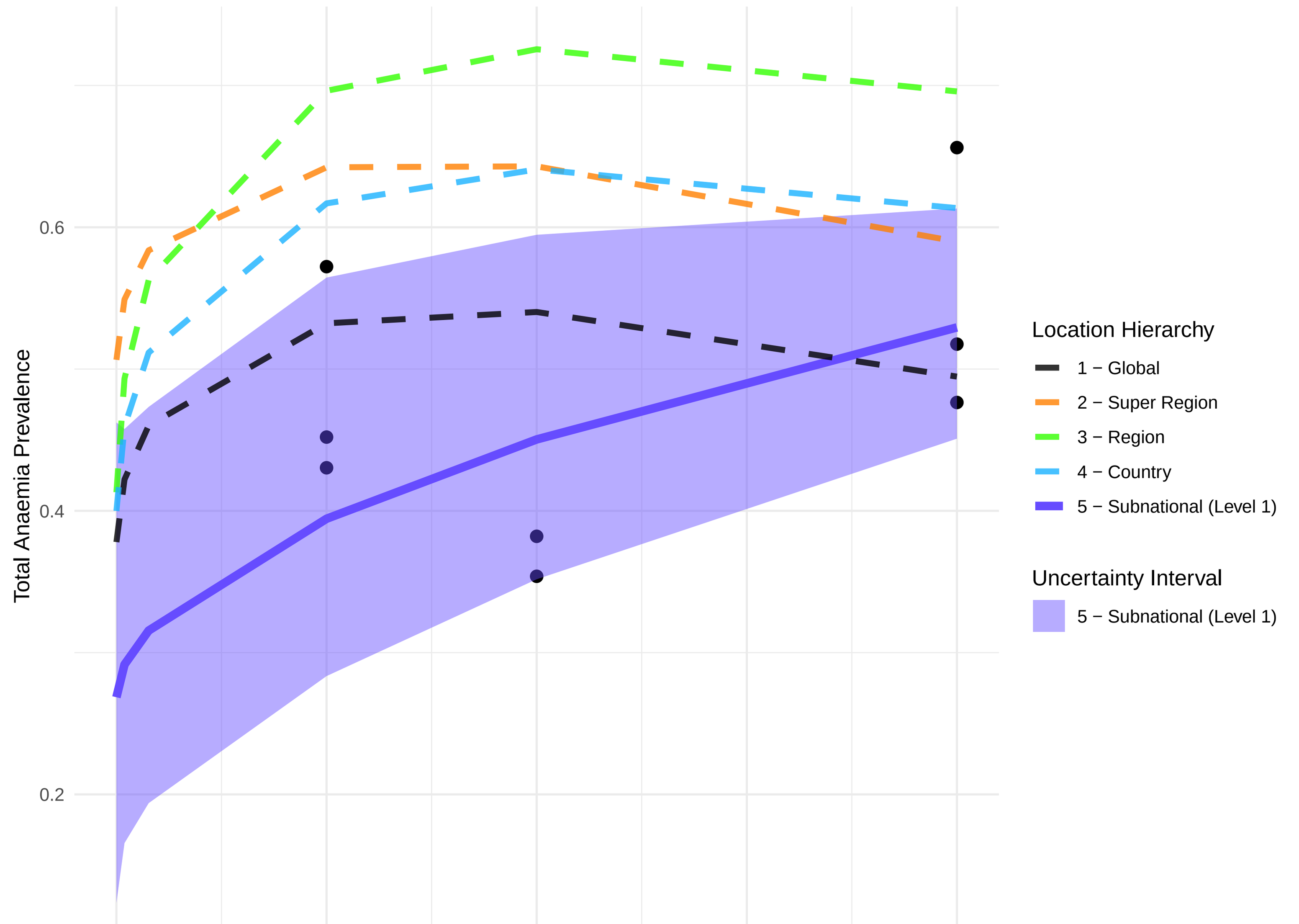

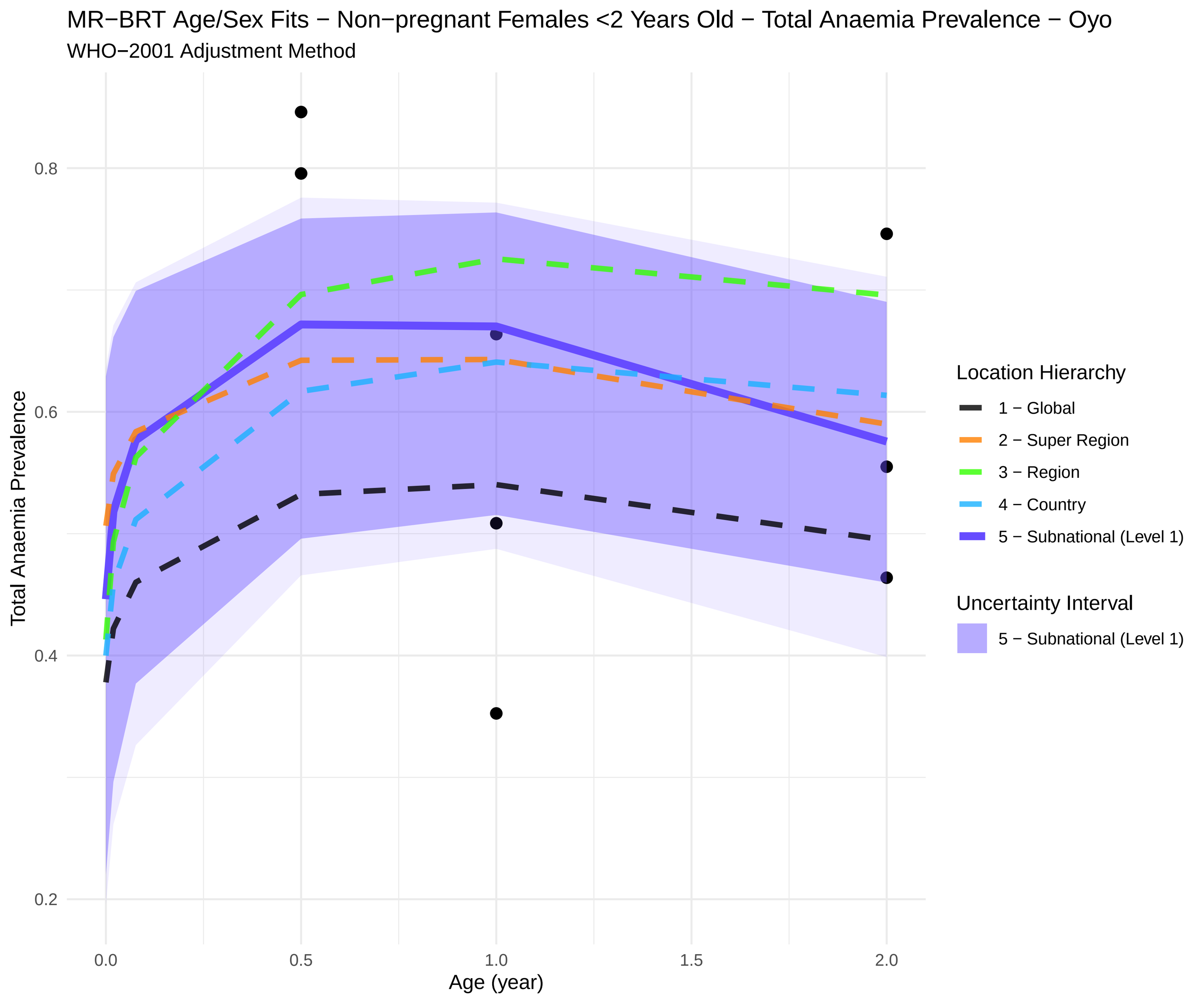
MR−BRT Age/Sex Fits − Non-pregnant Females <2 Years Old − Total Anaemia Prevalence − Oyo
WHO−2001 Adjustment Method
Total Anaemia Prevalence
Age (year)
Location Hierarchy
1 − Global
2 − Super Region
3 − Region
4 − Country
5 − Subnational (Level 1)
Uncertainty Interval
5 − Subnational (Level 1)

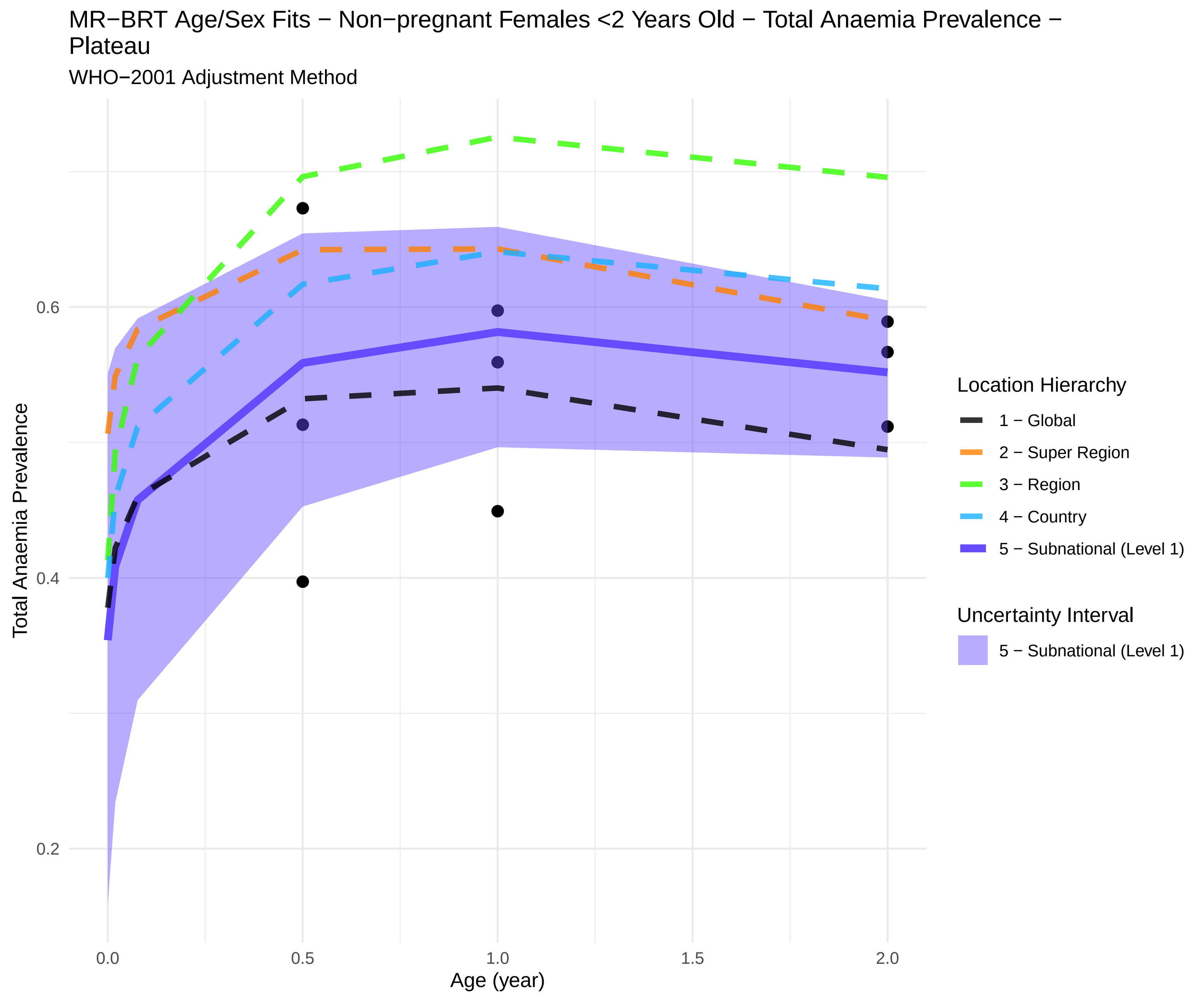
MR−BRT Age/Sex Fits − Non-pregnant Females <2 Years Old − Total Anaemia Prevalence − Plateau
WHO−2001 Adjustment Method
Total Anaemia Prevalence
Age (year)
Location Hierarchy
1 − Global
2 − Super Region
3 − Region
4 − Country
5 − Subnational (Level 1)
Uncertainty Interval
5 − Subnational (Level 1)

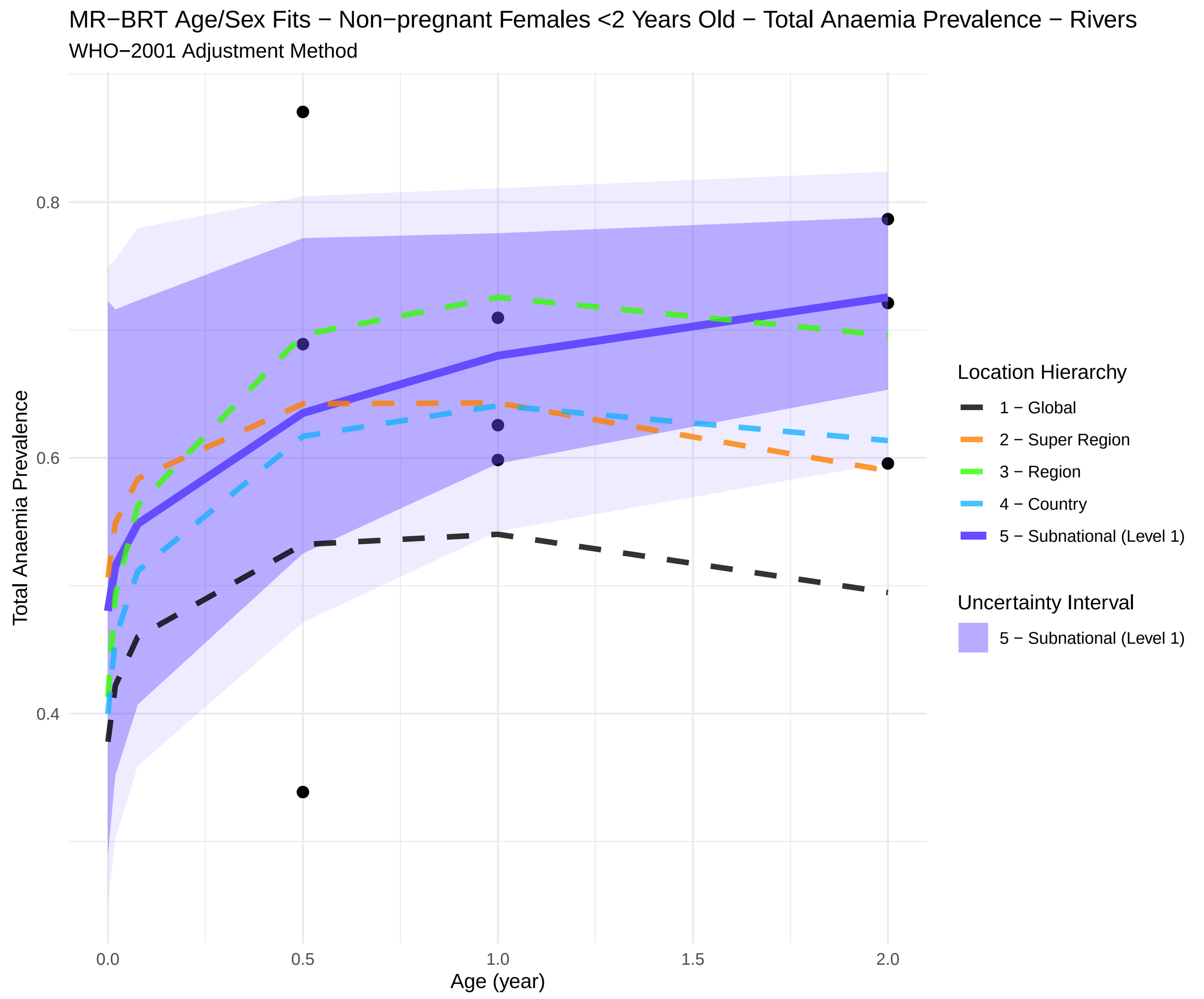
MR−BRT Age/Sex Fits − Non-pregnant Females <2 Years Old − Total Anaemia Prevalence − Rivers
WHO−2001 Adjustment Method
Total Anaemia Prevalence
Age (year)
Location Hierarchy
1 − Global
2 − Super Region
3 − Region
4 − Country
5 − Subnational (Level 1)
Uncertainty Interval
5 − Subnational (Level 1)

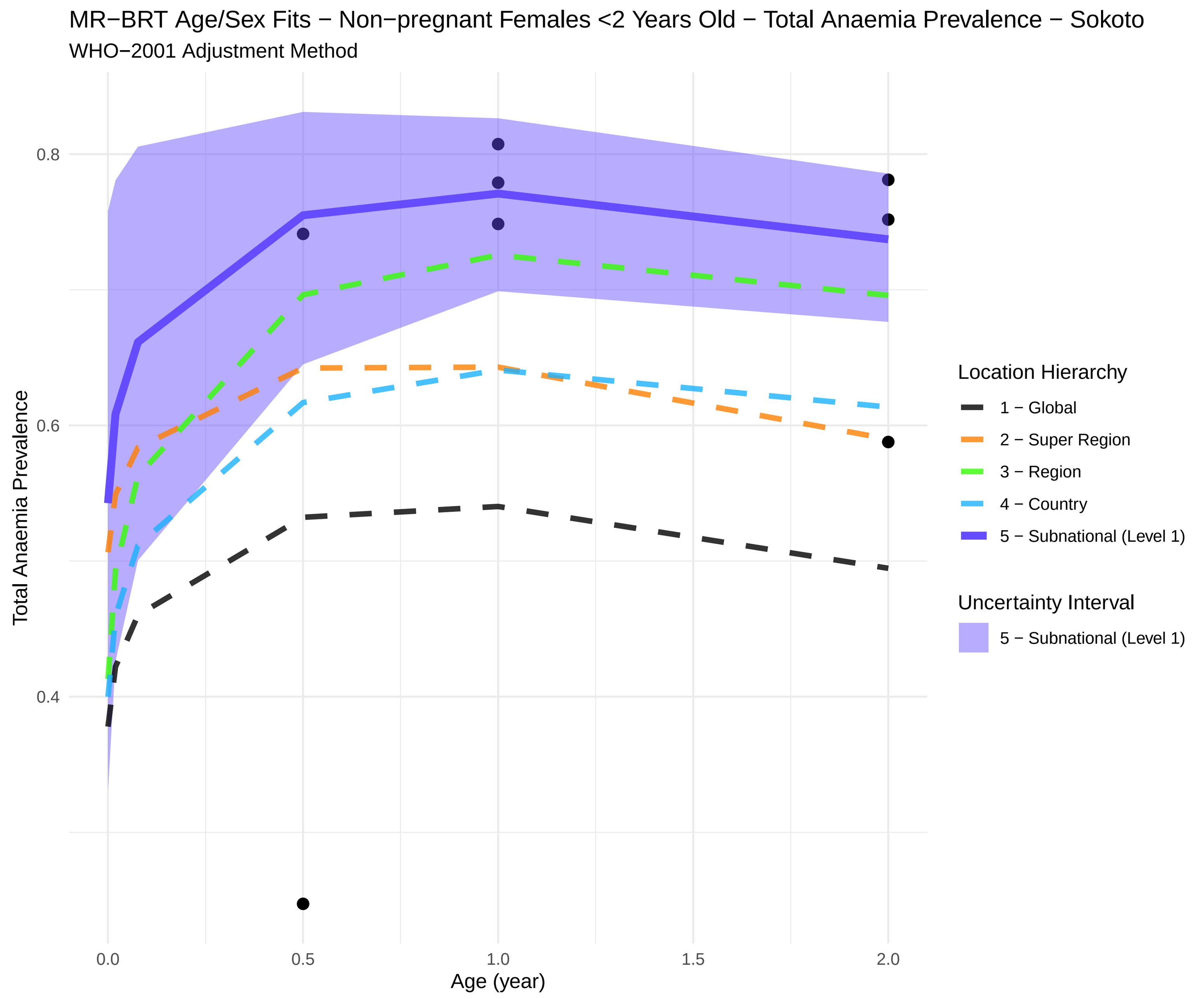
MR−BRT Age/Sex Fits − Non-pregnant Females <2 Years Old − Total Anaemia Prevalence − Sokoto
WHO−2001 Adjustment Method
Total Anaemia Prevalence
Age (year)
Location Hierarchy
1 − Global
2 − Super Region
3 − Region
4 − Country
5 − Subnational (Level 1)
Uncertainty Interval
5 − Subnational (Level 1)

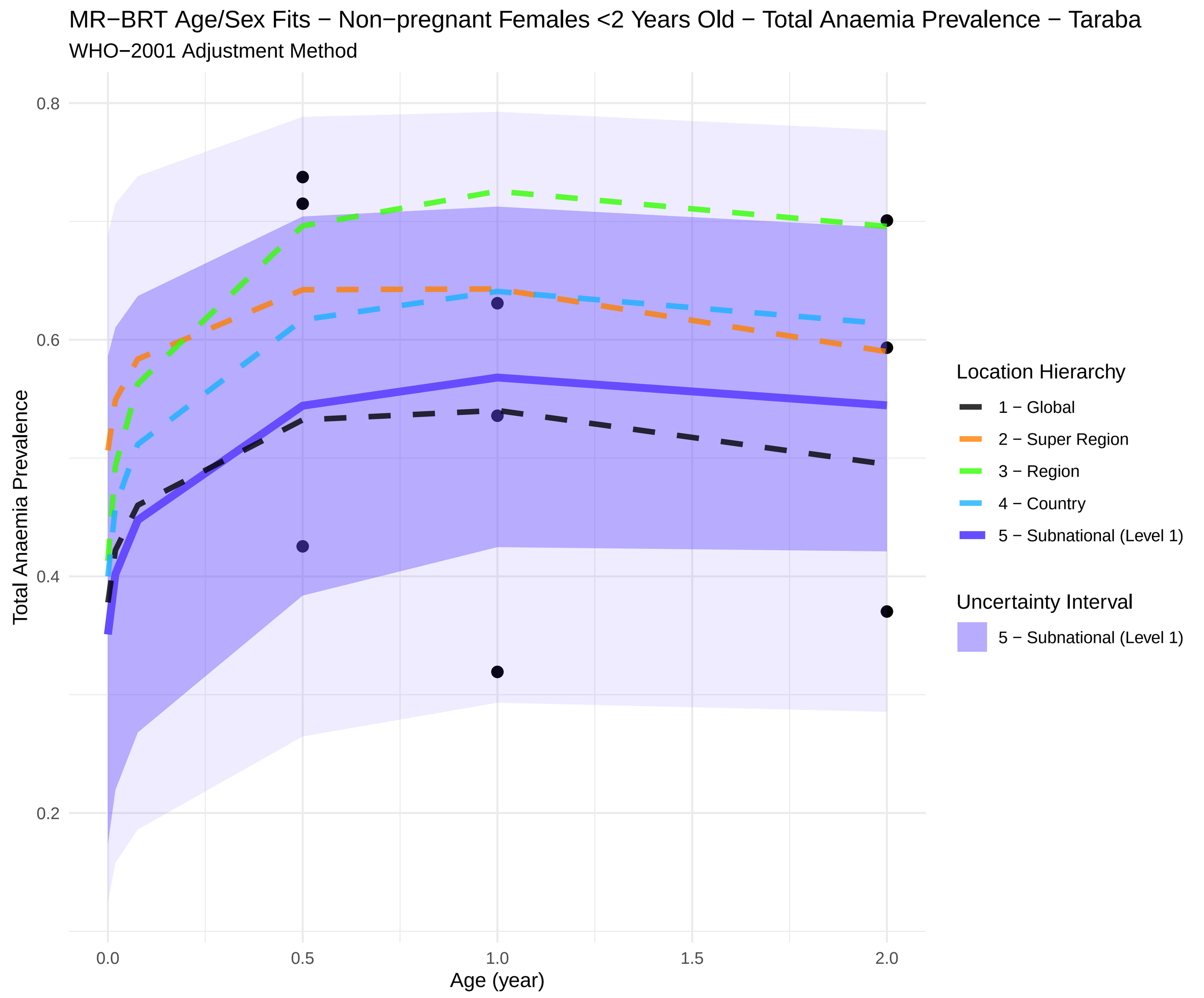
MR−BRT Age/Sex Fits − Non-pregnant Females <2 Years Old − Total Anaemia Prevalence − Taraba
WHO−2001 Adjustment Method
Total Anaemia Prevalence
Age (year)
Location Hierarchy
1 − Global
2 − Super Region
3 − Region
4 − Country
5 − Subnational (Level 1)
Uncertainty Interval
5 − Subnational (Level 1)

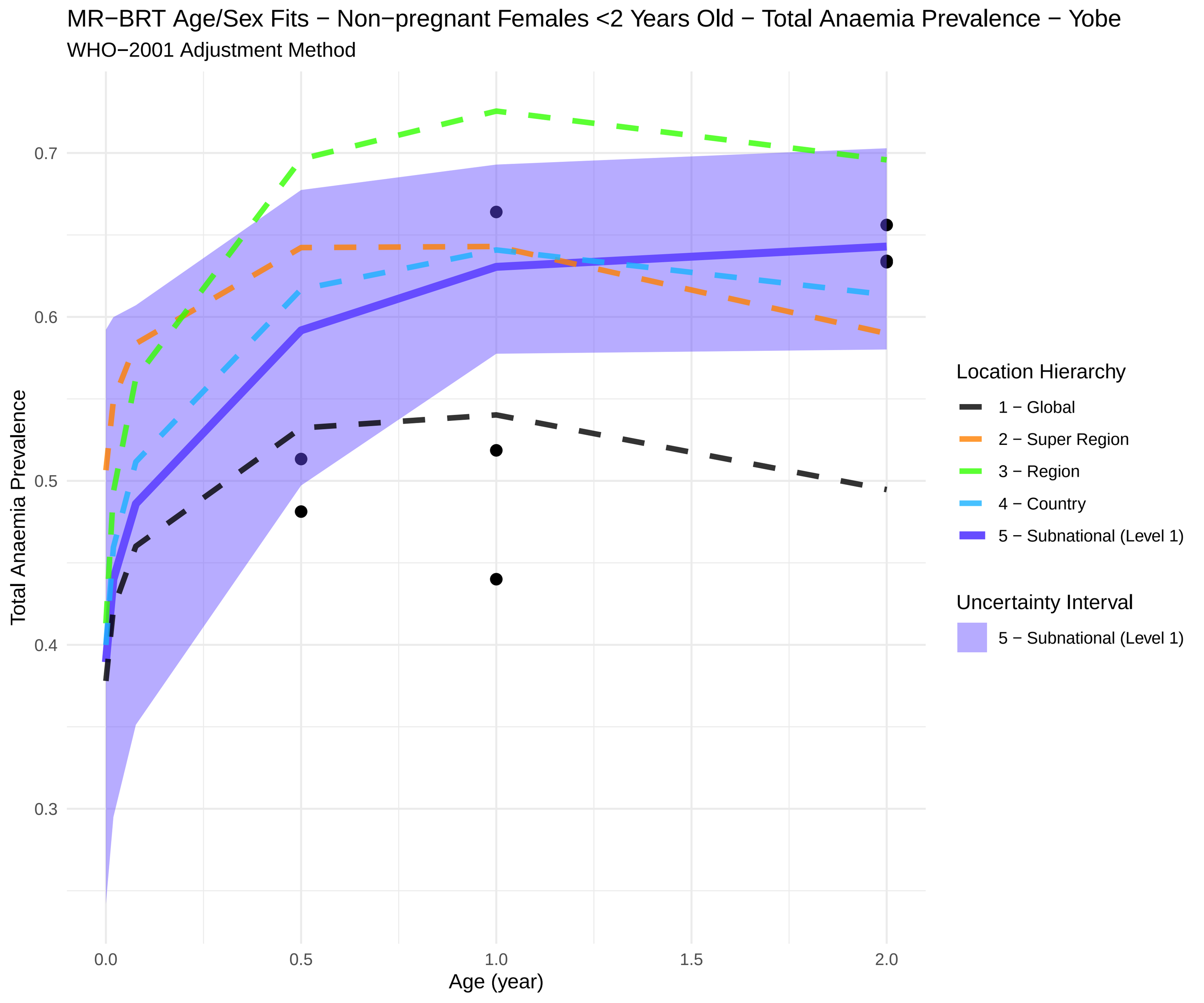
MR−BRT Age/Sex Fits − Non-pregnant Females <2 Years Old − Total Anaemia Prevalence − Yobe
WHO−2001 Adjustment Method
Total Anaemia Prevalence
Age (year)
Location Hierarchy
1 − Global
2 − Super Region
3 − Region
4 − Country
5 − Subnational (Level 1)
Uncertainty Interval
5 − Subnational (Level 1)

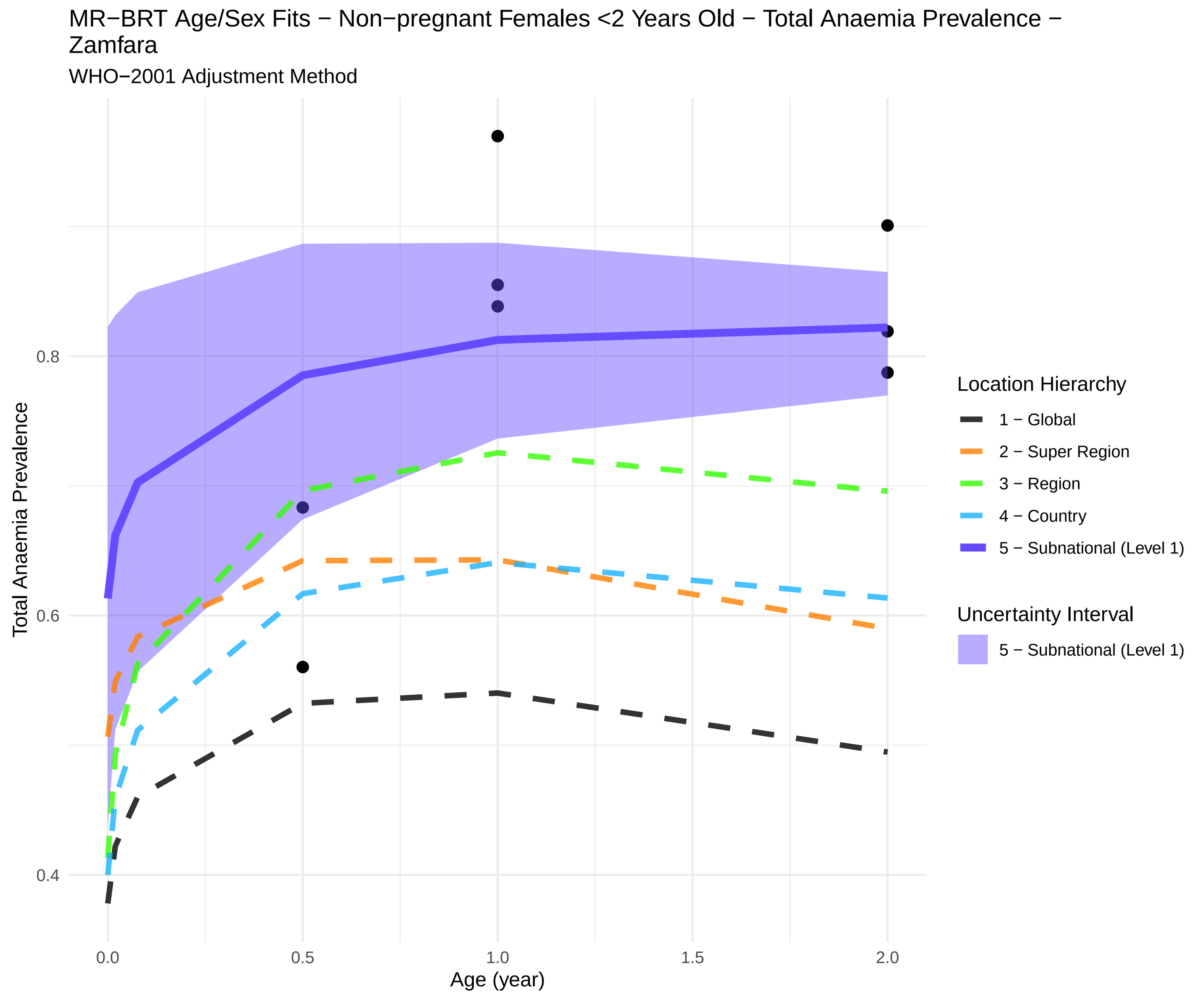
MR−BRT Age/Sex Fits − Non-pregnant Females <2 Years Old − Total Anaemia Prevalence − Zamfara
WHO−2001 Adjustment Method
Total Anaemia Prevalence
Age (year)
Location Hierarchy
1 − Global
2 − Super Region
3 − Region
4 − Country
5 − Subnational (Level 1)
Uncertainty Interval
5 − Subnational (Level 1)

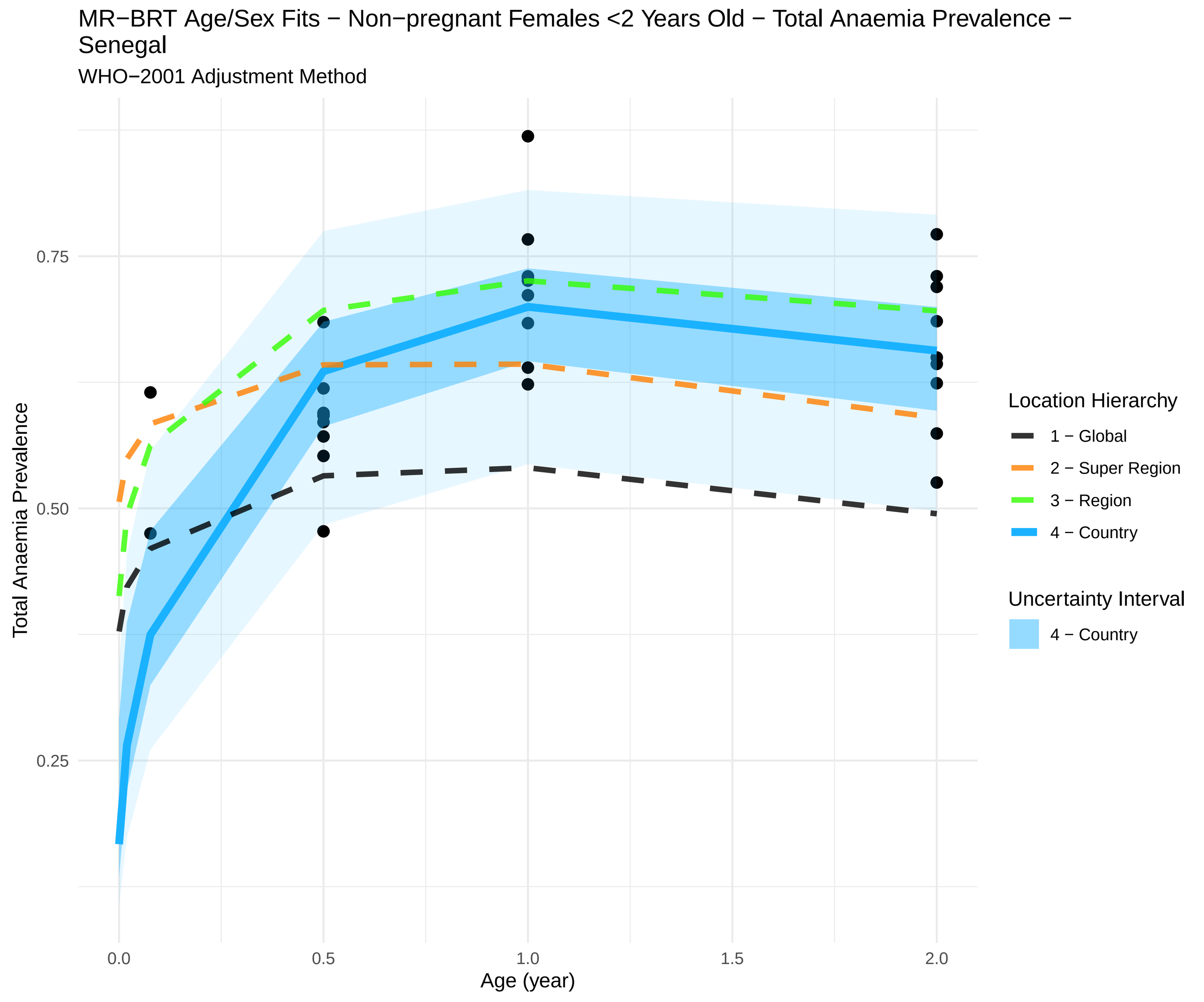
MR−BRT Age/Sex Fits − Non-pregnant Females <2 Years Old − Total Anaemia Prevalence − Senegal
WHO−2001 Adjustment Method
Total Anaemia Prevalence
Age (year)
Location Hierarchy
1 − Global
2 − Super Region
3 − Region
4 − Country
Uncertainty Interval
4 − Country

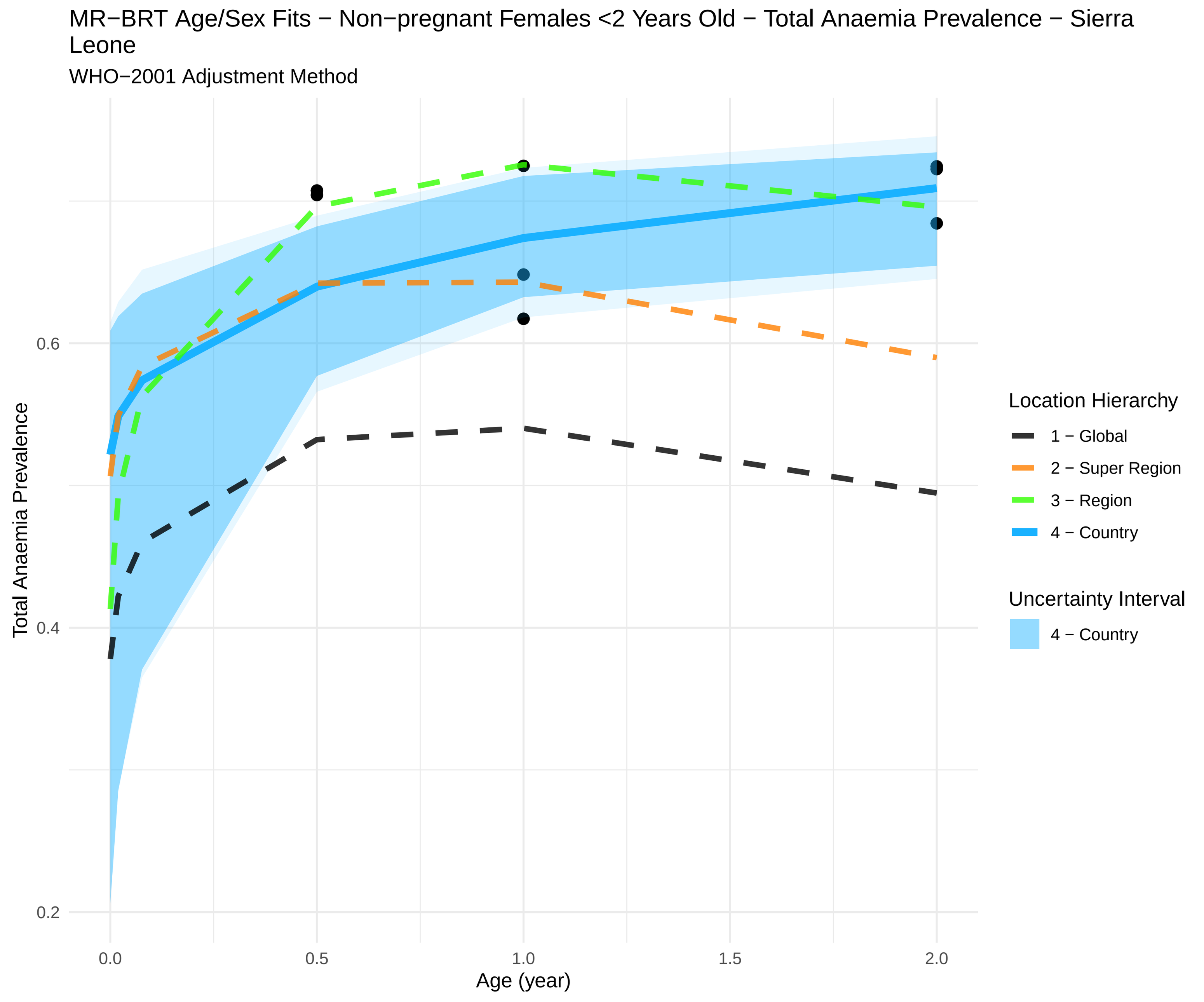
MR−BRT Age/Sex Fits − Non-pregnant Females <2 Years Old − Total Anaemia Prevalence − Sierra Leone
WHO−2001 Adjustment Method
Total Anaemia Prevalence
Age (year)
Location Hierarchy
1 − Global
2 − Super Region
3 − Region
4 − Country
Uncertainty Interval
4 − Country

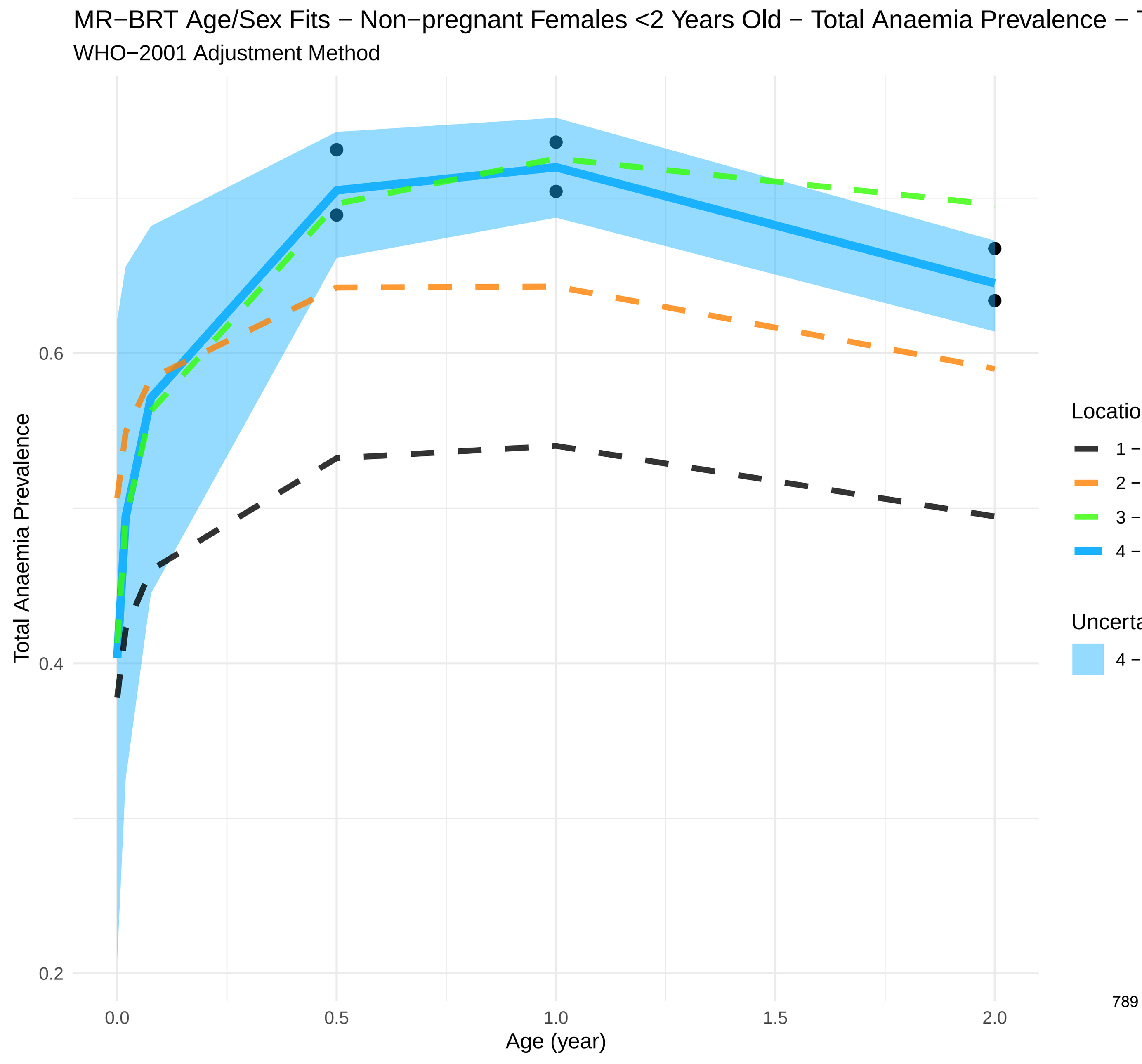
MR−BRT Age/Sex Fits − Non-pregnant Females <2 Years Old − Total Anaemia Prevalence − Togo
WHO−2001 Adjustment Method
Total Anaemia Prevalence
Age (year)
Location Hierarchy
1 − Global
2 − Super Region
3 − Region
4 − Country
Uncertainty Interval
4 − Country

MR−BRT Age/Sex Fits − Males <2 Years Old − Moderate+Severe Anaemia Prevalence − Global
WHO−2024 Adjustment Method

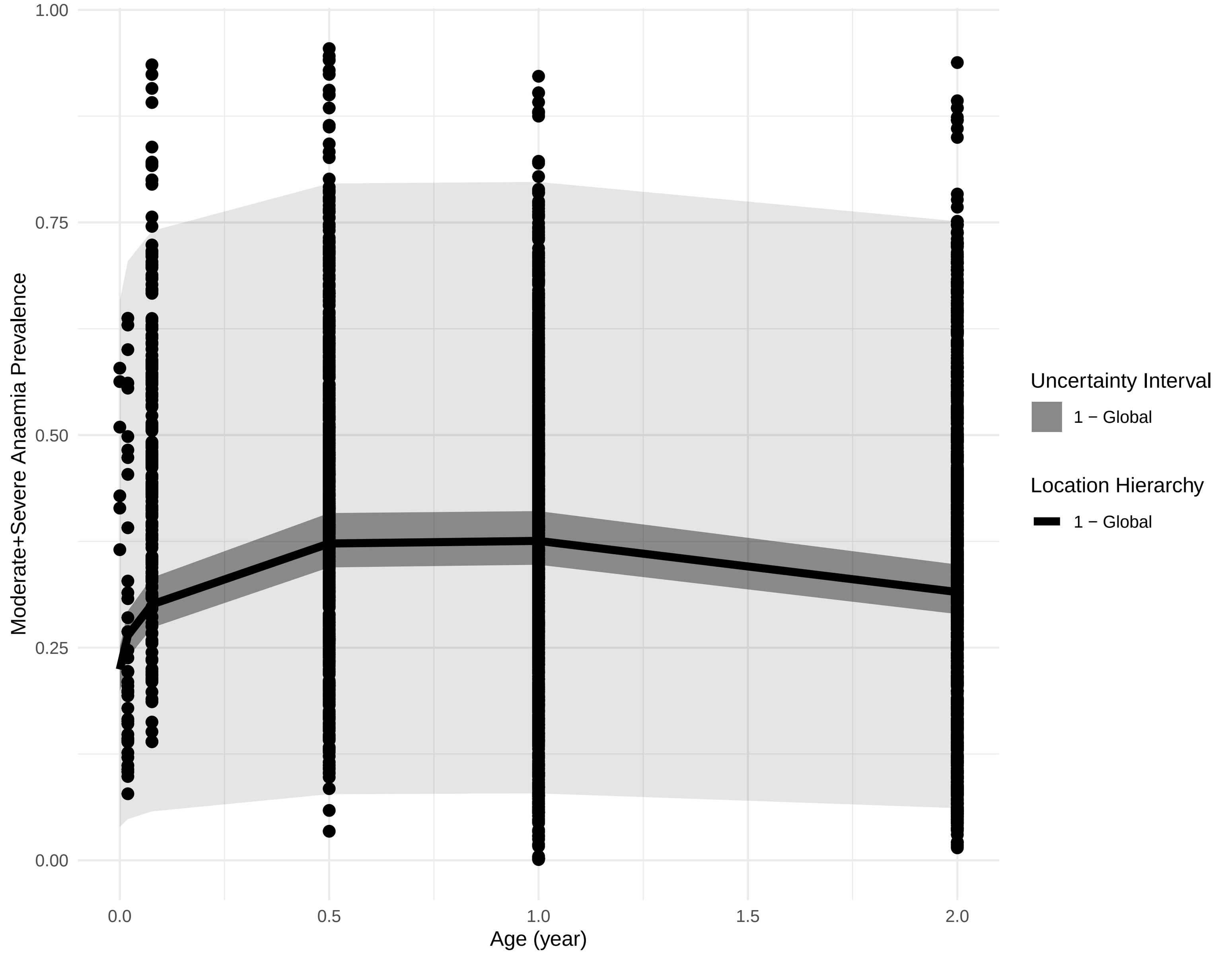

MR−BRT Age/Sex Fits − Males <2 Years Old − Moderate+Severe Anaemia Prevalence − Central Europe, Eastern Europe, and Central Asia
WHO−2024 Adjustment Method

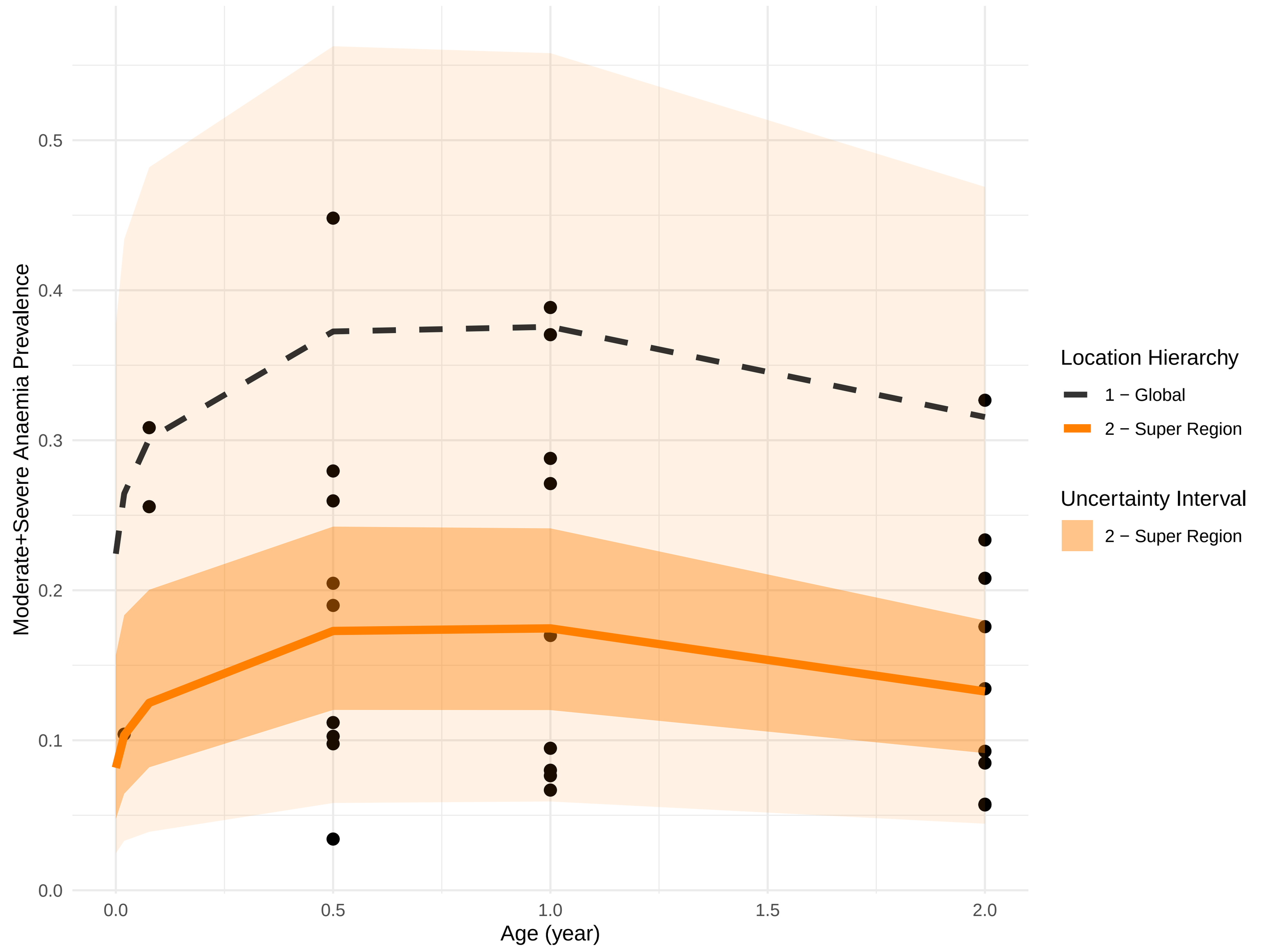

MR−BRT Age/Sex Fits − Males <2 Years Old − Moderate+Severe Anaemia Prevalence − Central Asia
WHO−2024 Adjustment Method

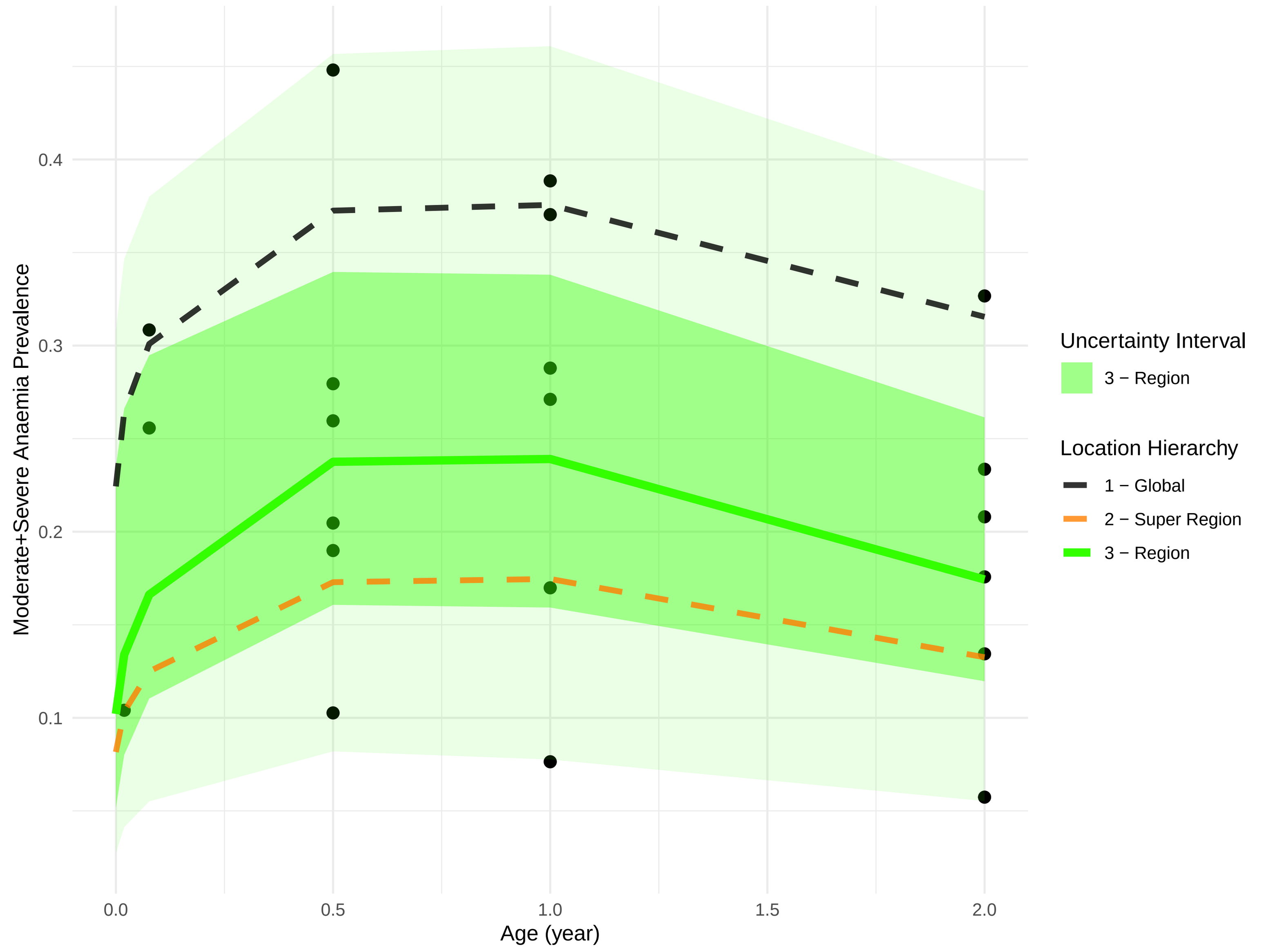

MR−BRT Age/Sex Fits − Males <2 Years Old − Moderate+Severe Anaemia Prevalence − Armenia
WHO−2024 Adjustment Method

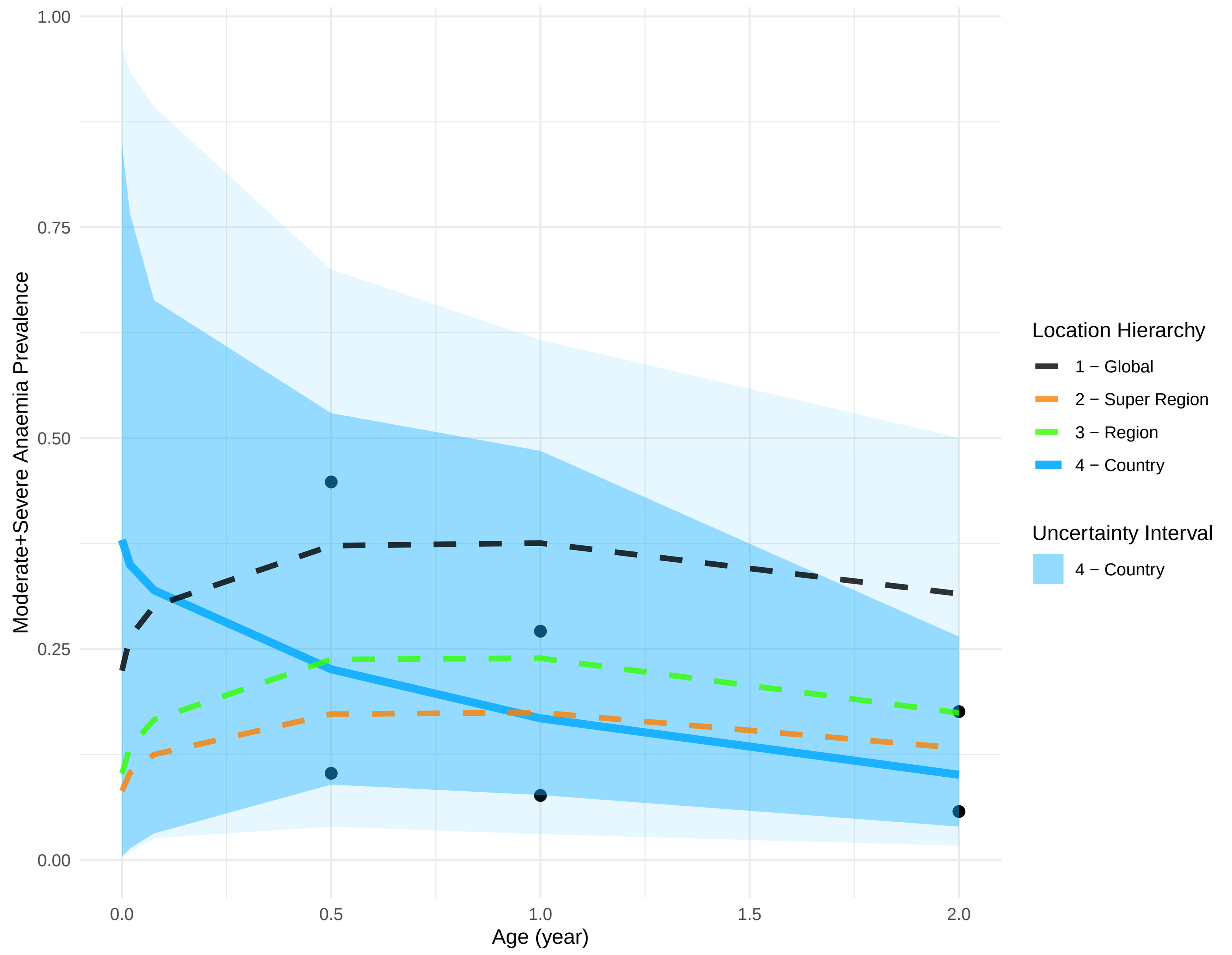

MR−BRT Age/Sex Fits − Males <2 Years Old − Moderate+Severe Anaemia Prevalence − Azerbaijan
WHO−2024 Adjustment Method

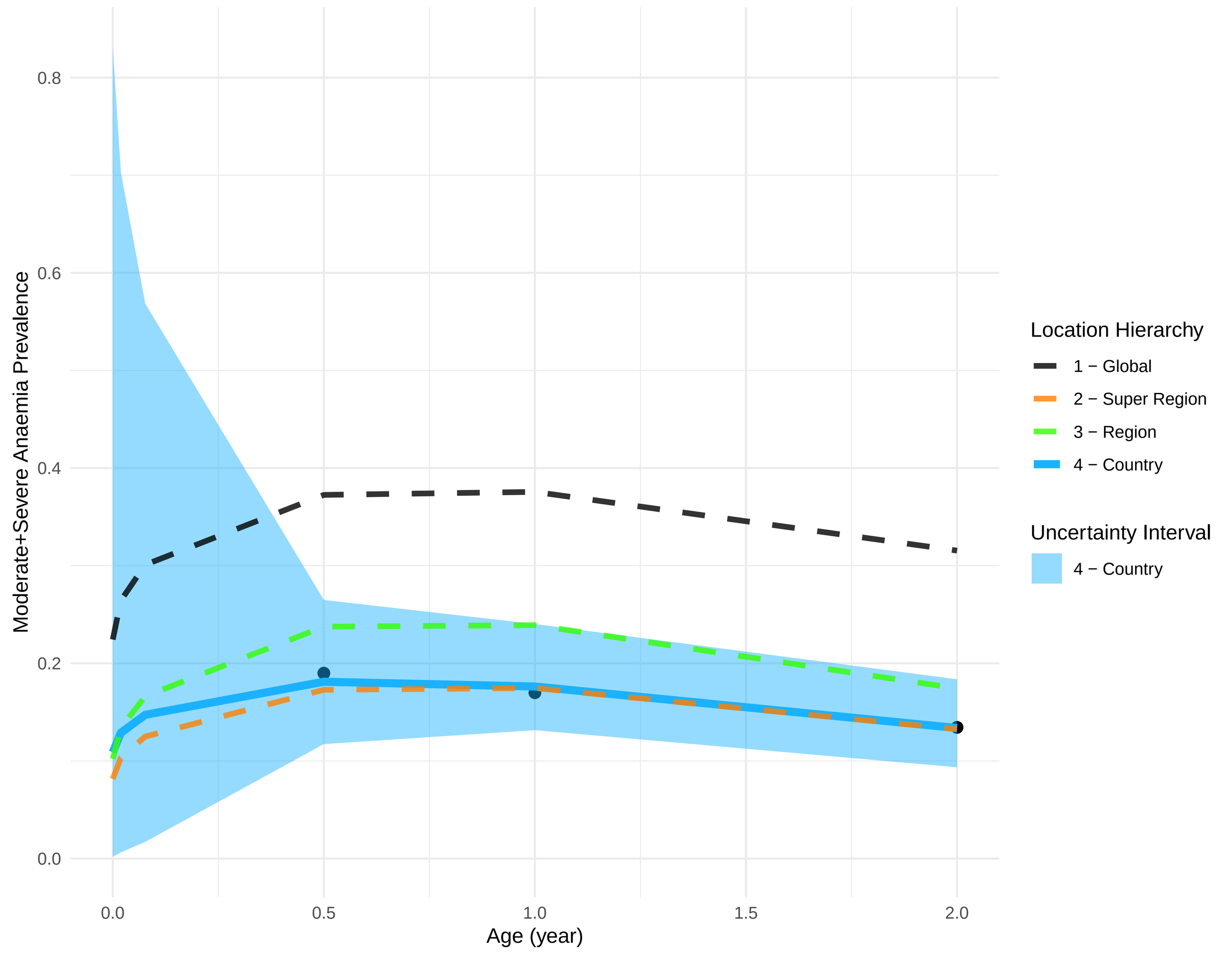

MR−BRT Age/Sex Fits − Males <2 Years Old − Moderate+Severe Anaemia Prevalence − Kyrgyzstan
WHO−2024 Adjustment Method

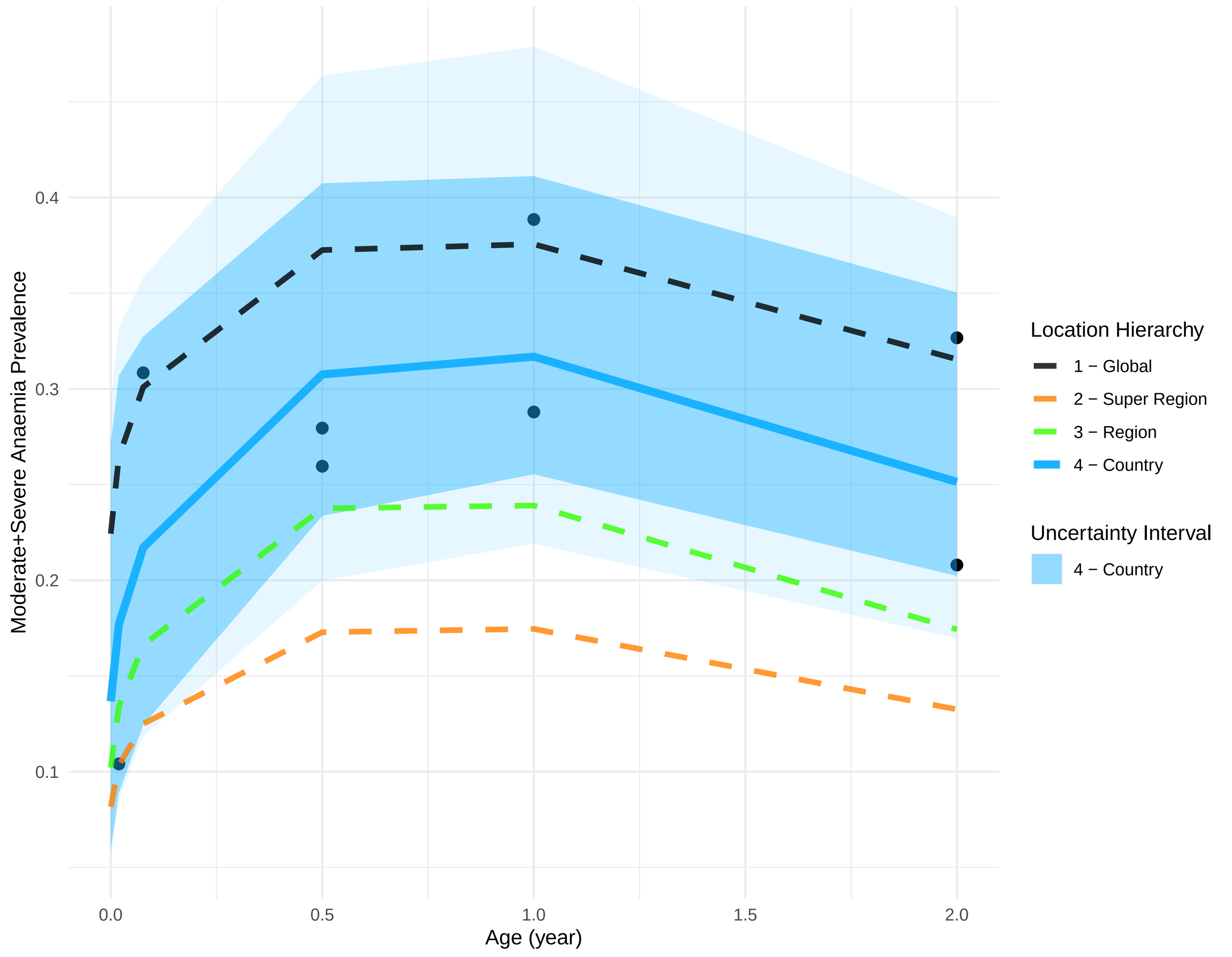

MR−BRT Age/Sex Fits − Males <2 Years Old − Moderate+Severe Anaemia Prevalence − Uzbekistan
WHO−2024 Adjustment Method

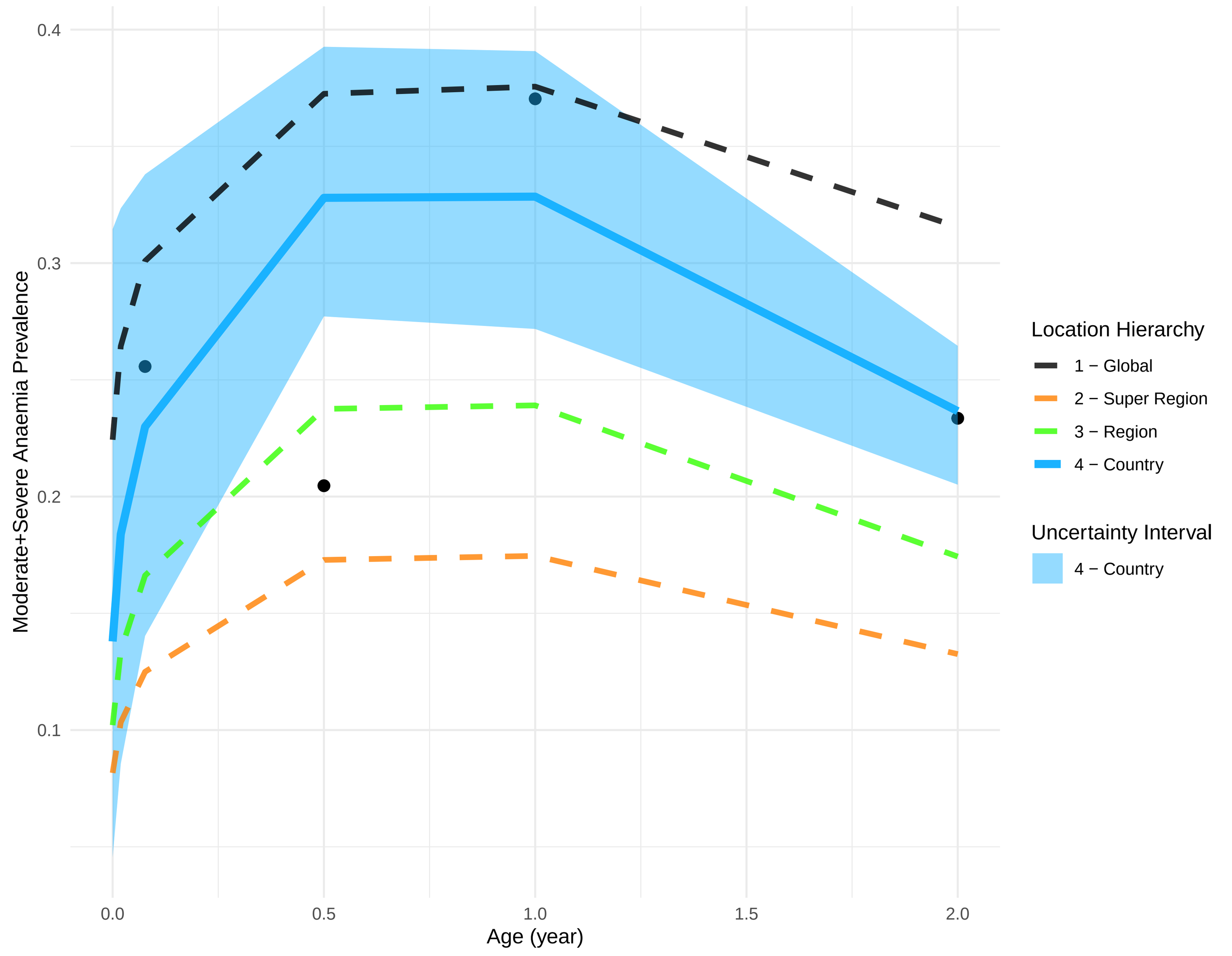

MR−BRT Age/Sex Fits − Males <2 Years Old − Moderate+Severe Anaemia Prevalence − Central Europe
WHO−2024 Adjustment Method

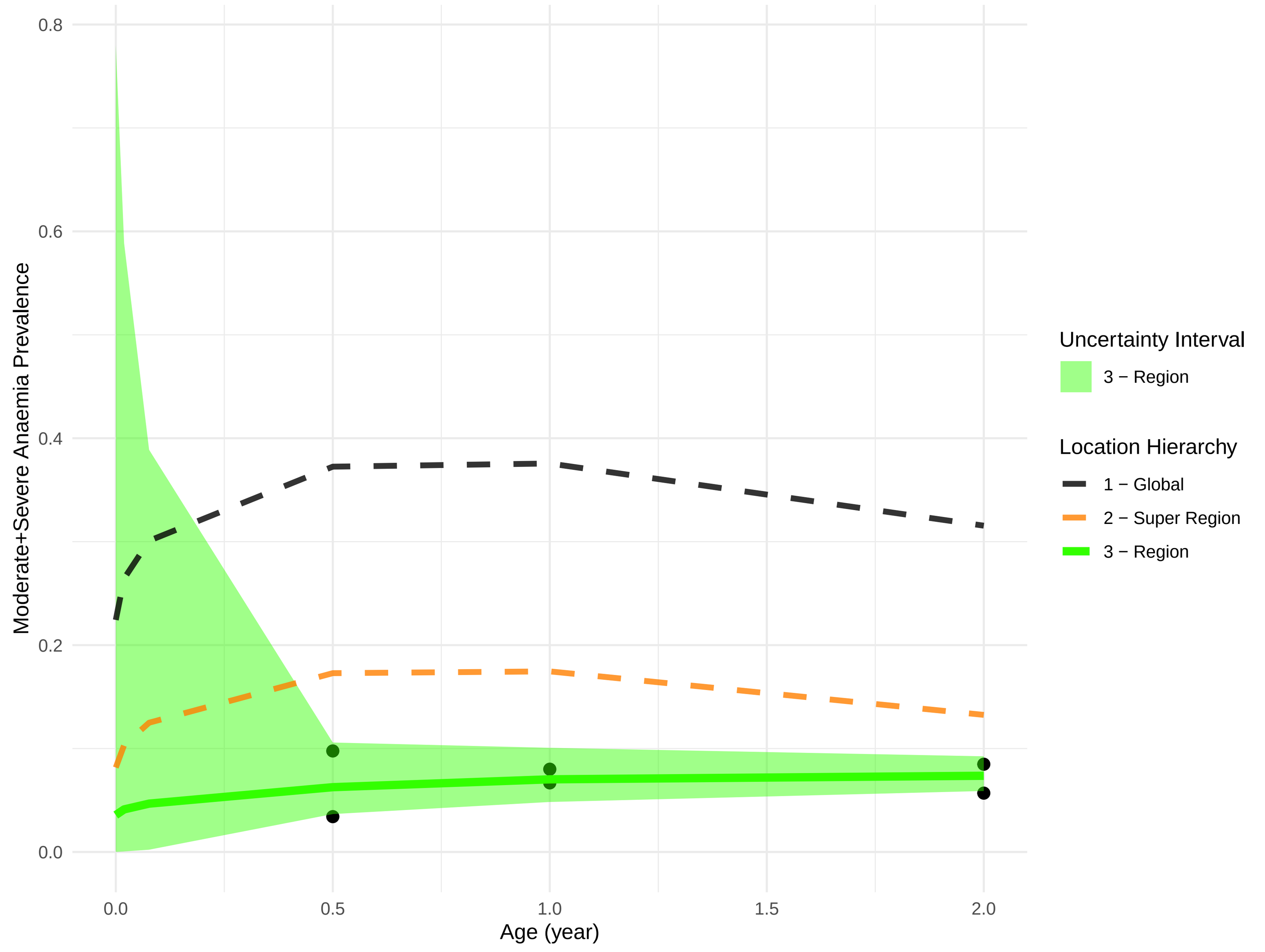

MR−BRT Age/Sex Fits − Males <2 Years Old − Moderate+Severe Anaemia Prevalence − Albania
WHO−2024 Adjustment Method

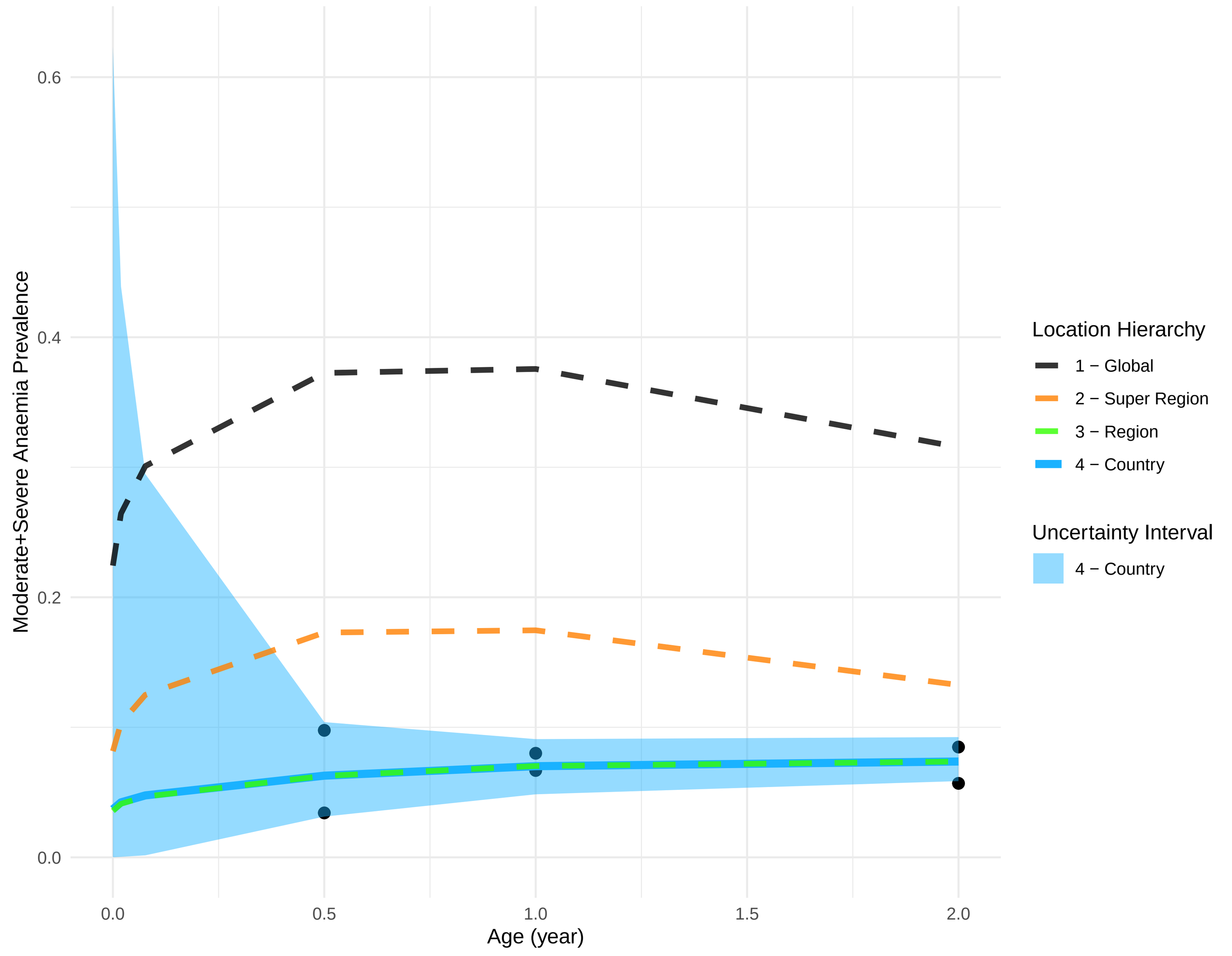

MR−BRT Age/Sex Fits − Males <2 Years Old − Moderate+Severe Anaemia Prevalence − Eastern Europe
WHO−2024 Adjustment Method

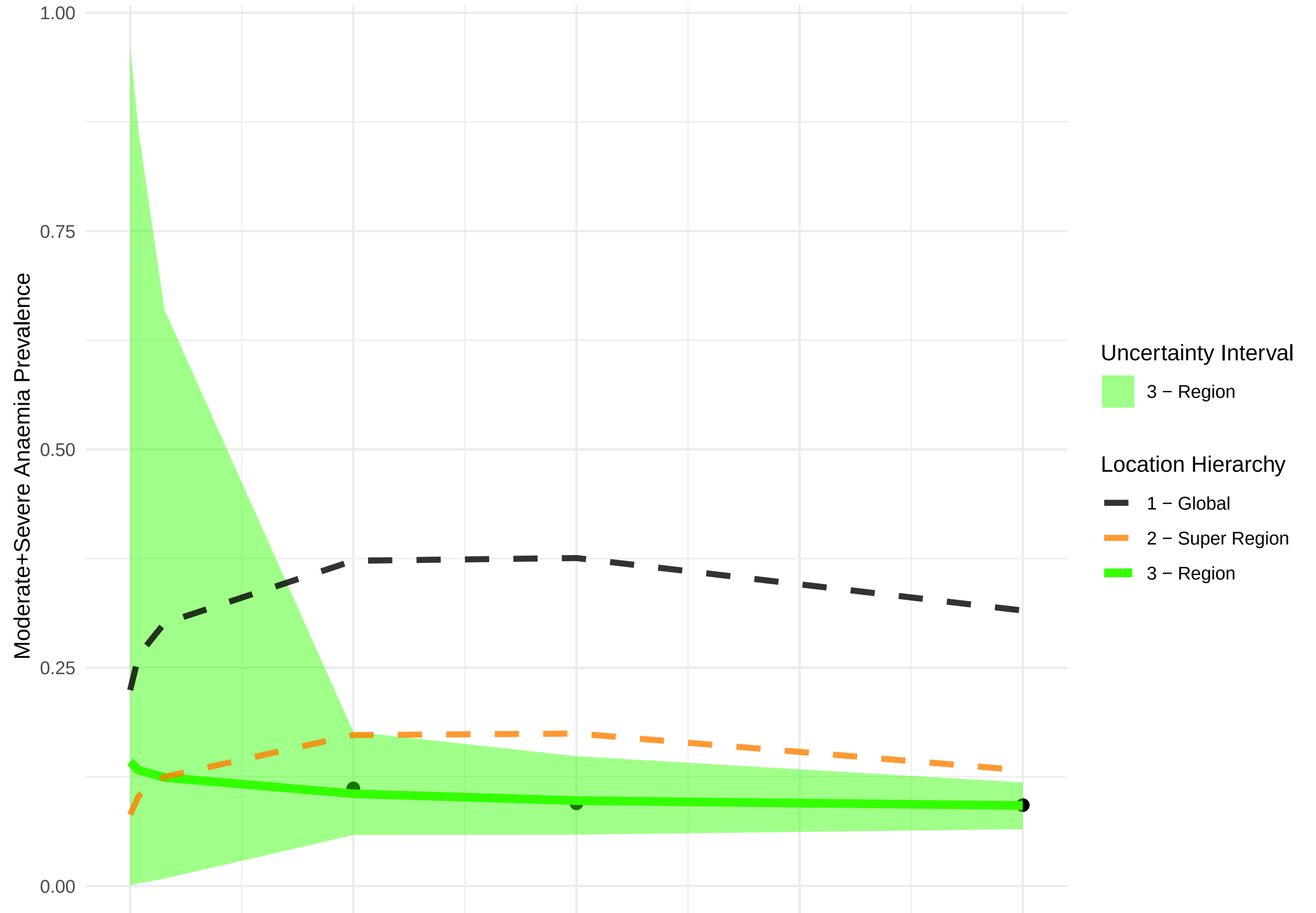

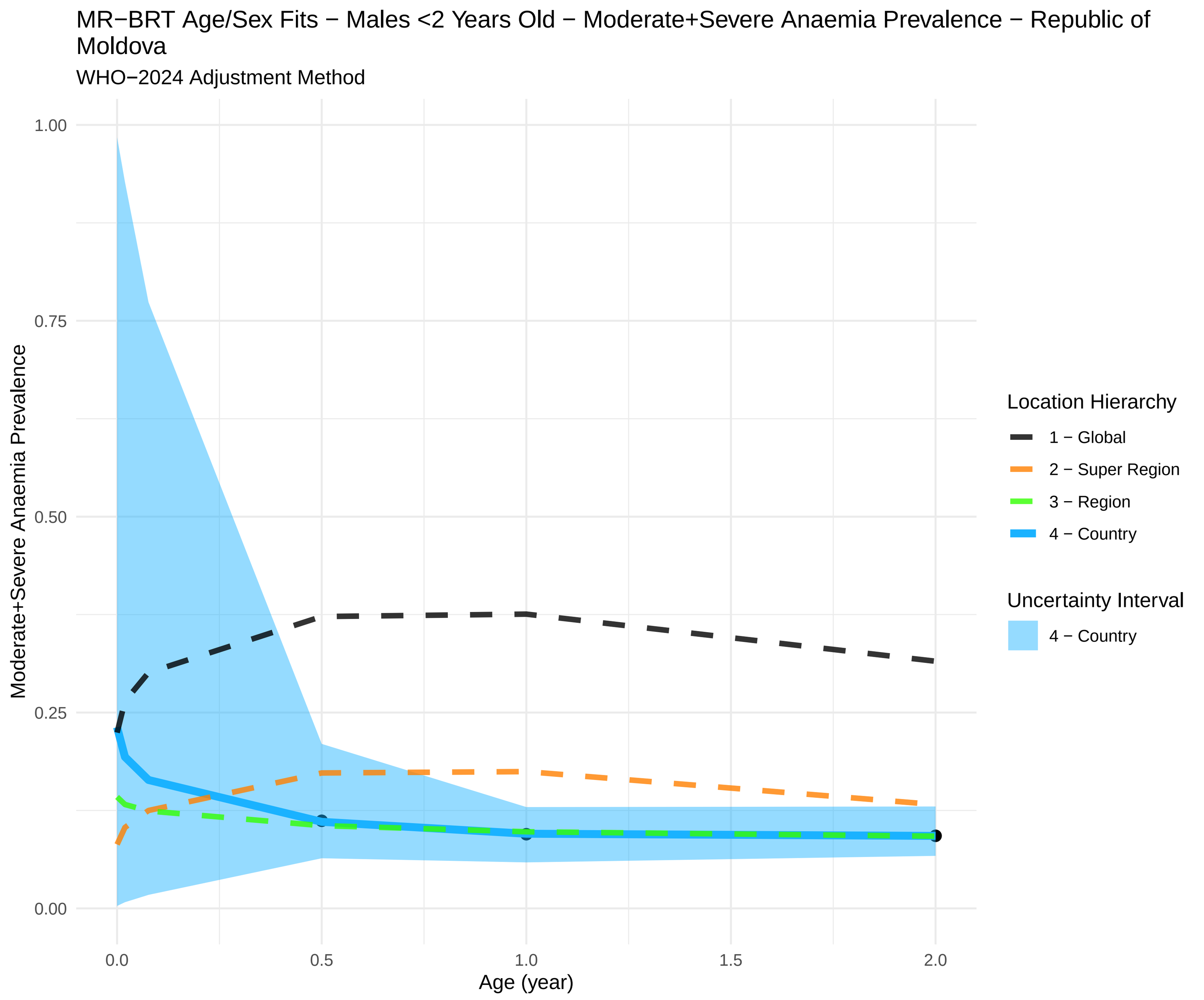
MR−BRT Age/Sex Fits − Males <2 Years Old − Moderate+Severe Anaemia Prevalence − Republic of Moldova
WHO−2024 Adjustment Method
Moderate+Severe Anaemia Prevalence
Age (year)
Location Hierarchy
1 − Global
2 − Super Region
3 − Region
4 − Country
Uncertainty Interval
4 − Country

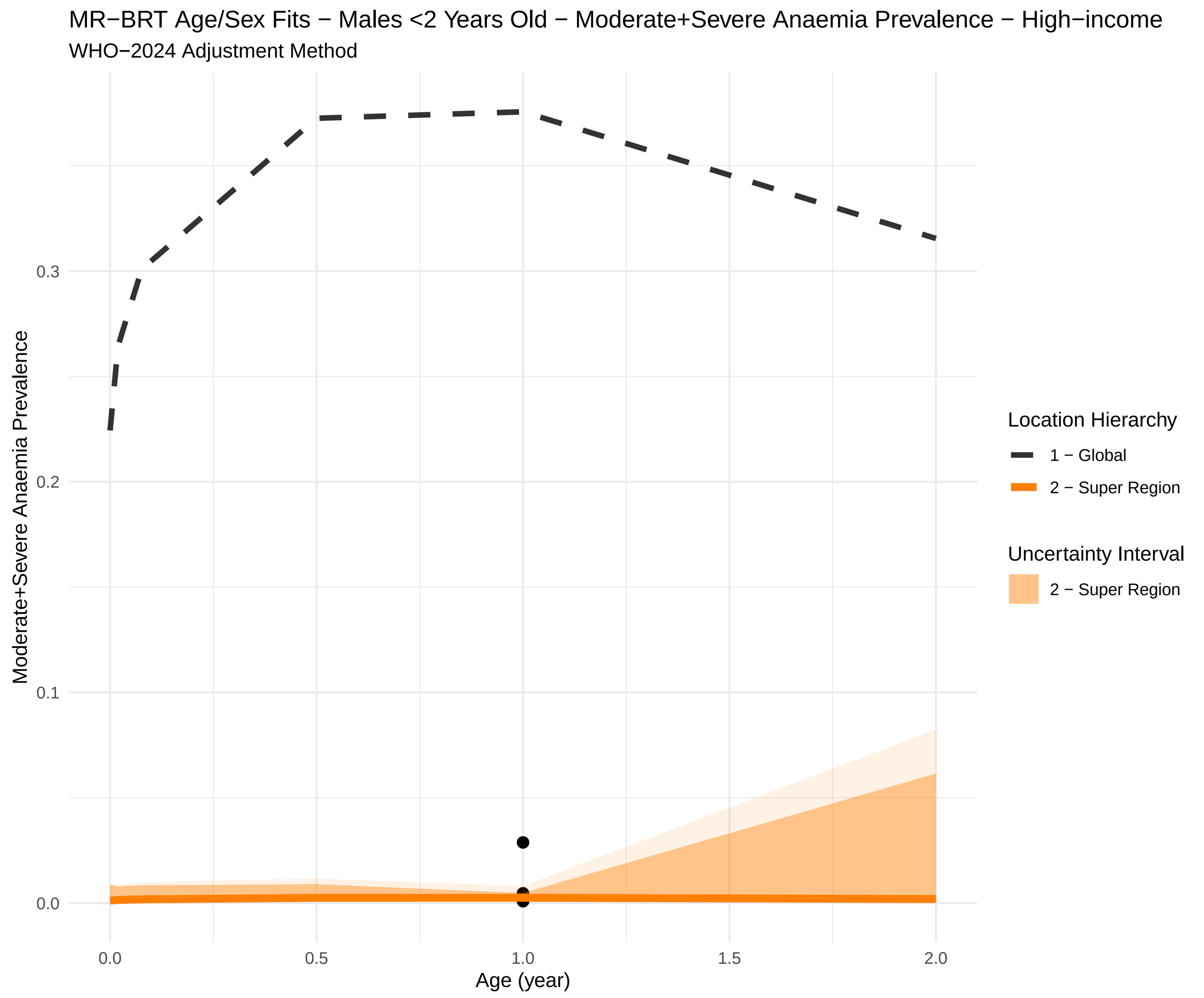
MR−BRT Age/Sex Fits − Males <2 Years Old − Moderate+Severe Anaemia Prevalence − High−income
WHO−2024 Adjustment Method
Moderate+Severe Anaemia Prevalence
Age (year)
Location Hierarchy
1 − Global
2 − Super Region
Uncertainty Interval
2 − Super Region

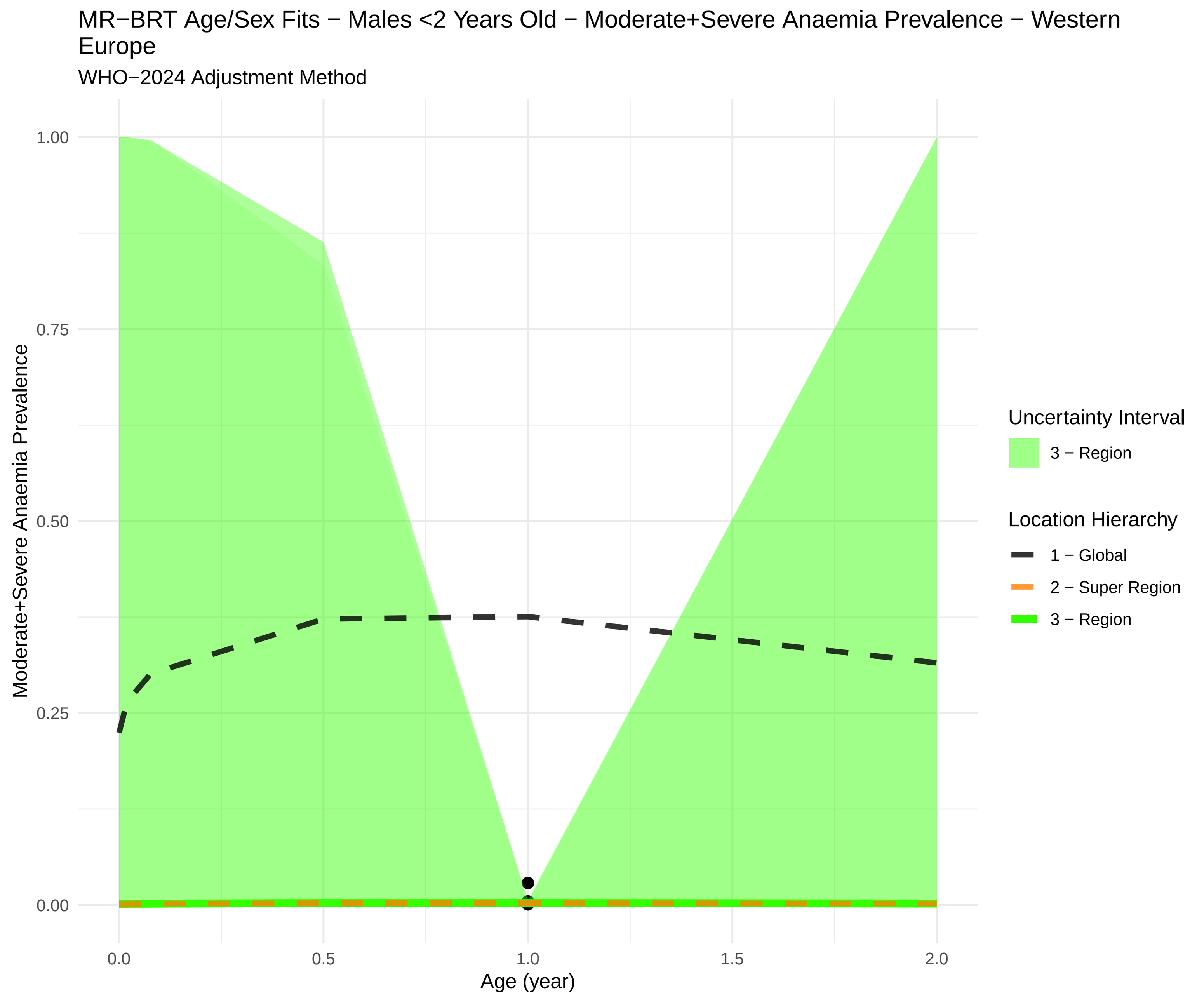
MR−BRT Age/Sex Fits − Males <2 Years Old − Moderate+Severe Anaemia Prevalence − Western Europe
WHO−2024 Adjustment Method
Moderate+Severe Anaemia Prevalence
Age (year)
Uncertainty Interval
3 − Region
Location Hierarchy
1 − Global
2 − Super Region
3 − Region

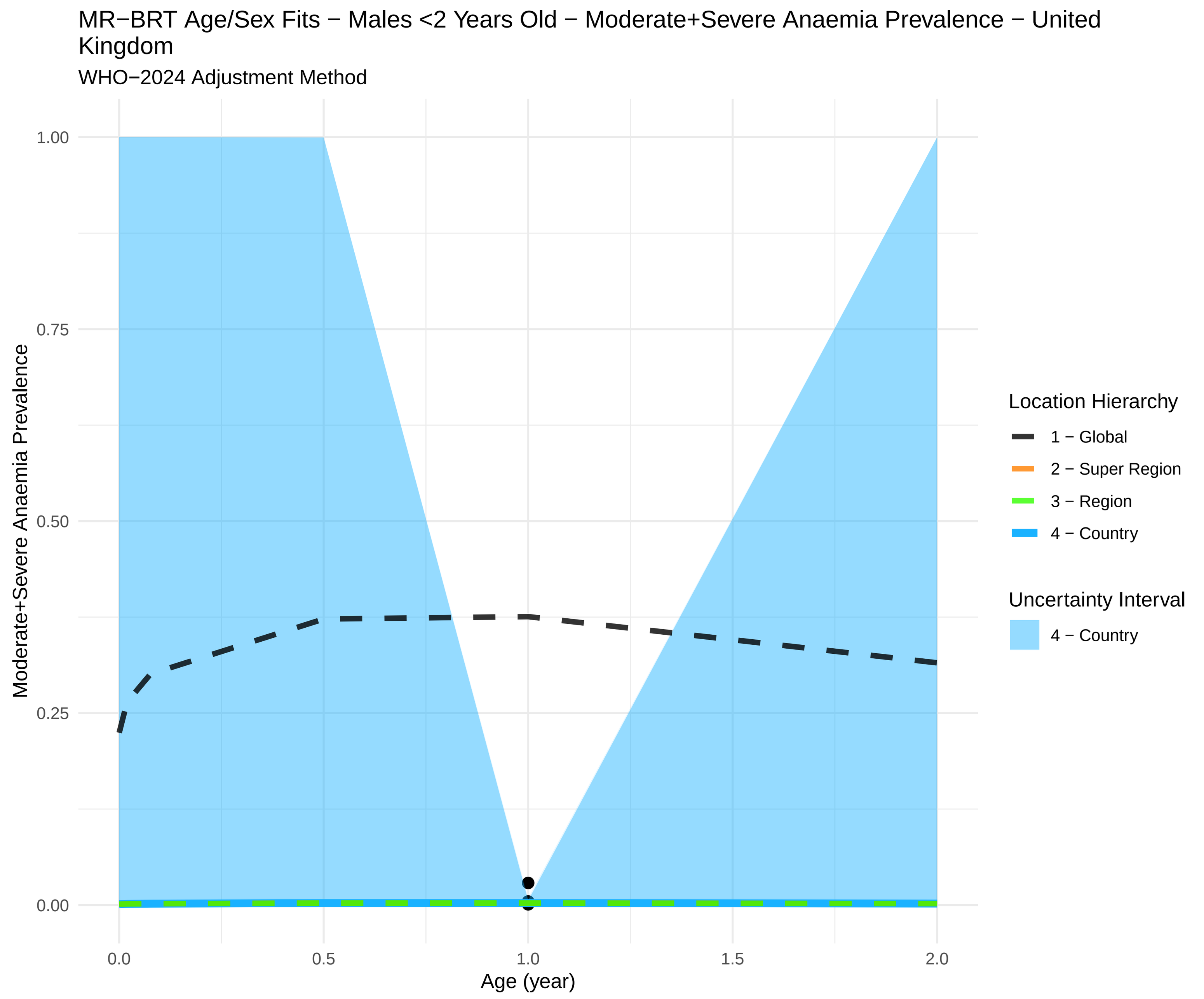
MR−BRT Age/Sex Fits − Males <2 Years Old − Moderate+Severe Anaemia Prevalence − United Kingdom
WHO−2024 Adjustment Method
Moderate+Severe Anaemia Prevalence
Age (year)
Location Hierarchy
1 − Global
2 − Super Region
3 − Region
4 − Country
Uncertainty Interval
4 − Country

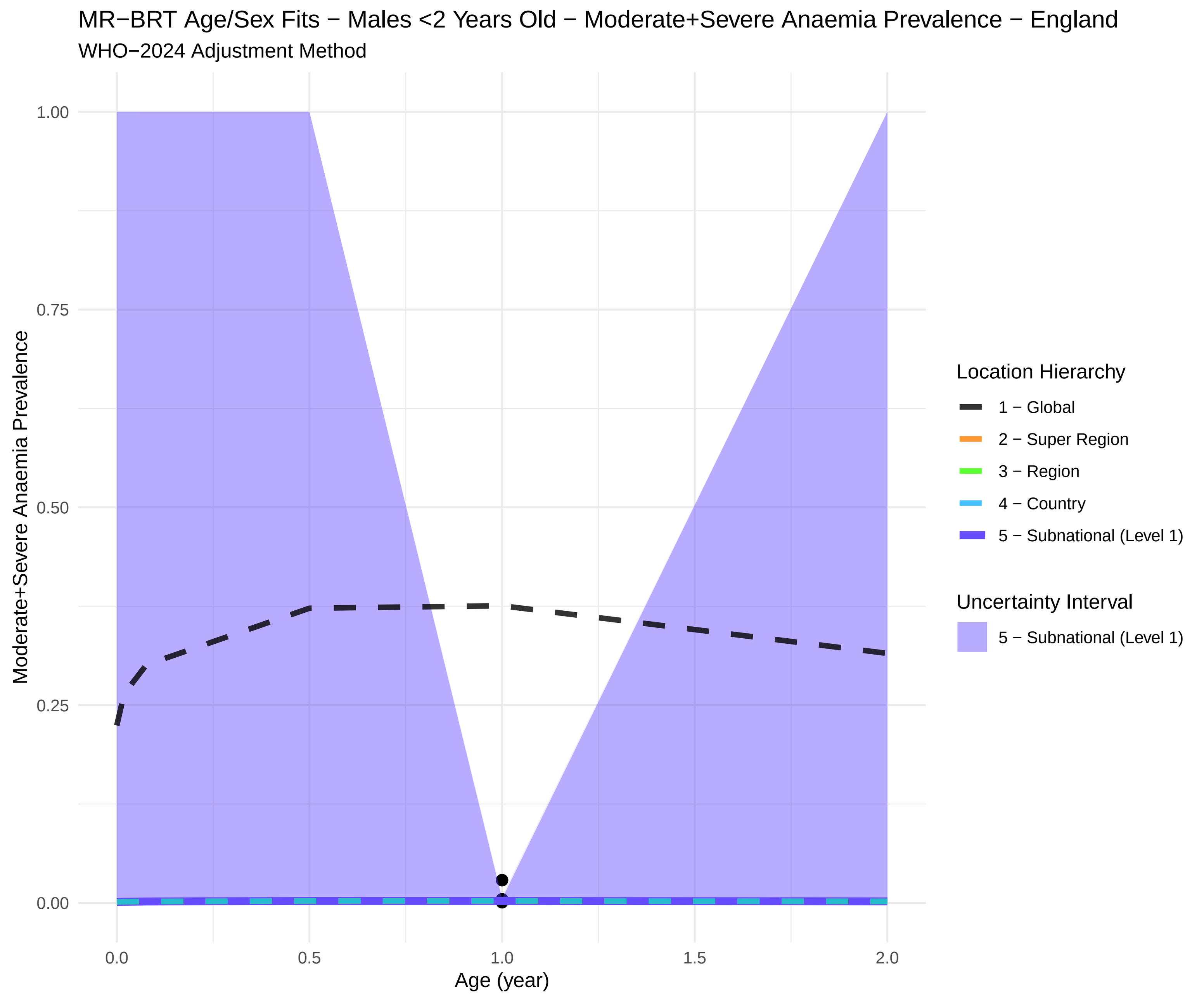
MR−BRT Age/Sex Fits − Males <2 Years Old − Moderate+Severe Anaemia Prevalence − England
WHO−2024 Adjustment Method
Moderate+Severe Anaemia Prevalence
Age (year)
Location Hierarchy
1 − Global
2 − Super Region
3 − Region
4 − Country
5 − Subnational (Level 1)
Uncertainty Interval
5 − Subnational (Level 1)

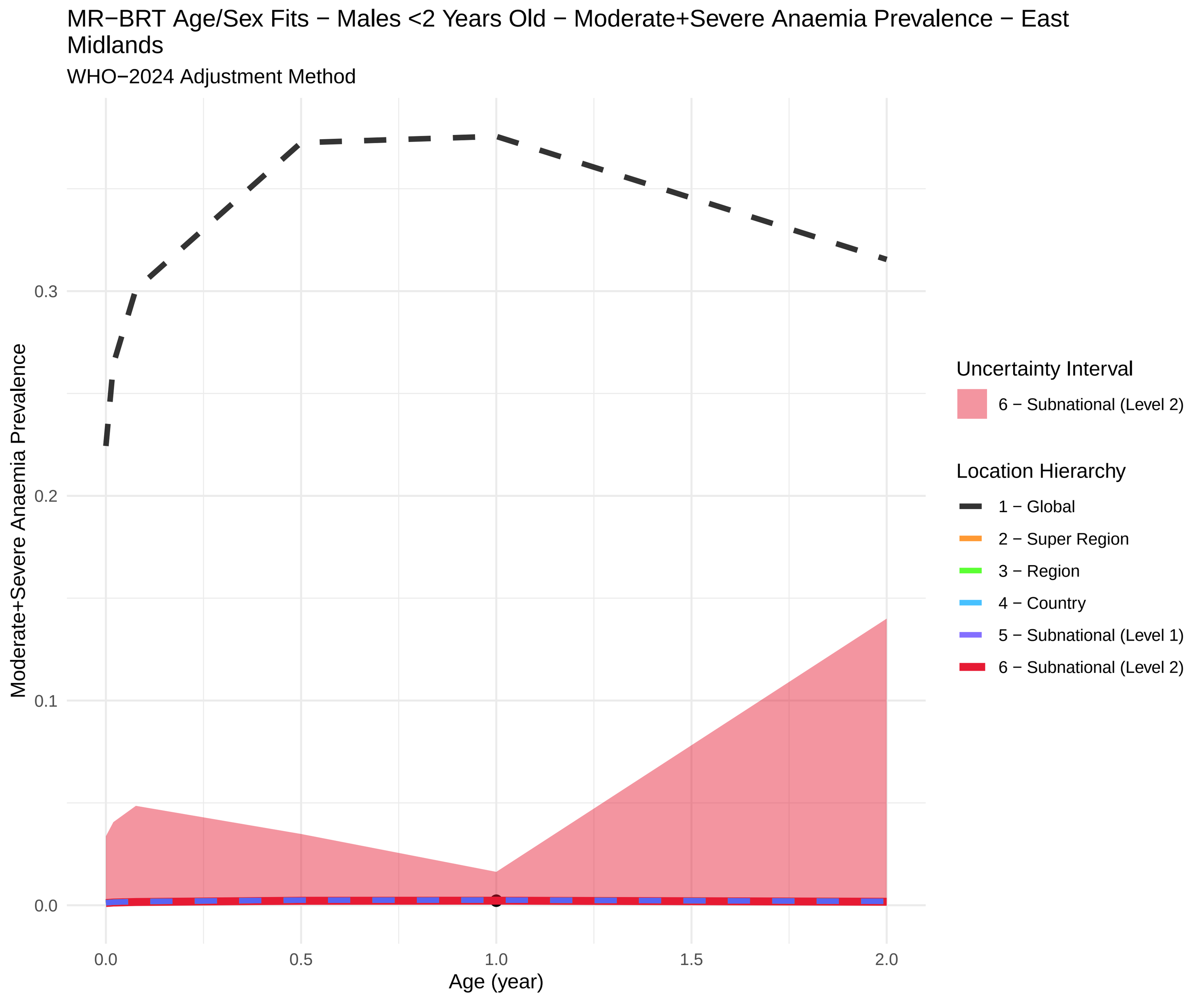
MR−BRT Age/Sex Fits − Males <2 Years Old − Moderate+Severe Anaemia Prevalence − East Midlands
WHO−2024 Adjustment Method
Moderate+Severe Anaemia Prevalence
Age (year)
Uncertainty Interval
6 − Subnational (Level 2)
Location Hierarchy
1 − Global
2 − Super Region
3 − Region
4 − Country
5 − Subnational (Level 1)
6 − Subnational (Level 2)

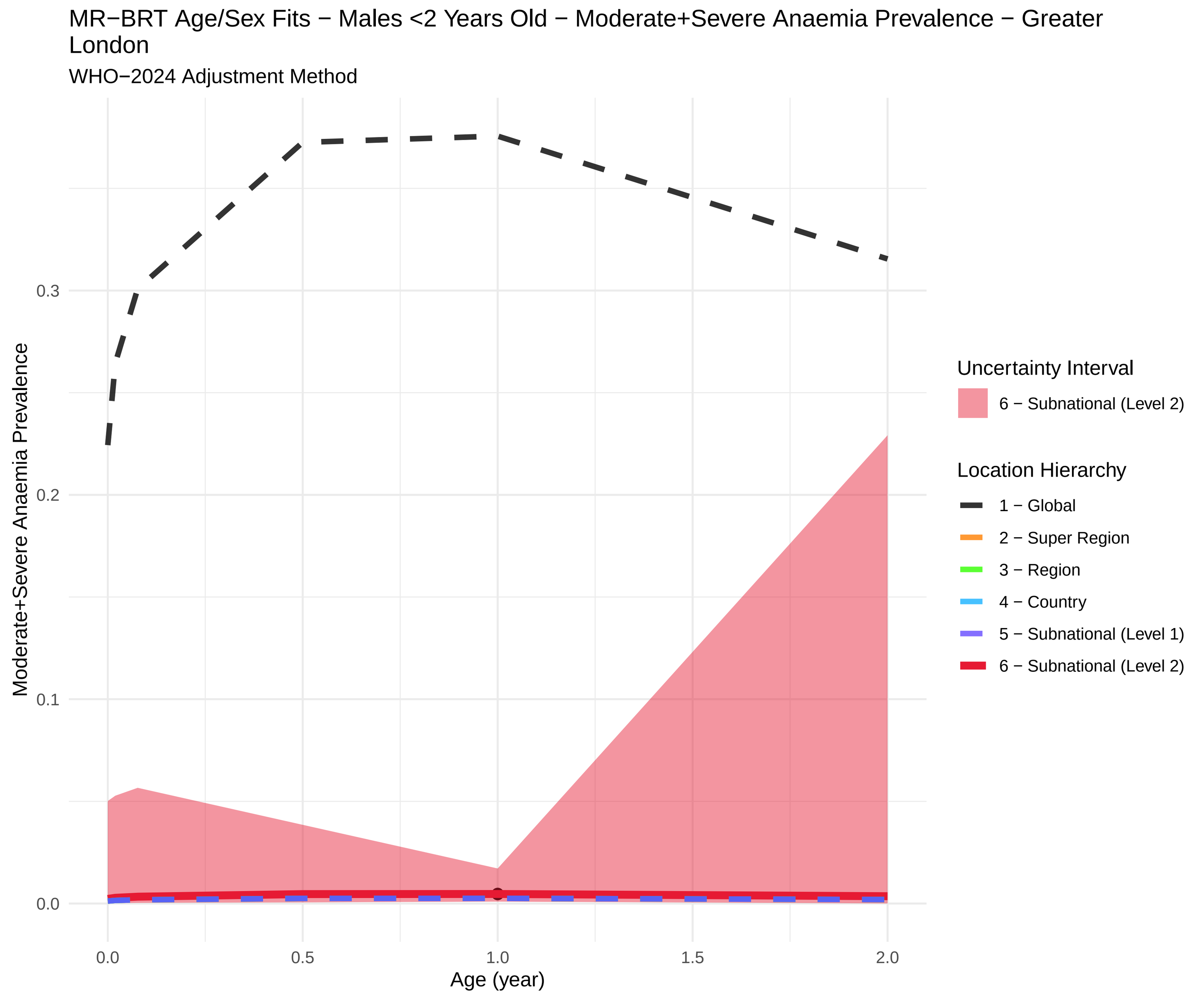
MR−BRT Age/Sex Fits − Males <2 Years Old − Moderate+Severe Anaemia Prevalence − Greater London
WHO−2024 Adjustment Method
Moderate+Severe Anaemia Prevalence
Age (year)
Uncertainty Interval
6 − Subnational (Level 2)
Location Hierarchy
1 − Global
2 − Super Region
3 − Region
4 − Country
5 − Subnational (Level 1)
6 − Subnational (Level 2)

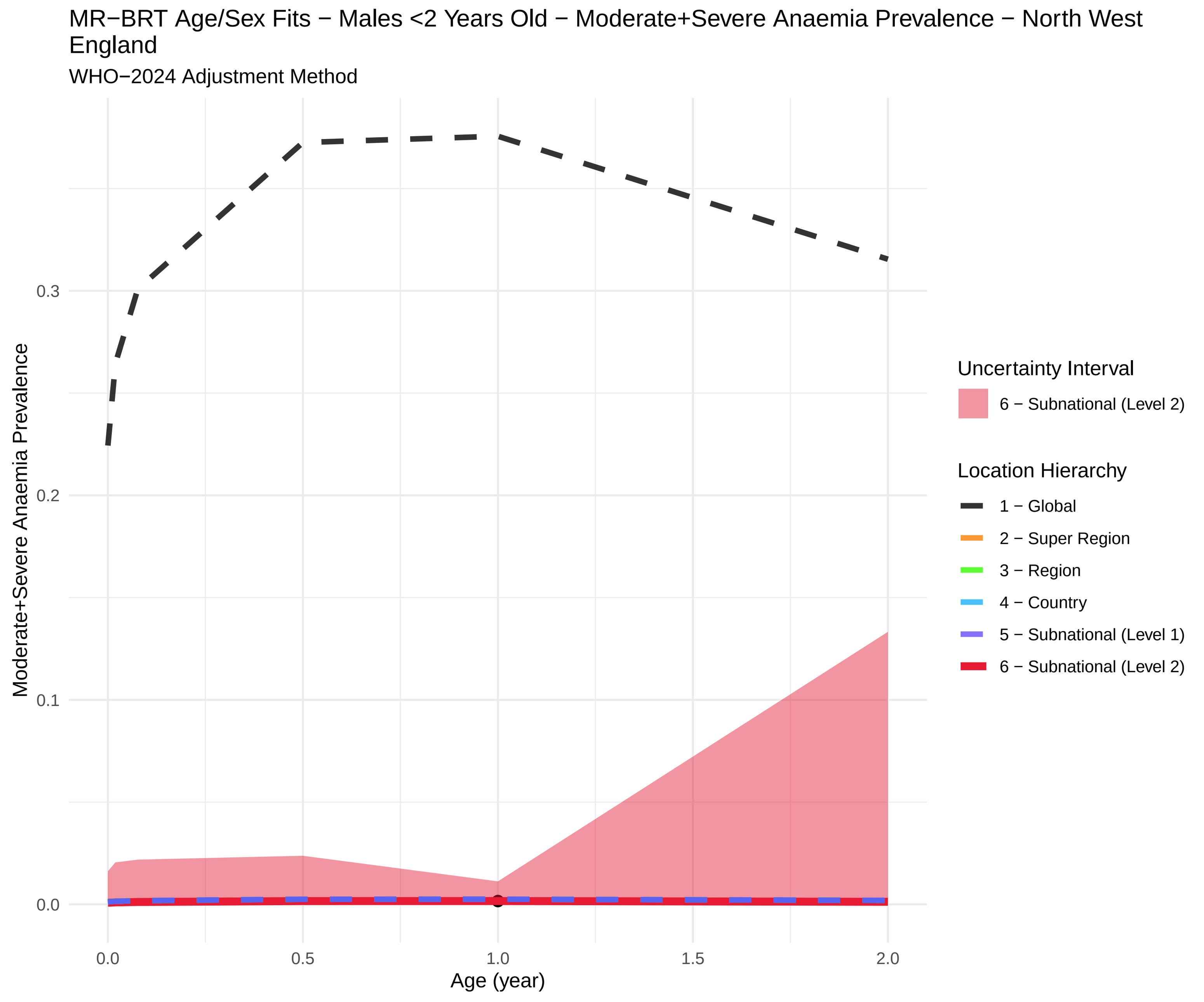
MR−BRT Age/Sex Fits − Males <2 Years Old − Moderate+Severe Anaemia Prevalence − North West England
WHO−2024 Adjustment Method
Moderate+Severe Anaemia Prevalence
Age (year)
Uncertainty Interval
6 − Subnational (Level 2)
Location Hierarchy
1 − Global
2 − Super Region
3 − Region
4 − Country
5 − Subnational (Level 1)
6 − Subnational (Level 2)

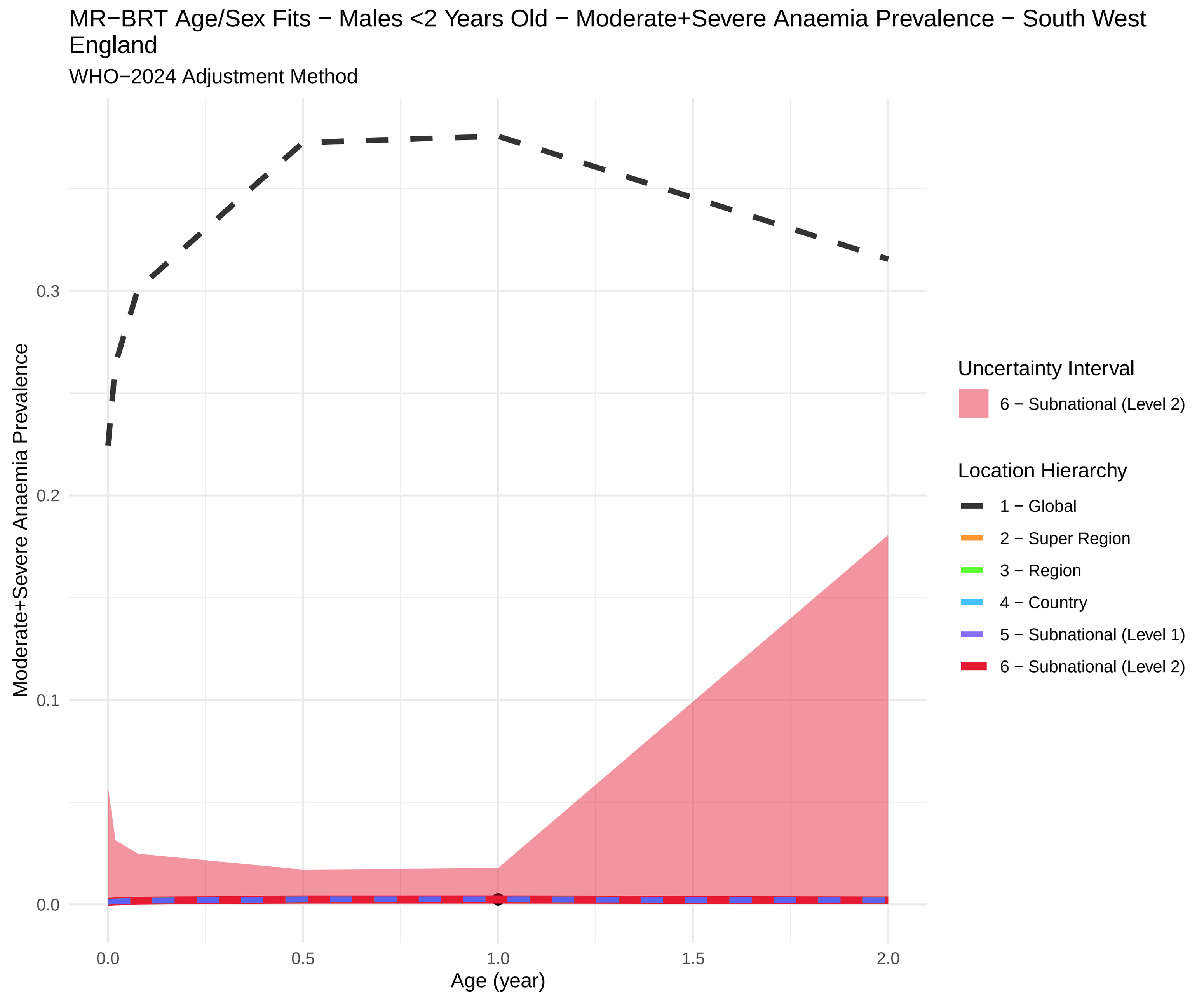
MR−BRT Age/Sex Fits − Males <2 Years Old − Moderate+Severe Anaemia Prevalence − South West England
WHO−2024 Adjustment Method
Moderate+Severe Anaemia Prevalence
Age (year)
Uncertainty Interval
6 − Subnational (Level 2)
Location Hierarchy
1 − Global
2 − Super Region
3 − Region
4 − Country
5 − Subnational (Level 1)
6 − Subnational (Level 2)

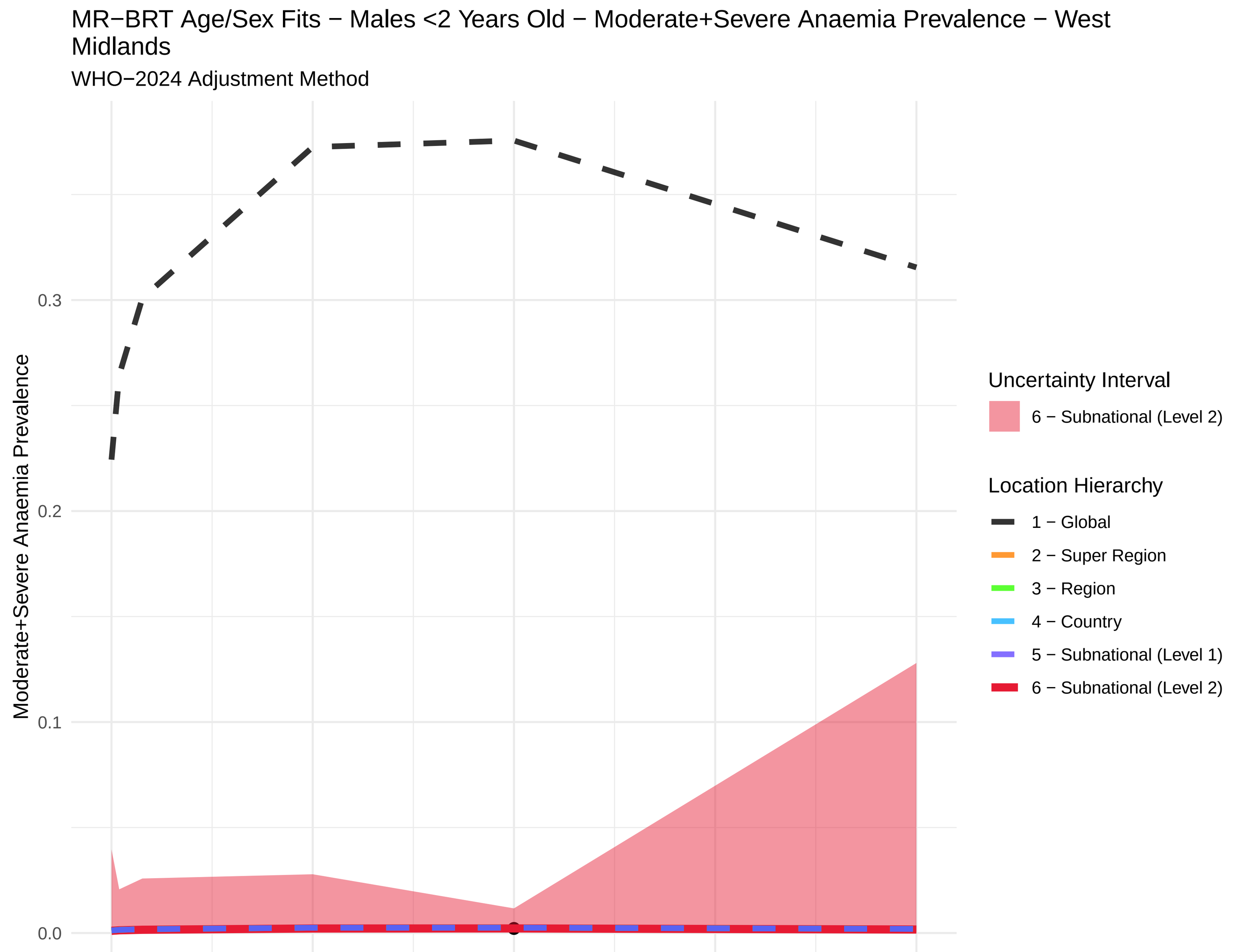
MR−BRT Age/Sex Fits − Males <2 Years Old − Moderate+Severe Anaemia Prevalence − West Midlands
WHO−2024 Adjustment Method
Moderate+Severe Anaemia Prevalence
Age (year)
Uncertainty Interval
6 − Subnational (Level 2)
Location Hierarchy
1 − Global
2 − Super Region
3 − Region
4 − Country
5 − Subnational (Level 1)
6 − Subnational (Level 2)

MR−BRT Age/Sex Fits − Males <2 Years Old − Moderate+Severe Anaemia Prevalence − Yorkshire and the Humber

WHO−2024 Adjustment Method

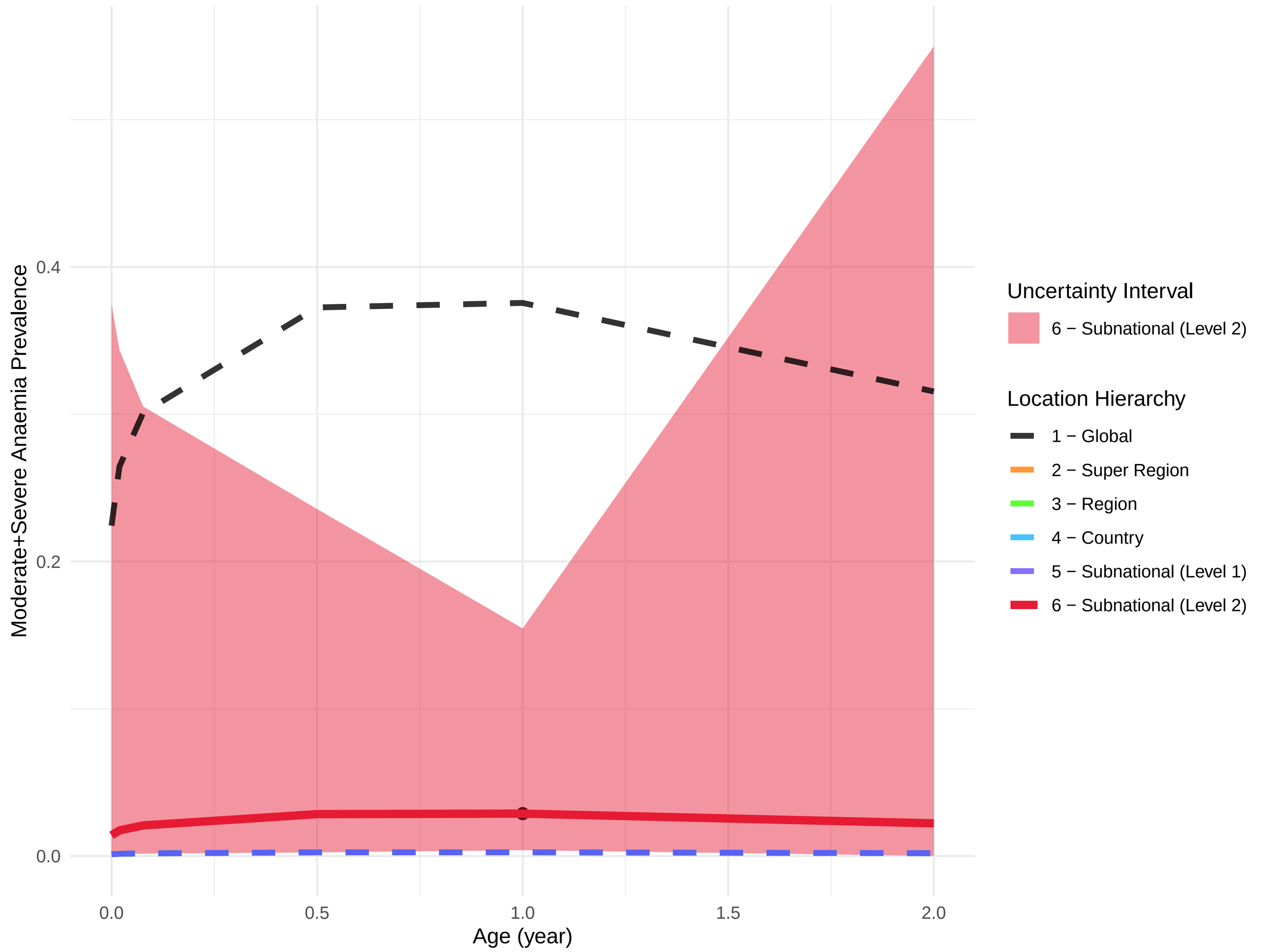

MR−BRT Age/Sex Fits − Males <2 Years Old − Moderate+Severe Anaemia Prevalence − Latin America and Caribbean

WHO−2024 Adjustment Method

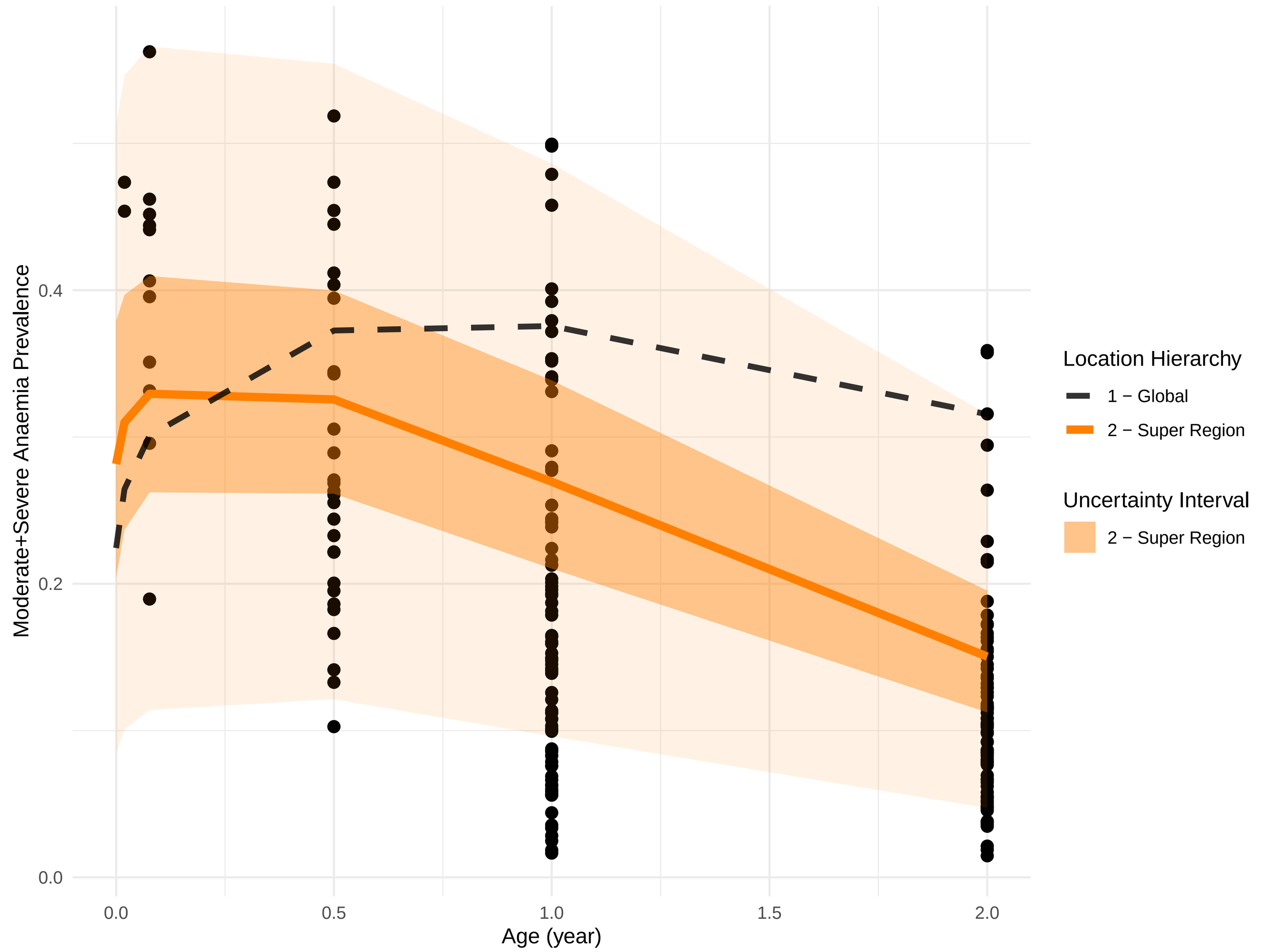

MR−BRT Age/Sex Fits − Males <2 Years Old − Moderate+Severe Anaemia Prevalence − Andean Latin America

WHO−2024 Adjustment Method

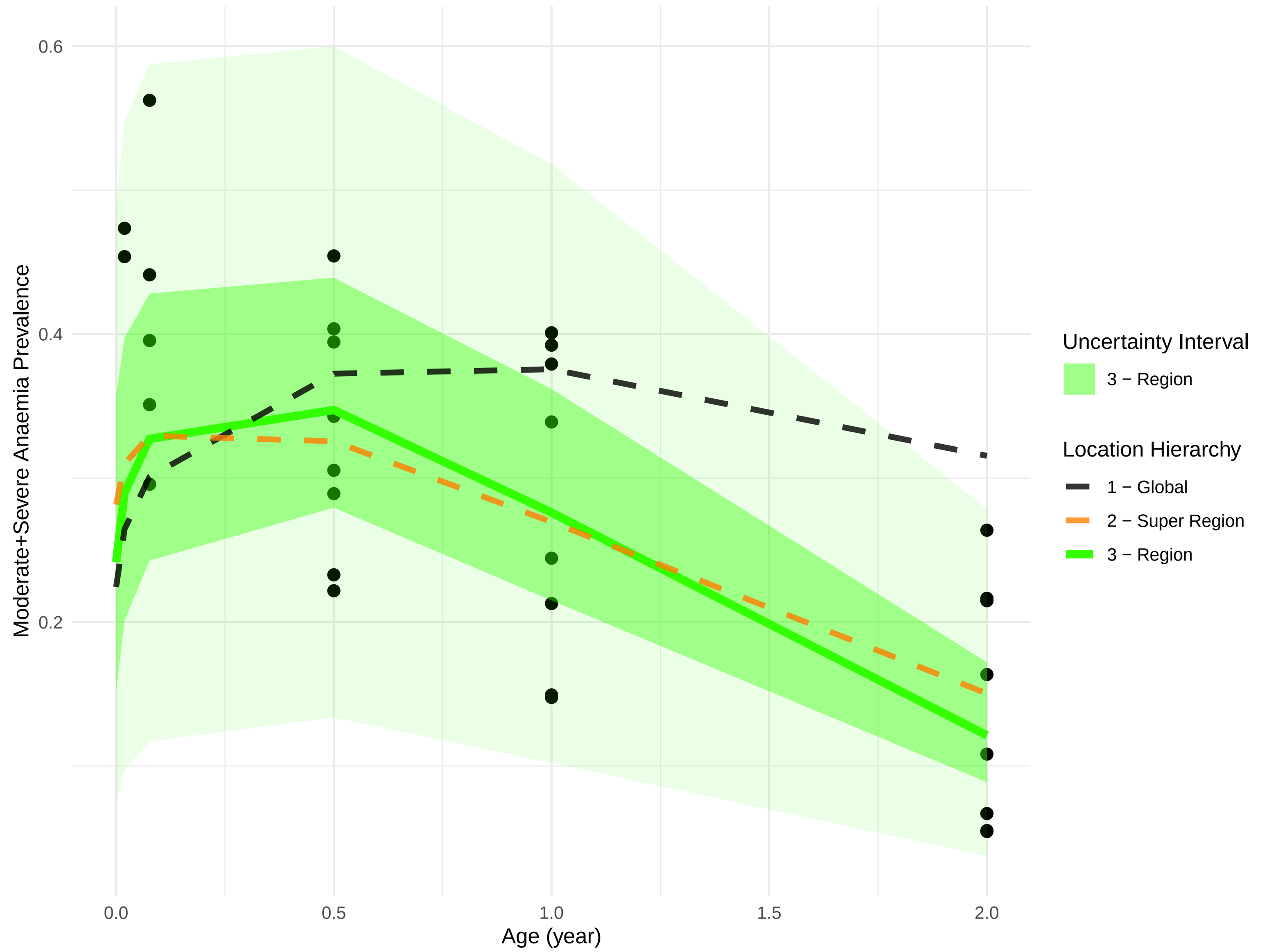

MR−BRT Age/Sex Fits − Males <2 Years Old − Moderate+Severe Anaemia Prevalence − Bolivia (Plurinational State of)

WHO−2024 Adjustment Method

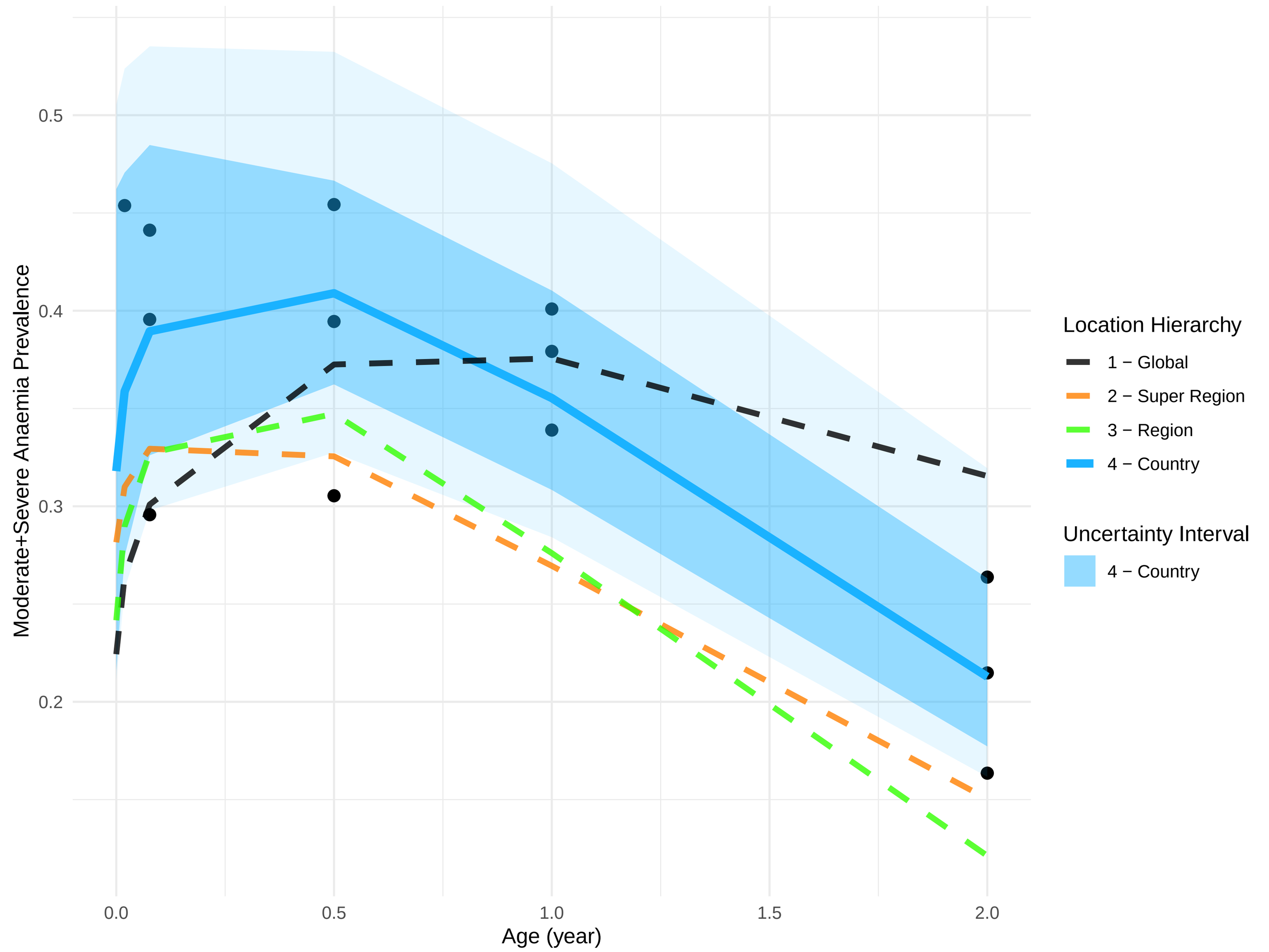

MR−BRT Age/Sex Fits − Males <2 Years Old − Moderate+Severe Anaemia Prevalence − Peru

WHO−2024 Adjustment Method

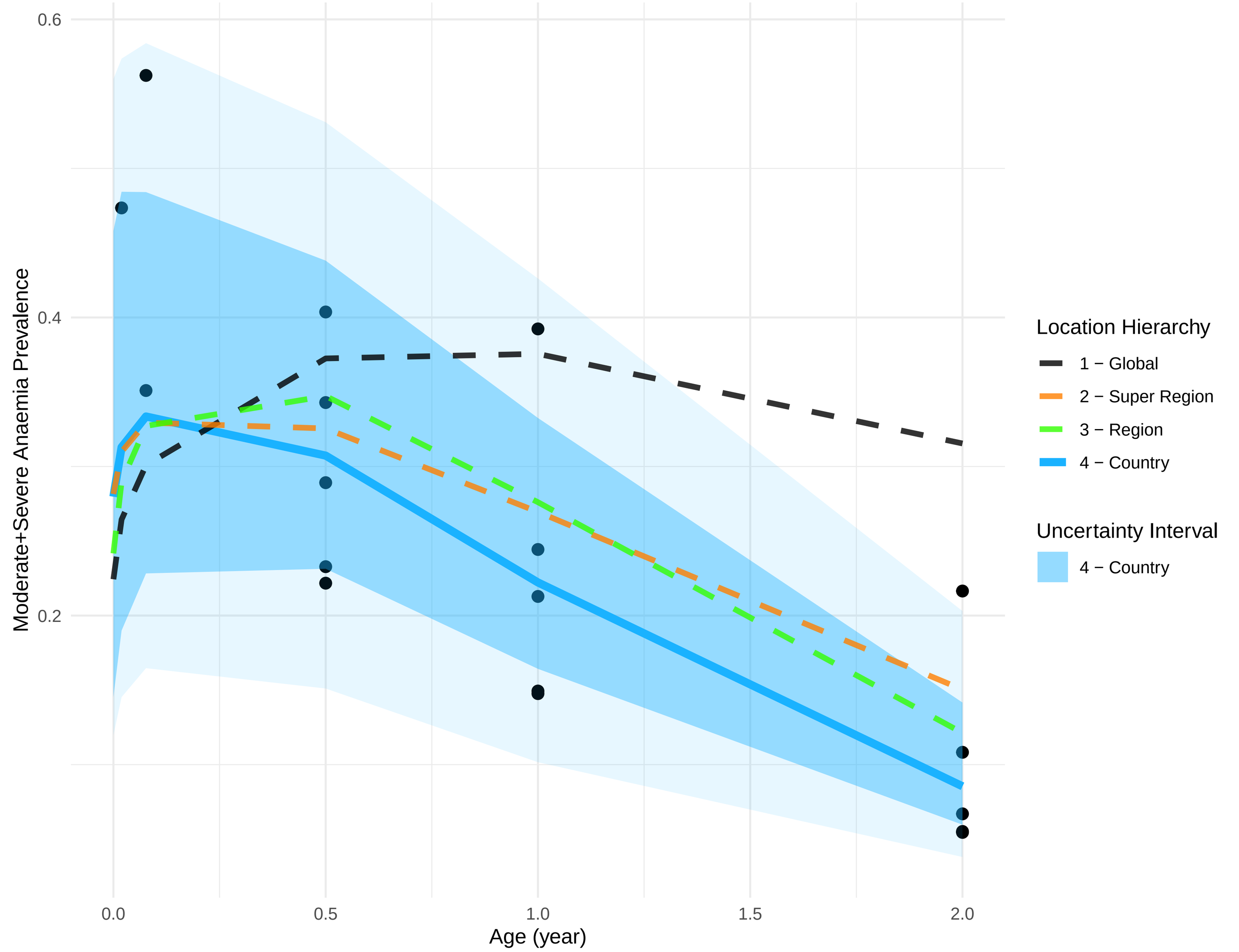

MR−BRT Age/Sex Fits − Males <2 Years Old − Moderate+Severe Anaemia Prevalence − Caribbean

WHO−2024 Adjustment Method

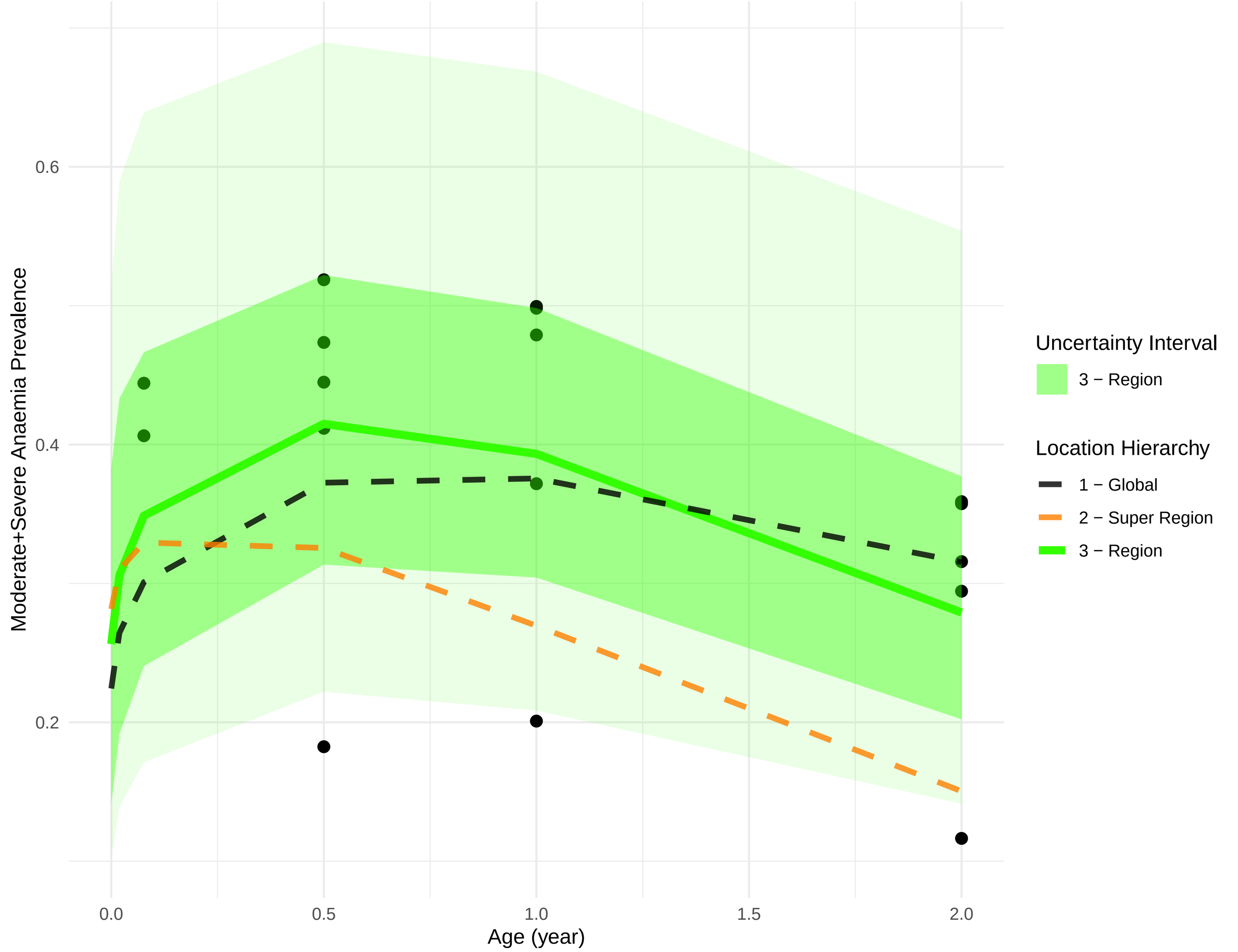

MR−BRT Age/Sex Fits − Males <2 Years Old − Moderate+Severe Anaemia Prevalence − Guyana

WHO−2024 Adjustment Method

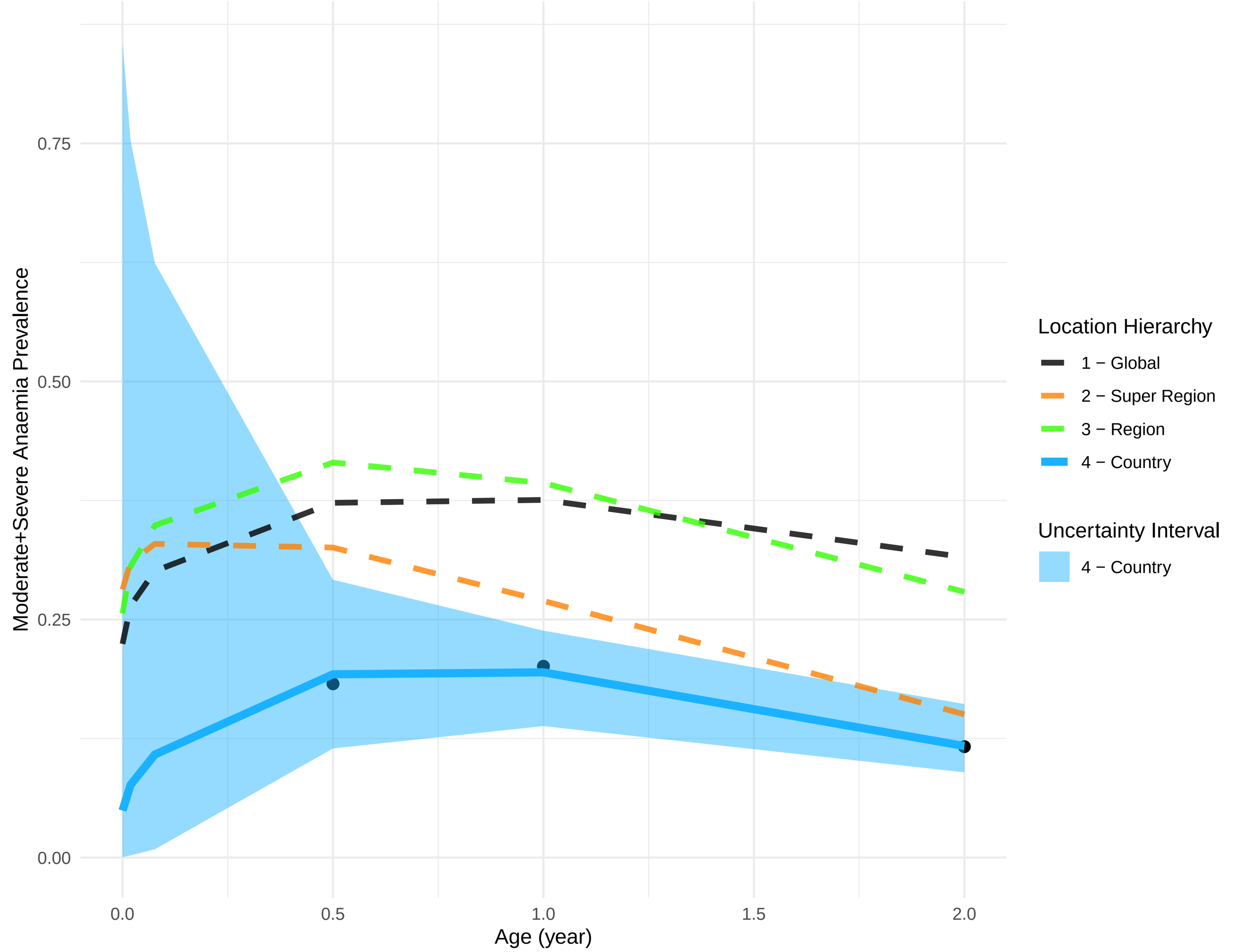

MR−BRT Age/Sex Fits − Males <2 Years Old − Moderate+Severe Anaemia Prevalence − Haiti

WHO−2024 Adjustment Method

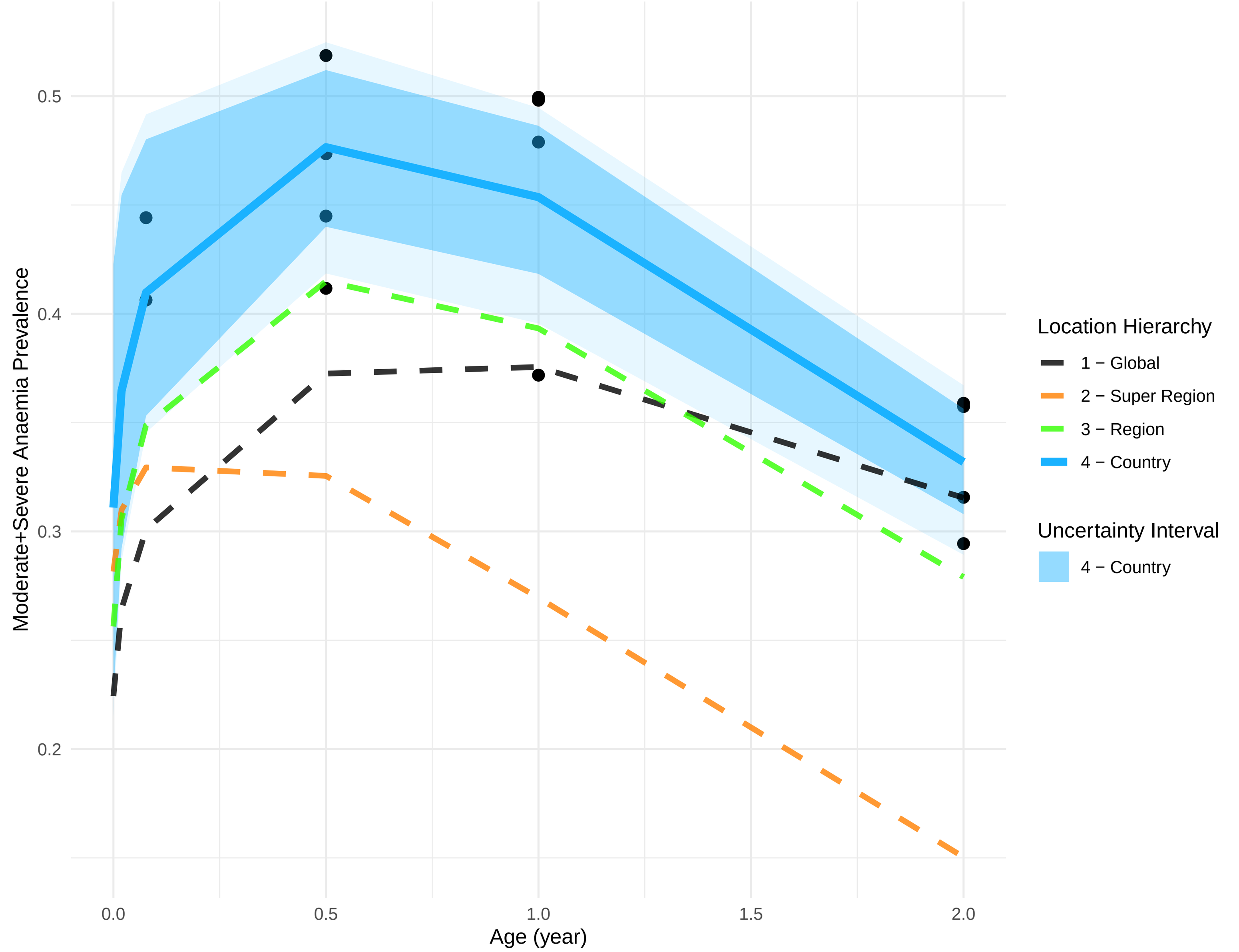

MR−BRT Age/Sex Fits − Males <2 Years Old − Moderate+Severe Anaemia Prevalence − Central Latin America

WHO−2024 Adjustment Method

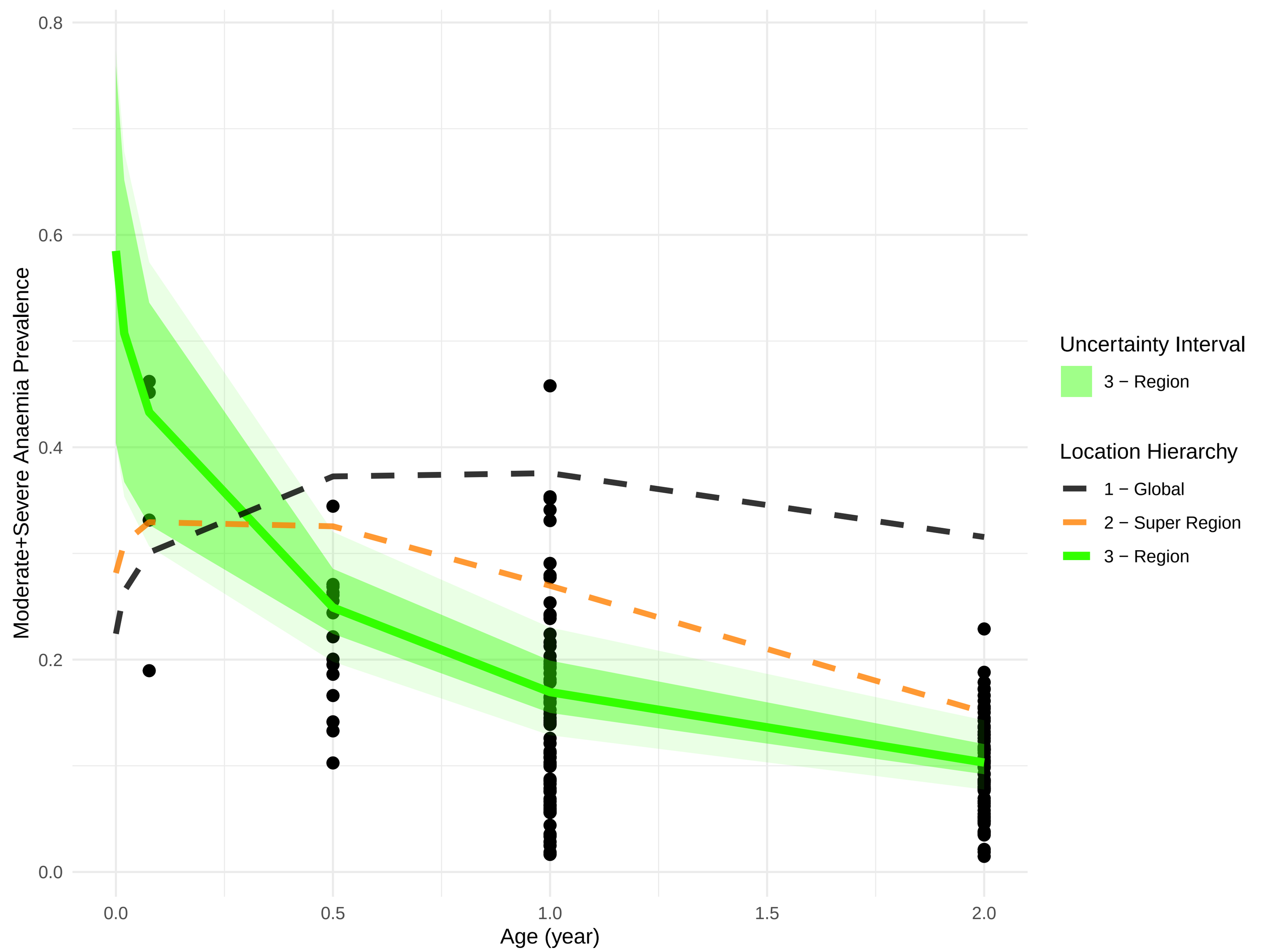

MR−BRT Age/Sex Fits − Males <2 Years Old − Moderate+Severe Anaemia Prevalence − Guatemala

WHO−2024 Adjustment Method

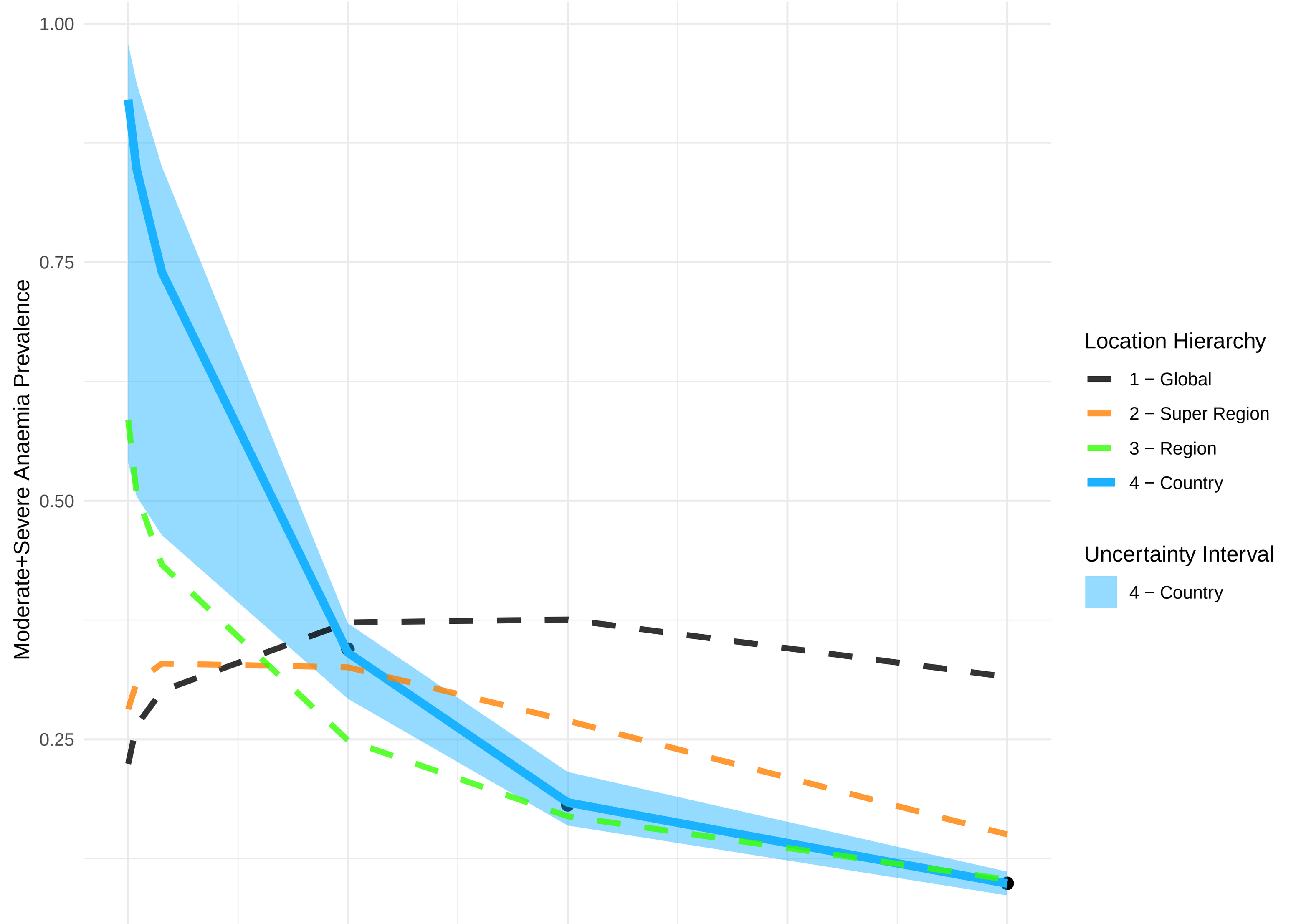

MR−BRT Age/Sex Fits − Males <2 Years Old − Moderate+Severe Anaemia Prevalence − Honduras
WHO−2024 Adjustment Method

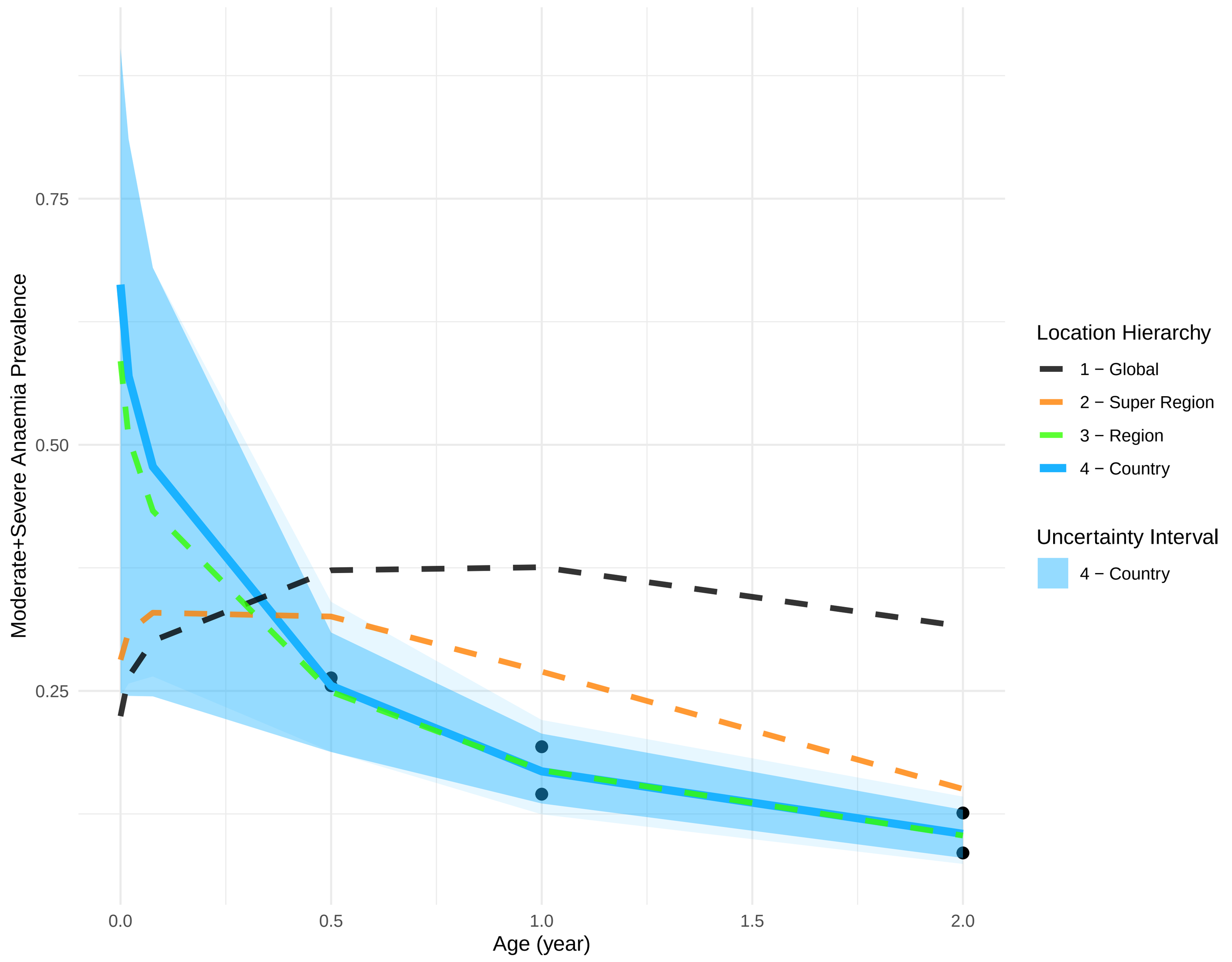

MR−BRT Age/Sex Fits − Males <2 Years Old − Moderate+Severe Anaemia Prevalence − Mexico
WHO−2024 Adjustment Method

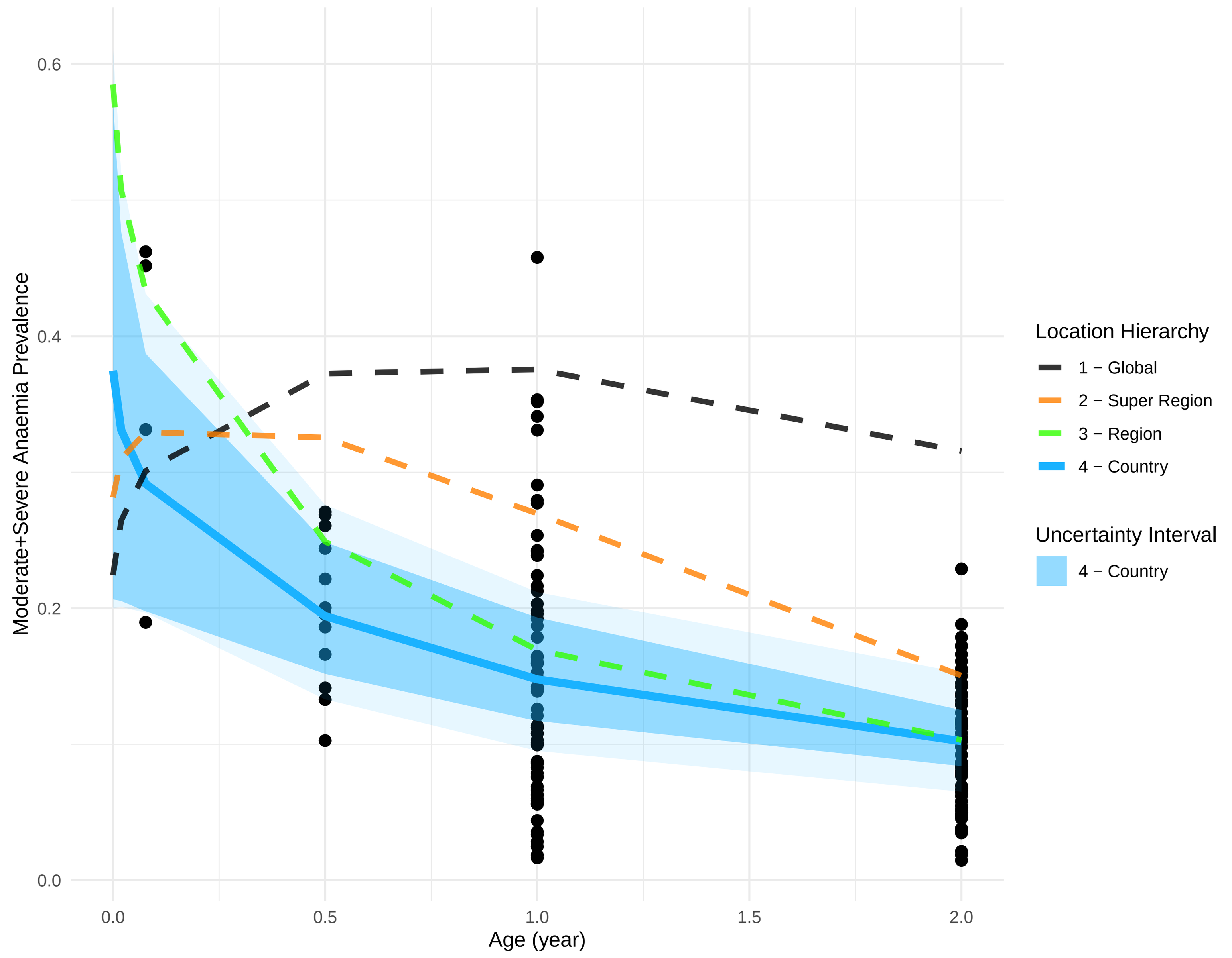

MR−BRT Age/Sex Fits − Males <2 Years Old − Moderate+Severe Anaemia Prevalence − Aguascalientes
WHO−2024 Adjustment Method

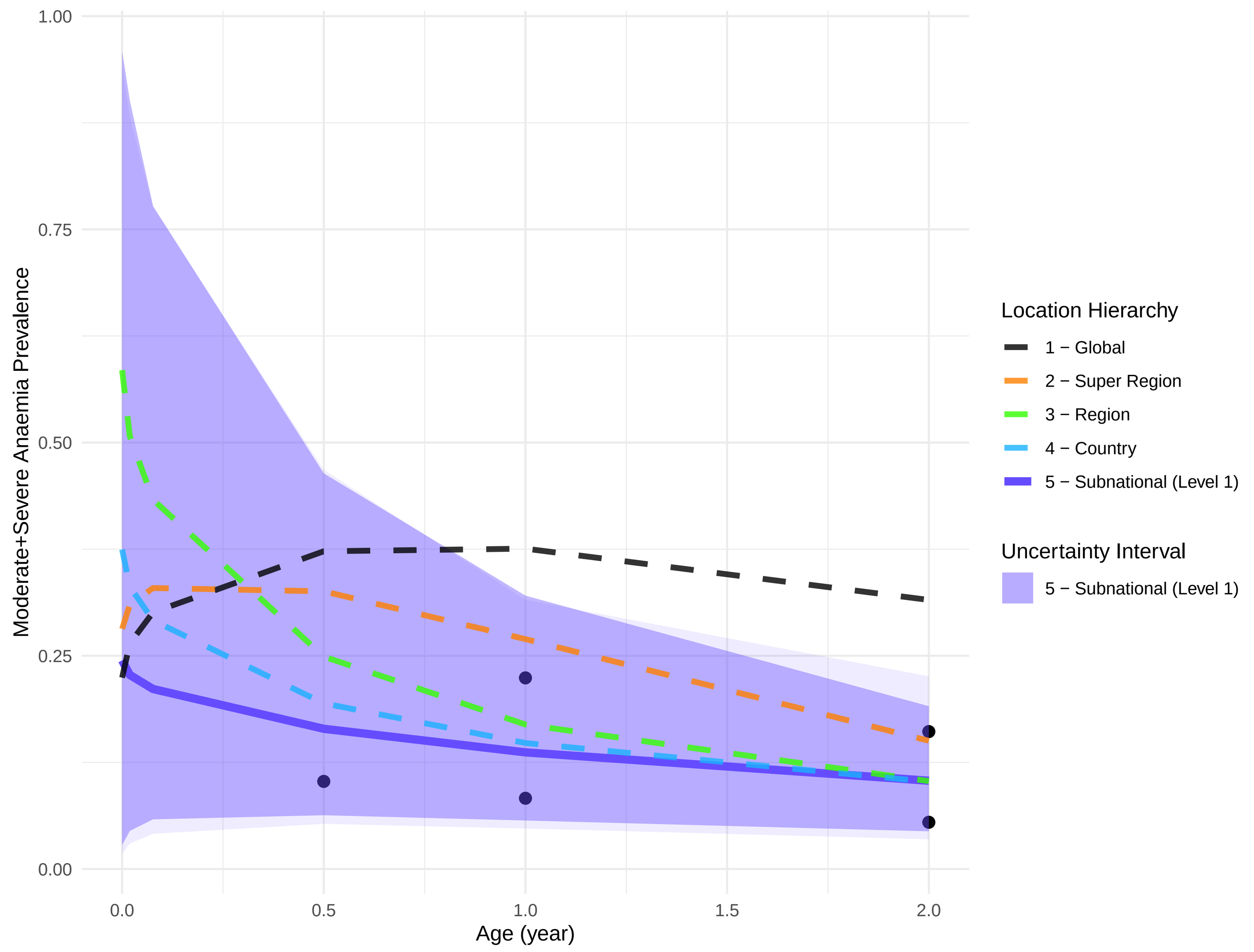

MR−BRT Age/Sex Fits − Males <2 Years Old − Moderate+Severe Anaemia Prevalence − Baja California
WHO−2024 Adjustment Method

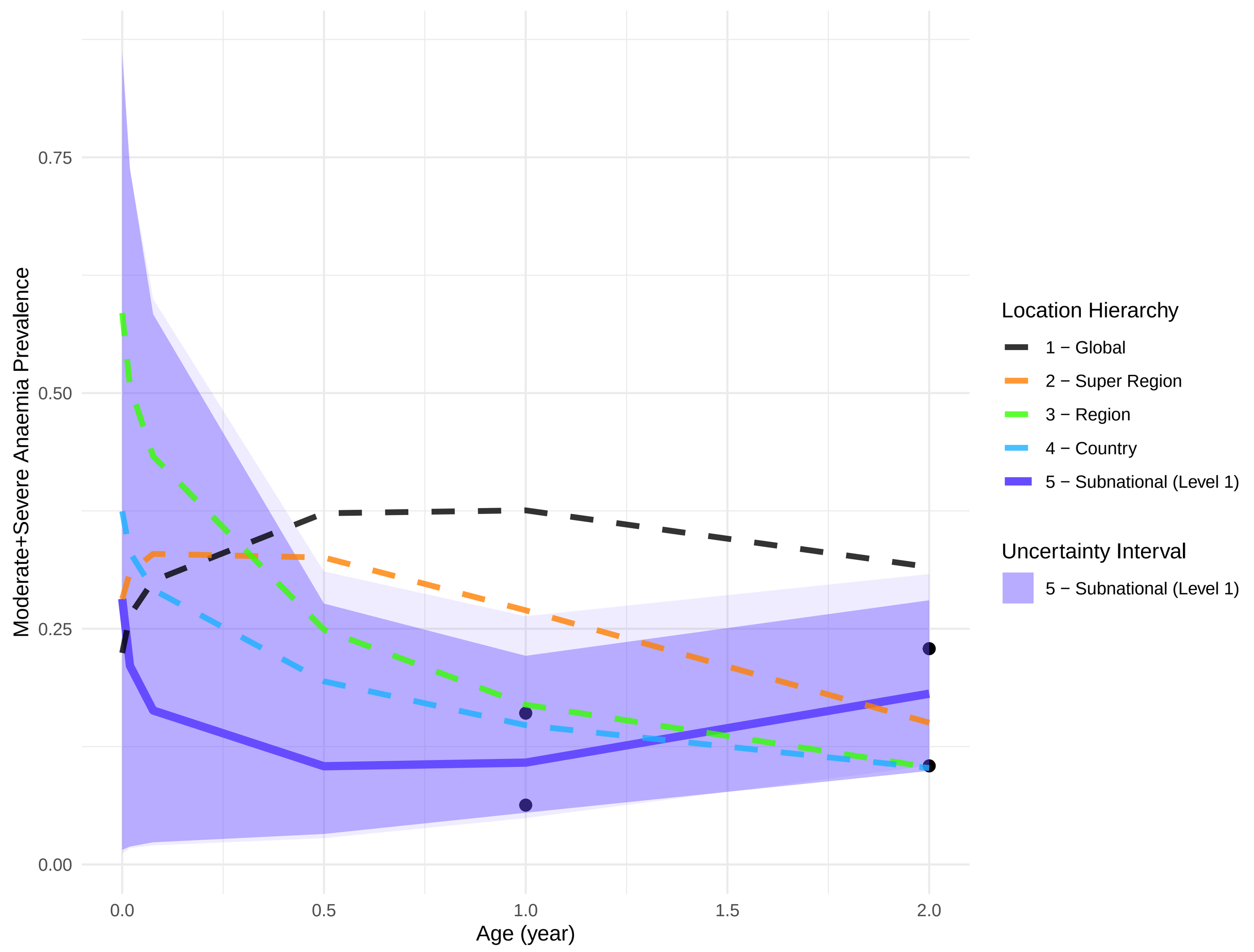

MR−BRT Age/Sex Fits − Males <2 Years Old − Moderate+Severe Anaemia Prevalence − Baja California Sur
WHO−2024 Adjustment Method

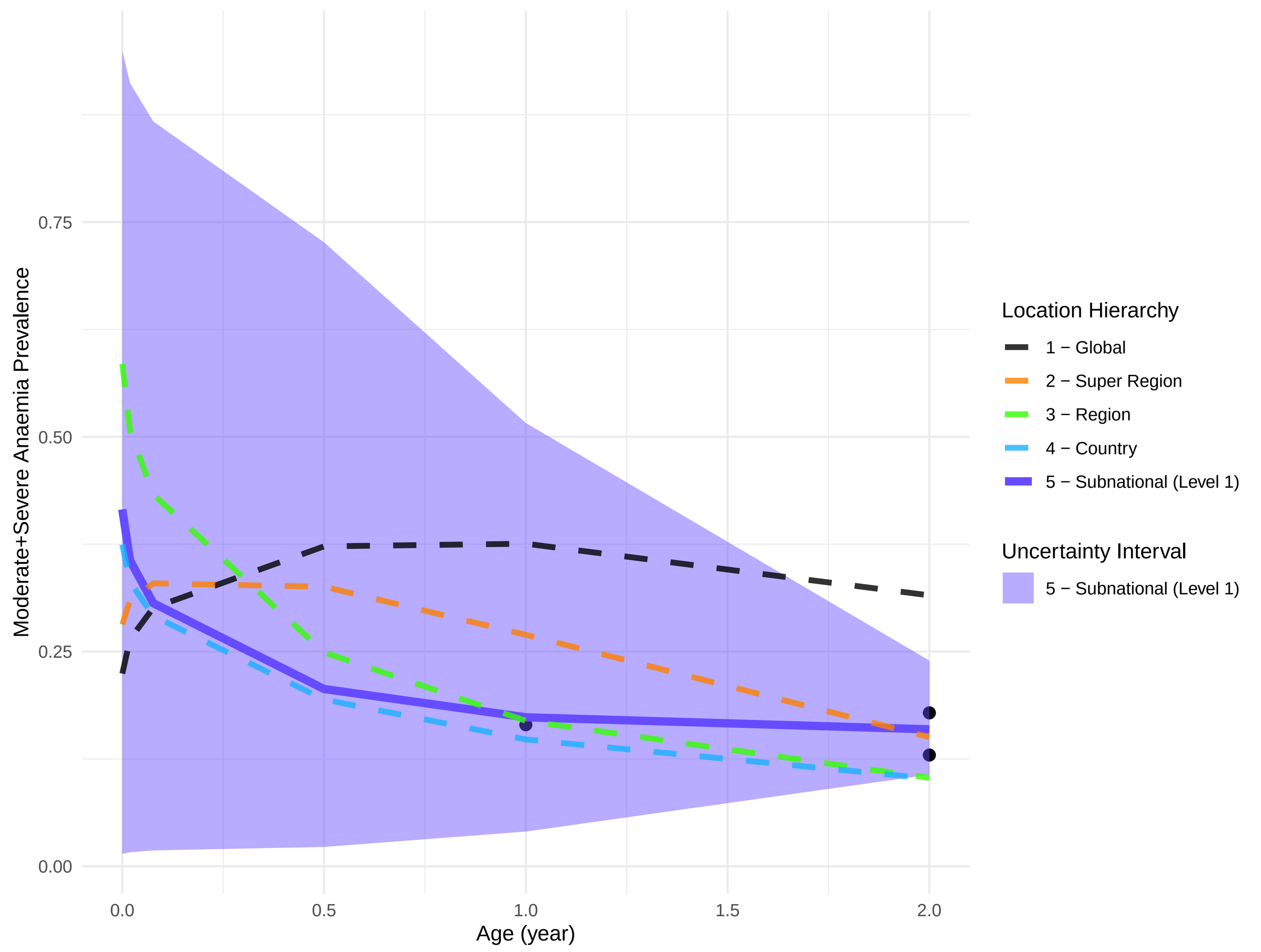

MR−BRT Age/Sex Fits − Males <2 Years Old − Moderate+Severe Anaemia Prevalence − Campeche
WHO−2024 Adjustment Method

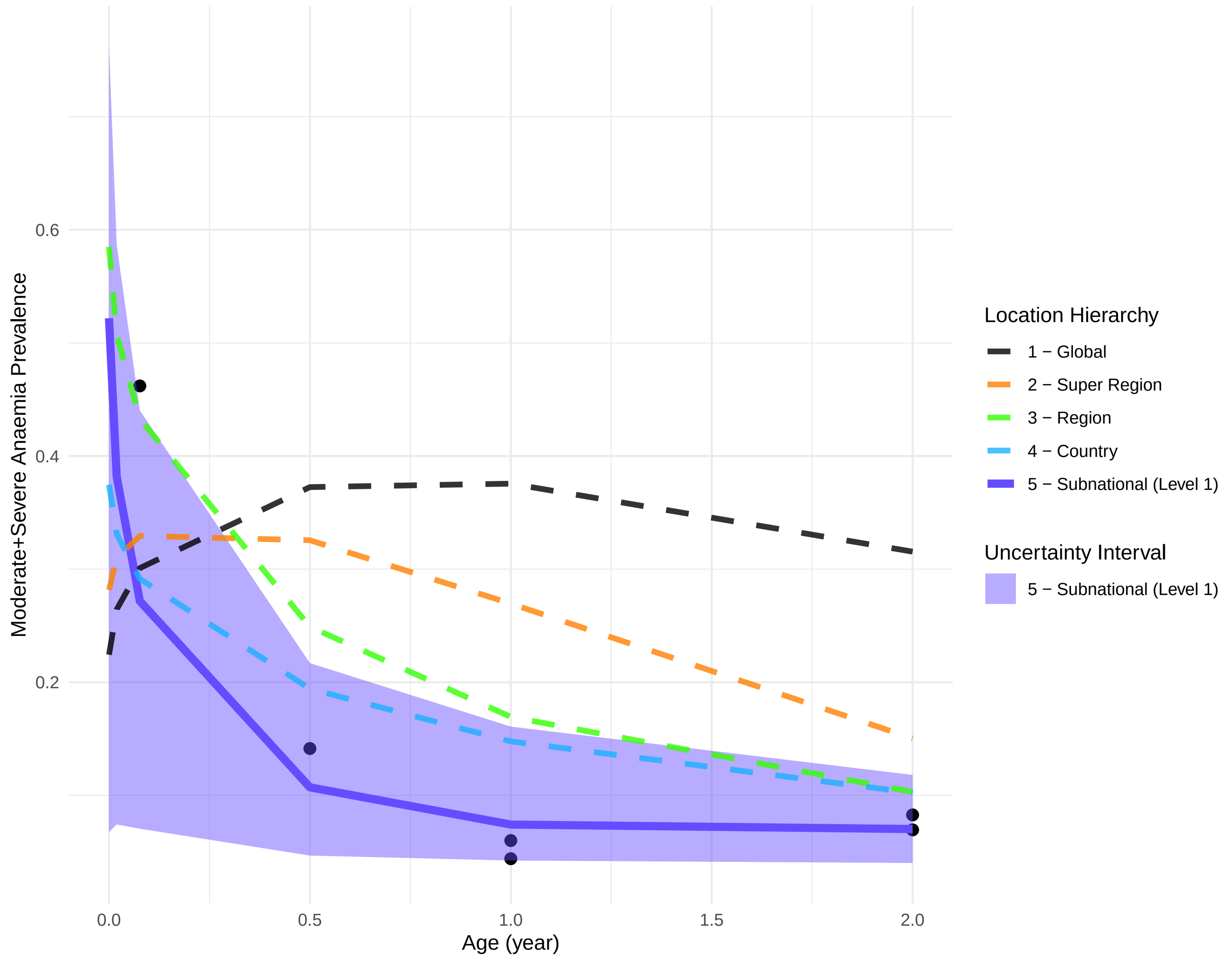

MR−BRT Age/Sex Fits − Males <2 Years Old − Moderate+Severe Anaemia Prevalence − Chiapas
WHO−2024 Adjustment Method

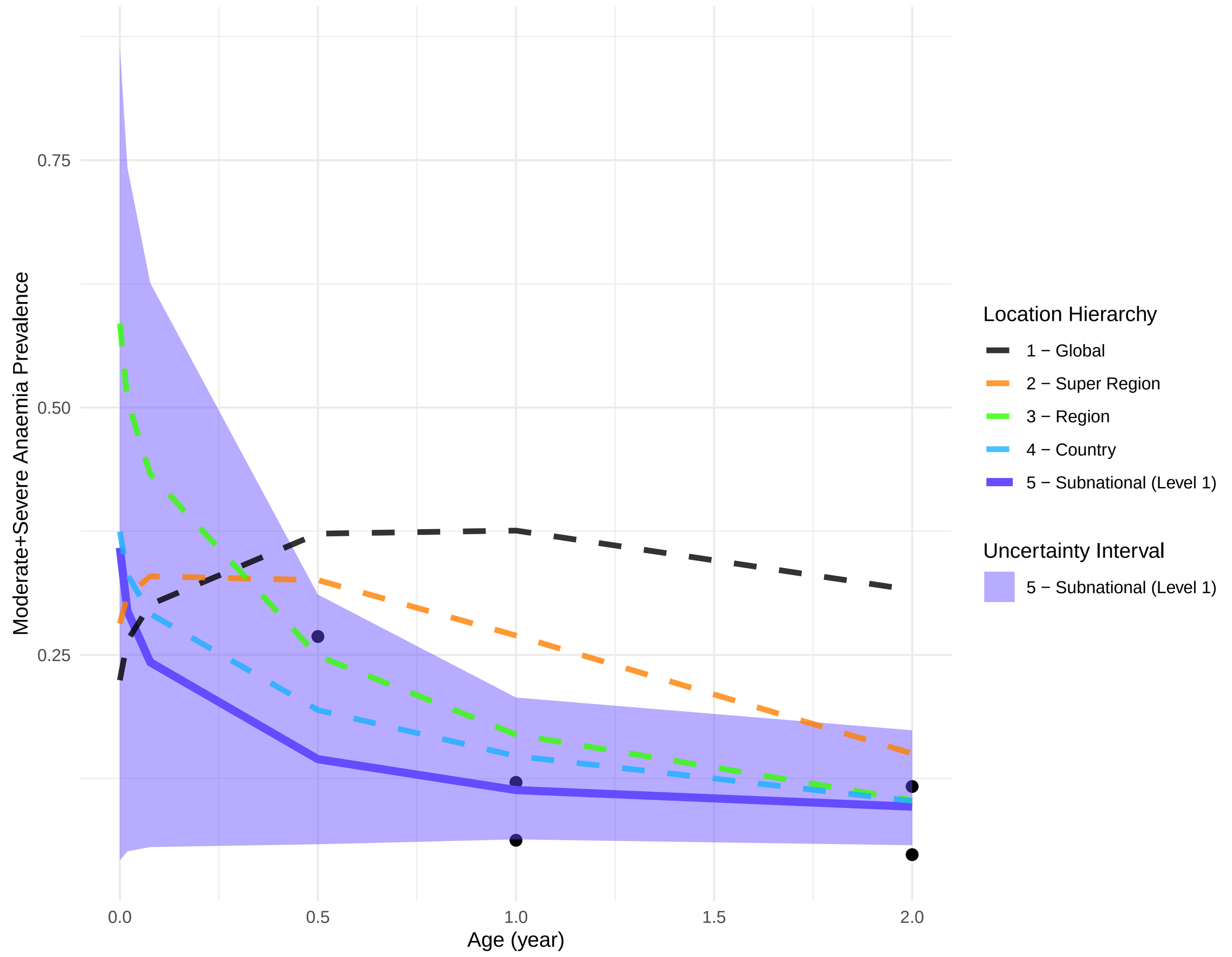

MR−BRT Age/Sex Fits − Males <2 Years Old − Moderate+Severe Anaemia Prevalence − Chihuahua
WHO−2024 Adjustment Method

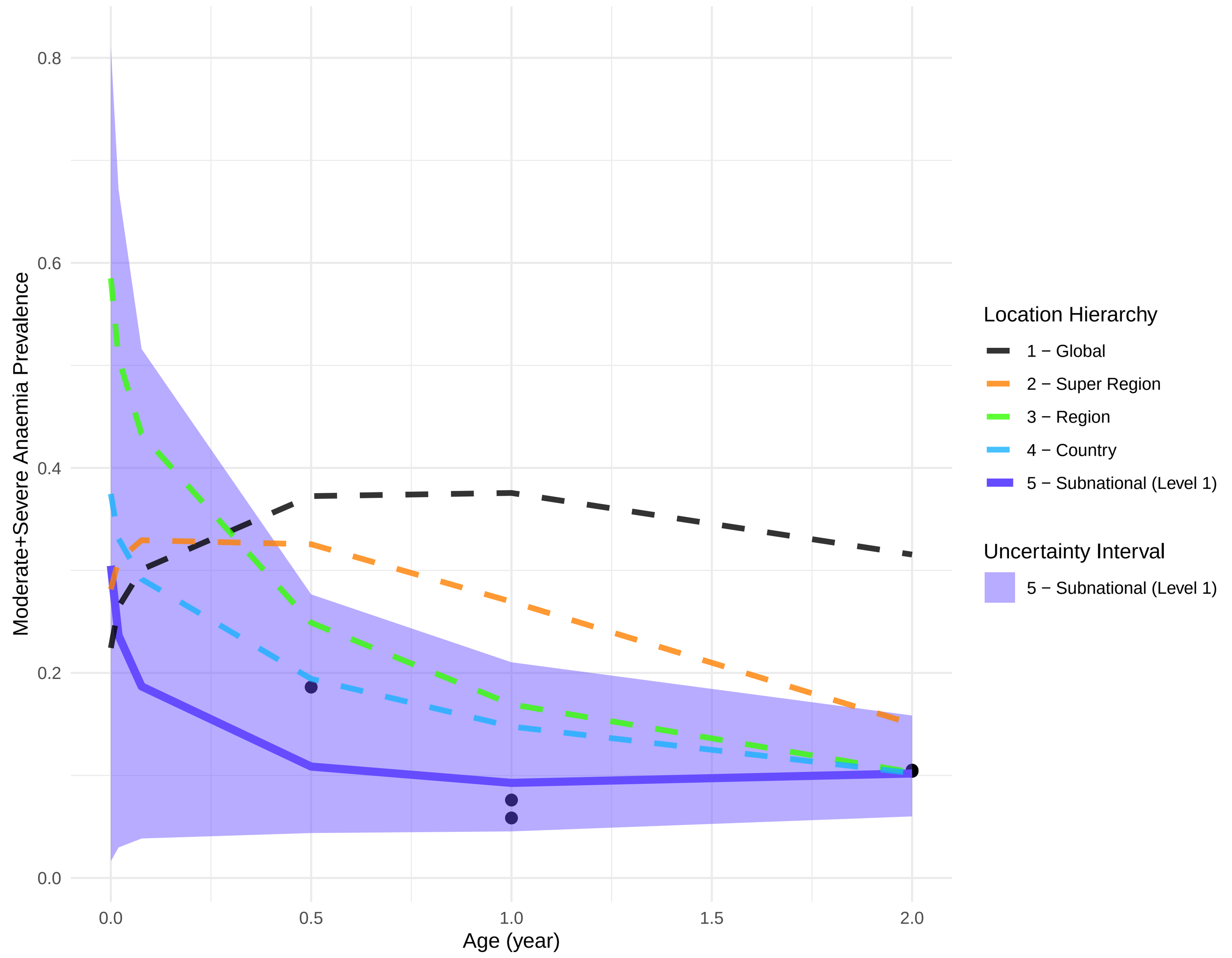

MR−BRT Age/Sex Fits − Males <2 Years Old − Moderate+Severe Anaemia Prevalence − Coahuila
WHO−2024 Adjustment Method

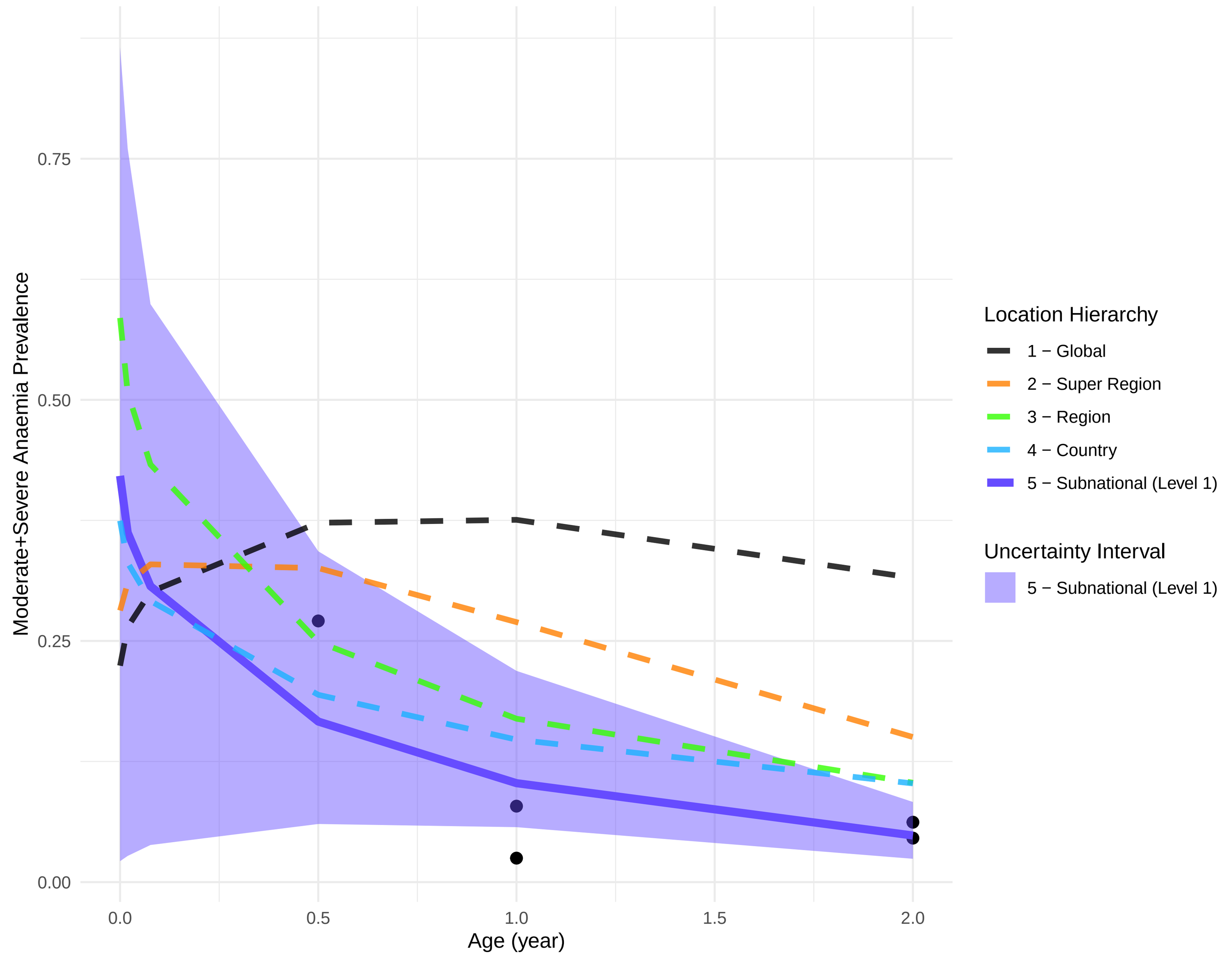

MR−BRT Age/Sex Fits − Males <2 Years Old − Moderate+Severe Anaemia Prevalence − Colima
WHO−2024 Adjustment Method

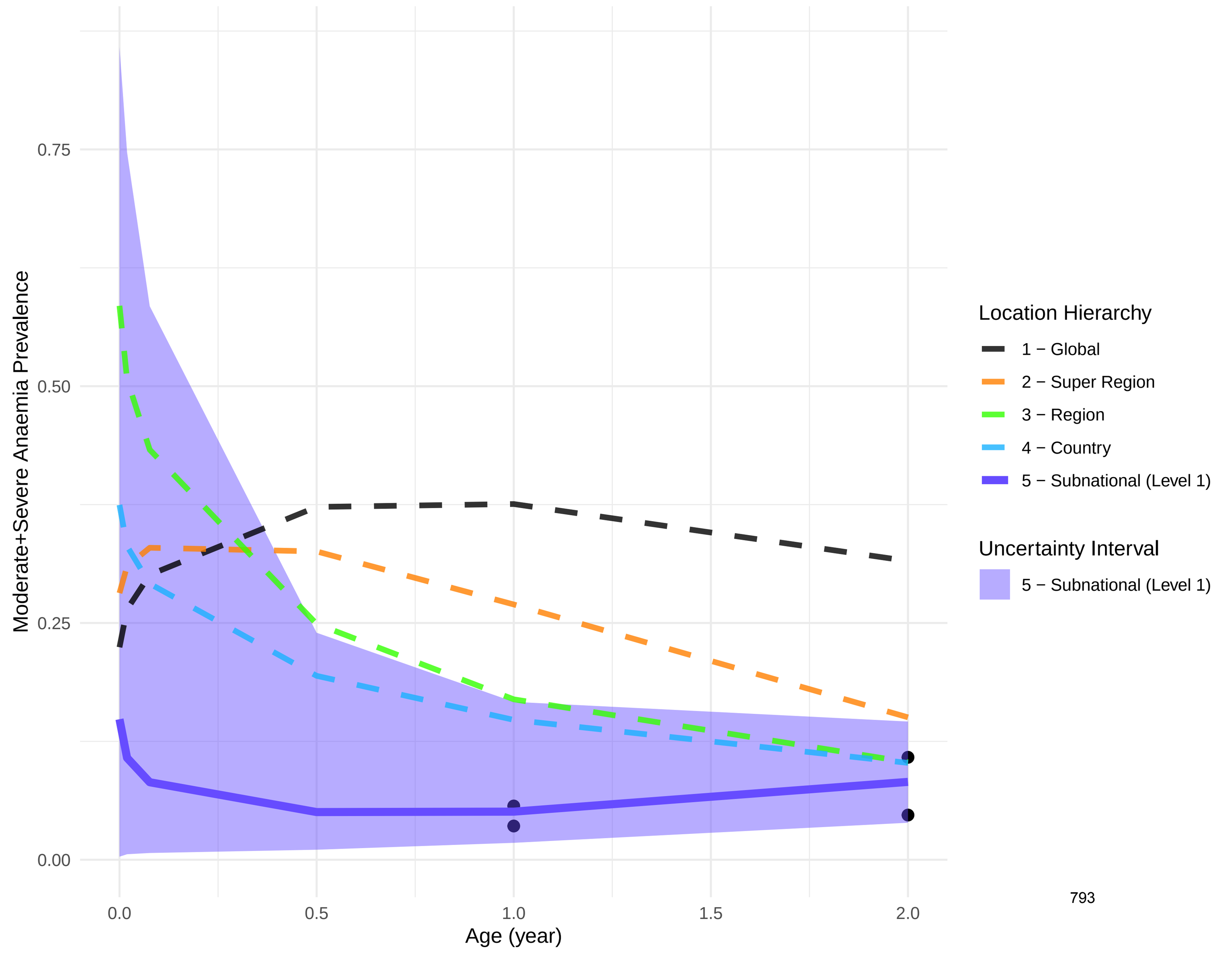

MR−BRT Age/Sex Fits − Males <2 Years Old − Moderate+Severe Anaemia Prevalence − Durango
WHO−2024 Adjustment Method

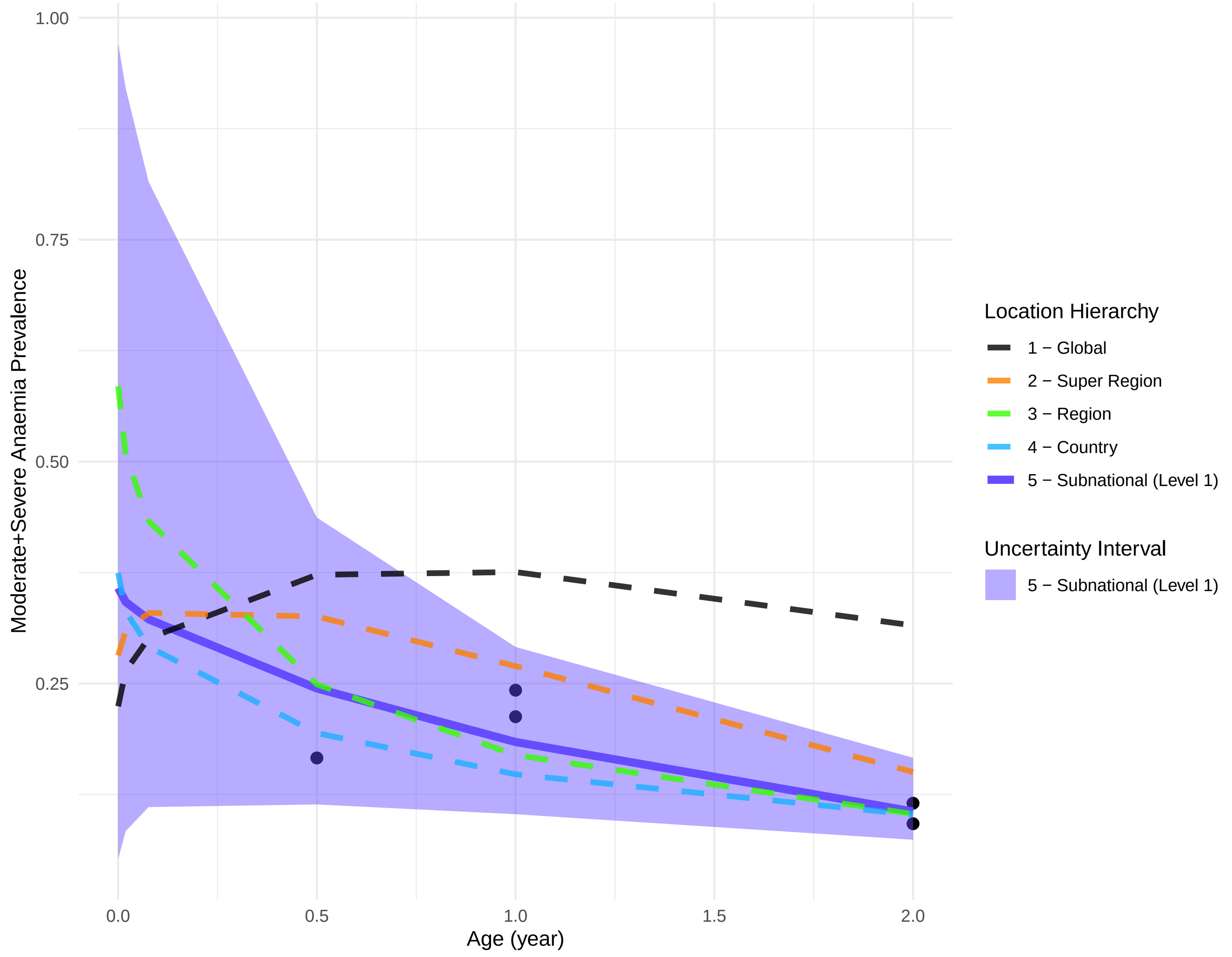

MR−BRT Age/Sex Fits − Males <2 Years Old − Moderate+Severe Anaemia Prevalence − Guanajuato
WHO−2024 Adjustment Method

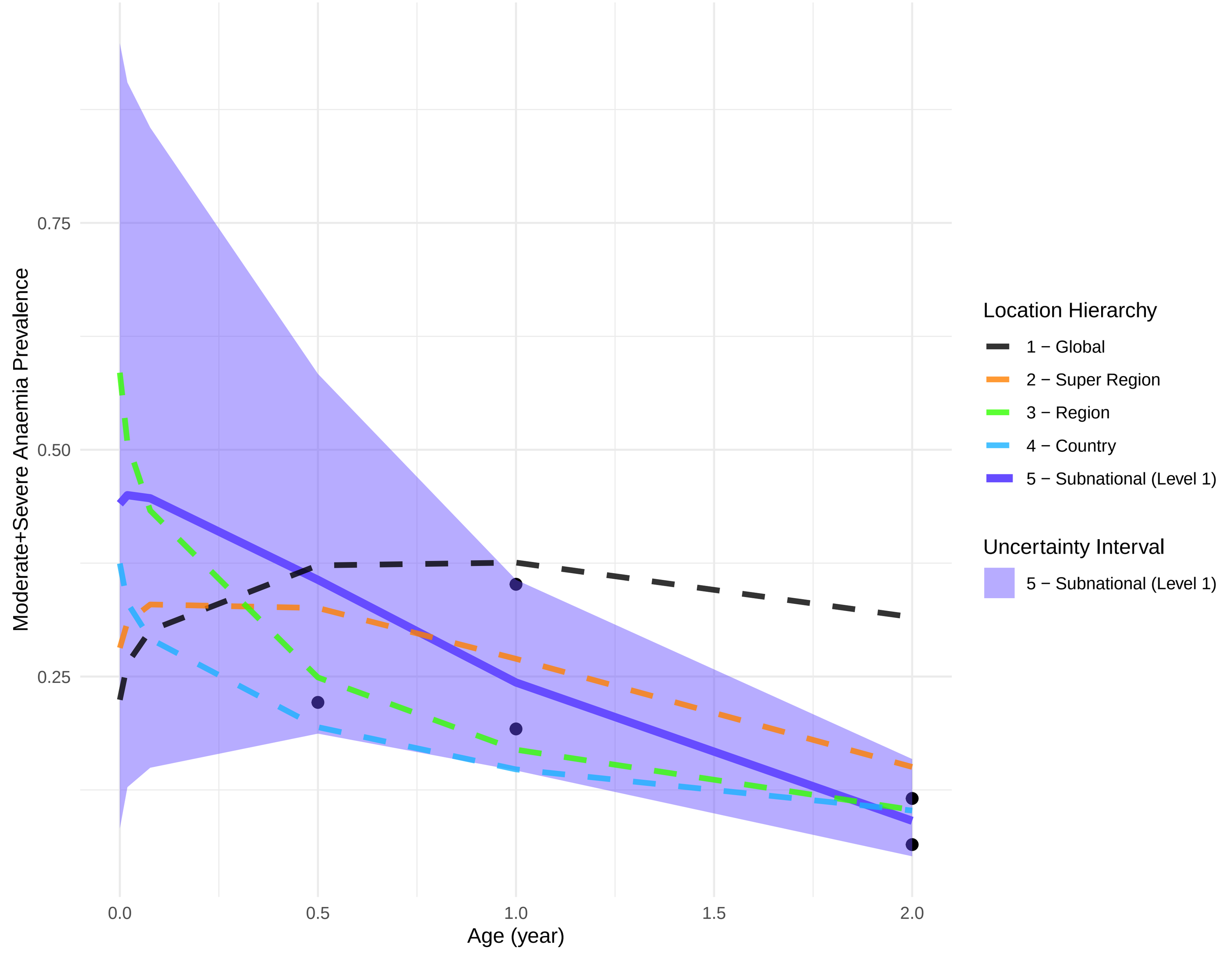

MR−BRT Age/Sex Fits − Males <2 Years Old − Moderate+Severe Anaemia Prevalence − Guerrero
WHO−2024 Adjustment Method

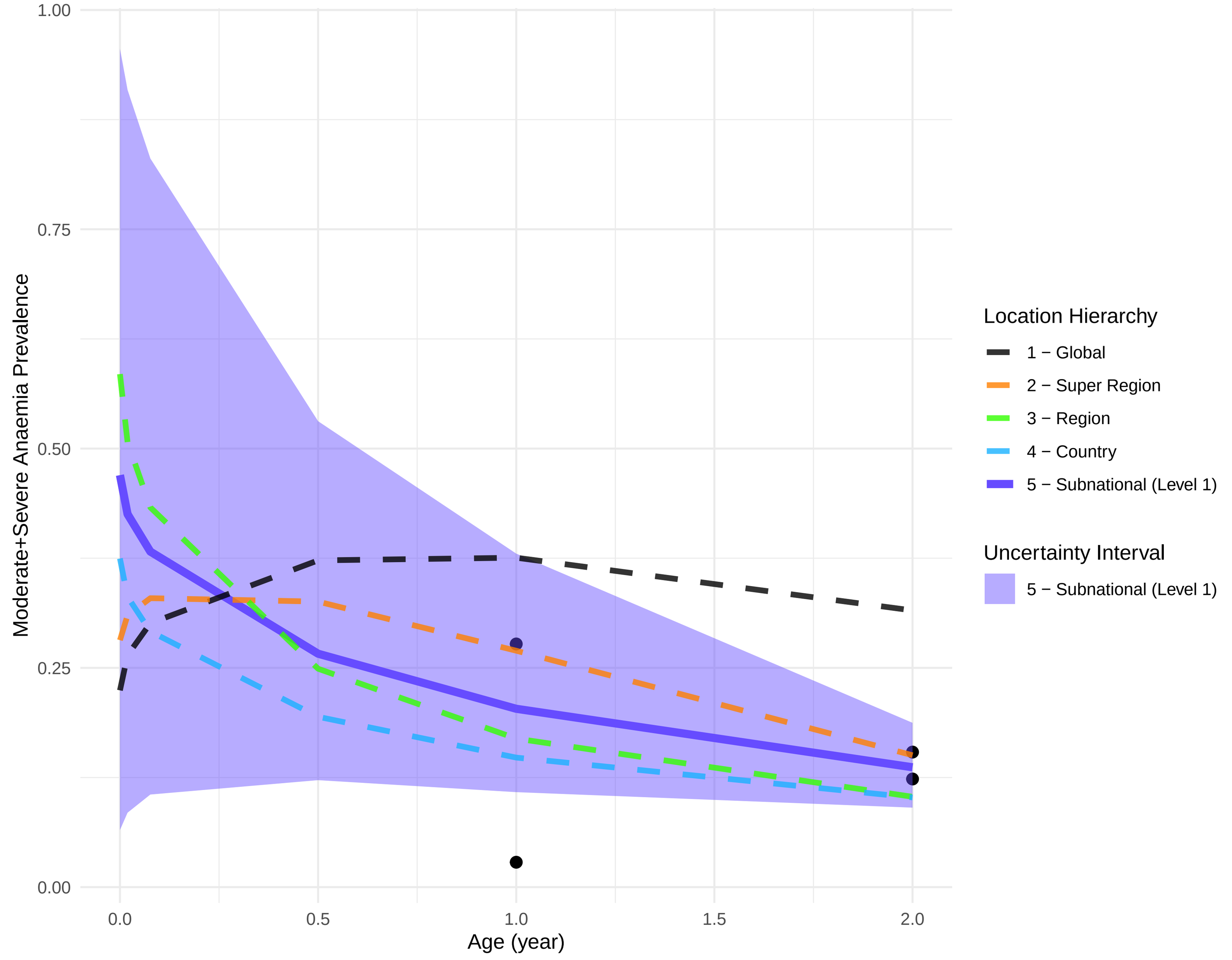

MR−BRT Age/Sex Fits − Males <2 Years Old − Moderate+Severe Anaemia Prevalence − Hidalgo
WHO−2024 Adjustment Method

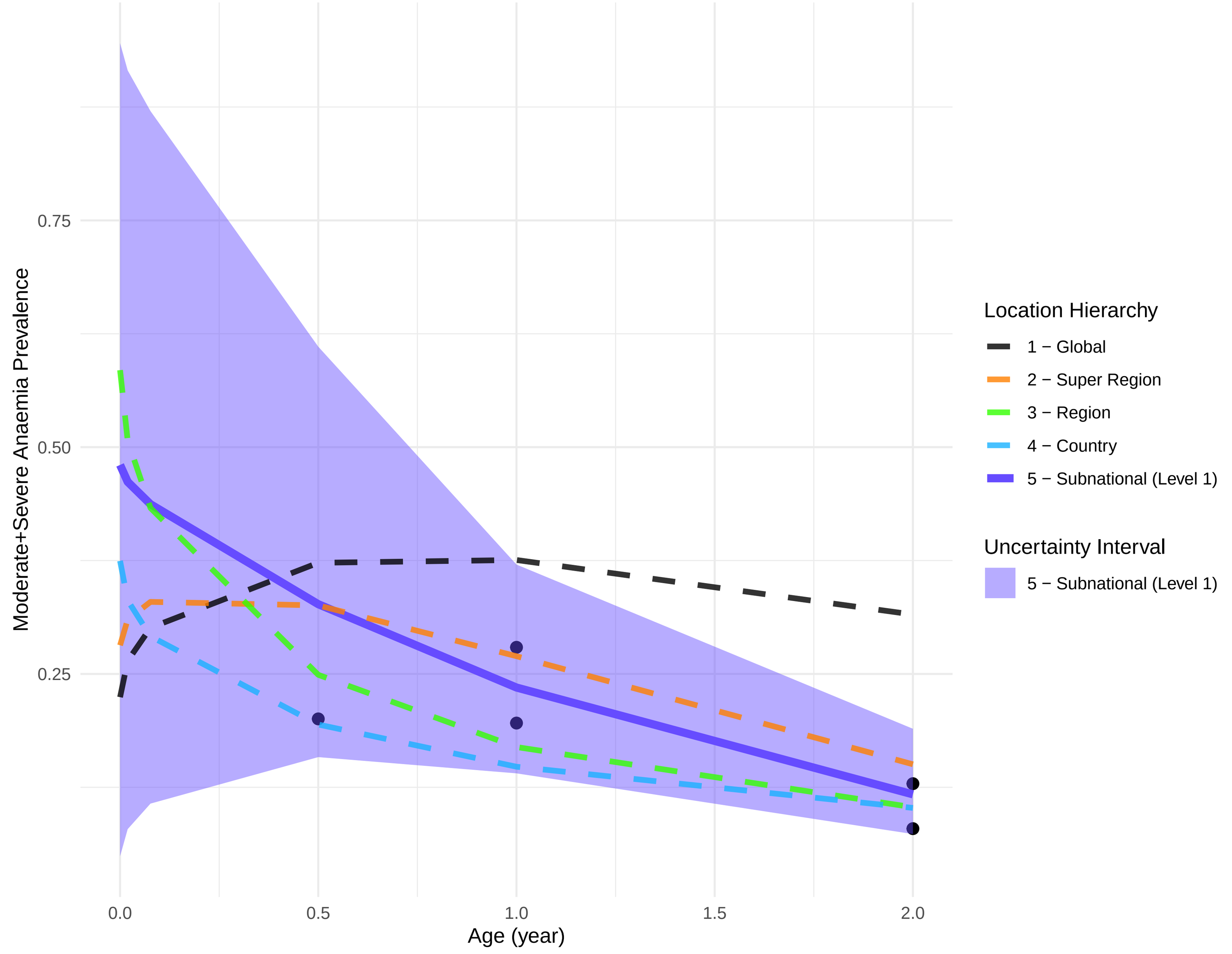

MR−BRT Age/Sex Fits − Males <2 Years Old − Moderate+Severe Anaemia Prevalence − Jalisco
WHO−2024 Adjustment Method

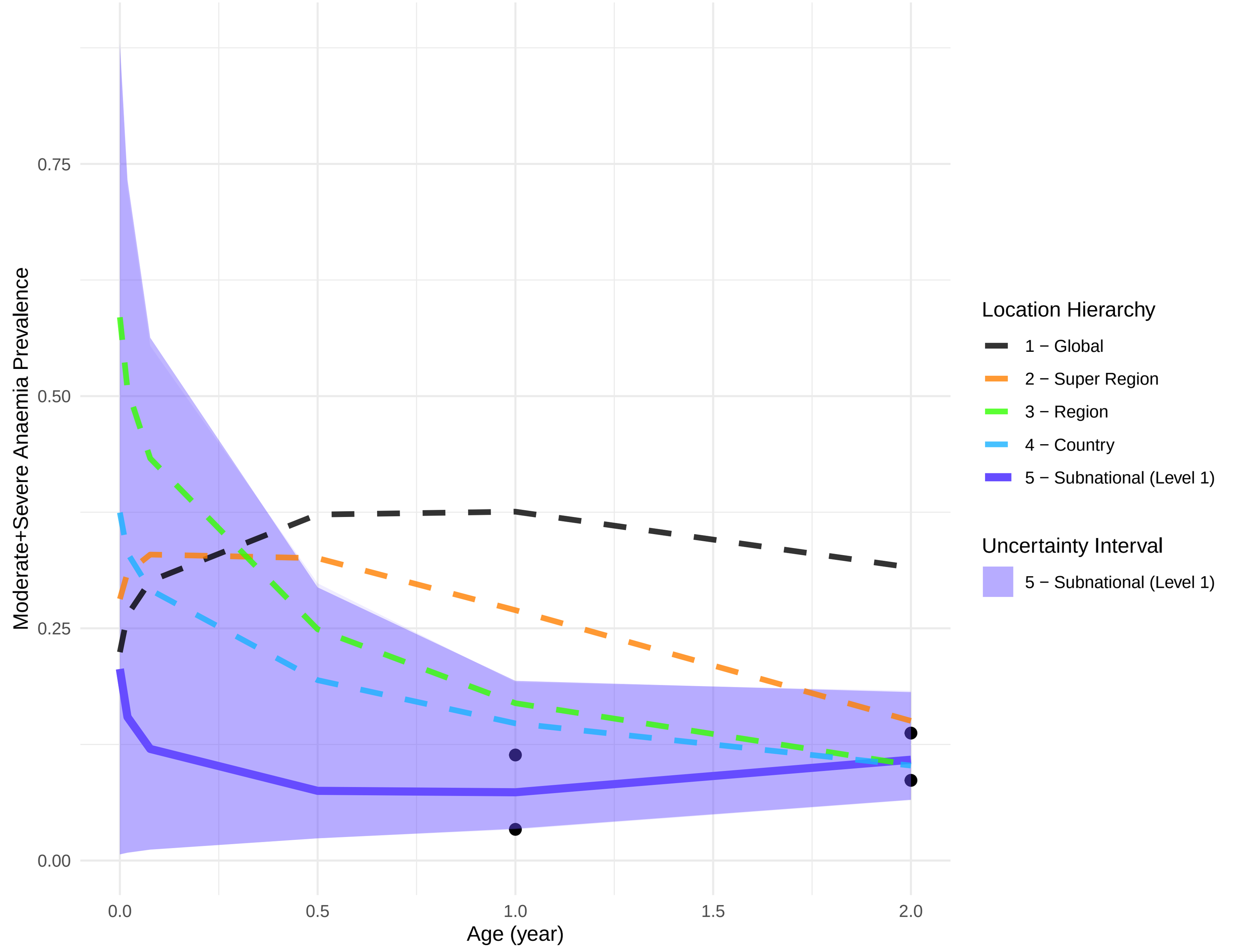

MR−BRT Age/Sex Fits − Males <2 Years Old − Moderate+Severe Anaemia Prevalence − México
WHO−2024 Adjustment Method

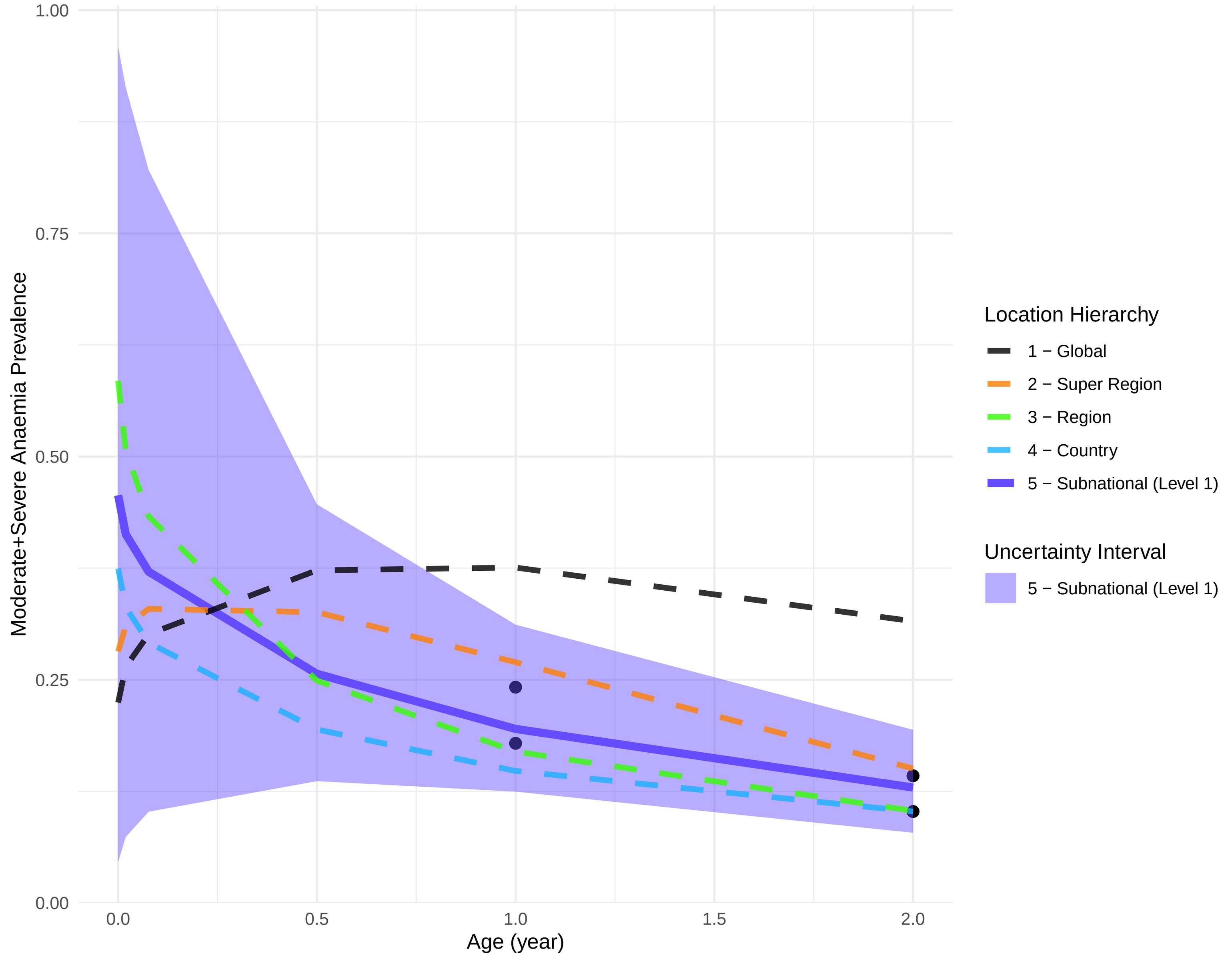

MR−BRT Age/Sex Fits − Males <2 Years Old − Moderate+Severe Anaemia Prevalence − Mexico City
WHO−2024 Adjustment Method

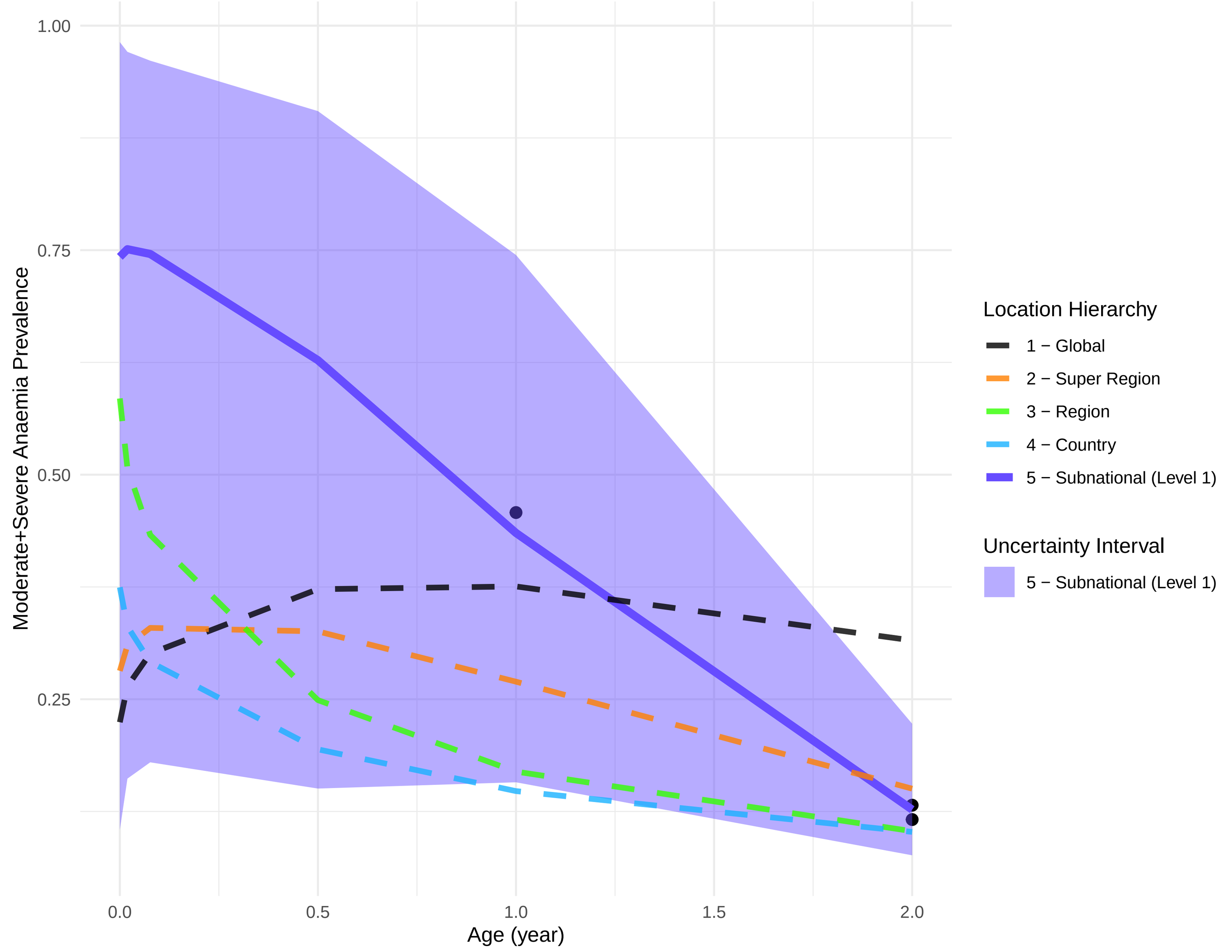

MR−BRT Age/Sex Fits − Males <2 Years Old − Moderate+Severe Anaemia Prevalence − Michoacán de Ocampo
WHO−2024 Adjustment Method

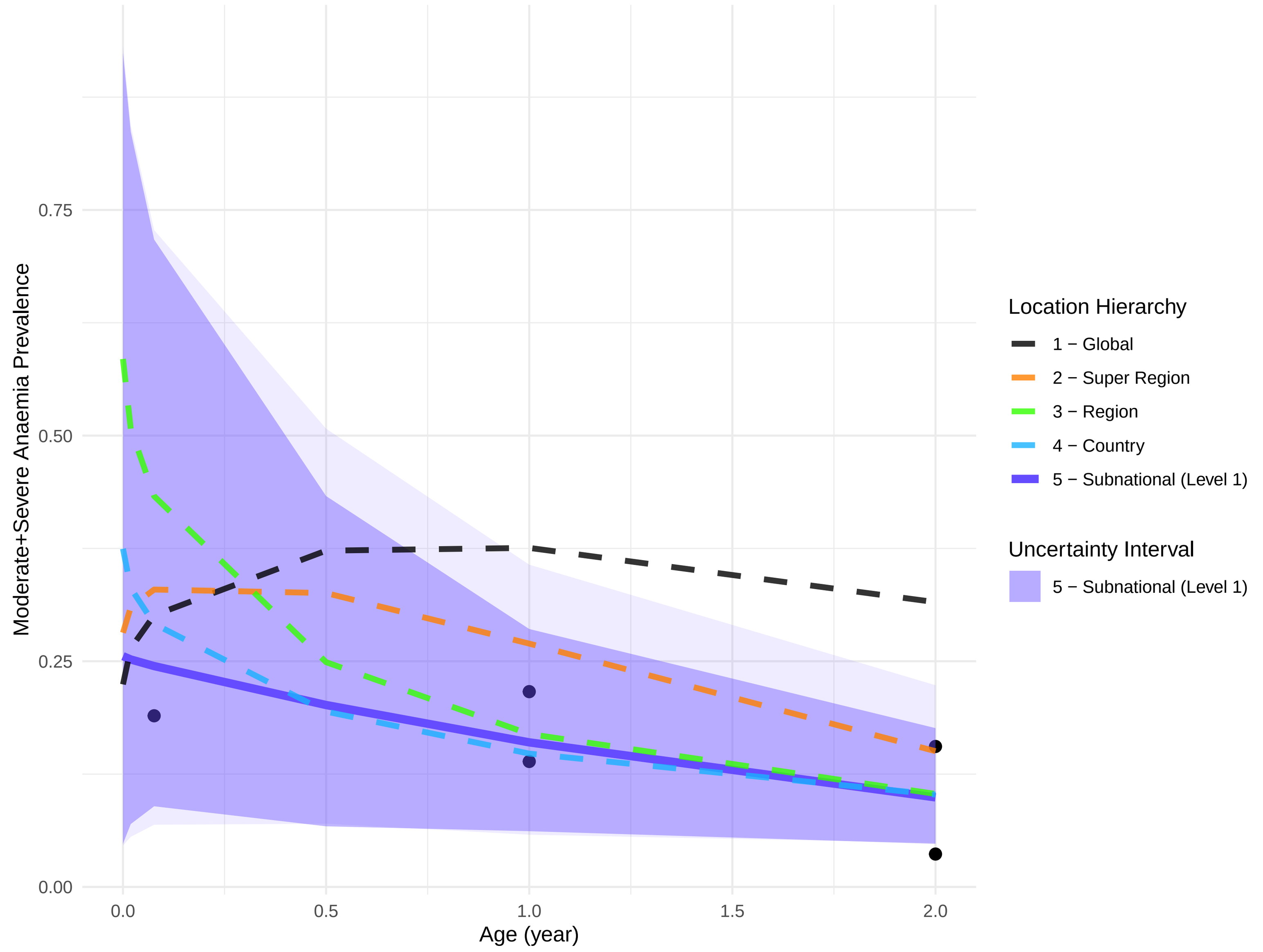

MR−BRT Age/Sex Fits − Males <2 Years Old − Moderate+Severe Anaemia Prevalence − Morelos
WHO−2024 Adjustment Method

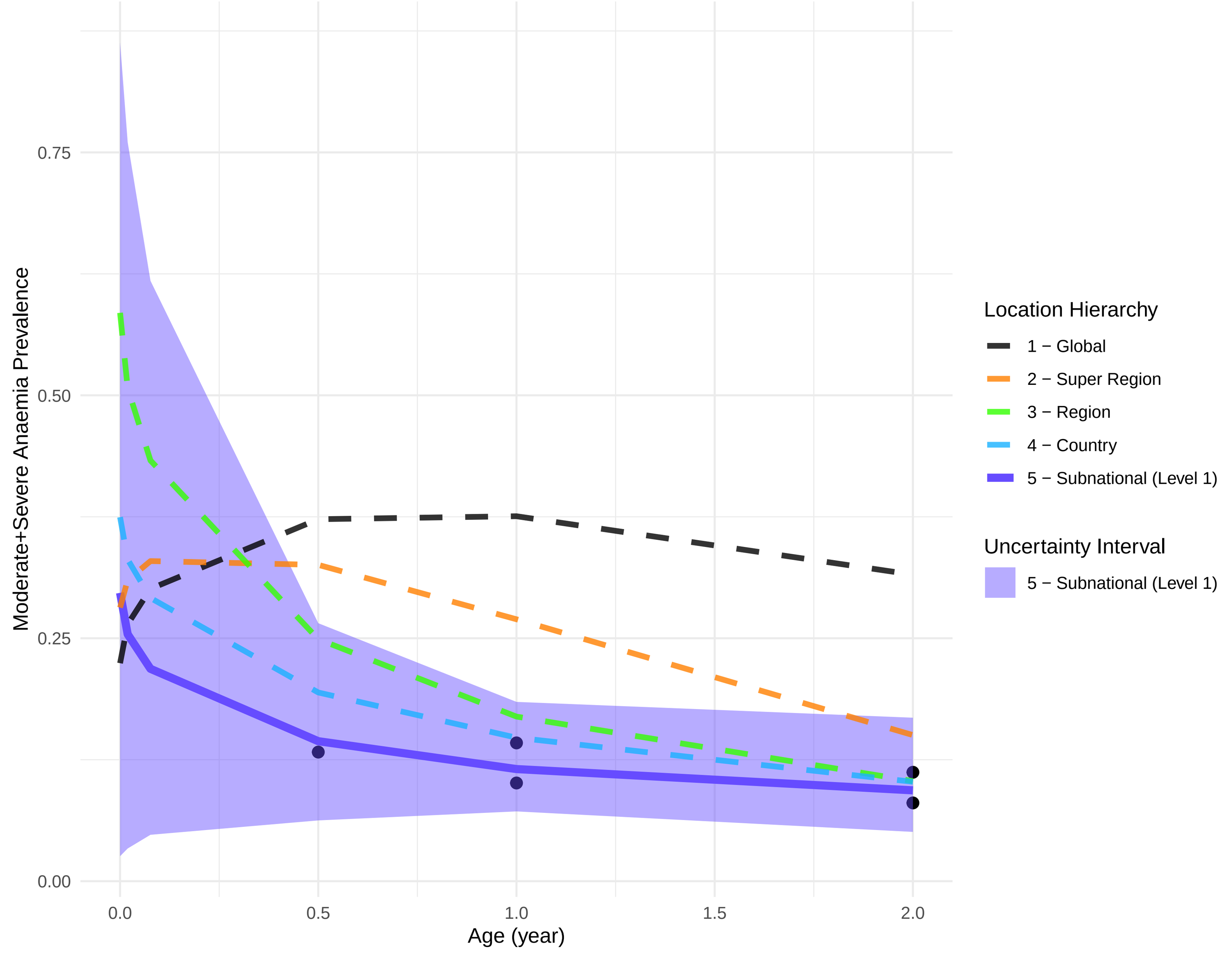

MR−BRT Age/Sex Fits − Males <2 Years Old − Moderate+Severe Anaemia Prevalence − Nayarit
WHO−2024 Adjustment Method

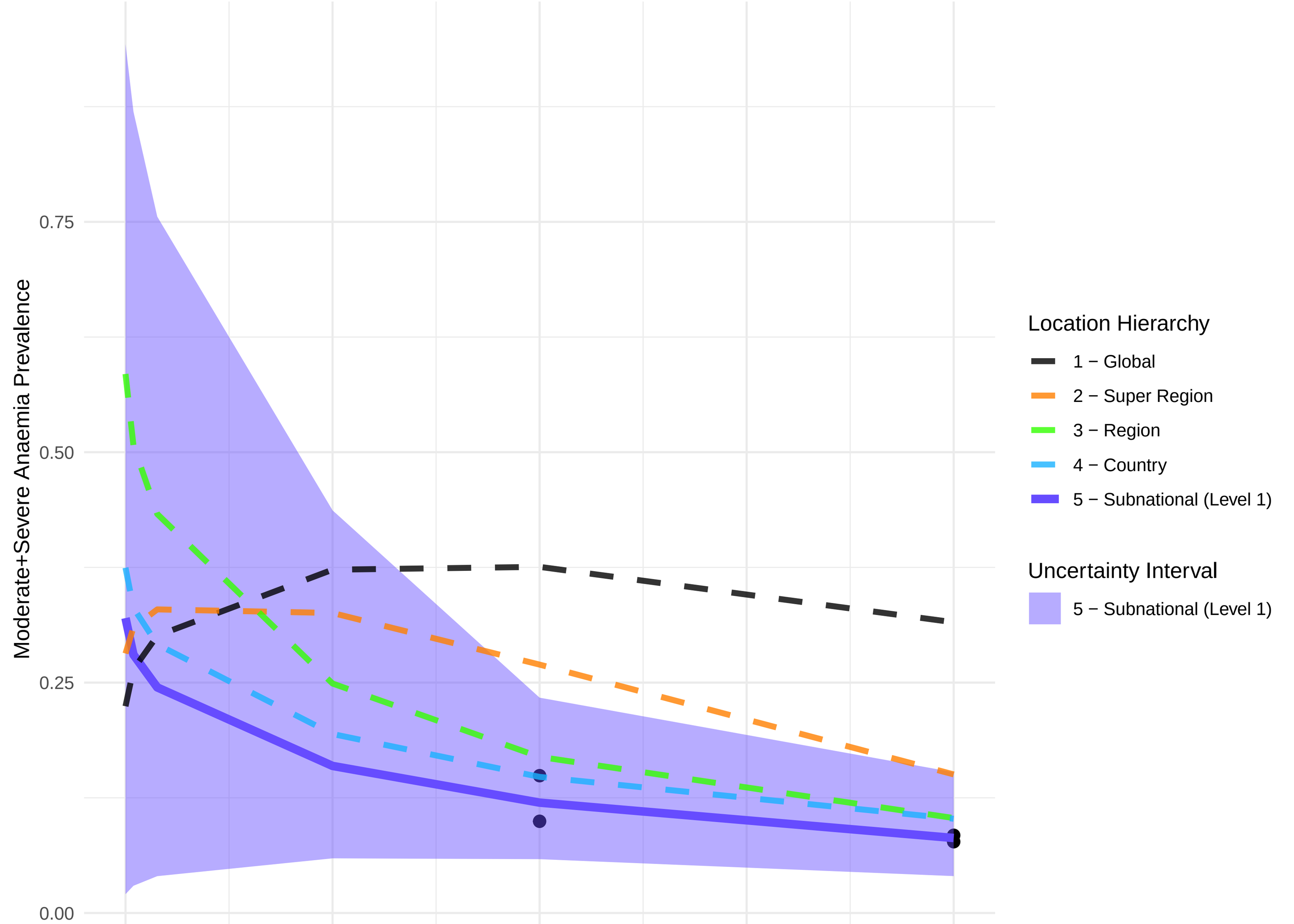

MR−BRT Age/Sex Fits − Males <2 Years Old − Moderate+Severe Anaemia Prevalence − Nuevo León
WHO−2024 Adjustment Method

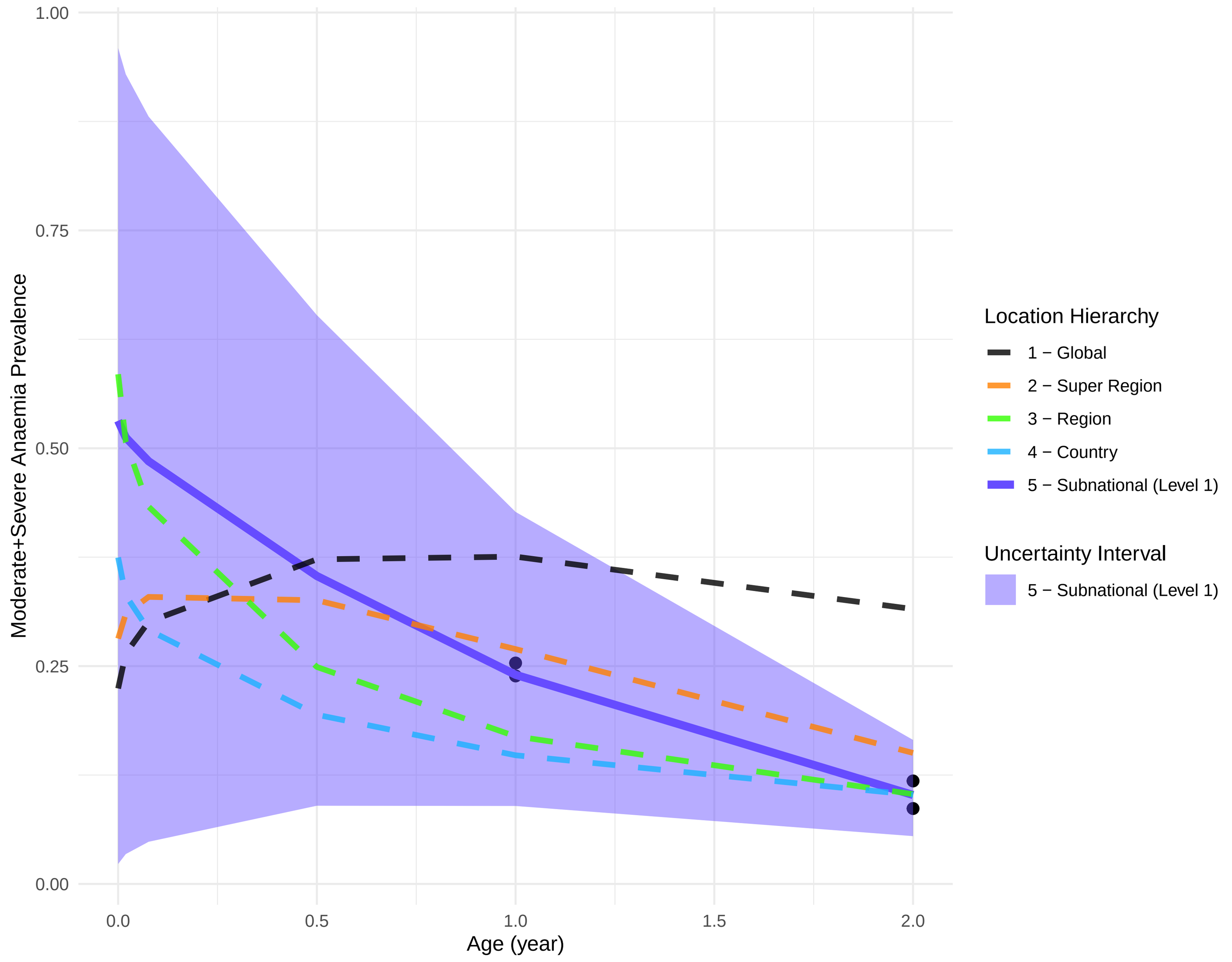

MR−BRT Age/Sex Fits − Males <2 Years Old − Moderate+Severe Anaemia Prevalence − Oaxaca
WHO−2024 Adjustment Method

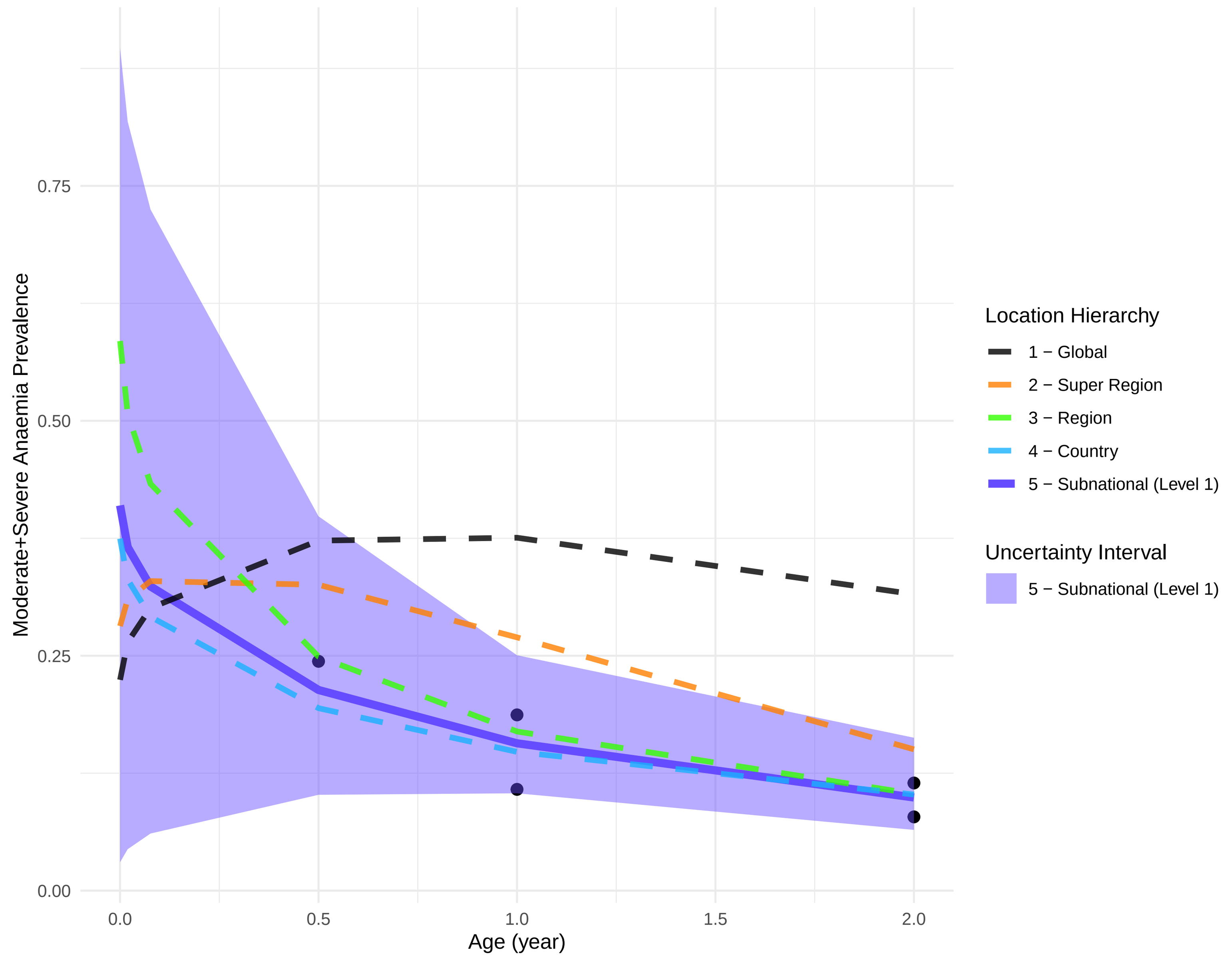

MR−BRT Age/Sex Fits − Males <2 Years Old − Moderate+Severe Anaemia Prevalence − Puebla
WHO−2024 Adjustment Method

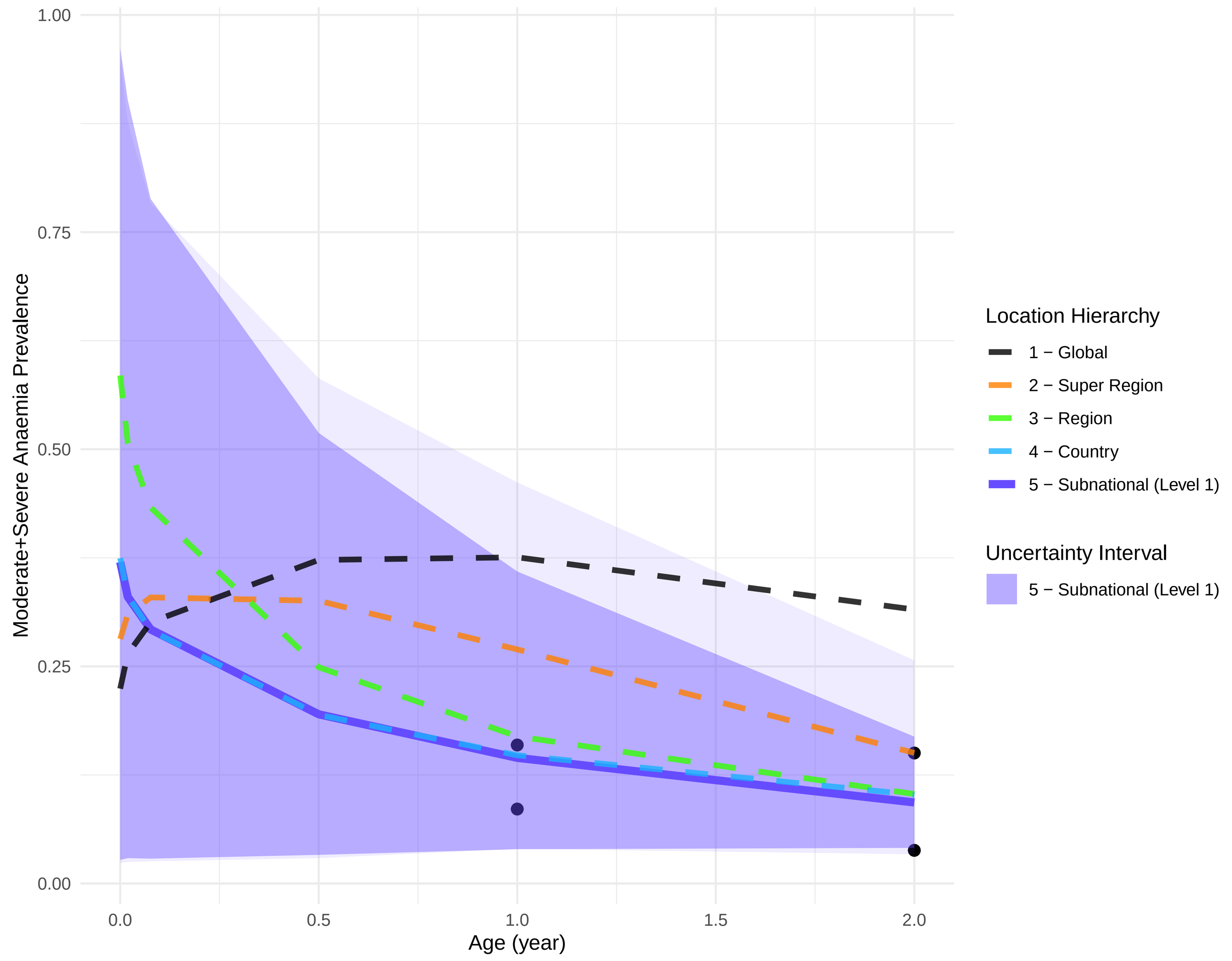

MR−BRT Age/Sex Fits − Males <2 Years Old − Moderate+Severe Anaemia Prevalence − Querétaro
WHO−2024 Adjustment Method

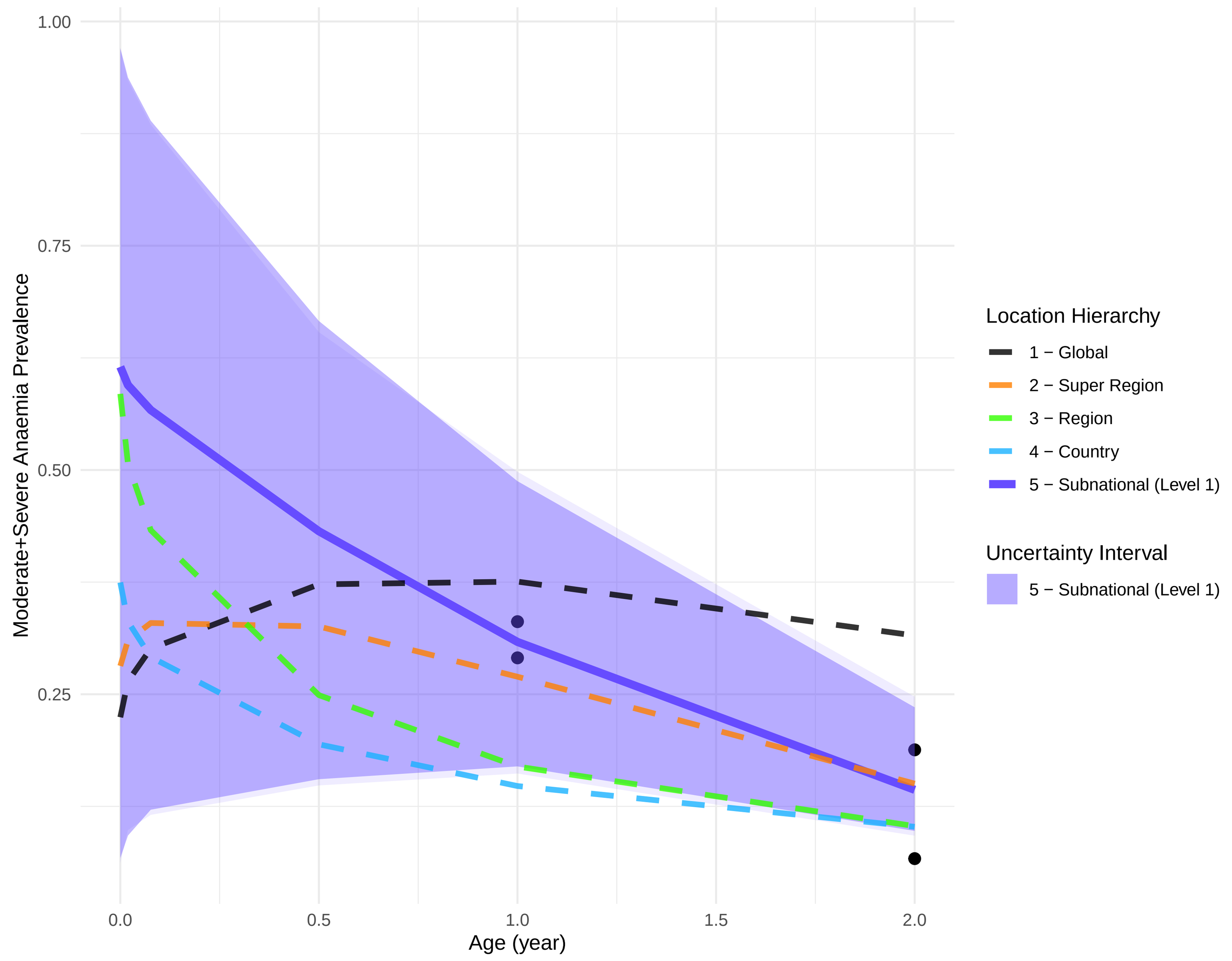

MR−BRT Age/Sex Fits − Males <2 Years Old − Moderate+Severe Anaemia Prevalence − Quintana Roo
WHO−2024 Adjustment Method

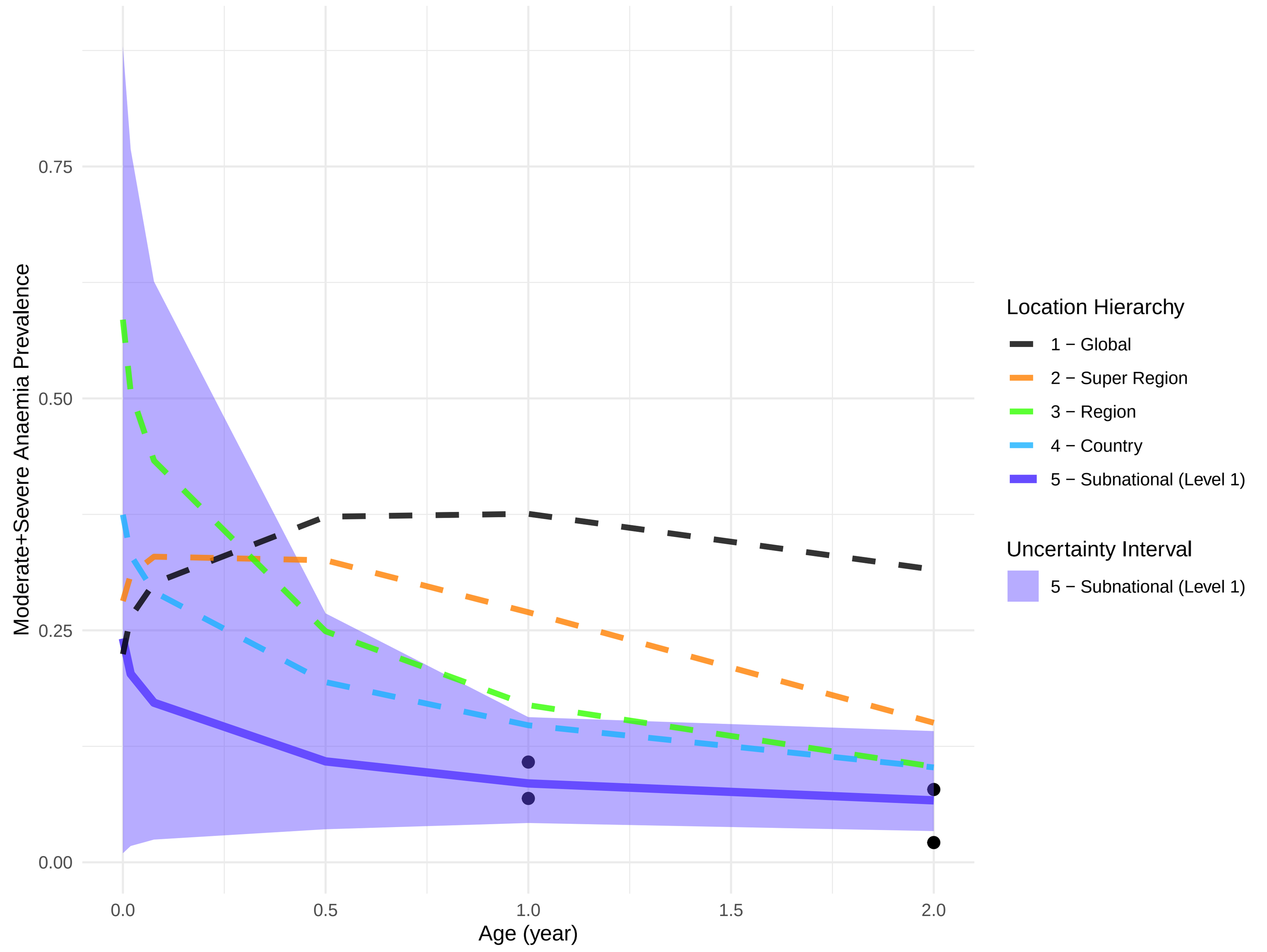

MR−BRT Age/Sex Fits − Males <2 Years Old − Moderate+Severe Anaemia Prevalence − San Luis Potosí
WHO−2024 Adjustment Method

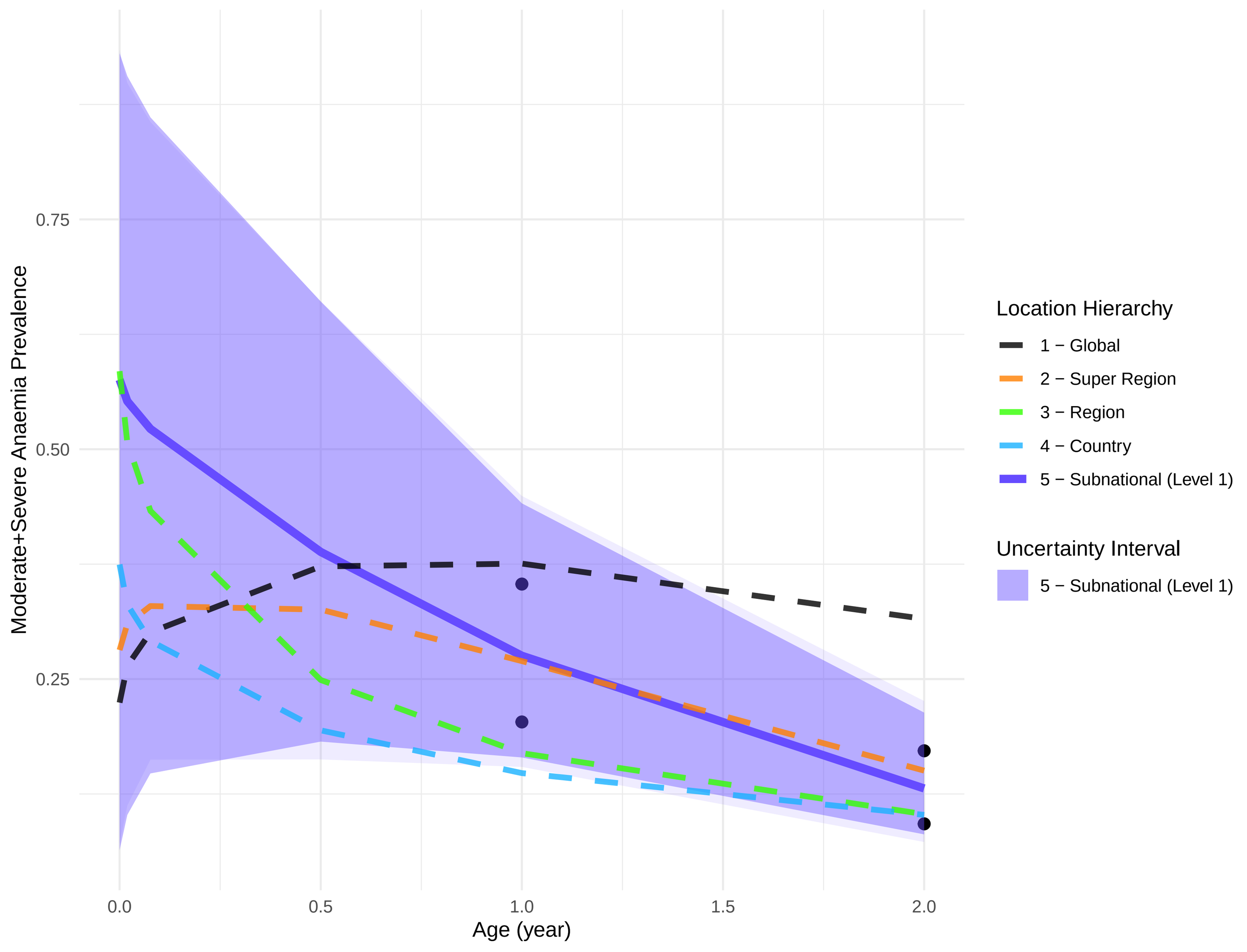

MR−BRT Age/Sex Fits − Males <2 Years Old − Moderate+Severe Anaemia Prevalence − Sinaloa
WHO−2024 Adjustment Method

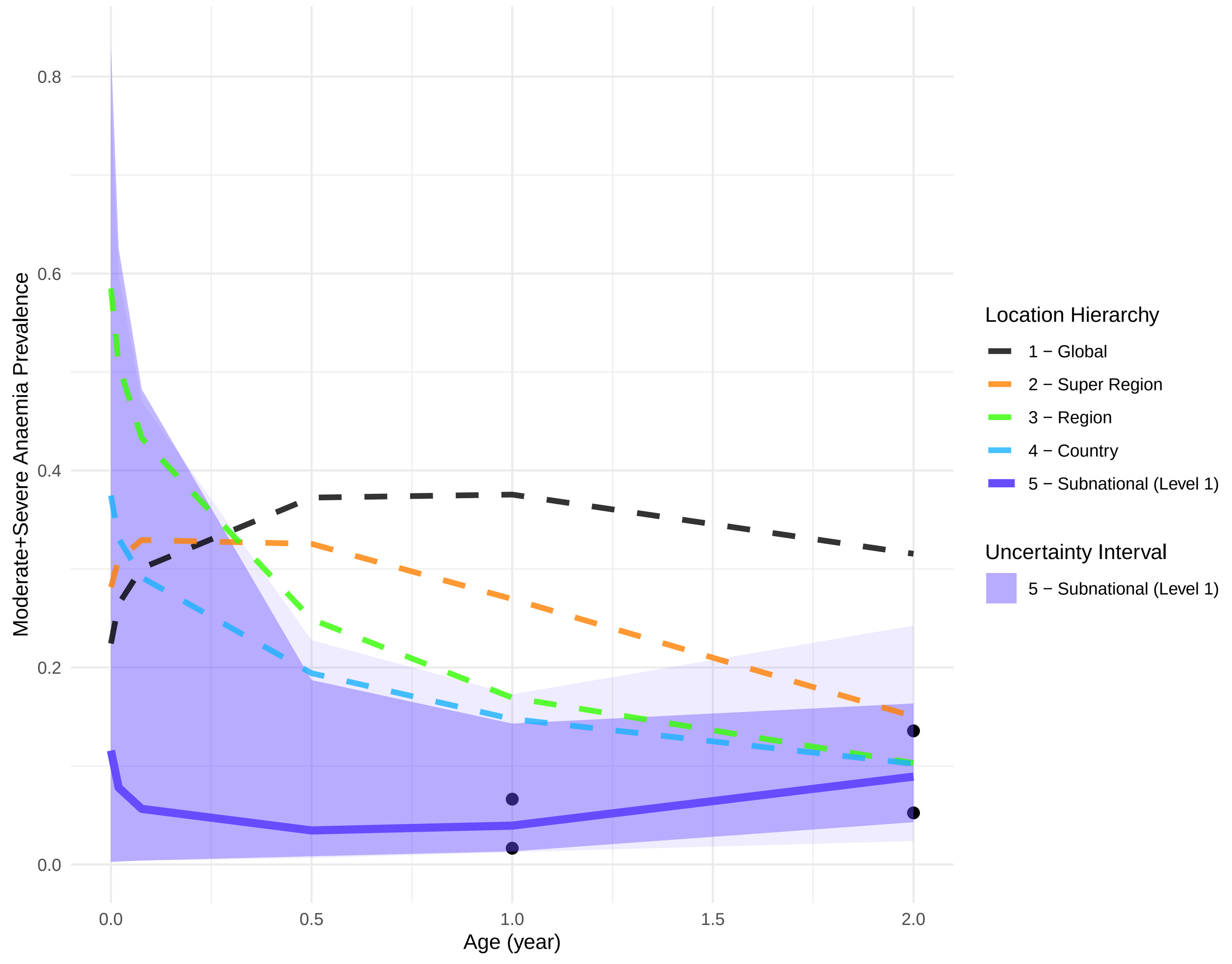

MR−BRT Age/Sex Fits − Males <2 Years Old − Moderate+Severe Anaemia Prevalence − Sonora
WHO−2024 Adjustment Method

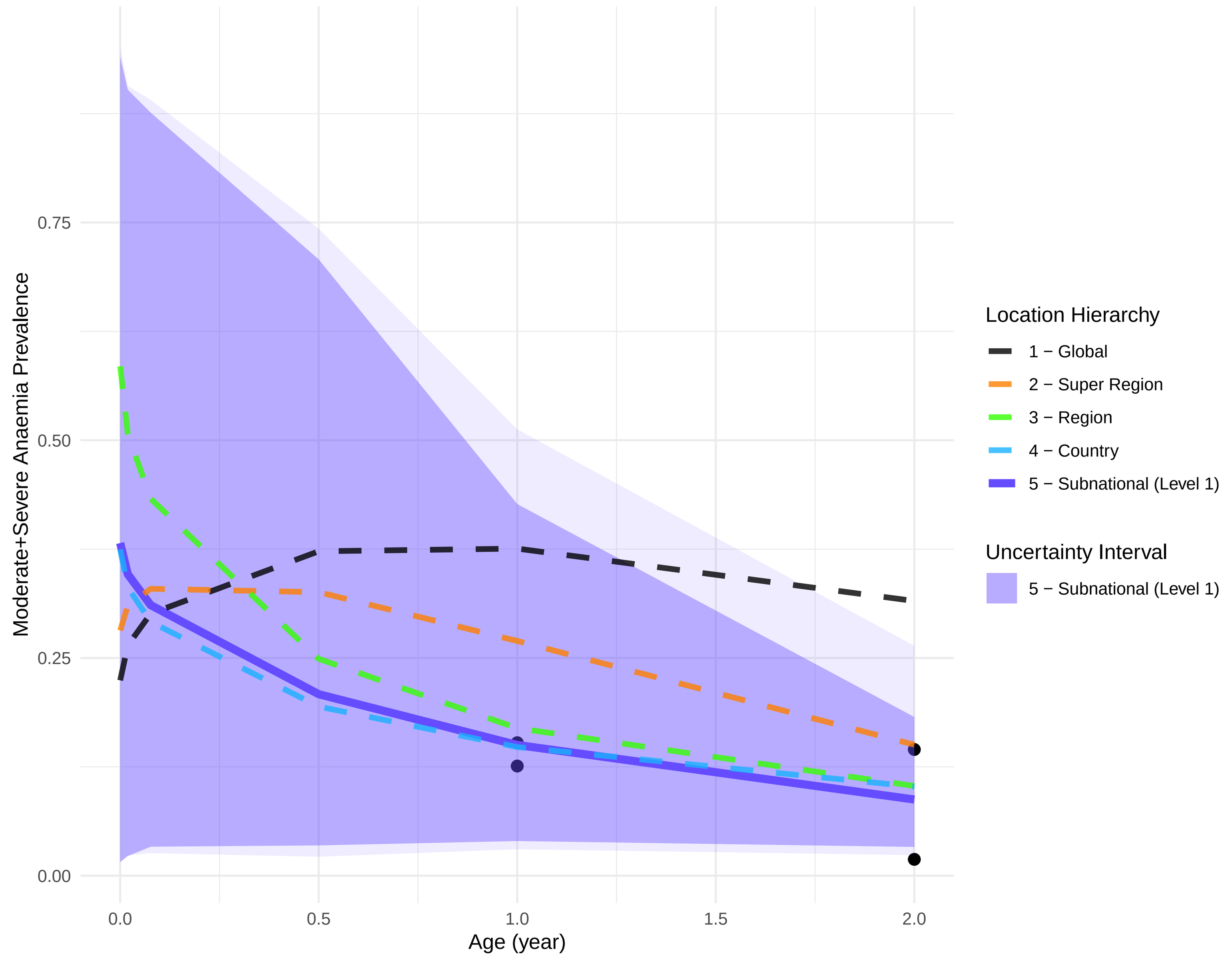

MR−BRT Age/Sex Fits − Males <2 Years Old − Moderate+Severe Anaemia Prevalence − Tabasco
WHO−2024 Adjustment Method

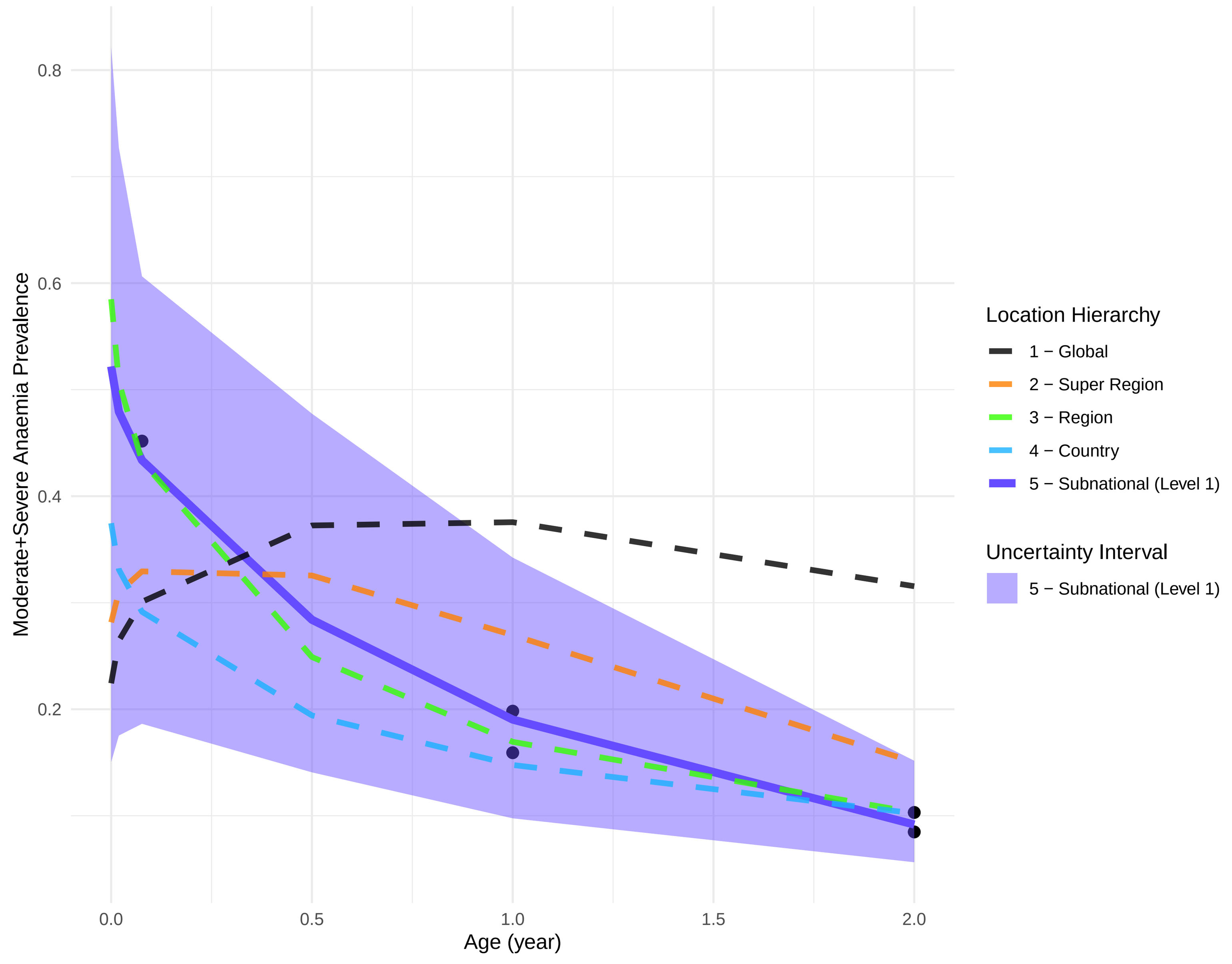

MR−BRT Age/Sex Fits − Males <2 Years Old − Moderate+Severe Anaemia Prevalence − Tamaulipas
WHO−2024 Adjustment Method

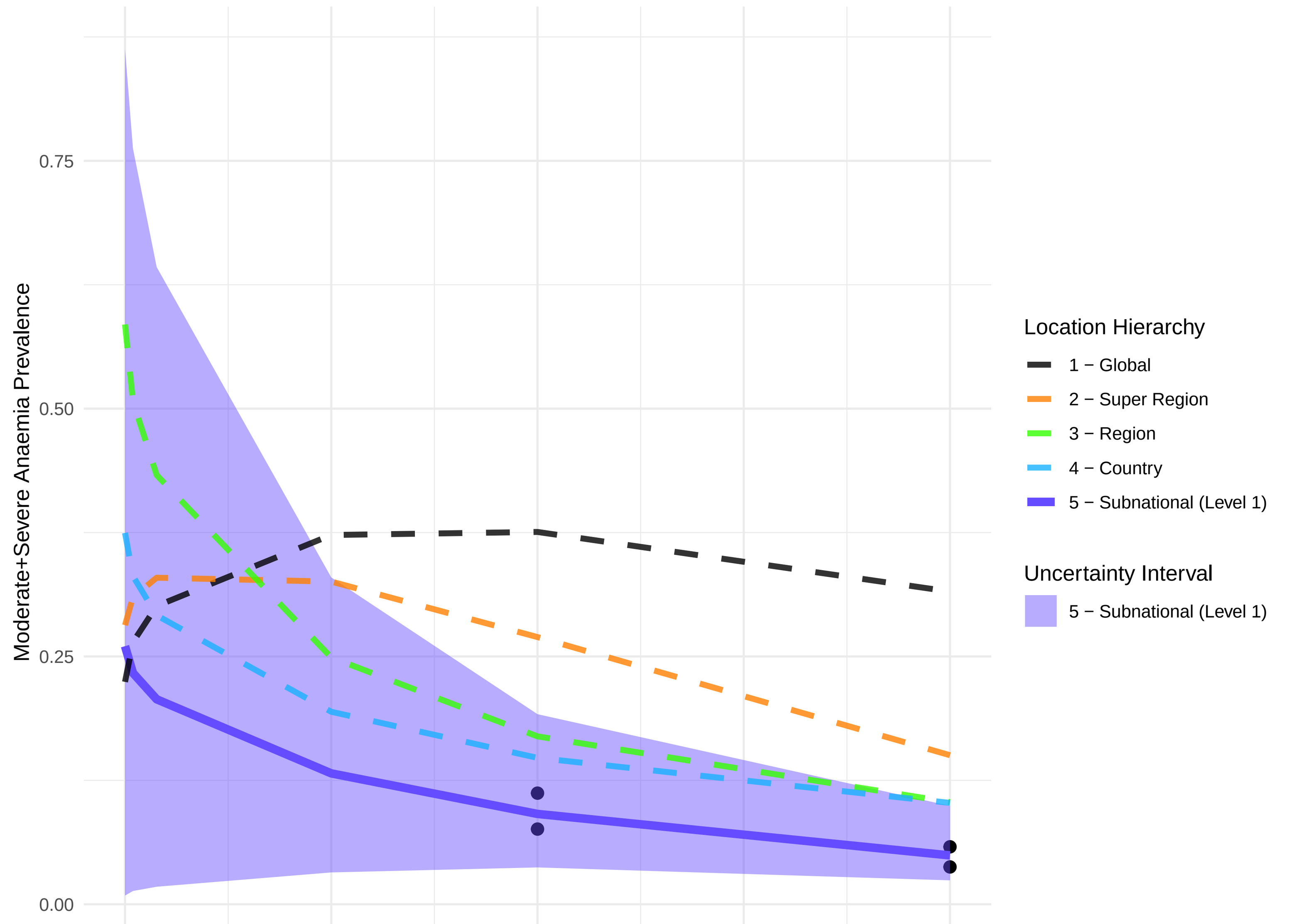

MR−BRT Age/Sex Fits − Males <2 Years Old − Moderate+Severe Anaemia Prevalence − Tlaxcala
WHO−2024 Adjustment Method

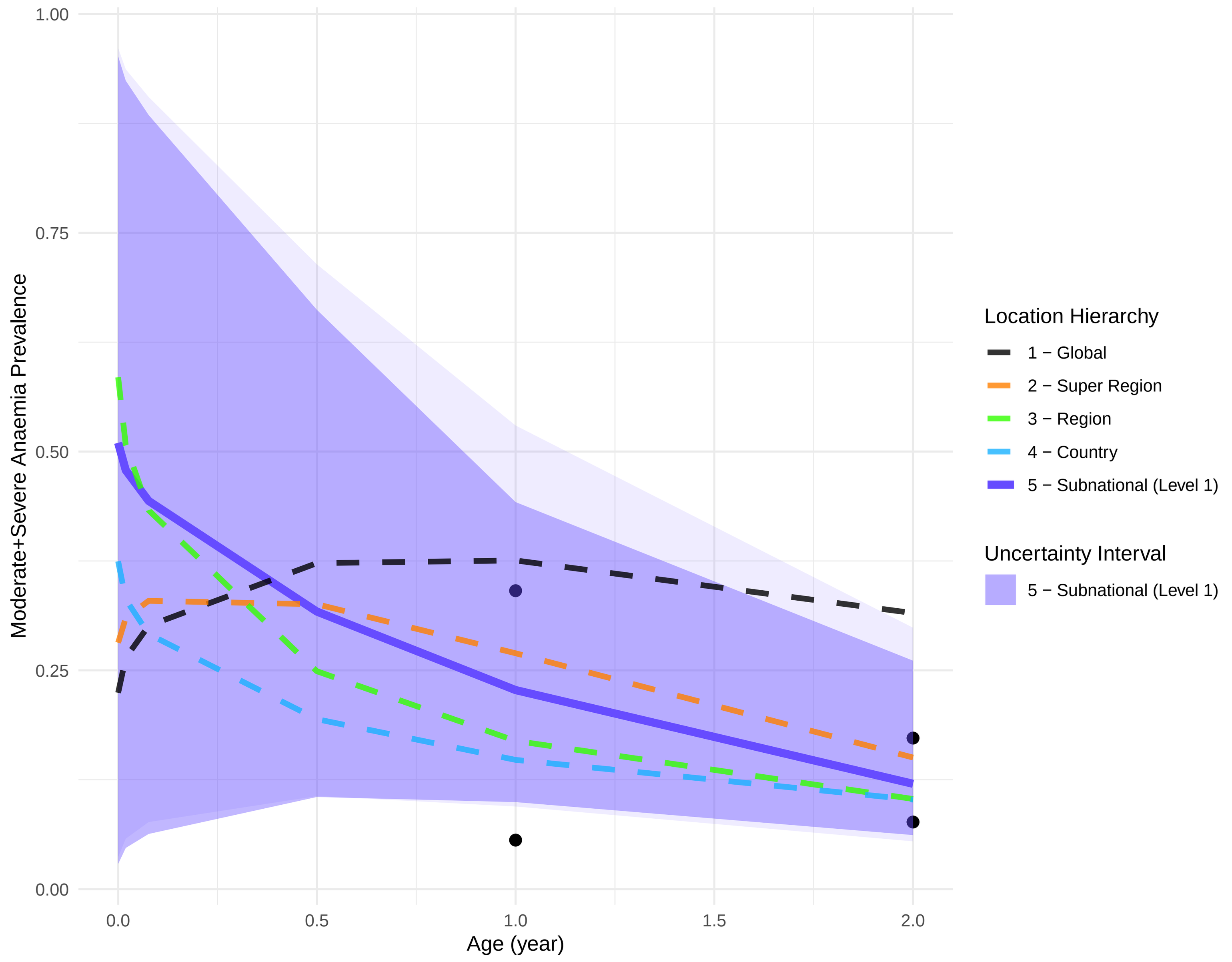

MR−BRT Age/Sex Fits − Males <2 Years Old − Moderate+Severe Anaemia Prevalence − Veracruz de Ignacio de la Llave
WHO−2024 Adjustment Method

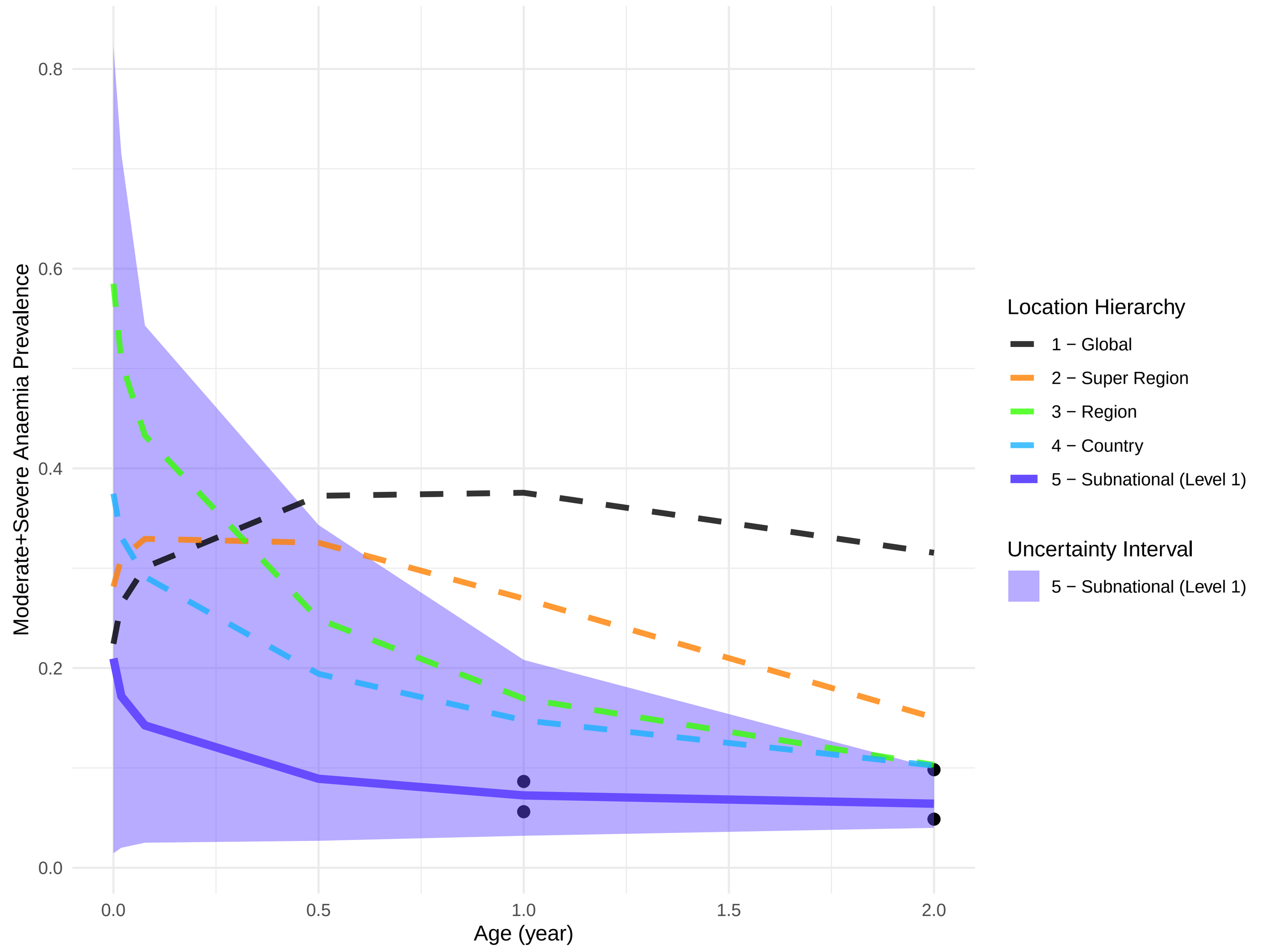

MR−BRT Age/Sex Fits − Males <2 Years Old − Moderate+Severe Anaemia Prevalence − Yucatán
WHO−2024 Adjustment Method

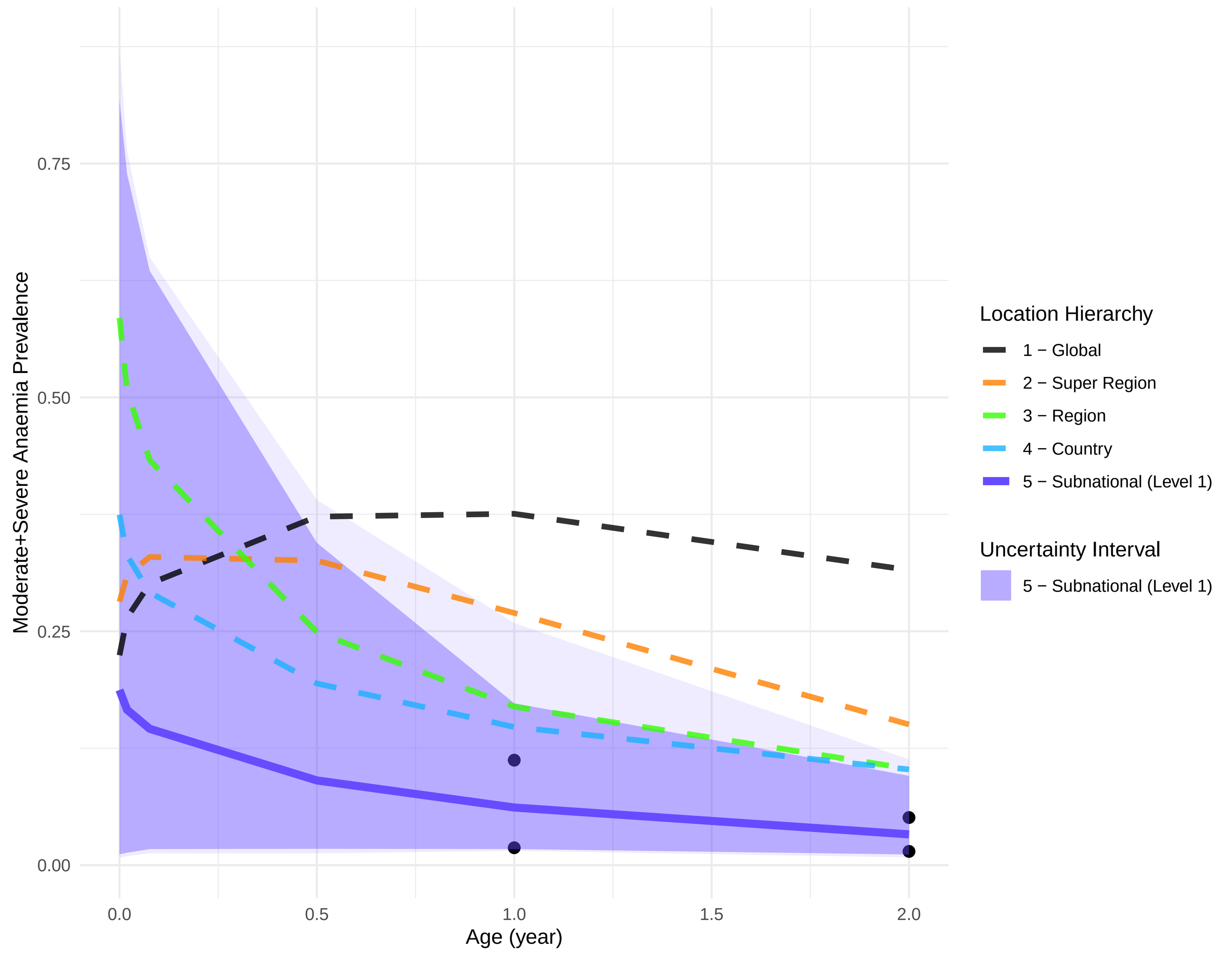

MR−BRT Age/Sex Fits − Males <2 Years Old − Moderate+Severe Anaemia Prevalence − Zacatecas
WHO−2024 Adjustment Method

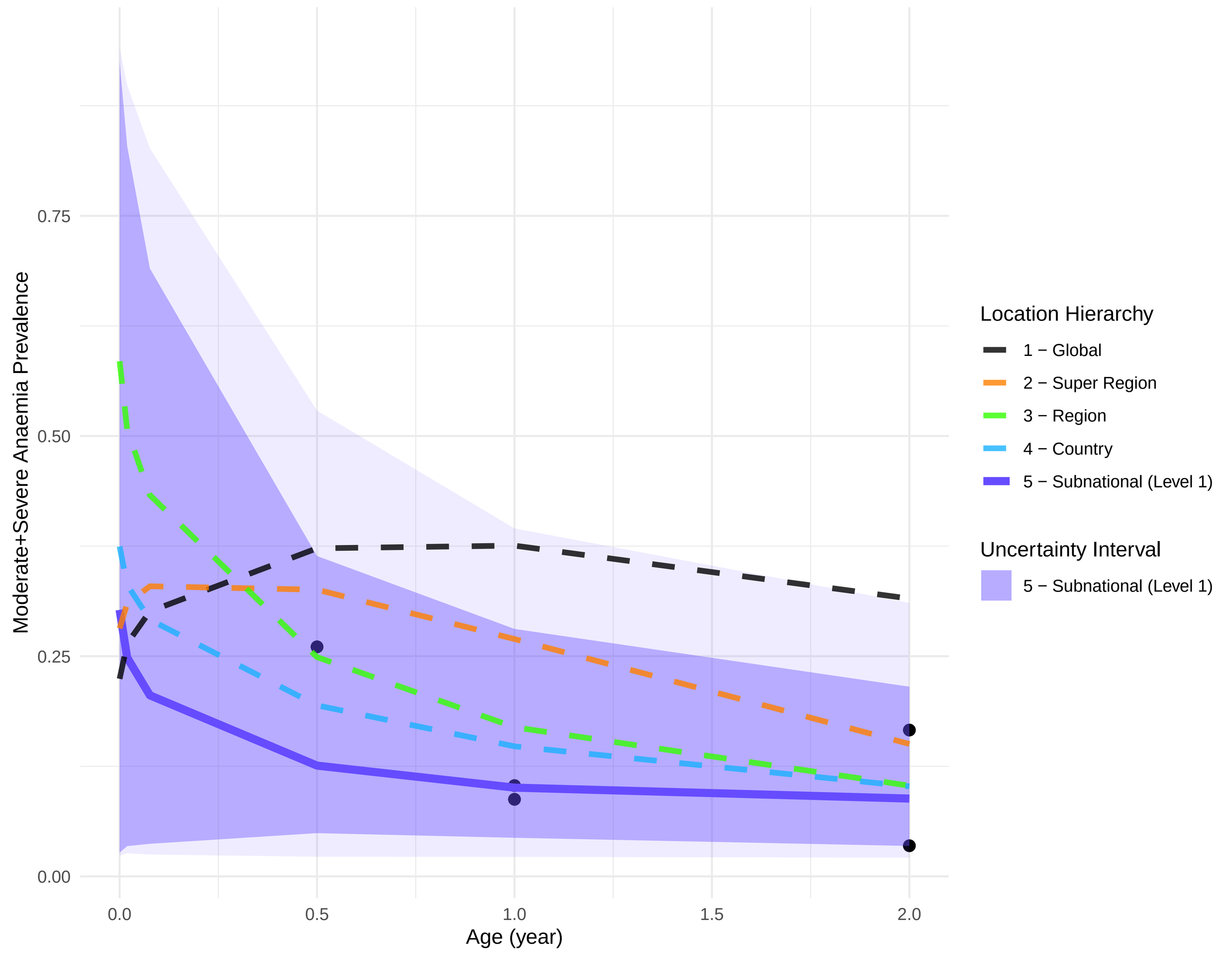

MR−BRT Age/Sex Fits − Males <2 Years Old − Moderate+Severe Anaemia Prevalence − North Africa and Middle East
WHO−2024 Adjustment Method

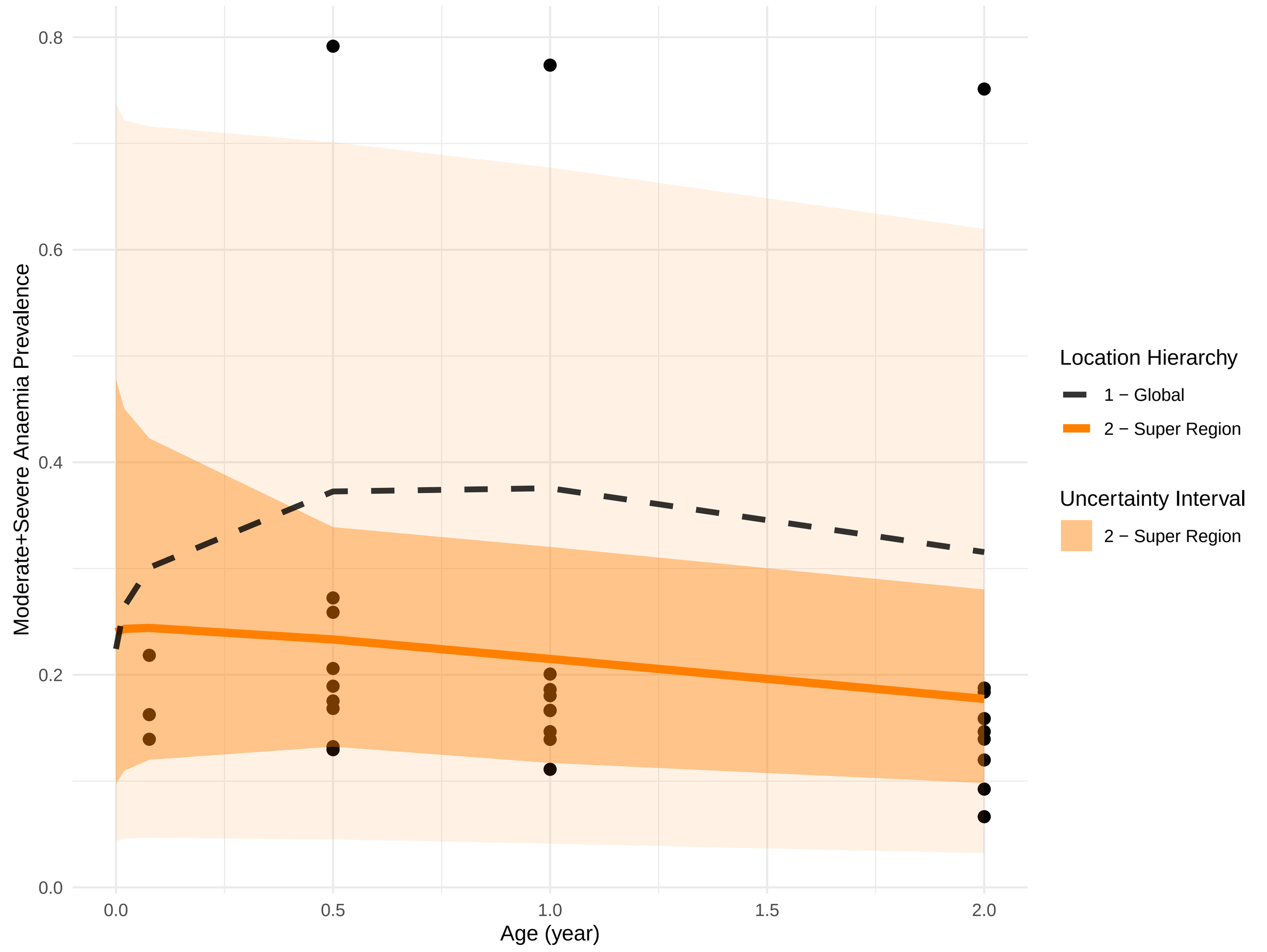

MR−BRT Age/Sex Fits − Males <2 Years Old − Moderate+Severe Anaemia Prevalence − North Africa and Middle East
WHO−2024 Adjustment Method

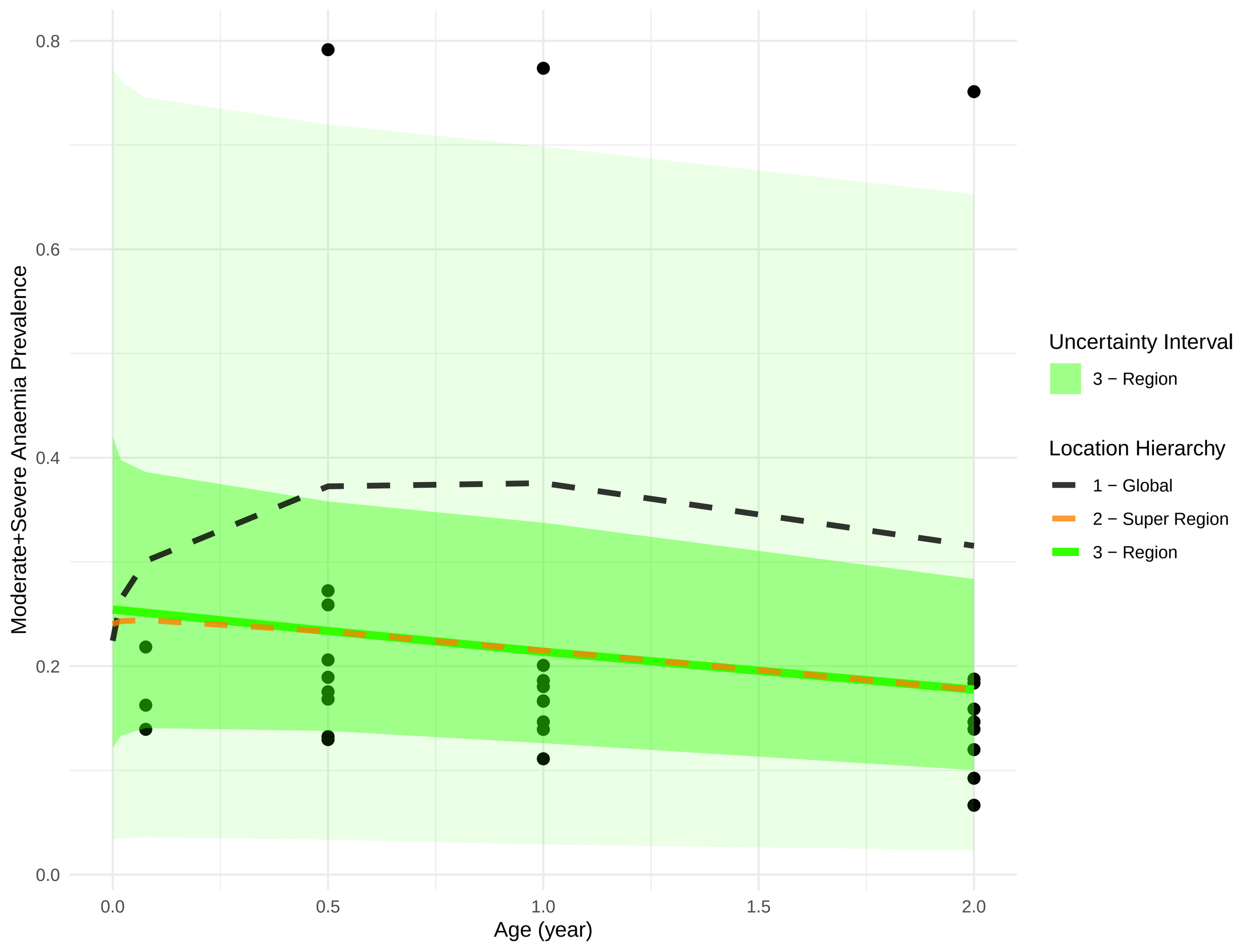

MR−BRT Age/Sex Fits − Males <2 Years Old − Moderate+Severe Anaemia Prevalence − Egypt
WHO−2024 Adjustment Method

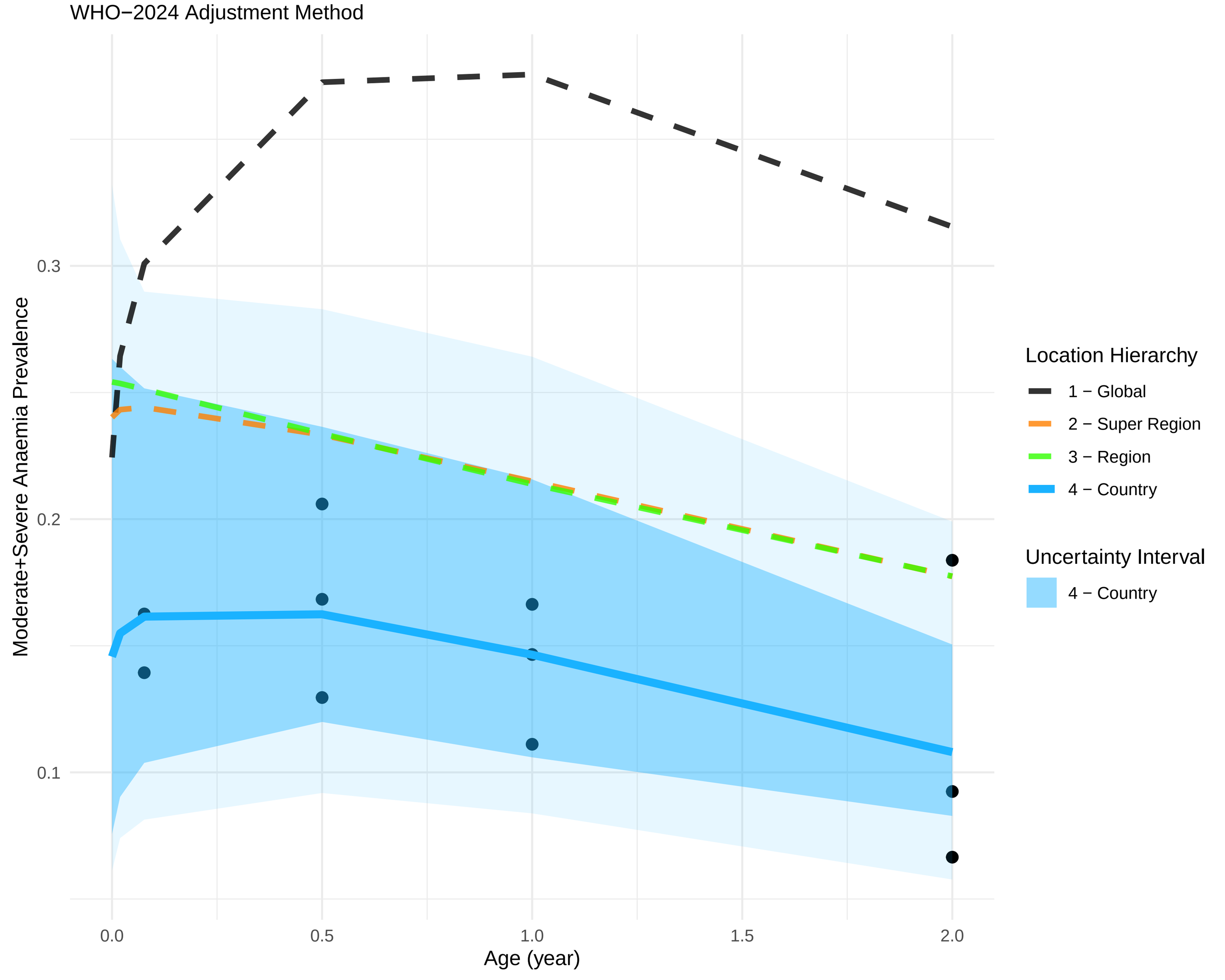

MR−BRT Age/Sex Fits − Males <2 Years Old − Moderate+Severe Anaemia Prevalence − Jordan
WHO−2024 Adjustment Method

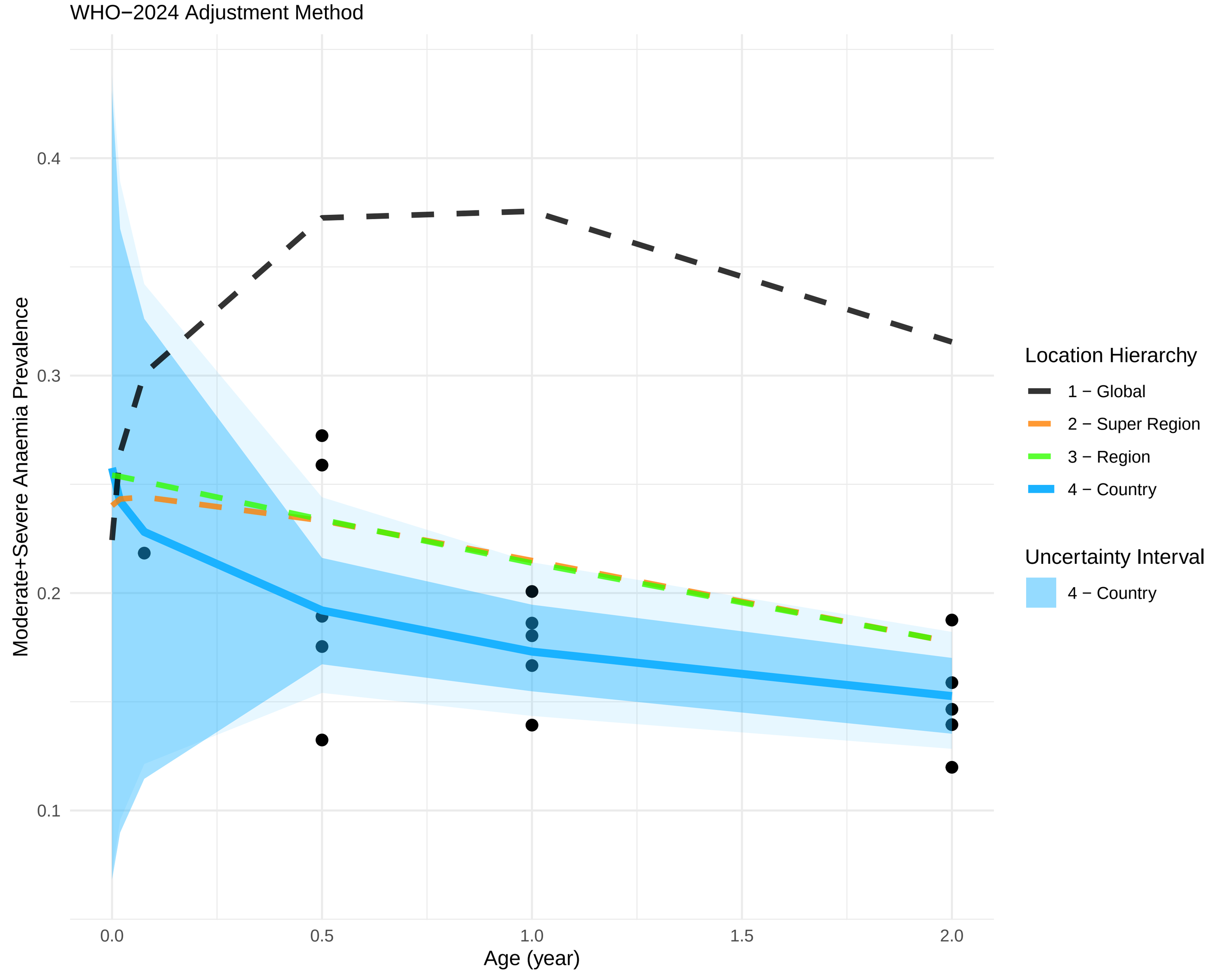

MR−BRT Age/Sex Fits − Males <2 Years Old − Moderate+Severe Anaemia Prevalence − Yemen
WHO−2024 Adjustment Method

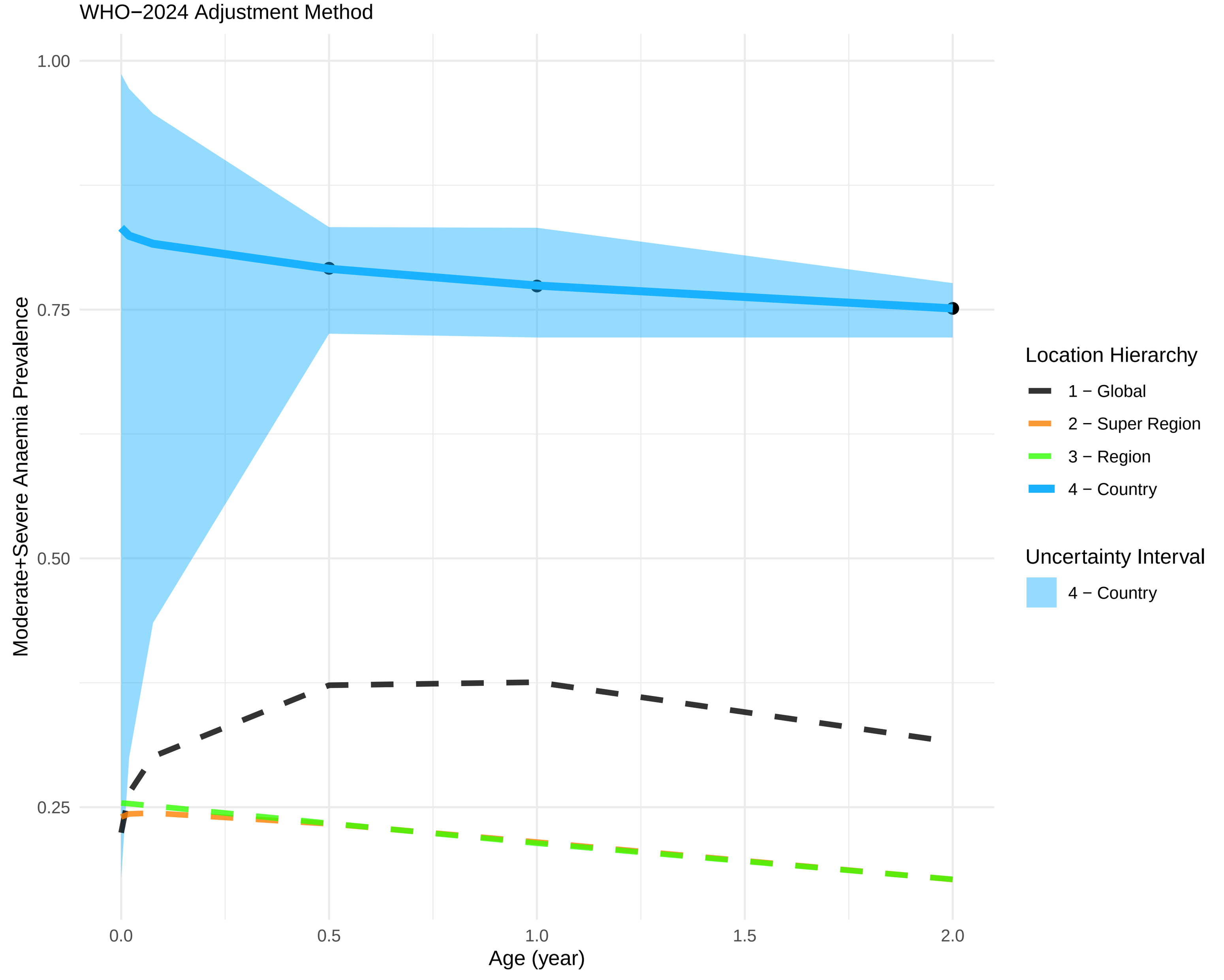

MR−BRT Age/Sex Fits − Males <2 Years Old − Moderate+Severe Anaemia Prevalence − South Asia
WHO−2024 Adjustment Method

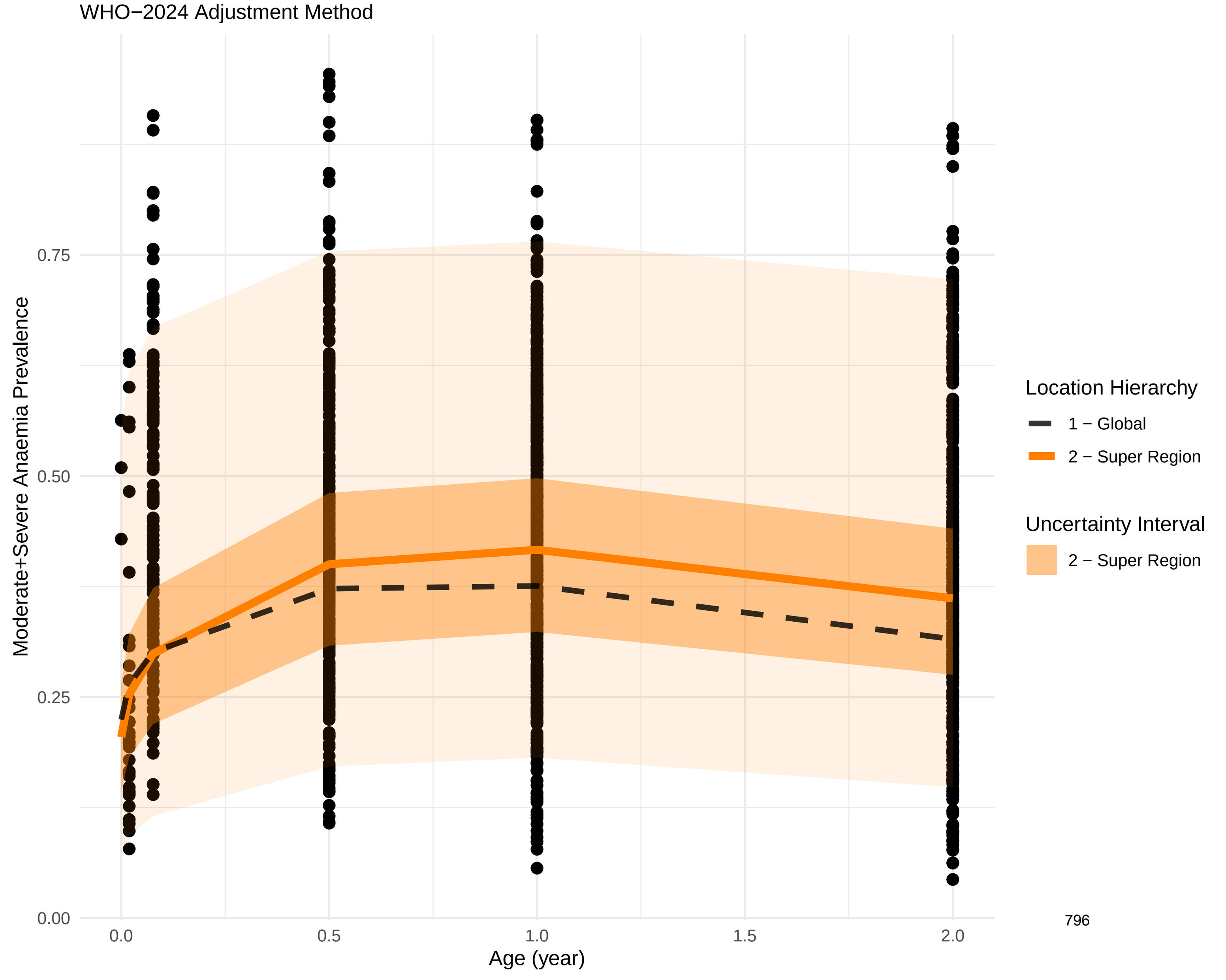

MR−BRT Age/Sex Fits − Males <2 Years Old − Moderate+Severe Anaemia Prevalence − South Asia
WHO−2024 Adjustment Method

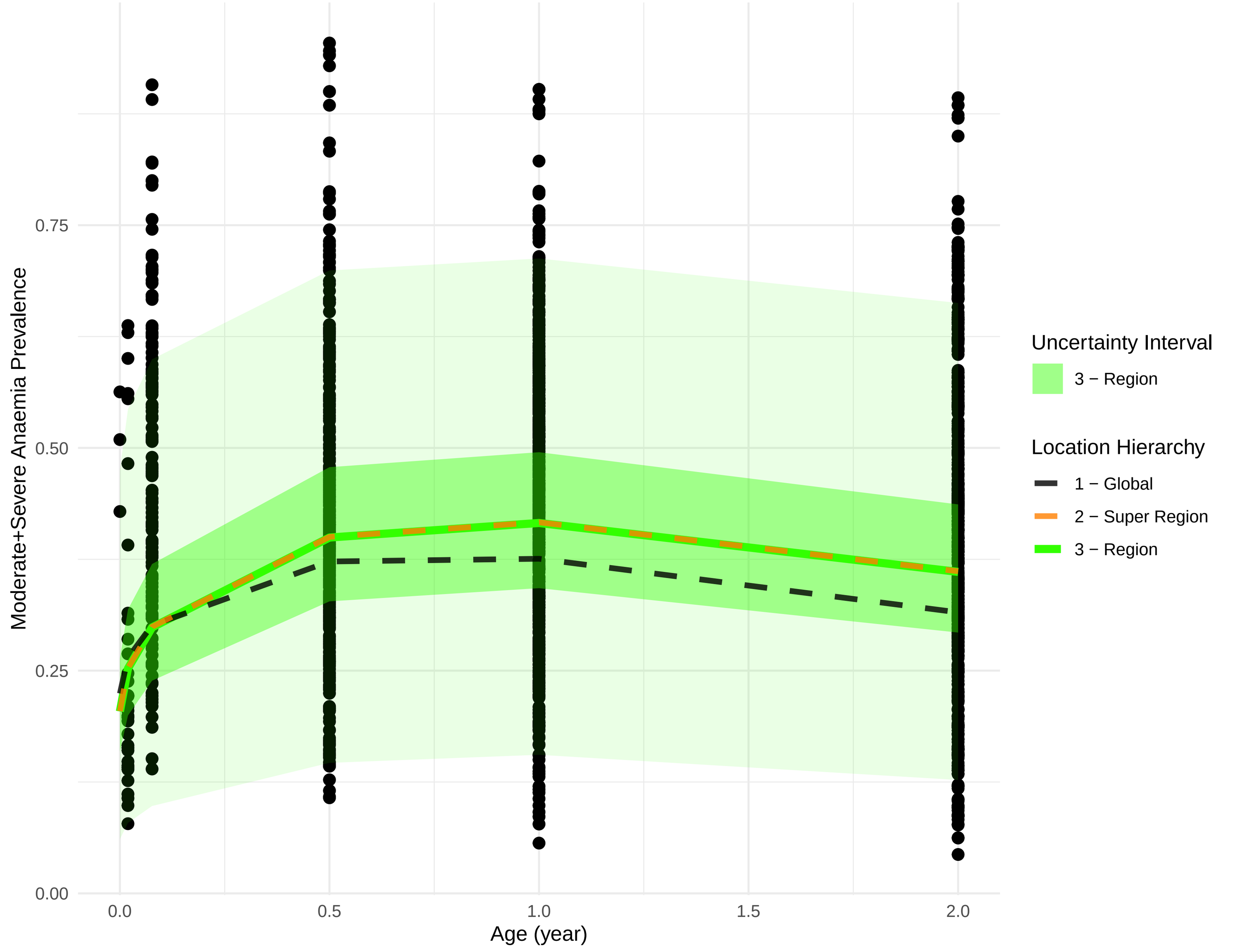

MR−BRT Age/Sex Fits − Males <2 Years Old − Moderate+Severe Anaemia Prevalence − Bangladesh
WHO−2024 Adjustment Method

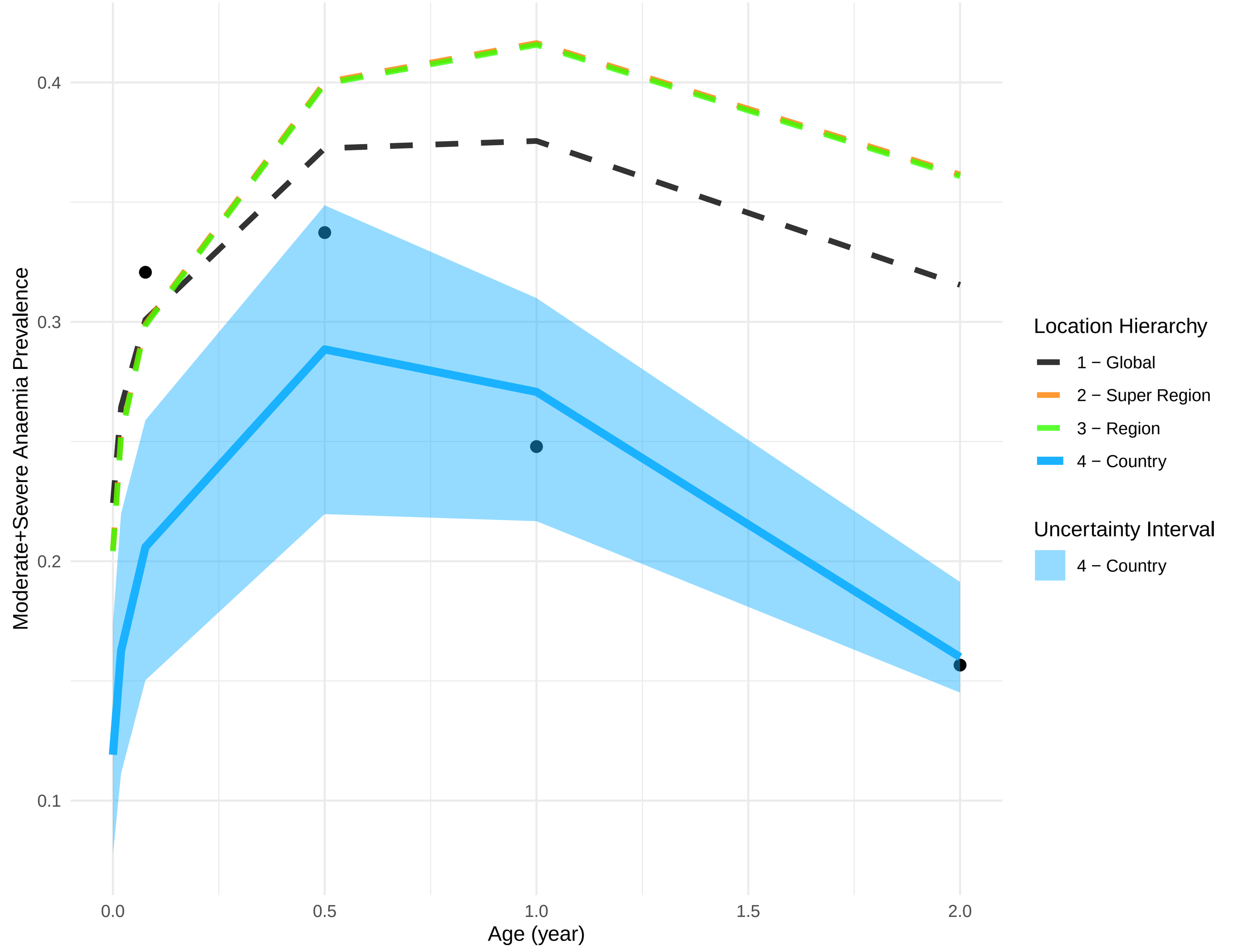

MR−BRT Age/Sex Fits − Males <2 Years Old − Moderate+Severe Anaemia Prevalence − India
WHO−2024 Adjustment Method

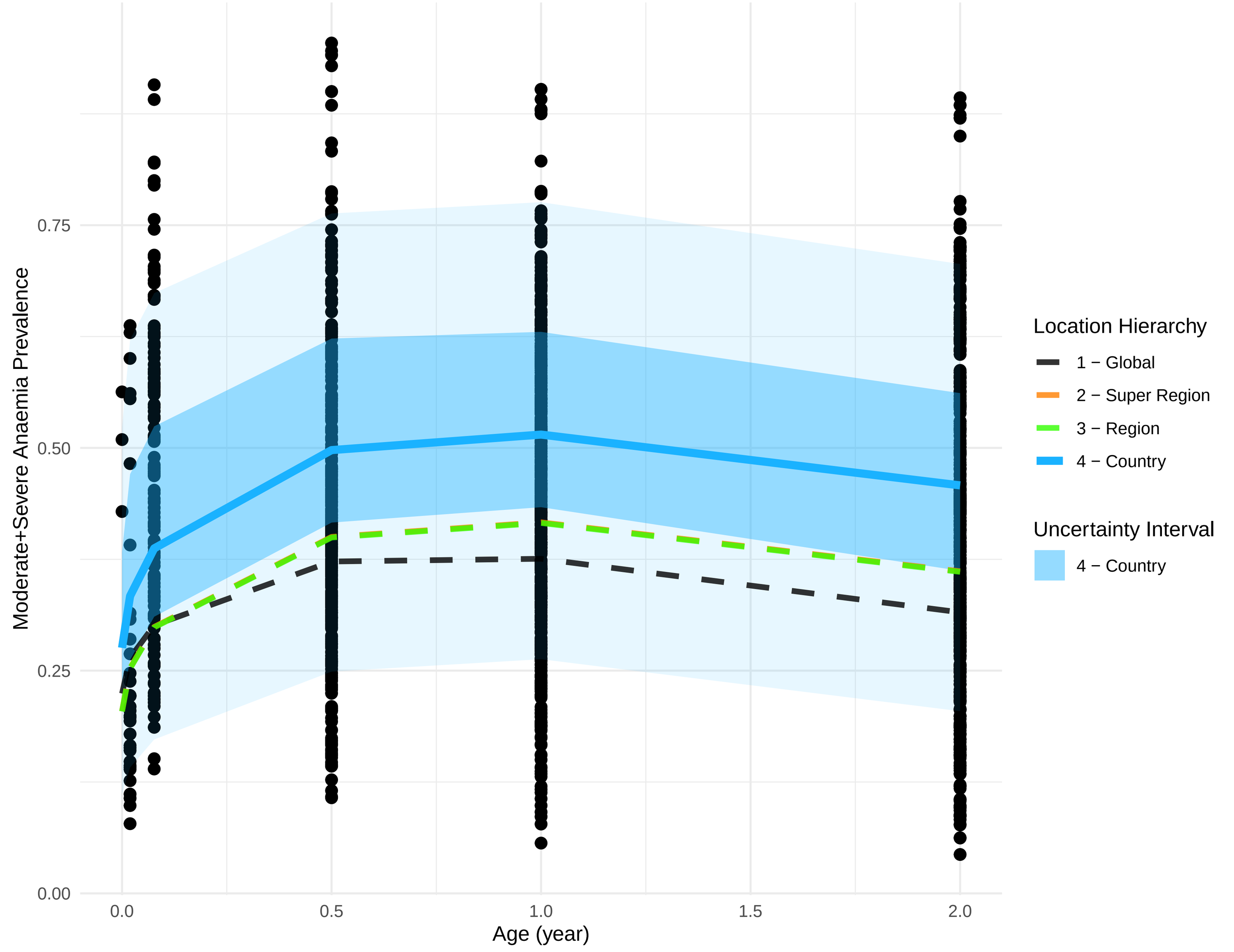

MR−BRT Age/Sex Fits − Males <2 Years Old − Moderate+Severe Anaemia Prevalence − Andhra Pradesh
WHO−2024 Adjustment Method

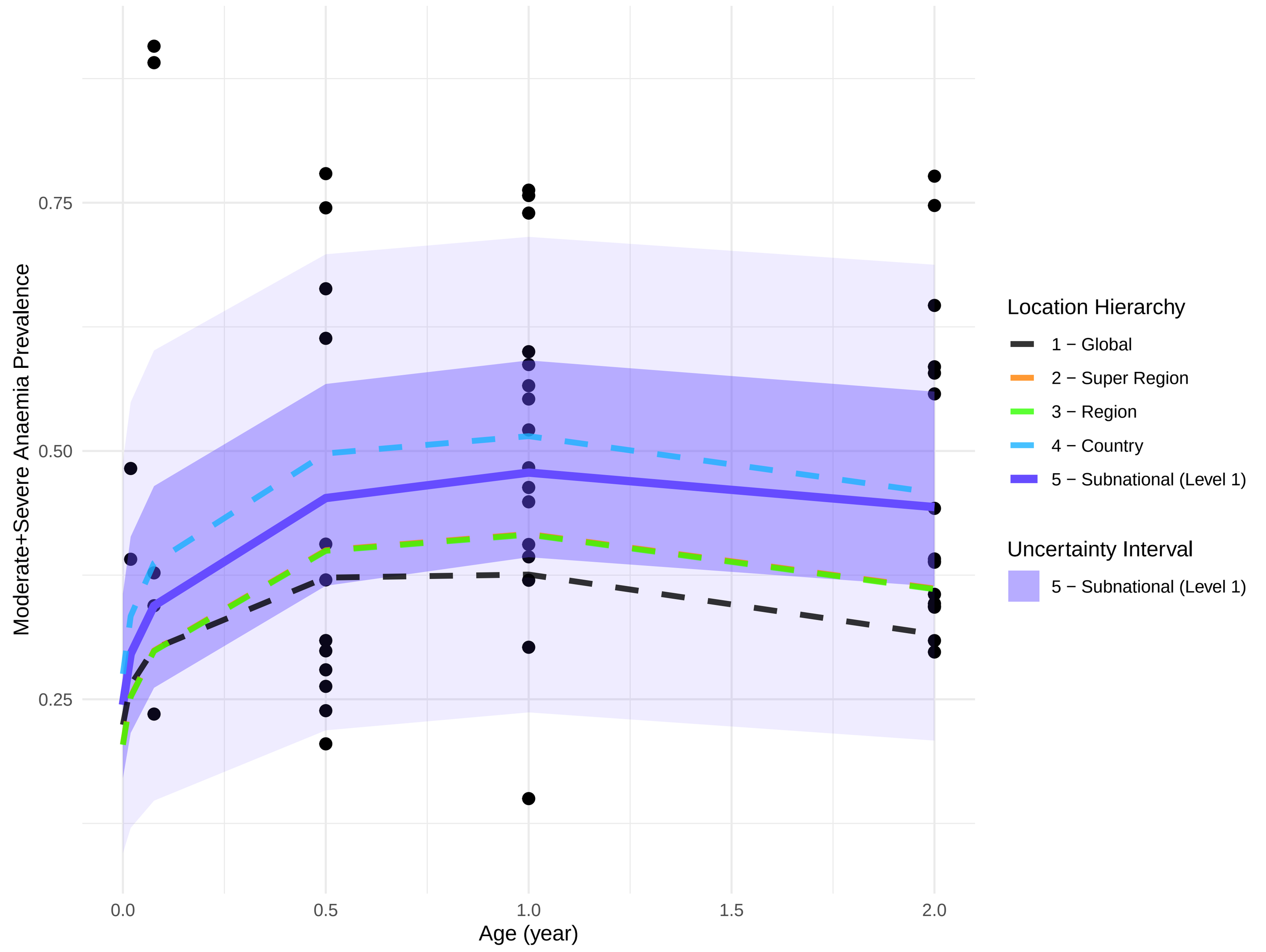

MR−BRT Age/Sex Fits − Males <2 Years Old − Moderate+Severe Anaemia Prevalence − Andhra Pradesh, Rural
WHO−2024 Adjustment Method

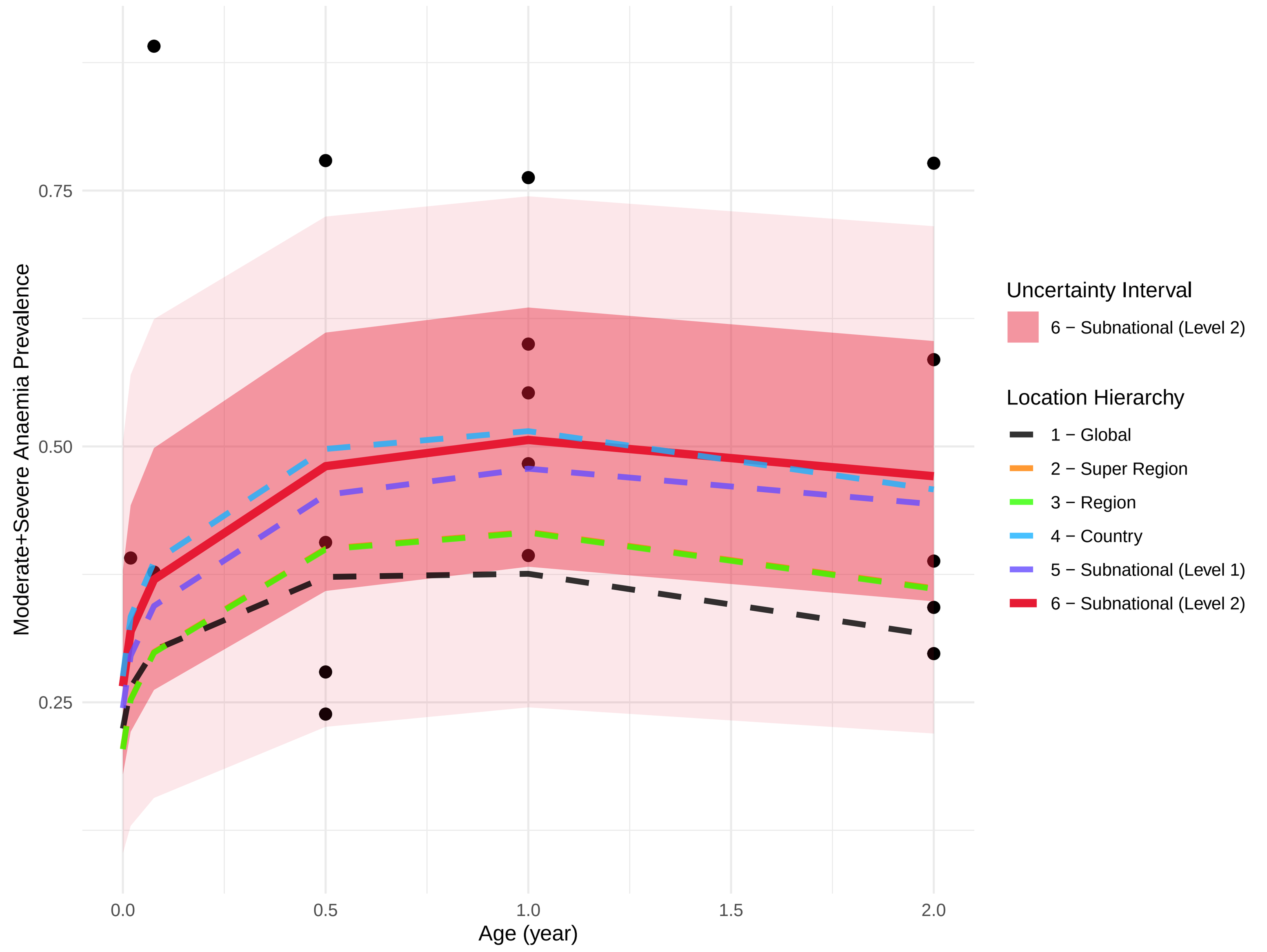

MR−BRT Age/Sex Fits − Males <2 Years Old − Moderate+Severe Anaemia Prevalence − Andhra Pradesh, Urban
WHO−2024 Adjustment Method

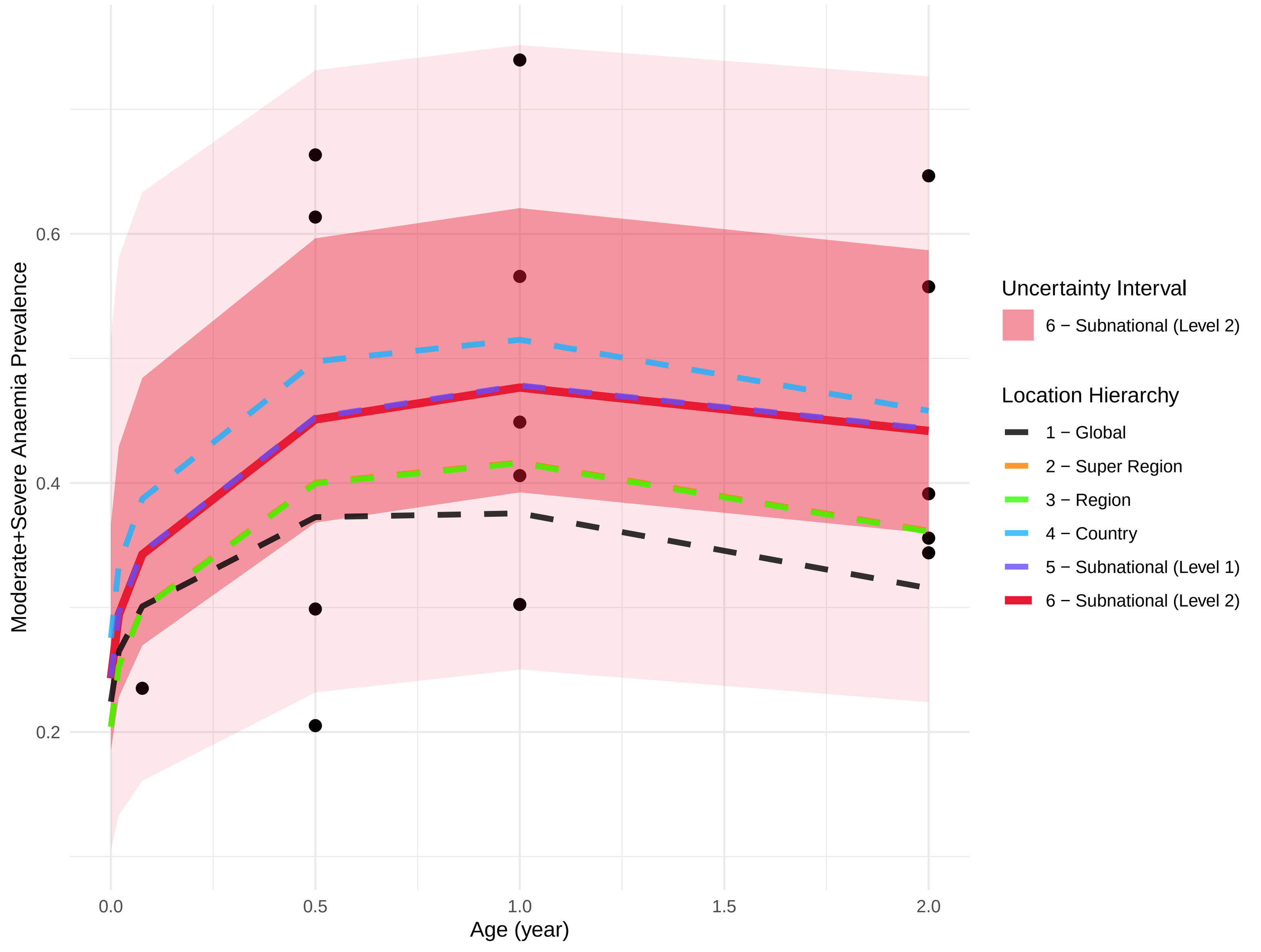

MR−BRT Age/Sex Fits − Males <2 Years Old − Moderate+Severe Anaemia Prevalence − Arunachal Pradesh
WHO−2024 Adjustment Method

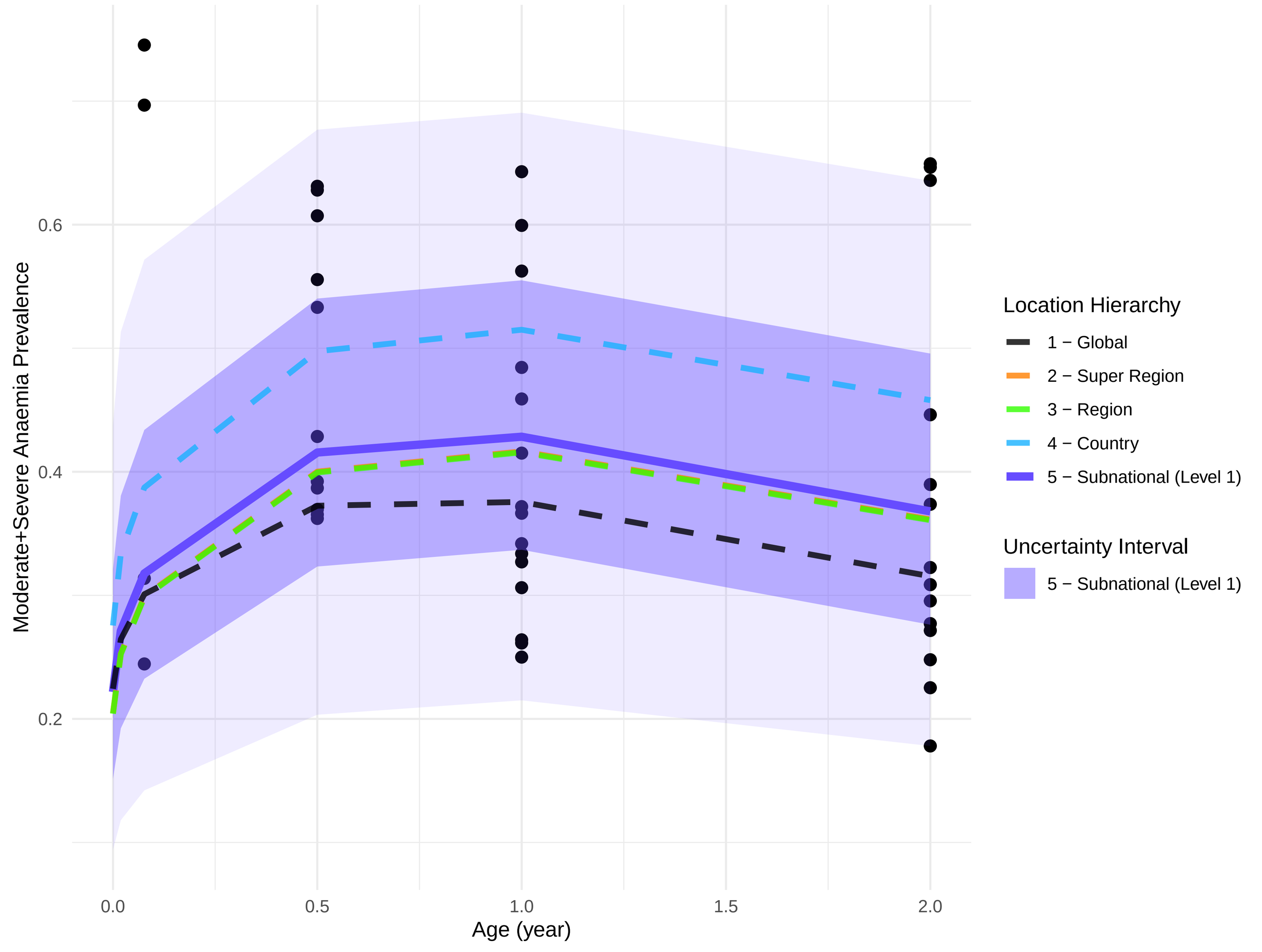

MR−BRT Age/Sex Fits − Males <2 Years Old − Moderate+Severe Anaemia Prevalence − Arunachal Pradesh, Rural
WHO−2024 Adjustment Method

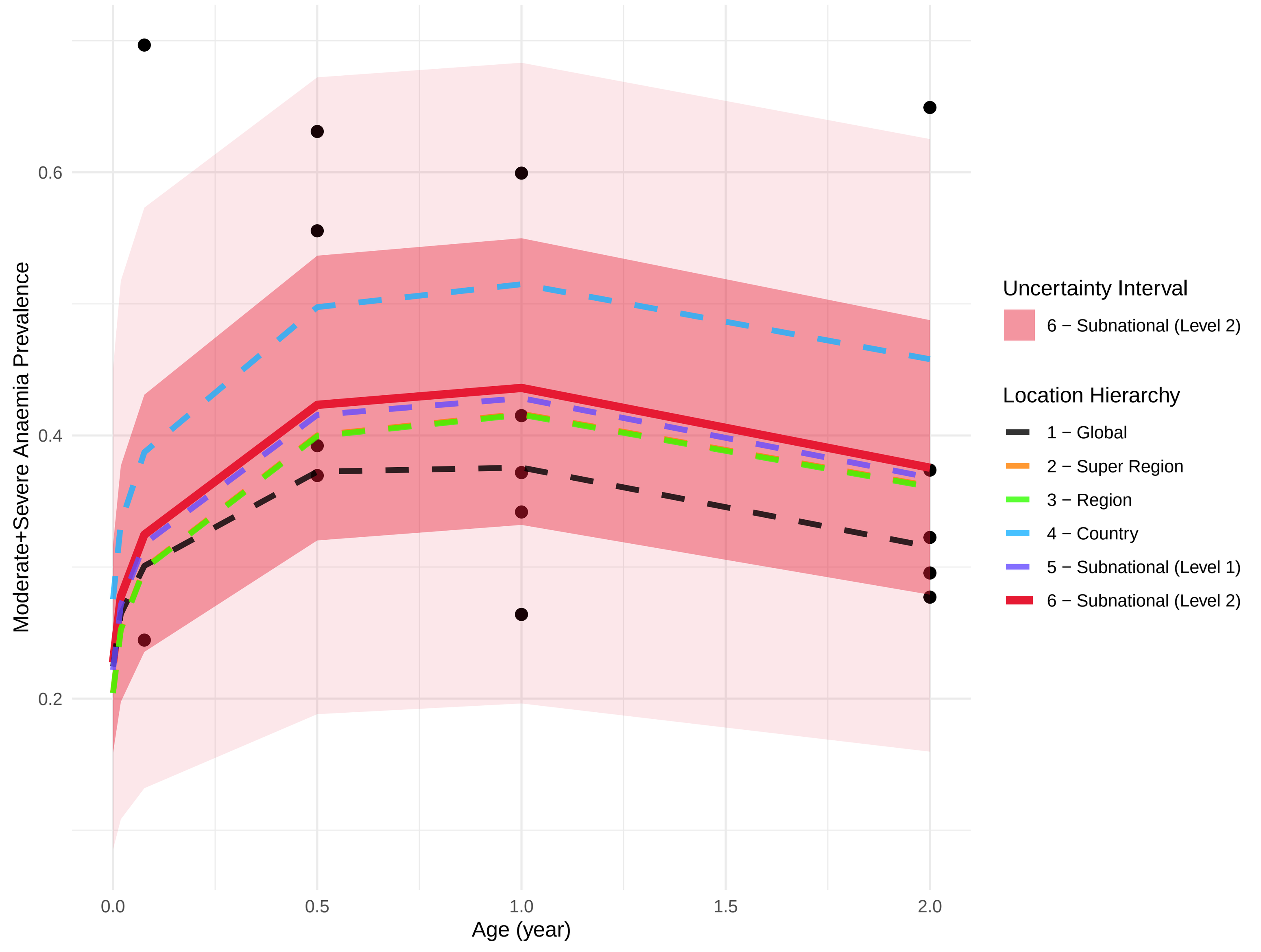

MR−BRT Age/Sex Fits − Males <2 Years Old − Moderate+Severe Anaemia Prevalence − Arunachal Pradesh, Urban
WHO−2024 Adjustment Method

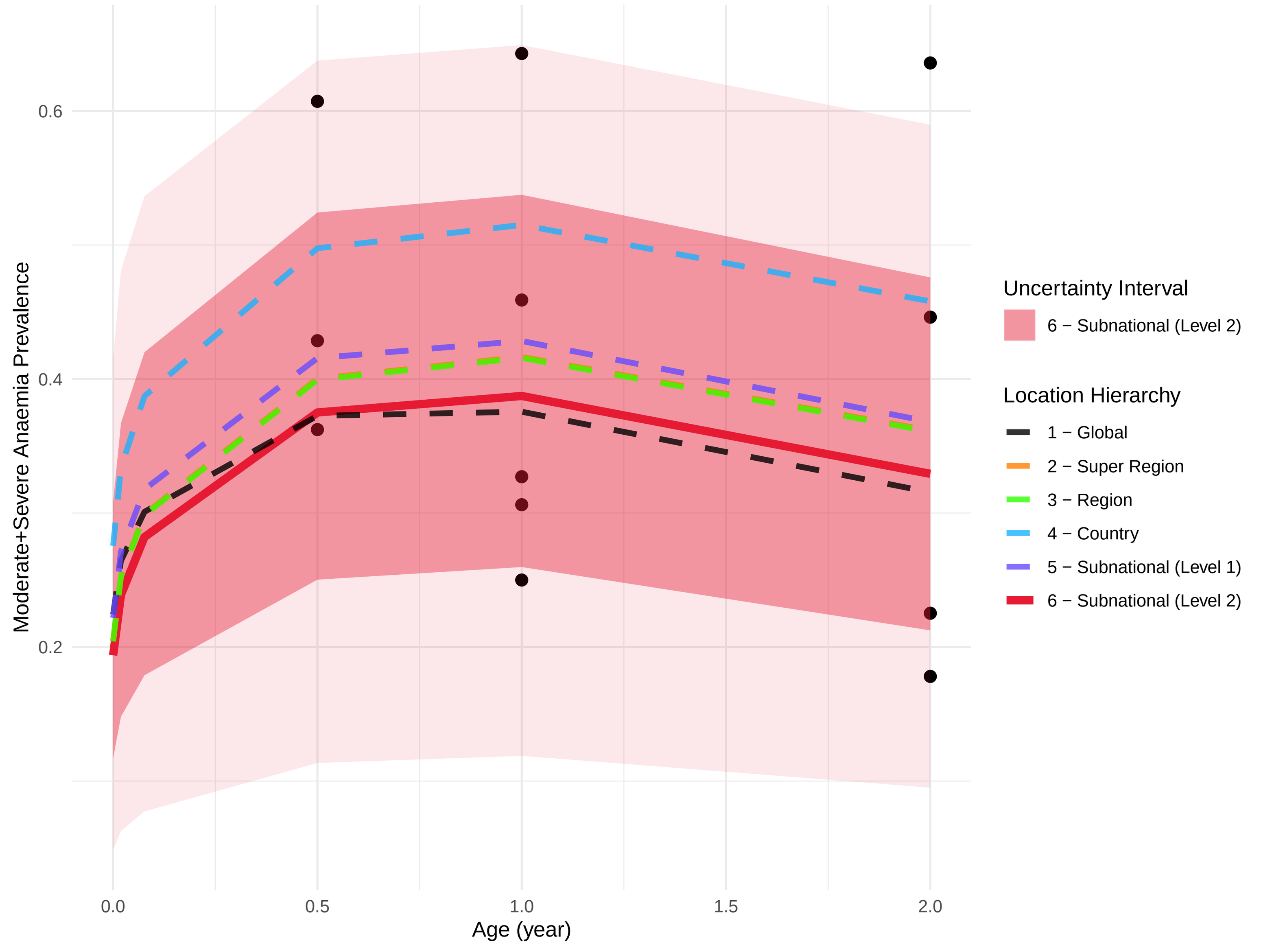

MR−BRT Age/Sex Fits − Males <2 Years Old − Moderate+Severe Anaemia Prevalence − Assam
WHO−2024 Adjustment Method

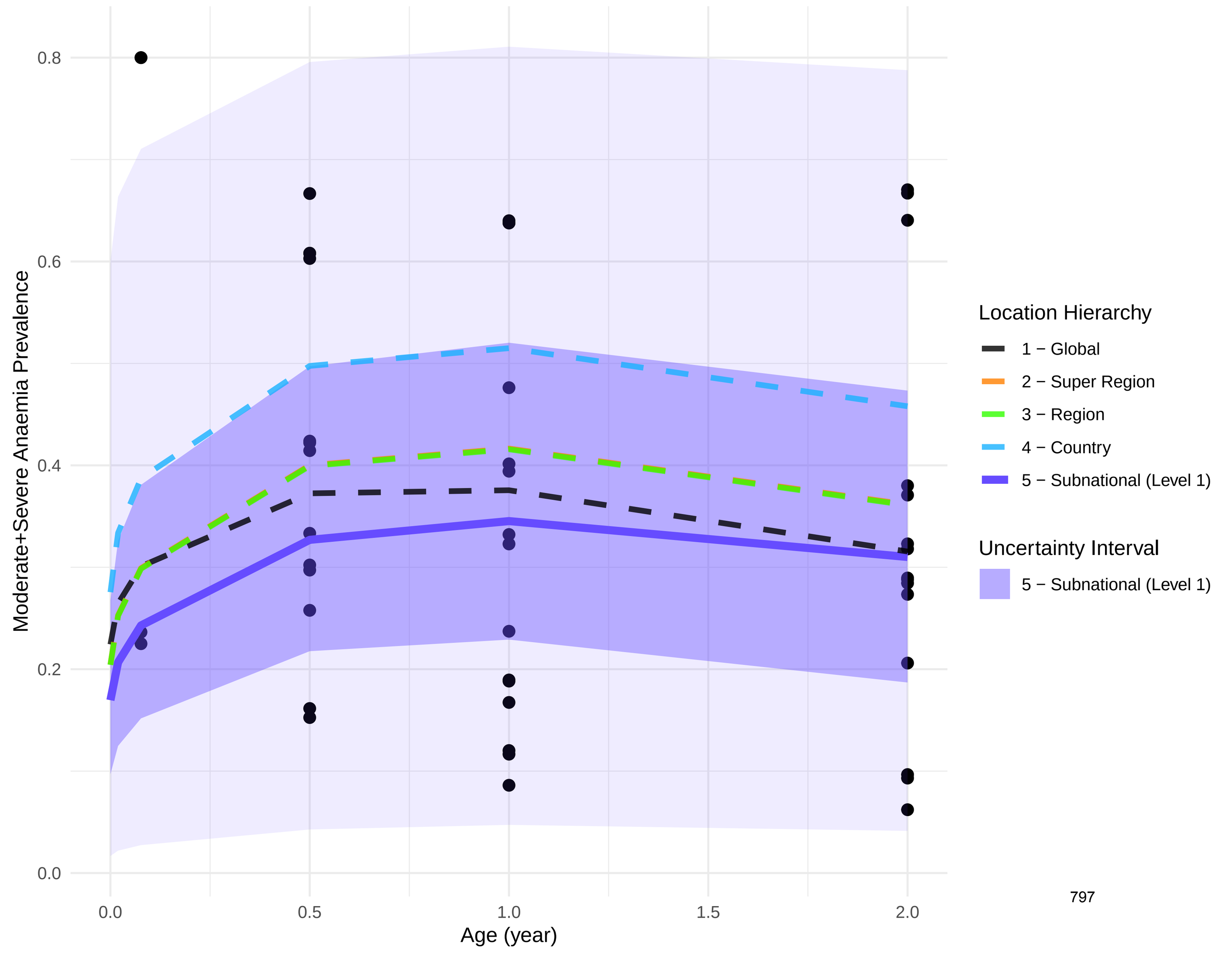

MR−BRT Age/Sex Fits − Males <2 Years Old − Moderate+Severe Anaemia Prevalence − Assam, Rural
WHO−2024 Adjustment Method

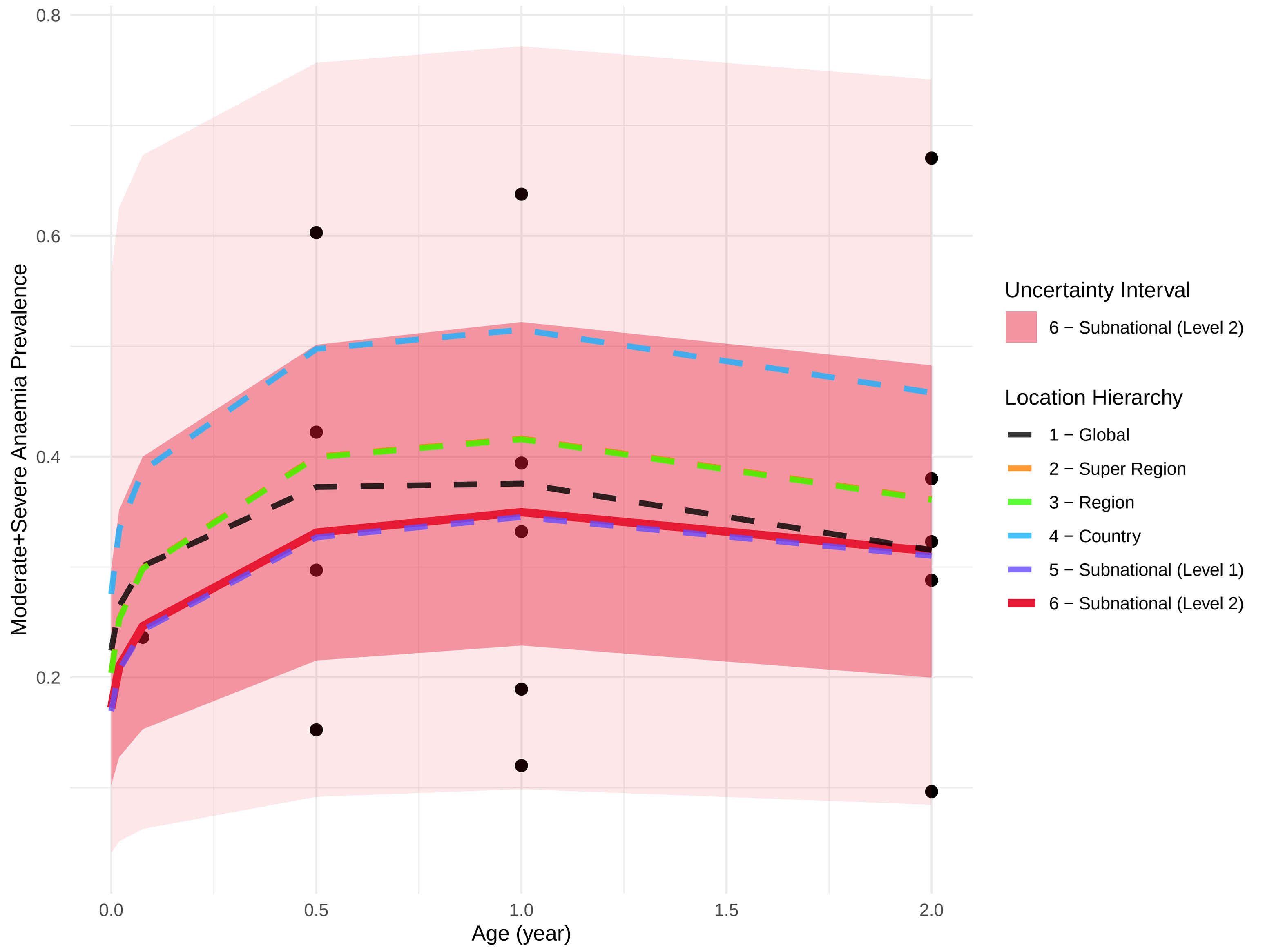

MR−BRT Age/Sex Fits − Males <2 Years Old − Moderate+Severe Anaemia Prevalence − Assam, Urban
WHO−2024 Adjustment Method

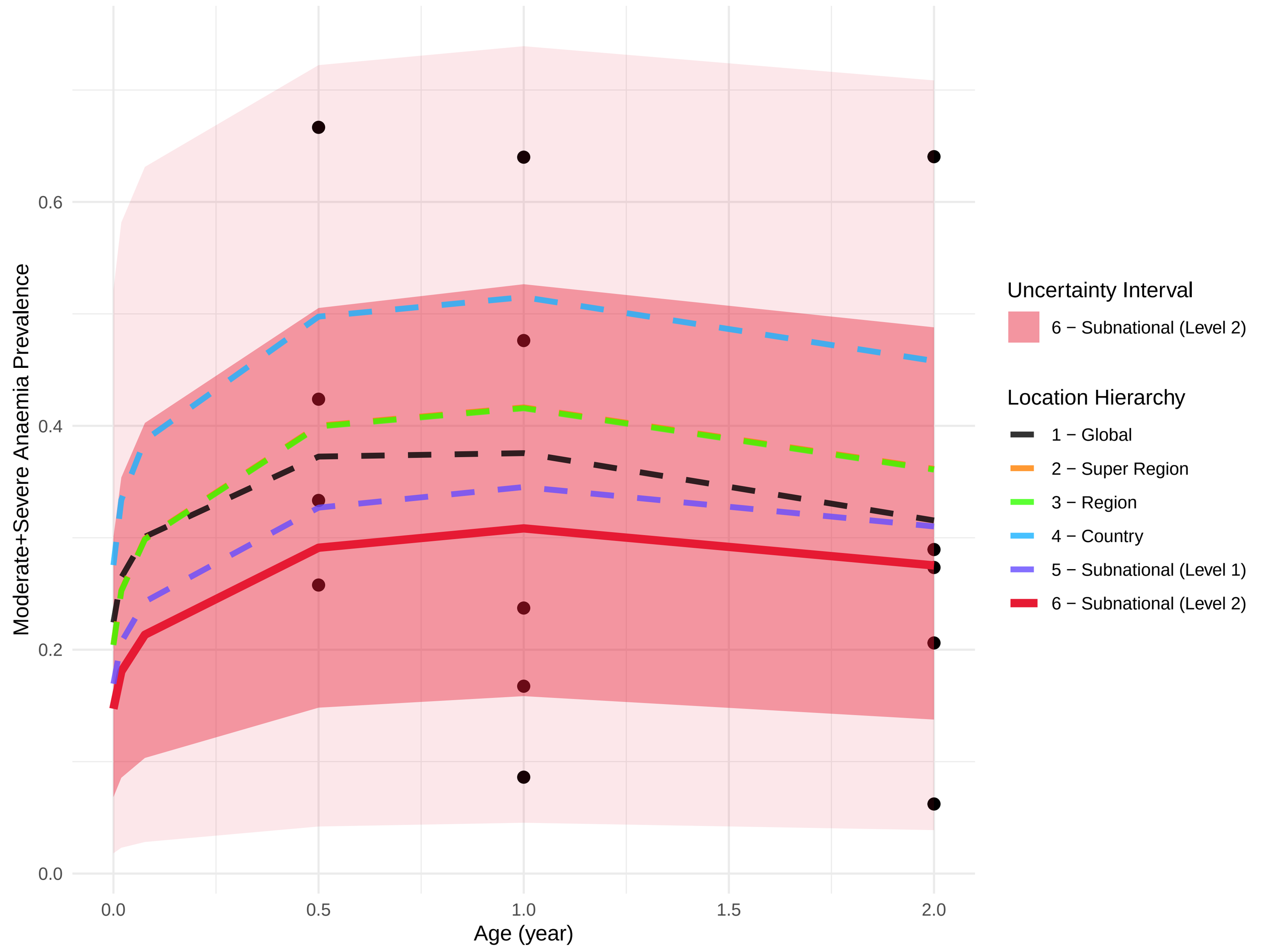

MR−BRT Age/Sex Fits − Males <2 Years Old − Moderate+Severe Anaemia Prevalence − Bihar
WHO−2024 Adjustment Method

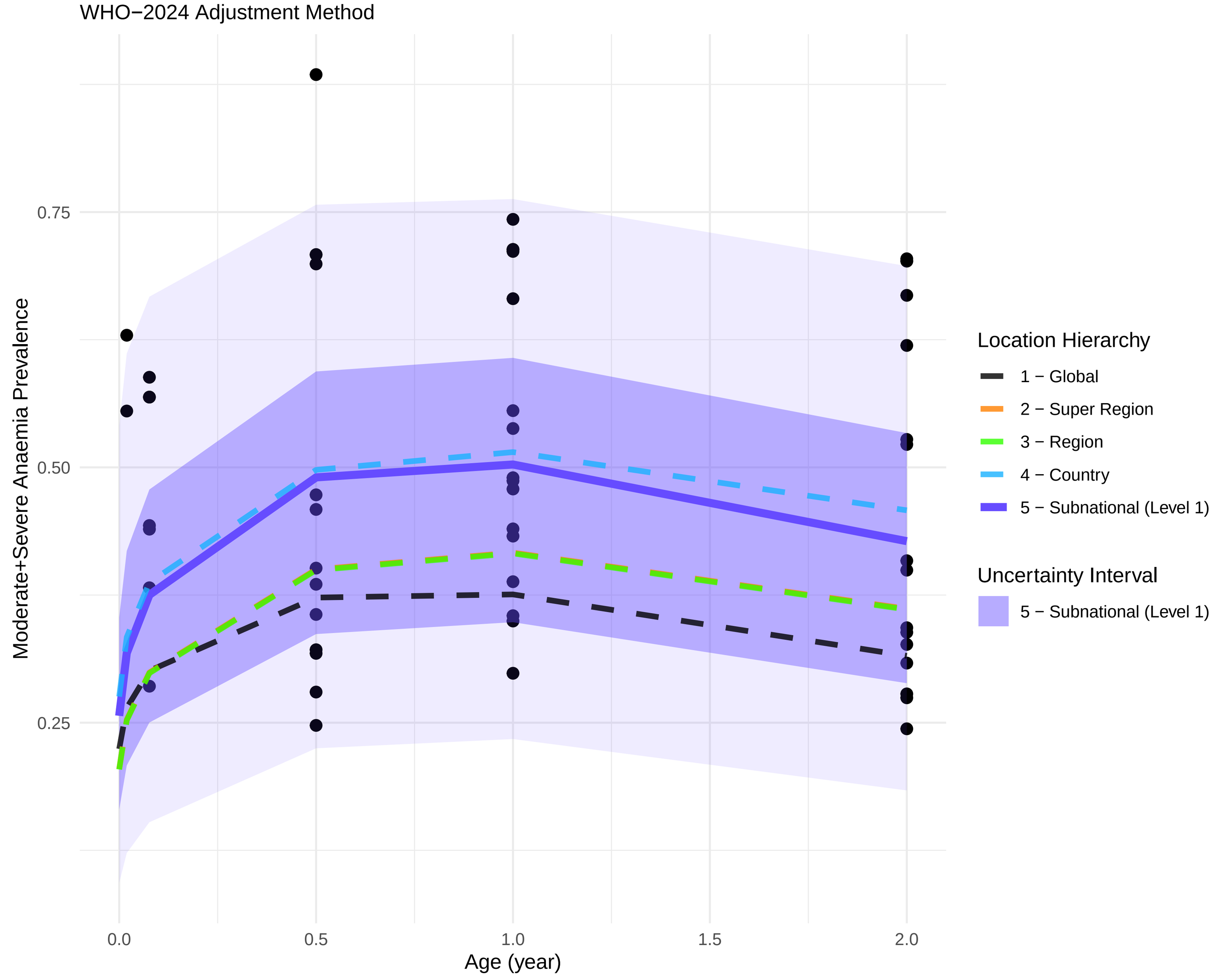

MR−BRT Age/Sex Fits − Males <2 Years Old − Moderate+Severe Anaemia Prevalence − Bihar, Rural
WHO−2024 Adjustment Method

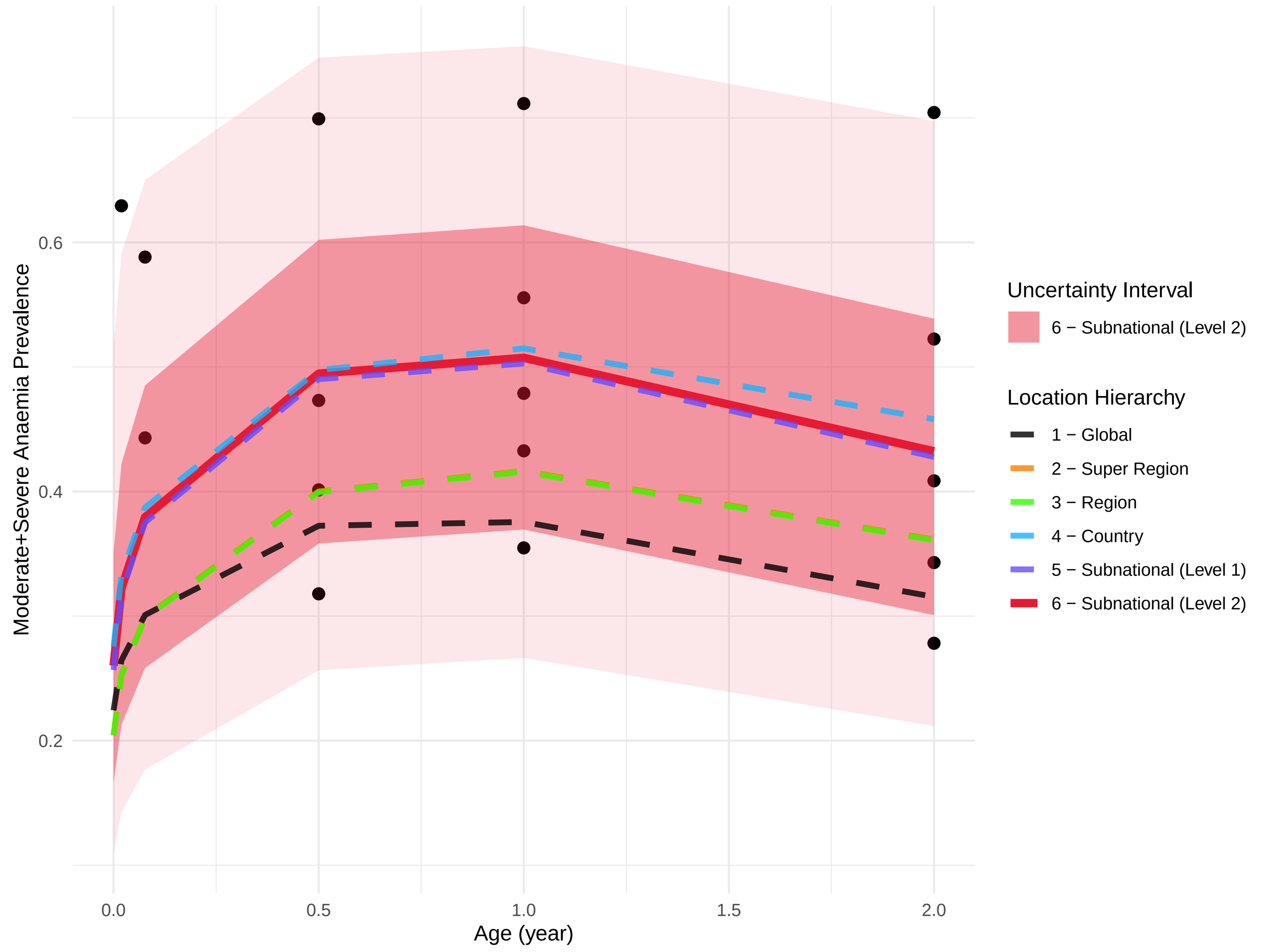

MR−BRT Age/Sex Fits − Males <2 Years Old − Moderate+Severe Anaemia Prevalence − Bihar, Urban
WHO−2024 Adjustment Method

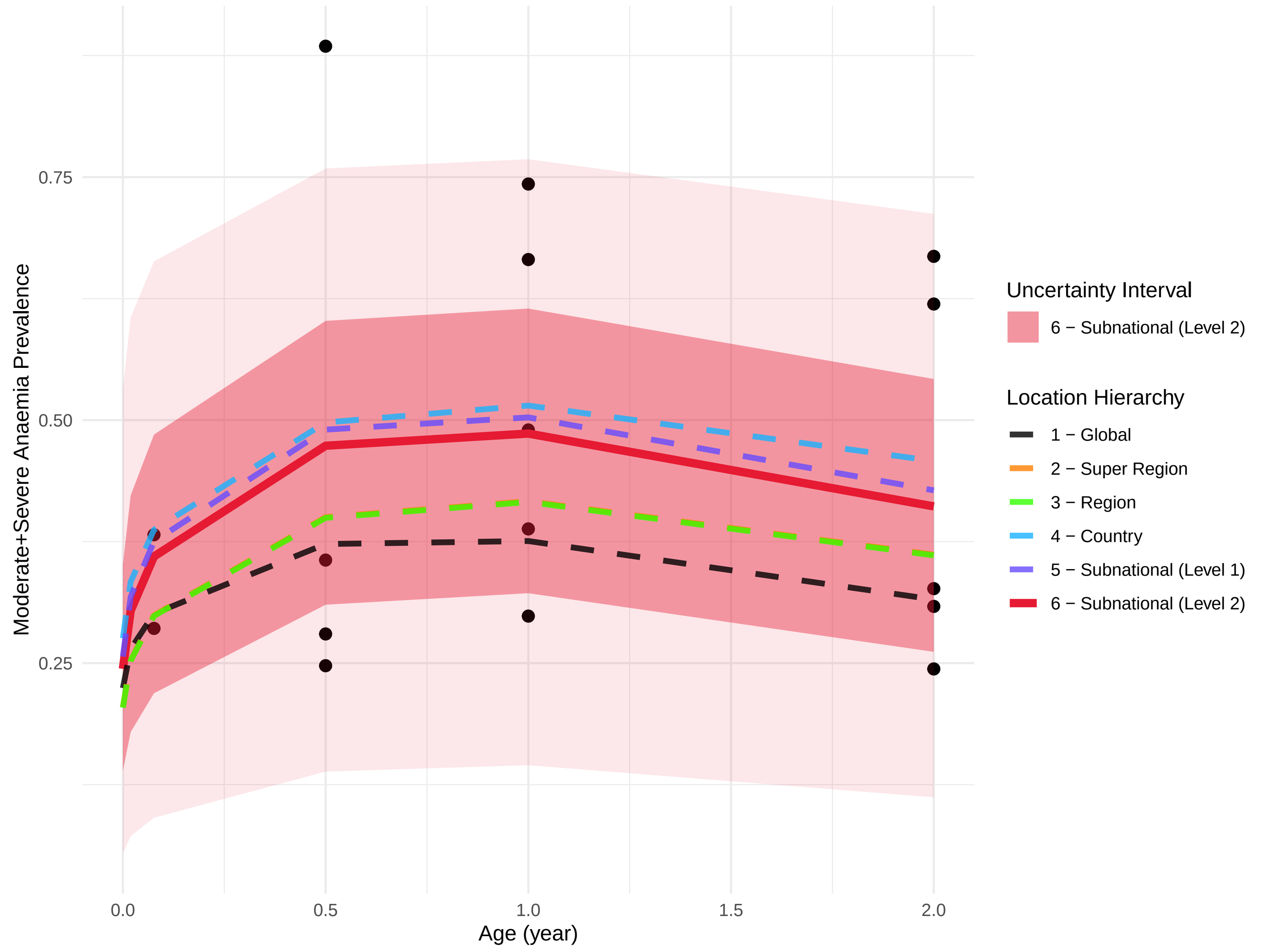

MR−BRT Age/Sex Fits − Males <2 Years Old − Moderate+Severe Anaemia Prevalence − Chhattisgarh
WHO−2024 Adjustment Method

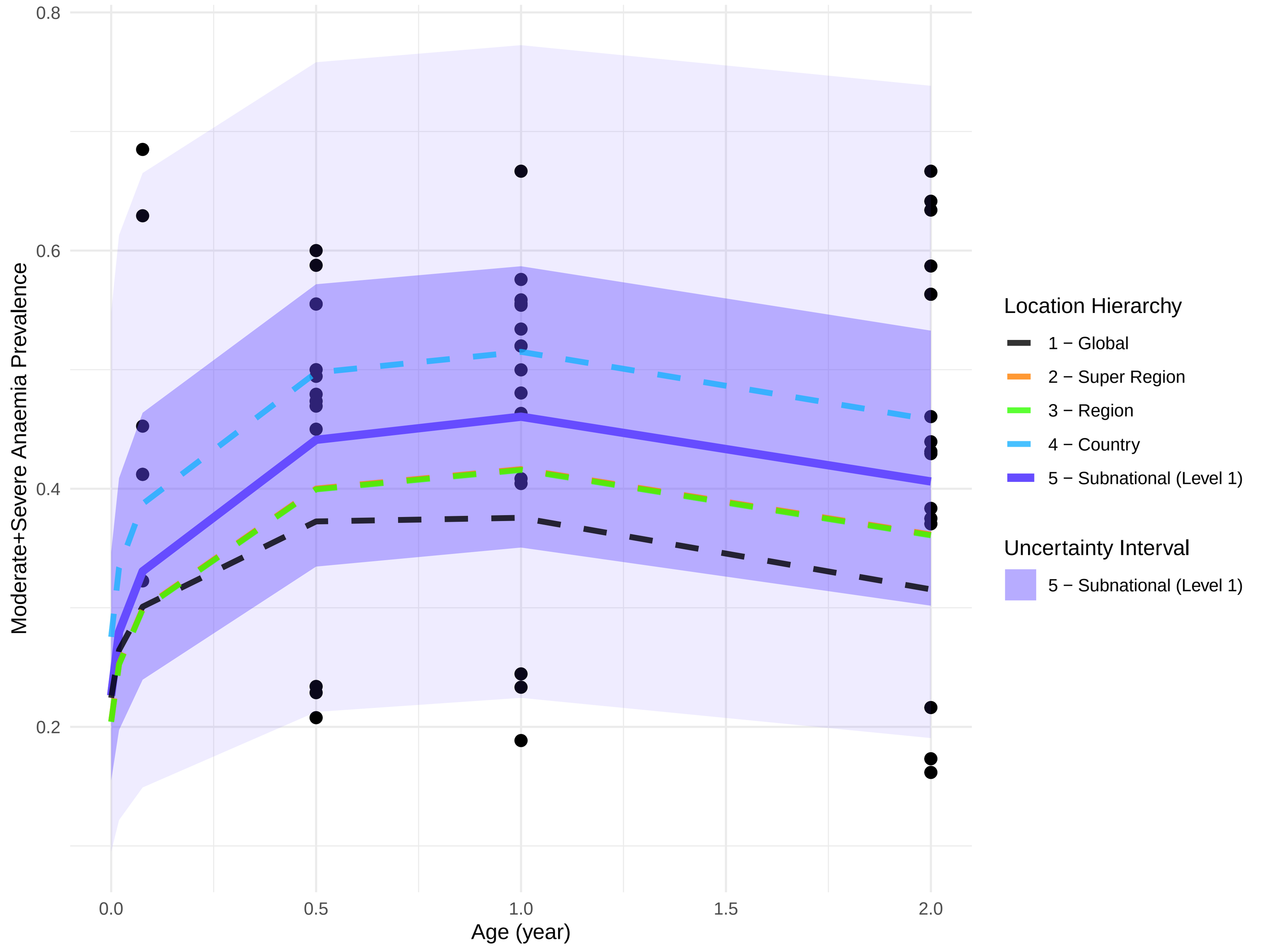

MR−BRT Age/Sex Fits − Males <2 Years Old − Moderate+Severe Anaemia Prevalence − Chhattisgarh, Rural
WHO−2024 Adjustment Method

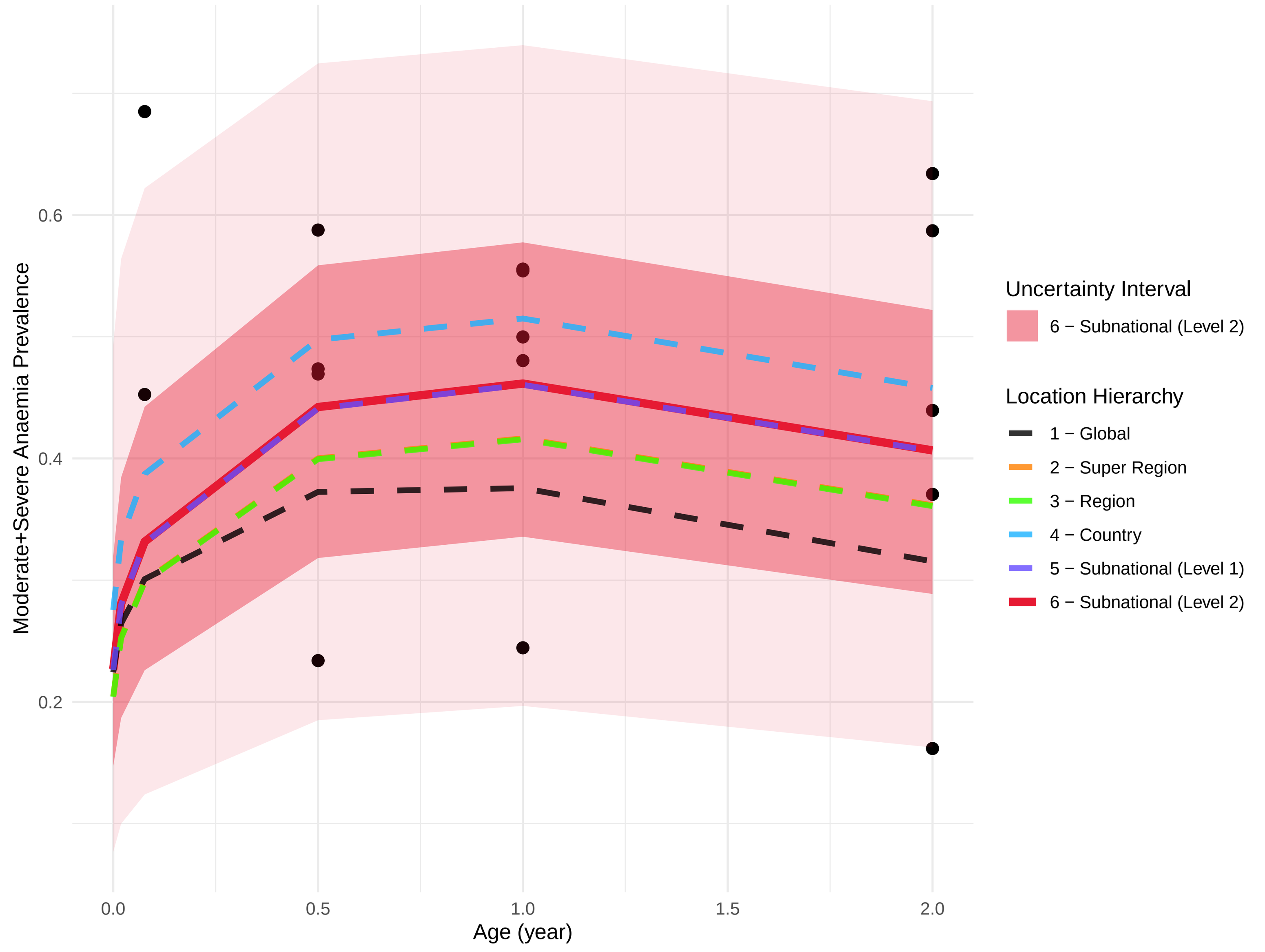

MR−BRT Age/Sex Fits − Males <2 Years Old − Moderate+Severe Anaemia Prevalence − Chhattisgarh, Urban
WHO−2024 Adjustment Method

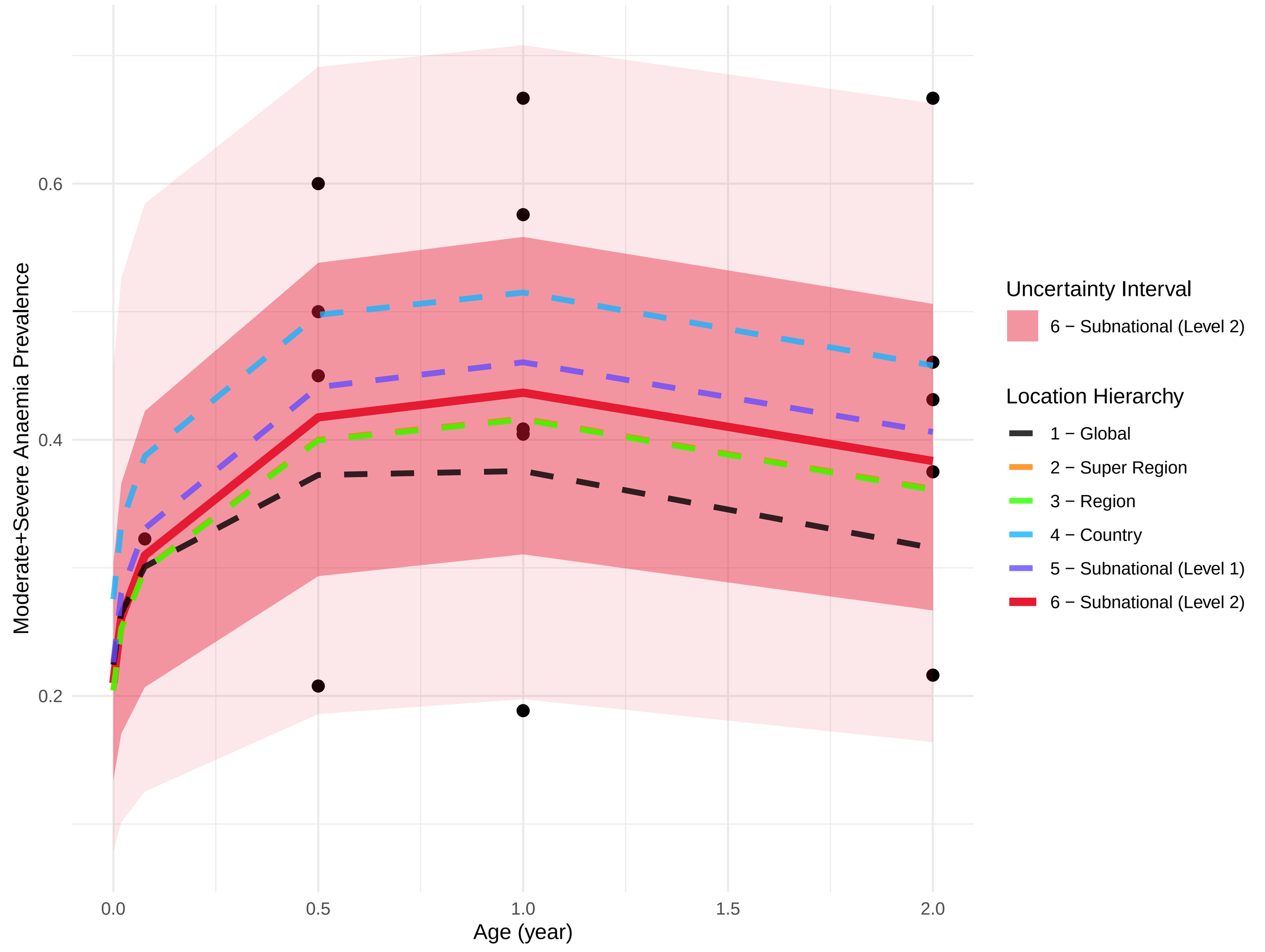

MR−BRT Age/Sex Fits − Males <2 Years Old − Moderate+Severe Anaemia Prevalence − Delhi
WHO−2024 Adjustment Method

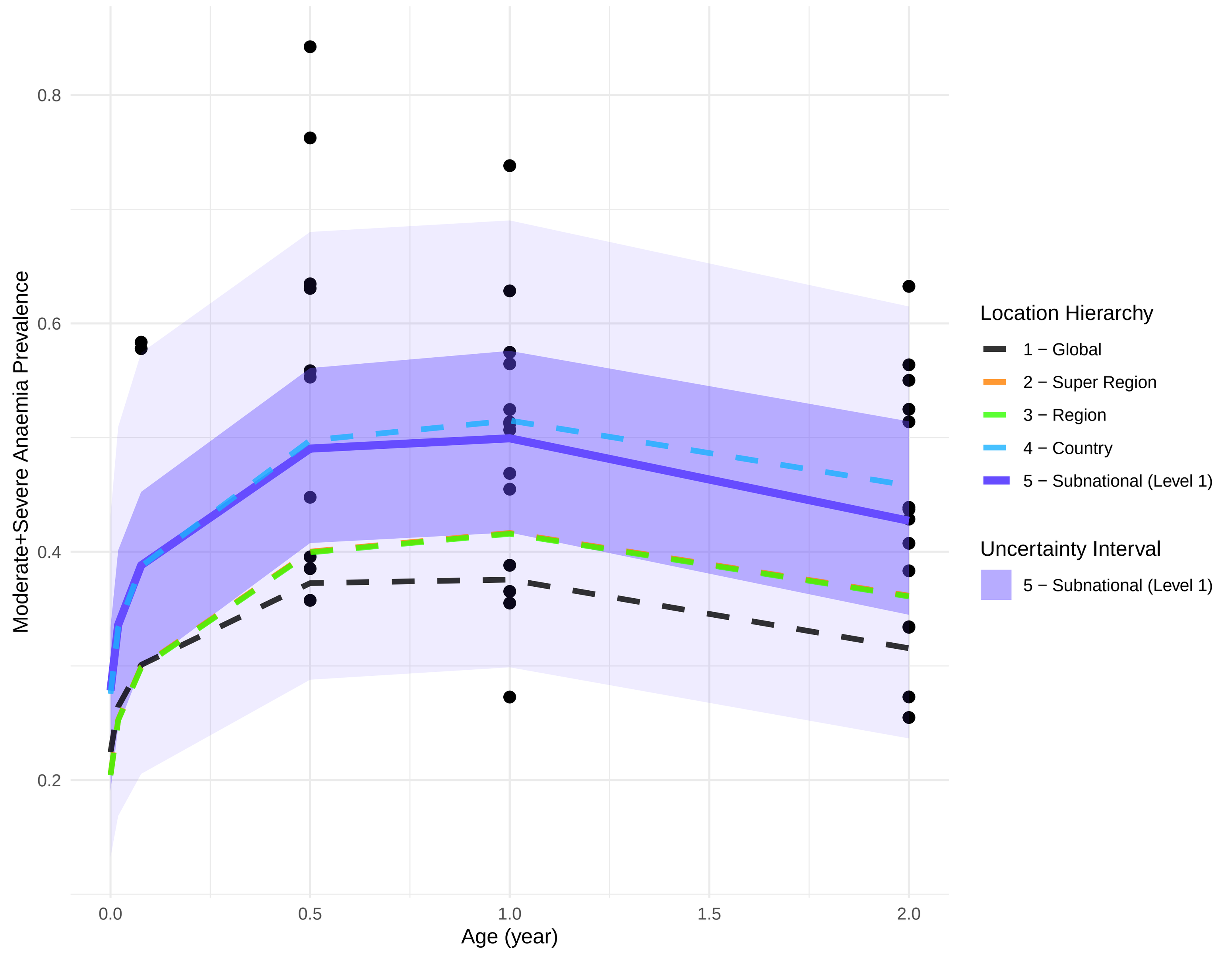

MR−BRT Age/Sex Fits − Males <2 Years Old − Moderate+Severe Anaemia Prevalence − Delhi, Rural
WHO−2024 Adjustment Method

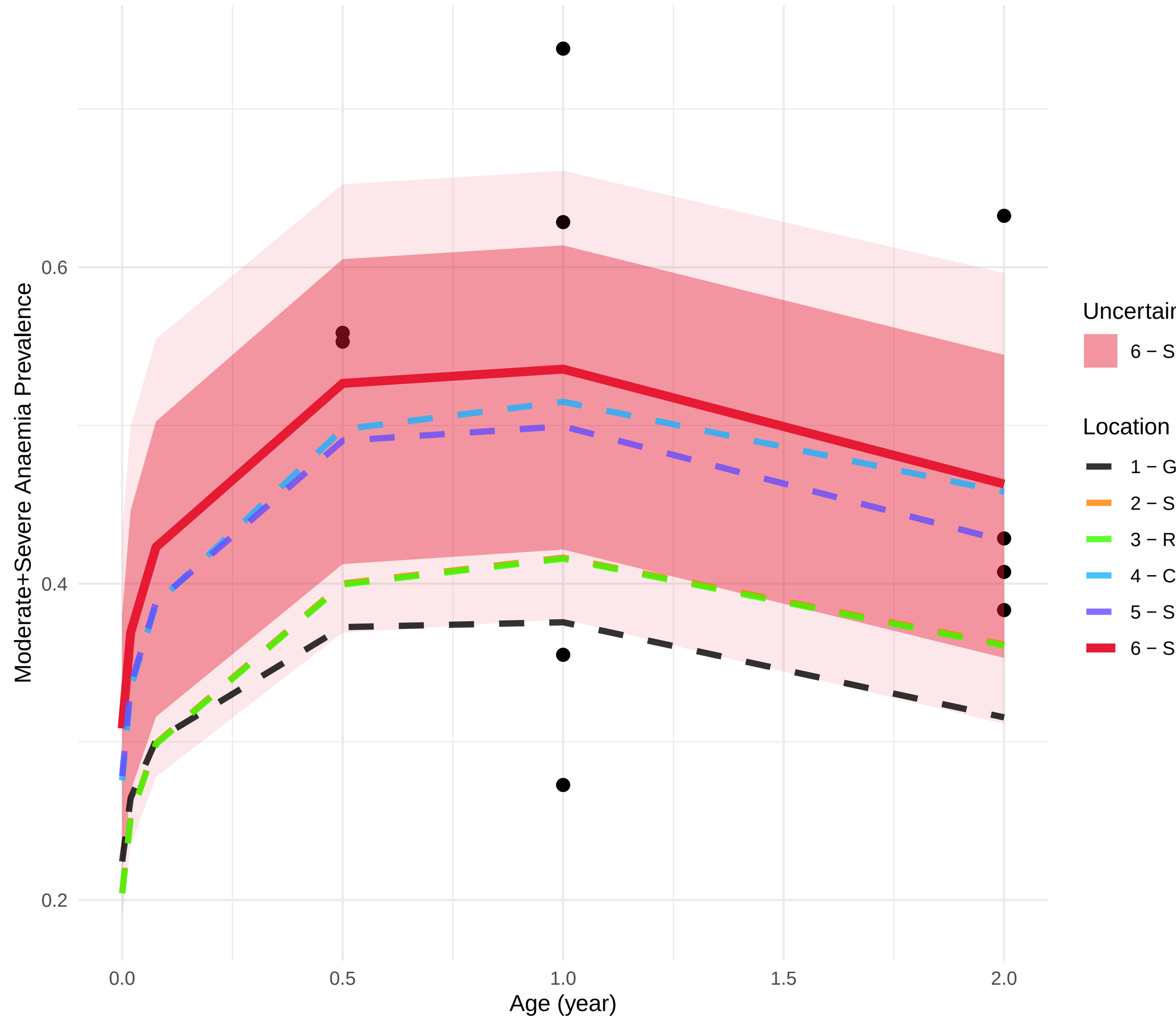

MR−BRT Age/Sex Fits − Males <2 Years Old − Moderate+Severe Anaemia Prevalence − Delhi, Urban
WHO−2024 Adjustment Method

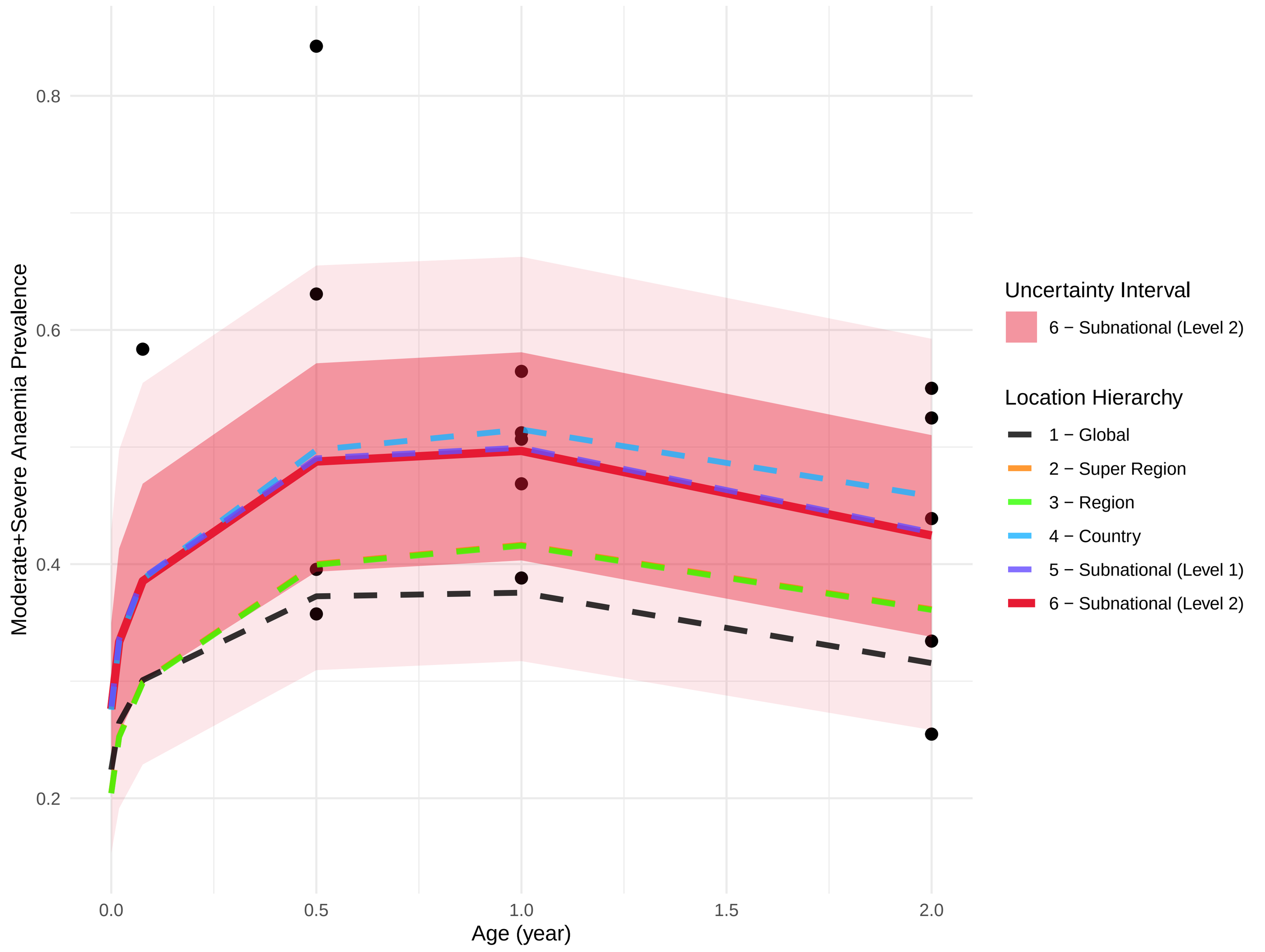

MR−BRT Age/Sex Fits − Males <2 Years Old − Moderate+Severe Anaemia Prevalence − Goa
WHO−2024 Adjustment Method

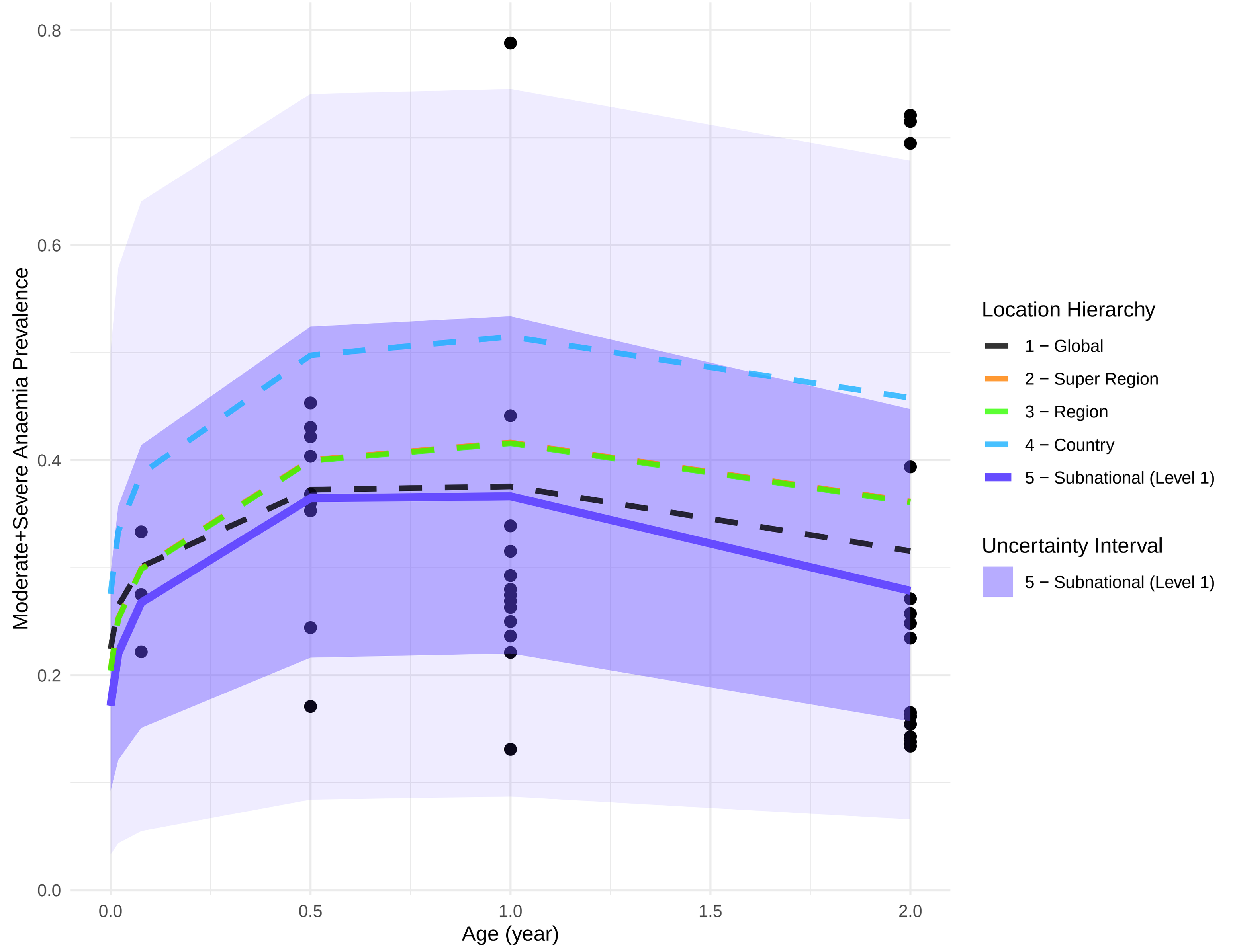

MR−BRT Age/Sex Fits − Males <2 Years Old − Moderate+Severe Anaemia Prevalence − Goa, Rural
WHO−2024 Adjustment Method

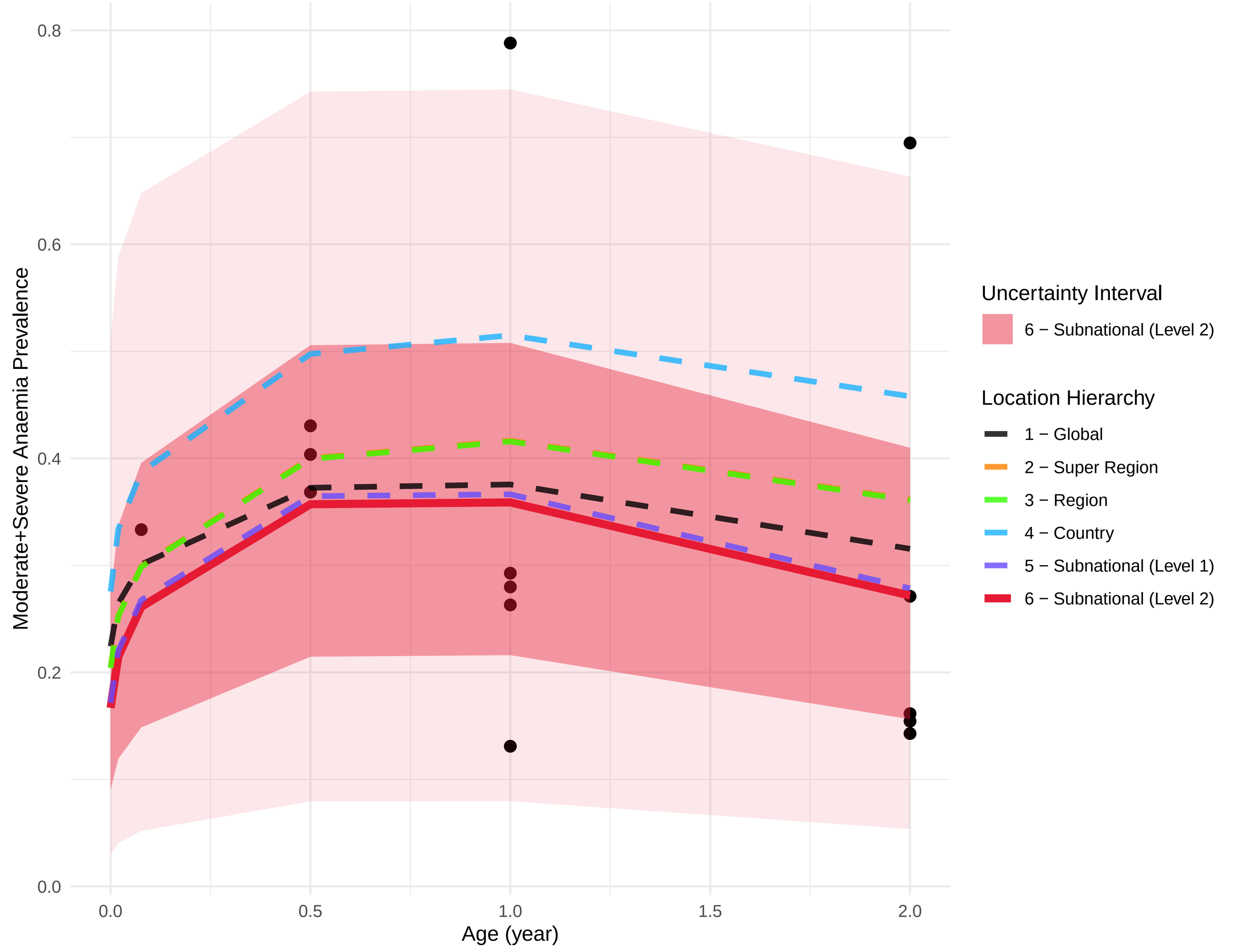

MR−BRT Age/Sex Fits − Males <2 Years Old − Moderate+Severe Anaemia Prevalence − Goa, Urban
WHO−2024 Adjustment Method

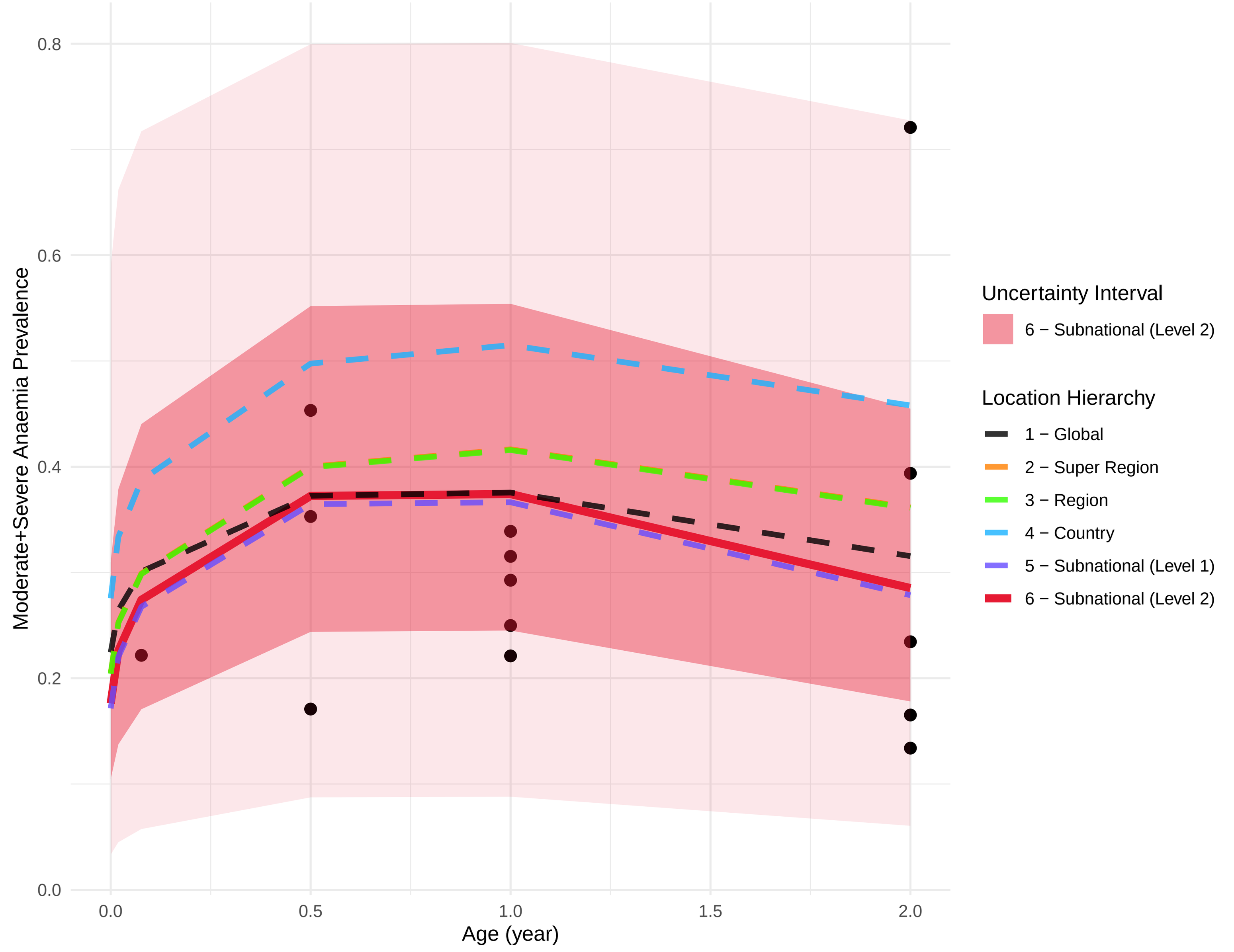

MR−BRT Age/Sex Fits − Males <2 Years Old − Moderate+Severe Anaemia Prevalence − Gujarat
WHO−2024 Adjustment Method

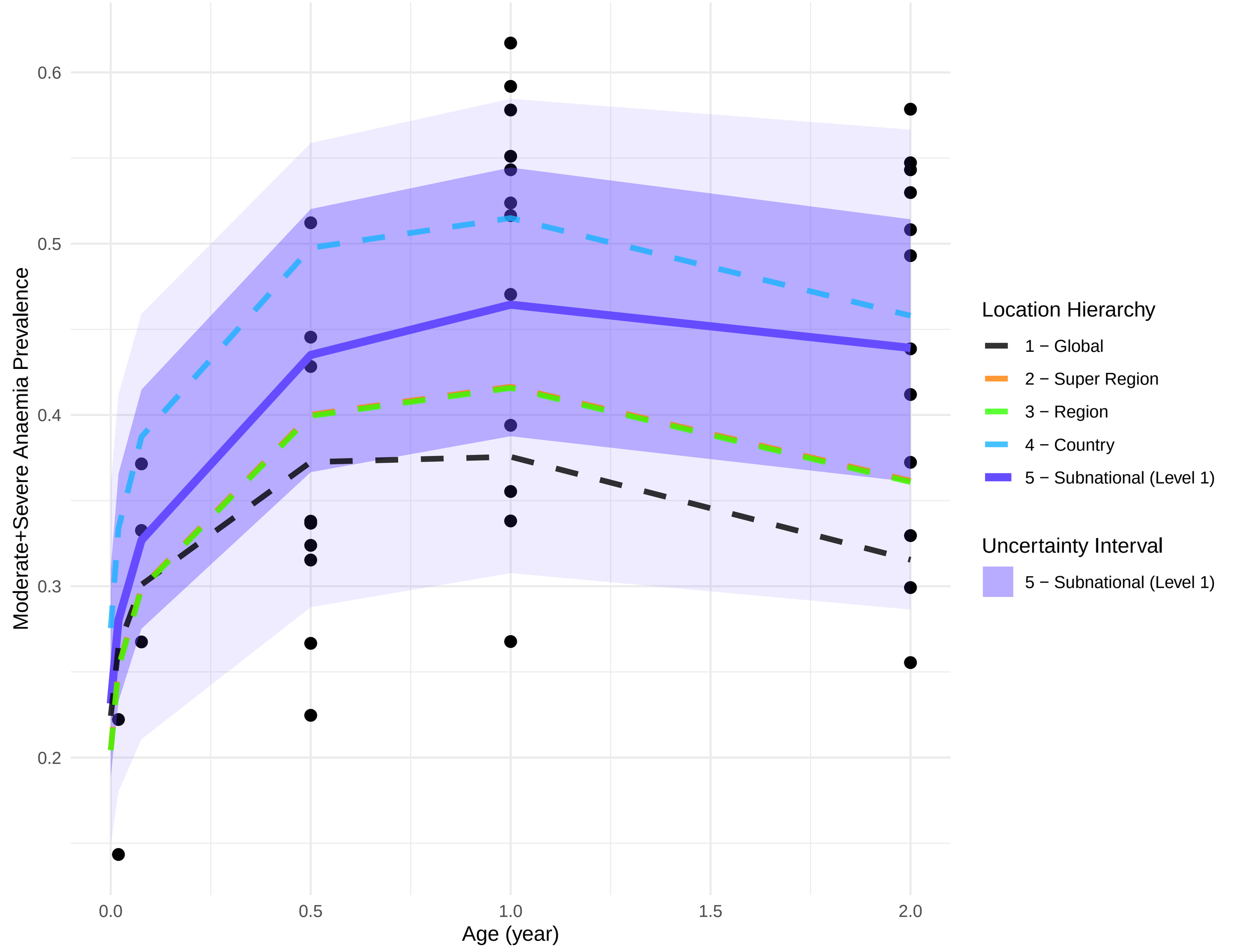

MR−BRT Age/Sex Fits − Males <2 Years Old − Moderate+Severe Anaemia Prevalence − Gujarat, Rural
WHO−2024 Adjustment Method

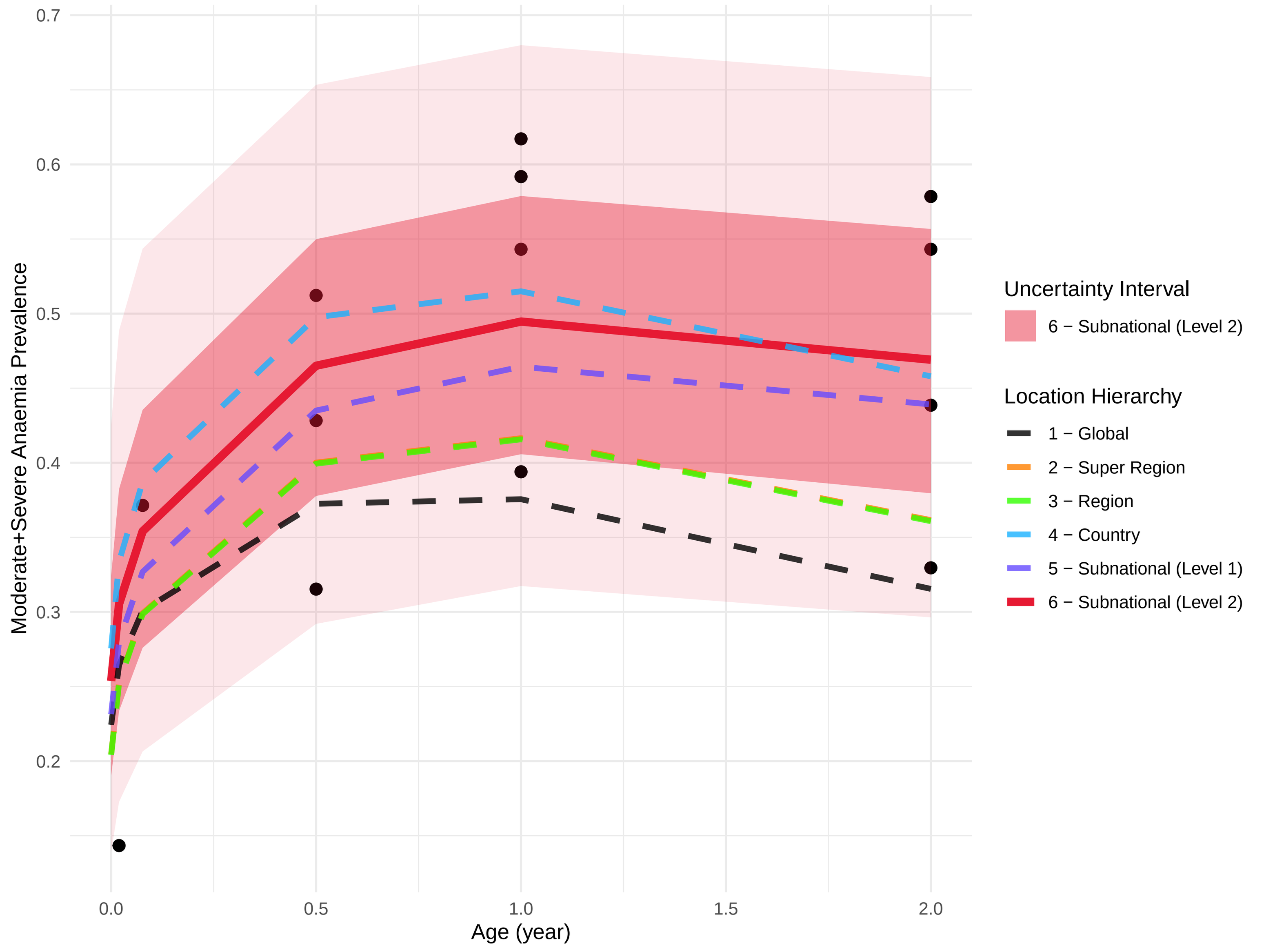

MR−BRT Age/Sex Fits − Males <2 Years Old − Moderate+Severe Anaemia Prevalence − Gujarat, Urban
WHO−2024 Adjustment Method

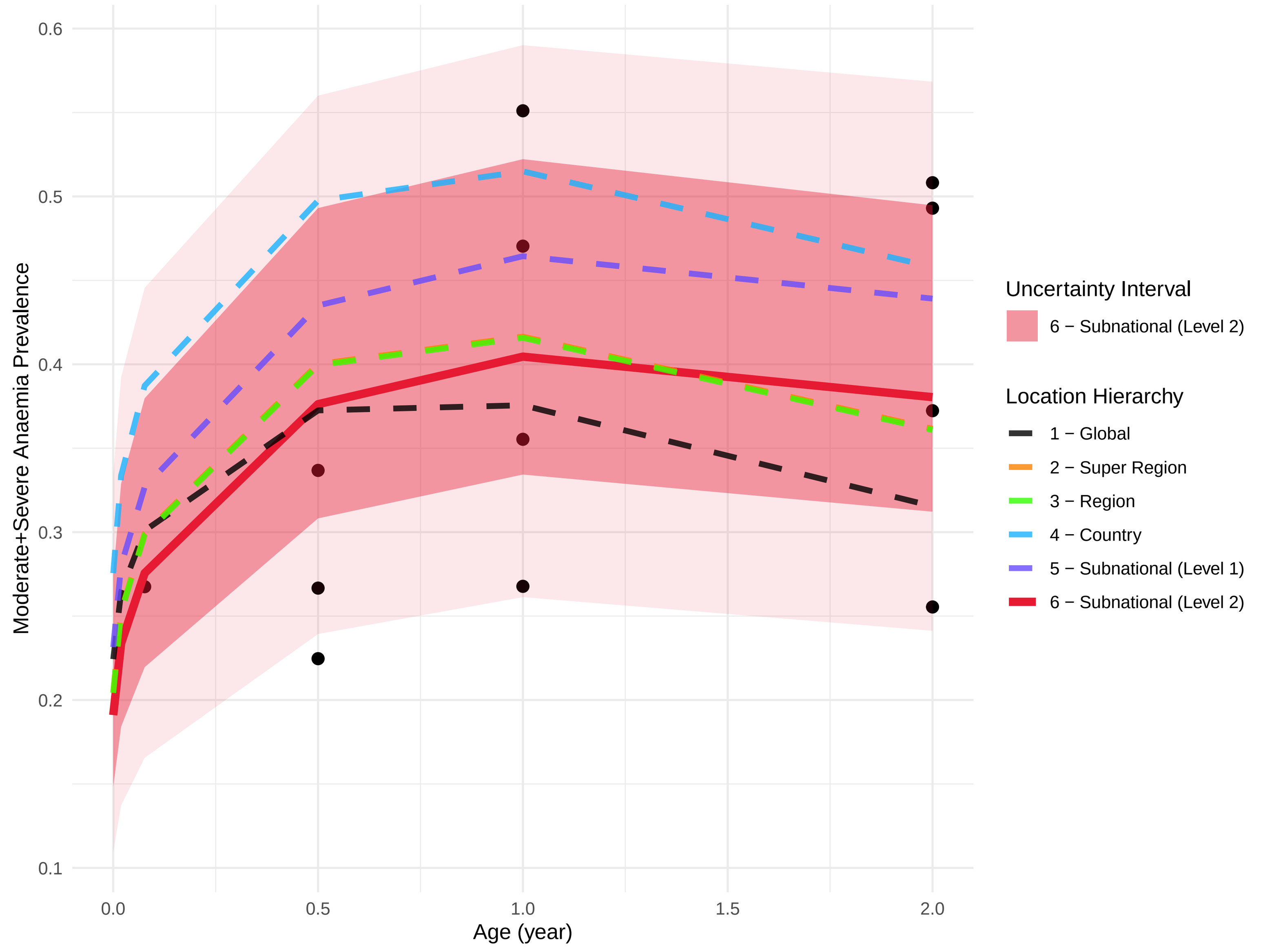

MR−BRT Age/Sex Fits − Males <2 Years Old − Moderate+Severe Anaemia Prevalence − Haryana
WHO−2024 Adjustment Method

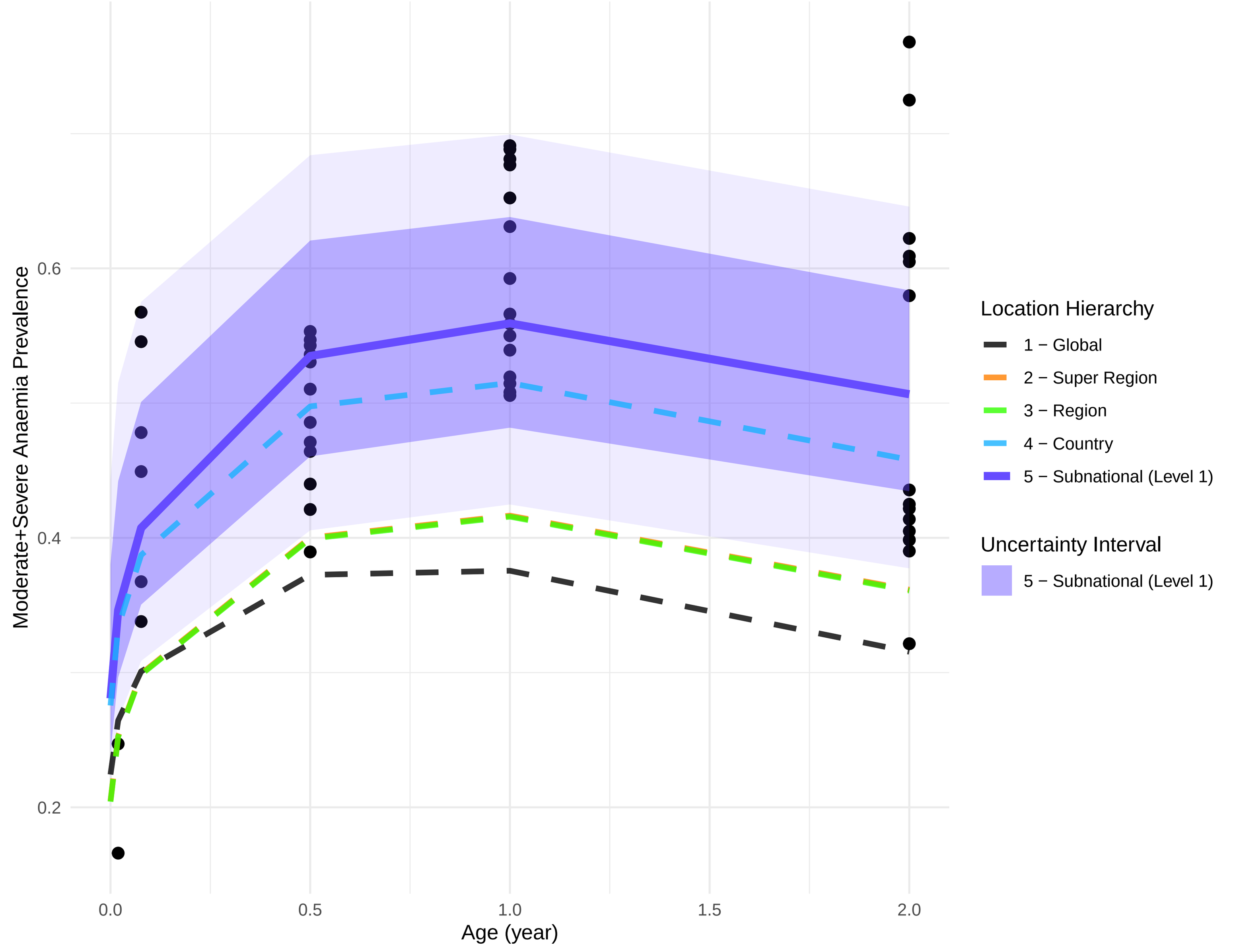

MR−BRT Age/Sex Fits − Males <2 Years Old − Moderate+Severe Anaemia Prevalence − Haryana, Rural
WHO−2024 Adjustment Method

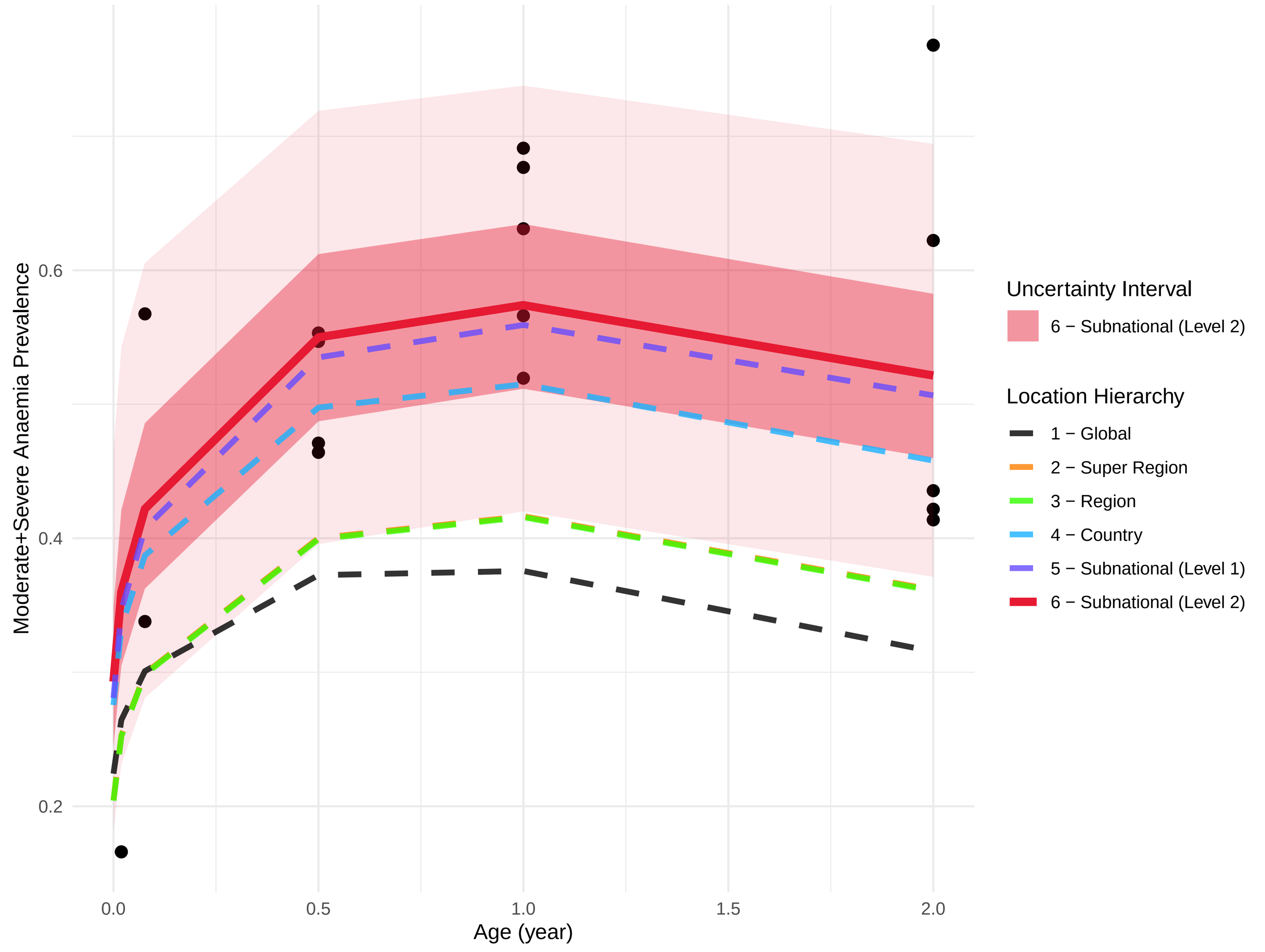

MR−BRT Age/Sex Fits − Males <2 Years Old − Moderate+Severe Anaemia Prevalence − Haryana, Urban
WHO−2024 Adjustment Method

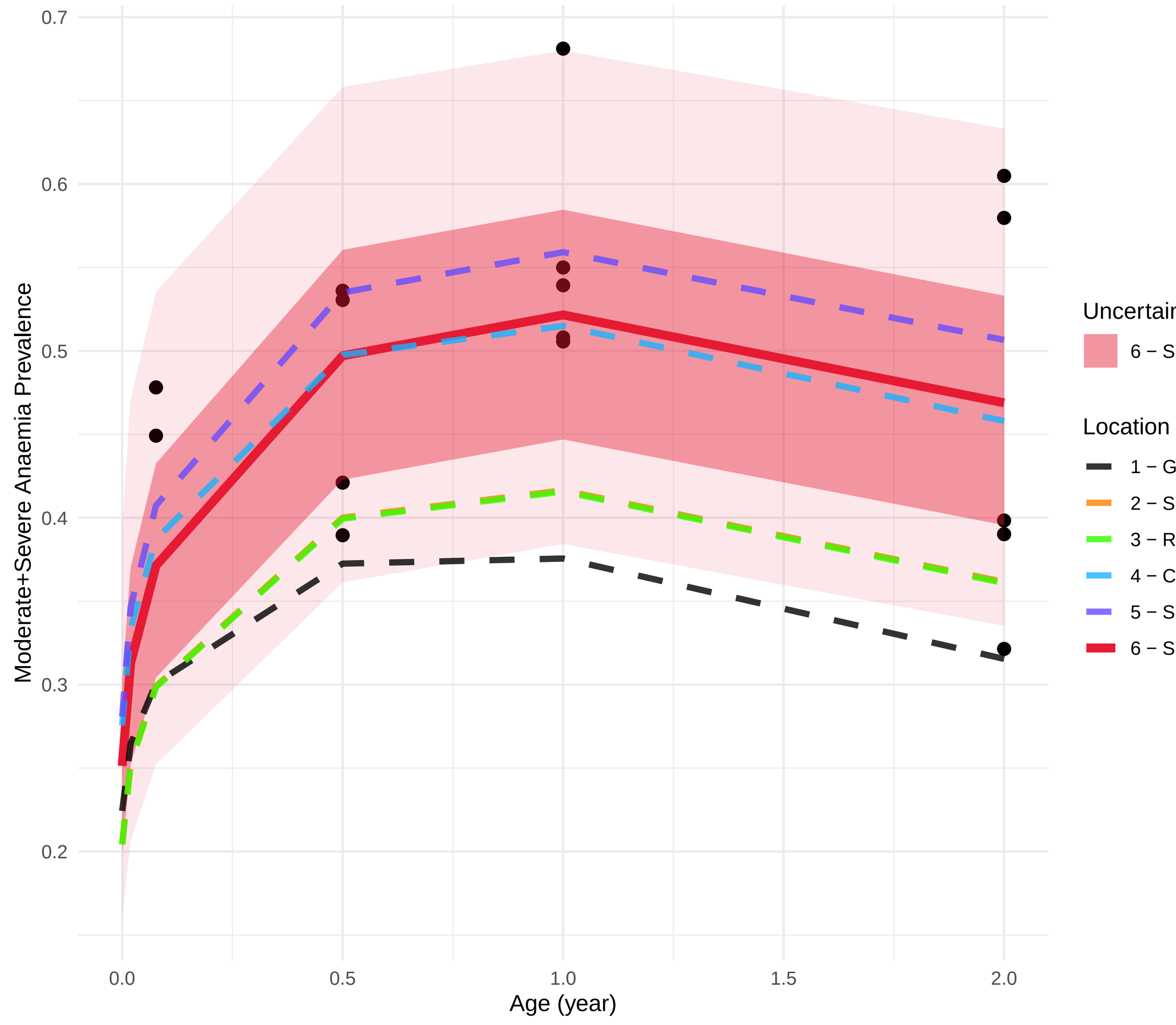

MR−BRT Age/Sex Fits − Males <2 Years Old − Moderate+Severe Anaemia Prevalence − Himachal Pradesh
WHO−2024 Adjustment Method

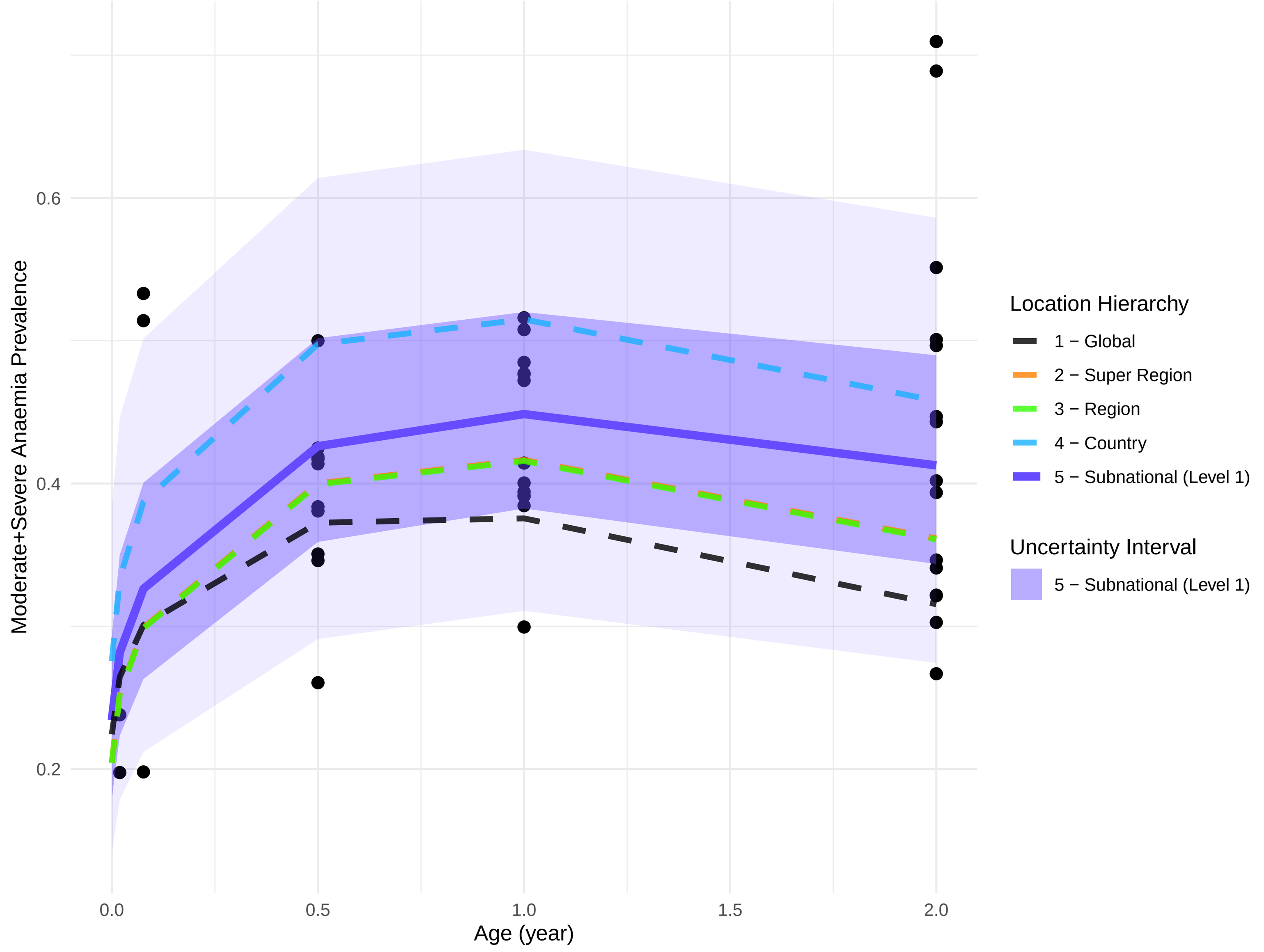

MR−BRT Age/Sex Fits − Males <2 Years Old − Moderate+Severe Anaemia Prevalence − Himachal Pradesh, Rural
WHO−2024 Adjustment Method

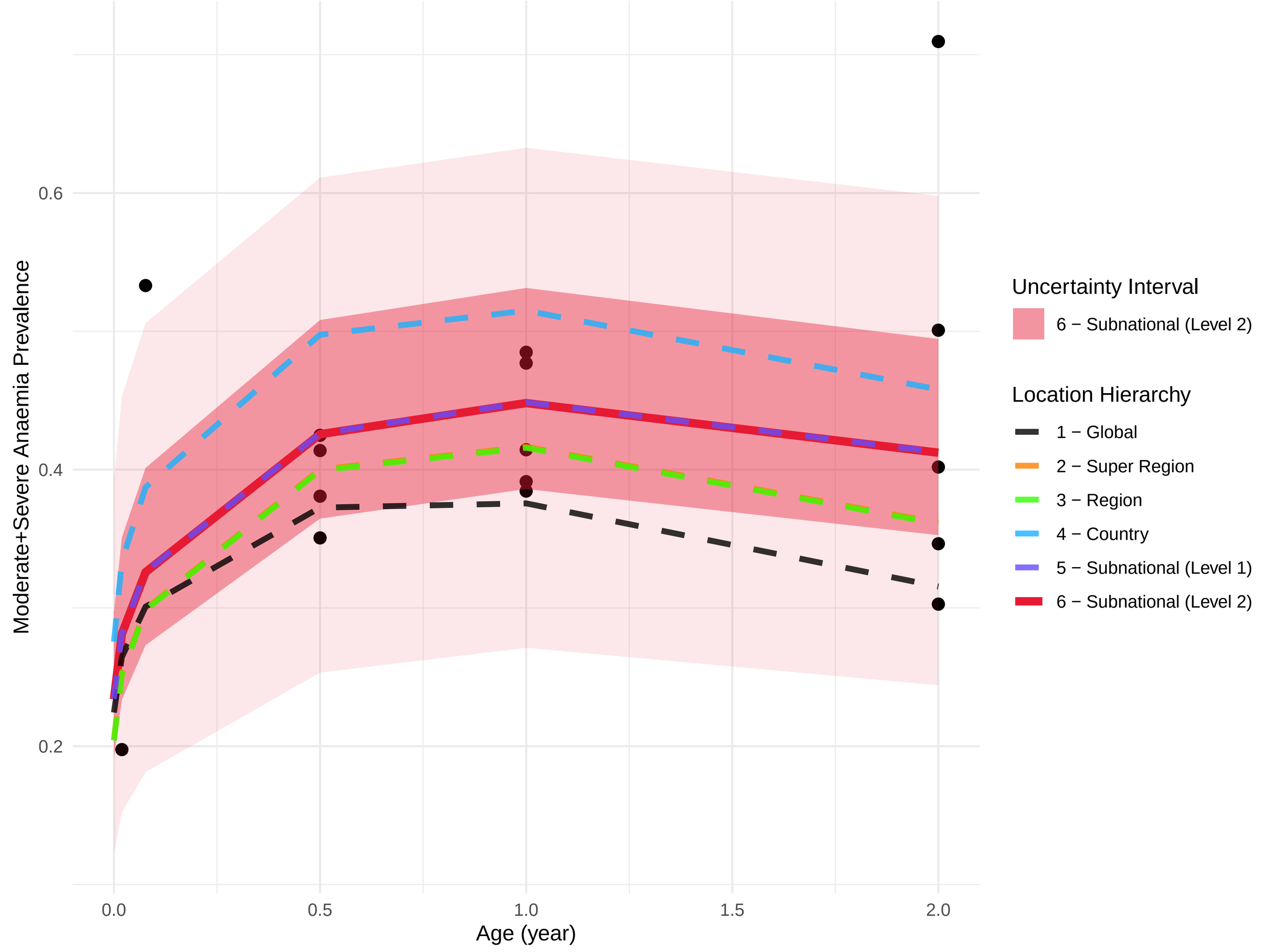

MR−BRT Age/Sex Fits − Males <2 Years Old − Moderate+Severe Anaemia Prevalence − Himachal Pradesh, Urban
WHO−2024 Adjustment Method

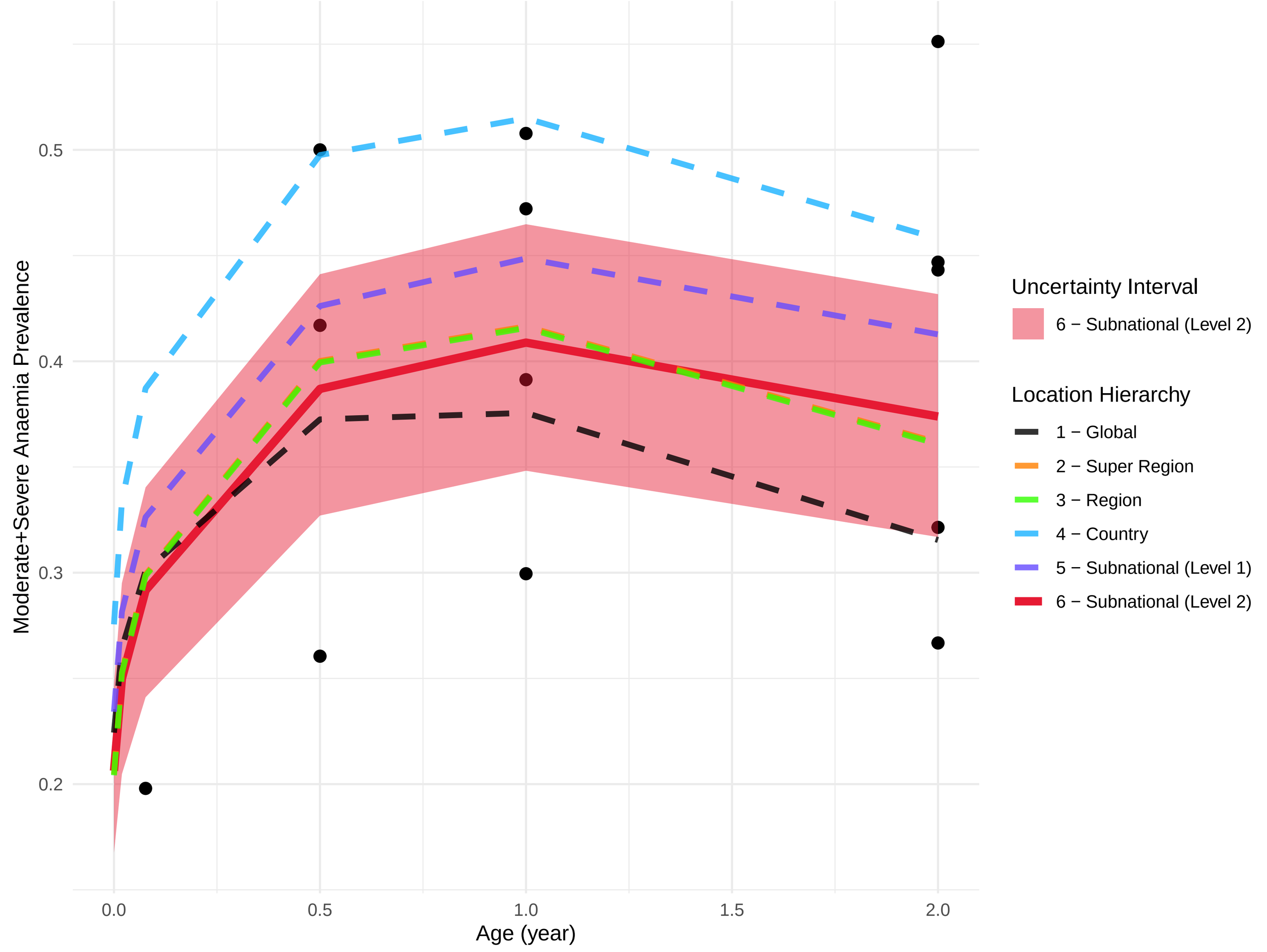

MR−BRT Age/Sex Fits − Males <2 Years Old − Moderate+Severe Anaemia Prevalence − Jammu & Kashmir and Ladakh
WHO−2024 Adjustment Method

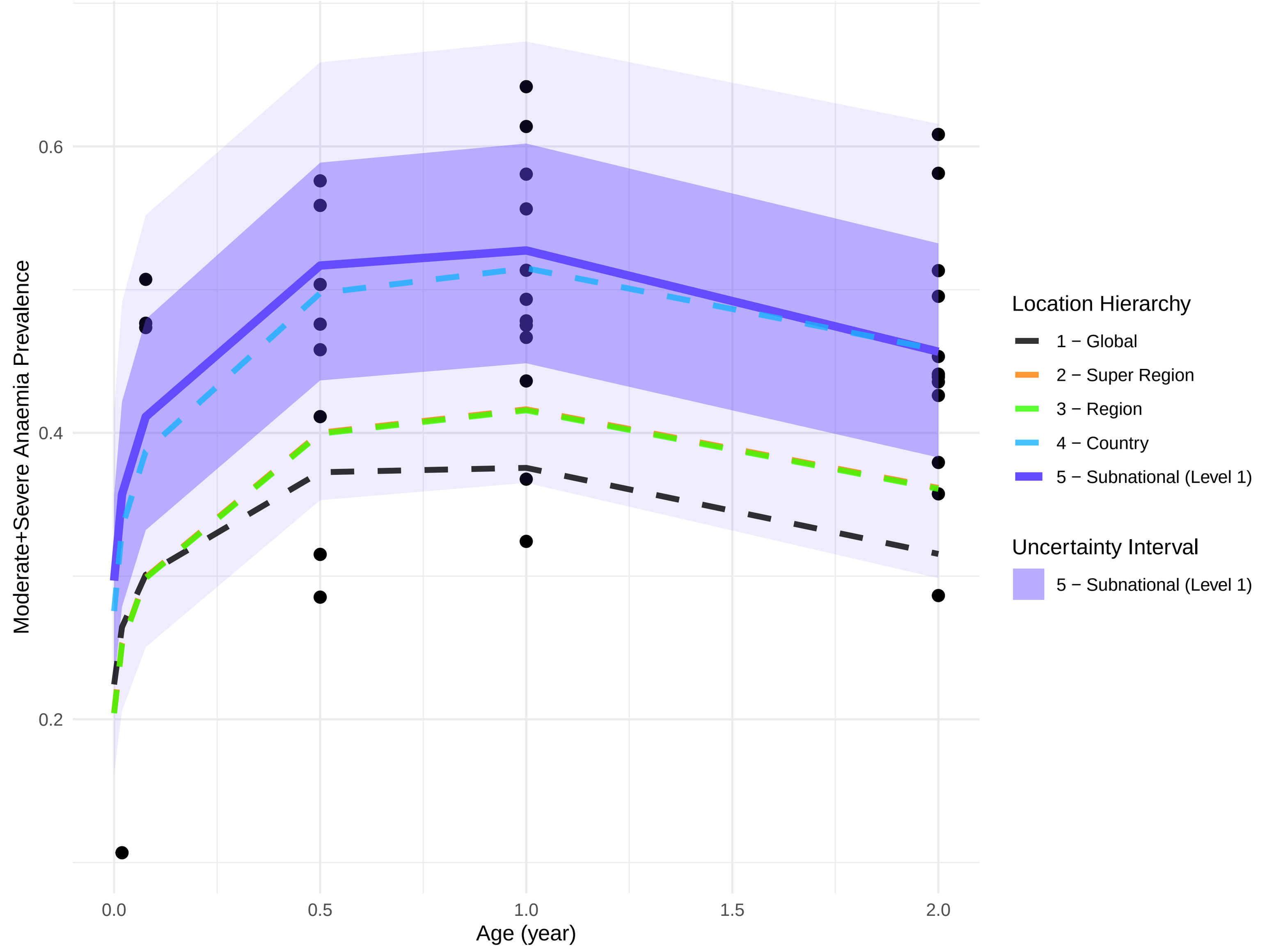

MR−BRT Age/Sex Fits − Males <2 Years Old − Moderate+Severe Anaemia Prevalence − Jammu & Kashmir and Ladakh, Rural
WHO−2024 Adjustment Method

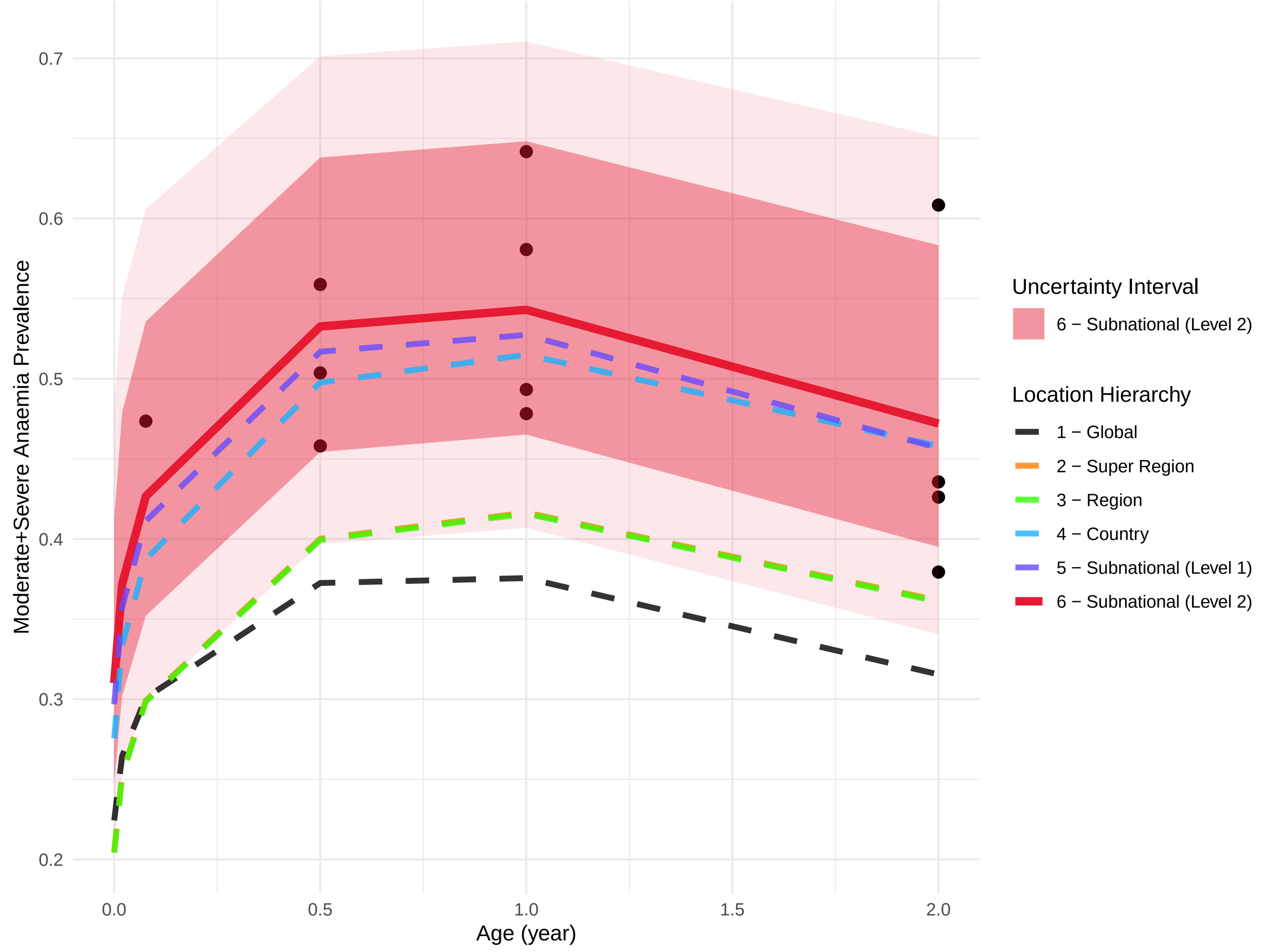

MR−BRT Age/Sex Fits − Males <2 Years Old − Moderate+Severe Anaemia Prevalence − Jammu & Kashmir and Ladakh, Urban
WHO−2024 Adjustment Method

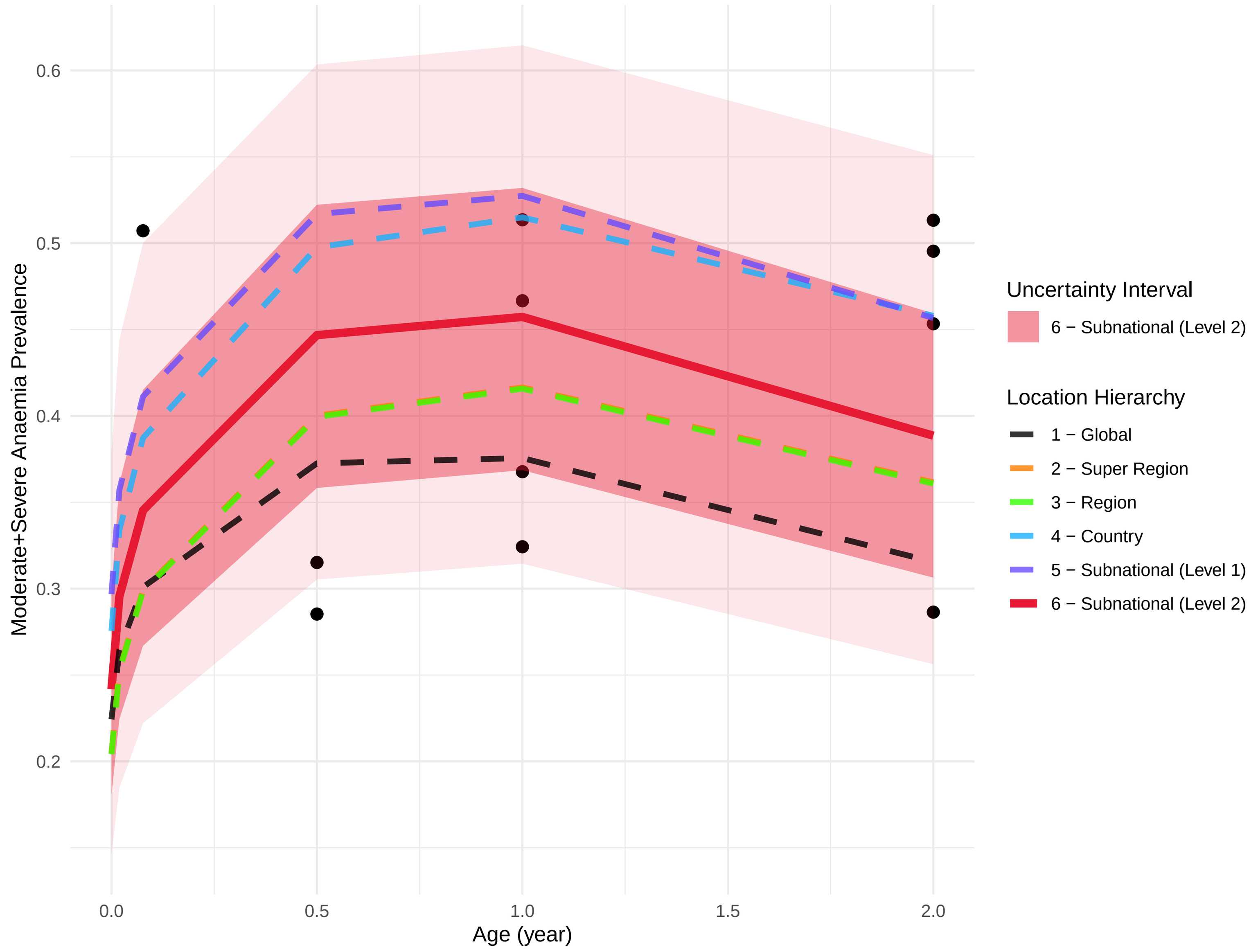

MR−BRT Age/Sex Fits − Males <2 Years Old − Moderate+Severe Anaemia Prevalence − Jharkhand
WHO−2024 Adjustment Method

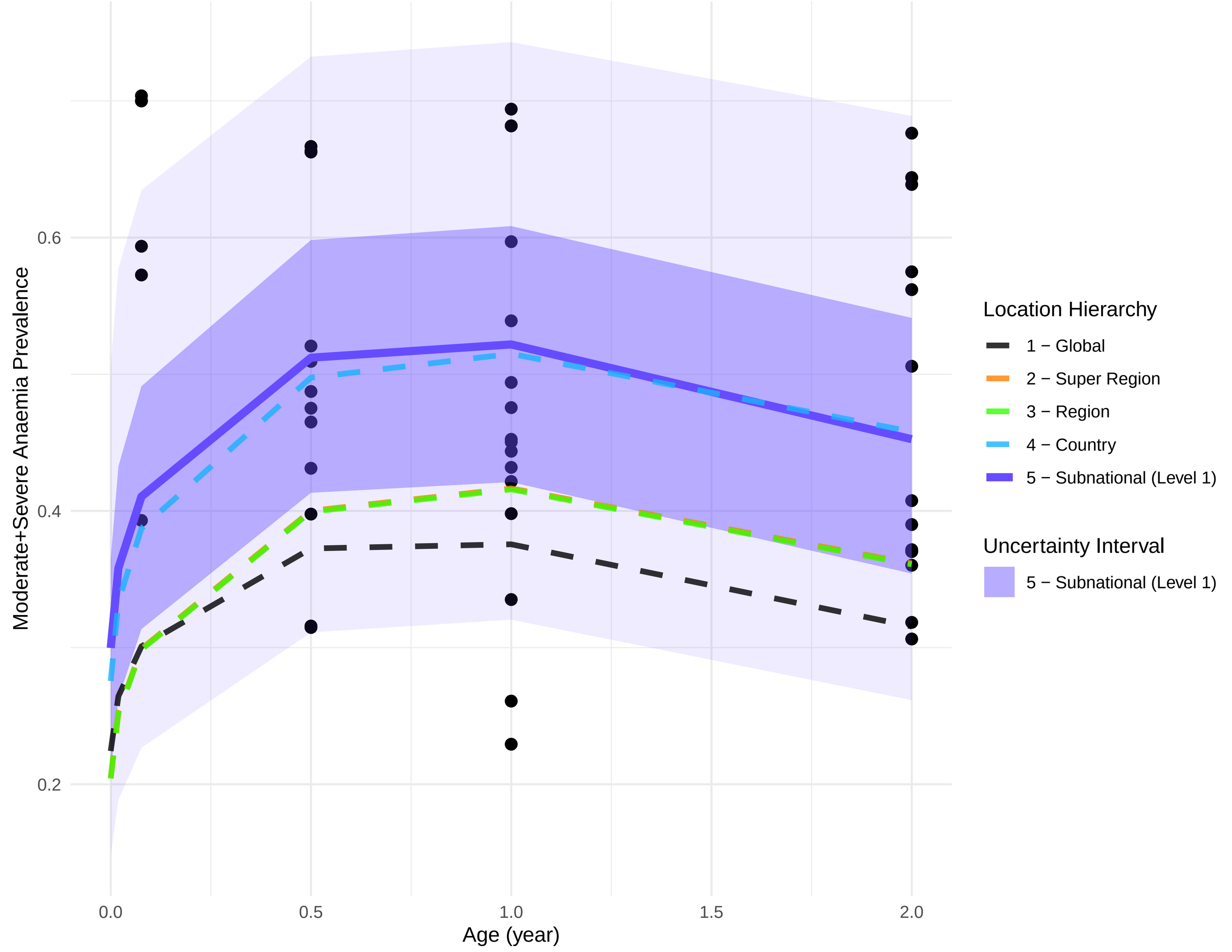

MR−BRT Age/Sex Fits − Males <2 Years Old − Moderate+Severe Anaemia Prevalence − Jharkhand, Rural
WHO−2024 Adjustment Method

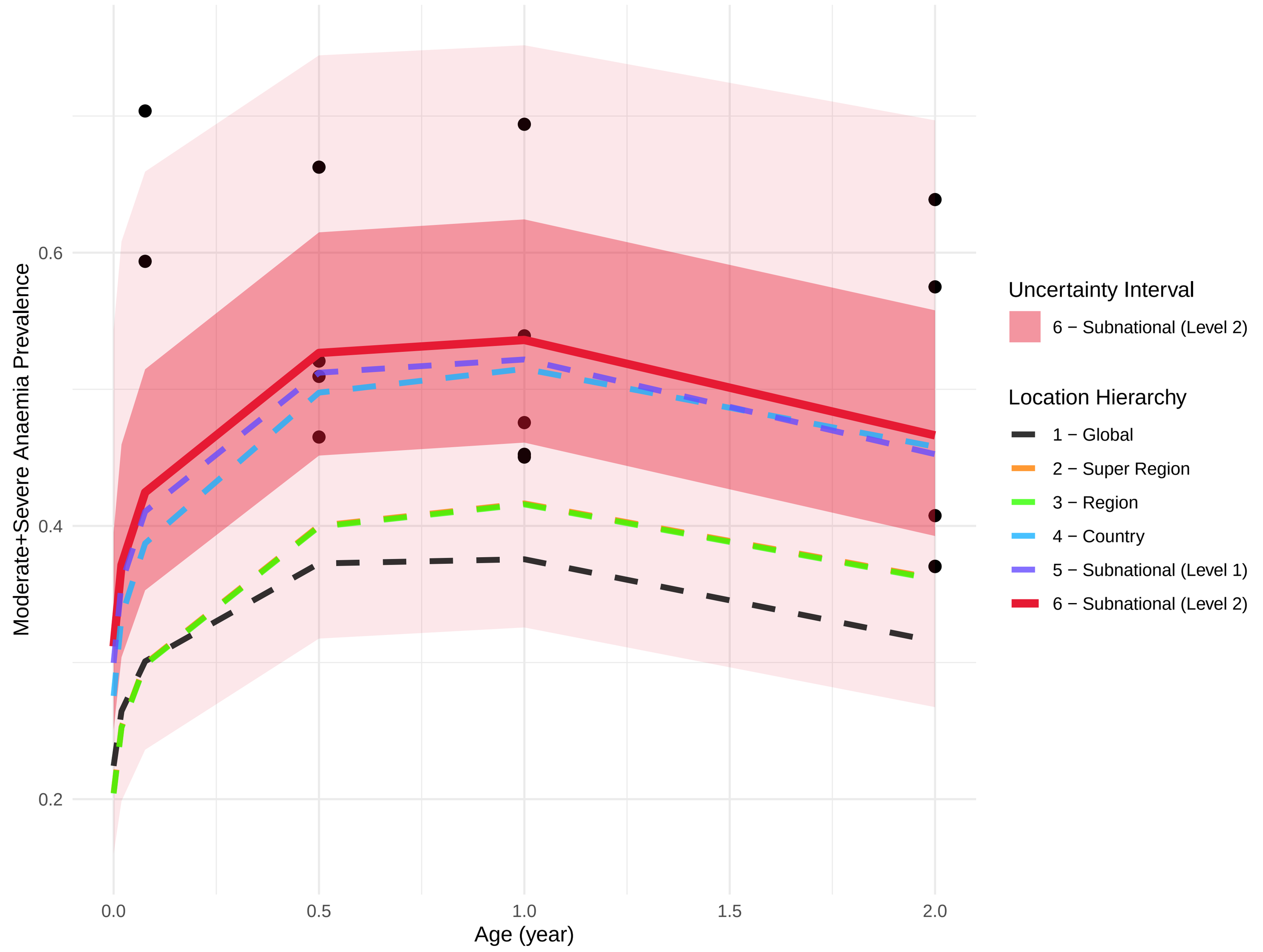

MR−BRT Age/Sex Fits − Males <2 Years Old − Moderate+Severe Anaemia Prevalence − Jharkhand, Urban
WHO−2024 Adjustment Method

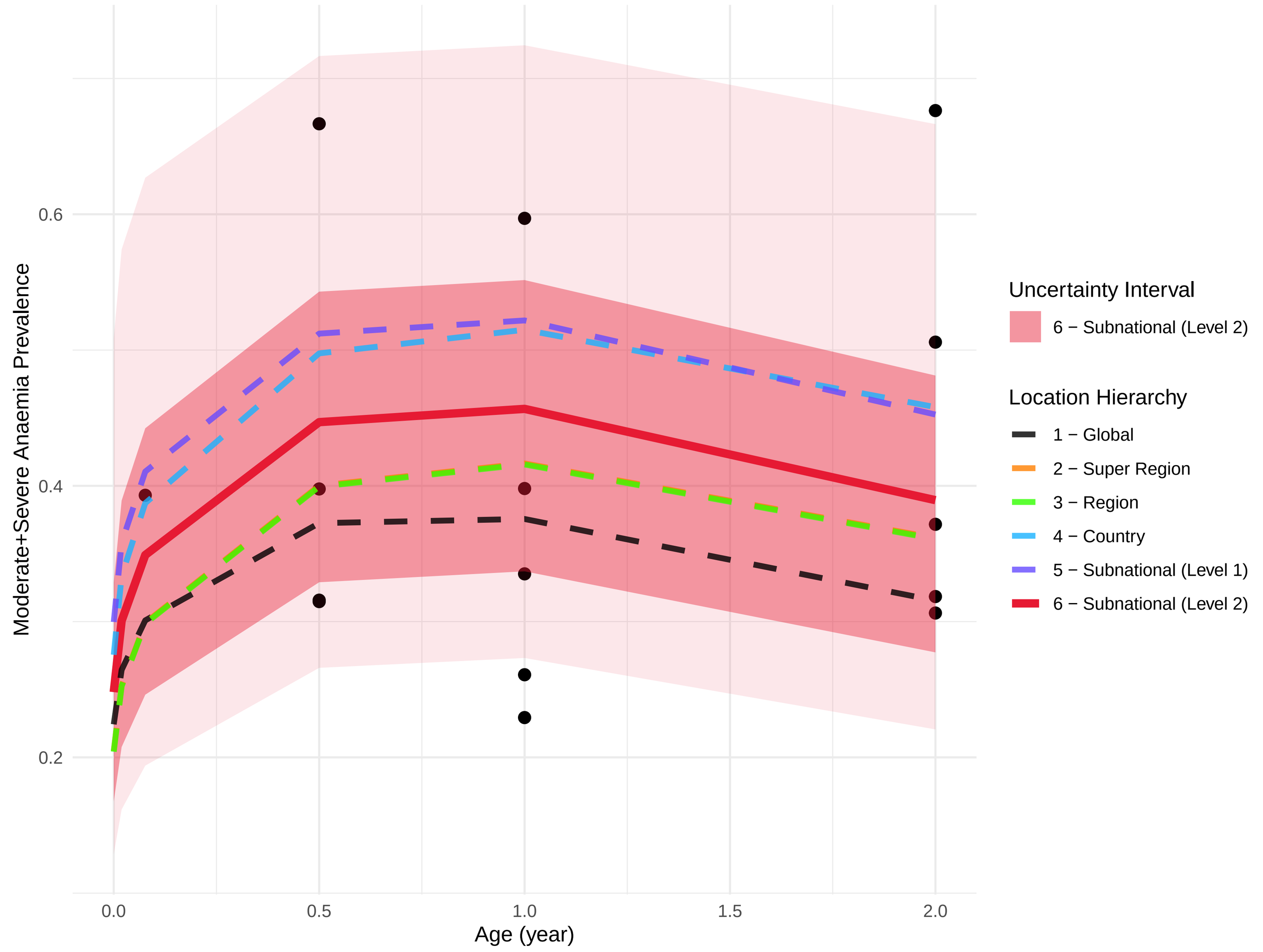

MR−BRT Age/Sex Fits − Males <2 Years Old − Moderate+Severe Anaemia Prevalence − Karnataka
WHO−2024 Adjustment Method

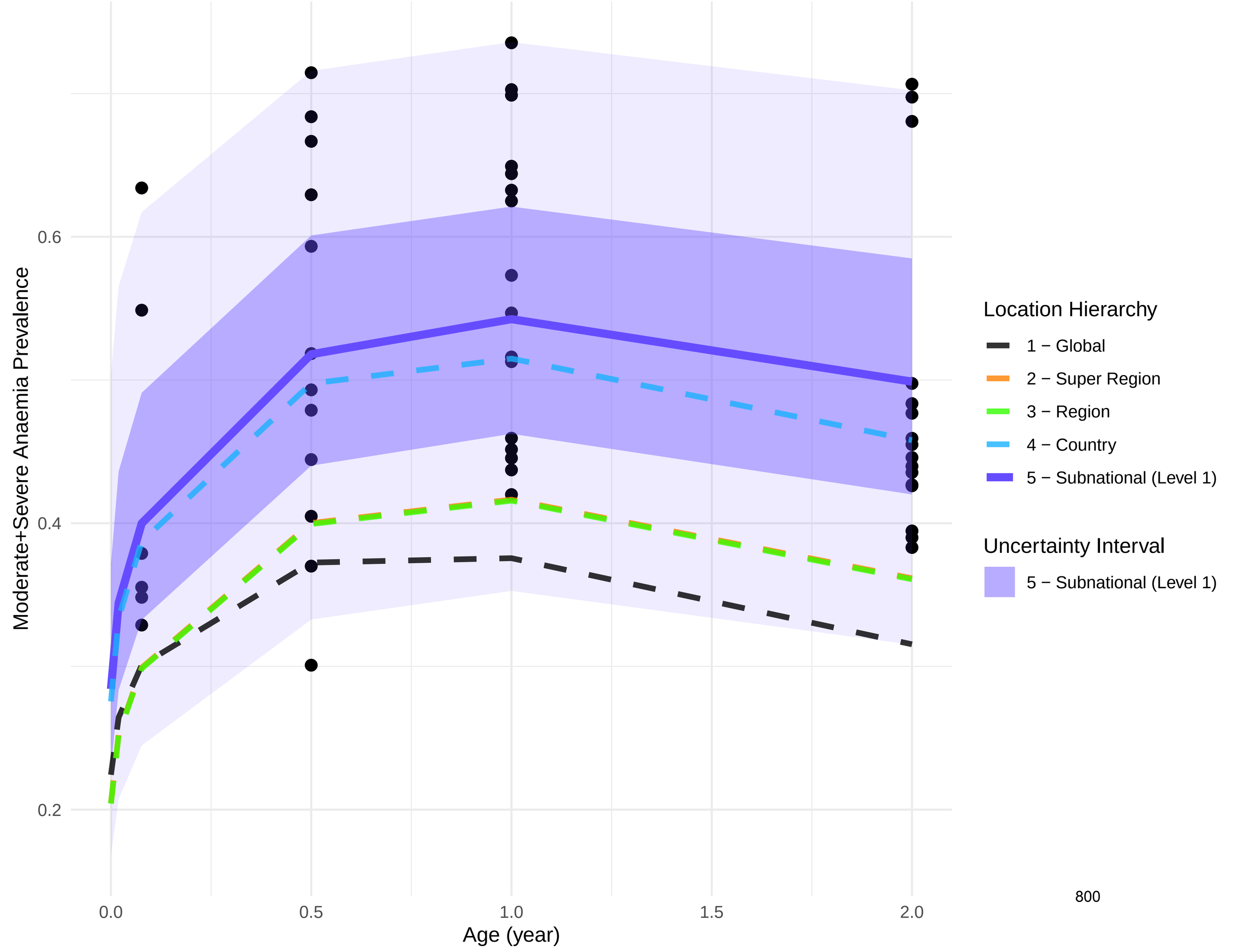

MR−BRT Age/Sex Fits − Males <2 Years Old − Moderate+Severe Anaemia Prevalence − Karnataka, Rural
WHO−2024 Adjustment Method

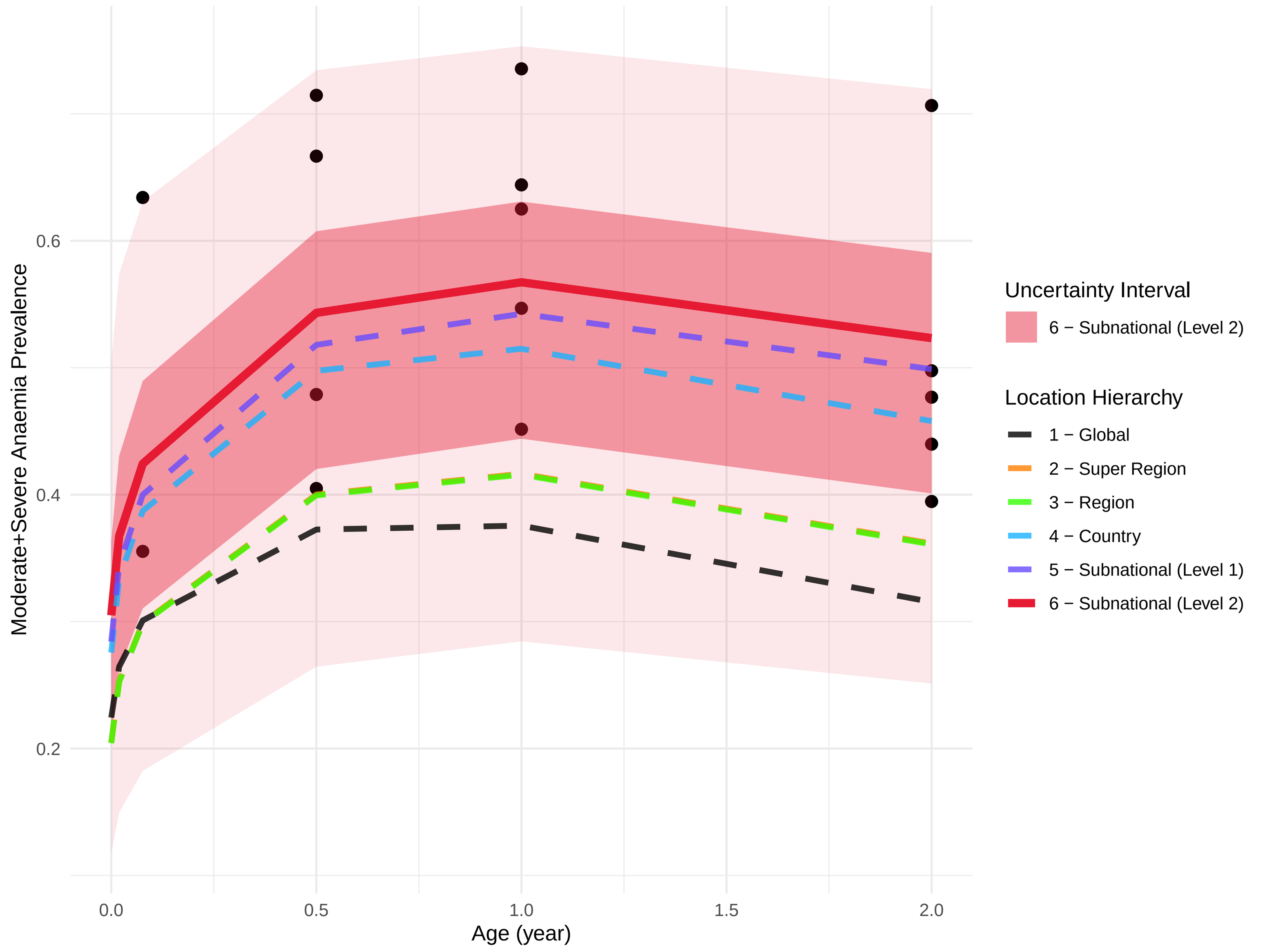

MR−BRT Age/Sex Fits − Males <2 Years Old − Moderate+Severe Anaemia Prevalence − Karnataka, Urban
WHO−2024 Adjustment Method

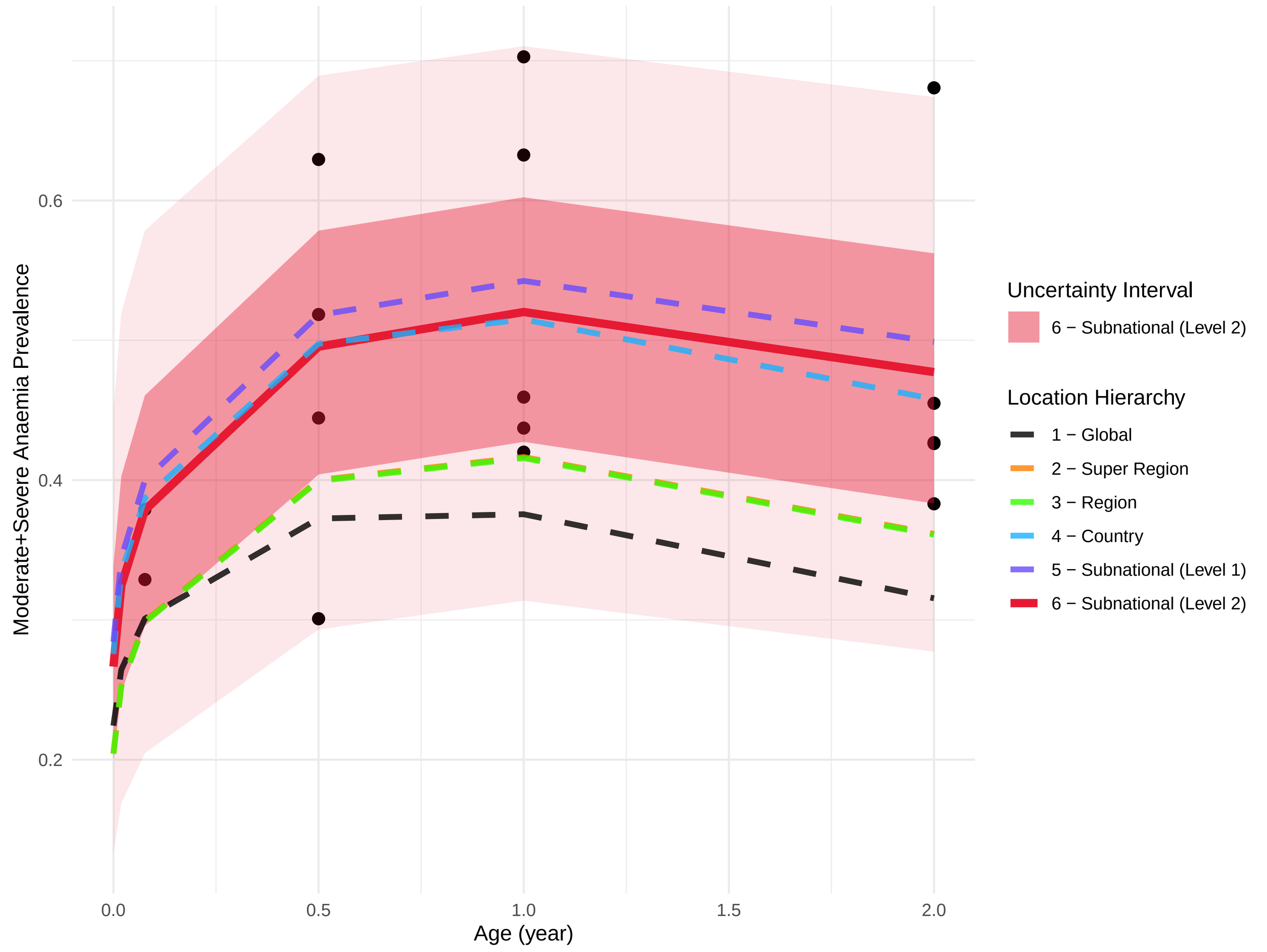

MR−BRT Age/Sex Fits − Males <2 Years Old − Moderate+Severe Anaemia Prevalence − Kerala
WHO−2024 Adjustment Method

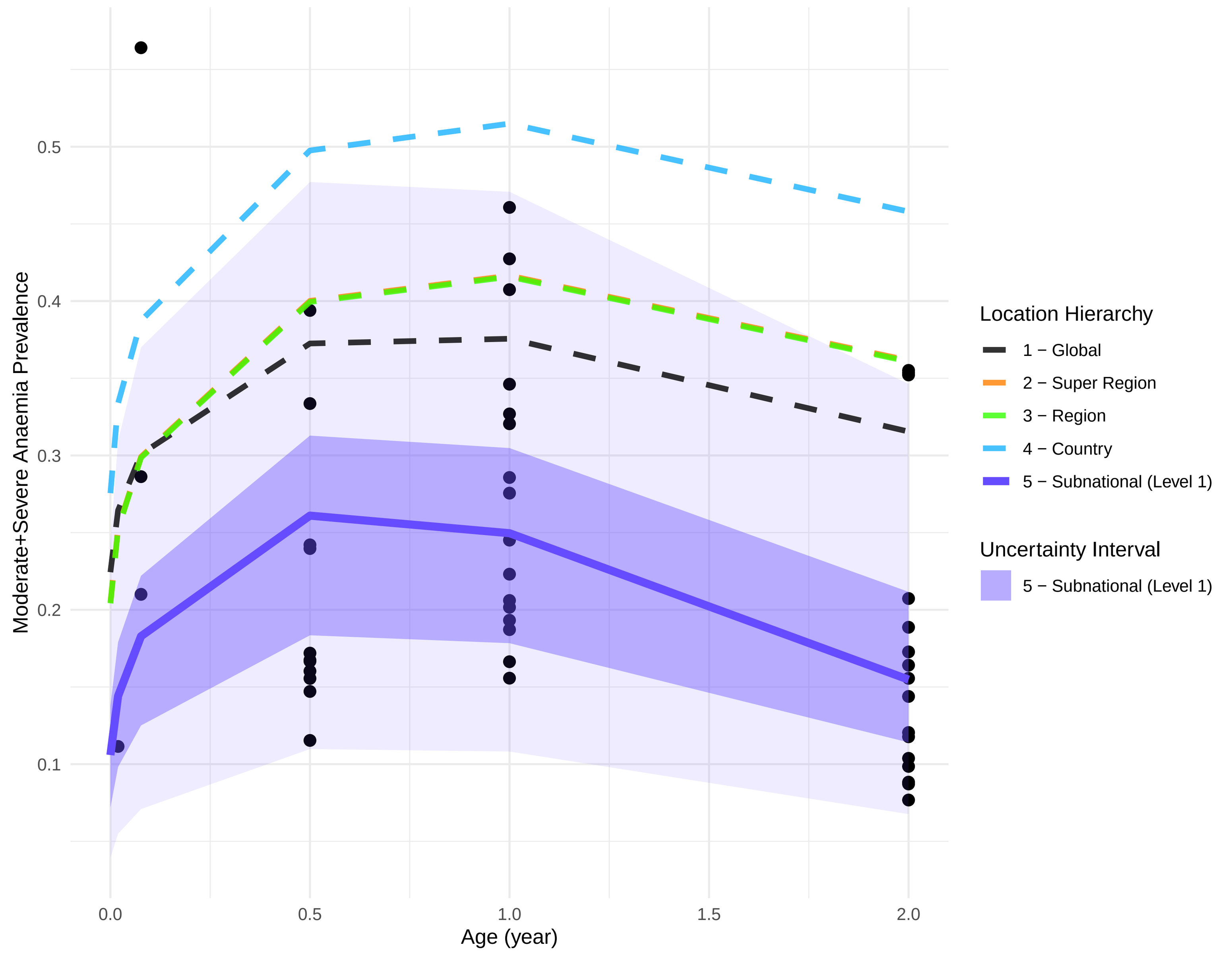

MR−BRT Age/Sex Fits − Males <2 Years Old − Moderate+Severe Anaemia Prevalence − Kerala, Rural
WHO−2024 Adjustment Method

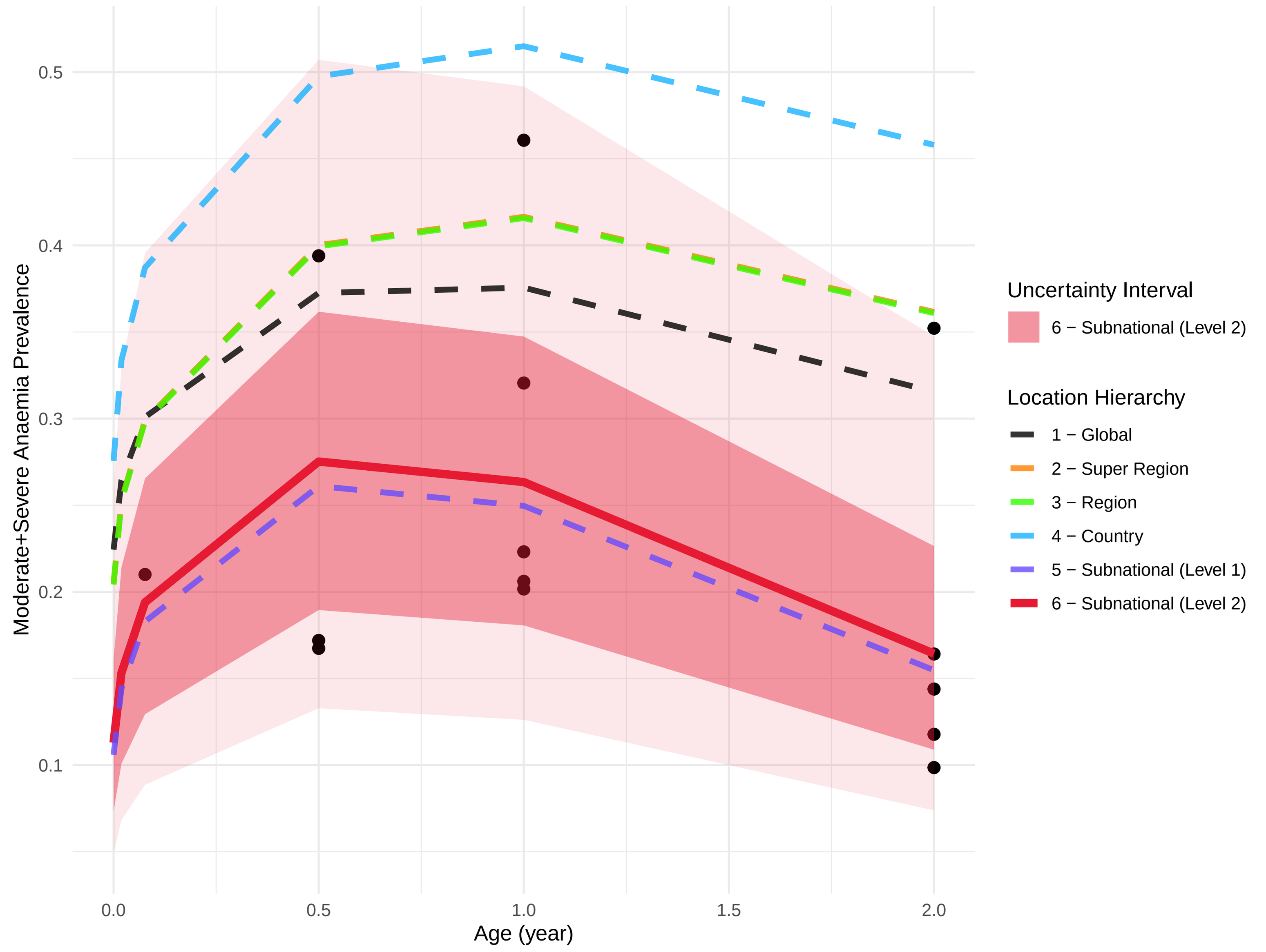

MR−BRT Age/Sex Fits − Males <2 Years Old − Moderate+Severe Anaemia Prevalence − Kerala, Urban
WHO−2024 Adjustment Method

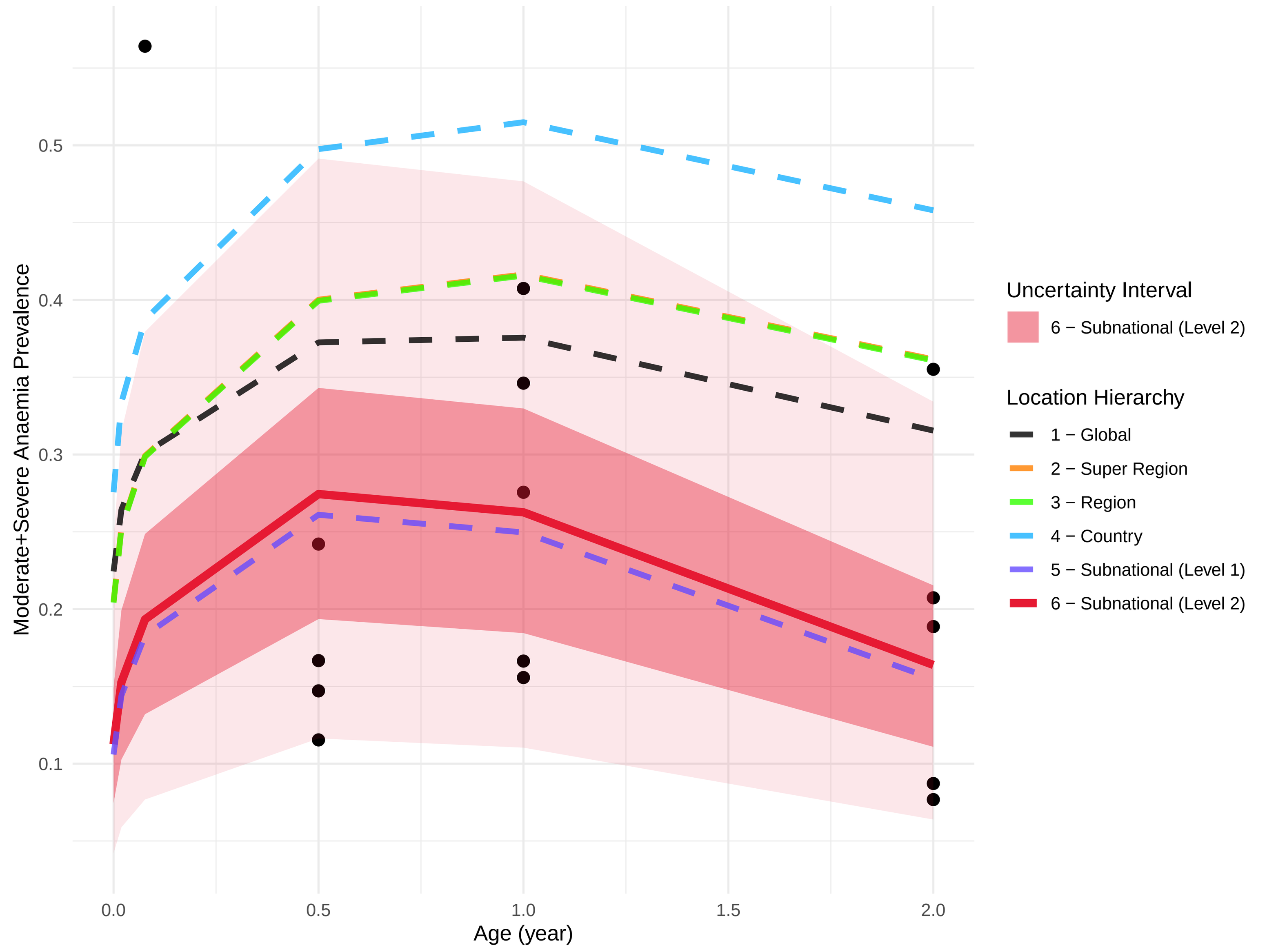

MR−BRT Age/Sex Fits − Males <2 Years Old − Moderate+Severe Anaemia Prevalence − Madhya Pradesh
WHO−2024 Adjustment Method

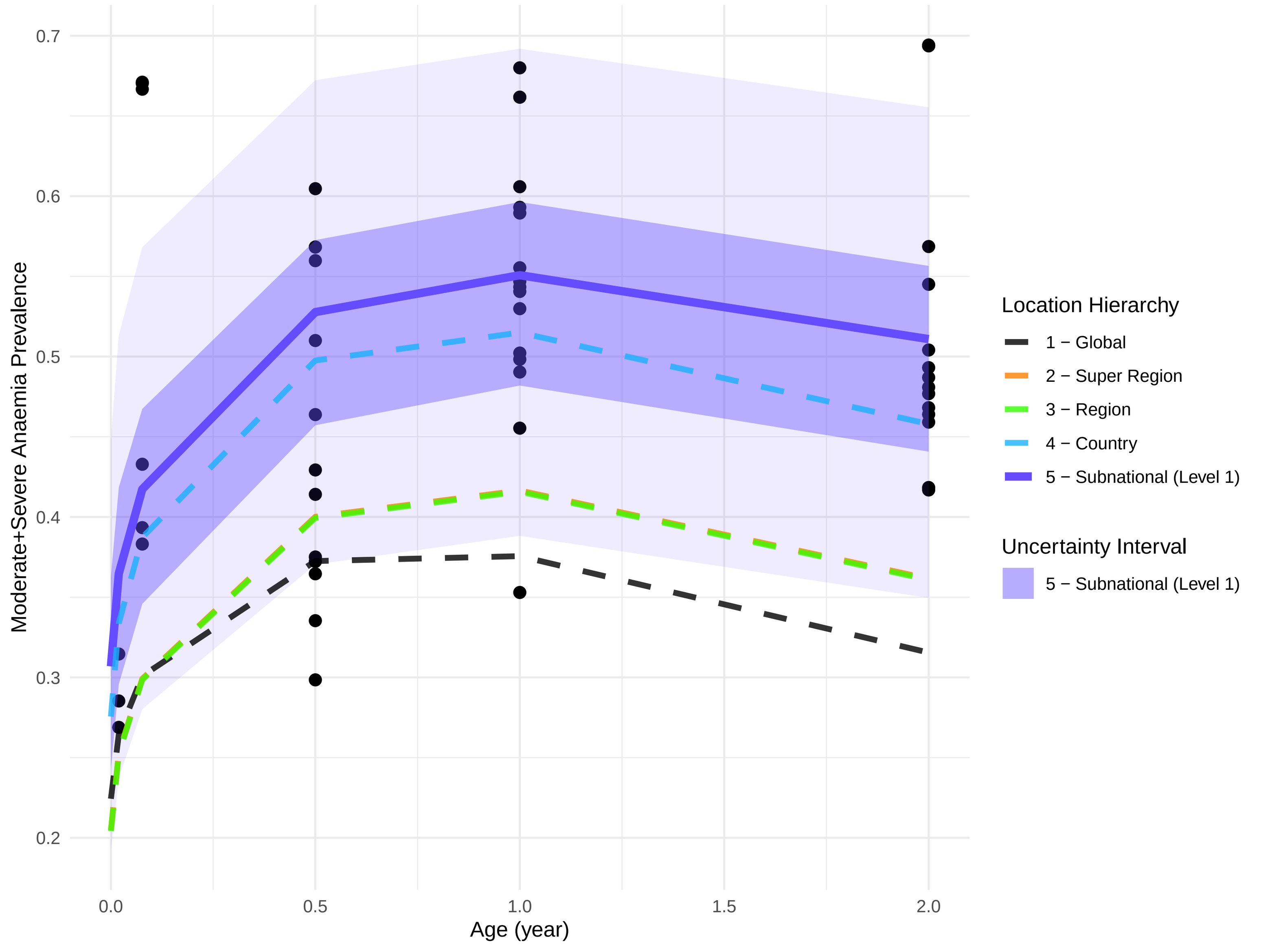

MR−BRT Age/Sex Fits − Males <2 Years Old − Moderate+Severe Anaemia Prevalence − Madhya Pradesh, Rural
WHO−2024 Adjustment Method

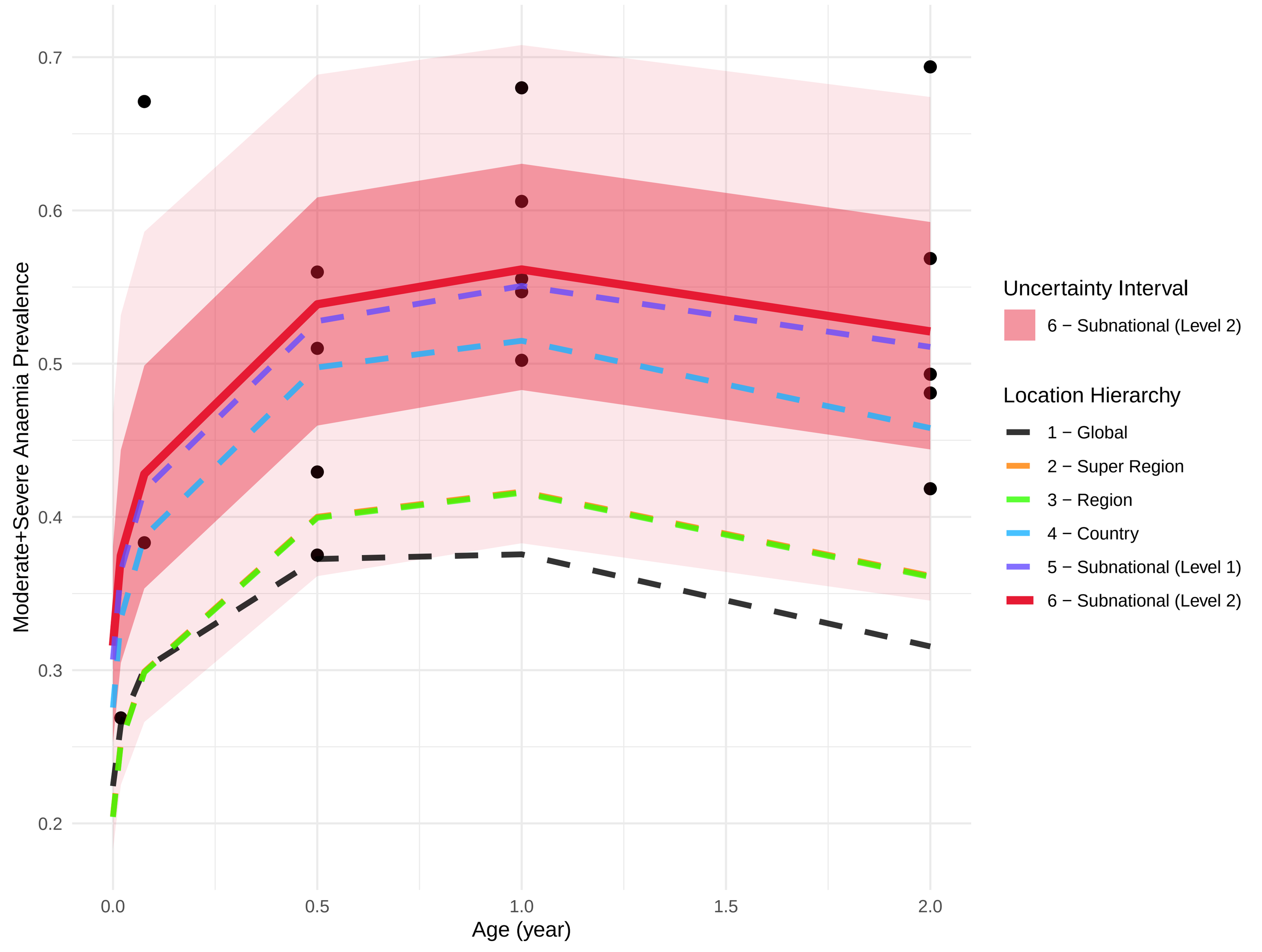

MR−BRT Age/Sex Fits − Males <2 Years Old − Moderate+Severe Anaemia Prevalence − Madhya Pradesh, Urban
WHO−2024 Adjustment Method

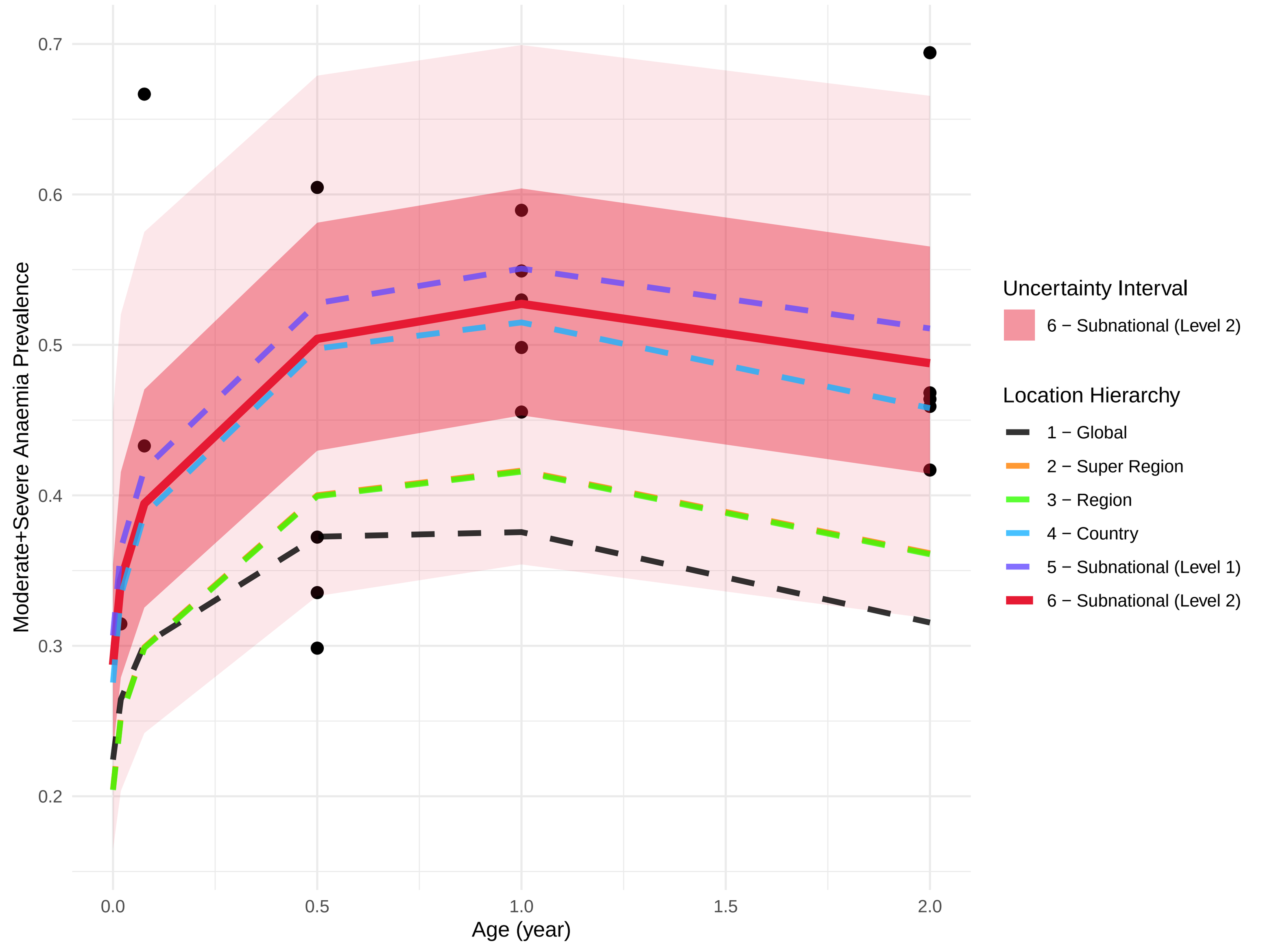

MR−BRT Age/Sex Fits − Males <2 Years Old − Moderate+Severe Anaemia Prevalence − Maharashtra
WHO−2024 Adjustment Method

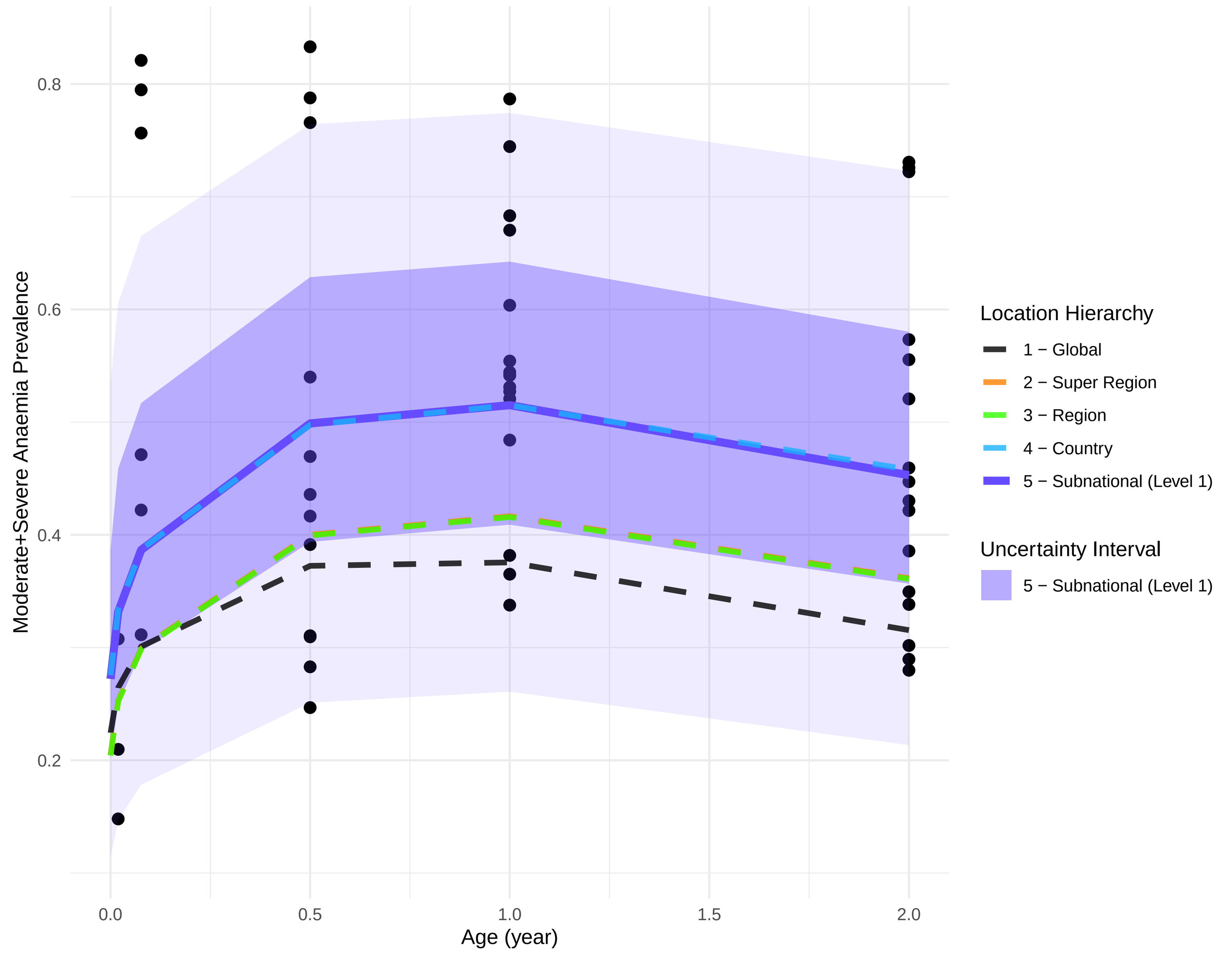

MR−BRT Age/Sex Fits − Males <2 Years Old − Moderate+Severe Anaemia Prevalence − Maharashtra, Rural
WHO−2024 Adjustment Method

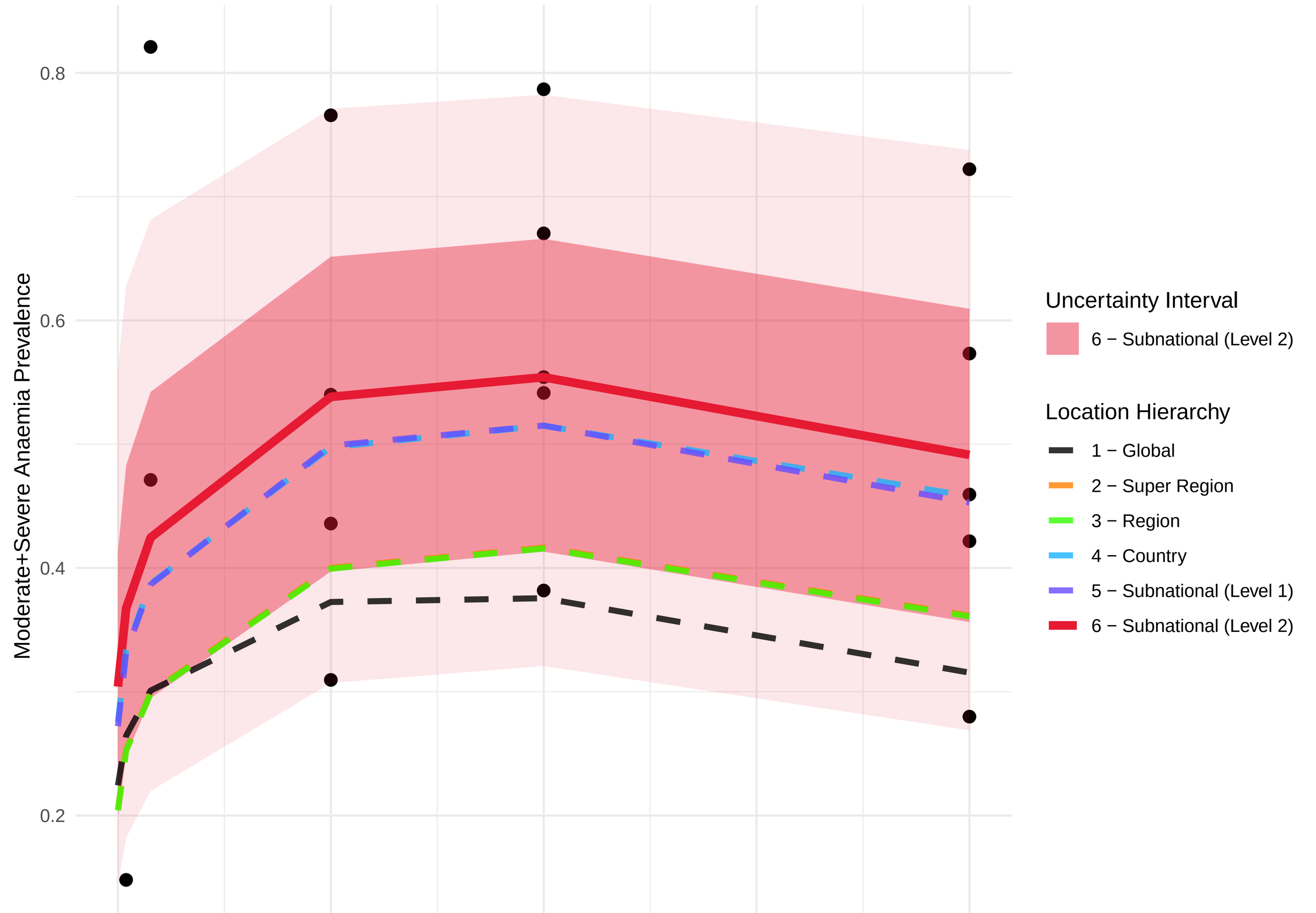

MR−BRT Age/Sex Fits − Males <2 Years Old − Moderate+Severe Anaemia Prevalence − Maharashtra, Urban
WHO−2024 Adjustment Method

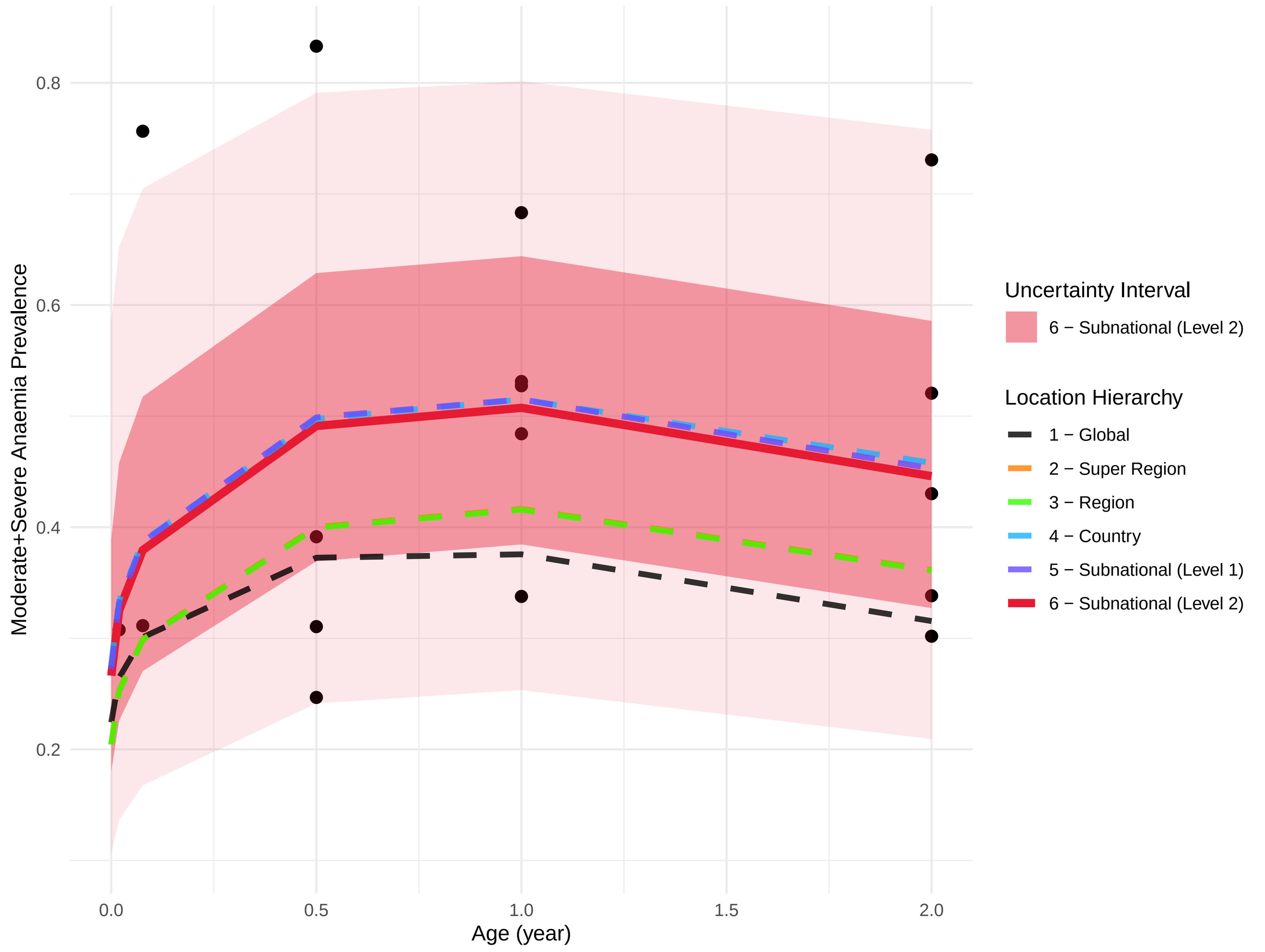

MR−BRT Age/Sex Fits − Males <2 Years Old − Moderate+Severe Anaemia Prevalence − Manipur
WHO−2024 Adjustment Method

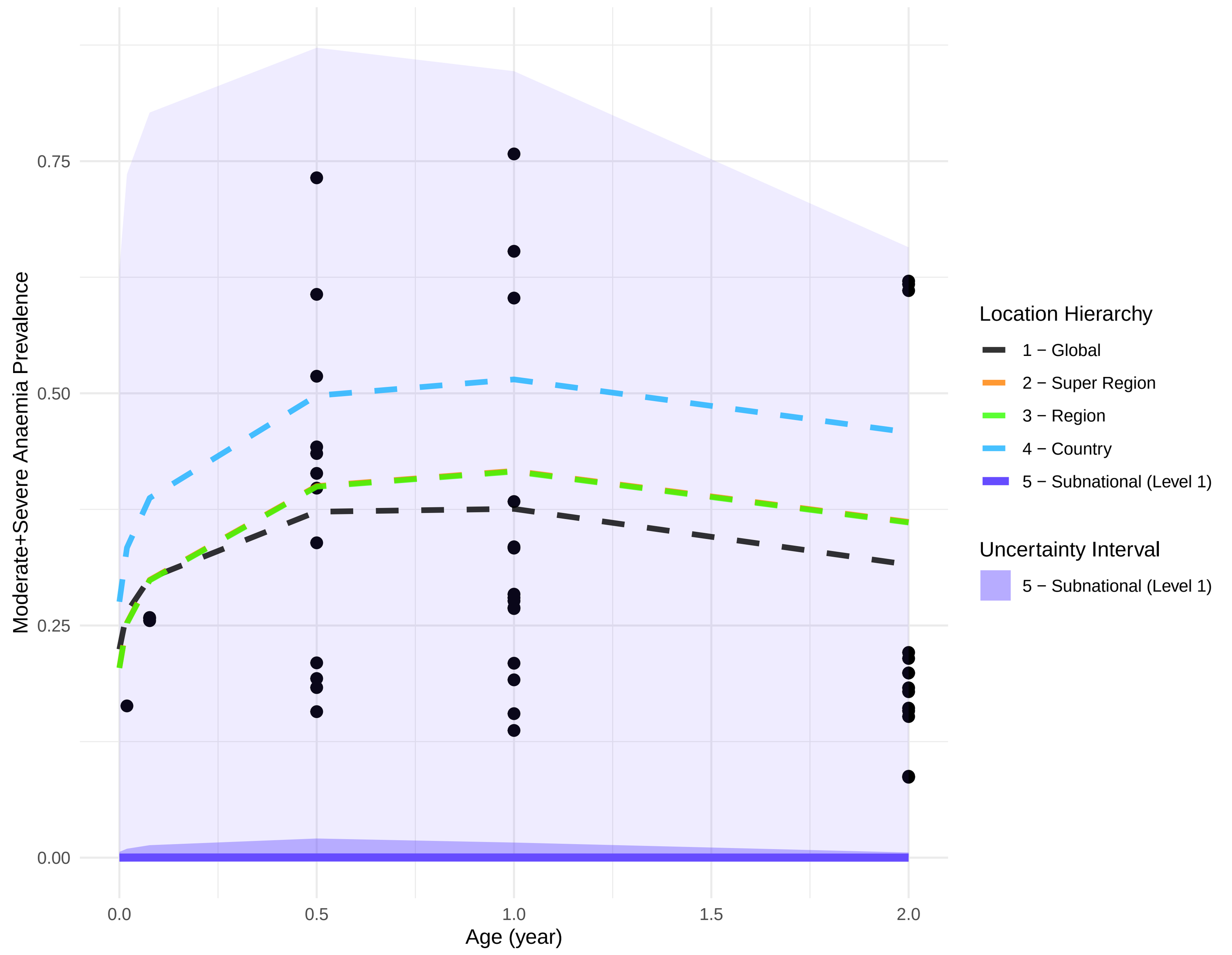

MR−BRT Age/Sex Fits − Males <2 Years Old − Moderate+Severe Anaemia Prevalence − Manipur, Rural
WHO−2024 Adjustment Method

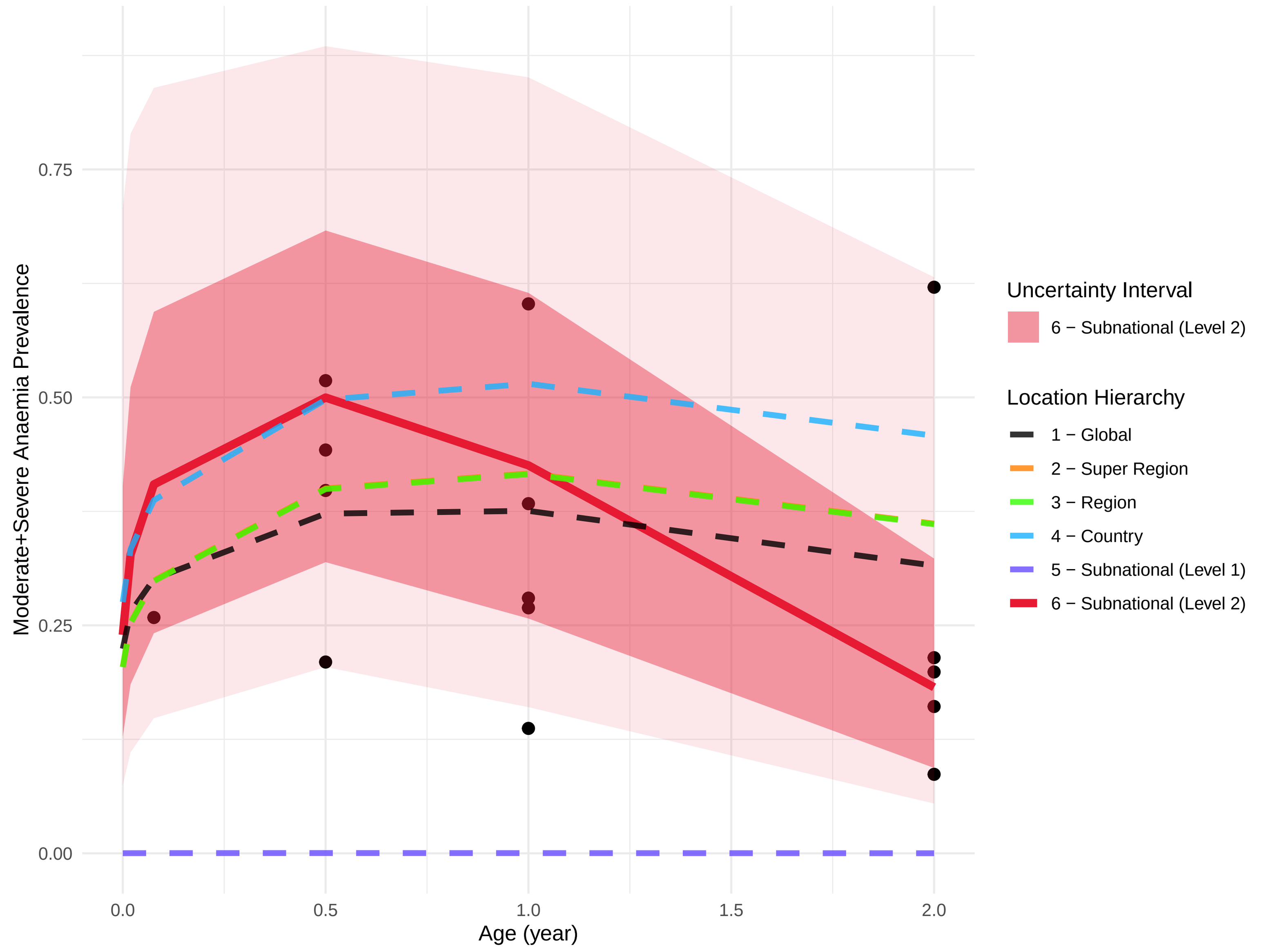

MR−BRT Age/Sex Fits − Males <2 Years Old − Moderate+Severe Anaemia Prevalence − Manipur, Urban
WHO−2024 Adjustment Method

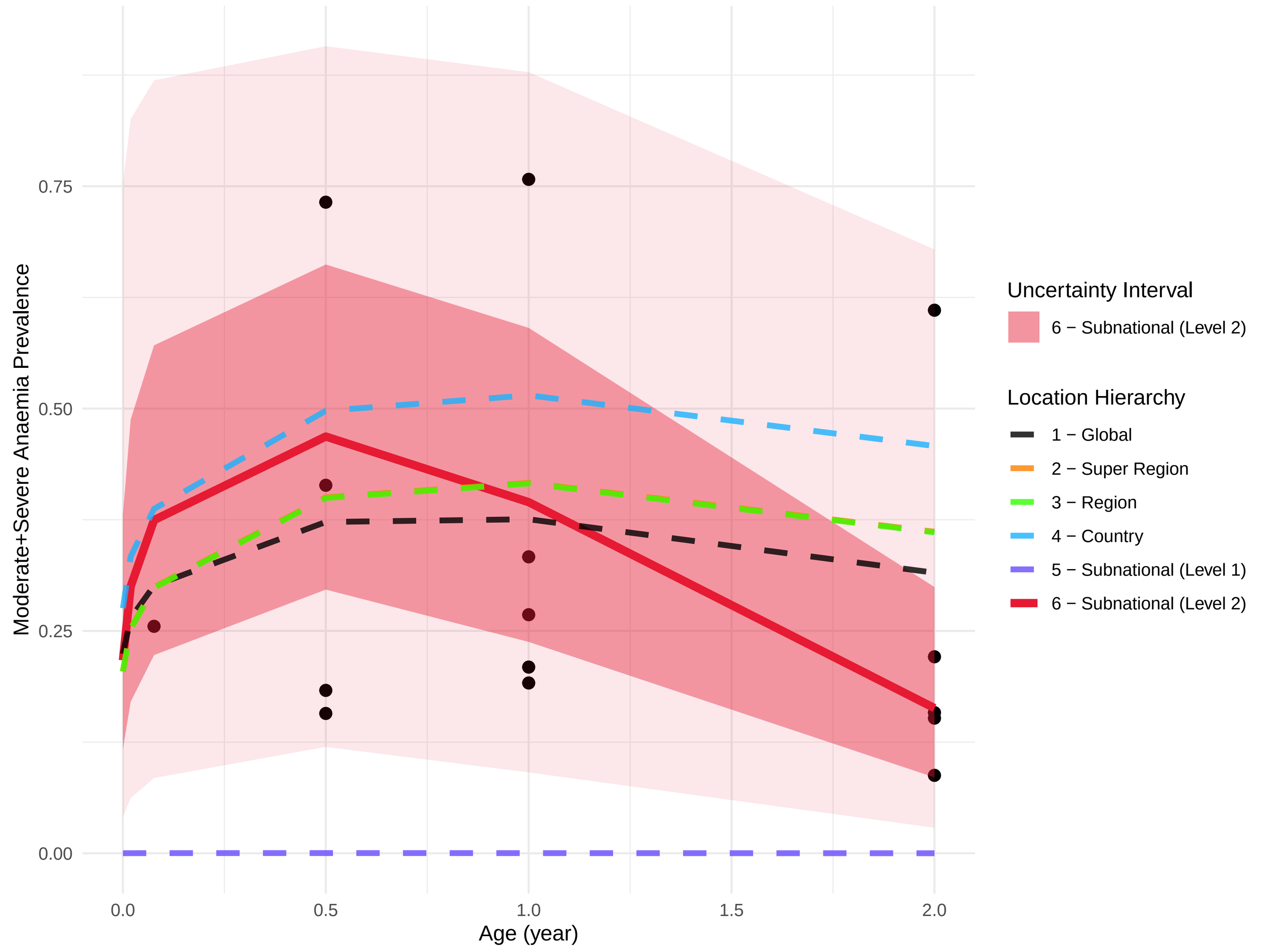

MR−BRT Age/Sex Fits − Males <2 Years Old − Moderate+Severe Anaemia Prevalence − Meghalaya
WHO−2024 Adjustment Method

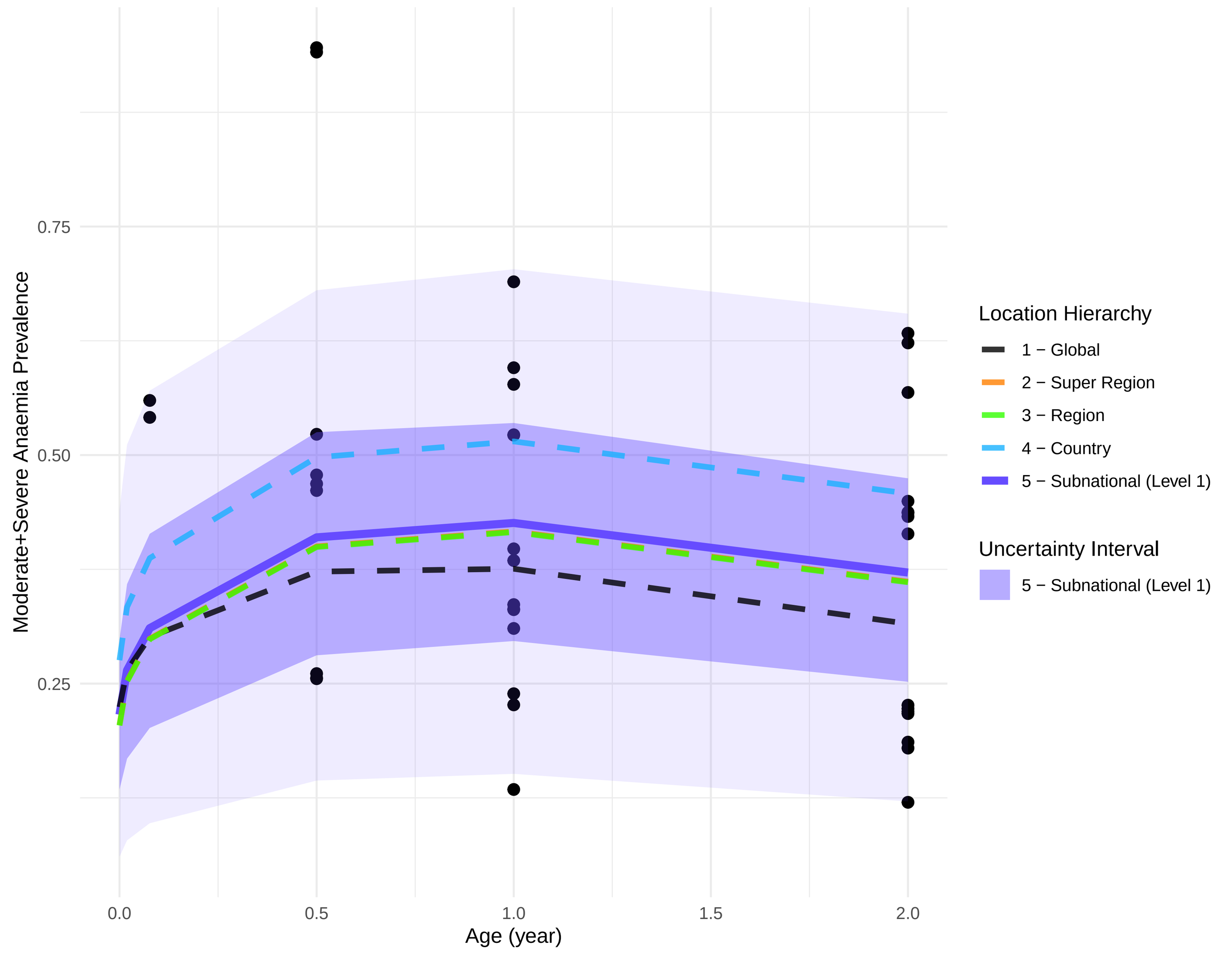

MR−BRT Age/Sex Fits − Males <2 Years Old − Moderate+Severe Anaemia Prevalence − Meghalaya, Rural
WHO−2024 Adjustment Method

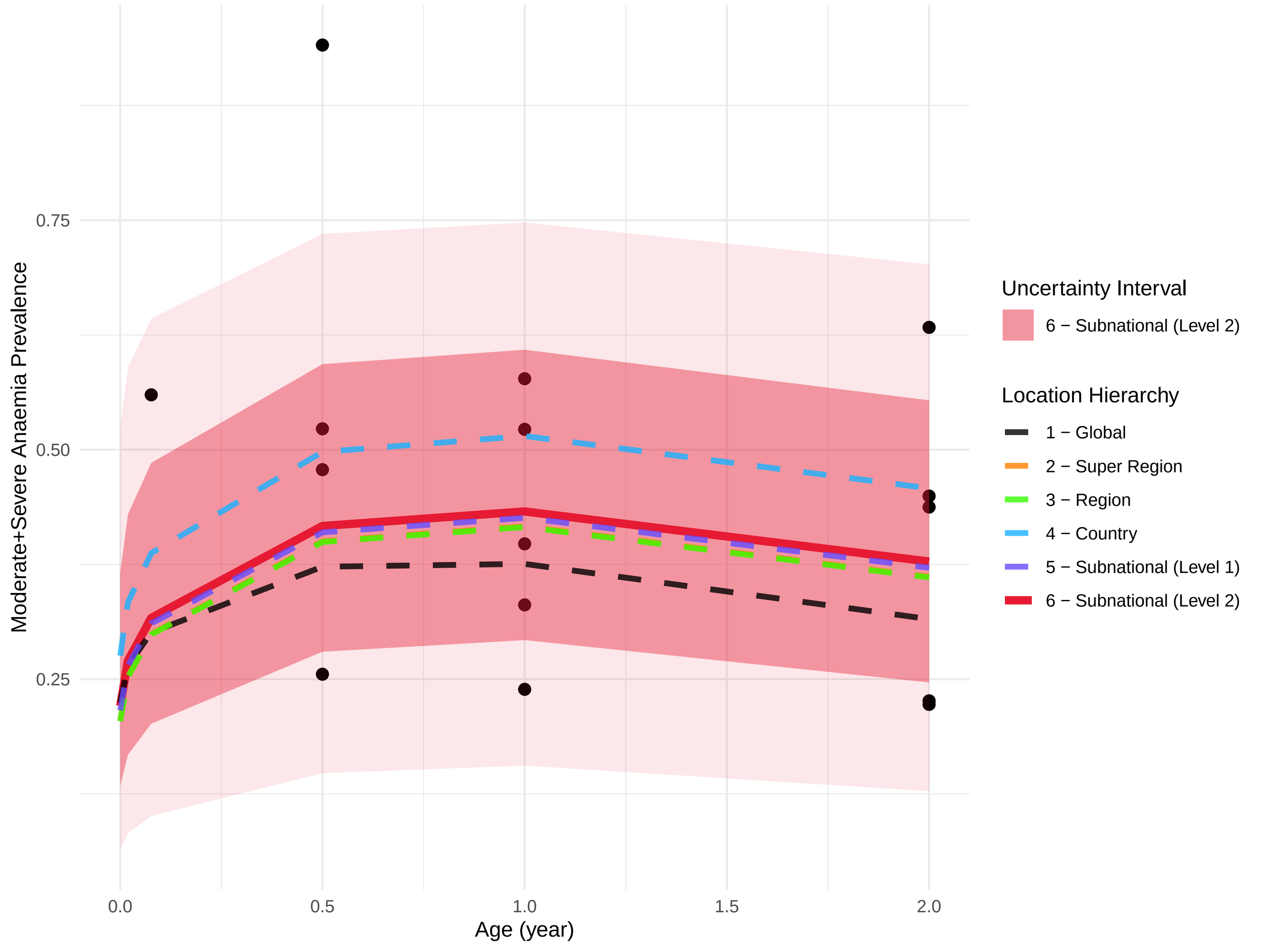

MR−BRT Age/Sex Fits − Males <2 Years Old − Moderate+Severe Anaemia Prevalence − Meghalaya, Urban
WHO−2024 Adjustment Method

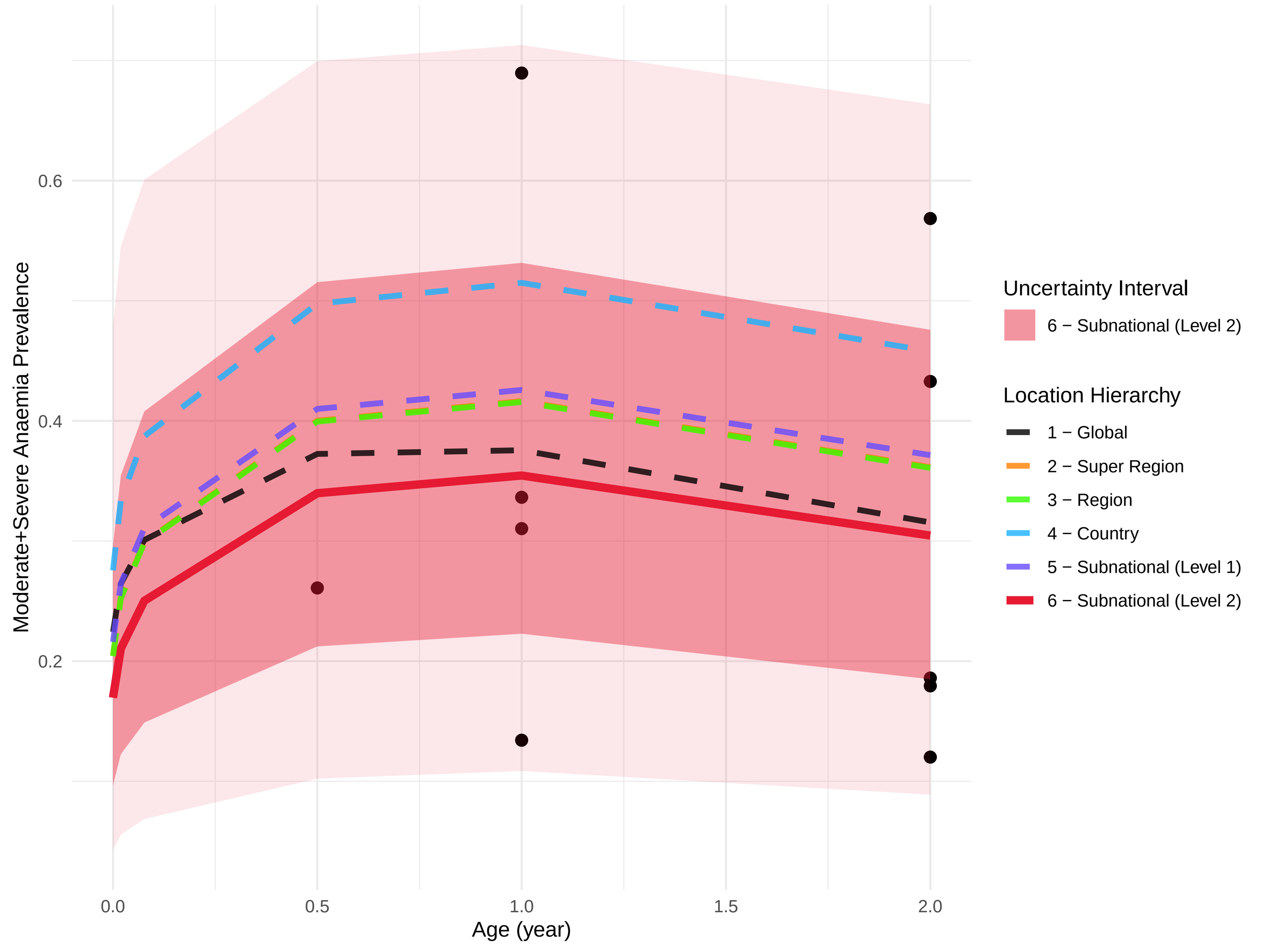

MR−BRT Age/Sex Fits − Males <2 Years Old − Moderate+Severe Anaemia Prevalence − Mizoram
WHO−2024 Adjustment Method

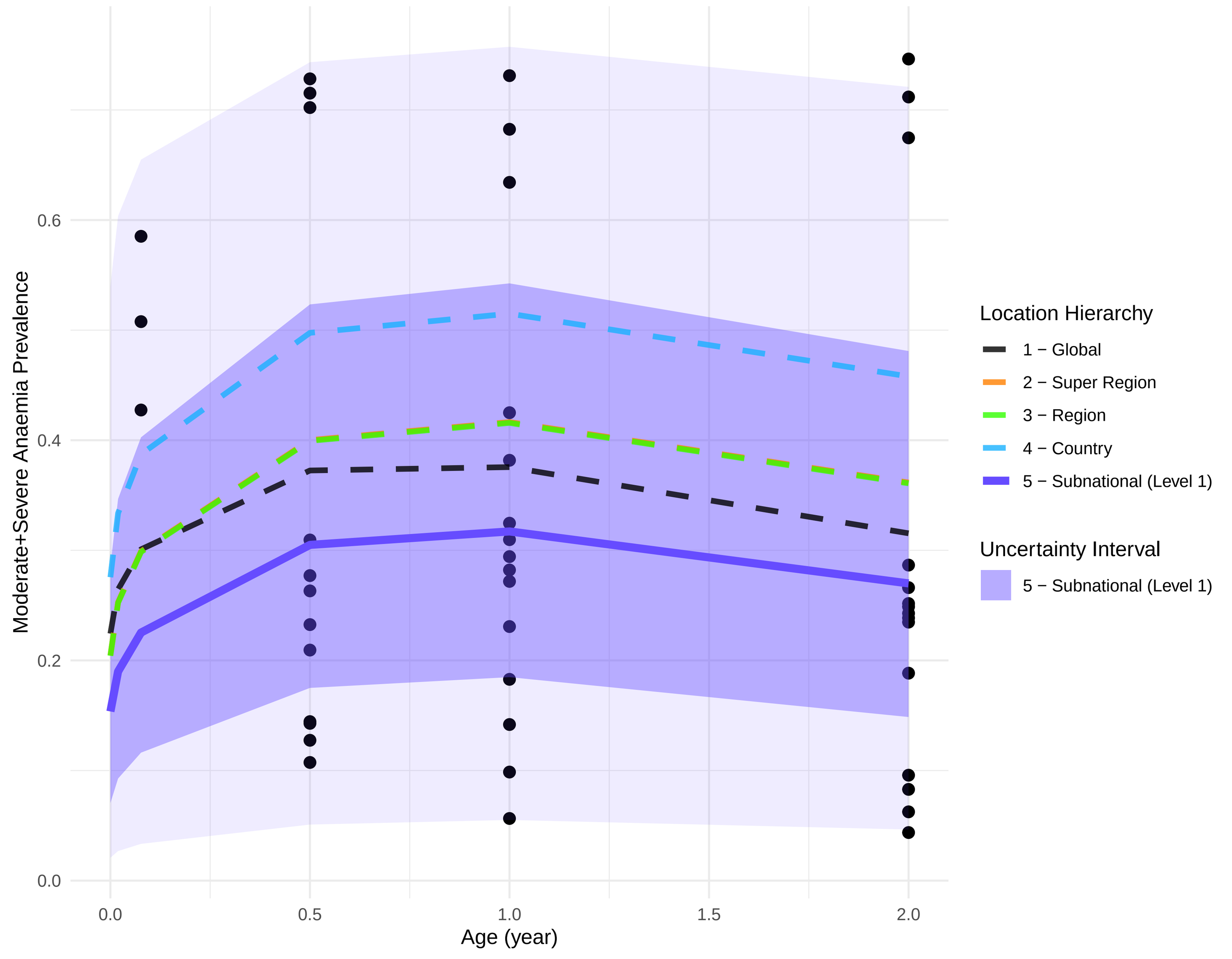

MR−BRT Age/Sex Fits − Males <2 Years Old − Moderate+Severe Anaemia Prevalence − Mizoram, Rural
WHO−2024 Adjustment Method

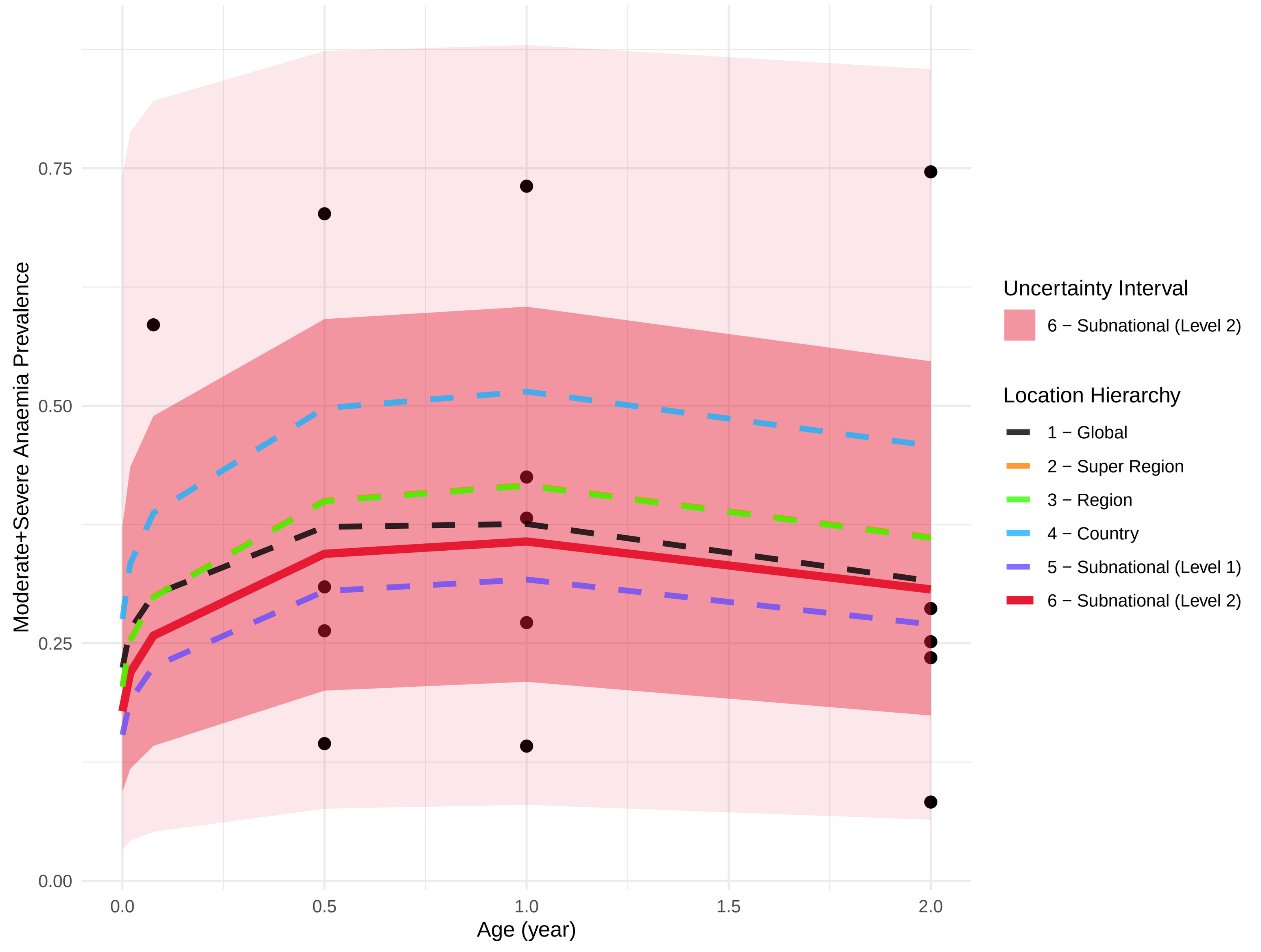

MR−BRT Age/Sex Fits − Males <2 Years Old − Moderate+Severe Anaemia Prevalence − Mizoram, Urban
WHO−2024 Adjustment Method

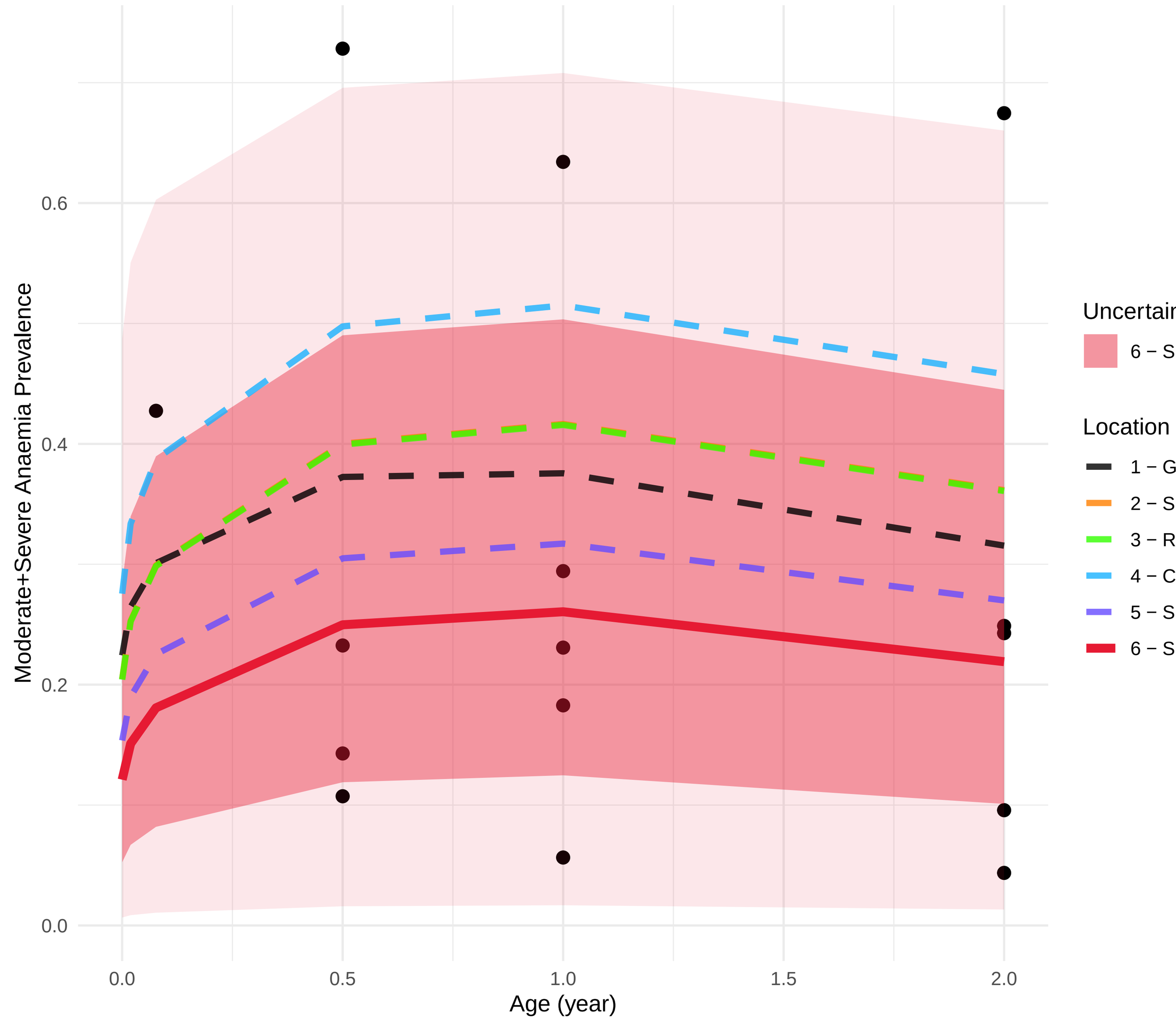

MR−BRT Age/Sex Fits − Males <2 Years Old − Moderate+Severe Anaemia Prevalence − Nagaland
WHO−2024 Adjustment Method

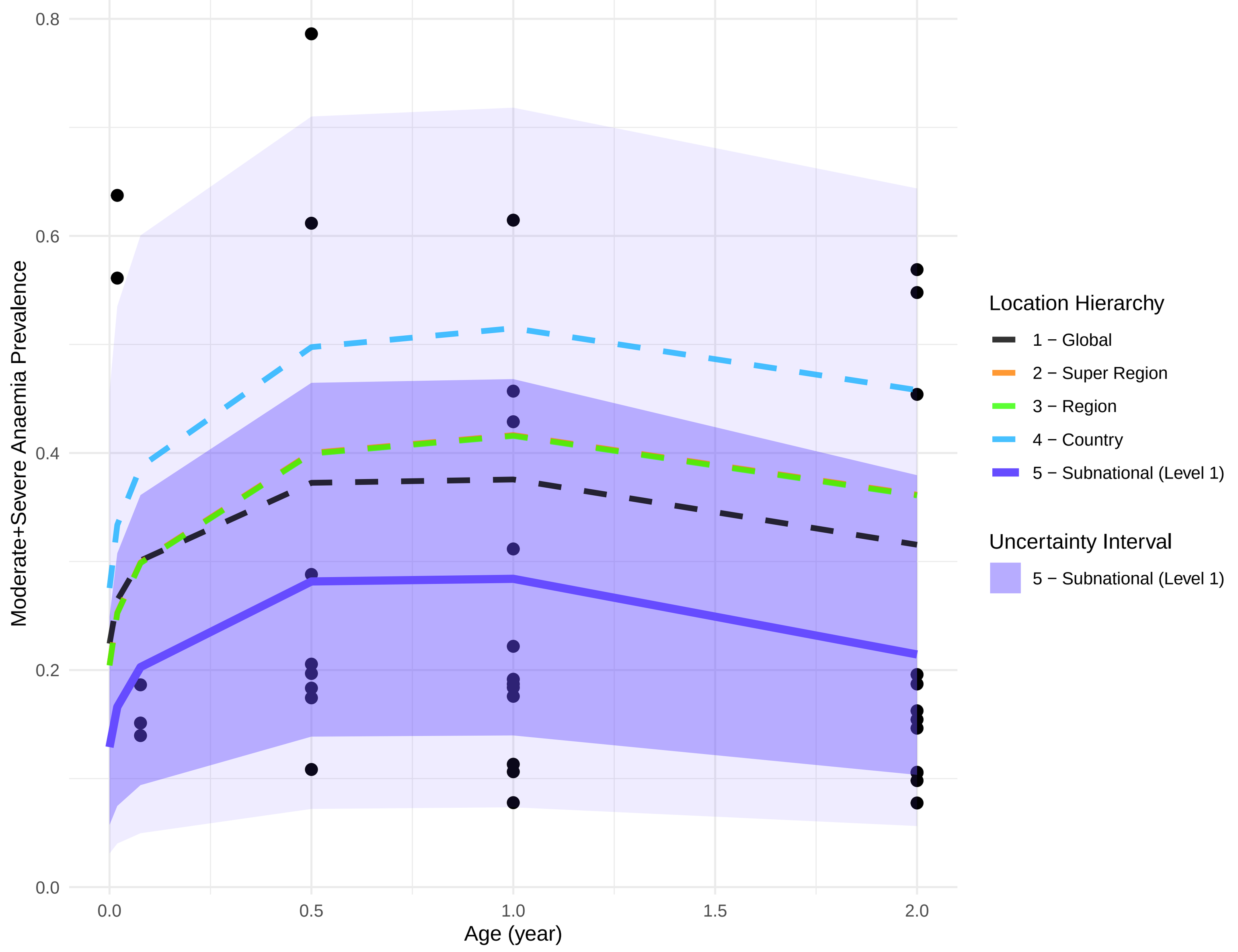

MR−BRT Age/Sex Fits − Males <2 Years Old − Moderate+Severe Anaemia Prevalence − Nagaland, Rural
WHO−2024 Adjustment Method

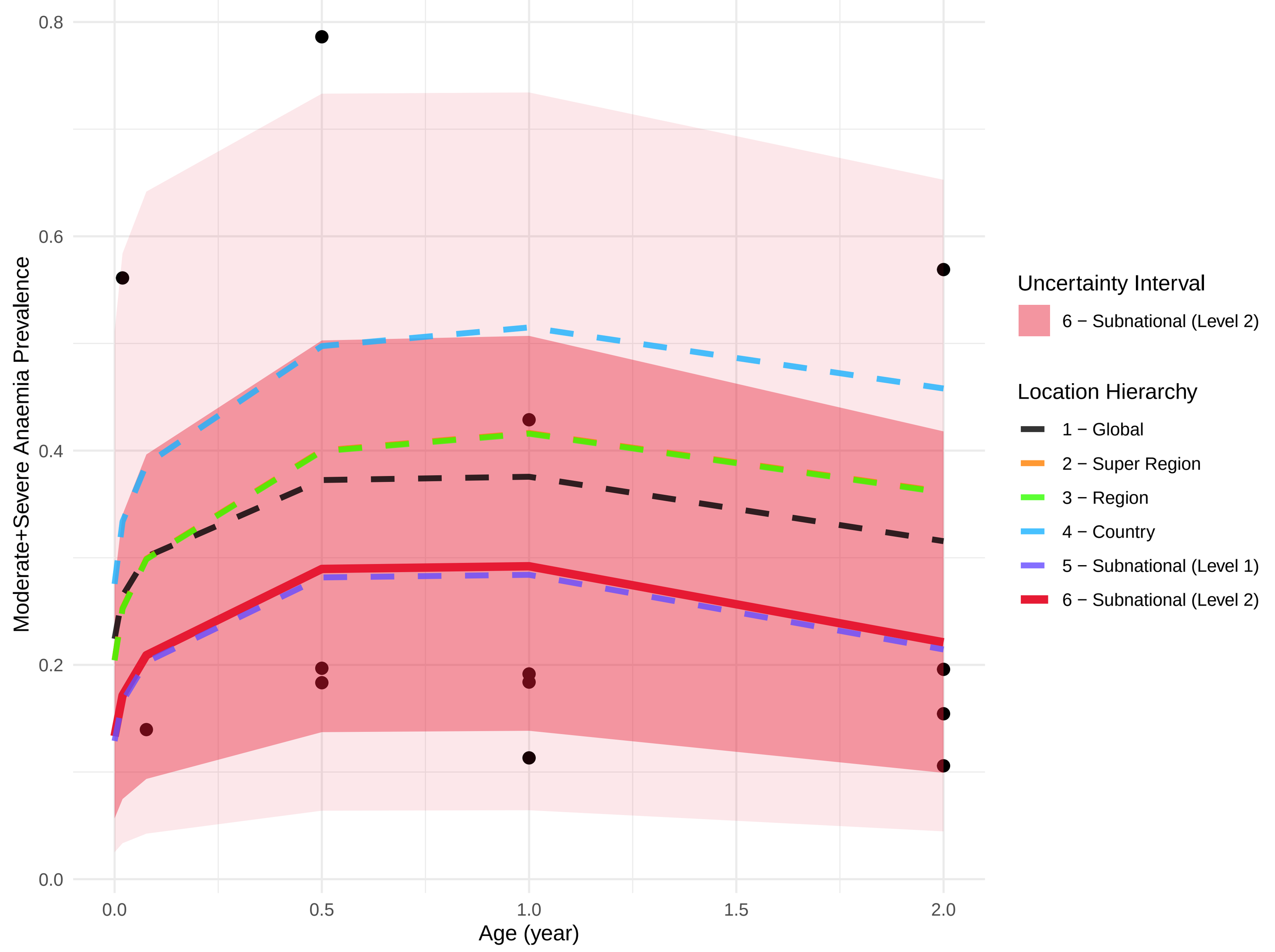

MR−BRT Age/Sex Fits − Males <2 Years Old − Moderate+Severe Anaemia Prevalence − Nagaland, Urban
WHO−2024 Adjustment Method

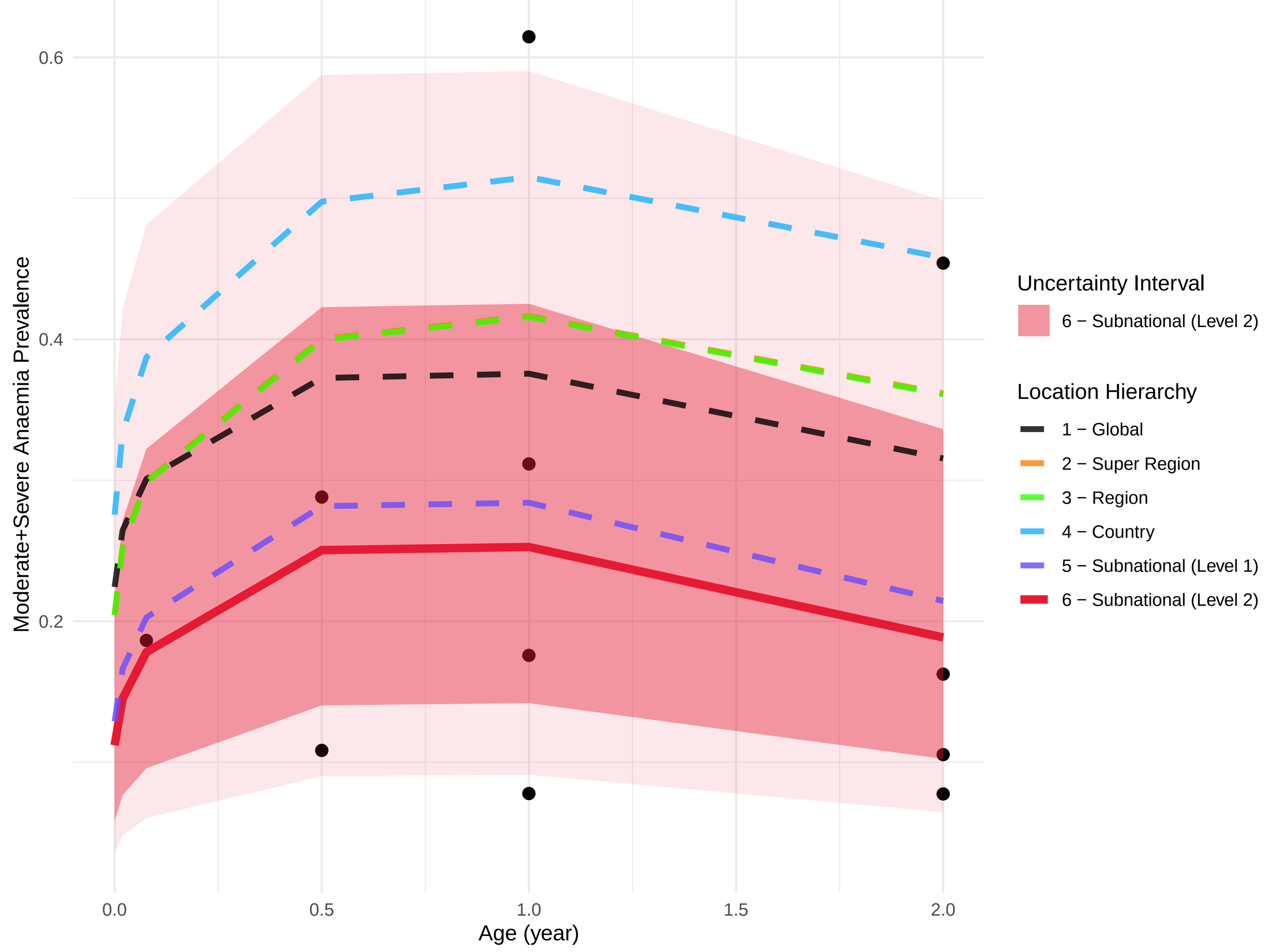

MR−BRT Age/Sex Fits − Males <2 Years Old − Moderate+Severe Anaemia Prevalence − Odisha
WHO−2024 Adjustment Method

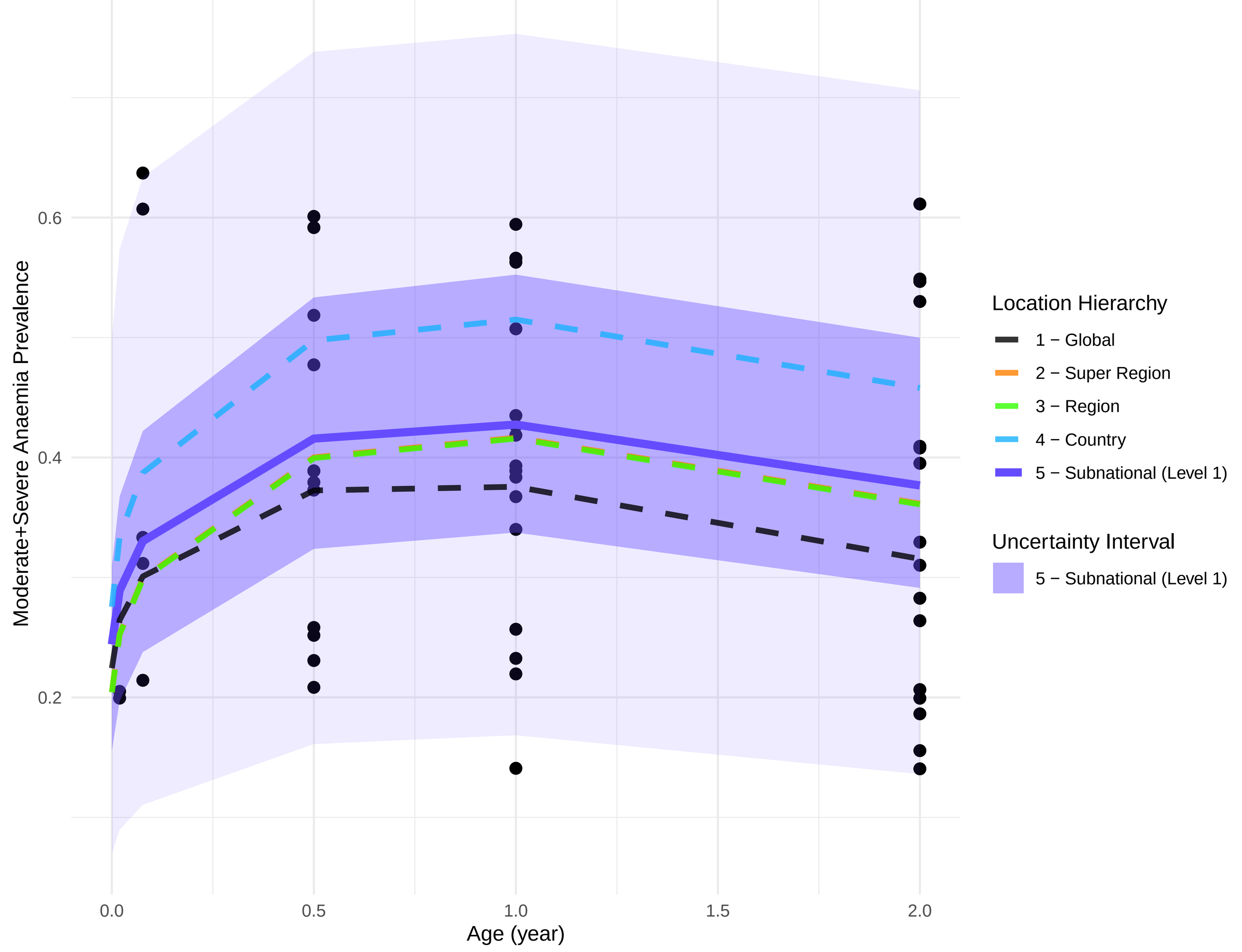

MR−BRT Age/Sex Fits − Males <2 Years Old − Moderate+Severe Anaemia Prevalence − Odisha, Rural
WHO−2024 Adjustment Method

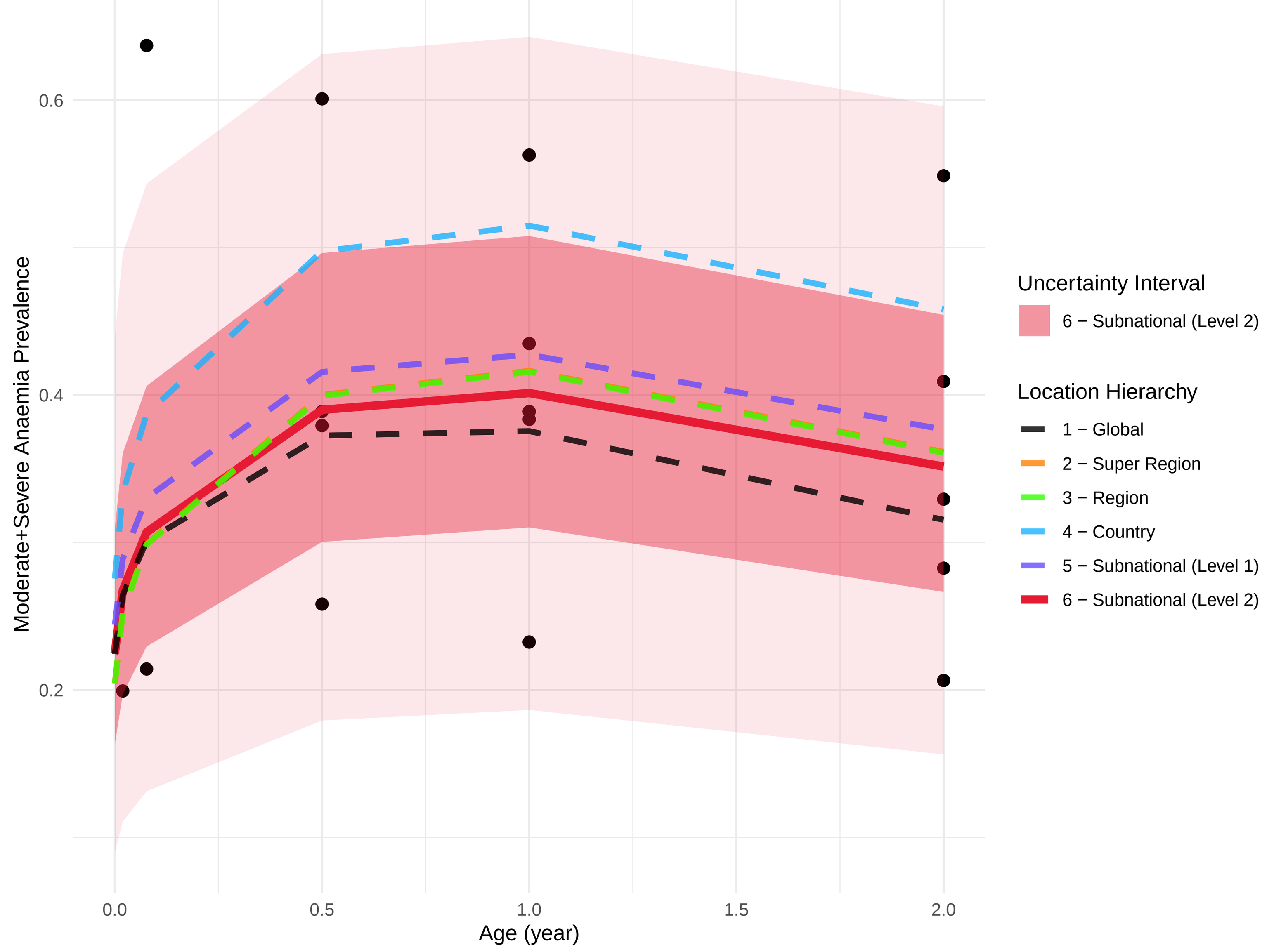

MR−BRT Age/Sex Fits − Males <2 Years Old − Moderate+Severe Anaemia Prevalence − Odisha, Urban
WHO−2024 Adjustment Method

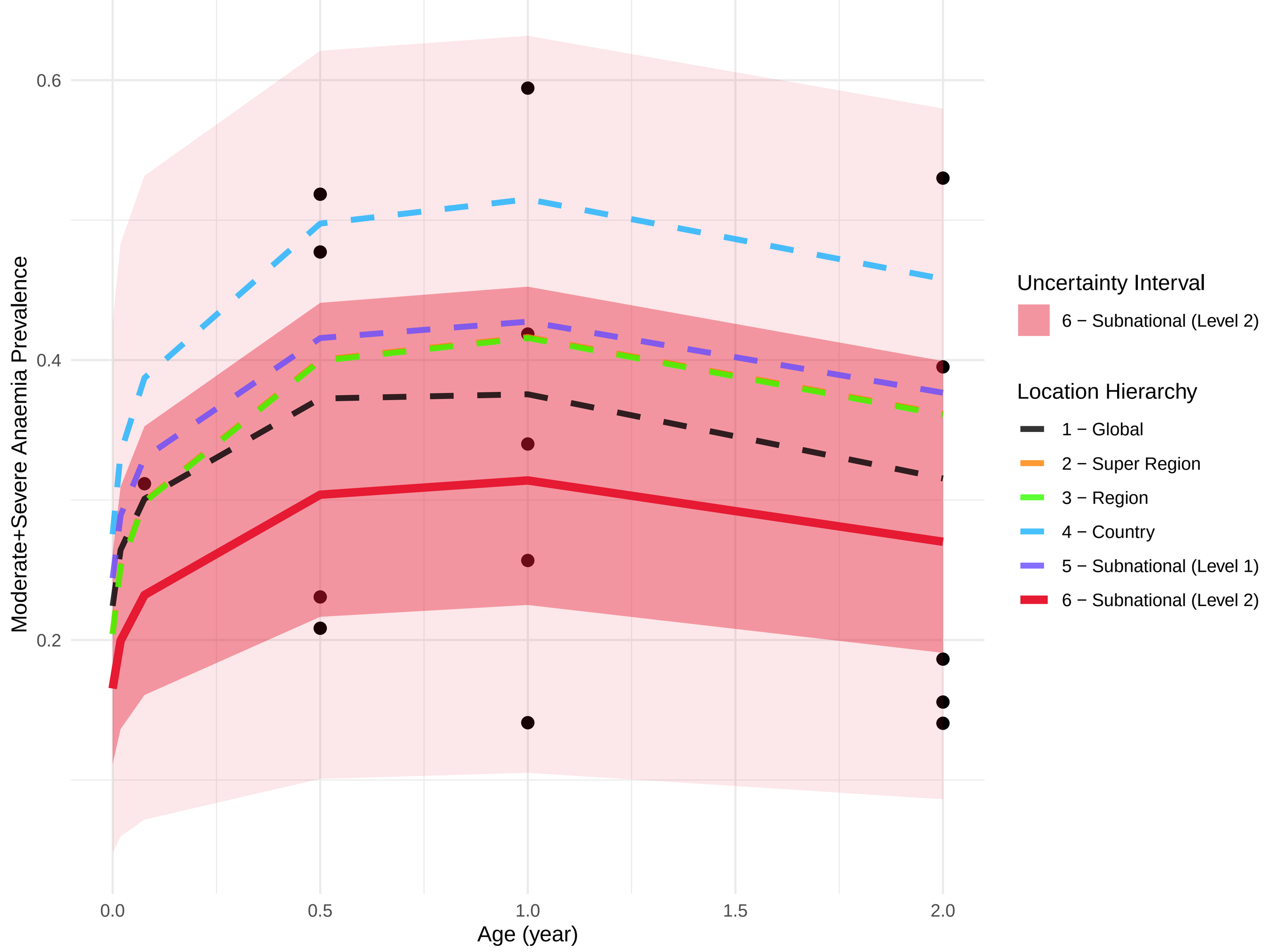

MR−BRT Age/Sex Fits − Males <2 Years Old − Moderate+Severe Anaemia Prevalence − Other Union Territories
WHO−2024 Adjustment Method

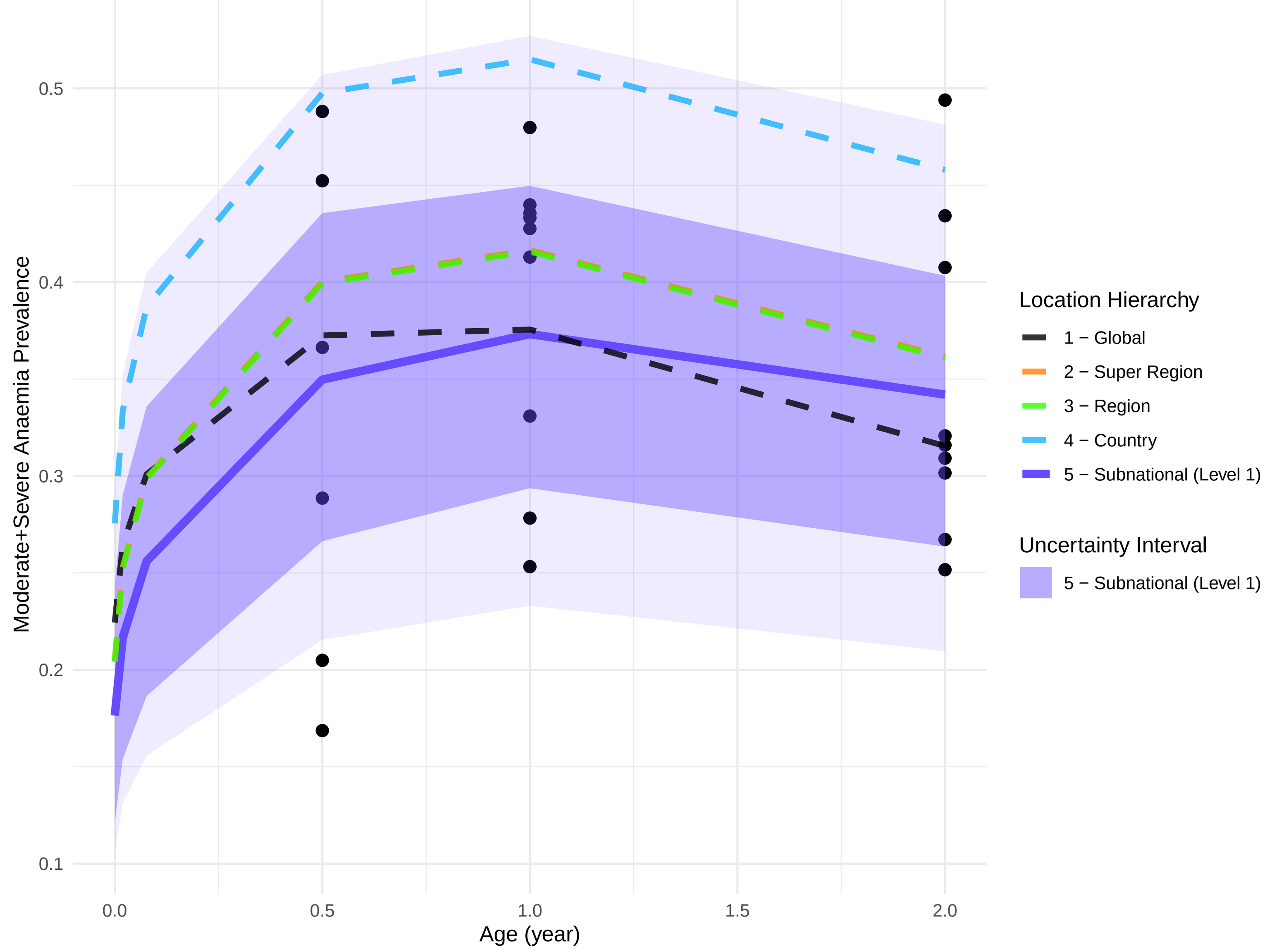

MR−BRT Age/Sex Fits − Males <2 Years Old − Moderate+Severe Anaemia Prevalence − Other Union Territories, Rural
WHO−2024 Adjustment Method

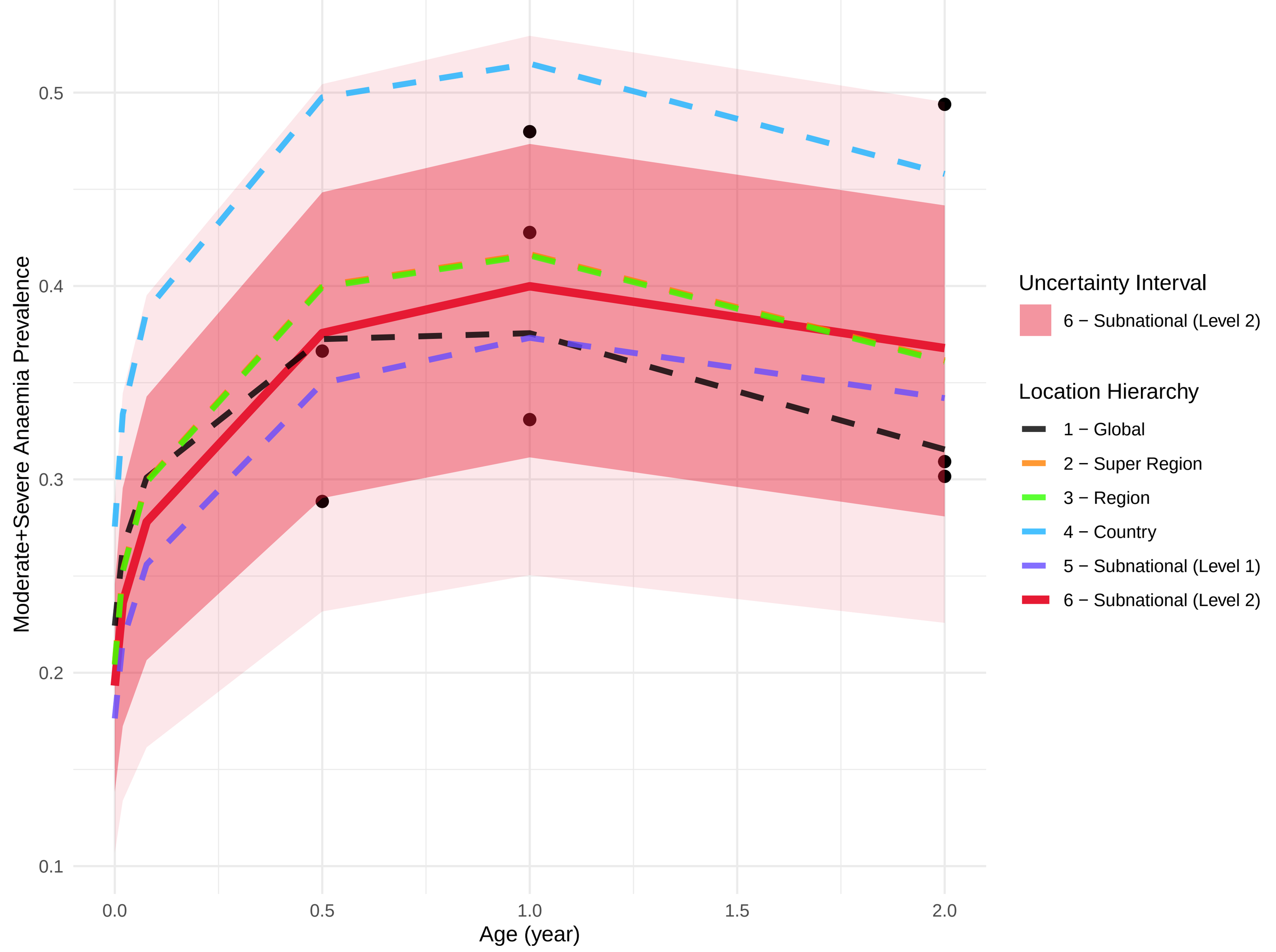

MR−BRT Age/Sex Fits − Males <2 Years Old − Moderate+Severe Anaemia Prevalence − Other Union Territories, Urban
WHO−2024 Adjustment Method

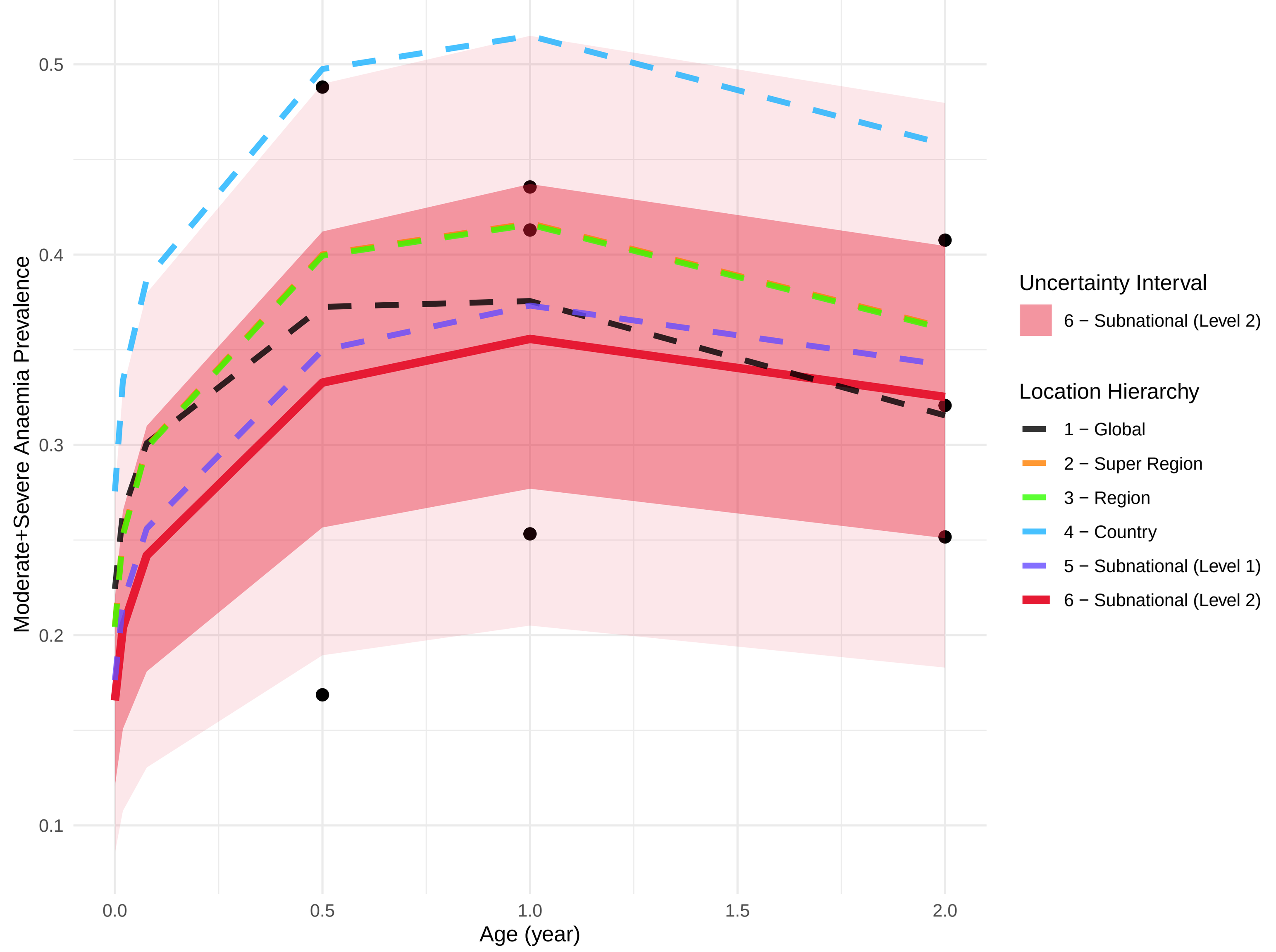

MR−BRT Age/Sex Fits − Males <2 Years Old − Moderate+Severe Anaemia Prevalence − Punjab
WHO−2024 Adjustment Method

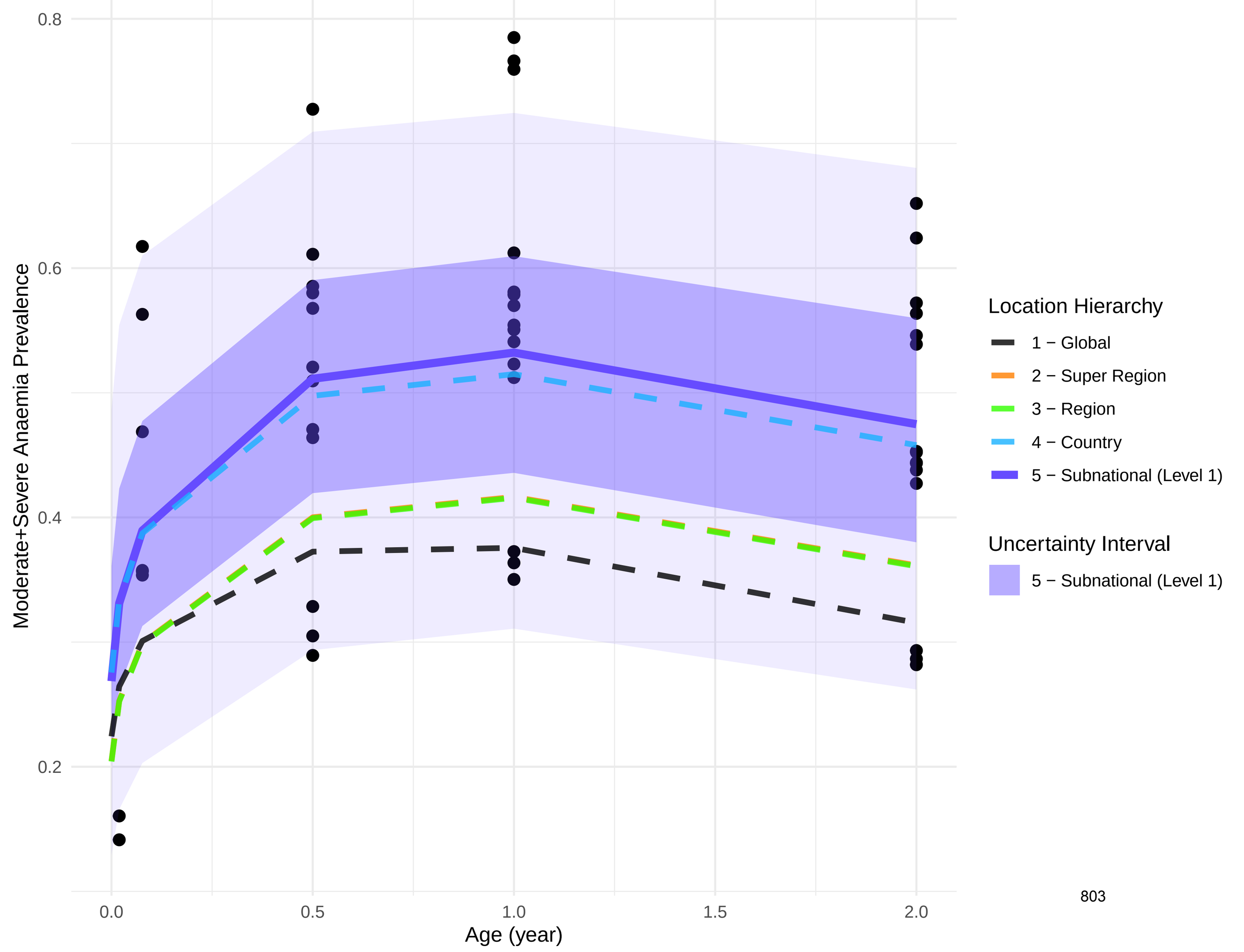

MR−BRT Age/Sex Fits − Males <2 Years Old − Moderate+Severe Anaemia Prevalence − Punjab, Rural
WHO−2024 Adjustment Method

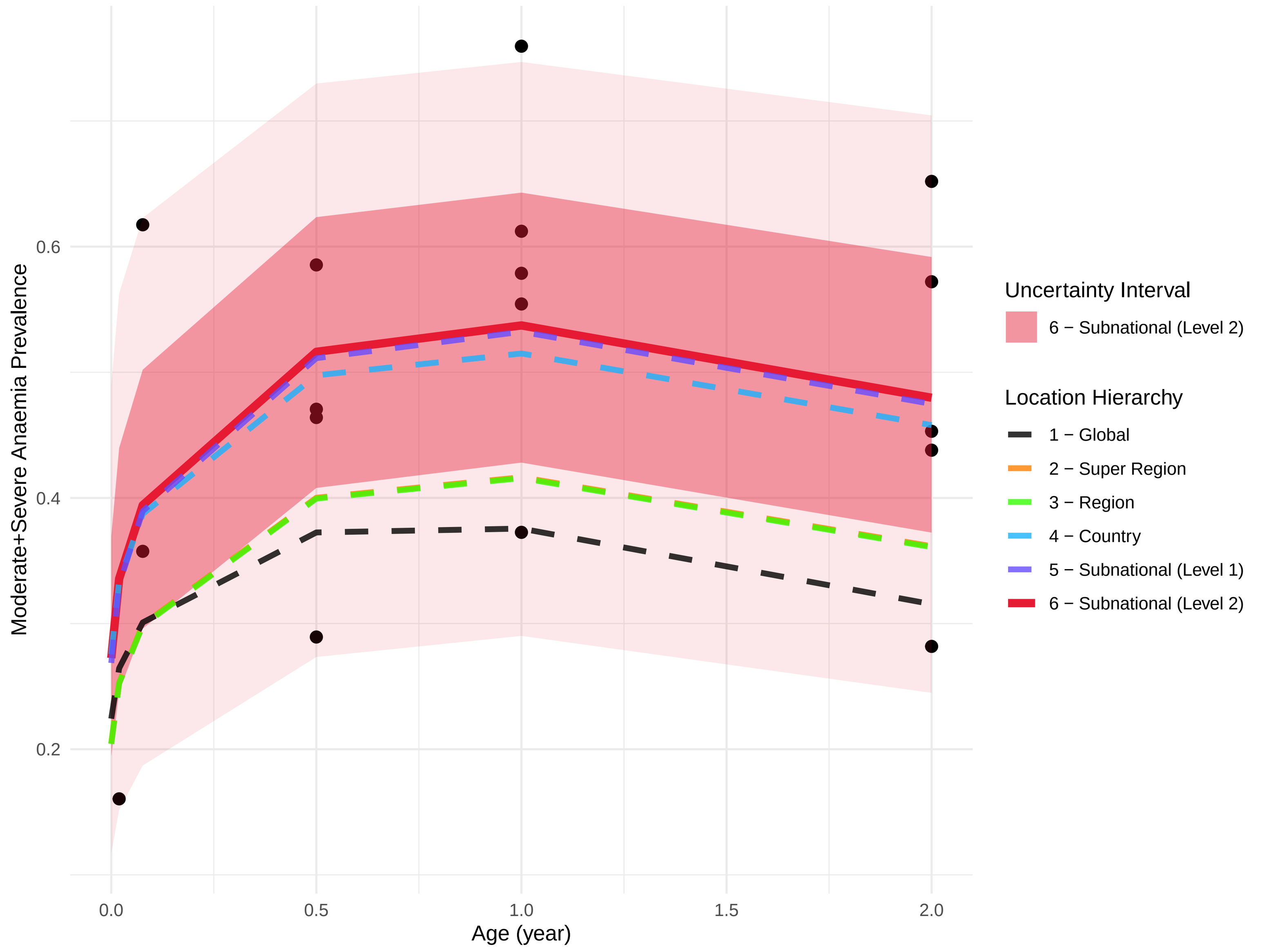

MR−BRT Age/Sex Fits − Males <2 Years Old − Moderate+Severe Anaemia Prevalence − Punjab, Urban
WHO−2024 Adjustment Method

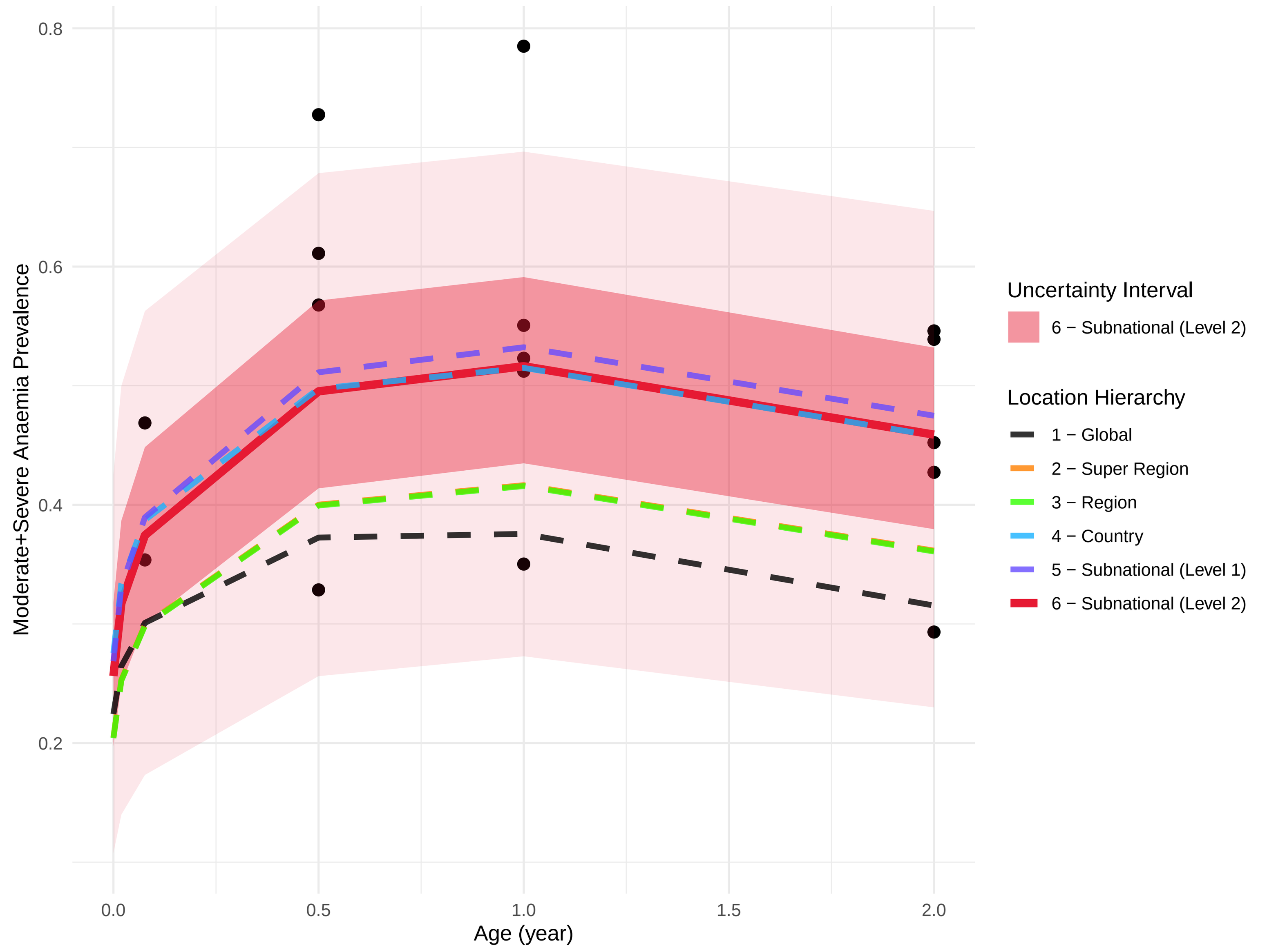

MR−BRT Age/Sex Fits − Males <2 Years Old − Moderate+Severe Anaemia Prevalence − Rajasthan
WHO−2024 Adjustment Method

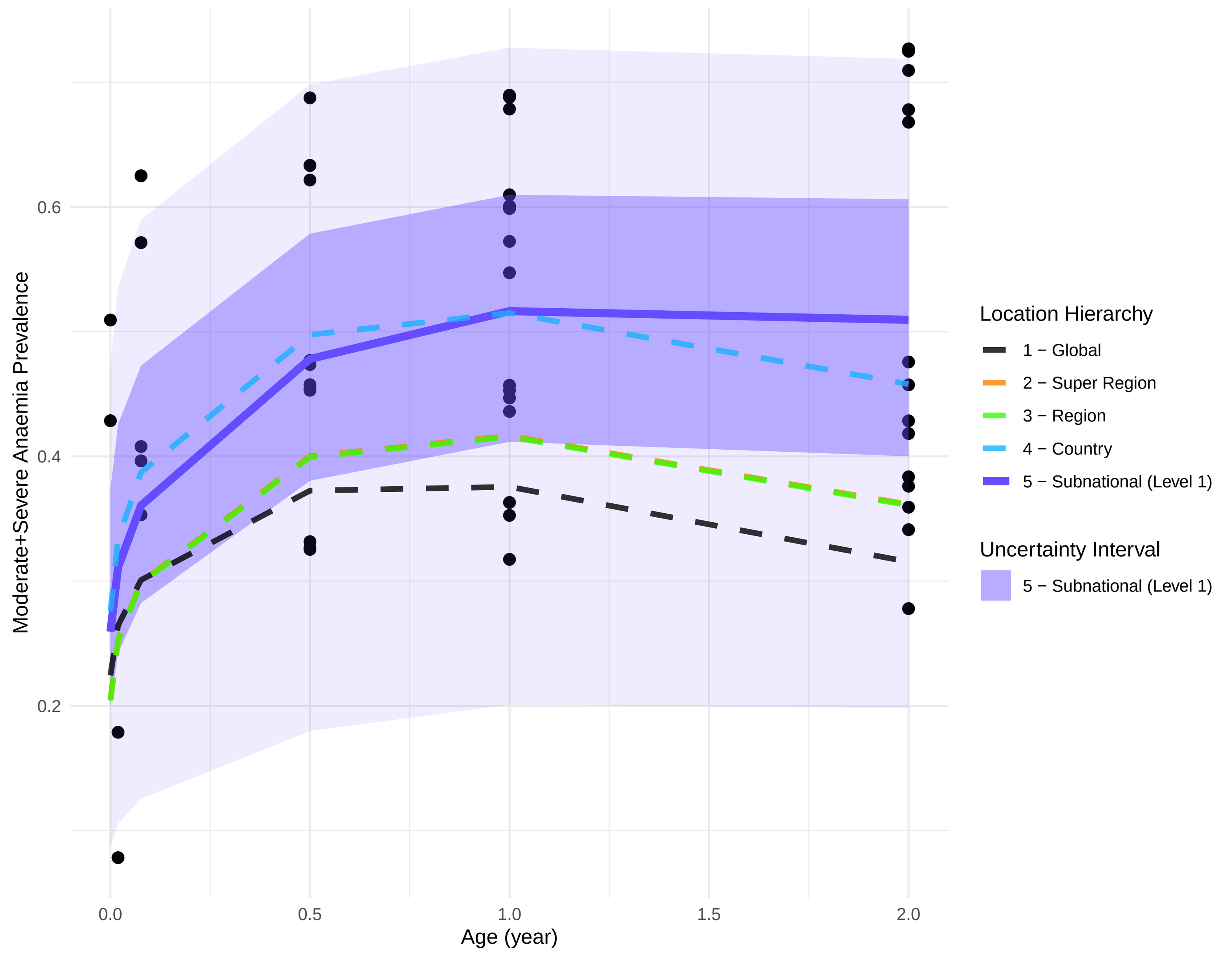

MR−BRT Age/Sex Fits − Males <2 Years Old − Moderate+Severe Anaemia Prevalence − Rajasthan, Rural
WHO−2024 Adjustment Method

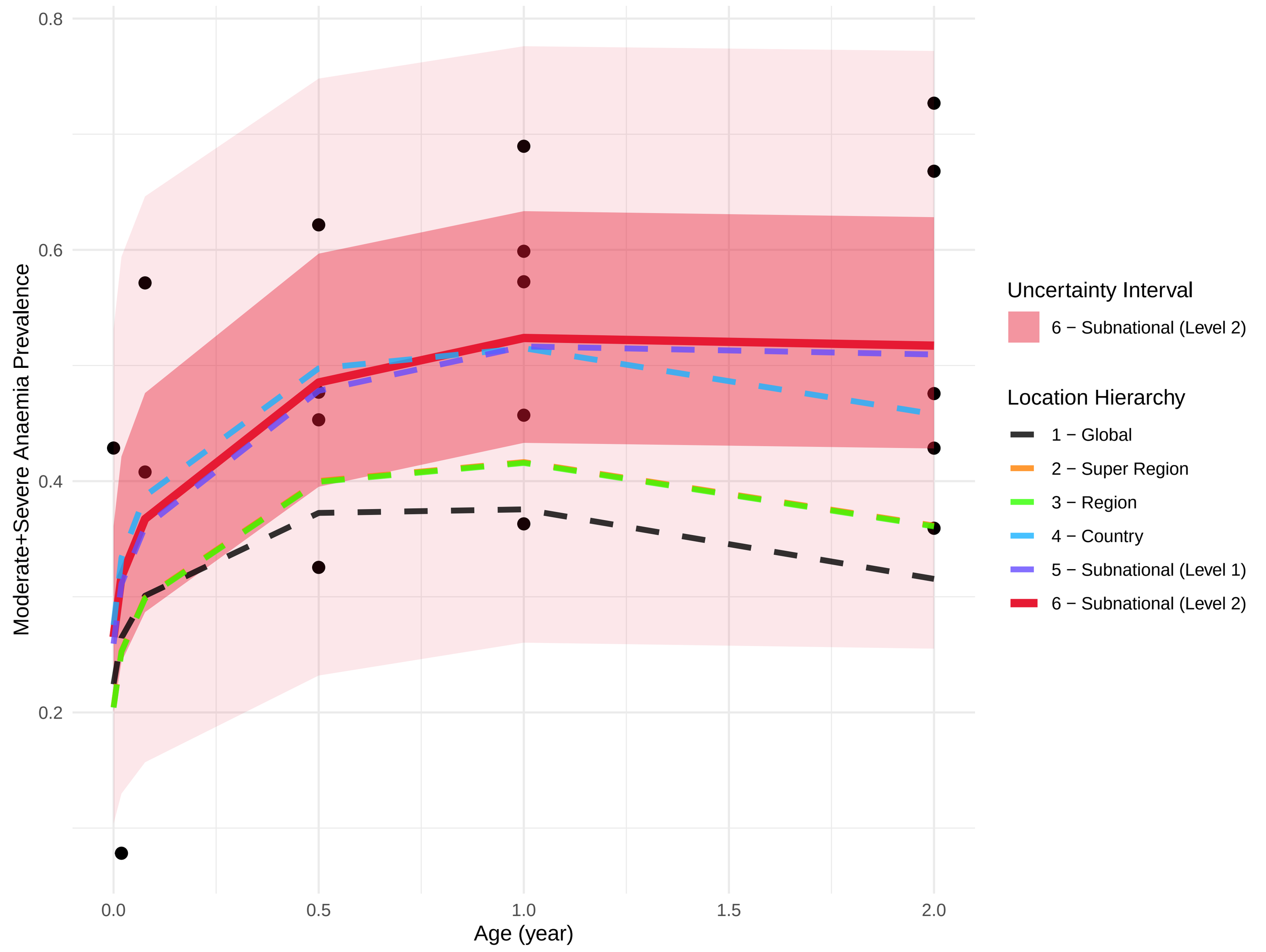

MR−BRT Age/Sex Fits − Males <2 Years Old − Moderate+Severe Anaemia Prevalence − Rajasthan, Urban
WHO−2024 Adjustment Method

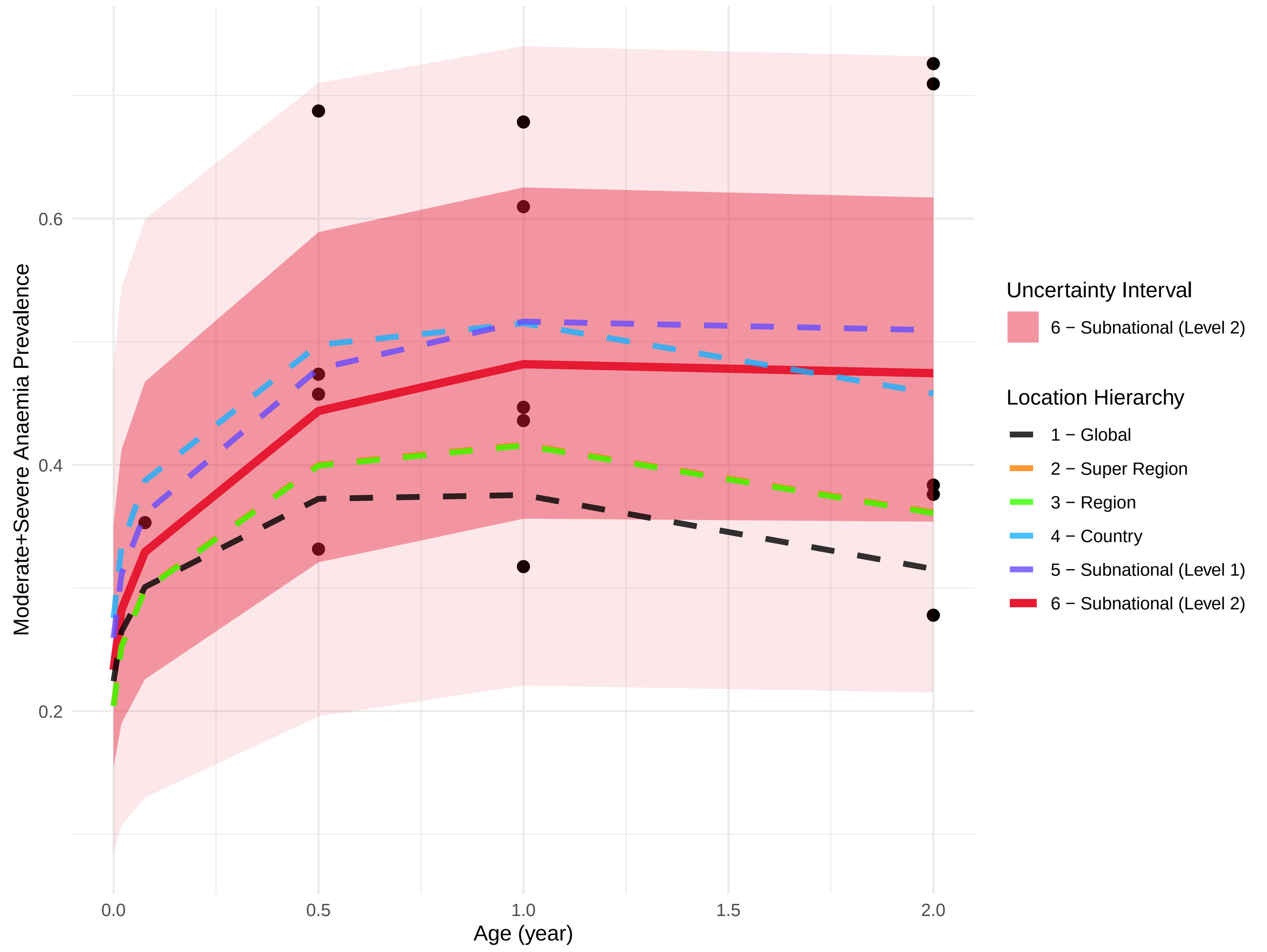

MR−BRT Age/Sex Fits − Males <2 Years Old − Moderate+Severe Anaemia Prevalence − Sikkim
WHO−2024 Adjustment Method

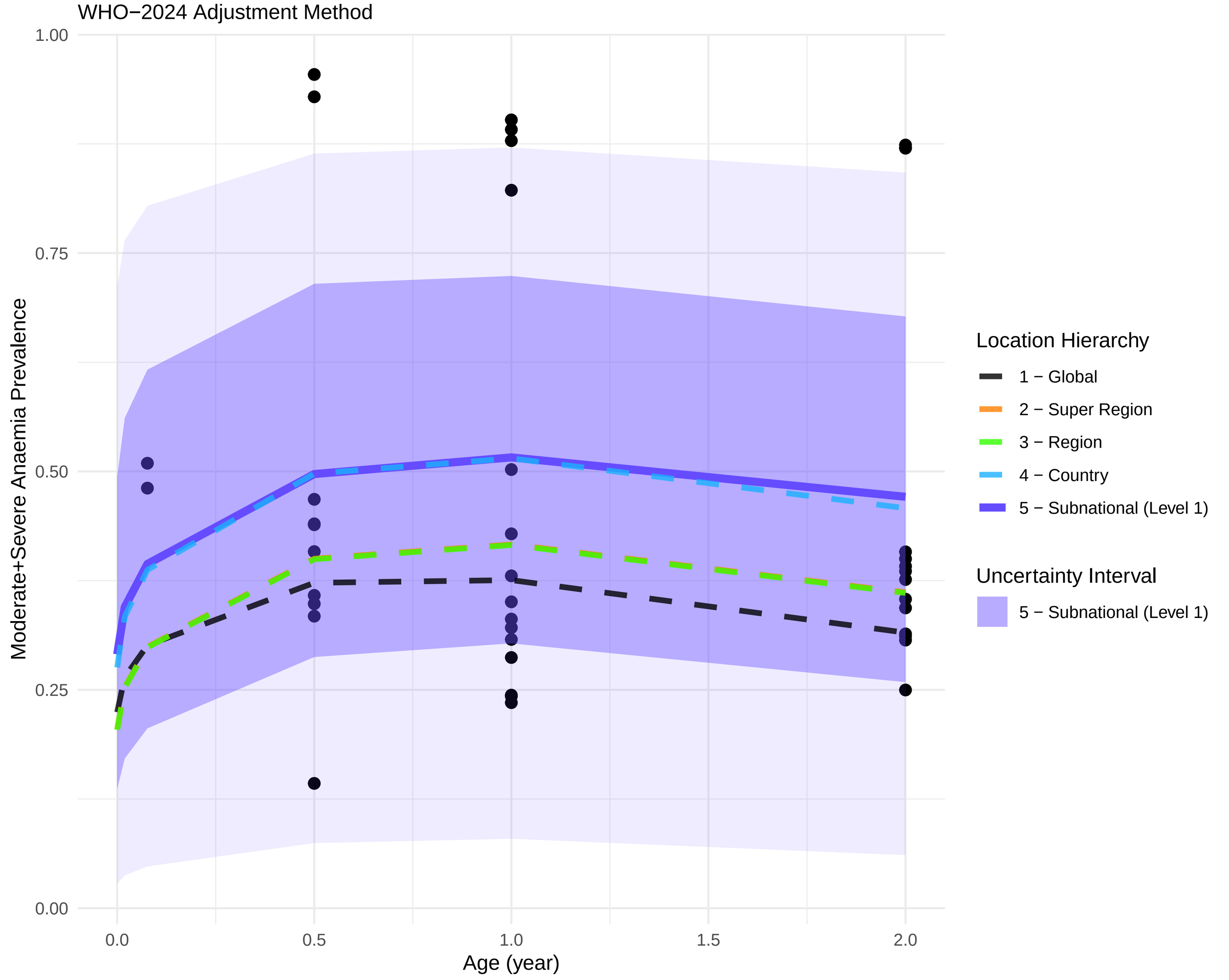

MR−BRT Age/Sex Fits − Males <2 Years Old − Moderate+Severe Anaemia Prevalence − Sikkim, Rural
WHO−2024 Adjustment Method

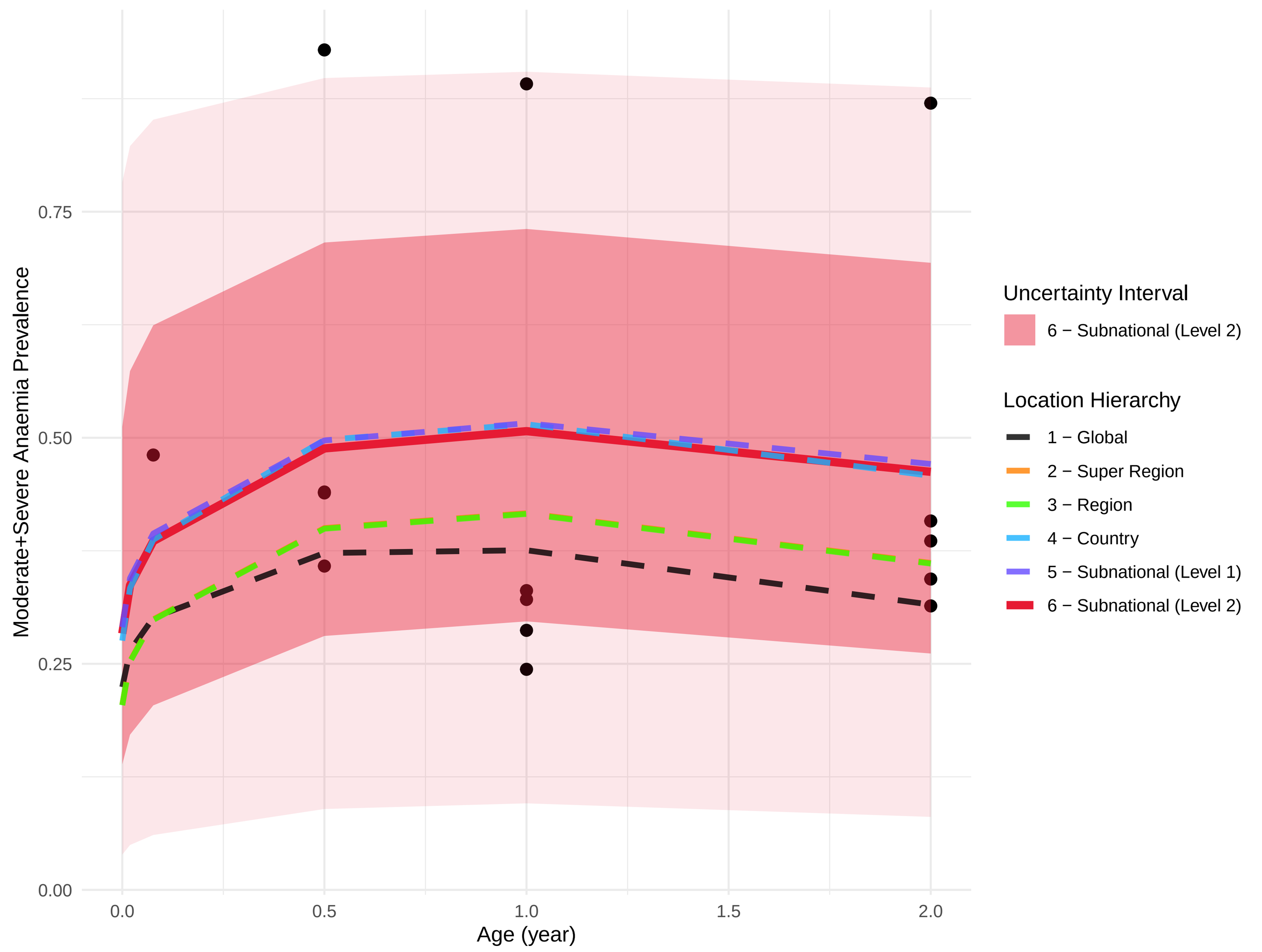

MR−BRT Age/Sex Fits − Males <2 Years Old − Moderate+Severe Anaemia Prevalence − Sikkim, Urban
WHO−2024 Adjustment Method

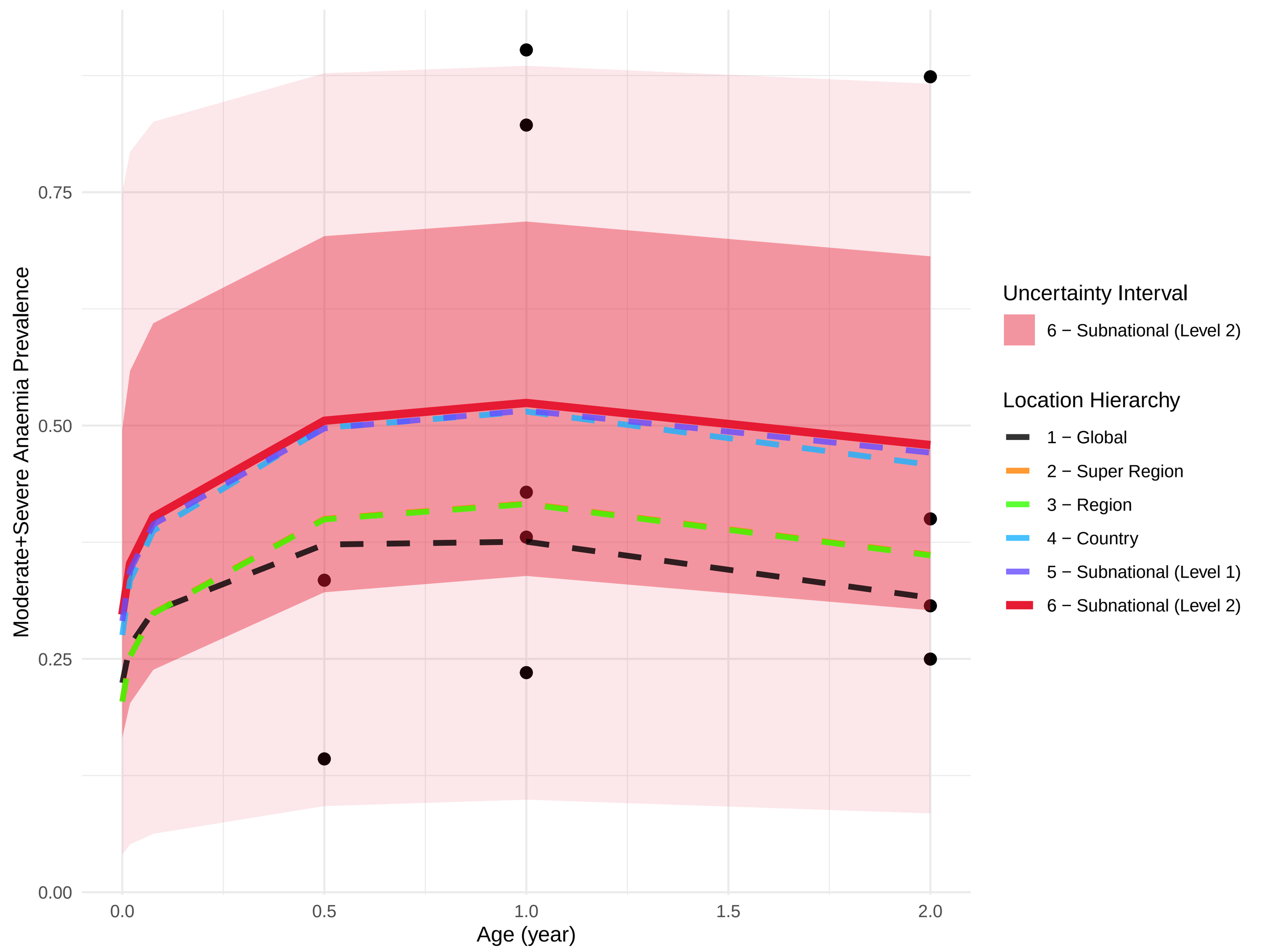

MR−BRT Age/Sex Fits − Males <2 Years Old − Moderate+Severe Anaemia Prevalence − Tamil Nadu
WHO−2024 Adjustment Method

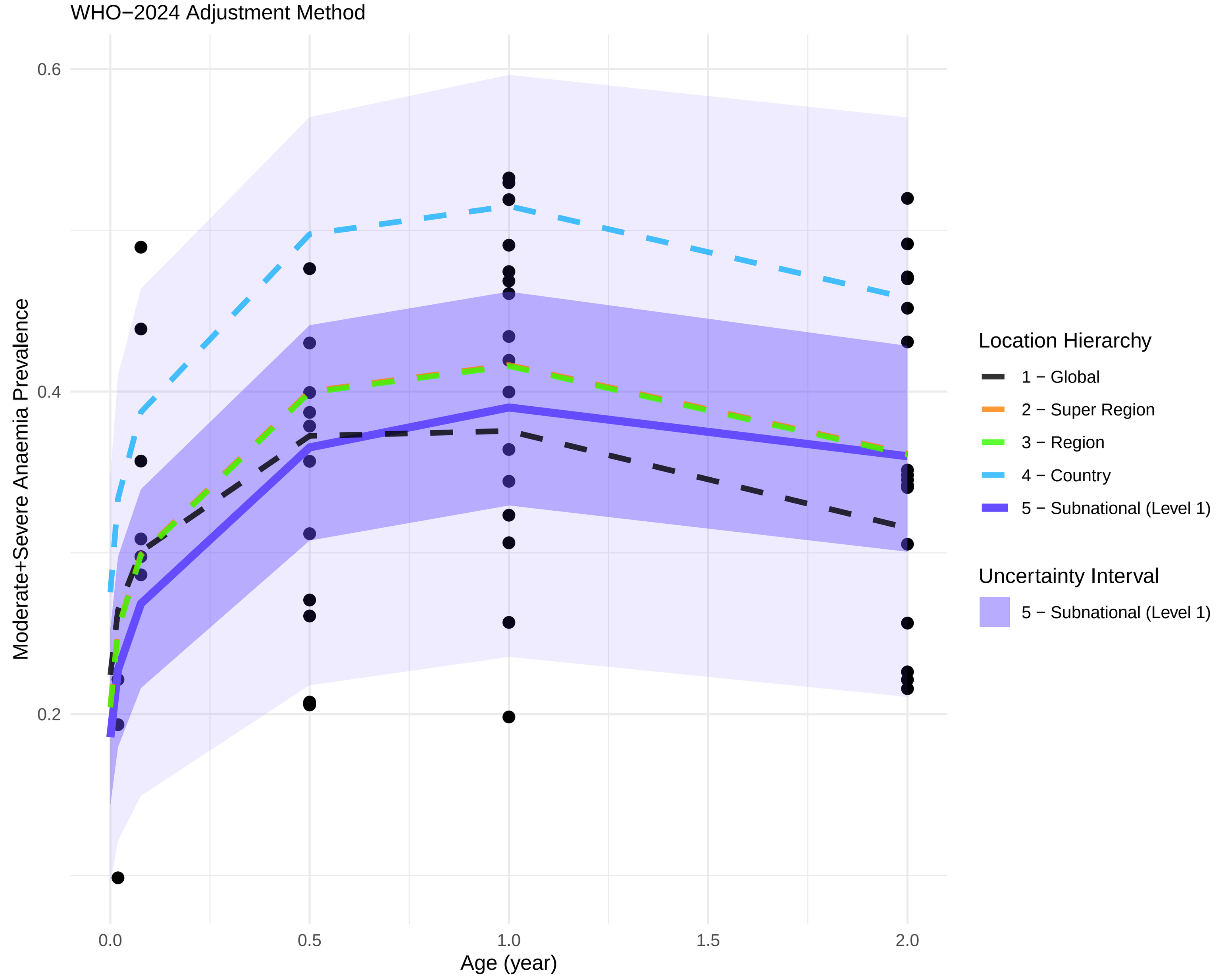

MR−BRT Age/Sex Fits − Males <2 Years Old − Moderate+Severe Anaemia Prevalence − Tamil Nadu, Rural
WHO−2024 Adjustment Method

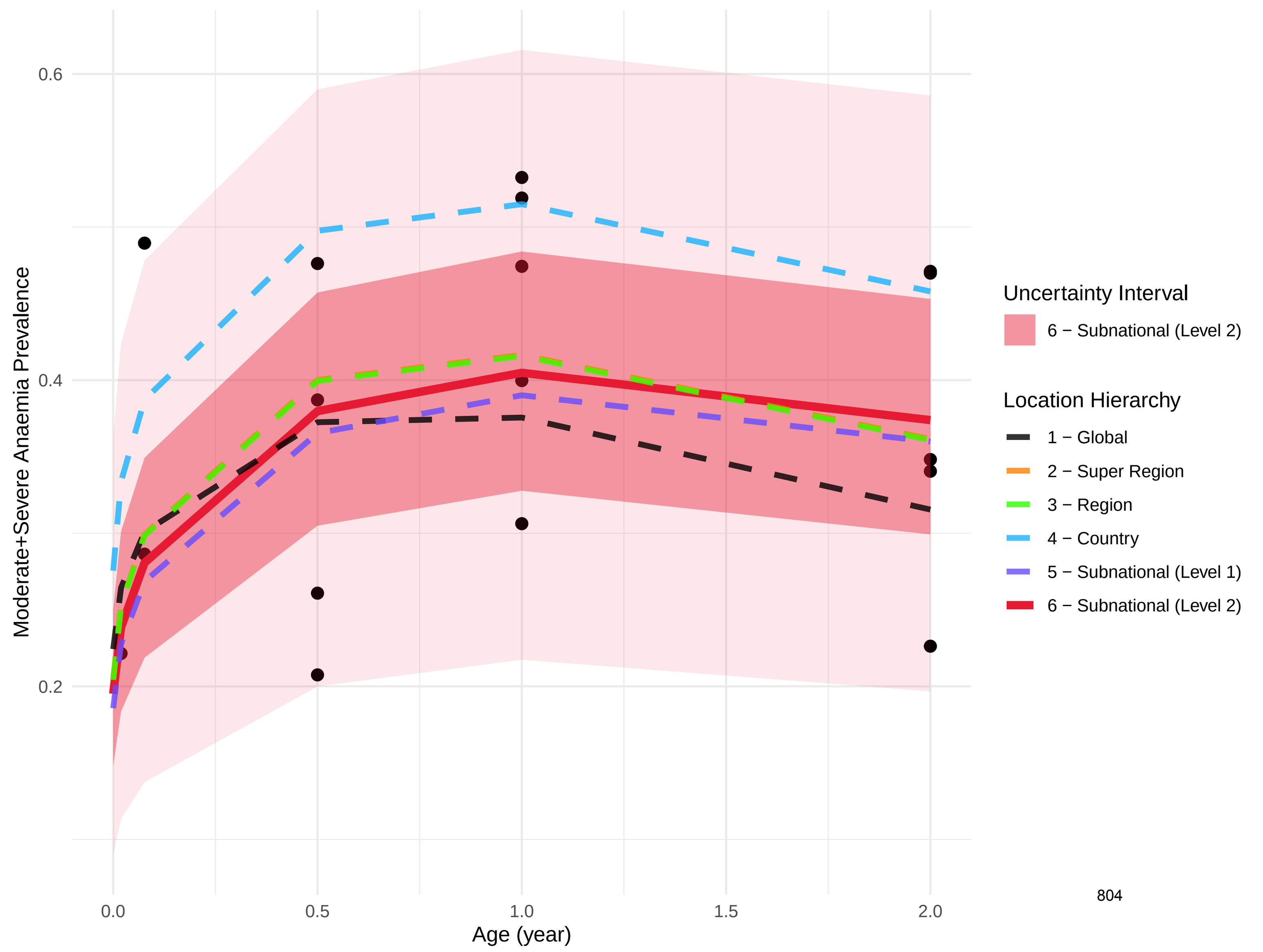

MR−BRT Age/Sex Fits − Males <2 Years Old − Moderate+Severe Anaemia Prevalence − Tamil Nadu, Urban
WHO−2024 Adjustment Method

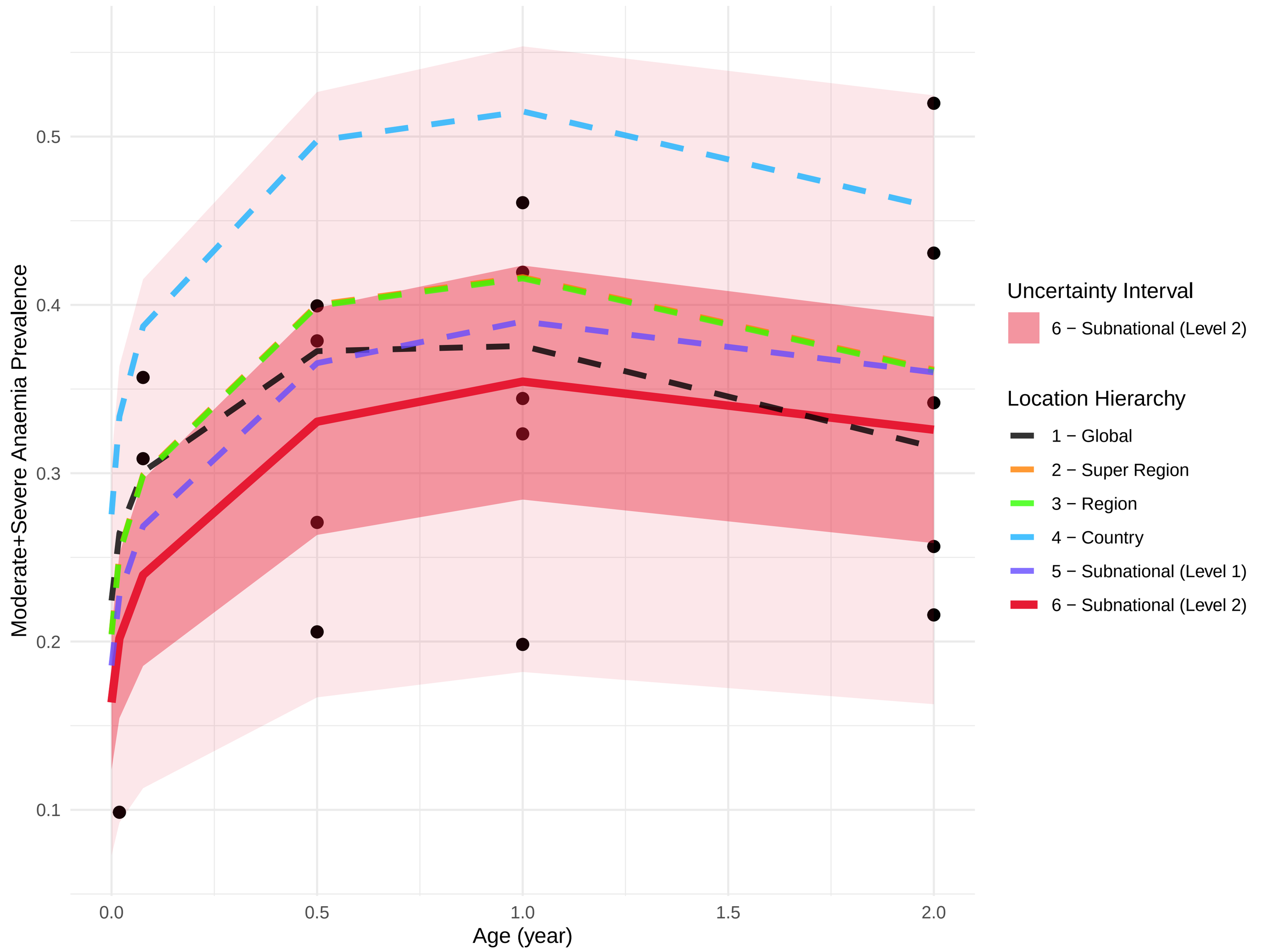

MR−BRT Age/Sex Fits − Males <2 Years Old − Moderate+Severe Anaemia Prevalence − Telangana
WHO−2024 Adjustment Method

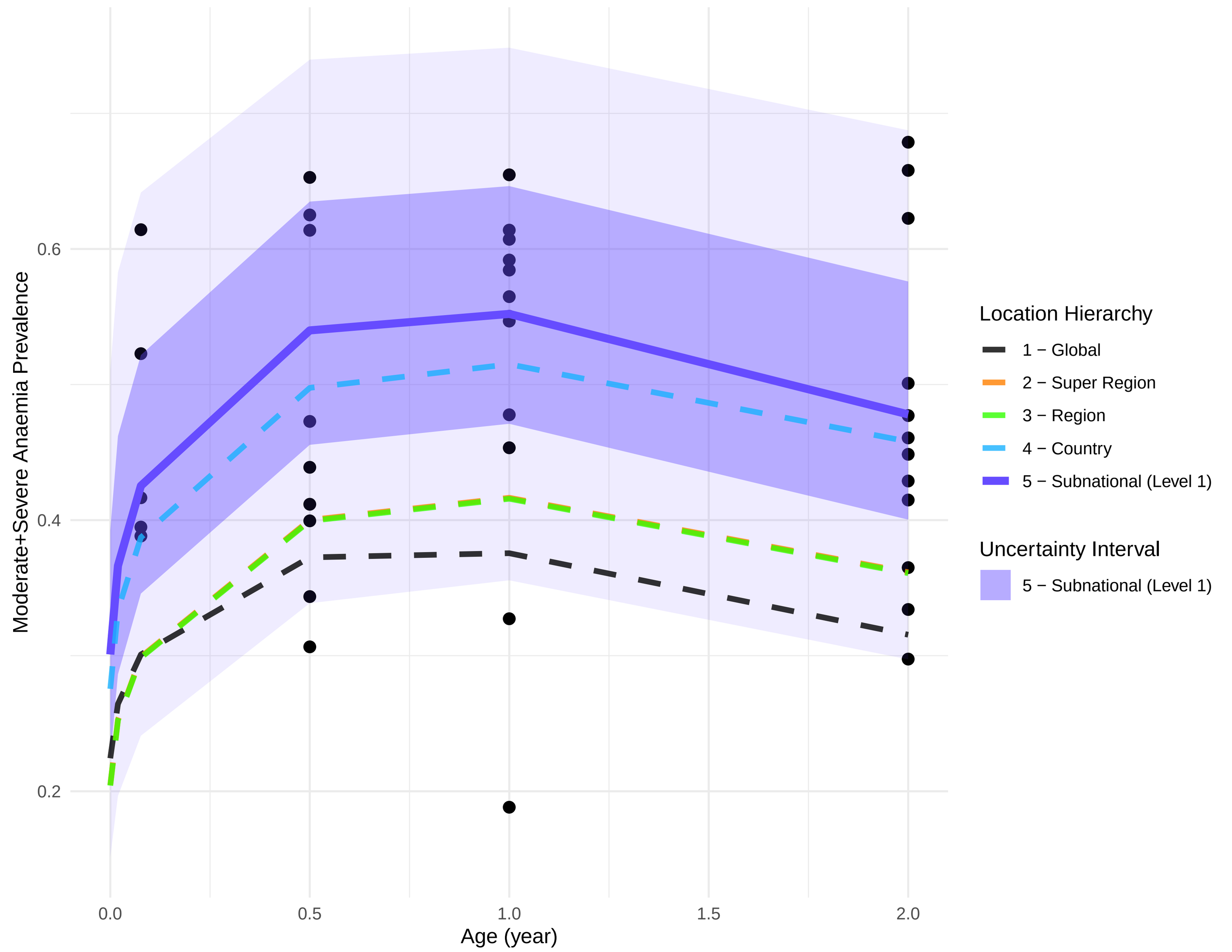

MR−BRT Age/Sex Fits − Males <2 Years Old − Moderate+Severe Anaemia Prevalence − Telangana, Rural
WHO−2024 Adjustment Method

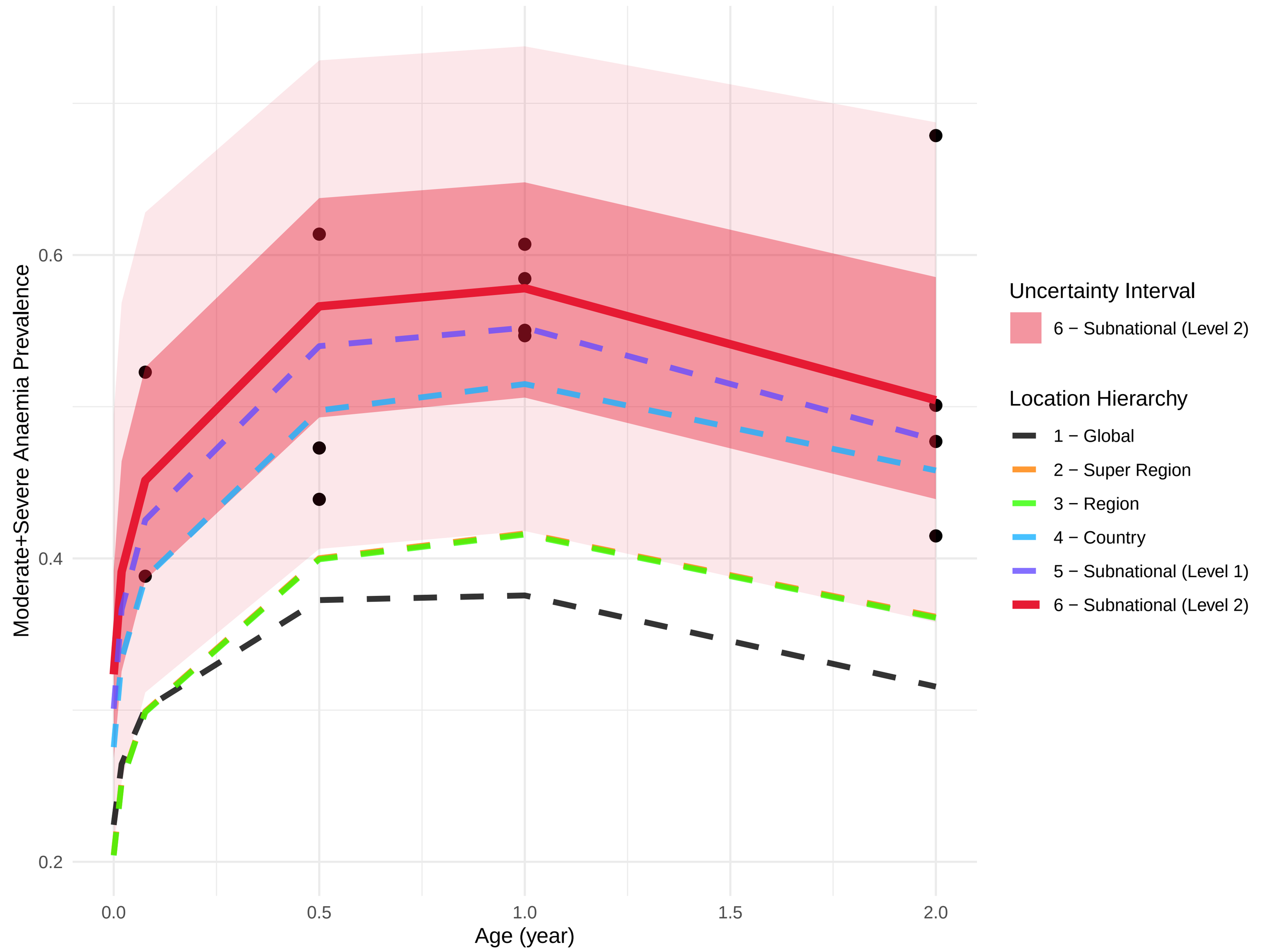

MR−BRT Age/Sex Fits − Males <2 Years Old − Moderate+Severe Anaemia Prevalence − Telangana, Urban
WHO−2024 Adjustment Method

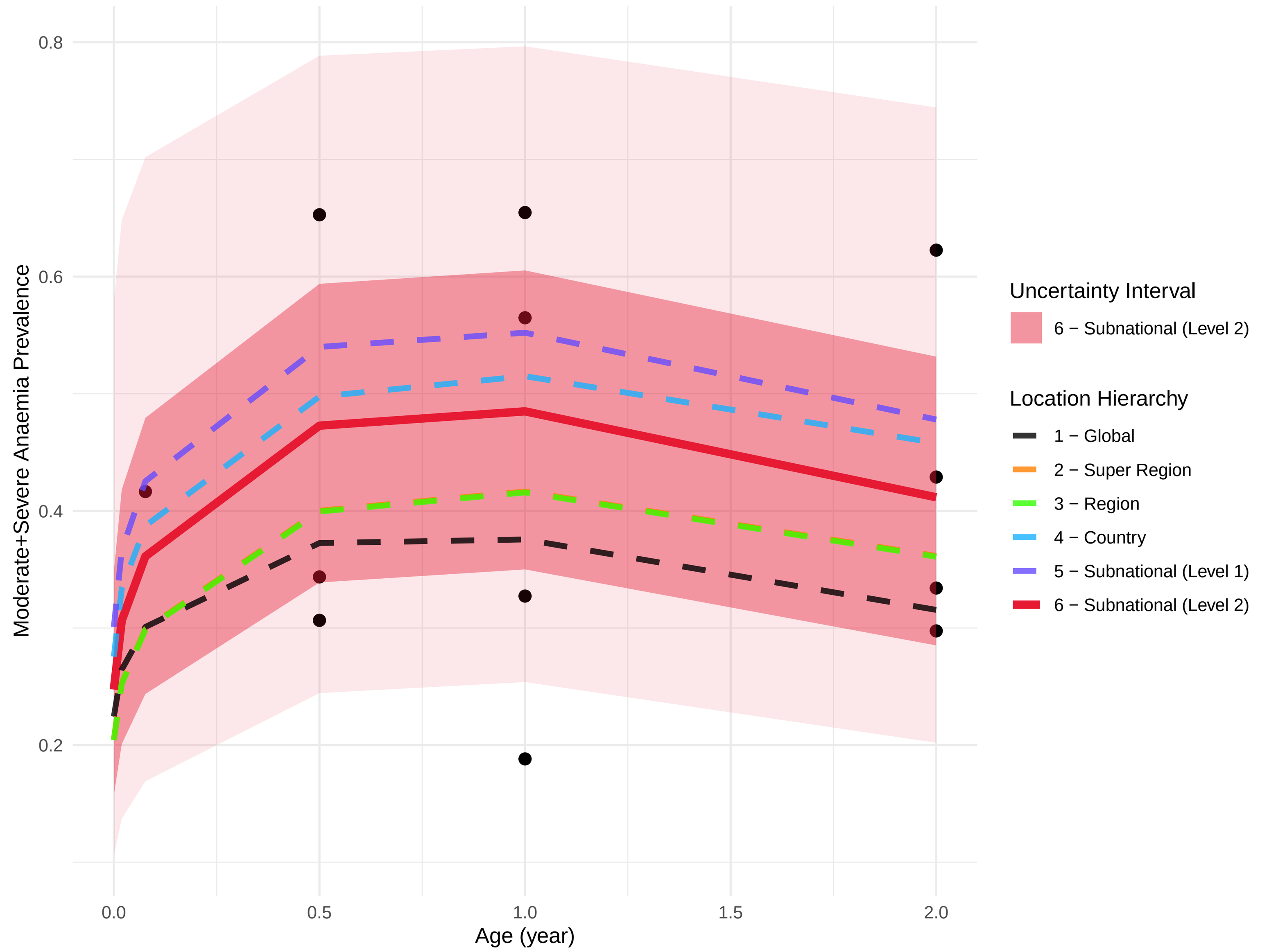

MR−BRT Age/Sex Fits − Males <2 Years Old − Moderate+Severe Anaemia Prevalence − Tripura
WHO−2024 Adjustment Method

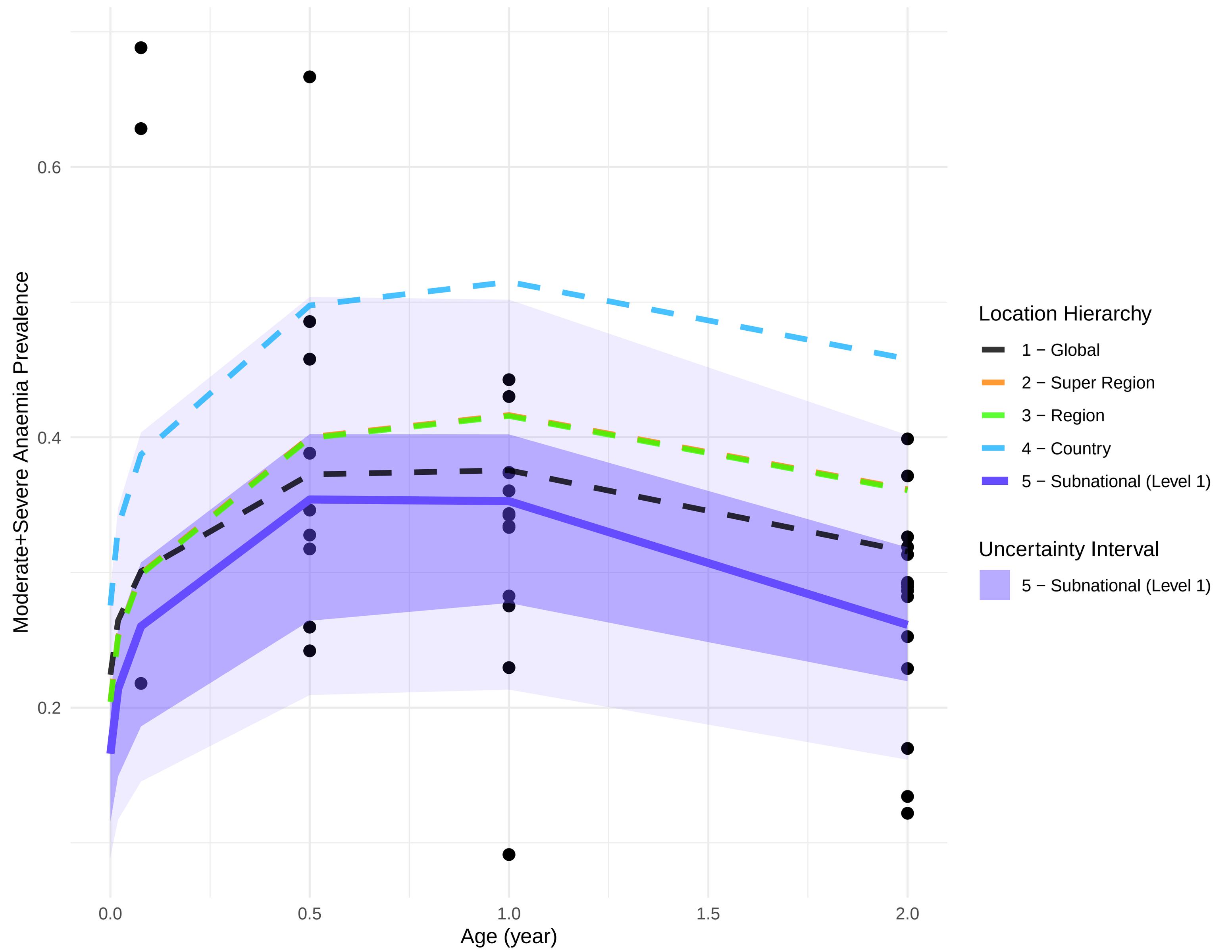

MR−BRT Age/Sex Fits − Males <2 Years Old − Moderate+Severe Anaemia Prevalence − Tripura, Rural
WHO−2024 Adjustment Method

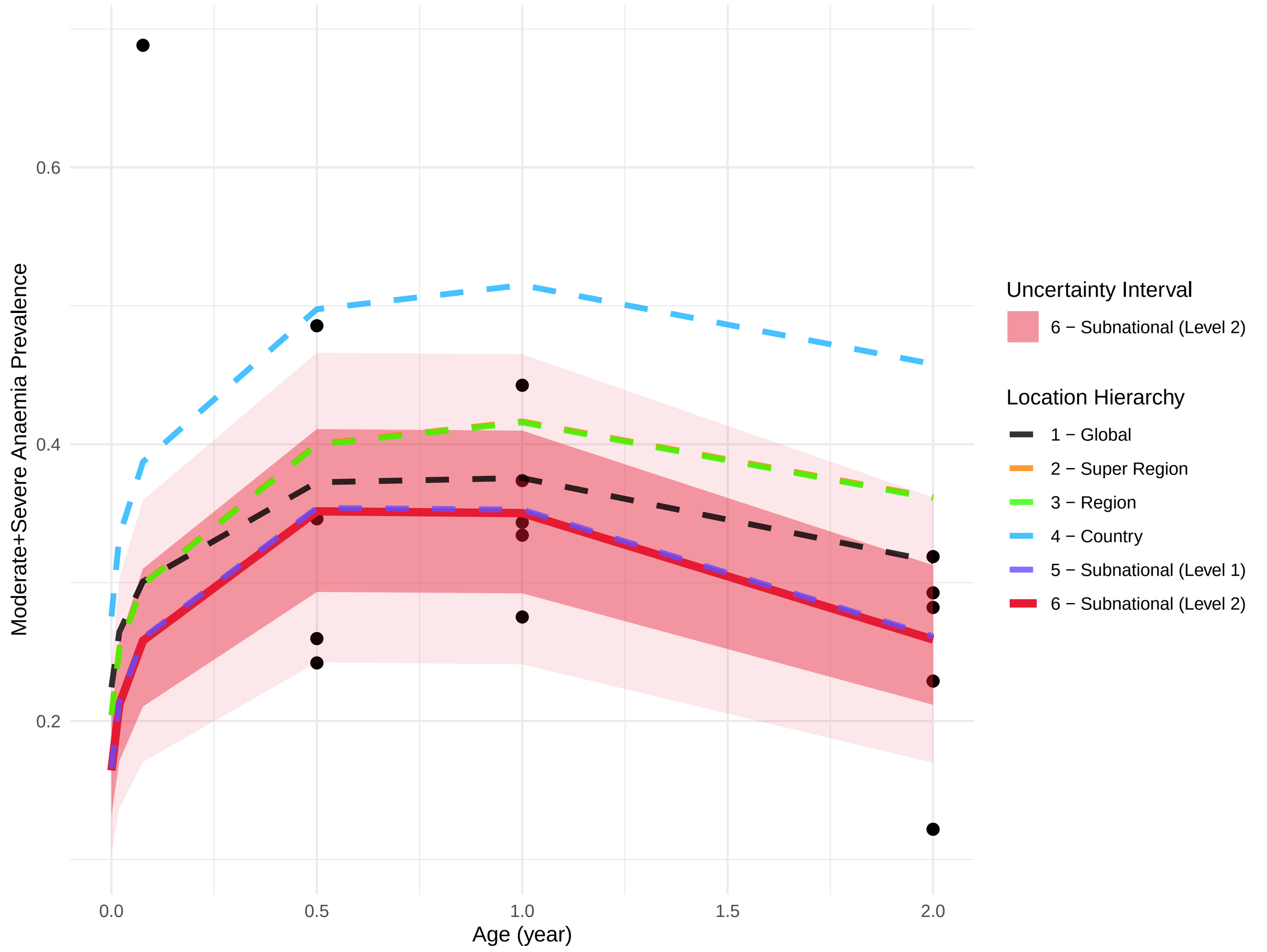

MR−BRT Age/Sex Fits − Males <2 Years Old − Moderate+Severe Anaemia Prevalence − Tripura, Urban
WHO−2024 Adjustment Method

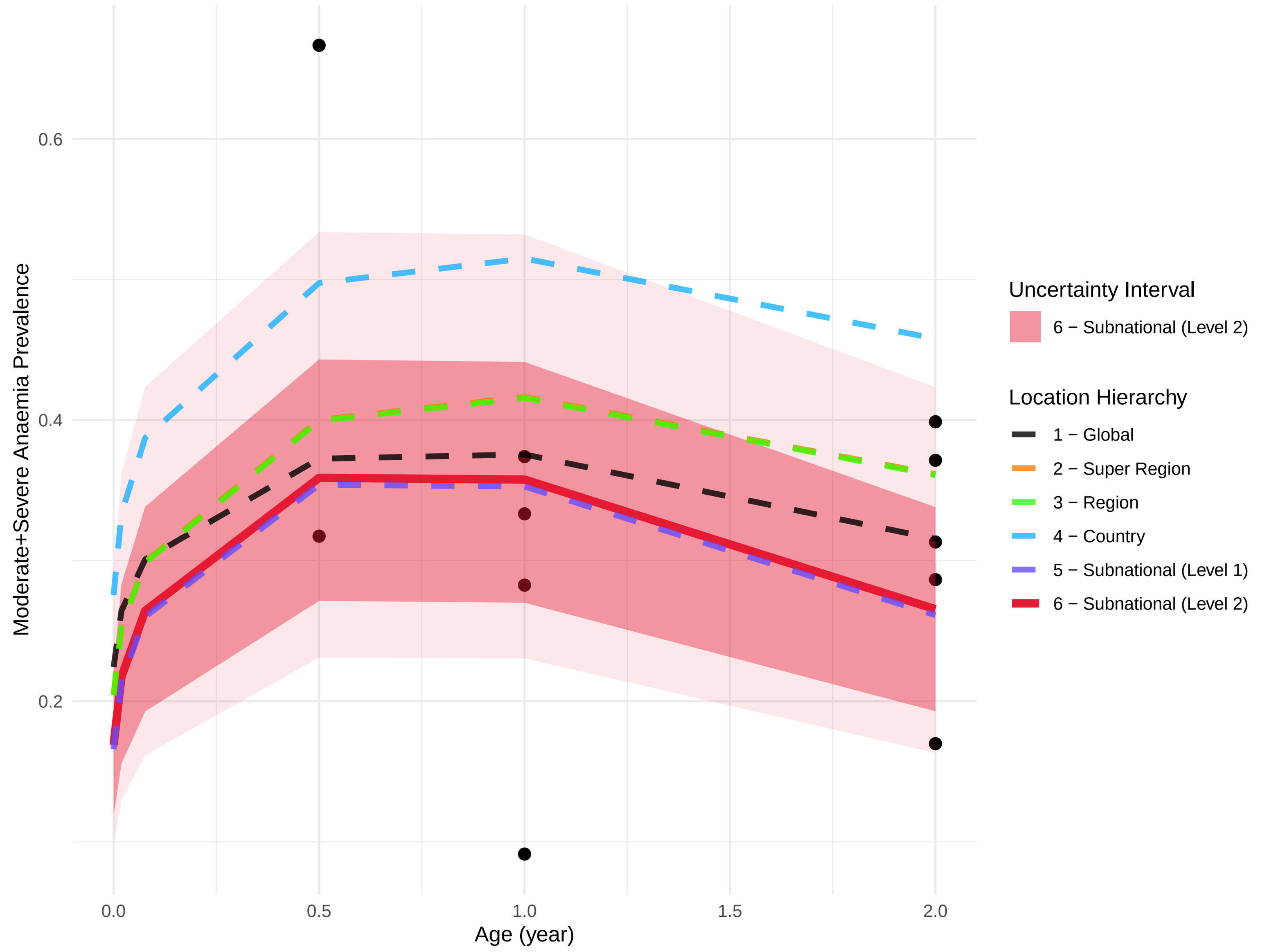

MR−BRT Age/Sex Fits − Males <2 Years Old − Moderate+Severe Anaemia Prevalence − Uttar Pradesh
WHO−2024 Adjustment Method

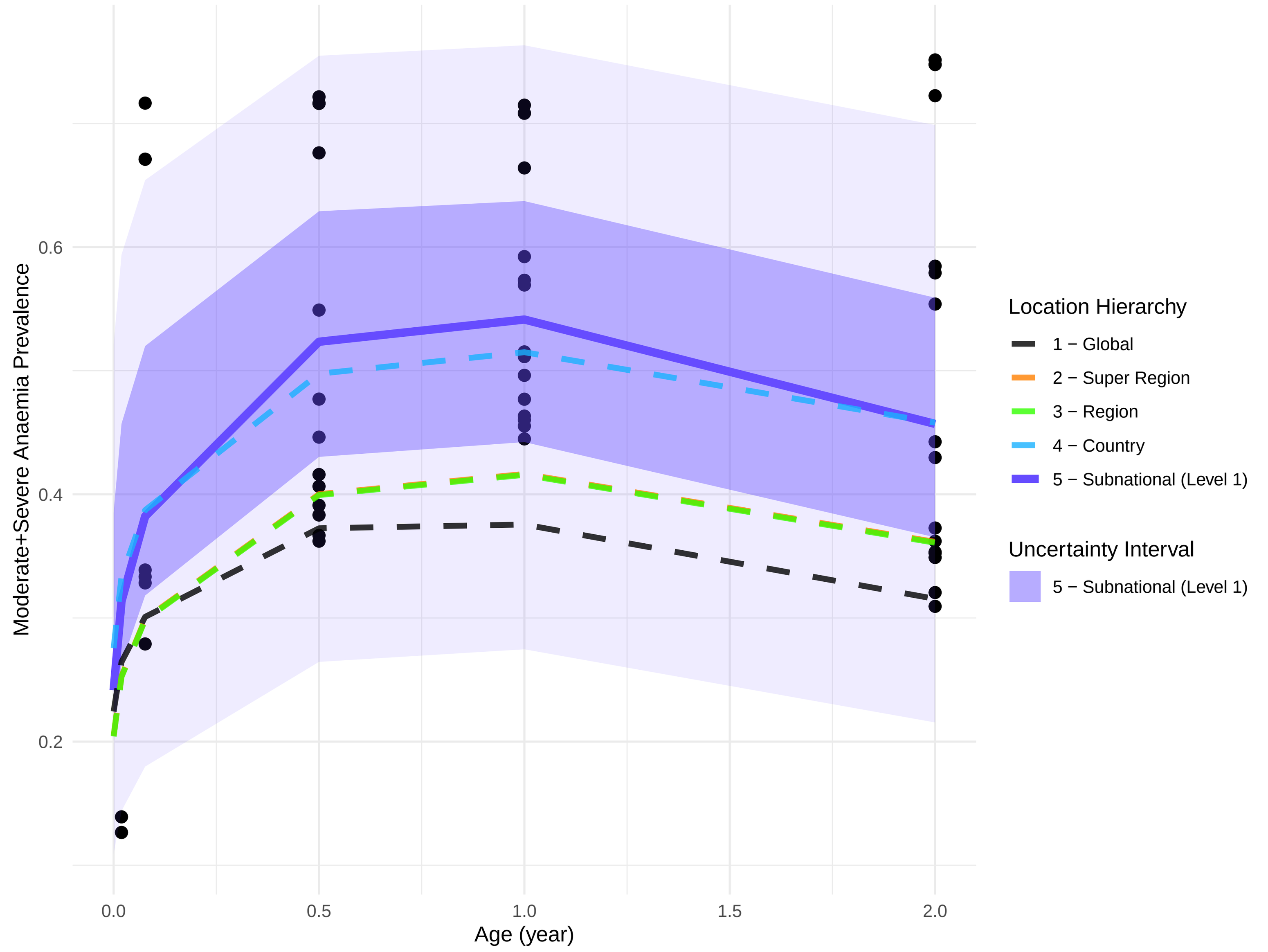

MR−BRT Age/Sex Fits − Males <2 Years Old − Moderate+Severe Anaemia Prevalence − Uttar Pradesh, Rural
WHO−2024 Adjustment Method

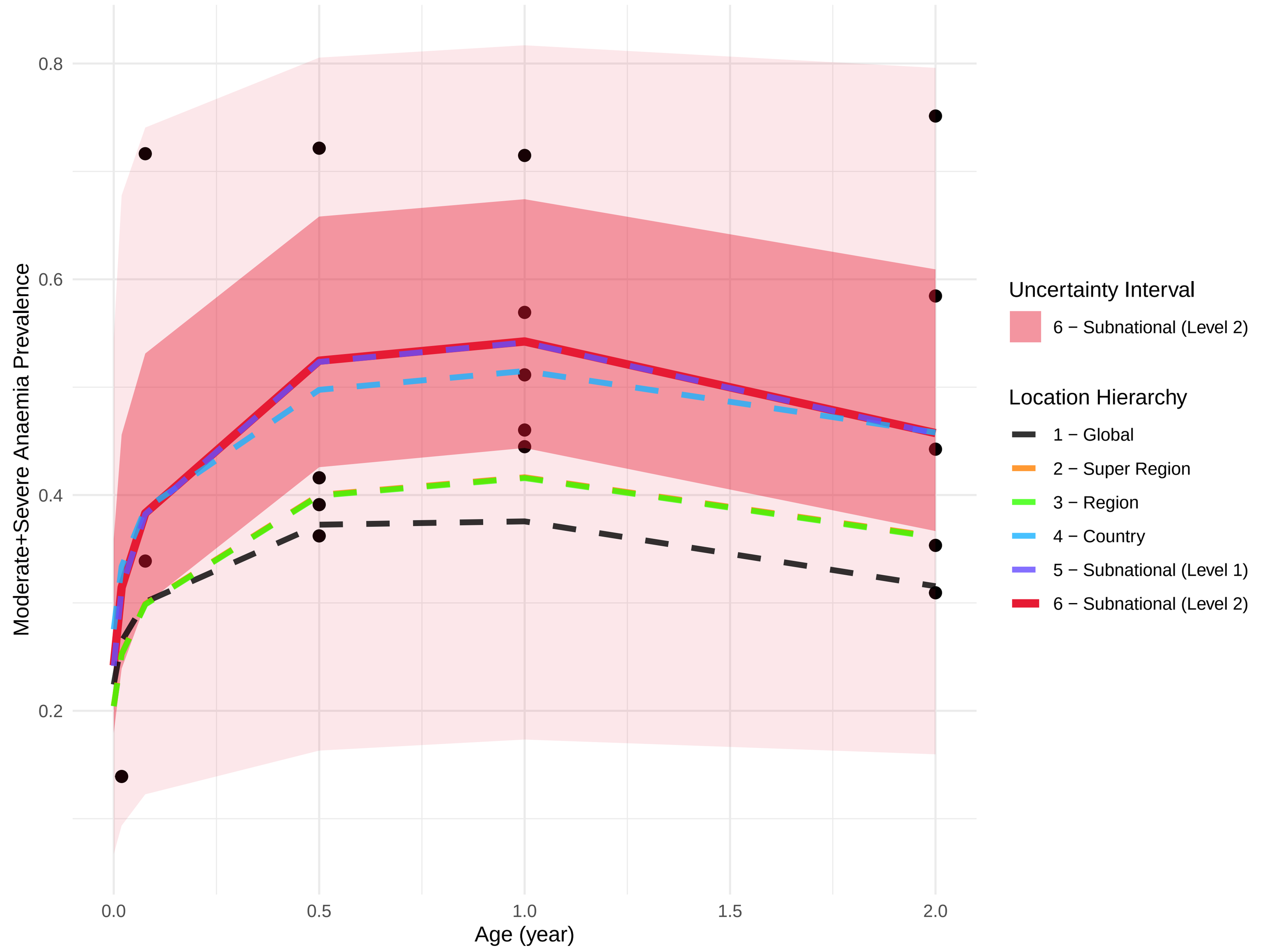

MR−BRT Age/Sex Fits − Males <2 Years Old − Moderate+Severe Anaemia Prevalence − Uttar Pradesh, Urban
WHO−2024 Adjustment Method

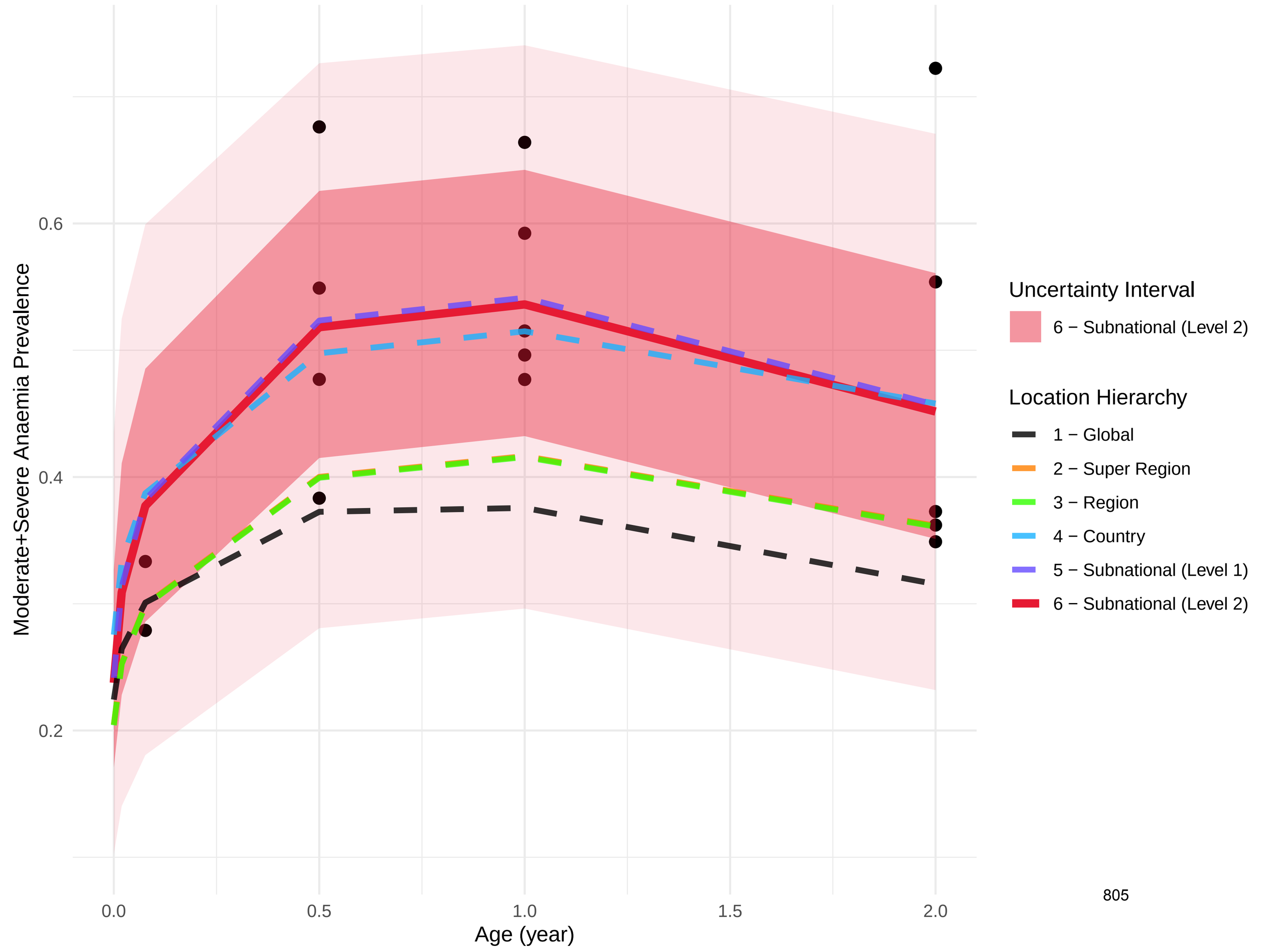

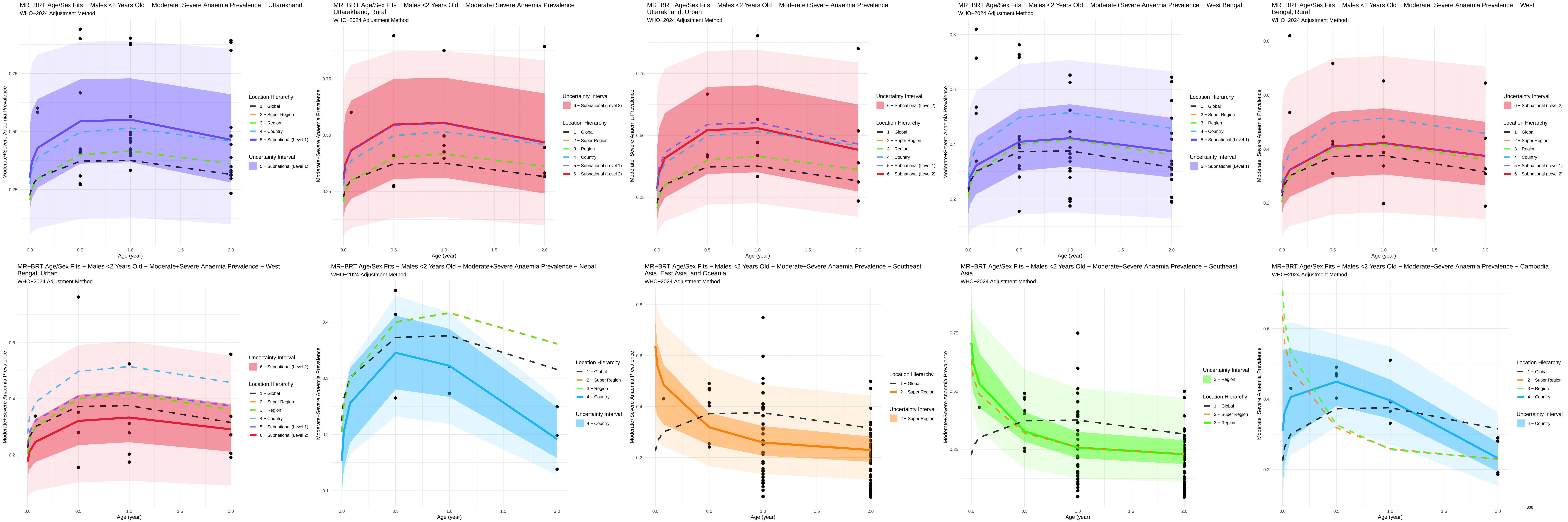
MR−BRT Age/Sex Fits − Males <2 Years Old − Moderate+Severe Anaemia Prevalence − Uttarakhand
WHO−2024 Adjustment Method
Location Hierarchy
1 − Global
2 − Super Region
3 − Region
4 − Country
5 − Subnational (Level 1)
Uncertainty Interval
5 − Subnational (Level 1)
MR−BRT Age/Sex Fits − Males <2 Years Old − Moderate+Severe Anaemia Prevalence − Uttarakhand, Rural
WHO−2024 Adjustment Method
Uncertainty Interval
6 − Subnational (Level 2)
Location Hierarchy
1 − Global
2 − Super Region
3 − Region
4 − Country
5 − Subnational (Level 1)
6 − Subnational (Level 2)
MR−BRT Age/Sex Fits − Males <2 Years Old − Moderate+Severe Anaemia Prevalence − Uttarakhand, Urban
WHO−2024 Adjustment Method
MR−BRT Age/Sex Fits − Males <2 Years Old − Moderate+Severe Anaemia Prevalence − West Bengal
WHO−2024 Adjustment Method
MR−BRT Age/Sex Fits − Males <2 Years Old − Moderate+Severe Anaemia Prevalence − West Bengal, Rural
WHO−2024 Adjustment Method
MR−BRT Age/Sex Fits − Males <2 Years Old − Moderate+Severe Anaemia Prevalence − West Bengal, Urban
WHO−2024 Adjustment Method
MR−BRT Age/Sex Fits − Males <2 Years Old − Moderate+Severe Anaemia Prevalence − Nepal
WHO−2024 Adjustment Method
Location Hierarchy
1 − Global
2 − Super Region
3 − Region
4 − Country
Uncertainty Interval
4 − Country
MR−BRT Age/Sex Fits − Males <2 Years Old − Moderate+Severe Anaemia Prevalence − Southeast Asia, East Asia, and Oceania
WHO−2024 Adjustment Method
Location Hierarchy
1 − Global
2 − Super Region
Uncertainty Interval
2 − Super Region
MR−BRT Age/Sex Fits − Males <2 Years Old − Moderate+Severe Anaemia Prevalence − Southeast Asia
WHO−2024 Adjustment Method
Uncertainty Interval
3 − Region
Location Hierarchy
1 − Global
2 − Super Region
3 − Region
MR−BRT Age/Sex Fits − Males <2 Years Old − Moderate+Severe Anaemia Prevalence − Cambodia
WHO−2024 Adjustment Method
Moderate+Severe Anaemia Prevalence
Age (year)

MR−BRT Age/Sex Fits − Males <2 Years Old − Moderate+Severe Anaemia Prevalence − Indonesia
WHO−2024 Adjustment Method

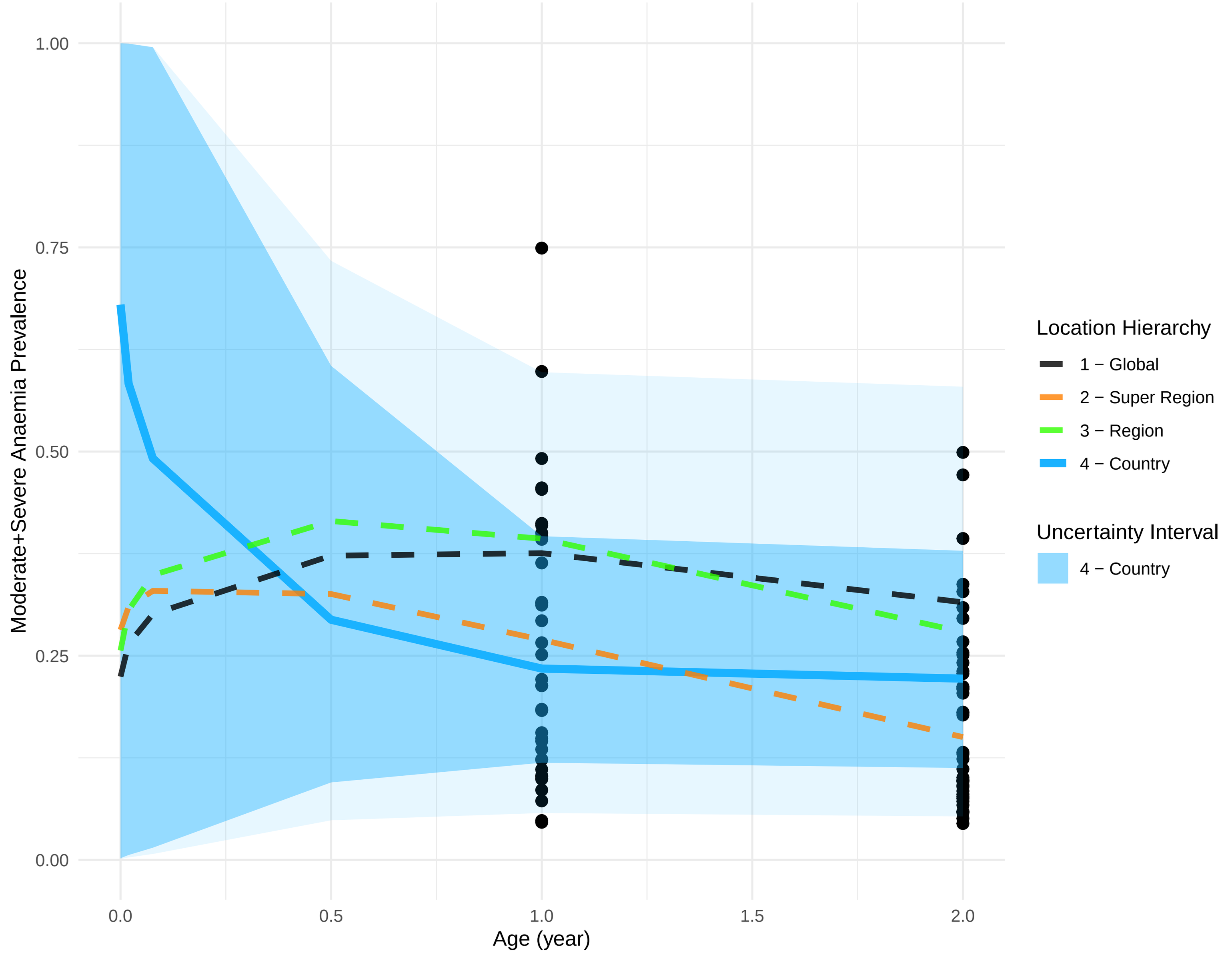

MR−BRT Age/Sex Fits − Males <2 Years Old − Moderate+Severe Anaemia Prevalence − Bali
WHO−2024 Adjustment Method

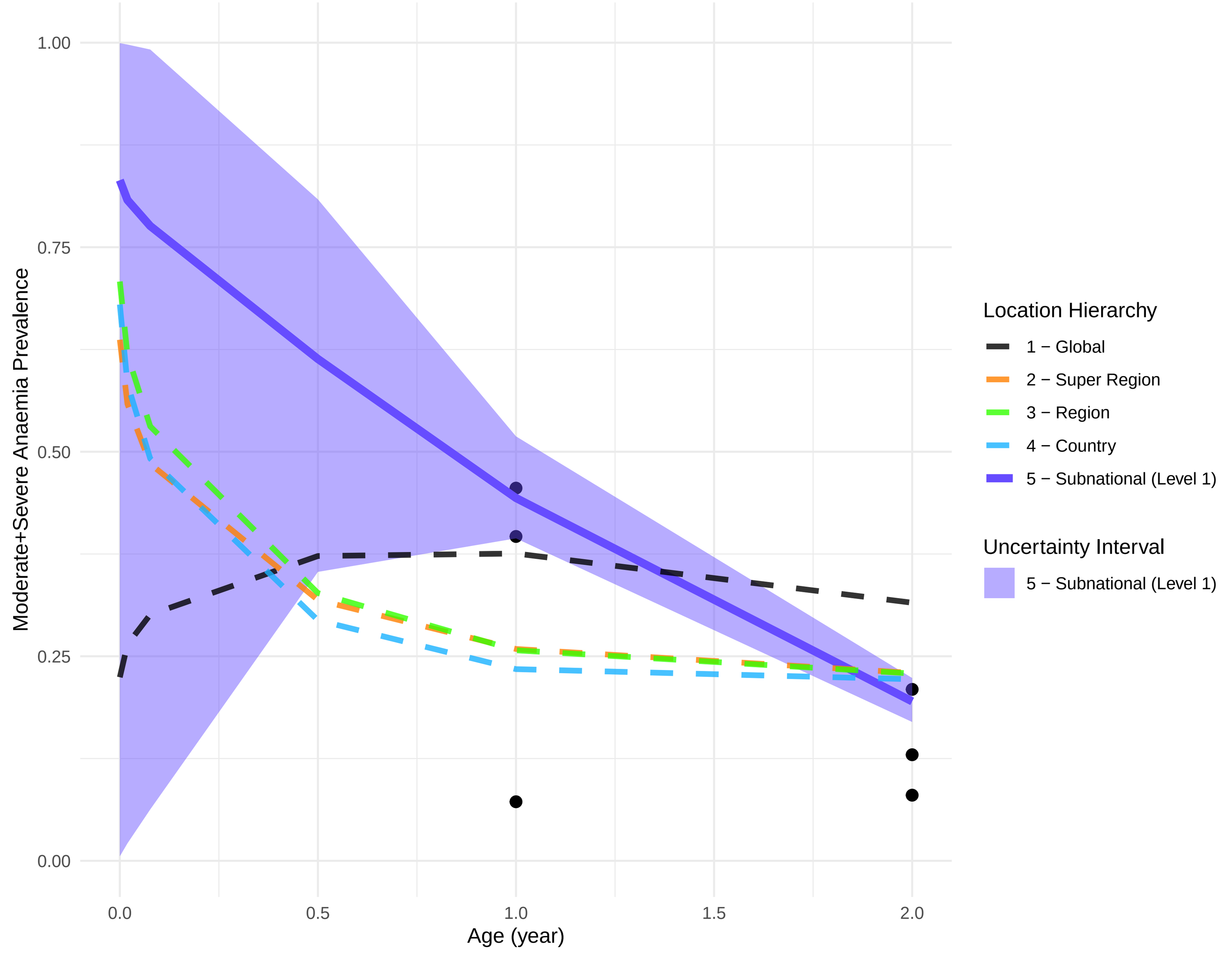

MR−BRT Age/Sex Fits − Males <2 Years Old − Moderate+Severe Anaemia Prevalence − Bangka−Belitung Islands
WHO−2024 Adjustment Method

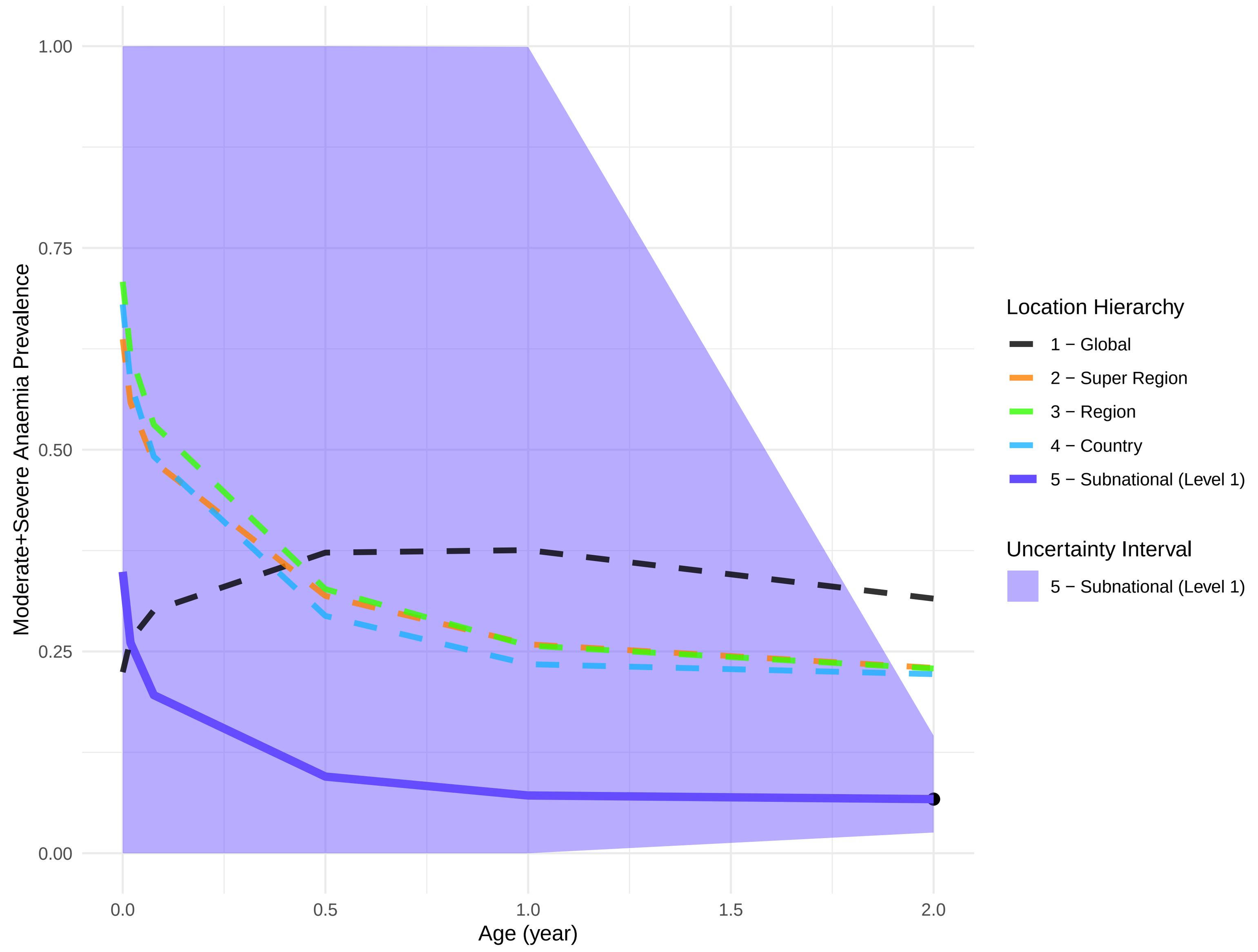

MR−BRT Age/Sex Fits − Males <2 Years Old − Moderate+Severe Anaemia Prevalence − Banten
WHO−2024 Adjustment Method

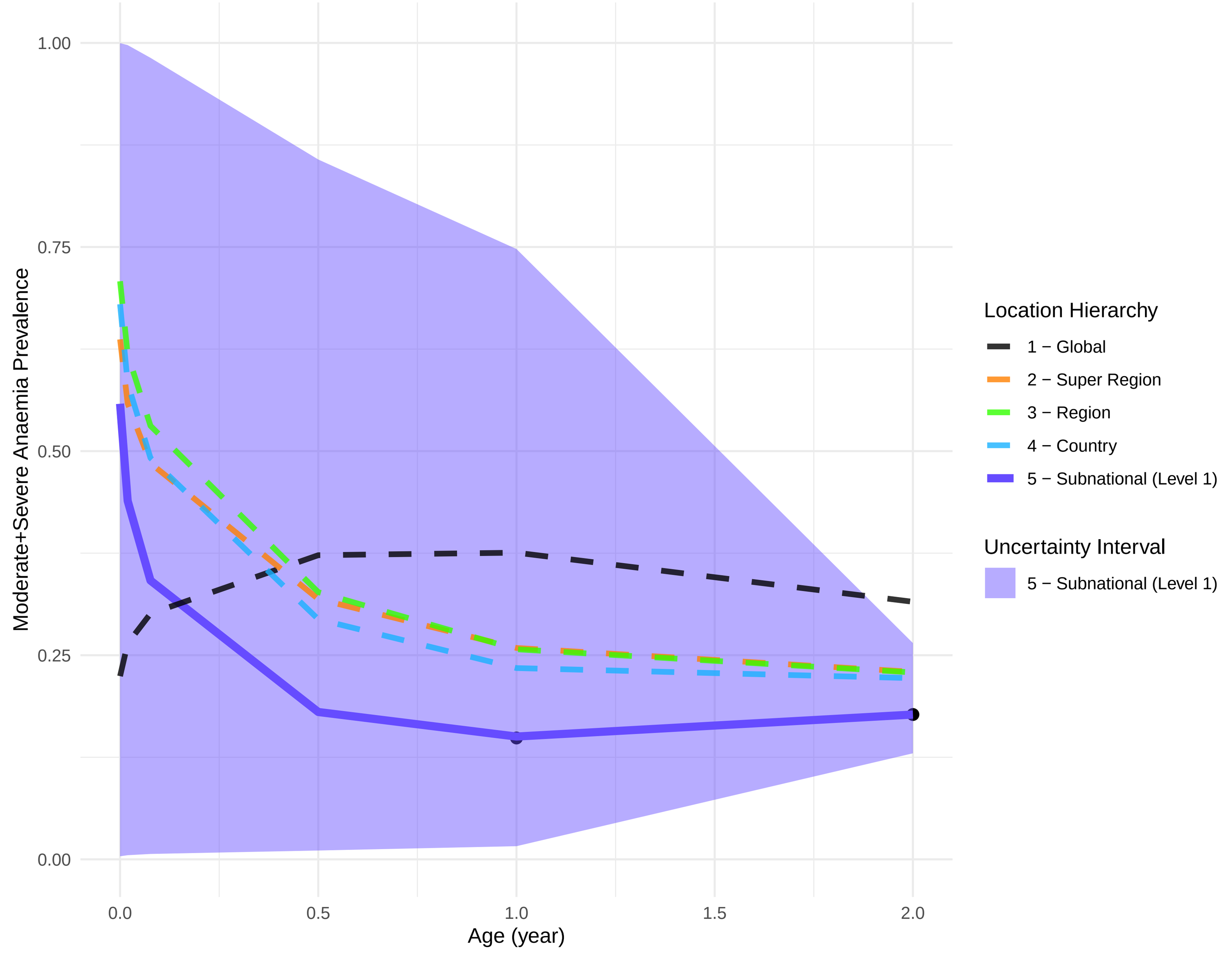

MR−BRT Age/Sex Fits − Males <2 Years Old − Moderate+Severe Anaemia Prevalence − Jakarta
WHO−2024 Adjustment Method

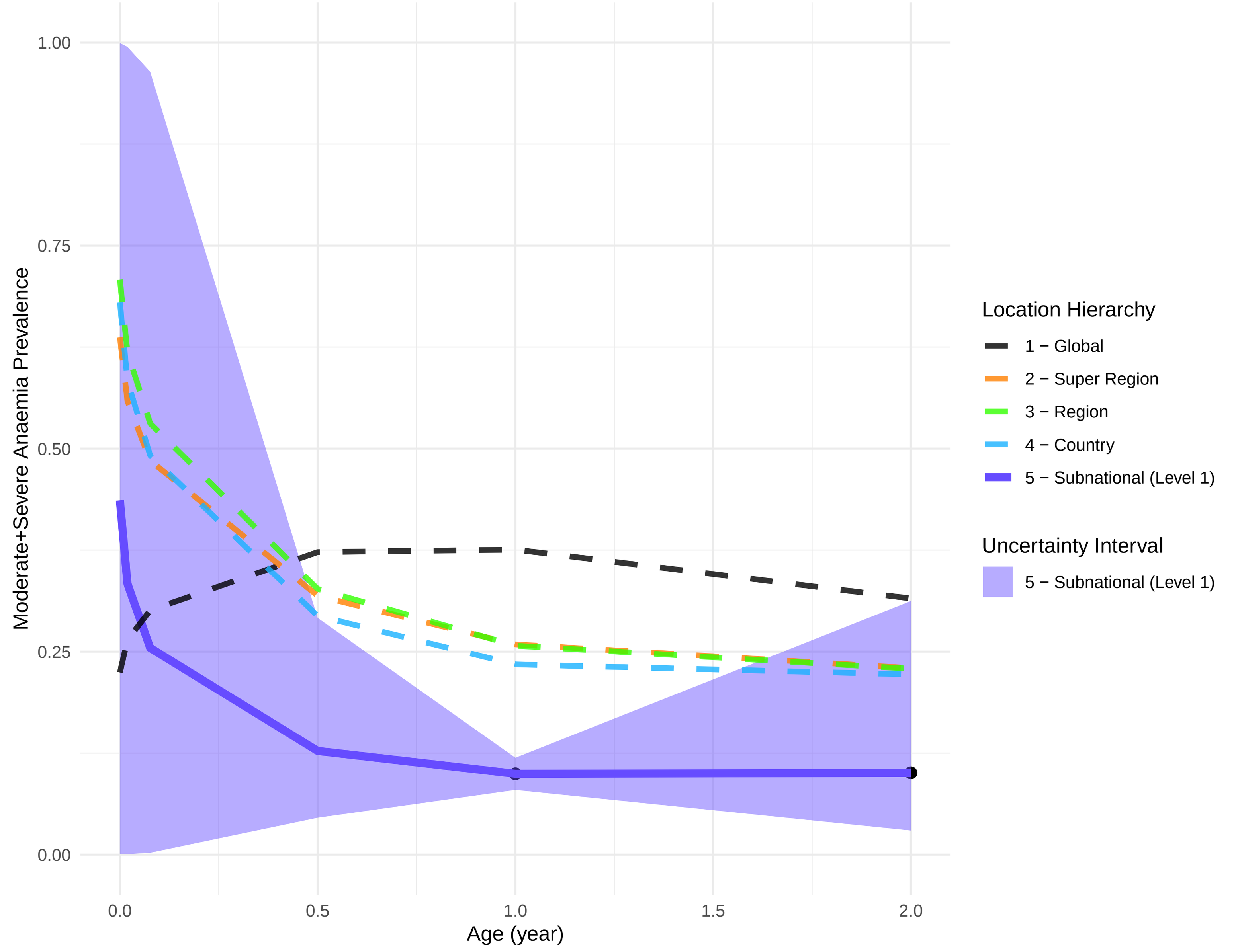

MR−BRT Age/Sex Fits − Males <2 Years Old − Moderate+Severe Anaemia Prevalence − West Java
WHO−2024 Adjustment Method

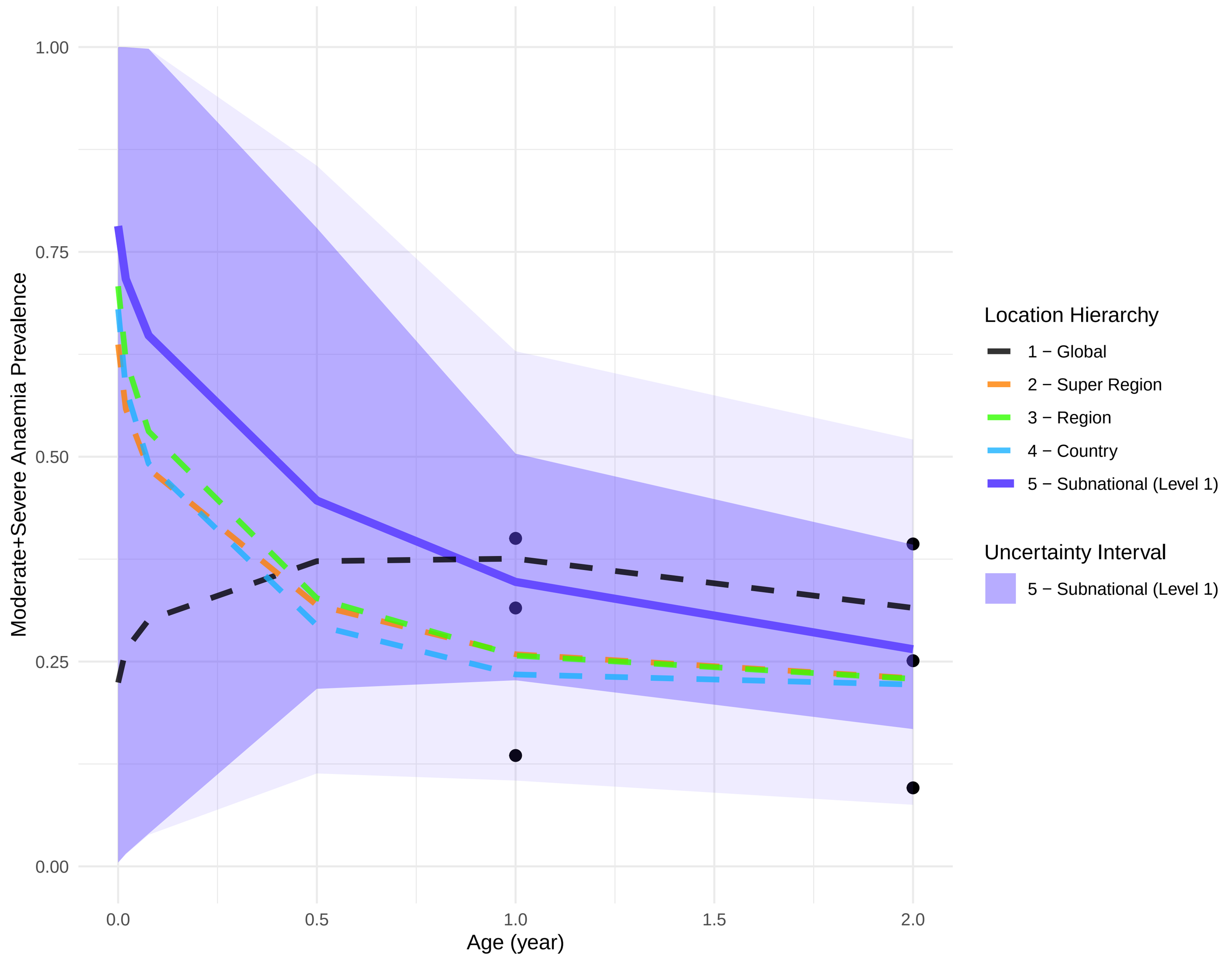

MR−BRT Age/Sex Fits − Males <2 Years Old − Moderate+Severe Anaemia Prevalence − Central Java
WHO−2024 Adjustment Method

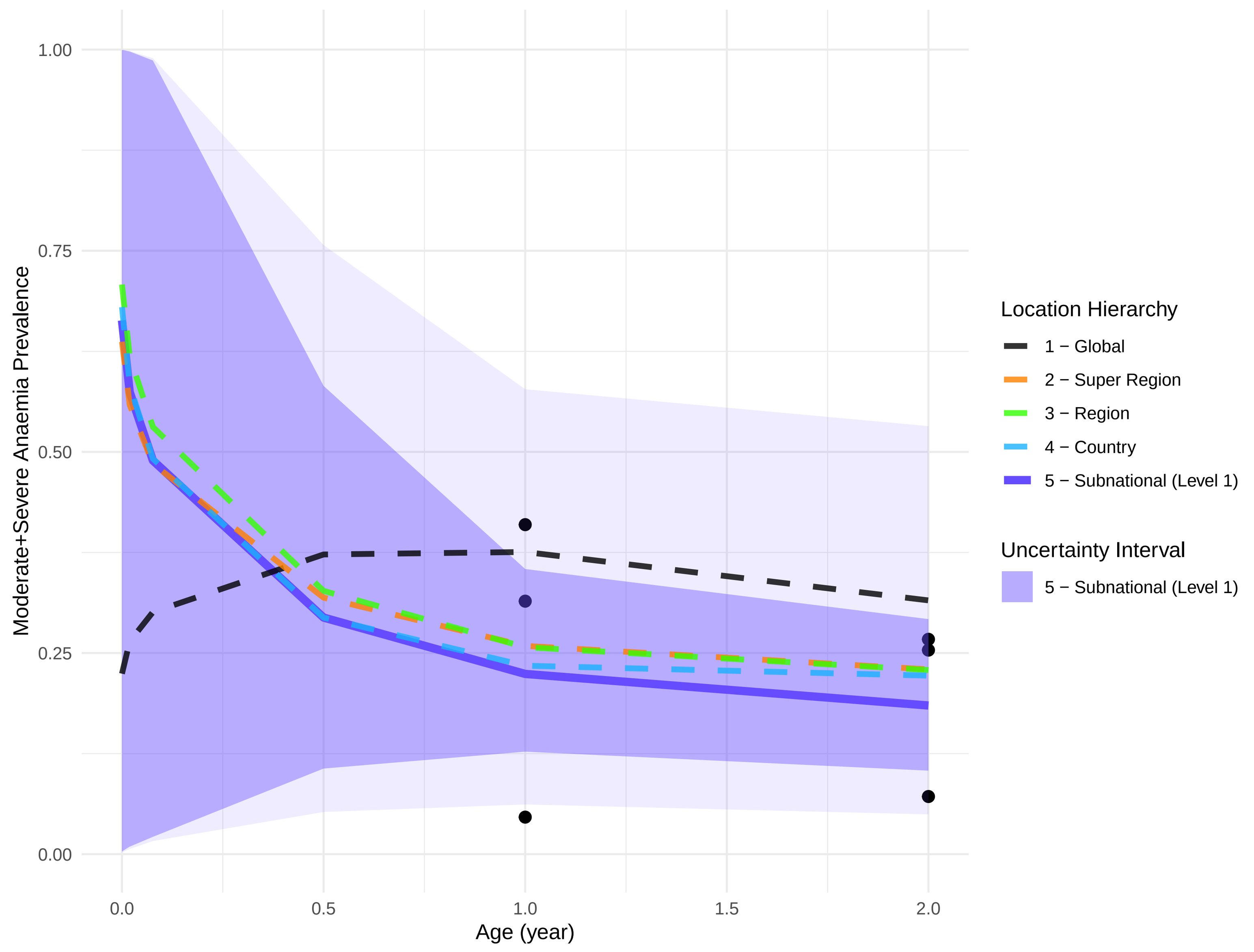

MR−BRT Age/Sex Fits − Males <2 Years Old − Moderate+Severe Anaemia Prevalence − East Java
WHO−2024 Adjustment Method

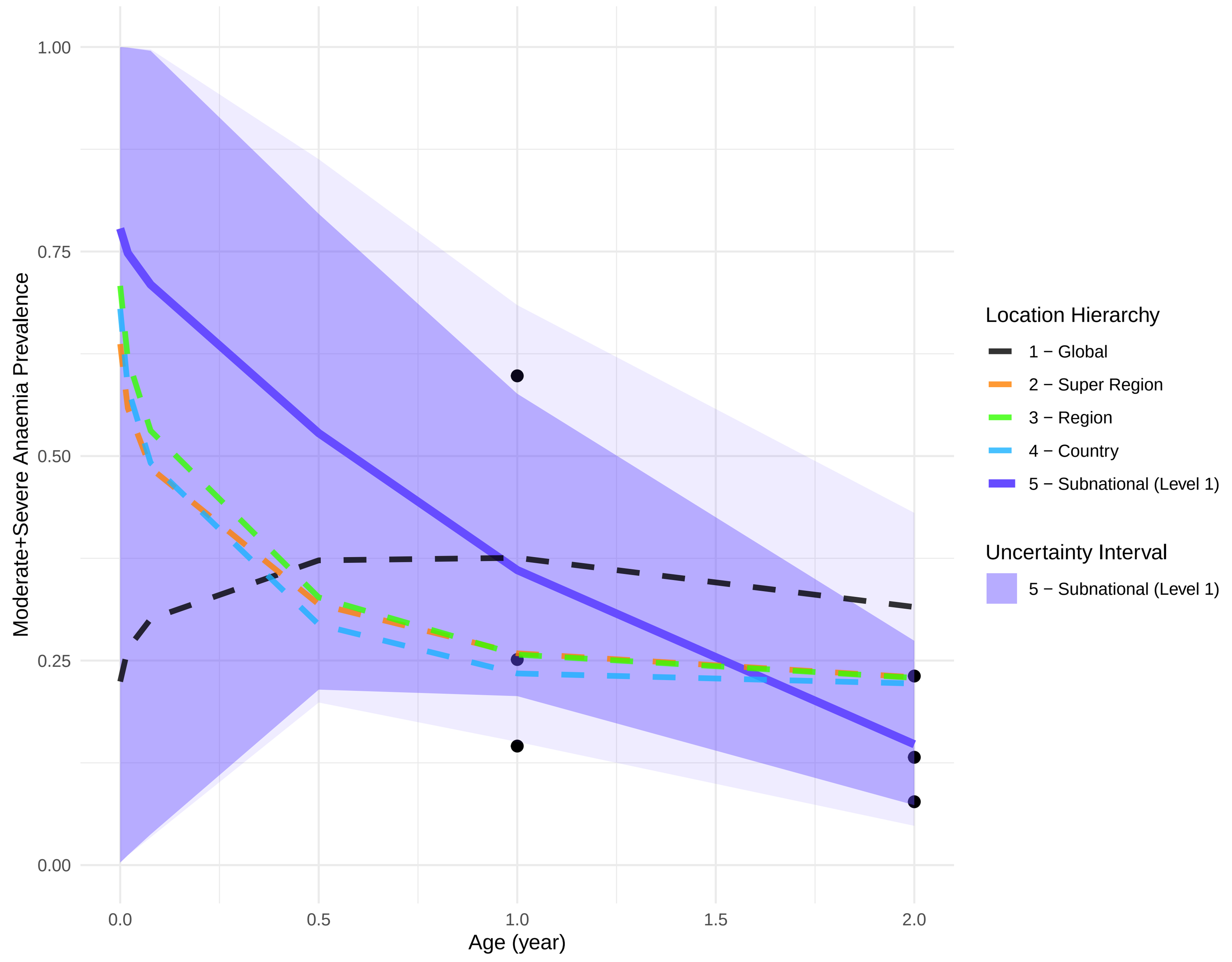

MR−BRT Age/Sex Fits − Males <2 Years Old − Moderate+Severe Anaemia Prevalence − South Kalimantan
WHO−2024 Adjustment Method

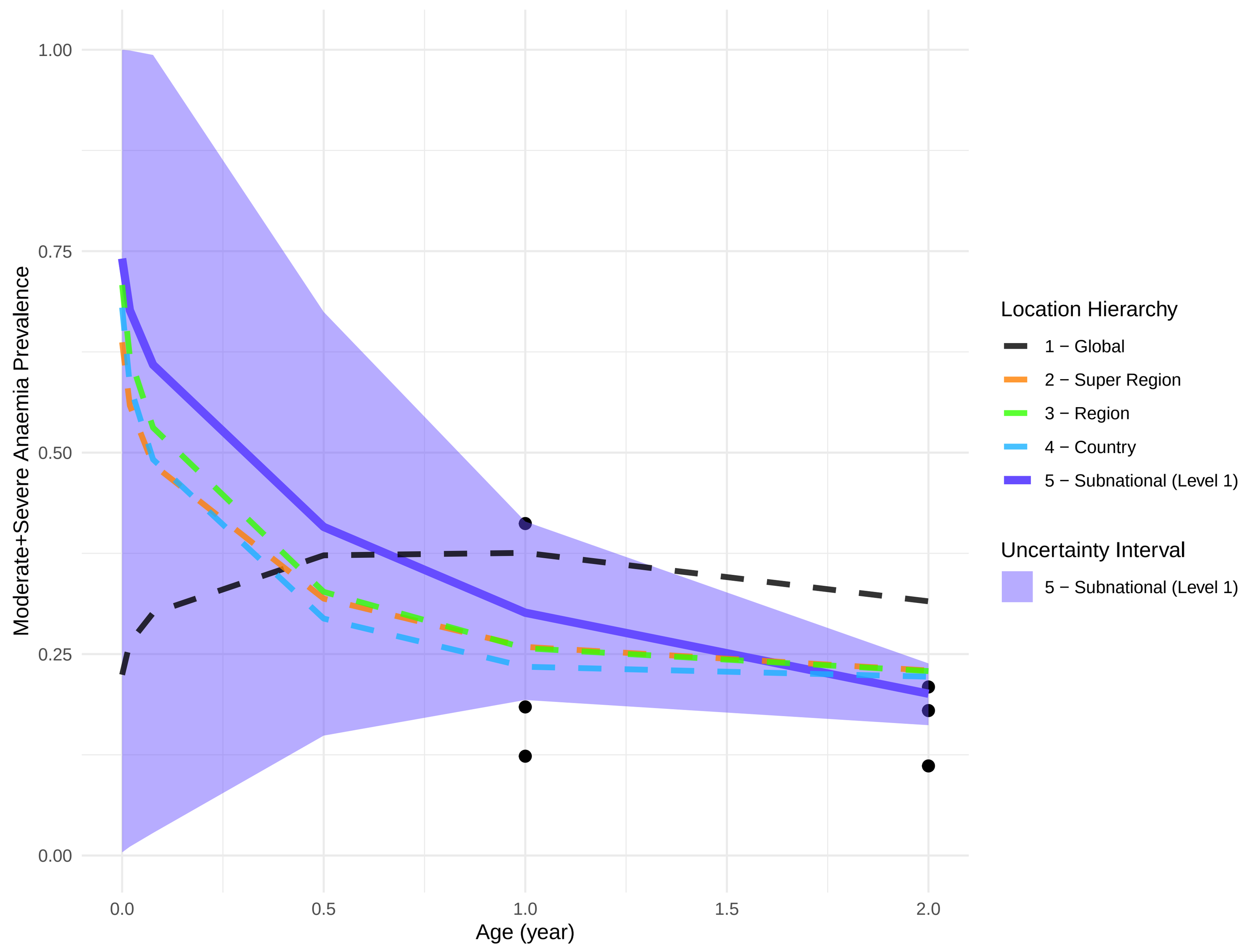

MR−BRT Age/Sex Fits − Males <2 Years Old − Moderate+Severe Anaemia Prevalence − Lampung
WHO−2024 Adjustment Method

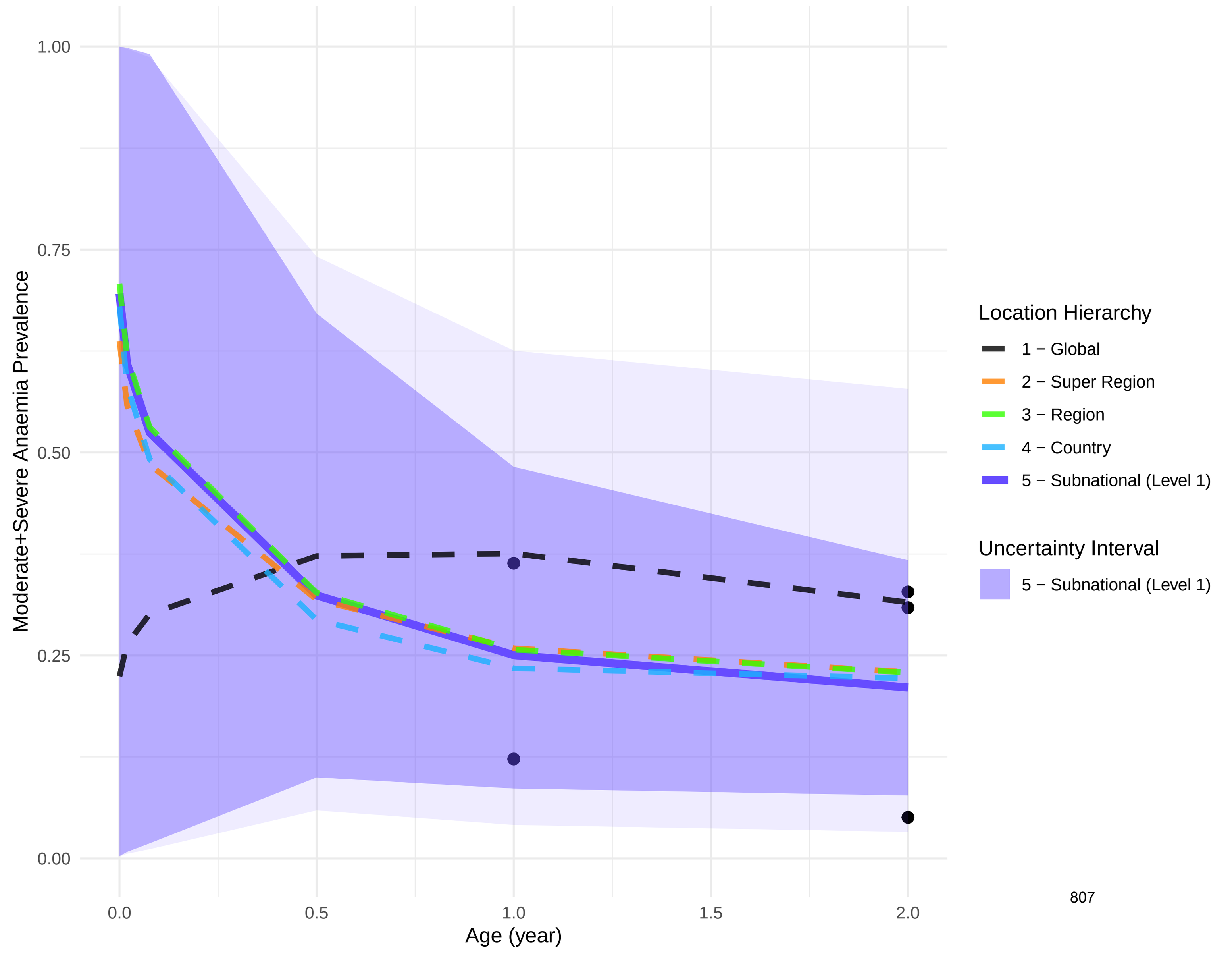

MR−BRT Age/Sex Fits − Males <2 Years Old − Moderate+Severe Anaemia Prevalence − West Nusa Tenggara
WHO−2024 Adjustment Method

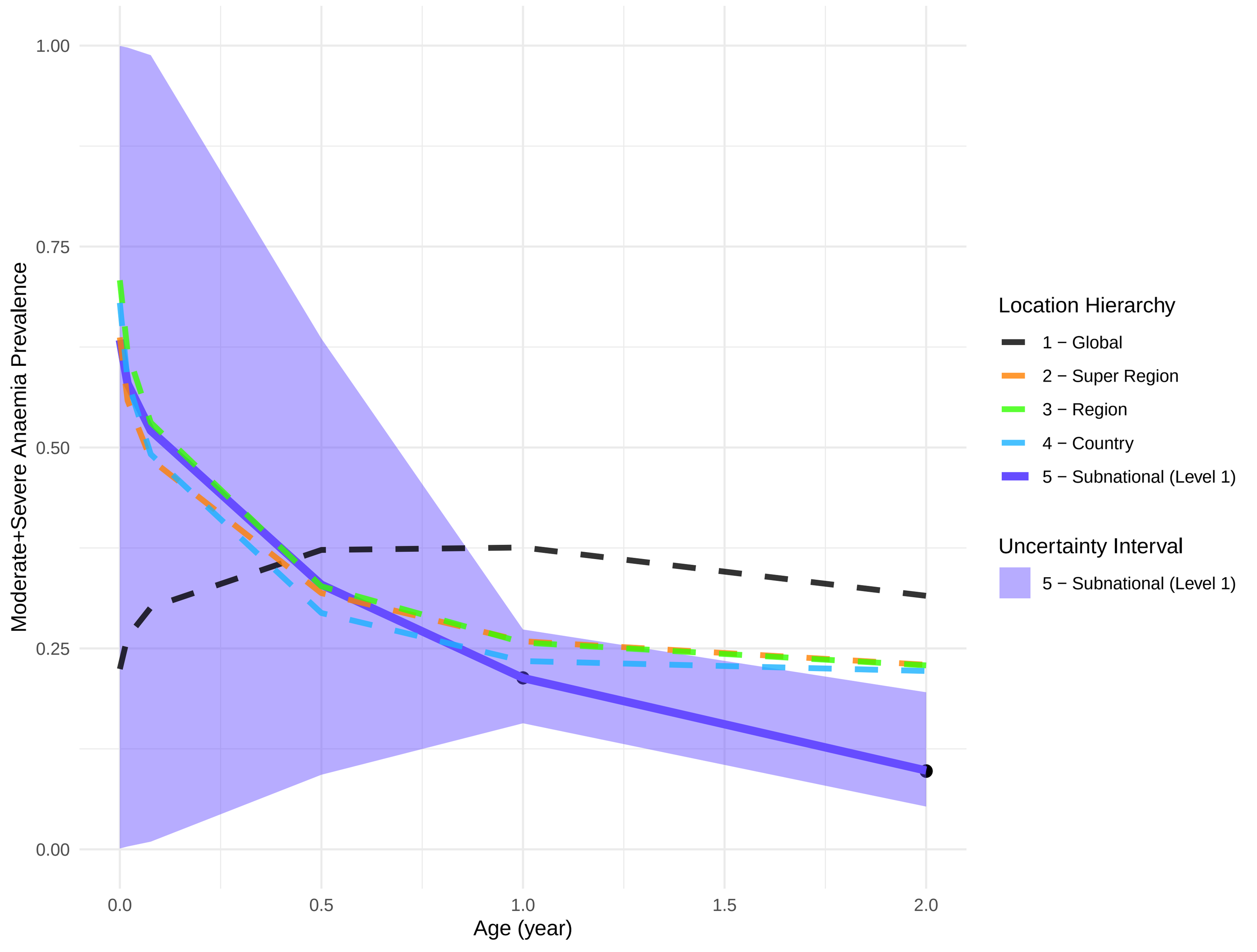

MR−BRT Age/Sex Fits − Males <2 Years Old − Moderate+Severe Anaemia Prevalence − Riau
WHO−2024 Adjustment Method

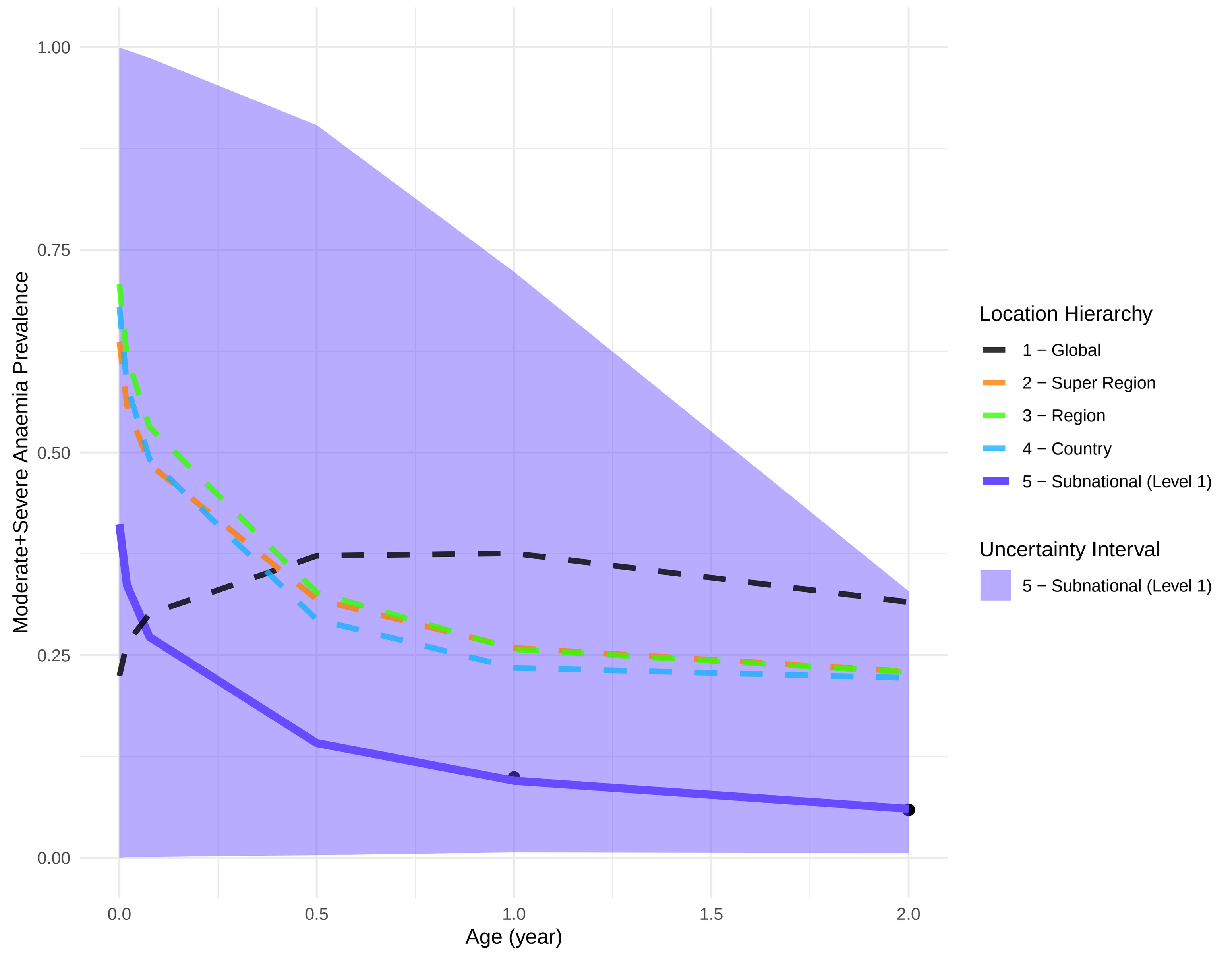

MR−BRT Age/Sex Fits − Males <2 Years Old − Moderate+Severe Anaemia Prevalence − South Sulawesi
WHO−2024 Adjustment Method

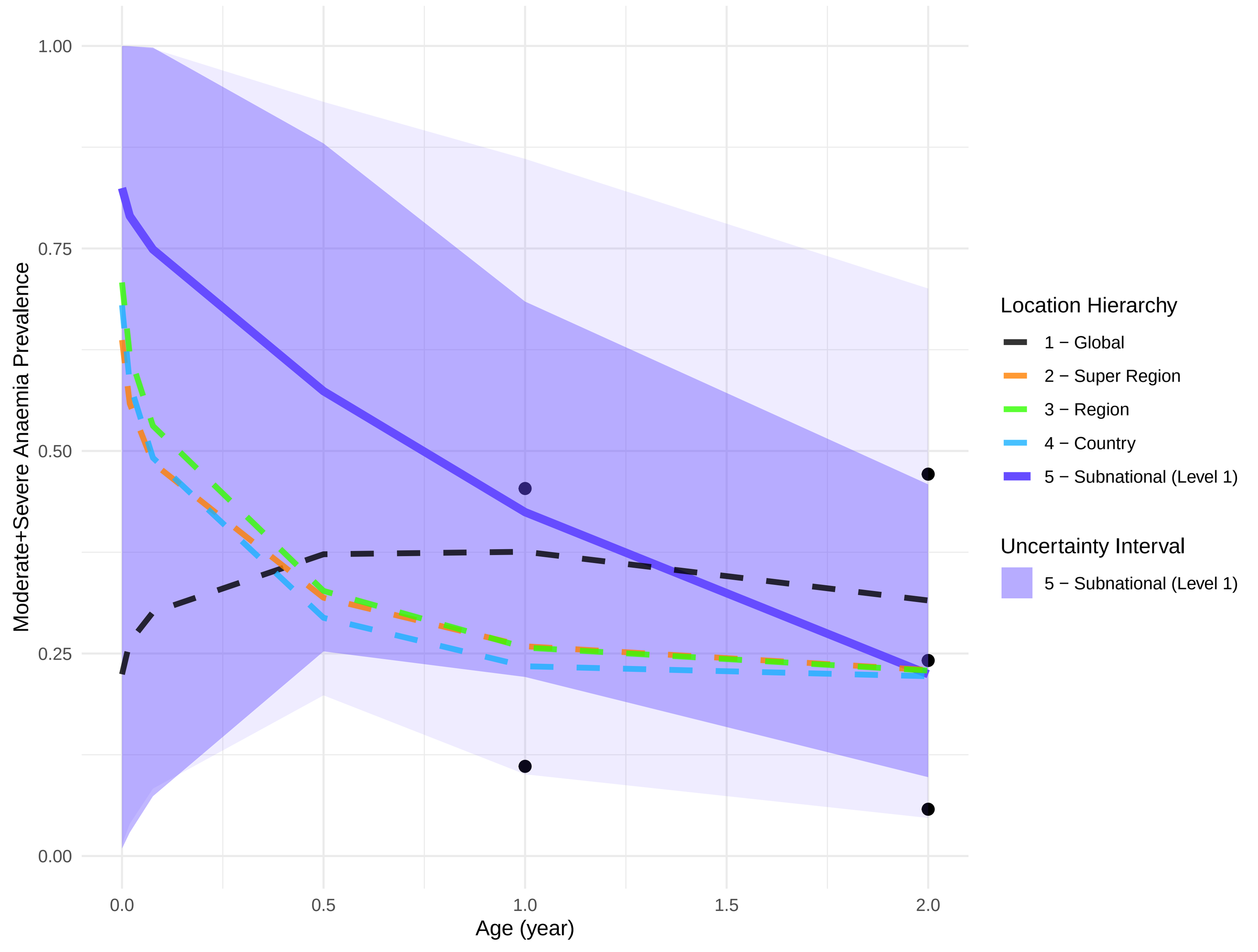

MR−BRT Age/Sex Fits − Males <2 Years Old − Moderate+Severe Anaemia Prevalence − West Sumatra
WHO−2024 Adjustment Method

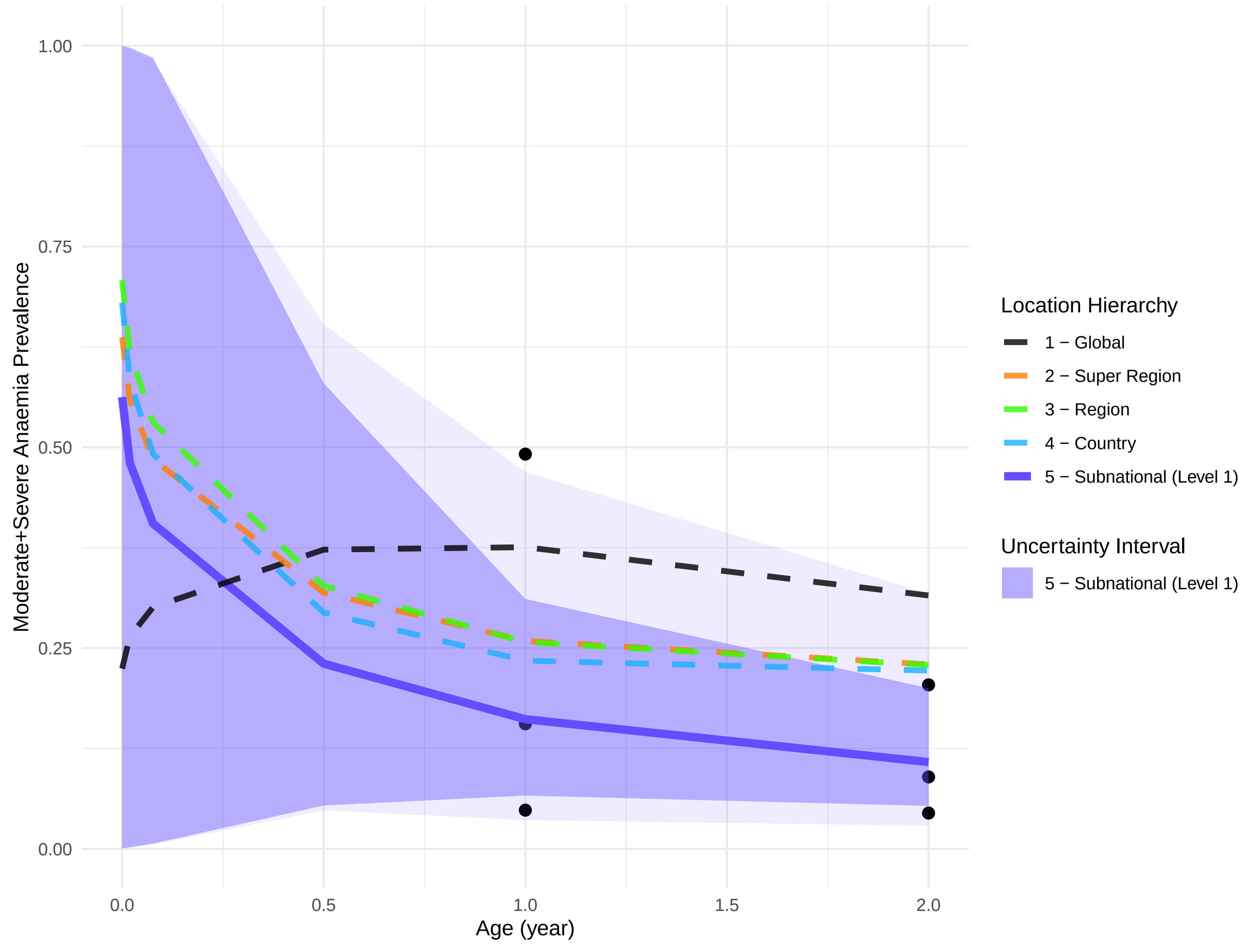

MR−BRT Age/Sex Fits − Males <2 Years Old − Moderate+Severe Anaemia Prevalence − South Sumatra
WHO−2024 Adjustment Method

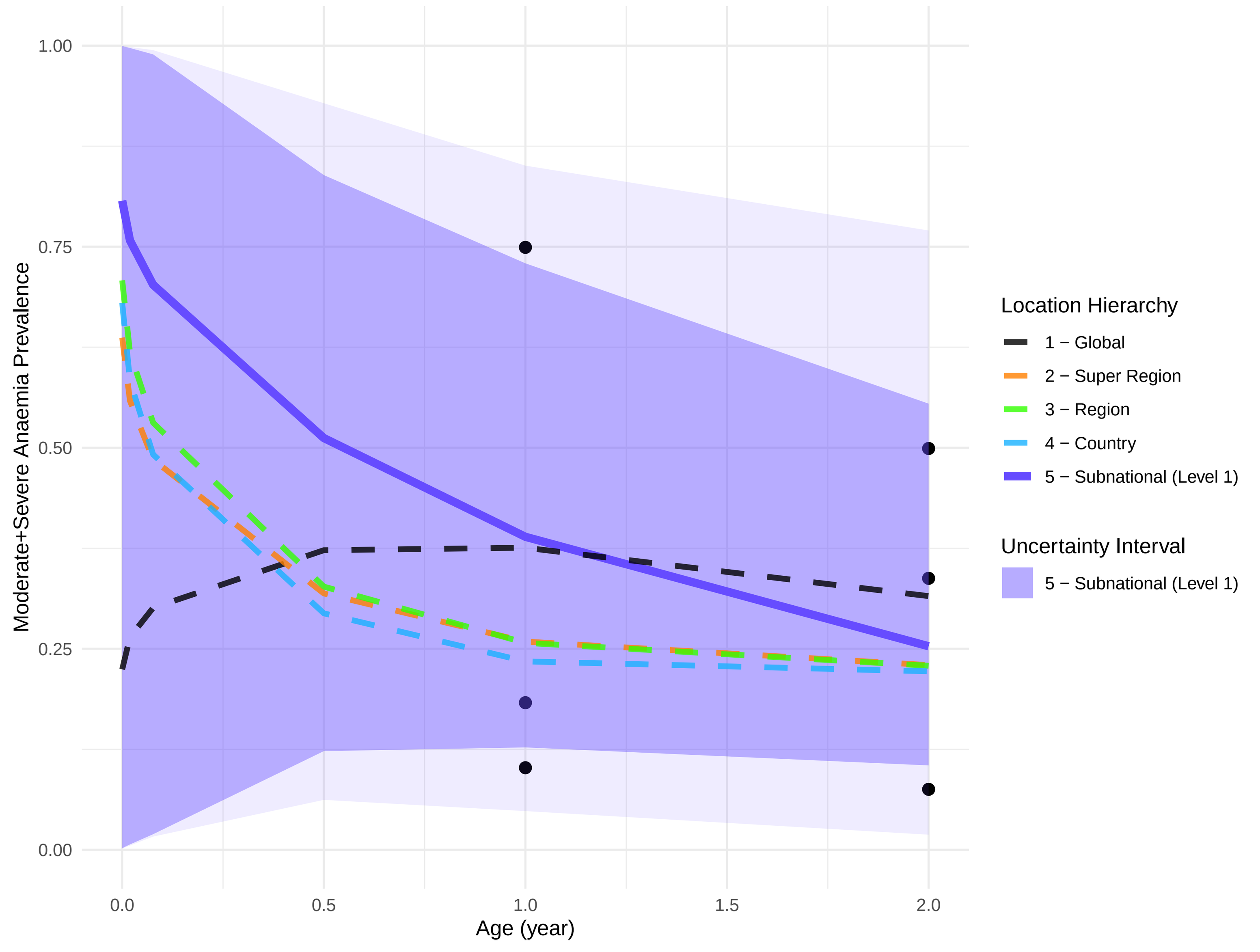

MR−BRT Age/Sex Fits − Males <2 Years Old − Moderate+Severe Anaemia Prevalence − North Sumatra
WHO−2024 Adjustment Method

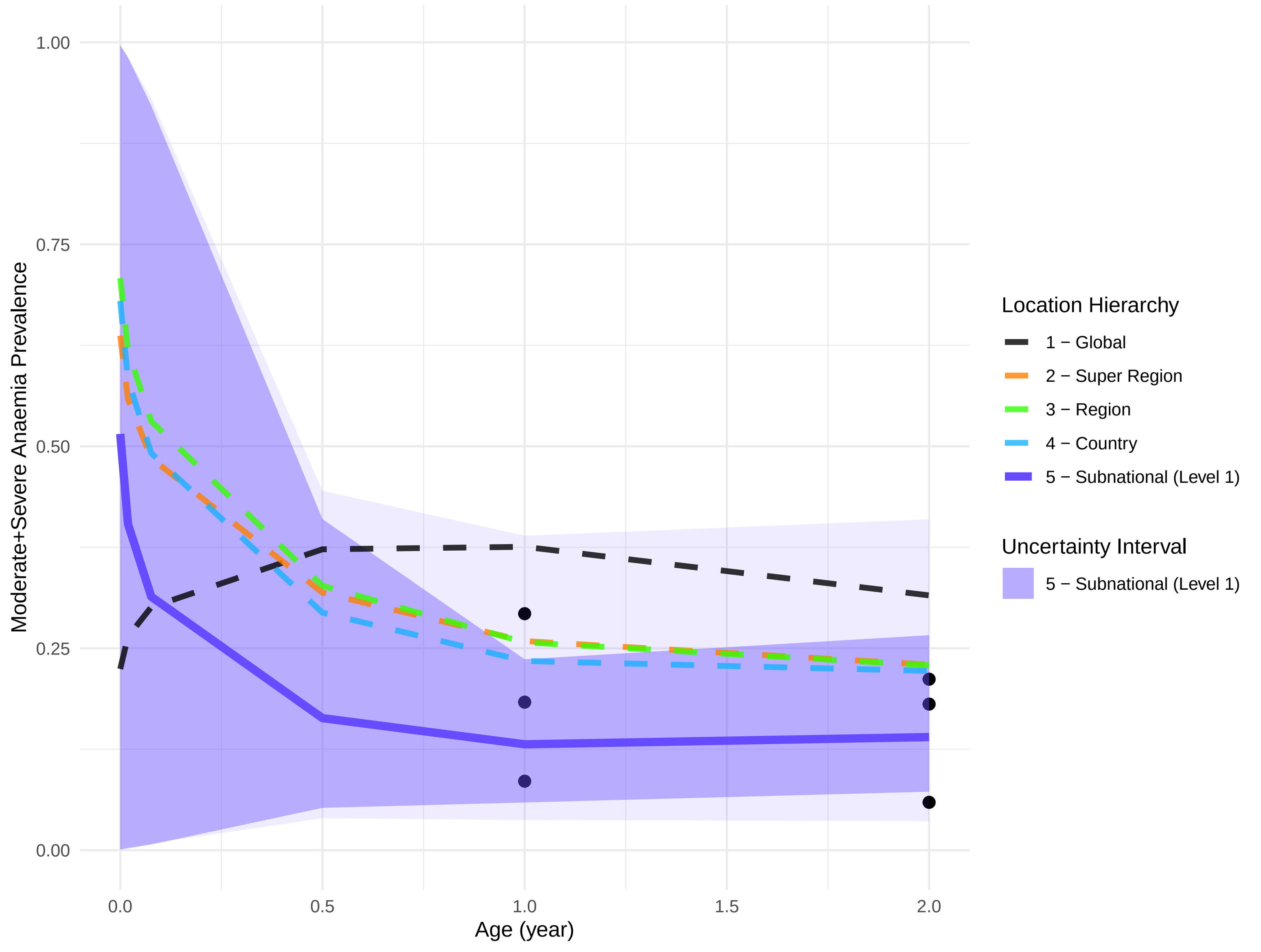

MR−BRT Age/Sex Fits − Males <2 Years Old − Moderate+Severe Anaemia Prevalence − Yogyakarta
WHO−2024 Adjustment Method

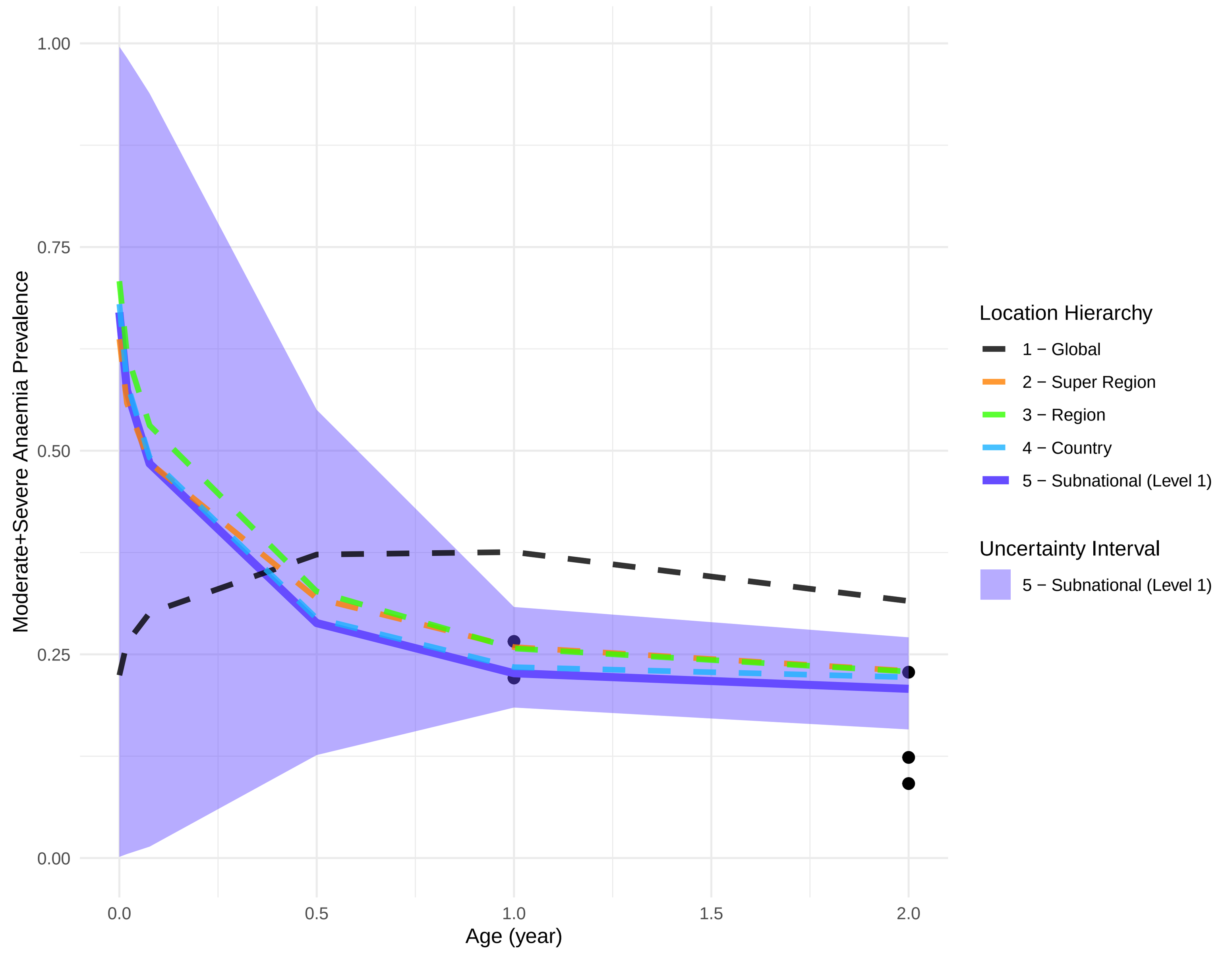

MR−BRT Age/Sex Fits − Males <2 Years Old − Moderate+Severe Anaemia Prevalence − Myanmar
WHO−2024 Adjustment Method

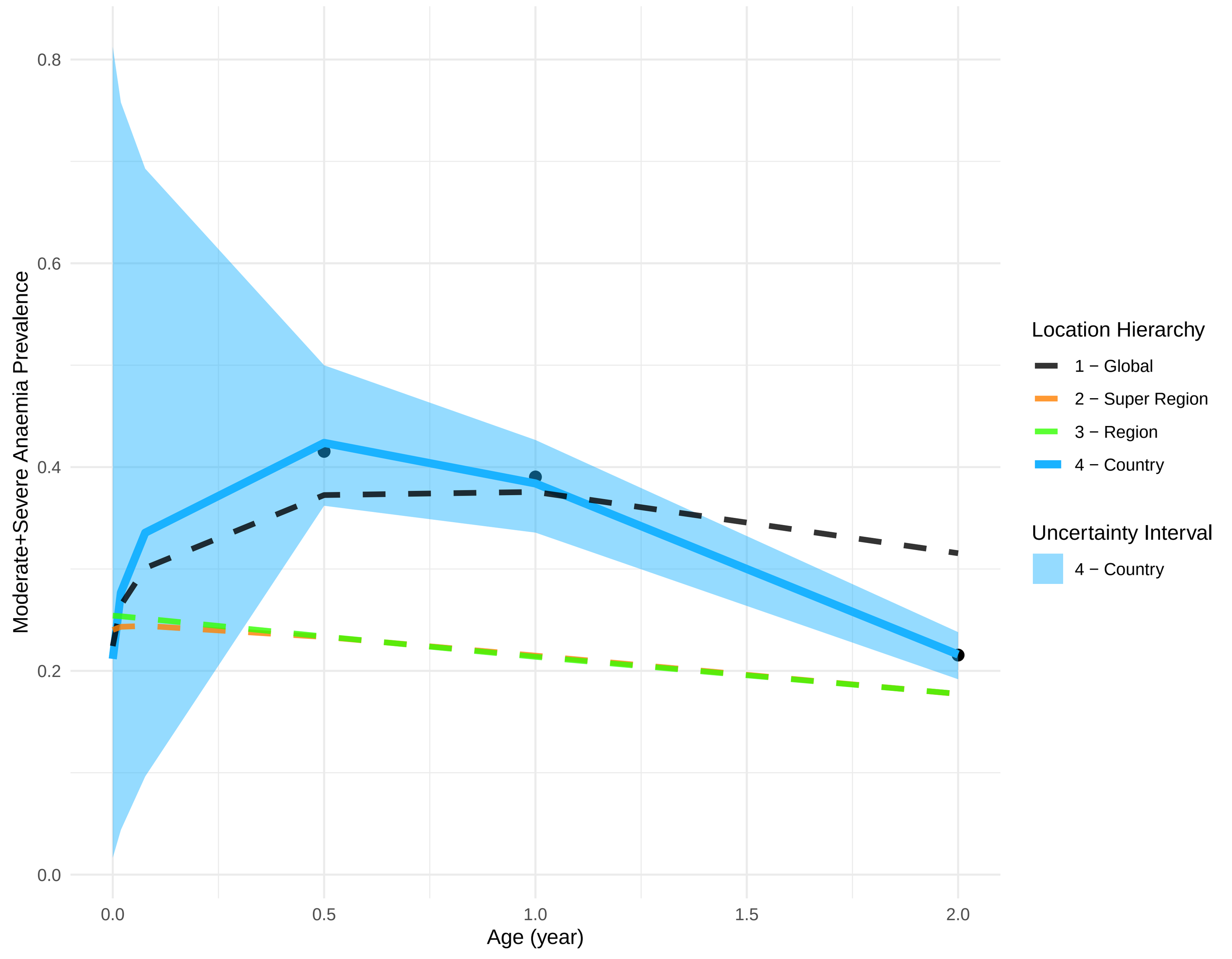

MR−BRT Age/Sex Fits − Males <2 Years Old − Moderate+Severe Anaemia Prevalence − Timor−Leste
WHO−2024 Adjustment Method

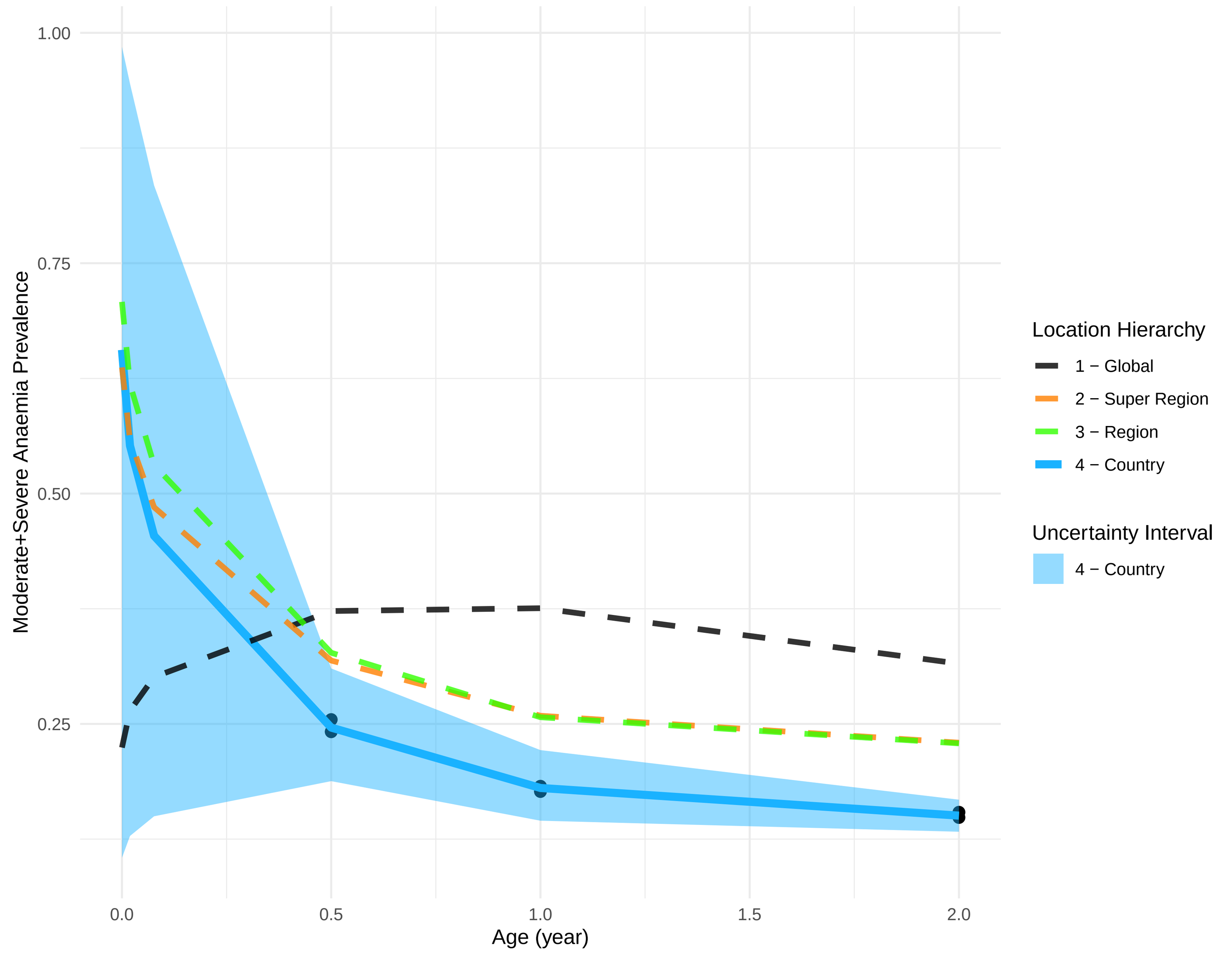

MR−BRT Age/Sex Fits − Males <2 Years Old − Moderate+Severe Anaemia Prevalence − Sub−Saharan Africa
WHO−2024 Adjustment Method

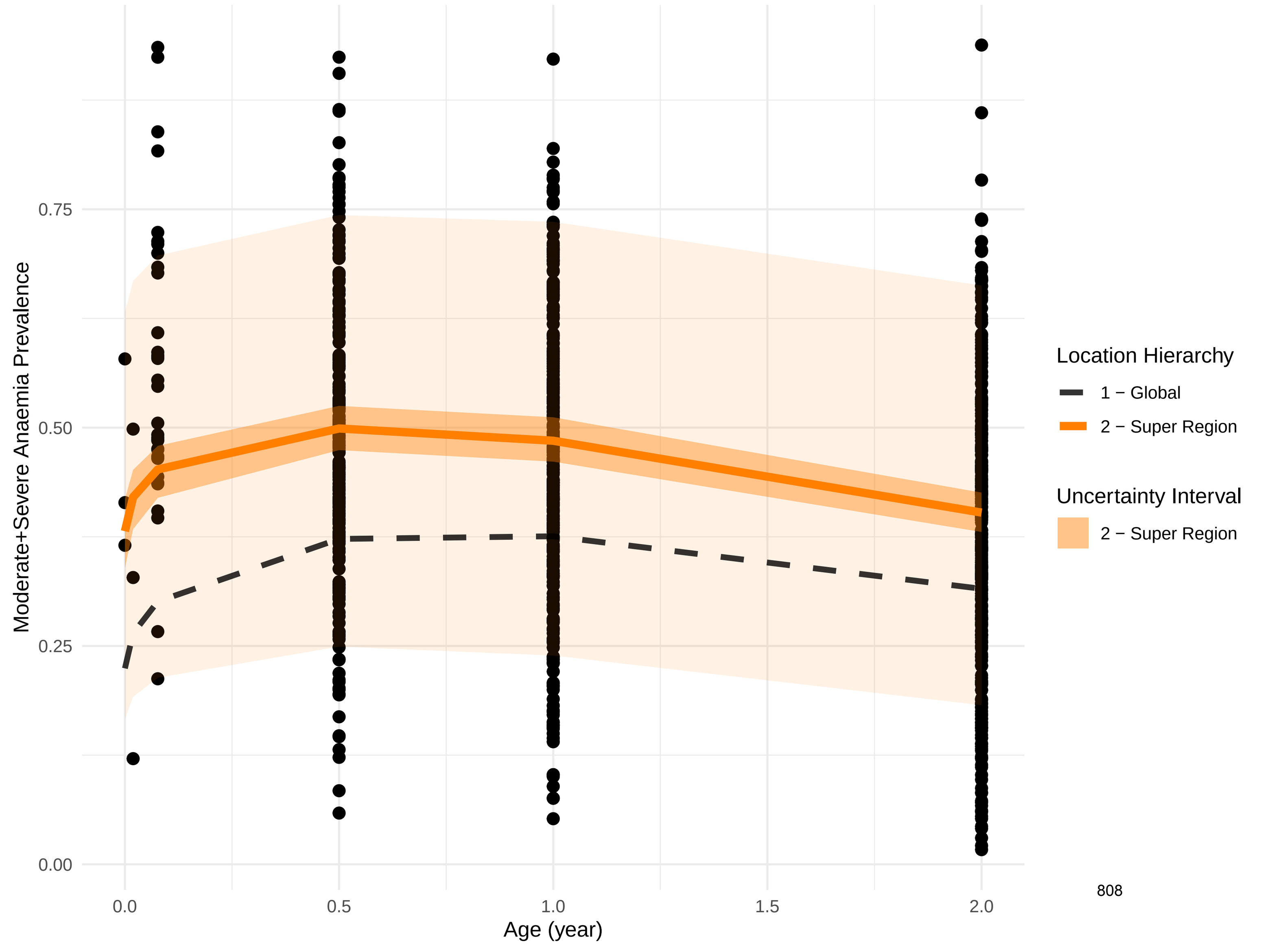

MR−BRT Age/Sex Fits − Males <2 Years Old − Moderate+Severe Anaemia Prevalence − Central Sub−Saharan Africa
WHO−2024 Adjustment Method

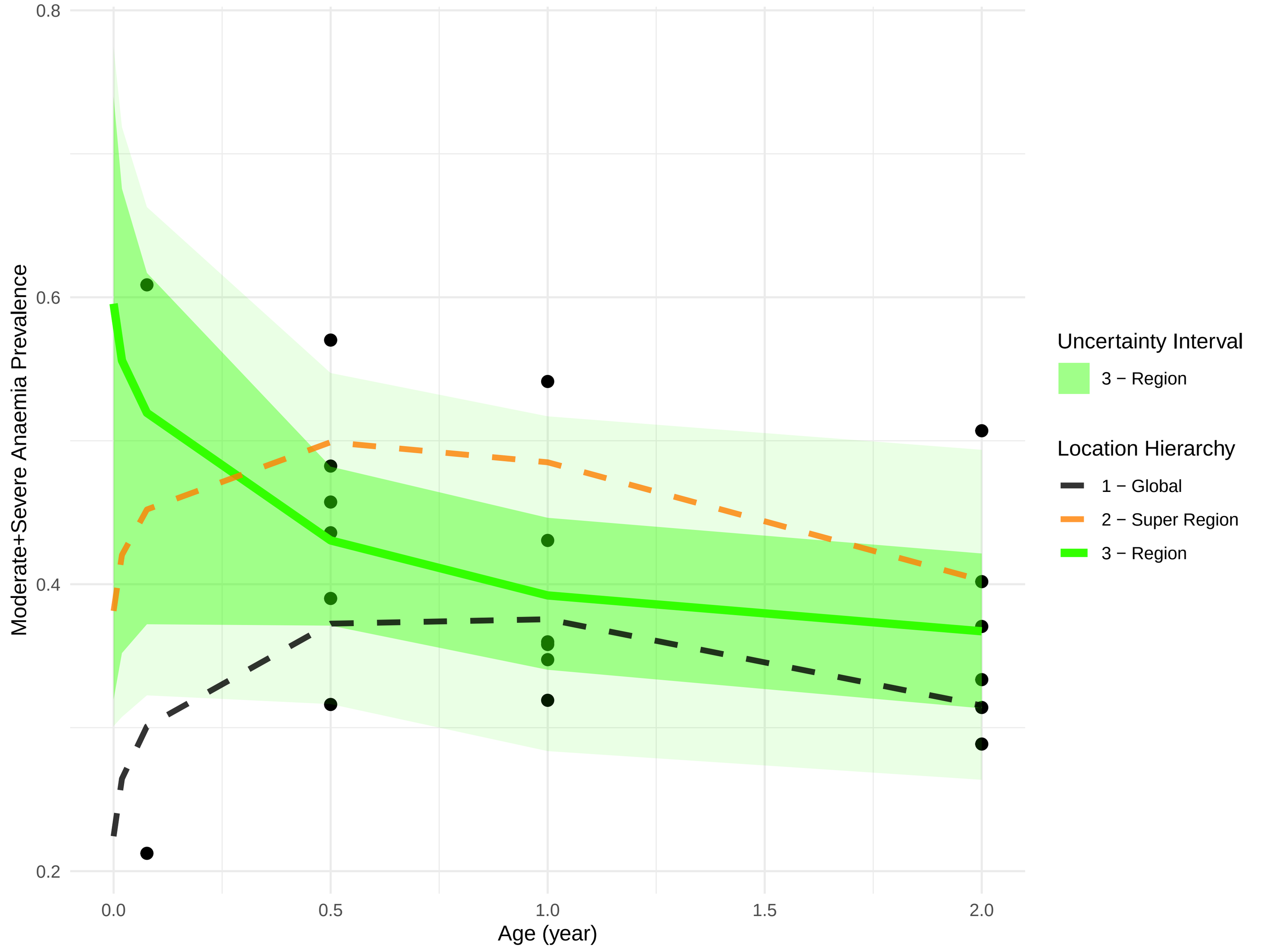

MR−BRT Age/Sex Fits − Males <2 Years Old − Moderate+Severe Anaemia Prevalence − Angola
WHO−2024 Adjustment Method

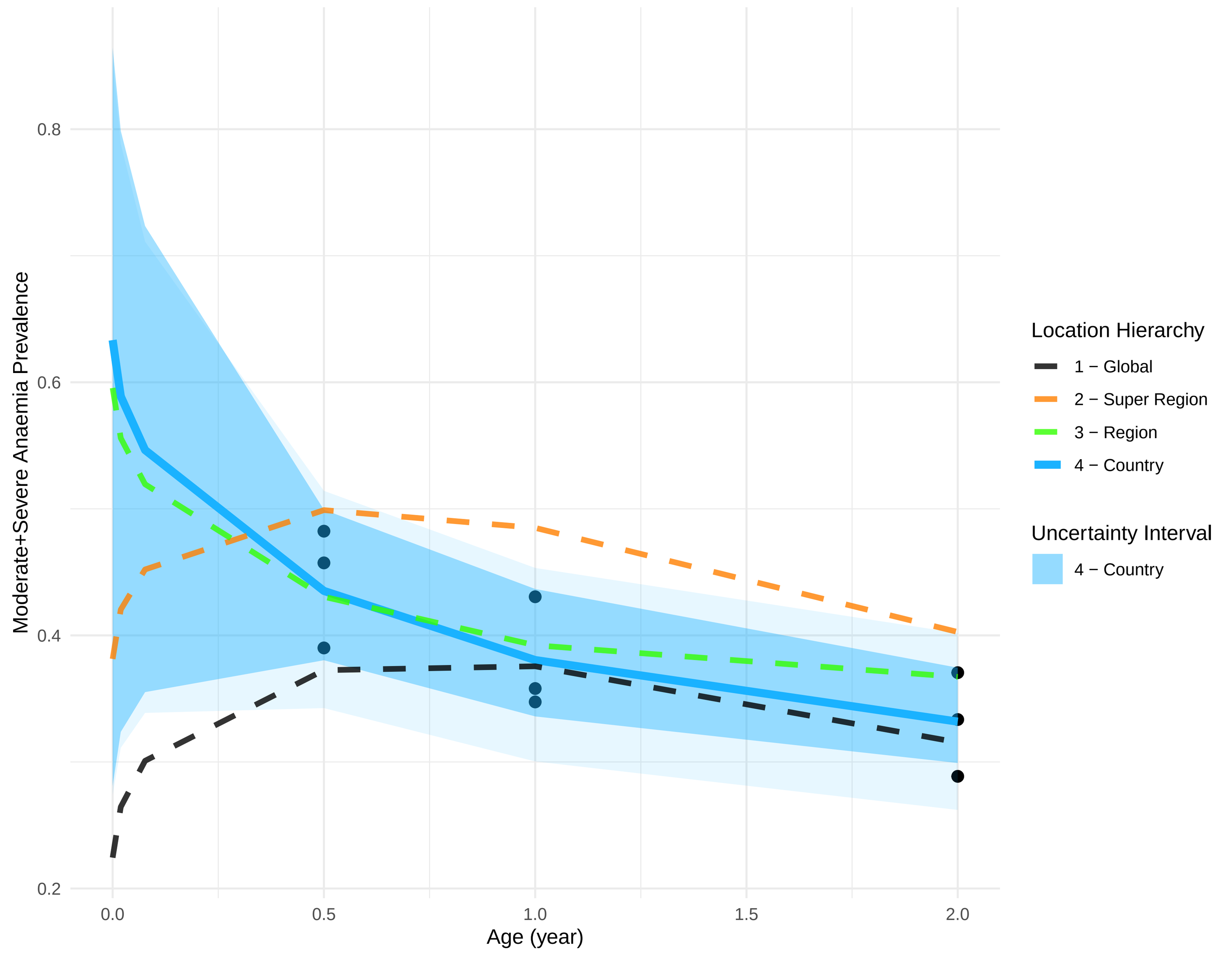

MR−BRT Age/Sex Fits − Males <2 Years Old − Moderate+Severe Anaemia Prevalence − Democratic Republic of the Congo
WHO−2024 Adjustment Method

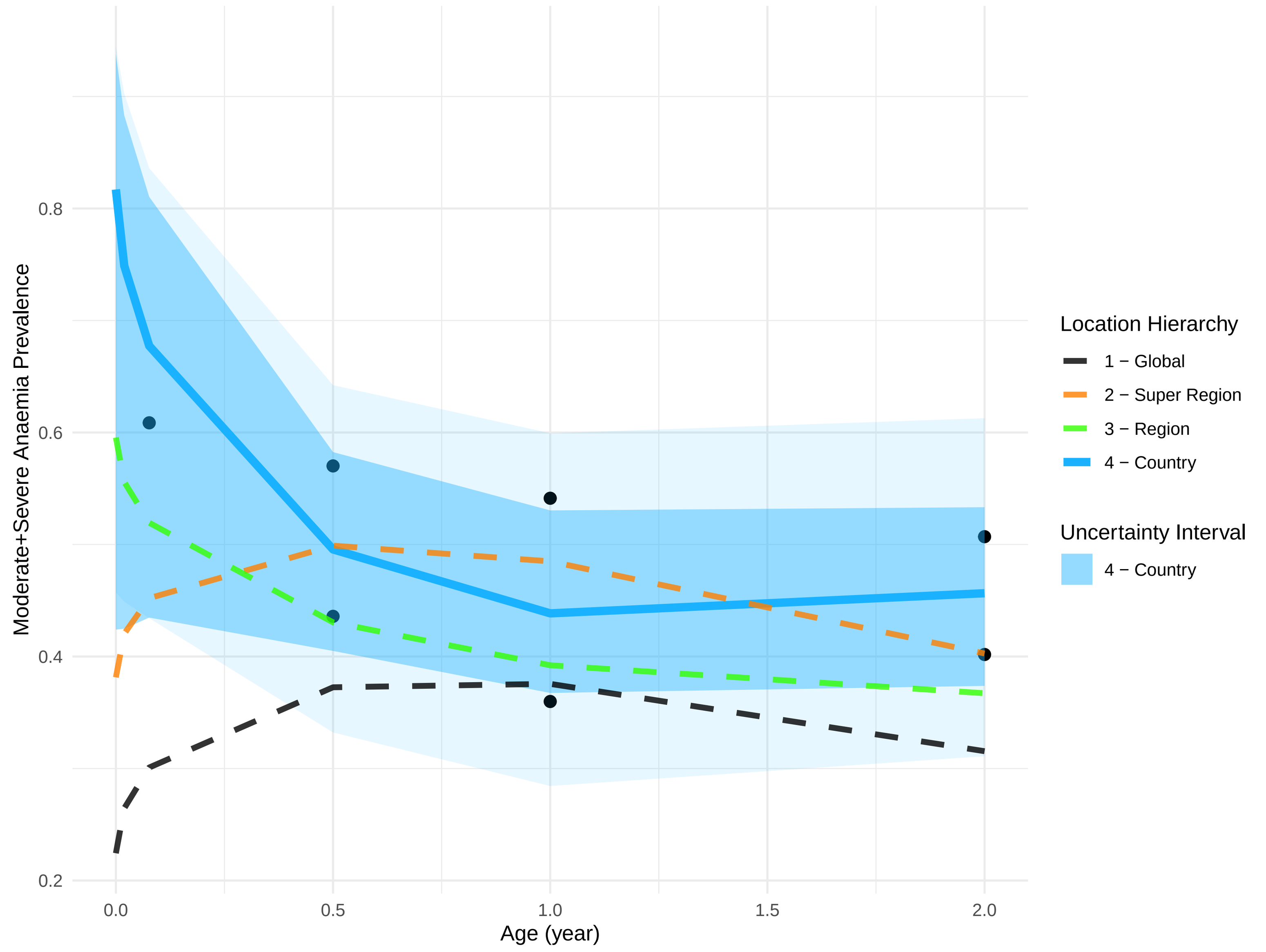

MR−BRT Age/Sex Fits − Males <2 Years Old − Moderate+Severe Anaemia Prevalence − Gabon
WHO−2024 Adjustment Method

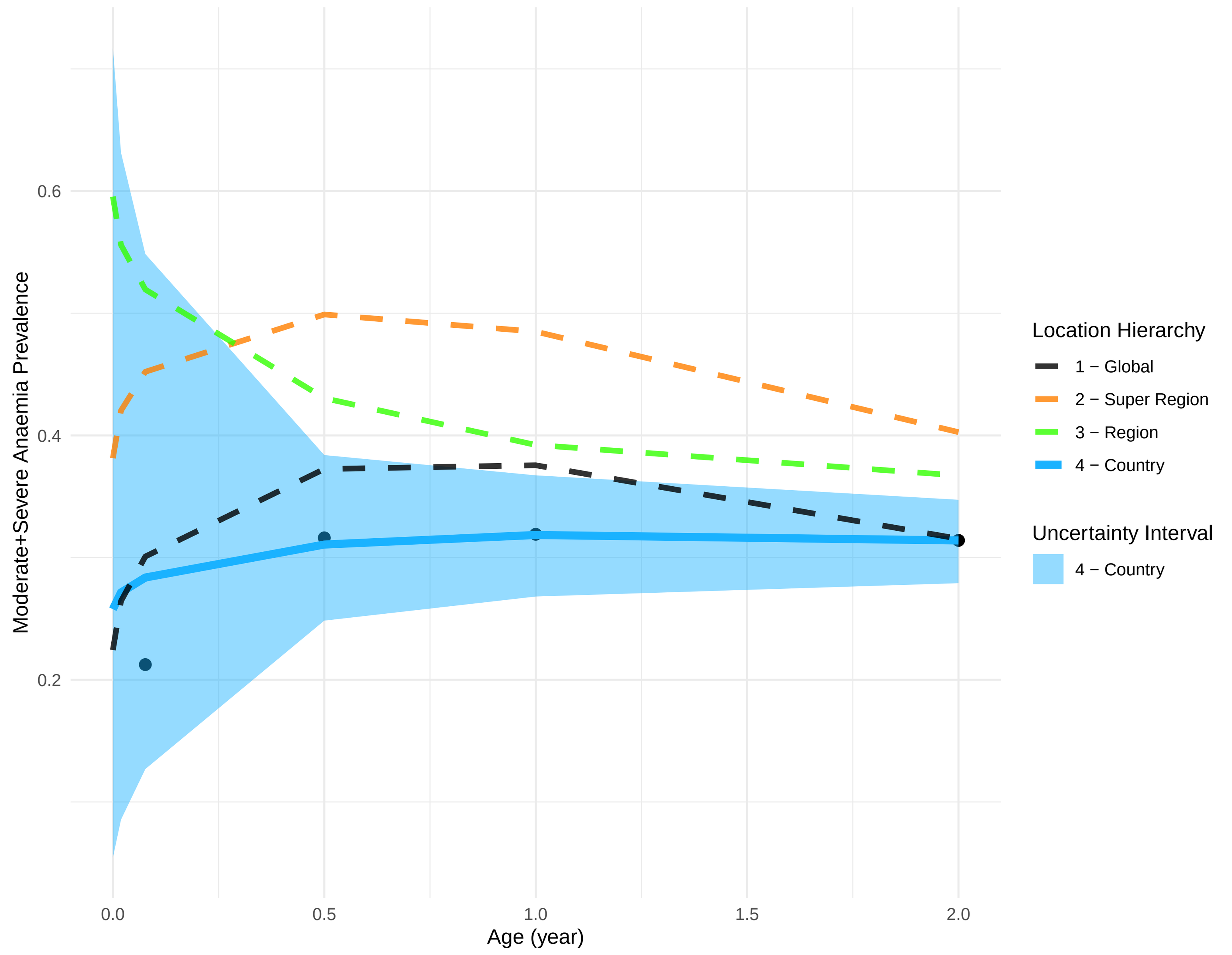

MR−BRT Age/Sex Fits − Males <2 Years Old − Moderate+Severe Anaemia Prevalence − Eastern Sub−Saharan Africa
WHO−2024 Adjustment Method

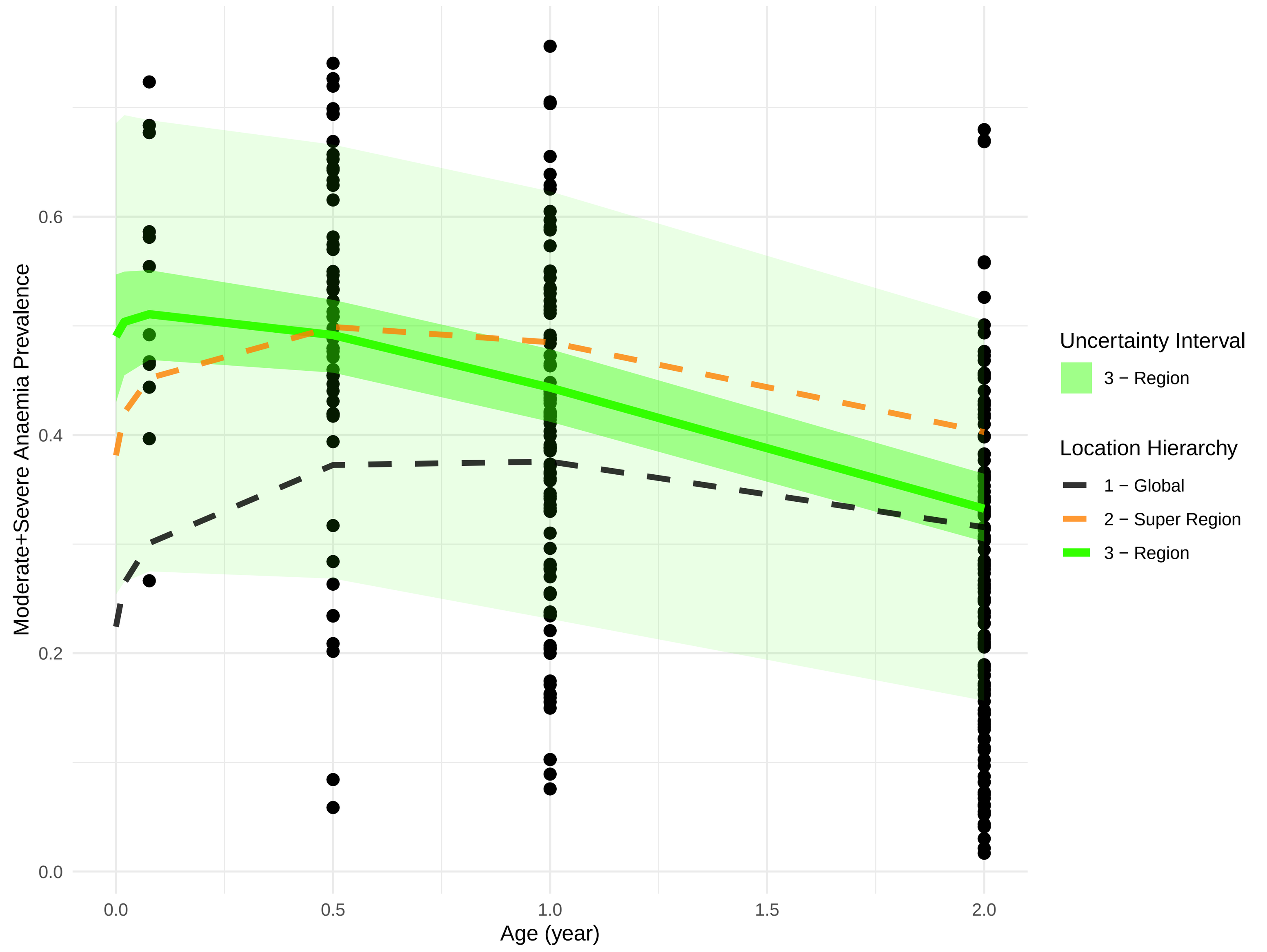

MR−BRT Age/Sex Fits − Males <2 Years Old − Moderate+Severe Anaemia Prevalence − Burundi
WHO−2024 Adjustment Method

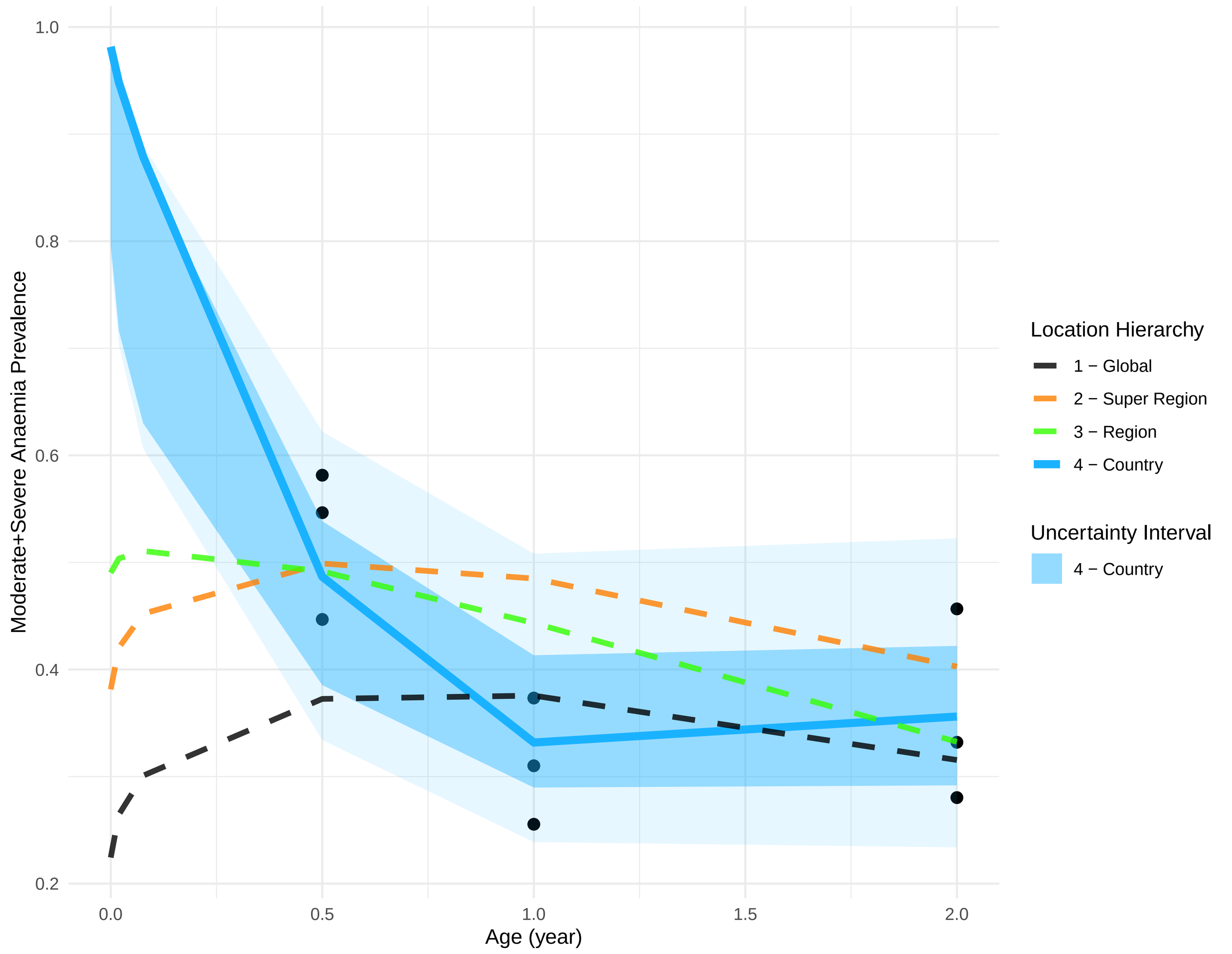

MR−BRT Age/Sex Fits − Males <2 Years Old − Moderate+Severe Anaemia Prevalence − Ethiopia
WHO−2024 Adjustment Method

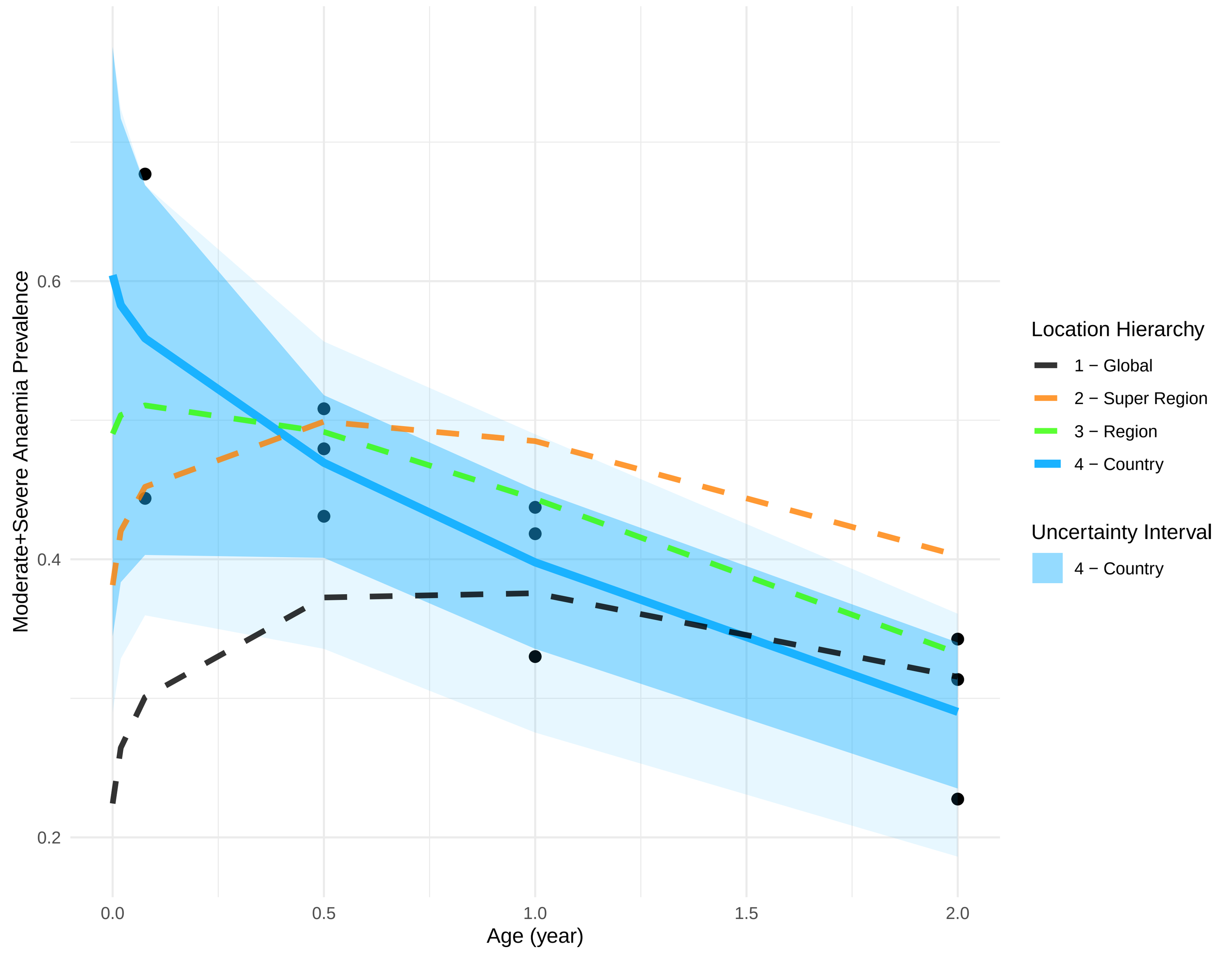

MR−BRT Age/Sex Fits − Males <2 Years Old − Moderate+Severe Anaemia Prevalence − Kenya
WHO−2024 Adjustment Method

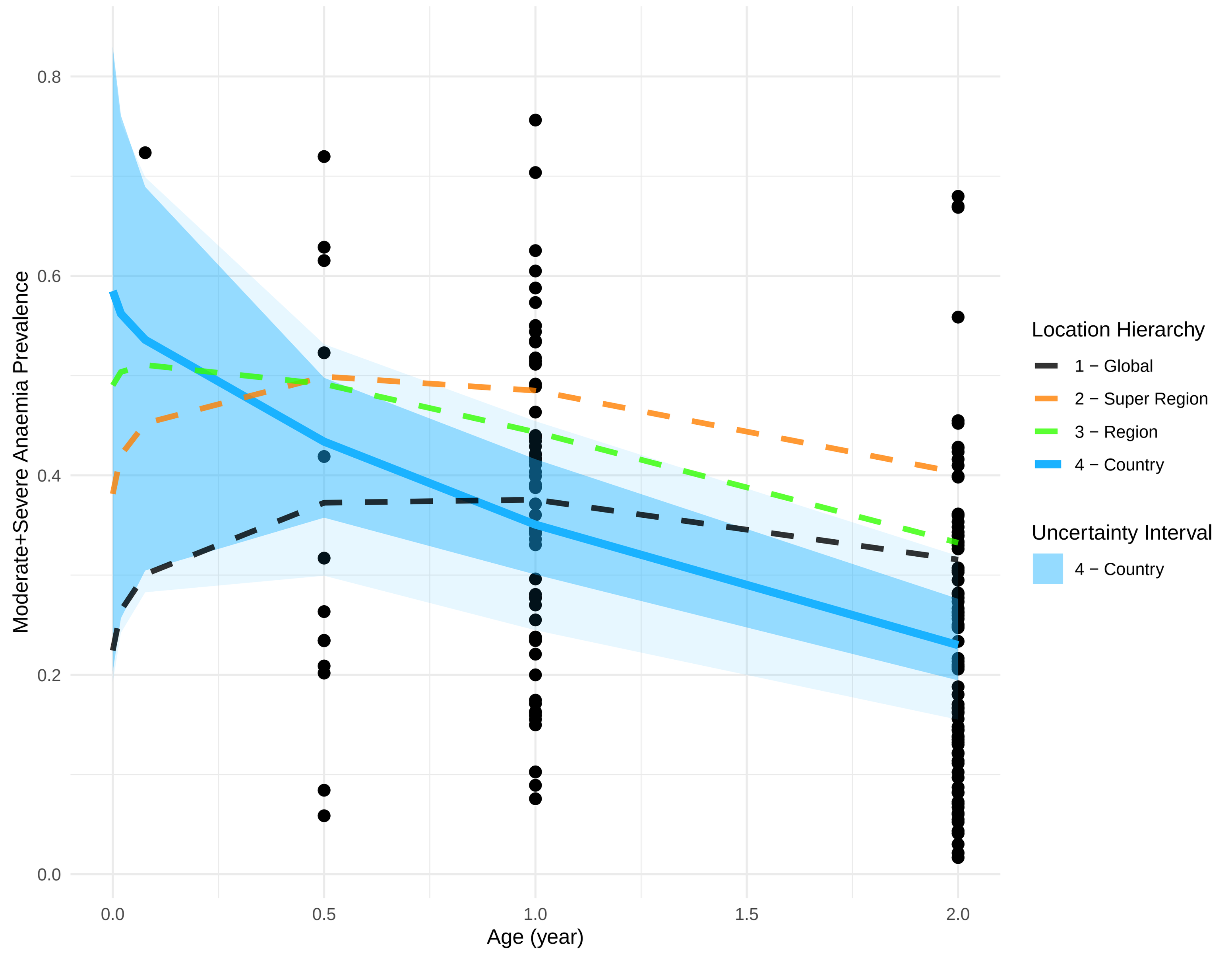

MR−BRT Age/Sex Fits − Males <2 Years Old − Moderate+Severe Anaemia Prevalence − Baringo
WHO−2024 Adjustment Method

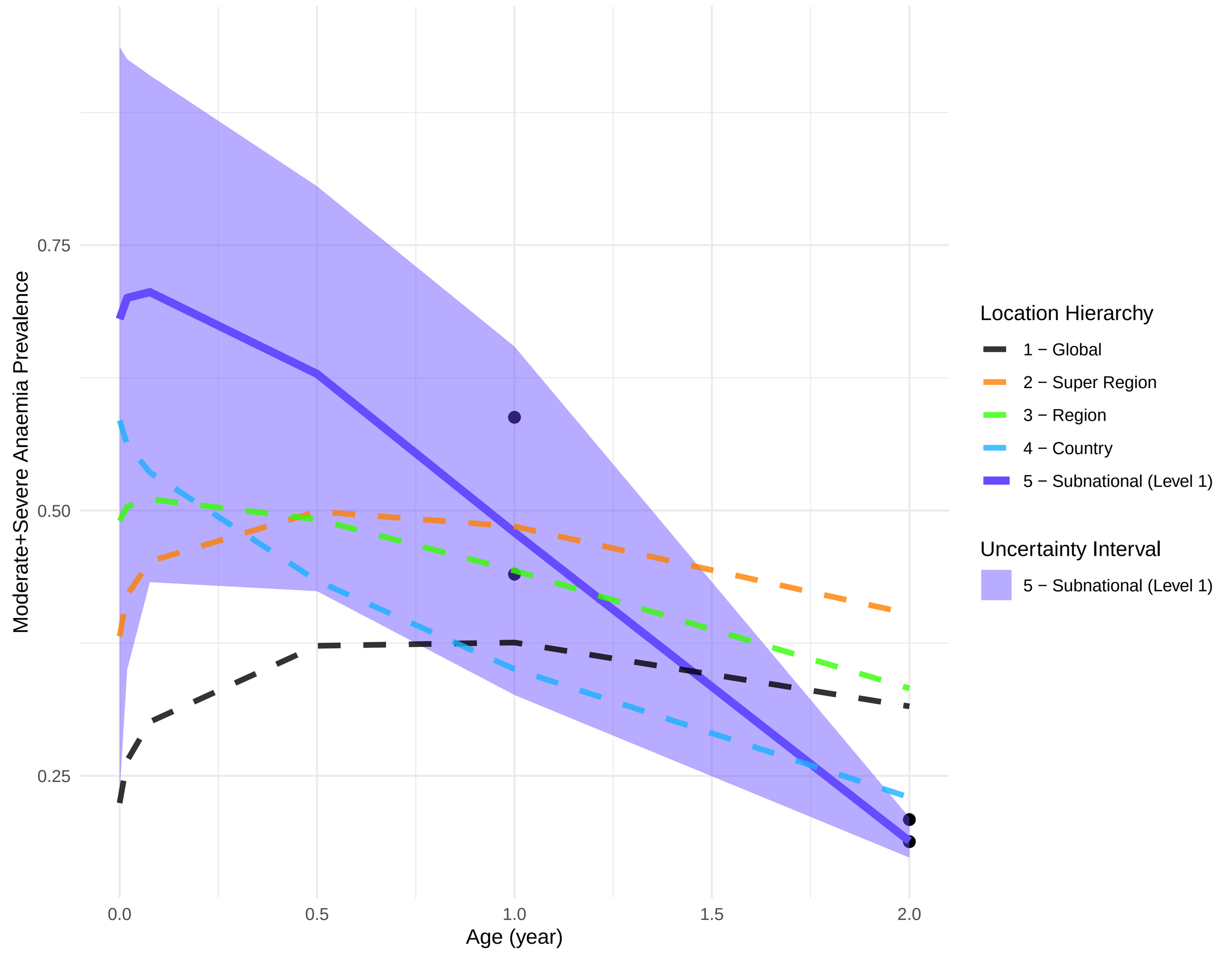

MR−BRT Age/Sex Fits − Males <2 Years Old − Moderate+Severe Anaemia Prevalence − Bomet
WHO−2024 Adjustment Method

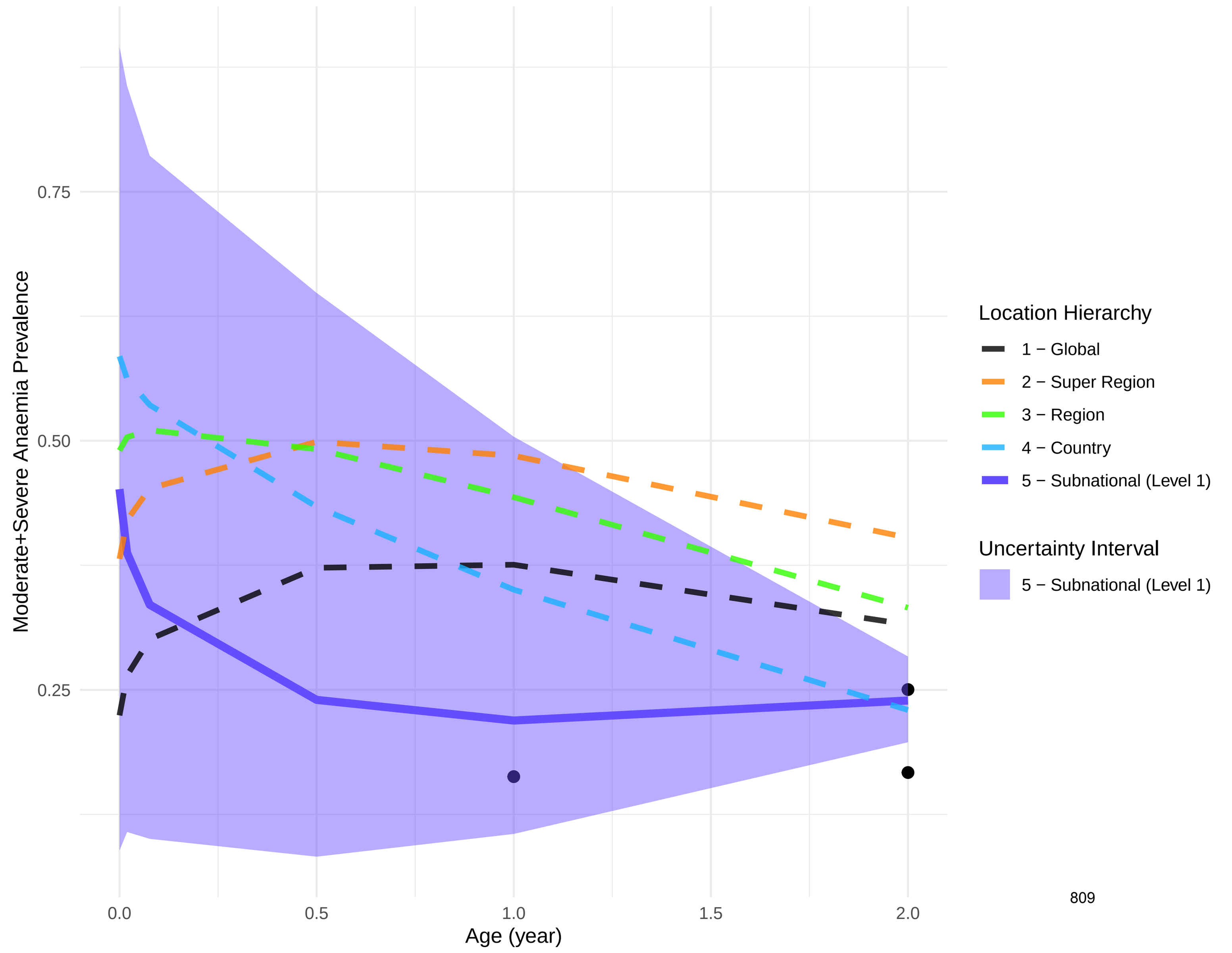

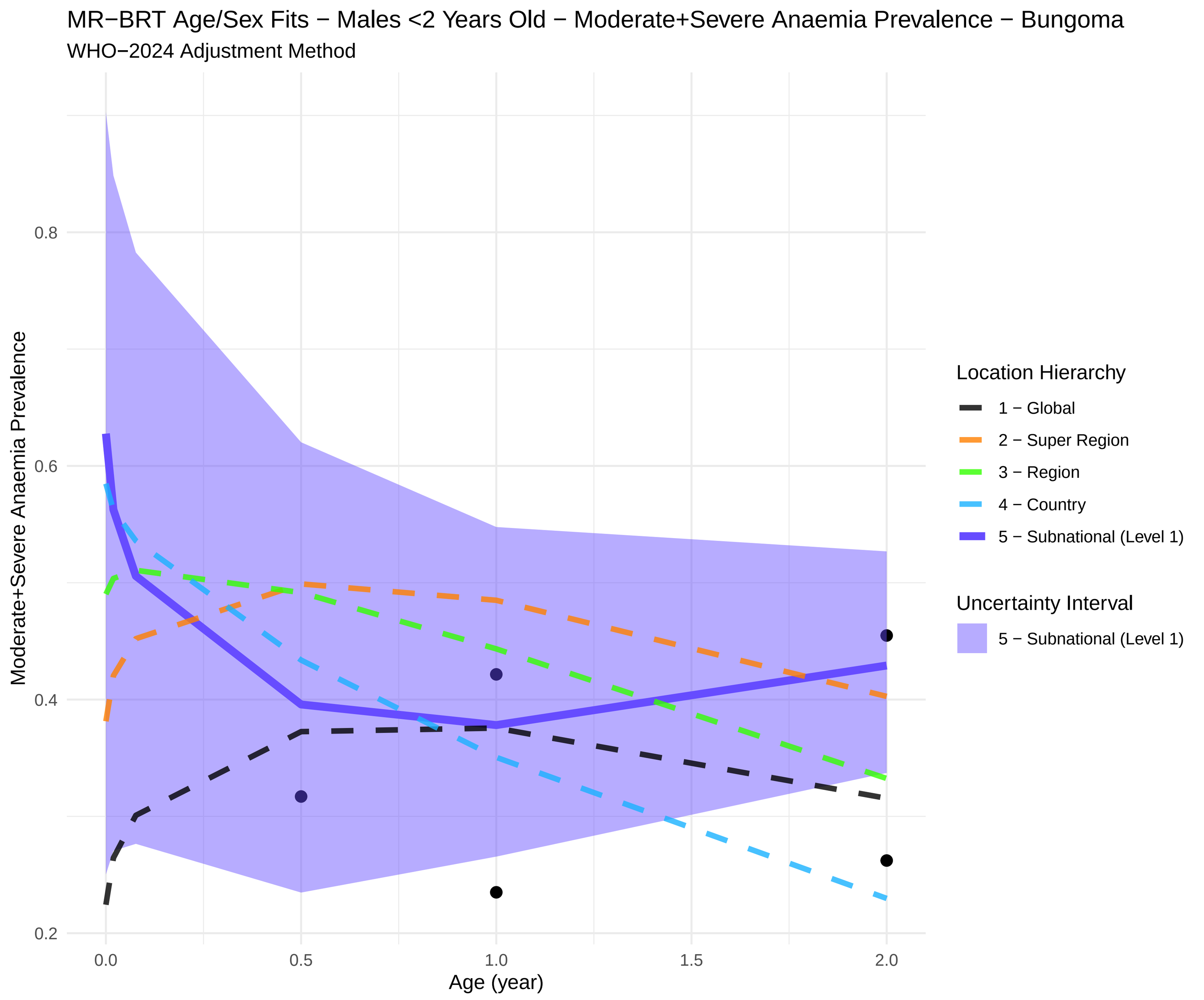
MR−BRT Age/Sex Fits − Males <2 Years Old − Moderate+Severe Anaemia Prevalence − Bungoma
WHO−2024 Adjustment Method
Moderate+Severe Anaemia Prevalence
Age (year)
Location Hierarchy
1 − Global
2 − Super Region
3 − Region
4 − Country
5 − Subnational (Level 1)
Uncertainty Interval
5 − Subnational (Level 1)

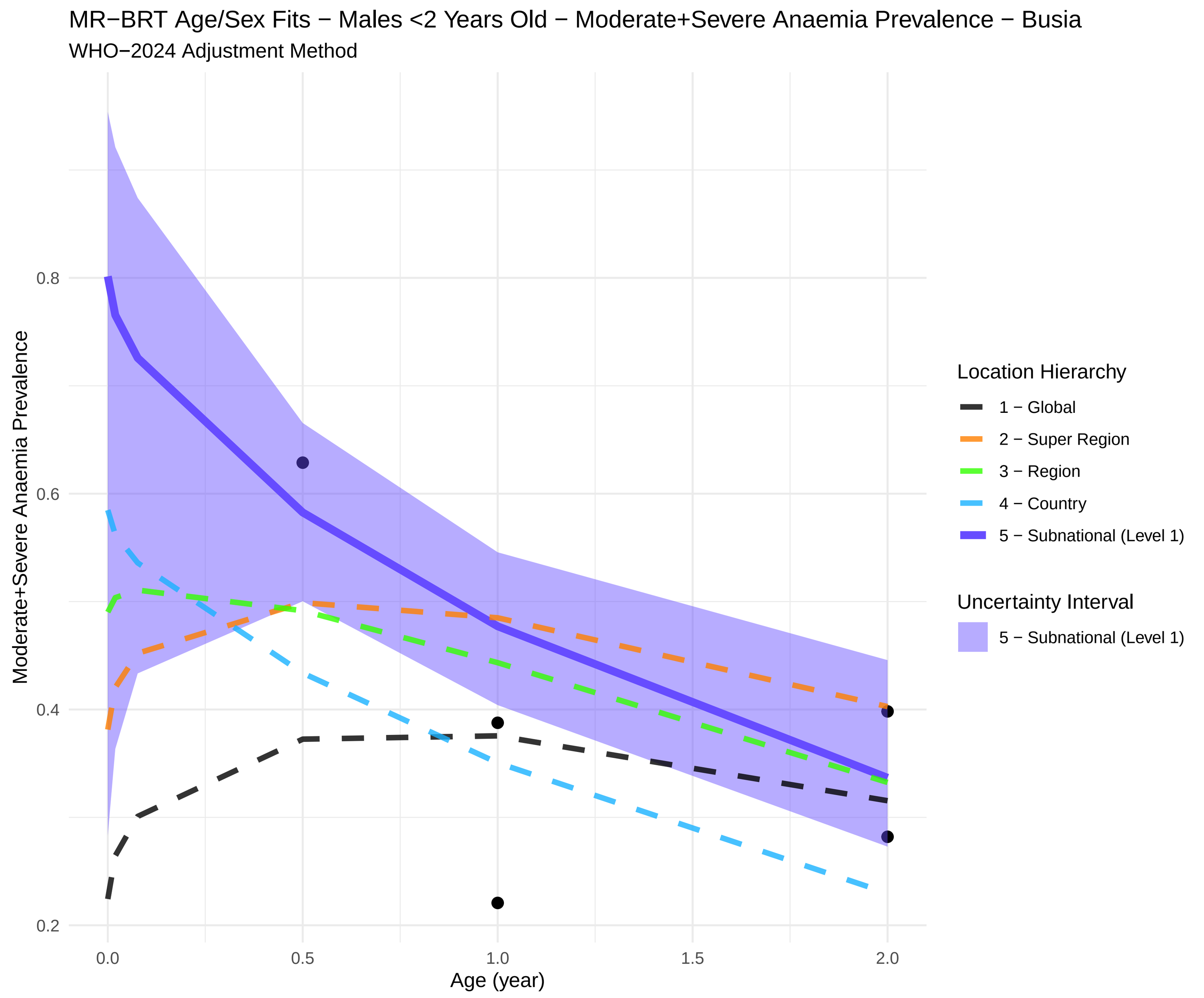
MR−BRT Age/Sex Fits − Males <2 Years Old − Moderate+Severe Anaemia Prevalence − Busia
WHO−2024 Adjustment Method
Moderate+Severe Anaemia Prevalence
Age (year)
Location Hierarchy
1 − Global
2 − Super Region
3 − Region
4 − Country
5 − Subnational (Level 1)
Uncertainty Interval
5 − Subnational (Level 1)

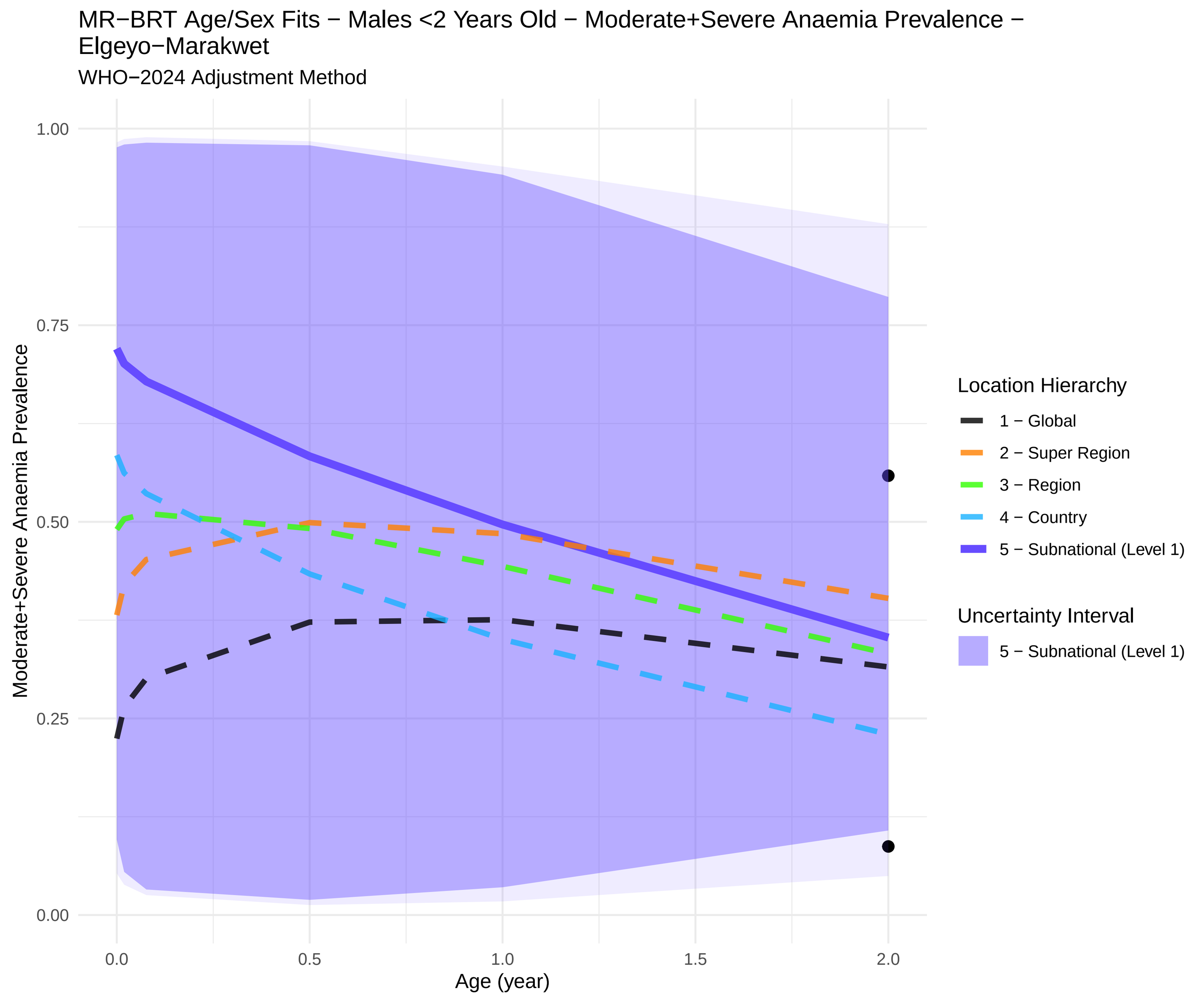
MR−BRT Age/Sex Fits − Males <2 Years Old − Moderate+Severe Anaemia Prevalence − Elgeyo−Marakwet
WHO−2024 Adjustment Method
Moderate+Severe Anaemia Prevalence
Age (year)
Location Hierarchy
1 − Global
2 − Super Region
3 − Region
4 − Country
5 − Subnational (Level 1)
Uncertainty Interval
5 − Subnational (Level 1)

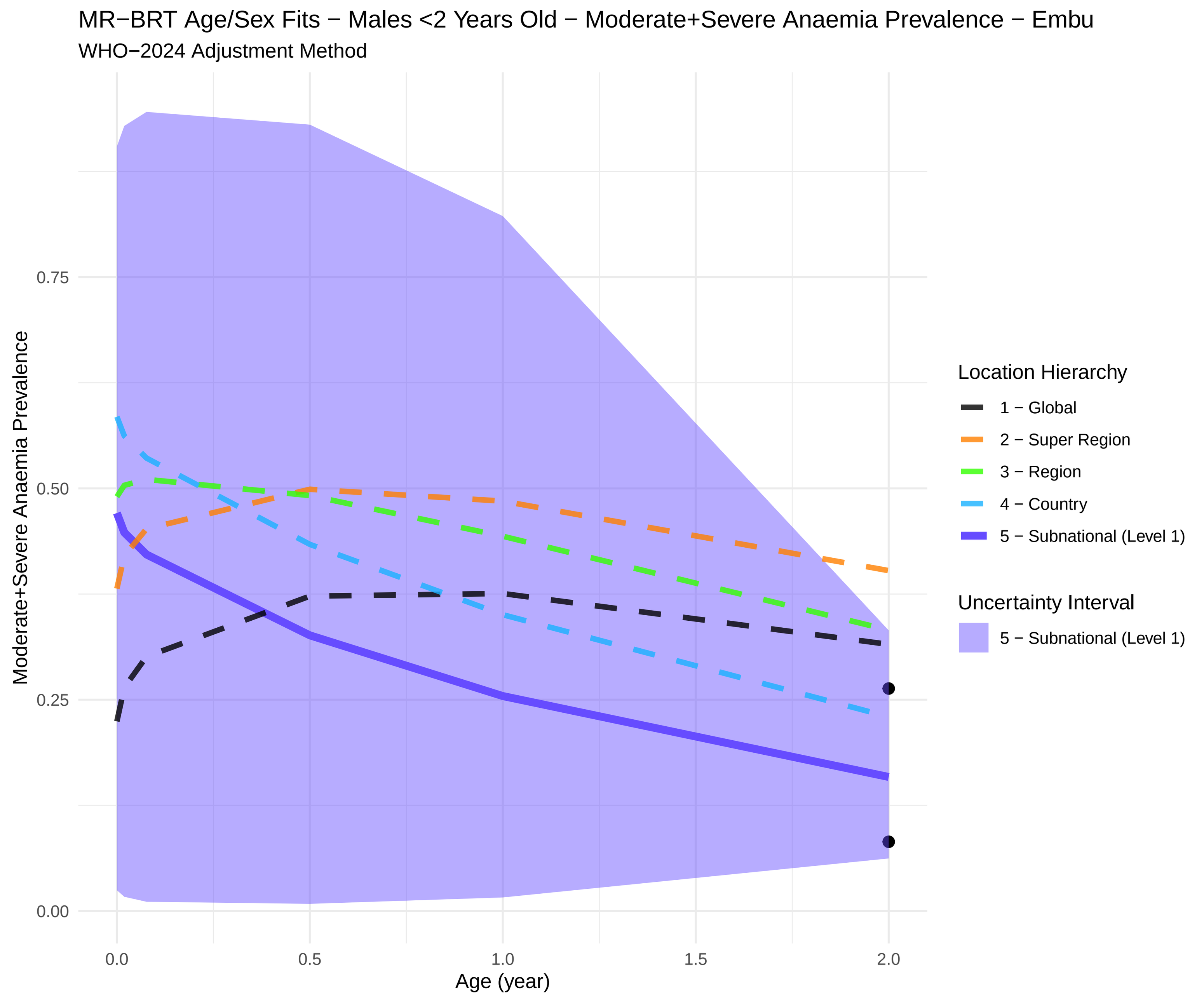
MR−BRT Age/Sex Fits − Males <2 Years Old − Moderate+Severe Anaemia Prevalence − Embu
WHO−2024 Adjustment Method
Moderate+Severe Anaemia Prevalence
Age (year)
Location Hierarchy
1 − Global
2 − Super Region
3 − Region
4 − Country
5 − Subnational (Level 1)
Uncertainty Interval
5 − Subnational (Level 1)

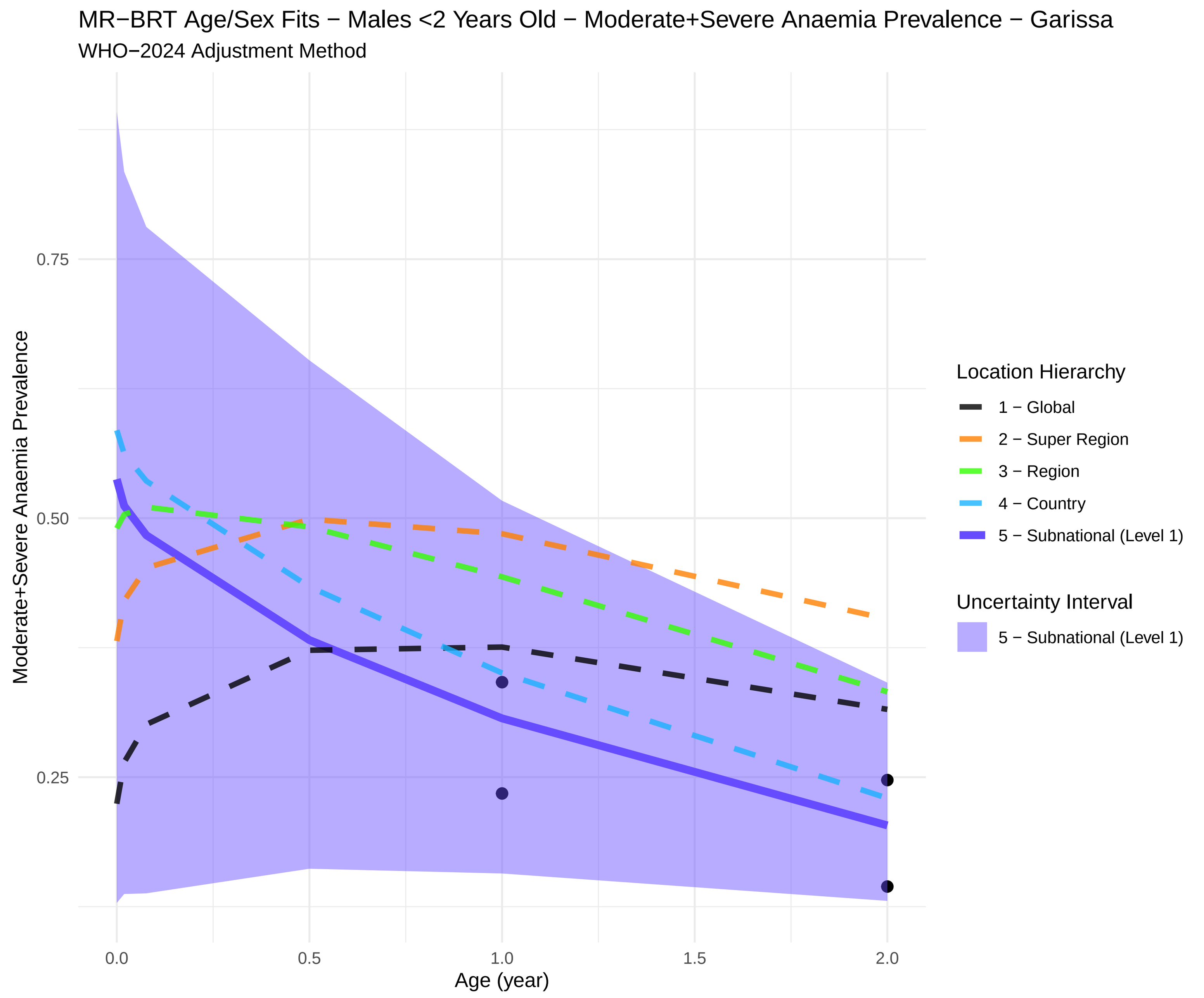
MR−BRT Age/Sex Fits − Males <2 Years Old − Moderate+Severe Anaemia Prevalence − Garissa
WHO−2024 Adjustment Method
Moderate+Severe Anaemia Prevalence
Age (year)
Location Hierarchy
1 − Global
2 − Super Region
3 − Region
4 − Country
5 − Subnational (Level 1)
Uncertainty Interval
5 − Subnational (Level 1)

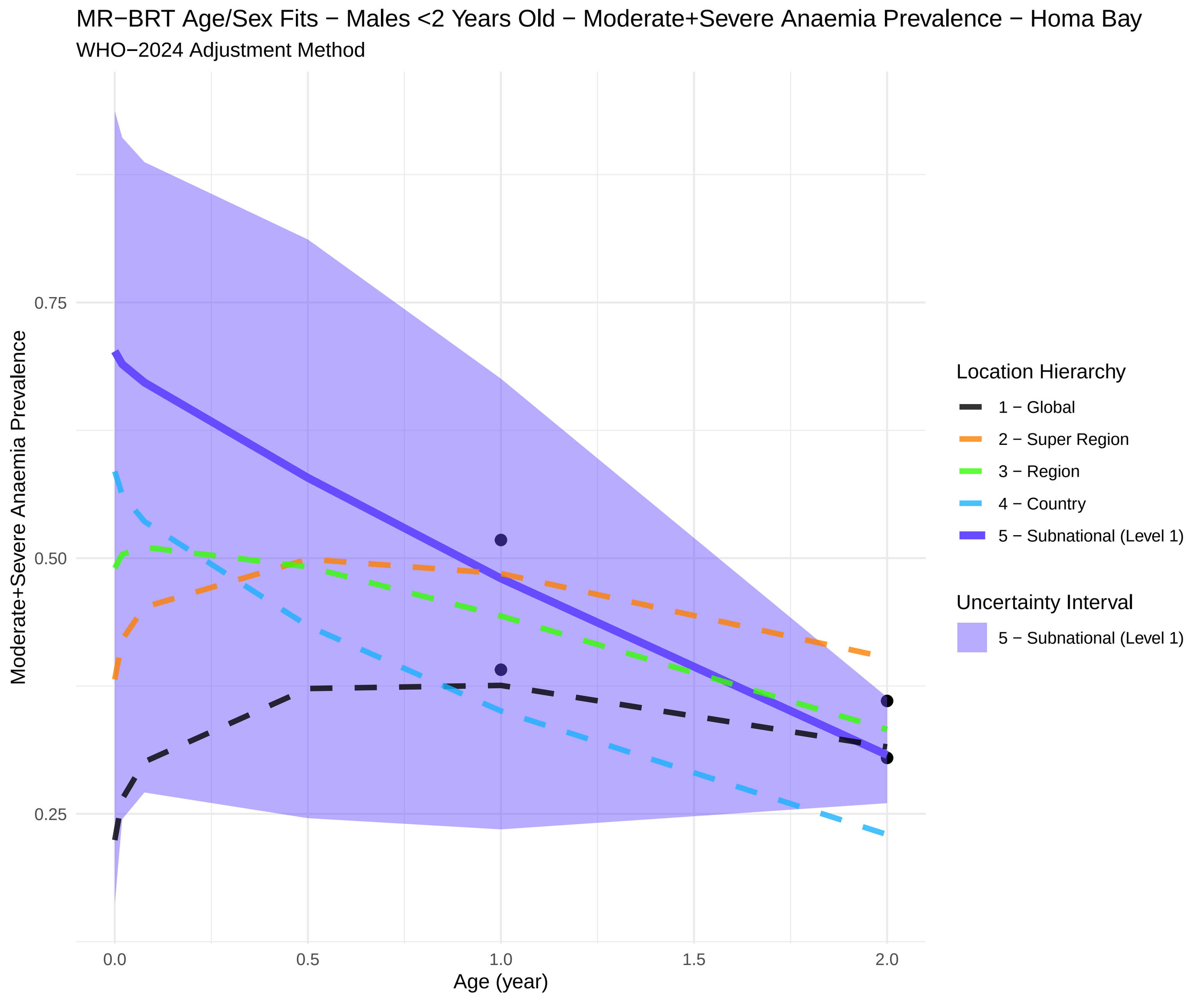
MR−BRT Age/Sex Fits − Males <2 Years Old − Moderate+Severe Anaemia Prevalence − Homa Bay
WHO−2024 Adjustment Method
Moderate+Severe Anaemia Prevalence
Age (year)
Location Hierarchy
1 − Global
2 − Super Region
3 − Region
4 − Country
5 − Subnational (Level 1)
Uncertainty Interval
5 − Subnational (Level 1)

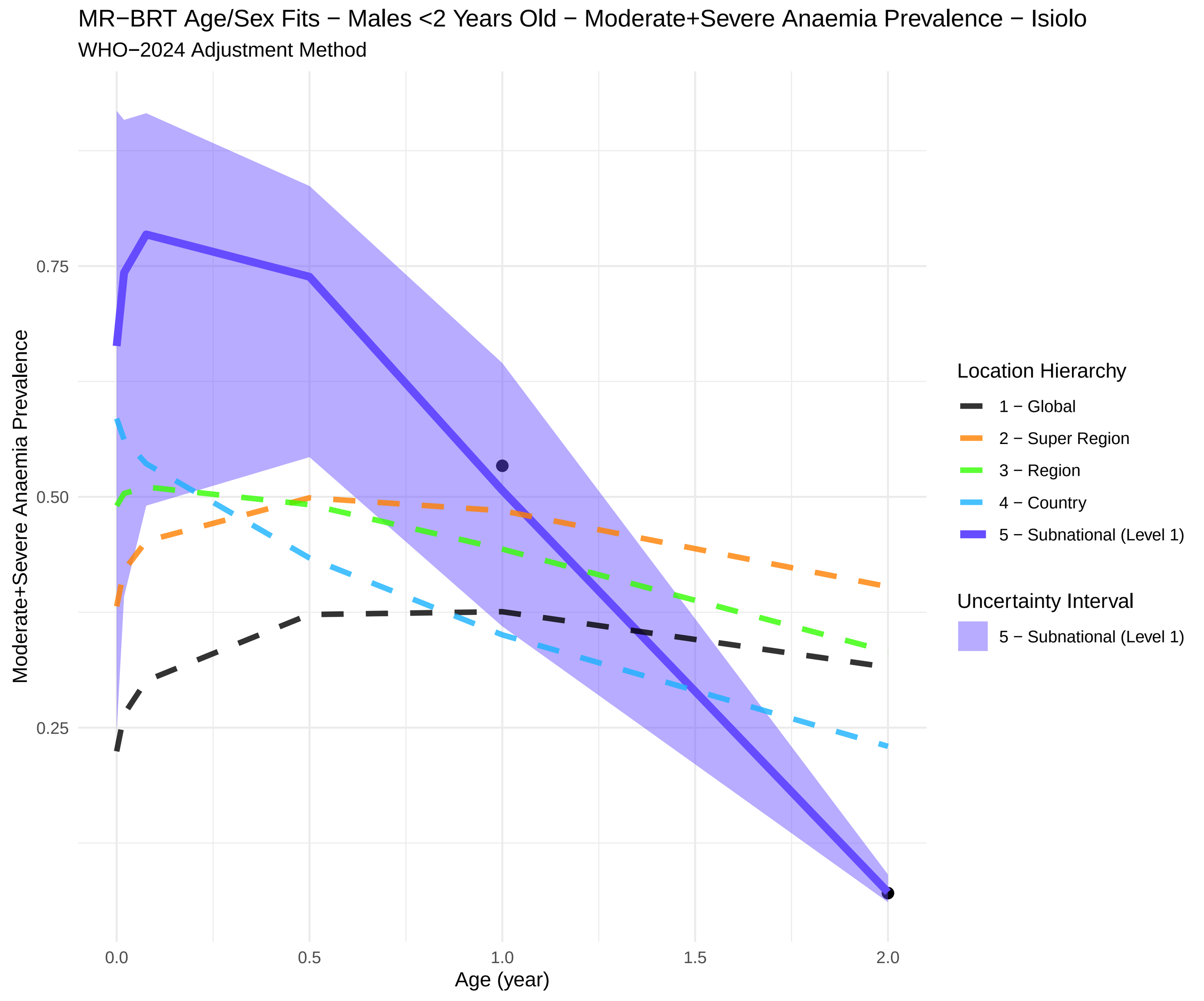
MR−BRT Age/Sex Fits − Males <2 Years Old − Moderate+Severe Anaemia Prevalence − Isiolo
WHO−2024 Adjustment Method
Moderate+Severe Anaemia Prevalence
Age (year)
Location Hierarchy
1 − Global
2 − Super Region
3 − Region
4 − Country
5 − Subnational (Level 1)
Uncertainty Interval
5 − Subnational (Level 1)

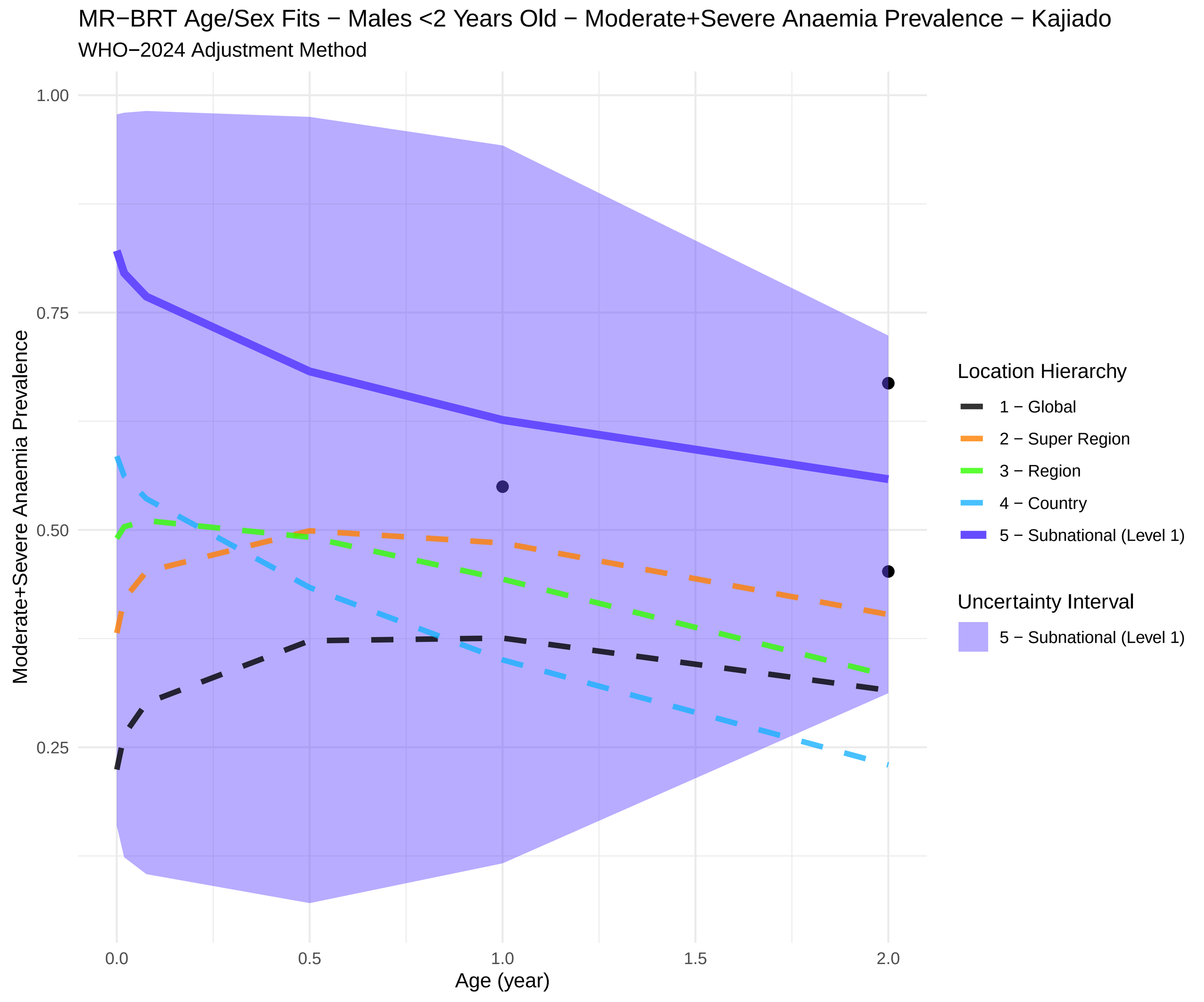
MR−BRT Age/Sex Fits − Males <2 Years Old − Moderate+Severe Anaemia Prevalence − Kajiado
WHO−2024 Adjustment Method
Moderate+Severe Anaemia Prevalence
Age (year)
Location Hierarchy
1 − Global
2 − Super Region
3 − Region
4 − Country
5 − Subnational (Level 1)
Uncertainty Interval
5 − Subnational (Level 1)

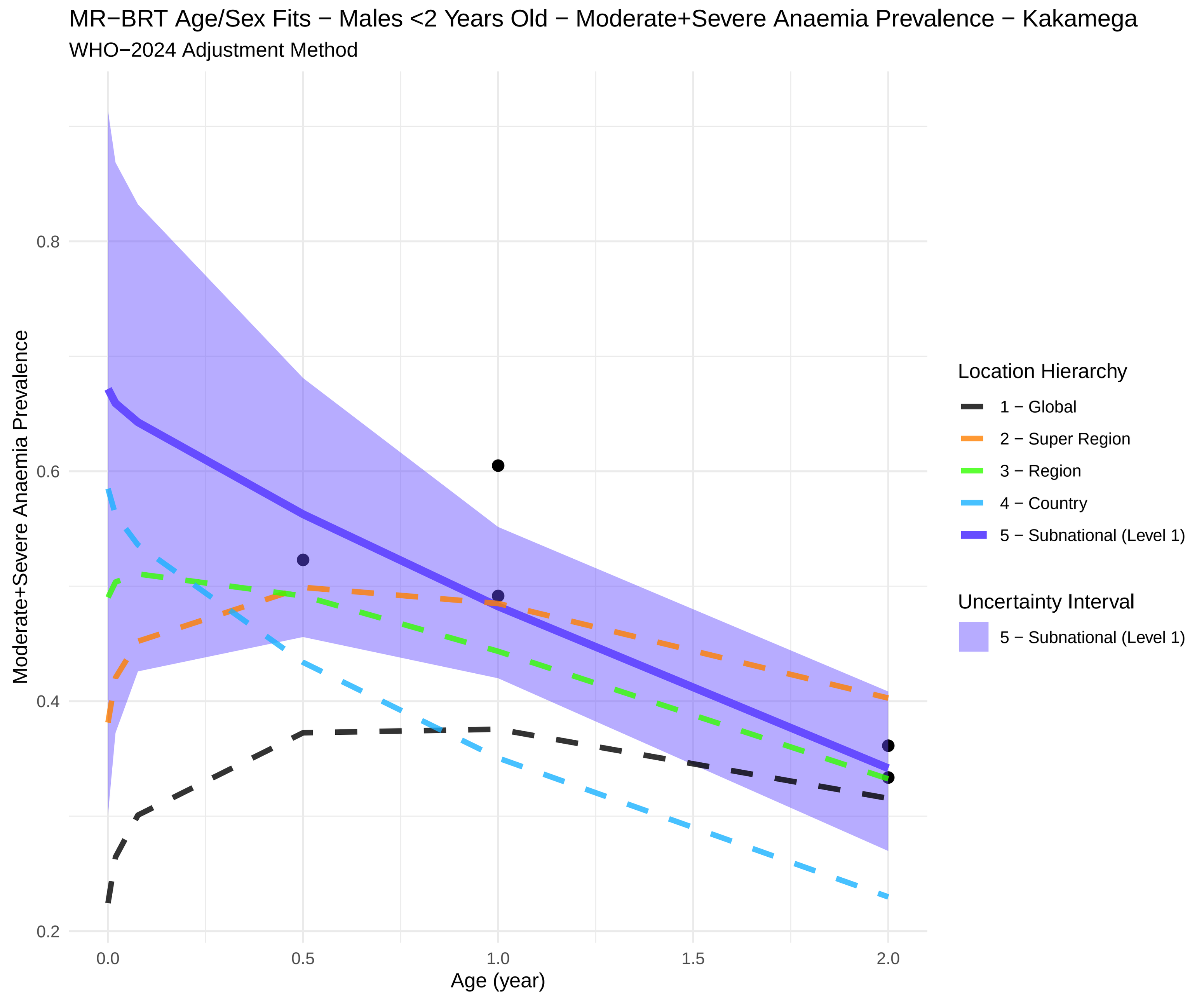
MR−BRT Age/Sex Fits − Males <2 Years Old − Moderate+Severe Anaemia Prevalence − Kakamega
WHO−2024 Adjustment Method
Moderate+Severe Anaemia Prevalence
Age (year)
Location Hierarchy
1 − Global
2 − Super Region
3 − Region
4 − Country
5 − Subnational (Level 1)
Uncertainty Interval
5 − Subnational (Level 1)

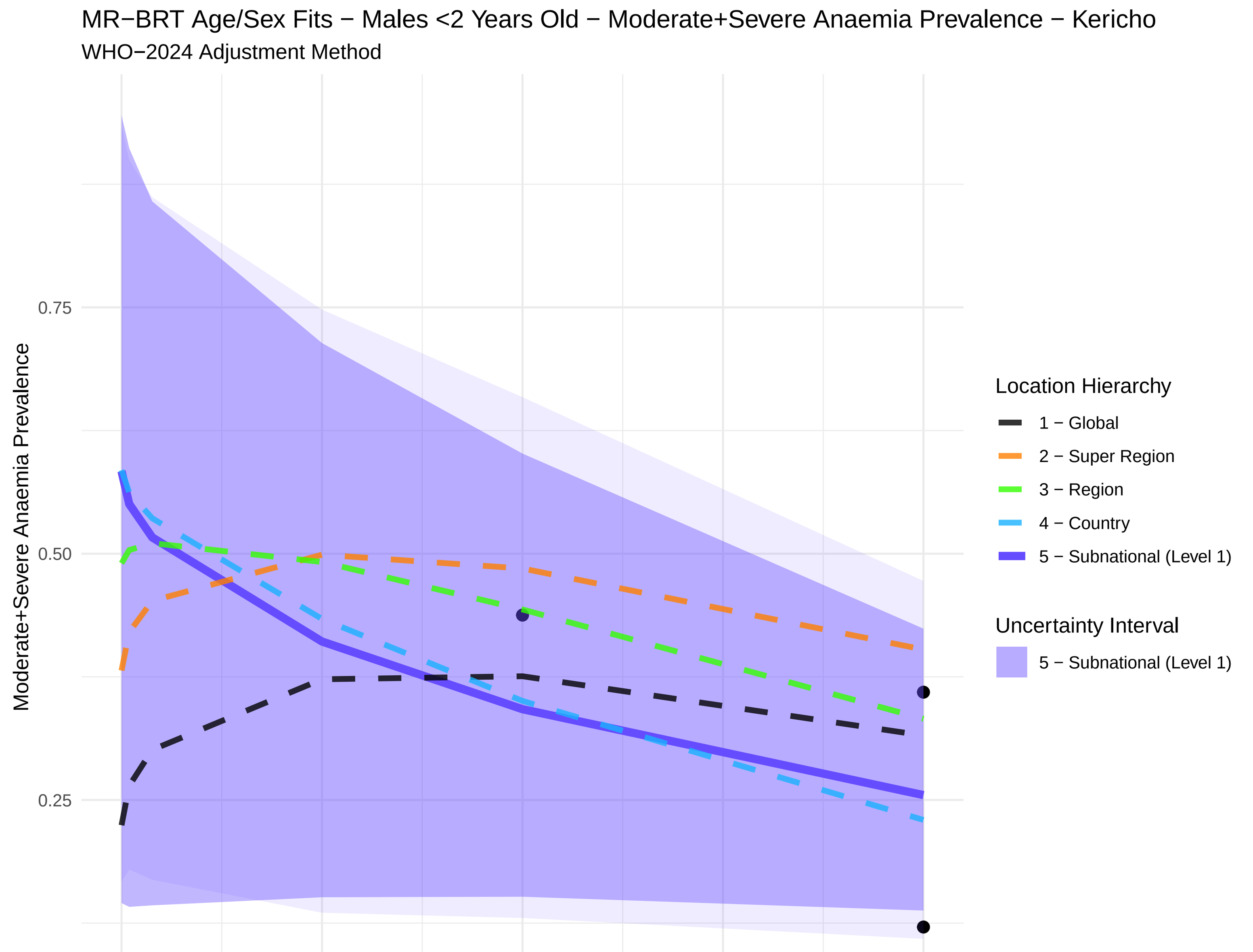
MR−BRT Age/Sex Fits − Males <2 Years Old − Moderate+Severe Anaemia Prevalence − Kericho
WHO−2024 Adjustment Method
Moderate+Severe Anaemia Prevalence
Age (year)
Location Hierarchy
1 − Global
2 − Super Region
3 − Region
4 − Country
5 − Subnational (Level 1)
Uncertainty Interval
5 − Subnational (Level 1)

MR−BRT Age/Sex Fits − Males <2 Years Old − Moderate+Severe Anaemia Prevalence − Kiambu
WHO−2024 Adjustment Method

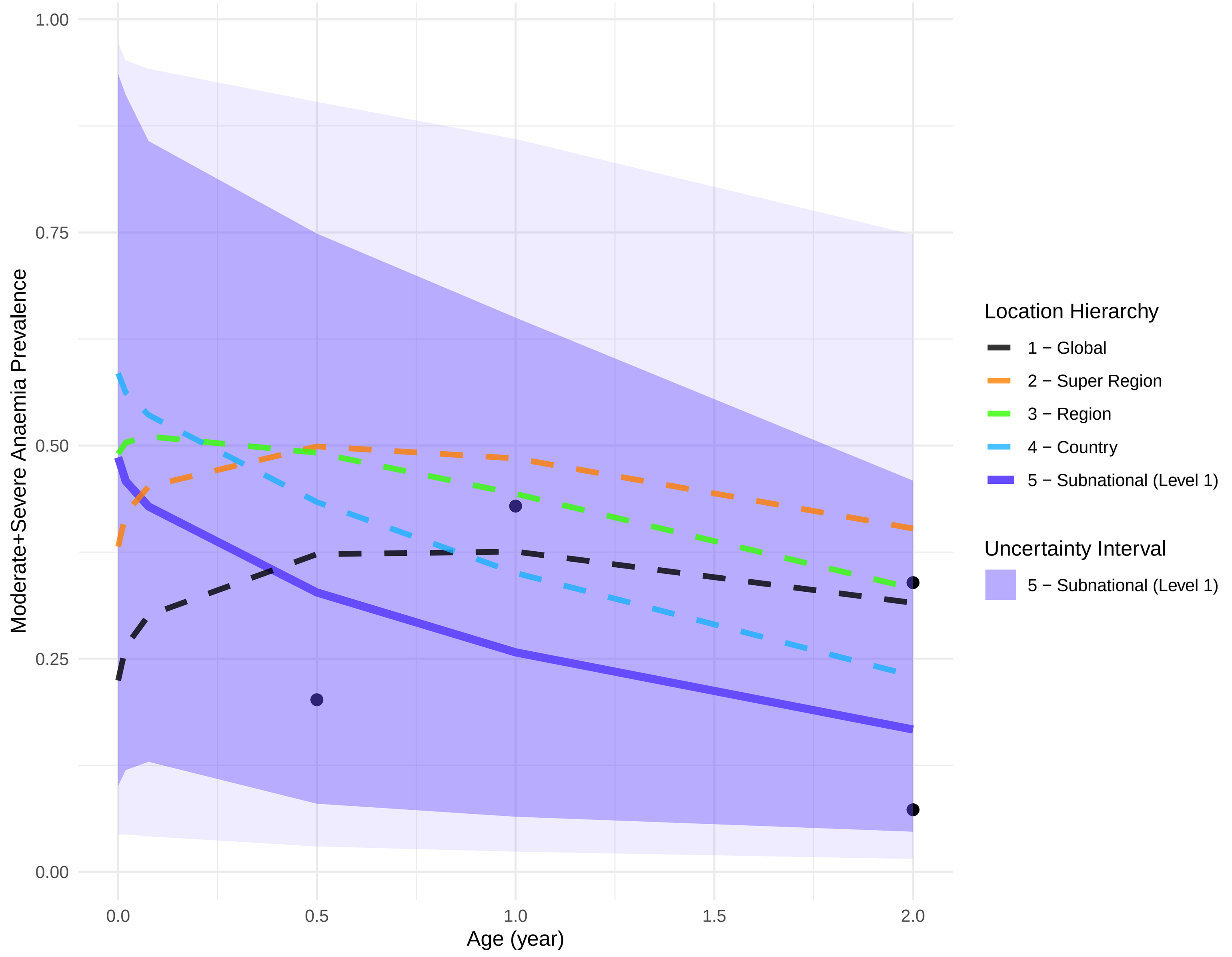

MR−BRT Age/Sex Fits − Males <2 Years Old − Moderate+Severe Anaemia Prevalence − Kilifi
WHO−2024 Adjustment Method

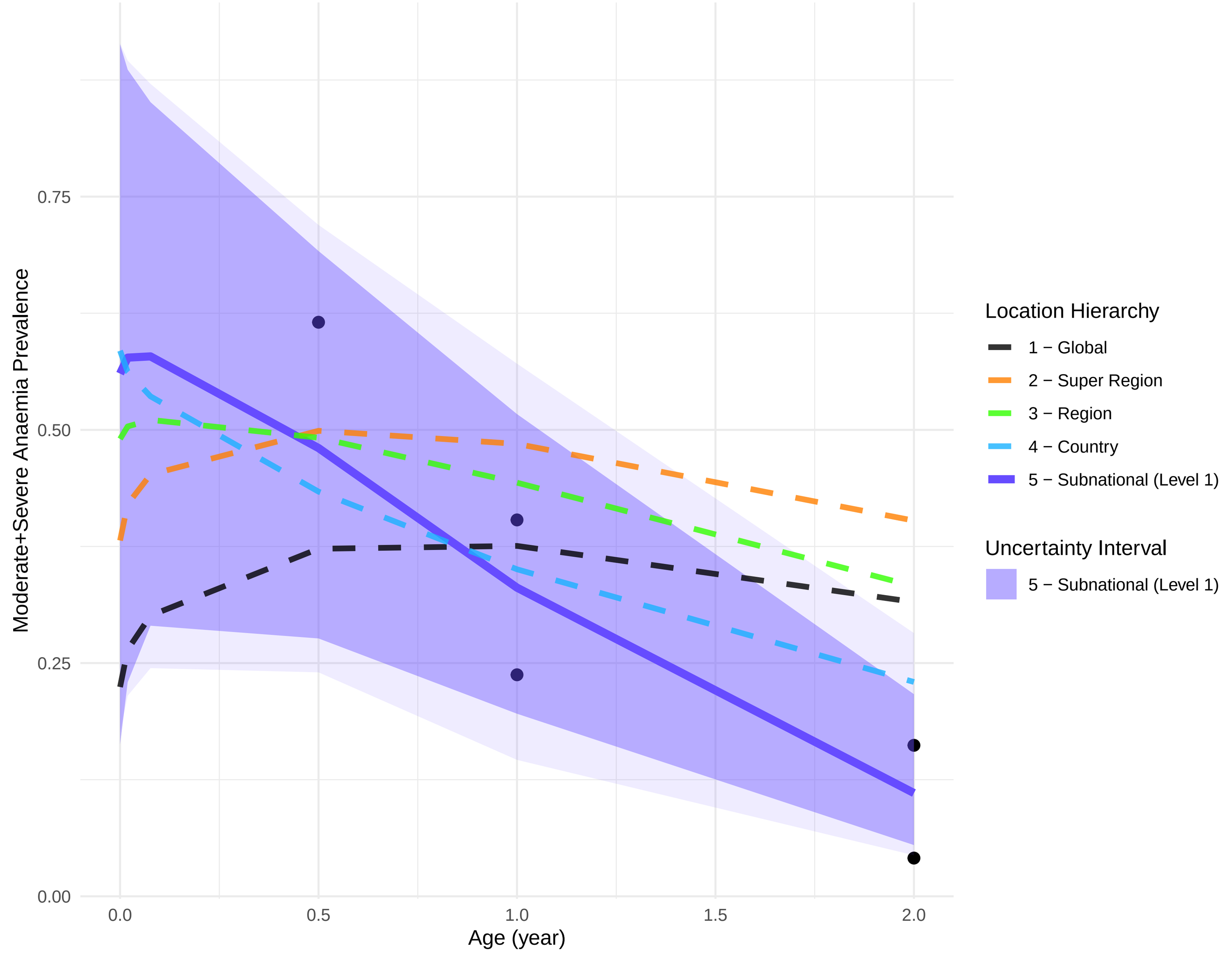

MR−BRT Age/Sex Fits − Males <2 Years Old − Moderate+Severe Anaemia Prevalence − Kirinyaga
WHO−2024 Adjustment Method

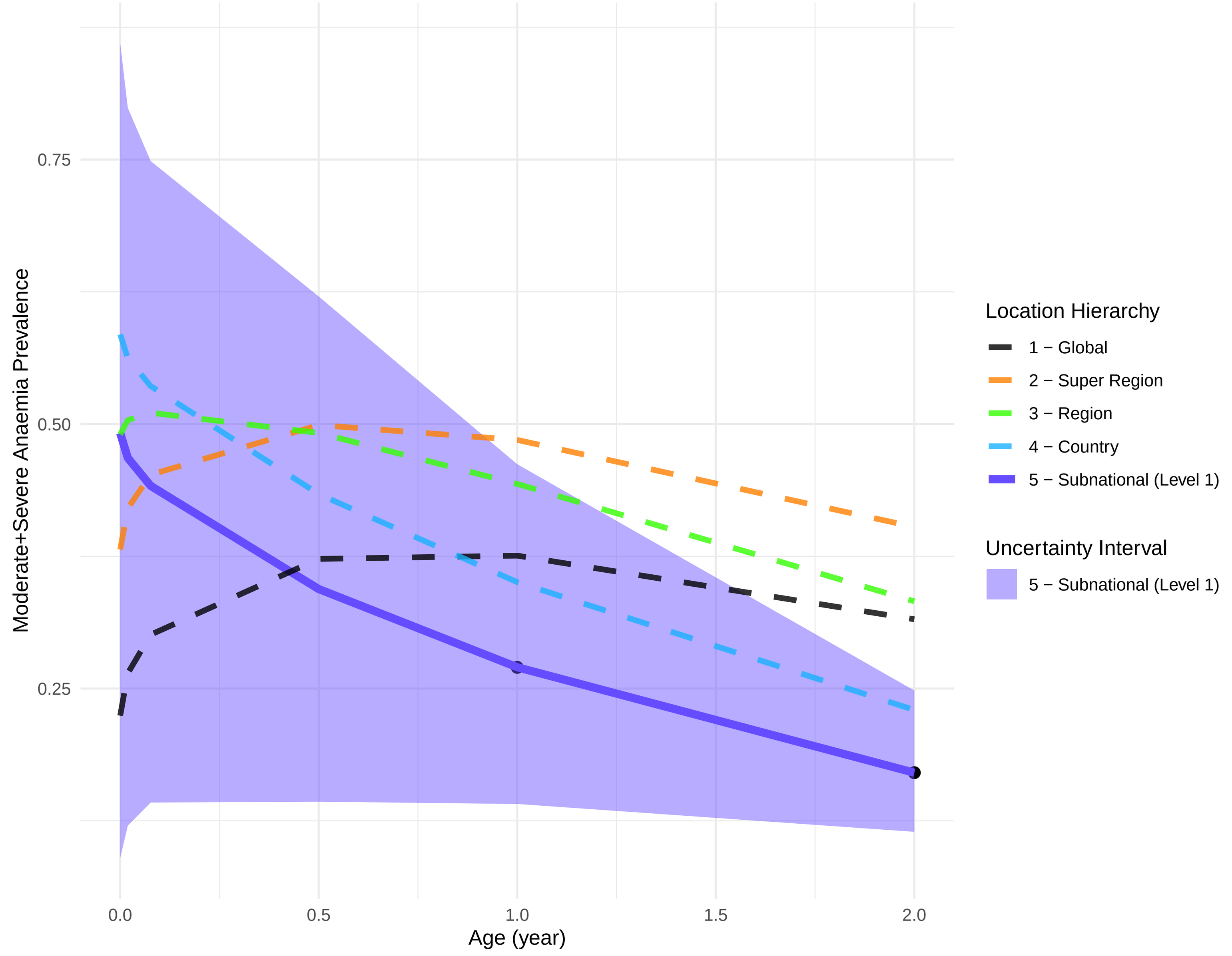

MR−BRT Age/Sex Fits − Males <2 Years Old − Moderate+Severe Anaemia Prevalence − Kisii
WHO−2024 Adjustment Method

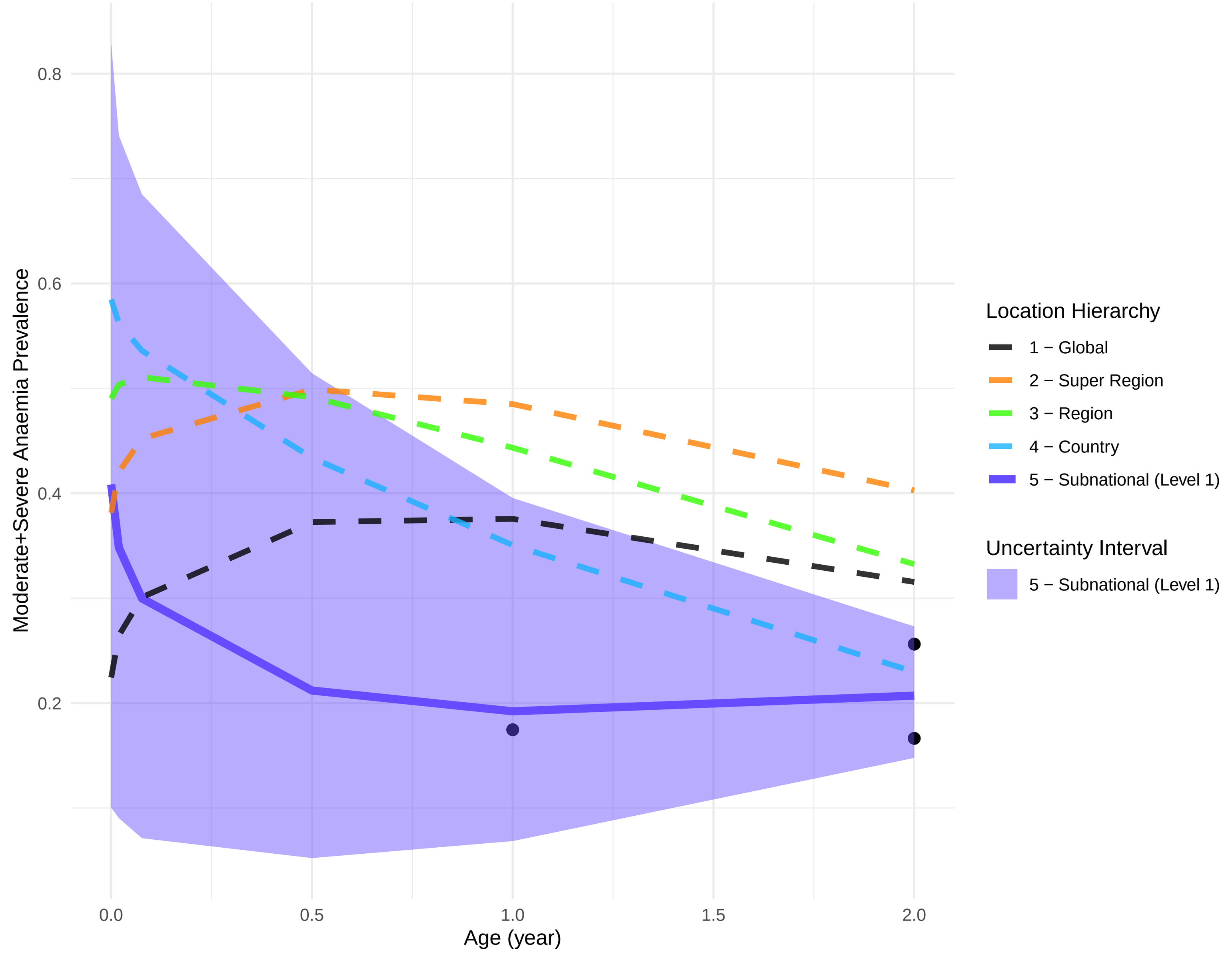

MR−BRT Age/Sex Fits − Males <2 Years Old − Moderate+Severe Anaemia Prevalence − Kisumu
WHO−2024 Adjustment Method

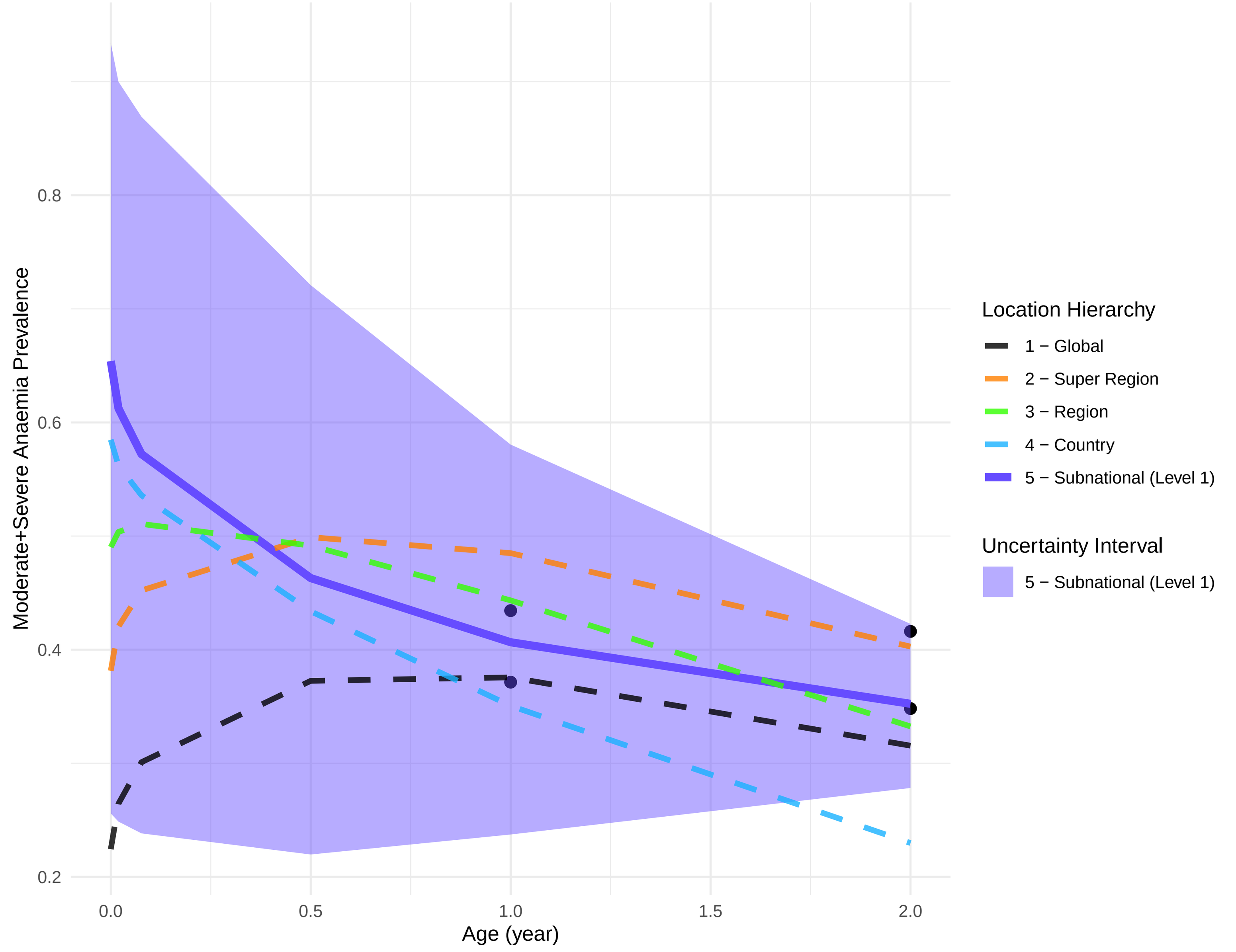

MR−BRT Age/Sex Fits − Males <2 Years Old − Moderate+Severe Anaemia Prevalence − Kitui
WHO−2024 Adjustment Method

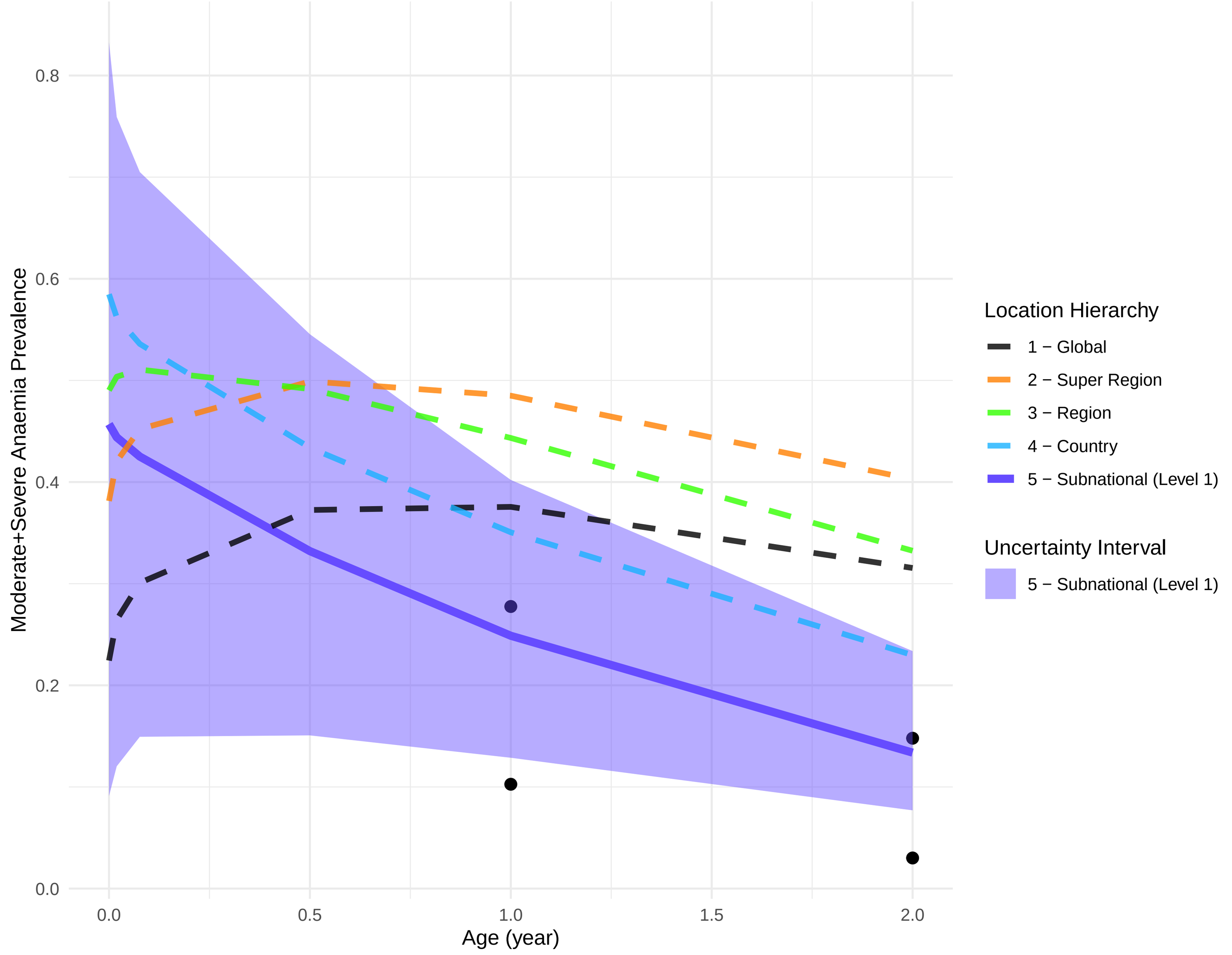

MR−BRT Age/Sex Fits − Males <2 Years Old − Moderate+Severe Anaemia Prevalence − Kwale
WHO−2024 Adjustment Method

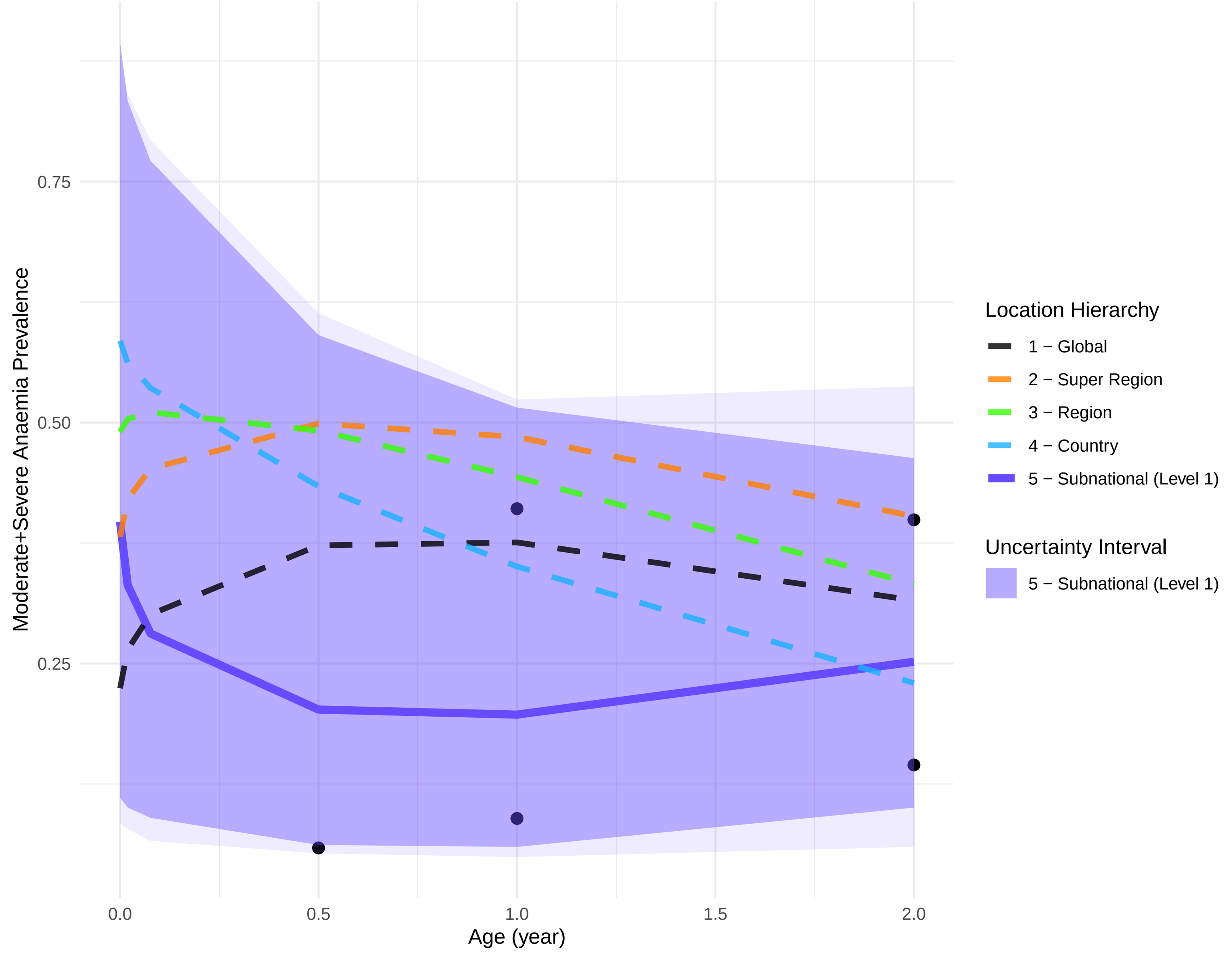

MR−BRT Age/Sex Fits − Males <2 Years Old − Moderate+Severe Anaemia Prevalence − Laikipia
WHO−2024 Adjustment Method

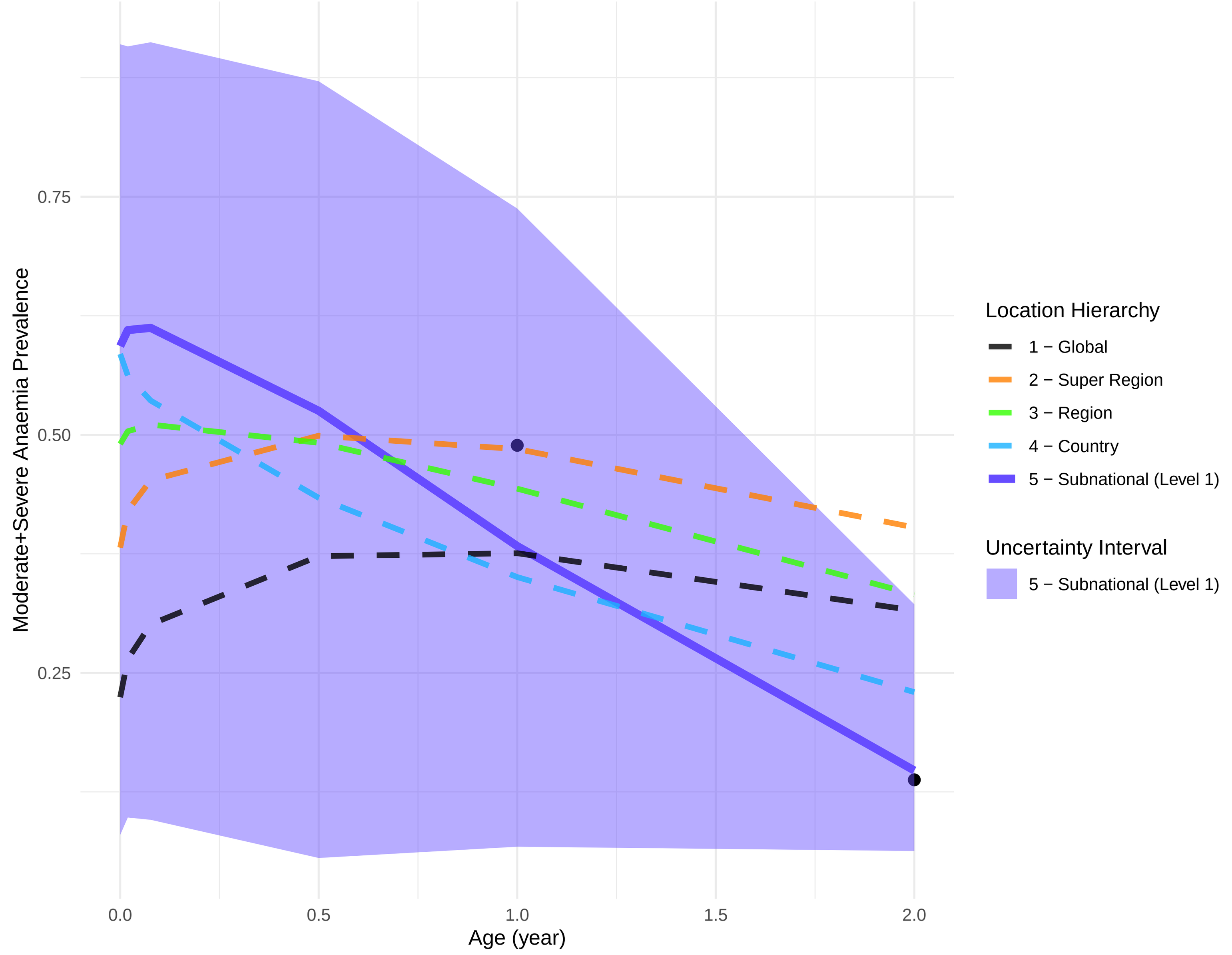

MR−BRT Age/Sex Fits − Males <2 Years Old − Moderate+Severe Anaemia Prevalence − Lamu
WHO−2024 Adjustment Method

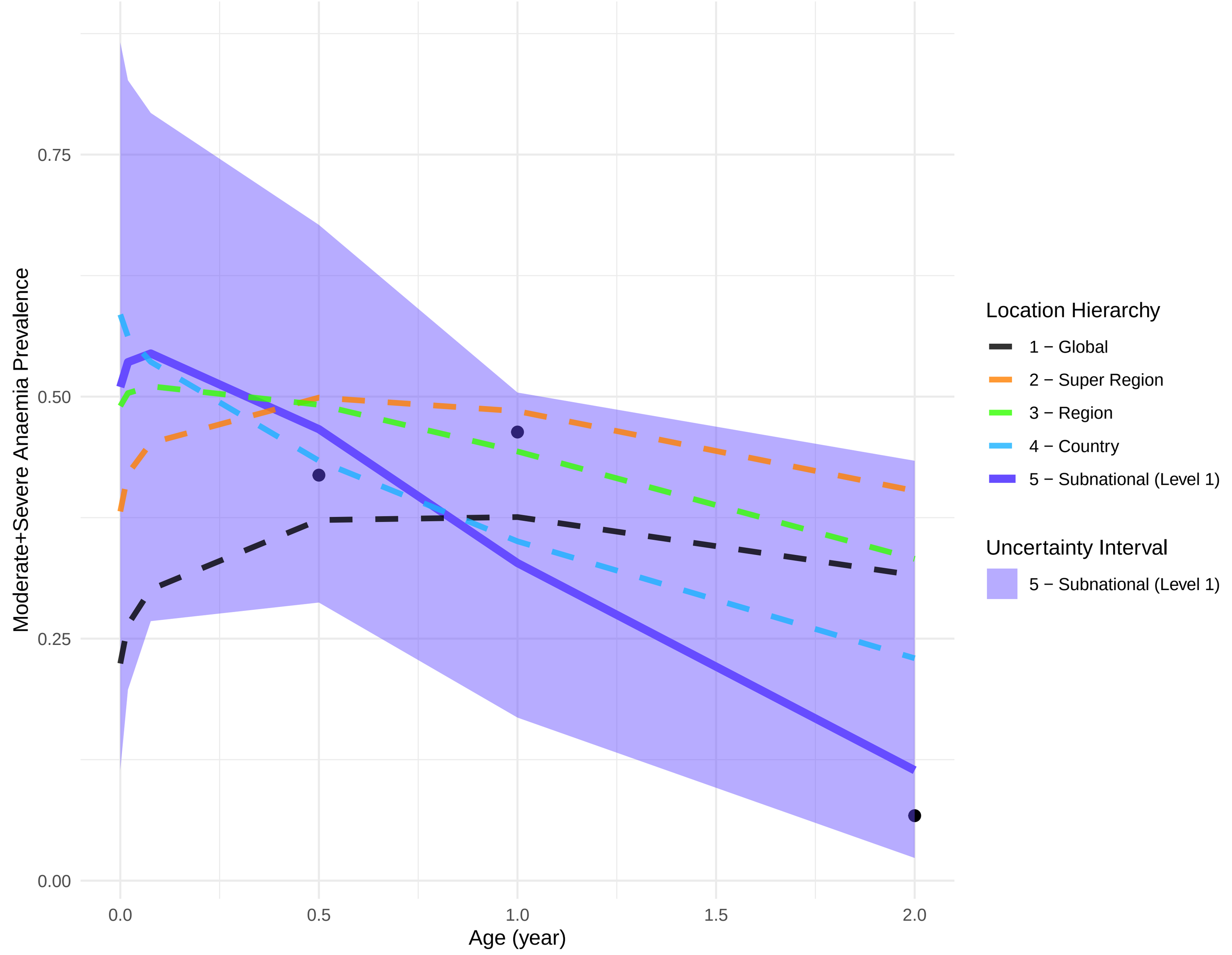

MR−BRT Age/Sex Fits − Males <2 Years Old − Moderate+Severe Anaemia Prevalence − Machakos
WHO−2024 Adjustment Method

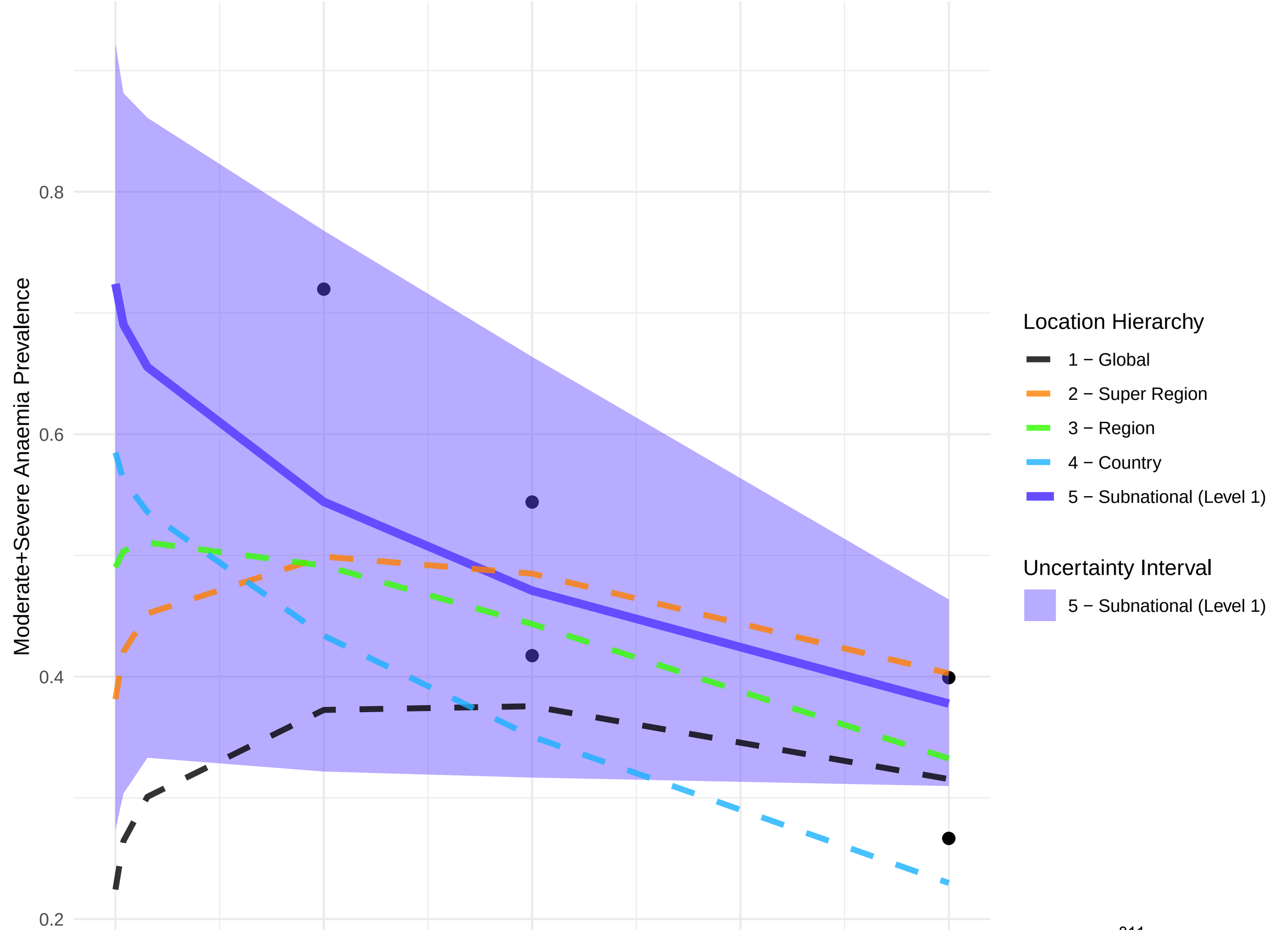

MR−BRT Age/Sex Fits − Males <2 Years Old − Moderate+Severe Anaemia Prevalence − Makueni
WHO−2024 Adjustment Method

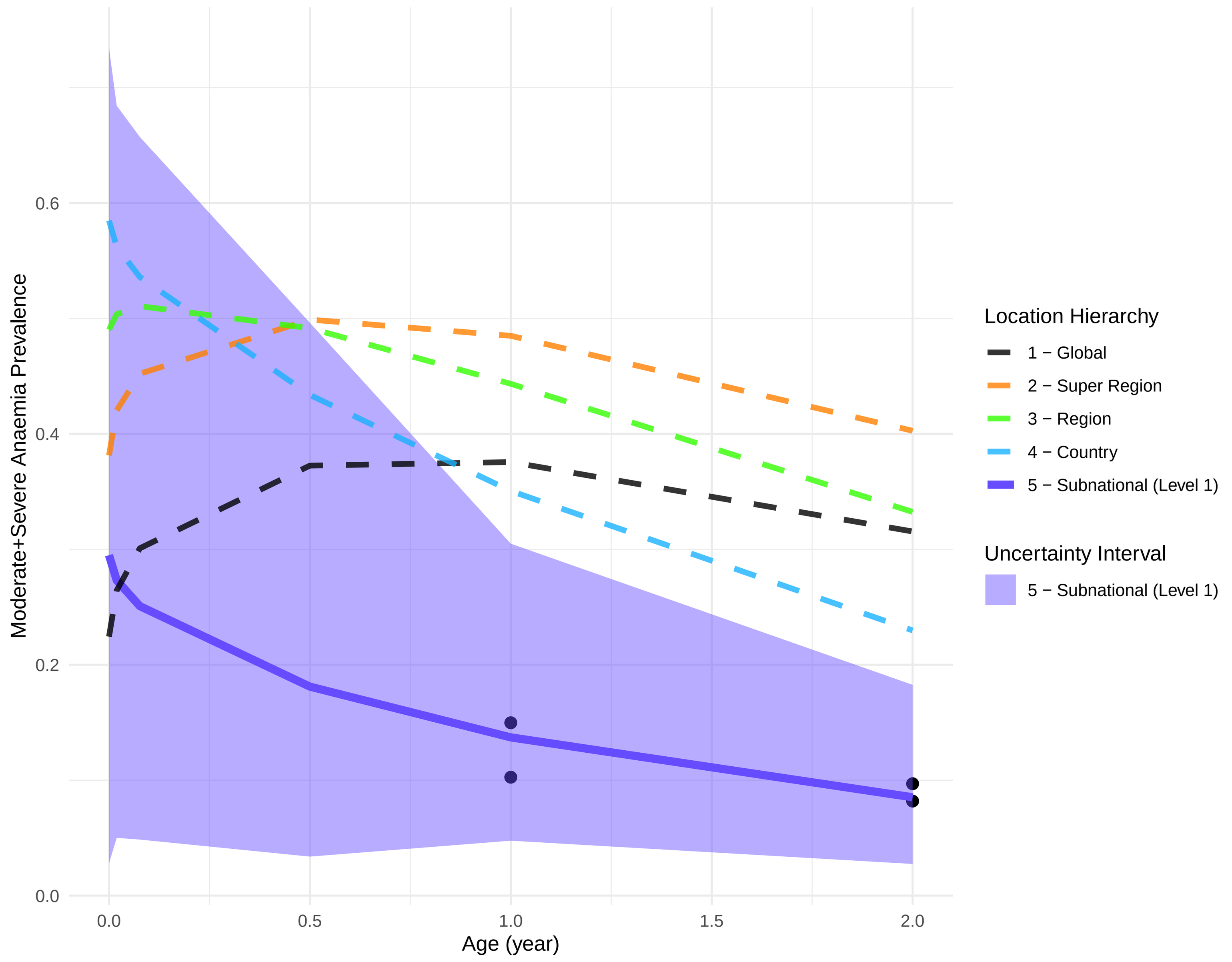

MR−BRT Age/Sex Fits − Males <2 Years Old − Moderate+Severe Anaemia Prevalence − Mandera
WHO−2024 Adjustment Method

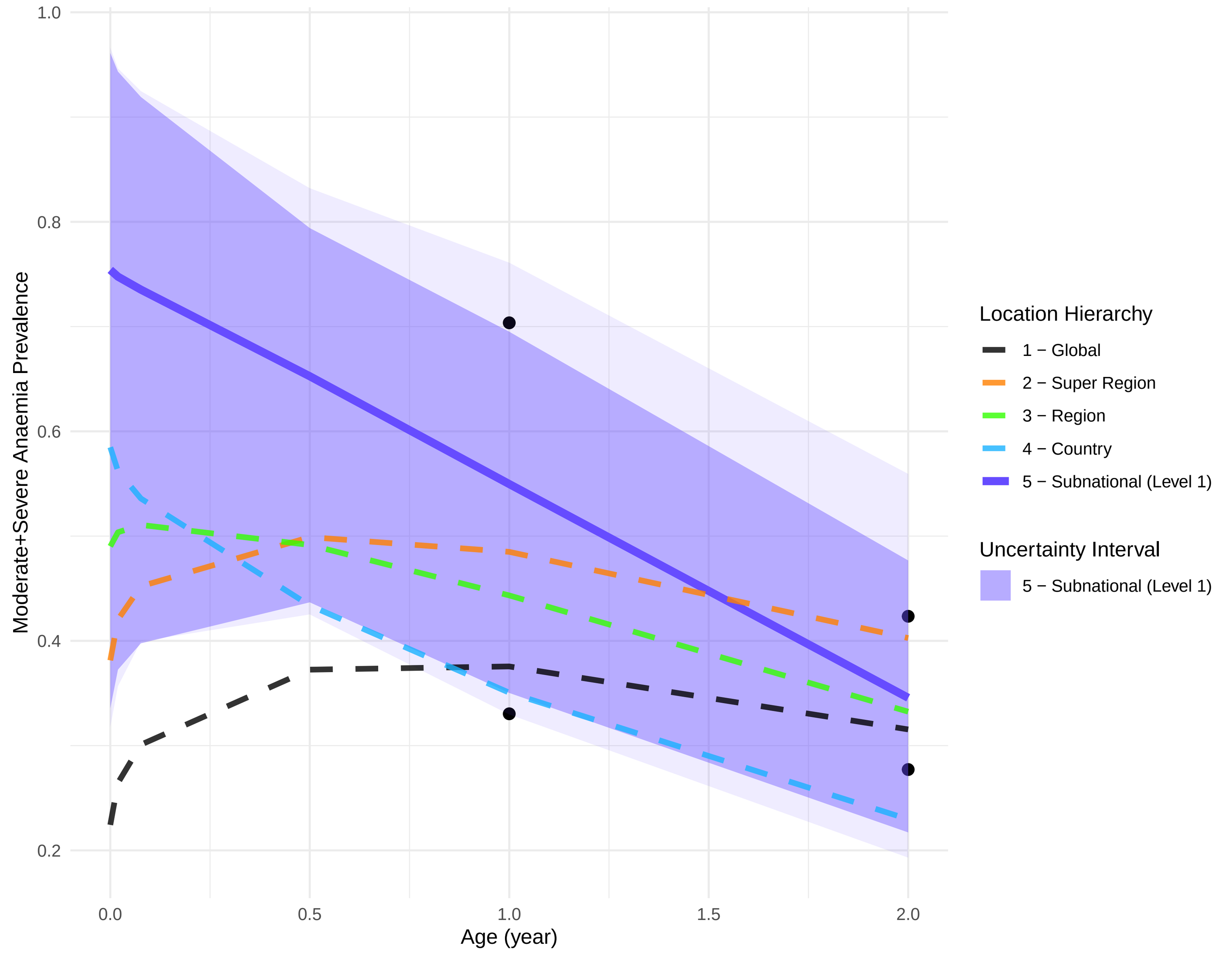

MR−BRT Age/Sex Fits − Males <2 Years Old − Moderate+Severe Anaemia Prevalence − Marsabit
WHO−2024 Adjustment Method

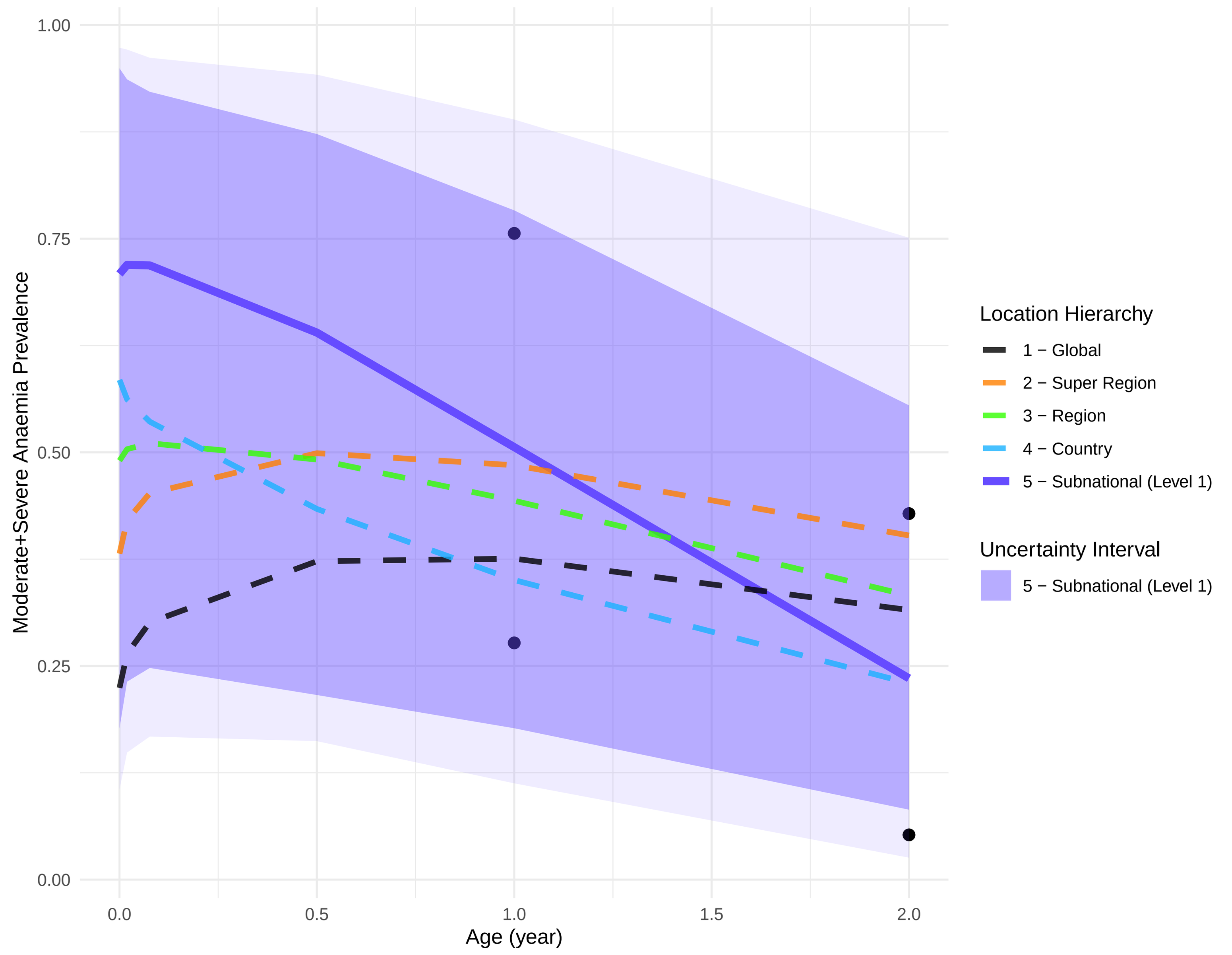

MR−BRT Age/Sex Fits − Males <2 Years Old − Moderate+Severe Anaemia Prevalence − Meru
WHO−2024 Adjustment Method

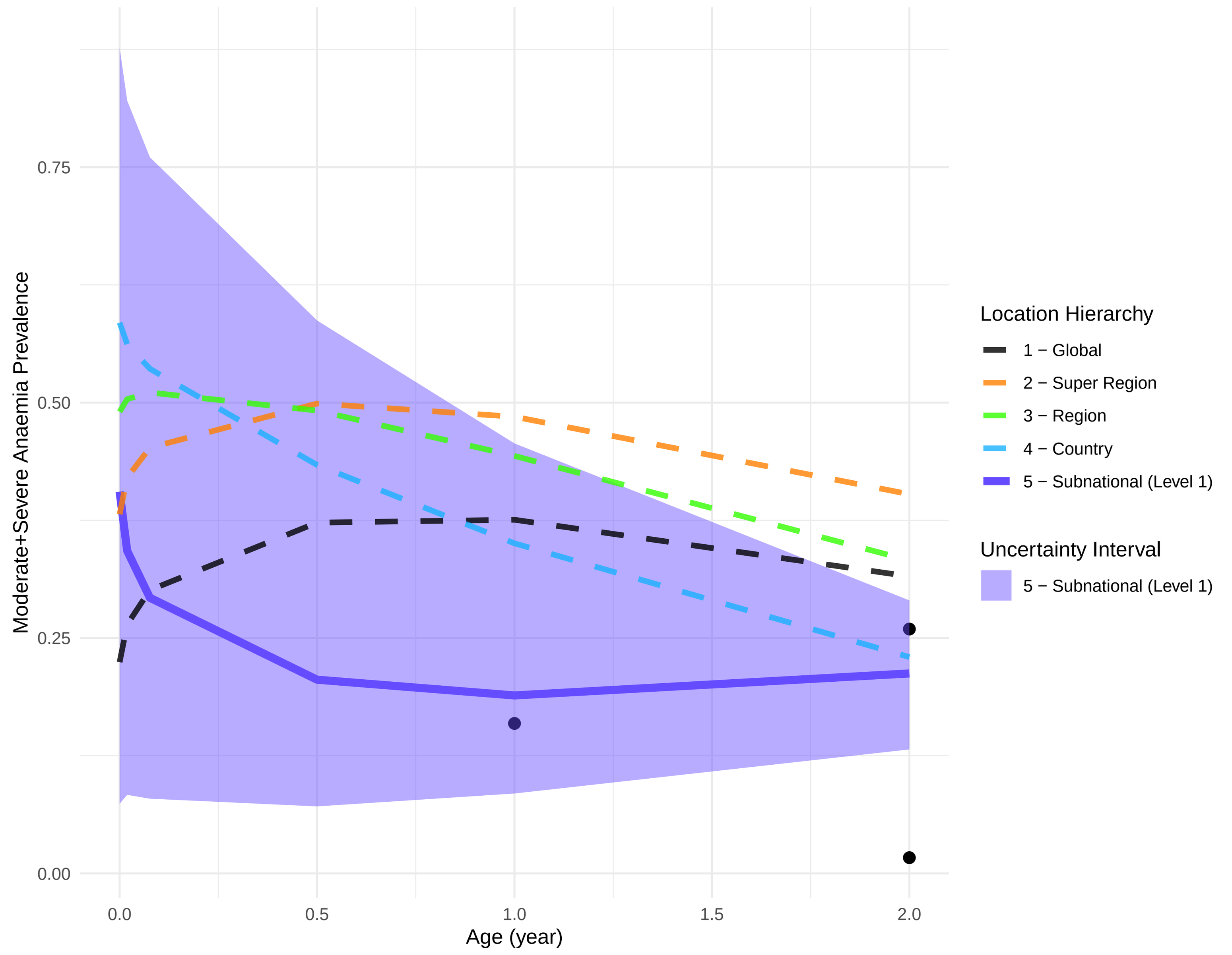

MR−BRT Age/Sex Fits − Males <2 Years Old − Moderate+Severe Anaemia Prevalence − Migori
WHO−2024 Adjustment Method

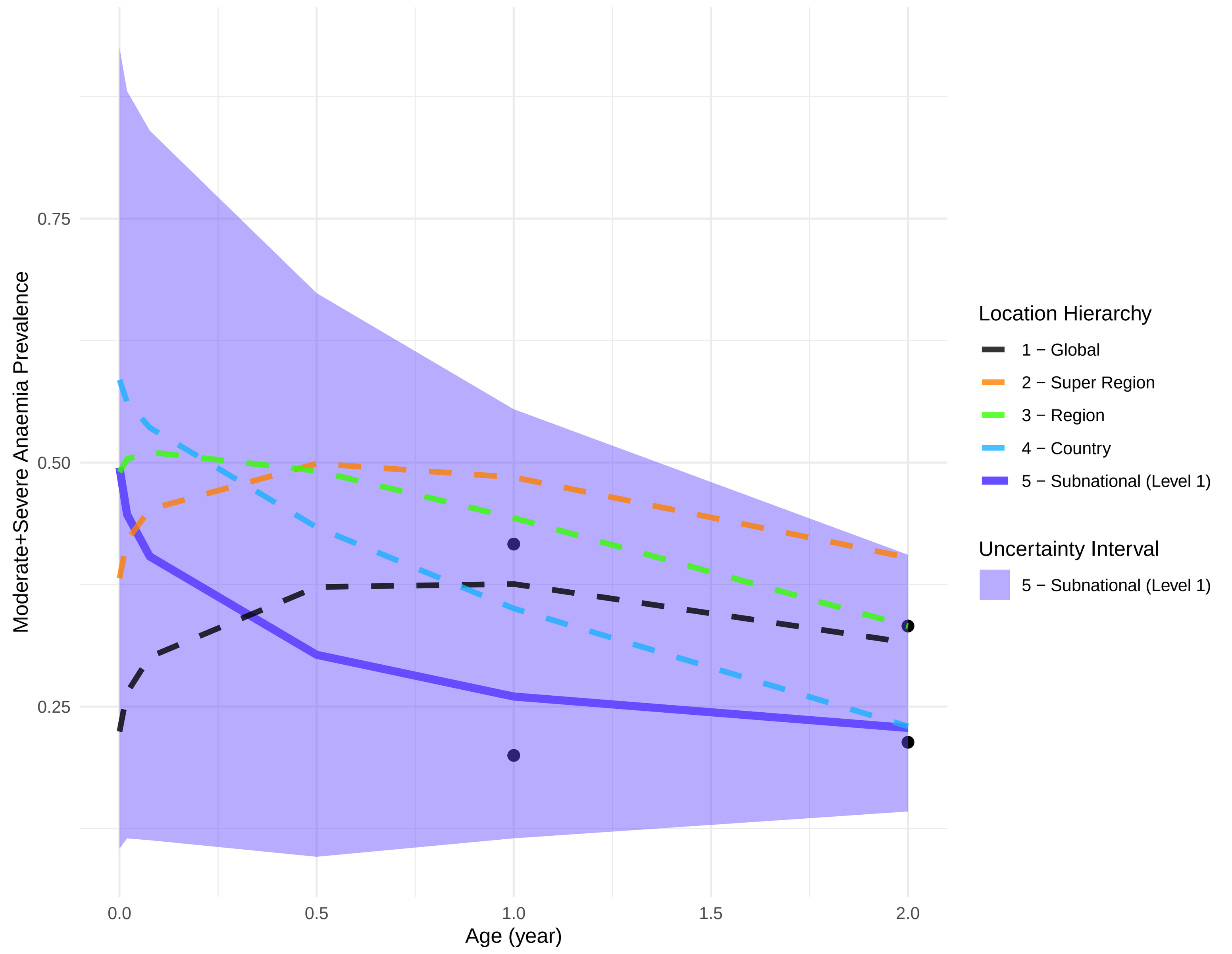

MR−BRT Age/Sex Fits − Males <2 Years Old − Moderate+Severe Anaemia Prevalence − Mombasa
WHO−2024 Adjustment Method

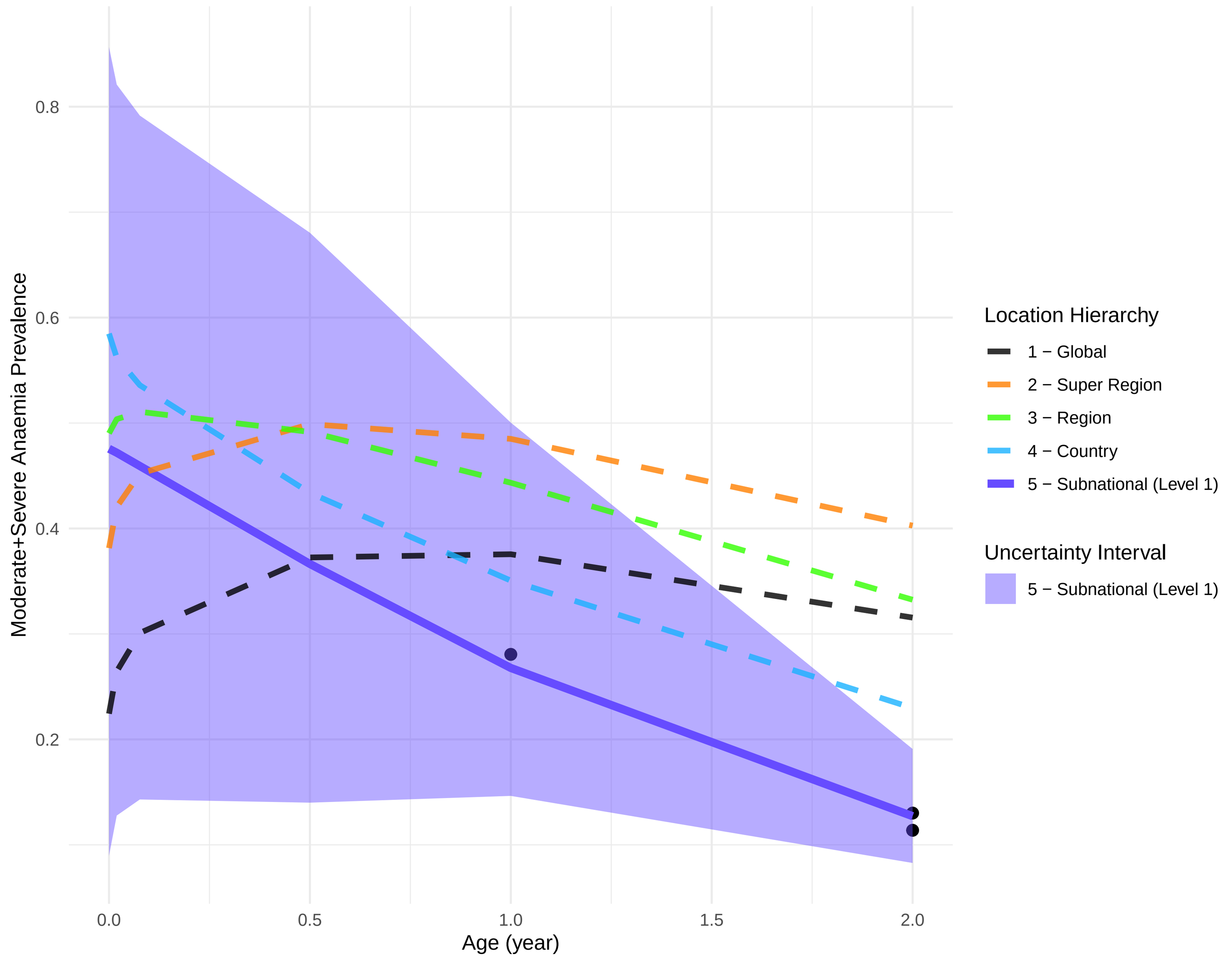

MR−BRT Age/Sex Fits − Males <2 Years Old − Moderate+Severe Anaemia Prevalence − Murang'a
WHO−2024 Adjustment Method

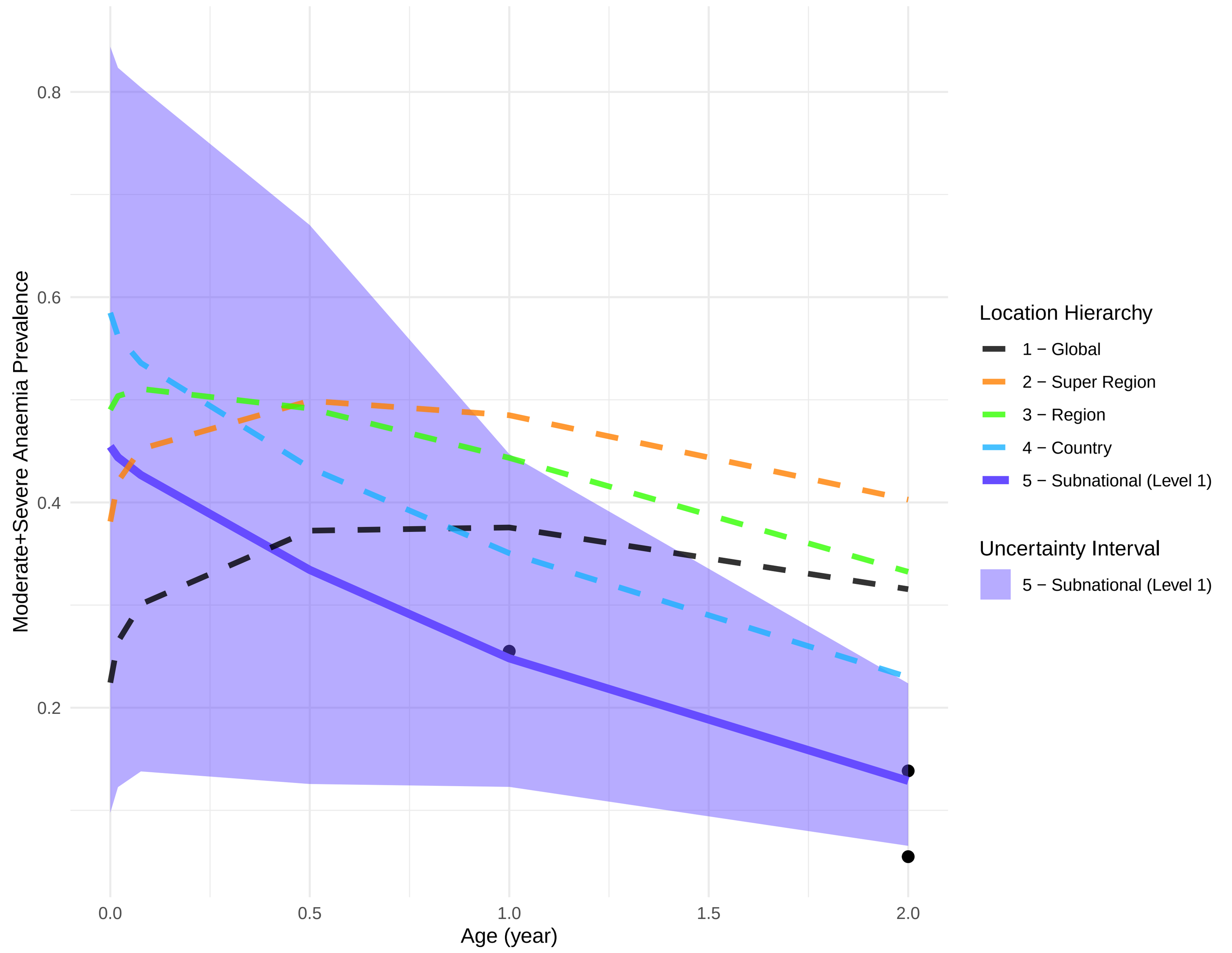

MR−BRT Age/Sex Fits − Males <2 Years Old − Moderate+Severe Anaemia Prevalence − Nairobi
WHO−2024 Adjustment Method

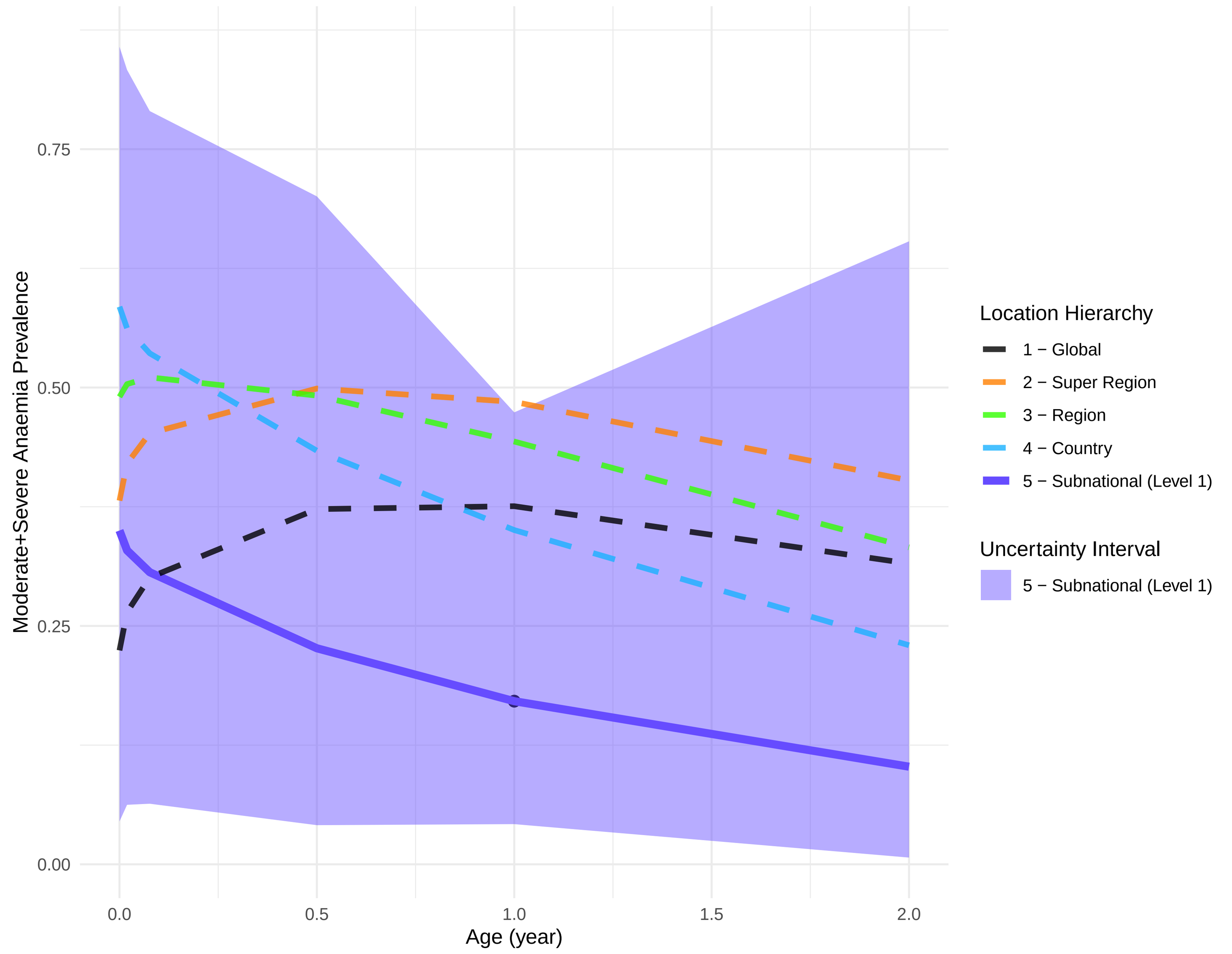

MR−BRT Age/Sex Fits − Males <2 Years Old − Moderate+Severe Anaemia Prevalence − Nakuru
WHO−2024 Adjustment Method

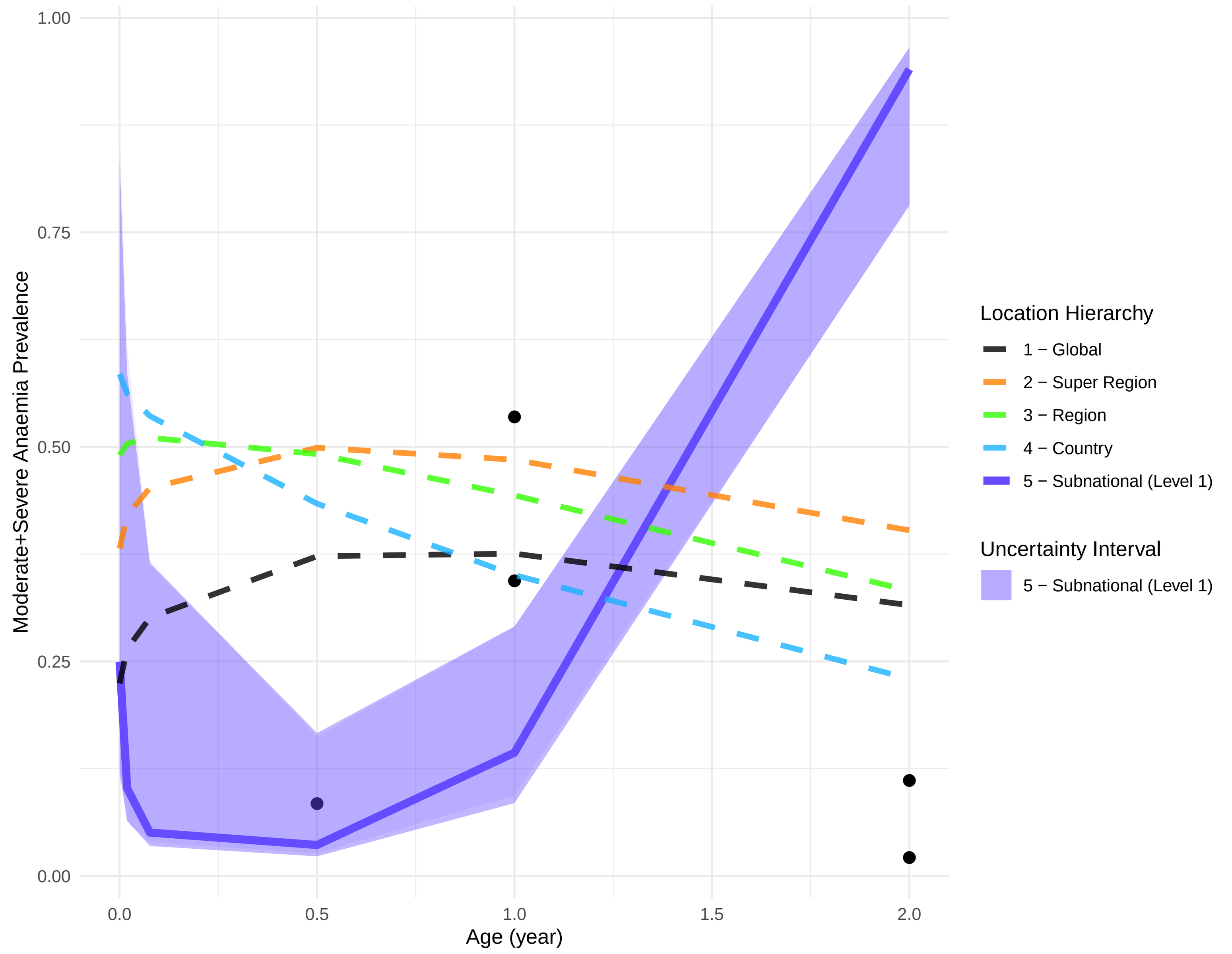

MR−BRT Age/Sex Fits − Males <2 Years Old − Moderate+Severe Anaemia Prevalence − Nandi
WHO−2024 Adjustment Method

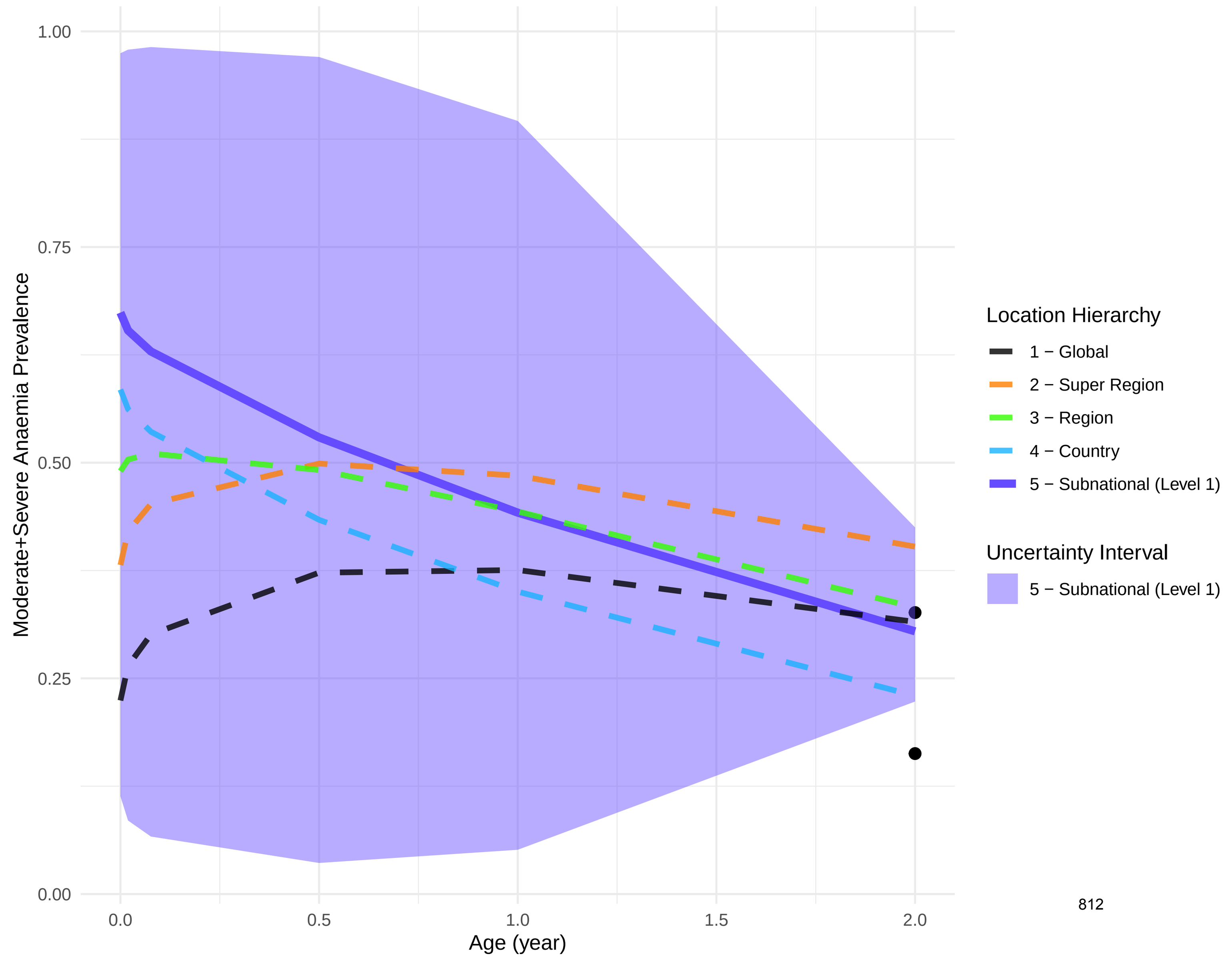

MR−BRT Age/Sex Fits − Males <2 Years Old − Moderate+Severe Anaemia Prevalence − Narok
WHO−2024 Adjustment Method

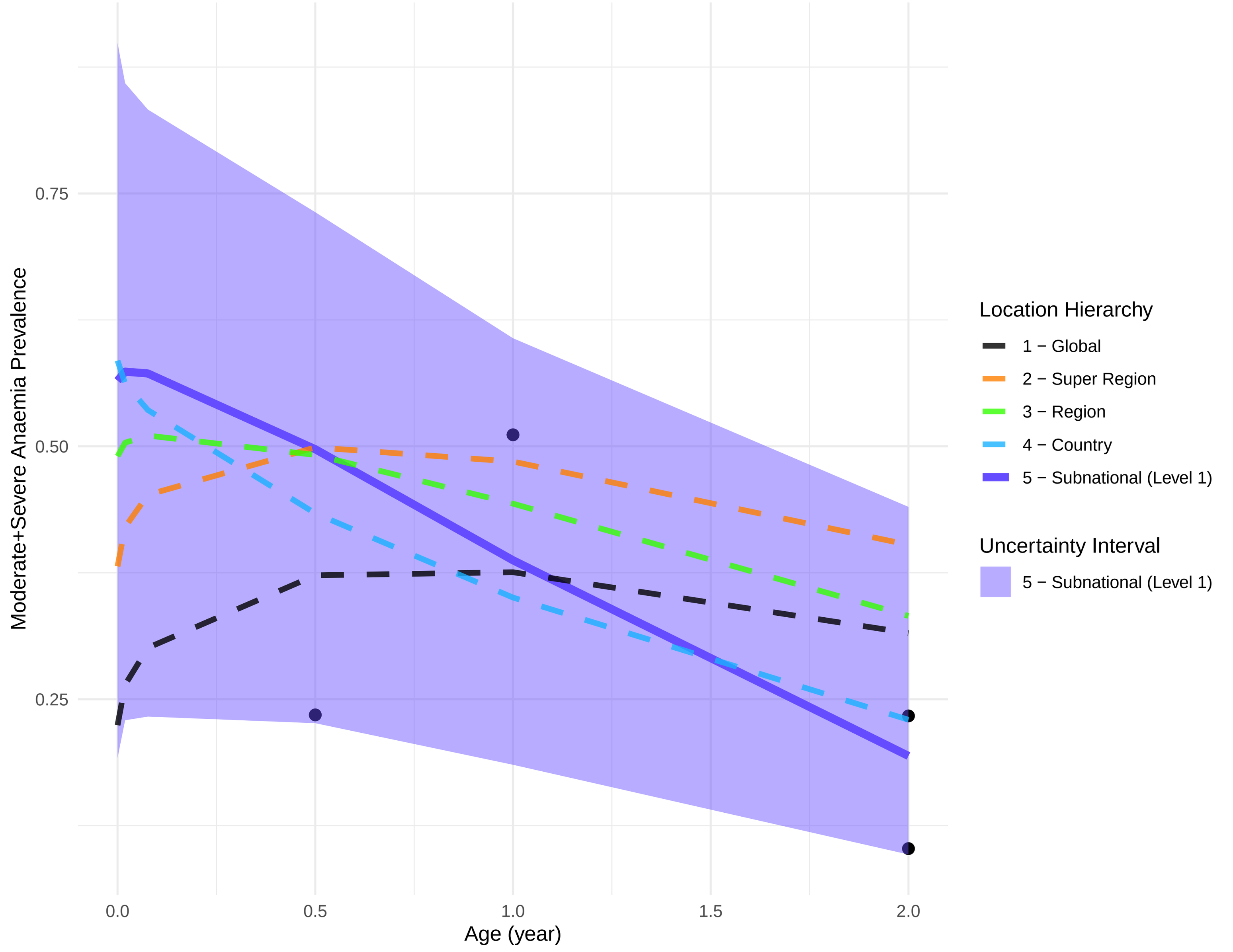

MR−BRT Age/Sex Fits − Males <2 Years Old − Moderate+Severe Anaemia Prevalence − Nyamira
WHO−2024 Adjustment Method

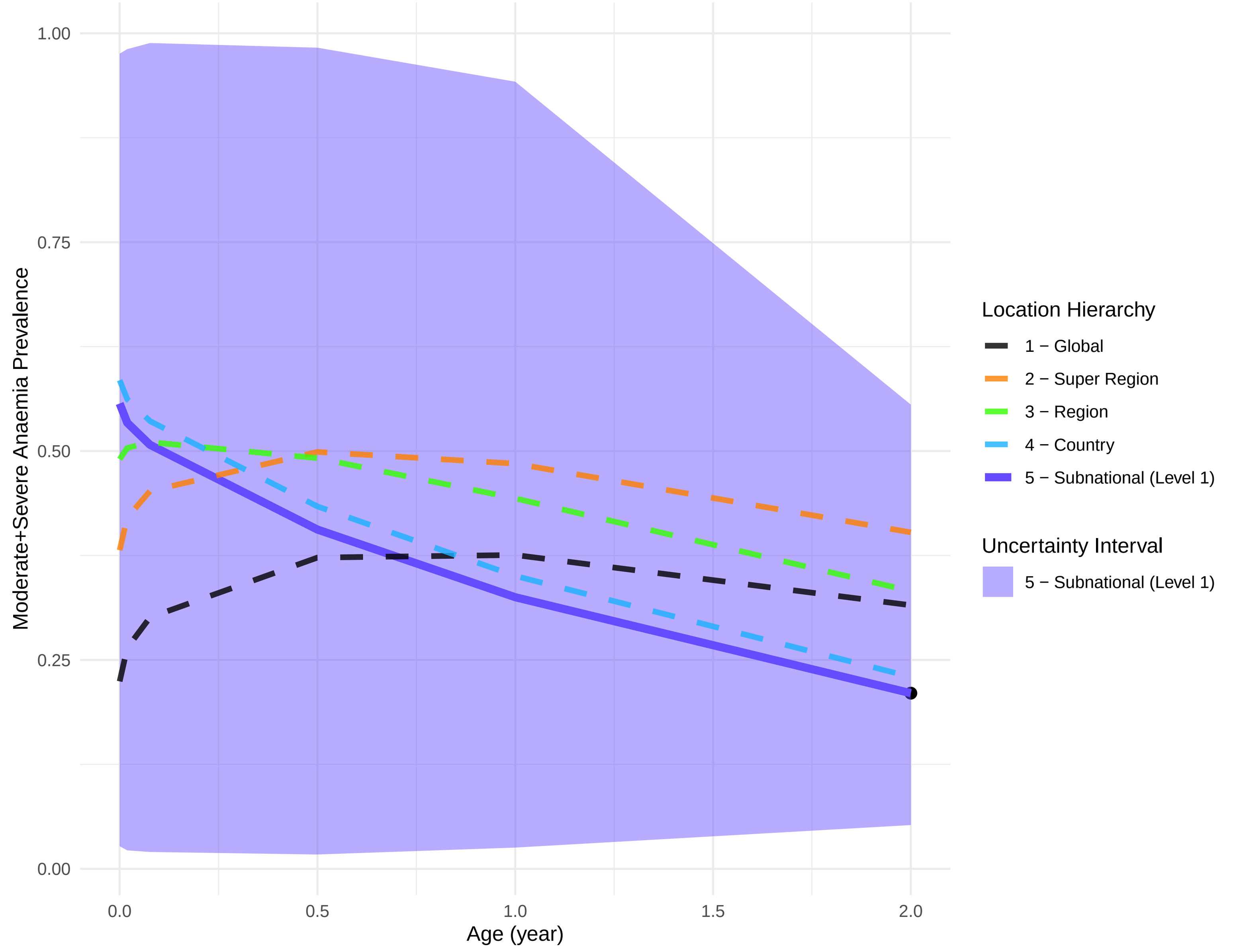

MR−BRT Age/Sex Fits − Males <2 Years Old − Moderate+Severe Anaemia Prevalence − Nyandarua
WHO−2024 Adjustment Method

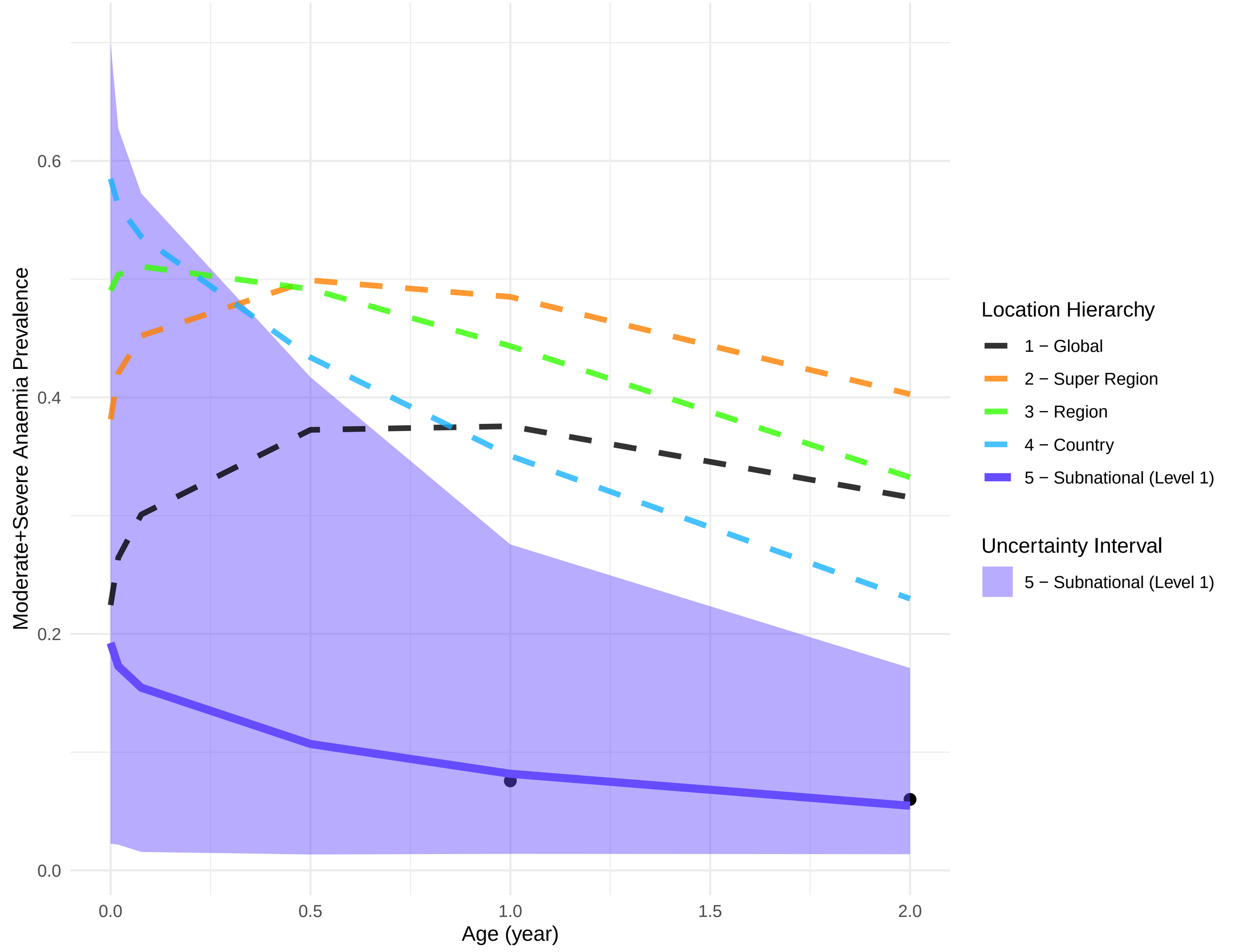

MR−BRT Age/Sex Fits − Males <2 Years Old − Moderate+Severe Anaemia Prevalence − Nyeri
WHO−2024 Adjustment Method

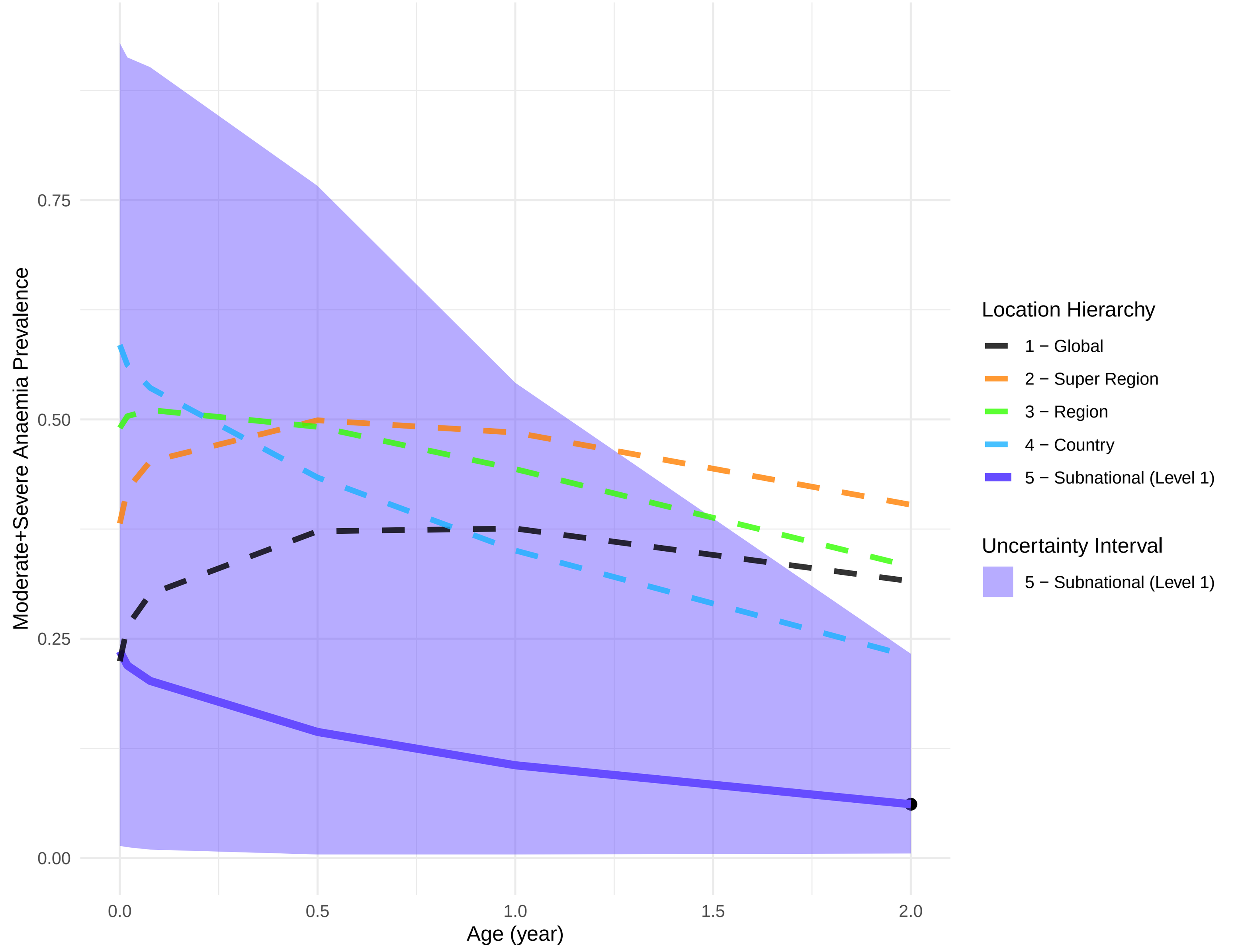

MR−BRT Age/Sex Fits − Males <2 Years Old − Moderate+Severe Anaemia Prevalence − Samburu
WHO−2024 Adjustment Method

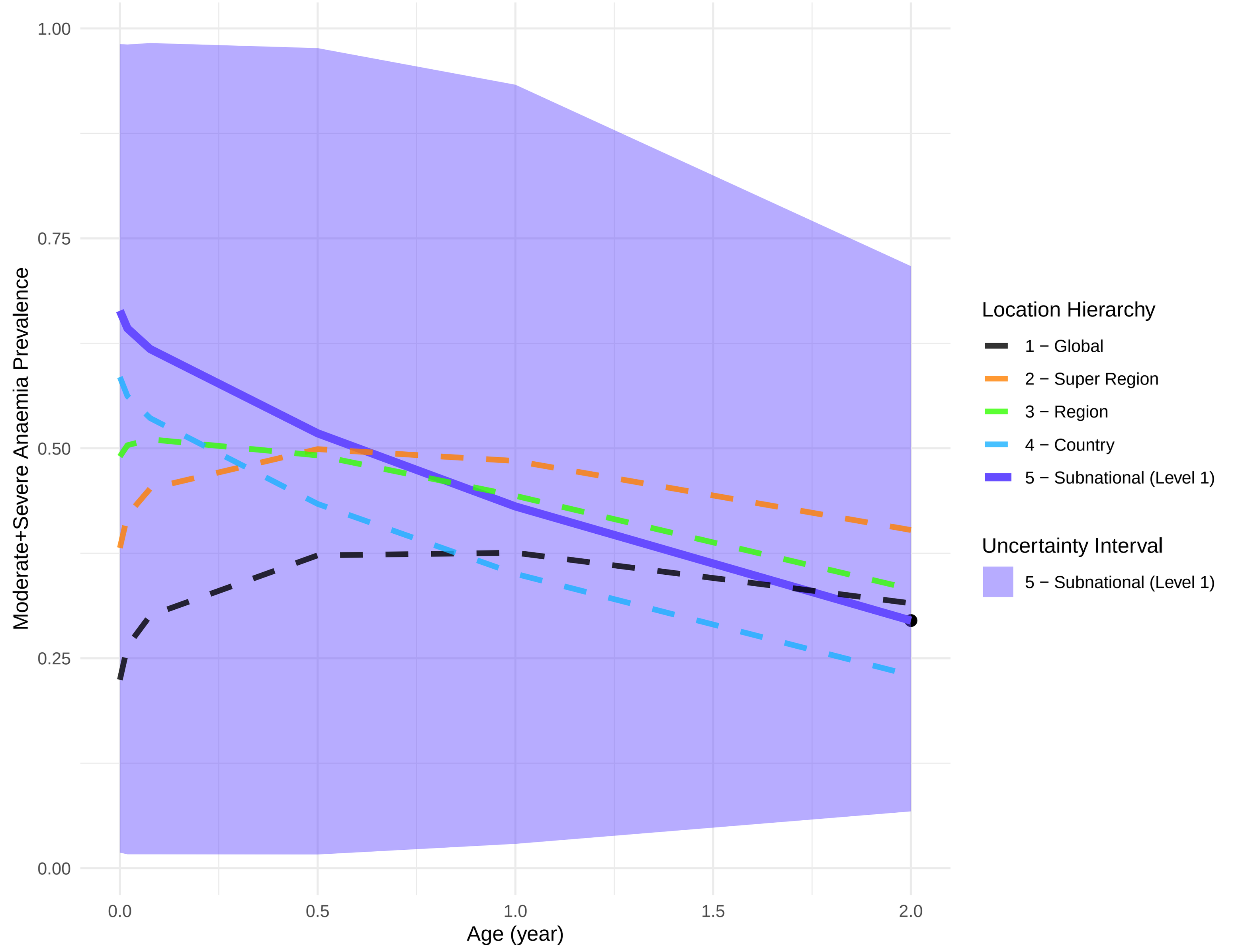

MR−BRT Age/Sex Fits − Males <2 Years Old − Moderate+Severe Anaemia Prevalence − Siaya
WHO−2024 Adjustment Method

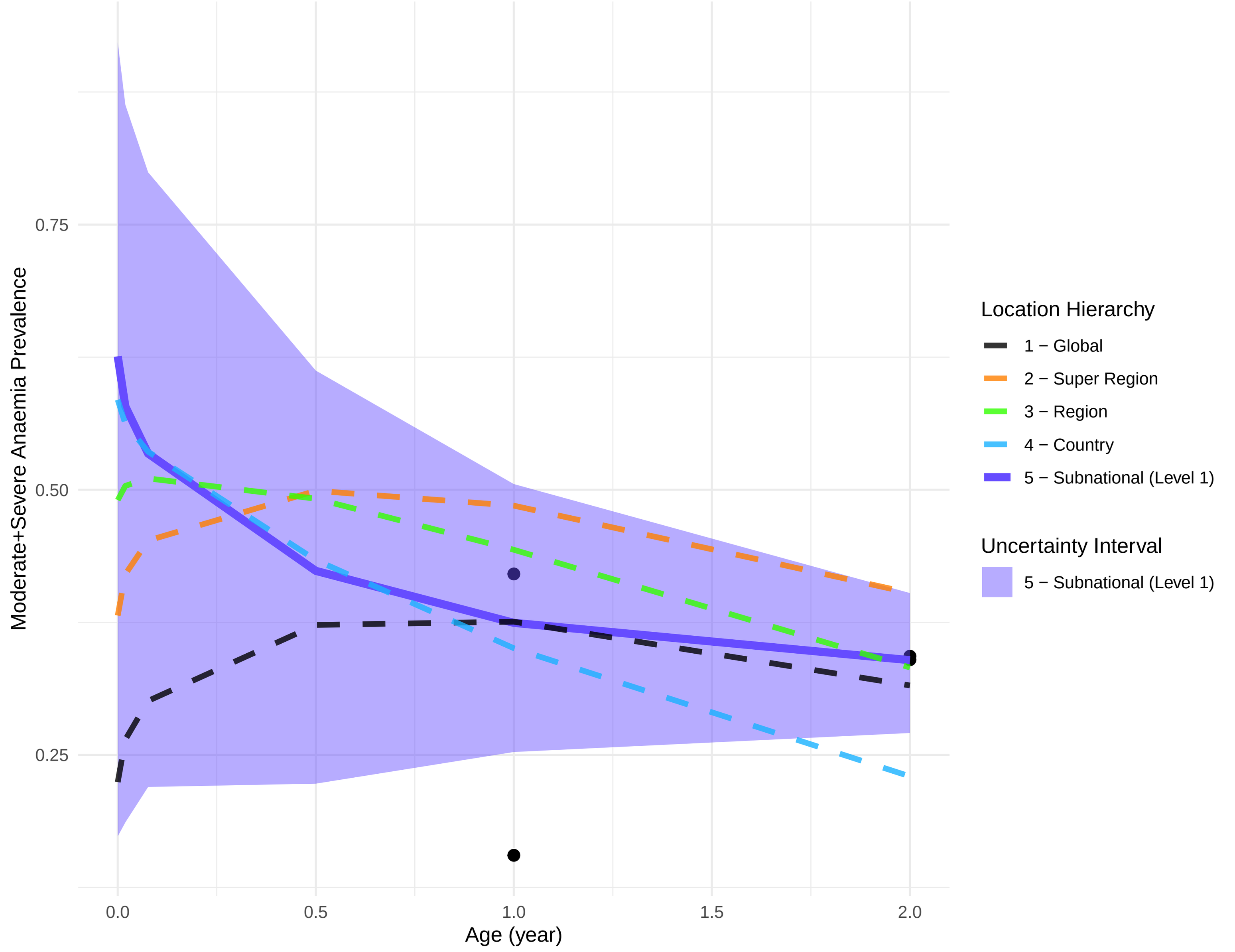

MR−BRT Age/Sex Fits − Males <2 Years Old − Moderate+Severe Anaemia Prevalence − Taita Taveta
WHO−2024 Adjustment Method

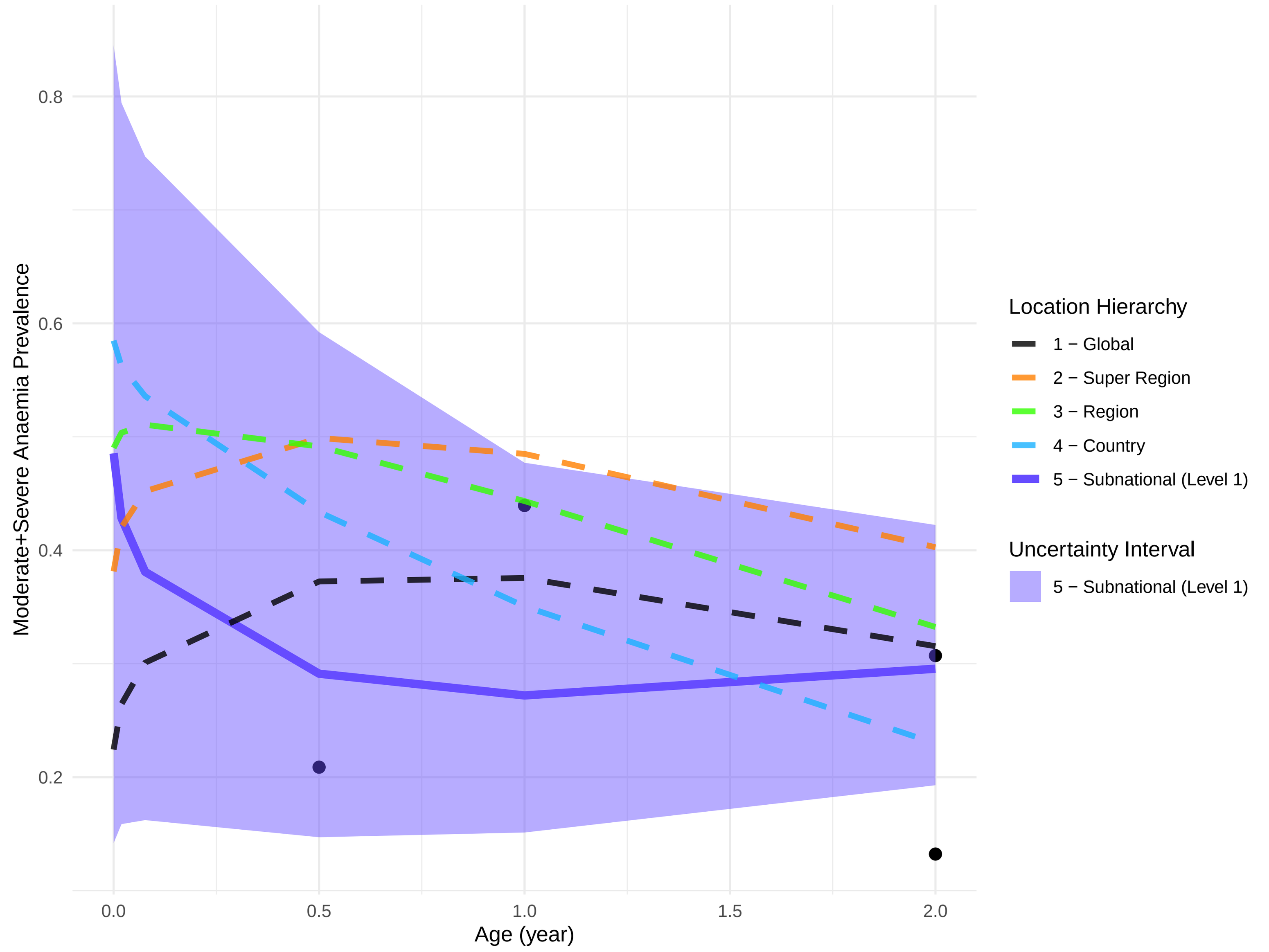

MR−BRT Age/Sex Fits − Males <2 Years Old − Moderate+Severe Anaemia Prevalence − Tana River
WHO−2024 Adjustment Method

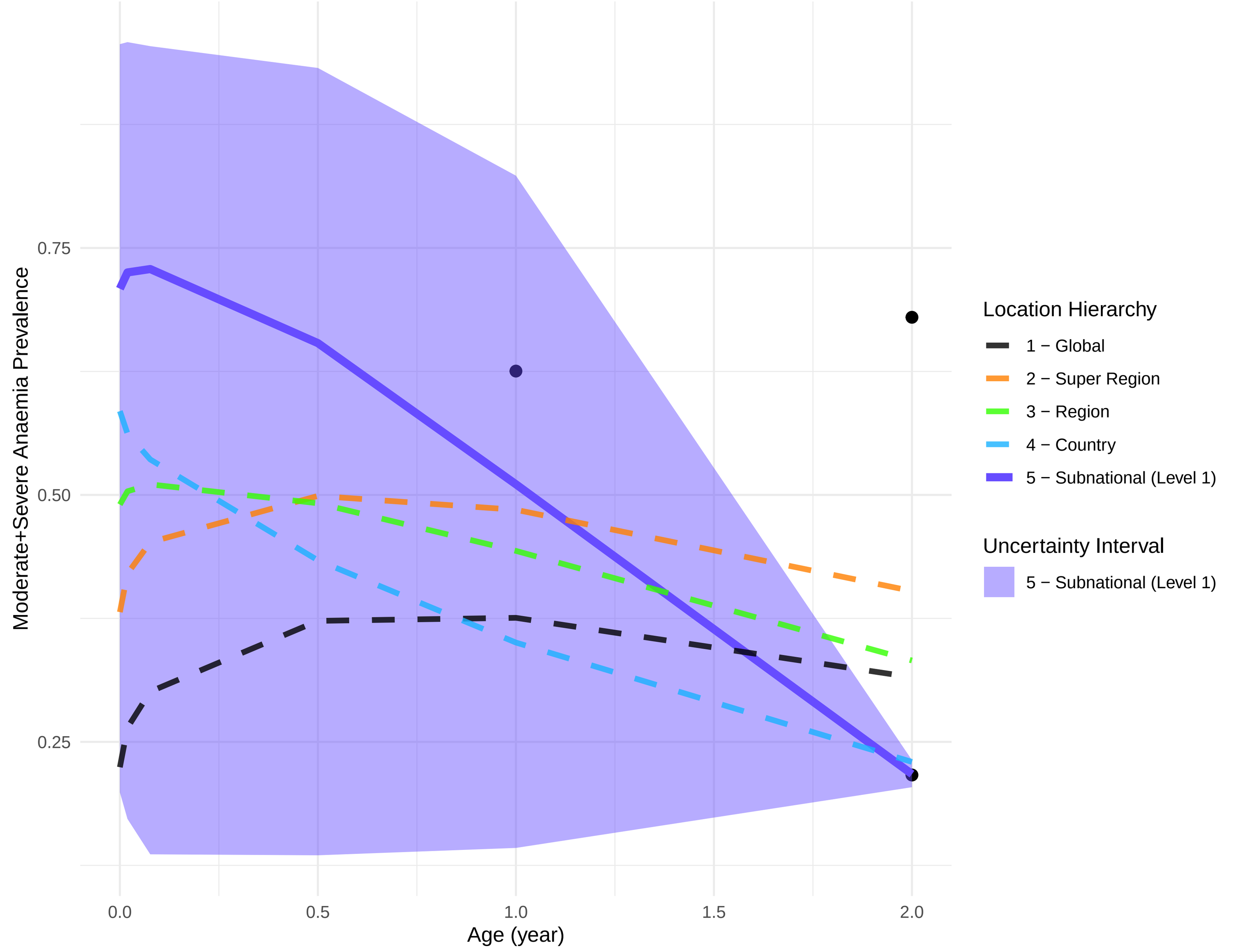

MR−BRT Age/Sex Fits − Males <2 Years Old − Moderate+Severe Anaemia Prevalence − Tharaka Nithi
WHO−2024 Adjustment Method

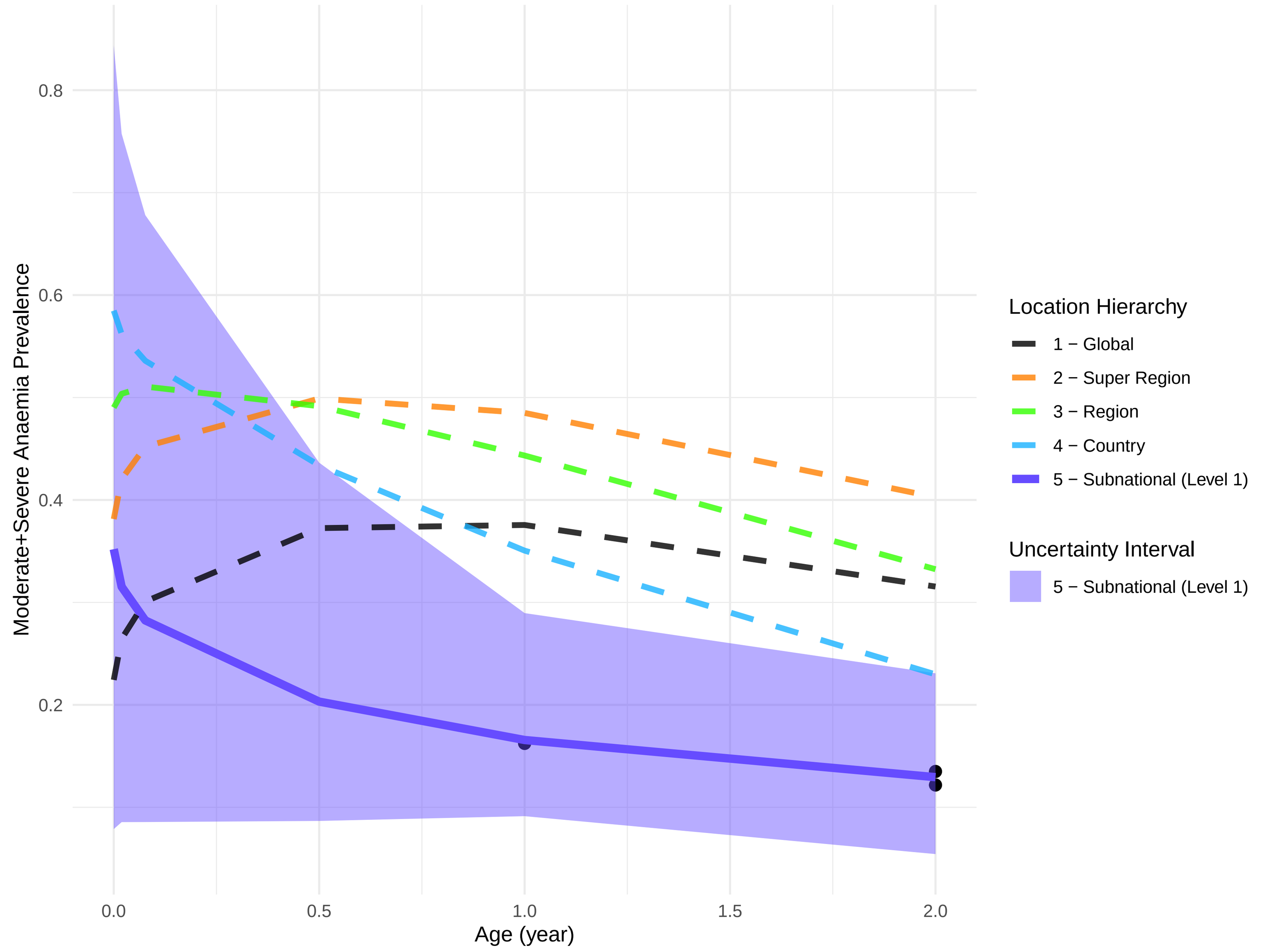

MR−BRT Age/Sex Fits − Males <2 Years Old − Moderate+Severe Anaemia Prevalence − Trans Nzoia
WHO−2024 Adjustment Method

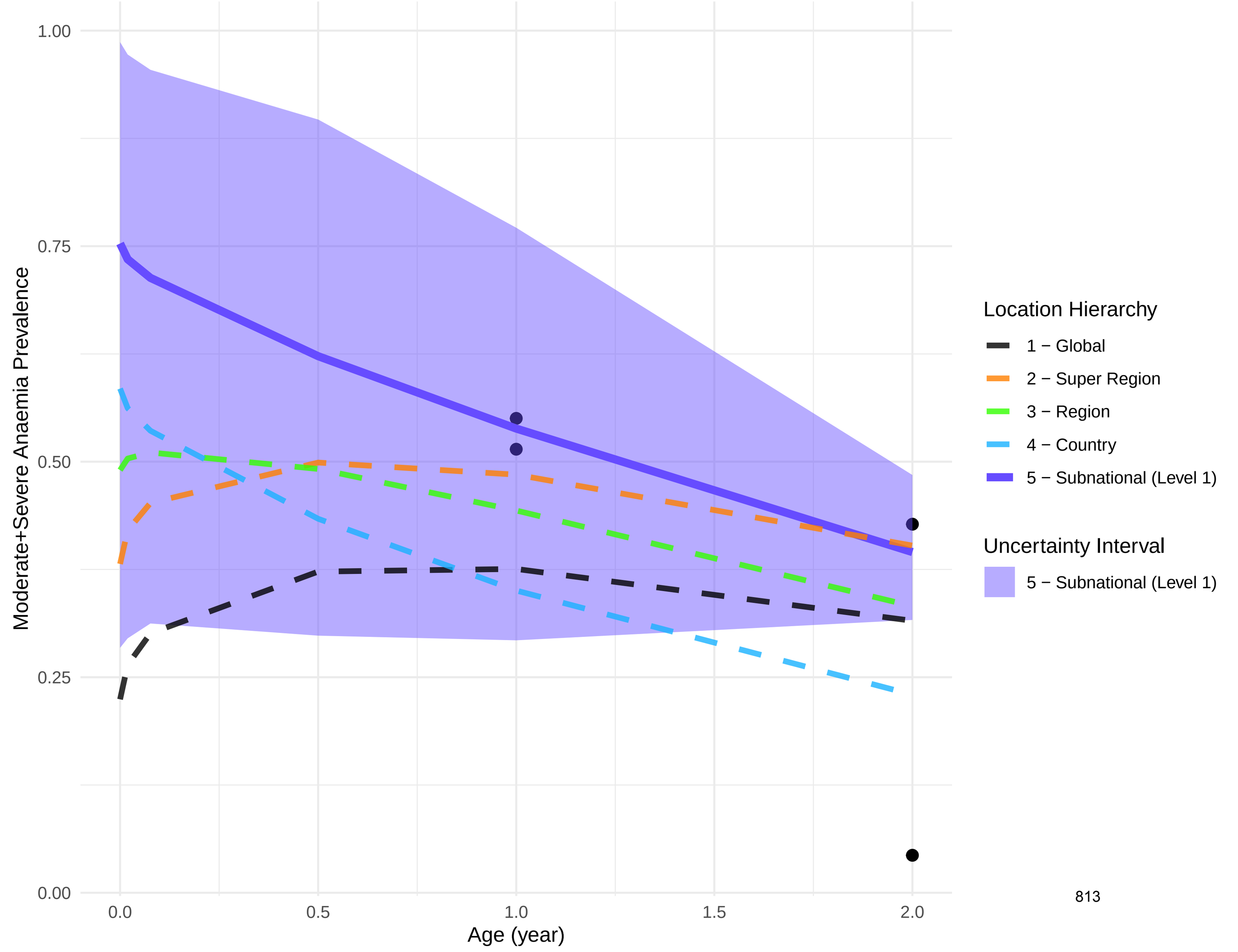

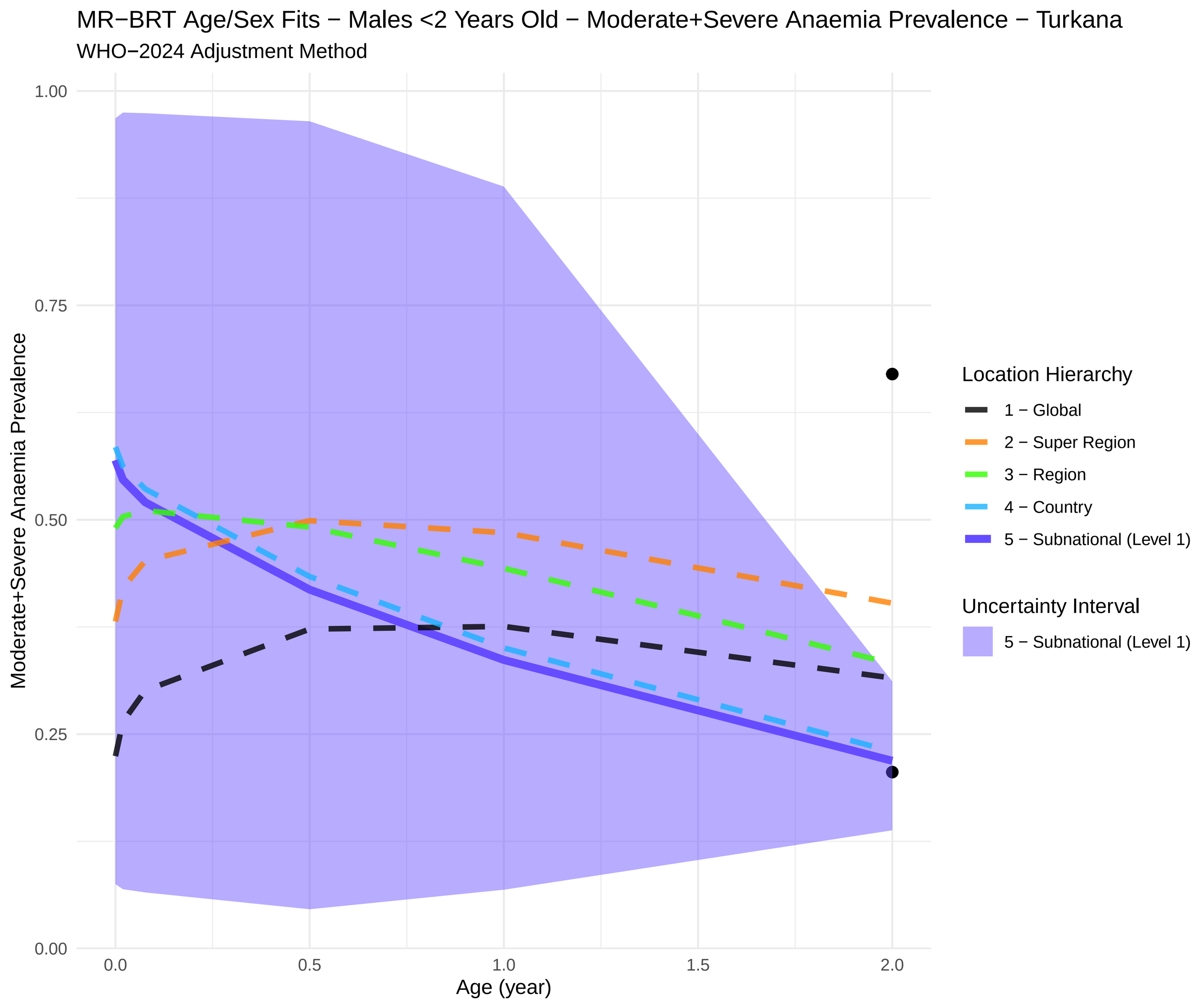
MR−BRT Age/Sex Fits − Males <2 Years Old − Moderate+Severe Anaemia Prevalence − Turkana
WHO−2024 Adjustment Method
Moderate+Severe Anaemia Prevalence
Age (year)
Location Hierarchy
1 − Global
2 − Super Region
3 − Region
4 − Country
5 − Subnational (Level 1)
Uncertainty Interval
5 − Subnational (Level 1)

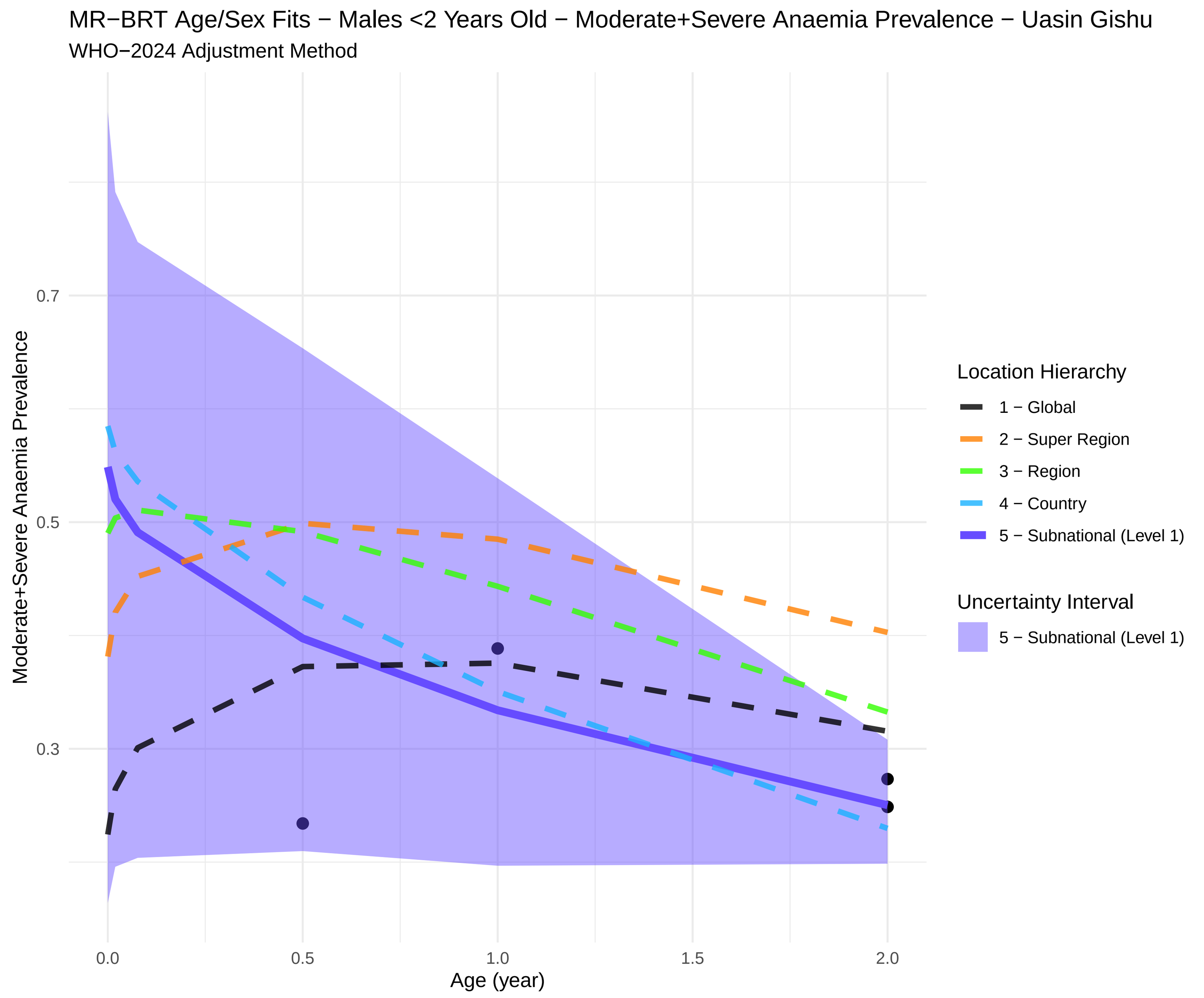
MR−BRT Age/Sex Fits − Males <2 Years Old − Moderate+Severe Anaemia Prevalence − Uasin Gishu
WHO−2024 Adjustment Method
Moderate+Severe Anaemia Prevalence
Age (year)
Location Hierarchy
1 − Global
2 − Super Region
3 − Region
4 − Country
5 − Subnational (Level 1)
Uncertainty Interval
5 − Subnational (Level 1)

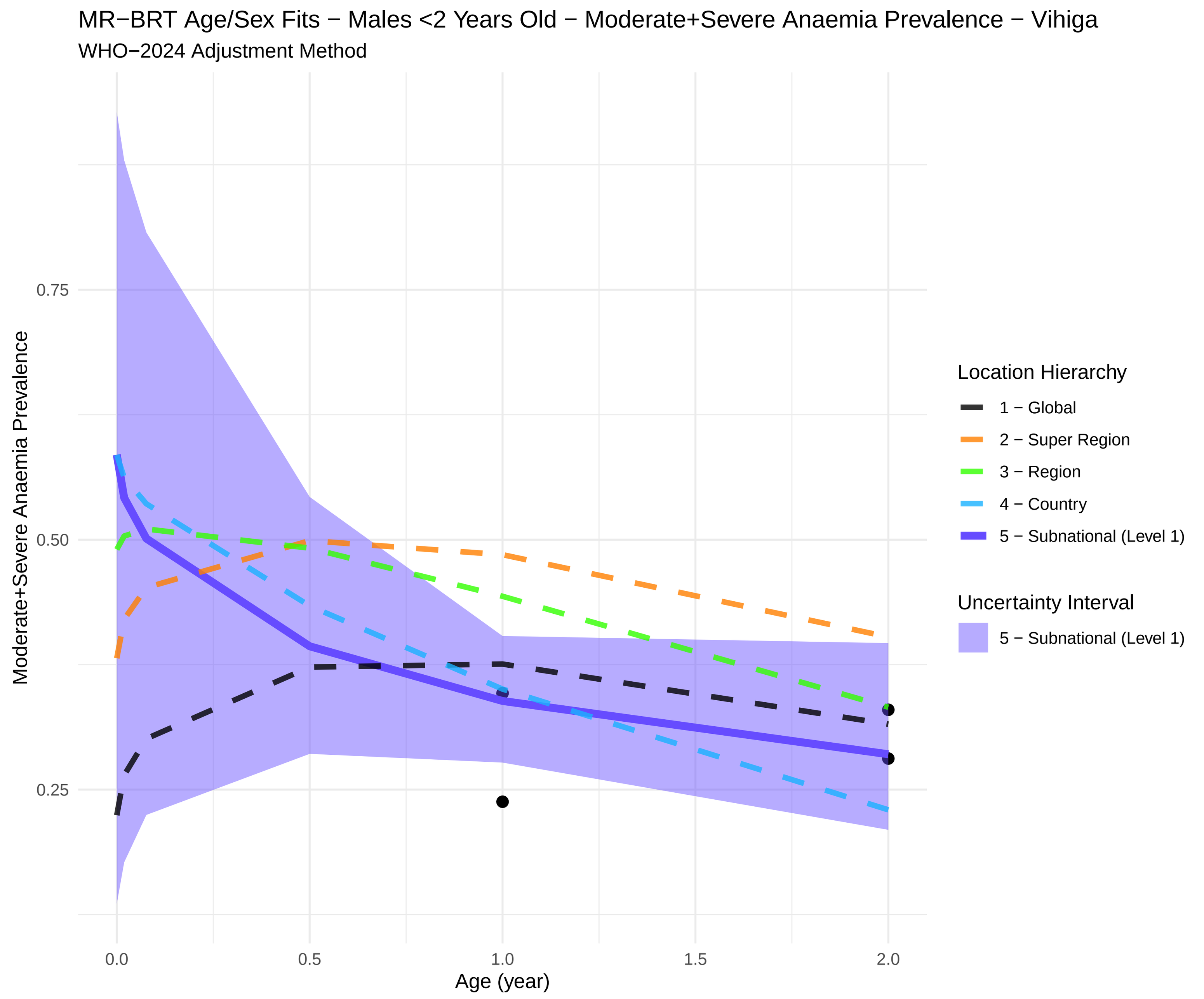
MR−BRT Age/Sex Fits − Males <2 Years Old − Moderate+Severe Anaemia Prevalence − Vihiga
WHO−2024 Adjustment Method
Moderate+Severe Anaemia Prevalence
Age (year)
Location Hierarchy
1 − Global
2 − Super Region
3 − Region
4 − Country
5 − Subnational (Level 1)
Uncertainty Interval
5 − Subnational (Level 1)

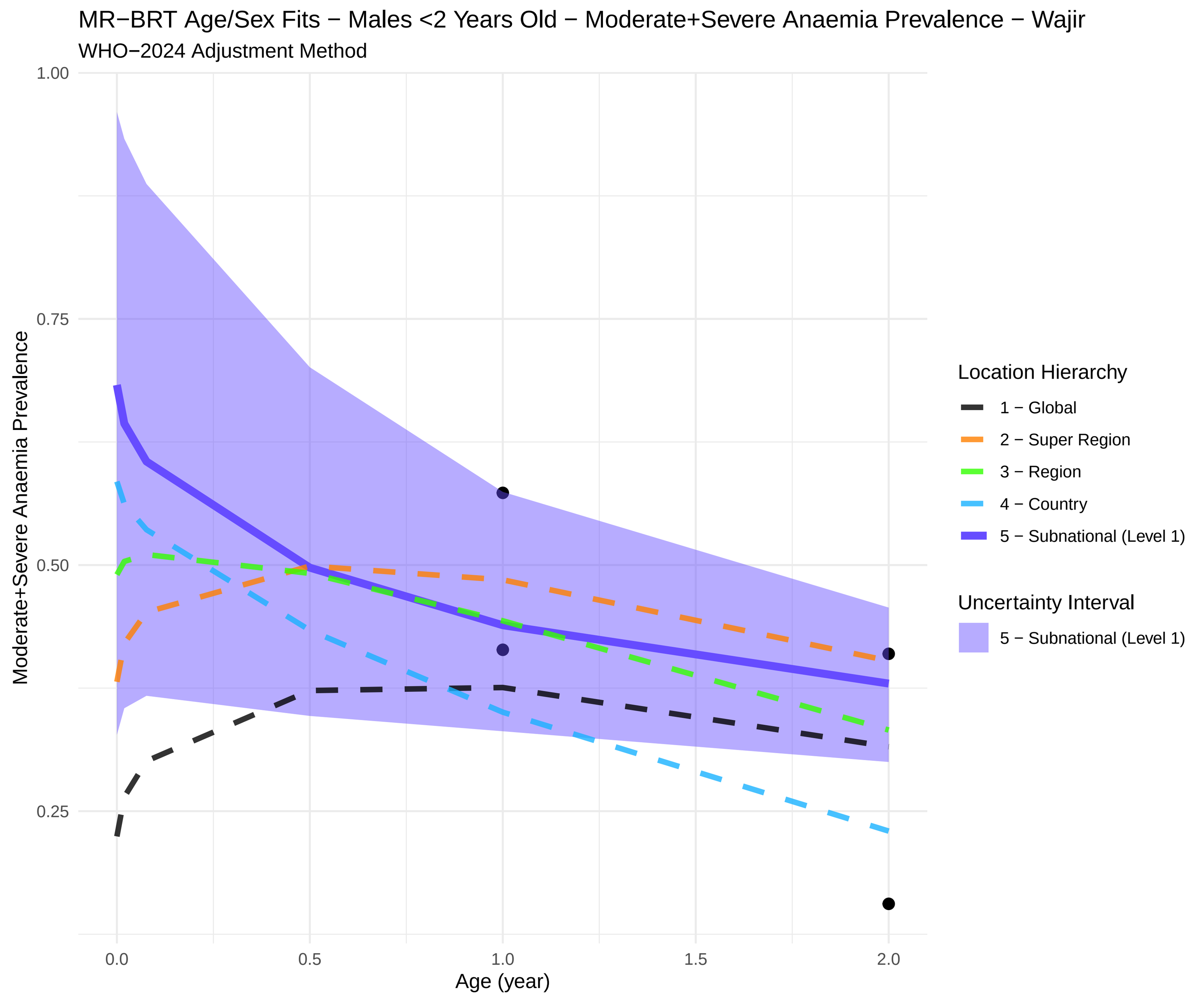
MR−BRT Age/Sex Fits − Males <2 Years Old − Moderate+Severe Anaemia Prevalence − Wajir
WHO−2024 Adjustment Method
Moderate+Severe Anaemia Prevalence
Age (year)
Location Hierarchy
1 − Global
2 − Super Region
3 − Region
4 − Country
5 − Subnational (Level 1)
Uncertainty Interval
5 − Subnational (Level 1)

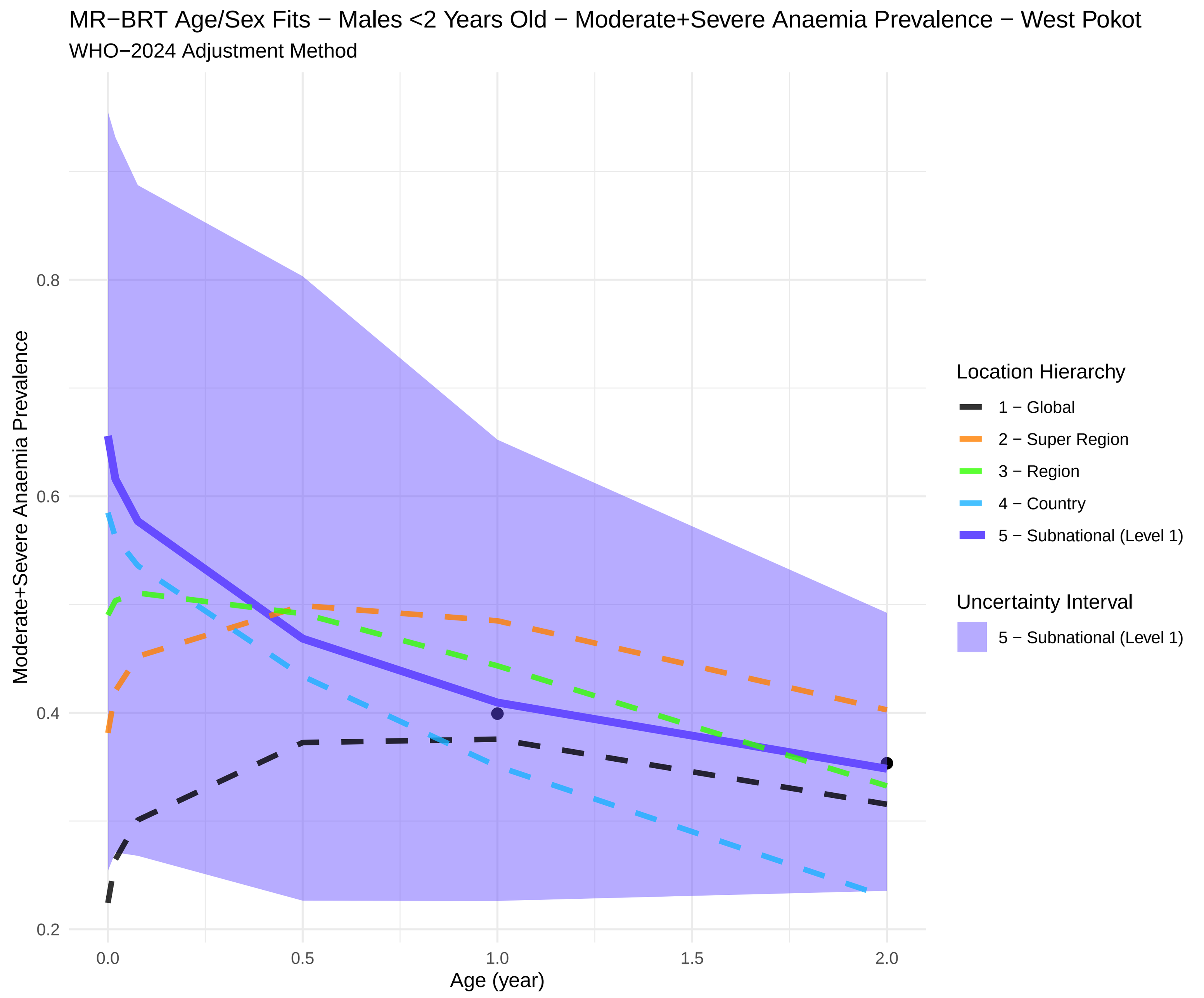
MR−BRT Age/Sex Fits − Males <2 Years Old − Moderate+Severe Anaemia Prevalence − West Pokot
WHO−2024 Adjustment Method
Moderate+Severe Anaemia Prevalence
Age (year)
Location Hierarchy
1 − Global
2 − Super Region
3 − Region
4 − Country
5 − Subnational (Level 1)
Uncertainty Interval
5 − Subnational (Level 1)

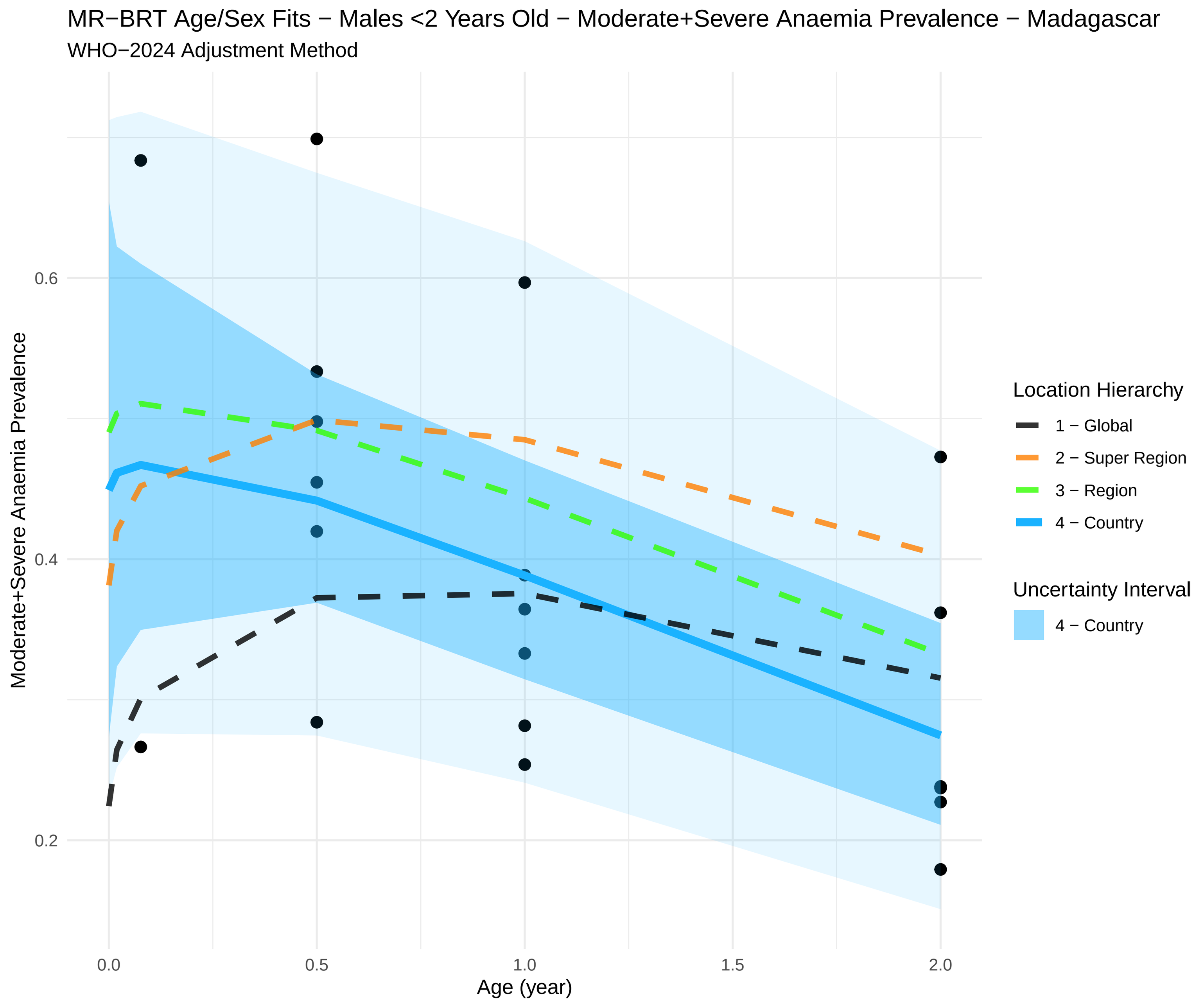
MR−BRT Age/Sex Fits − Males <2 Years Old − Moderate+Severe Anaemia Prevalence − Madagascar
WHO−2024 Adjustment Method
Moderate+Severe Anaemia Prevalence
Age (year)
Location Hierarchy
1 − Global
2 − Super Region
3 − Region
4 − Country
Uncertainty Interval
4 − Country

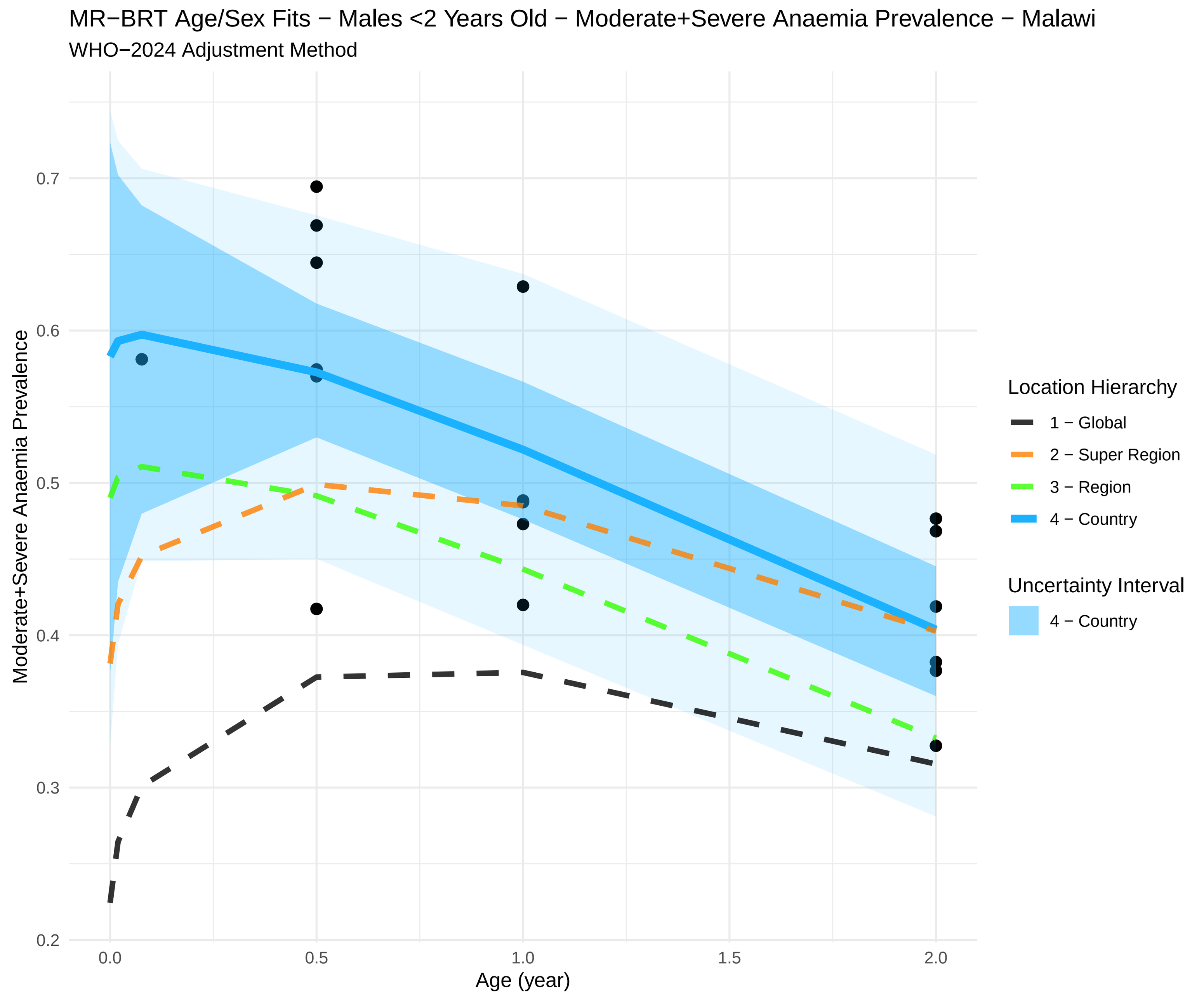
MR−BRT Age/Sex Fits − Males <2 Years Old − Moderate+Severe Anaemia Prevalence − Malawi
WHO−2024 Adjustment Method
Moderate+Severe Anaemia Prevalence
Age (year)
Location Hierarchy
1 − Global
2 − Super Region
3 − Region
4 − Country
Uncertainty Interval
4 − Country

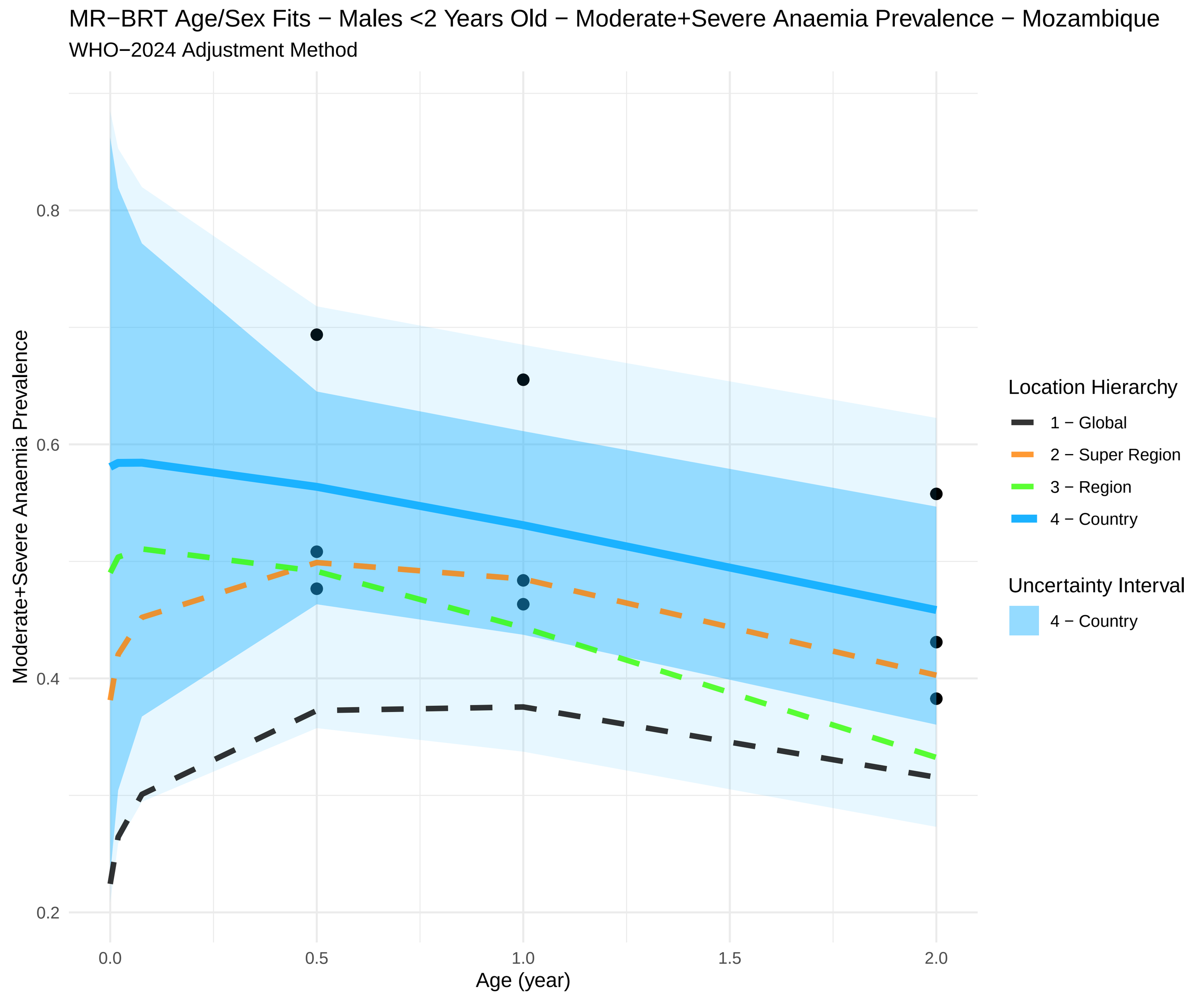
MR−BRT Age/Sex Fits − Males <2 Years Old − Moderate+Severe Anaemia Prevalence − Mozambique
WHO−2024 Adjustment Method
Moderate+Severe Anaemia Prevalence
Age (year)
Location Hierarchy
1 − Global
2 − Super Region
3 − Region
4 − Country
Uncertainty Interval
4 − Country

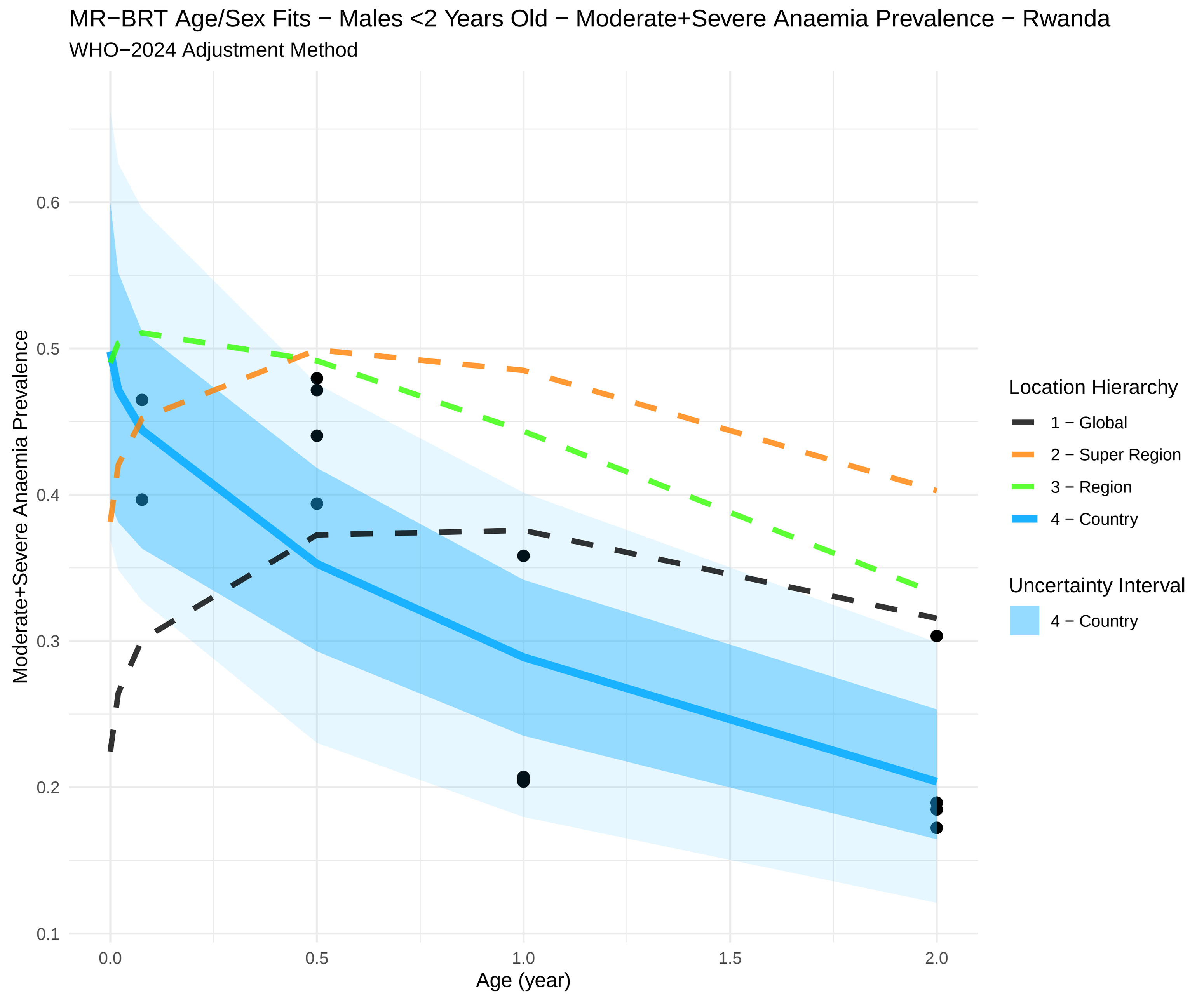
MR−BRT Age/Sex Fits − Males <2 Years Old − Moderate+Severe Anaemia Prevalence − Rwanda
WHO−2024 Adjustment Method
Moderate+Severe Anaemia Prevalence
Age (year)
Location Hierarchy
1 − Global
2 − Super Region
3 − Region
4 − Country
Uncertainty Interval
4 − Country

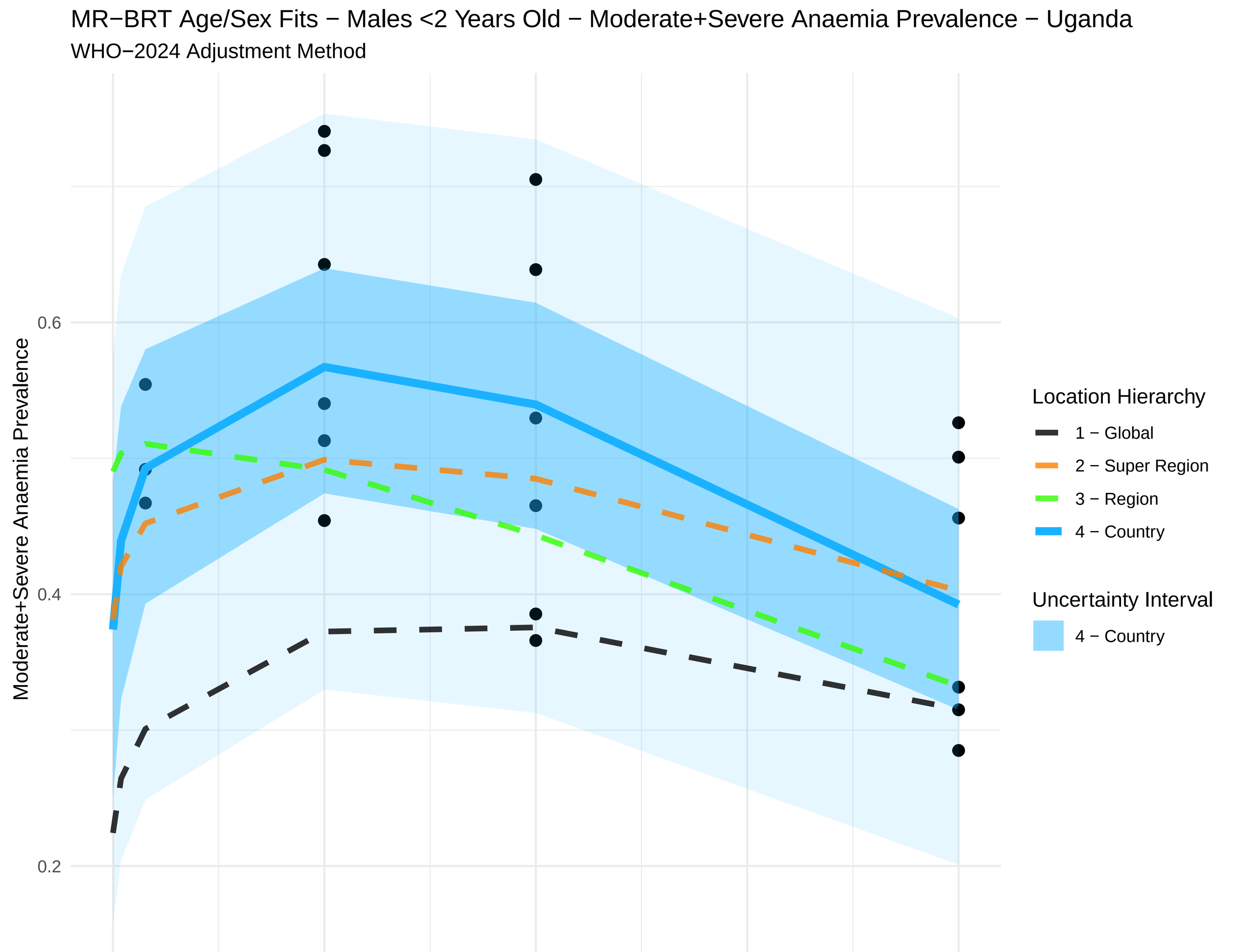
MR−BRT Age/Sex Fits − Males <2 Years Old − Moderate+Severe Anaemia Prevalence − Uganda
WHO−2024 Adjustment Method
Moderate+Severe Anaemia Prevalence
Age (year)
Location Hierarchy
1 − Global
2 − Super Region
3 − Region
4 − Country
Uncertainty Interval
4 − Country

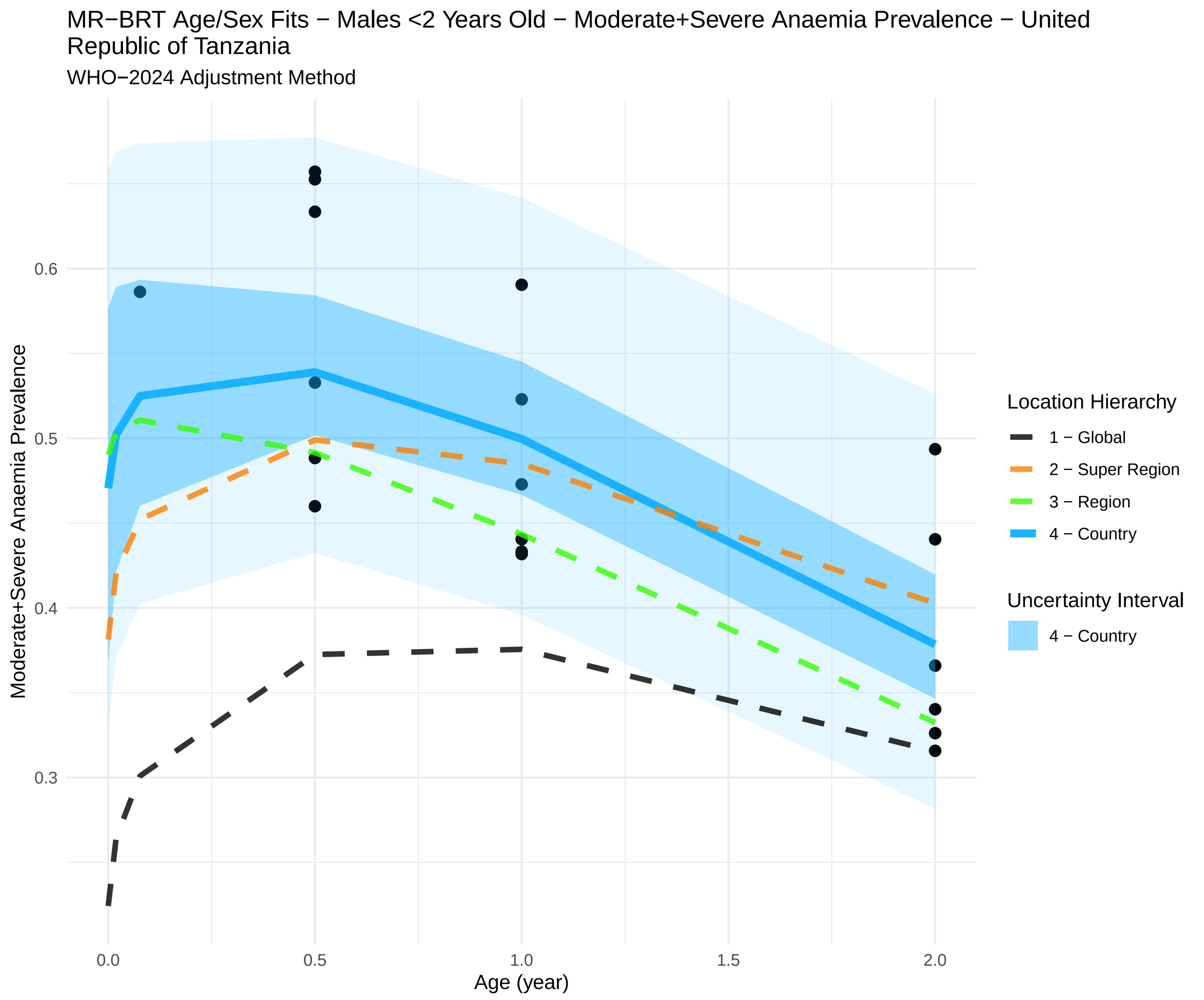
MR−BRT Age/Sex Fits − Males <2 Years Old − Moderate+Severe Anaemia Prevalence − United Republic of Tanzania
WHO−2024 Adjustment Method
Moderate+Severe Anaemia Prevalence
Age (year)
Location Hierarchy
1 − Global
2 − Super Region
3 − Region
4 − Country
Uncertainty Interval
4 − Country

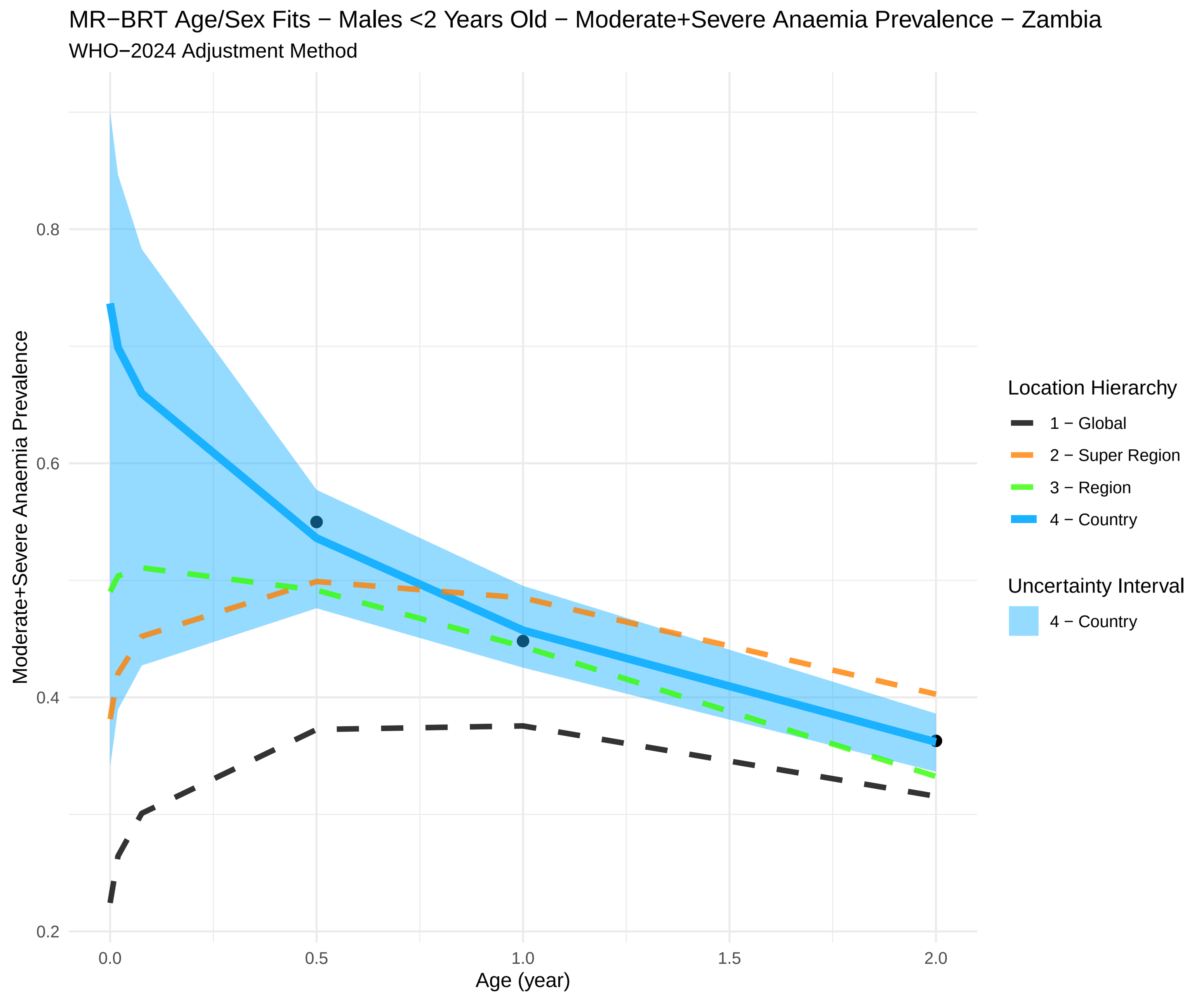
MR−BRT Age/Sex Fits − Males <2 Years Old − Moderate+Severe Anaemia Prevalence − Zambia
WHO−2024 Adjustment Method
Moderate+Severe Anaemia Prevalence
Age (year)
Location Hierarchy
1 − Global
2 − Super Region
3 − Region
4 − Country
Uncertainty Interval
4 − Country

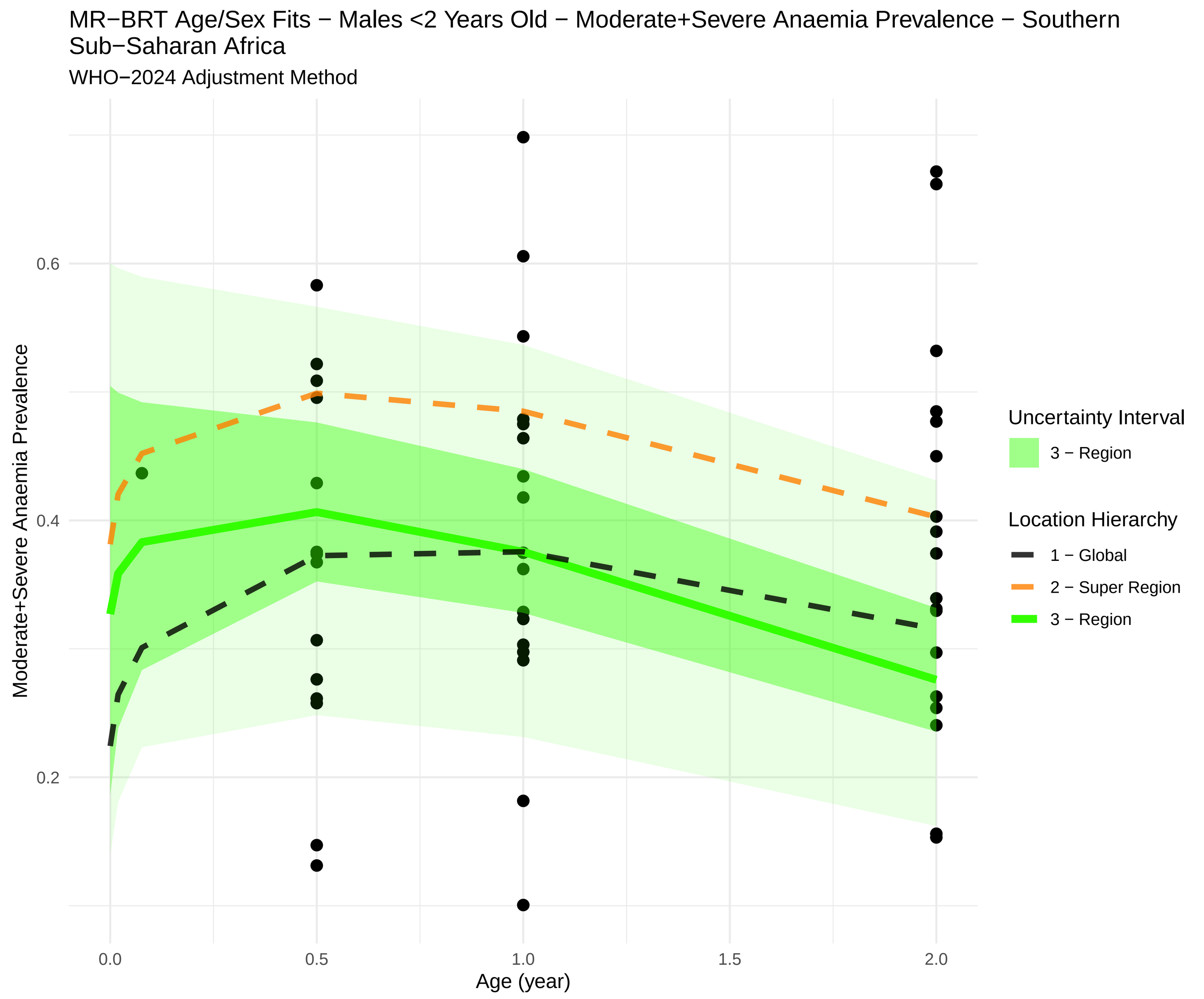
MR−BRT Age/Sex Fits − Males <2 Years Old − Moderate+Severe Anaemia Prevalence − Southern Sub−Saharan Africa
WHO−2024 Adjustment Method
Moderate+Severe Anaemia Prevalence
Age (year)
Uncertainty Interval
3 − Region
Location Hierarchy
1 − Global
2 − Super Region
3 − Region

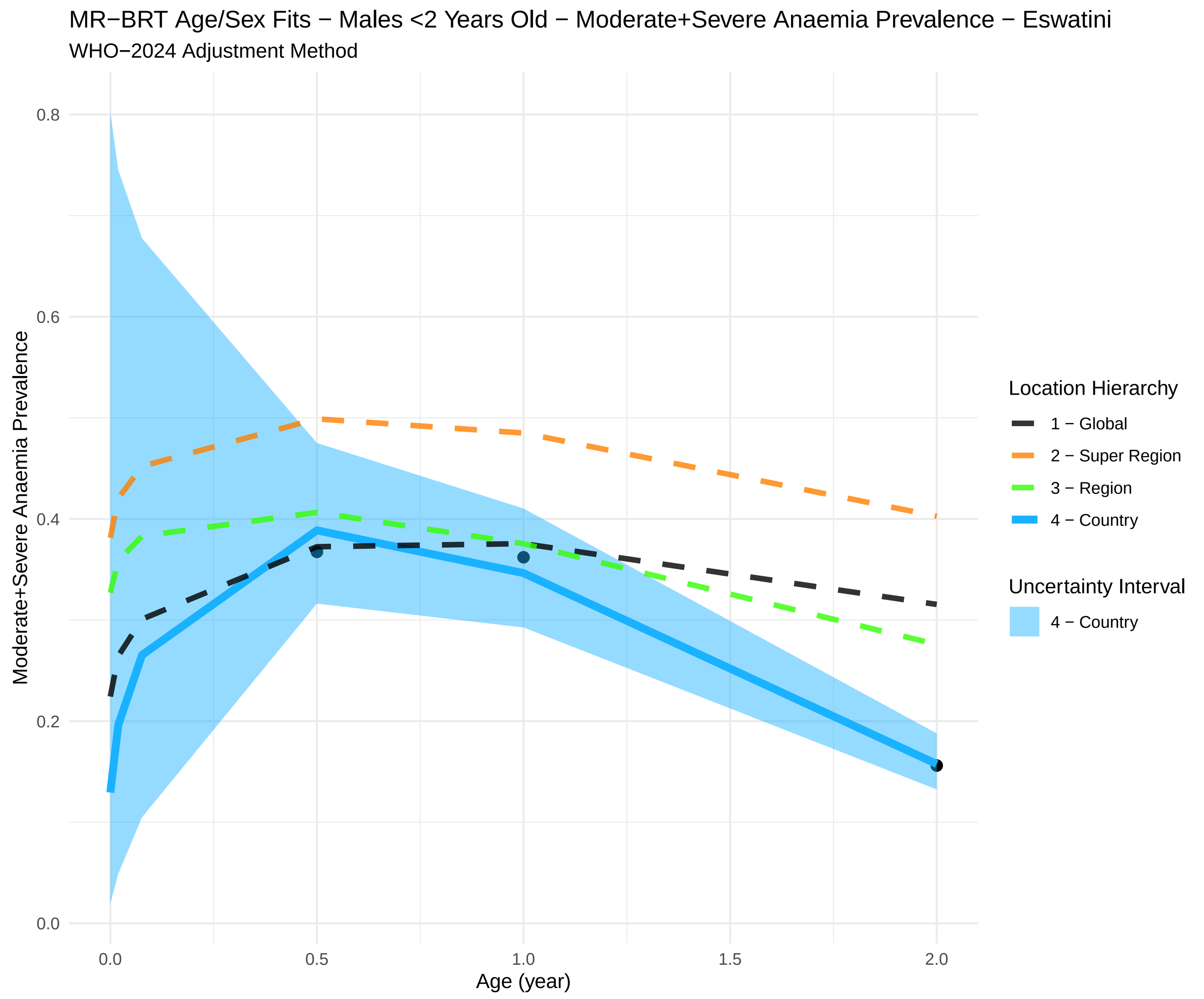
MR−BRT Age/Sex Fits − Males <2 Years Old − Moderate+Severe Anaemia Prevalence − Eswatini
WHO−2024 Adjustment Method
Moderate+Severe Anaemia Prevalence
Age (year)
Location Hierarchy
1 − Global
2 − Super Region
3 − Region
4 − Country
Uncertainty Interval
4 − Country

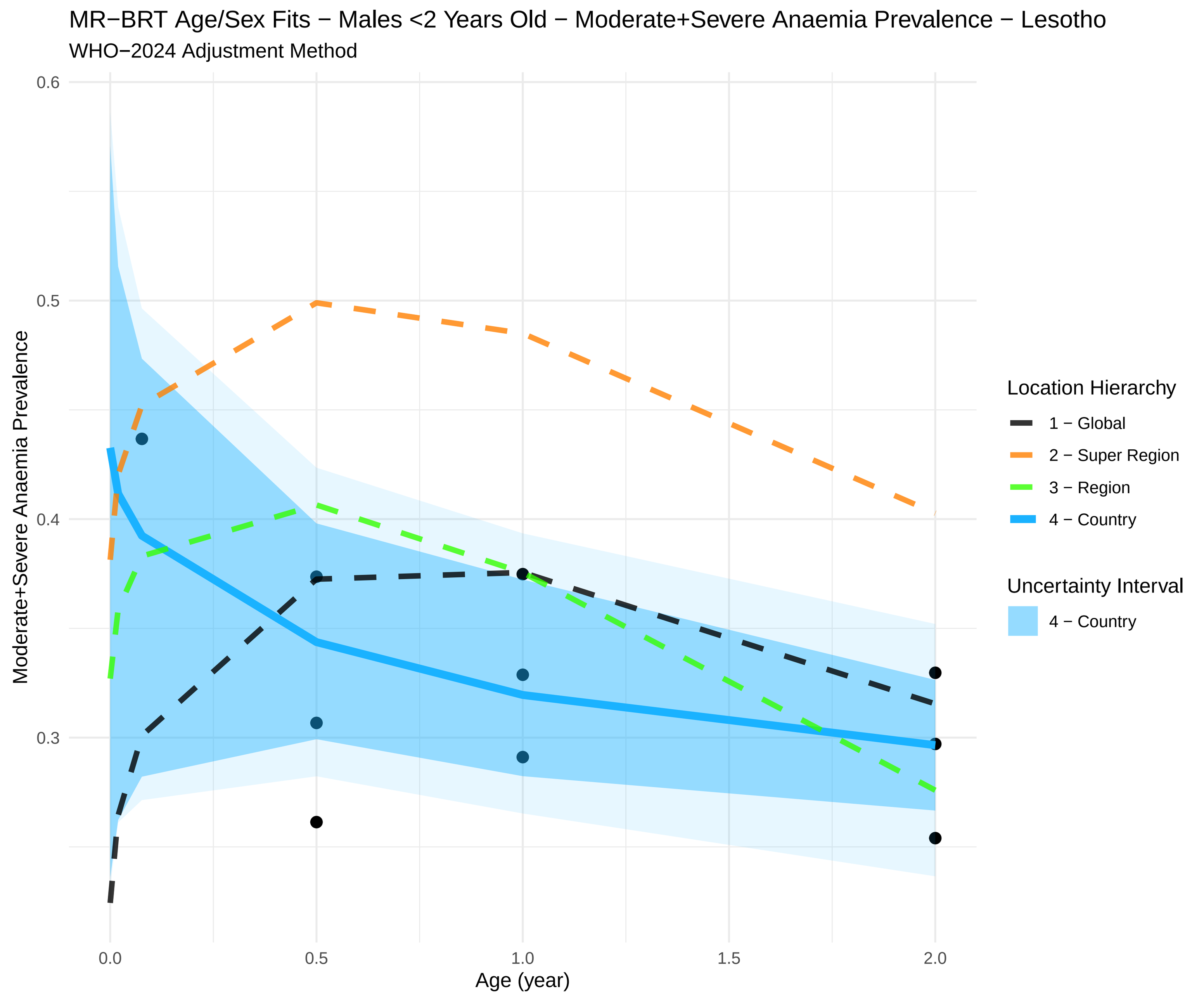
MR−BRT Age/Sex Fits − Males <2 Years Old − Moderate+Severe Anaemia Prevalence − Lesotho
WHO−2024 Adjustment Method
Moderate+Severe Anaemia Prevalence
Age (year)
Location Hierarchy
1 − Global
2 − Super Region
3 − Region
4 − Country
Uncertainty Interval
4 − Country

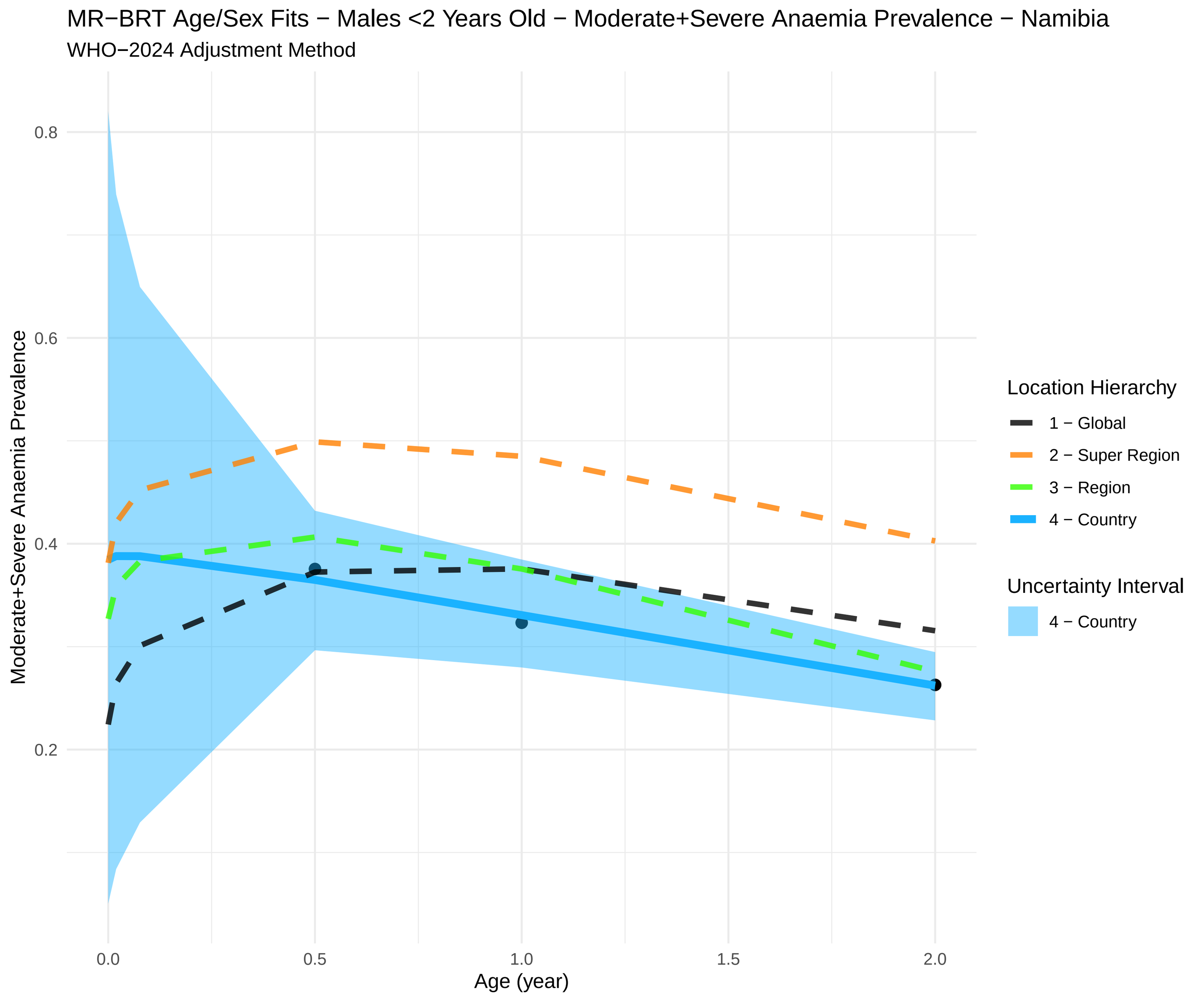
MR−BRT Age/Sex Fits − Males <2 Years Old − Moderate+Severe Anaemia Prevalence − Namibia
WHO−2024 Adjustment Method
Moderate+Severe Anaemia Prevalence
Age (year)
Location Hierarchy
1 − Global
2 − Super Region
3 − Region
4 − Country
Uncertainty Interval
4 − Country

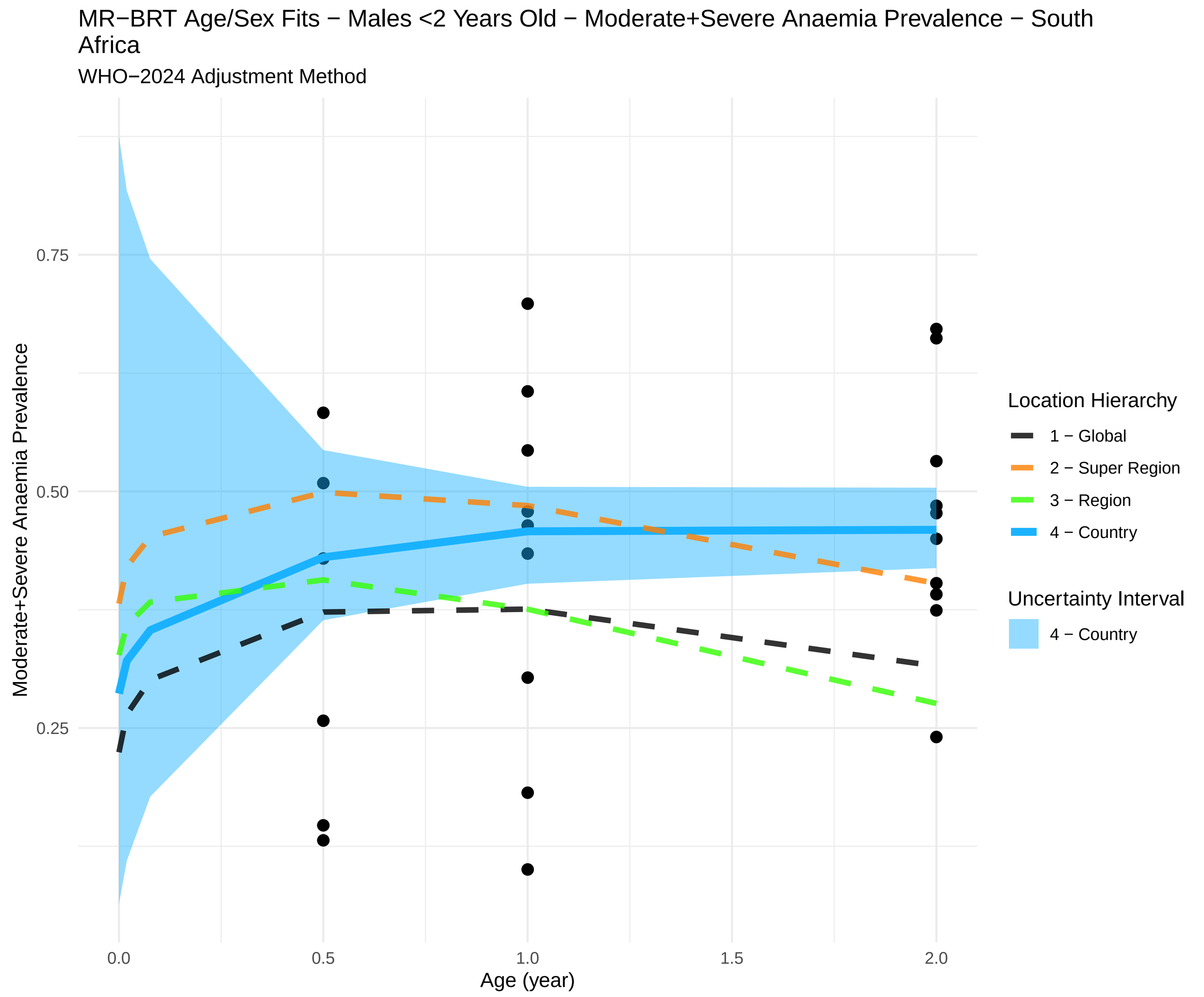
MR−BRT Age/Sex Fits − Males <2 Years Old − Moderate+Severe Anaemia Prevalence − South Africa
WHO−2024 Adjustment Method
Moderate+Severe Anaemia Prevalence
Age (year)
Location Hierarchy
1 − Global
2 − Super Region
3 − Region
4 − Country
Uncertainty Interval
4 − Country

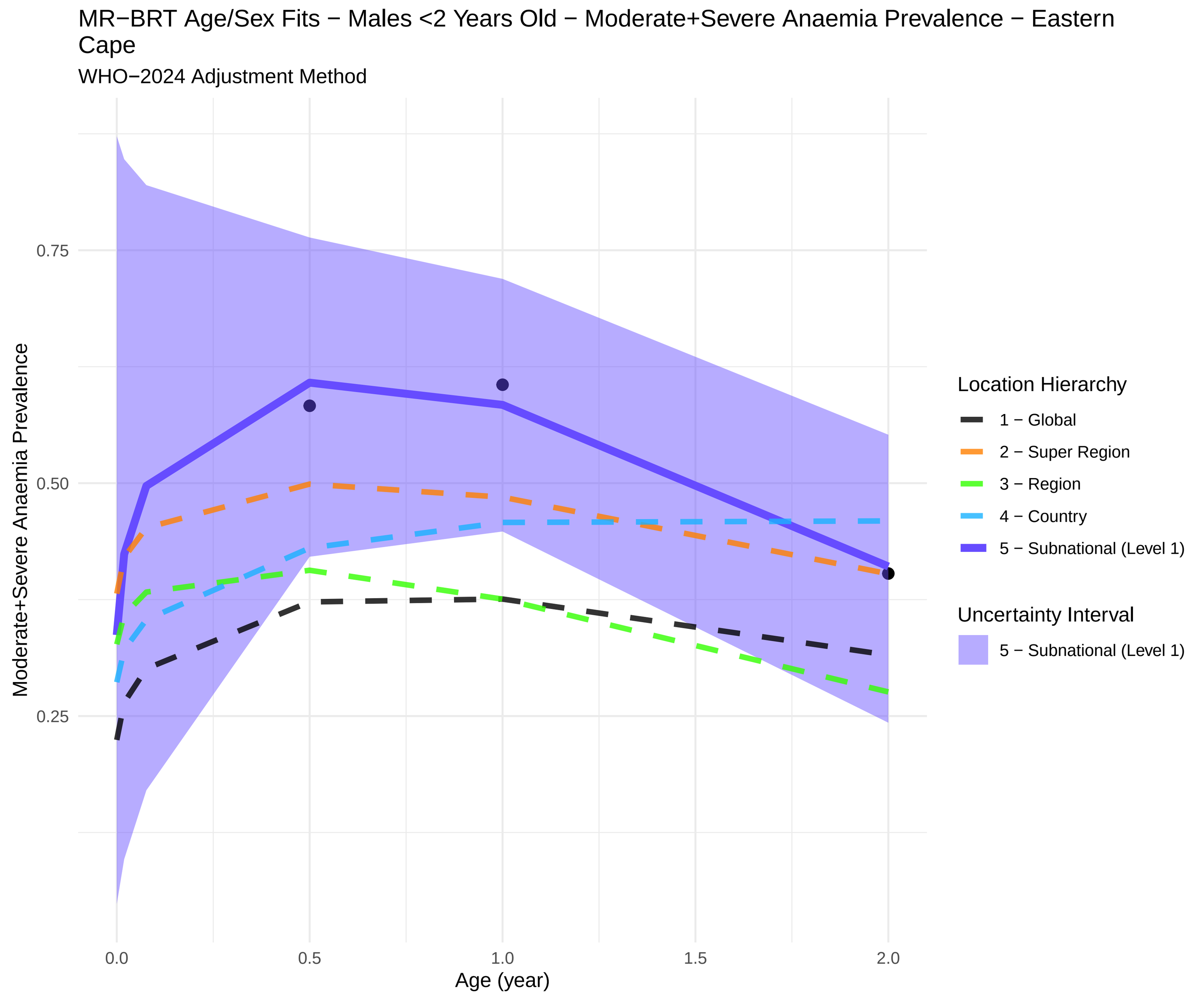
MR−BRT Age/Sex Fits − Males <2 Years Old − Moderate+Severe Anaemia Prevalence − Eastern Cape
WHO−2024 Adjustment Method
Moderate+Severe Anaemia Prevalence
Age (year)
Location Hierarchy
1 − Global
2 − Super Region
3 − Region
4 − Country
5 − Subnational (Level 1)
Uncertainty Interval
5 − Subnational (Level 1)

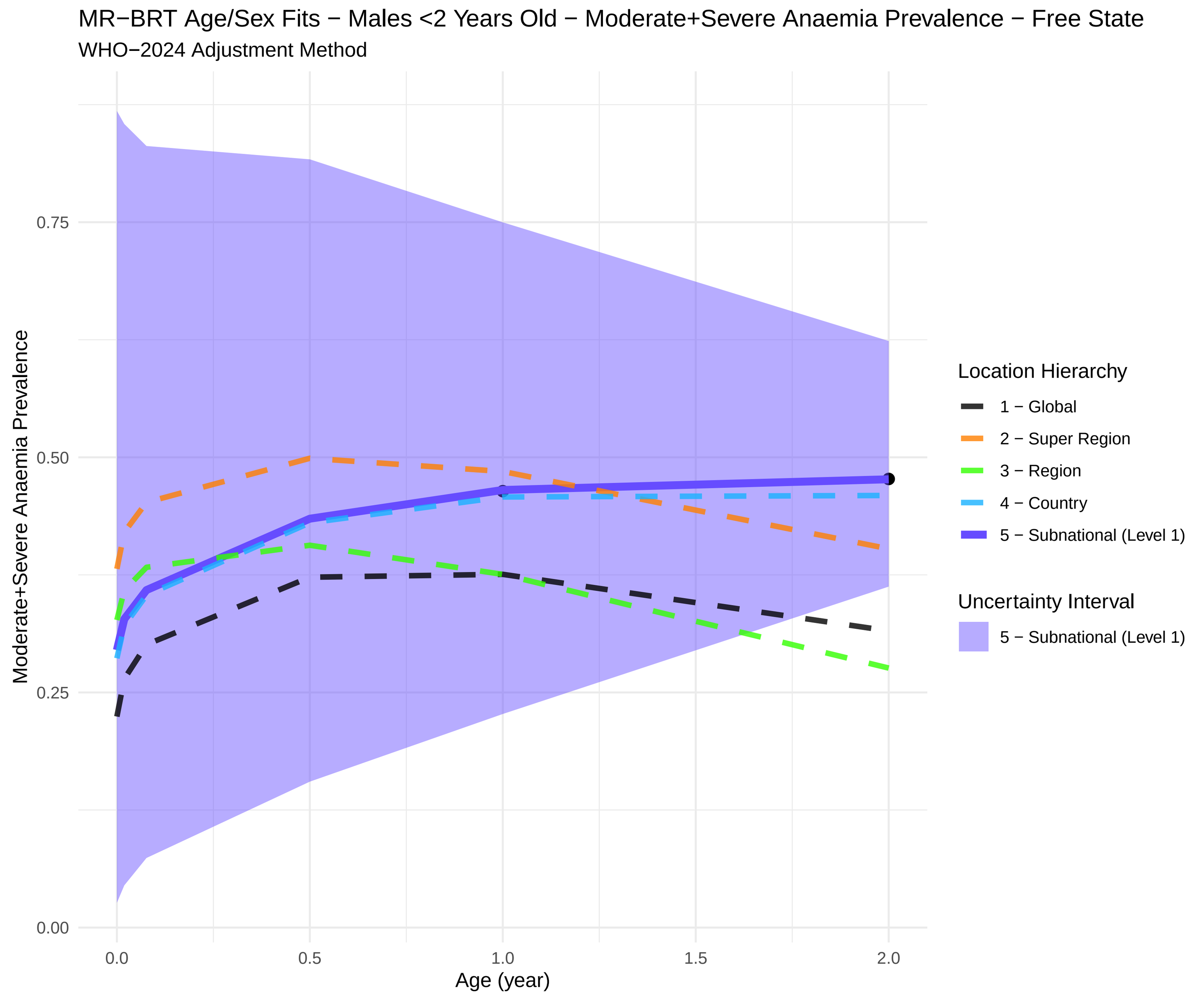
MR−BRT Age/Sex Fits − Males <2 Years Old − Moderate+Severe Anaemia Prevalence − Free State
WHO−2024 Adjustment Method
Moderate+Severe Anaemia Prevalence
Age (year)
Location Hierarchy
1 − Global
2 − Super Region
3 − Region
4 − Country
5 − Subnational (Level 1)
Uncertainty Interval
5 − Subnational (Level 1)

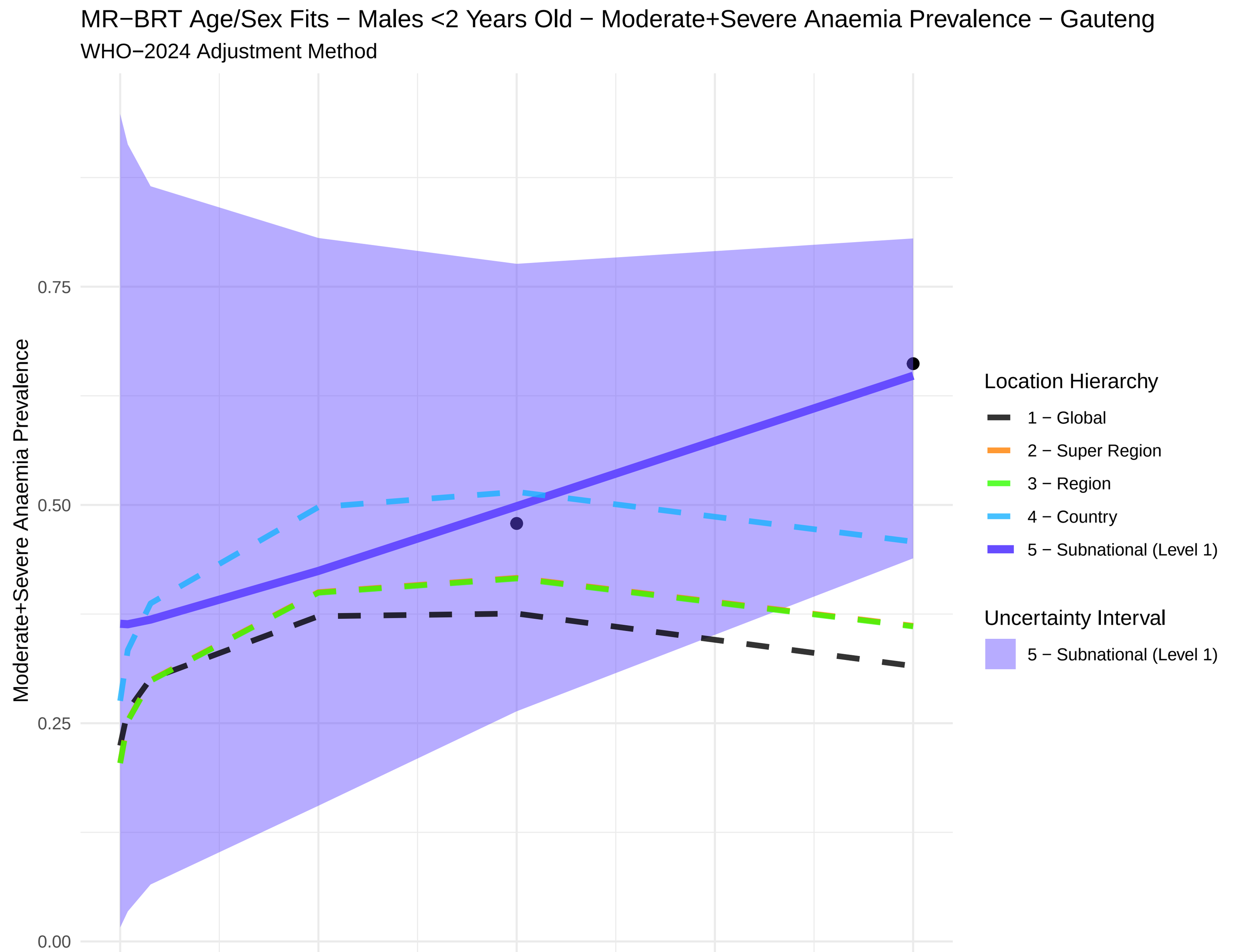
MR−BRT Age/Sex Fits − Males <2 Years Old − Moderate+Severe Anaemia Prevalence − Gauteng
WHO−2024 Adjustment Method
Moderate+Severe Anaemia Prevalence
Age (year)
Location Hierarchy
1 − Global
2 − Super Region
3 − Region
4 − Country
5 − Subnational (Level 1)
Uncertainty Interval
5 − Subnational (Level 1)

MR−BRT Age/Sex Fits − Males <2 Years Old − Moderate+Severe Anaemia Prevalence − KwaZulu−Natal
WHO−2024 Adjustment Method

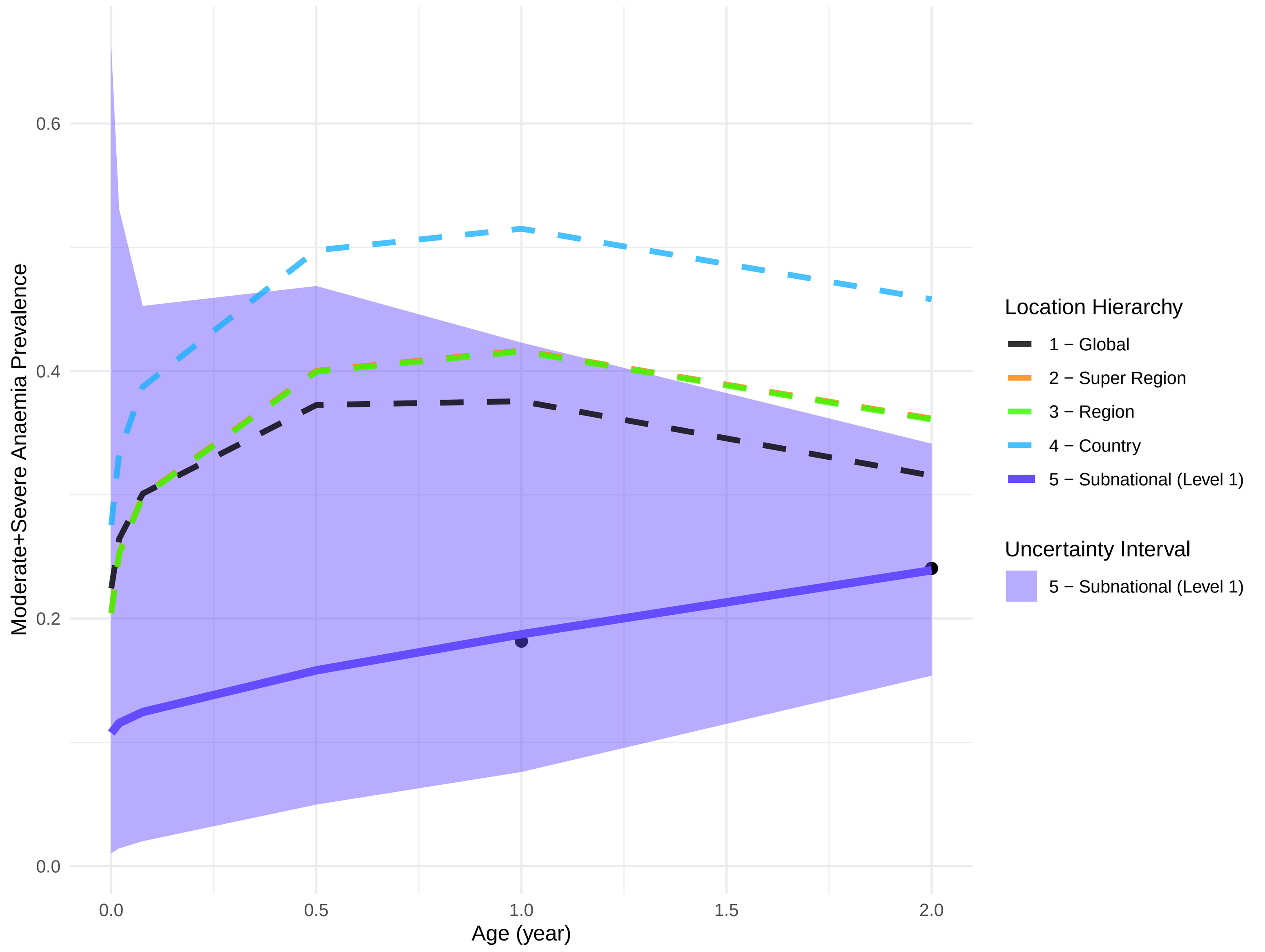

MR−BRT Age/Sex Fits − Males <2 Years Old − Moderate+Severe Anaemia Prevalence − Limpopo
WHO−2024 Adjustment Method

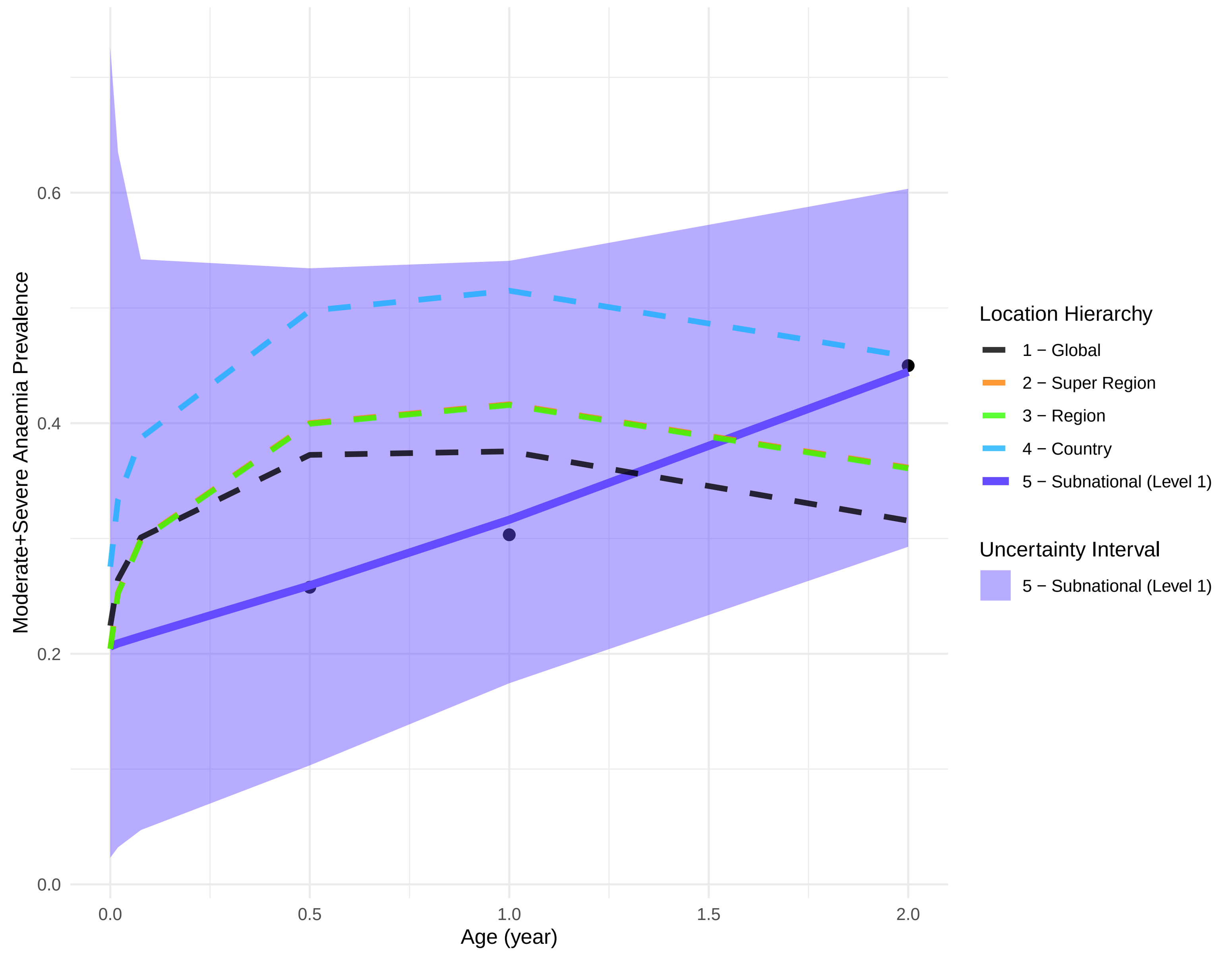

MR−BRT Age/Sex Fits − Males <2 Years Old − Moderate+Severe Anaemia Prevalence − Mpumalanga
WHO−2024 Adjustment Method

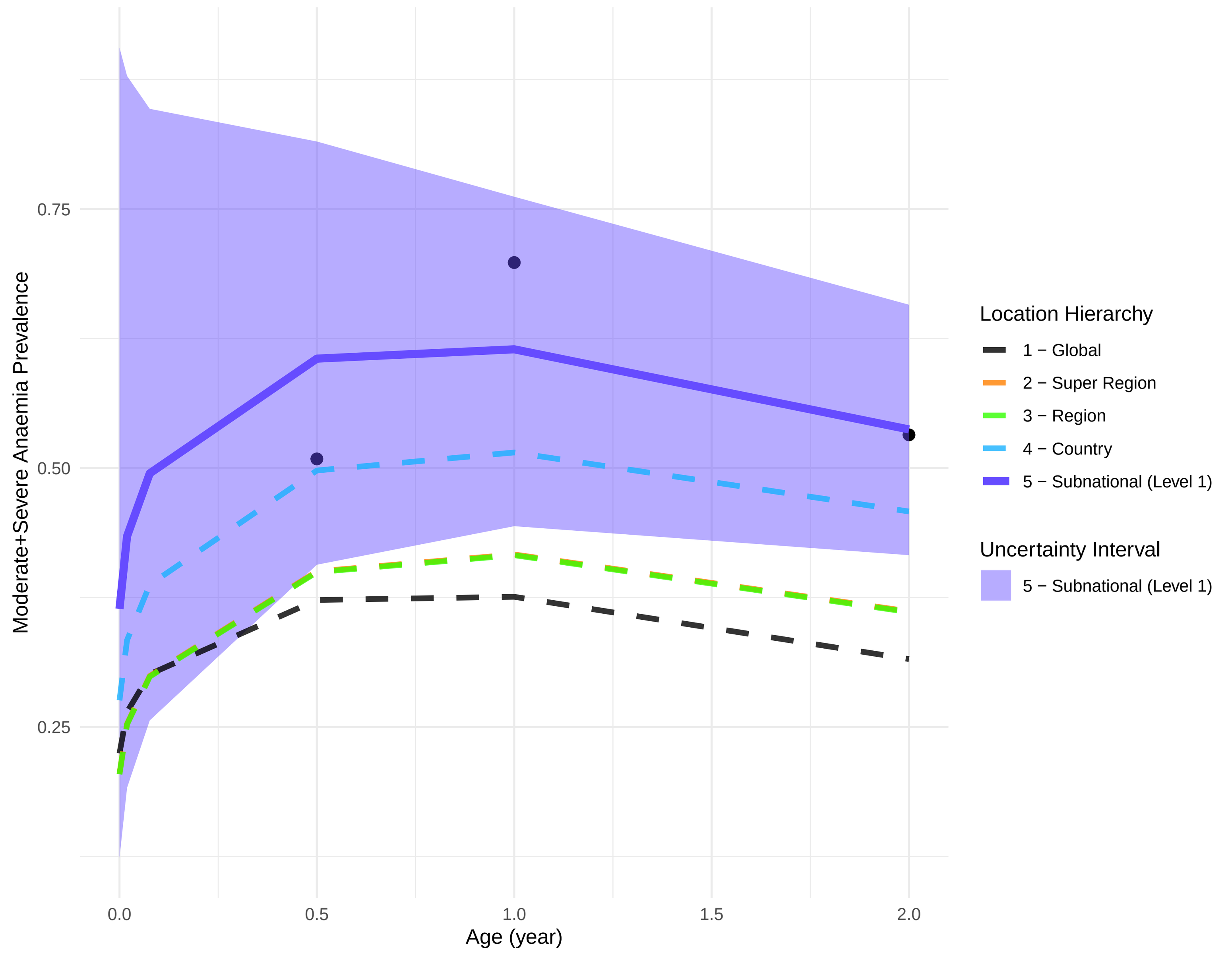

MR−BRT Age/Sex Fits − Males <2 Years Old − Moderate+Severe Anaemia Prevalence − North−West
WHO−2024 Adjustment Method

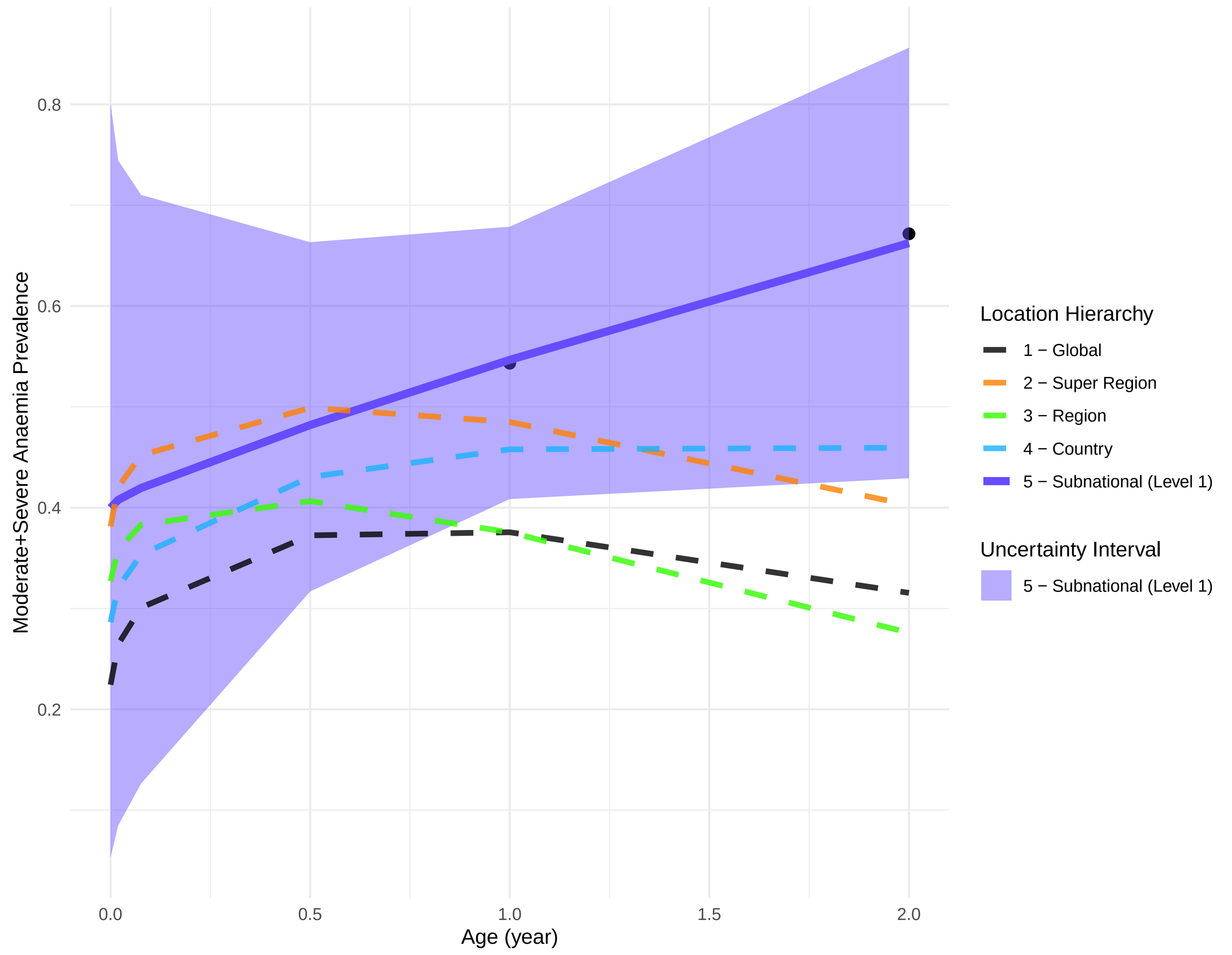

MR−BRT Age/Sex Fits − Males <2 Years Old − Moderate+Severe Anaemia Prevalence − Northern Cape
WHO−2024 Adjustment Method

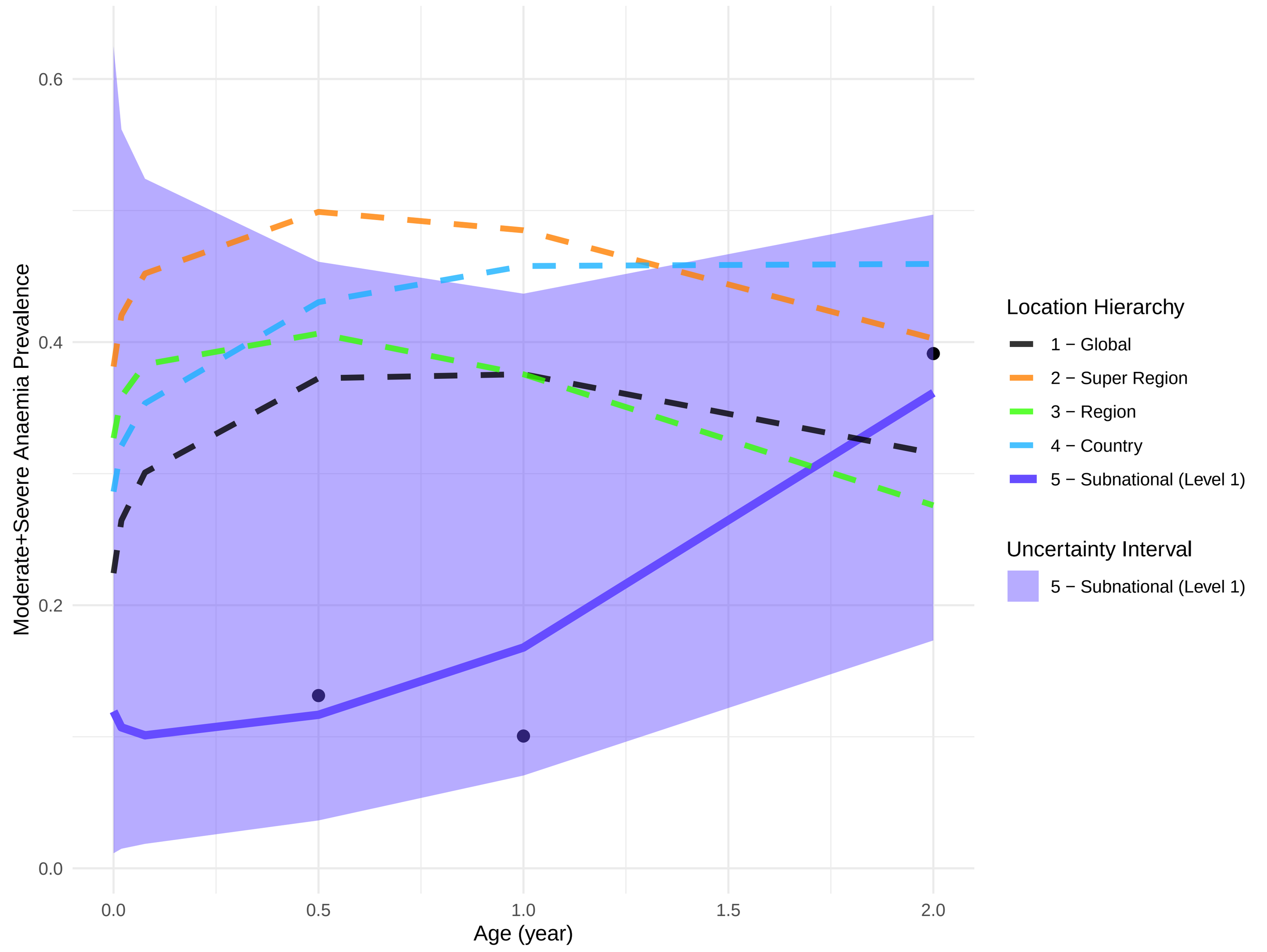

MR−BRT Age/Sex Fits − Males <2 Years Old − Moderate+Severe Anaemia Prevalence − Western Cape
WHO−2024 Adjustment Method

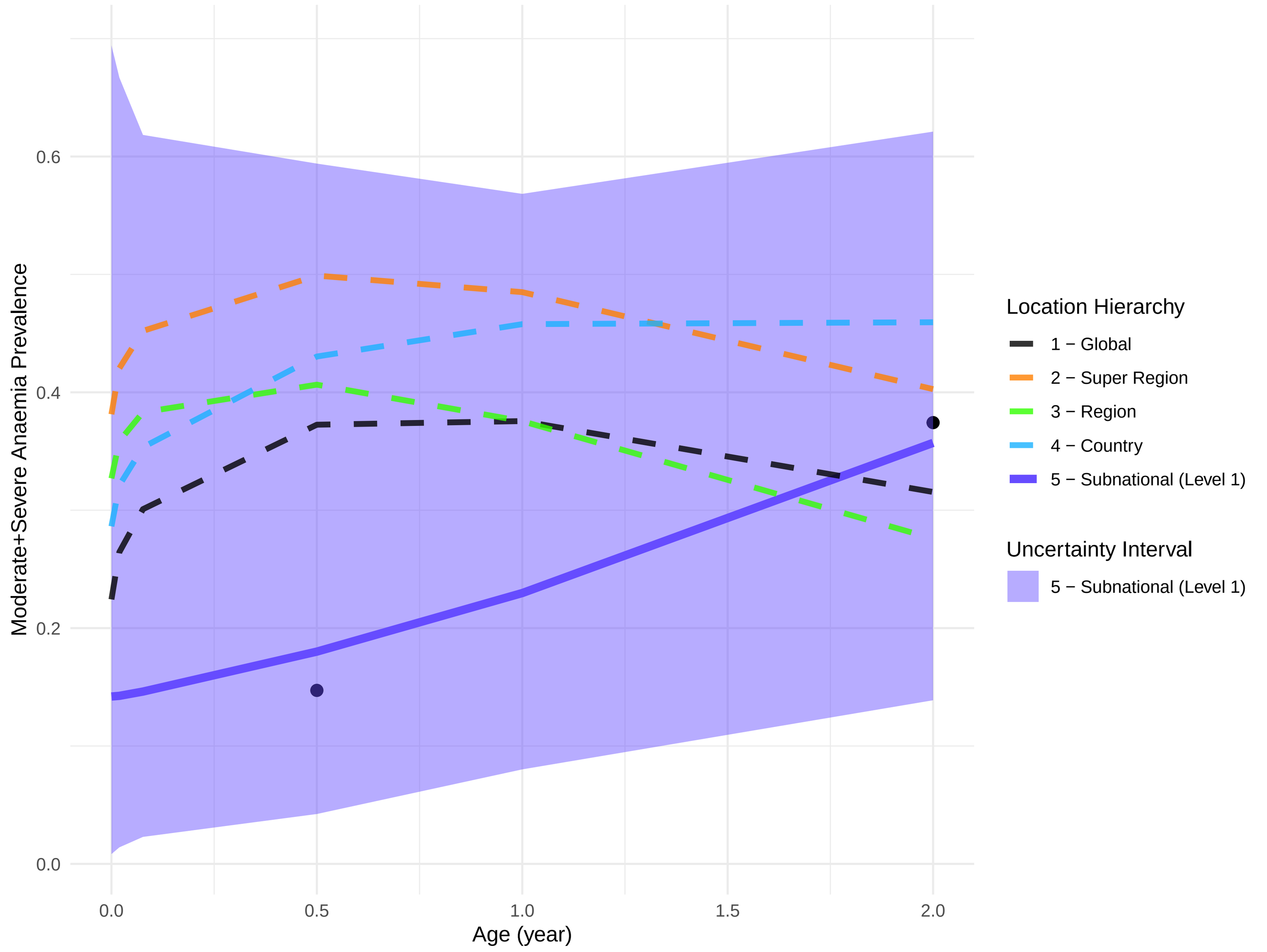

MR−BRT Age/Sex Fits − Males <2 Years Old − Moderate+Severe Anaemia Prevalence − Zimbabwe
WHO−2024 Adjustment Method

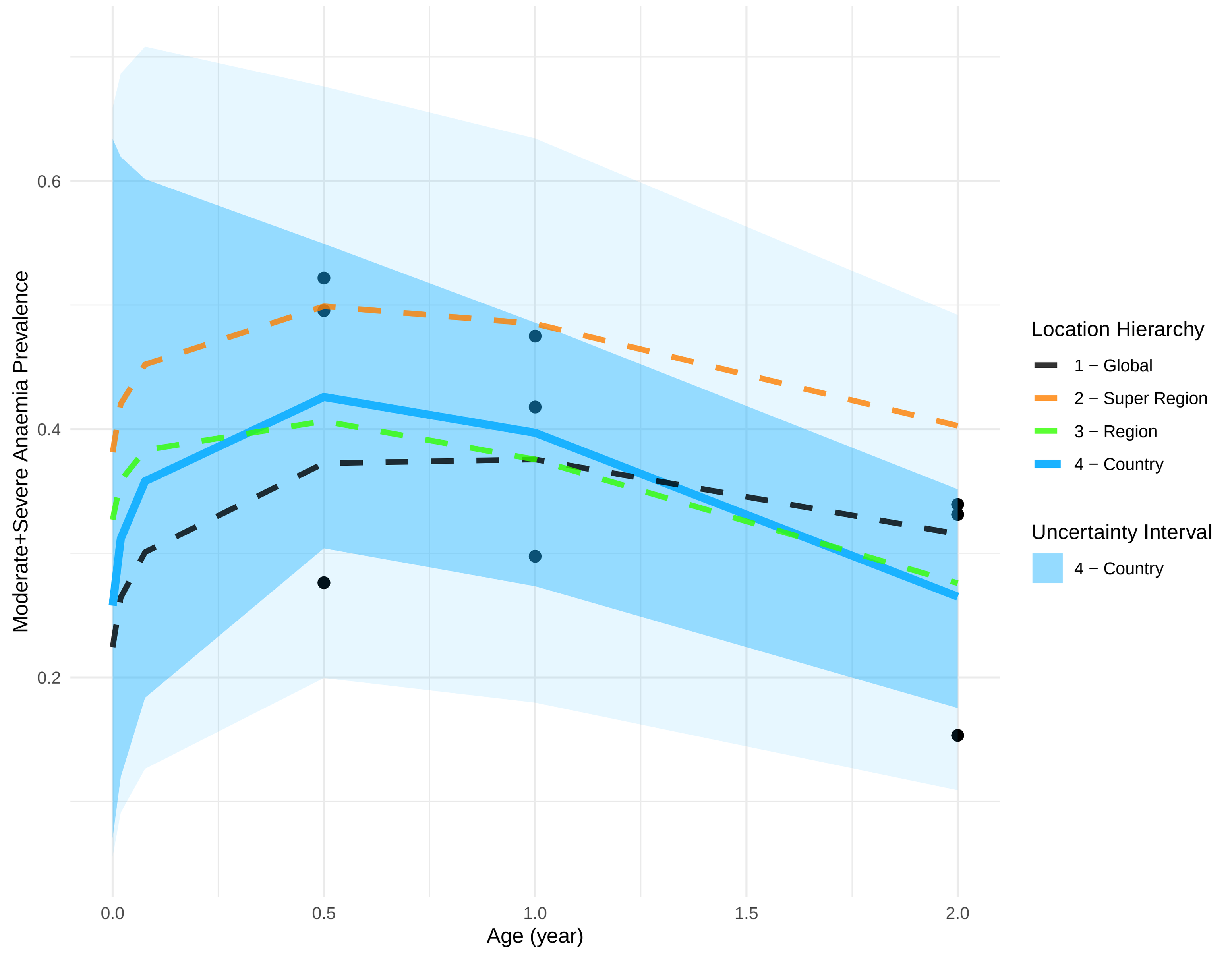

MR−BRT Age/Sex Fits − Males <2 Years Old − Moderate+Severe Anaemia Prevalence − Western Sub−Saharan Africa
WHO−2024 Adjustment Method

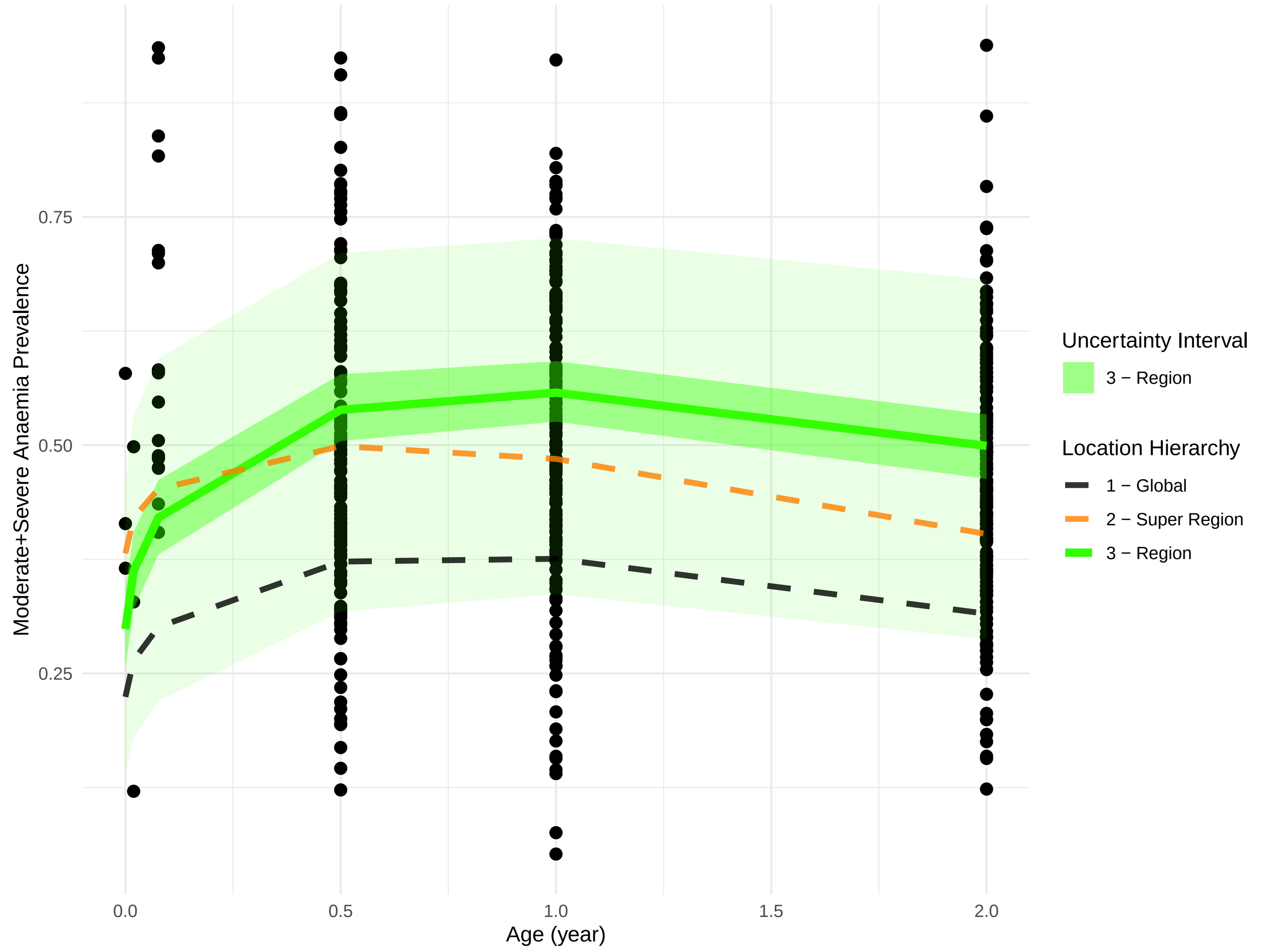

MR−BRT Age/Sex Fits − Males <2 Years Old − Moderate+Severe Anaemia Prevalence − Benin
WHO−2024 Adjustment Method

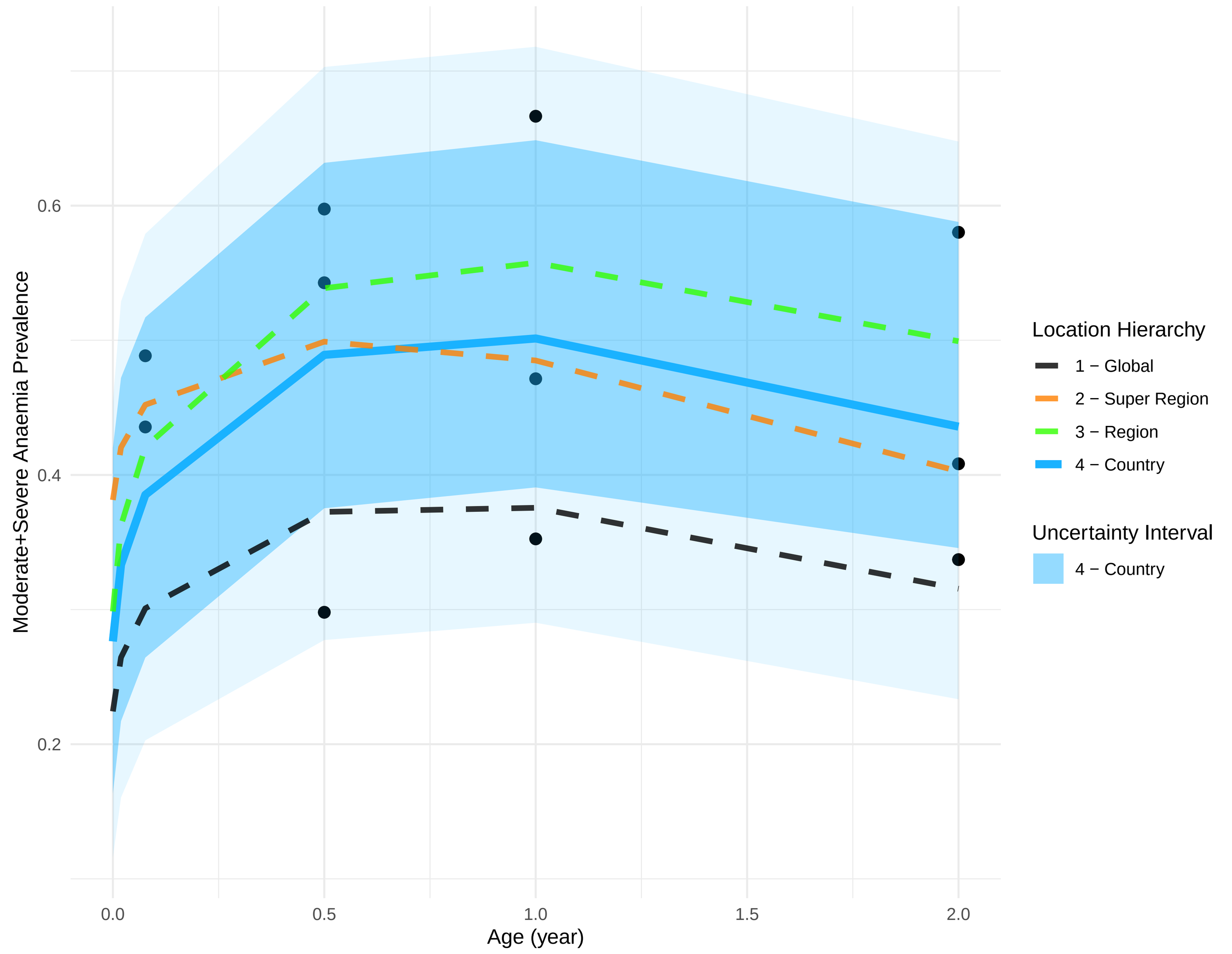

MR−BRT Age/Sex Fits − Males <2 Years Old − Moderate+Severe Anaemia Prevalence − Burkina Faso
WHO−2024 Adjustment Method

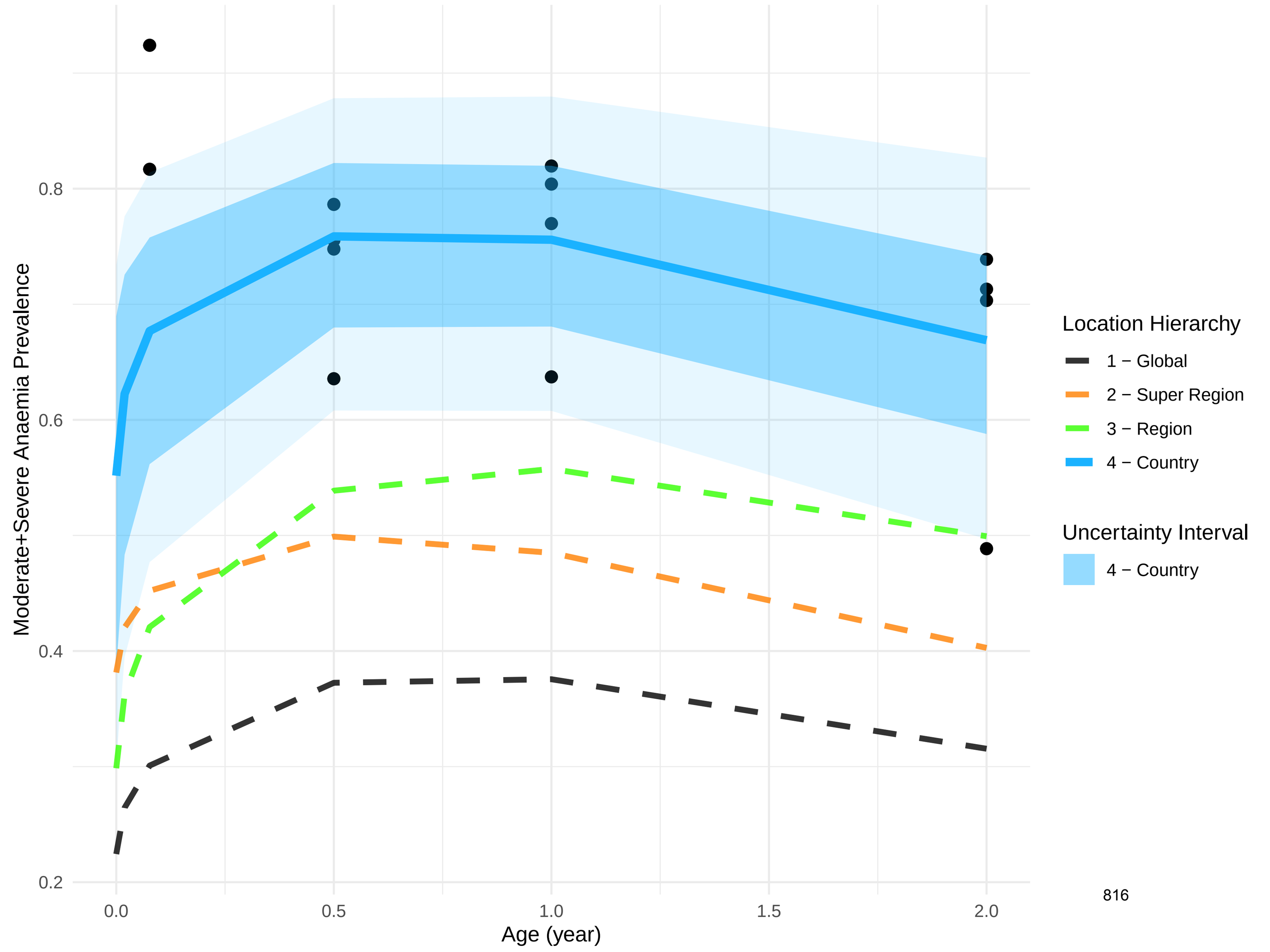

MR−BRT Age/Sex Fits − Males <2 Years Old − Moderate+Severe Anaemia Prevalence − Cameroon
WHO−2024 Adjustment Method

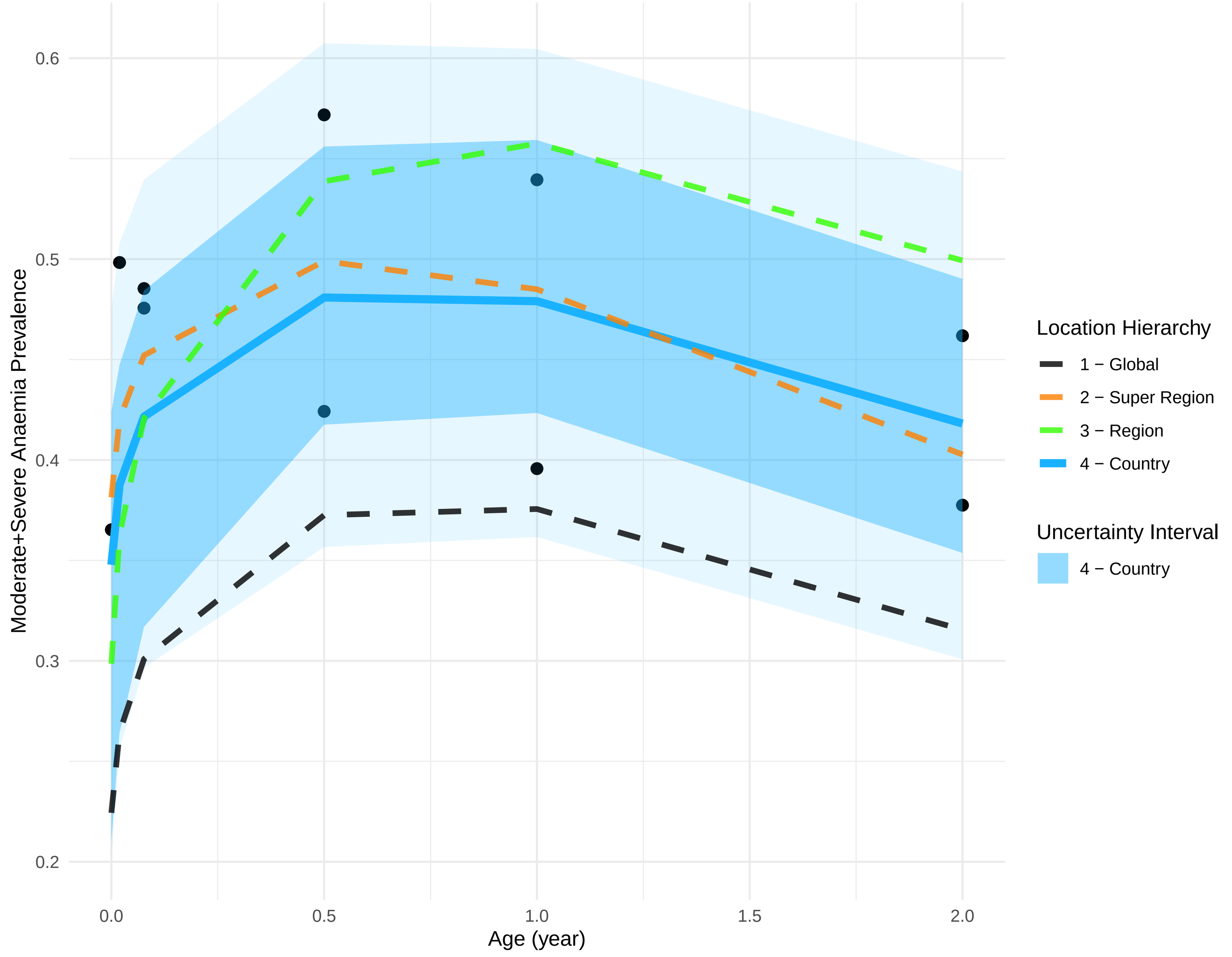

MR−BRT Age/Sex Fits − Males <2 Years Old − Moderate+Severe Anaemia Prevalence − Côte d'Ivoire
WHO−2024 Adjustment Method

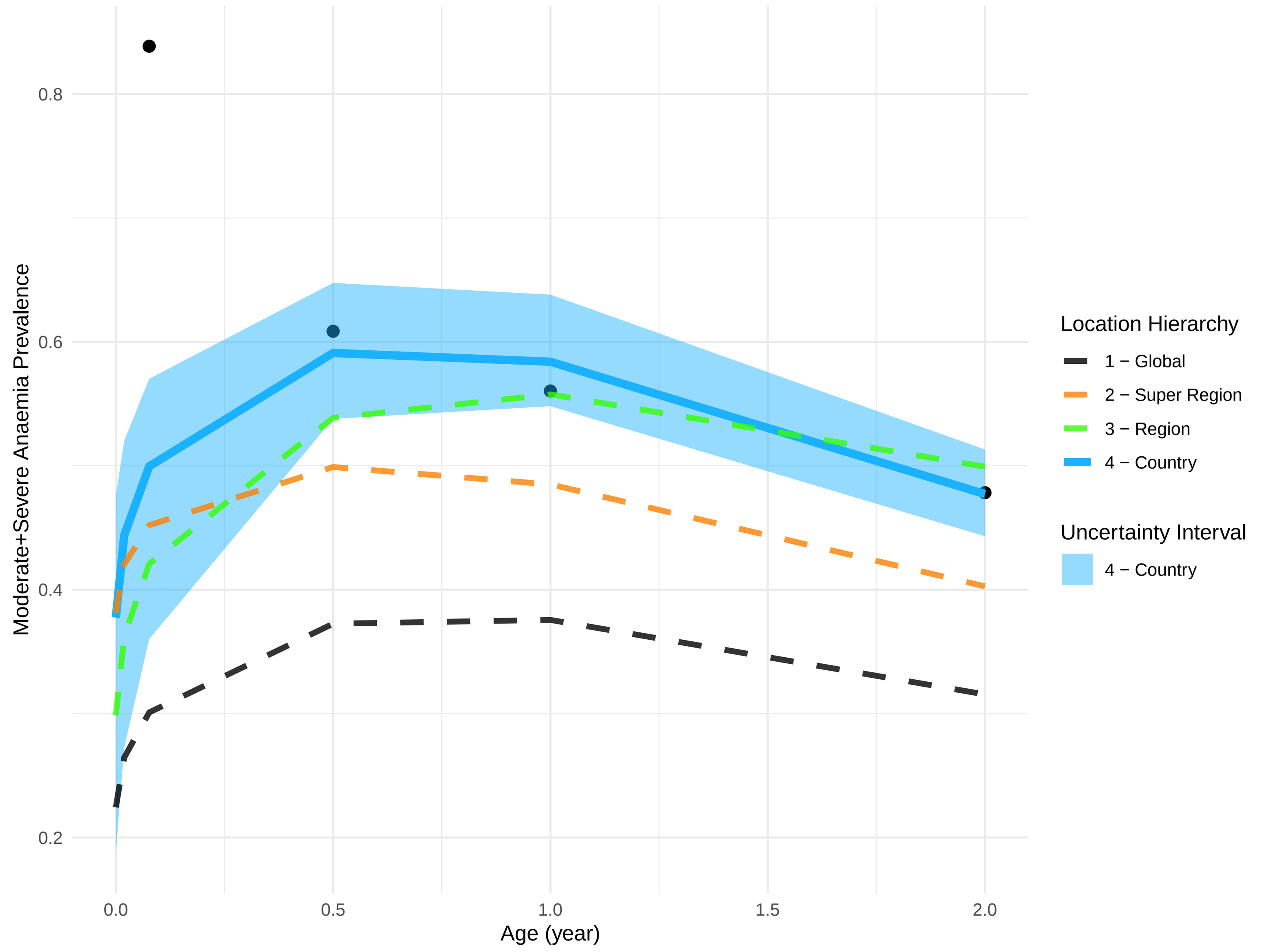

MR−BRT Age/Sex Fits − Males <2 Years Old − Moderate+Severe Anaemia Prevalence − Gambia
WHO−2024 Adjustment Method

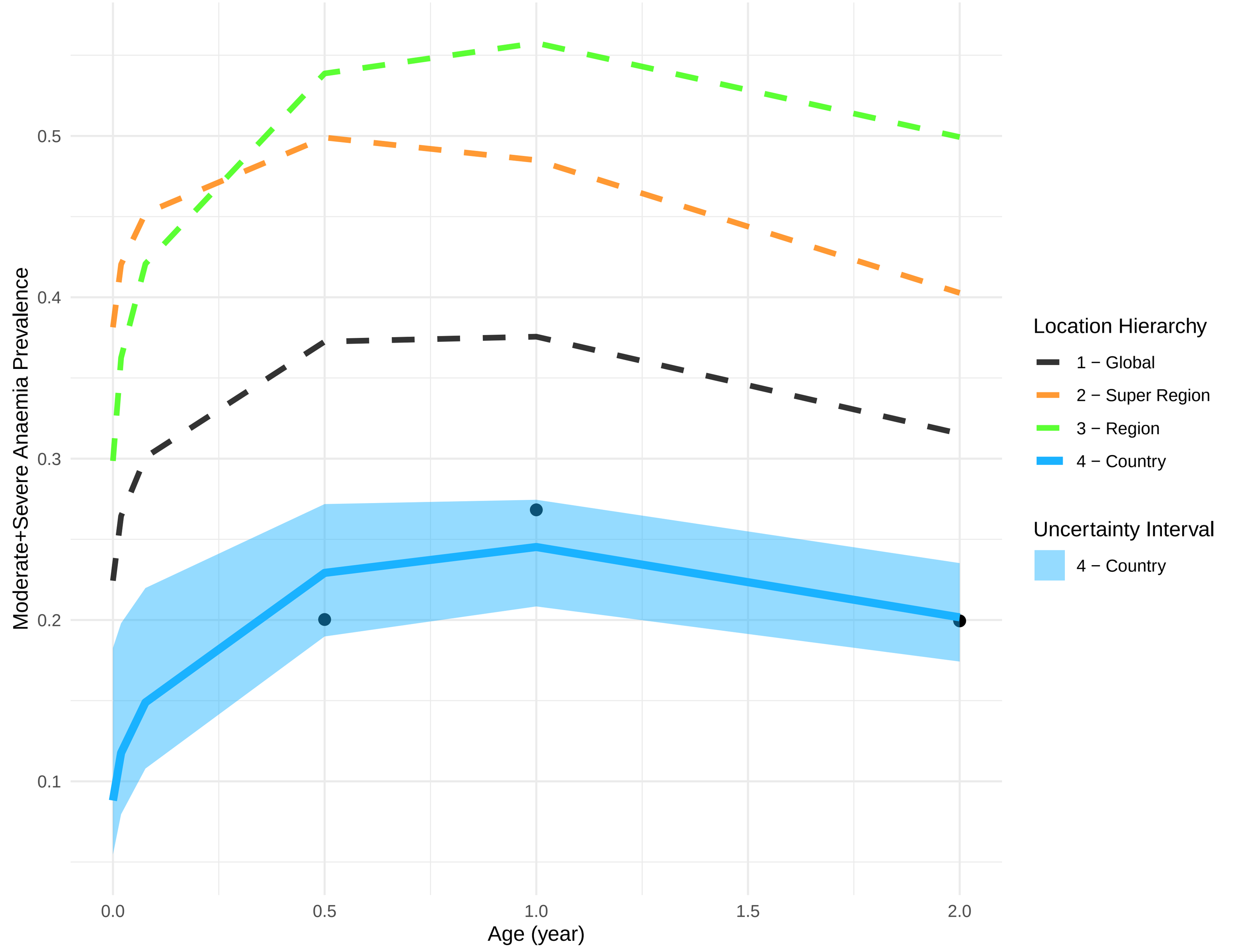

MR−BRT Age/Sex Fits − Males <2 Years Old − Moderate+Severe Anaemia Prevalence − Ghana
WHO−2024 Adjustment Method

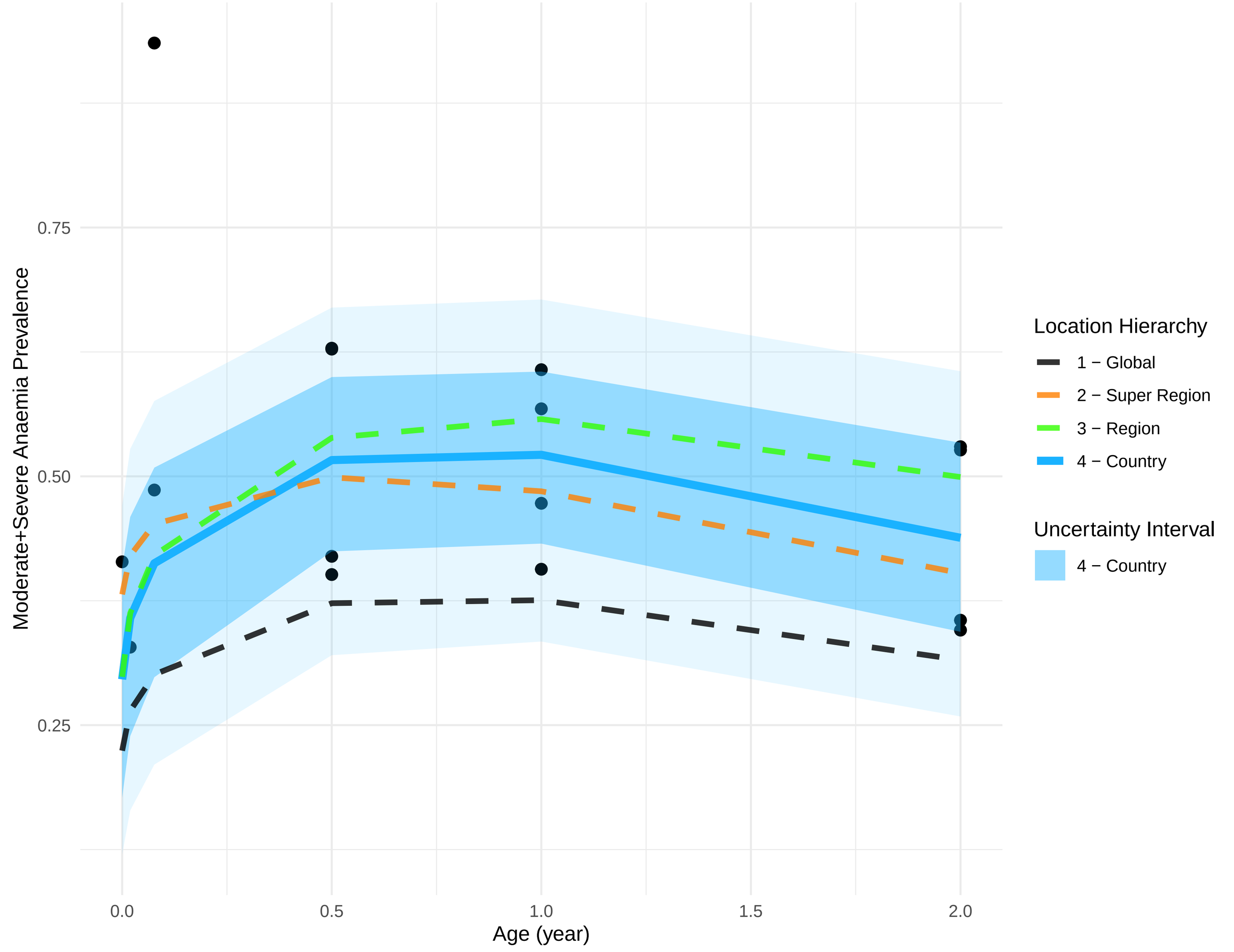

MR−BRT Age/Sex Fits − Males <2 Years Old − Moderate+Severe Anaemia Prevalence − Guinea
WHO−2024 Adjustment Method

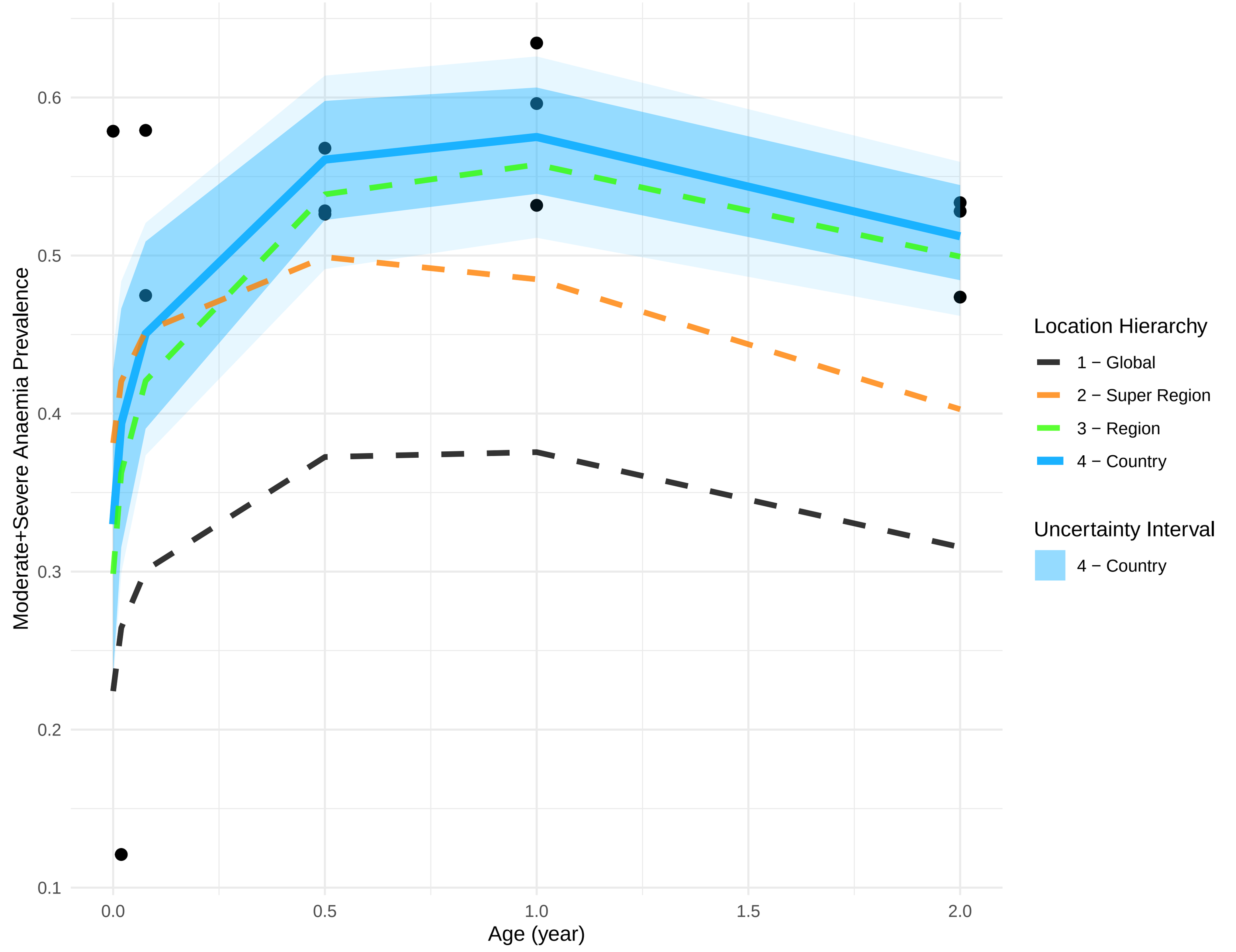

MR−BRT Age/Sex Fits − Males <2 Years Old − Moderate+Severe Anaemia Prevalence − Liberia
WHO−2024 Adjustment Method

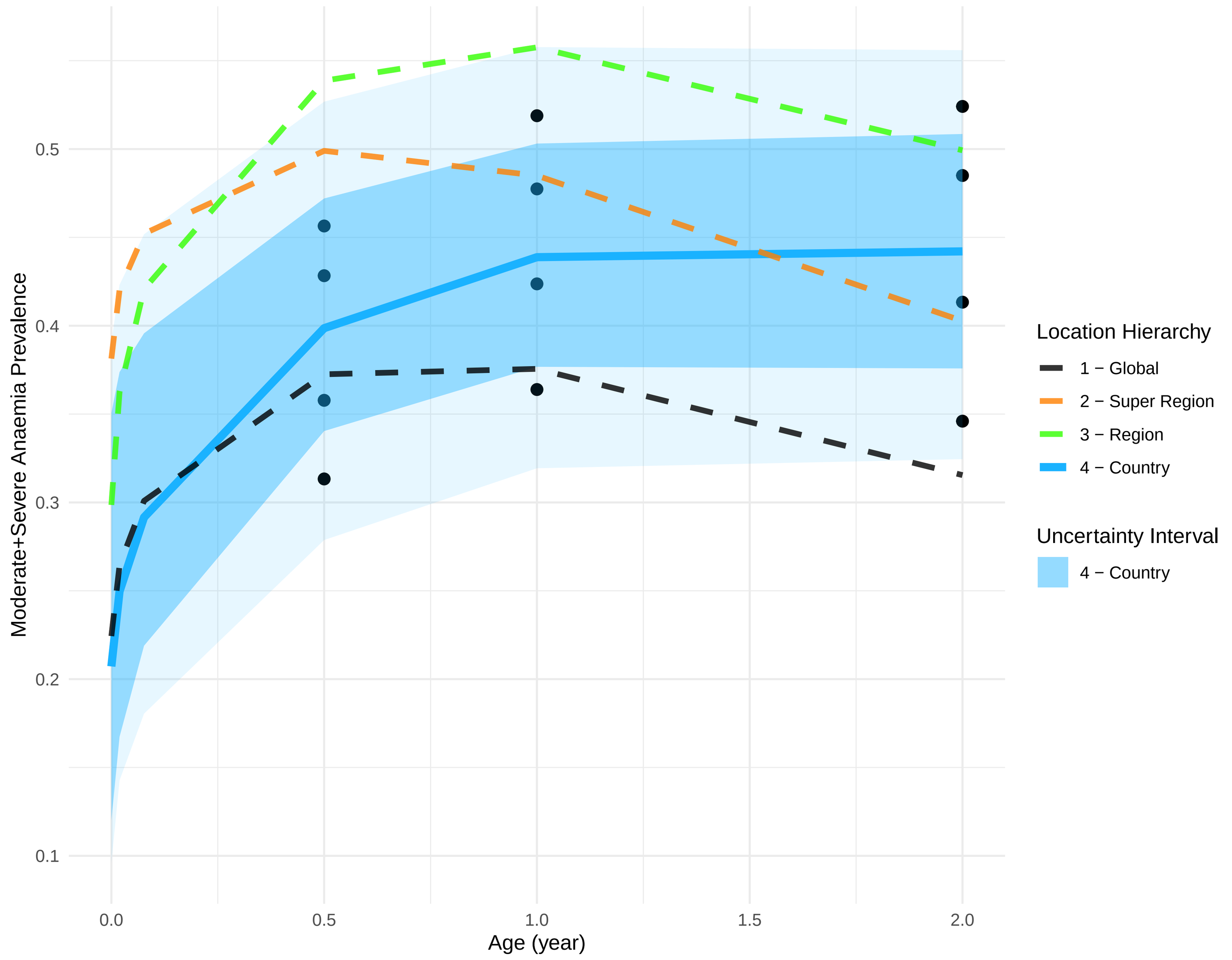

MR−BRT Age/Sex Fits − Males <2 Years Old − Moderate+Severe Anaemia Prevalence − Mali
WHO−2024 Adjustment Method

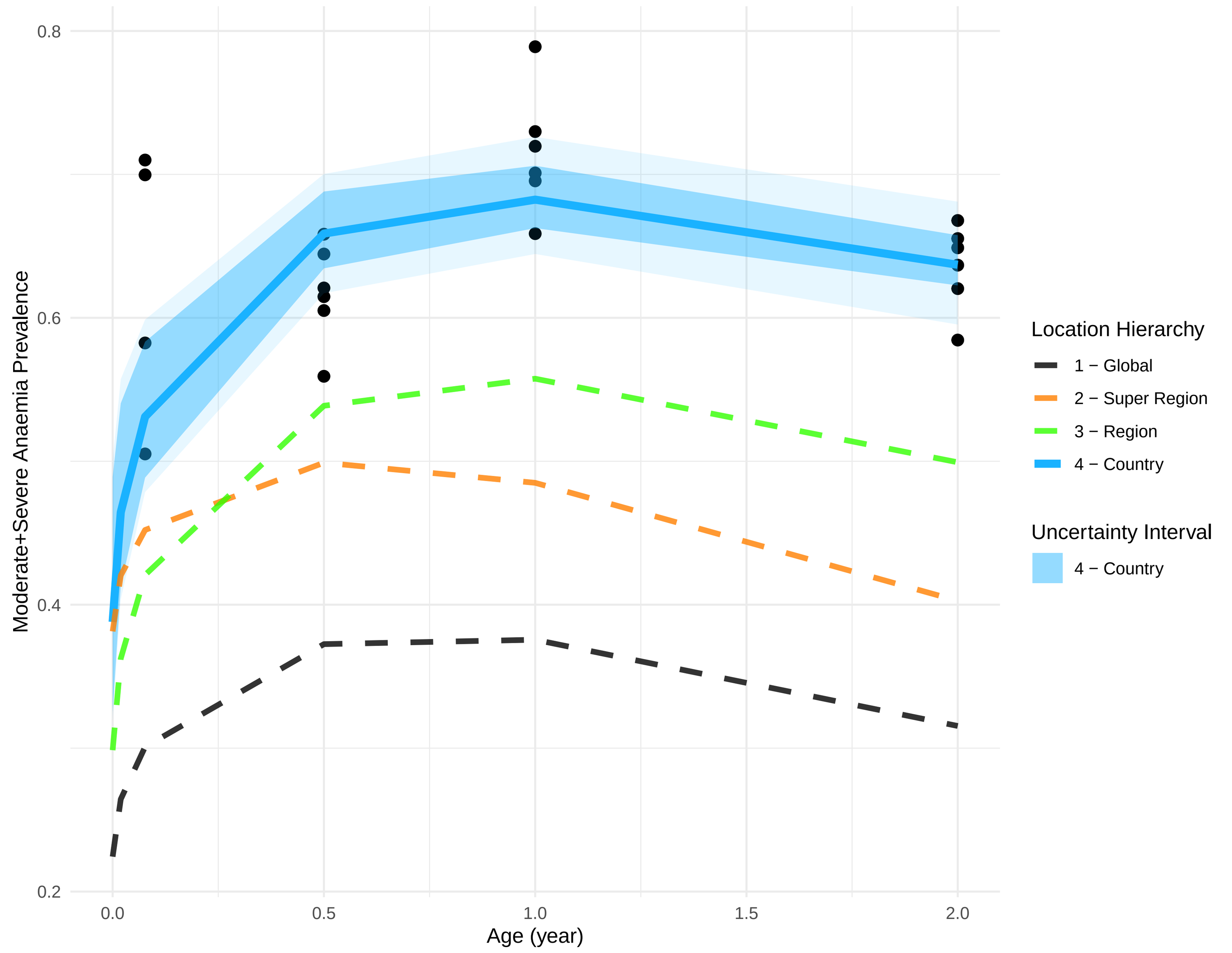

MR−BRT Age/Sex Fits − Males <2 Years Old − Moderate+Severe Anaemia Prevalence − Niger
WHO−2024 Adjustment Method

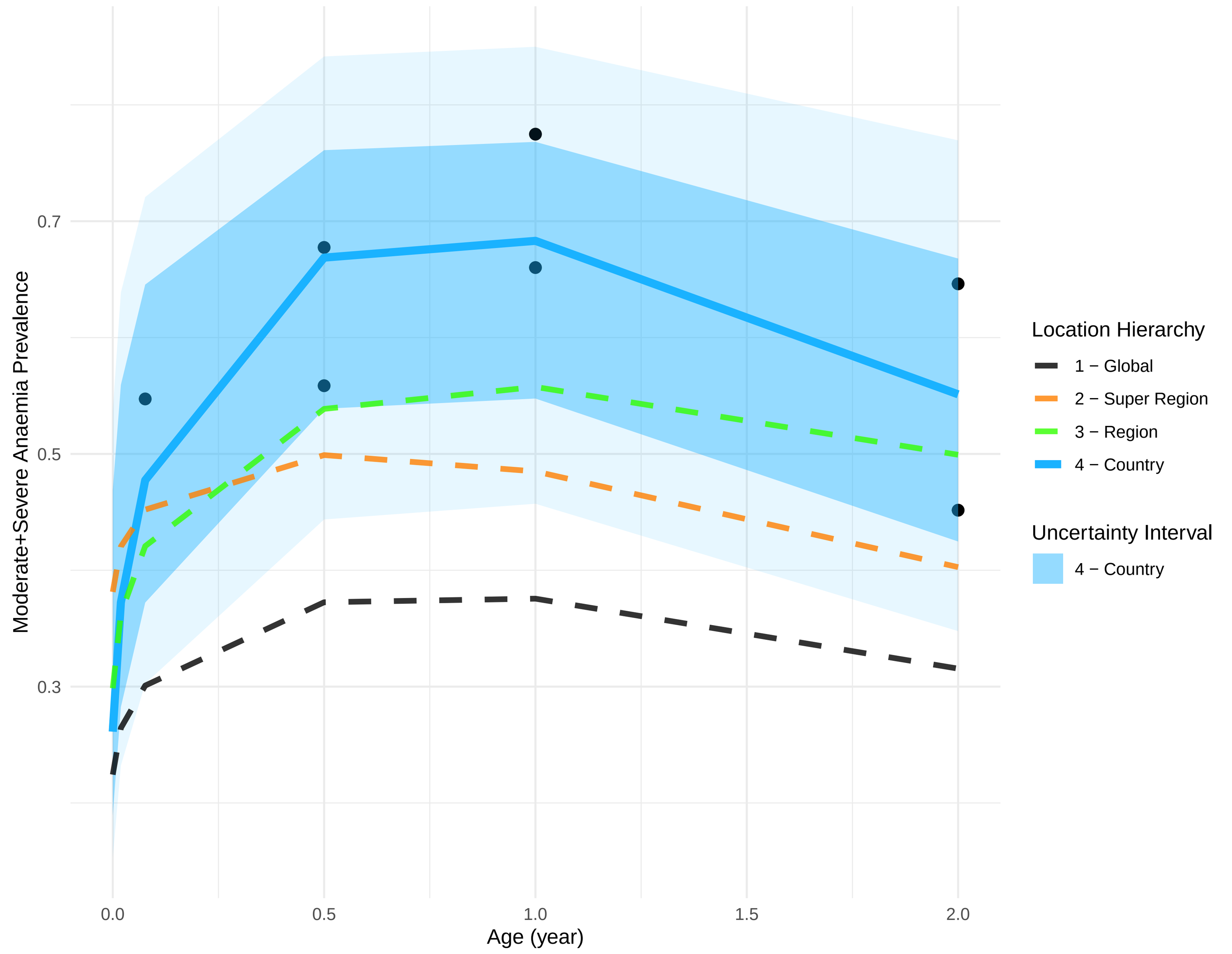

MR−BRT Age/Sex Fits − Males <2 Years Old − Moderate+Severe Anaemia Prevalence − Nigeria
WHO−2024 Adjustment Method

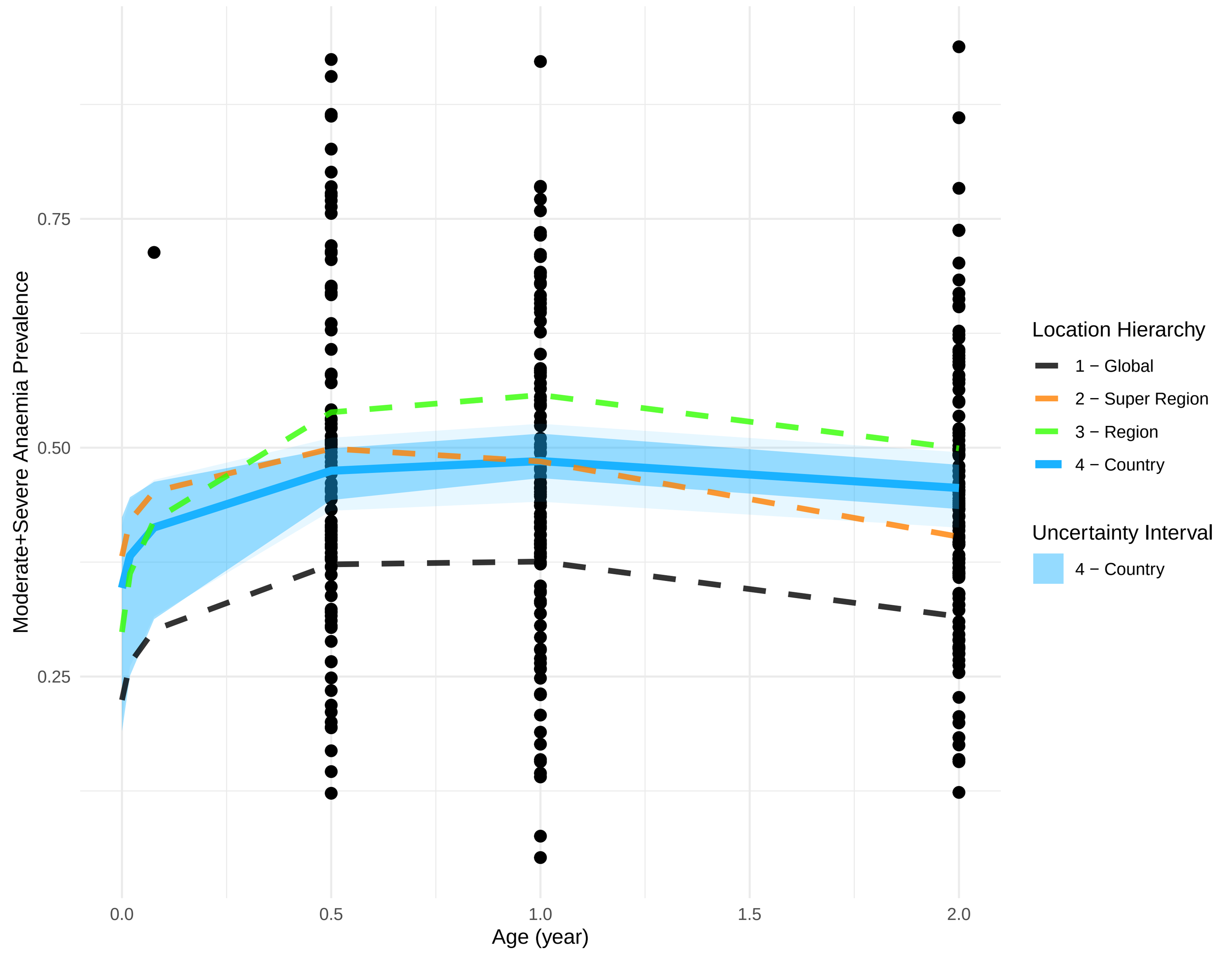

MR−BRT Age/Sex Fits − Males <2 Years Old − Moderate+Severe Anaemia Prevalence − Abia
WHO−2024 Adjustment Method

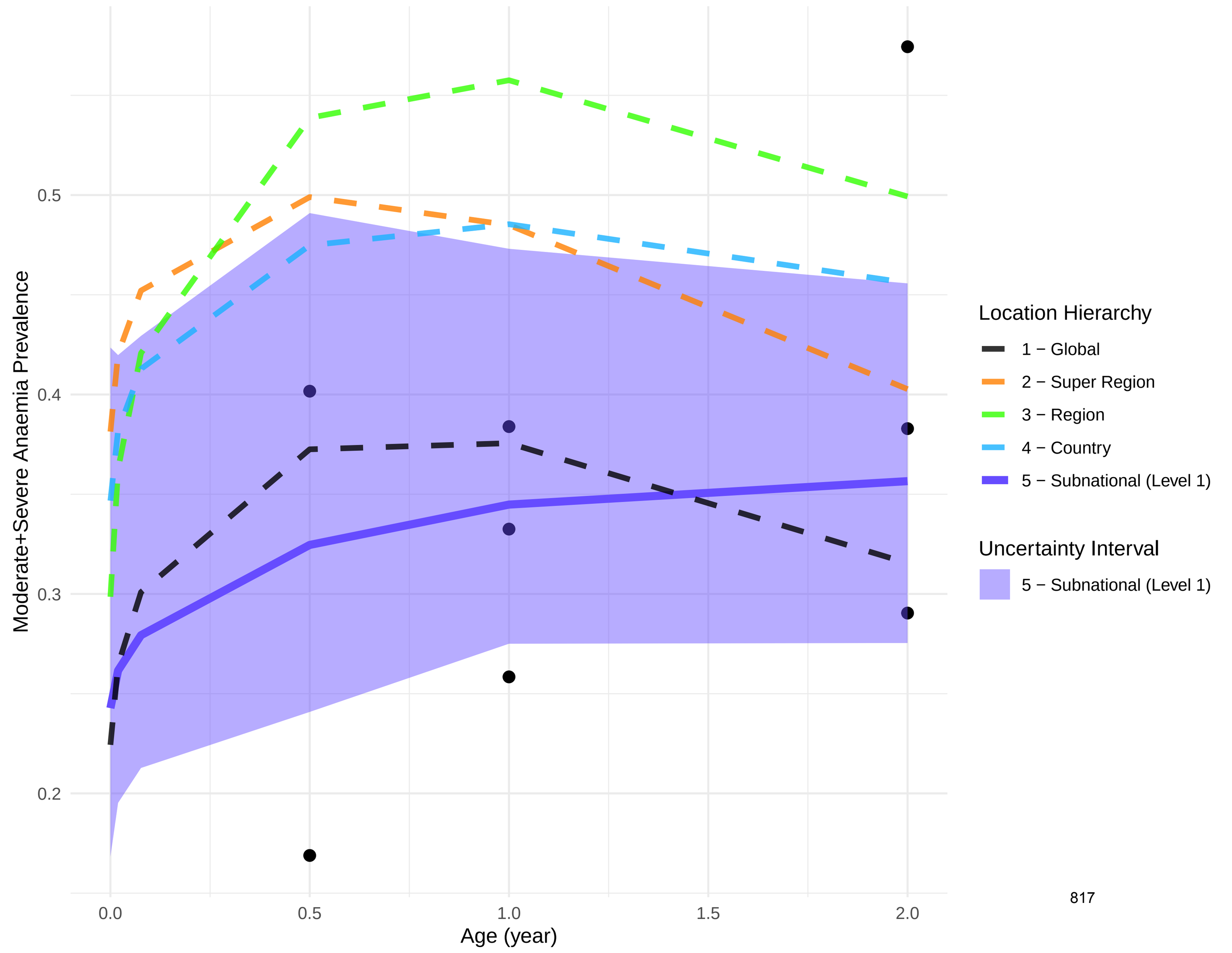

MR−BRT Age/Sex Fits − Males <2 Years Old − Moderate+Severe Anaemia Prevalence − Adamawa
WHO−2024 Adjustment Method

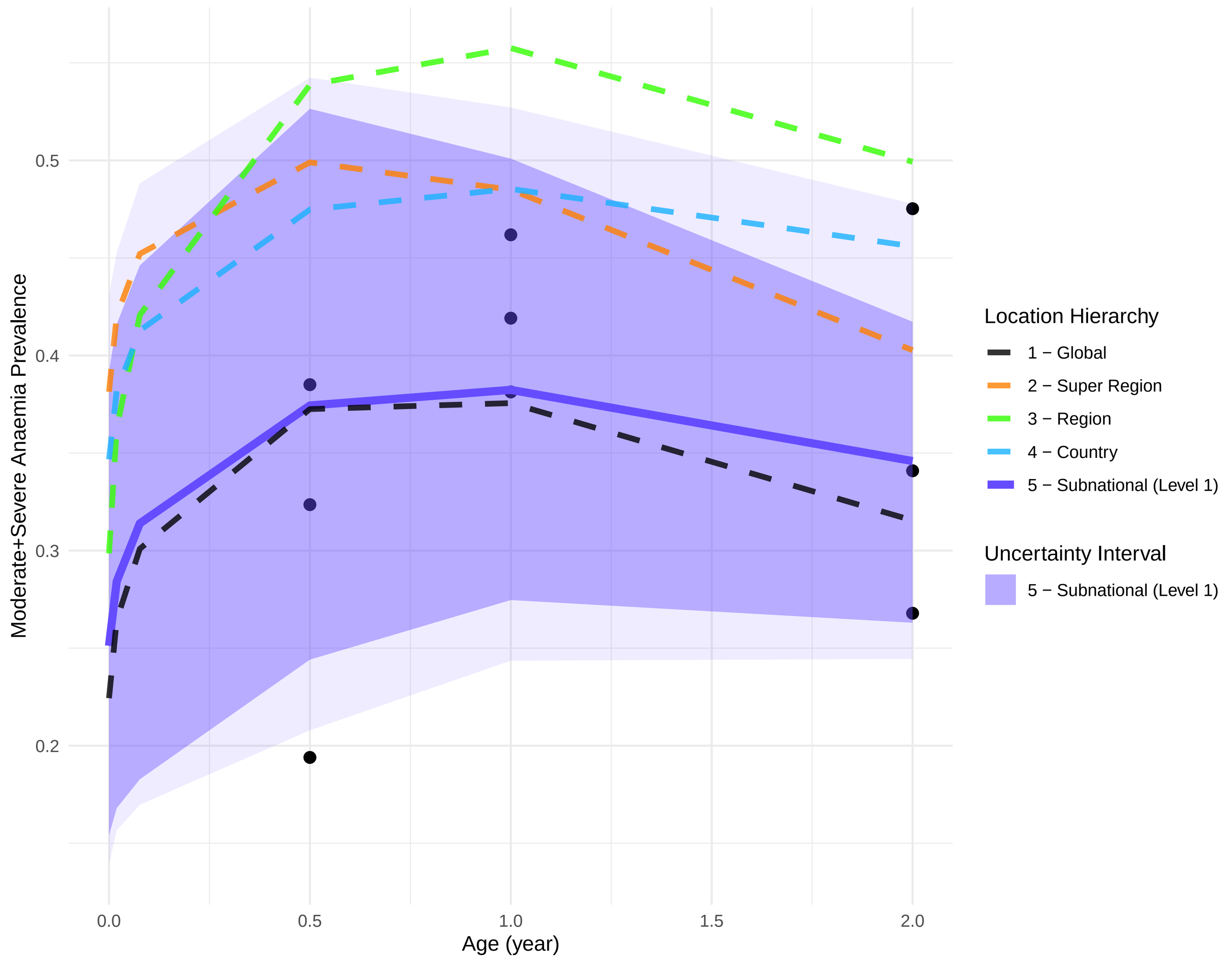

MR−BRT Age/Sex Fits − Males <2 Years Old − Moderate+Severe Anaemia Prevalence − Akwa Ibom
WHO−2024 Adjustment Method

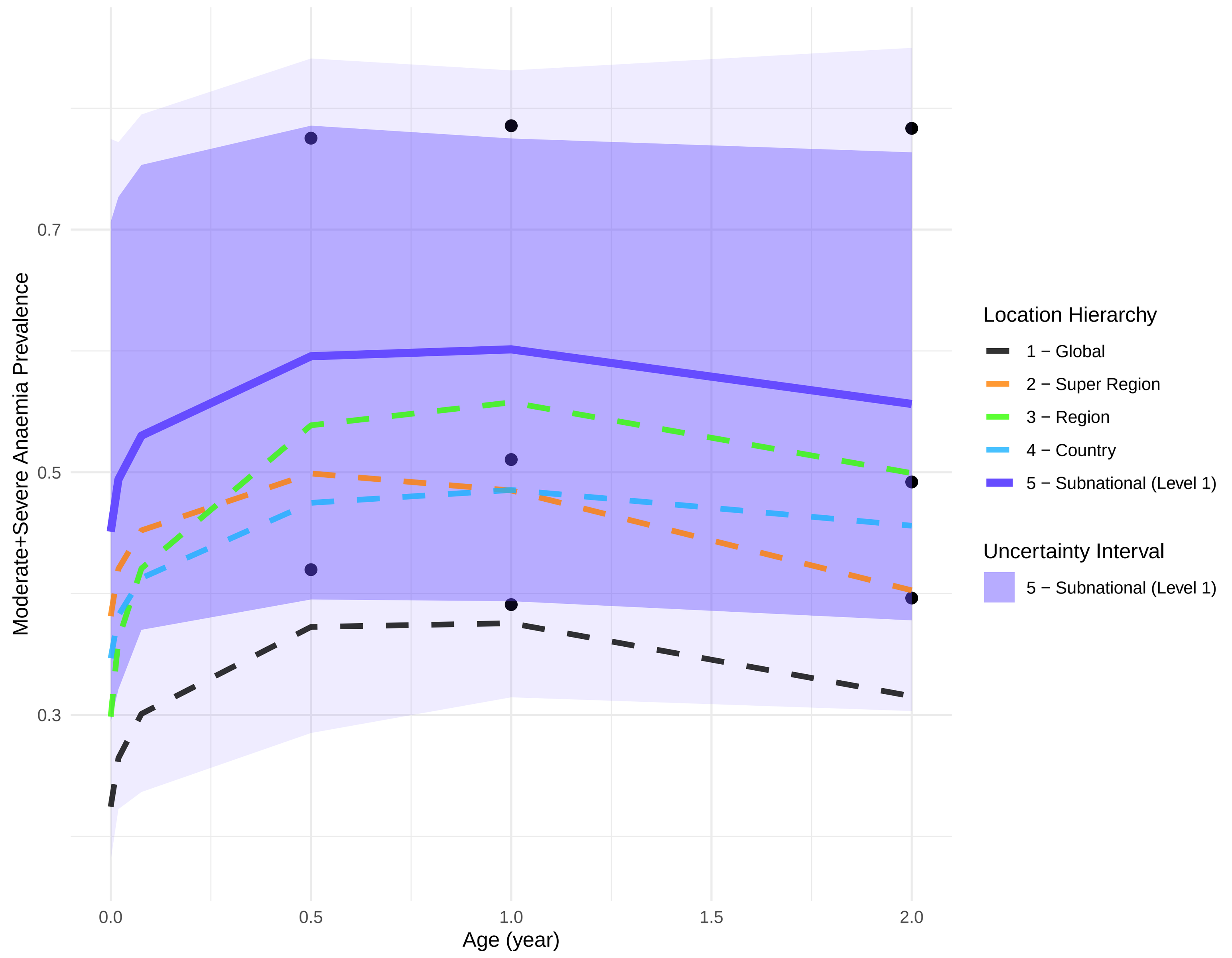

MR−BRT Age/Sex Fits − Males <2 Years Old − Moderate+Severe Anaemia Prevalence − Anambra
WHO−2024 Adjustment Method

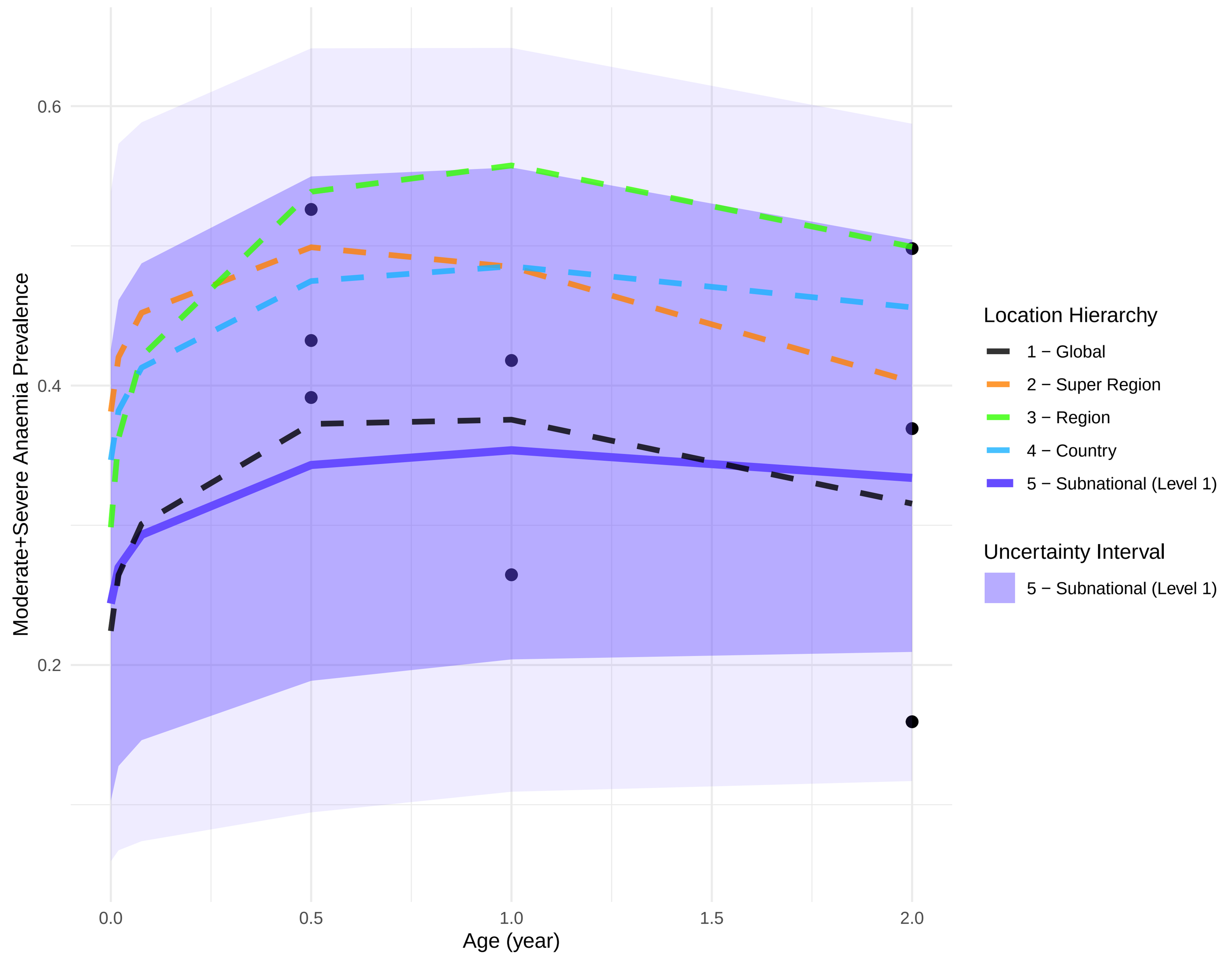

MR−BRT Age/Sex Fits − Males <2 Years Old − Moderate+Severe Anaemia Prevalence − Bauchi
WHO−2024 Adjustment Method

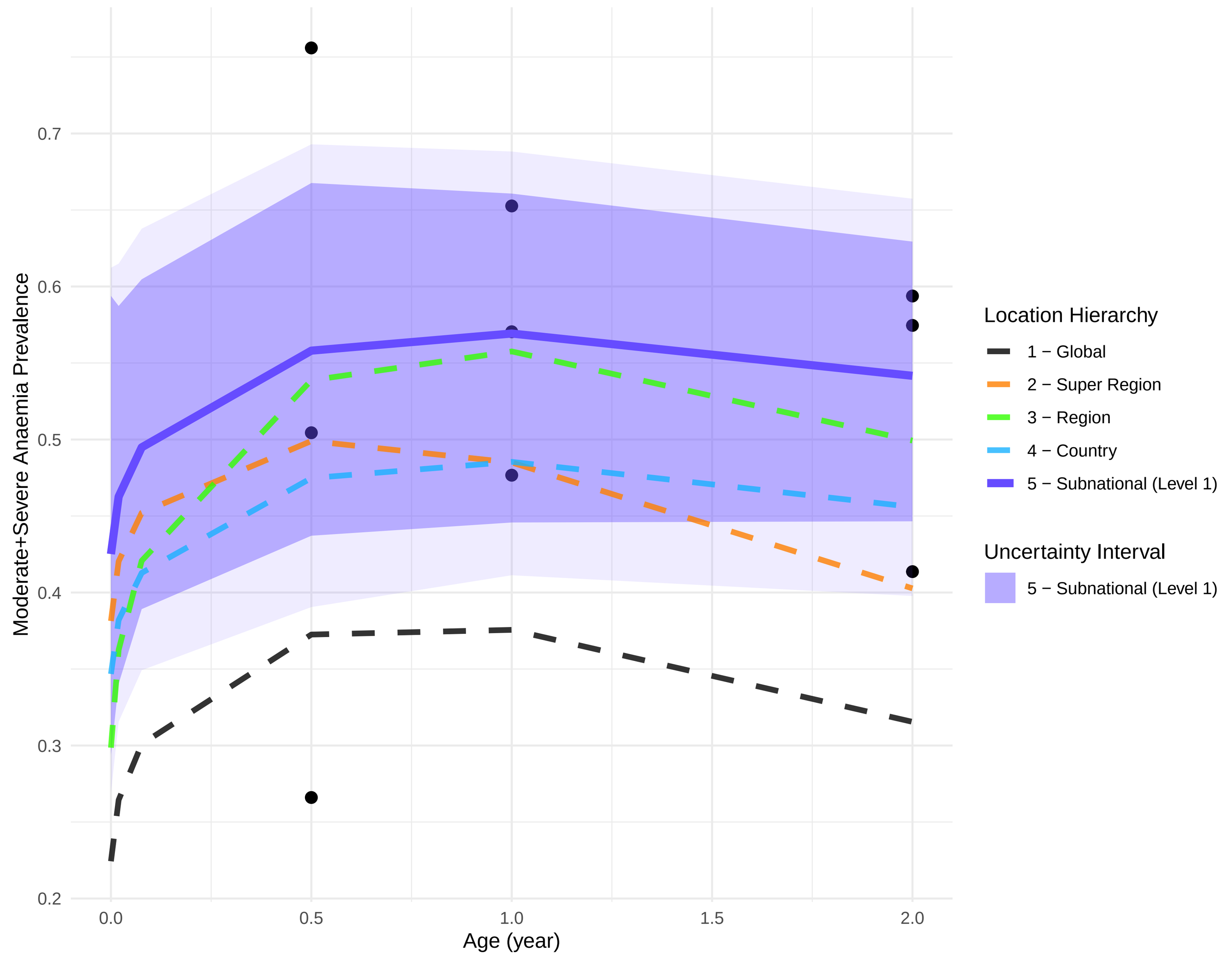

MR−BRT Age/Sex Fits − Males <2 Years Old − Moderate+Severe Anaemia Prevalence − Bayelsa
WHO−2024 Adjustment Method

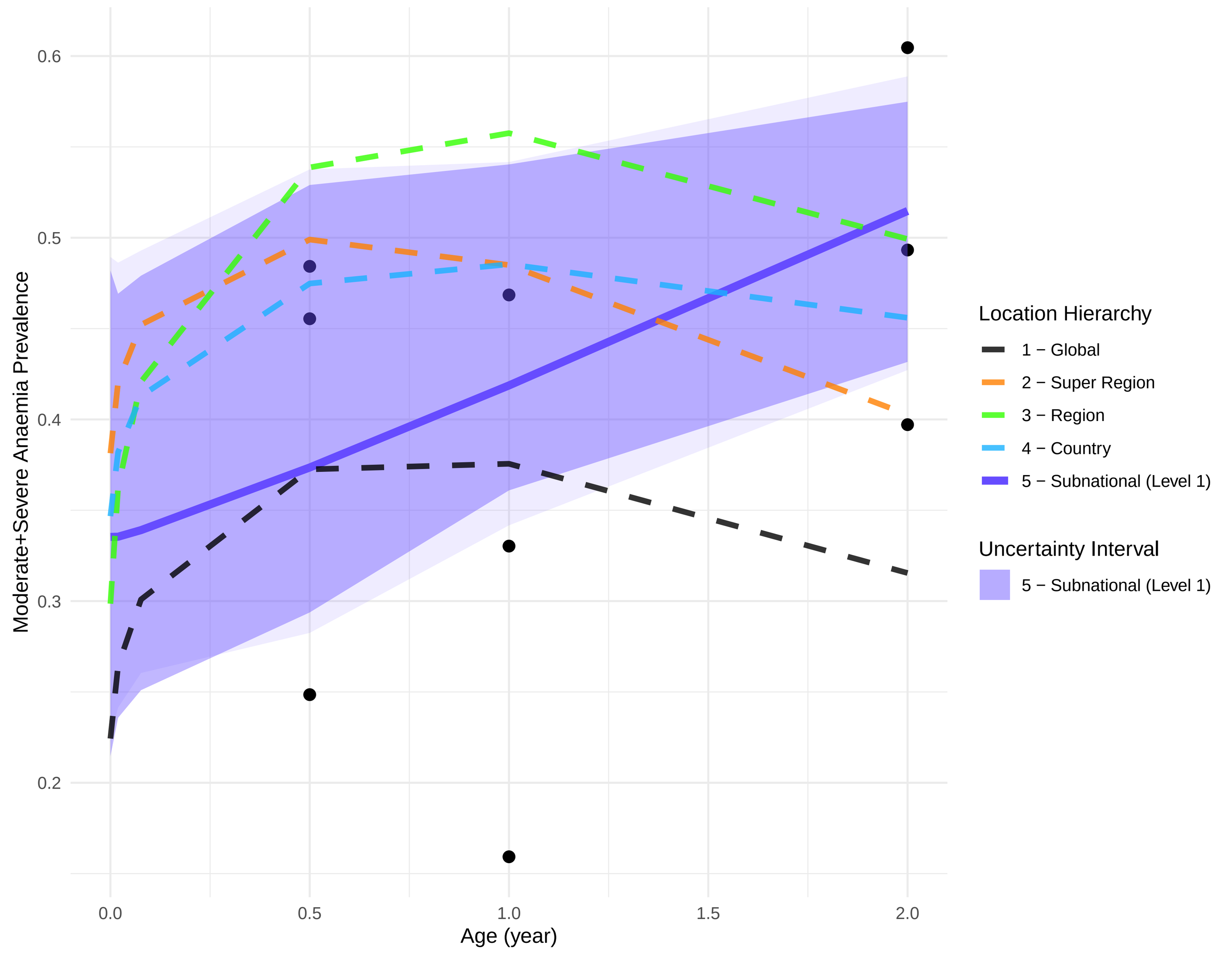

MR−BRT Age/Sex Fits − Males <2 Years Old − Moderate+Severe Anaemia Prevalence − Benue
WHO−2024 Adjustment Method

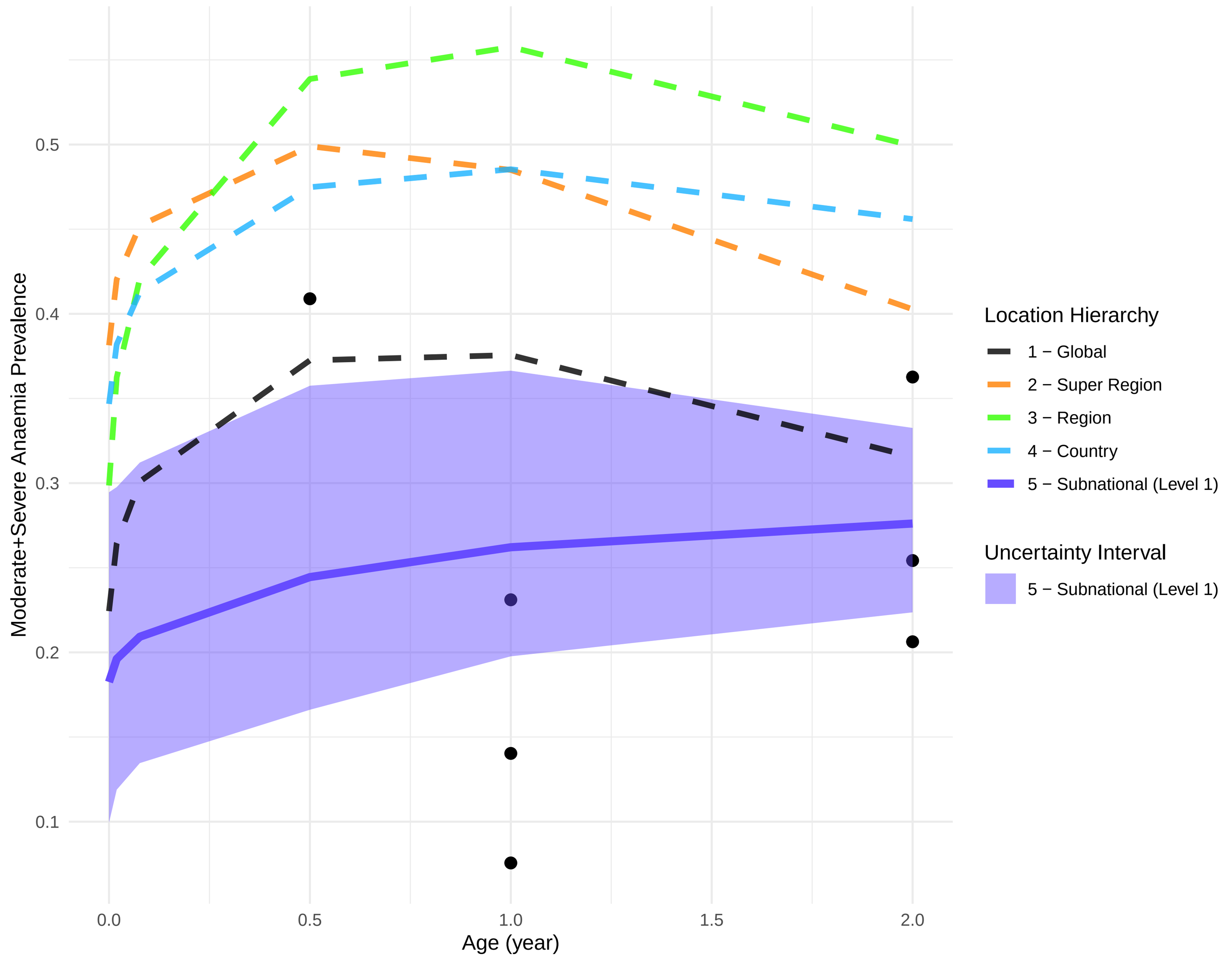

MR−BRT Age/Sex Fits − Males <2 Years Old − Moderate+Severe Anaemia Prevalence − Borno
WHO−2024 Adjustment Method

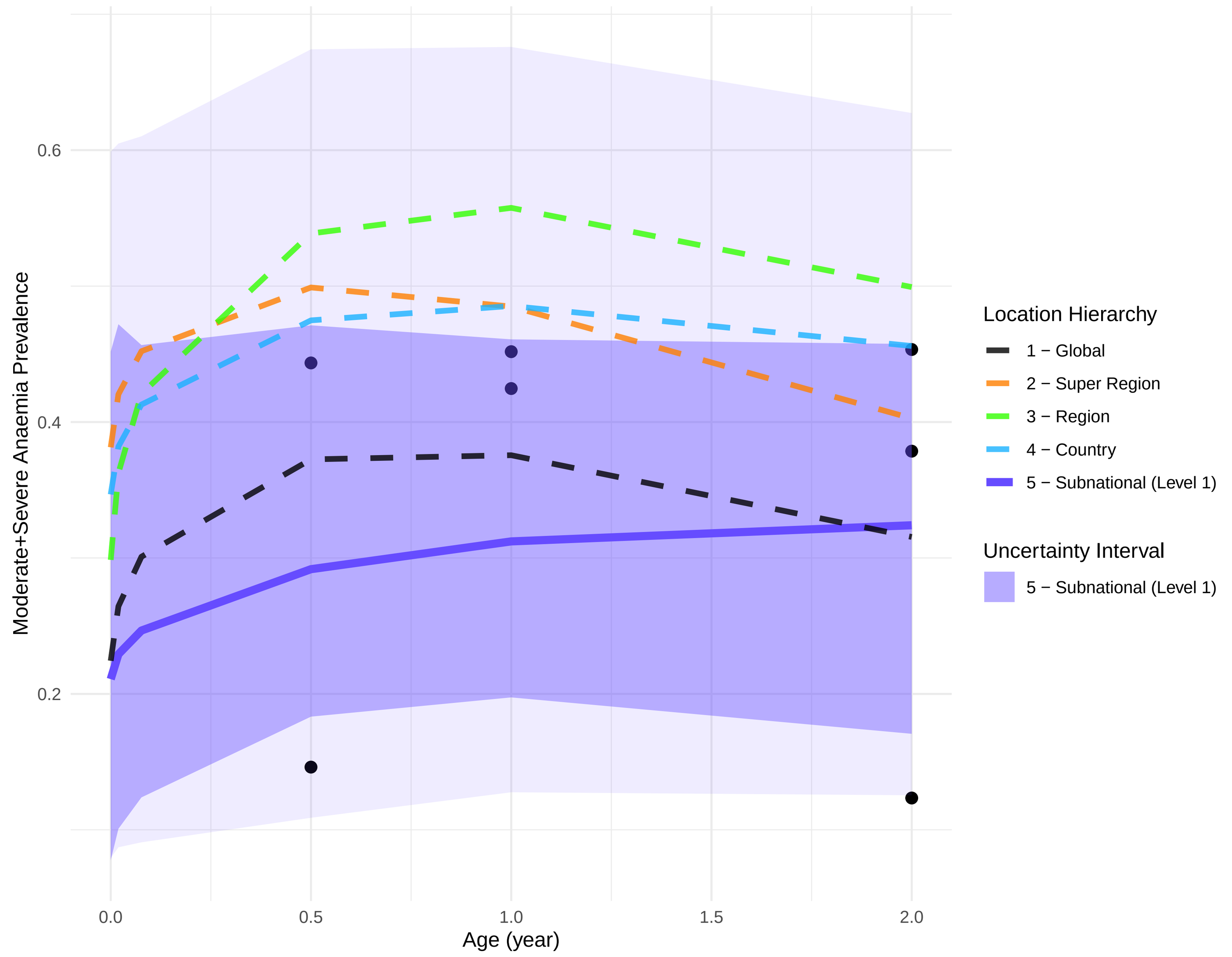

MR−BRT Age/Sex Fits − Males <2 Years Old − Moderate+Severe Anaemia Prevalence − Cross River
WHO−2024 Adjustment Method

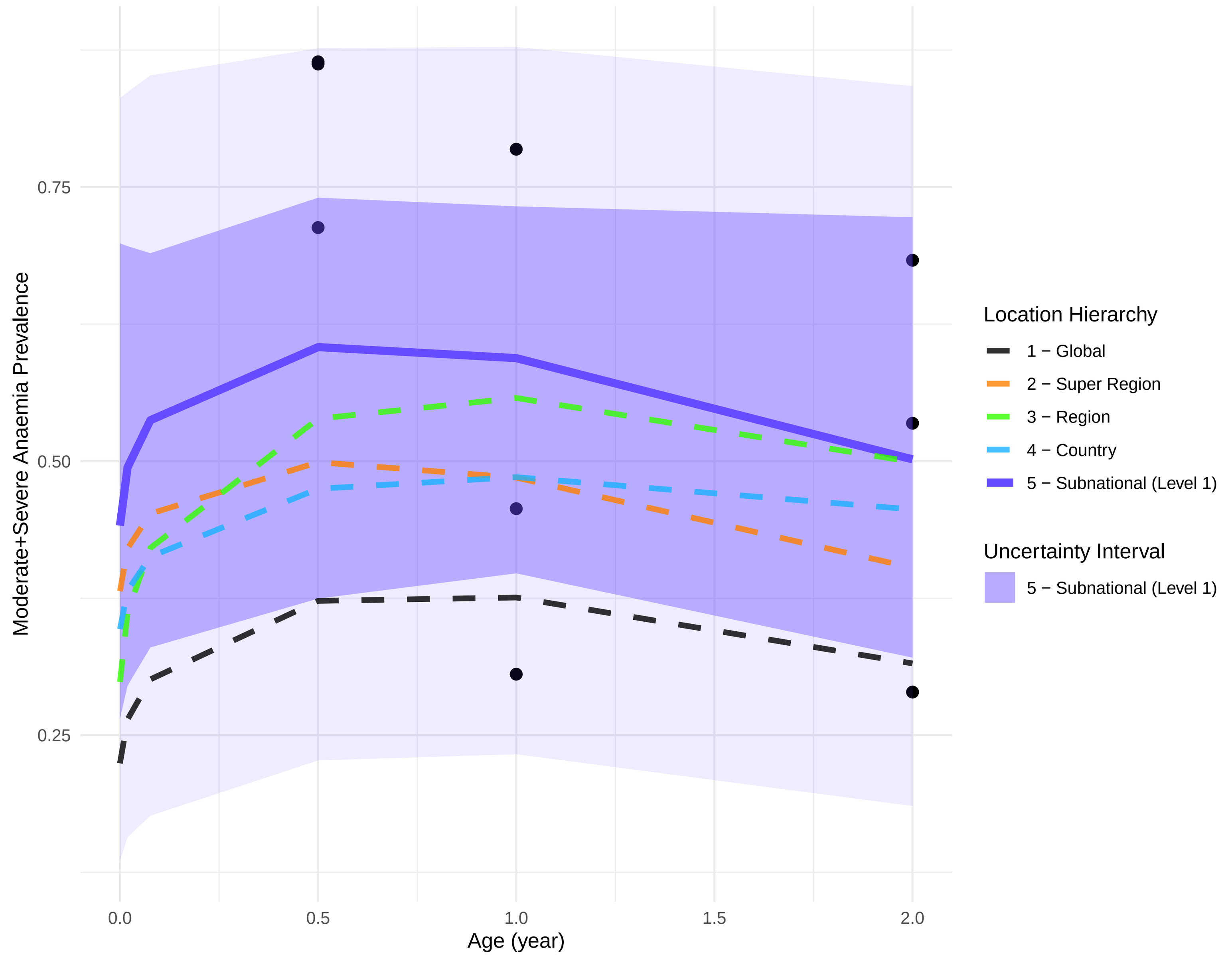

MR−BRT Age/Sex Fits − Males <2 Years Old − Moderate+Severe Anaemia Prevalence − Delta
WHO−2024 Adjustment Method

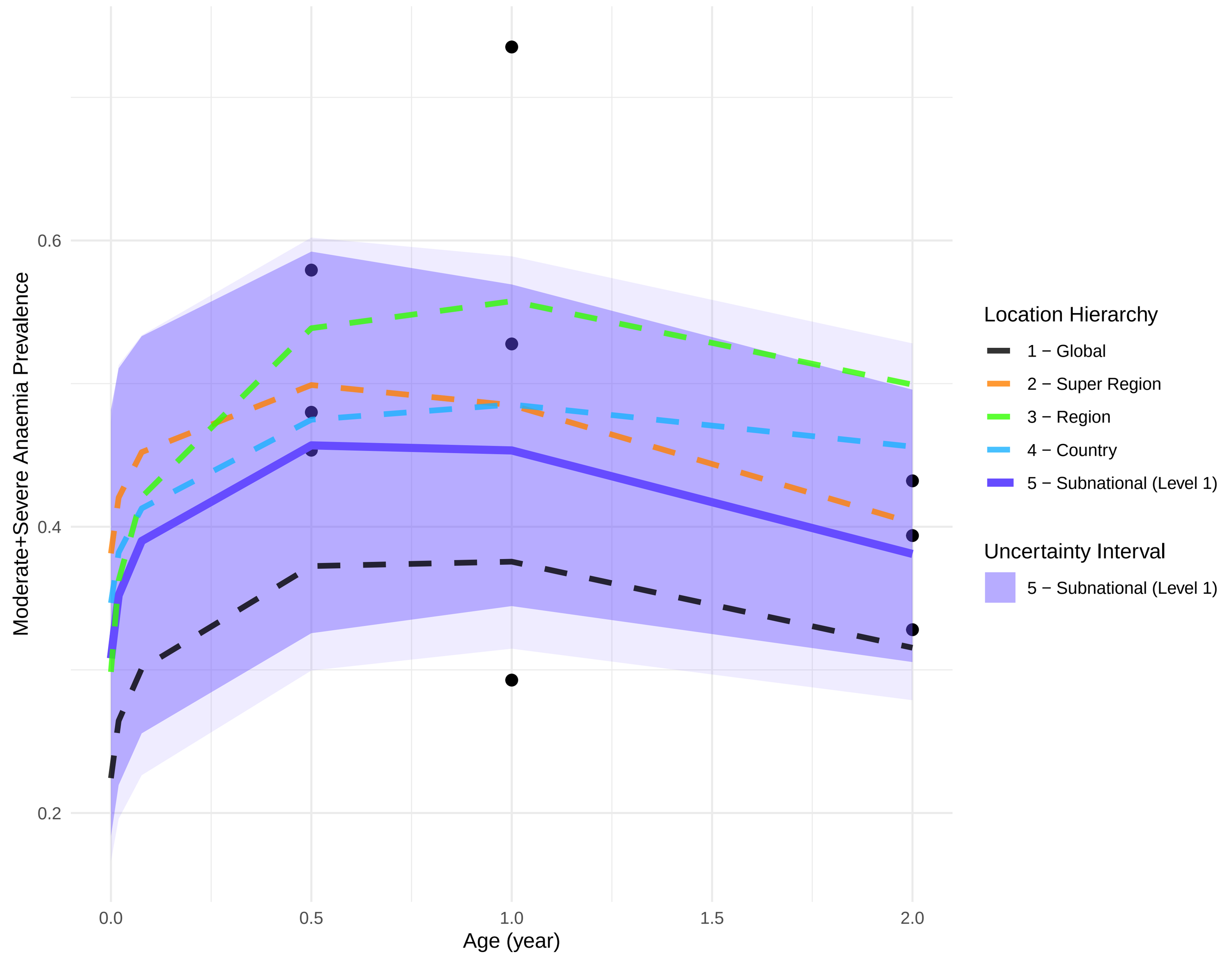

MR−BRT Age/Sex Fits − Males <2 Years Old − Moderate+Severe Anaemia Prevalence − Ebonyi
WHO−2024 Adjustment Method

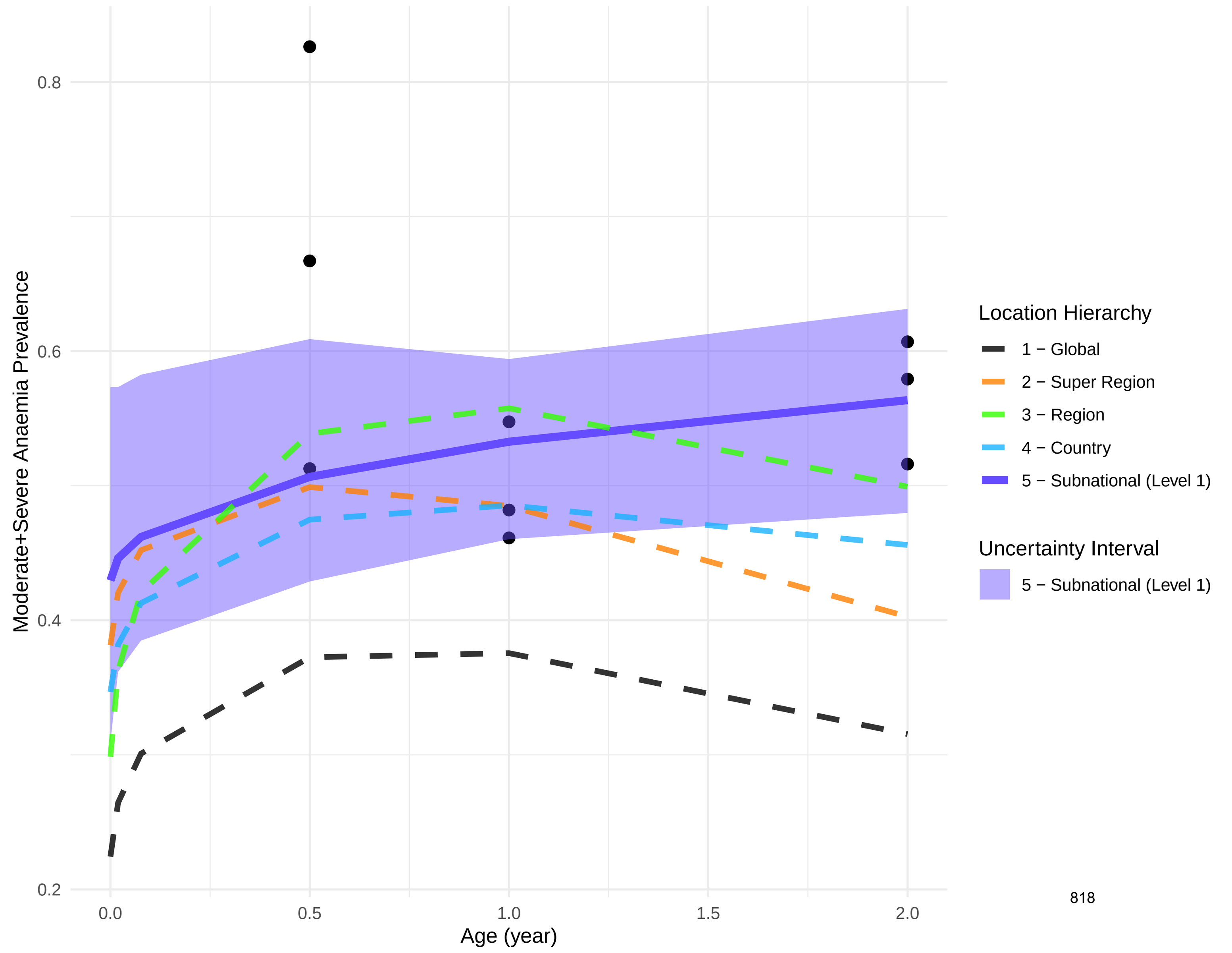

MR−BRT Age/Sex Fits − Males <2 Years Old − Moderate+Severe Anaemia Prevalence − Edo
WHO−2024 Adjustment Method

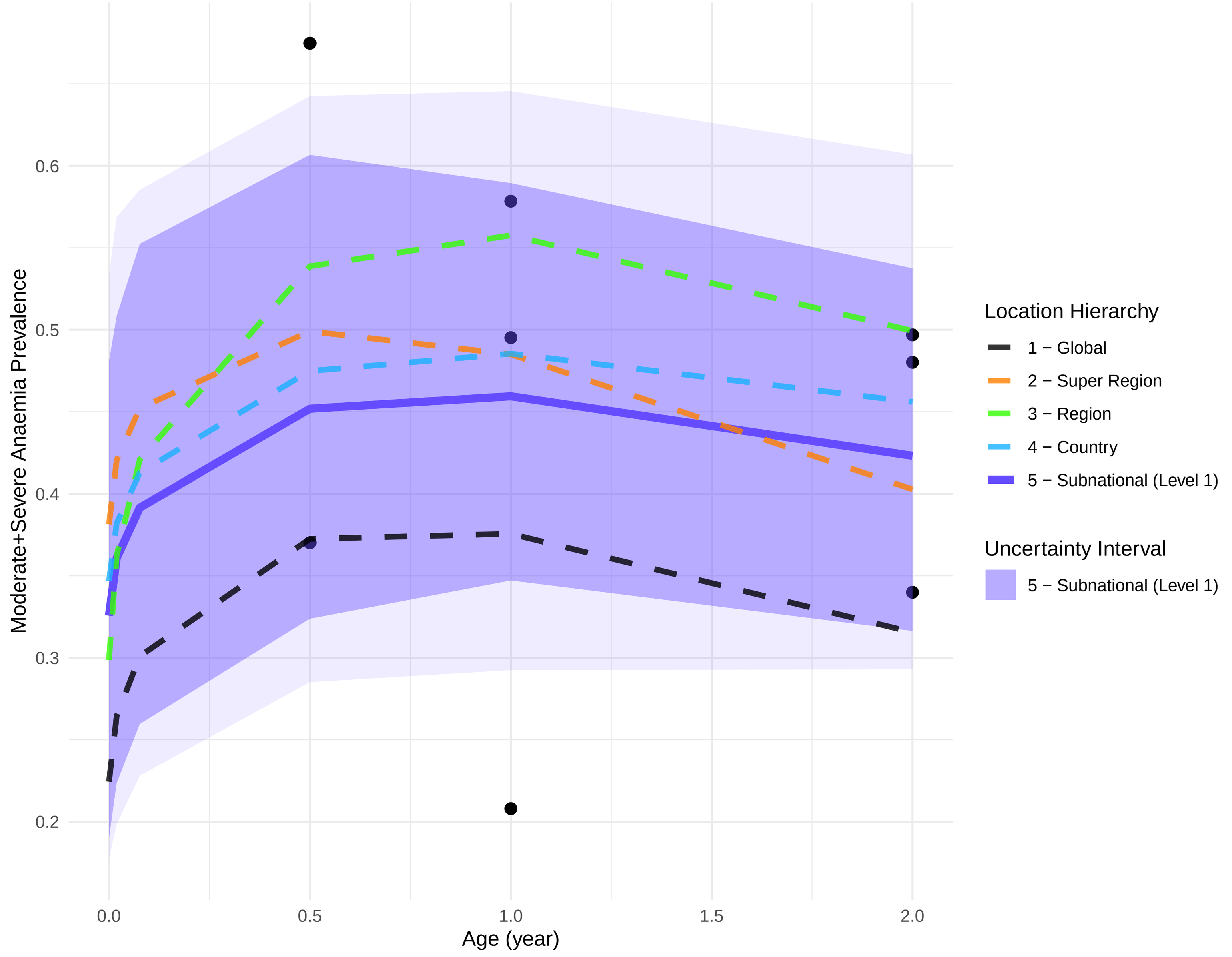

MR−BRT Age/Sex Fits − Males <2 Years Old − Moderate+Severe Anaemia Prevalence − Ekiti
WHO−2024 Adjustment Method

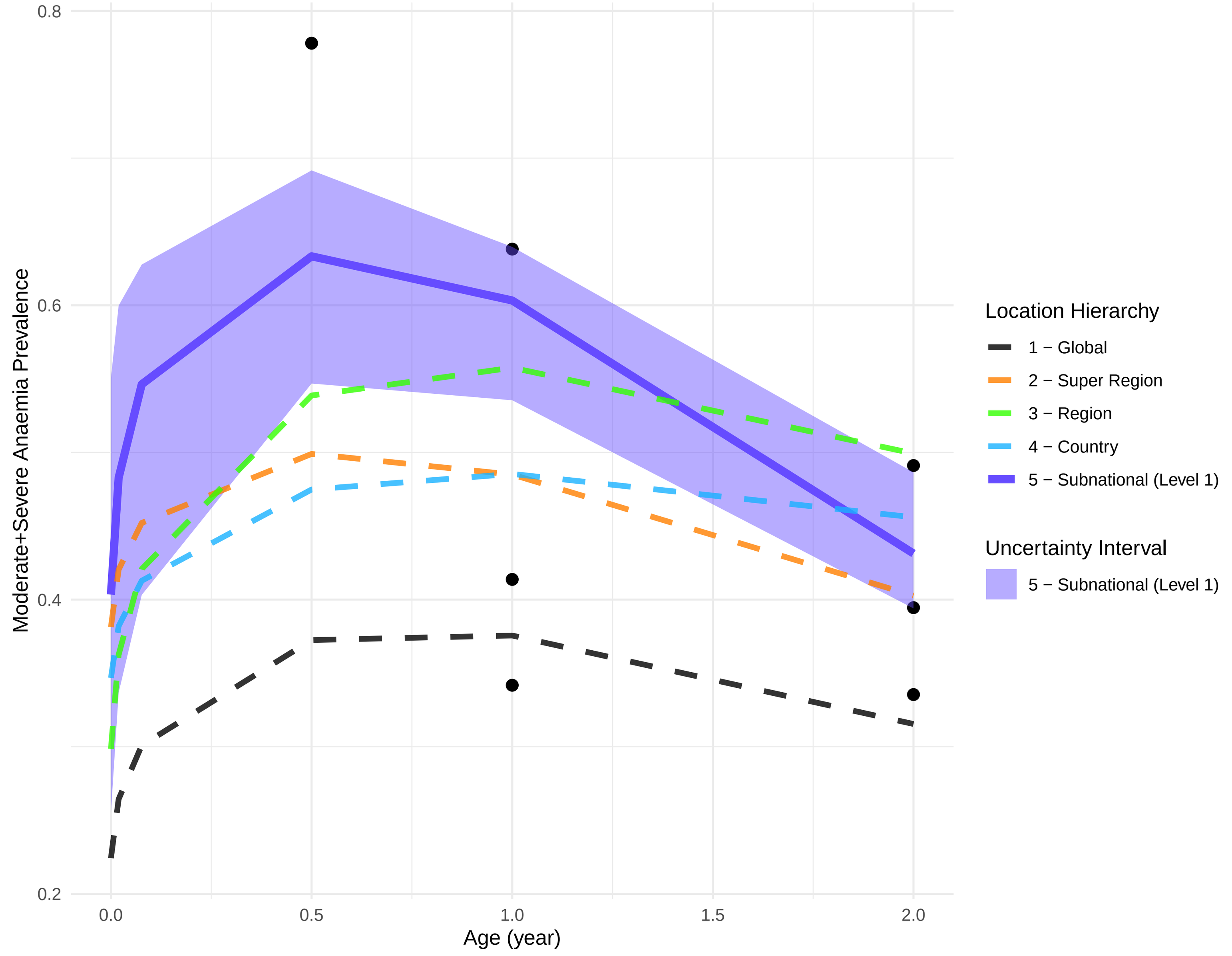

MR−BRT Age/Sex Fits − Males <2 Years Old − Moderate+Severe Anaemia Prevalence − Enugu
WHO−2024 Adjustment Method

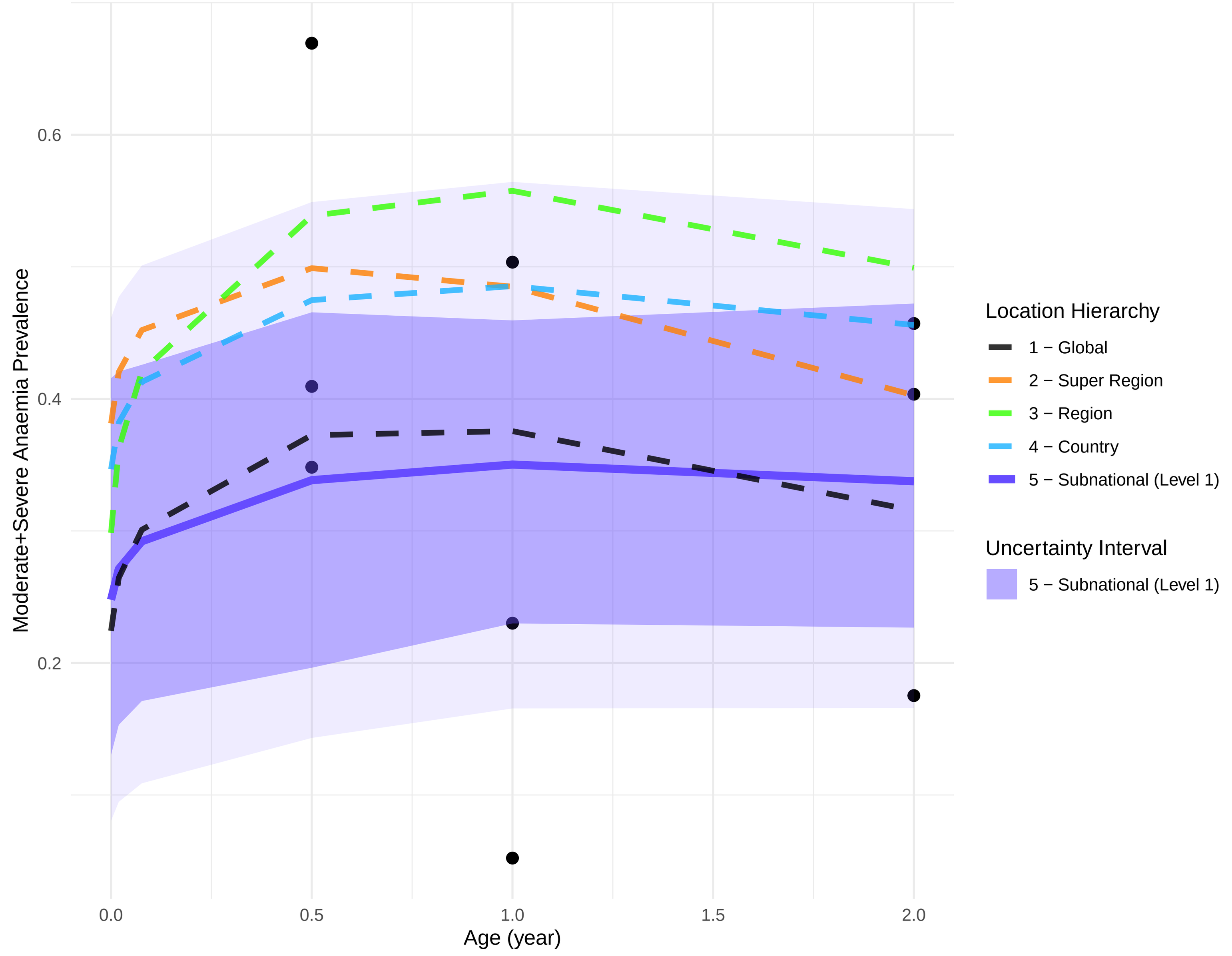

MR−BRT Age/Sex Fits − Males <2 Years Old − Moderate+Severe Anaemia Prevalence − FCT (Abuja)
WHO−2024 Adjustment Method

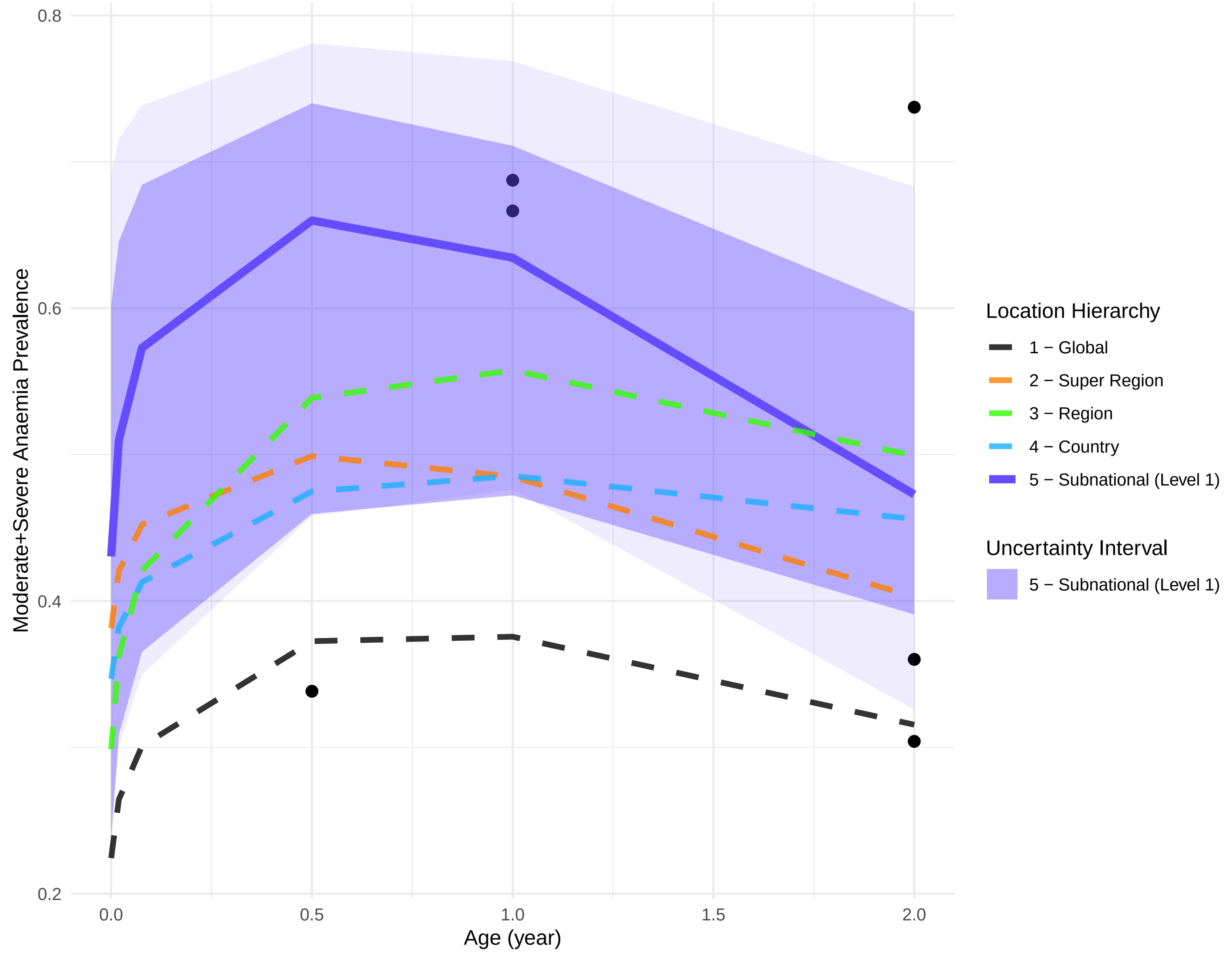

MR−BRT Age/Sex Fits − Males <2 Years Old − Moderate+Severe Anaemia Prevalence − Gombe
WHO−2024 Adjustment Method

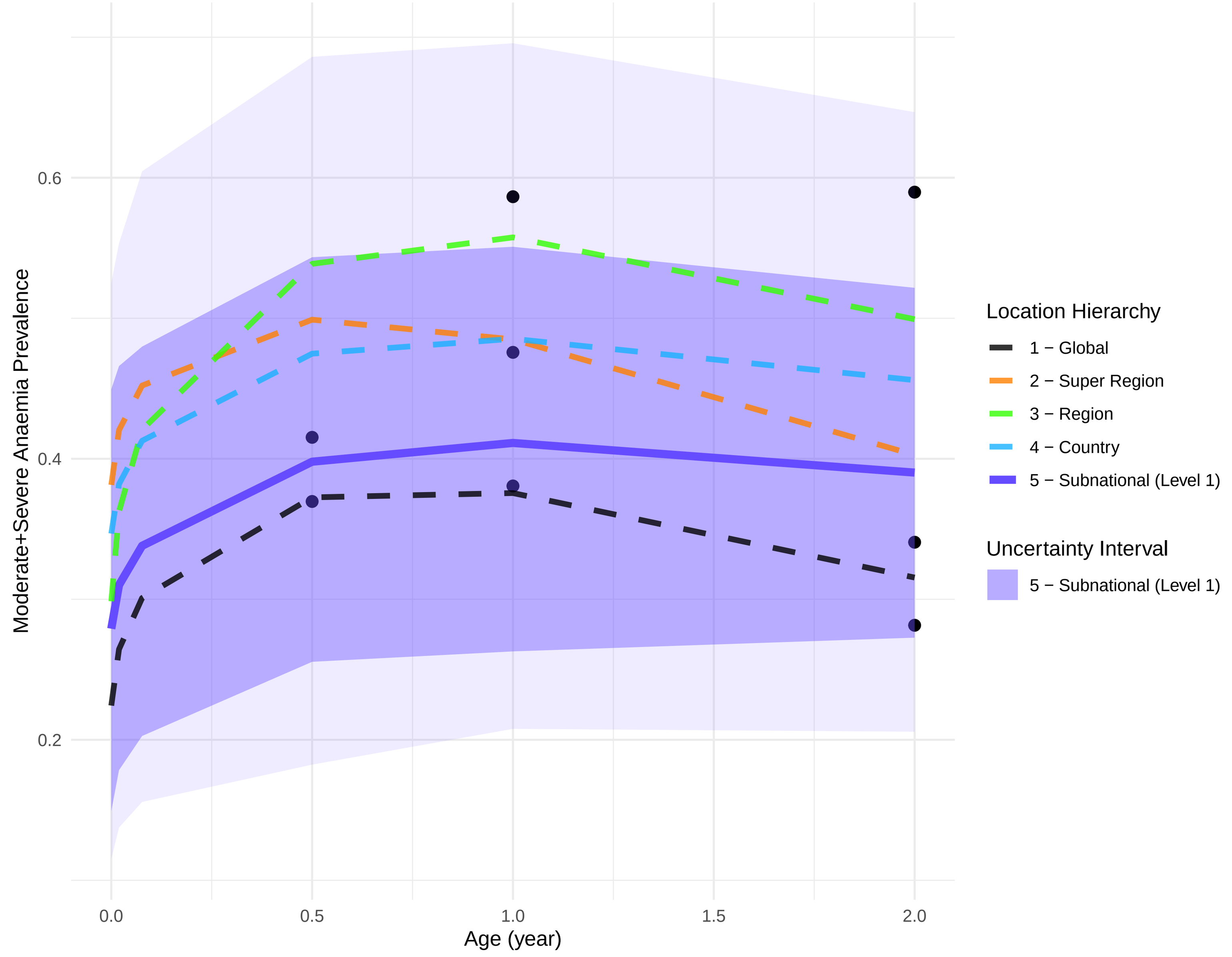

MR−BRT Age/Sex Fits − Males <2 Years Old − Moderate+Severe Anaemia Prevalence − Imo
WHO−2024 Adjustment Method

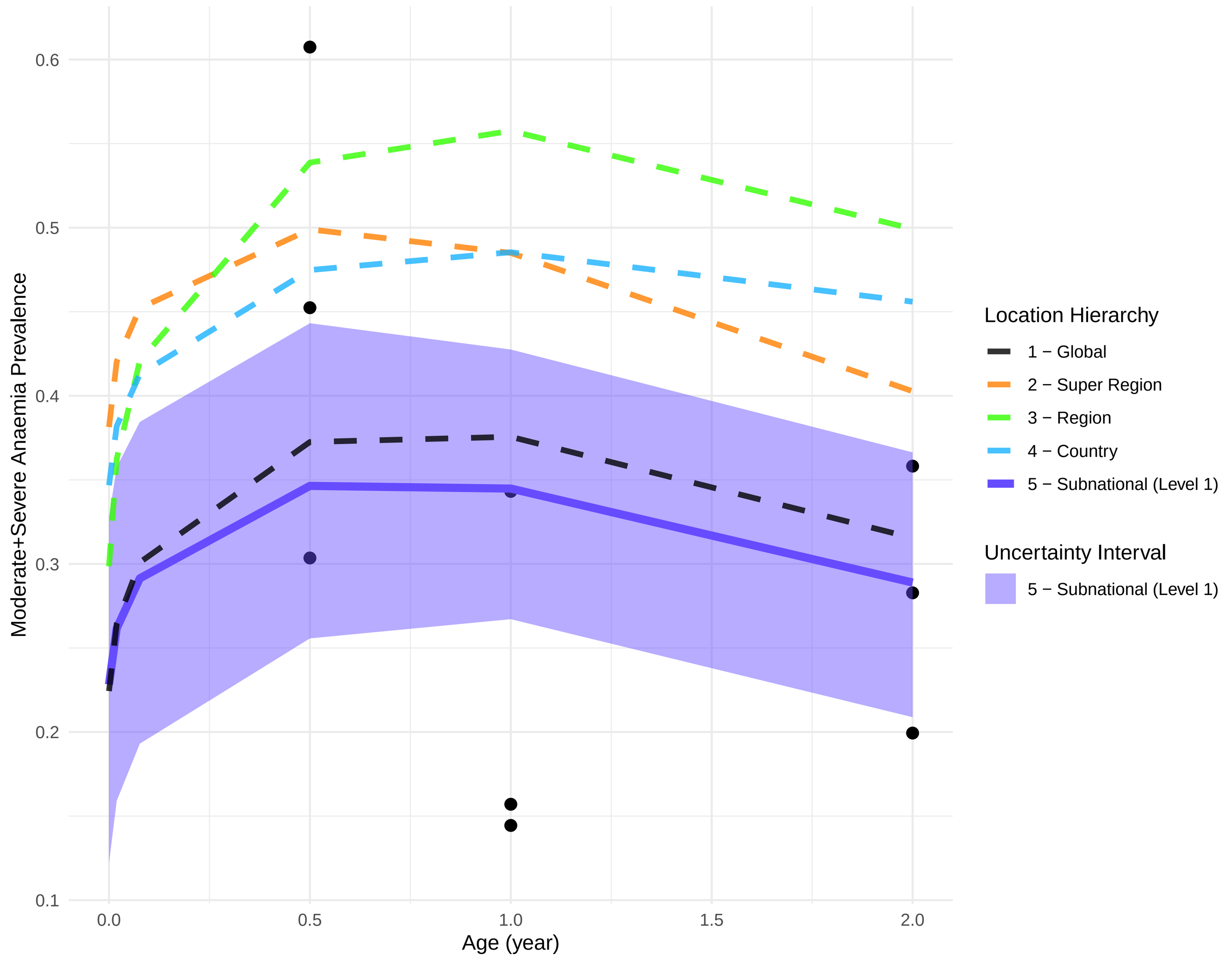

MR−BRT Age/Sex Fits − Males <2 Years Old − Moderate+Severe Anaemia Prevalence − Jigawa
WHO−2024 Adjustment Method

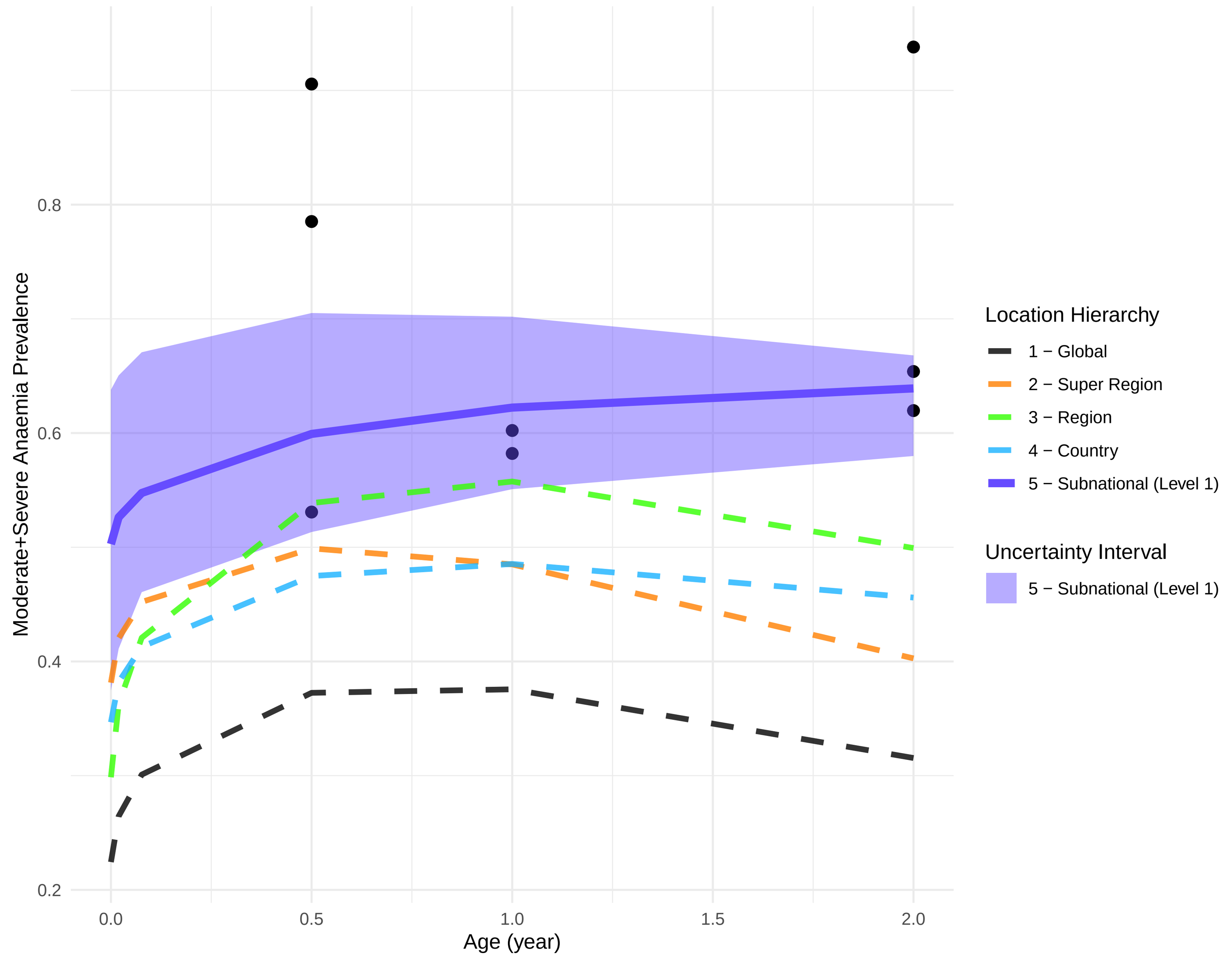

MR−BRT Age/Sex Fits − Males <2 Years Old − Moderate+Severe Anaemia Prevalence − Kaduna
WHO−2024 Adjustment Method

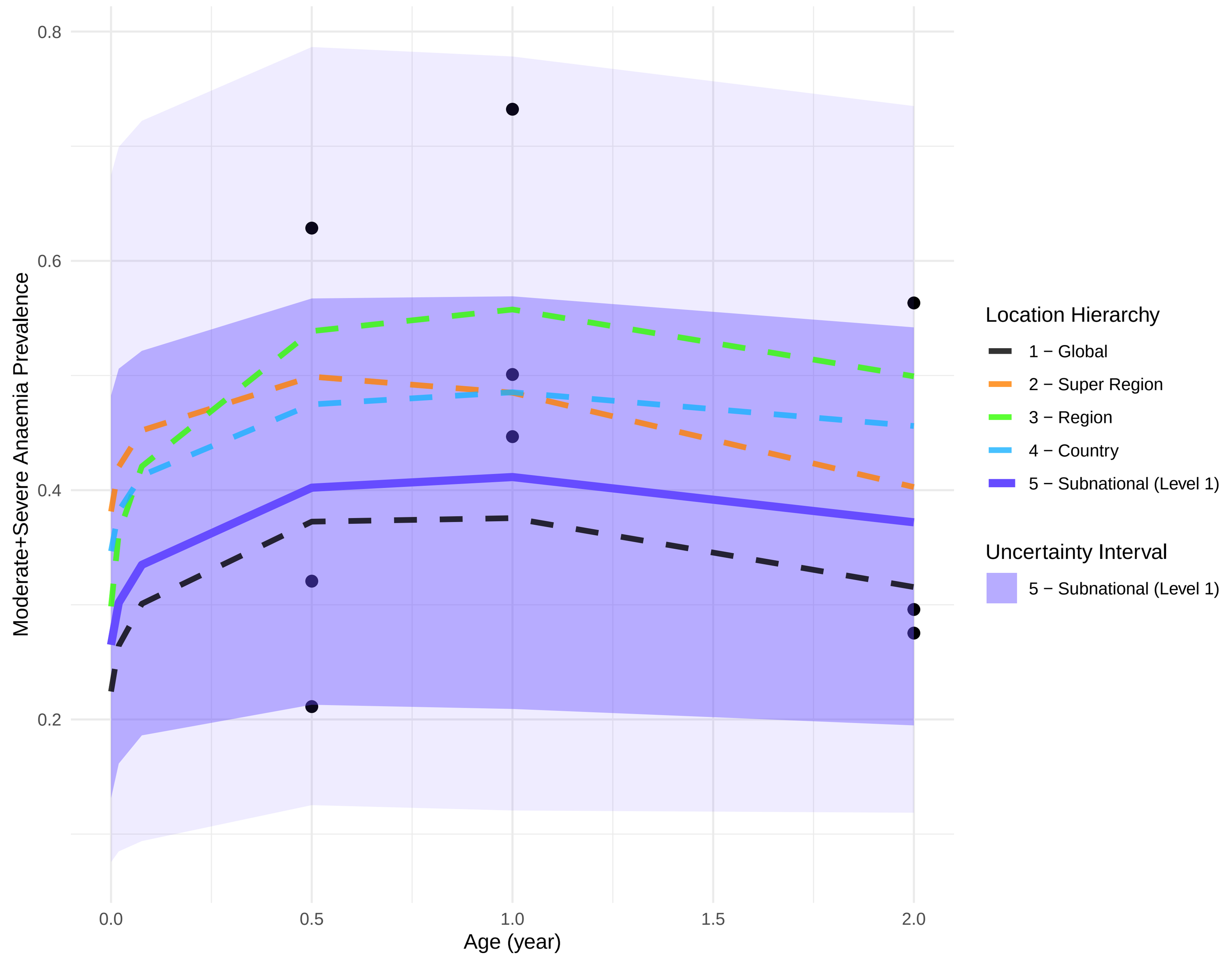

MR−BRT Age/Sex Fits − Males <2 Years Old − Moderate+Severe Anaemia Prevalence − Kano
WHO−2024 Adjustment Method

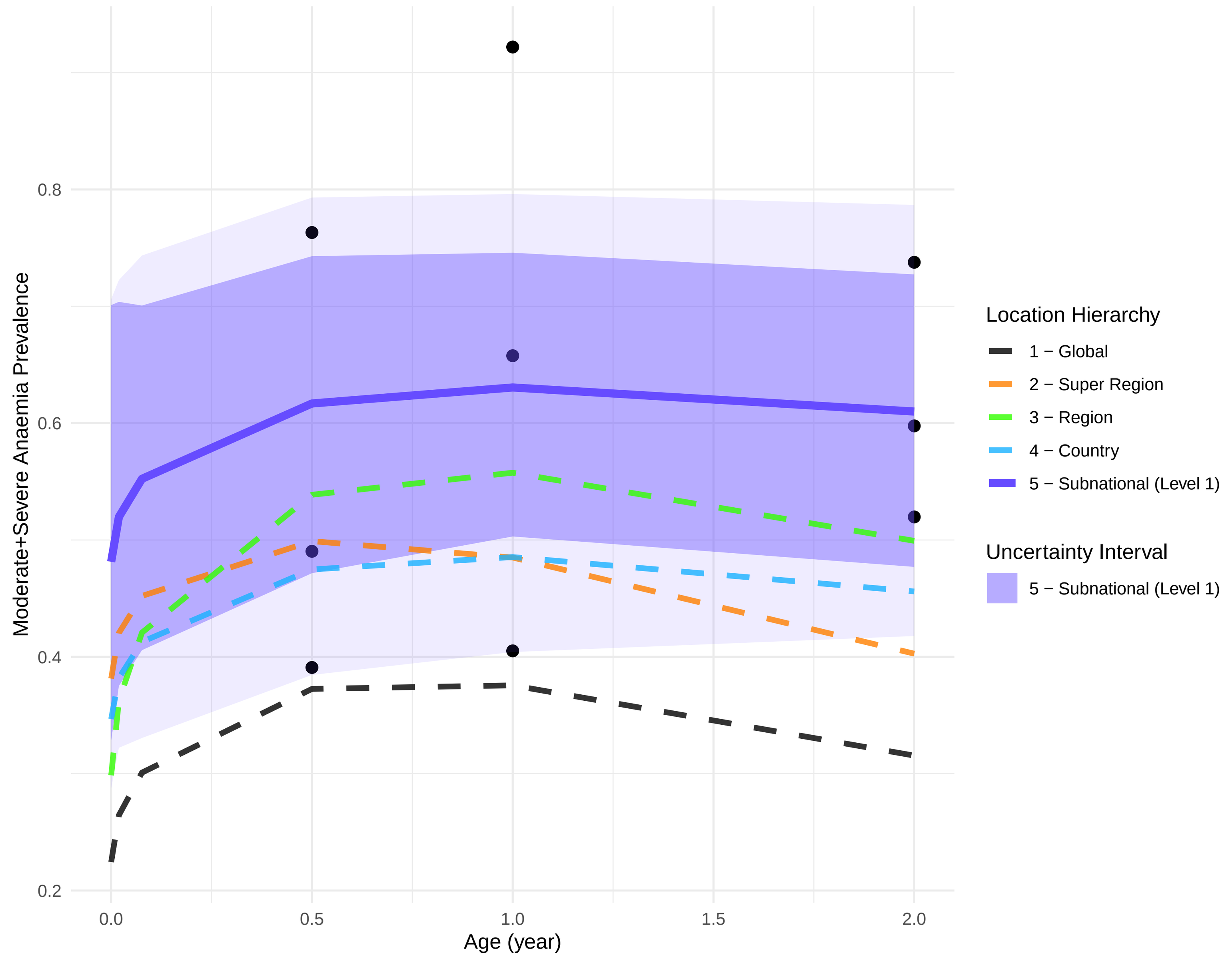

MR−BRT Age/Sex Fits − Males <2 Years Old − Moderate+Severe Anaemia Prevalence − Katsina
WHO−2024 Adjustment Method

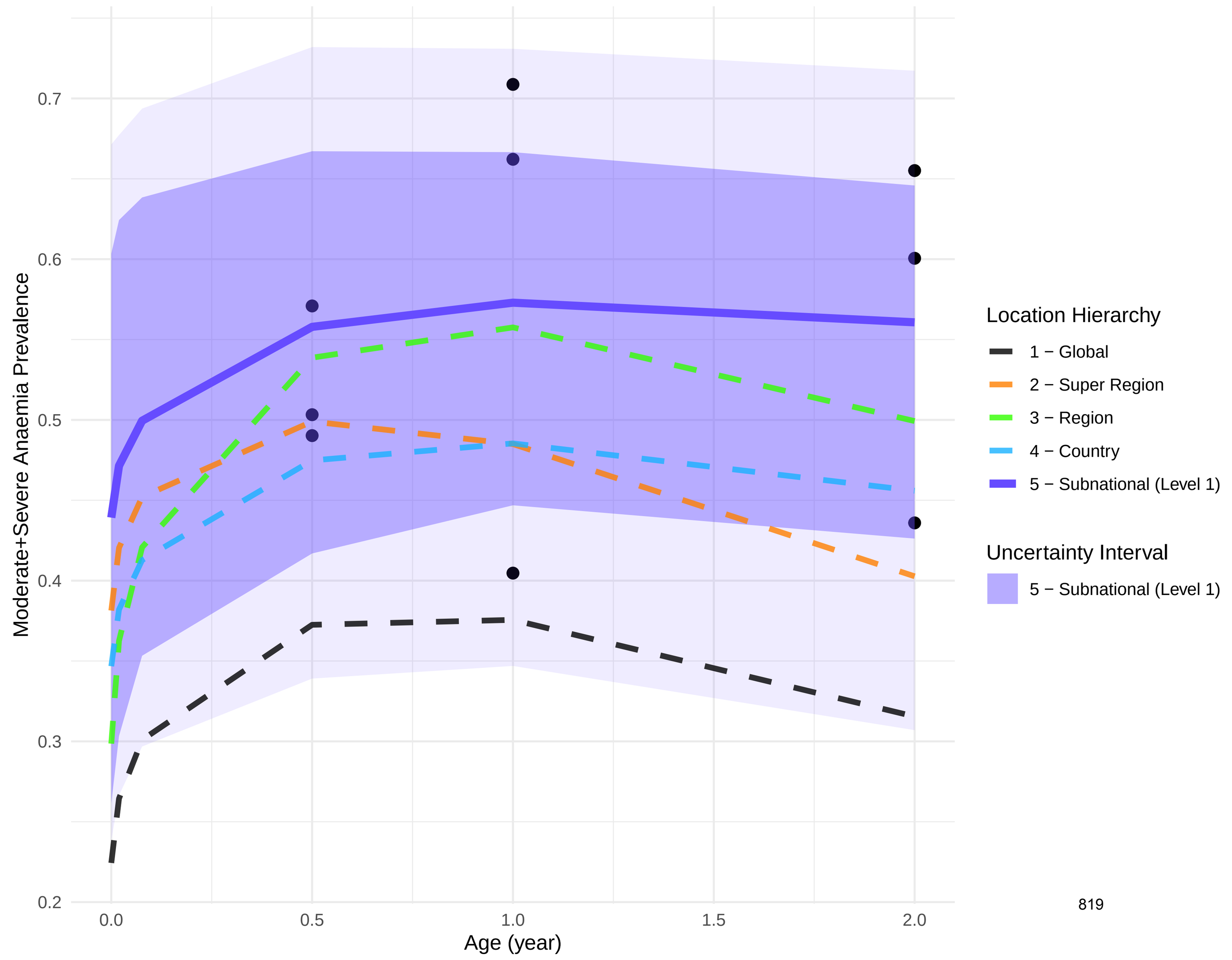

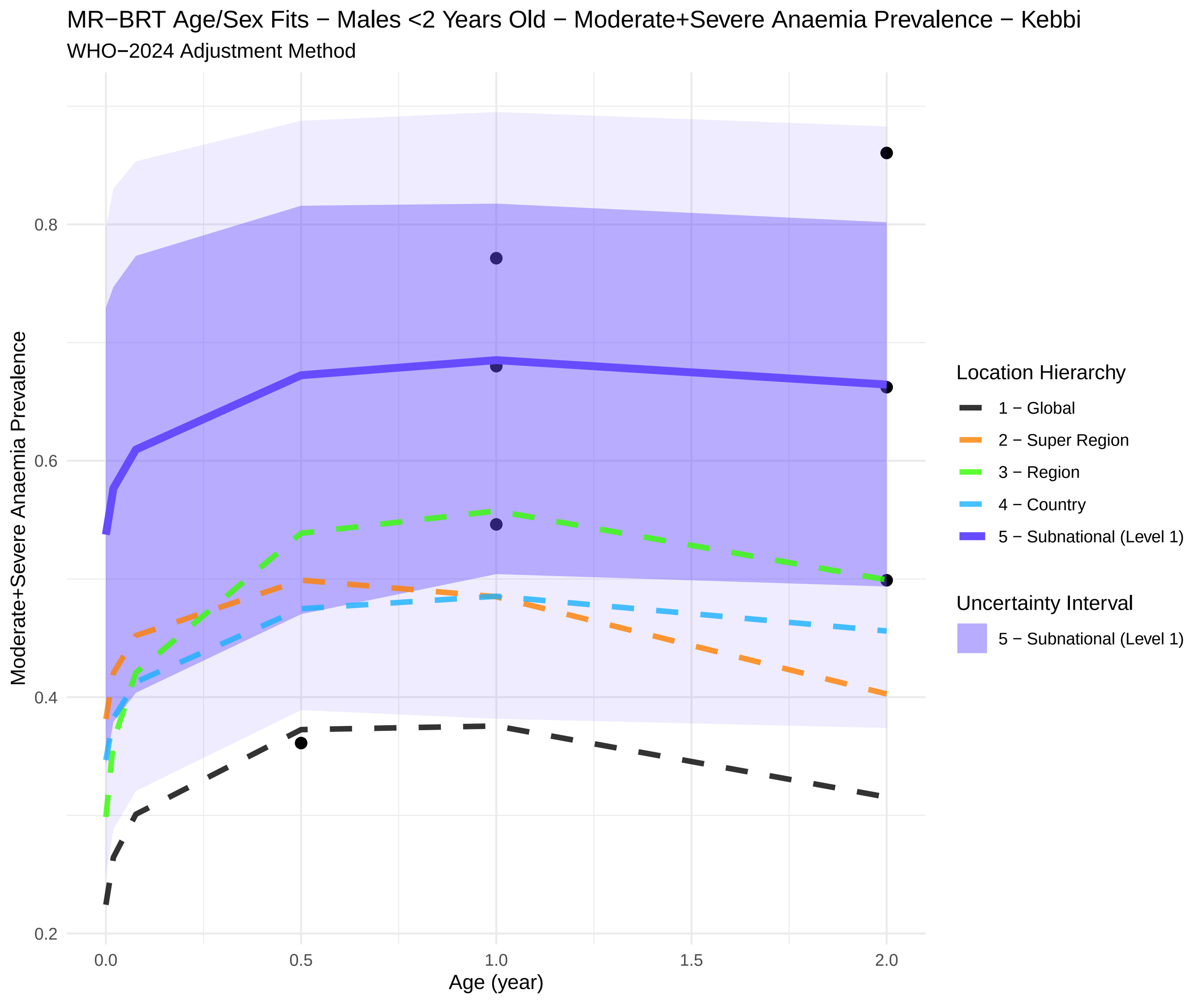
MR−BRT Age/Sex Fits − Males <2 Years Old − Moderate+Severe Anaemia Prevalence − Kebbi
WHO−2024 Adjustment Method
Moderate+Severe Anaemia Prevalence
Age (year)
Location Hierarchy
1 − Global
2 − Super Region
3 − Region
4 − Country
5 − Subnational (Level 1)
Uncertainty Interval
5 − Subnational (Level 1)

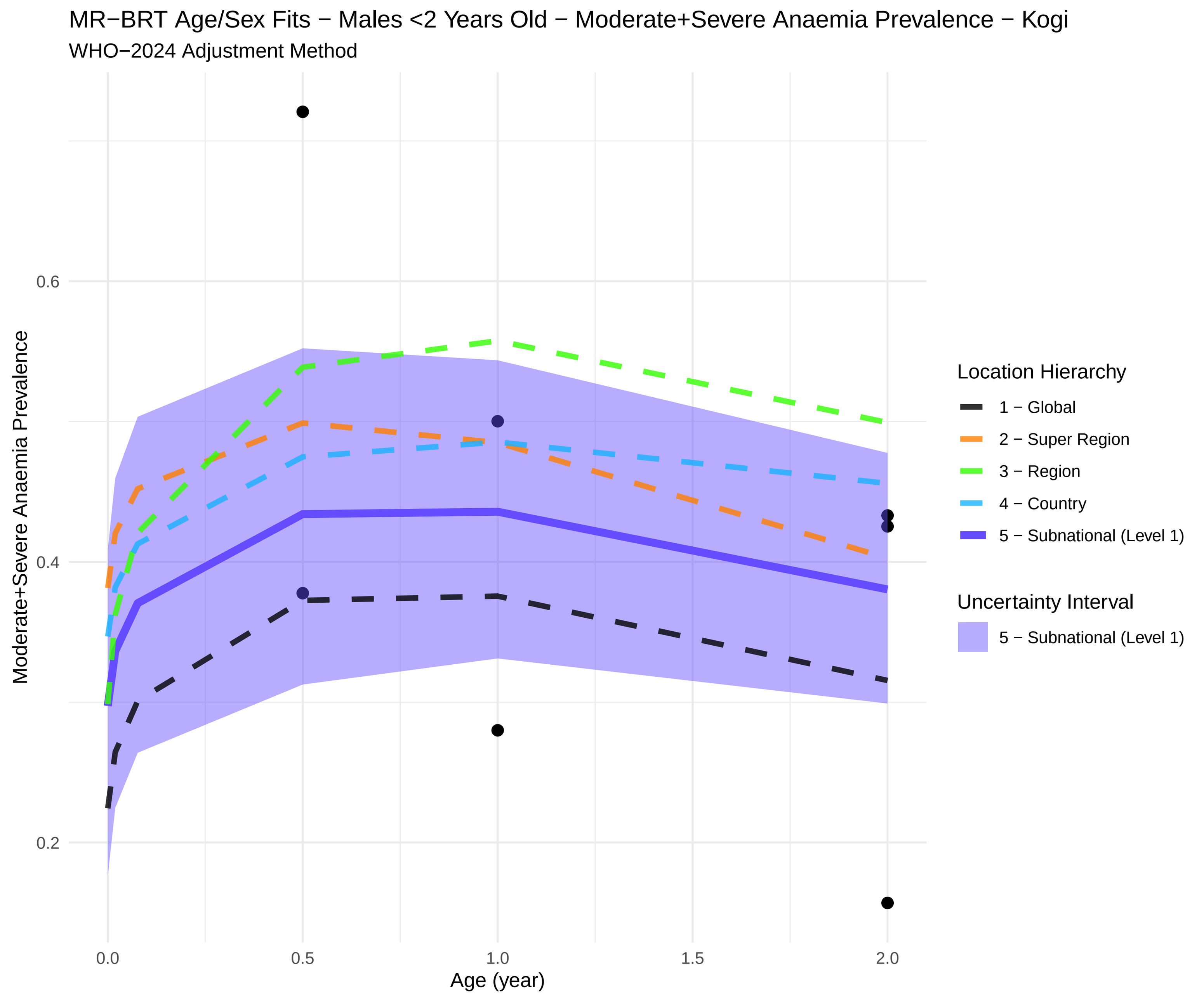
MR−BRT Age/Sex Fits − Males <2 Years Old − Moderate+Severe Anaemia Prevalence − Kogi
WHO−2024 Adjustment Method
Moderate+Severe Anaemia Prevalence
Age (year)
Location Hierarchy
1 − Global
2 − Super Region
3 − Region
4 − Country
5 − Subnational (Level 1)
Uncertainty Interval
5 − Subnational (Level 1)

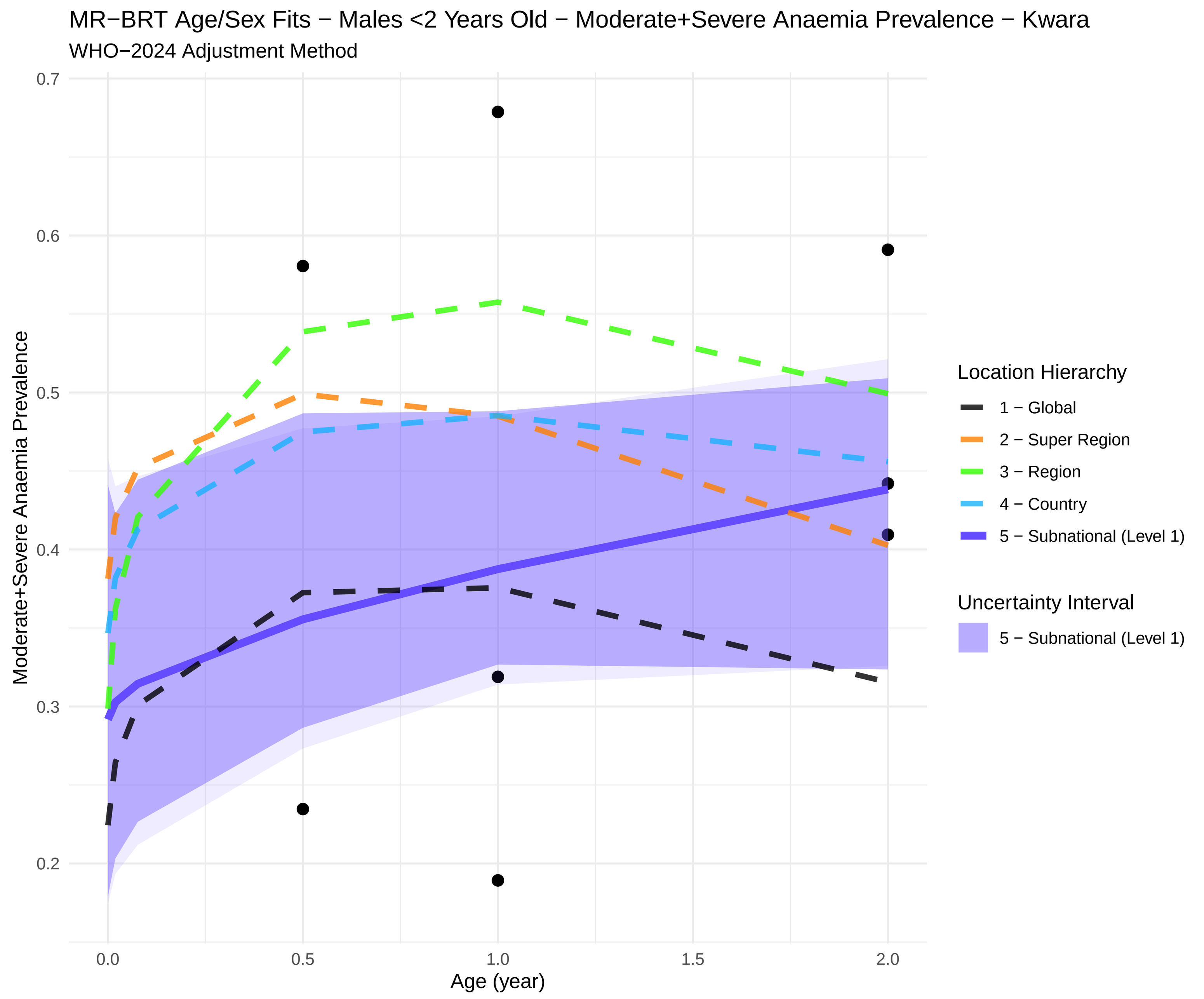
MR−BRT Age/Sex Fits − Males <2 Years Old − Moderate+Severe Anaemia Prevalence − Kwara
WHO−2024 Adjustment Method
Moderate+Severe Anaemia Prevalence
Age (year)
Location Hierarchy
1 − Global
2 − Super Region
3 − Region
4 − Country
5 − Subnational (Level 1)
Uncertainty Interval
5 − Subnational (Level 1)

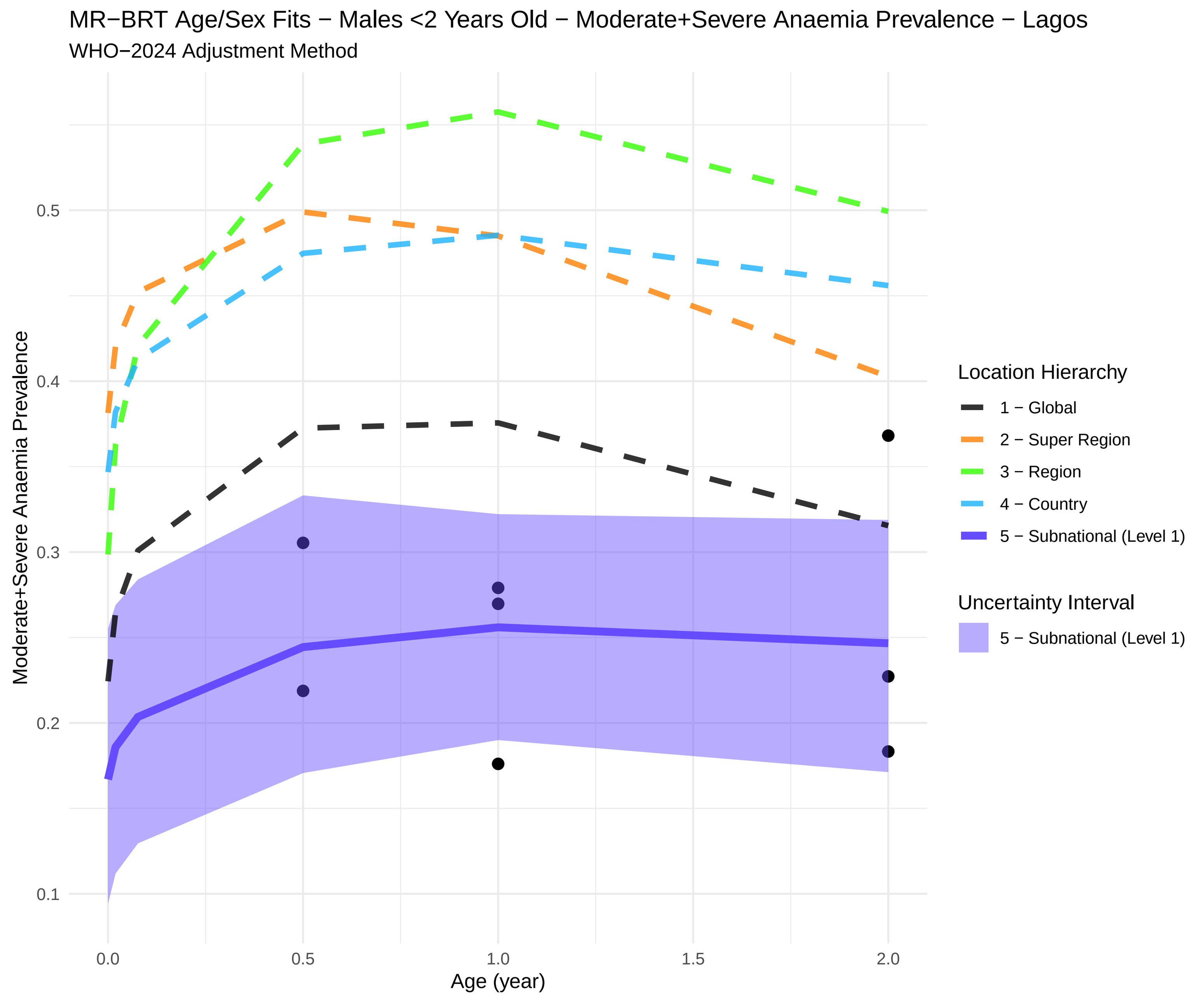
MR−BRT Age/Sex Fits − Males <2 Years Old − Moderate+Severe Anaemia Prevalence − Lagos
WHO−2024 Adjustment Method
Moderate+Severe Anaemia Prevalence
Age (year)
Location Hierarchy
1 − Global
2 − Super Region
3 − Region
4 − Country
5 − Subnational (Level 1)
Uncertainty Interval
5 − Subnational (Level 1)

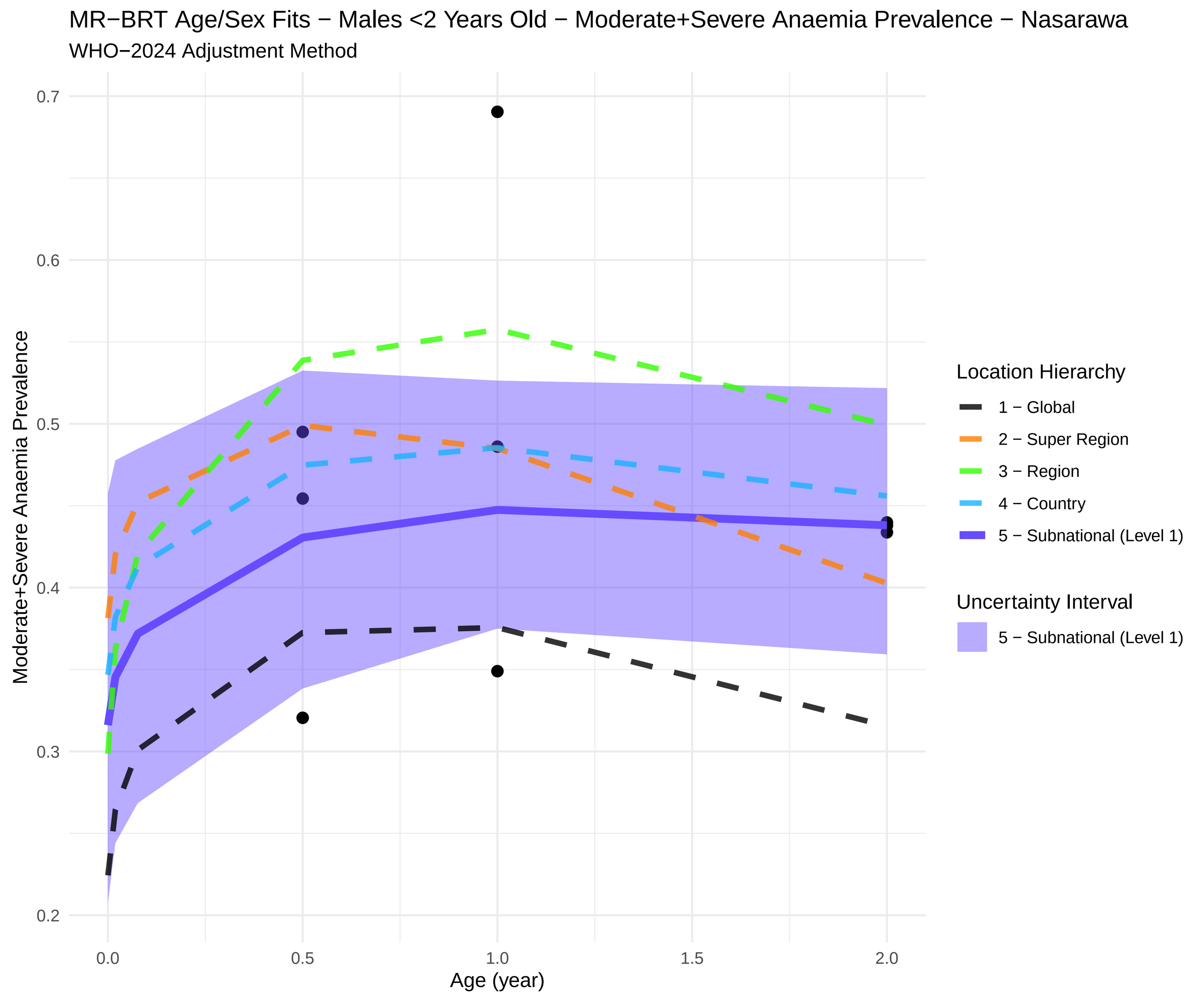
MR−BRT Age/Sex Fits − Males <2 Years Old − Moderate+Severe Anaemia Prevalence − Nasarawa
WHO−2024 Adjustment Method
Moderate+Severe Anaemia Prevalence
Age (year)
Location Hierarchy
1 − Global
2 − Super Region
3 − Region
4 − Country
5 − Subnational (Level 1)
Uncertainty Interval
5 − Subnational (Level 1)

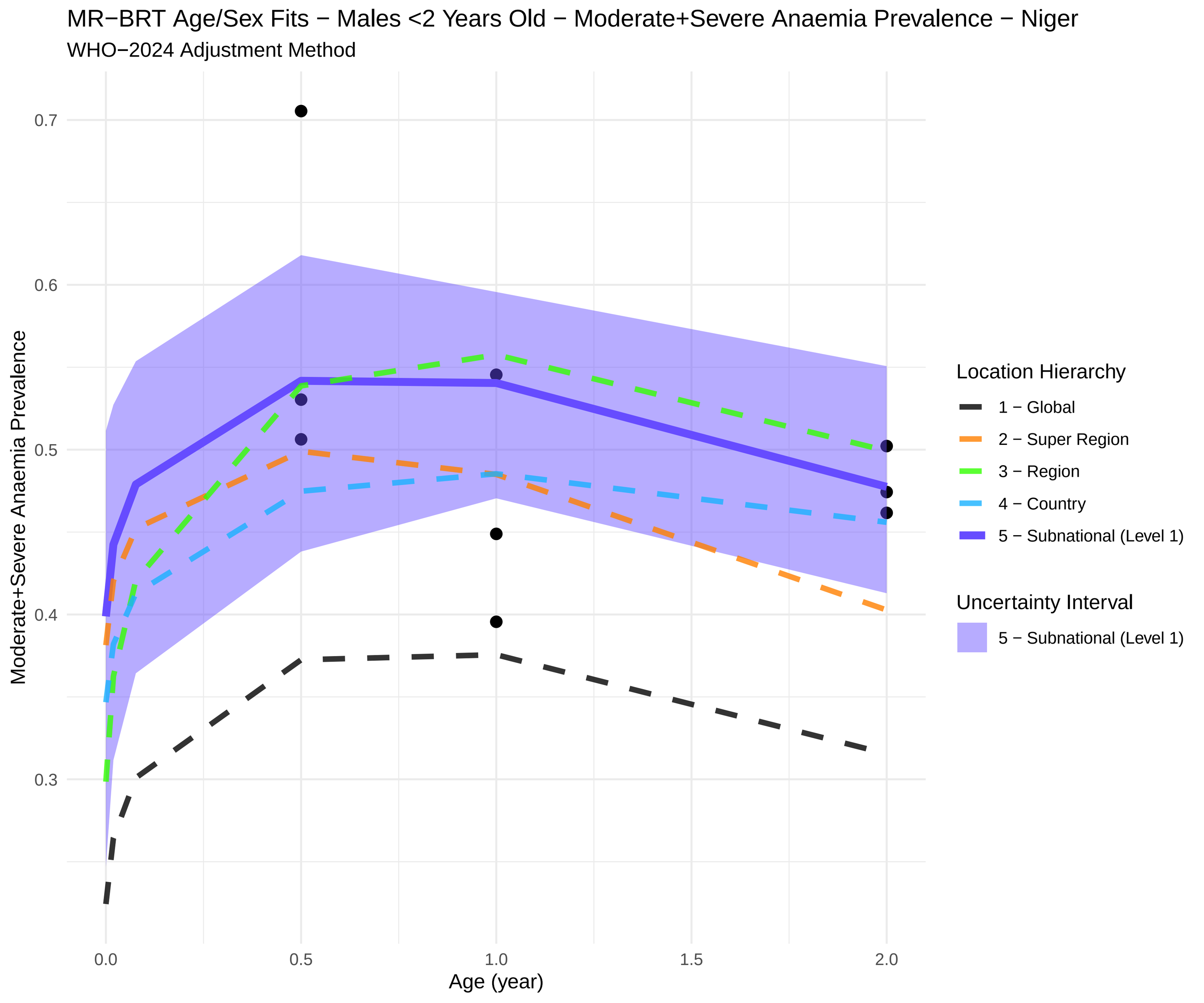
MR−BRT Age/Sex Fits − Males <2 Years Old − Moderate+Severe Anaemia Prevalence − Niger
WHO−2024 Adjustment Method
Moderate+Severe Anaemia Prevalence
Age (year)
Location Hierarchy
1 − Global
2 − Super Region
3 − Region
4 − Country
5 − Subnational (Level 1)
Uncertainty Interval
5 − Subnational (Level 1)

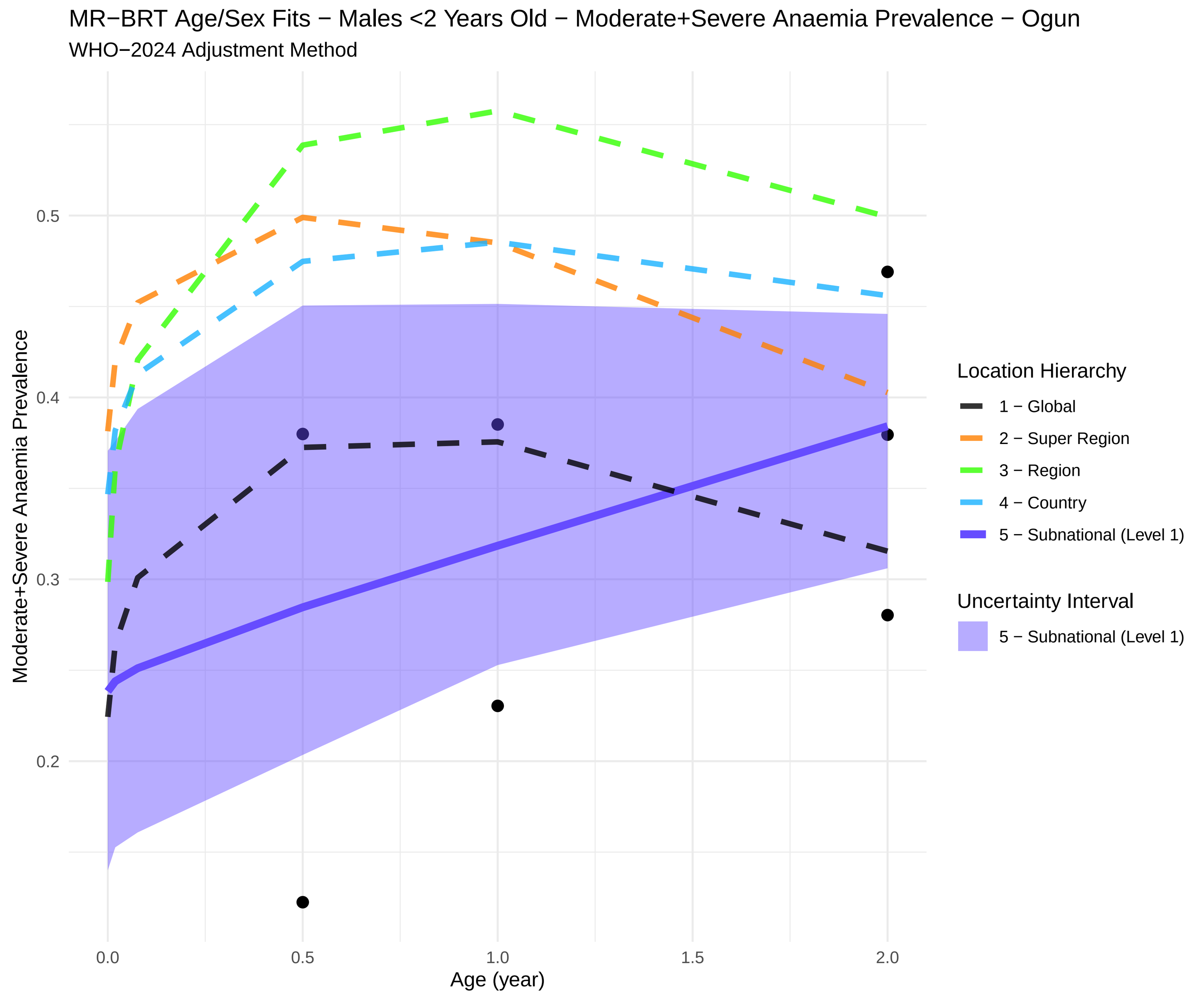
MR−BRT Age/Sex Fits − Males <2 Years Old − Moderate+Severe Anaemia Prevalence − Ogun
WHO−2024 Adjustment Method
Moderate+Severe Anaemia Prevalence
Age (year)
Location Hierarchy
1 − Global
2 − Super Region
3 − Region
4 − Country
5 − Subnational (Level 1)
Uncertainty Interval
5 − Subnational (Level 1)

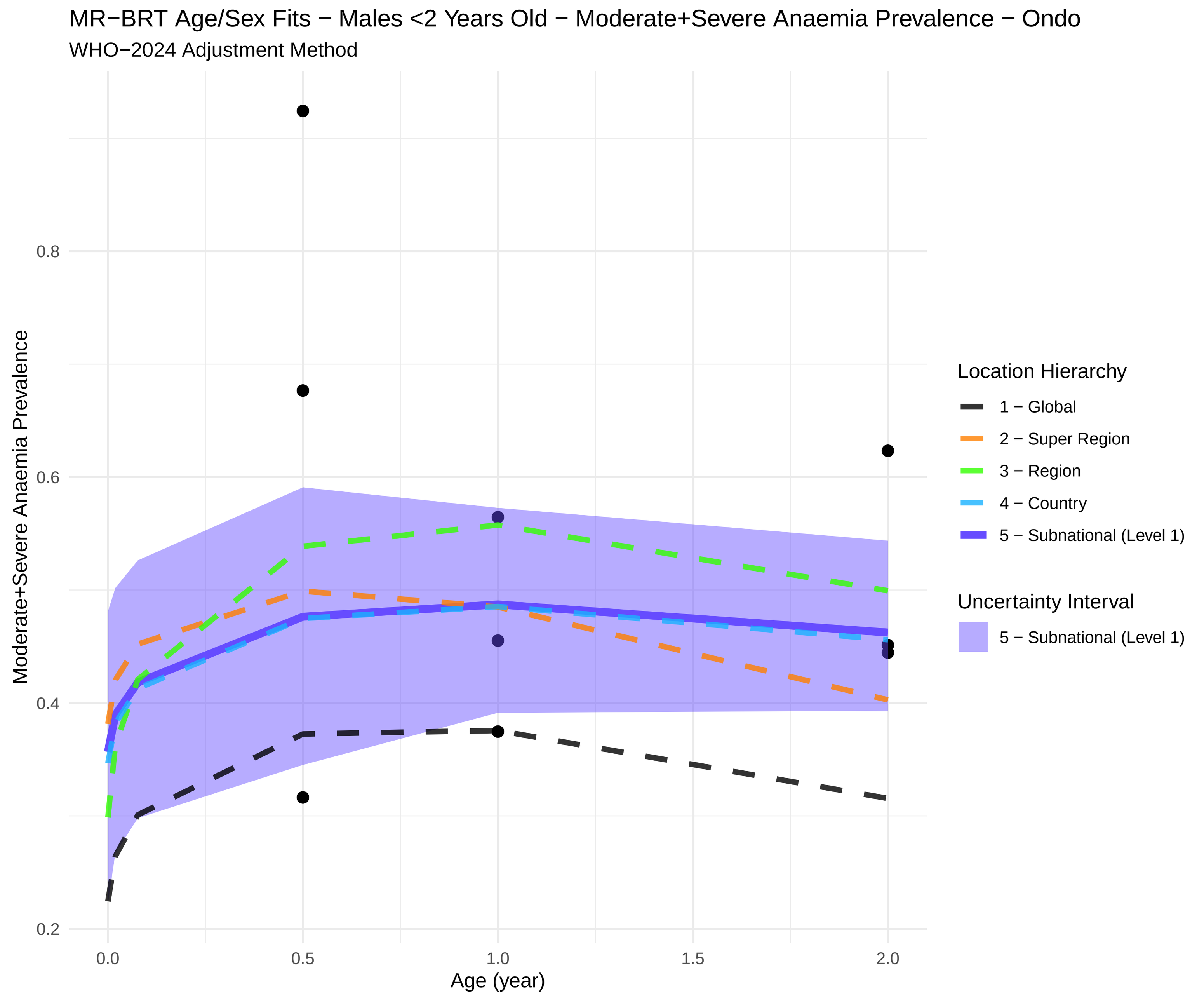
MR−BRT Age/Sex Fits − Males <2 Years Old − Moderate+Severe Anaemia Prevalence − Ondo
WHO−2024 Adjustment Method
Moderate+Severe Anaemia Prevalence
Age (year)
Location Hierarchy
1 − Global
2 − Super Region
3 − Region
4 − Country
5 − Subnational (Level 1)
Uncertainty Interval
5 − Subnational (Level 1)

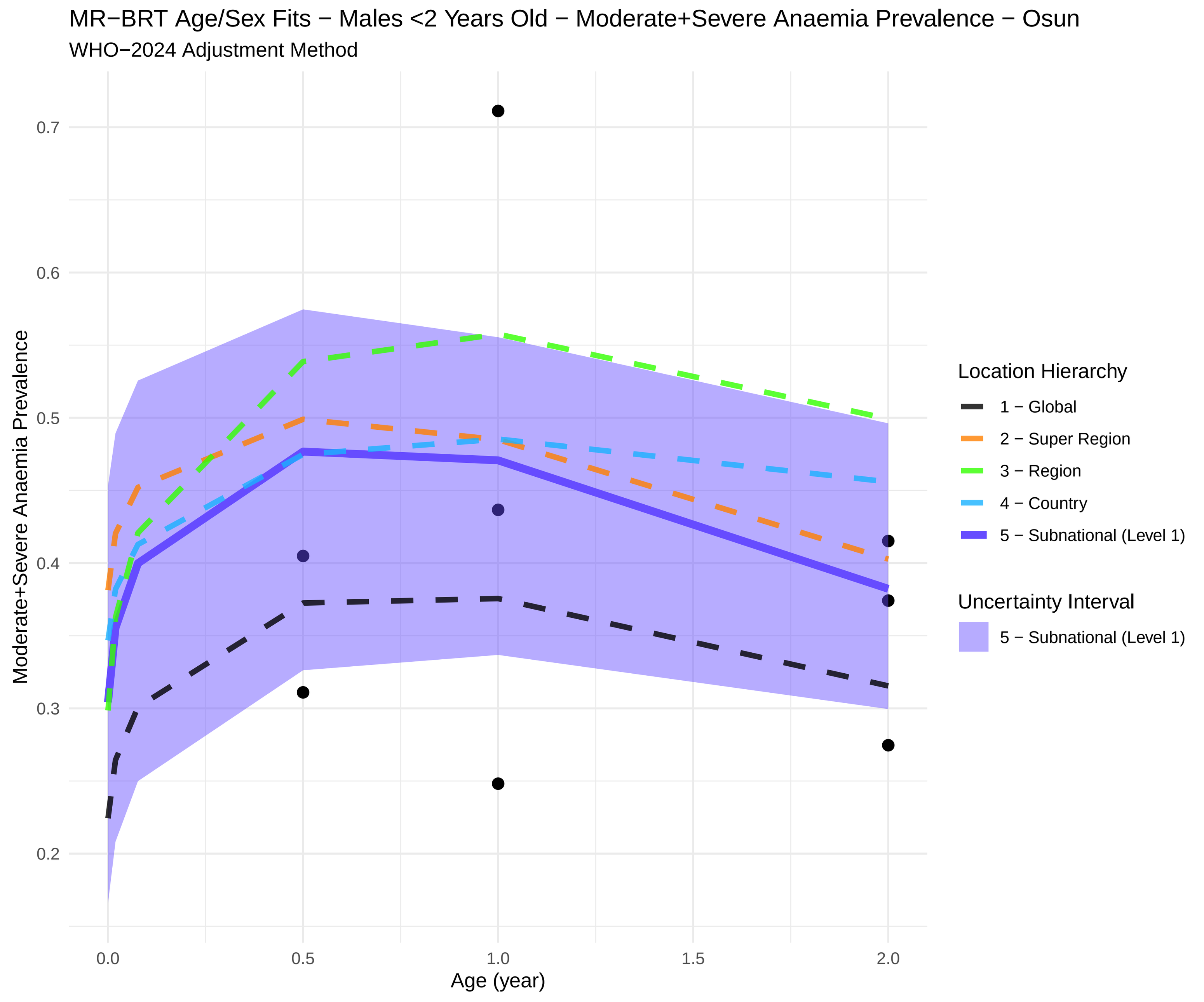
MR−BRT Age/Sex Fits − Males <2 Years Old − Moderate+Severe Anaemia Prevalence − Osun
WHO−2024 Adjustment Method
Moderate+Severe Anaemia Prevalence
Age (year)
Location Hierarchy
1 − Global
2 − Super Region
3 − Region
4 − Country
5 − Subnational (Level 1)
Uncertainty Interval
5 − Subnational (Level 1)

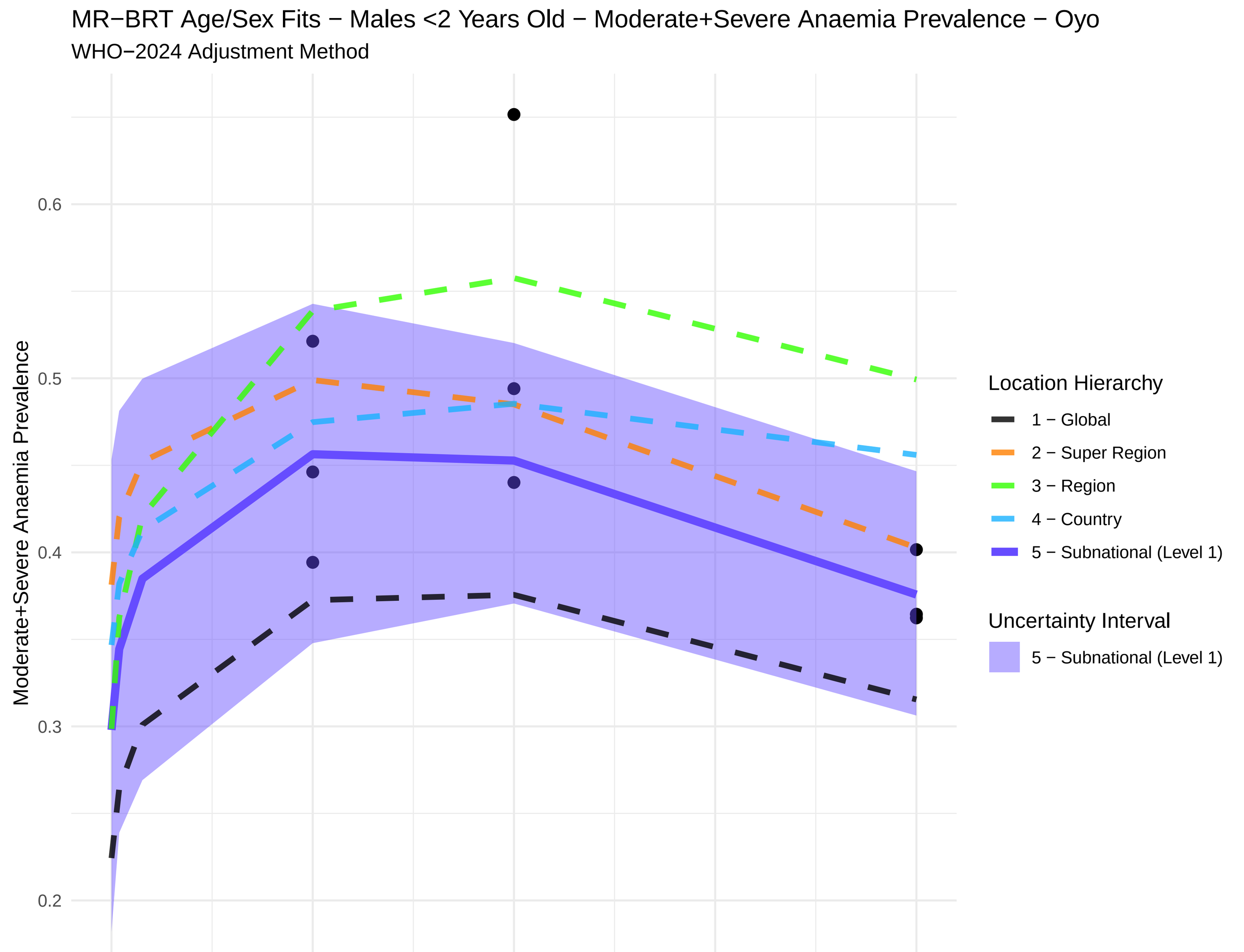
MR−BRT Age/Sex Fits − Males <2 Years Old − Moderate+Severe Anaemia Prevalence − Oyo
WHO−2024 Adjustment Method
Moderate+Severe Anaemia Prevalence
Age (year)
Location Hierarchy
1 − Global
2 − Super Region
3 − Region
4 − Country
5 − Subnational (Level 1)
Uncertainty Interval
5 − Subnational (Level 1)

MR−BRT Age/Sex Fits − Males <2 Years Old − Moderate+Severe Anaemia Prevalence − Plateau
WHO−2024 Adjustment Method

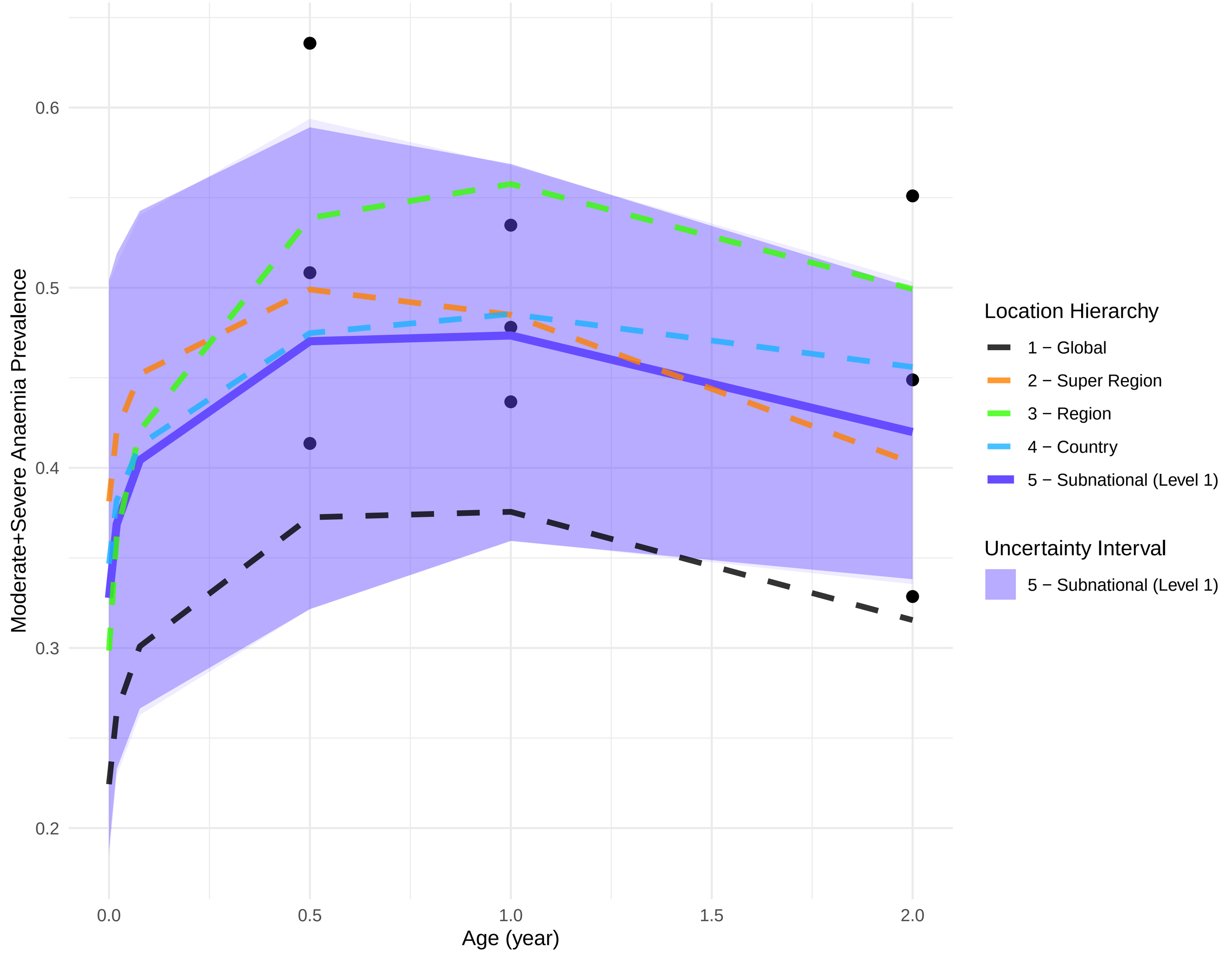

MR−BRT Age/Sex Fits − Males <2 Years Old − Moderate+Severe Anaemia Prevalence − Rivers
WHO−2024 Adjustment Method

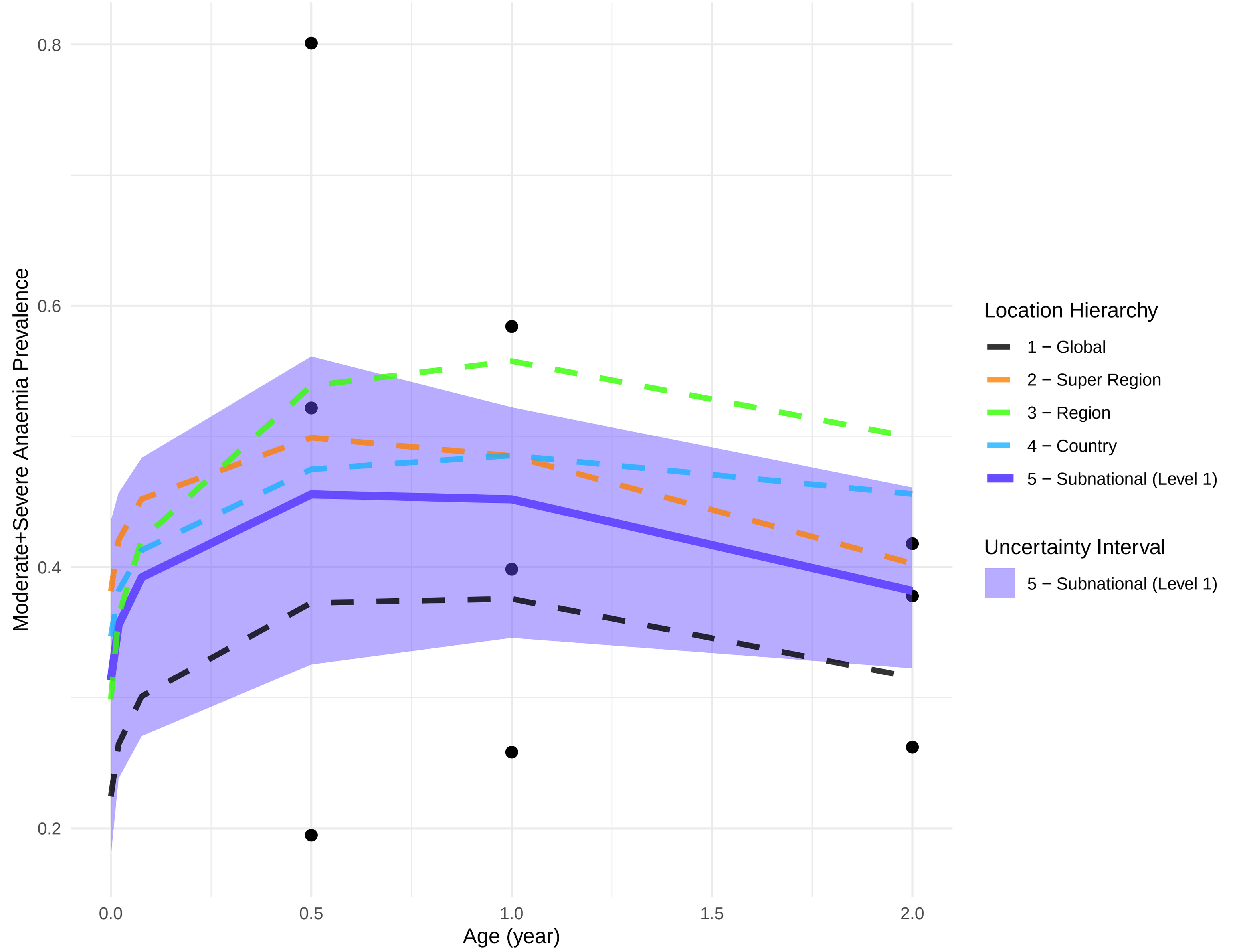

MR−BRT Age/Sex Fits − Males <2 Years Old − Moderate+Severe Anaemia Prevalence − Sokoto
WHO−2024 Adjustment Method

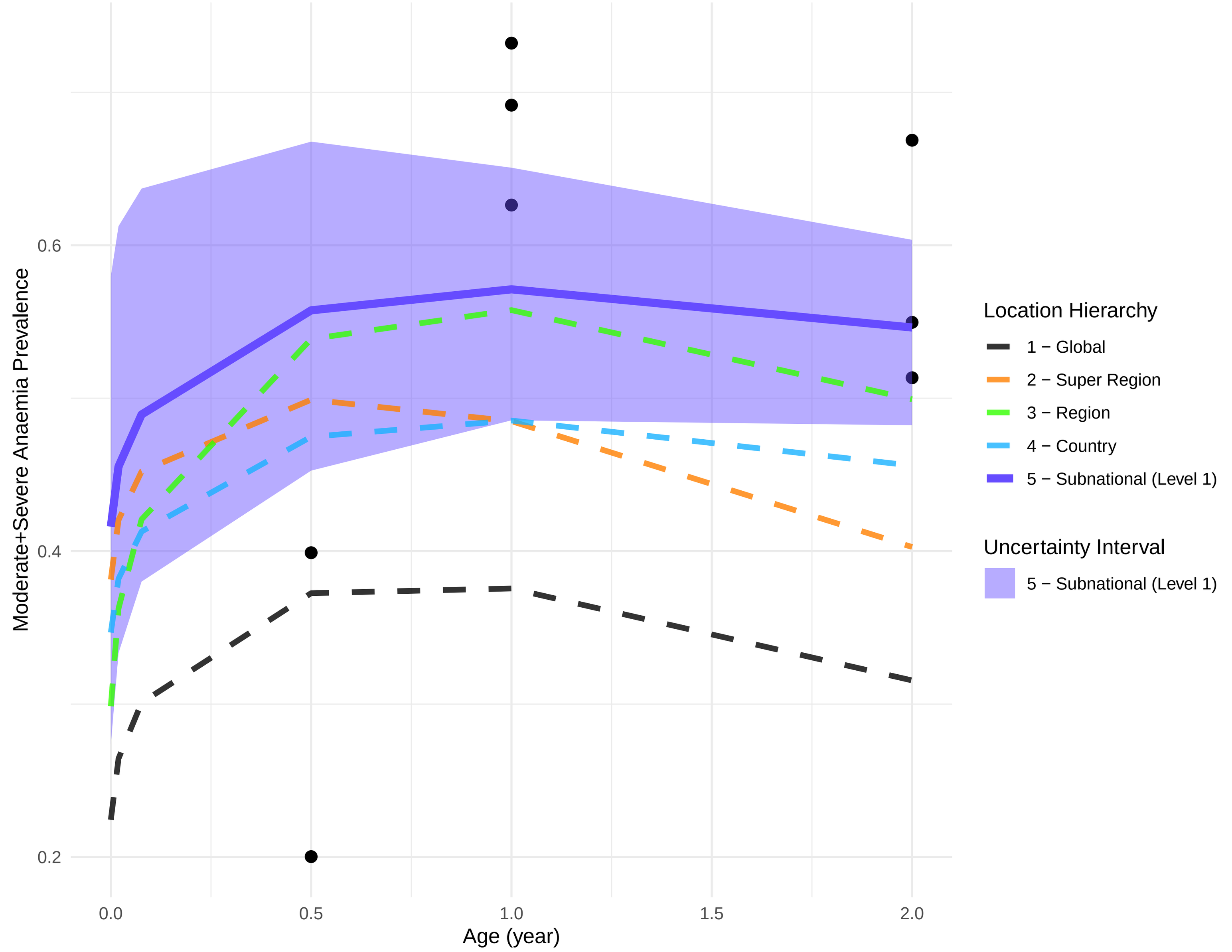

MR−BRT Age/Sex Fits − Males <2 Years Old − Moderate+Severe Anaemia Prevalence − Taraba
WHO−2024 Adjustment Method

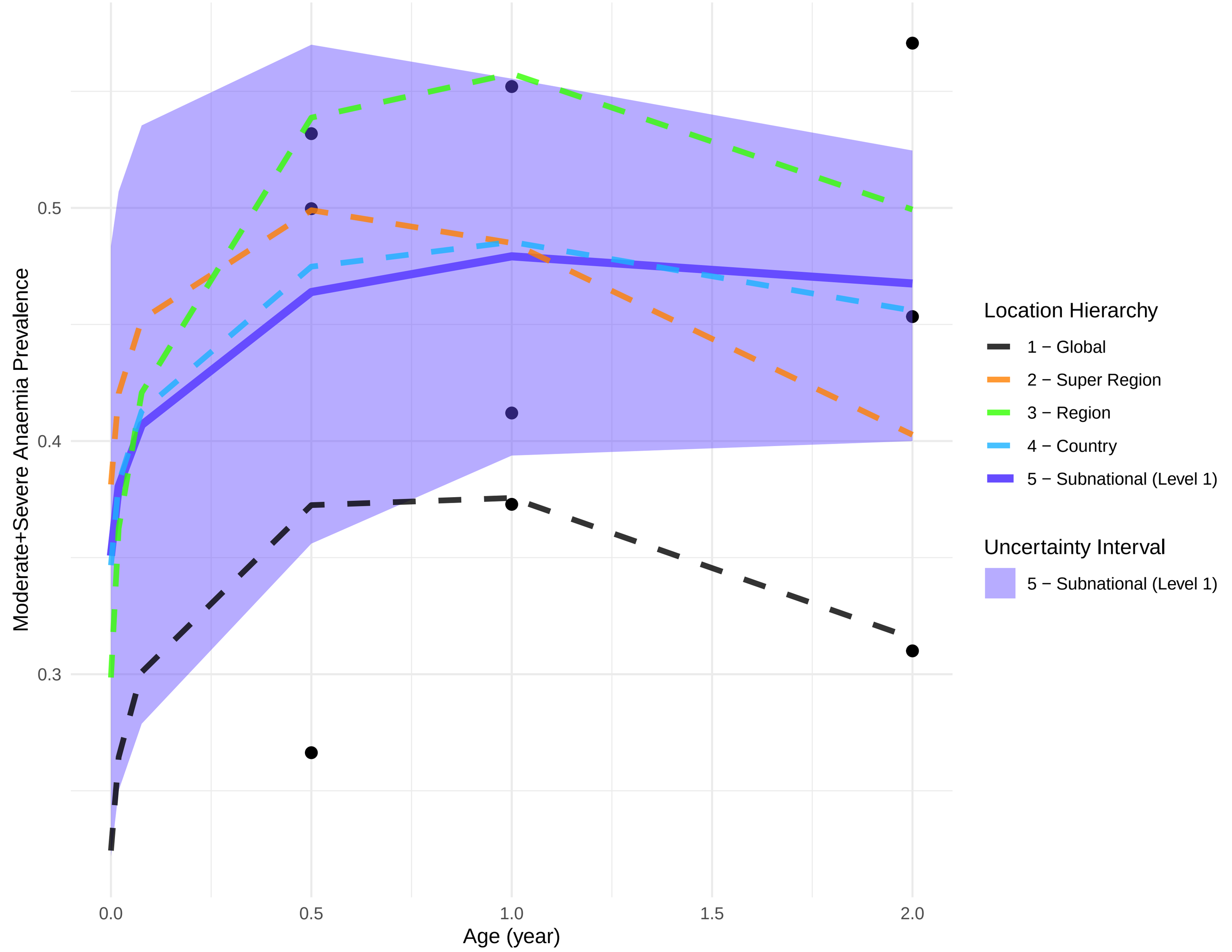

MR−BRT Age/Sex Fits − Males <2 Years Old − Moderate+Severe Anaemia Prevalence − Yobe
WHO−2024 Adjustment Method

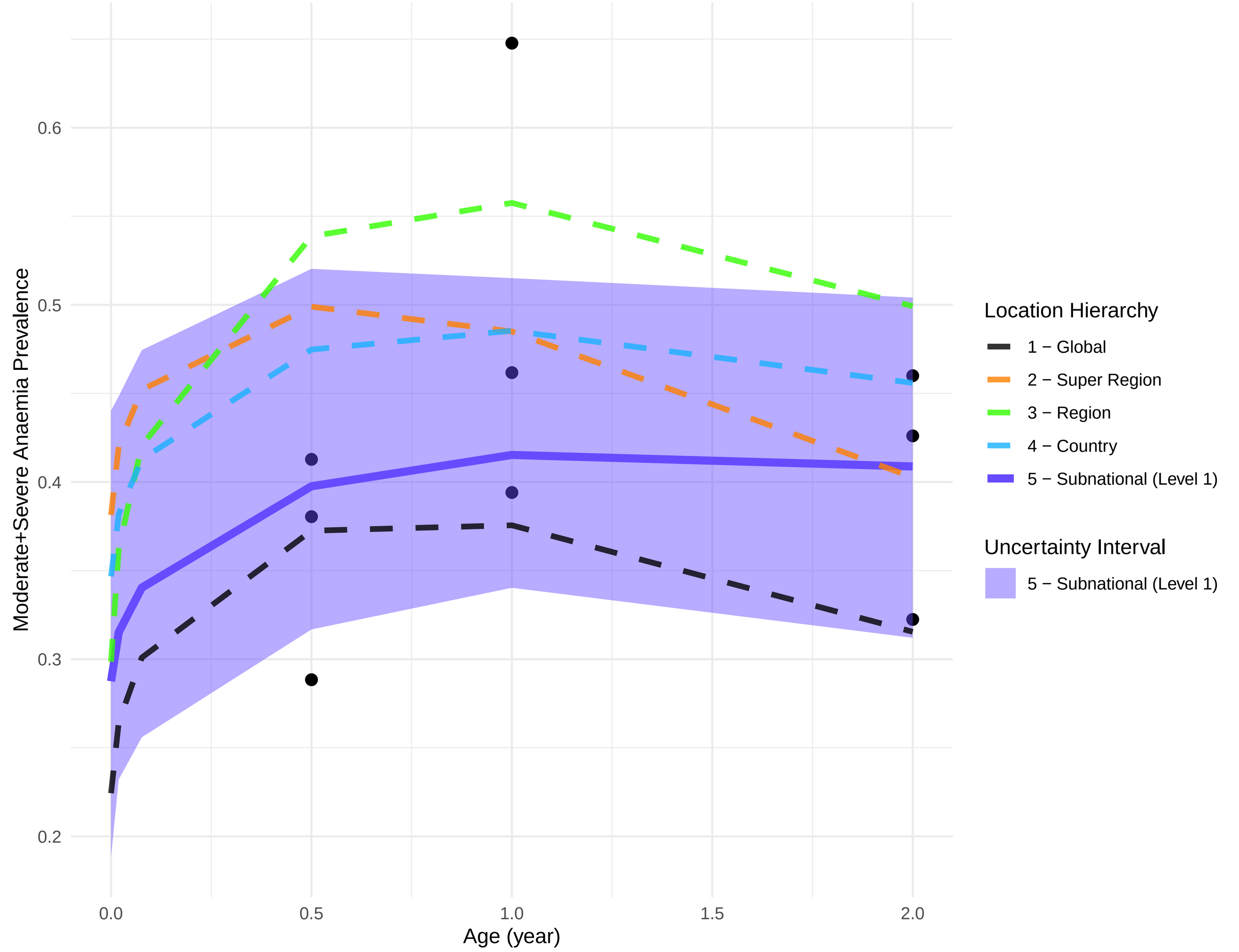

MR−BRT Age/Sex Fits − Males <2 Years Old − Moderate+Severe Anaemia Prevalence − Zamfara
WHO−2024 Adjustment Method

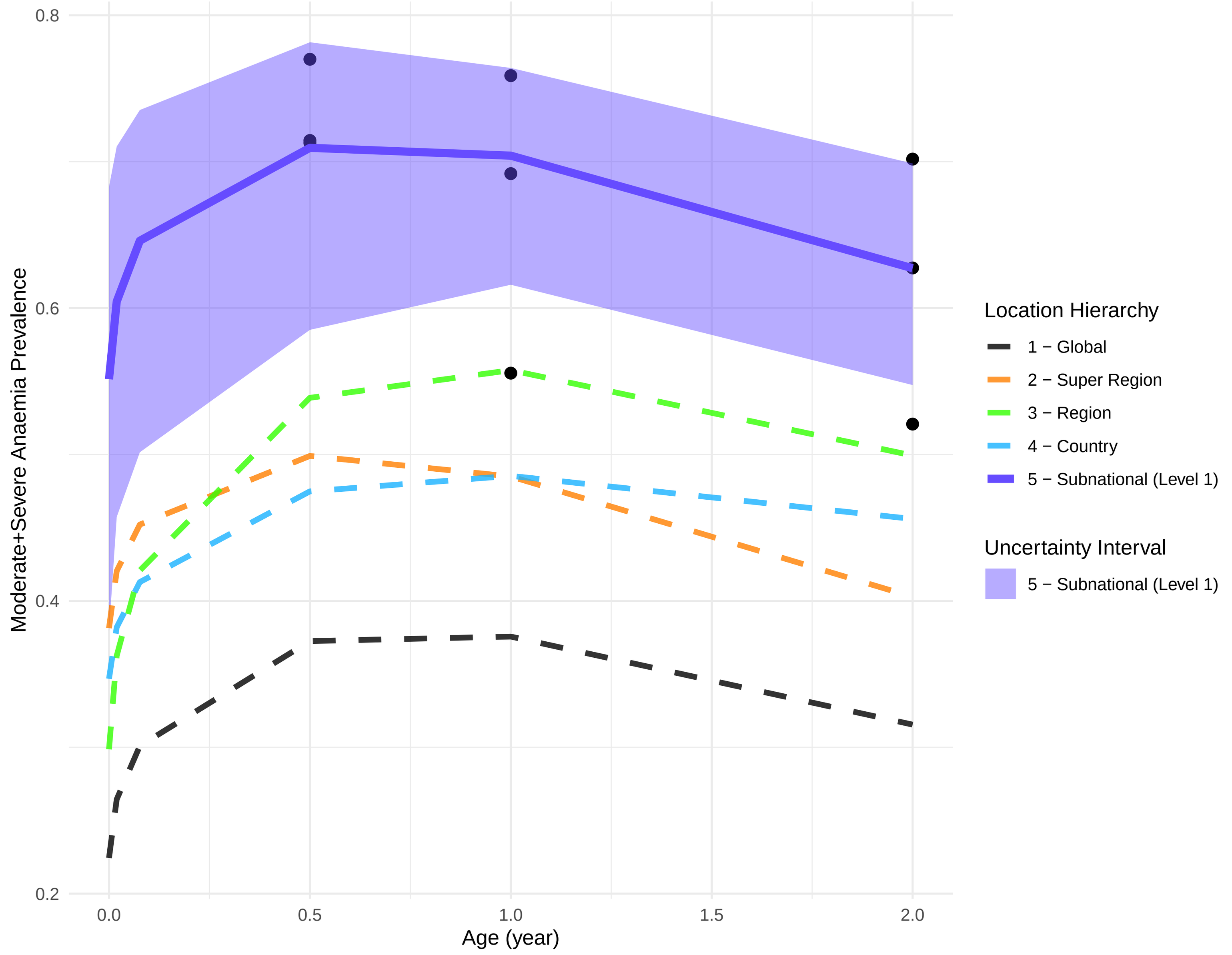

MR−BRT Age/Sex Fits − Males <2 Years Old − Moderate+Severe Anaemia Prevalence − Senegal
WHO−2024 Adjustment Method

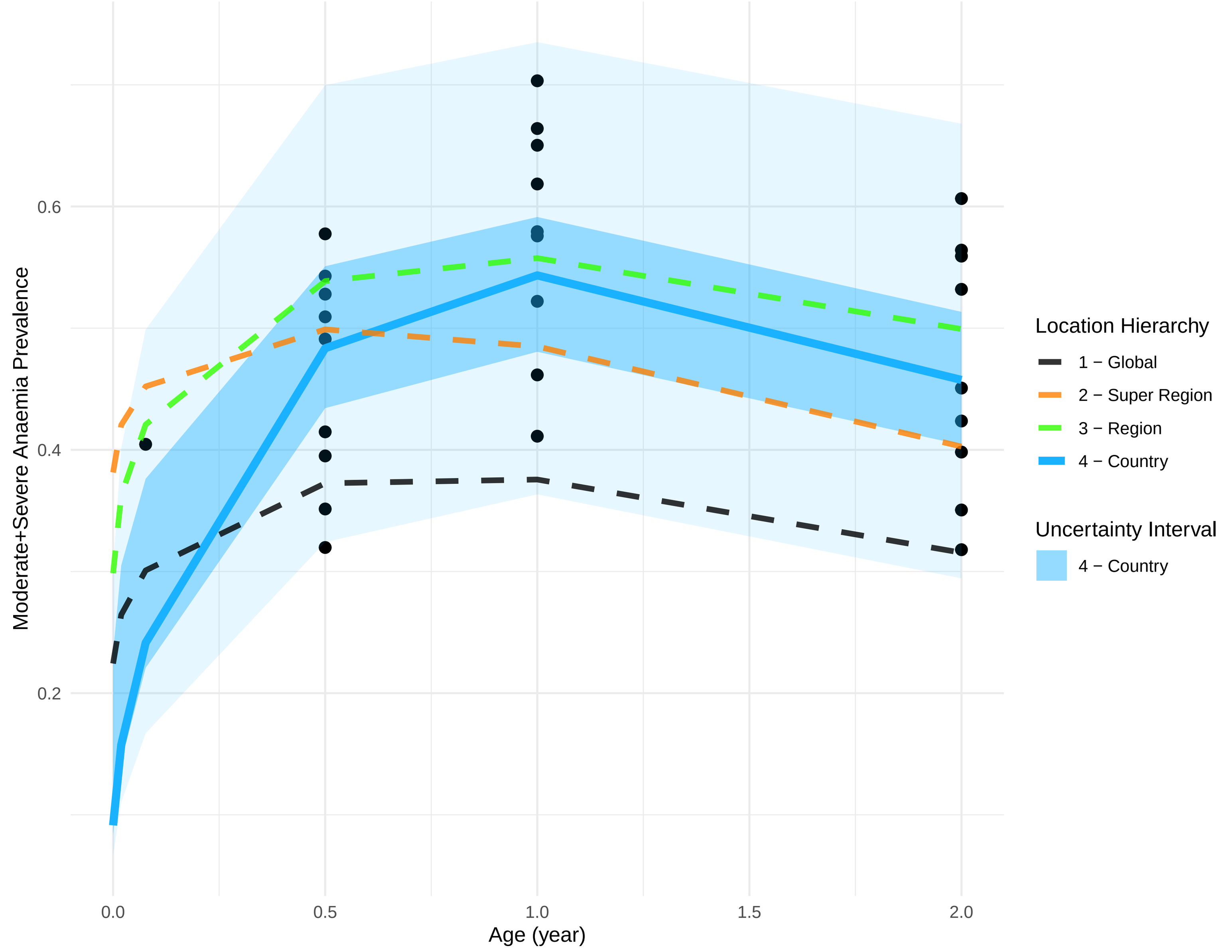

MR−BRT Age/Sex Fits − Males <2 Years Old − Moderate+Severe Anaemia Prevalence − Sierra Leone
WHO−2024 Adjustment Method

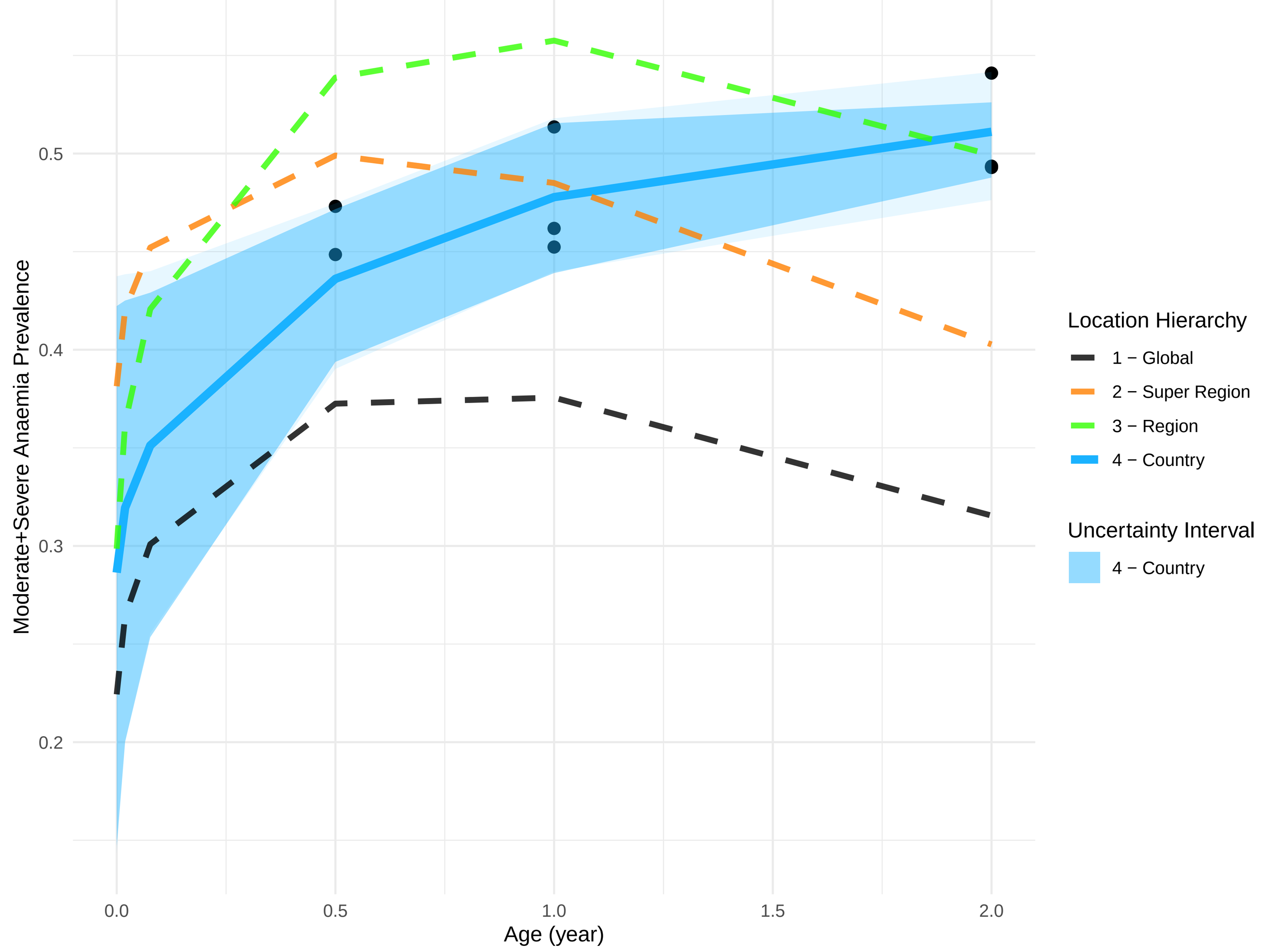

MR−BRT Age/Sex Fits − Males <2 Years Old − Moderate+Severe Anaemia Prevalence − Togo
WHO−2024 Adjustment Method

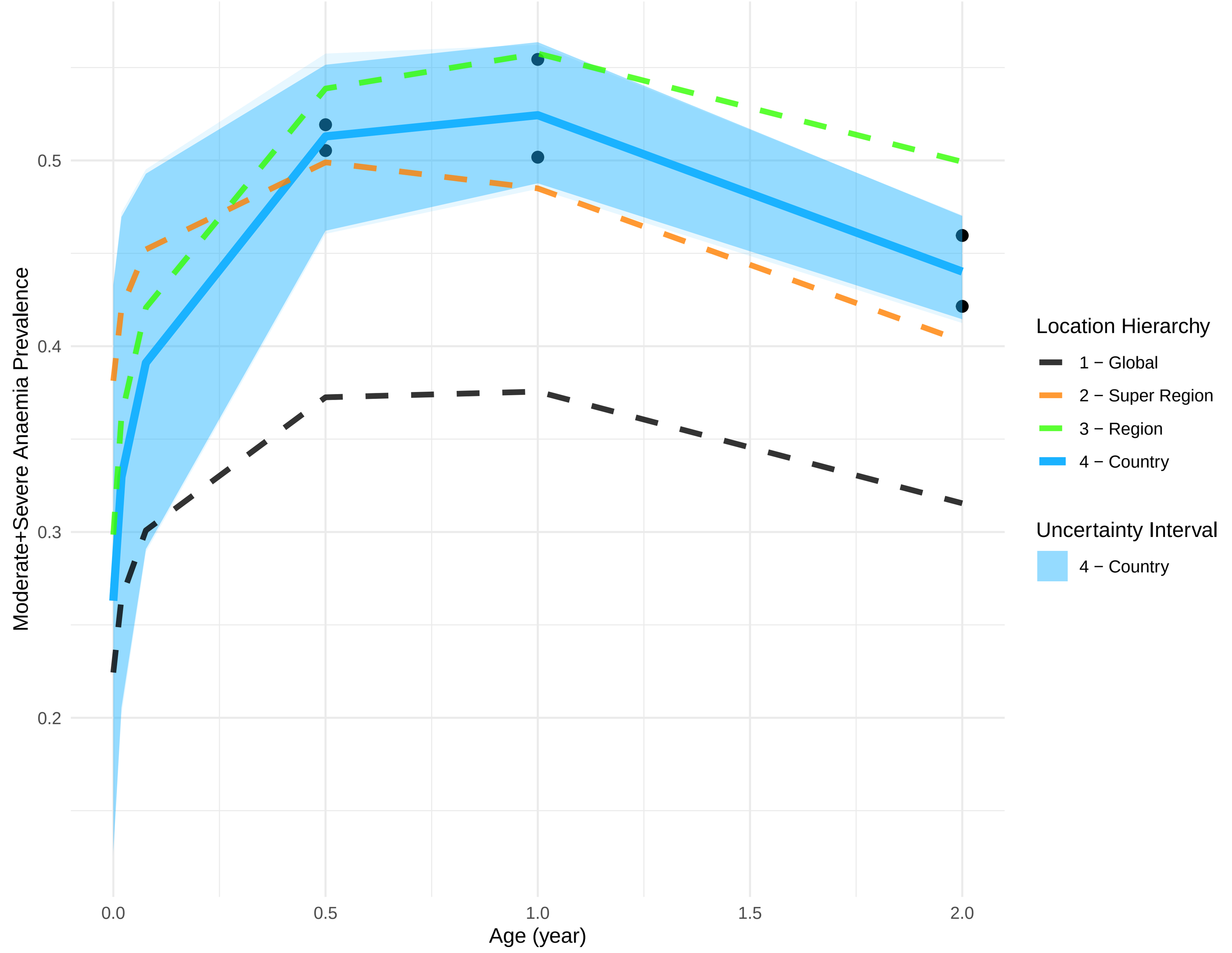

MR−BRT Age/Sex Fits − Non−pregnant Females <2 Years Old − Moderate+Severe Anaemia Prevalence − Global
WHO−2024 Adjustment Method

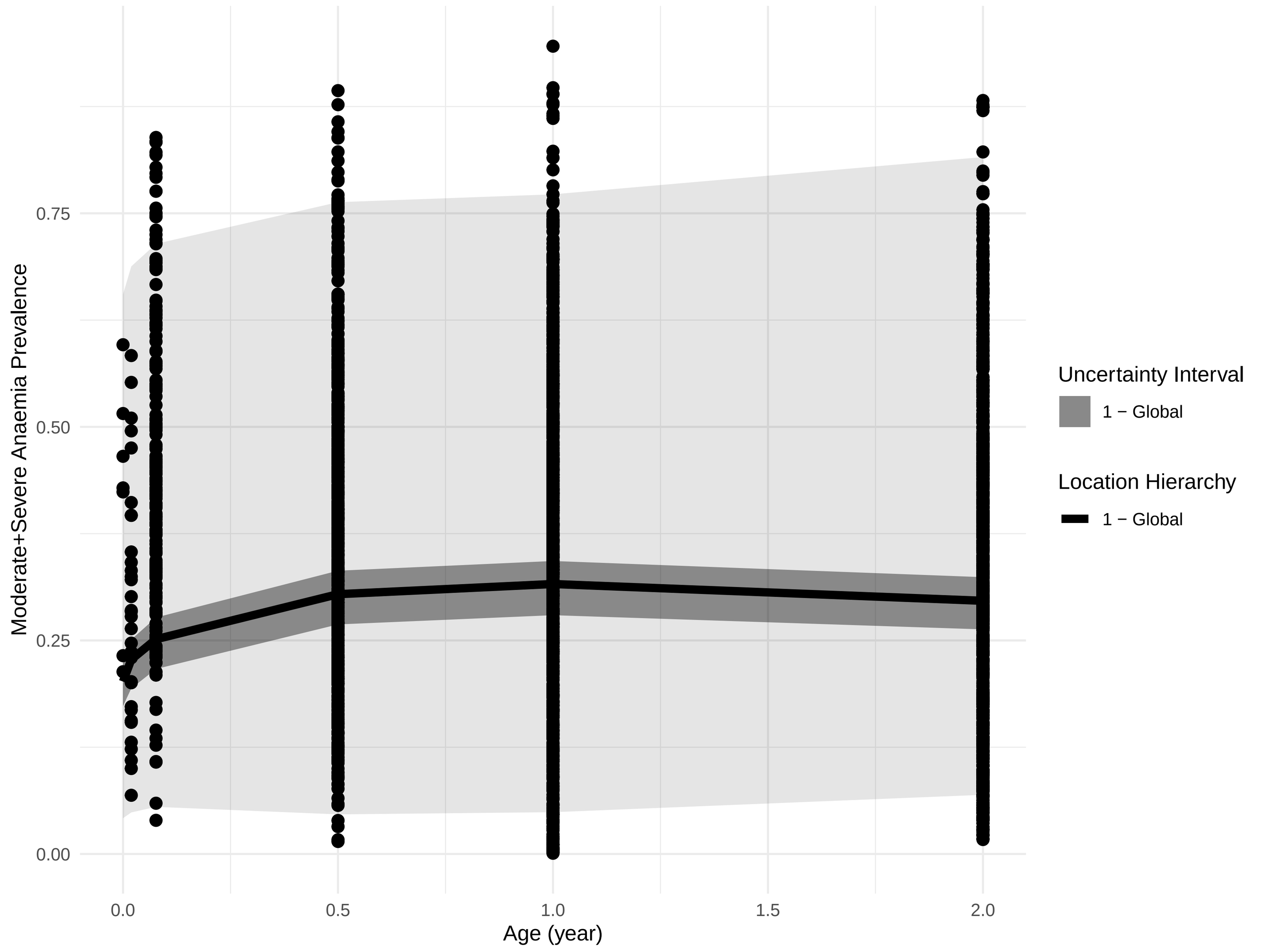

MR−BRT Age/Sex Fits − Non−pregnant Females <2 Years Old − Moderate+Severe Anaemia Prevalence − Central Europe, Eastern Europe, and Central Asia
WHO−2024 Adjustment Method

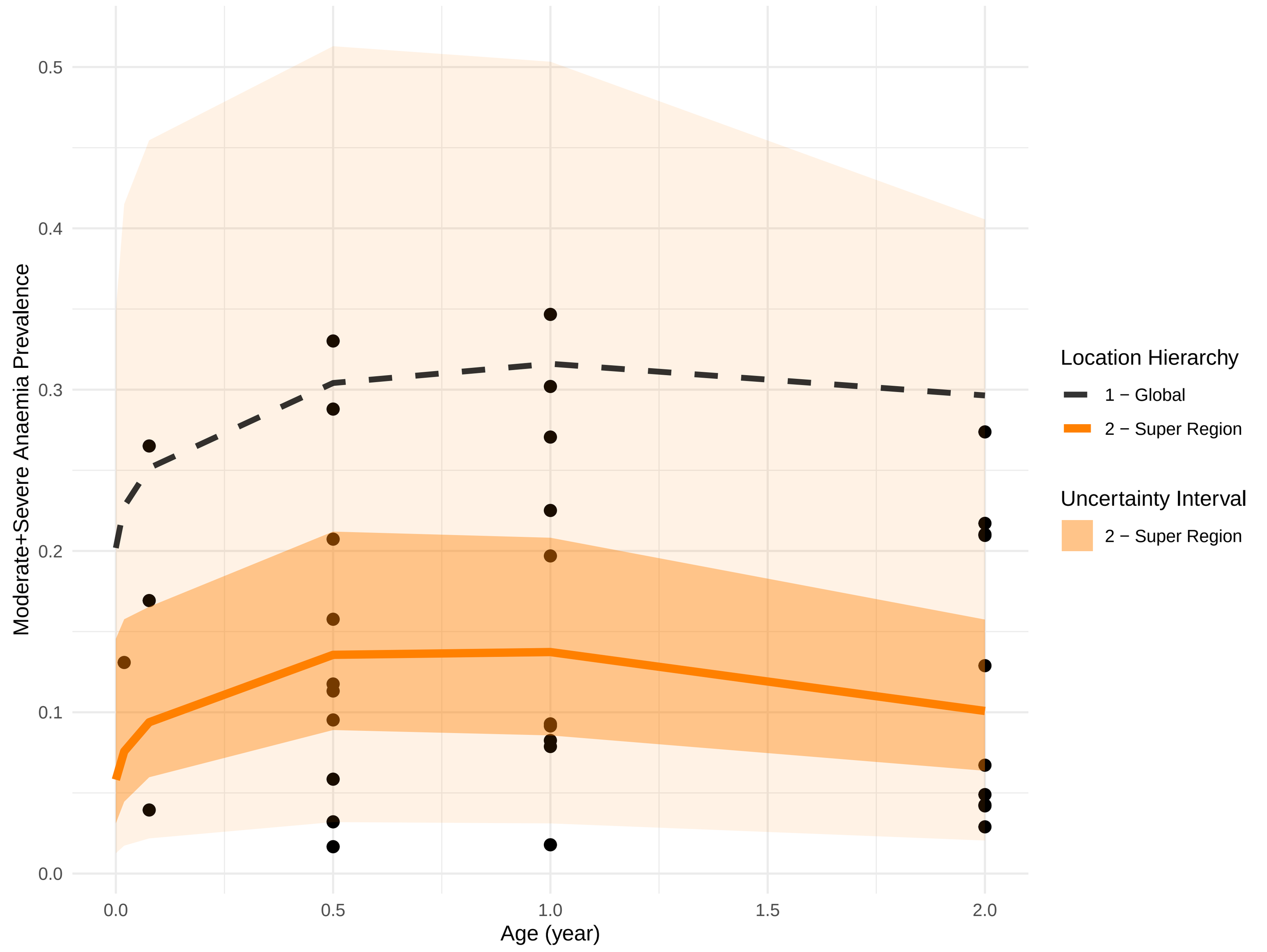

MR−BRT Age/Sex Fits − Non−pregnant Females <2 Years Old − Moderate+Severe Anaemia Prevalence − Central Asia
WHO−2024 Adjustment Method

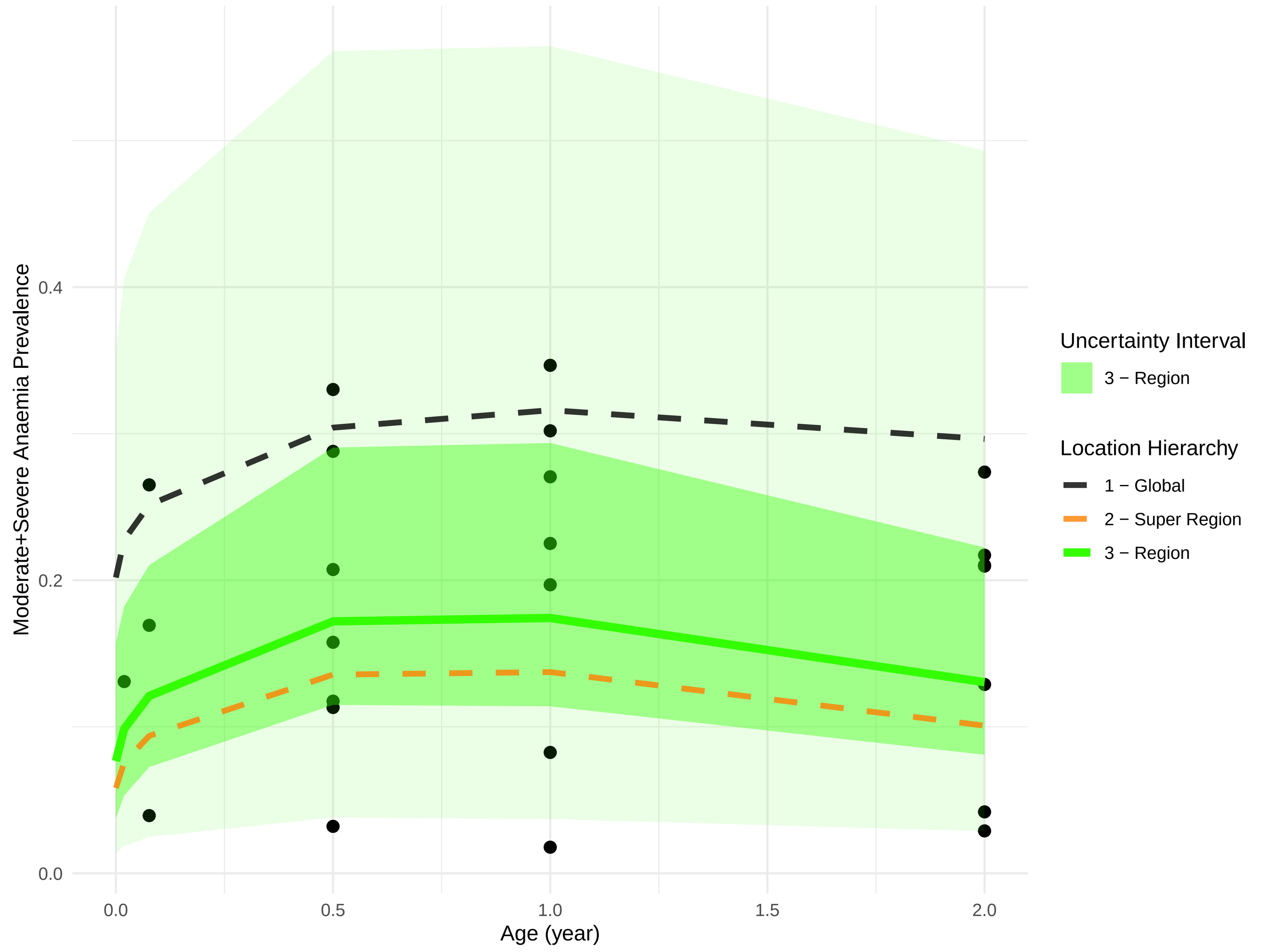

MR−BRT Age/Sex Fits − Non−pregnant Females <2 Years Old − Moderate+Severe Anaemia Prevalence − Armenia
WHO−2024 Adjustment Method

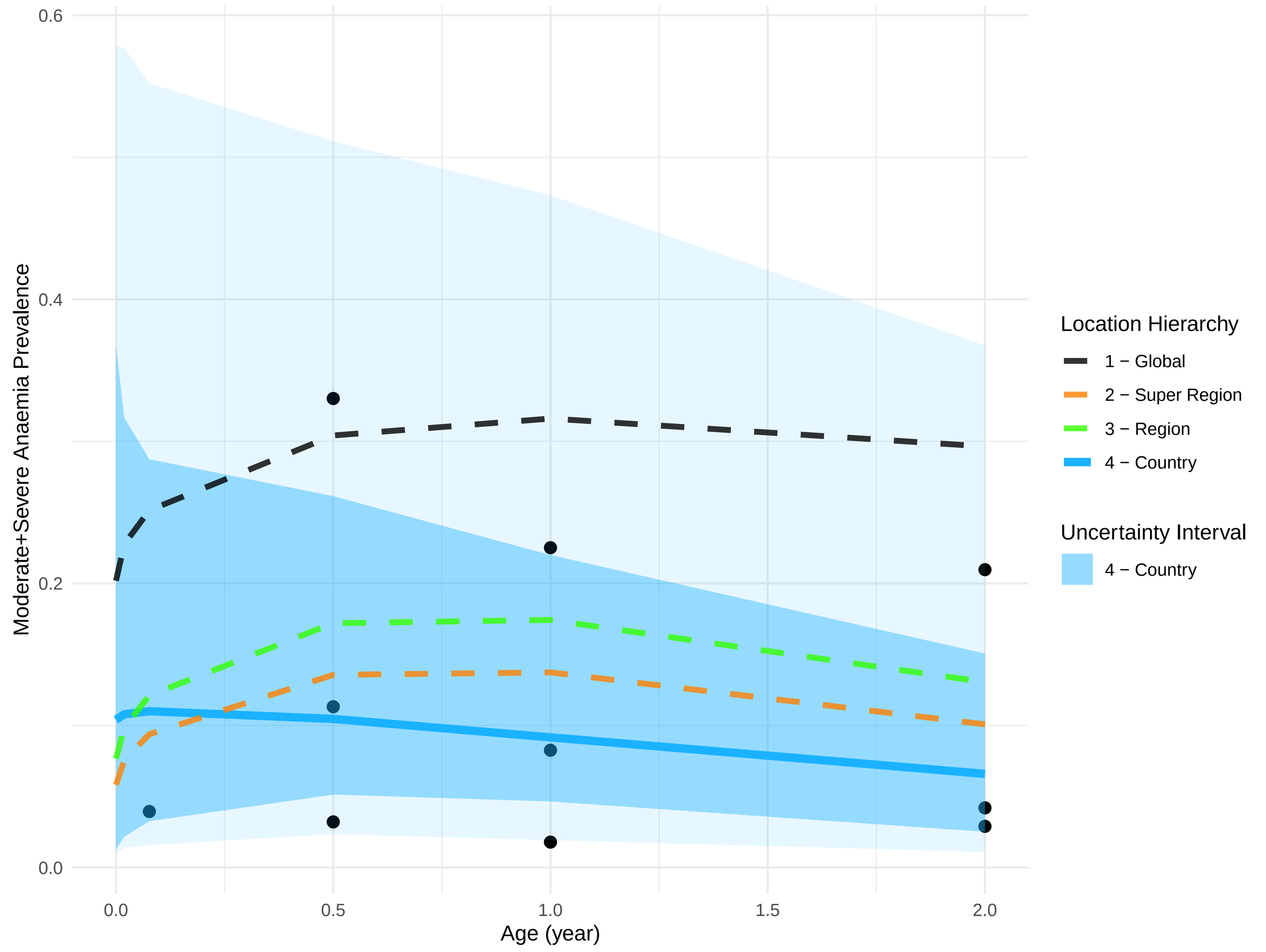

MR−BRT Age/Sex Fits − Non−pregnant Females <2 Years Old − Moderate+Severe Anaemia Prevalence − Azerbaijan
WHO−2024 Adjustment Method

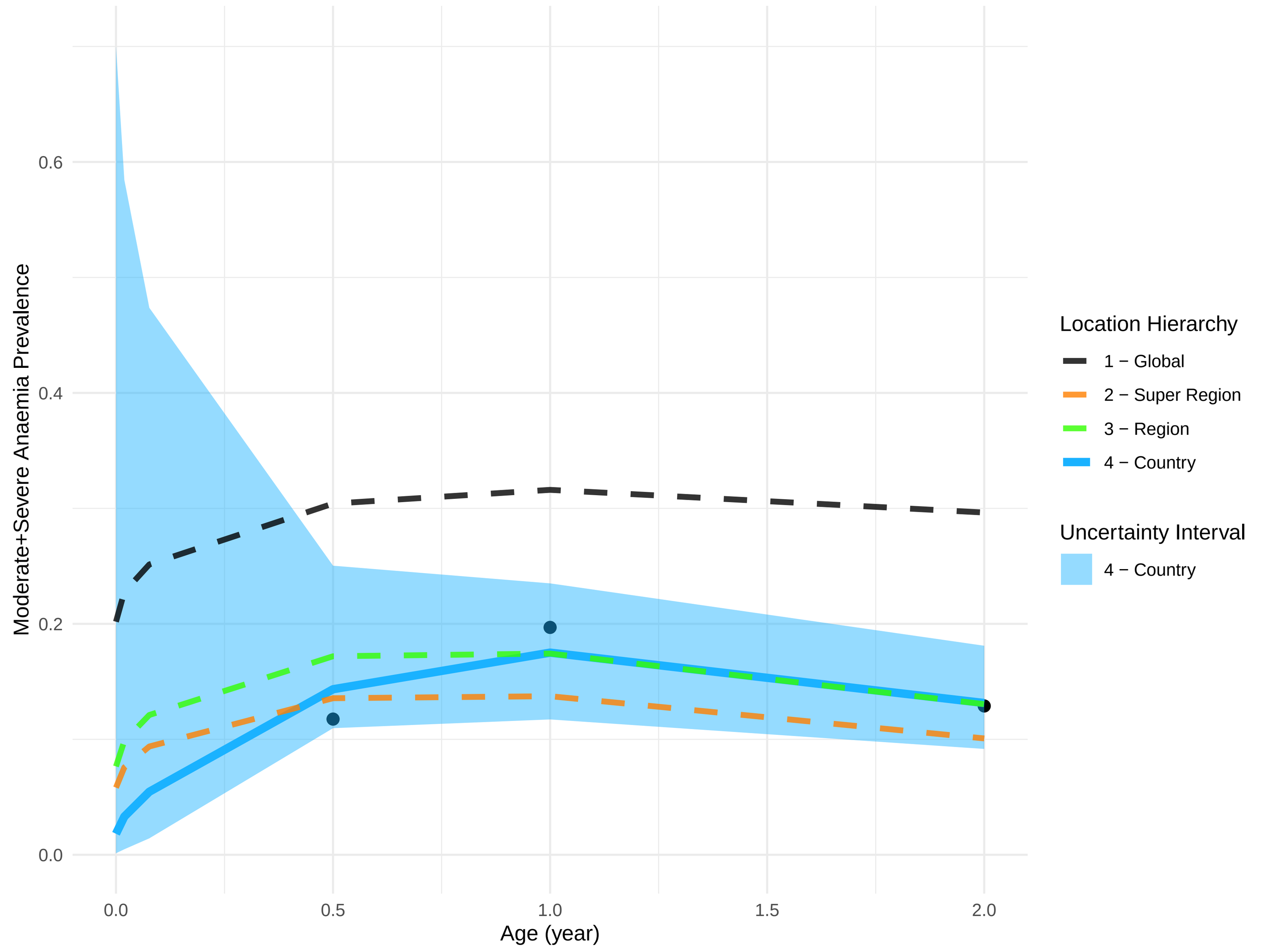

MR−BRT Age/Sex Fits − Non−pregnant Females <2 Years Old − Moderate+Severe Anaemia Prevalence − Kyrgyzstan
WHO−2024 Adjustment Method

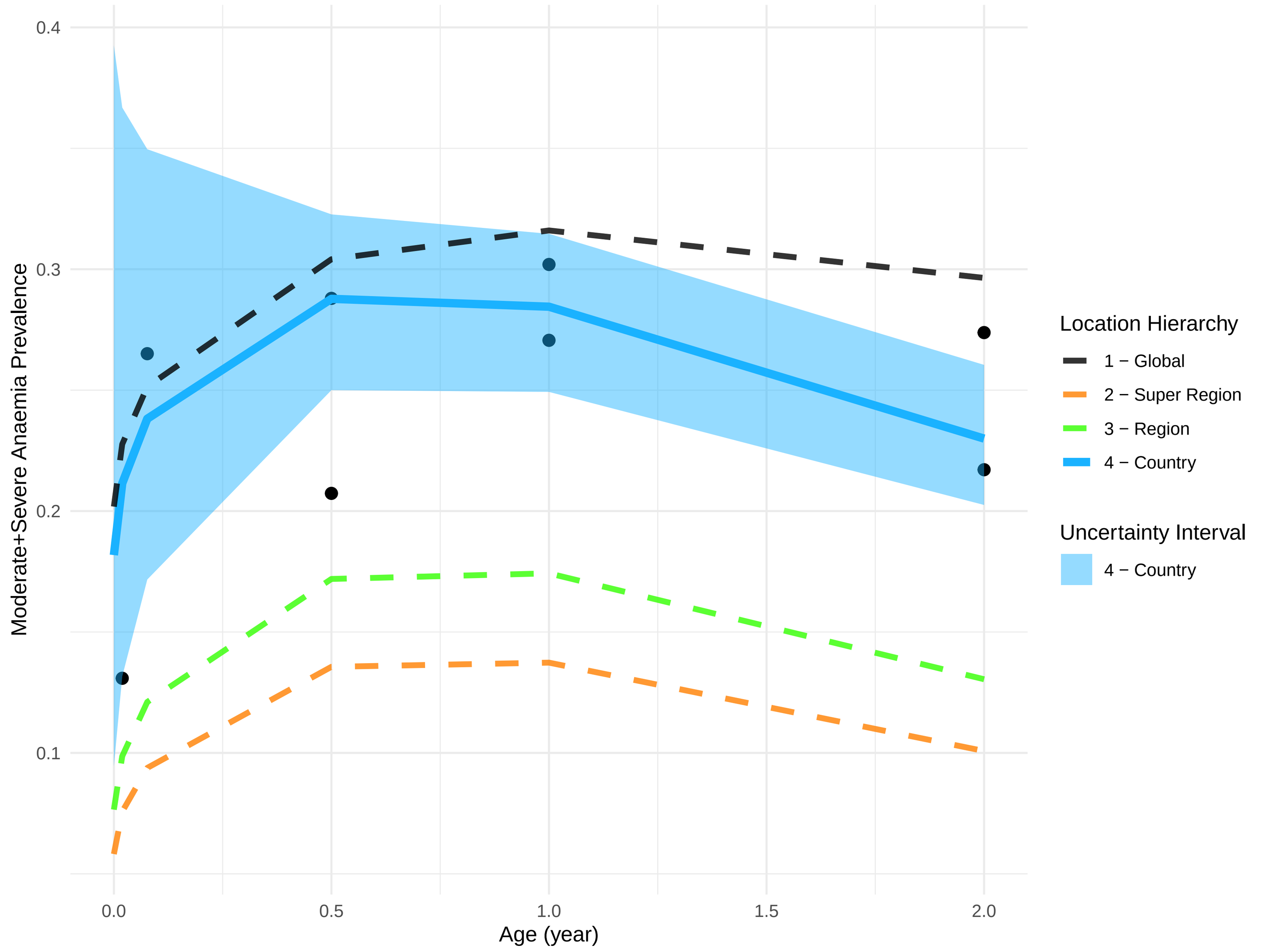

MR−BRT Age/Sex Fits − Non−pregnant Females <2 Years Old − Moderate+Severe Anaemia Prevalence − Uzbekistan
WHO−2024 Adjustment Method

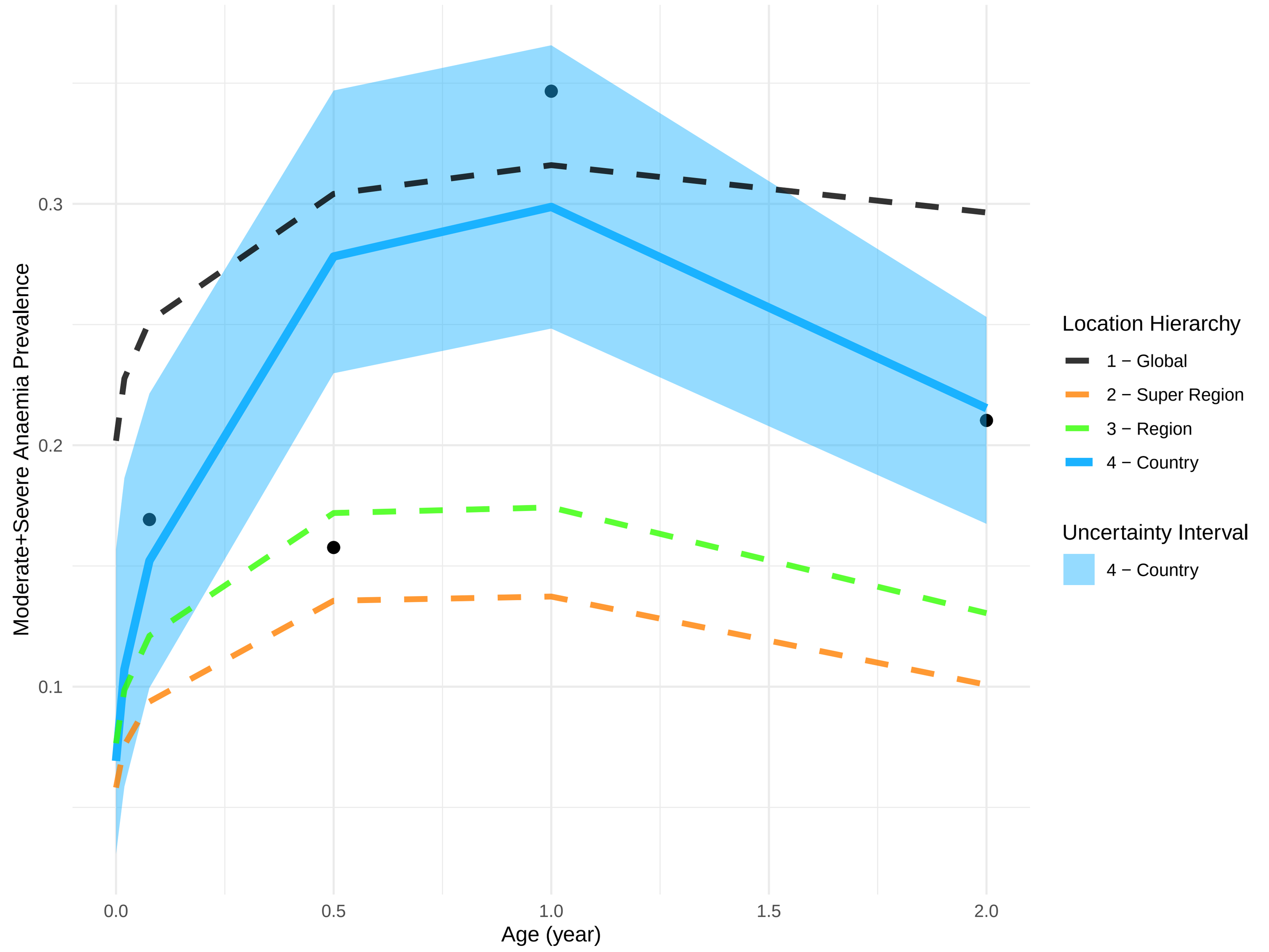

MR−BRT Age/Sex Fits − Non−pregnant Females <2 Years Old − Moderate+Severe Anaemia Prevalence − Central Europe
WHO−2024 Adjustment Method

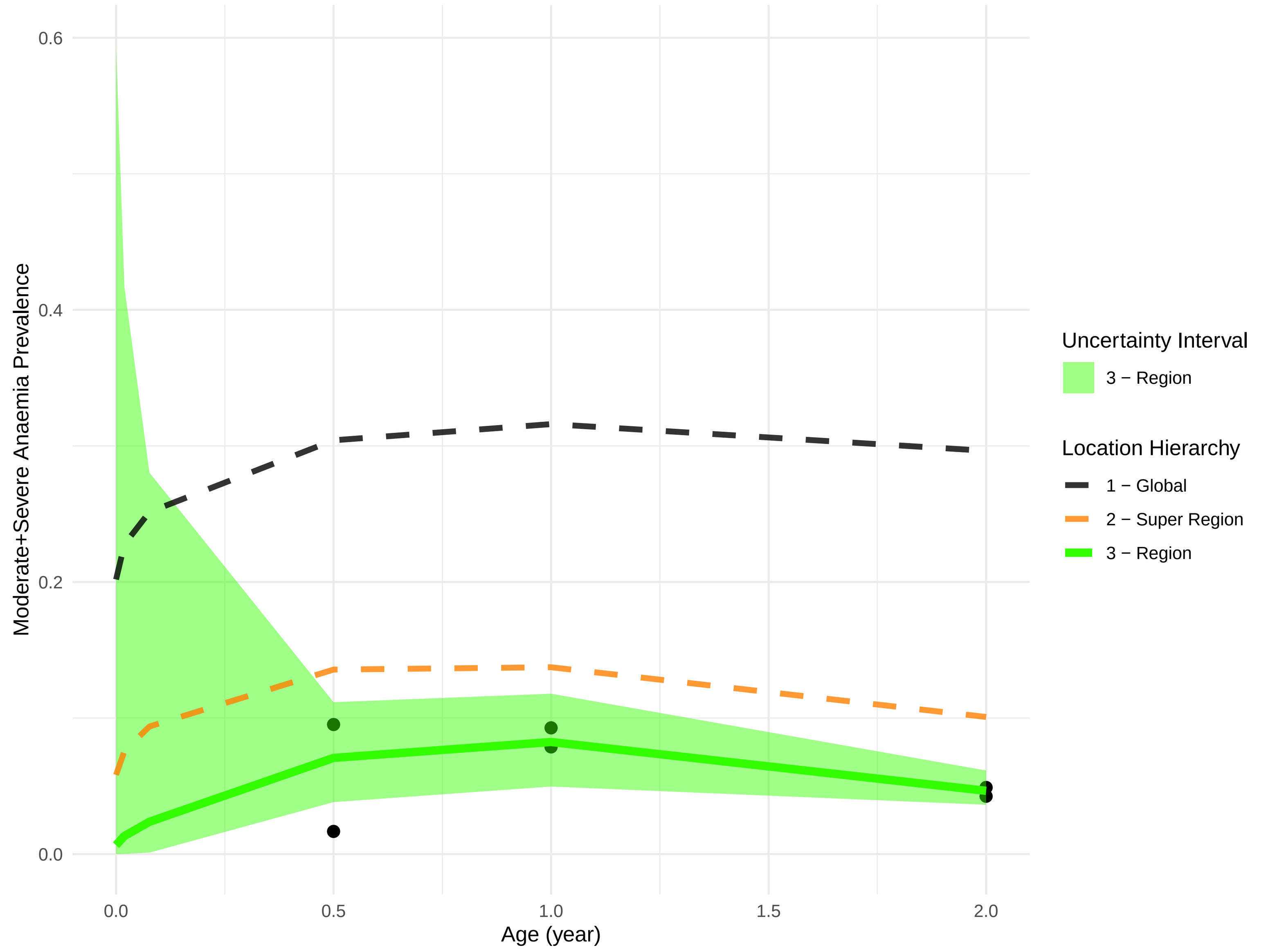

MR−BRT Age/Sex Fits − Non−pregnant Females <2 Years Old − Moderate+Severe Anaemia Prevalence − Albania
WHO−2024 Adjustment Method

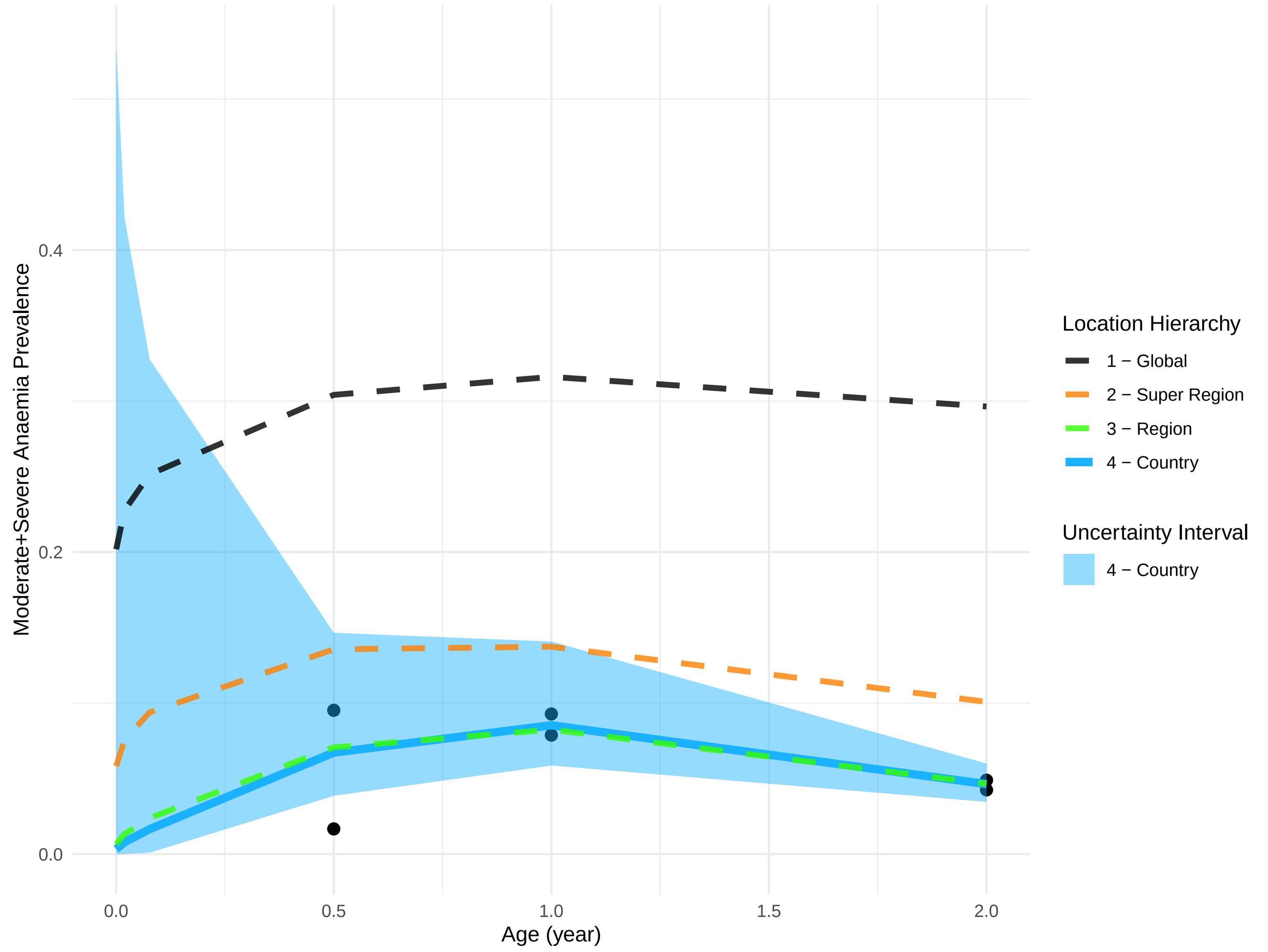

MR−BRT Age/Sex Fits − Non−pregnant Females <2 Years Old − Moderate+Severe Anaemia Prevalence − Eastern Europe
WHO−2024 Adjustment Method

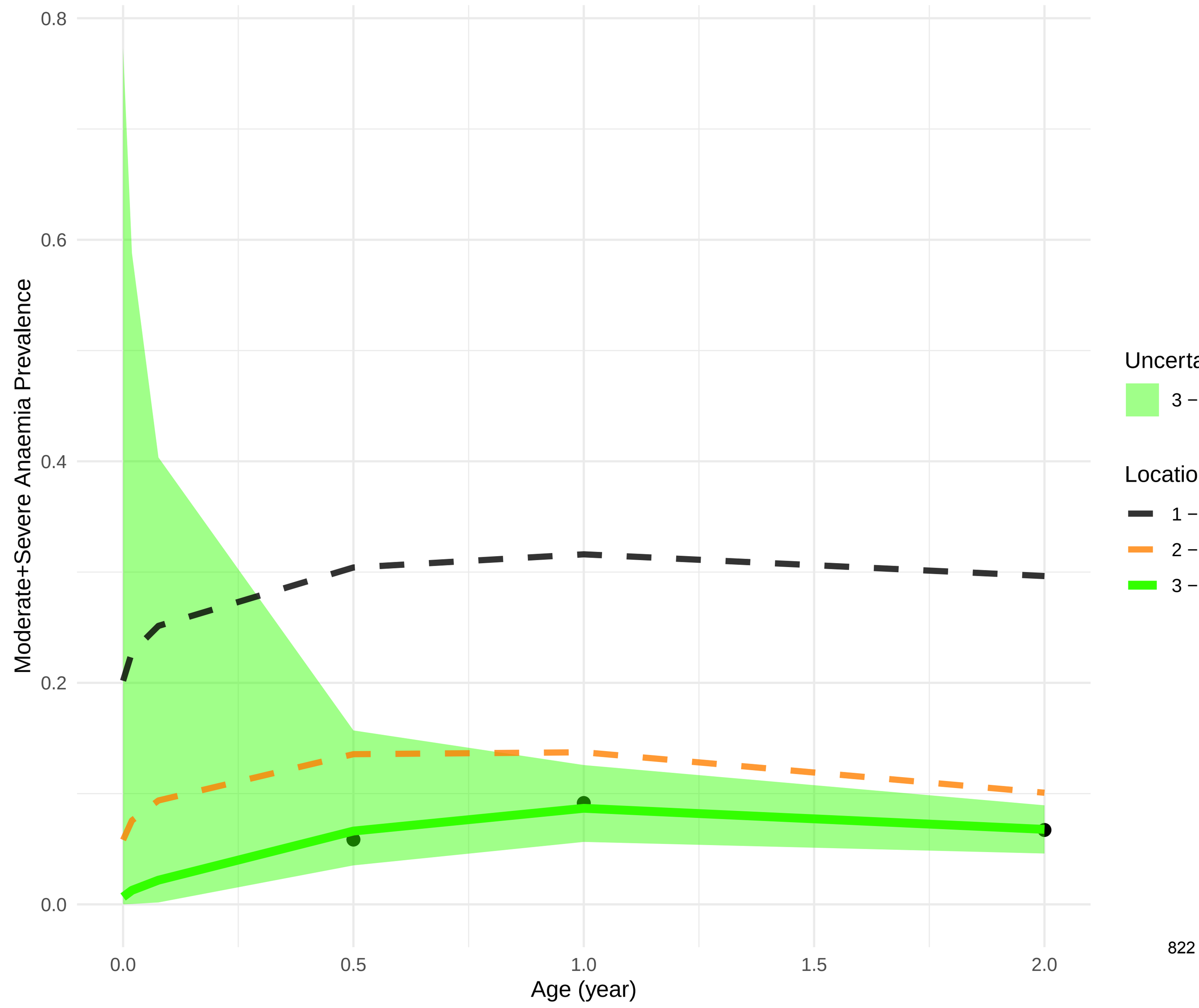

MR−BRT Age/Sex Fits − Non−pregnant Females <2 Years Old − Moderate+Severe Anaemia Prevalence − Republic of Moldova
WHO−2024 Adjustment Method

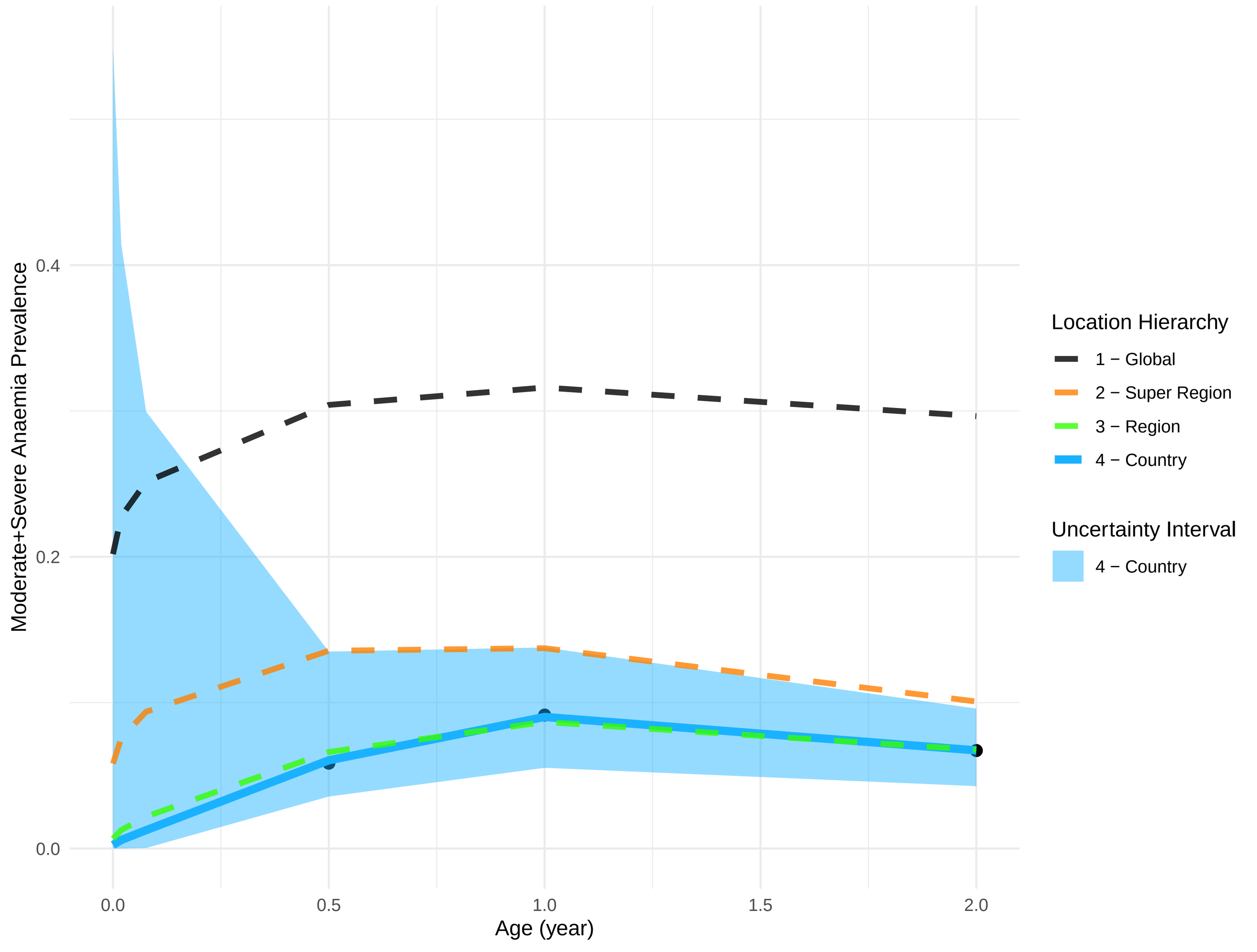

MR−BRT Age/Sex Fits − Non−pregnant Females <2 Years Old − Moderate+Severe Anaemia Prevalence − High−income
WHO−2024 Adjustment Method

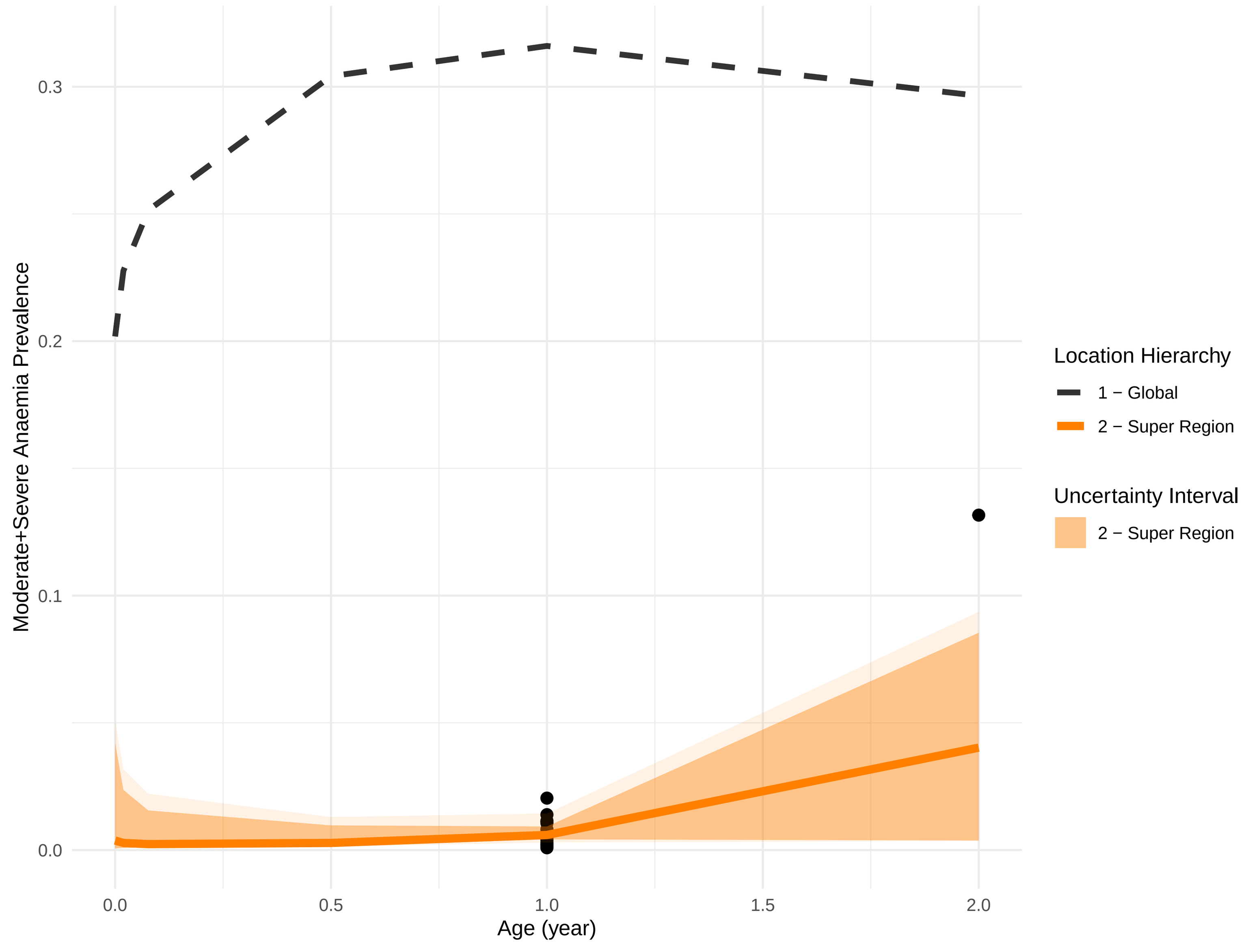

MR−BRT Age/Sex Fits − Non−pregnant Females <2 Years Old − Moderate+Severe Anaemia Prevalence − Latin America and Caribbean
WHO−2024 Adjustment Method

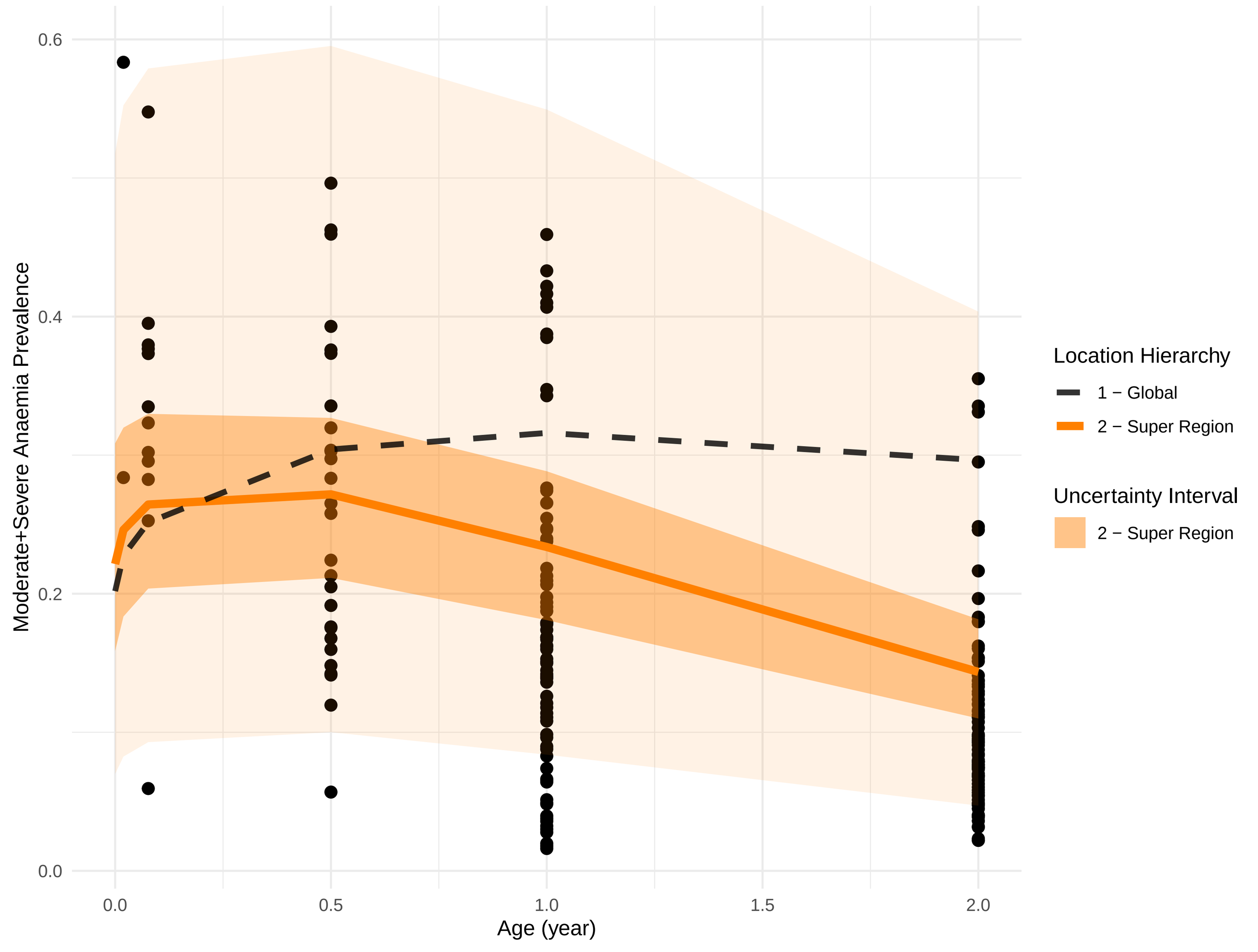

MR−BRT Age/Sex Fits − Non−pregnant Females <2 Years Old − Moderate+Severe Anaemia Prevalence − Andean Latin America
WHO−2024 Adjustment Method

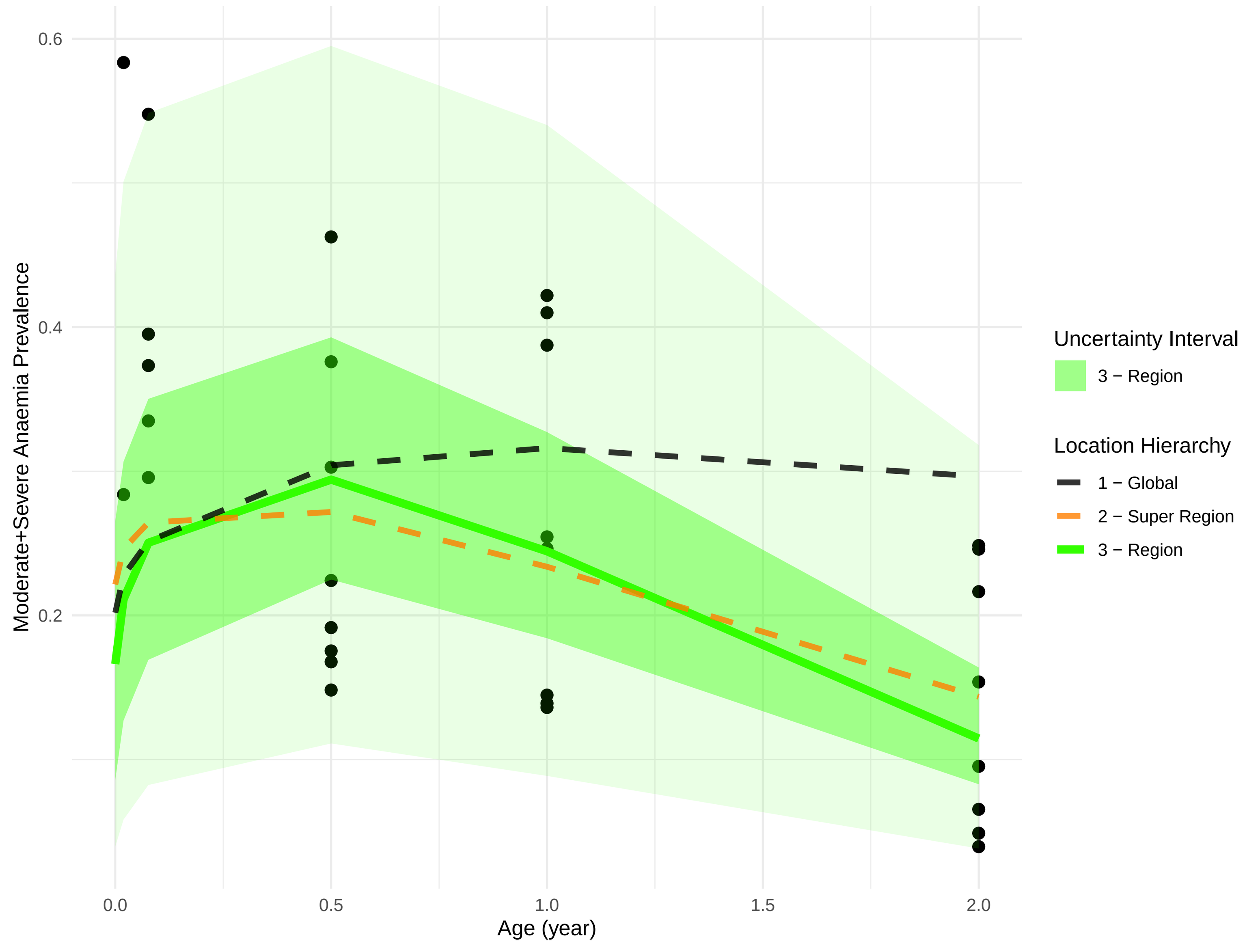

MR−BRT Age/Sex Fits − Non−pregnant Females <2 Years Old − Moderate+Severe Anaemia Prevalence − Bolivia (Plurinational State of)
WHO−2024 Adjustment Method

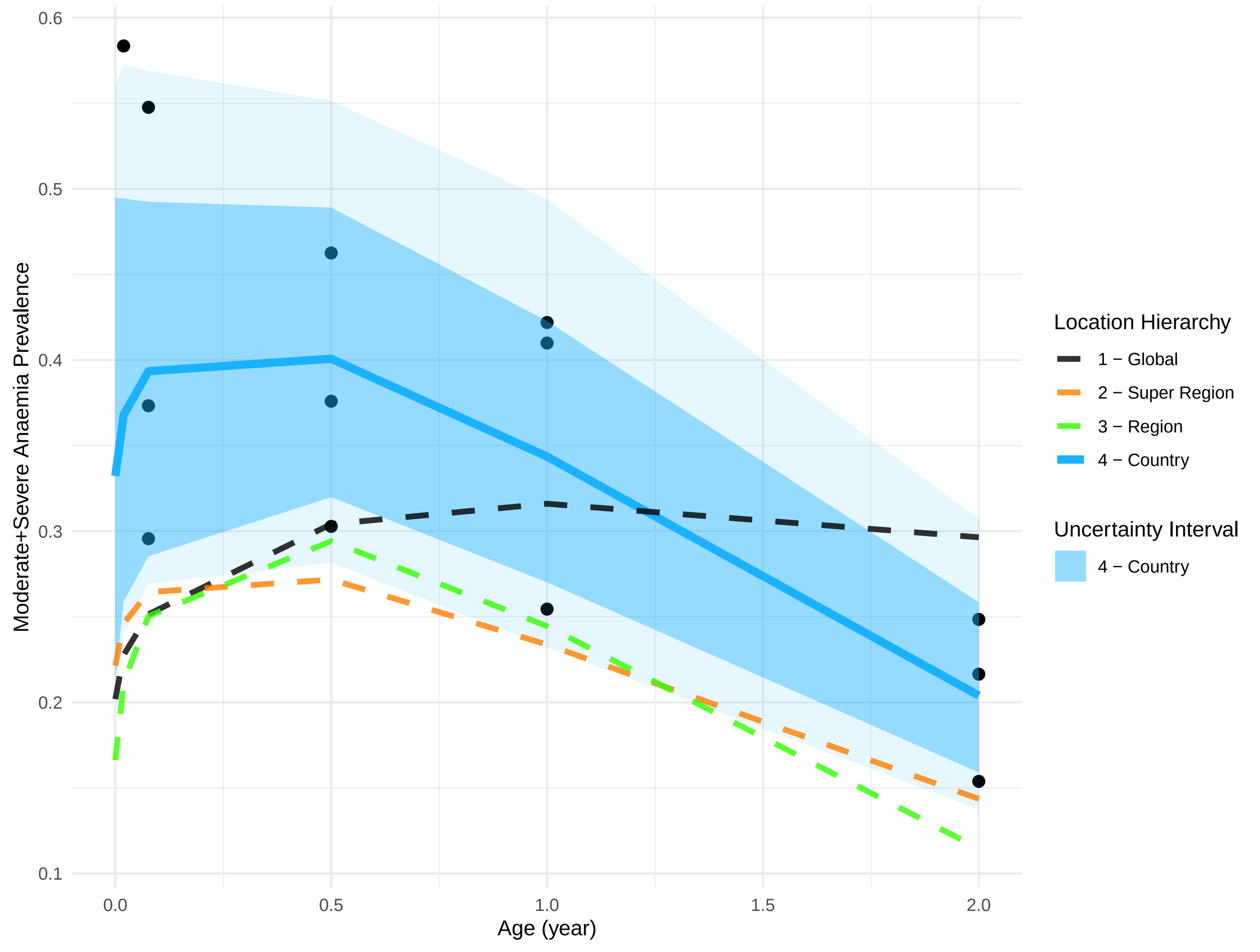

MR−BRT Age/Sex Fits − Non−pregnant Females <2 Years Old − Moderate+Severe Anaemia Prevalence − Peru
WHO−2024 Adjustment Method

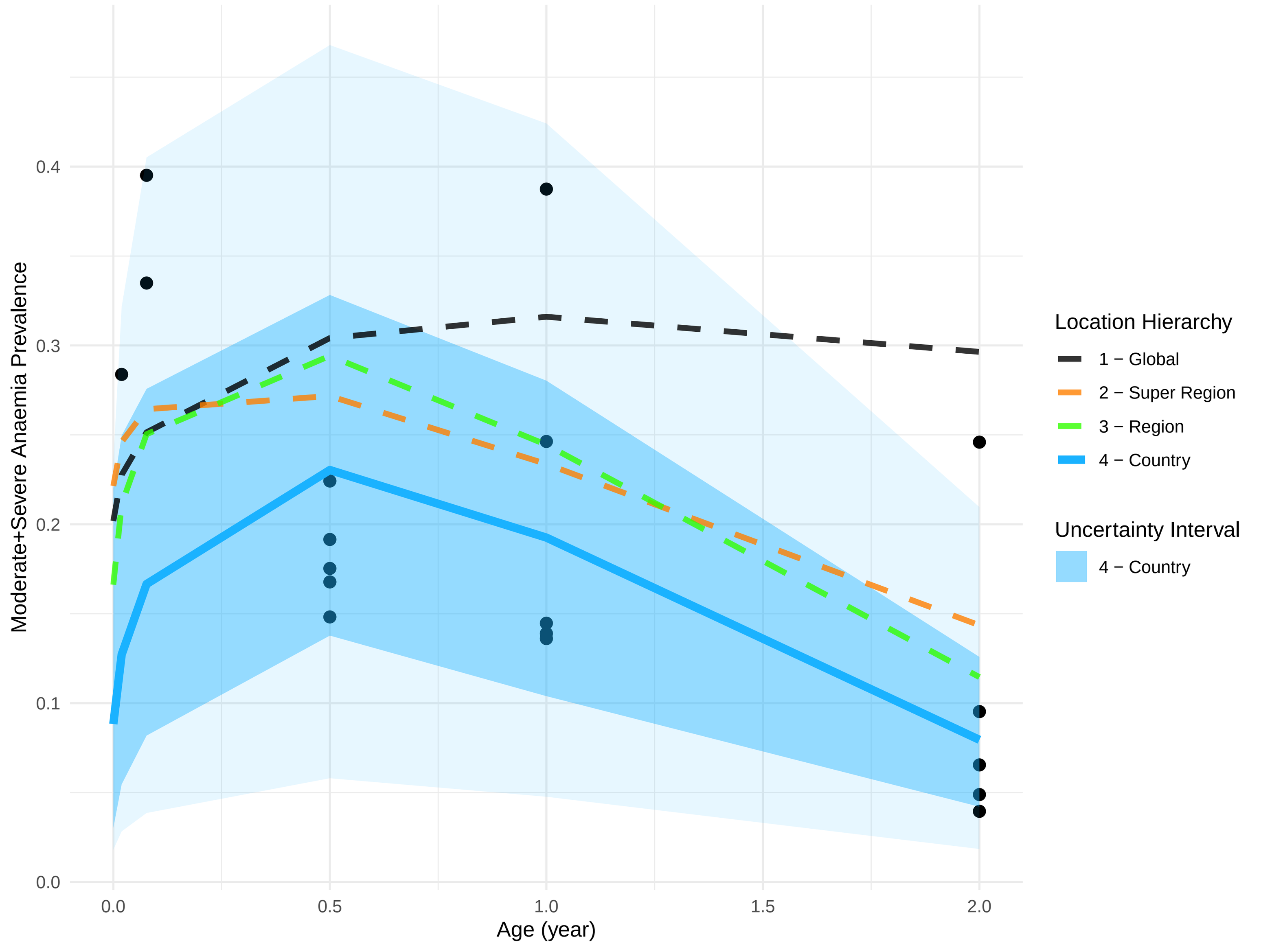

MR−BRT Age/Sex Fits − Non−pregnant Females <2 Years Old − Moderate+Severe Anaemia Prevalence − Caribbean
WHO−2024 Adjustment Method

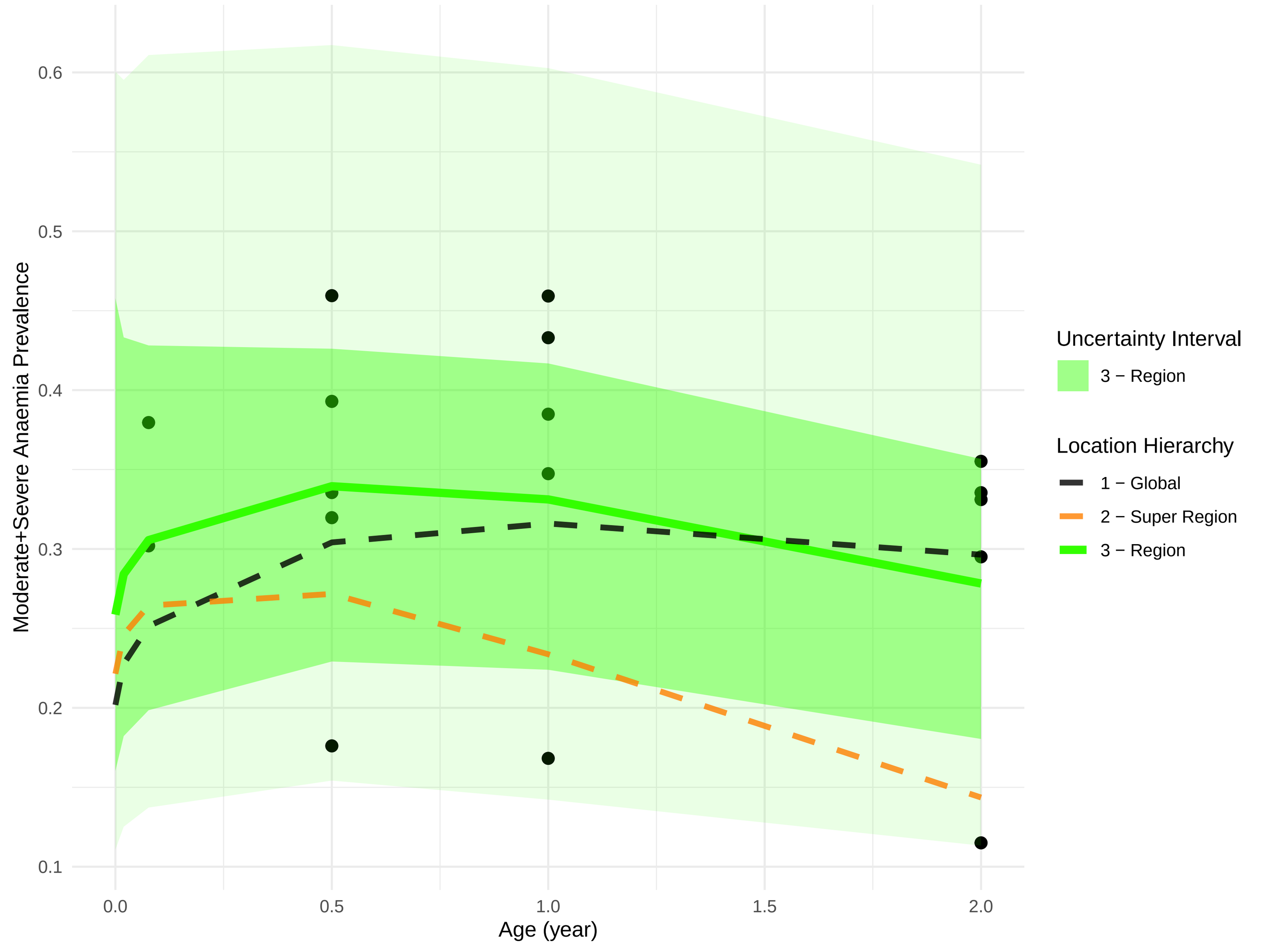

MR−BRT Age/Sex Fits − Non−pregnant Females <2 Years Old − Moderate+Severe Anaemia Prevalence − Guyana
WHO−2024 Adjustment Method

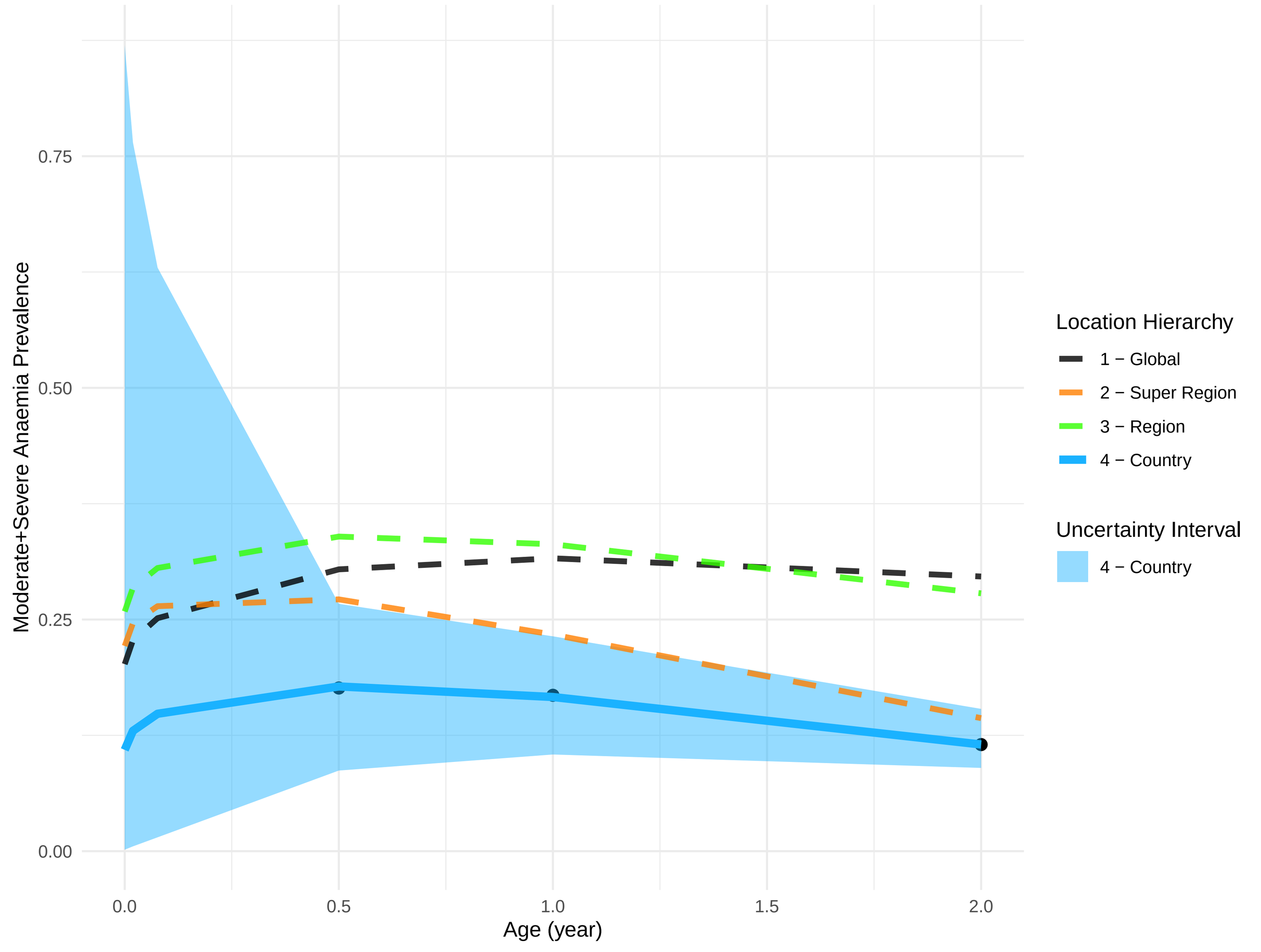

MR−BRT Age/Sex Fits − Non−pregnant Females <2 Years Old − Moderate+Severe Anaemia Prevalence − Haiti
WHO−2024 Adjustment Method

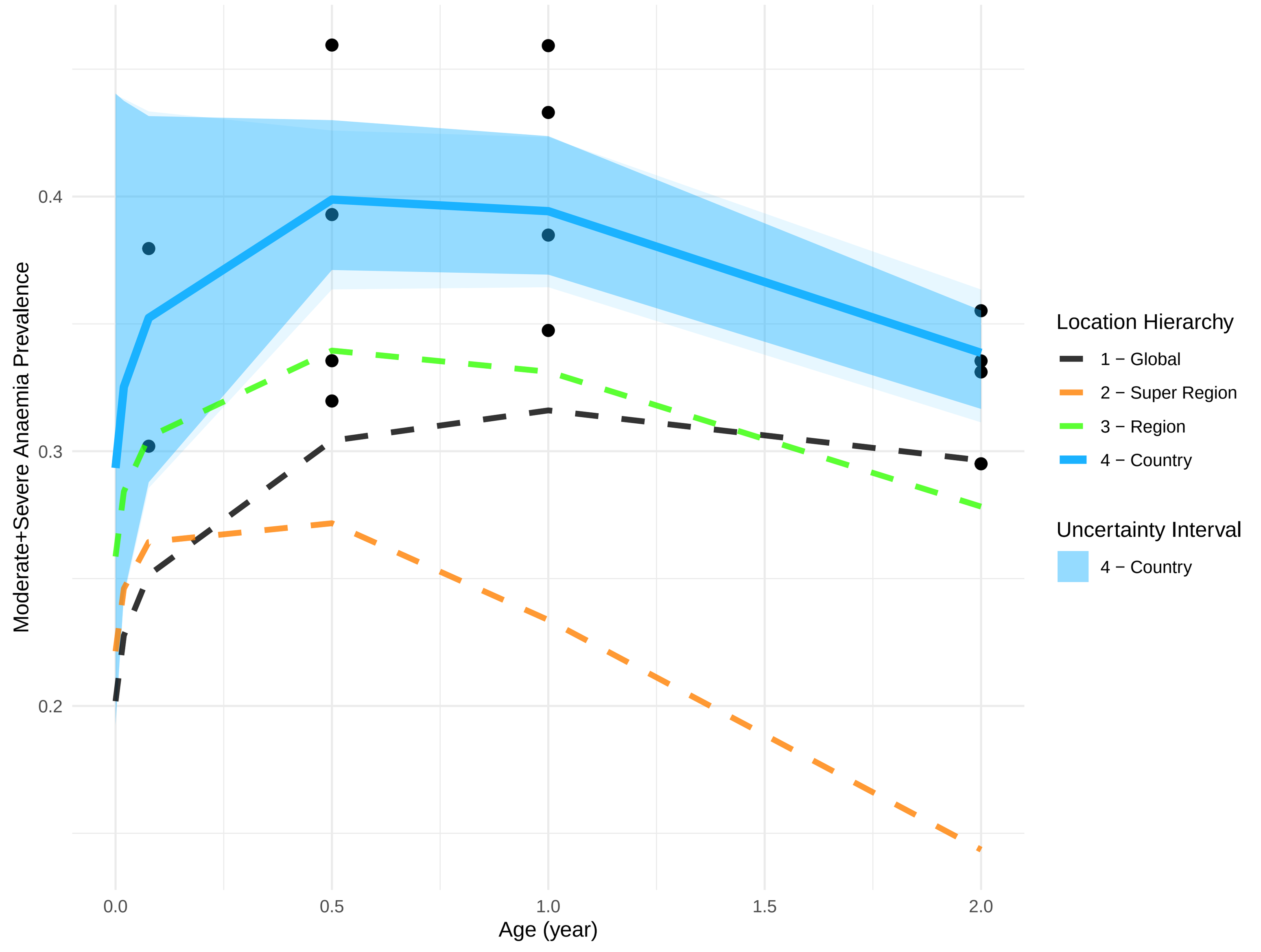

MR−BRT Age/Sex Fits − Non−pregnant Females <2 Years Old − Moderate+Severe Anaemia Prevalence − Central Latin America
WHO−2024 Adjustment Method

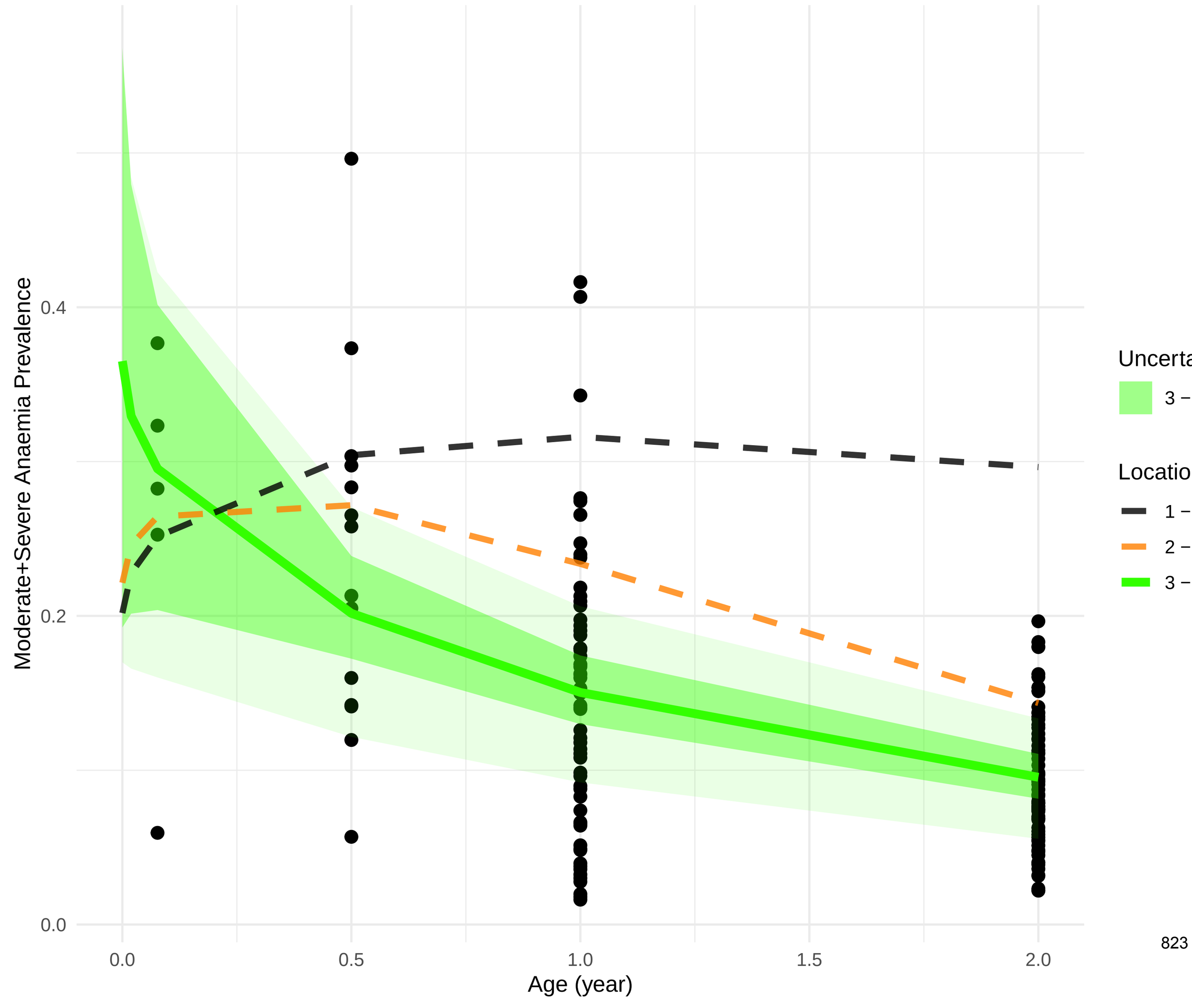

MR−BRT Age/Sex Fits − Non−pregnant Females <2 Years Old − Moderate+Severe Anaemia Prevalence − Guatemala
WHO−2024 Adjustment Method

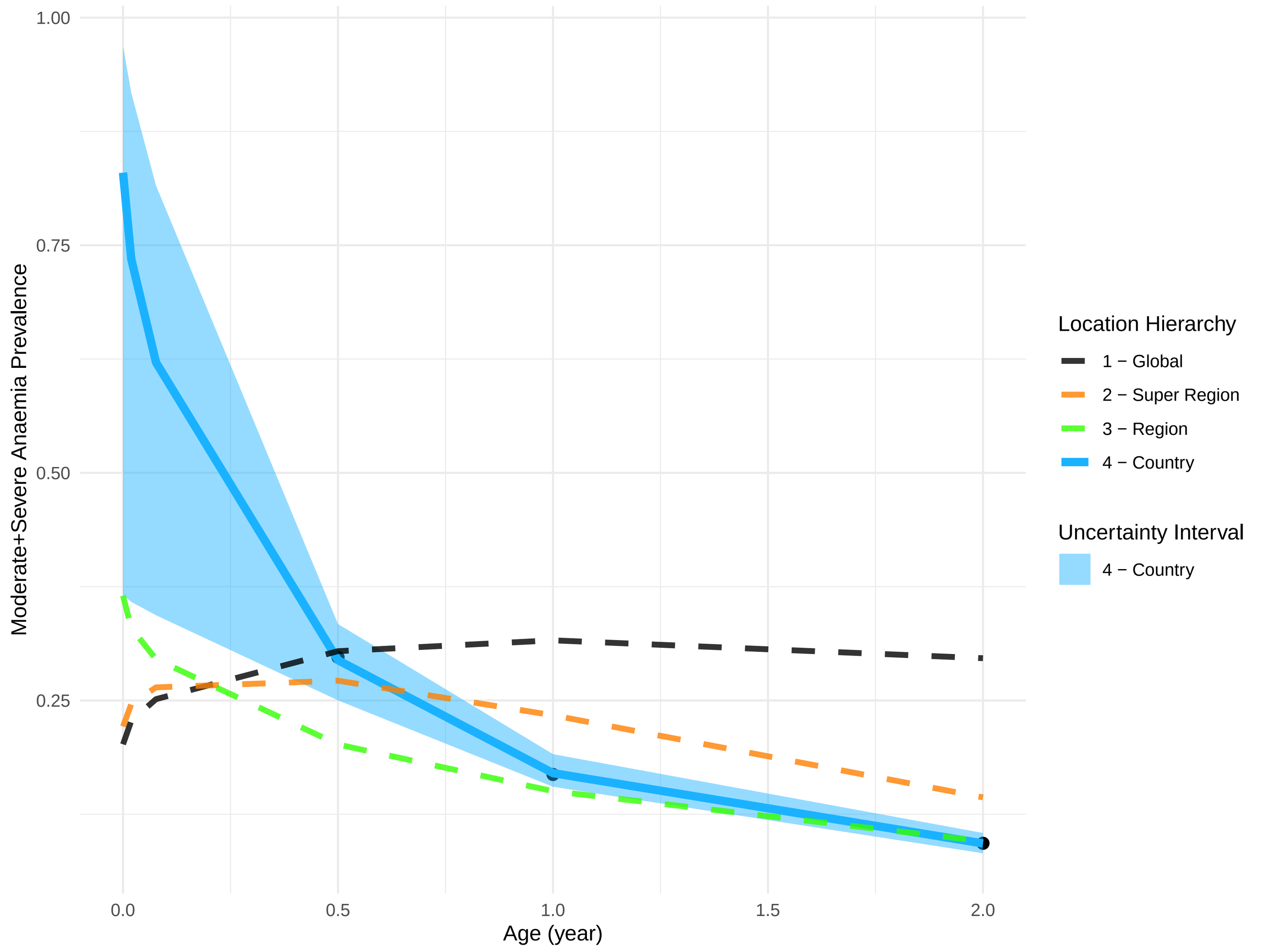

MR−BRT Age/Sex Fits − Non−pregnant Females <2 Years Old − Moderate+Severe Anaemia Prevalence − Honduras
WHO−2024 Adjustment Method

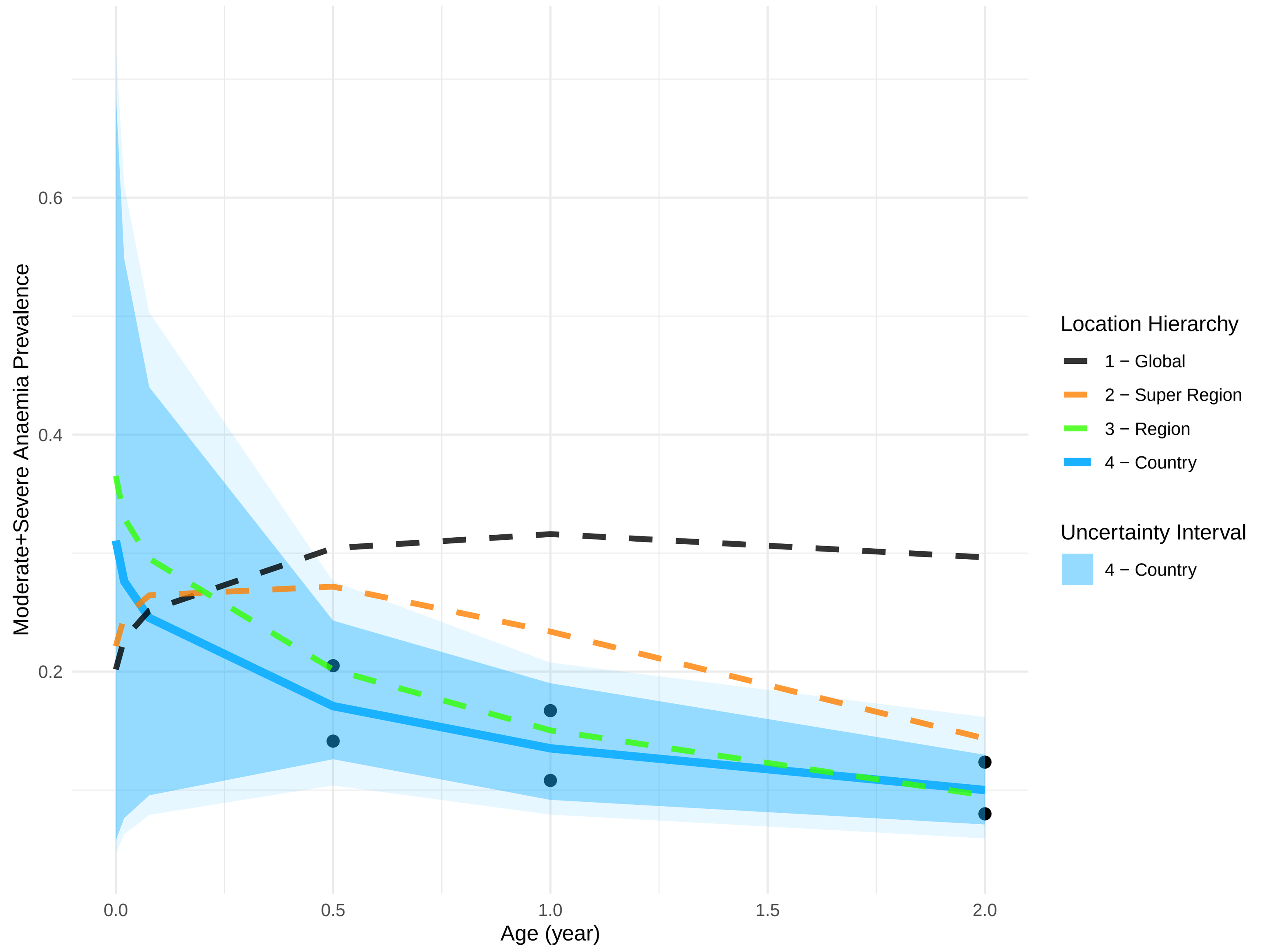

MR−BRT Age/Sex Fits − Non−pregnant Females <2 Years Old − Moderate+Severe Anaemia Prevalence − Mexico
WHO−2024 Adjustment Method

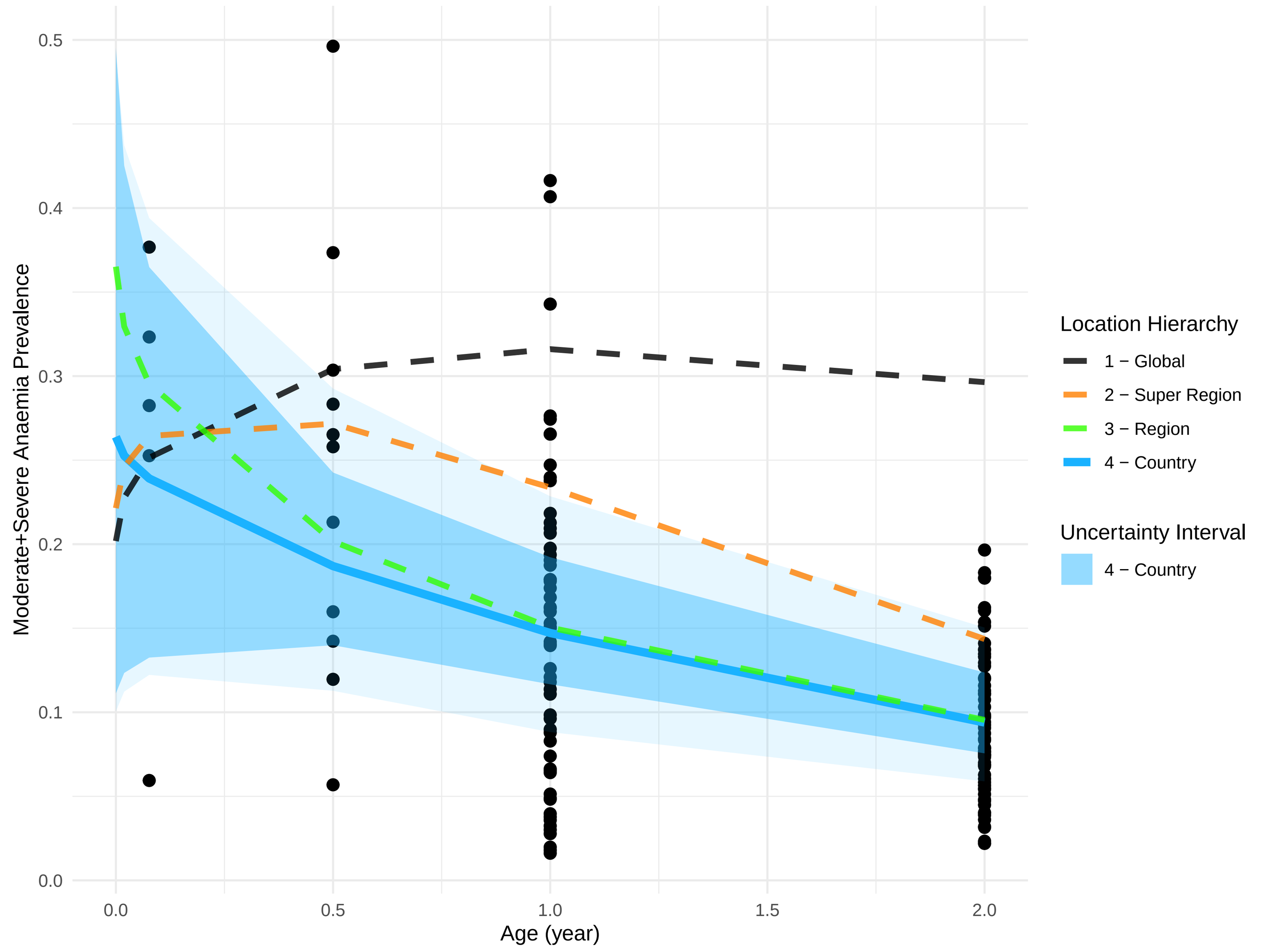

MR−BRT Age/Sex Fits − Non−pregnant Females <2 Years Old − Moderate+Severe Anaemia Prevalence − Aguascalientes
WHO−2024 Adjustment Method

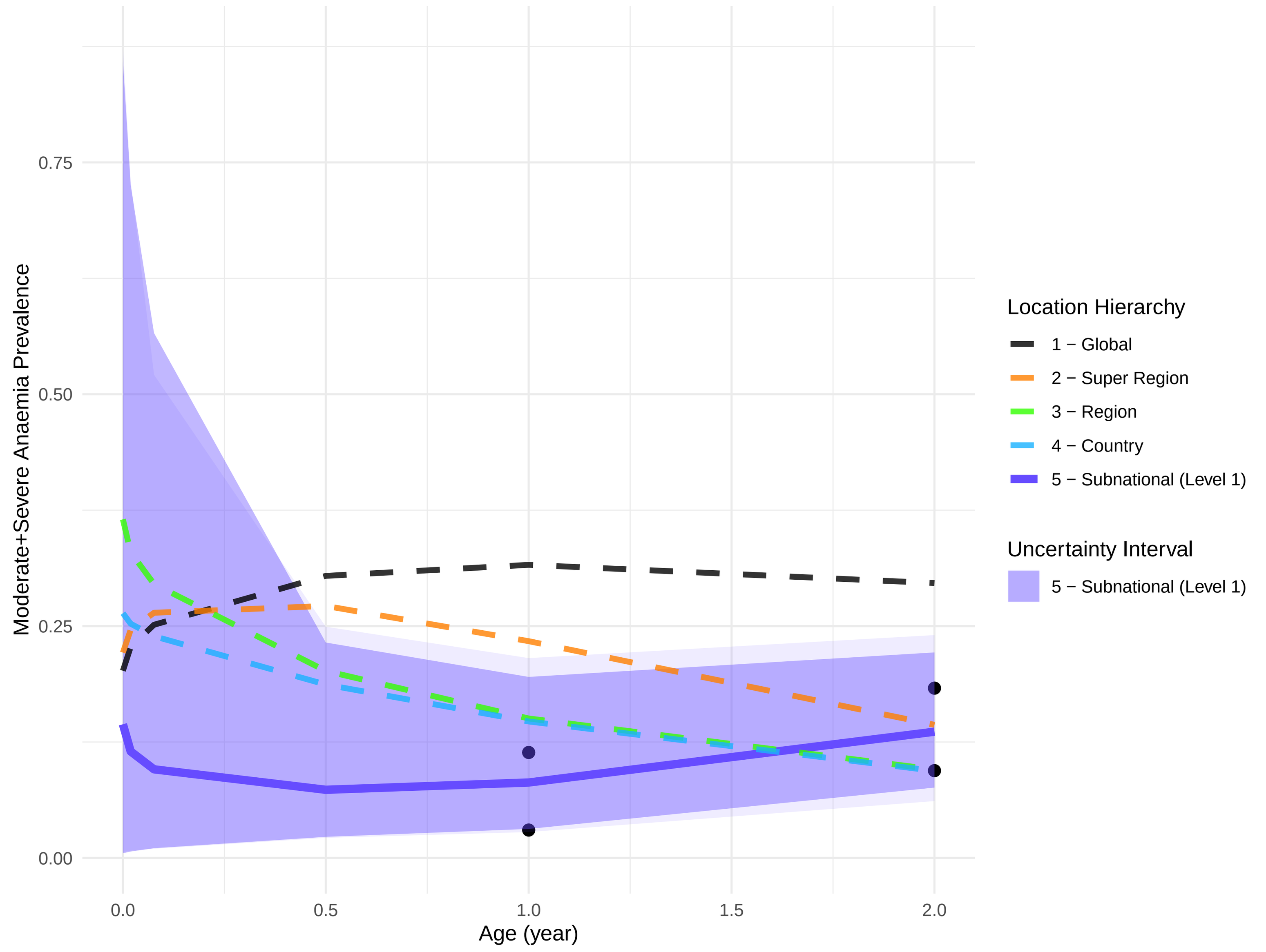

MR−BRT Age/Sex Fits − Non−pregnant Females <2 Years Old − Moderate+Severe Anaemia Prevalence − Baja California
WHO−2024 Adjustment Method

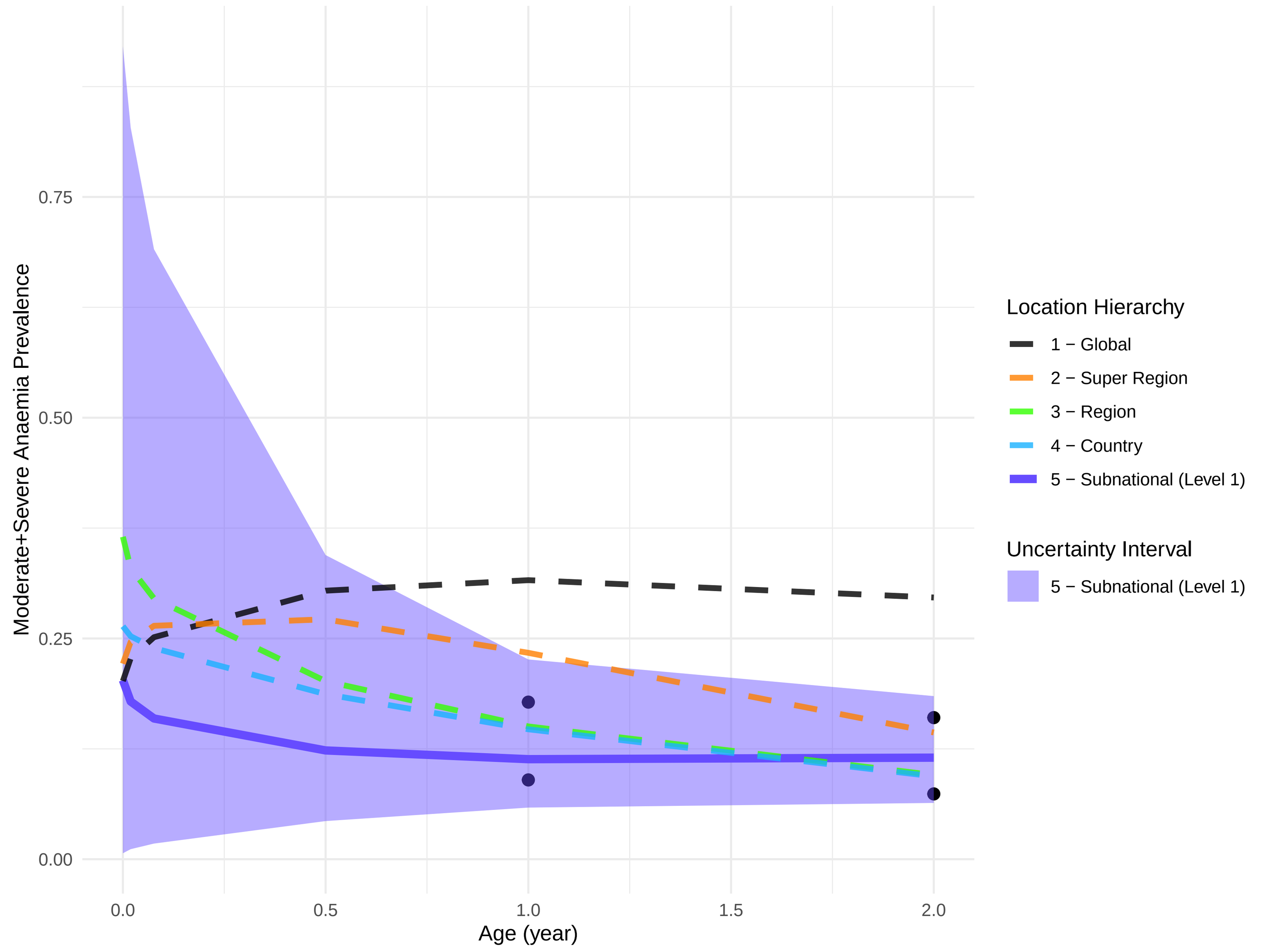

MR−BRT Age/Sex Fits − Non−pregnant Females <2 Years Old − Moderate+Severe Anaemia Prevalence − Baja California Sur
WHO−2024 Adjustment Method

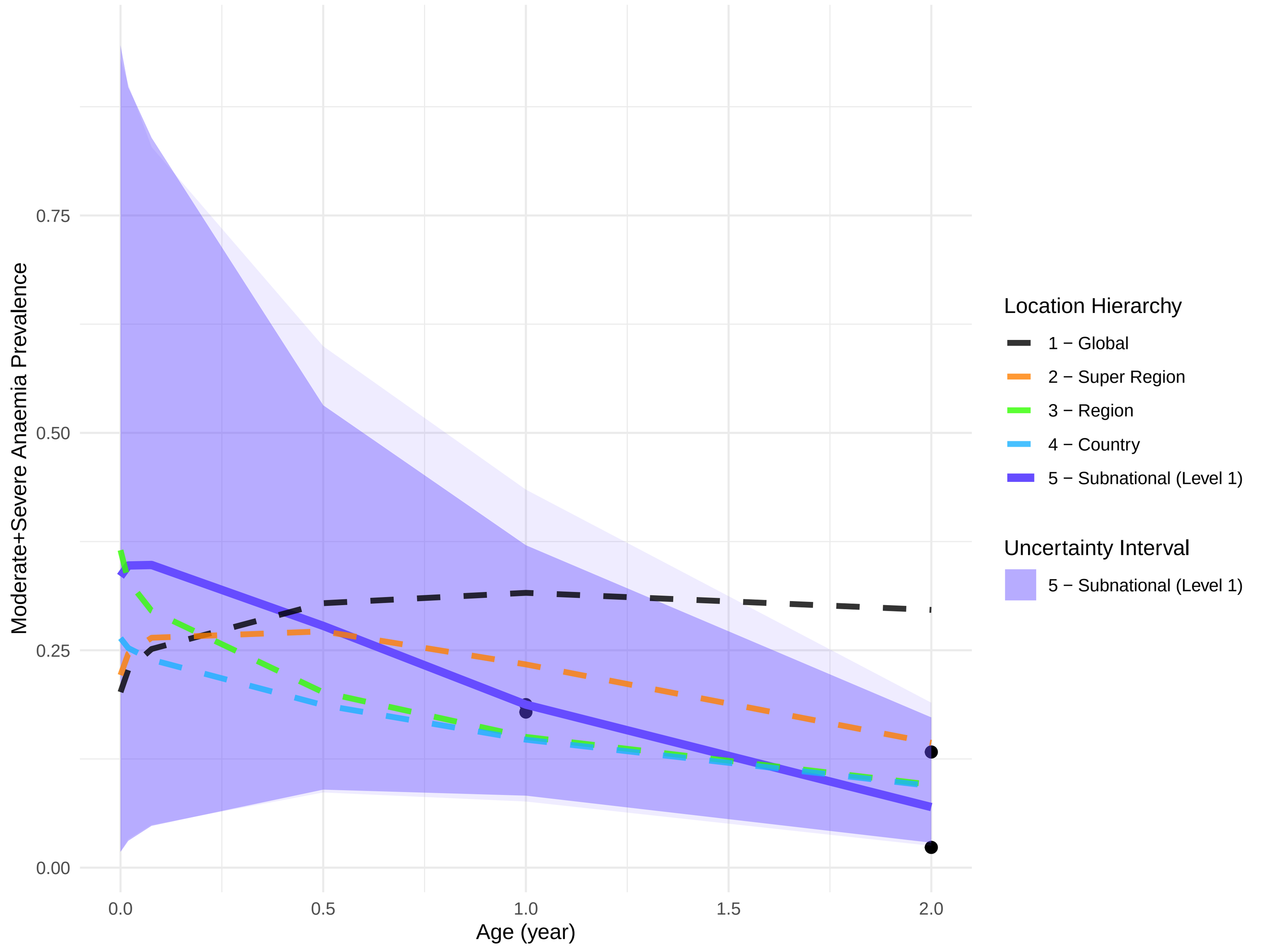

MR−BRT Age/Sex Fits − Non−pregnant Females <2 Years Old − Moderate+Severe Anaemia Prevalence − Campeche
WHO−2024 Adjustment Method

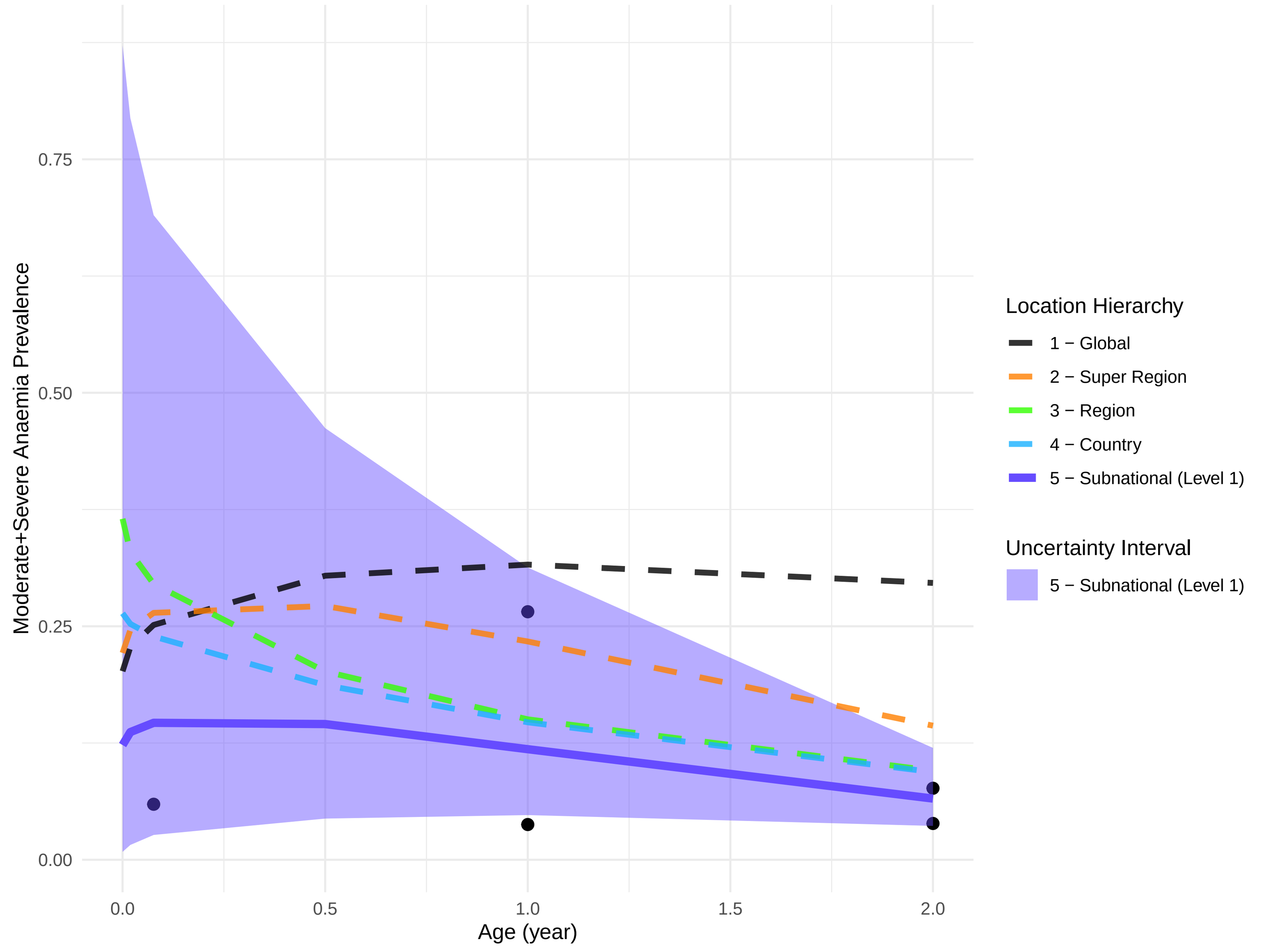

MR−BRT Age/Sex Fits − Non−pregnant Females <2 Years Old − Moderate+Severe Anaemia Prevalence − Chiapas
WHO−2024 Adjustment Method

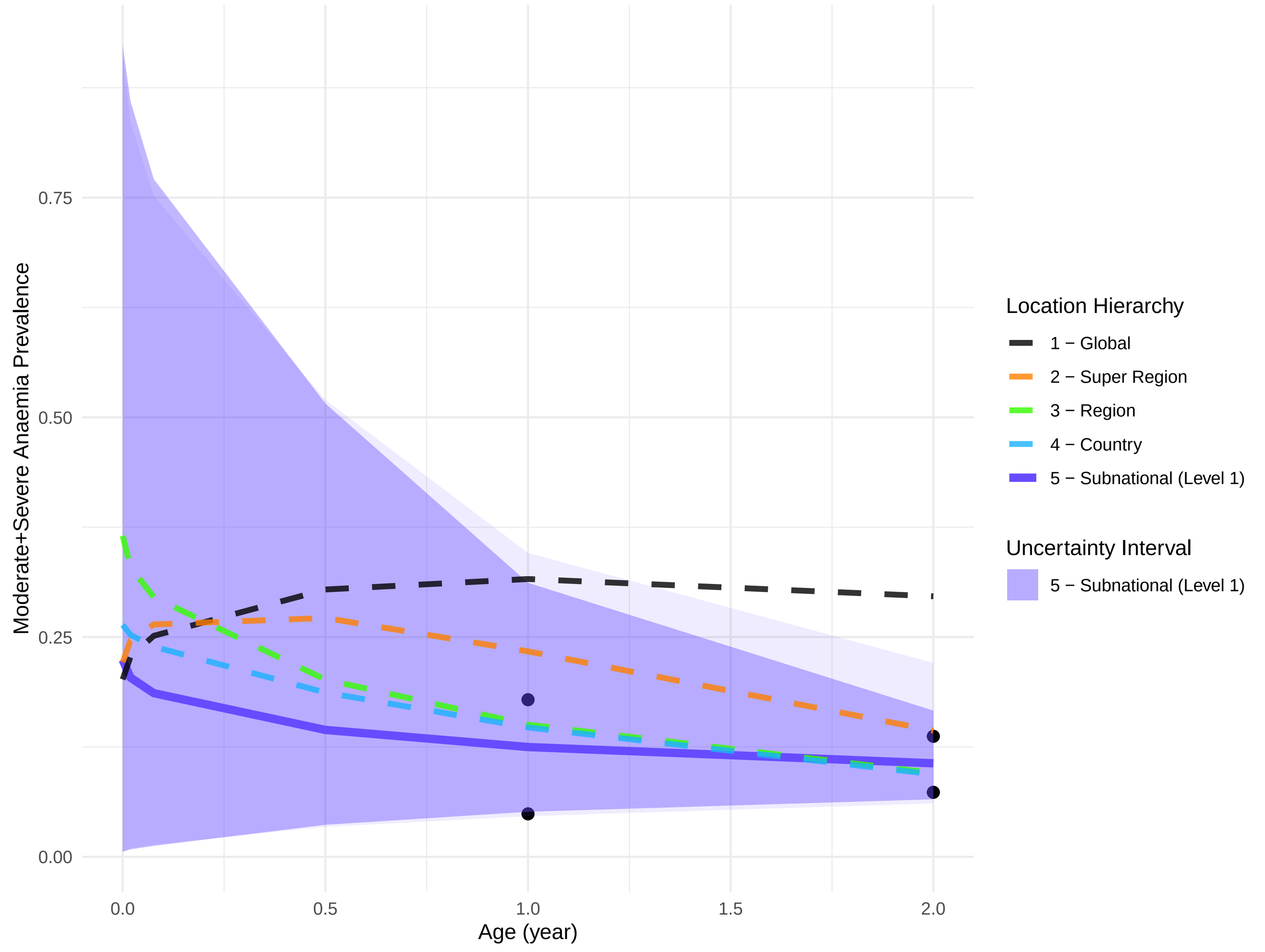

MR−BRT Age/Sex Fits − Non−pregnant Females <2 Years Old − Moderate+Severe Anaemia Prevalence − Chihuahua
WHO−2024 Adjustment Method

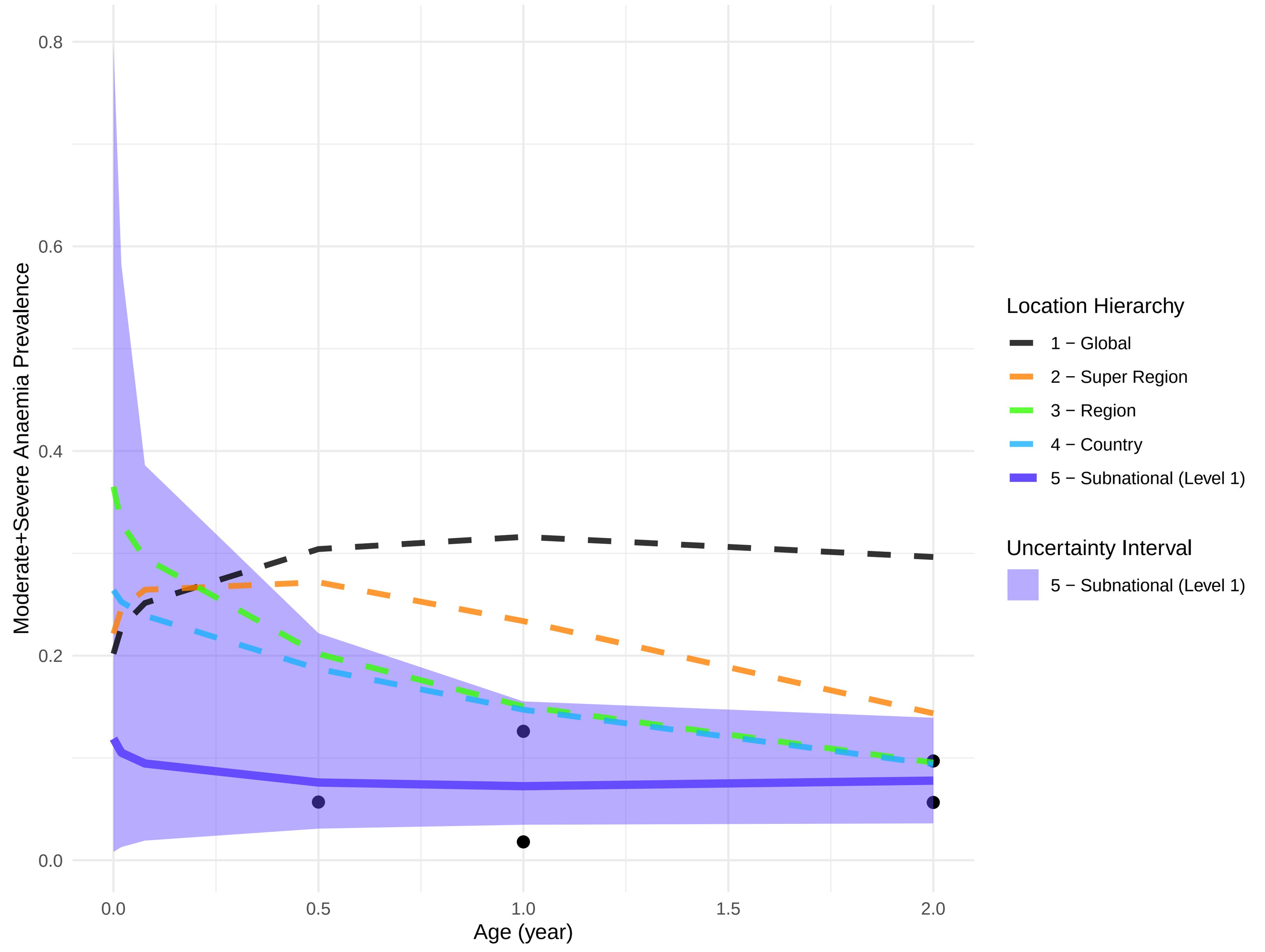

MR−BRT Age/Sex Fits − Non−pregnant Females <2 Years Old − Moderate+Severe Anaemia Prevalence − Coahuila
WHO−2024 Adjustment Method

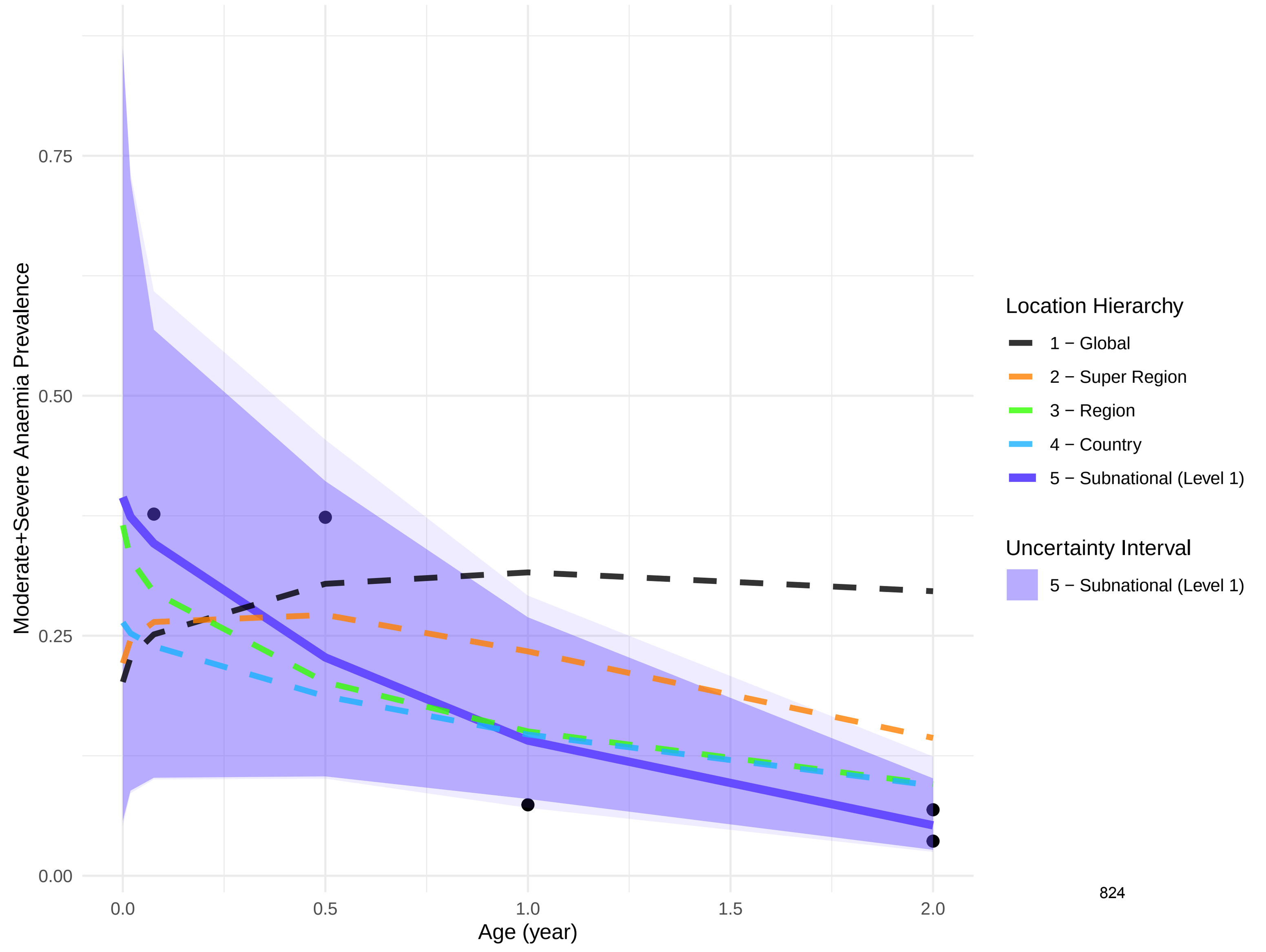

MR−BRT Age/Sex Fits − Non−pregnant Females <2 Years Old − Moderate+Severe Anaemia Prevalence − Colima
WHO−2024 Adjustment Method

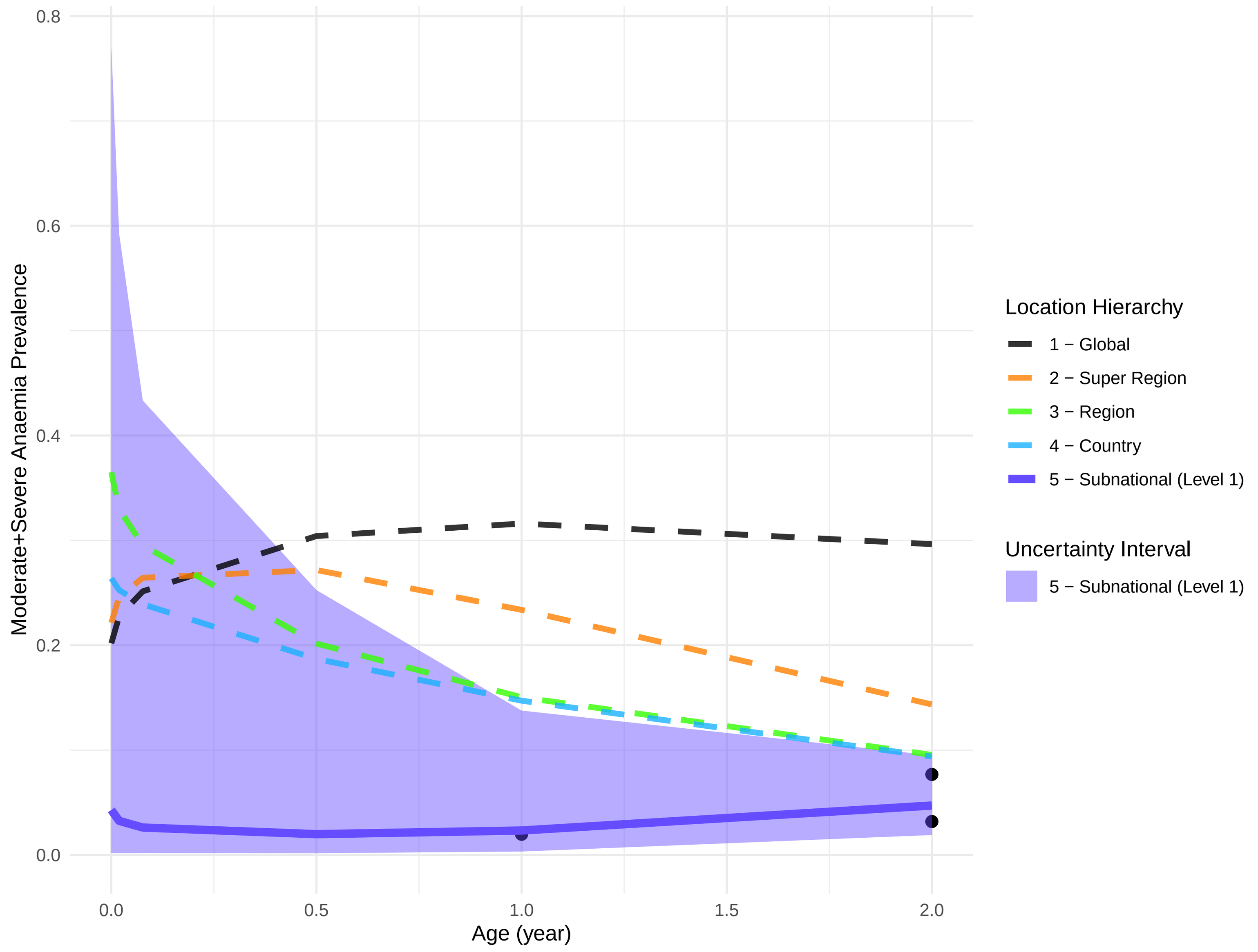

MR−BRT Age/Sex Fits − Non−pregnant Females <2 Years Old − Moderate+Severe Anaemia Prevalence − Durango
WHO−2024 Adjustment Method

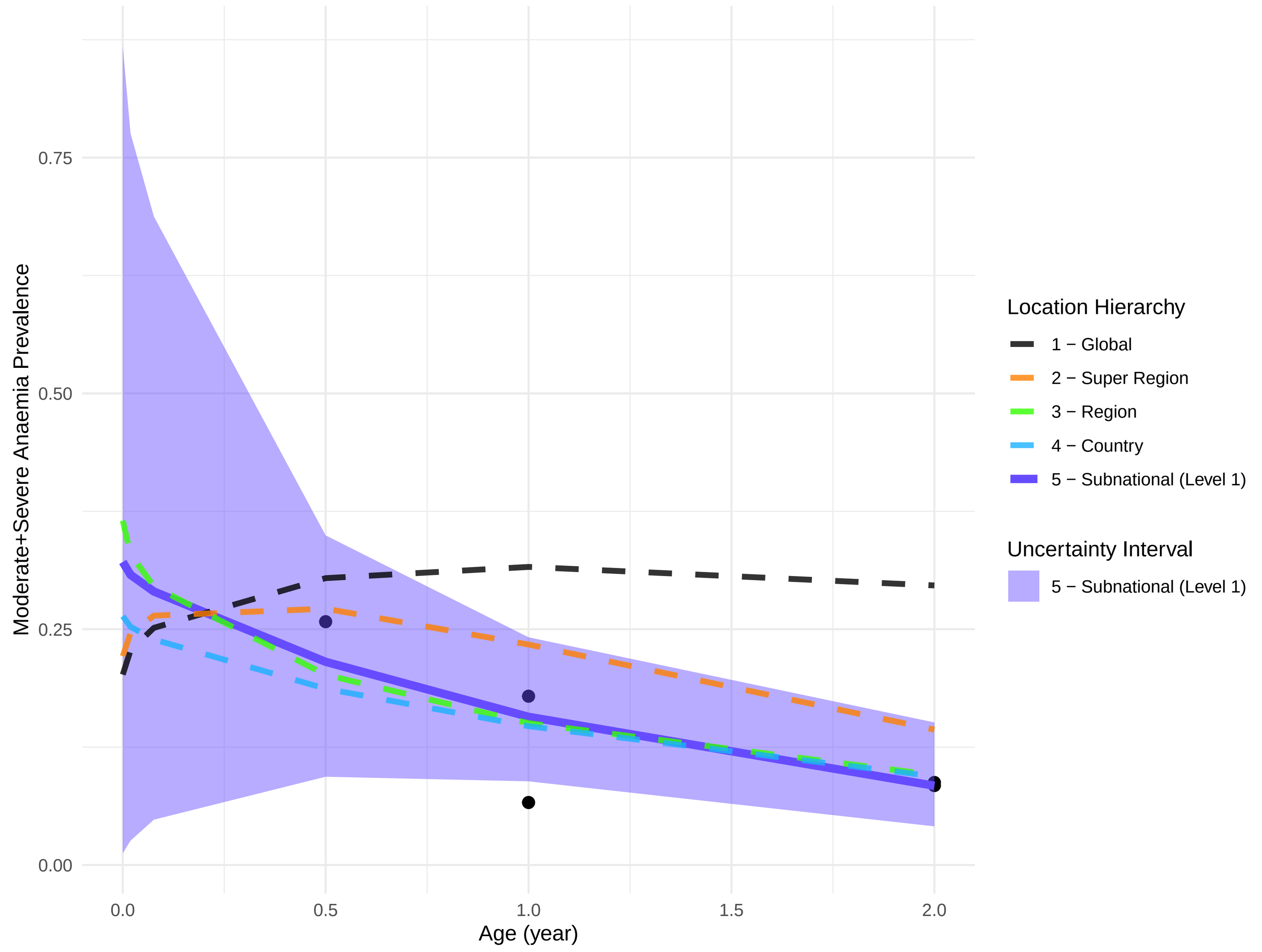

MR−BRT Age/Sex Fits − Non−pregnant Females <2 Years Old − Moderate+Severe Anaemia Prevalence − Guanajuato
WHO−2024 Adjustment Method

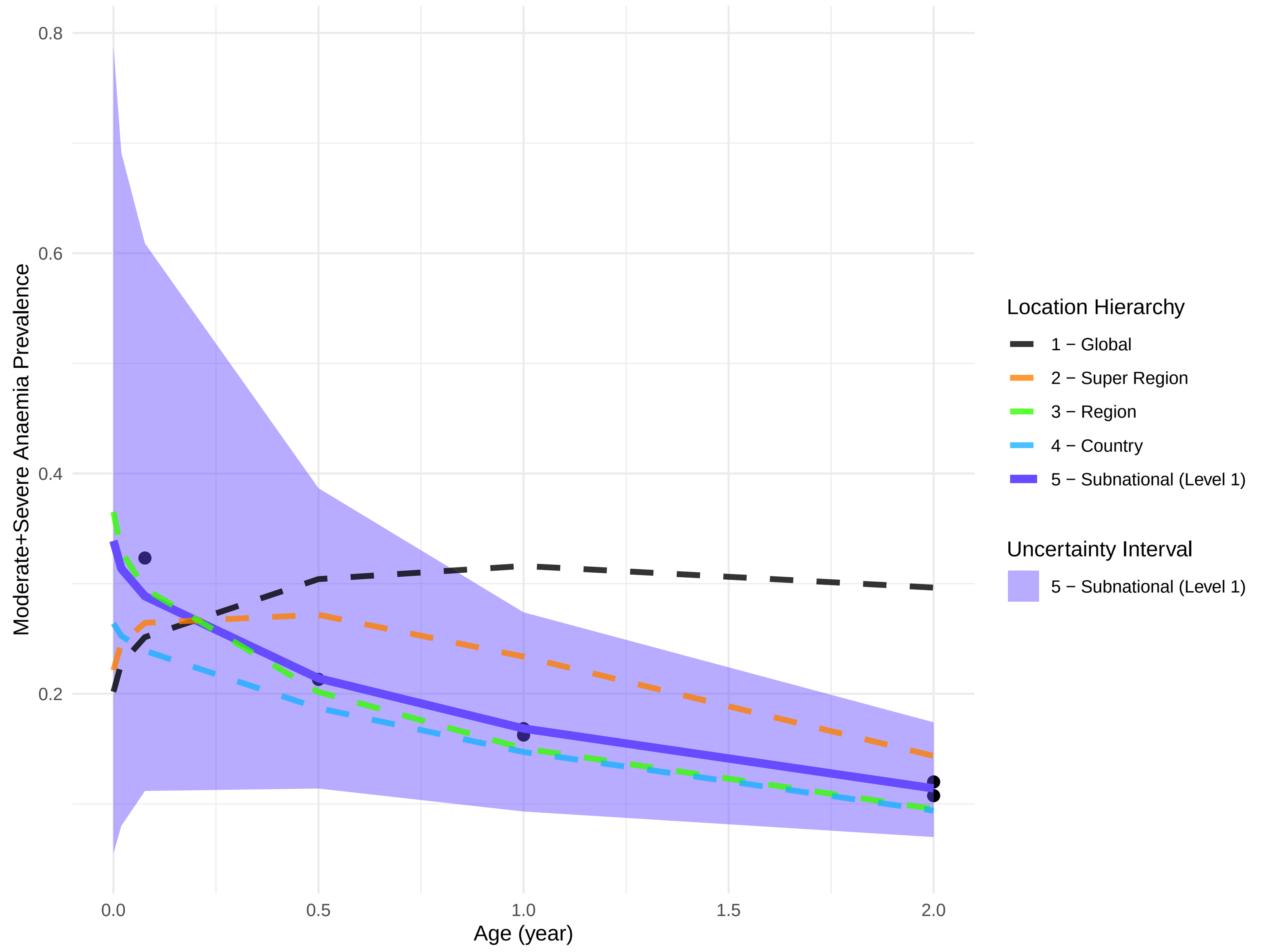

MR−BRT Age/Sex Fits − Non−pregnant Females <2 Years Old − Moderate+Severe Anaemia Prevalence − Guerrero
WHO−2024 Adjustment Method

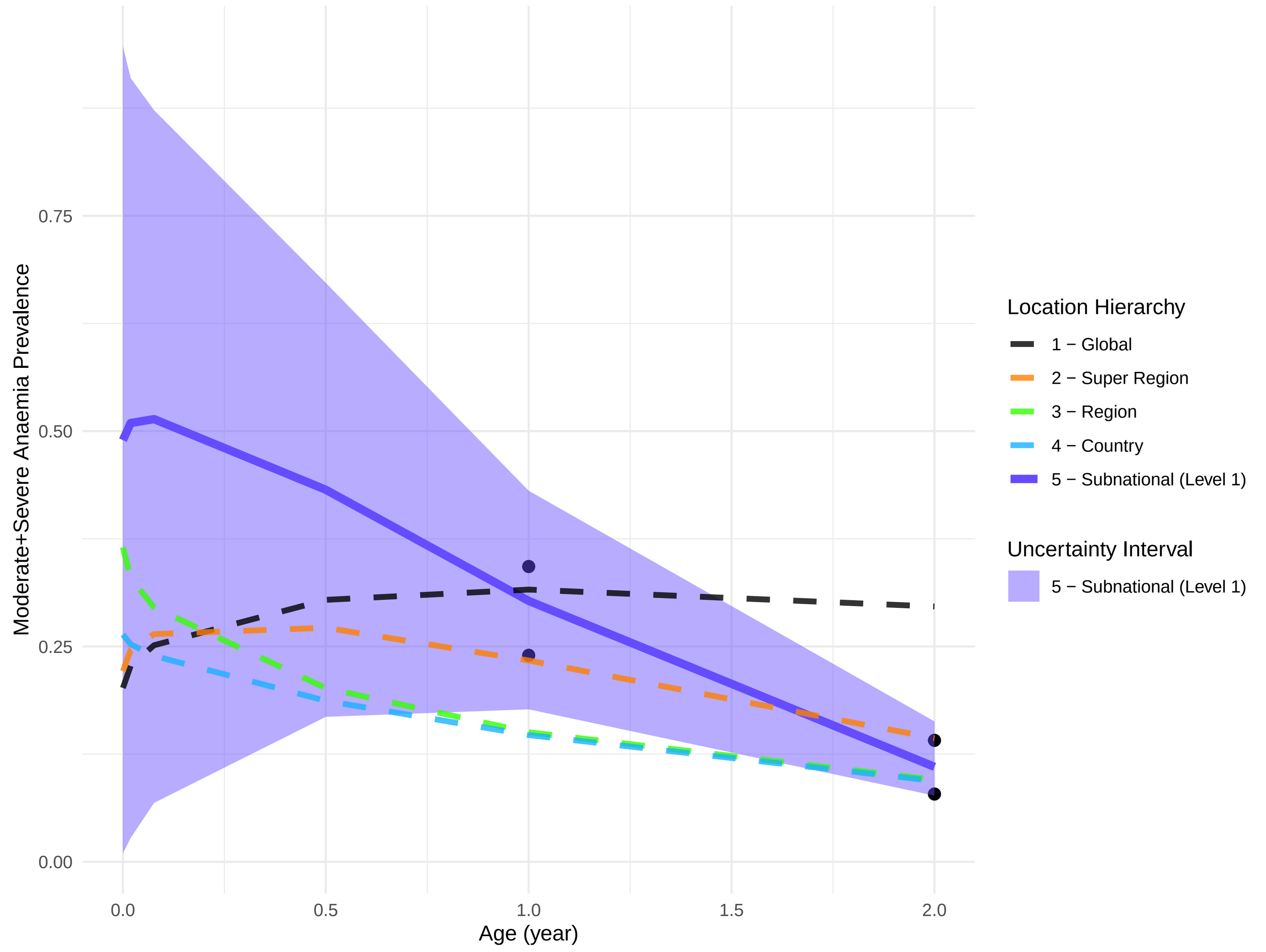

MR−BRT Age/Sex Fits − Non−pregnant Females <2 Years Old − Moderate+Severe Anaemia Prevalence − Hidalgo
WHO−2024 Adjustment Method

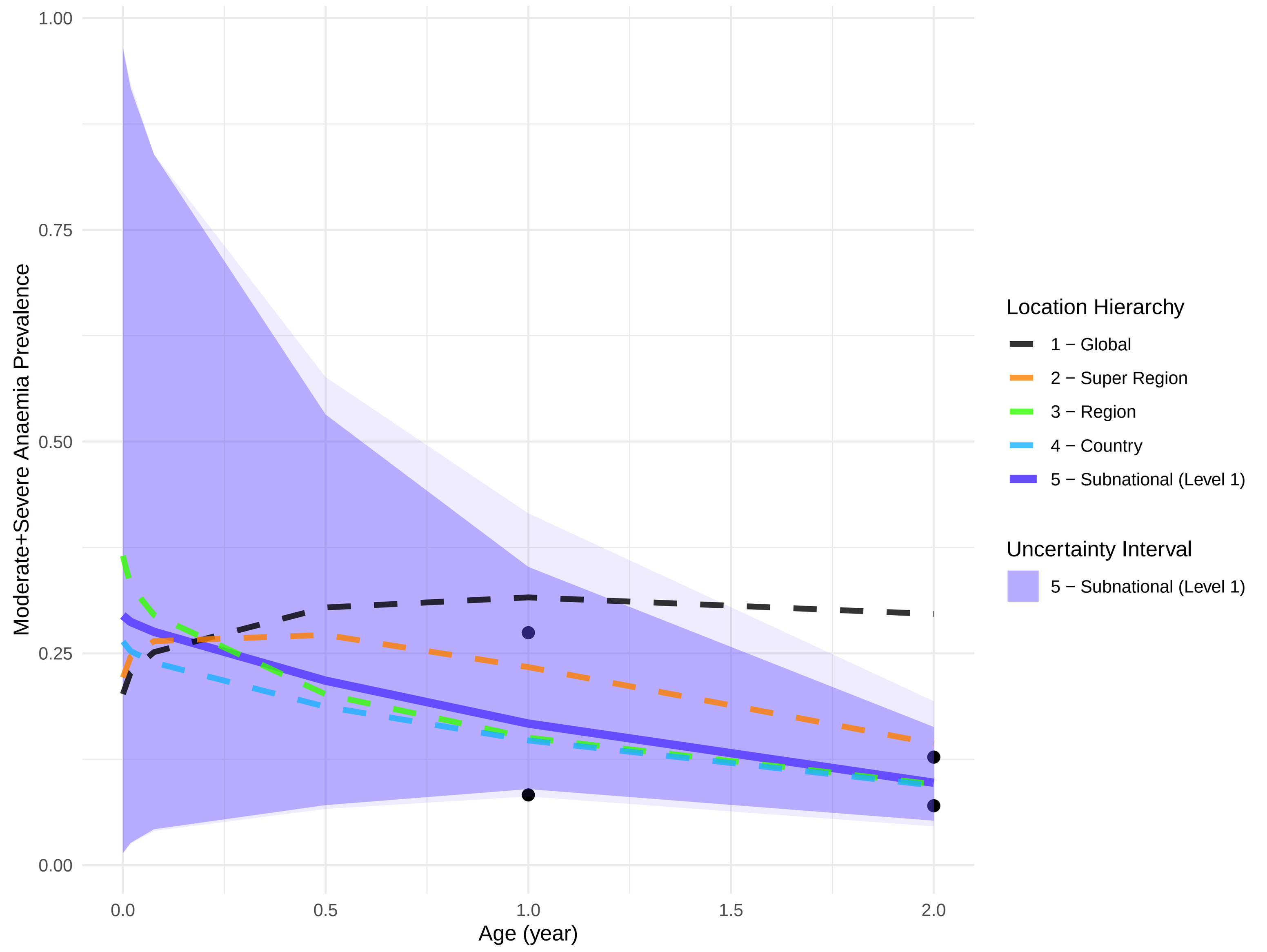

MR−BRT Age/Sex Fits − Non−pregnant Females <2 Years Old − Moderate+Severe Anaemia Prevalence − Jalisco
WHO−2024 Adjustment Method

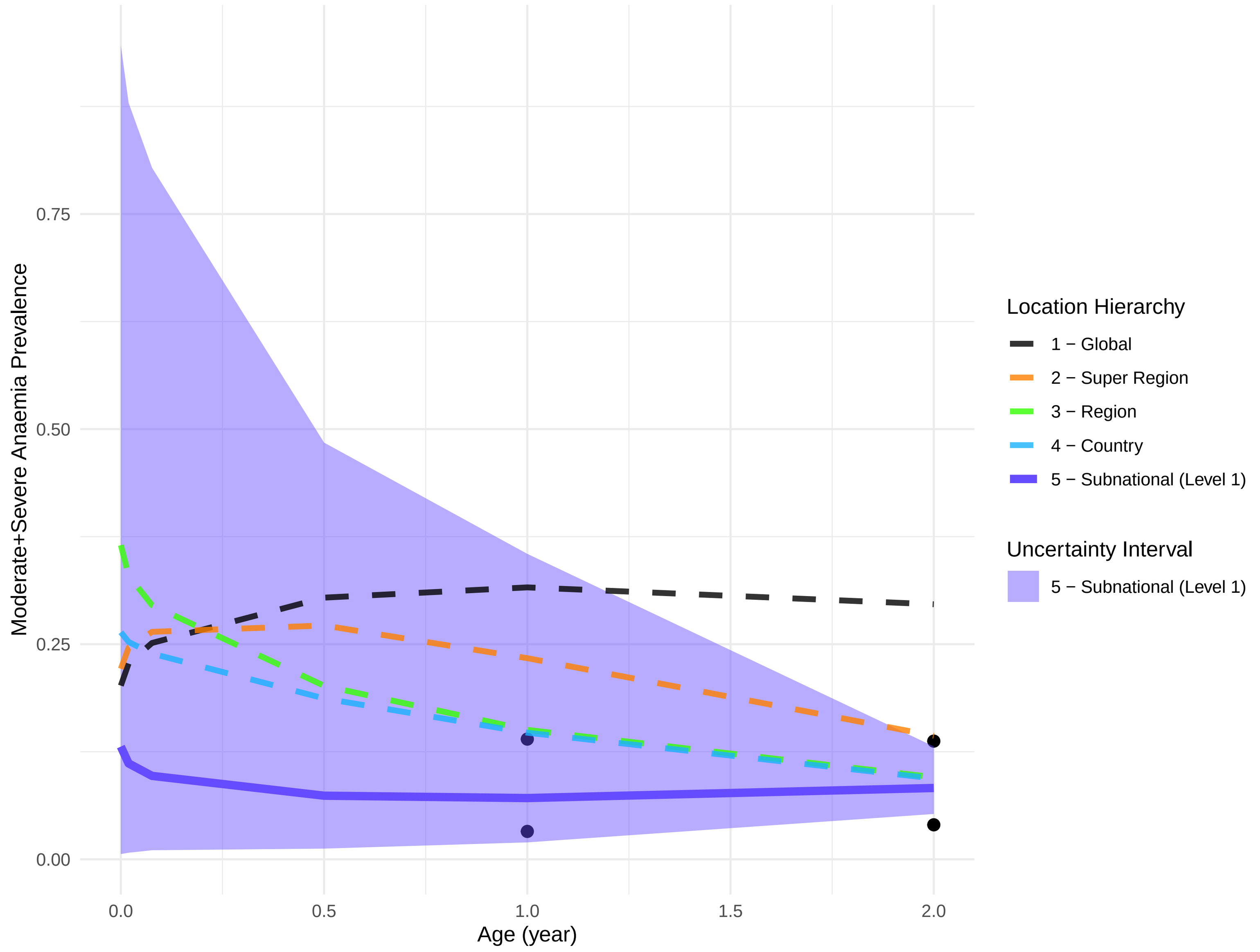

MR−BRT Age/Sex Fits − Non−pregnant Females <2 Years Old − Moderate+Severe Anaemia Prevalence − México
WHO−2024 Adjustment Method

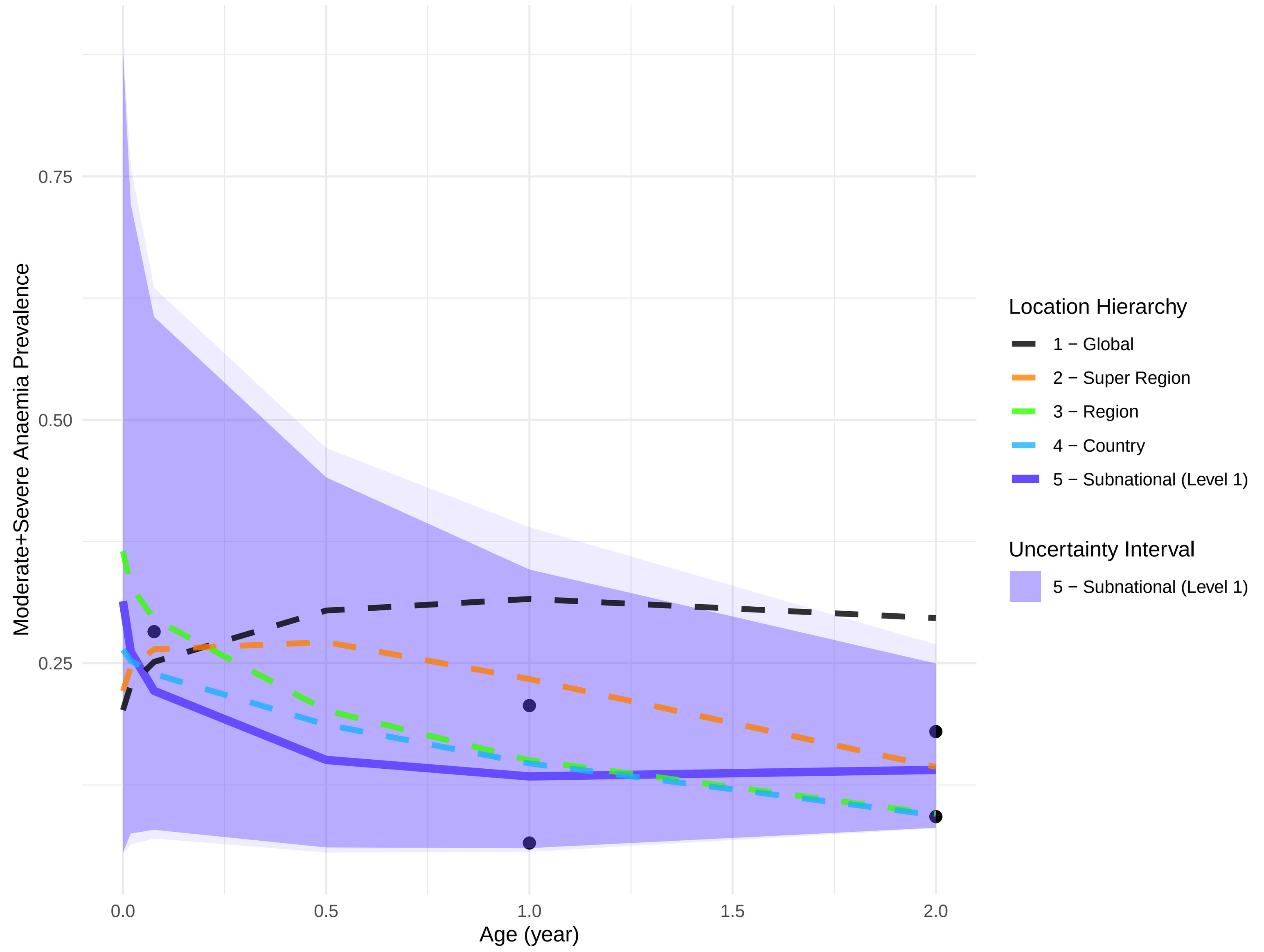

MR−BRT Age/Sex Fits − Non−pregnant Females <2 Years Old − Moderate+Severe Anaemia Prevalence − Mexico City
WHO−2024 Adjustment Method

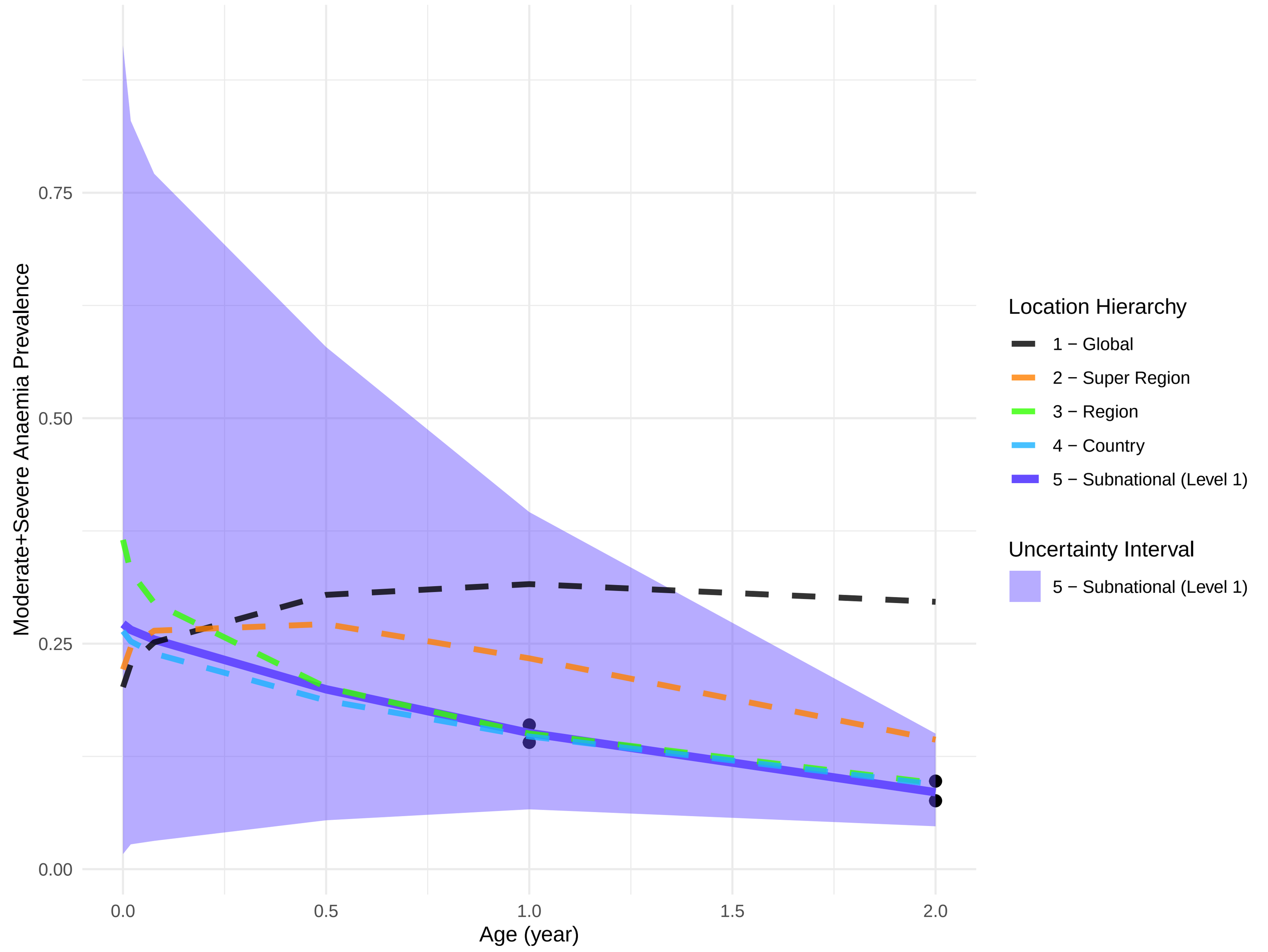

MR−BRT Age/Sex Fits − Non−pregnant Females <2 Years Old − Moderate+Severe Anaemia Prevalence − Michoacán de Ocampo
WHO−2024 Adjustment Method

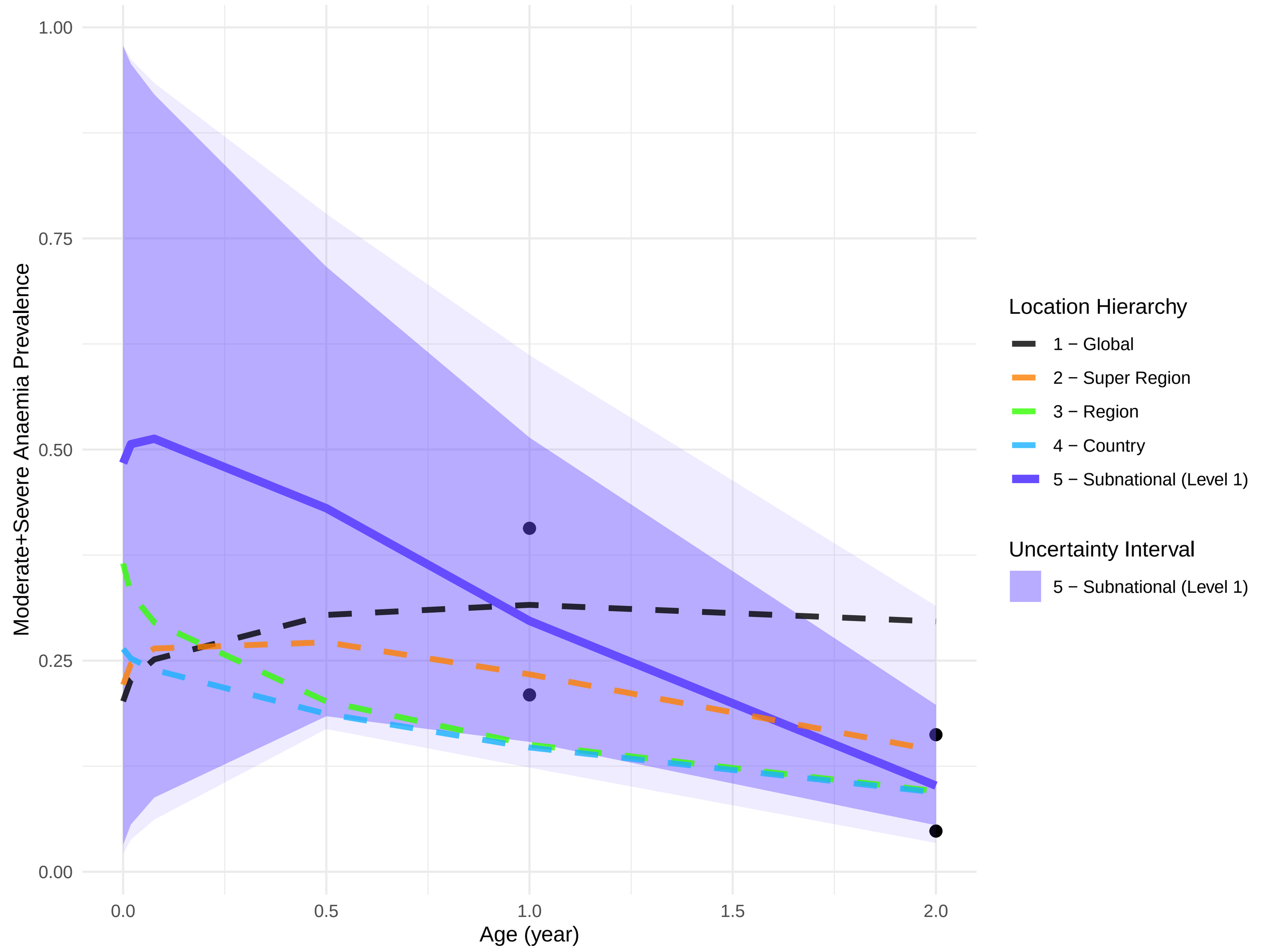

MR−BRT Age/Sex Fits − Non−pregnant Females <2 Years Old − Moderate+Severe Anaemia Prevalence − Morelos
WHO−2024 Adjustment Method

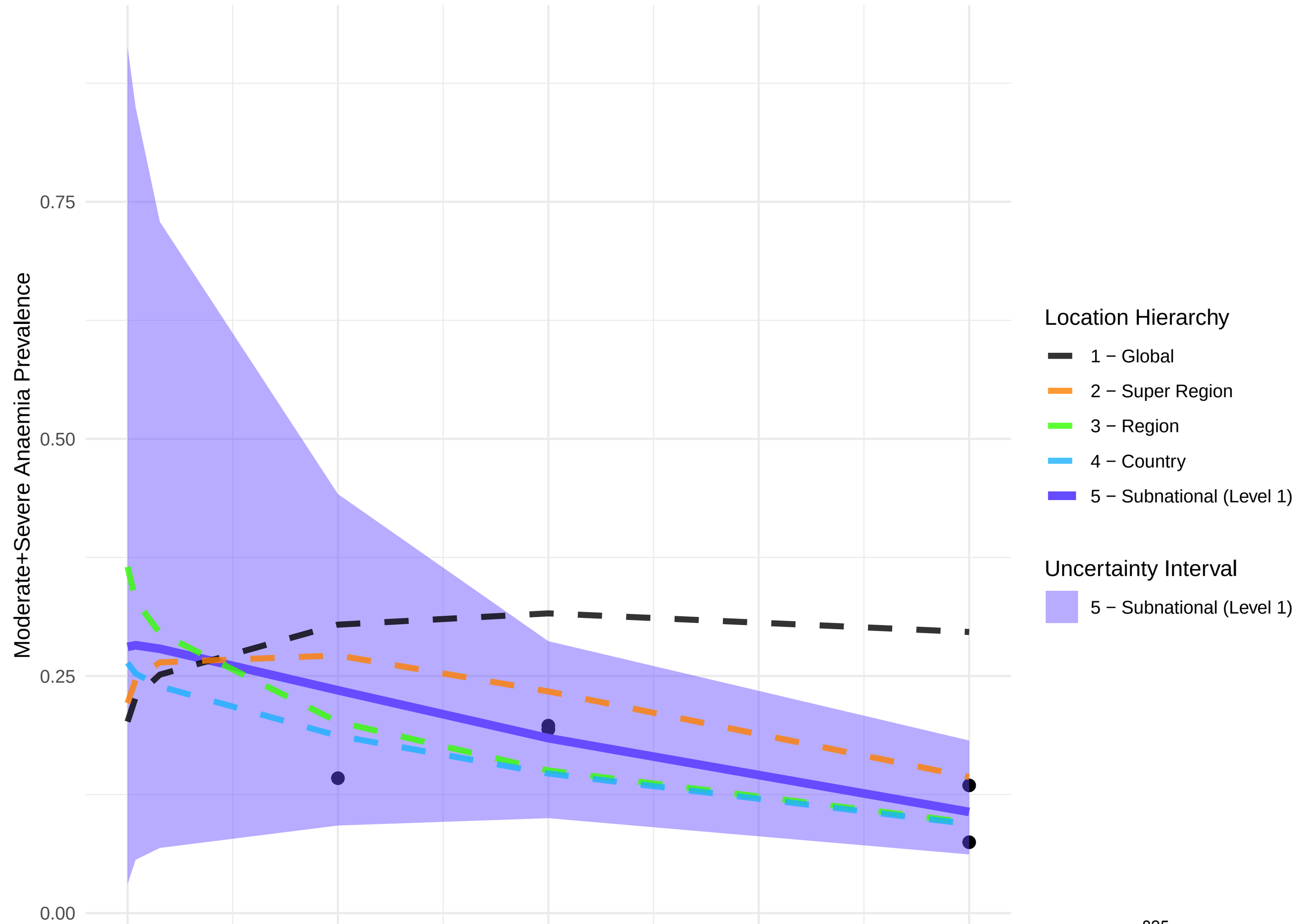

MR−BRT Age/Sex Fits − Non−pregnant Females <2 Years Old − Moderate+Severe Anaemia Prevalence − Nayarit
WHO−2024 Adjustment Method

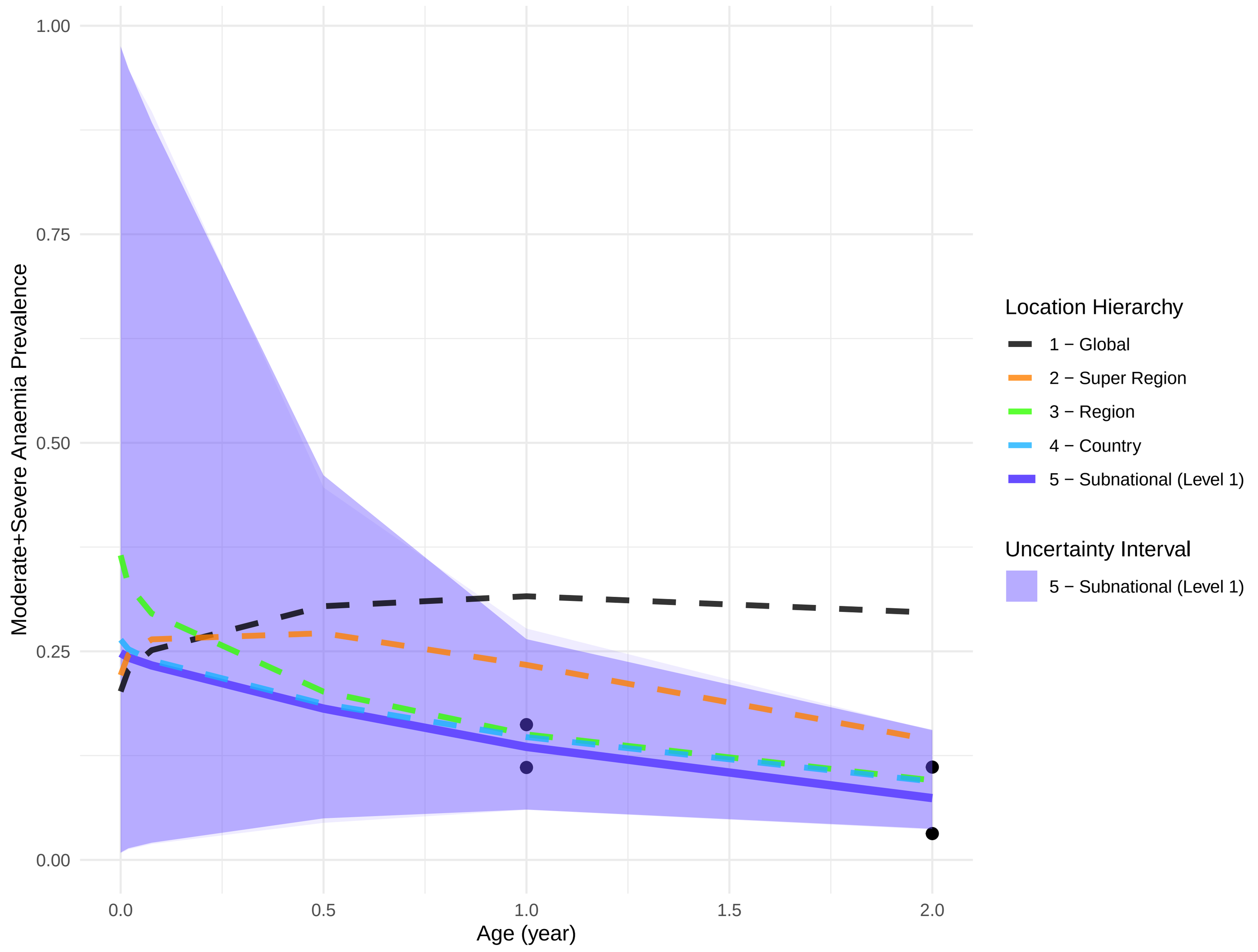

MR−BRT Age/Sex Fits − Non−pregnant Females <2 Years Old − Moderate+Severe Anaemia Prevalence − Nuevo León
WHO−2024 Adjustment Method

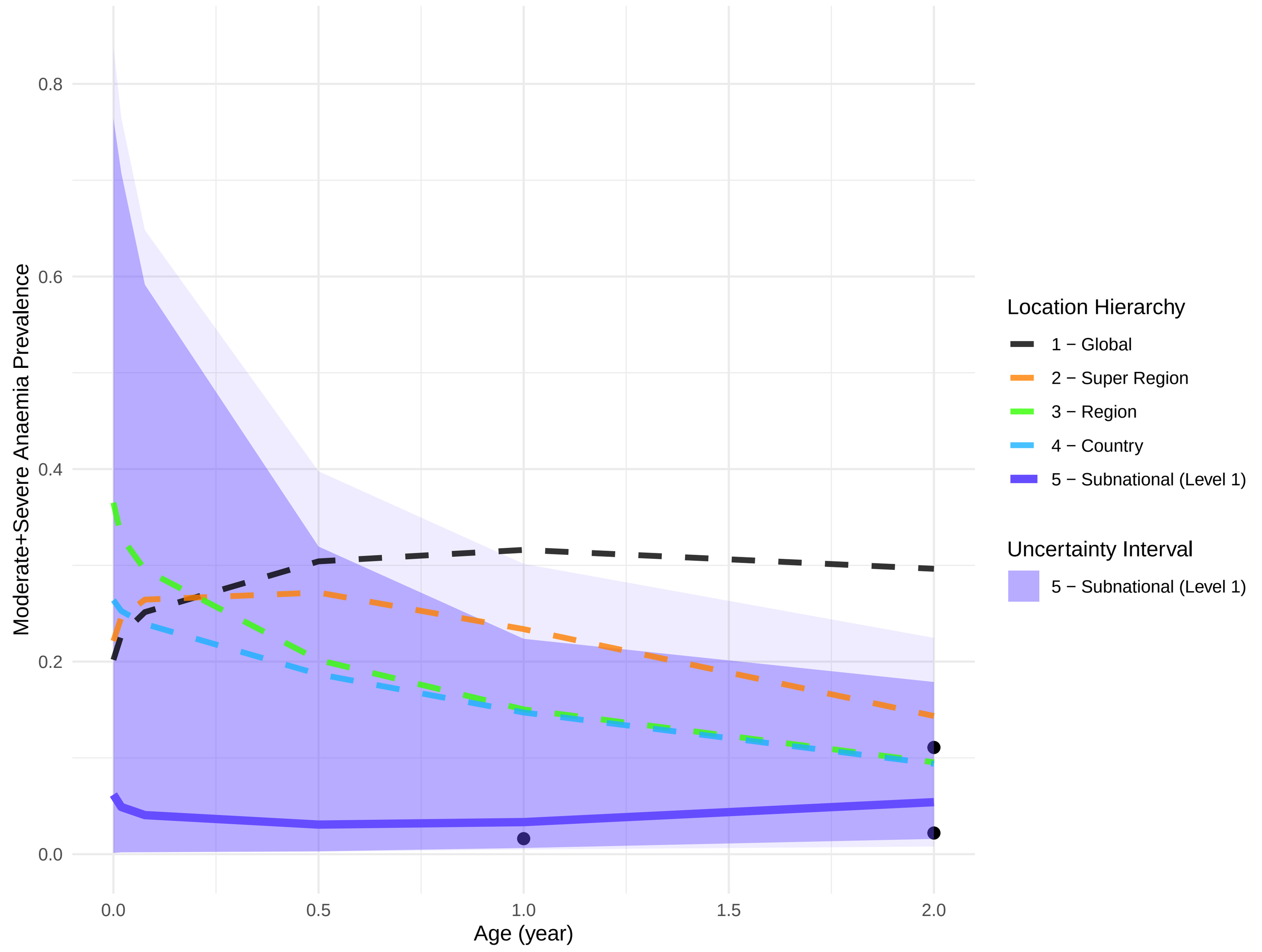

MR−BRT Age/Sex Fits − Non−pregnant Females <2 Years Old − Moderate+Severe Anaemia Prevalence − Oaxaca
WHO−2024 Adjustment Method

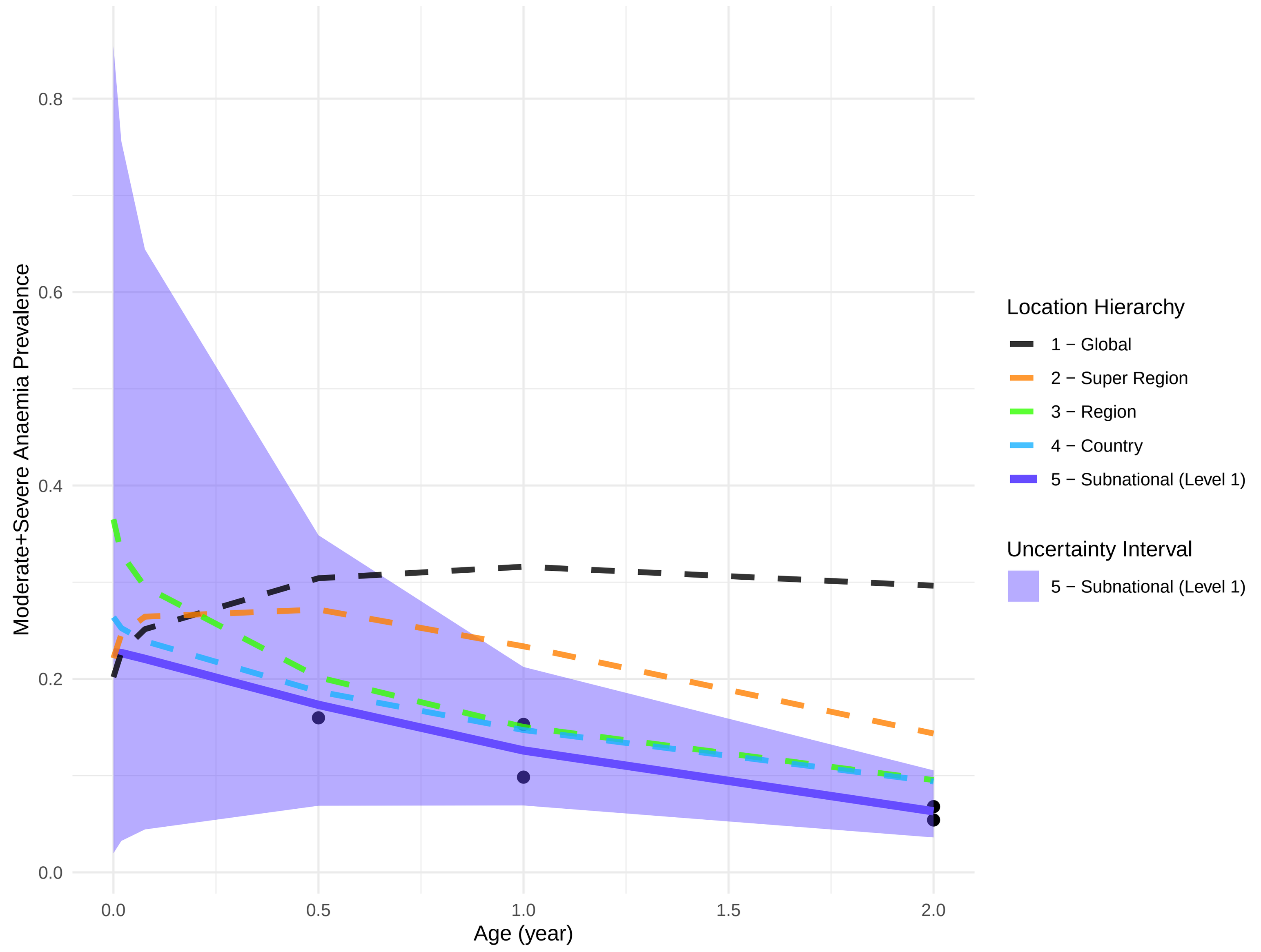

MR−BRT Age/Sex Fits − Non−pregnant Females <2 Years Old − Moderate+Severe Anaemia Prevalence − Puebla
WHO−2024 Adjustment Method

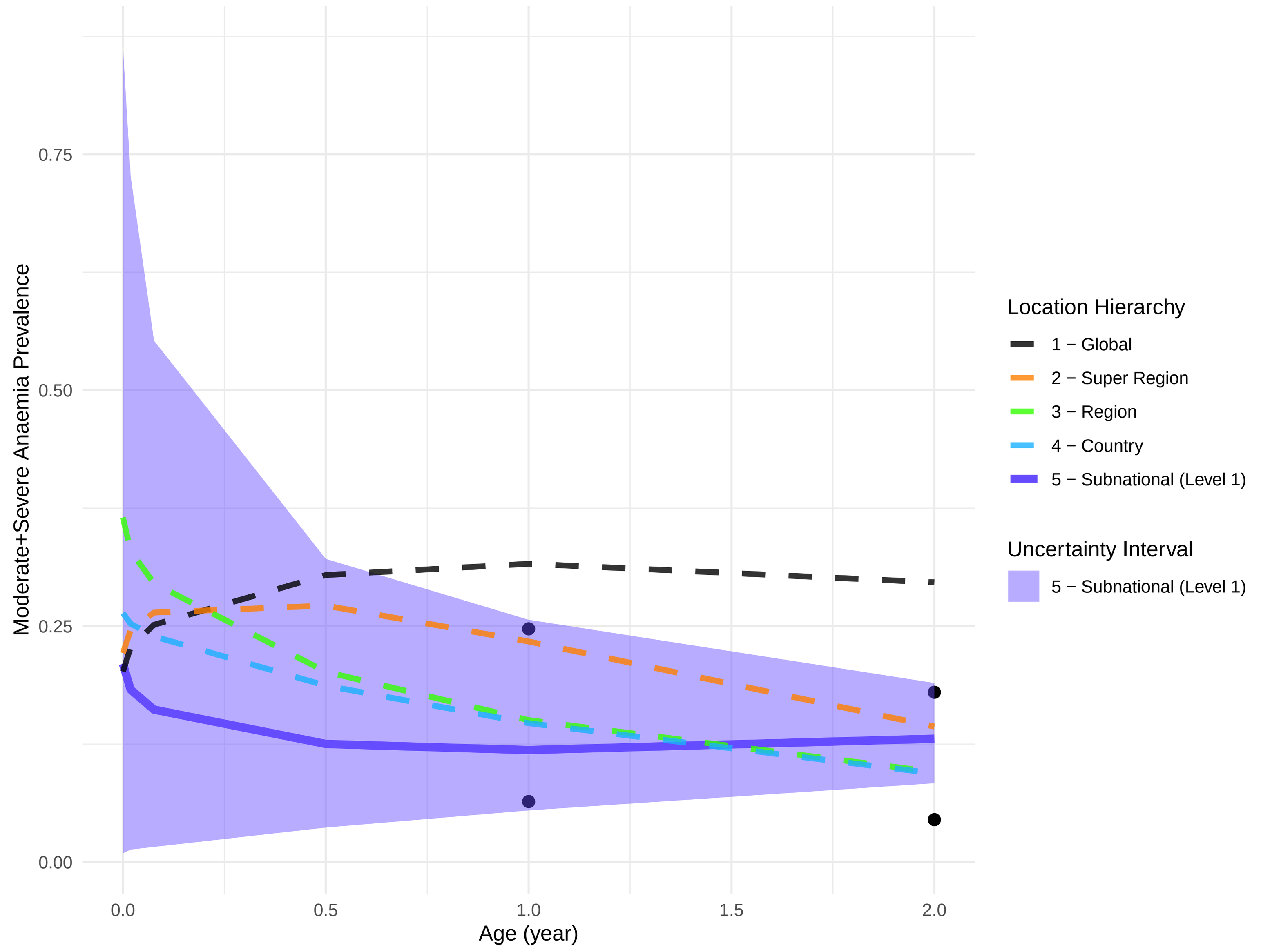

MR−BRT Age/Sex Fits − Non−pregnant Females <2 Years Old − Moderate+Severe Anaemia Prevalence − Querétaro
WHO−2024 Adjustment Method

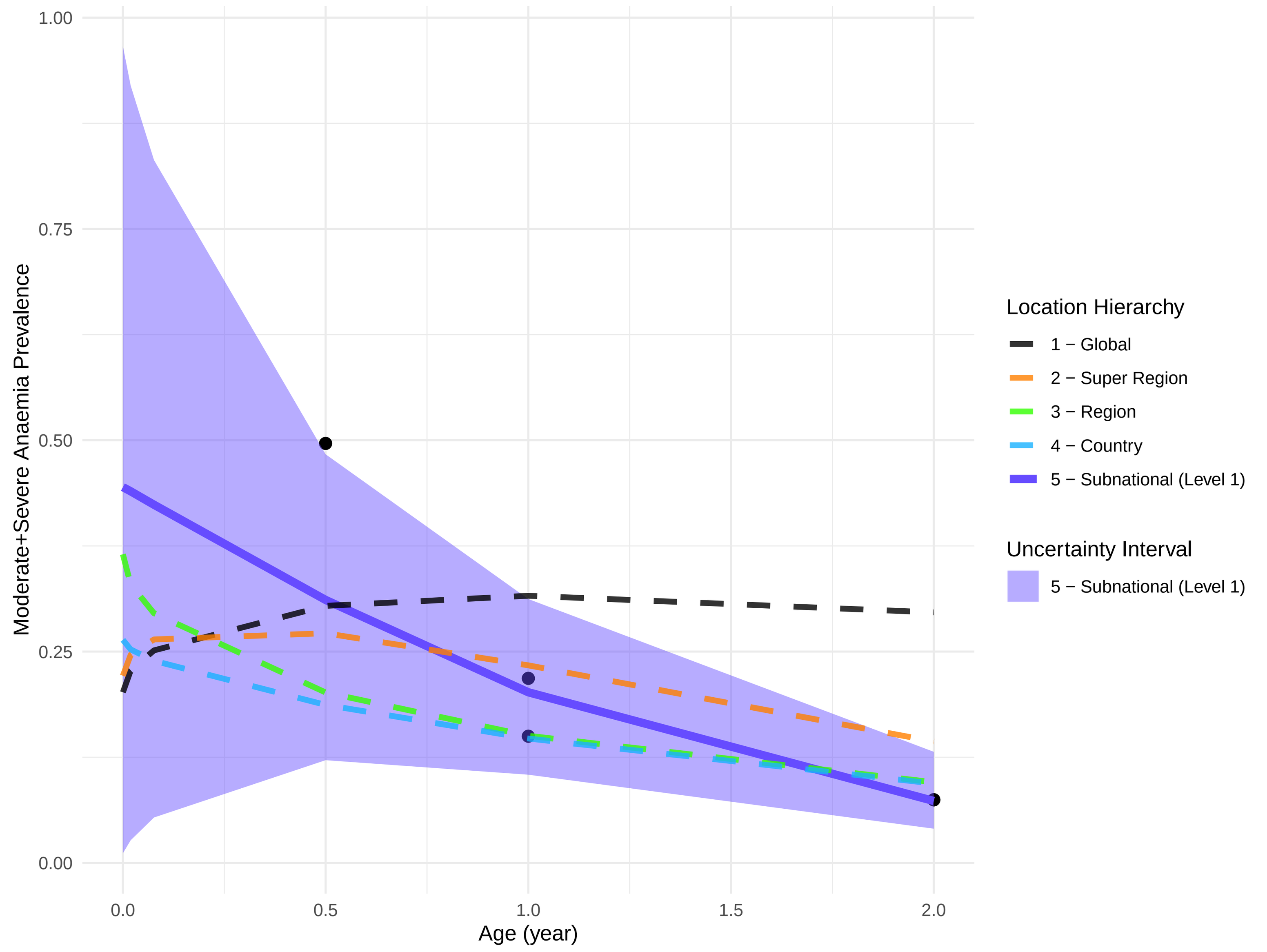

MR−BRT Age/Sex Fits − Non−pregnant Females <2 Years Old − Moderate+Severe Anaemia Prevalence − Quintana Roo
WHO−2024 Adjustment Method

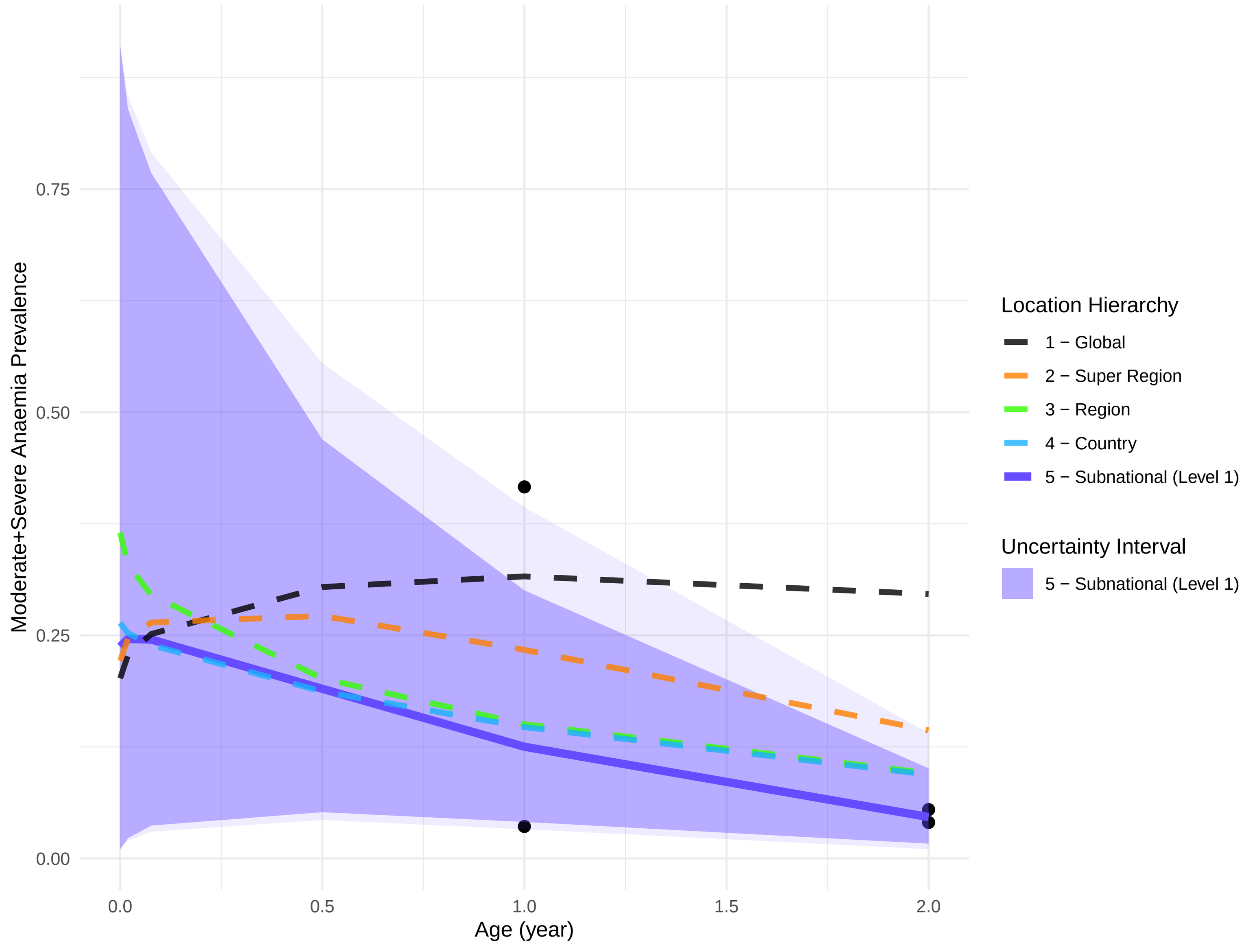

MR−BRT Age/Sex Fits − Non−pregnant Females <2 Years Old − Moderate+Severe Anaemia Prevalence − San Luis Potosí
WHO−2024 Adjustment Method

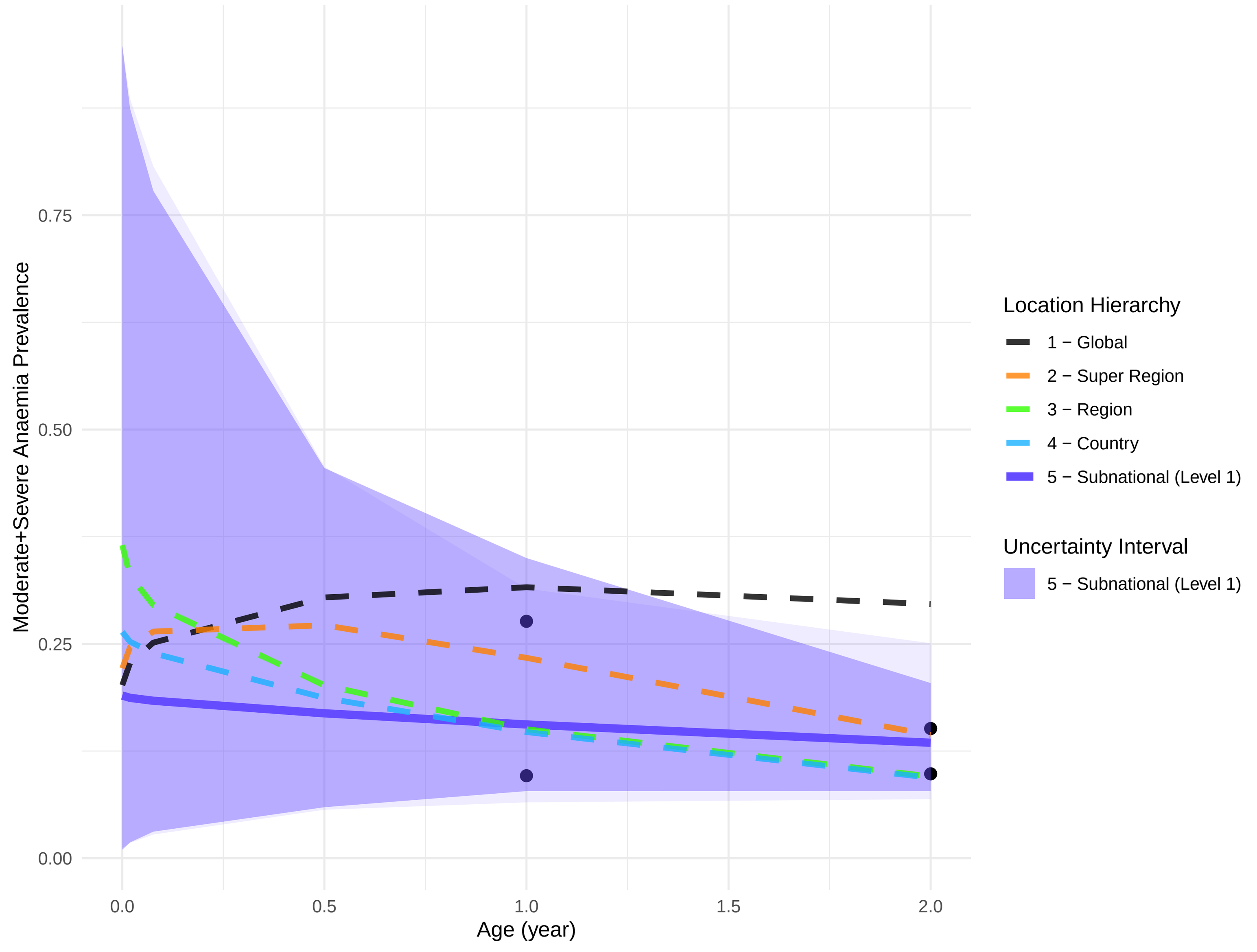

MR−BRT Age/Sex Fits − Non−pregnant Females <2 Years Old − Moderate+Severe Anaemia Prevalence − Sinaloa
WHO−2024 Adjustment Method

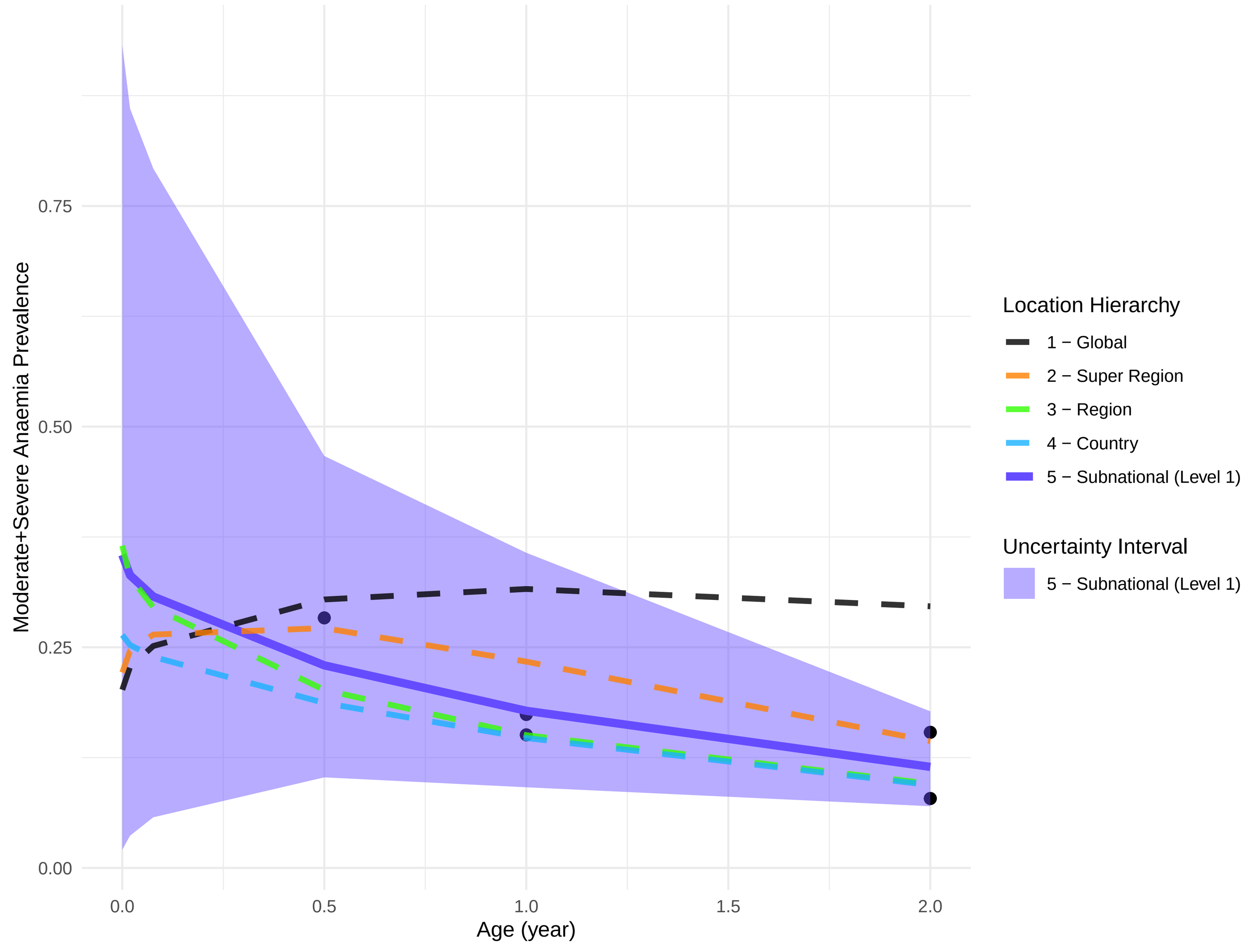

MR−BRT Age/Sex Fits − Non−pregnant Females <2 Years Old − Moderate+Severe Anaemia Prevalence − Sonora
WHO−2024 Adjustment Method

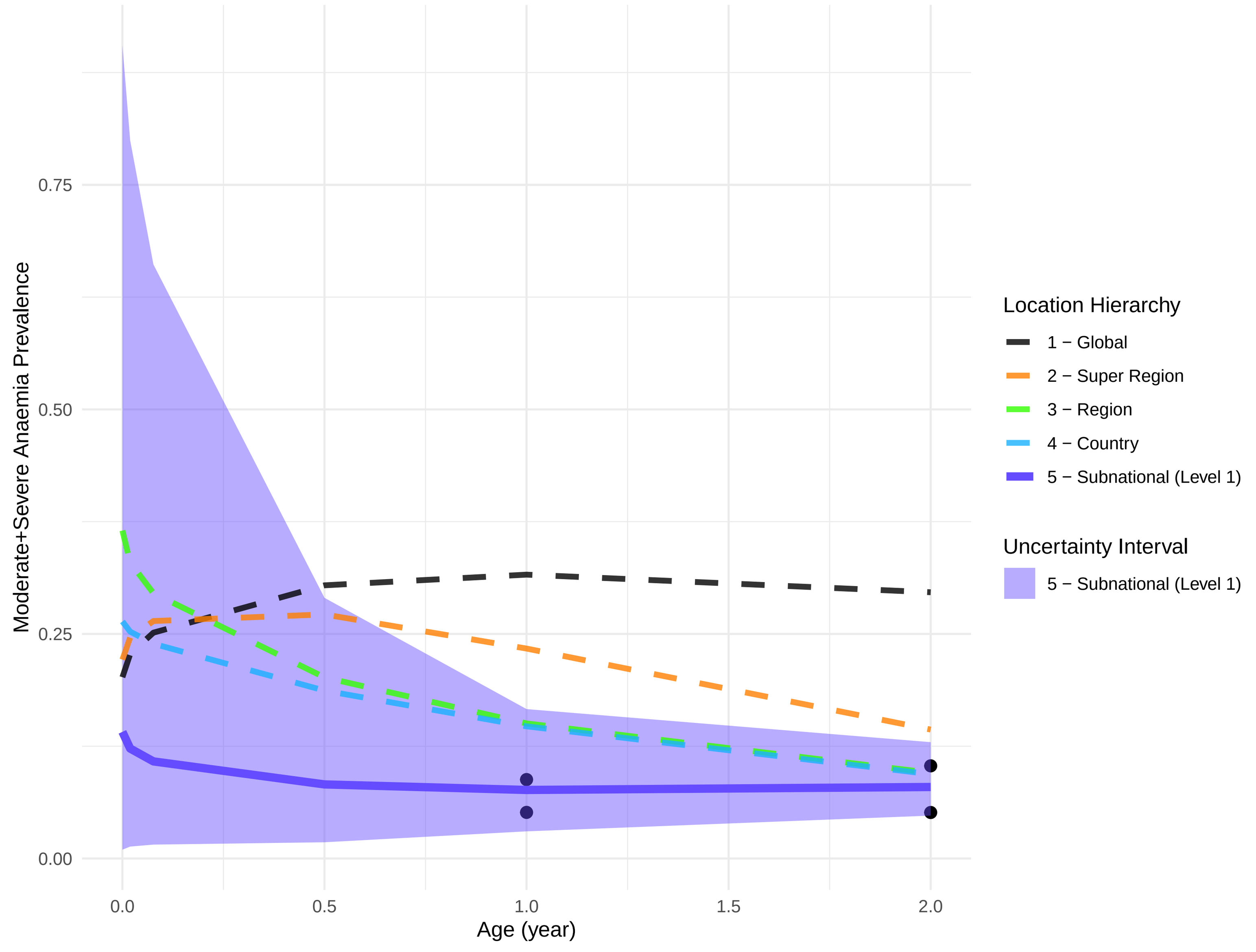

MR−BRT Age/Sex Fits − Non−pregnant Females <2 Years Old − Moderate+Severe Anaemia Prevalence − Tabasco
WHO−2024 Adjustment Method

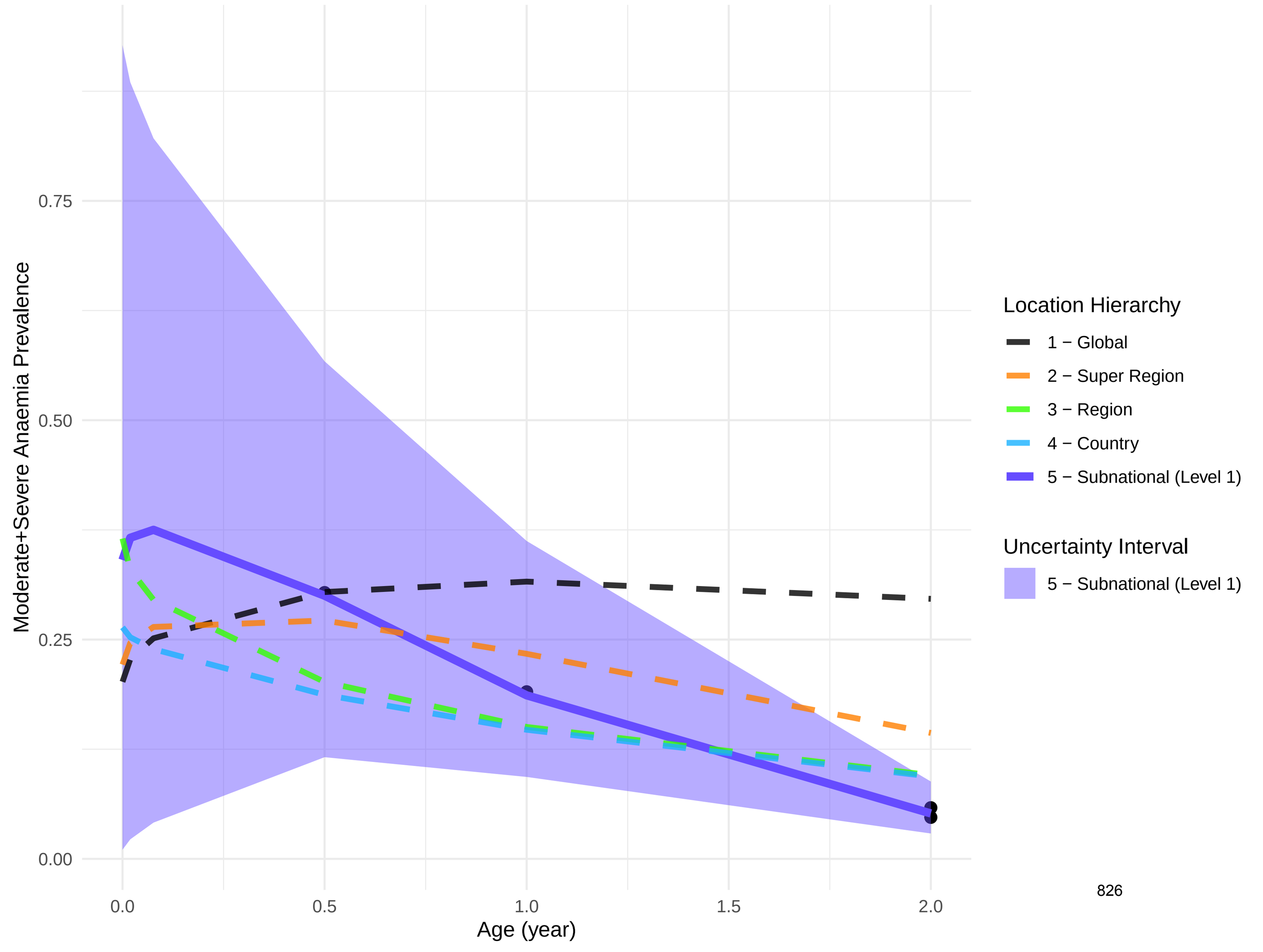

MR−BRT Age/Sex Fits − Non−pregnant Females <2 Years Old − Moderate+Severe Anaemia Prevalence − Tamaulipas
WHO−2024 Adjustment Method

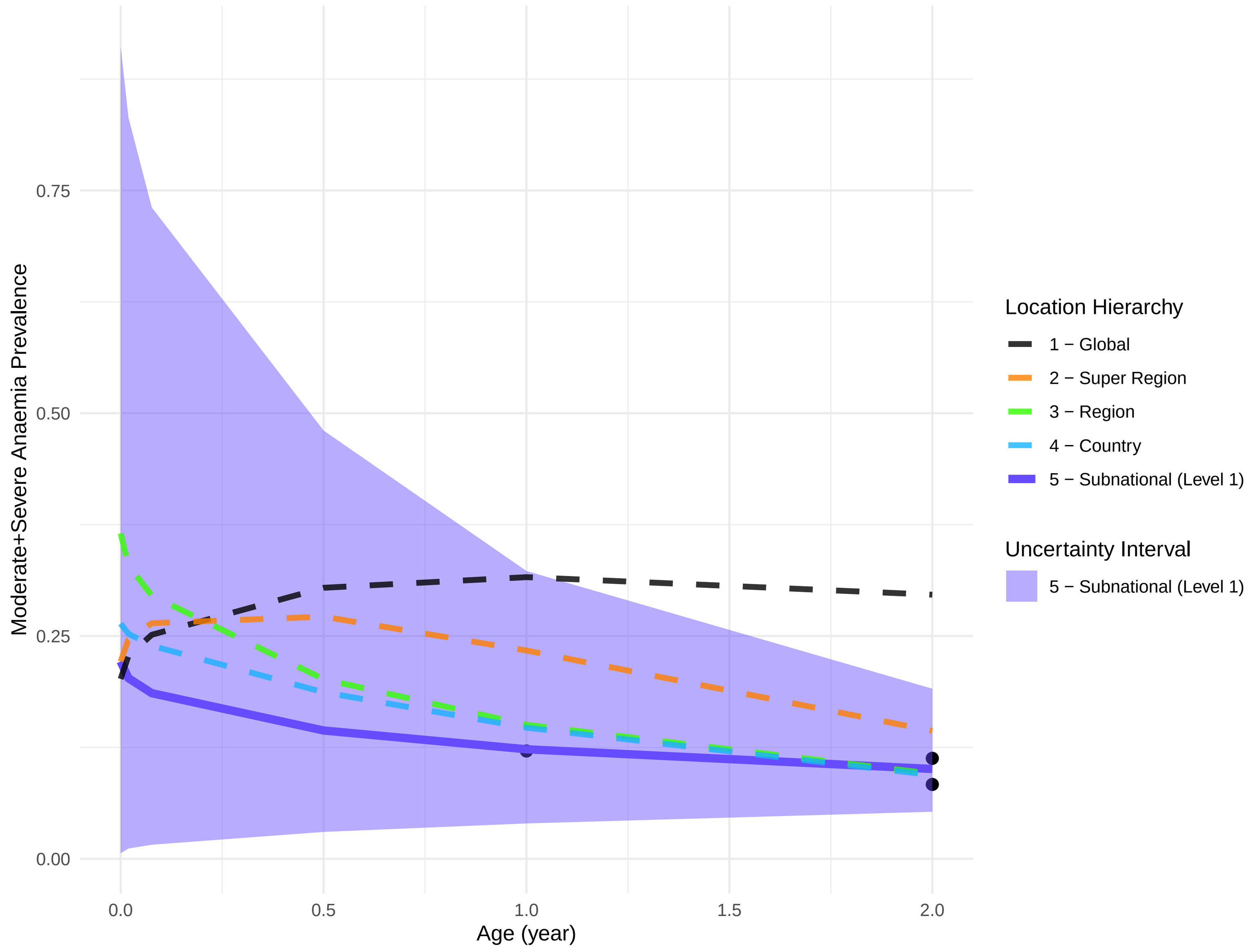

MR−BRT Age/Sex Fits − Non−pregnant Females <2 Years Old − Moderate+Severe Anaemia Prevalence − Tlaxcala
WHO−2024 Adjustment Method

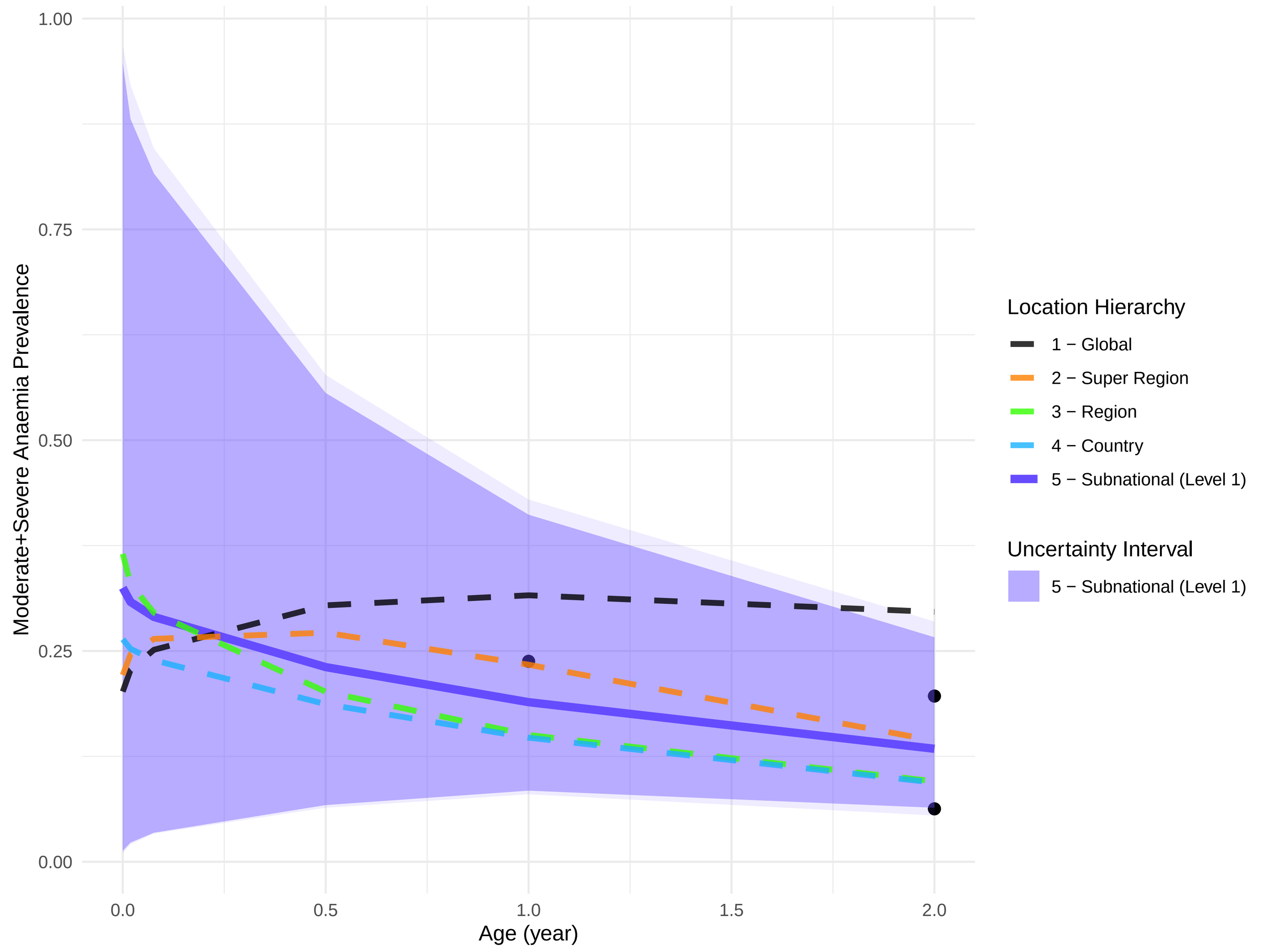

MR−BRT Age/Sex Fits − Non−pregnant Females <2 Years Old − Moderate+Severe Anaemia Prevalence − Veracruz de Ignacio de la Llave
WHO−2024 Adjustment Method

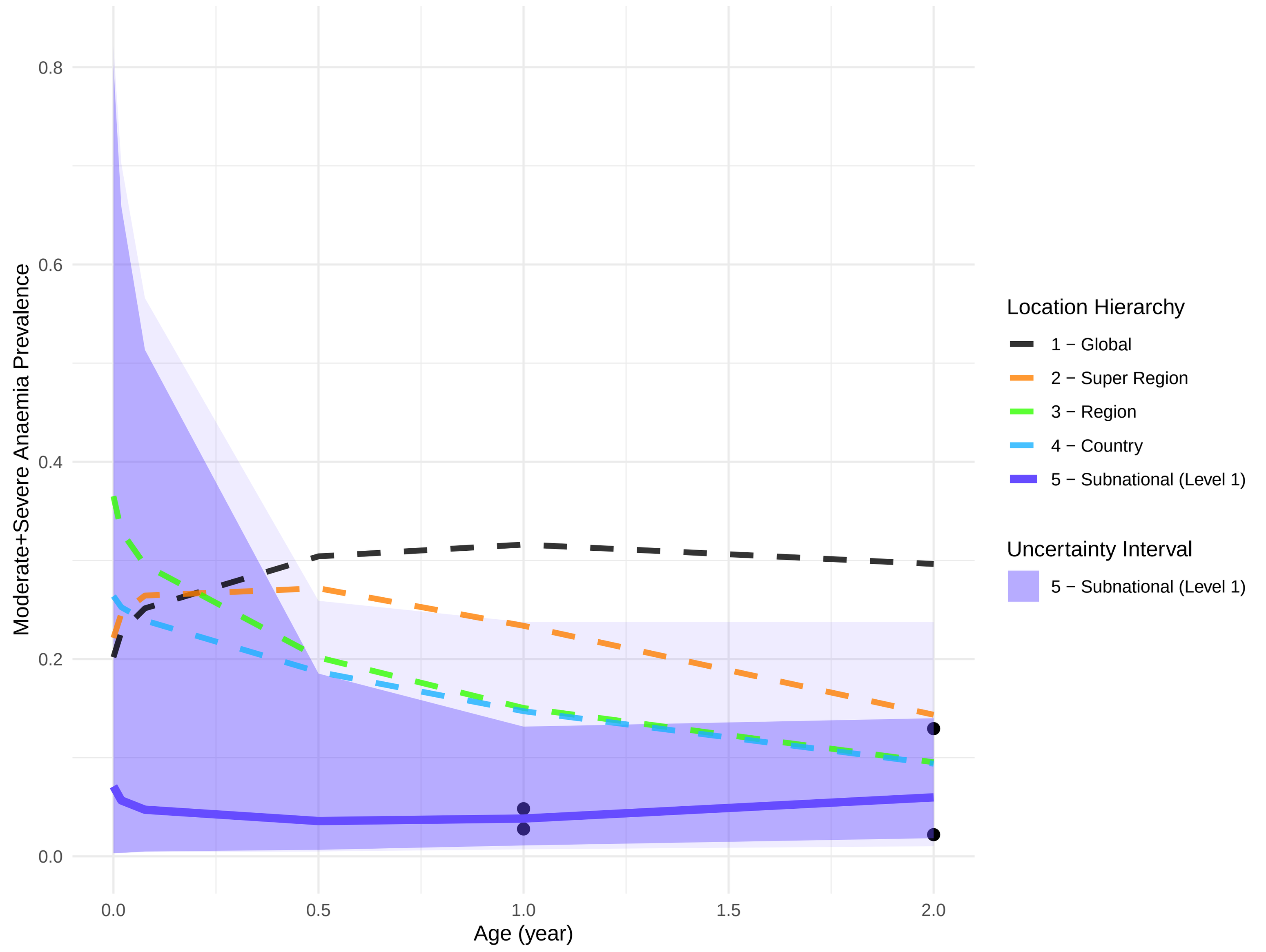

MR−BRT Age/Sex Fits − Non−pregnant Females <2 Years Old − Moderate+Severe Anaemia Prevalence − Yucatán
WHO−2024 Adjustment Method

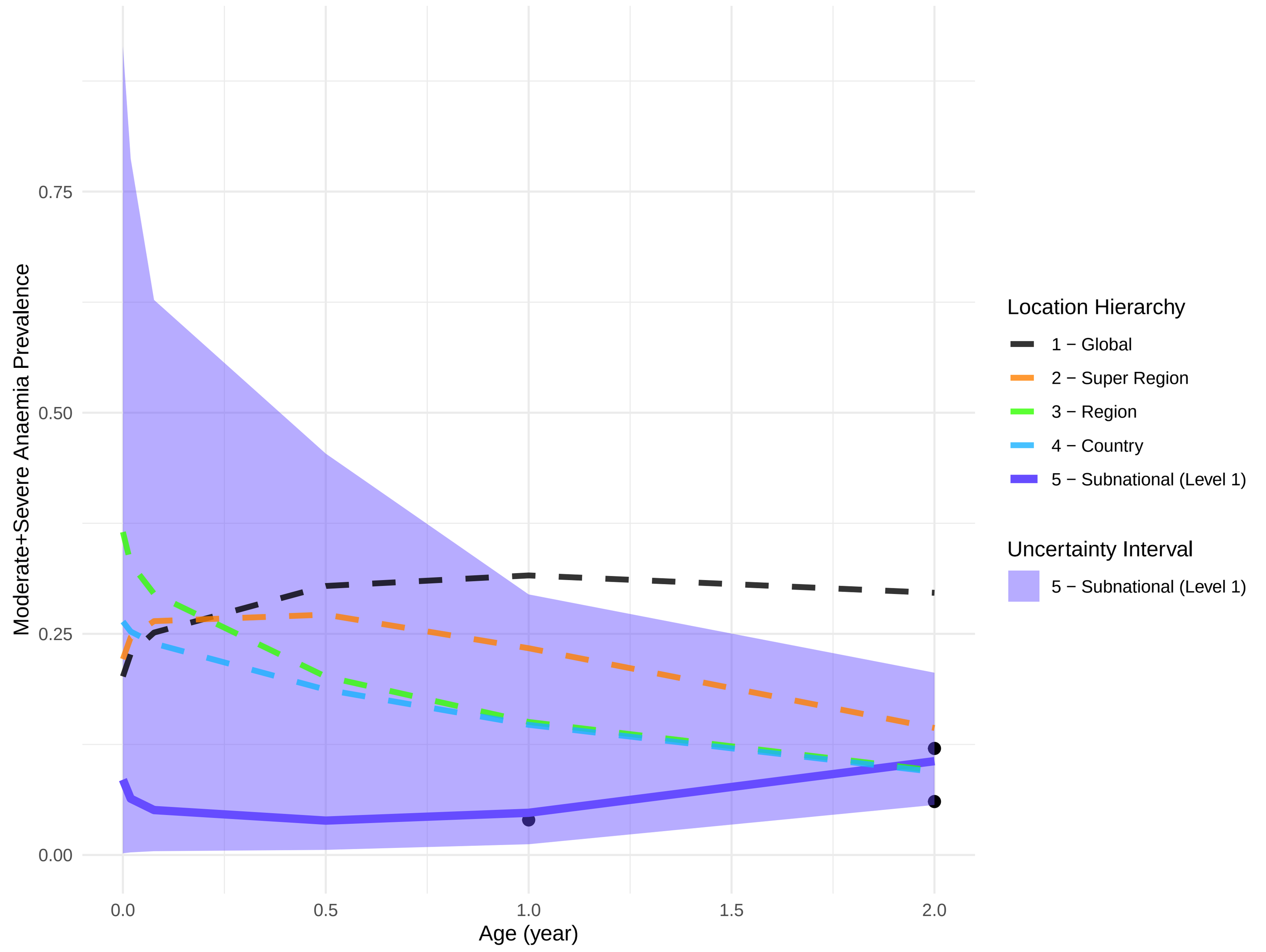

MR−BRT Age/Sex Fits − Non−pregnant Females <2 Years Old − Moderate+Severe Anaemia Prevalence − Zacatecas
WHO−2024 Adjustment Method

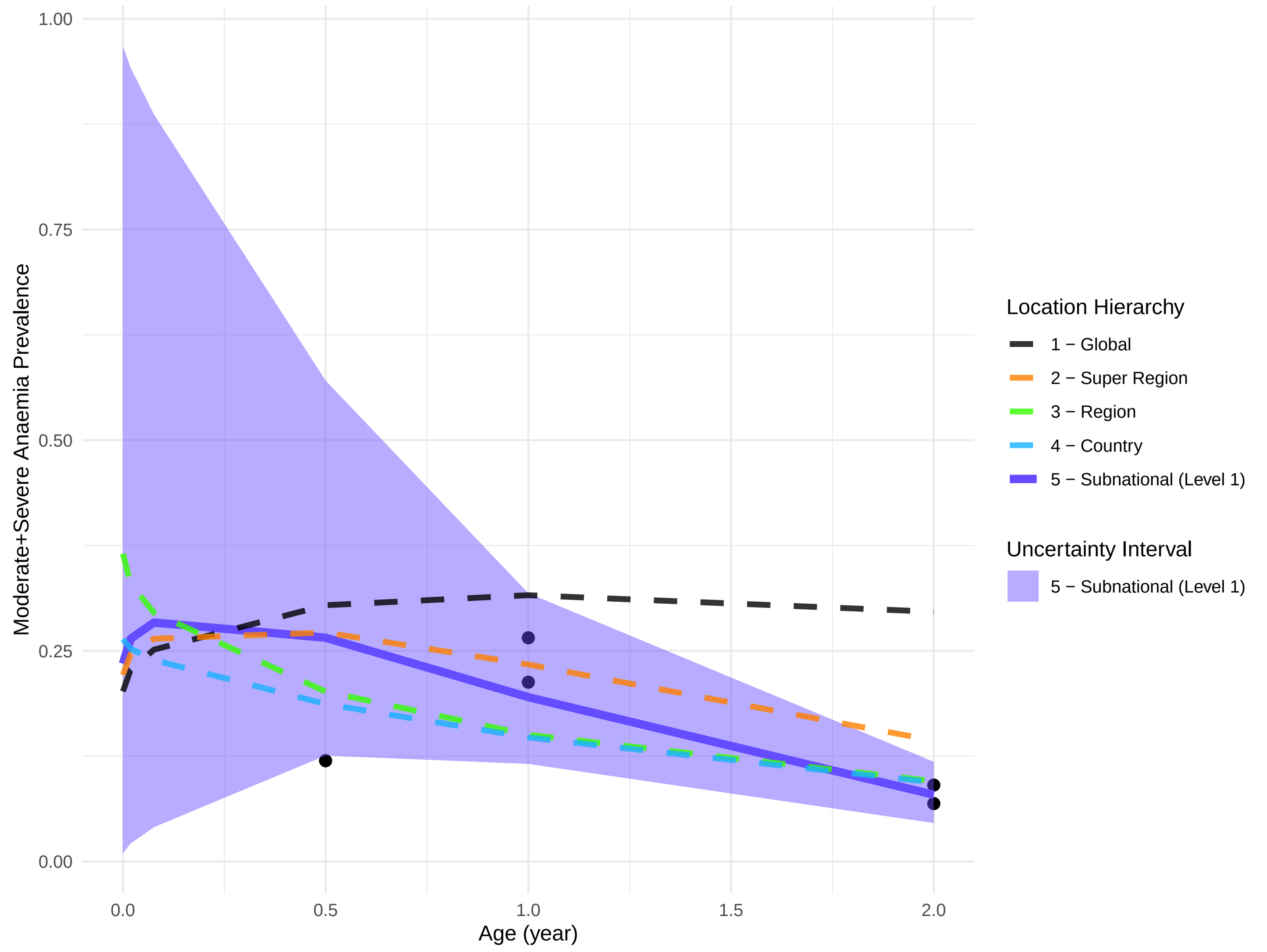

MR−BRT Age/Sex Fits − Non−pregnant Females <2 Years Old − Moderate+Severe Anaemia Prevalence − North Africa and Middle East
WHO−2024 Adjustment Method

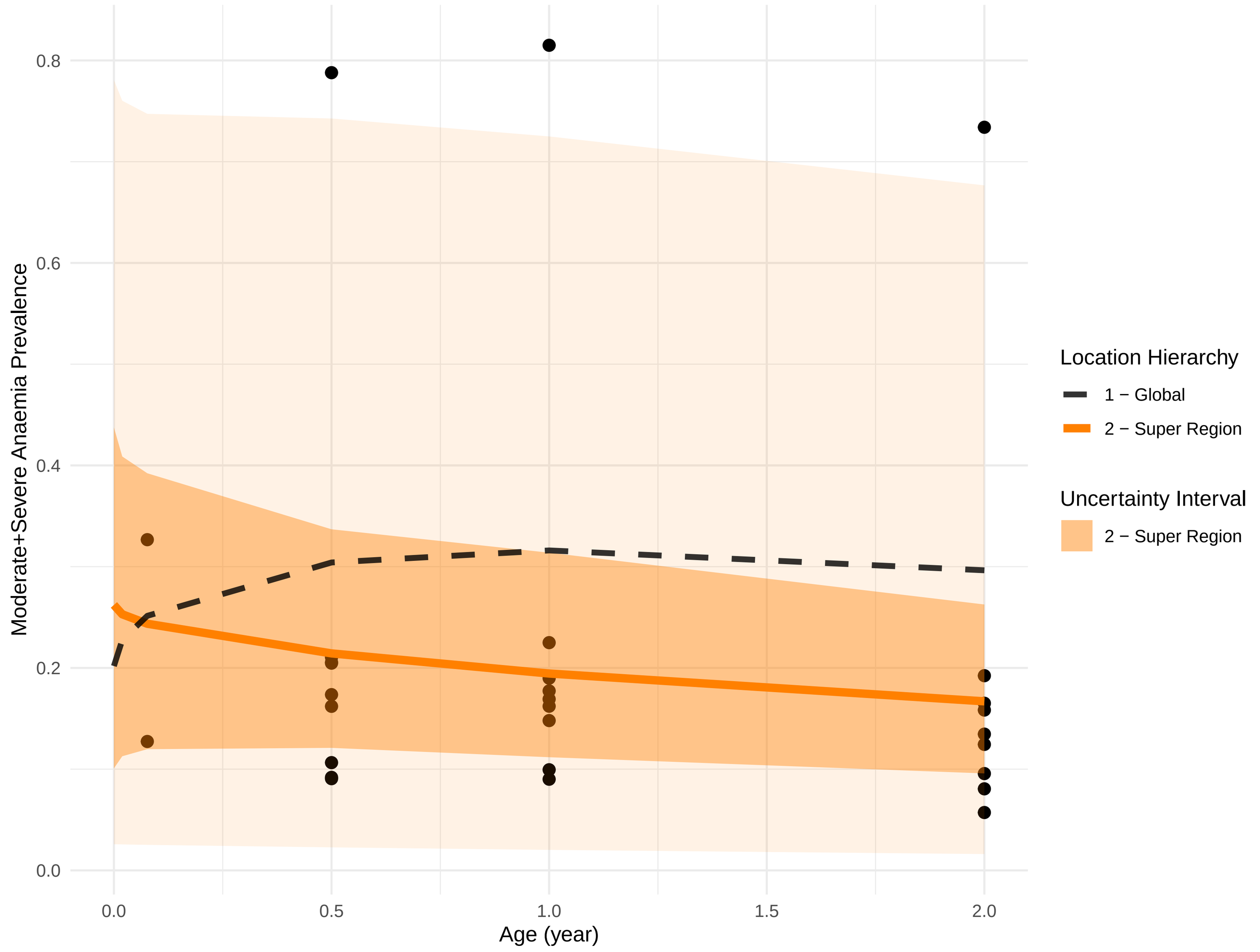

MR−BRT Age/Sex Fits − Non−pregnant Females <2 Years Old − Moderate+Severe Anaemia Prevalence − North Africa and Middle East
WHO−2024 Adjustment Method

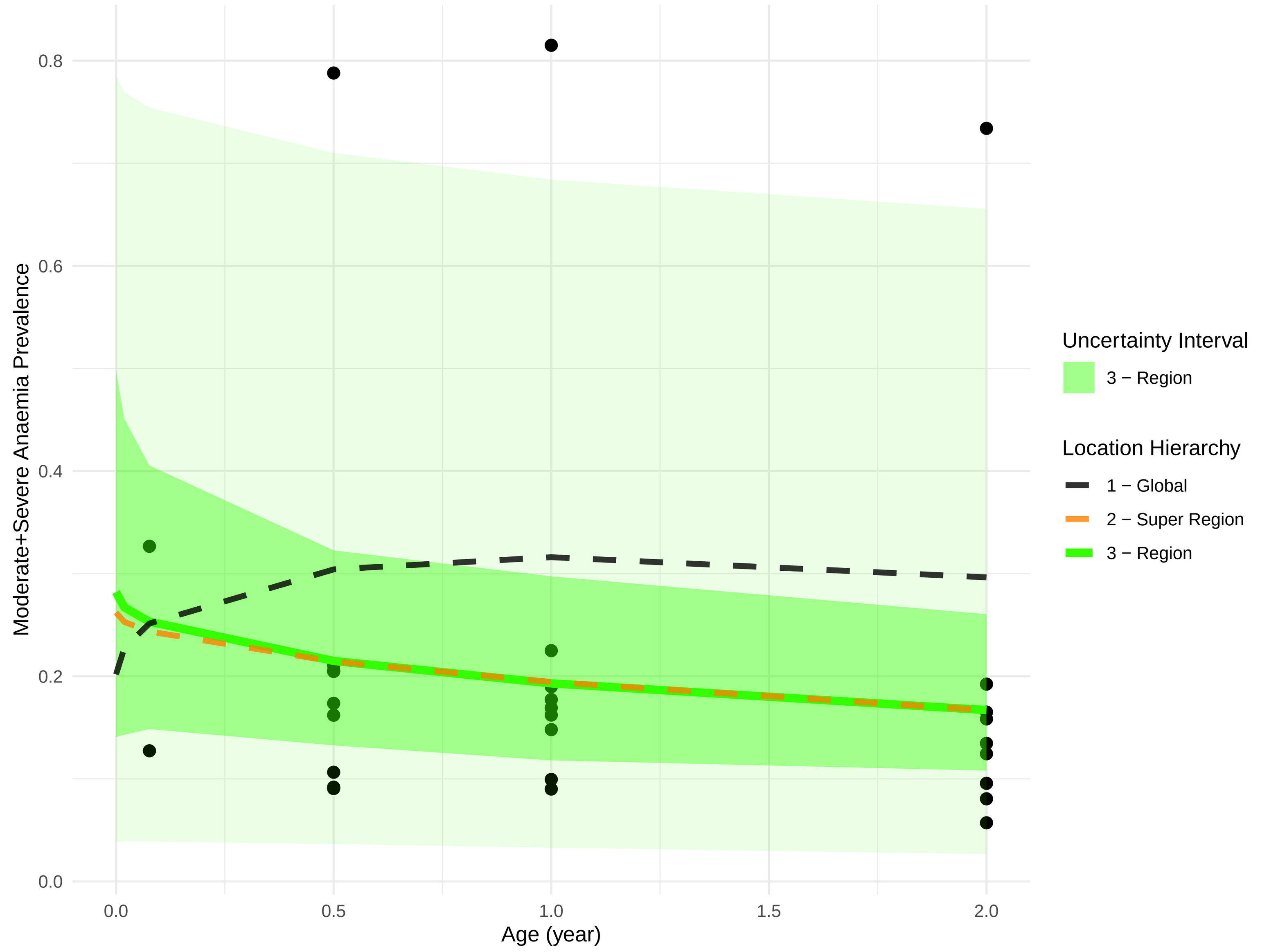

MR−BRT Age/Sex Fits − Non−pregnant Females <2 Years Old − Moderate+Severe Anaemia Prevalence − Egypt
WHO−2024 Adjustment Method

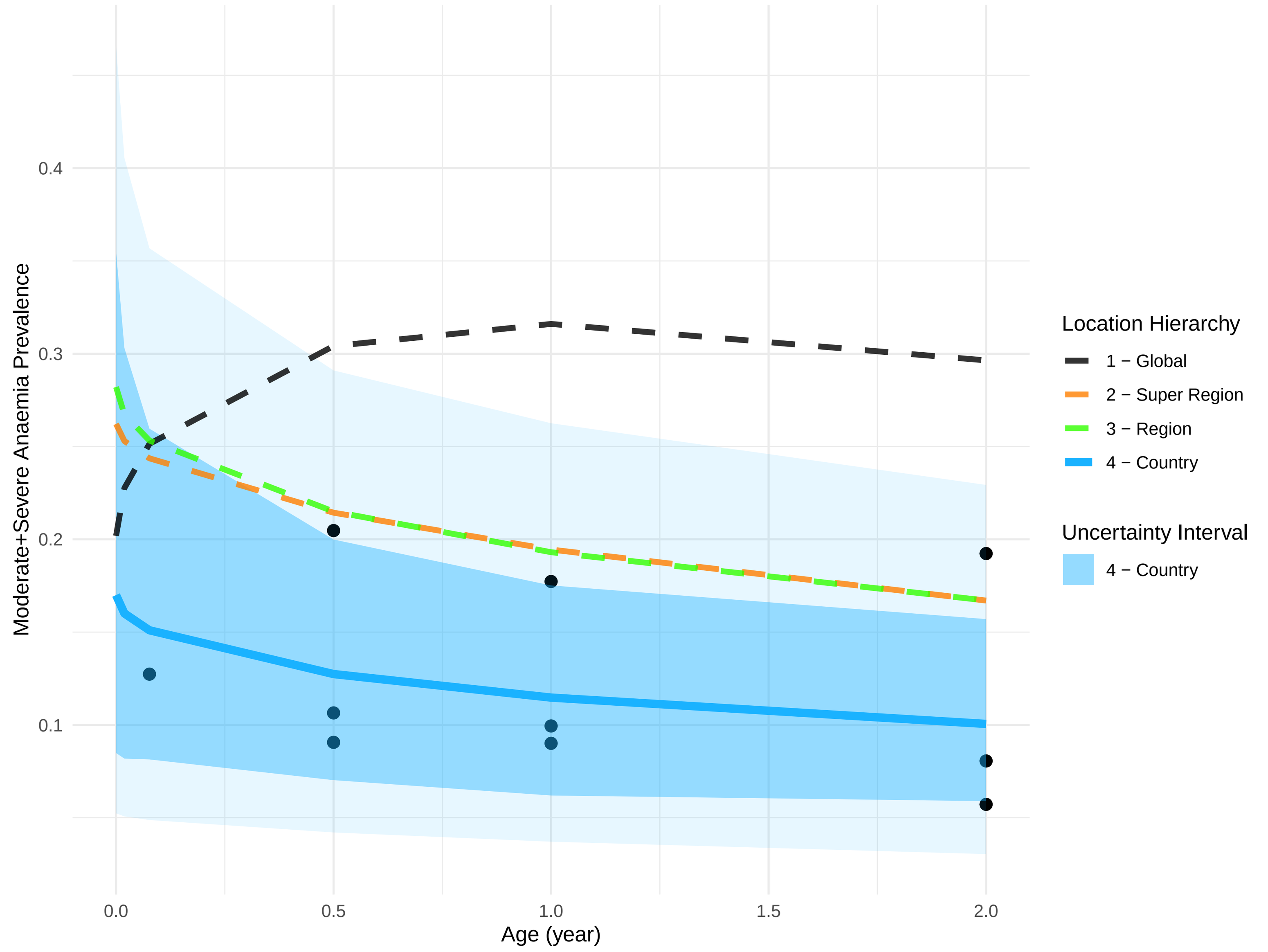

MR−BRT Age/Sex Fits − Non−pregnant Females <2 Years Old − Moderate+Severe Anaemia Prevalence − Jordan
WHO−2024 Adjustment Method

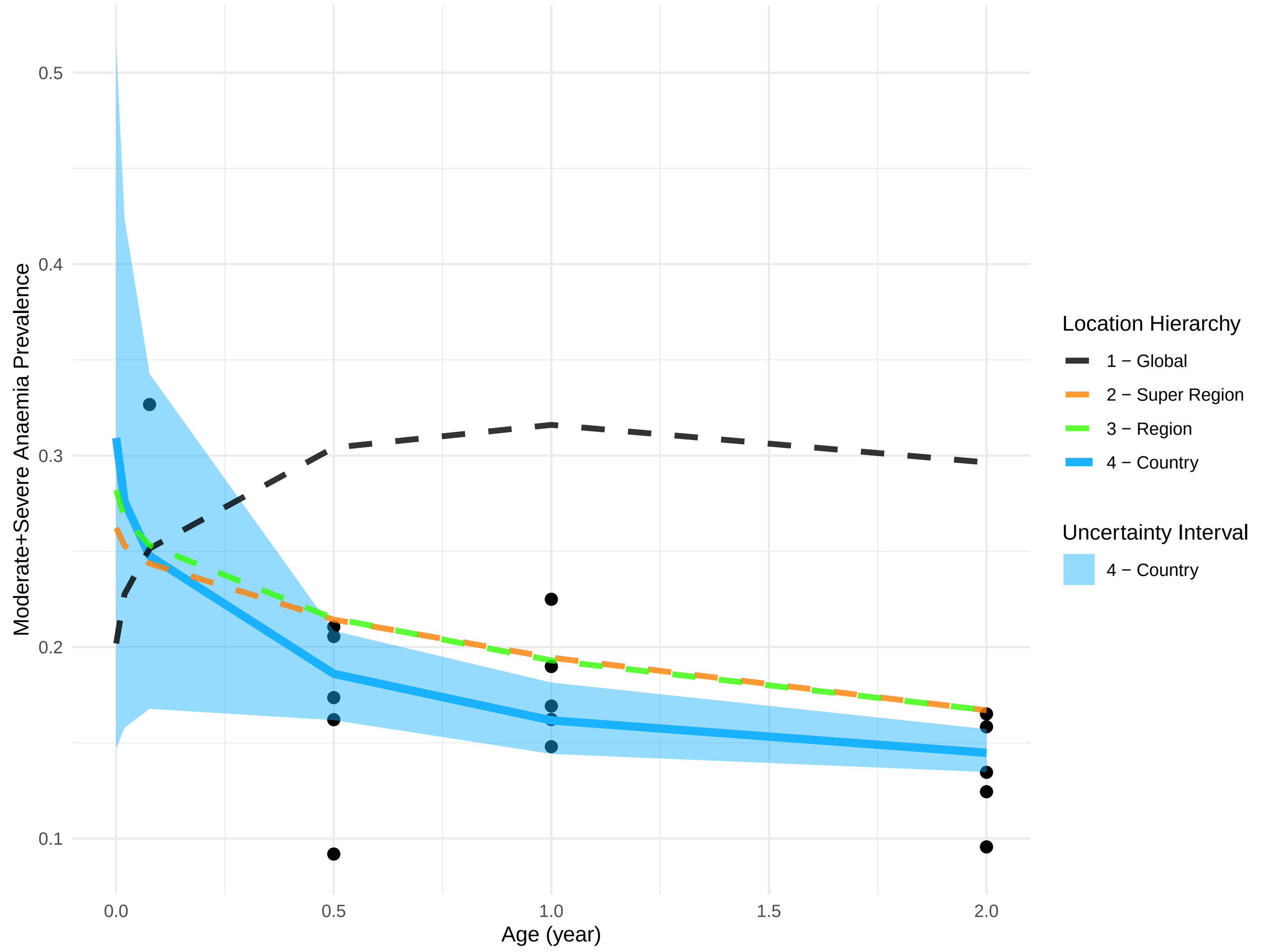

MR−BRT Age/Sex Fits − Non−pregnant Females <2 Years Old − Moderate+Severe Anaemia Prevalence − Yemen
WHO−2024 Adjustment Method

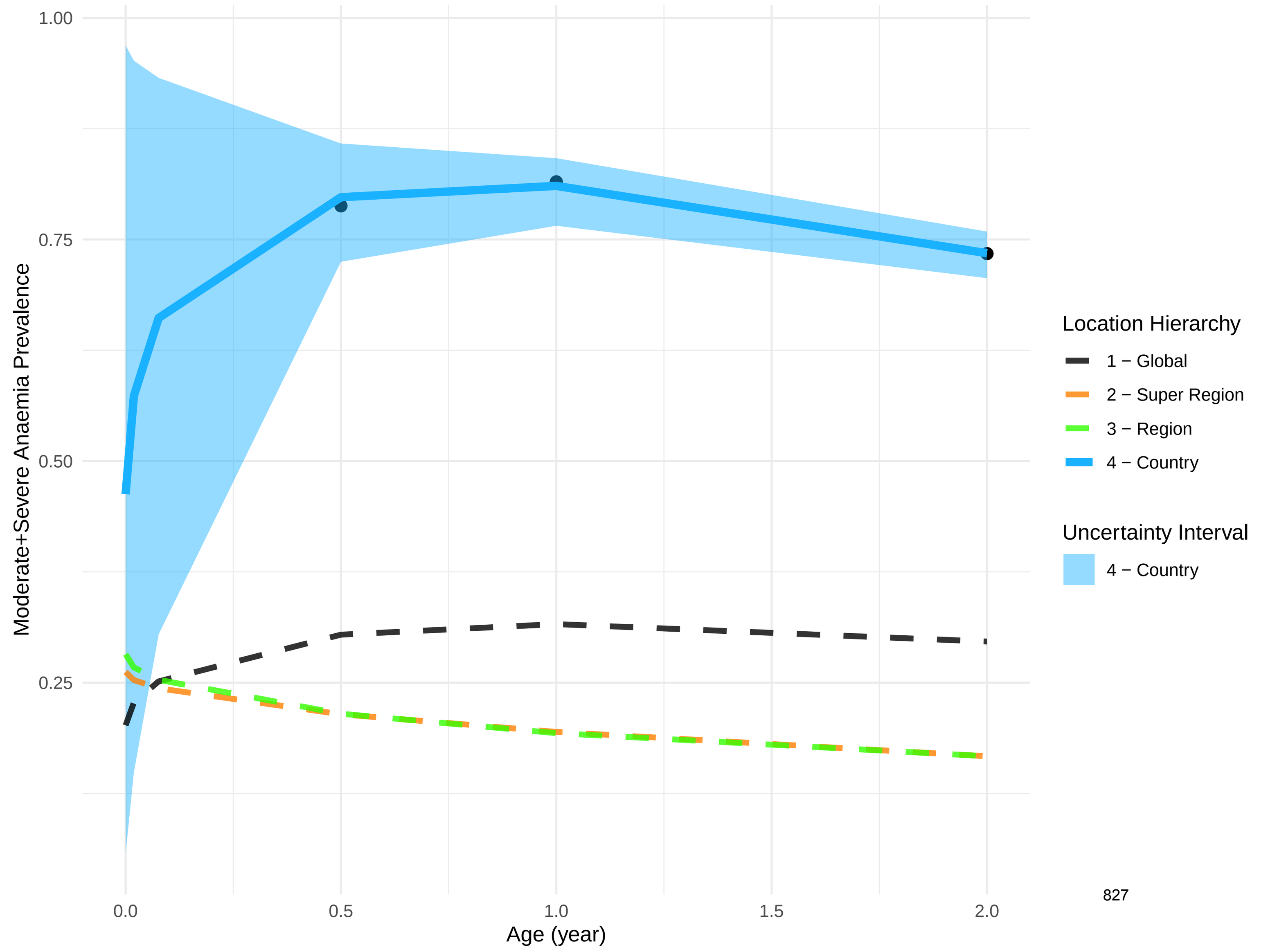

MR−BRT Age/Sex Fits − Non−pregnant Females <2 Years Old − Moderate+Severe Anaemia Prevalence − South Asia
WHO−2024 Adjustment Method

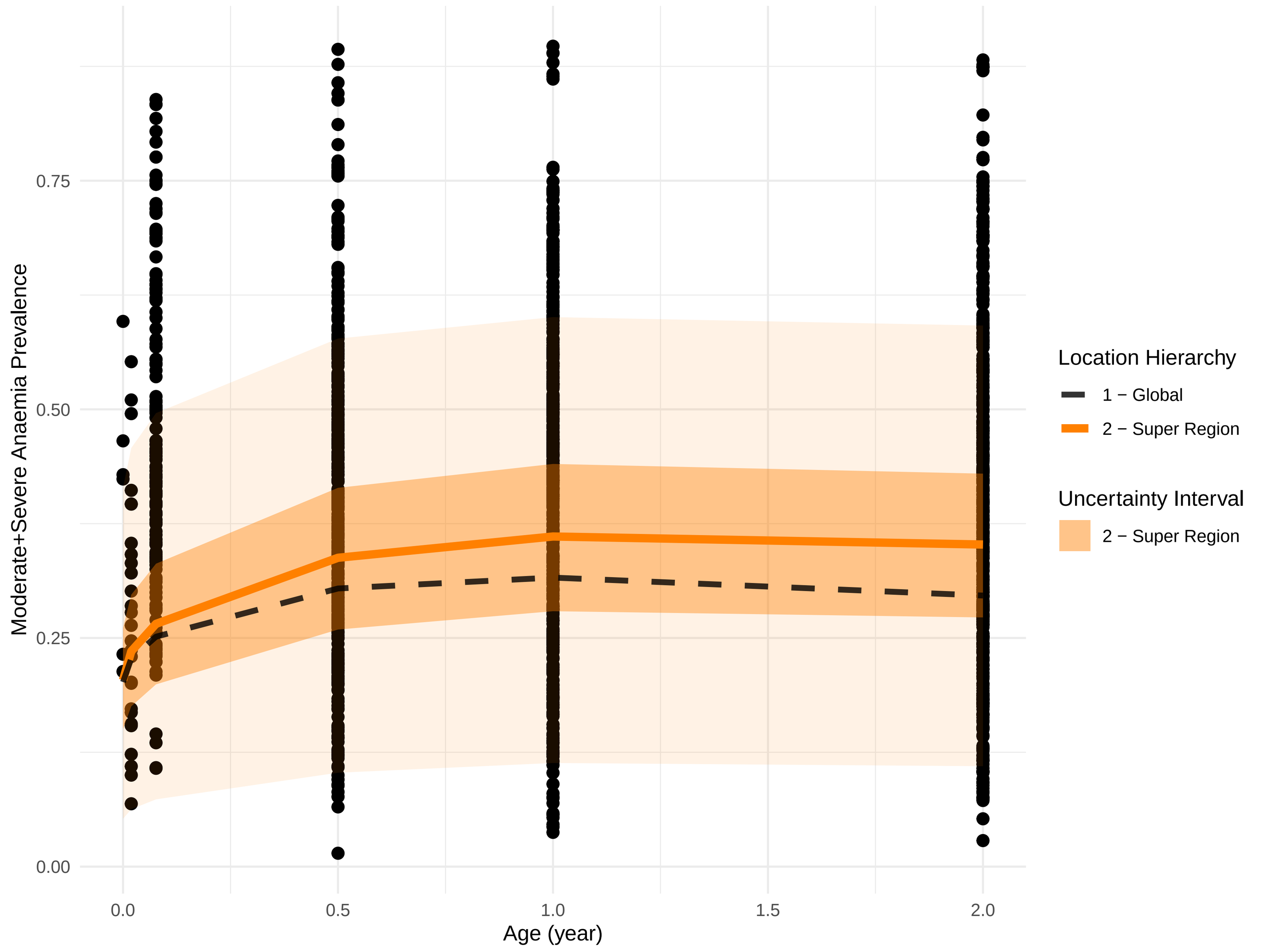

MR−BRT Age/Sex Fits − Non−pregnant Females <2 Years Old − Moderate+Severe Anaemia Prevalence − South Asia
WHO−2024 Adjustment Method

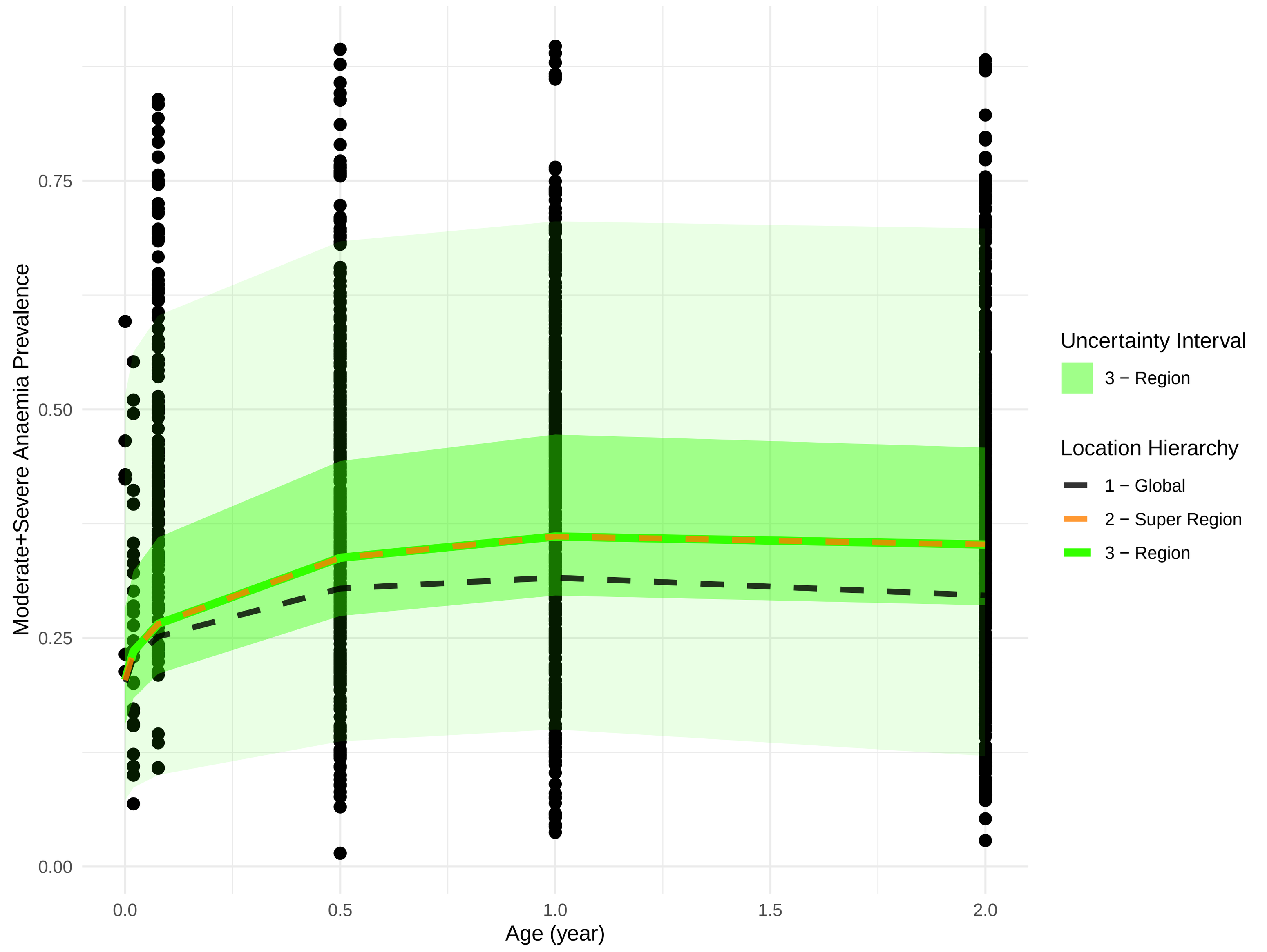

MR−BRT Age/Sex Fits − Non−pregnant Females <2 Years Old − Moderate+Severe Anaemia Prevalence − Bangladesh
WHO−2024 Adjustment Method

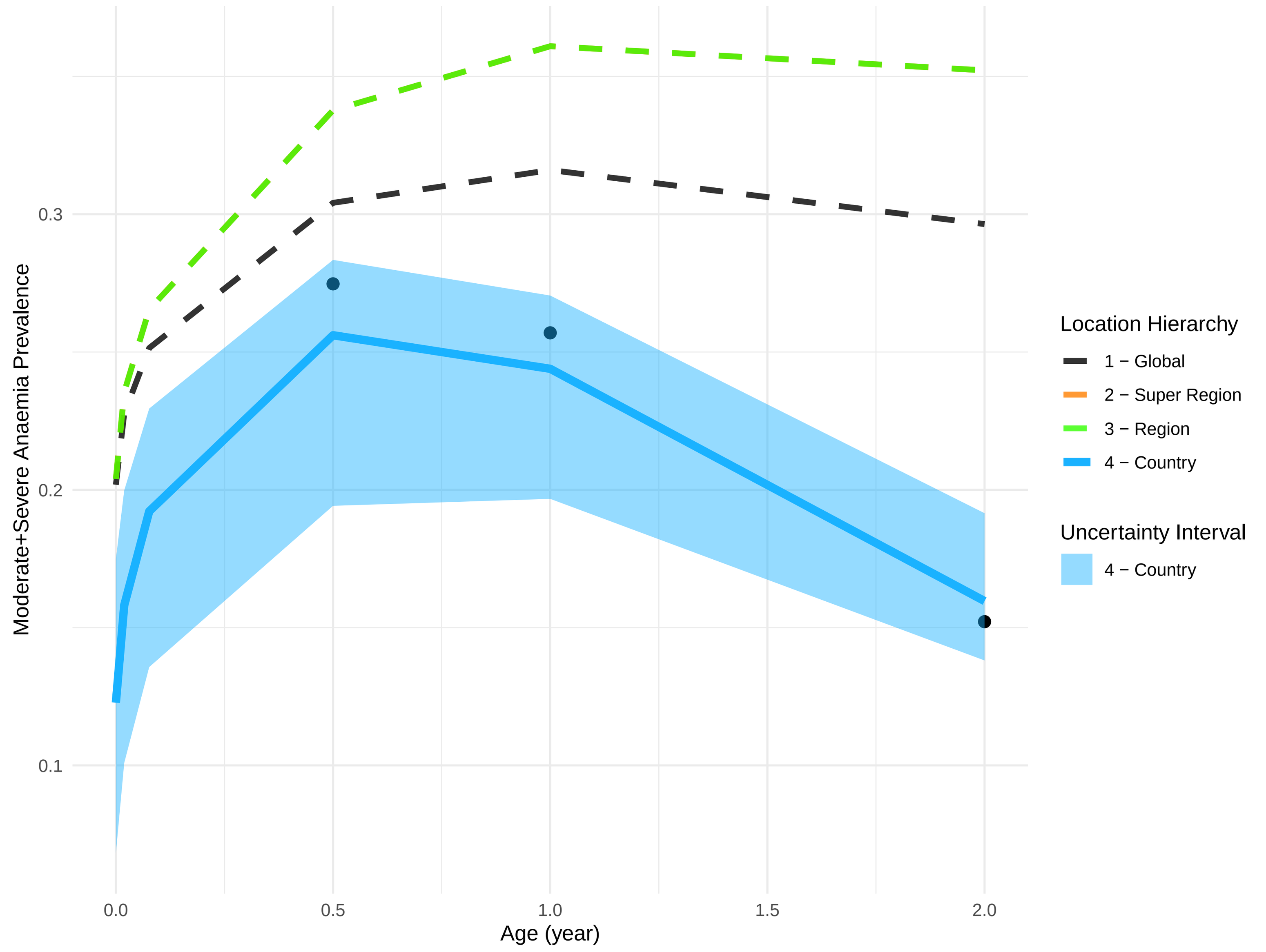

MR−BRT Age/Sex Fits − Non−pregnant Females <2 Years Old − Moderate+Severe Anaemia Prevalence − India
WHO−2024 Adjustment Method

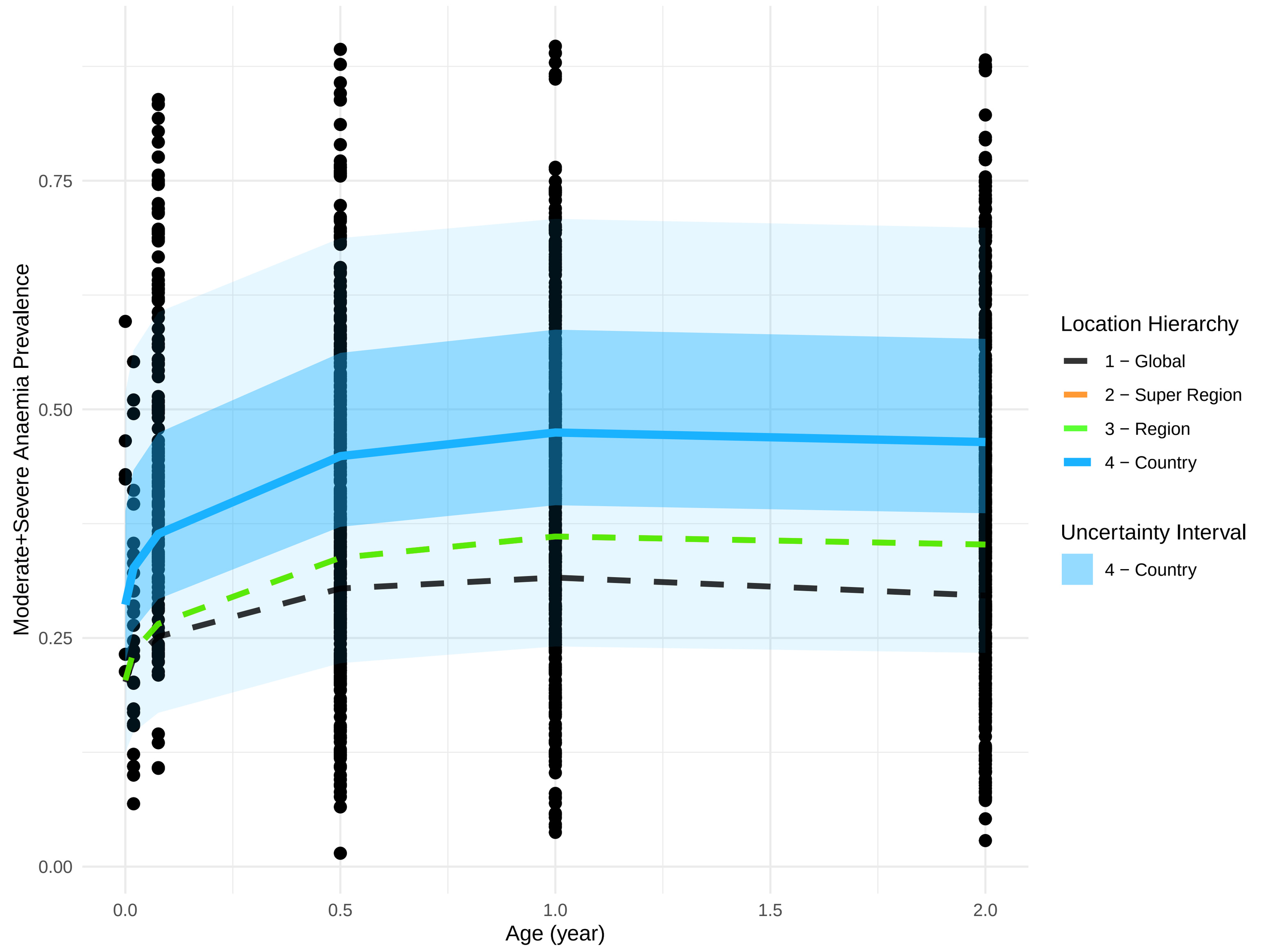

MR−BRT Age/Sex Fits − Non−pregnant Females <2 Years Old − Moderate+Severe Anaemia Prevalence − Andhra Pradesh
WHO−2024 Adjustment Method

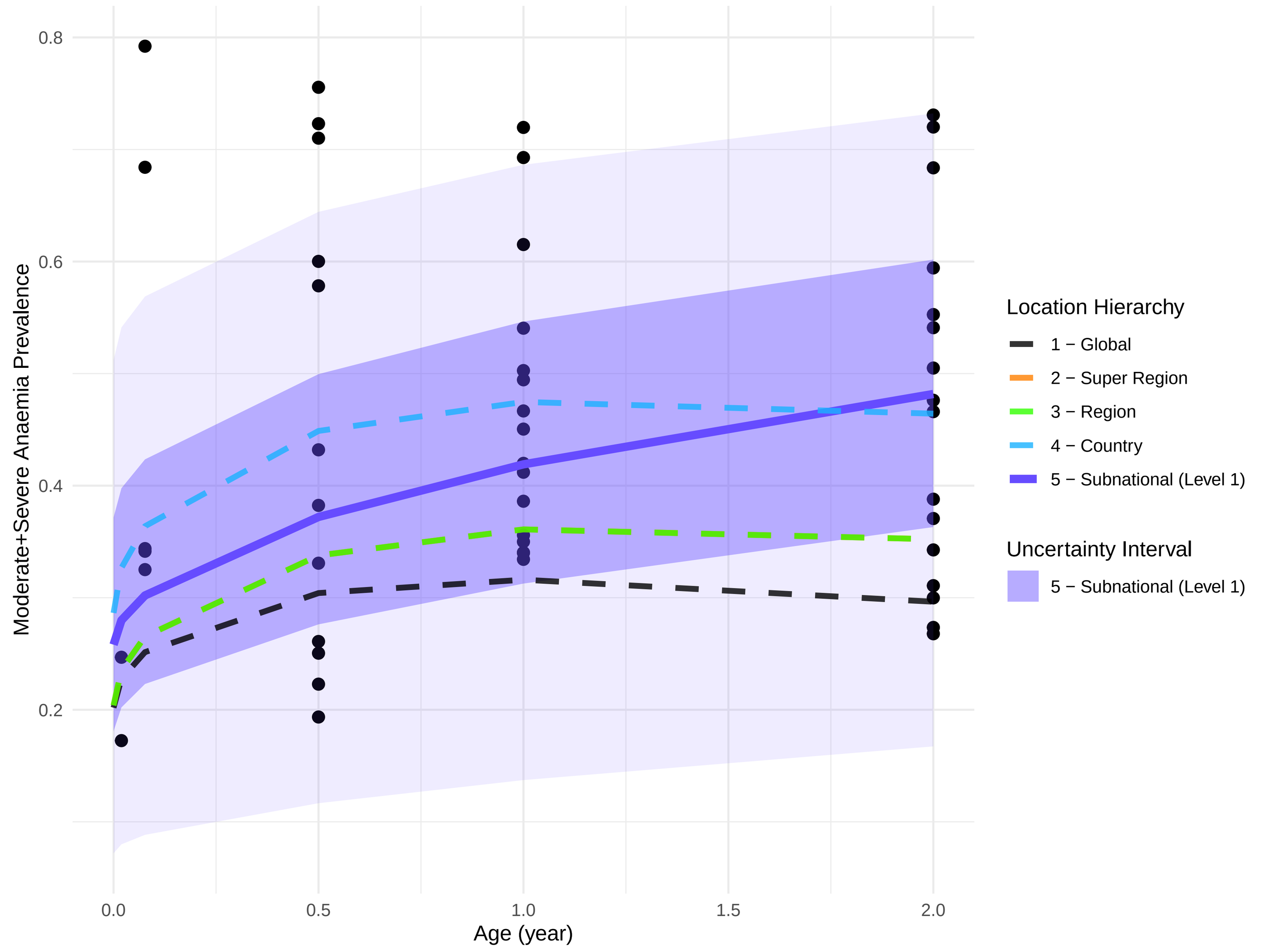

MR−BRT Age/Sex Fits − Non−pregnant Females <2 Years Old − Moderate+Severe Anaemia Prevalence − Andhra Pradesh, Rural
WHO−2024 Adjustment Method

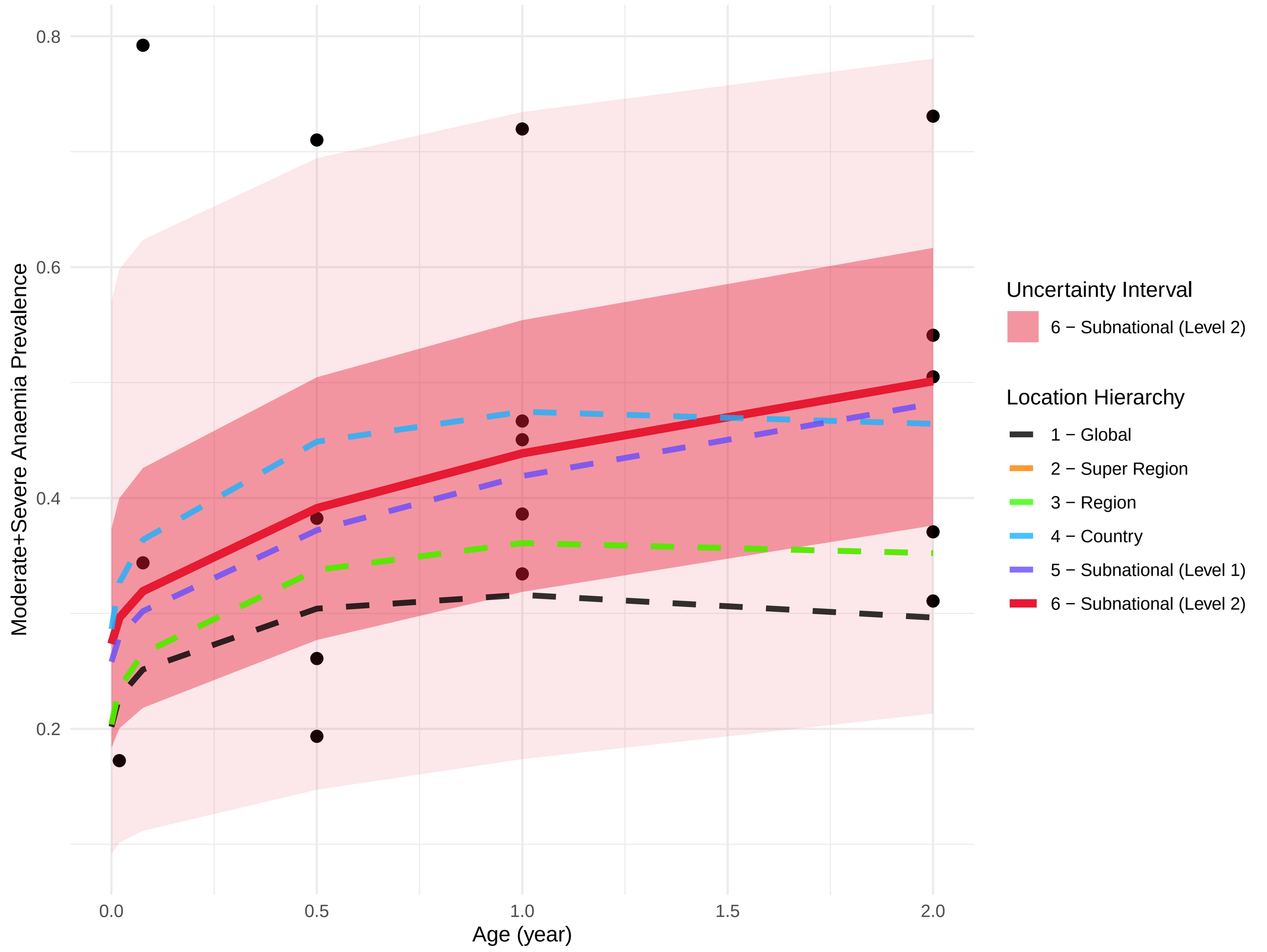

MR−BRT Age/Sex Fits − Non−pregnant Females <2 Years Old − Moderate+Severe Anaemia Prevalence − Andhra Pradesh, Urban
WHO−2024 Adjustment Method

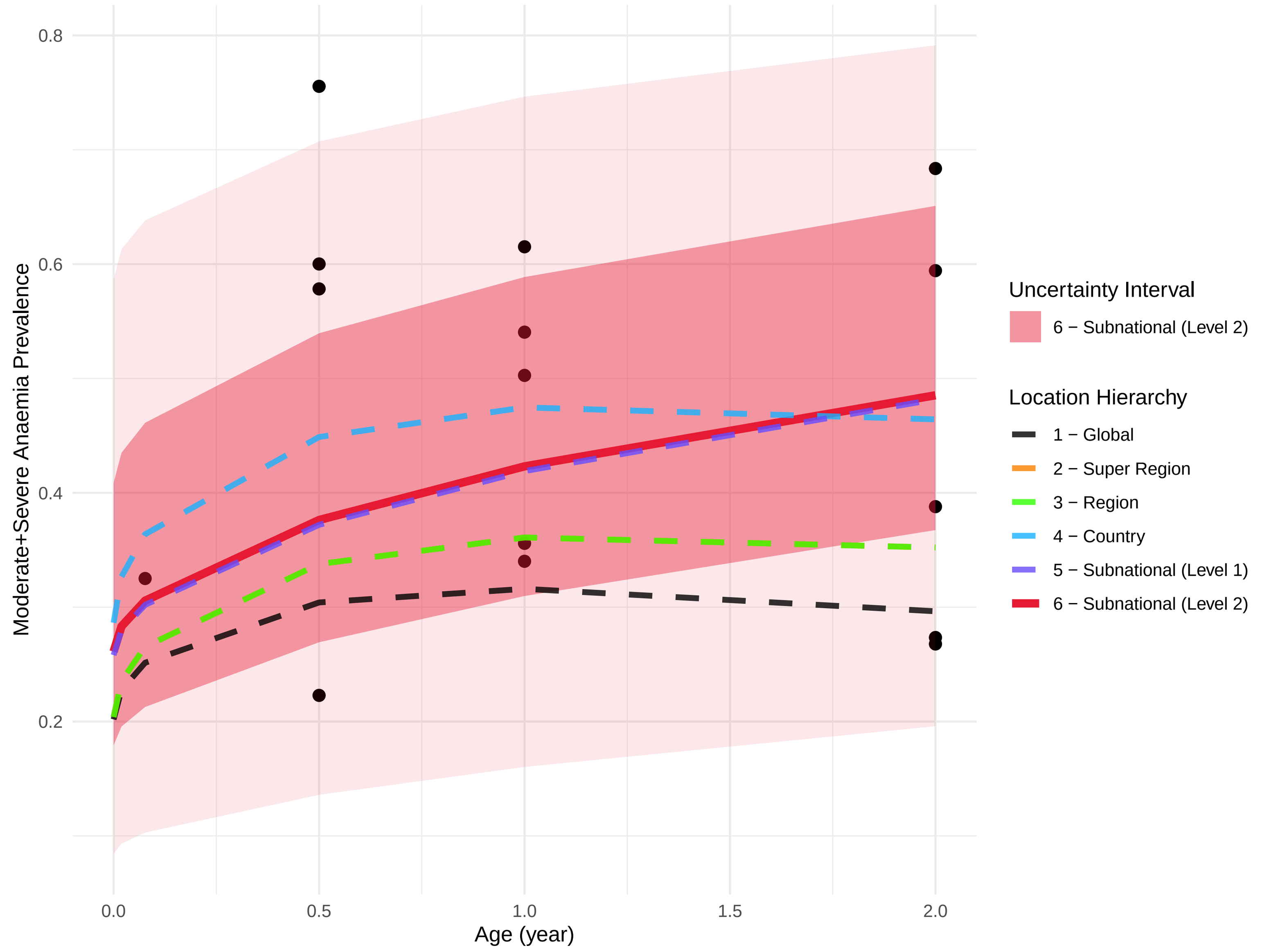

MR−BRT Age/Sex Fits − Non−pregnant Females <2 Years Old − Moderate+Severe Anaemia Prevalence − Arunachal Pradesh
WHO−2024 Adjustment Method

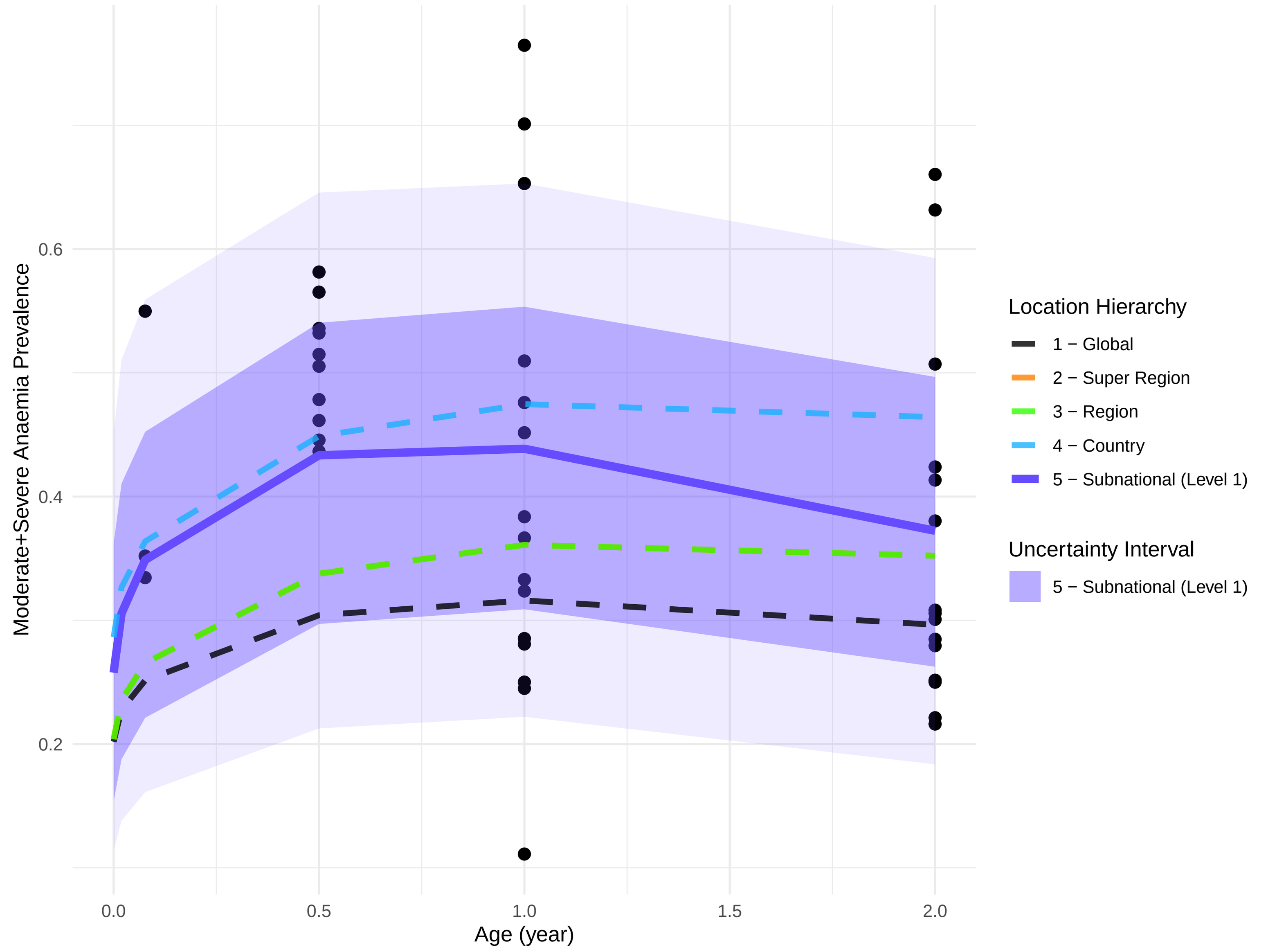

MR−BRT Age/Sex Fits − Non−pregnant Females <2 Years Old − Moderate+Severe Anaemia Prevalence − Arunachal Pradesh, Rural
WHO−2024 Adjustment Method

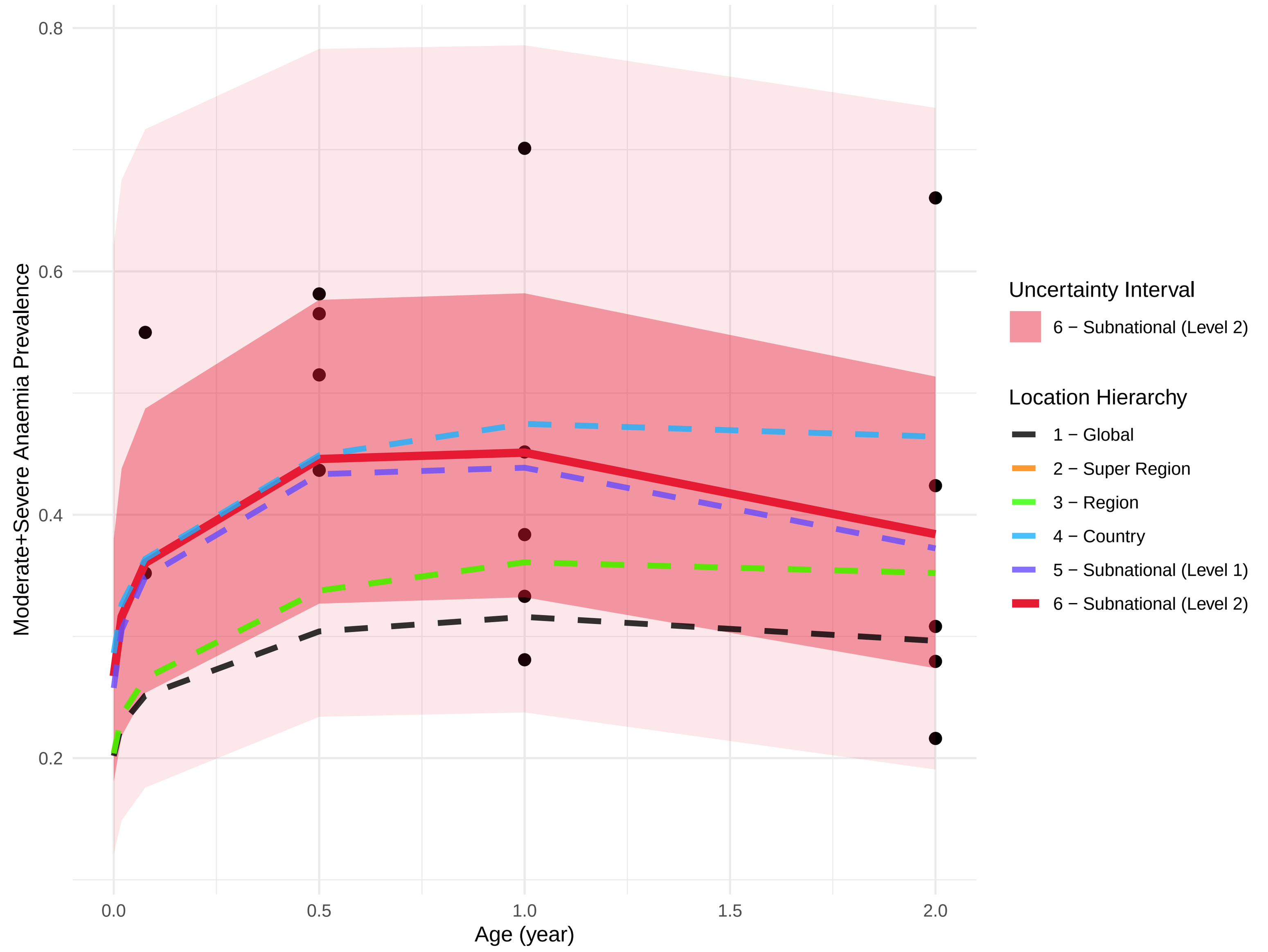

MR−BRT Age/Sex Fits − Non−pregnant Females <2 Years Old − Moderate+Severe Anaemia Prevalence − Arunachal Pradesh, Urban
WHO−2024 Adjustment Method

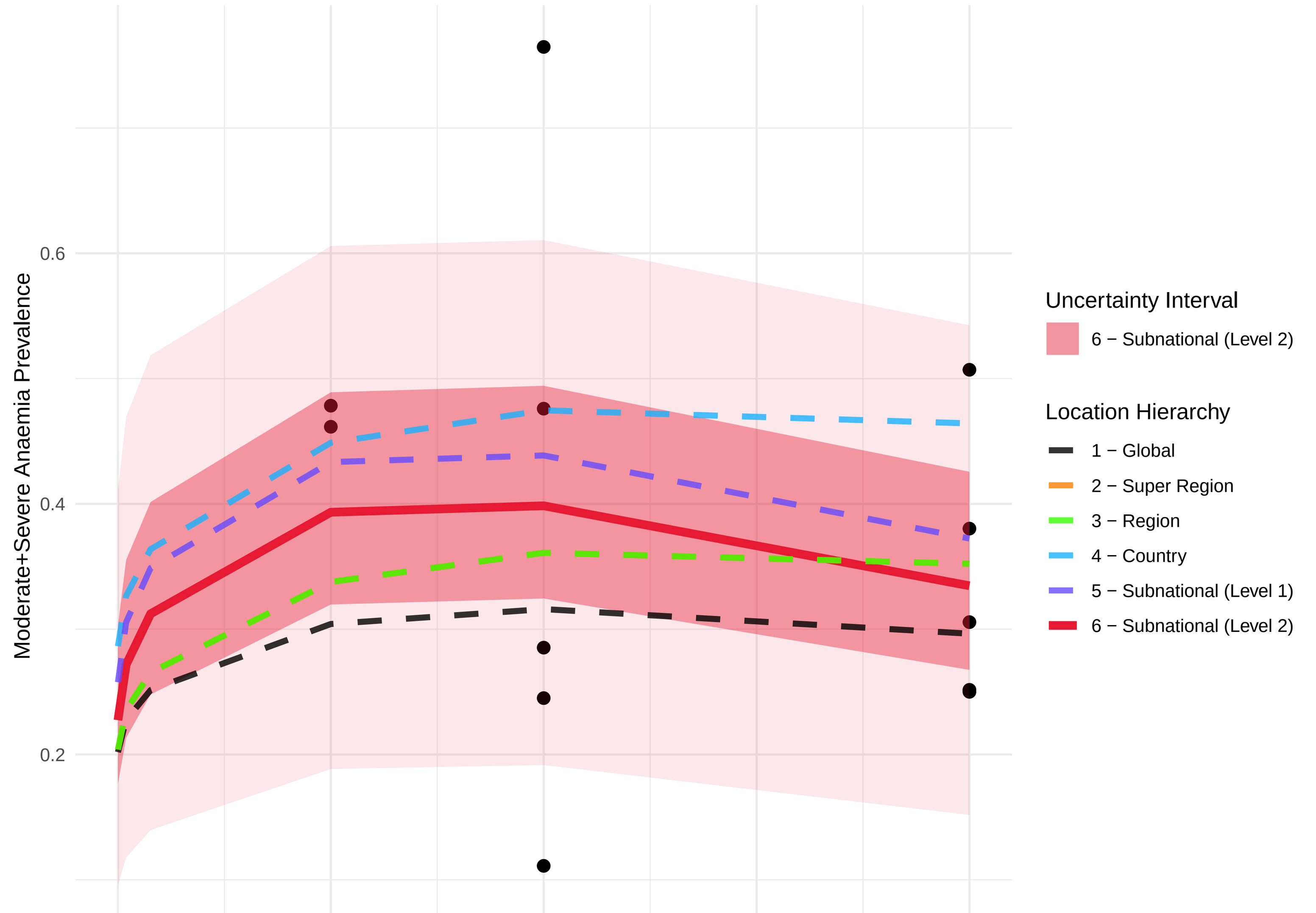

MR−BRT Age/Sex Fits − Non−pregnant Females <2 Years Old − Moderate+Severe Anaemia Prevalence − Assam
WHO−2024 Adjustment Method

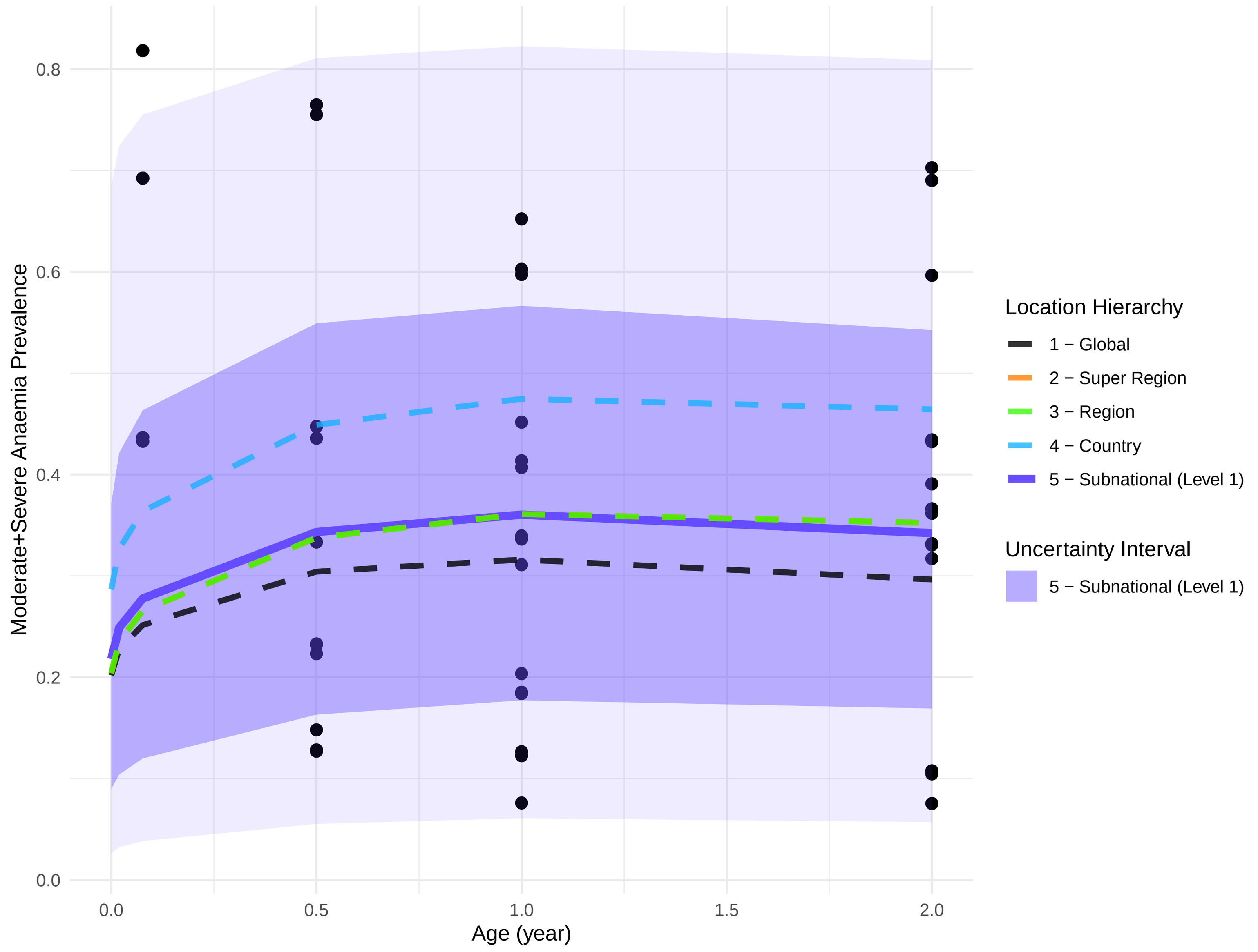

MR−BRT Age/Sex Fits − Non−pregnant Females <2 Years Old − Moderate+Severe Anaemia Prevalence − Assam, Rural
WHO−2024 Adjustment Method

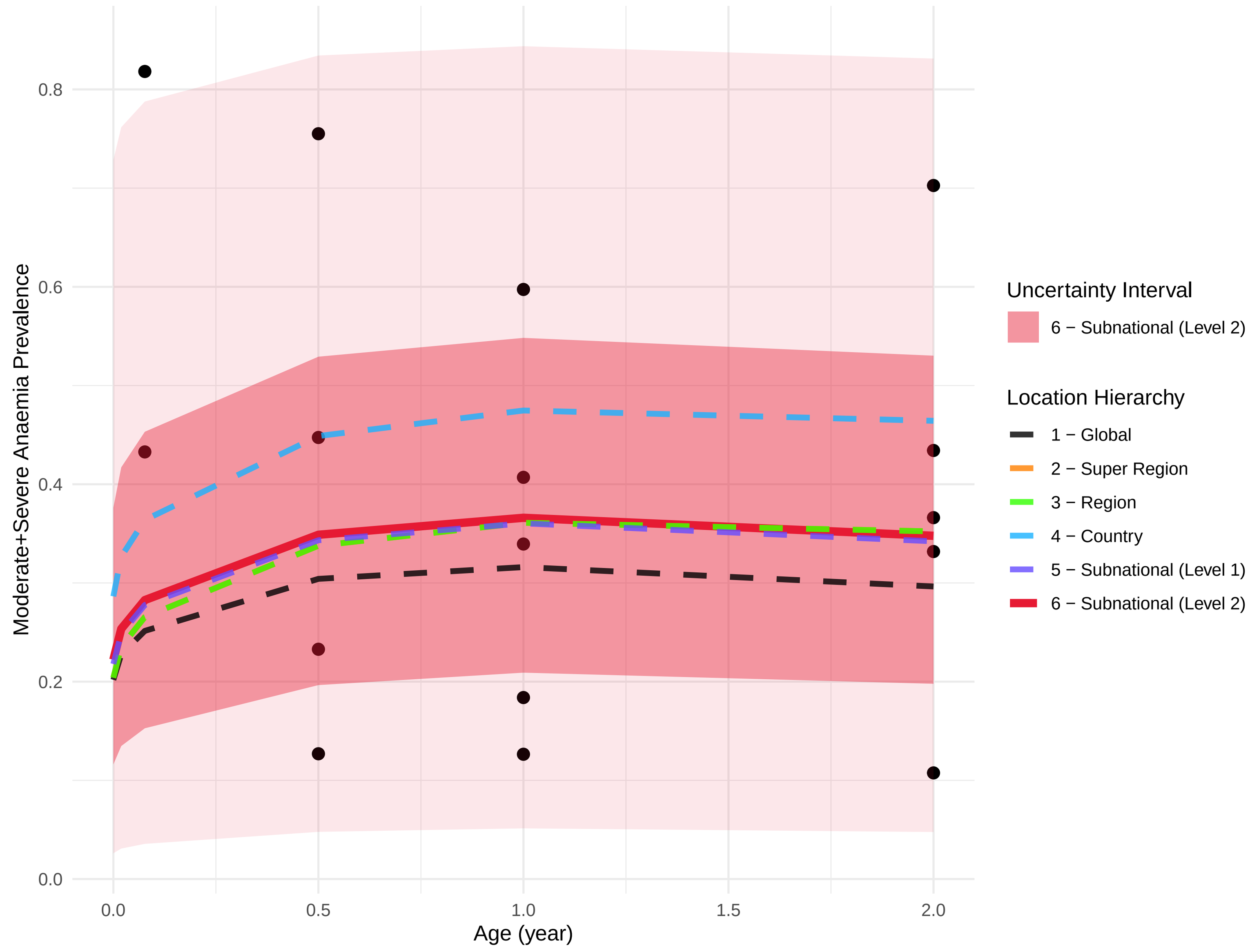

MR−BRT Age/Sex Fits − Non−pregnant Females <2 Years Old − Moderate+Severe Anaemia Prevalence − Assam, Urban
WHO−2024 Adjustment Method

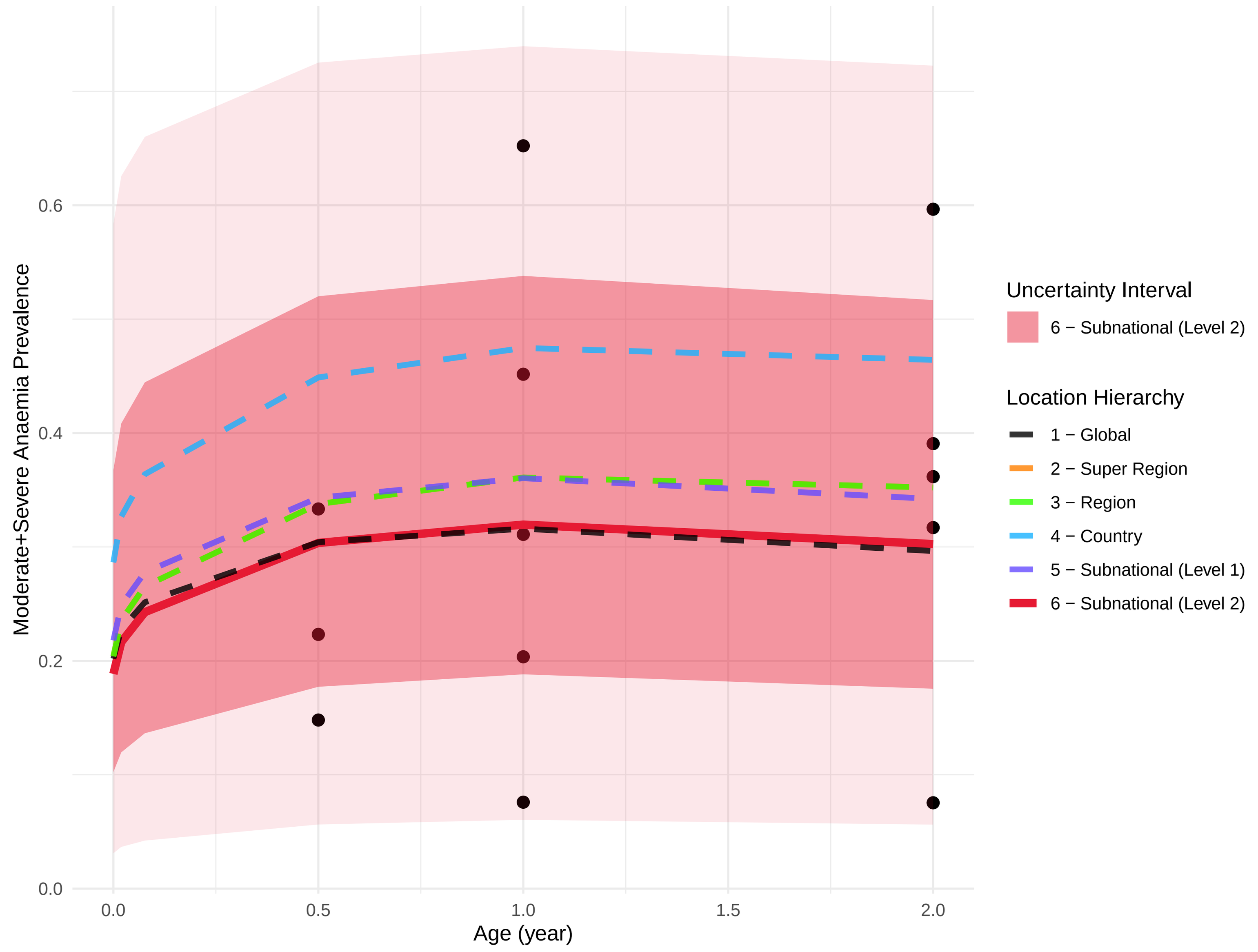

MR−BRT Age/Sex Fits − Non−pregnant Females <2 Years Old − Moderate+Severe Anaemia Prevalence − Bihar
WHO−2024 Adjustment Method

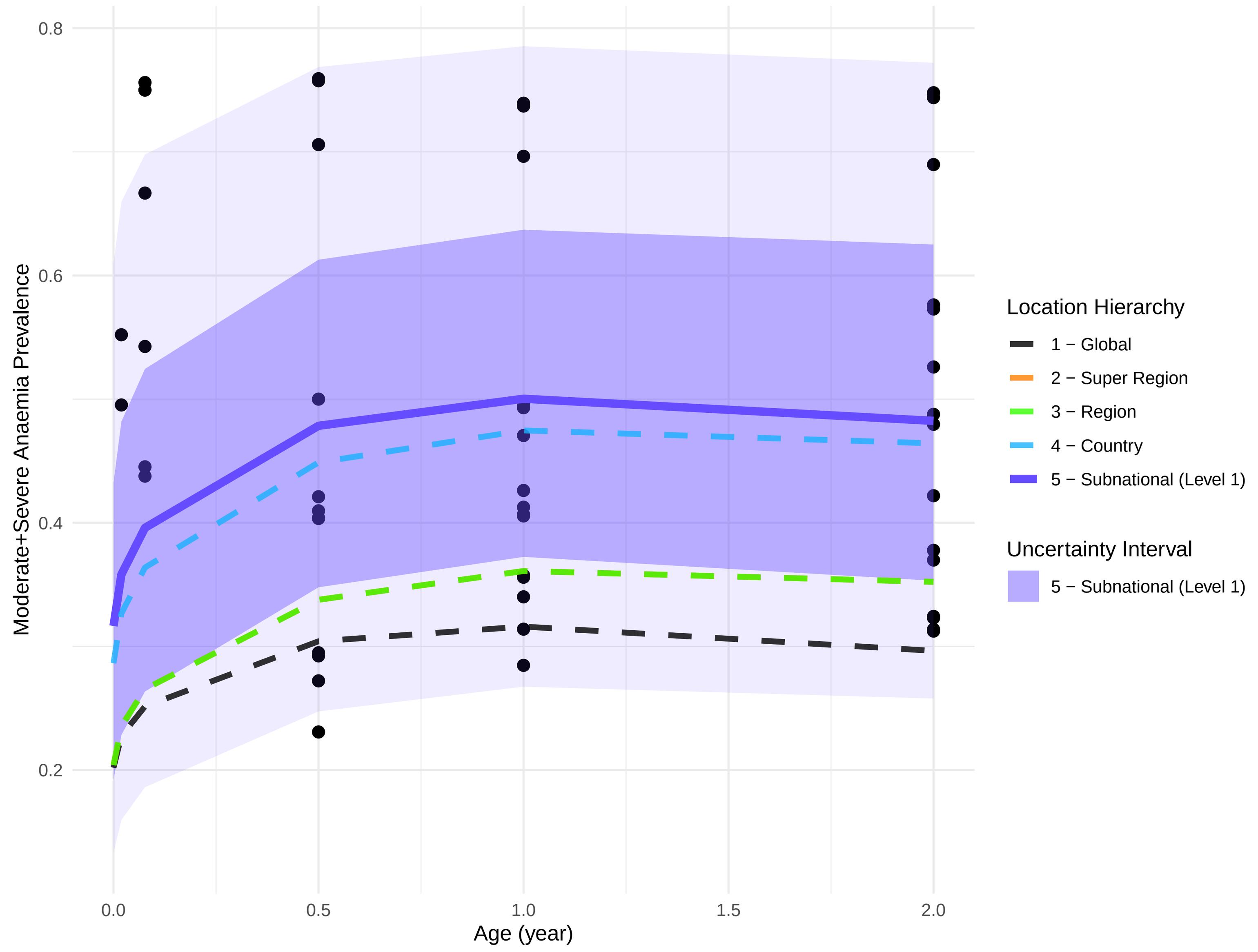

MR−BRT Age/Sex Fits − Non−pregnant Females <2 Years Old − Moderate+Severe Anaemia Prevalence − Bihar, Rural
WHO−2024 Adjustment Method

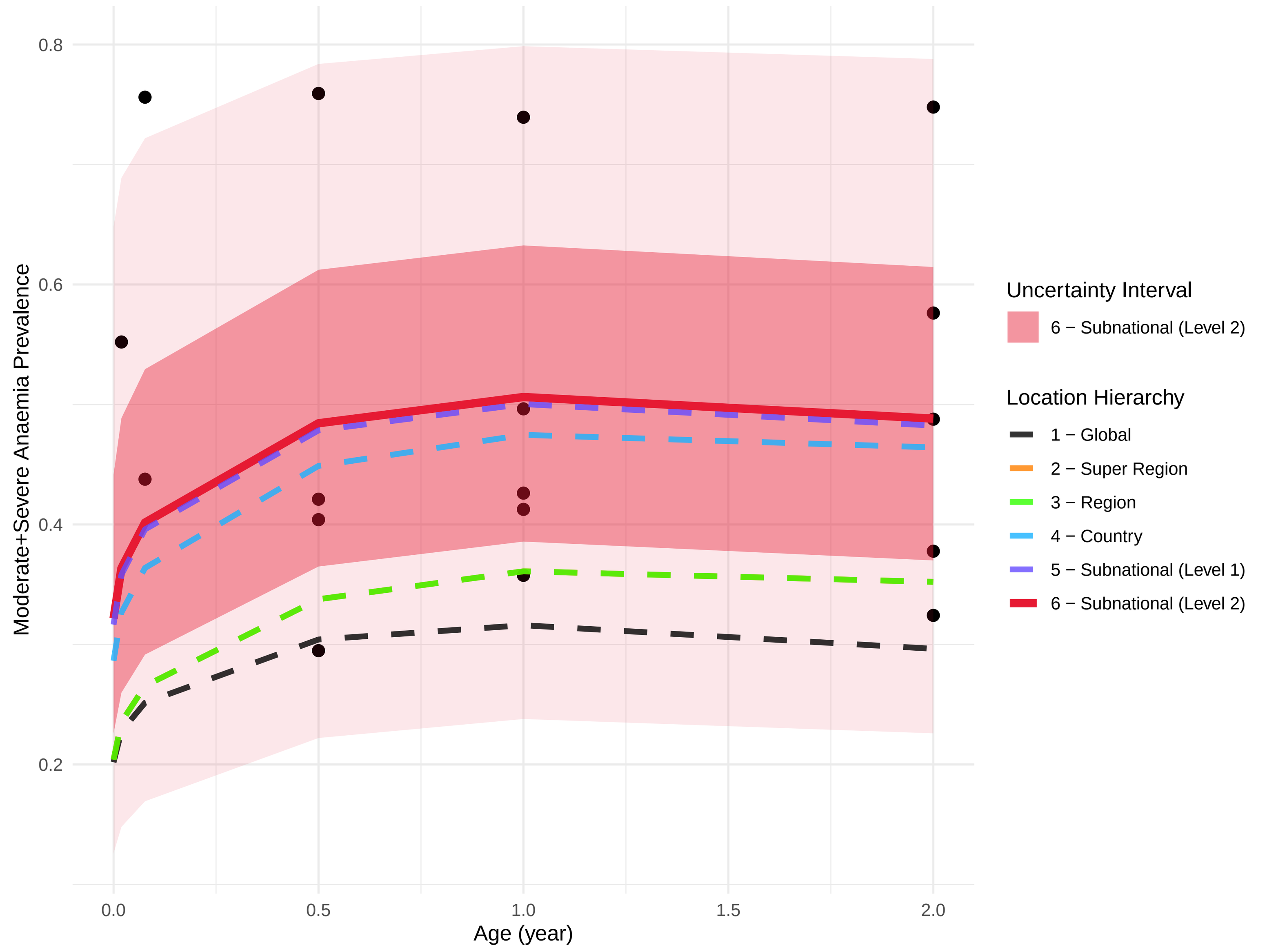

MR−BRT Age/Sex Fits − Non−pregnant Females <2 Years Old − Moderate+Severe Anaemia Prevalence − Bihar, Urban
WHO−2024 Adjustment Method

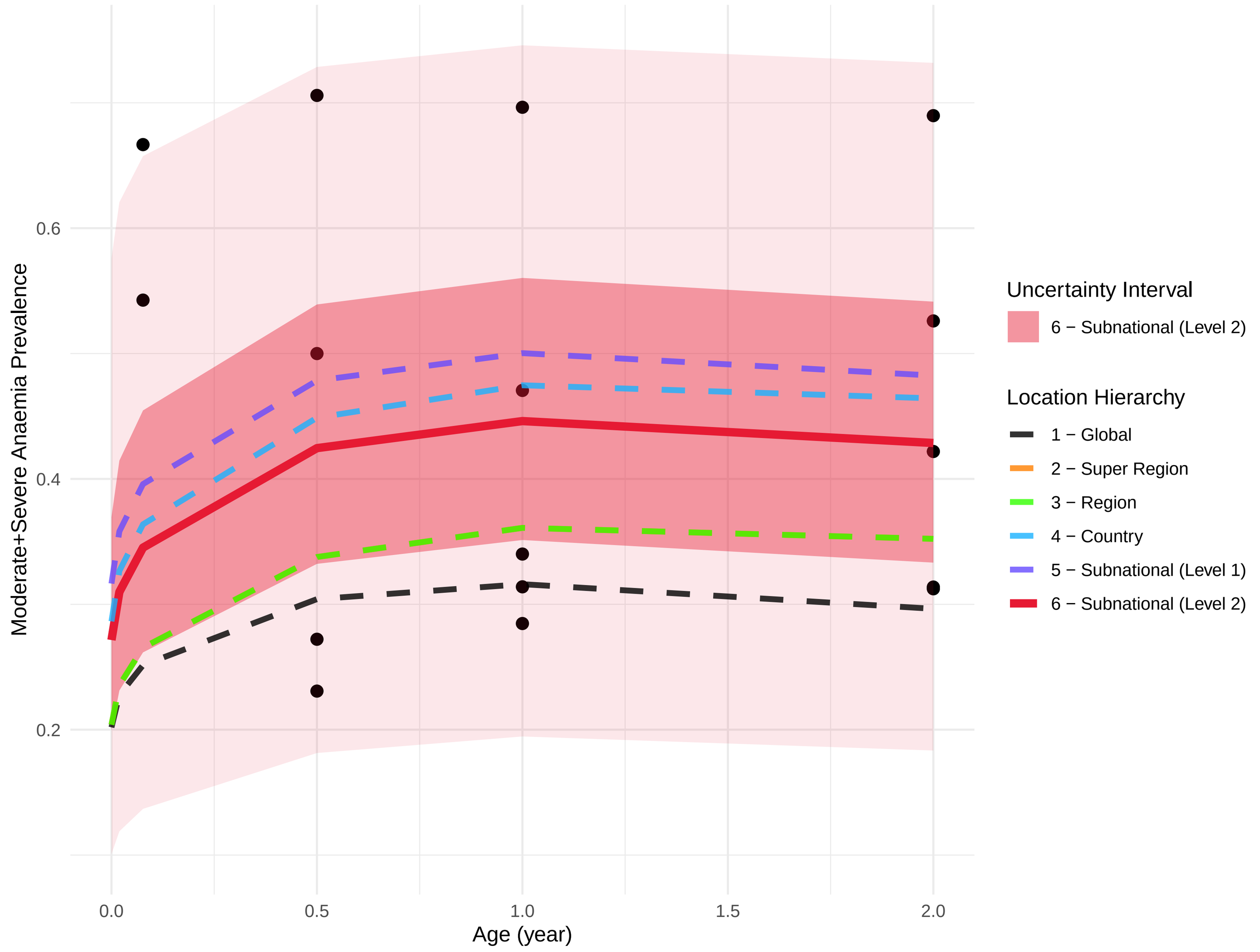

MR−BRT Age/Sex Fits − Non−pregnant Females <2 Years Old − Moderate+Severe Anaemia Prevalence − Chhattisgarh
WHO−2024 Adjustment Method

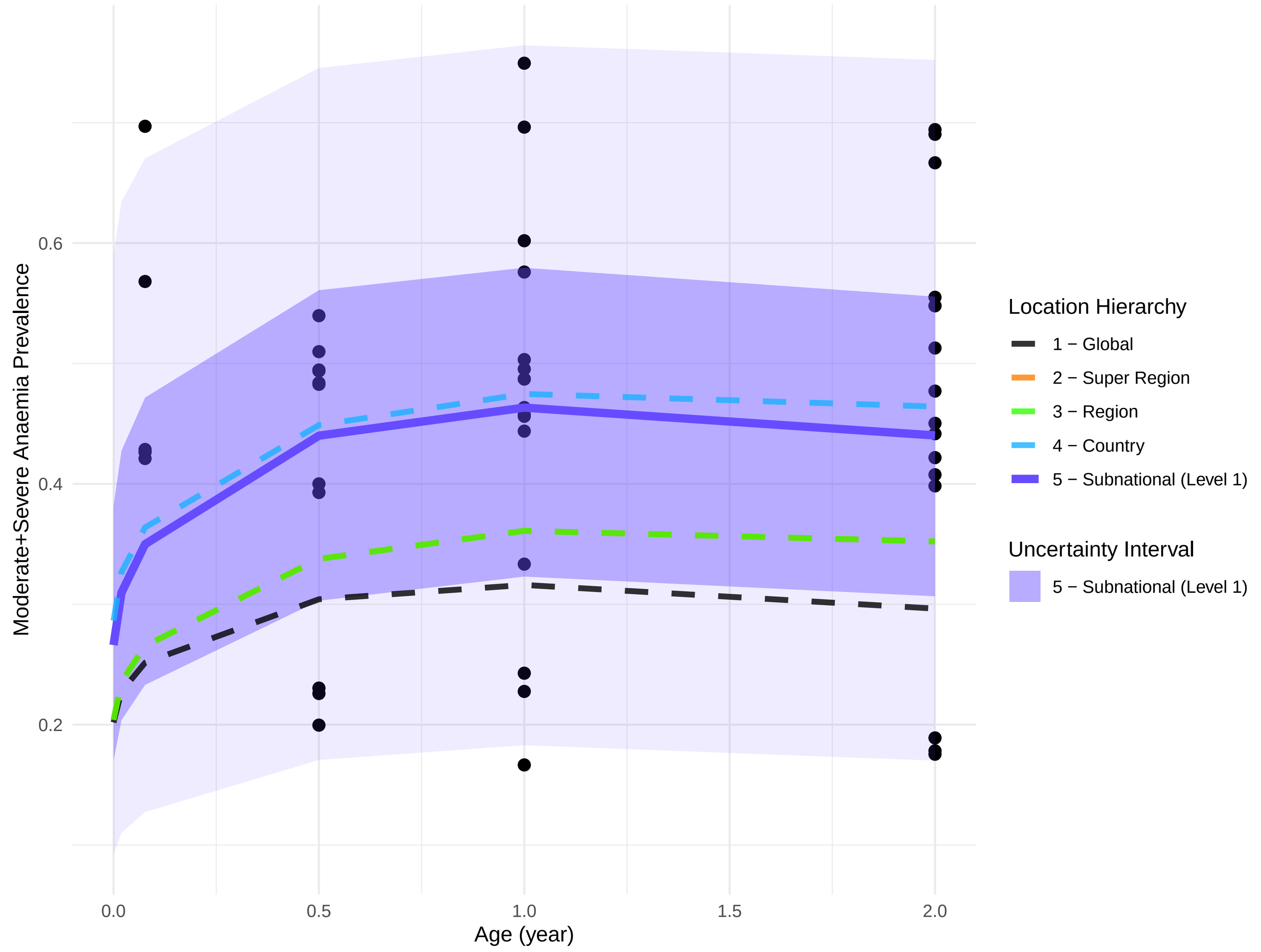

MR−BRT Age/Sex Fits − Non−pregnant Females <2 Years Old − Moderate+Severe Anaemia Prevalence − Chhattisgarh, Rural
WHO−2024 Adjustment Method

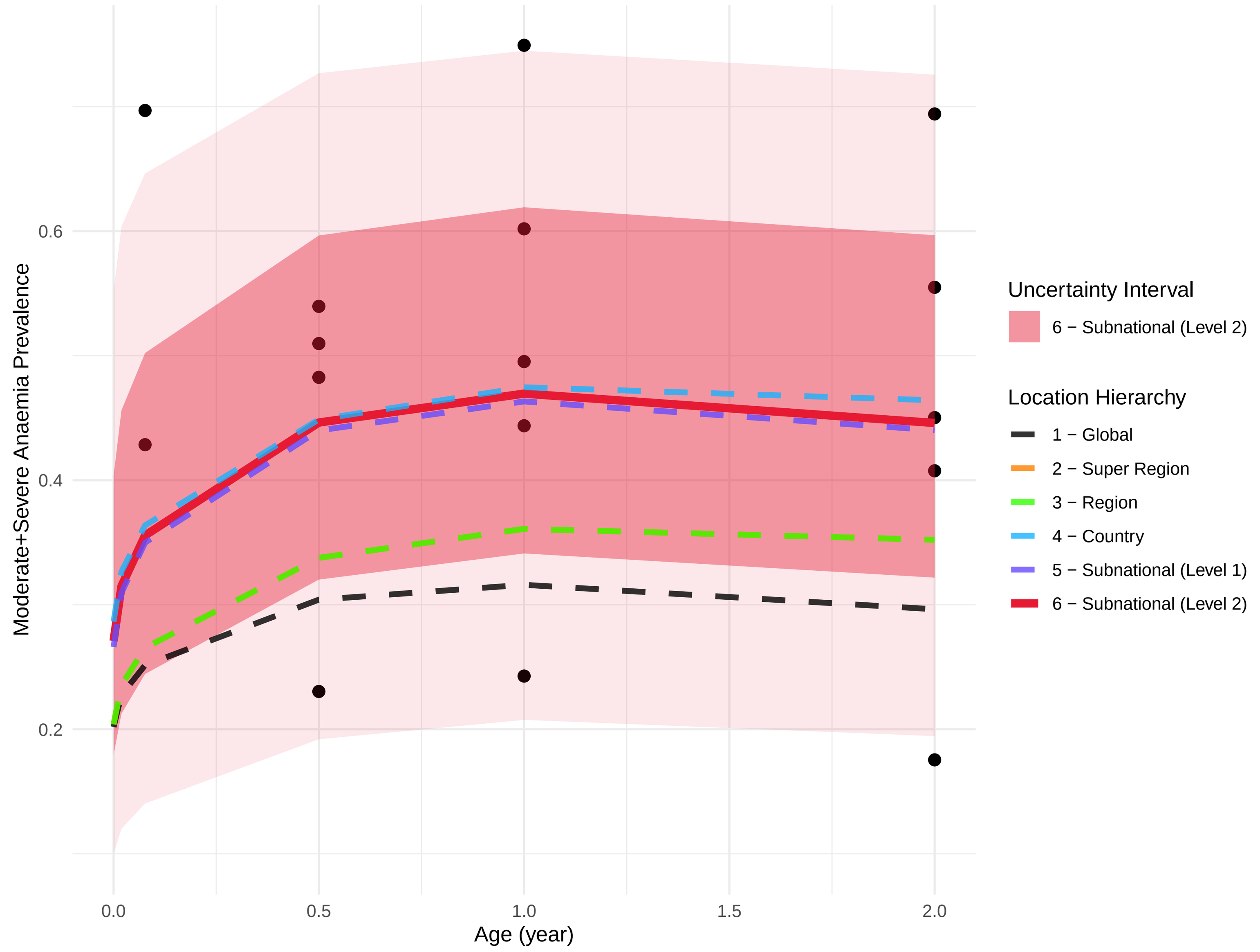

MR−BRT Age/Sex Fits − Non−pregnant Females <2 Years Old − Moderate+Severe Anaemia Prevalence − Chhattisgarh, Urban
WHO−2024 Adjustment Method

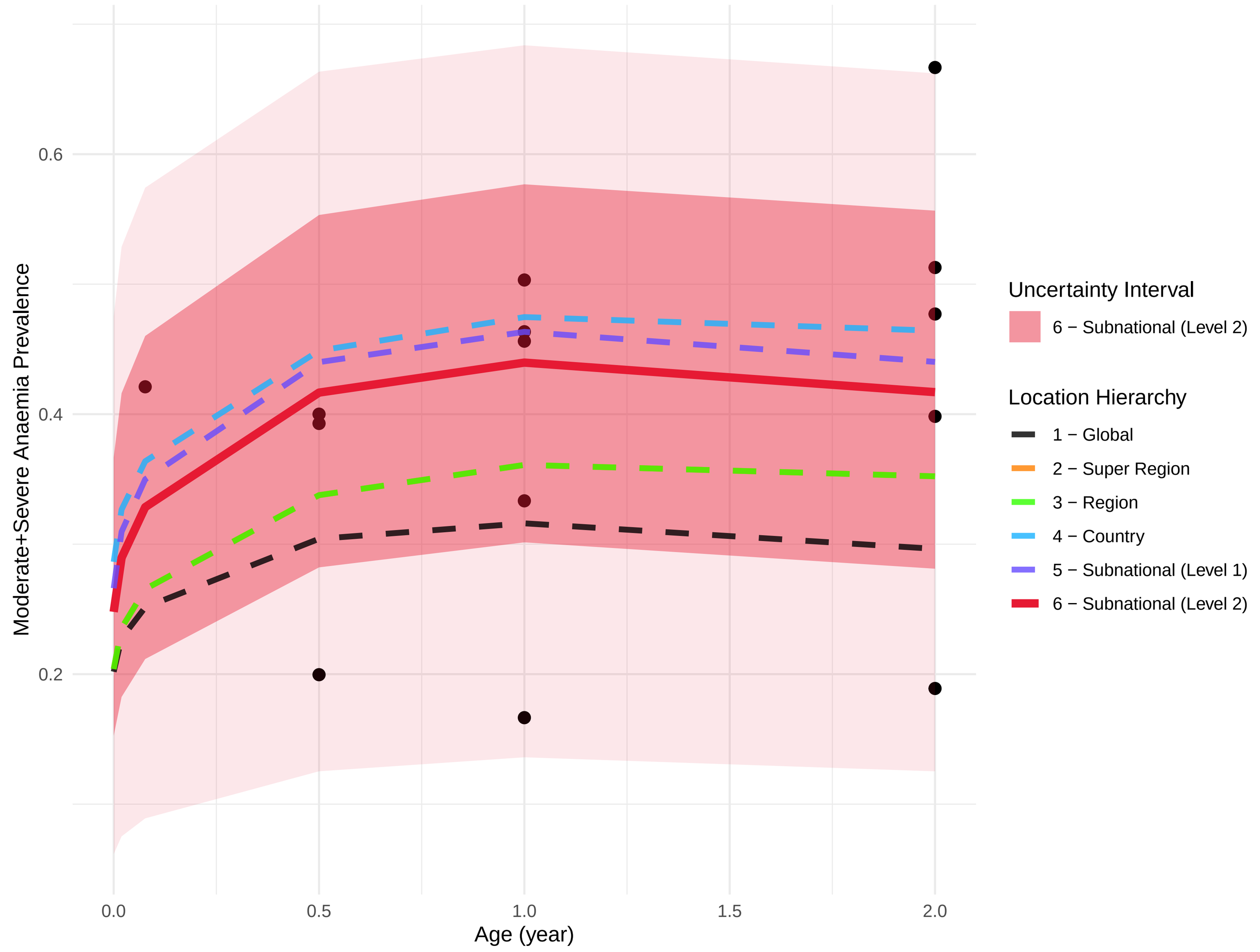

MR−BRT Age/Sex Fits − Non−pregnant Females <2 Years Old − Moderate+Severe Anaemia Prevalence − Delhi
WHO−2024 Adjustment Method

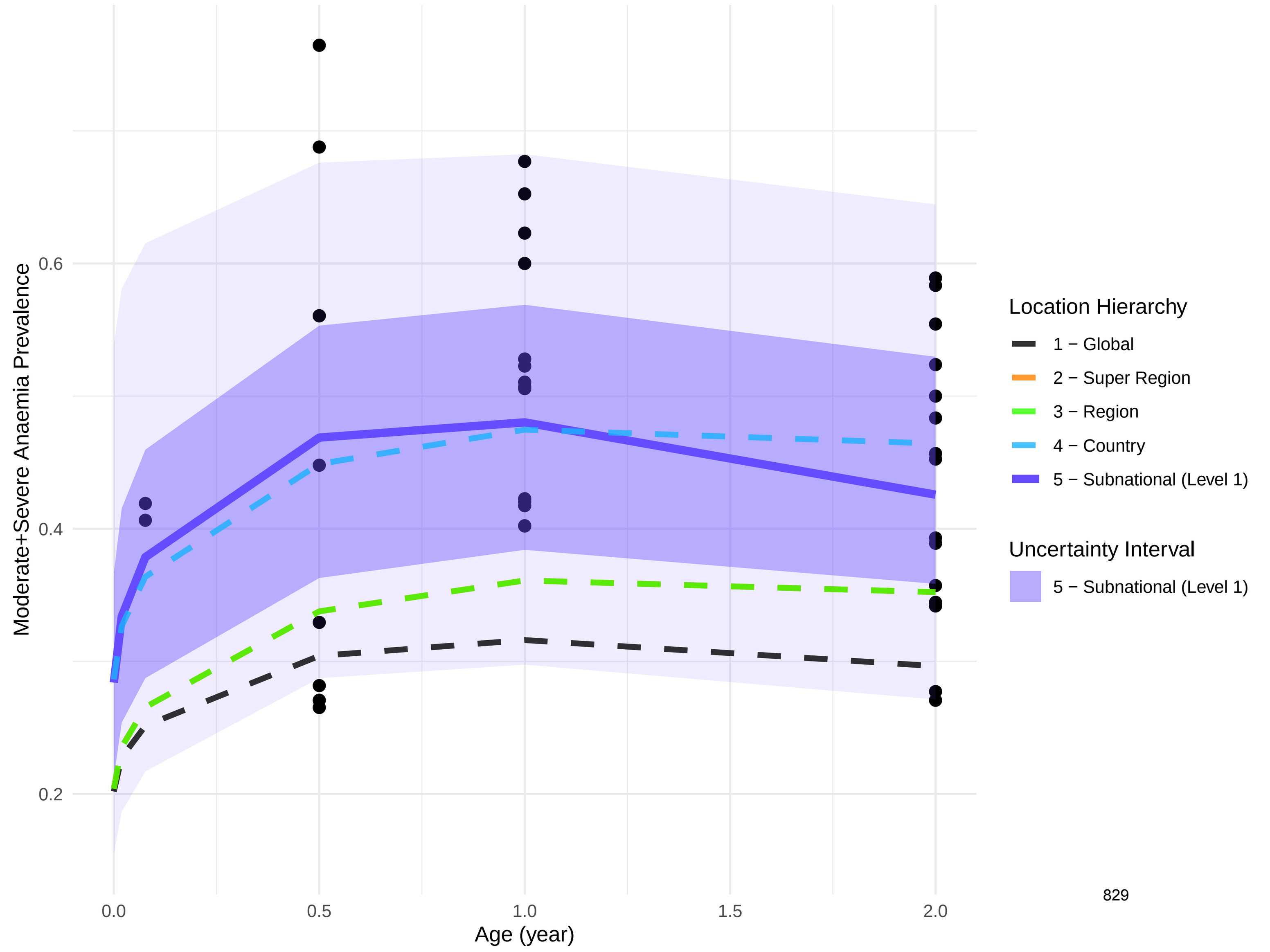

MR−BRT Age/Sex Fits − Non−pregnant Females <2 Years Old − Moderate+Severe Anaemia Prevalence − Delhi, Rural
WHO−2024 Adjustment Method

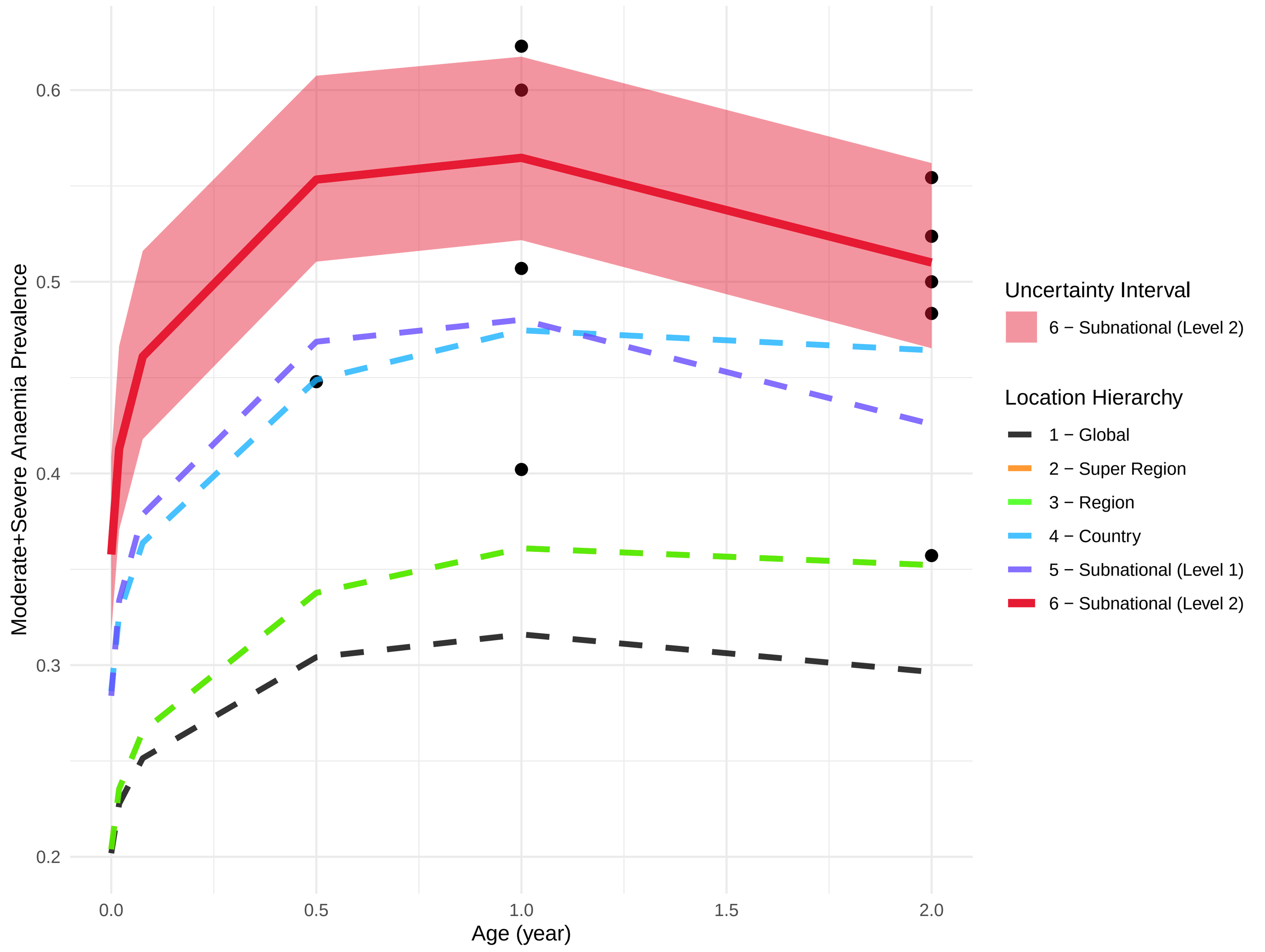

MR−BRT Age/Sex Fits − Non−pregnant Females <2 Years Old − Moderate+Severe Anaemia Prevalence − Delhi, Urban
WHO−2024 Adjustment Method

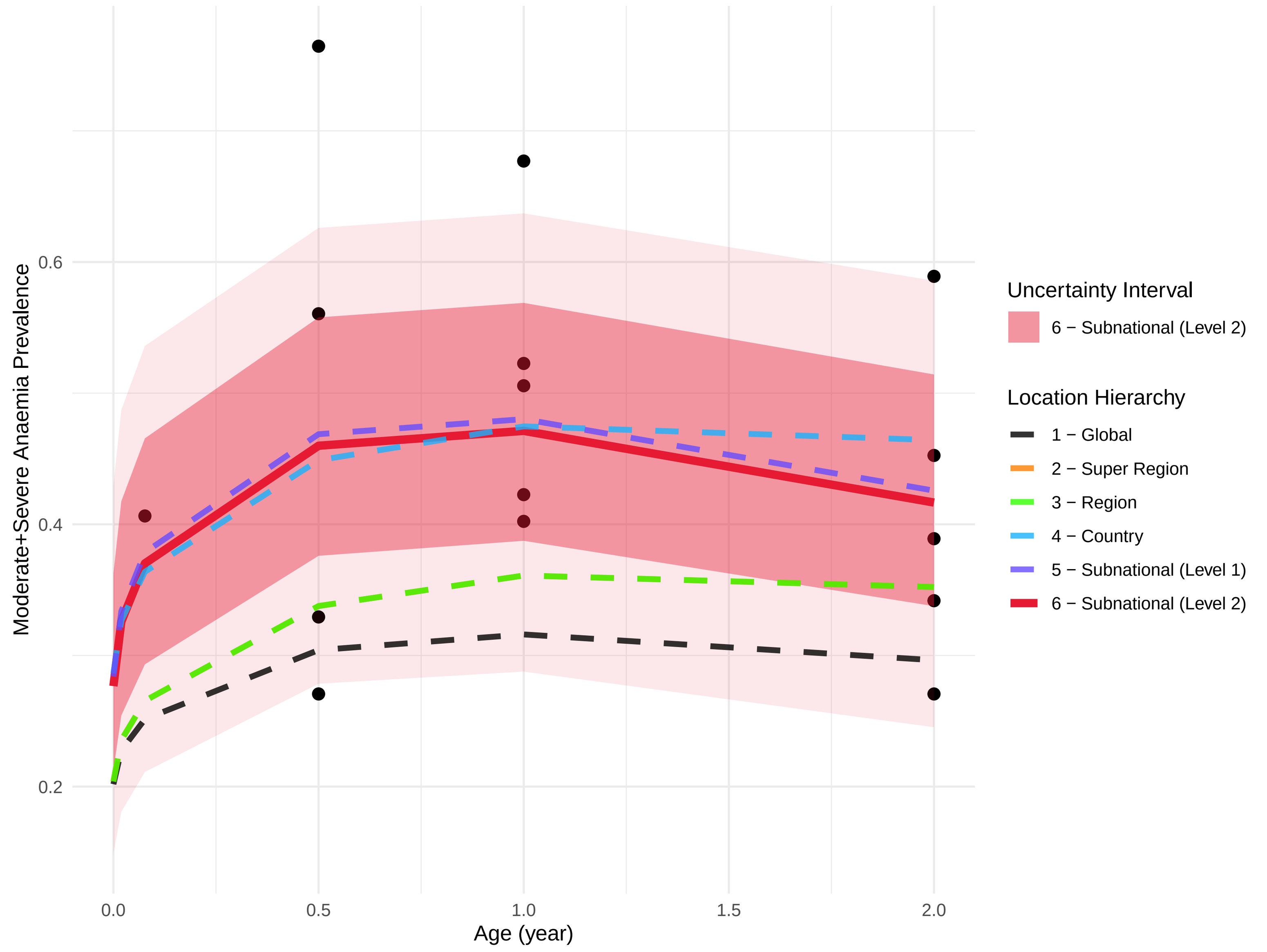

MR−BRT Age/Sex Fits − Non−pregnant Females <2 Years Old − Moderate+Severe Anaemia Prevalence − Goa
WHO−2024 Adjustment Method

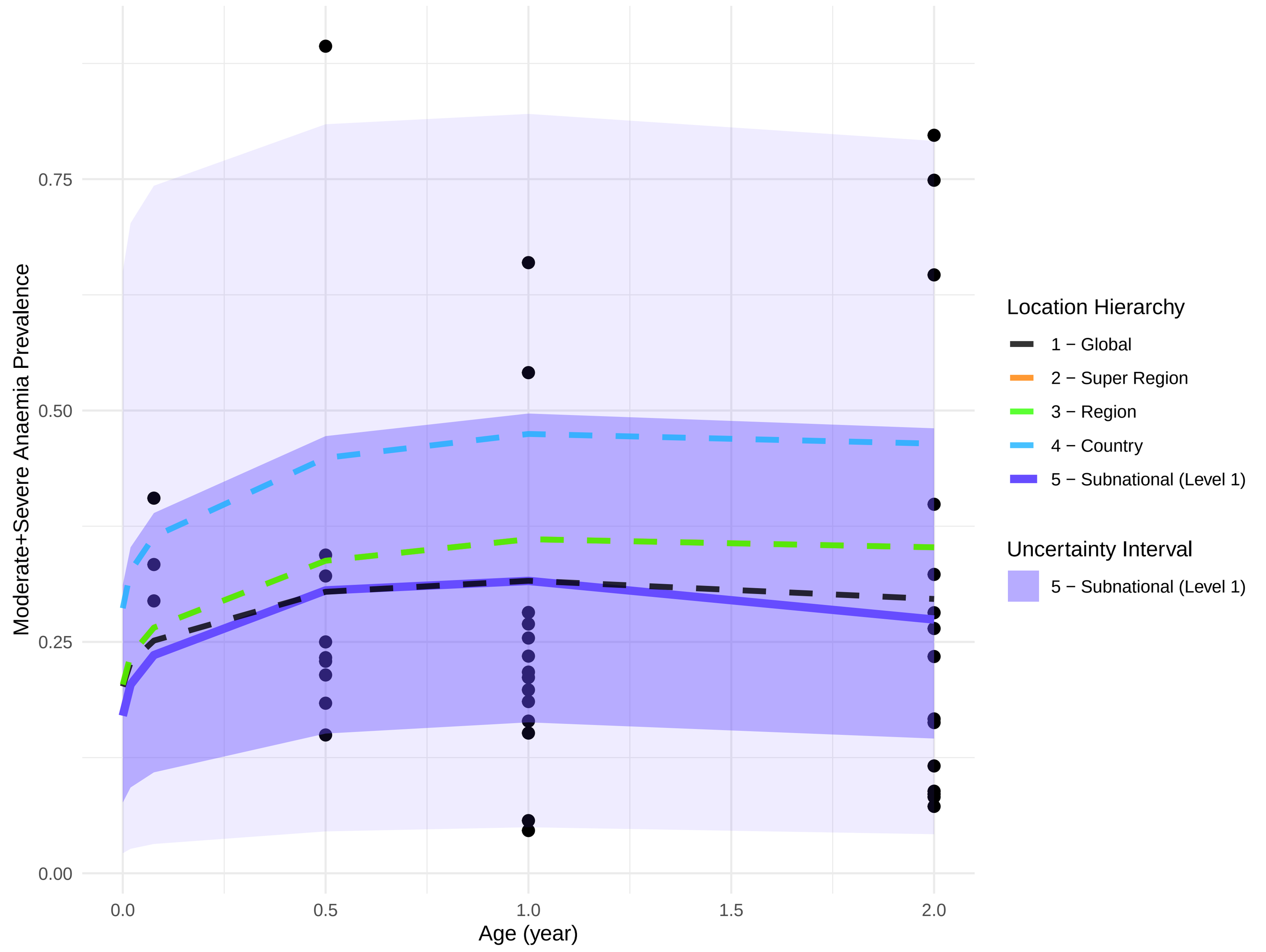

MR−BRT Age/Sex Fits − Non−pregnant Females <2 Years Old − Moderate+Severe Anaemia Prevalence − Goa, Rural
WHO−2024 Adjustment Method

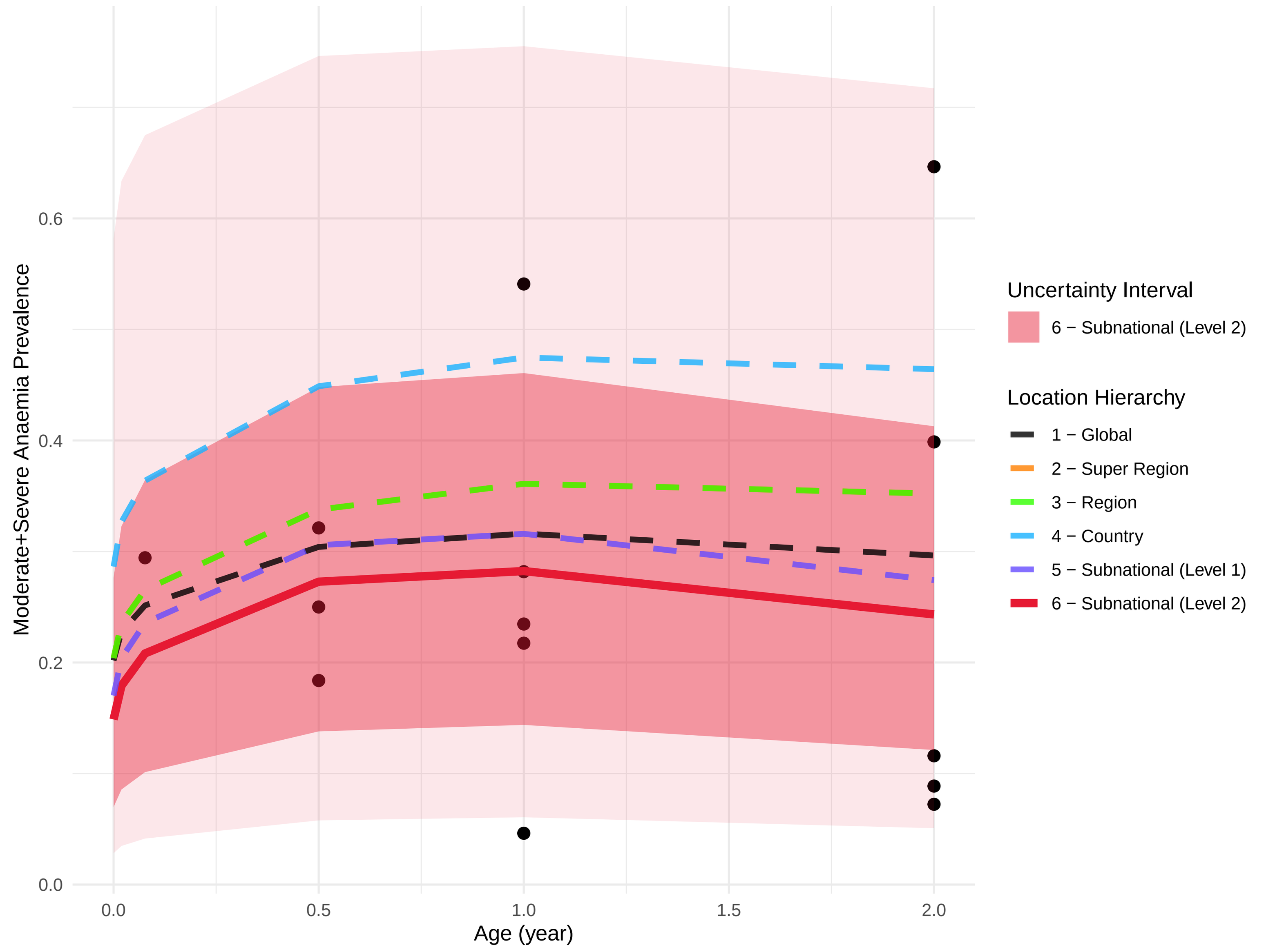

MR−BRT Age/Sex Fits − Non−pregnant Females <2 Years Old − Moderate+Severe Anaemia Prevalence − Goa, Urban
WHO−2024 Adjustment Method

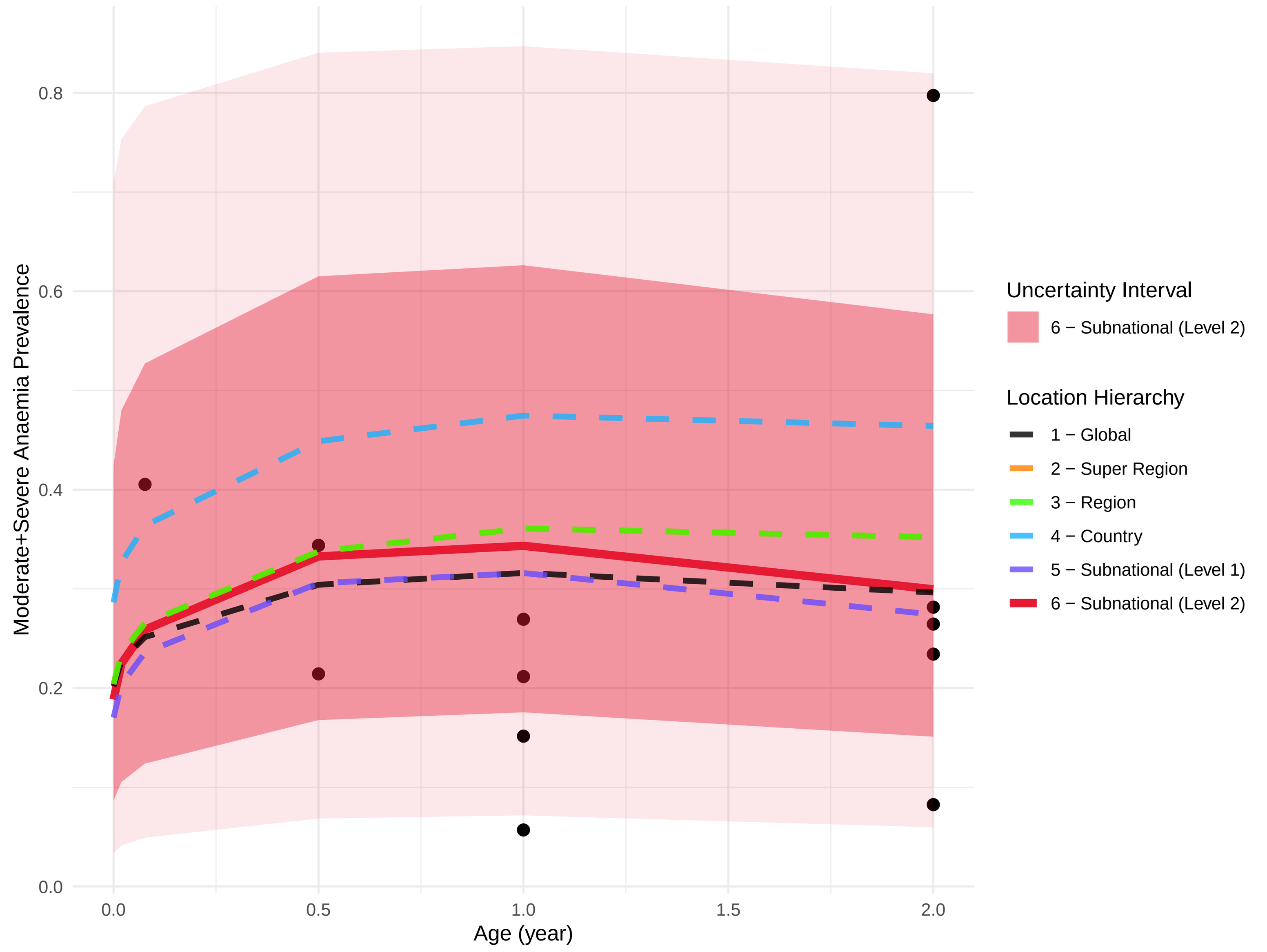

MR−BRT Age/Sex Fits − Non−pregnant Females <2 Years Old − Moderate+Severe Anaemia Prevalence − Gujarat
WHO−2024 Adjustment Method

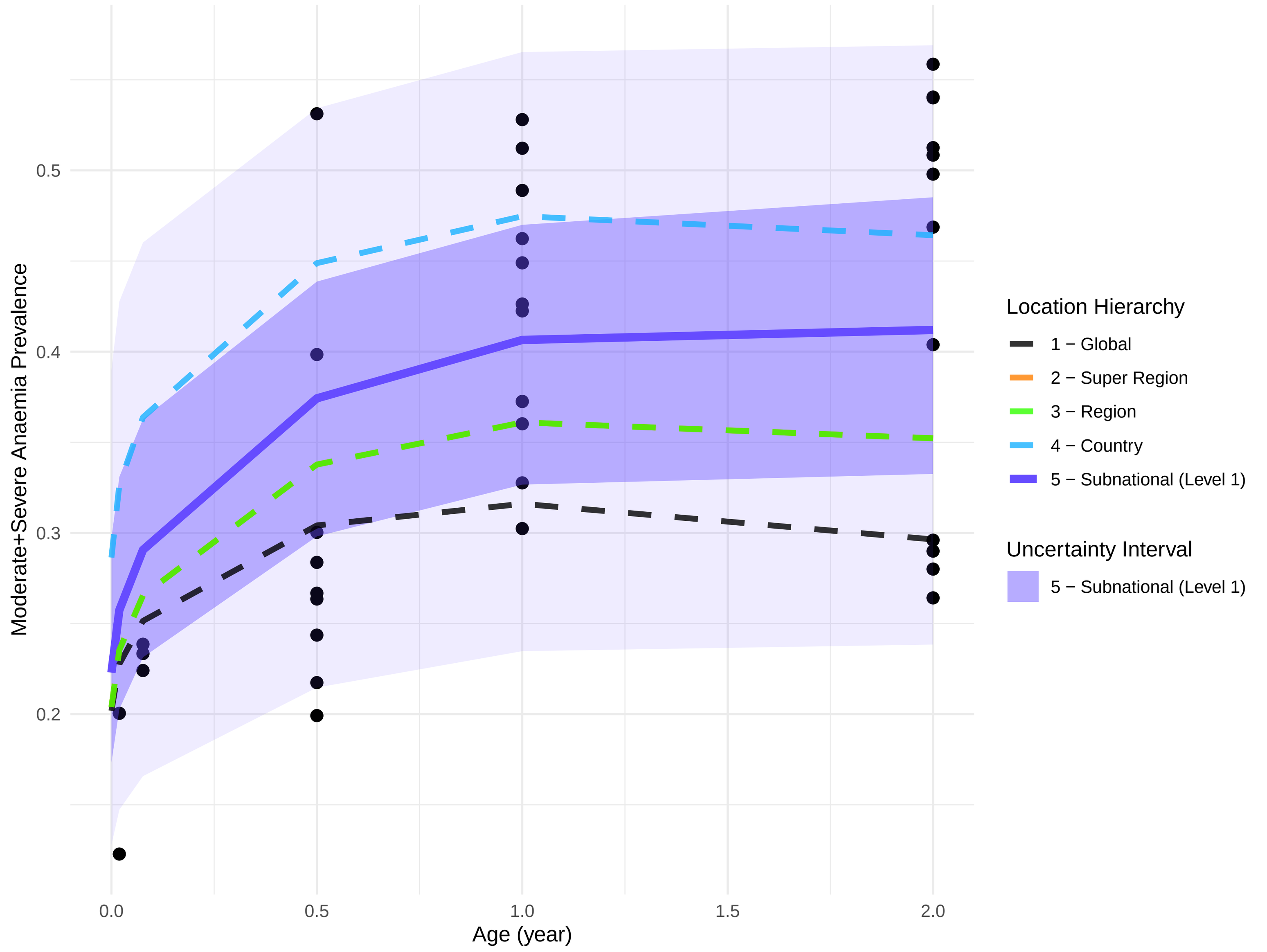

MR−BRT Age/Sex Fits − Non−pregnant Females <2 Years Old − Moderate+Severe Anaemia Prevalence − Gujarat, Rural
WHO−2024 Adjustment Method

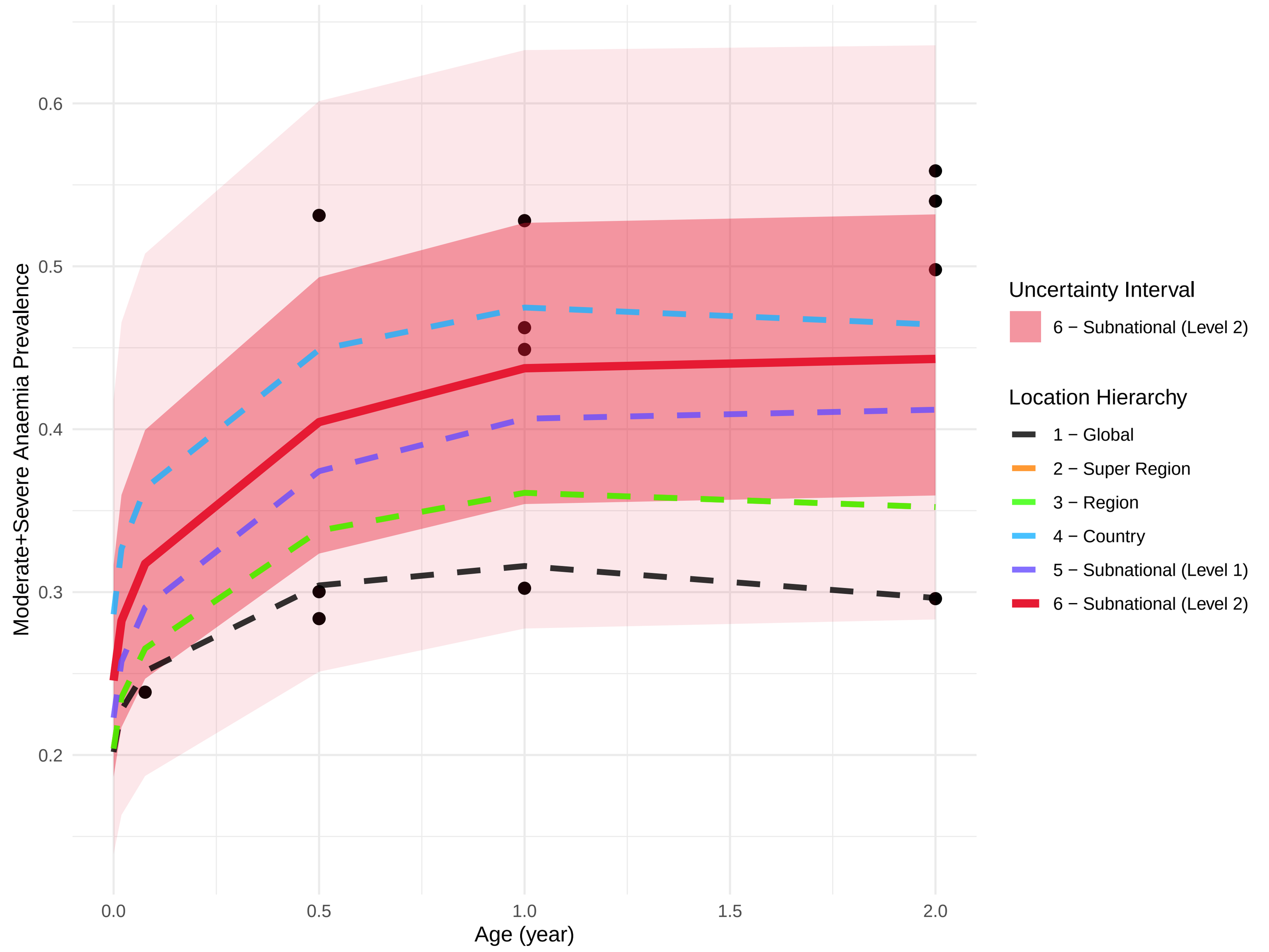

MR−BRT Age/Sex Fits − Non−pregnant Females <2 Years Old − Moderate+Severe Anaemia Prevalence − Gujarat, Urban
WHO−2024 Adjustment Method

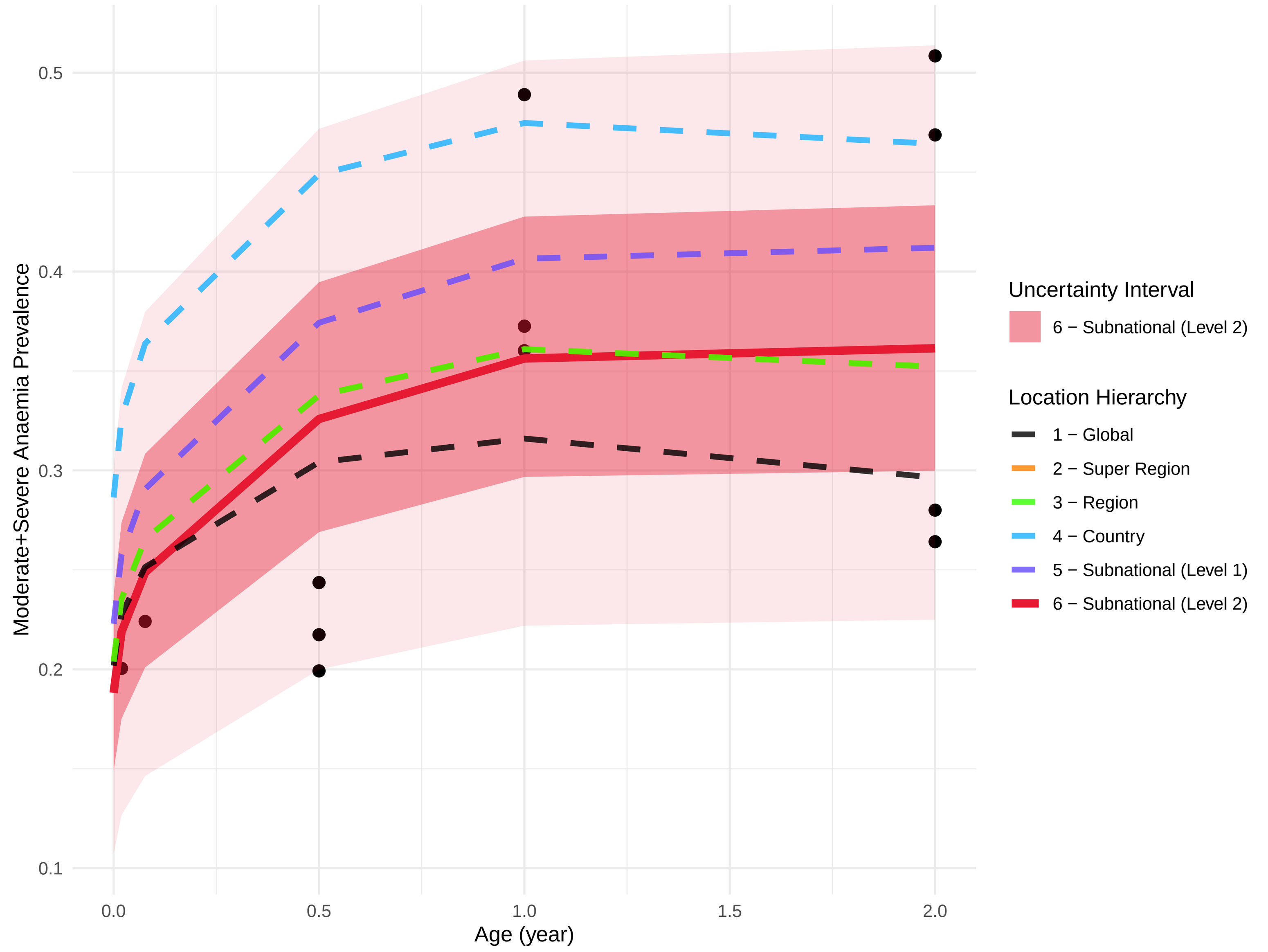

MR−BRT Age/Sex Fits − Non−pregnant Females <2 Years Old − Moderate+Severe Anaemia Prevalence − Haryana
WHO−2024 Adjustment Method

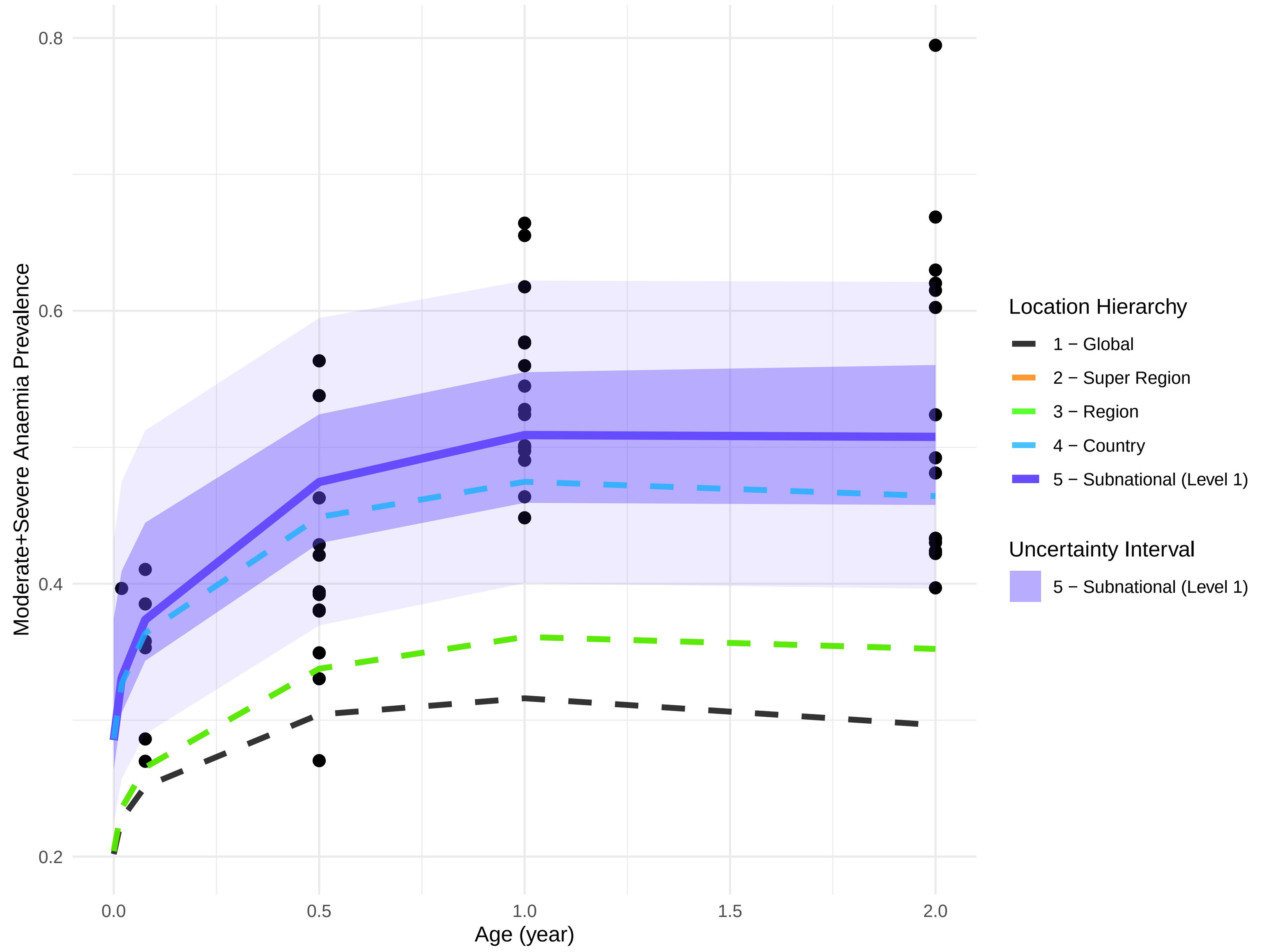

MR−BRT Age/Sex Fits − Non−pregnant Females <2 Years Old − Moderate+Severe Anaemia Prevalence − Haryana, Rural
WHO−2024 Adjustment Method

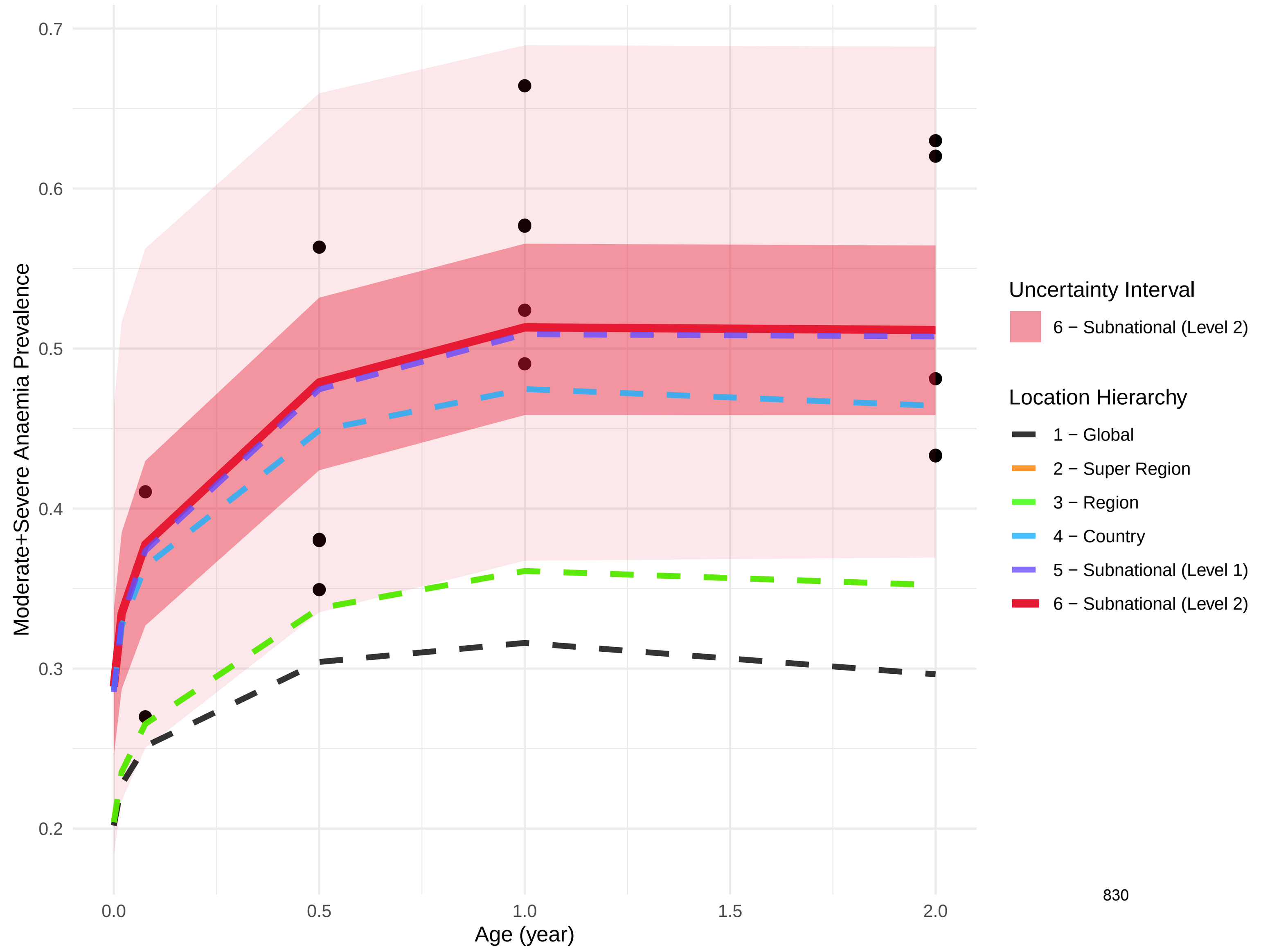

MR−BRT Age/Sex Fits − Non−pregnant Females <2 Years Old − Moderate+Severe Anaemia Prevalence − Haryana, Urban
WHO−2024 Adjustment Method

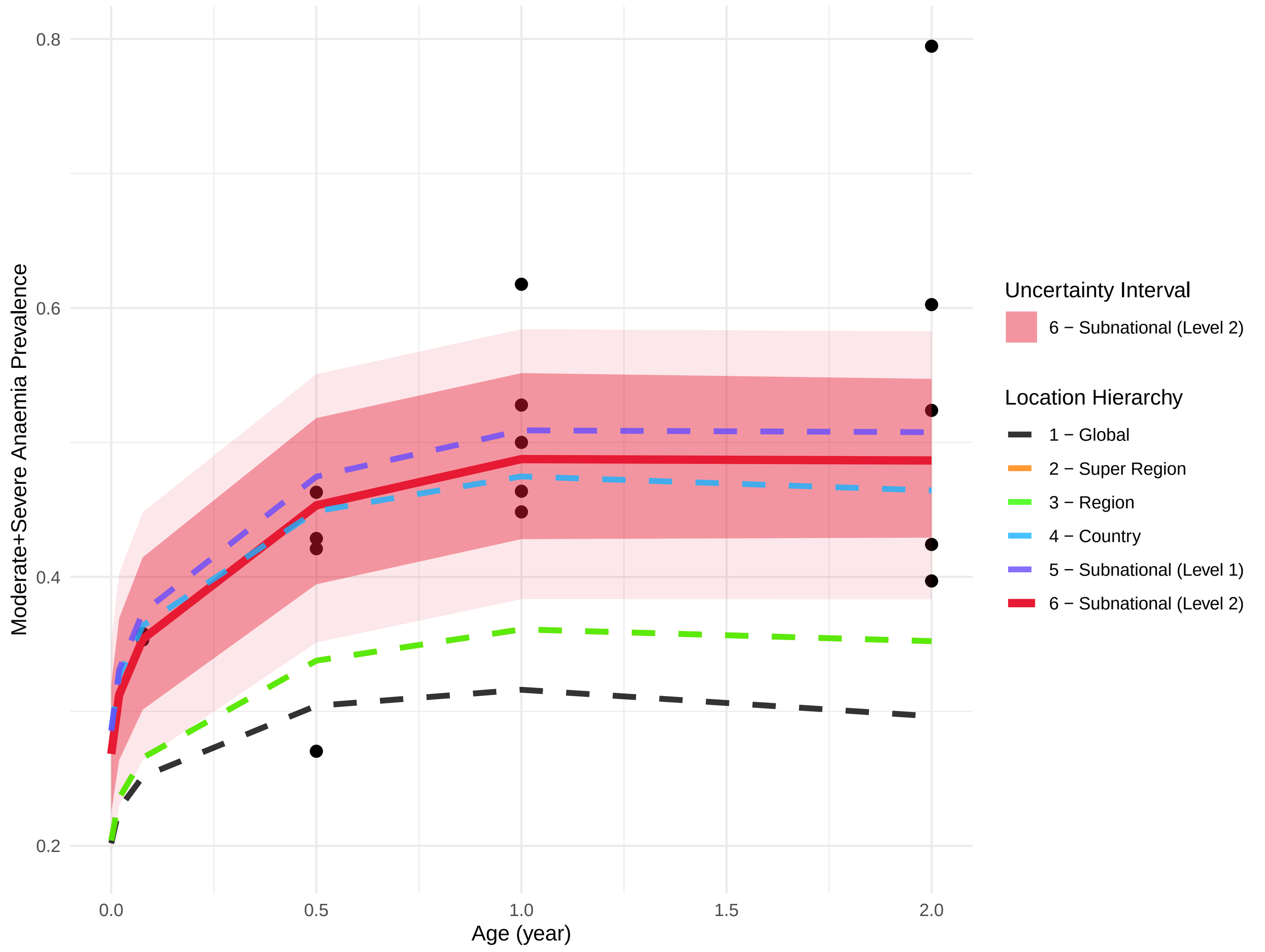

MR−BRT Age/Sex Fits − Non−pregnant Females <2 Years Old − Moderate+Severe Anaemia Prevalence − Himachal Pradesh
WHO−2024 Adjustment Method

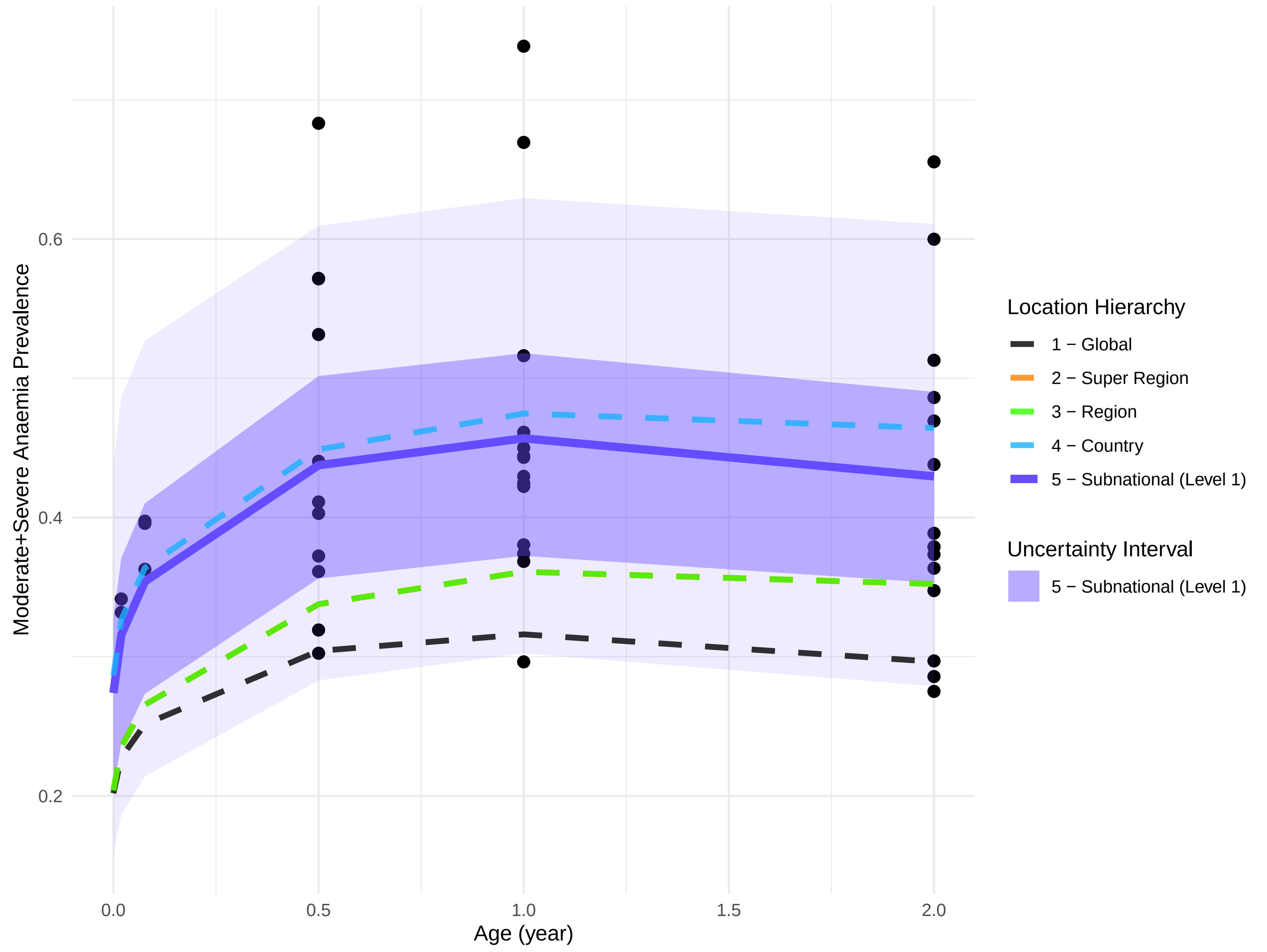

MR−BRT Age/Sex Fits − Non−pregnant Females <2 Years Old − Moderate+Severe Anaemia Prevalence − Himachal Pradesh, Rural
WHO−2024 Adjustment Method

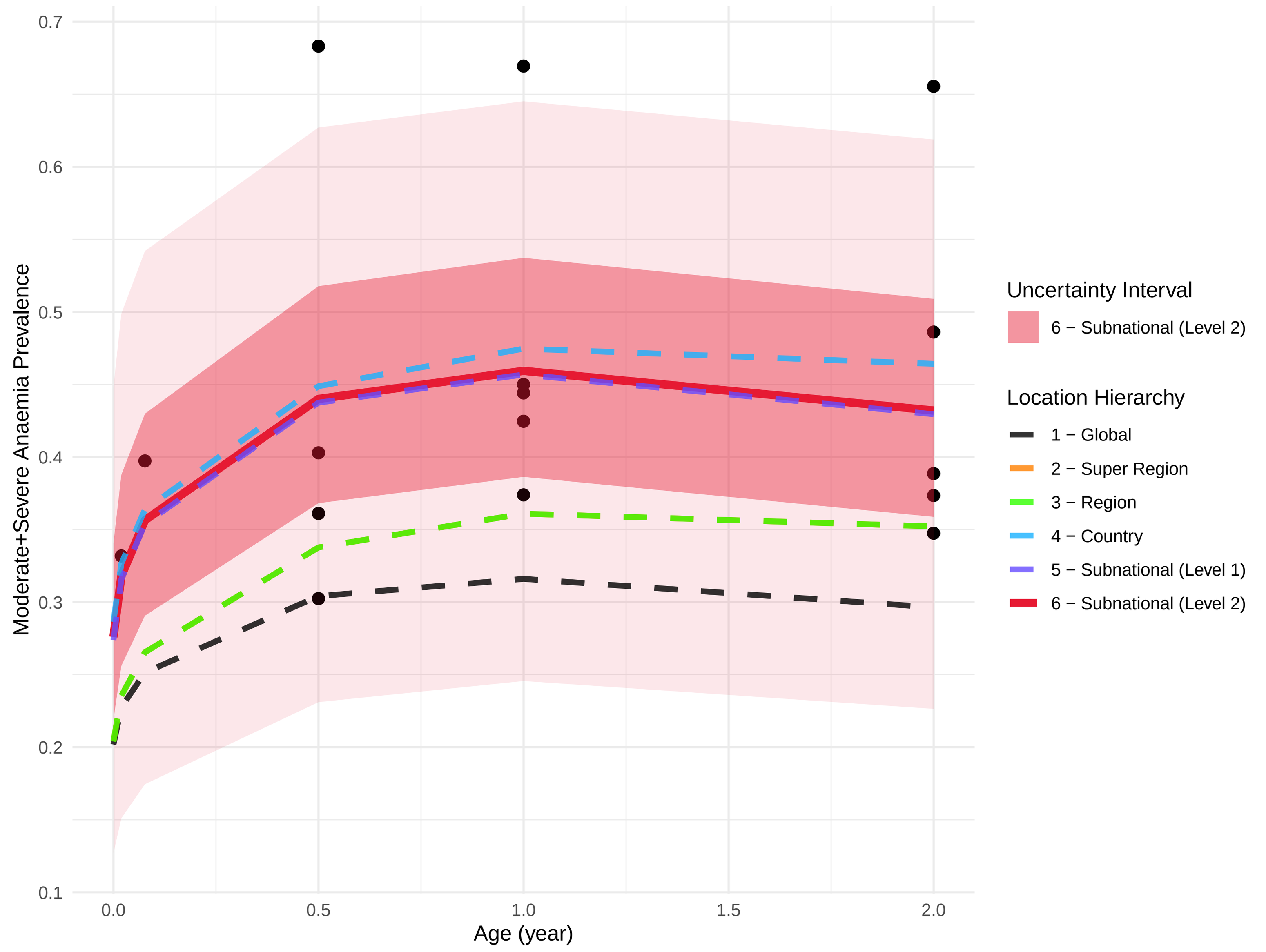

MR−BRT Age/Sex Fits − Non−pregnant Females <2 Years Old − Moderate+Severe Anaemia Prevalence − Himachal Pradesh, Urban
WHO−2024 Adjustment Method

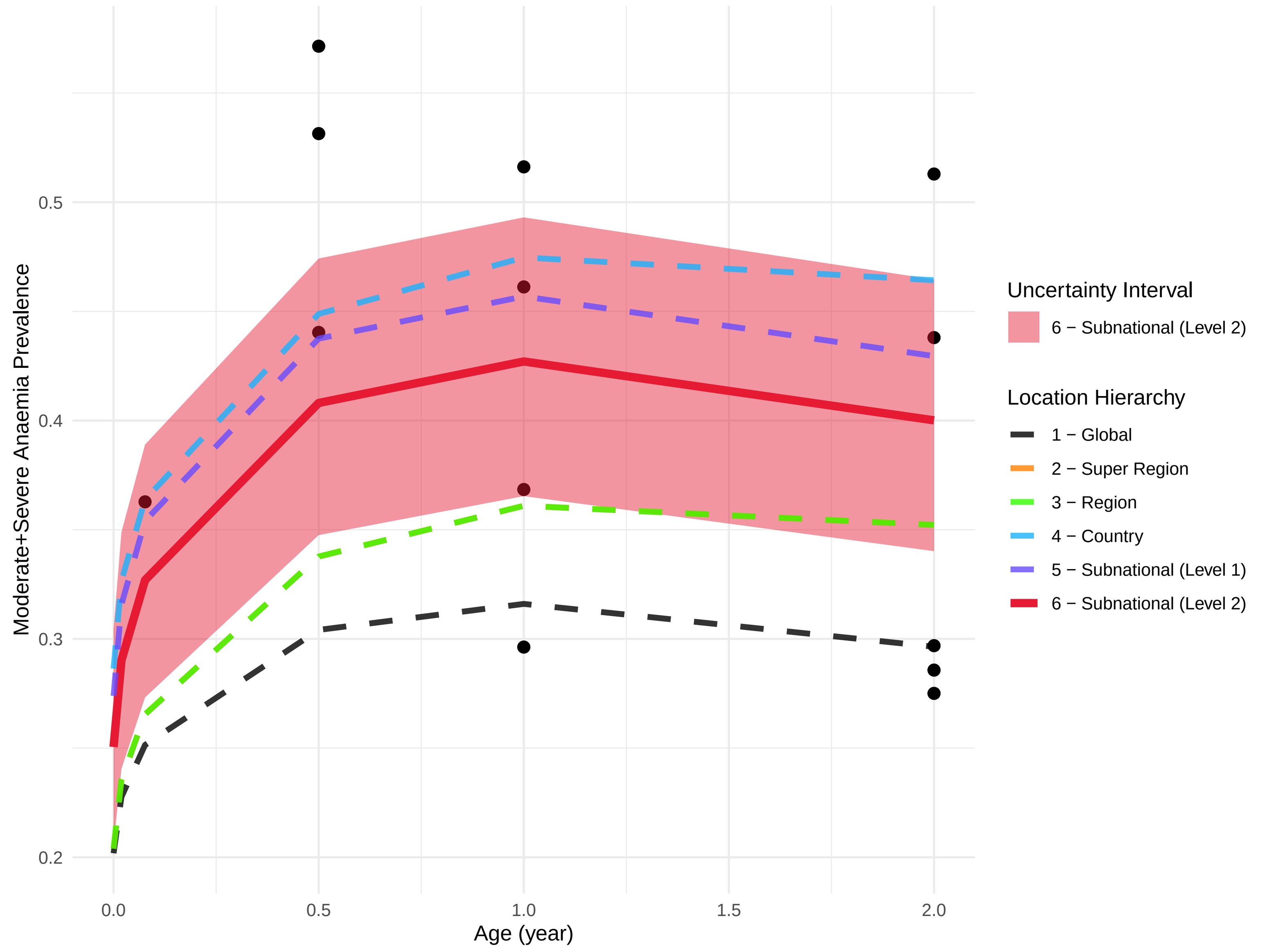

MR−BRT Age/Sex Fits − Non−pregnant Females <2 Years Old − Moderate+Severe Anaemia Prevalence − Jammu & Kashmir and Ladakh
WHO−2024 Adjustment Method

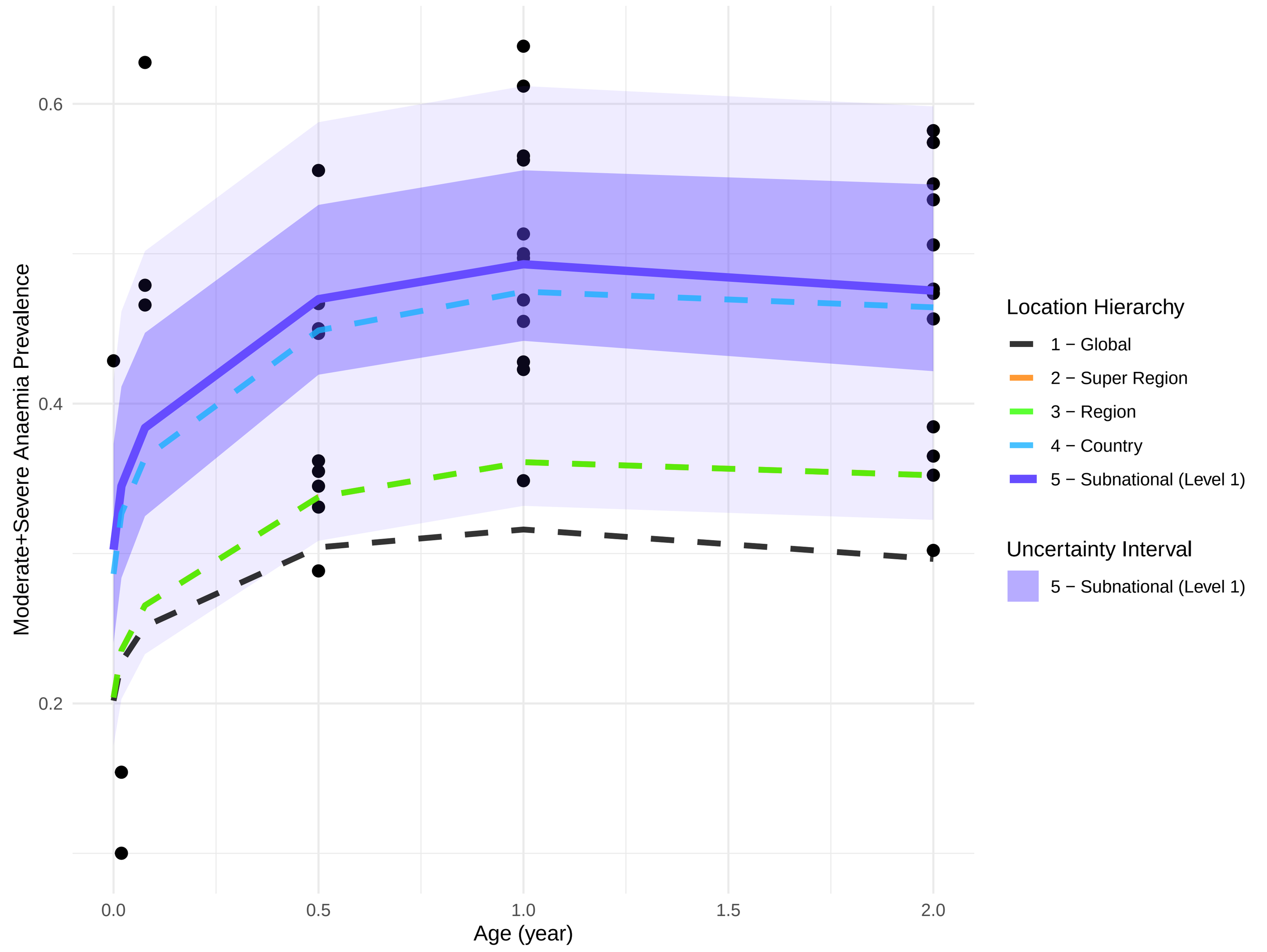

MR−BRT Age/Sex Fits − Non−pregnant Females <2 Years Old − Moderate+Severe Anaemia Prevalence − Jammu & Kashmir and Ladakh, Rural
WHO−2024 Adjustment Method

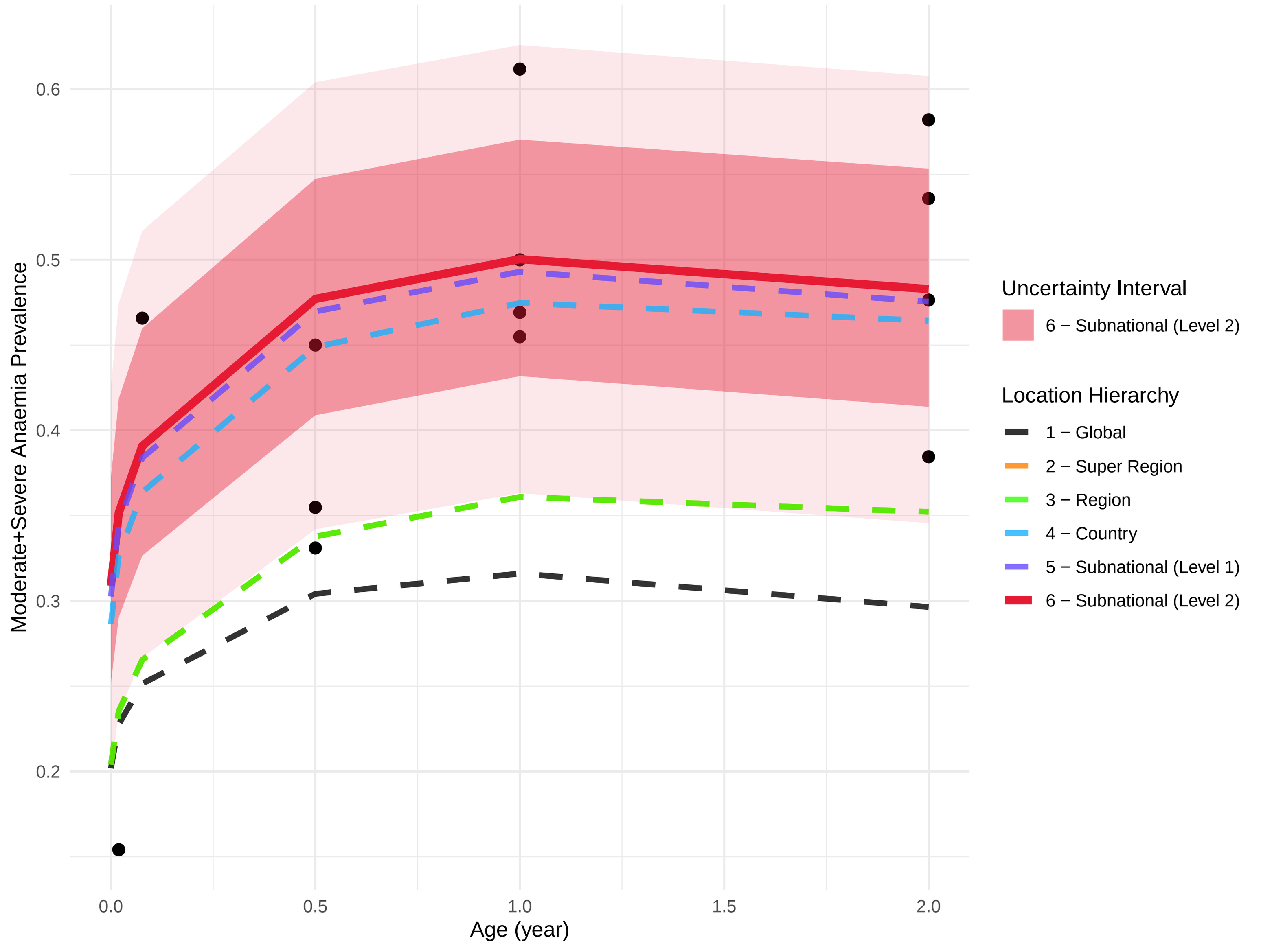

MR−BRT Age/Sex Fits − Non−pregnant Females <2 Years Old − Moderate+Severe Anaemia Prevalence − Jammu & Kashmir and Ladakh, Urban
WHO−2024 Adjustment Method

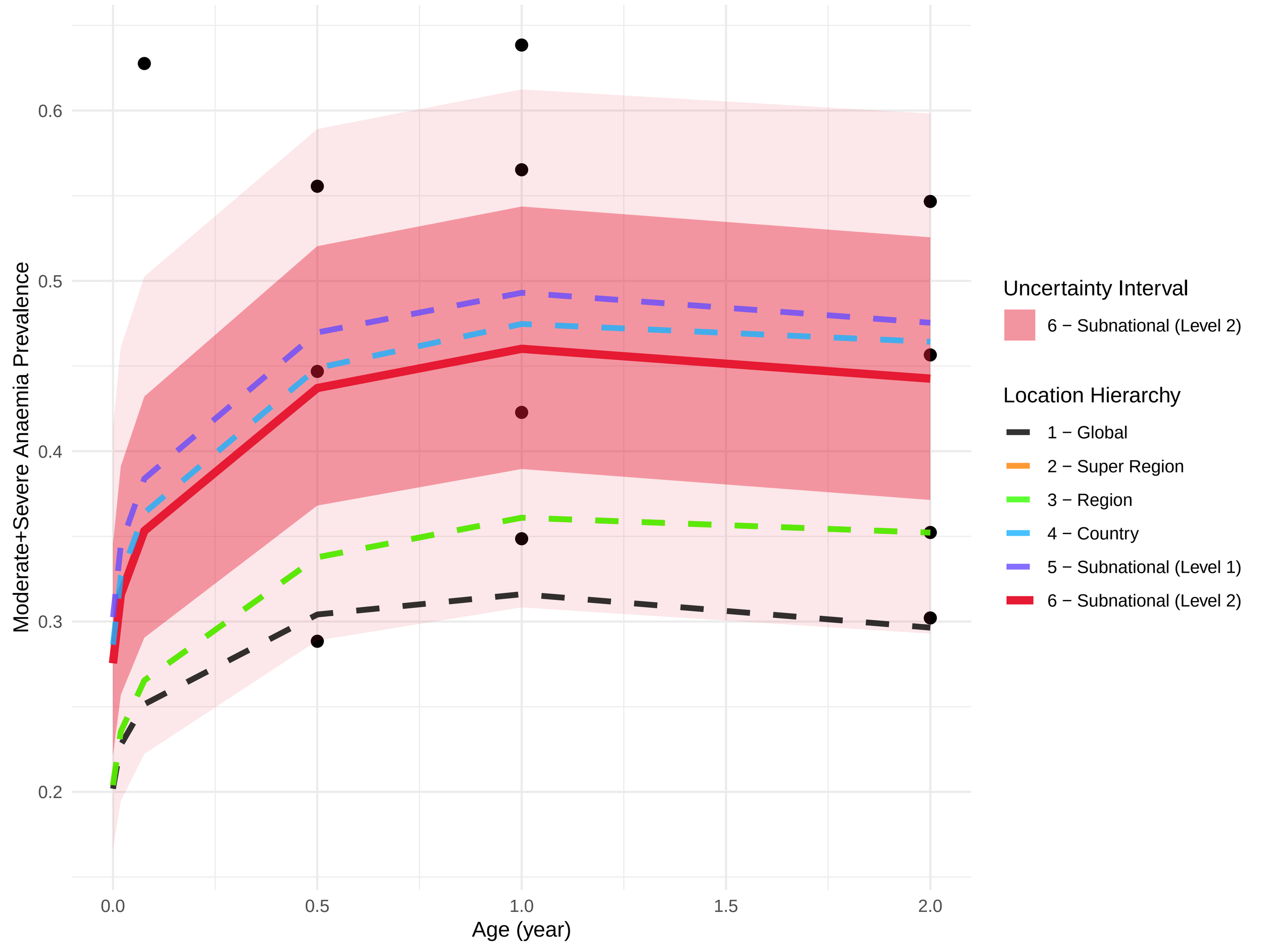

MR−BRT Age/Sex Fits − Non−pregnant Females <2 Years Old − Moderate+Severe Anaemia Prevalence − Jharkhand
WHO−2024 Adjustment Method

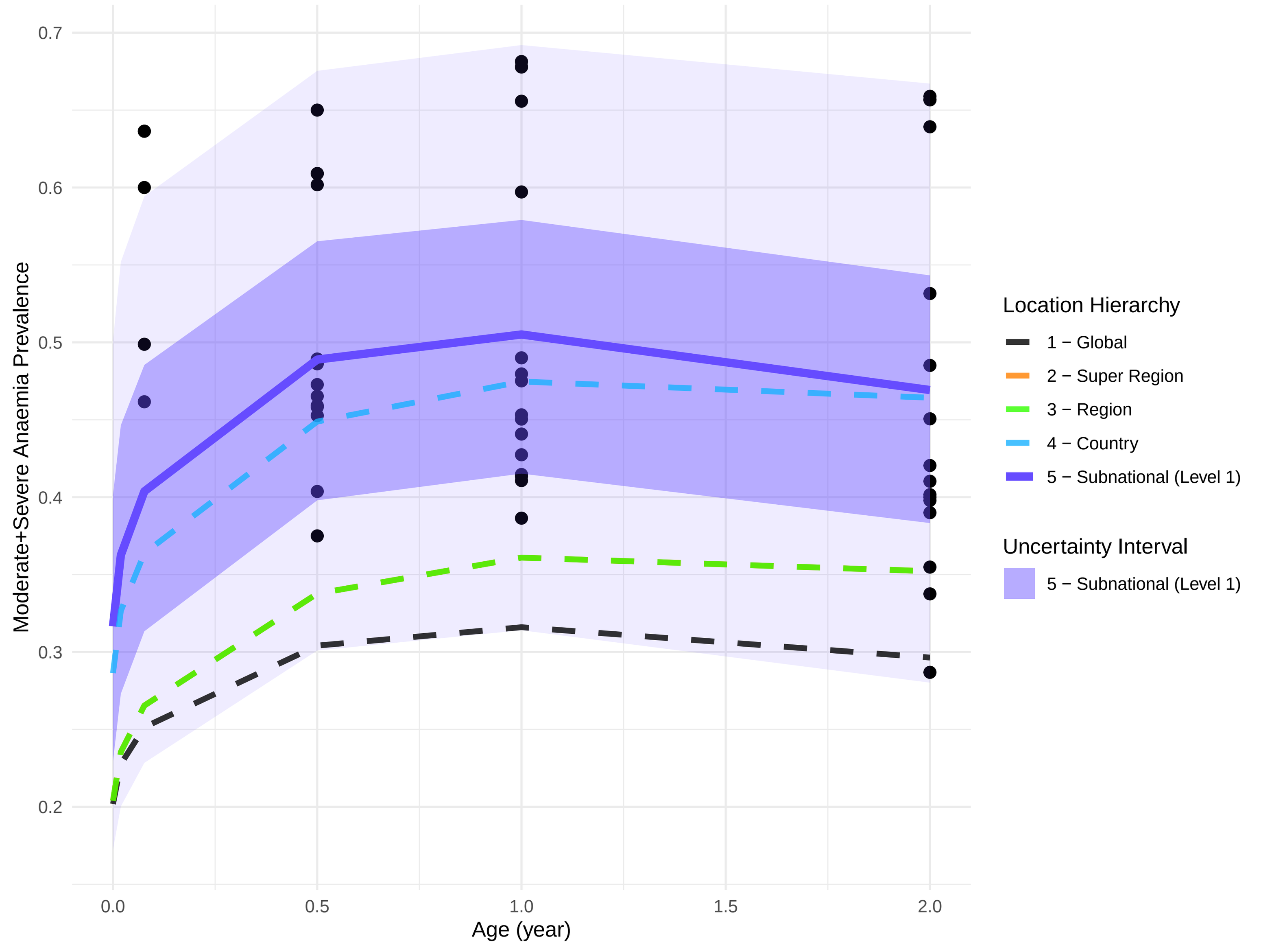

MR−BRT Age/Sex Fits − Non−pregnant Females <2 Years Old − Moderate+Severe Anaemia Prevalence − Jharkhand, Rural
WHO−2024 Adjustment Method

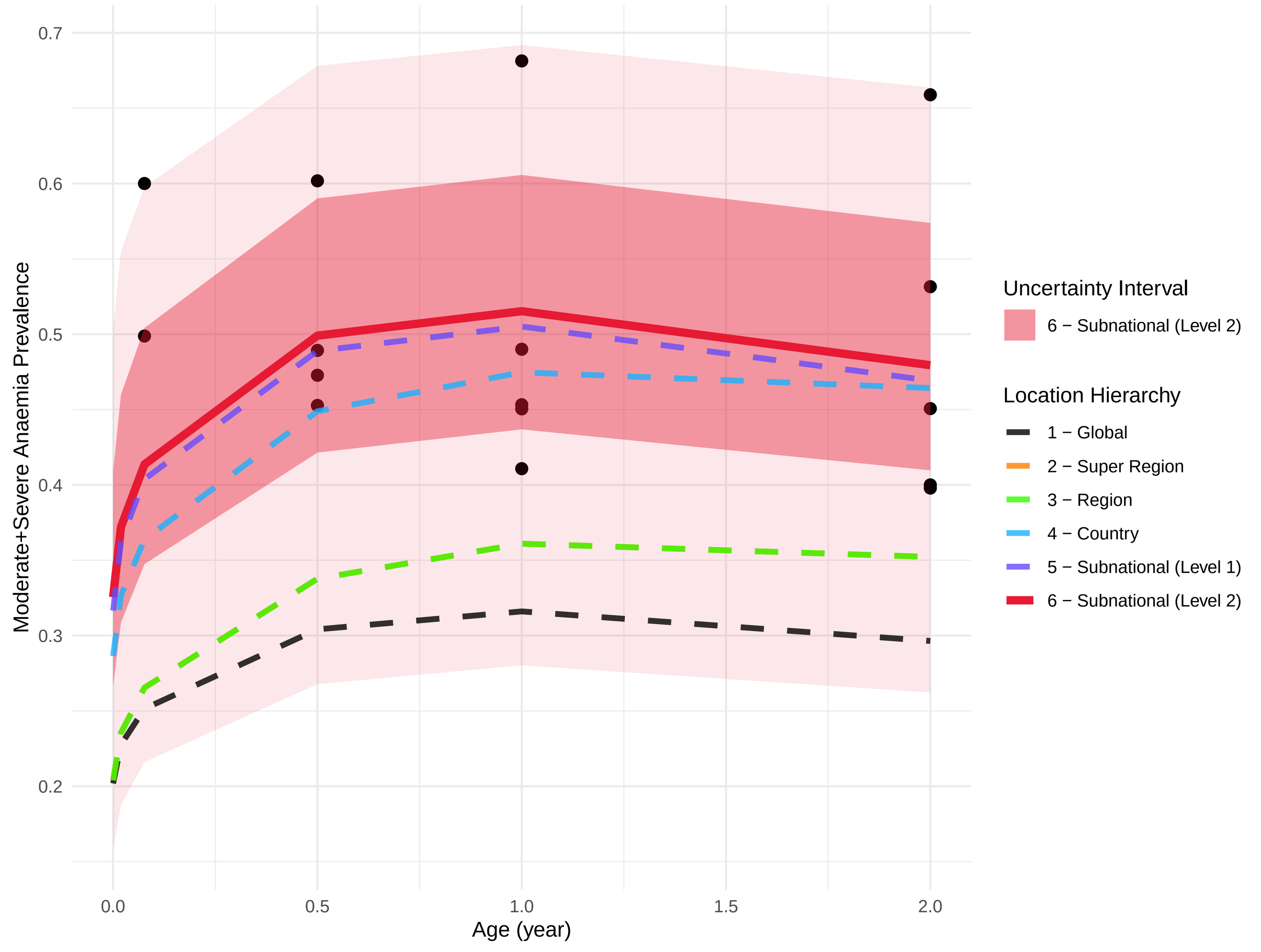

MR−BRT Age/Sex Fits − Non−pregnant Females <2 Years Old − Moderate+Severe Anaemia Prevalence − Jharkhand, Urban
WHO−2024 Adjustment Method

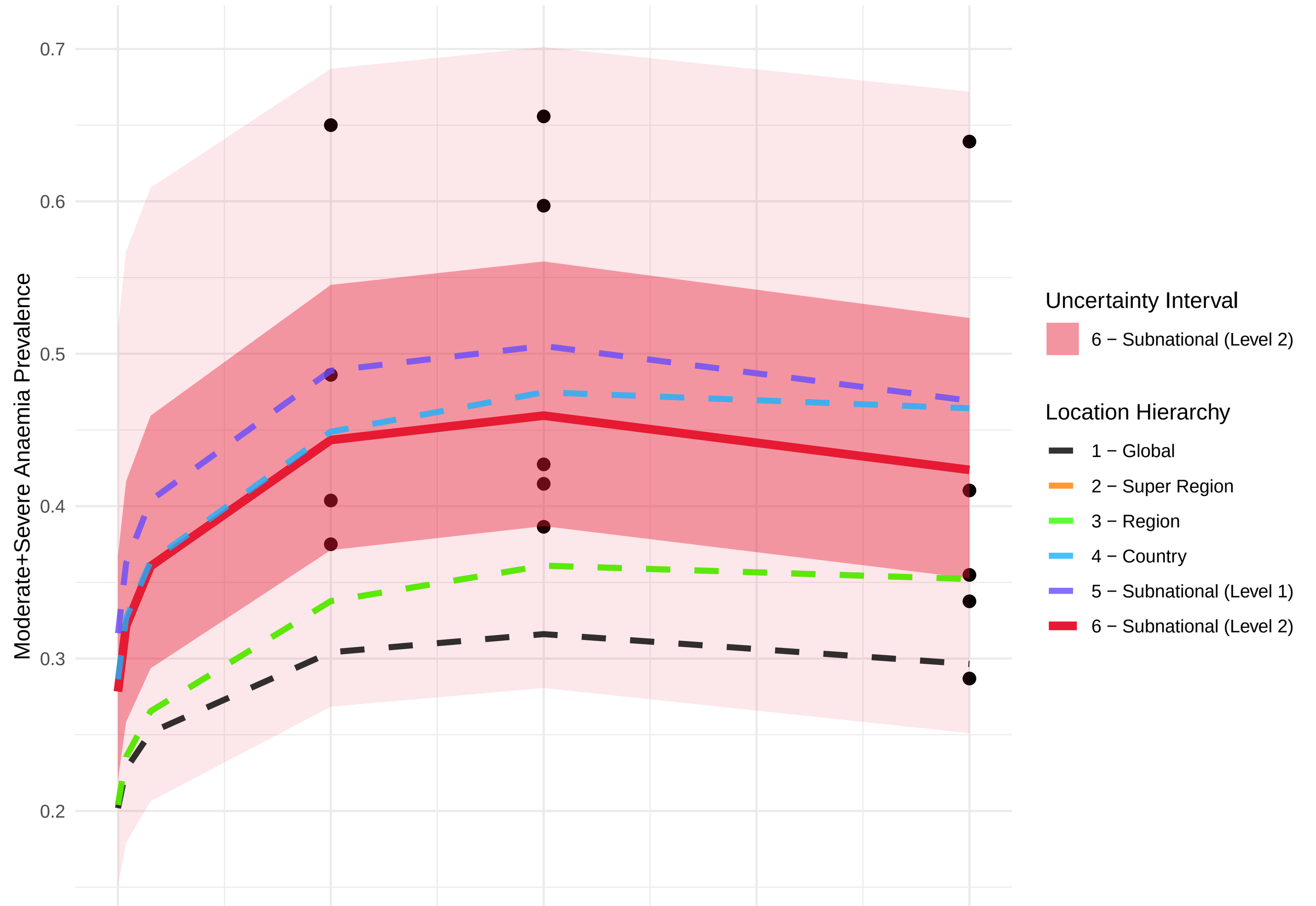

MR−BRT Age/Sex Fits − Non−pregnant Females <2 Years Old − Moderate+Severe Anaemia Prevalence − Karnataka
WHO−2024 Adjustment Method

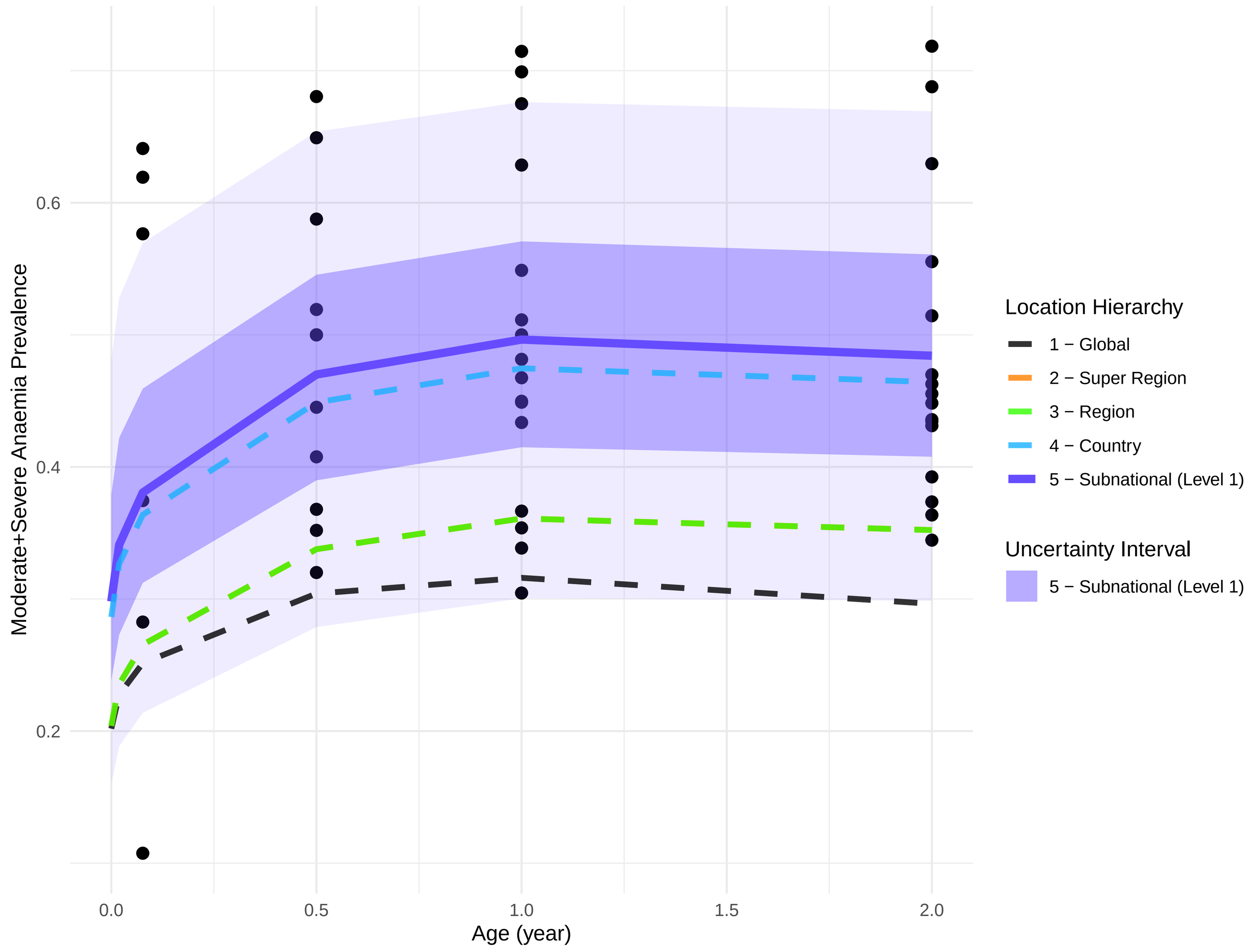

MR−BRT Age/Sex Fits − Non−pregnant Females <2 Years Old − Moderate+Severe Anaemia Prevalence − Karnataka, Rural
WHO−2024 Adjustment Method

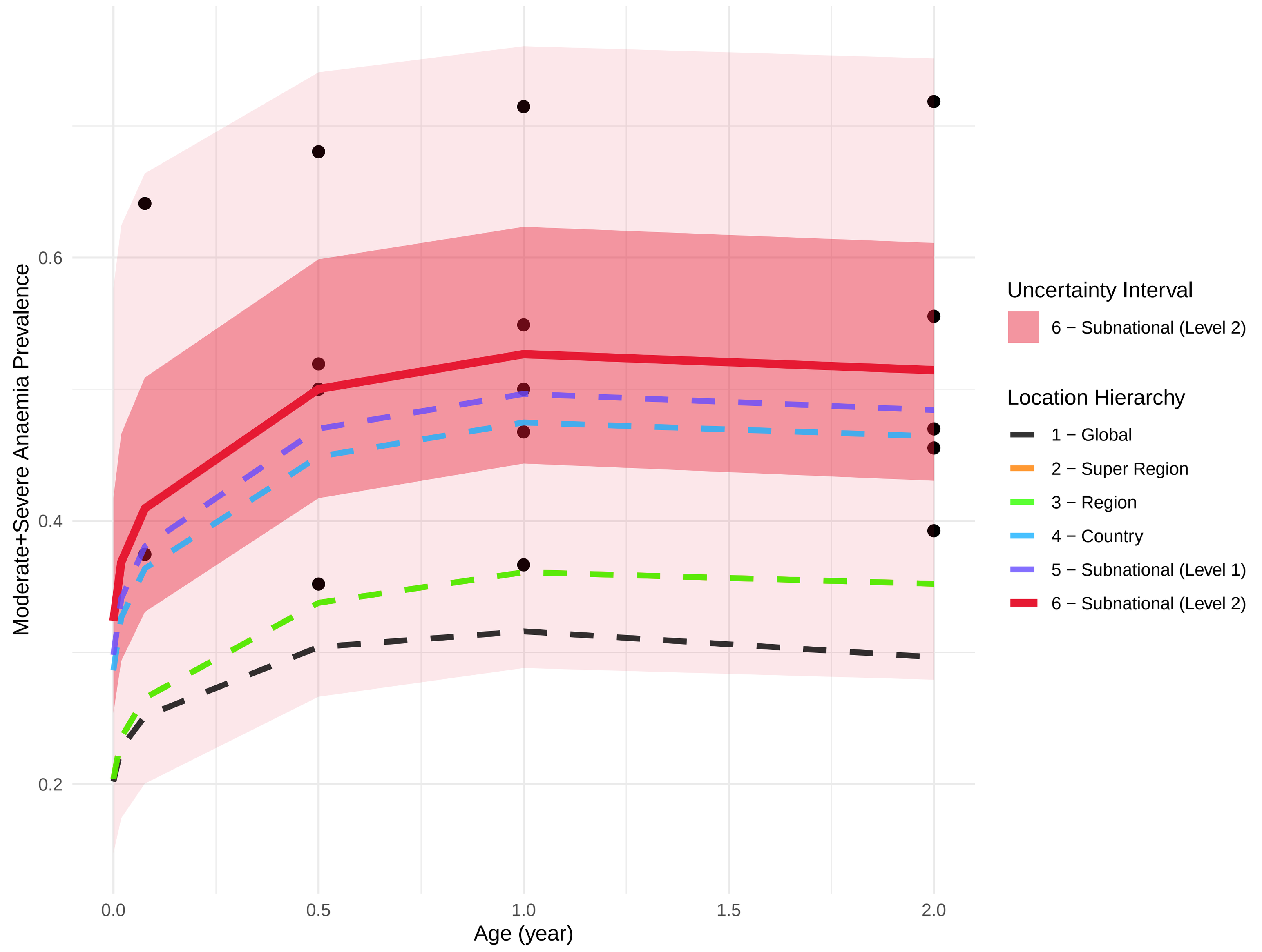

MR−BRT Age/Sex Fits − Non−pregnant Females <2 Years Old − Moderate+Severe Anaemia Prevalence − Karnataka, Urban
WHO−2024 Adjustment Method

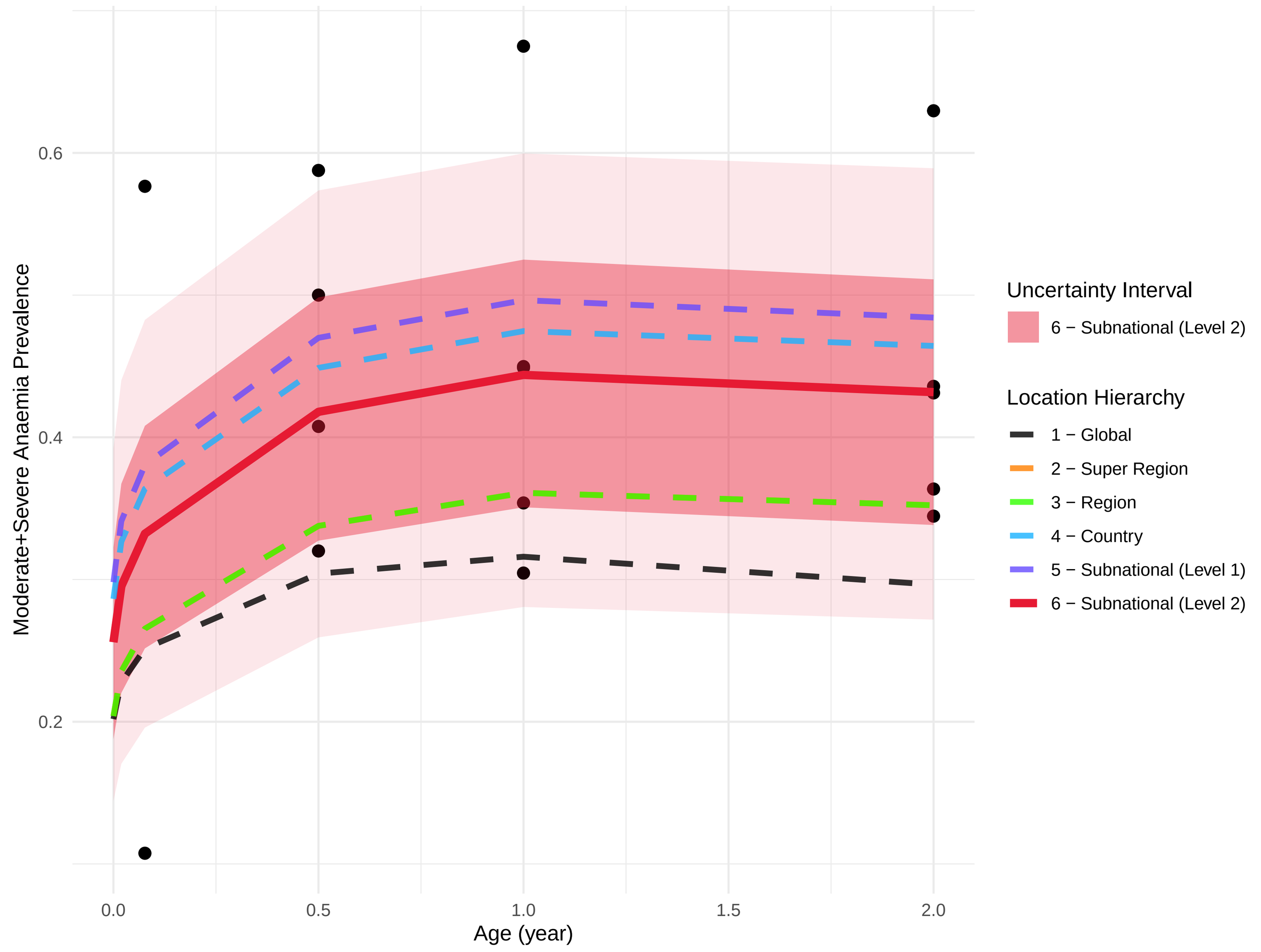

MR−BRT Age/Sex Fits − Non−pregnant Females <2 Years Old − Moderate+Severe Anaemia Prevalence − Kerala
WHO−2024 Adjustment Method

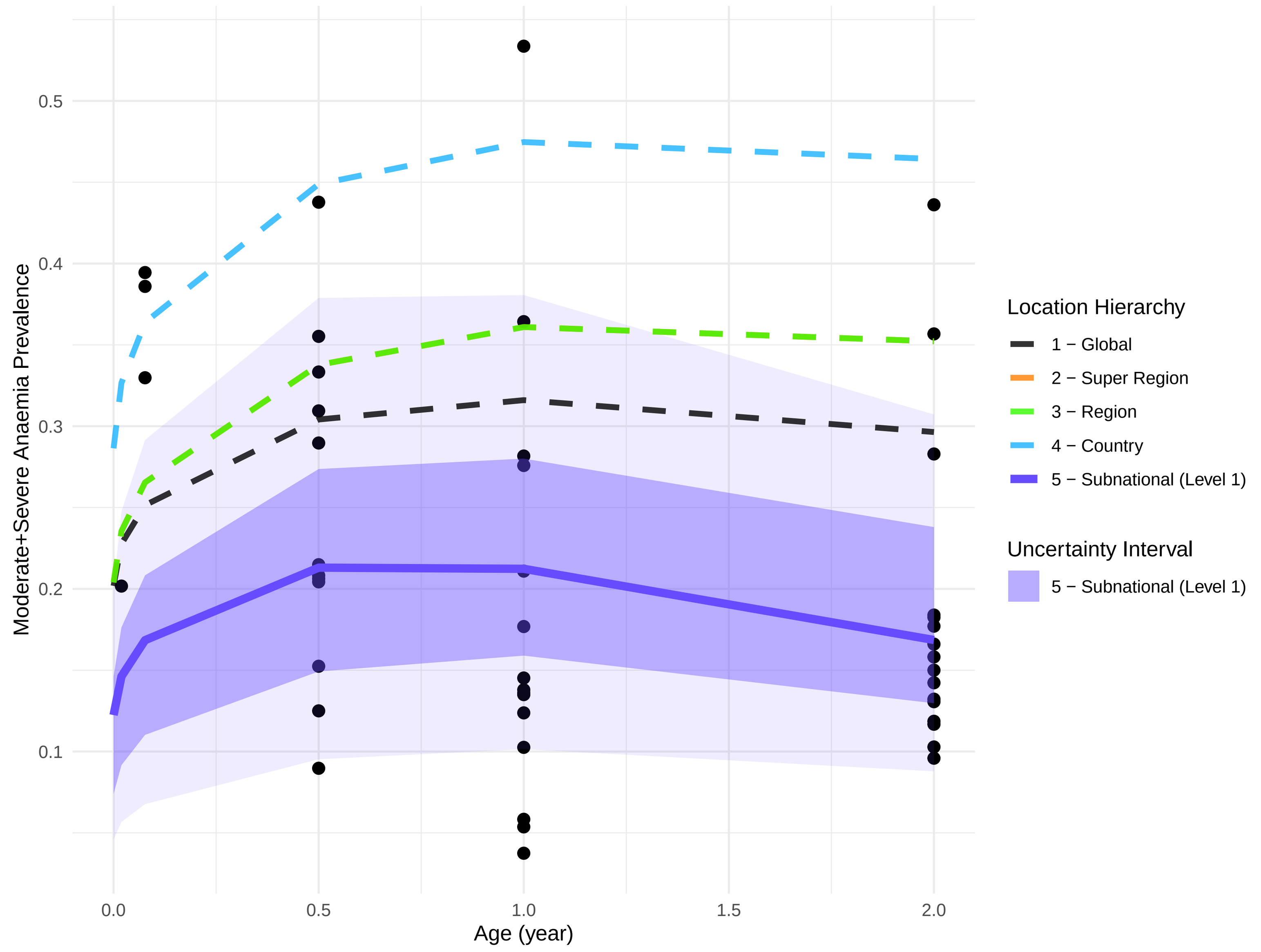

MR−BRT Age/Sex Fits − Non−pregnant Females <2 Years Old − Moderate+Severe Anaemia Prevalence − Kerala, Rural
WHO−2024 Adjustment Method

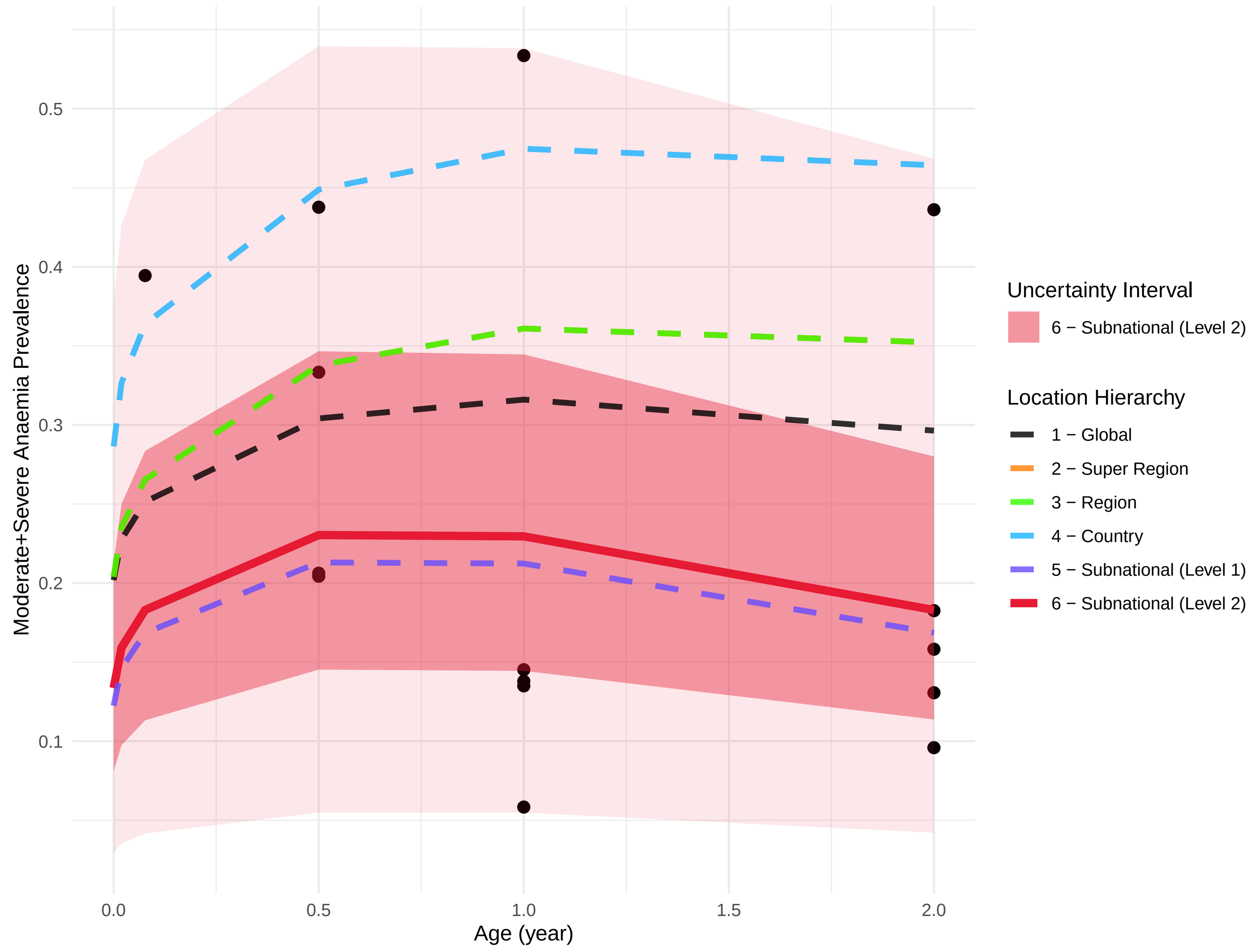

MR−BRT Age/Sex Fits − Non−pregnant Females <2 Years Old − Moderate+Severe Anaemia Prevalence − Kerala, Urban
WHO−2024 Adjustment Method

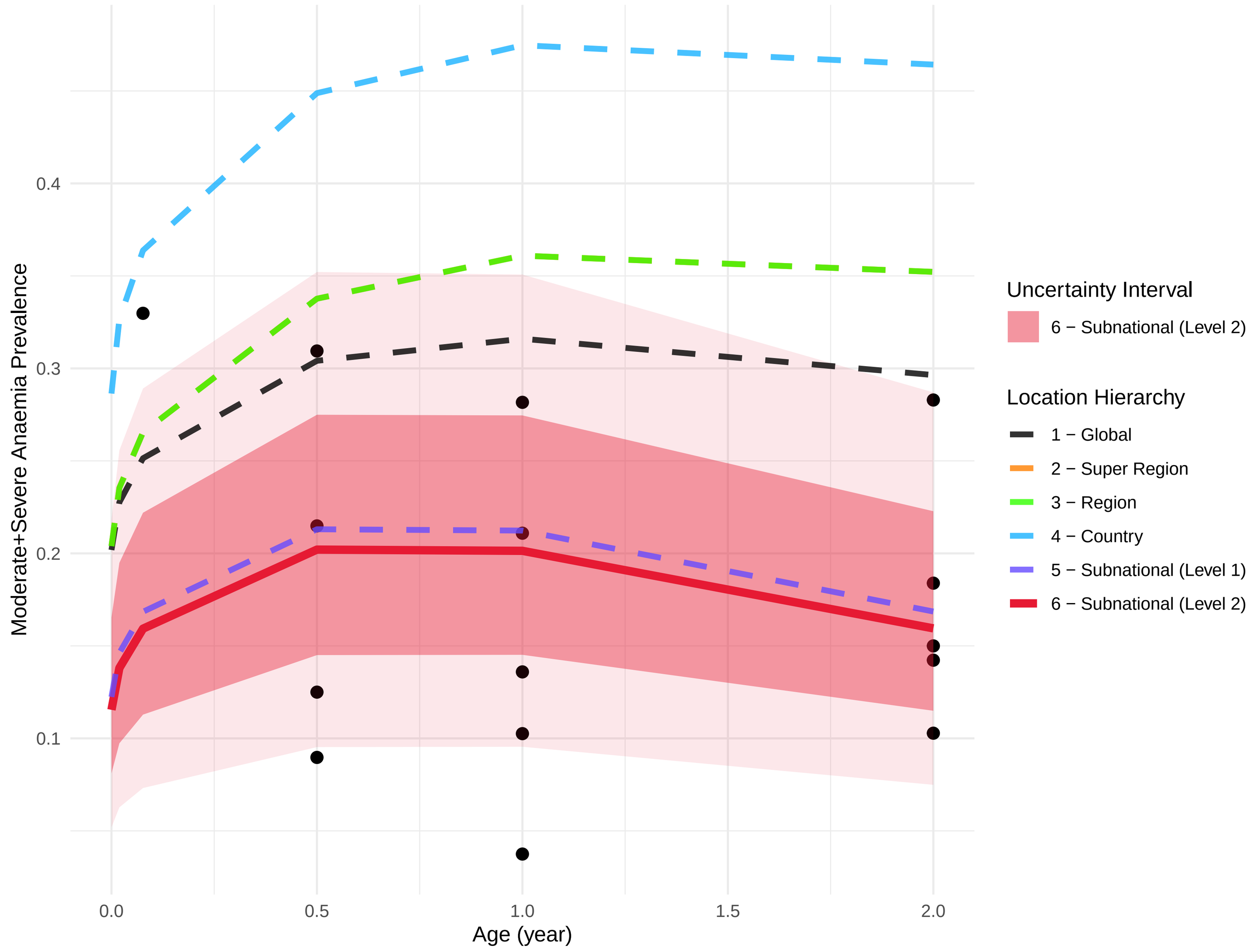

MR−BRT Age/Sex Fits − Non−pregnant Females <2 Years Old − Moderate+Severe Anaemia Prevalence − Madhya Pradesh
WHO−2024 Adjustment Method

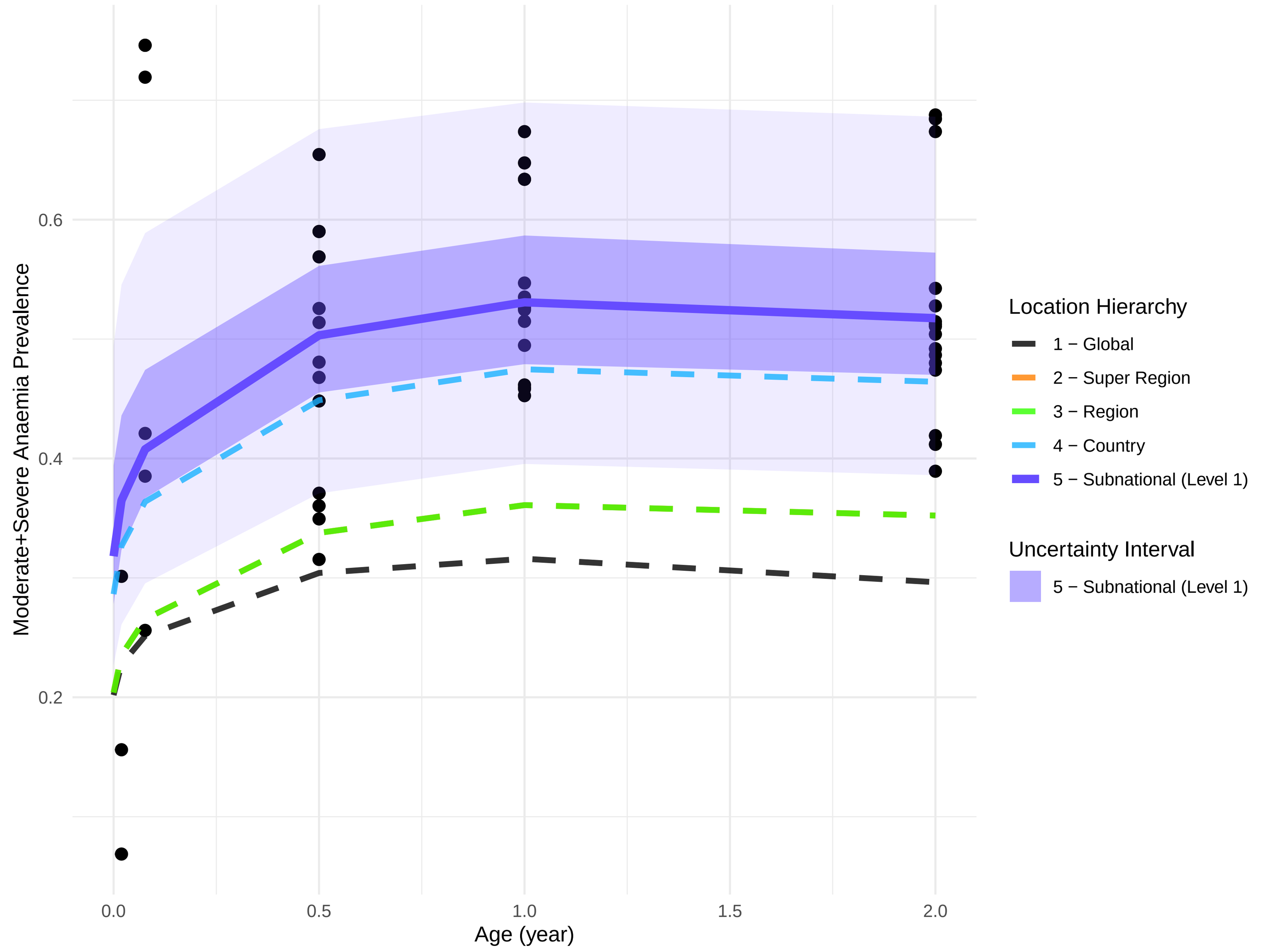

MR−BRT Age/Sex Fits − Non−pregnant Females <2 Years Old − Moderate+Severe Anaemia Prevalence − Madhya Pradesh, Rural
WHO−2024 Adjustment Method

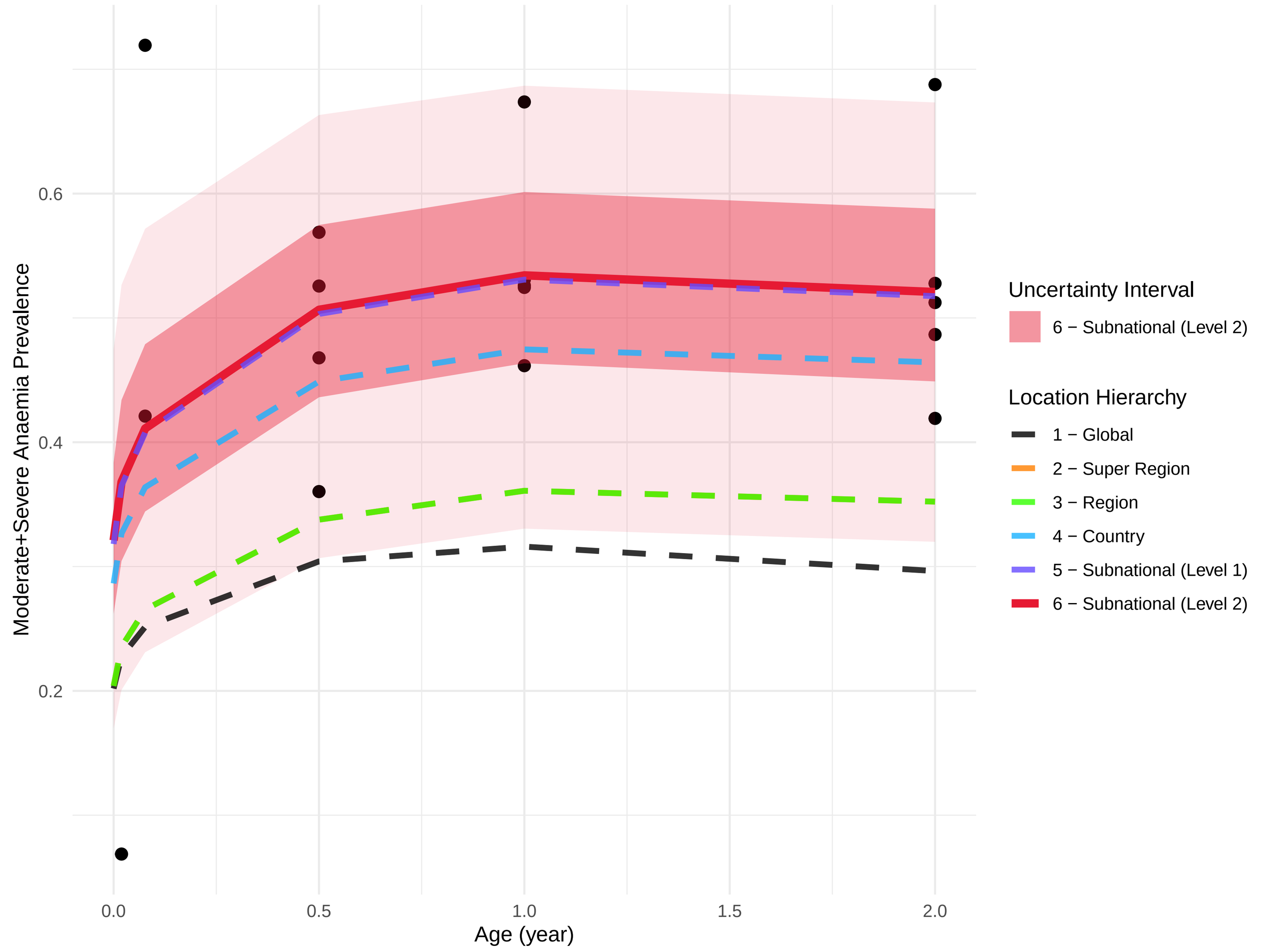

MR−BRT Age/Sex Fits − Non−pregnant Females <2 Years Old − Moderate+Severe Anaemia Prevalence − Madhya Pradesh, Urban
WHO−2024 Adjustment Method

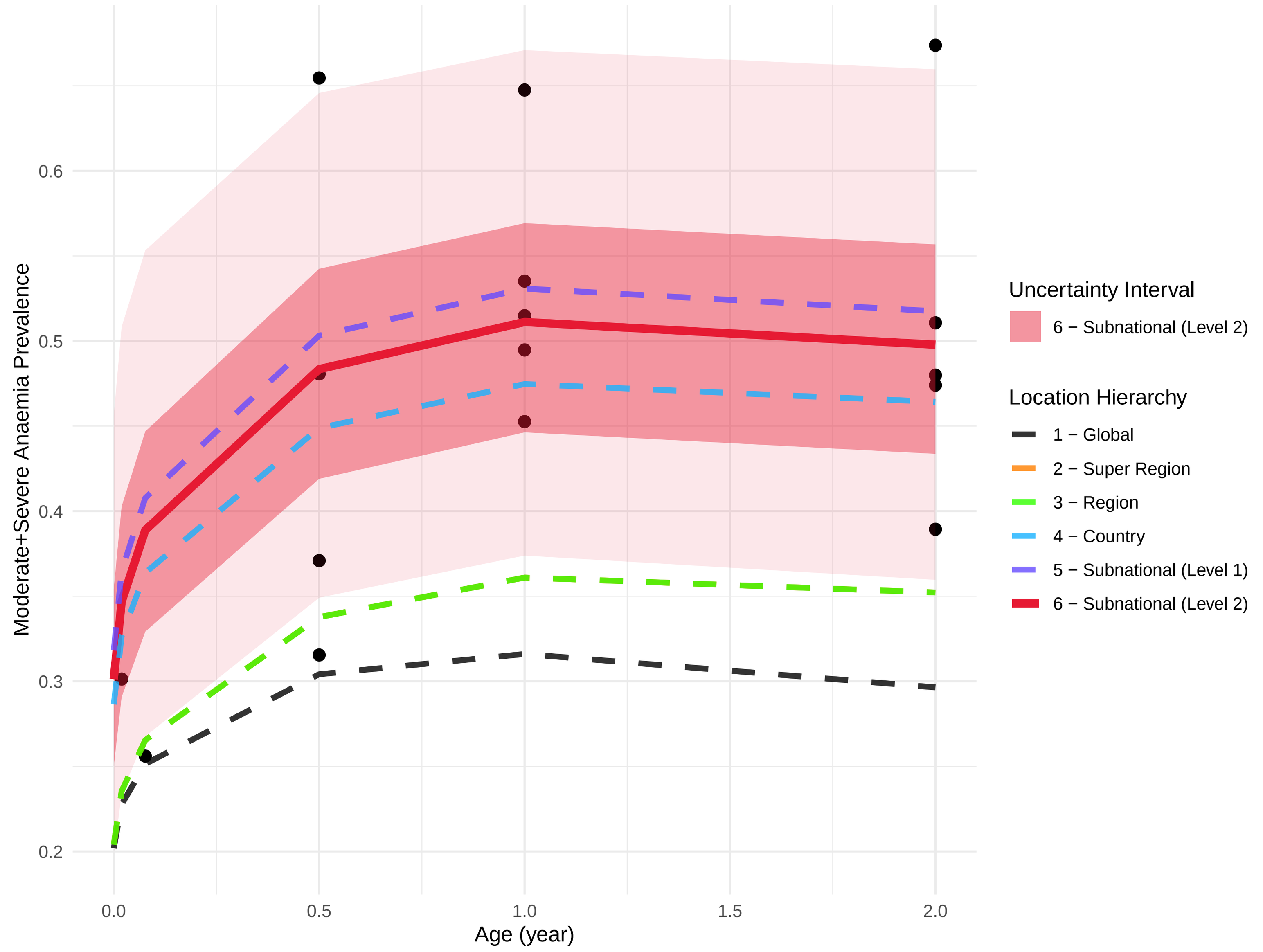

MR−BRT Age/Sex Fits − Non−pregnant Females <2 Years Old − Moderate+Severe Anaemia Prevalence − Maharashtra
WHO−2024 Adjustment Method

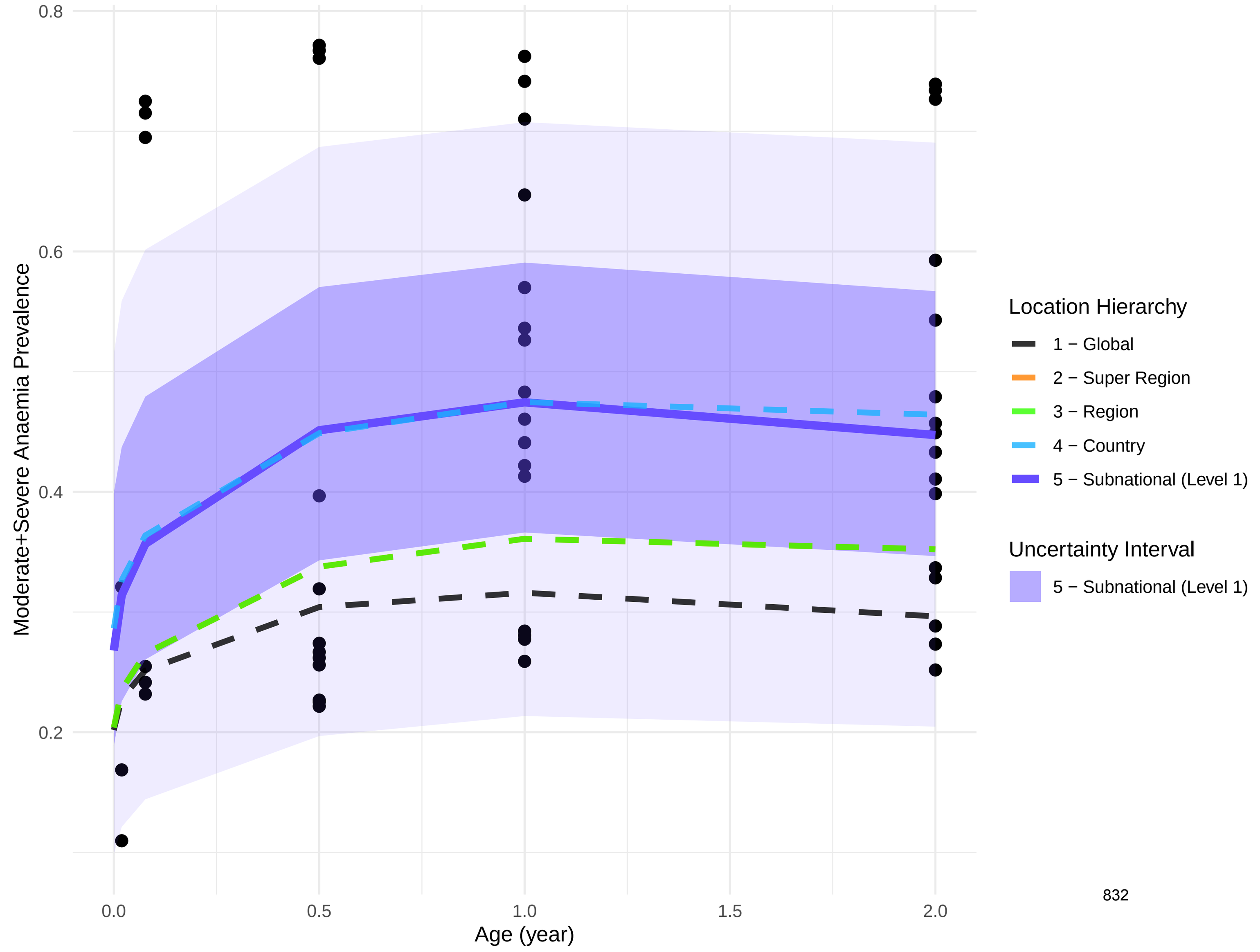

MR−BRT Age/Sex Fits − Non−pregnant Females <2 Years Old − Moderate+Severe Anaemia Prevalence − Maharashtra, Rural
WHO−2024 Adjustment Method

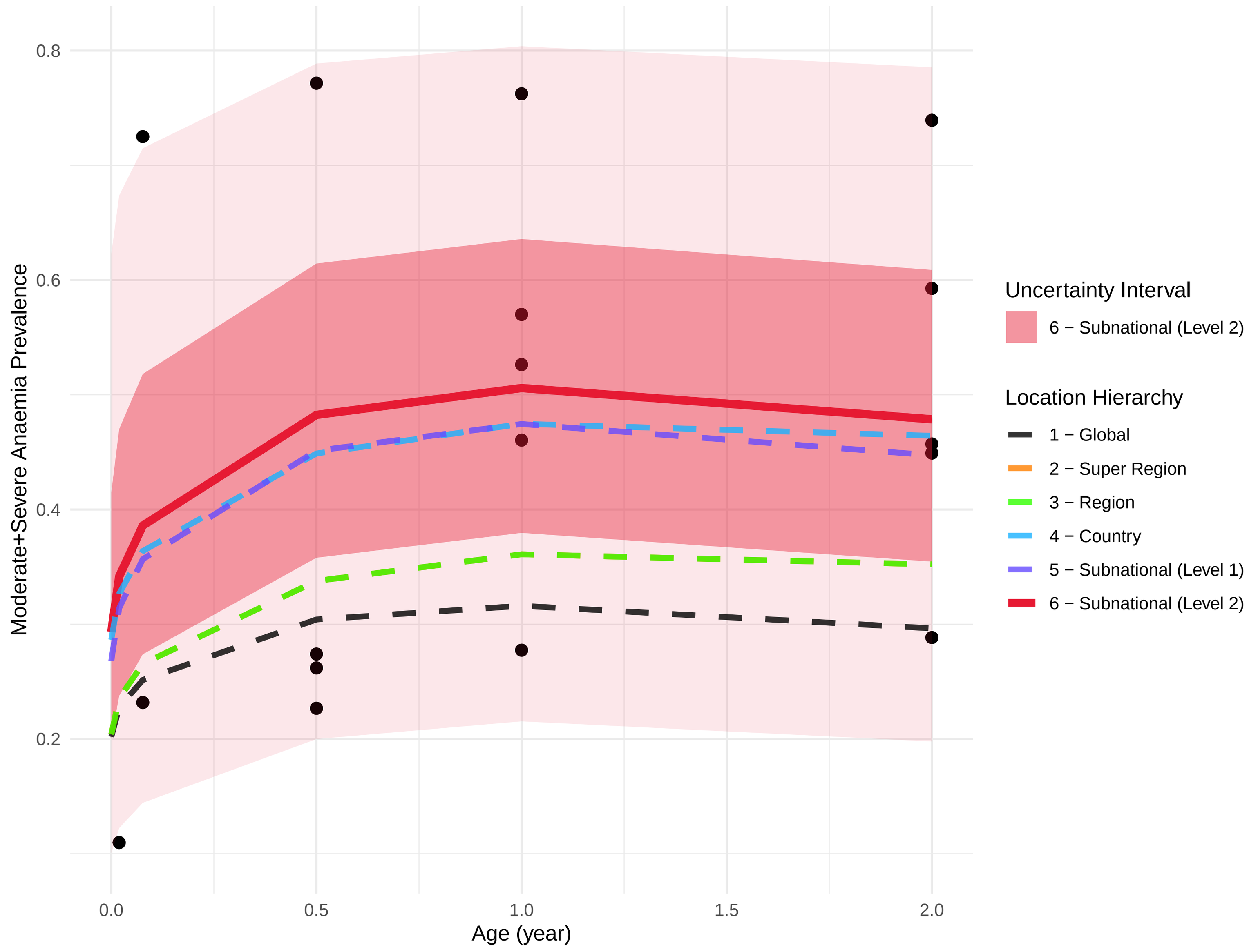

MR−BRT Age/Sex Fits − Non−pregnant Females <2 Years Old − Moderate+Severe Anaemia Prevalence − Maharashtra, Urban
WHO−2024 Adjustment Method

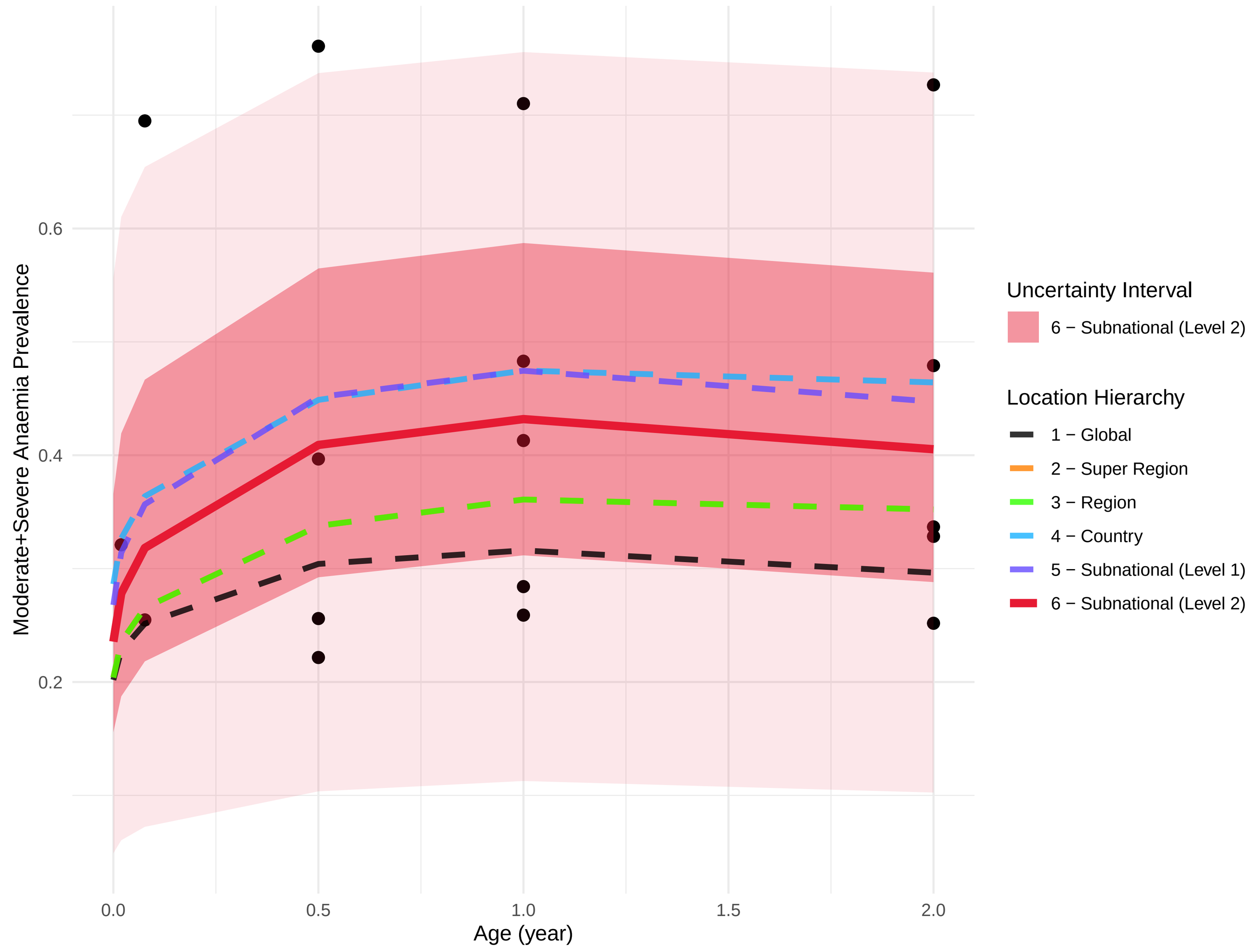

MR−BRT Age/Sex Fits − Non−pregnant Females <2 Years Old − Moderate+Severe Anaemia Prevalence − Manipur
WHO−2024 Adjustment Method

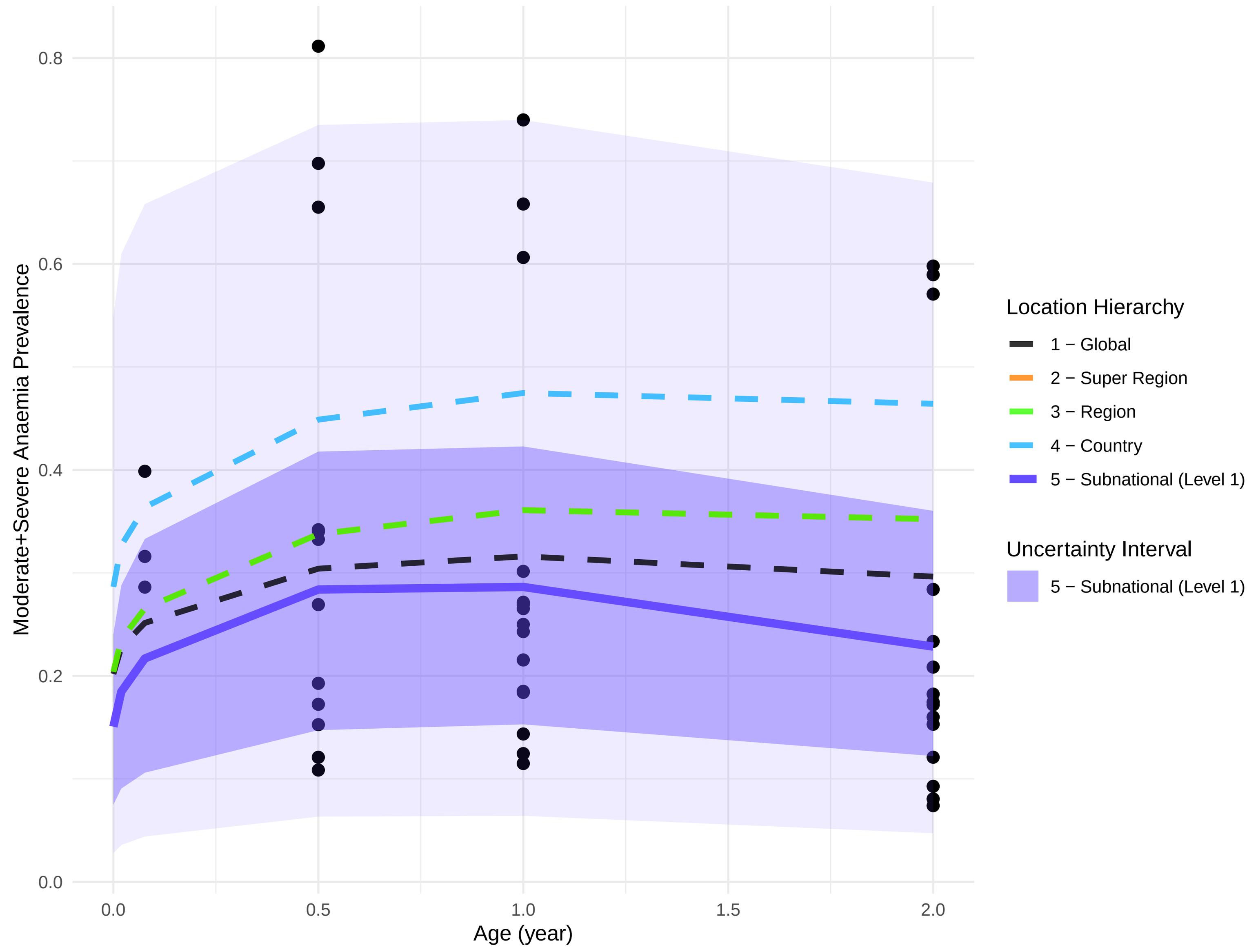

MR−BRT Age/Sex Fits − Non−pregnant Females <2 Years Old − Moderate+Severe Anaemia Prevalence − Manipur, Rural
WHO−2024 Adjustment Method

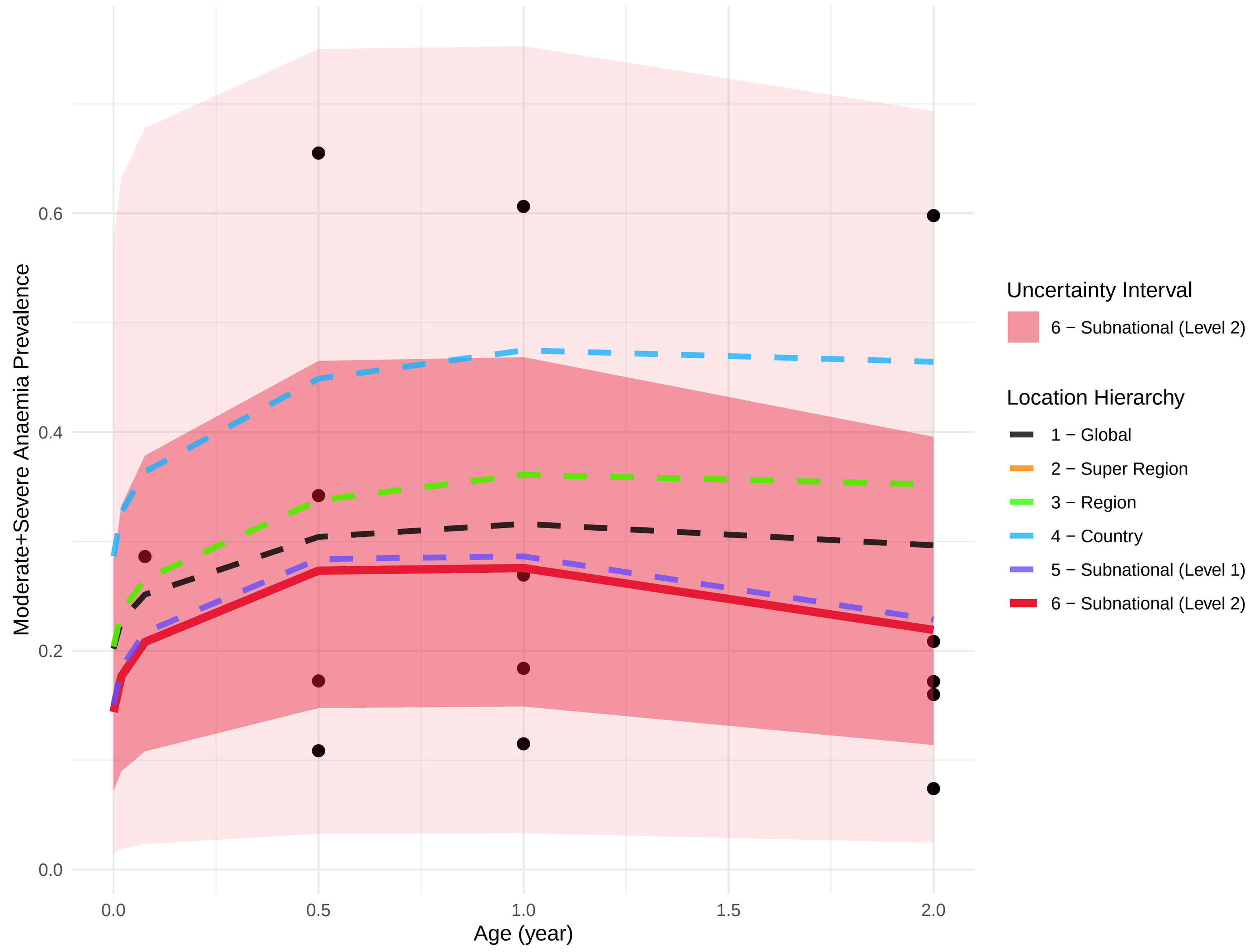

MR−BRT Age/Sex Fits − Non−pregnant Females <2 Years Old − Moderate+Severe Anaemia Prevalence − Manipur, Urban
WHO−2024 Adjustment Method

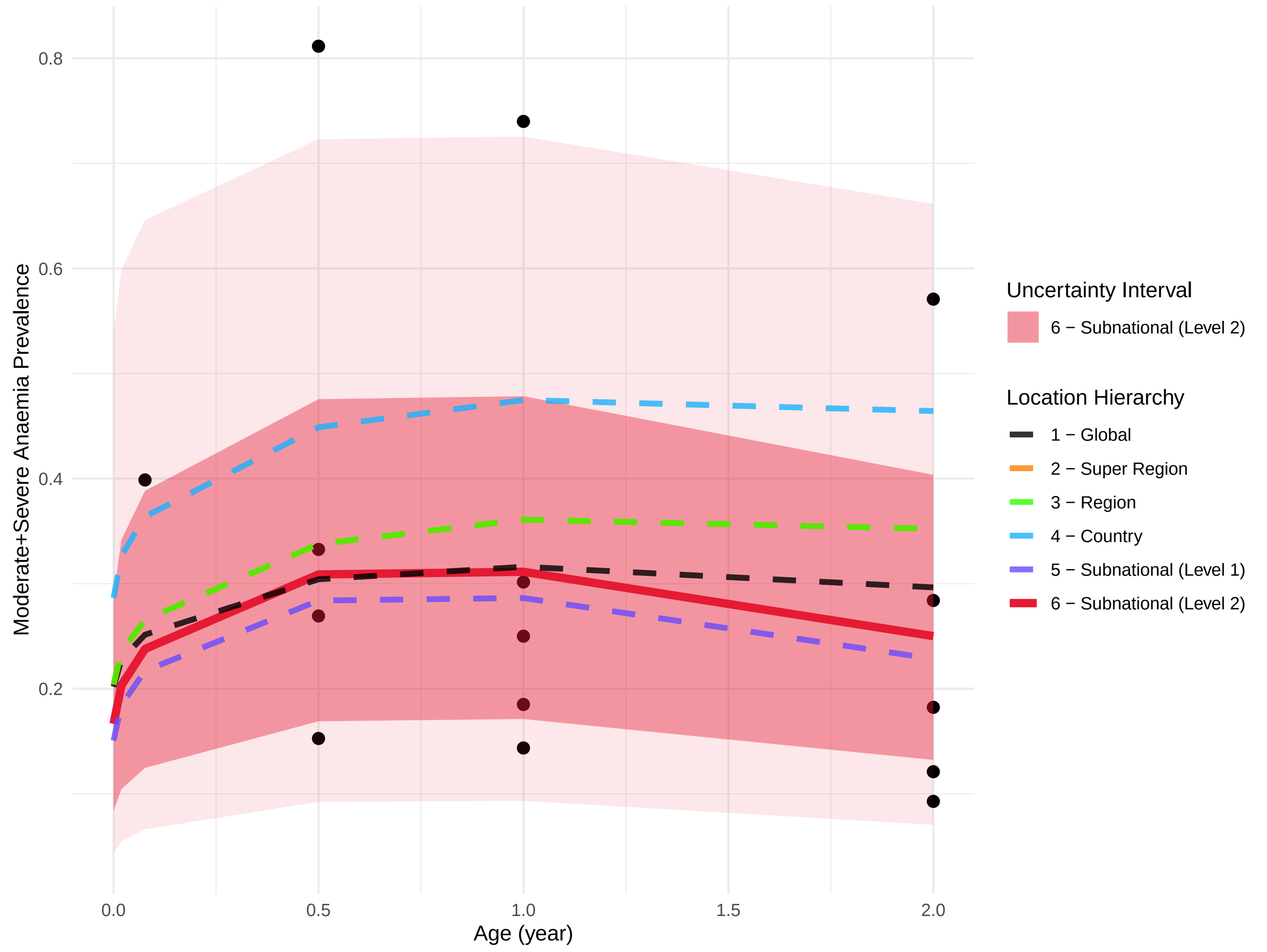

MR−BRT Age/Sex Fits − Non−pregnant Females <2 Years Old − Moderate+Severe Anaemia Prevalence − Meghalaya
WHO−2024 Adjustment Method

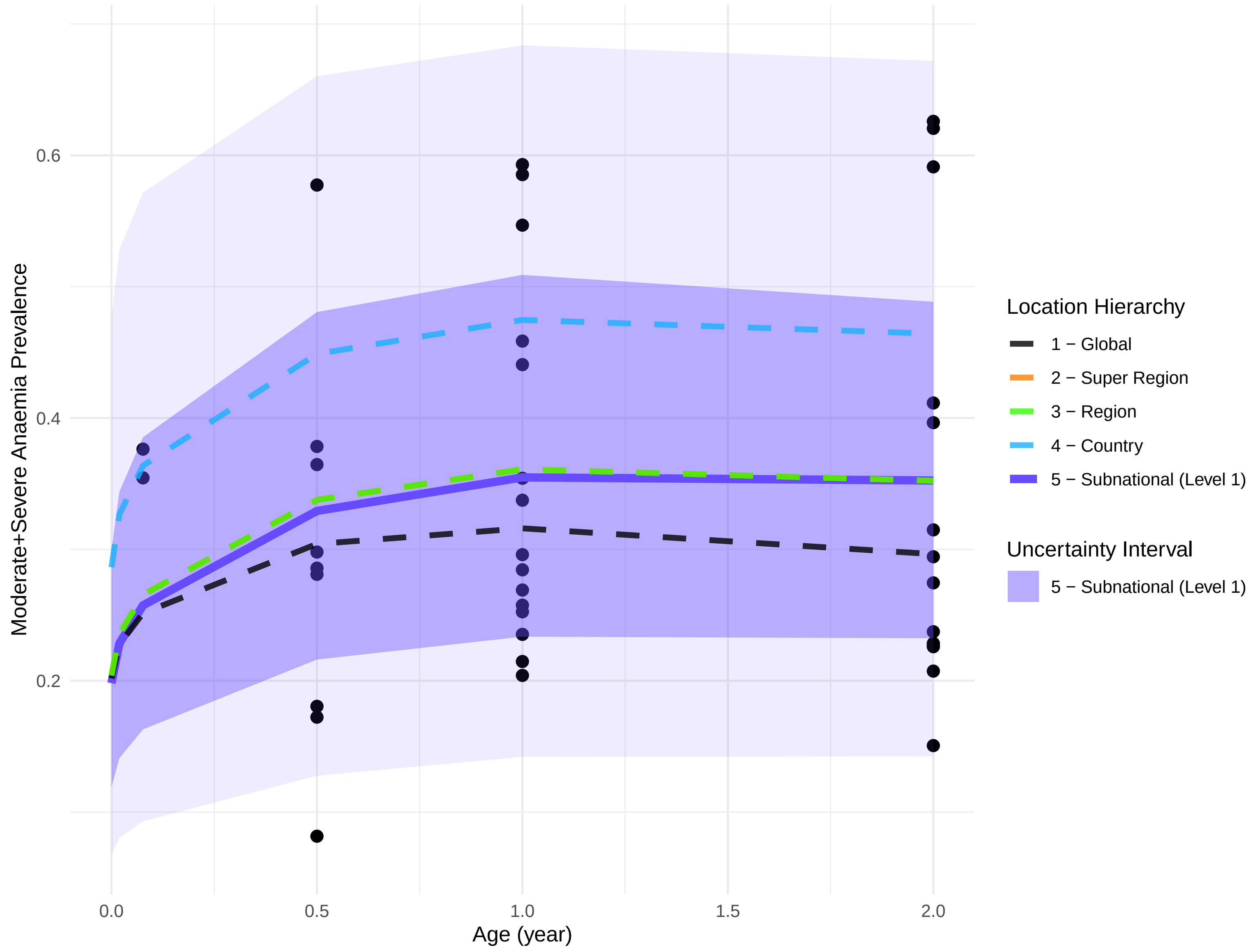

MR−BRT Age/Sex Fits − Non−pregnant Females <2 Years Old − Moderate+Severe Anaemia Prevalence − Meghalaya, Rural
WHO−2024 Adjustment Method

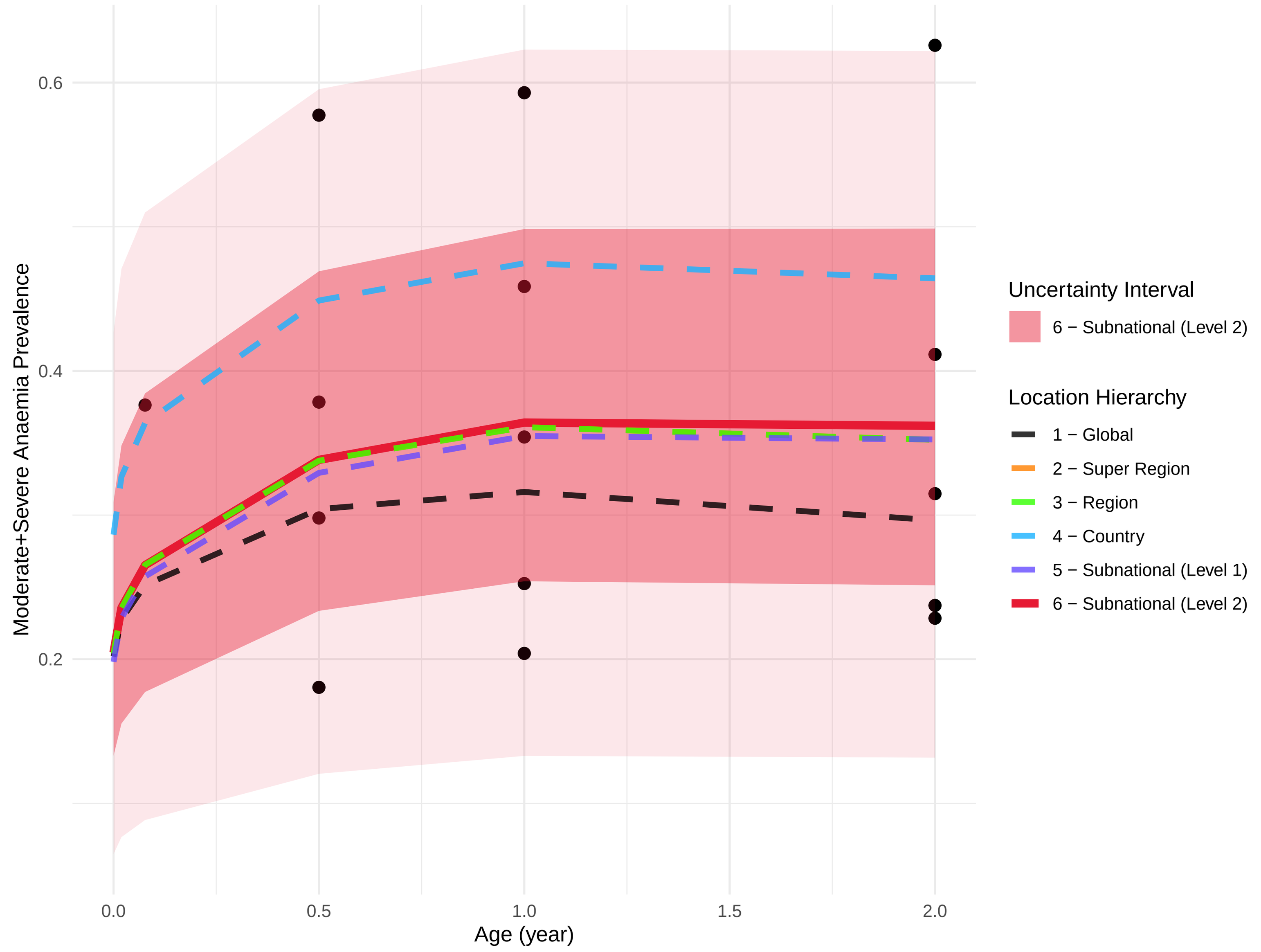

MR−BRT Age/Sex Fits − Non−pregnant Females <2 Years Old − Moderate+Severe Anaemia Prevalence − Meghalaya, Urban
WHO−2024 Adjustment Method

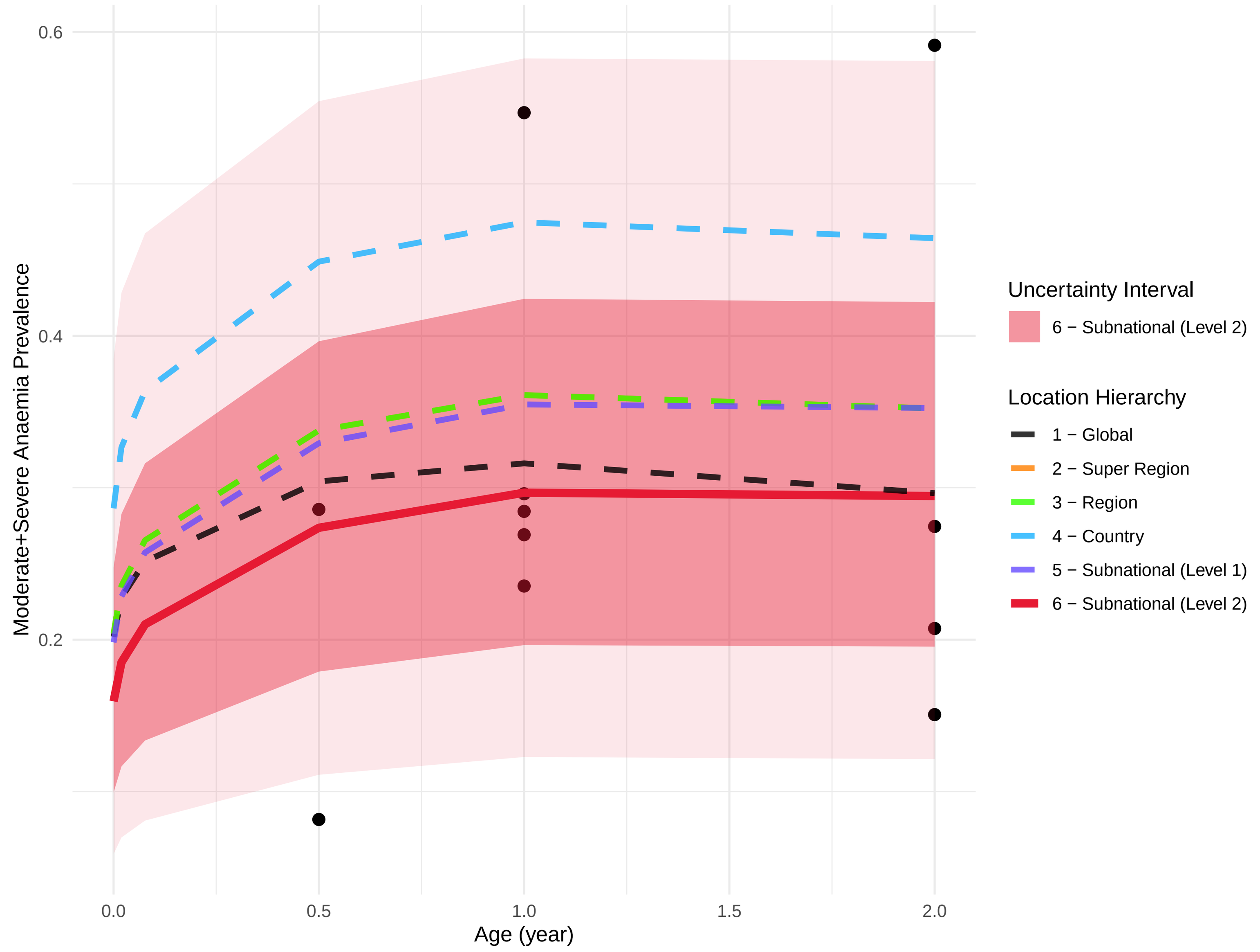

MR−BRT Age/Sex Fits − Non−pregnant Females <2 Years Old − Moderate+Severe Anaemia Prevalence − Mizoram
WHO−2024 Adjustment Method

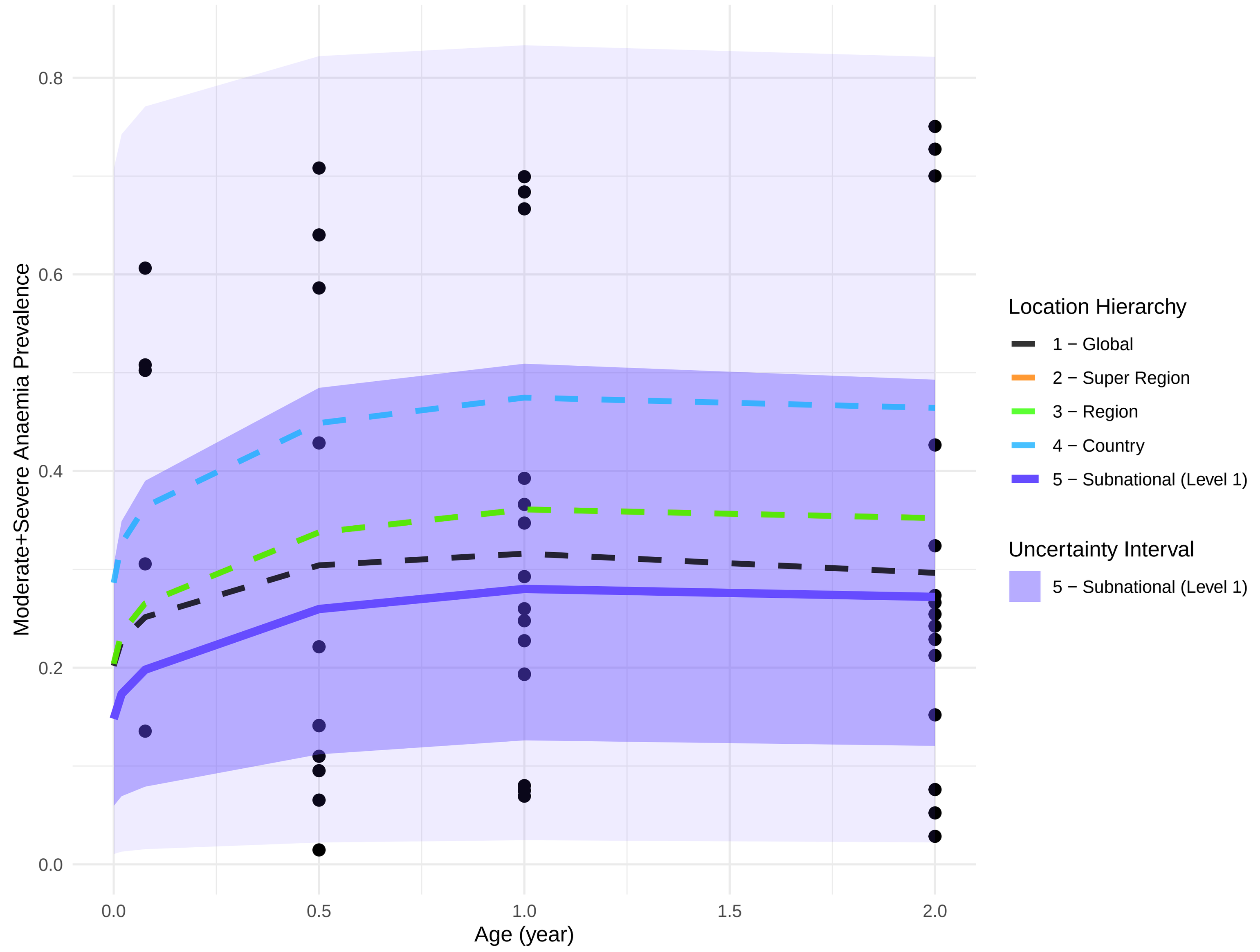

MR−BRT Age/Sex Fits − Non−pregnant Females <2 Years Old − Moderate+Severe Anaemia Prevalence − Mizoram, Rural
WHO−2024 Adjustment Method

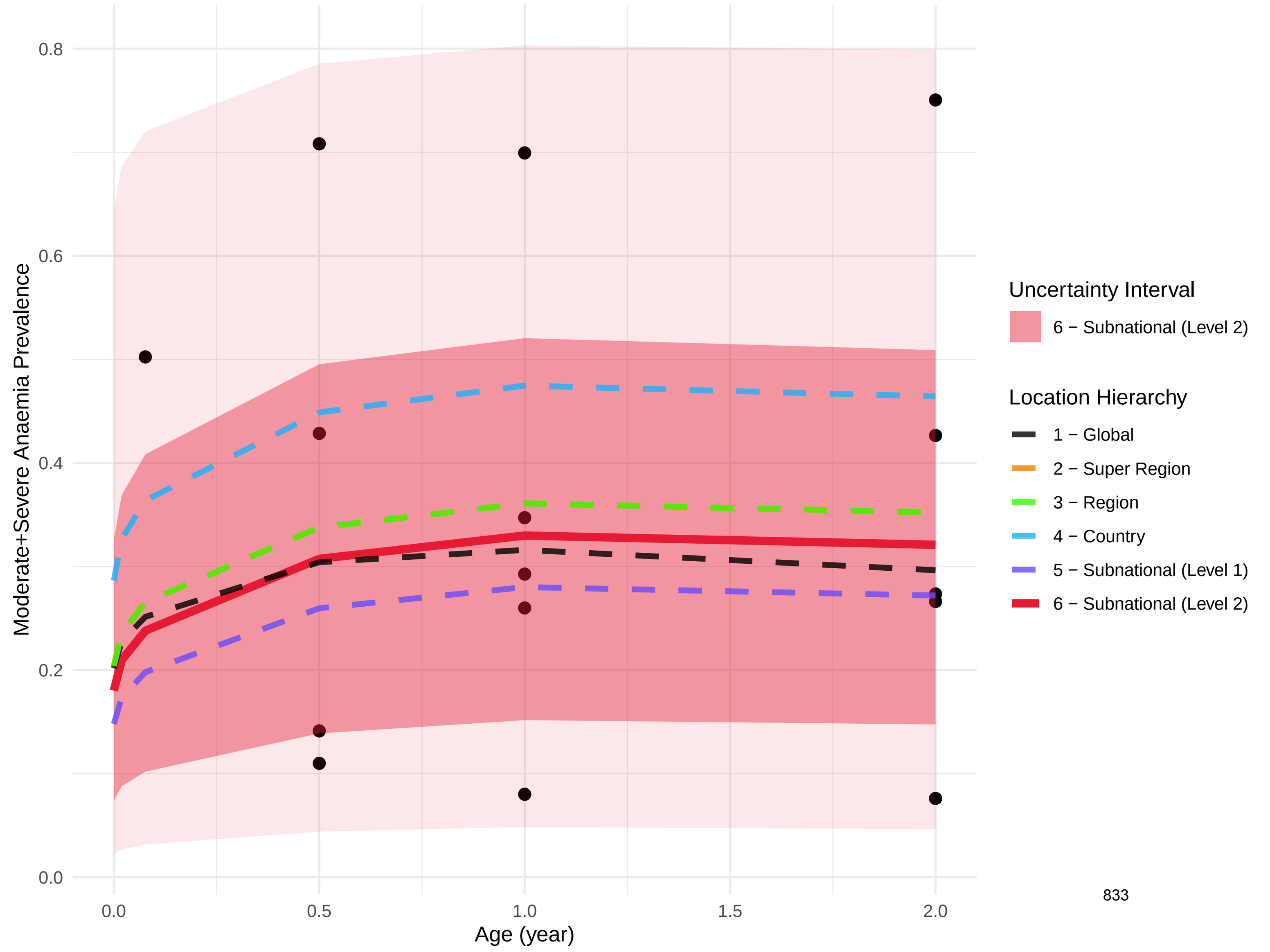

MR−BRT Age/Sex Fits − Non−pregnant Females <2 Years Old − Moderate+Severe Anaemia Prevalence − Mizoram, Urban
WHO−2024 Adjustment Method

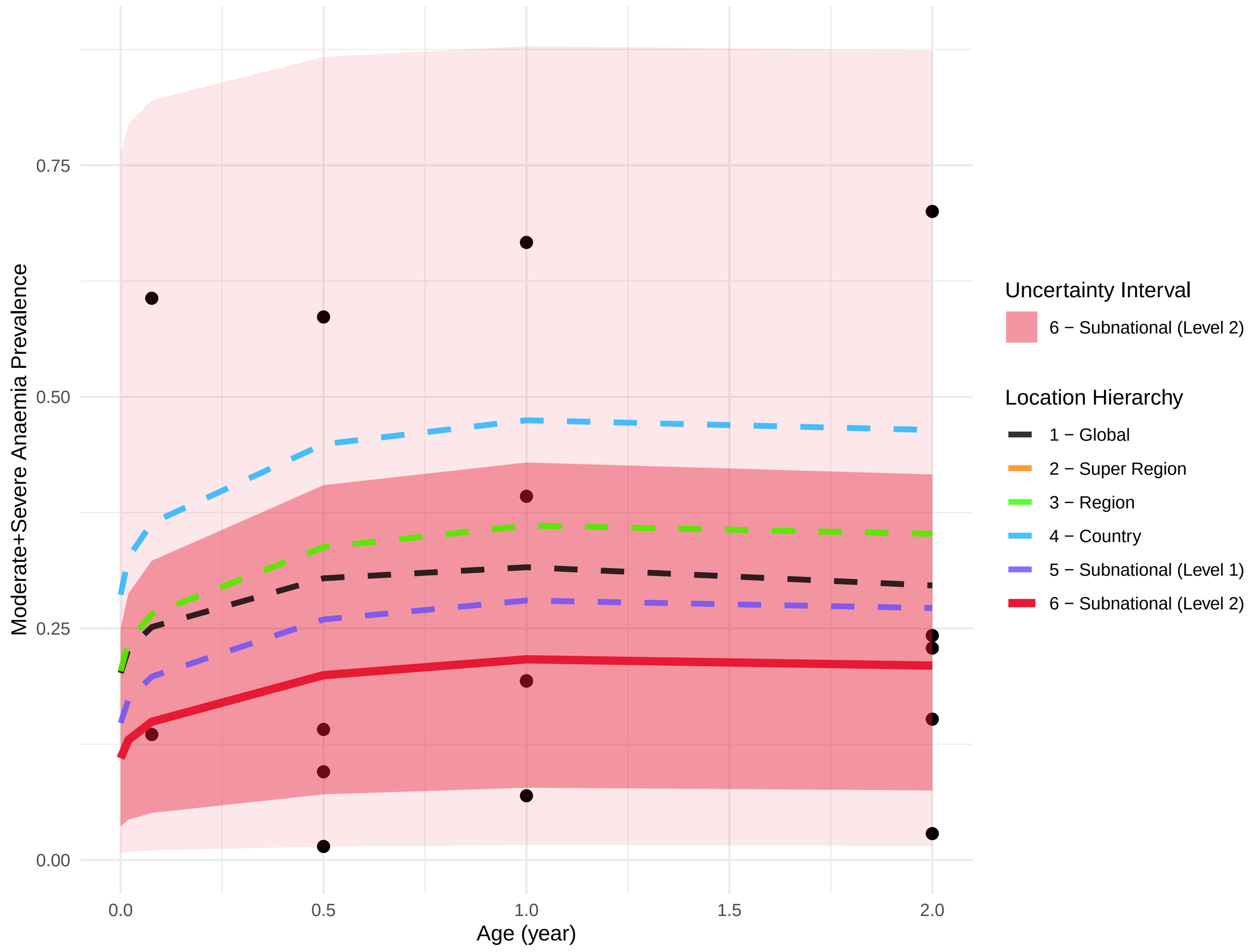

MR−BRT Age/Sex Fits − Non−pregnant Females <2 Years Old − Moderate+Severe Anaemia Prevalence − Nagaland
WHO−2024 Adjustment Method

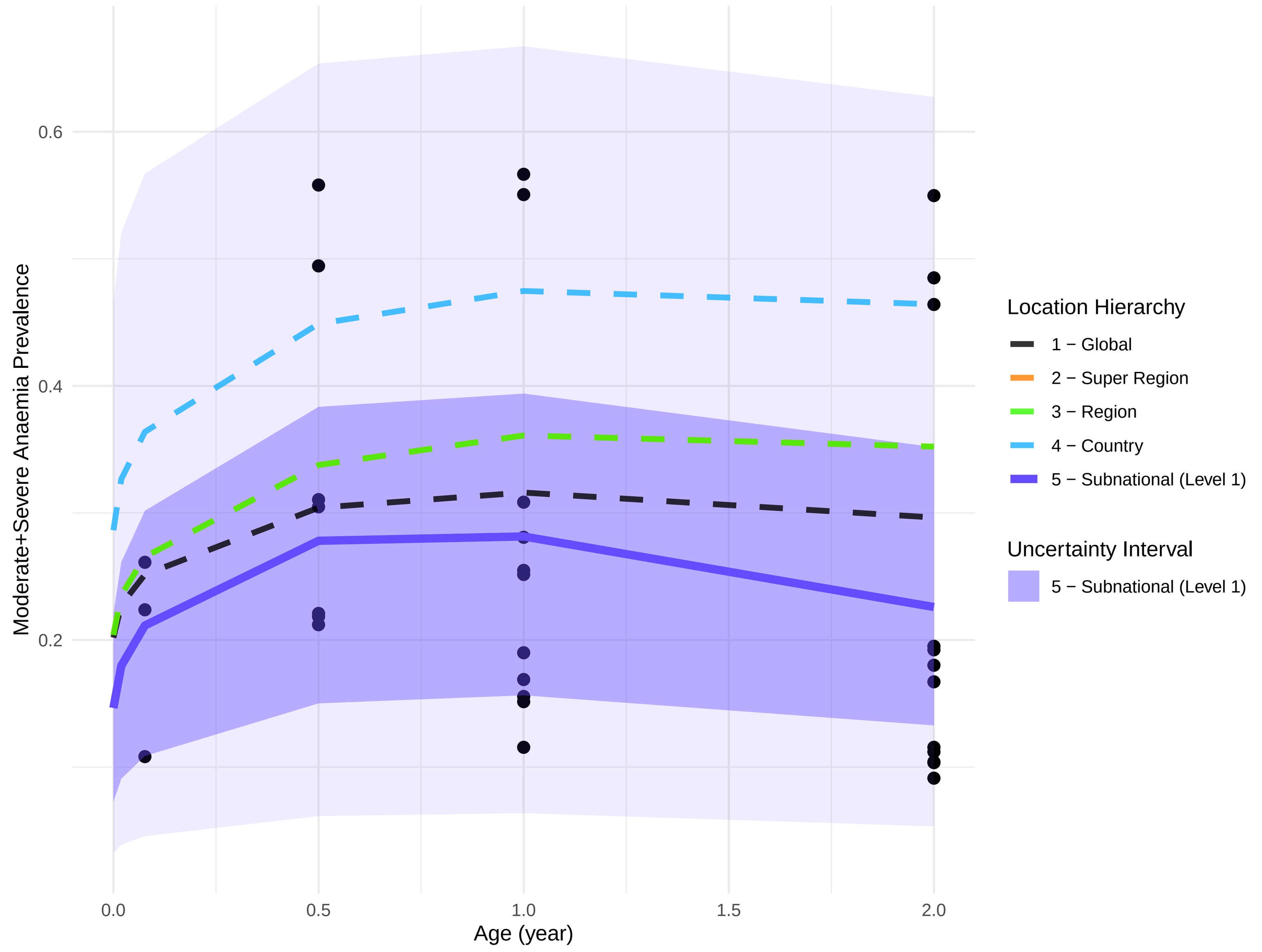

MR−BRT Age/Sex Fits − Non−pregnant Females <2 Years Old − Moderate+Severe Anaemia Prevalence − Nagaland, Rural
WHO−2024 Adjustment Method

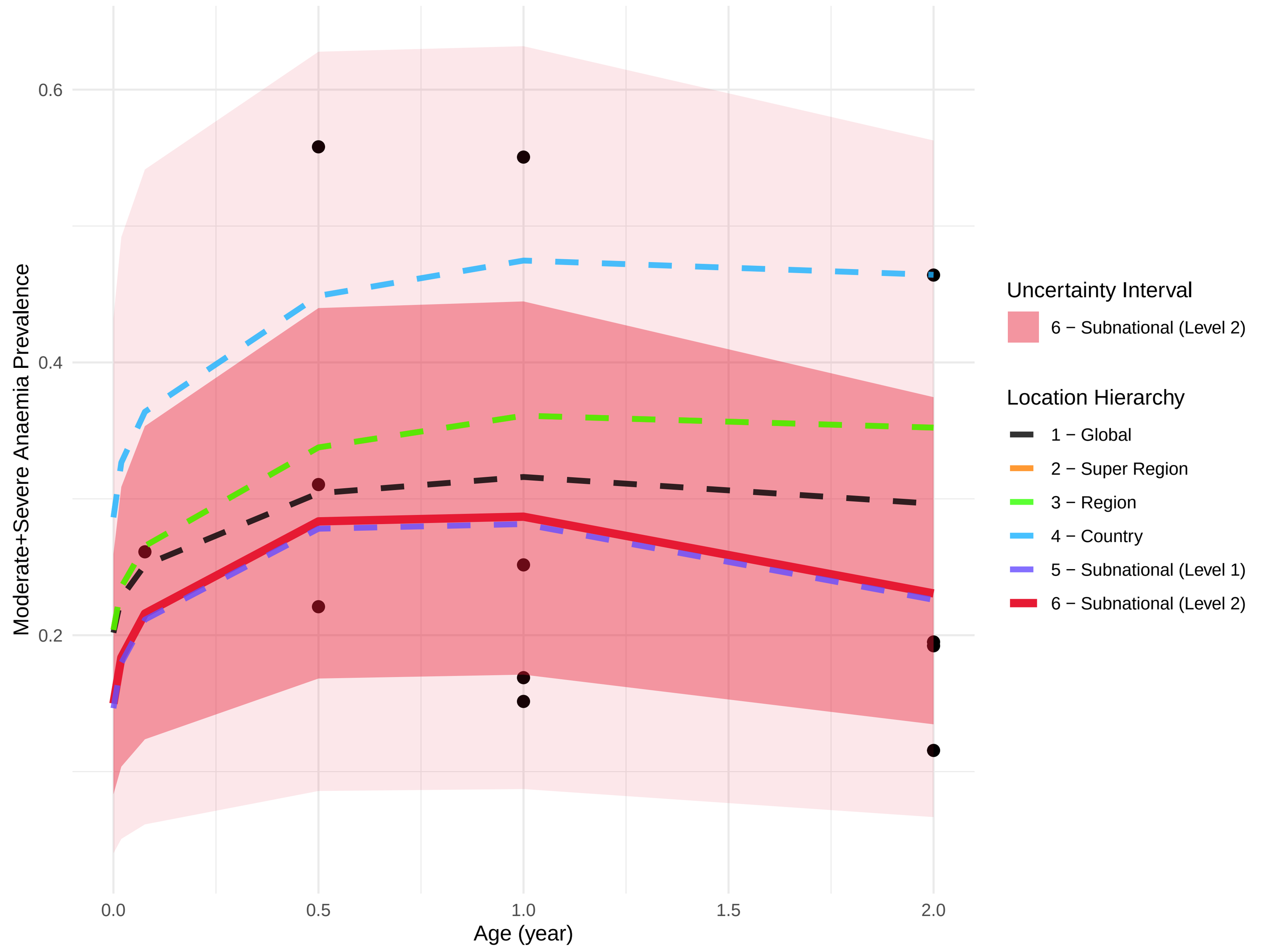

MR−BRT Age/Sex Fits − Non−pregnant Females <2 Years Old − Moderate+Severe Anaemia Prevalence − Nagaland, Urban
WHO−2024 Adjustment Method

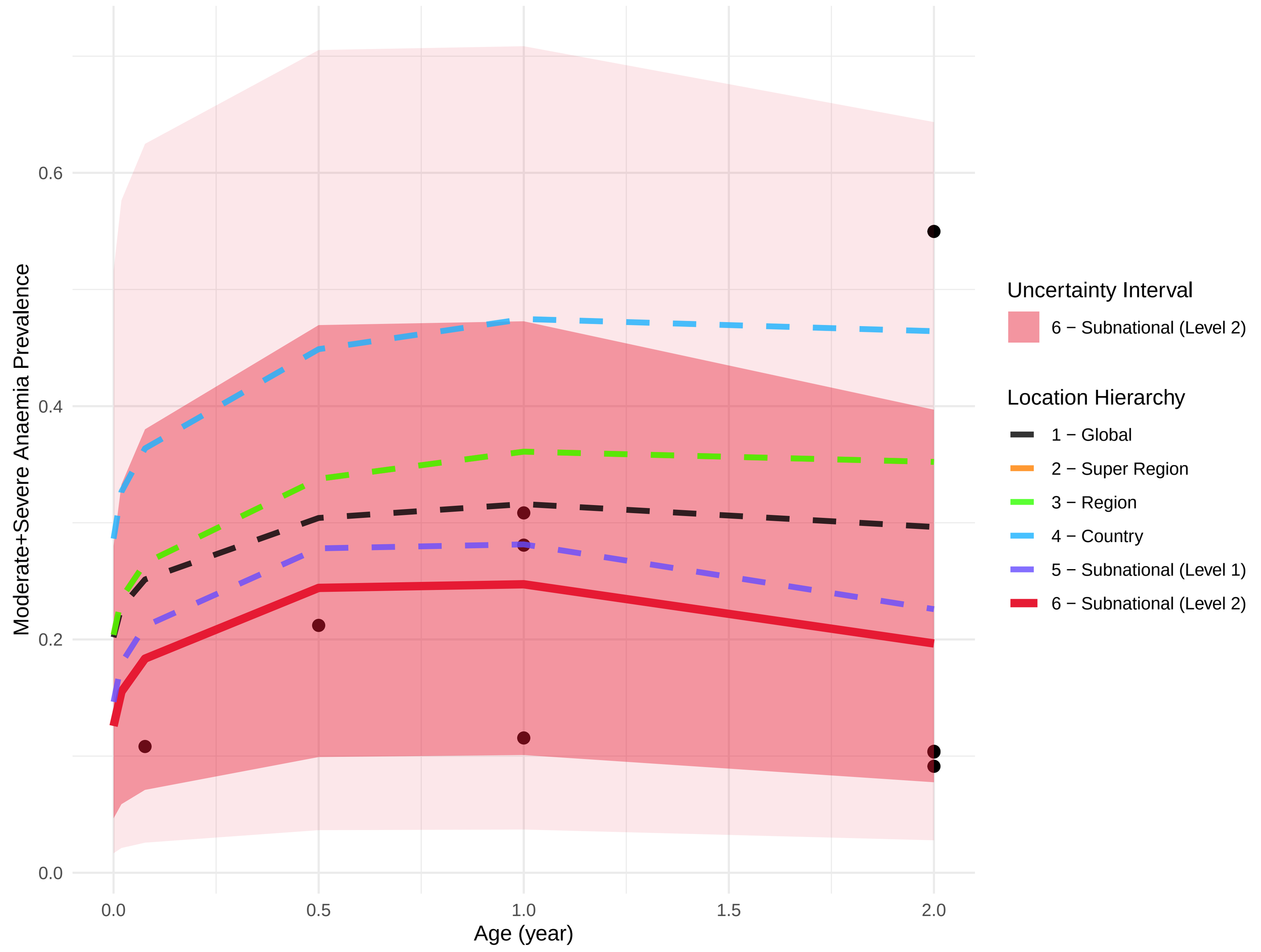

MR−BRT Age/Sex Fits − Non−pregnant Females <2 Years Old − Moderate+Severe Anaemia Prevalence − Odisha
WHO−2024 Adjustment Method

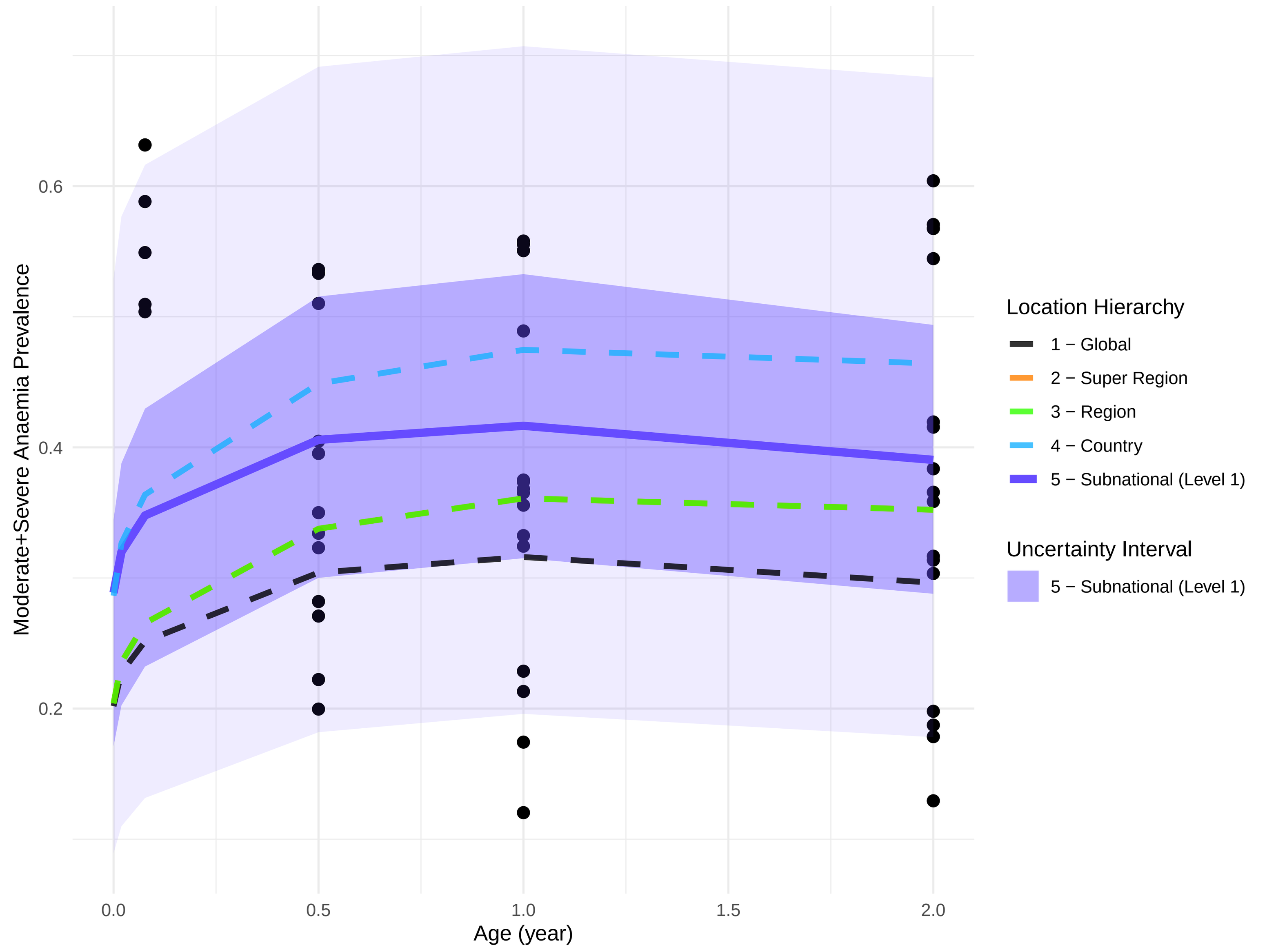

MR−BRT Age/Sex Fits − Non−pregnant Females <2 Years Old − Moderate+Severe Anaemia Prevalence − Odisha, Rural
WHO−2024 Adjustment Method

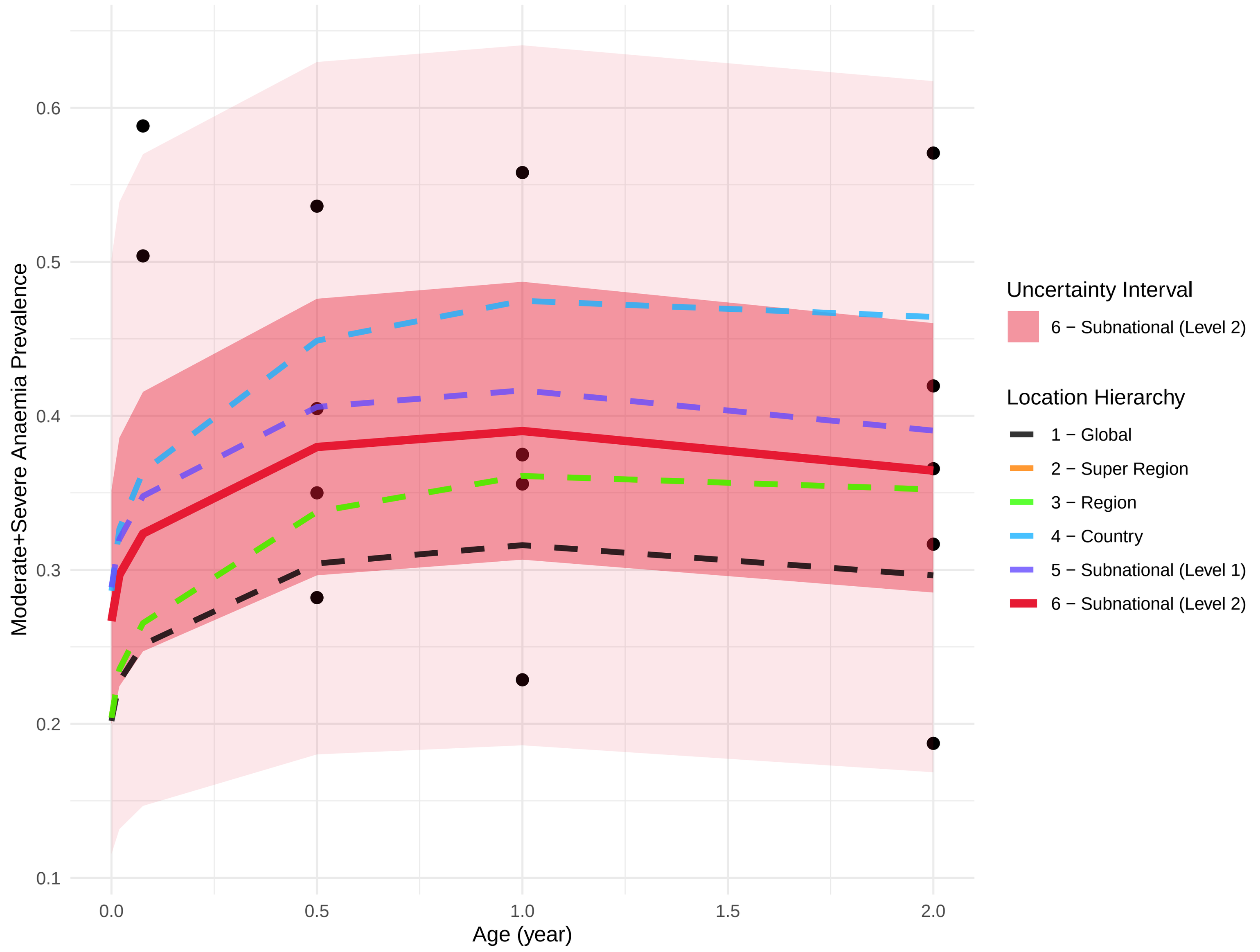

MR−BRT Age/Sex Fits − Non−pregnant Females <2 Years Old − Moderate+Severe Anaemia Prevalence − Odisha, Urban
WHO−2024 Adjustment Method

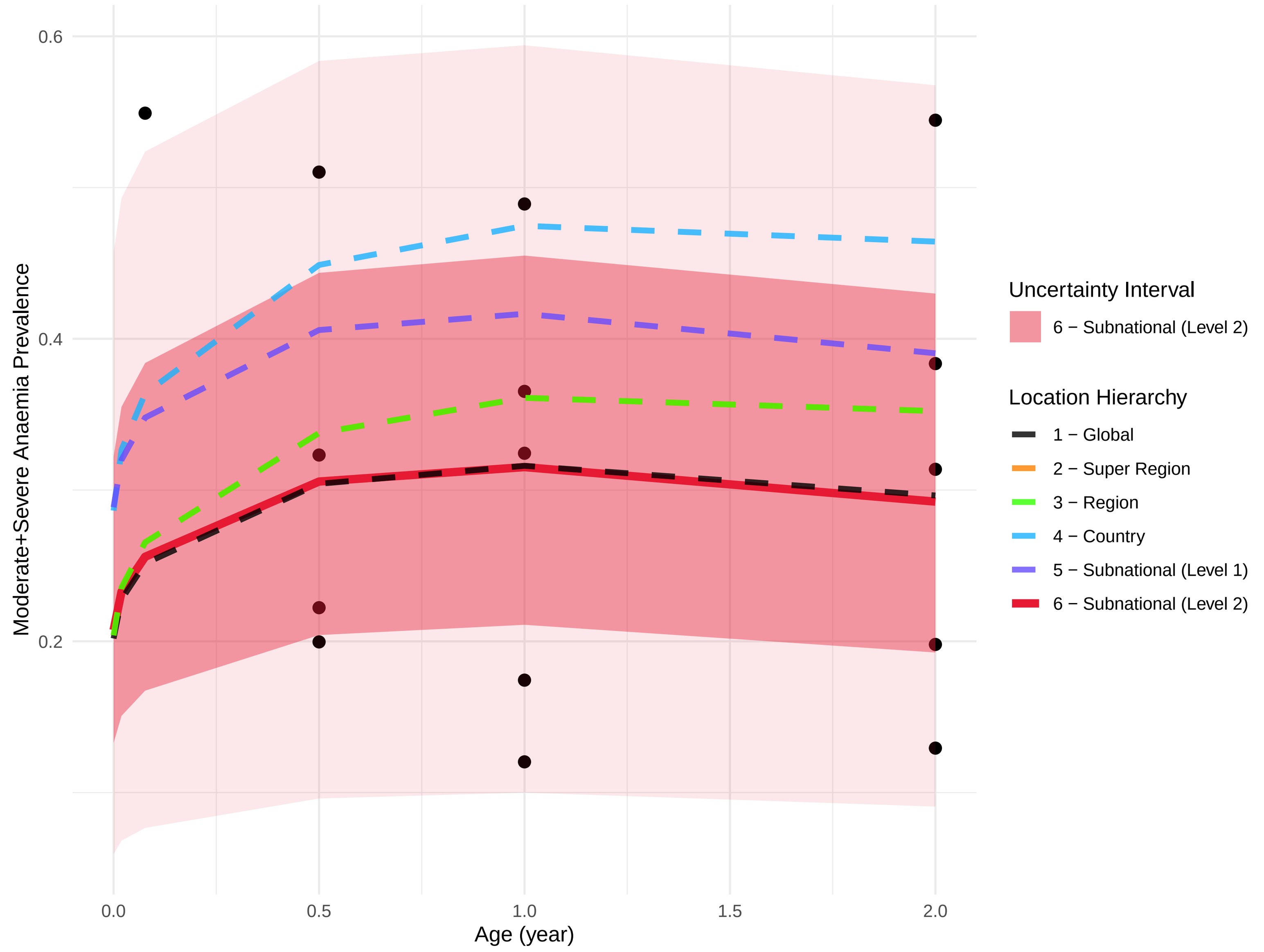

MR−BRT Age/Sex Fits − Non−pregnant Females <2 Years Old − Moderate+Severe Anaemia Prevalence − Other Union Territories
WHO−2024 Adjustment Method

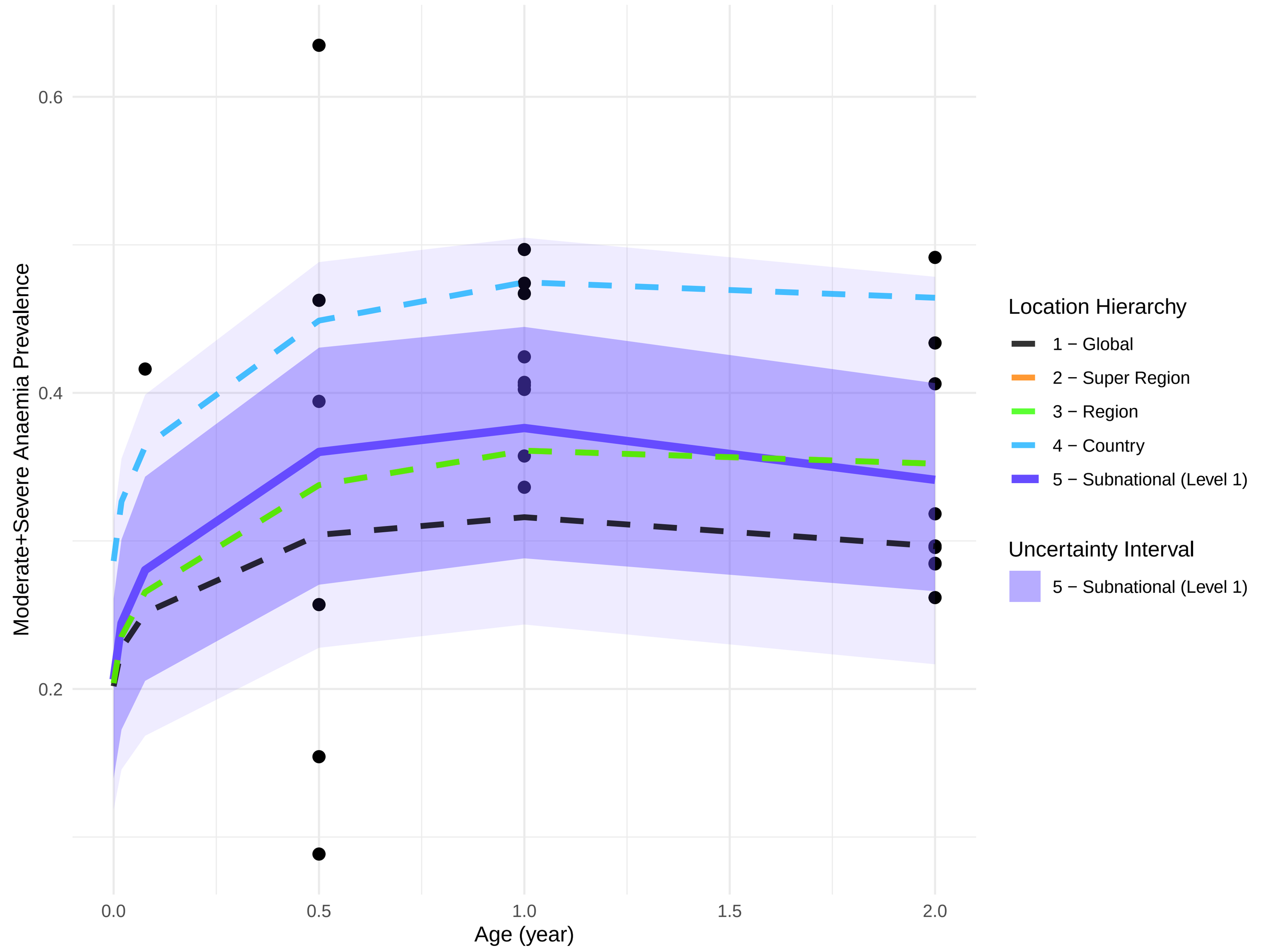

MR−BRT Age/Sex Fits − Non−pregnant Females <2 Years Old − Moderate+Severe Anaemia Prevalence − Other Union Territories, Rural
WHO−2024 Adjustment Method

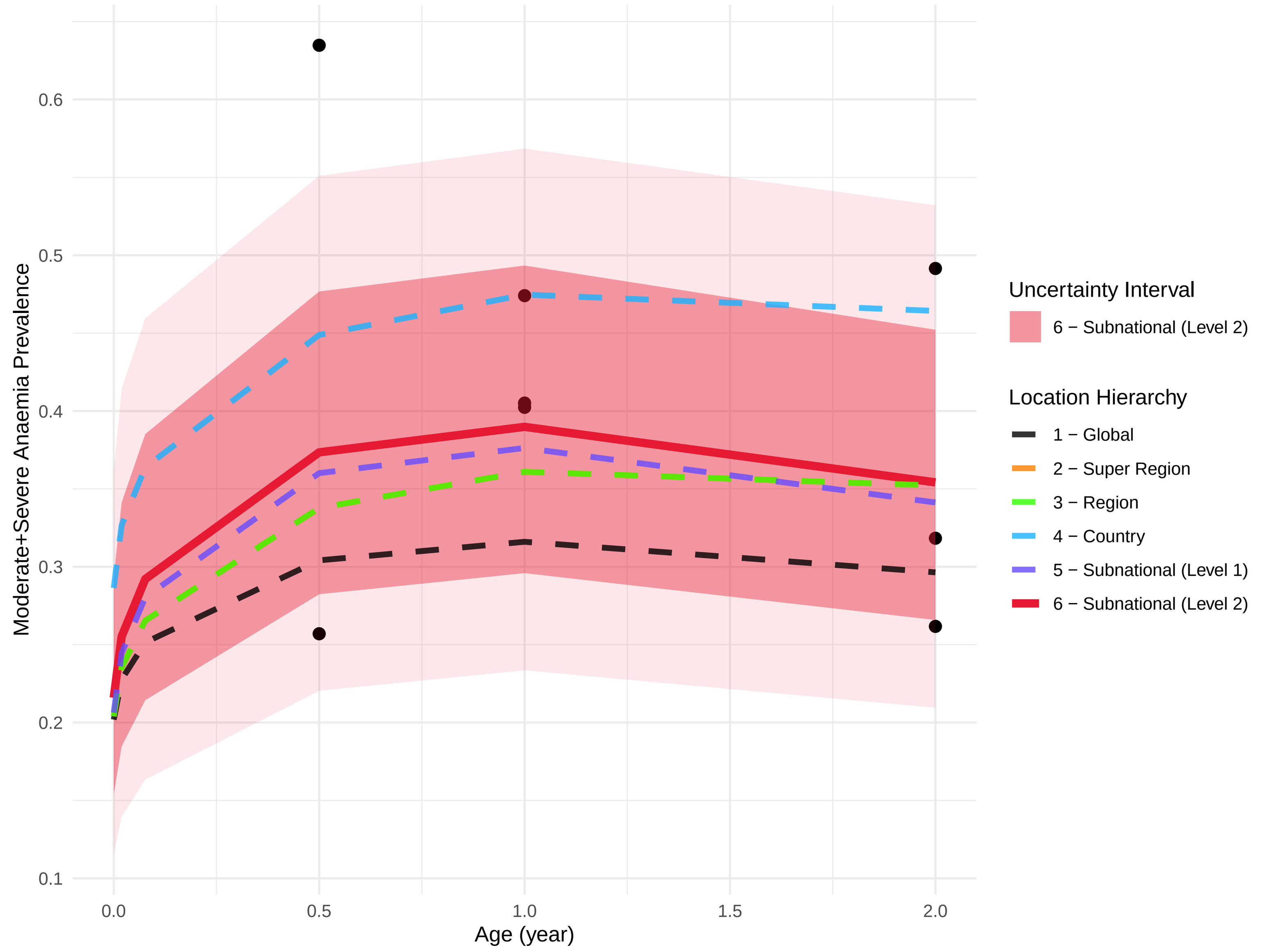

MR−BRT Age/Sex Fits − Non−pregnant Females <2 Years Old − Moderate+Severe Anaemia Prevalence − Other Union Territories, Urban
WHO−2024 Adjustment Method

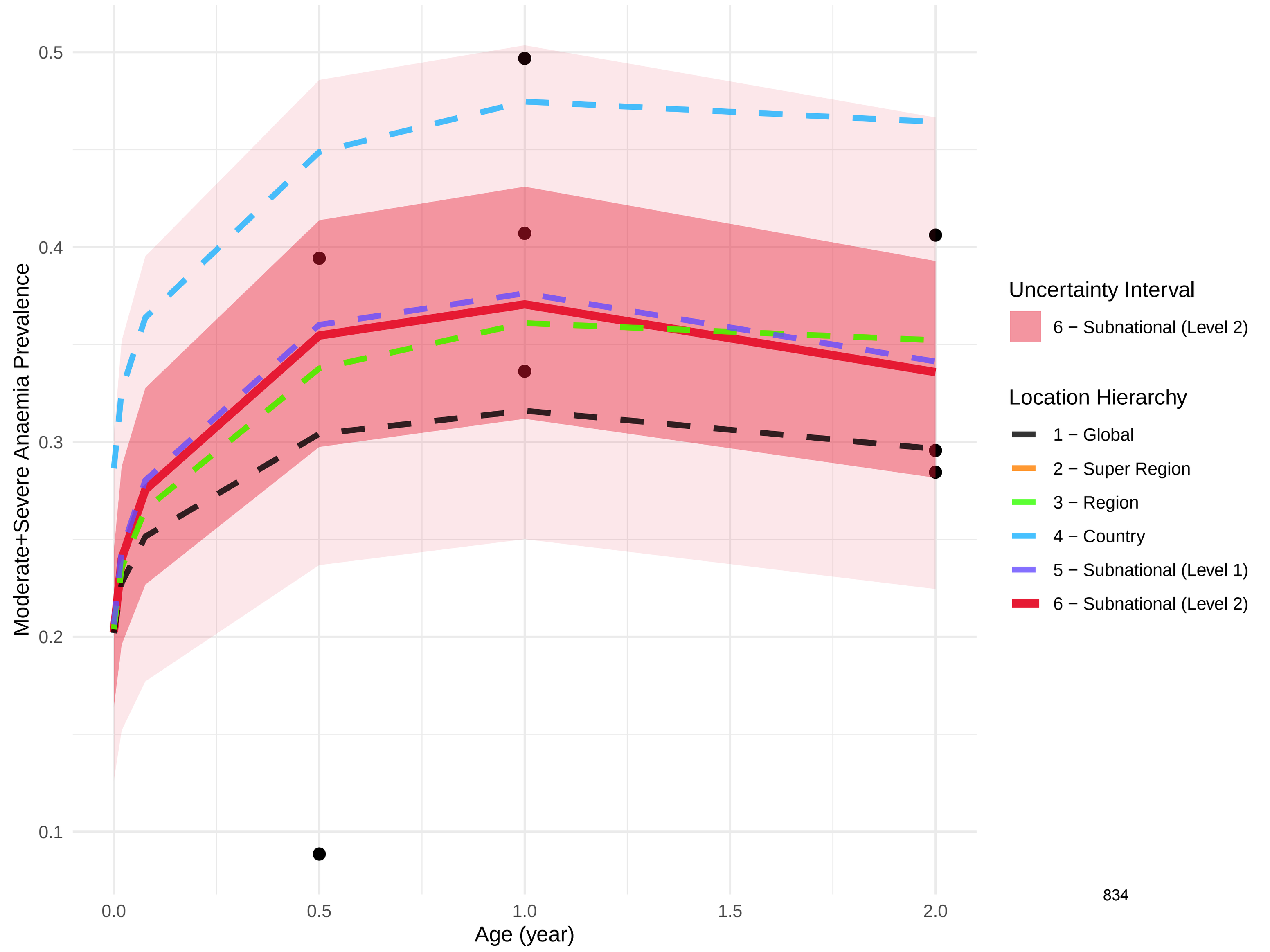

MR−BRT Age/Sex Fits − Non−pregnant Females <2 Years Old − Moderate+Severe Anaemia Prevalence − Punjab
WHO−2024 Adjustment Method

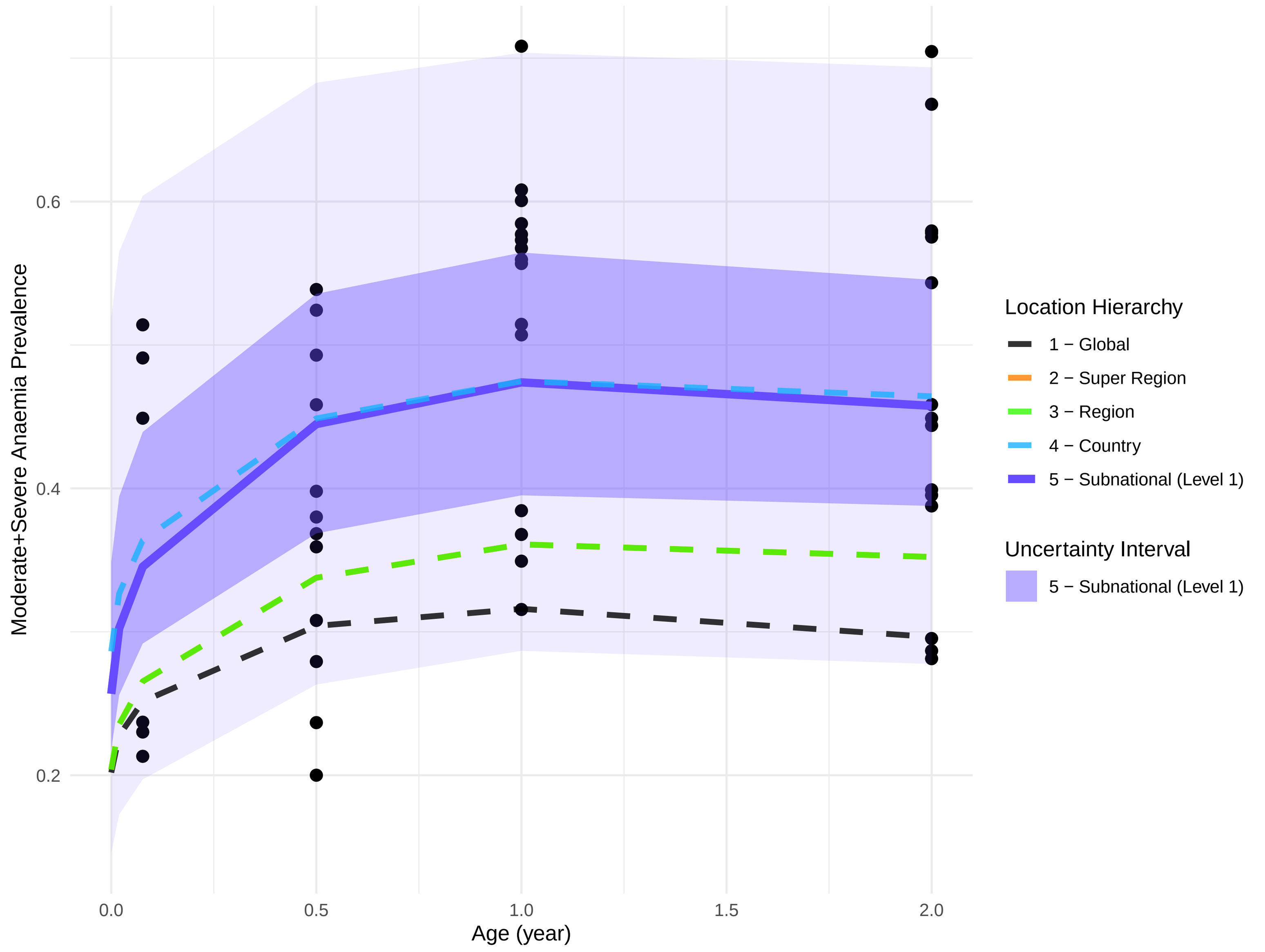

MR−BRT Age/Sex Fits − Non−pregnant Females <2 Years Old − Moderate+Severe Anaemia Prevalence − Punjab, Rural
WHO−2024 Adjustment Method

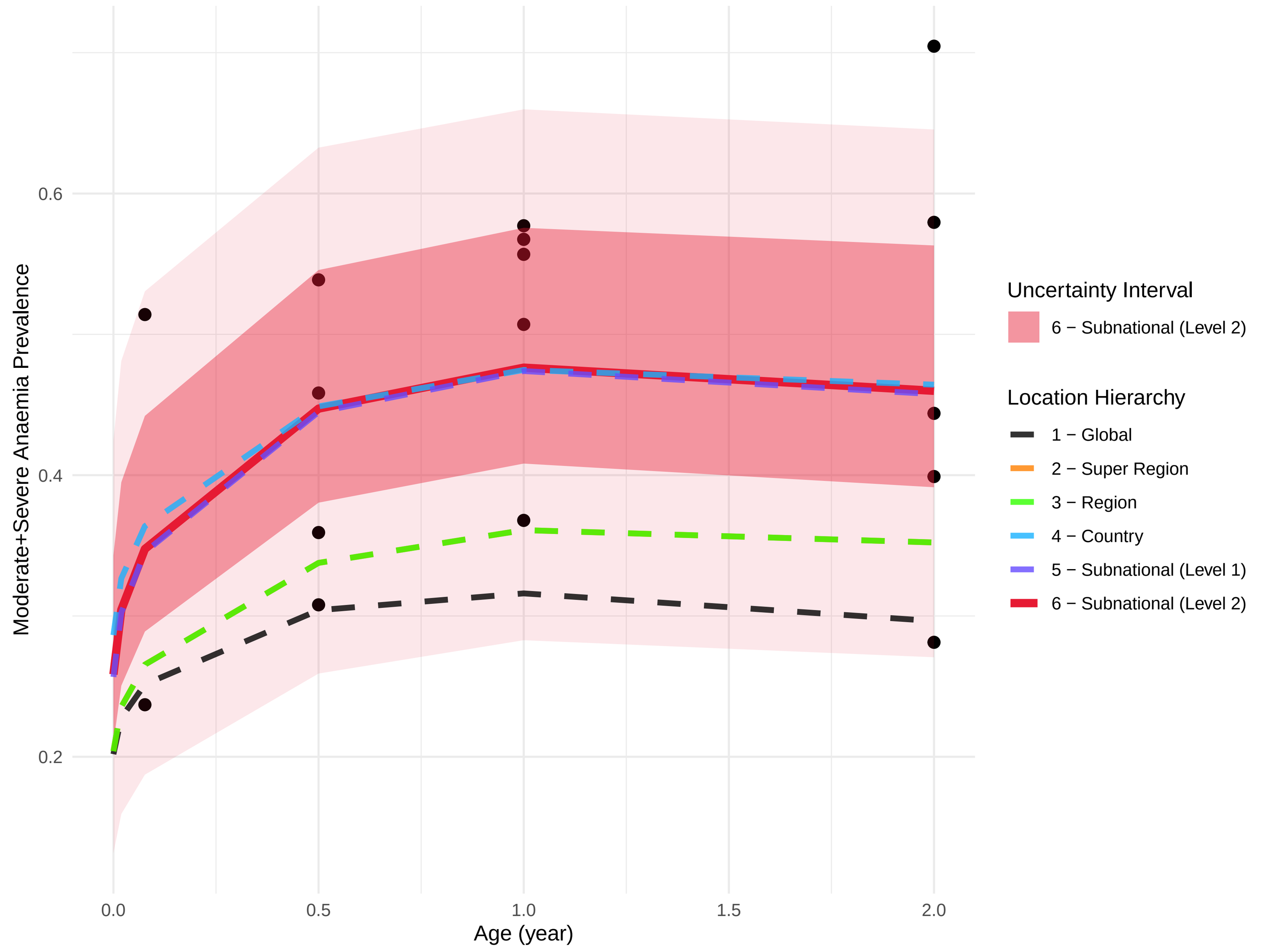

MR−BRT Age/Sex Fits − Non−pregnant Females <2 Years Old − Moderate+Severe Anaemia Prevalence − Punjab, Urban
WHO−2024 Adjustment Method

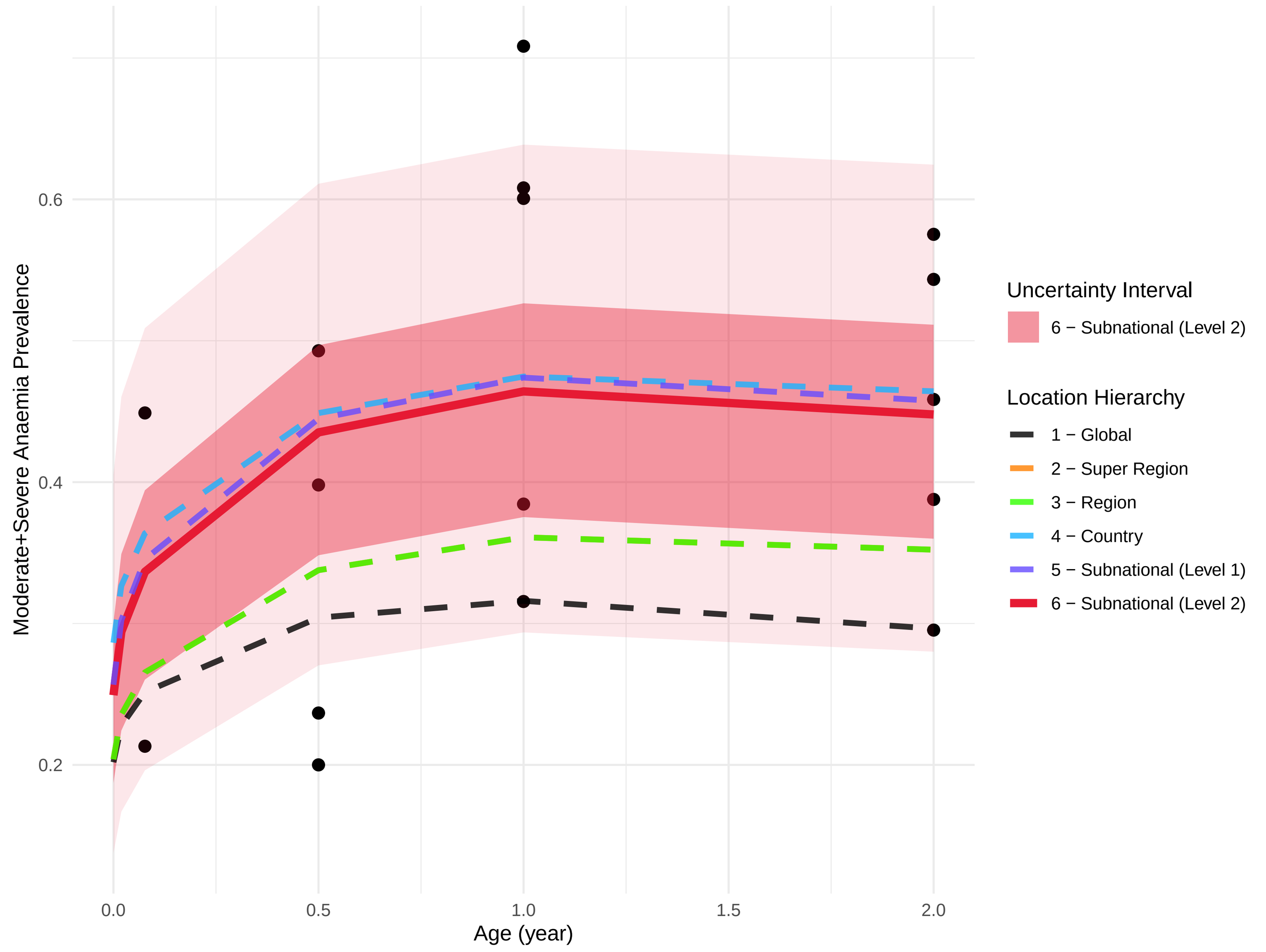

MR−BRT Age/Sex Fits − Non−pregnant Females <2 Years Old − Moderate+Severe Anaemia Prevalence − Rajasthan
WHO−2024 Adjustment Method

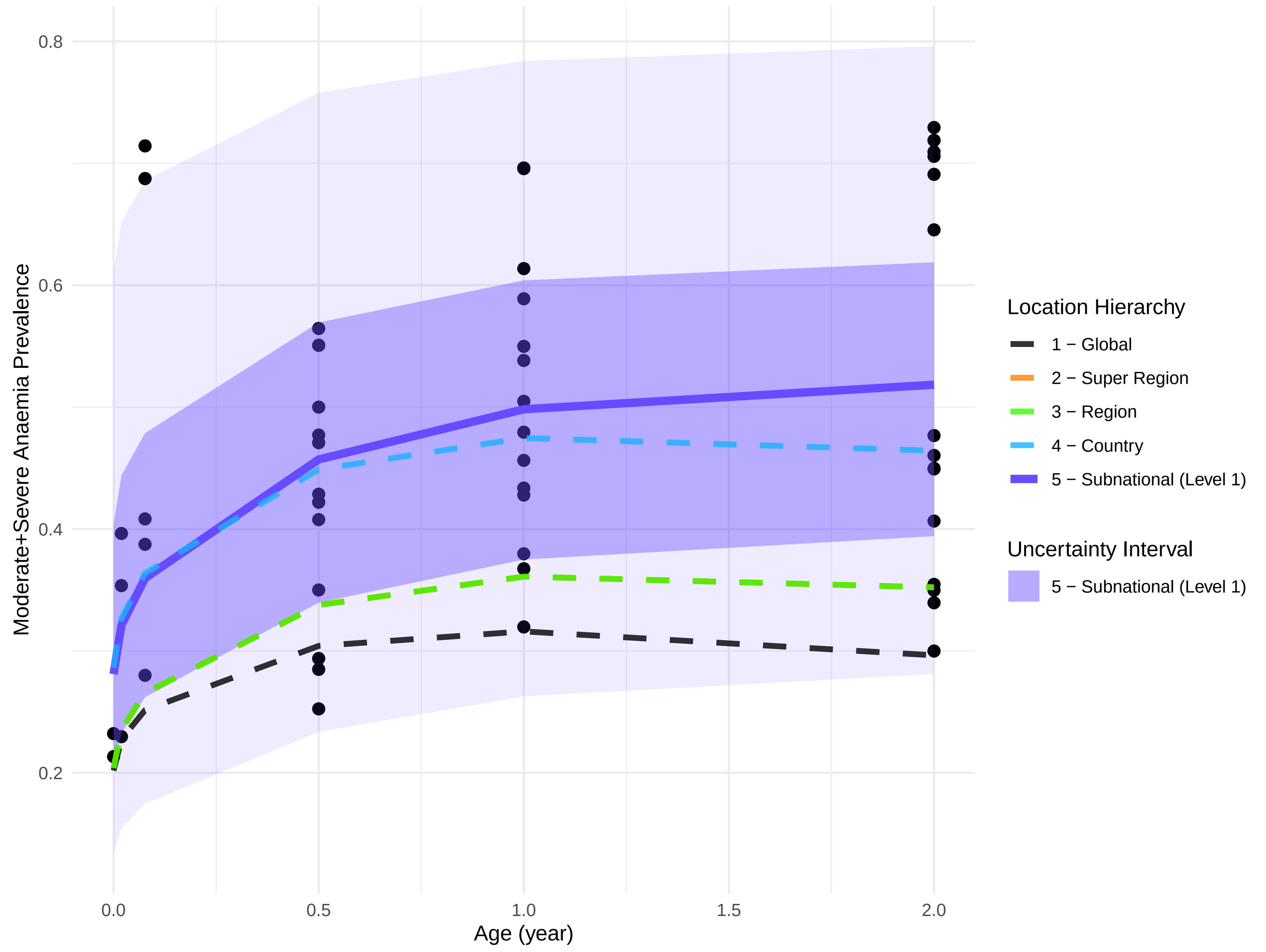

MR−BRT Age/Sex Fits − Non−pregnant Females <2 Years Old − Moderate+Severe Anaemia Prevalence − Rajasthan, Rural
WHO−2024 Adjustment Method

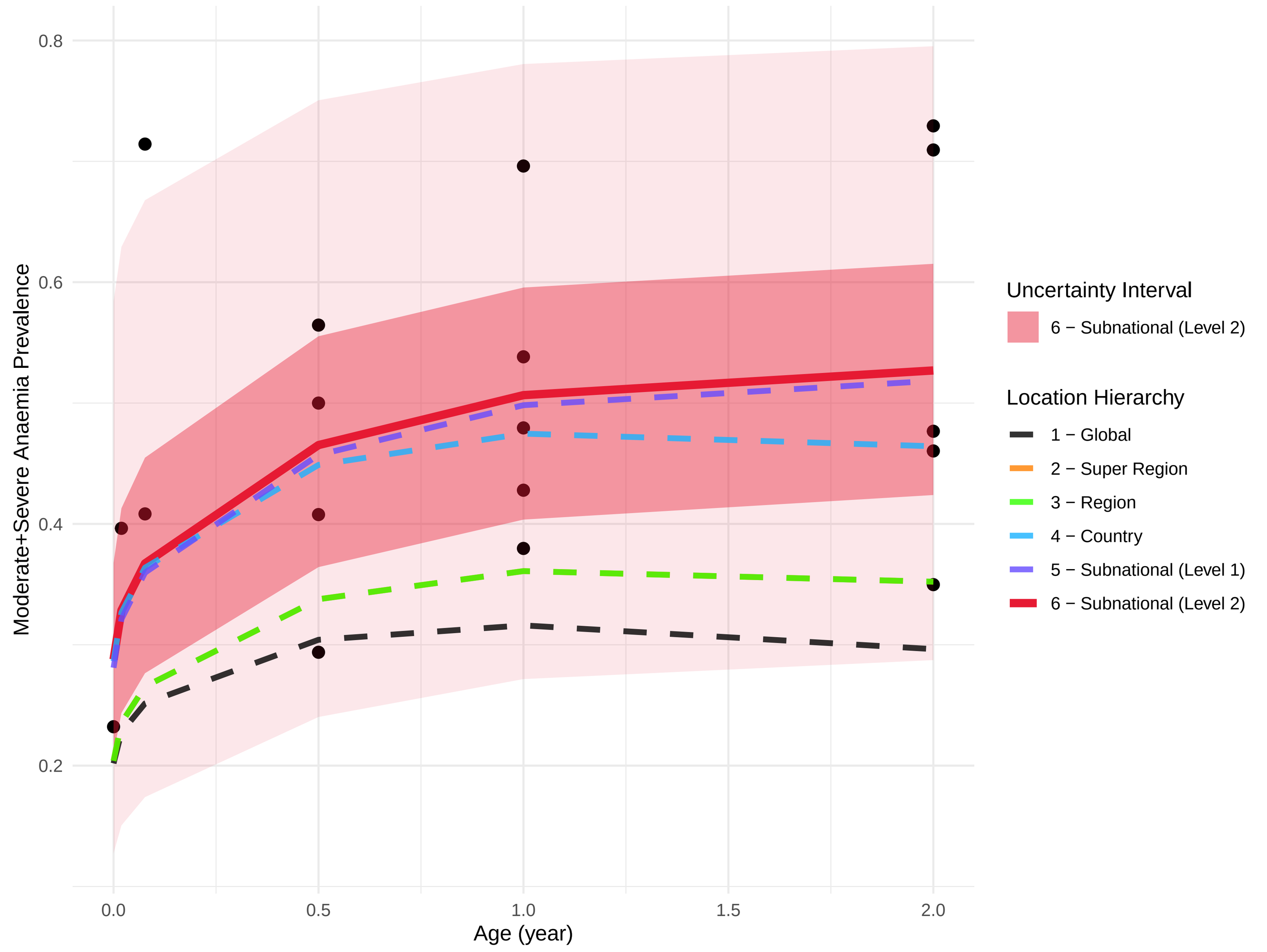

MR−BRT Age/Sex Fits − Non−pregnant Females <2 Years Old − Moderate+Severe Anaemia Prevalence − Rajasthan, Urban
WHO−2024 Adjustment Method

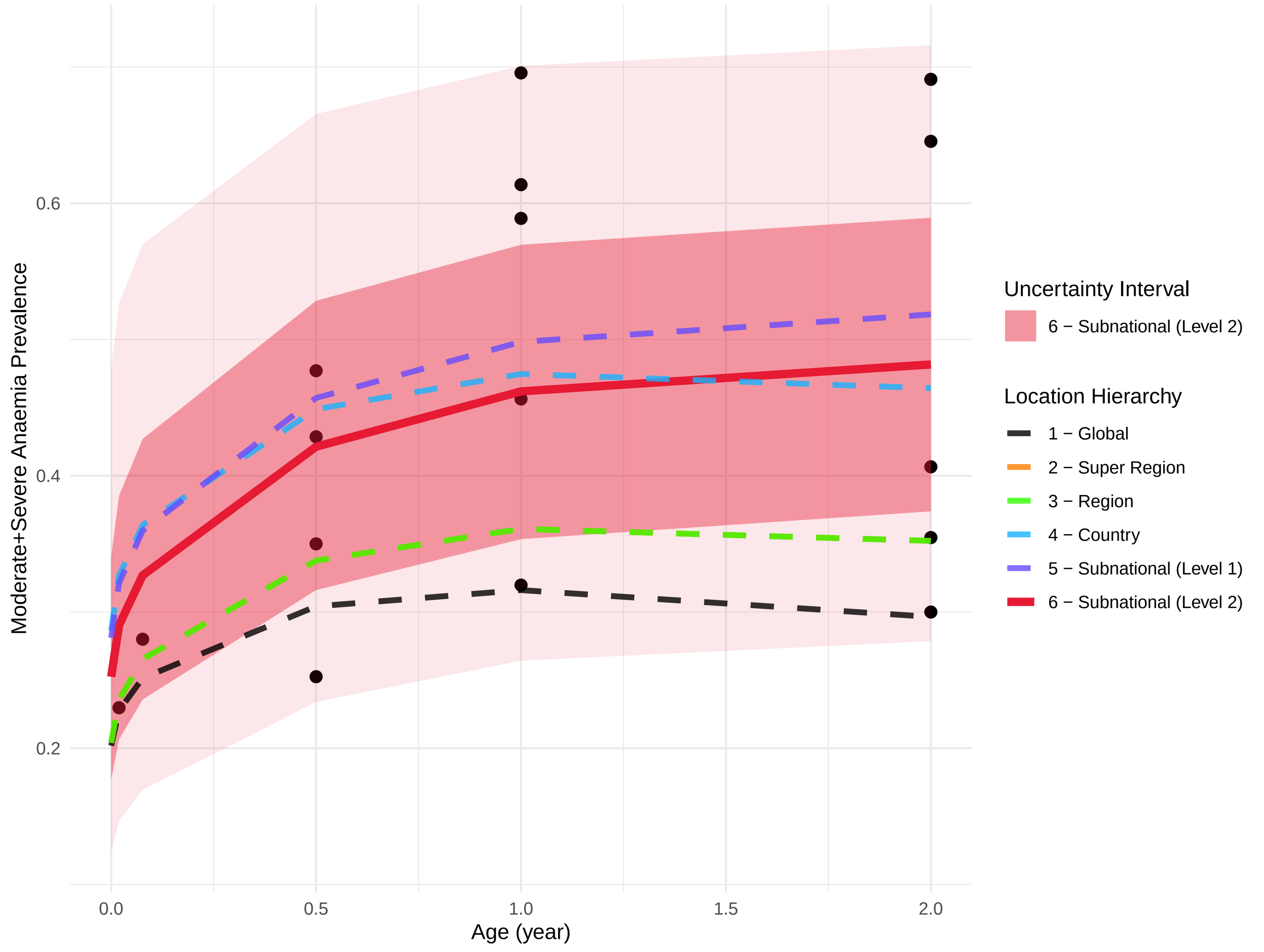

MR−BRT Age/Sex Fits − Non−pregnant Females <2 Years Old − Moderate+Severe Anaemia Prevalence − Sikkim
WHO−2024 Adjustment Method

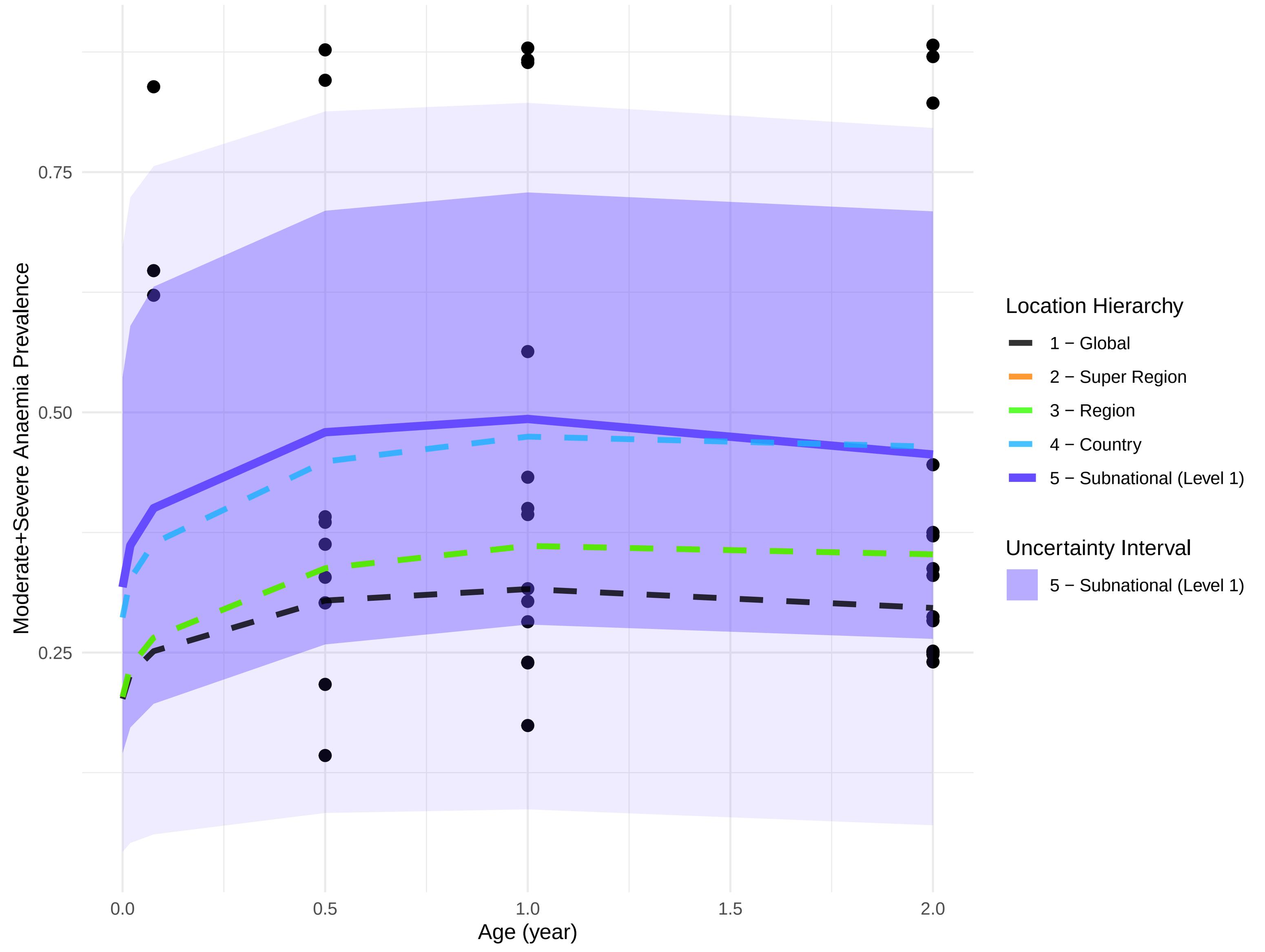

MR−BRT Age/Sex Fits − Non−pregnant Females <2 Years Old − Moderate+Severe Anaemia Prevalence − Sikkim, Rural
WHO−2024 Adjustment Method

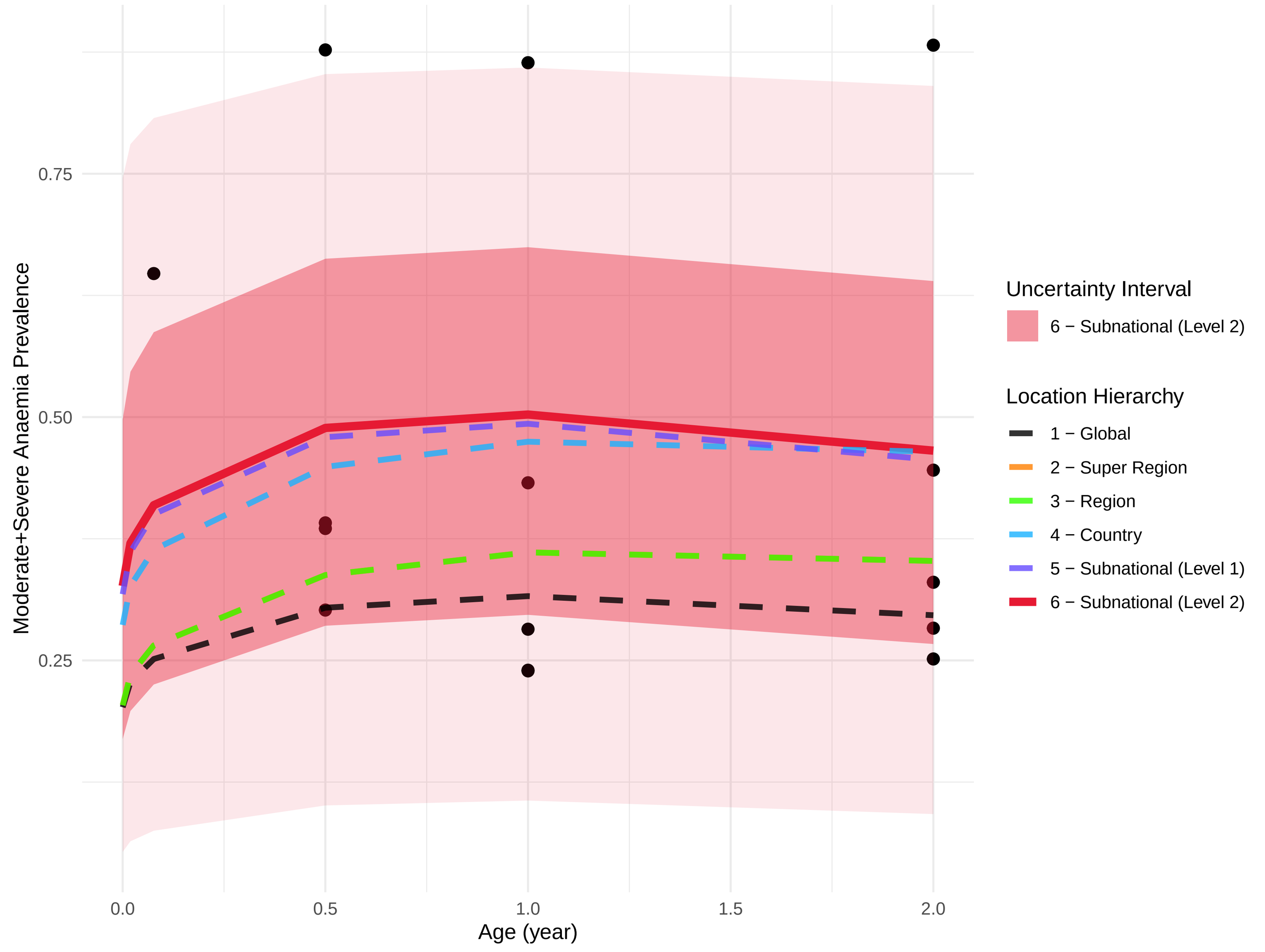

MR−BRT Age/Sex Fits − Non−pregnant Females <2 Years Old − Moderate+Severe Anaemia Prevalence − Sikkim, Urban
WHO−2024 Adjustment Method

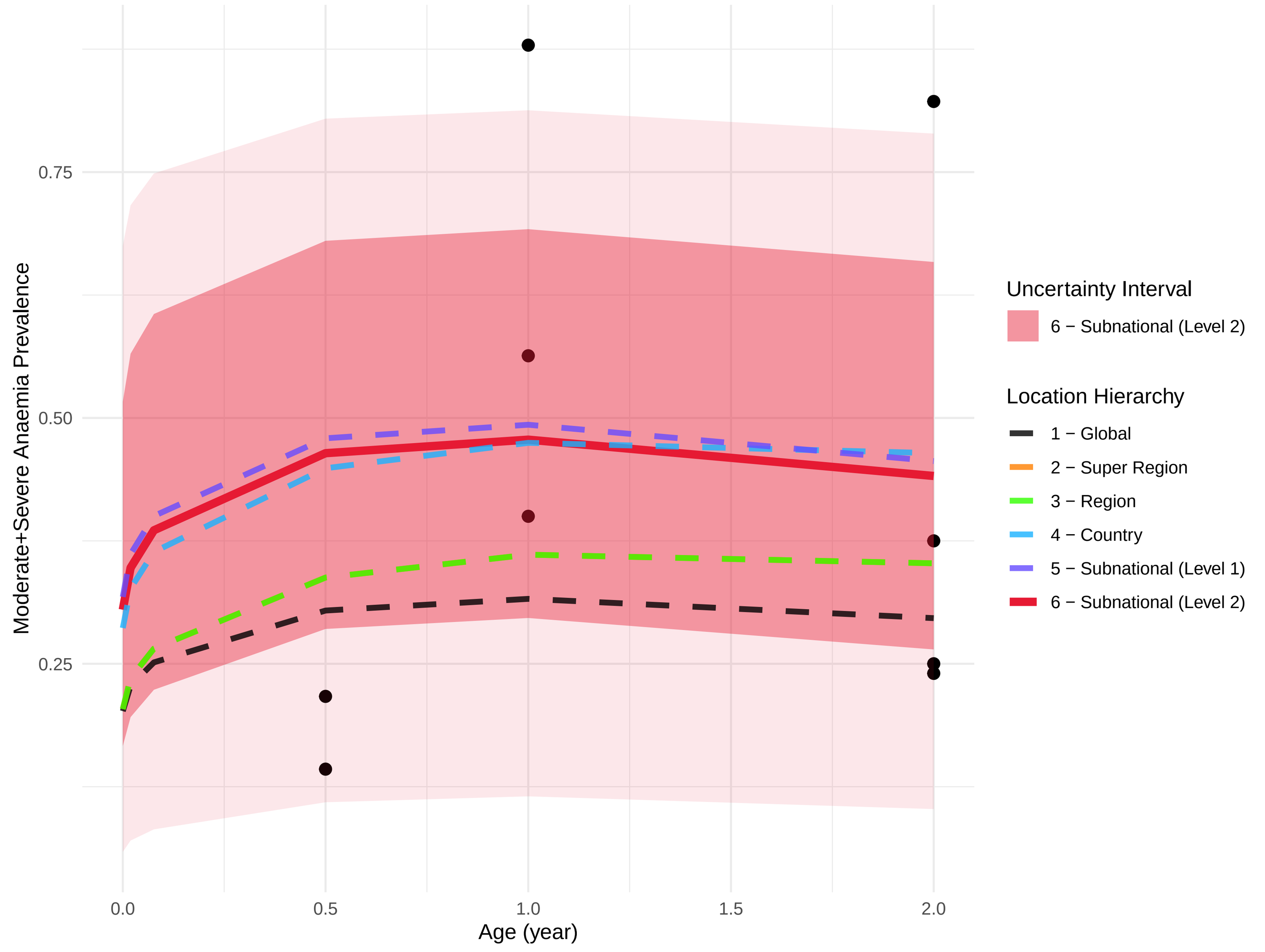

MR−BRT Age/Sex Fits − Non−pregnant Females <2 Years Old − Moderate+Severe Anaemia Prevalence − Tamil Nadu
WHO−2024 Adjustment Method

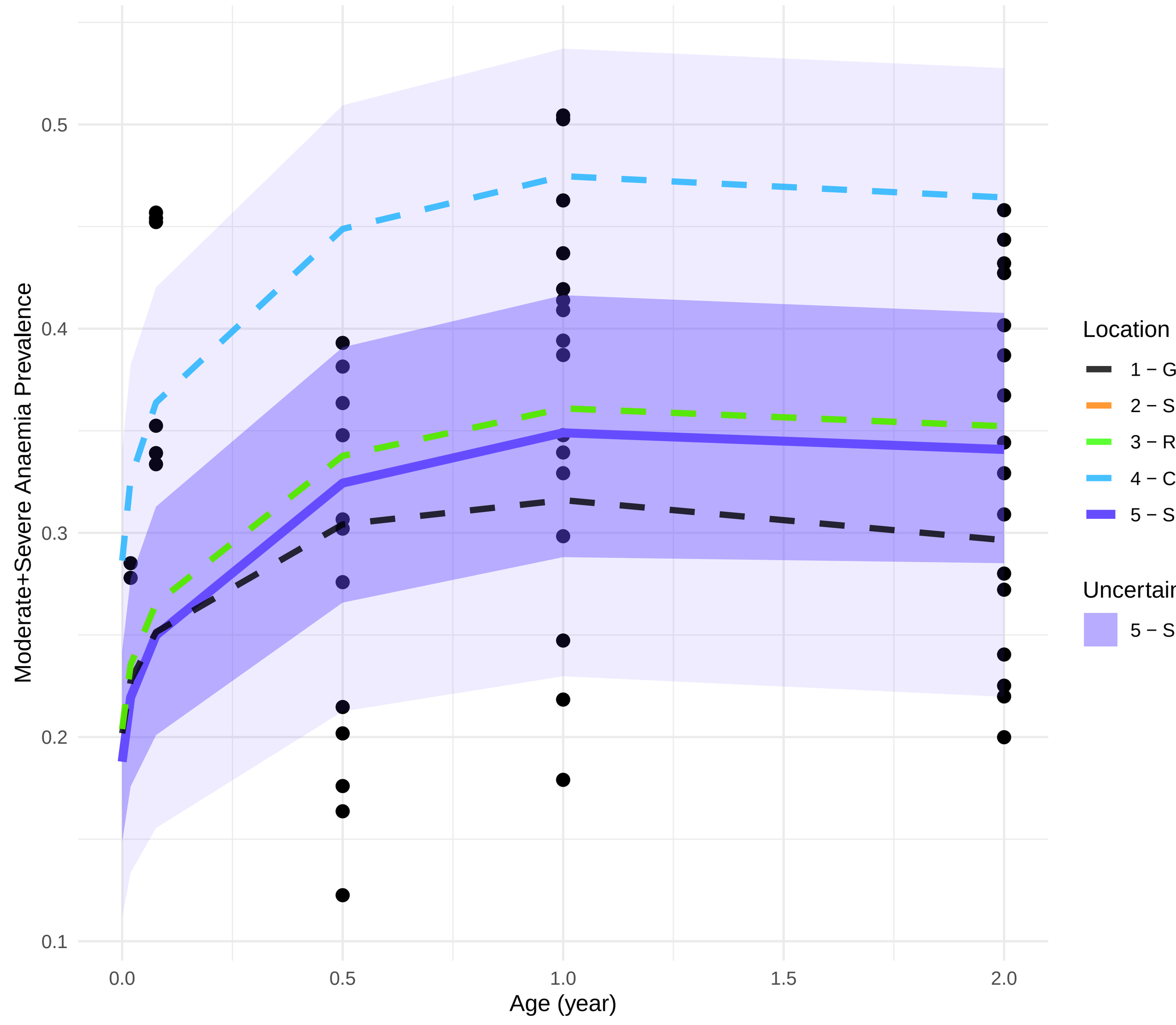

MR−BRT Age/Sex Fits − Non−pregnant Females <2 Years Old − Moderate+Severe Anaemia Prevalence − Tamil Nadu, Rural
WHO−2024 Adjustment Method

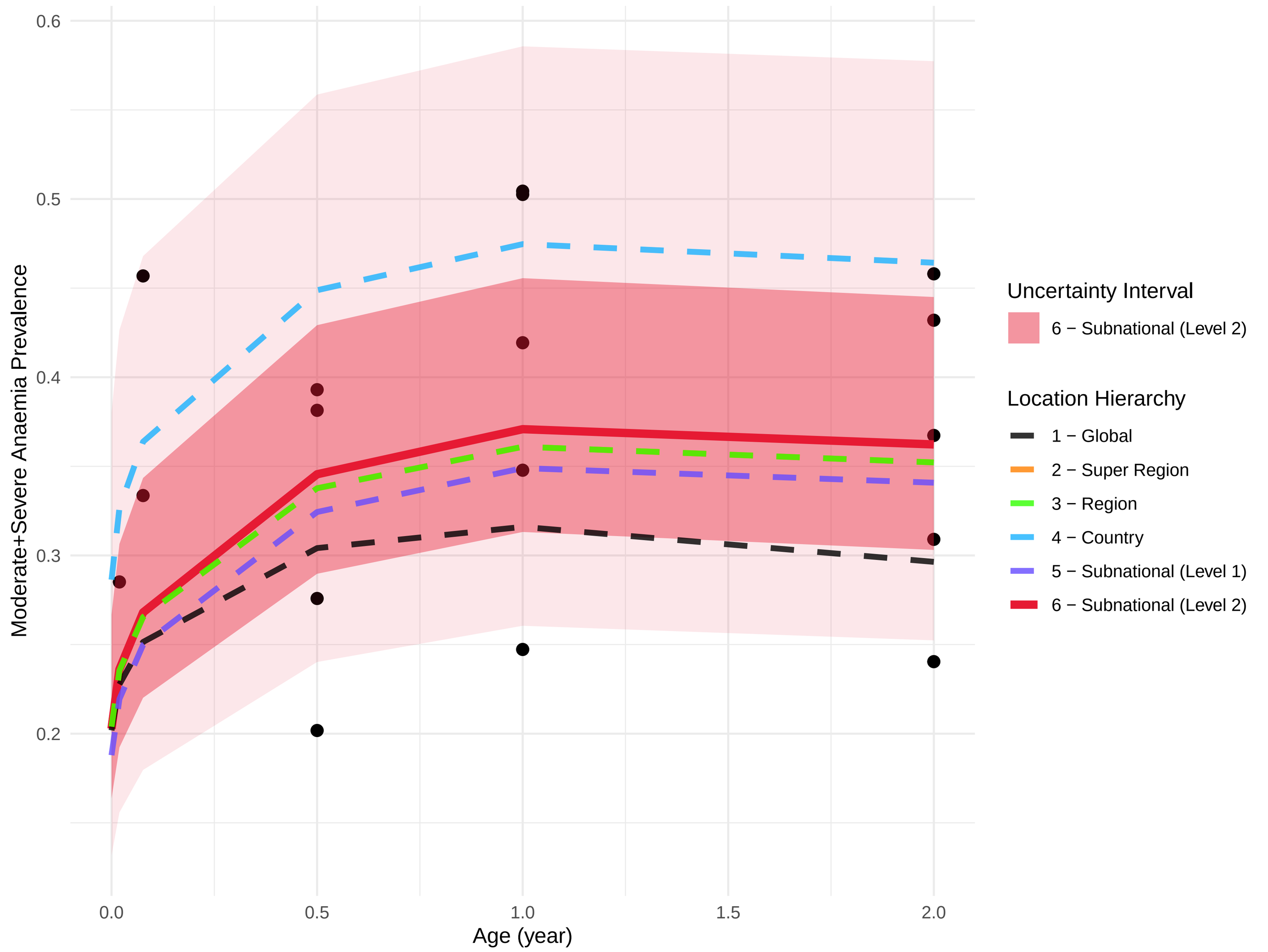

MR−BRT Age/Sex Fits − Non−pregnant Females <2 Years Old − Moderate+Severe Anaemia Prevalence − Tamil Nadu, Urban
WHO−2024 Adjustment Method

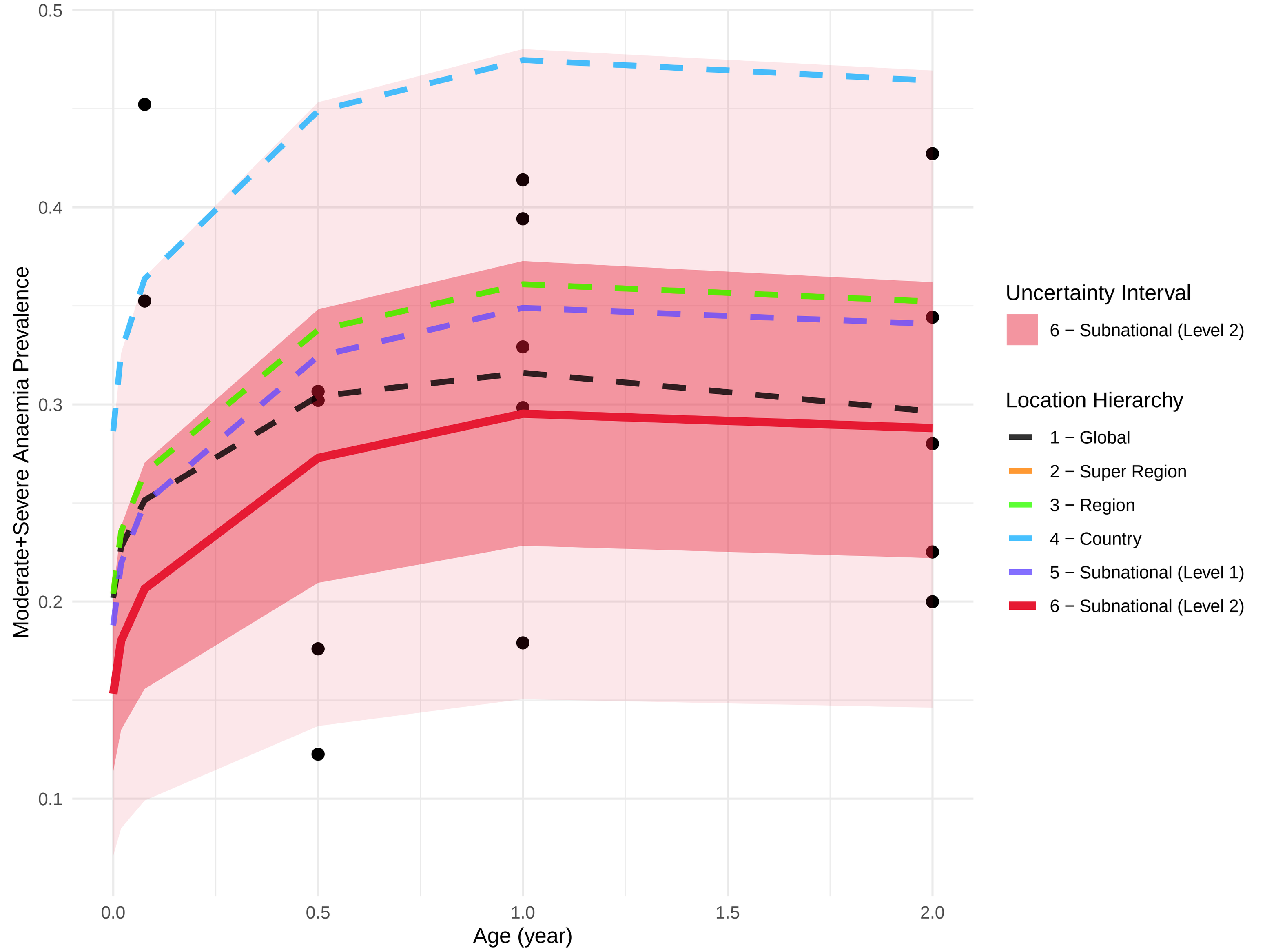

MR−BRT Age/Sex Fits − Non−pregnant Females <2 Years Old − Moderate+Severe Anaemia Prevalence − Telangana
WHO−2024 Adjustment Method

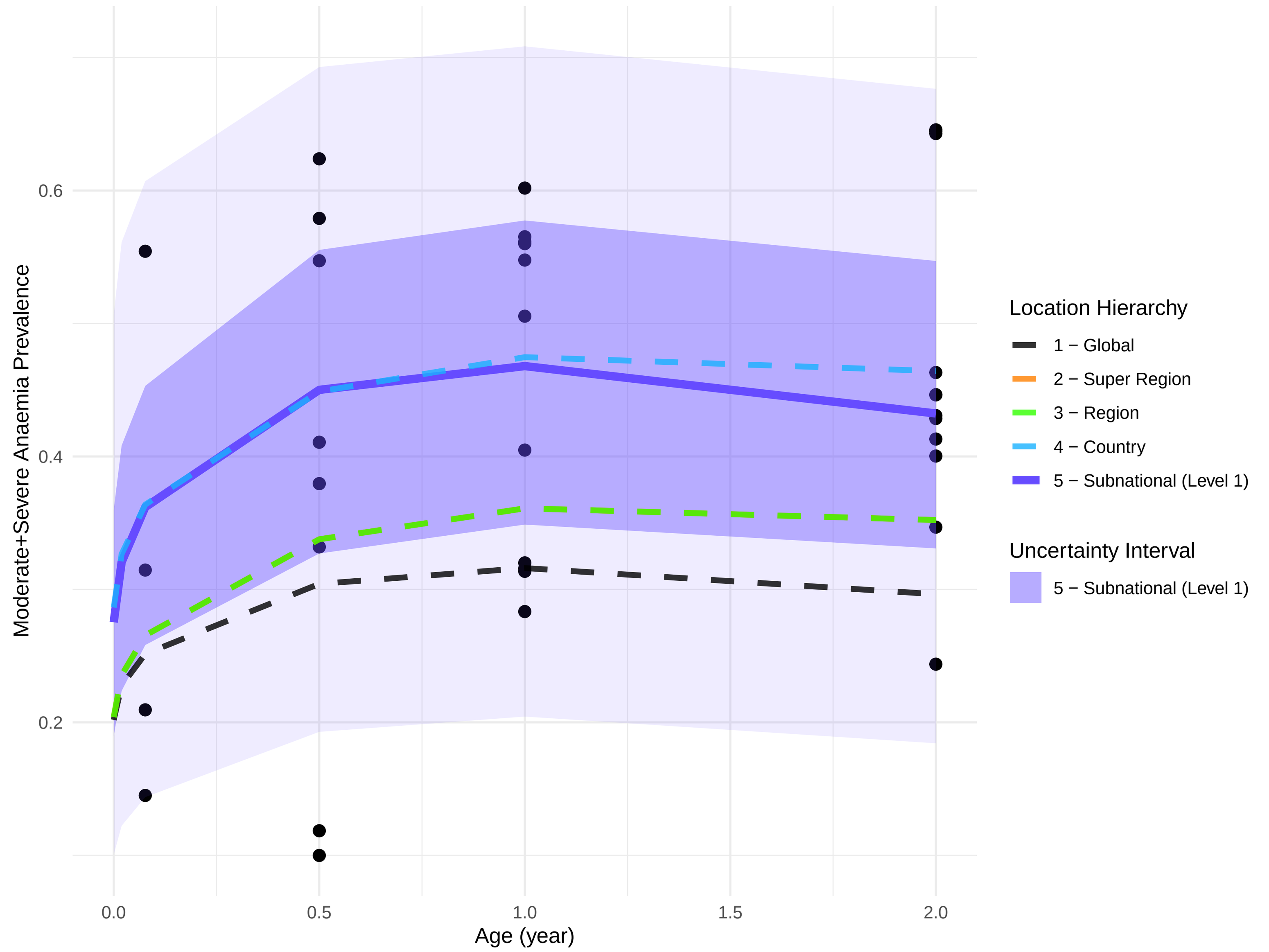

MR−BRT Age/Sex Fits − Non−pregnant Females <2 Years Old − Moderate+Severe Anaemia Prevalence − Telangana, Rural
WHO−2024 Adjustment Method

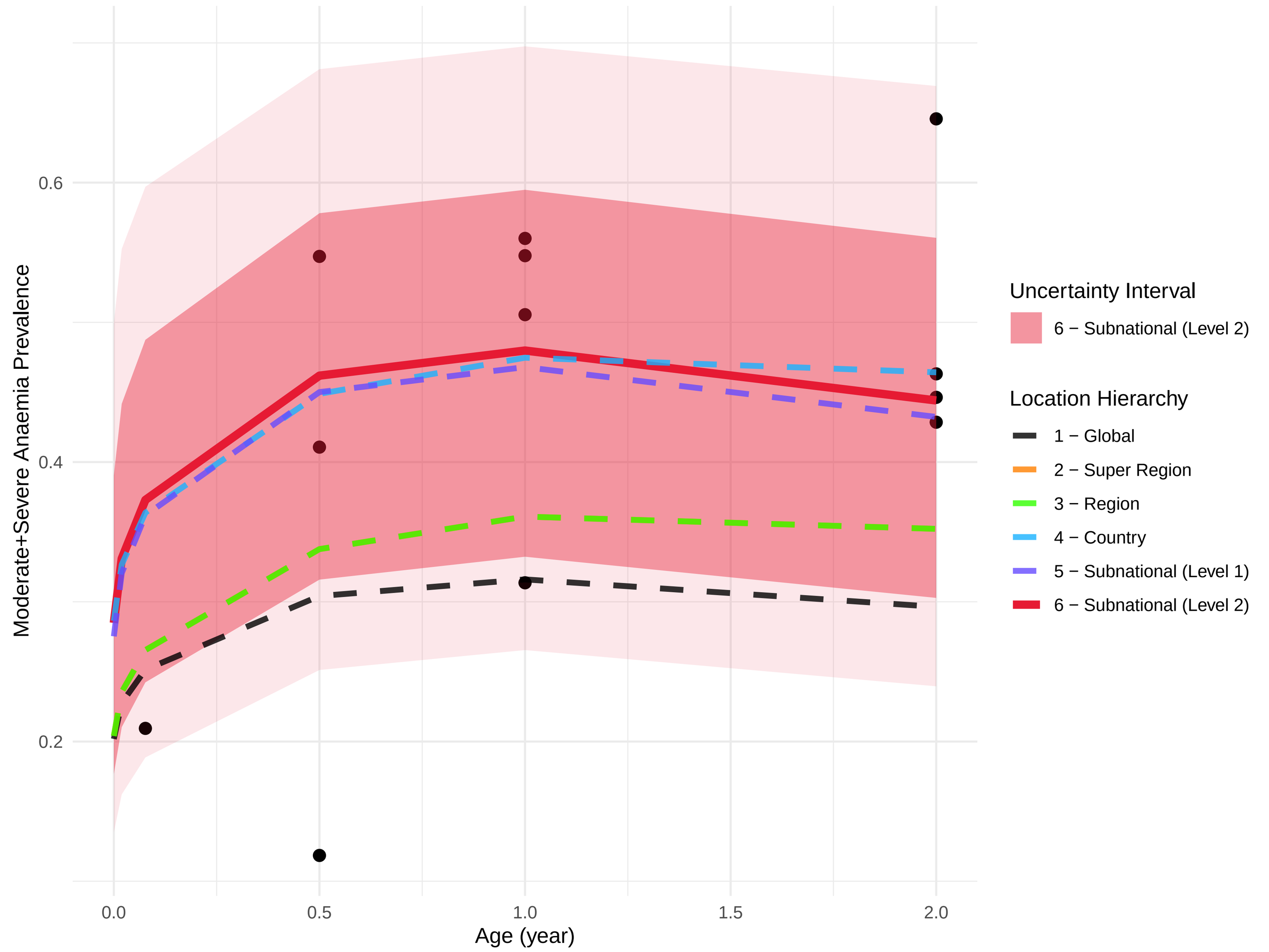

MR−BRT Age/Sex Fits − Non−pregnant Females <2 Years Old − Moderate+Severe Anaemia Prevalence − Telangana, Urban
WHO−2024 Adjustment Method

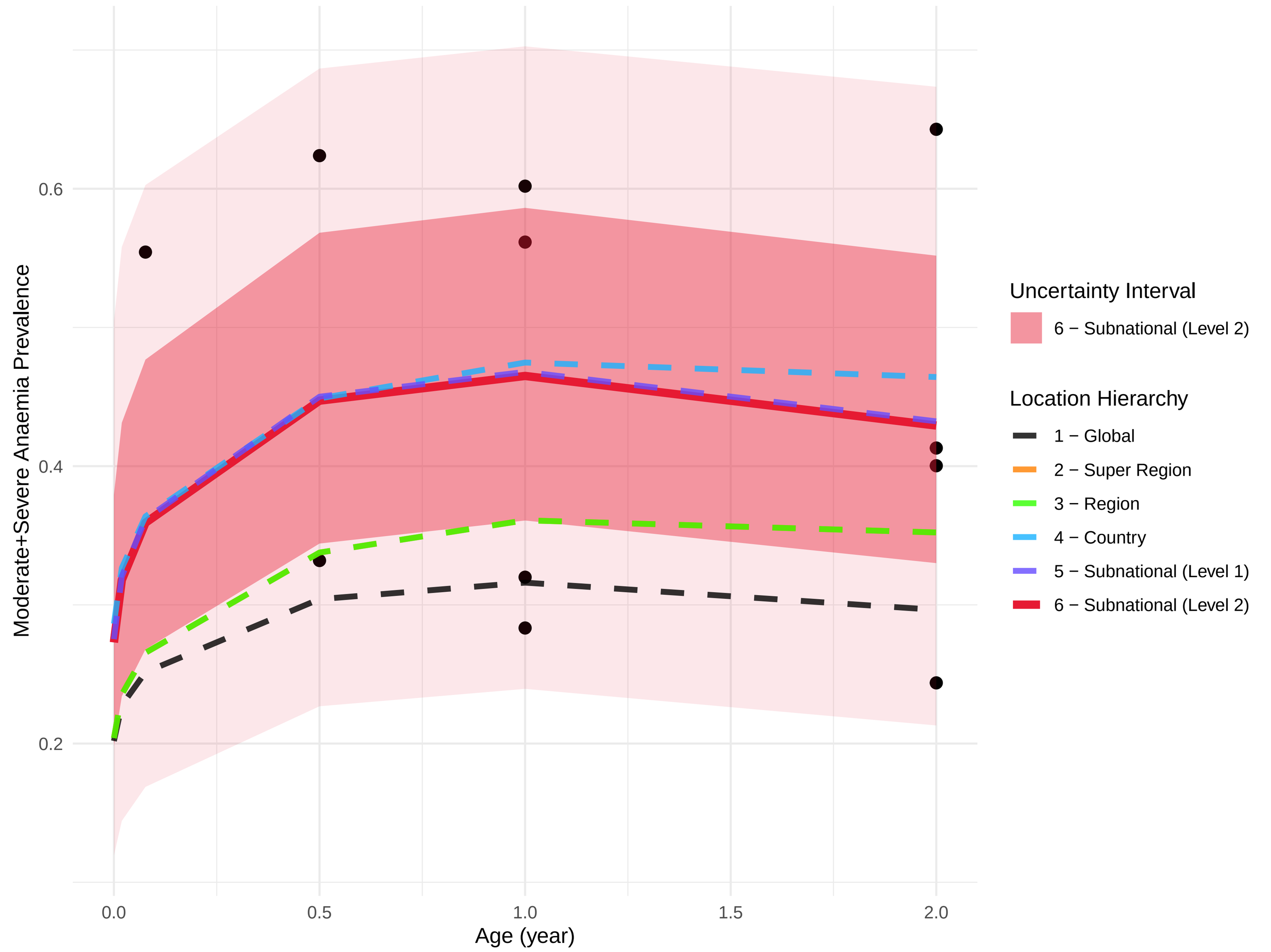

MR−BRT Age/Sex Fits − Non−pregnant Females <2 Years Old − Moderate+Severe Anaemia Prevalence − Tripura
WHO−2024 Adjustment Method

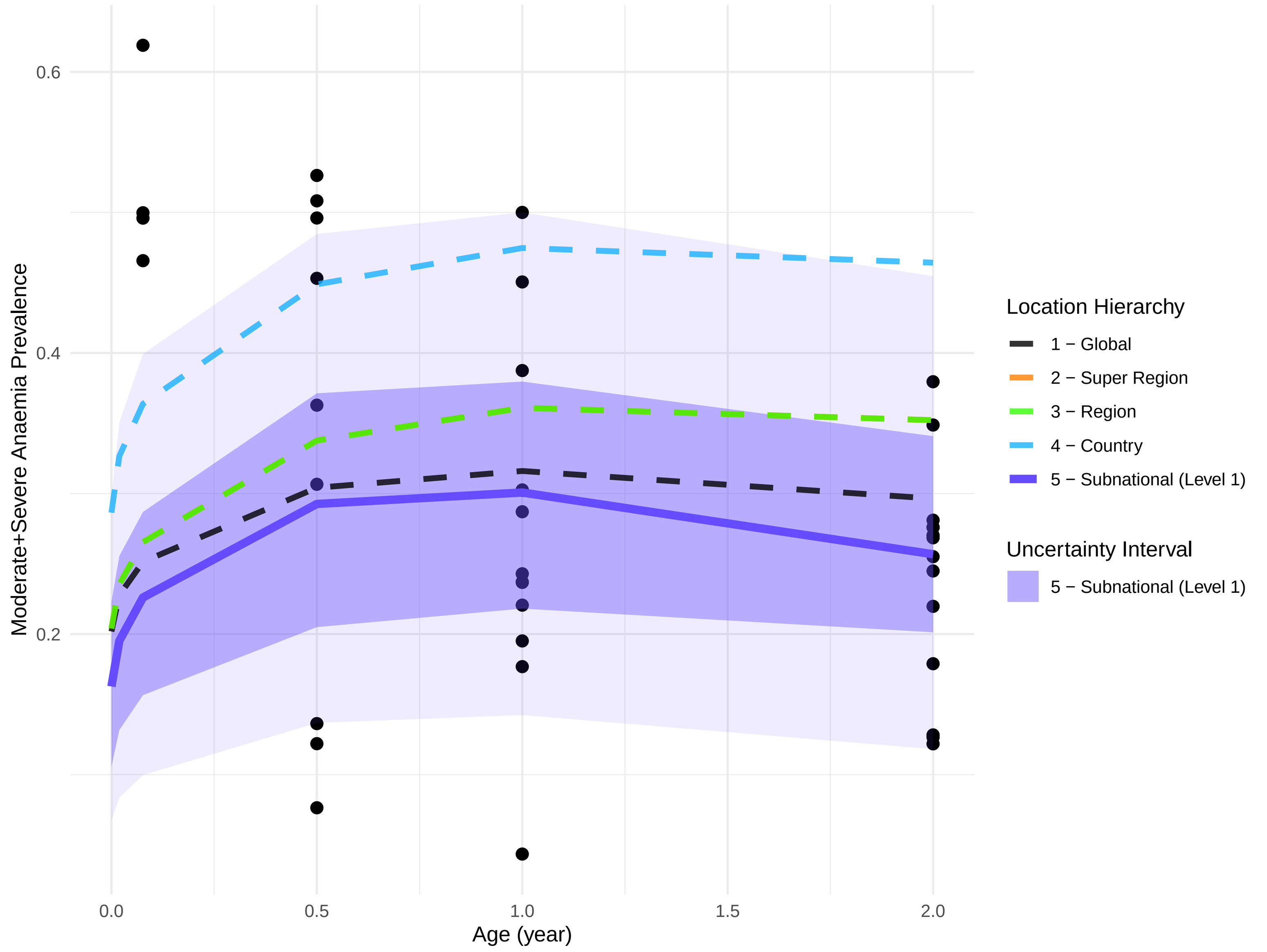

MR−BRT Age/Sex Fits − Non−pregnant Females <2 Years Old − Moderate+Severe Anaemia Prevalence − Tripura, Rural
WHO−2024 Adjustment Method

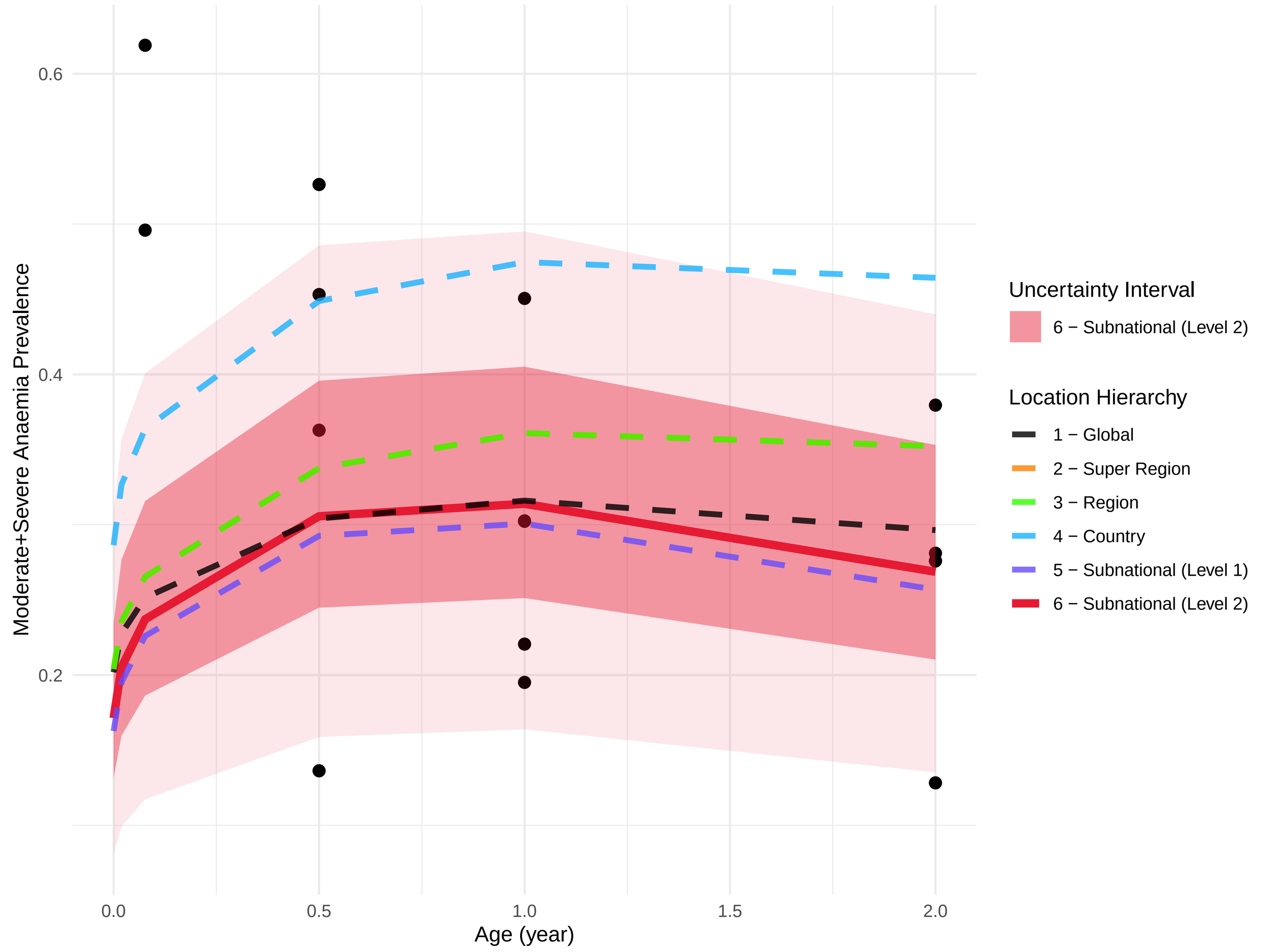

MR−BRT Age/Sex Fits − Non−pregnant Females <2 Years Old − Moderate+Severe Anaemia Prevalence − Tripura, Urban
WHO−2024 Adjustment Method

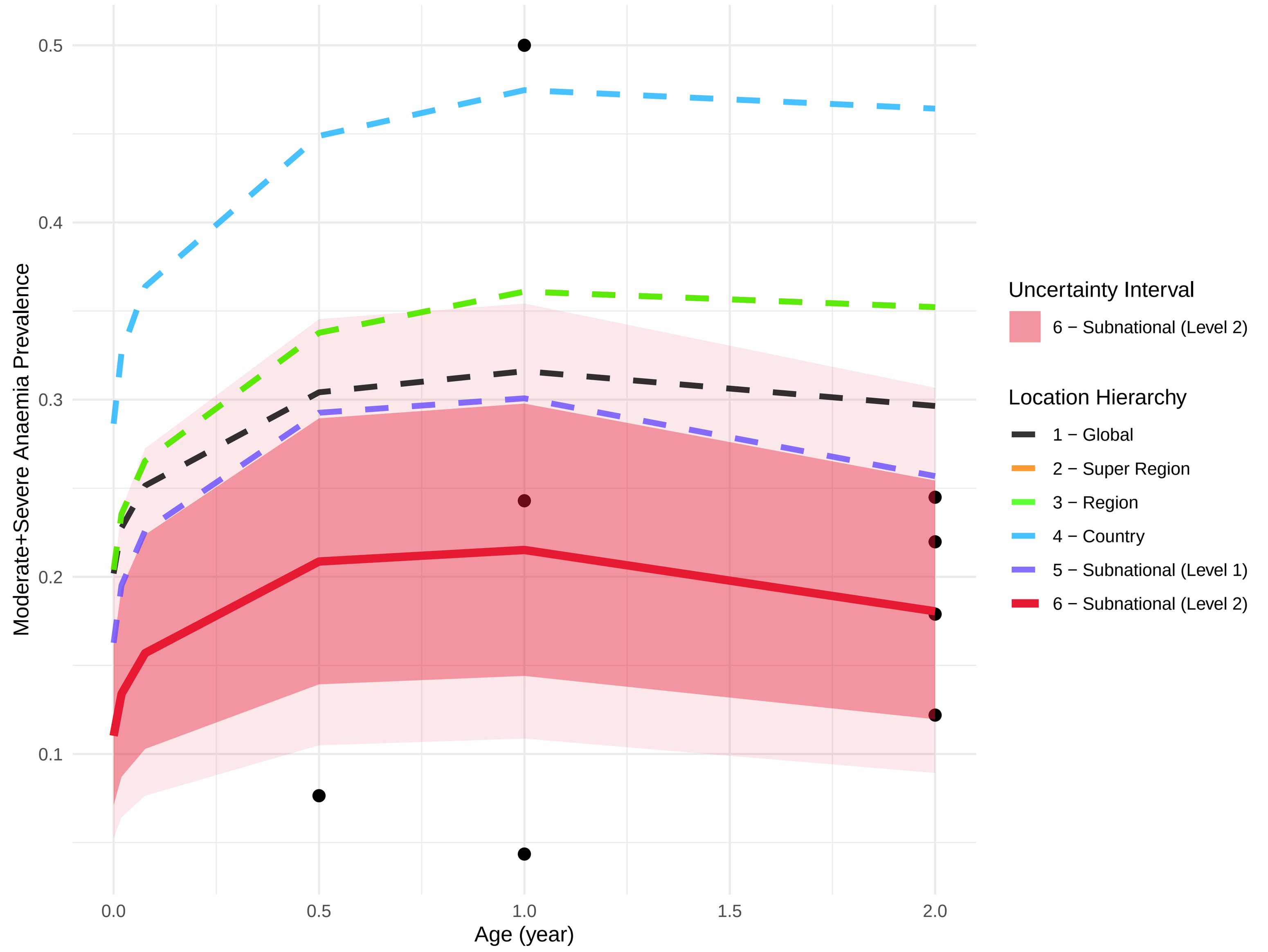

MR−BRT Age/Sex Fits − Non−pregnant Females <2 Years Old − Moderate+Severe Anaemia Prevalence − Uttar Pradesh
WHO−2024 Adjustment Method

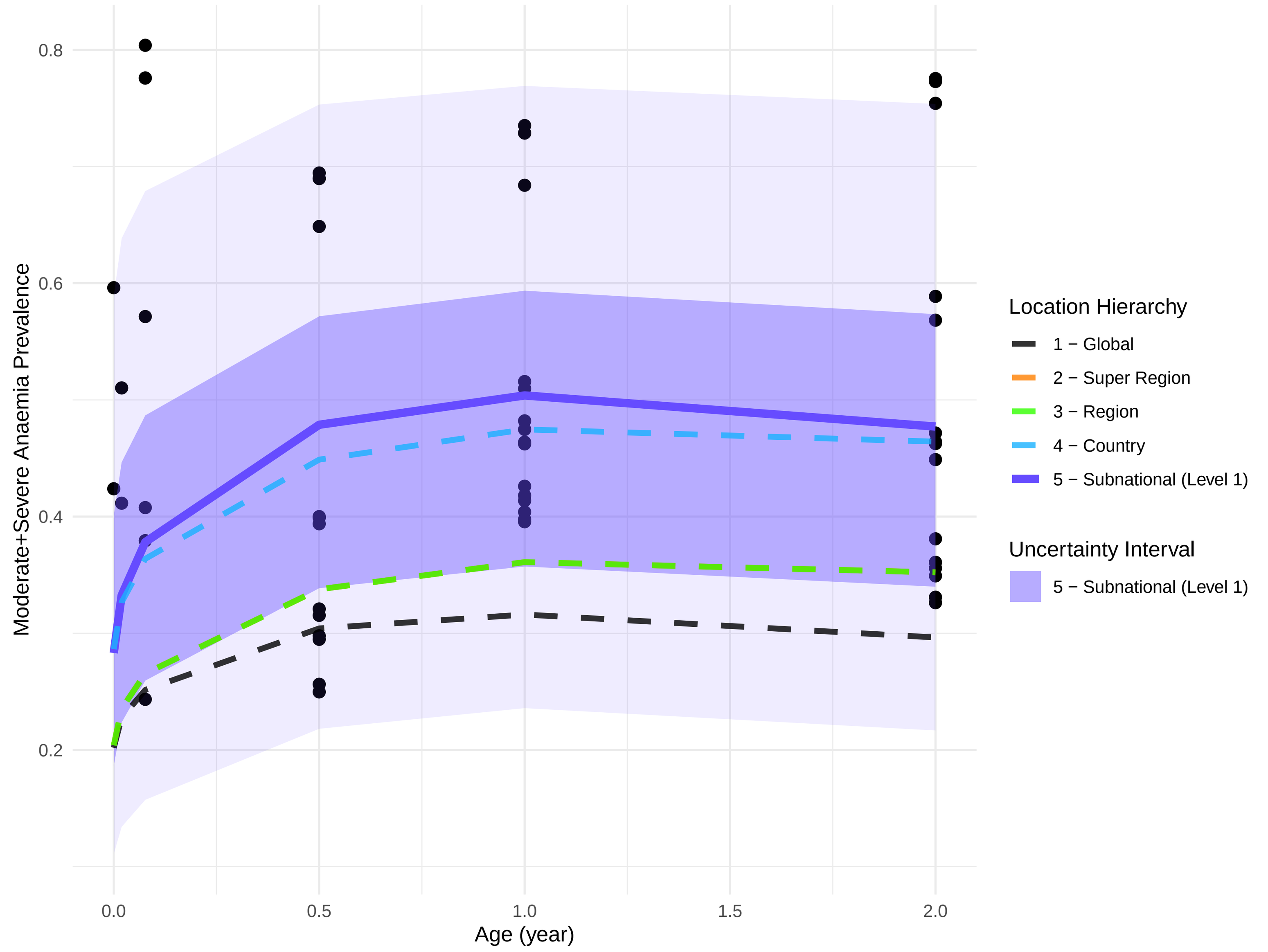

MR−BRT Age/Sex Fits − Non−pregnant Females <2 Years Old − Moderate+Severe Anaemia Prevalence − Uttar Pradesh, Rural
WHO−2024 Adjustment Method

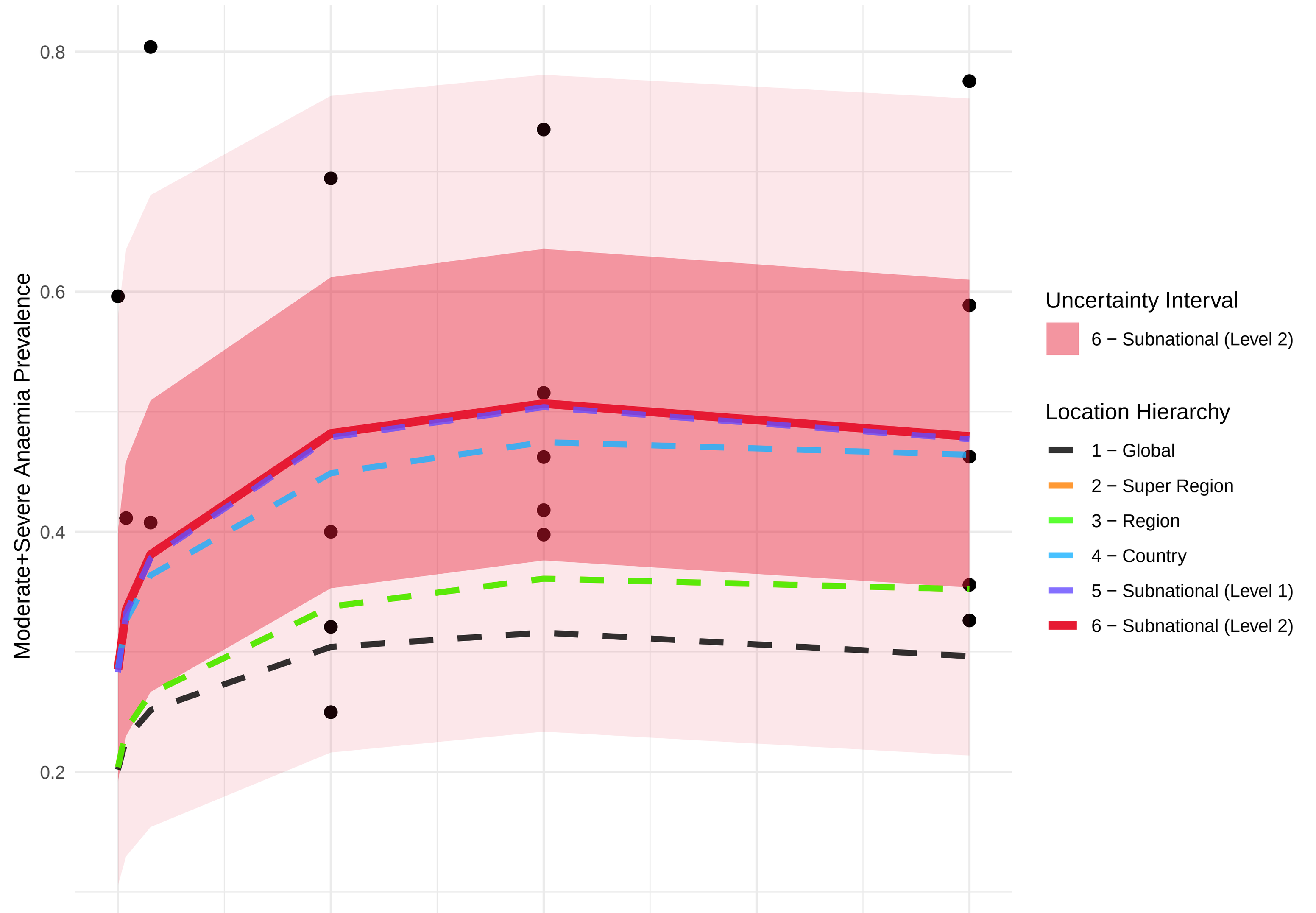

MR−BRT Age/Sex Fits − Non−pregnant Females <2 Years Old − Moderate+Severe Anaemia Prevalence − Uttar Pradesh, Urban
WHO−2024 Adjustment Method

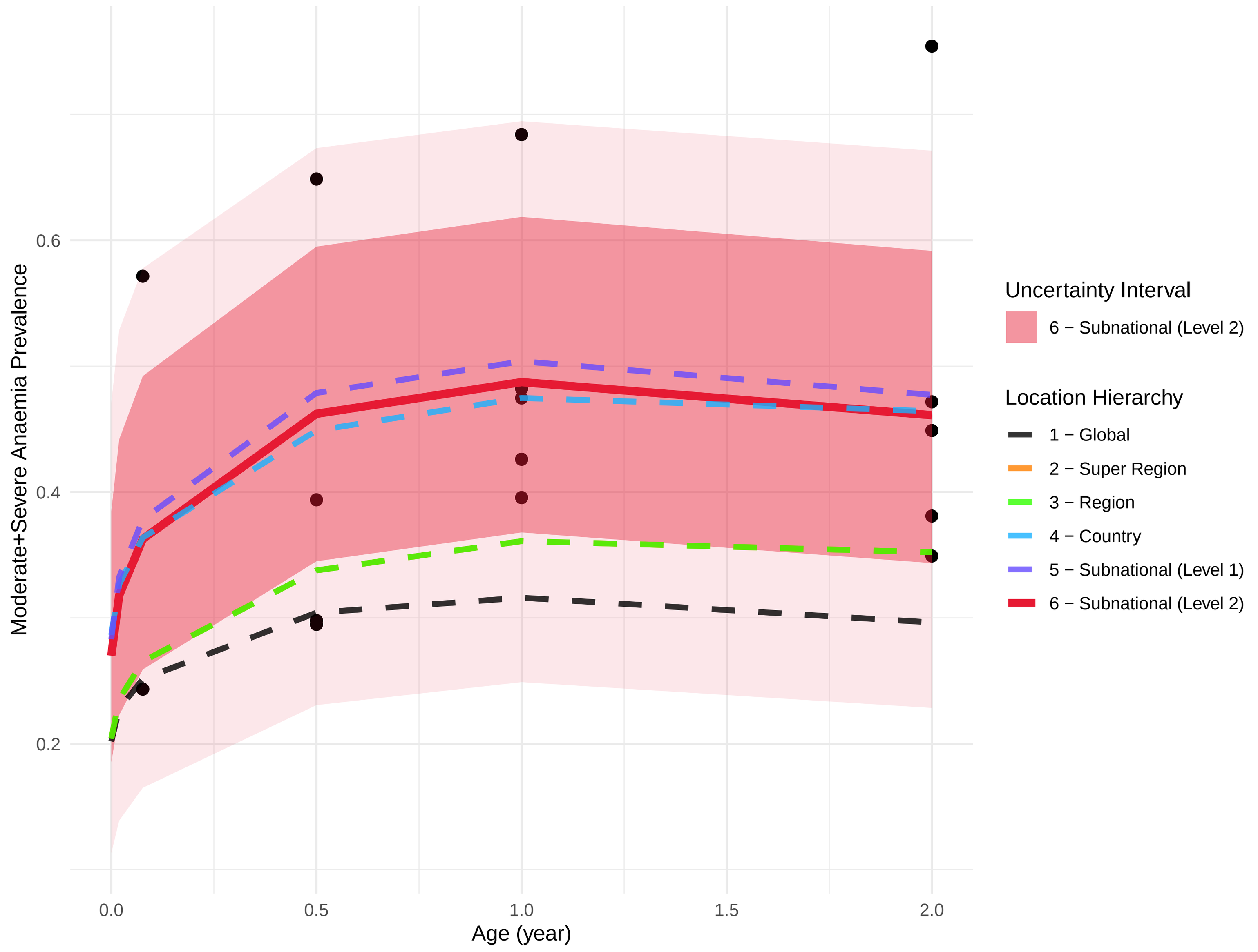

MR−BRT Age/Sex Fits − Non−pregnant Females <2 Years Old − Moderate+Severe Anaemia Prevalence − Uttarakhand
WHO−2024 Adjustment Method

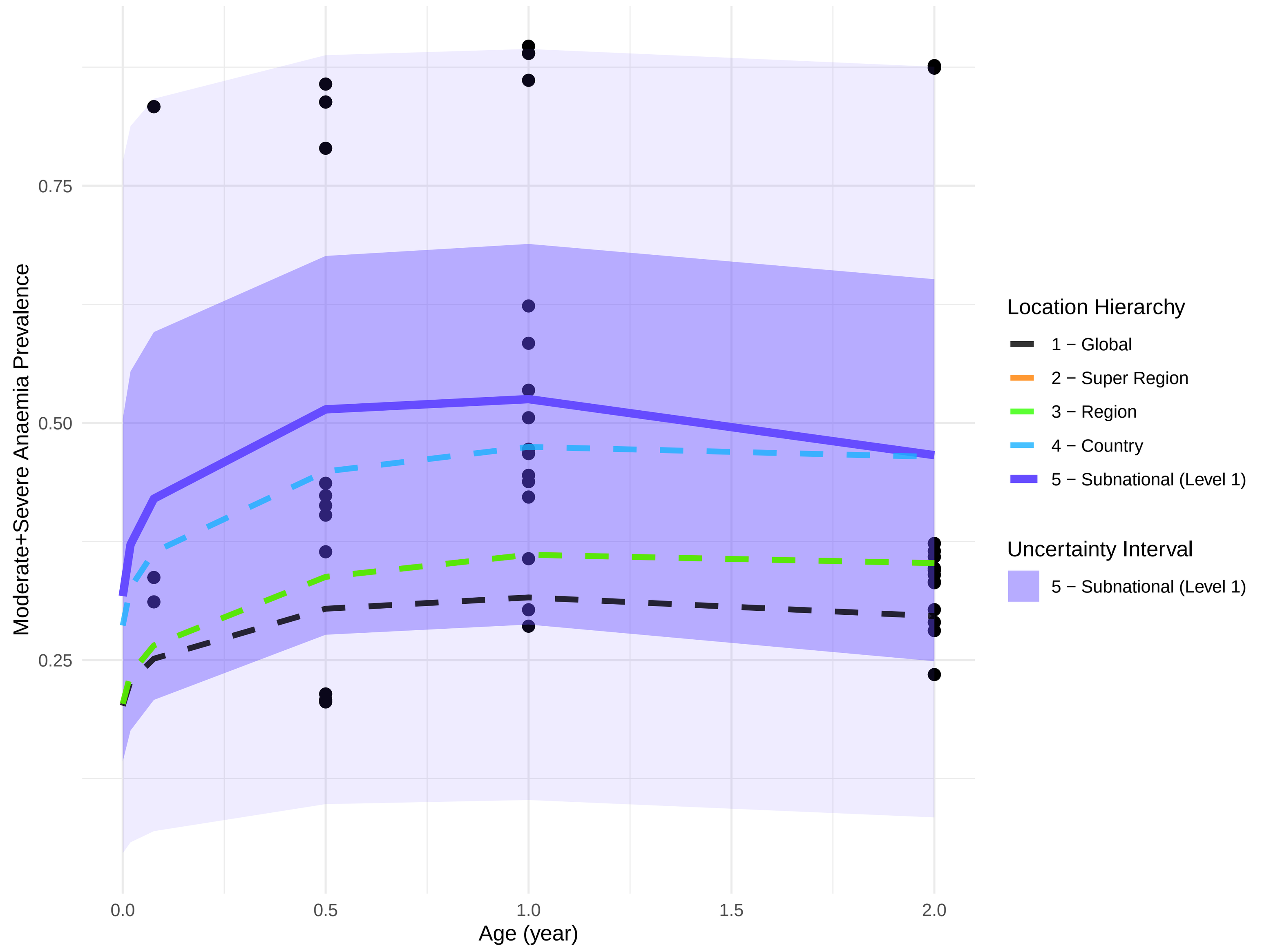

MR−BRT Age/Sex Fits − Non−pregnant Females <2 Years Old − Moderate+Severe Anaemia Prevalence − Uttarakhand, Rural
WHO−2024 Adjustment Method

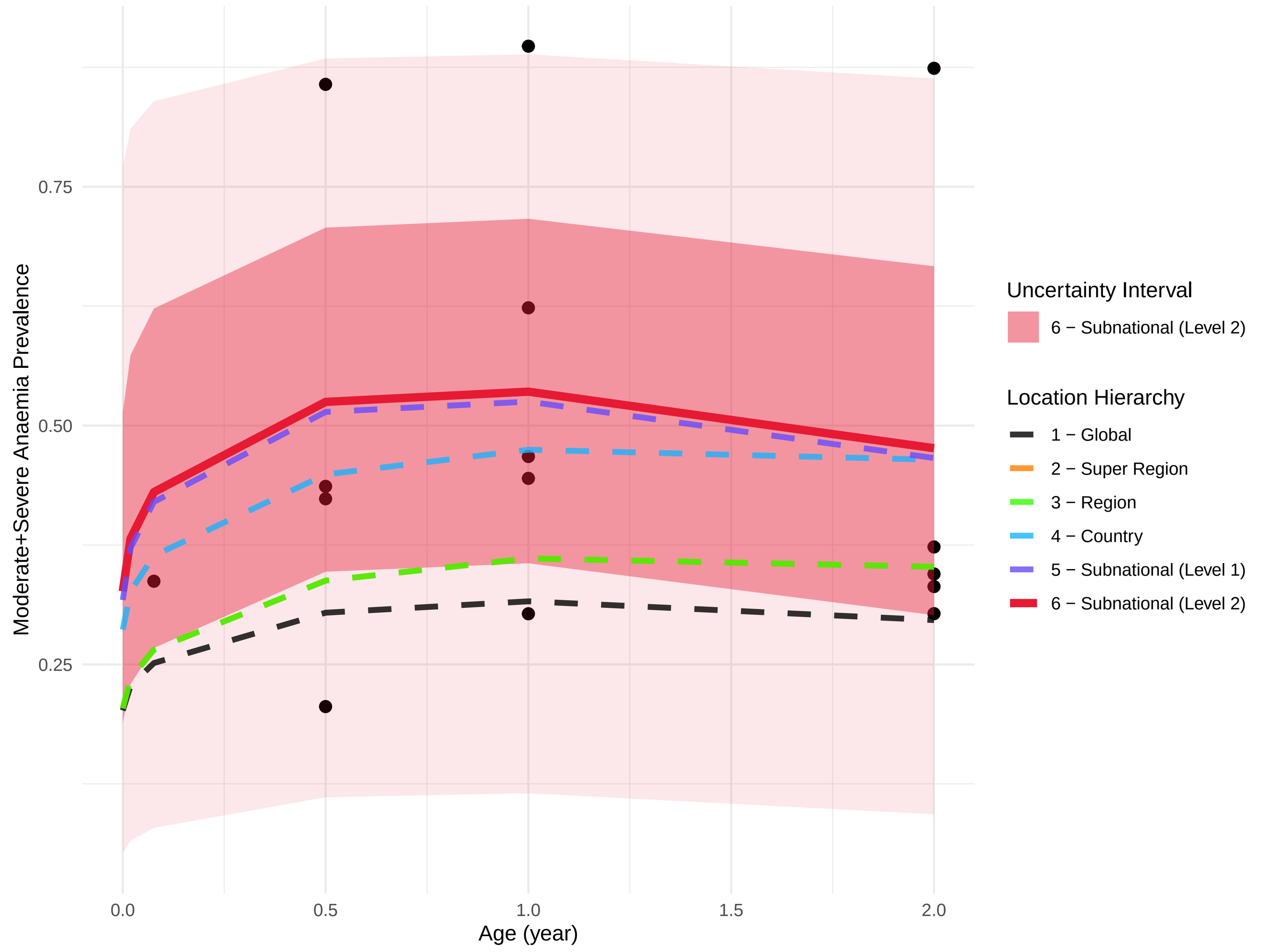

MR−BRT Age/Sex Fits − Non−pregnant Females <2 Years Old − Moderate+Severe Anaemia Prevalence − Uttarakhand, Urban
WHO−2024 Adjustment Method

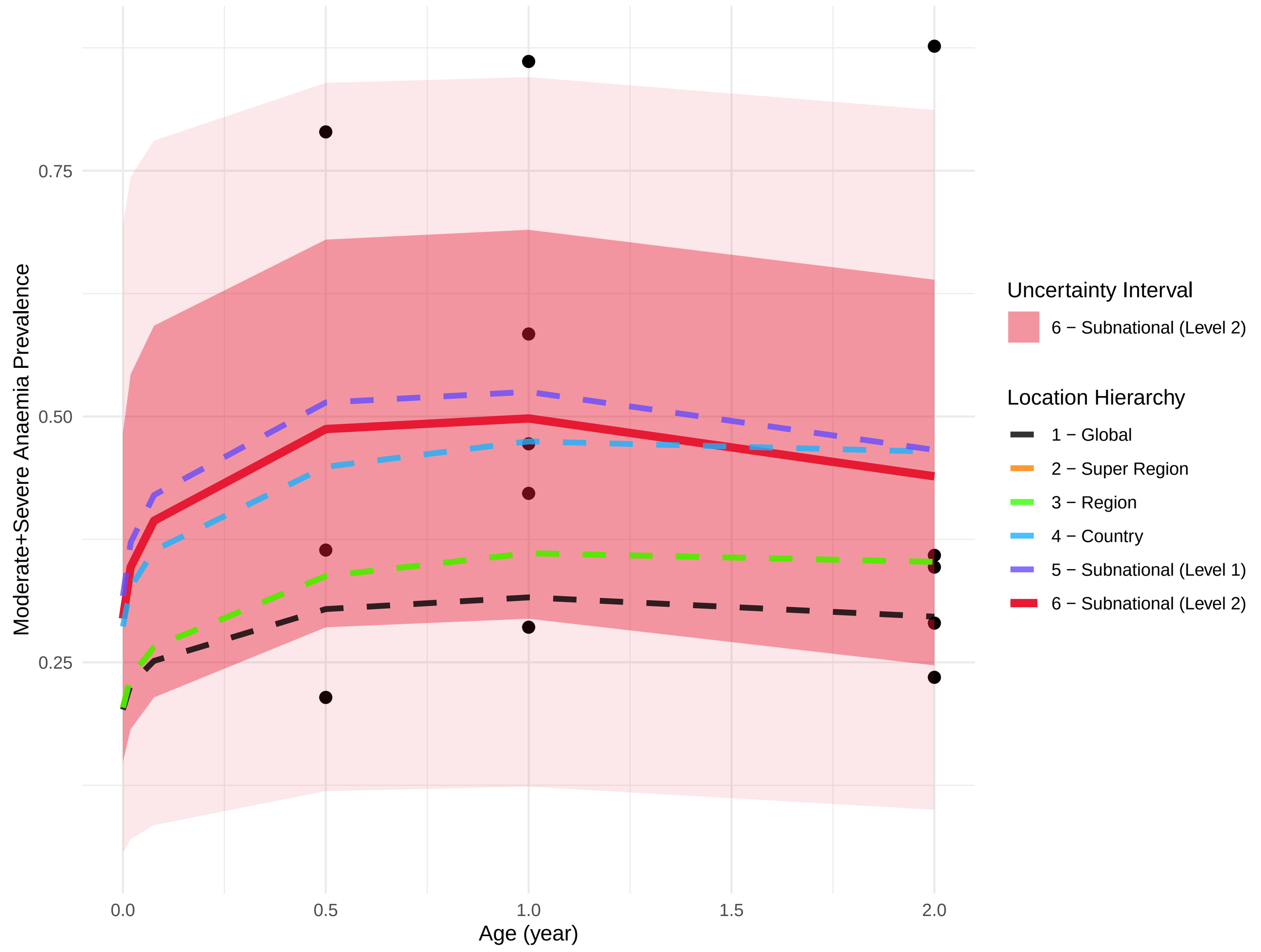

MR−BRT Age/Sex Fits − Non−pregnant Females <2 Years Old − Moderate+Severe Anaemia Prevalence − West Bengal
WHO−2024 Adjustment Method

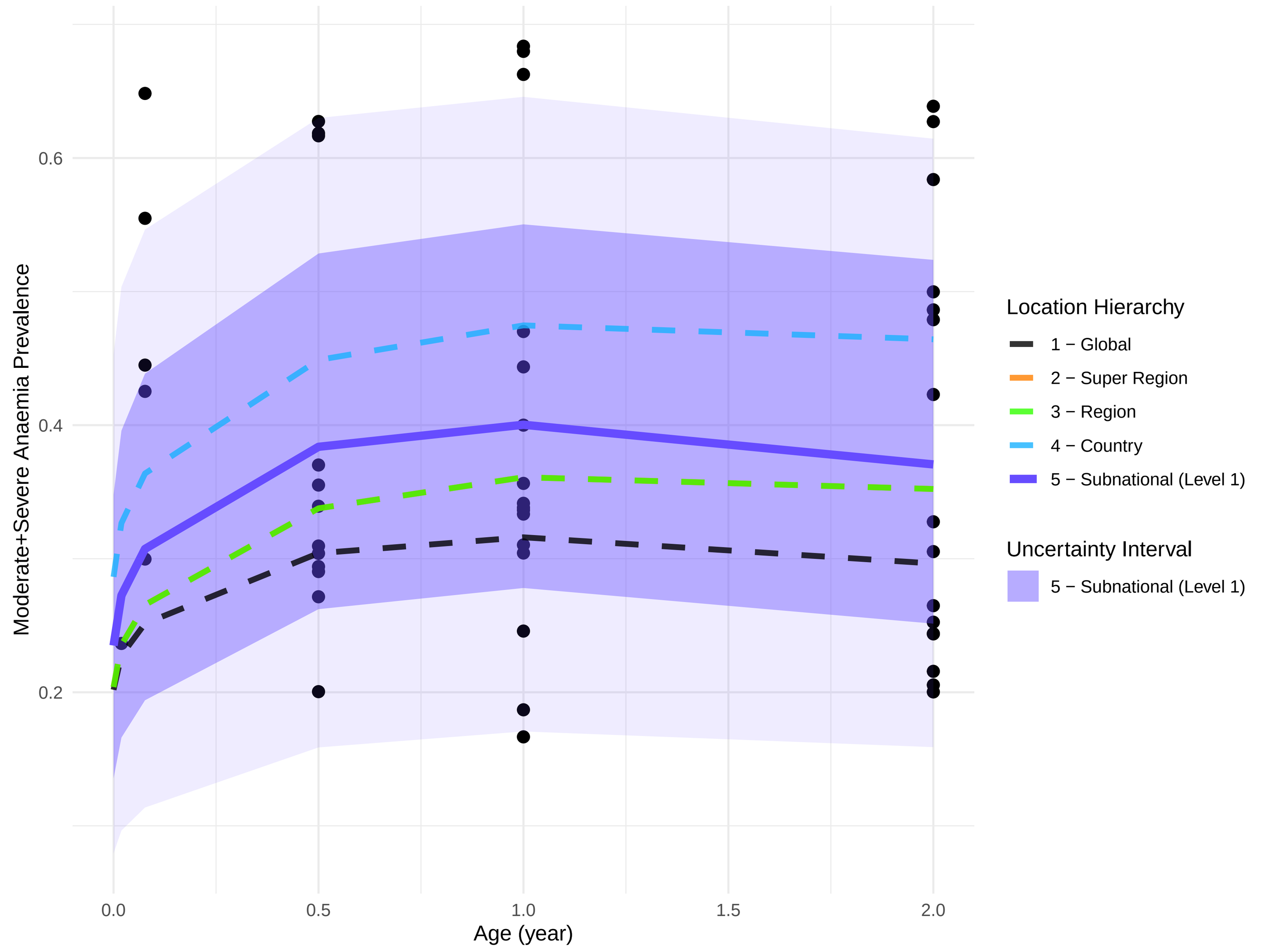

MR−BRT Age/Sex Fits − Non−pregnant Females <2 Years Old − Moderate+Severe Anaemia Prevalence − West Bengal, Rural
WHO−2024 Adjustment Method

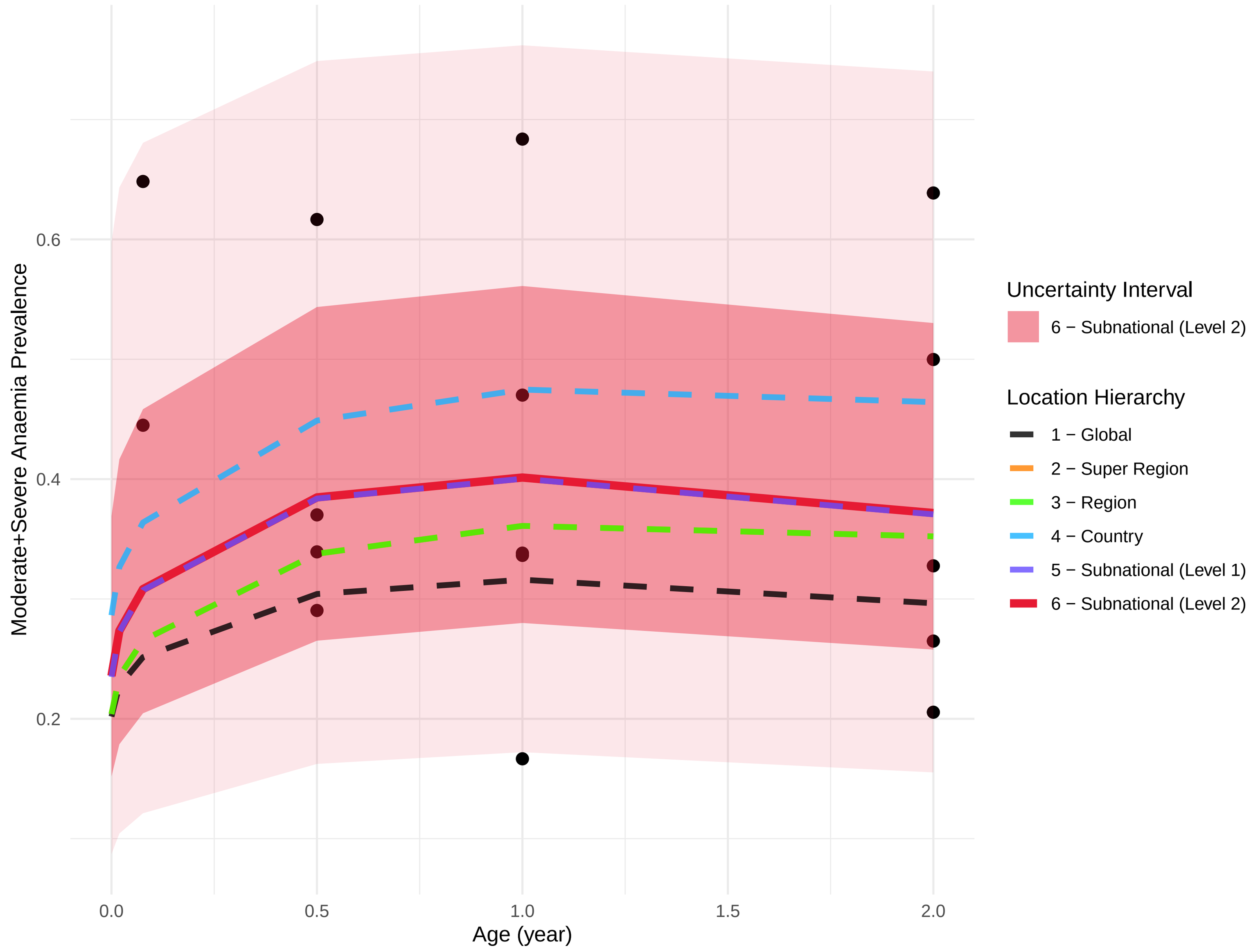

MR−BRT Age/Sex Fits − Non−pregnant Females <2 Years Old − Moderate+Severe Anaemia Prevalence − West Bengal, Urban
WHO−2024 Adjustment Method

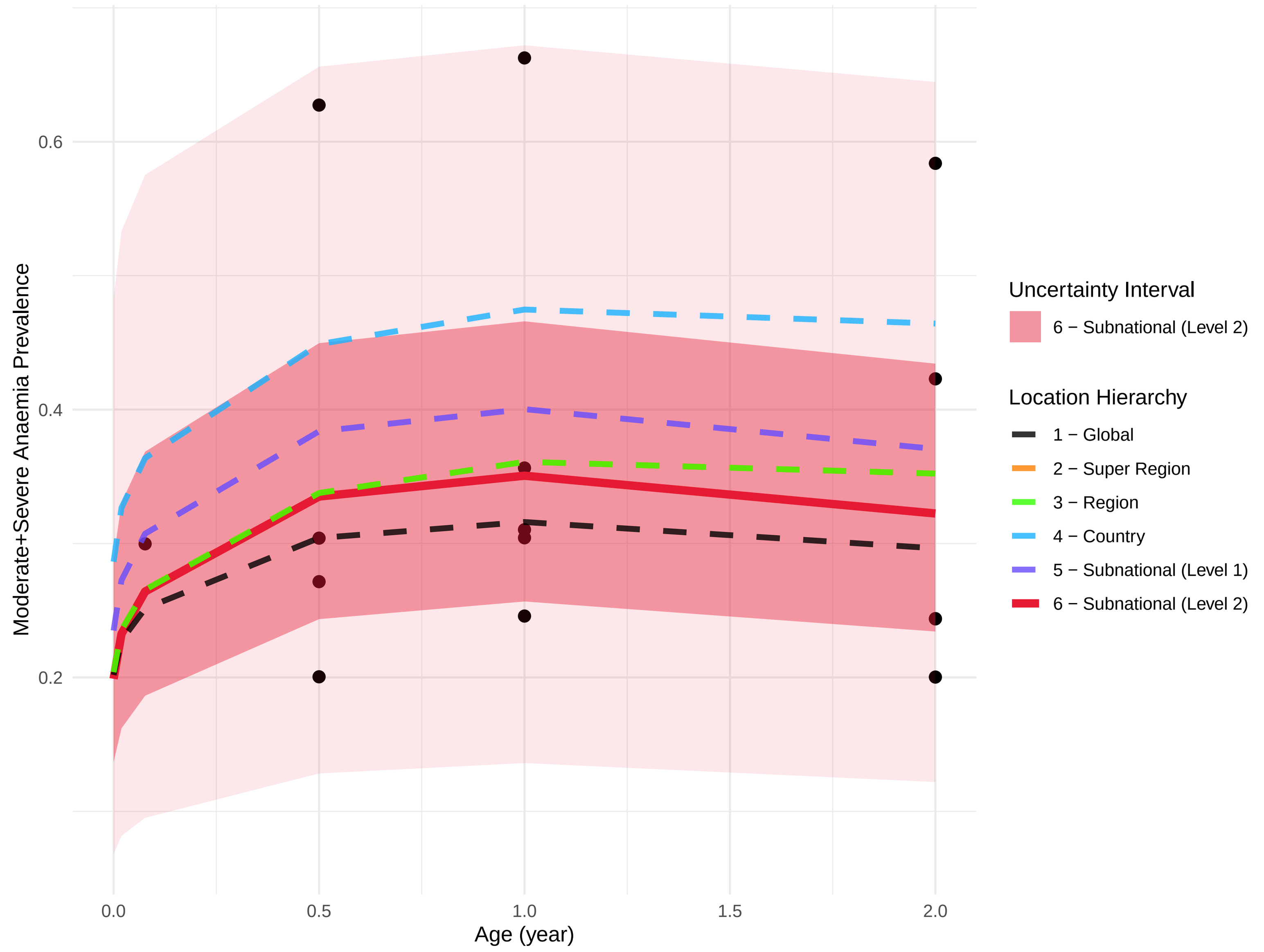

MR−BRT Age/Sex Fits − Non−pregnant Females <2 Years Old − Moderate+Severe Anaemia Prevalence − Nepal
WHO−2024 Adjustment Method

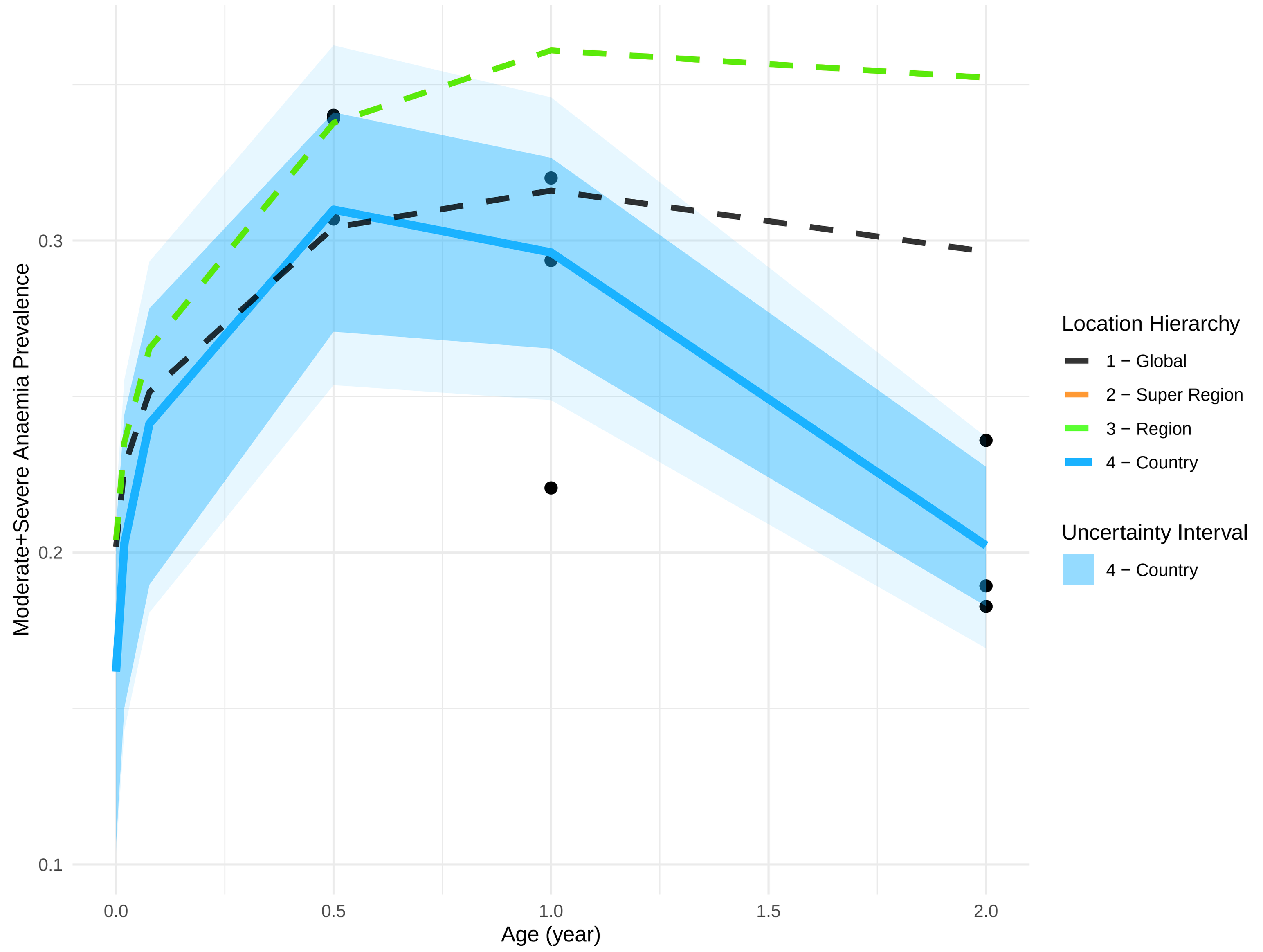

MR−BRT Age/Sex Fits − Non−pregnant Females <2 Years Old − Moderate+Severe Anaemia Prevalence − Pakistan
WHO−2024 Adjustment Method

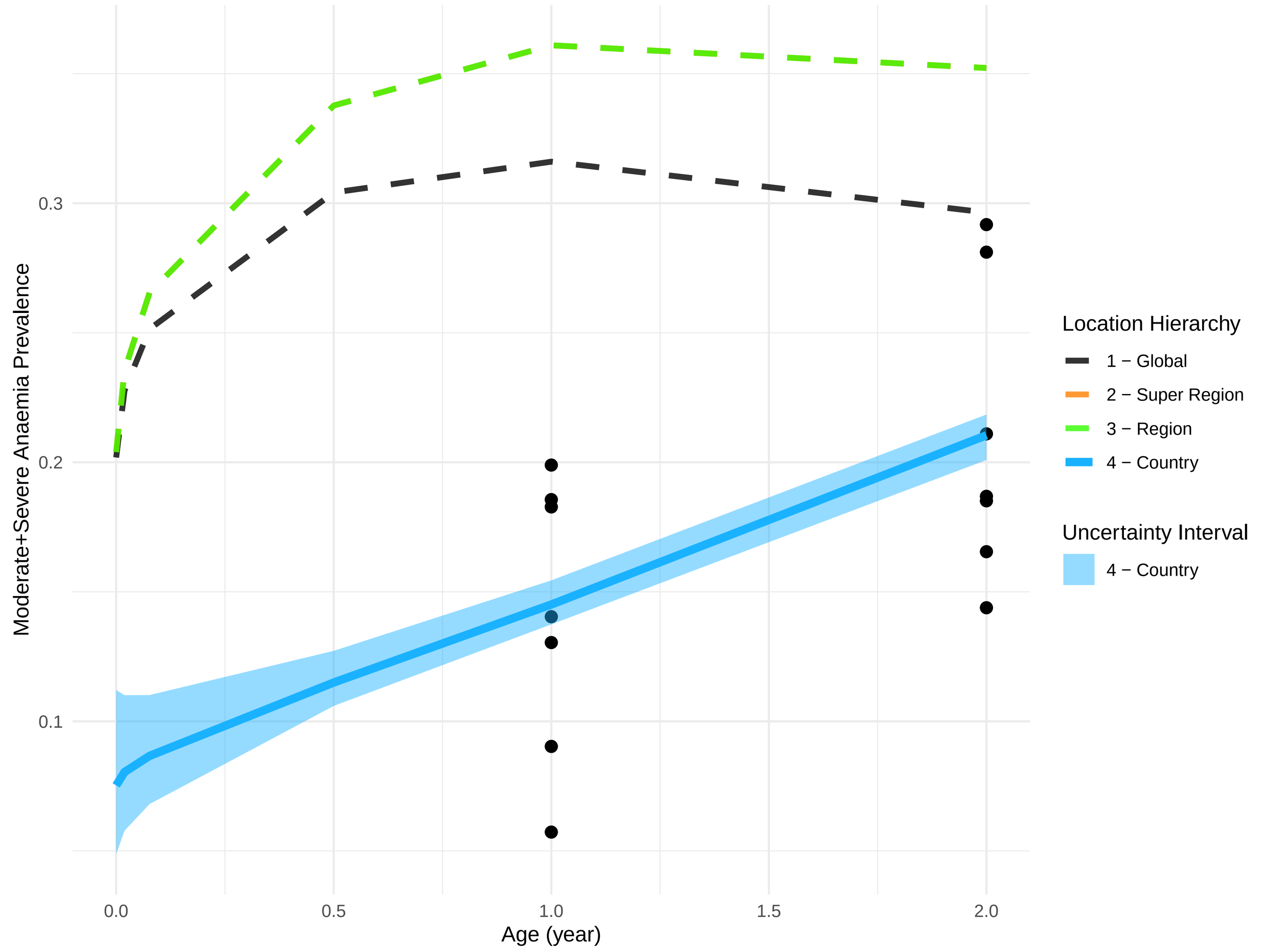

MR−BRT Age/Sex Fits − Non−pregnant Females <2 Years Old − Moderate+Severe Anaemia Prevalence − Azad Jammu & Kashmir

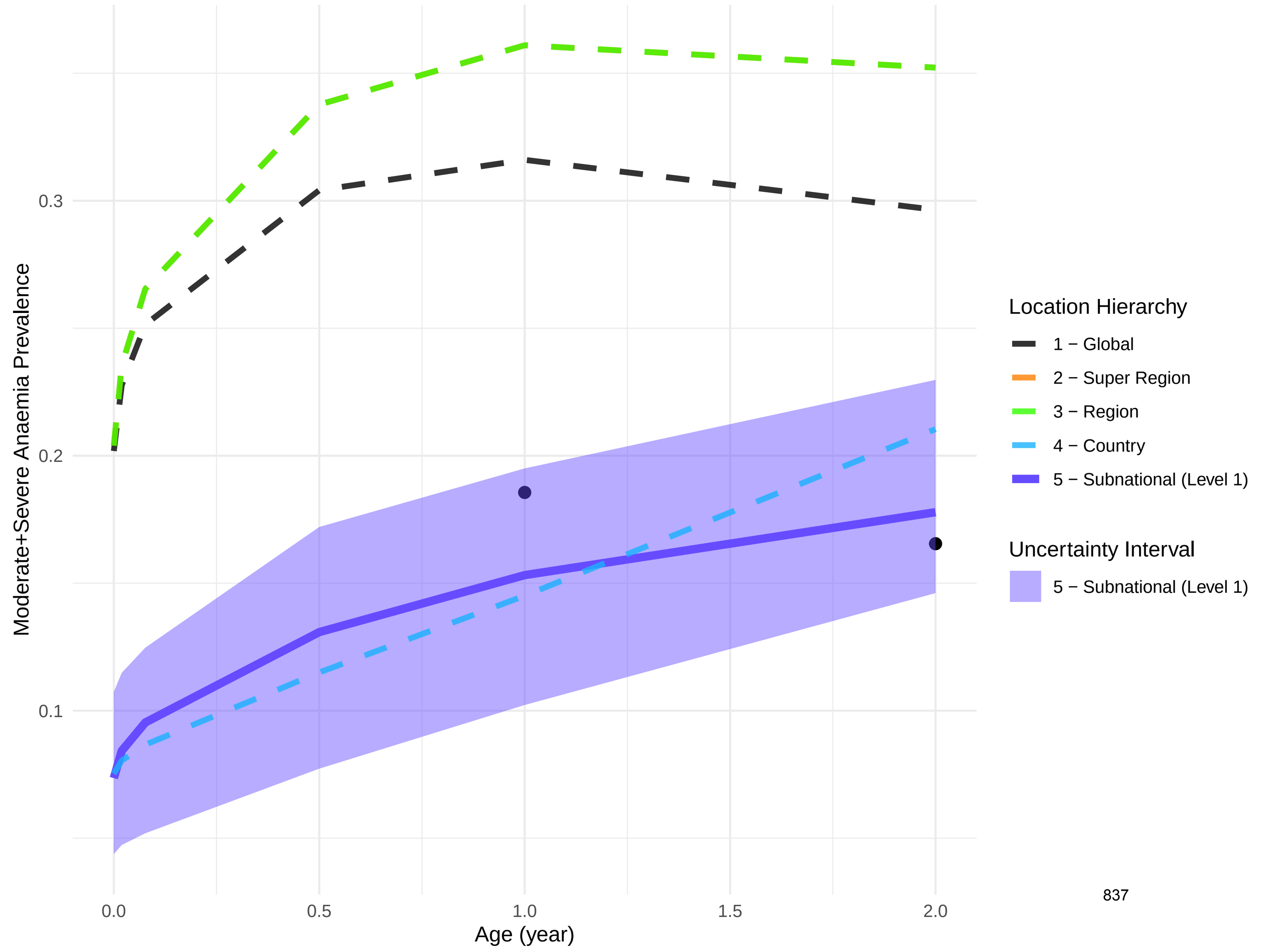

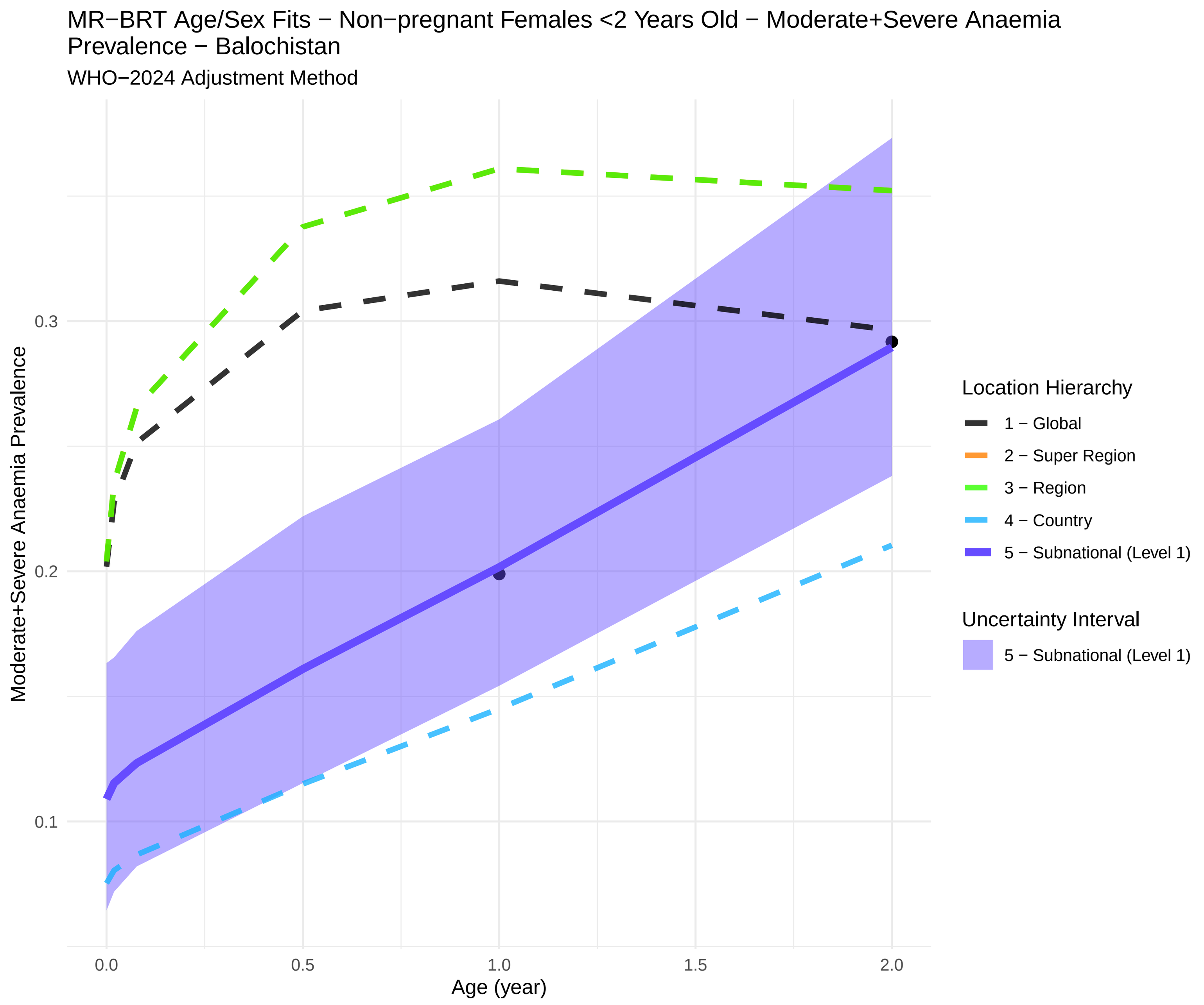
MR−BRT Age/Sex Fits − Non−pregnant Females <2 Years Old − Moderate+Severe Anaemia Prevalence − Balochistan
WHO−2024 Adjustment Method
Moderate+Severe Anaemia Prevalence
Age (year)
Location Hierarchy
1 − Global
2 − Super Region
3 − Region
4 − Country
5 − Subnational (Level 1)
Uncertainty Interval
5 − Subnational (Level 1)

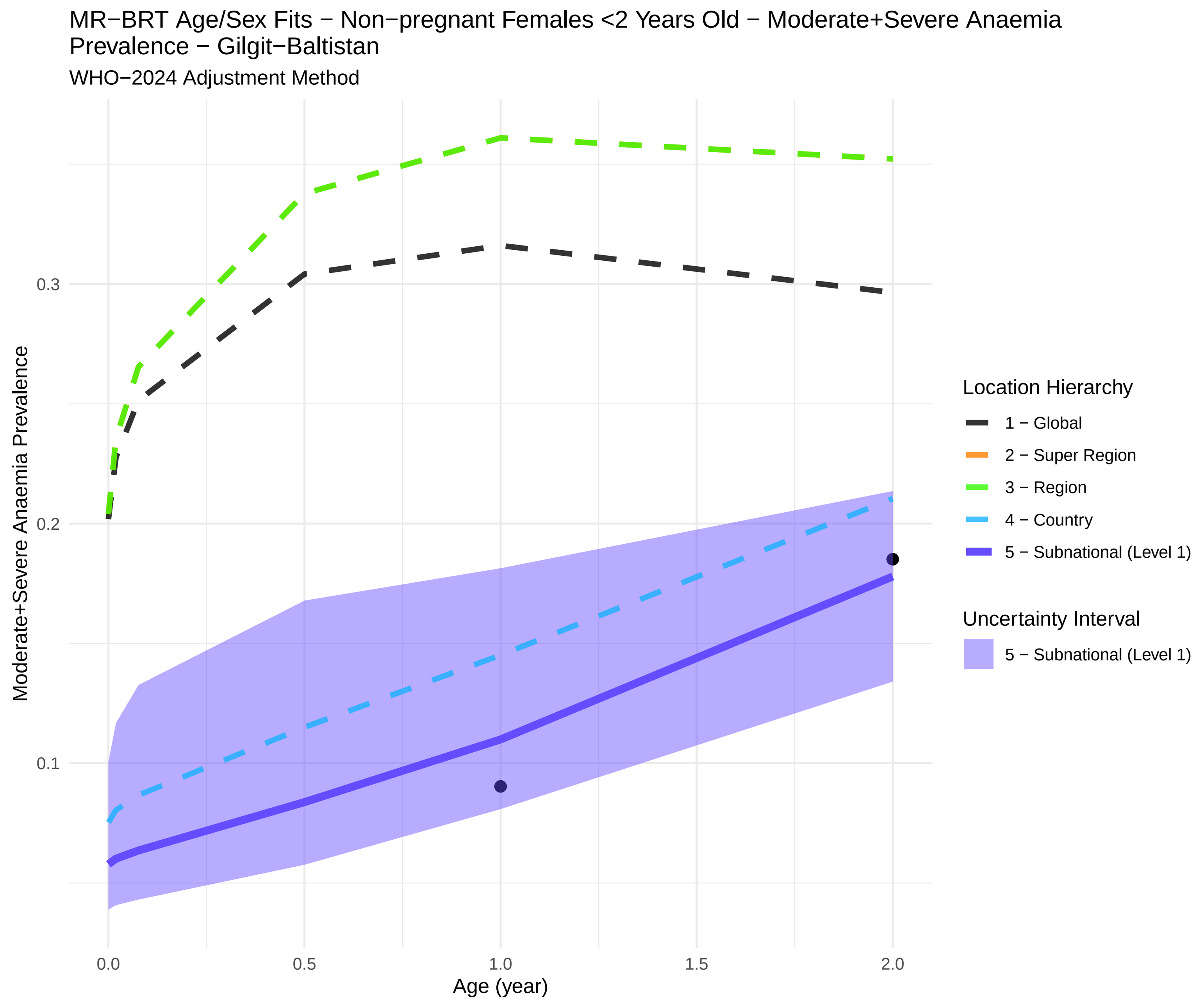
MR−BRT Age/Sex Fits − Non−pregnant Females <2 Years Old − Moderate+Severe Anaemia Prevalence − Gilgit−Baltistan
WHO−2024 Adjustment Method
Moderate+Severe Anaemia Prevalence
Age (year)
Location Hierarchy
1 − Global
2 − Super Region
3 − Region
4 − Country
5 − Subnational (Level 1)
Uncertainty Interval
5 − Subnational (Level 1)

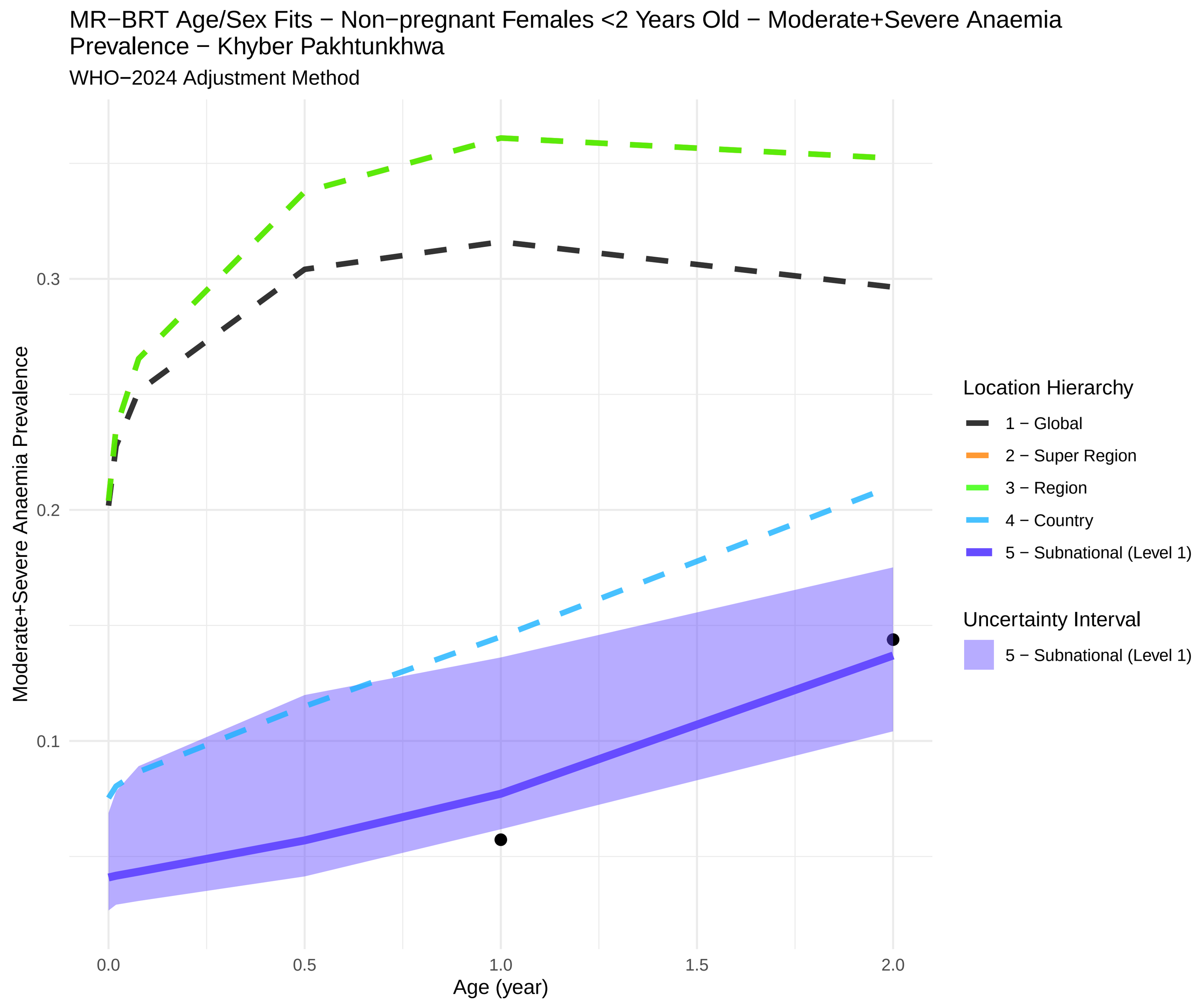
MR−BRT Age/Sex Fits − Non−pregnant Females <2 Years Old − Moderate+Severe Anaemia Prevalence − Khyber Pakhtunkhwa
WHO−2024 Adjustment Method
Moderate+Severe Anaemia Prevalence
Age (year)
Location Hierarchy
1 − Global
2 − Super Region
3 − Region
4 − Country
5 − Subnational (Level 1)
Uncertainty Interval
5 − Subnational (Level 1)

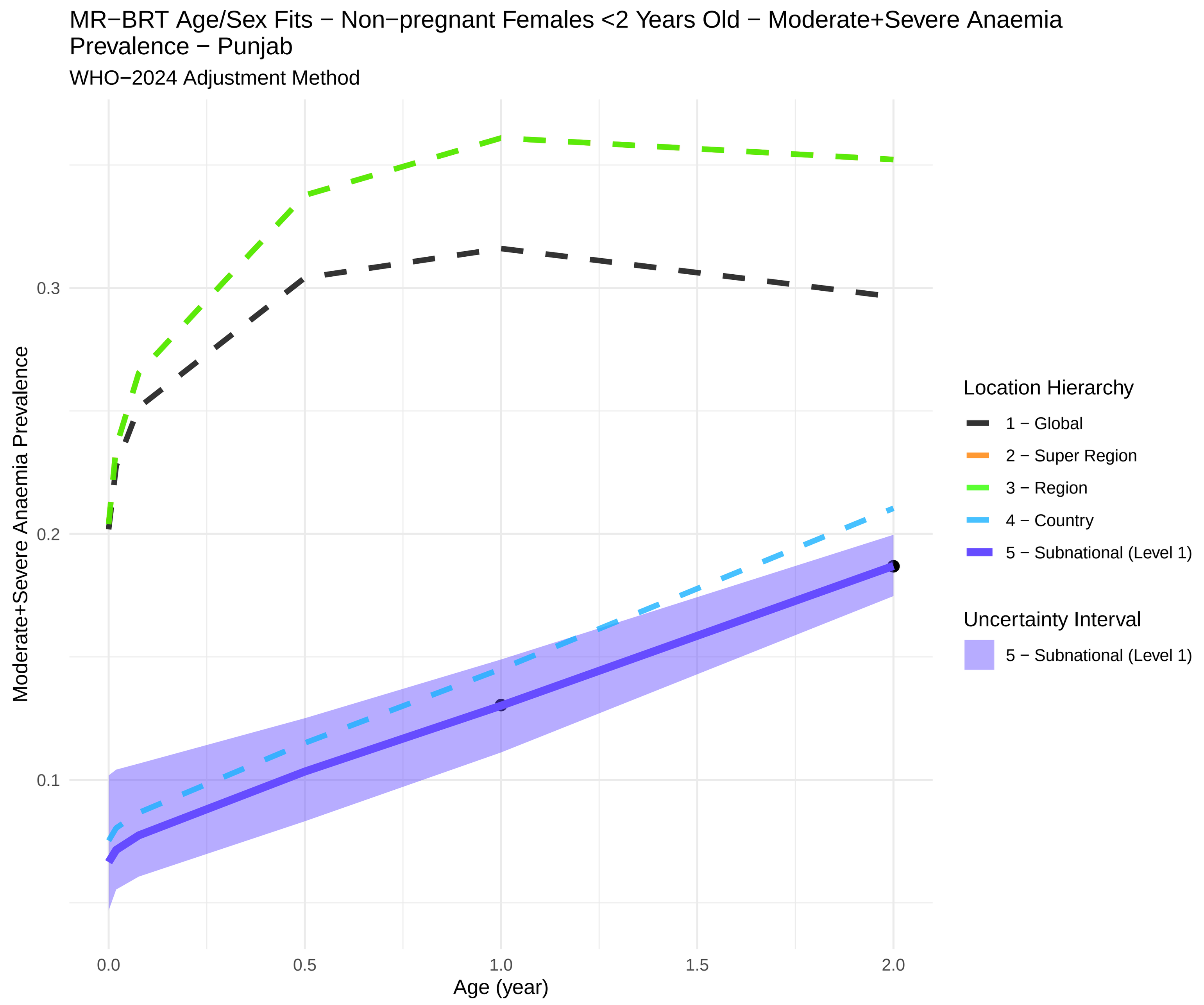
MR−BRT Age/Sex Fits − Non−pregnant Females <2 Years Old − Moderate+Severe Anaemia Prevalence − Punjab
WHO−2024 Adjustment Method
Moderate+Severe Anaemia Prevalence
Age (year)
Location Hierarchy
1 − Global
2 − Super Region
3 − Region
4 − Country
5 − Subnational (Level 1)
Uncertainty Interval
5 − Subnational (Level 1)

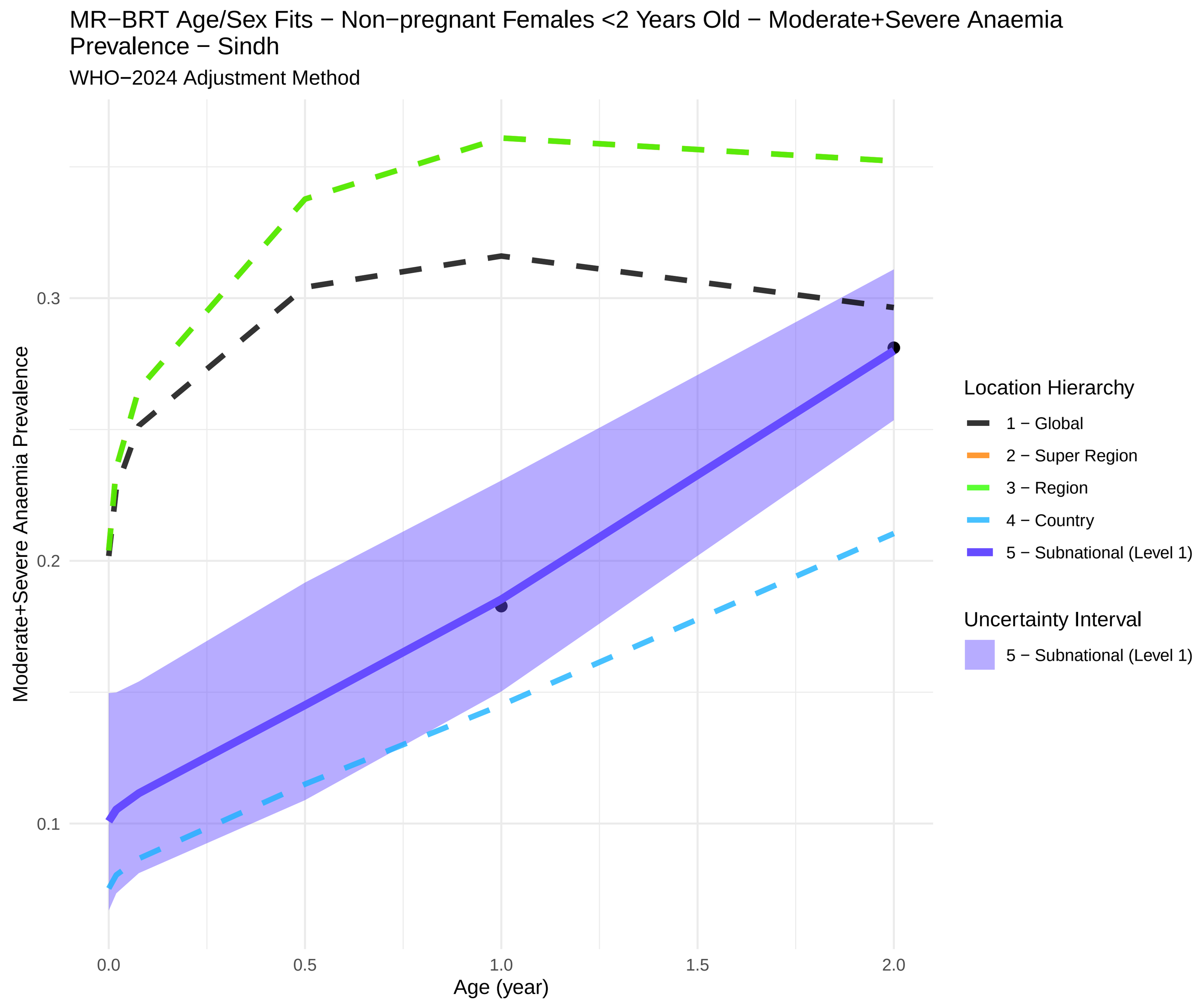
MR−BRT Age/Sex Fits − Non−pregnant Females <2 Years Old − Moderate+Severe Anaemia Prevalence − Sindh
WHO−2024 Adjustment Method
Moderate+Severe Anaemia Prevalence
Age (year)
Location Hierarchy
1 − Global
2 − Super Region
3 − Region
4 − Country
5 − Subnational (Level 1)
Uncertainty Interval
5 − Subnational (Level 1)

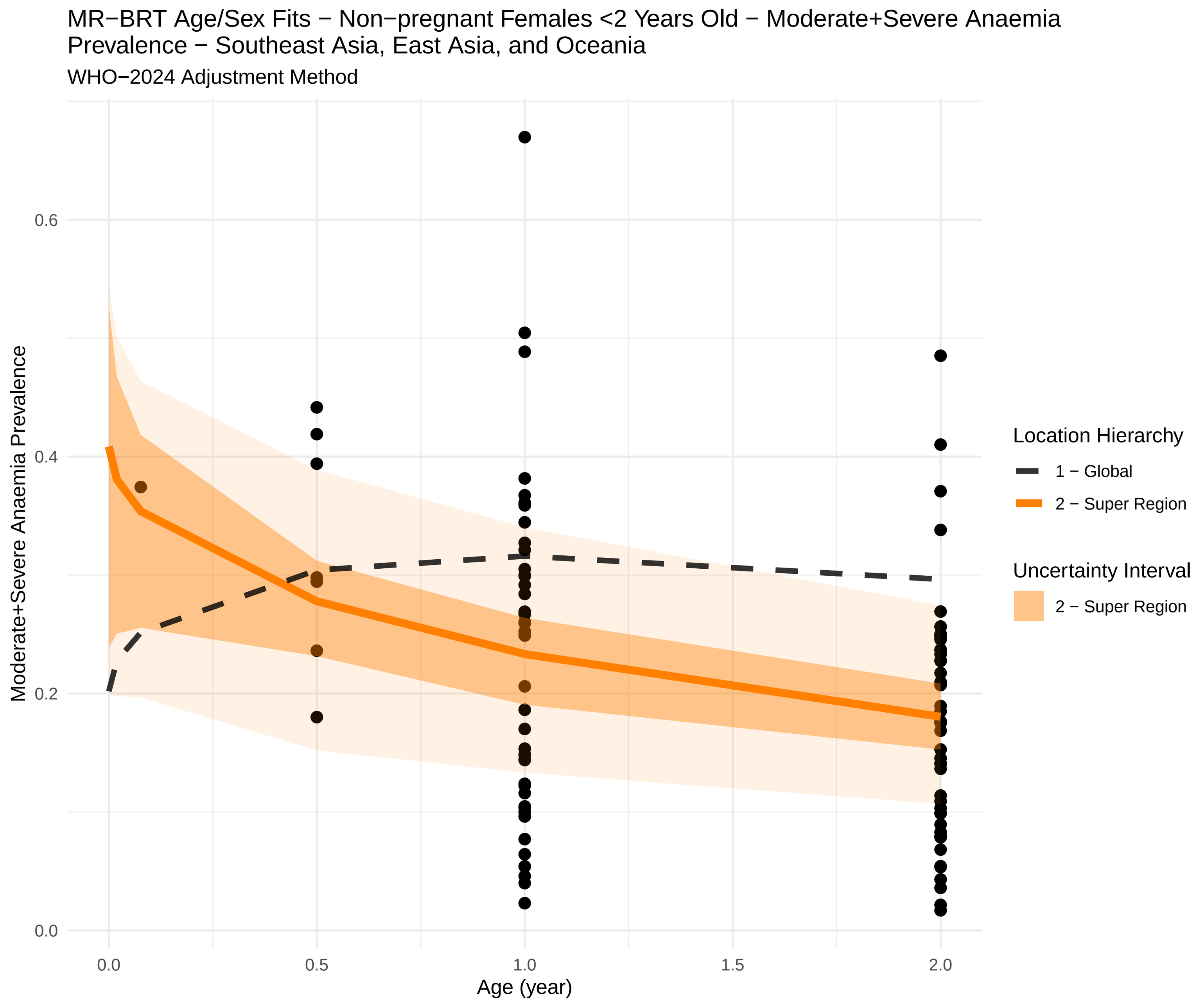
MR−BRT Age/Sex Fits − Non−pregnant Females <2 Years Old − Moderate+Severe Anaemia Prevalence − Southeast Asia, East Asia, and Oceania
WHO−2024 Adjustment Method
Moderate+Severe Anaemia Prevalence
Age (year)
Location Hierarchy
1 − Global
2 − Super Region
Uncertainty Interval
2 − Super Region

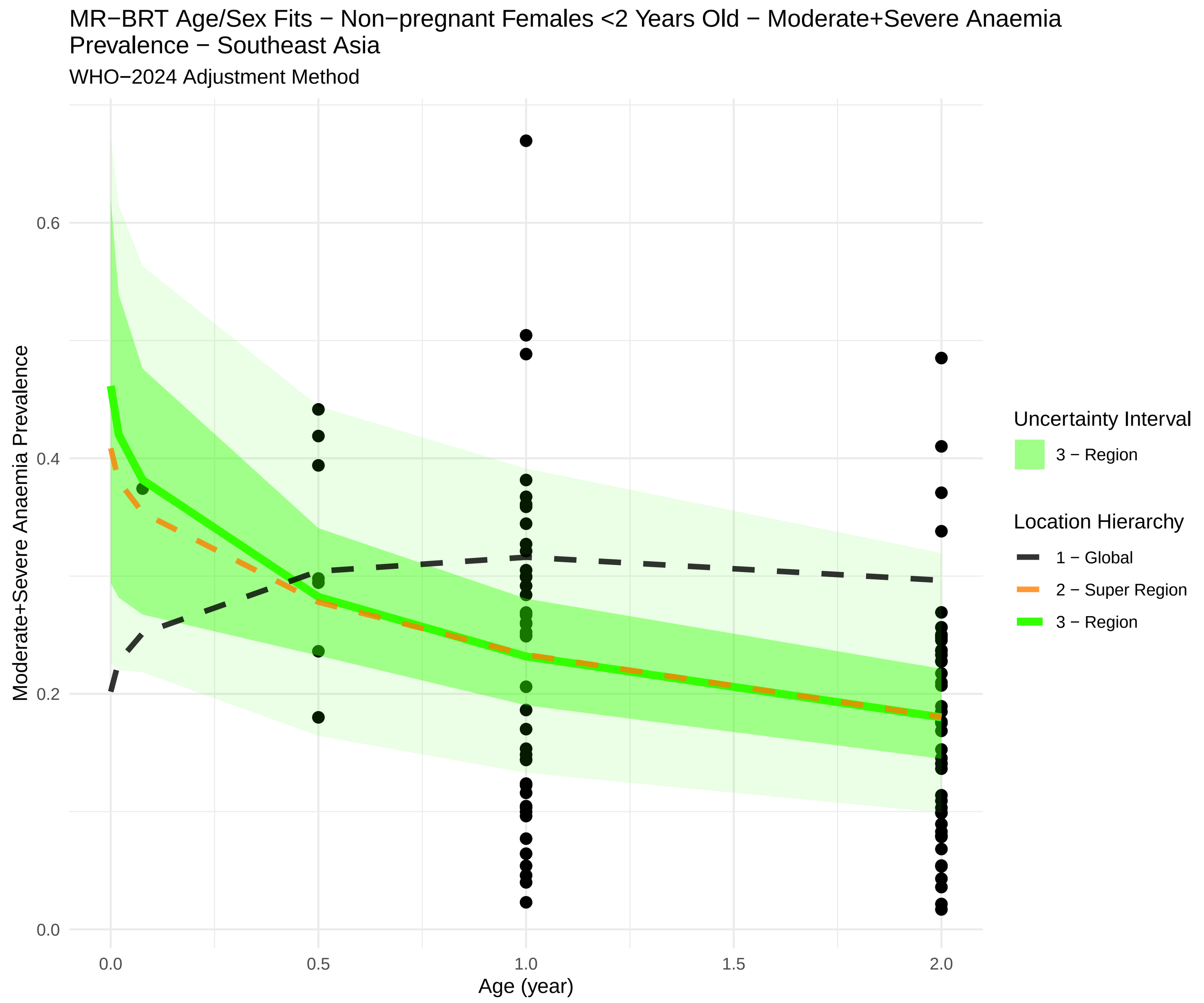
MR−BRT Age/Sex Fits − Non−pregnant Females <2 Years Old − Moderate+Severe Anaemia Prevalence − Southeast Asia
WHO−2024 Adjustment Method
Moderate+Severe Anaemia Prevalence
Age (year)
Uncertainty Interval
3 − Region
Location Hierarchy
1 − Global
2 − Super Region
3 − Region

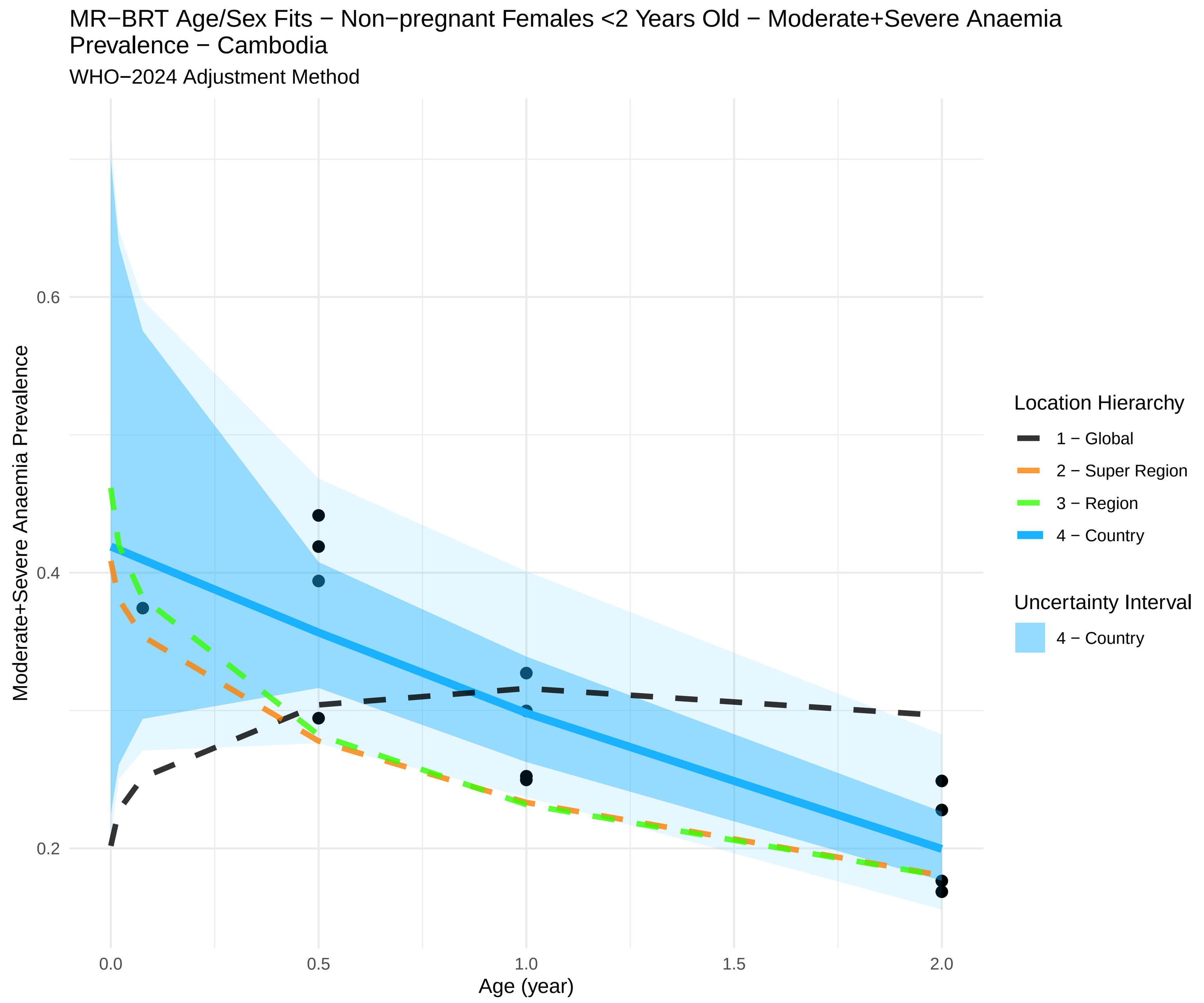
MR−BRT Age/Sex Fits − Non−pregnant Females <2 Years Old − Moderate+Severe Anaemia Prevalence − Cambodia
WHO−2024 Adjustment Method
Moderate+Severe Anaemia Prevalence
Age (year)
Location Hierarchy
1 − Global
2 − Super Region
3 − Region
4 − Country
Uncertainty Interval
4 − Country

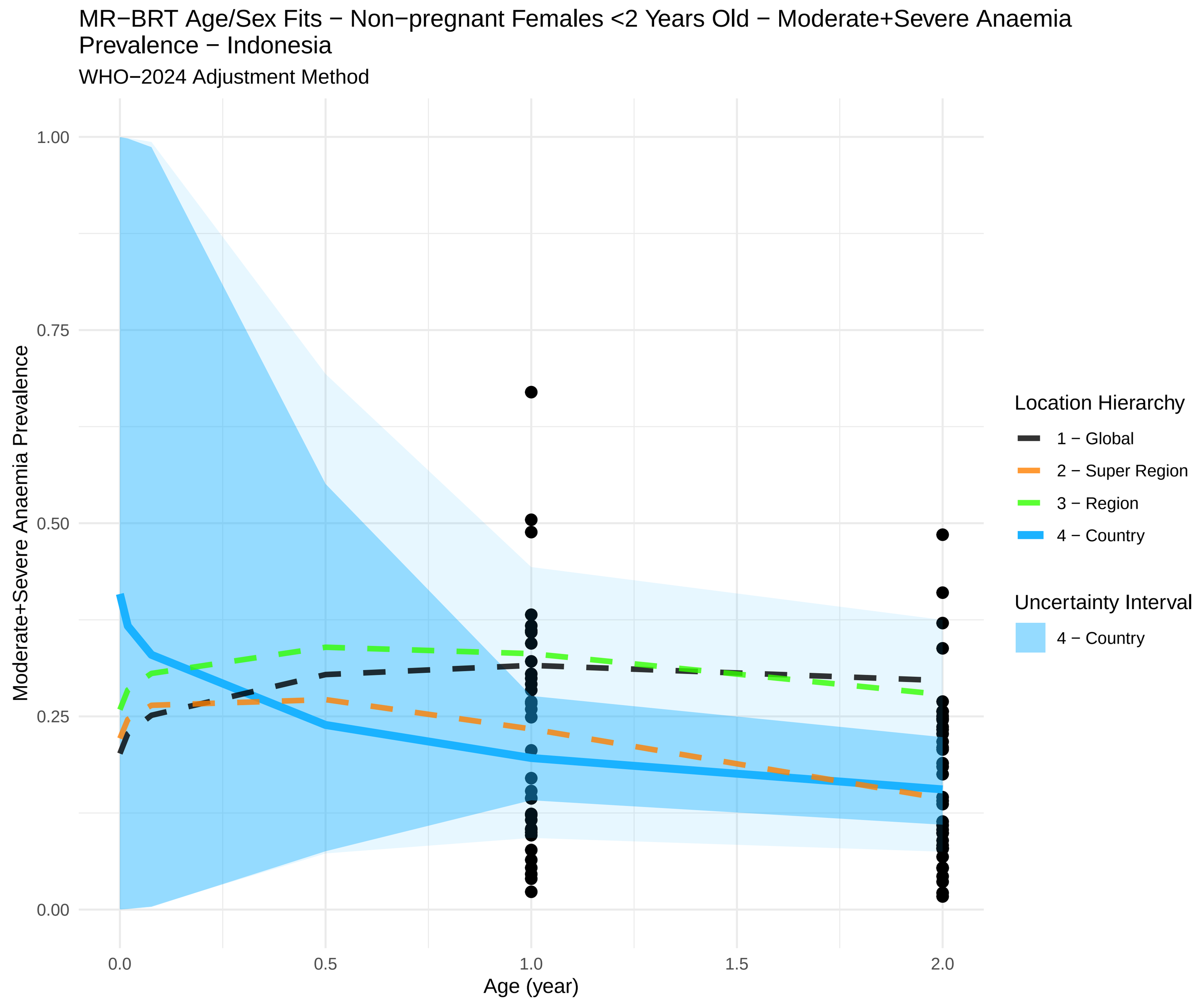
MR−BRT Age/Sex Fits − Non−pregnant Females <2 Years Old − Moderate+Severe Anaemia Prevalence − Indonesia
WHO−2024 Adjustment Method
Moderate+Severe Anaemia Prevalence
Age (year)
Location Hierarchy
1 − Global
2 − Super Region
3 − Region
4 − Country
Uncertainty Interval
4 − Country

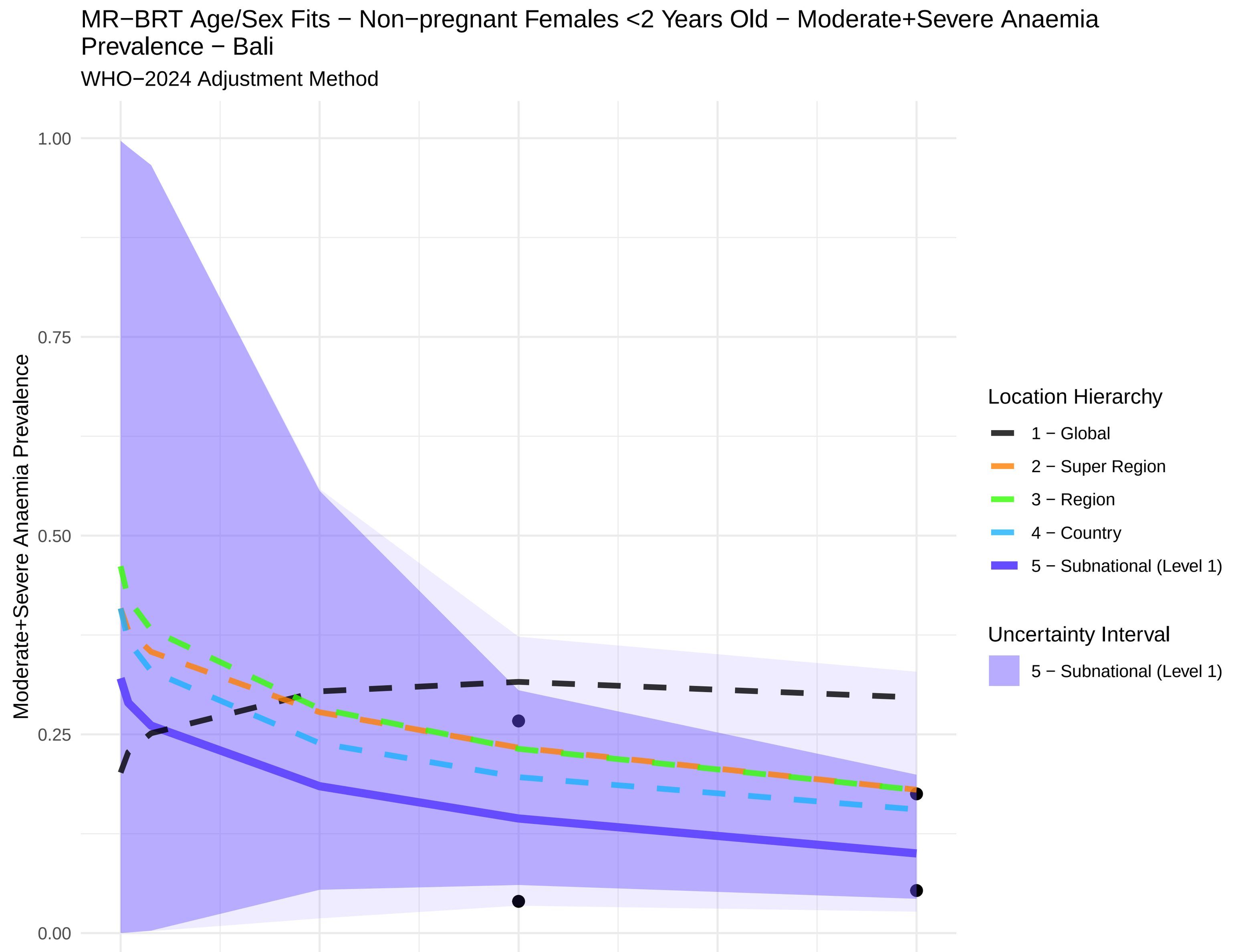
MR−BRT Age/Sex Fits − Non−pregnant Females <2 Years Old − Moderate+Severe Anaemia Prevalence − Bali
WHO−2024 Adjustment Method
Moderate+Severe Anaemia Prevalence
Age (year)
Location Hierarchy
1 − Global
2 − Super Region
3 − Region
4 − Country
5 − Subnational (Level 1)
Uncertainty Interval
5 − Subnational (Level 1)

MR−BRT Age/Sex Fits − Non−pregnant Females <2 Years Old − Moderate+Severe Anaemia Prevalence − Banten
WHO−2024 Adjustment Method

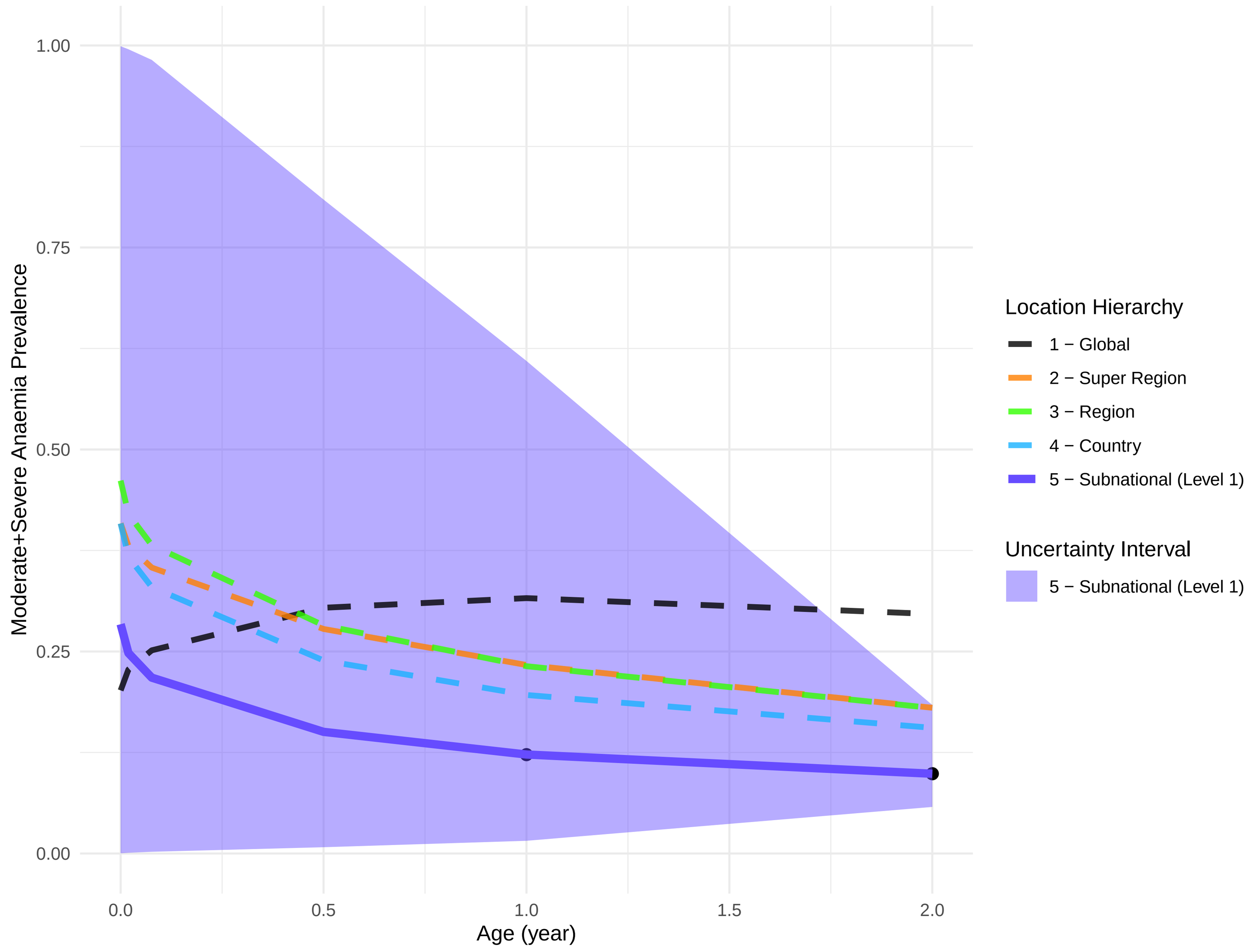

MR−BRT Age/Sex Fits − Non−pregnant Females <2 Years Old − Moderate+Severe Anaemia Prevalence − Jakarta
WHO−2024 Adjustment Method

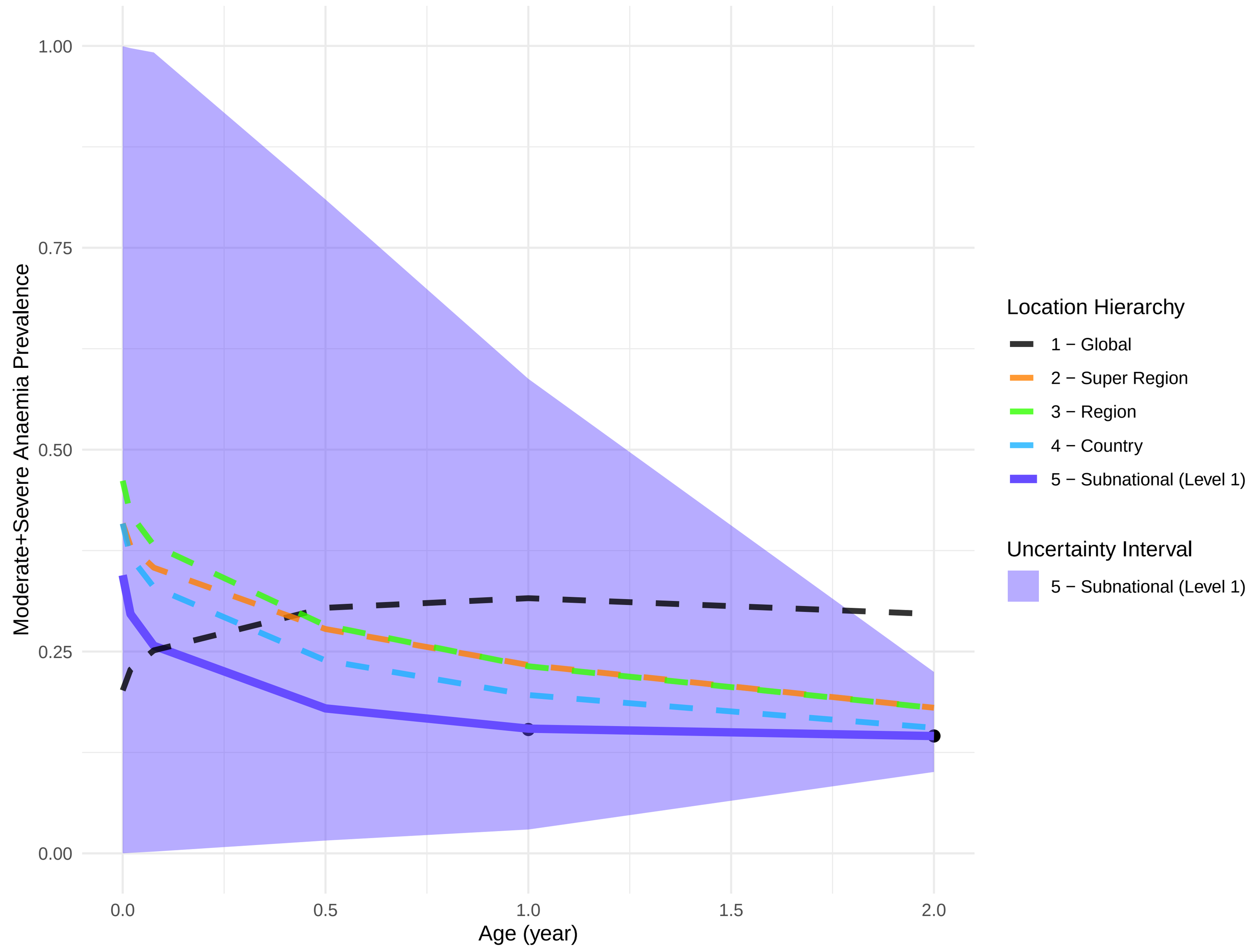

MR−BRT Age/Sex Fits − Non−pregnant Females <2 Years Old − Moderate+Severe Anaemia Prevalence − West Java
WHO−2024 Adjustment Method

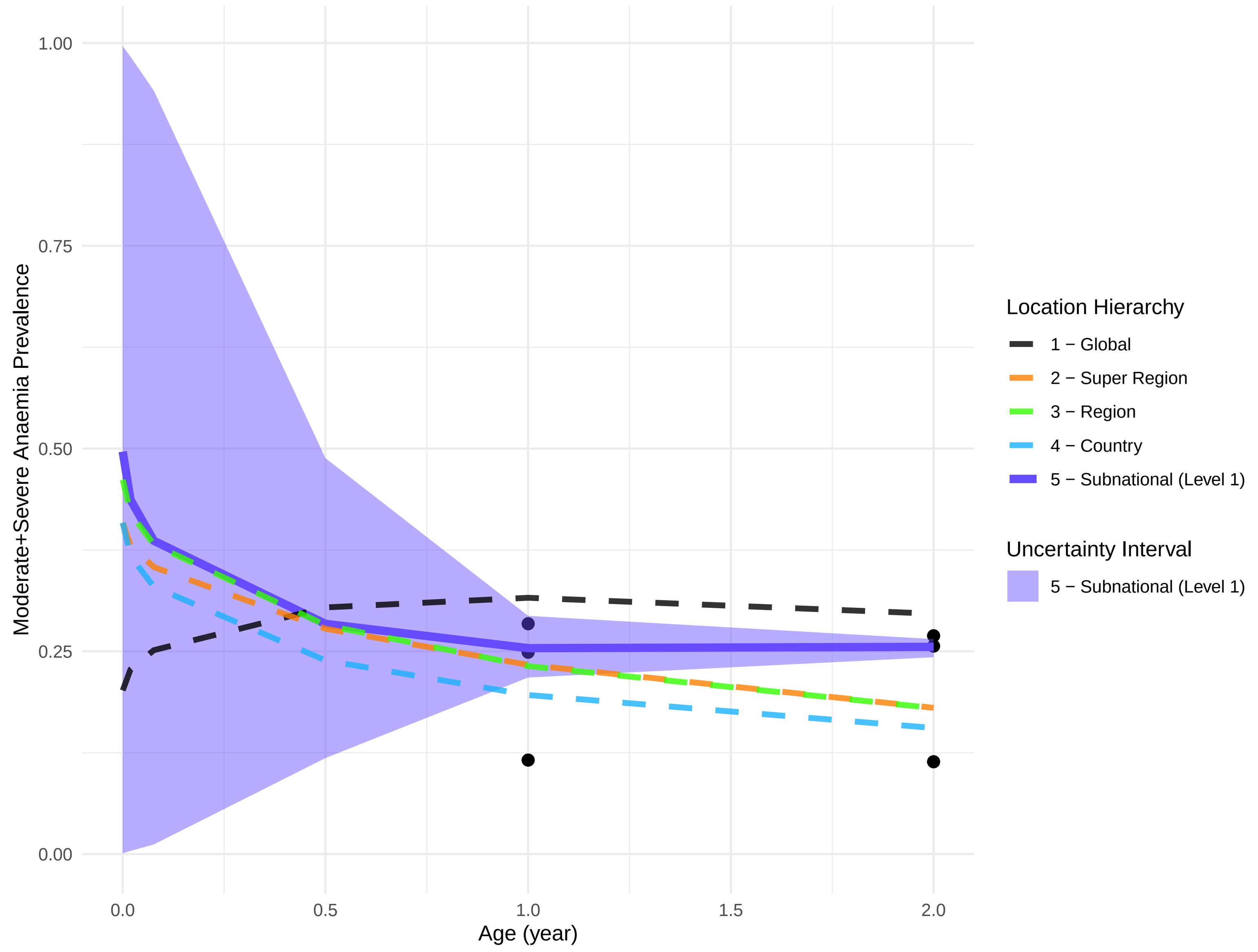

MR−BRT Age/Sex Fits − Non−pregnant Females <2 Years Old − Moderate+Severe Anaemia Prevalence − Central Java
WHO−2024 Adjustment Method

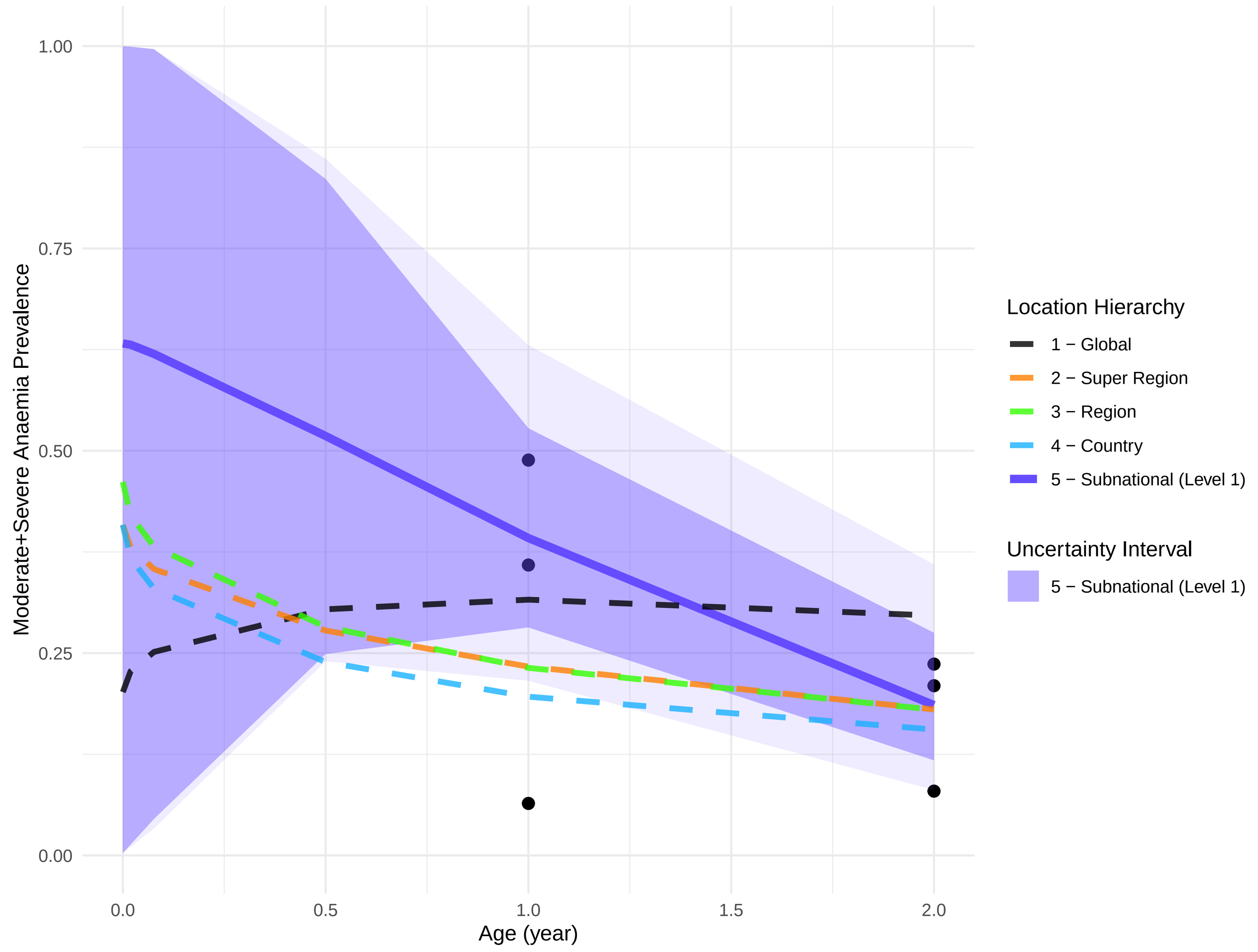

MR−BRT Age/Sex Fits − Non−pregnant Females <2 Years Old − Moderate+Severe Anaemia Prevalence − East Java
WHO−2024 Adjustment Method

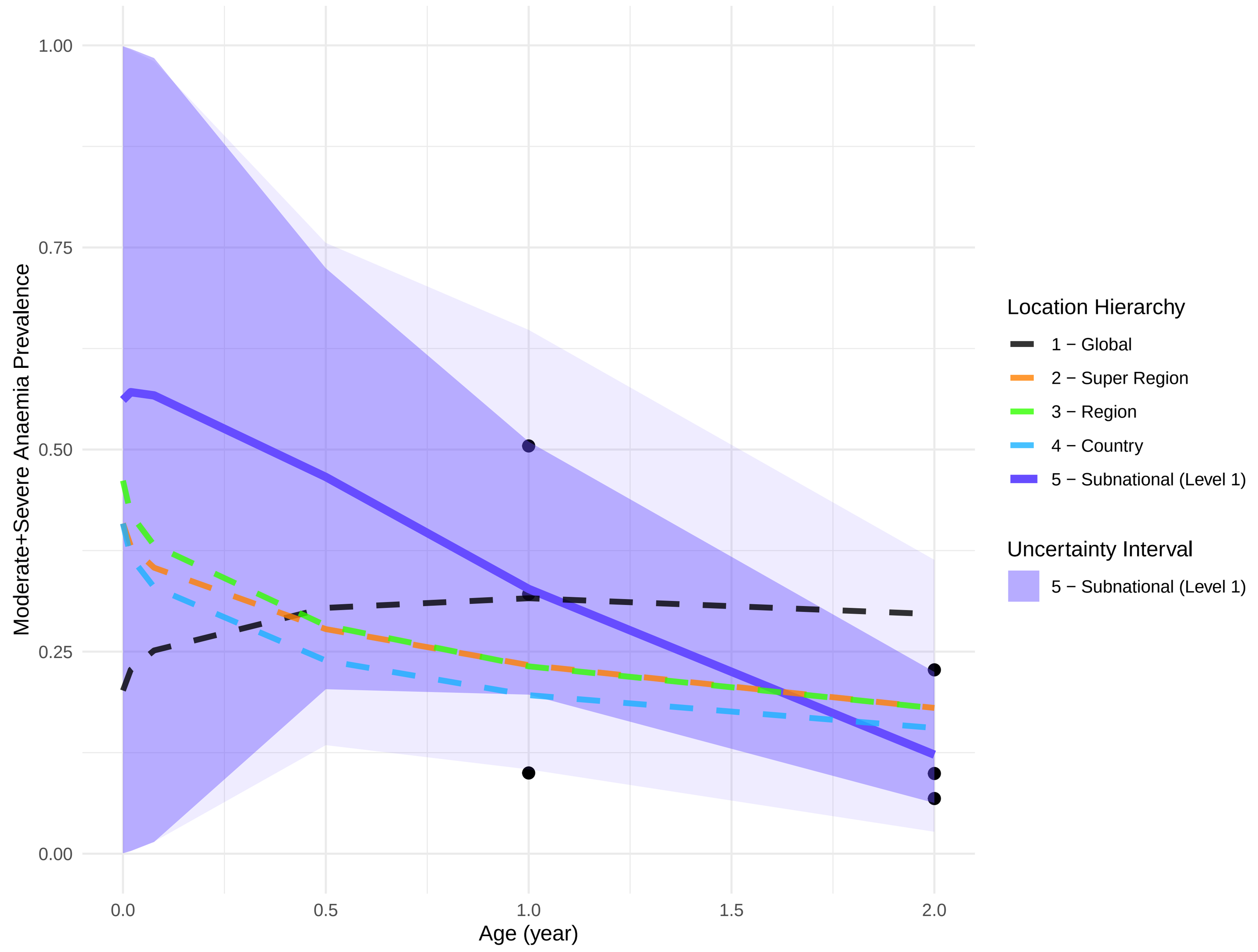

MR−BRT Age/Sex Fits − Non−pregnant Females <2 Years Old − Moderate+Severe Anaemia Prevalence − South Kalimantan
WHO−2024 Adjustment Method

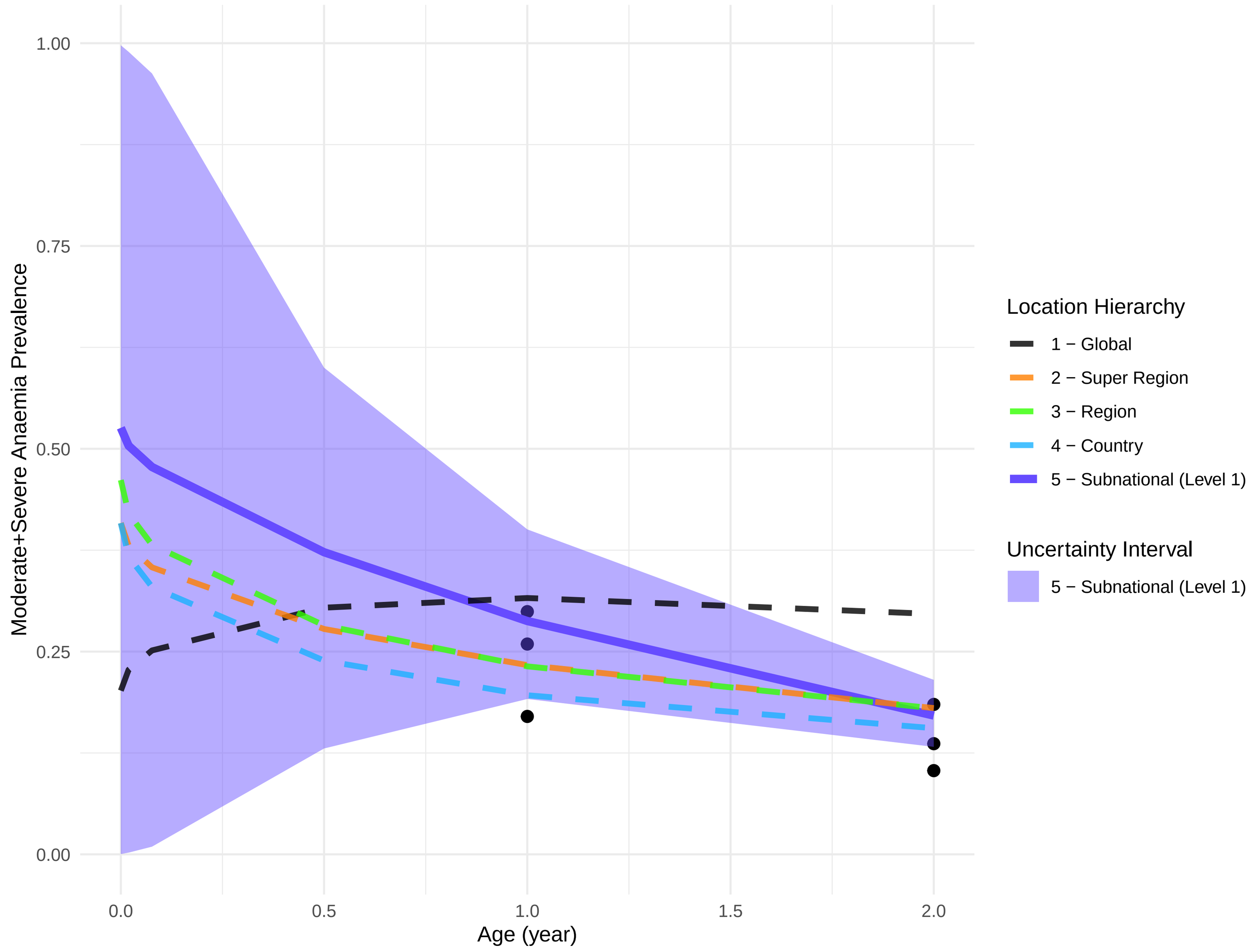

MR−BRT Age/Sex Fits − Non−pregnant Females <2 Years Old − Moderate+Severe Anaemia Prevalence − Lampung
WHO−2024 Adjustment Method

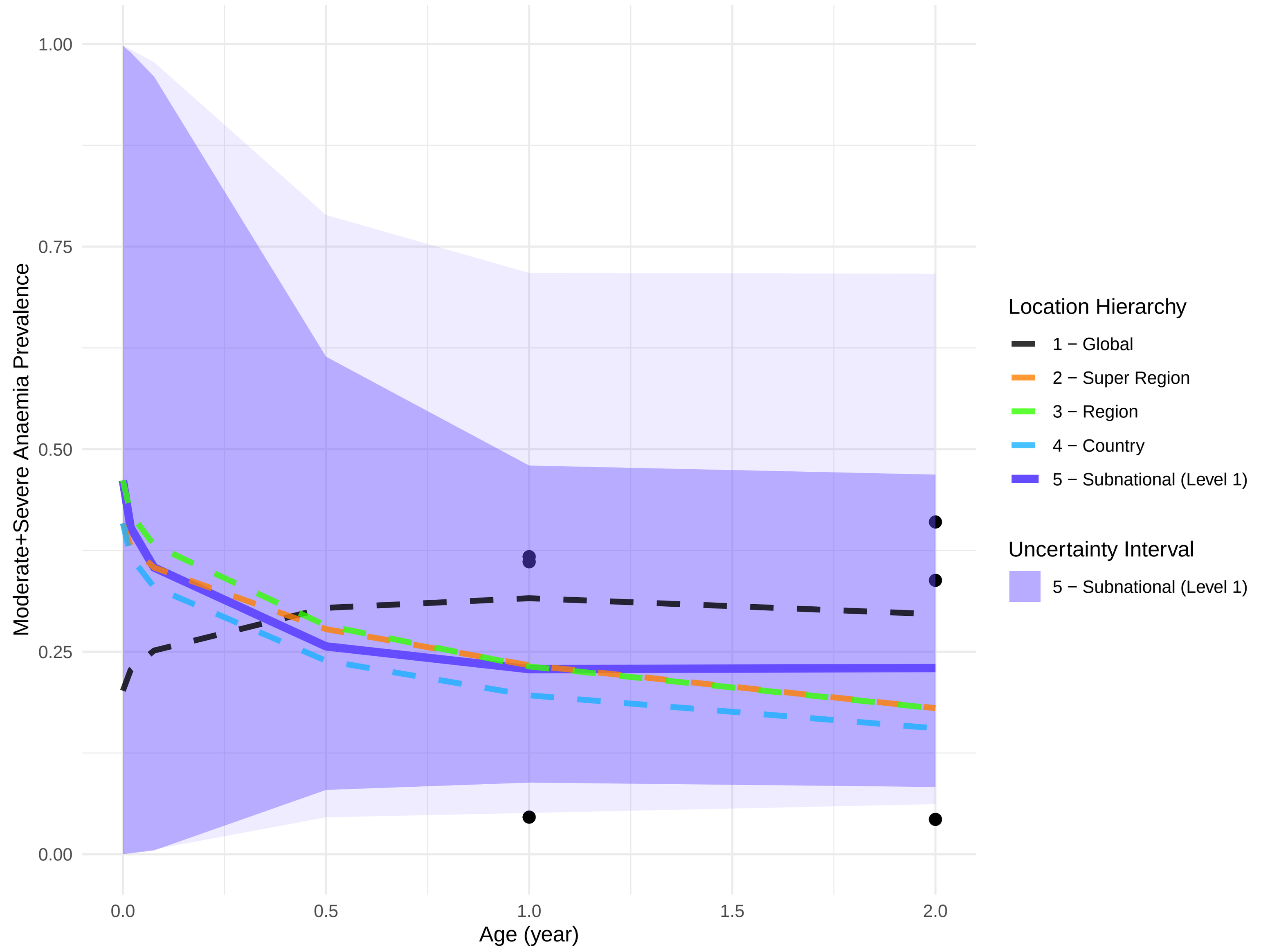

MR−BRT Age/Sex Fits − Non−pregnant Females <2 Years Old − Moderate+Severe Anaemia Prevalence − West Nusa Tenggara
WHO−2024 Adjustment Method

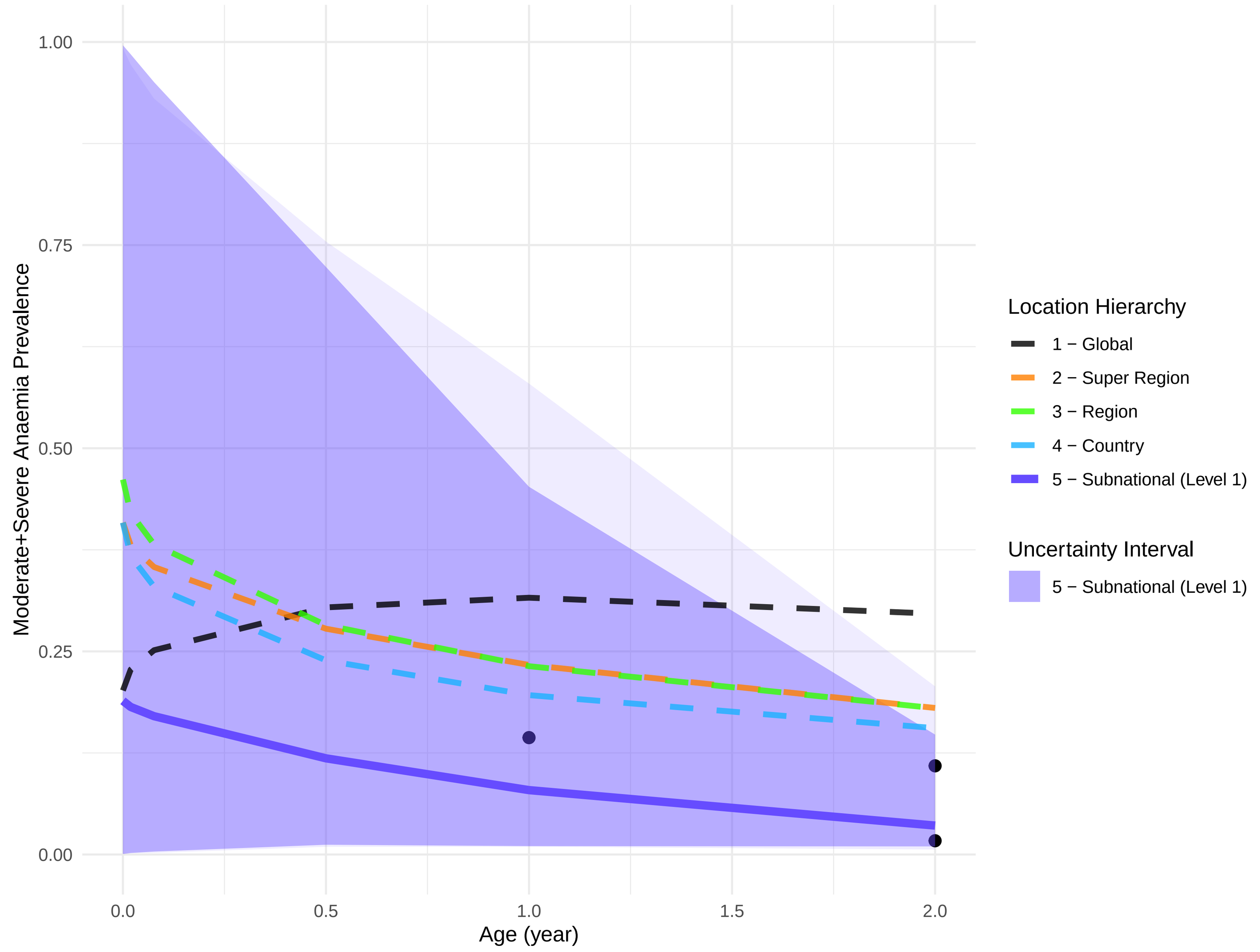

MR−BRT Age/Sex Fits − Non−pregnant Females <2 Years Old − Moderate+Severe Anaemia Prevalence − South Sulawesi
WHO−2024 Adjustment Method

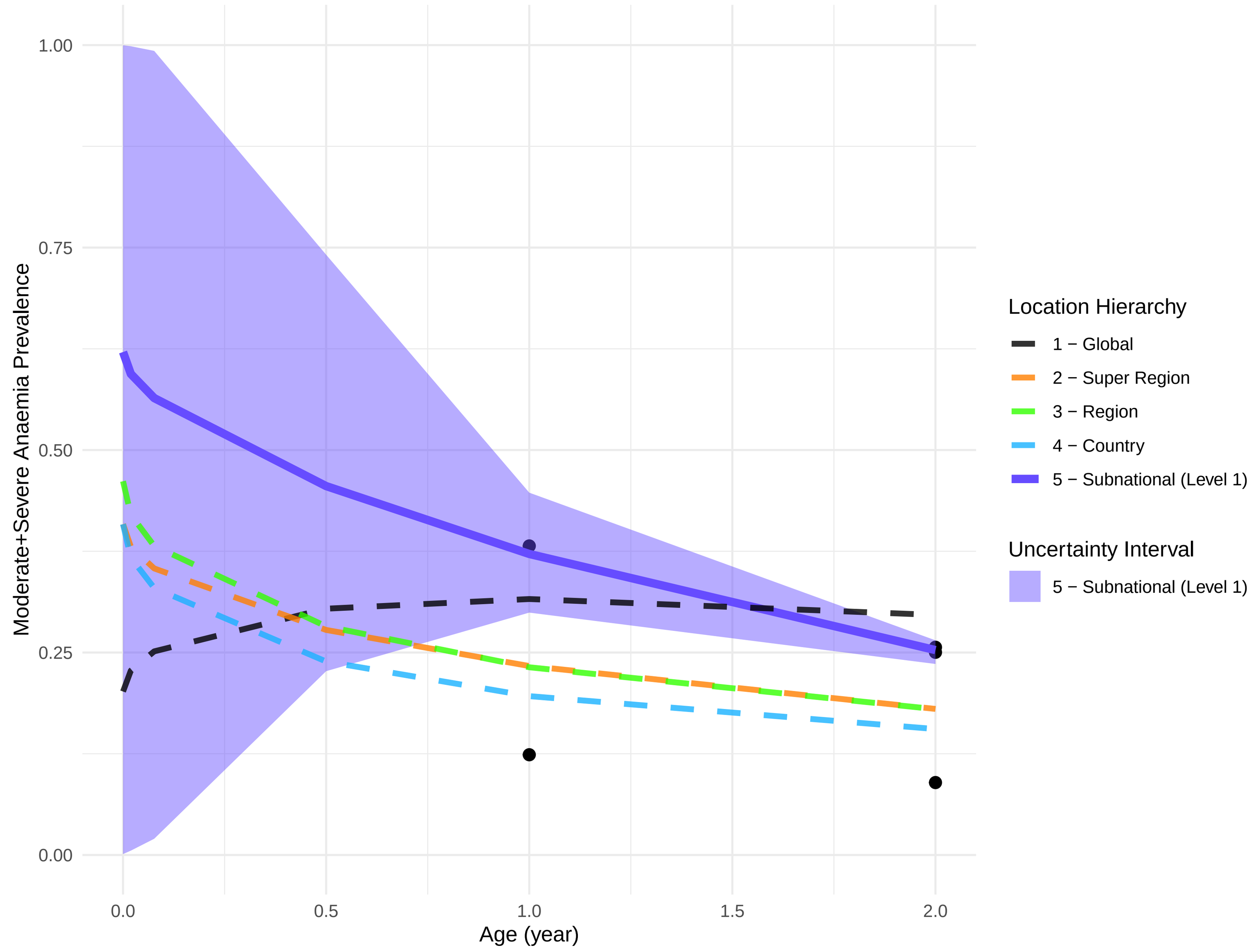

MR−BRT Age/Sex Fits − Non−pregnant Females <2 Years Old − Moderate+Severe Anaemia Prevalence − West Sumatra
WHO−2024 Adjustment Method

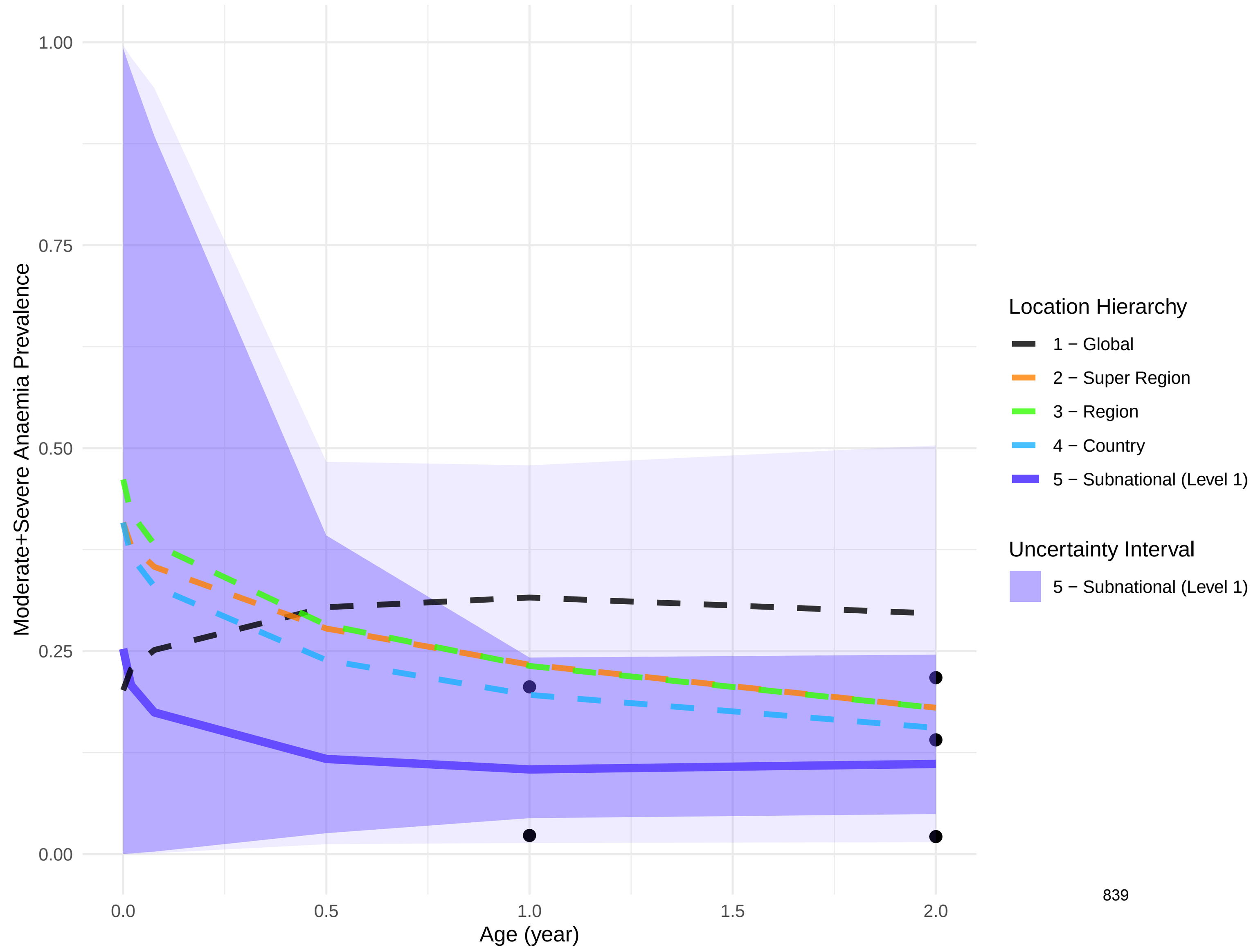

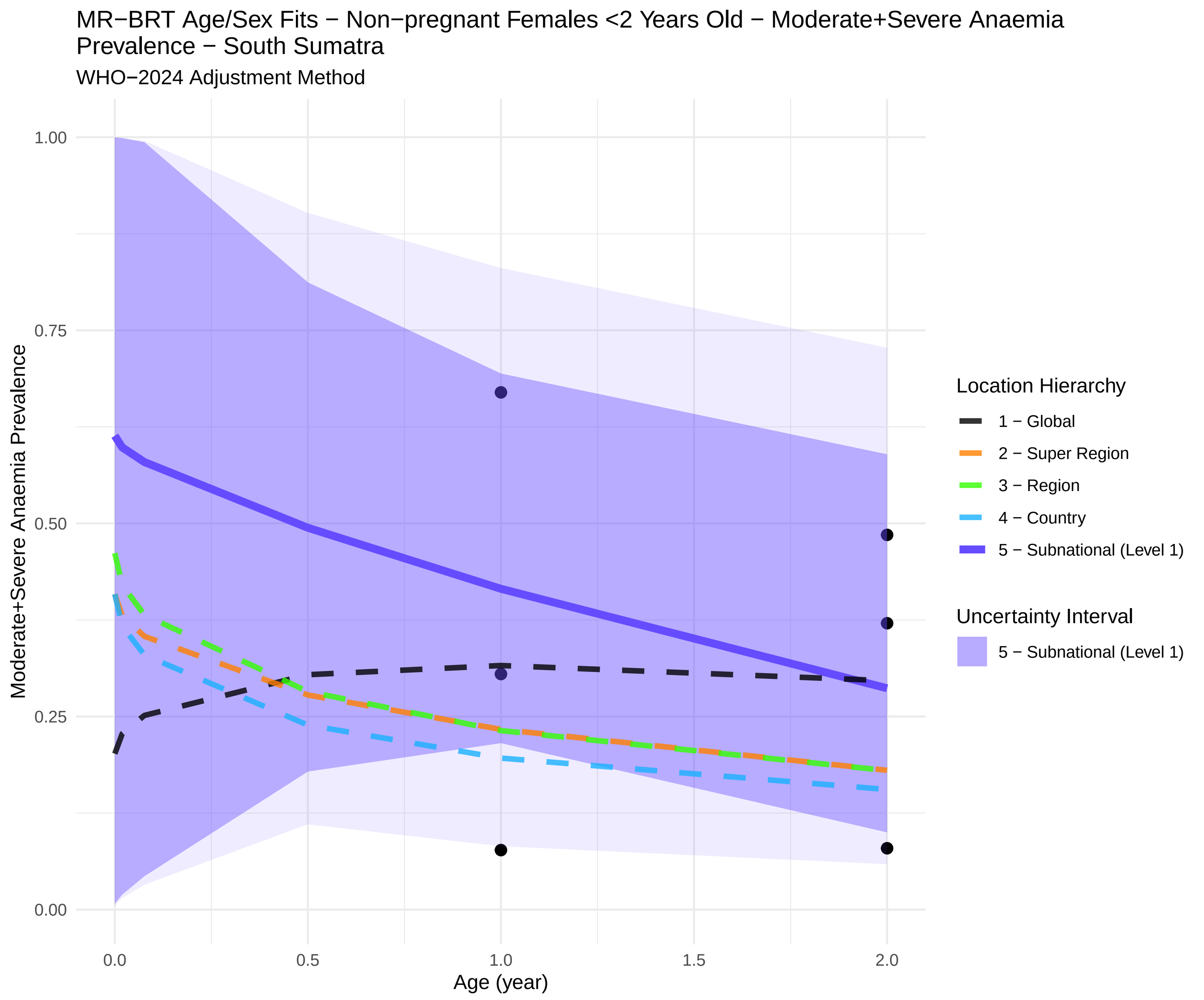
MR−BRT Age/Sex Fits − Non−pregnant Females <2 Years Old − Moderate+Severe Anaemia Prevalence − South Sumatra
WHO−2024 Adjustment Method
Moderate+Severe Anaemia Prevalence
Age (year)
Location Hierarchy
1 − Global
2 − Super Region
3 − Region
4 − Country
5 − Subnational (Level 1)
Uncertainty Interval
5 − Subnational (Level 1)

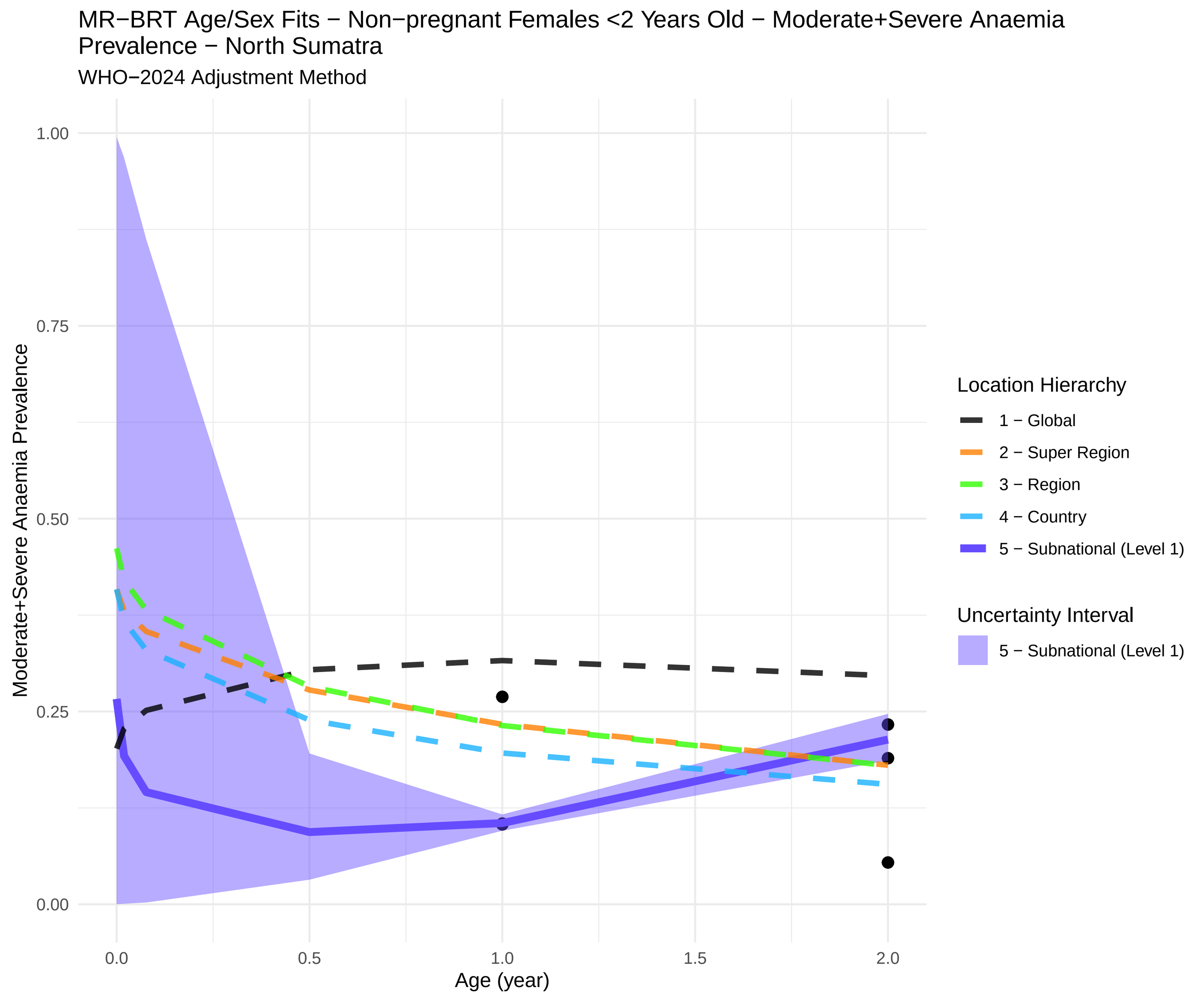
MR−BRT Age/Sex Fits − Non−pregnant Females <2 Years Old − Moderate+Severe Anaemia Prevalence − North Sumatra
WHO−2024 Adjustment Method
Moderate+Severe Anaemia Prevalence
Age (year)
Location Hierarchy
1 − Global
2 − Super Region
3 − Region
4 − Country
5 − Subnational (Level 1)
Uncertainty Interval
5 − Subnational (Level 1)

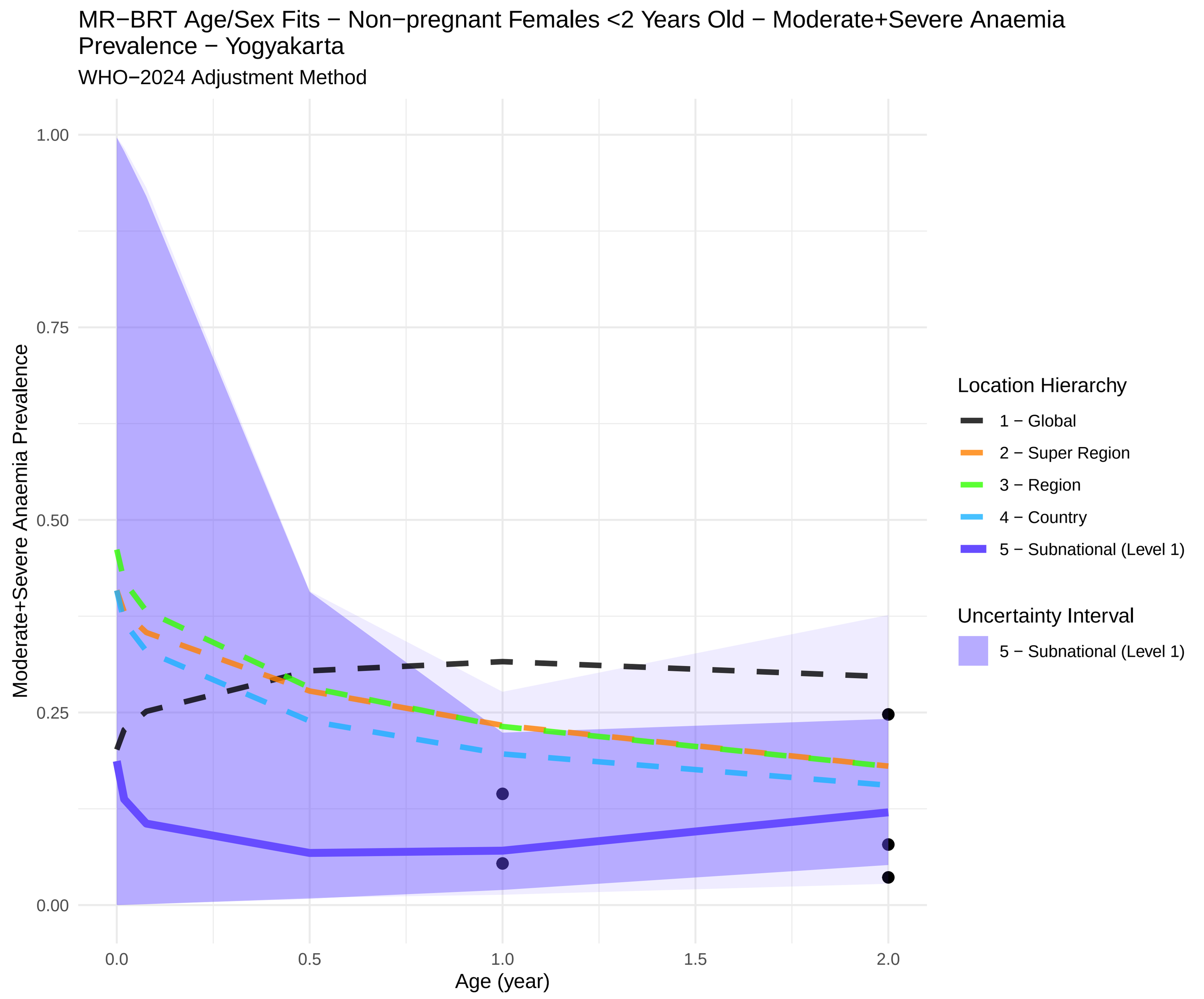
MR−BRT Age/Sex Fits − Non−pregnant Females <2 Years Old − Moderate+Severe Anaemia Prevalence − Yogyakarta
WHO−2024 Adjustment Method
Moderate+Severe Anaemia Prevalence
Age (year)
Location Hierarchy
1 − Global
2 − Super Region
3 − Region
4 − Country
5 − Subnational (Level 1)
Uncertainty Interval
5 − Subnational (Level 1)

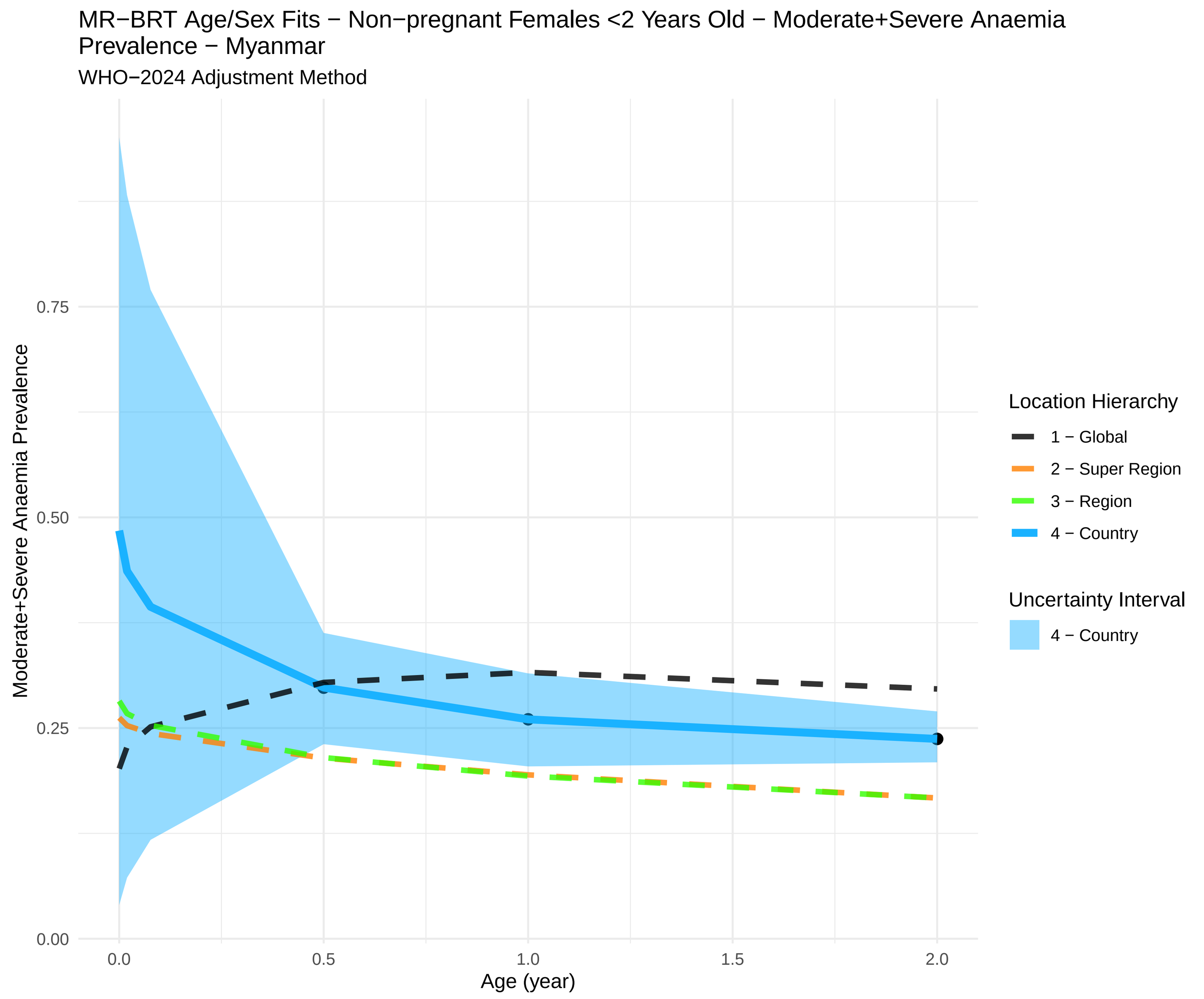
MR−BRT Age/Sex Fits − Non−pregnant Females <2 Years Old − Moderate+Severe Anaemia Prevalence − Myanmar
WHO−2024 Adjustment Method
Moderate+Severe Anaemia Prevalence
Age (year)
Location Hierarchy
1 − Global
2 − Super Region
3 − Region
4 − Country
Uncertainty Interval
4 − Country

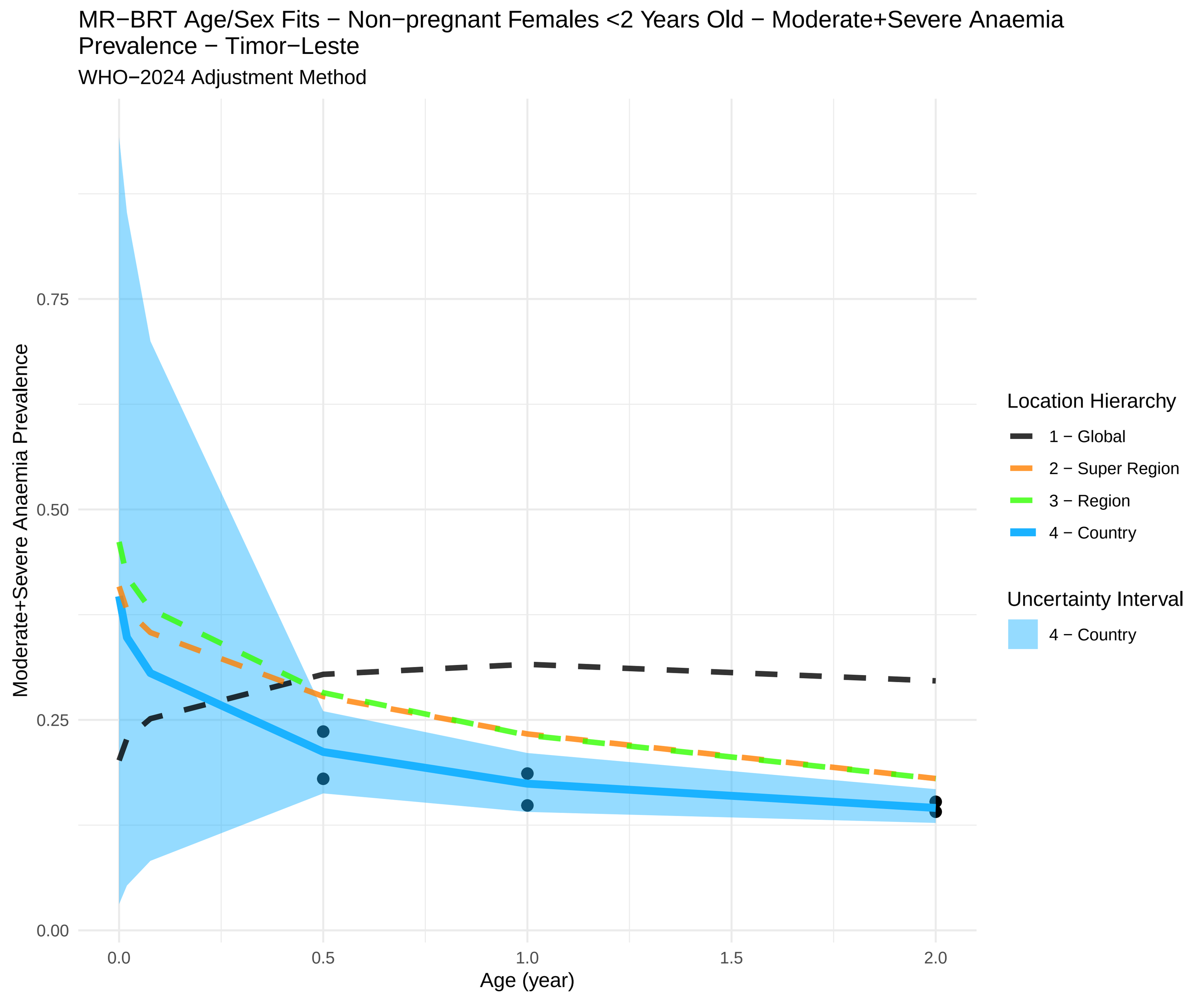
MR−BRT Age/Sex Fits − Non−pregnant Females <2 Years Old − Moderate+Severe Anaemia Prevalence − Timor−Leste
WHO−2024 Adjustment Method
Moderate+Severe Anaemia Prevalence
Age (year)
Location Hierarchy
1 − Global
2 − Super Region
3 − Region
4 − Country
Uncertainty Interval
4 − Country

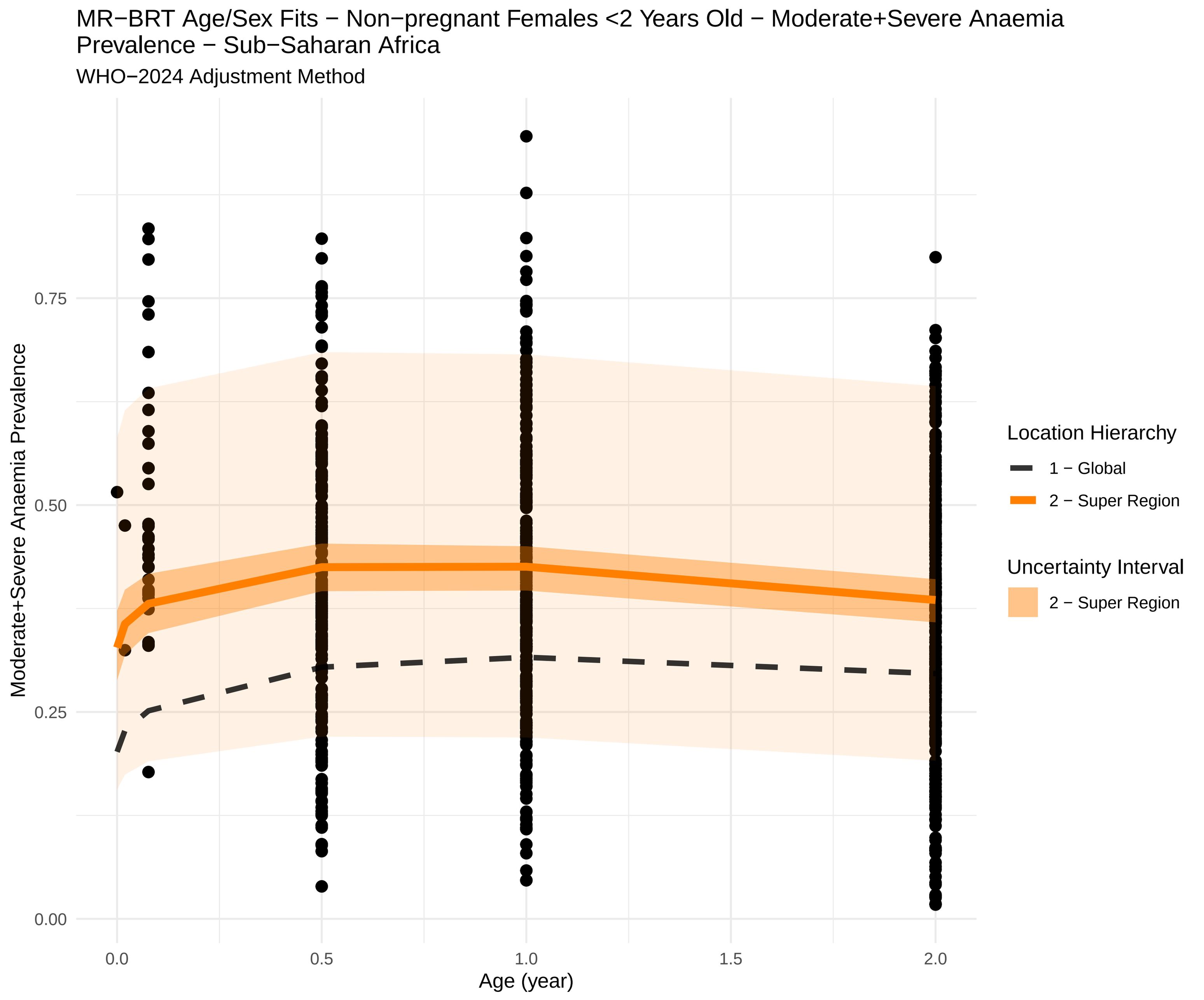
MR−BRT Age/Sex Fits − Non−pregnant Females <2 Years Old − Moderate+Severe Anaemia Prevalence − Sub−Saharan Africa
WHO−2024 Adjustment Method
Moderate+Severe Anaemia Prevalence
Age (year)
Location Hierarchy
1 − Global
2 − Super Region
Uncertainty Interval
2 − Super Region

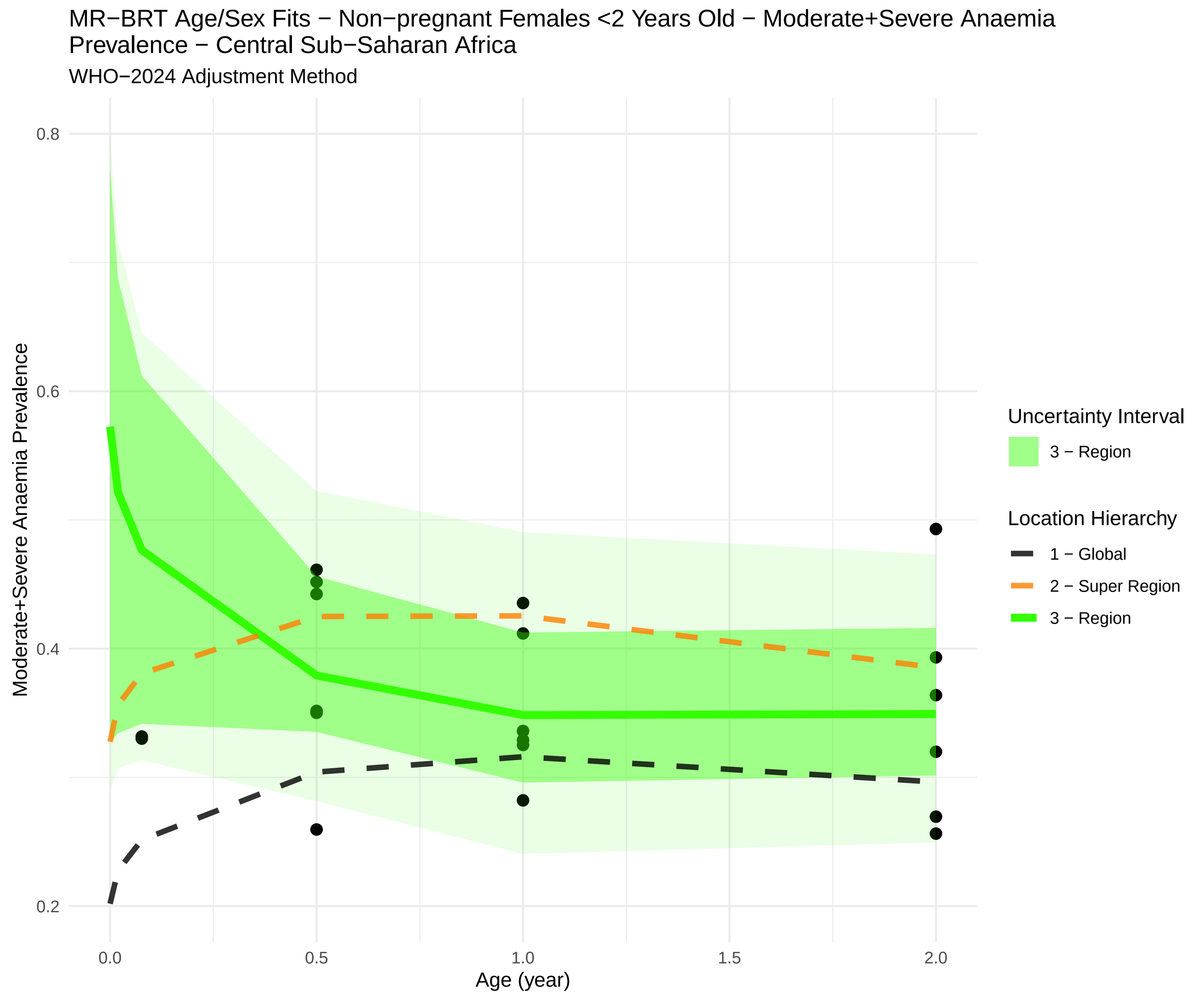
MR−BRT Age/Sex Fits − Non−pregnant Females <2 Years Old − Moderate+Severe Anaemia Prevalence − Central Sub−Saharan Africa
WHO−2024 Adjustment Method
Moderate+Severe Anaemia Prevalence
Age (year)
Uncertainty Interval
3 − Region
Location Hierarchy
1 − Global
2 − Super Region
3 − Region

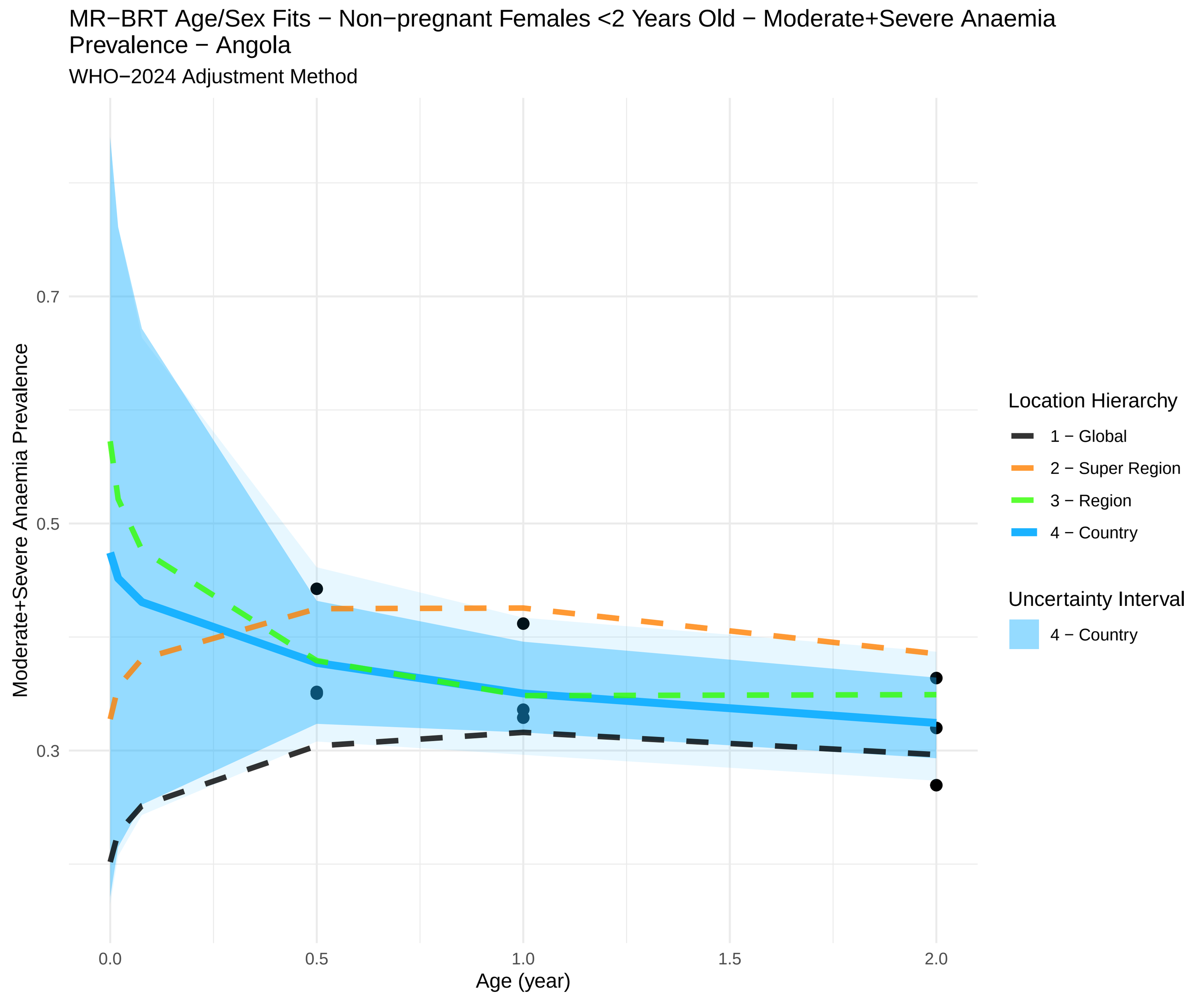
MR−BRT Age/Sex Fits − Non−pregnant Females <2 Years Old − Moderate+Severe Anaemia Prevalence − Angola
WHO−2024 Adjustment Method
Moderate+Severe Anaemia Prevalence
Age (year)
Location Hierarchy
1 − Global
2 − Super Region
3 − Region
4 − Country
Uncertainty Interval
4 − Country

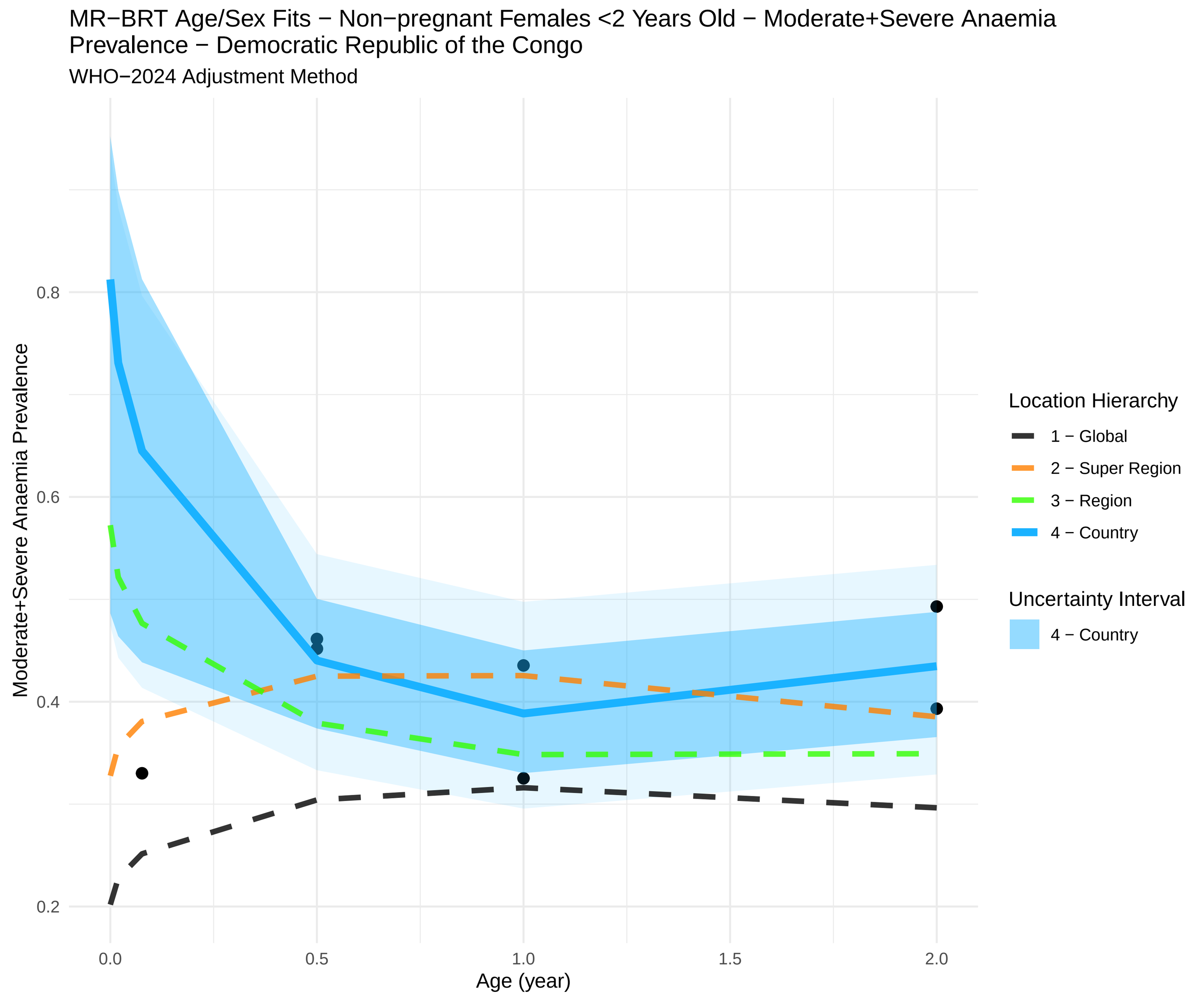
MR−BRT Age/Sex Fits − Non−pregnant Females <2 Years Old − Moderate+Severe Anaemia Prevalence − Democratic Republic of the Congo
WHO−2024 Adjustment Method
Moderate+Severe Anaemia Prevalence
Age (year)
Location Hierarchy
1 − Global
2 − Super Region
3 − Region
4 − Country
Uncertainty Interval
4 − Country

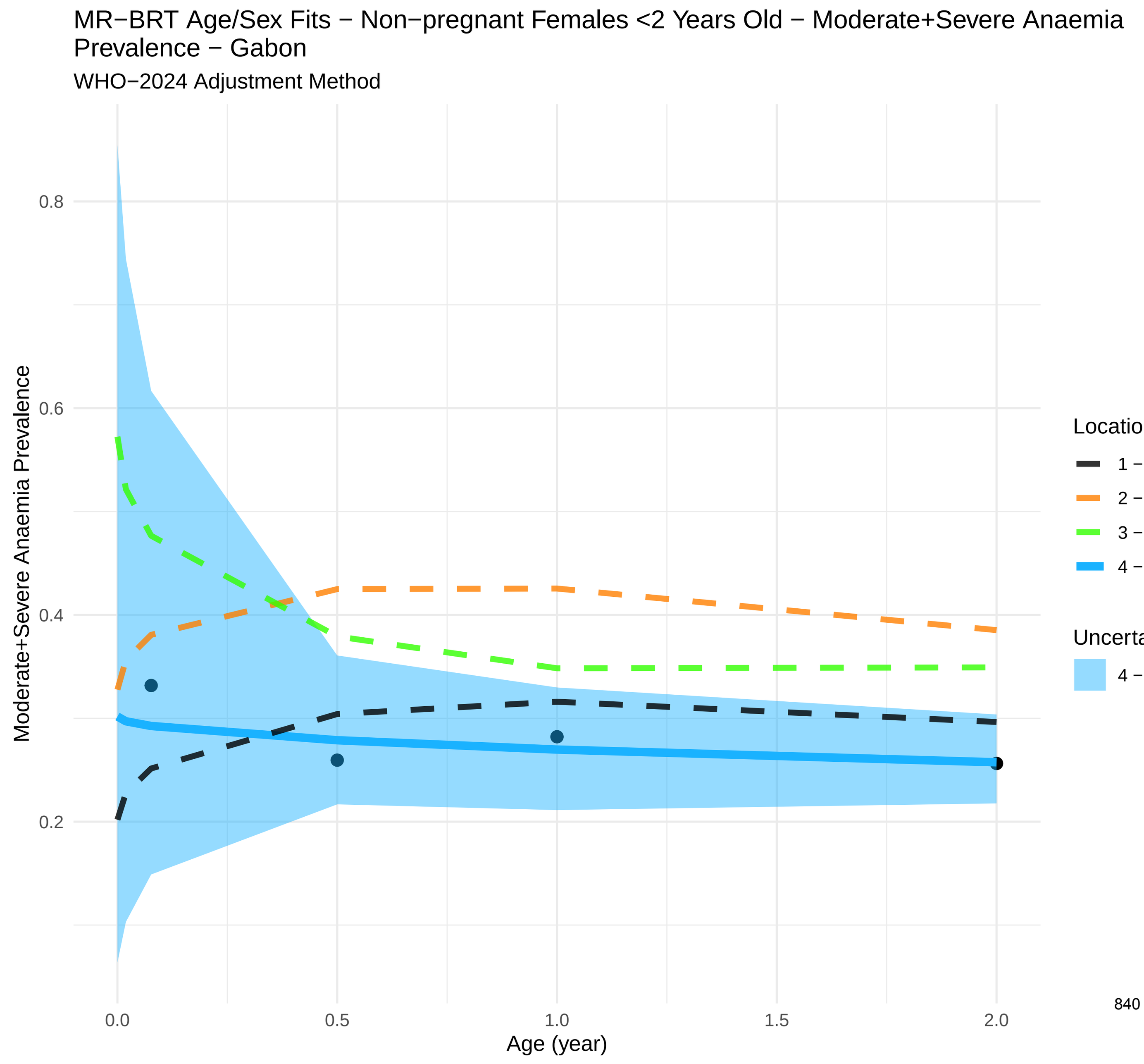
MR−BRT Age/Sex Fits − Non−pregnant Females <2 Years Old − Moderate+Severe Anaemia Prevalence − Gabon
WHO−2024 Adjustment Method
Moderate+Severe Anaemia Prevalence
Age (year)
Location Hierarchy
1 − Global
2 − Super Region
3 − Region
4 − Country
Uncertainty Interval
4 − Country

MR−BRT Age/Sex Fits − Non-pregnant Females <2 Years Old − Moderate+Severe Anaemia Prevalence − Eastern Sub-Saharan Africa
WHO−2024 Adjustment Method

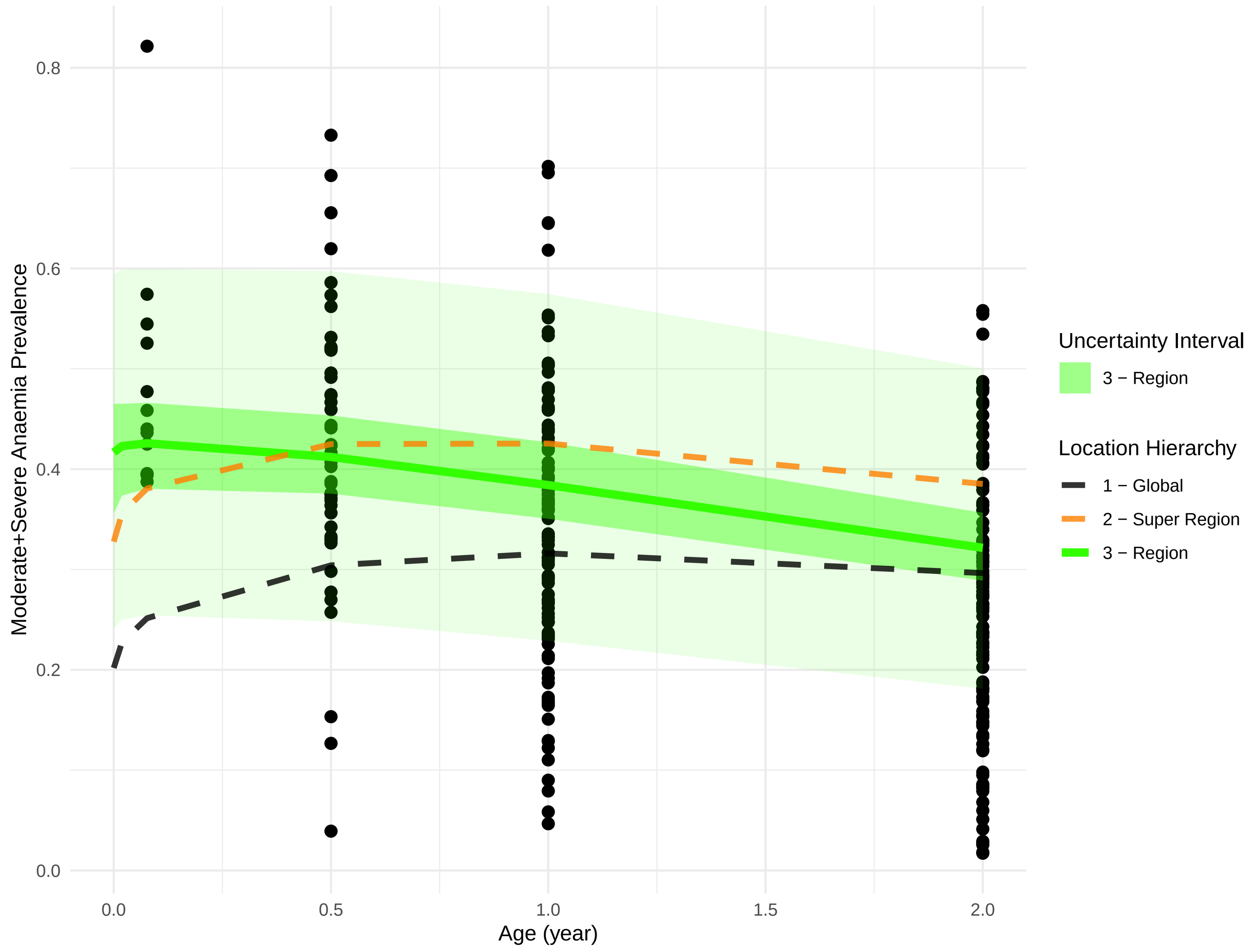

MR−BRT Age/Sex Fits − Non-pregnant Females <2 Years Old − Moderate+Severe Anaemia Prevalence − Burundi
WHO−2024 Adjustment Method

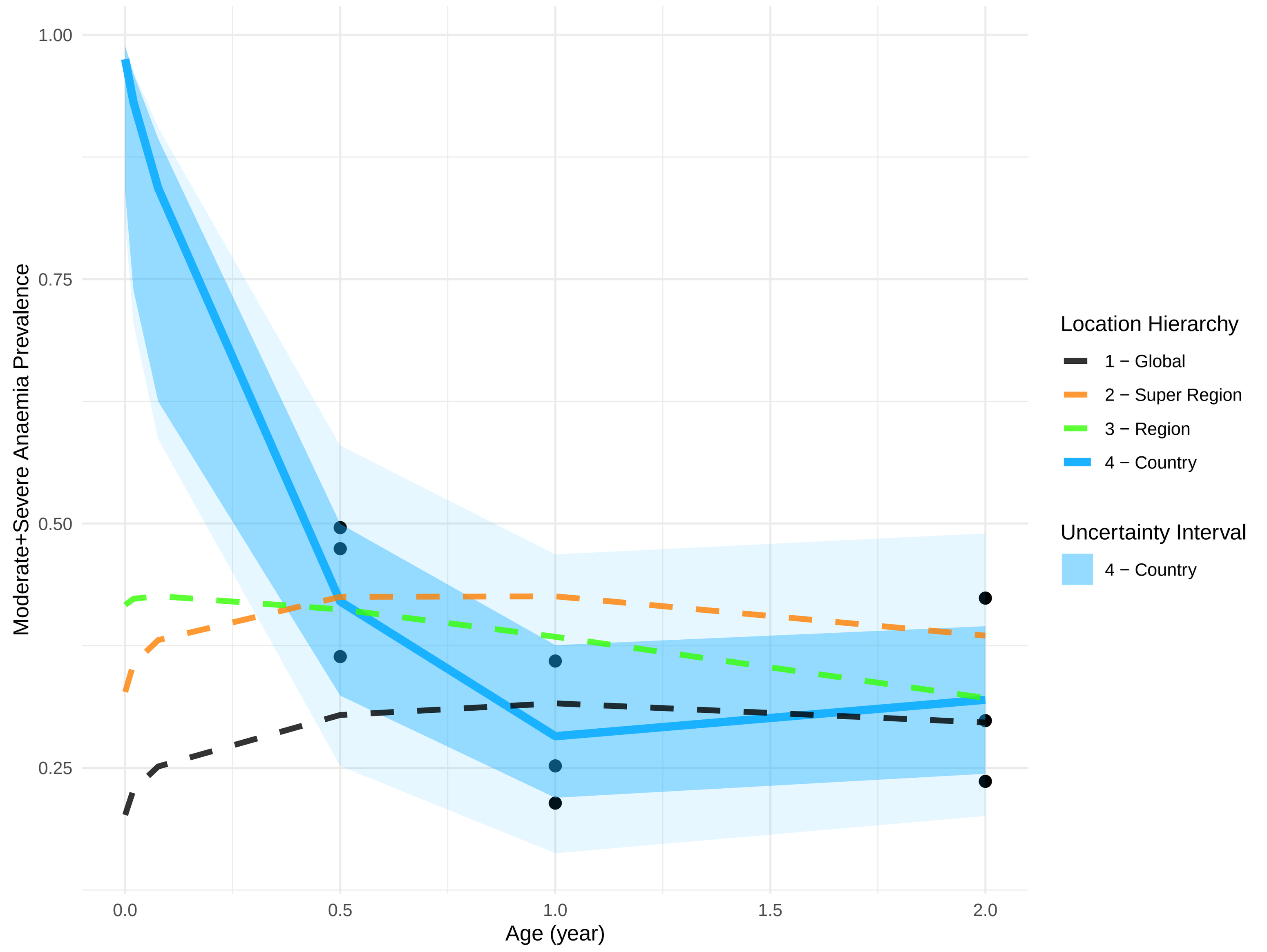

MR−BRT Age/Sex Fits − Non-pregnant Females <2 Years Old − Moderate+Severe Anaemia Prevalence − Ethiopia
WHO−2024 Adjustment Method

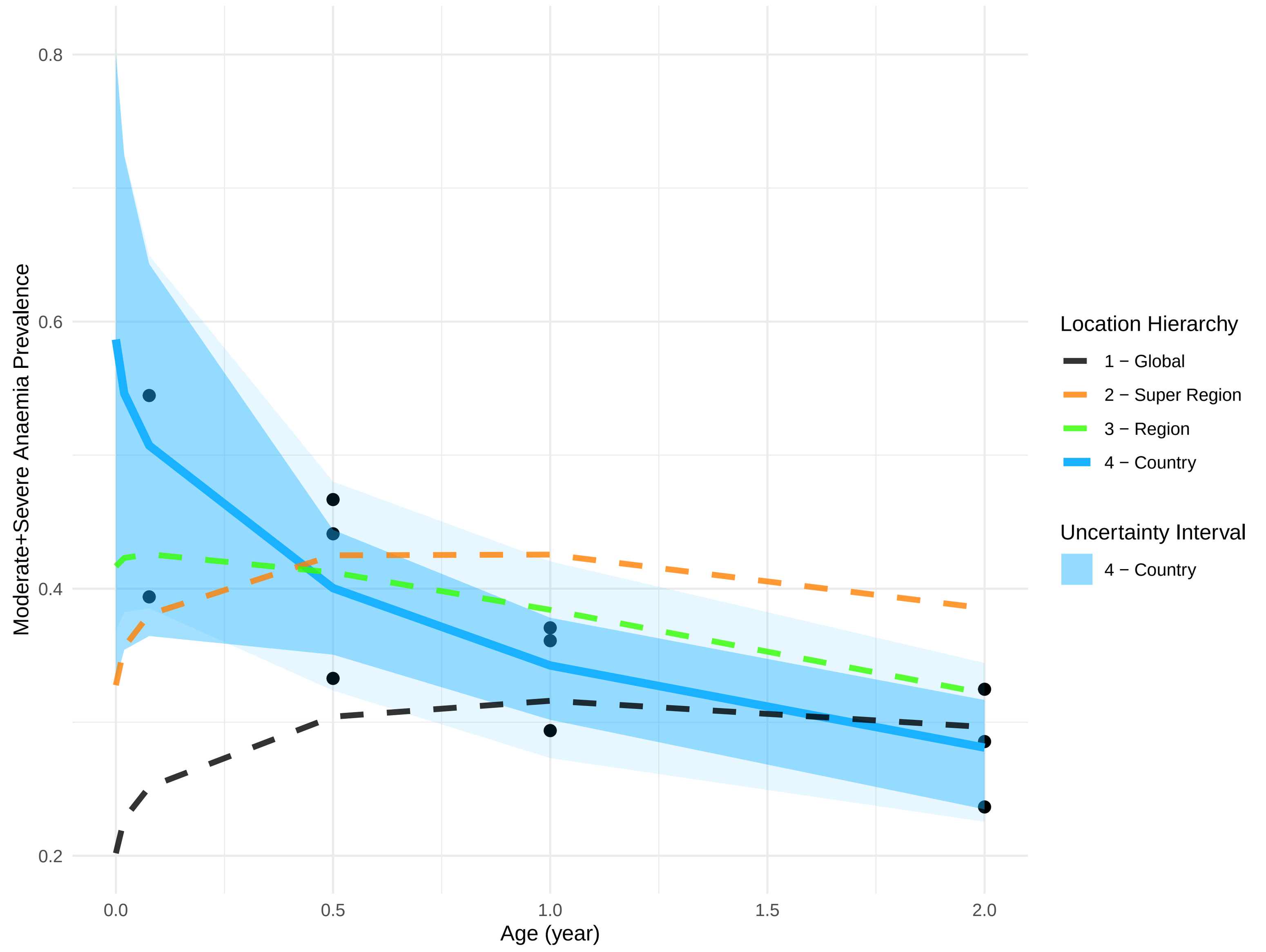

MR−BRT Age/Sex Fits − Non-pregnant Females <2 Years Old − Moderate+Severe Anaemia Prevalence − Kenya
WHO−2024 Adjustment Method

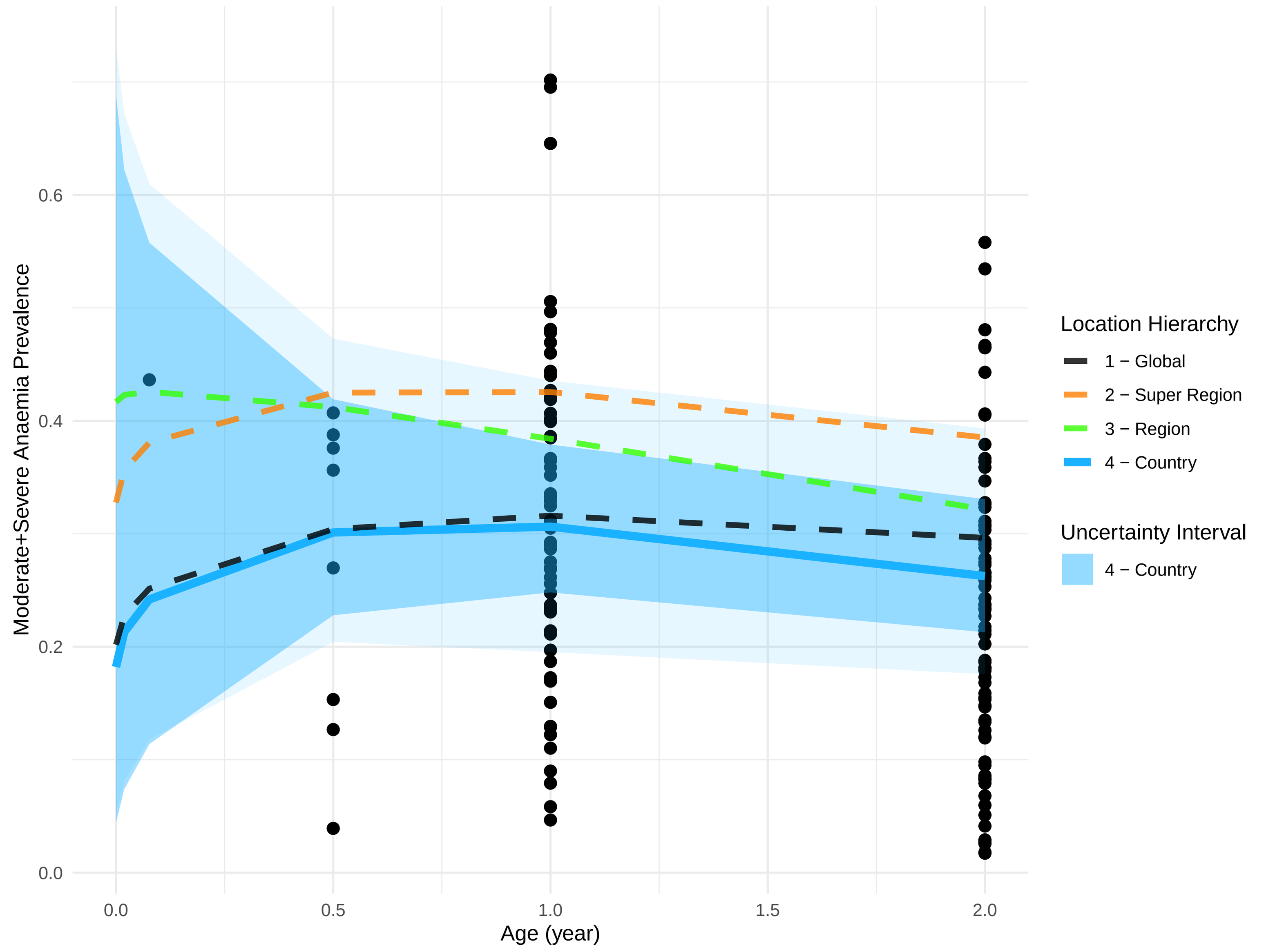

MR−BRT Age/Sex Fits − Non-pregnant Females <2 Years Old − Moderate+Severe Anaemia Prevalence − Baringo
WHO−2024 Adjustment Method

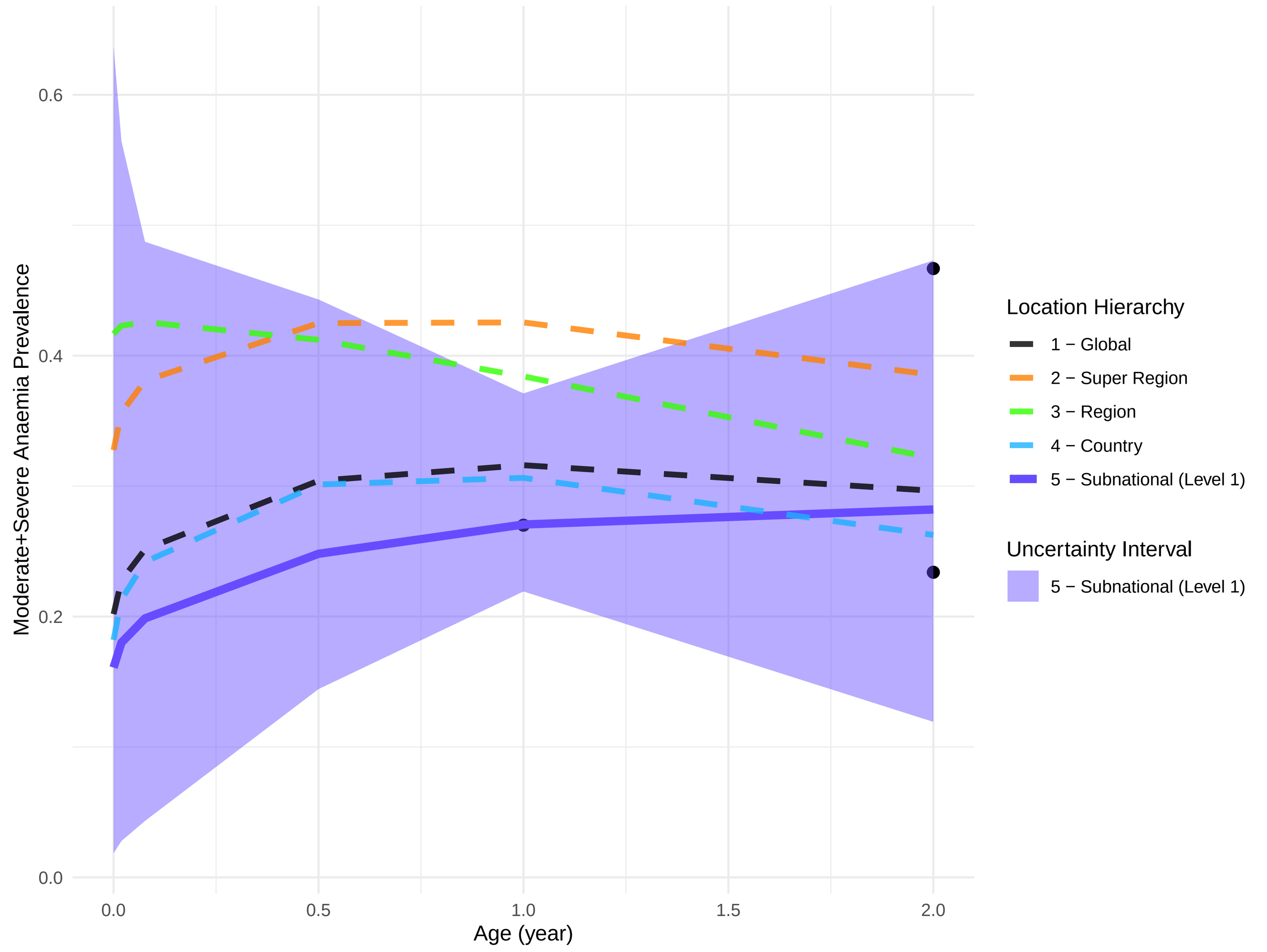

MR−BRT Age/Sex Fits − Non-pregnant Females <2 Years Old − Moderate+Severe Anaemia Prevalence − Bomet
WHO−2024 Adjustment Method

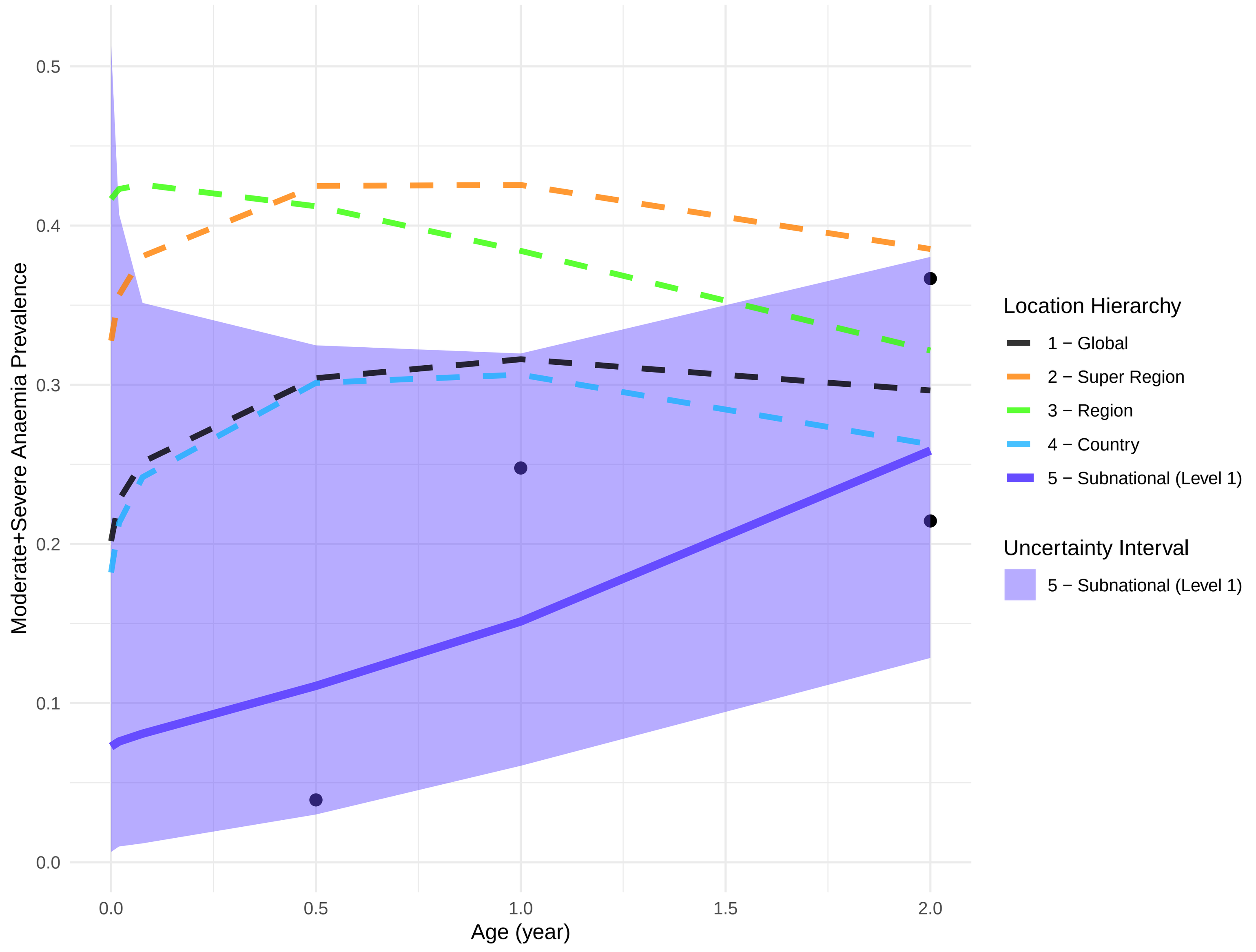

MR−BRT Age/Sex Fits − Non-pregnant Females <2 Years Old − Moderate+Severe Anaemia Prevalence − Bungoma
WHO−2024 Adjustment Method

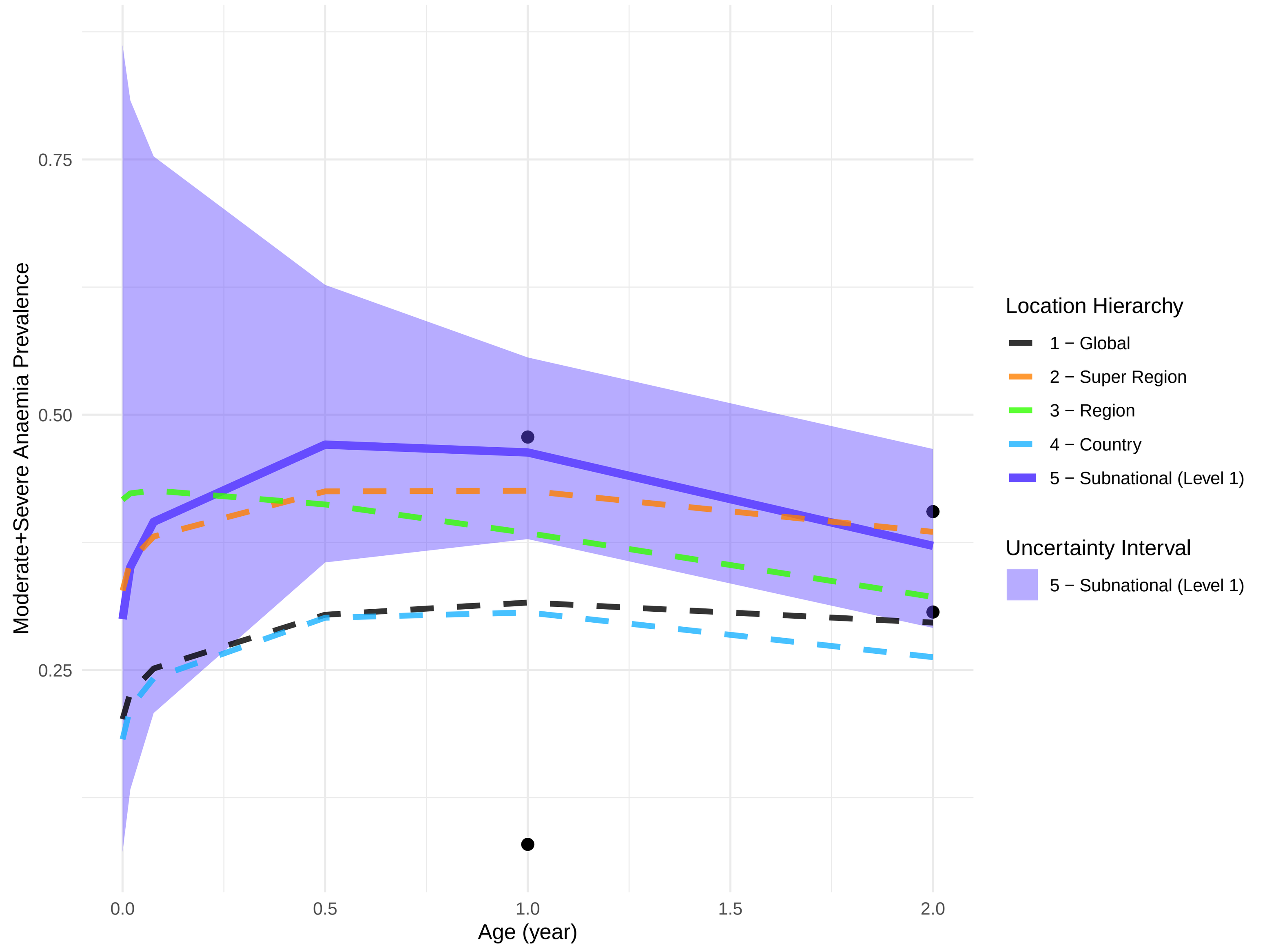

MR−BRT Age/Sex Fits − Non-pregnant Females <2 Years Old − Moderate+Severe Anaemia Prevalence − Busia
WHO−2024 Adjustment Method

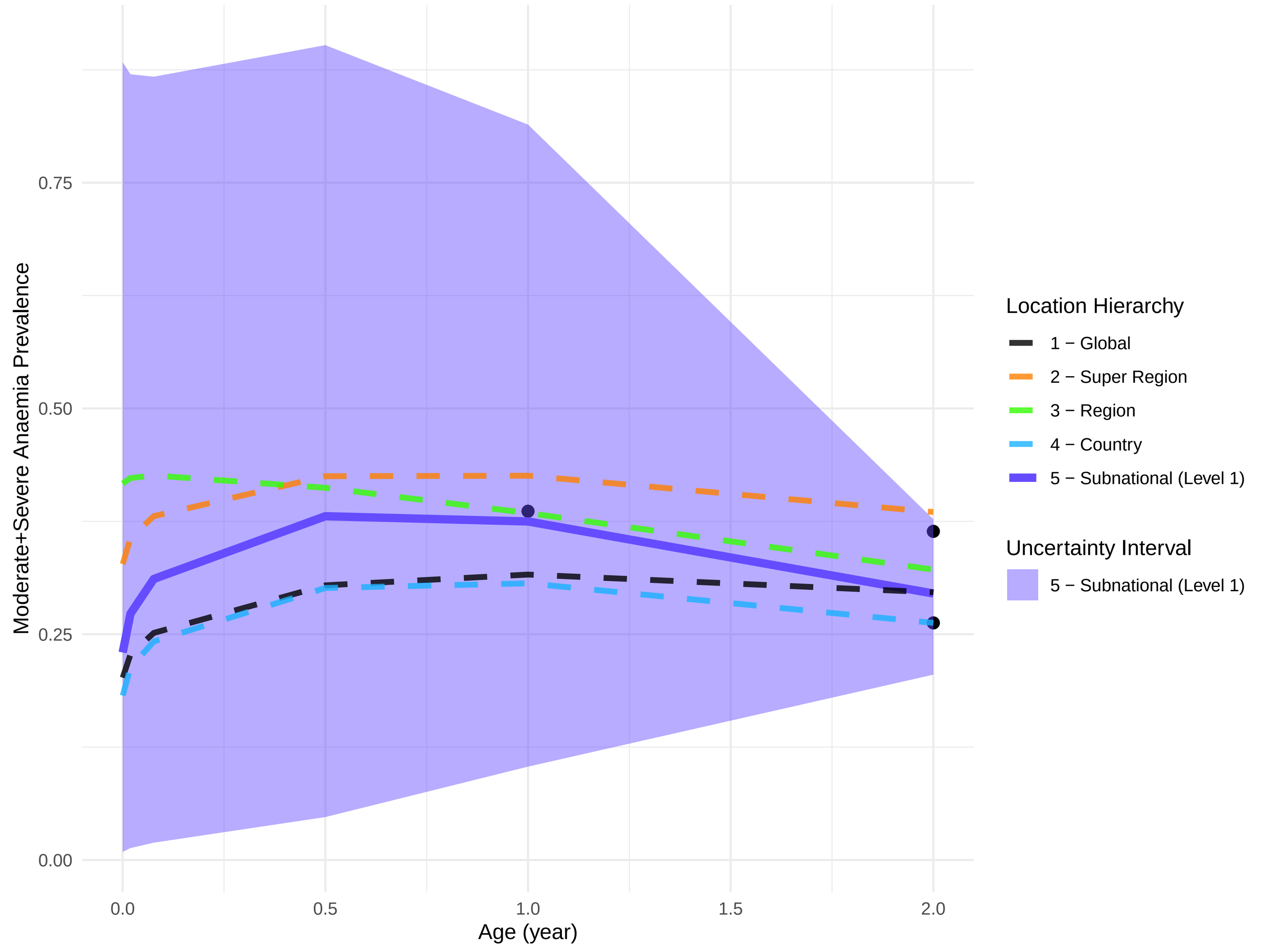

MR−BRT Age/Sex Fits − Non-pregnant Females <2 Years Old − Moderate+Severe Anaemia Prevalence − Elgeyo-Marakwet
WHO−2024 Adjustment Method

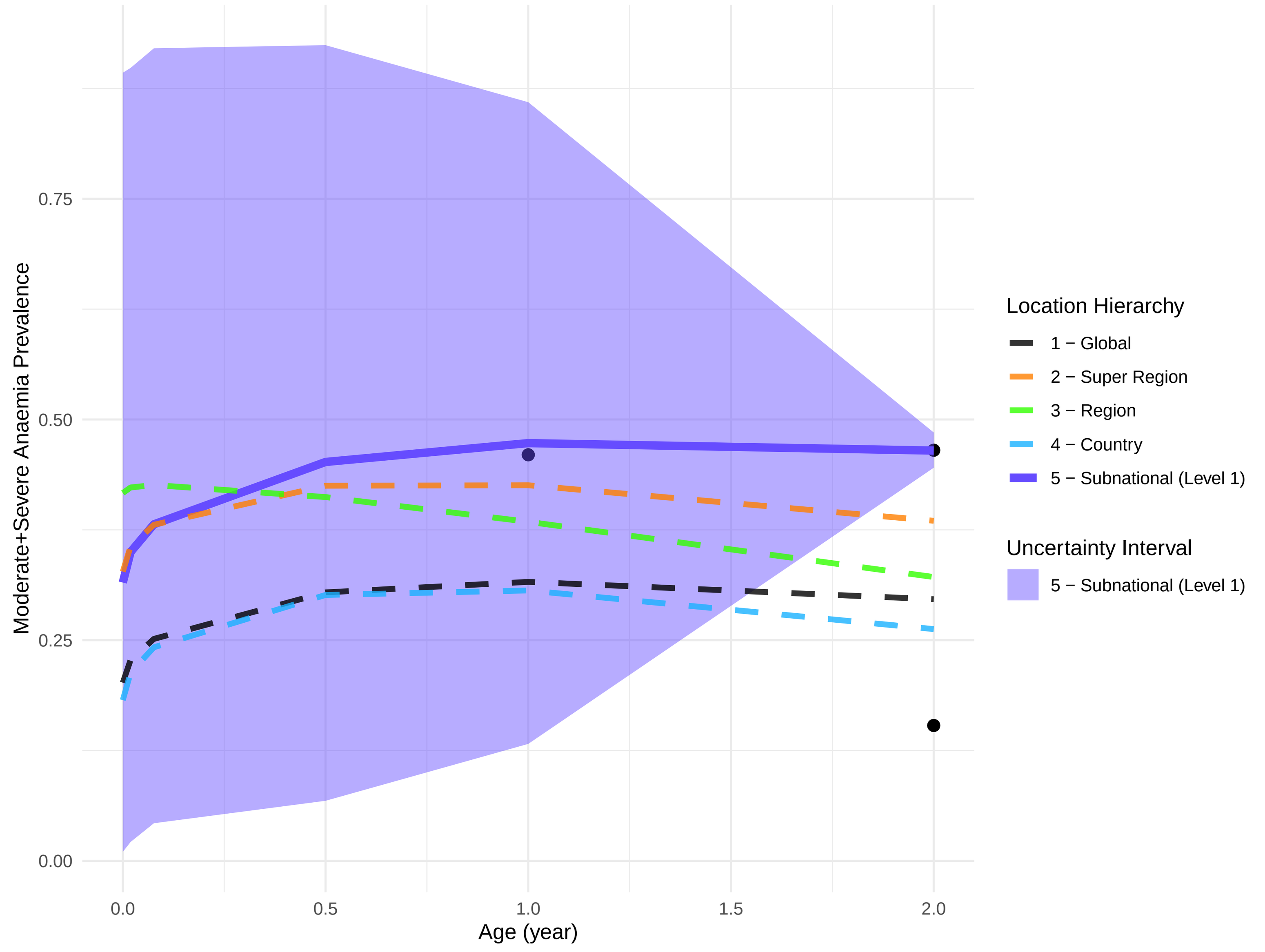

MR−BRT Age/Sex Fits − Non-pregnant Females <2 Years Old − Moderate+Severe Anaemia Prevalence − Embu
WHO−2024 Adjustment Method

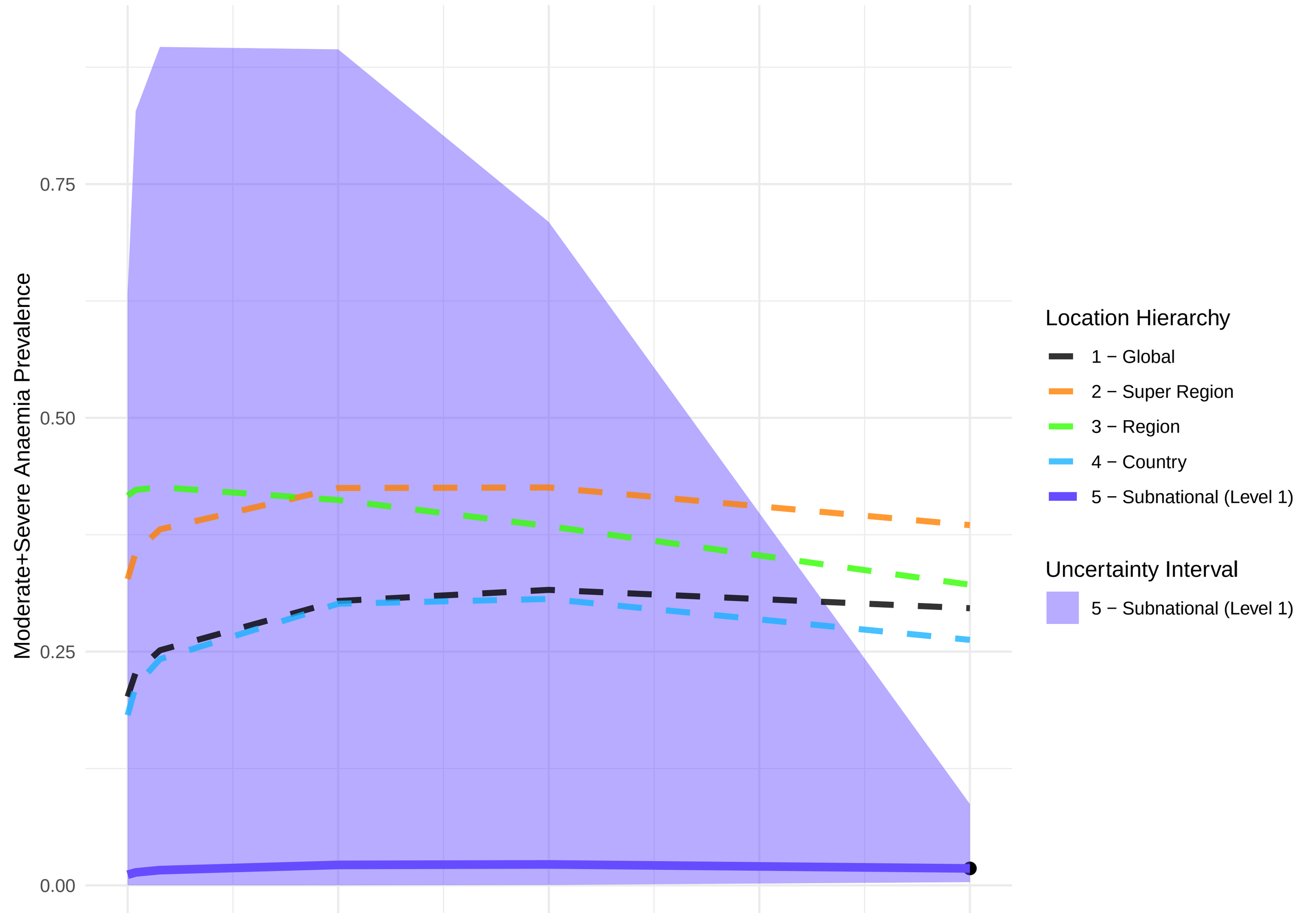

MR−BRT Age/Sex Fits − Non−pregnant Females <2 Years Old − Moderate+Severe Anaemia Prevalence − Garissa
WHO−2024 Adjustment Method

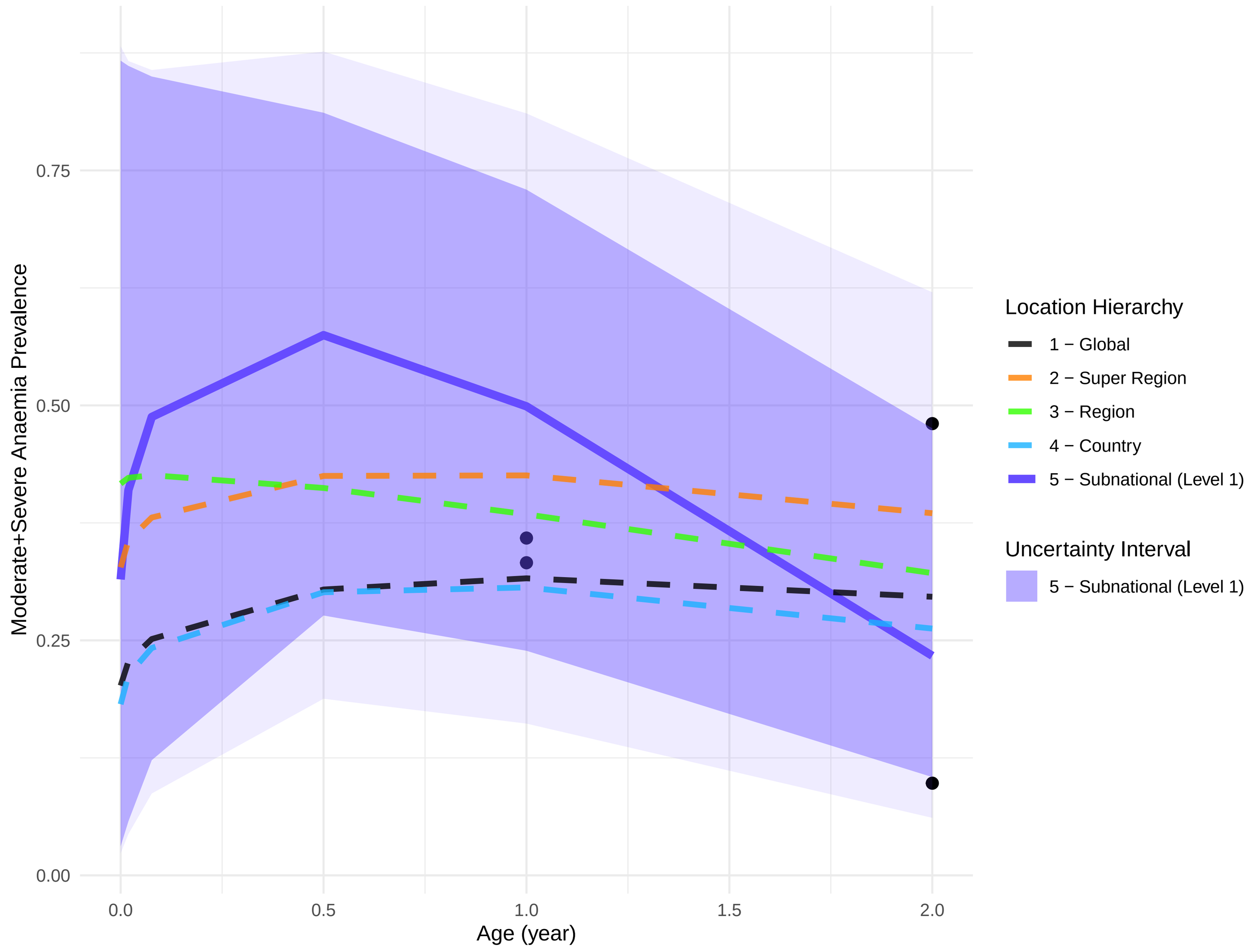

MR−BRT Age/Sex Fits − Non−pregnant Females <2 Years Old − Moderate+Severe Anaemia Prevalence − Homa Bay
WHO−2024 Adjustment Method

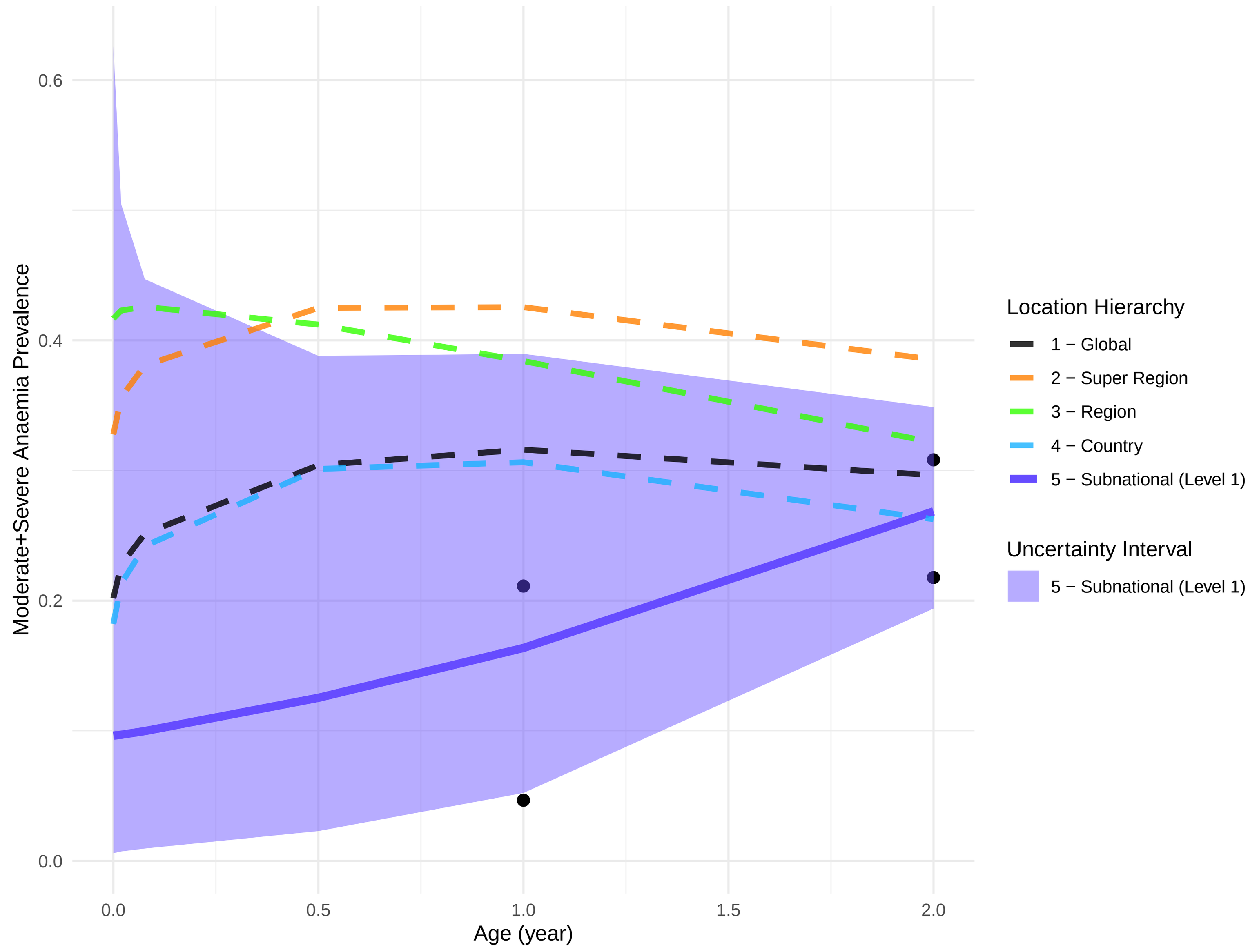

MR−BRT Age/Sex Fits − Non−pregnant Females <2 Years Old − Moderate+Severe Anaemia Prevalence − Isiolo
WHO−2024 Adjustment Method

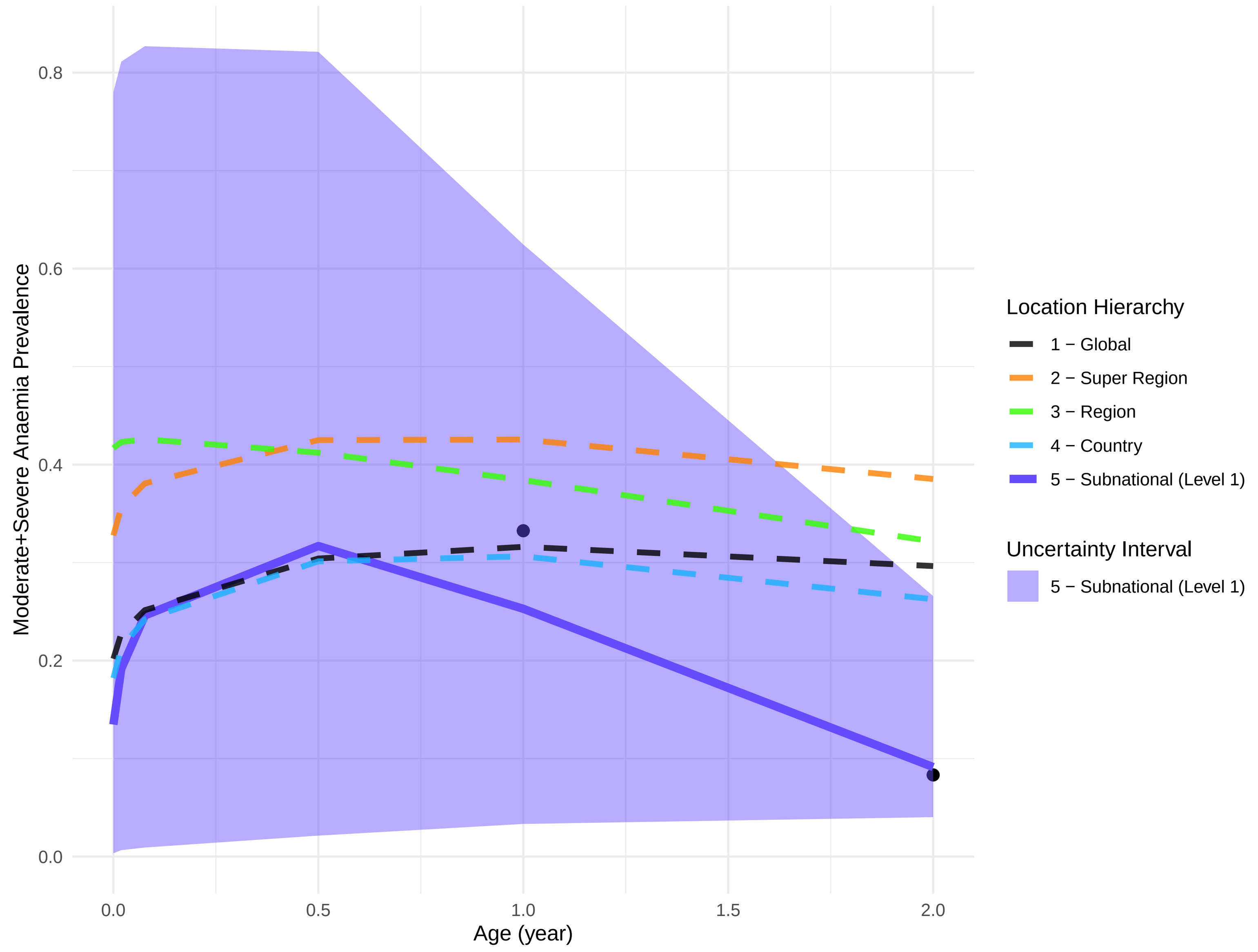

MR−BRT Age/Sex Fits − Non−pregnant Females <2 Years Old − Moderate+Severe Anaemia Prevalence − Kajiado
WHO−2024 Adjustment Method

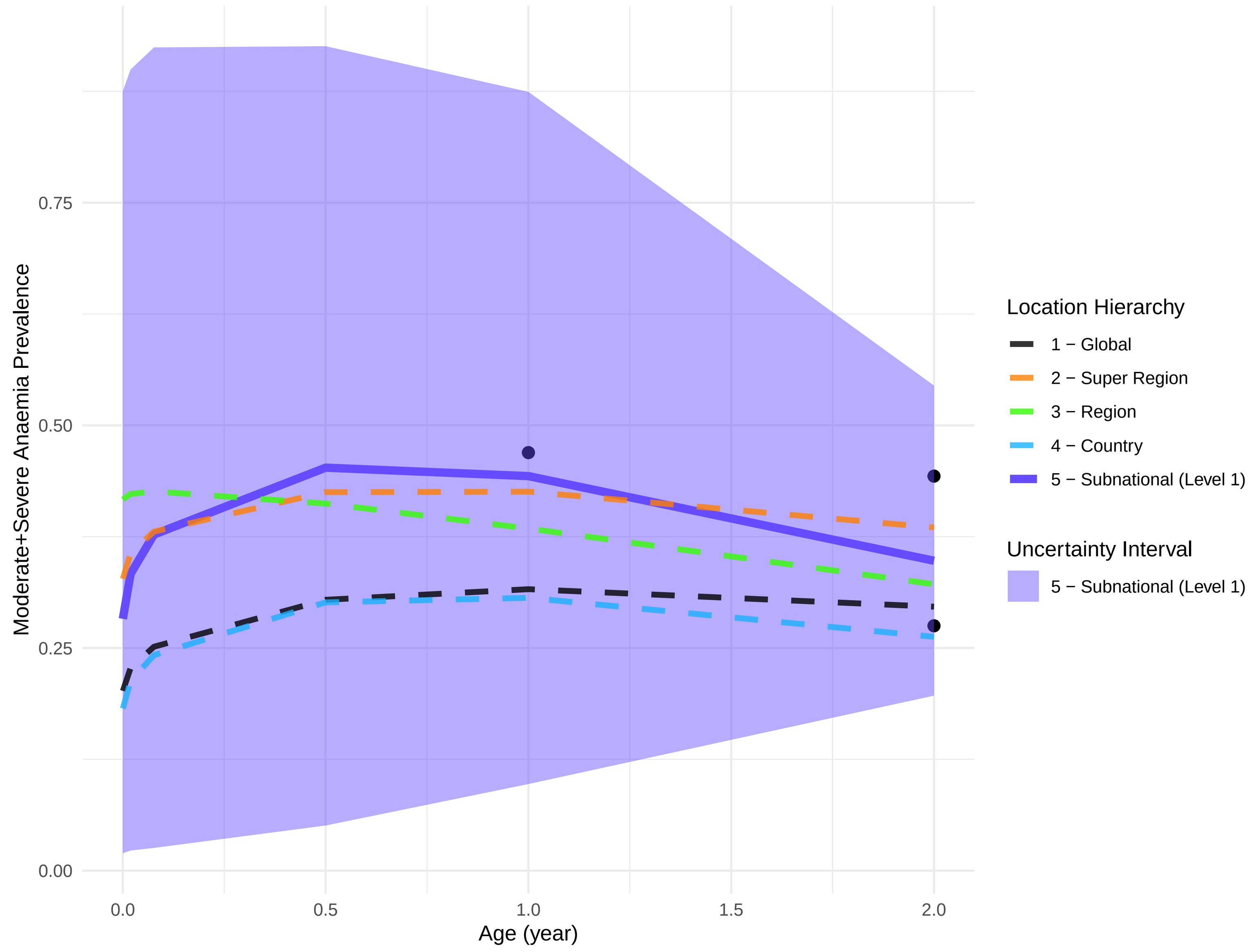

MR−BRT Age/Sex Fits − Non−pregnant Females <2 Years Old − Moderate+Severe Anaemia Prevalence − Kakamega
WHO−2024 Adjustment Method

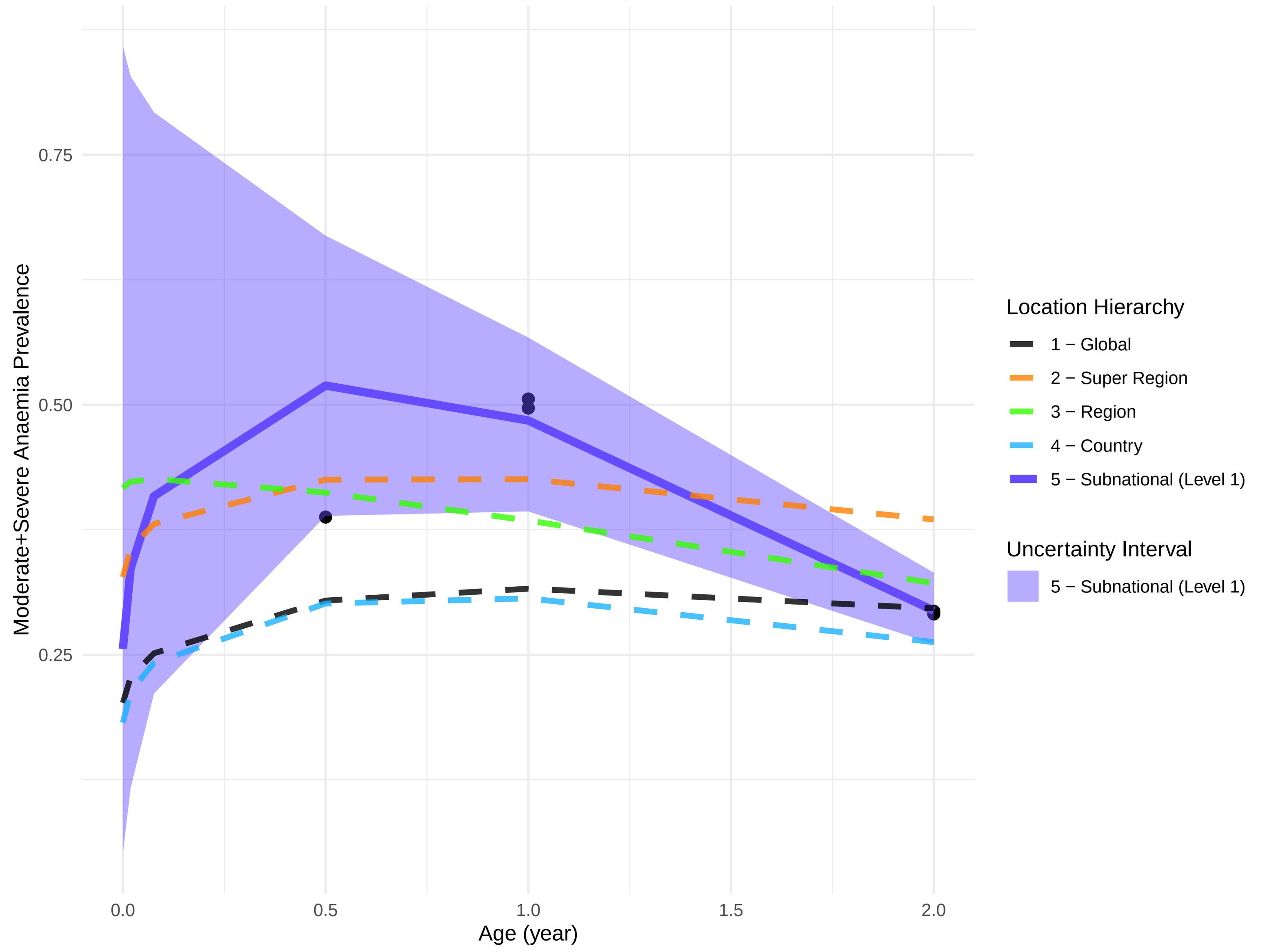

MR−BRT Age/Sex Fits − Non−pregnant Females <2 Years Old − Moderate+Severe Anaemia Prevalence − Kericho
WHO−2024 Adjustment Method

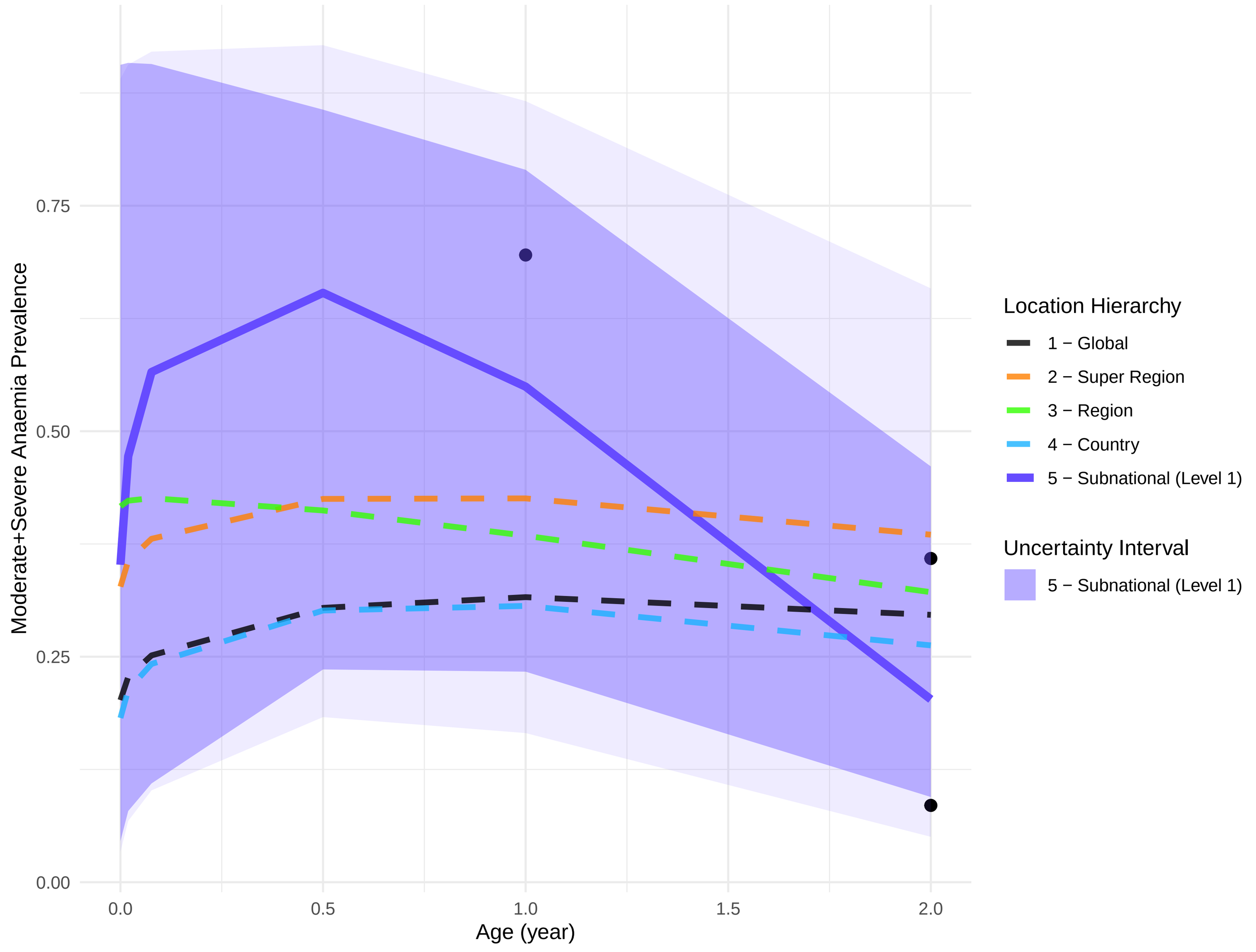

MR−BRT Age/Sex Fits − Non−pregnant Females <2 Years Old − Moderate+Severe Anaemia Prevalence − Kiambu
WHO−2024 Adjustment Method

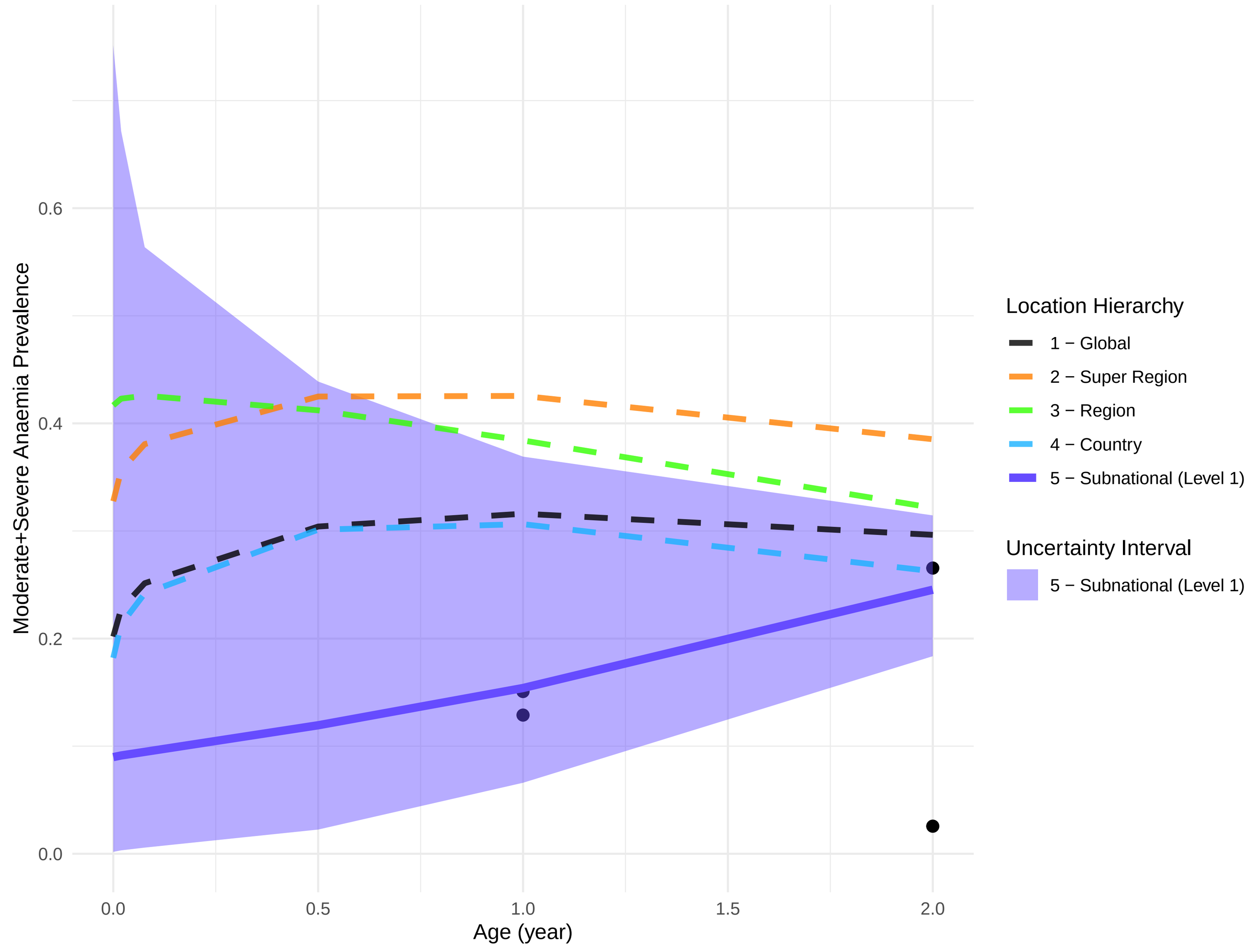

MR−BRT Age/Sex Fits − Non−pregnant Females <2 Years Old − Moderate+Severe Anaemia Prevalence − Kilifi
WHO−2024 Adjustment Method

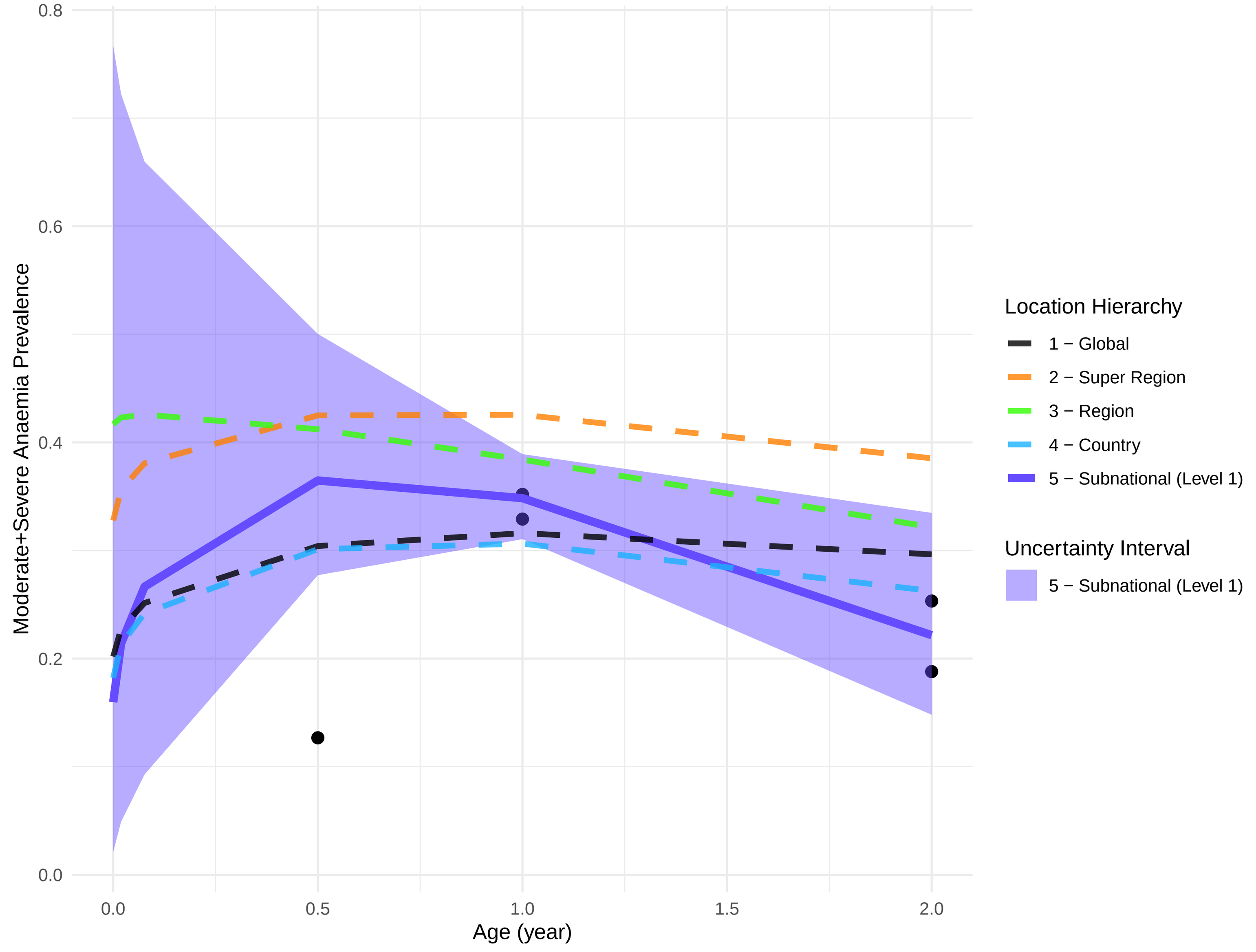

MR−BRT Age/Sex Fits − Non−pregnant Females <2 Years Old − Moderate+Severe Anaemia Prevalence − Kirinyaga
WHO−2024 Adjustment Method

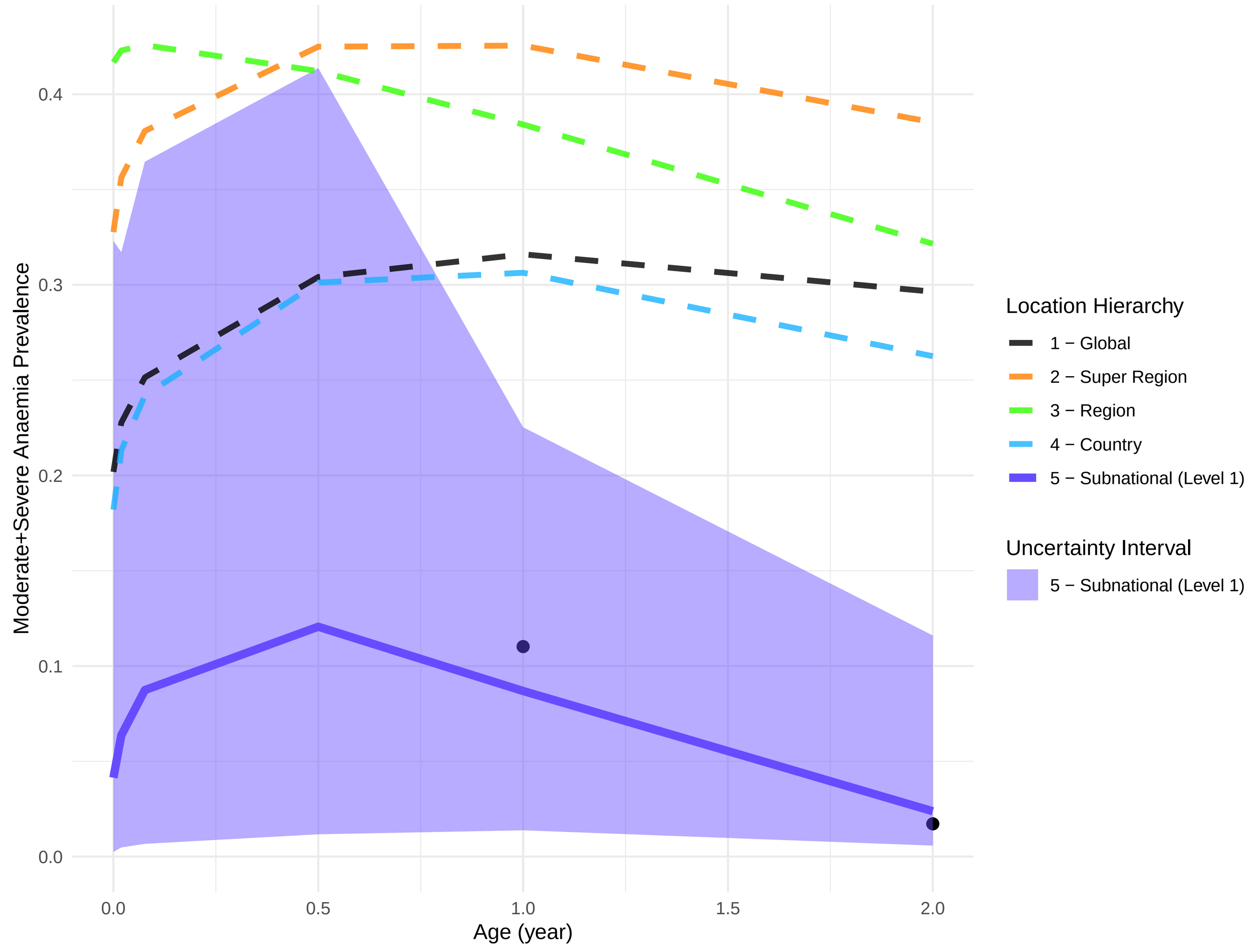

MR−BRT Age/Sex Fits − Non−pregnant Females <2 Years Old − Moderate+Severe Anaemia Prevalence − Kisii
WHO−2024 Adjustment Method

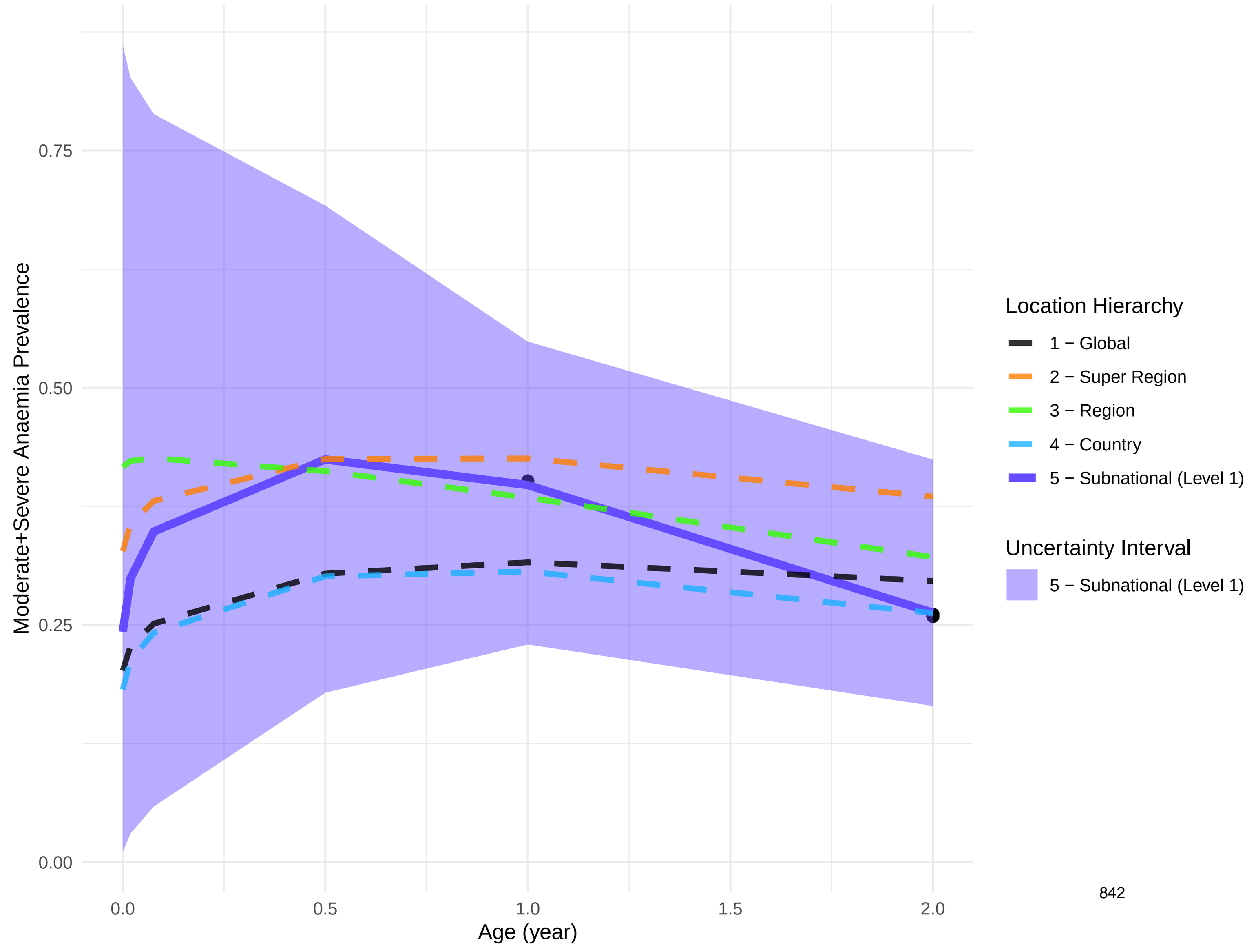

MR−BRT Age/Sex Fits − Non−pregnant Females <2 Years Old − Moderate+Severe Anaemia Prevalence − Kisumu
WHO−2024 Adjustment Method

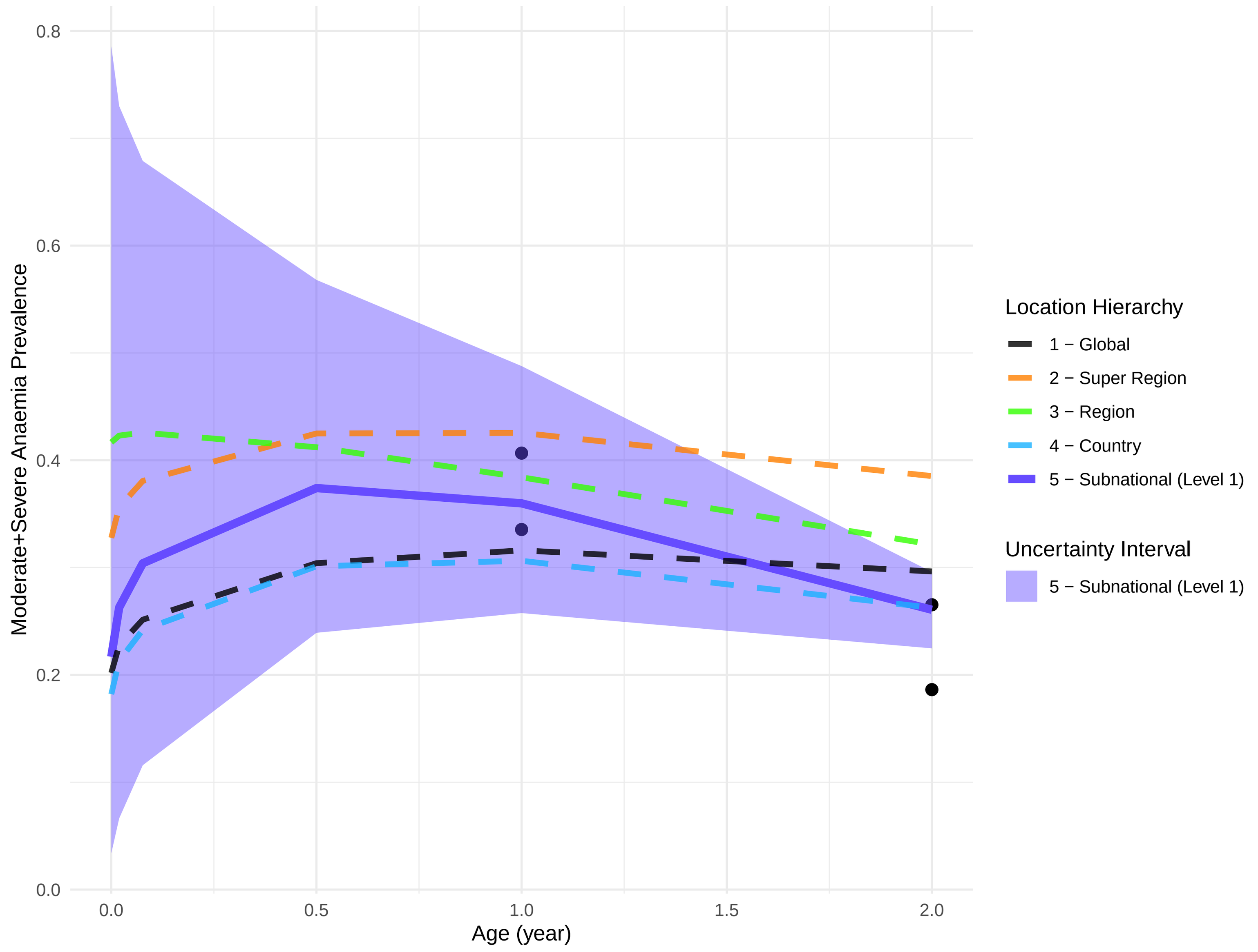

MR−BRT Age/Sex Fits − Non−pregnant Females <2 Years Old − Moderate+Severe Anaemia Prevalence − Kitui
WHO−2024 Adjustment Method

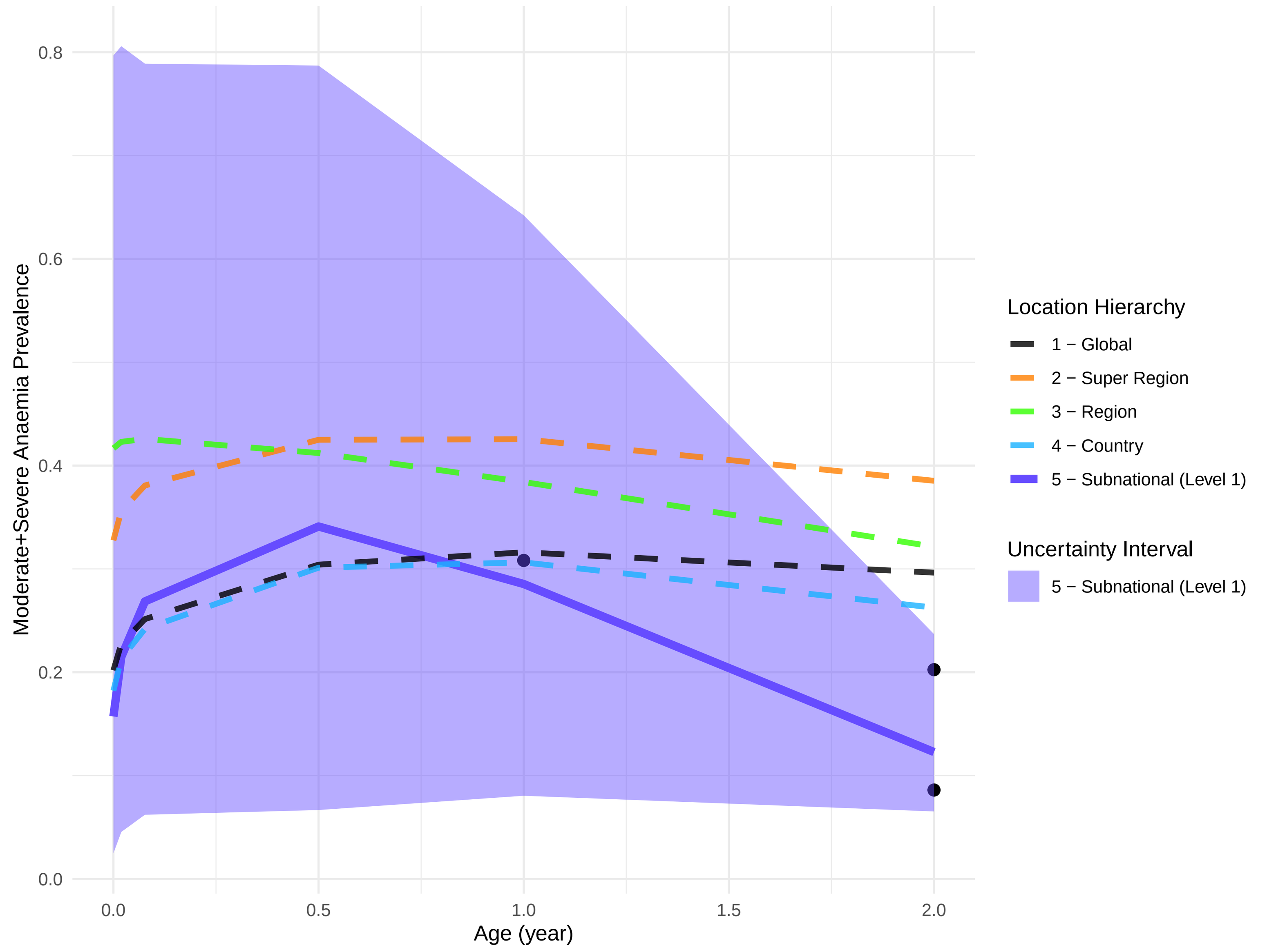

MR−BRT Age/Sex Fits − Non−pregnant Females <2 Years Old − Moderate+Severe Anaemia Prevalence − Kwale
WHO−2024 Adjustment Method

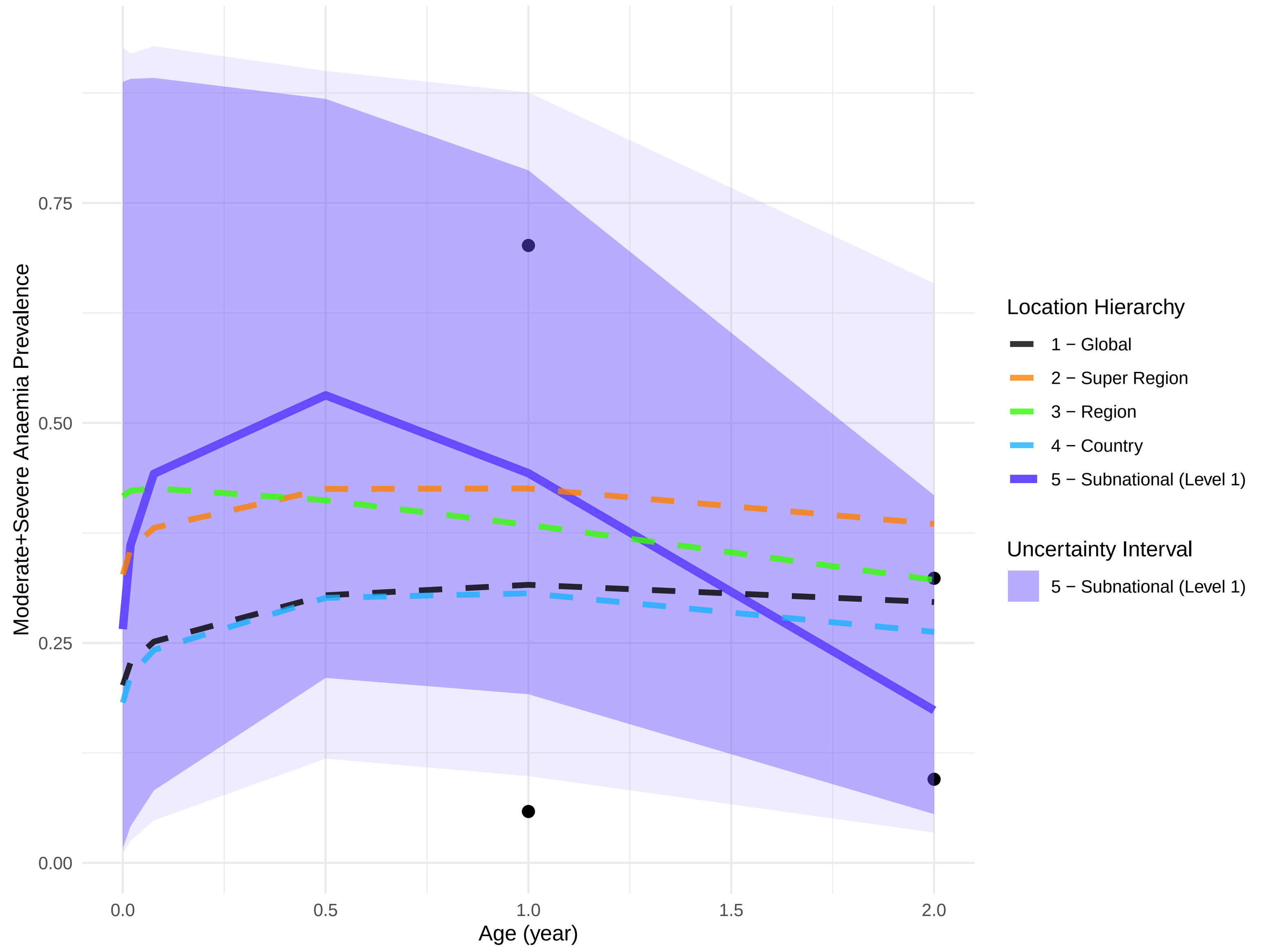

MR−BRT Age/Sex Fits − Non−pregnant Females <2 Years Old − Moderate+Severe Anaemia Prevalence − Laikipia
WHO−2024 Adjustment Method

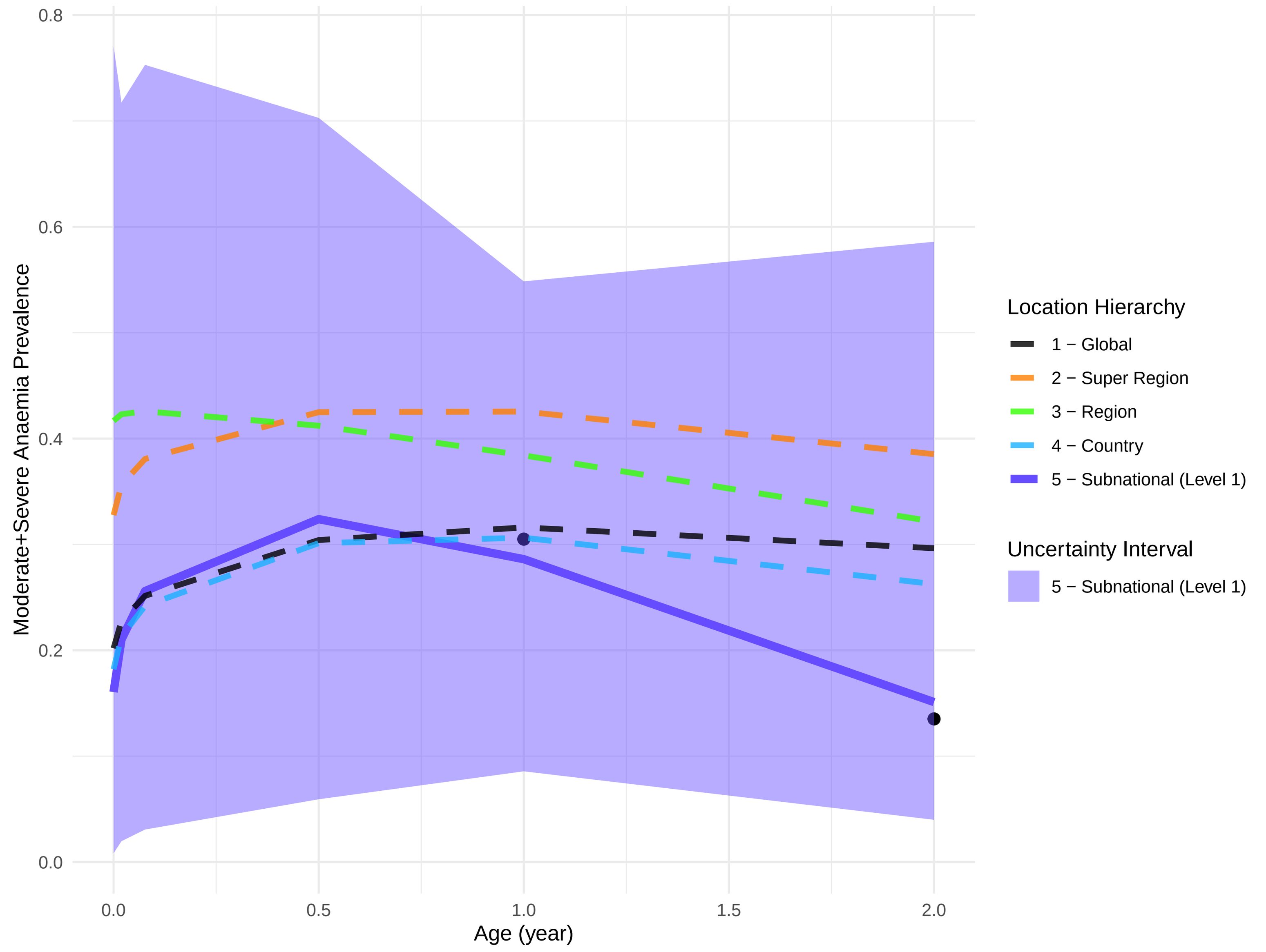

MR−BRT Age/Sex Fits − Non−pregnant Females <2 Years Old − Moderate+Severe Anaemia Prevalence − Lamu
WHO−2024 Adjustment Method

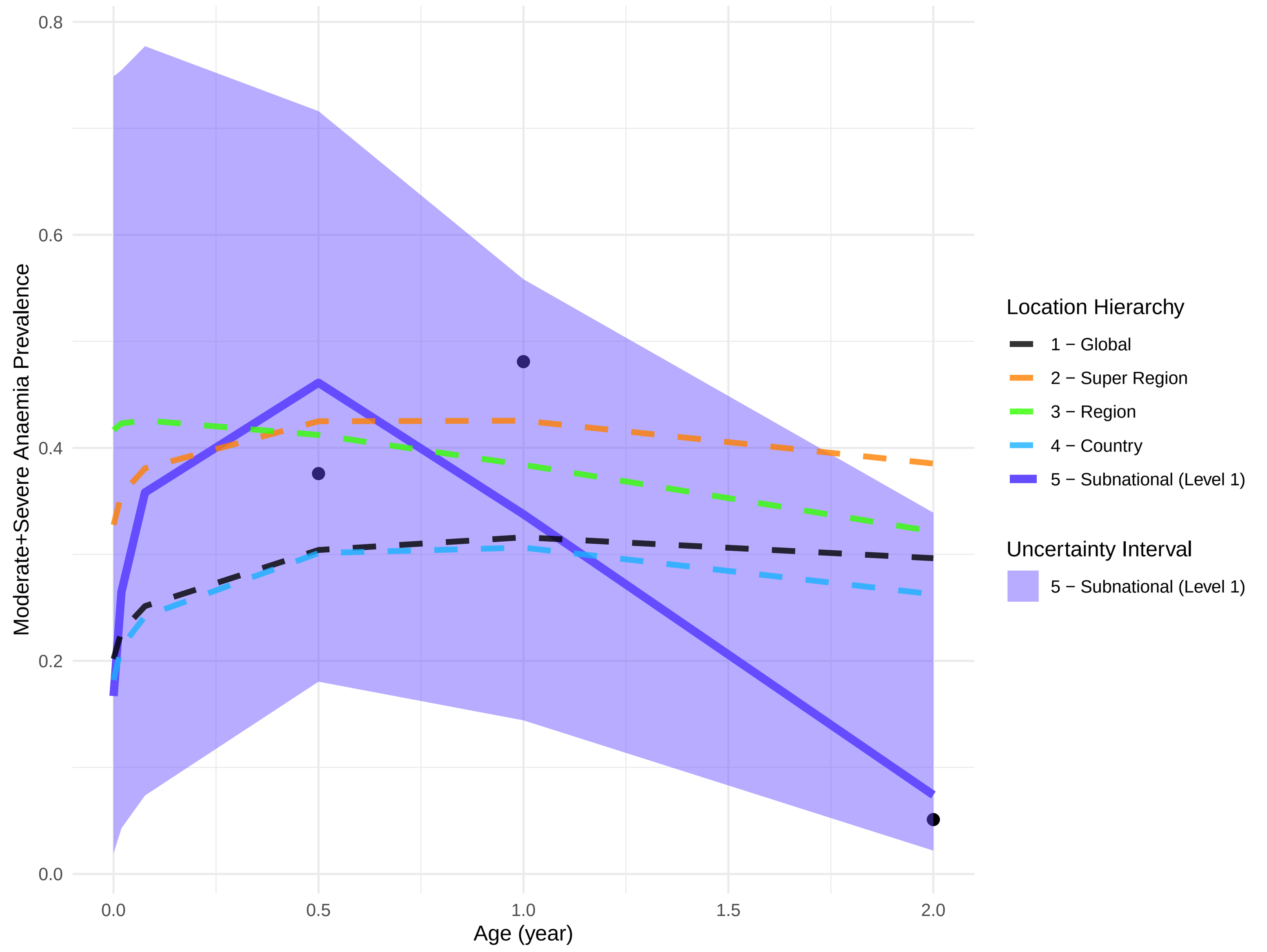

MR−BRT Age/Sex Fits − Non−pregnant Females <2 Years Old − Moderate+Severe Anaemia Prevalence − Machakos
WHO−2024 Adjustment Method

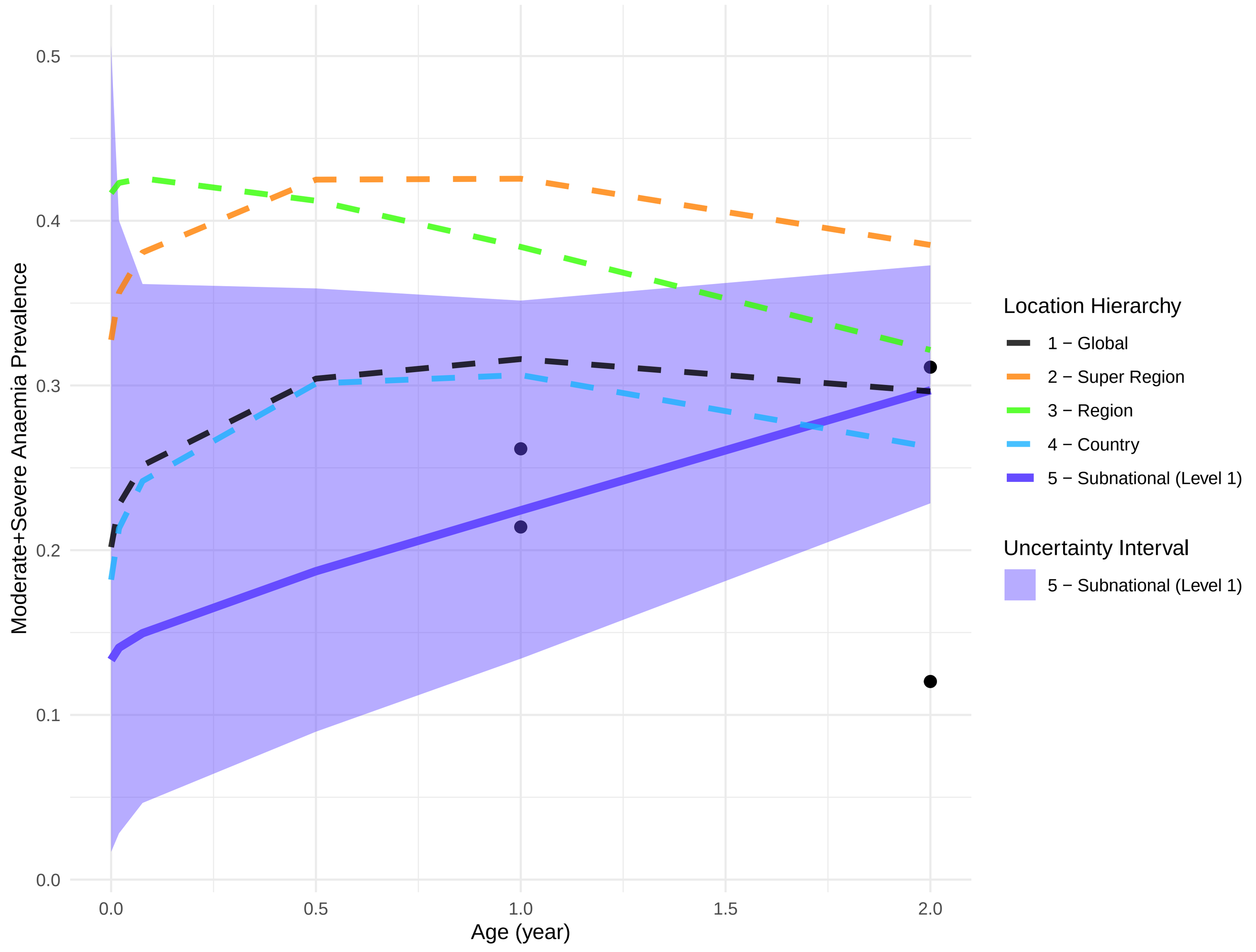

MR−BRT Age/Sex Fits − Non−pregnant Females <2 Years Old − Moderate+Severe Anaemia Prevalence − Makueni
WHO−2024 Adjustment Method

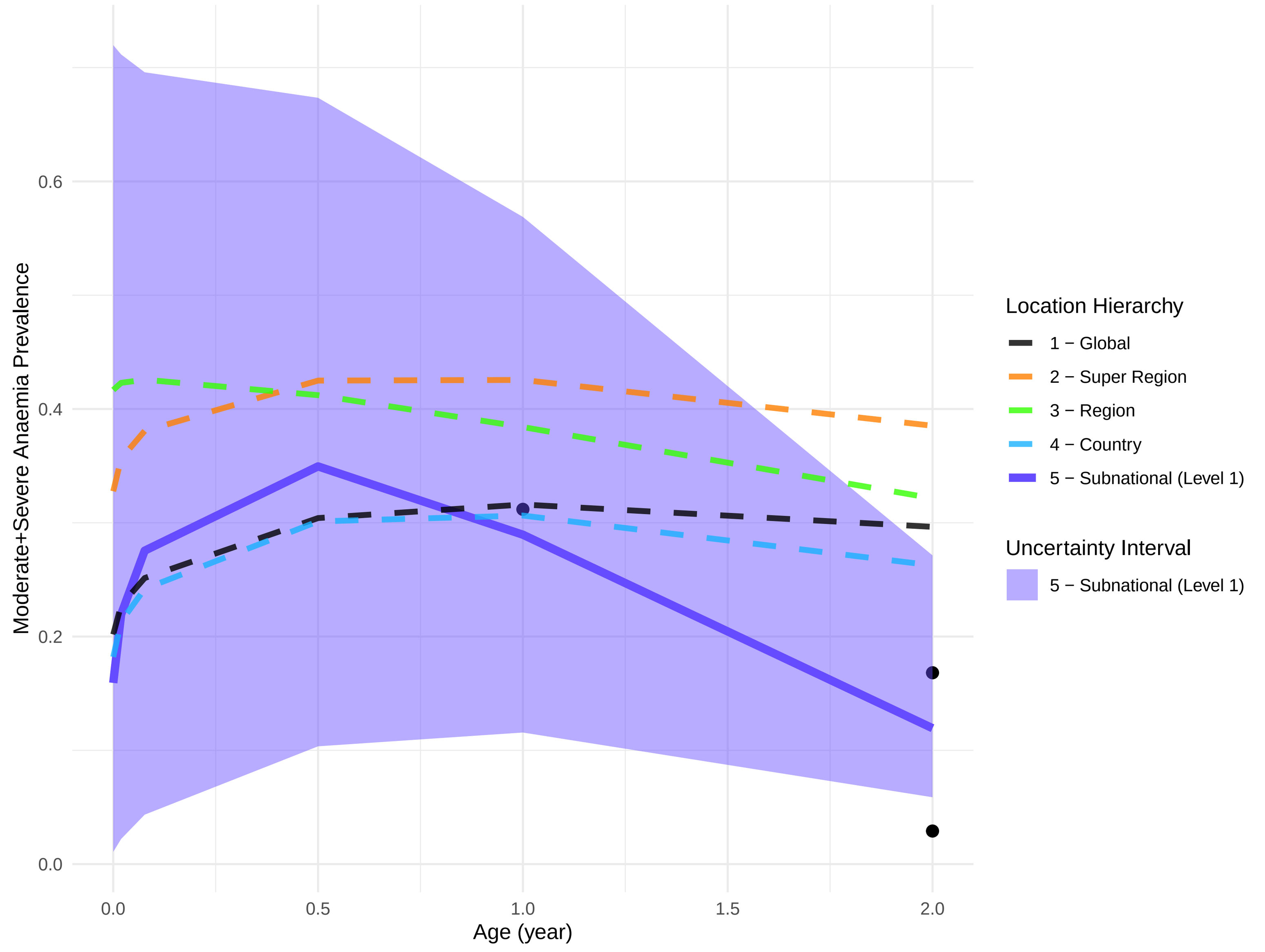

MR−BRT Age/Sex Fits − Non−pregnant Females <2 Years Old − Moderate+Severe Anaemia Prevalence − Mandera
WHO−2024 Adjustment Method

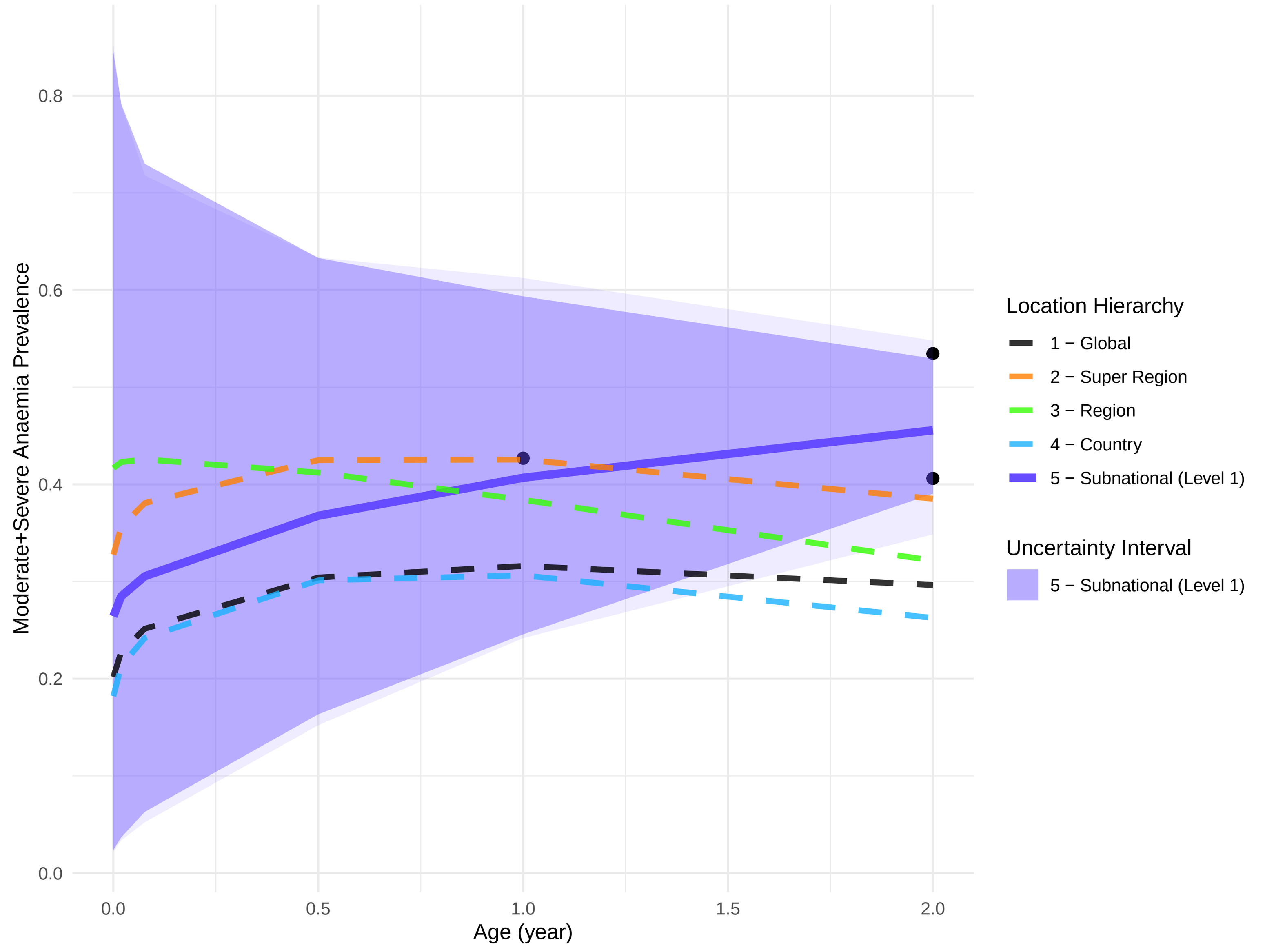

MR−BRT Age/Sex Fits − Non−pregnant Females <2 Years Old − Moderate+Severe Anaemia Prevalence − Marsabit
WHO−2024 Adjustment Method

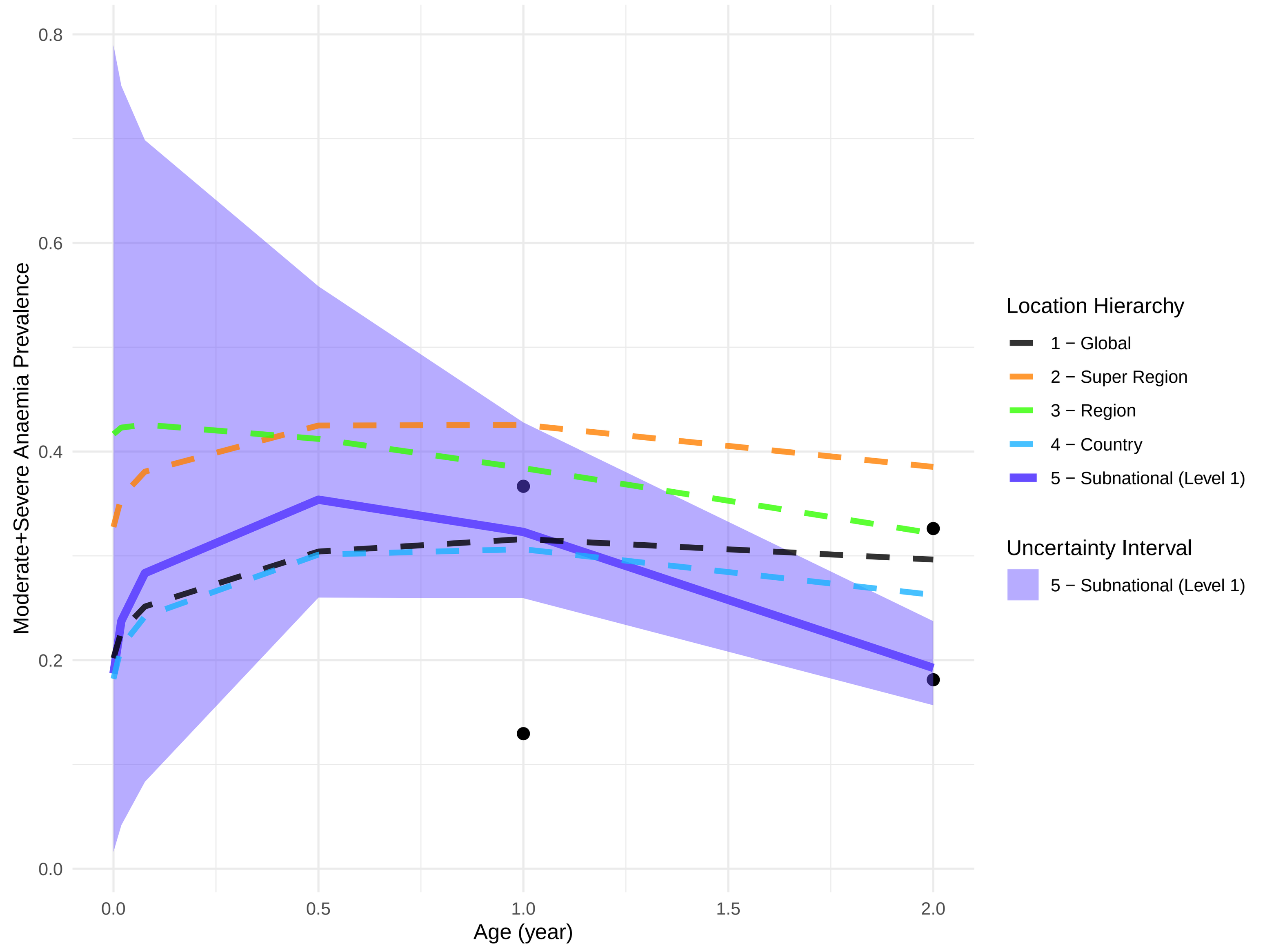

MR−BRT Age/Sex Fits − Non−pregnant Females <2 Years Old − Moderate+Severe Anaemia Prevalence − Meru
WHO−2024 Adjustment Method

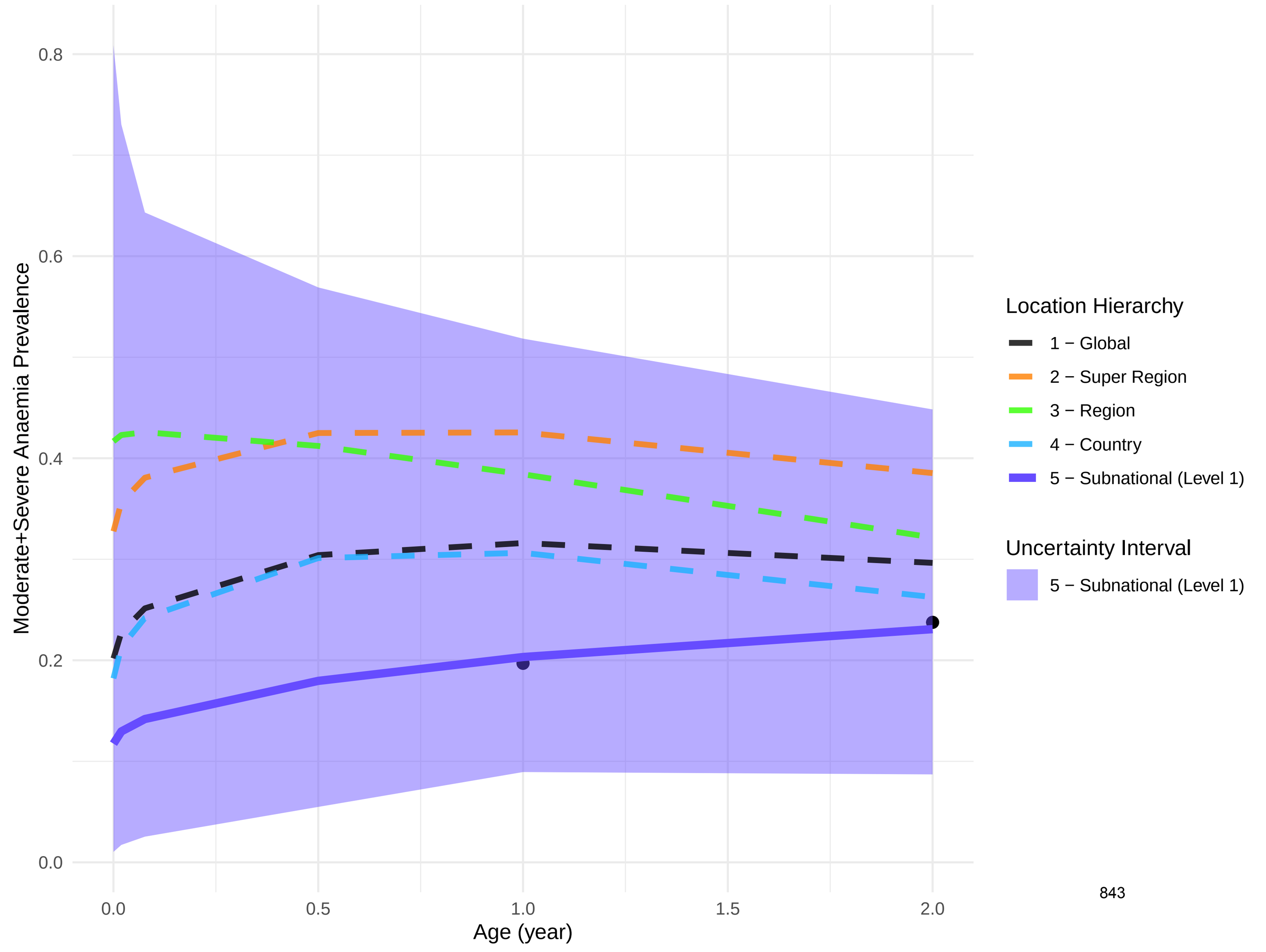

MR−BRT Age/Sex Fits − Non−pregnant Females <2 Years Old − Moderate+Severe Anaemia Prevalence − Migori
WHO−2024 Adjustment Method

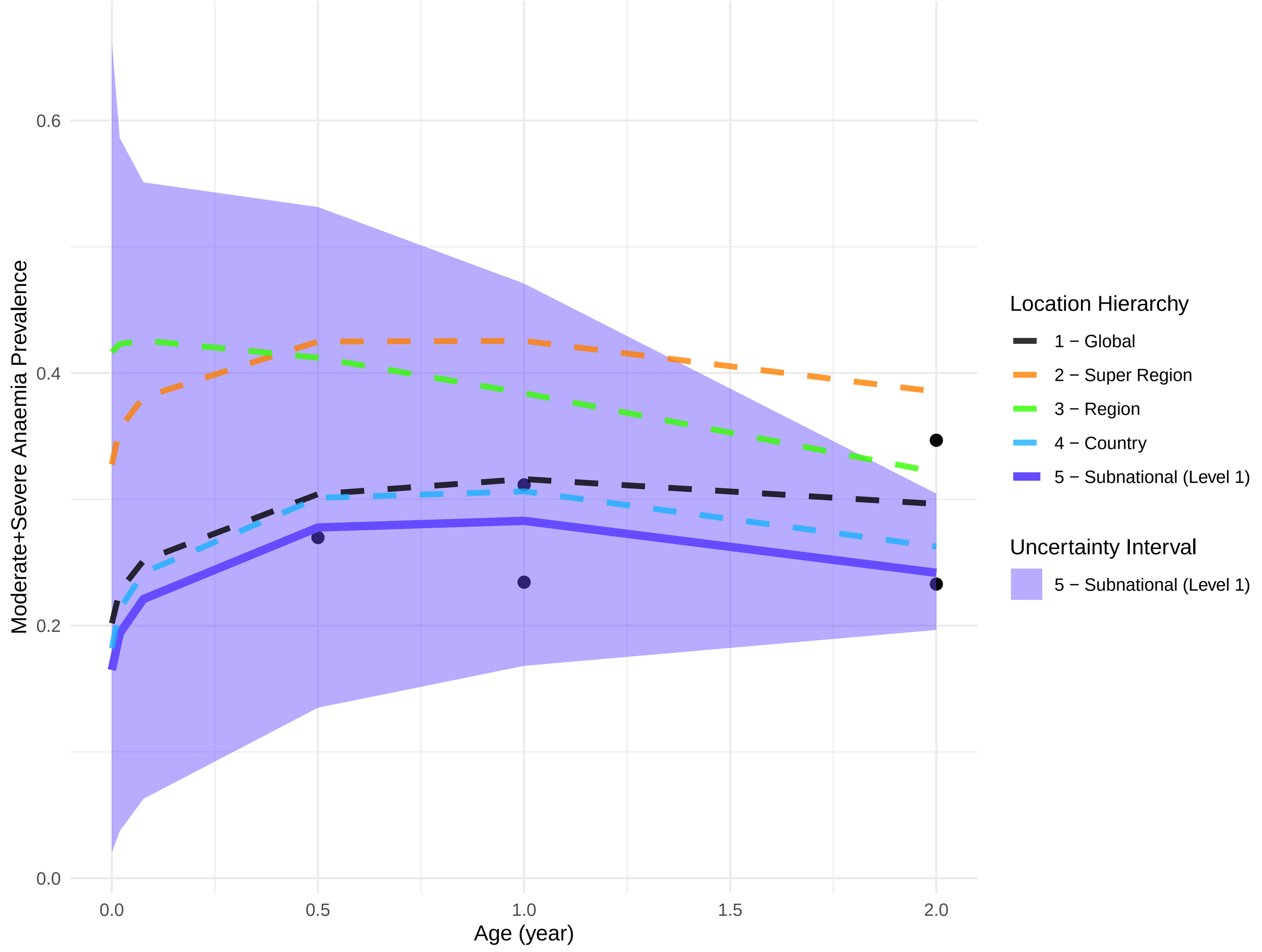

MR−BRT Age/Sex Fits − Non−pregnant Females <2 Years Old − Moderate+Severe Anaemia Prevalence − Mombasa
WHO−2024 Adjustment Method

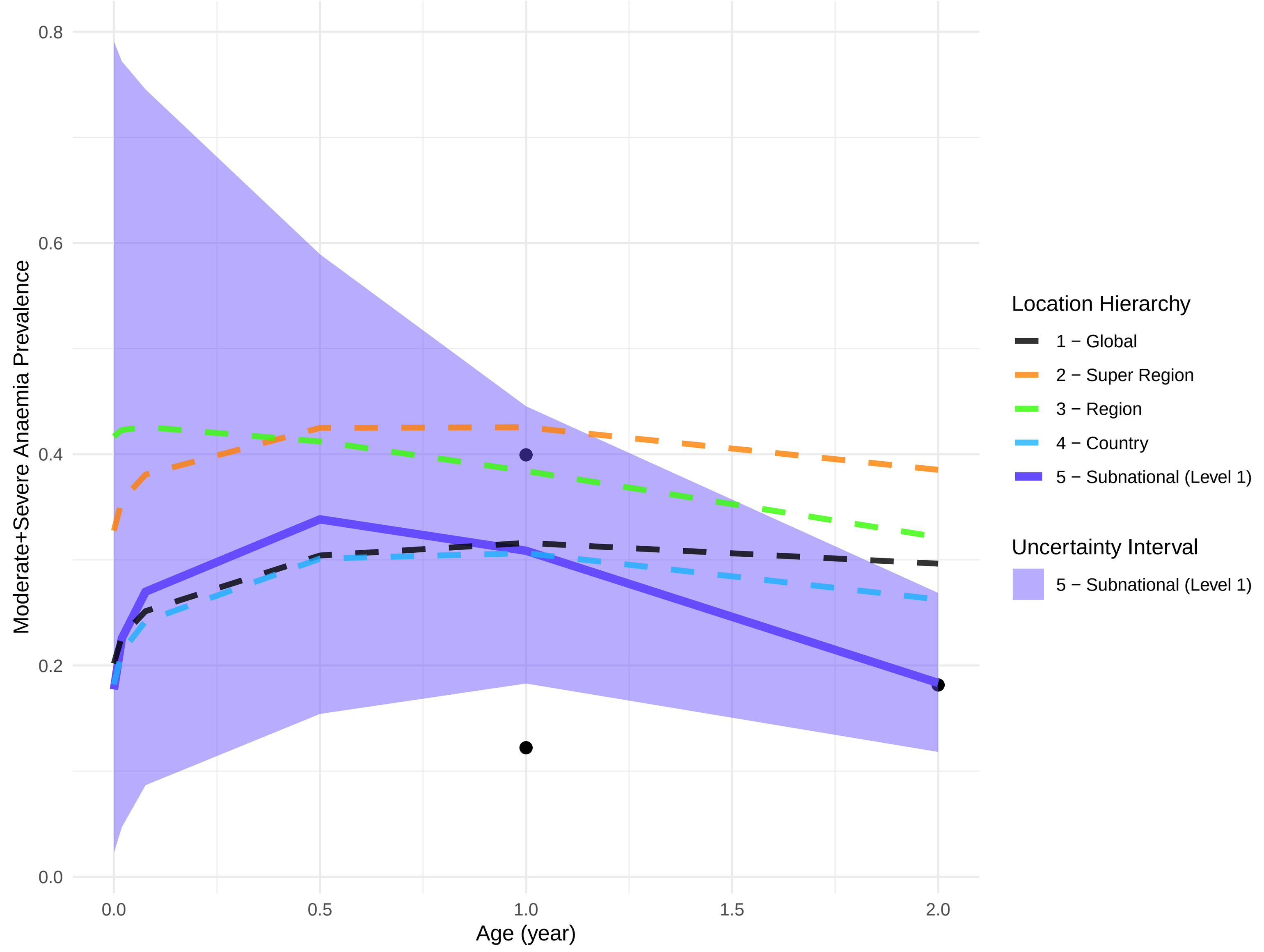

MR−BRT Age/Sex Fits − Non−pregnant Females <2 Years Old − Moderate+Severe Anaemia Prevalence − Murang'a
WHO−2024 Adjustment Method

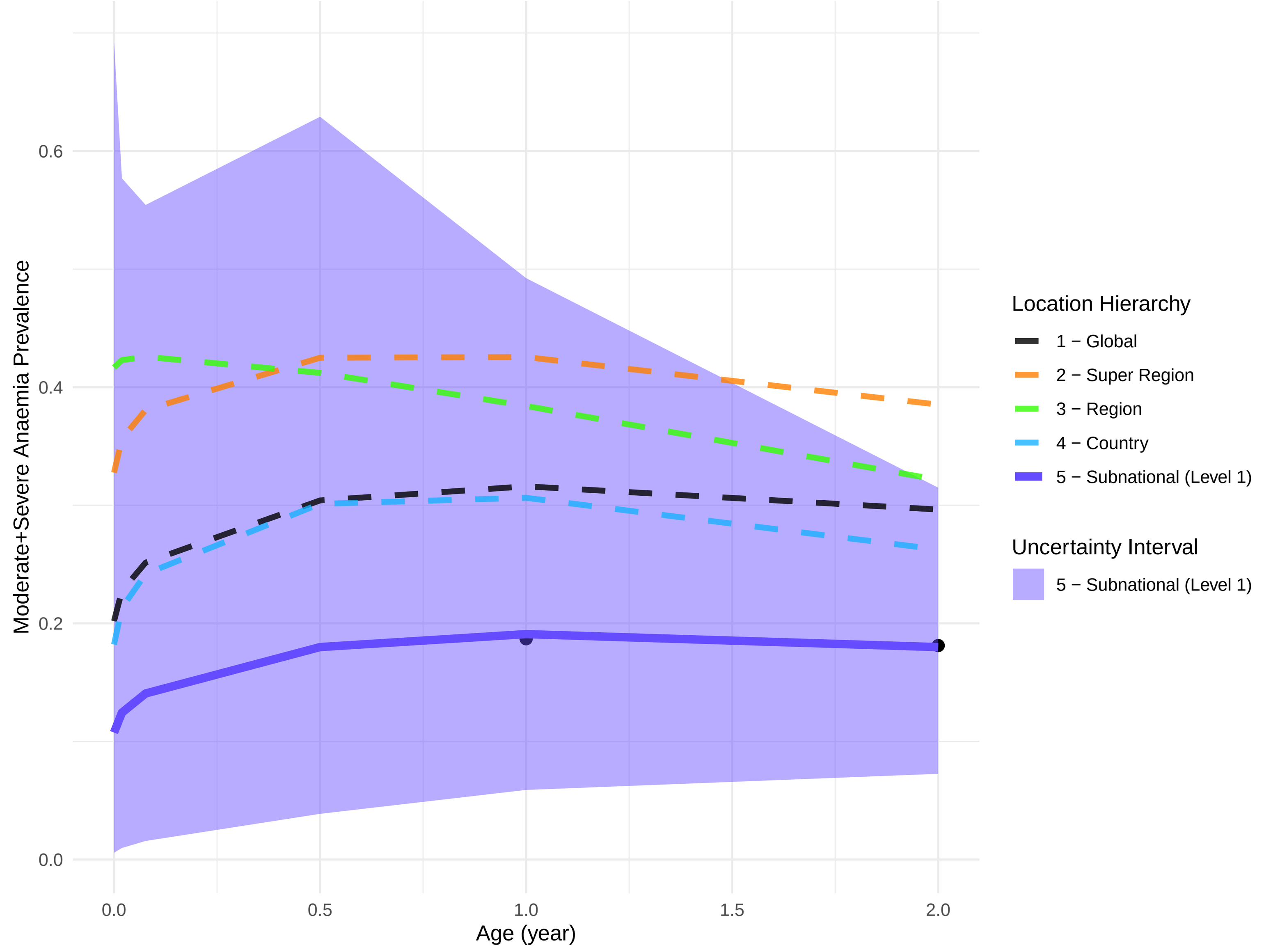

MR−BRT Age/Sex Fits − Non−pregnant Females <2 Years Old − Moderate+Severe Anaemia Prevalence − Nakuru
WHO−2024 Adjustment Method

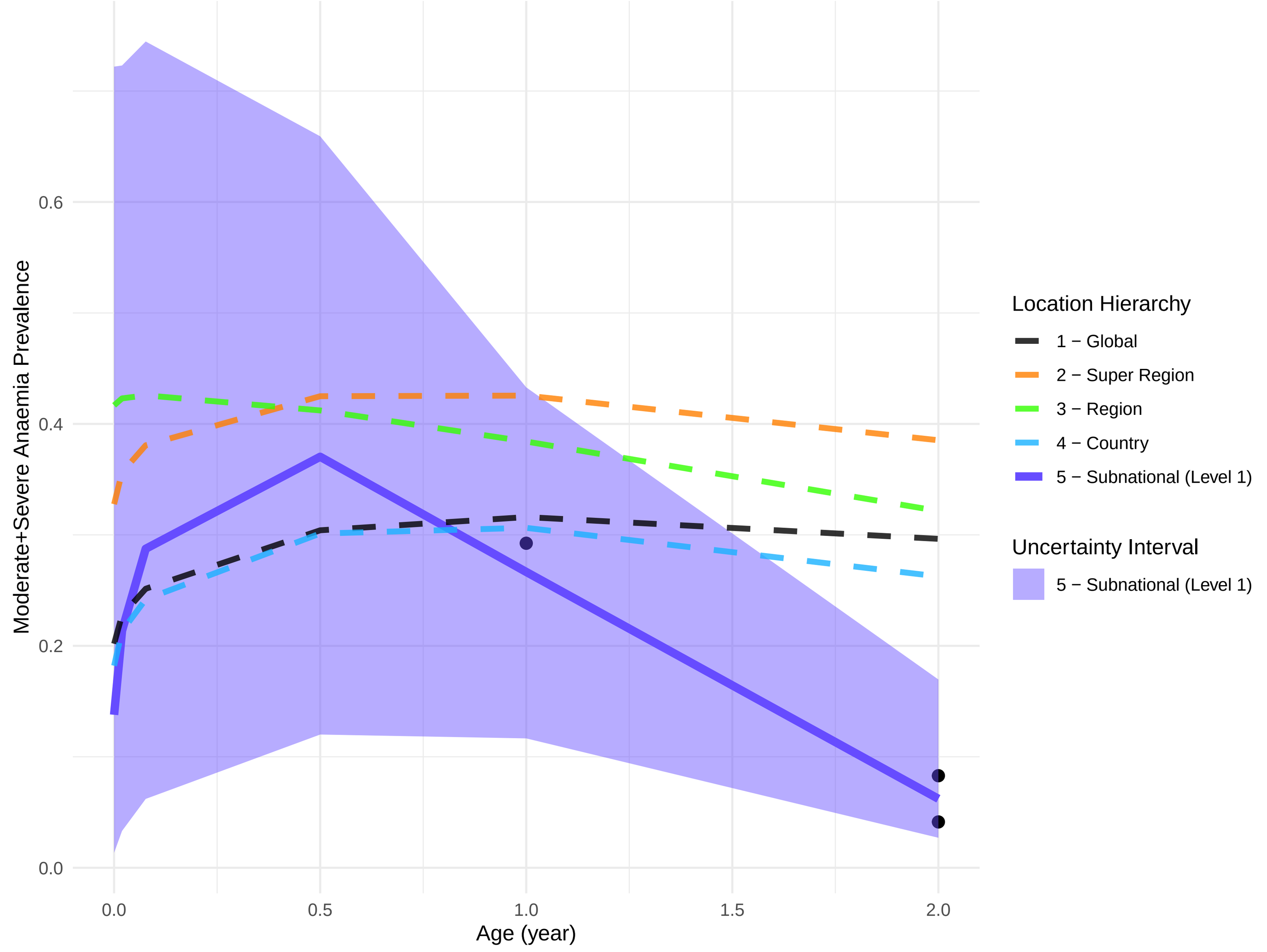

MR−BRT Age/Sex Fits − Non−pregnant Females <2 Years Old − Moderate+Severe Anaemia Prevalence − Nandi
WHO−2024 Adjustment Method

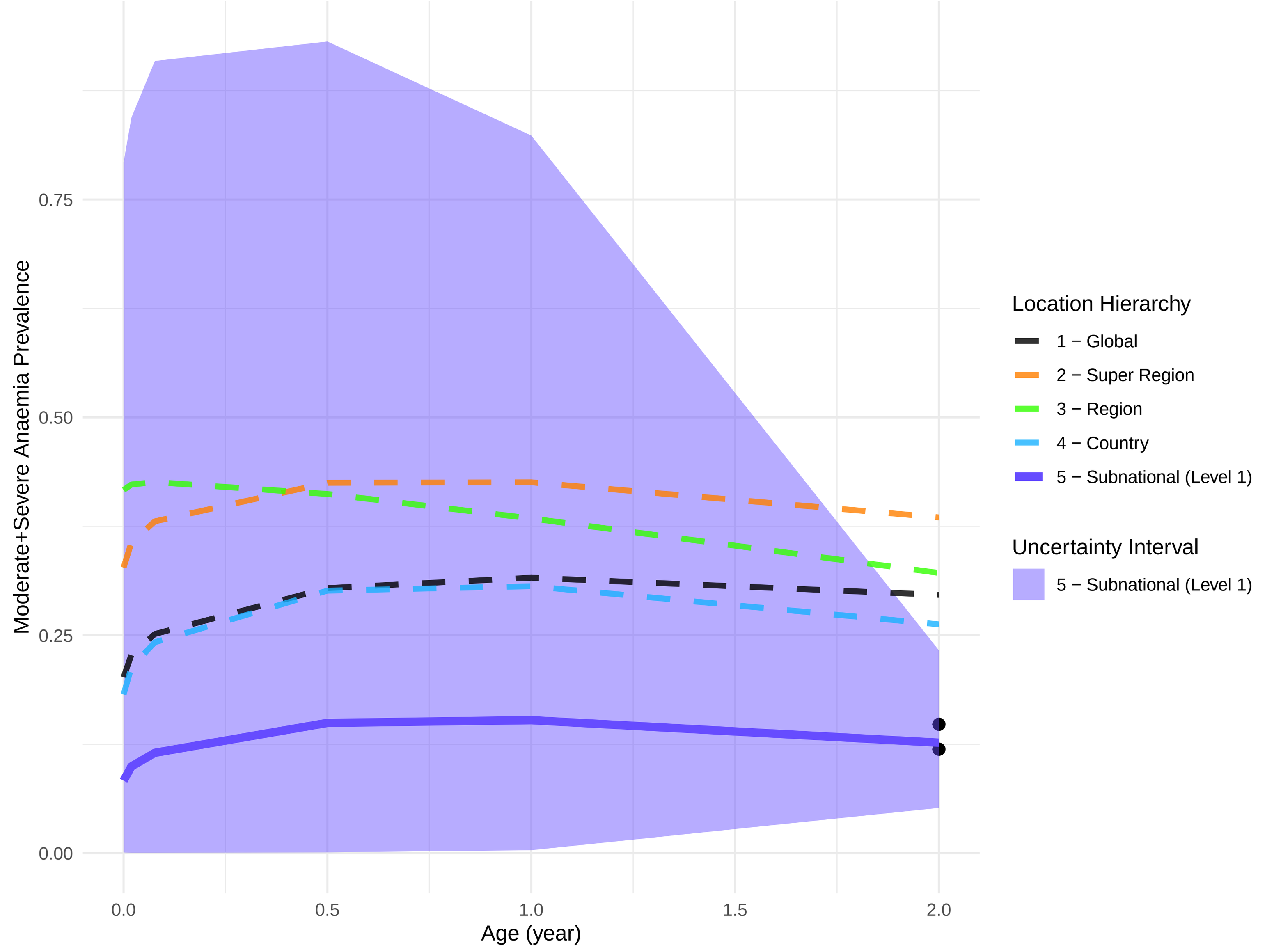

MR−BRT Age/Sex Fits − Non−pregnant Females <2 Years Old − Moderate+Severe Anaemia Prevalence − Narok
WHO−2024 Adjustment Method

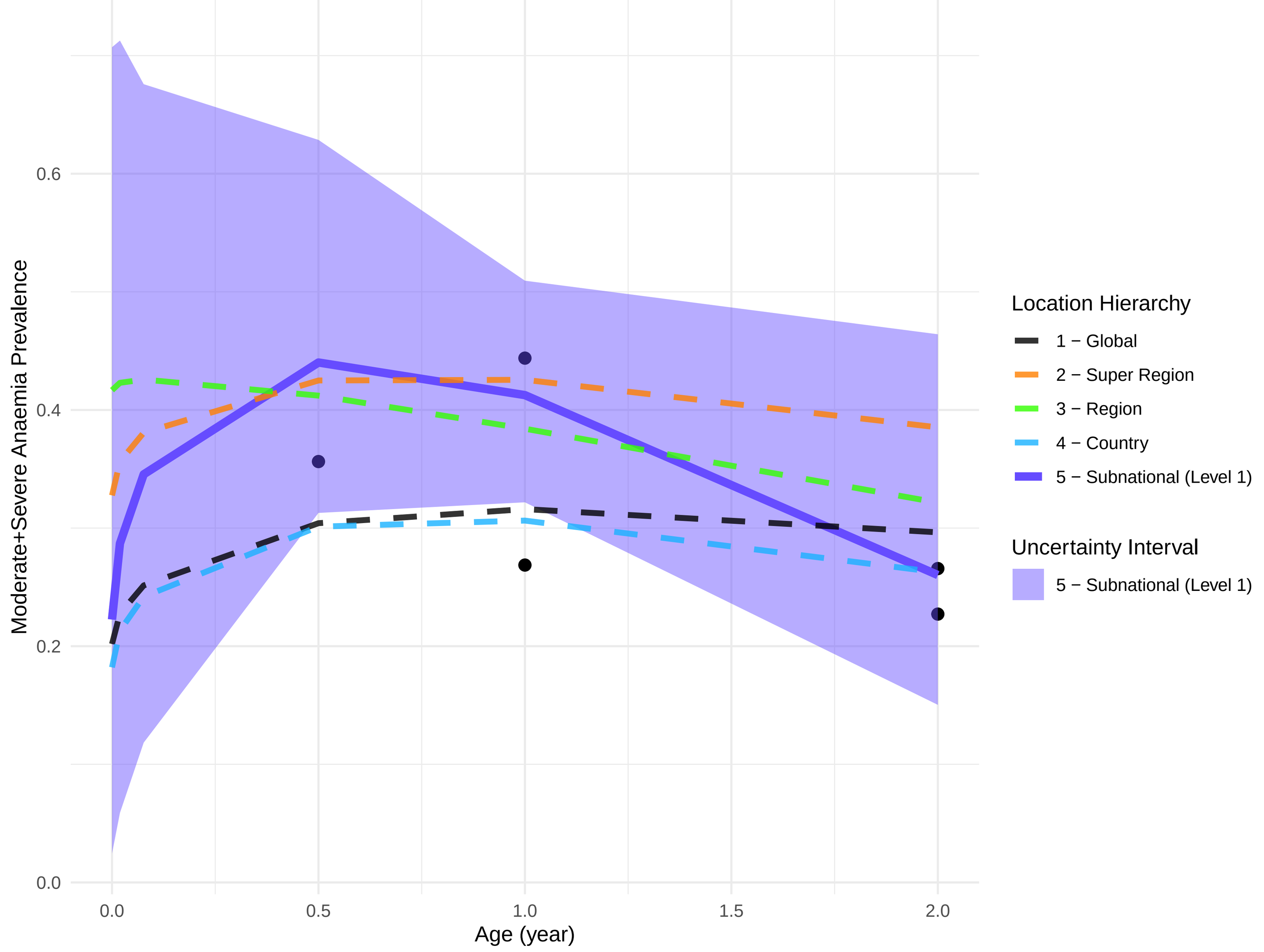

MR−BRT Age/Sex Fits − Non−pregnant Females <2 Years Old − Moderate+Severe Anaemia Prevalence − Nyamira
WHO−2024 Adjustment Method

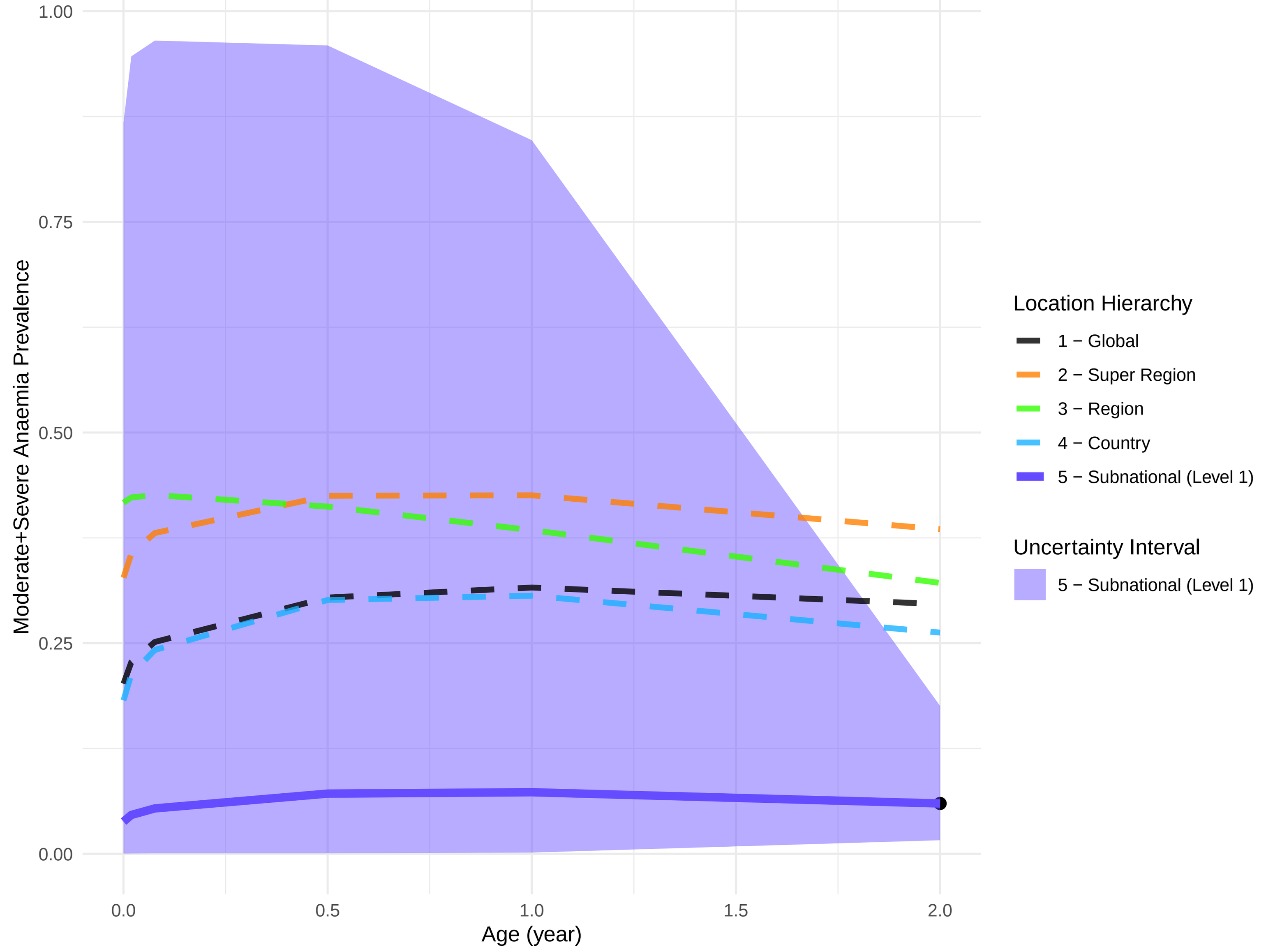

MR−BRT Age/Sex Fits − Non−pregnant Females <2 Years Old − Moderate+Severe Anaemia Prevalence − Nyandarua
WHO−2024 Adjustment Method

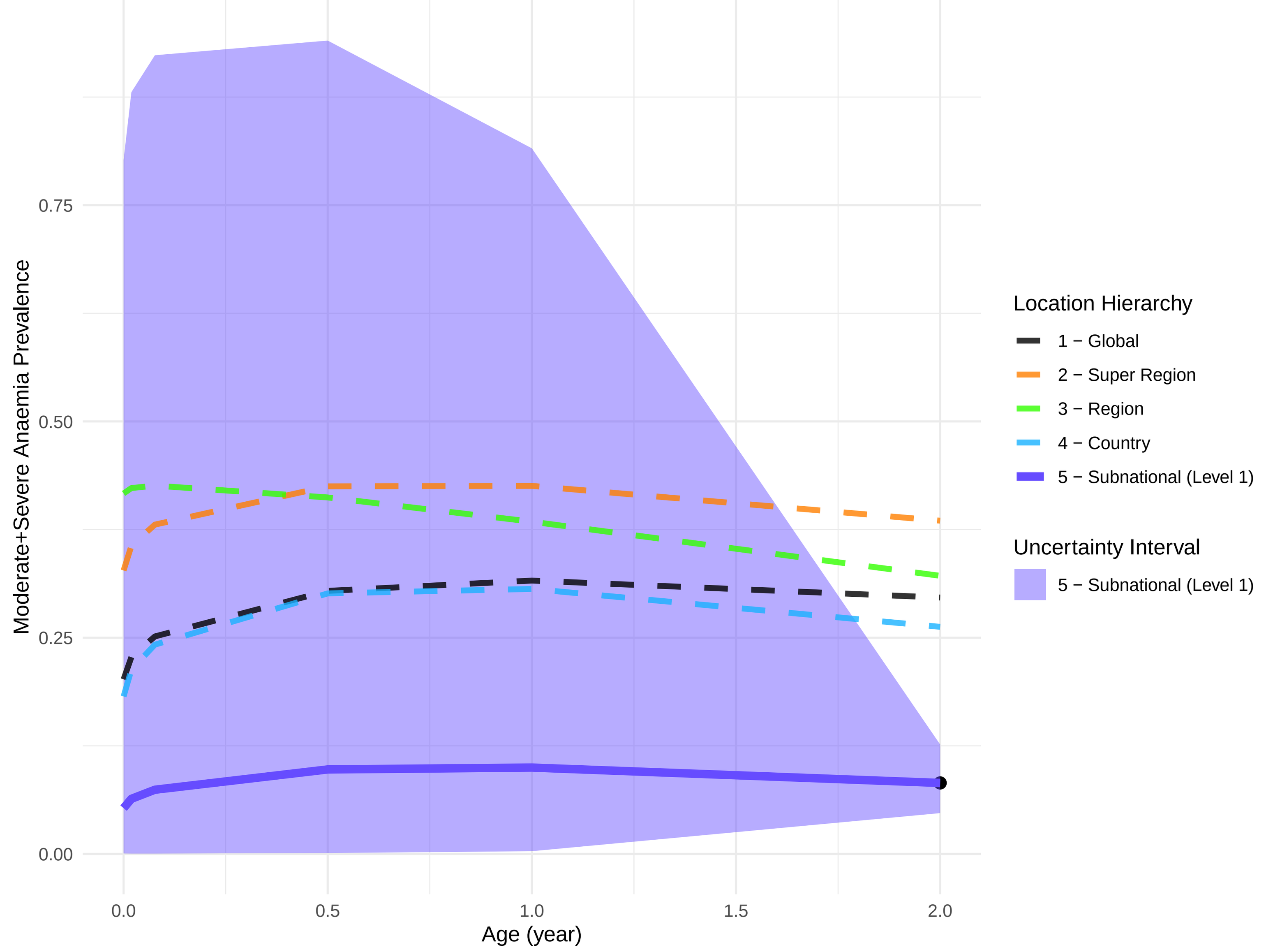

MR−BRT Age/Sex Fits − Non−pregnant Females <2 Years Old − Moderate+Severe Anaemia Prevalence − Nyeri
WHO−2024 Adjustment Method

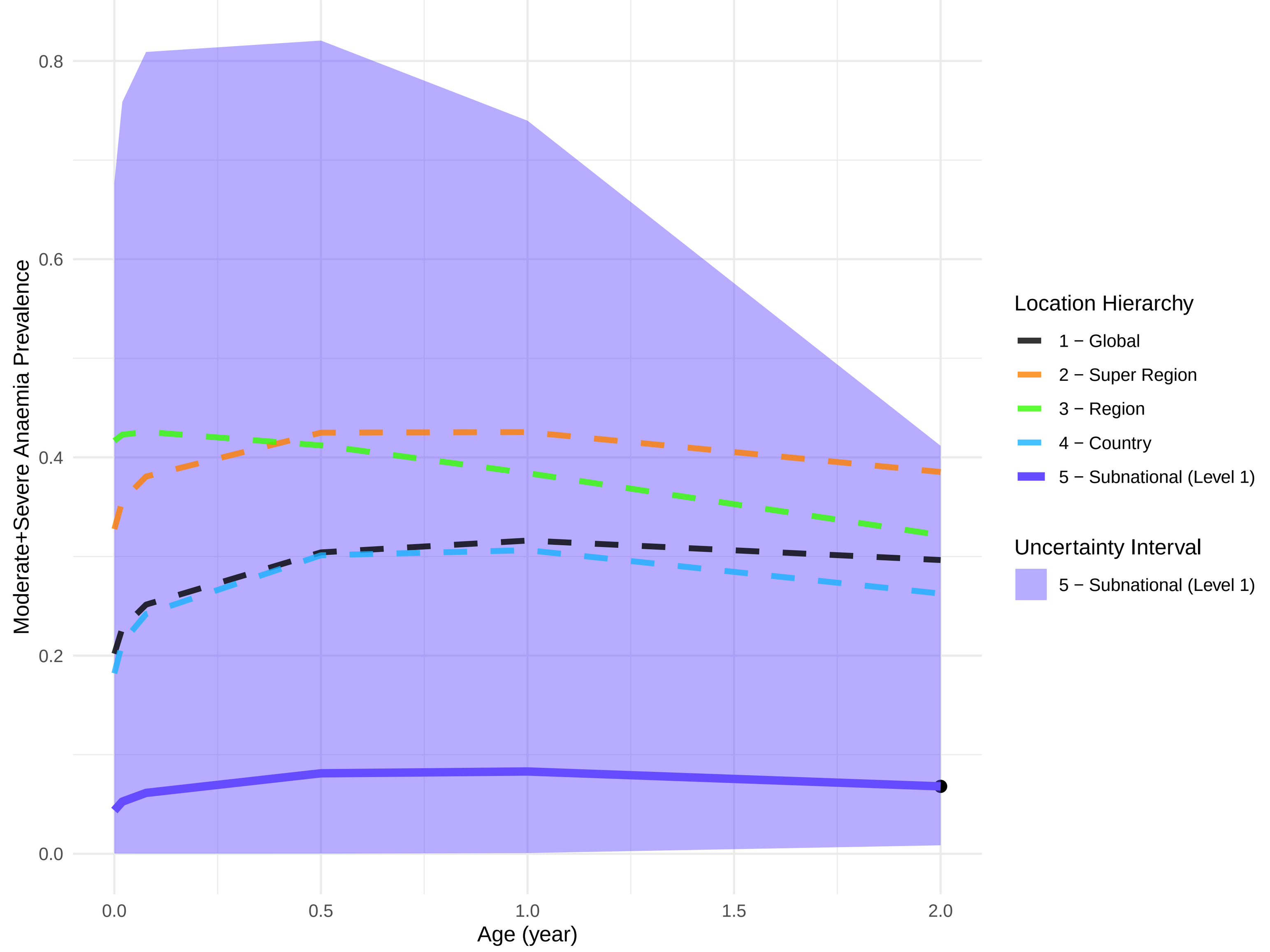

MR−BRT Age/Sex Fits − Non−pregnant Females <2 Years Old − Moderate+Severe Anaemia Prevalence − Samburu
WHO−2024 Adjustment Method

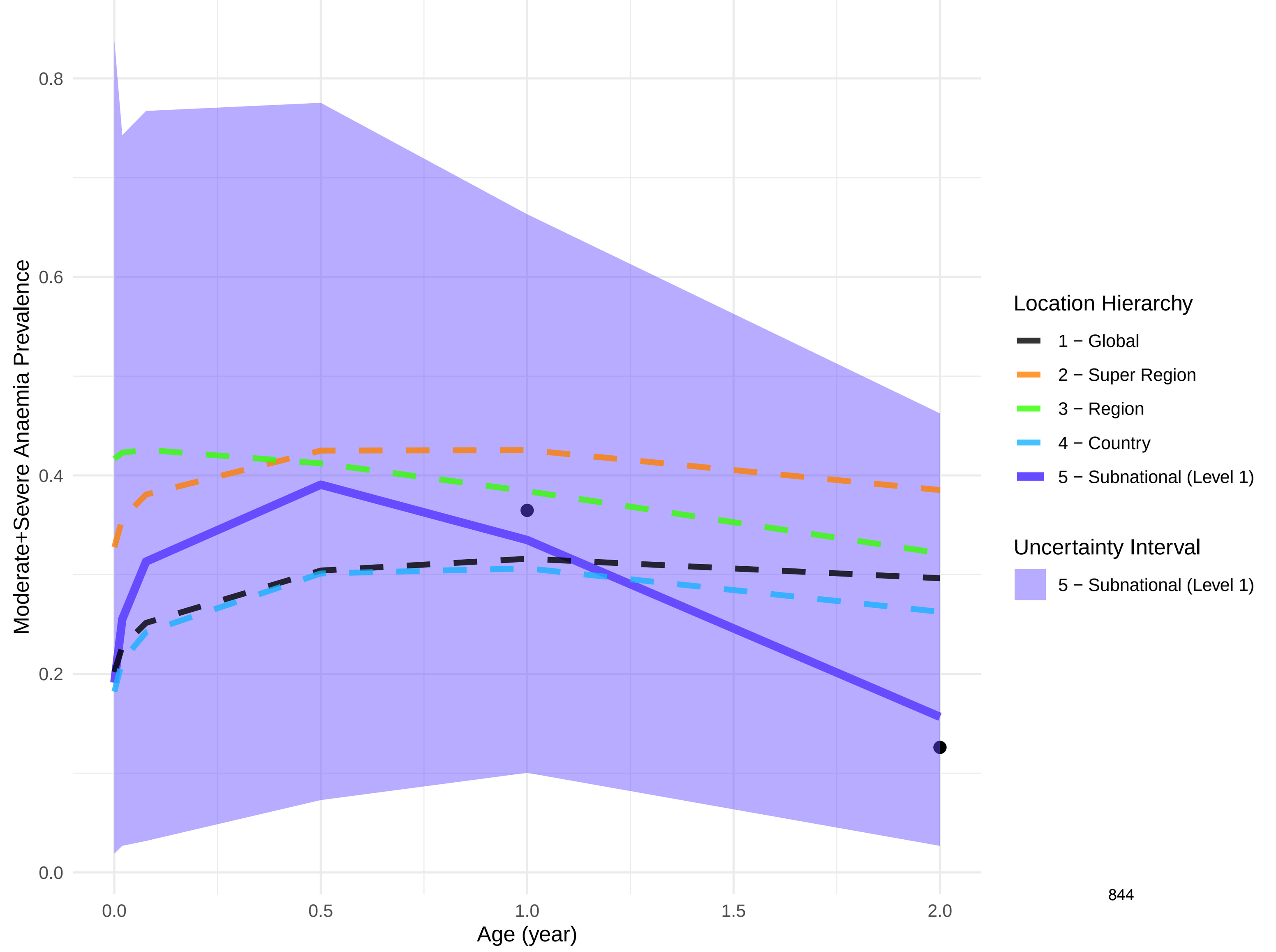

MR−BRT Age/Sex Fits − Non−pregnant Females <2 Years Old − Moderate+Severe Anaemia Prevalence − Siaya
WHO−2024 Adjustment Method

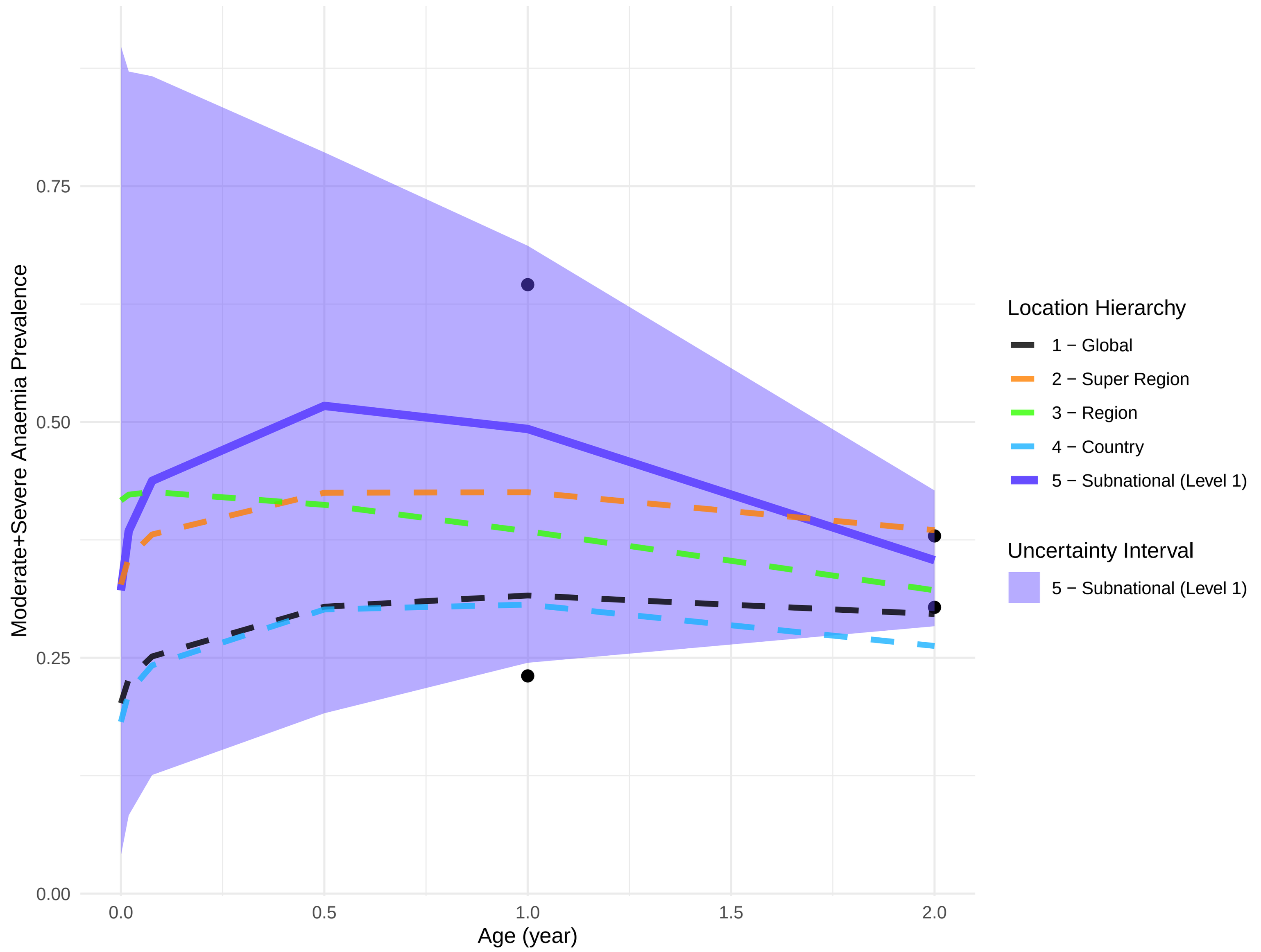

MR−BRT Age/Sex Fits − Non−pregnant Females <2 Years Old − Moderate+Severe Anaemia Prevalence − Taita Taveta
WHO−2024 Adjustment Method

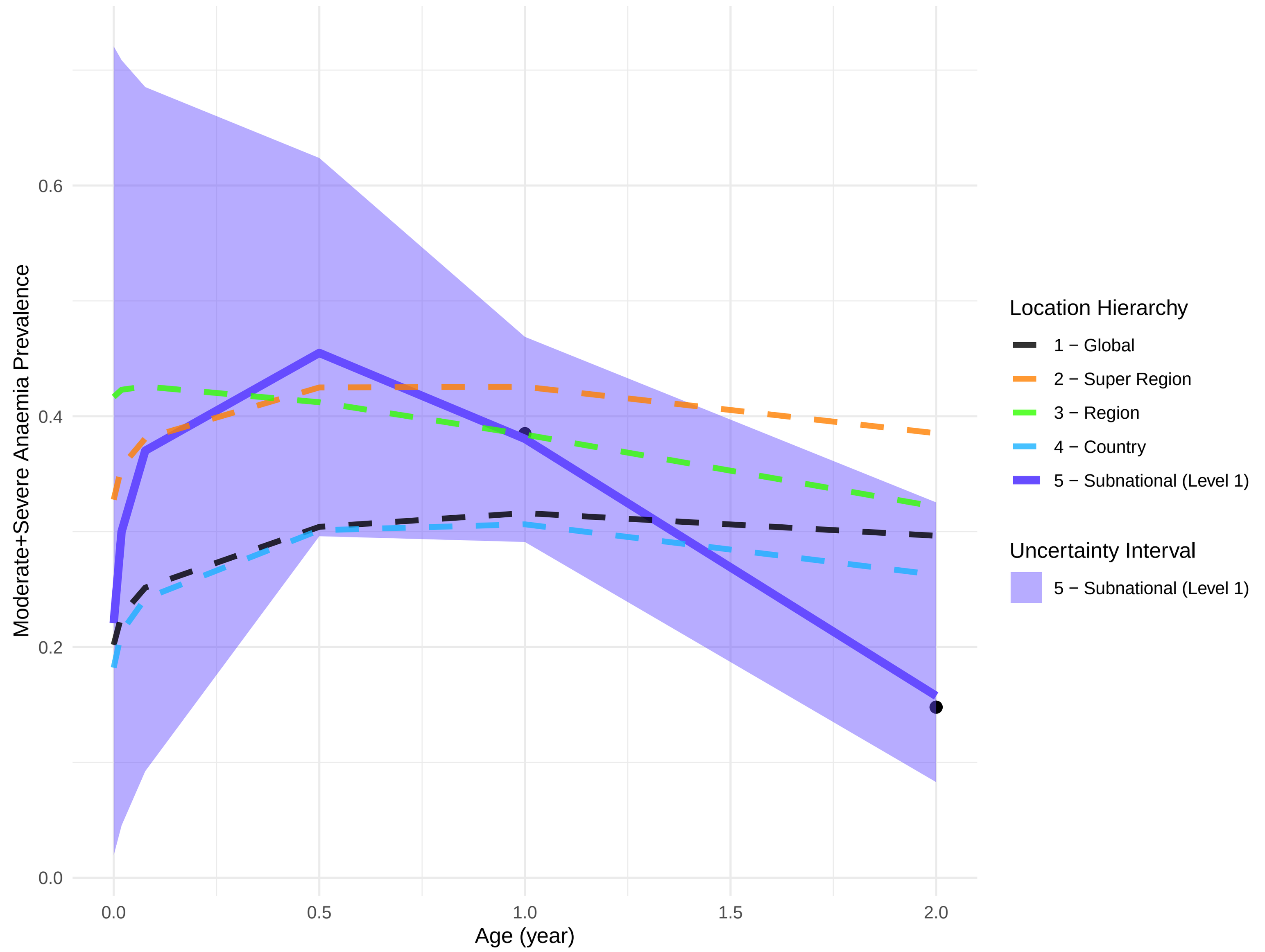

MR−BRT Age/Sex Fits − Non−pregnant Females <2 Years Old − Moderate+Severe Anaemia Prevalence − Tana River
WHO−2024 Adjustment Method

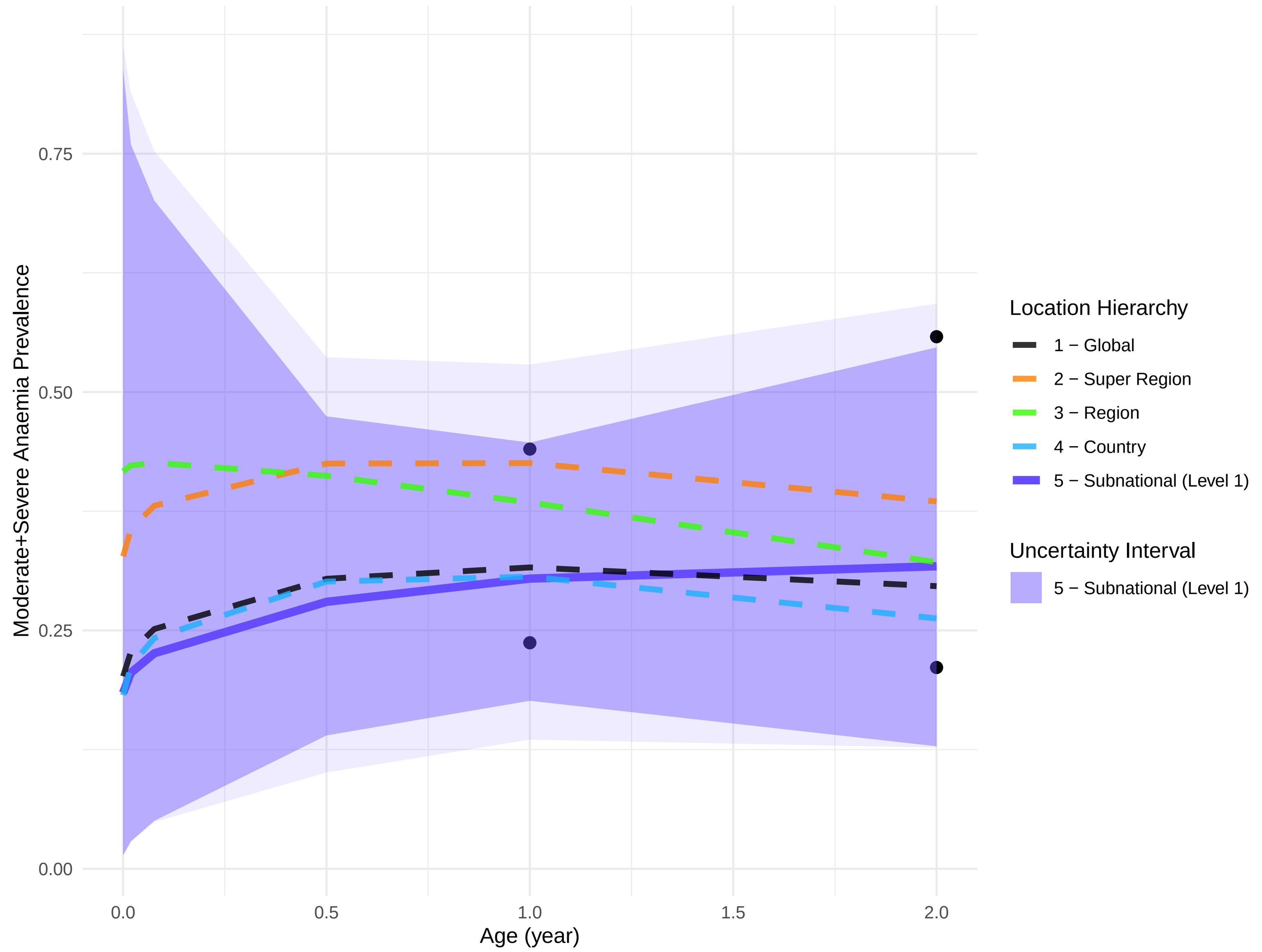

MR−BRT Age/Sex Fits − Non−pregnant Females <2 Years Old − Moderate+Severe Anaemia Prevalence − Tharaka Nithi
WHO−2024 Adjustment Method

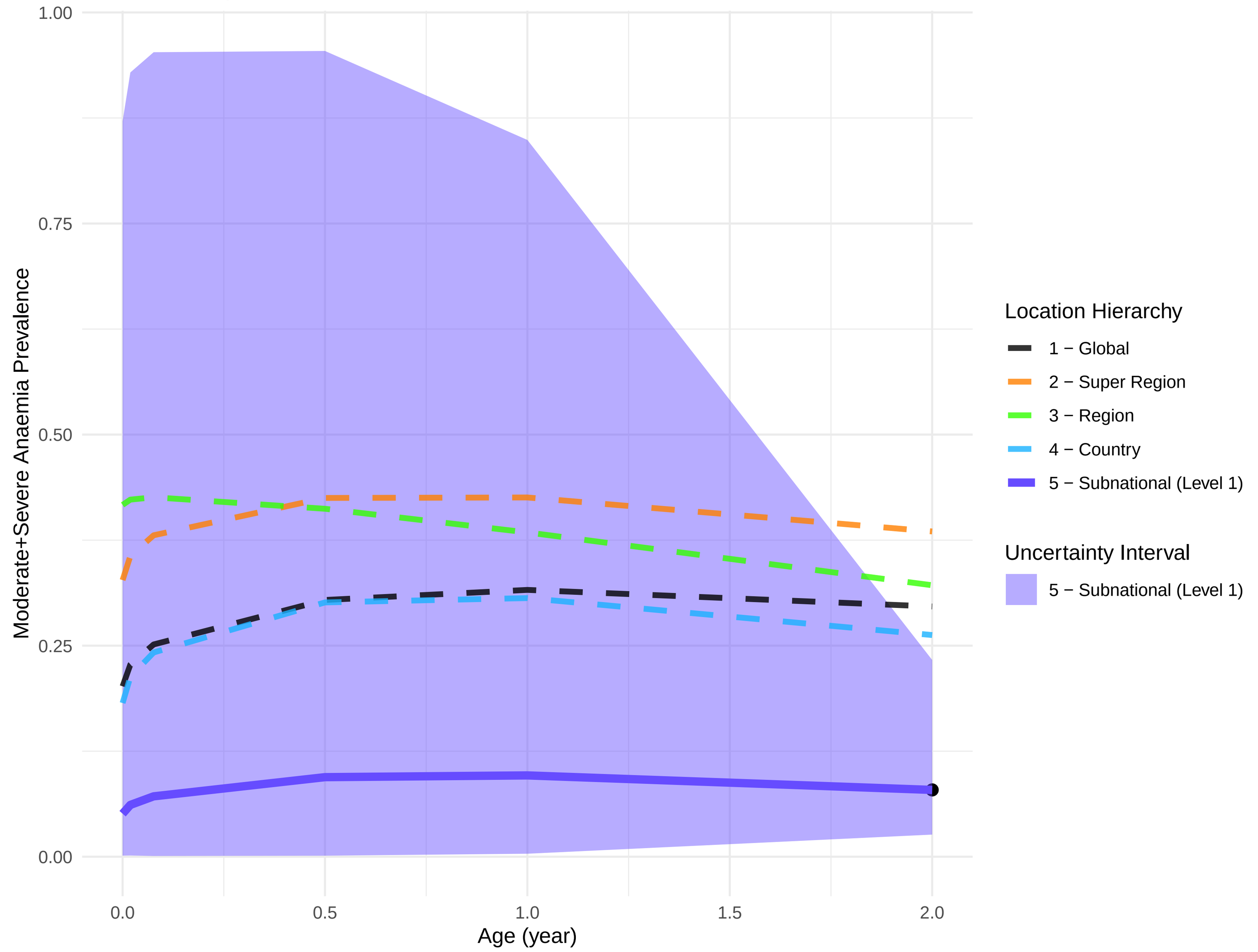

MR−BRT Age/Sex Fits − Non−pregnant Females <2 Years Old − Moderate+Severe Anaemia Prevalence − Trans Nzoia
WHO−2024 Adjustment Method

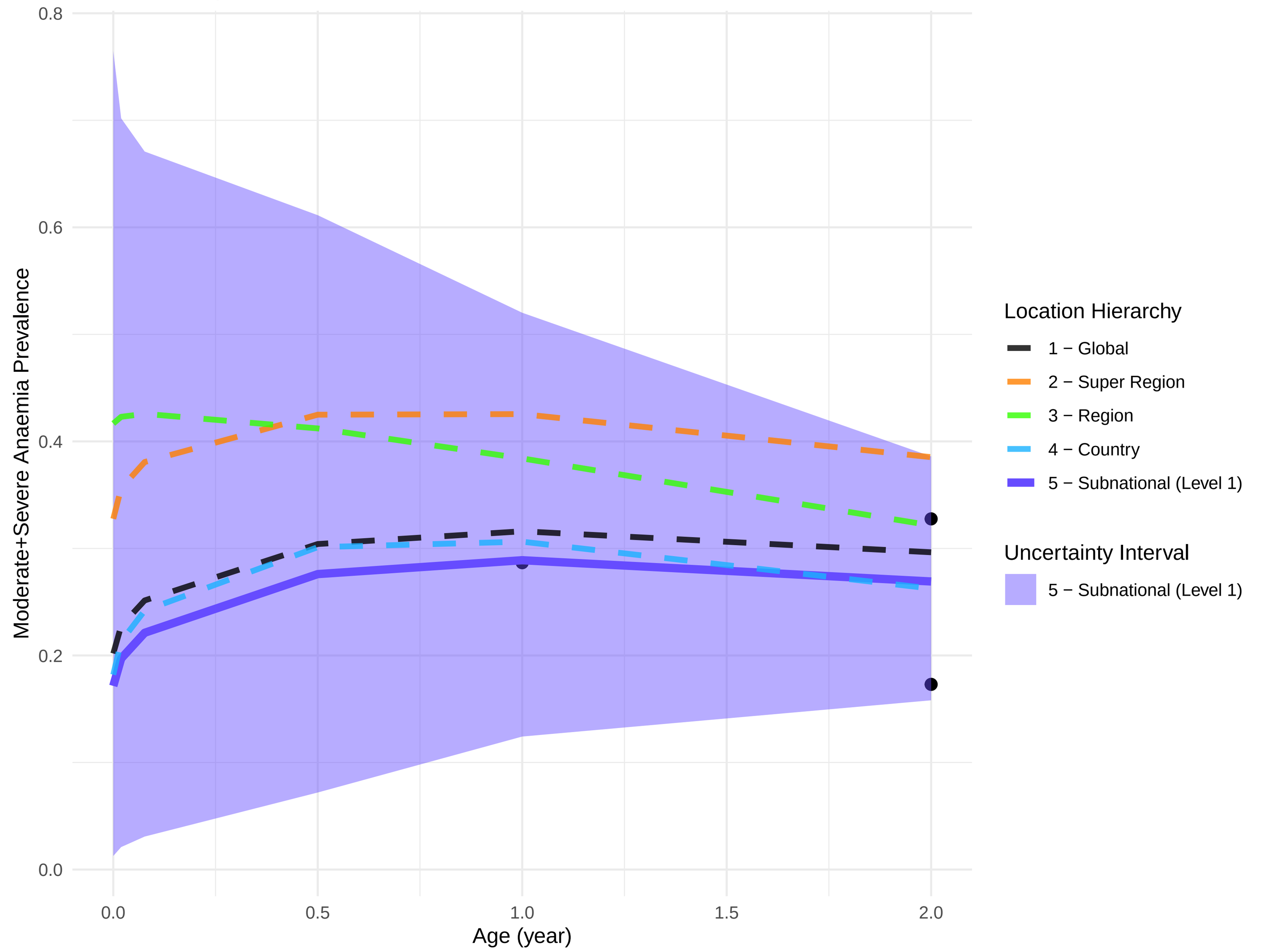

MR−BRT Age/Sex Fits − Non−pregnant Females <2 Years Old − Moderate+Severe Anaemia Prevalence − Turkana
WHO−2024 Adjustment Method

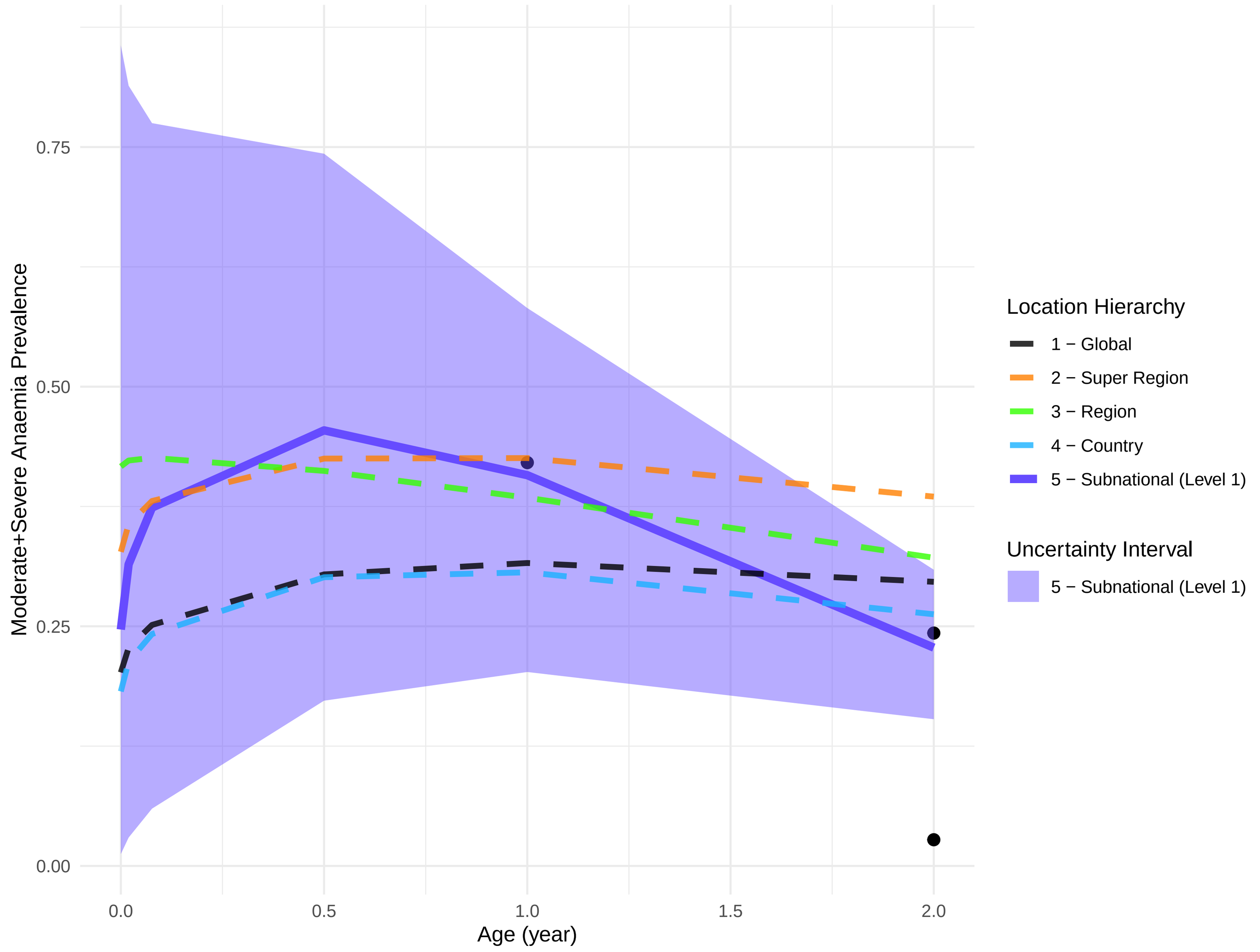

MR−BRT Age/Sex Fits − Non−pregnant Females <2 Years Old − Moderate+Severe Anaemia Prevalence − Uasin Gishu
WHO−2024 Adjustment Method

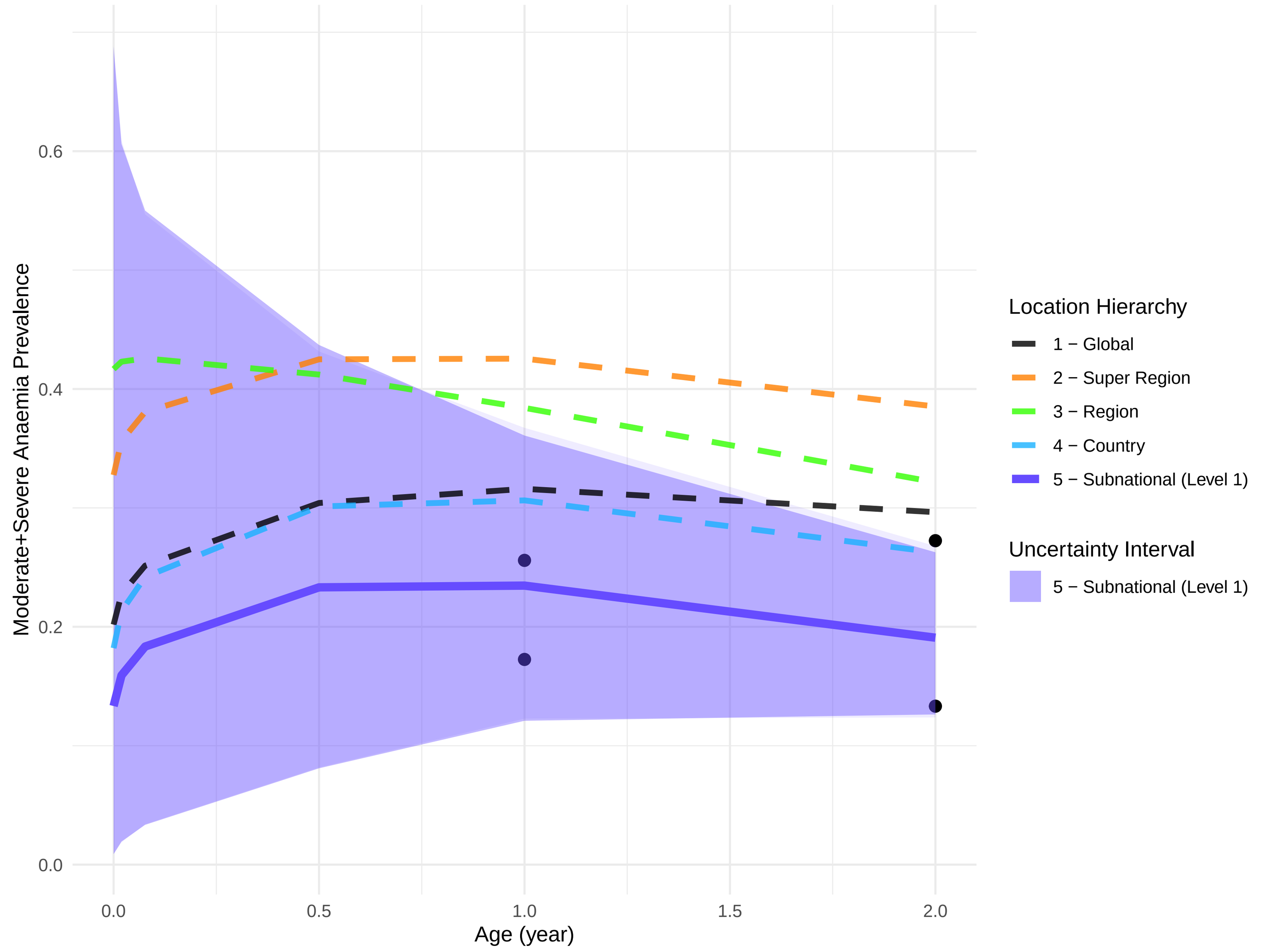

MR−BRT Age/Sex Fits − Non−pregnant Females <2 Years Old − Moderate+Severe Anaemia Prevalence − Vihiga
WHO−2024 Adjustment Method

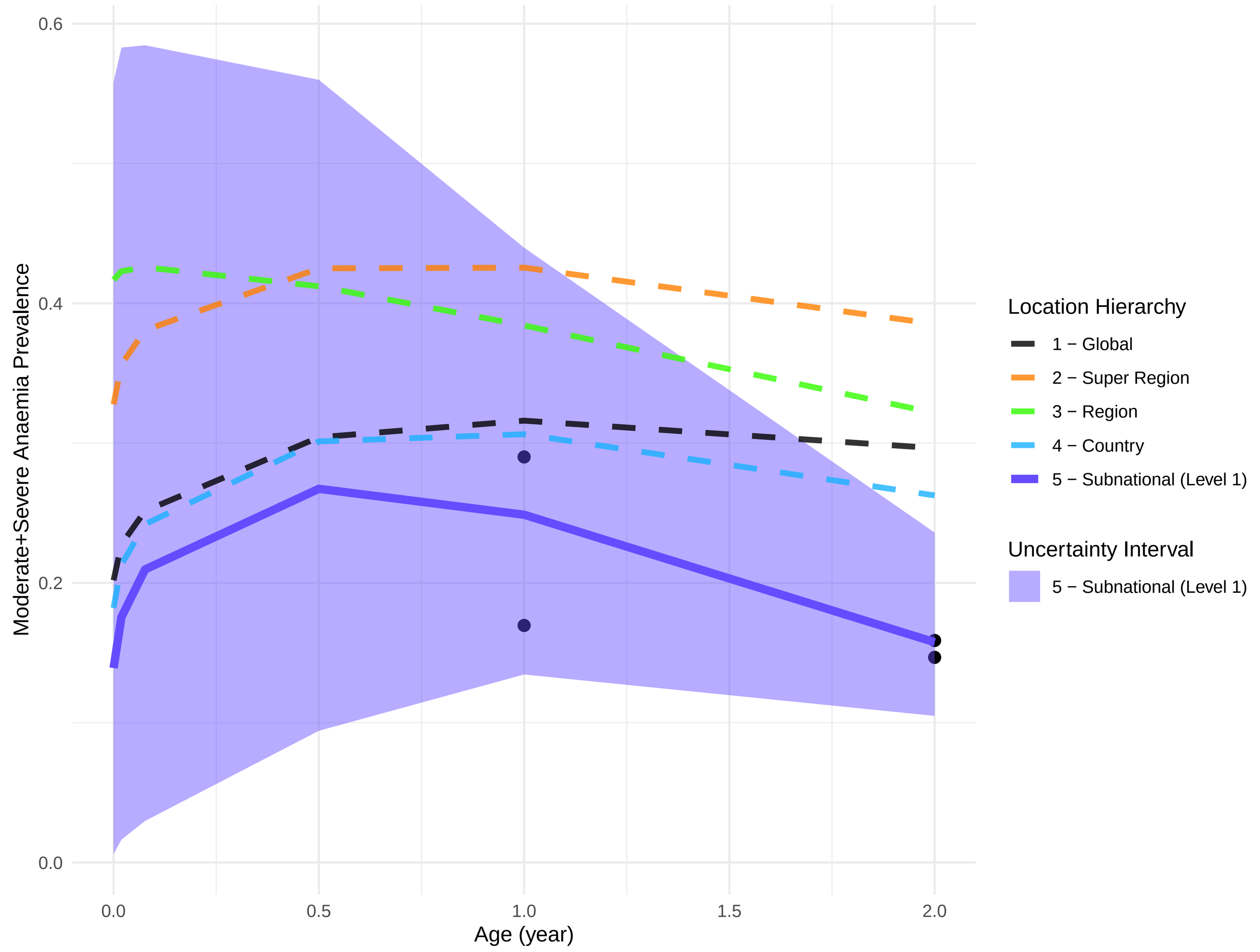

MR−BRT Age/Sex Fits − Non−pregnant Females <2 Years Old − Moderate+Severe Anaemia Prevalence − Wajir
WHO−2024 Adjustment Method

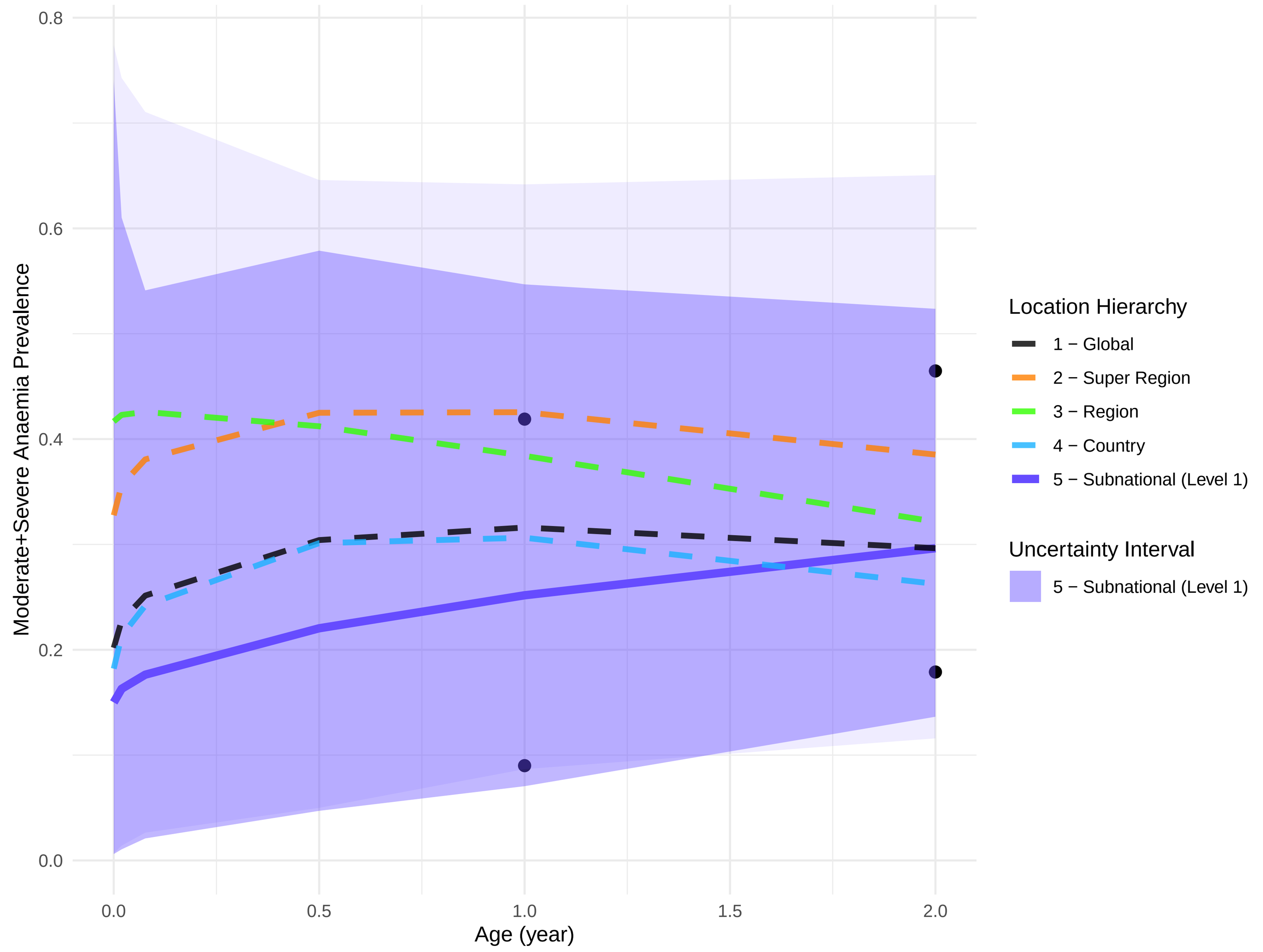

MR−BRT Age/Sex Fits − Non−pregnant Females <2 Years Old − Moderate+Severe Anaemia Prevalence − West Pokot
WHO−2024 Adjustment Method

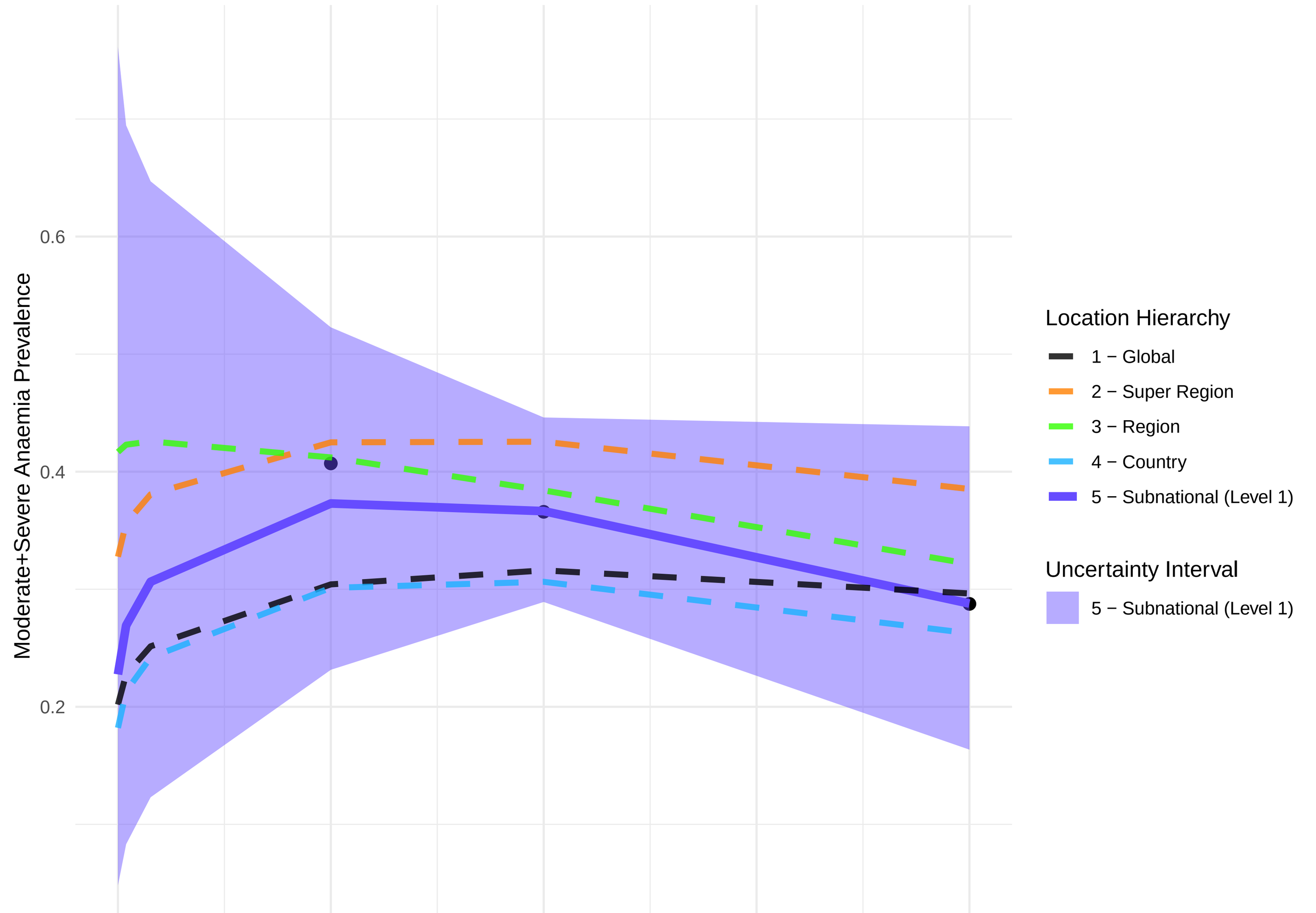

MR−BRT Age/Sex Fits − Non−pregnant Females <2 Years Old − Moderate+Severe Anaemia Prevalence − Madagascar
WHO−2024 Adjustment Method

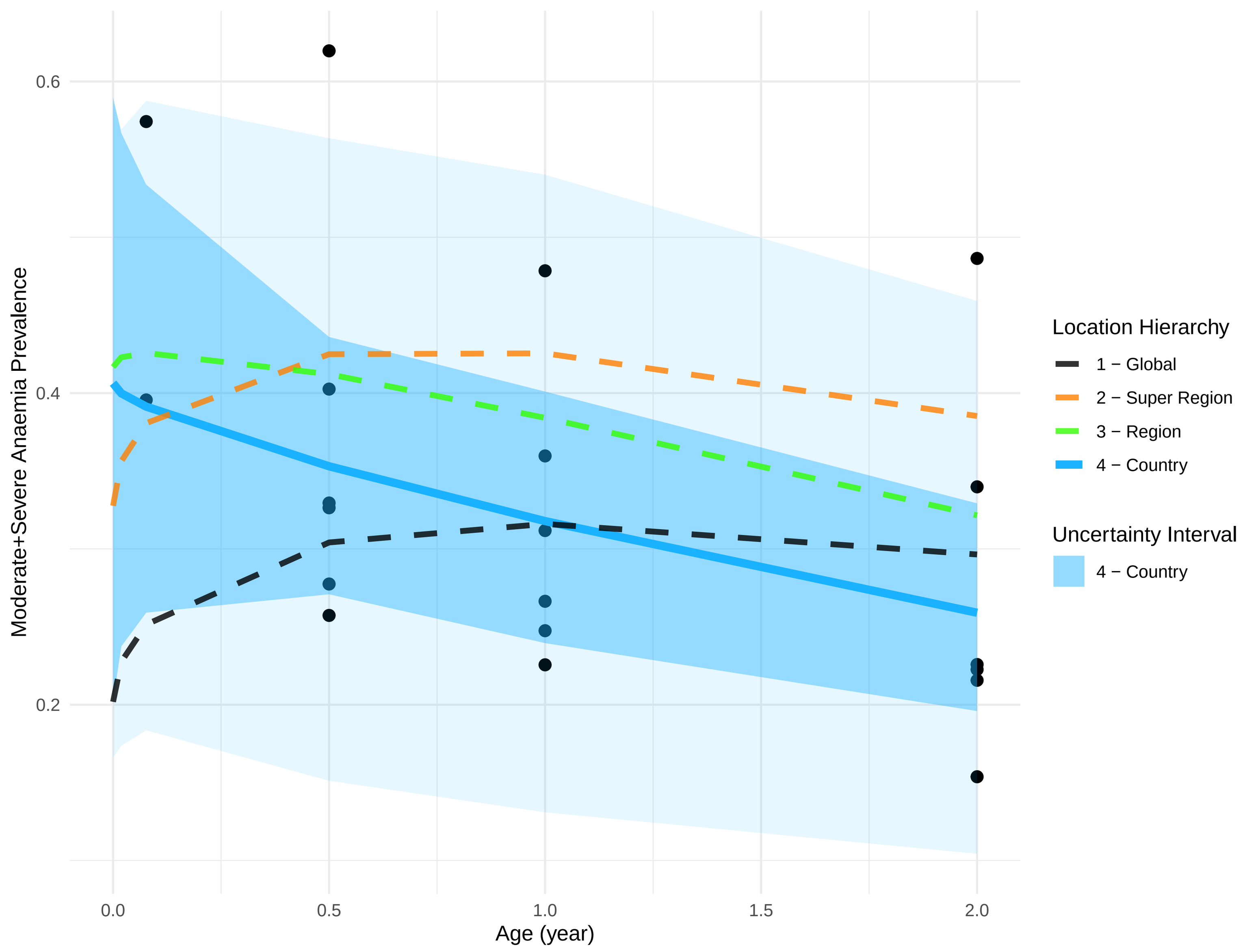

MR−BRT Age/Sex Fits − Non−pregnant Females <2 Years Old − Moderate+Severe Anaemia Prevalence − Malawi
WHO−2024 Adjustment Method

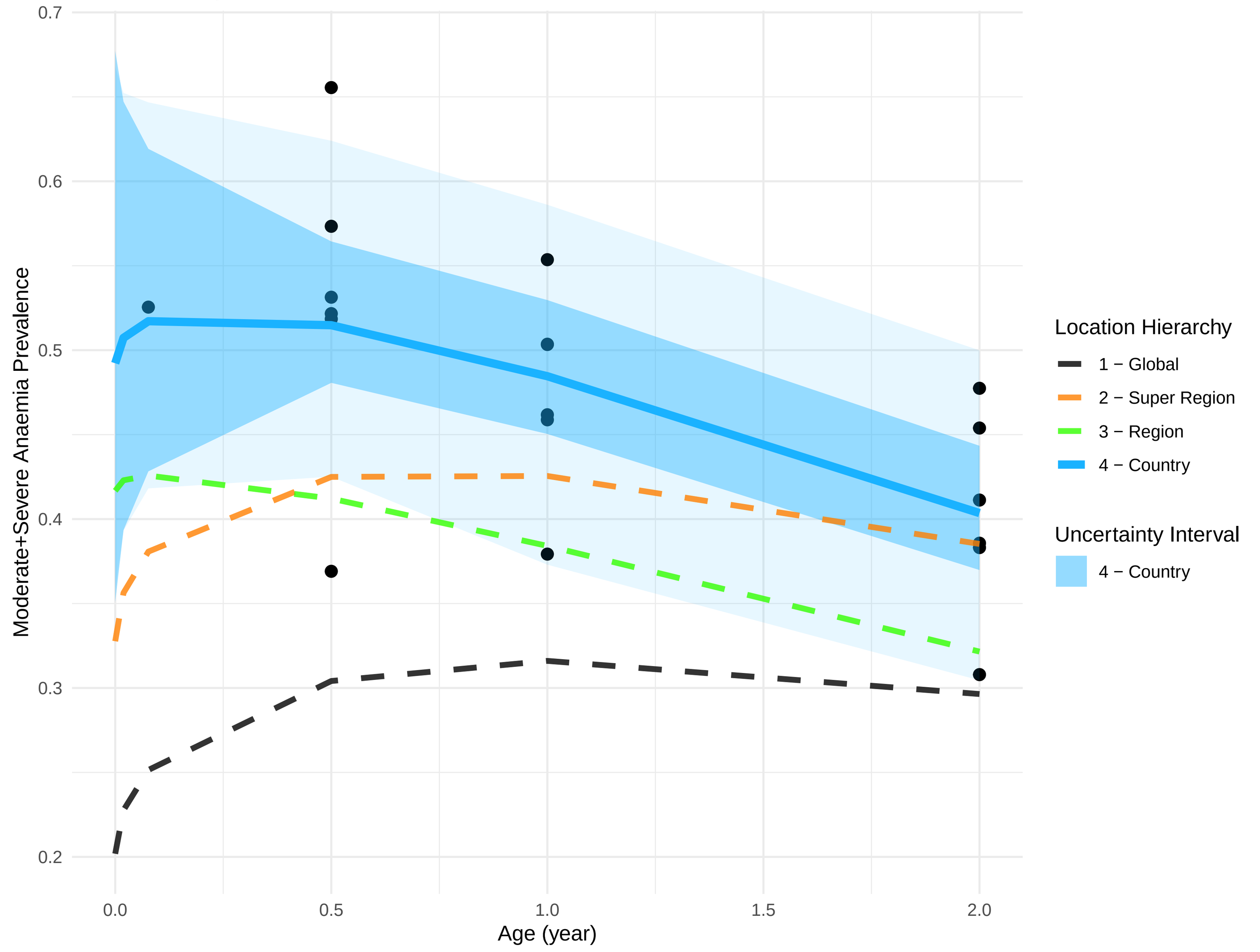

MR−BRT Age/Sex Fits − Non−pregnant Females <2 Years Old − Moderate+Severe Anaemia Prevalence − Mozambique
WHO−2024 Adjustment Method

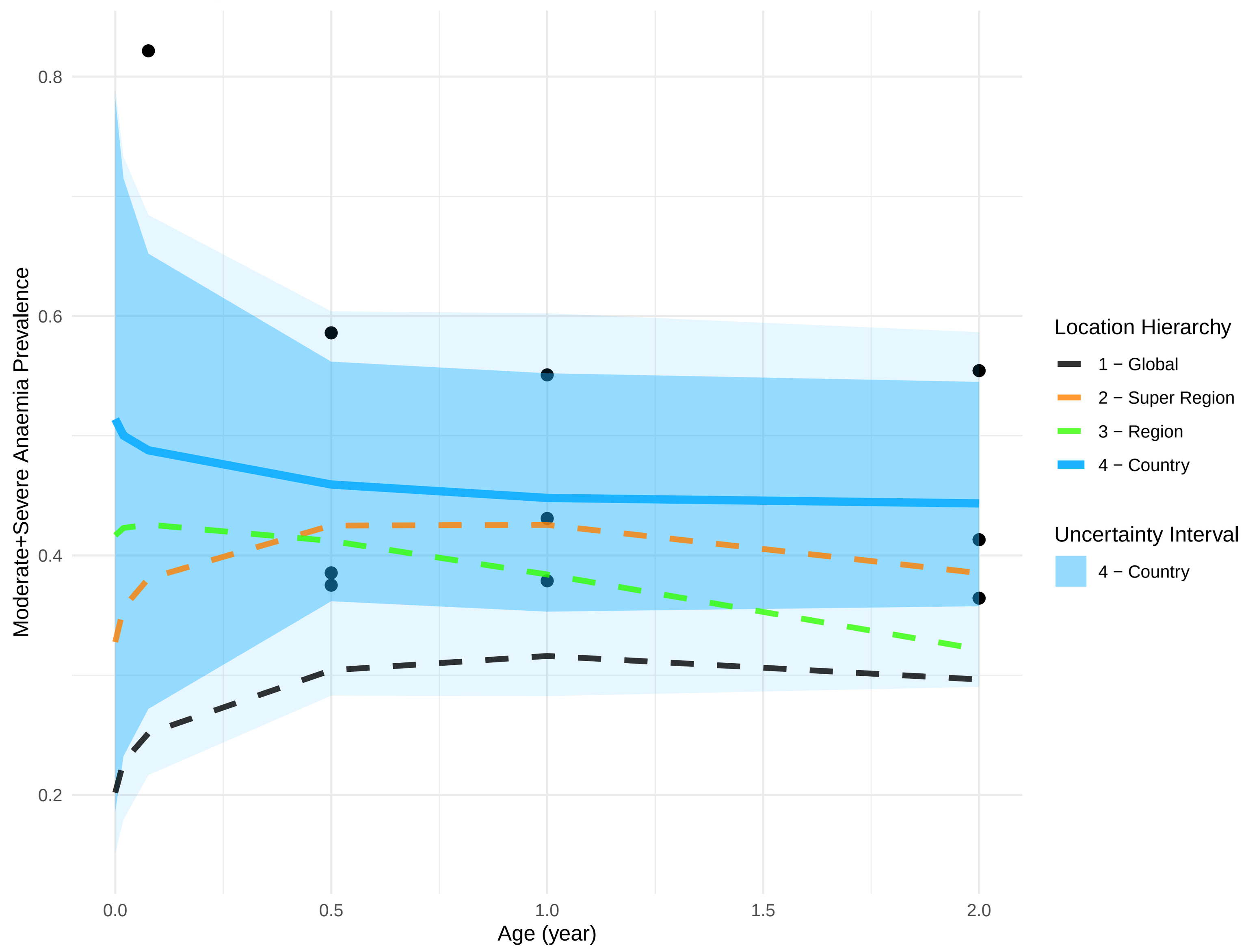

MR−BRT Age/Sex Fits − Non−pregnant Females <2 Years Old − Moderate+Severe Anaemia Prevalence − Rwanda
WHO−2024 Adjustment Method

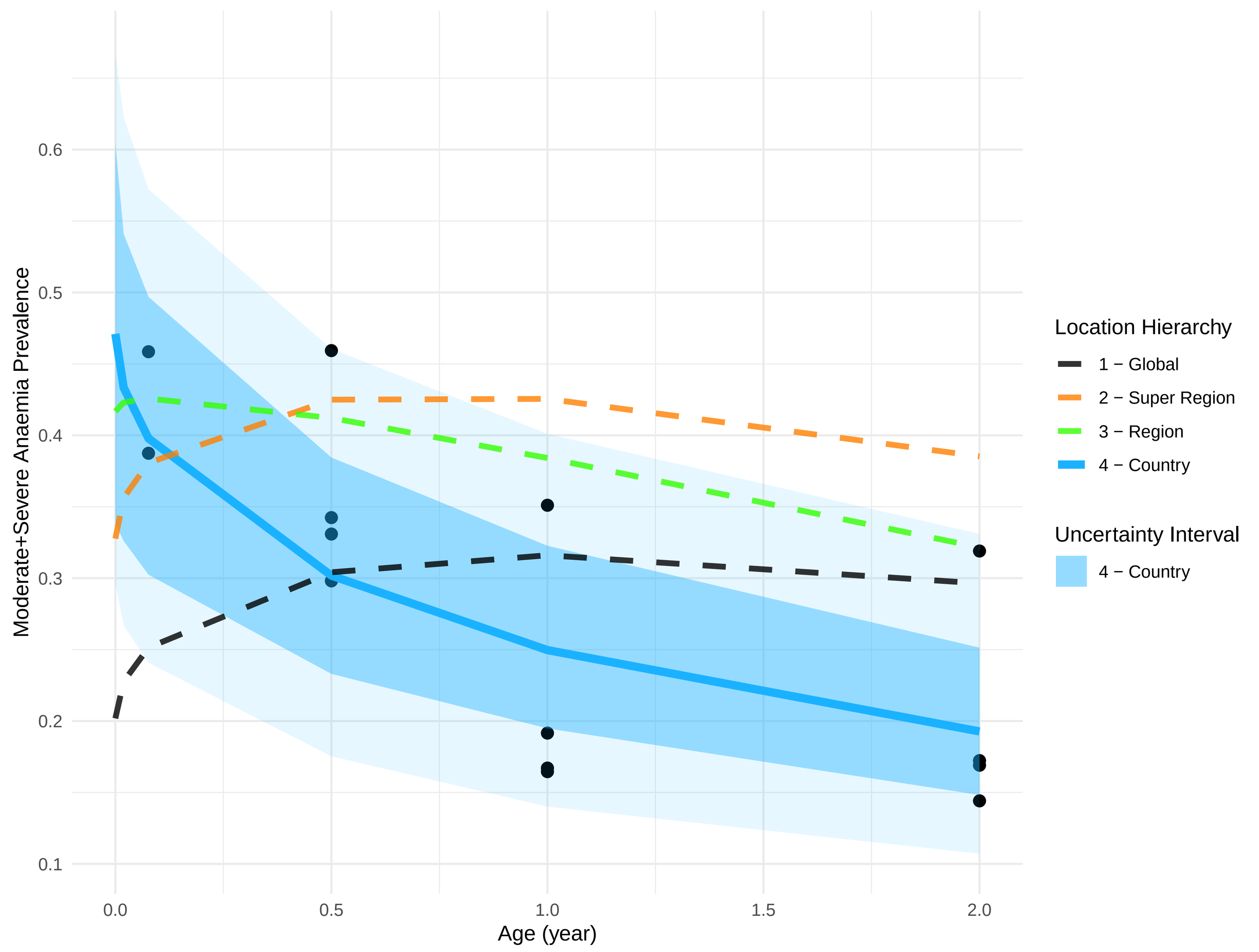

MR−BRT Age/Sex Fits − Non−pregnant Females <2 Years Old − Moderate+Severe Anaemia Prevalence − Uganda
WHO−2024 Adjustment Method

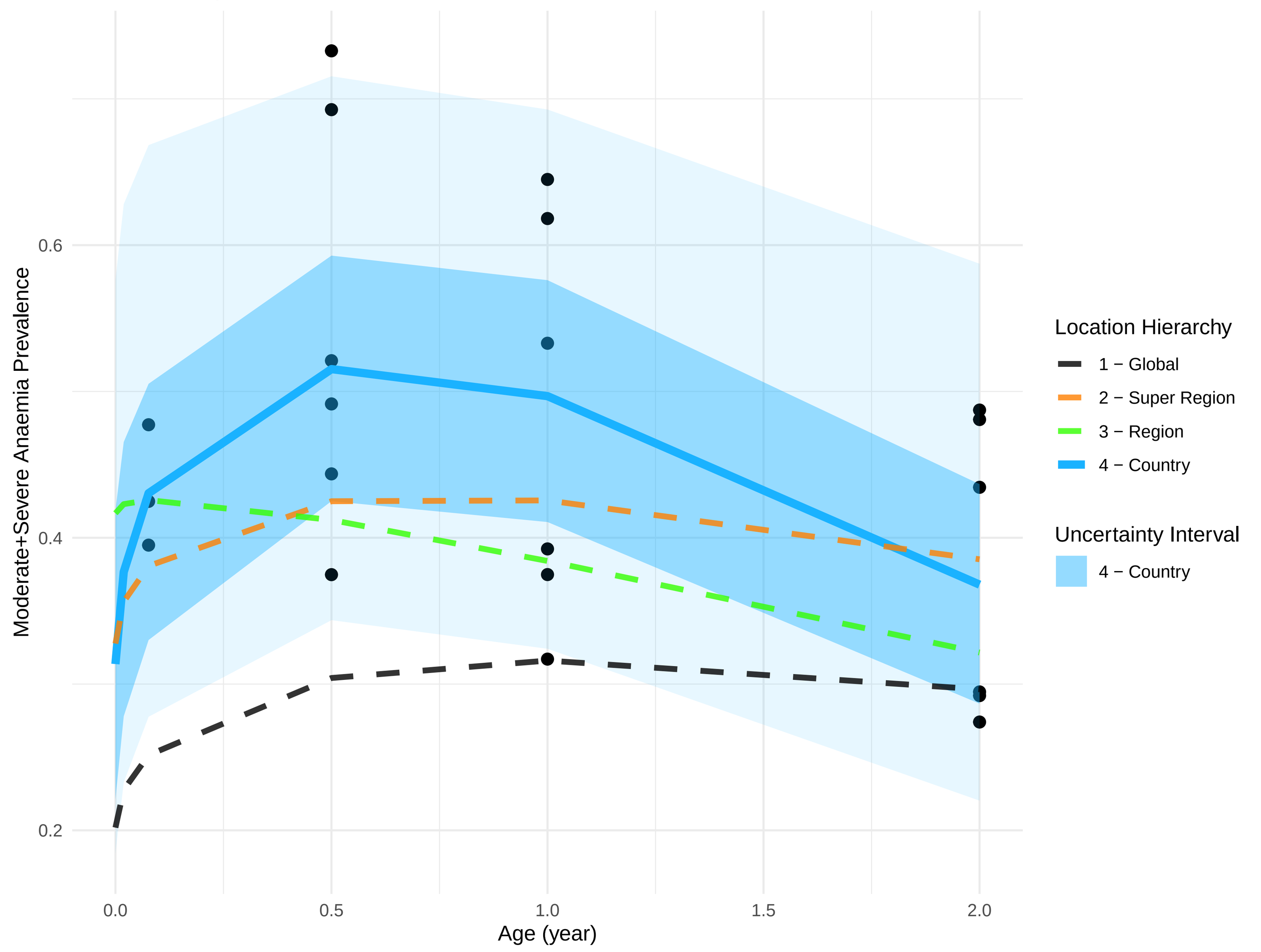

MR−BRT Age/Sex Fits − Non−pregnant Females <2 Years Old − Moderate+Severe Anaemia Prevalence − United Republic of Tanzania
WHO−2024 Adjustment Method

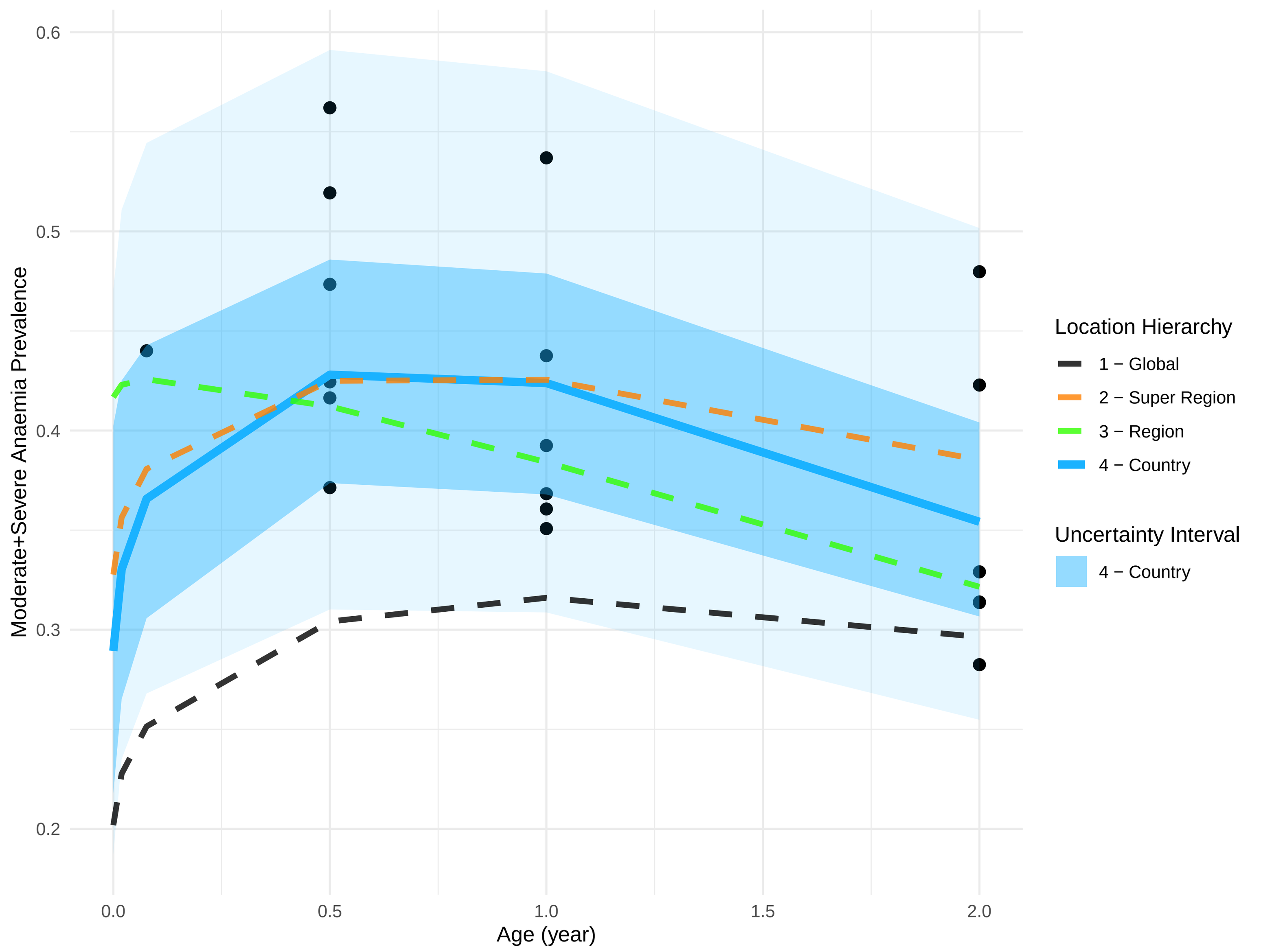

MR−BRT Age/Sex Fits − Non−pregnant Females <2 Years Old − Moderate+Severe Anaemia Prevalence − Zambia
WHO−2024 Adjustment Method

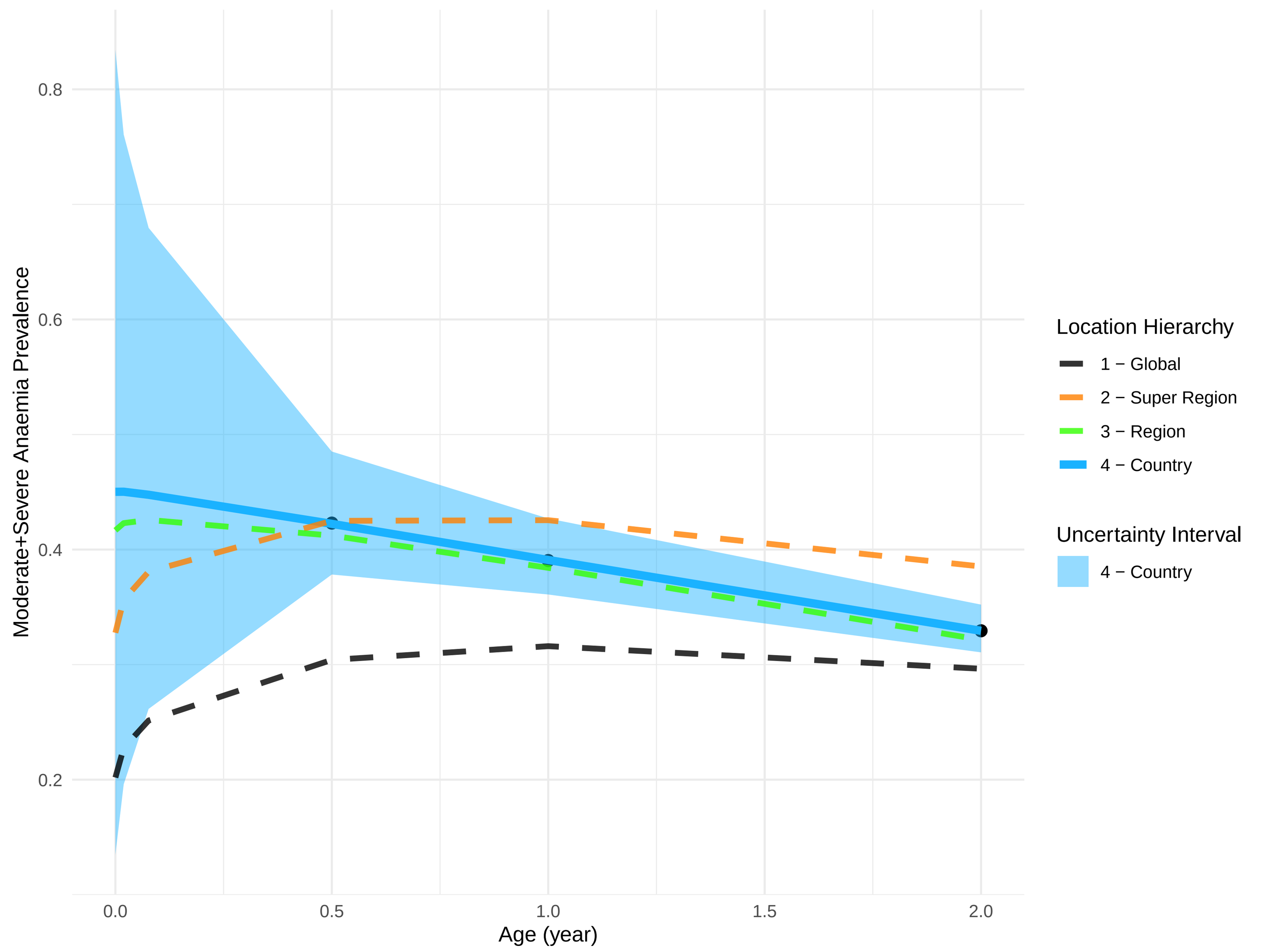

MR−BRT Age/Sex Fits − Non−pregnant Females <2 Years Old − Moderate+Severe Anaemia Prevalence − Southern Sub−Saharan Africa
WHO−2024 Adjustment Method

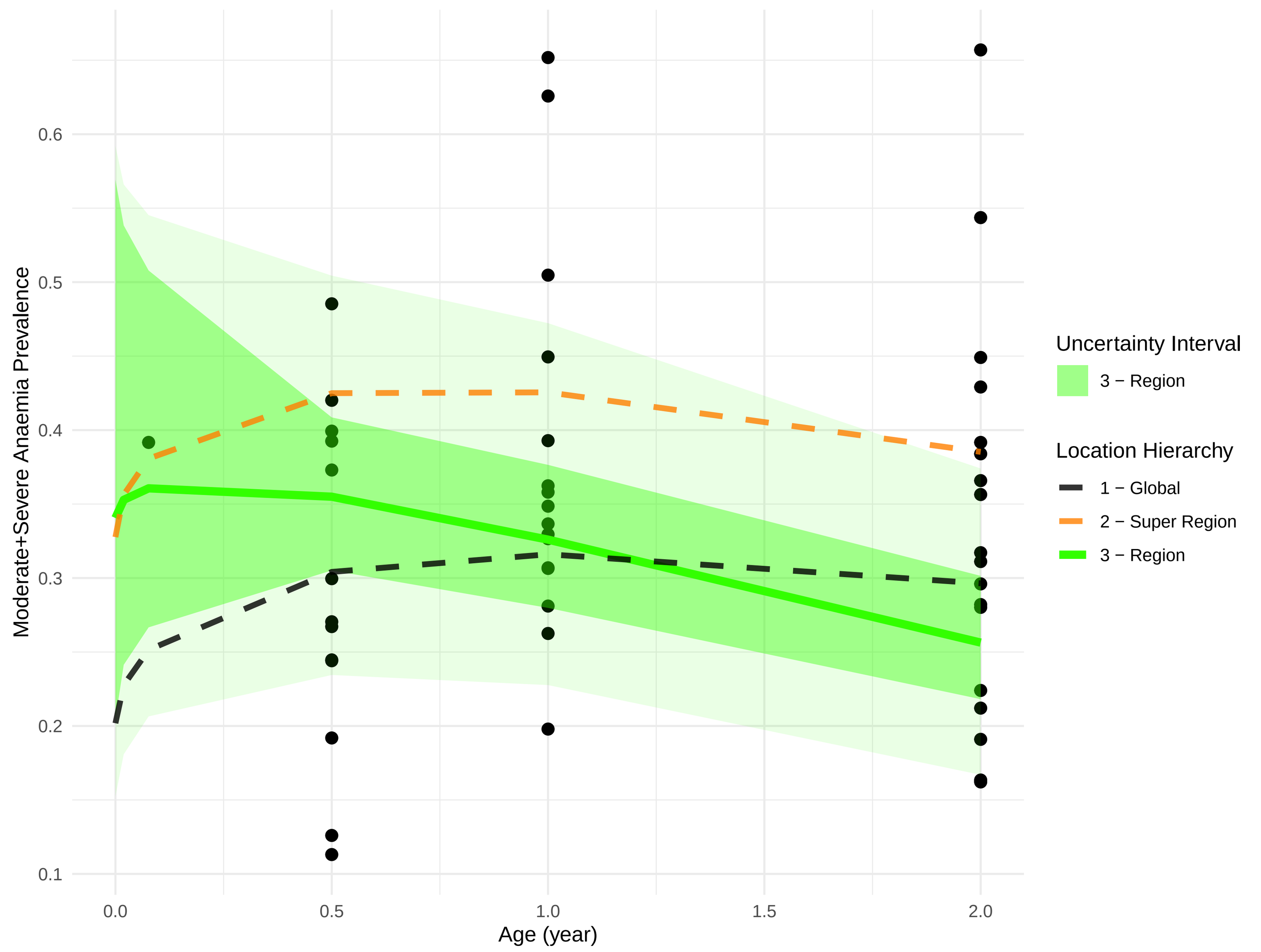

MR−BRT Age/Sex Fits − Non−pregnant Females <2 Years Old − Moderate+Severe Anaemia Prevalence − Eswatini
WHO−2024 Adjustment Method

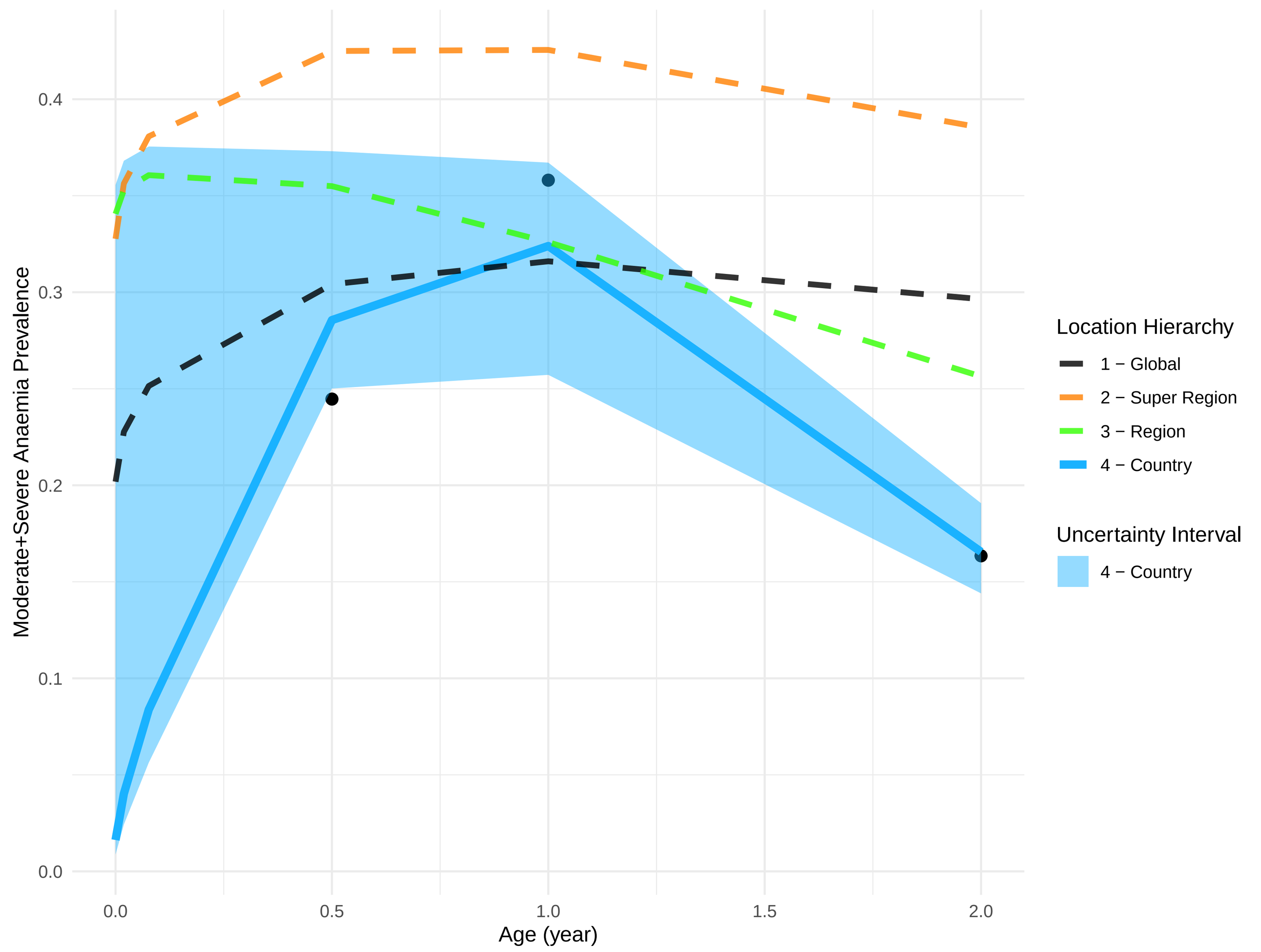

MR−BRT Age/Sex Fits − Non−pregnant Females <2 Years Old − Moderate+Severe Anaemia Prevalence − Lesotho
WHO−2024 Adjustment Method

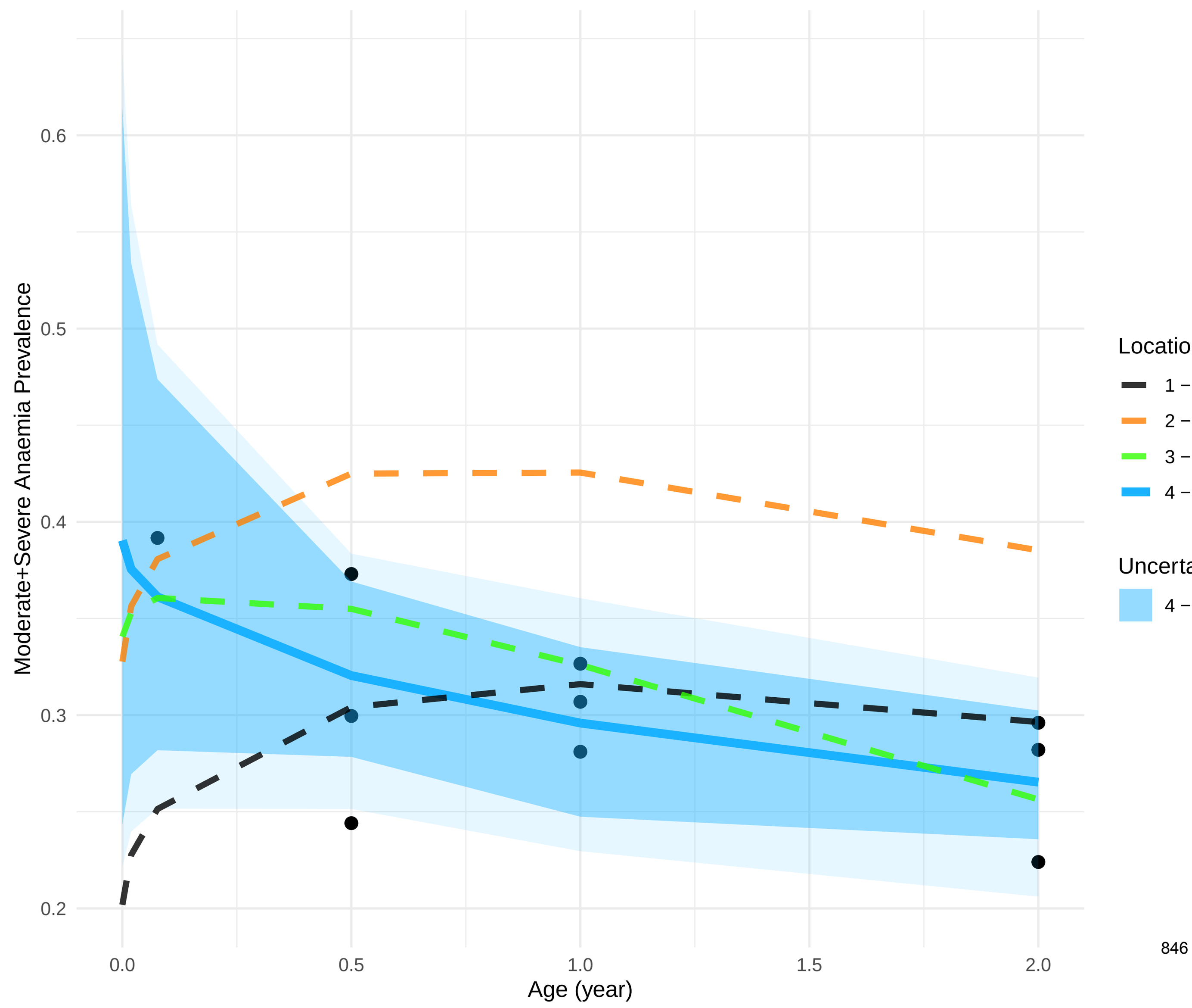

MR−BRT Age/Sex Fits − Non−pregnant Females <2 Years Old − Moderate+Severe Anaemia Prevalence − Namibia
WHO−2024 Adjustment Method

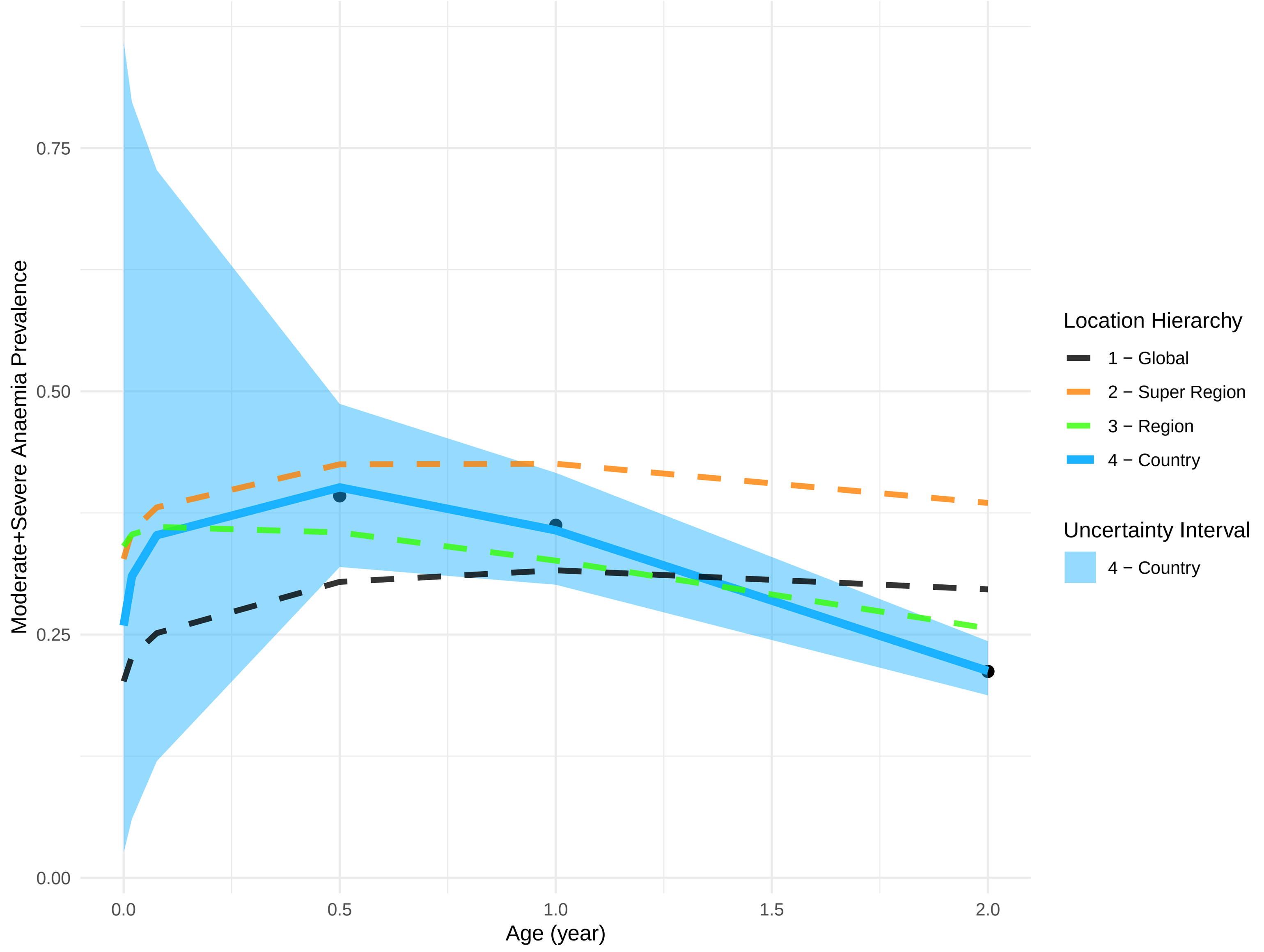

MR−BRT Age/Sex Fits − Non−pregnant Females <2 Years Old − Moderate+Severe Anaemia Prevalence − South Africa
WHO−2024 Adjustment Method

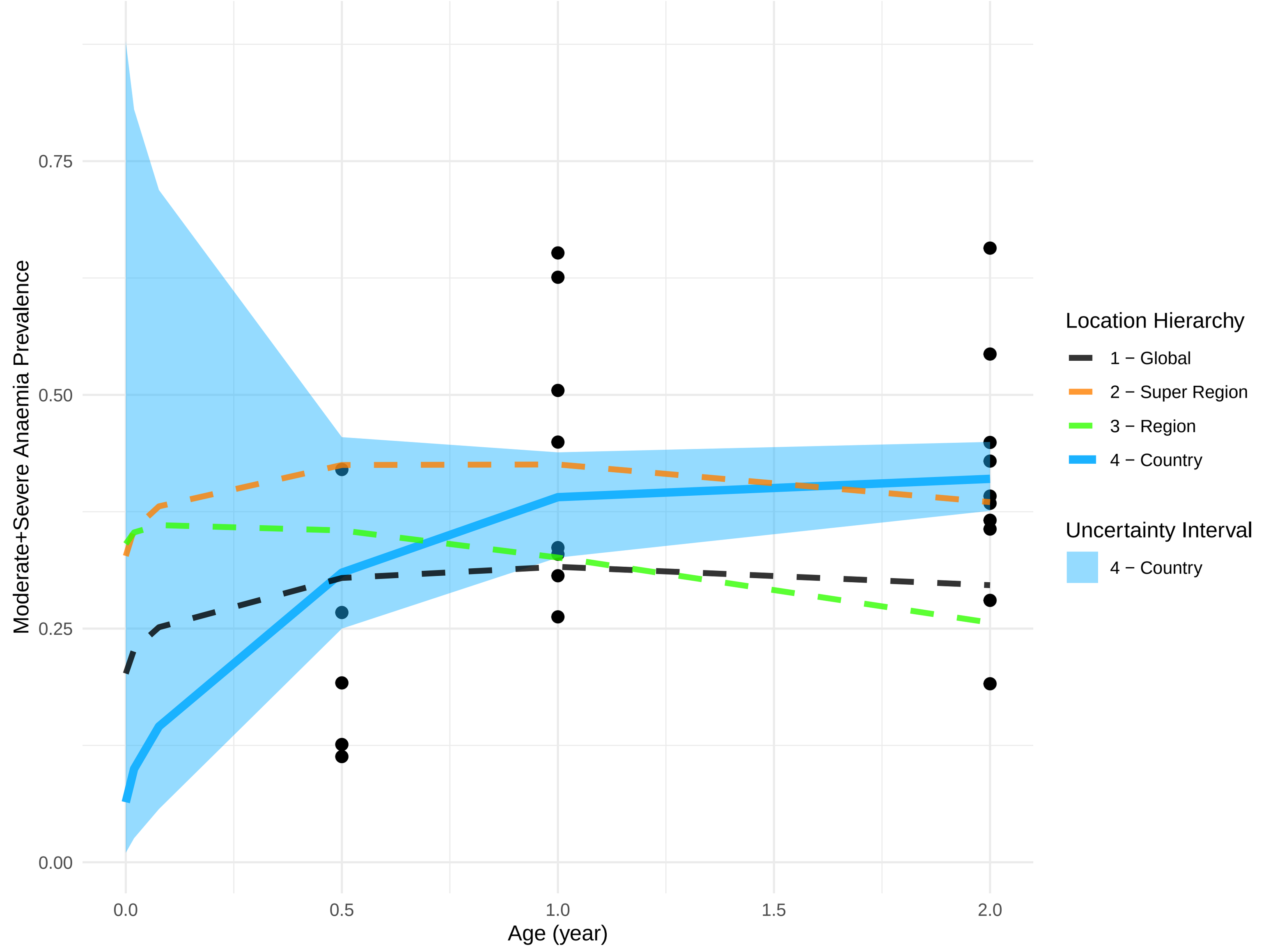

MR−BRT Age/Sex Fits − Non−pregnant Females <2 Years Old − Moderate+Severe Anaemia Prevalence − Eastern Cape
WHO−2024 Adjustment Method

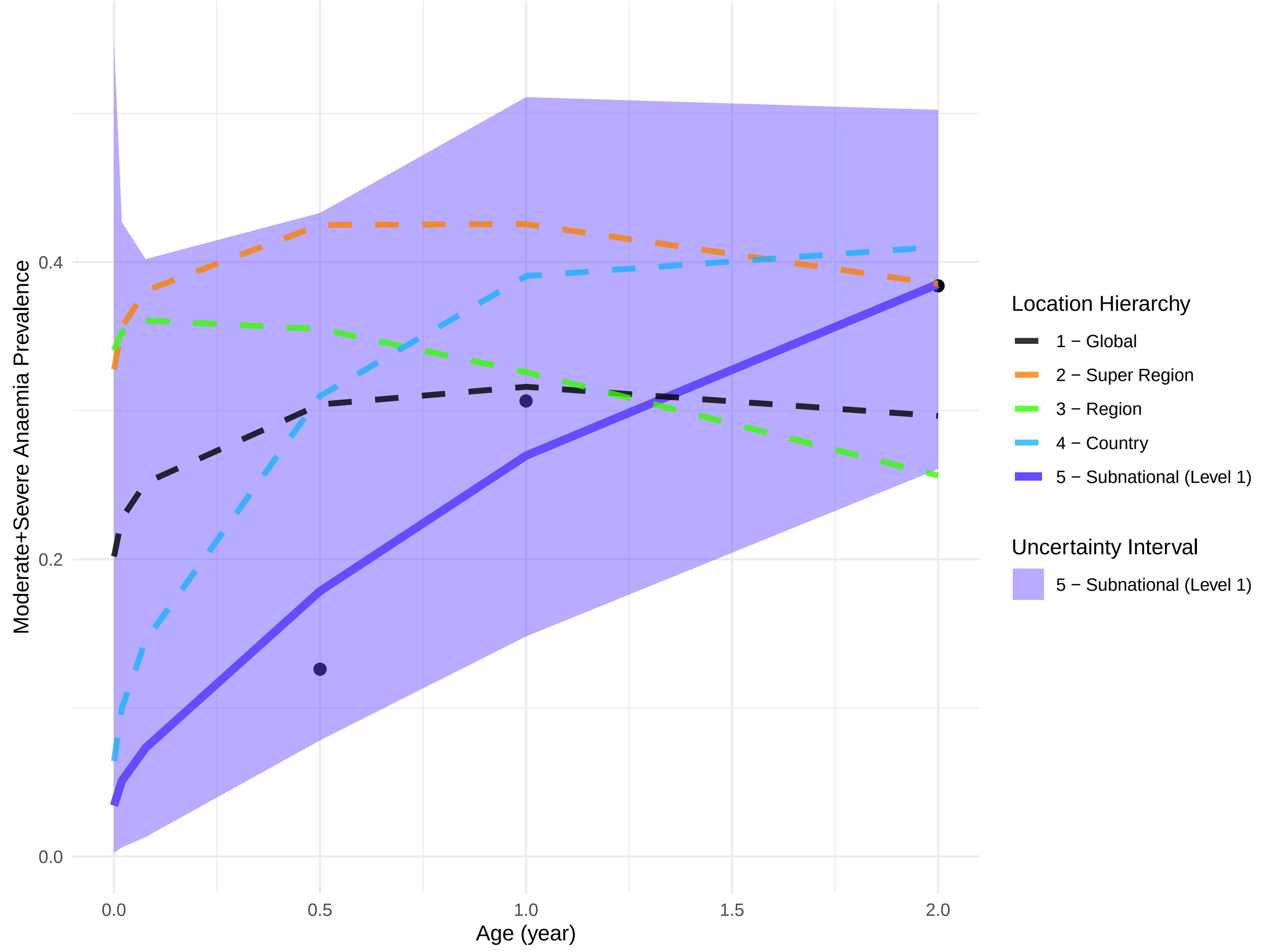

MR−BRT Age/Sex Fits − Non−pregnant Females <2 Years Old − Moderate+Severe Anaemia Prevalence − Free State
WHO−2024 Adjustment Method

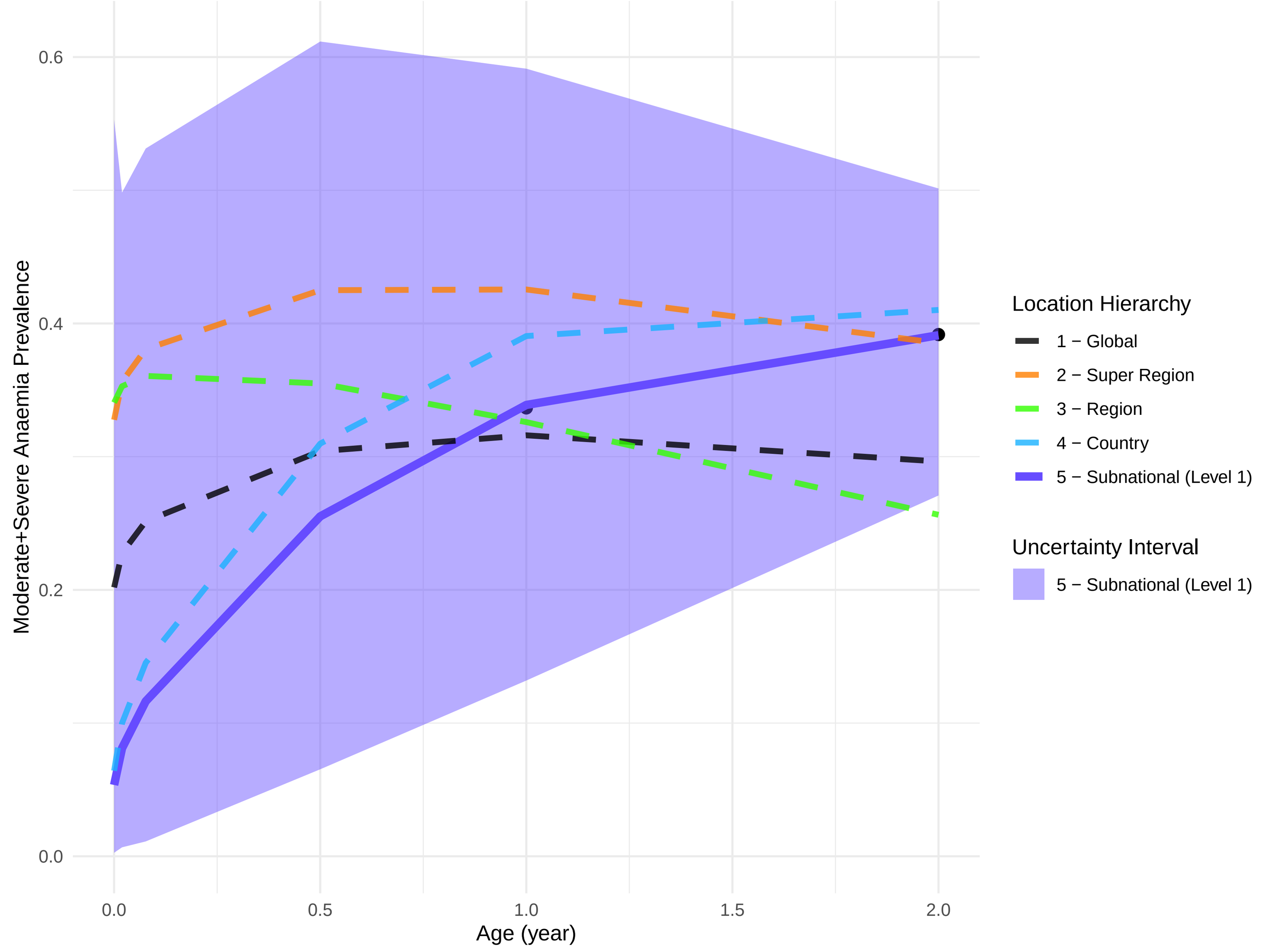

MR−BRT Age/Sex Fits − Non−pregnant Females <2 Years Old − Moderate+Severe Anaemia Prevalence − Gauteng
WHO−2024 Adjustment Method

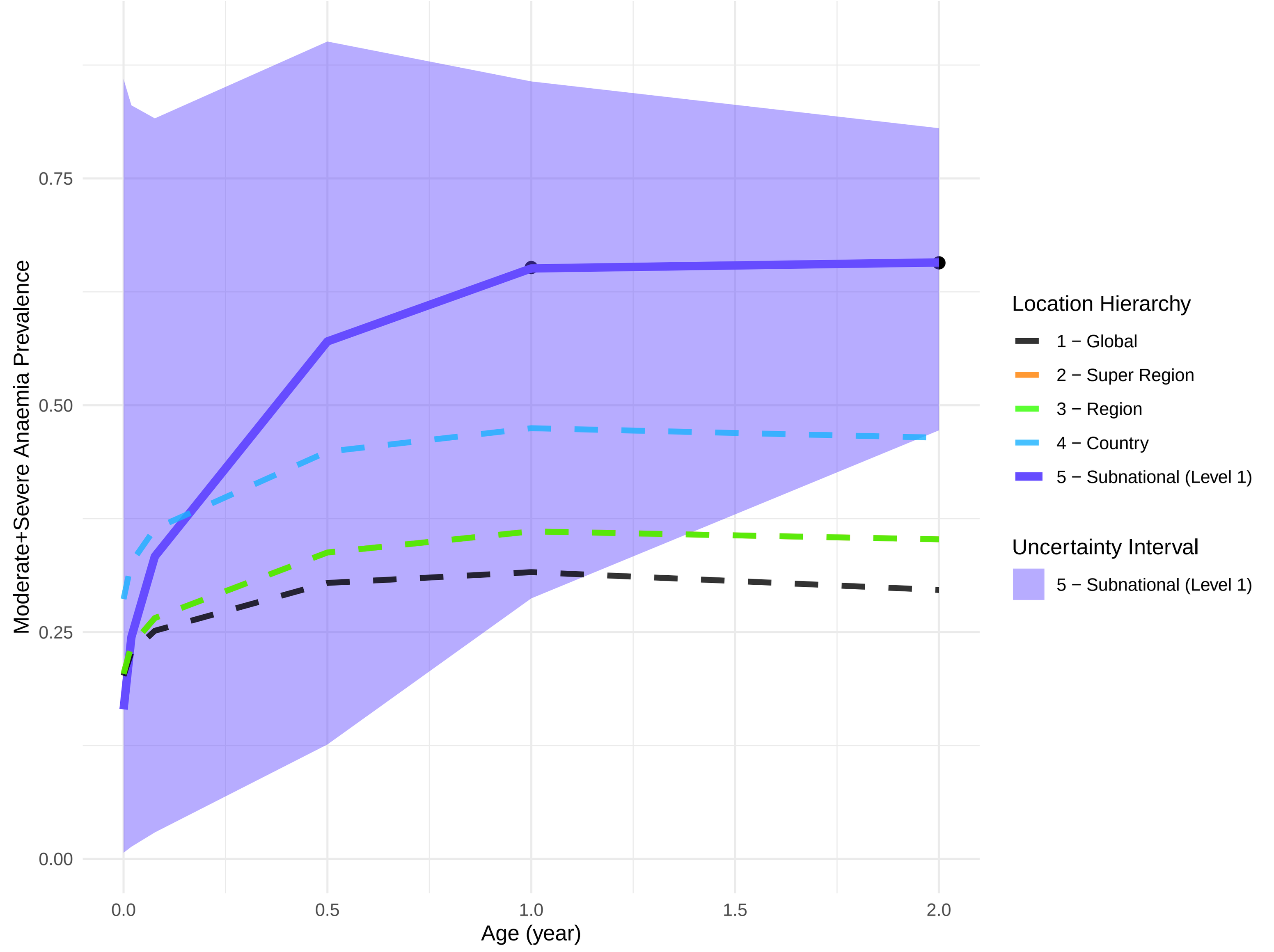

MR−BRT Age/Sex Fits − Non−pregnant Females <2 Years Old − Moderate+Severe Anaemia Prevalence − KwaZulu−Natal
WHO−2024 Adjustment Method

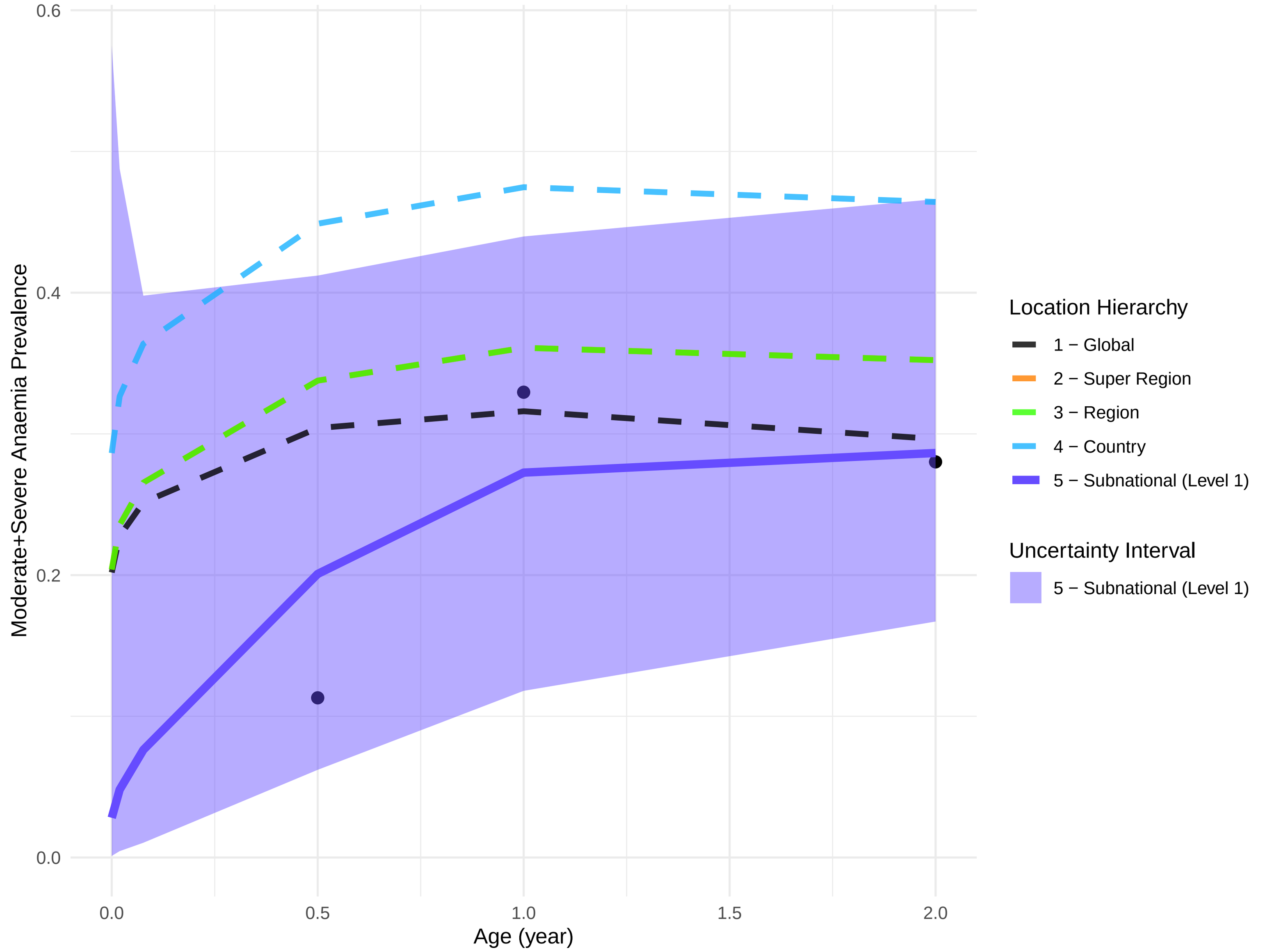

MR−BRT Age/Sex Fits − Non−pregnant Females <2 Years Old − Moderate+Severe Anaemia Prevalence − Limpopo
WHO−2024 Adjustment Method

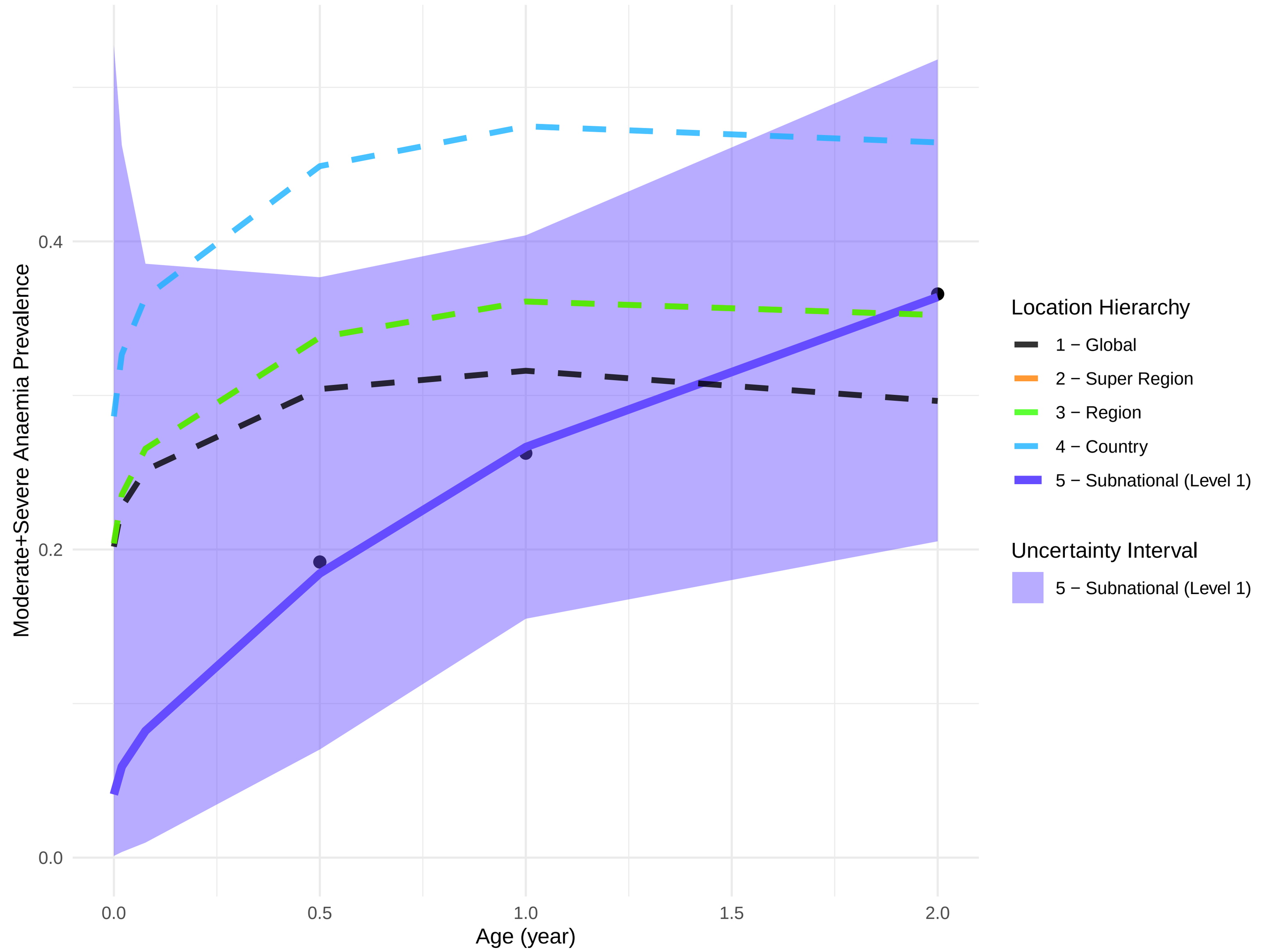

MR−BRT Age/Sex Fits − Non−pregnant Females <2 Years Old − Moderate+Severe Anaemia Prevalence − Mpumalanga
WHO−2024 Adjustment Method

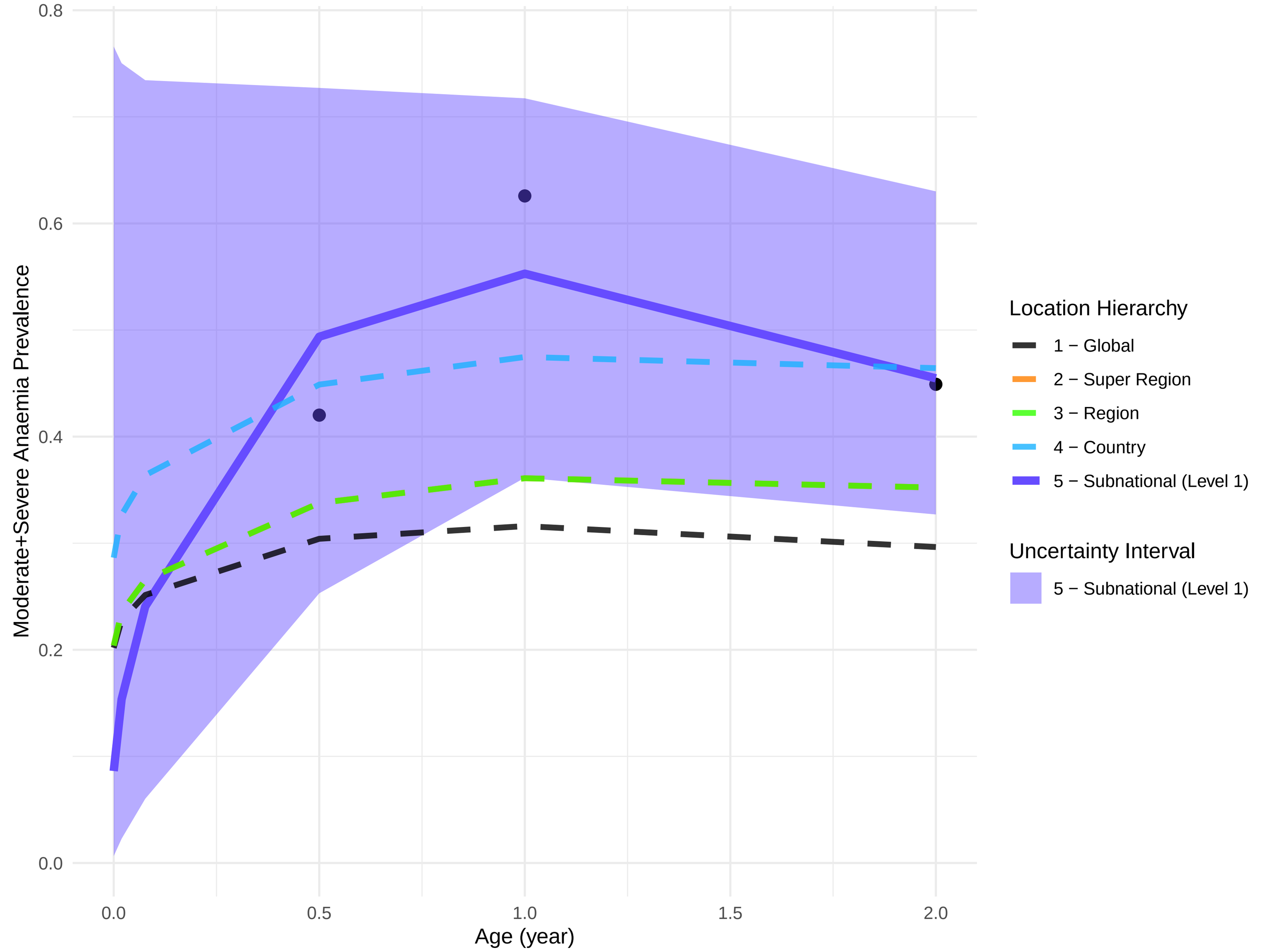

MR−BRT Age/Sex Fits − Non−pregnant Females <2 Years Old − Moderate+Severe Anaemia Prevalence − North−West
WHO−2024 Adjustment Method

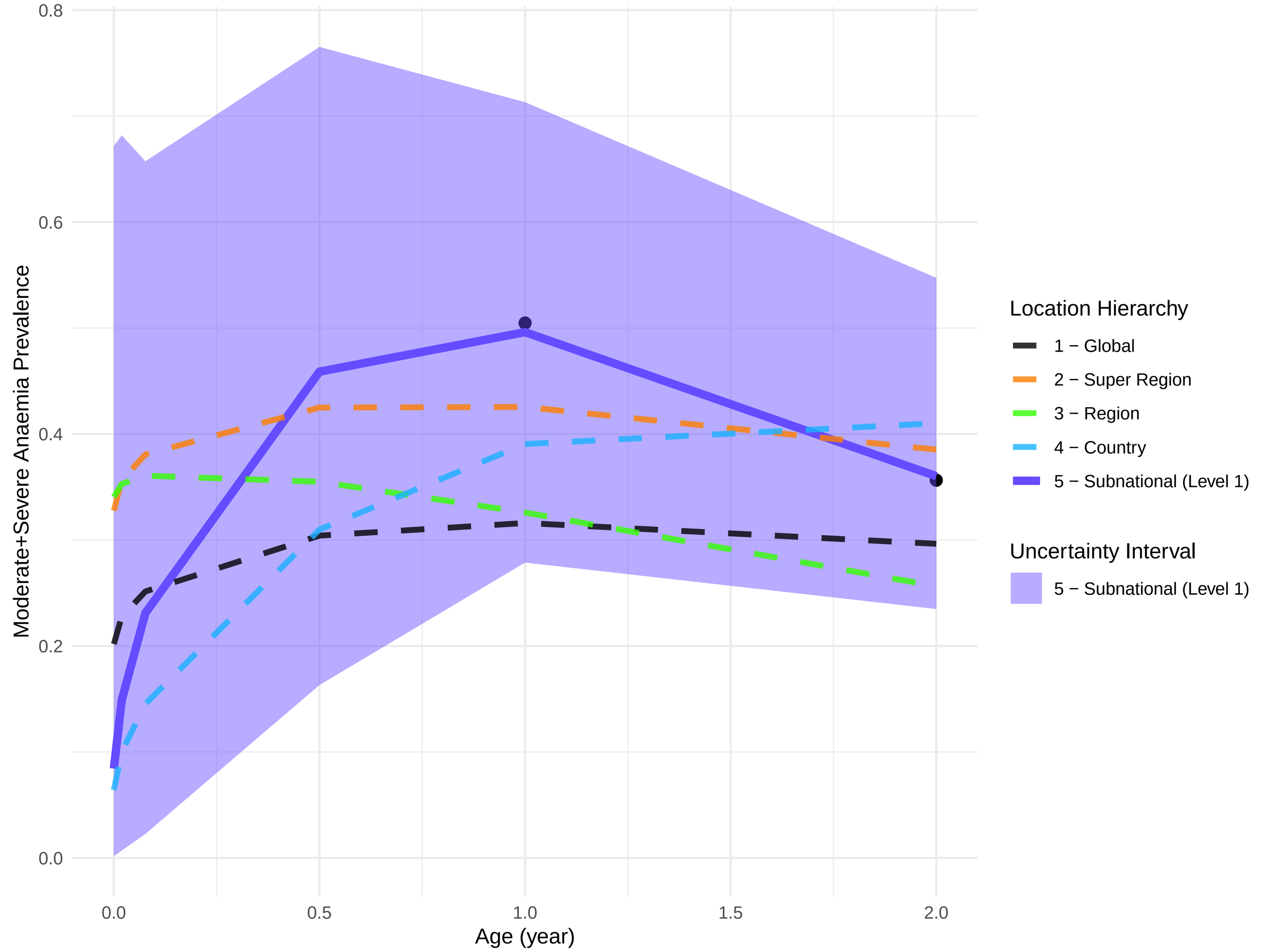

MR−BRT Age/Sex Fits − Non−pregnant Females <2 Years Old − Moderate+Severe Anaemia Prevalence − Northern Cape
WHO−2024 Adjustment Method

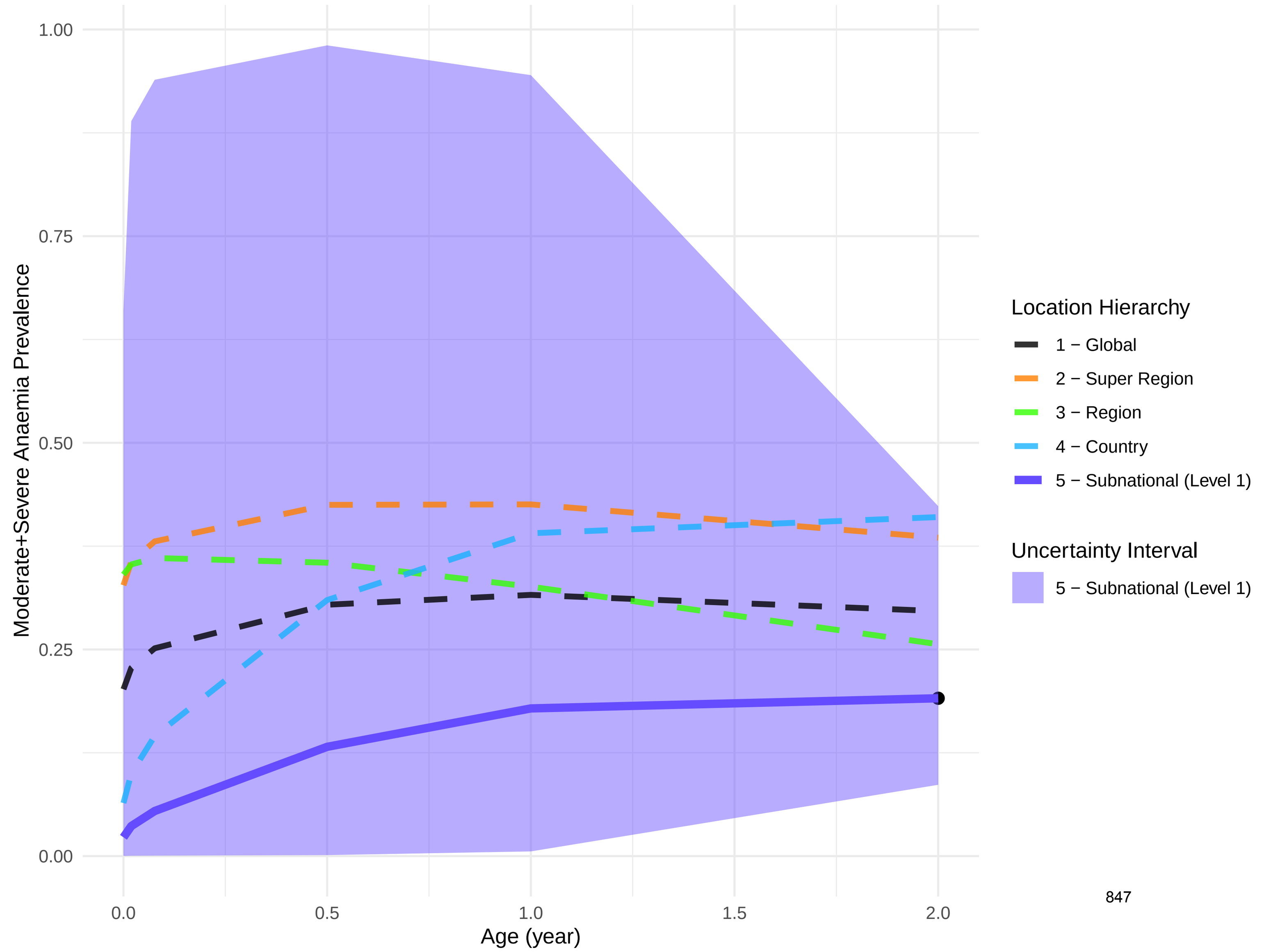

MR−BRT Age/Sex Fits − Non−pregnant Females <2 Years Old − Moderate+Severe Anaemia Prevalence − Western Cape
WHO−2024 Adjustment Method

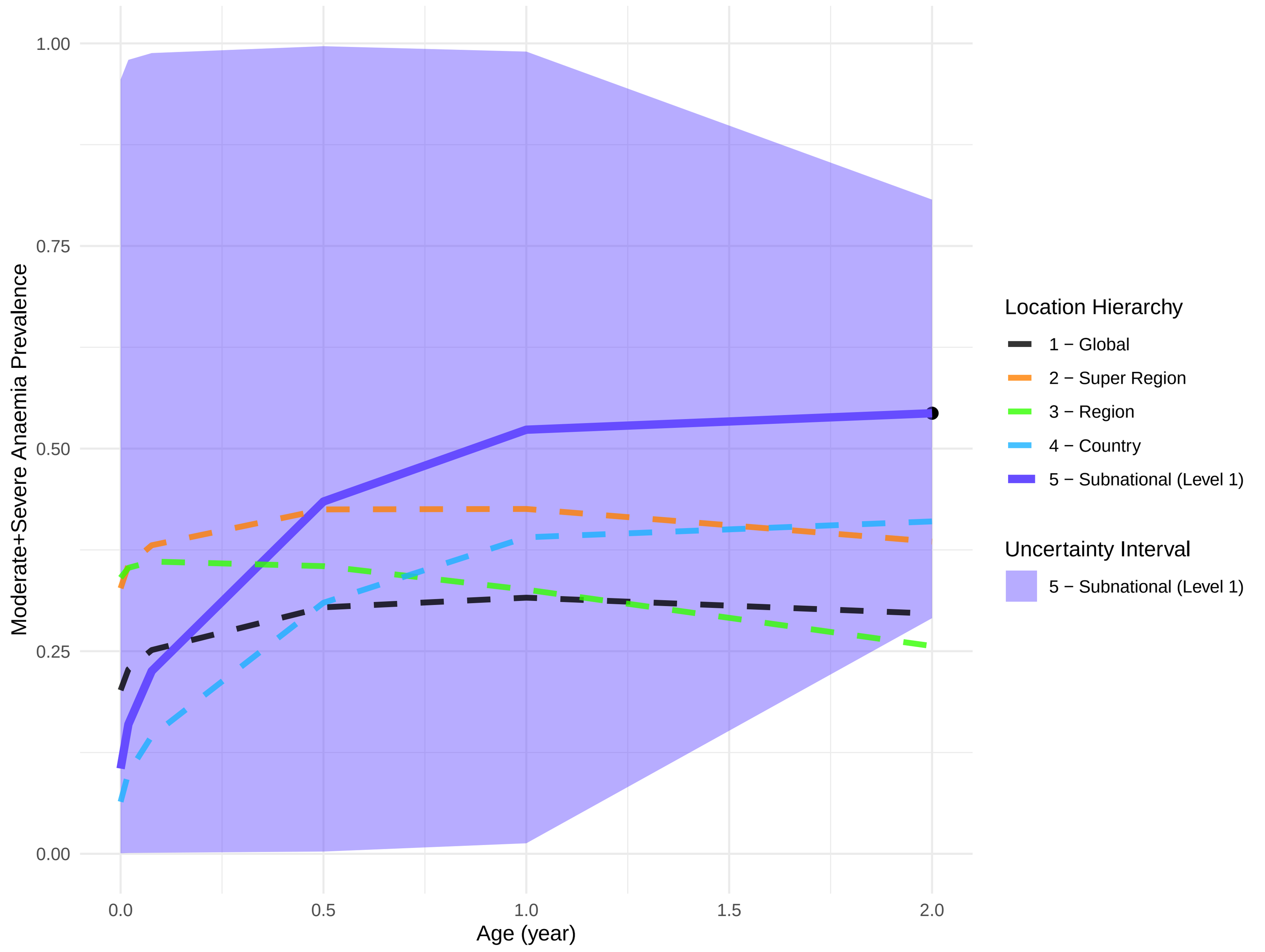

MR−BRT Age/Sex Fits − Non−pregnant Females <2 Years Old − Moderate+Severe Anaemia Prevalence − Zimbabwe
WHO−2024 Adjustment Method

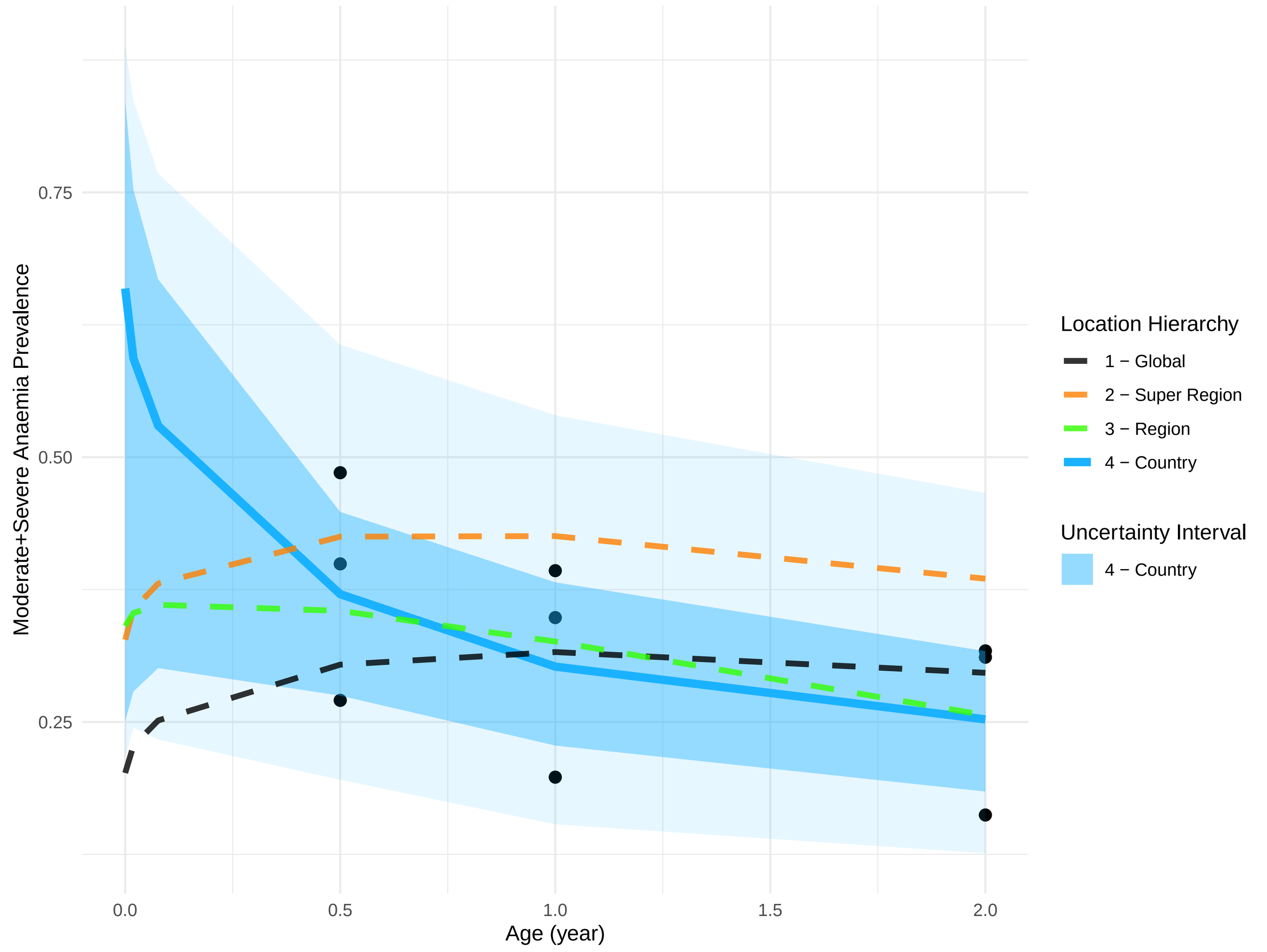

MR−BRT Age/Sex Fits − Non−pregnant Females <2 Years Old − Moderate+Severe Anaemia Prevalence − Western Sub−Saharan Africa
WHO−2024 Adjustment Method

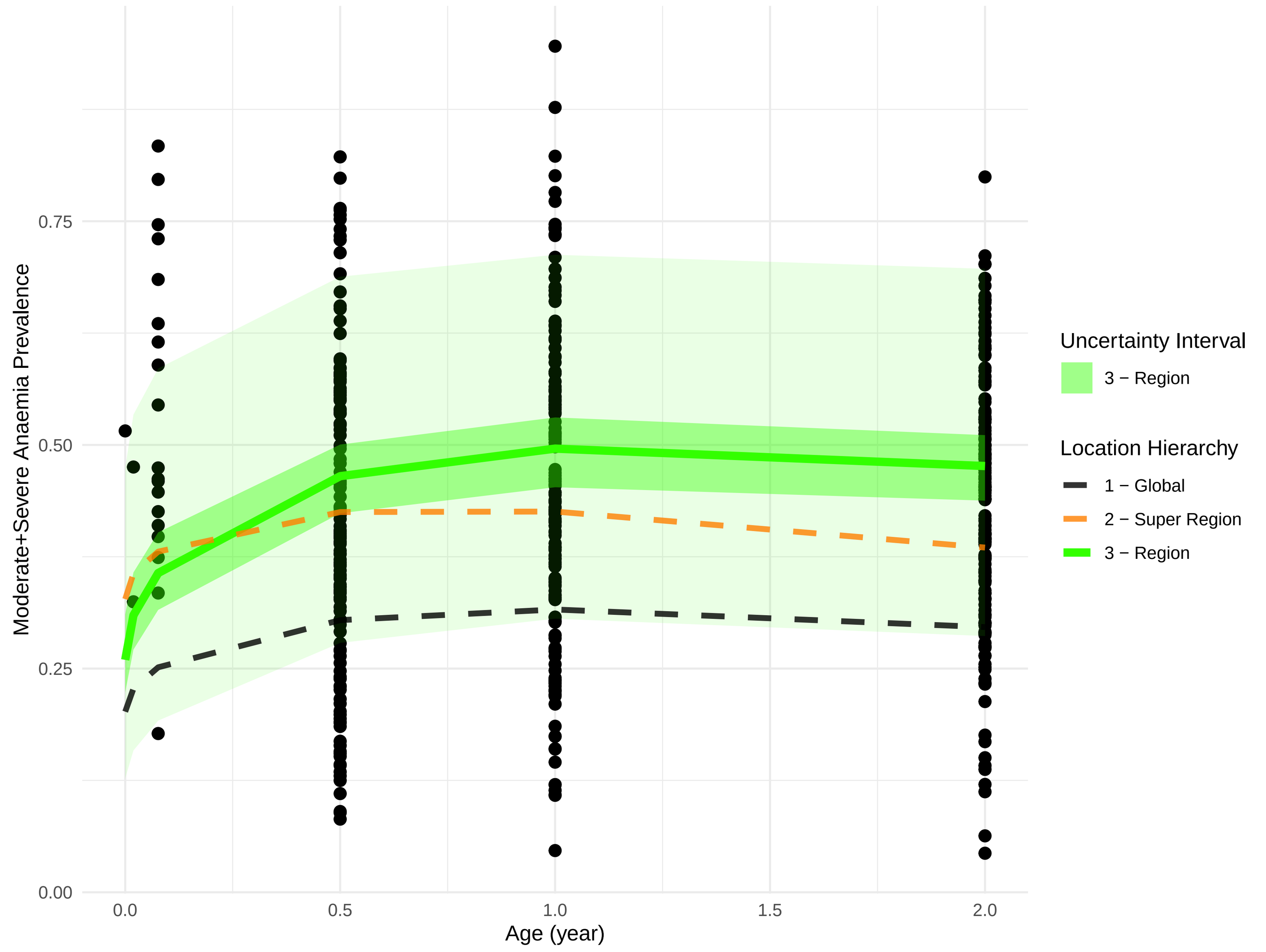

MR−BRT Age/Sex Fits − Non−pregnant Females <2 Years Old − Moderate+Severe Anaemia Prevalence − Benin
WHO−2024 Adjustment Method

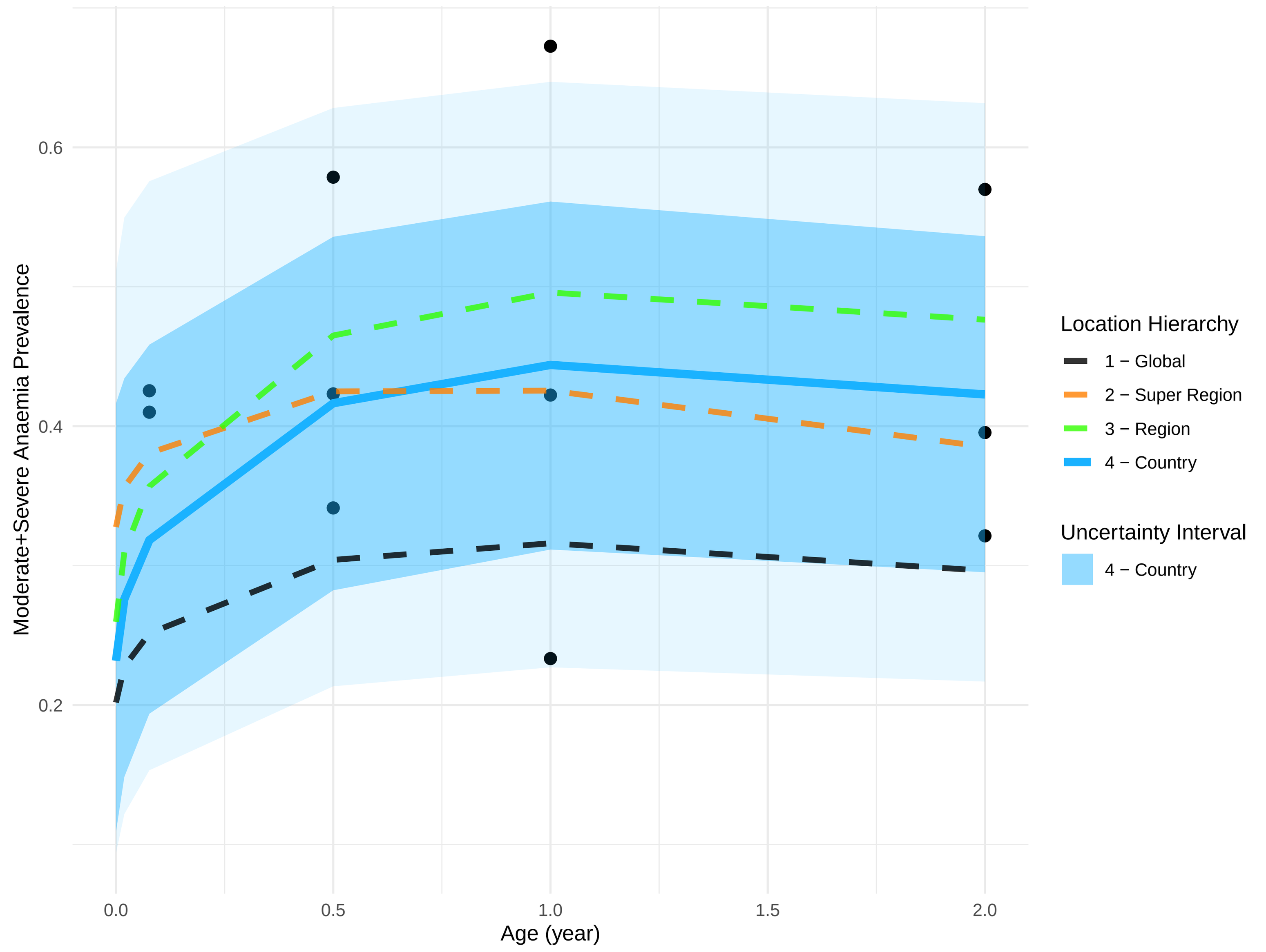

MR−BRT Age/Sex Fits − Non−pregnant Females <2 Years Old − Moderate+Severe Anaemia Prevalence − Burkina Faso
WHO−2024 Adjustment Method

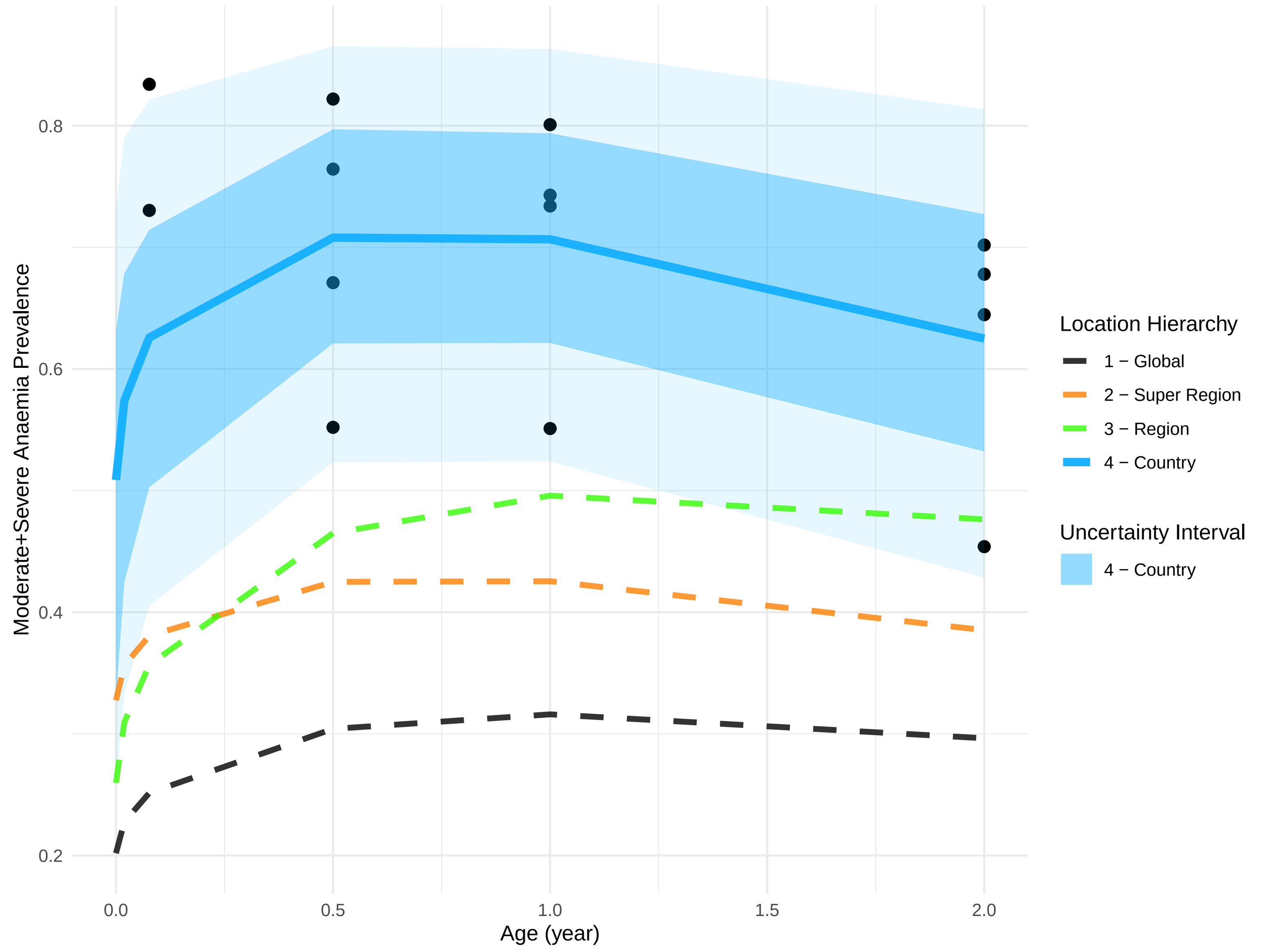

MR−BRT Age/Sex Fits − Non−pregnant Females <2 Years Old − Moderate+Severe Anaemia Prevalence − Cameroon
WHO−2024 Adjustment Method

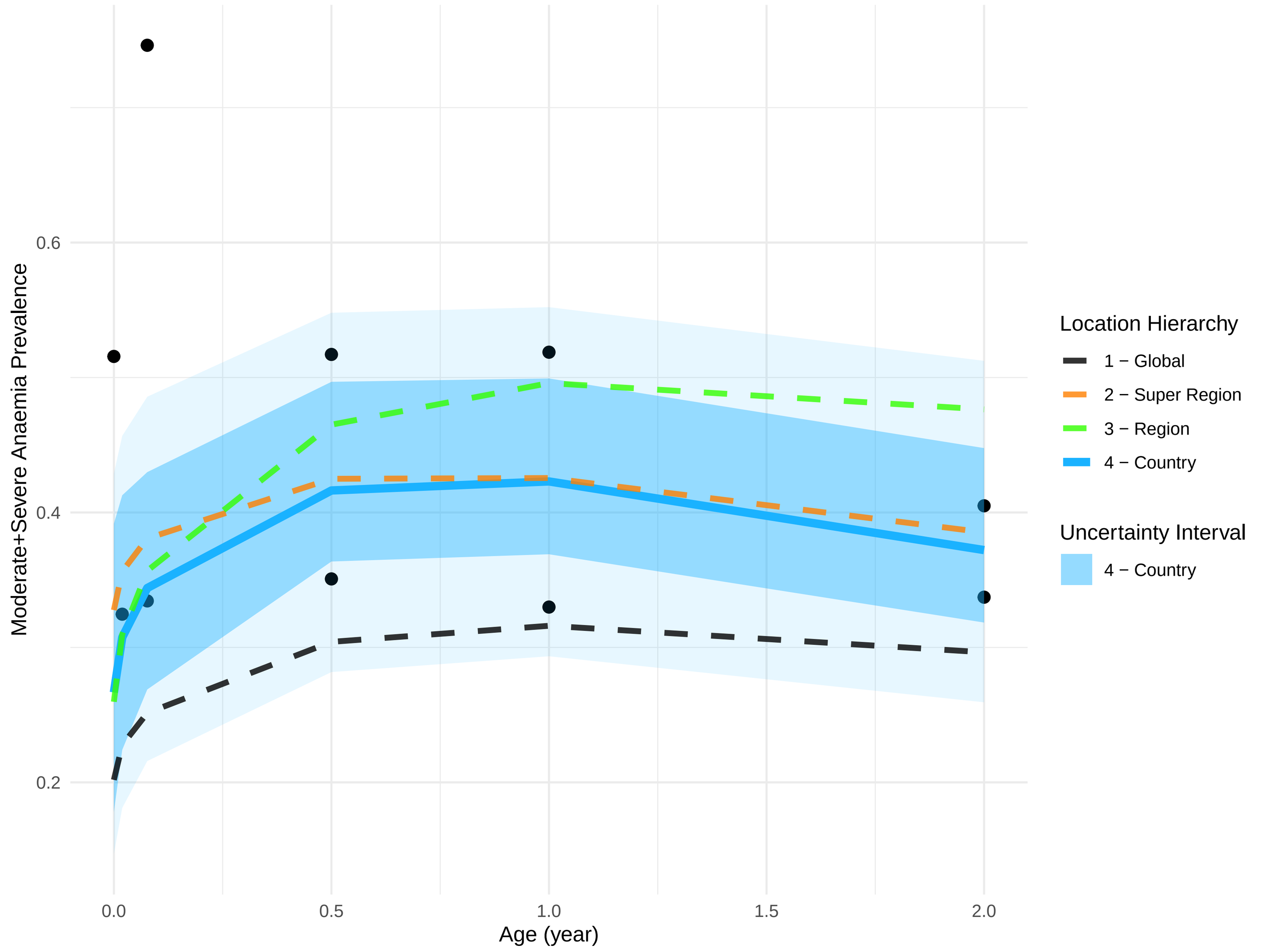

MR−BRT Age/Sex Fits − Non−pregnant Females <2 Years Old − Moderate+Severe Anaemia Prevalence − Côte d'Ivoire
WHO−2024 Adjustment Method

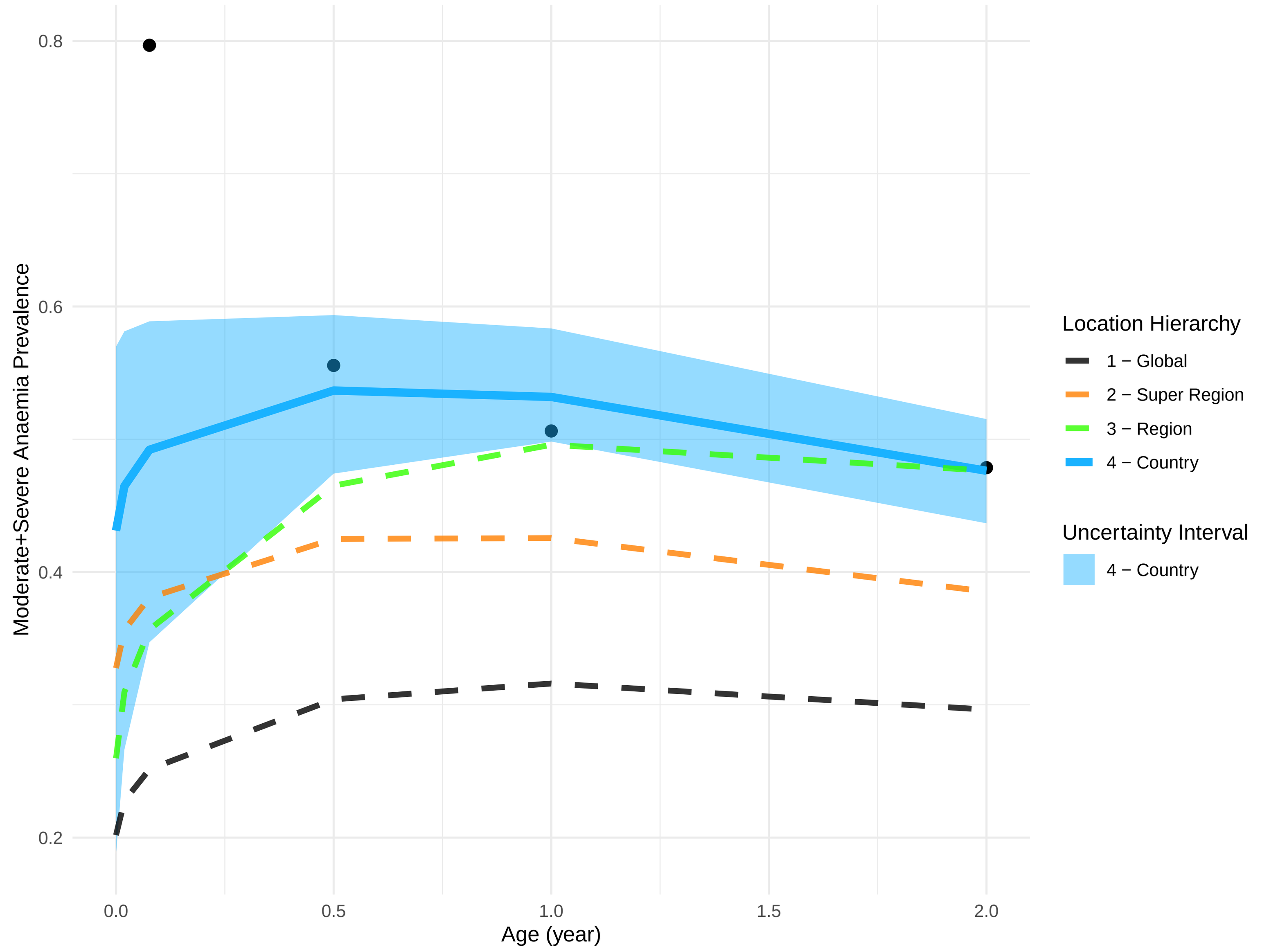

MR−BRT Age/Sex Fits − Non−pregnant Females <2 Years Old − Moderate+Severe Anaemia Prevalence − Gambia
WHO−2024 Adjustment Method

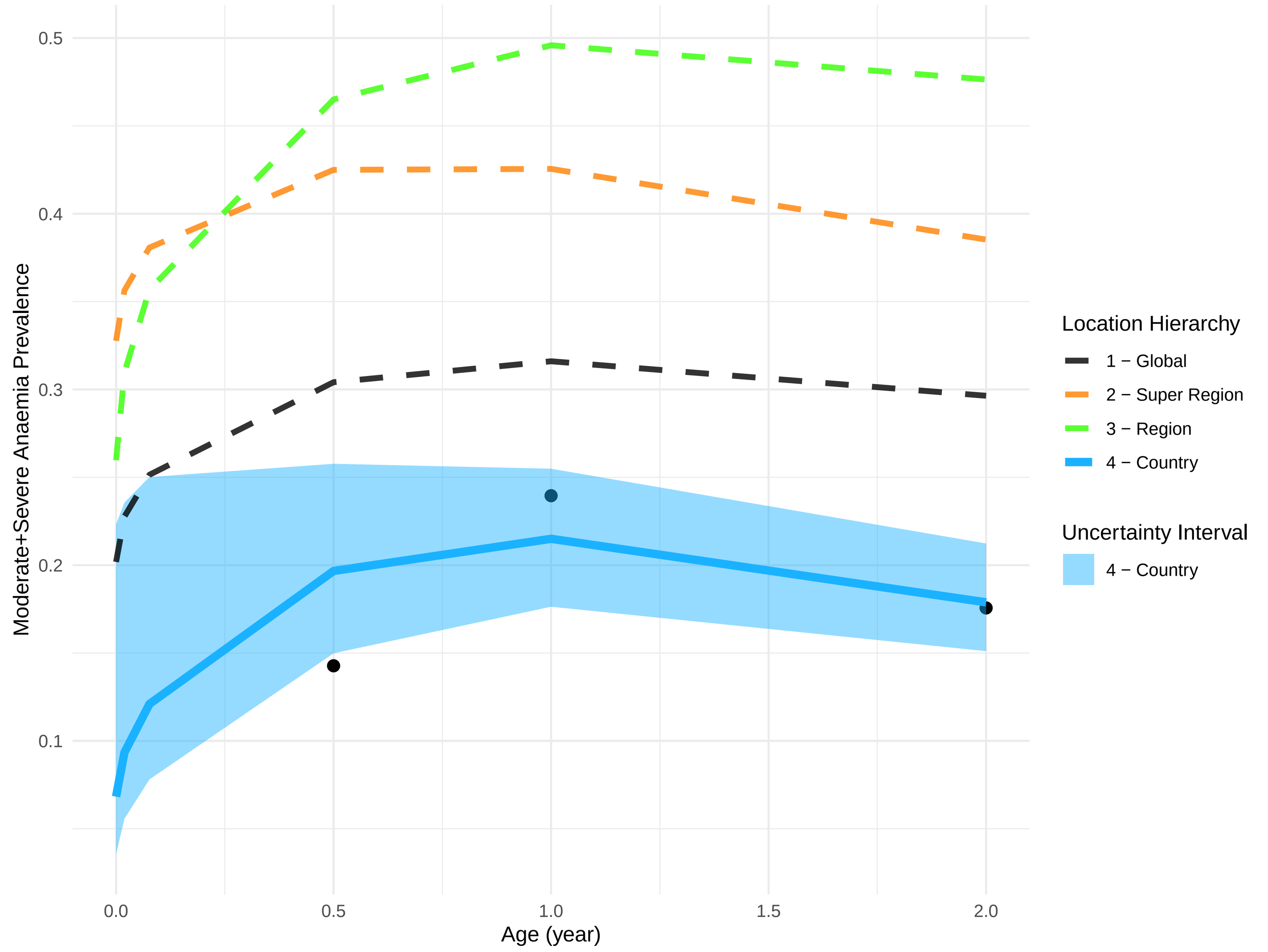

MR−BRT Age/Sex Fits − Non−pregnant Females <2 Years Old − Moderate+Severe Anaemia Prevalence − Ghana
WHO−2024 Adjustment Method

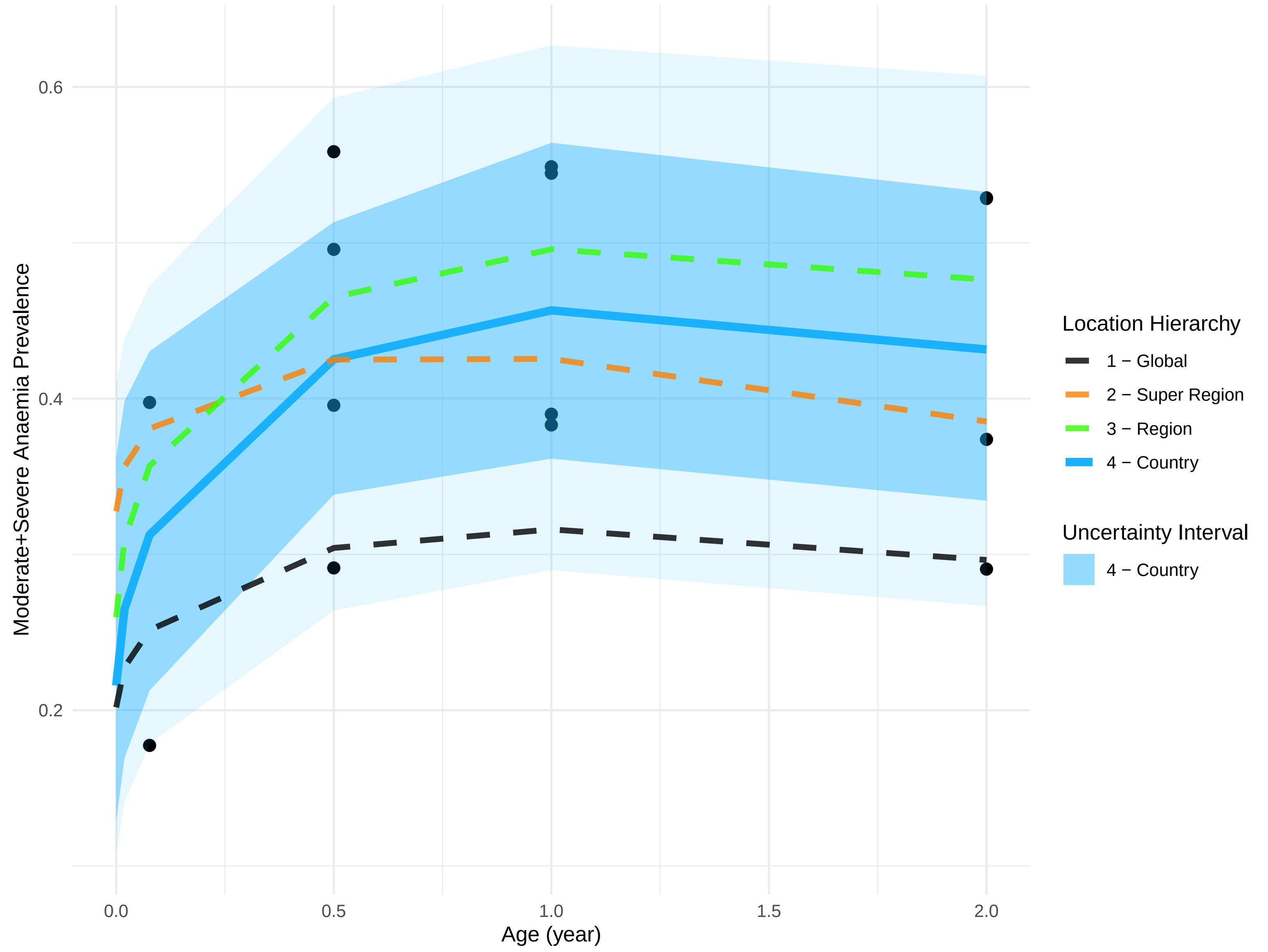

MR−BRT Age/Sex Fits − Non−pregnant Females <2 Years Old − Moderate+Severe Anaemia Prevalence − Guinea
WHO−2024 Adjustment Method

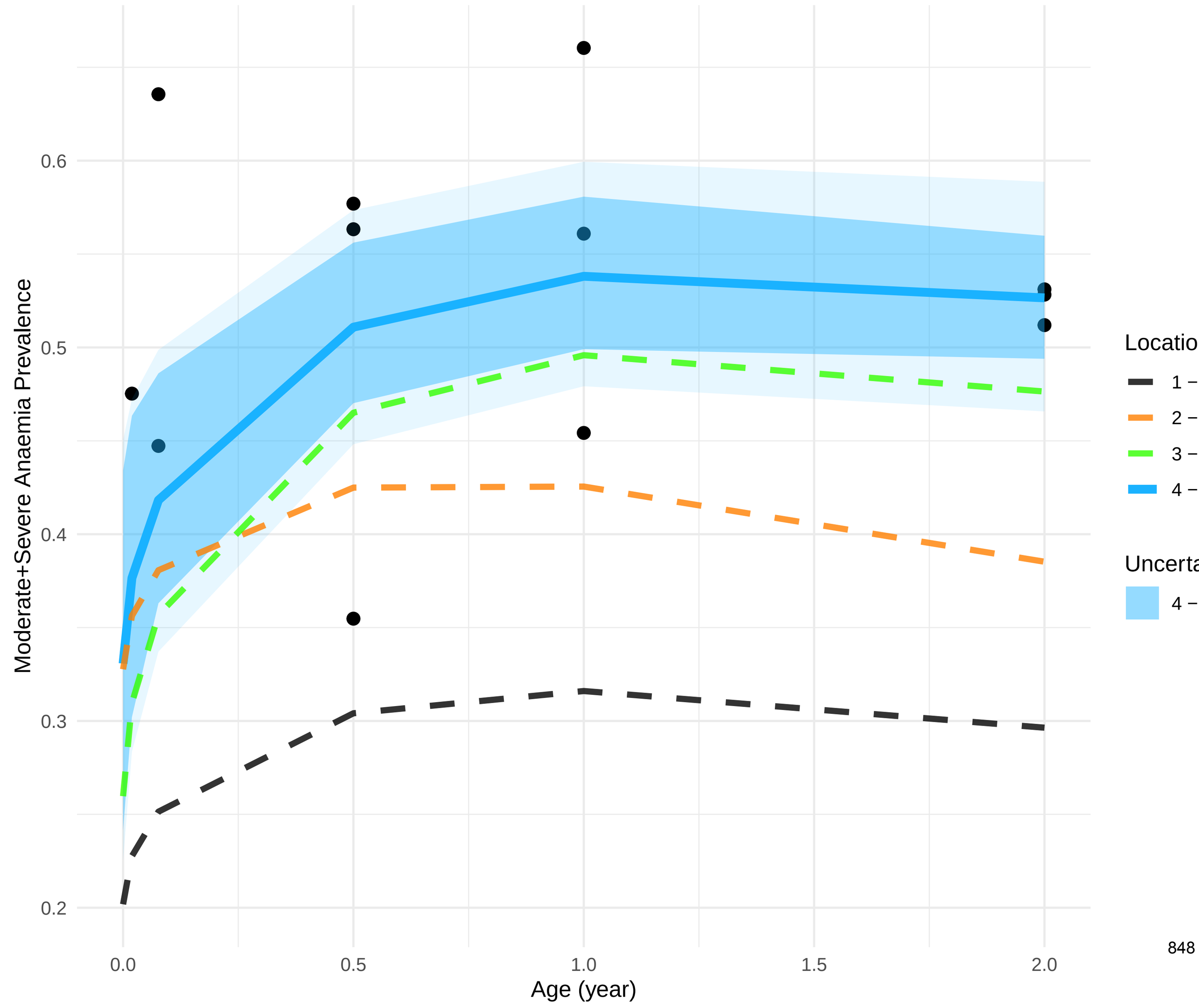

MR−BRT Age/Sex Fits − Non−pregnant Females <2 Years Old − Moderate+Severe Anaemia Prevalence − Liberia
WHO−2024 Adjustment Method

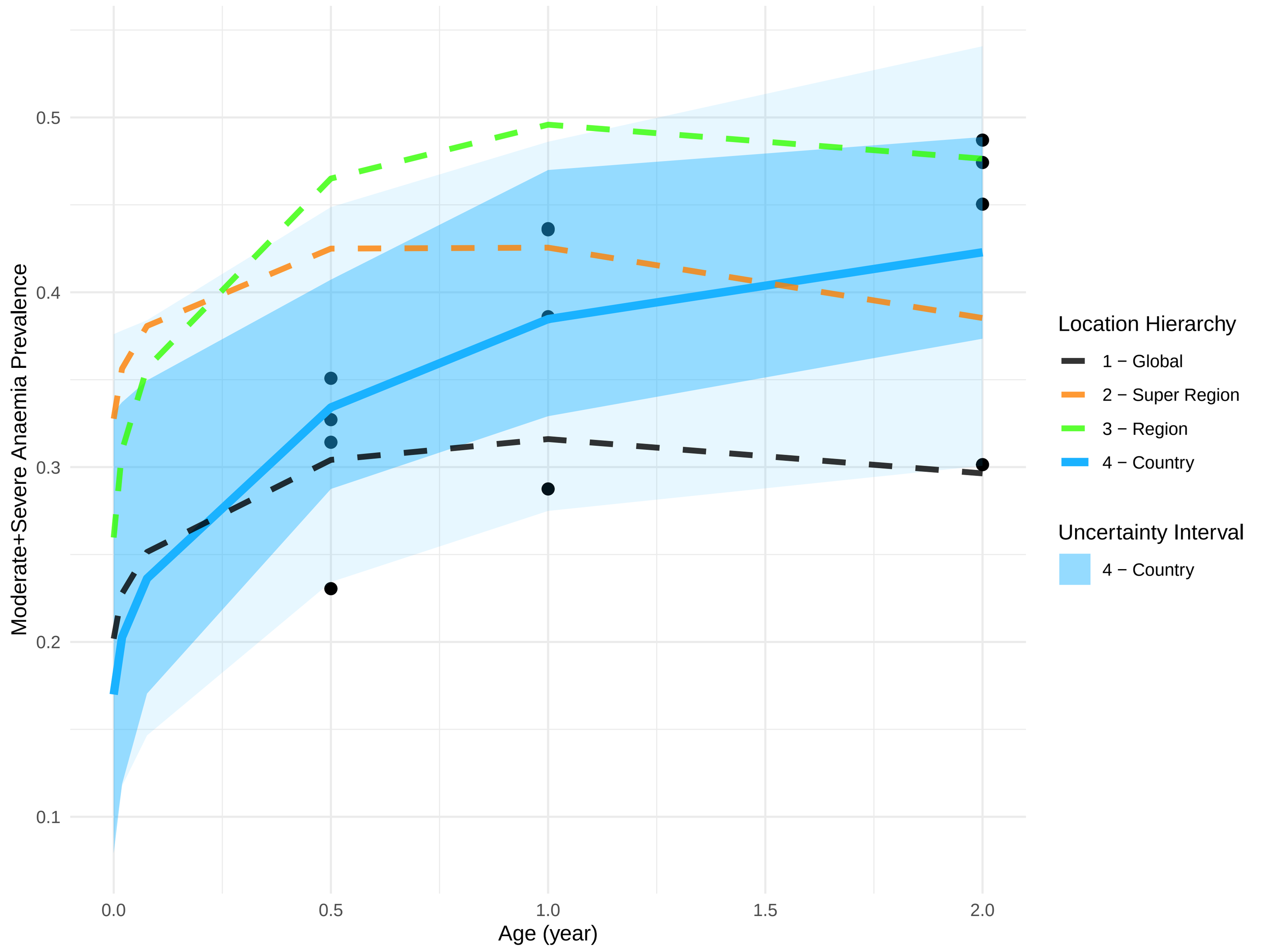

MR−BRT Age/Sex Fits − Non−pregnant Females <2 Years Old − Moderate+Severe Anaemia Prevalence − Mali
WHO−2024 Adjustment Method

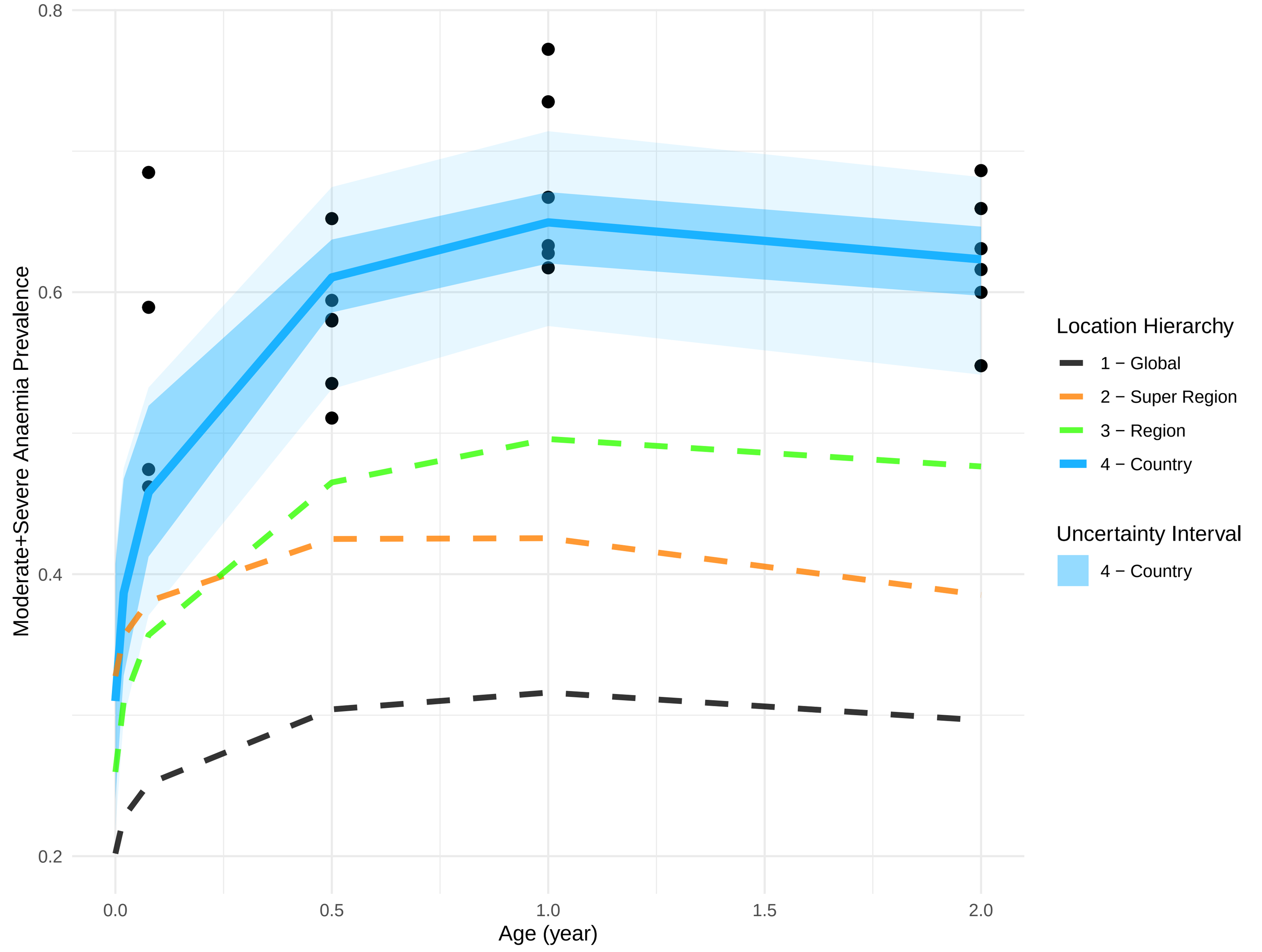

MR−BRT Age/Sex Fits − Non−pregnant Females <2 Years Old − Moderate+Severe Anaemia Prevalence − Niger
WHO−2024 Adjustment Method

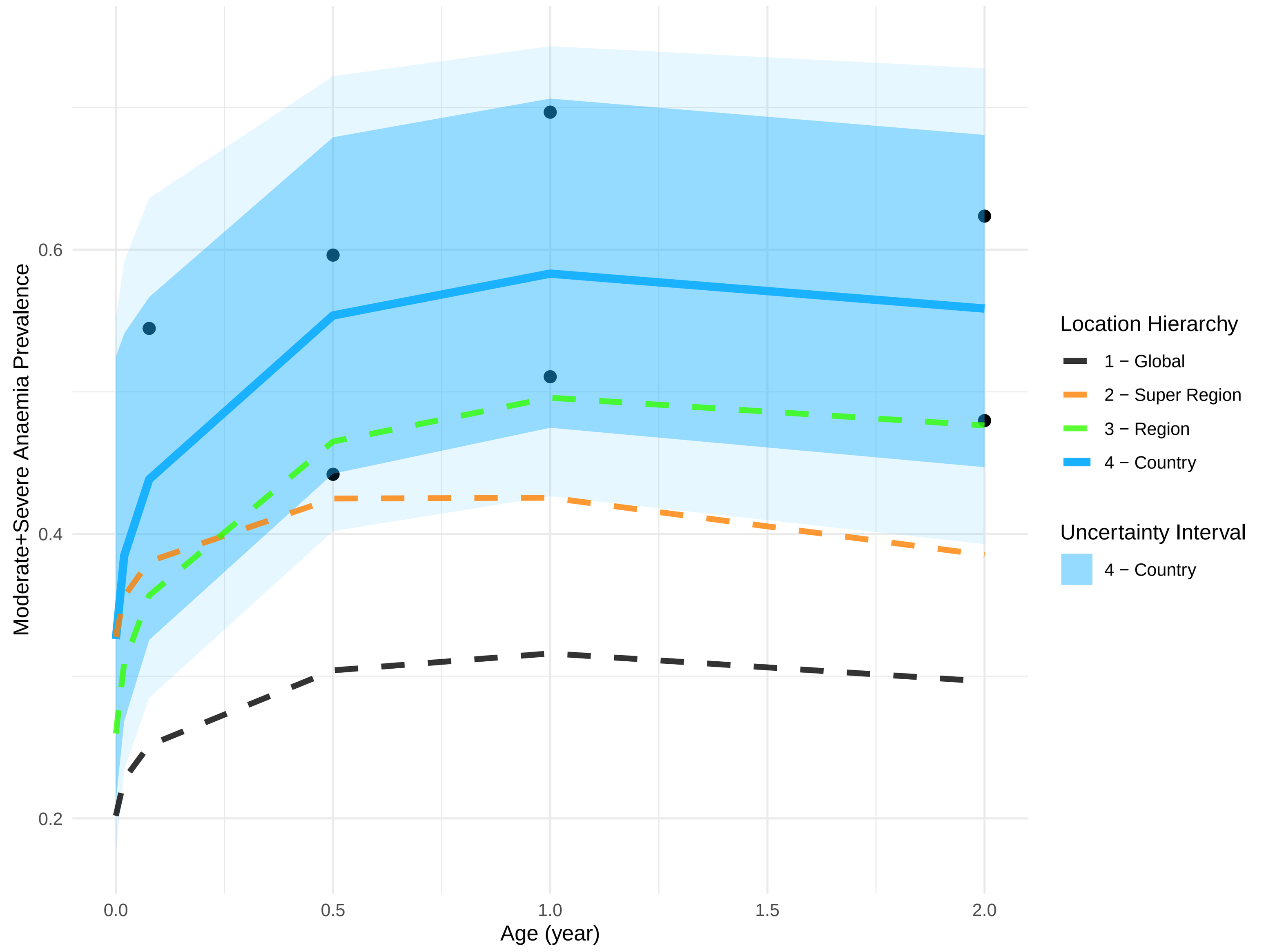

MR−BRT Age/Sex Fits − Non−pregnant Females <2 Years Old − Moderate+Severe Anaemia Prevalence − Nigeria
WHO−2024 Adjustment Method

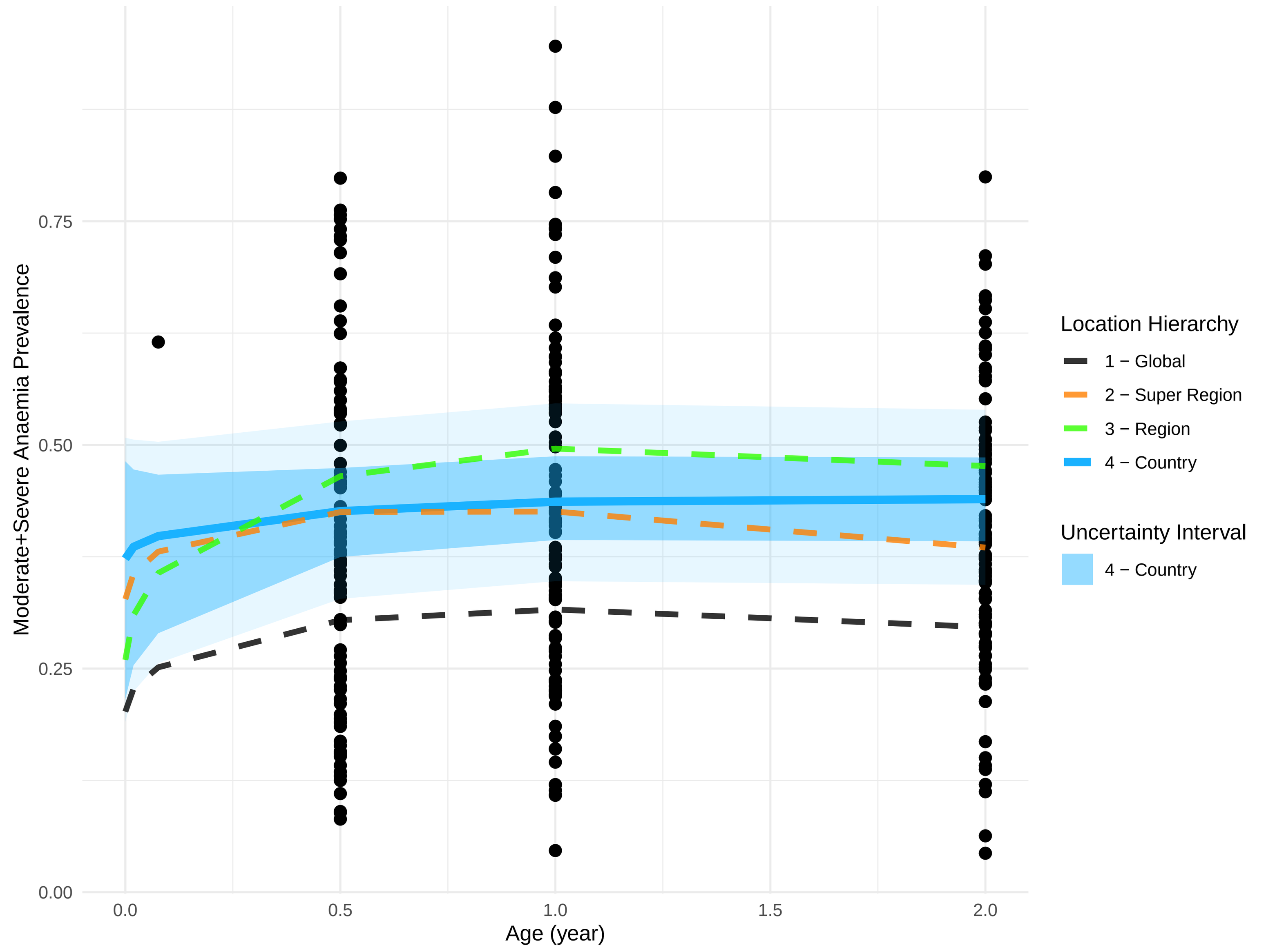

MR−BRT Age/Sex Fits − Non−pregnant Females <2 Years Old − Moderate+Severe Anaemia Prevalence − Abia
WHO−2024 Adjustment Method

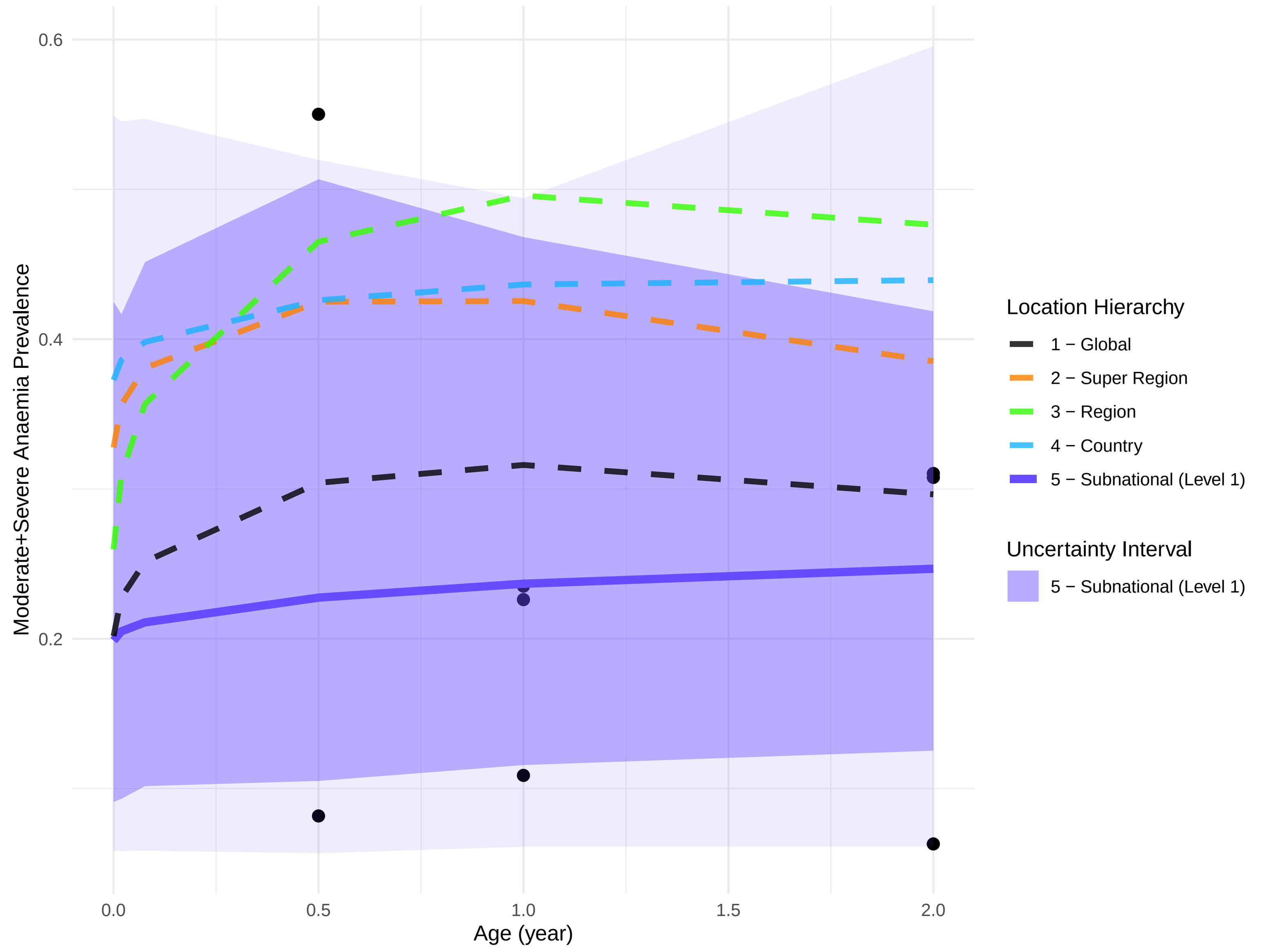

MR−BRT Age/Sex Fits − Non−pregnant Females <2 Years Old − Moderate+Severe Anaemia Prevalence − Adamawa
WHO−2024 Adjustment Method

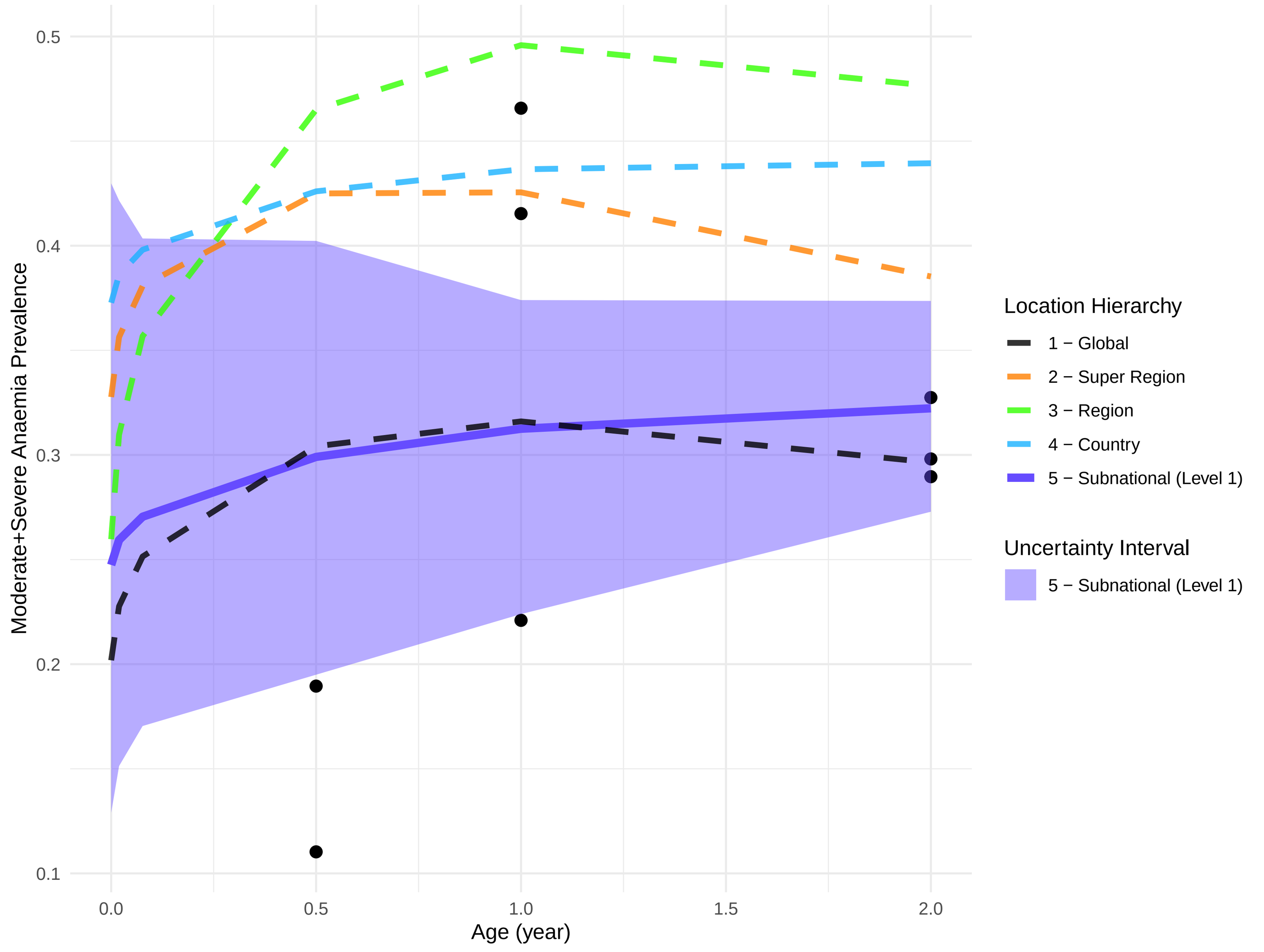

MR−BRT Age/Sex Fits − Non−pregnant Females <2 Years Old − Moderate+Severe Anaemia Prevalence − Akwa Ibom
WHO−2024 Adjustment Method

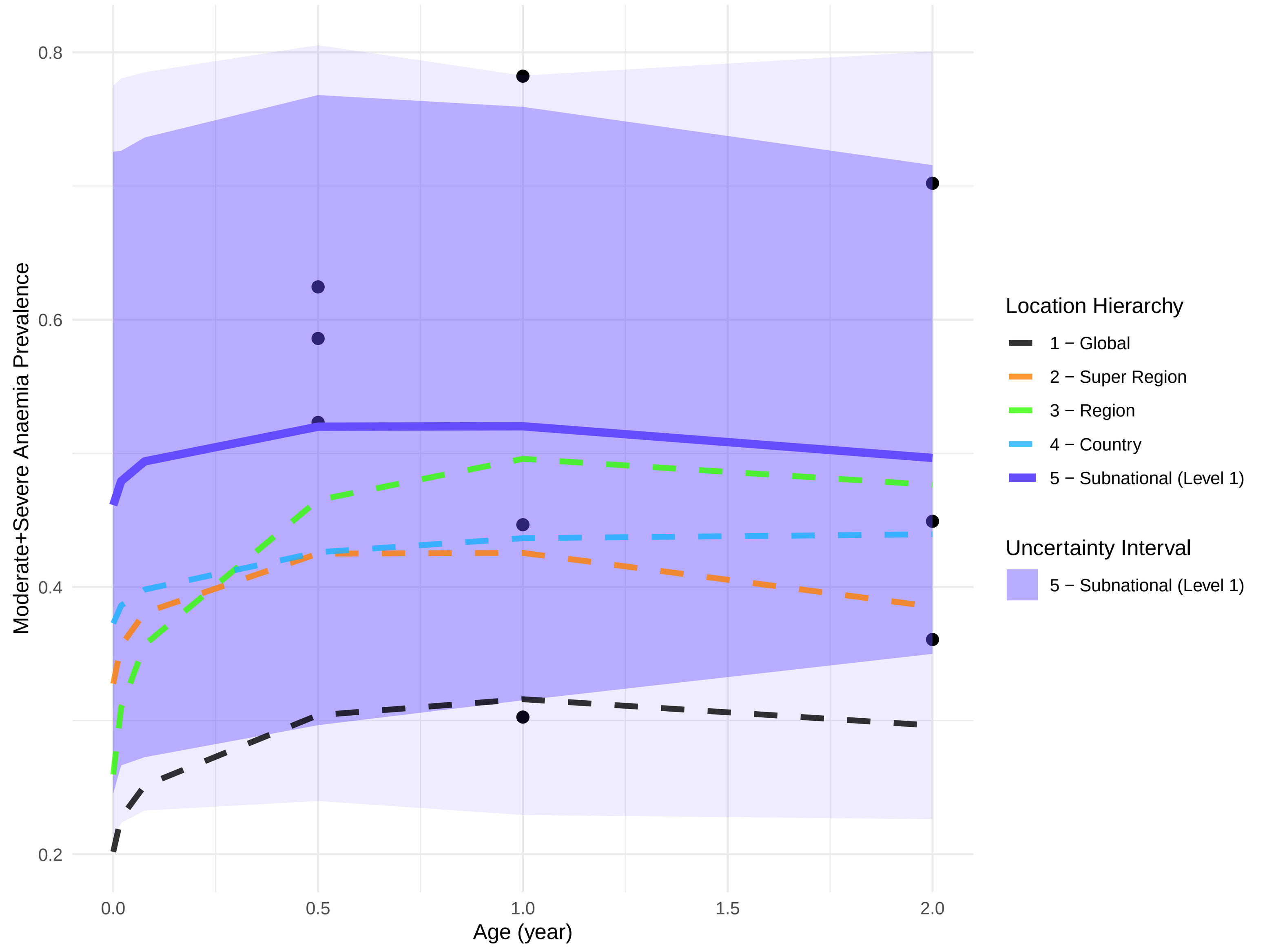

MR−BRT Age/Sex Fits − Non−pregnant Females <2 Years Old − Moderate+Severe Anaemia Prevalence − Anambra
WHO−2024 Adjustment Method

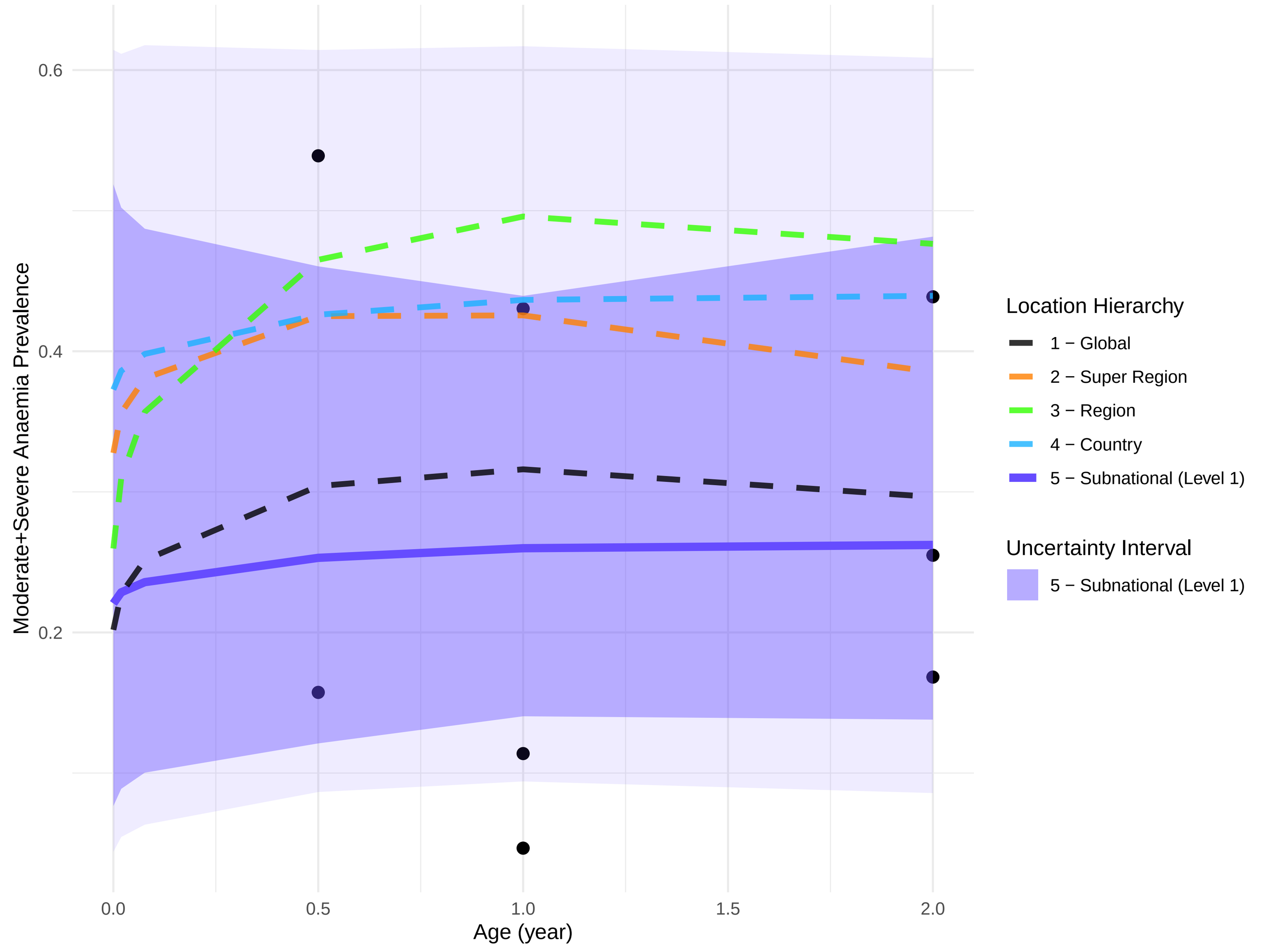

MR−BRT Age/Sex Fits − Non−pregnant Females <2 Years Old − Moderate+Severe Anaemia Prevalence − Bauchi
WHO−2024 Adjustment Method

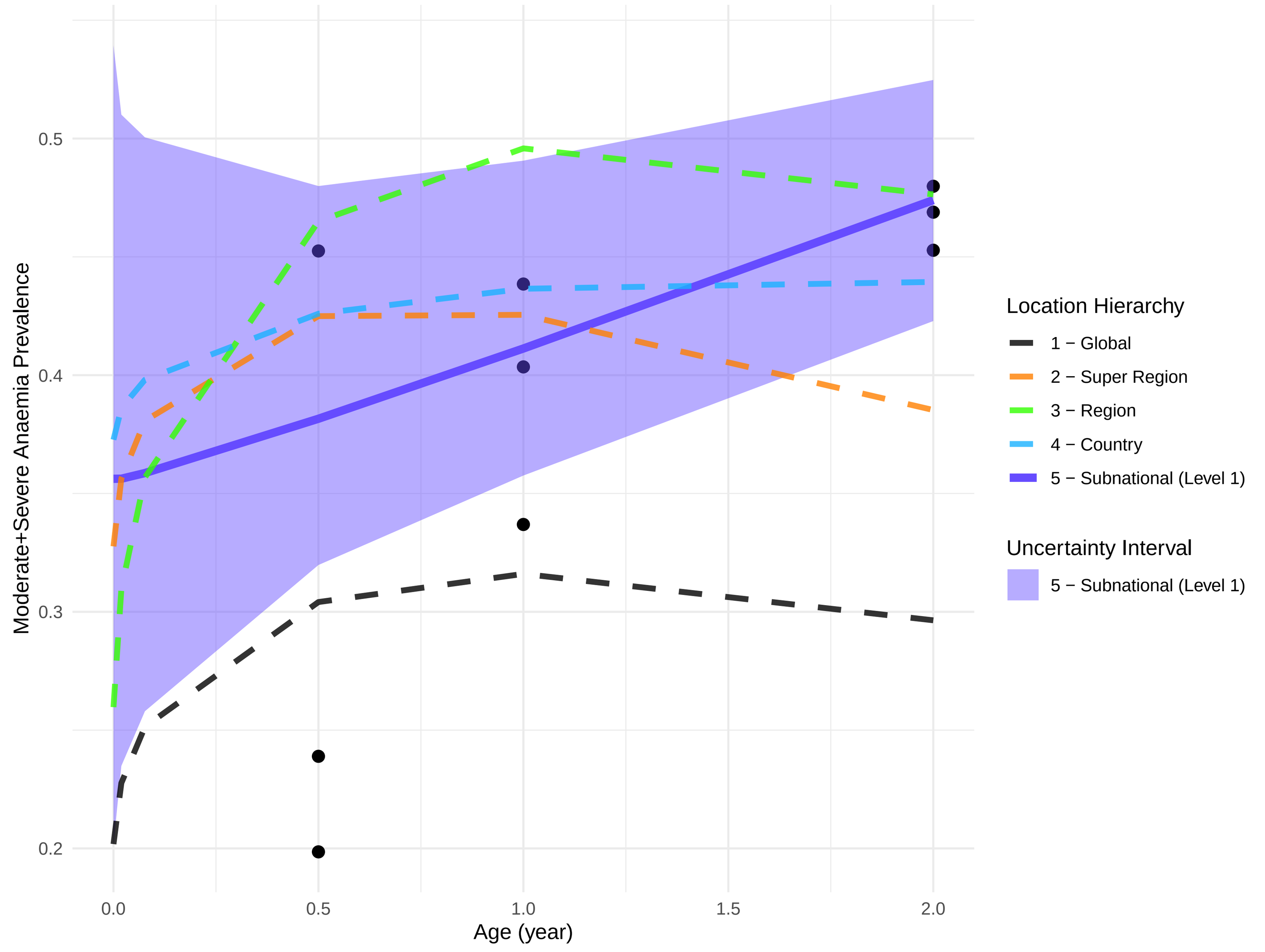

MR−BRT Age/Sex Fits − Non−pregnant Females <2 Years Old − Moderate+Severe Anaemia Prevalence − Bayelsa
WHO−2024 Adjustment Method

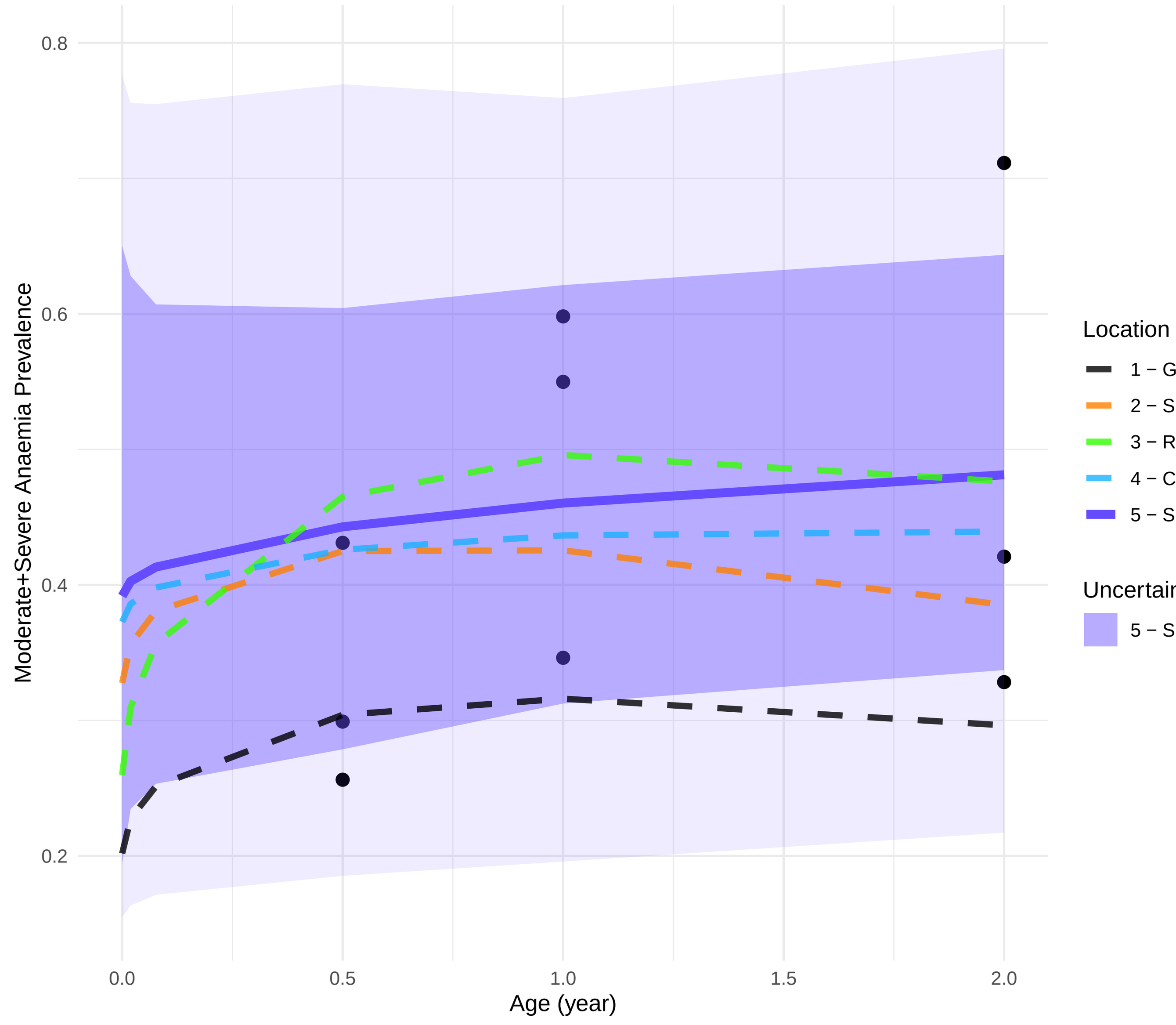

MR−BRT Age/Sex Fits − Non−pregnant Females <2 Years Old − Moderate+Severe Anaemia Prevalence − Benue
WHO−2024 Adjustment Method

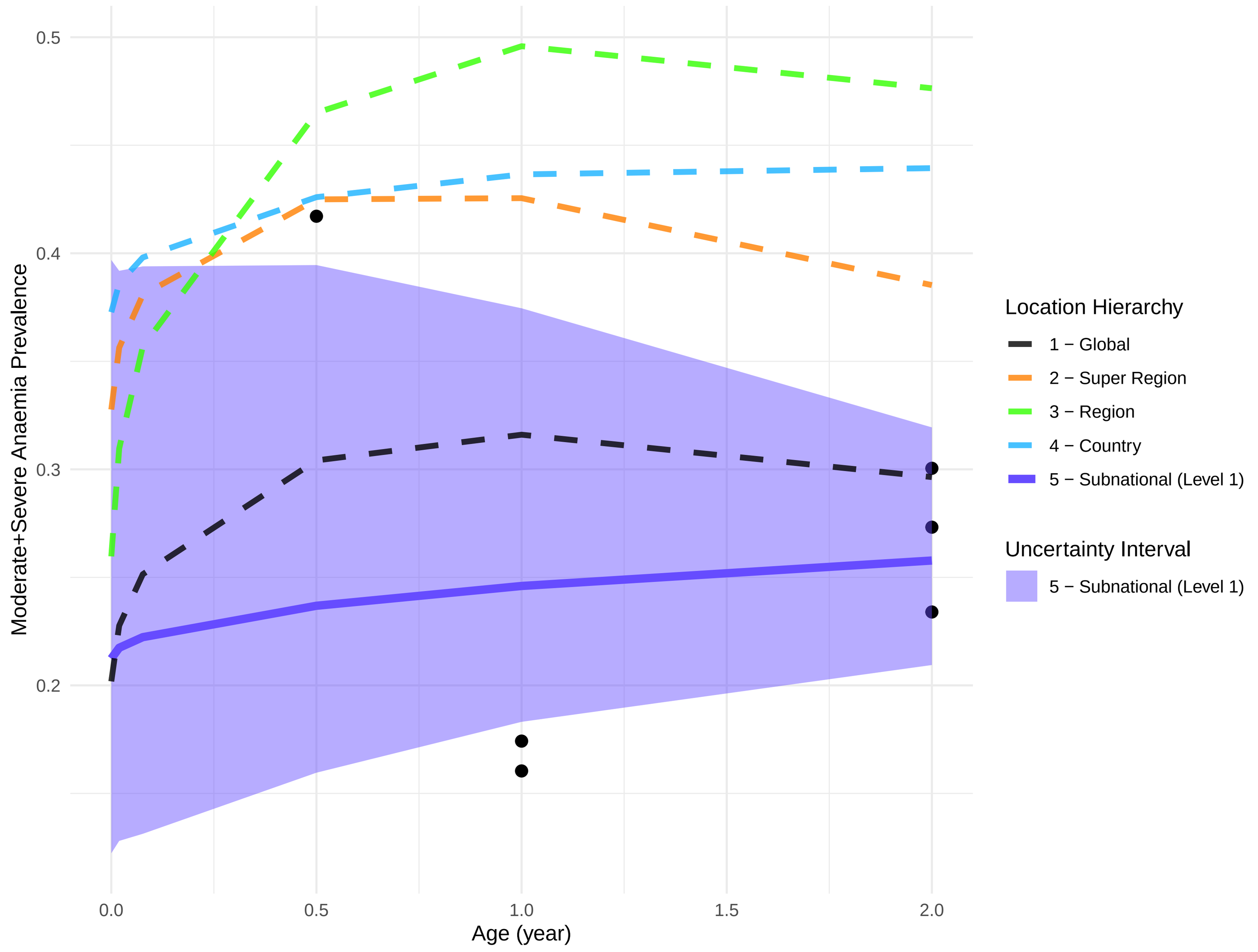

MR−BRT Age/Sex Fits − Non−pregnant Females <2 Years Old − Moderate+Severe Anaemia Prevalence − Borno
WHO−2024 Adjustment Method

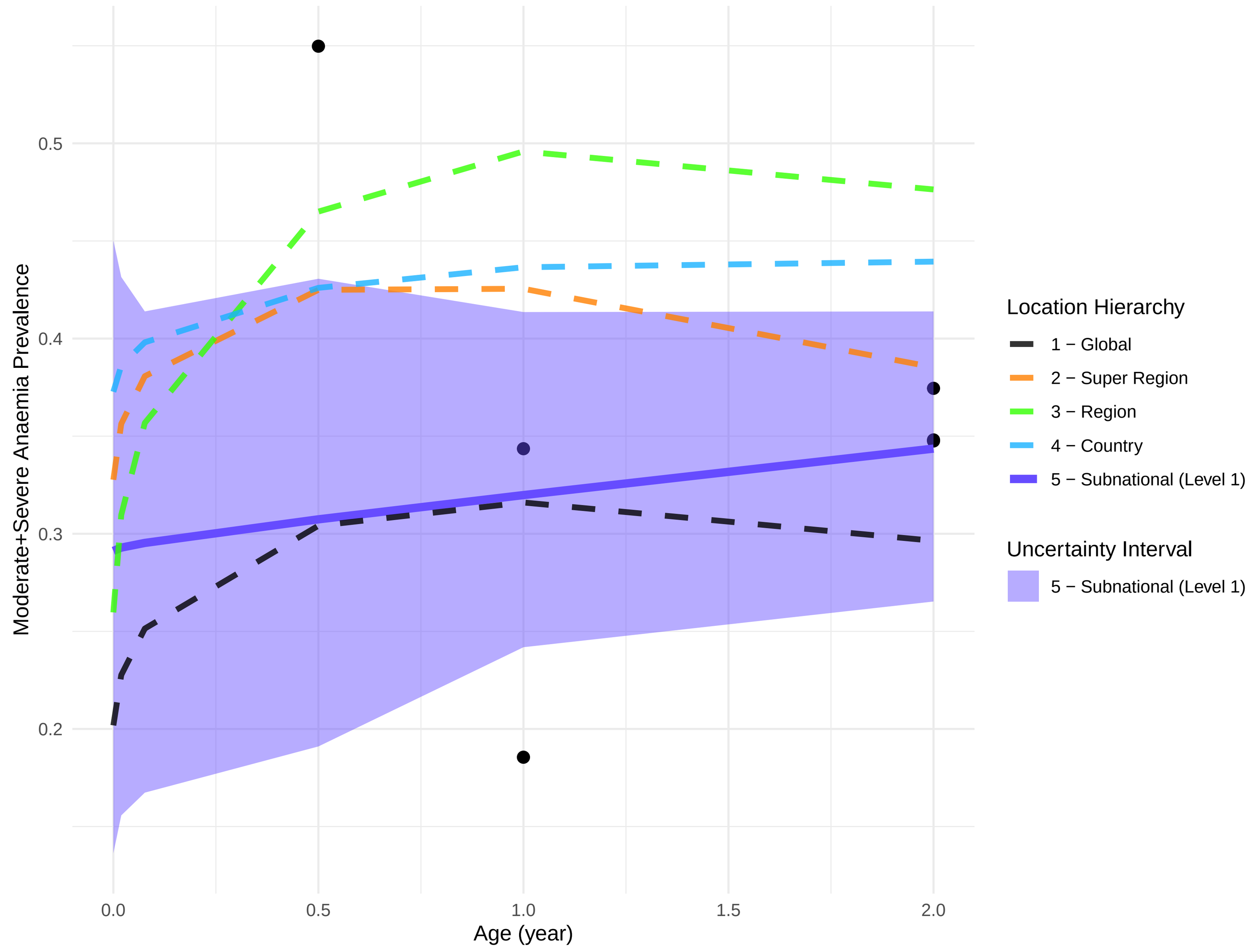

MR−BRT Age/Sex Fits − Non−pregnant Females <2 Years Old − Moderate+Severe Anaemia Prevalence − Cross River
WHO−2024 Adjustment Method

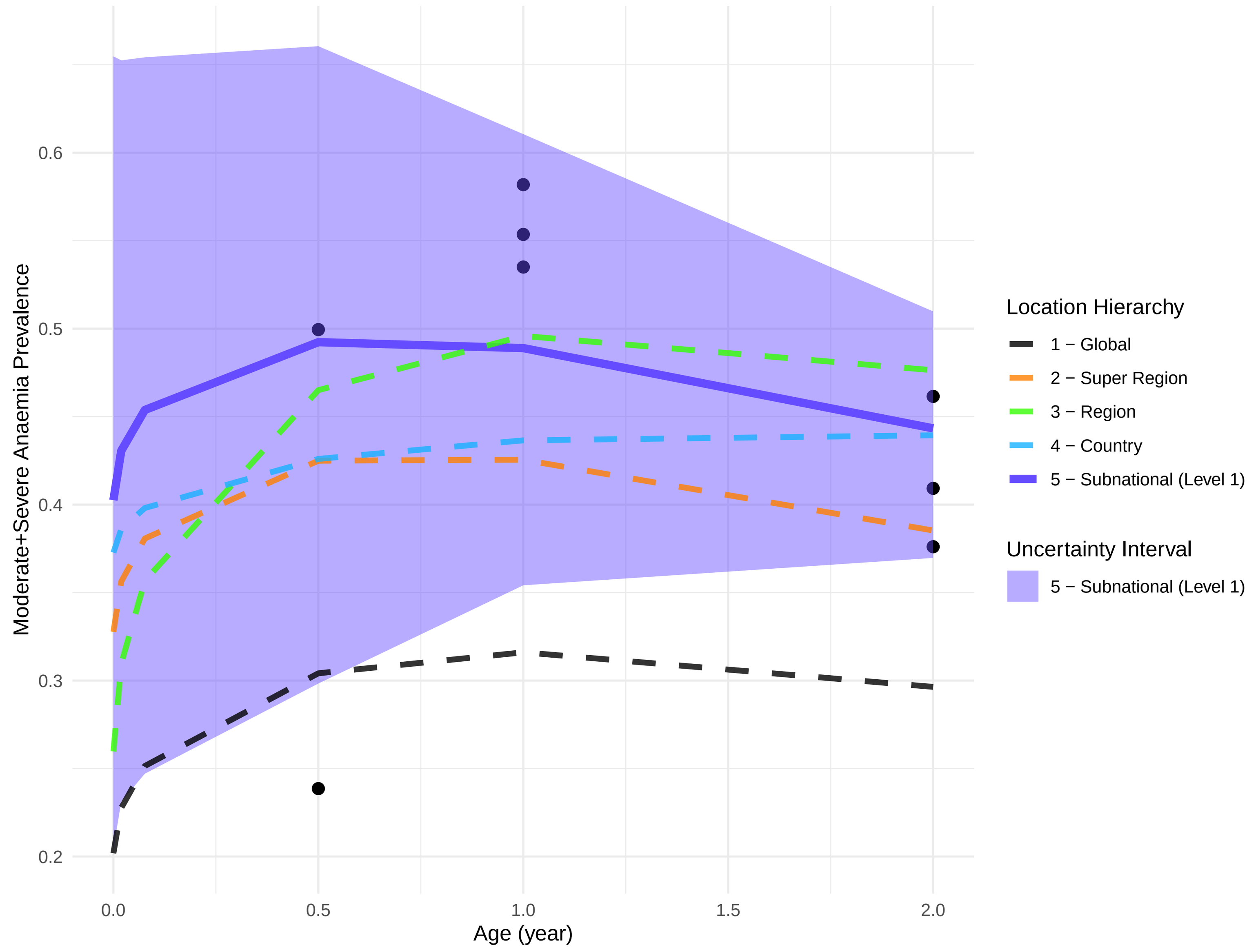

MR−BRT Age/Sex Fits − Non−pregnant Females <2 Years Old − Moderate+Severe Anaemia Prevalence − Delta
WHO−2024 Adjustment Method

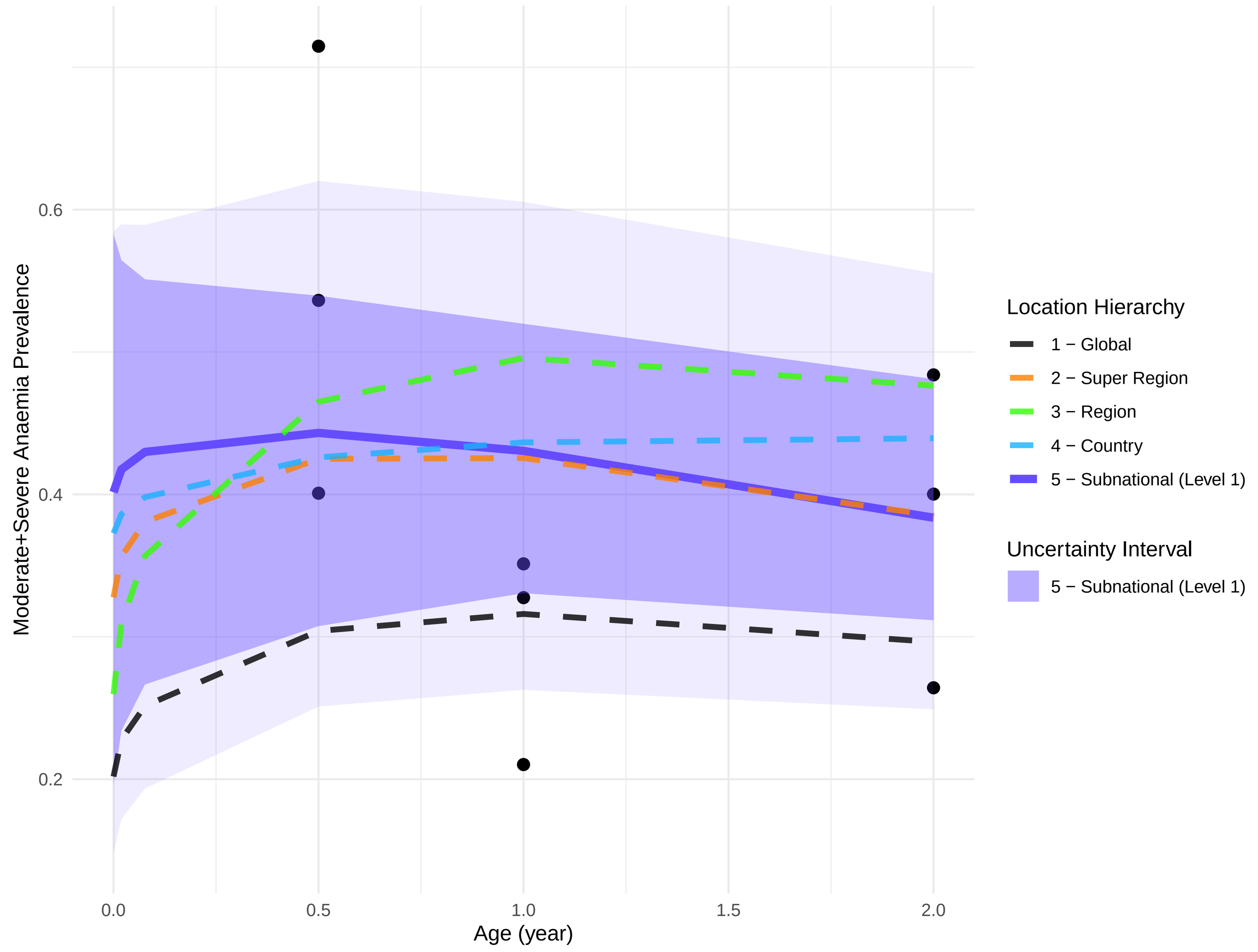

MR−BRT Age/Sex Fits − Non−pregnant Females <2 Years Old − Moderate+Severe Anaemia Prevalence − Ebonyi
WHO−2024 Adjustment Method

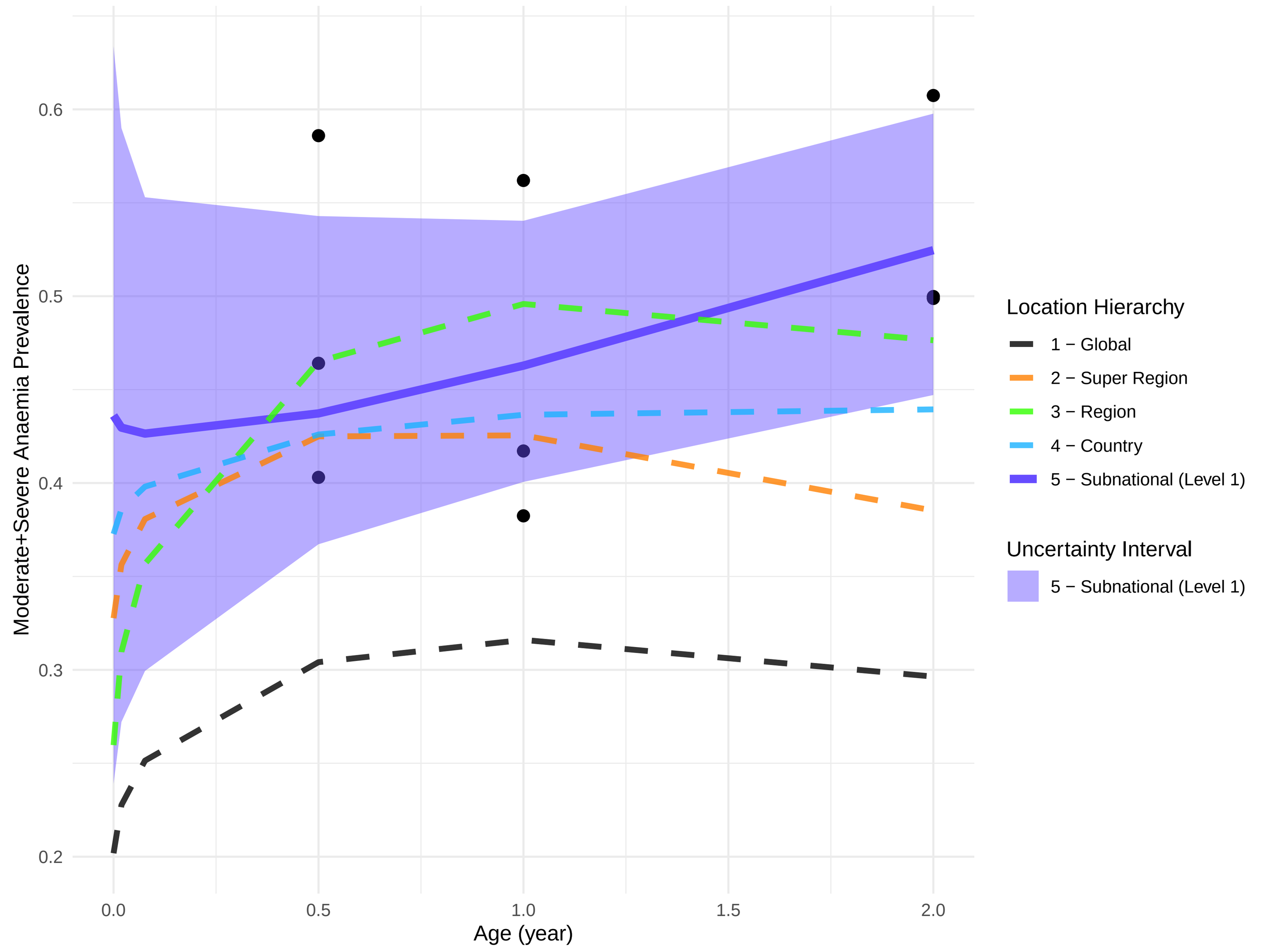

MR−BRT Age/Sex Fits − Non−pregnant Females <2 Years Old − Moderate+Severe Anaemia Prevalence − Edo
WHO−2024 Adjustment Method

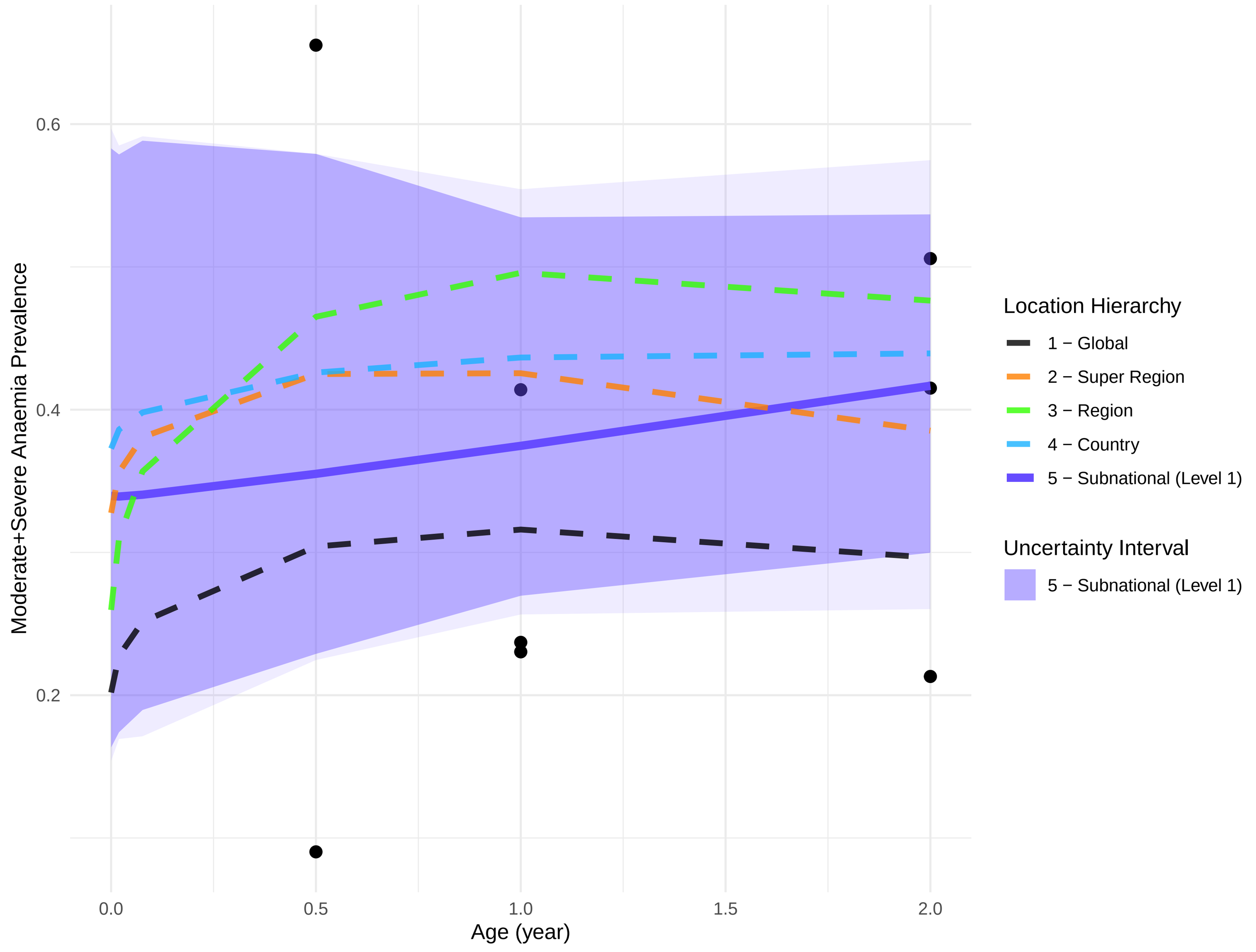

MR−BRT Age/Sex Fits − Non−pregnant Females <2 Years Old − Moderate+Severe Anaemia Prevalence − Ekiti
WHO−2024 Adjustment Method

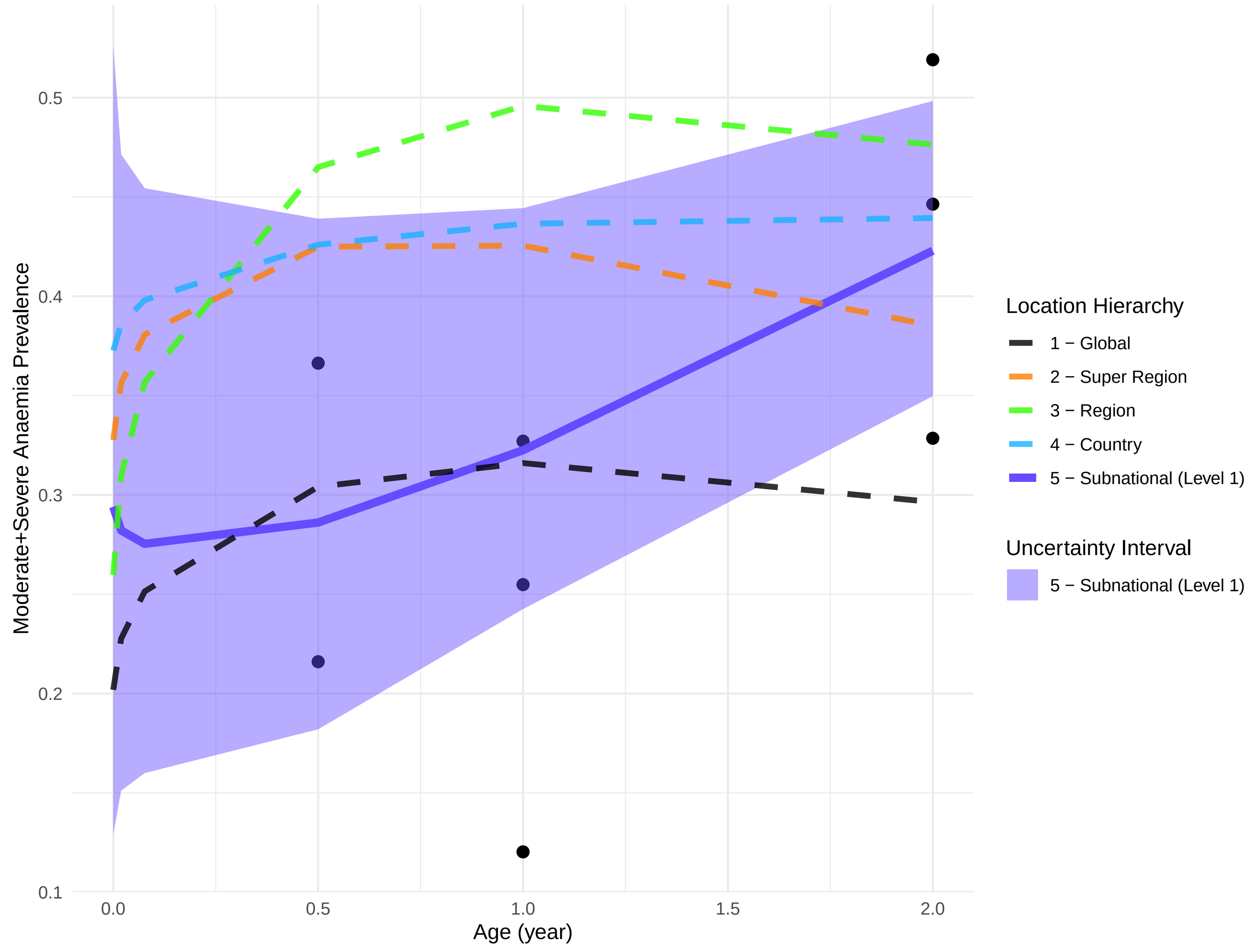

MR−BRT Age/Sex Fits − Non−pregnant Females <2 Years Old − Moderate+Severe Anaemia Prevalence − Enugu
WHO−2024 Adjustment Method

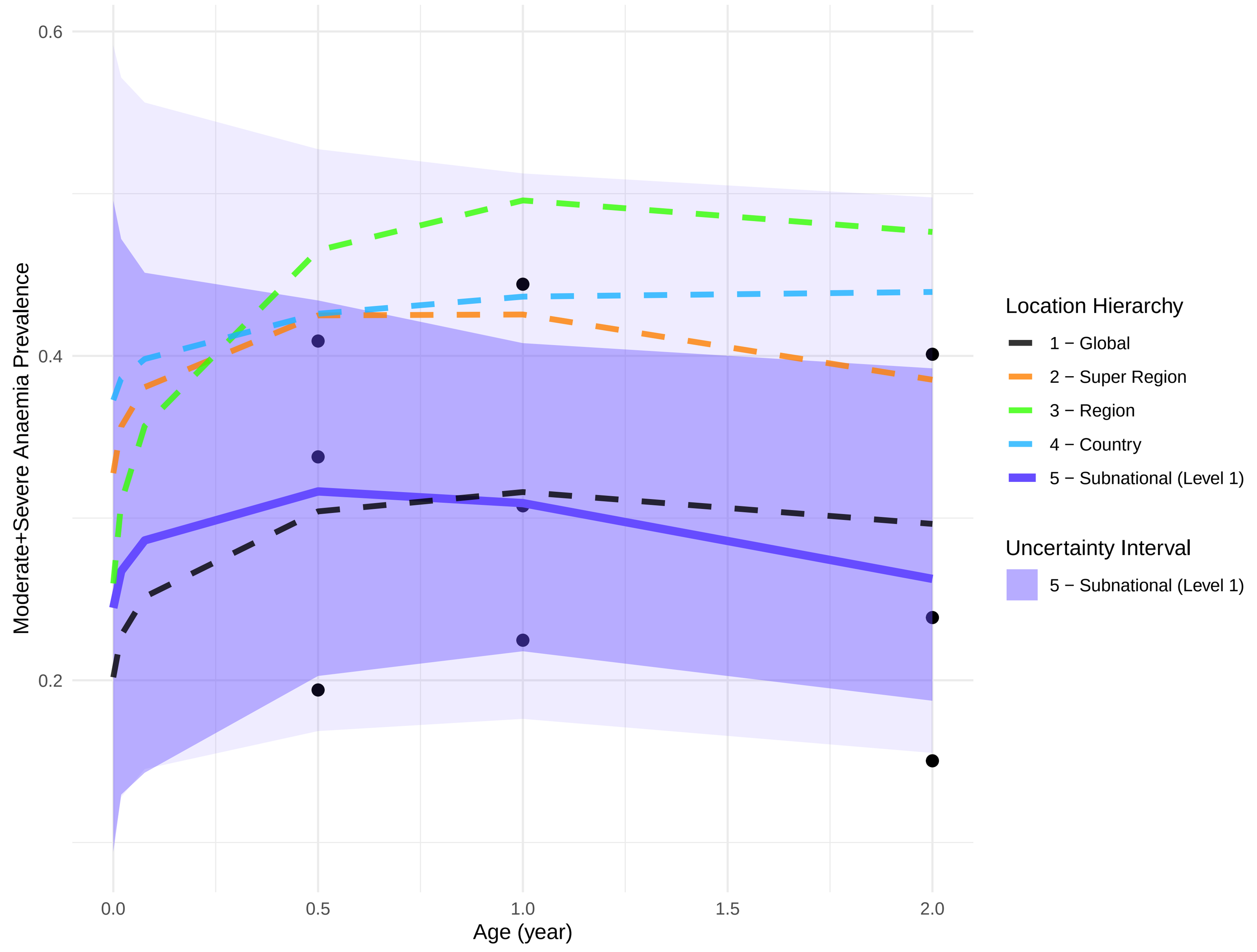

MR−BRT Age/Sex Fits − Non−pregnant Females <2 Years Old − Moderate+Severe Anaemia Prevalence − FCT (Abuja)
WHO−2024 Adjustment Method

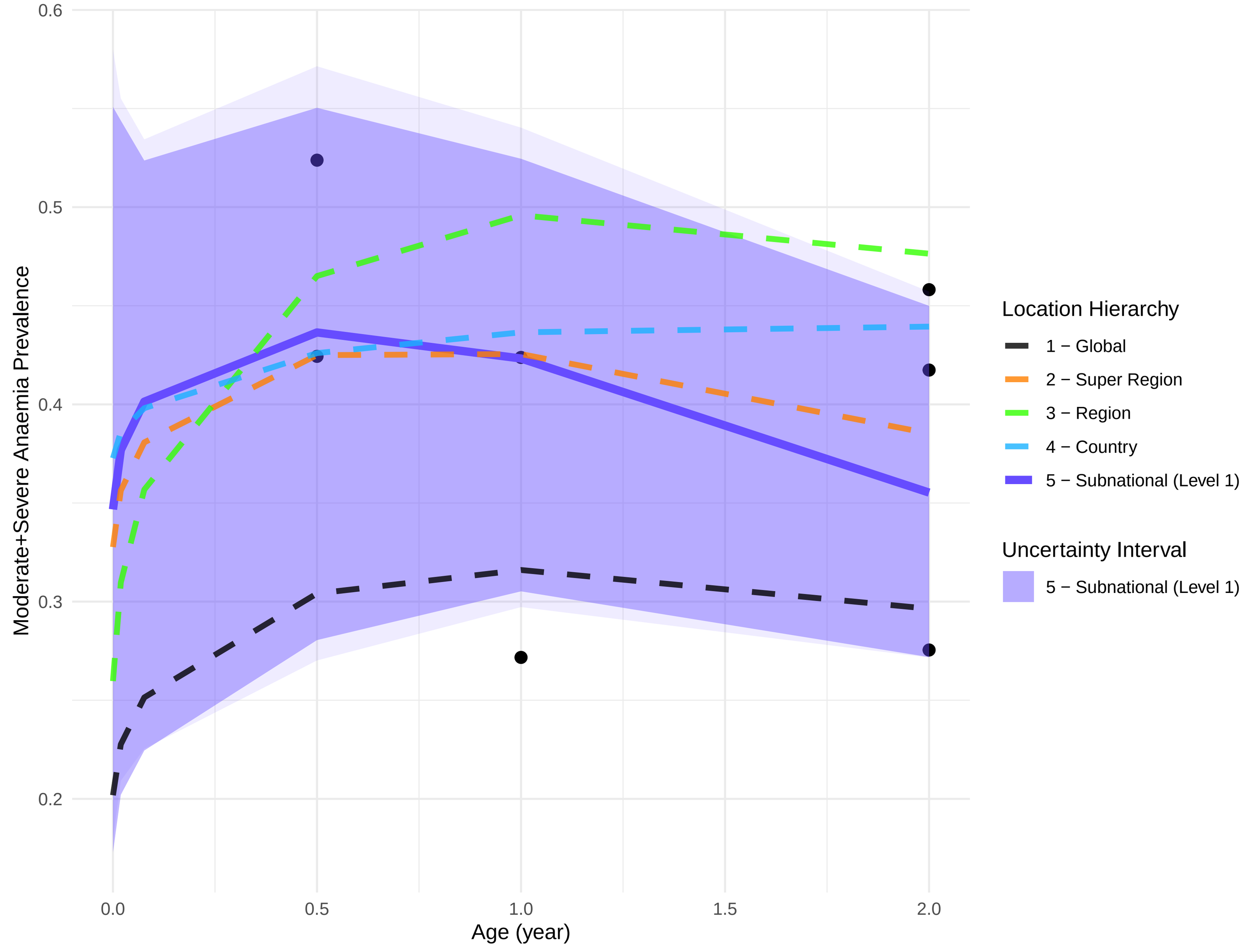

MR−BRT Age/Sex Fits − Non−pregnant Females <2 Years Old − Moderate+Severe Anaemia Prevalence − Gombe
WHO−2024 Adjustment Method

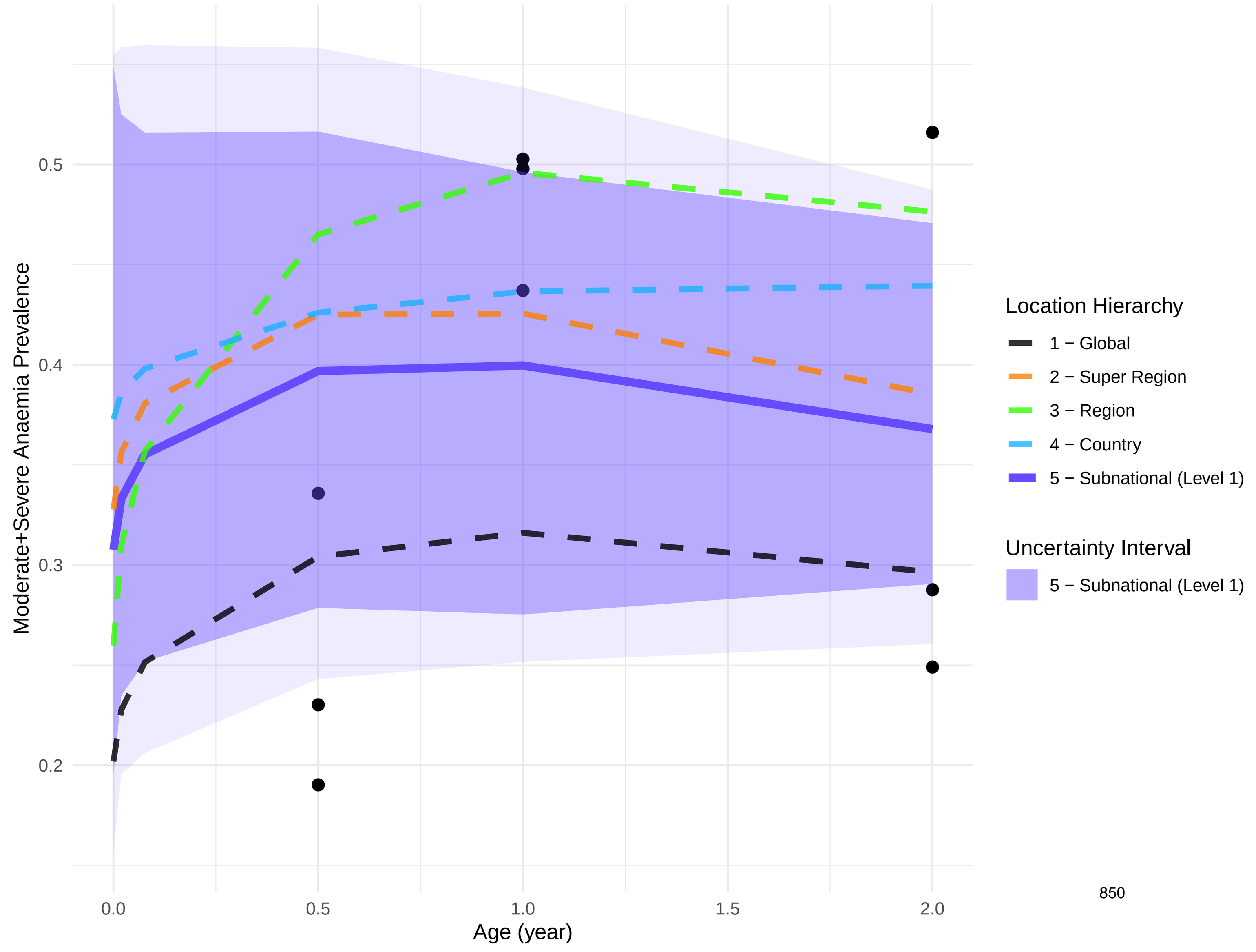

MR−BRT Age/Sex Fits − Non−pregnant Females <2 Years Old − Moderate+Severe Anaemia Prevalence − Imo
WHO−2024 Adjustment Method

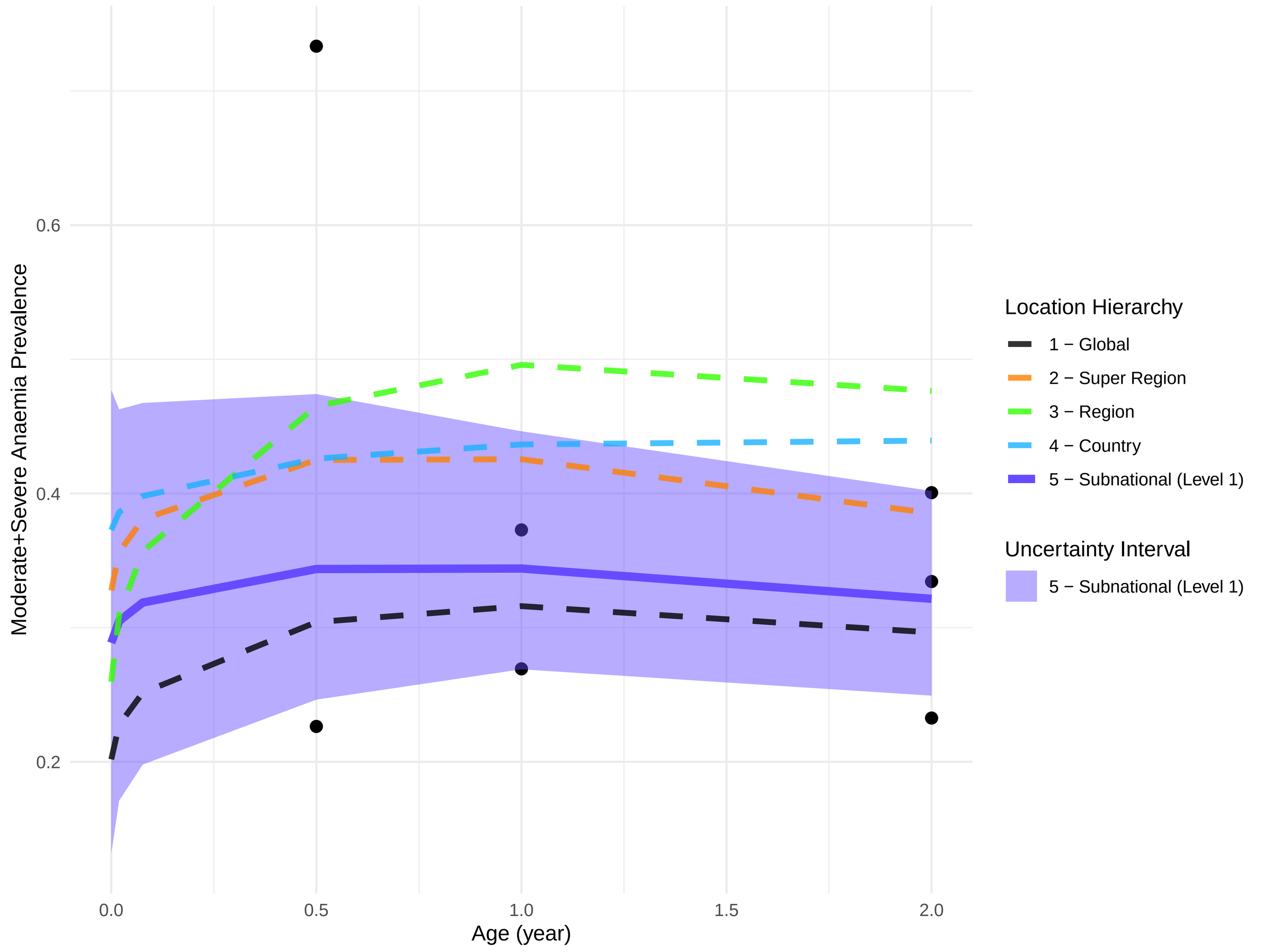

MR−BRT Age/Sex Fits − Non−pregnant Females <2 Years Old − Moderate+Severe Anaemia Prevalence − Jigawa
WHO−2024 Adjustment Method

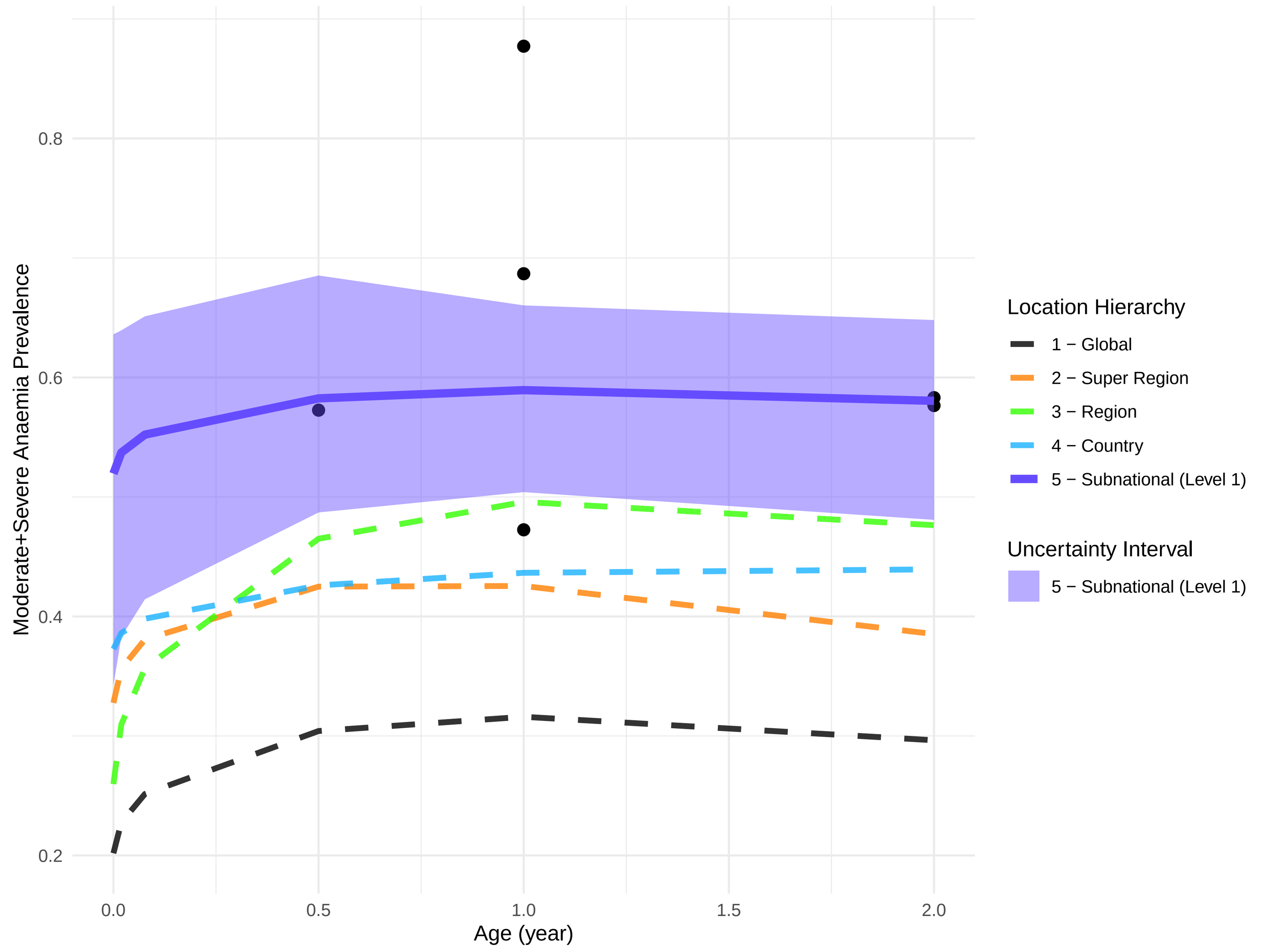

MR−BRT Age/Sex Fits − Non−pregnant Females <2 Years Old − Moderate+Severe Anaemia Prevalence − Kaduna
WHO−2024 Adjustment Method

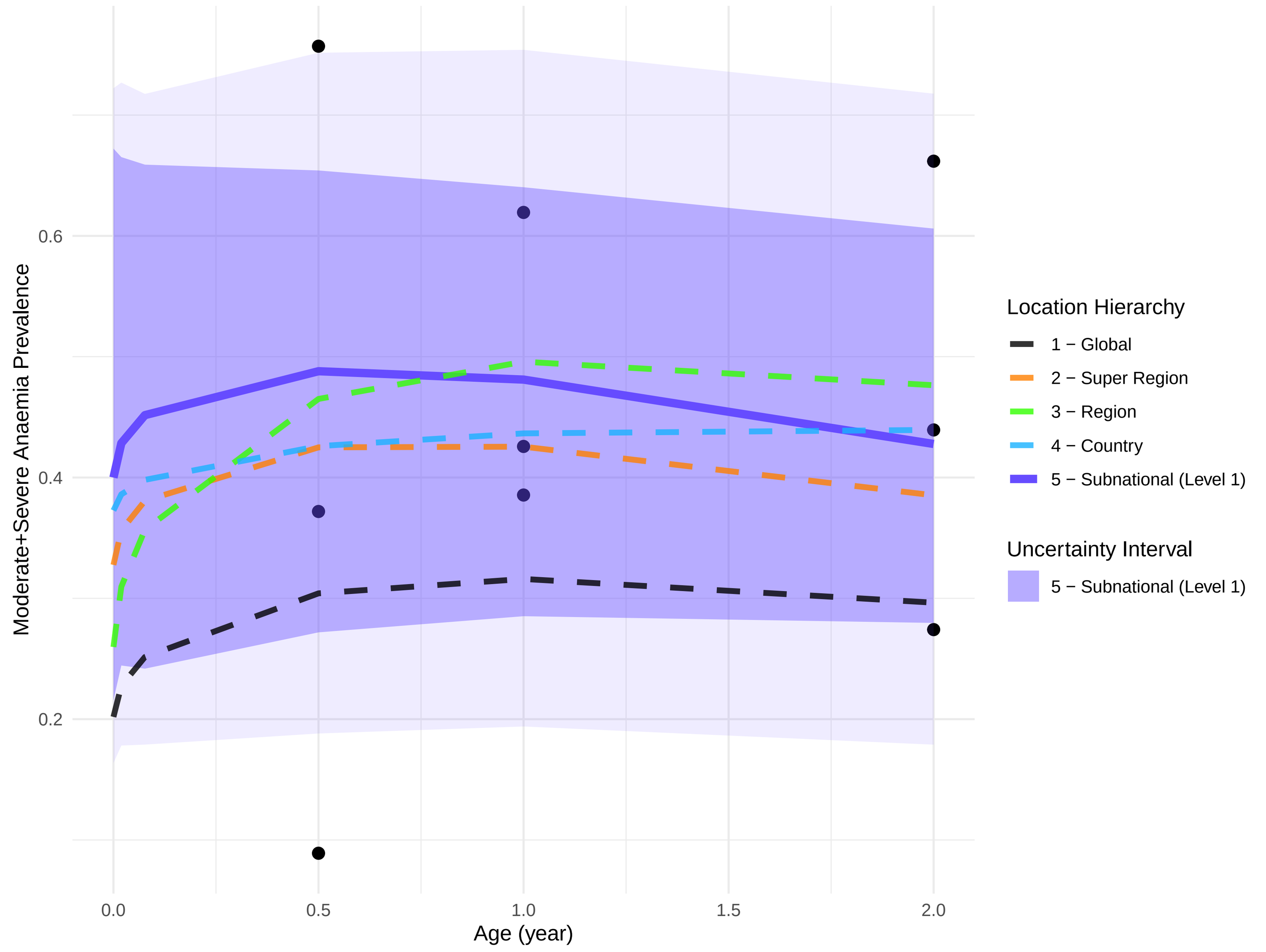

MR−BRT Age/Sex Fits − Non−pregnant Females <2 Years Old − Moderate+Severe Anaemia Prevalence − Kano
WHO−2024 Adjustment Method

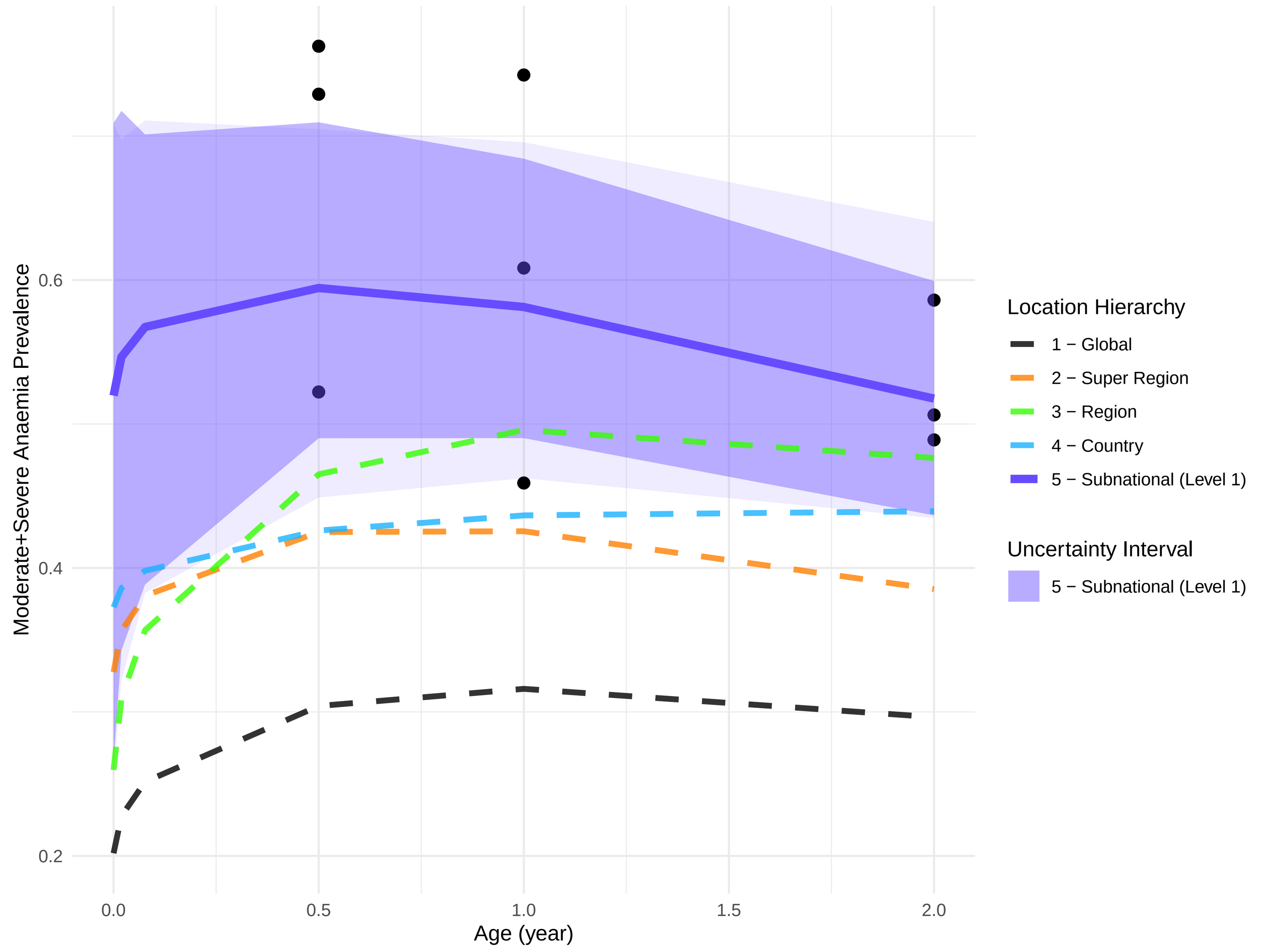

MR−BRT Age/Sex Fits − Non−pregnant Females <2 Years Old − Moderate+Severe Anaemia Prevalence − Katsina
WHO−2024 Adjustment Method

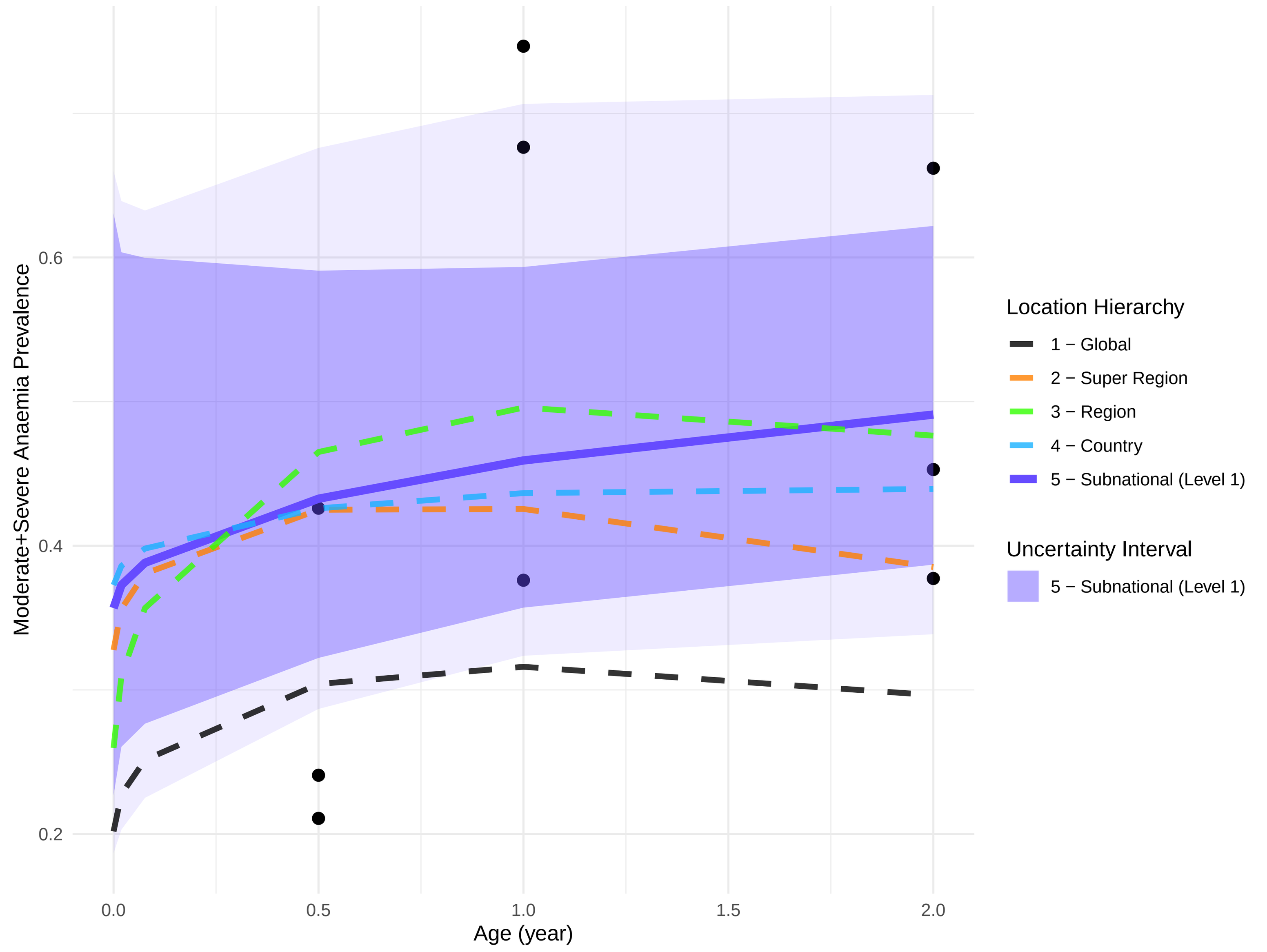

MR−BRT Age/Sex Fits − Non−pregnant Females <2 Years Old − Moderate+Severe Anaemia Prevalence − Kebbi
WHO−2024 Adjustment Method

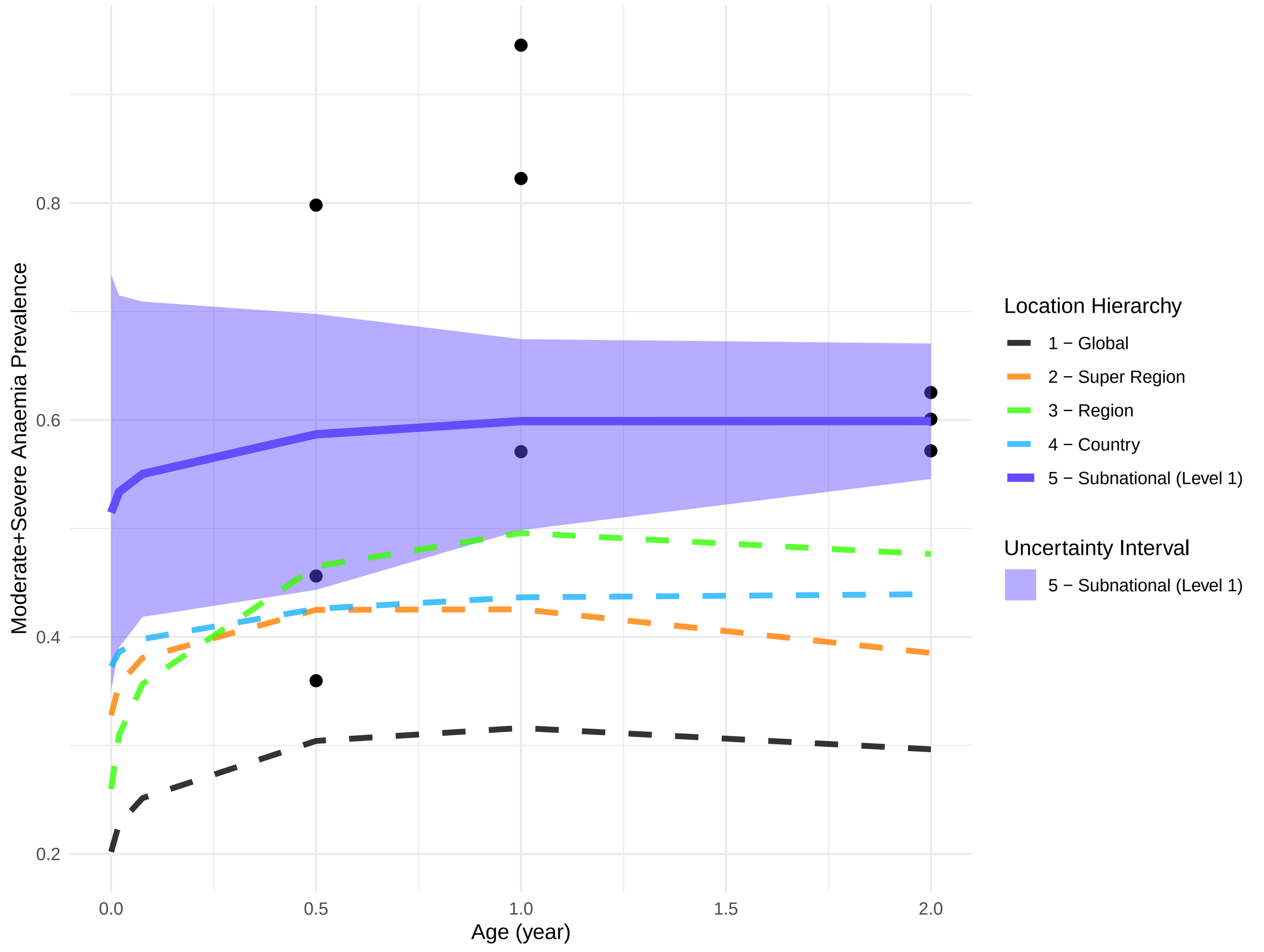

MR−BRT Age/Sex Fits − Non−pregnant Females <2 Years Old − Moderate+Severe Anaemia Prevalence − Kogi
WHO−2024 Adjustment Method

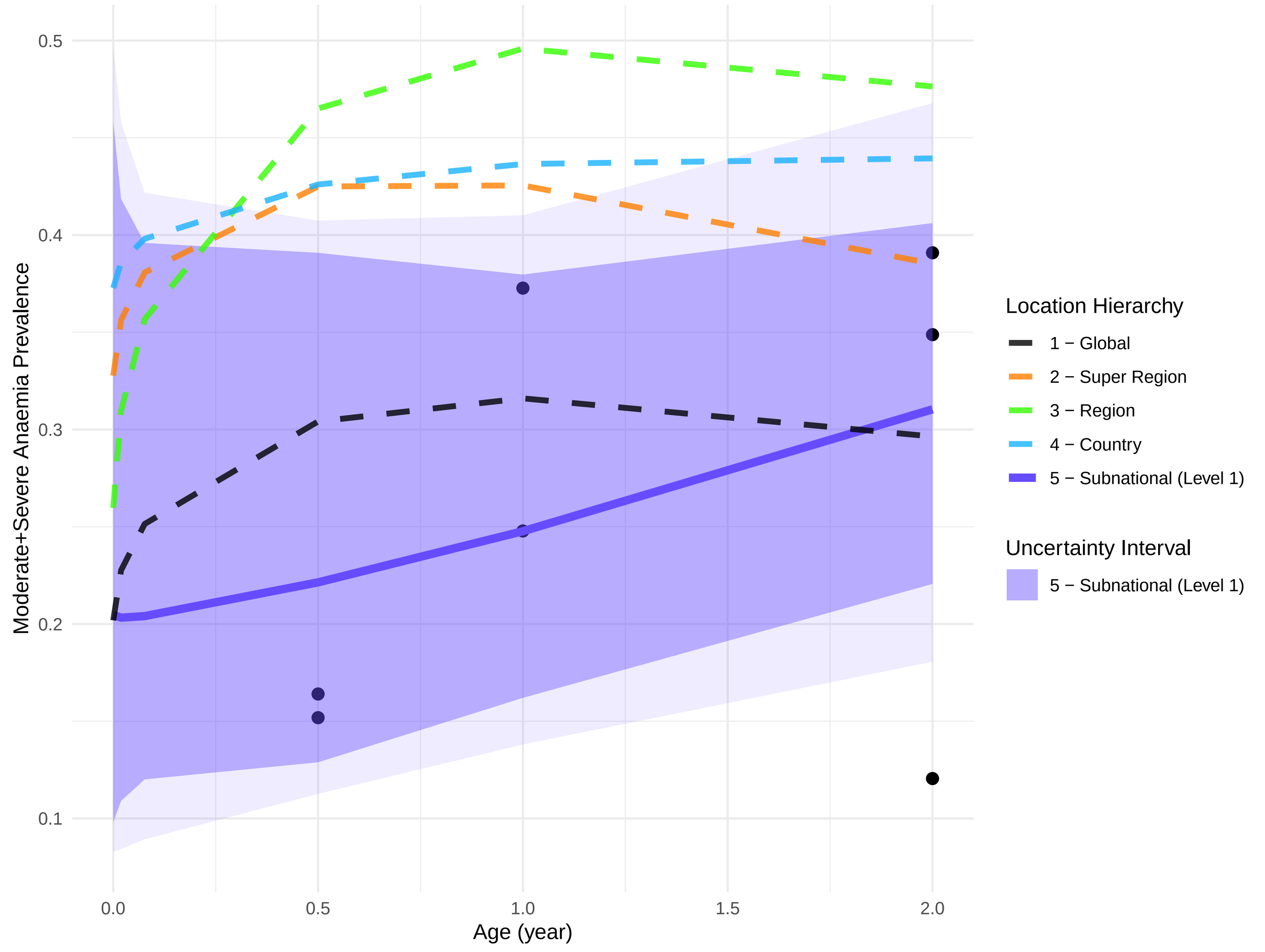

MR−BRT Age/Sex Fits − Non−pregnant Females <2 Years Old − Moderate+Severe Anaemia Prevalence − Kwara
WHO−2024 Adjustment Method

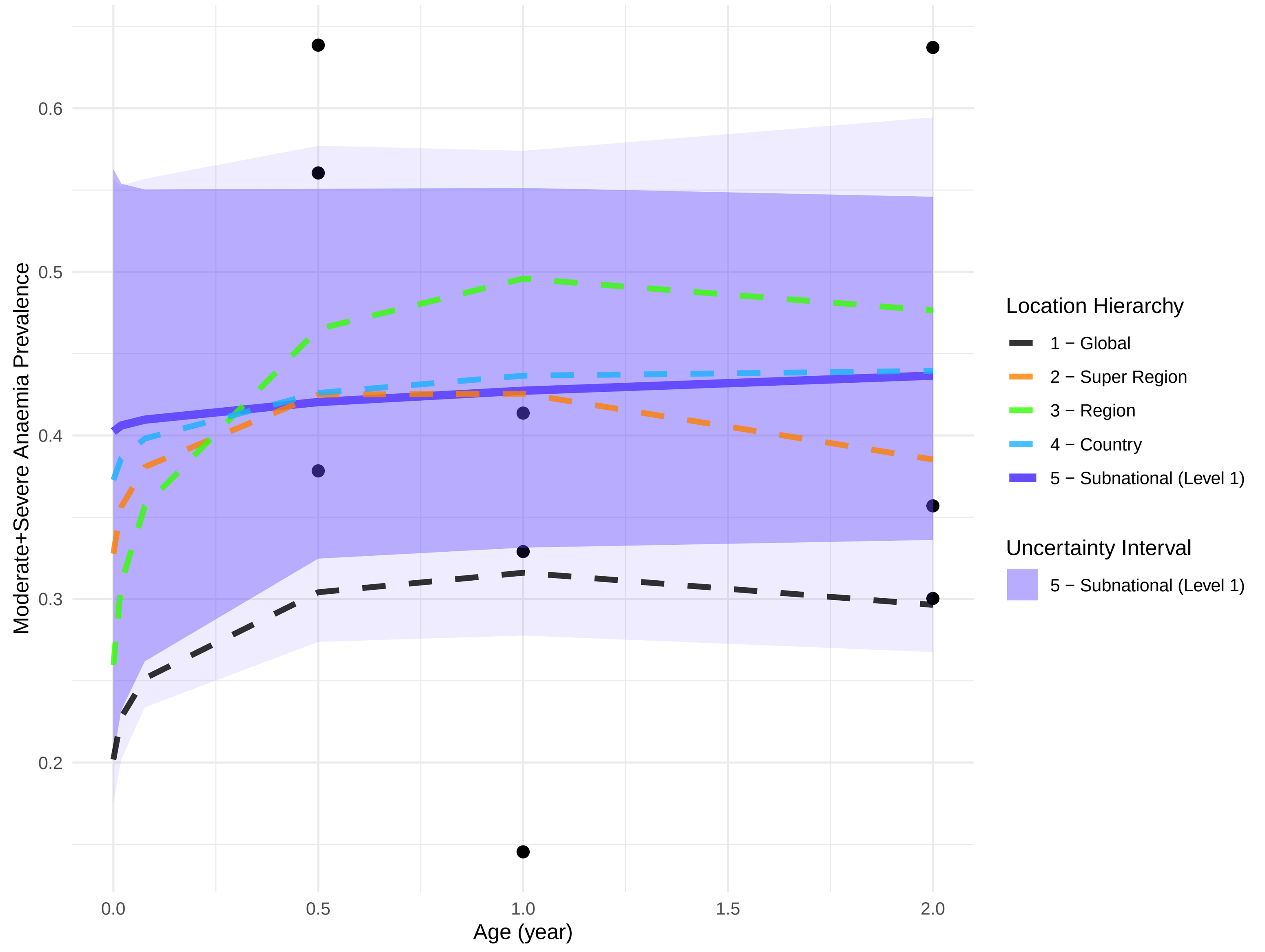

MR−BRT Age/Sex Fits − Non−pregnant Females <2 Years Old − Moderate+Severe Anaemia Prevalence − Lagos
WHO−2024 Adjustment Method

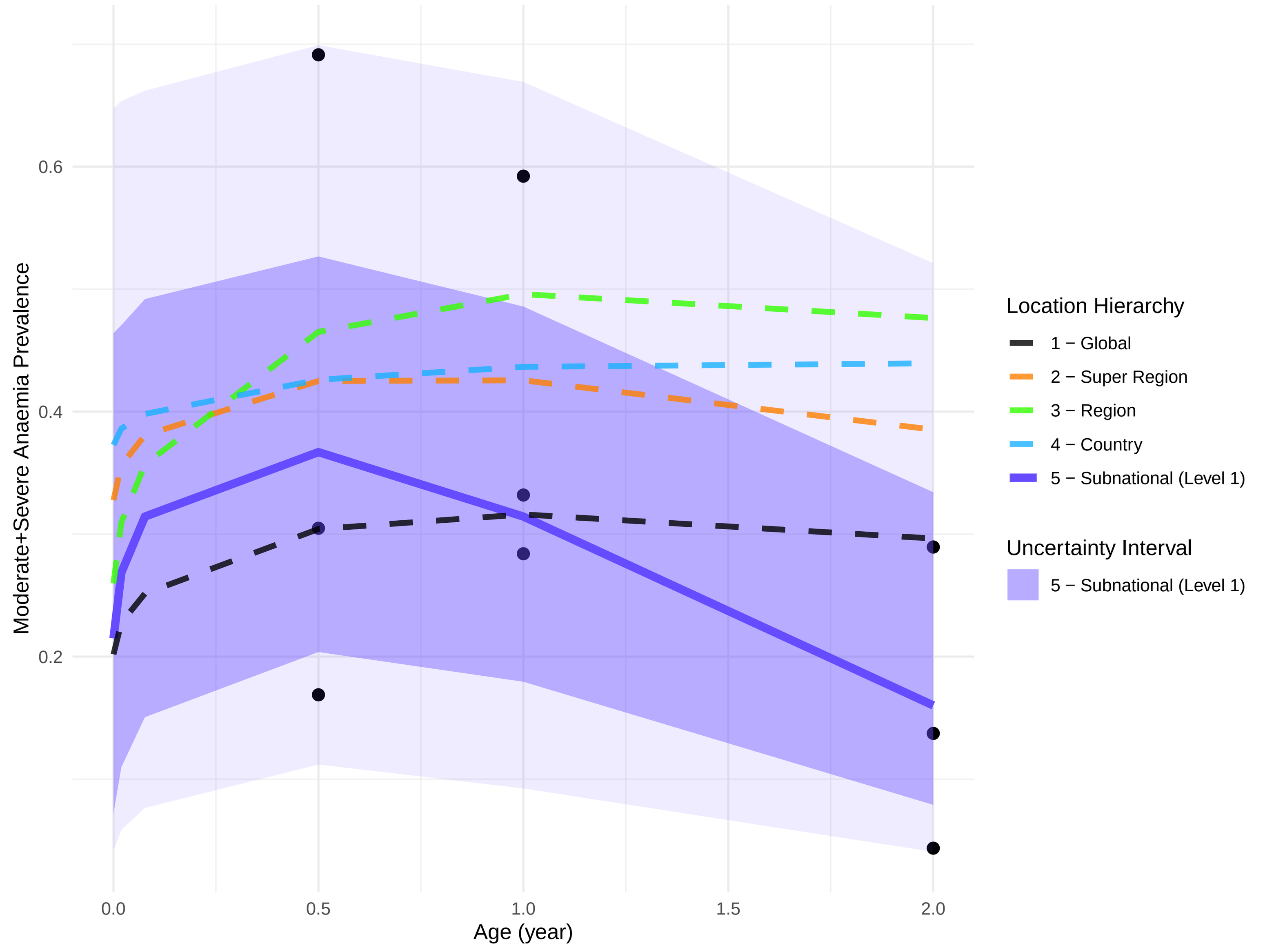

MR−BRT Age/Sex Fits − Non−pregnant Females <2 Years Old − Moderate+Severe Anaemia Prevalence − Nasarawa
WHO−2024 Adjustment Method

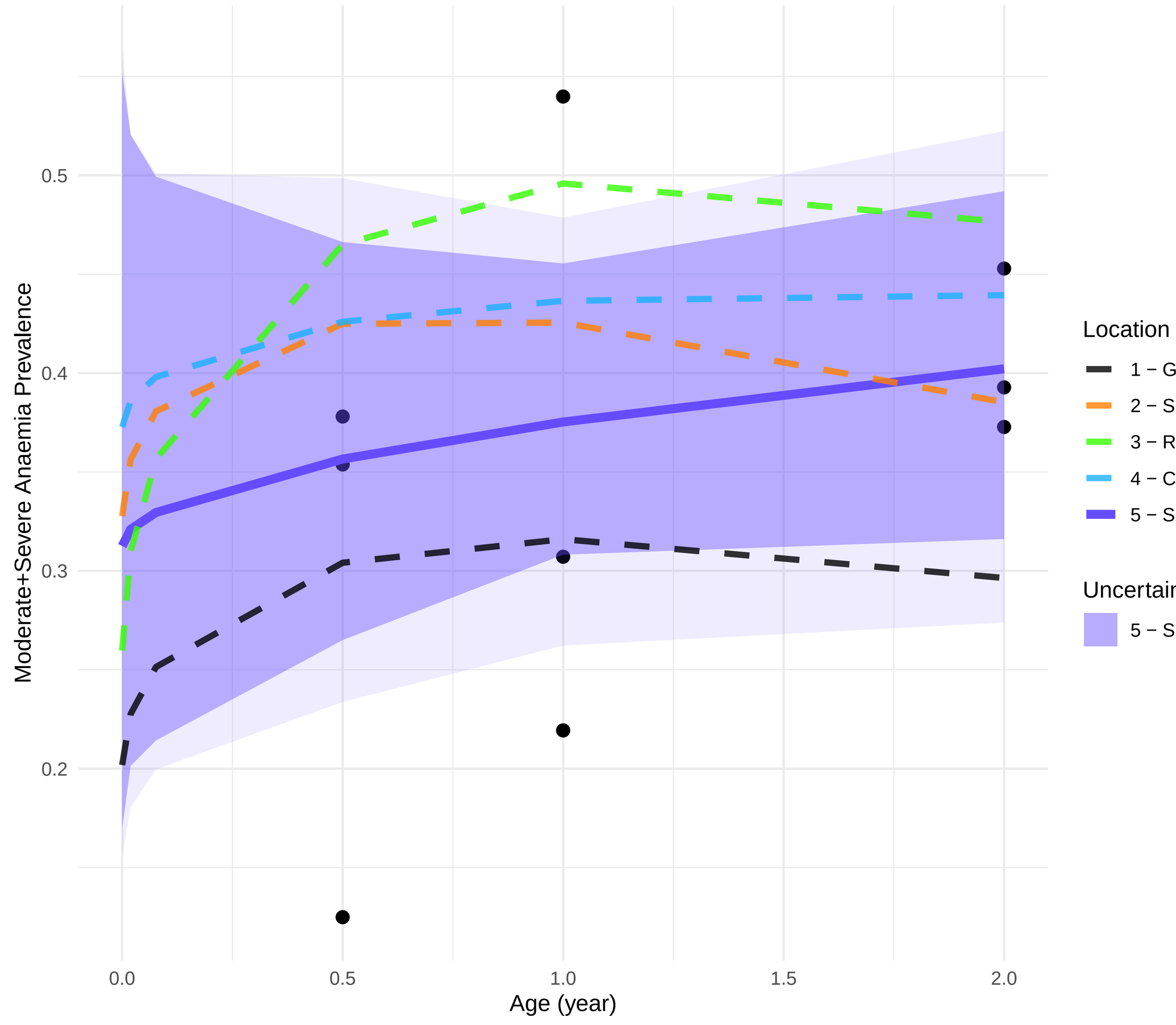

MR−BRT Age/Sex Fits − Non−pregnant Females <2 Years Old − Moderate+Severe Anaemia Prevalence − Niger
WHO−2024 Adjustment Method

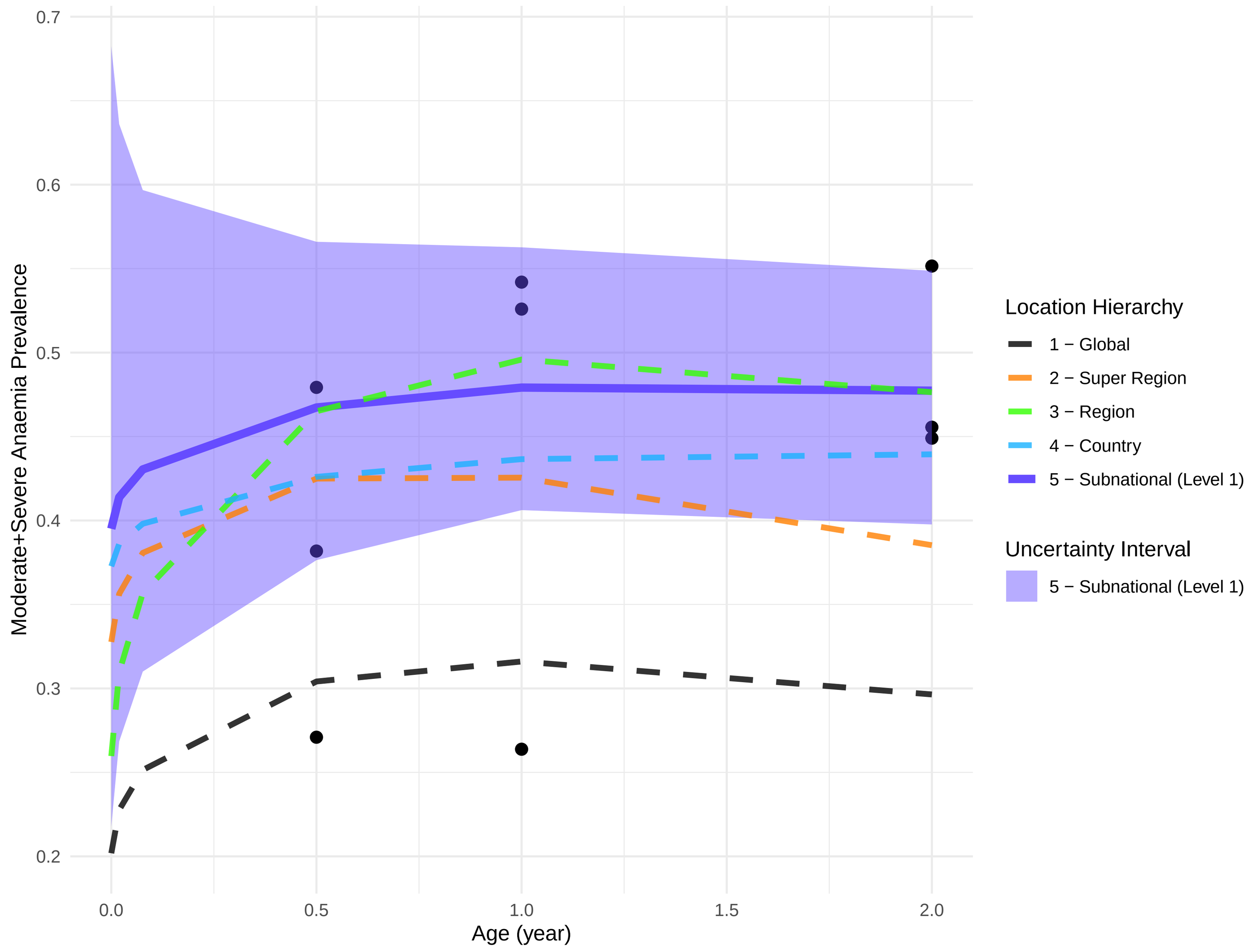

MR−BRT Age/Sex Fits − Non−pregnant Females <2 Years Old − Moderate+Severe Anaemia Prevalence − Ogun
WHO−2024 Adjustment Method

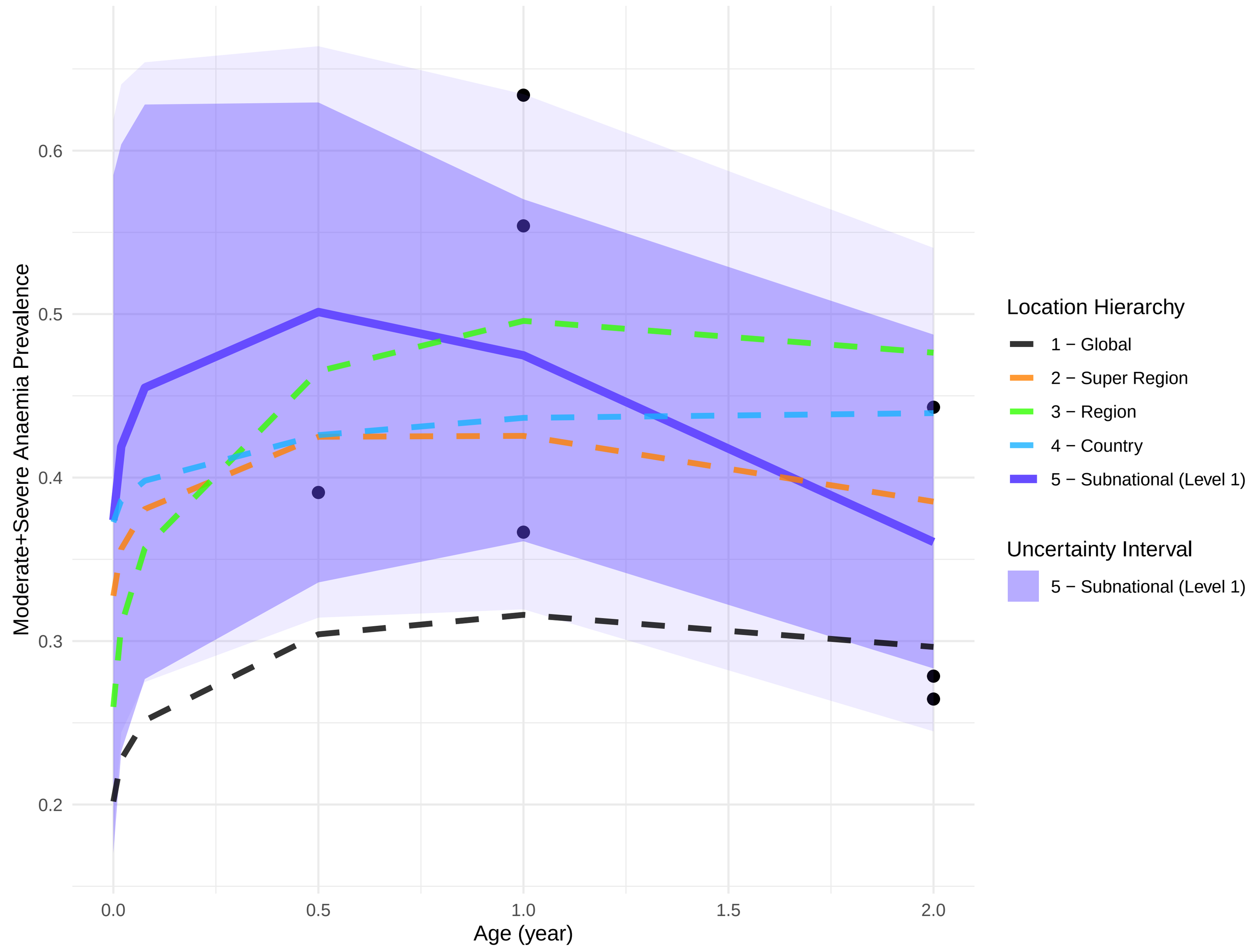

MR−BRT Age/Sex Fits − Non−pregnant Females <2 Years Old − Moderate+Severe Anaemia Prevalence − Ondo
WHO−2024 Adjustment Method

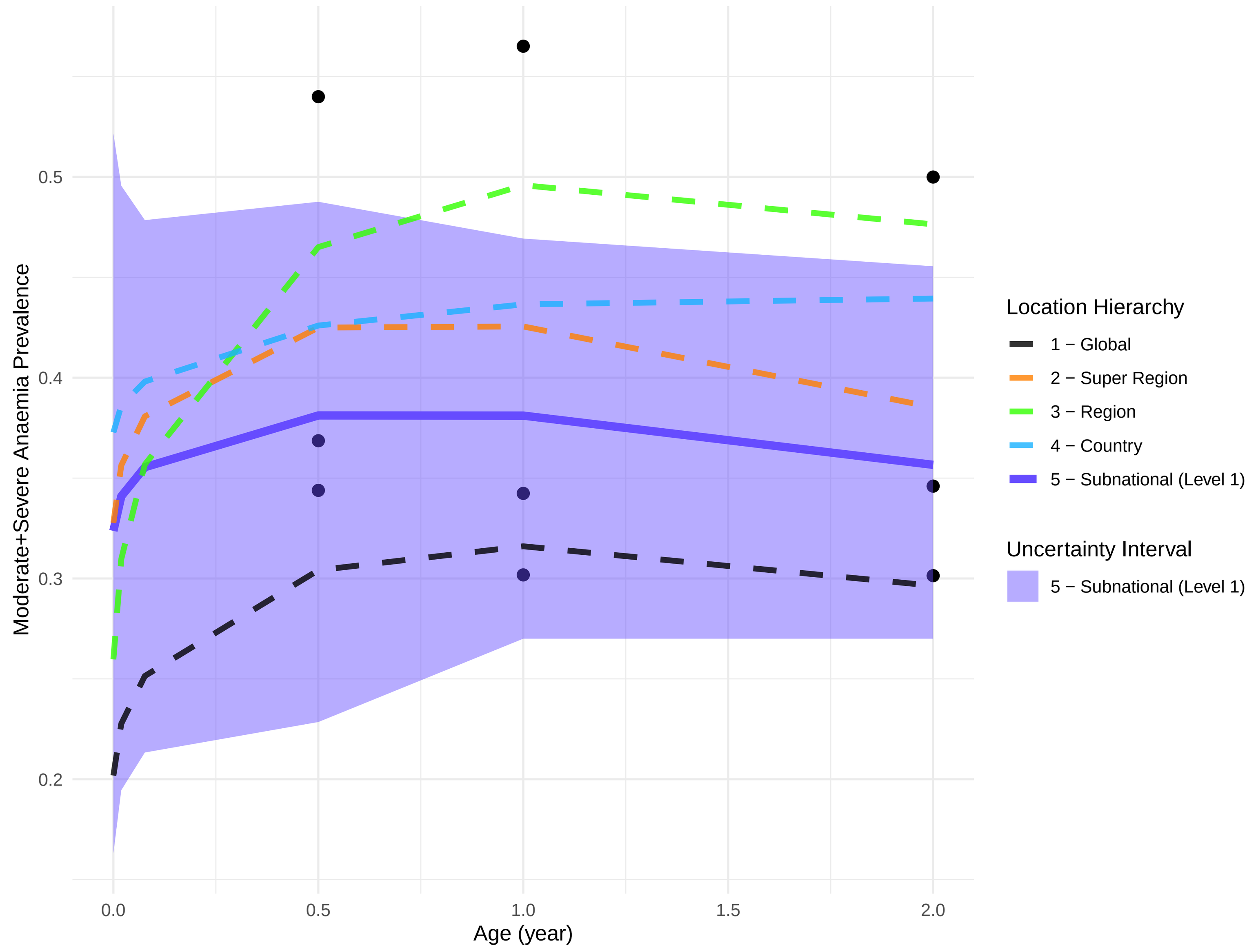

MR−BRT Age/Sex Fits − Non−pregnant Females <2 Years Old − Moderate+Severe Anaemia Prevalence − Osun
WHO−2024 Adjustment Method

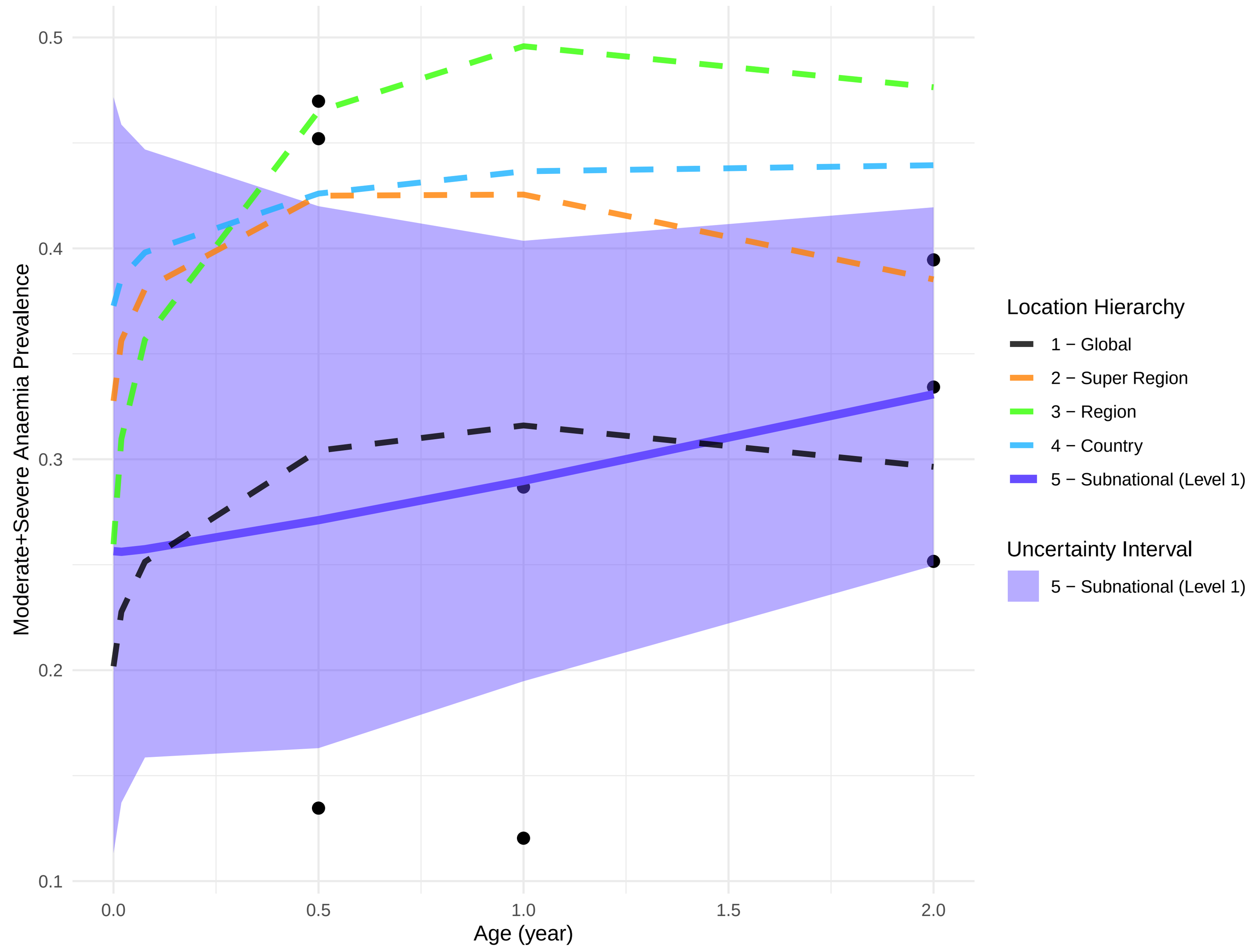

MR−BRT Age/Sex Fits − Non−pregnant Females <2 Years Old − Moderate+Severe Anaemia Prevalence − Oyo
WHO−2024 Adjustment Method

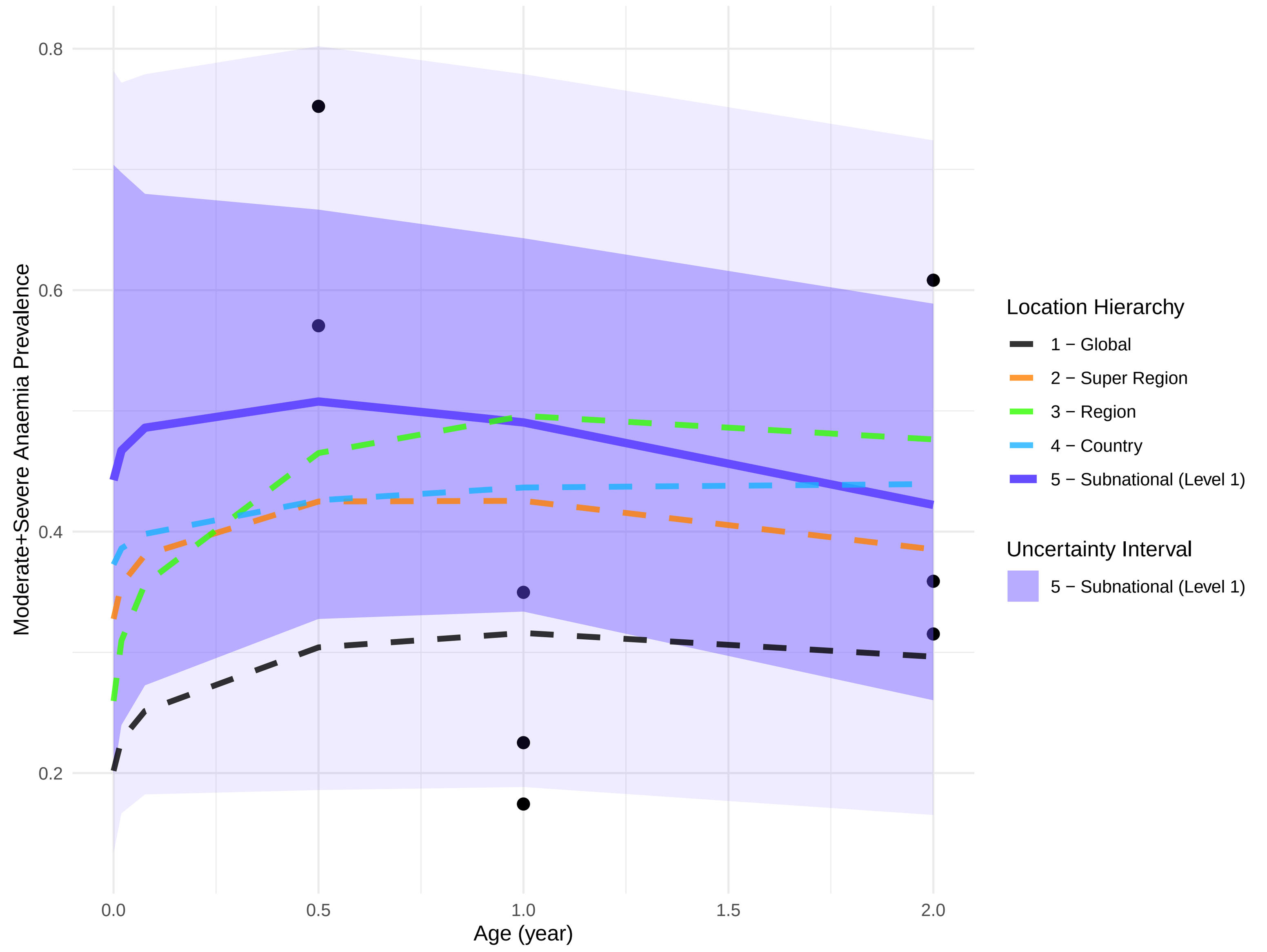

MR−BRT Age/Sex Fits − Non−pregnant Females <2 Years Old − Moderate+Severe Anaemia Prevalence − Plateau
WHO−2024 Adjustment Method

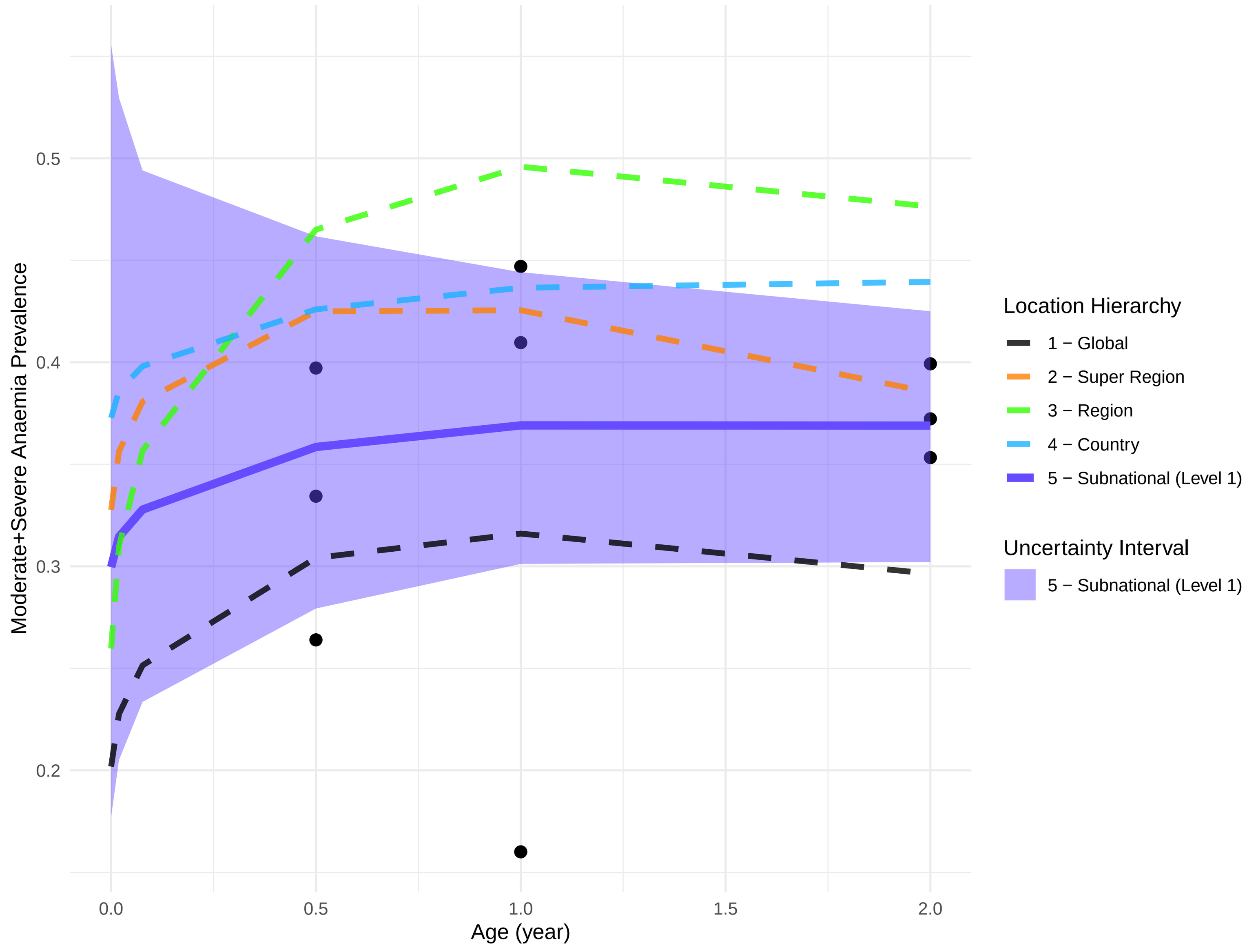

MR−BRT Age/Sex Fits − Non−pregnant Females <2 Years Old − Moderate+Severe Anaemia Prevalence − Rivers
WHO−2024 Adjustment Method

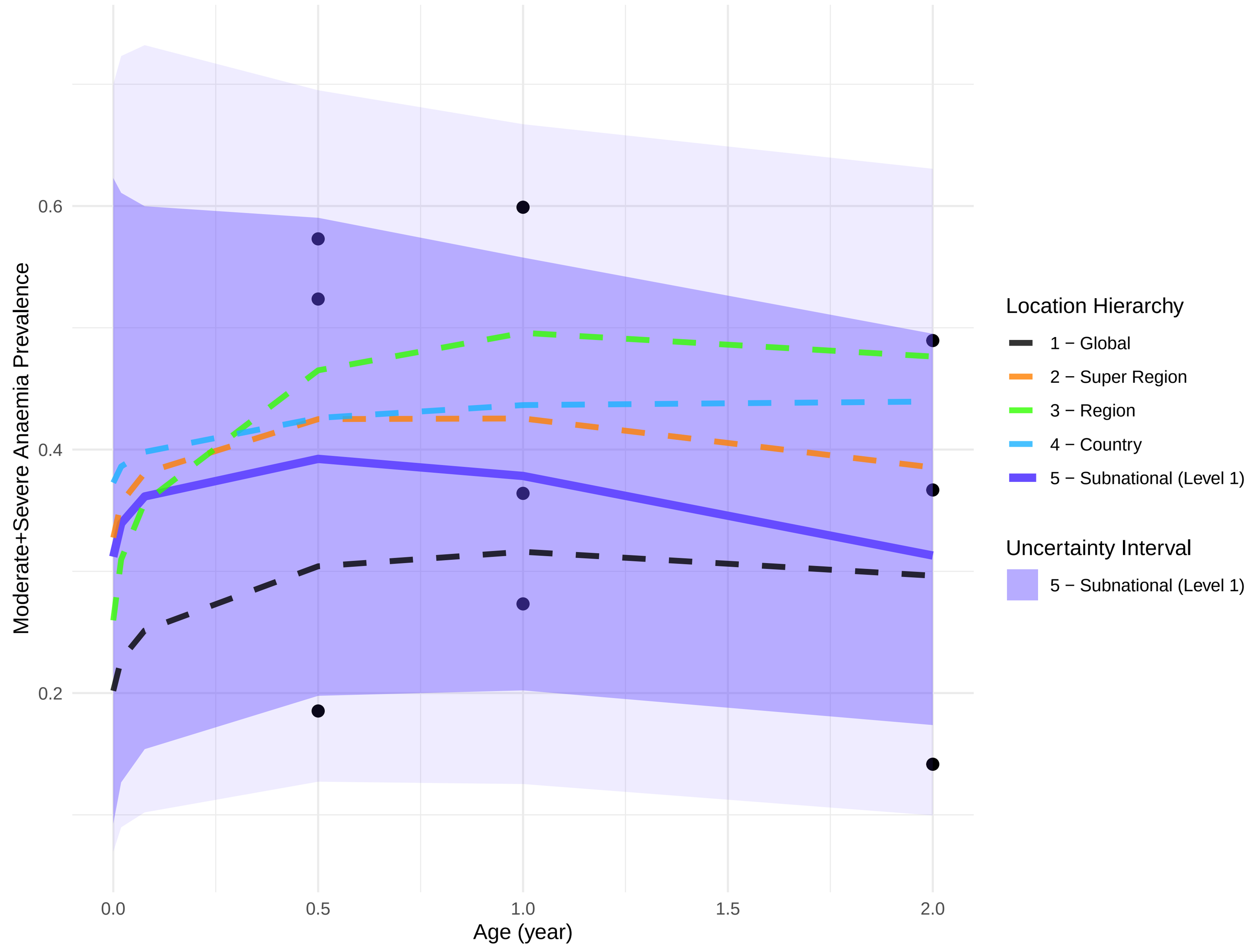

MR−BRT Age/Sex Fits − Non−pregnant Females <2 Years Old − Moderate+Severe Anaemia Prevalence − Sokoto
WHO−2024 Adjustment Method

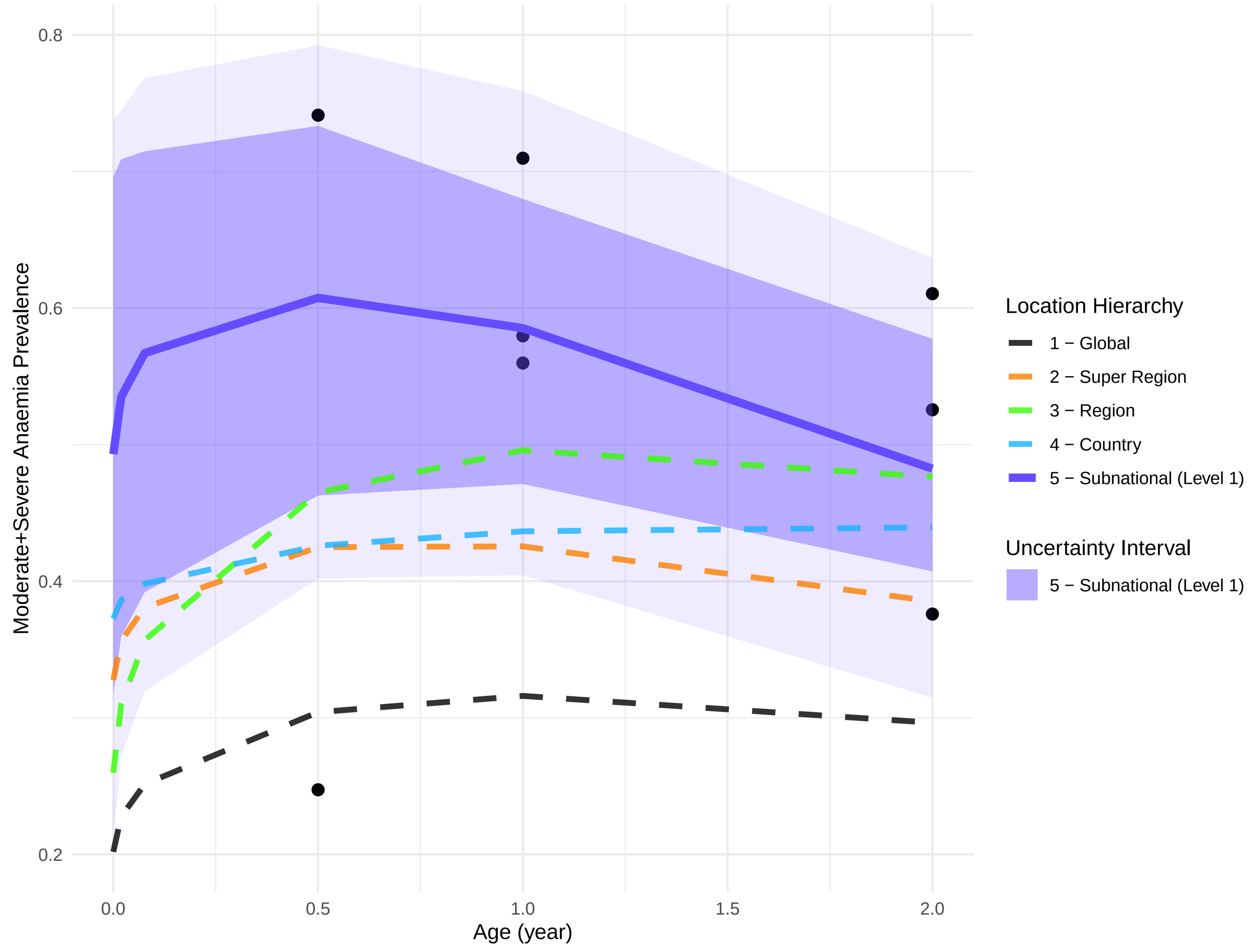

MR−BRT Age/Sex Fits − Non−pregnant Females <2 Years Old − Moderate+Severe Anaemia Prevalence − Taraba
WHO−2024 Adjustment Method

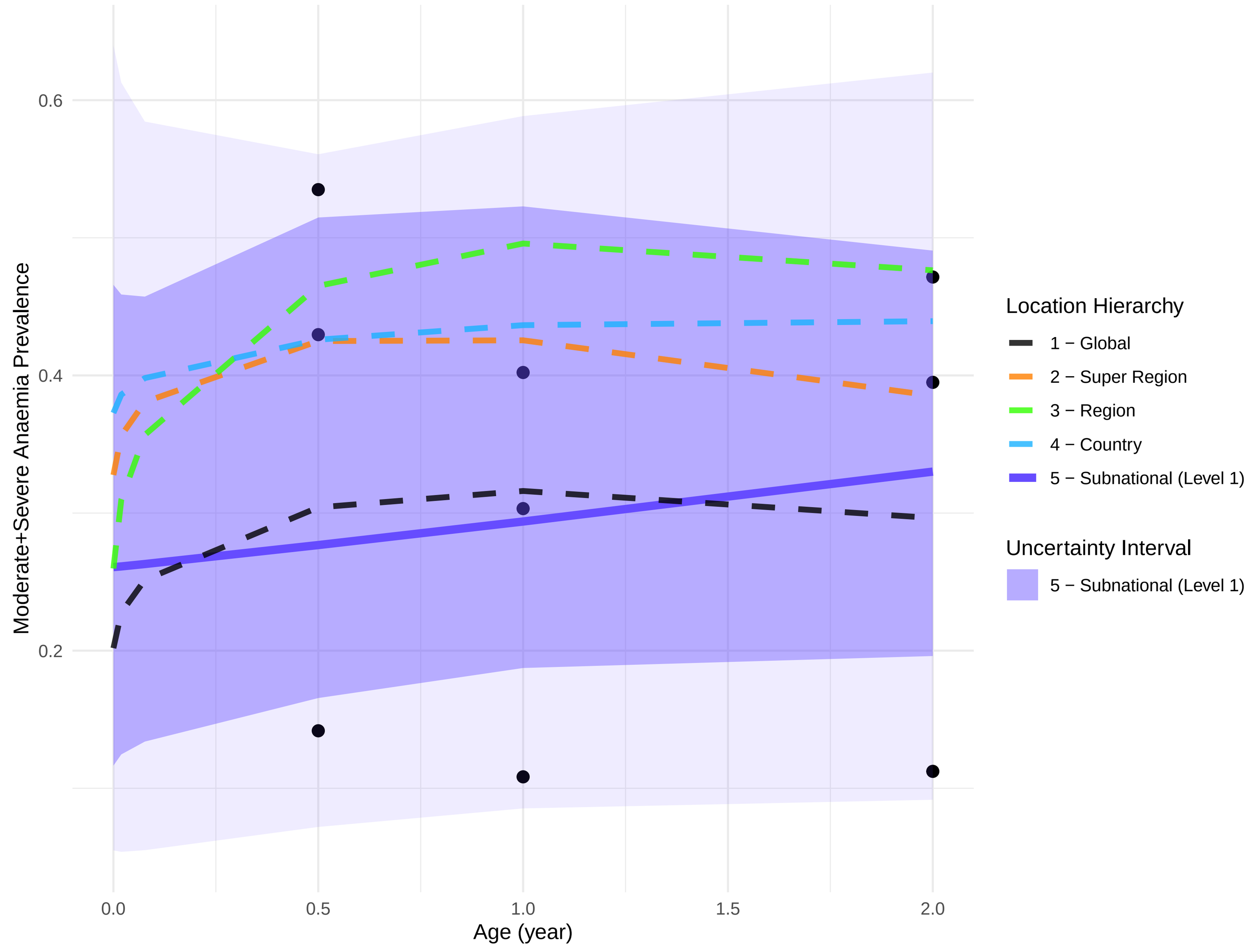

MR−BRT Age/Sex Fits − Non−pregnant Females <2 Years Old − Moderate+Severe Anaemia Prevalence − Yobe
WHO−2024 Adjustment Method

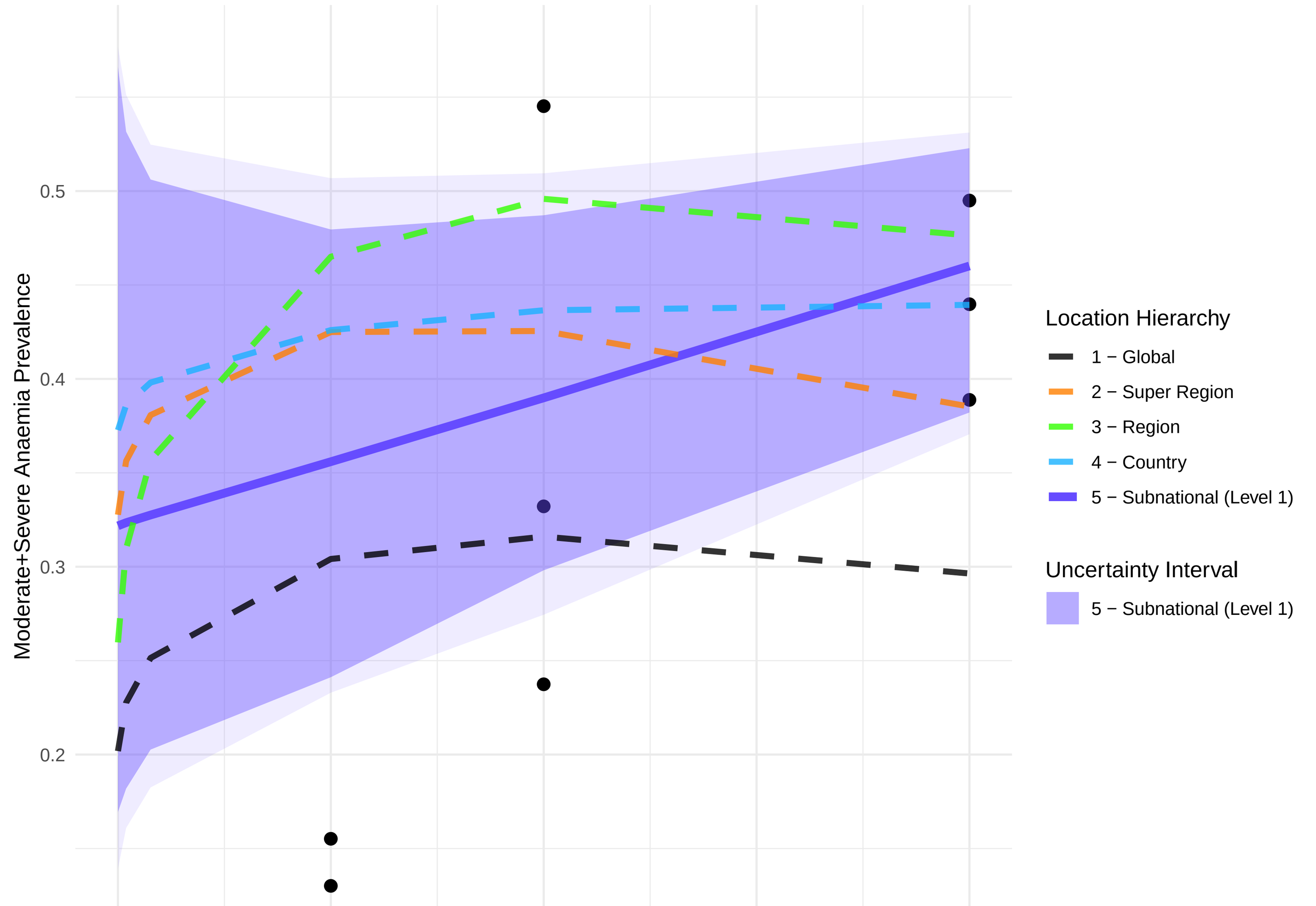

MR−BRT Age/Sex Fits − Non−pregnant Females <2 Years Old − Moderate+Severe Anaemia Prevalence − Zamfara
WHO−2024 Adjustment Method

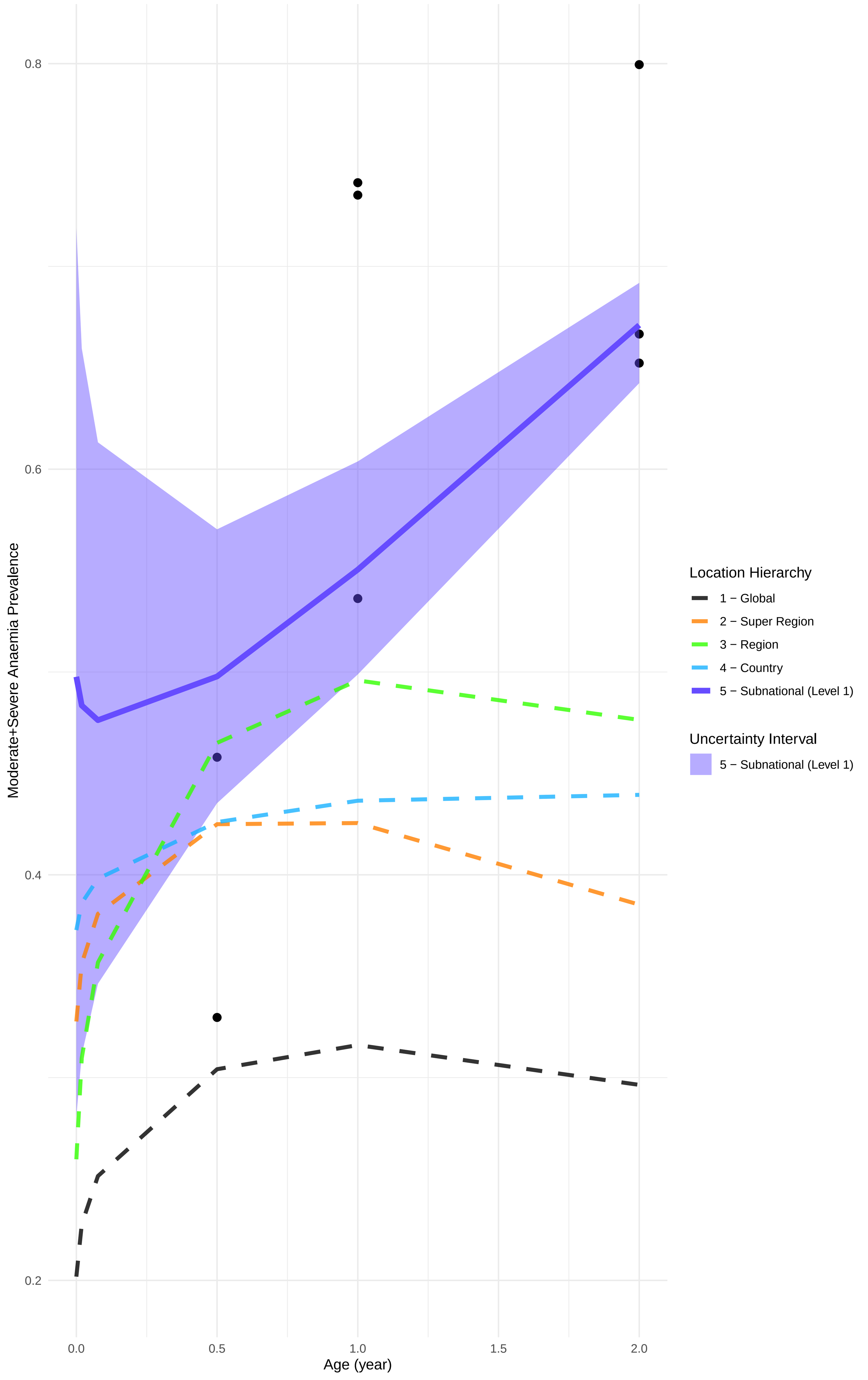

MR−BRT Age/Sex Fits − Non−pregnant Females <2 Years Old − Moderate+Severe Anaemia Prevalence − Senegal
WHO−2024 Adjustment Method

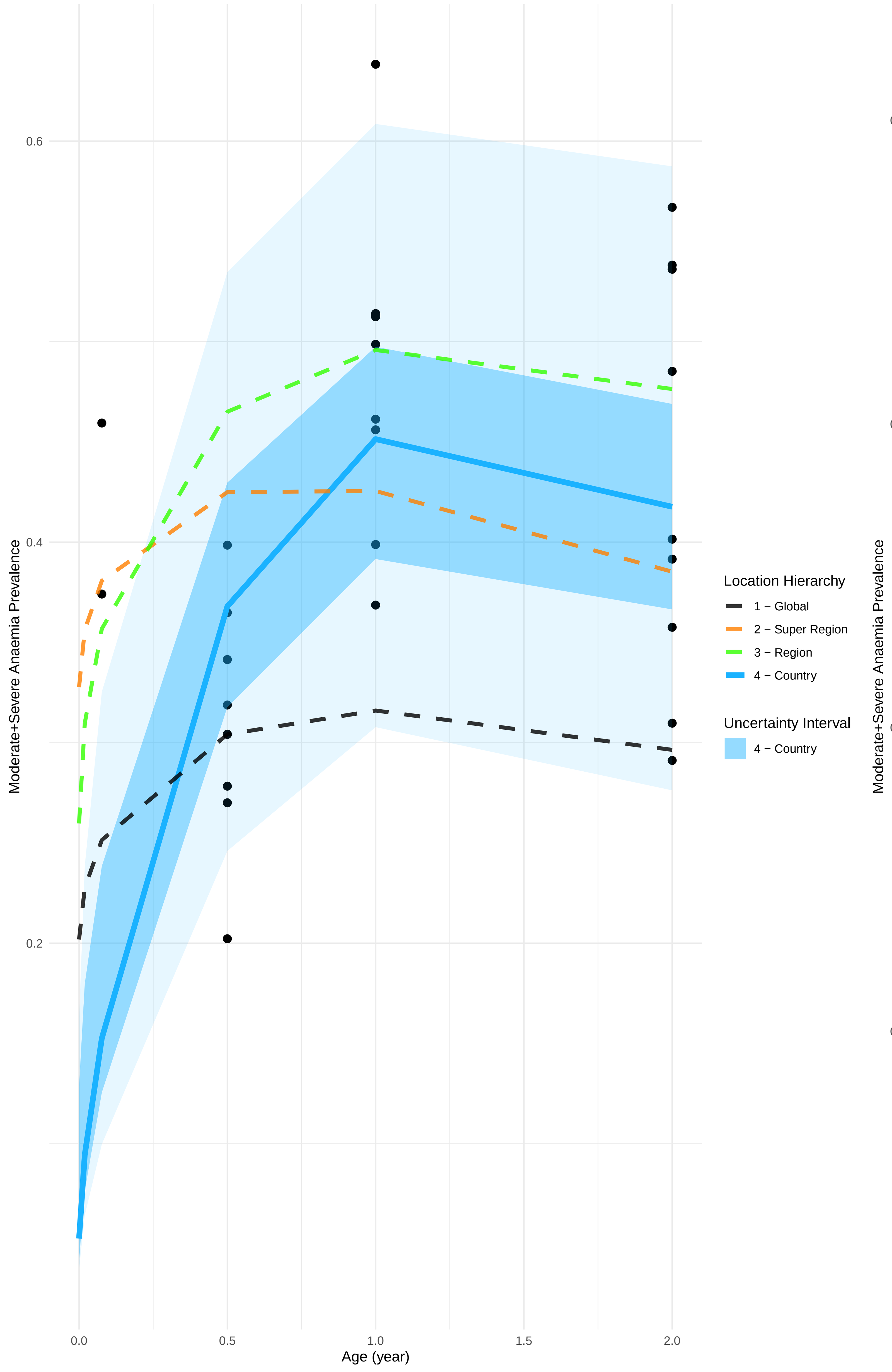

MR−BRT Age/Sex Fits − Non−pregnant Females <2 Years Old − Moderate+Severe Anaemia Prevalence − Sierra Leone
WHO−2024 Adjustment Method

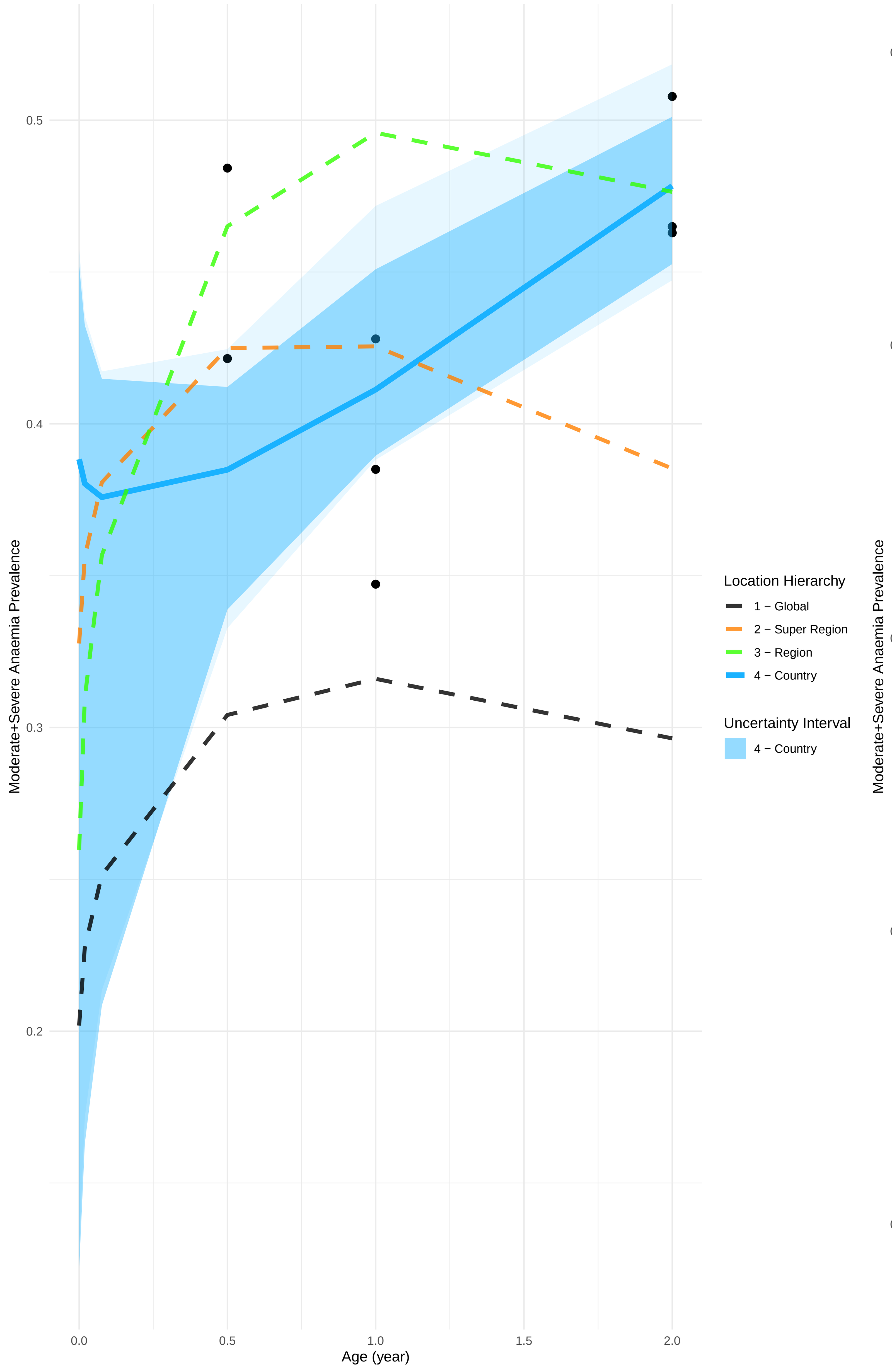

MR−BRT Age/Sex Fits − Non−pregnant Females <2 Years Old − Moderate+Severe Anaemia Prevalence − Togo
WHO−2024 Adjustment Method

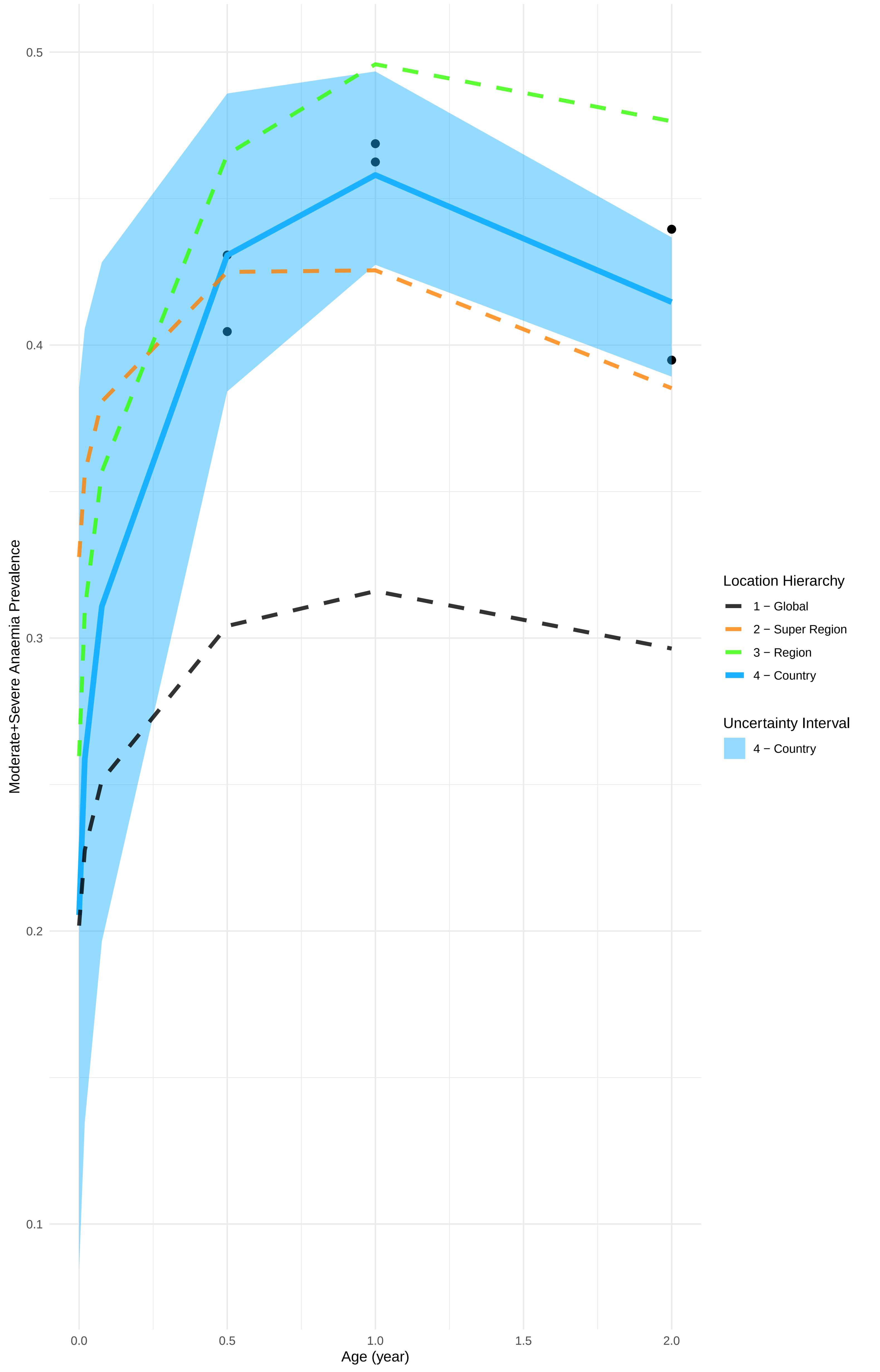

MR−BRT Age/Sex Fits − Males <2 Years Old − Moderate+Severe Anaemia Prevalence − Global
WHO−2001 Adjustment Method

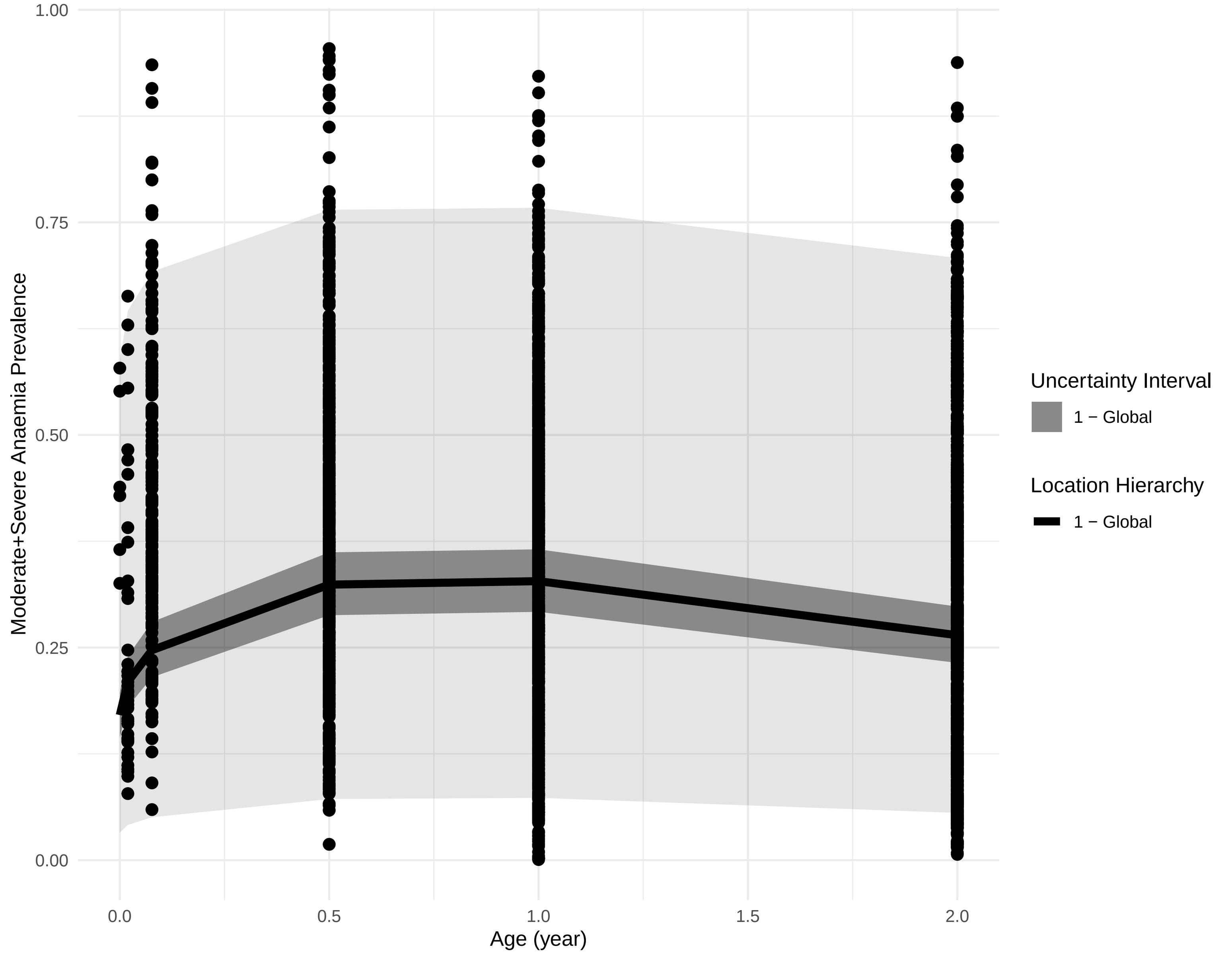

MR−BRT Age/Sex Fits − Males <2 Years Old − Moderate+Severe Anaemia Prevalence − Central Europe, Eastern Europe, and Central Asia
WHO−2001 Adjustment Method

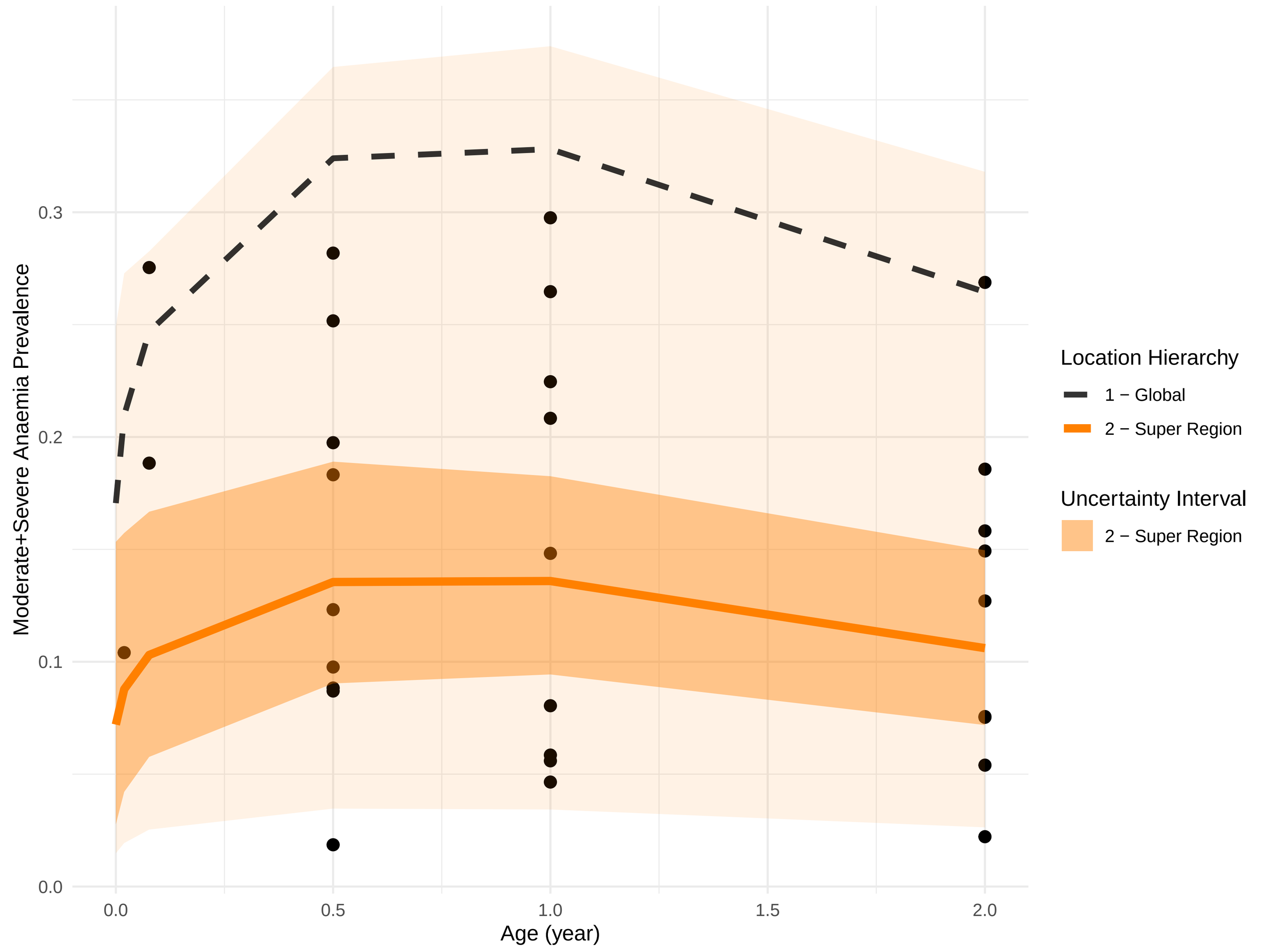

MR−BRT Age/Sex Fits − Males <2 Years Old − Moderate+Severe Anaemia Prevalence − Central Asia
WHO−2001 Adjustment Method

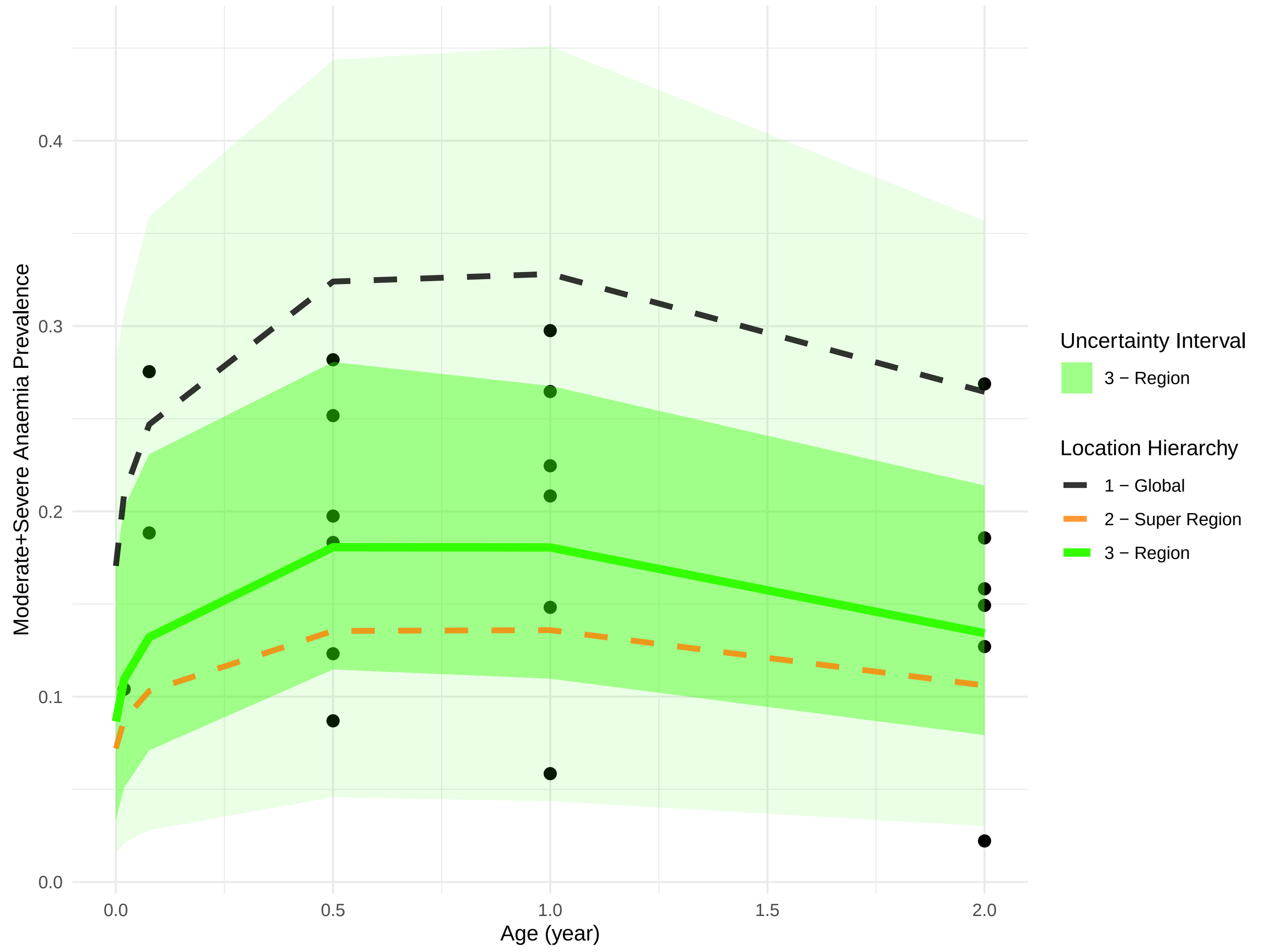

MR−BRT Age/Sex Fits − Males <2 Years Old − Moderate+Severe Anaemia Prevalence − Armenia
WHO−2001 Adjustment Method

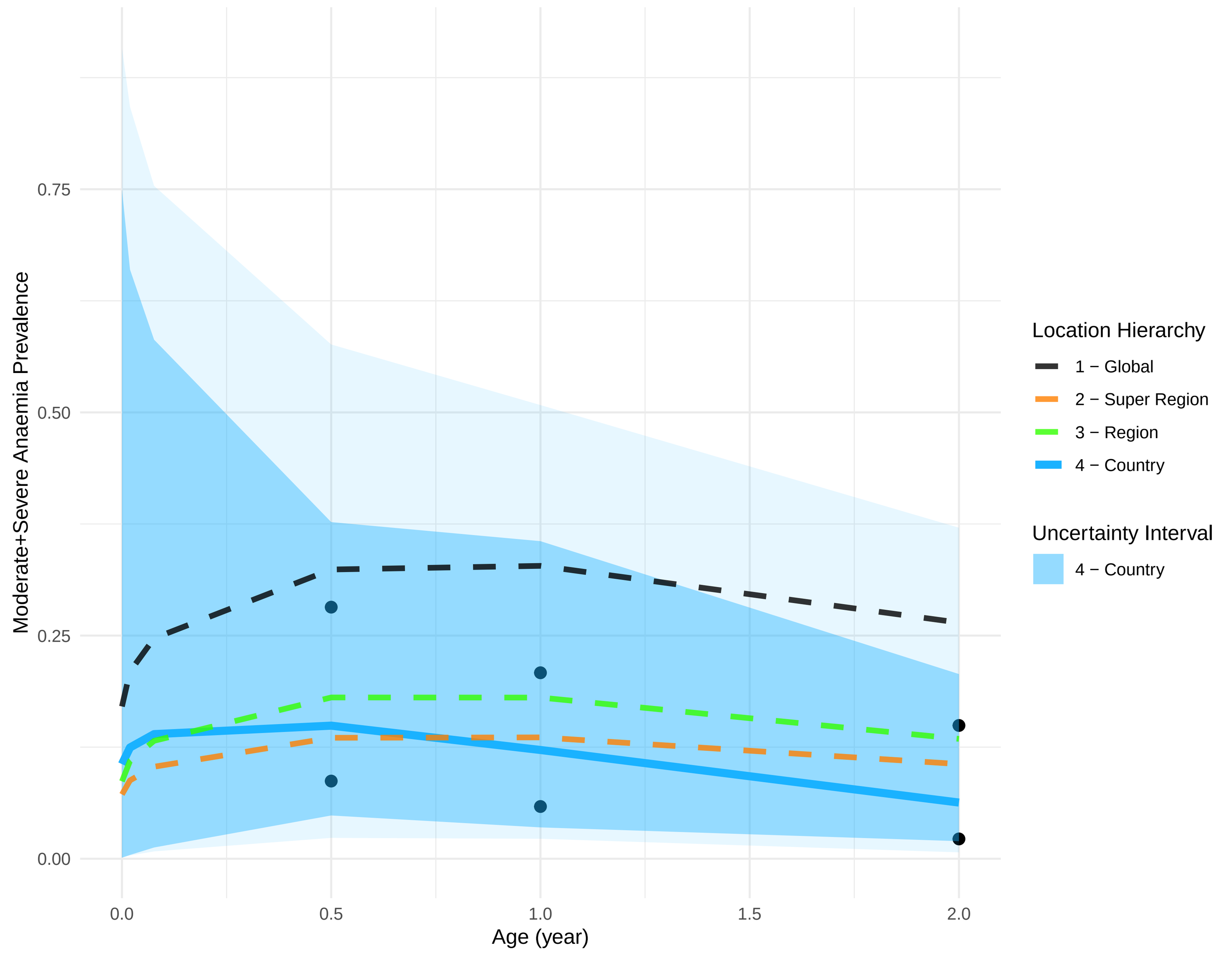

MR−BRT Age/Sex Fits − Males <2 Years Old − Moderate+Severe Anaemia Prevalence − Azerbaijan
WHO−2001 Adjustment Method

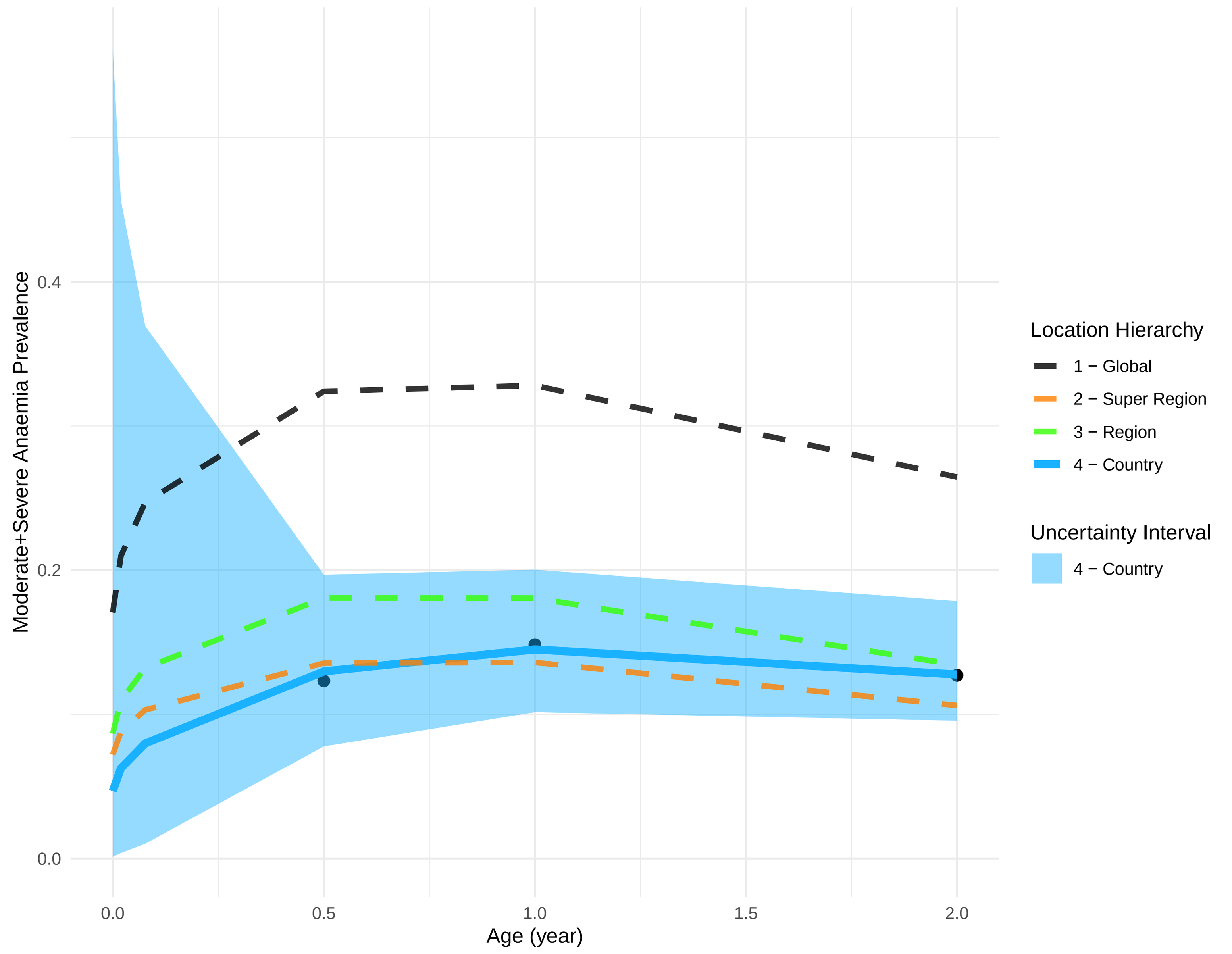

MR−BRT Age/Sex Fits − Males <2 Years Old − Moderate+Severe Anaemia Prevalence − Kyrgyzstan
WHO−2001 Adjustment Method

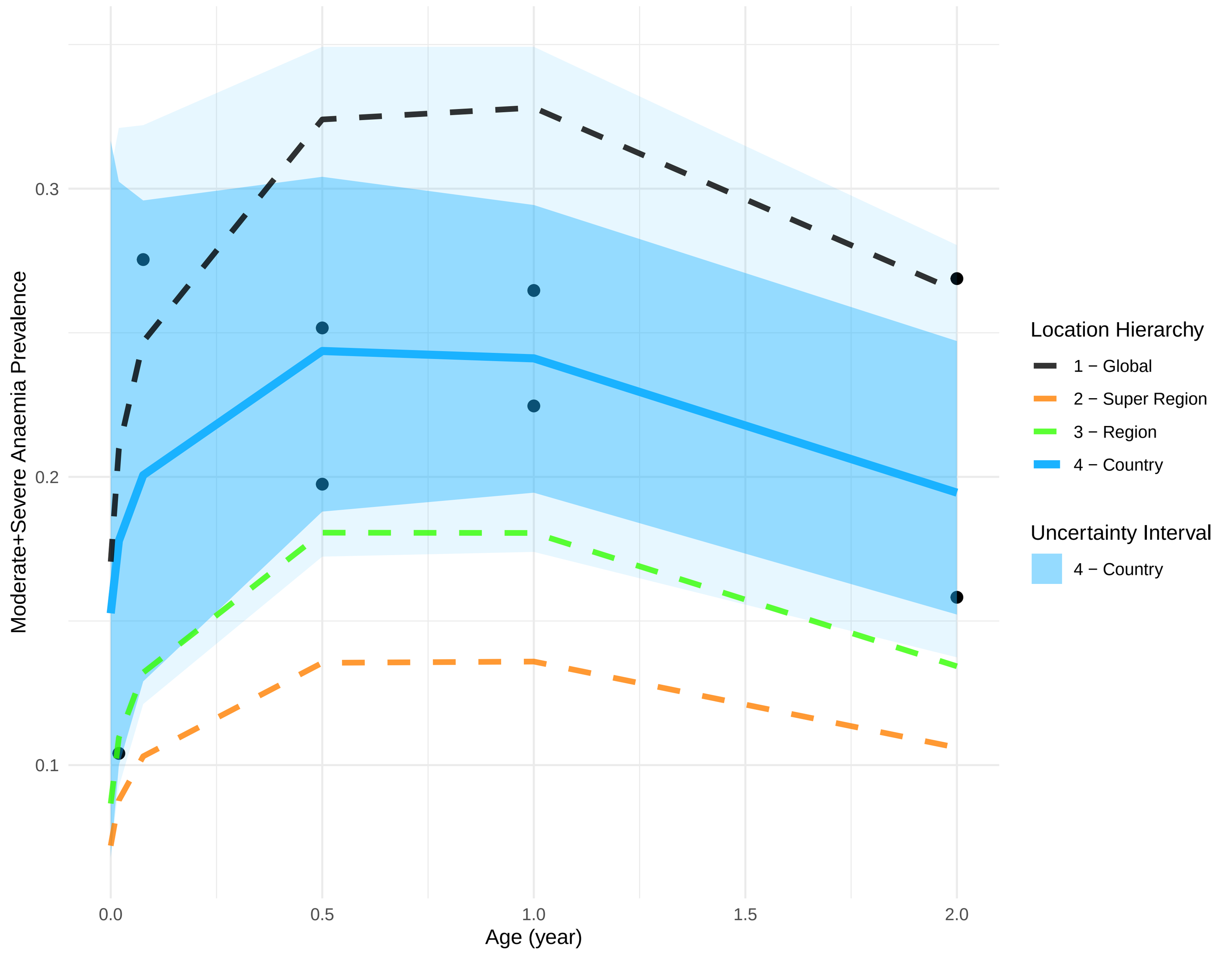

MR−BRT Age/Sex Fits − Males <2 Years Old − Moderate+Severe Anaemia Prevalence − Uzbekistan
WHO−2001 Adjustment Method

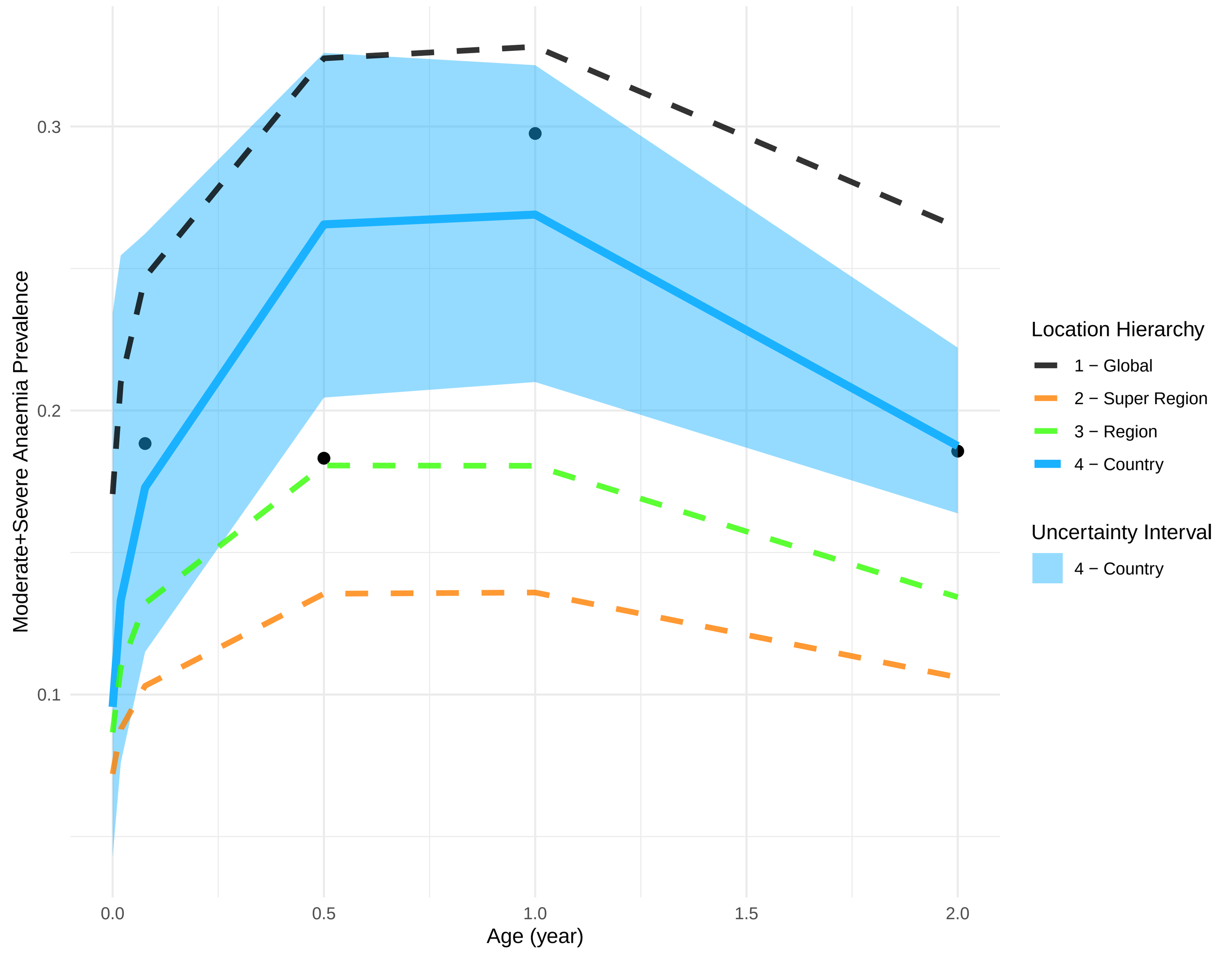

MR−BRT Age/Sex Fits − Males <2 Years Old − Moderate+Severe Anaemia Prevalence − Central Europe
WHO−2001 Adjustment Method

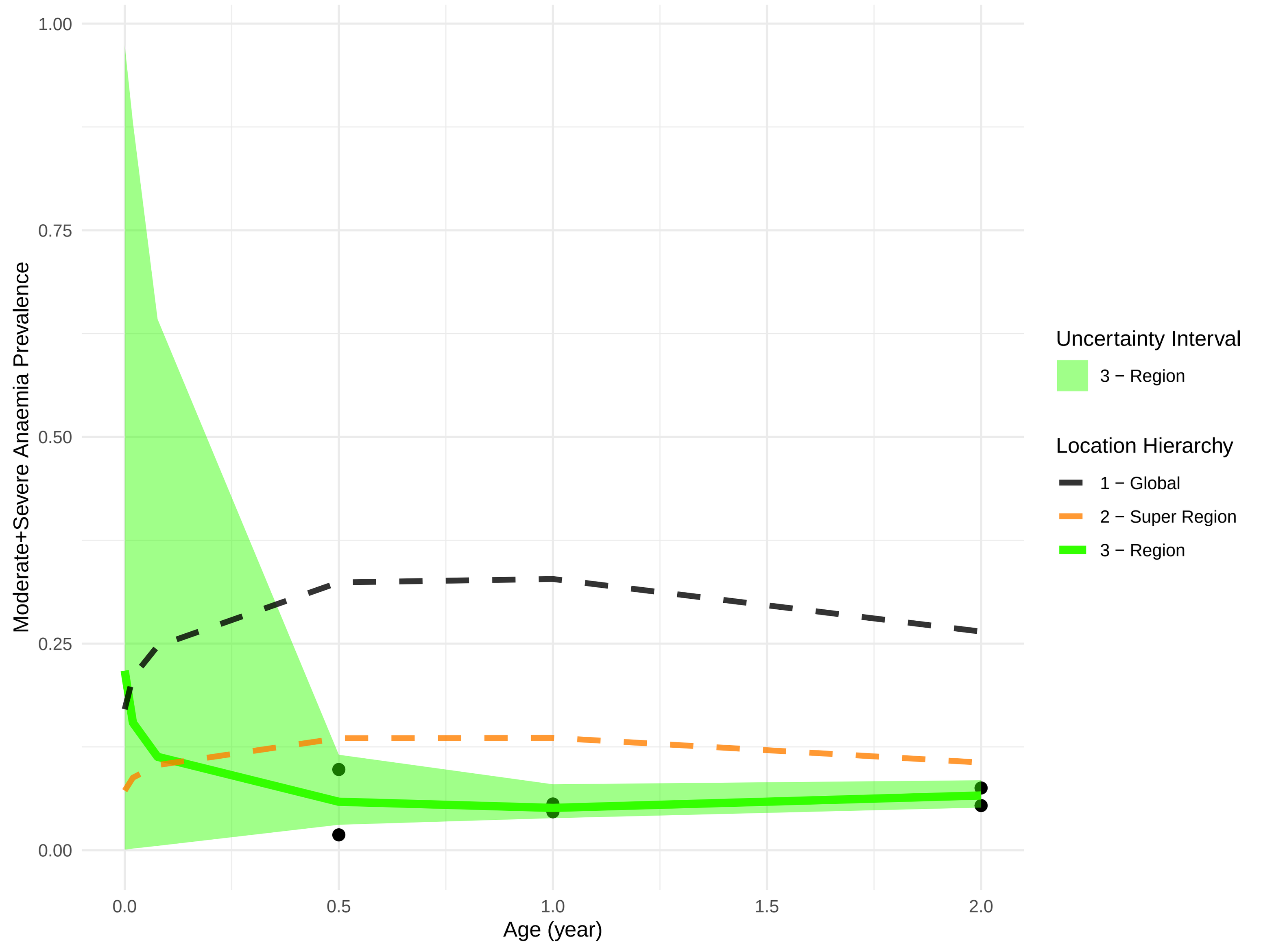

MR−BRT Age/Sex Fits − Males <2 Years Old − Moderate+Severe Anaemia Prevalence − Albania
WHO−2001 Adjustment Method

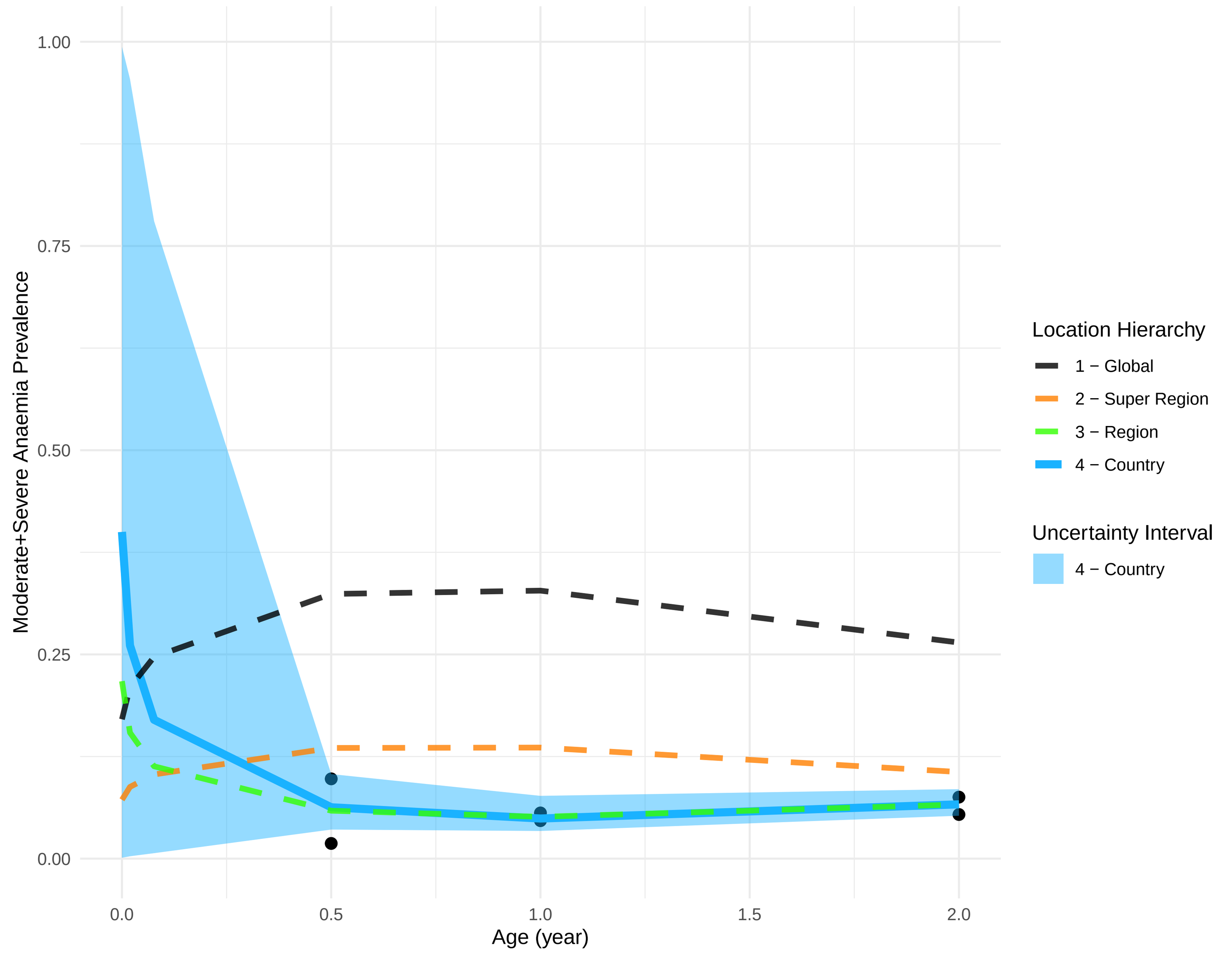

MR−BRT Age/Sex Fits − Males <2 Years Old − Moderate+Severe Anaemia Prevalence − Eastern Europe
WHO−2001 Adjustment Method

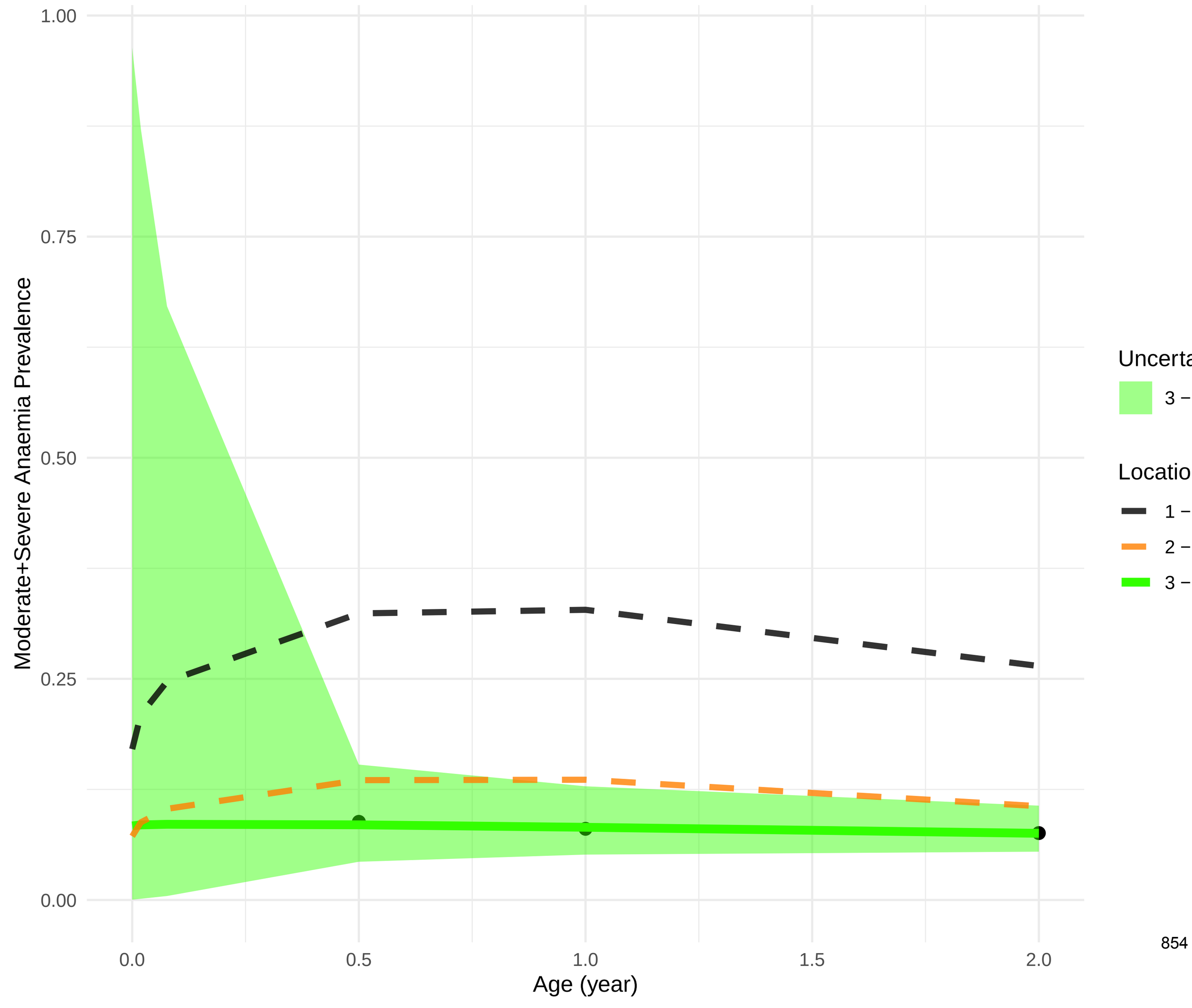

MR−BRT Age/Sex Fits − Males <2 Years Old − Moderate+Severe Anaemia Prevalence − Republic of Moldova
WHO−2001 Adjustment Method

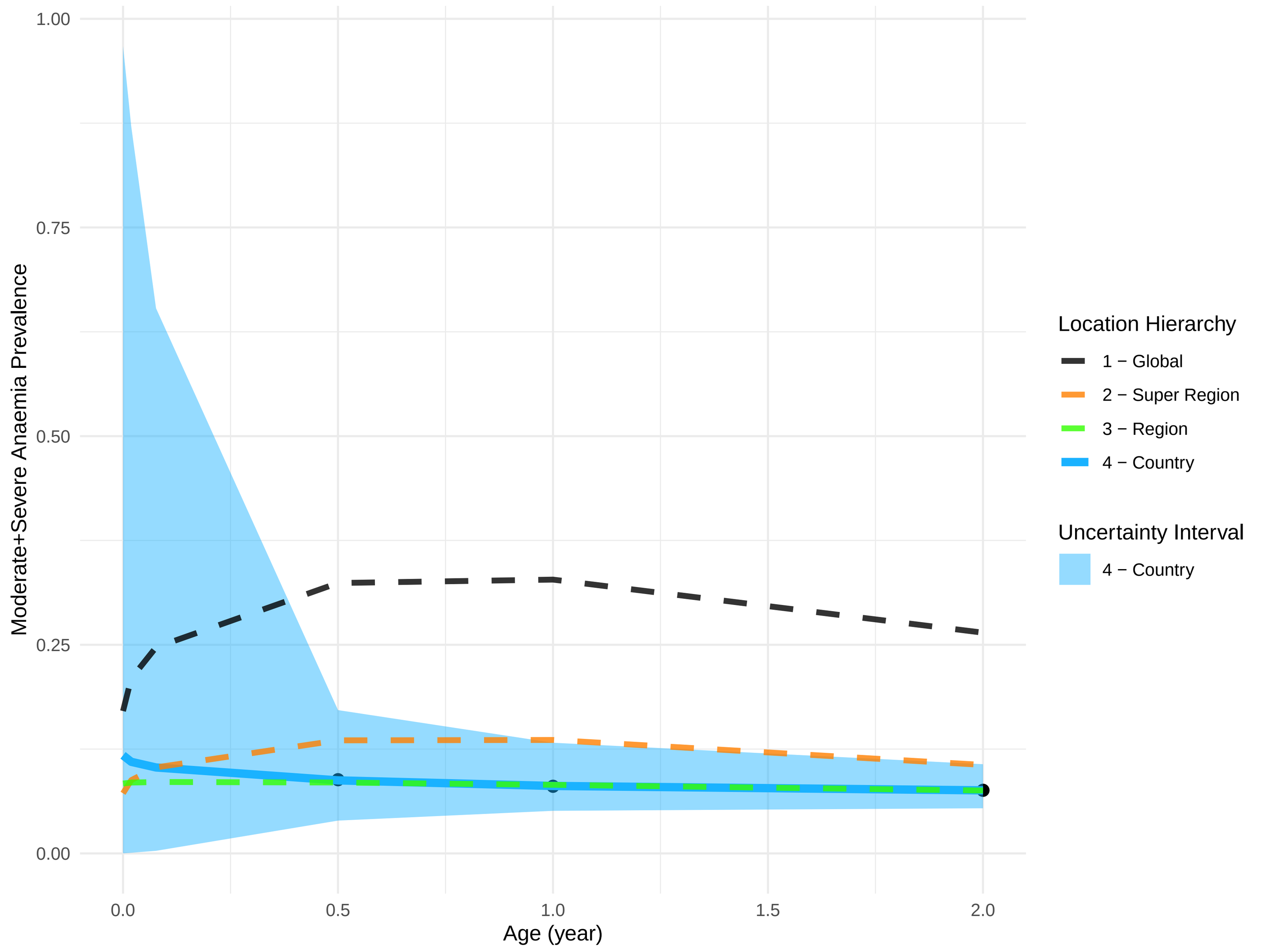

MR−BRT Age/Sex Fits − Males <2 Years Old − Moderate+Severe Anaemia Prevalence − High−income
WHO−2001 Adjustment Method

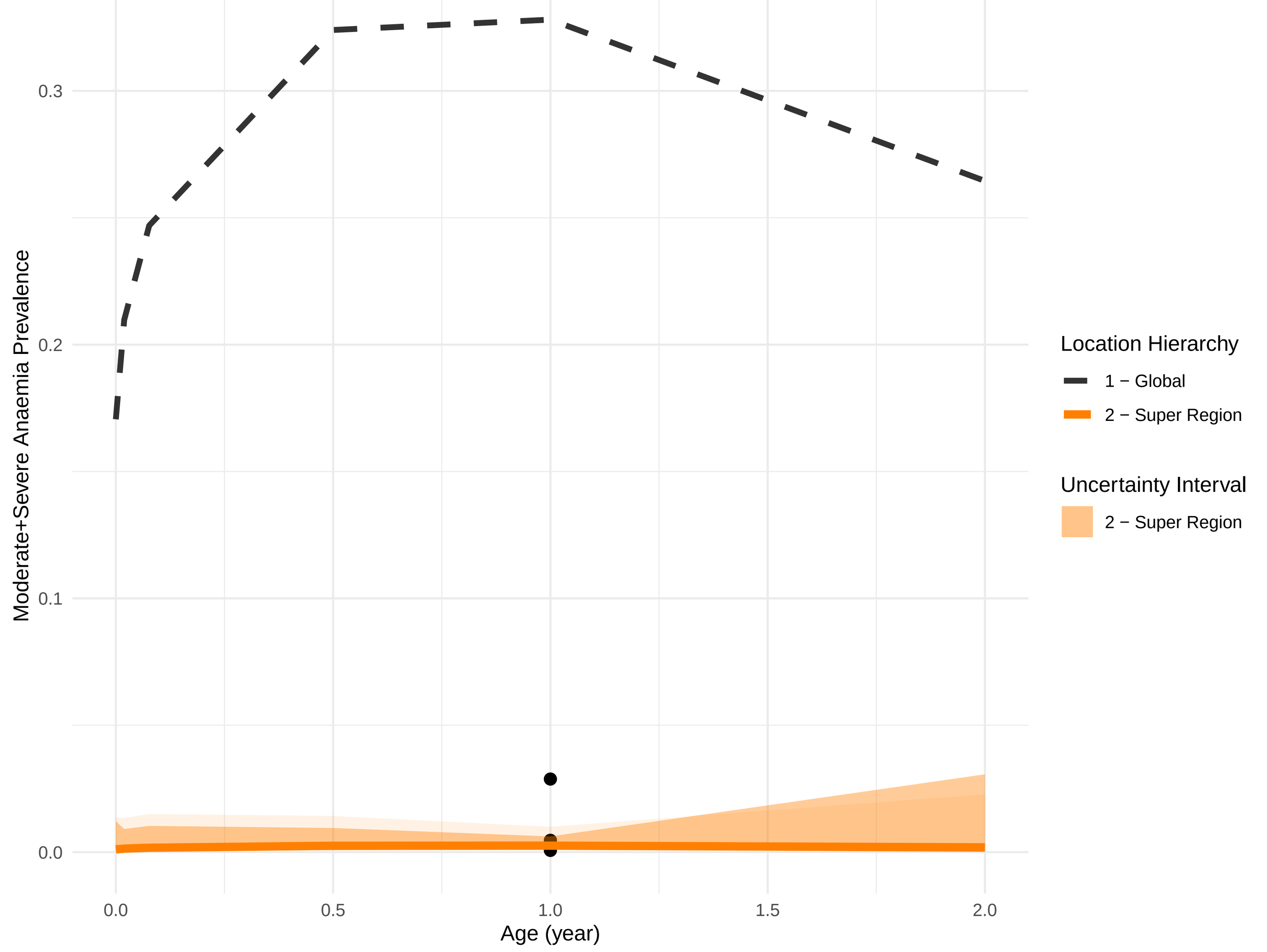

MR−BRT Age/Sex Fits − Males <2 Years Old − Moderate+Severe Anaemia Prevalence − Western Europe
WHO−2001 Adjustment Method

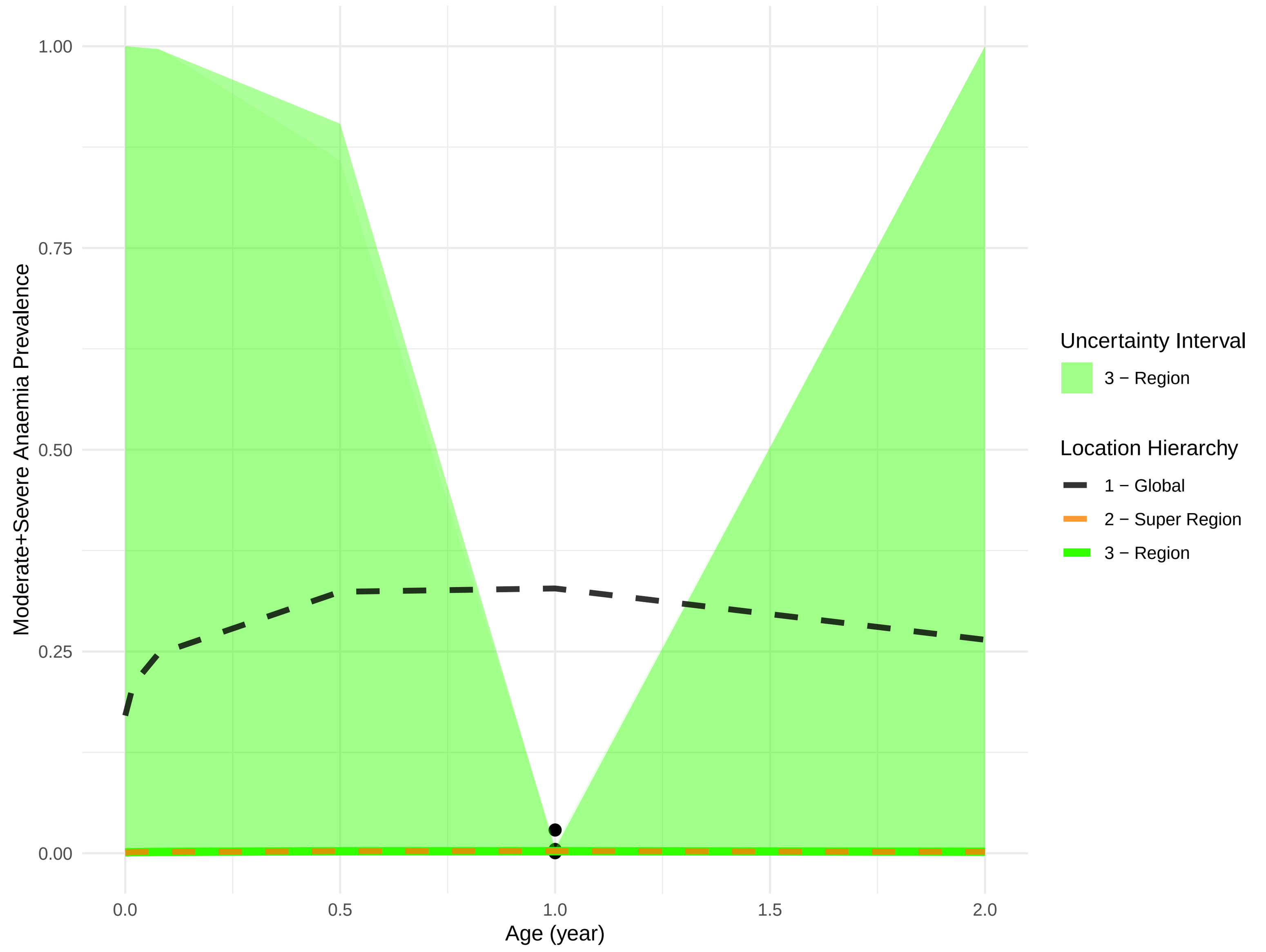

MR−BRT Age/Sex Fits − Males <2 Years Old − Moderate+Severe Anaemia Prevalence − United Kingdom
WHO−2001 Adjustment Method

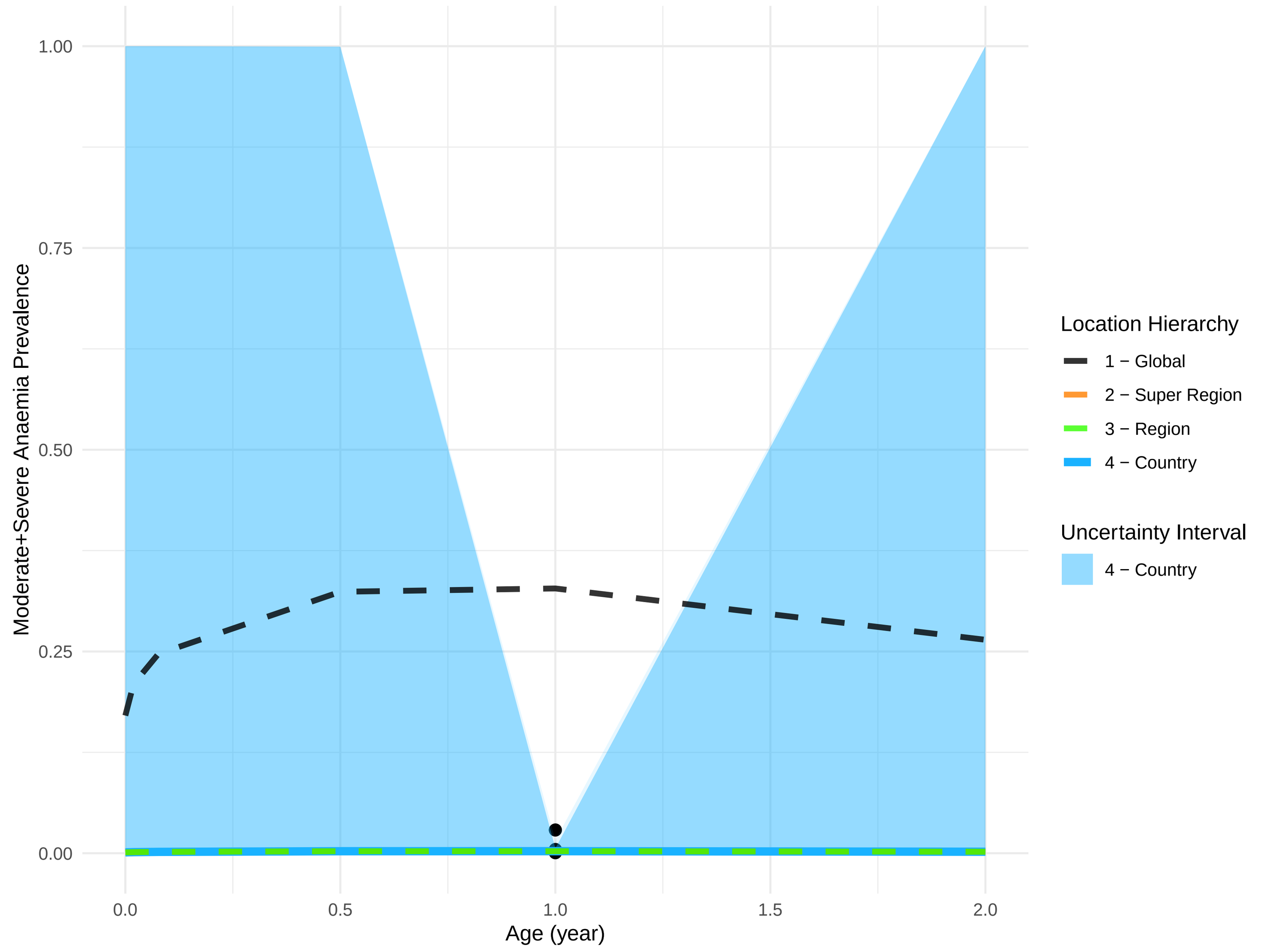

MR−BRT Age/Sex Fits − Males <2 Years Old − Moderate+Severe Anaemia Prevalence − England
WHO−2001 Adjustment Method

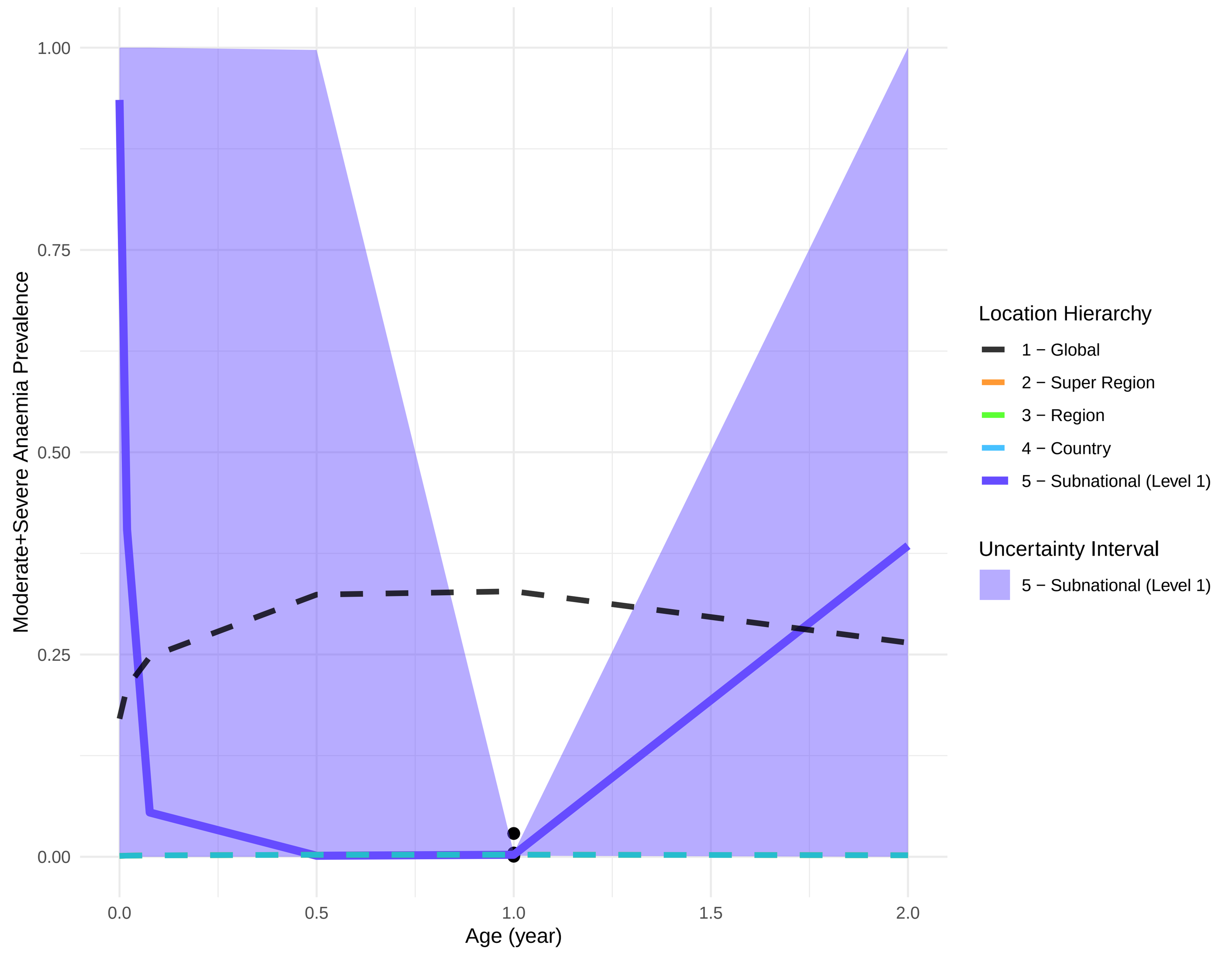

MR−BRT Age/Sex Fits − Males <2 Years Old − Moderate+Severe Anaemia Prevalence − East Midlands
WHO−2001 Adjustment Method

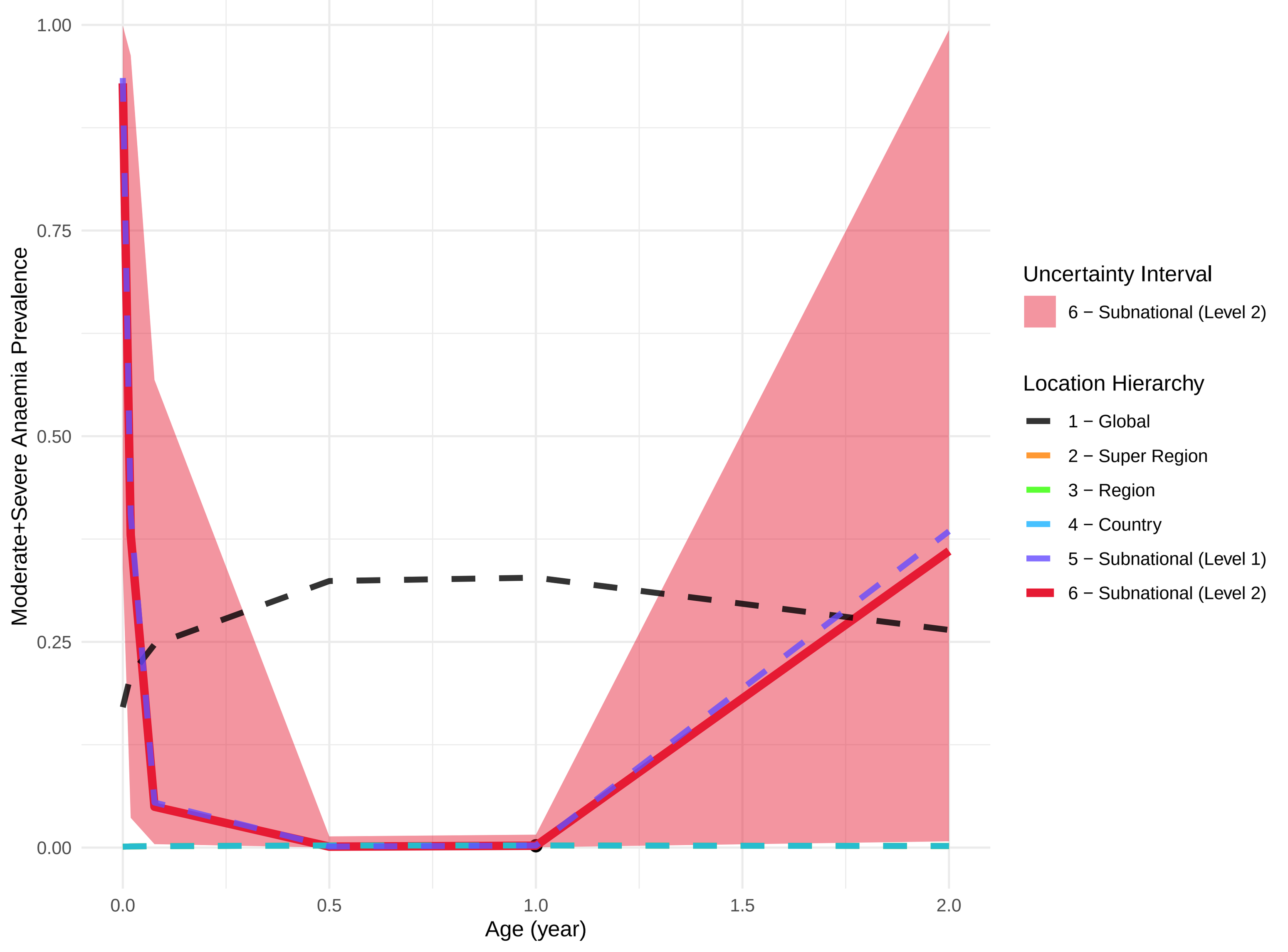

MR−BRT Age/Sex Fits − Males <2 Years Old − Moderate+Severe Anaemia Prevalence − Greater London
WHO−2001 Adjustment Method

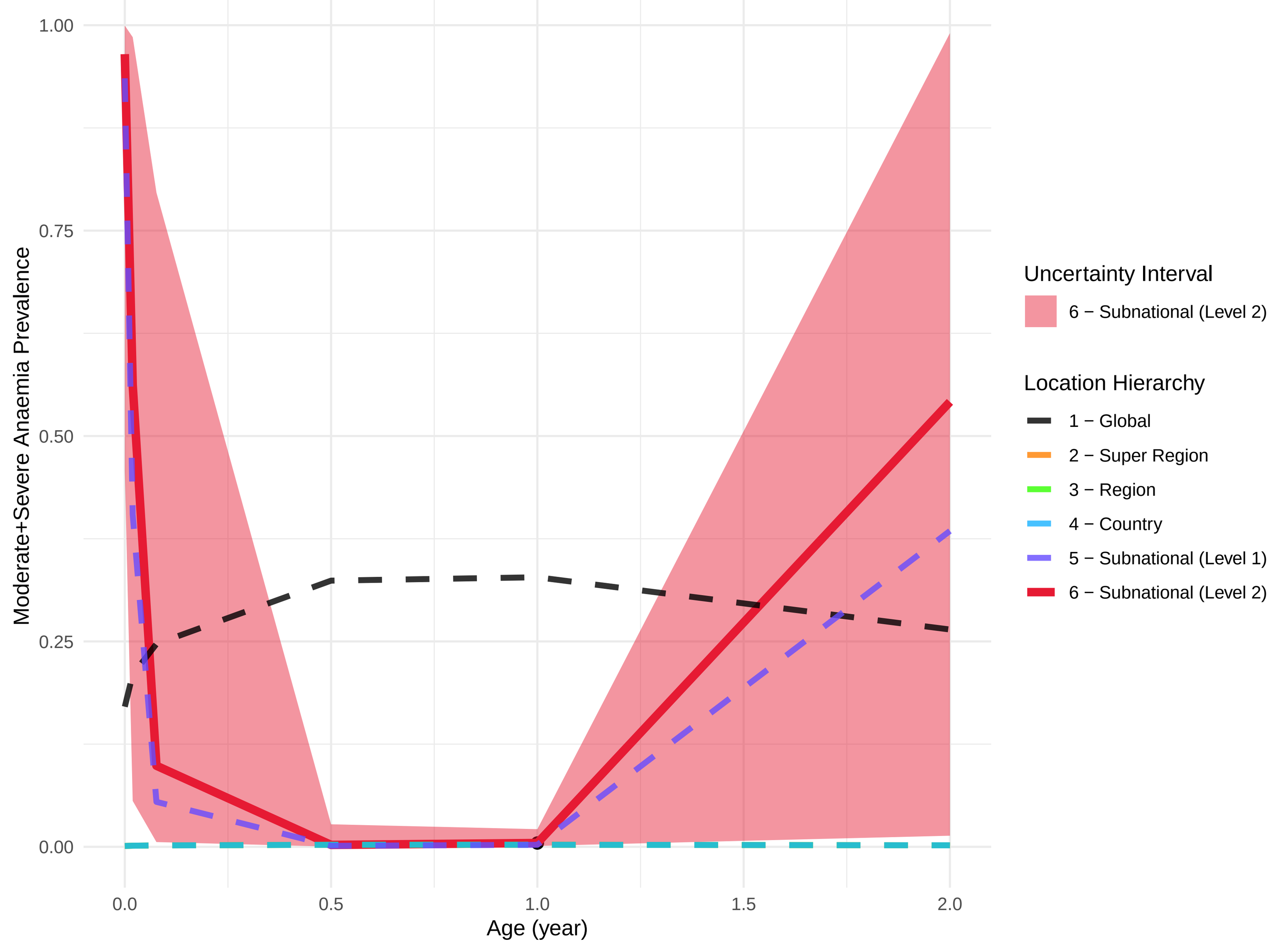

MR−BRT Age/Sex Fits − Males <2 Years Old − Moderate+Severe Anaemia Prevalence − South West England
WHO−2001 Adjustment Method

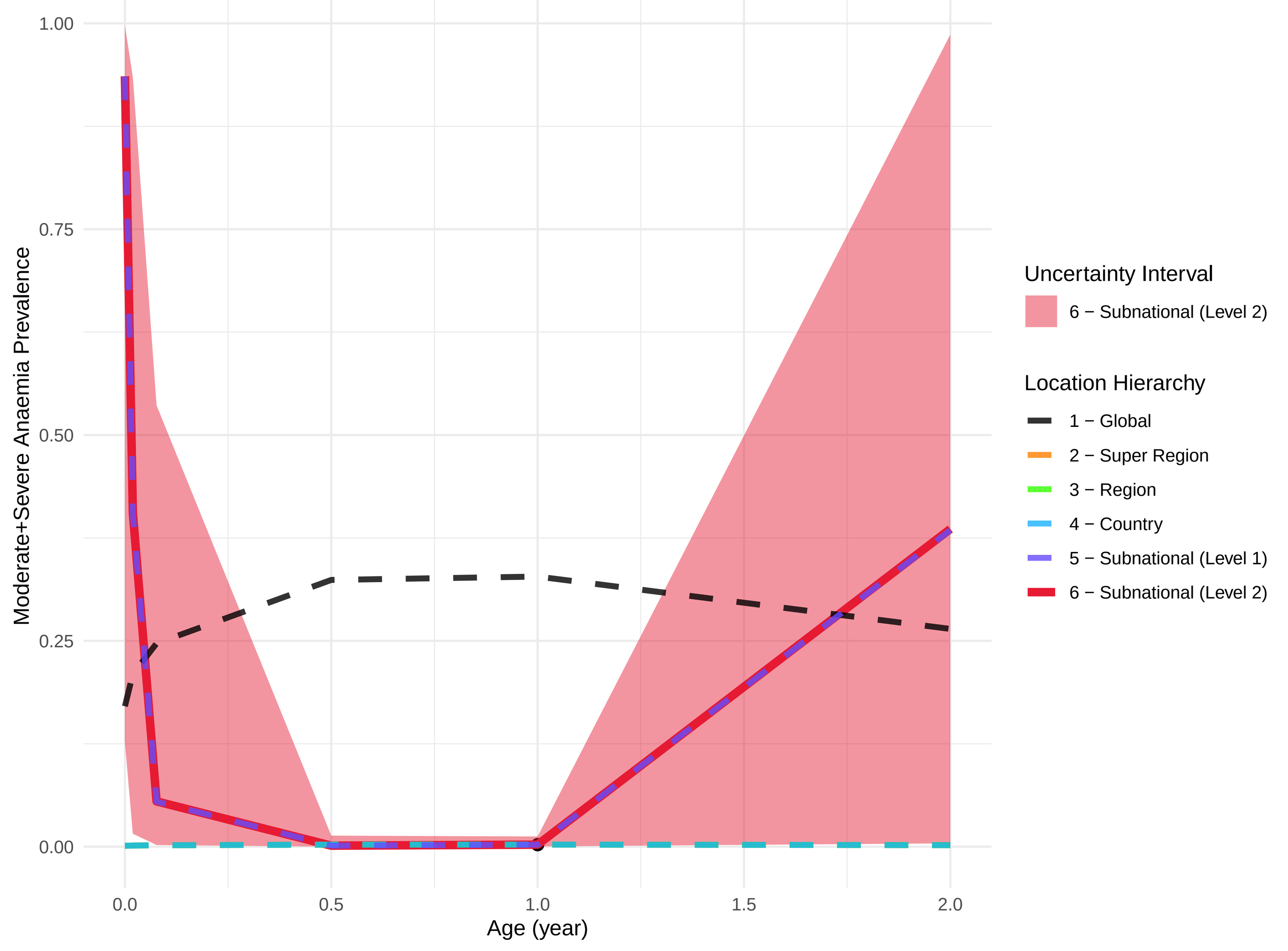

MR−BRT Age/Sex Fits − Males <2 Years Old − Moderate+Severe Anaemia Prevalence − West Midlands
WHO−2001 Adjustment Method

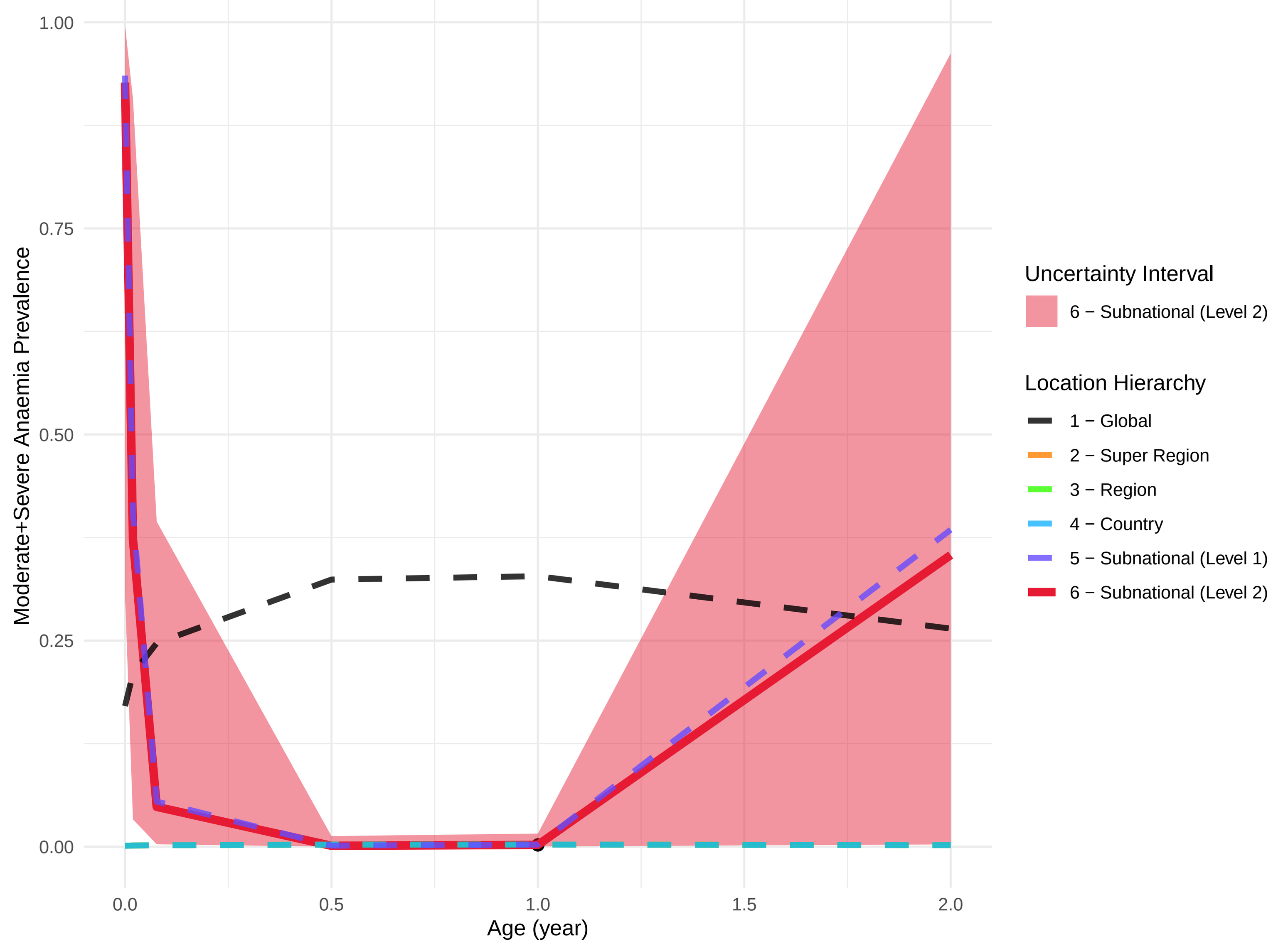

MR−BRT Age/Sex Fits − Males <2 Years Old − Moderate+Severe Anaemia Prevalence − Yorkshire and the Humber
WHO−2001 Adjustment Method

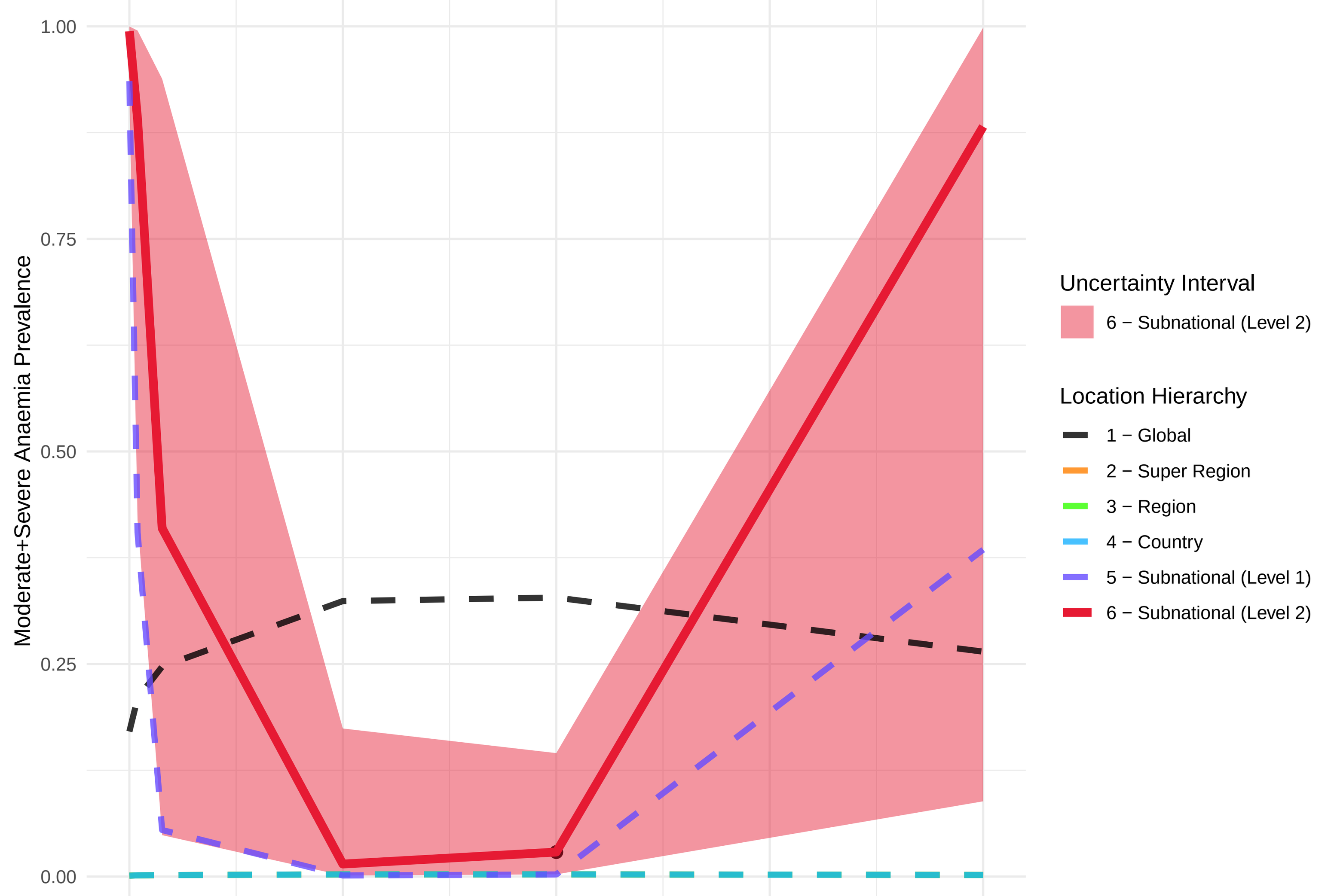

MR−BRT Age/Sex Fits − Males <2 Years Old − Moderate+Severe Anaemia Prevalence − Latin America and Caribbean
WHO−2001 Adjustment Method

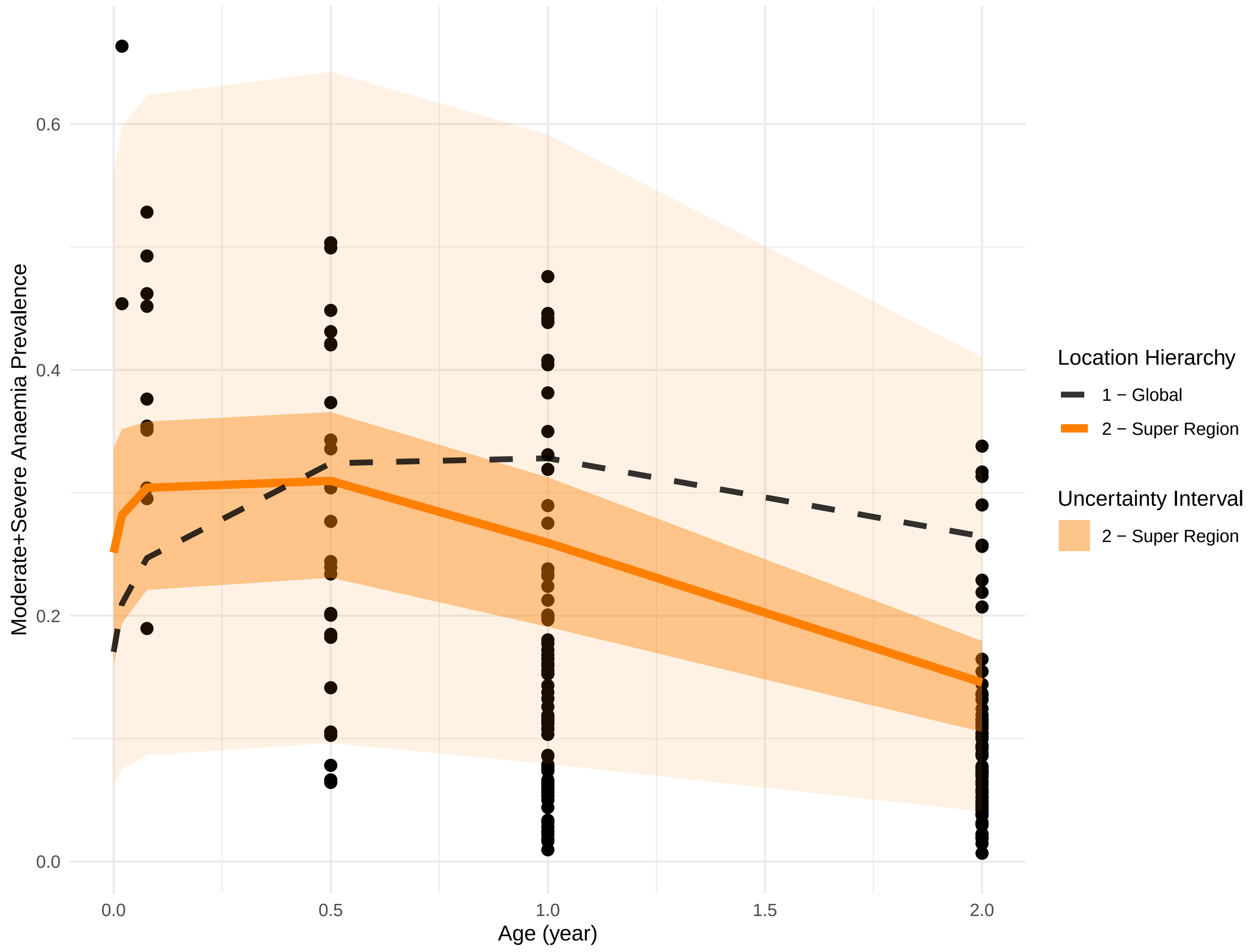

MR−BRT Age/Sex Fits − Males <2 Years Old − Moderate+Severe Anaemia Prevalence − Andean Latin America
WHO−2001 Adjustment Method

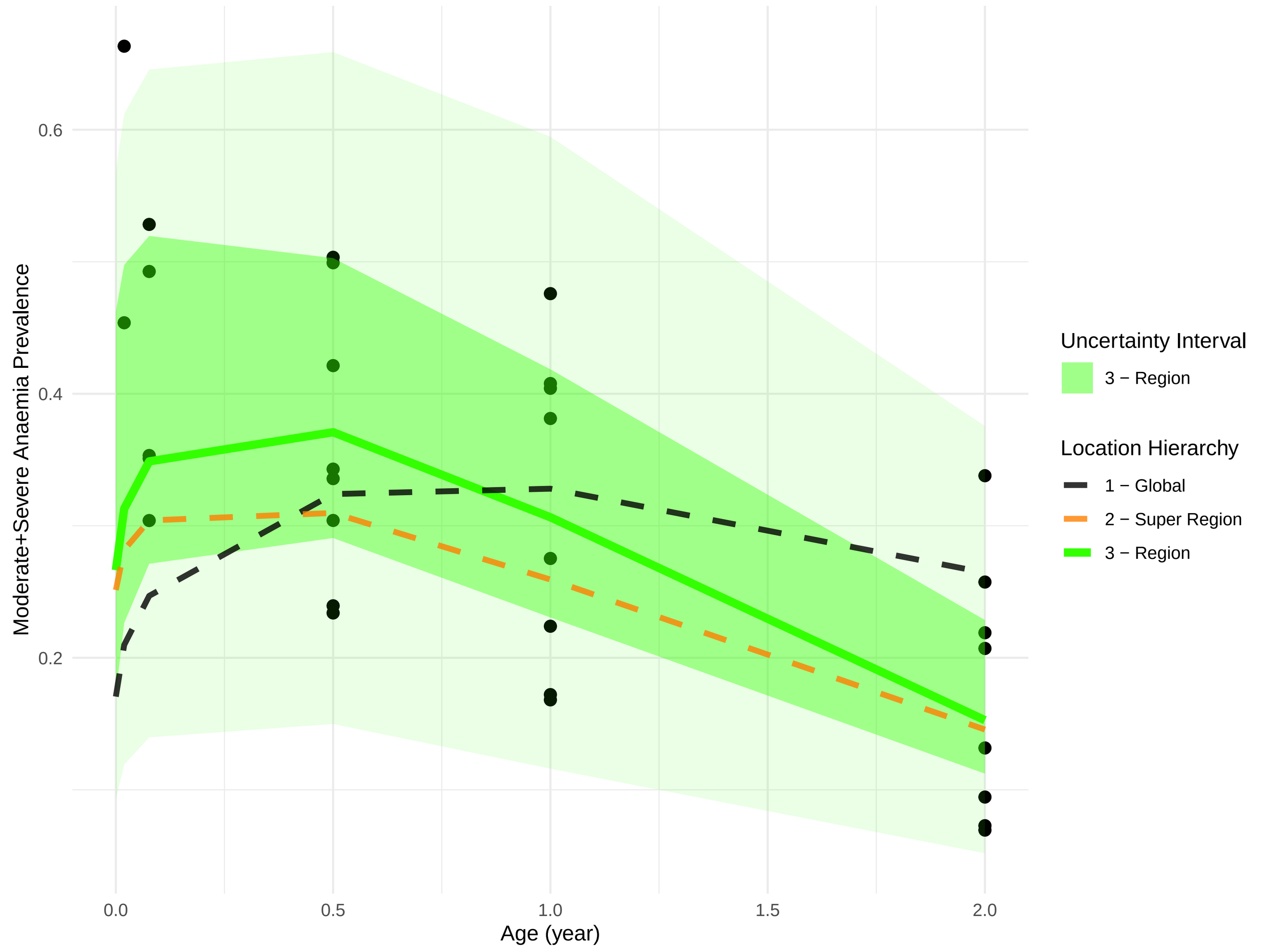

MR−BRT Age/Sex Fits − Males <2 Years Old − Moderate+Severe Anaemia Prevalence − Bolivia (Plurinational State of)
WHO−2001 Adjustment Method

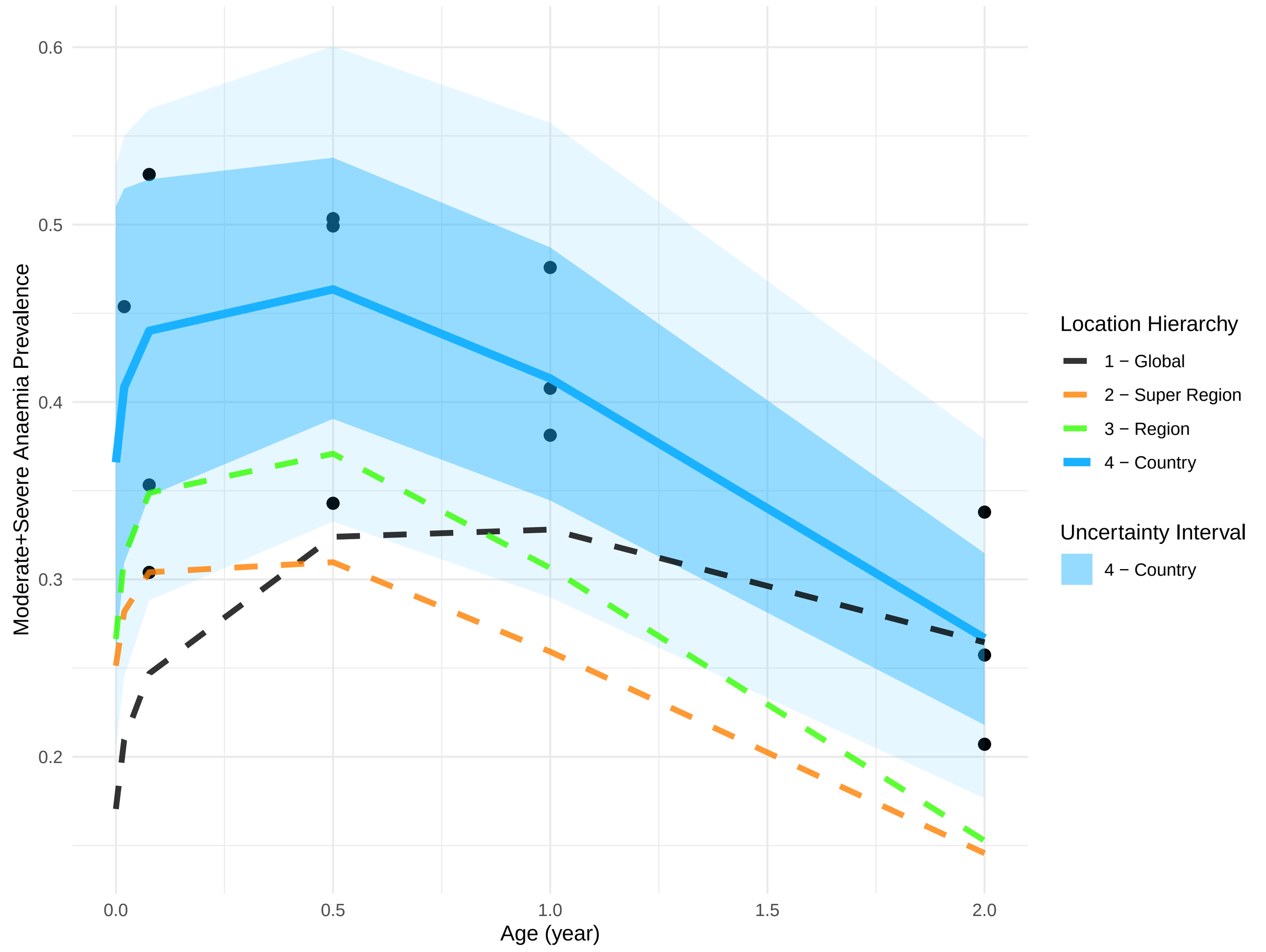

MR−BRT Age/Sex Fits − Males <2 Years Old − Moderate+Severe Anaemia Prevalence − Peru
WHO−2001 Adjustment Method

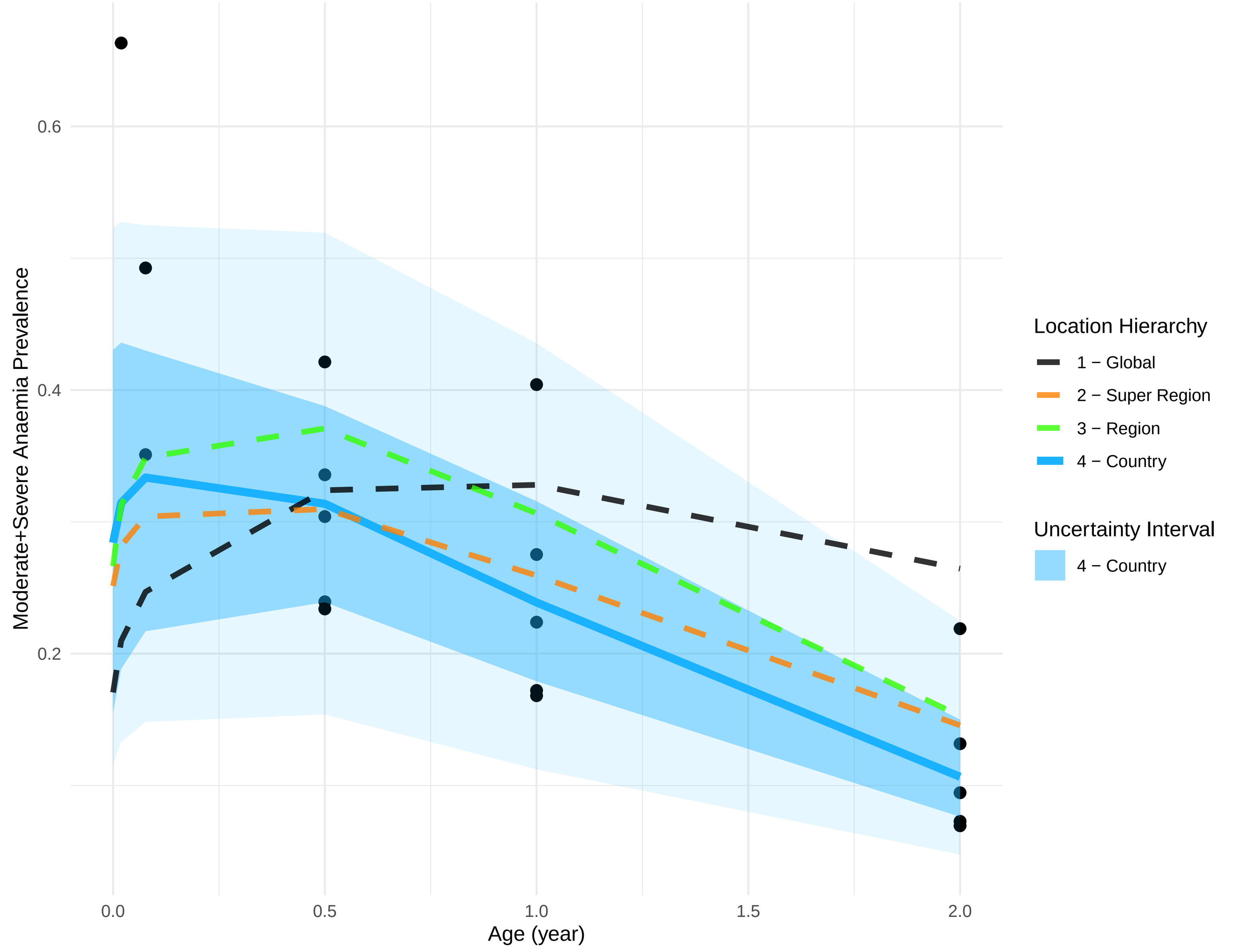

MR−BRT Age/Sex Fits − Males <2 Years Old − Moderate+Severe Anaemia Prevalence − Caribbean
WHO−2001 Adjustment Method

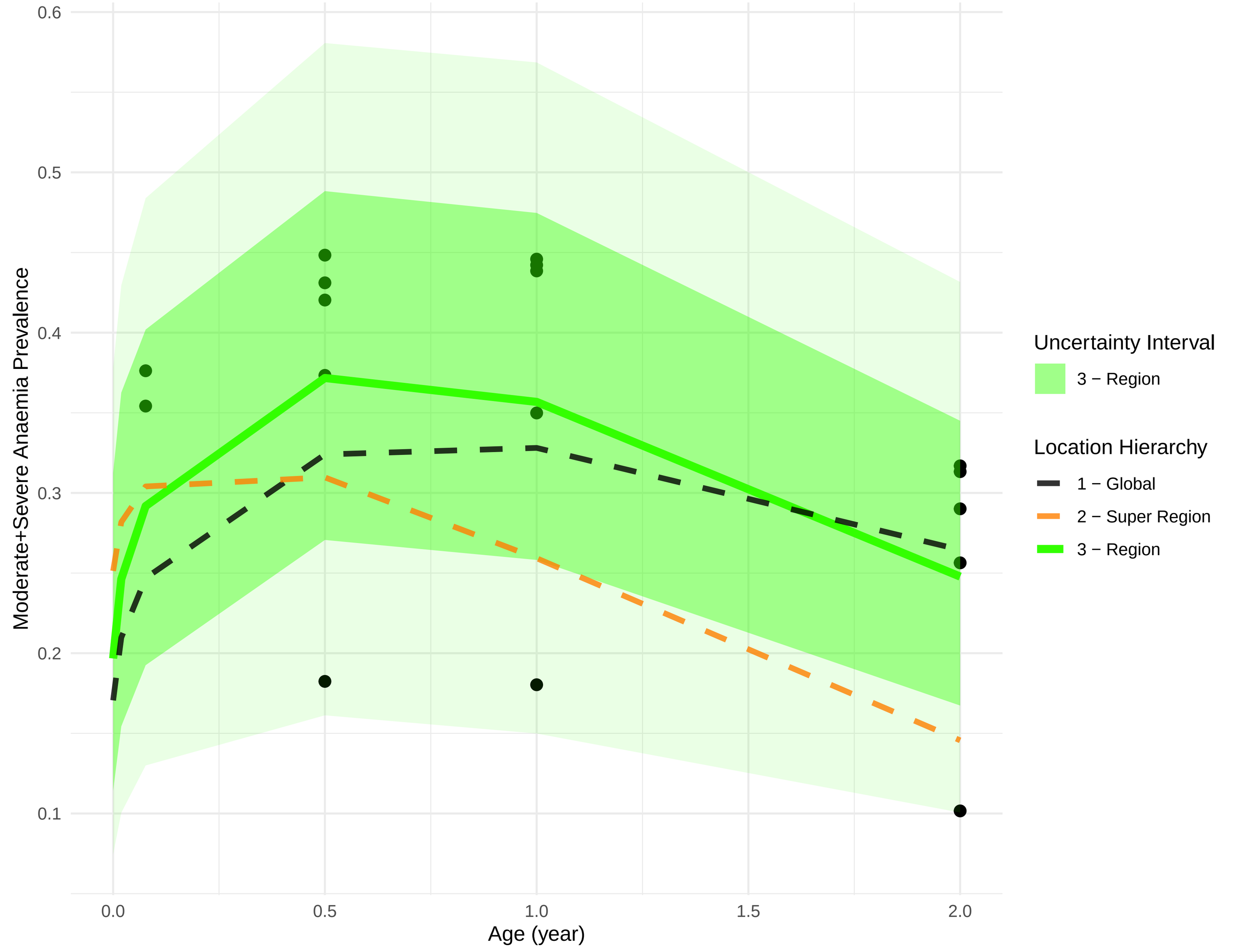

MR−BRT Age/Sex Fits − Males <2 Years Old − Moderate+Severe Anaemia Prevalence − Guyana
WHO−2001 Adjustment Method

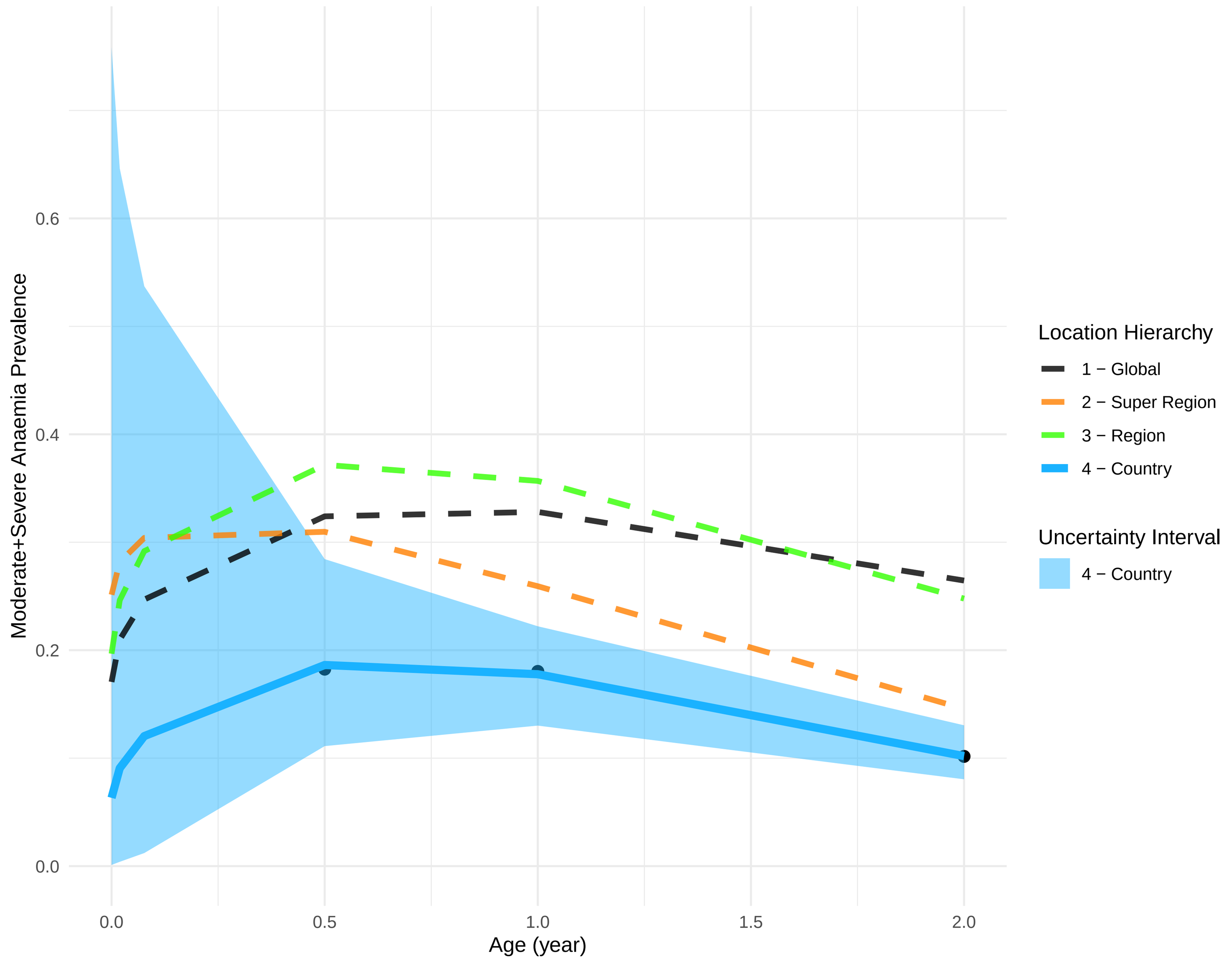

MR−BRT Age/Sex Fits − Males <2 Years Old − Moderate+Severe Anaemia Prevalence − Haiti
WHO−2001 Adjustment Method

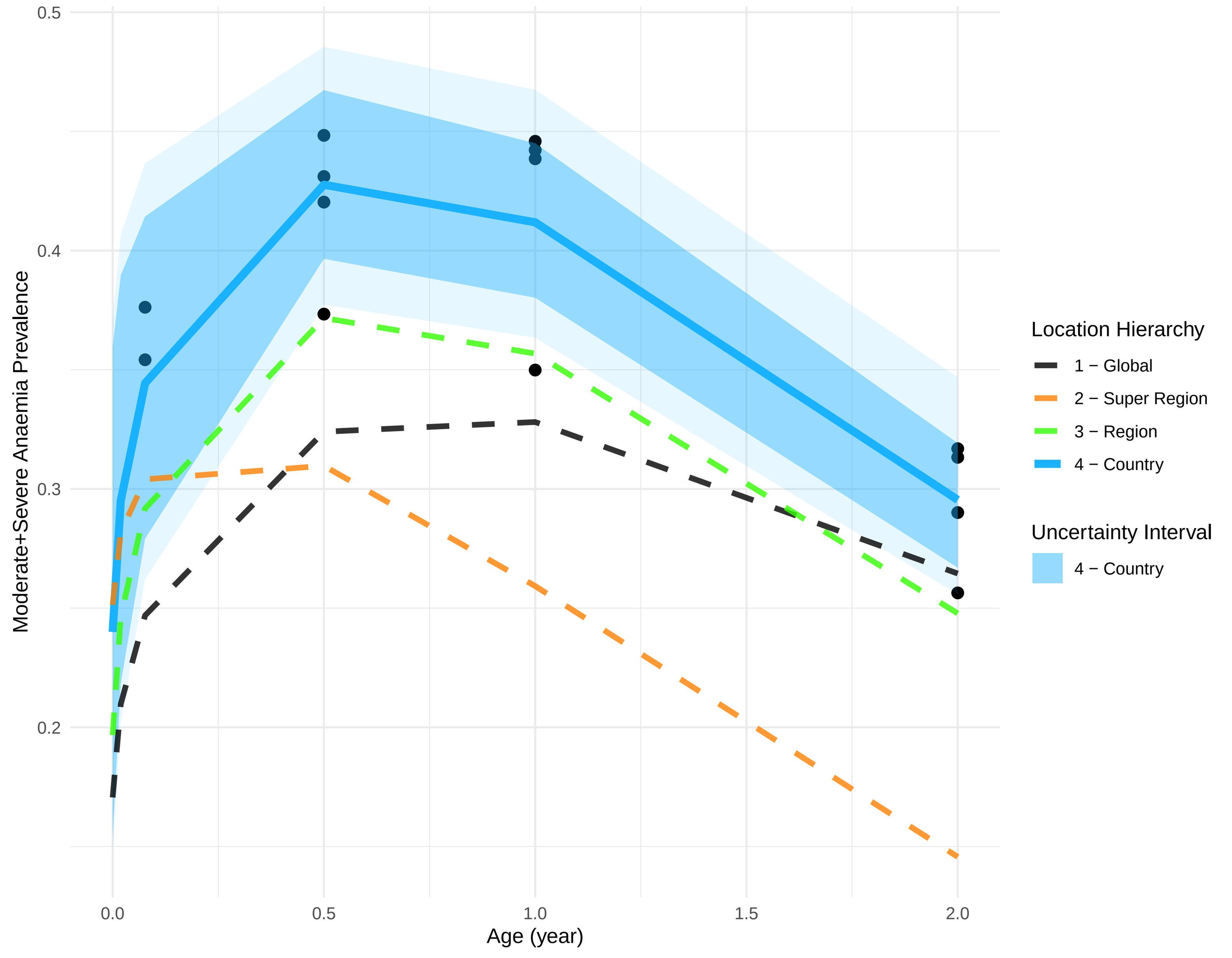

MR−BRT Age/Sex Fits − Males <2 Years Old − Moderate+Severe Anaemia Prevalence − Central Latin America
WHO−2001 Adjustment Method

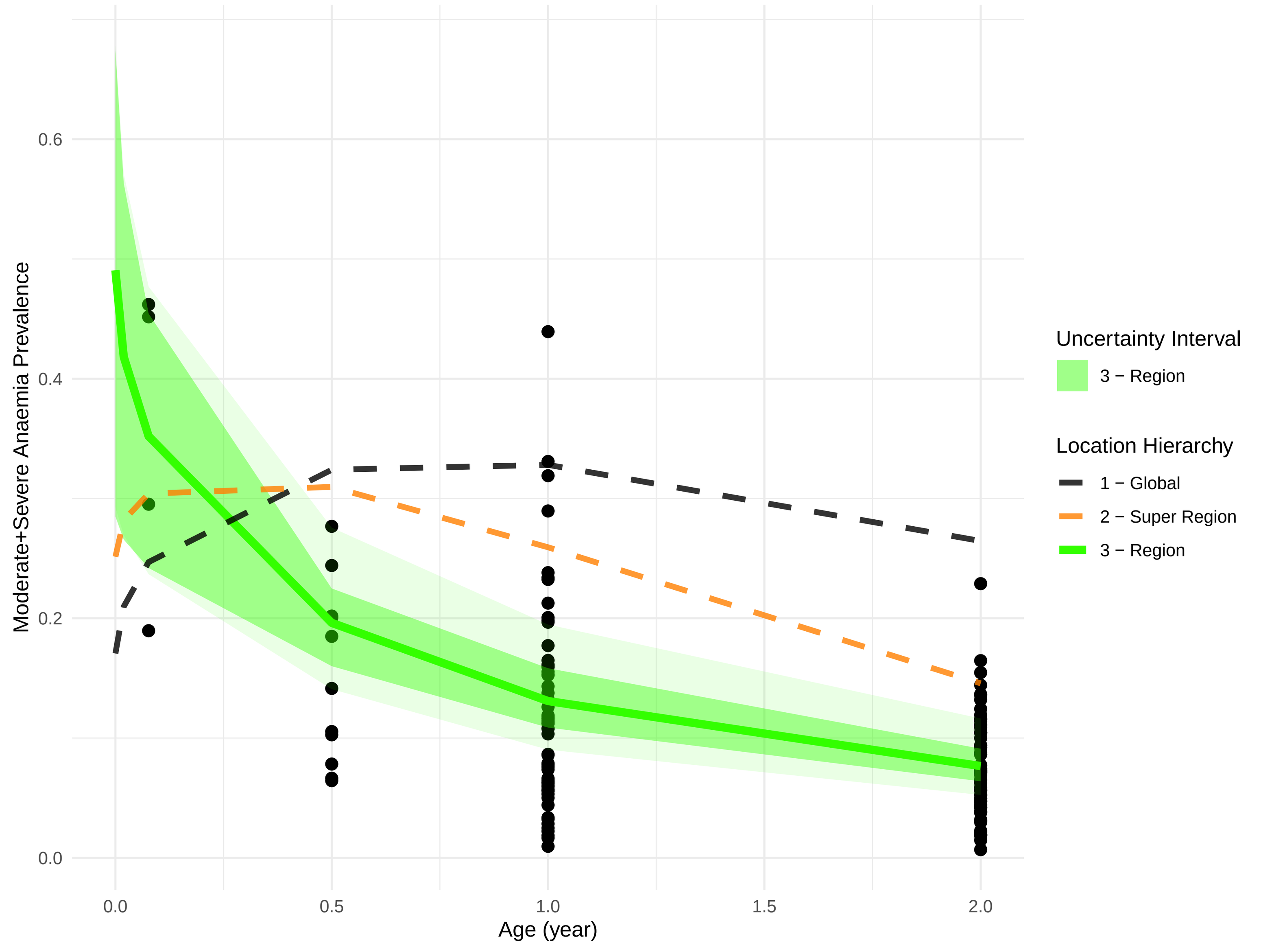

MR−BRT Age/Sex Fits − Males <2 Years Old − Moderate+Severe Anaemia Prevalence − Guatemala
WHO−2001 Adjustment Method

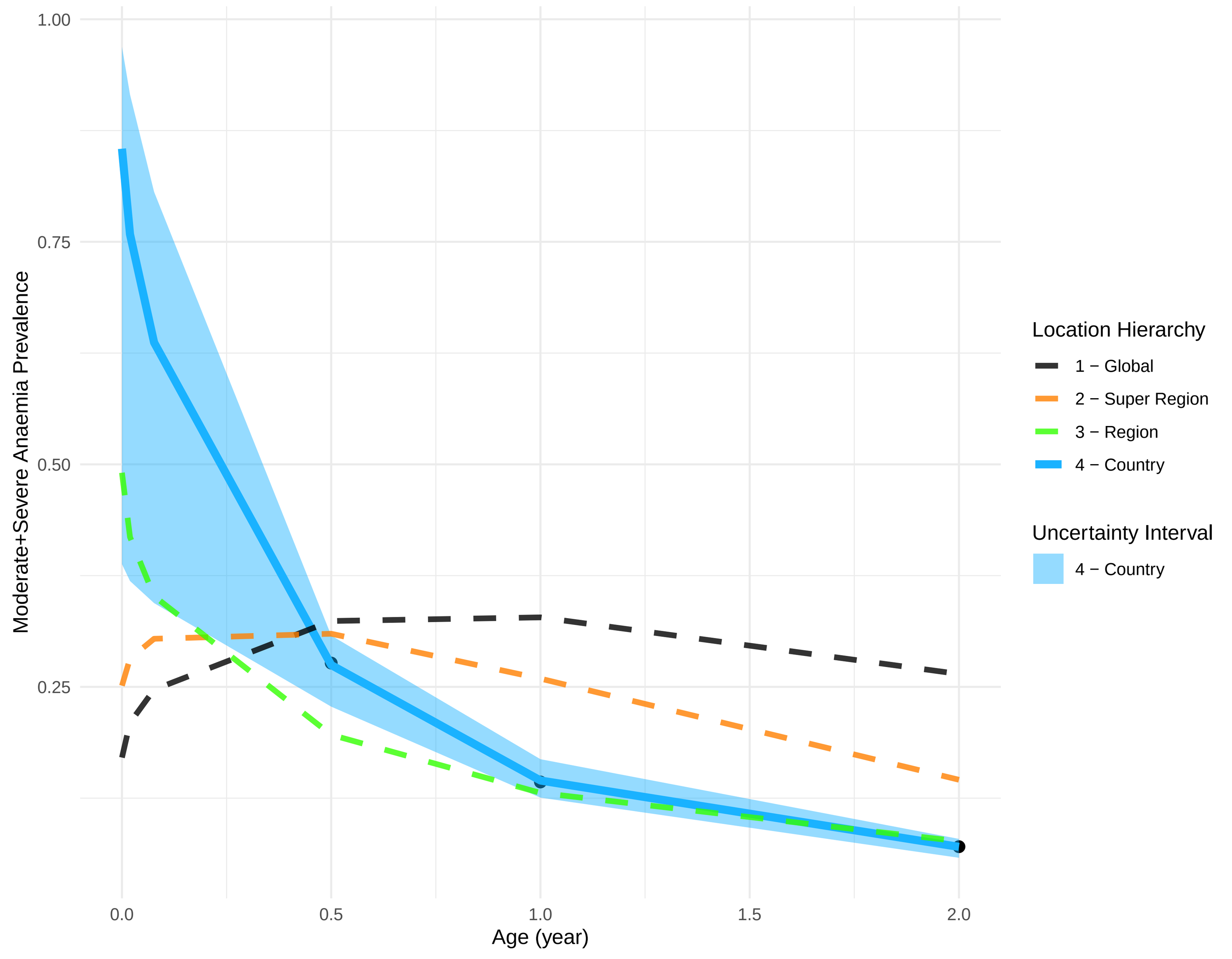

MR−BRT Age/Sex Fits − Males <2 Years Old − Moderate+Severe Anaemia Prevalence − Honduras
WHO−2001 Adjustment Method

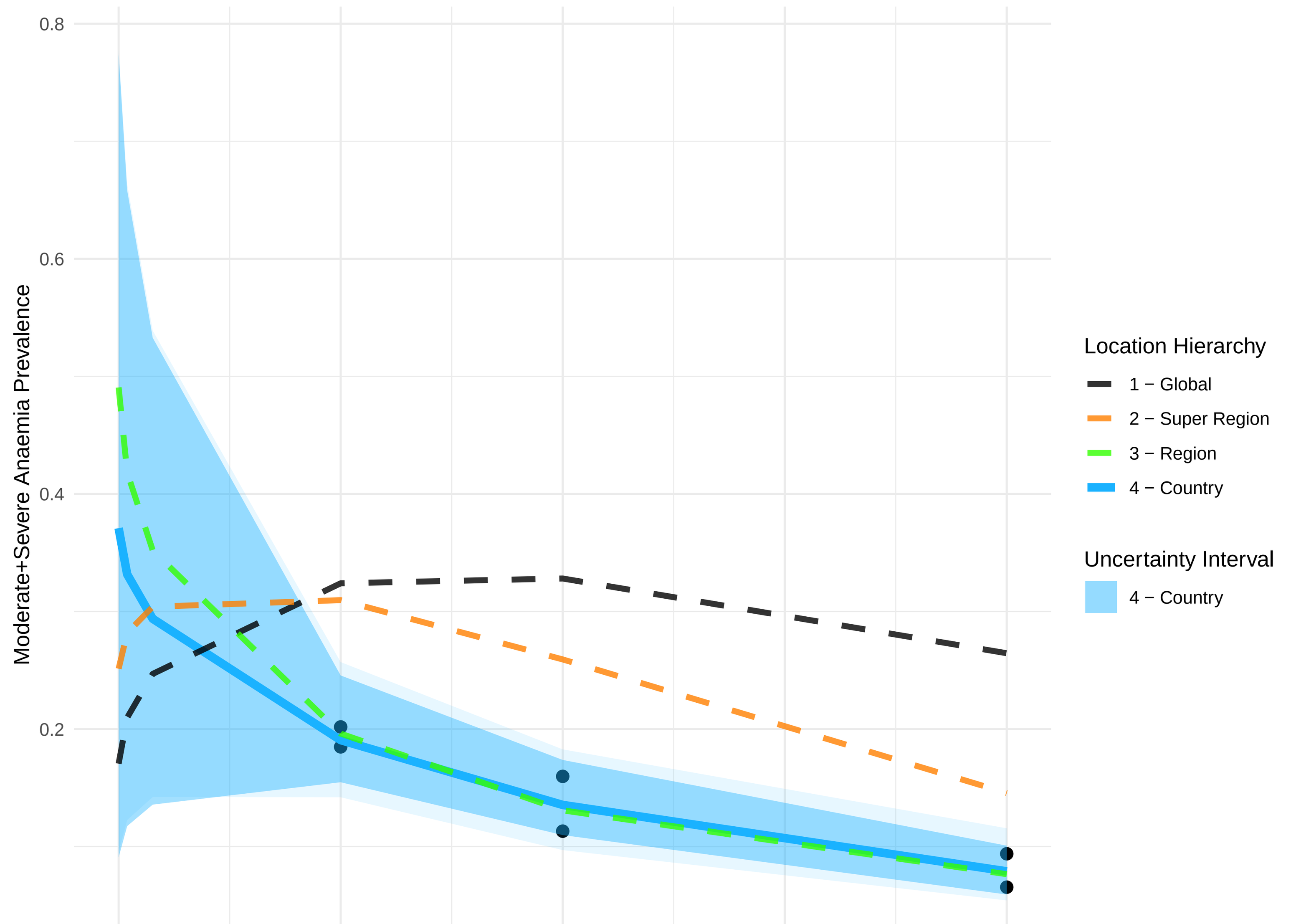

MR−BRT Age/Sex Fits − Males <2 Years Old − Moderate+Severe Anaemia Prevalence − Mexico
WHO−2001 Adjustment Method

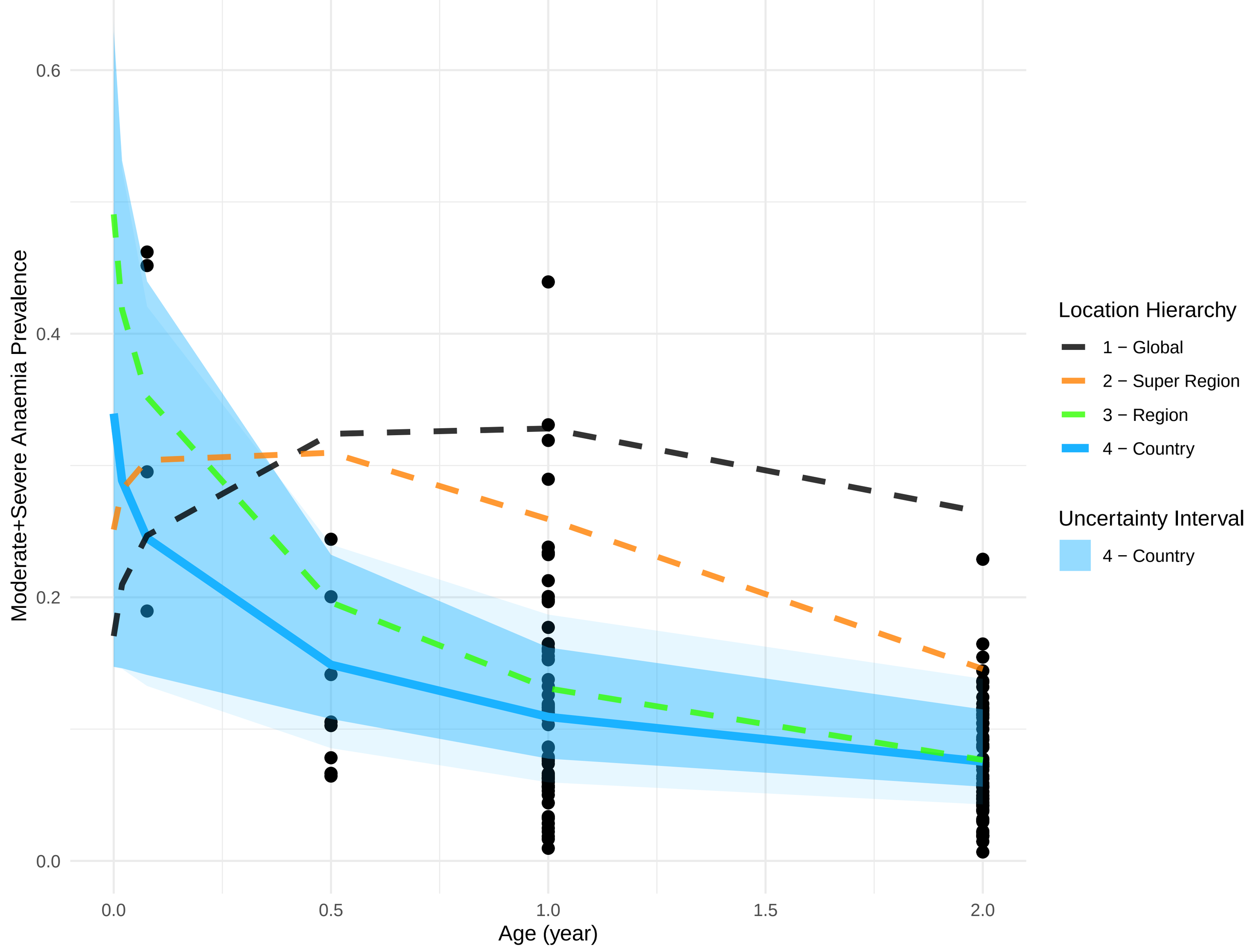

MR−BRT Age/Sex Fits − Males <2 Years Old − Moderate+Severe Anaemia Prevalence − Aguascalientes
WHO−2001 Adjustment Method

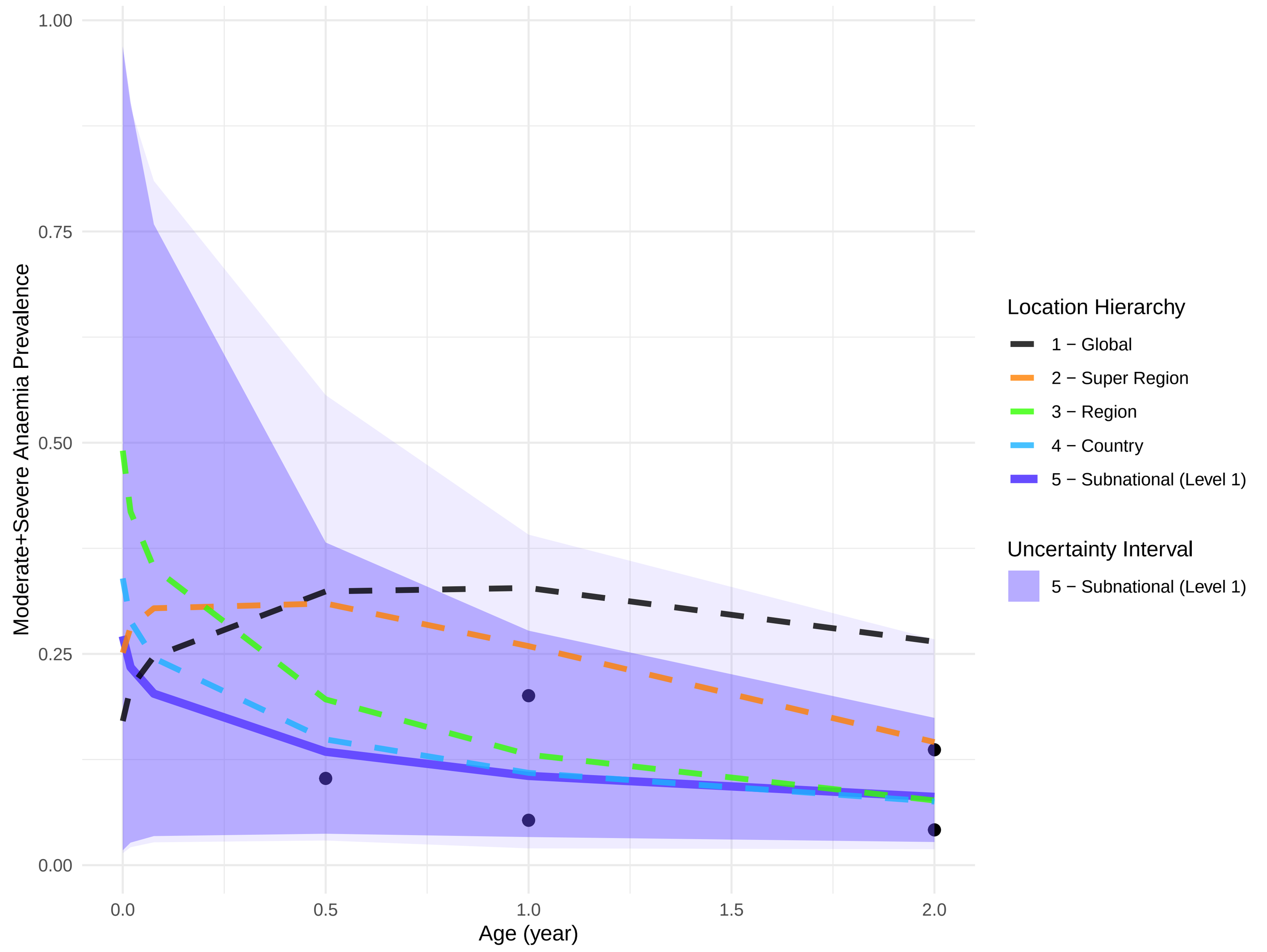

MR−BRT Age/Sex Fits − Males <2 Years Old − Moderate+Severe Anaemia Prevalence − Baja California
WHO−2001 Adjustment Method

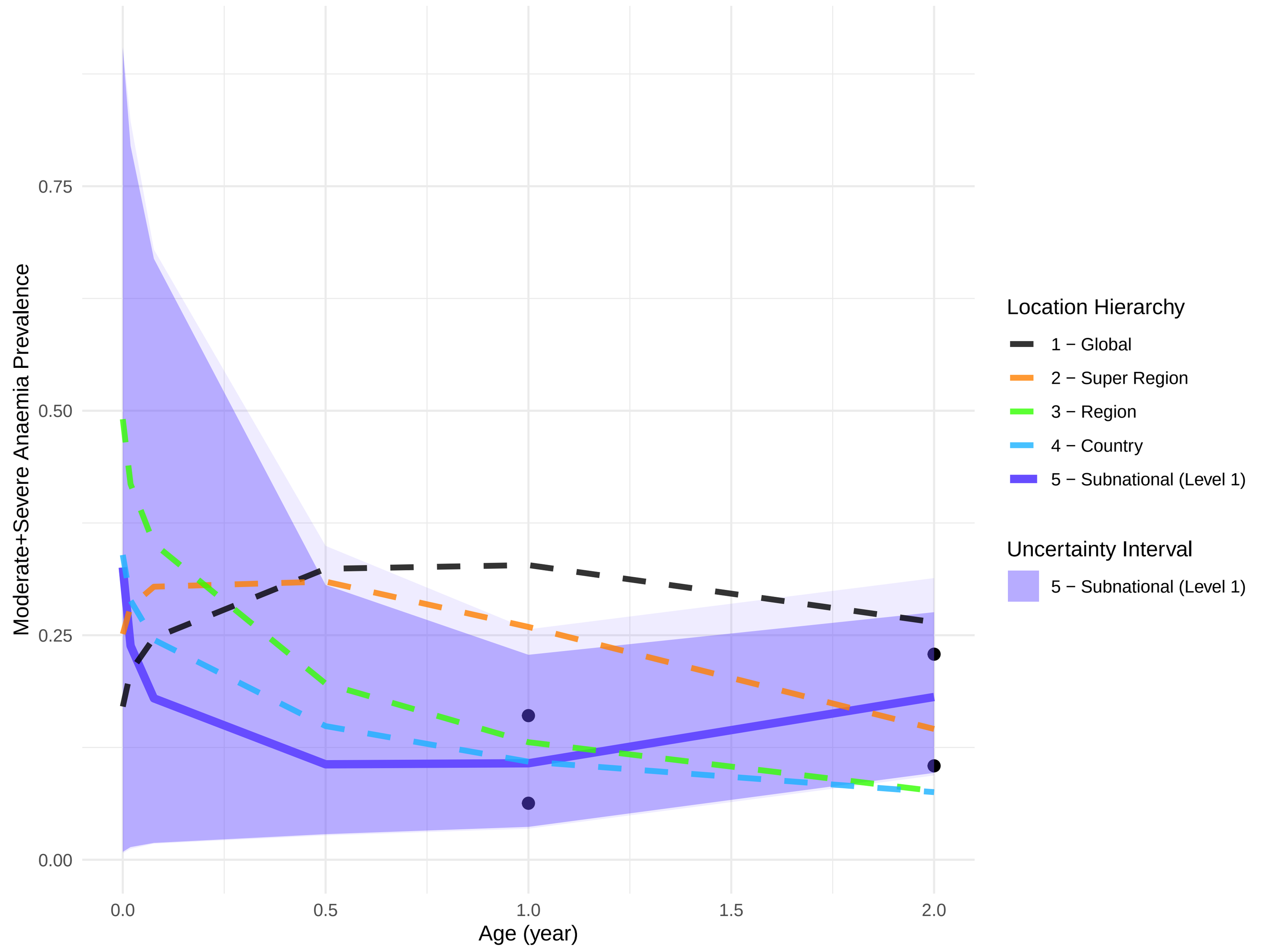

MR−BRT Age/Sex Fits − Males <2 Years Old − Moderate+Severe Anaemia Prevalence − Baja California Sur
WHO−2001 Adjustment Method

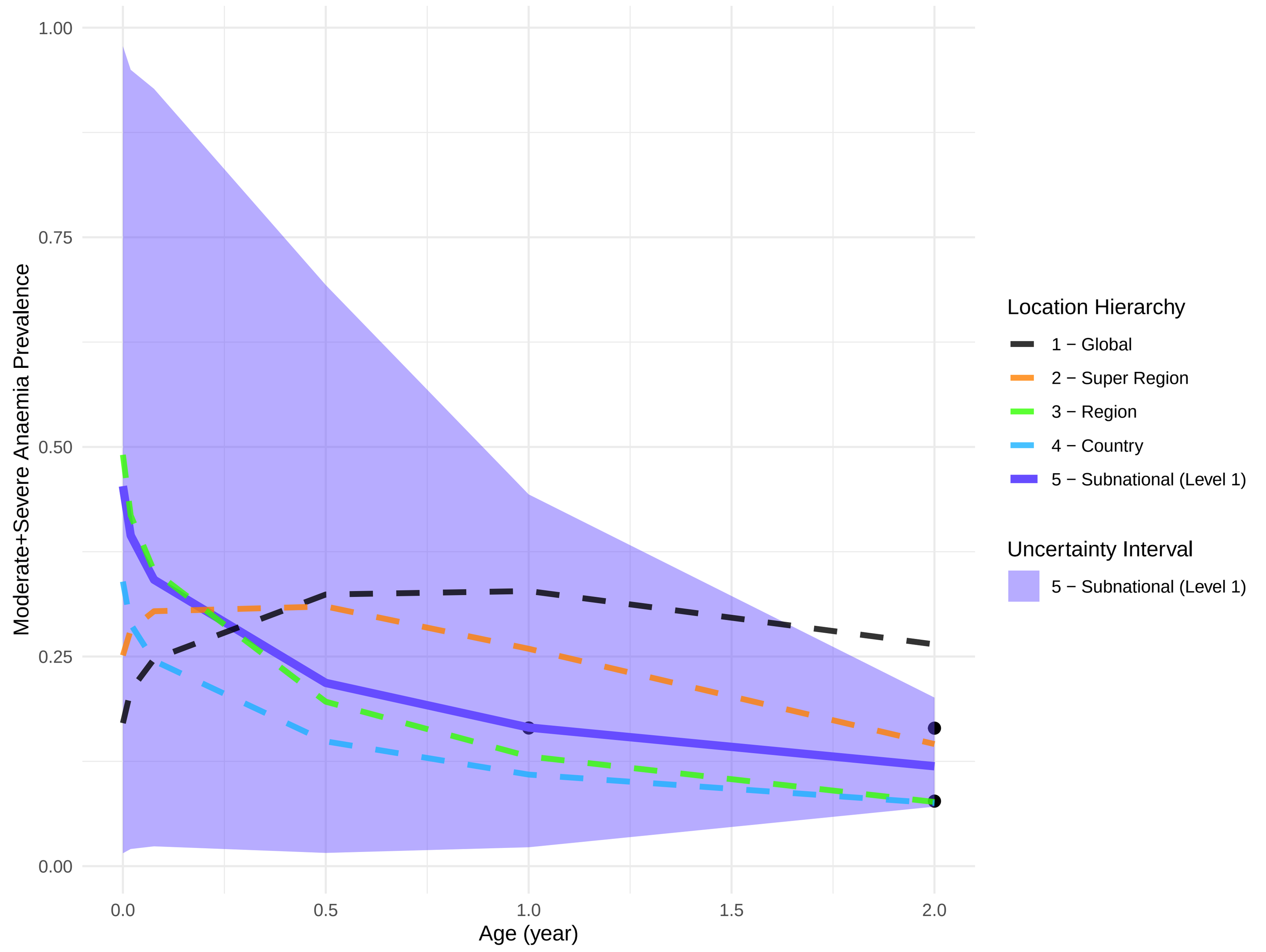

MR−BRT Age/Sex Fits − Males <2 Years Old − Moderate+Severe Anaemia Prevalence − Campeche
WHO−2001 Adjustment Method

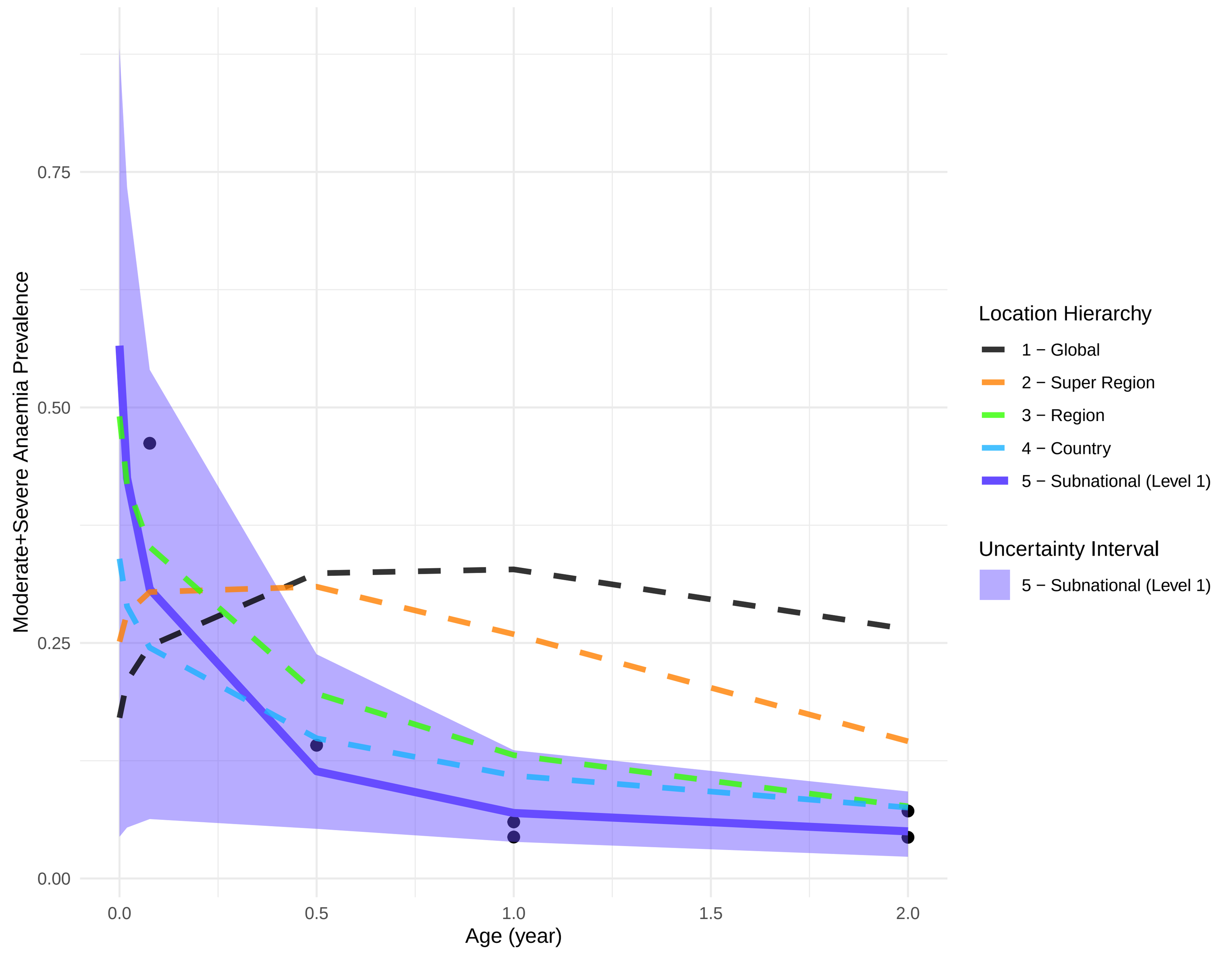

MR−BRT Age/Sex Fits − Males <2 Years Old − Moderate+Severe Anaemia Prevalence − Chiapas
WHO−2001 Adjustment Method

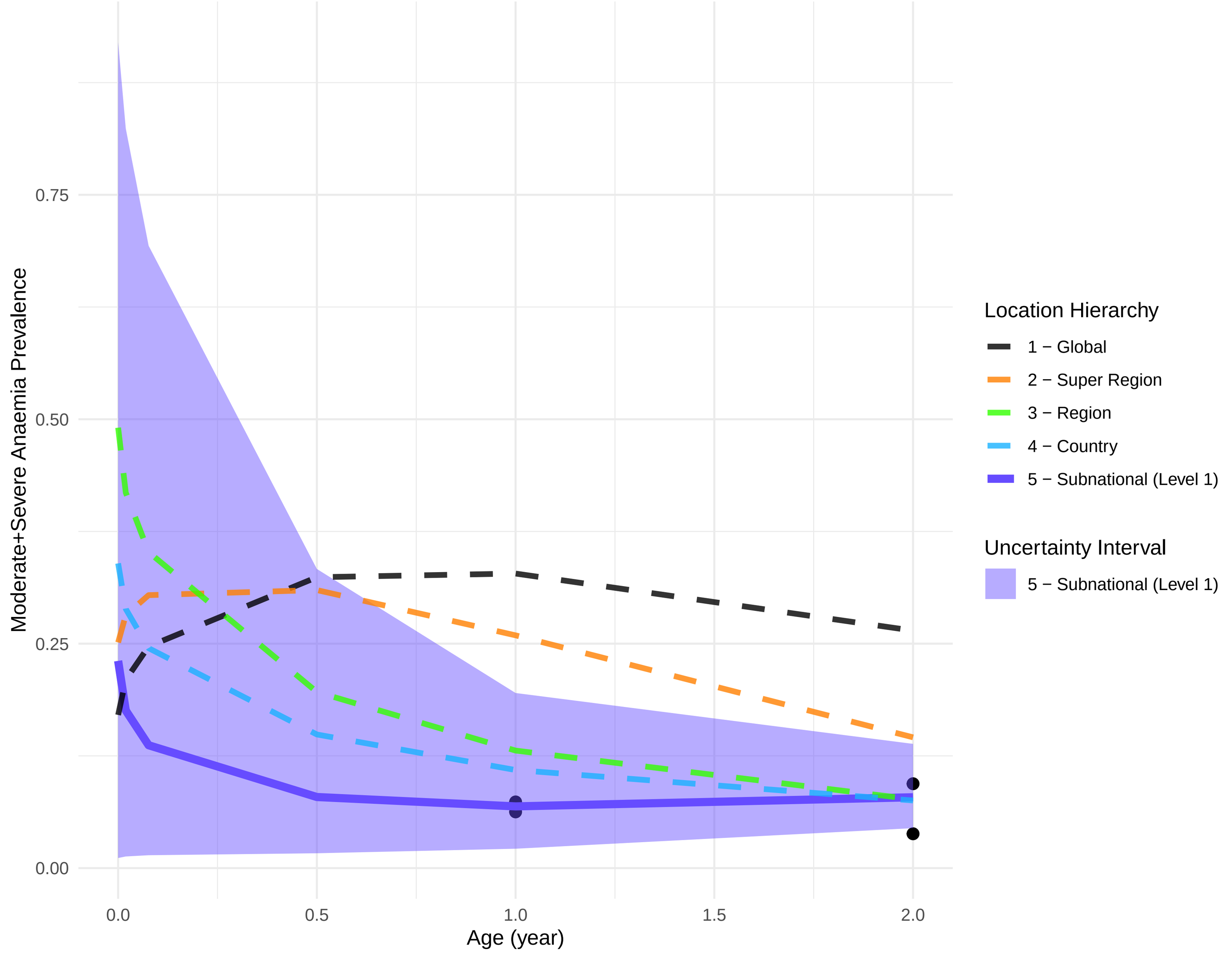

MR−BRT Age/Sex Fits − Males <2 Years Old − Moderate+Severe Anaemia Prevalence − Chihuahua
WHO−2001 Adjustment Method

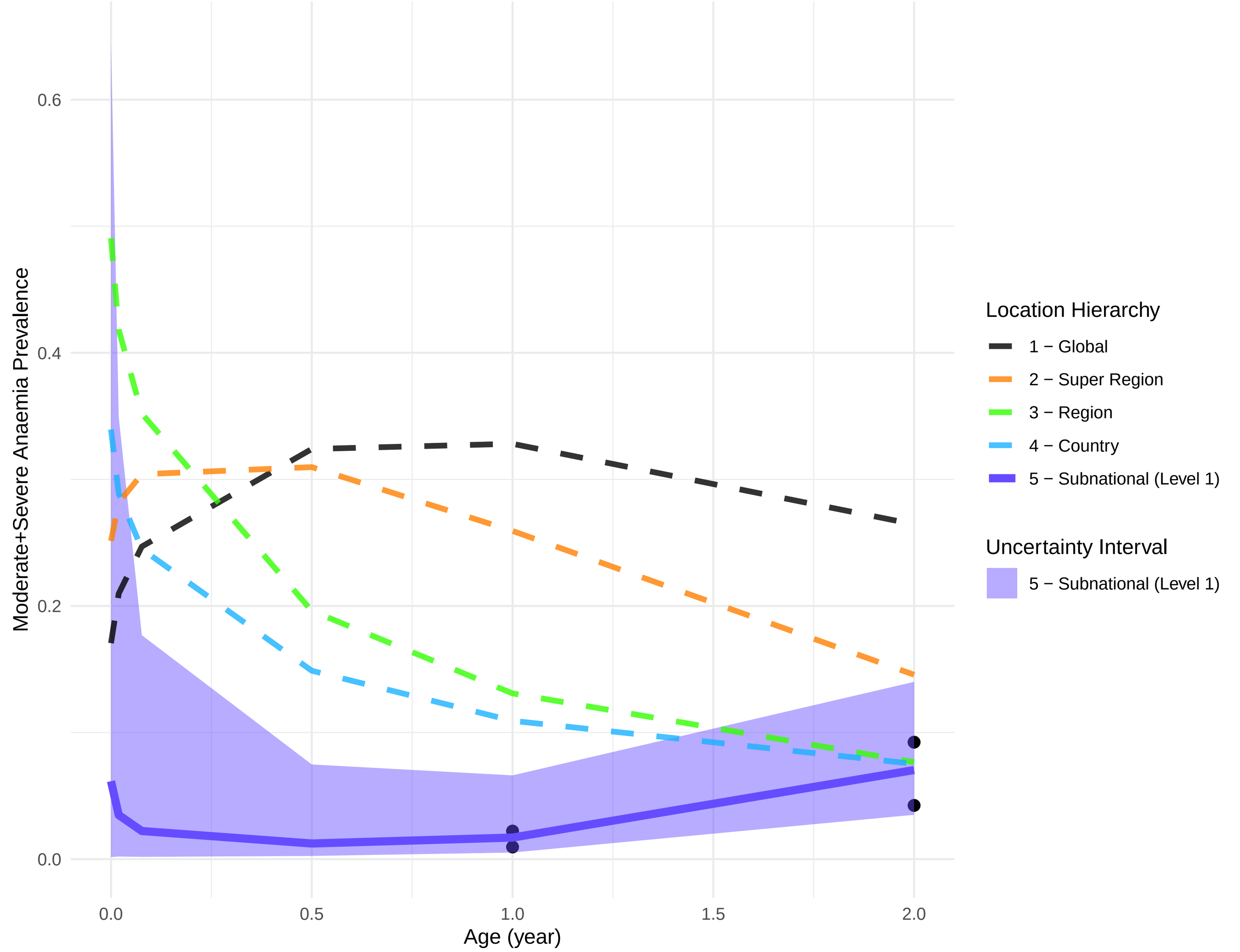

MR−BRT Age/Sex Fits − Males <2 Years Old − Moderate+Severe Anaemia Prevalence − Coahuila
WHO−2001 Adjustment Method

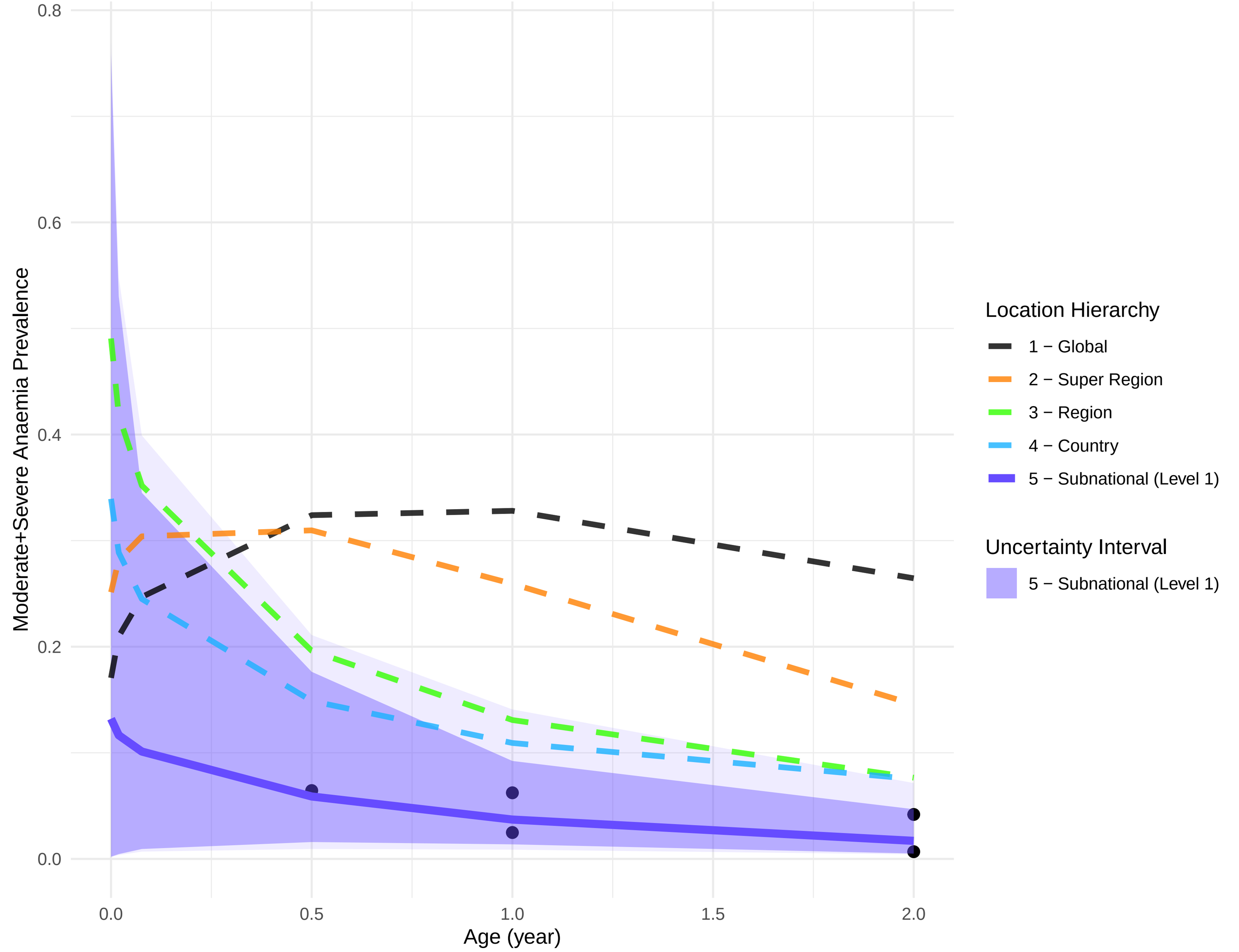

MR−BRT Age/Sex Fits − Males <2 Years Old − Moderate+Severe Anaemia Prevalence − Colima
WHO−2001 Adjustment Method

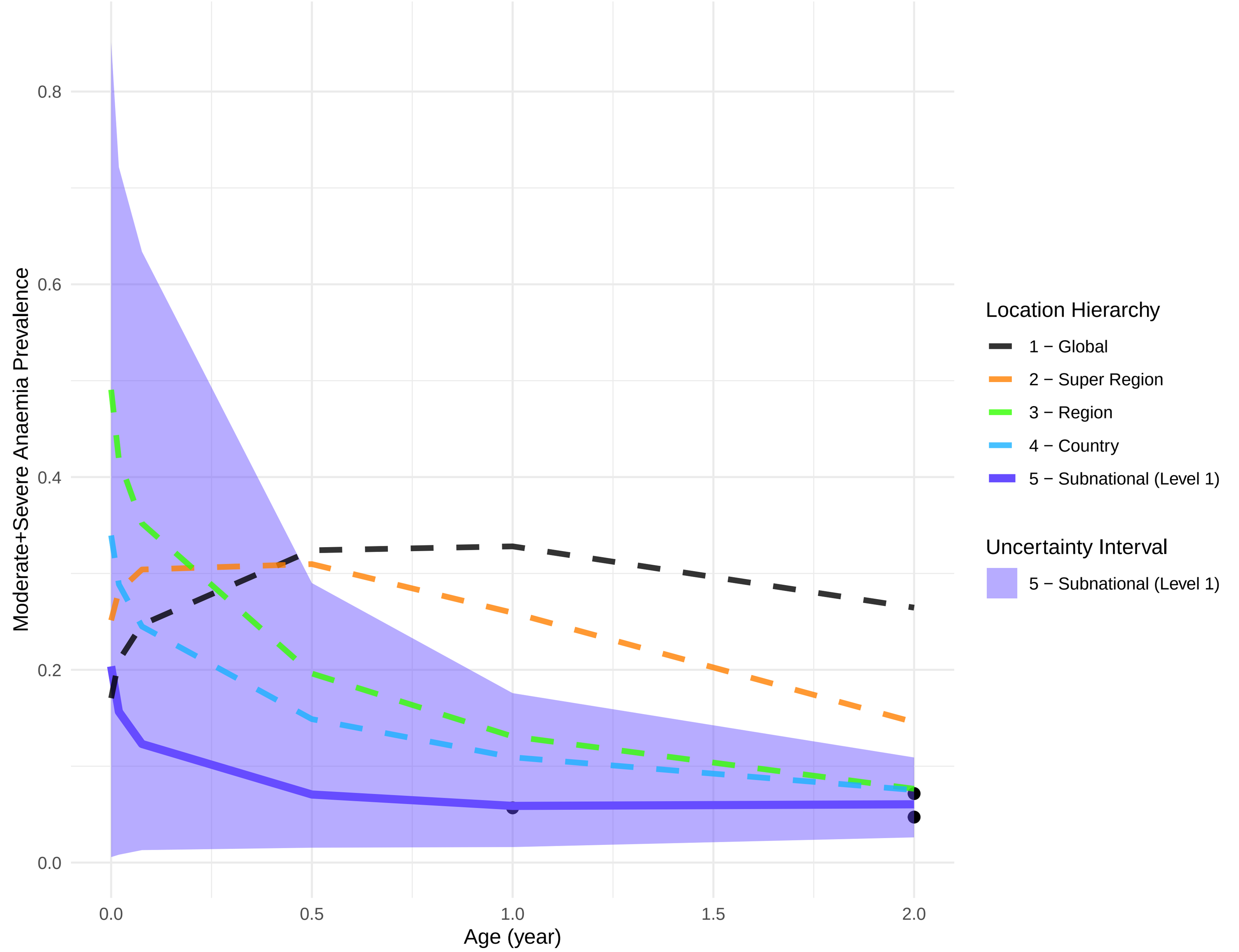

MR−BRT Age/Sex Fits − Males <2 Years Old − Moderate+Severe Anaemia Prevalence − Durango
WHO−2001 Adjustment Method

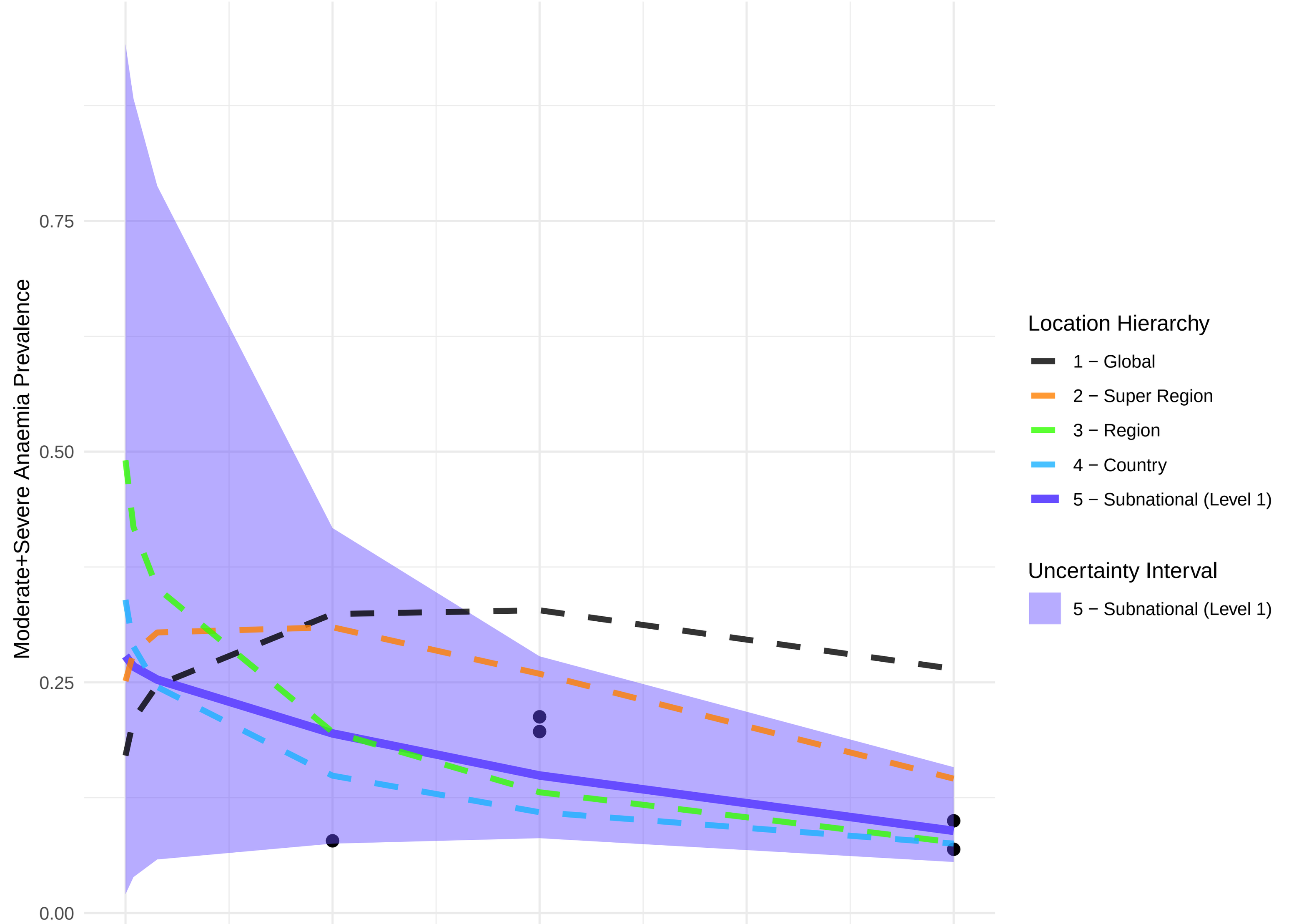

MR−BRT Age/Sex Fits − Males <2 Years Old − Moderate+Severe Anaemia Prevalence − Guanajuato
WHO−2001 Adjustment Method

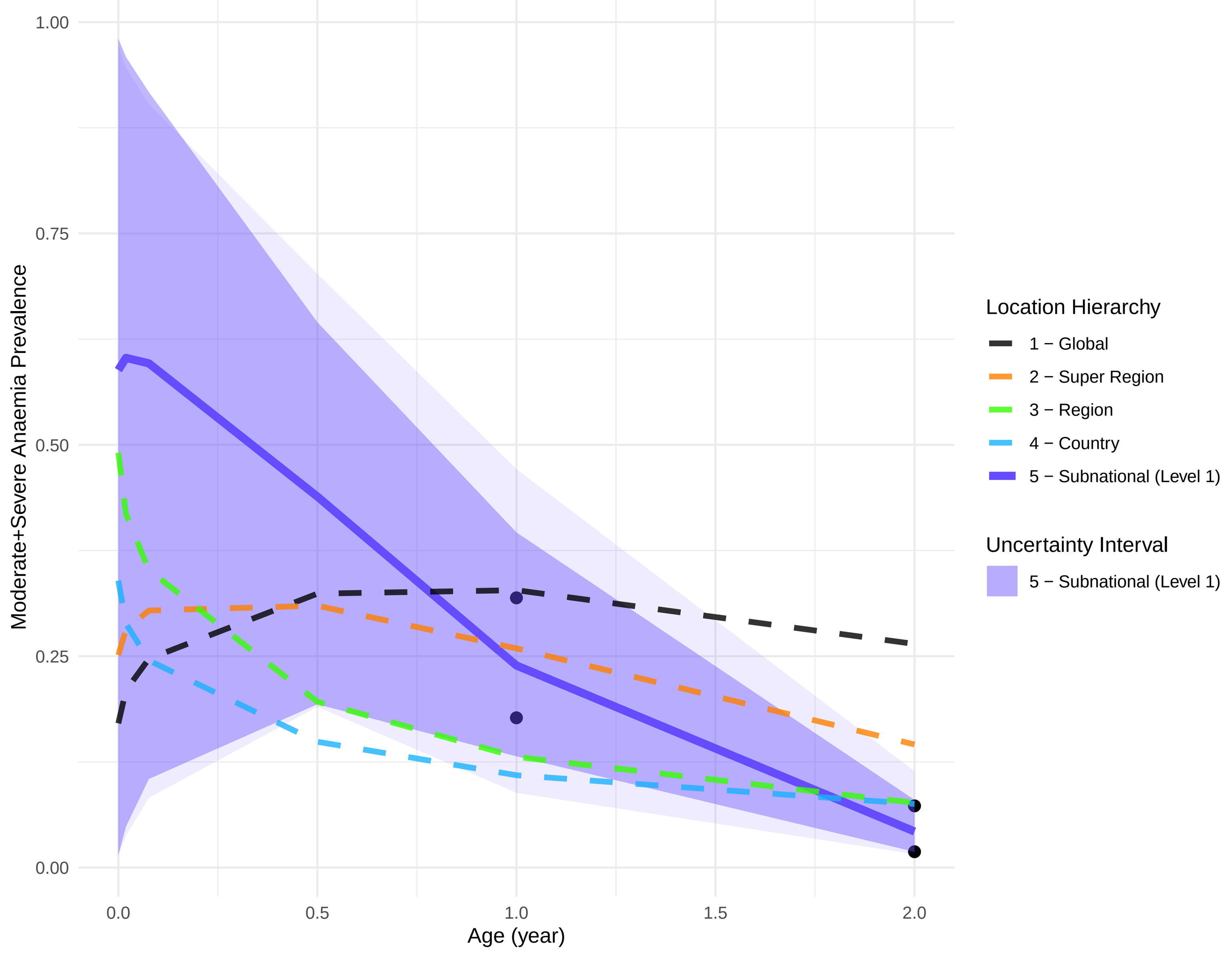

MR−BRT Age/Sex Fits − Males <2 Years Old − Moderate+Severe Anaemia Prevalence − Guerrero
WHO−2001 Adjustment Method

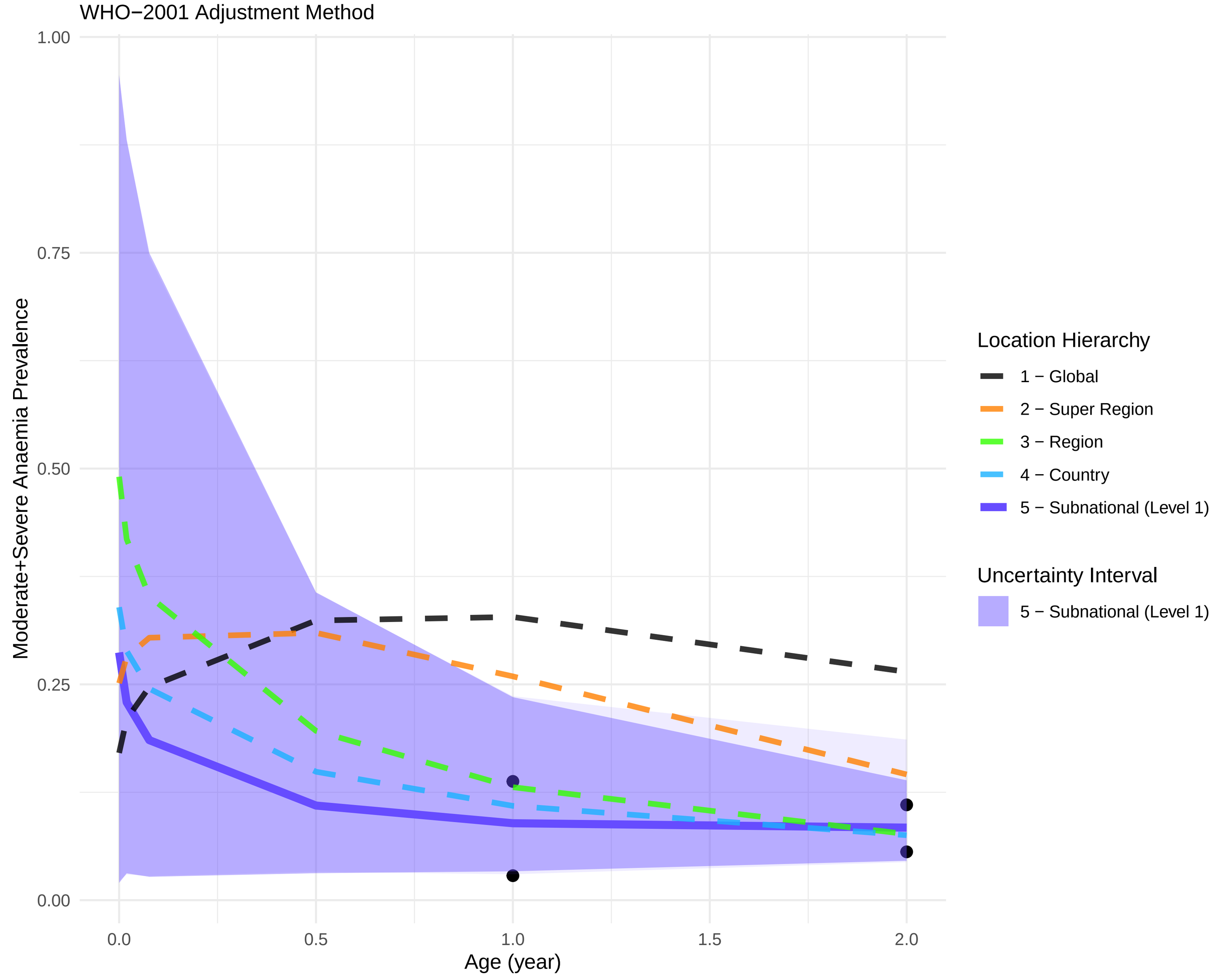

MR−BRT Age/Sex Fits − Males <2 Years Old − Moderate+Severe Anaemia Prevalence − Hidalgo
WHO−2001 Adjustment Method

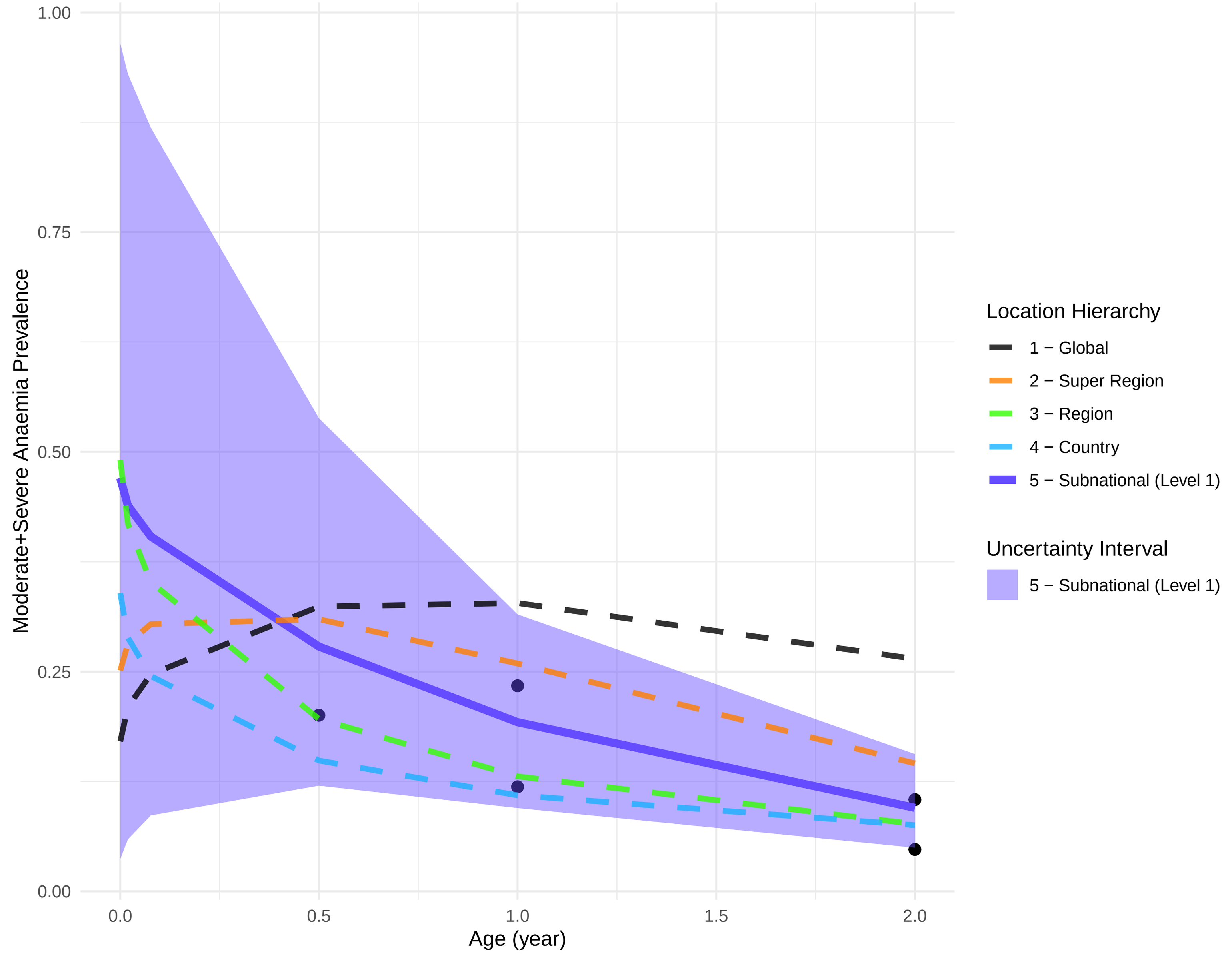

MR−BRT Age/Sex Fits − Males <2 Years Old − Moderate+Severe Anaemia Prevalence − Jalisco
WHO−2001 Adjustment Method

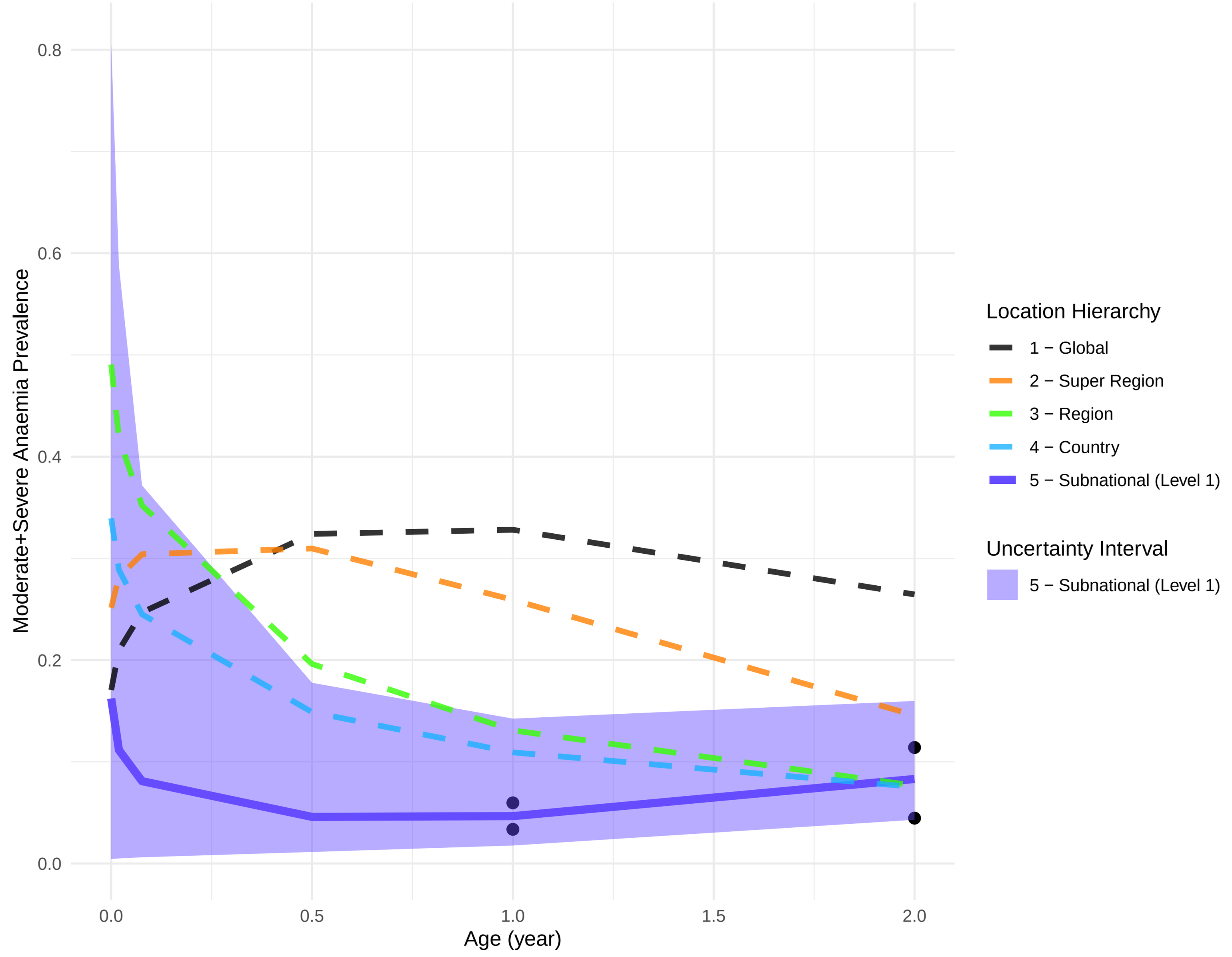

MR−BRT Age/Sex Fits − Males <2 Years Old − Moderate+Severe Anaemia Prevalence − México
WHO−2001 Adjustment Method

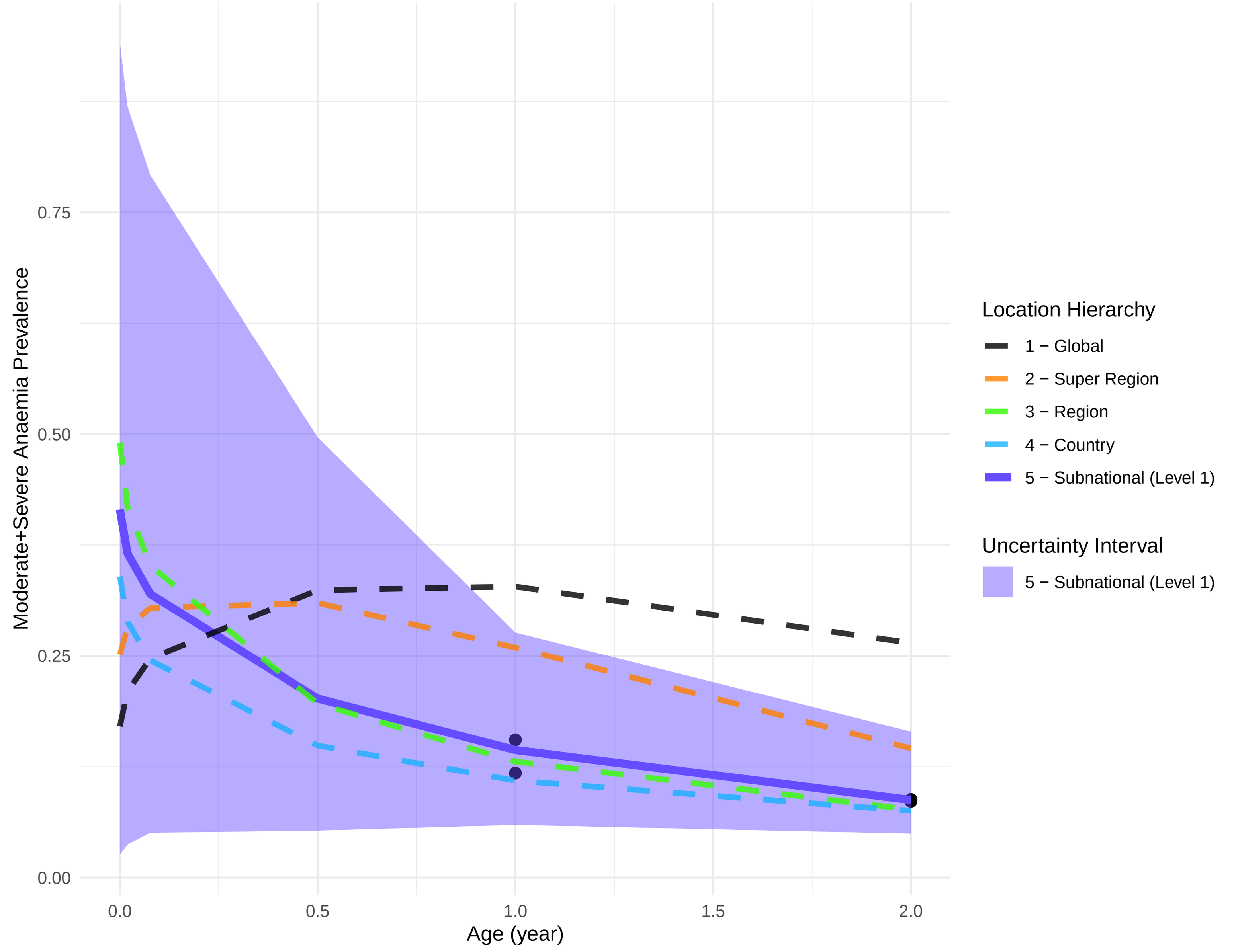

MR−BRT Age/Sex Fits − Males <2 Years Old − Moderate+Severe Anaemia Prevalence − Mexico City
WHO−2001 Adjustment Method

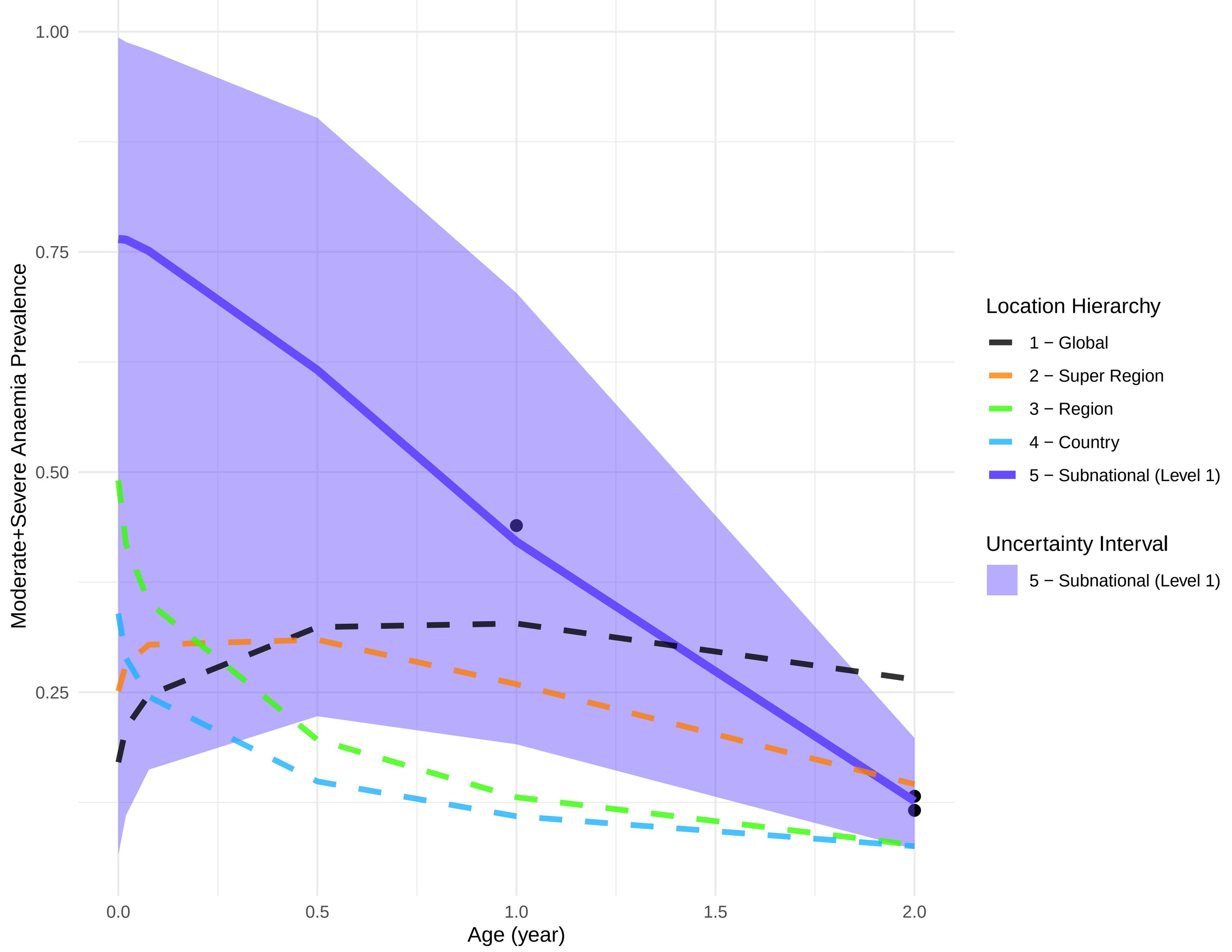

MR−BRT Age/Sex Fits − Males <2 Years Old − Moderate+Severe Anaemia Prevalence − Michoacán de Ocampo
WHO−2001 Adjustment Method

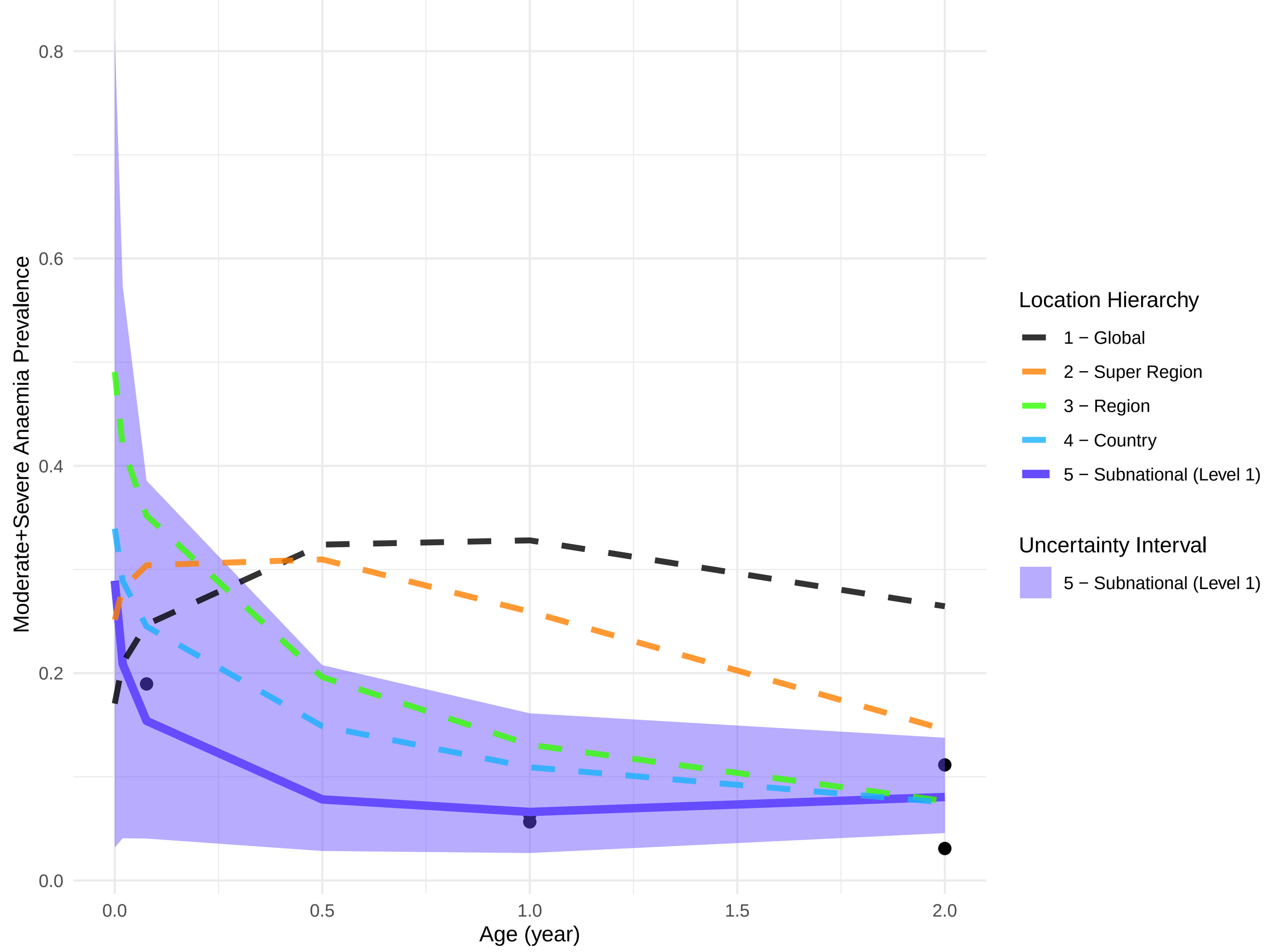

MR−BRT Age/Sex Fits − Males <2 Years Old − Moderate+Severe Anaemia Prevalence − Morelos
WHO−2001 Adjustment Method

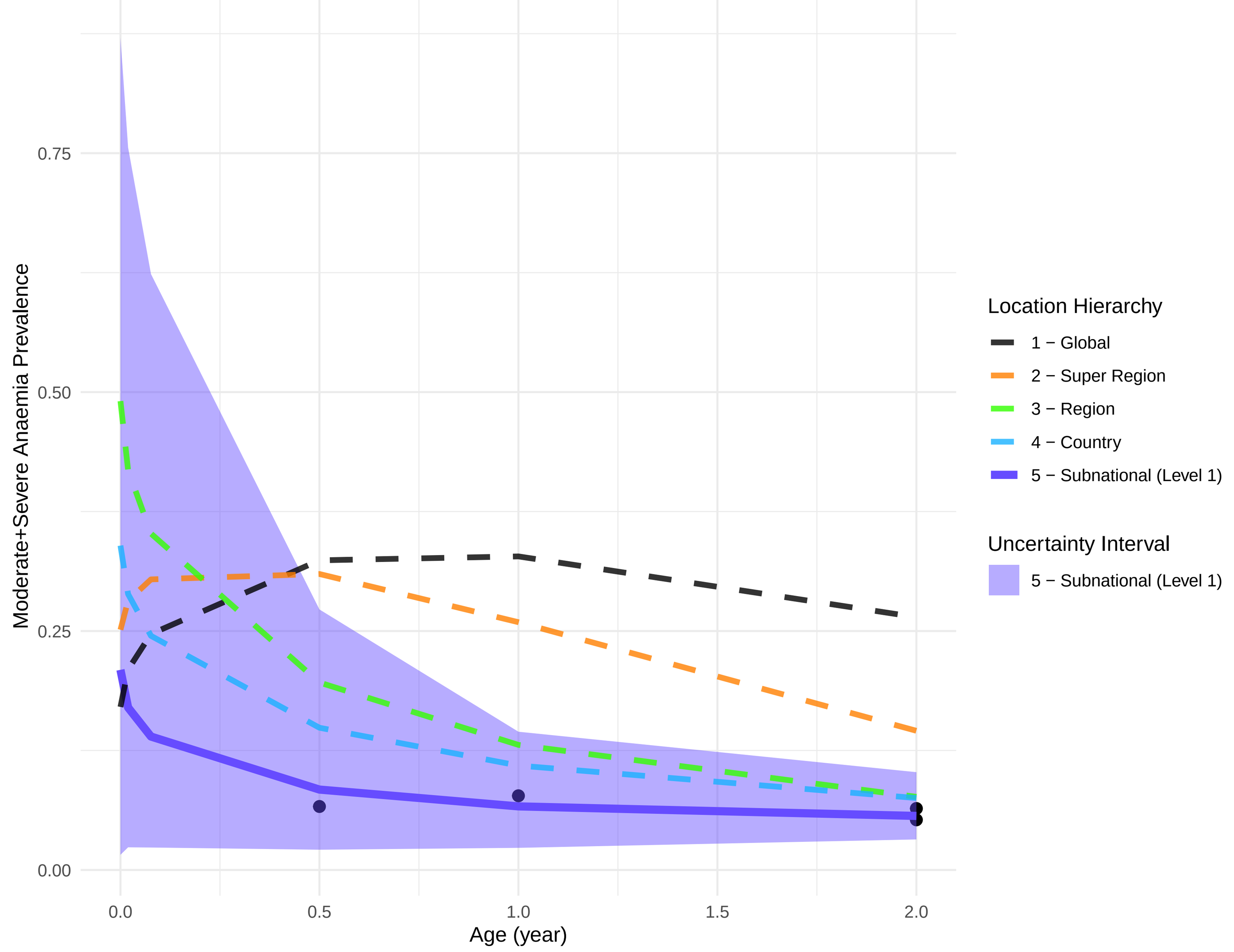

MR−BRT Age/Sex Fits − Males <2 Years Old − Moderate+Severe Anaemia Prevalence − Nayarit
WHO−2001 Adjustment Method

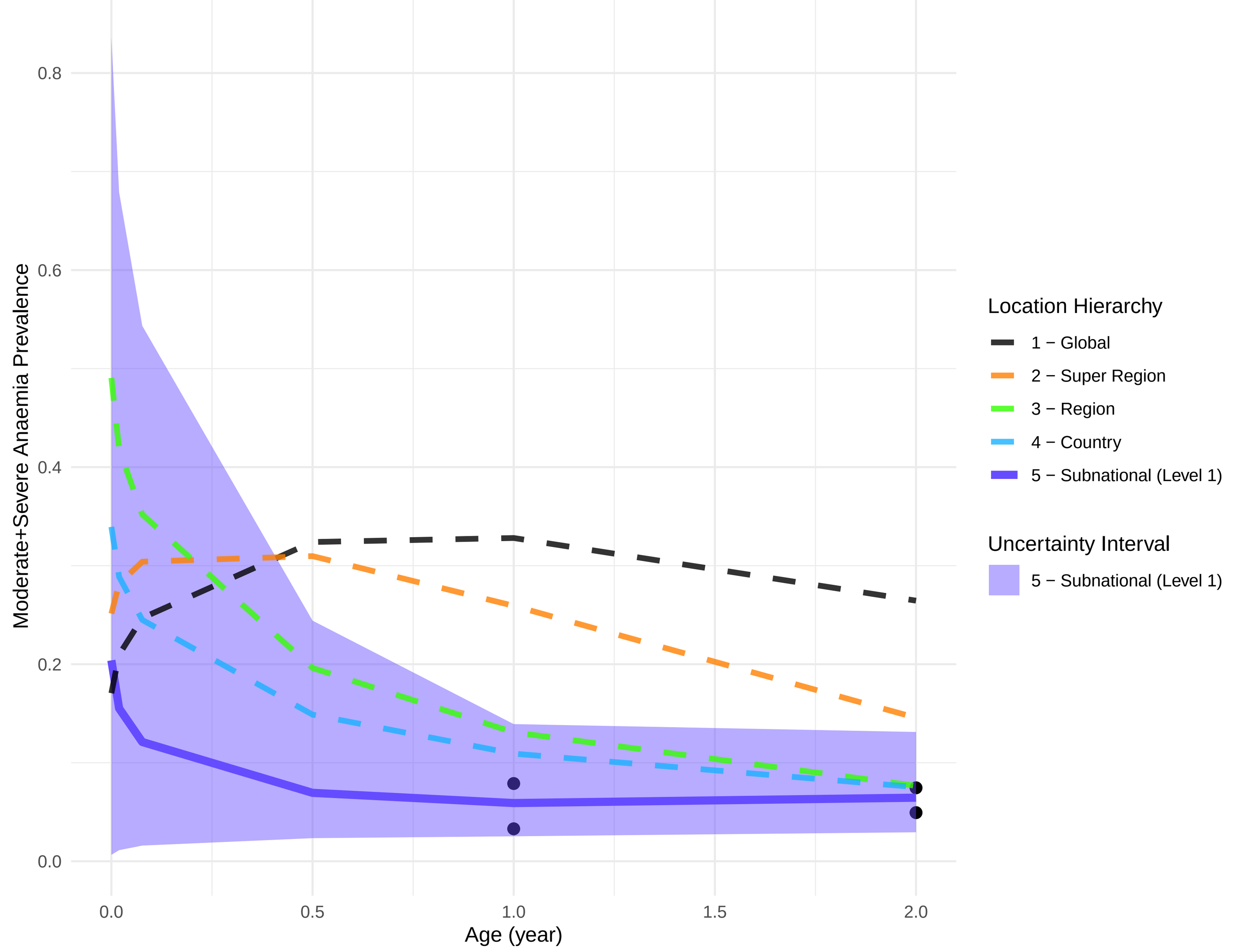

MR−BRT Age/Sex Fits − Males <2 Years Old − Moderate+Severe Anaemia Prevalence − Nuevo León
WHO−2001 Adjustment Method

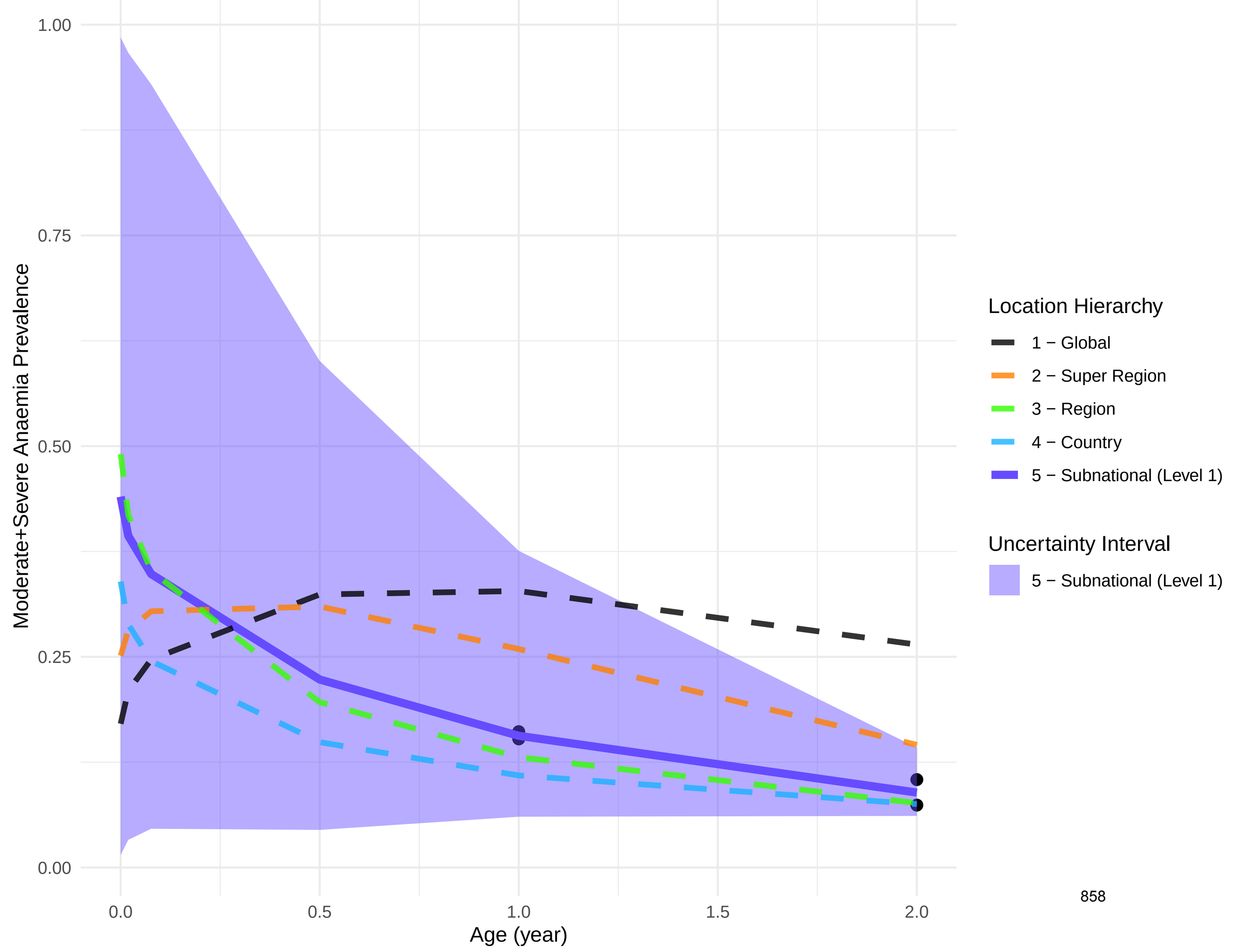

MR−BRT Age/Sex Fits − Males <2 Years Old − Moderate+Severe Anaemia Prevalence − Oaxaca
WHO−2001 Adjustment Method

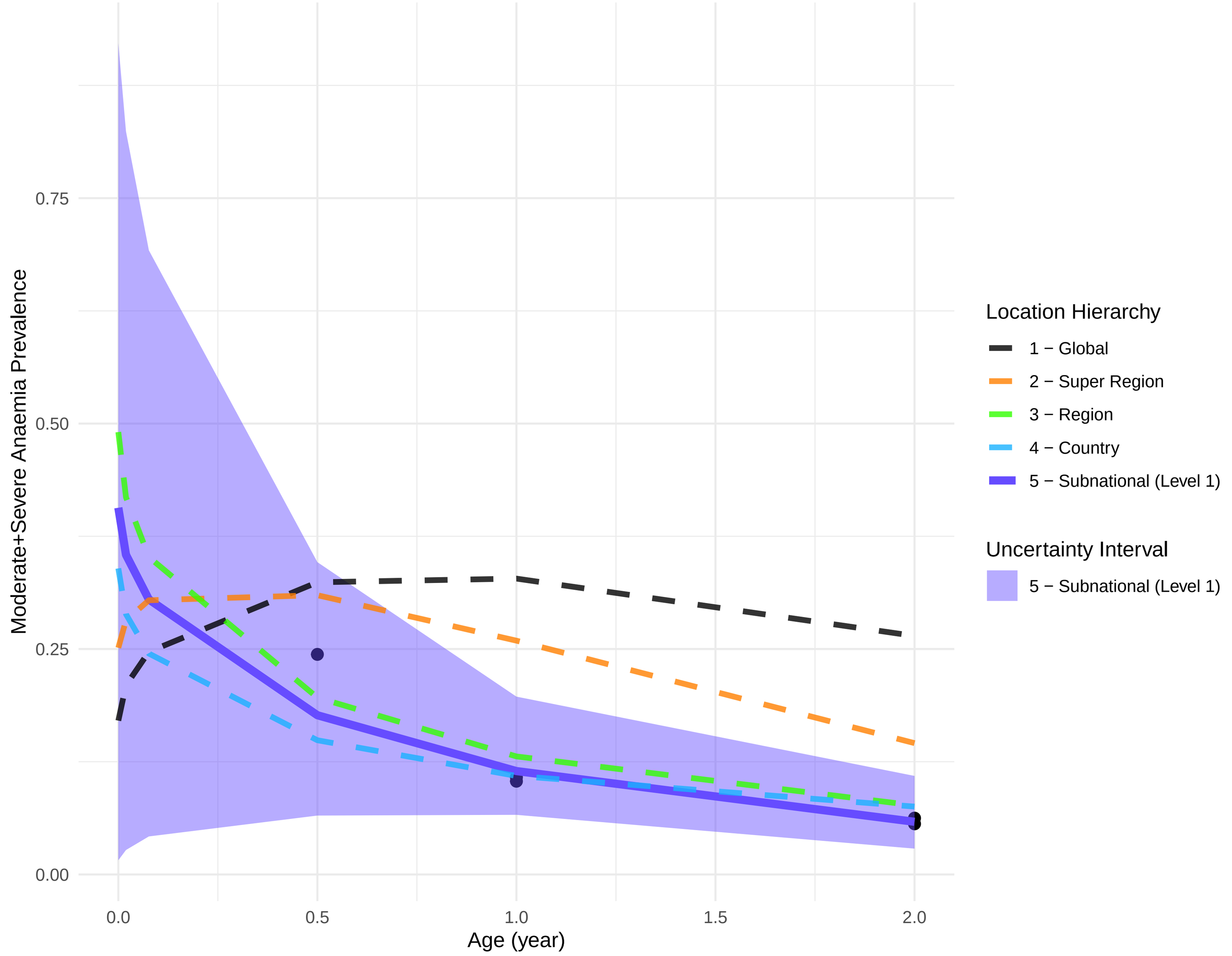

MR−BRT Age/Sex Fits − Males <2 Years Old − Moderate+Severe Anaemia Prevalence − Puebla
WHO−2001 Adjustment Method

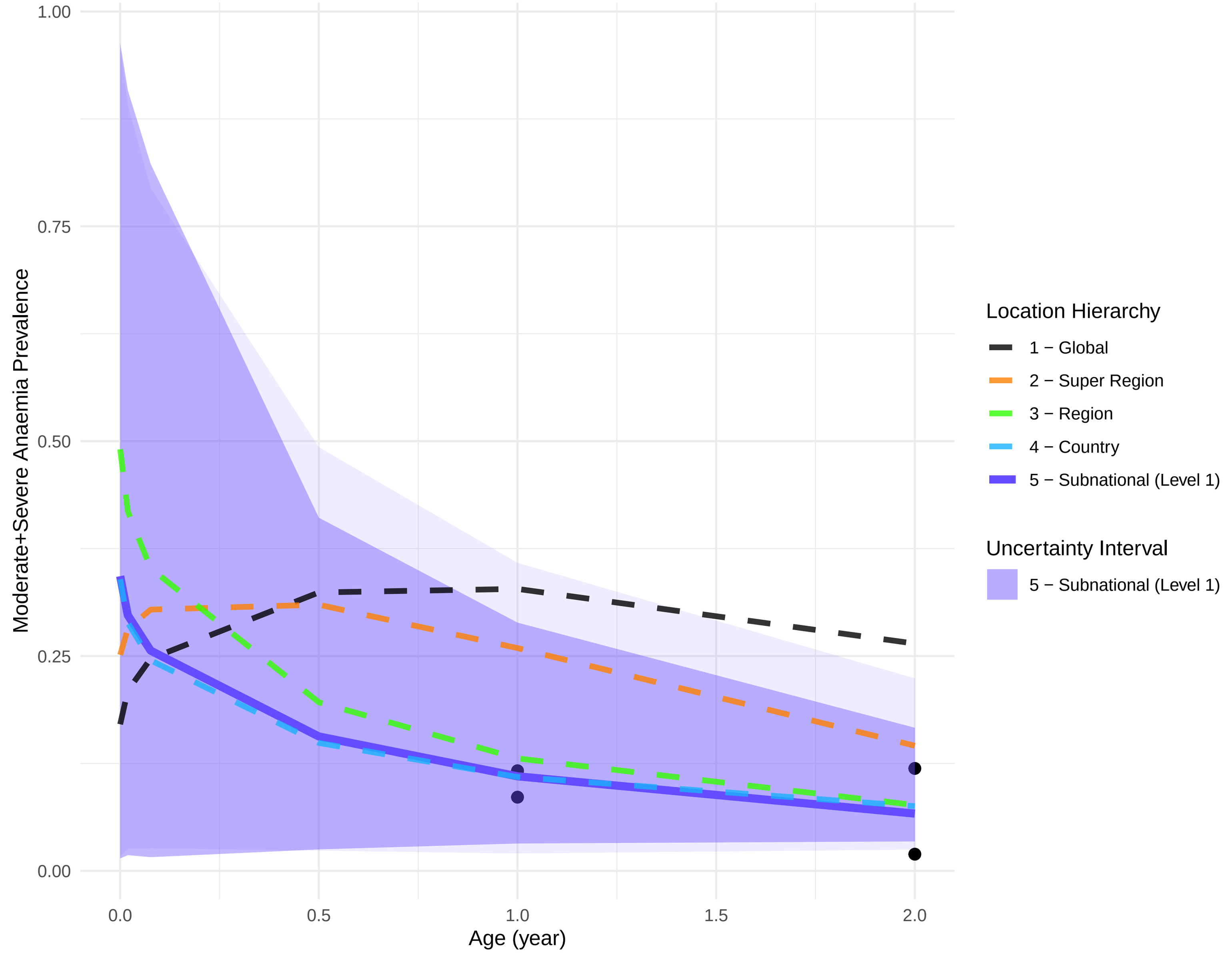

MR−BRT Age/Sex Fits − Males <2 Years Old − Moderate+Severe Anaemia Prevalence − Querétaro
WHO−2001 Adjustment Method

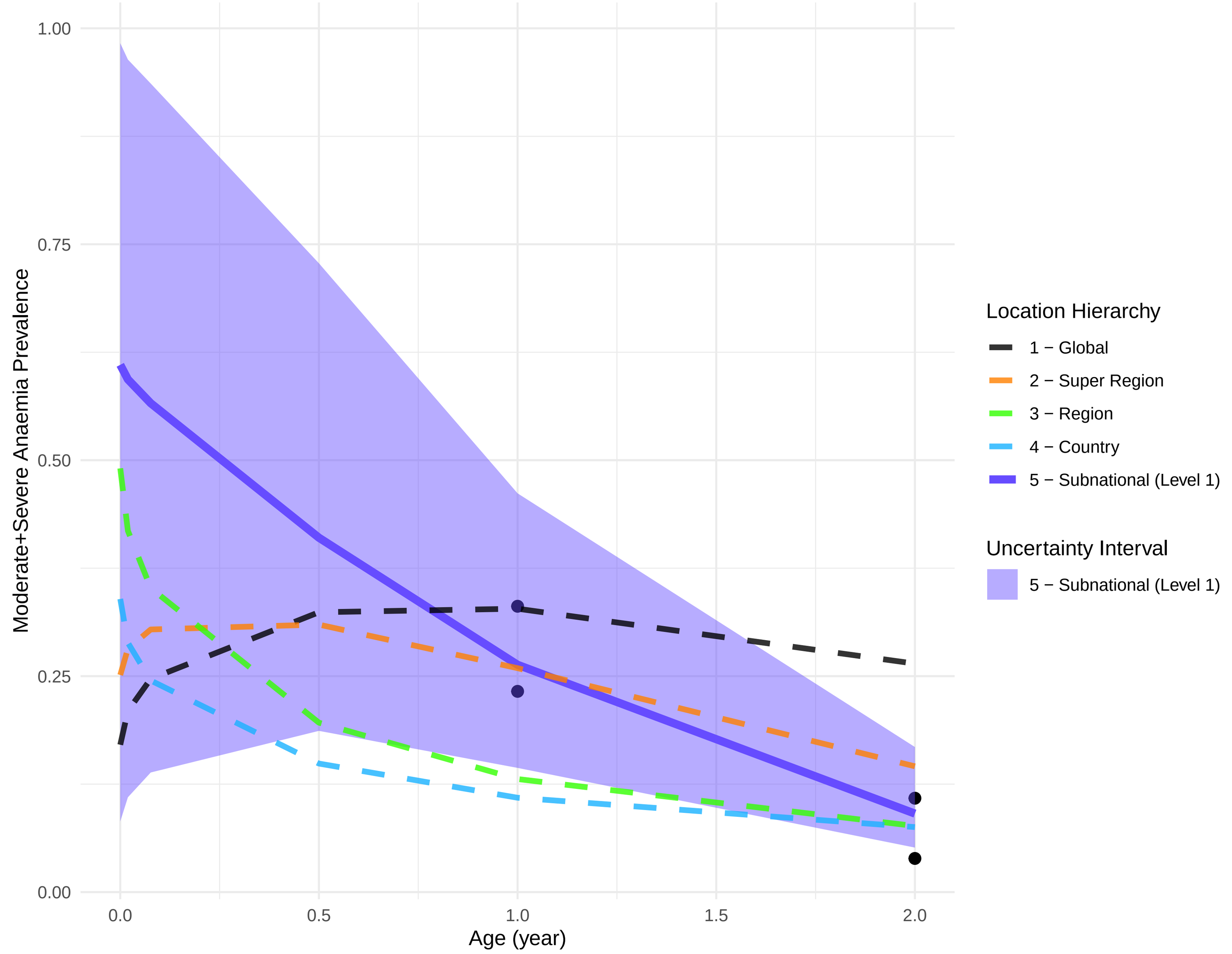

MR−BRT Age/Sex Fits − Males <2 Years Old − Moderate+Severe Anaemia Prevalence − Quintana Roo
WHO−2001 Adjustment Method

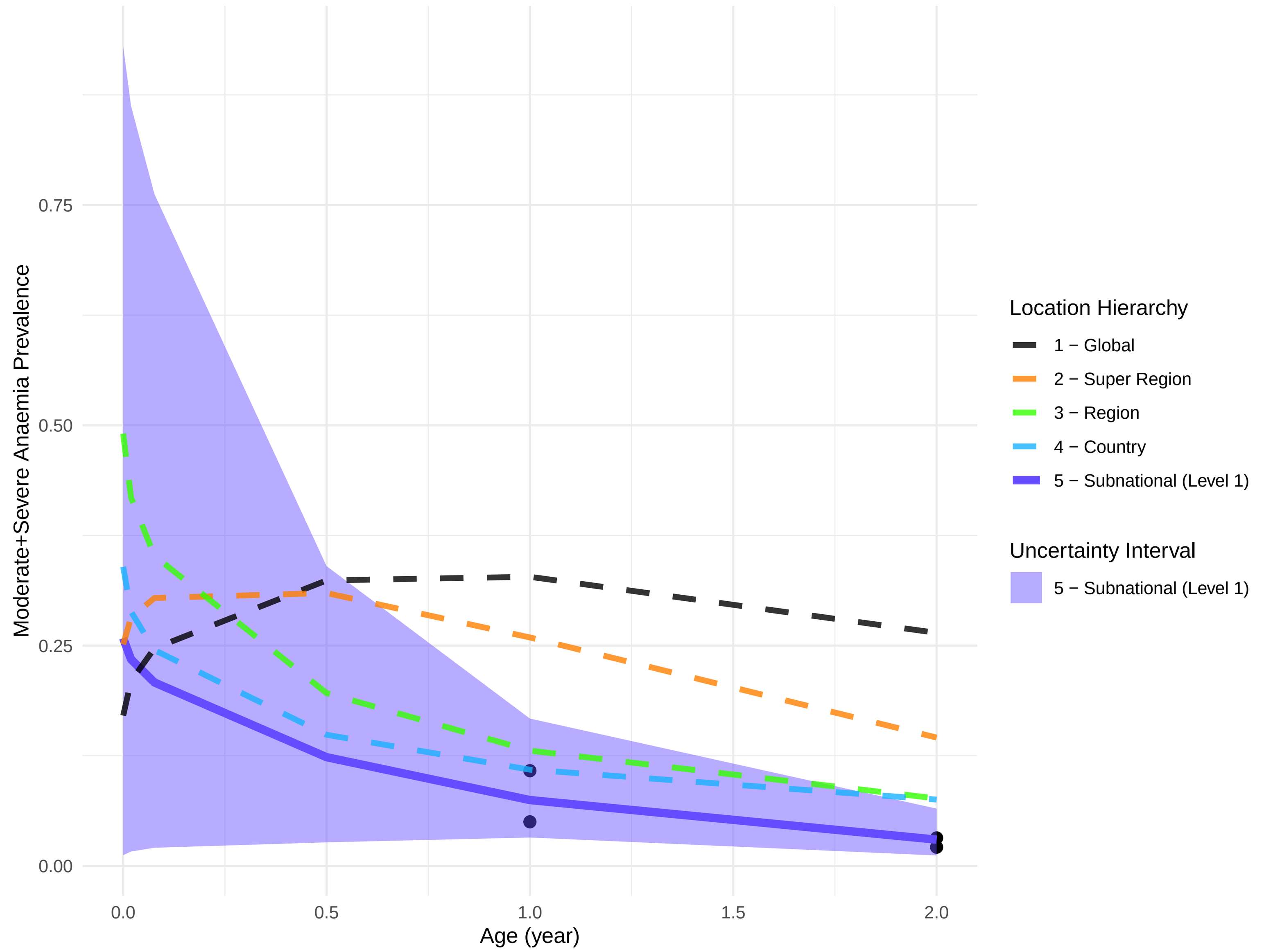

MR−BRT Age/Sex Fits − Males <2 Years Old − Moderate+Severe Anaemia Prevalence − San Luis Potosí
WHO−2001 Adjustment Method

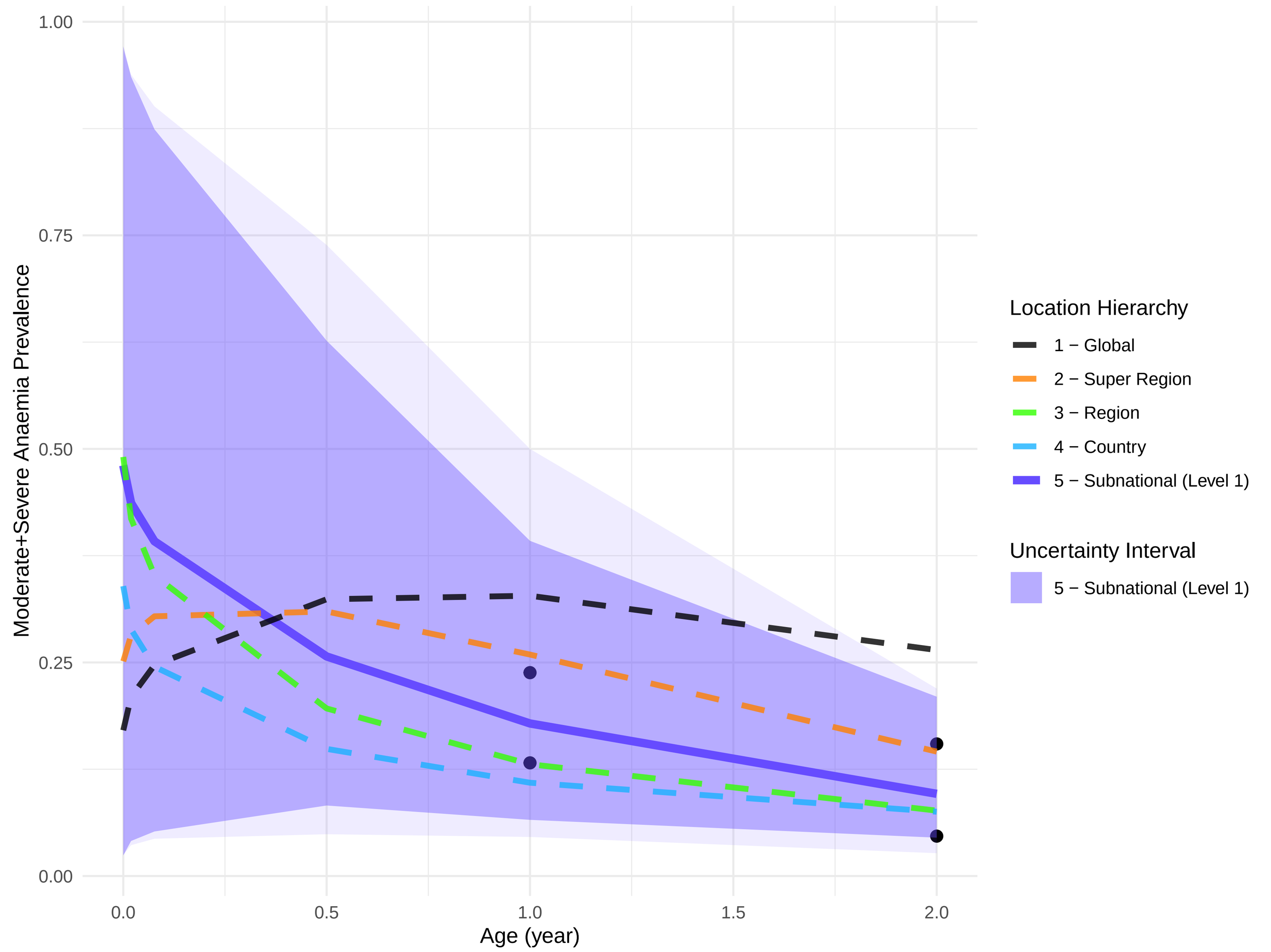

MR−BRT Age/Sex Fits − Males <2 Years Old − Moderate+Severe Anaemia Prevalence − Sinaloa
WHO−2001 Adjustment Method

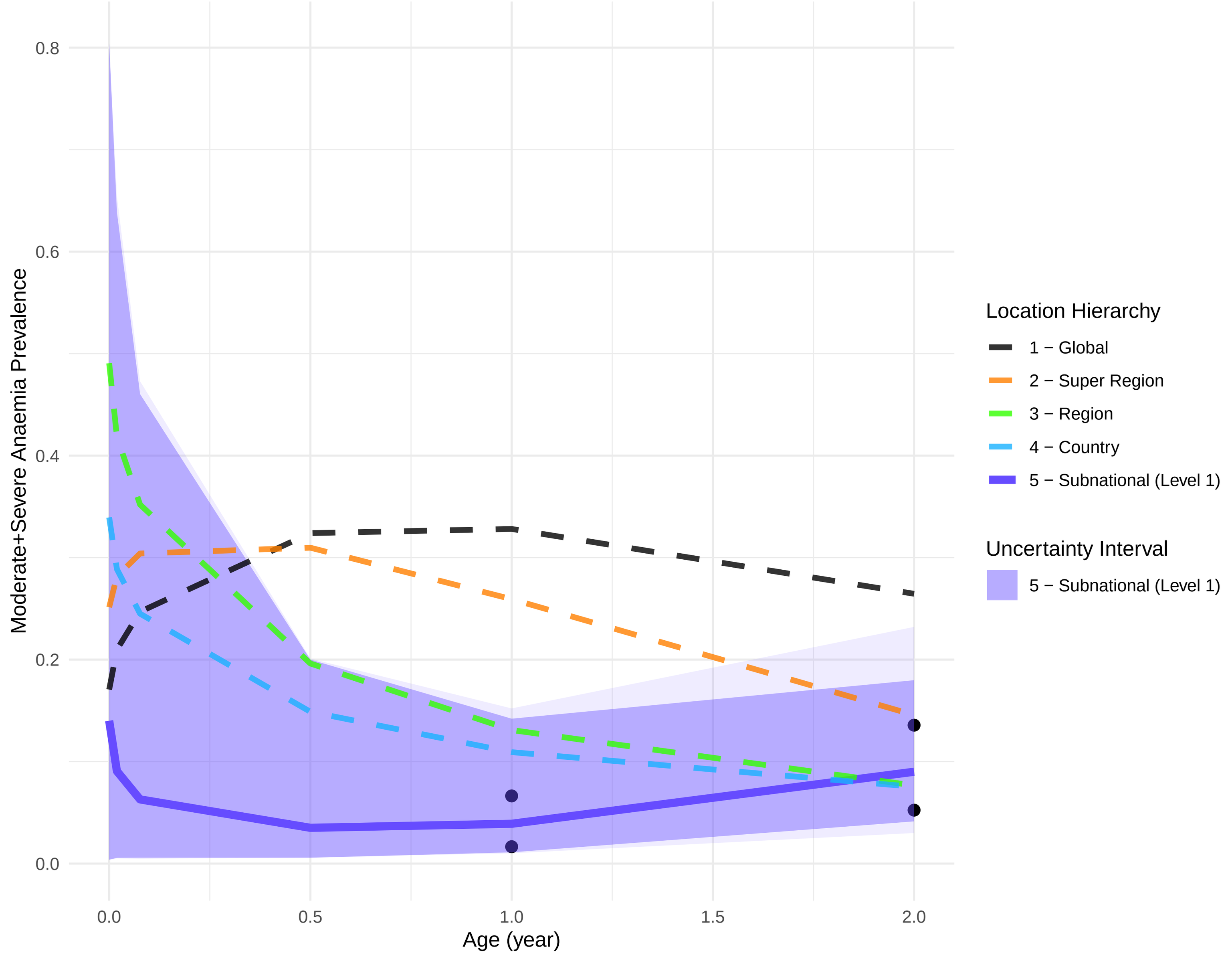

MR−BRT Age/Sex Fits − Males <2 Years Old − Moderate+Severe Anaemia Prevalence − Sonora
WHO−2001 Adjustment Method

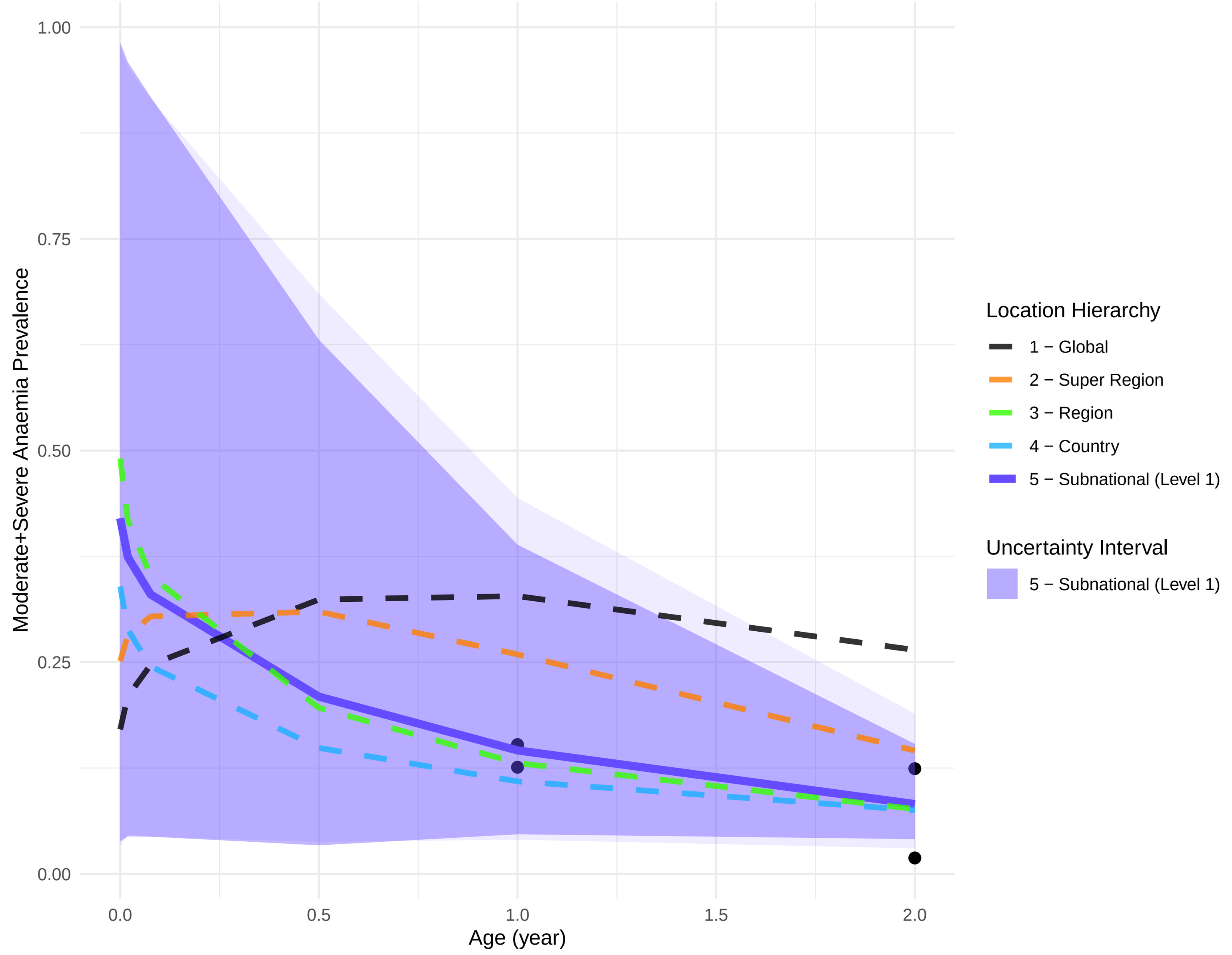

MR−BRT Age/Sex Fits − Males <2 Years Old − Moderate+Severe Anaemia Prevalence − Tabasco
WHO−2001 Adjustment Method

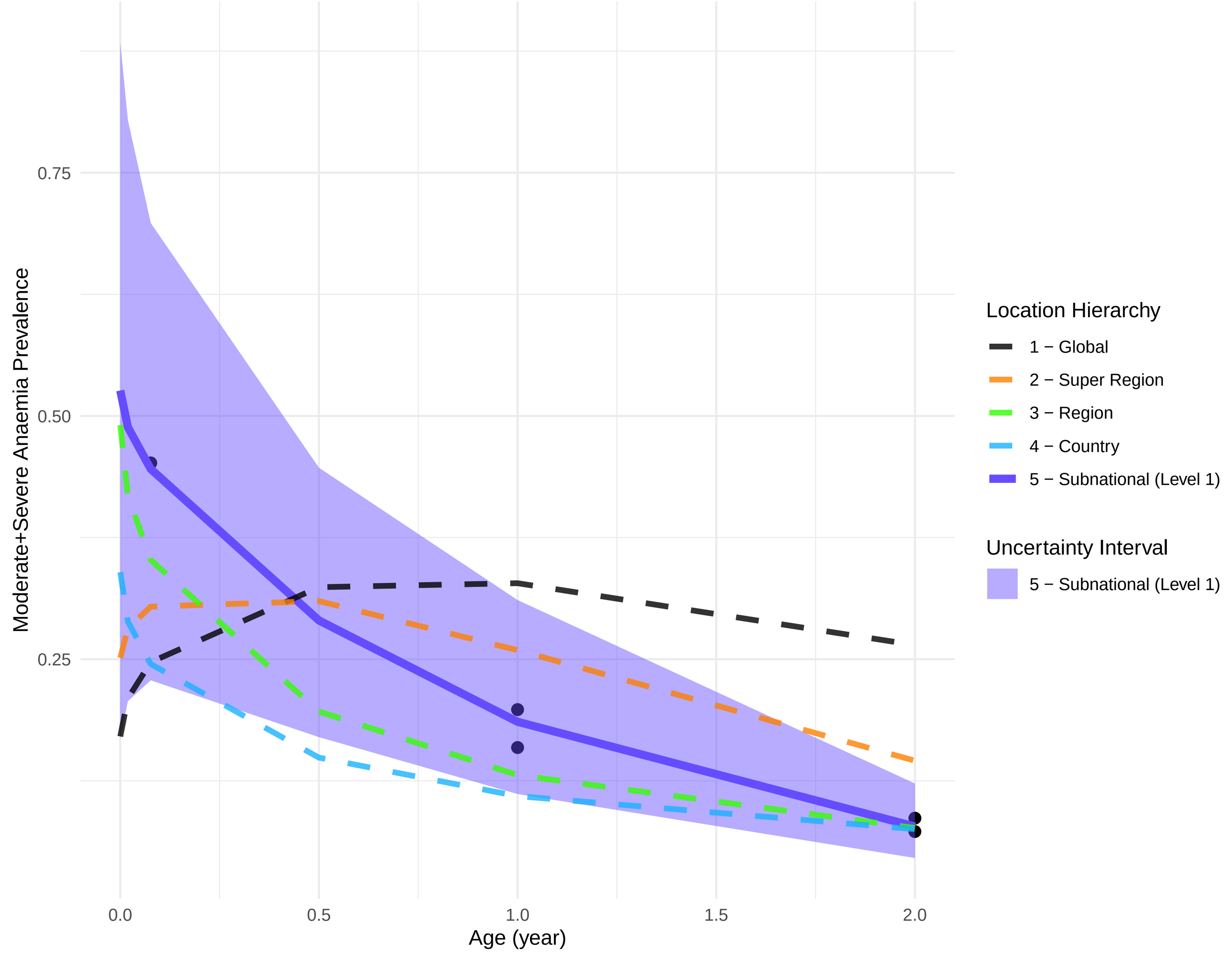

MR−BRT Age/Sex Fits − Males <2 Years Old − Moderate+Severe Anaemia Prevalence − Tamaulipas
WHO−2001 Adjustment Method

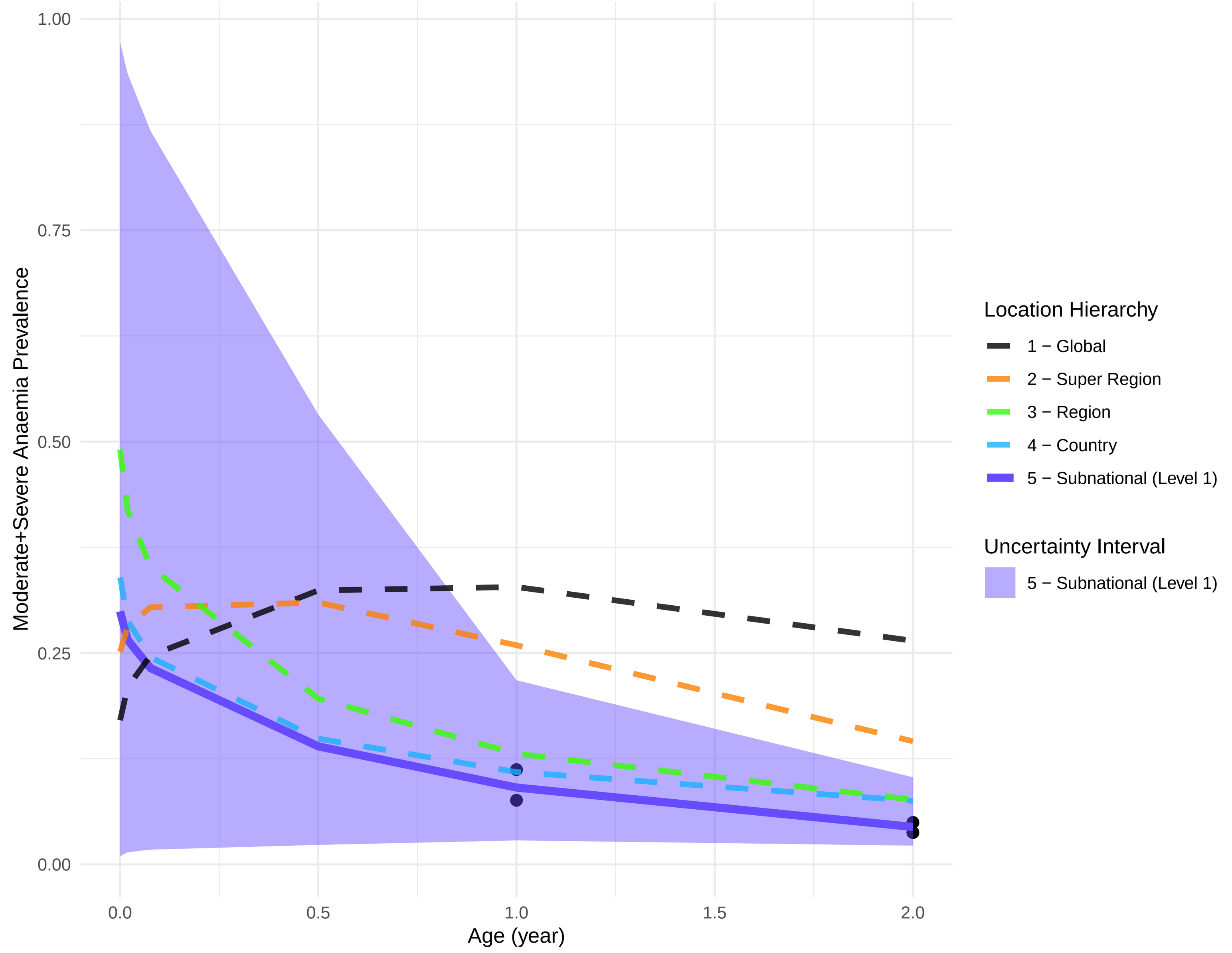

MR−BRT Age/Sex Fits − Males <2 Years Old − Moderate+Severe Anaemia Prevalence − Tlaxcala
WHO−2001 Adjustment Method

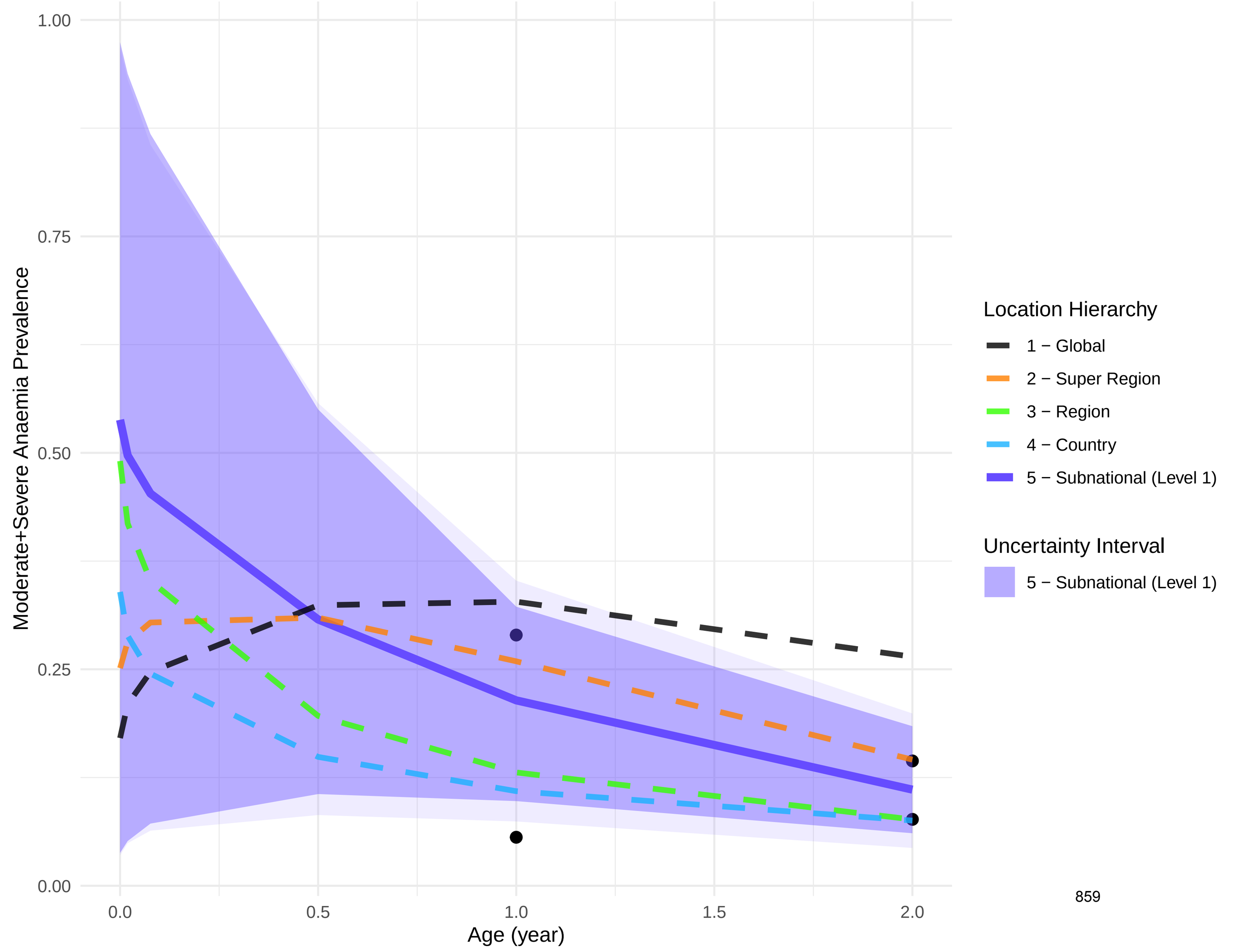

MR−BRT Age/Sex Fits − Males <2 Years Old − Moderate+Severe Anaemia Prevalence − Veracruz de Ignacio de la Llave
WHO−2001 Adjustment Method

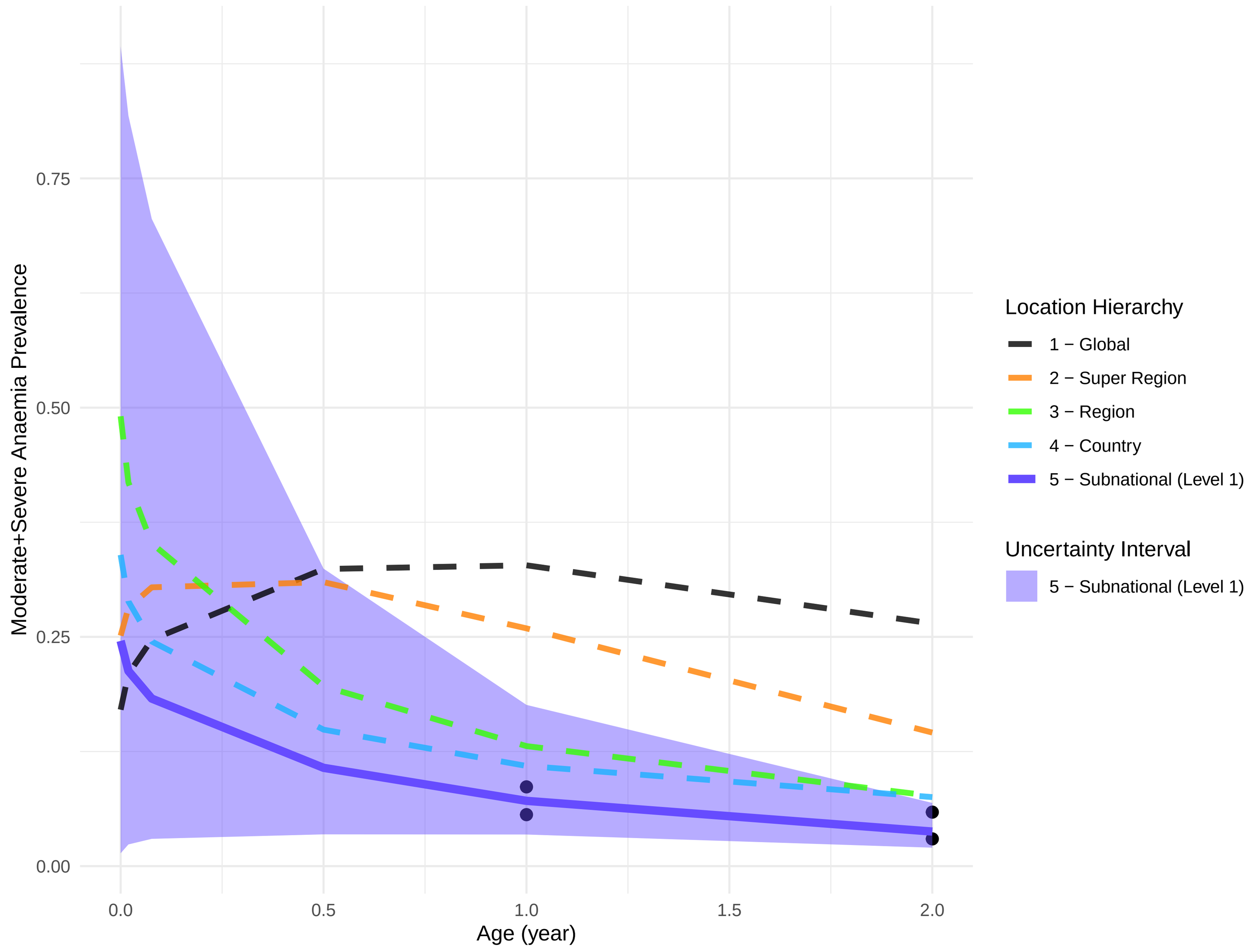

MR−BRT Age/Sex Fits − Males <2 Years Old − Moderate+Severe Anaemia Prevalence − Yucatán
WHO−2001 Adjustment Method

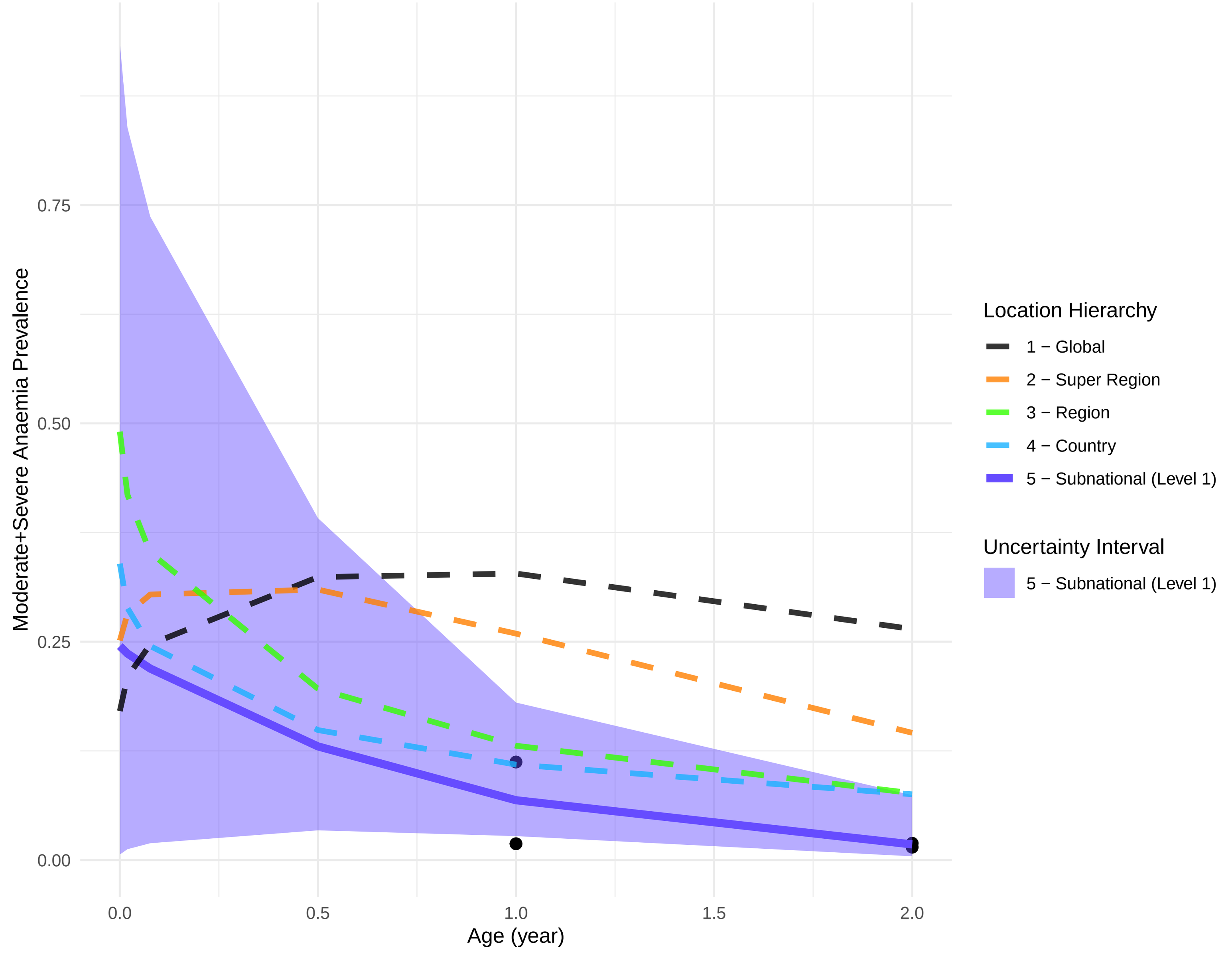

MR−BRT Age/Sex Fits − Males <2 Years Old − Moderate+Severe Anaemia Prevalence − Zacatecas
WHO−2001 Adjustment Method

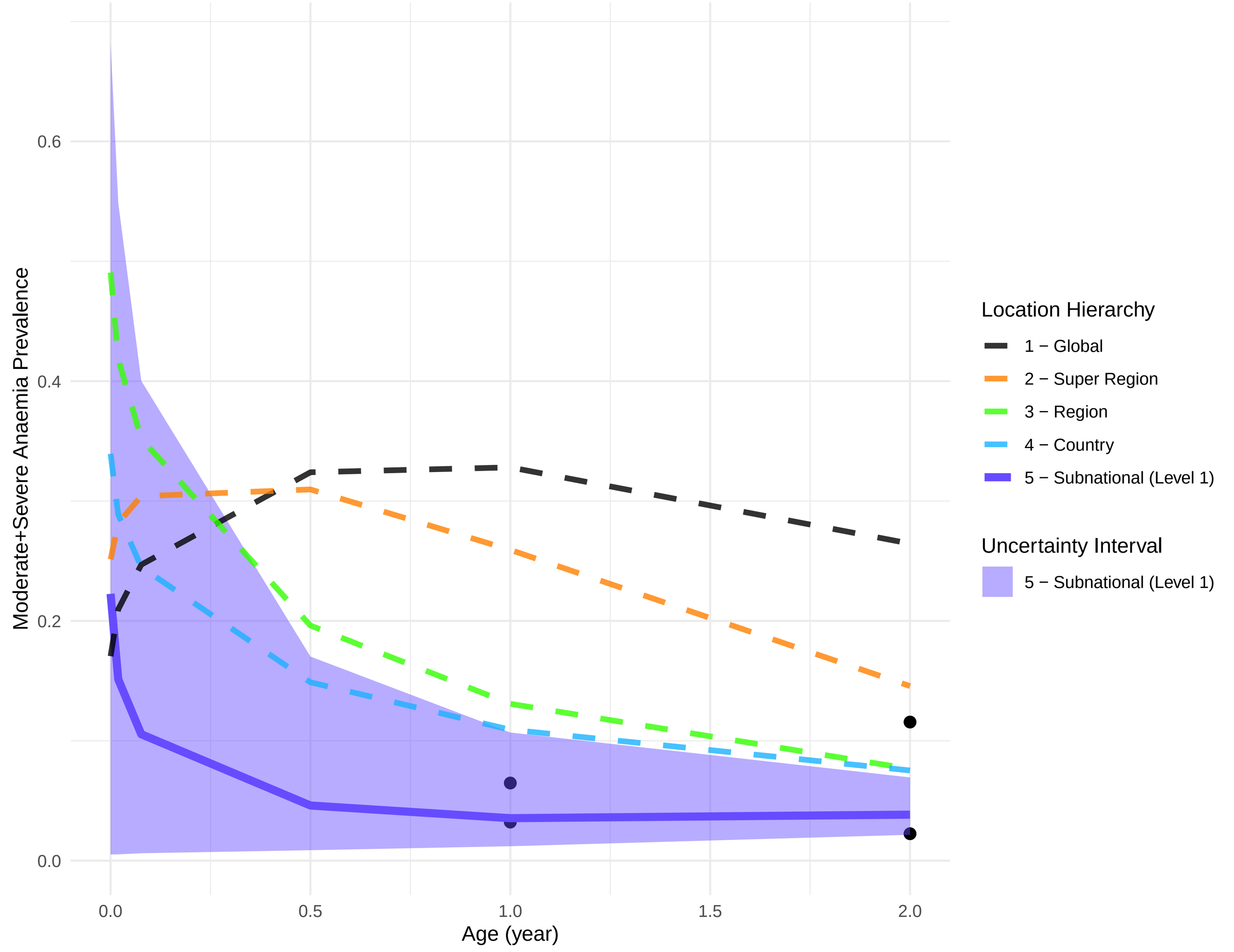

MR−BRT Age/Sex Fits − Males <2 Years Old − Moderate+Severe Anaemia Prevalence − North Africa and Middle East
WHO−2001 Adjustment Method

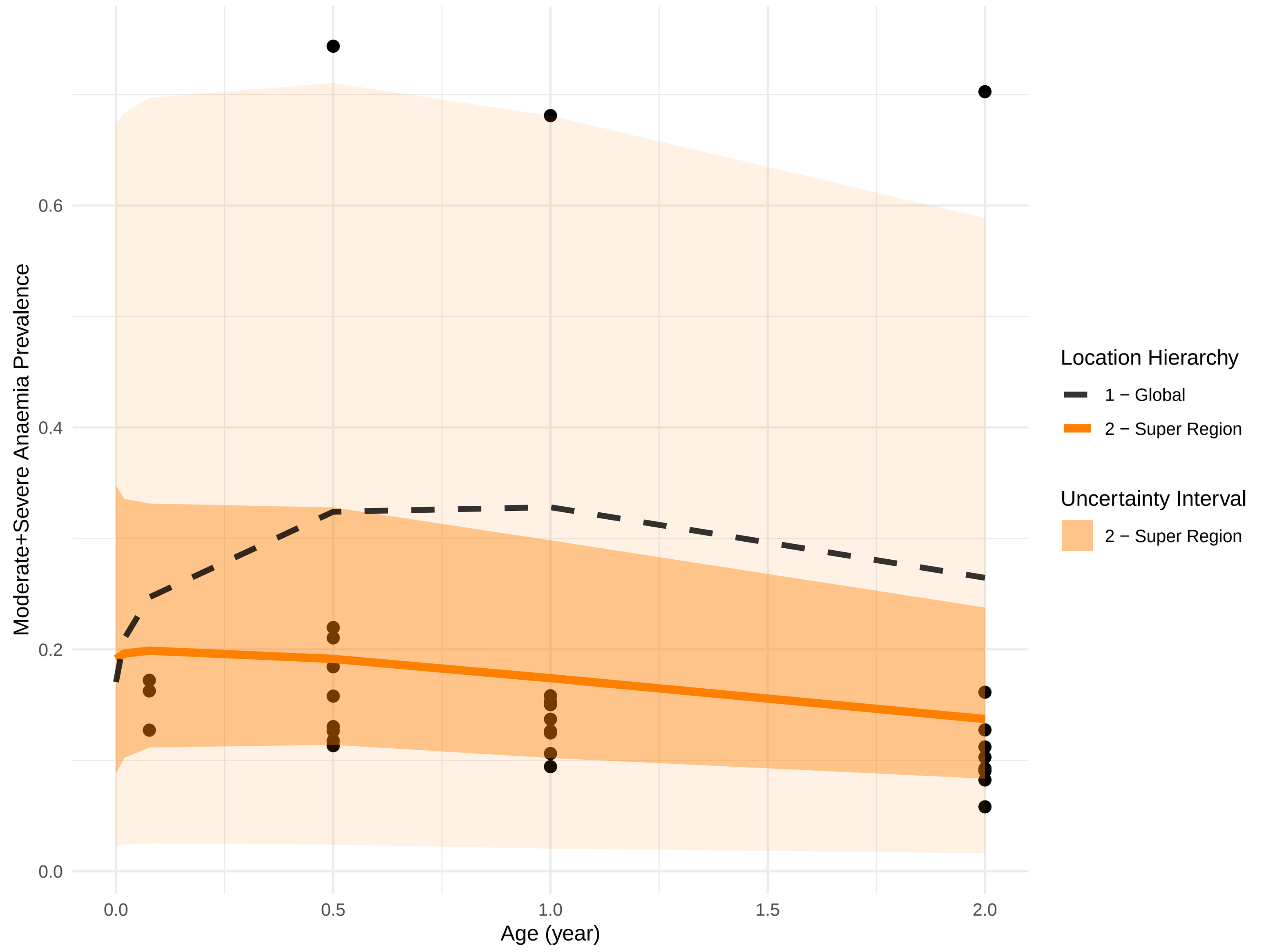

MR−BRT Age/Sex Fits − Males <2 Years Old − Moderate+Severe Anaemia Prevalence − North Africa and Middle East
WHO−2001 Adjustment Method

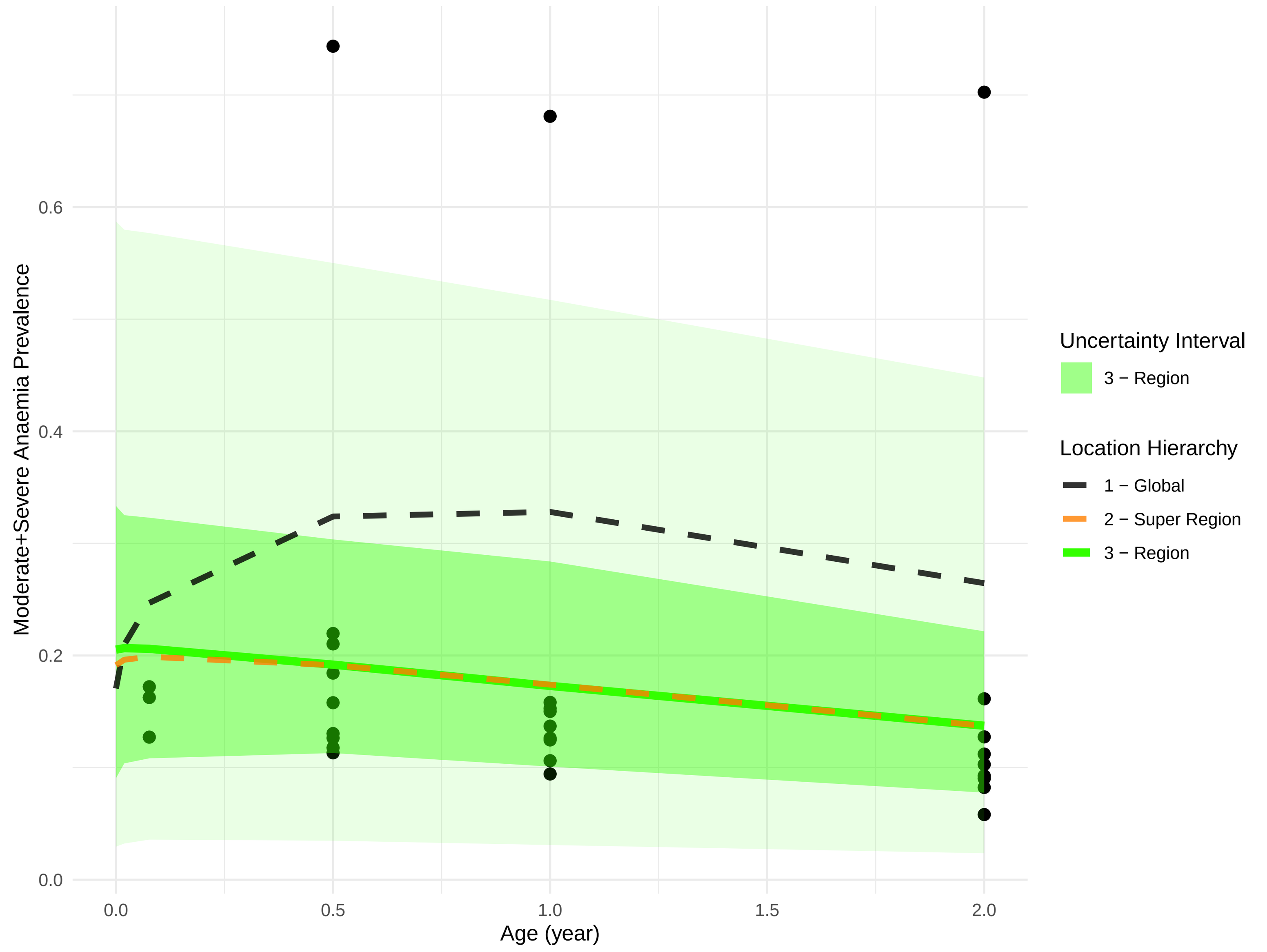

MR−BRT Age/Sex Fits − Males <2 Years Old − Moderate+Severe Anaemia Prevalence − Egypt
WHO−2001 Adjustment Method

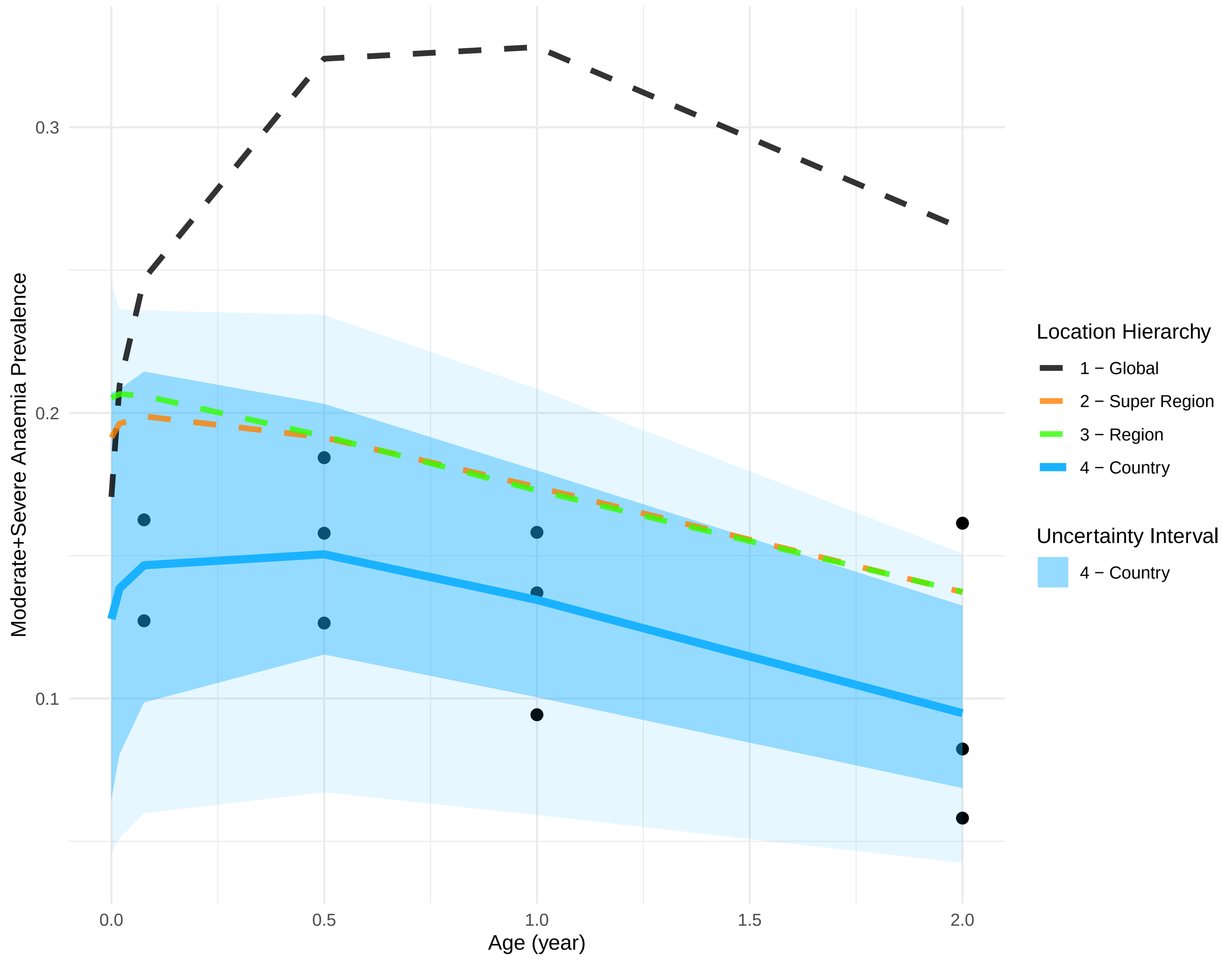

MR−BRT Age/Sex Fits − Males <2 Years Old − Moderate+Severe Anaemia Prevalence − Jordan
WHO−2001 Adjustment Method

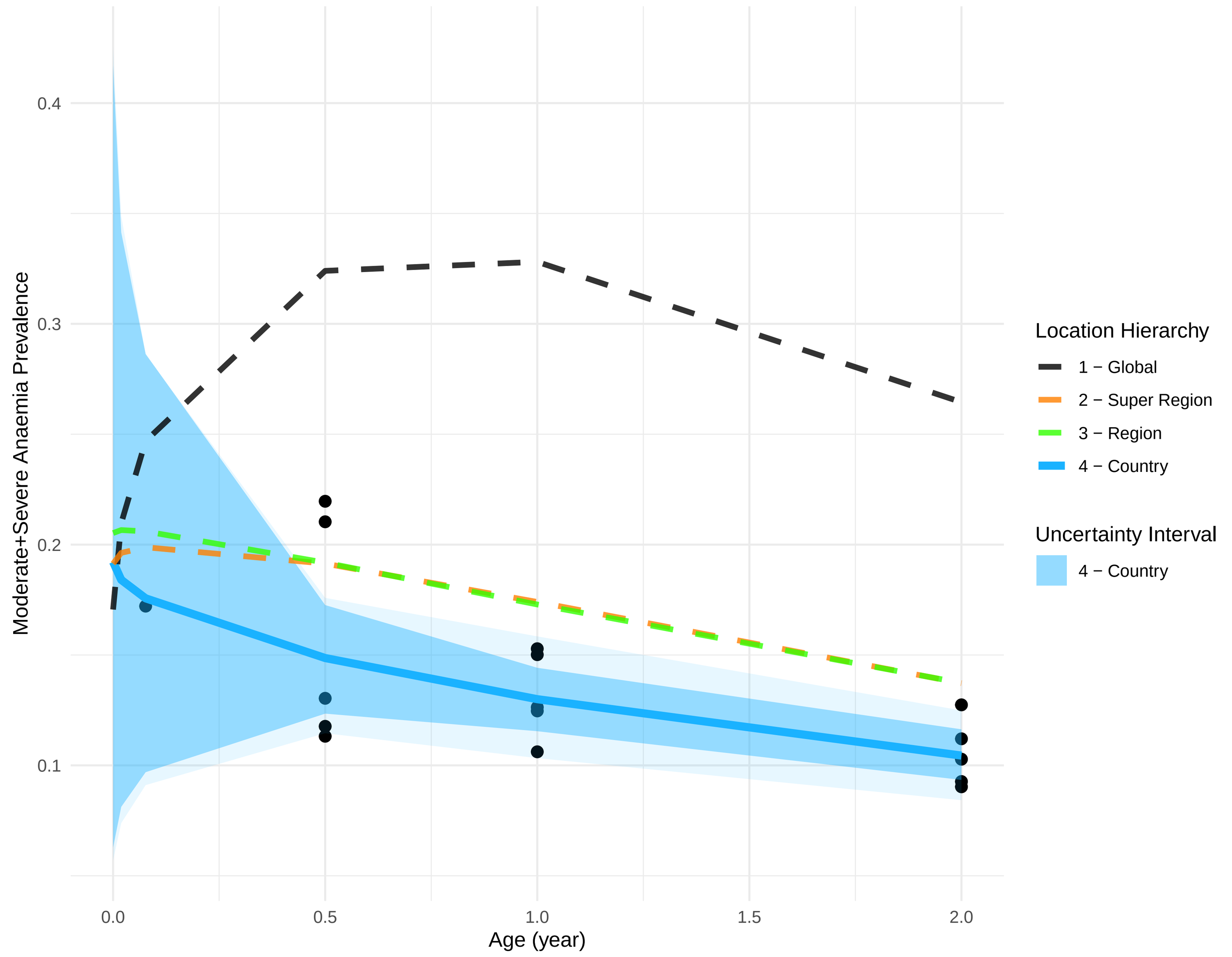

MR−BRT Age/Sex Fits − Males <2 Years Old − Moderate+Severe Anaemia Prevalence − Yemen
WHO−2001 Adjustment Method

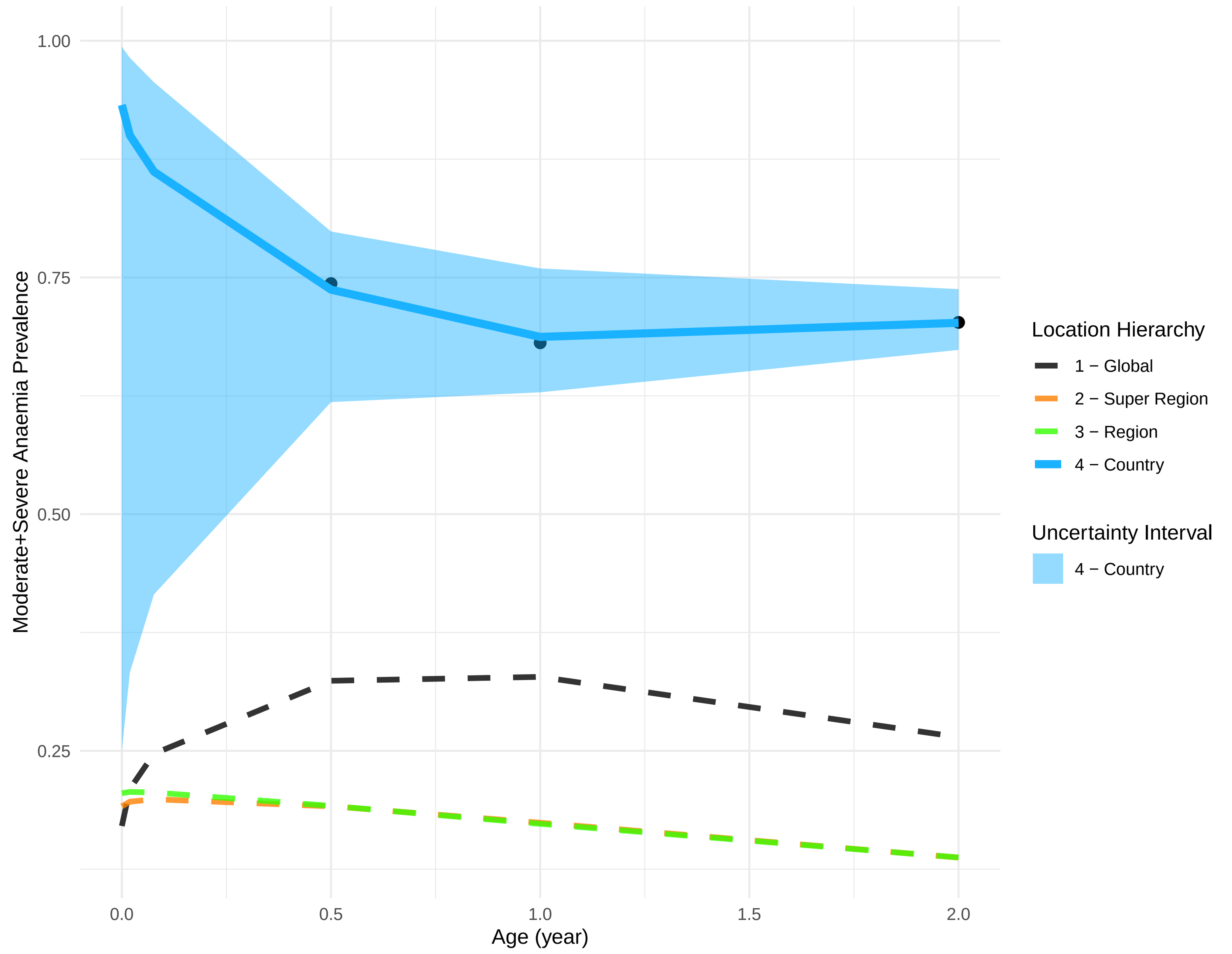

MR−BRT Age/Sex Fits − Males <2 Years Old − Moderate+Severe Anaemia Prevalence − South Asia
WHO−2001 Adjustment Method

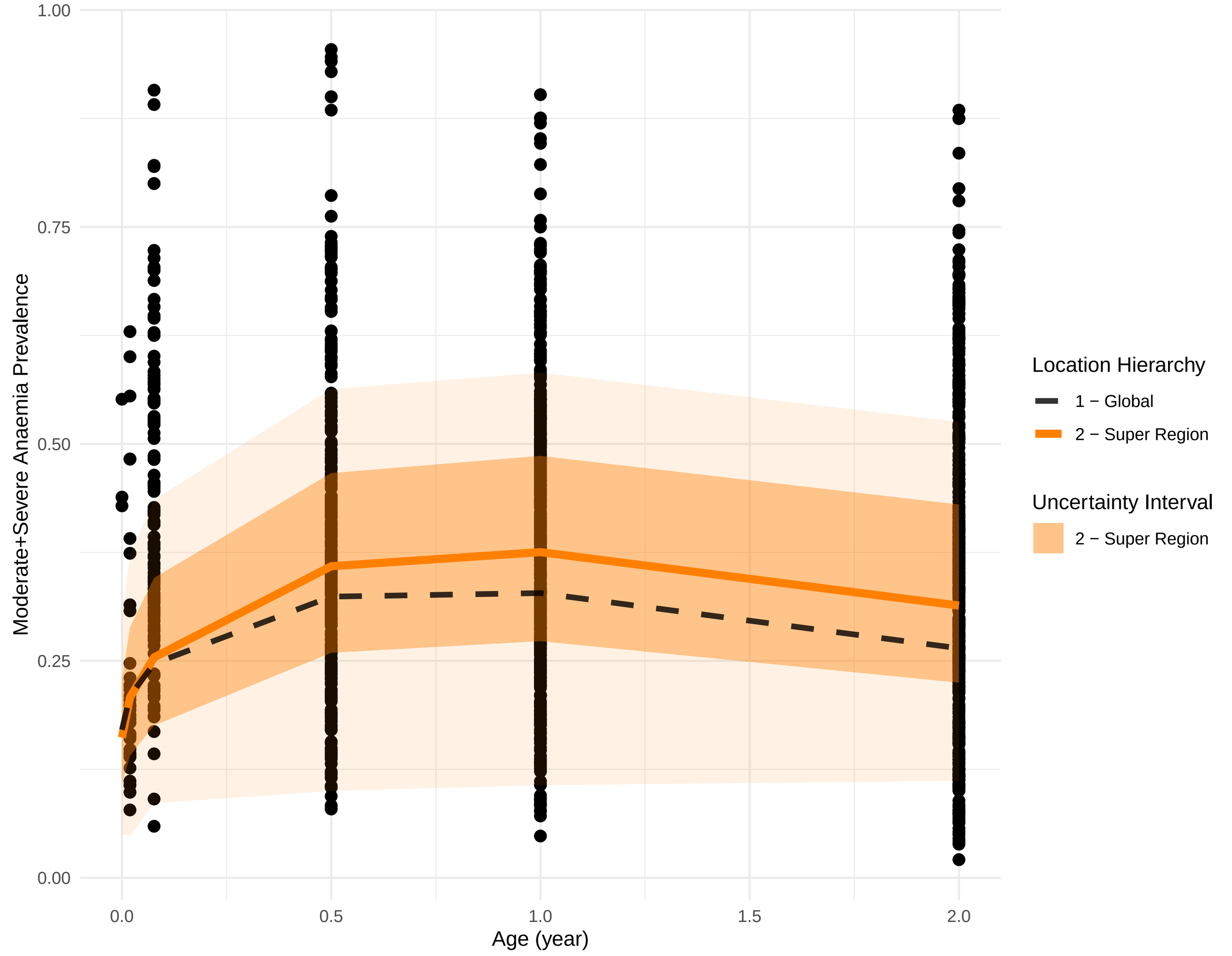

MR−BRT Age/Sex Fits − Males <2 Years Old − Moderate+Severe Anaemia Prevalence − South Asia
WHO−2001 Adjustment Method

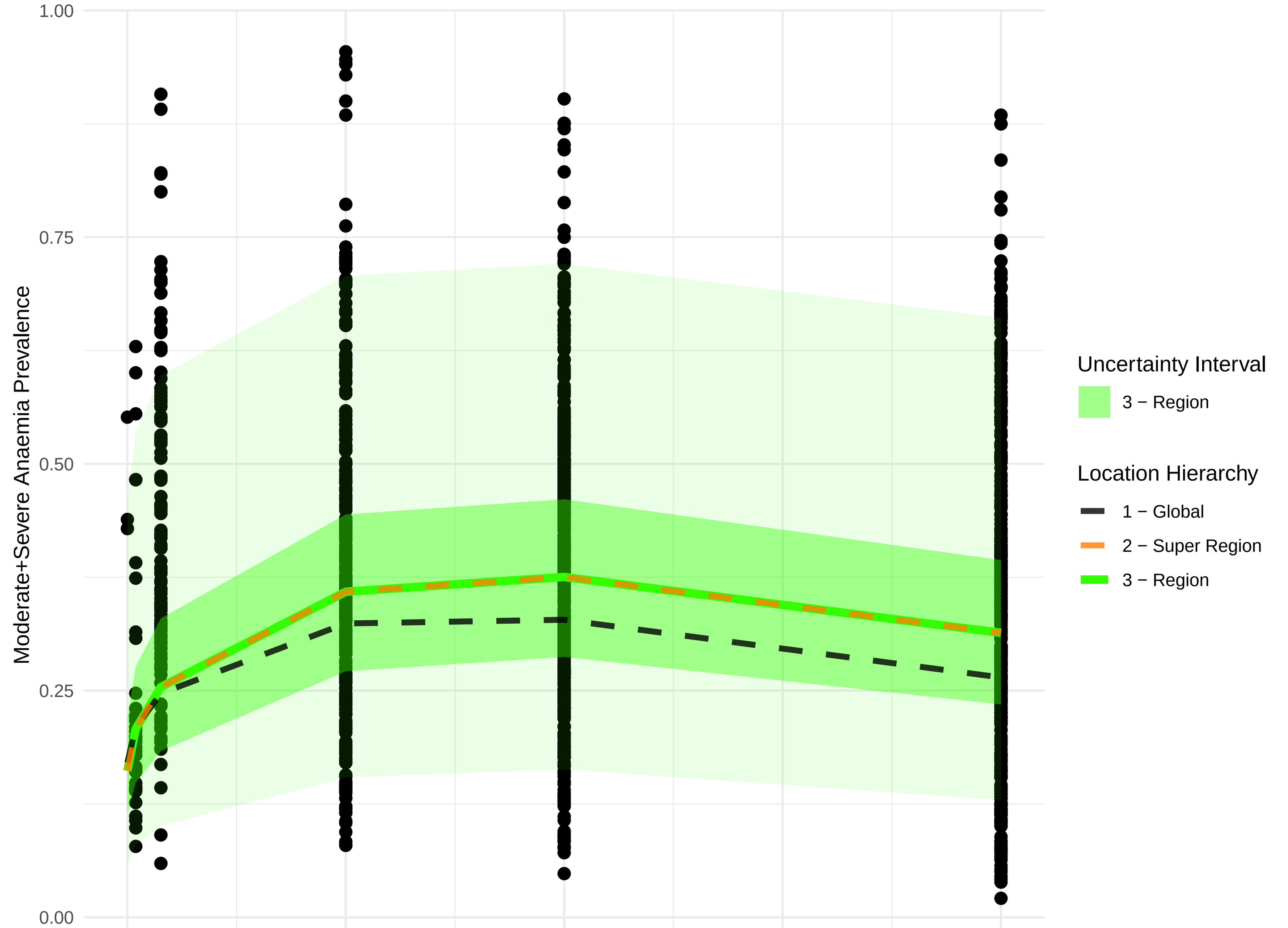

MR−BRT Age/Sex Fits − Males <2 Years Old − Moderate+Severe Anaemia Prevalence − Bangladesh
WHO−2001 Adjustment Method

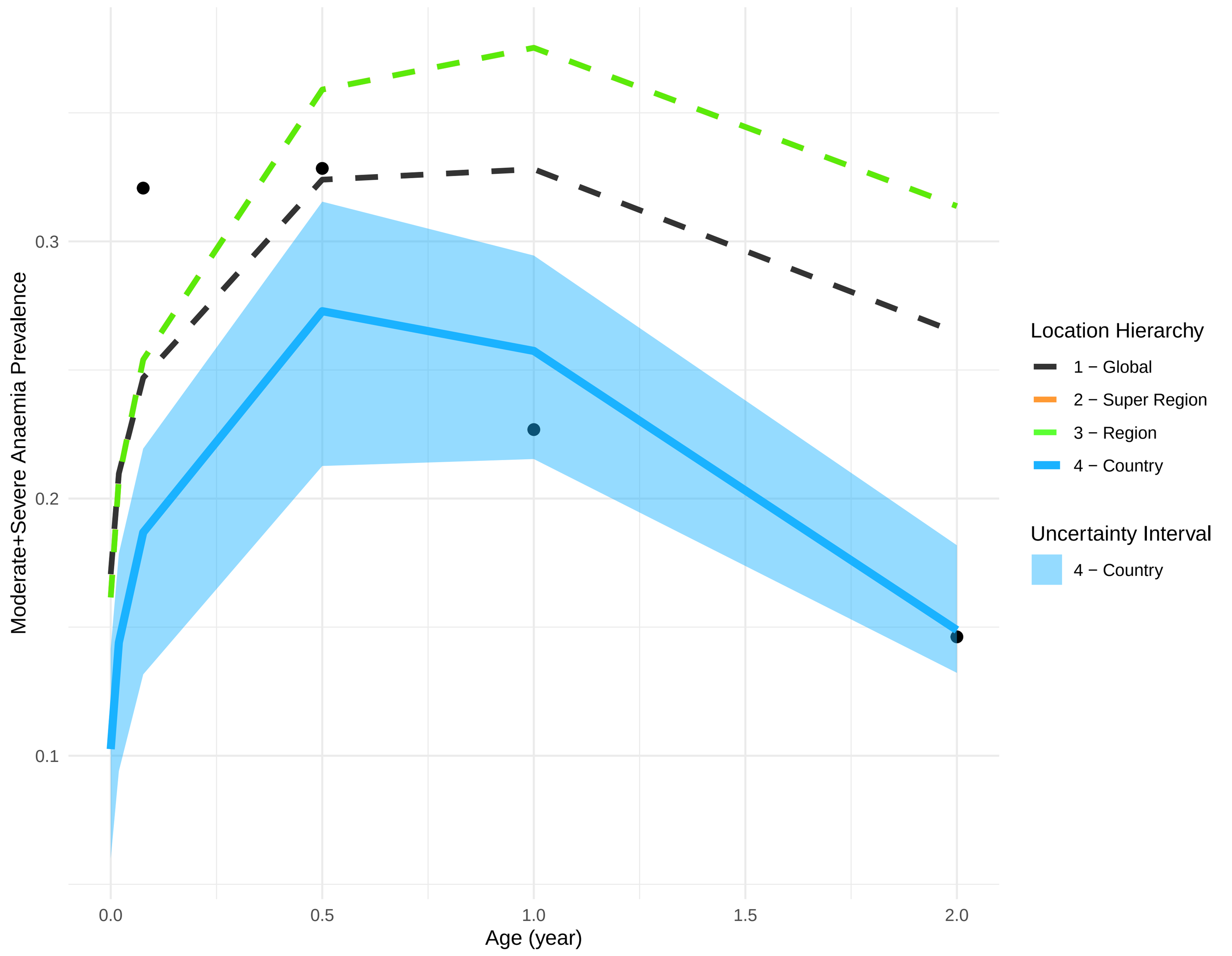

MR−BRT Age/Sex Fits − Males <2 Years Old − Moderate+Severe Anaemia Prevalence − India
WHO−2001 Adjustment Method

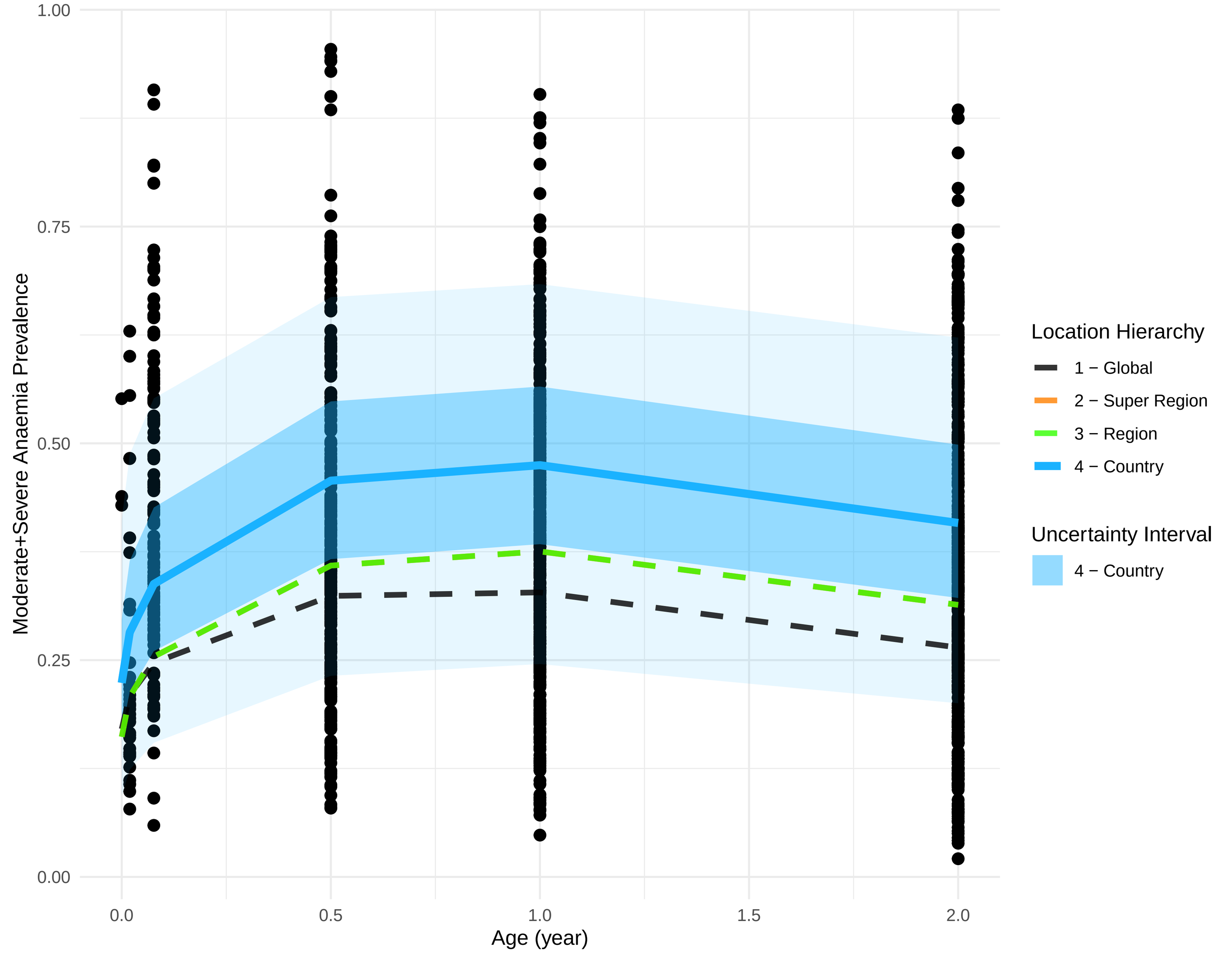

MR−BRT Age/Sex Fits − Males <2 Years Old − Moderate+Severe Anaemia Prevalence − Andhra Pradesh
WHO−2001 Adjustment Method

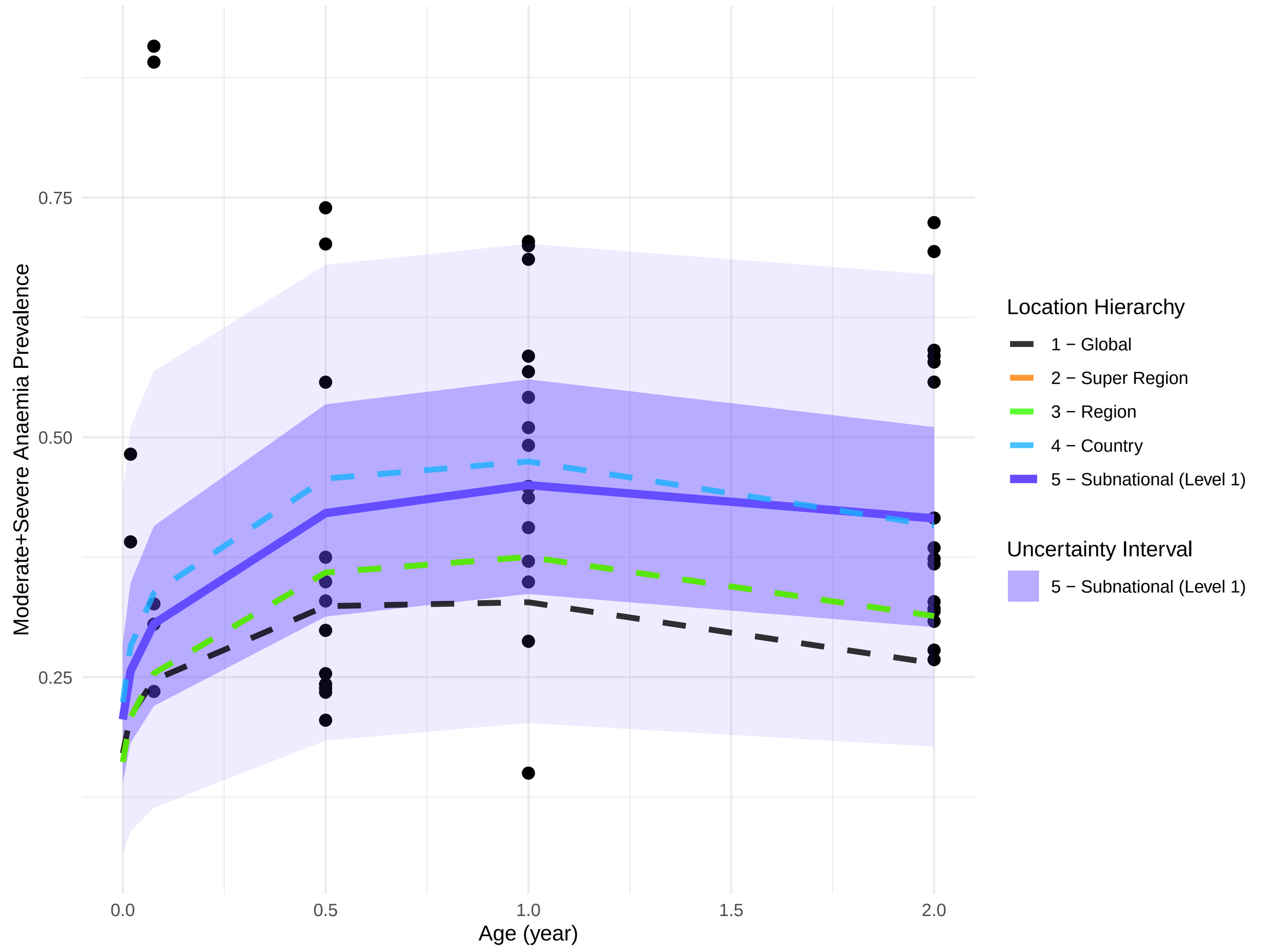

MR−BRT Age/Sex Fits − Males <2 Years Old − Moderate+Severe Anaemia Prevalence − Andhra Pradesh, Rural
WHO−2001 Adjustment Method

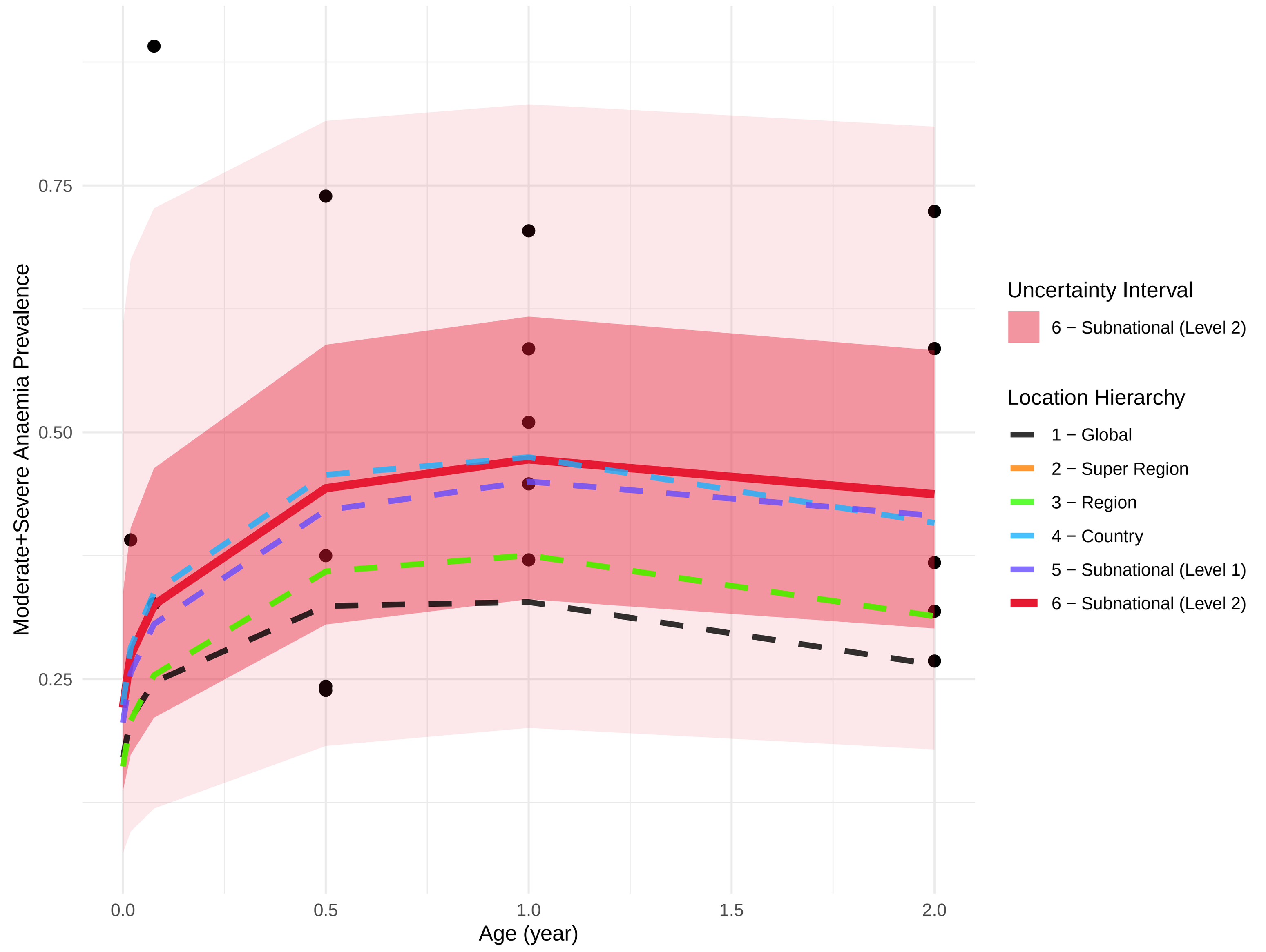

MR−BRT Age/Sex Fits − Males <2 Years Old − Moderate+Severe Anaemia Prevalence − Andhra Pradesh, Urban
WHO−2001 Adjustment Method

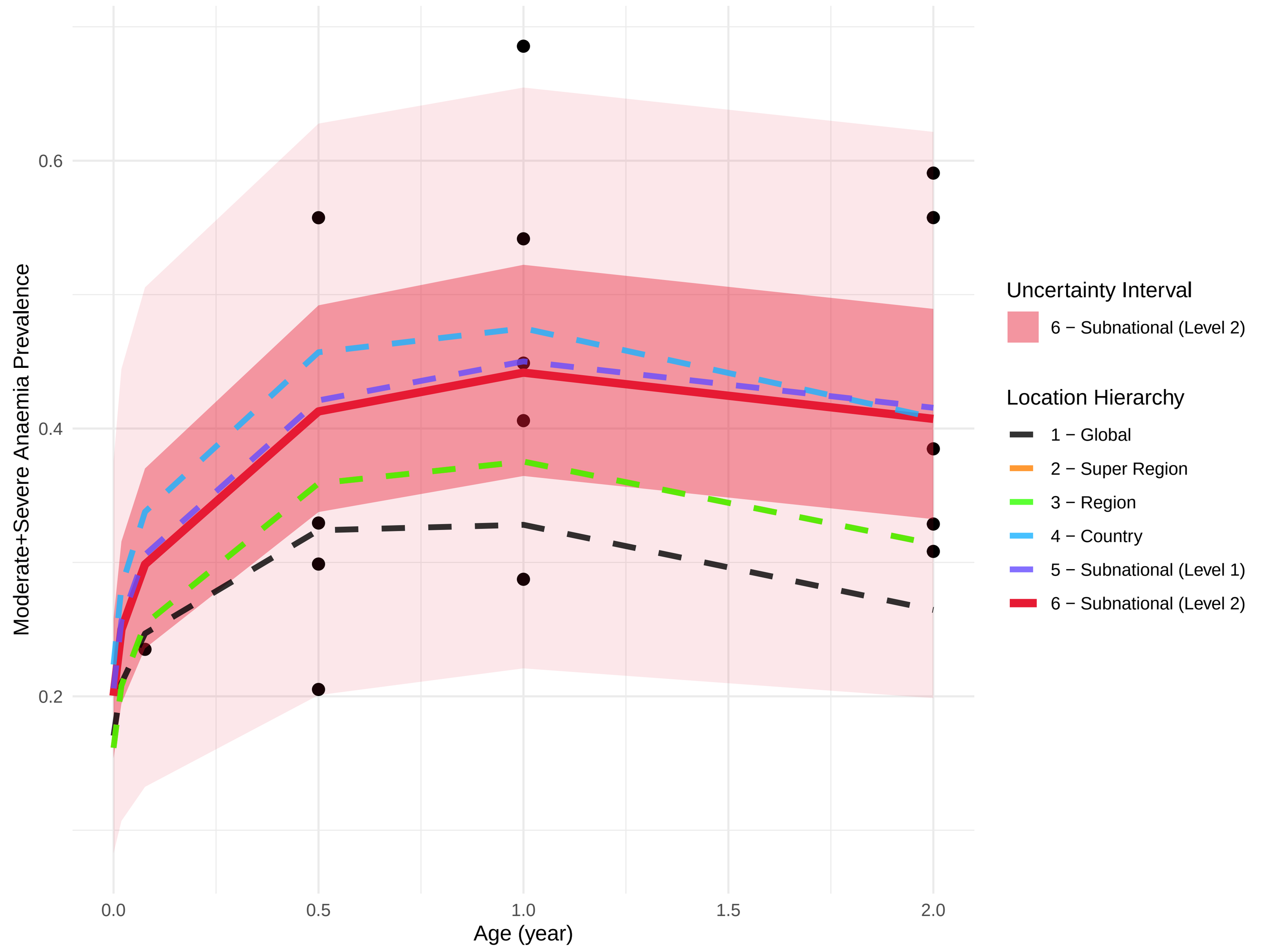

MR−BRT Age/Sex Fits − Males <2 Years Old − Moderate+Severe Anaemia Prevalence − Arunachal Pradesh
WHO−2001 Adjustment Method

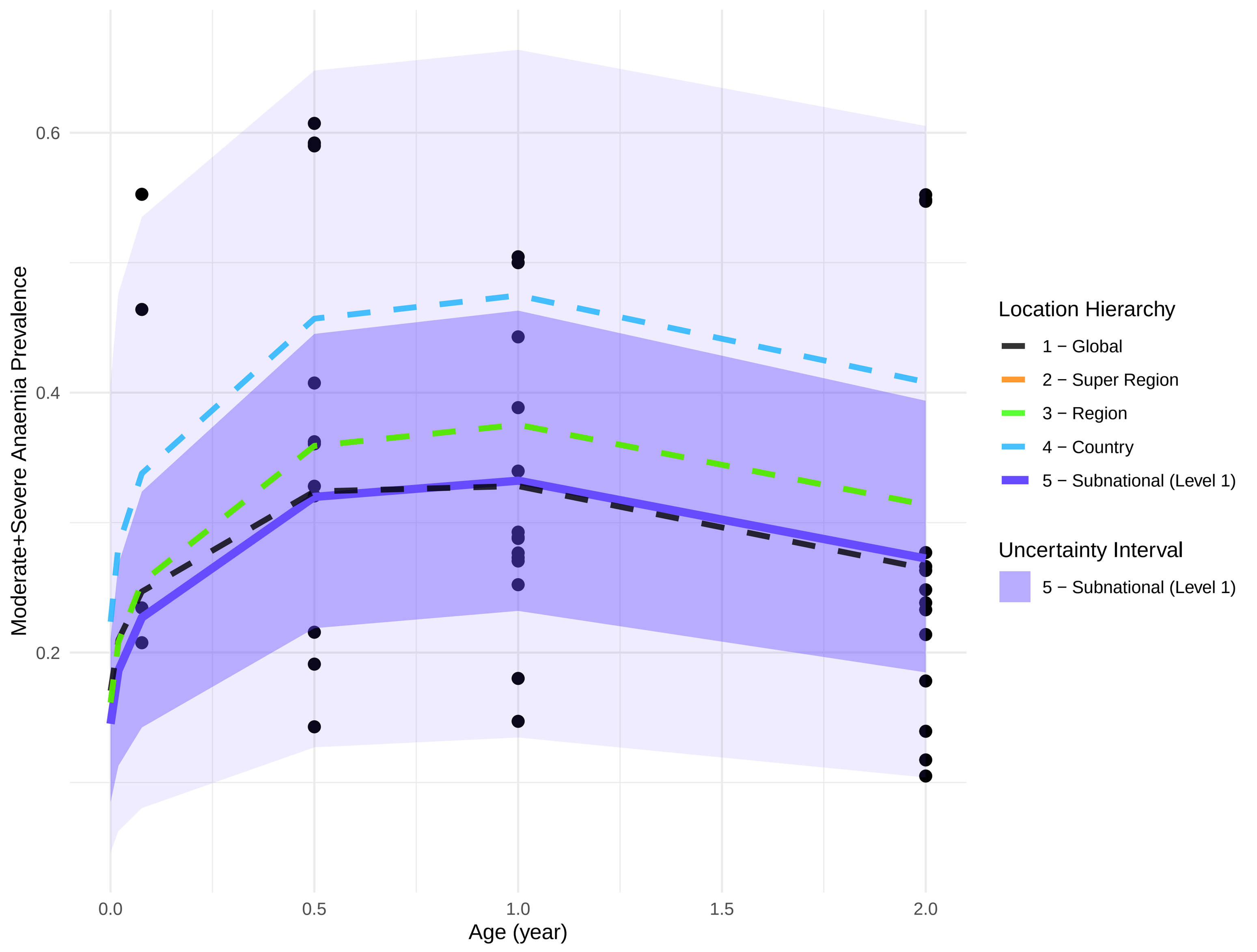

MR−BRT Age/Sex Fits − Males <2 Years Old − Moderate+Severe Anaemia Prevalence − Arunachal Pradesh, Rural
WHO−2001 Adjustment Method

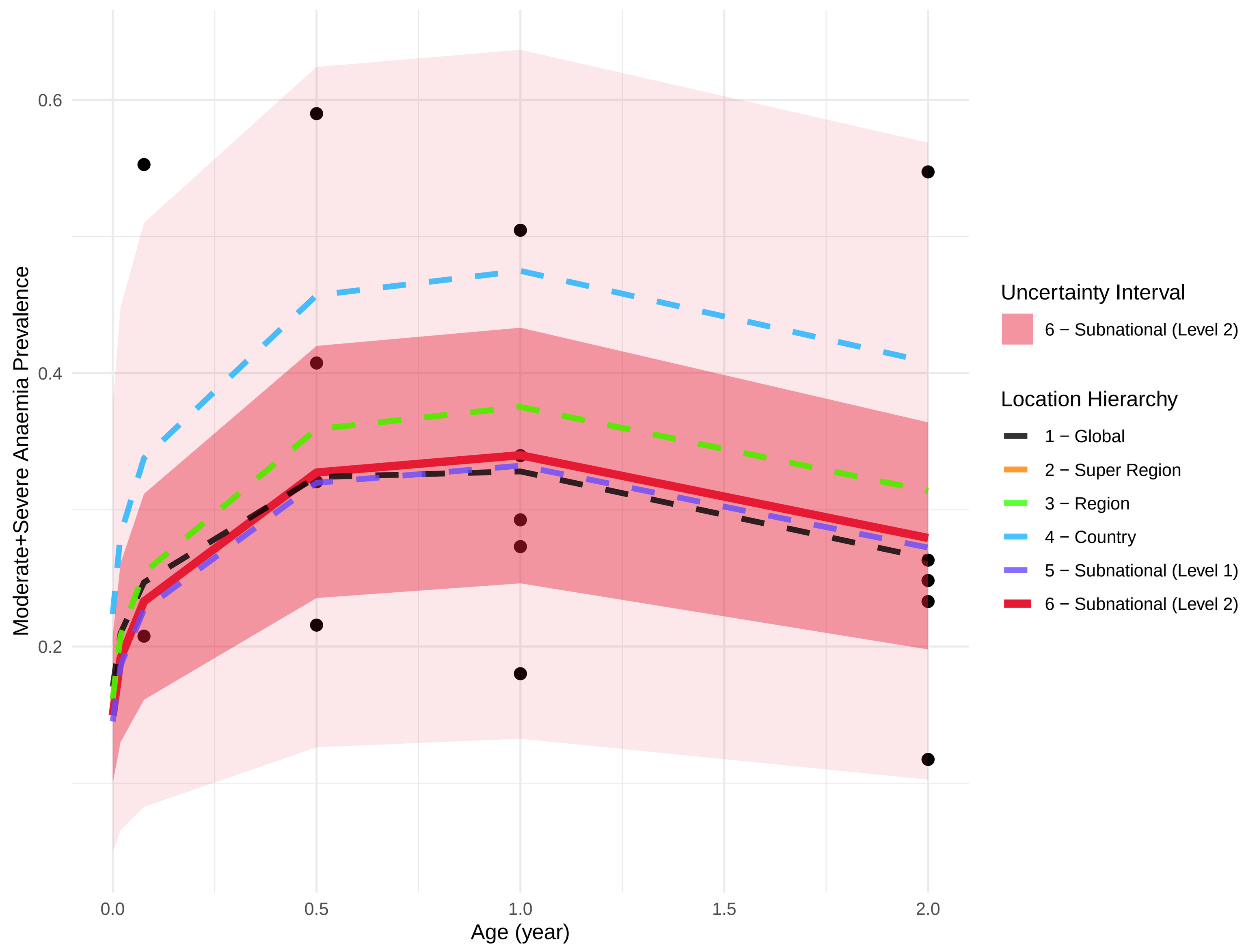

MR−BRT Age/Sex Fits − Males <2 Years Old − Moderate+Severe Anaemia Prevalence − Arunachal Pradesh, Urban
WHO−2001 Adjustment Method

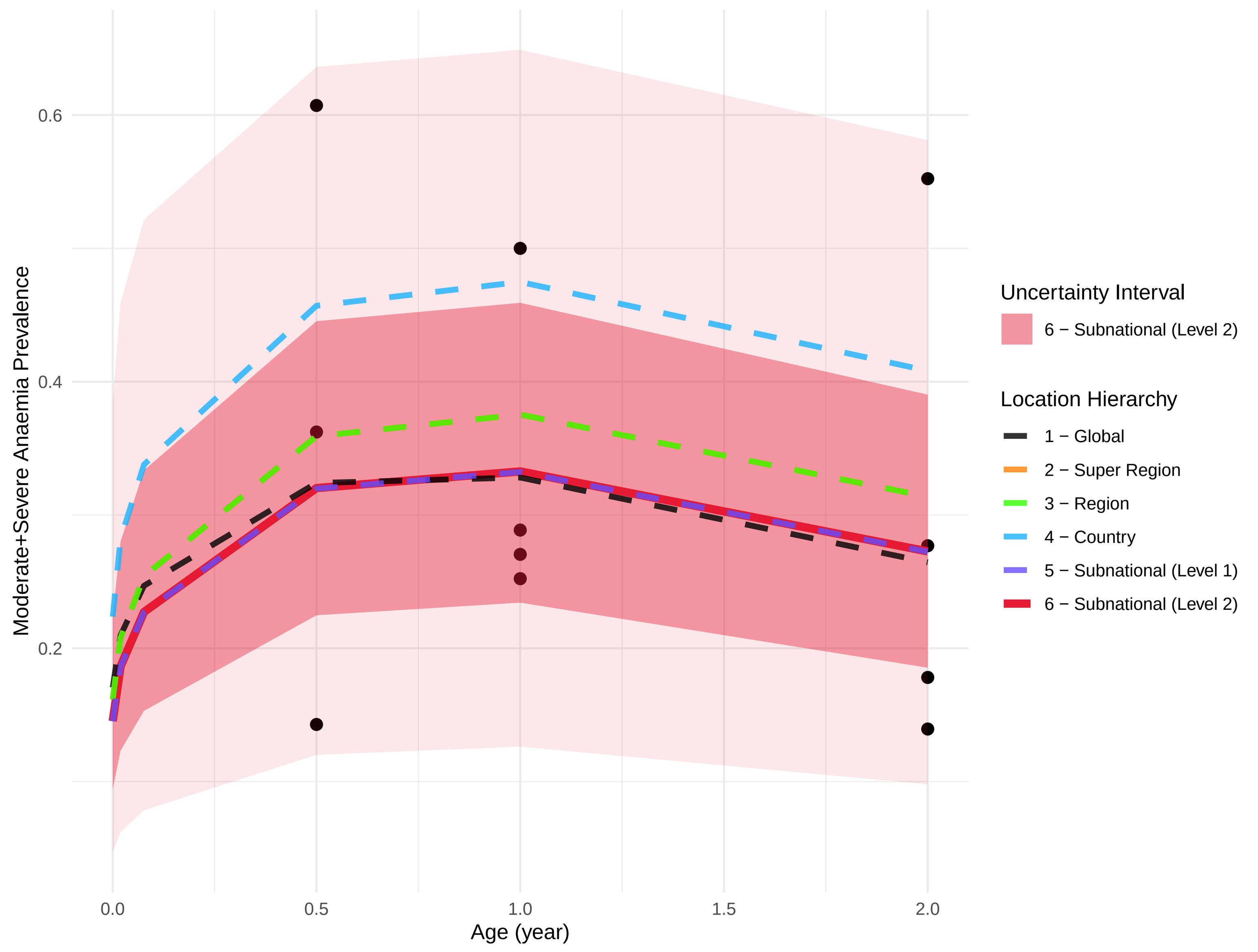

MR−BRT Age/Sex Fits − Males <2 Years Old − Moderate+Severe Anaemia Prevalence − Assam
WHO−2001 Adjustment Method

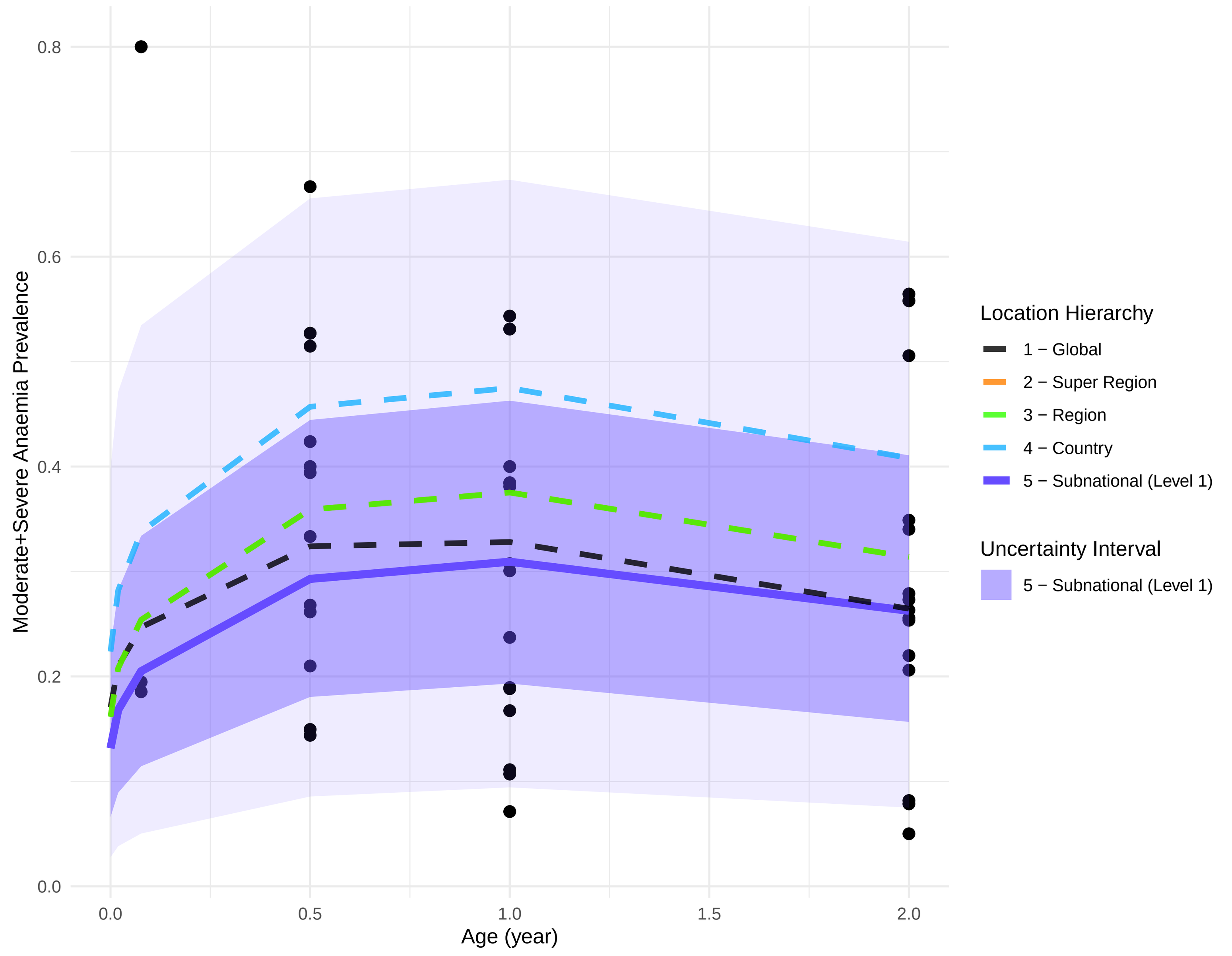

MR−BRT Age/Sex Fits − Males <2 Years Old − Moderate+Severe Anaemia Prevalence − Assam, Rural
WHO−2001 Adjustment Method

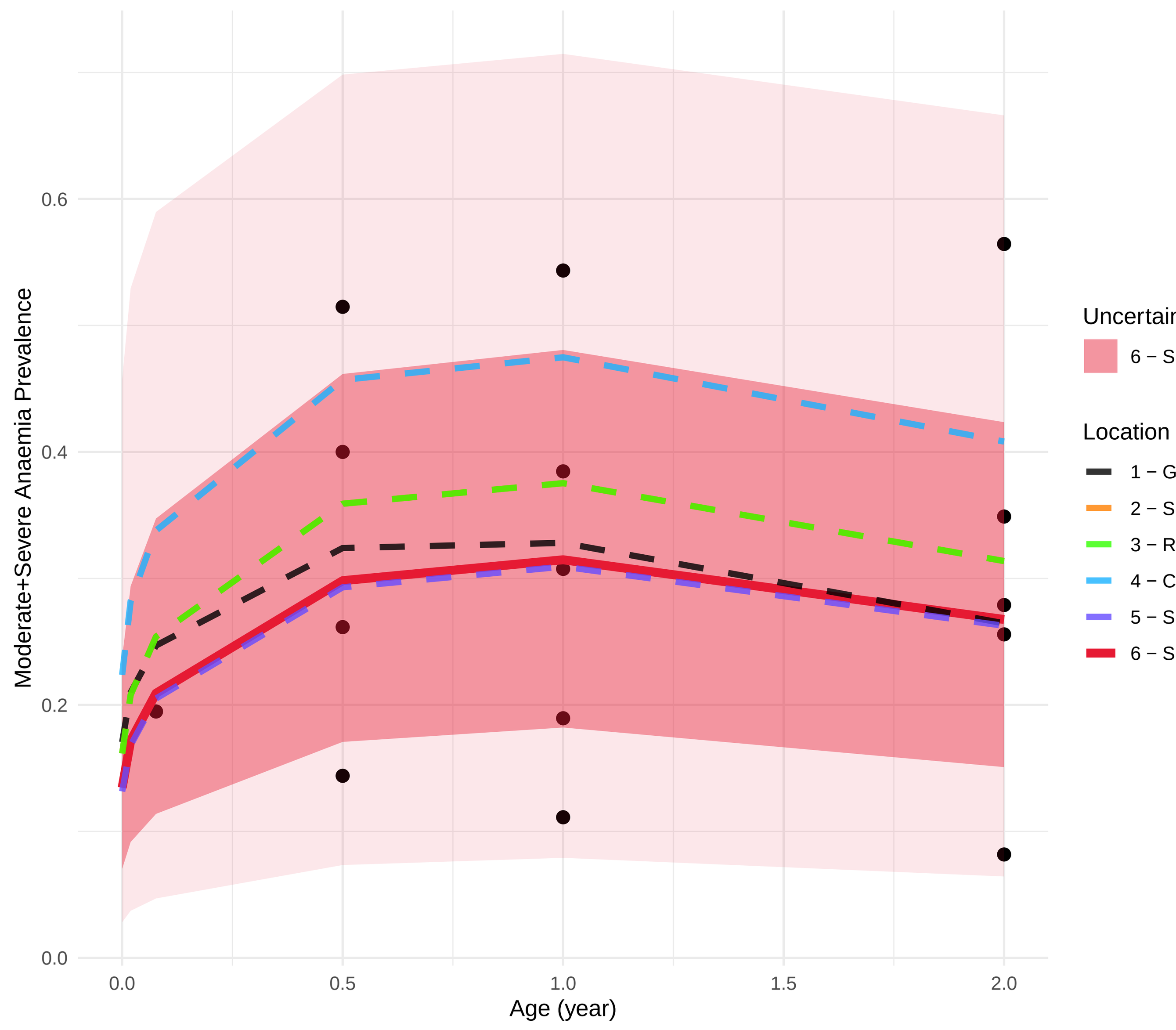

MR−BRT Age/Sex Fits − Males <2 Years Old − Moderate+Severe Anaemia Prevalence − Assam, Urban
WHO−2001 Adjustment Method

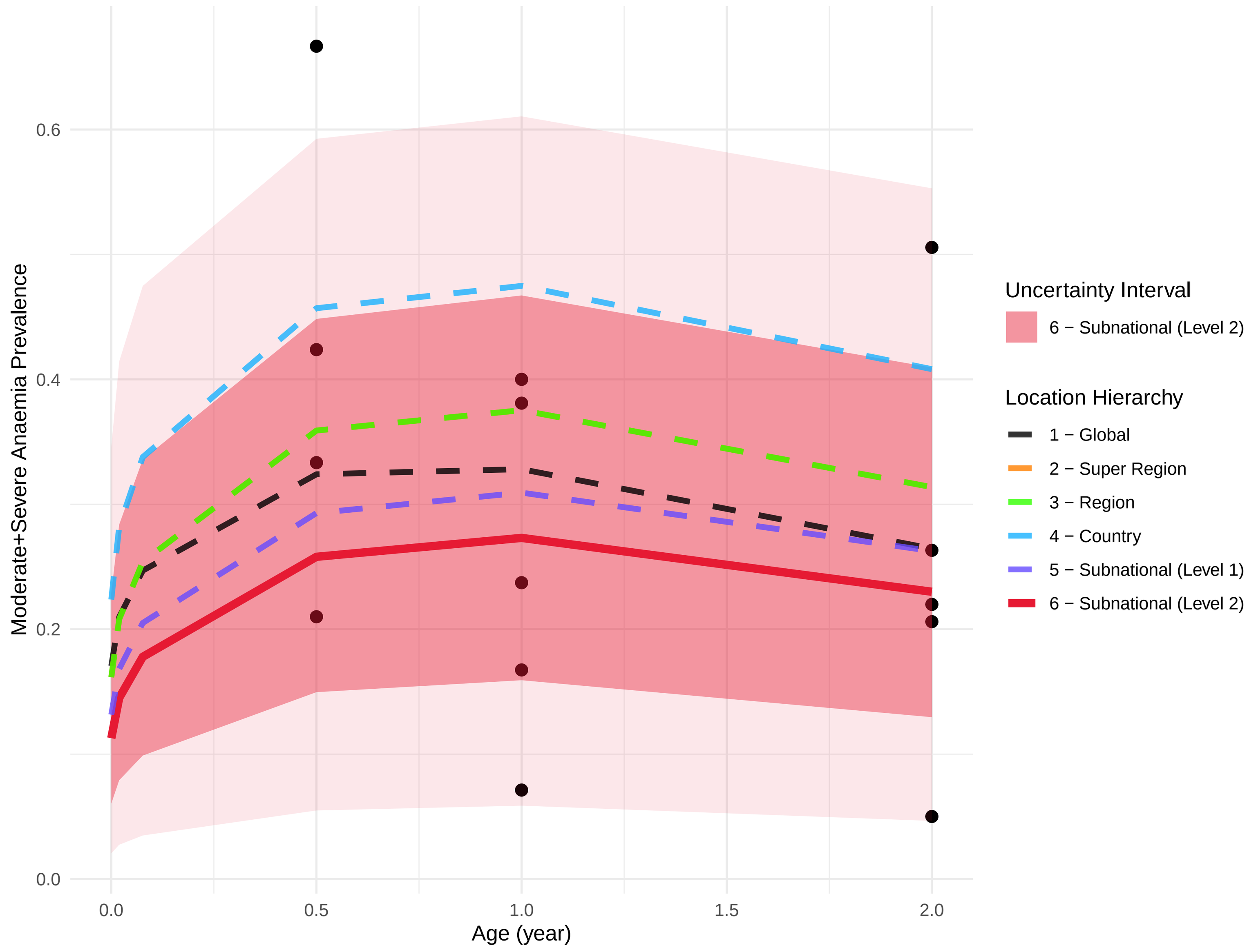

MR−BRT Age/Sex Fits − Males <2 Years Old − Moderate+Severe Anaemia Prevalence − Bihar
WHO−2001 Adjustment Method

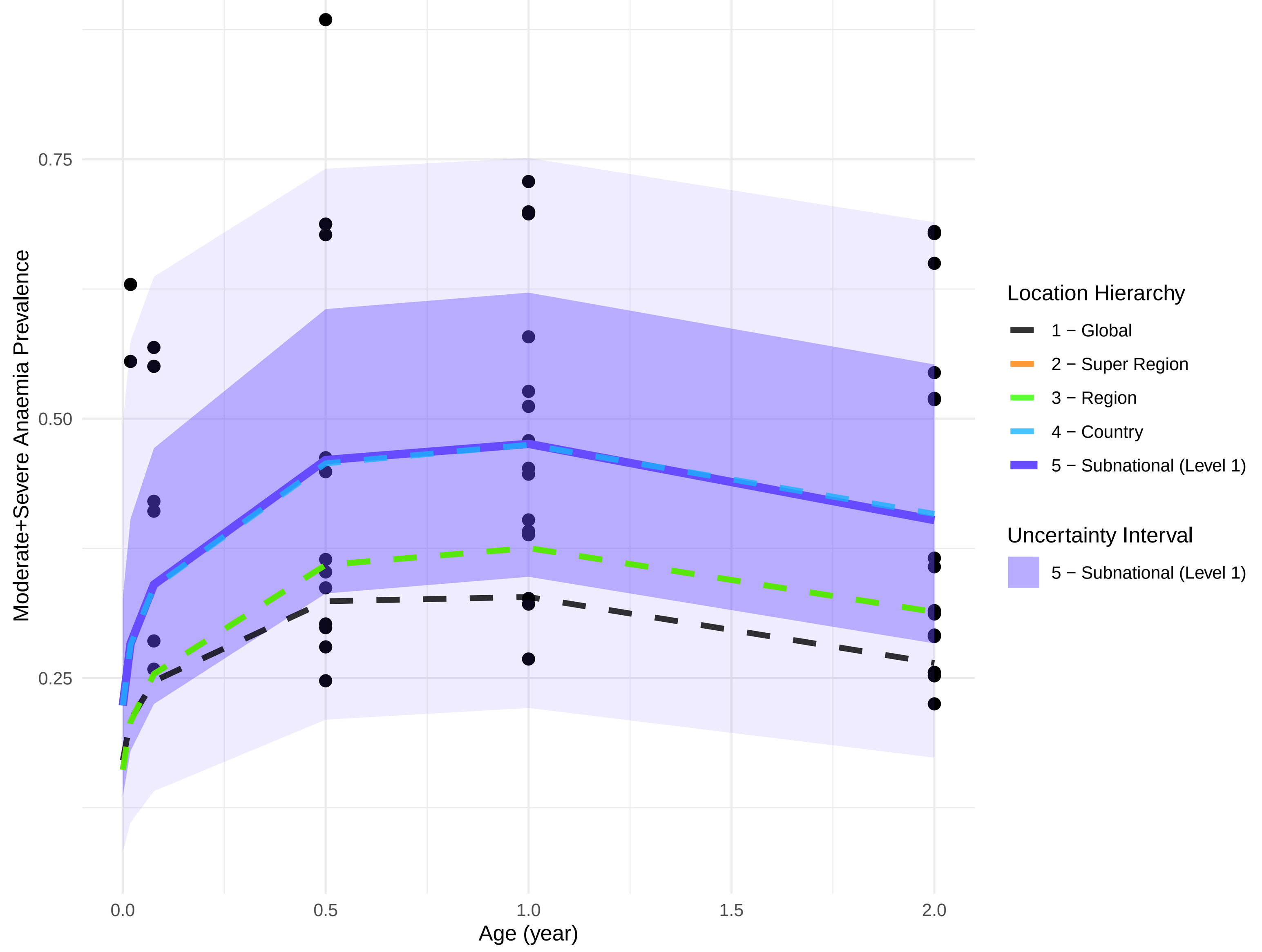

MR−BRT Age/Sex Fits − Males <2 Years Old − Moderate+Severe Anaemia Prevalence − Bihar, Rural
WHO−2001 Adjustment Method

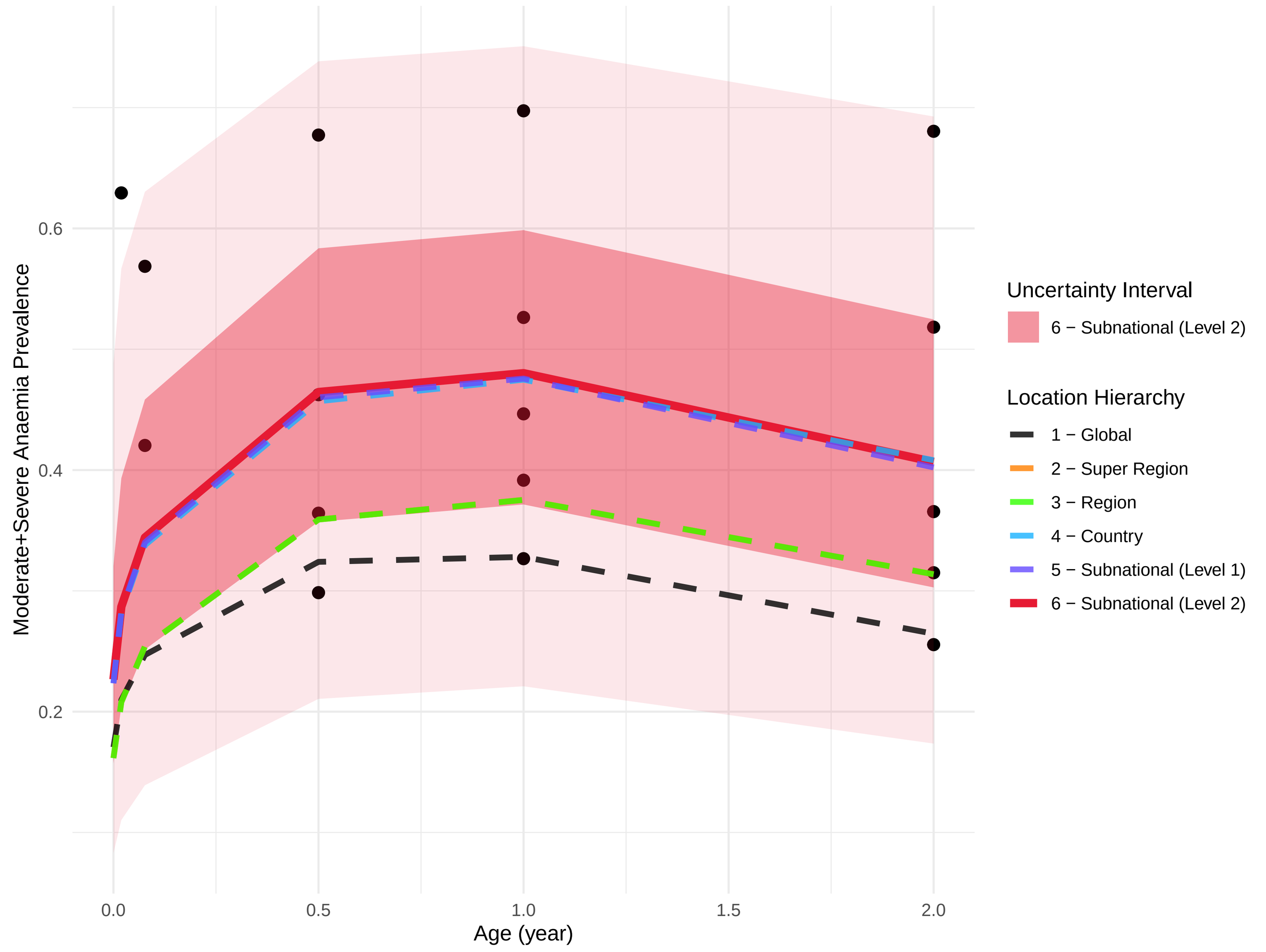

MR−BRT Age/Sex Fits − Males <2 Years Old − Moderate+Severe Anaemia Prevalence − Bihar, Urban
WHO−2001 Adjustment Method

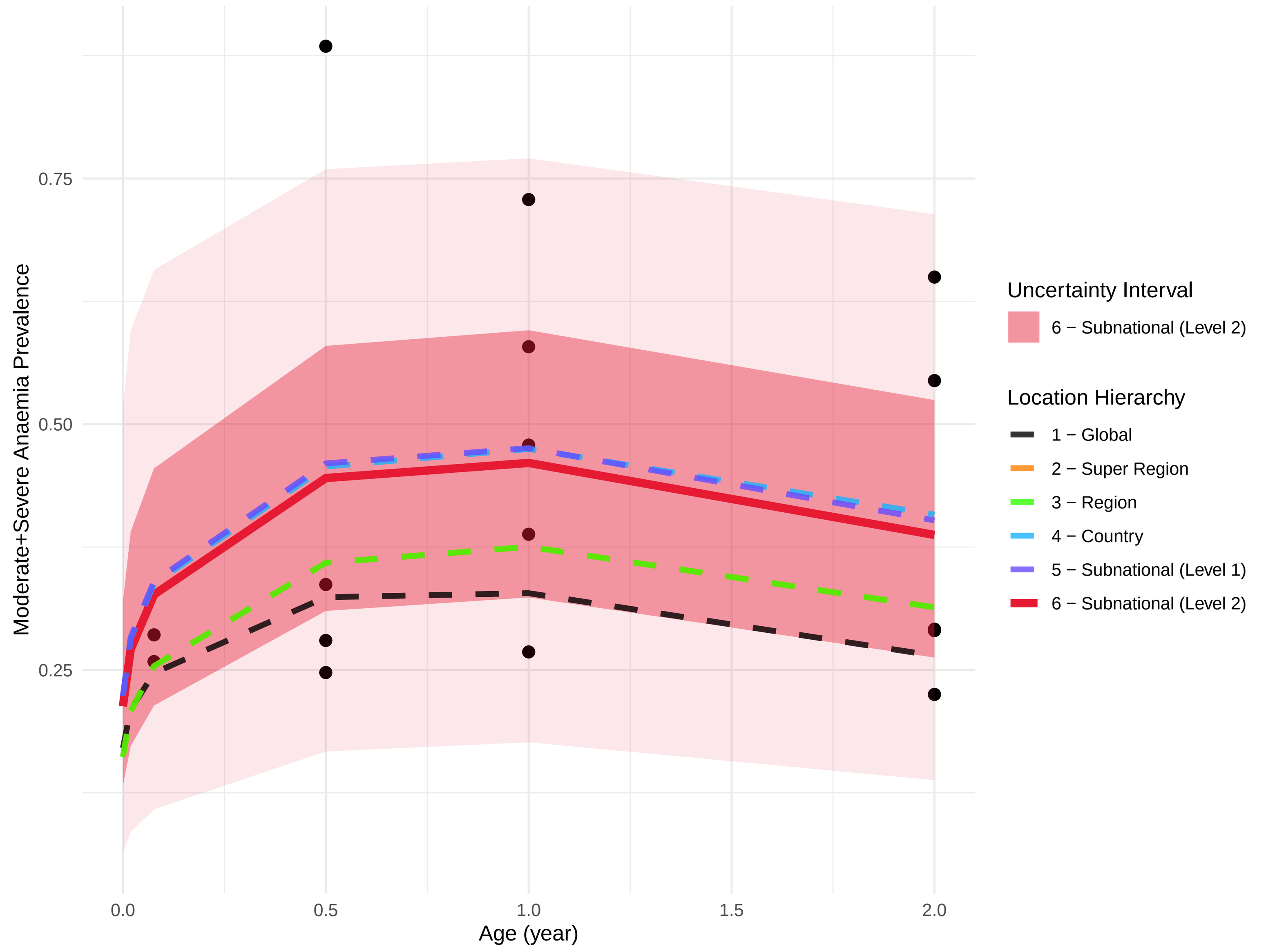

MR−BRT Age/Sex Fits − Males <2 Years Old − Moderate+Severe Anaemia Prevalence − Chhattisgarh
WHO−2001 Adjustment Method

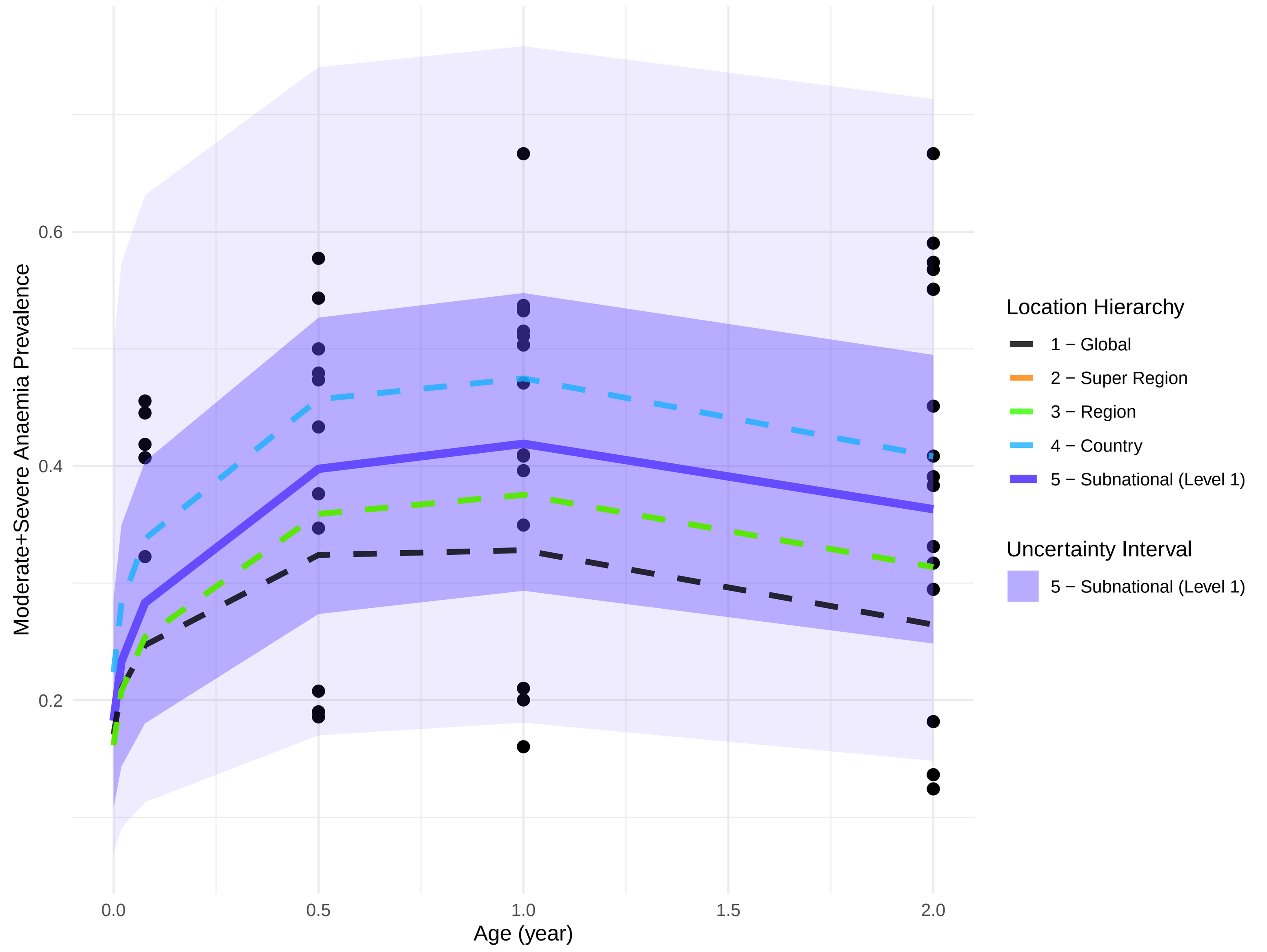

MR−BRT Age/Sex Fits − Males <2 Years Old − Moderate+Severe Anaemia Prevalence − Chhattisgarh, Rural
WHO−2001 Adjustment Method

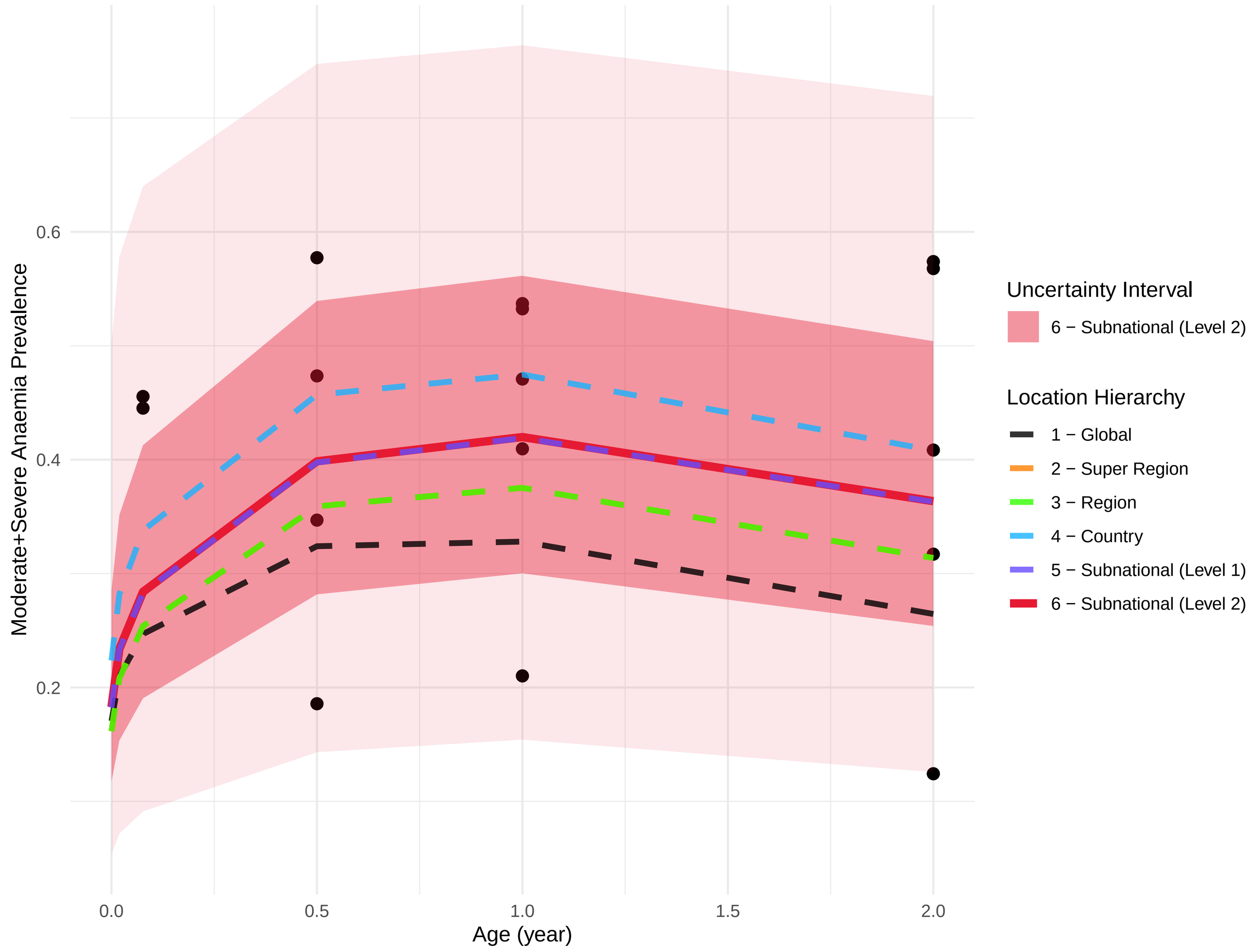

MR−BRT Age/Sex Fits − Males <2 Years Old − Moderate+Severe Anaemia Prevalence − Chhattisgarh, Urban
WHO−2001 Adjustment Method

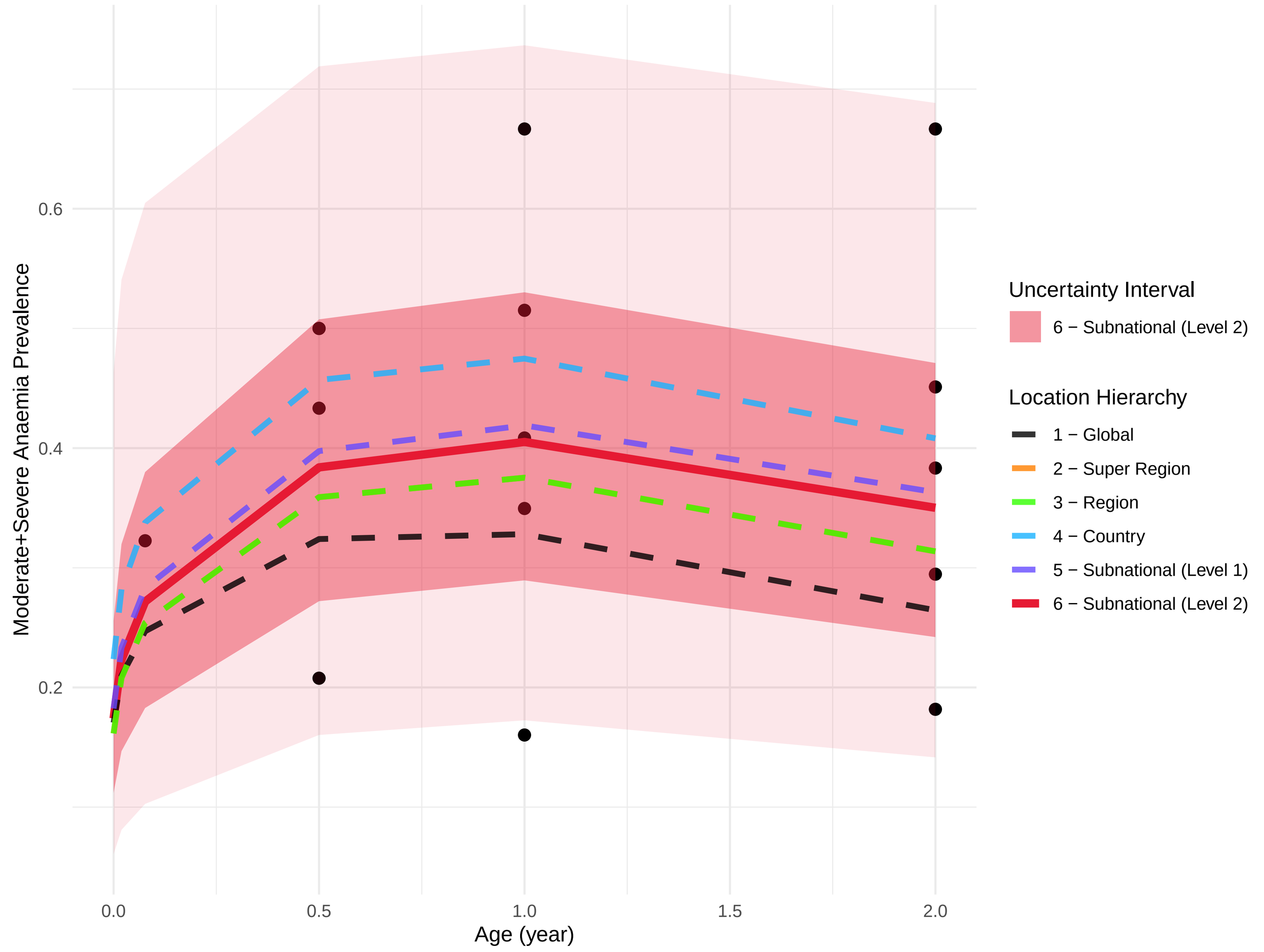

MR−BRT Age/Sex Fits − Males <2 Years Old − Moderate+Severe Anaemia Prevalence − Delhi
WHO−2001 Adjustment Method

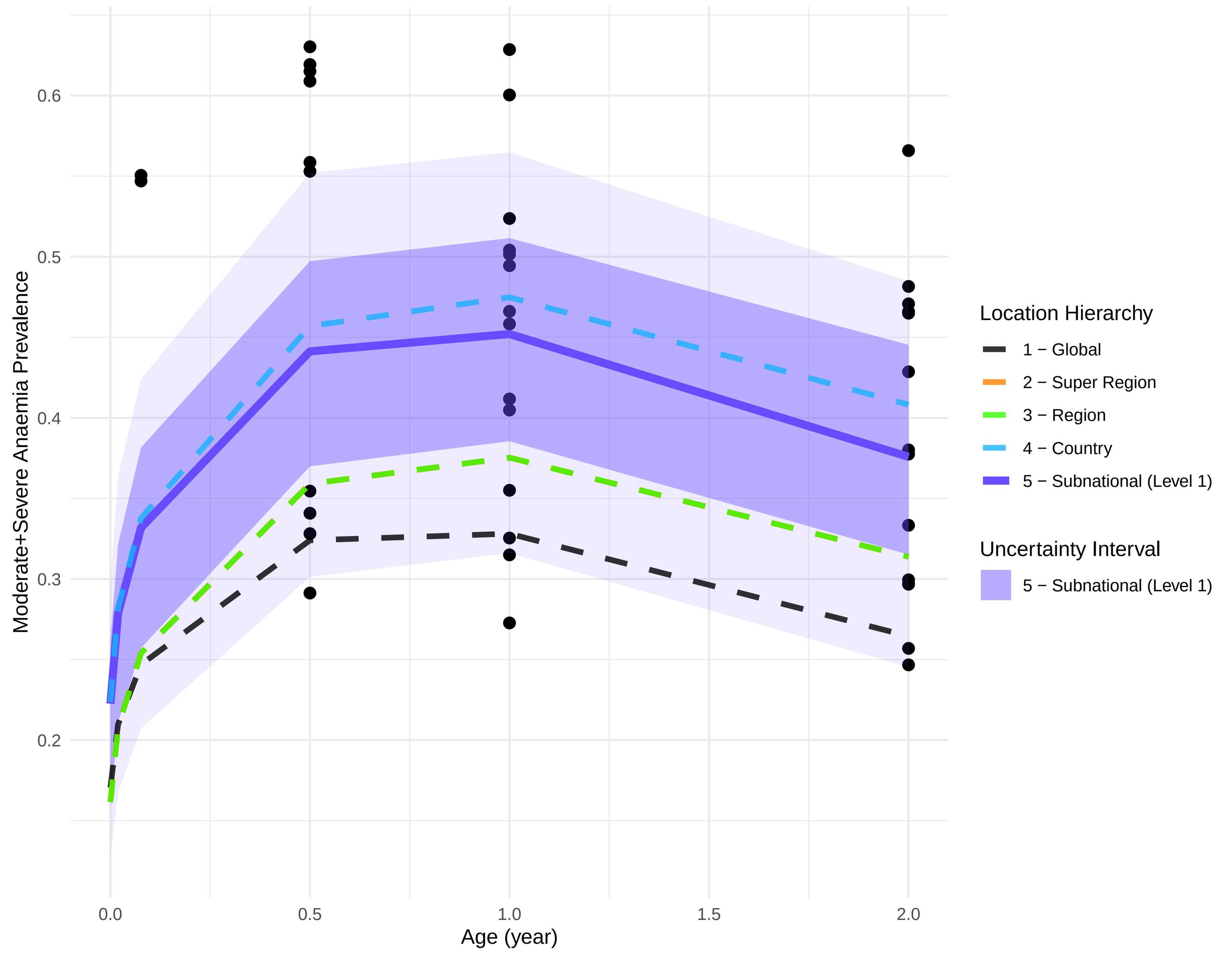

MR−BRT Age/Sex Fits − Males <2 Years Old − Moderate+Severe Anaemia Prevalence − Delhi, Rural
WHO−2001 Adjustment Method

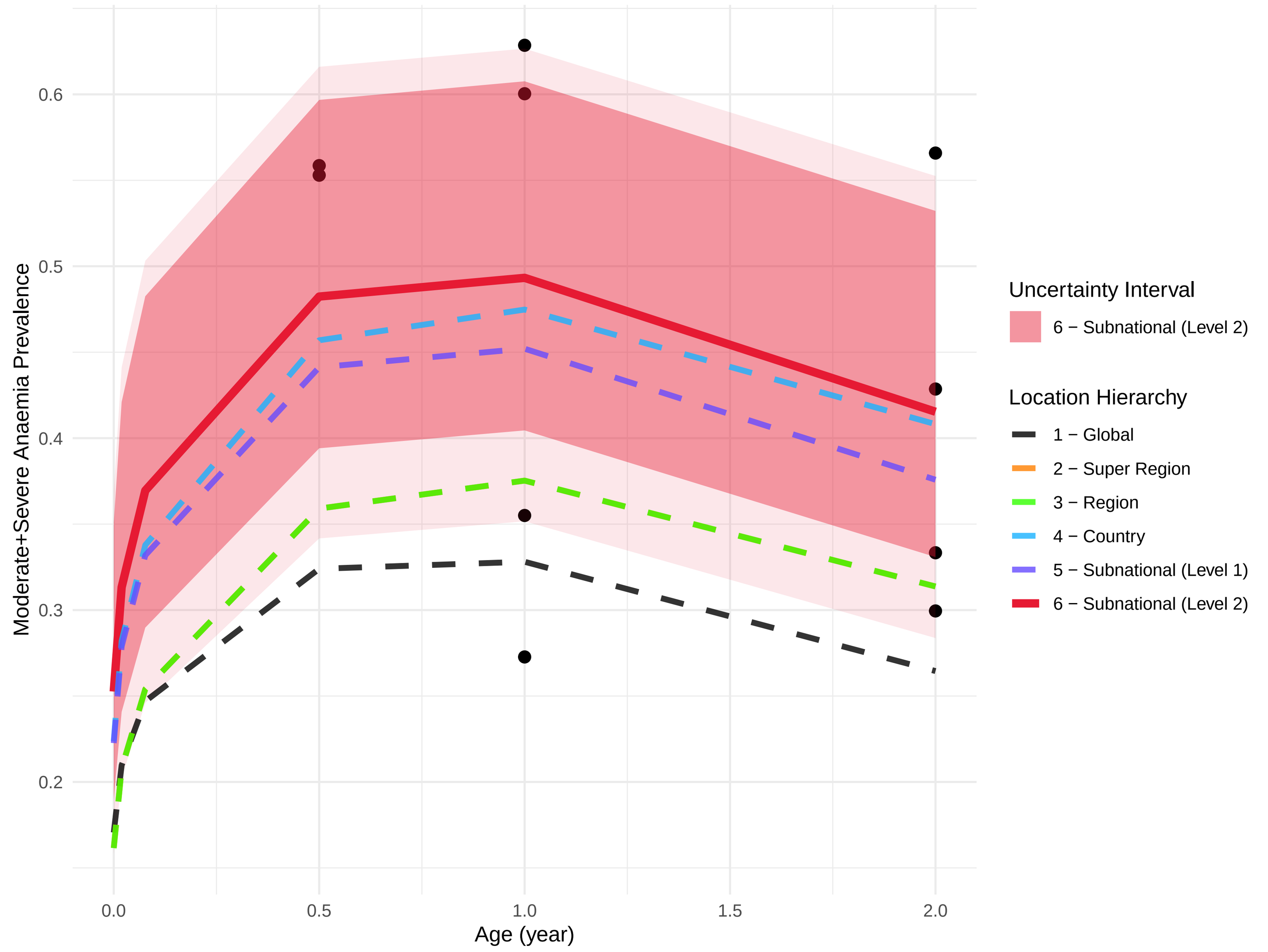

MR−BRT Age/Sex Fits − Males <2 Years Old − Moderate+Severe Anaemia Prevalence − Delhi, Urban
WHO−2001 Adjustment Method

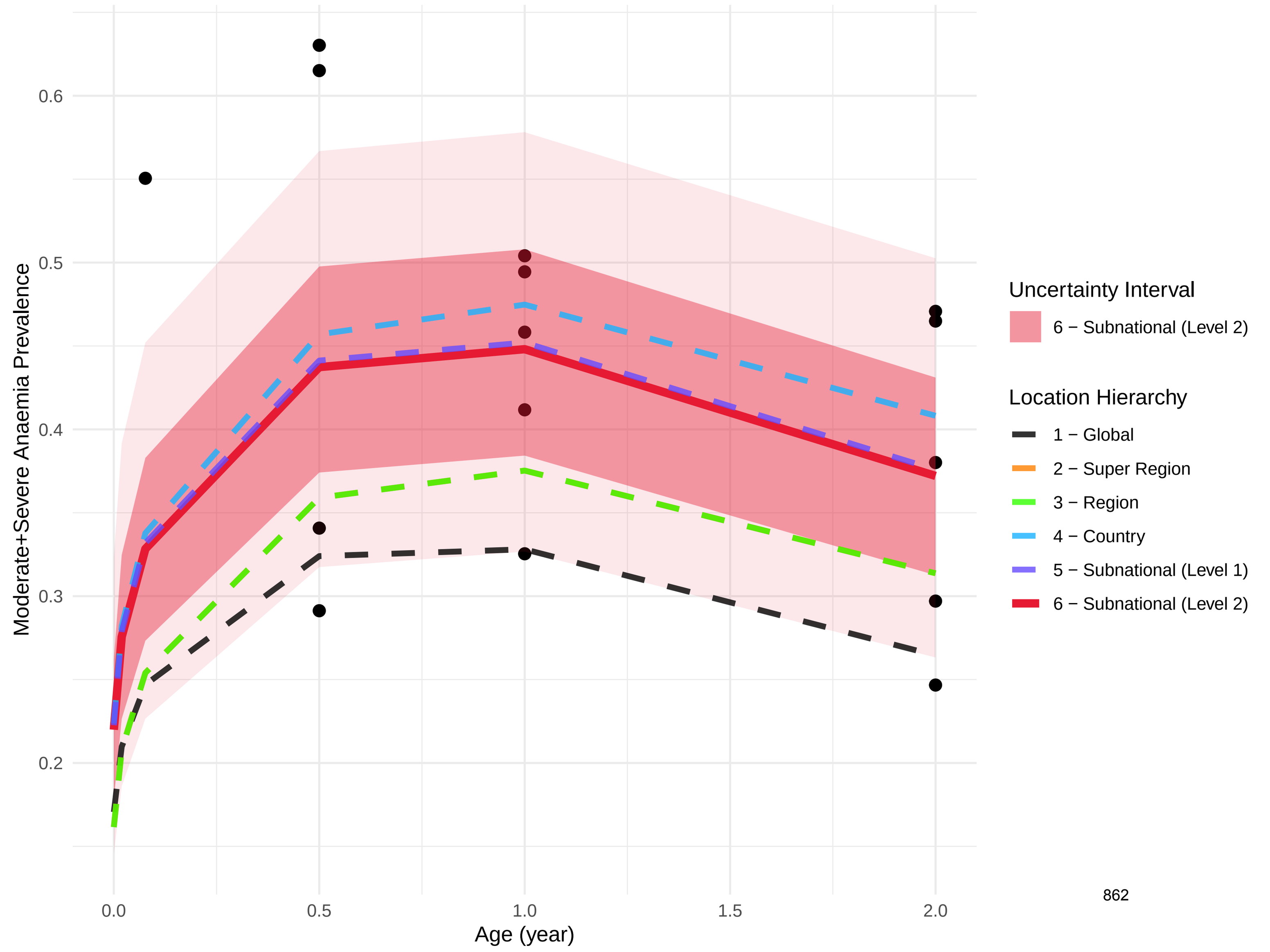

MR−BRT Age/Sex Fits − Males <2 Years Old − Moderate+Severe Anaemia Prevalence − Goa
WHO−2001 Adjustment Method

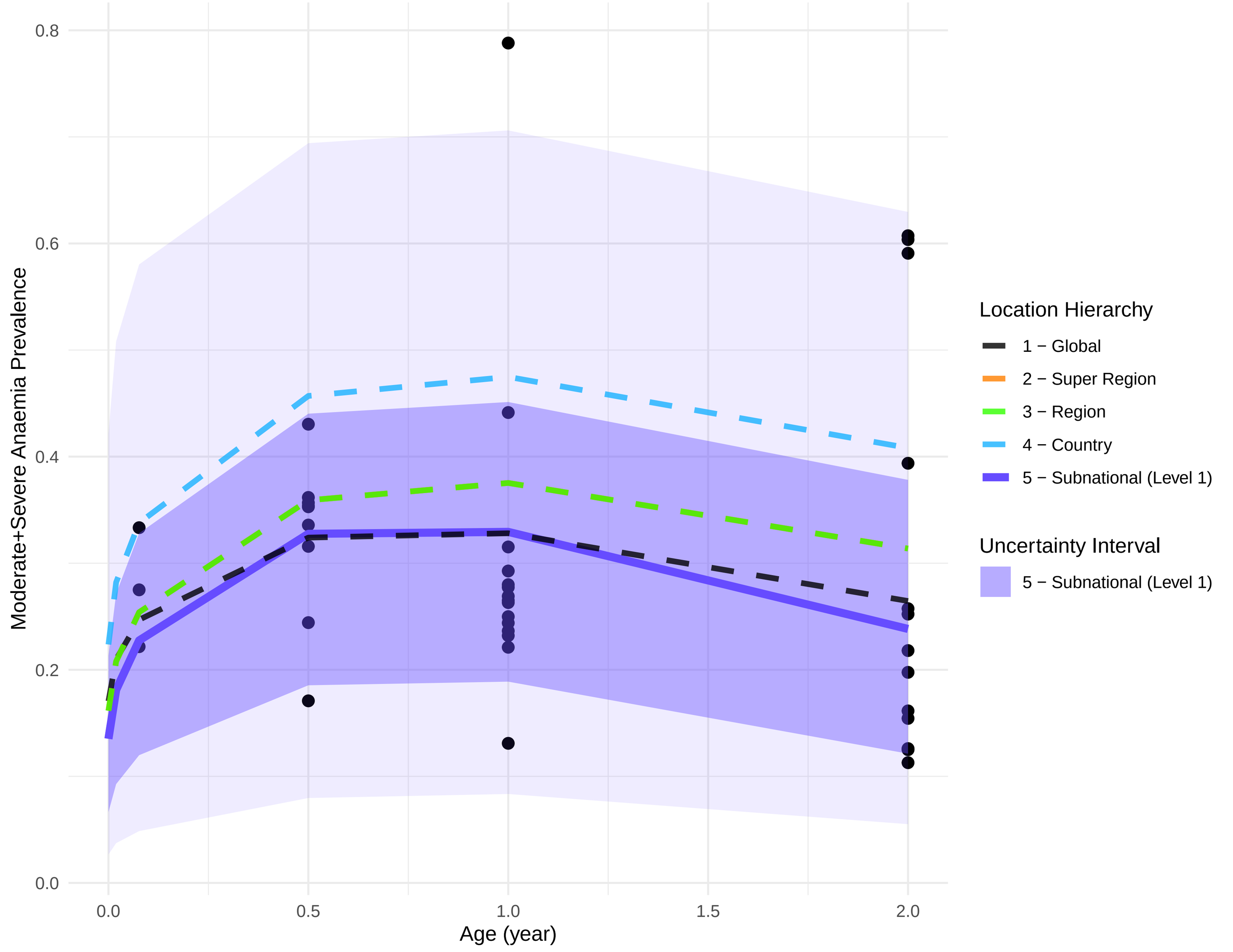

MR−BRT Age/Sex Fits − Males <2 Years Old − Moderate+Severe Anaemia Prevalence − Goa, Rural
WHO−2001 Adjustment Method

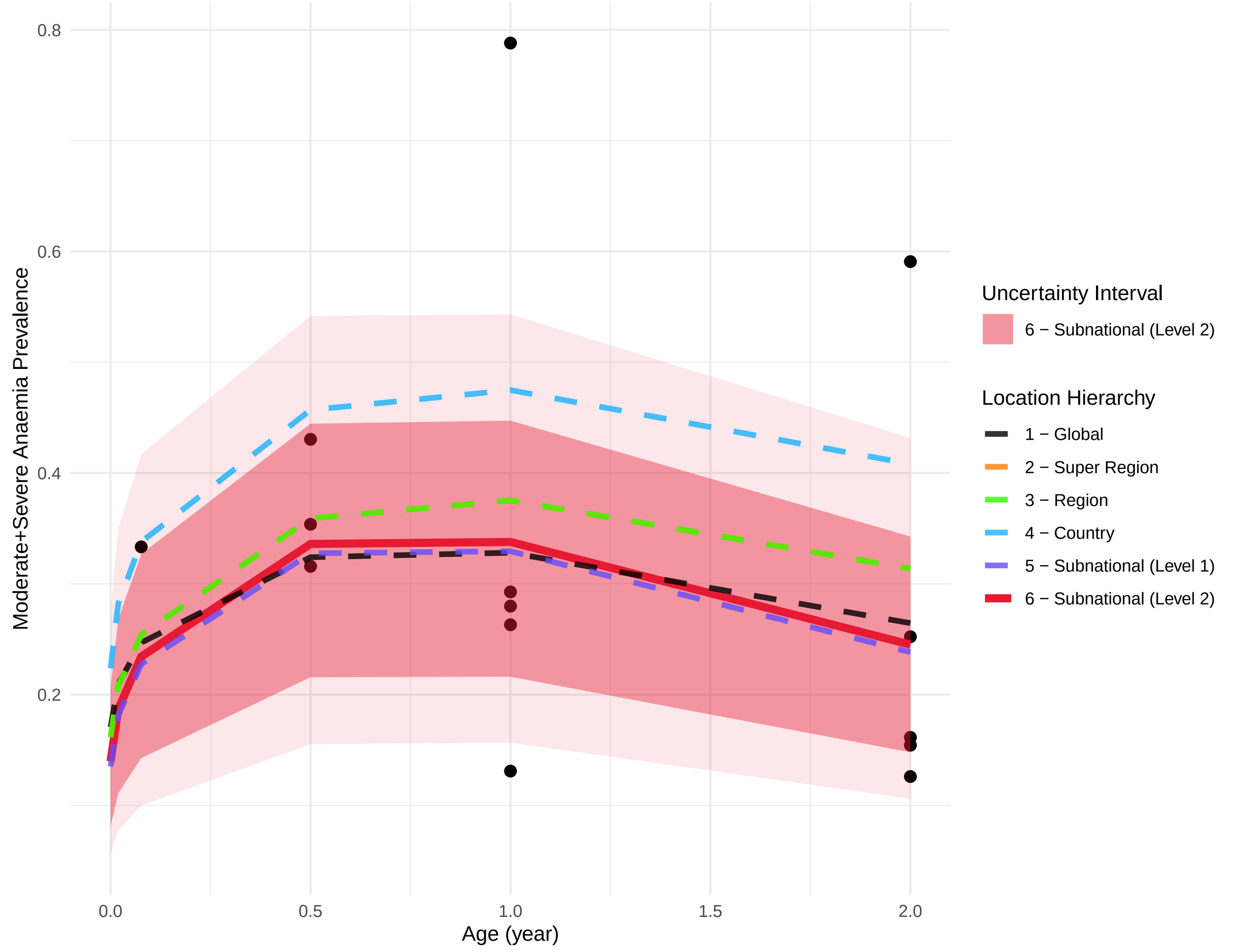

MR−BRT Age/Sex Fits − Males <2 Years Old − Moderate+Severe Anaemia Prevalence − Goa, Urban
WHO−2001 Adjustment Method

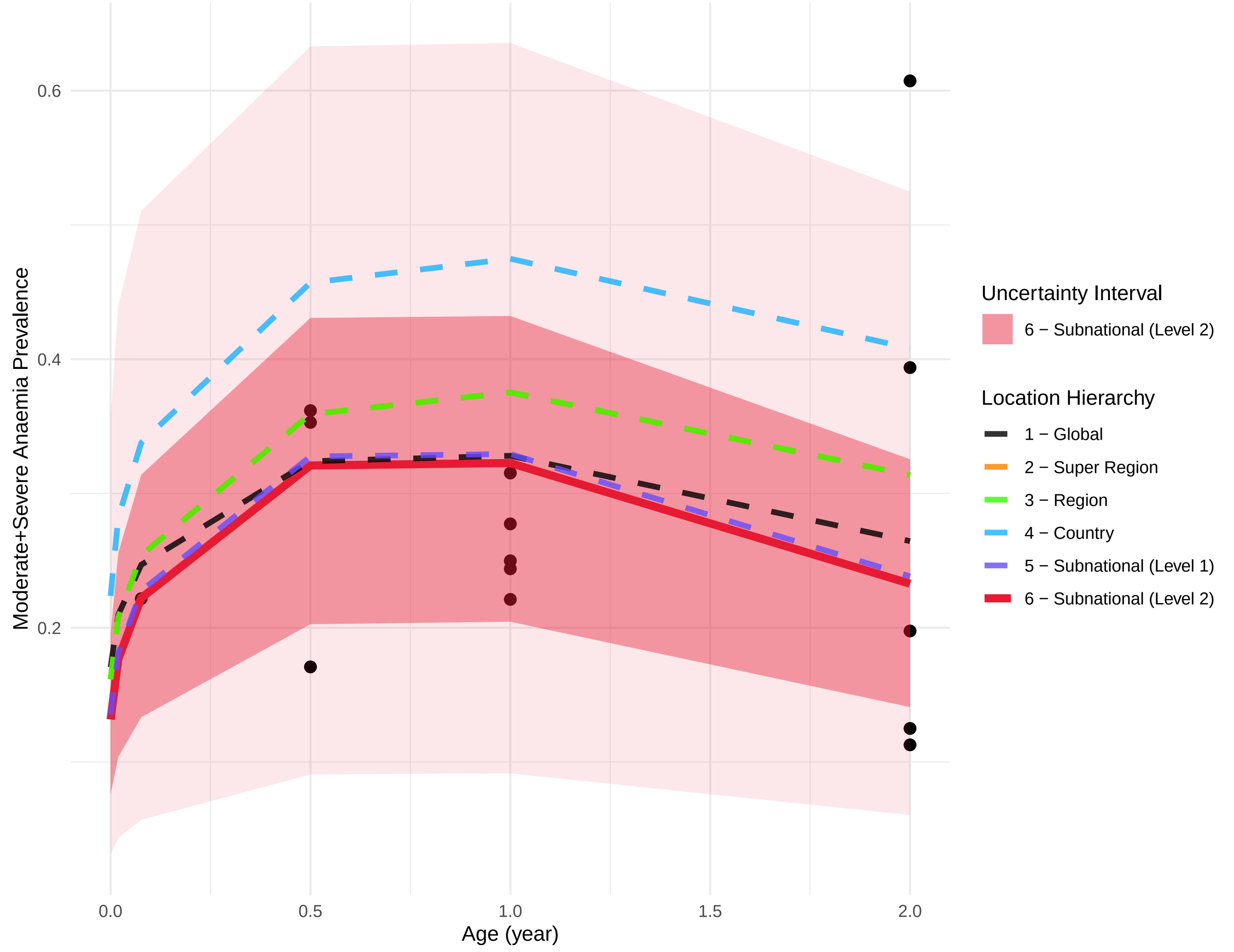

MR−BRT Age/Sex Fits − Males <2 Years Old − Moderate+Severe Anaemia Prevalence − Gujarat
WHO−2001 Adjustment Method

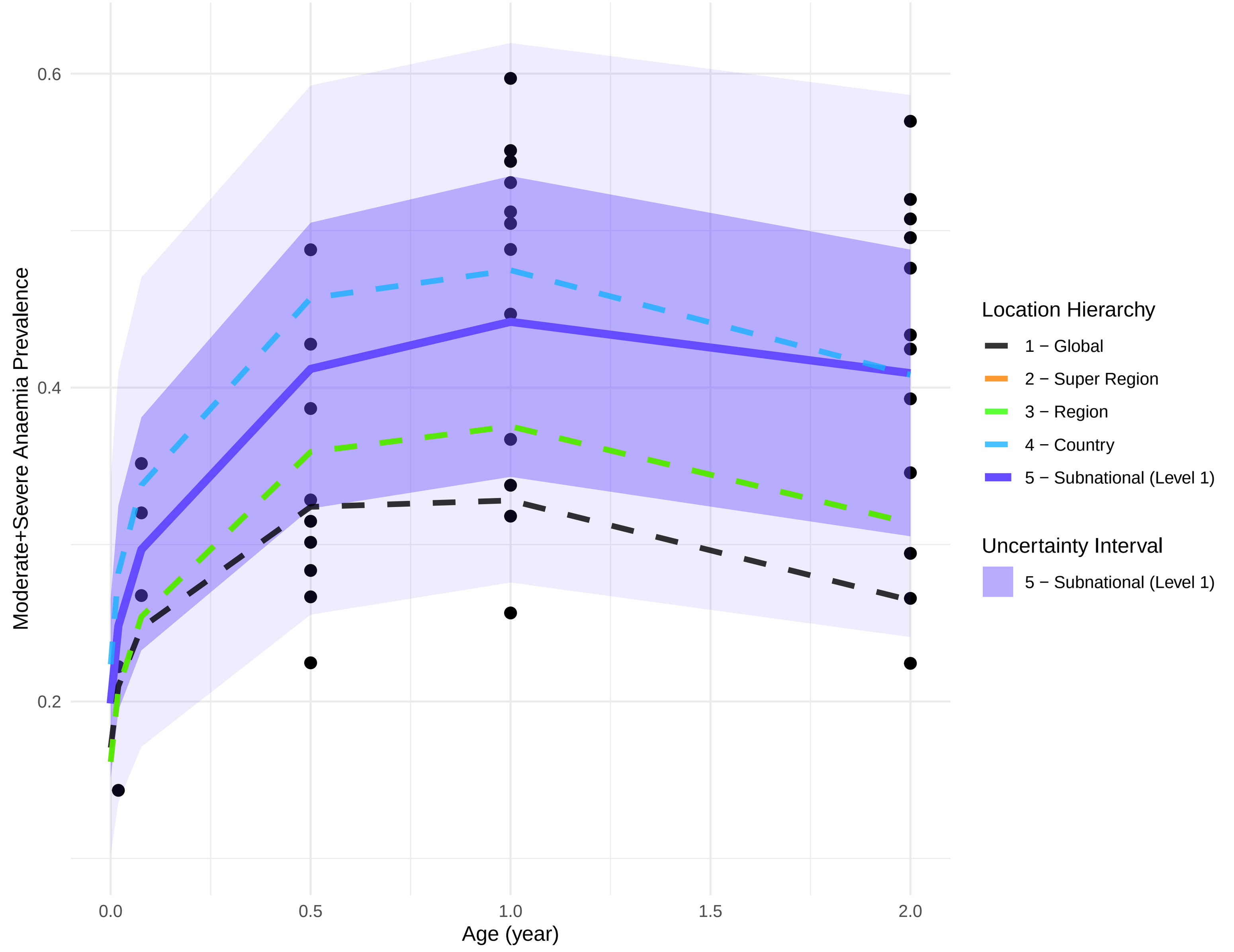

MR−BRT Age/Sex Fits − Males <2 Years Old − Moderate+Severe Anaemia Prevalence − Gujarat, Rural
WHO−2001 Adjustment Method

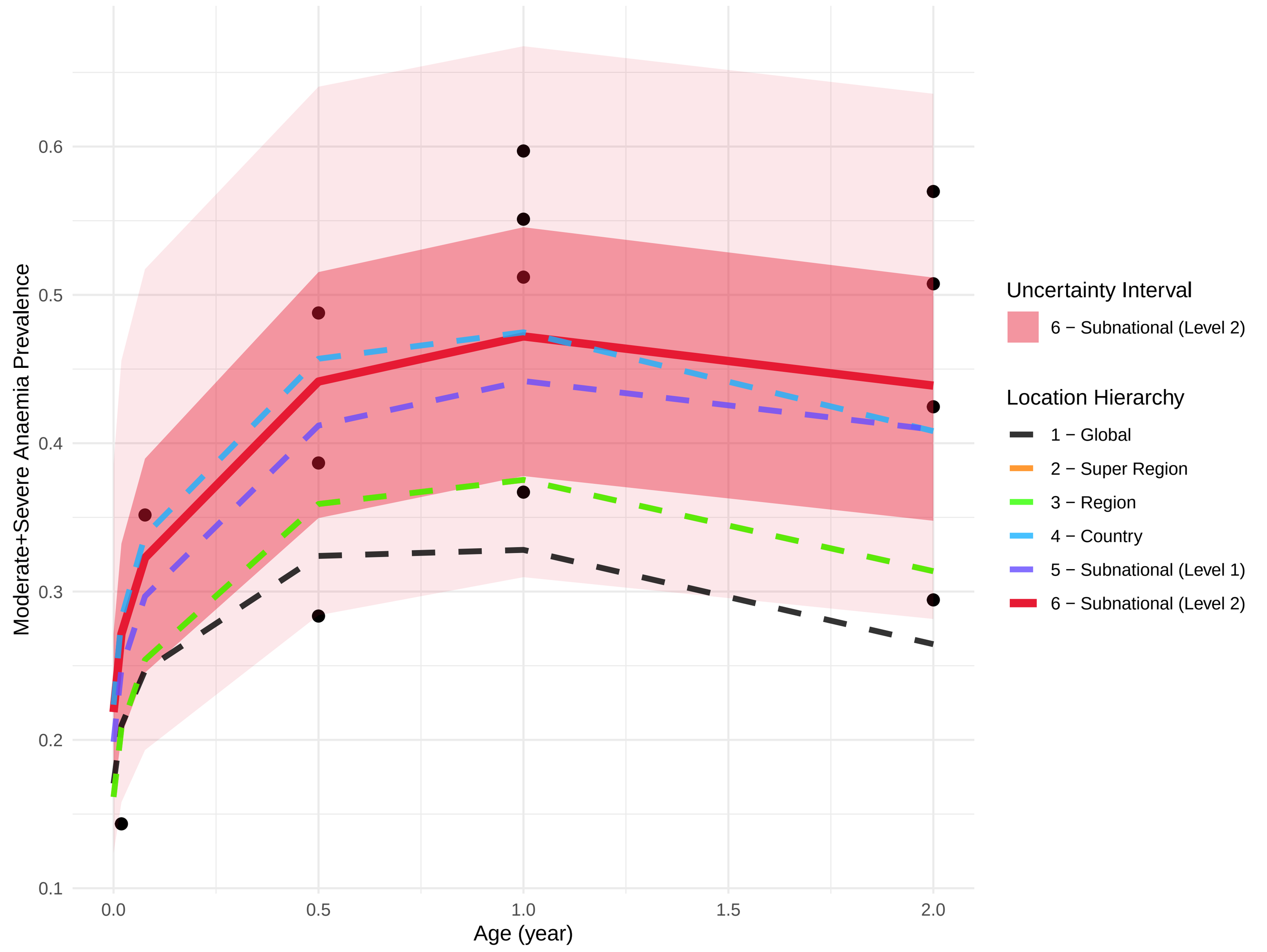

MR−BRT Age/Sex Fits − Males <2 Years Old − Moderate+Severe Anaemia Prevalence − Gujarat, Urban
WHO−2001 Adjustment Method

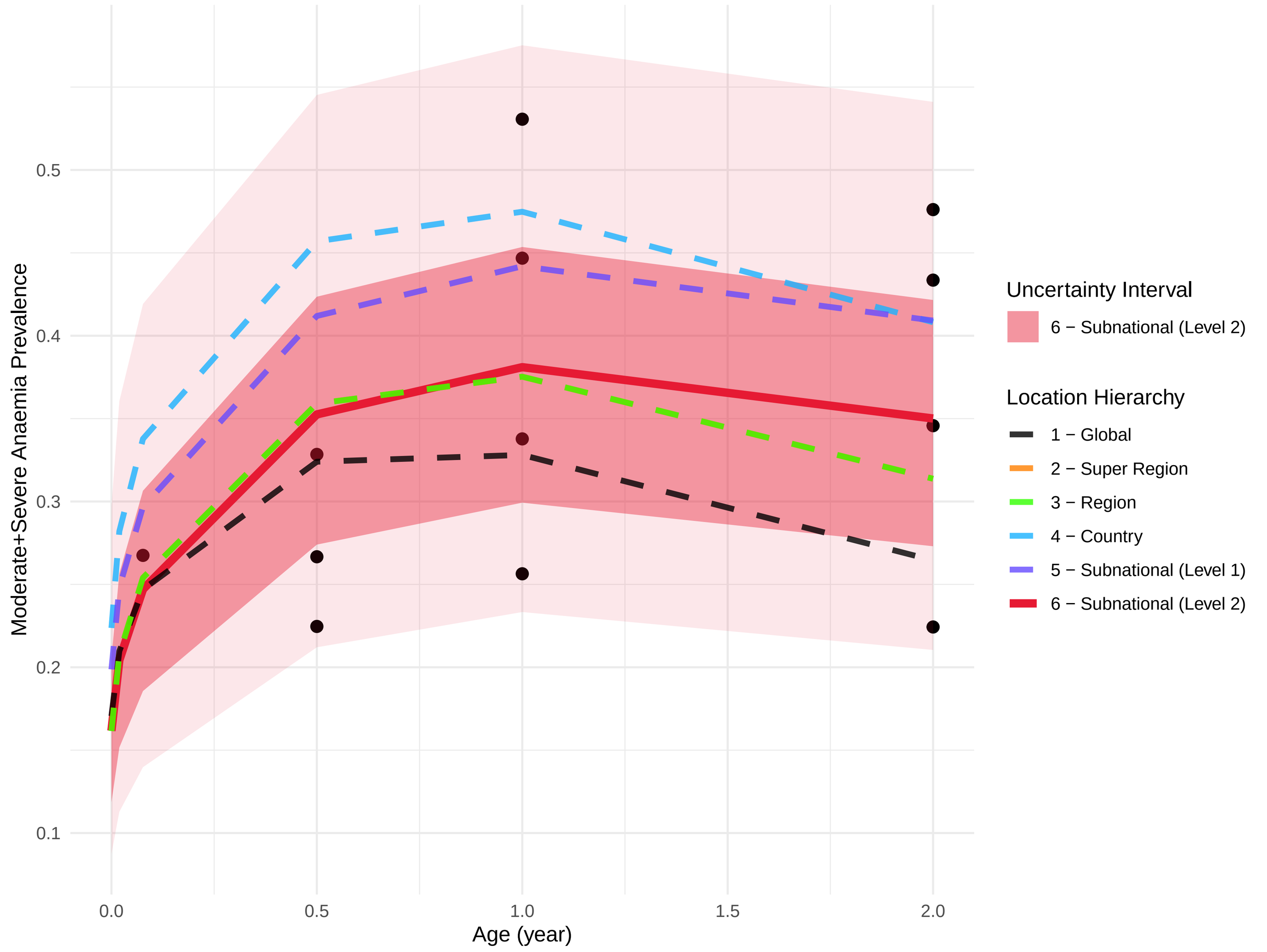

MR−BRT Age/Sex Fits − Males <2 Years Old − Moderate+Severe Anaemia Prevalence − Haryana
WHO−2001 Adjustment Method

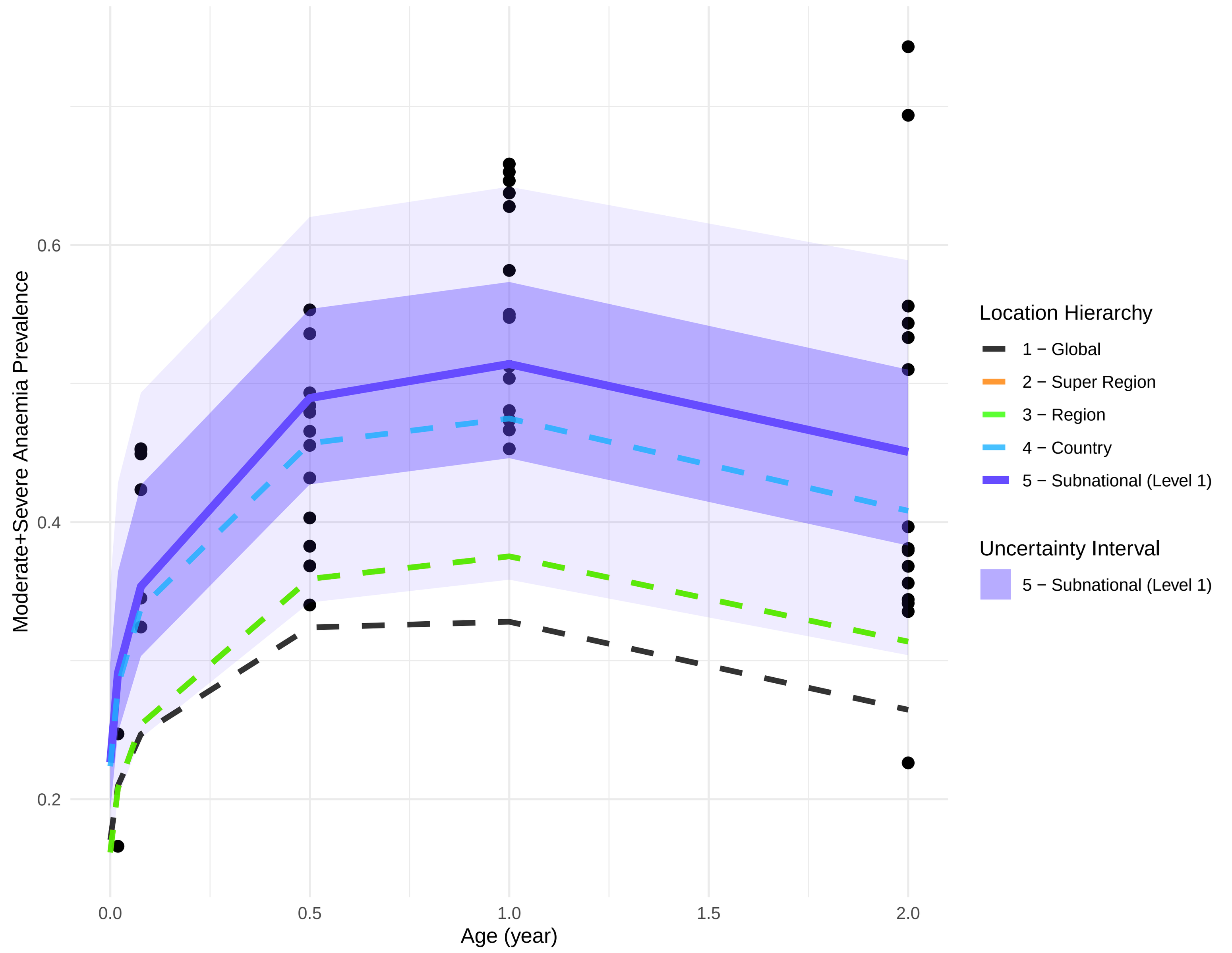

MR−BRT Age/Sex Fits − Males <2 Years Old − Moderate+Severe Anaemia Prevalence − Haryana, Rural
WHO−2001 Adjustment Method

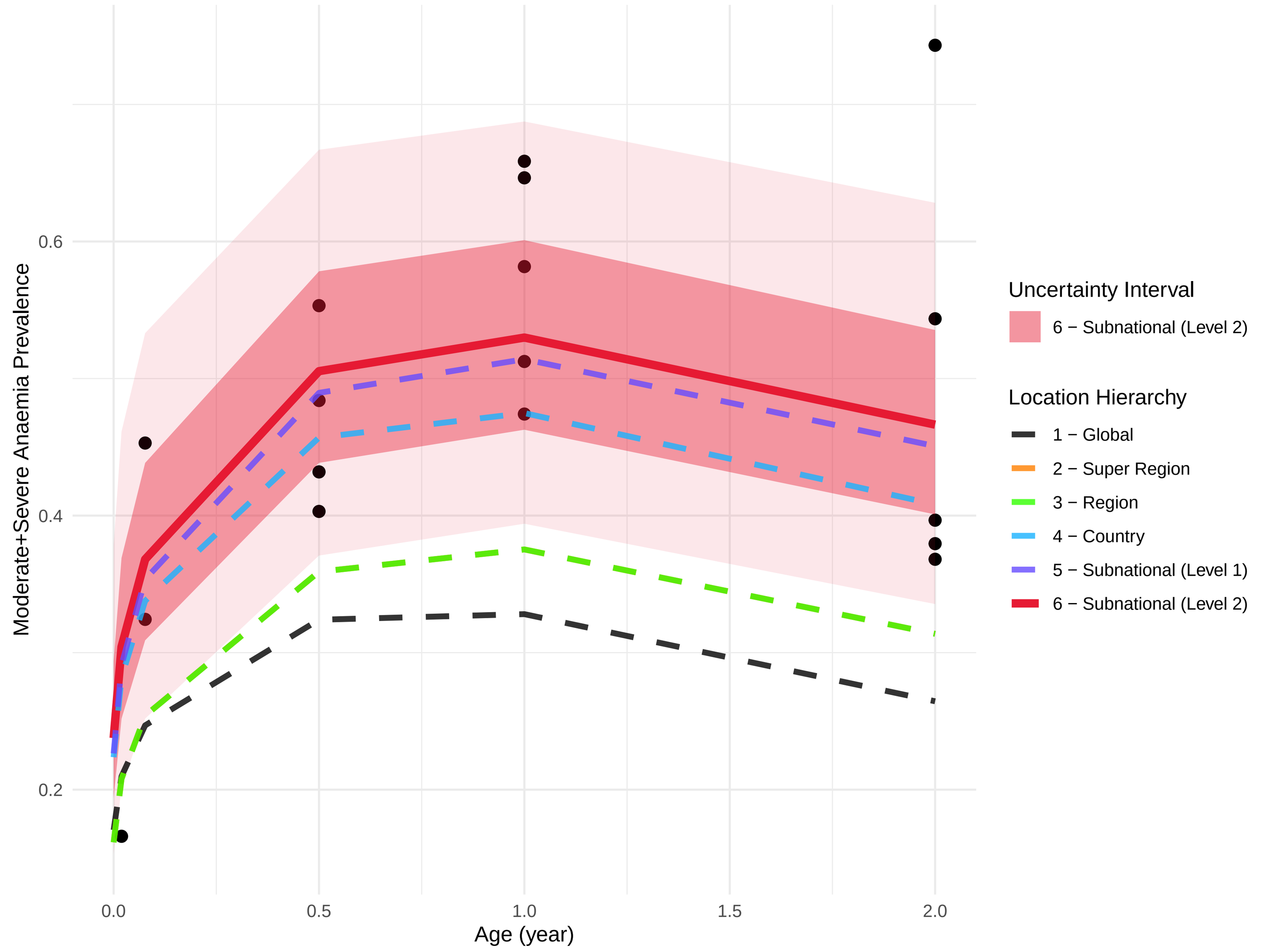

MR−BRT Age/Sex Fits − Males <2 Years Old − Moderate+Severe Anaemia Prevalence − Haryana, Urban
WHO−2001 Adjustment Method

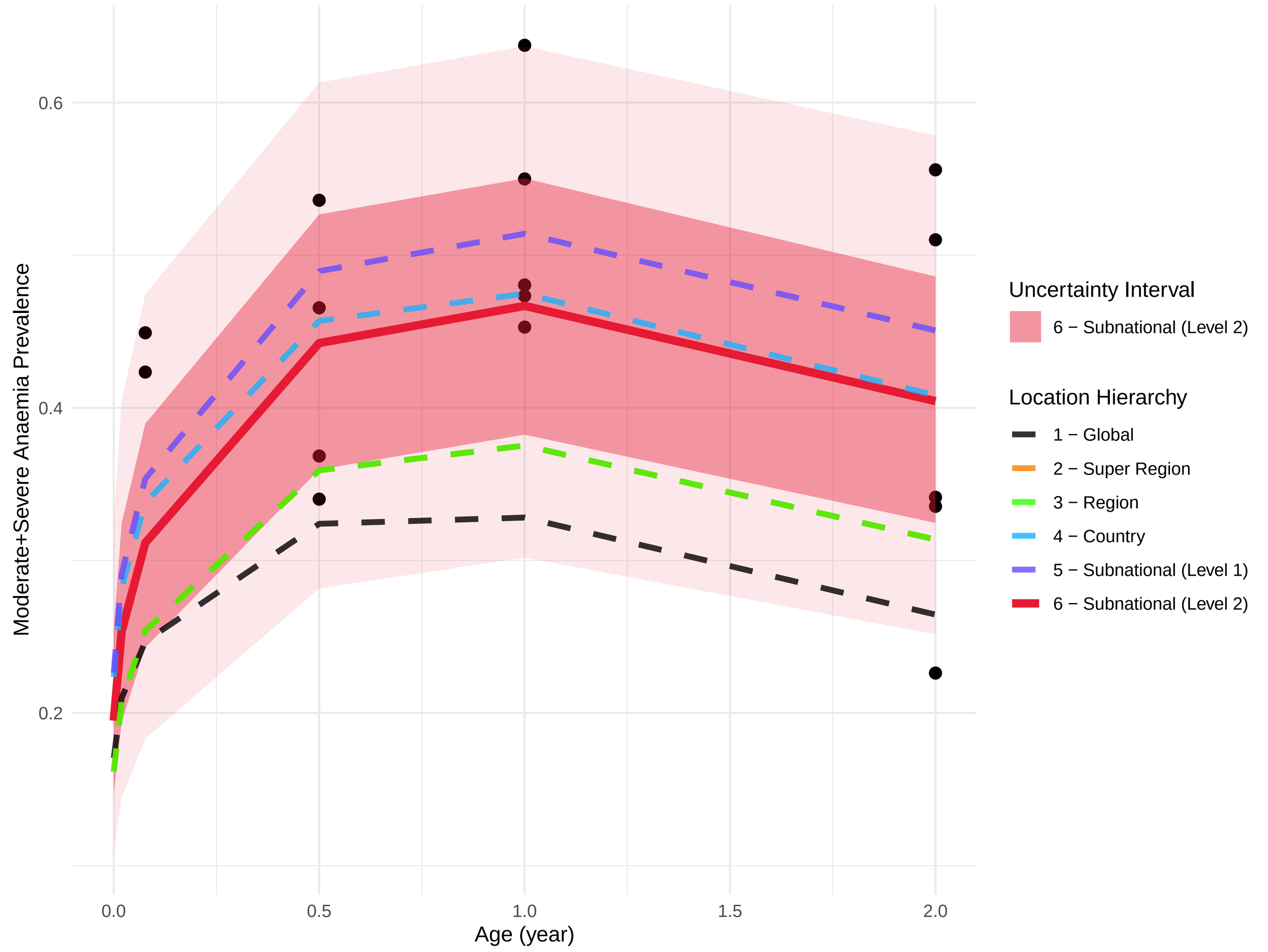

MR−BRT Age/Sex Fits − Males <2 Years Old − Moderate+Severe Anaemia Prevalence − Himachal Pradesh
WHO−2001 Adjustment Method

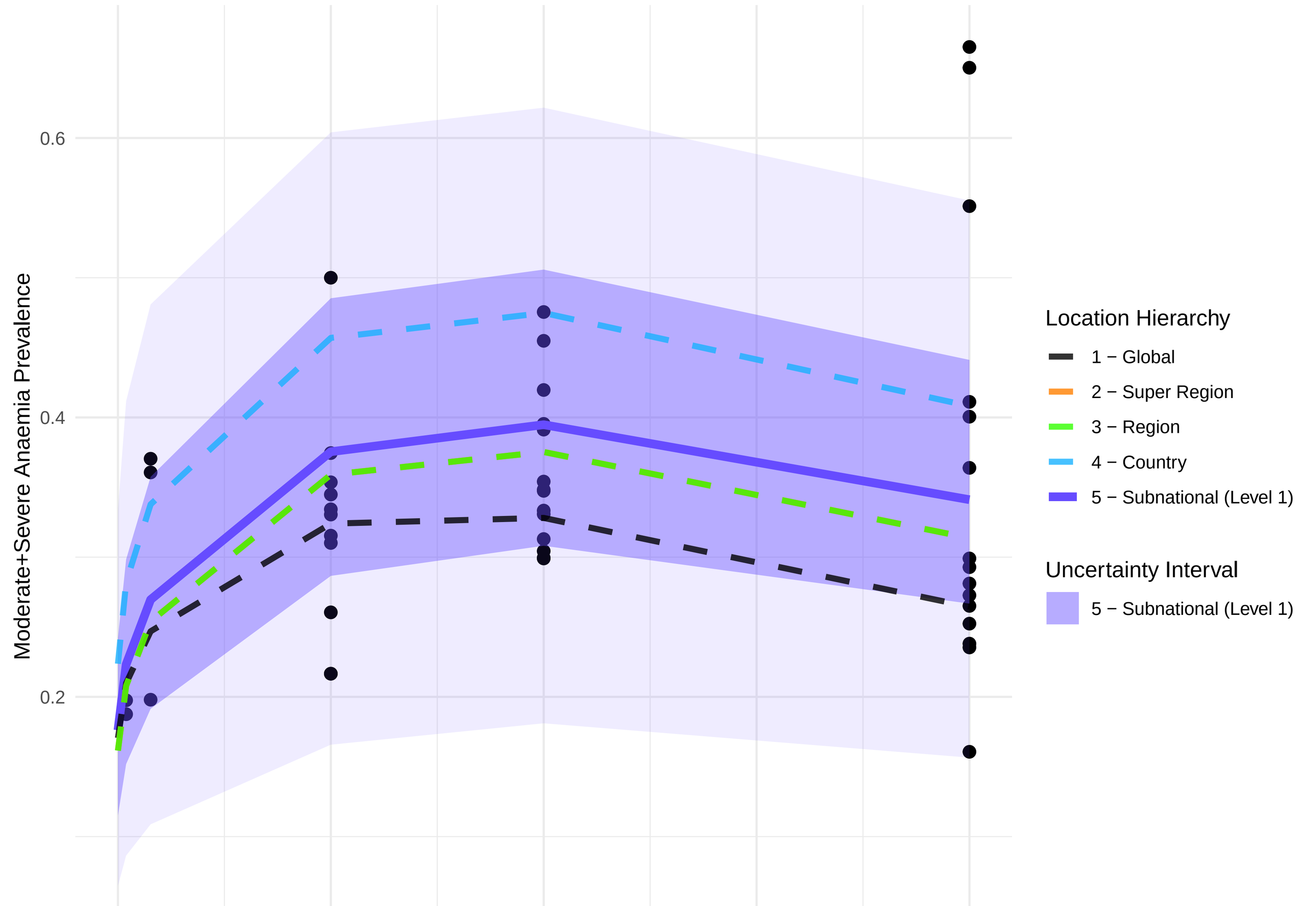

MR−BRT Age/Sex Fits − Males <2 Years Old − Moderate+Severe Anaemia Prevalence − Himachal Pradesh, Rural
WHO−2001 Adjustment Method

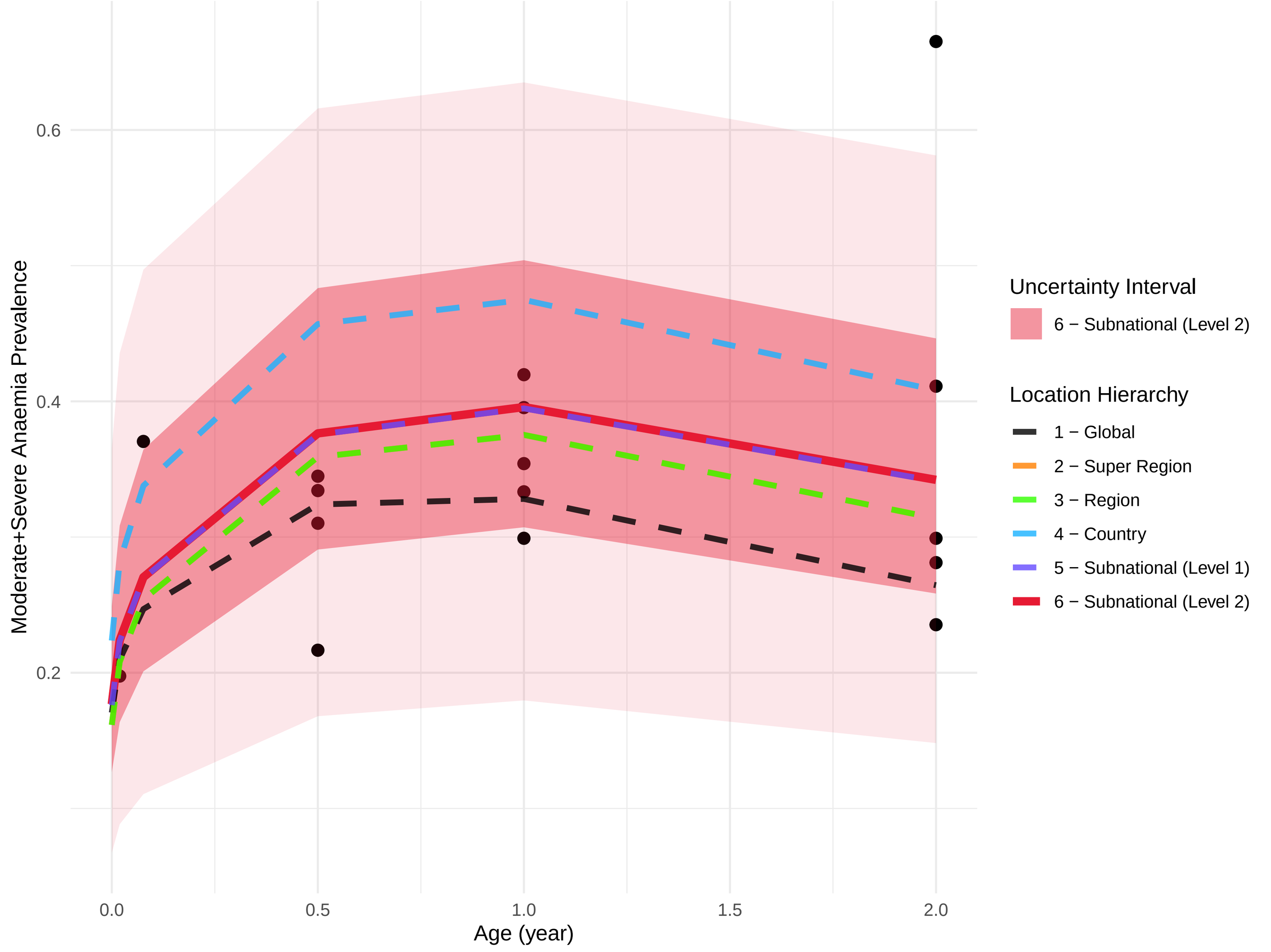

MR−BRT Age/Sex Fits − Males <2 Years Old − Moderate+Severe Anaemia Prevalence − Himachal Pradesh, Urban
WHO−2001 Adjustment Method

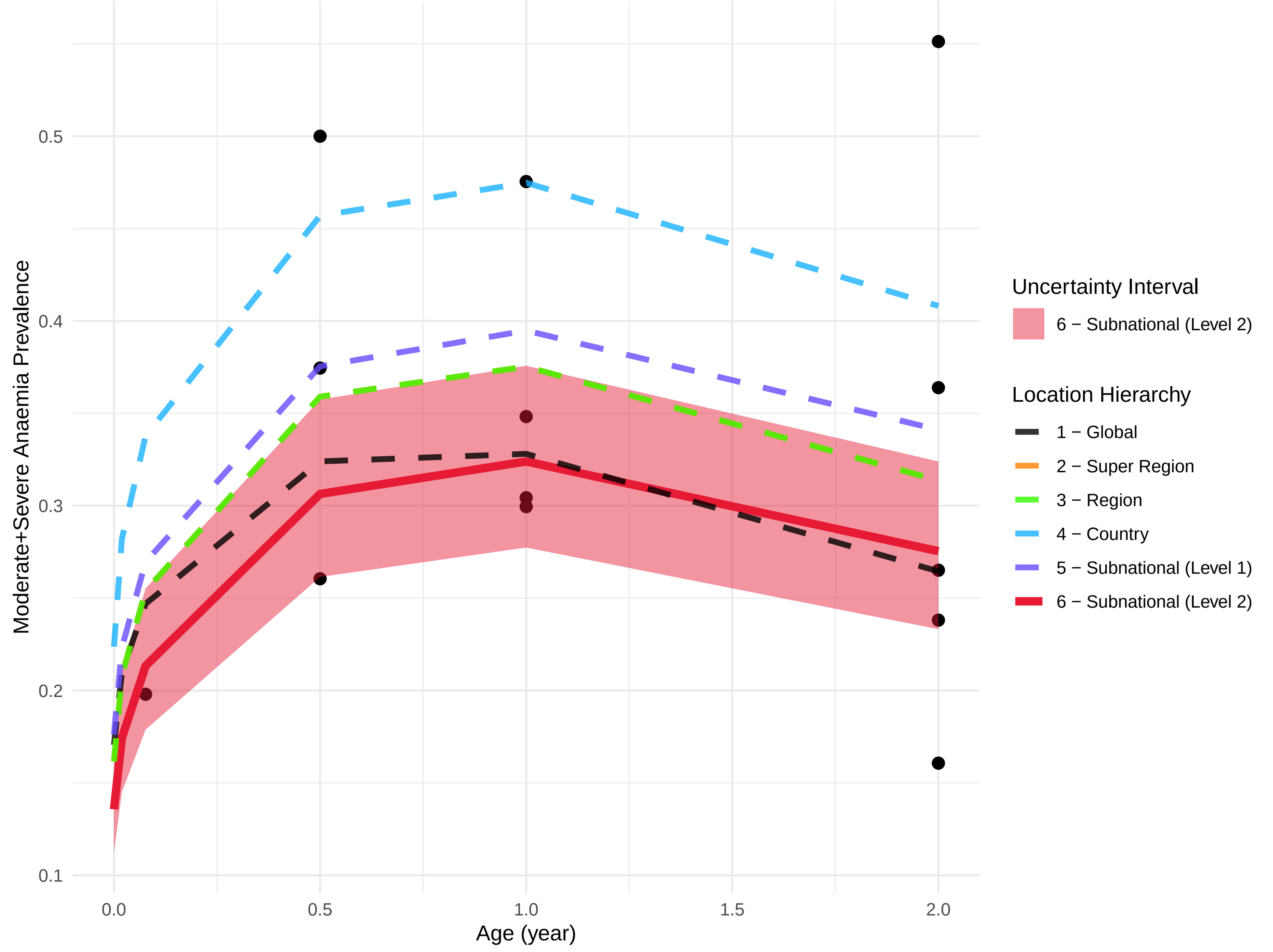

MR−BRT Age/Sex Fits − Males <2 Years Old − Moderate+Severe Anaemia Prevalence − Jammu & Kashmir and Ladakh
WHO−2001 Adjustment Method

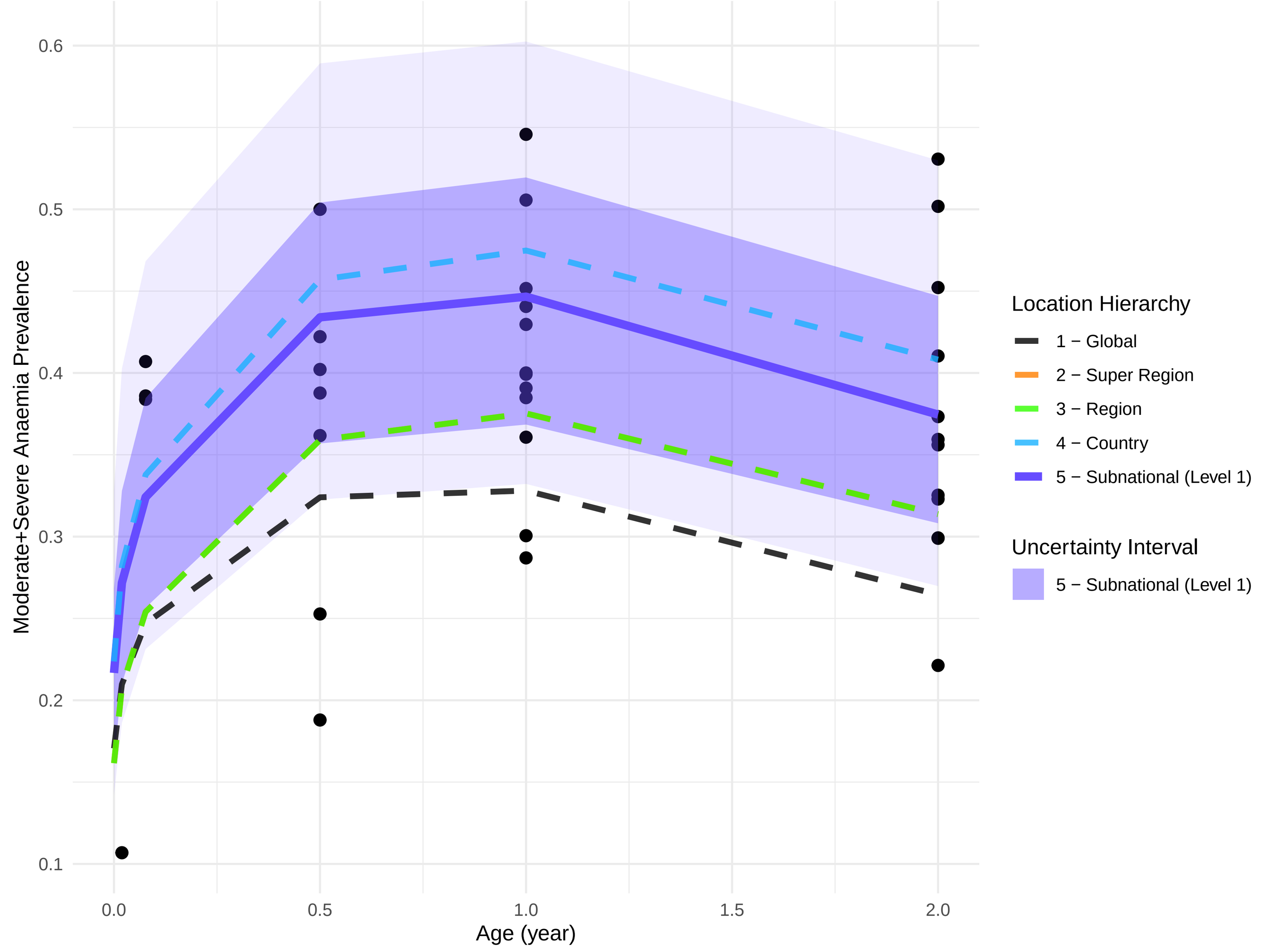

MR−BRT Age/Sex Fits − Males <2 Years Old − Moderate+Severe Anaemia Prevalence − Jammu & Kashmir and Ladakh, Rural
WHO−2001 Adjustment Method

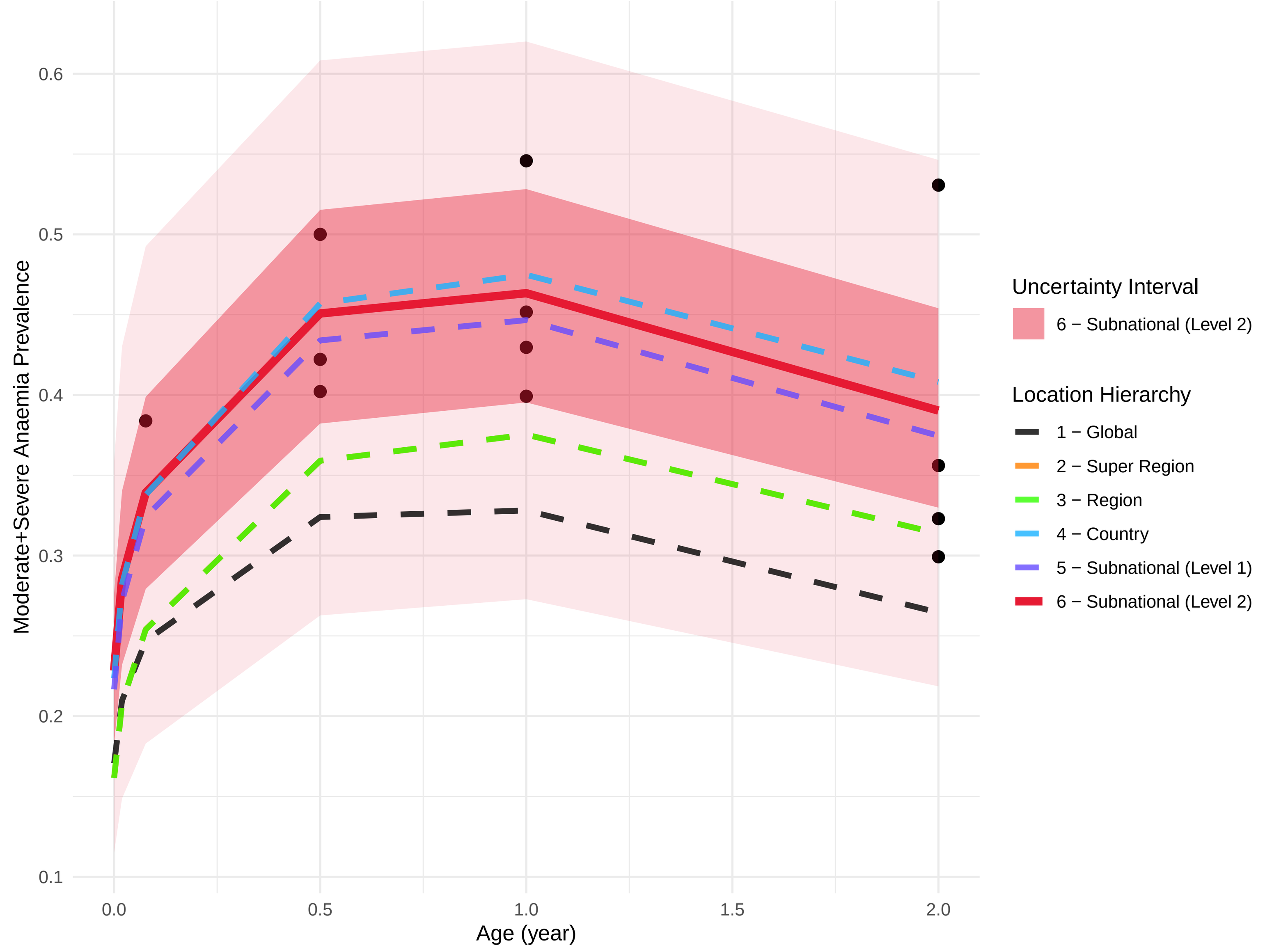

MR−BRT Age/Sex Fits − Males <2 Years Old − Moderate+Severe Anaemia Prevalence − Jammu & Kashmir and Ladakh, Urban
WHO−2001 Adjustment Method

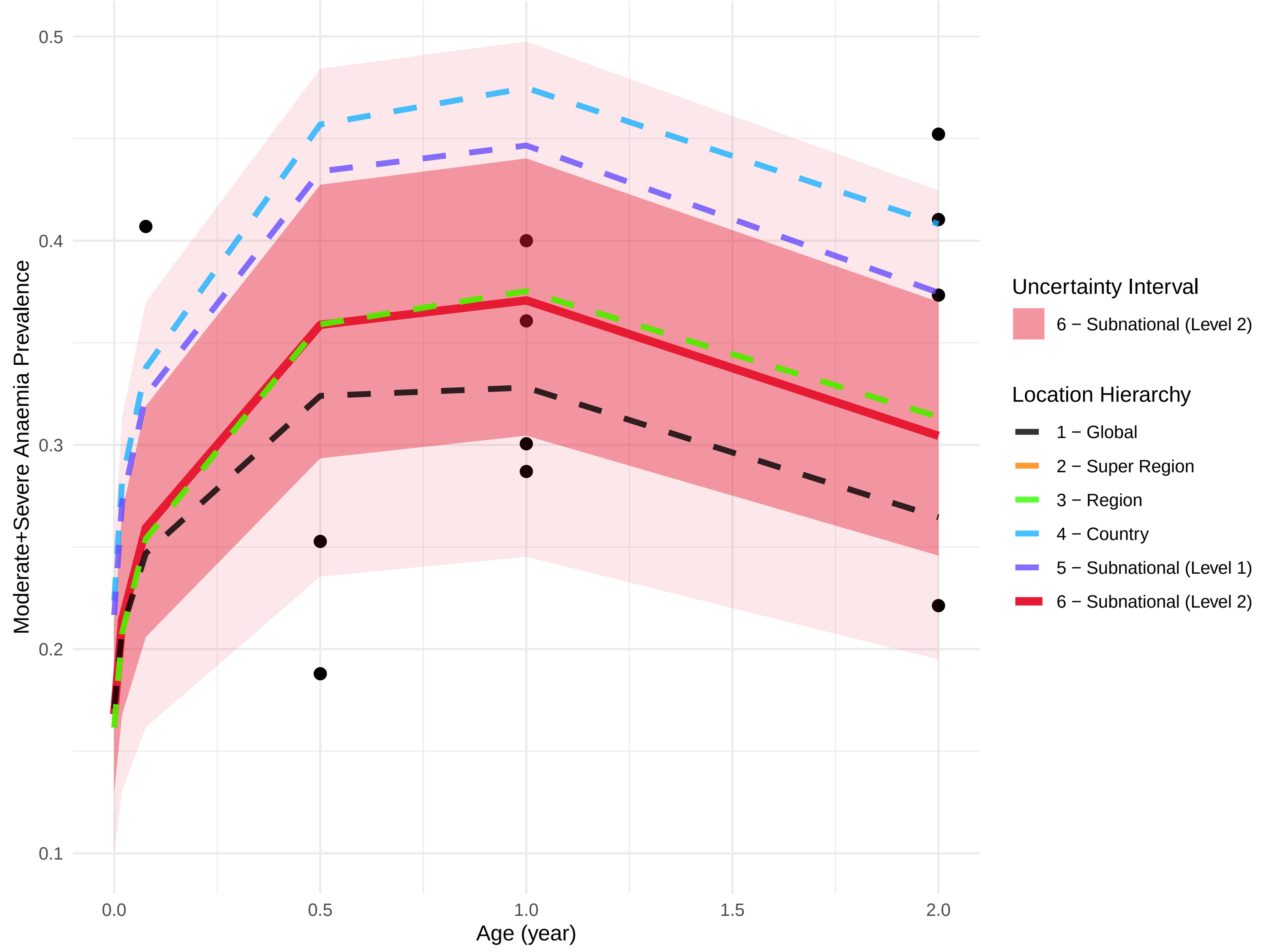

MR−BRT Age/Sex Fits − Males <2 Years Old − Moderate+Severe Anaemia Prevalence − Jharkhand
WHO−2001 Adjustment Method

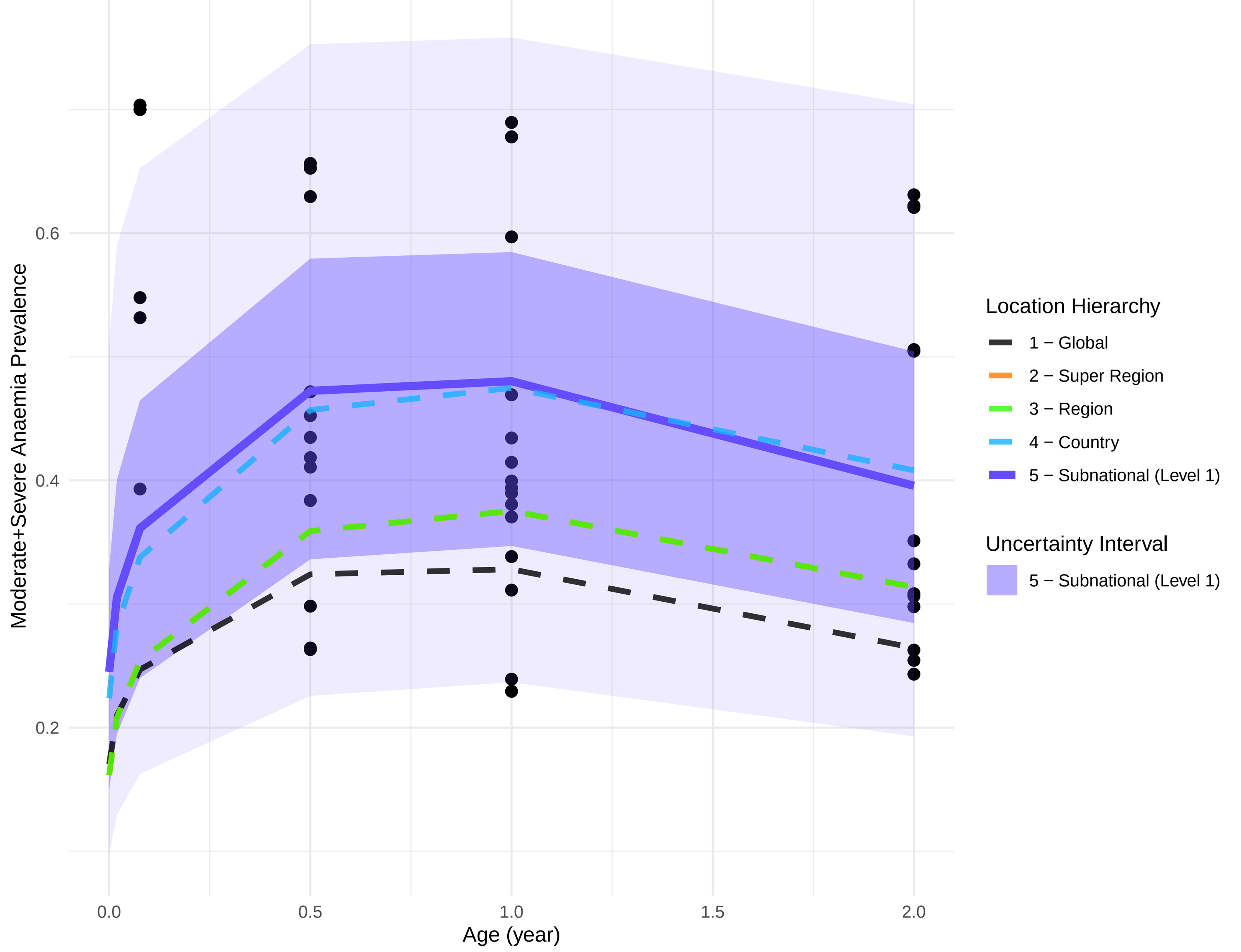

MR−BRT Age/Sex Fits − Males <2 Years Old − Moderate+Severe Anaemia Prevalence − Jharkhand, Rural
WHO−2001 Adjustment Method

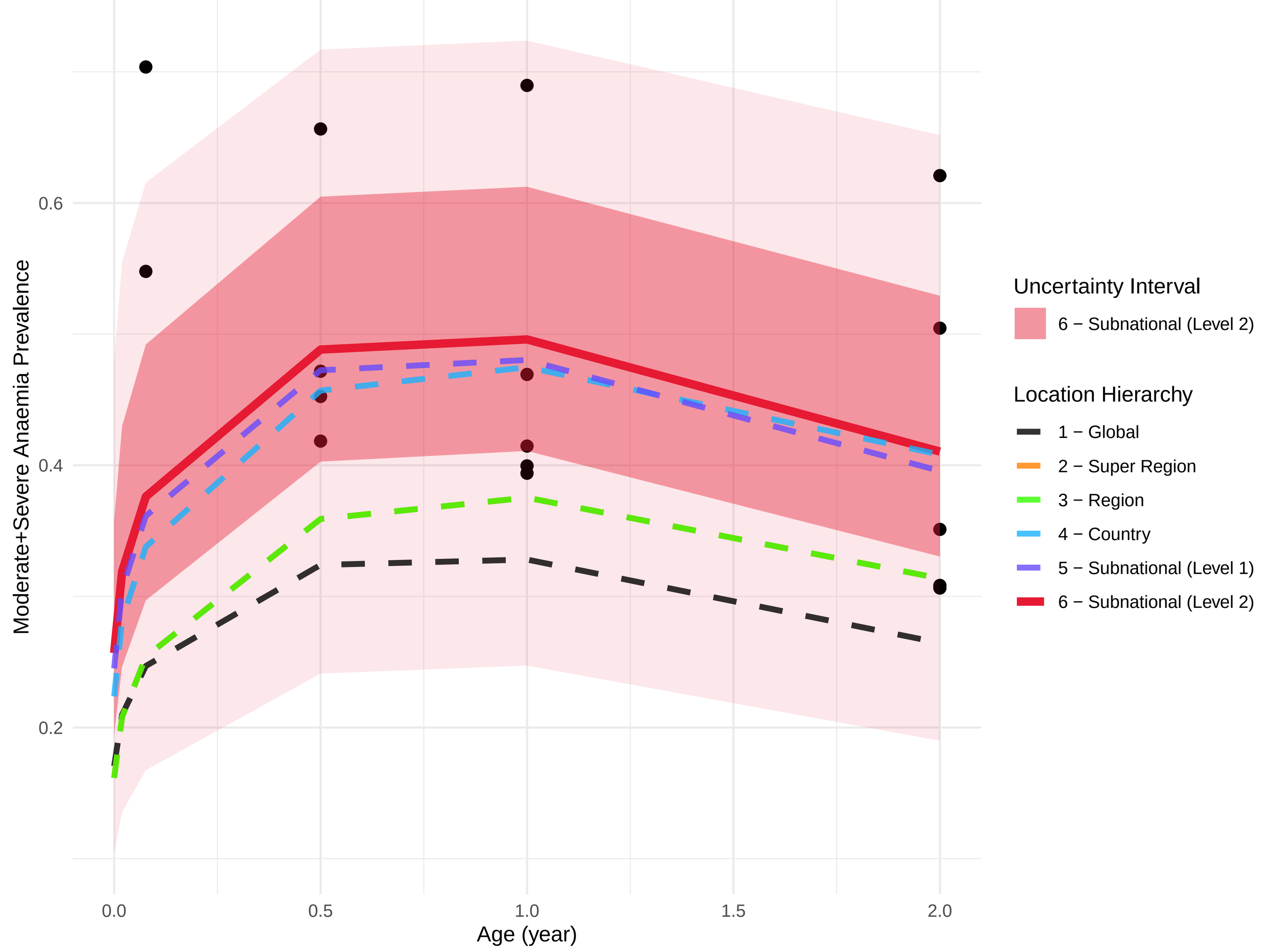

MR−BRT Age/Sex Fits − Males <2 Years Old − Moderate+Severe Anaemia Prevalence − Jharkhand, Urban
WHO−2001 Adjustment Method

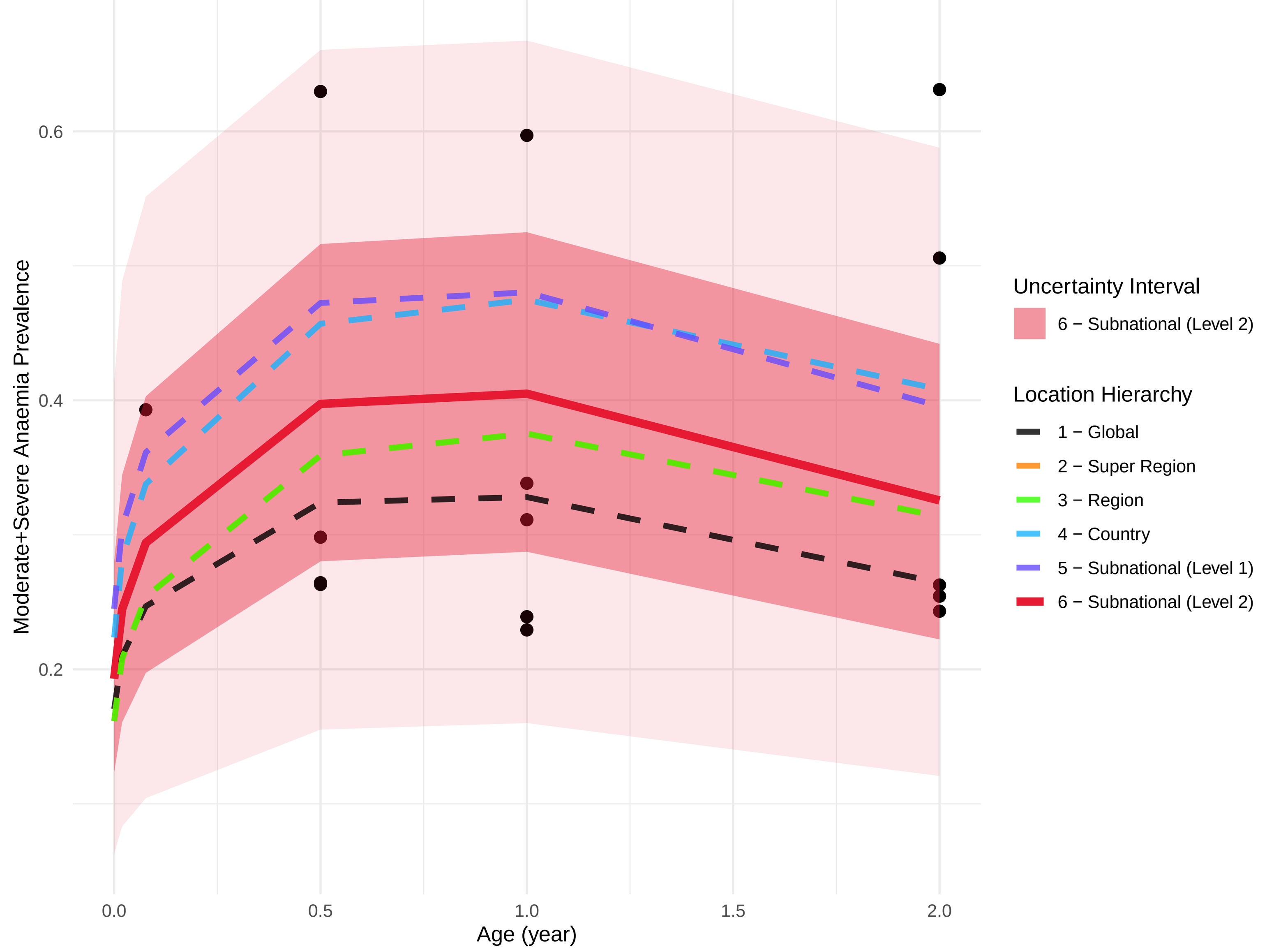

MR−BRT Age/Sex Fits − Males <2 Years Old − Moderate+Severe Anaemia Prevalence − Karnataka
WHO−2001 Adjustment Method

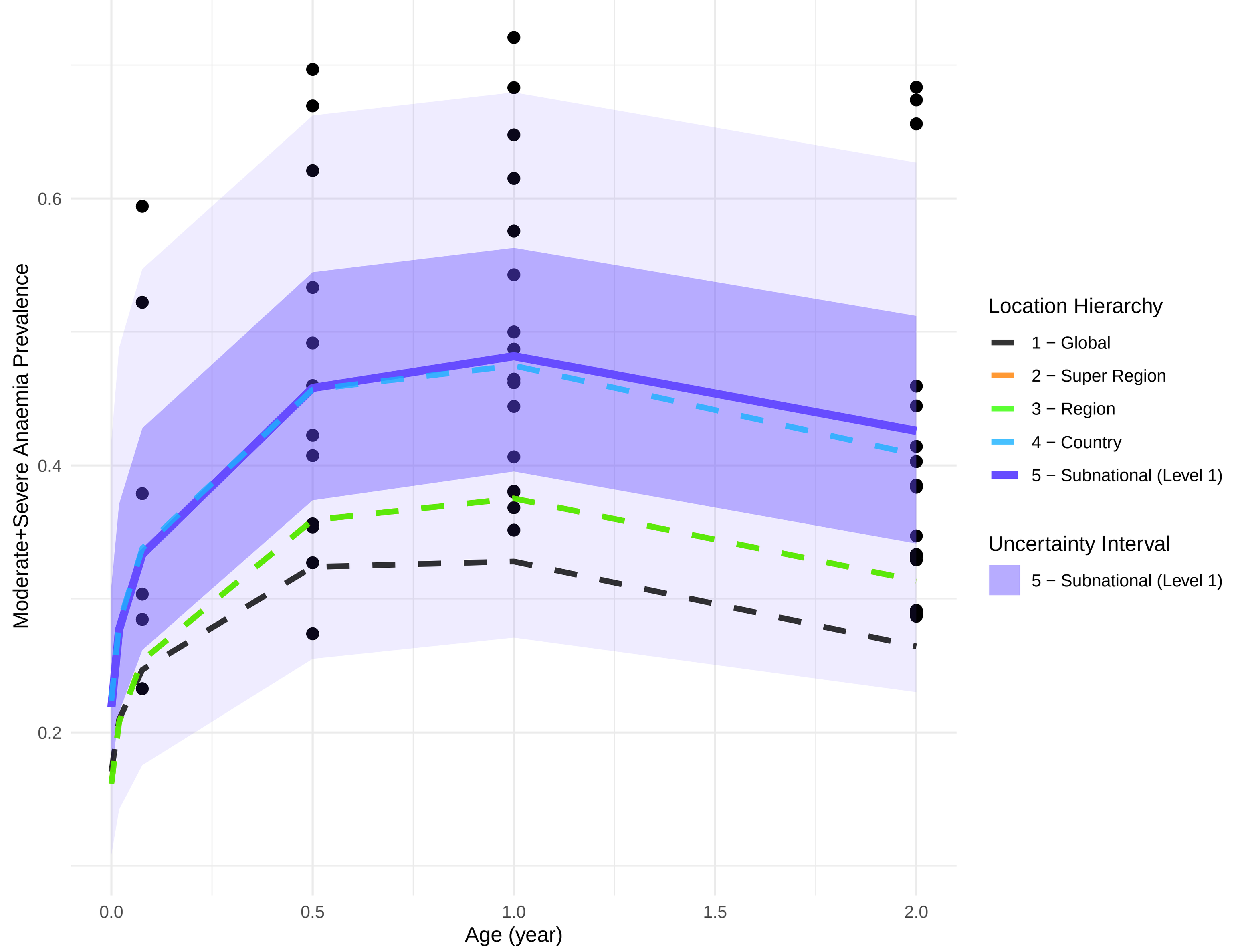

MR−BRT Age/Sex Fits − Males <2 Years Old − Moderate+Severe Anaemia Prevalence − Karnataka, Rural
WHO−2001 Adjustment Method

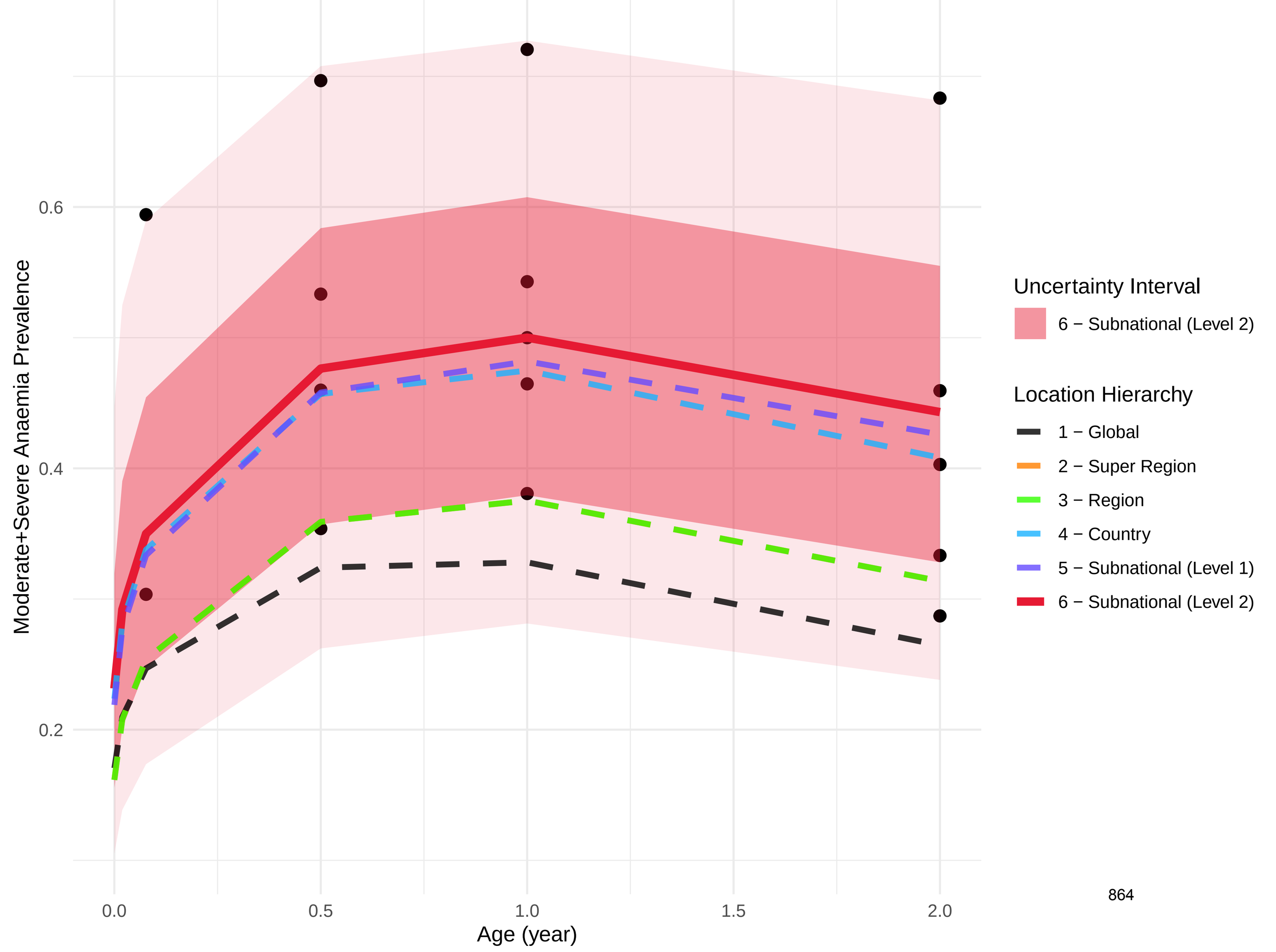

MR−BRT Age/Sex Fits − Males <2 Years Old − Moderate+Severe Anaemia Prevalence − Karnataka, Urban
WHO−2001 Adjustment Method

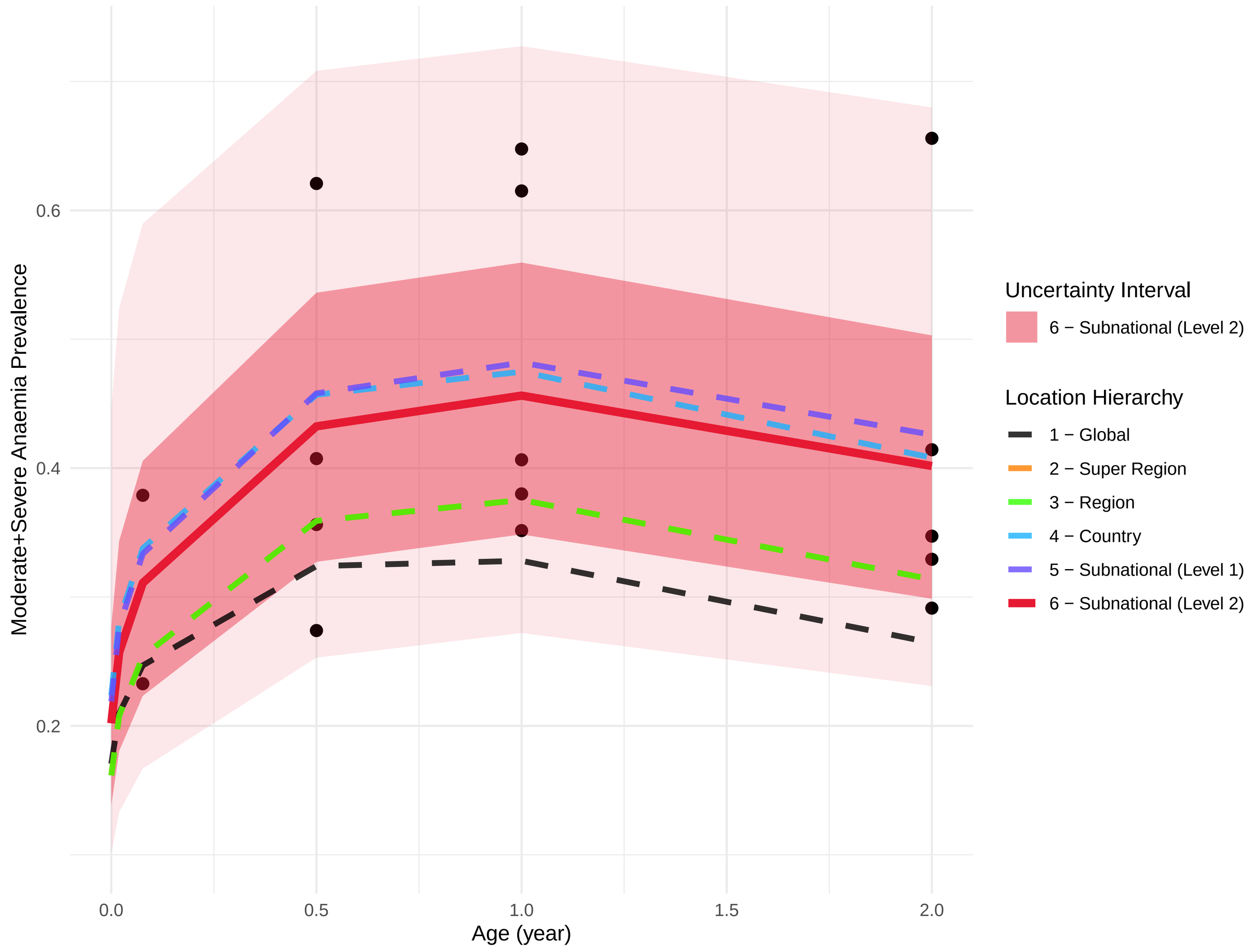

MR−BRT Age/Sex Fits − Males <2 Years Old − Moderate+Severe Anaemia Prevalence − Kerala
WHO−2001 Adjustment Method

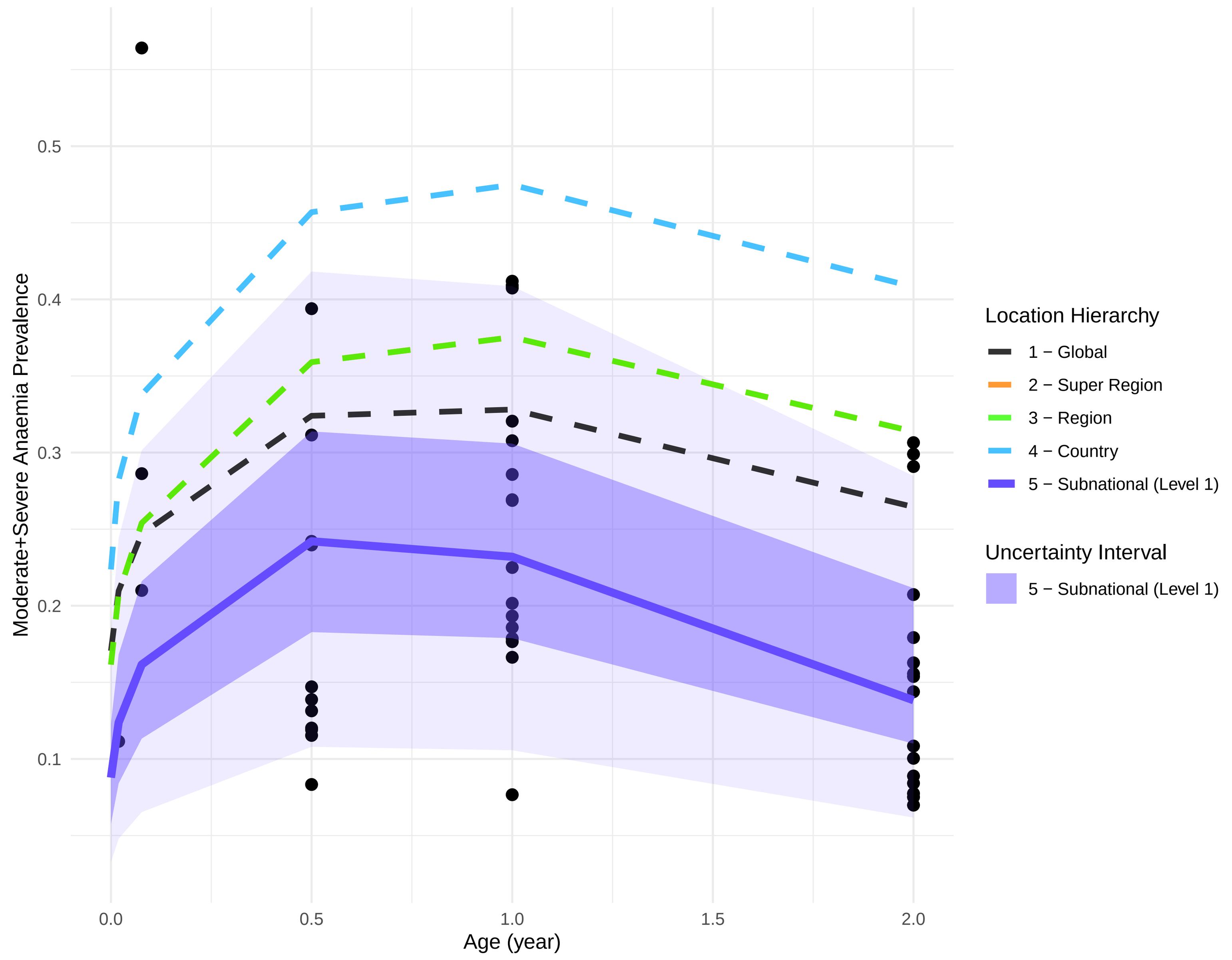

MR−BRT Age/Sex Fits − Males <2 Years Old − Moderate+Severe Anaemia Prevalence − Kerala, Rural
WHO−2001 Adjustment Method

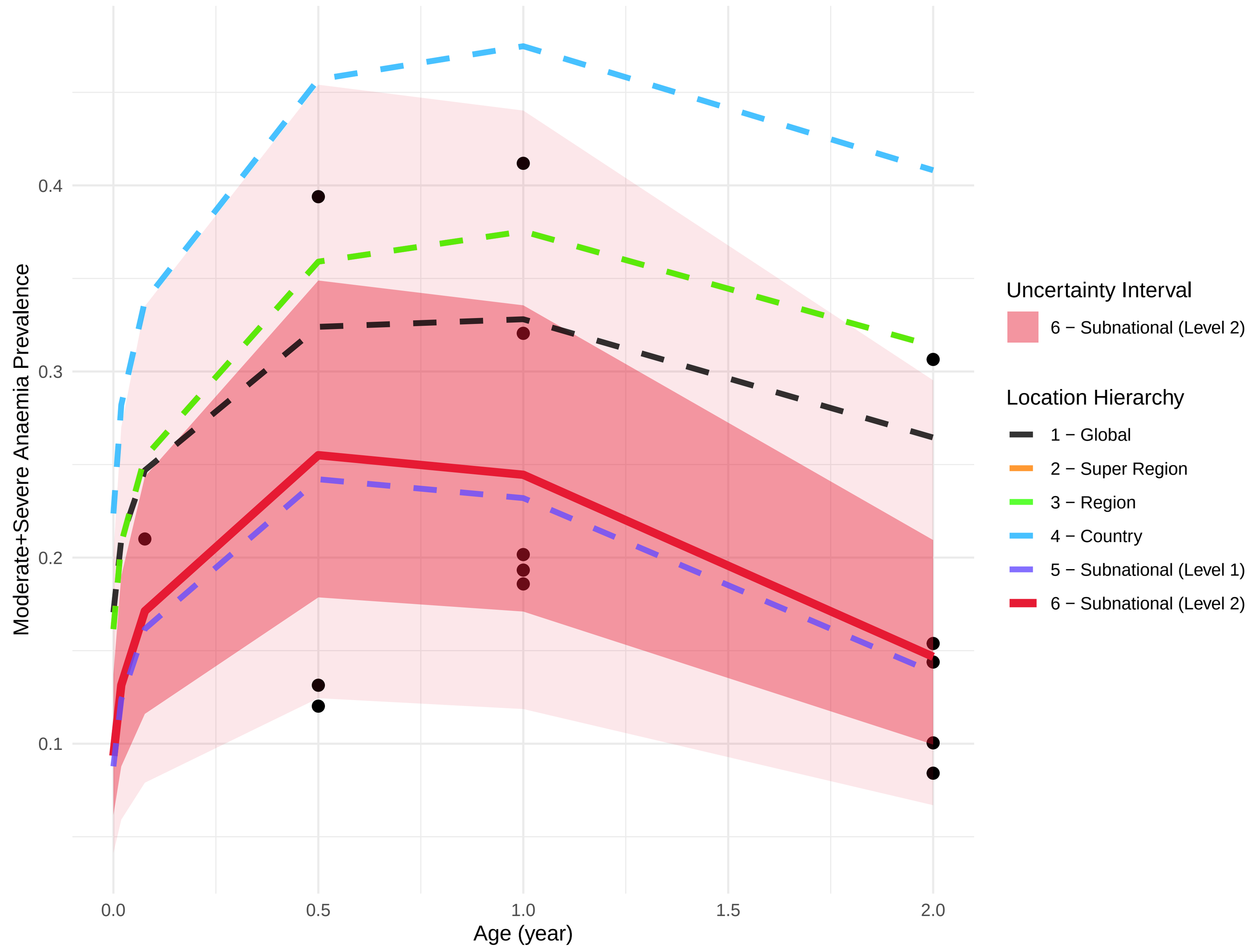

MR−BRT Age/Sex Fits − Males <2 Years Old − Moderate+Severe Anaemia Prevalence − Kerala, Urban
WHO−2001 Adjustment Method

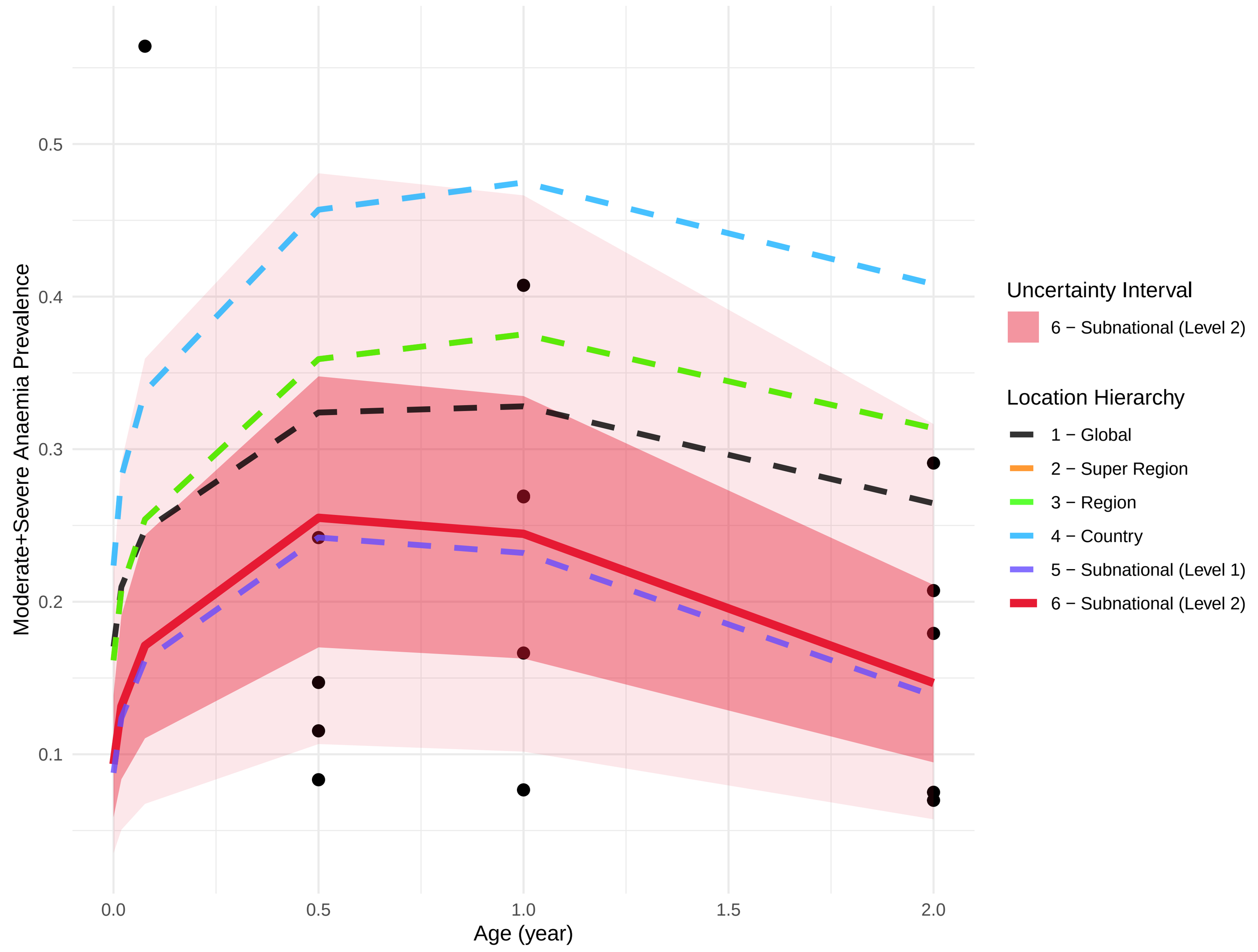

MR−BRT Age/Sex Fits − Males <2 Years Old − Moderate+Severe Anaemia Prevalence − Madhya Pradesh
WHO−2001 Adjustment Method

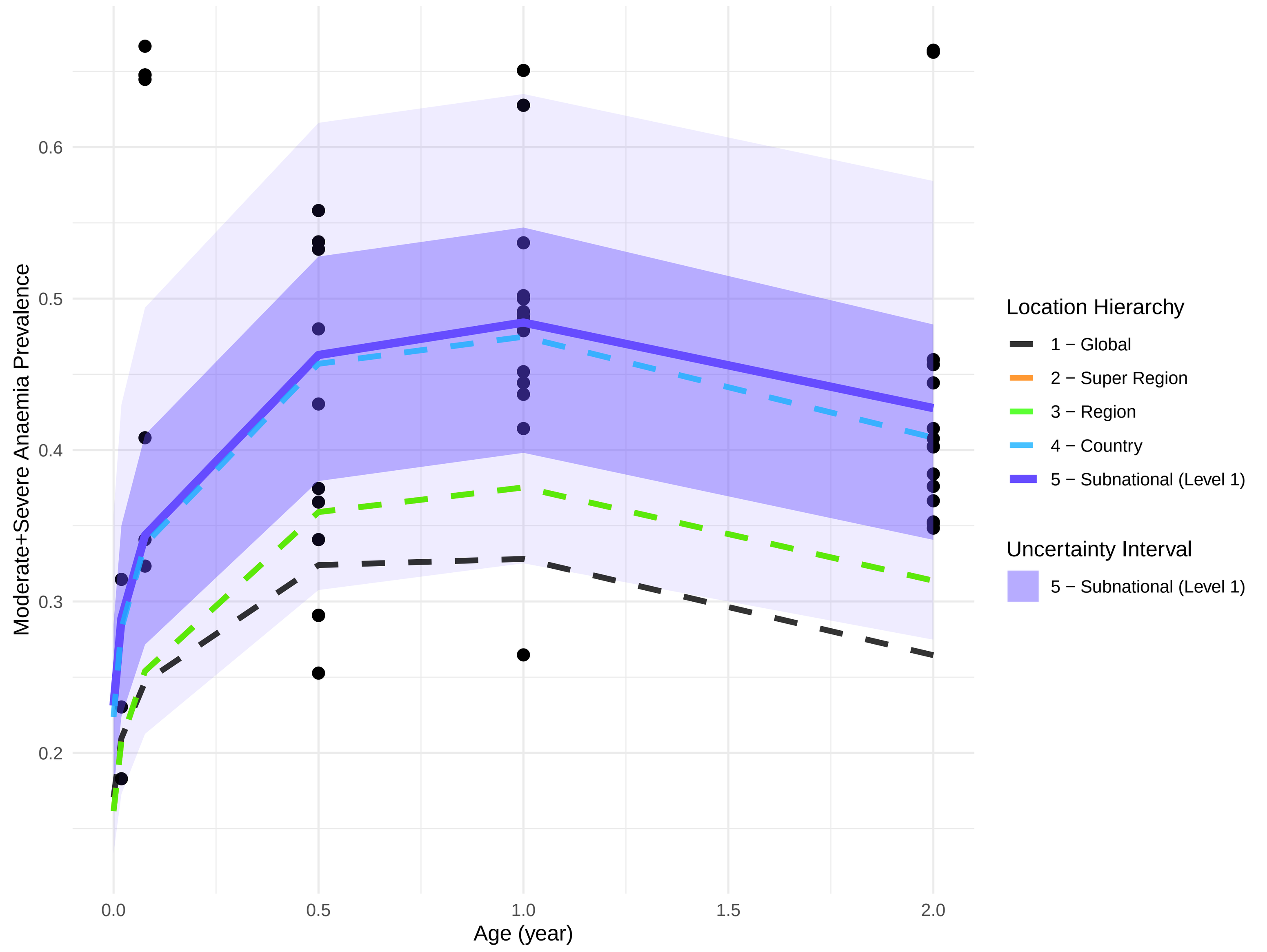

MR−BRT Age/Sex Fits − Males <2 Years Old − Moderate+Severe Anaemia Prevalence − Madhya Pradesh, Rural
WHO−2001 Adjustment Method

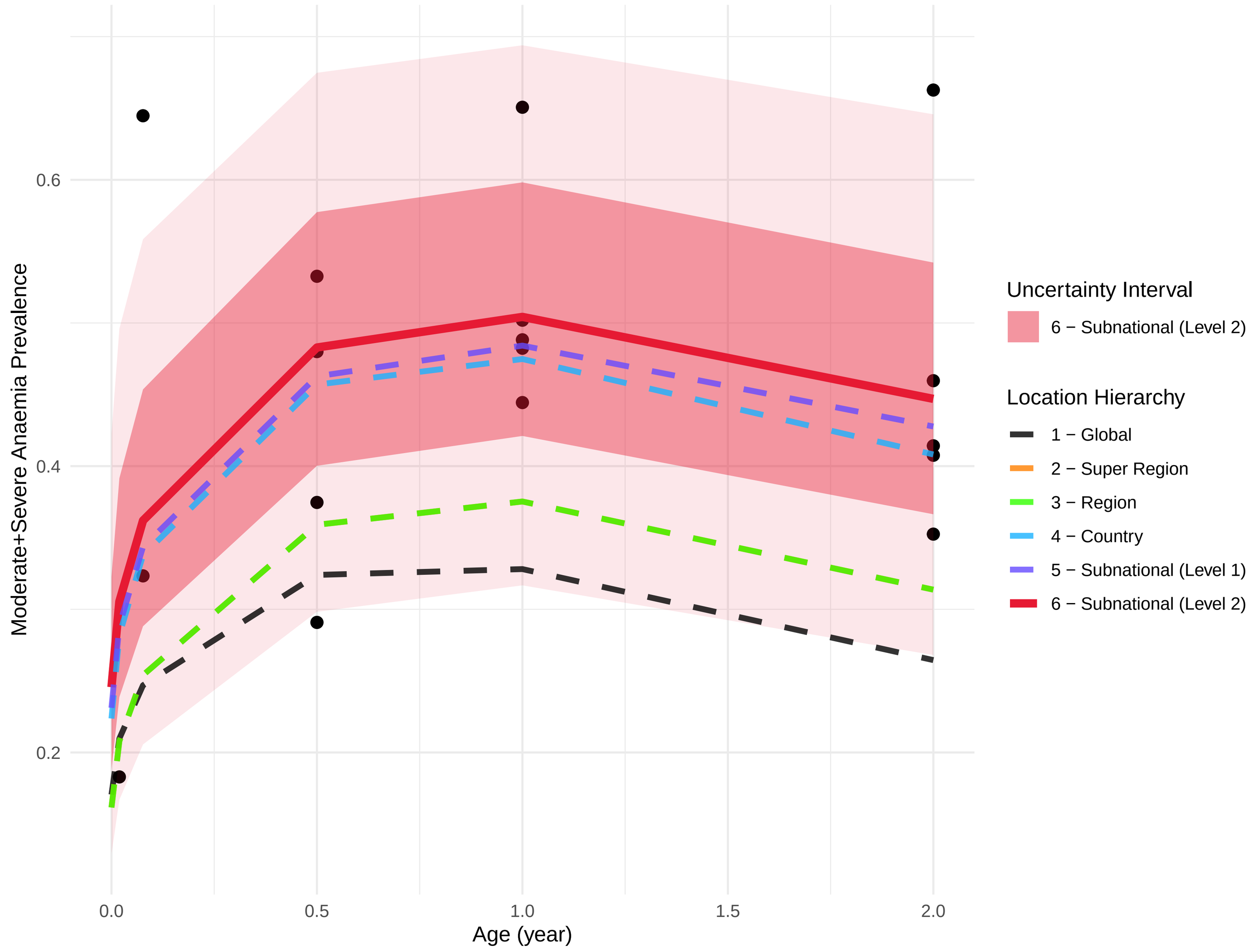

MR−BRT Age/Sex Fits − Males <2 Years Old − Moderate+Severe Anaemia Prevalence − Madhya Pradesh, Urban
WHO−2001 Adjustment Method

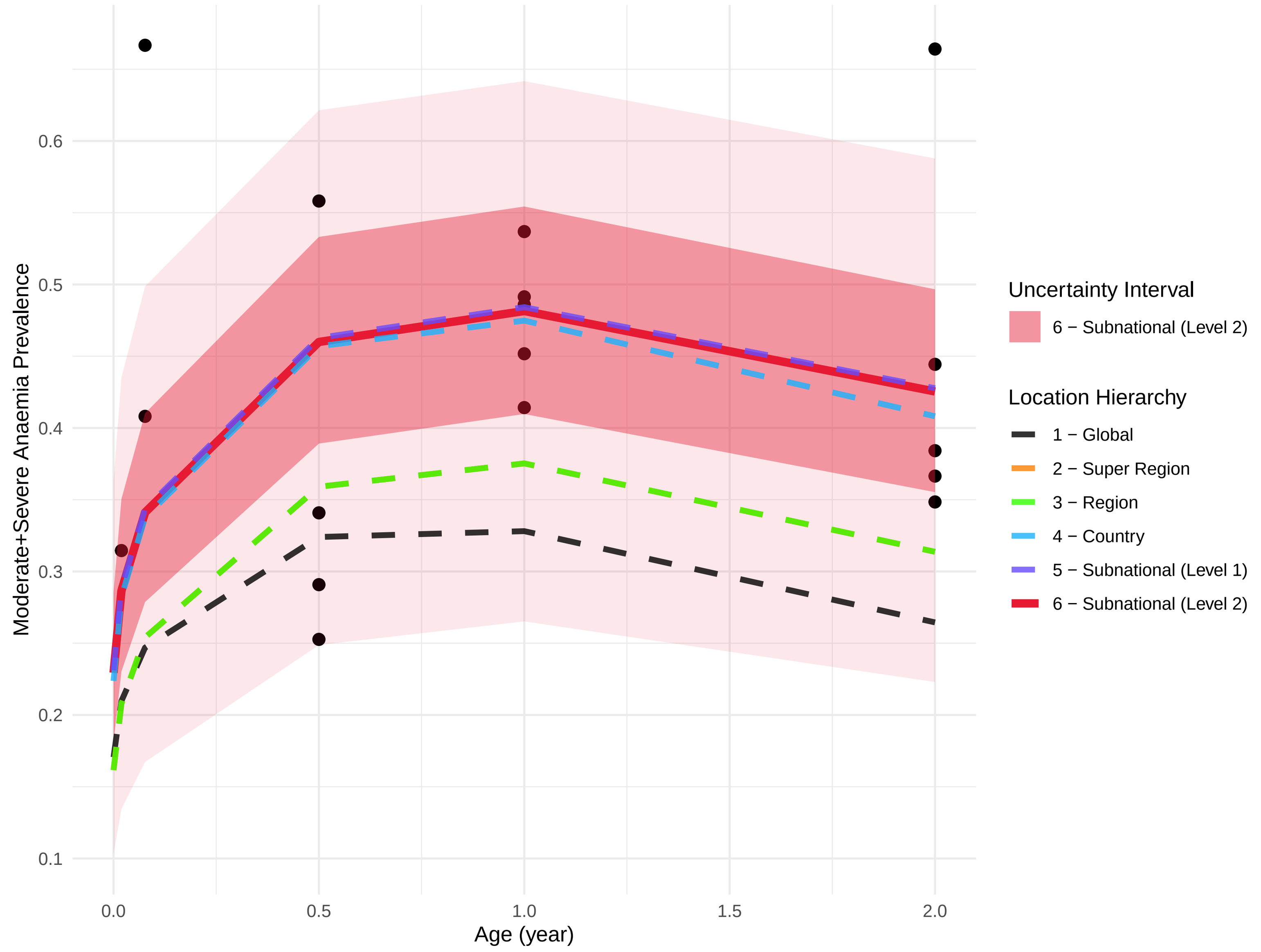

MR−BRT Age/Sex Fits − Males <2 Years Old − Moderate+Severe Anaemia Prevalence − Maharashtra
WHO−2001 Adjustment Method

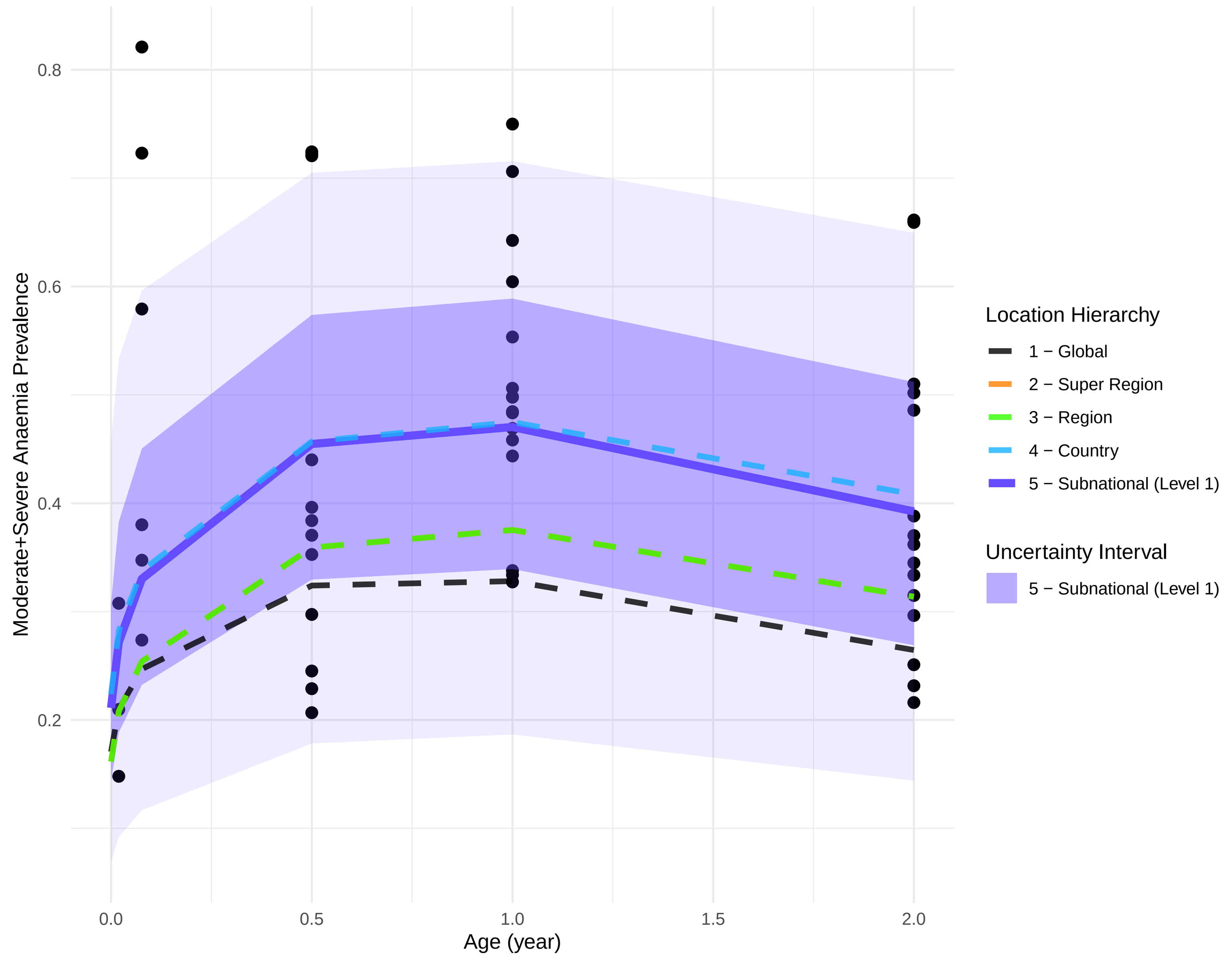

MR−BRT Age/Sex Fits − Males <2 Years Old − Moderate+Severe Anaemia Prevalence − Maharashtra, Rural
WHO−2001 Adjustment Method

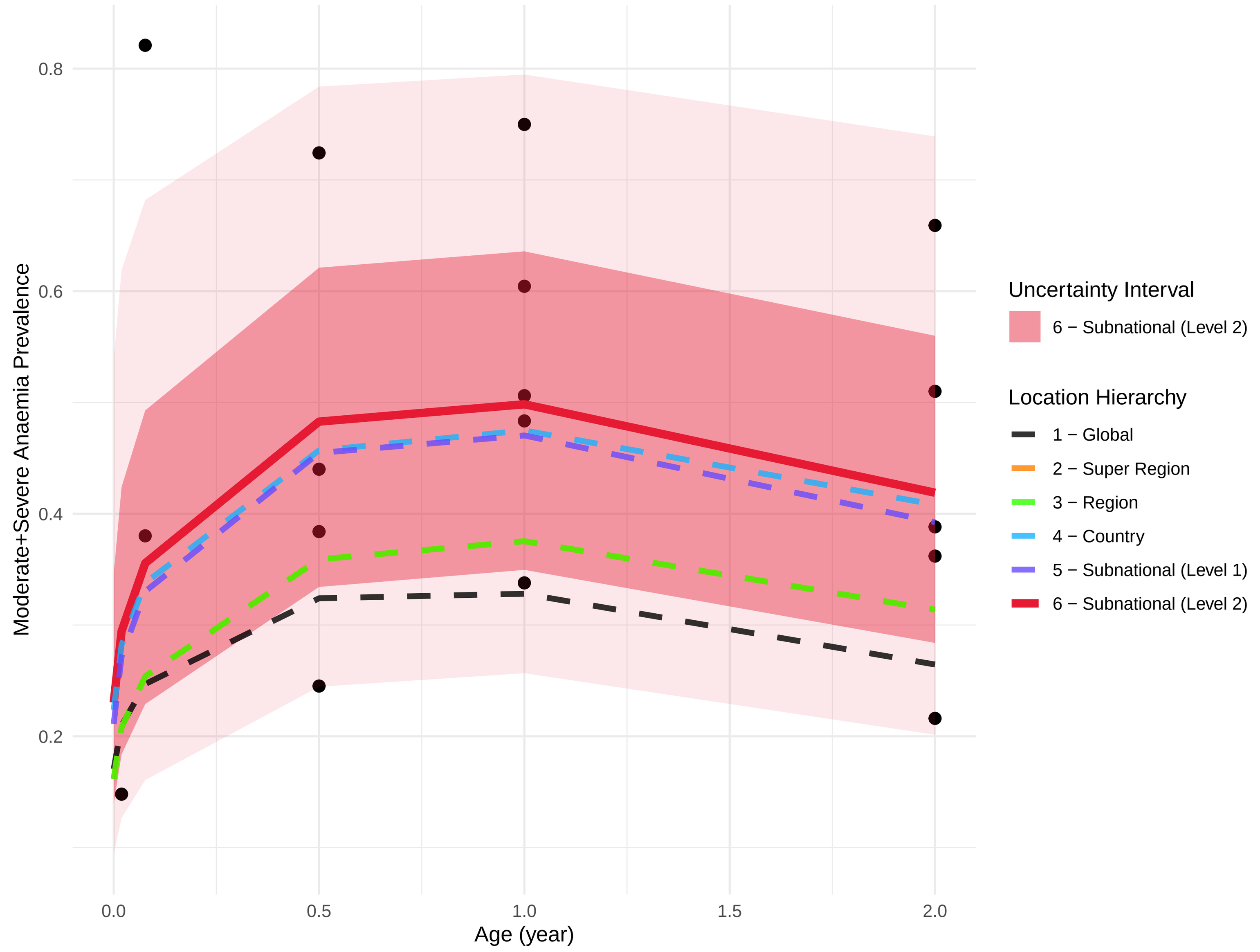

MR−BRT Age/Sex Fits − Males <2 Years Old − Moderate+Severe Anaemia Prevalence − Maharashtra, Urban
WHO−2001 Adjustment Method

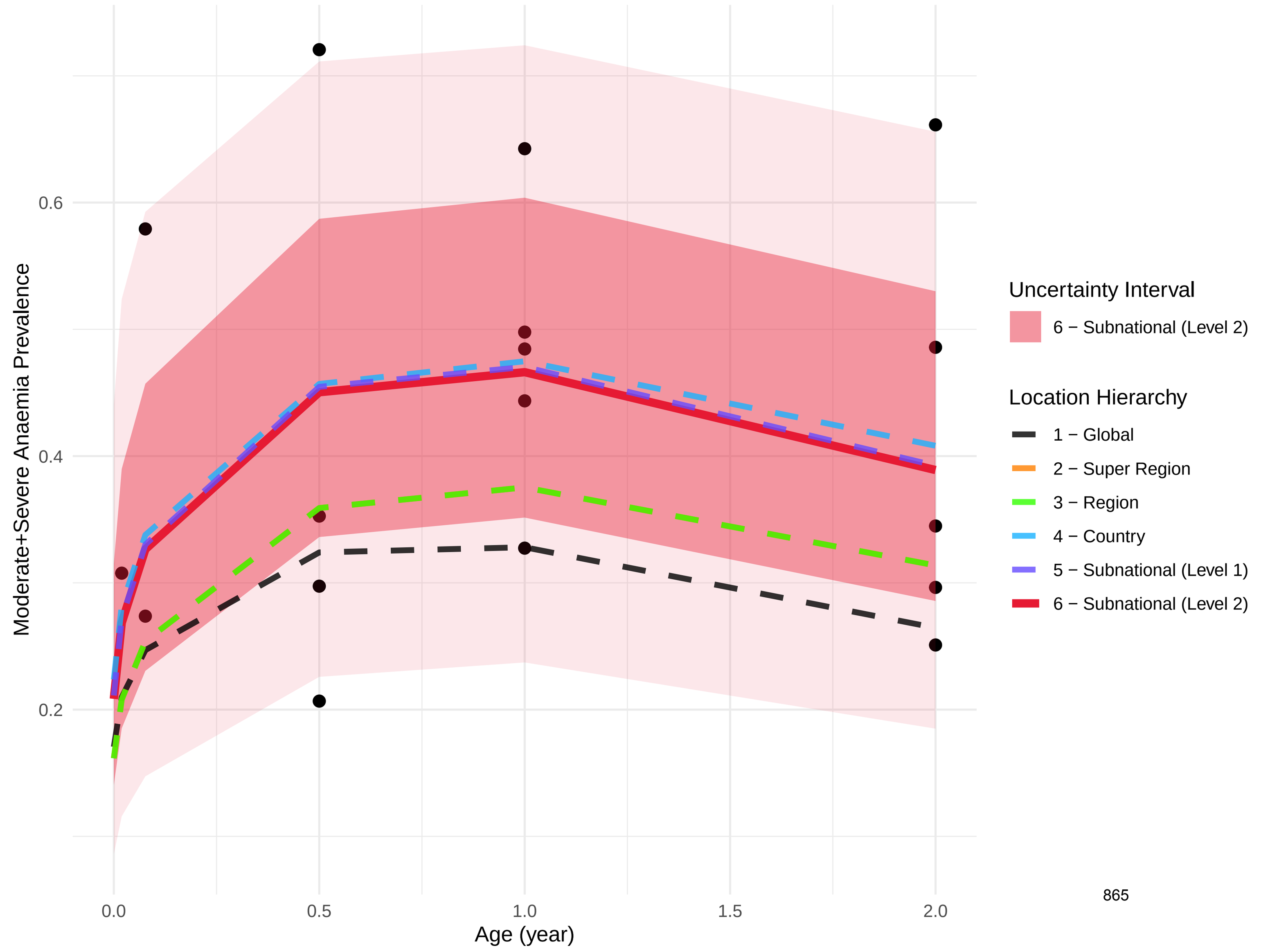

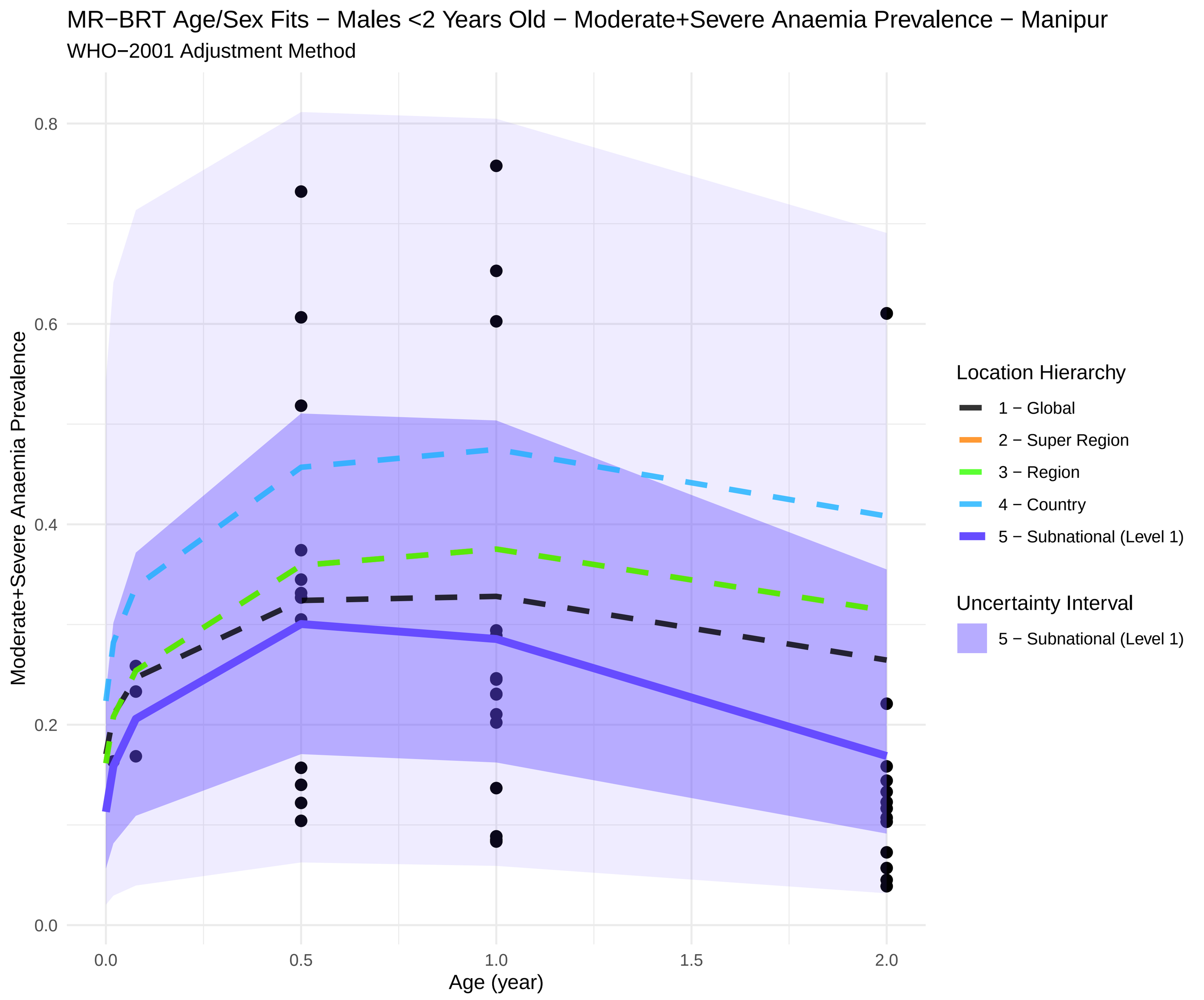
MR−BRT Age/Sex Fits − Males <2 Years Old − Moderate+Severe Anaemia Prevalence − Manipur
WHO−2001 Adjustment Method
Moderate+Severe Anaemia Prevalence
Age (year)
Location Hierarchy
1 − Global
2 − Super Region
3 − Region
4 − Country
5 − Subnational (Level 1)
Uncertainty Interval
5 − Subnational (Level 1)

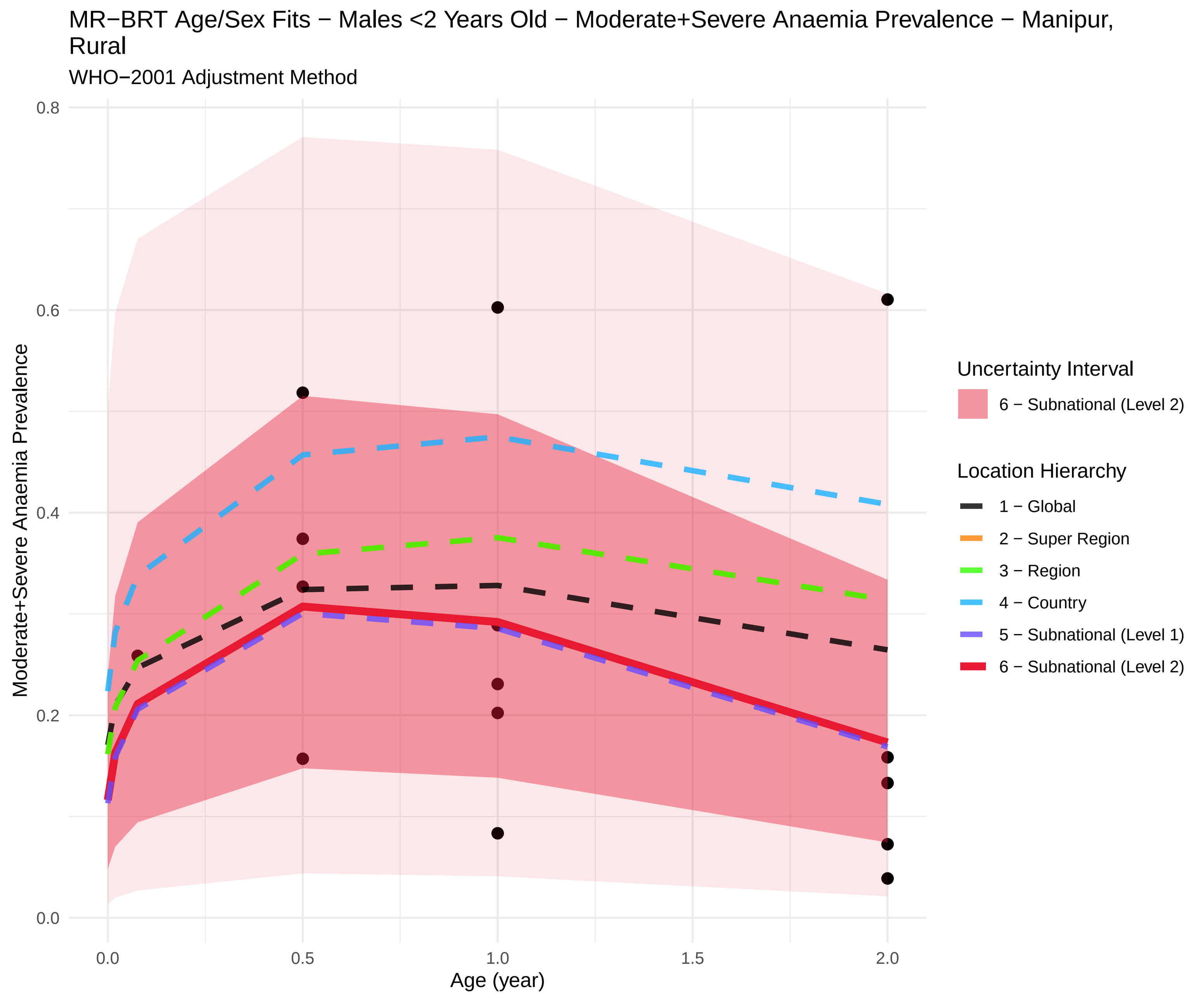
MR−BRT Age/Sex Fits − Males <2 Years Old − Moderate+Severe Anaemia Prevalence − Manipur, Rural
WHO−2001 Adjustment Method
Moderate+Severe Anaemia Prevalence
Age (year)
Uncertainty Interval
6 − Subnational (Level 2)
Location Hierarchy
1 − Global
2 − Super Region
3 − Region
4 − Country
5 − Subnational (Level 1)
6 − Subnational (Level 2)

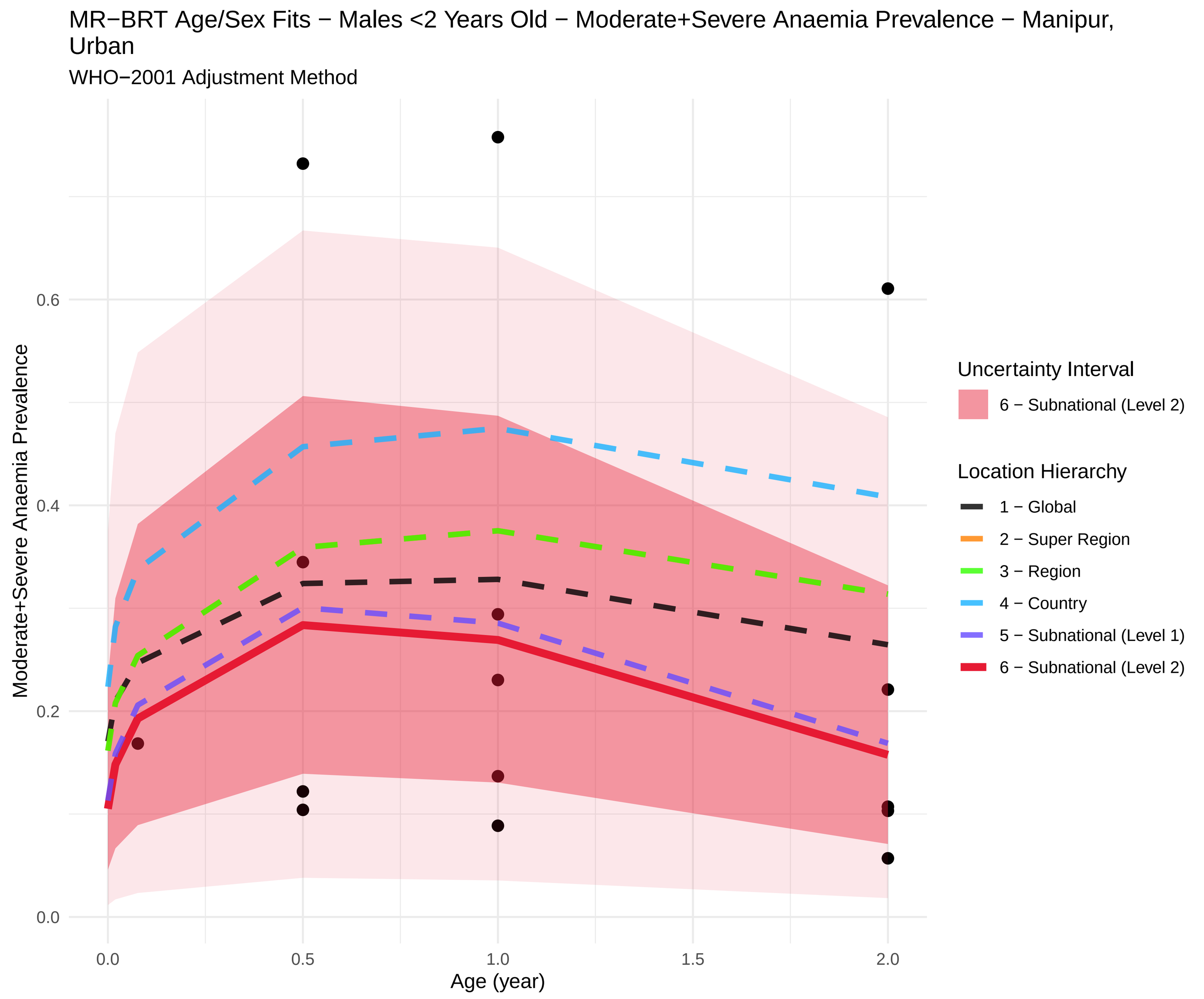
MR−BRT Age/Sex Fits − Males <2 Years Old − Moderate+Severe Anaemia Prevalence − Manipur, Urban
WHO−2001 Adjustment Method
Moderate+Severe Anaemia Prevalence
Age (year)
Uncertainty Interval
6 − Subnational (Level 2)
Location Hierarchy
1 − Global
2 − Super Region
3 − Region
4 − Country
5 − Subnational (Level 1)
6 − Subnational (Level 2)

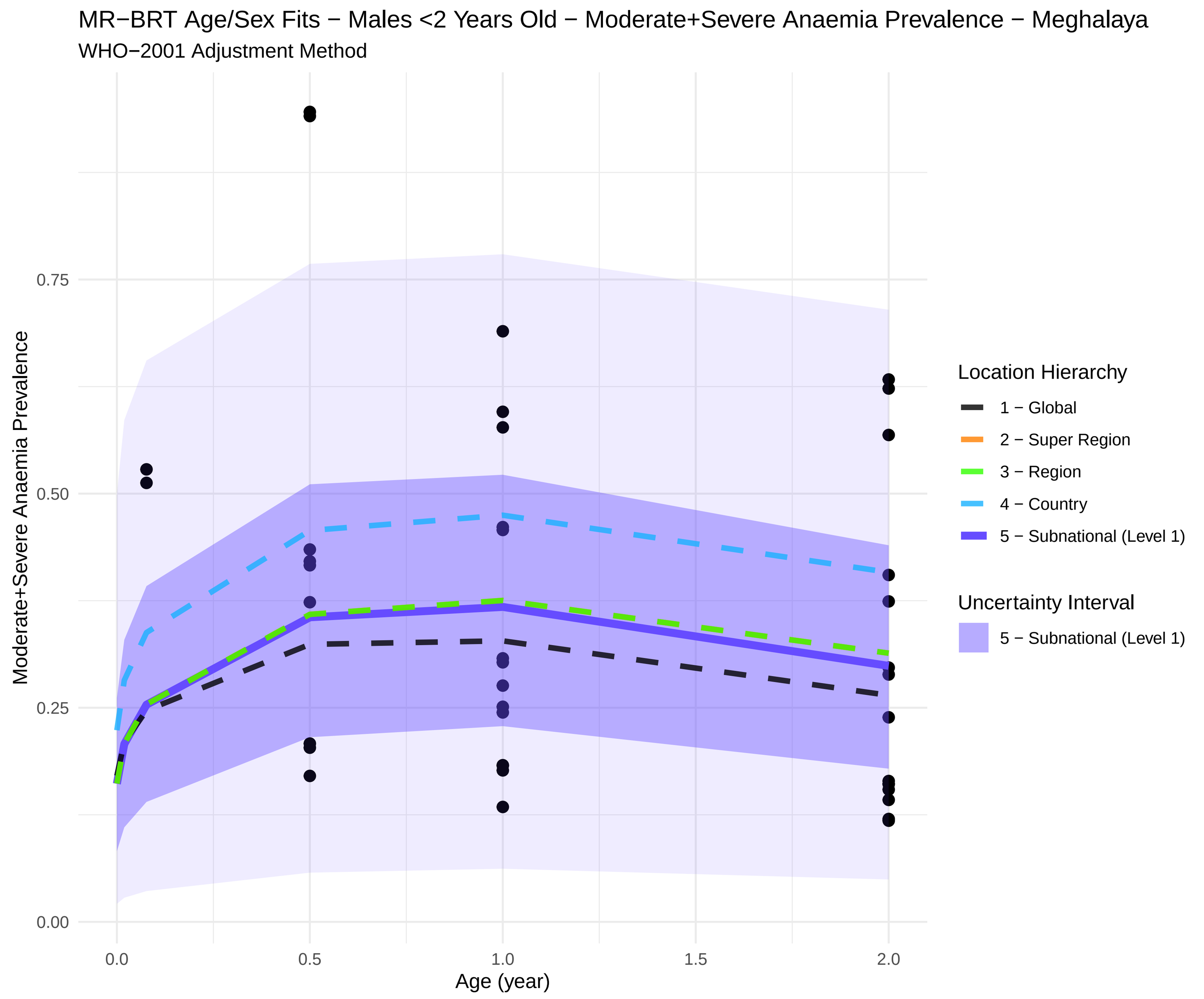
MR−BRT Age/Sex Fits − Males <2 Years Old − Moderate+Severe Anaemia Prevalence − Meghalaya
WHO−2001 Adjustment Method
Moderate+Severe Anaemia Prevalence
Age (year)
Location Hierarchy
1 − Global
2 − Super Region
3 − Region
4 − Country
5 − Subnational (Level 1)
Uncertainty Interval
5 − Subnational (Level 1)

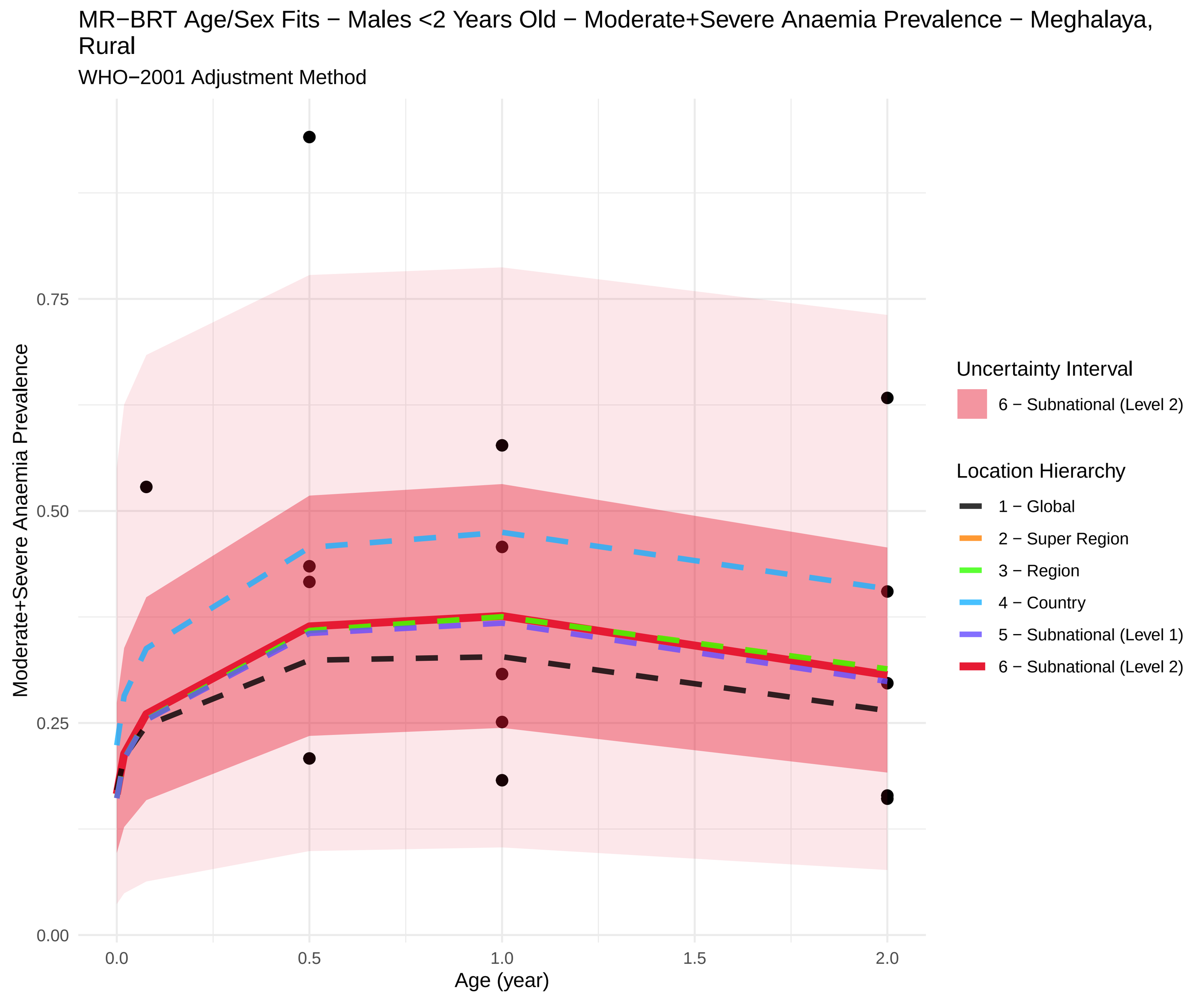
MR−BRT Age/Sex Fits − Males <2 Years Old − Moderate+Severe Anaemia Prevalence − Meghalaya, Rural
WHO−2001 Adjustment Method
Moderate+Severe Anaemia Prevalence
Age (year)
Uncertainty Interval
6 − Subnational (Level 2)
Location Hierarchy
1 − Global
2 − Super Region
3 − Region
4 − Country
5 − Subnational (Level 1)
6 − Subnational (Level 2)

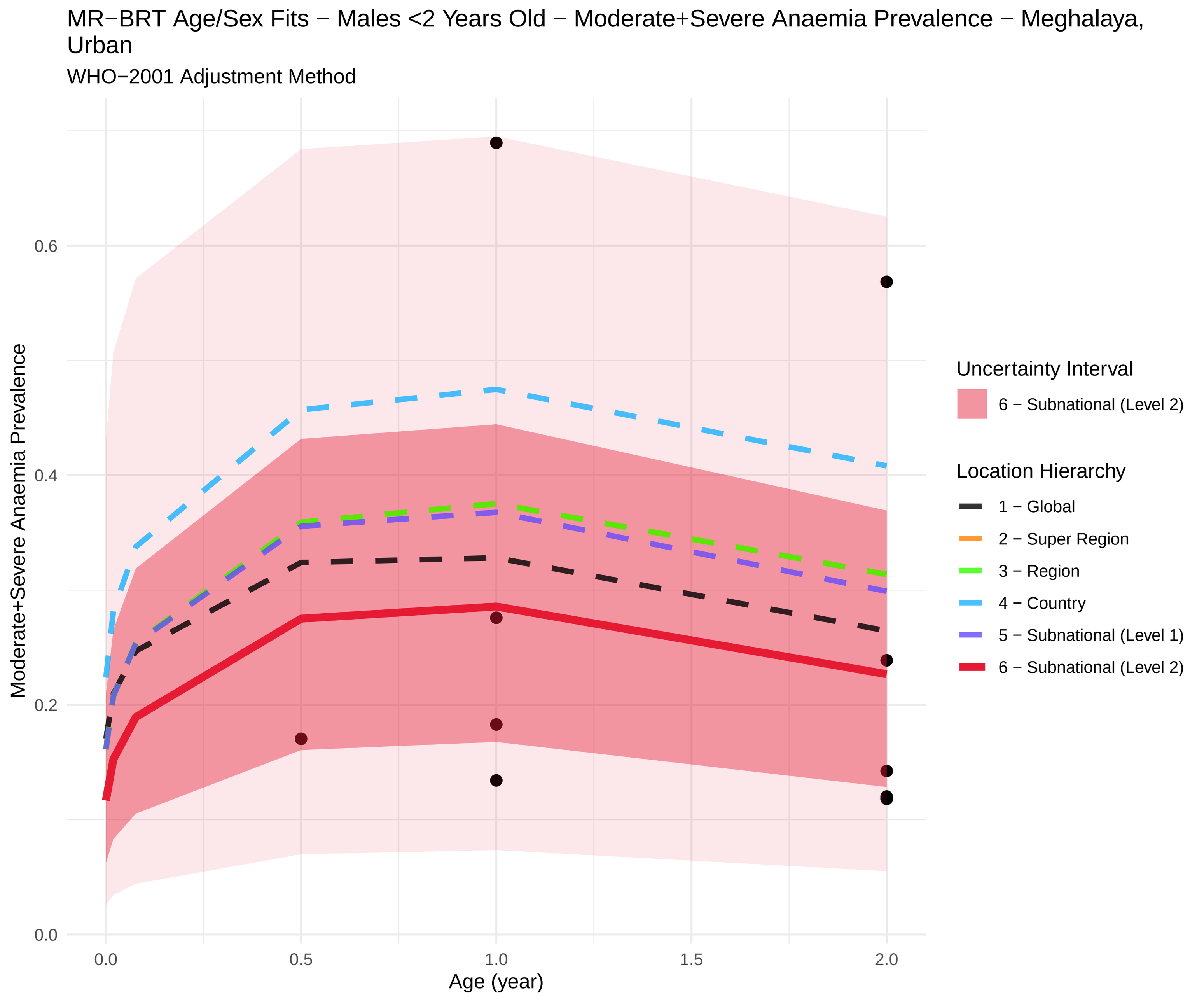
MR−BRT Age/Sex Fits − Males <2 Years Old − Moderate+Severe Anaemia Prevalence − Meghalaya, Urban
WHO−2001 Adjustment Method
Moderate+Severe Anaemia Prevalence
Age (year)
Uncertainty Interval
6 − Subnational (Level 2)
Location Hierarchy
1 − Global
2 − Super Region
3 − Region
4 − Country
5 − Subnational (Level 1)
6 − Subnational (Level 2)

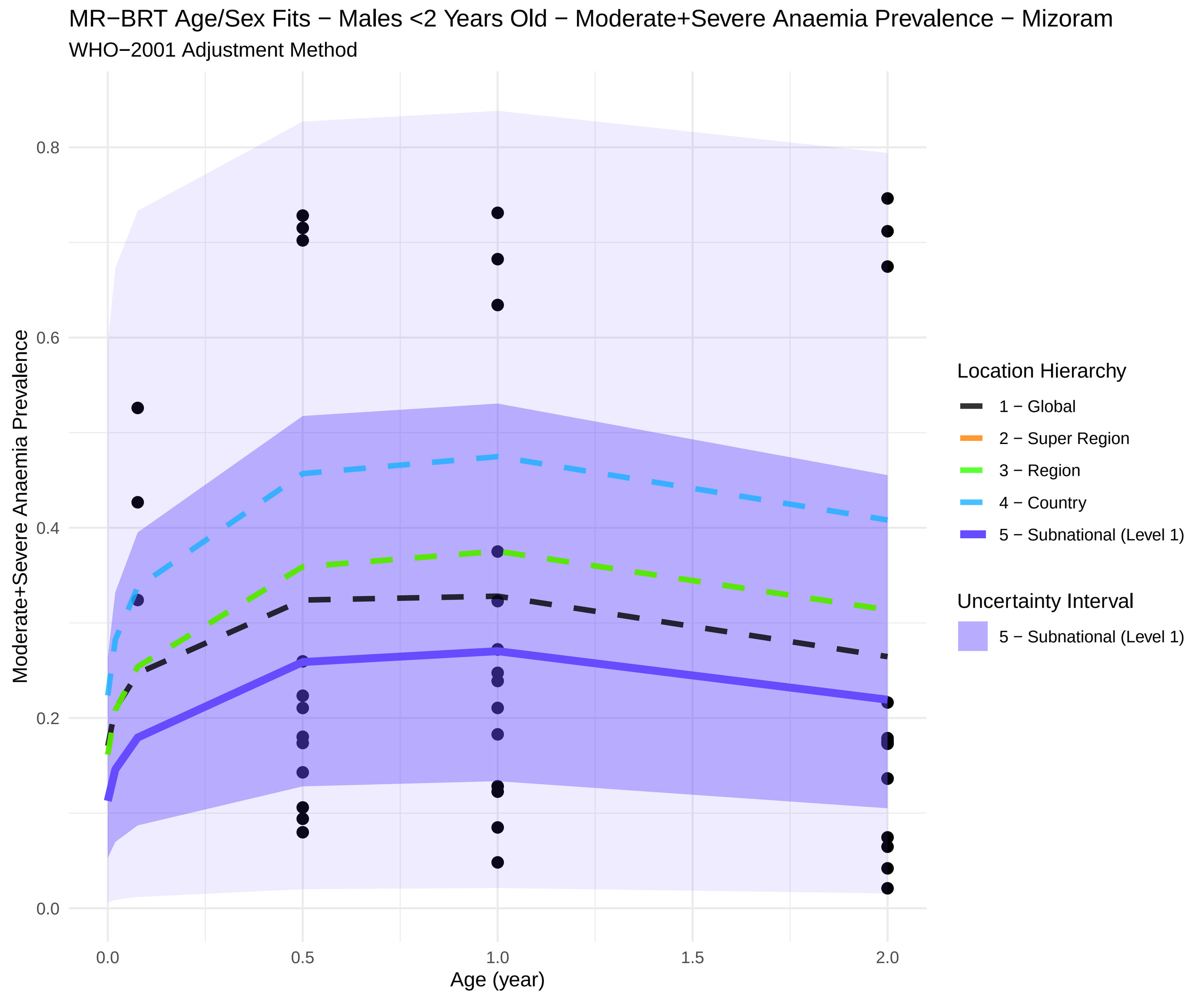
MR−BRT Age/Sex Fits − Males <2 Years Old − Moderate+Severe Anaemia Prevalence − Mizoram
WHO−2001 Adjustment Method
Moderate+Severe Anaemia Prevalence
Age (year)
Location Hierarchy
1 − Global
2 − Super Region
3 − Region
4 − Country
5 − Subnational (Level 1)
Uncertainty Interval
5 − Subnational (Level 1)

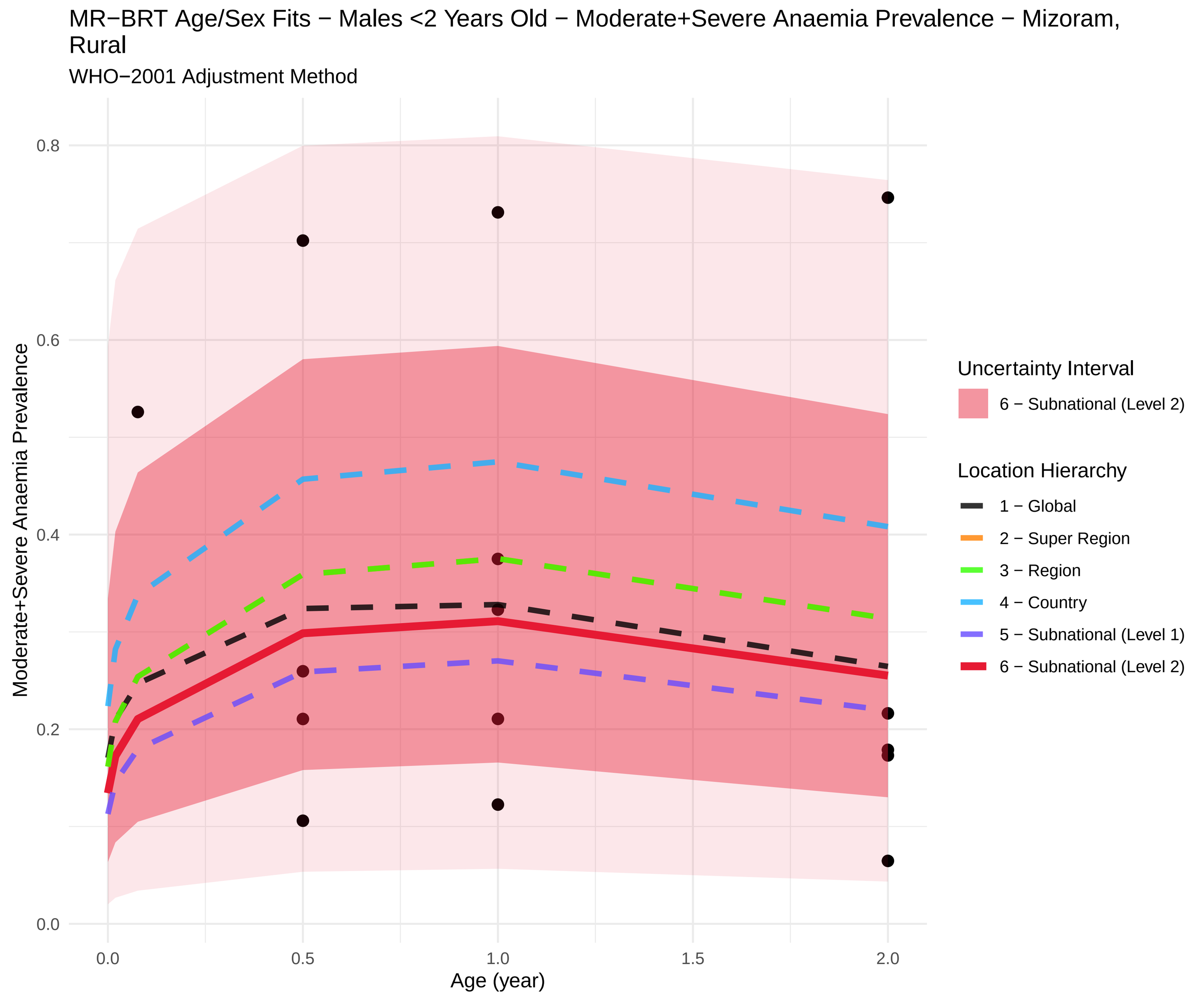
MR−BRT Age/Sex Fits − Males <2 Years Old − Moderate+Severe Anaemia Prevalence − Mizoram, Rural
WHO−2001 Adjustment Method
Moderate+Severe Anaemia Prevalence
Age (year)
Uncertainty Interval
6 − Subnational (Level 2)
Location Hierarchy
1 − Global
2 − Super Region
3 − Region
4 − Country
5 − Subnational (Level 1)
6 − Subnational (Level 2)

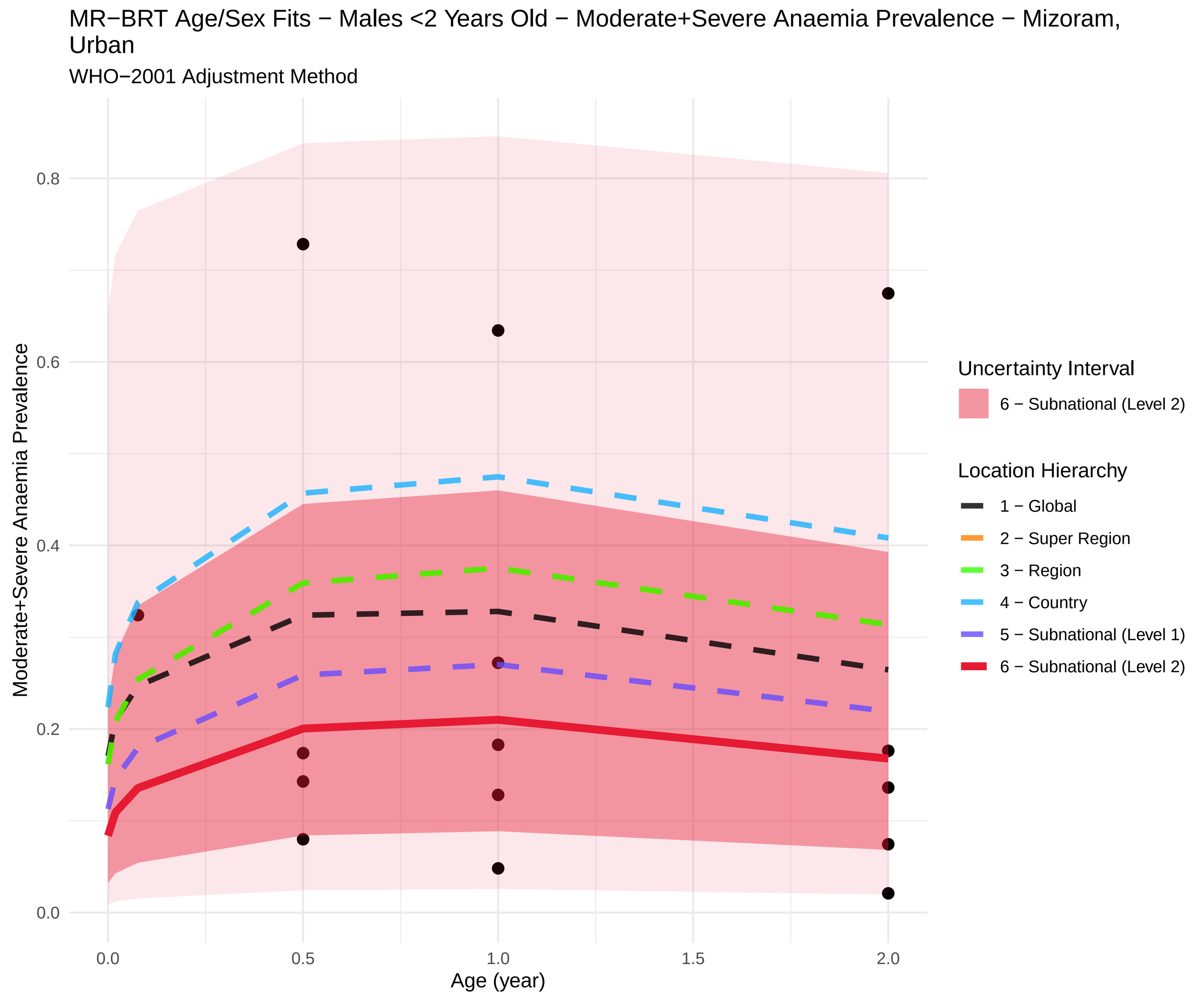
MR−BRT Age/Sex Fits − Males <2 Years Old − Moderate+Severe Anaemia Prevalence − Mizoram, Urban
WHO−2001 Adjustment Method
Moderate+Severe Anaemia Prevalence
Age (year)
Uncertainty Interval
6 − Subnational (Level 2)
Location Hierarchy
1 − Global
2 − Super Region
3 − Region
4 − Country
5 − Subnational (Level 1)
6 − Subnational (Level 2)

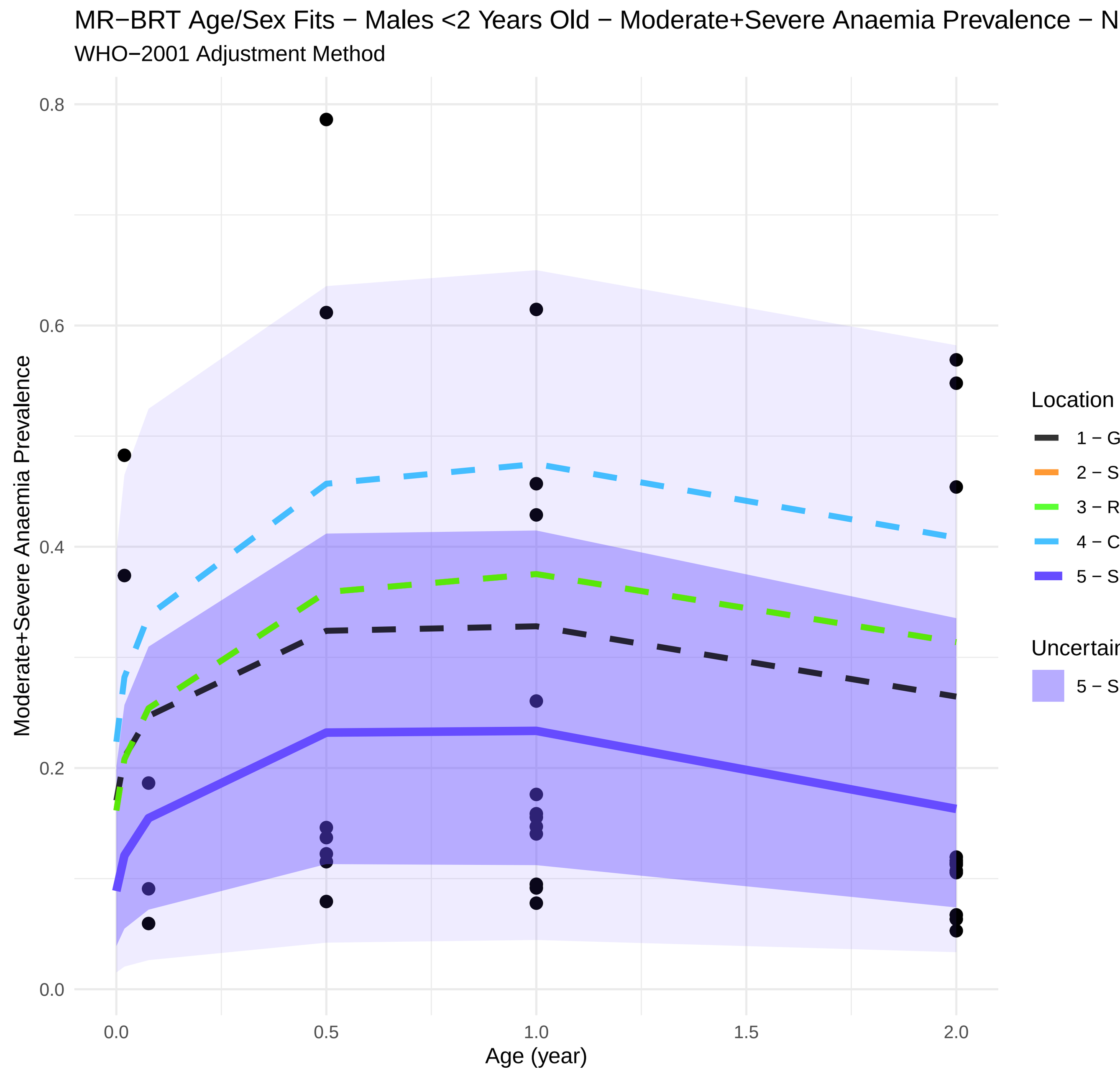
MR−BRT Age/Sex Fits − Males <2 Years Old − Moderate+Severe Anaemia Prevalence − Nagaland
WHO−2001 Adjustment Method
Moderate+Severe Anaemia Prevalence
Age (year)
Location Hierarchy
1 − Global
2 − Super Region
3 − Region
4 − Country
5 − Subnational (Level 1)
Uncertainty Interval
5 − Subnational (Level 1)

MR−BRT Age/Sex Fits − Males <2 Years Old − Moderate+Severe Anaemia Prevalence − Nagaland, Rural
WHO−2001 Adjustment Method

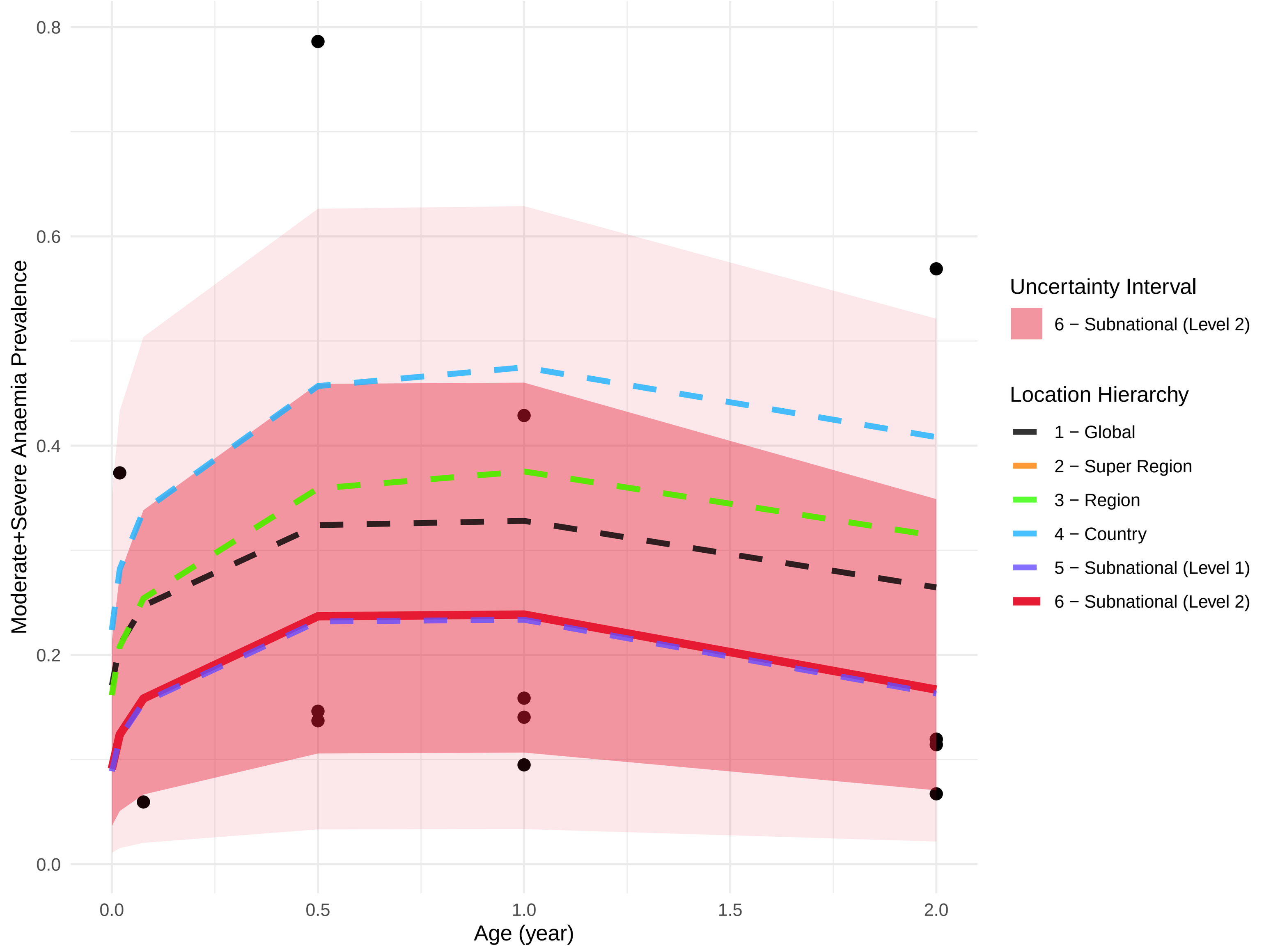

MR−BRT Age/Sex Fits − Males <2 Years Old − Moderate+Severe Anaemia Prevalence − Nagaland, Urban
WHO−2001 Adjustment Method

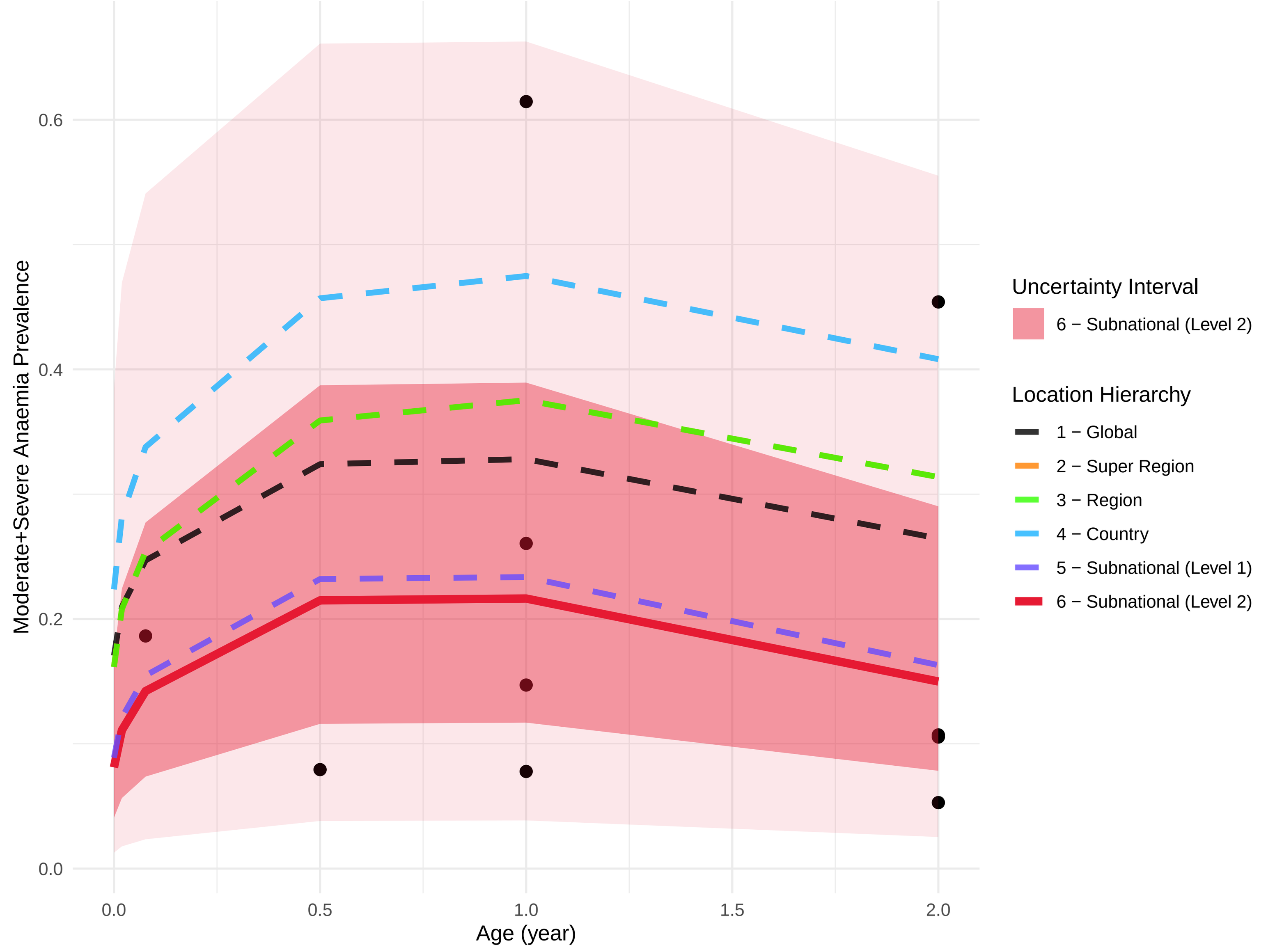

MR−BRT Age/Sex Fits − Males <2 Years Old − Moderate+Severe Anaemia Prevalence − Odisha
WHO−2001 Adjustment Method

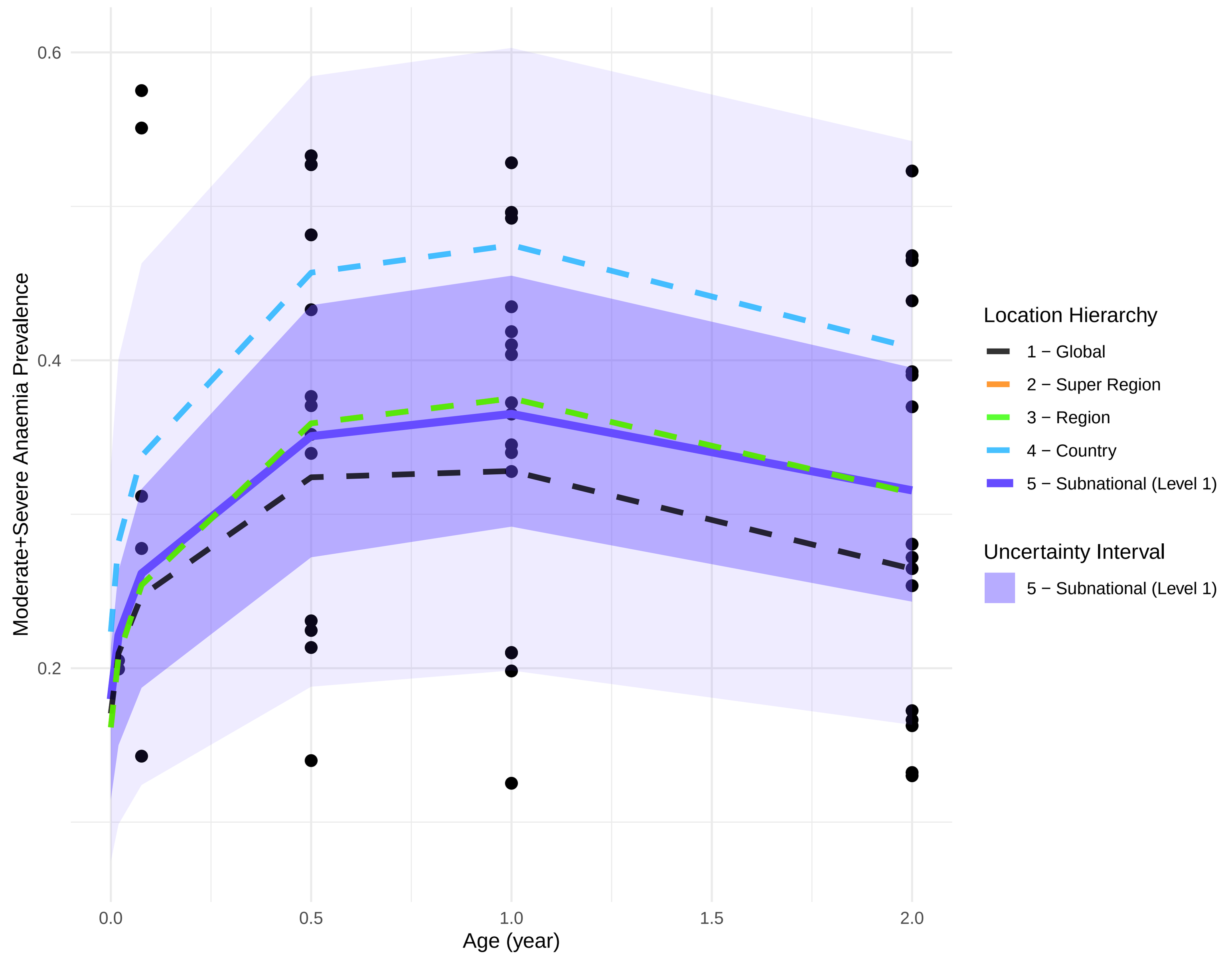

MR−BRT Age/Sex Fits − Males <2 Years Old − Moderate+Severe Anaemia Prevalence − Odisha, Rural
WHO−2001 Adjustment Method

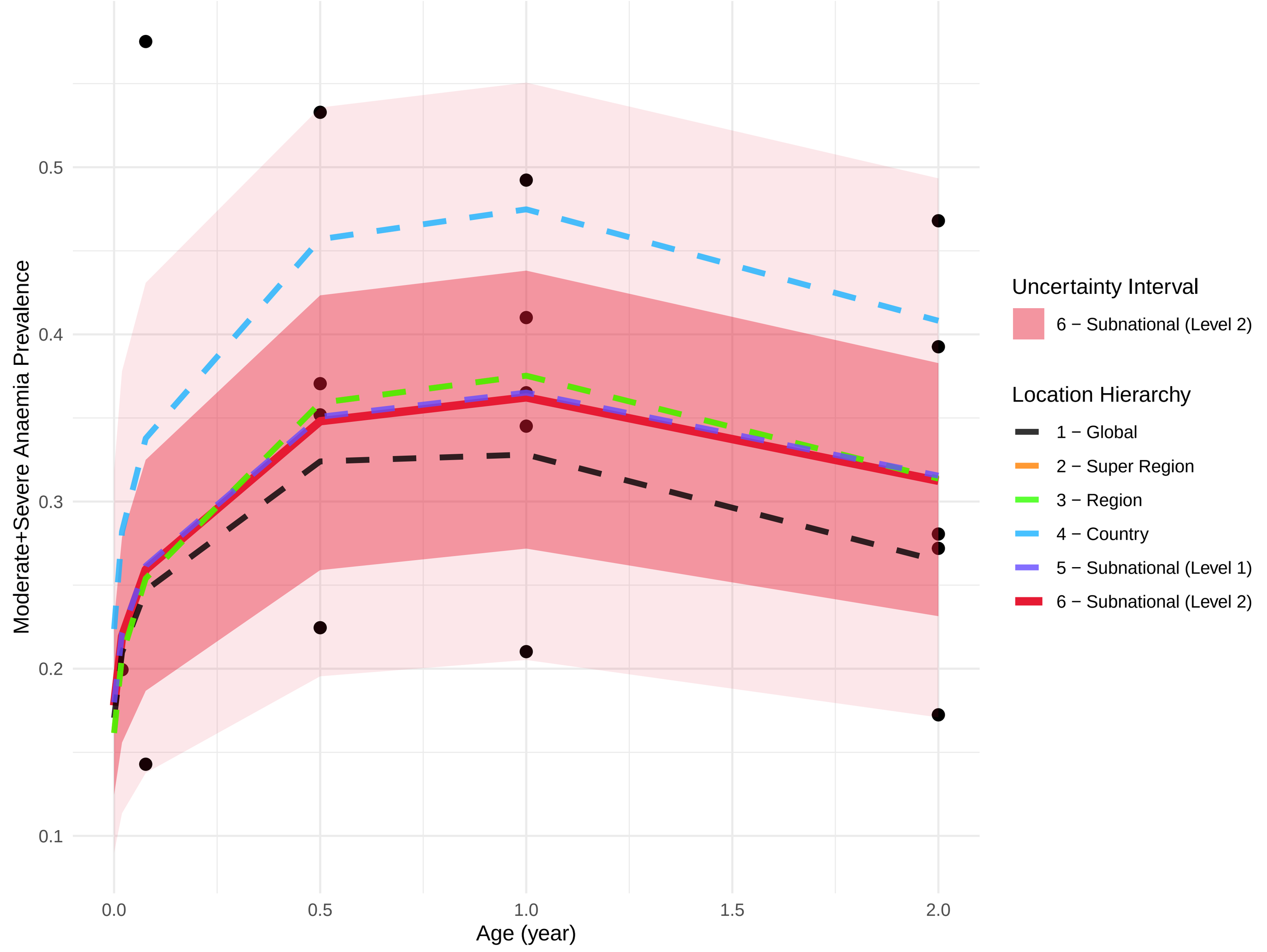

MR−BRT Age/Sex Fits − Males <2 Years Old − Moderate+Severe Anaemia Prevalence − Odisha, Urban
WHO−2001 Adjustment Method

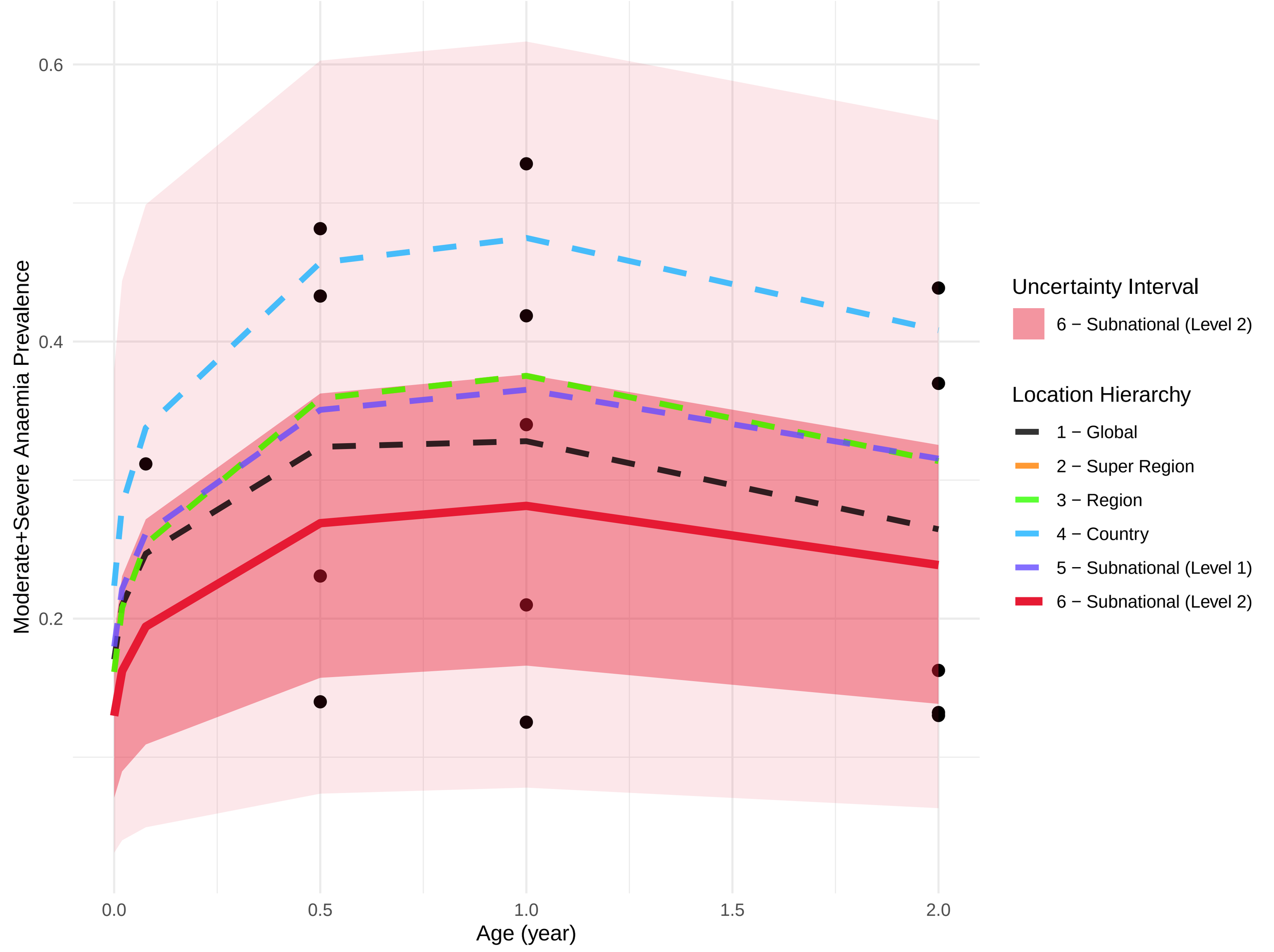

MR−BRT Age/Sex Fits − Males <2 Years Old − Moderate+Severe Anaemia Prevalence − Other Union Territories
WHO−2001 Adjustment Method

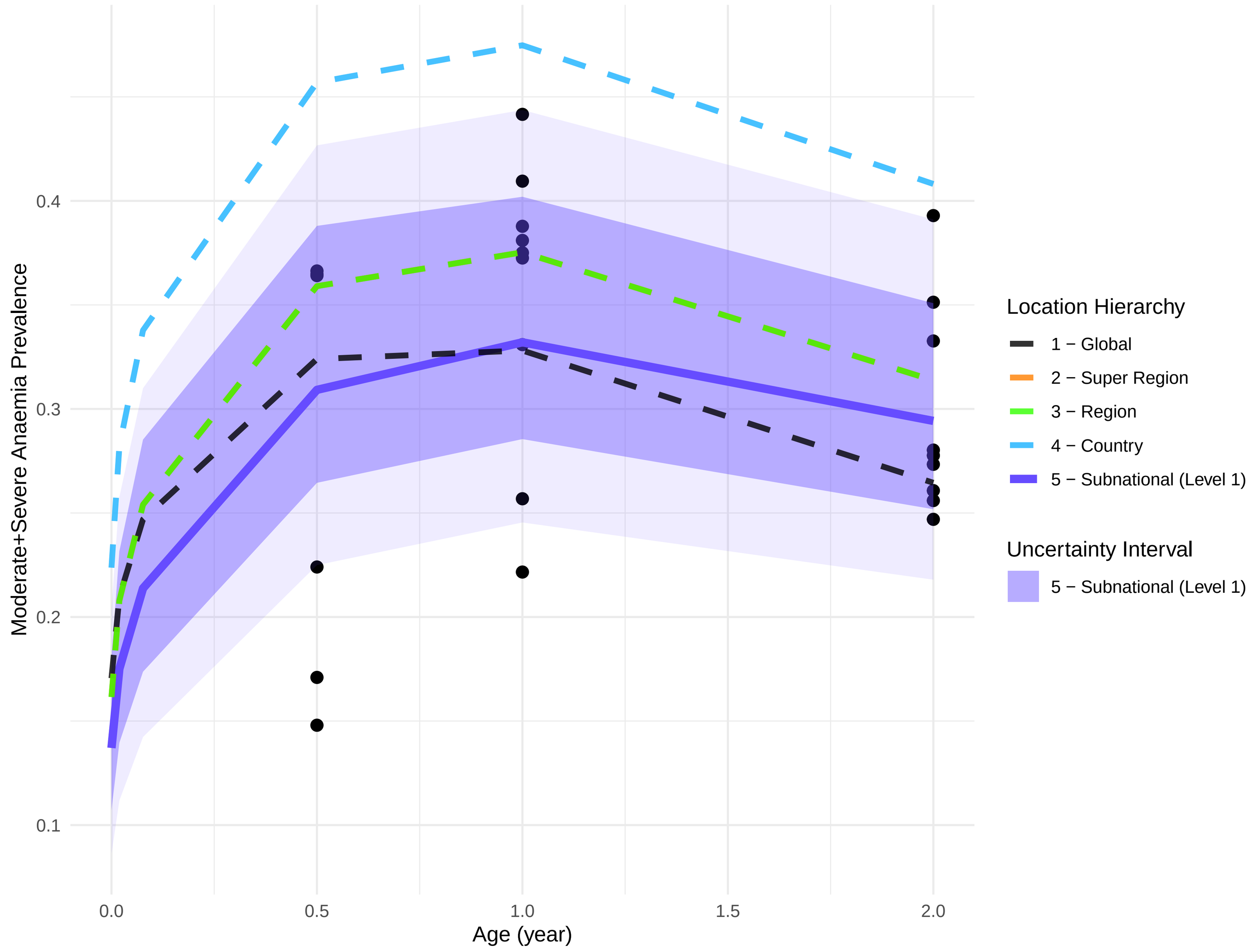

MR−BRT Age/Sex Fits − Males <2 Years Old − Moderate+Severe Anaemia Prevalence − Other Union Territories, Rural
WHO−2001 Adjustment Method

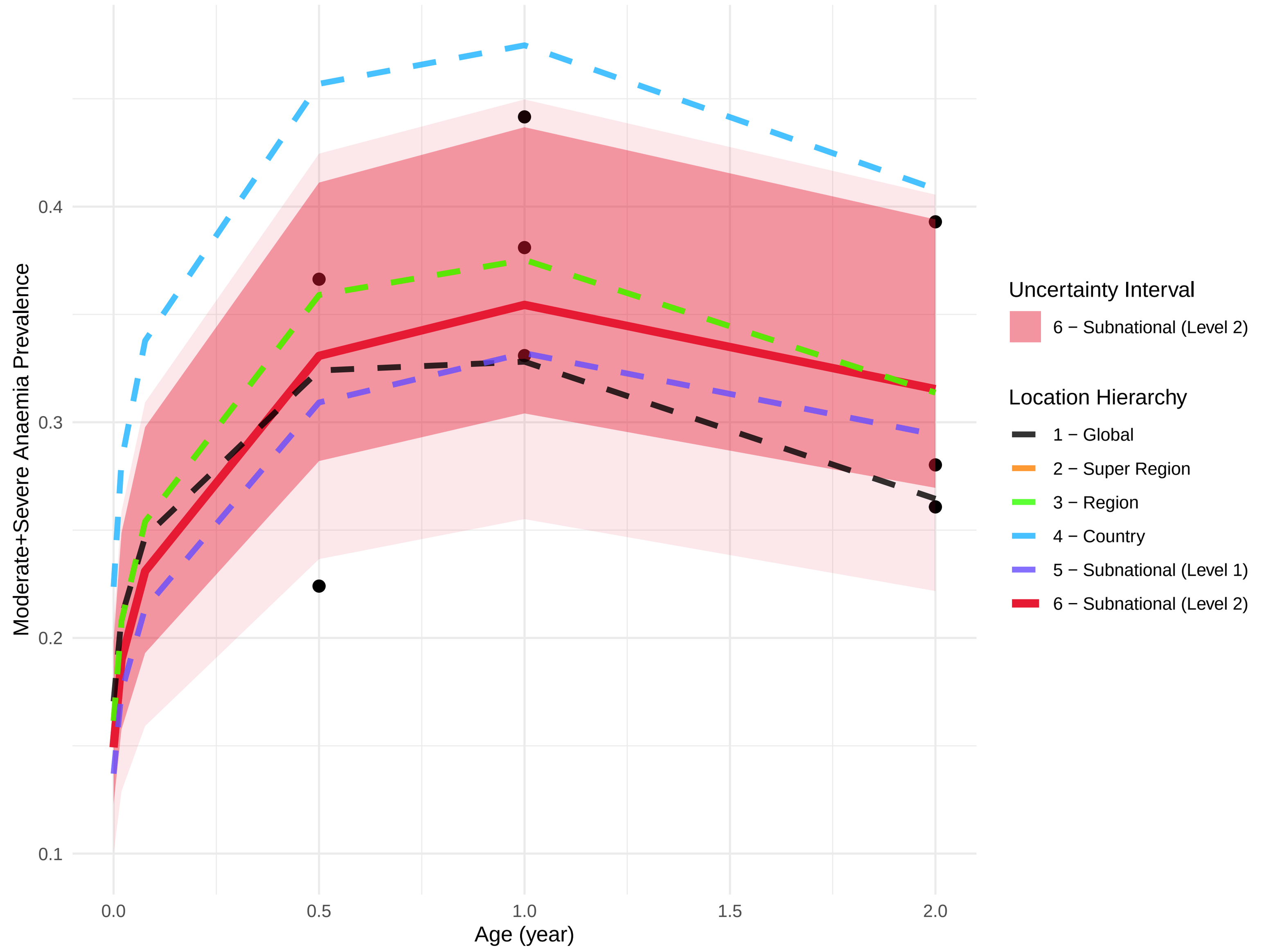

MR−BRT Age/Sex Fits − Males <2 Years Old − Moderate+Severe Anaemia Prevalence − Other Union Territories, Urban
WHO−2001 Adjustment Method

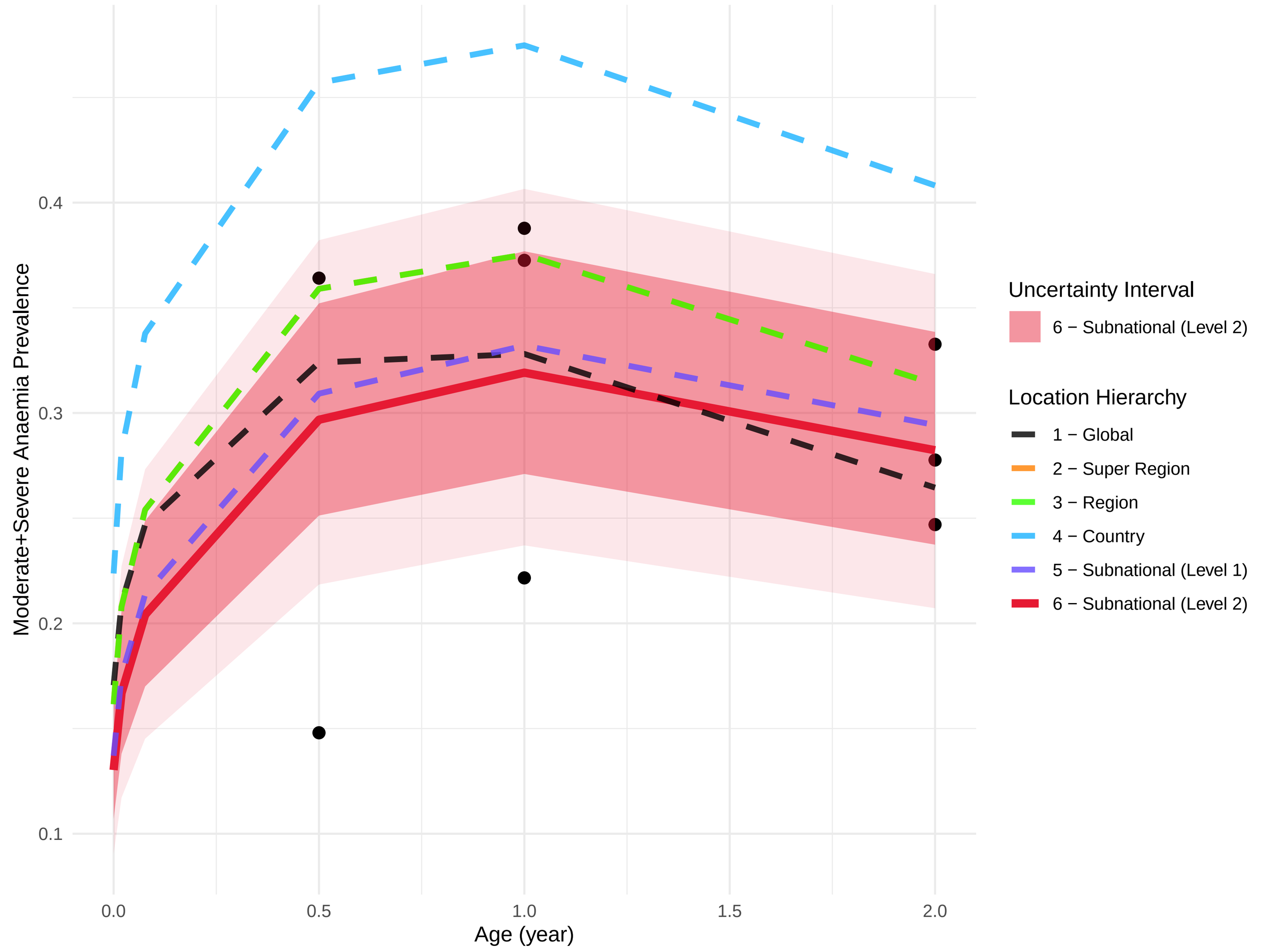

MR−BRT Age/Sex Fits − Males <2 Years Old − Moderate+Severe Anaemia Prevalence − Punjab
WHO−2001 Adjustment Method

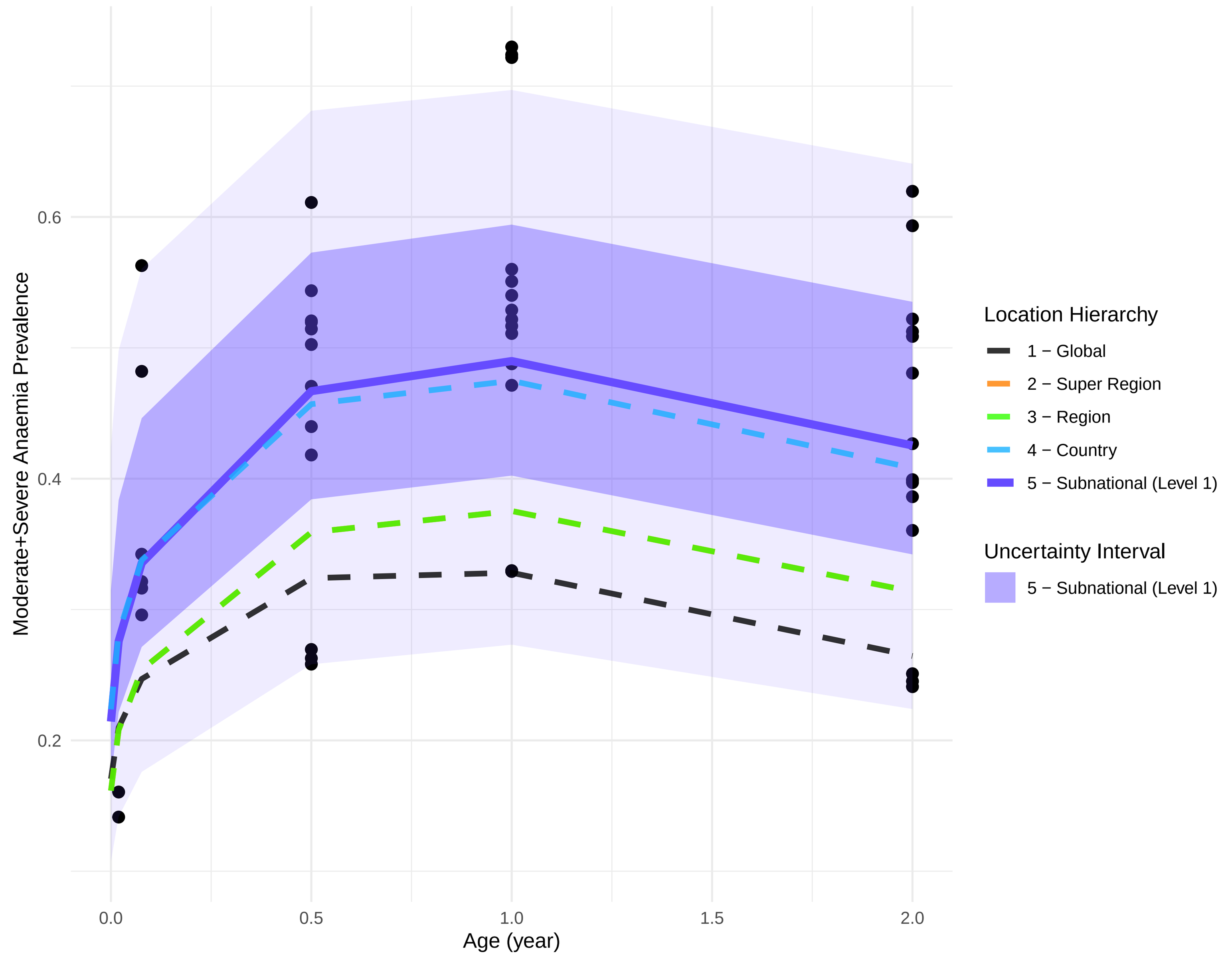

MR−BRT Age/Sex Fits − Males <2 Years Old − Moderate+Severe Anaemia Prevalence − Punjab, Rural
WHO−2001 Adjustment Method

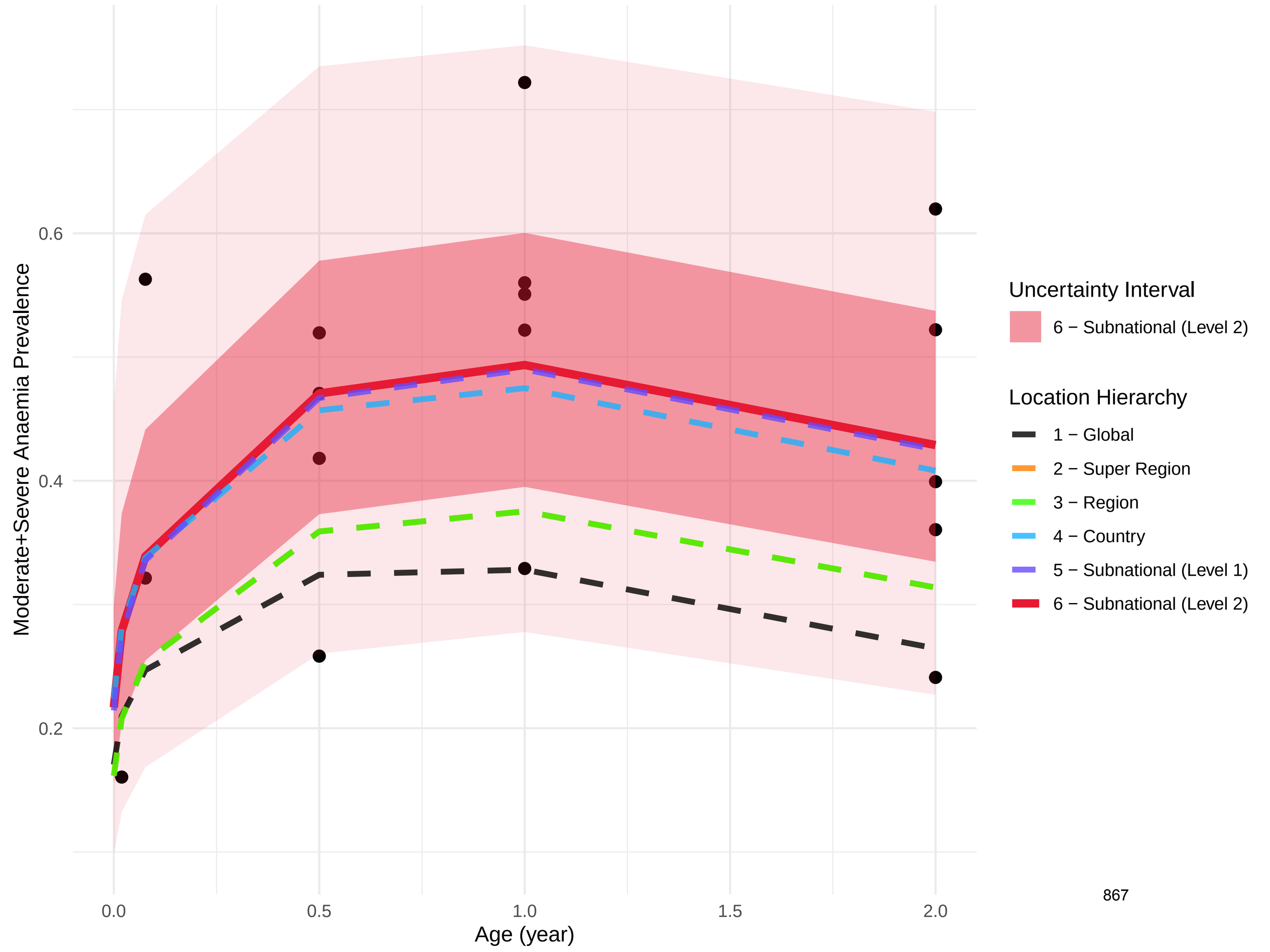

MR−BRT Age/Sex Fits − Males <2 Years Old − Moderate+Severe Anaemia Prevalence − Punjab, Urban
WHO−2001 Adjustment Method

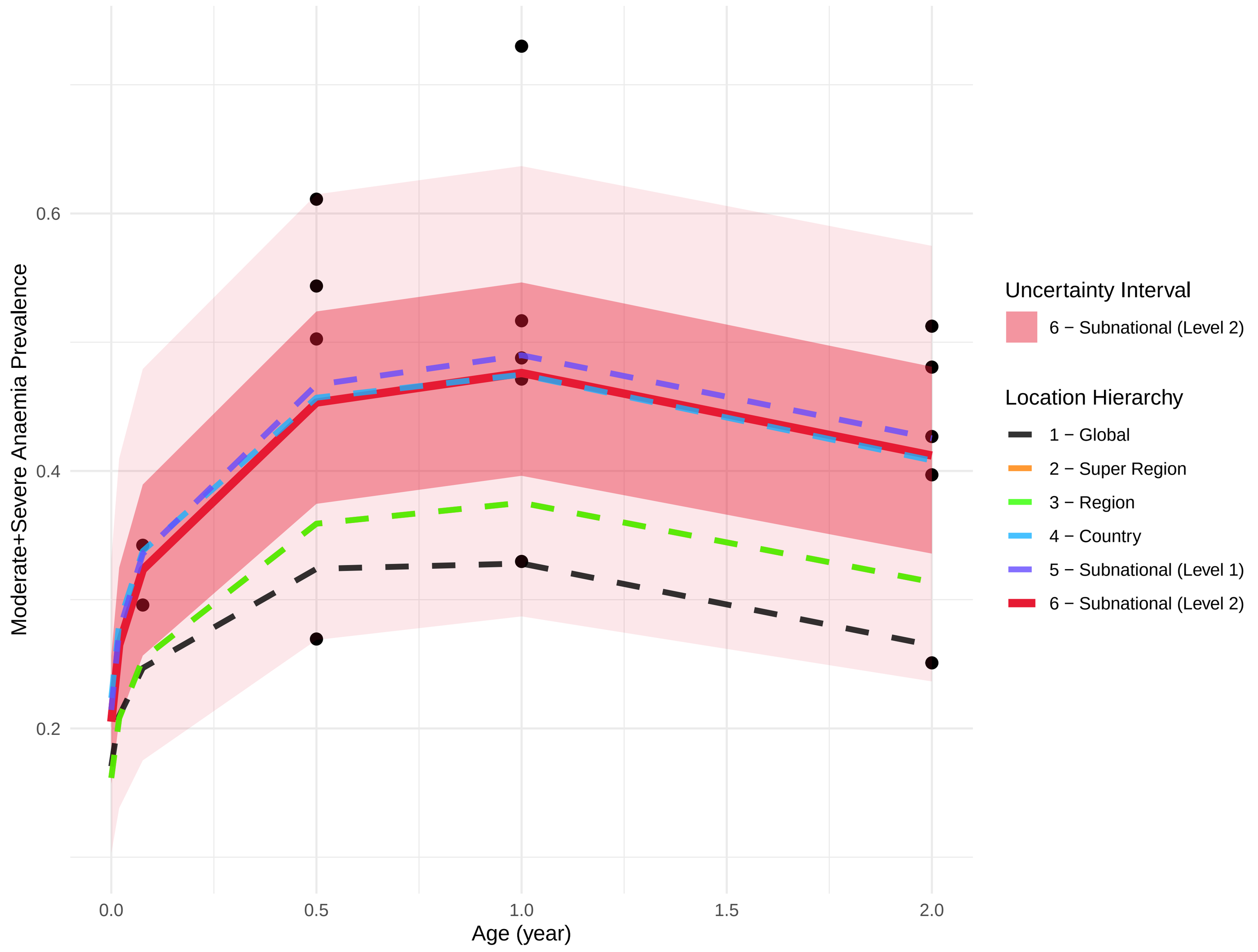

MR−BRT Age/Sex Fits − Males <2 Years Old − Moderate+Severe Anaemia Prevalence − Rajasthan
WHO−2001 Adjustment Method

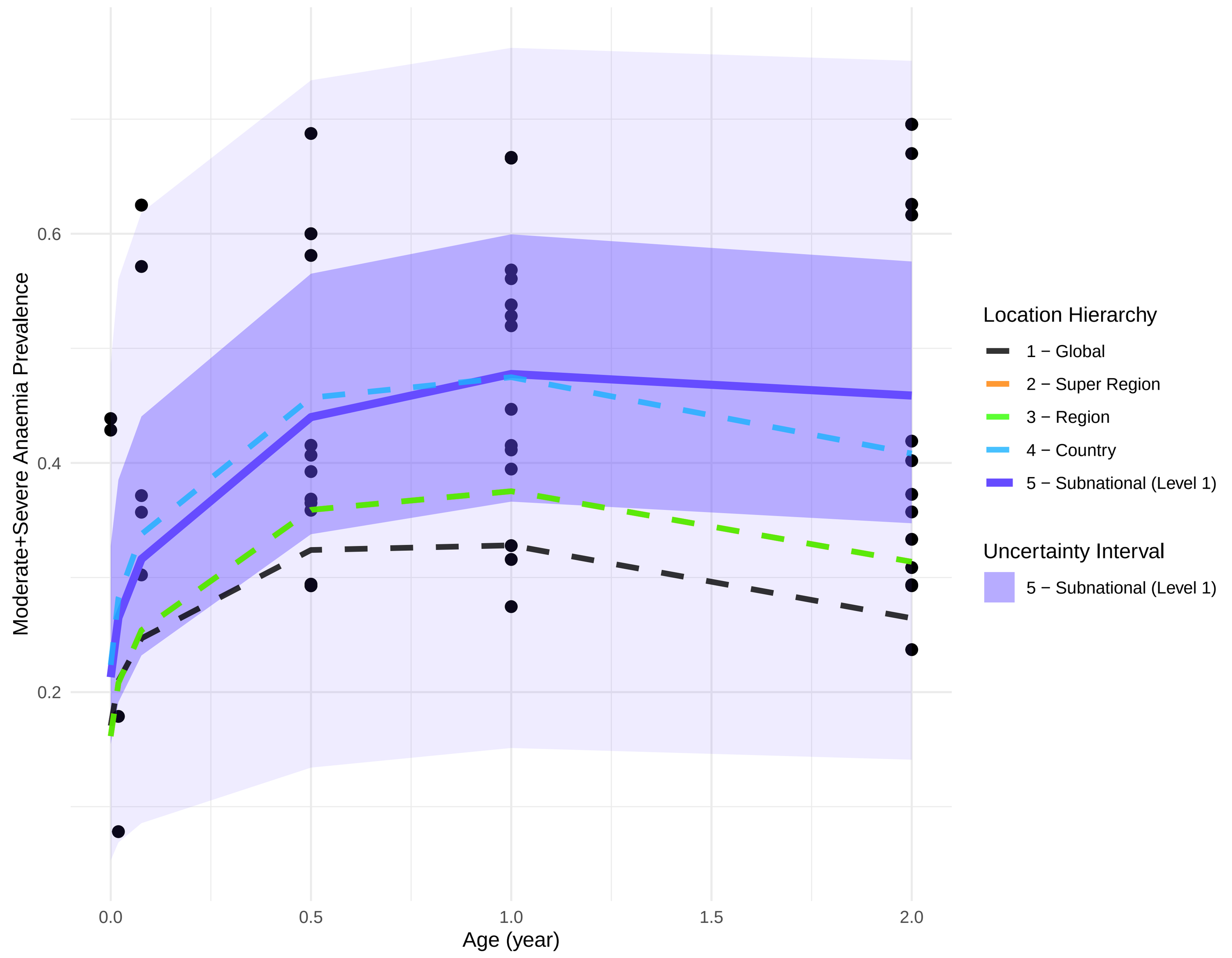

MR−BRT Age/Sex Fits − Males <2 Years Old − Moderate+Severe Anaemia Prevalence − Rajasthan, Rural
WHO−2001 Adjustment Method

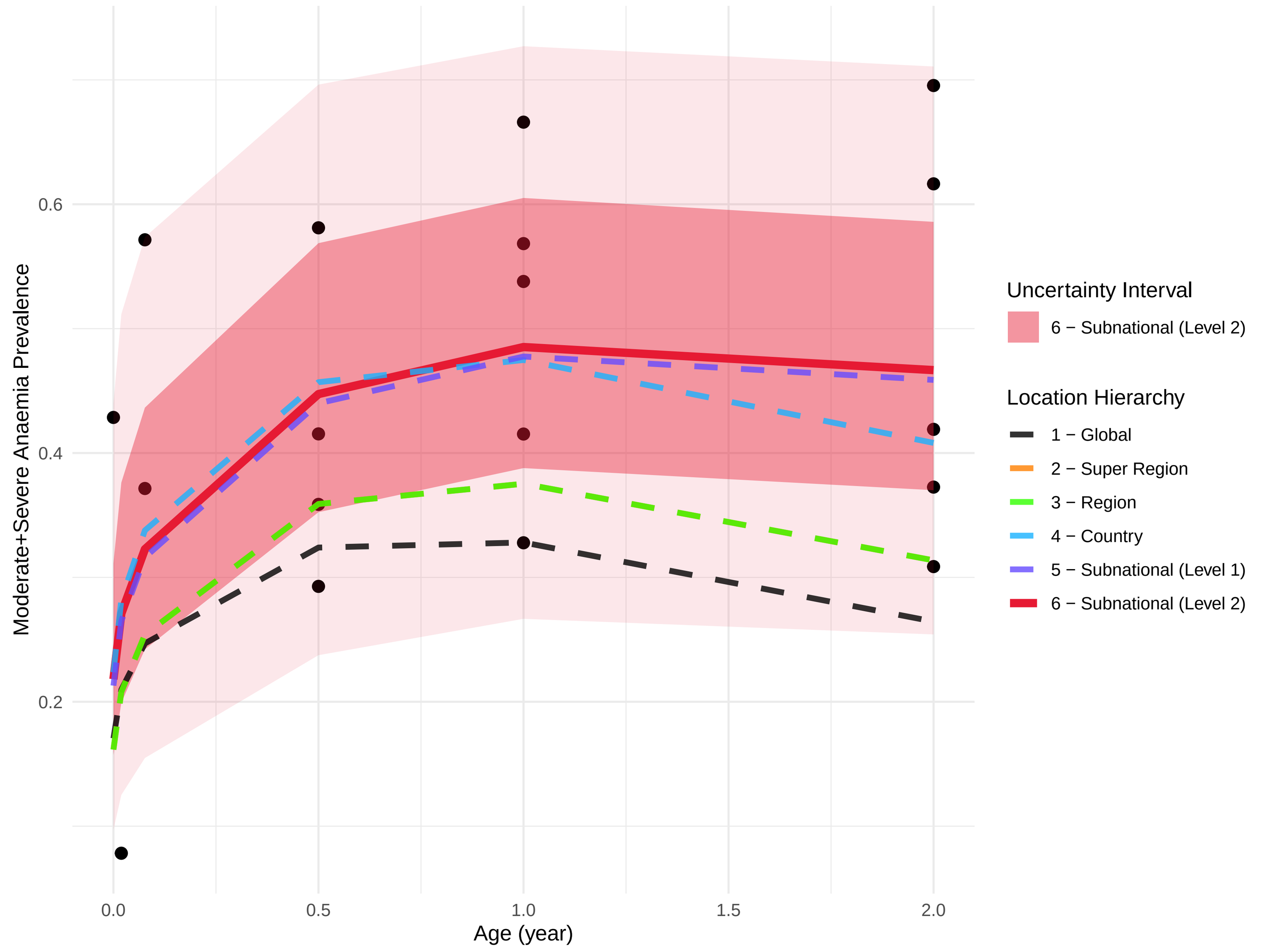

MR−BRT Age/Sex Fits − Males <2 Years Old − Moderate+Severe Anaemia Prevalence − Rajasthan, Urban
WHO−2001 Adjustment Method

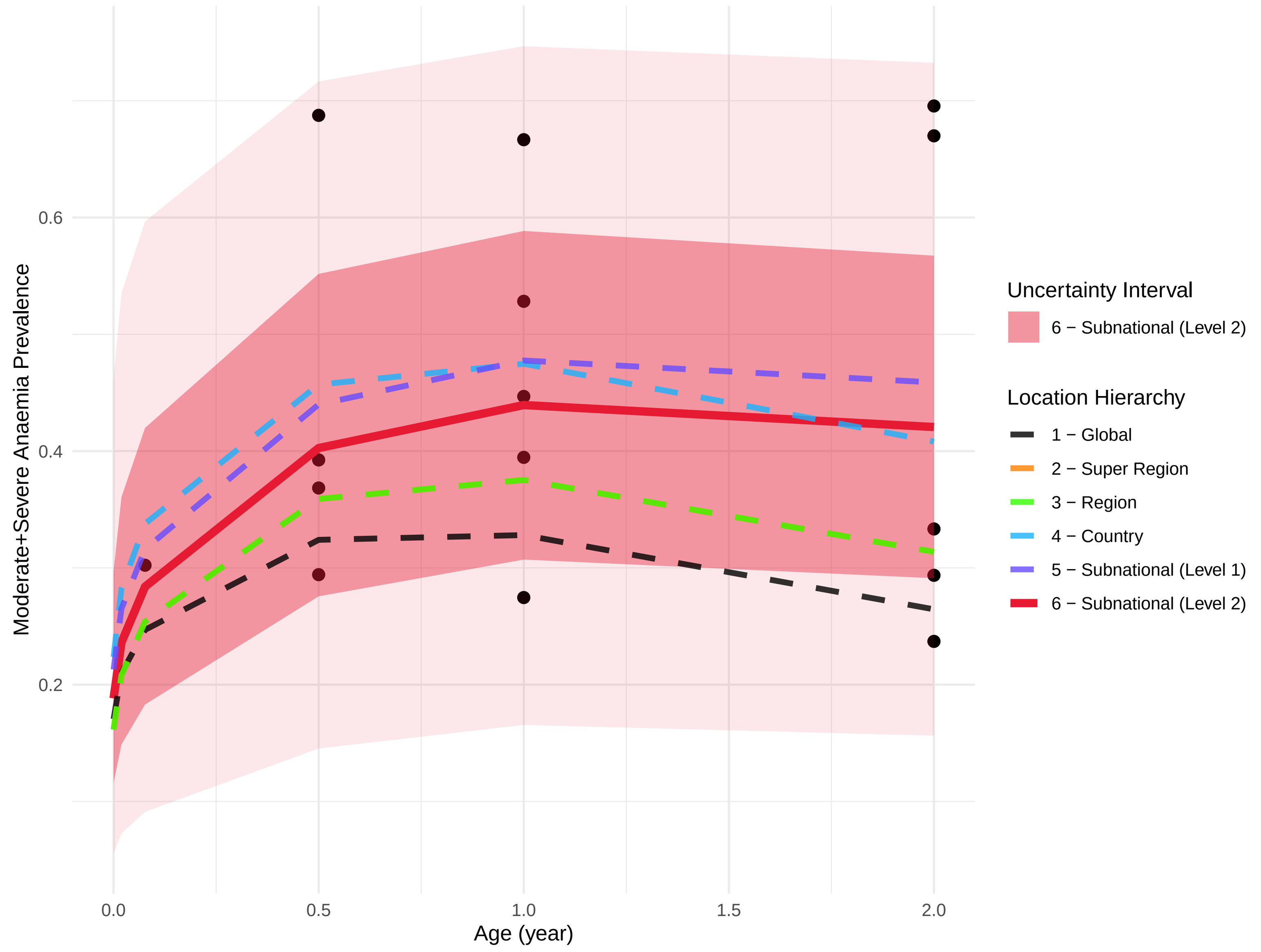

MR−BRT Age/Sex Fits − Males <2 Years Old − Moderate+Severe Anaemia Prevalence − Sikkim
WHO−2001 Adjustment Method

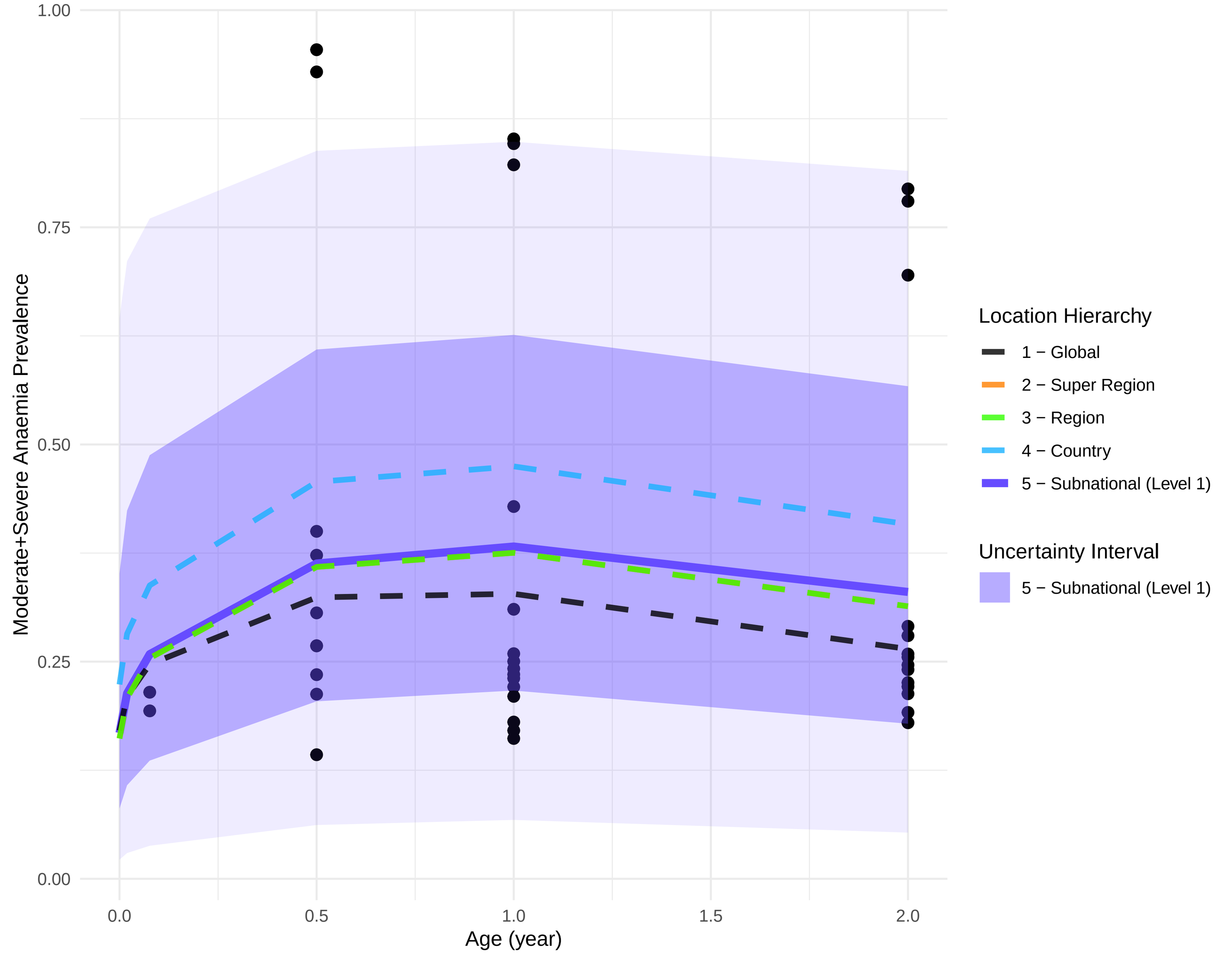

MR−BRT Age/Sex Fits − Males <2 Years Old − Moderate+Severe Anaemia Prevalence − Sikkim, Rural
WHO−2001 Adjustment Method

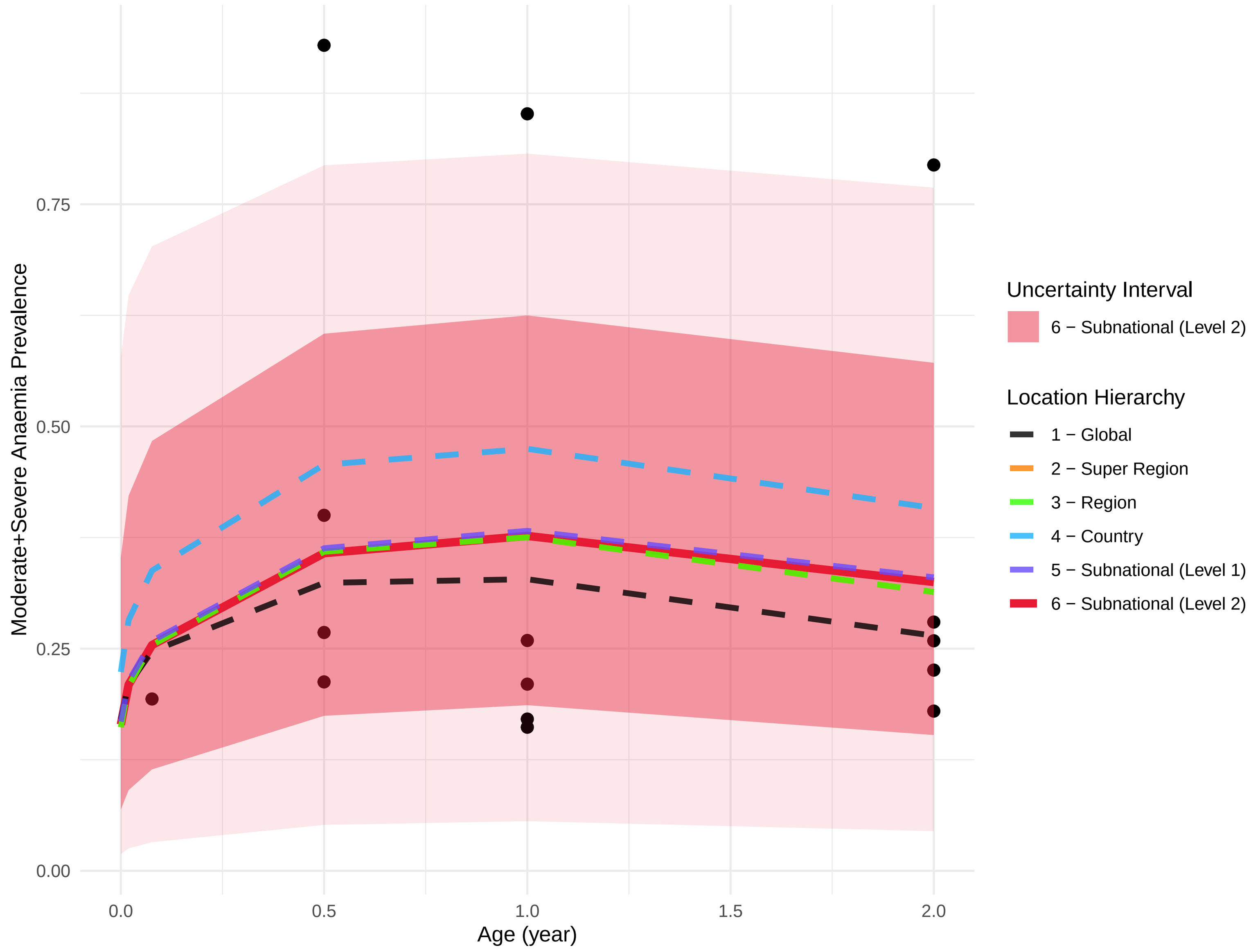

MR−BRT Age/Sex Fits − Males <2 Years Old − Moderate+Severe Anaemia Prevalence − Sikkim, Urban
WHO−2001 Adjustment Method

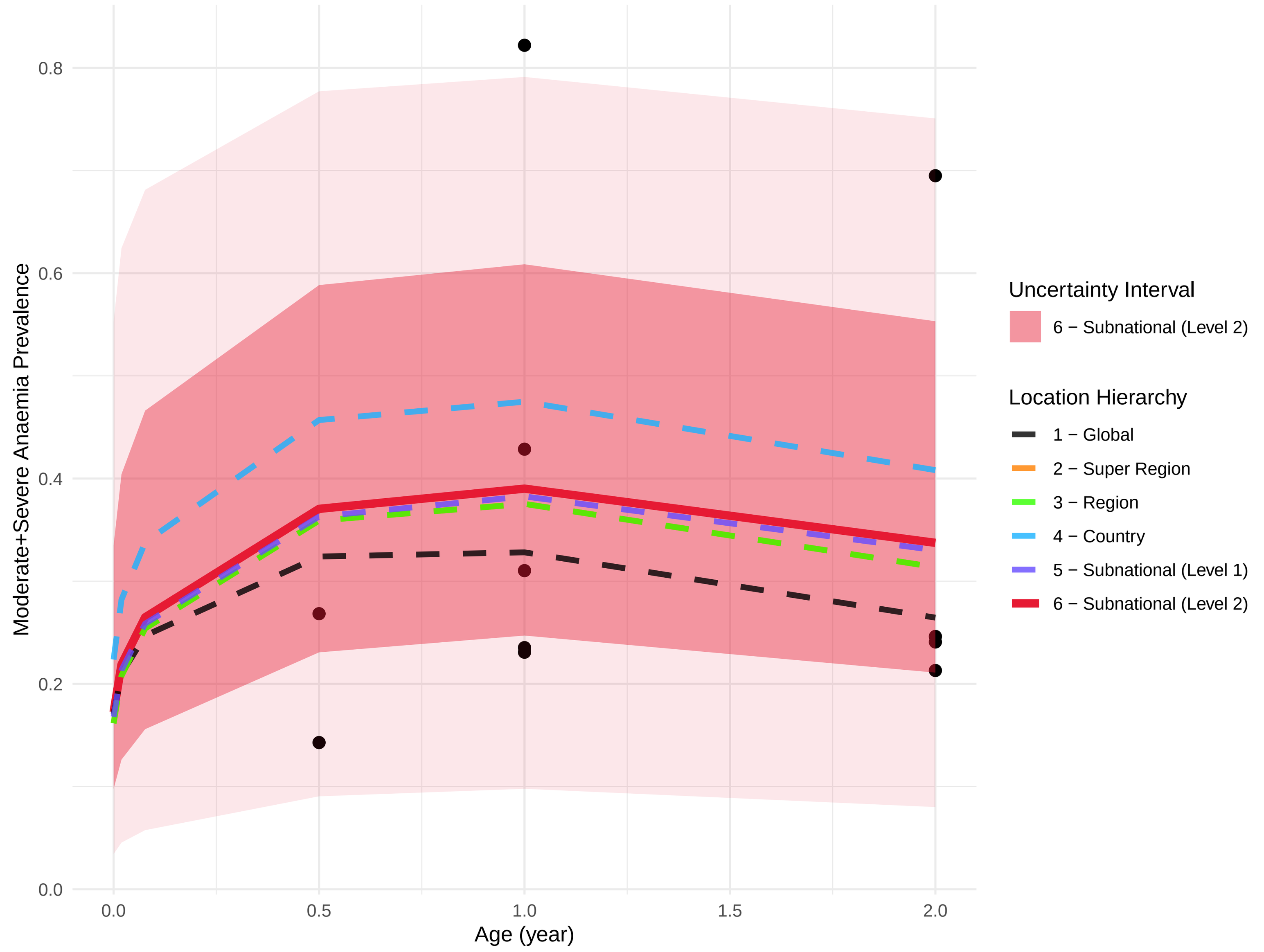

MR−BRT Age/Sex Fits − Males <2 Years Old − Moderate+Severe Anaemia Prevalence − Tamil Nadu
WHO−2001 Adjustment Method

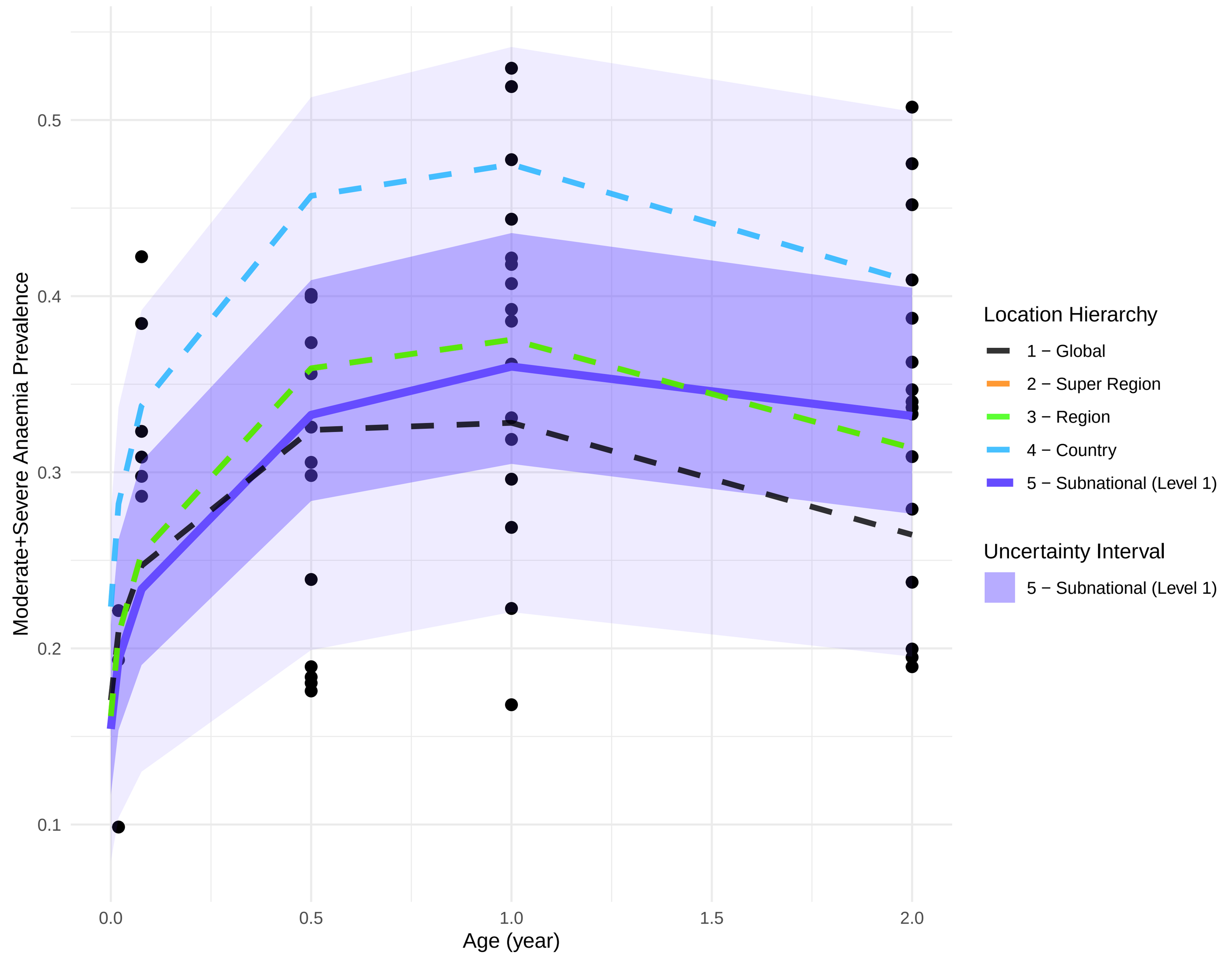

MR−BRT Age/Sex Fits − Males <2 Years Old − Moderate+Severe Anaemia Prevalence − Tamil Nadu, Rural
WHO−2001 Adjustment Method

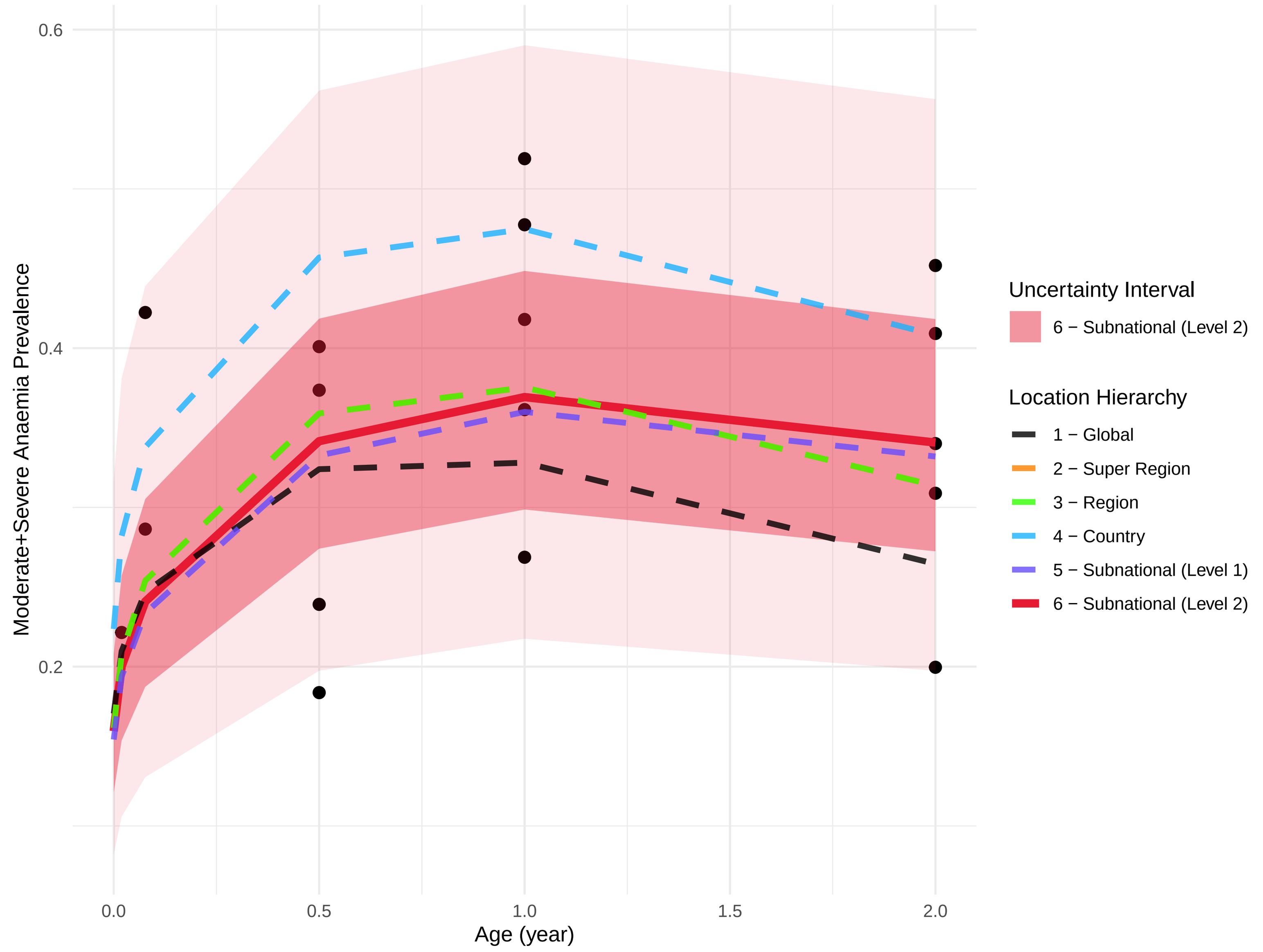

MR−BRT Age/Sex Fits − Males <2 Years Old − Moderate+Severe Anaemia Prevalence − Tamil Nadu, Urban
WHO−2001 Adjustment Method

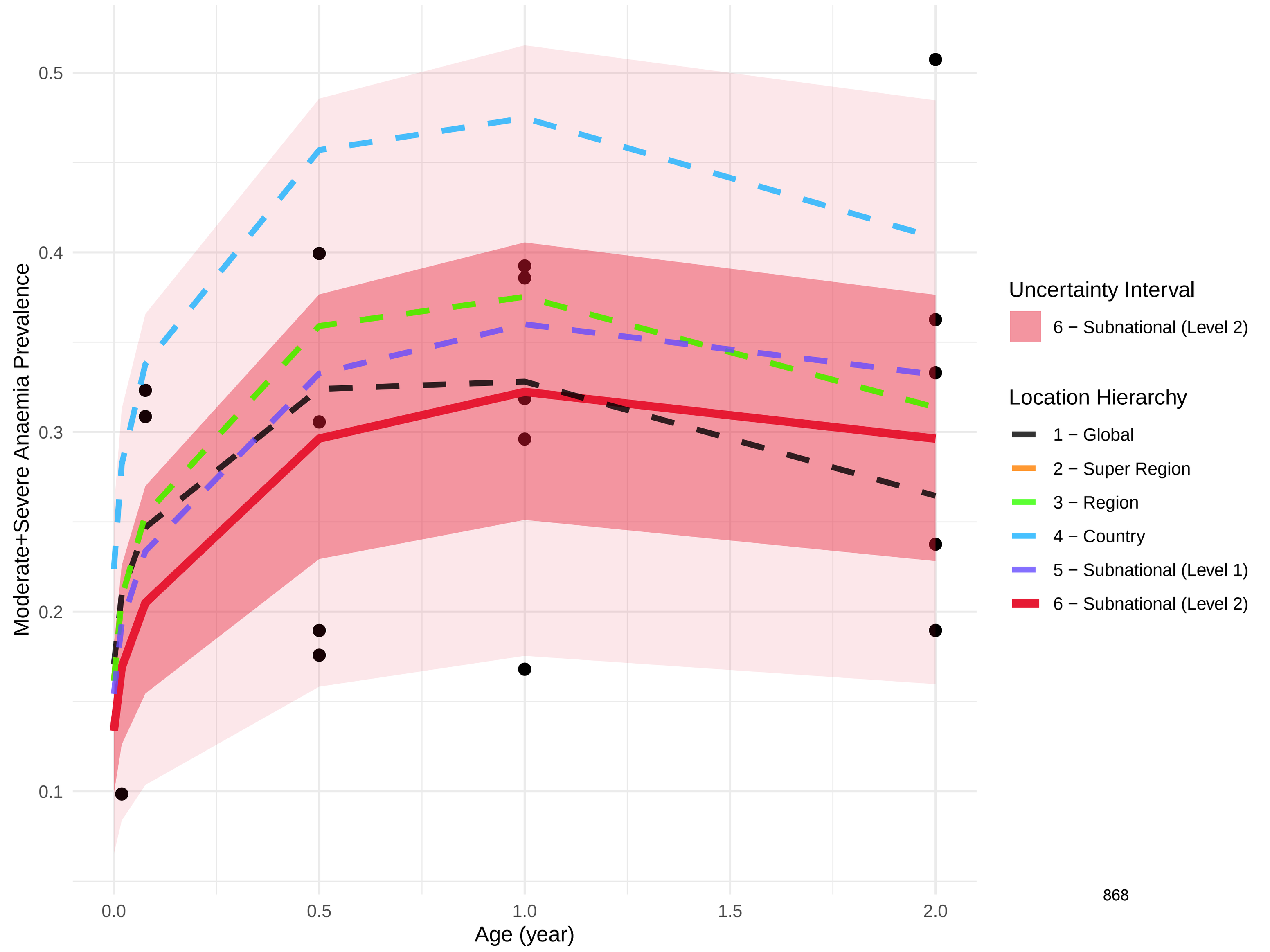

MR−BRT Age/Sex Fits − Males <2 Years Old − Moderate+Severe Anaemia Prevalence − Telangana
WHO−2001 Adjustment Method

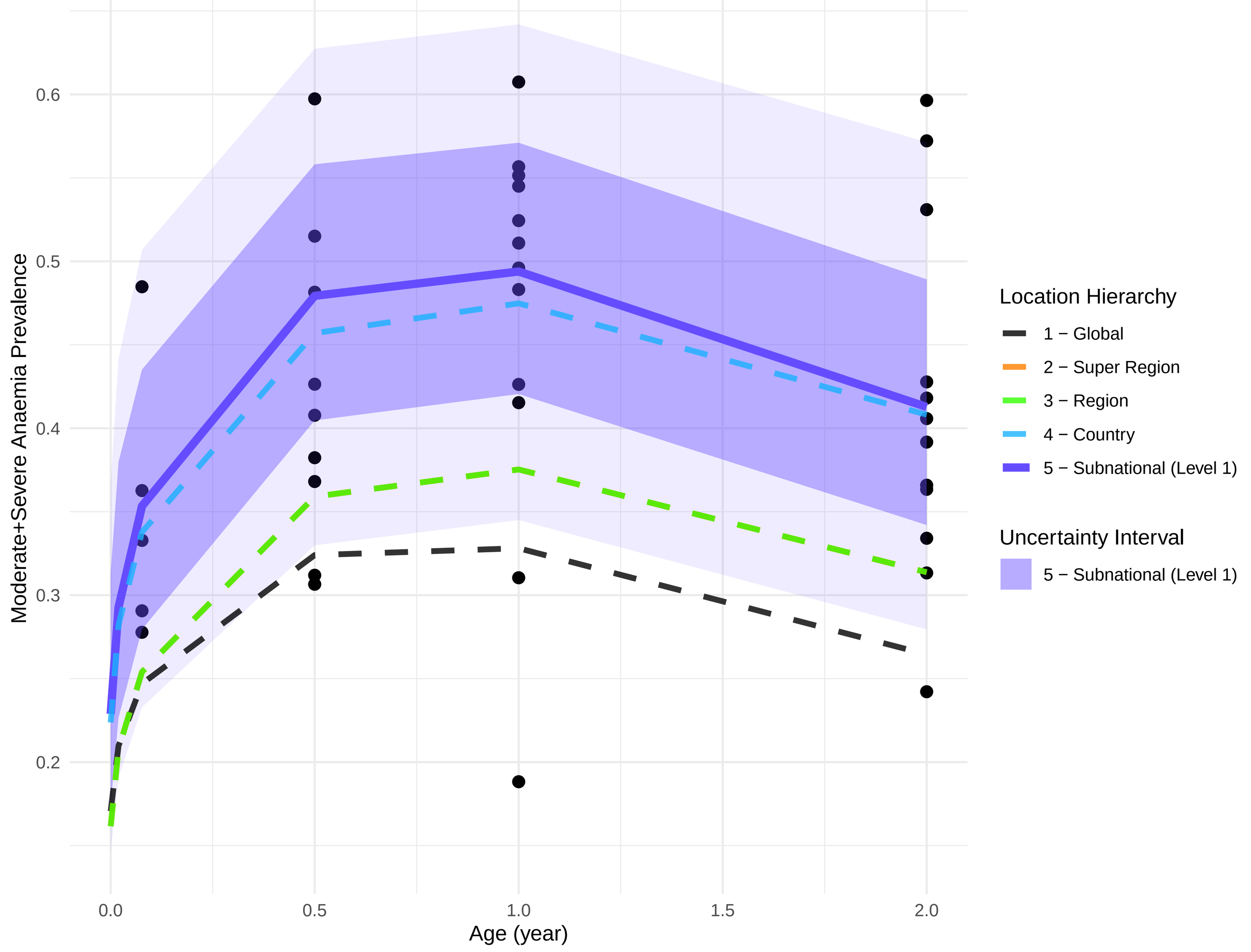

MR−BRT Age/Sex Fits − Males <2 Years Old − Moderate+Severe Anaemia Prevalence − Telangana, Rural
WHO−2001 Adjustment Method

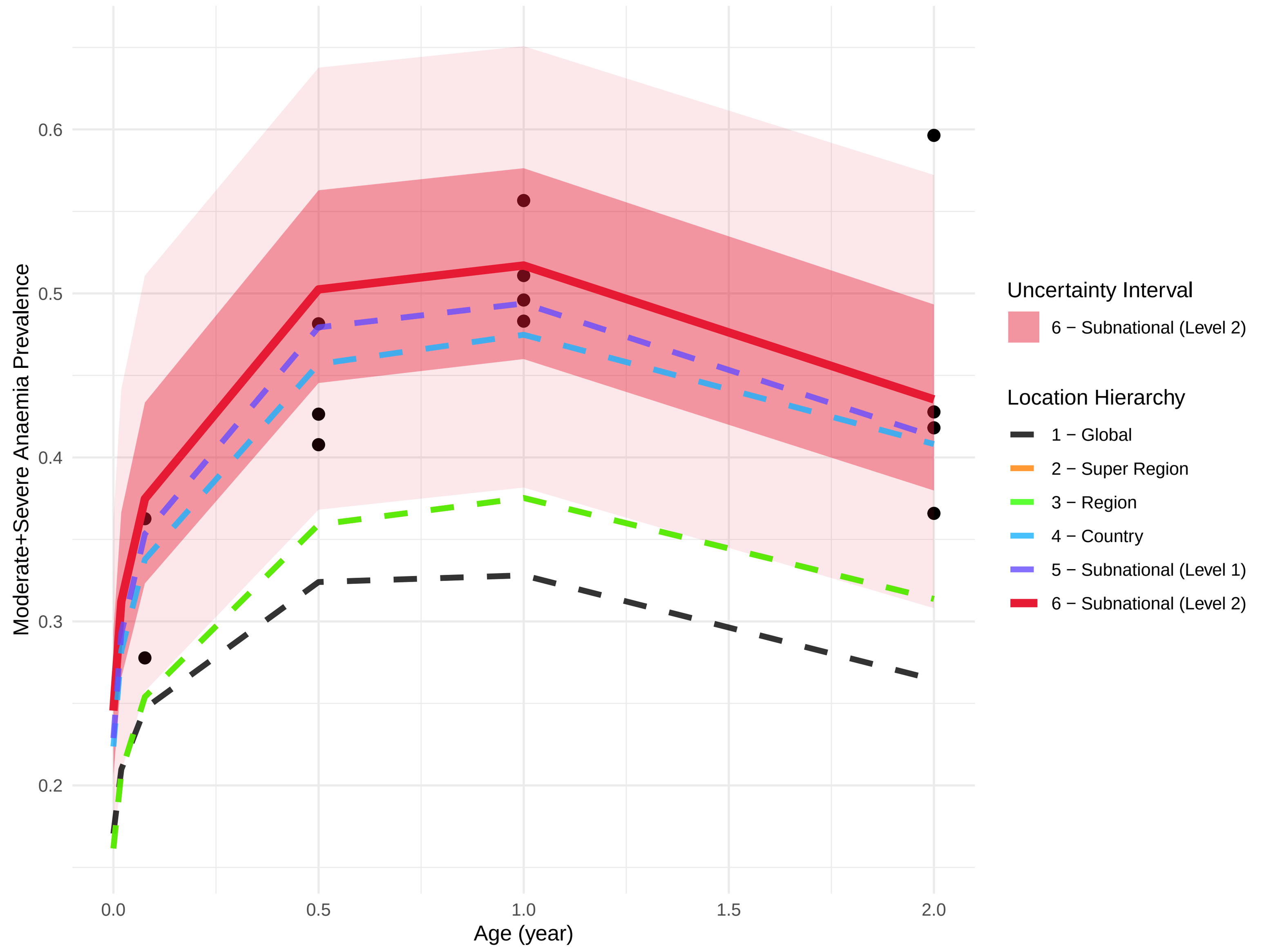

MR−BRT Age/Sex Fits − Males <2 Years Old − Moderate+Severe Anaemia Prevalence − Telangana, Urban
WHO−2001 Adjustment Method

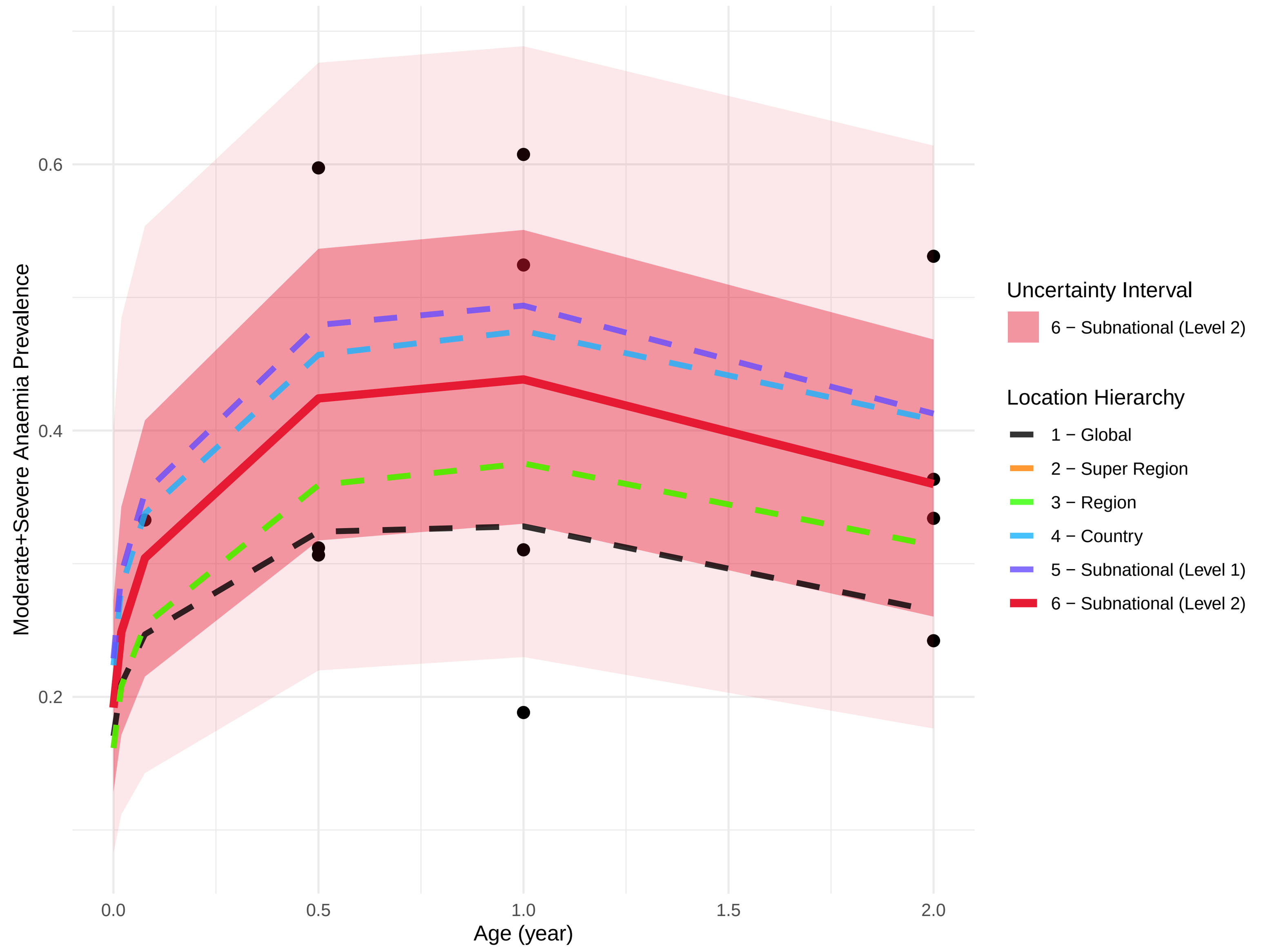

MR−BRT Age/Sex Fits − Males <2 Years Old − Moderate+Severe Anaemia Prevalence − Tripura
WHO−2001 Adjustment Method

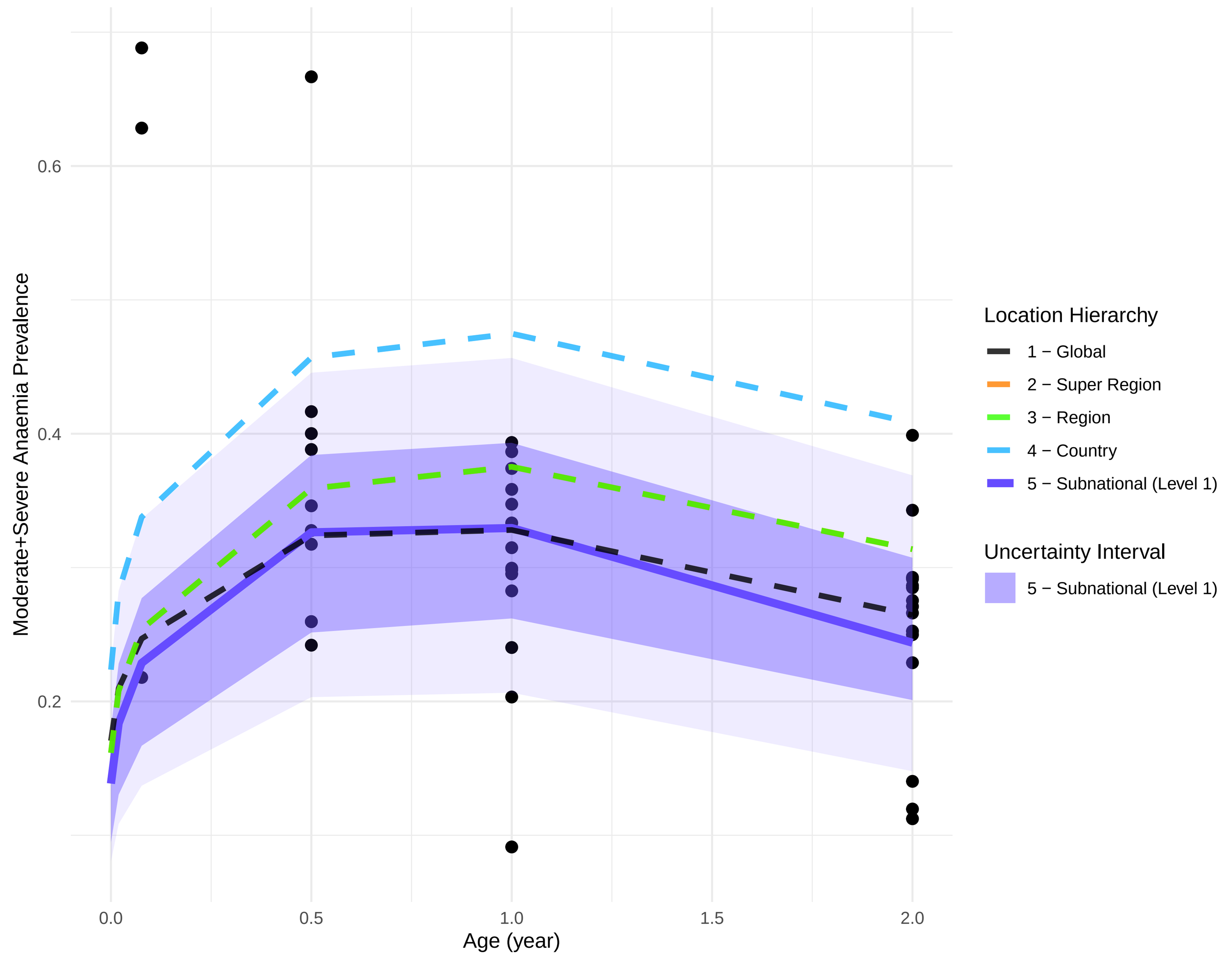

MR−BRT Age/Sex Fits − Males <2 Years Old − Moderate+Severe Anaemia Prevalence − Tripura, Rural
WHO−2001 Adjustment Method

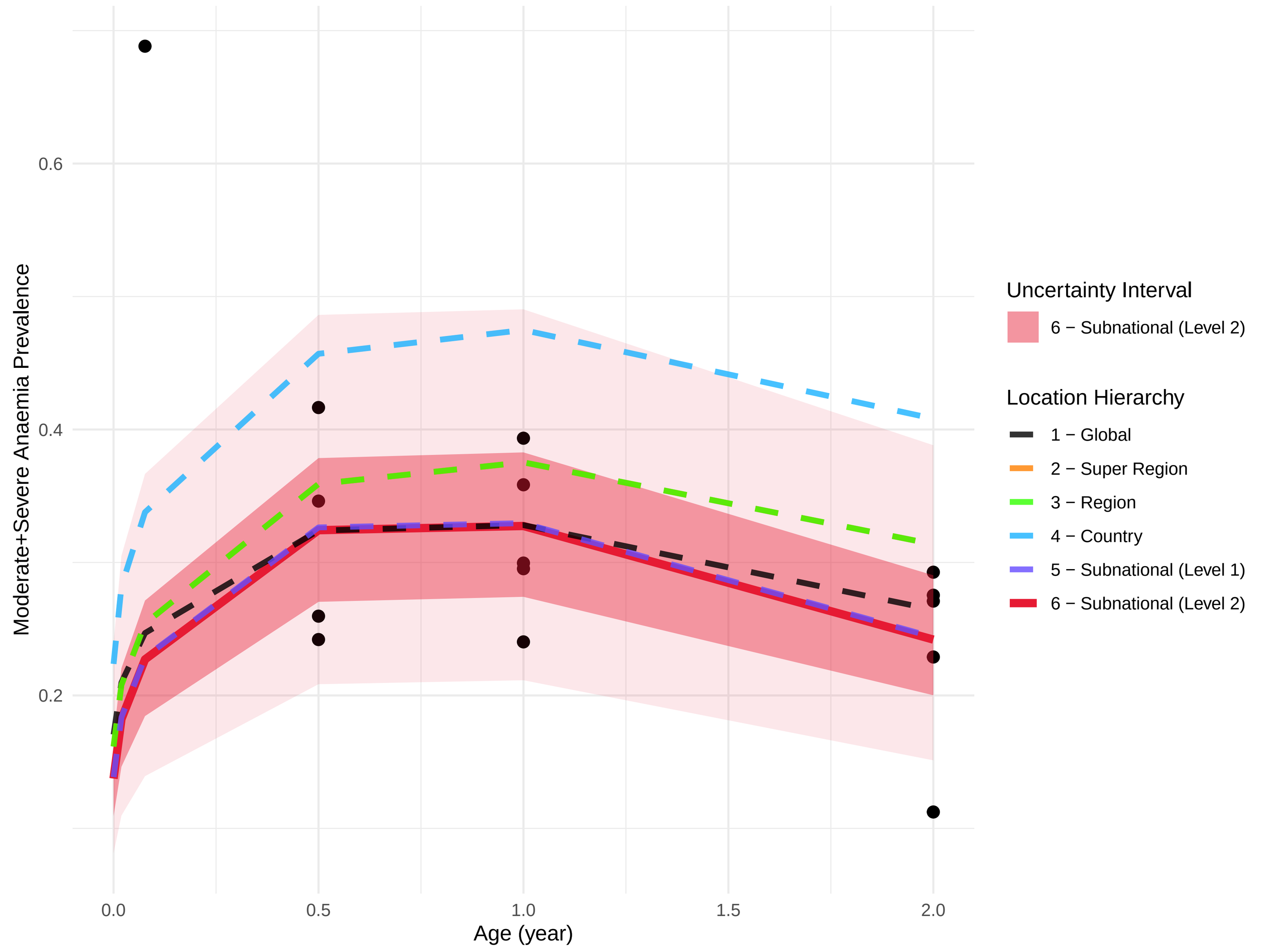

MR−BRT Age/Sex Fits − Males <2 Years Old − Moderate+Severe Anaemia Prevalence − Tripura, Urban
WHO−2001 Adjustment Method

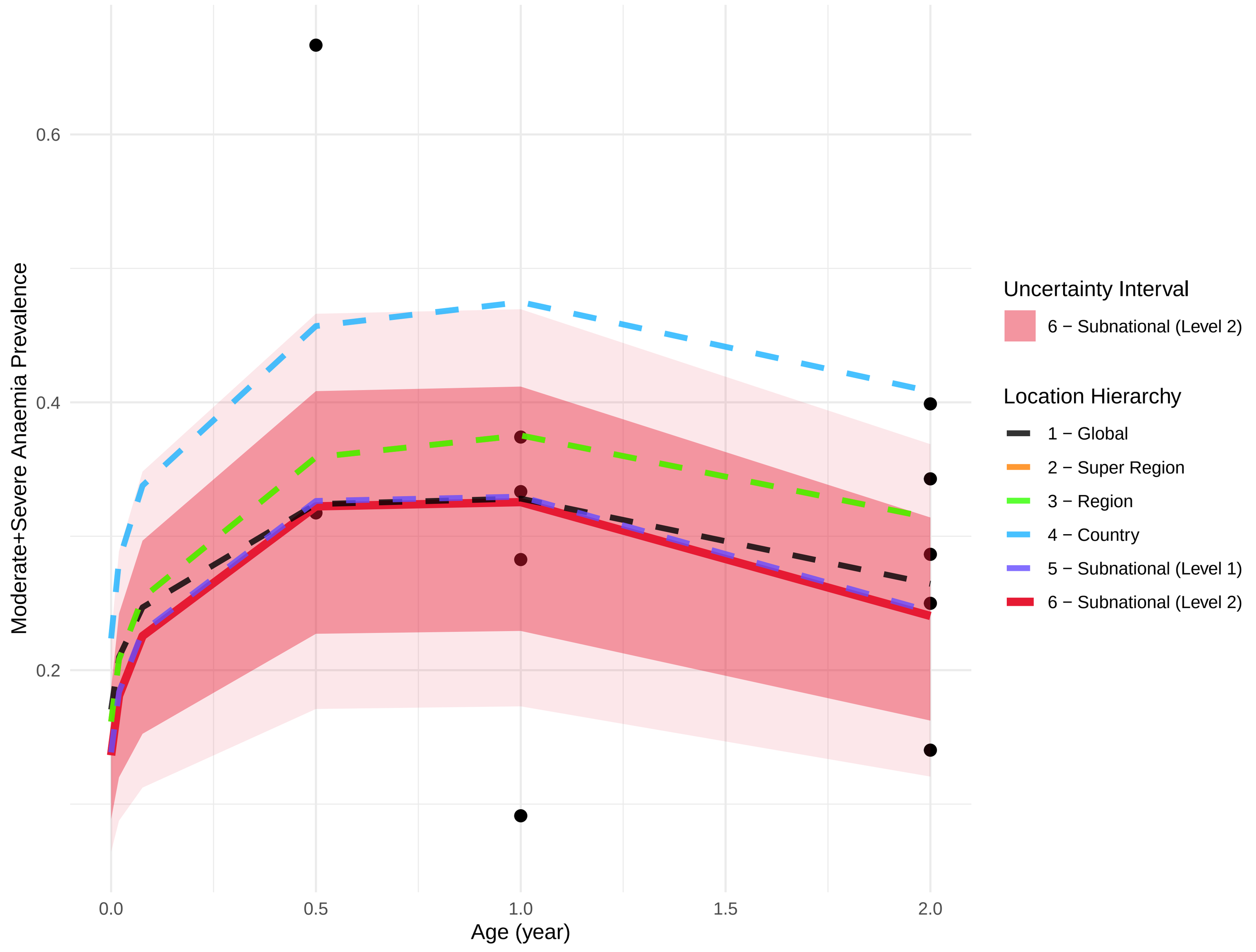

MR−BRT Age/Sex Fits − Males <2 Years Old − Moderate+Severe Anaemia Prevalence − Uttar Pradesh
WHO−2001 Adjustment Method

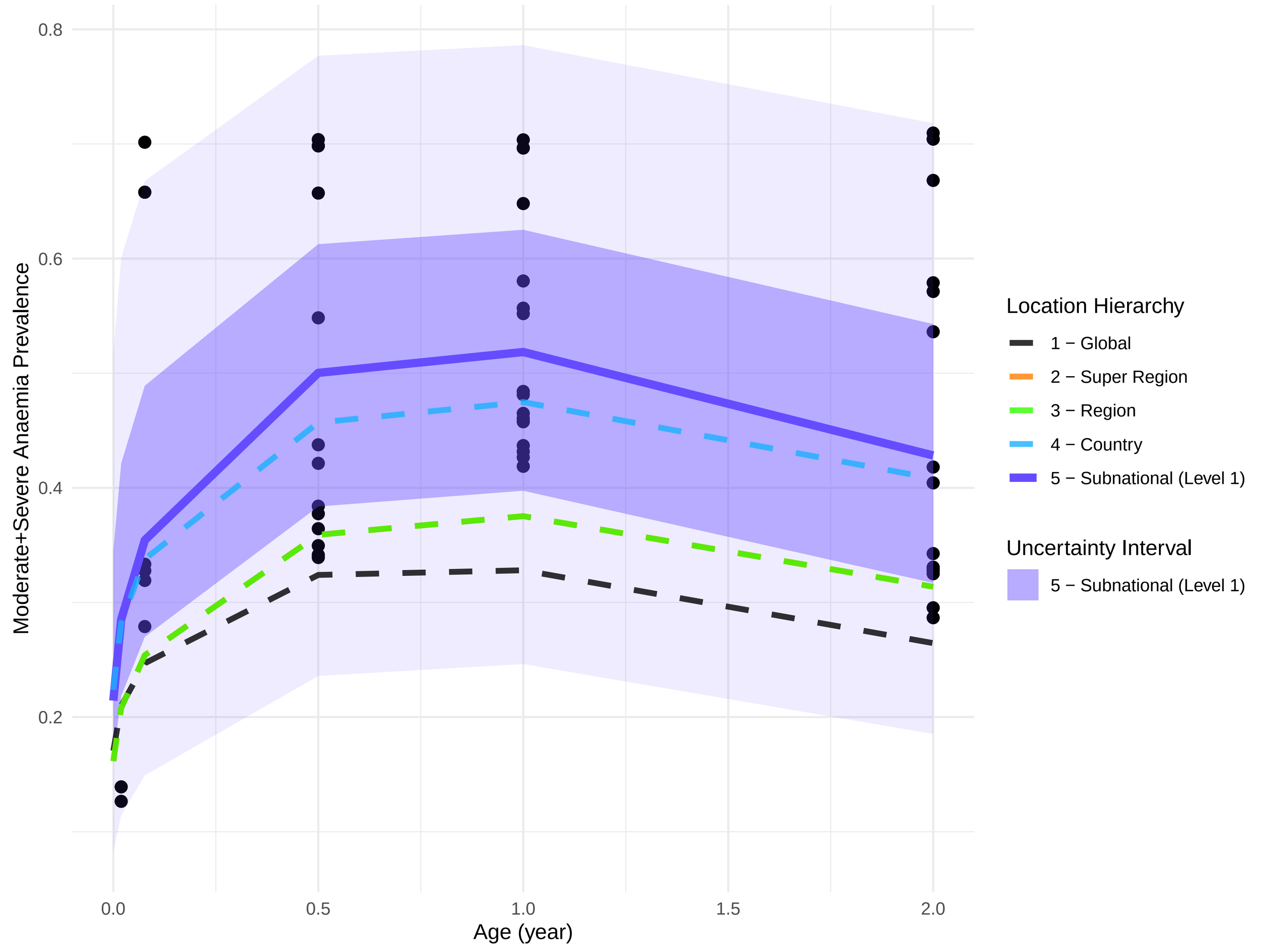

MR−BRT Age/Sex Fits − Males <2 Years Old − Moderate+Severe Anaemia Prevalence − Uttar Pradesh, Rural
WHO−2001 Adjustment Method

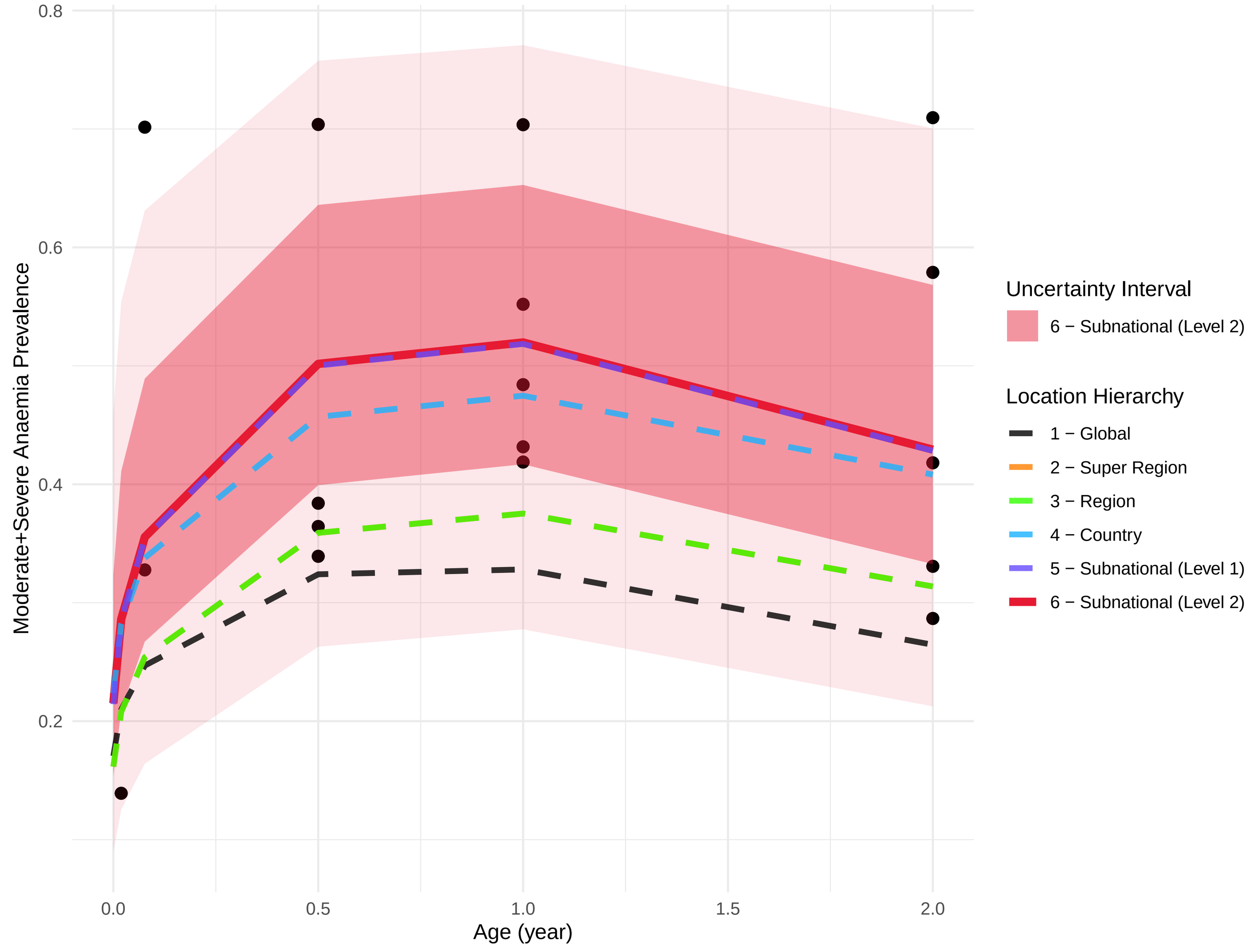

MR−BRT Age/Sex Fits − Males <2 Years Old − Moderate+Severe Anaemia Prevalence − Uttar Pradesh, Urban
WHO−2001 Adjustment Method

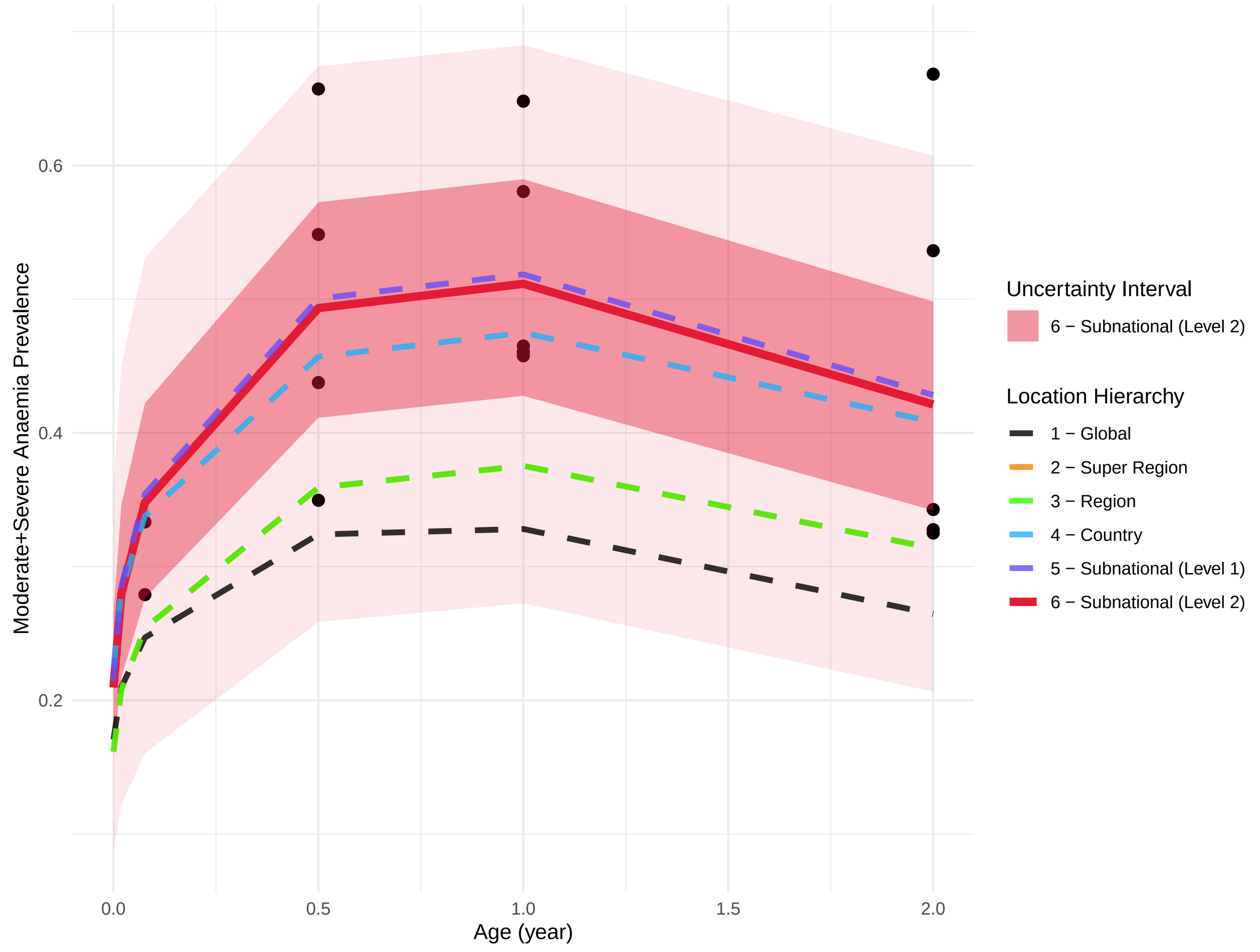

MR−BRT Age/Sex Fits − Males <2 Years Old − Moderate+Severe Anaemia Prevalence − Uttarakhand
WHO−2001 Adjustment Method

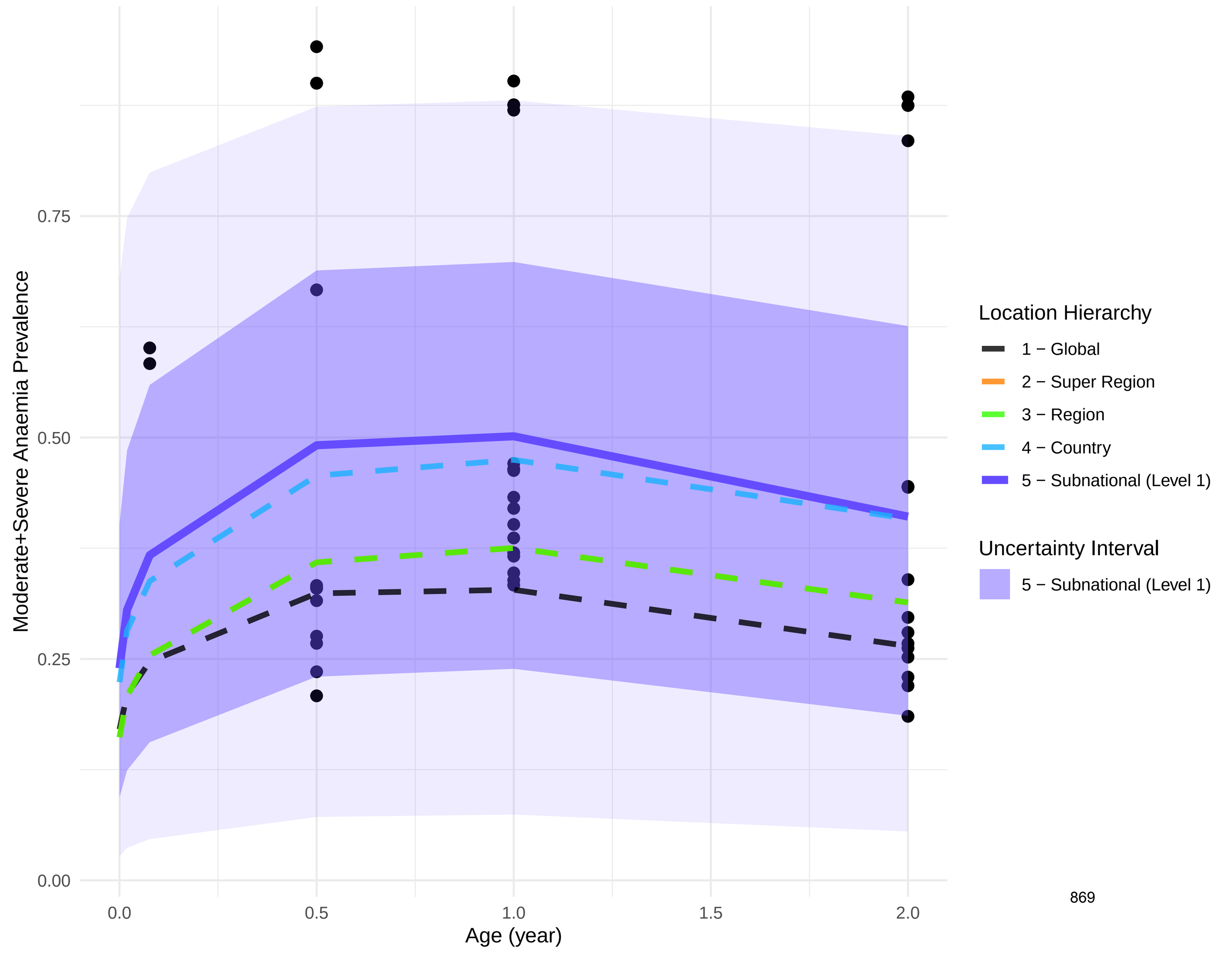

MR−BRT Age/Sex Fits − Males <2 Years Old − Moderate+Severe Anaemia Prevalence − Uttarakhand, Rural
WHO−2001 Adjustment Method

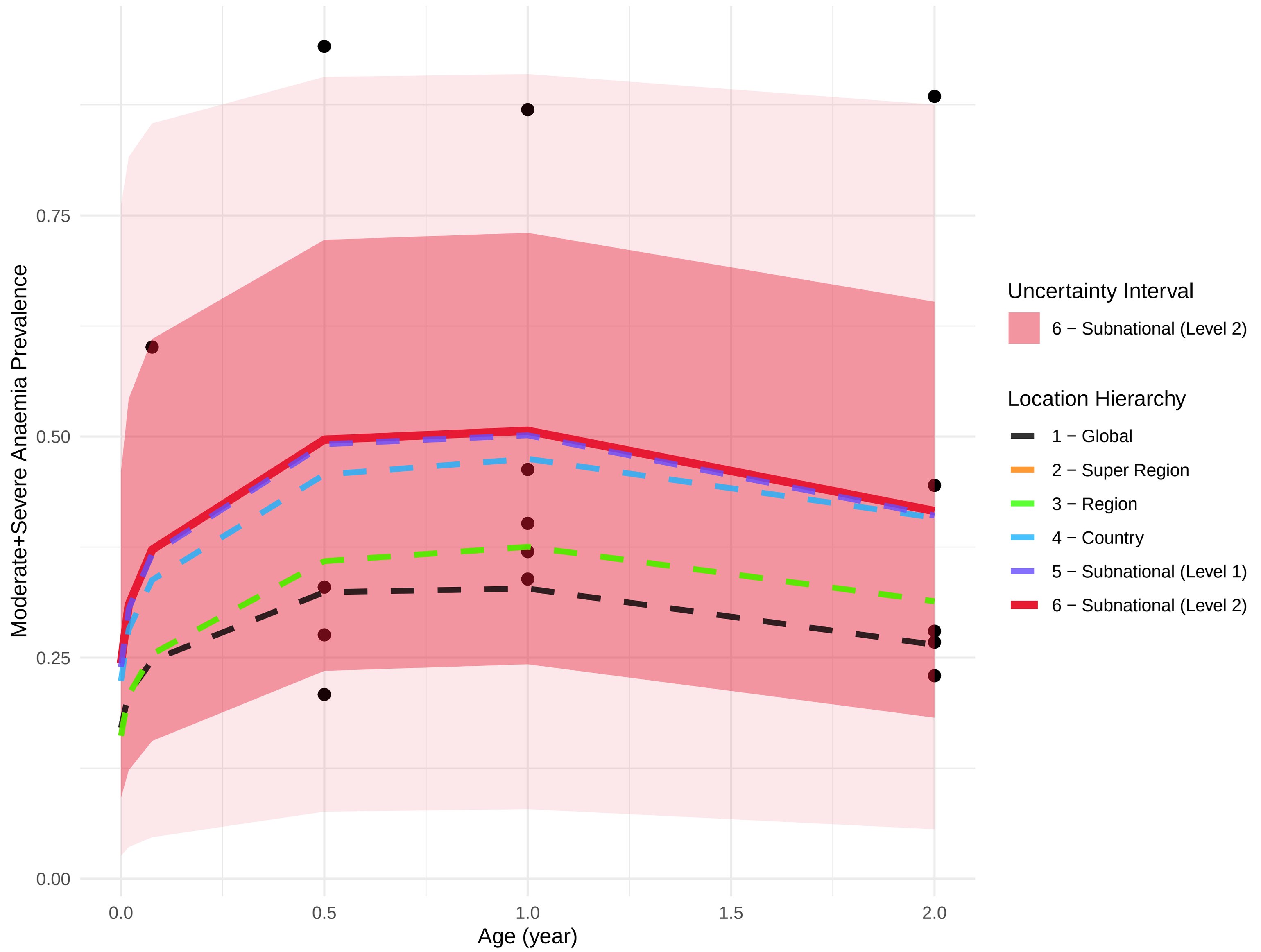

MR−BRT Age/Sex Fits − Males <2 Years Old − Moderate+Severe Anaemia Prevalence − Uttarakhand, Urban
WHO−2001 Adjustment Method

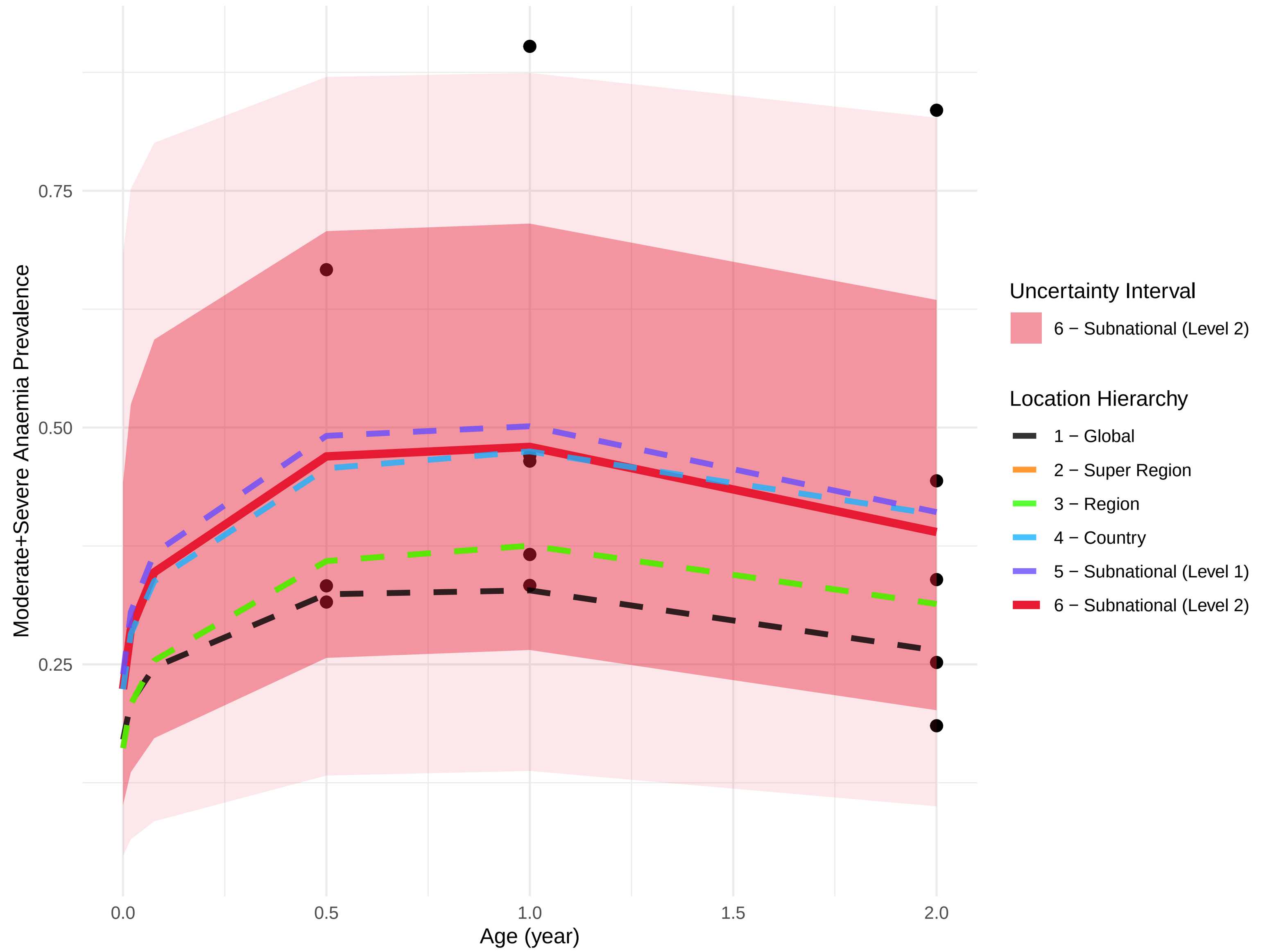

MR−BRT Age/Sex Fits − Males <2 Years Old − Moderate+Severe Anaemia Prevalence − West Bengal
WHO−2001 Adjustment Method

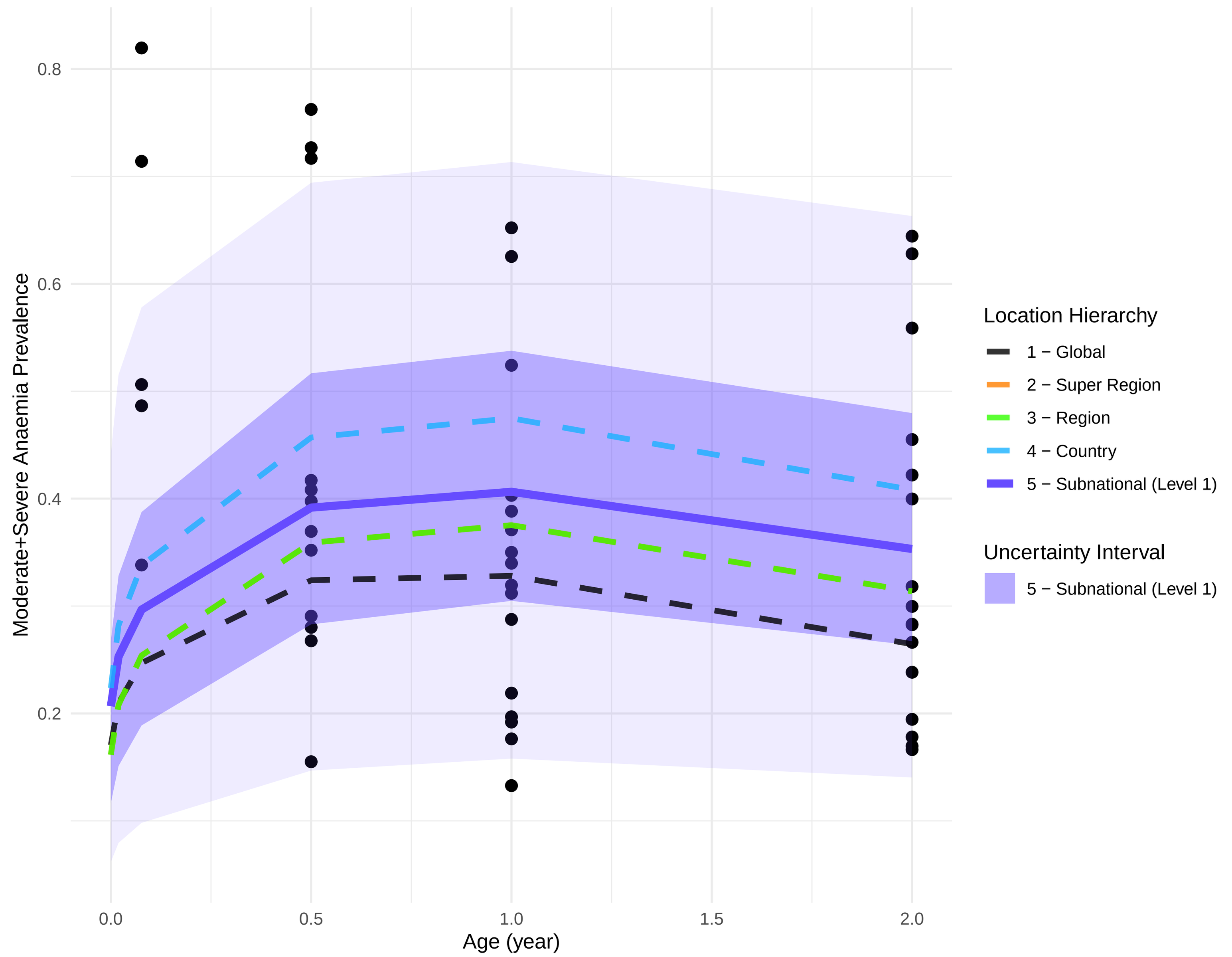

MR−BRT Age/Sex Fits − Males <2 Years Old − Moderate+Severe Anaemia Prevalence − West Bengal, Rural
WHO−2001 Adjustment Method

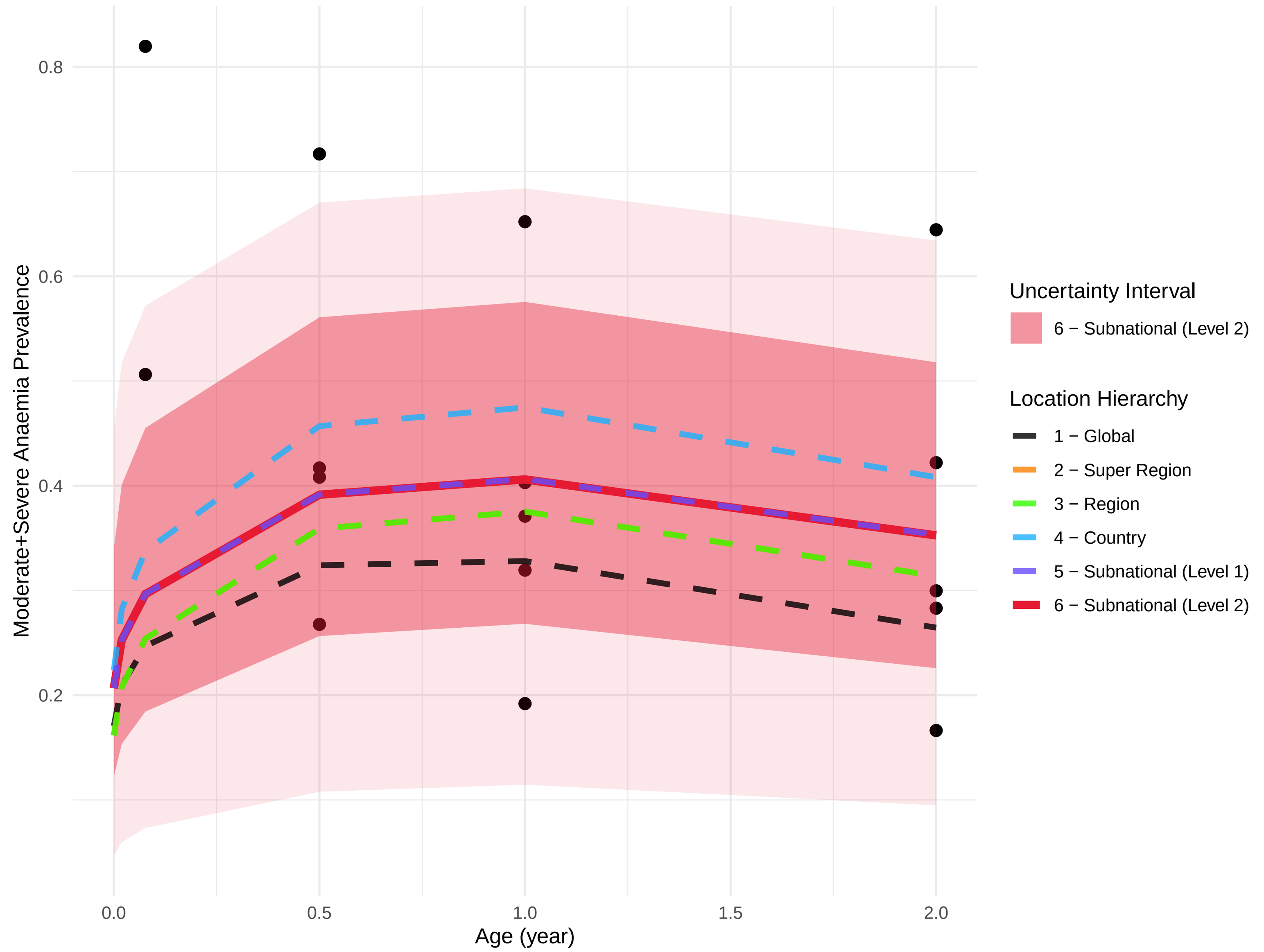

MR−BRT Age/Sex Fits − Males <2 Years Old − Moderate+Severe Anaemia Prevalence − West Bengal, Urban
WHO−2001 Adjustment Method

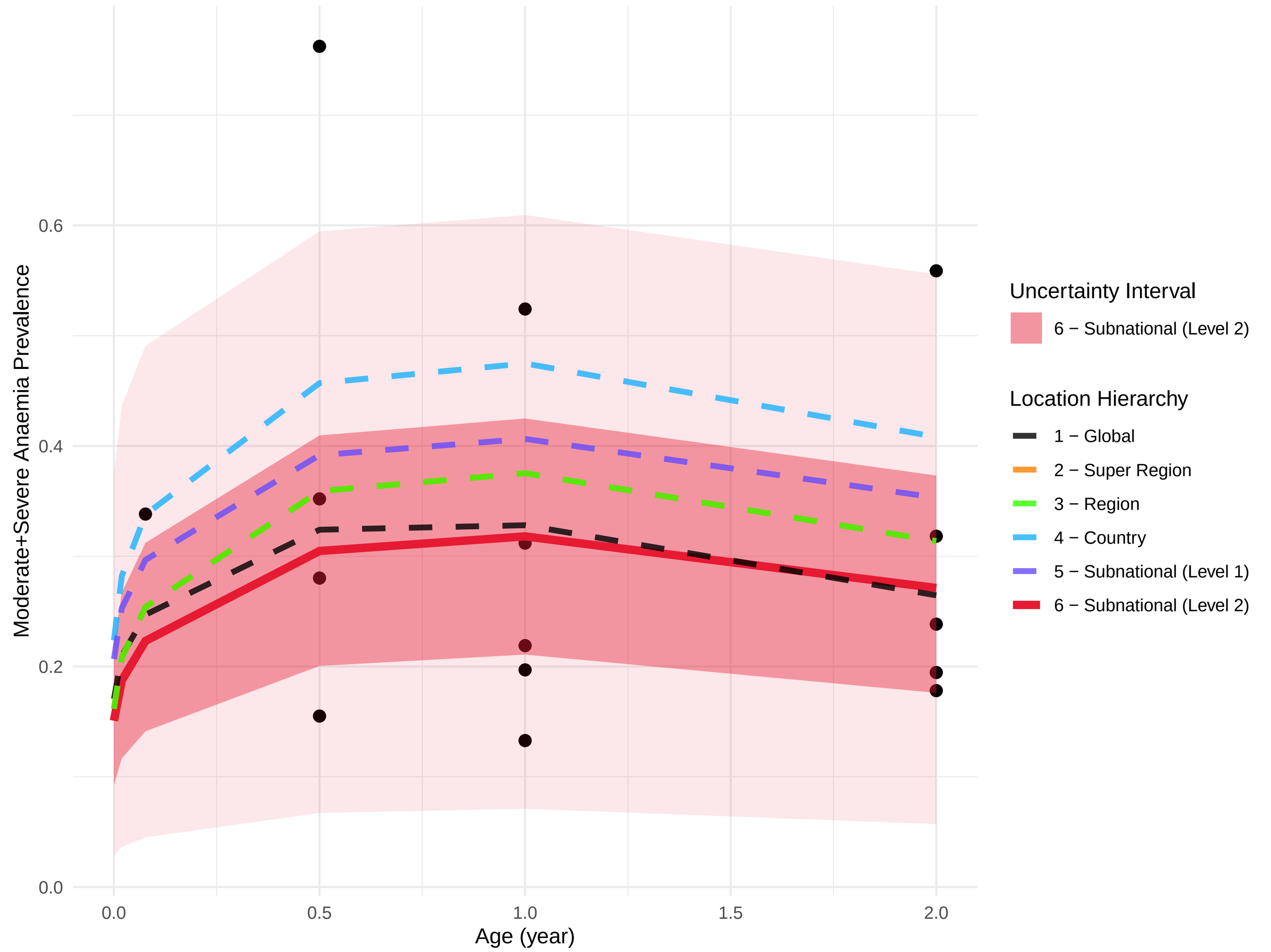

MR−BRT Age/Sex Fits − Males <2 Years Old − Moderate+Severe Anaemia Prevalence − Nepal
WHO−2001 Adjustment Method

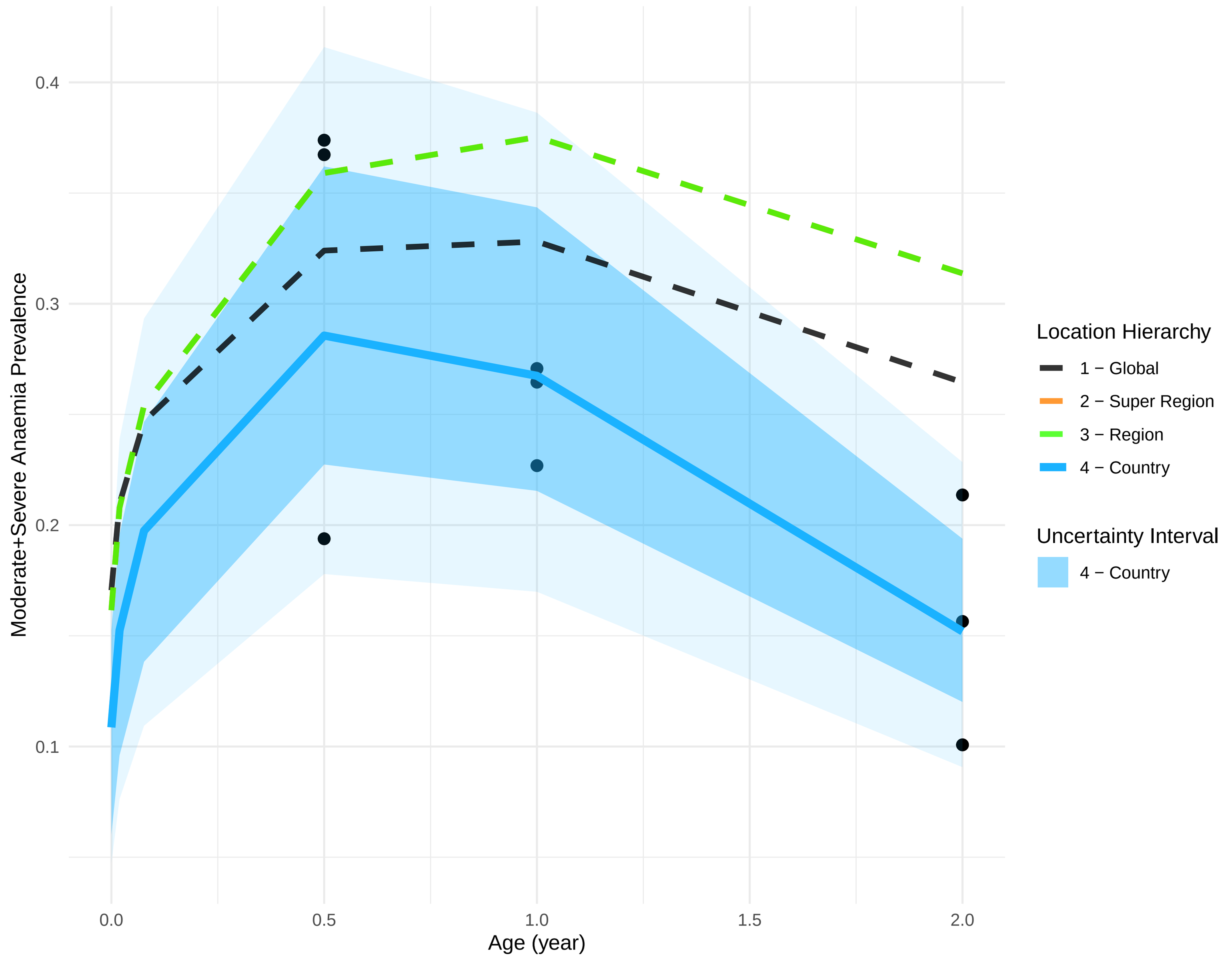

MR−BRT Age/Sex Fits − Males <2 Years Old − Moderate+Severe Anaemia Prevalence − Southeast Asia, East Asia, and Oceania
WHO−2001 Adjustment Method

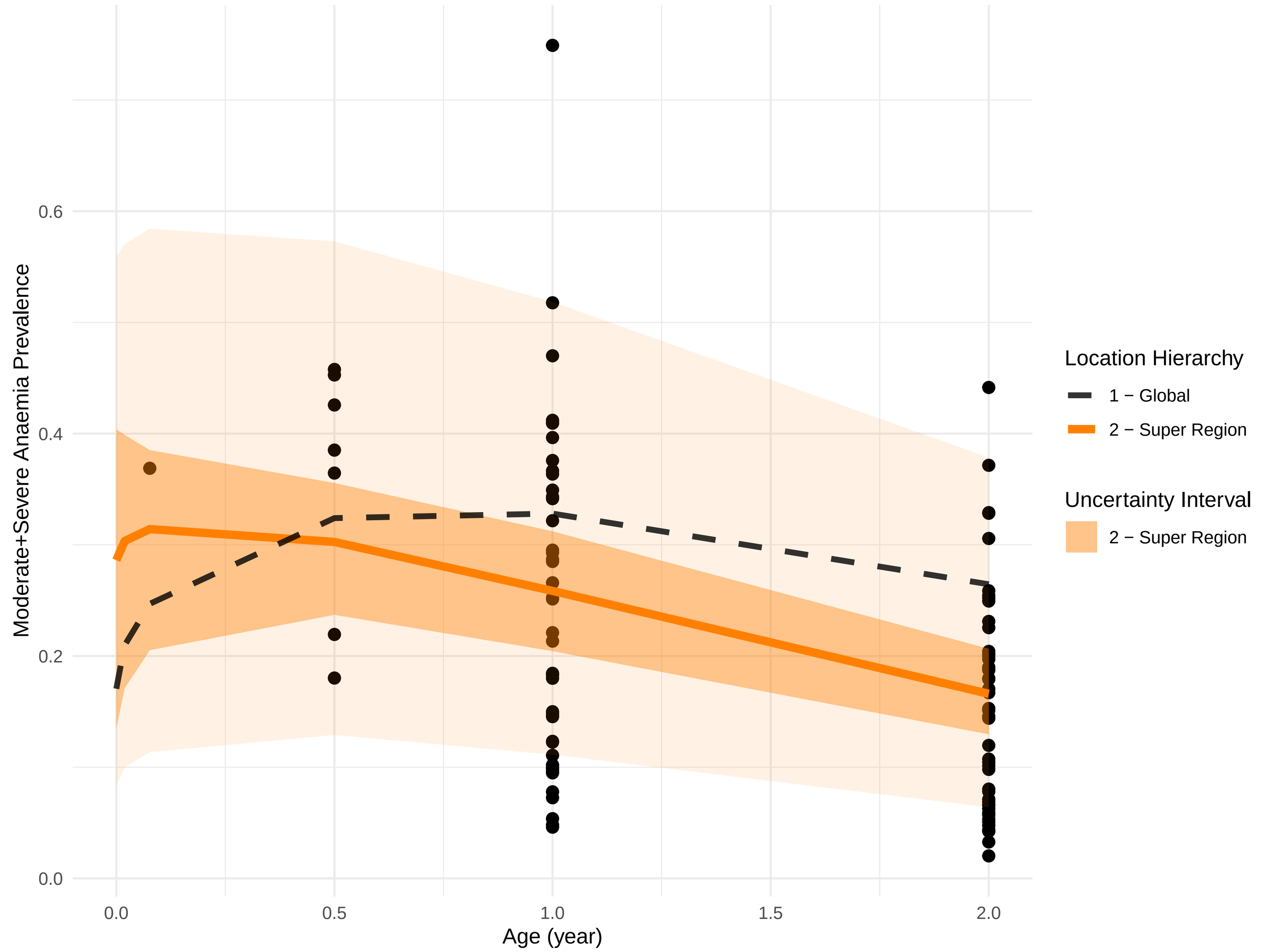

MR−BRT Age/Sex Fits − Males <2 Years Old − Moderate+Severe Anaemia Prevalence − Southeast Asia
WHO−2001 Adjustment Method

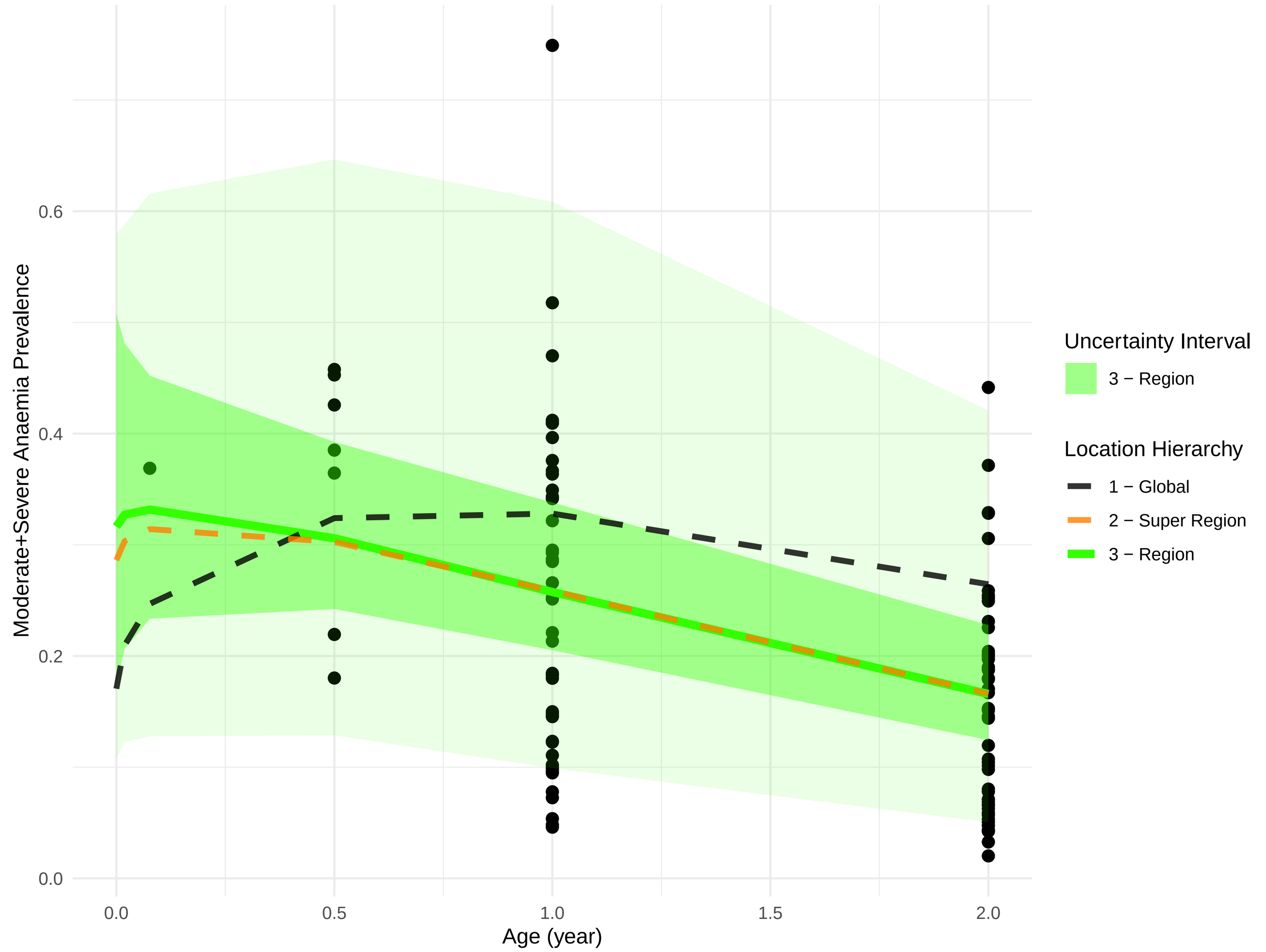

MR−BRT Age/Sex Fits − Males <2 Years Old − Moderate+Severe Anaemia Prevalence − Cambodia
WHO−2001 Adjustment Method

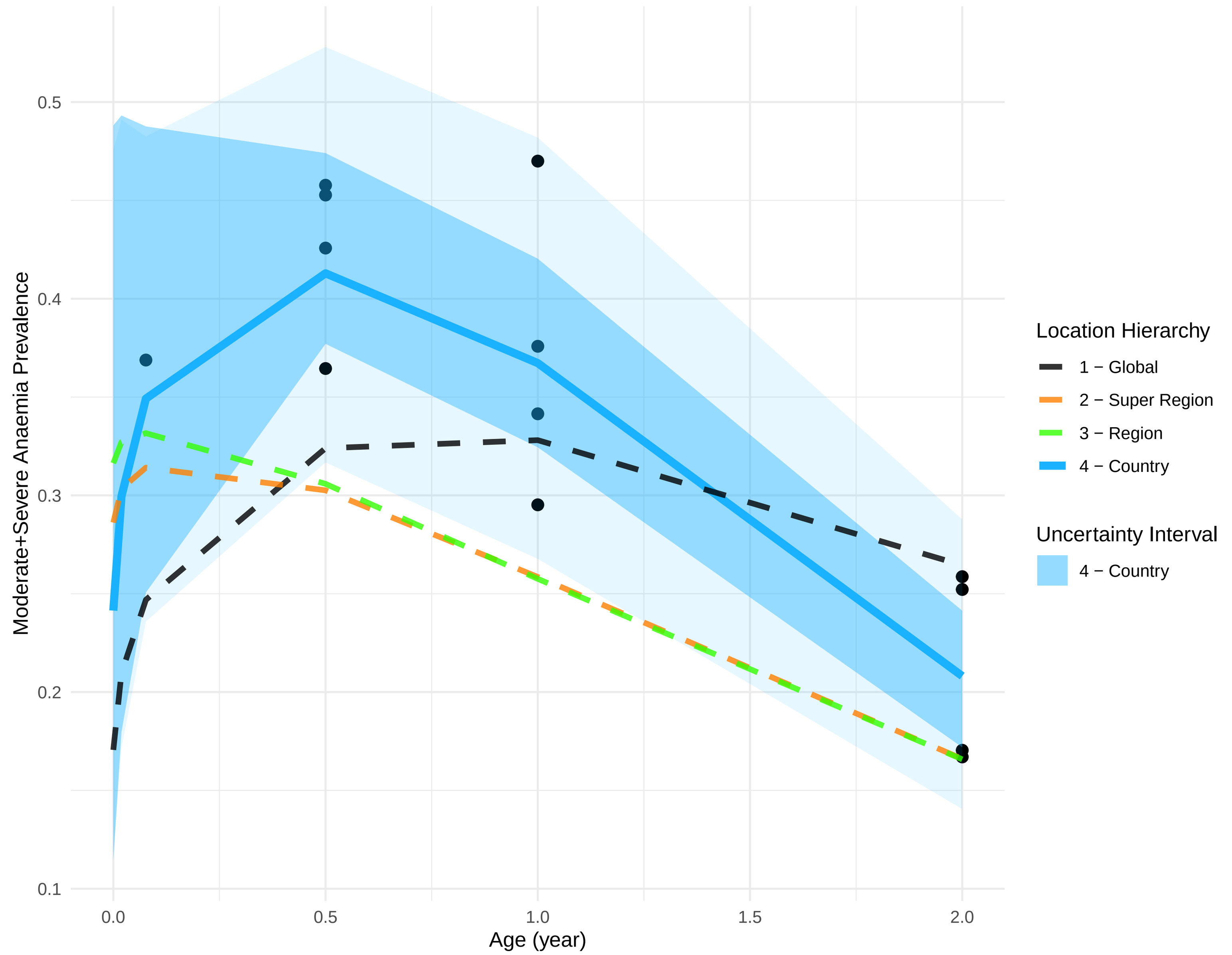

MR−BRT Age/Sex Fits − Males <2 Years Old − Moderate+Severe Anaemia Prevalence − Indonesia
WHO−2001 Adjustment Method

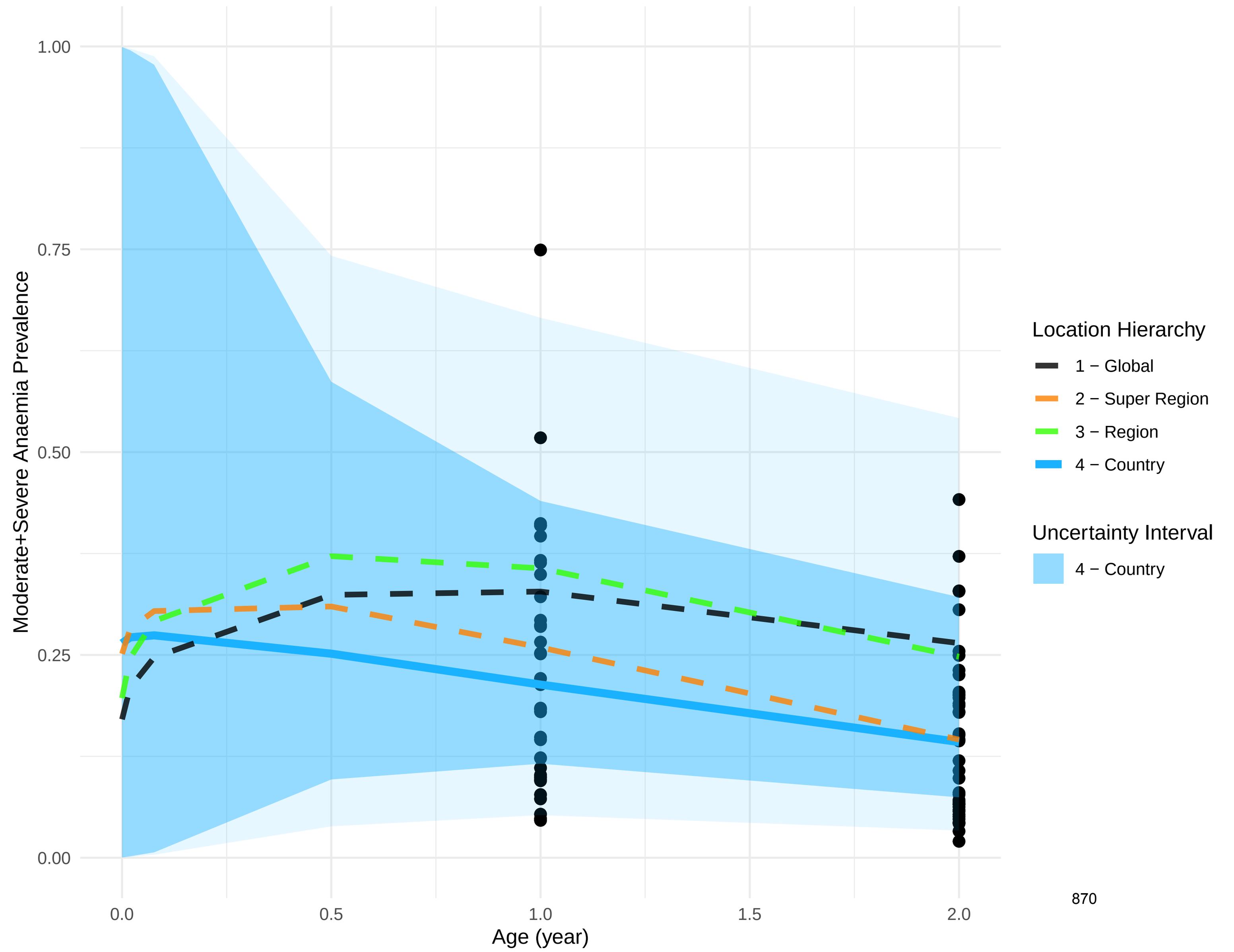

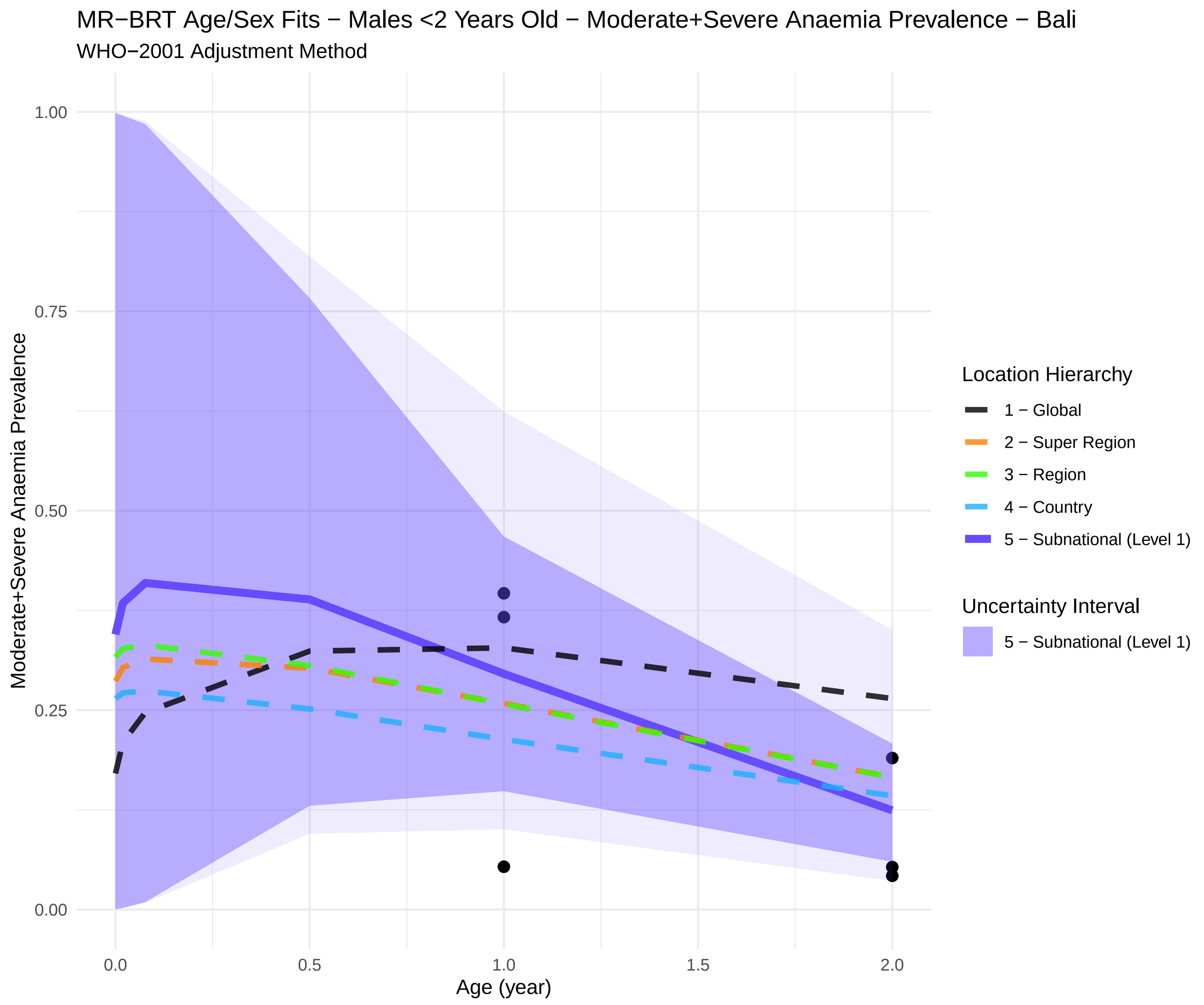
MR−BRT Age/Sex Fits − Males <2 Years Old − Moderate+Severe Anaemia Prevalence − Bali
WHO−2001 Adjustment Method
Moderate+Severe Anaemia Prevalence
Age (year)
Location Hierarchy
1 − Global
2 − Super Region
3 − Region
4 − Country
5 − Subnational (Level 1)
Uncertainty Interval
5 − Subnational (Level 1)

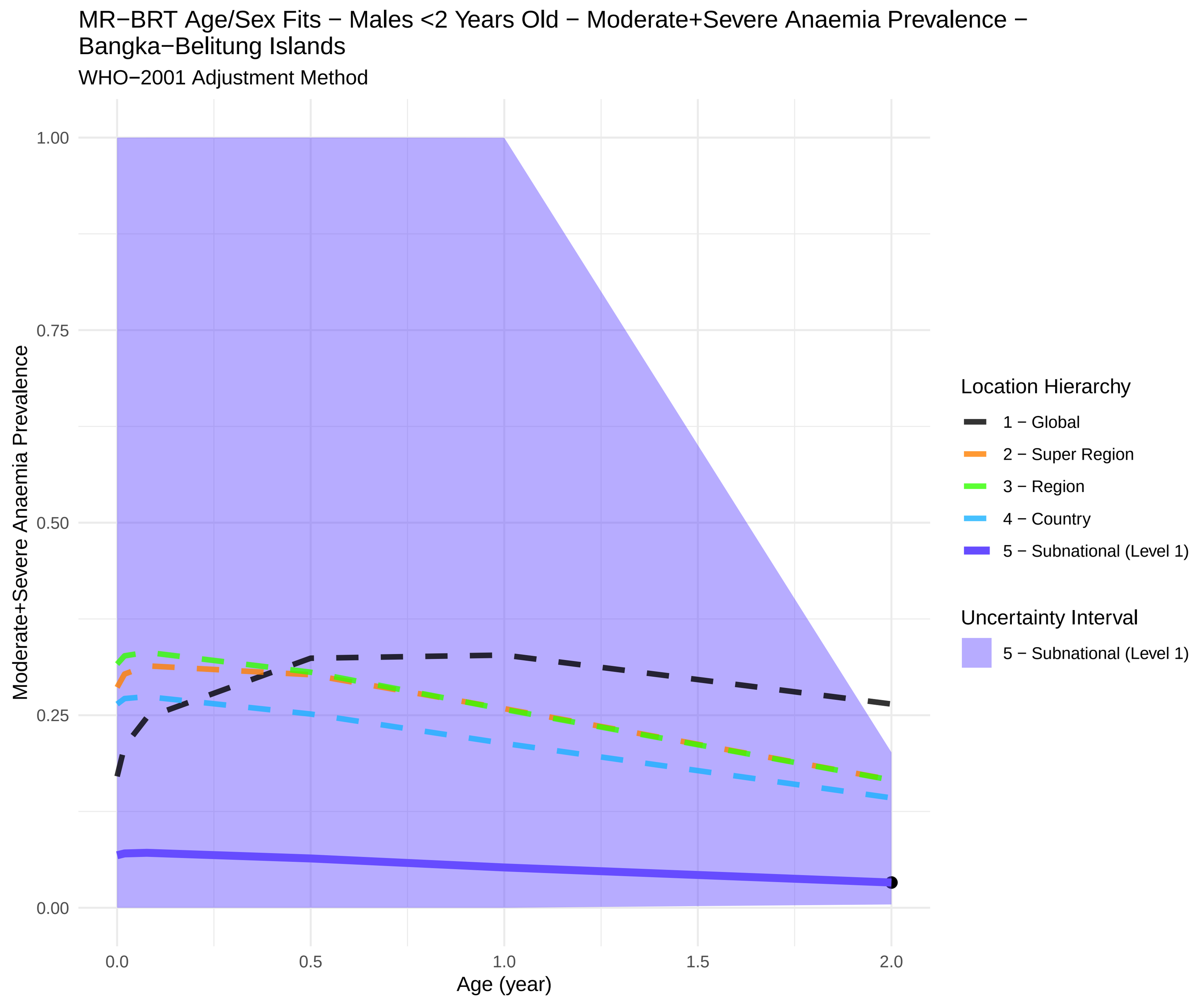
MR−BRT Age/Sex Fits − Males <2 Years Old − Moderate+Severe Anaemia Prevalence − Bangka−Belitung Islands
WHO−2001 Adjustment Method
Moderate+Severe Anaemia Prevalence
Age (year)
Location Hierarchy
1 − Global
2 − Super Region
3 − Region
4 − Country
5 − Subnational (Level 1)
Uncertainty Interval
5 − Subnational (Level 1)

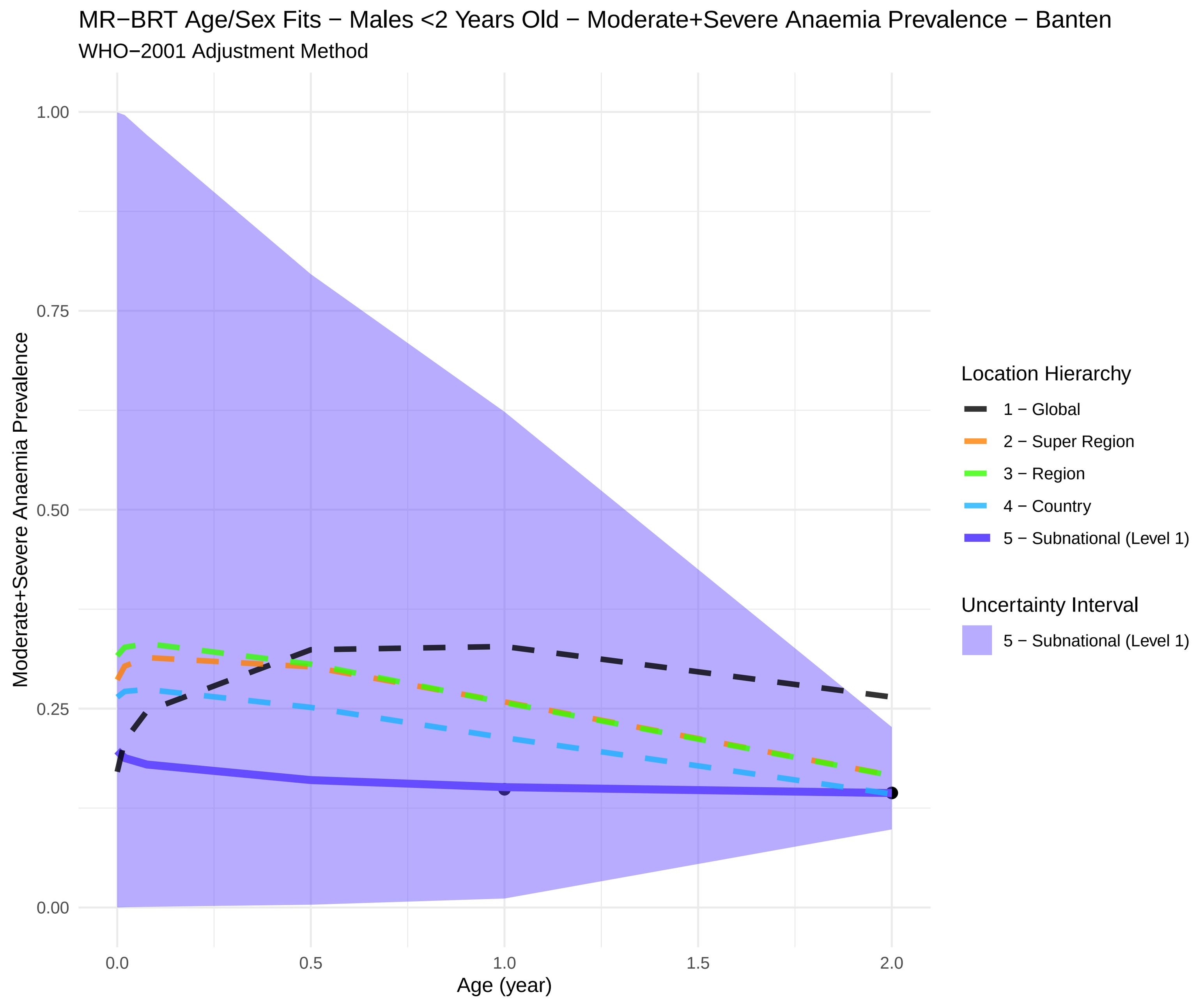
MR−BRT Age/Sex Fits − Males <2 Years Old − Moderate+Severe Anaemia Prevalence − Banten
WHO−2001 Adjustment Method
Moderate+Severe Anaemia Prevalence
Age (year)
Location Hierarchy
1 − Global
2 − Super Region
3 − Region
4 − Country
5 − Subnational (Level 1)
Uncertainty Interval
5 − Subnational (Level 1)

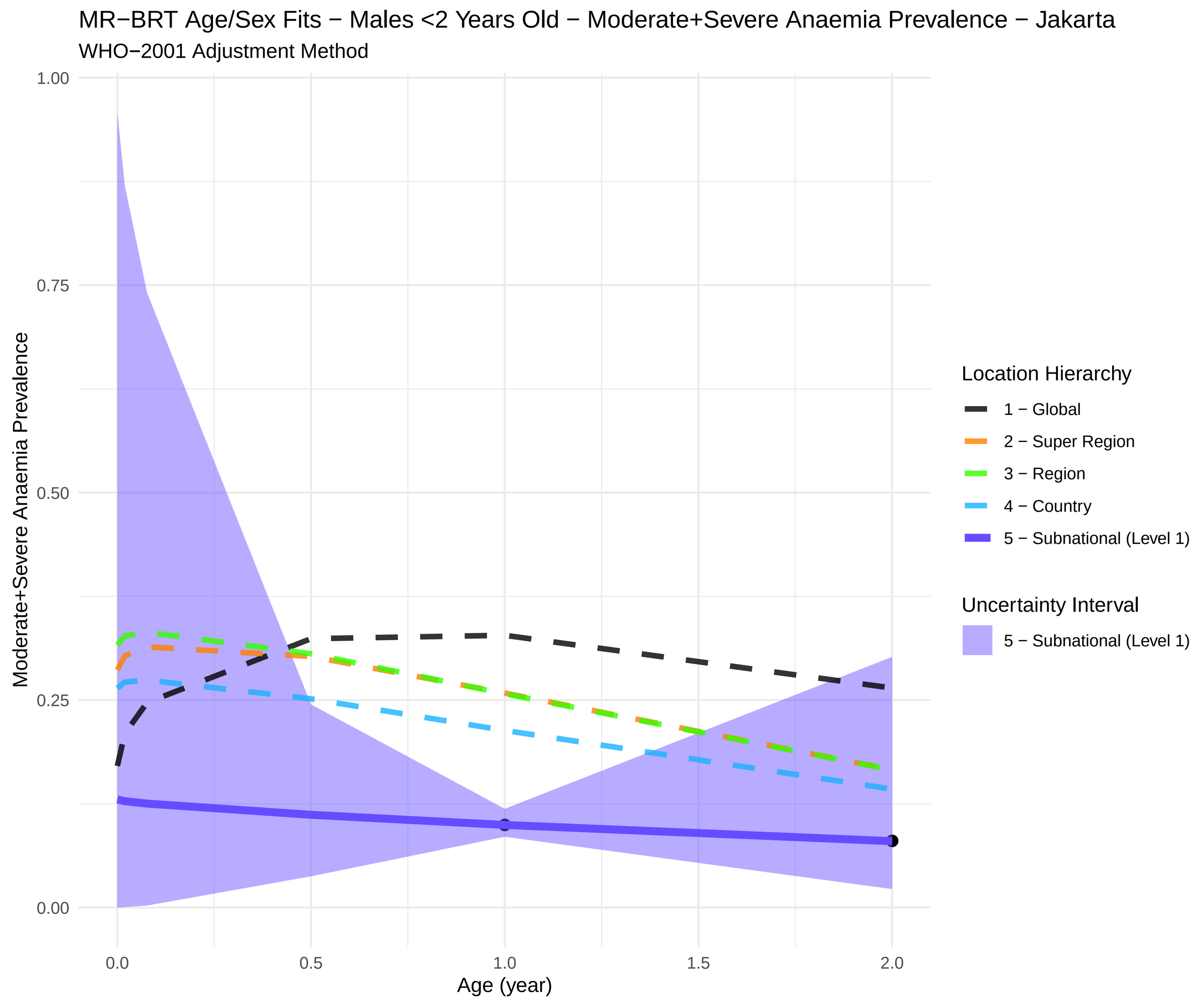
MR−BRT Age/Sex Fits − Males <2 Years Old − Moderate+Severe Anaemia Prevalence − Jakarta
WHO−2001 Adjustment Method
Moderate+Severe Anaemia Prevalence
Age (year)
Location Hierarchy
1 − Global
2 − Super Region
3 − Region
4 − Country
5 − Subnational (Level 1)
Uncertainty Interval
5 − Subnational (Level 1)

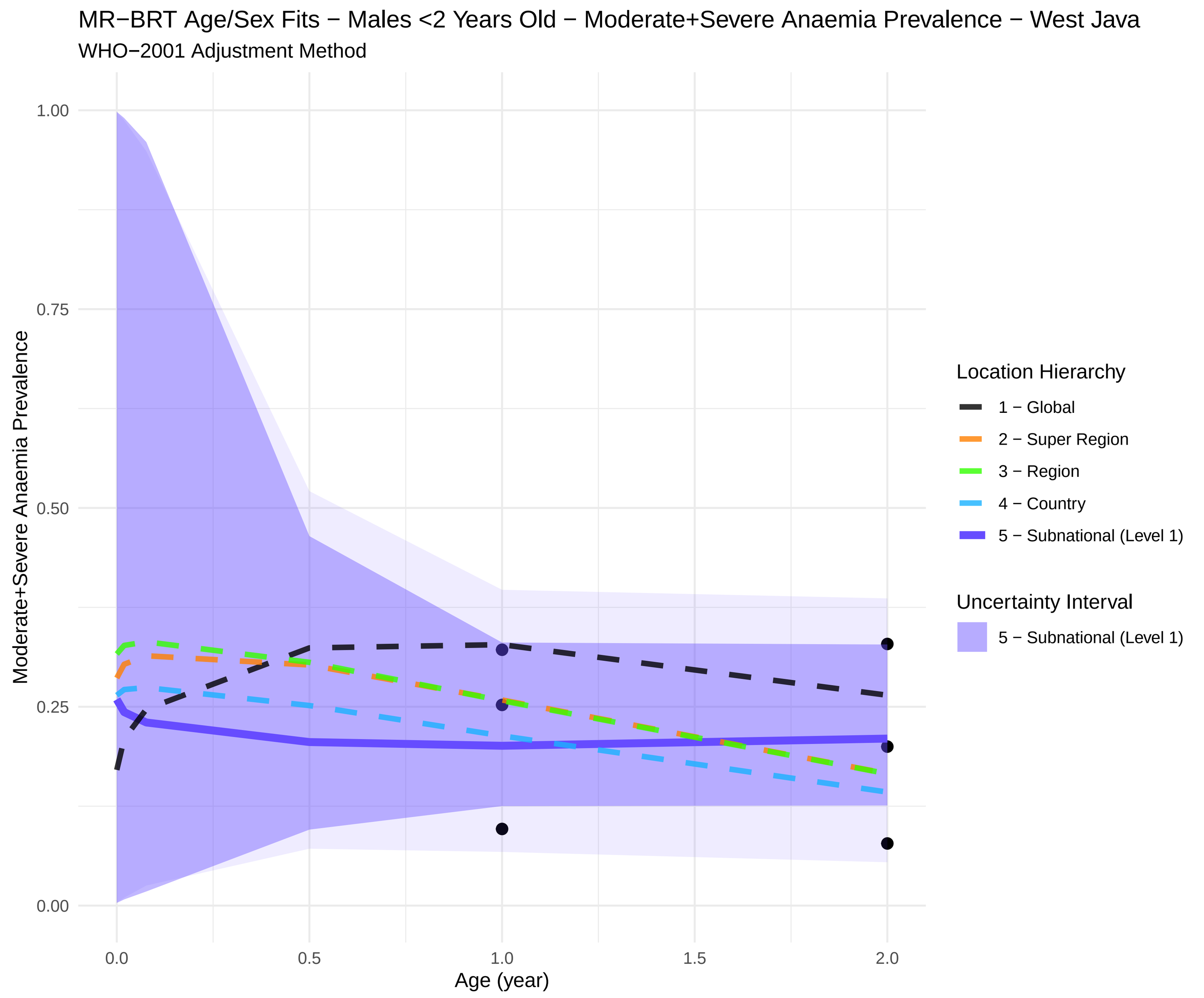
MR−BRT Age/Sex Fits − Males <2 Years Old − Moderate+Severe Anaemia Prevalence − West Java
WHO−2001 Adjustment Method
Moderate+Severe Anaemia Prevalence
Age (year)
Location Hierarchy
1 − Global
2 − Super Region
3 − Region
4 − Country
5 − Subnational (Level 1)
Uncertainty Interval
5 − Subnational (Level 1)

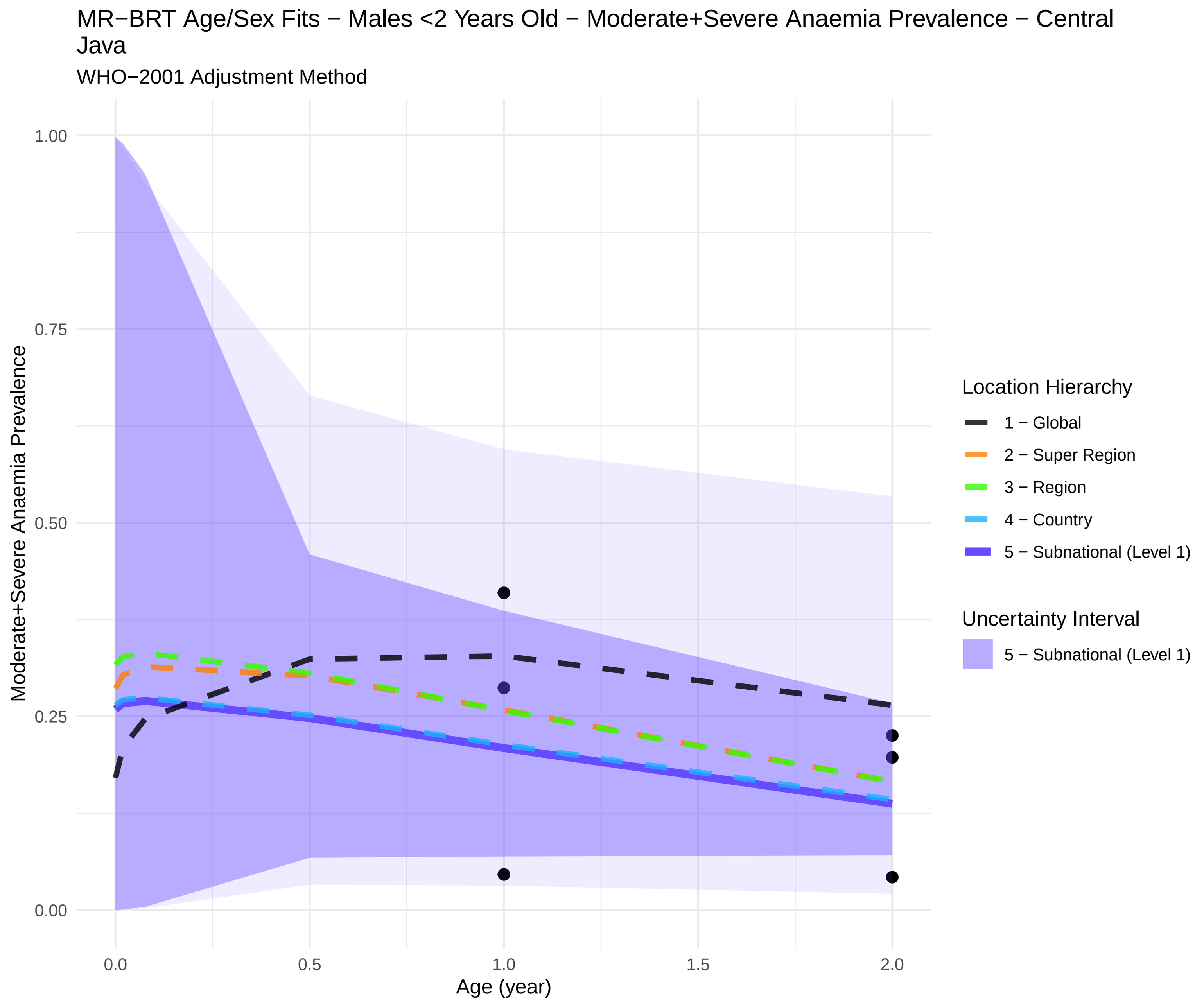
MR−BRT Age/Sex Fits − Males <2 Years Old − Moderate+Severe Anaemia Prevalence − Central Java
WHO−2001 Adjustment Method
Moderate+Severe Anaemia Prevalence
Age (year)
Location Hierarchy
1 − Global
2 − Super Region
3 − Region
4 − Country
5 − Subnational (Level 1)
Uncertainty Interval
5 − Subnational (Level 1)

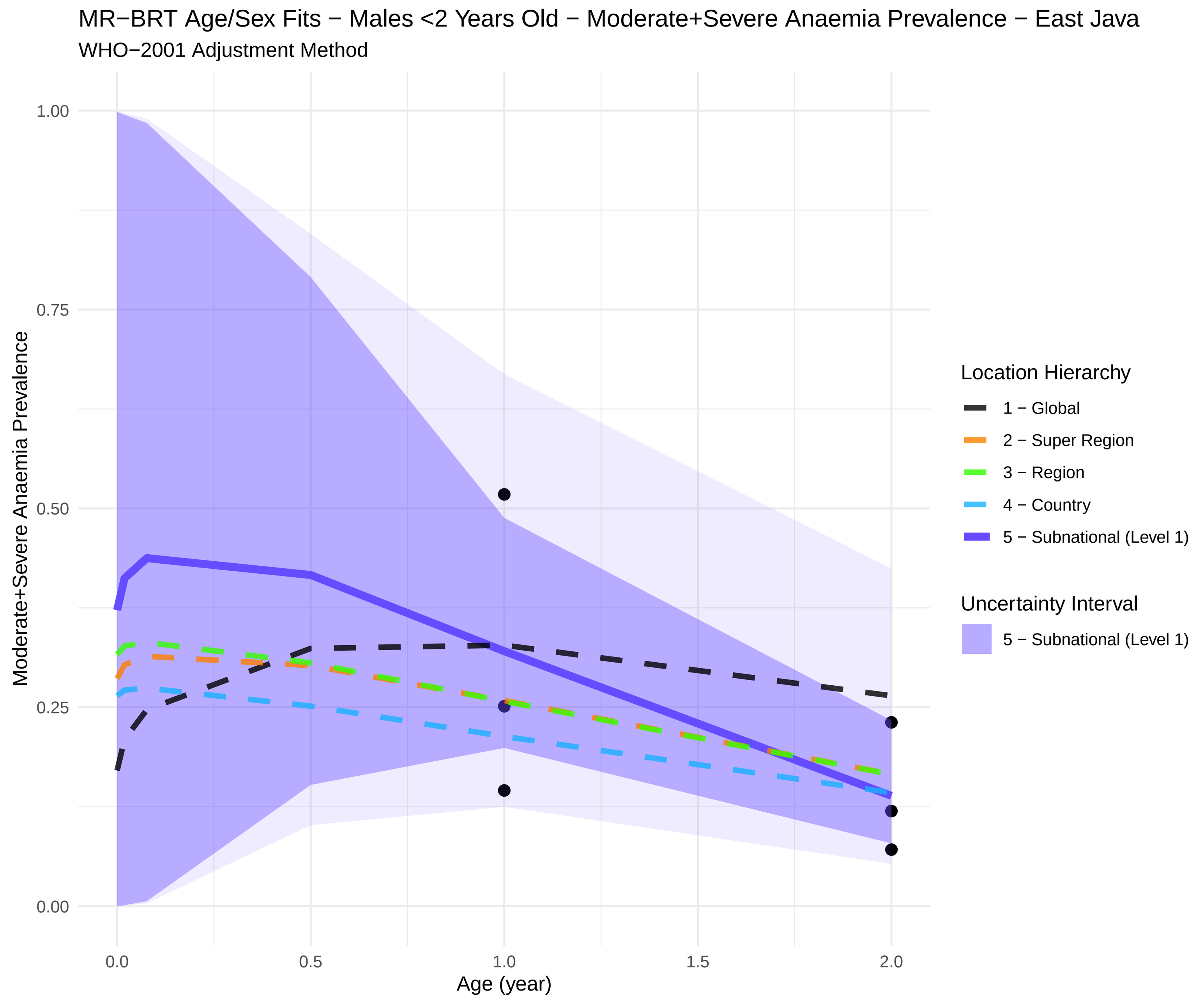
MR−BRT Age/Sex Fits − Males <2 Years Old − Moderate+Severe Anaemia Prevalence − East Java
WHO−2001 Adjustment Method
Moderate+Severe Anaemia Prevalence
Age (year)
Location Hierarchy
1 − Global
2 − Super Region
3 − Region
4 − Country
5 − Subnational (Level 1)
Uncertainty Interval
5 − Subnational (Level 1)

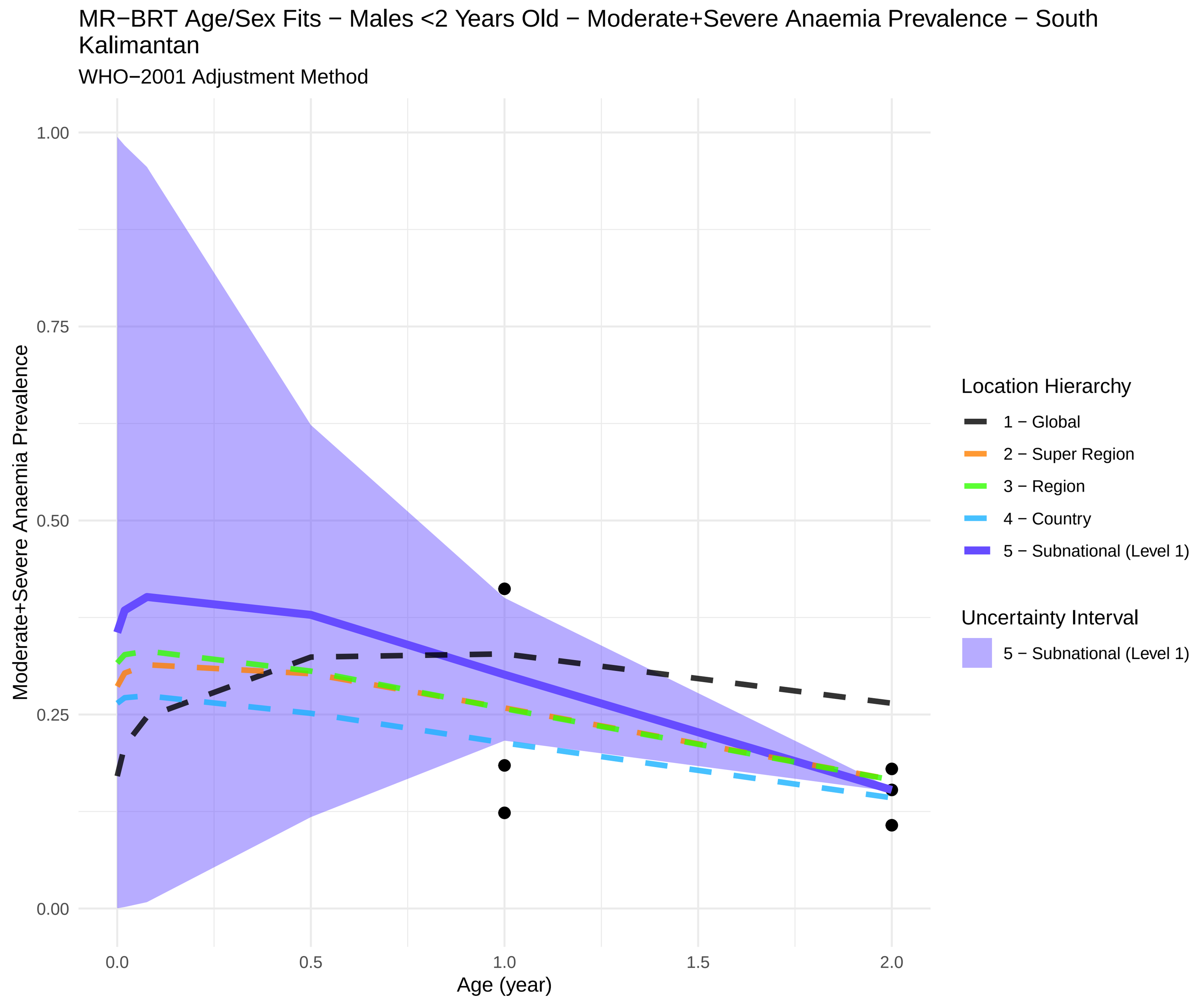
MR−BRT Age/Sex Fits − Males <2 Years Old − Moderate+Severe Anaemia Prevalence − South Kalimantan
WHO−2001 Adjustment Method
Moderate+Severe Anaemia Prevalence
Age (year)
Location Hierarchy
1 − Global
2 − Super Region
3 − Region
4 − Country
5 − Subnational (Level 1)
Uncertainty Interval
5 − Subnational (Level 1)

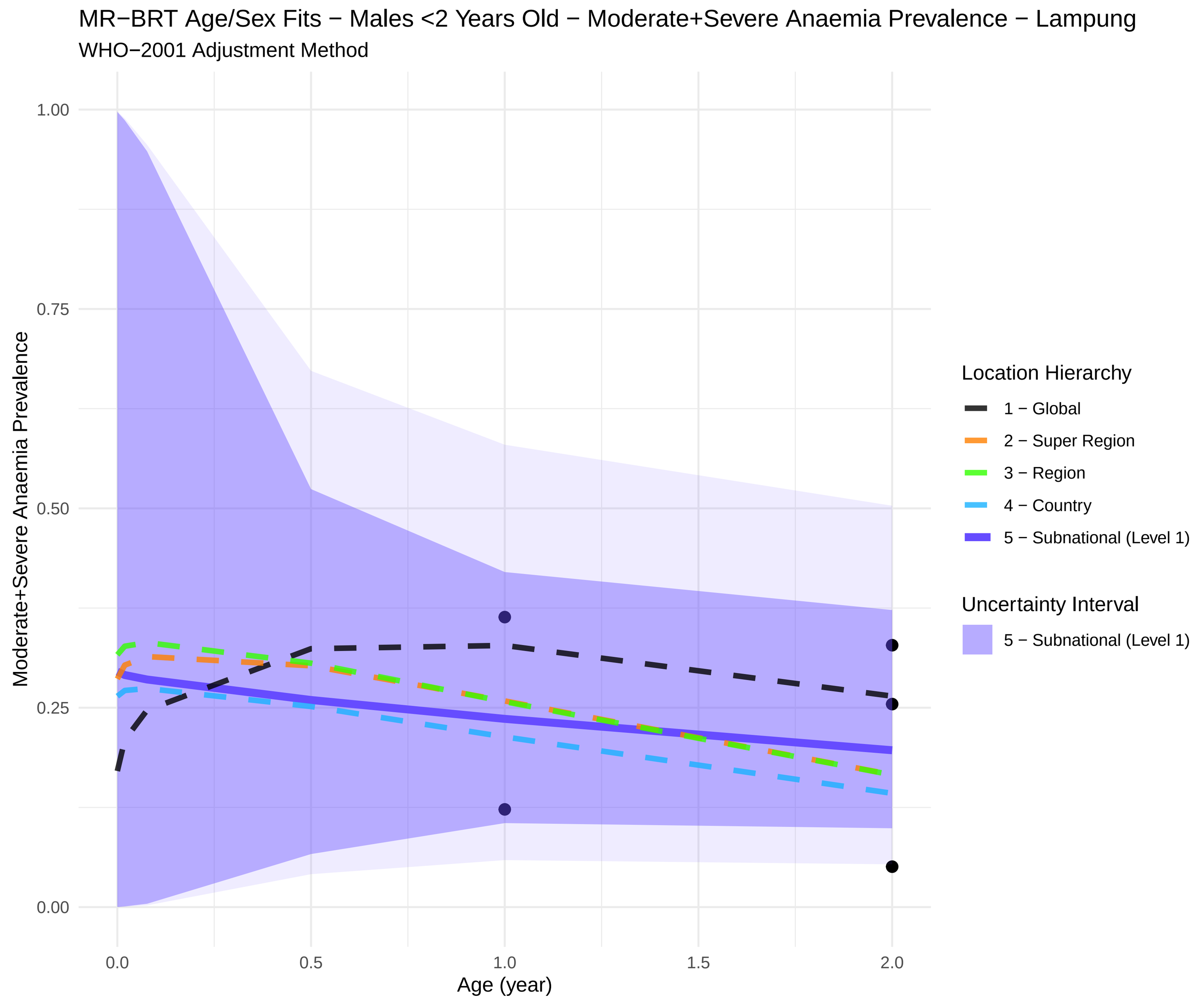
MR−BRT Age/Sex Fits − Males <2 Years Old − Moderate+Severe Anaemia Prevalence − Lampung
WHO−2001 Adjustment Method
Moderate+Severe Anaemia Prevalence
Age (year)
Location Hierarchy
1 − Global
2 − Super Region
3 − Region
4 − Country
5 − Subnational (Level 1)
Uncertainty Interval
5 − Subnational (Level 1)

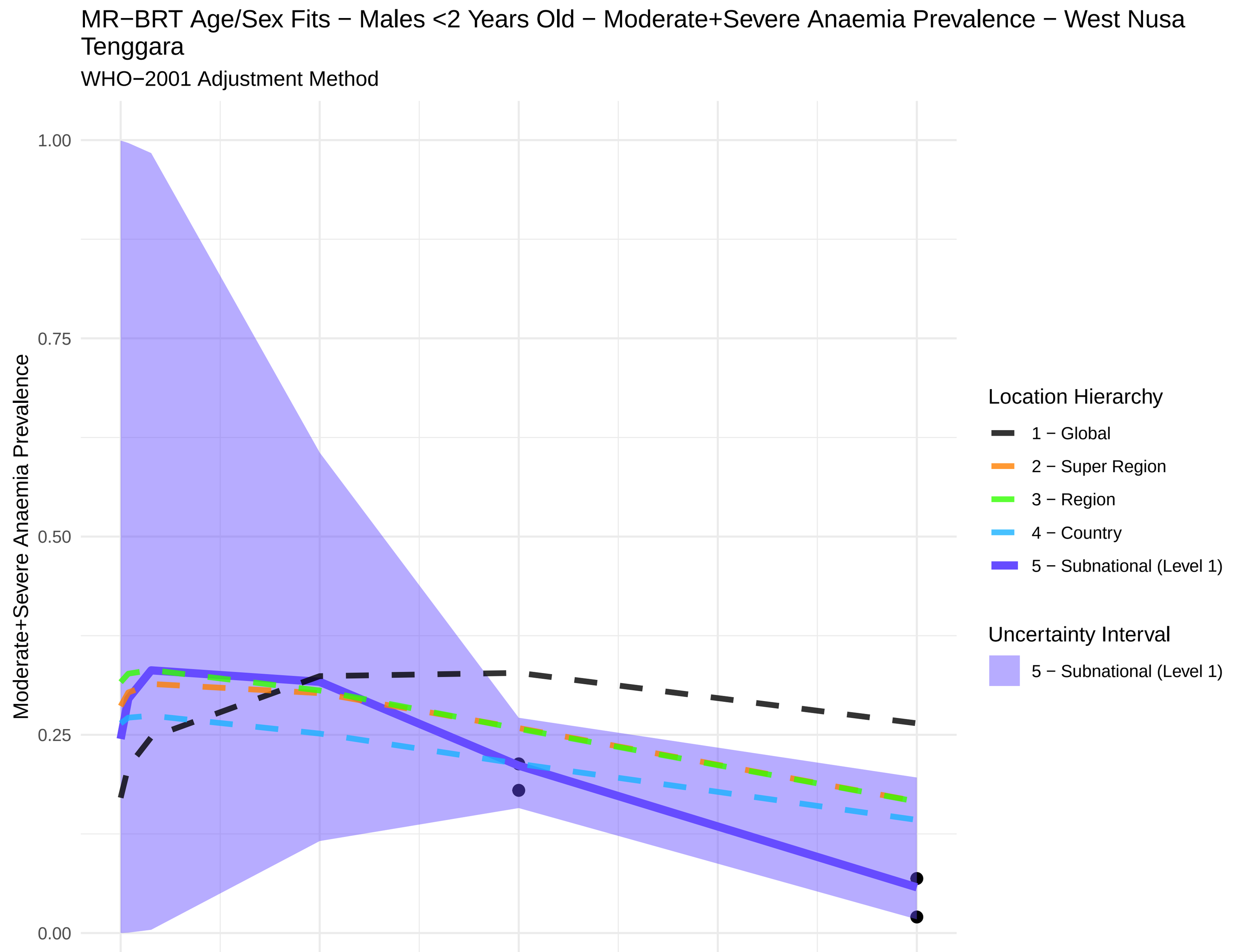
MR−BRT Age/Sex Fits − Males <2 Years Old − Moderate+Severe Anaemia Prevalence − West Nusa Tenggara
WHO−2001 Adjustment Method
Moderate+Severe Anaemia Prevalence
Age (year)
Location Hierarchy
1 − Global
2 − Super Region
3 − Region
4 − Country
5 − Subnational (Level 1)
Uncertainty Interval
5 − Subnational (Level 1)

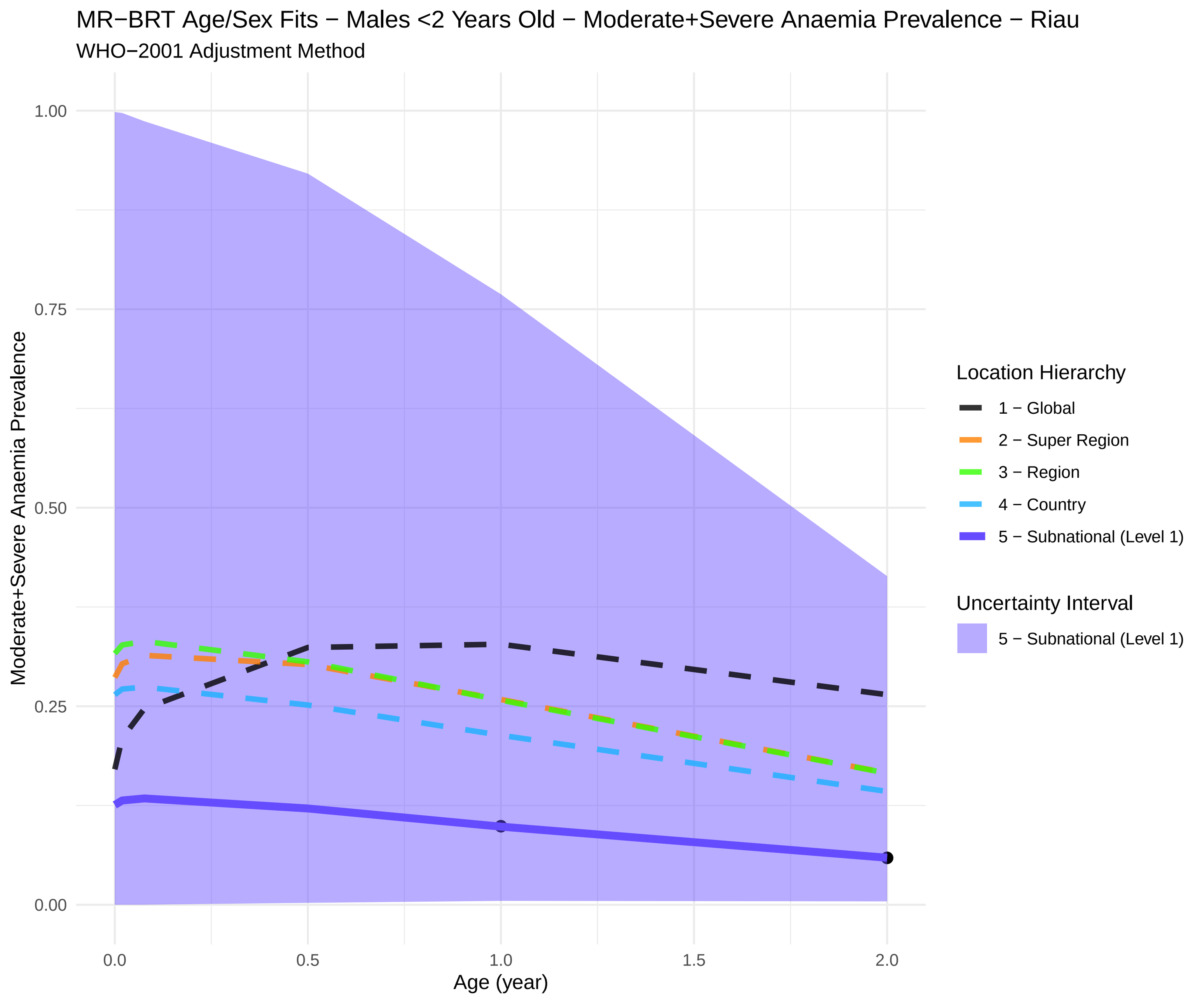
MR−BRT Age/Sex Fits − Males <2 Years Old − Moderate+Severe Anaemia Prevalence − Riau
WHO−2001 Adjustment Method
Moderate+Severe Anaemia Prevalence
Age (year)
Location Hierarchy
1 − Global
2 − Super Region
3 − Region
4 − Country
5 − Subnational (Level 1)
Uncertainty Interval
5 − Subnational (Level 1)

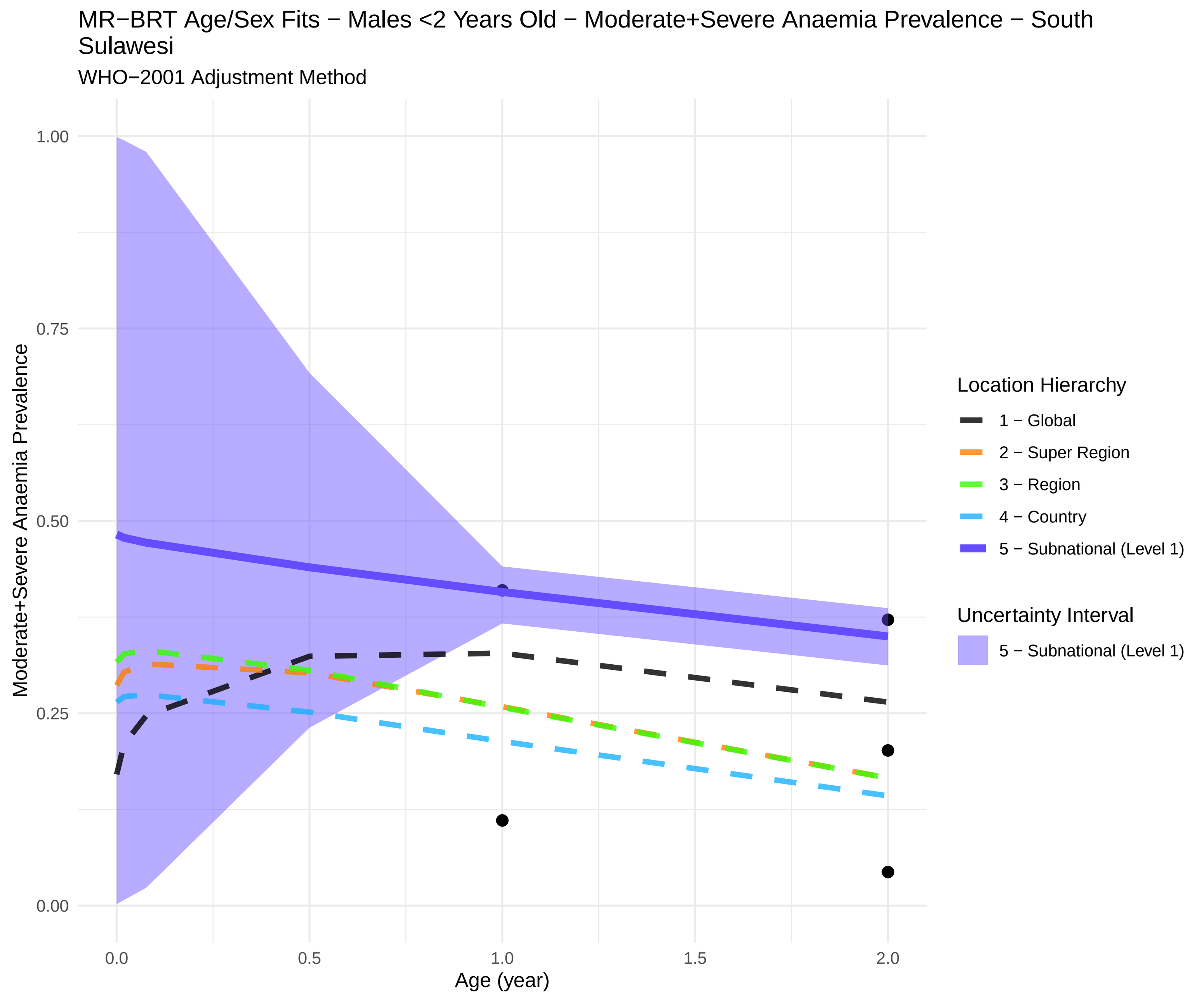
MR−BRT Age/Sex Fits − Males <2 Years Old − Moderate+Severe Anaemia Prevalence − South Sulawesi
WHO−2001 Adjustment Method
Moderate+Severe Anaemia Prevalence
Age (year)
Location Hierarchy
1 − Global
2 − Super Region
3 − Region
4 − Country
5 − Subnational (Level 1)
Uncertainty Interval
5 − Subnational (Level 1)

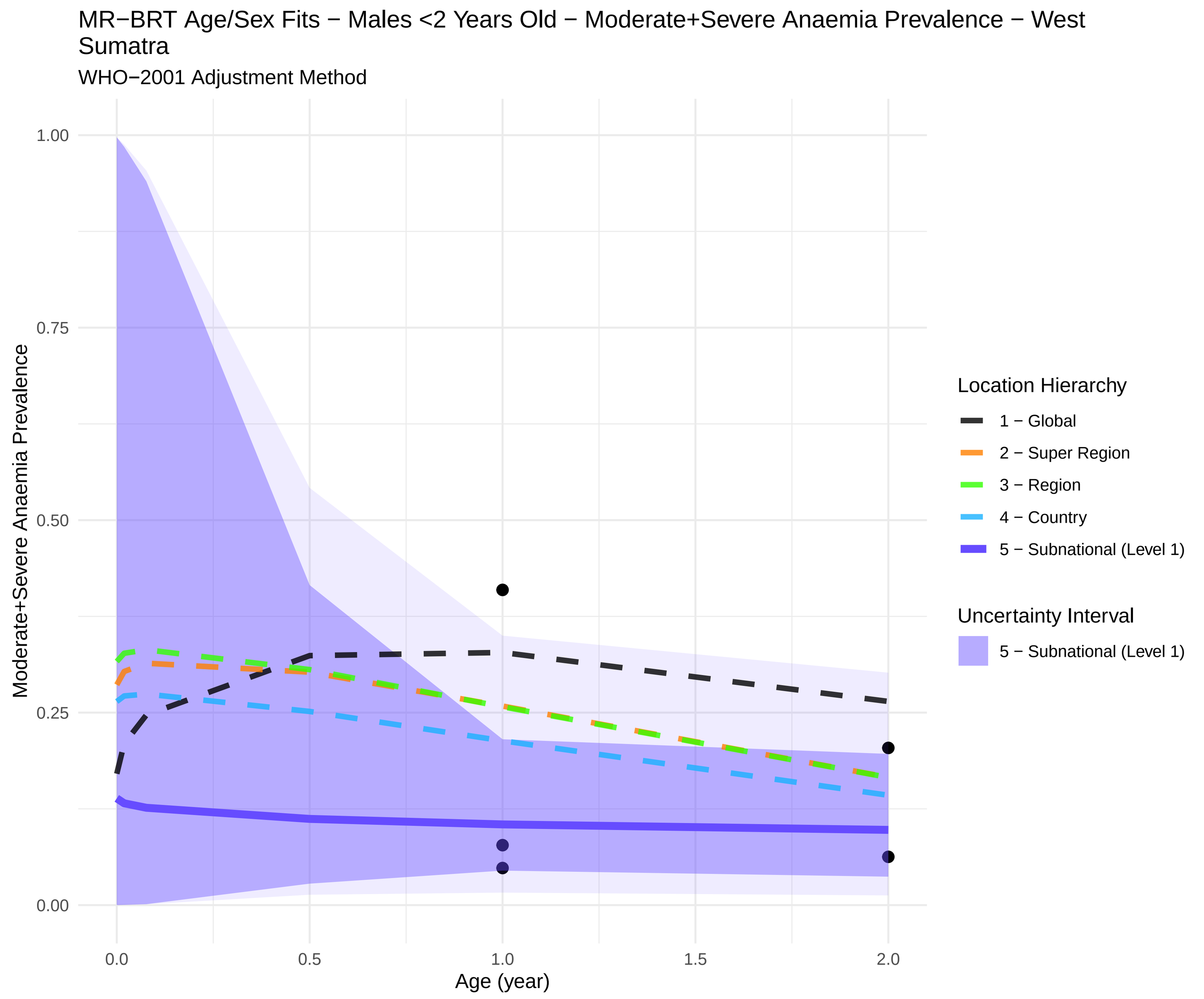
MR−BRT Age/Sex Fits − Males <2 Years Old − Moderate+Severe Anaemia Prevalence − West Sumatra
WHO−2001 Adjustment Method
Moderate+Severe Anaemia Prevalence
Age (year)
Location Hierarchy
1 − Global
2 − Super Region
3 − Region
4 − Country
5 − Subnational (Level 1)
Uncertainty Interval
5 − Subnational (Level 1)

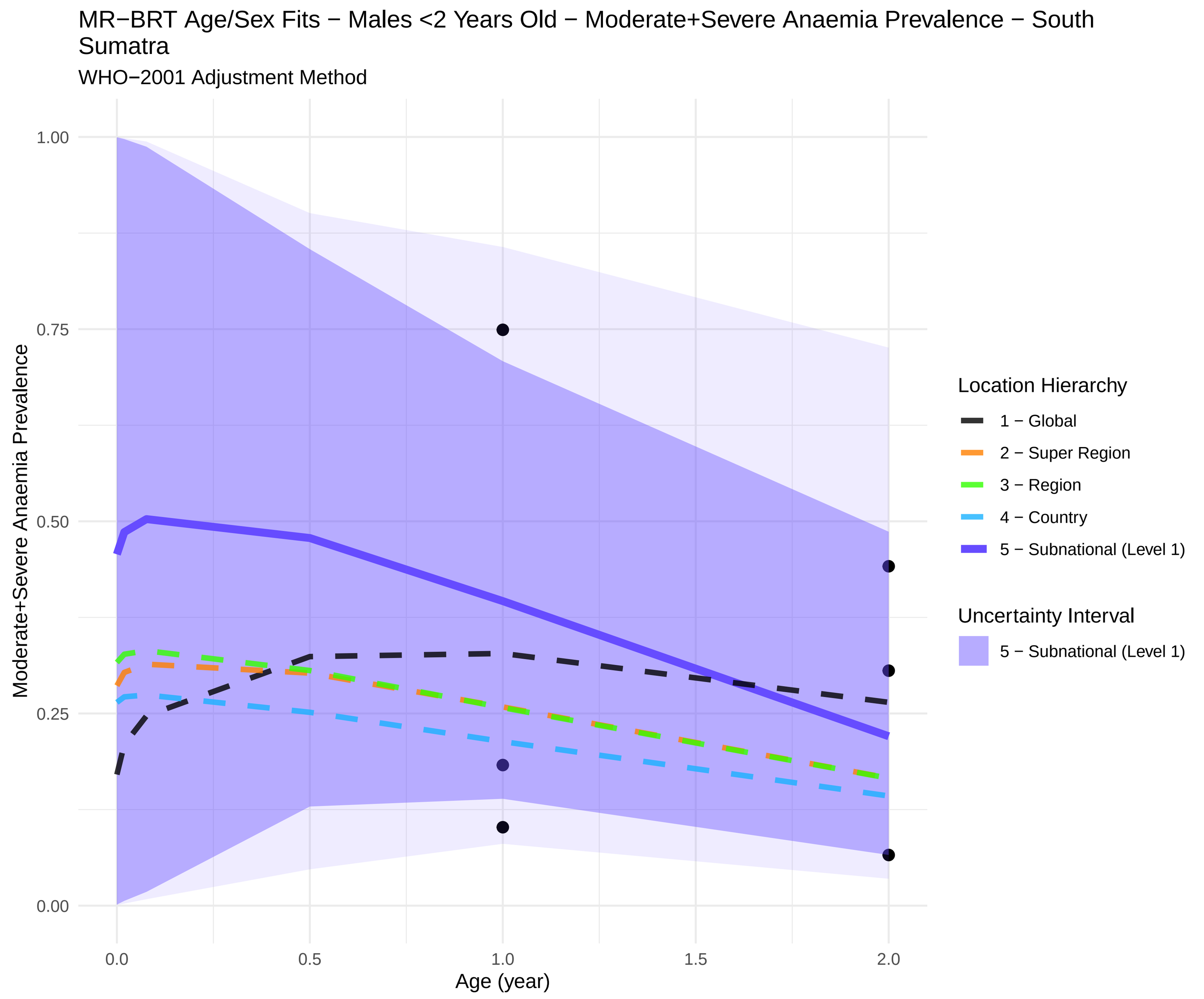
MR−BRT Age/Sex Fits − Males <2 Years Old − Moderate+Severe Anaemia Prevalence − South Sumatra
WHO−2001 Adjustment Method
Moderate+Severe Anaemia Prevalence
Age (year)
Location Hierarchy
1 − Global
2 − Super Region
3 − Region
4 − Country
5 − Subnational (Level 1)
Uncertainty Interval
5 − Subnational (Level 1)

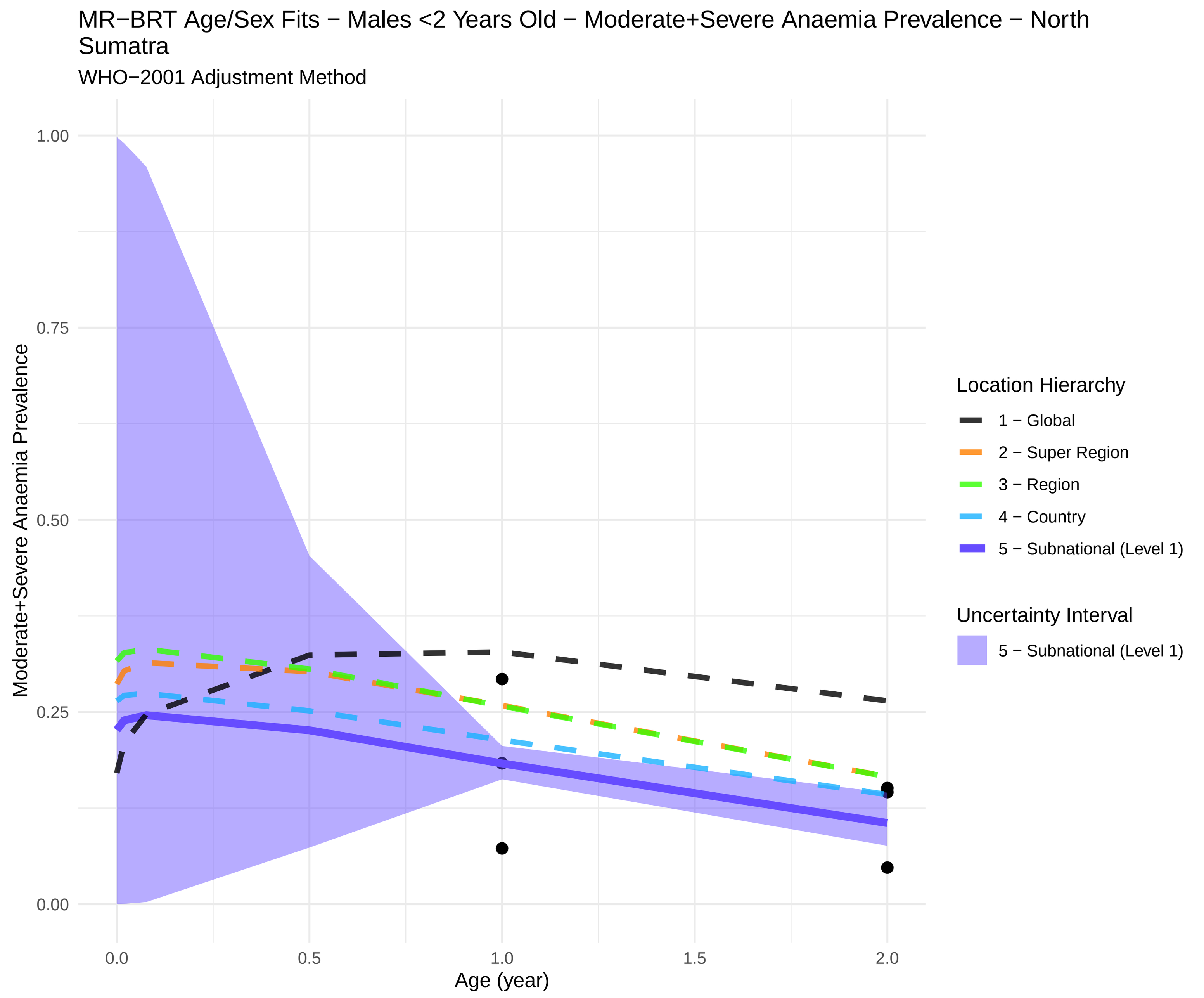
MR−BRT Age/Sex Fits − Males <2 Years Old − Moderate+Severe Anaemia Prevalence − North Sumatra
WHO−2001 Adjustment Method
Moderate+Severe Anaemia Prevalence
Age (year)
Location Hierarchy
1 − Global
2 − Super Region
3 − Region
4 − Country
5 − Subnational (Level 1)
Uncertainty Interval
5 − Subnational (Level 1)

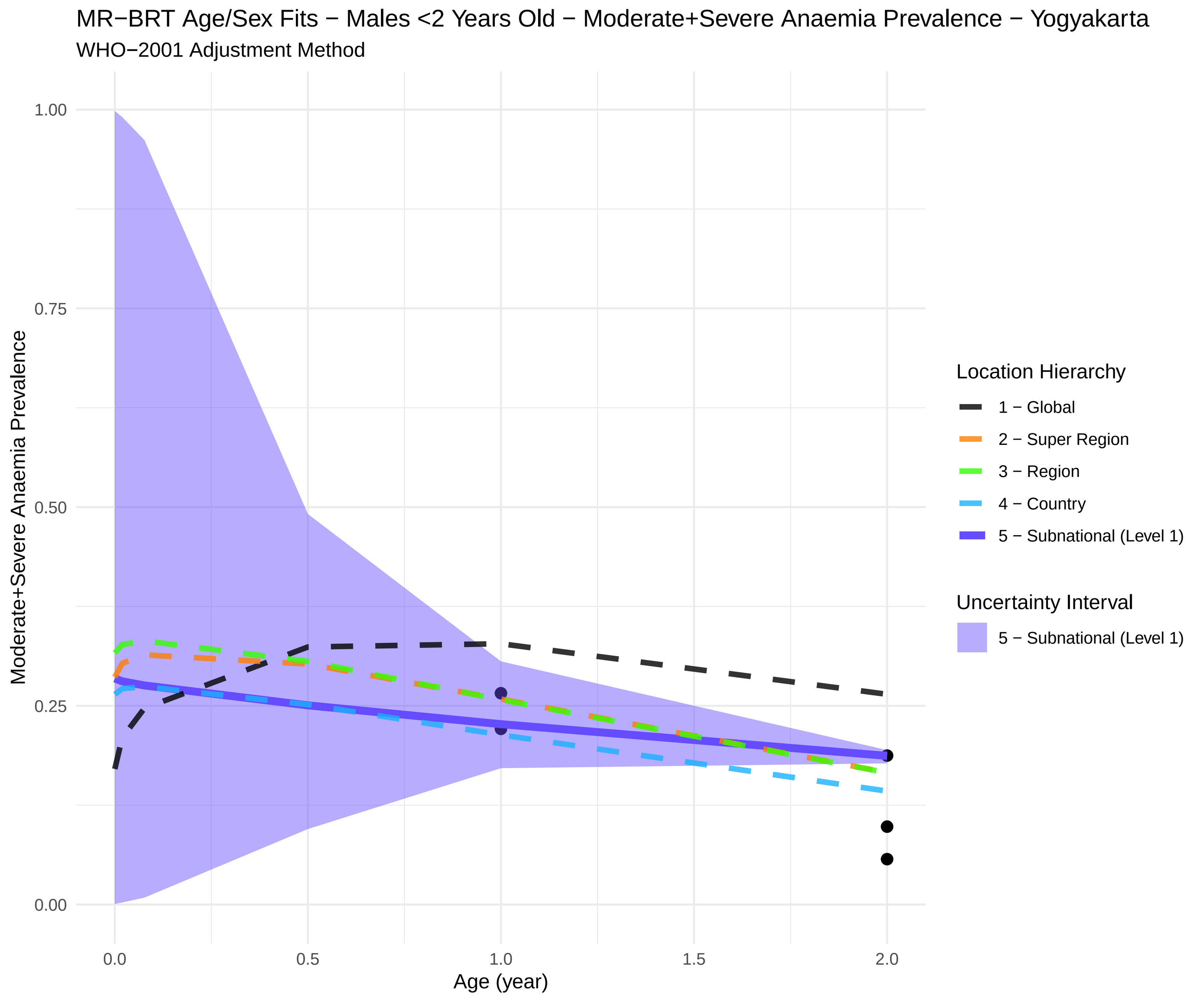
MR−BRT Age/Sex Fits − Males <2 Years Old − Moderate+Severe Anaemia Prevalence − Yogyakarta
WHO−2001 Adjustment Method
Moderate+Severe Anaemia Prevalence
Age (year)
Location Hierarchy
1 − Global
2 − Super Region
3 − Region
4 − Country
5 − Subnational (Level 1)
Uncertainty Interval
5 − Subnational (Level 1)

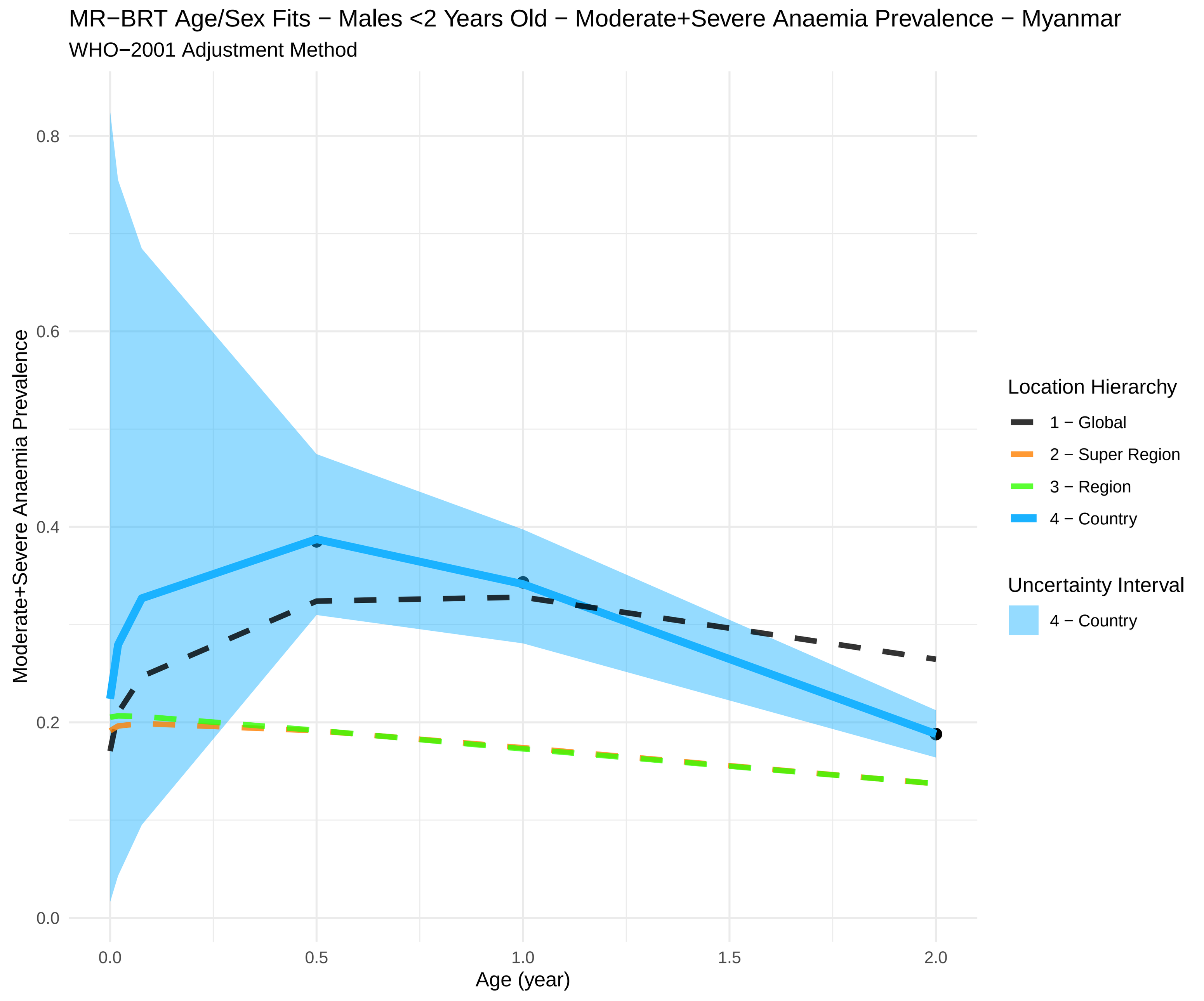
MR−BRT Age/Sex Fits − Males <2 Years Old − Moderate+Severe Anaemia Prevalence − Myanmar
WHO−2001 Adjustment Method
Moderate+Severe Anaemia Prevalence
Age (year)
Location Hierarchy
1 − Global
2 − Super Region
3 − Region
4 − Country
Uncertainty Interval
4 − Country

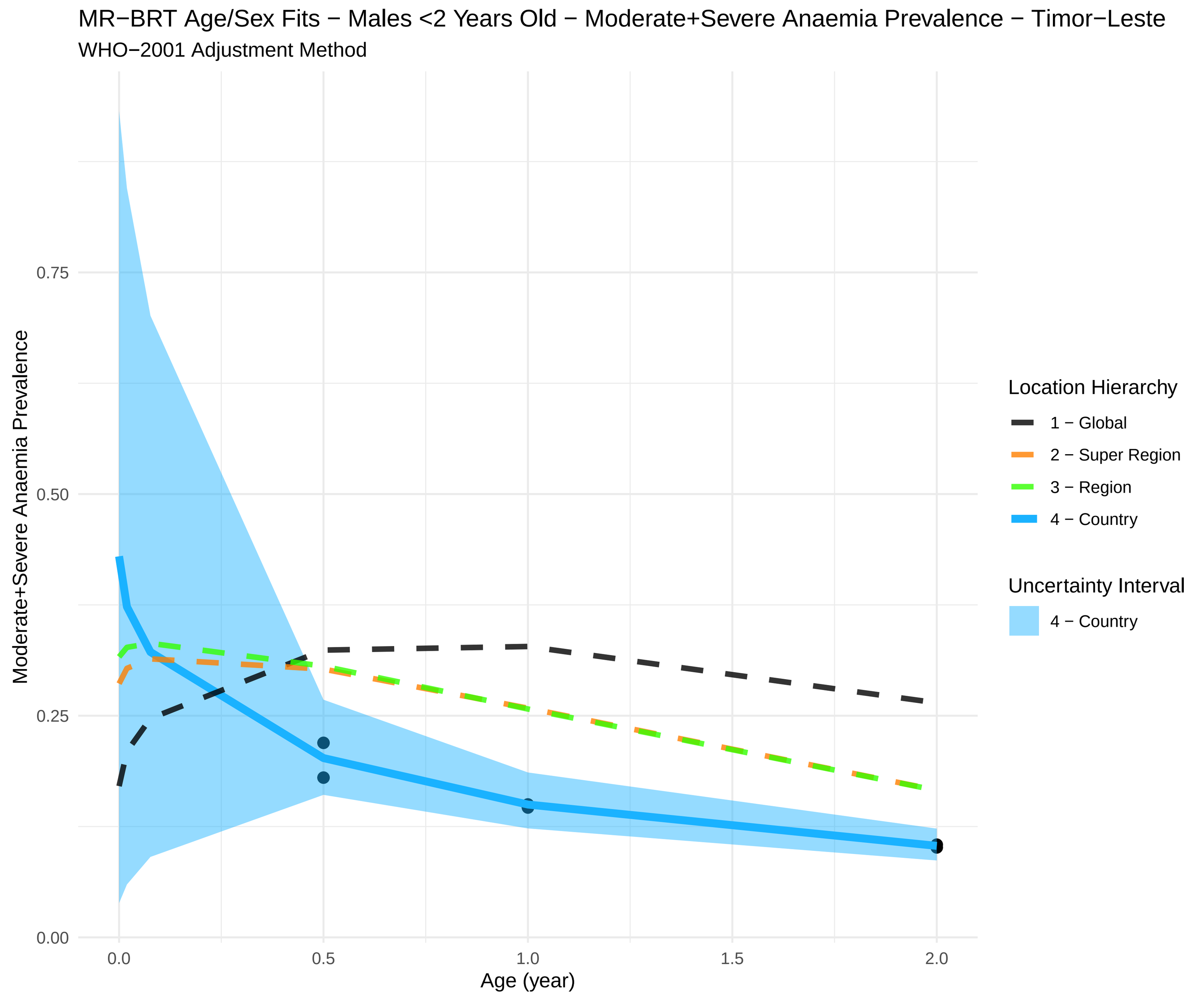
MR−BRT Age/Sex Fits − Males <2 Years Old − Moderate+Severe Anaemia Prevalence − Timor−Leste
WHO−2001 Adjustment Method
Moderate+Severe Anaemia Prevalence
Age (year)
Location Hierarchy
1 − Global
2 − Super Region
3 − Region
4 − Country
Uncertainty Interval
4 − Country

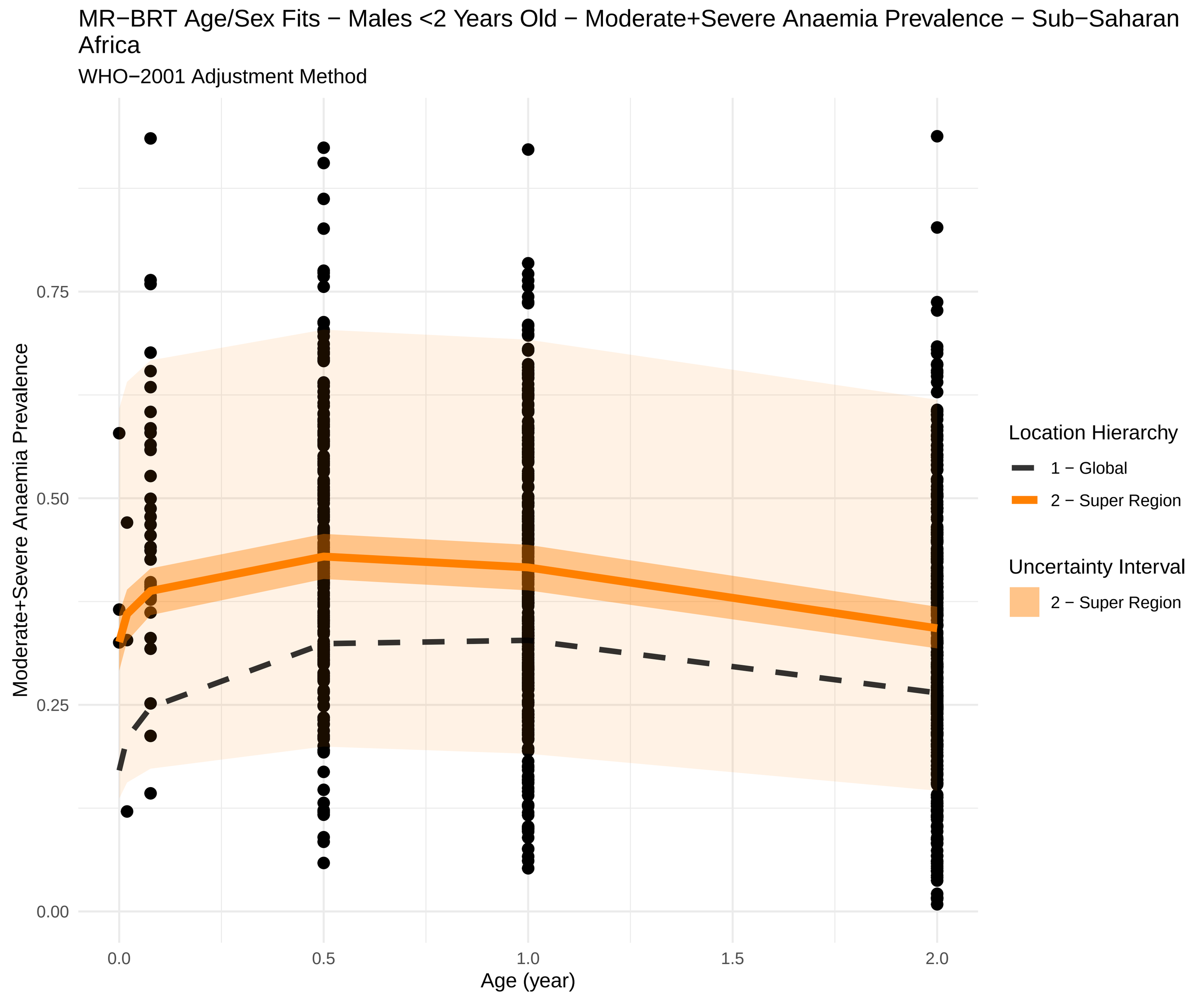
MR−BRT Age/Sex Fits − Males <2 Years Old − Moderate+Severe Anaemia Prevalence − Sub−Saharan Africa
WHO−2001 Adjustment Method
Moderate+Severe Anaemia Prevalence
Age (year)
Location Hierarchy
1 − Global
2 − Super Region
Uncertainty Interval
2 − Super Region

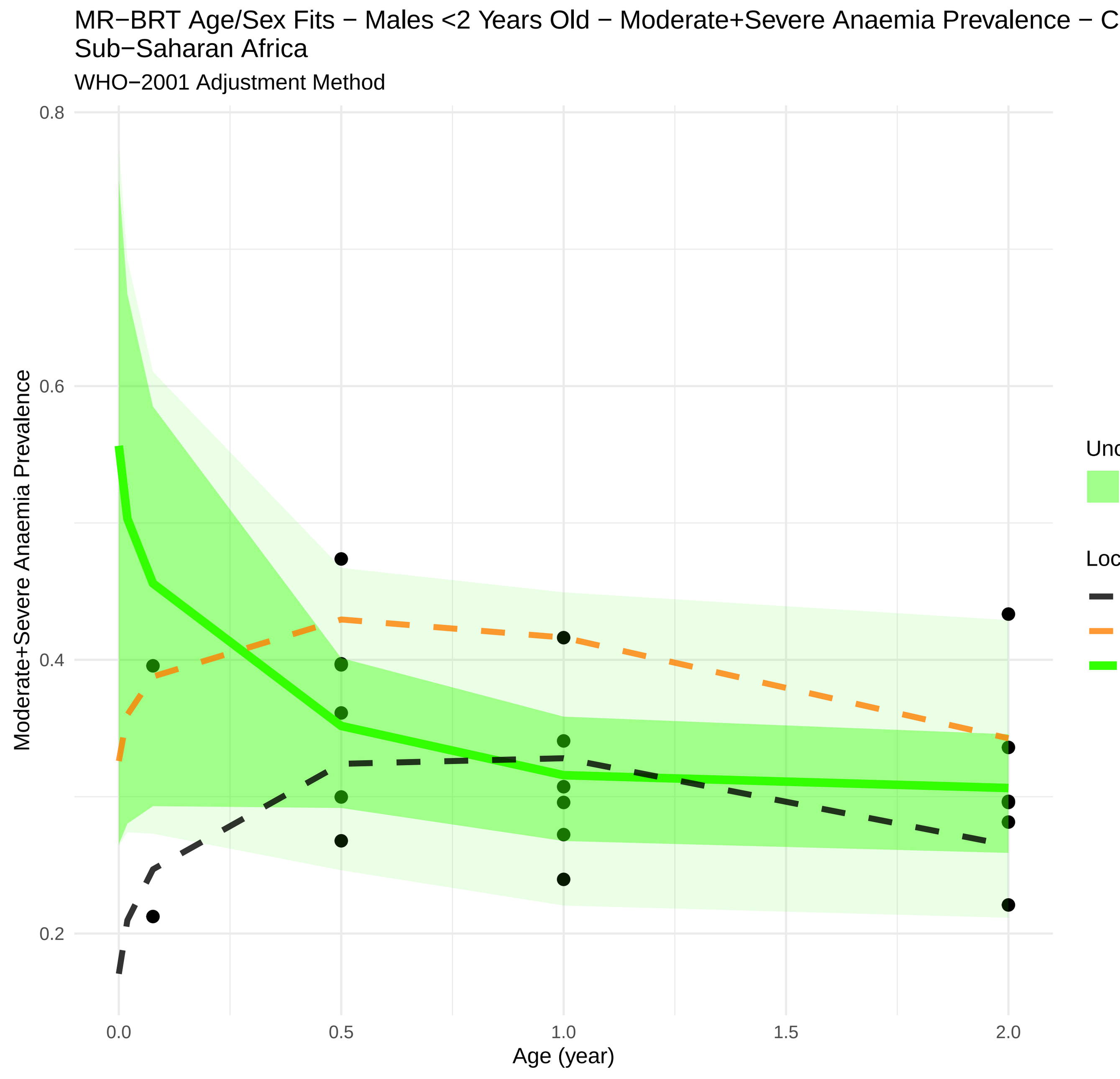
MR−BRT Age/Sex Fits − Males <2 Years Old − Moderate+Severe Anaemia Prevalence − Central Sub−Saharan Africa
WHO−2001 Adjustment Method
Moderate+Severe Anaemia Prevalence
Age (year)
Uncertainty Interval
3 − Region
Location Hierarchy
1 − Global
2 − Super Region
3 − Region

MR−BRT Age/Sex Fits − Males <2 Years Old − Moderate+Severe Anaemia Prevalence − Angola
WHO−2001 Adjustment Method

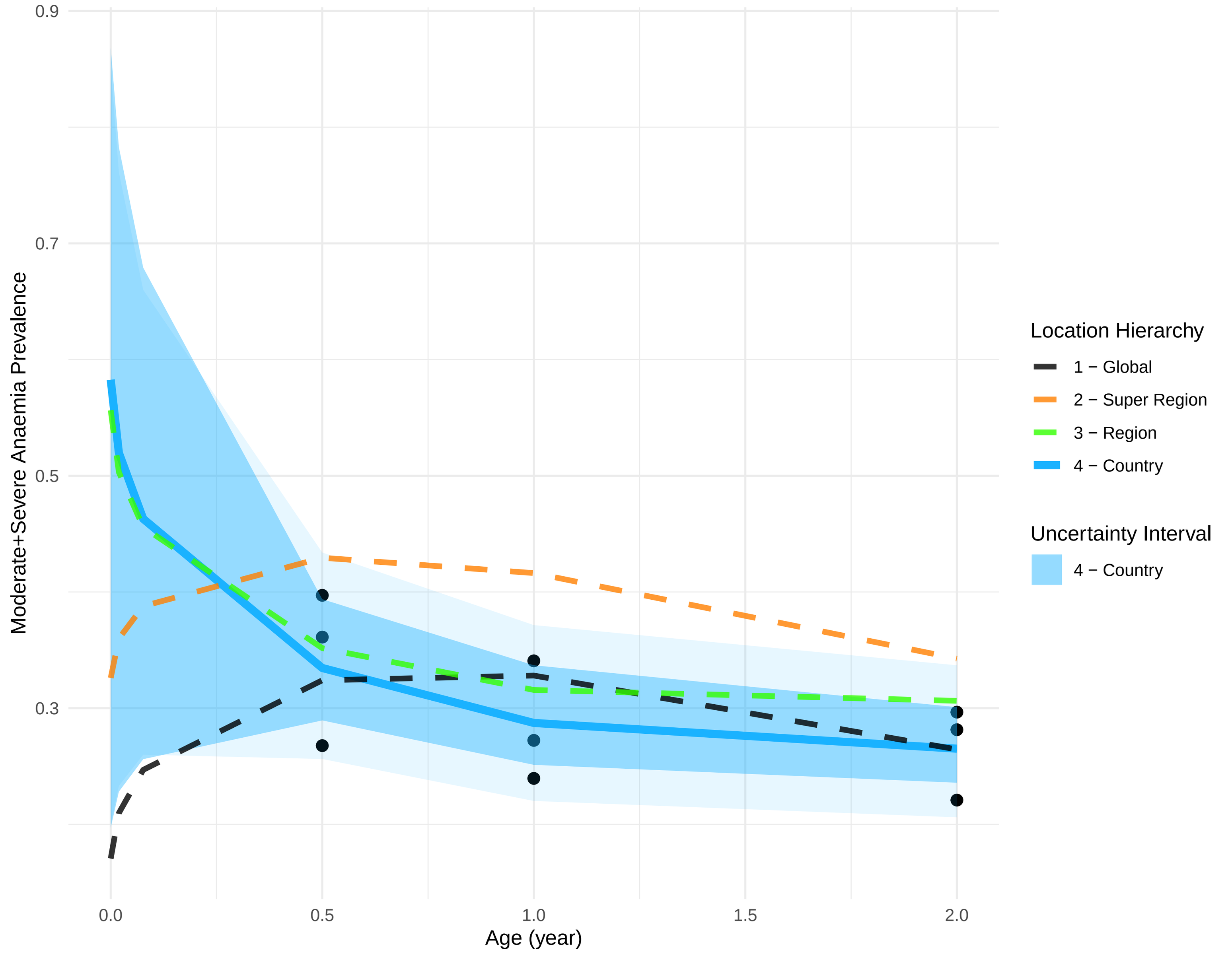

MR−BRT Age/Sex Fits − Males <2 Years Old − Moderate+Severe Anaemia Prevalence − Democratic Republic of the Congo
WHO−2001 Adjustment Method

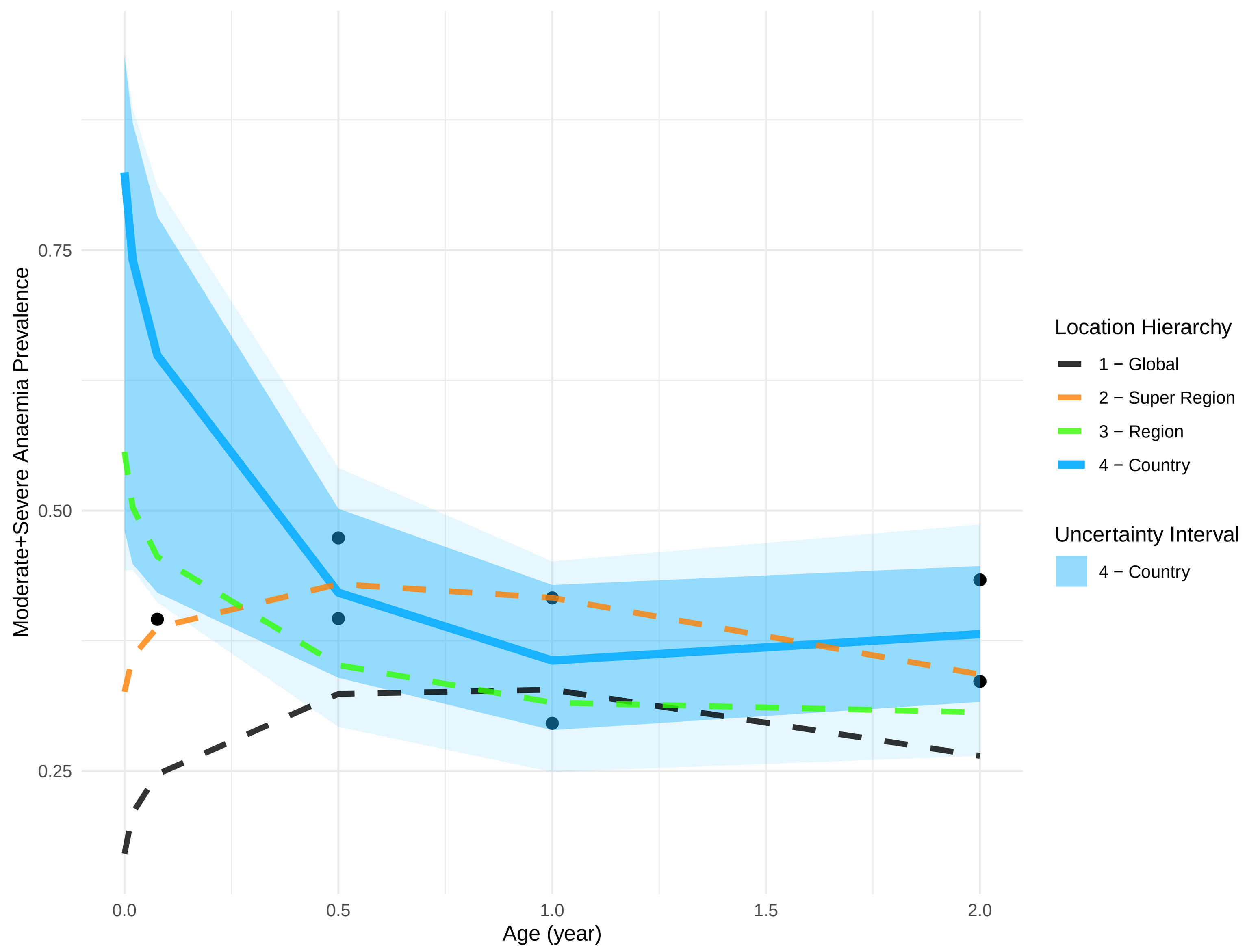

MR−BRT Age/Sex Fits − Males <2 Years Old − Moderate+Severe Anaemia Prevalence − Gabon
WHO−2001 Adjustment Method

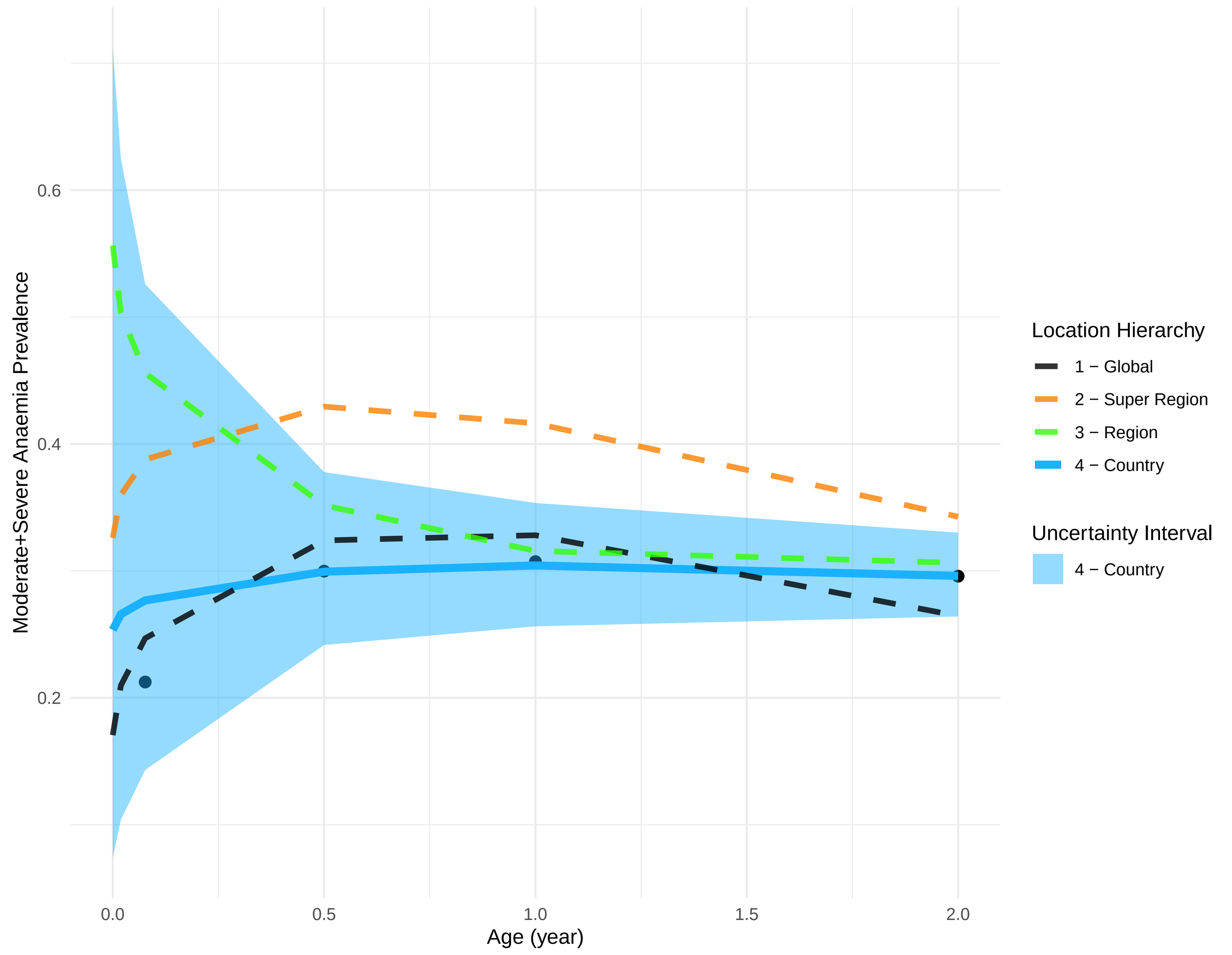

MR−BRT Age/Sex Fits − Males <2 Years Old − Moderate+Severe Anaemia Prevalence − Eastern Sub−Saharan Africa
WHO−2001 Adjustment Method

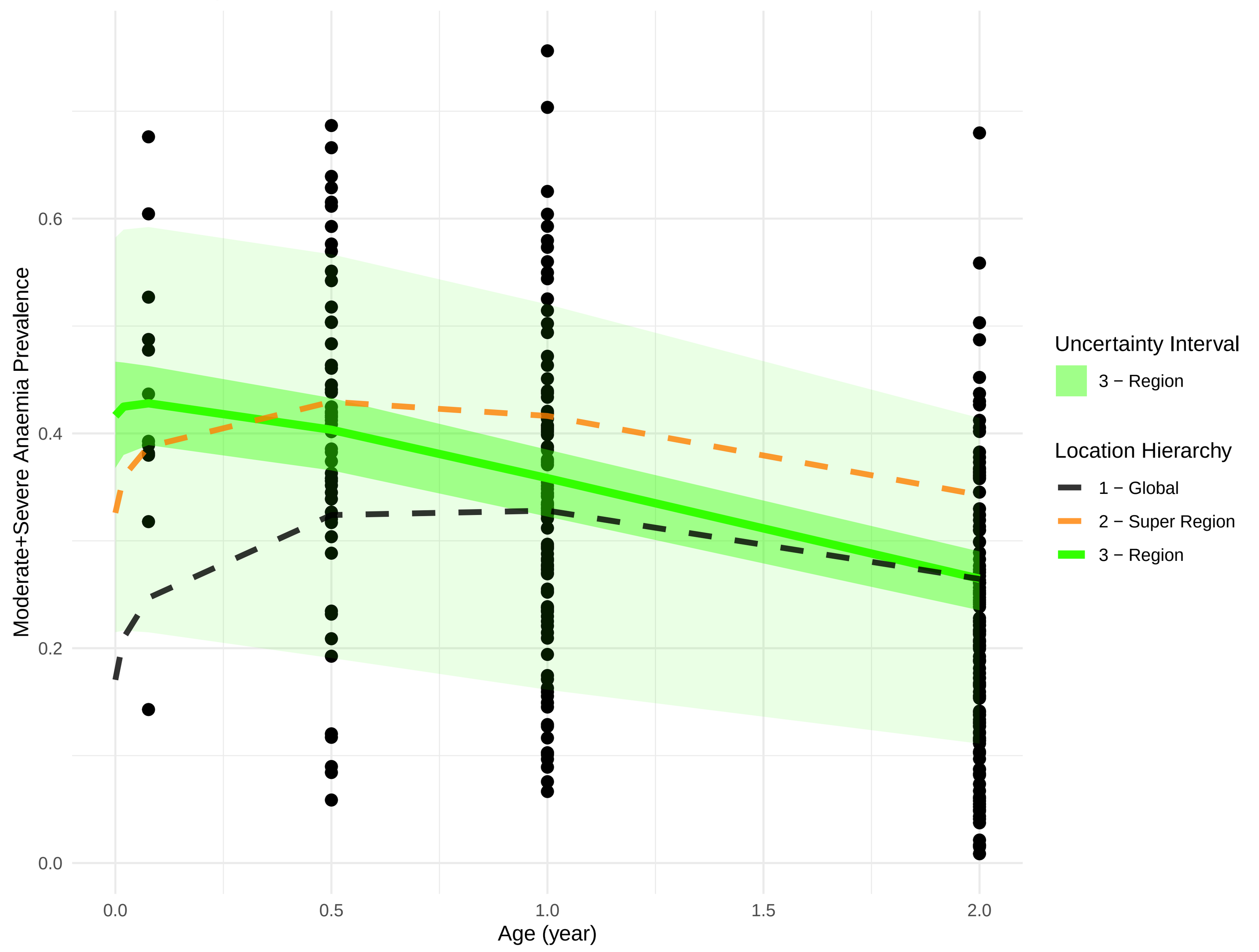

MR−BRT Age/Sex Fits − Males <2 Years Old − Moderate+Severe Anaemia Prevalence − Burundi
WHO−2001 Adjustment Method

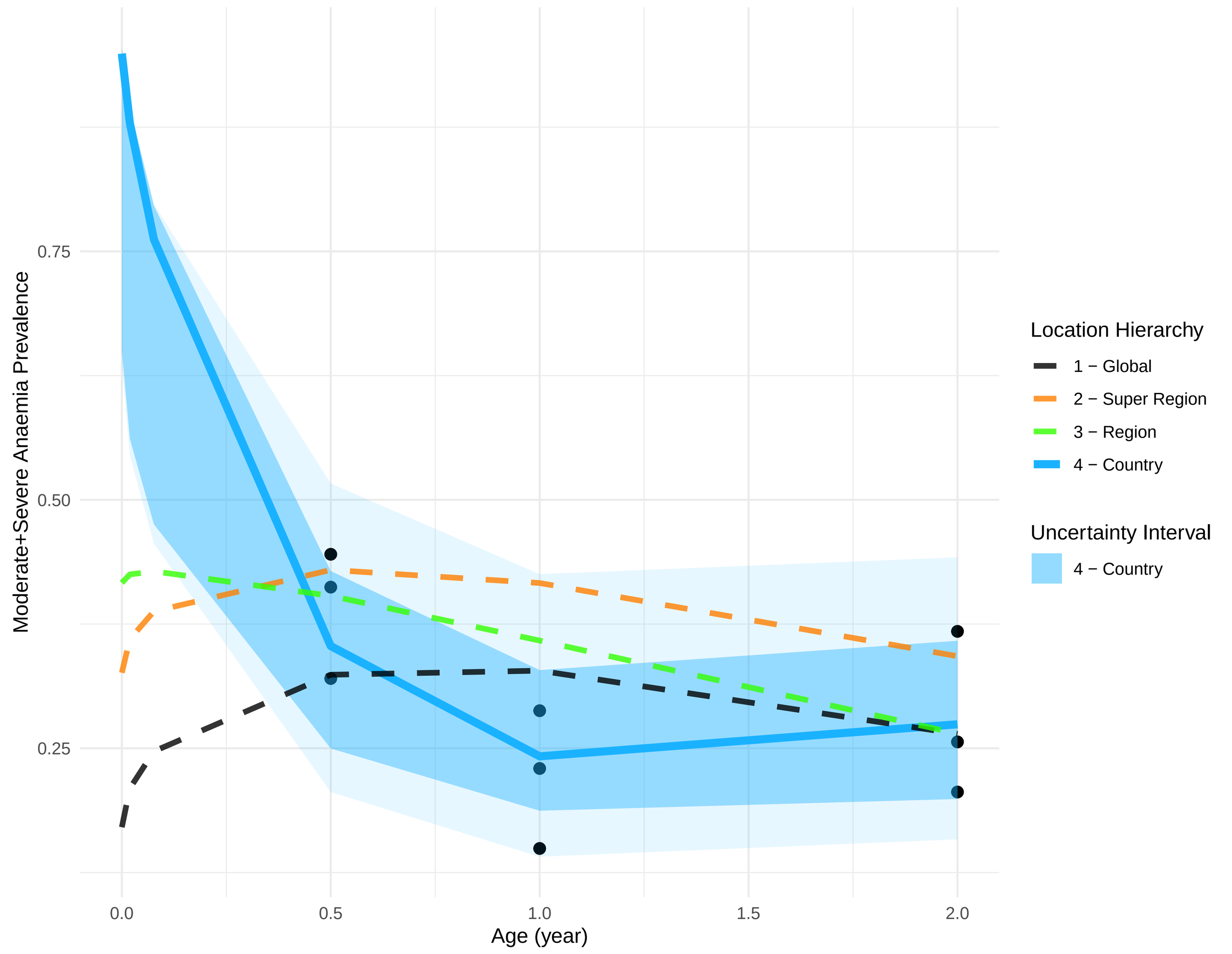

MR−BRT Age/Sex Fits − Males <2 Years Old − Moderate+Severe Anaemia Prevalence − Ethiopia
WHO−2001 Adjustment Method

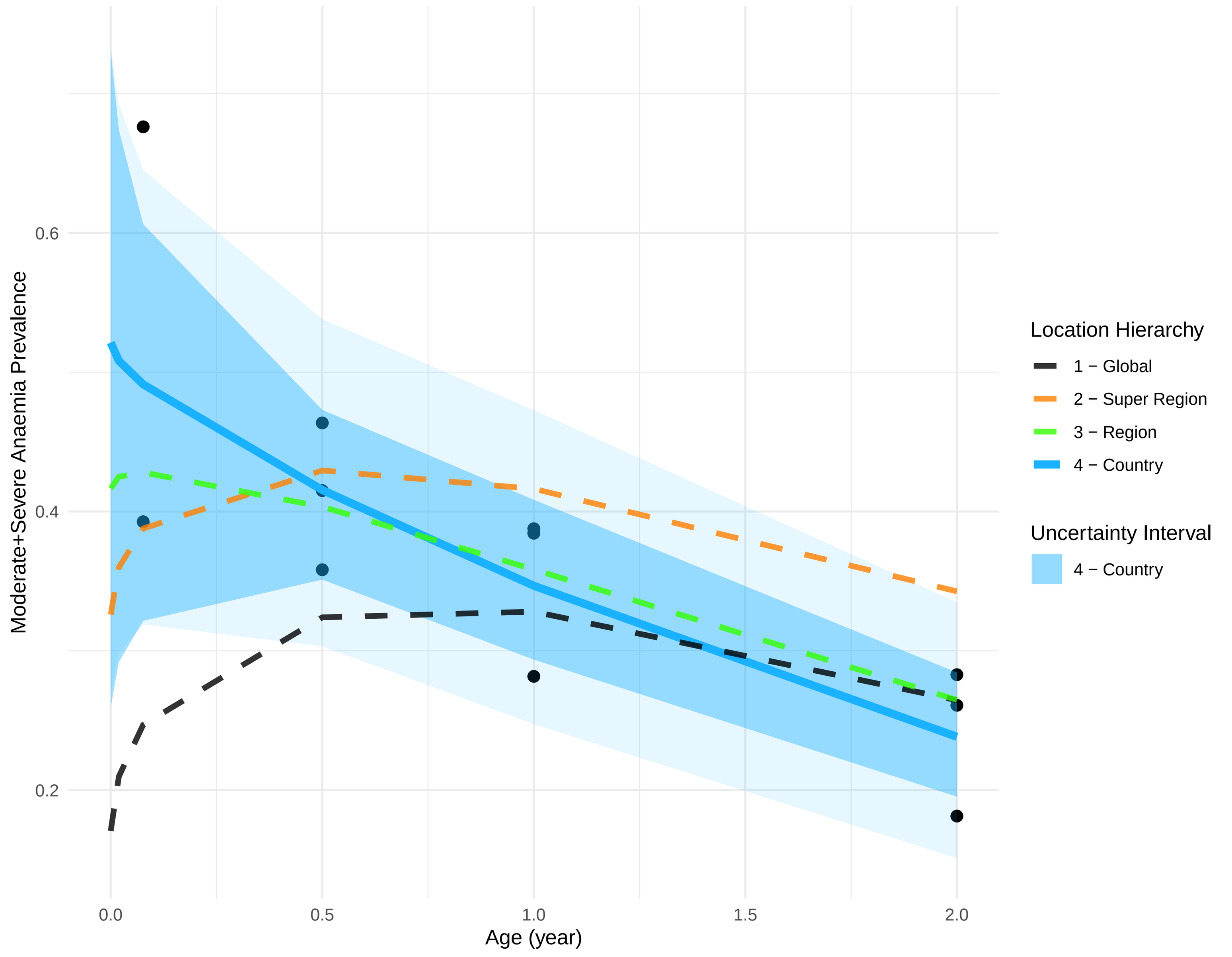

MR−BRT Age/Sex Fits − Males <2 Years Old − Moderate+Severe Anaemia Prevalence − Kenya
WHO−2001 Adjustment Method

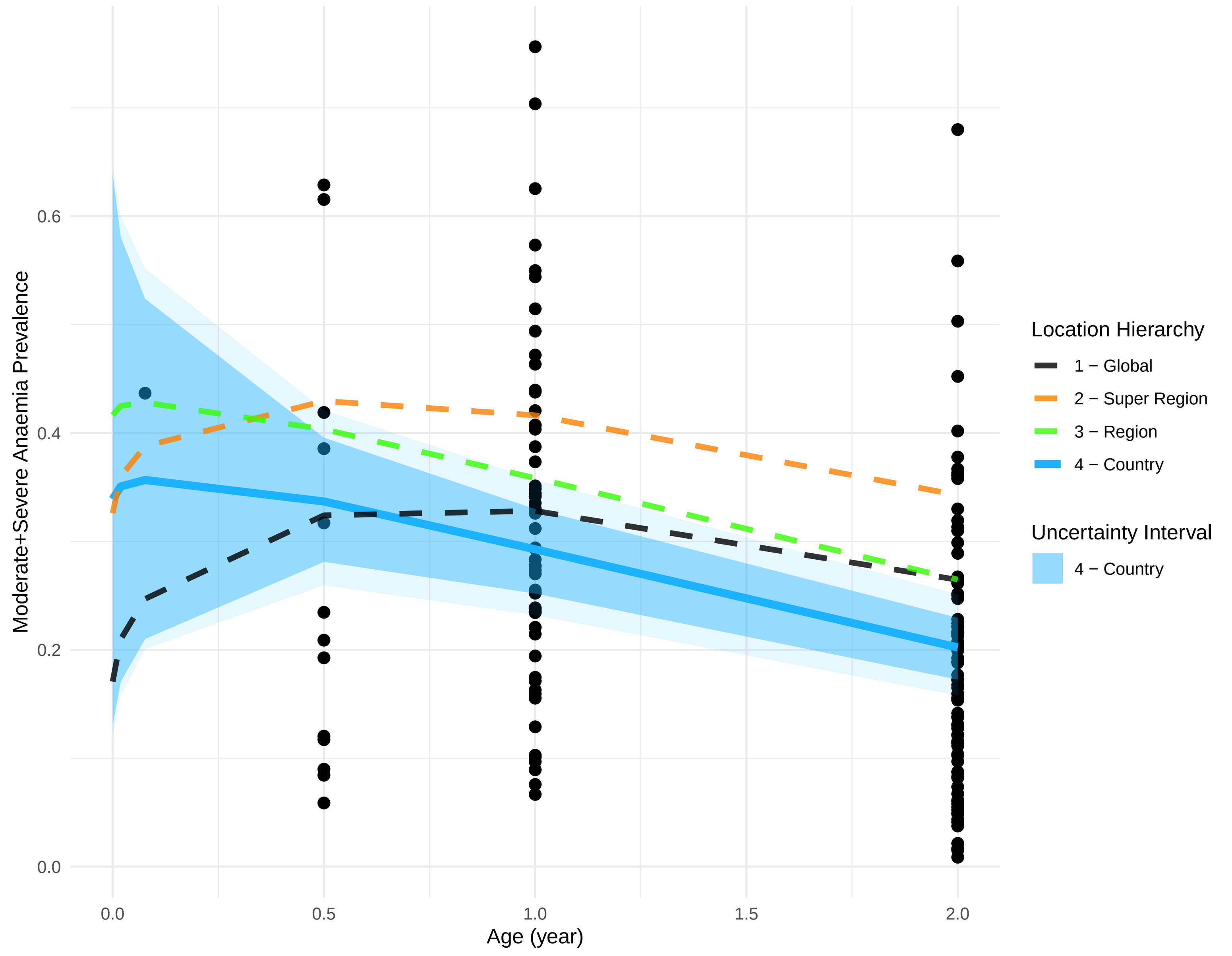

MR−BRT Age/Sex Fits − Males <2 Years Old − Moderate+Severe Anaemia Prevalence − Baringo
WHO−2001 Adjustment Method

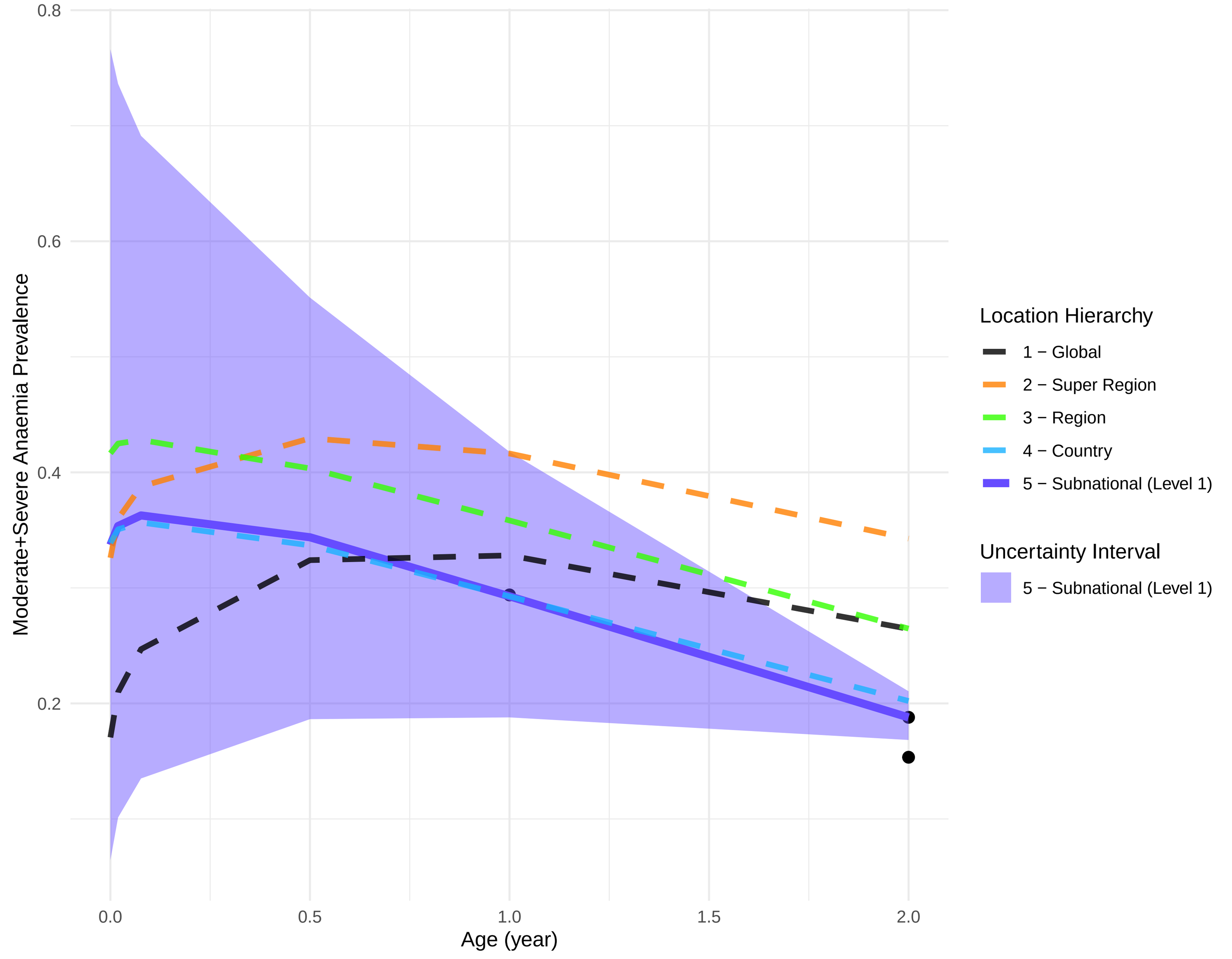

MR−BRT Age/Sex Fits − Males <2 Years Old − Moderate+Severe Anaemia Prevalence − Bomet
WHO−2001 Adjustment Method

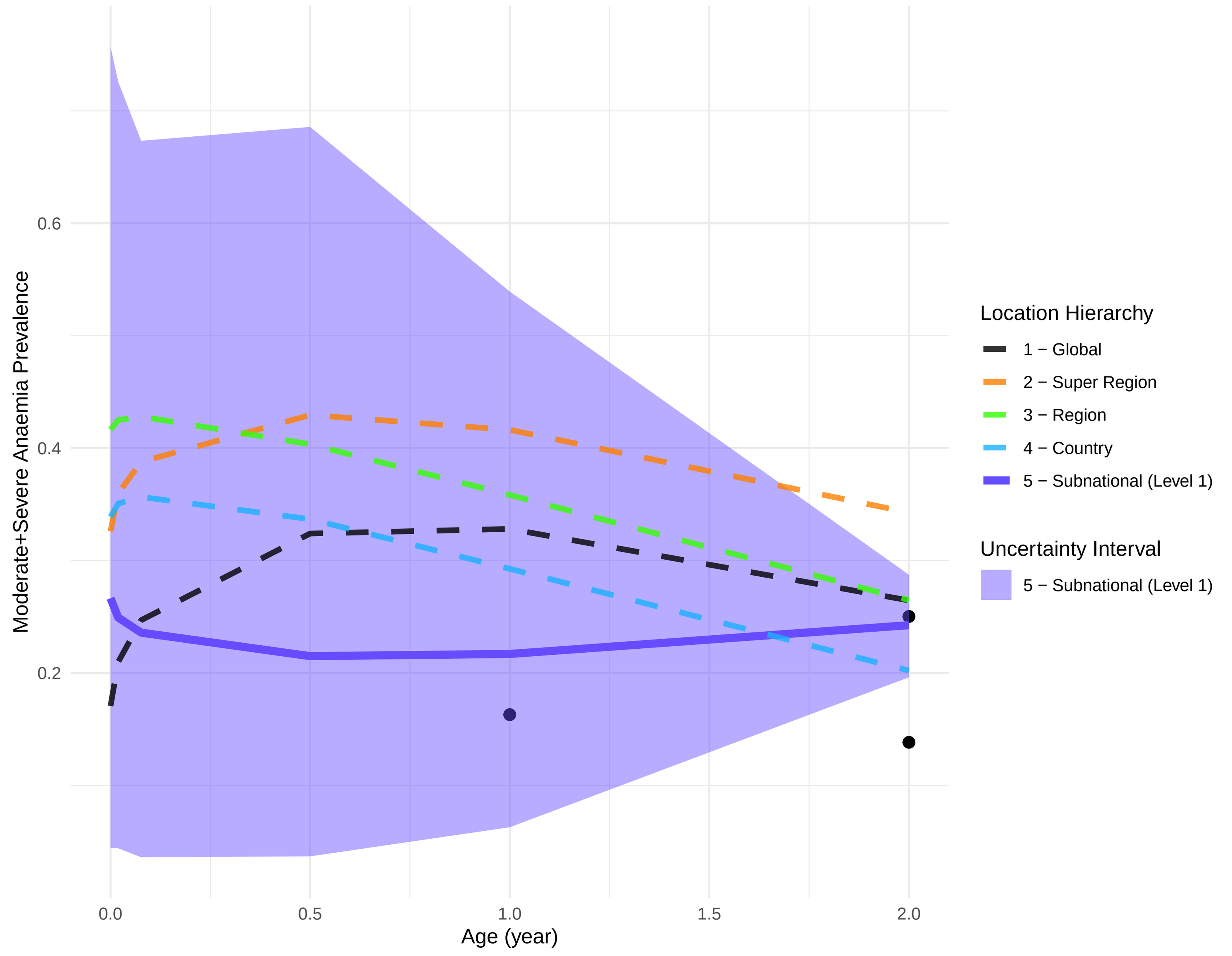

MR−BRT Age/Sex Fits − Males <2 Years Old − Moderate+Severe Anaemia Prevalence − Bungoma
WHO−2001 Adjustment Method

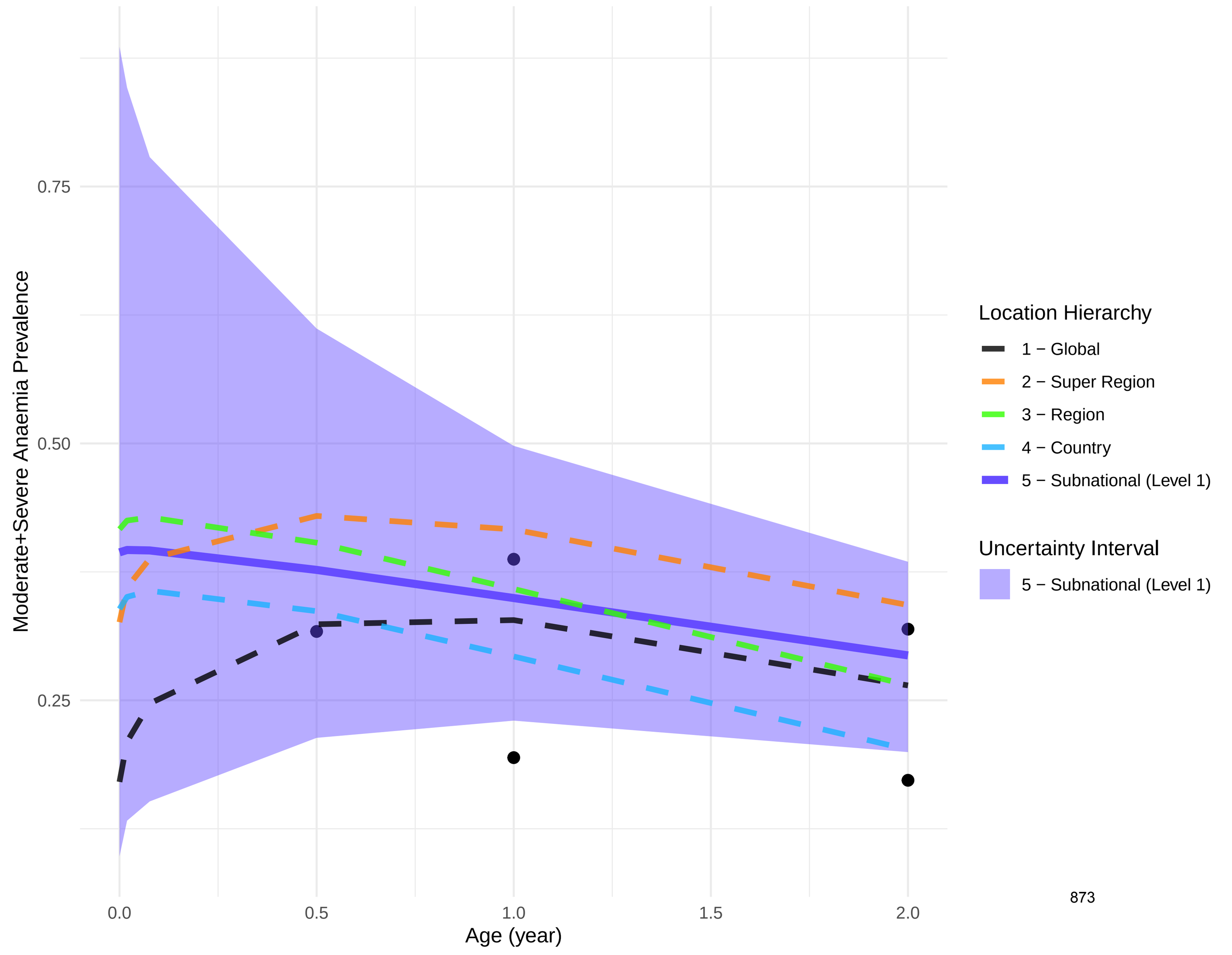

MR−BRT Age/Sex Fits − Males <2 Years Old − Moderate+Severe Anaemia Prevalence − Busia
WHO−2001 Adjustment Method

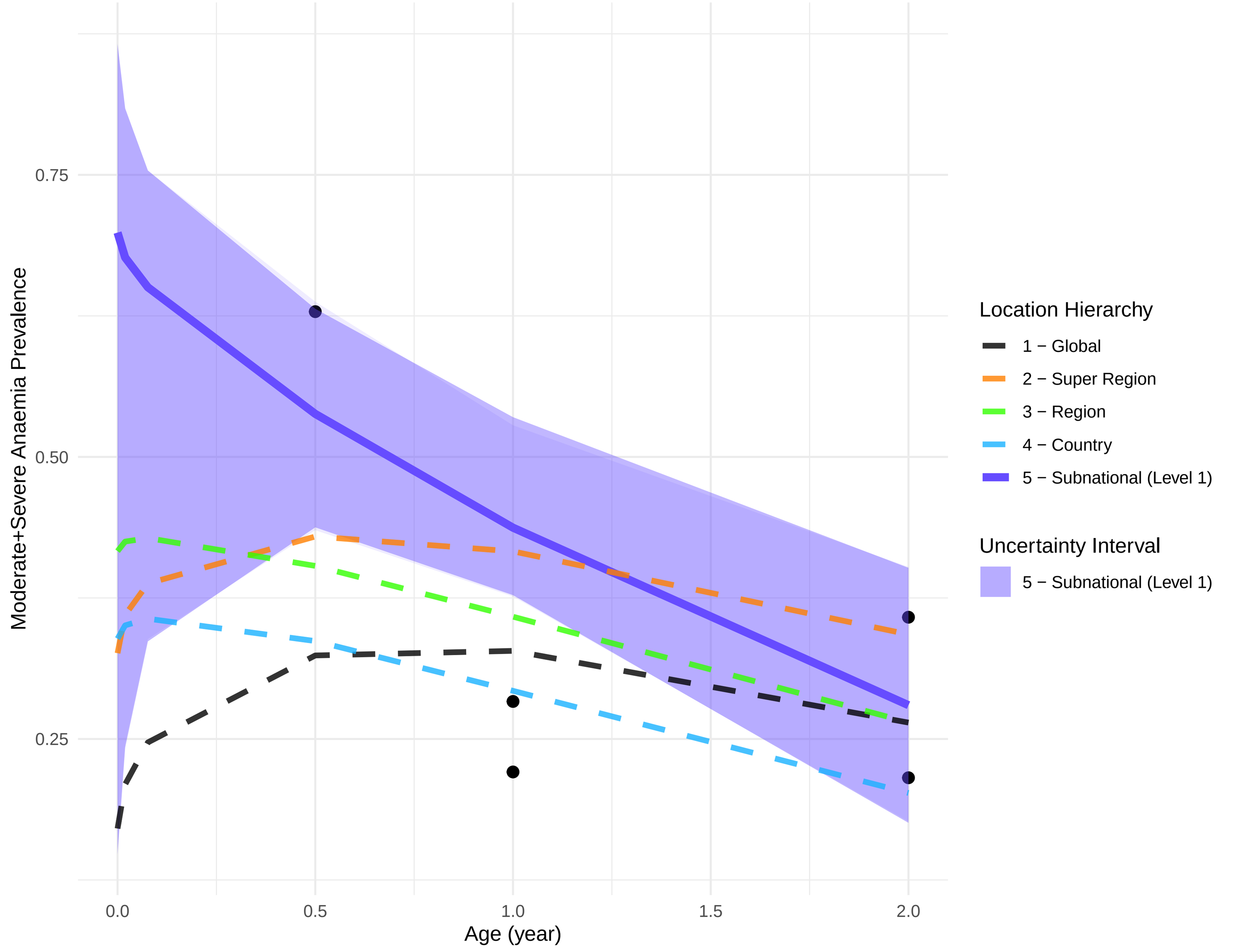

MR−BRT Age/Sex Fits − Males <2 Years Old − Moderate+Severe Anaemia Prevalence − Elgeyo−Marakwet
WHO−2001 Adjustment Method

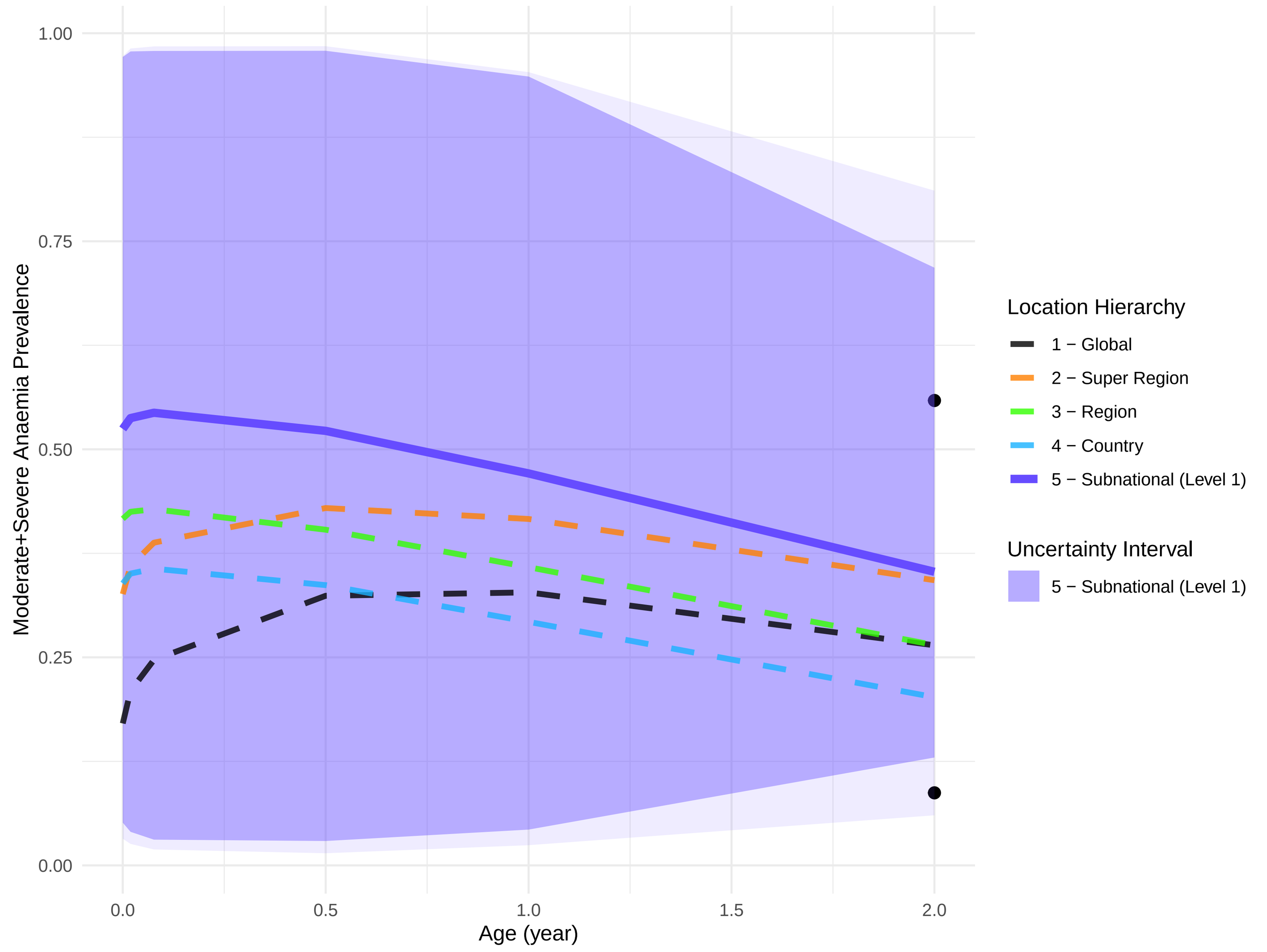

MR−BRT Age/Sex Fits − Males <2 Years Old − Moderate+Severe Anaemia Prevalence − Embu
WHO−2001 Adjustment Method

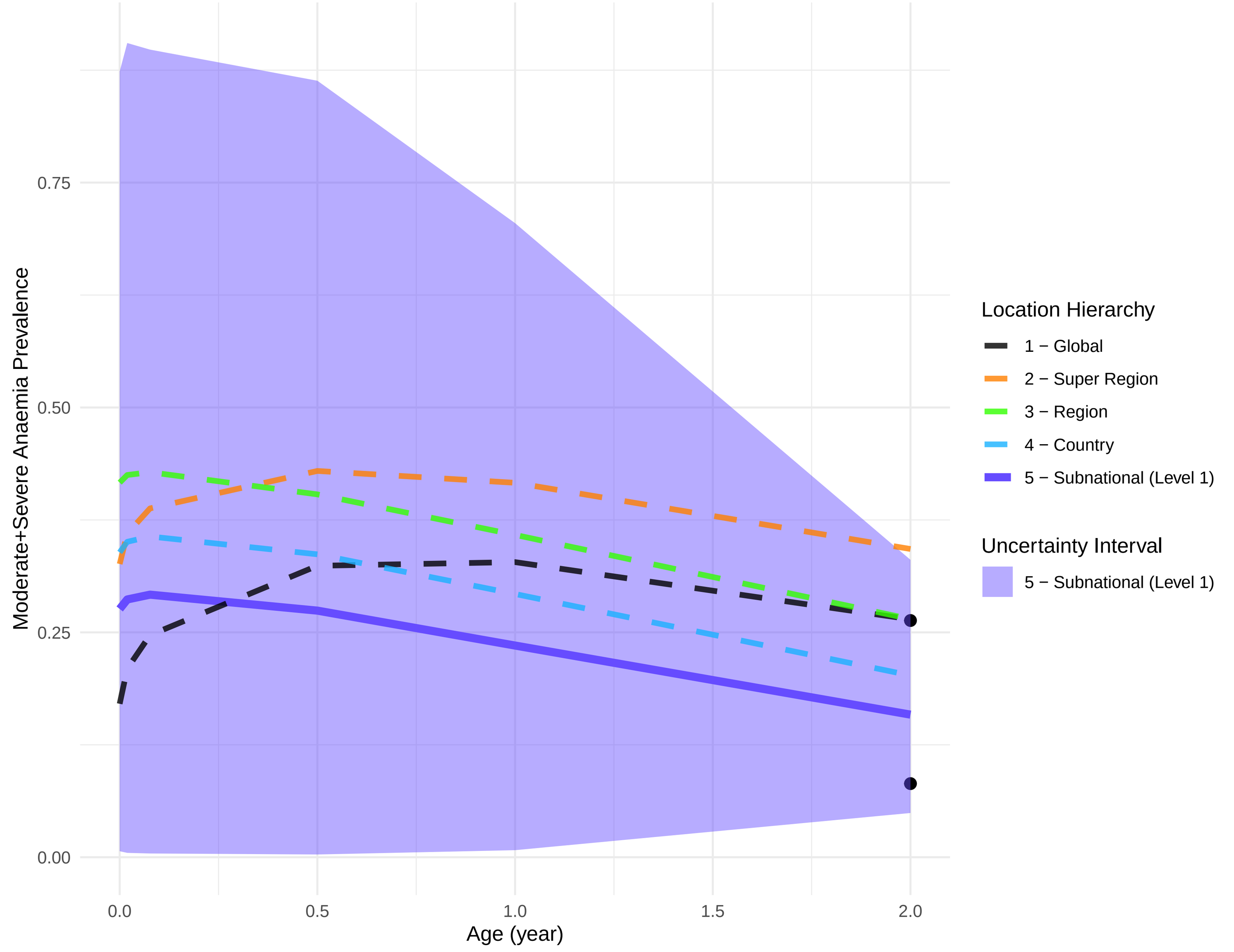

MR−BRT Age/Sex Fits − Males <2 Years Old − Moderate+Severe Anaemia Prevalence − Garissa
WHO−2001 Adjustment Method

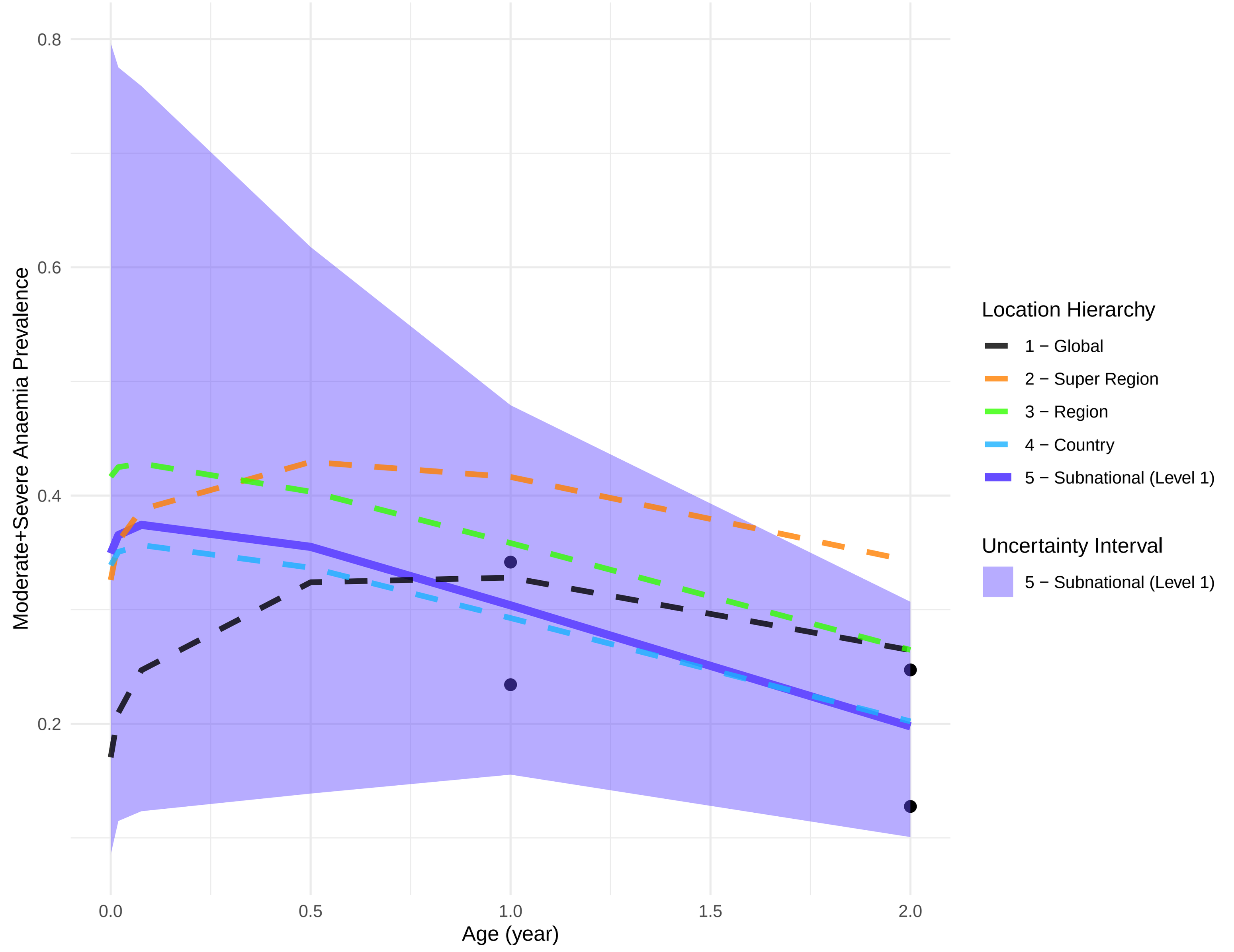

MR−BRT Age/Sex Fits − Males <2 Years Old − Moderate+Severe Anaemia Prevalence − Homa Bay
WHO−2001 Adjustment Method

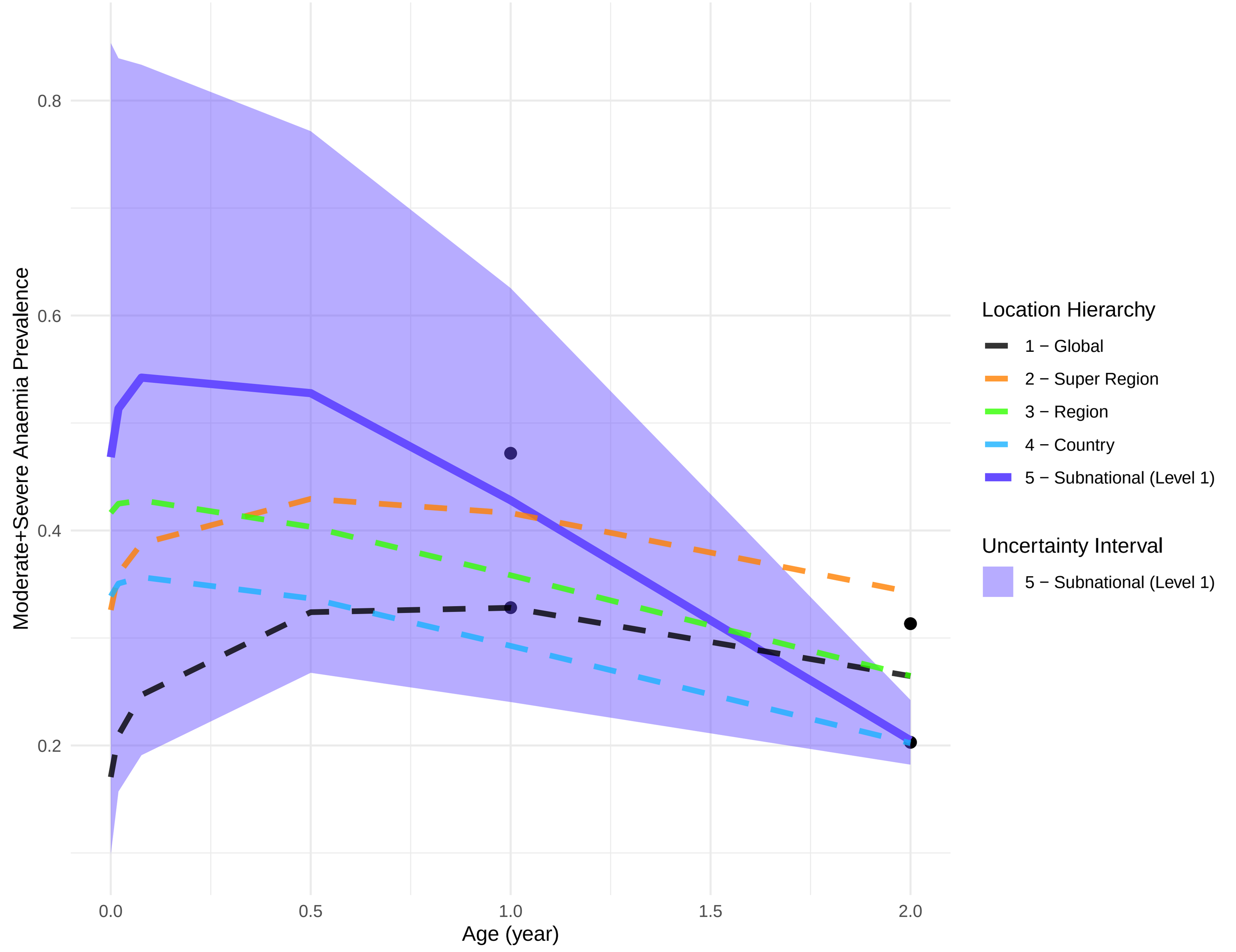

MR−BRT Age/Sex Fits − Males <2 Years Old − Moderate+Severe Anaemia Prevalence − Isiolo
WHO−2001 Adjustment Method

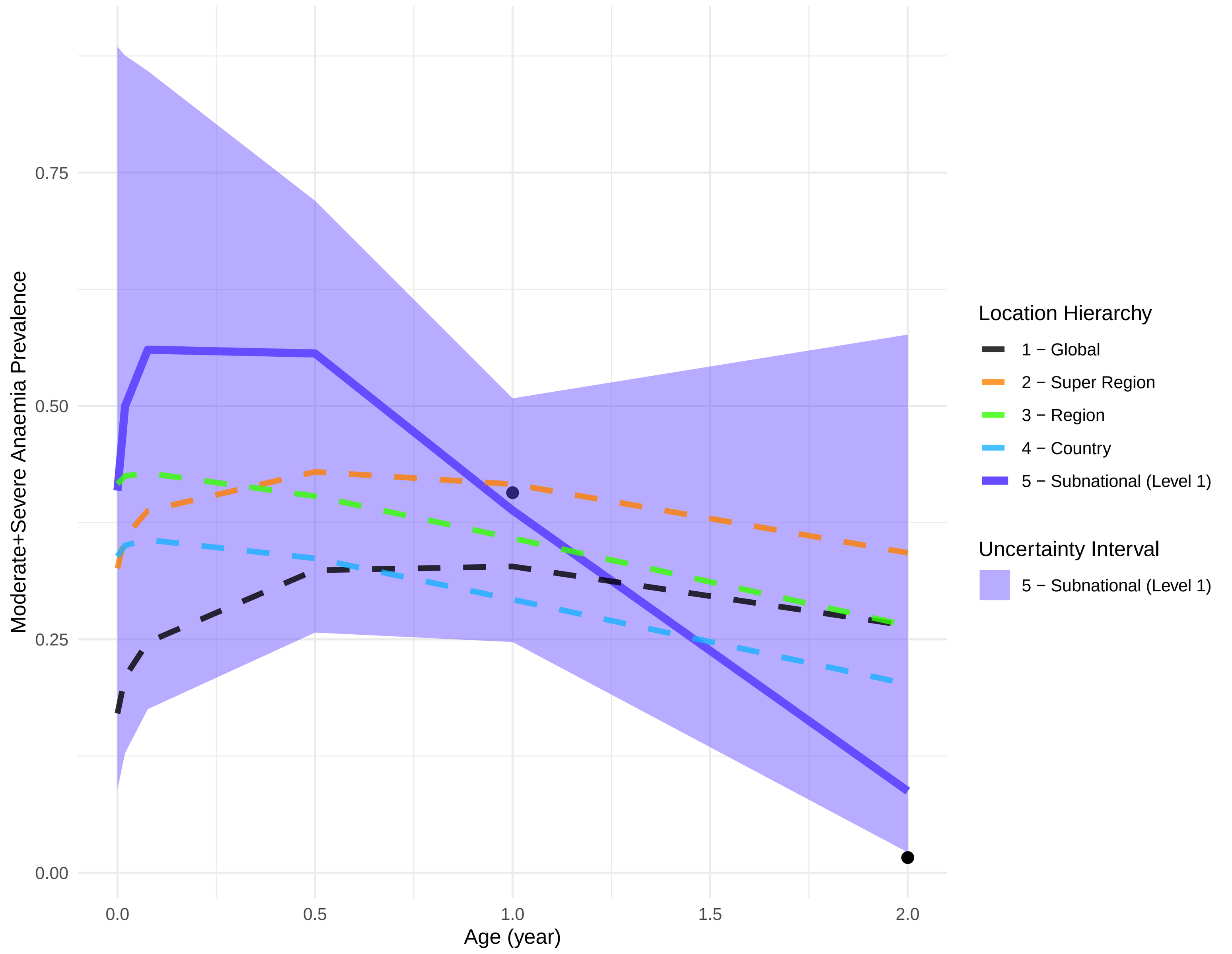

MR−BRT Age/Sex Fits − Males <2 Years Old − Moderate+Severe Anaemia Prevalence − Kajiado
WHO−2001 Adjustment Method

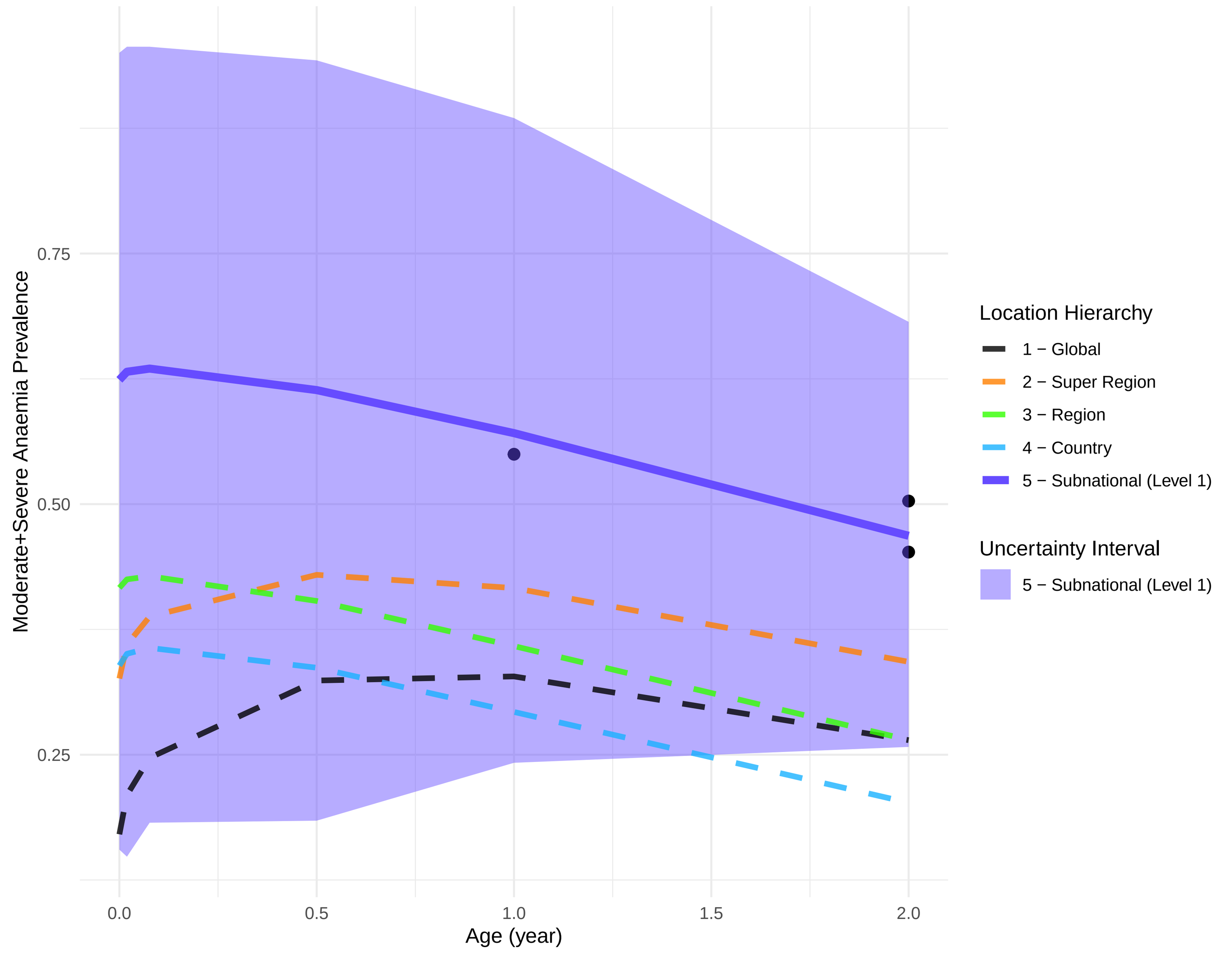

MR−BRT Age/Sex Fits − Males <2 Years Old − Moderate+Severe Anaemia Prevalence − Kakamega
WHO−2001 Adjustment Method

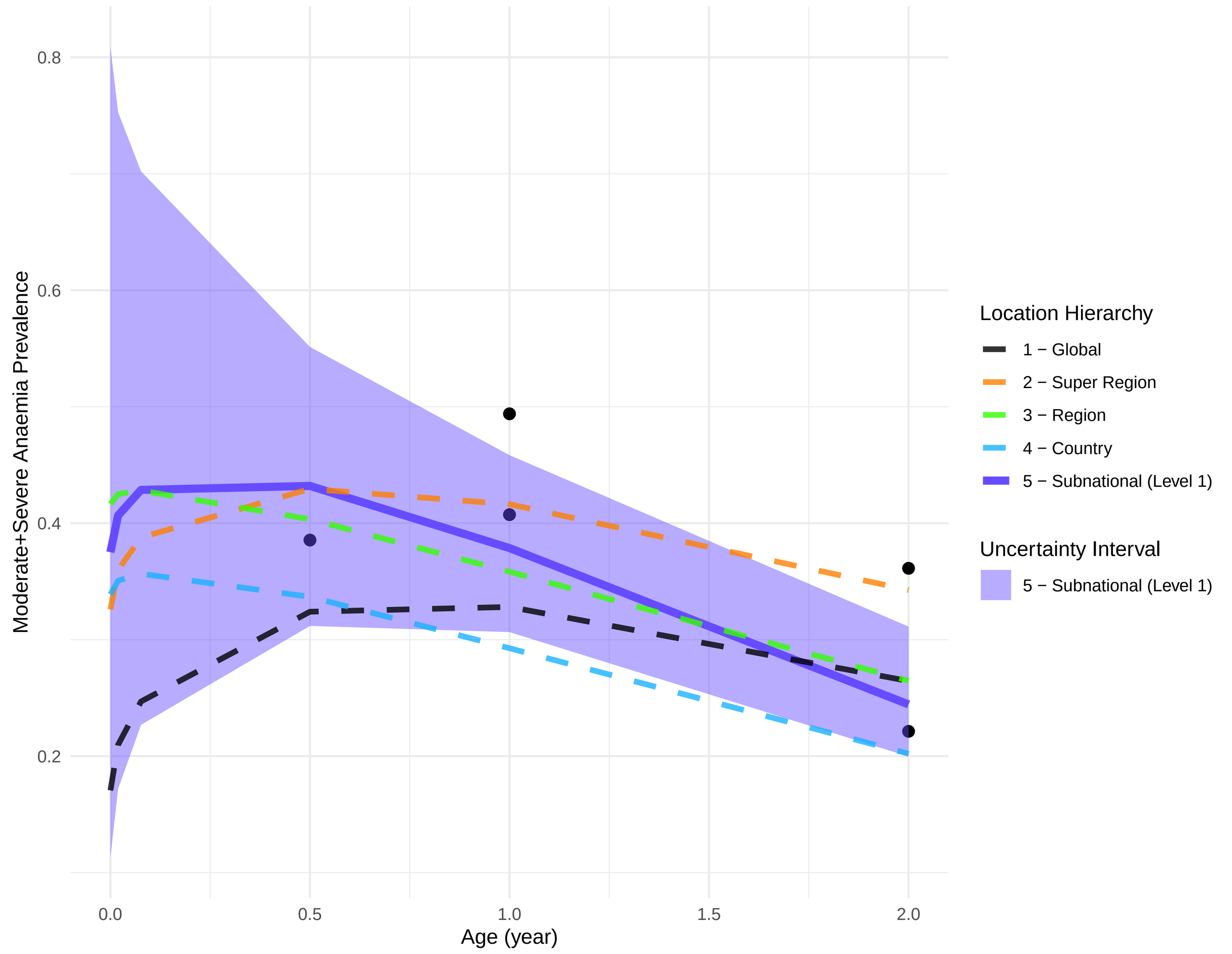

MR−BRT Age/Sex Fits − Males <2 Years Old − Moderate+Severe Anaemia Prevalence − Kericho
WHO−2001 Adjustment Method

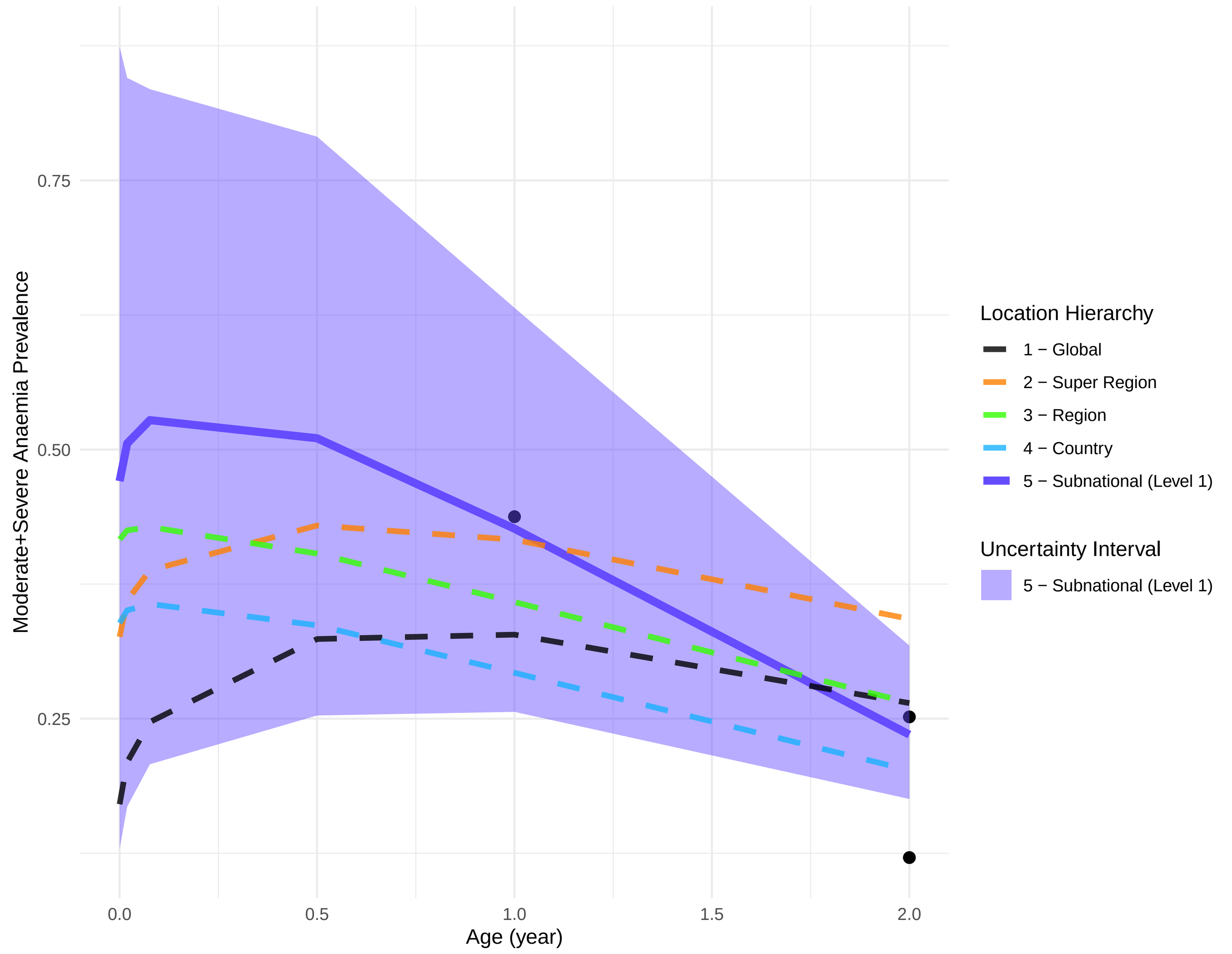

MR−BRT Age/Sex Fits − Males <2 Years Old − Moderate+Severe Anaemia Prevalence − Kiambu
WHO−2001 Adjustment Method

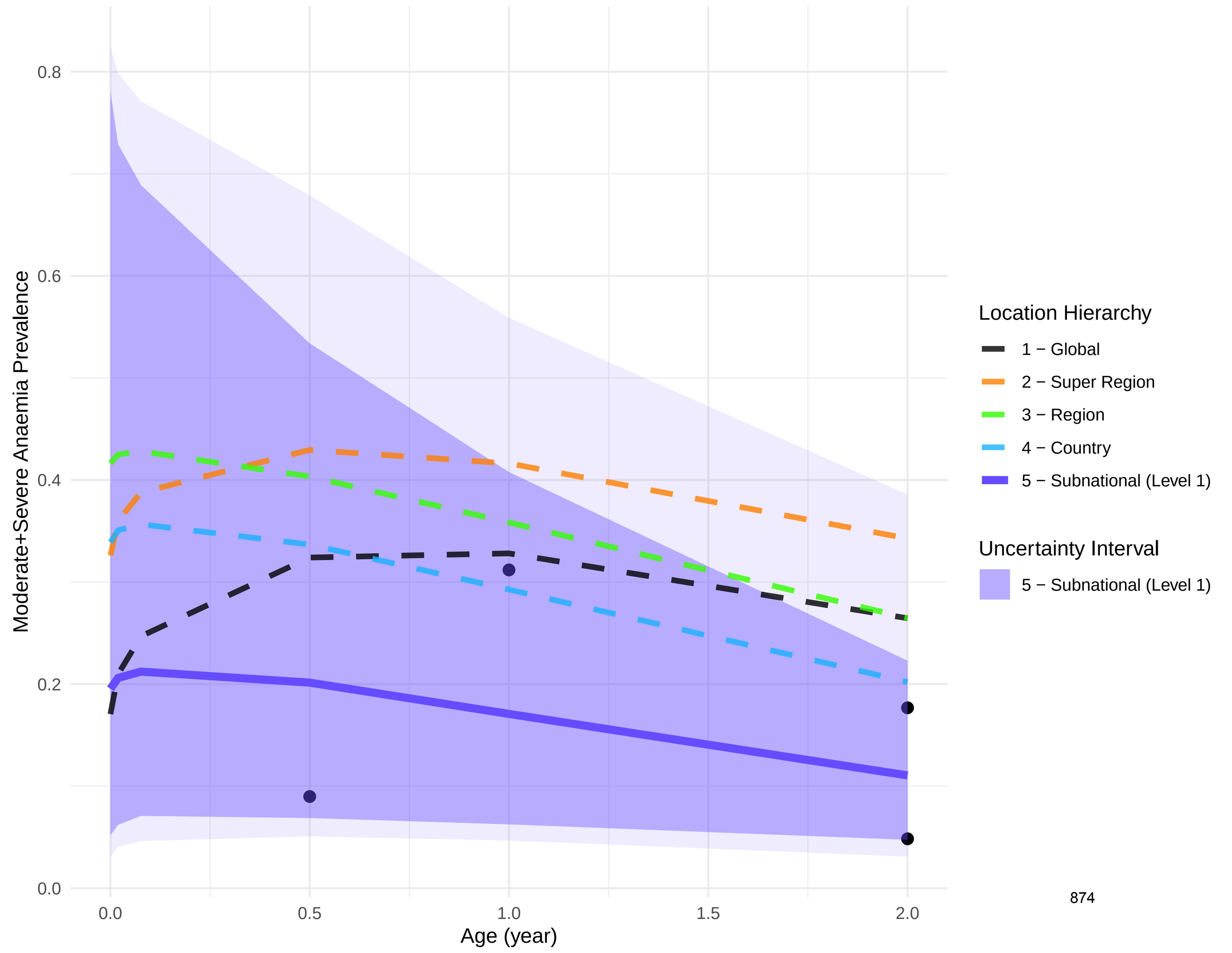

MR−BRT Age/Sex Fits − Males <2 Years Old − Moderate+Severe Anaemia Prevalence − Kilifi
WHO−2001 Adjustment Method

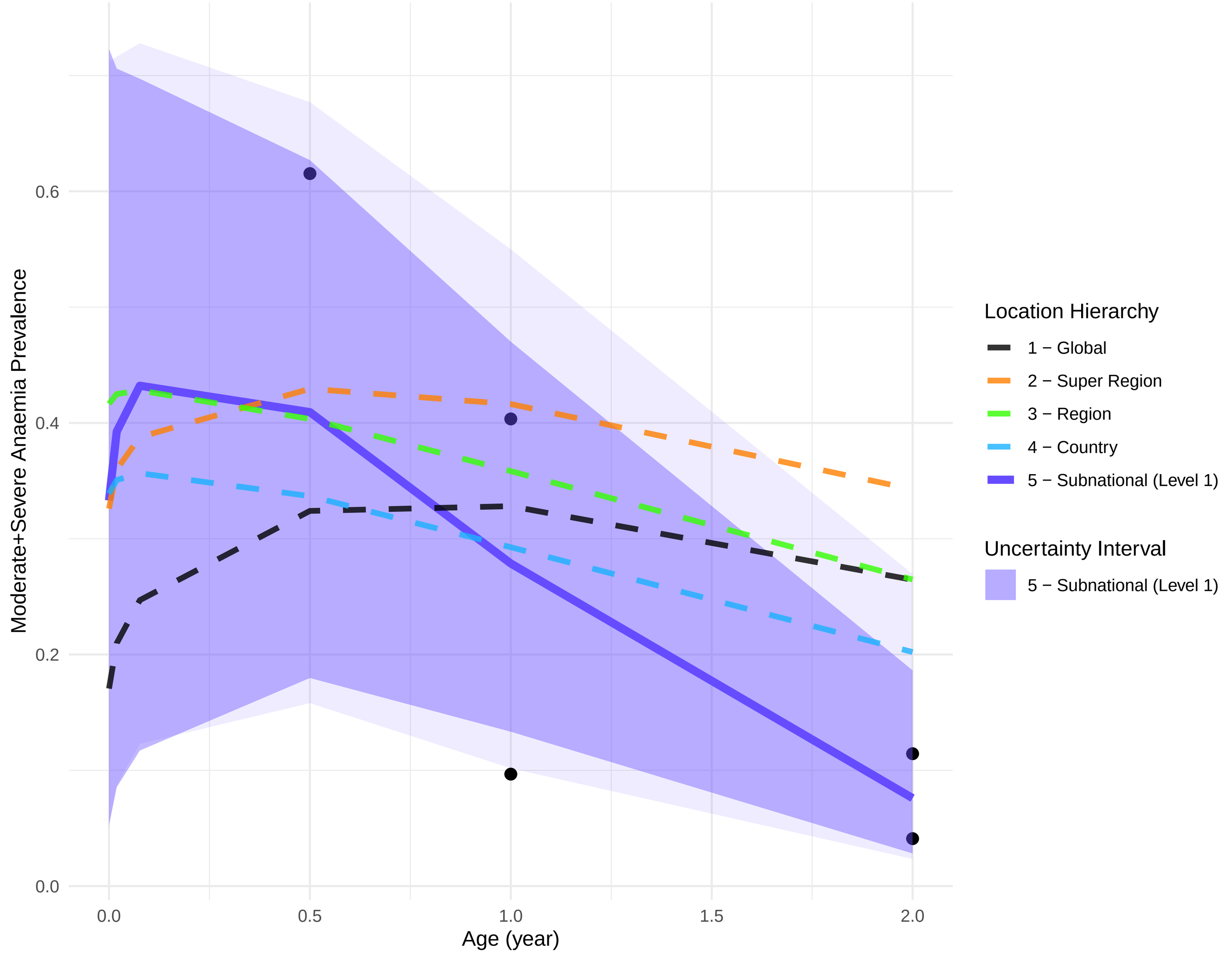

MR−BRT Age/Sex Fits − Males <2 Years Old − Moderate+Severe Anaemia Prevalence − Kirinyaga
WHO−2001 Adjustment Method

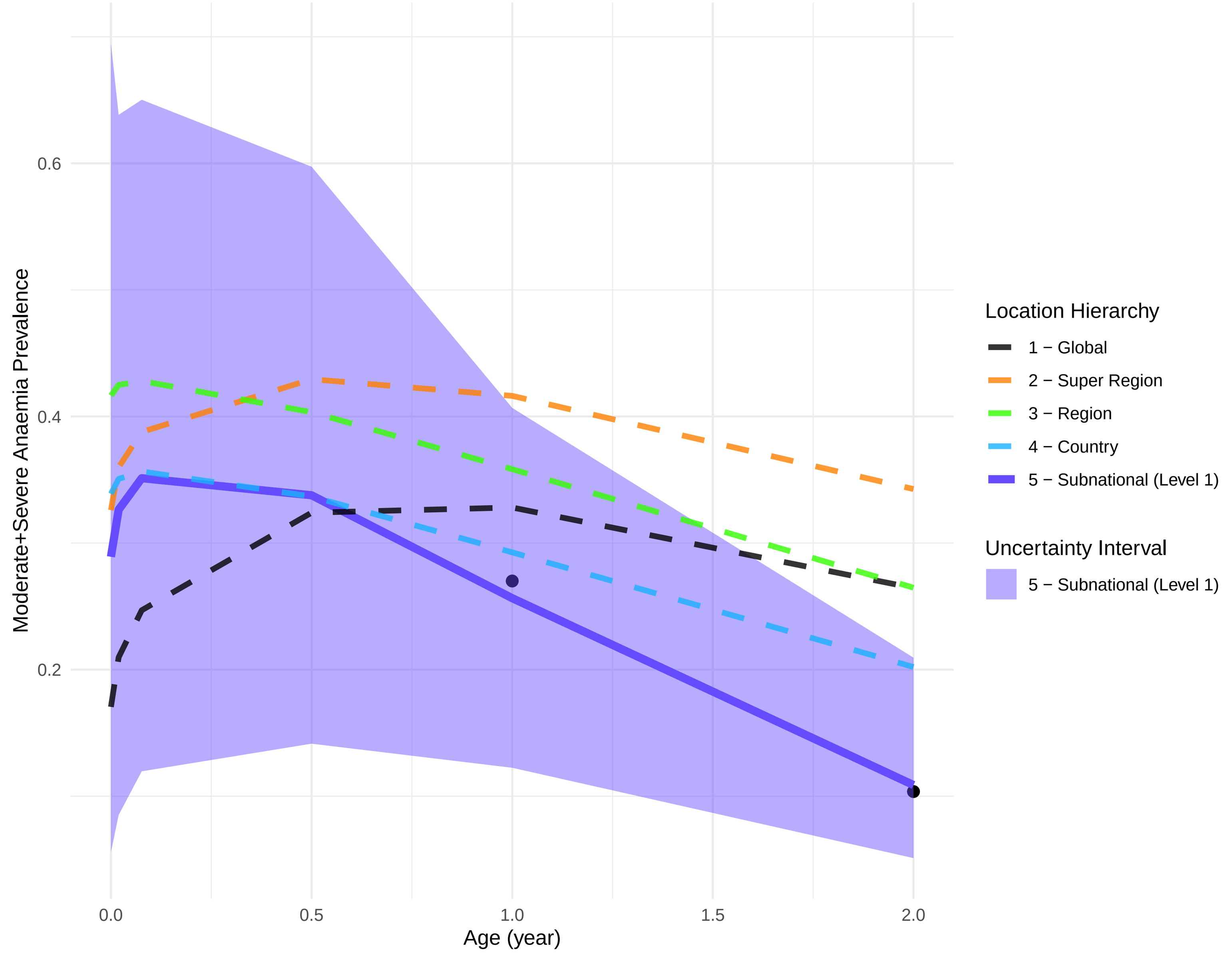

MR−BRT Age/Sex Fits − Males <2 Years Old − Moderate+Severe Anaemia Prevalence − Kisii
WHO−2001 Adjustment Method

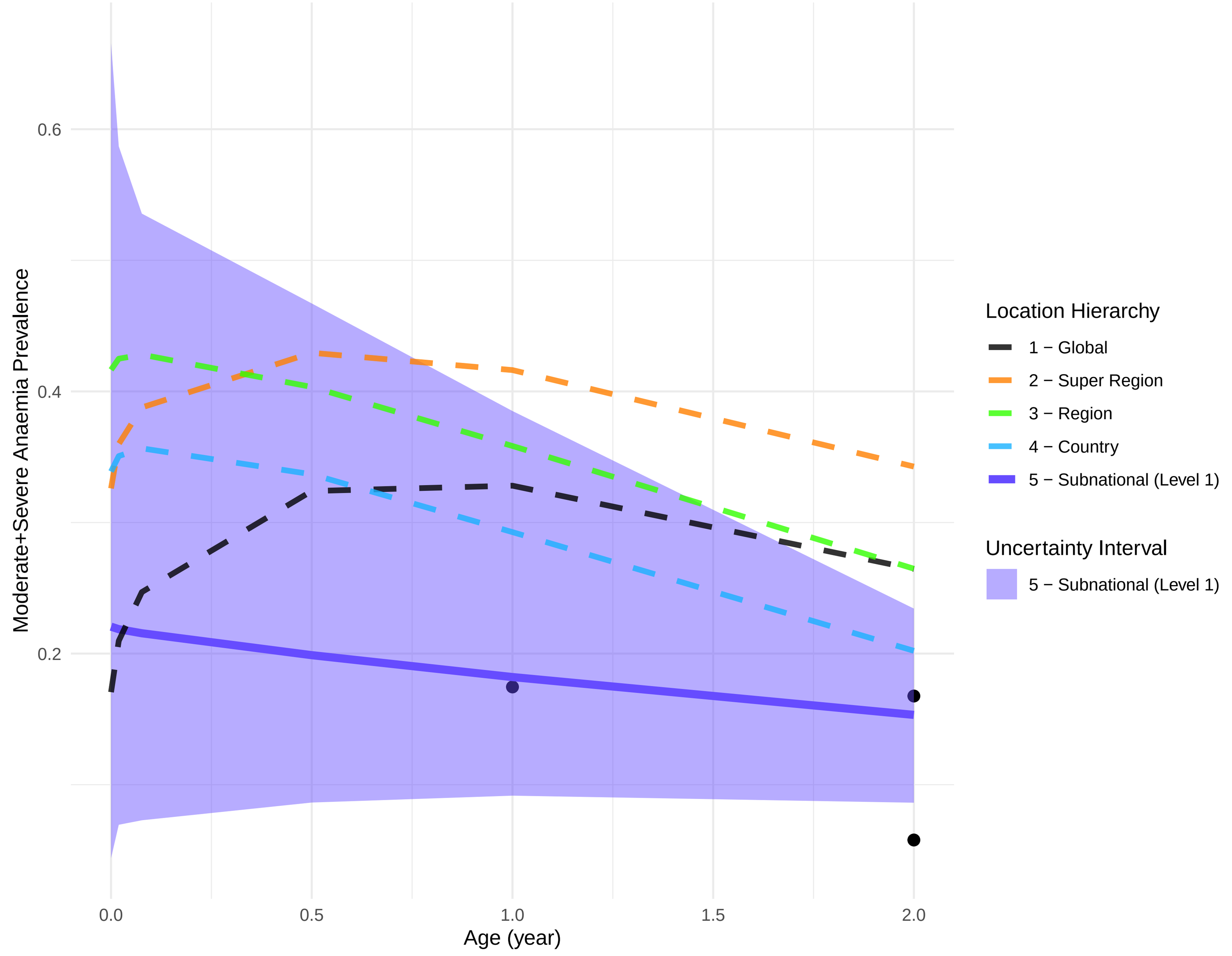

MR−BRT Age/Sex Fits − Males <2 Years Old − Moderate+Severe Anaemia Prevalence − Kisumu
WHO−2001 Adjustment Method

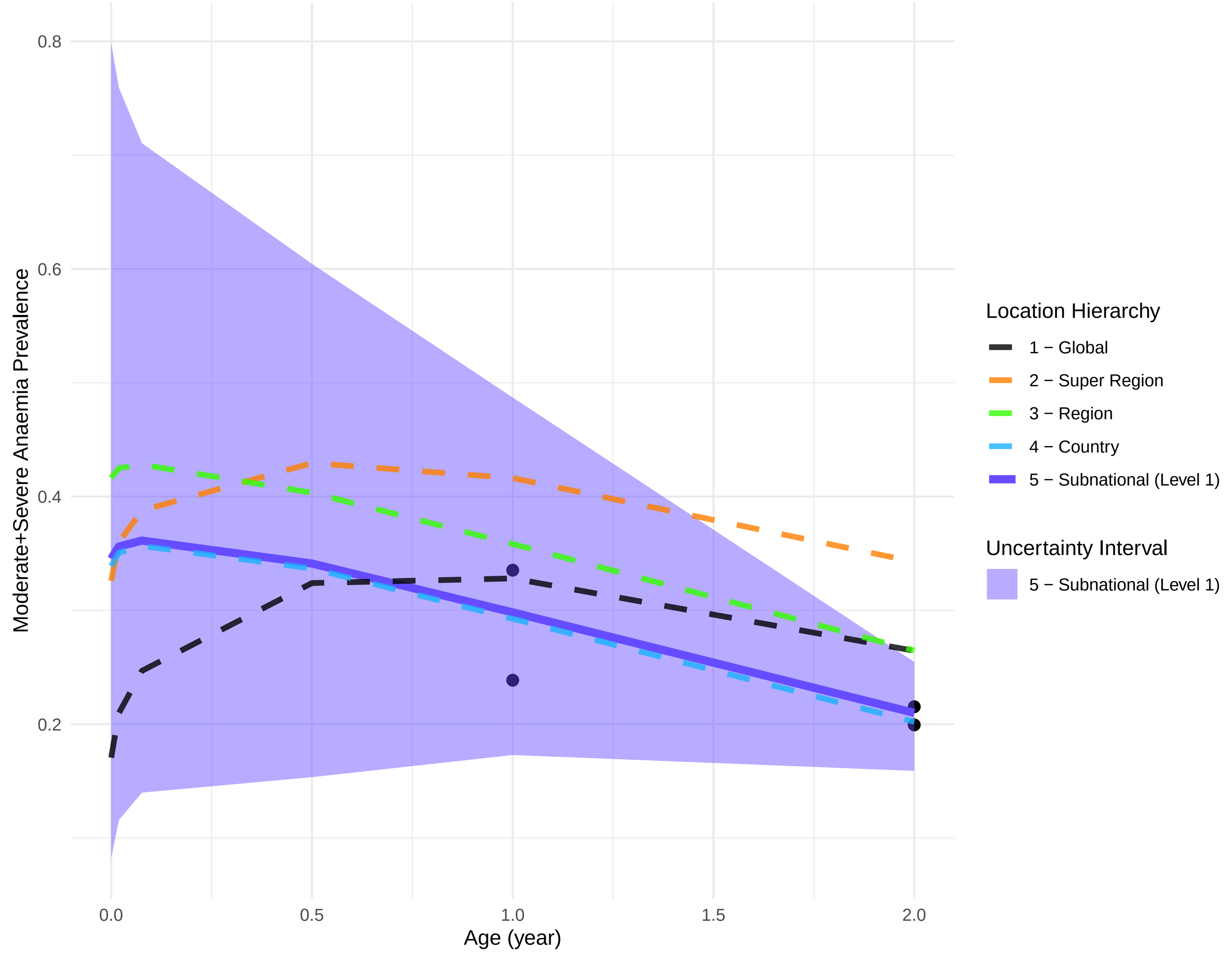

MR−BRT Age/Sex Fits − Males <2 Years Old − Moderate+Severe Anaemia Prevalence − Kitui
WHO−2001 Adjustment Method

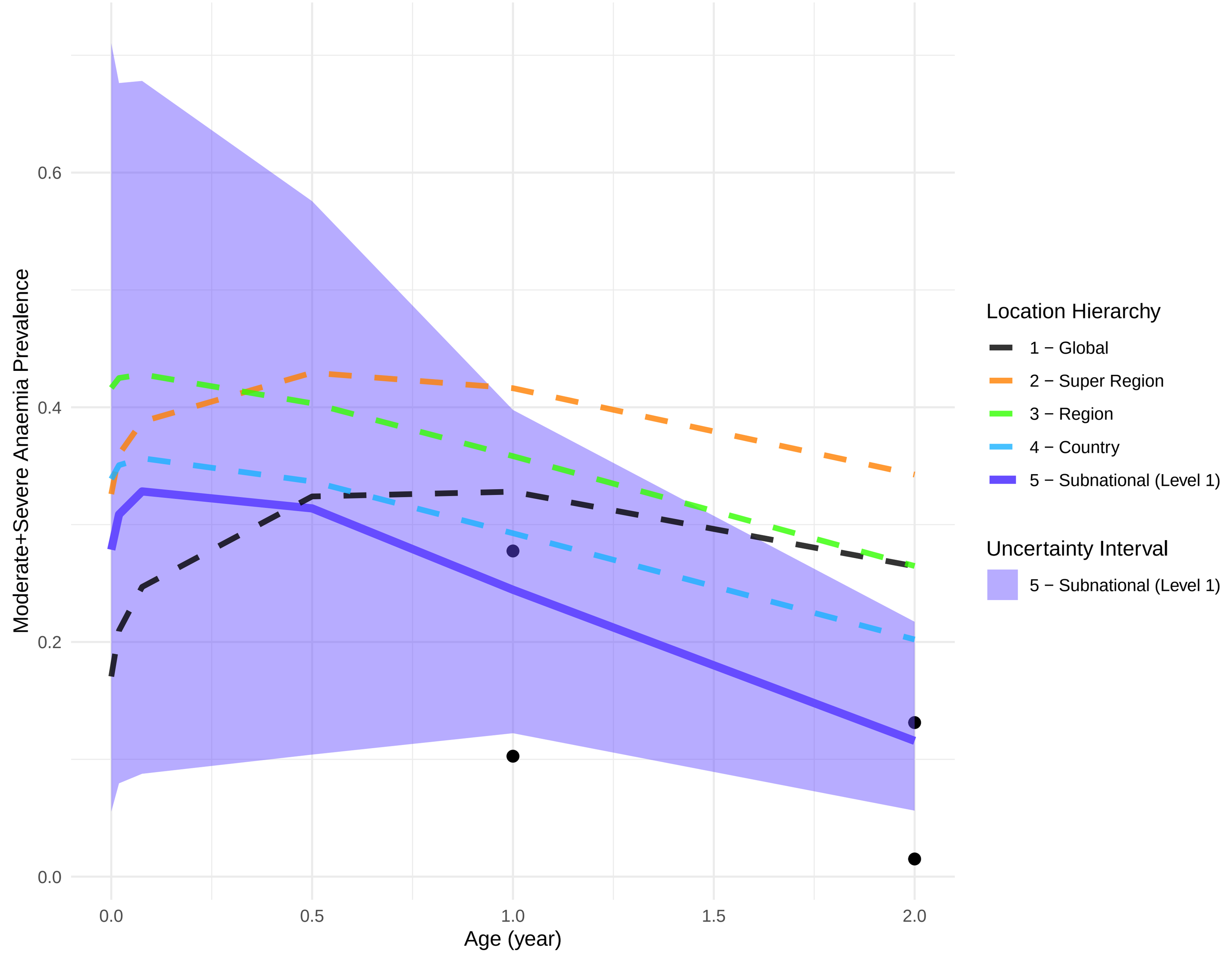

MR−BRT Age/Sex Fits − Males <2 Years Old − Moderate+Severe Anaemia Prevalence − Kwale
WHO−2001 Adjustment Method

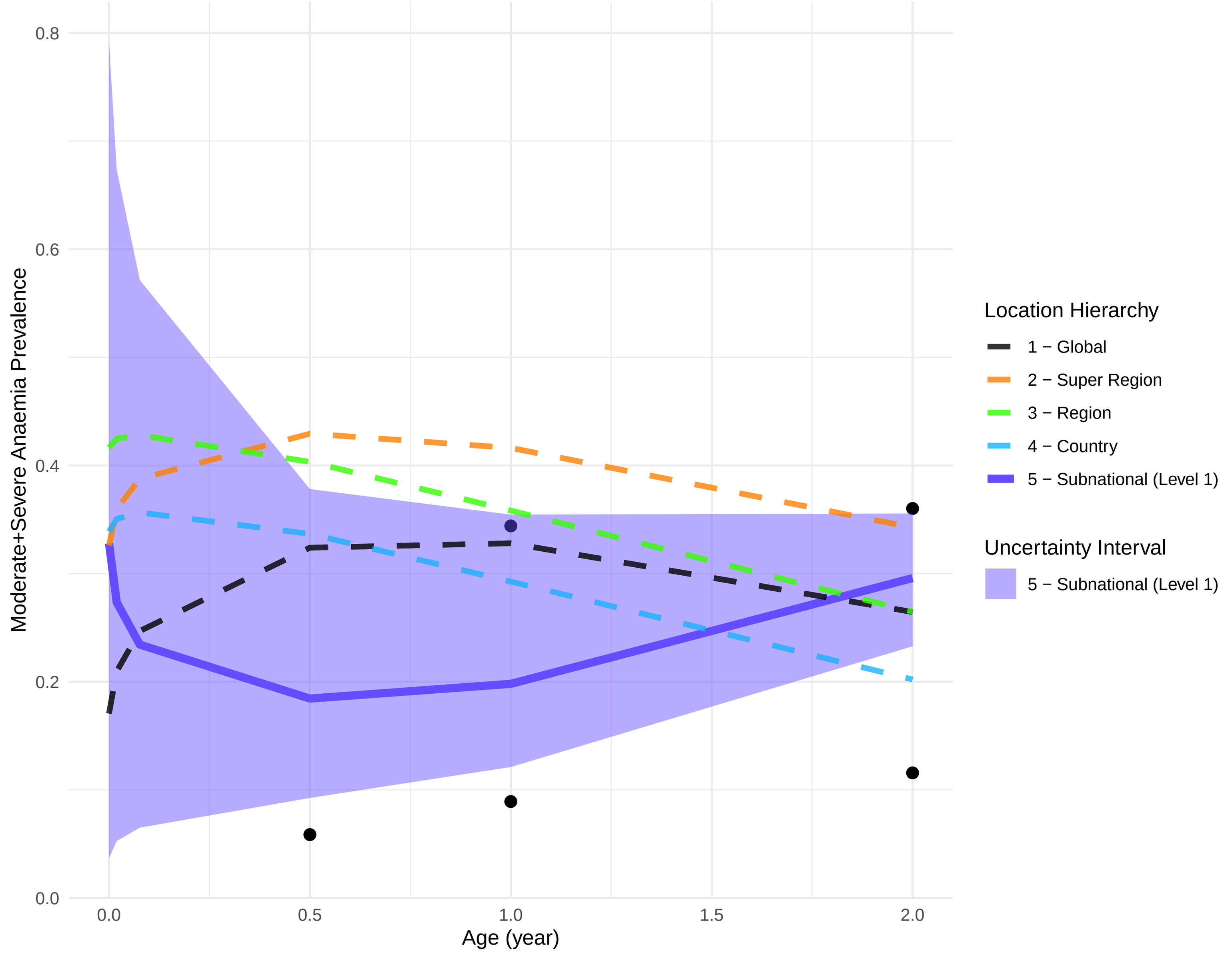

MR−BRT Age/Sex Fits − Males <2 Years Old − Moderate+Severe Anaemia Prevalence − Laikipia
WHO−2001 Adjustment Method

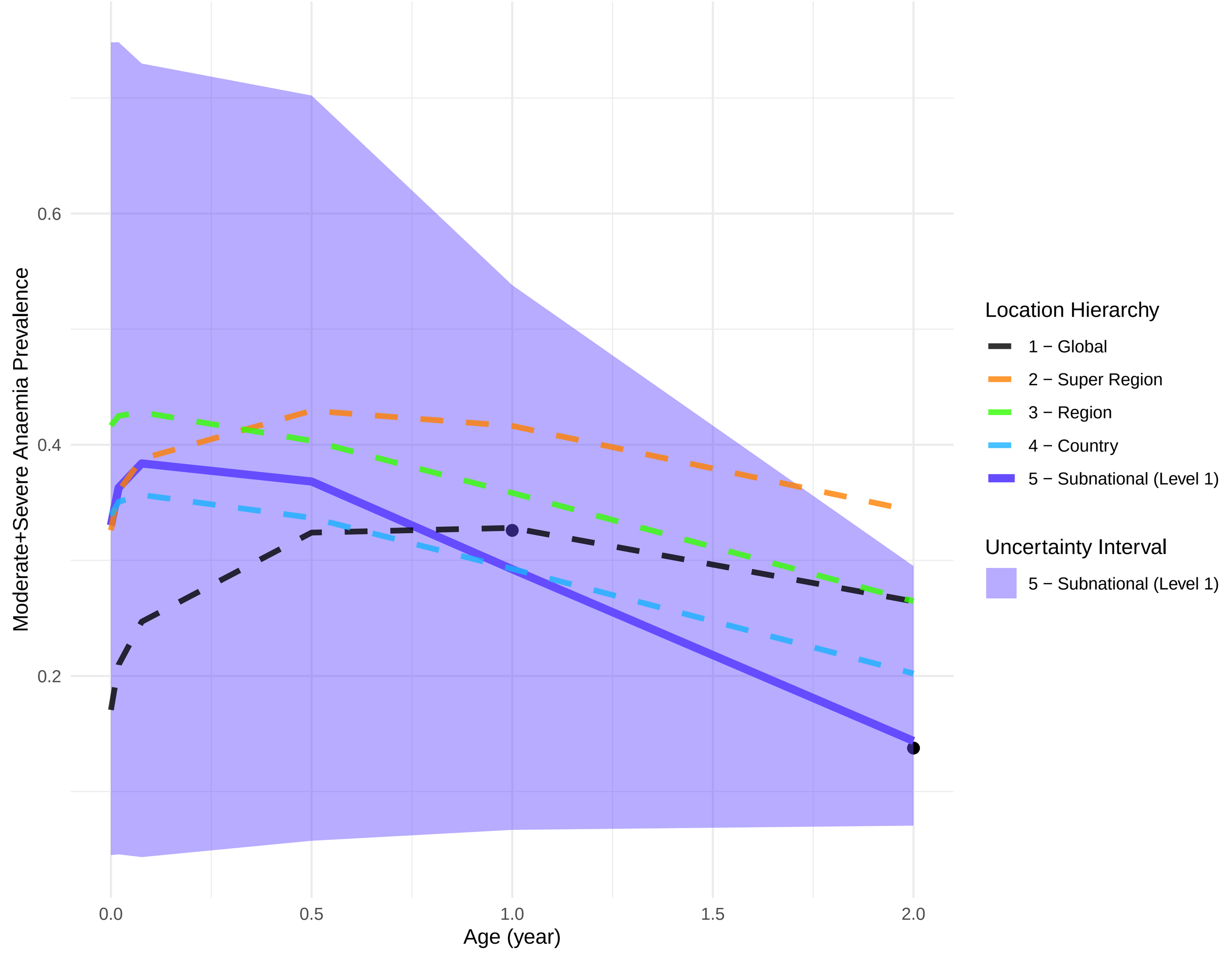

MR−BRT Age/Sex Fits − Males <2 Years Old − Moderate+Severe Anaemia Prevalence − Lamu
WHO−2001 Adjustment Method

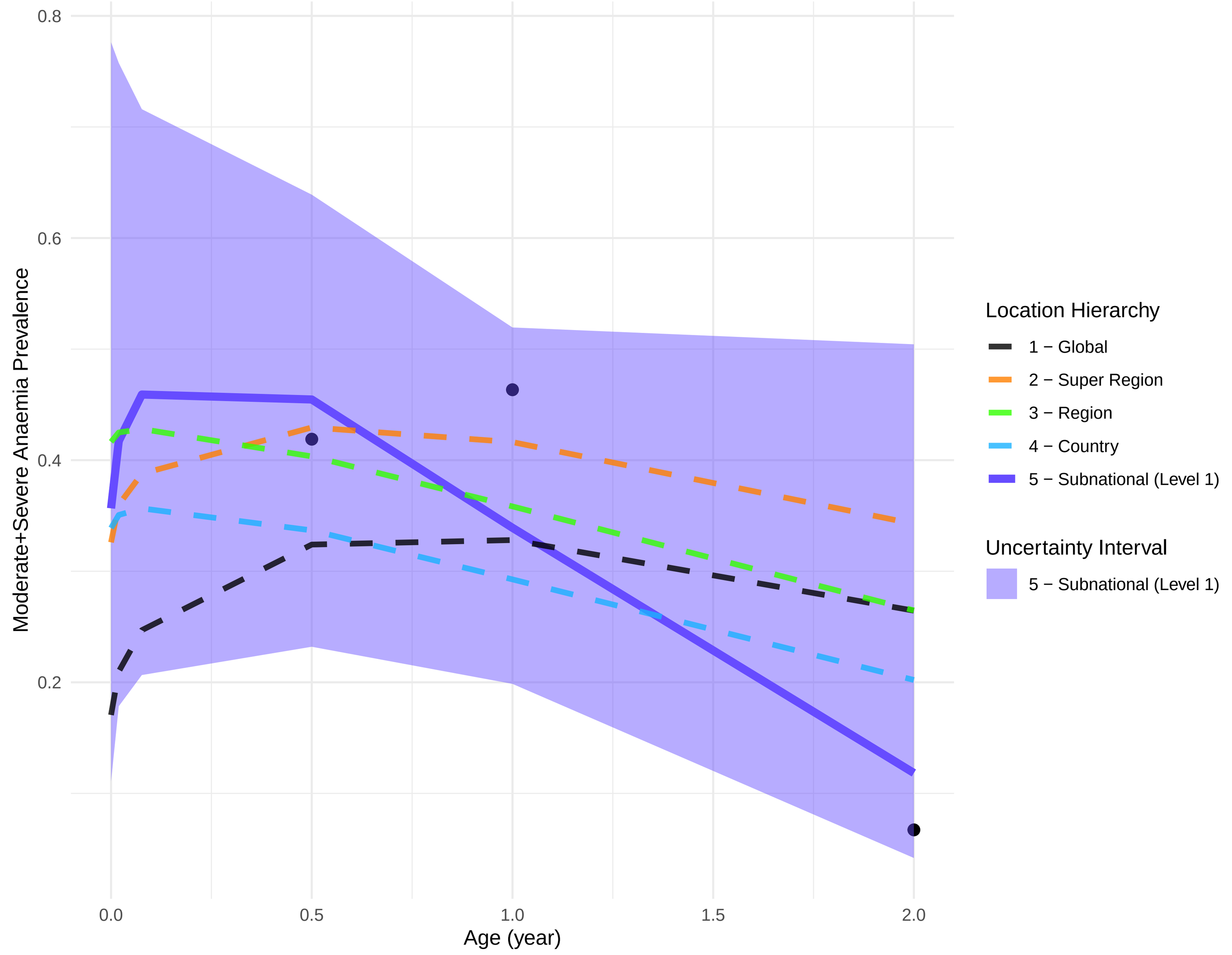

MR−BRT Age/Sex Fits − Males <2 Years Old − Moderate+Severe Anaemia Prevalence − Machakos
WHO−2001 Adjustment Method

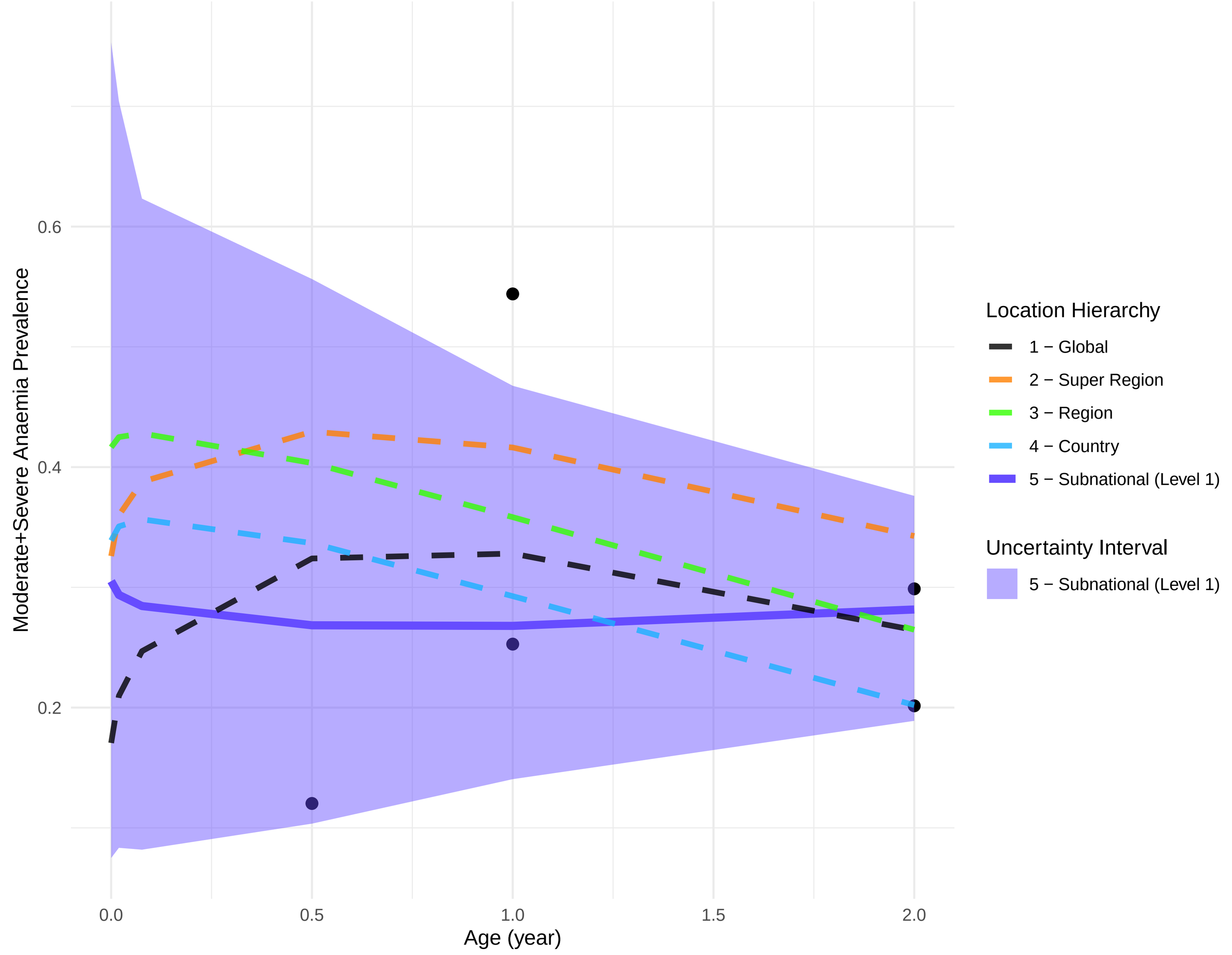

MR−BRT Age/Sex Fits − Males <2 Years Old − Moderate+Severe Anaemia Prevalence − Makueni
WHO−2001 Adjustment Method

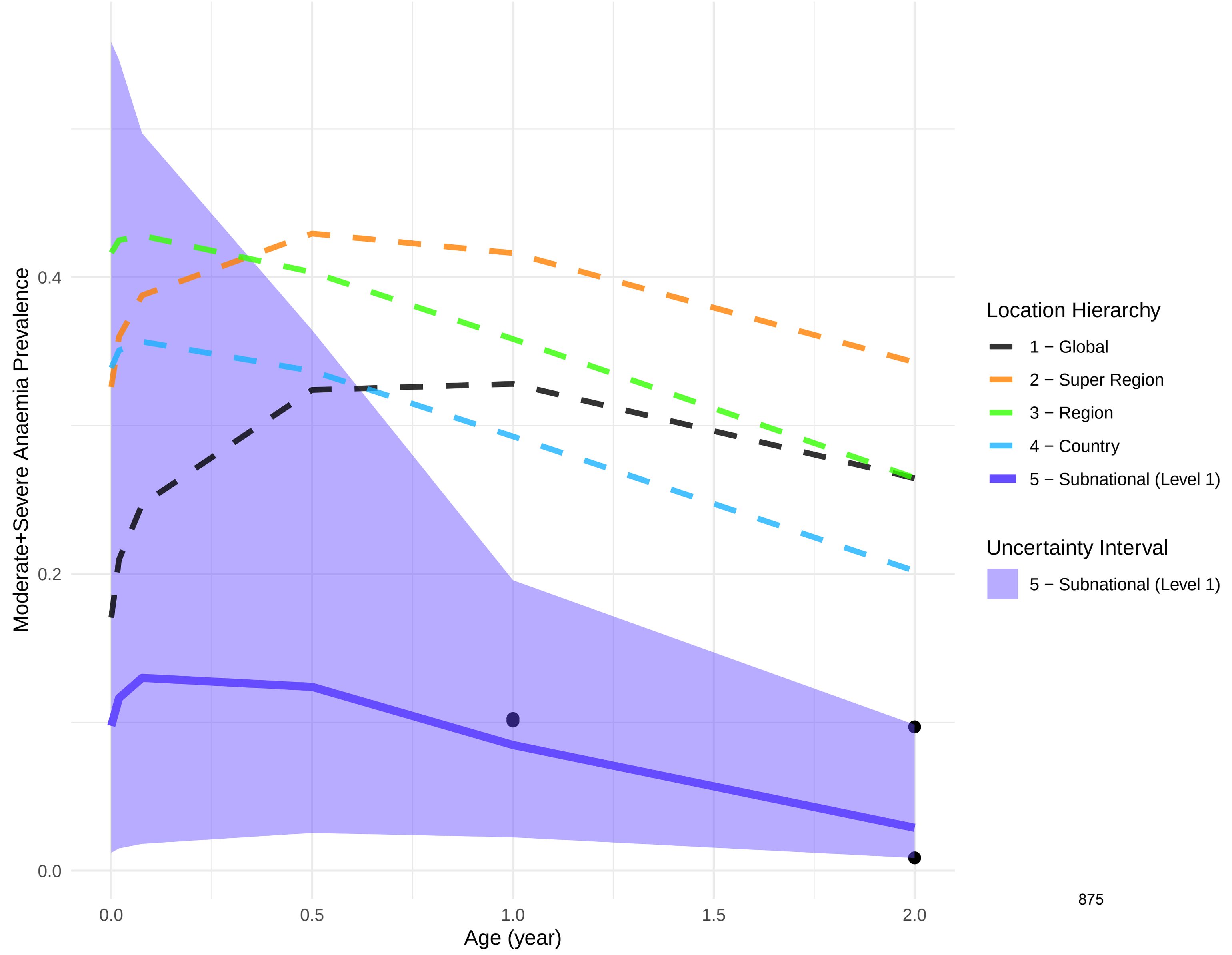

MR−BRT Age/Sex Fits − Males <2 Years Old − Moderate+Severe Anaemia Prevalence − Mandera
WHO−2001 Adjustment Method

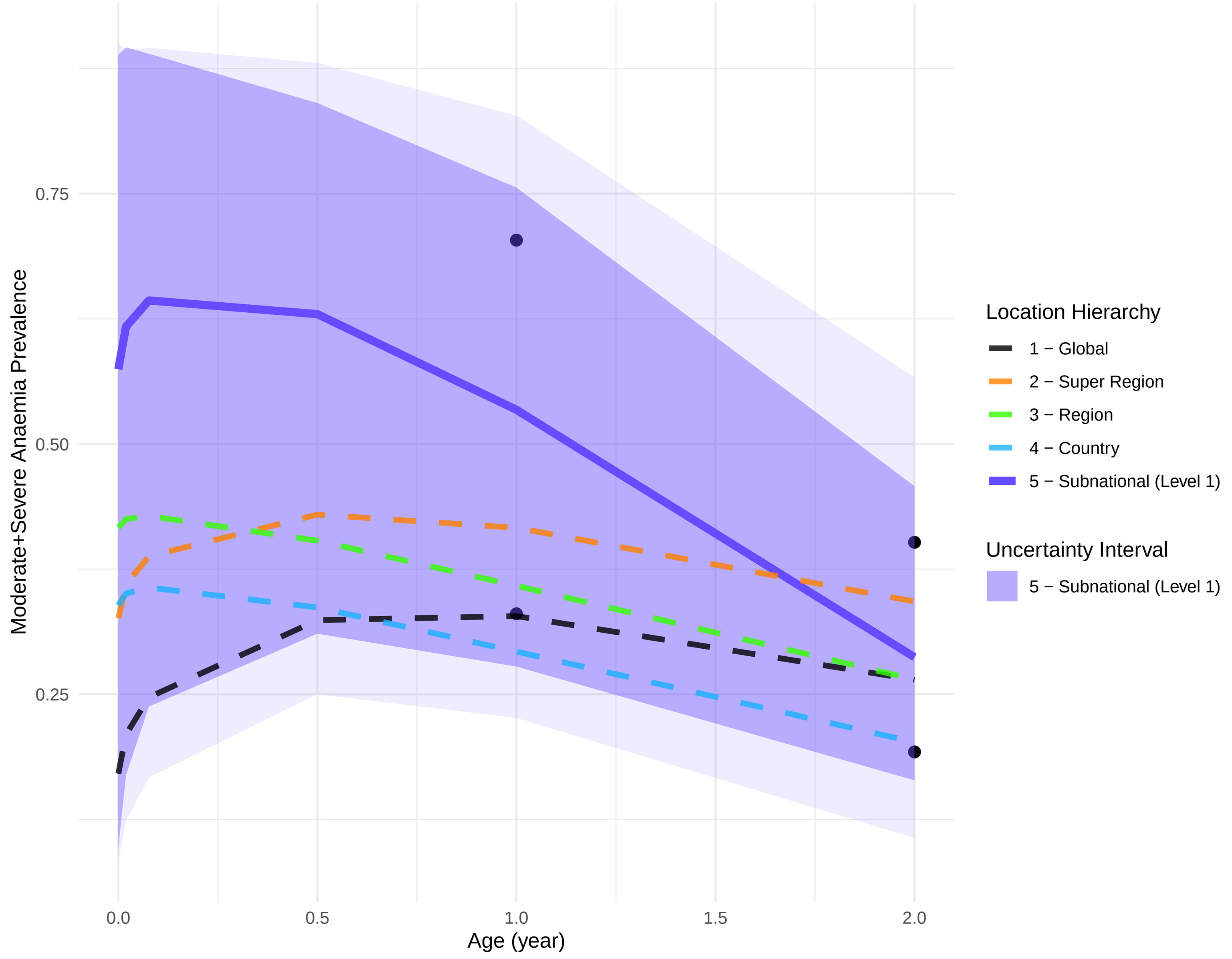

MR−BRT Age/Sex Fits − Males <2 Years Old − Moderate+Severe Anaemia Prevalence − Marsabit
WHO−2001 Adjustment Method

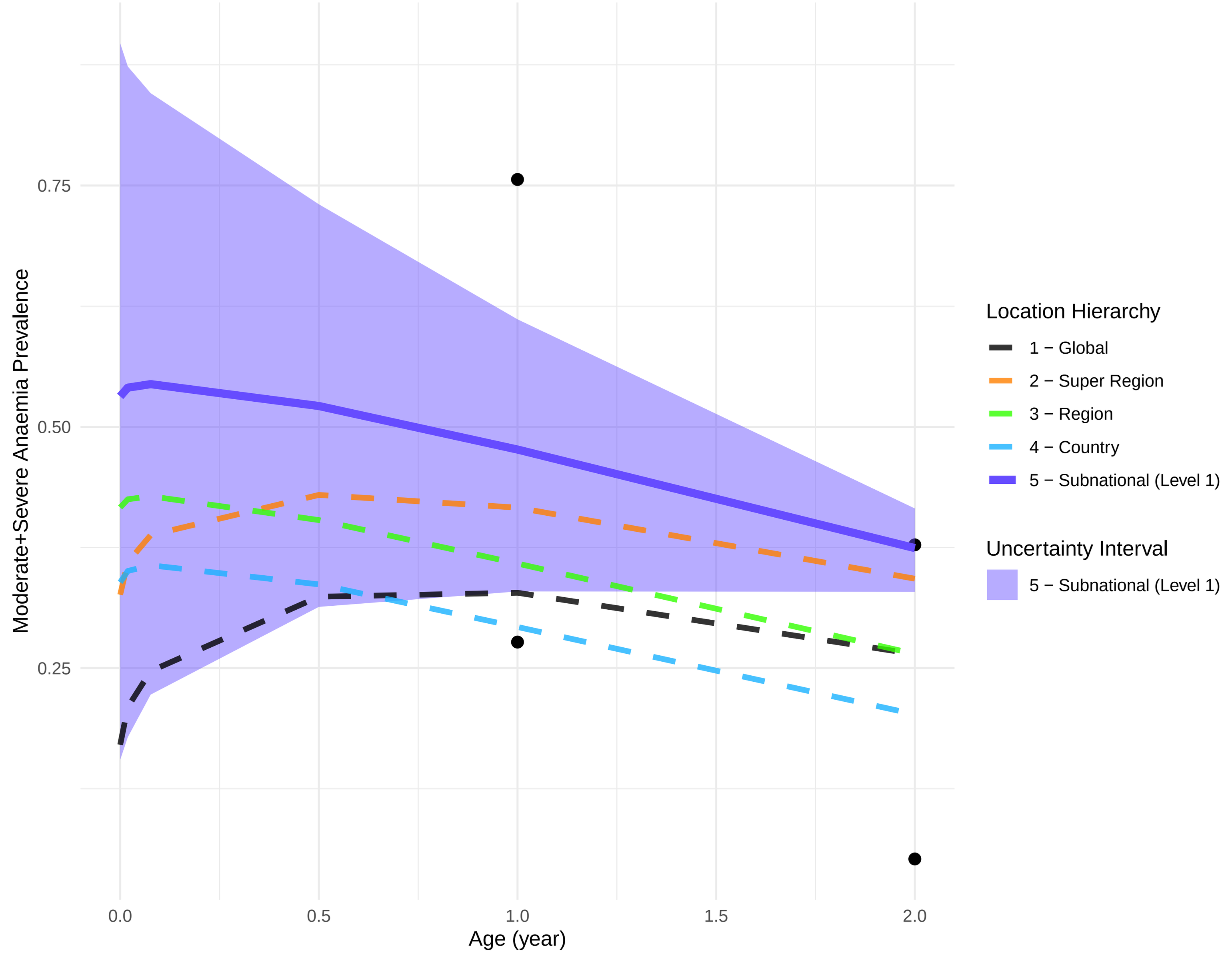

MR−BRT Age/Sex Fits − Males <2 Years Old − Moderate+Severe Anaemia Prevalence − Meru
WHO−2001 Adjustment Method

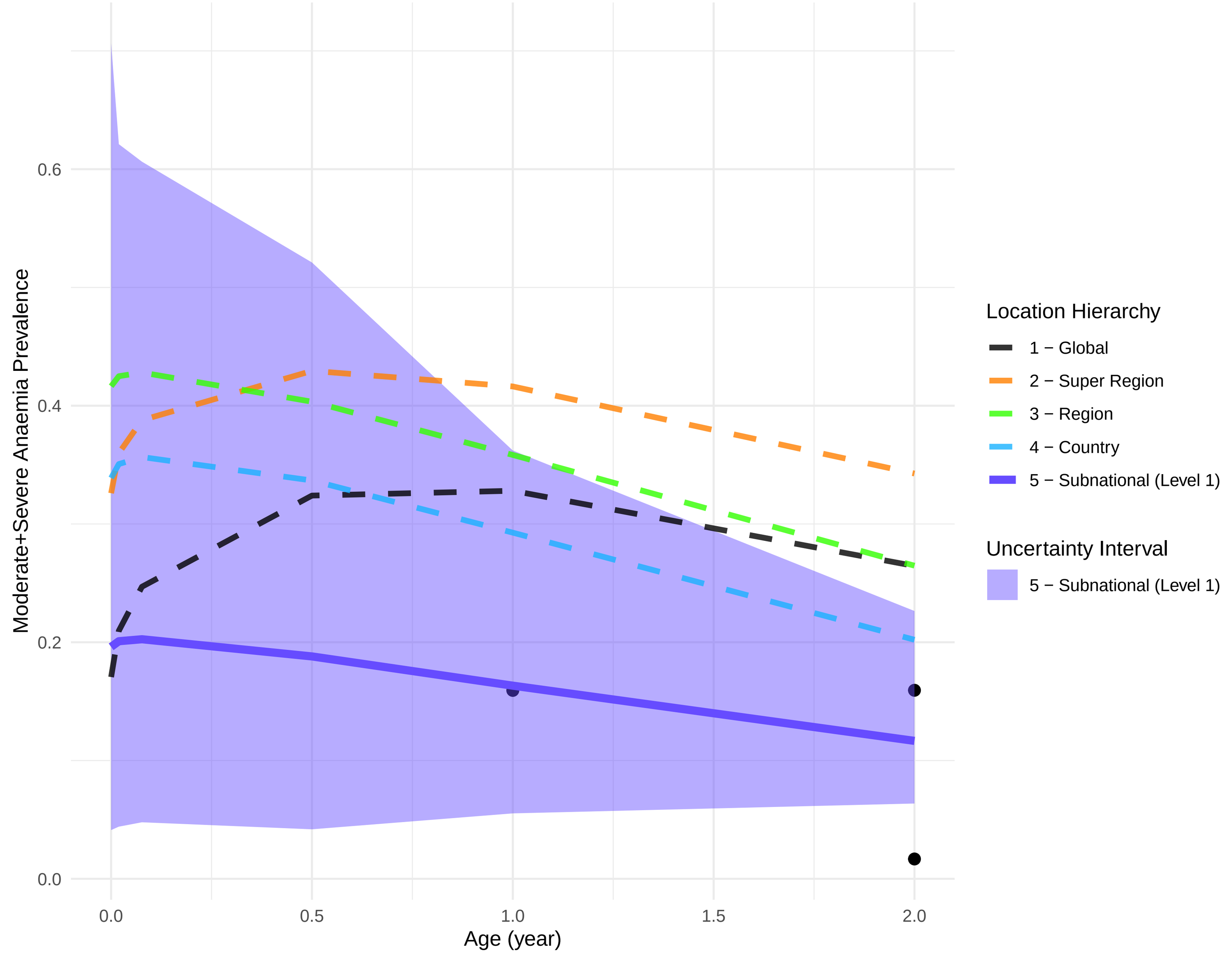

MR−BRT Age/Sex Fits − Males <2 Years Old − Moderate+Severe Anaemia Prevalence − Migori
WHO−2001 Adjustment Method

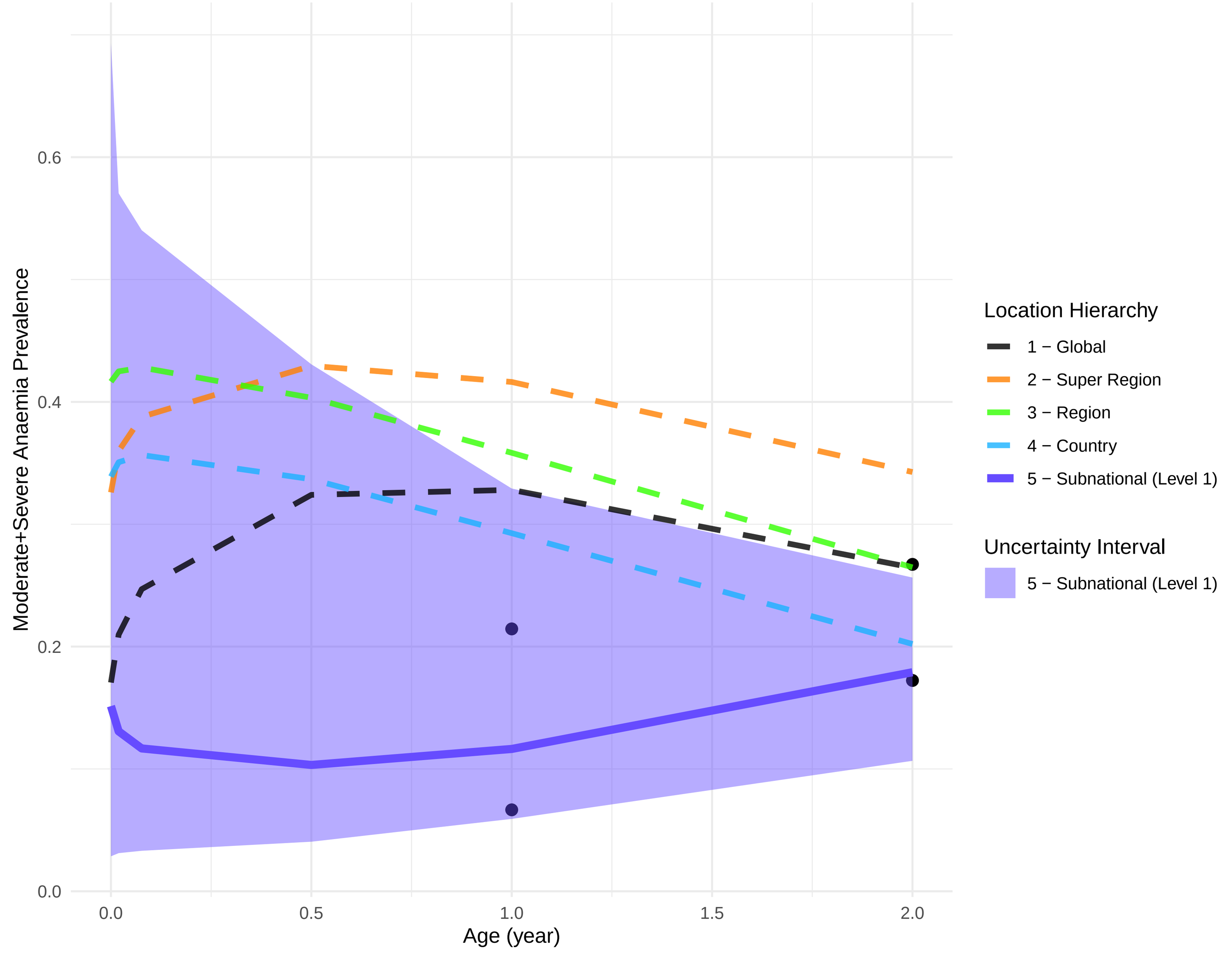

MR−BRT Age/Sex Fits − Males <2 Years Old − Moderate+Severe Anaemia Prevalence − Mombasa
WHO−2001 Adjustment Method

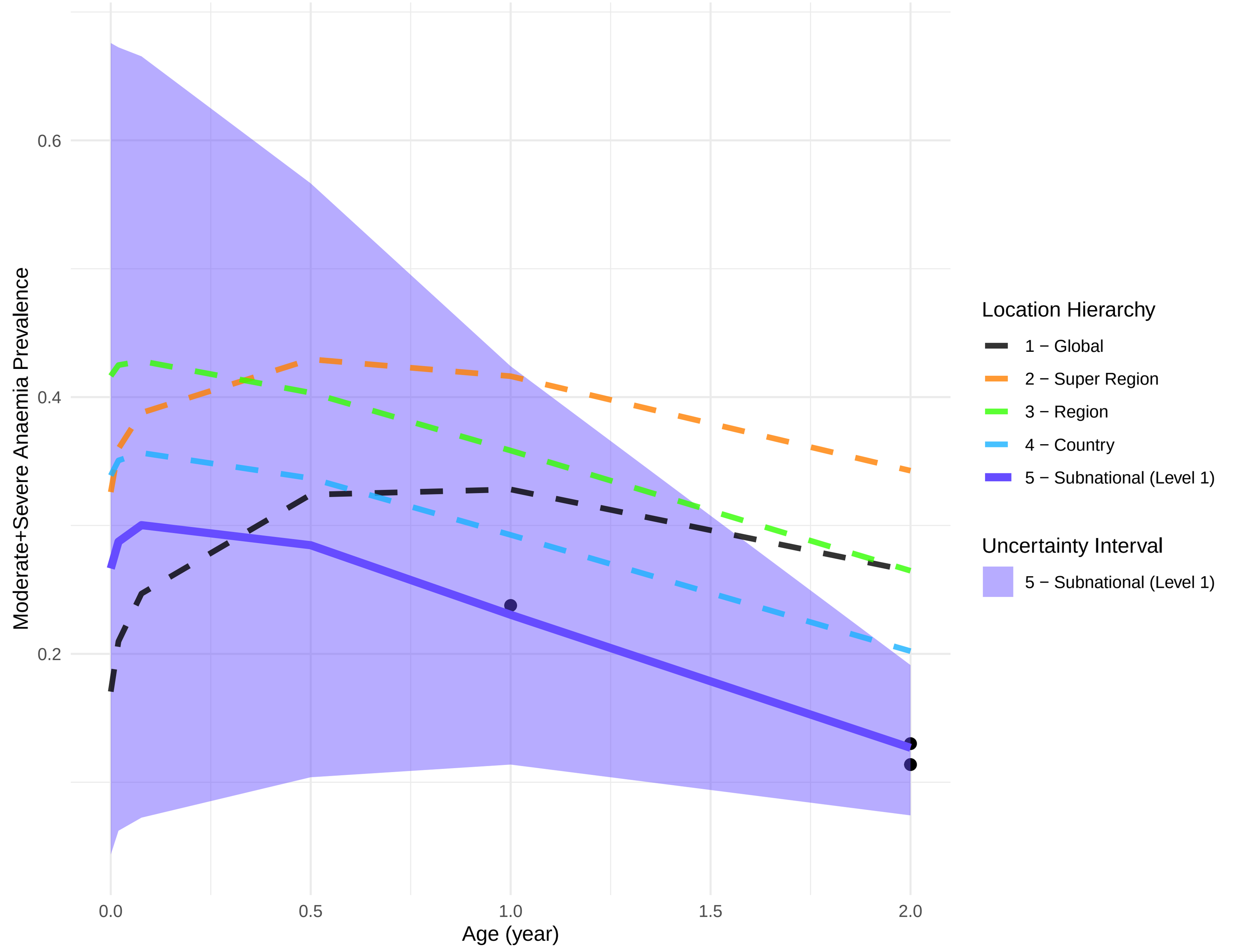

MR−BRT Age/Sex Fits − Males <2 Years Old − Moderate+Severe Anaemia Prevalence − Murang'a
WHO−2001 Adjustment Method

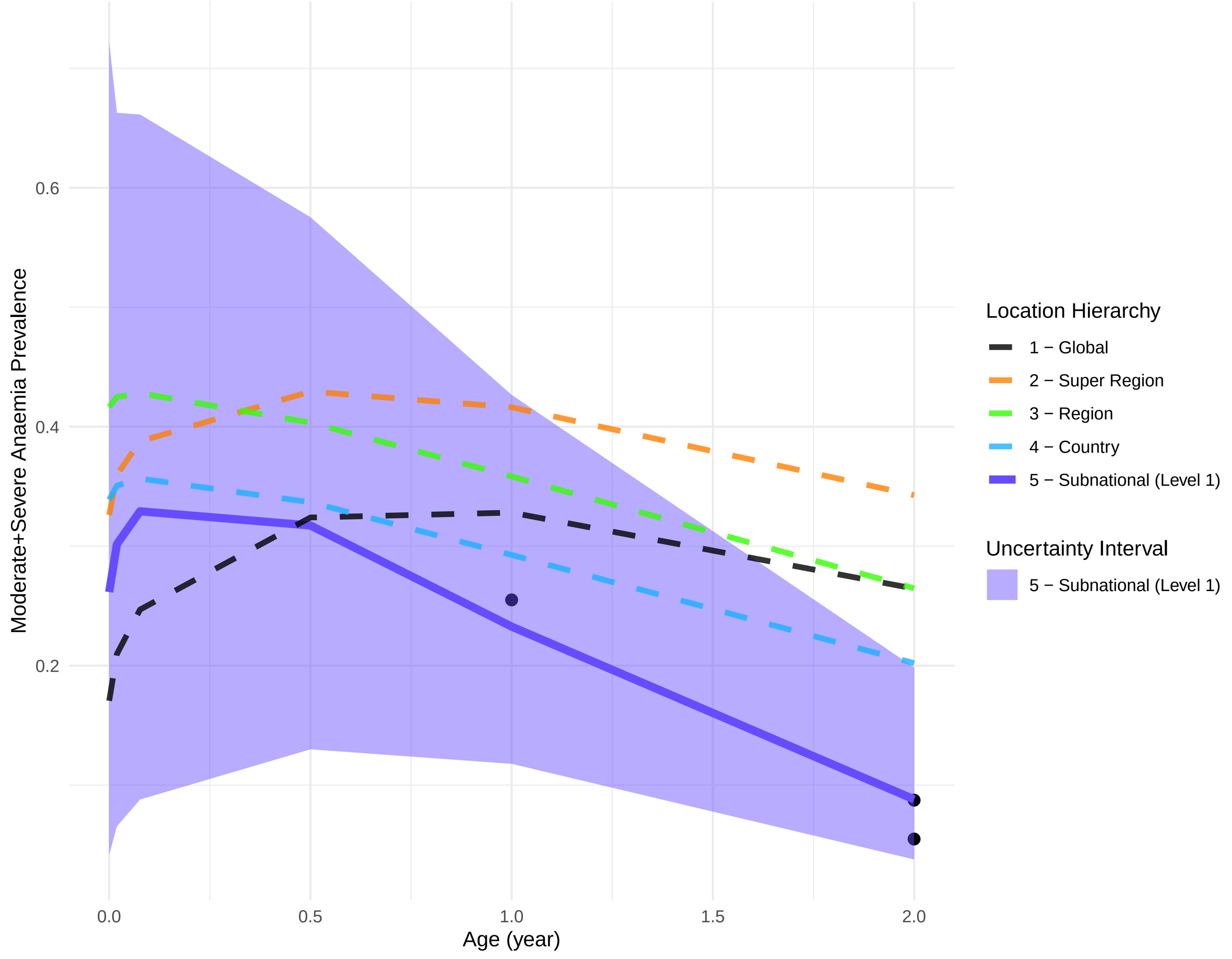

MR−BRT Age/Sex Fits − Males <2 Years Old − Moderate+Severe Anaemia Prevalence − Nairobi
WHO−2001 Adjustment Method

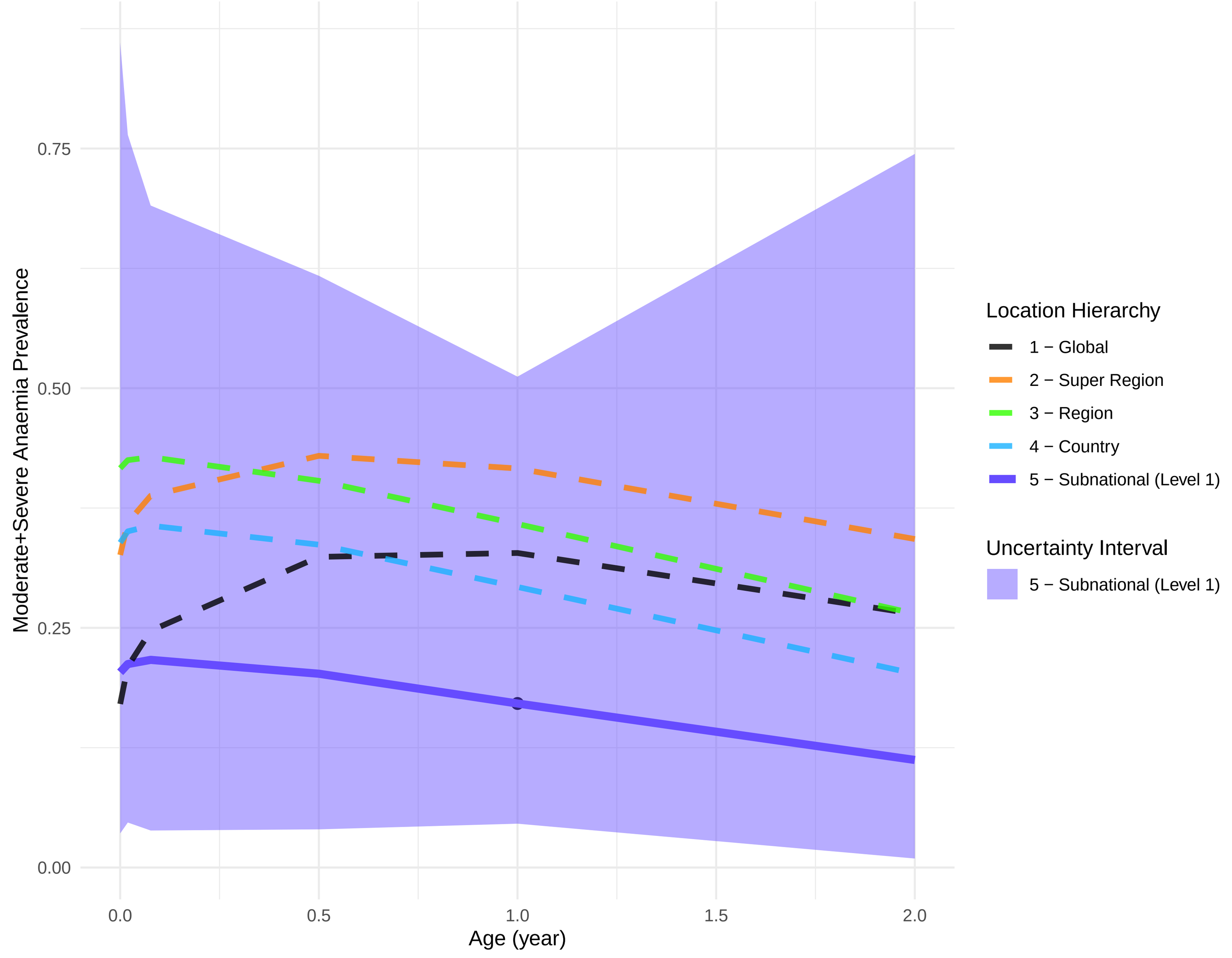

MR−BRT Age/Sex Fits − Males <2 Years Old − Moderate+Severe Anaemia Prevalence − Nakuru
WHO−2001 Adjustment Method

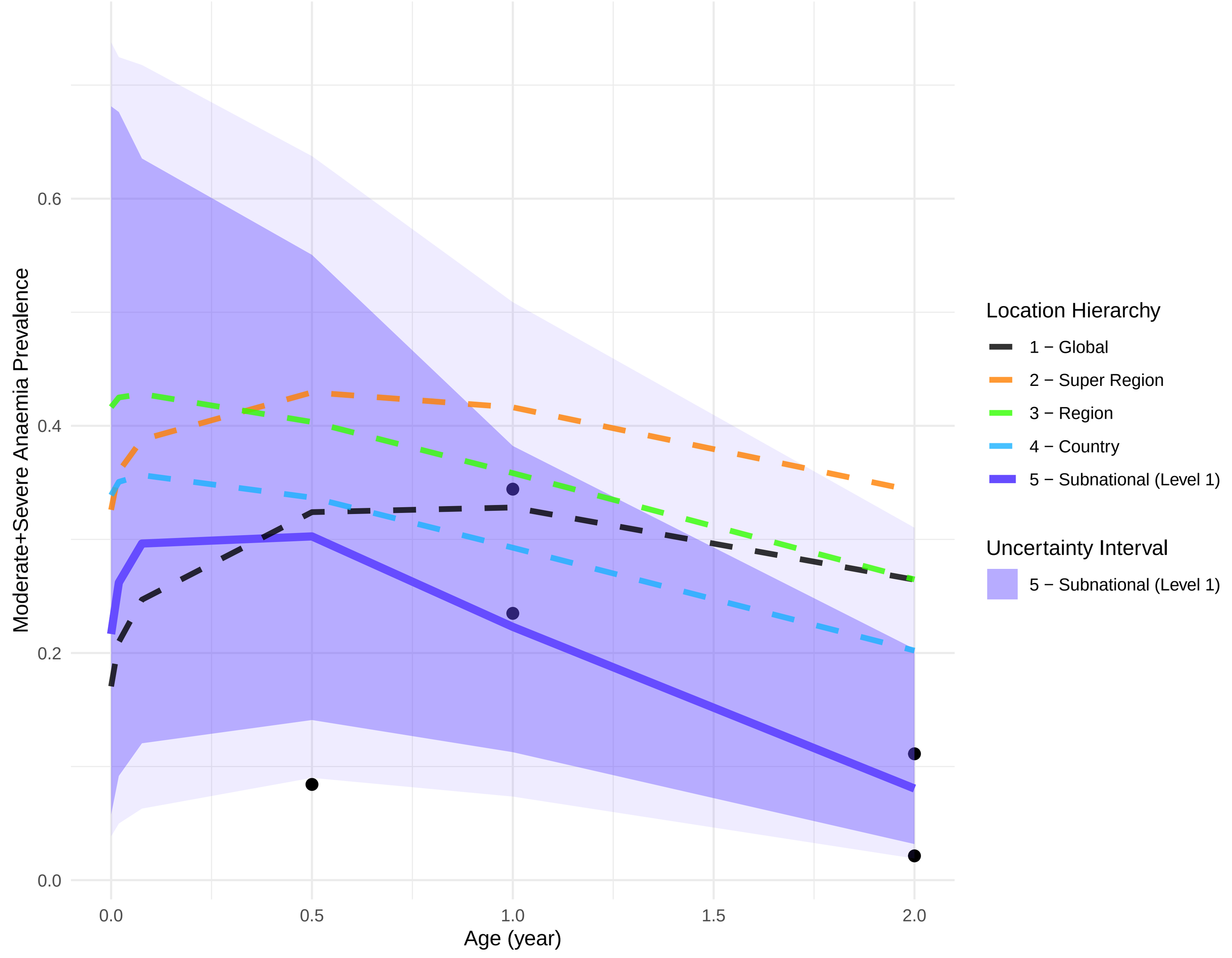

MR−BRT Age/Sex Fits − Males <2 Years Old − Moderate+Severe Anaemia Prevalence − Nandi
WHO−2001 Adjustment Method

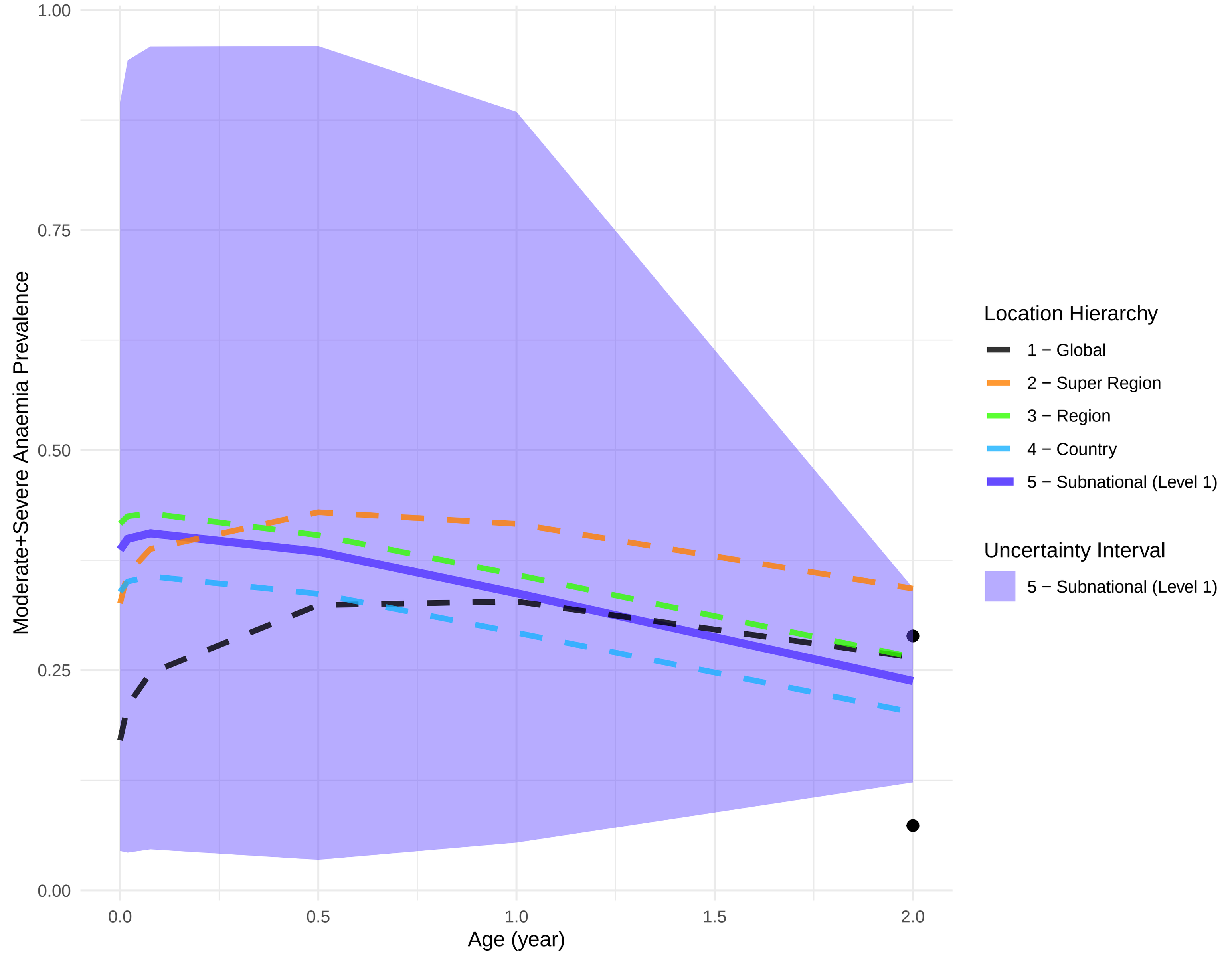

MR−BRT Age/Sex Fits − Males <2 Years Old − Moderate+Severe Anaemia Prevalence − Narok
WHO−2001 Adjustment Method

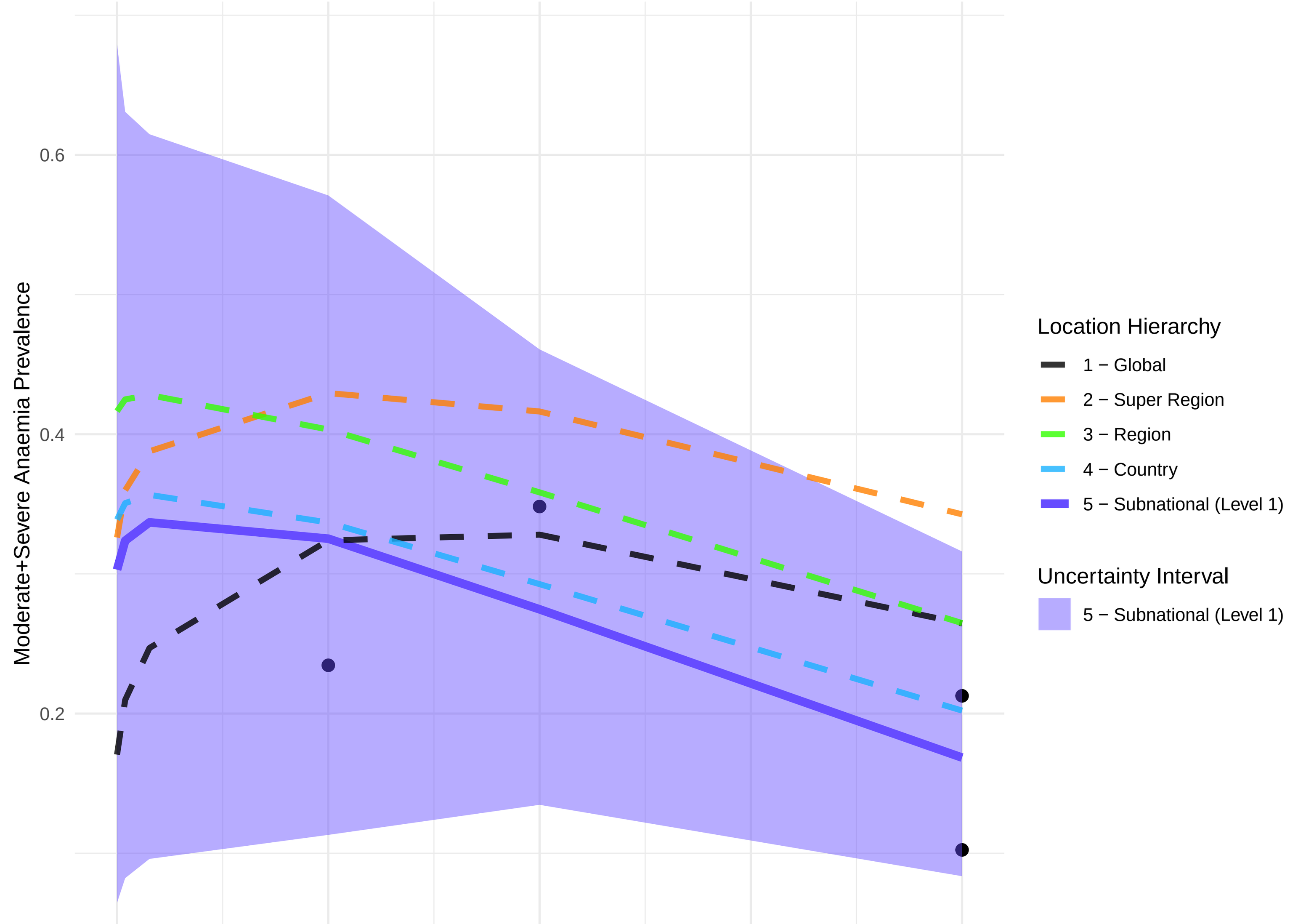

MR−BRT Age/Sex Fits − Males <2 Years Old − Moderate+Severe Anaemia Prevalence − Nyamira
WHO−2001 Adjustment Method

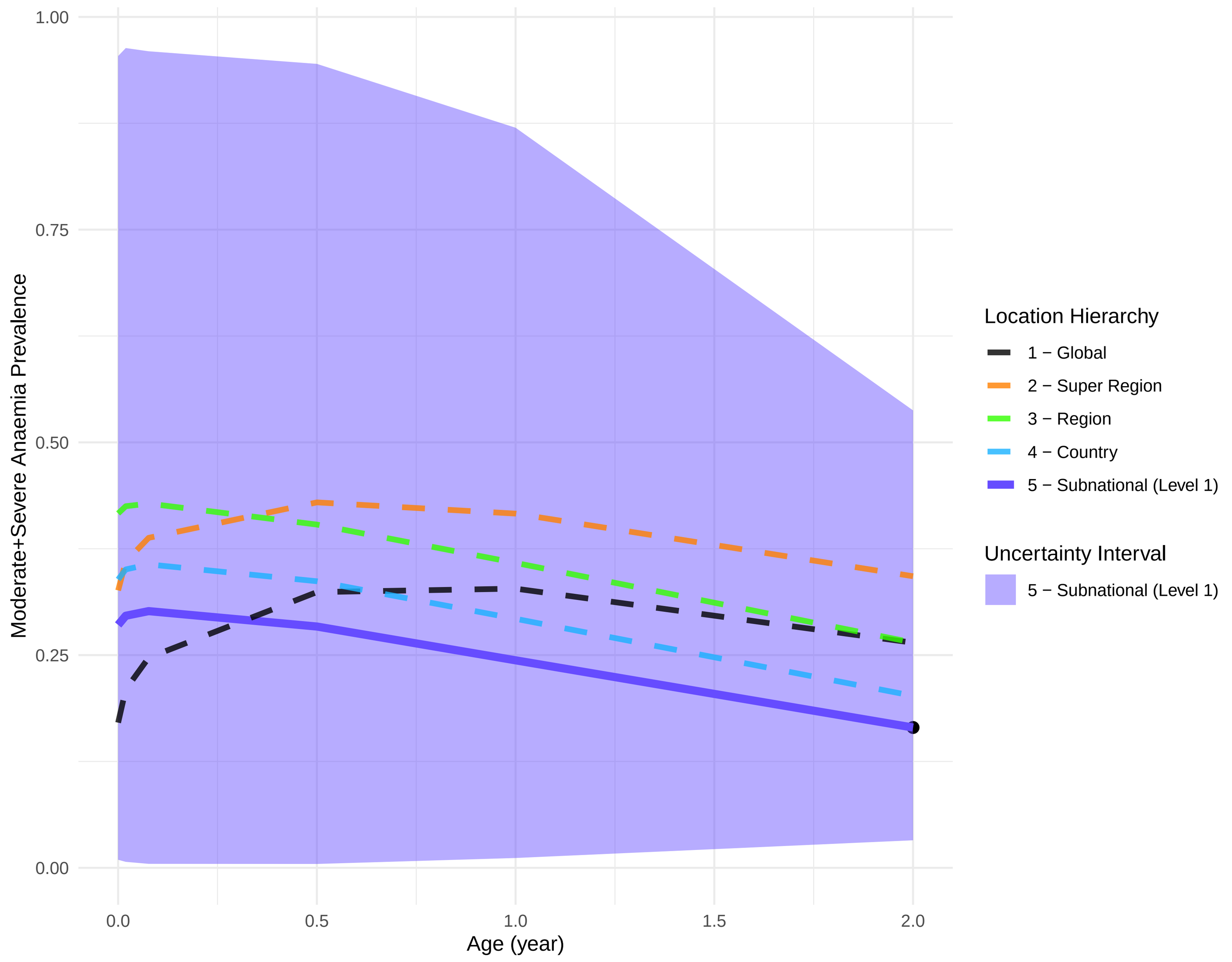

MR−BRT Age/Sex Fits − Males <2 Years Old − Moderate+Severe Anaemia Prevalence − Nyandarua
WHO−2001 Adjustment Method

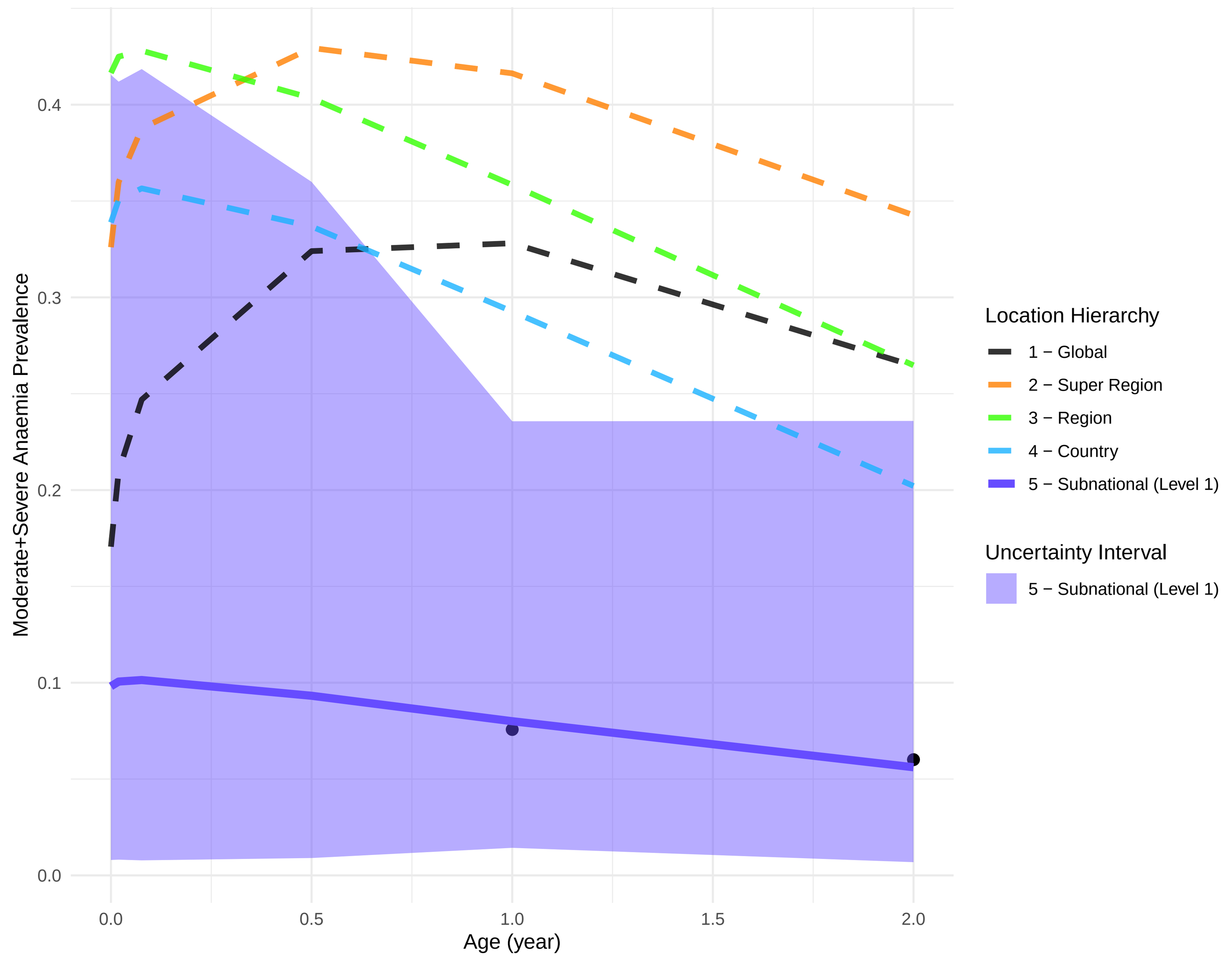

MR−BRT Age/Sex Fits − Males <2 Years Old − Moderate+Severe Anaemia Prevalence − Nyeri
WHO−2001 Adjustment Method

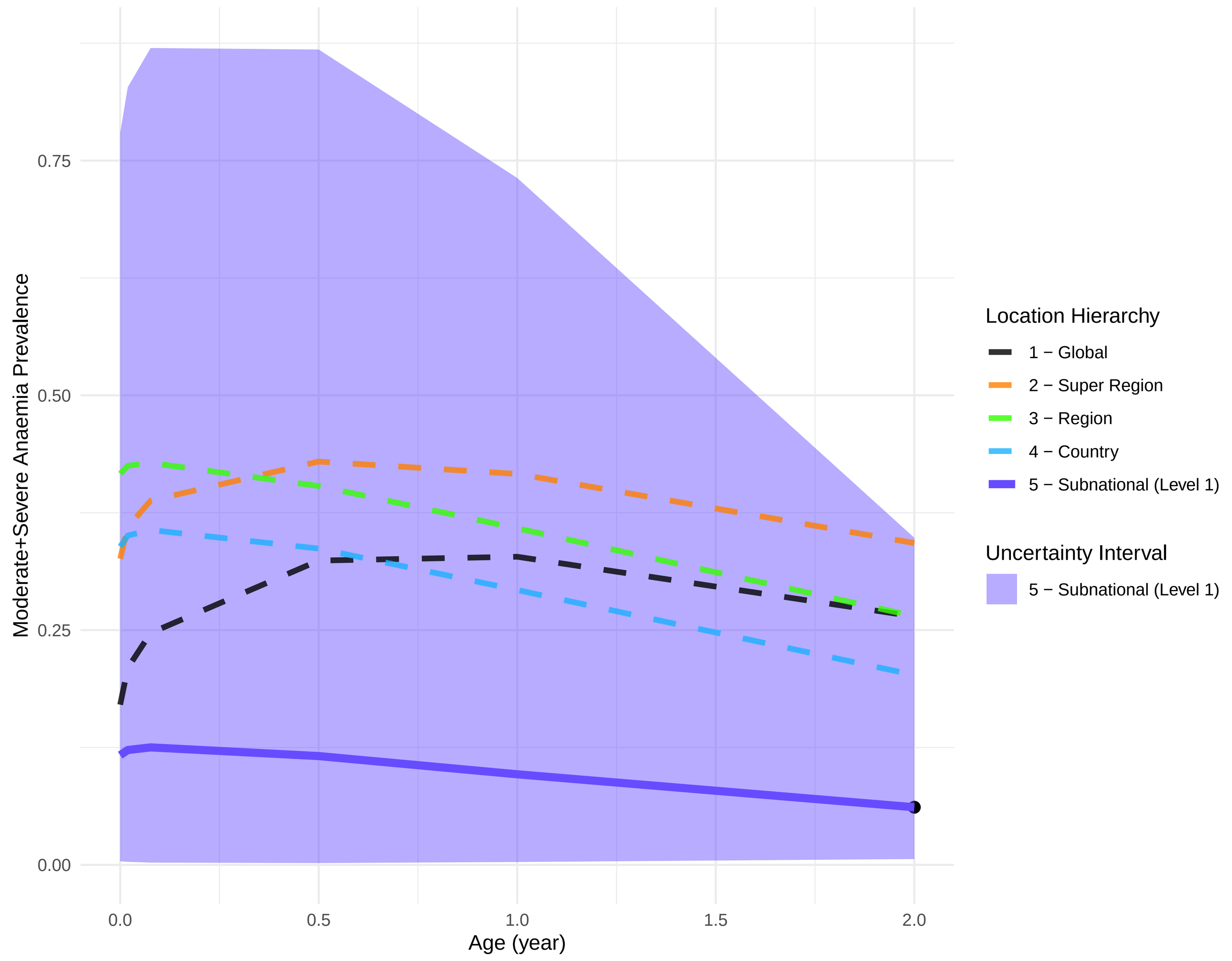

MR−BRT Age/Sex Fits − Males <2 Years Old − Moderate+Severe Anaemia Prevalence − Samburu
WHO−2001 Adjustment Method

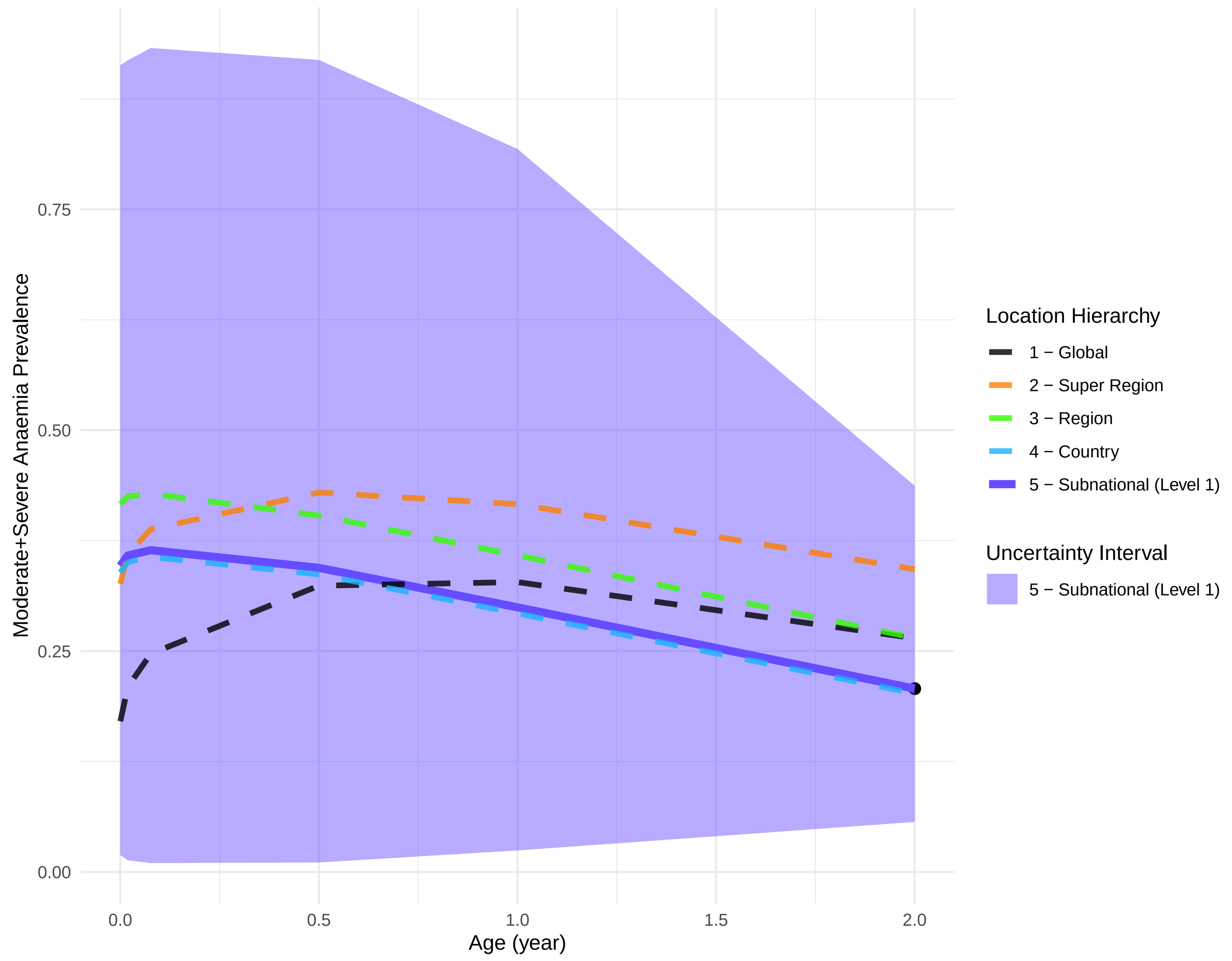

MR−BRT Age/Sex Fits − Males <2 Years Old − Moderate+Severe Anaemia Prevalence − Siaya
WHO−2001 Adjustment Method

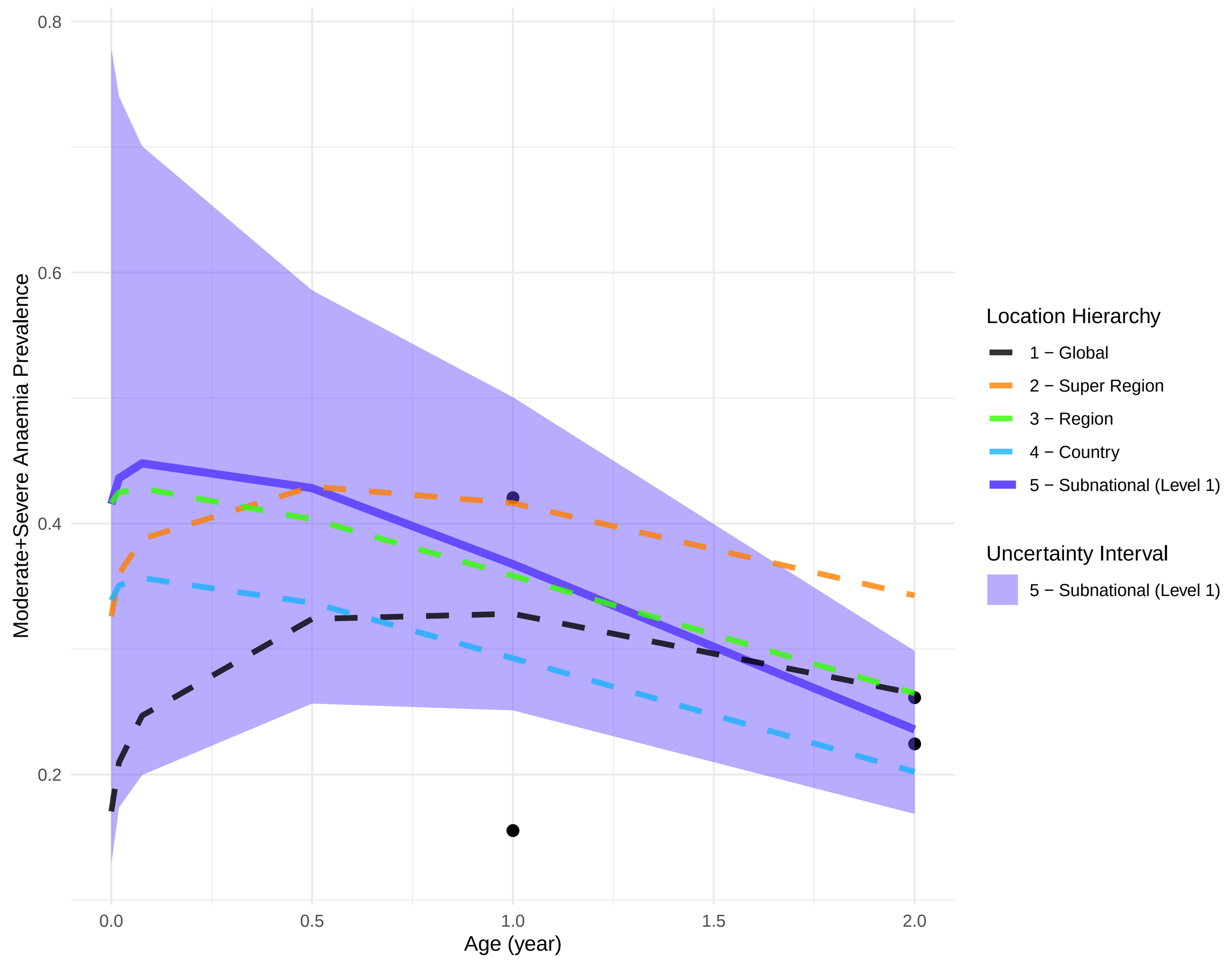

MR−BRT Age/Sex Fits − Males <2 Years Old − Moderate+Severe Anaemia Prevalence − Taita Taveta
WHO−2001 Adjustment Method

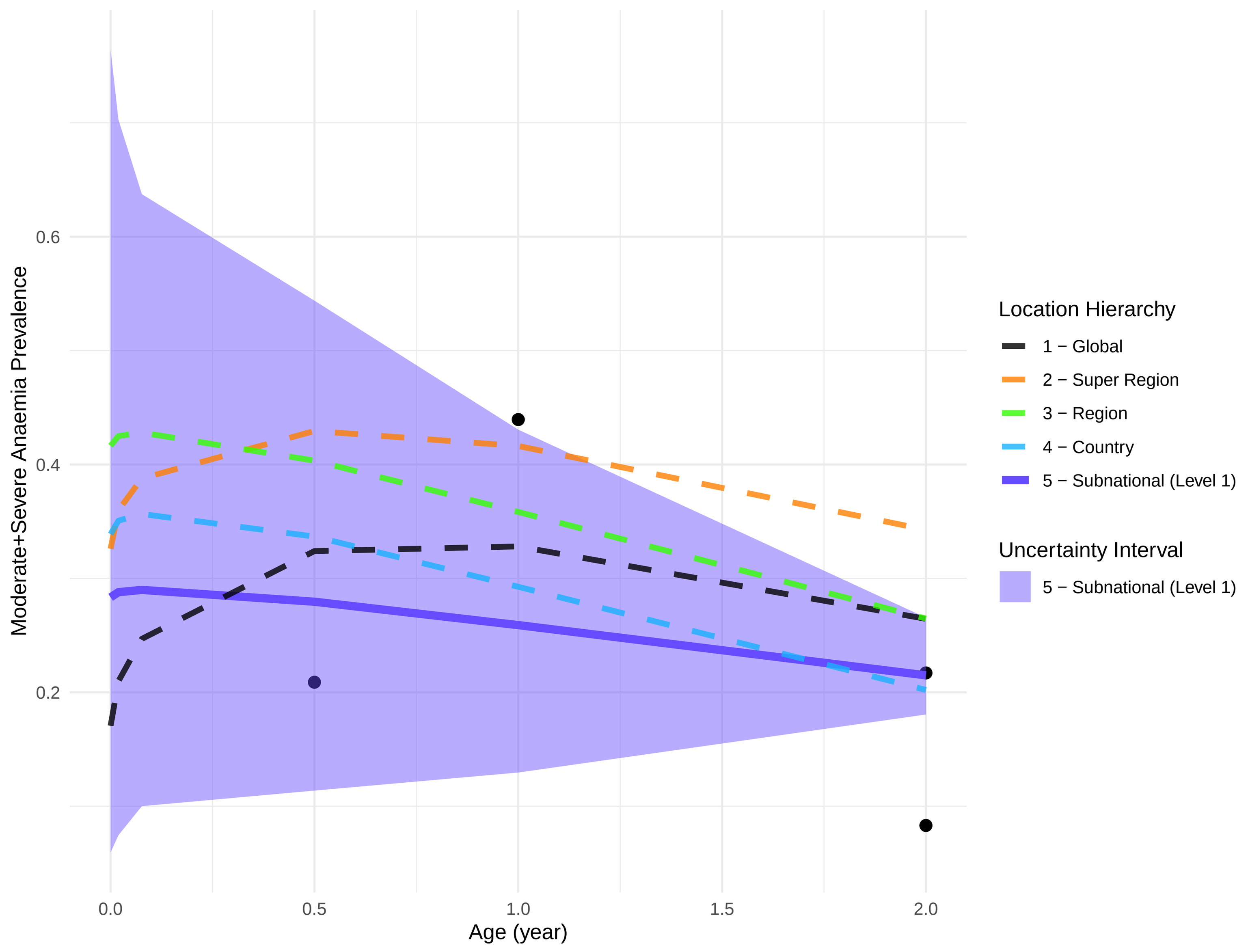

MR−BRT Age/Sex Fits − Males <2 Years Old − Moderate+Severe Anaemia Prevalence − Tana River
WHO−2001 Adjustment Method

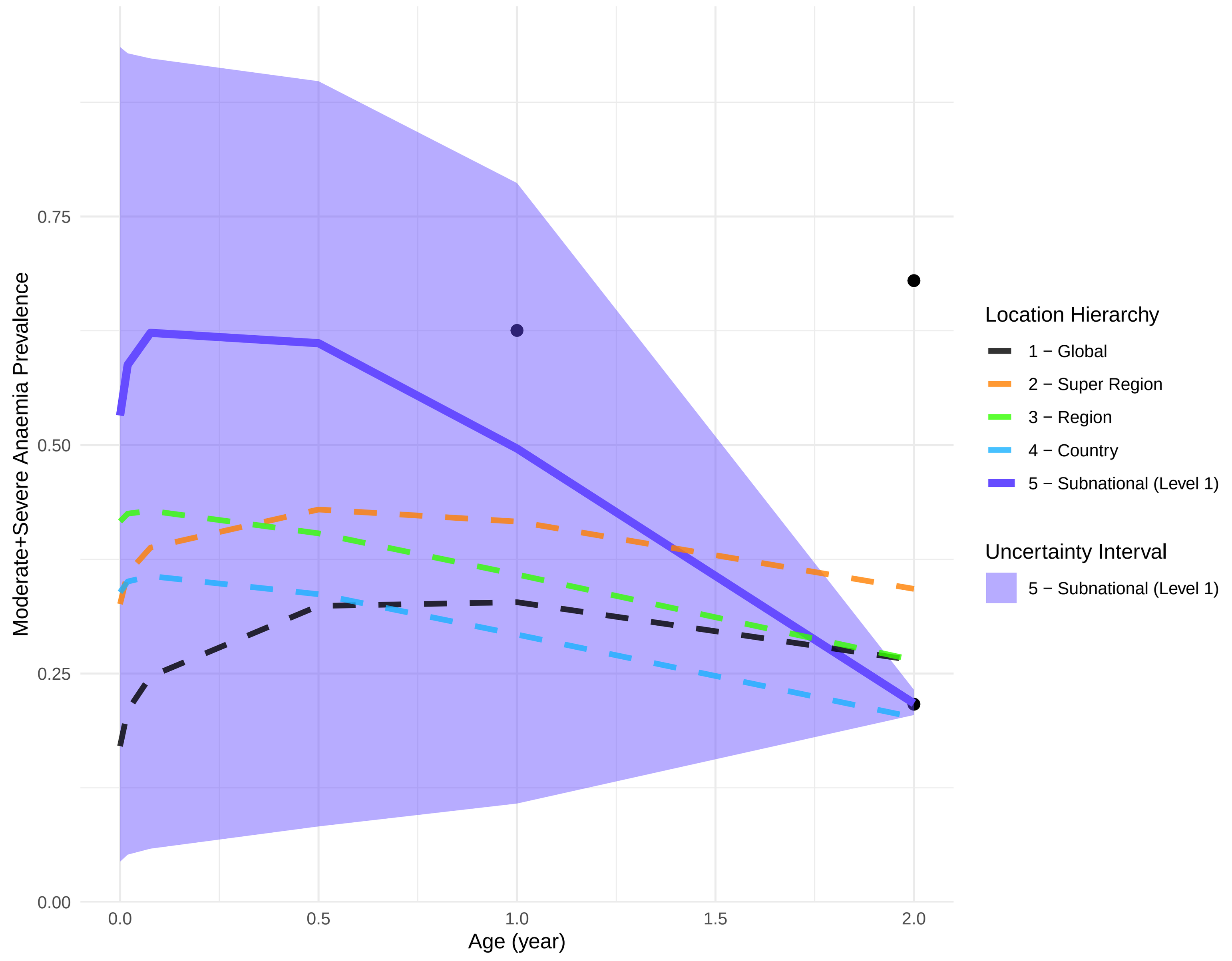

MR−BRT Age/Sex Fits − Males <2 Years Old − Moderate+Severe Anaemia Prevalence − Tharaka Nithi
WHO−2001 Adjustment Method

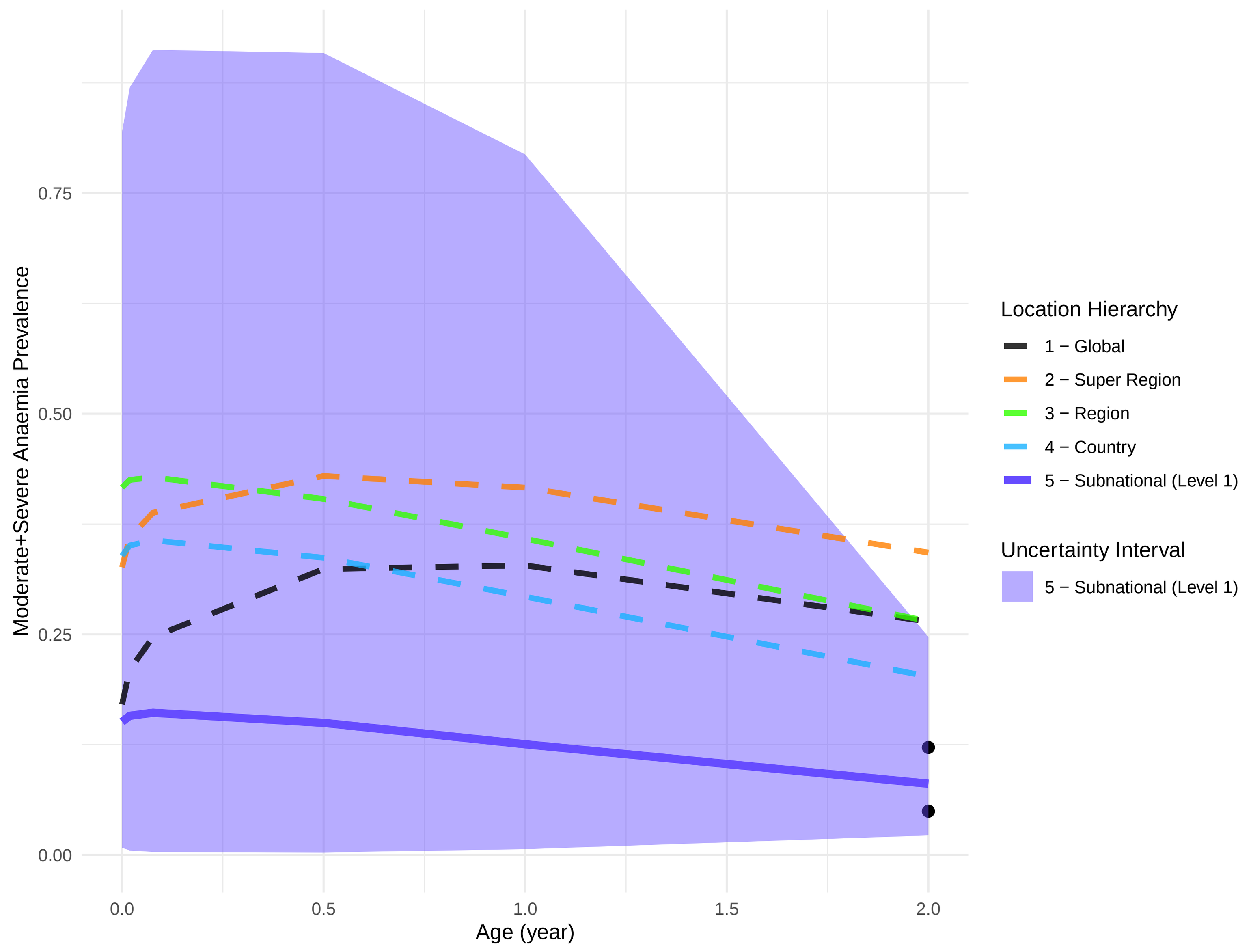

MR−BRT Age/Sex Fits − Males <2 Years Old − Moderate+Severe Anaemia Prevalence − Trans Nzoia
WHO−2001 Adjustment Method

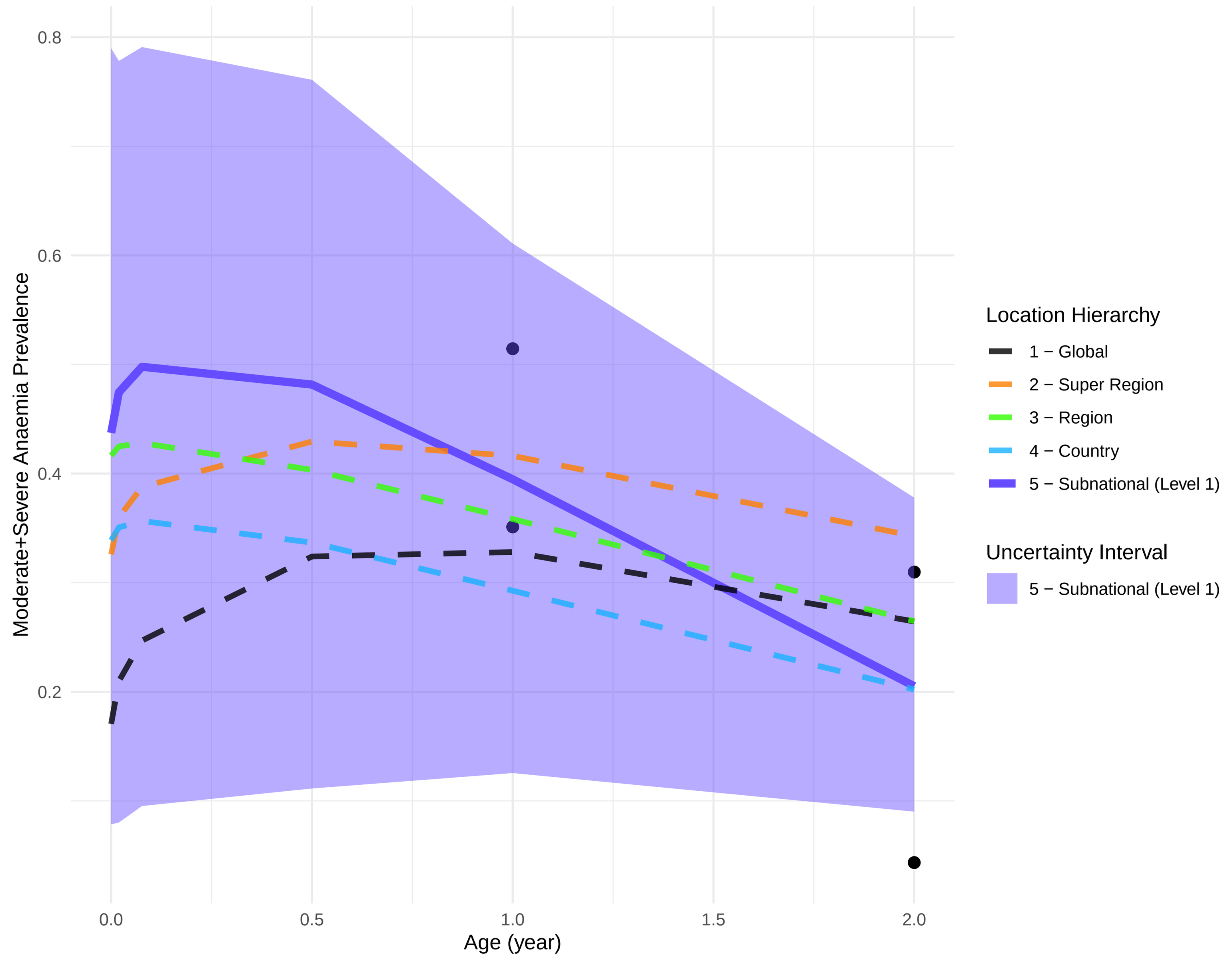

MR−BRT Age/Sex Fits − Males <2 Years Old − Moderate+Severe Anaemia Prevalence − Turkana
WHO−2001 Adjustment Method

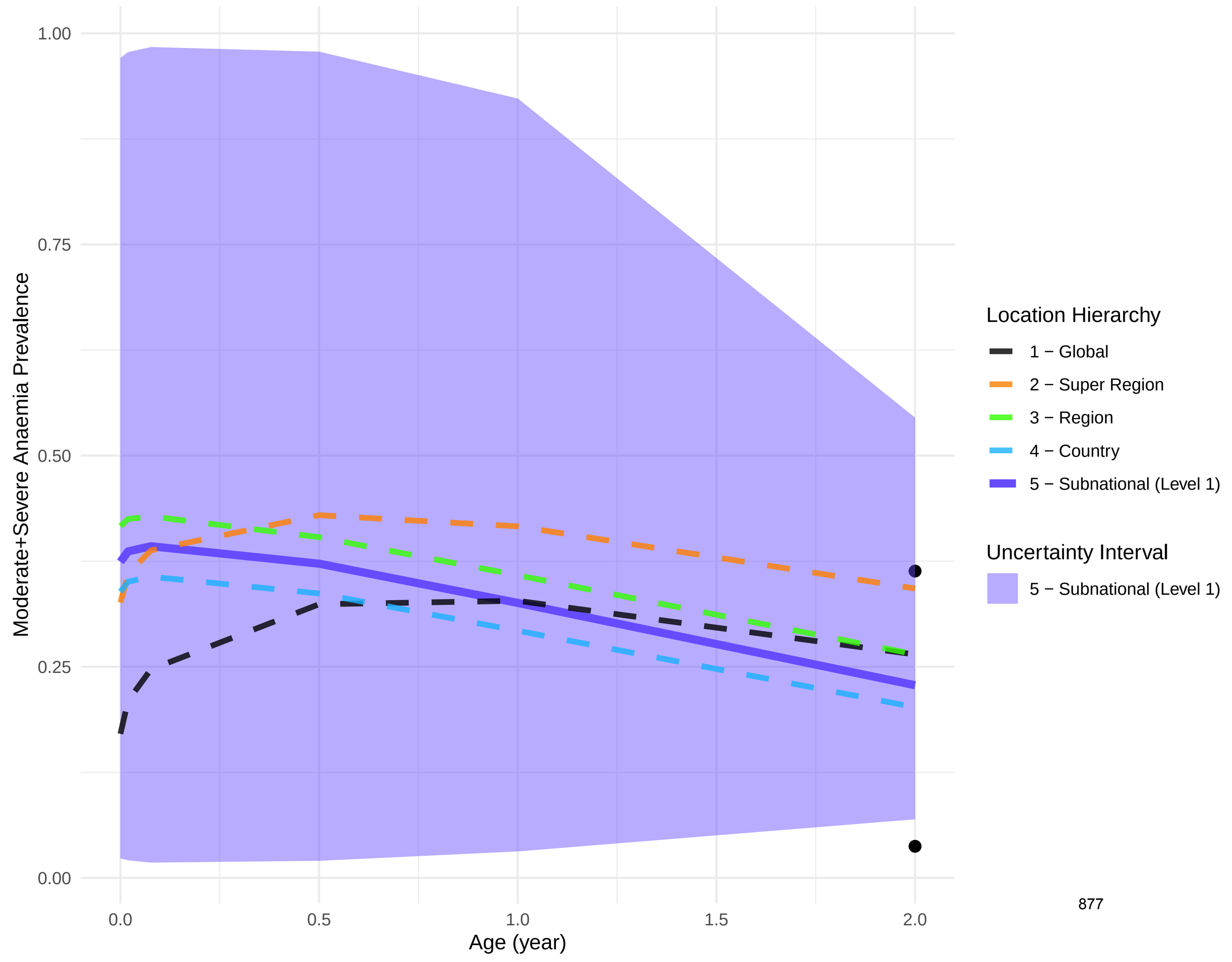

MR−BRT Age/Sex Fits − Males <2 Years Old − Moderate+Severe Anaemia Prevalence − Uasin Gishu
WHO−2001 Adjustment Method

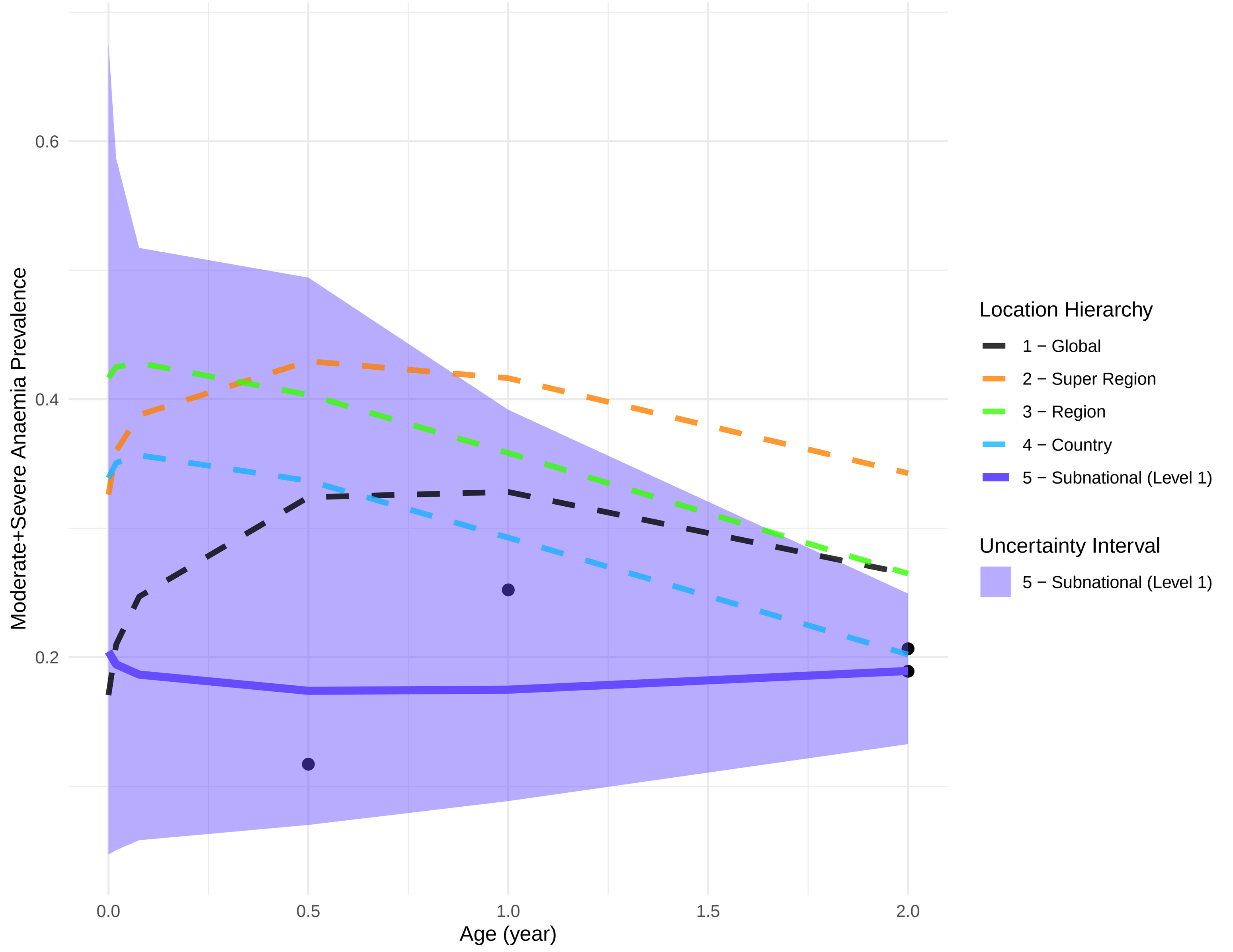

MR−BRT Age/Sex Fits − Males <2 Years Old − Moderate+Severe Anaemia Prevalence − Vihiga
WHO−2001 Adjustment Method

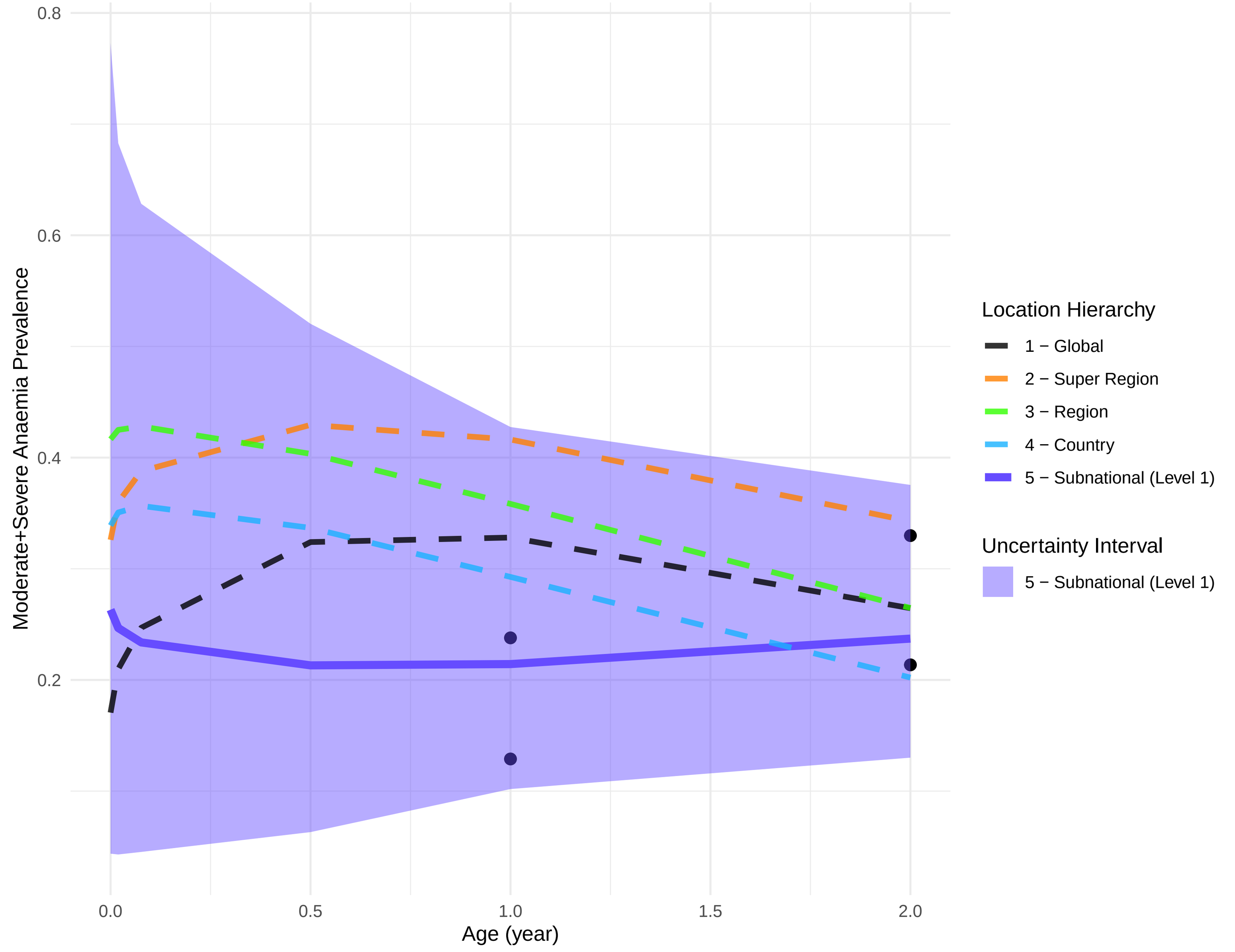

MR−BRT Age/Sex Fits − Males <2 Years Old − Moderate+Severe Anaemia Prevalence − Wajir
WHO−2001 Adjustment Method

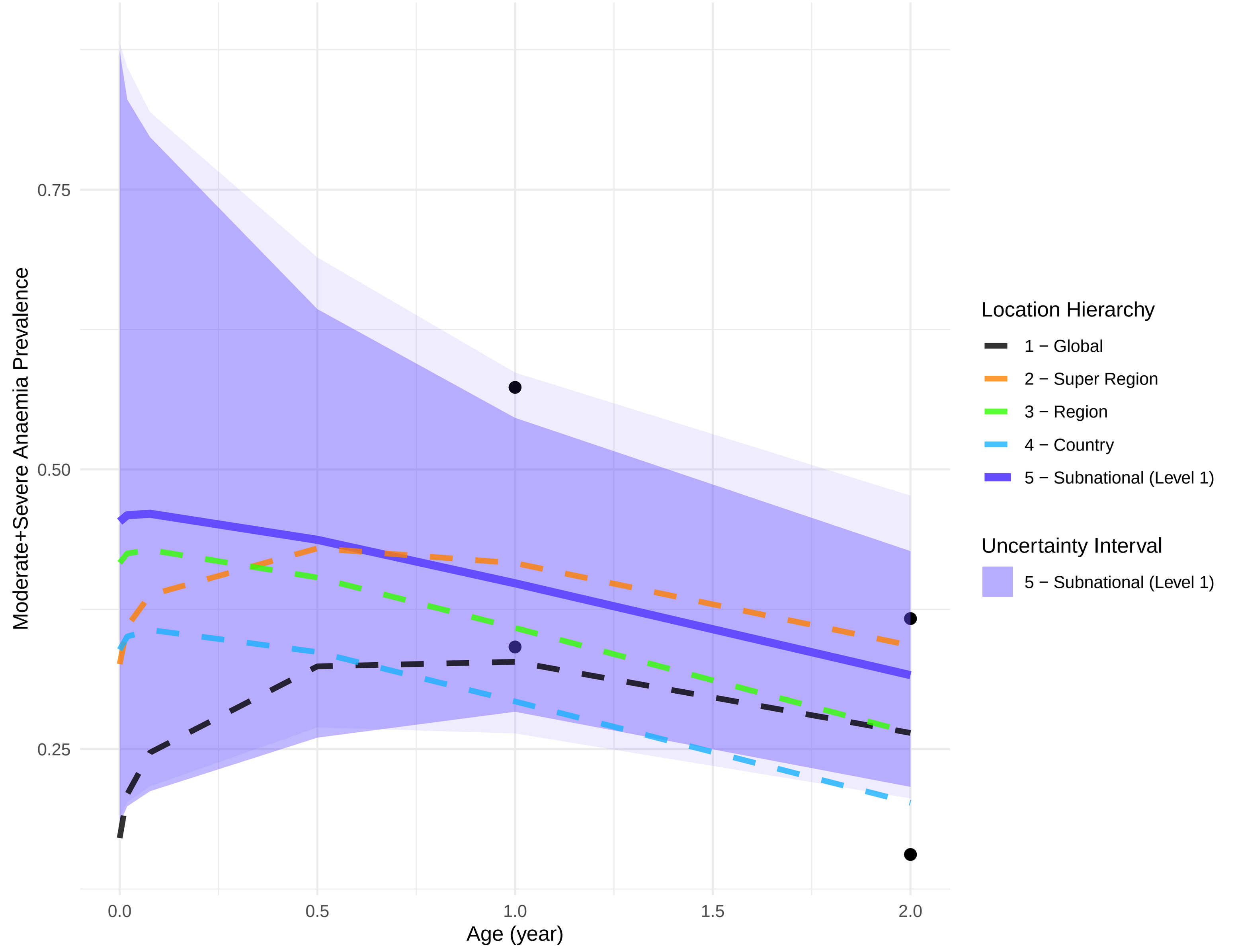

MR−BRT Age/Sex Fits − Males <2 Years Old − Moderate+Severe Anaemia Prevalence − West Pokot
WHO−2001 Adjustment Method

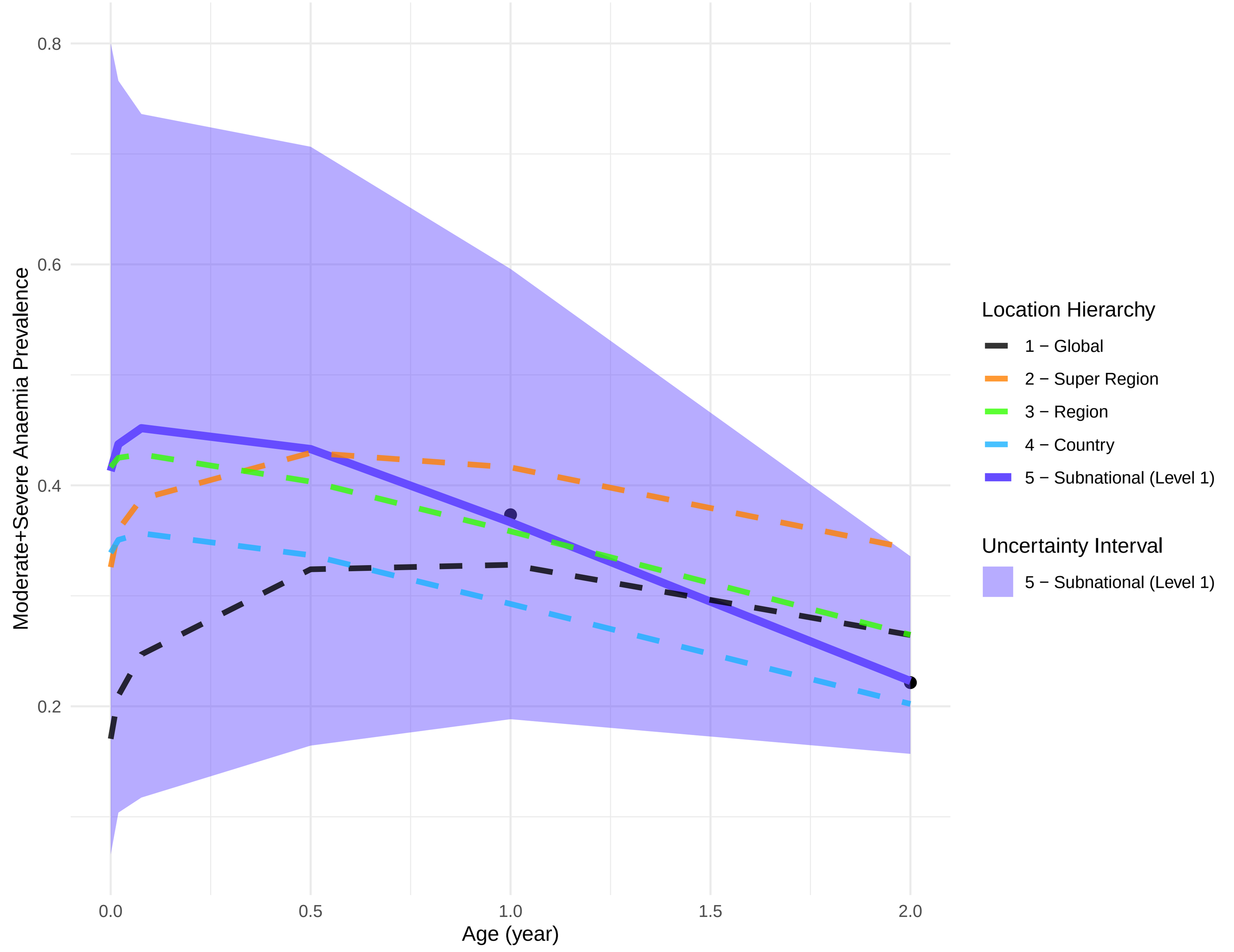

MR−BRT Age/Sex Fits − Males <2 Years Old − Moderate+Severe Anaemia Prevalence − Madagascar
WHO−2001 Adjustment Method

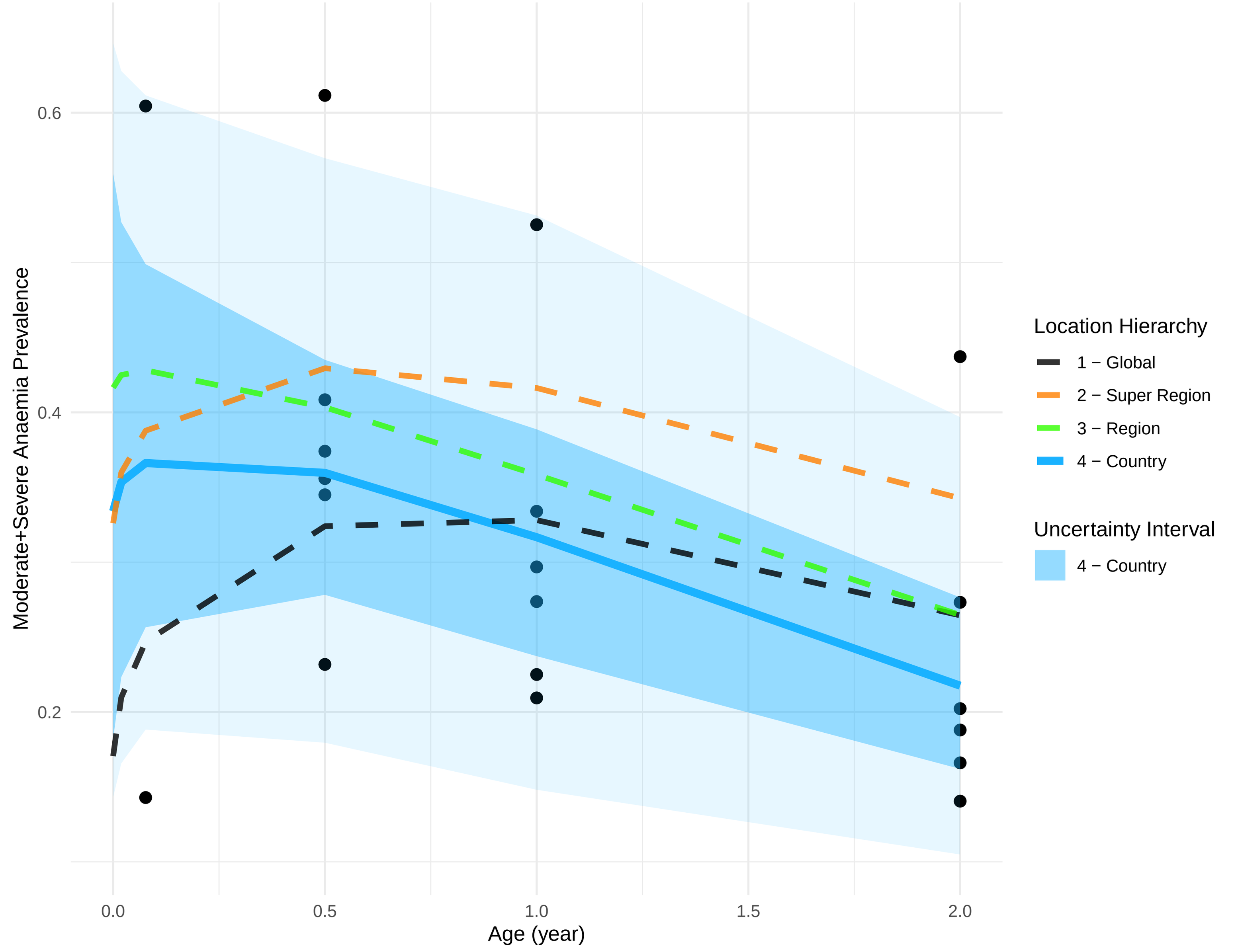

MR−BRT Age/Sex Fits − Males <2 Years Old − Moderate+Severe Anaemia Prevalence − Malawi
WHO−2001 Adjustment Method

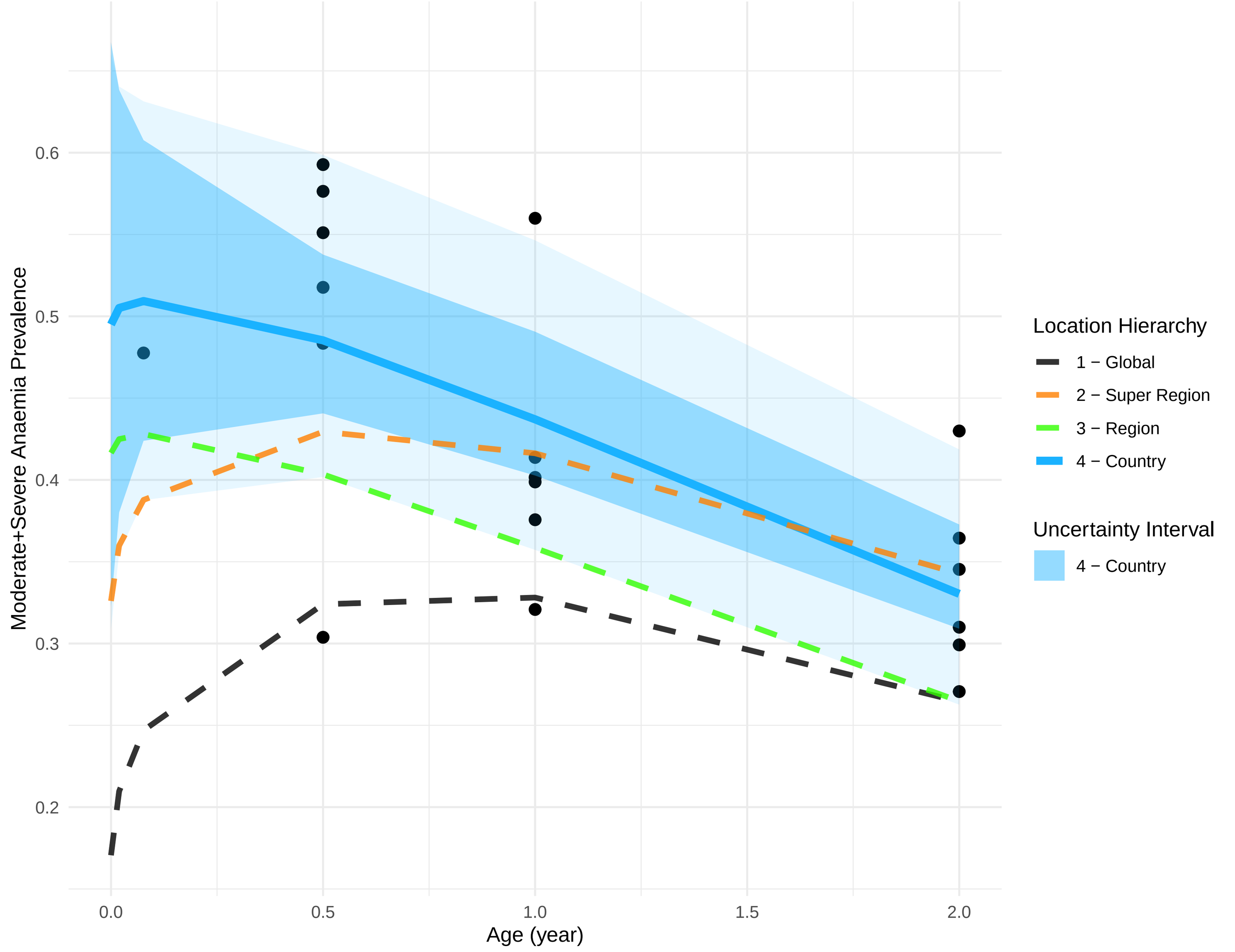

MR−BRT Age/Sex Fits − Males <2 Years Old − Moderate+Severe Anaemia Prevalence − Mozambique
WHO−2001 Adjustment Method

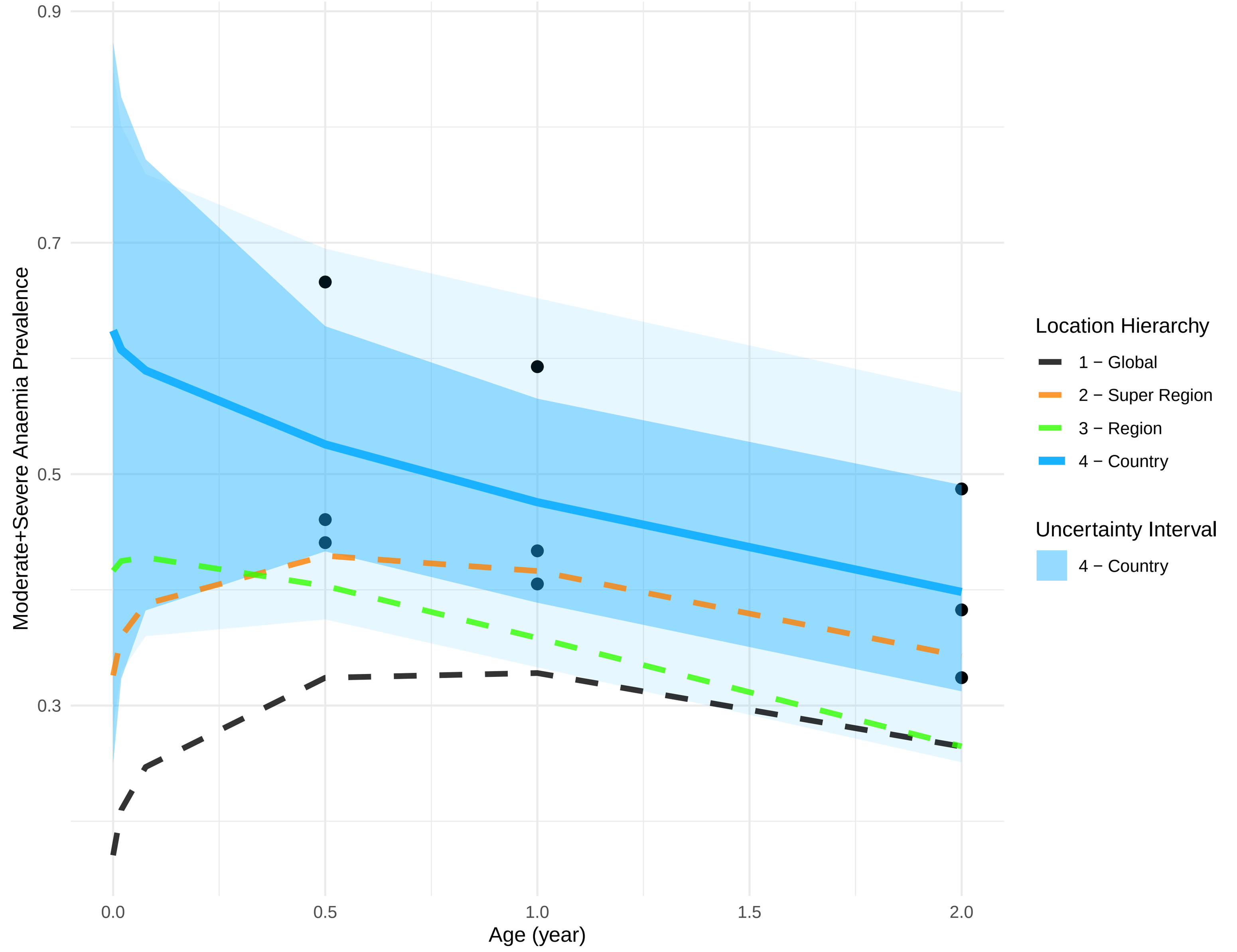

MR−BRT Age/Sex Fits − Males <2 Years Old − Moderate+Severe Anaemia Prevalence − Rwanda
WHO−2001 Adjustment Method

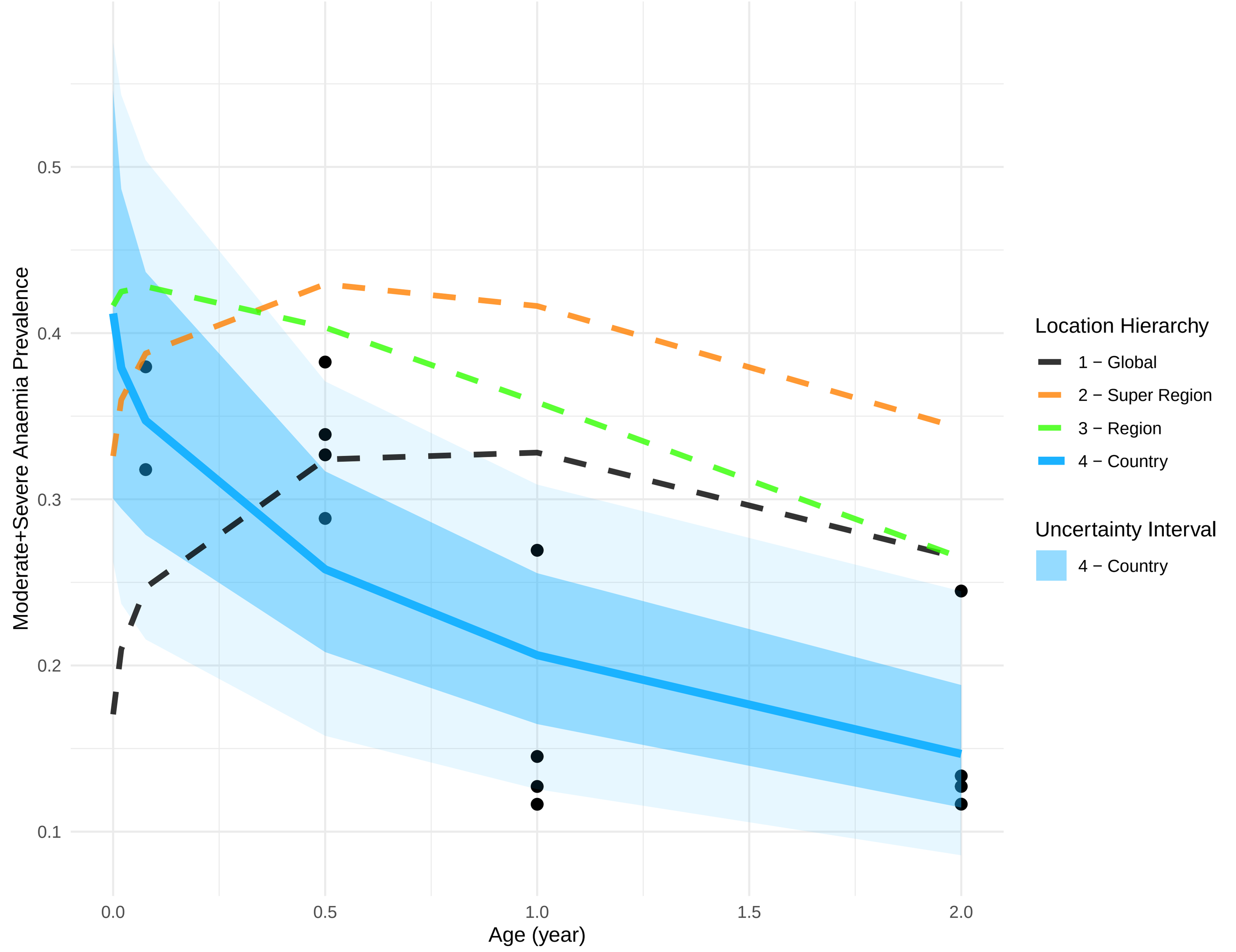

MR−BRT Age/Sex Fits − Males <2 Years Old − Moderate+Severe Anaemia Prevalence − Uganda
WHO−2001 Adjustment Method

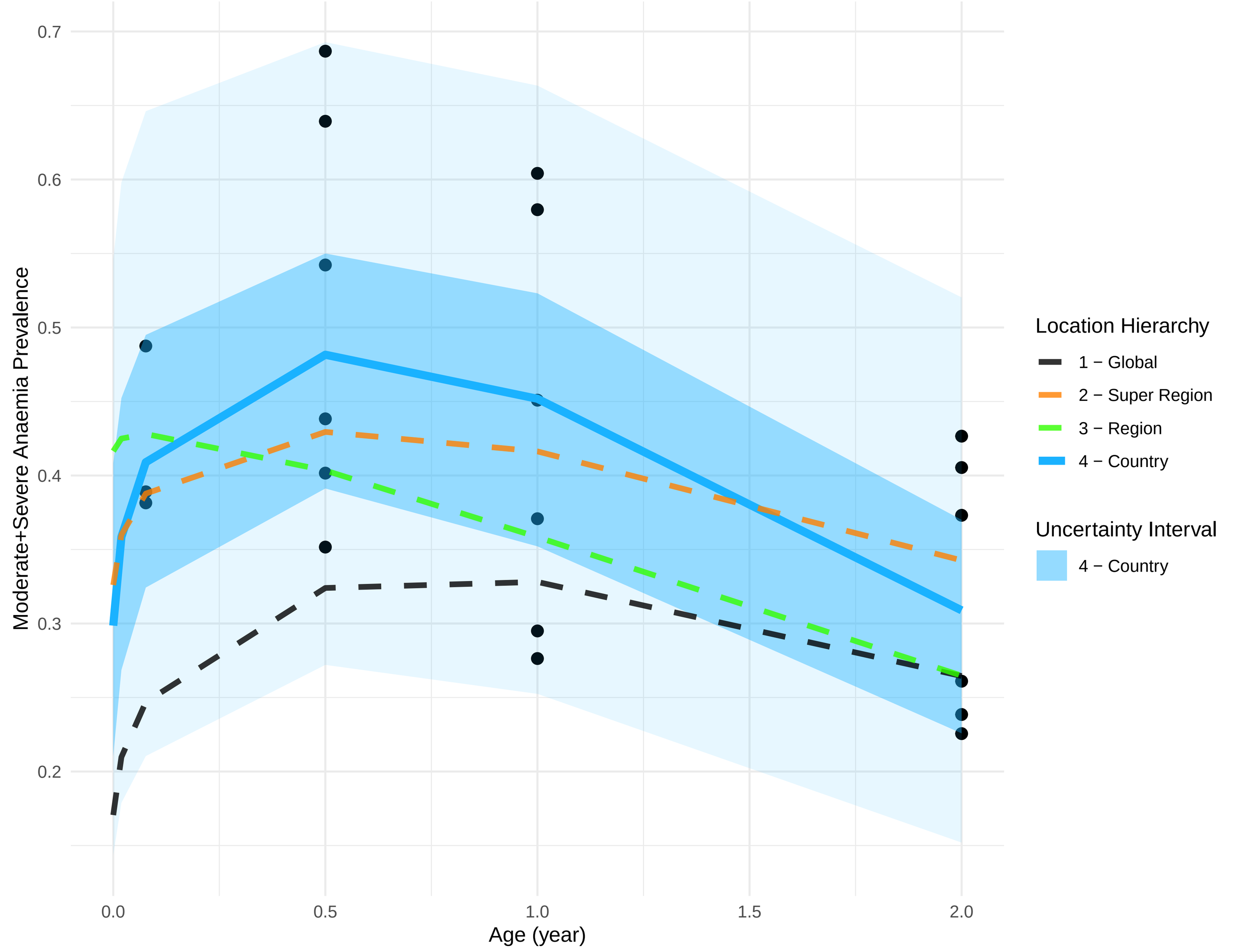

MR−BRT Age/Sex Fits − Males <2 Years Old − Moderate+Severe Anaemia Prevalence − United Republic of Tanzania
WHO−2001 Adjustment Method

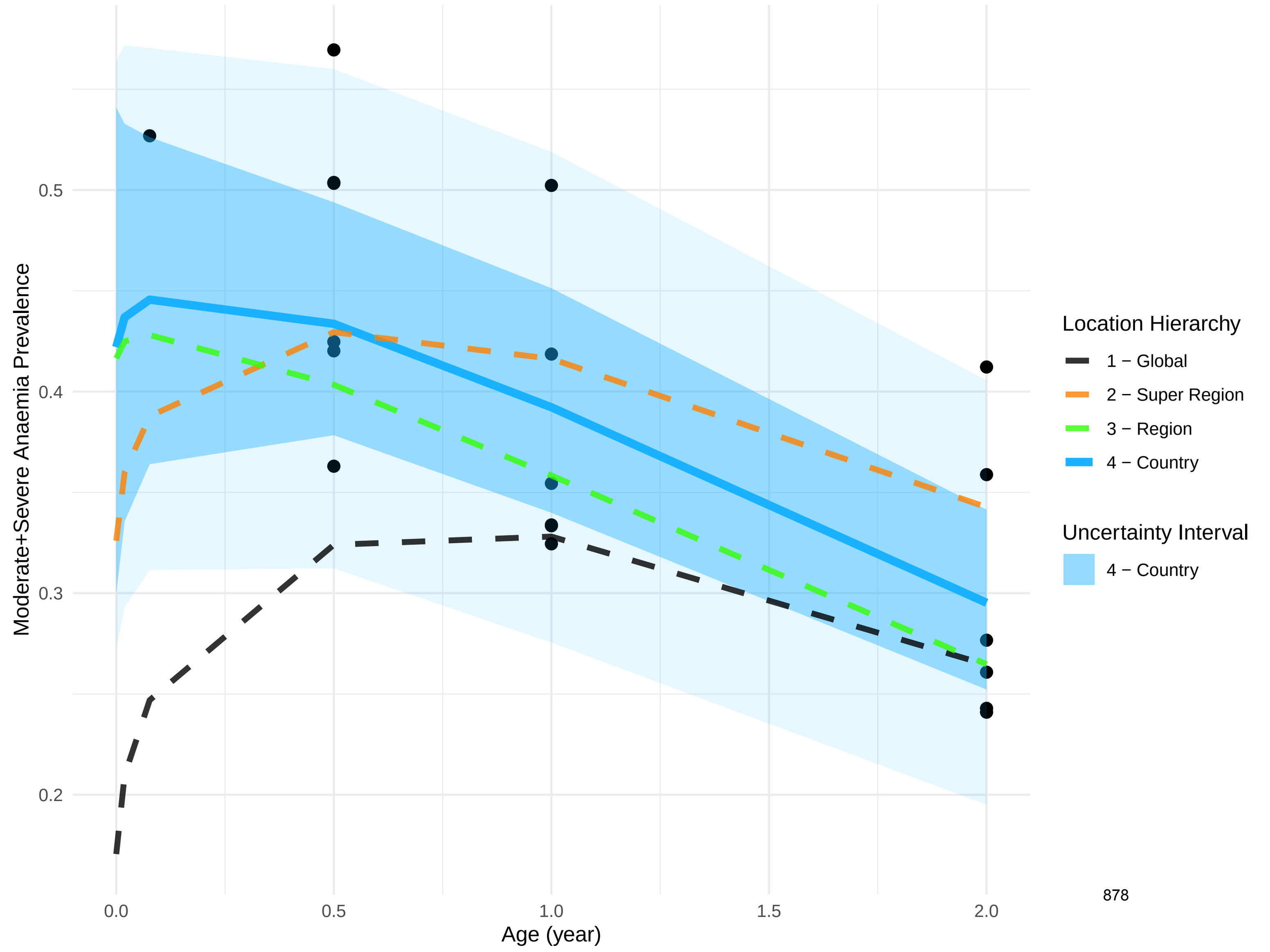

MR−BRT Age/Sex Fits − Males <2 Years Old − Moderate+Severe Anaemia Prevalence − Zambia
WHO−2001 Adjustment Method

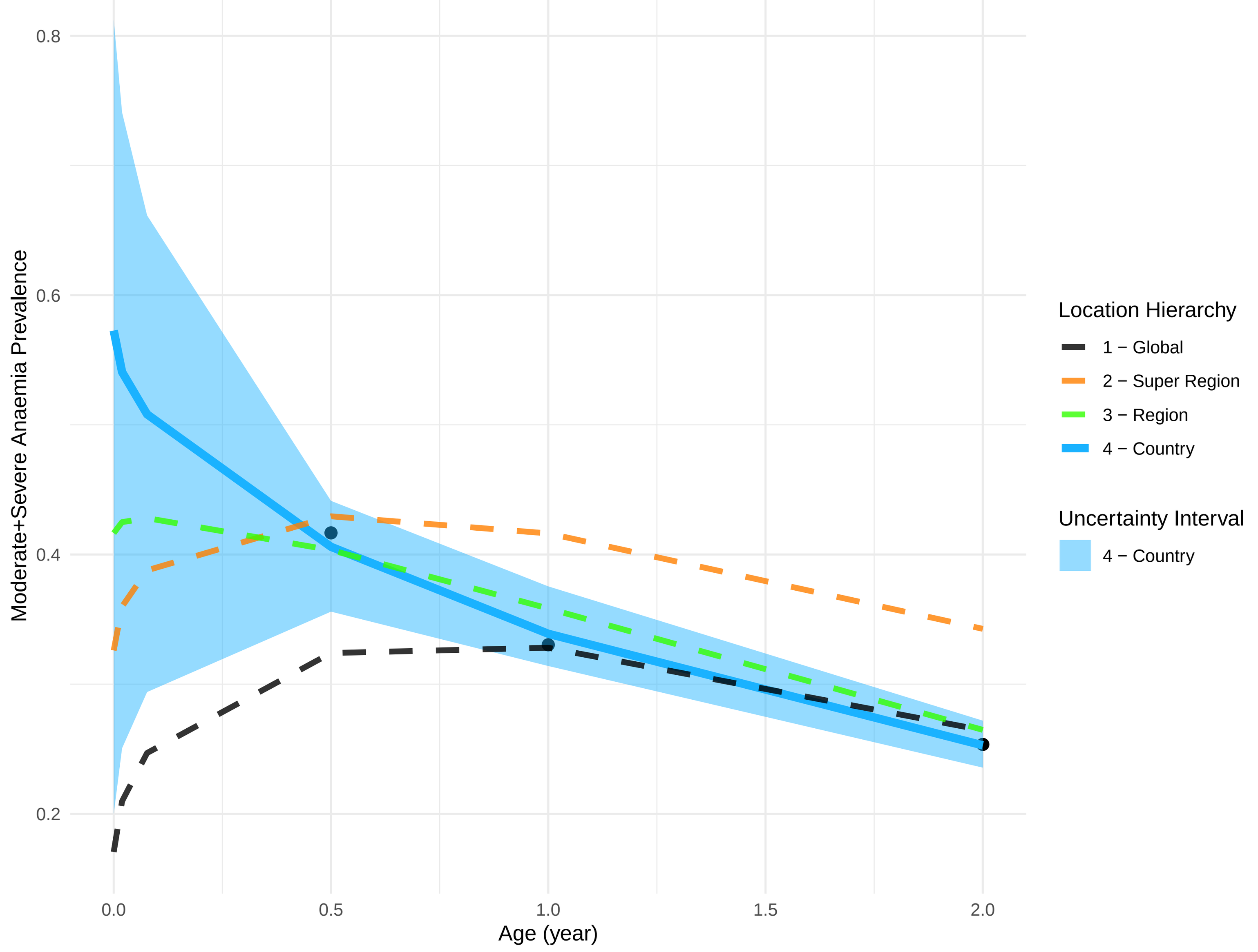

MR−BRT Age/Sex Fits − Males <2 Years Old − Moderate+Severe Anaemia Prevalence − Southern Sub−Saharan Africa
WHO−2001 Adjustment Method

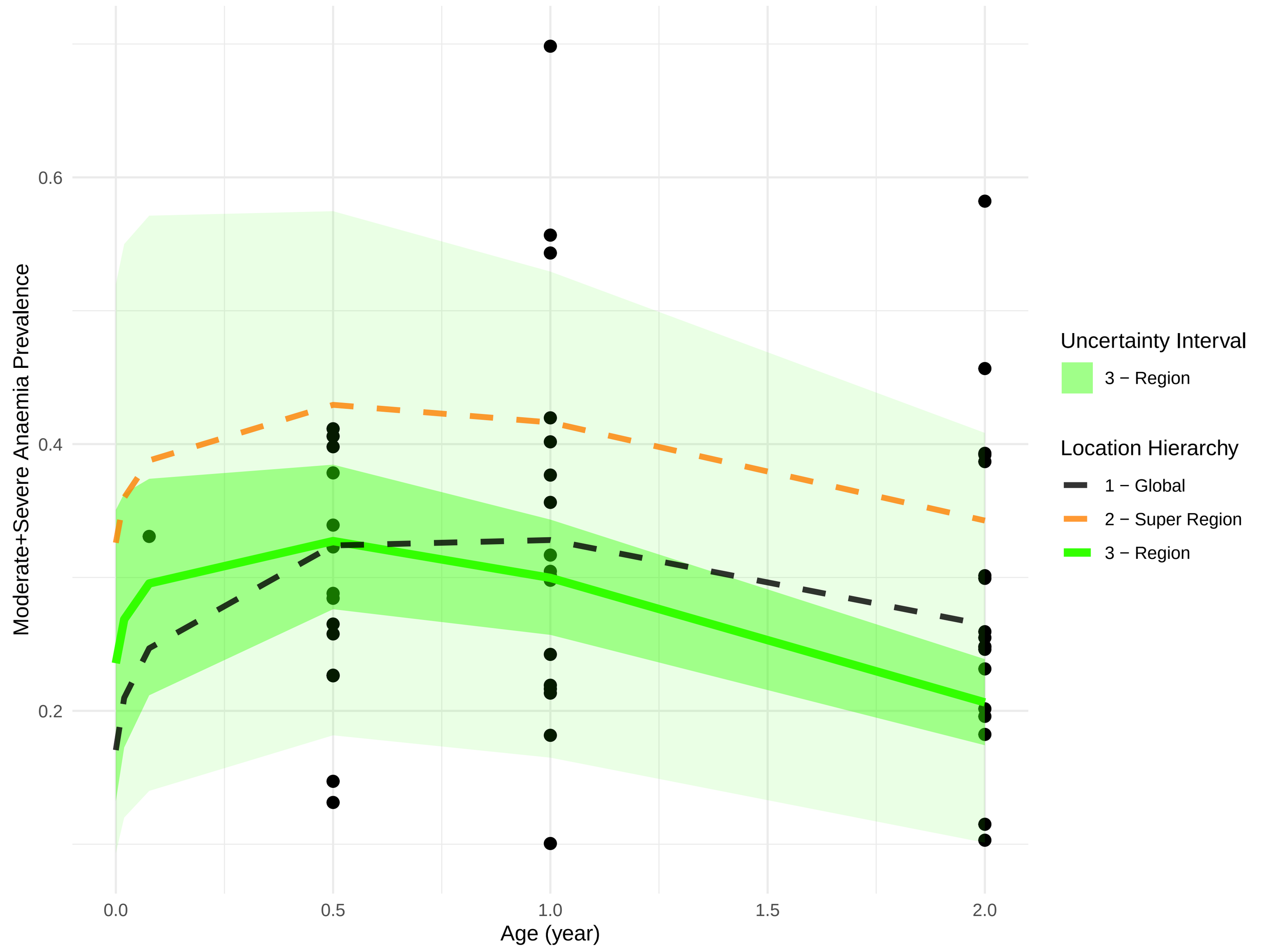

MR−BRT Age/Sex Fits − Males <2 Years Old − Moderate+Severe Anaemia Prevalence − Eswatini
WHO−2001 Adjustment Method

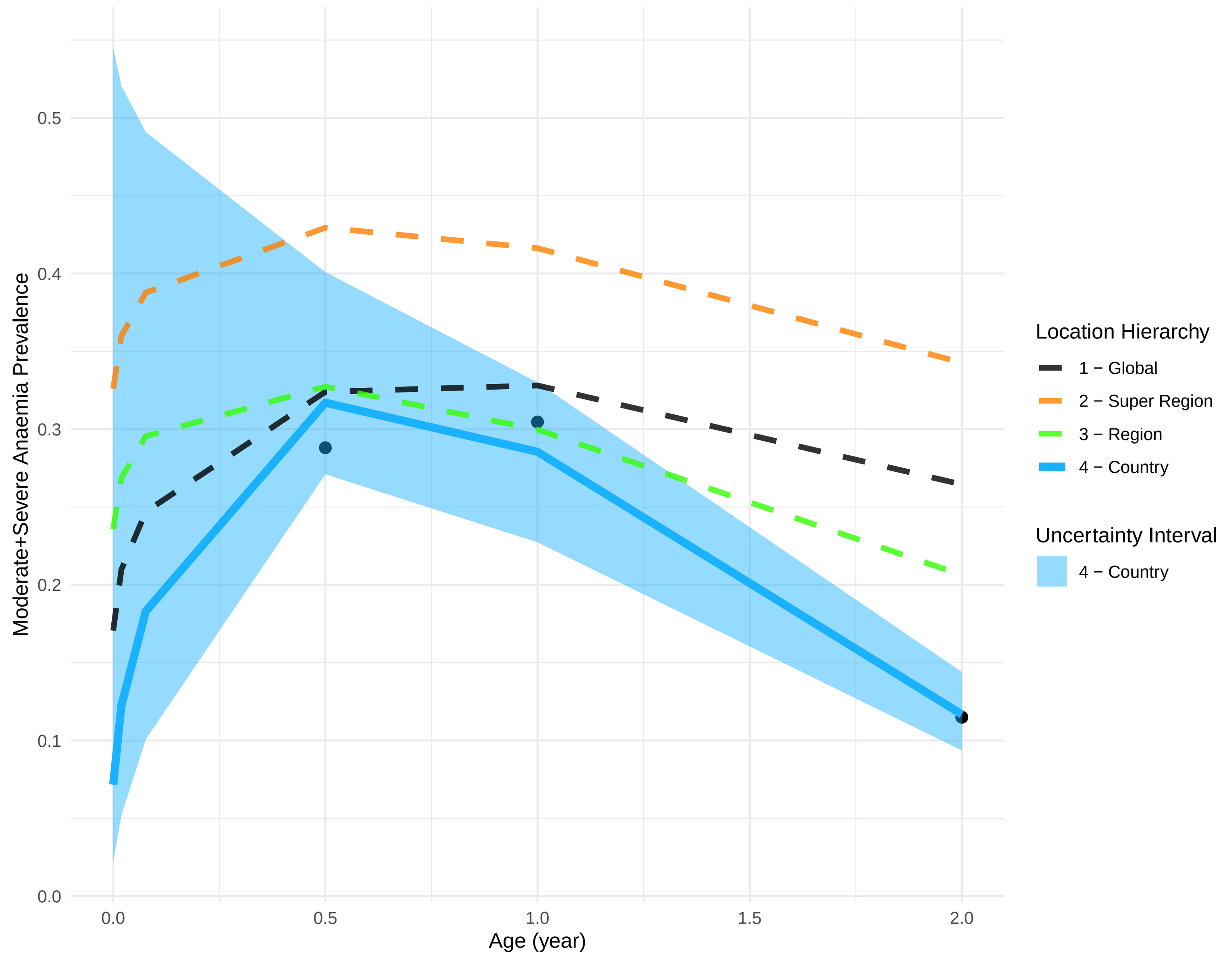

MR−BRT Age/Sex Fits − Males <2 Years Old − Moderate+Severe Anaemia Prevalence − Lesotho
WHO−2001 Adjustment Method

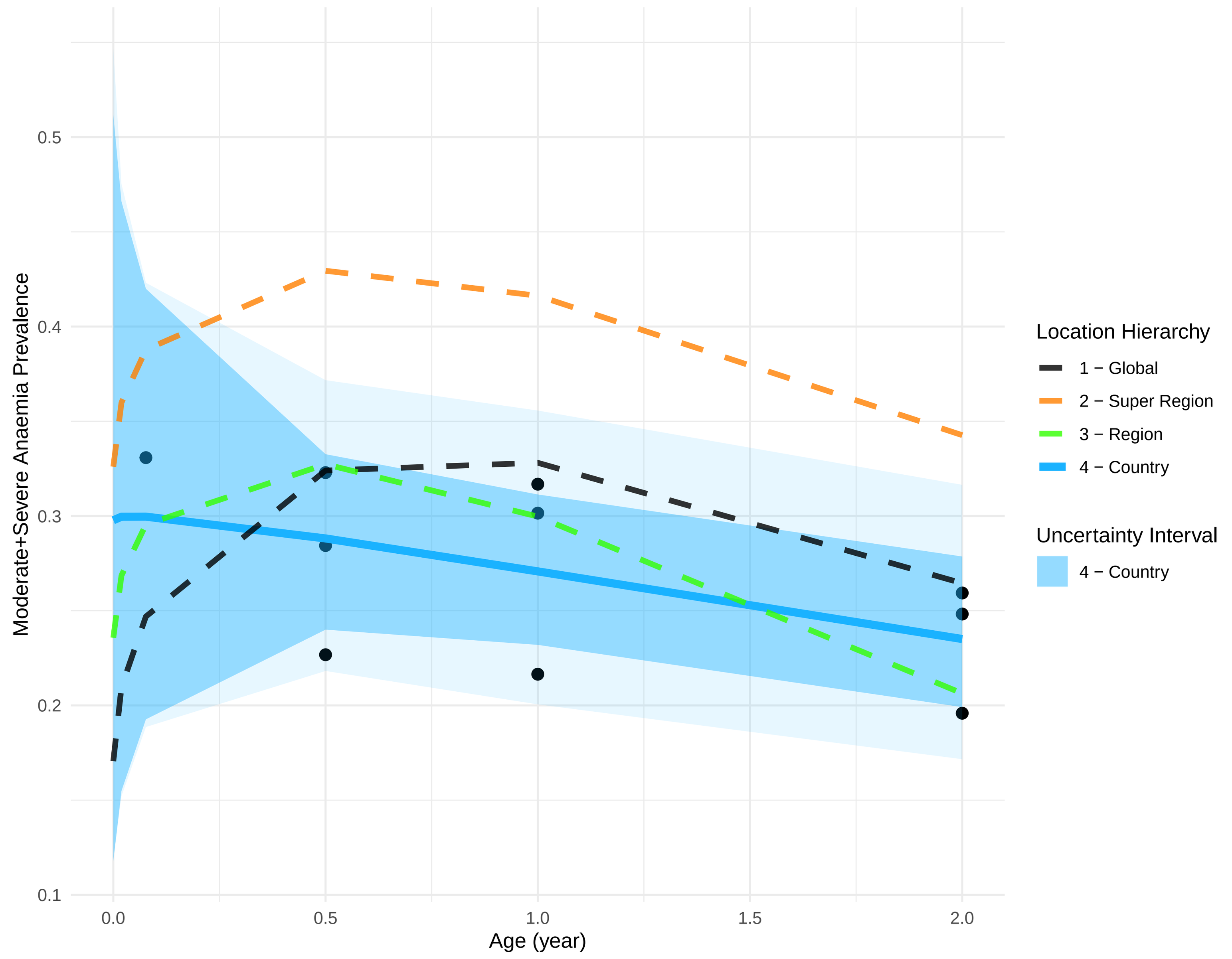

MR−BRT Age/Sex Fits − Males <2 Years Old − Moderate+Severe Anaemia Prevalence − Namibia
WHO−2001 Adjustment Method

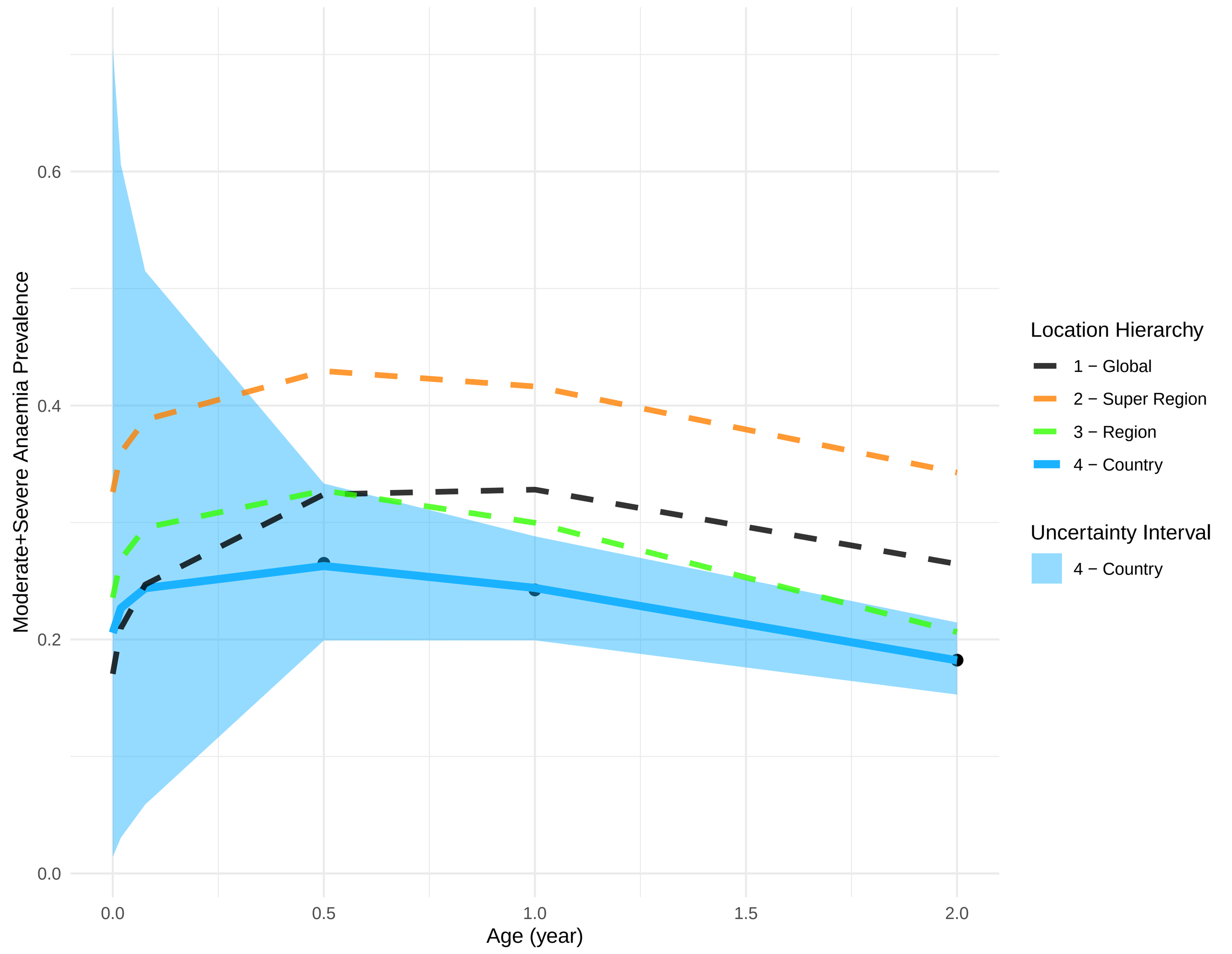

MR−BRT Age/Sex Fits − Males <2 Years Old − Moderate+Severe Anaemia Prevalence − South Africa
WHO−2001 Adjustment Method

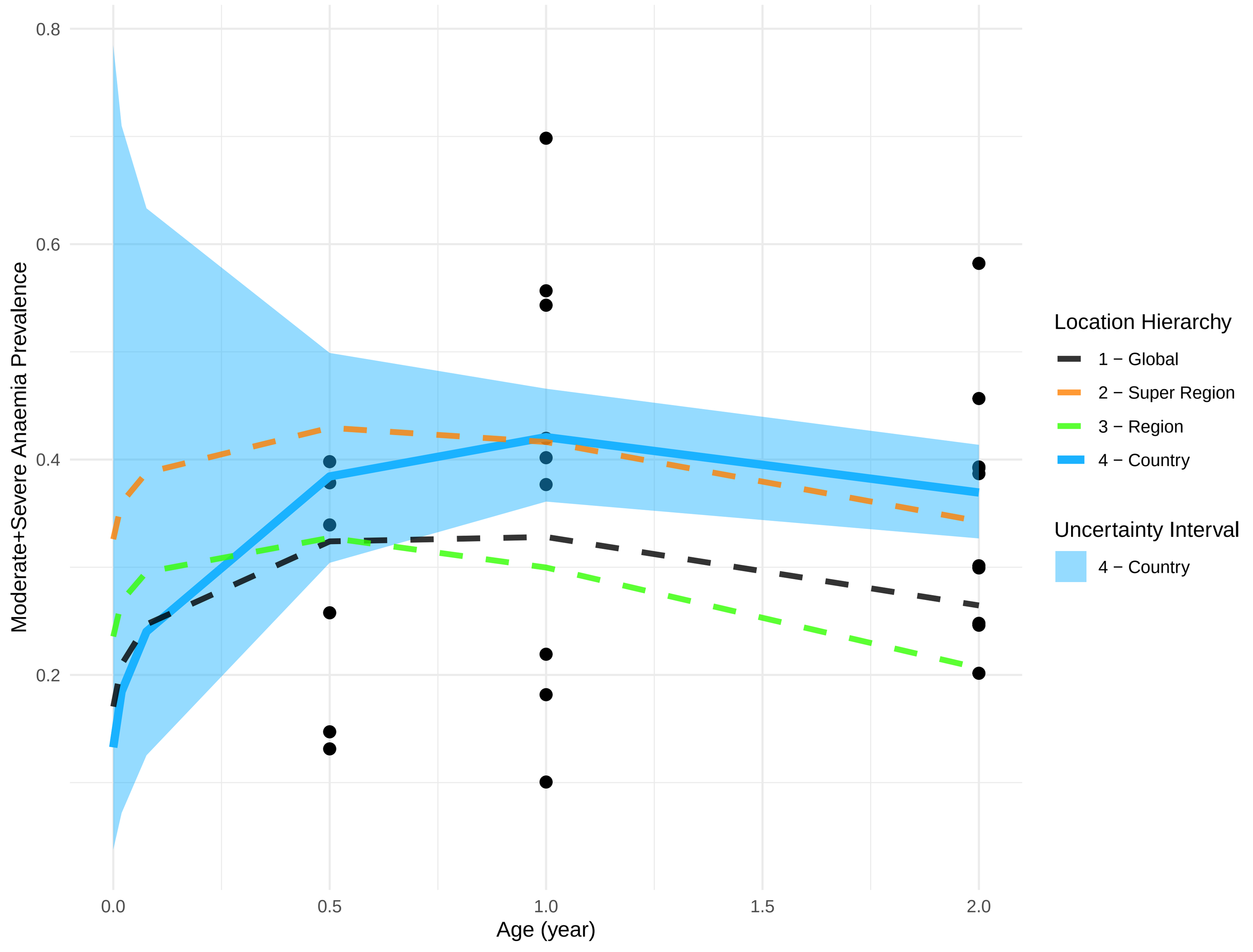

MR−BRT Age/Sex Fits − Males <2 Years Old − Moderate+Severe Anaemia Prevalence − Eastern Cape
WHO−2001 Adjustment Method

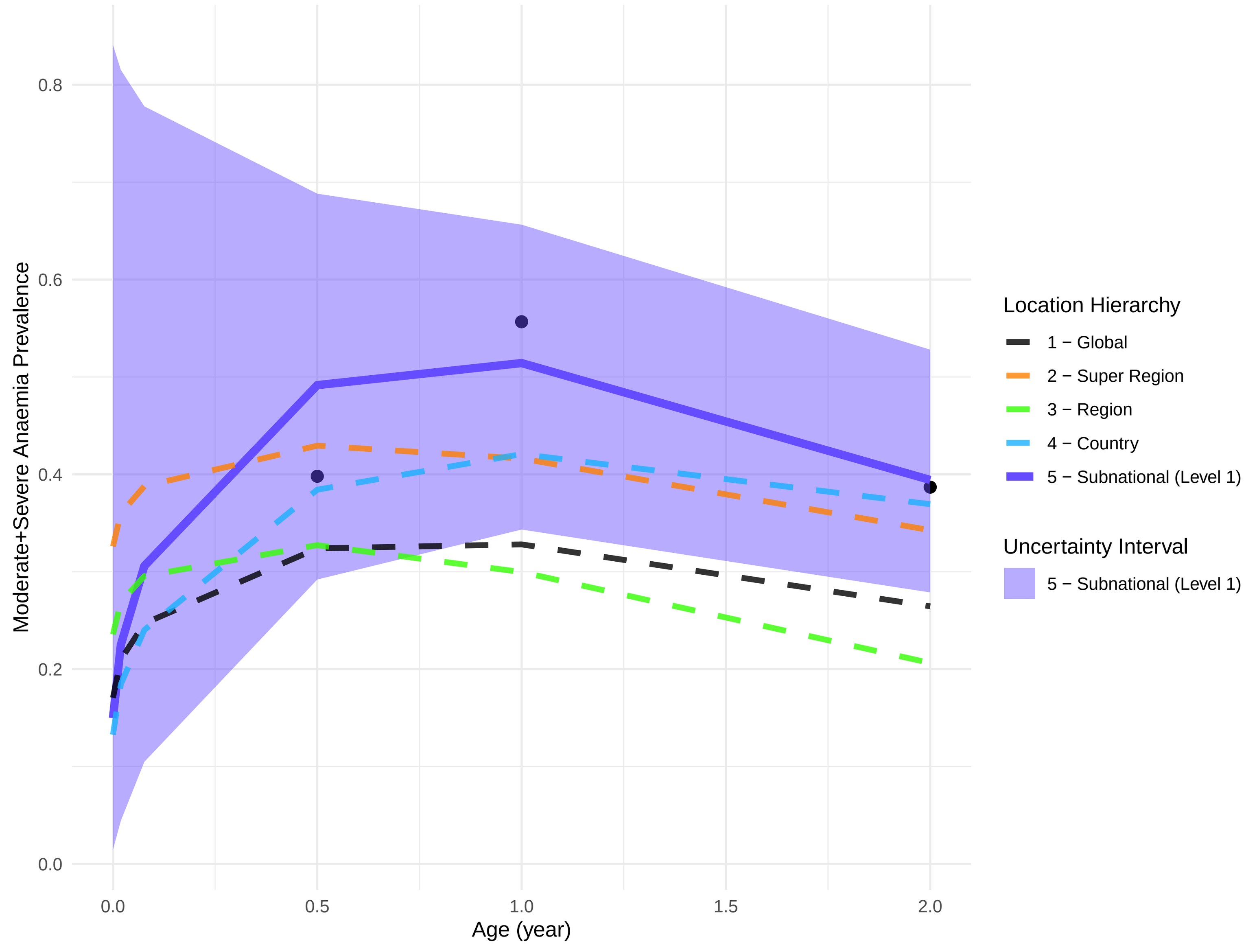

MR−BRT Age/Sex Fits − Males <2 Years Old − Moderate+Severe Anaemia Prevalence − Free State
WHO−2001 Adjustment Method

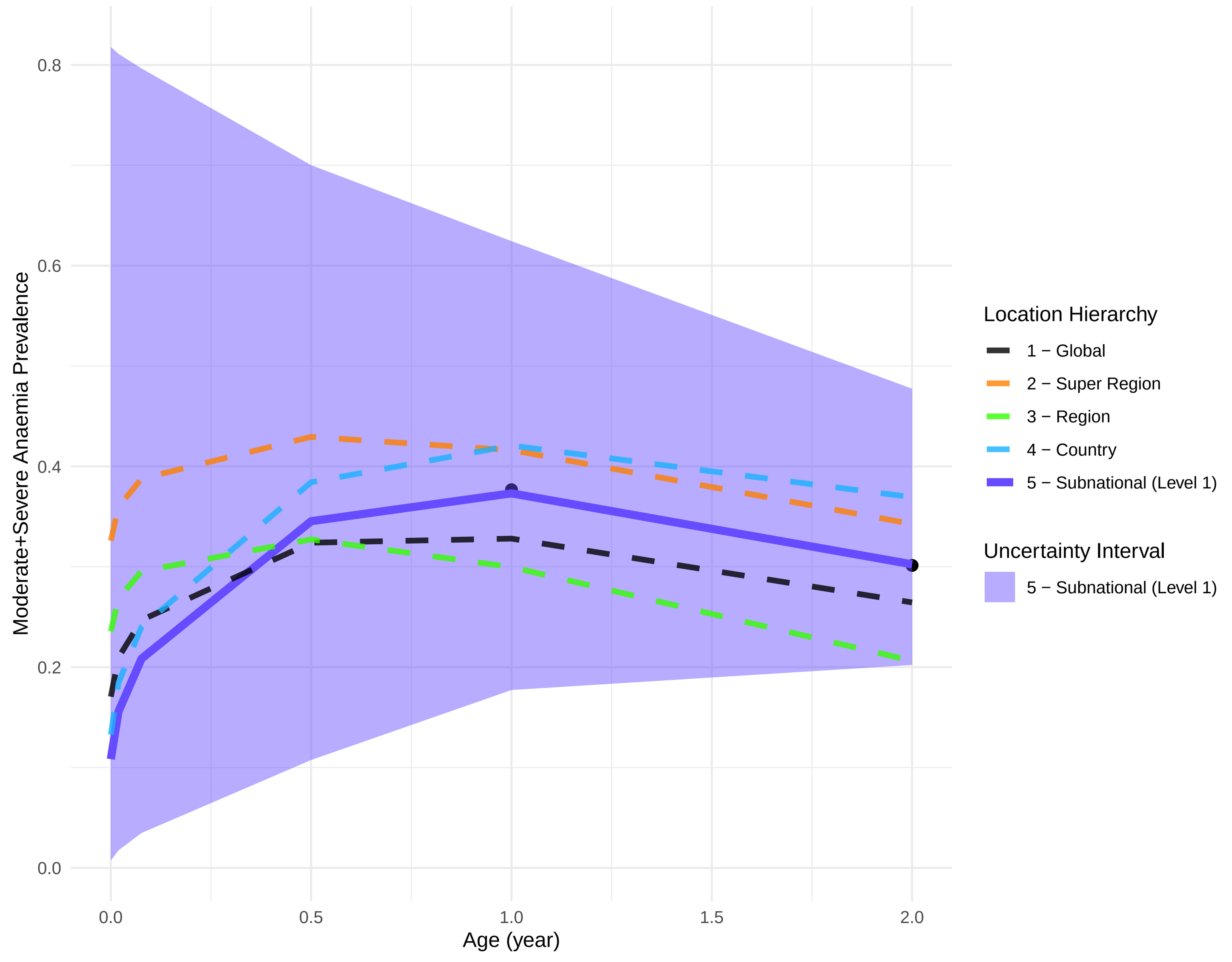

MR−BRT Age/Sex Fits − Males <2 Years Old − Moderate+Severe Anaemia Prevalence − Gauteng
WHO−2001 Adjustment Method

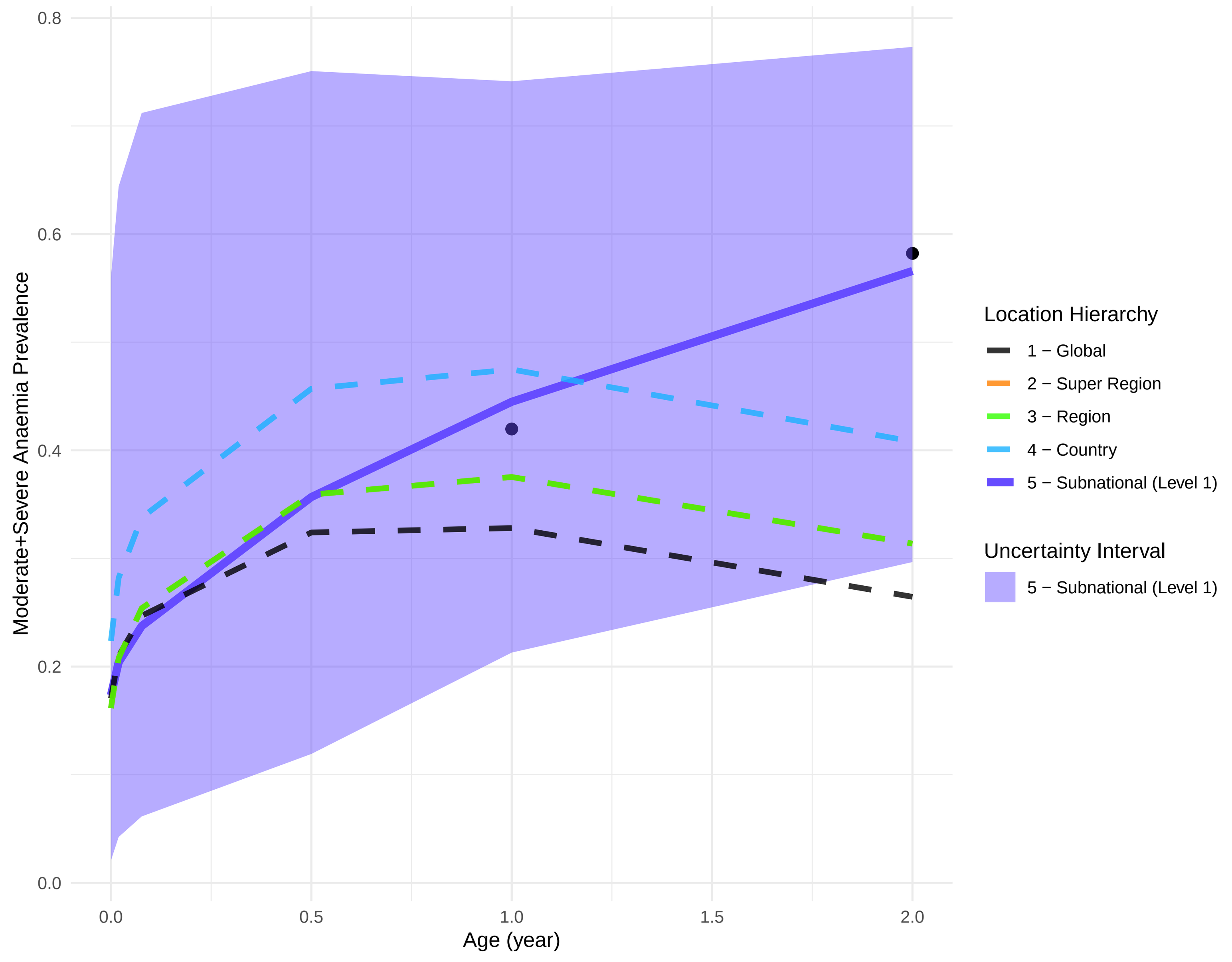

MR−BRT Age/Sex Fits − Males <2 Years Old − Moderate+Severe Anaemia Prevalence − KwaZulu−Natal
WHO−2001 Adjustment Method

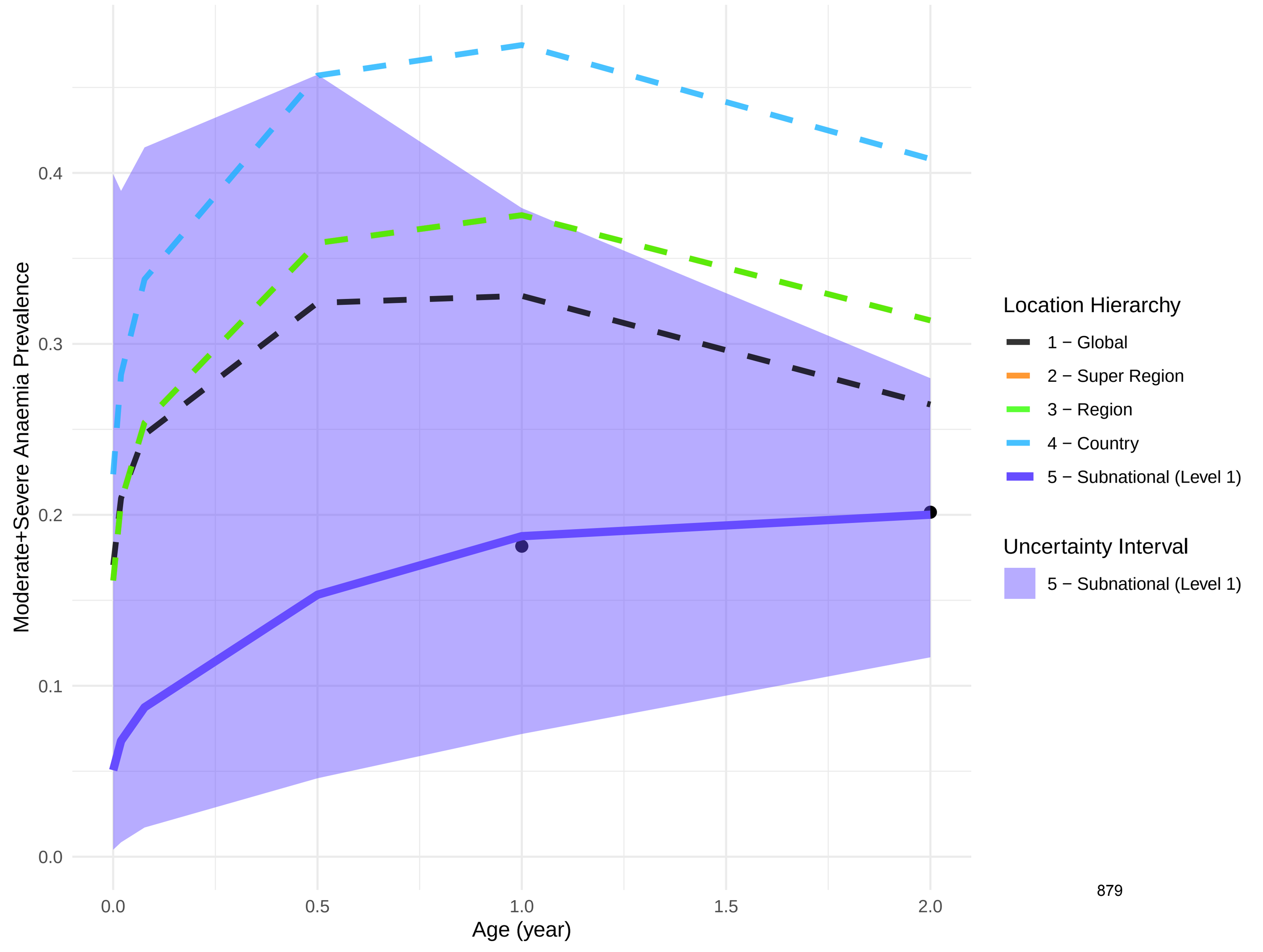

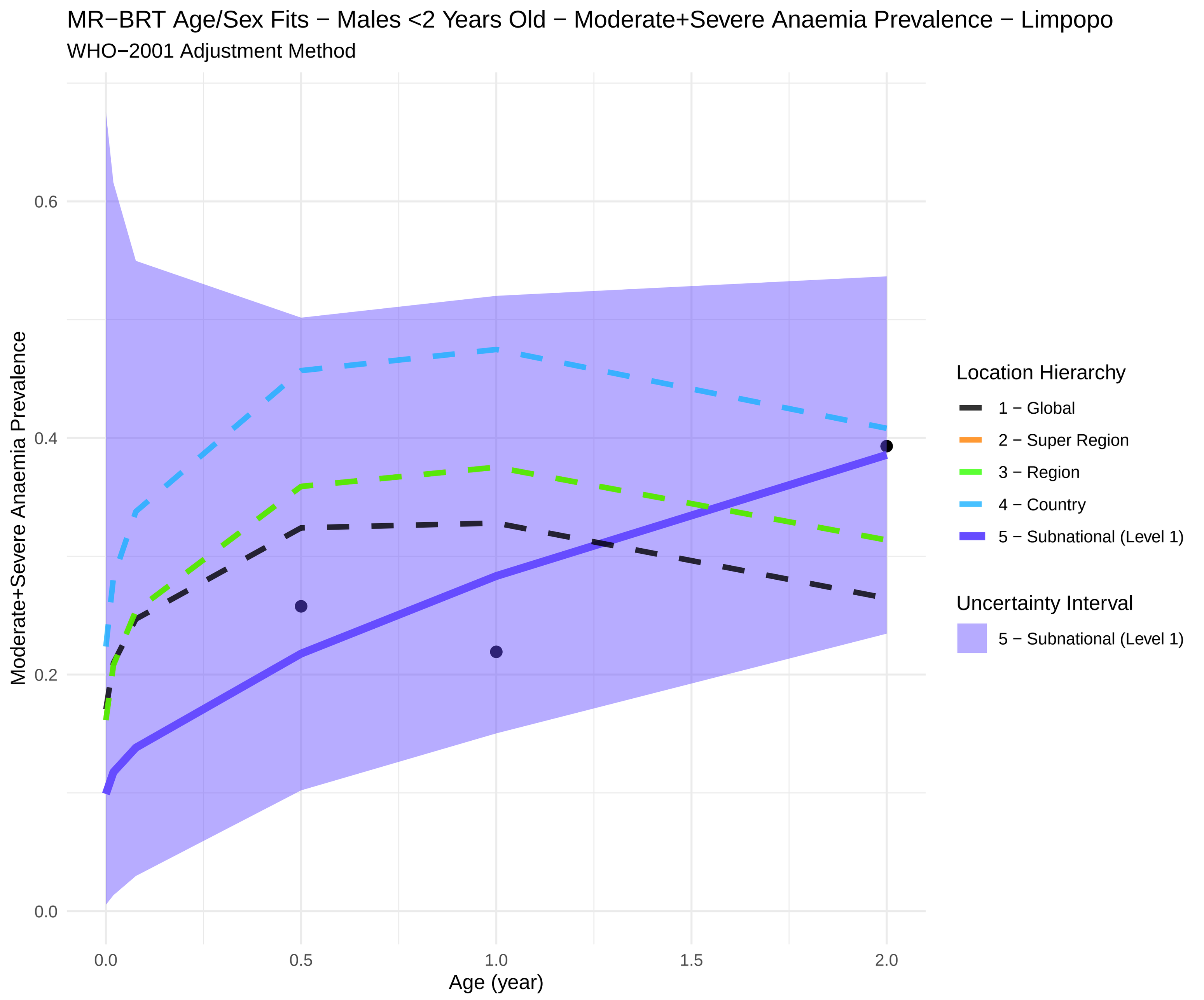
MR−BRT Age/Sex Fits − Males <2 Years Old − Moderate+Severe Anaemia Prevalence − Limpopo
WHO−2001 Adjustment Method
Moderate+Severe Anaemia Prevalence
Age (year)
Location Hierarchy
1 − Global
2 − Super Region
3 − Region
4 − Country
5 − Subnational (Level 1)
Uncertainty Interval
5 − Subnational (Level 1)

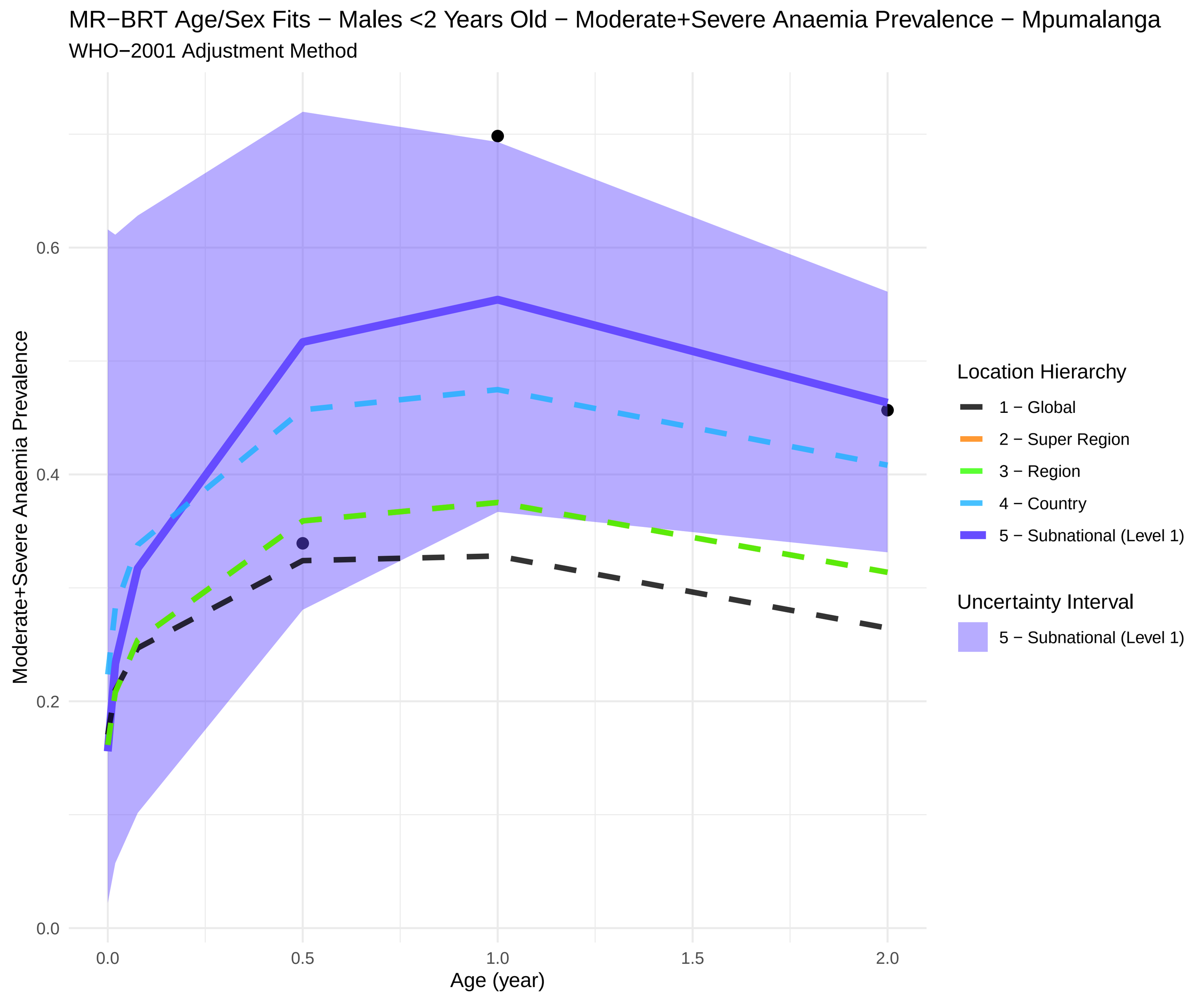
MR−BRT Age/Sex Fits − Males <2 Years Old − Moderate+Severe Anaemia Prevalence − Mpumalanga
WHO−2001 Adjustment Method
Moderate+Severe Anaemia Prevalence
Age (year)
Location Hierarchy
1 − Global
2 − Super Region
3 − Region
4 − Country
5 − Subnational (Level 1)
Uncertainty Interval
5 − Subnational (Level 1)

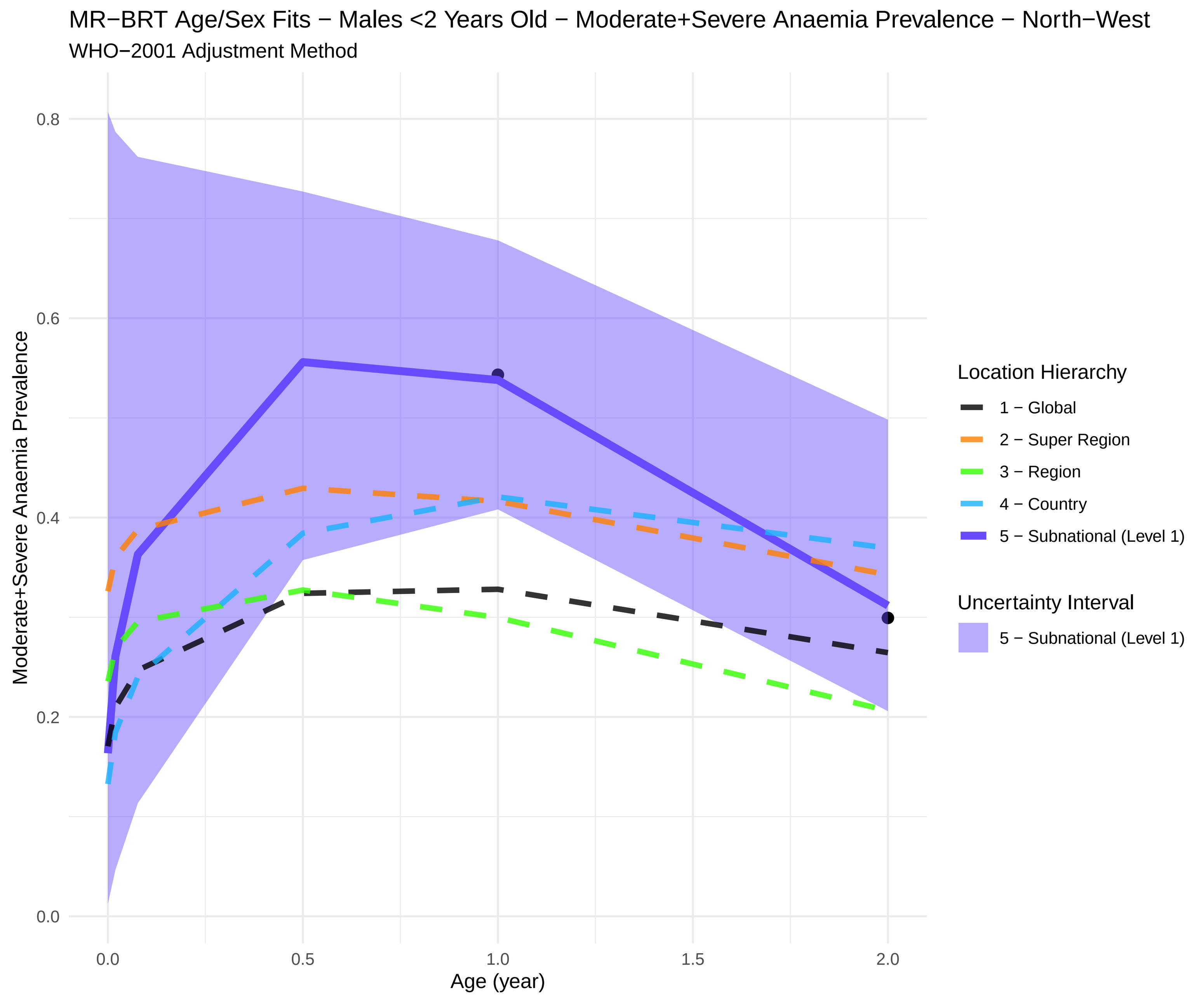
MR−BRT Age/Sex Fits − Males <2 Years Old − Moderate+Severe Anaemia Prevalence − North−West
WHO−2001 Adjustment Method
Moderate+Severe Anaemia Prevalence
Age (year)
Location Hierarchy
1 − Global
2 − Super Region
3 − Region
4 − Country
5 − Subnational (Level 1)
Uncertainty Interval
5 − Subnational (Level 1)

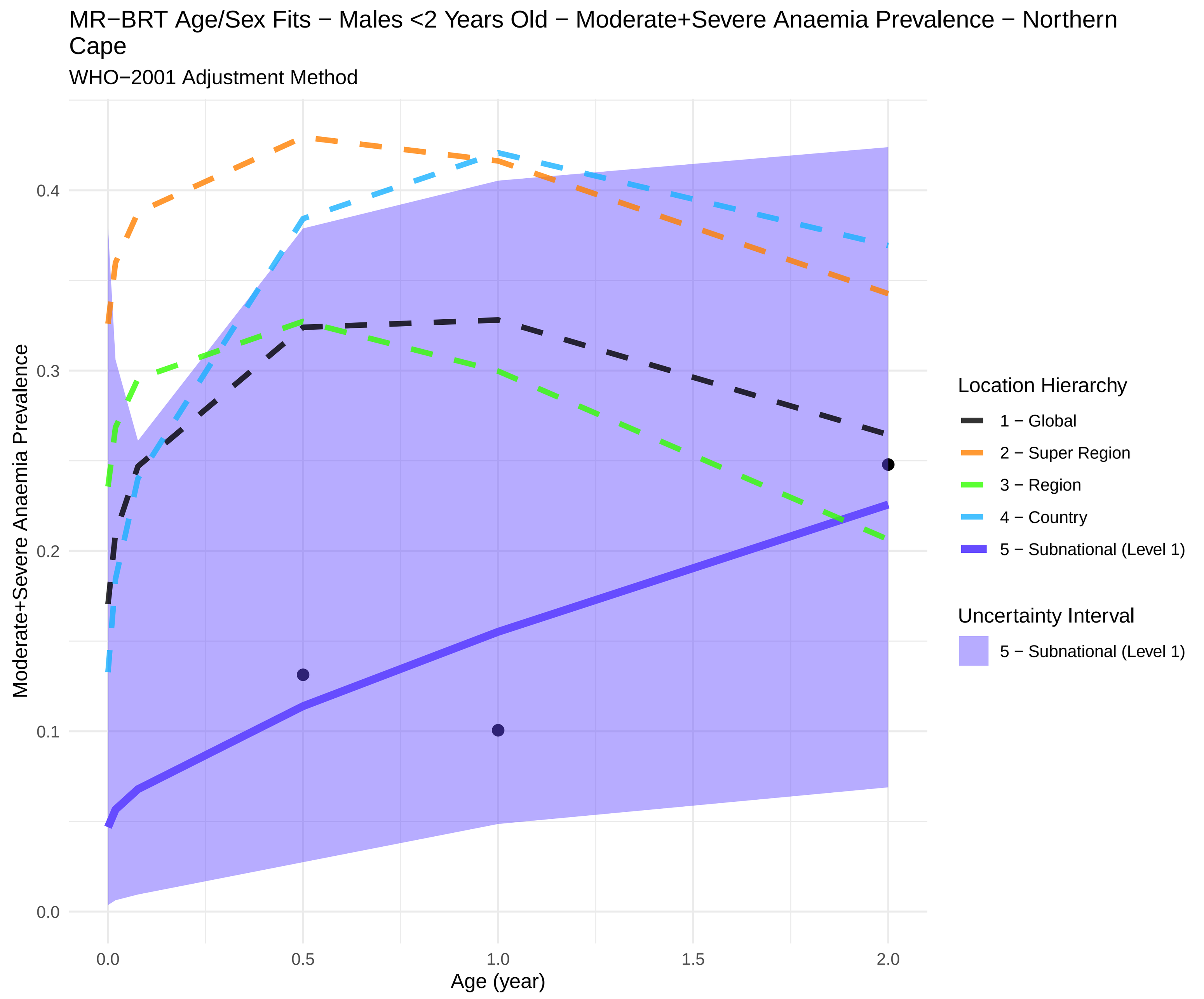
MR−BRT Age/Sex Fits − Males <2 Years Old − Moderate+Severe Anaemia Prevalence − Northern Cape
WHO−2001 Adjustment Method
Moderate+Severe Anaemia Prevalence
Age (year)
Location Hierarchy
1 − Global
2 − Super Region
3 − Region
4 − Country
5 − Subnational (Level 1)
Uncertainty Interval
5 − Subnational (Level 1)

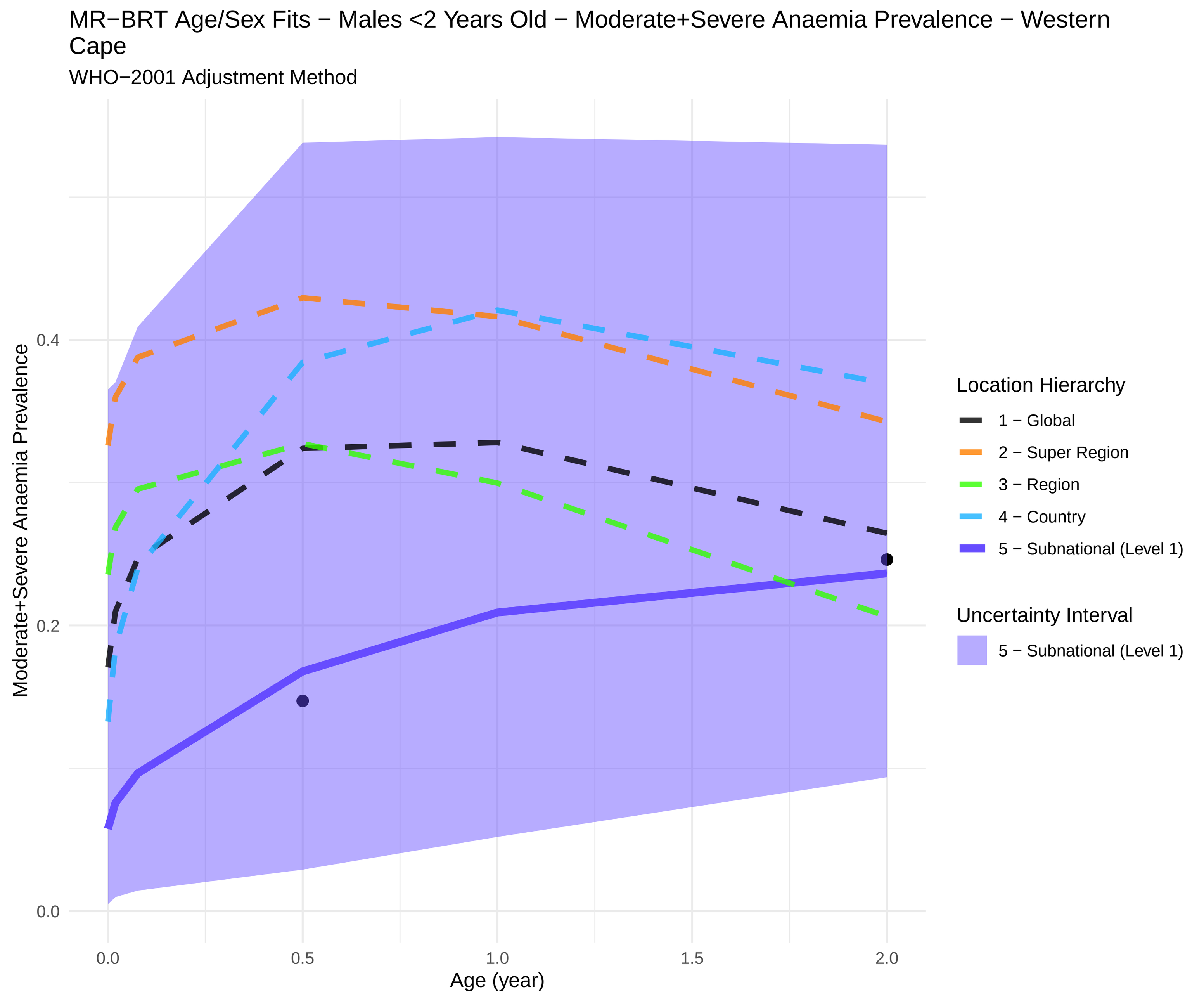
MR−BRT Age/Sex Fits − Males <2 Years Old − Moderate+Severe Anaemia Prevalence − Western Cape
WHO−2001 Adjustment Method
Moderate+Severe Anaemia Prevalence
Age (year)
Location Hierarchy
1 − Global
2 − Super Region
3 − Region
4 − Country
5 − Subnational (Level 1)
Uncertainty Interval
5 − Subnational (Level 1)

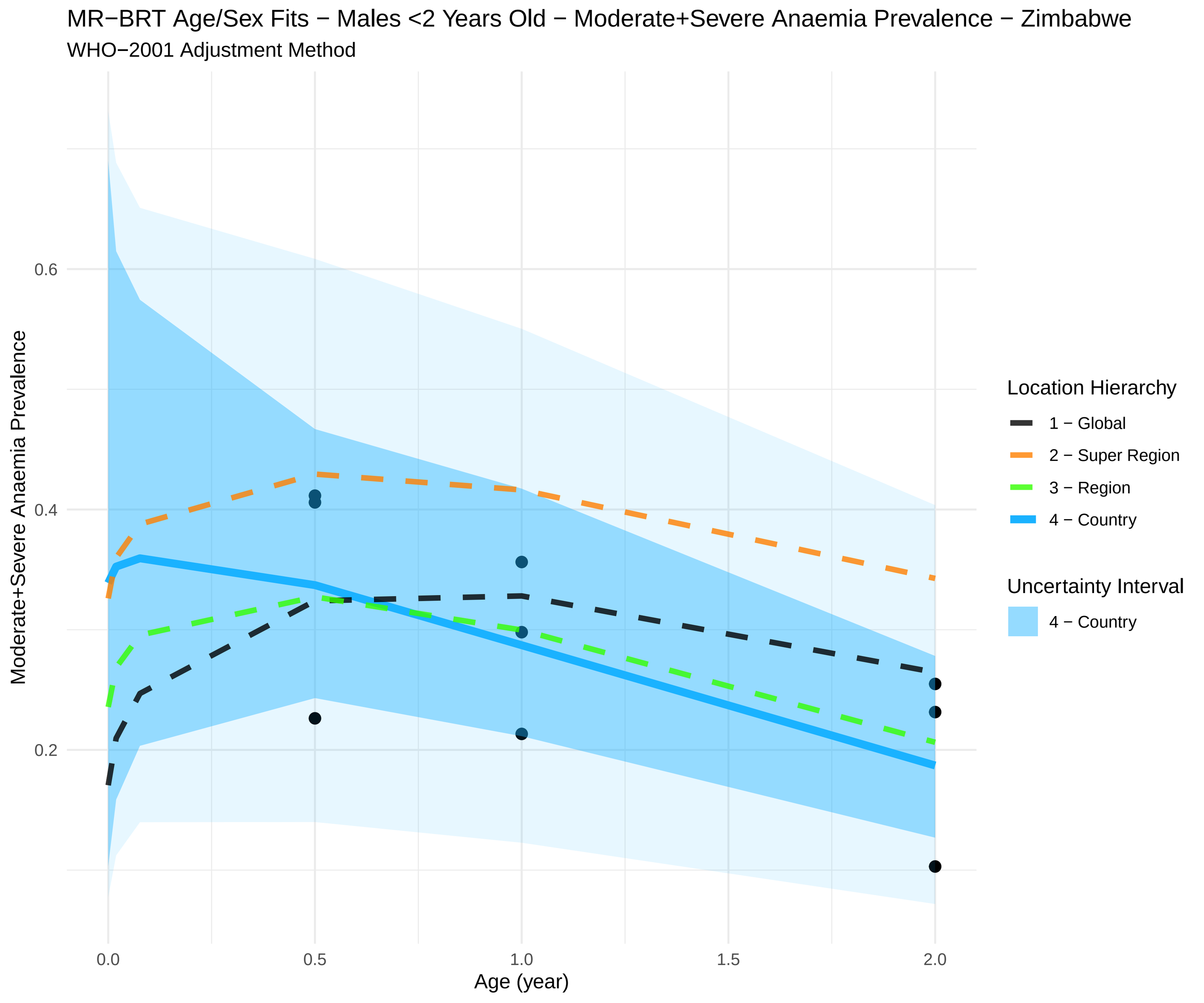
MR−BRT Age/Sex Fits − Males <2 Years Old − Moderate+Severe Anaemia Prevalence − Zimbabwe
WHO−2001 Adjustment Method
Moderate+Severe Anaemia Prevalence
Age (year)
Location Hierarchy
1 − Global
2 − Super Region
3 − Region
4 − Country
Uncertainty Interval
4 − Country

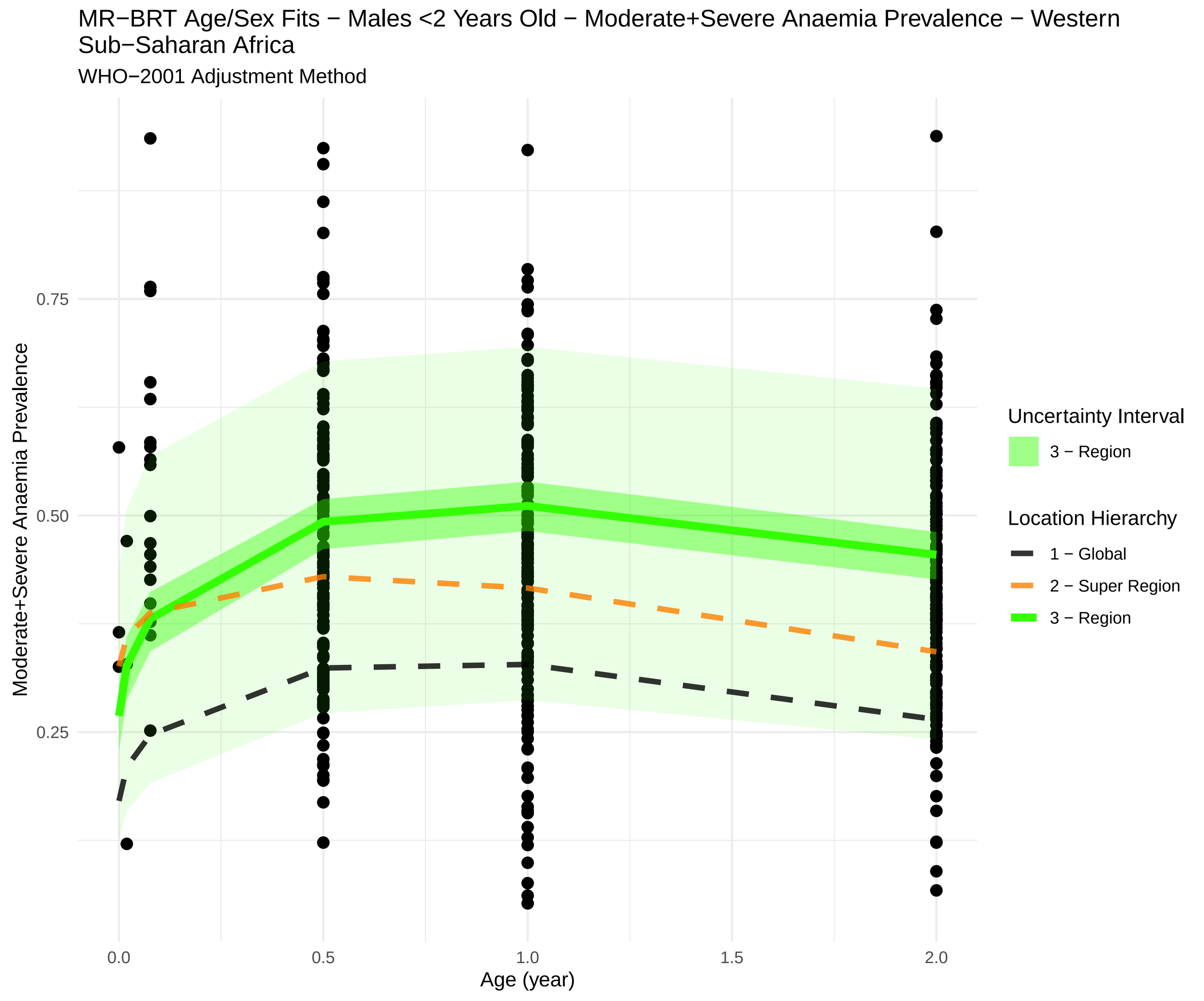
MR−BRT Age/Sex Fits − Males <2 Years Old − Moderate+Severe Anaemia Prevalence − Western Sub−Saharan Africa
WHO−2001 Adjustment Method
Moderate+Severe Anaemia Prevalence
Age (year)
Uncertainty Interval
3 − Region
Location Hierarchy
1 − Global
2 − Super Region
3 − Region

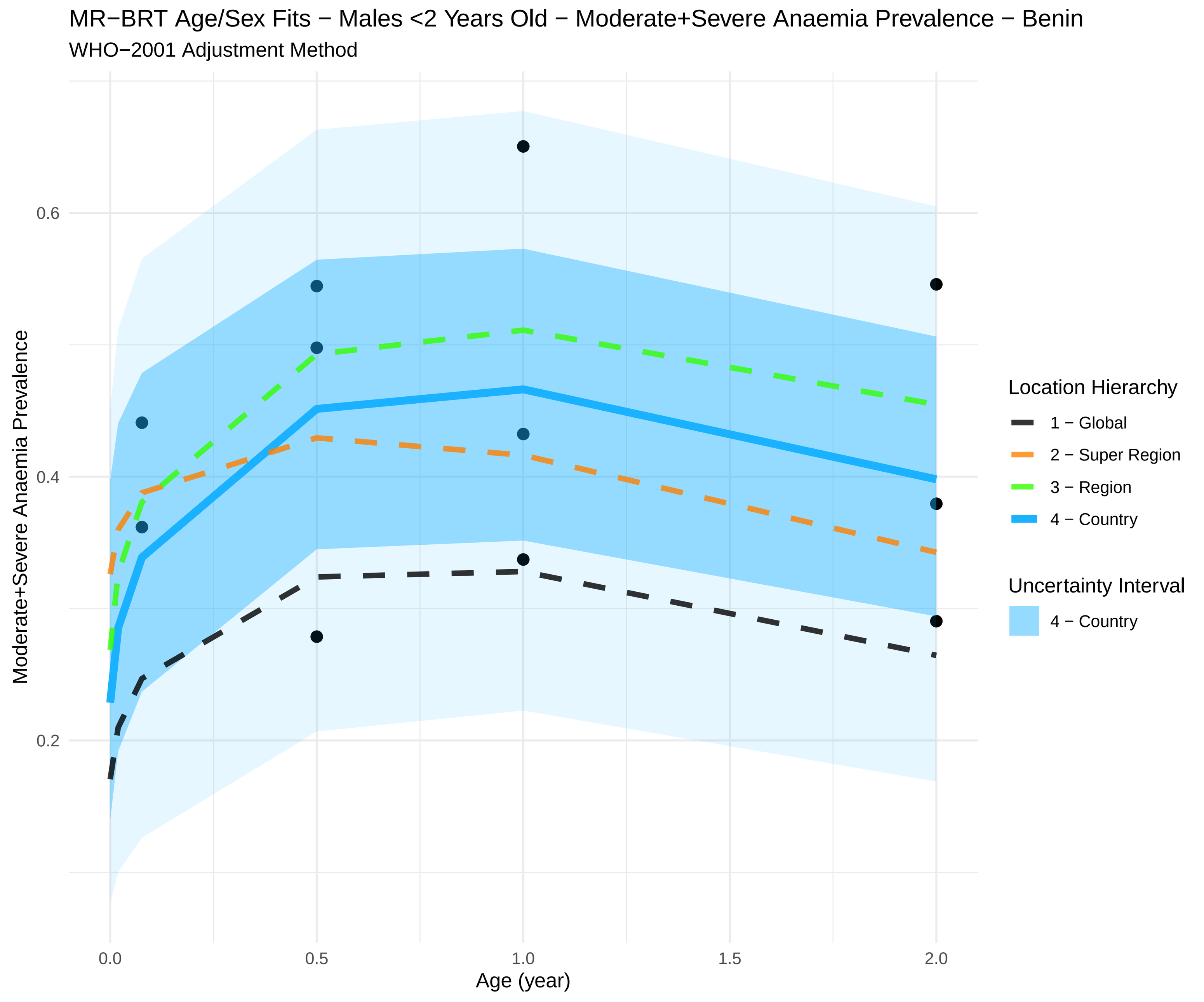
MR−BRT Age/Sex Fits − Males <2 Years Old − Moderate+Severe Anaemia Prevalence − Benin
WHO−2001 Adjustment Method
Moderate+Severe Anaemia Prevalence
Age (year)
Location Hierarchy
1 − Global
2 − Super Region
3 − Region
4 − Country
Uncertainty Interval
4 − Country

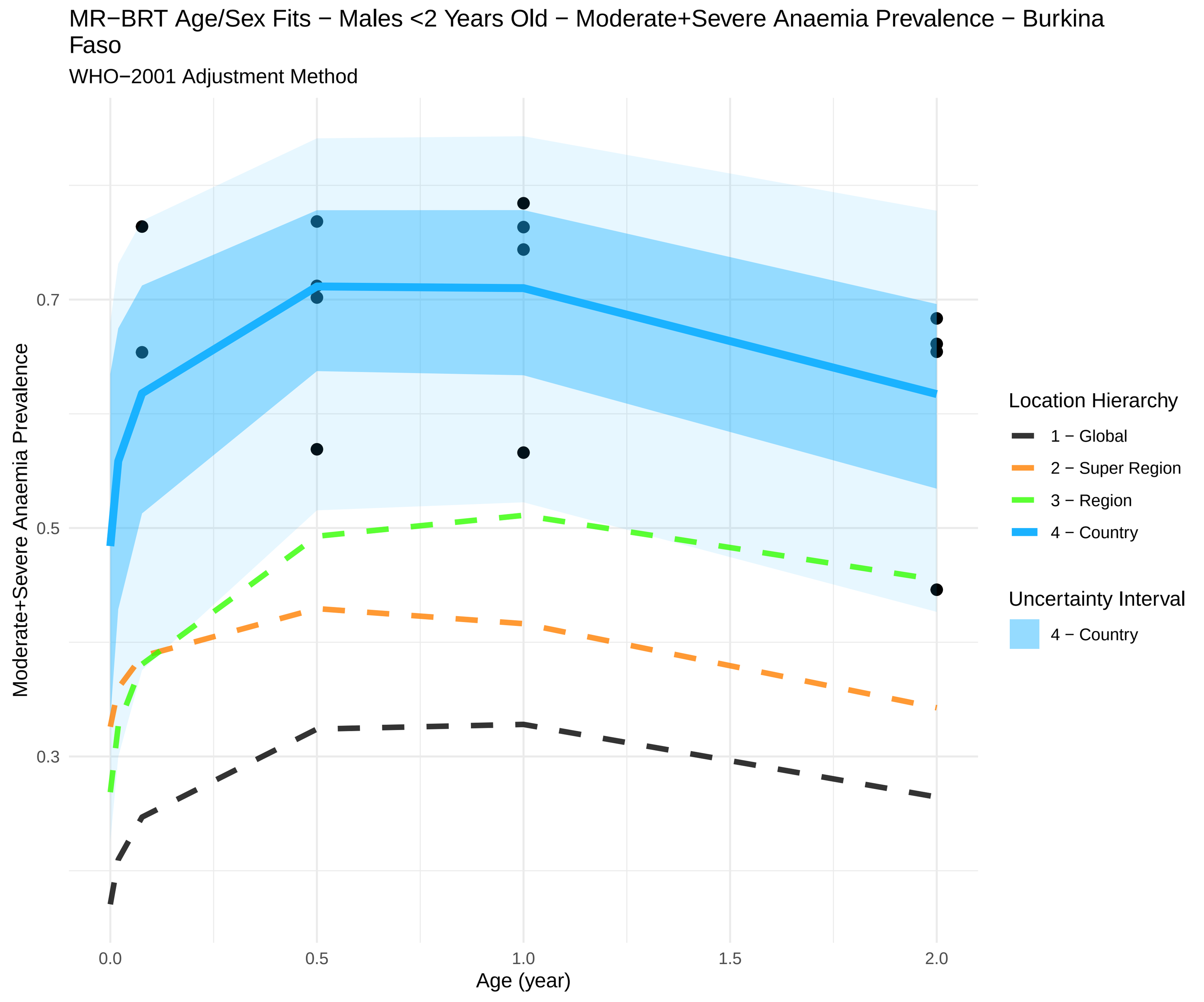
MR−BRT Age/Sex Fits − Males <2 Years Old − Moderate+Severe Anaemia Prevalence − Burkina Faso
WHO−2001 Adjustment Method
Moderate+Severe Anaemia Prevalence
Age (year)
Location Hierarchy
1 − Global
2 − Super Region
3 − Region
4 − Country
Uncertainty Interval
4 − Country

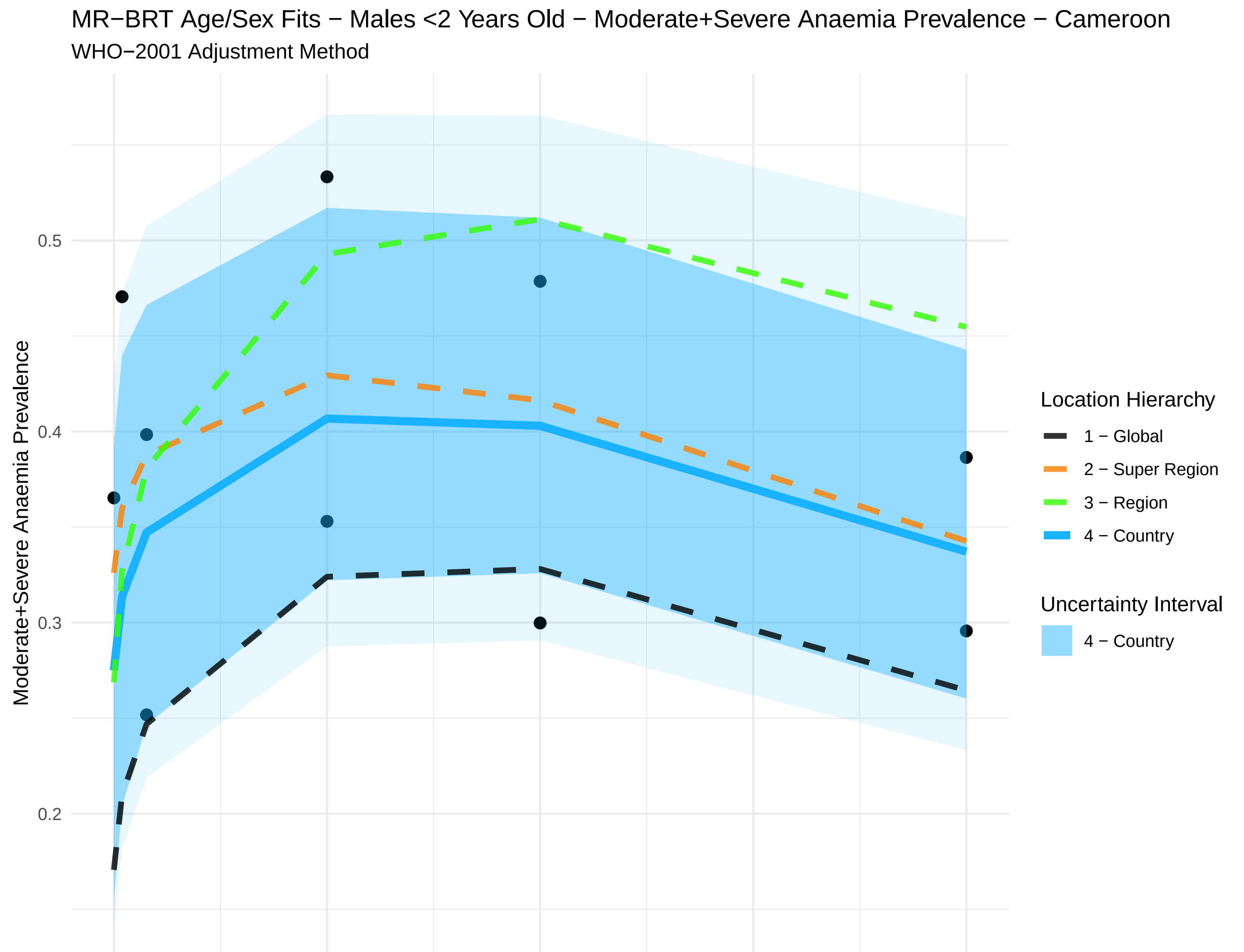
MR−BRT Age/Sex Fits − Males <2 Years Old − Moderate+Severe Anaemia Prevalence − Cameroon
WHO−2001 Adjustment Method
Moderate+Severe Anaemia Prevalence
Age (year)
Location Hierarchy
1 − Global
2 − Super Region
3 − Region
4 − Country
Uncertainty Interval
4 − Country

MR−BRT Age/Sex Fits − Males <2 Years Old − Moderate+Severe Anaemia Prevalence − Côte d'Ivoire
WHO−2001 Adjustment Method

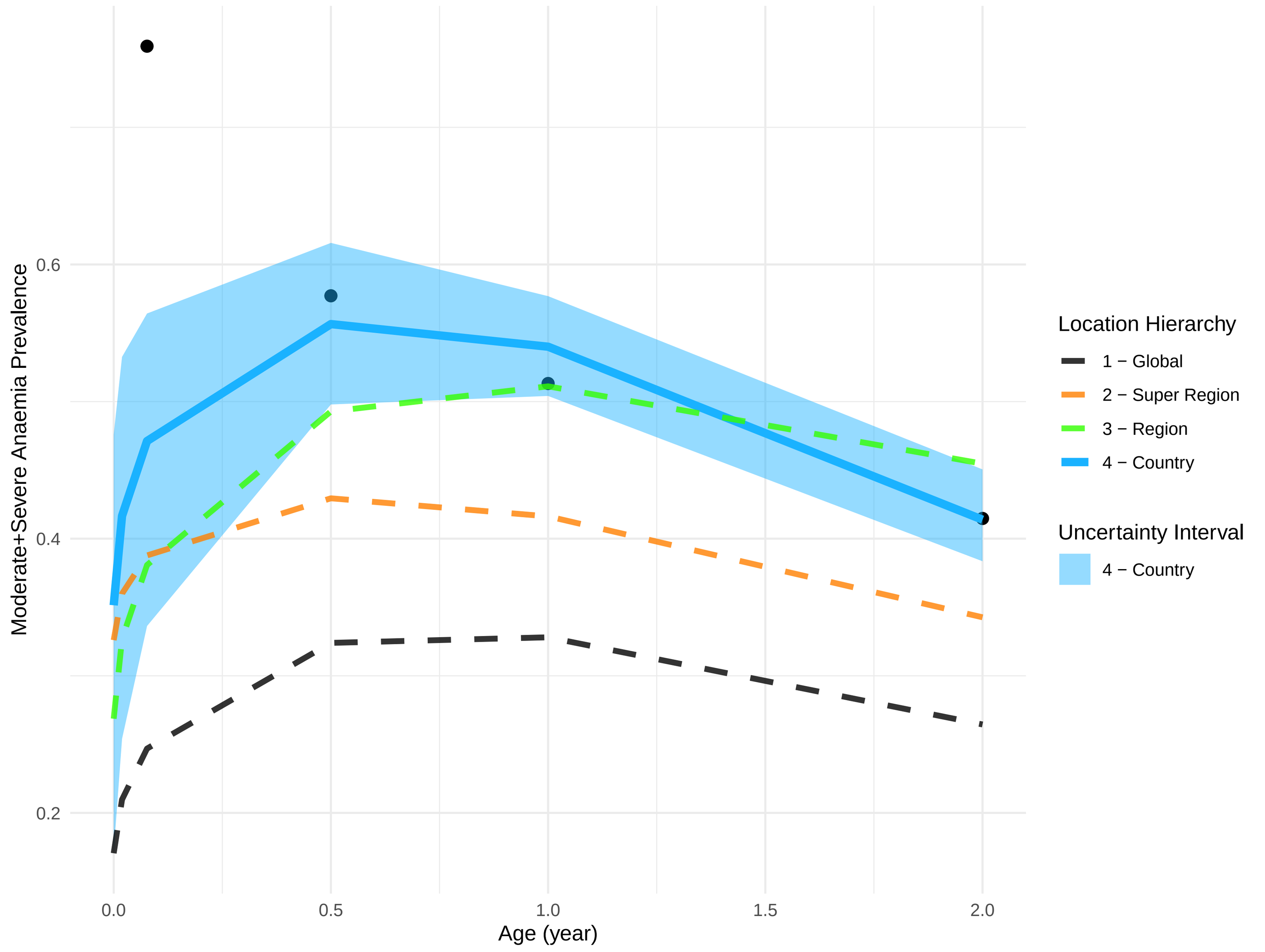

MR−BRT Age/Sex Fits − Males <2 Years Old − Moderate+Severe Anaemia Prevalence − Gambia
WHO−2001 Adjustment Method

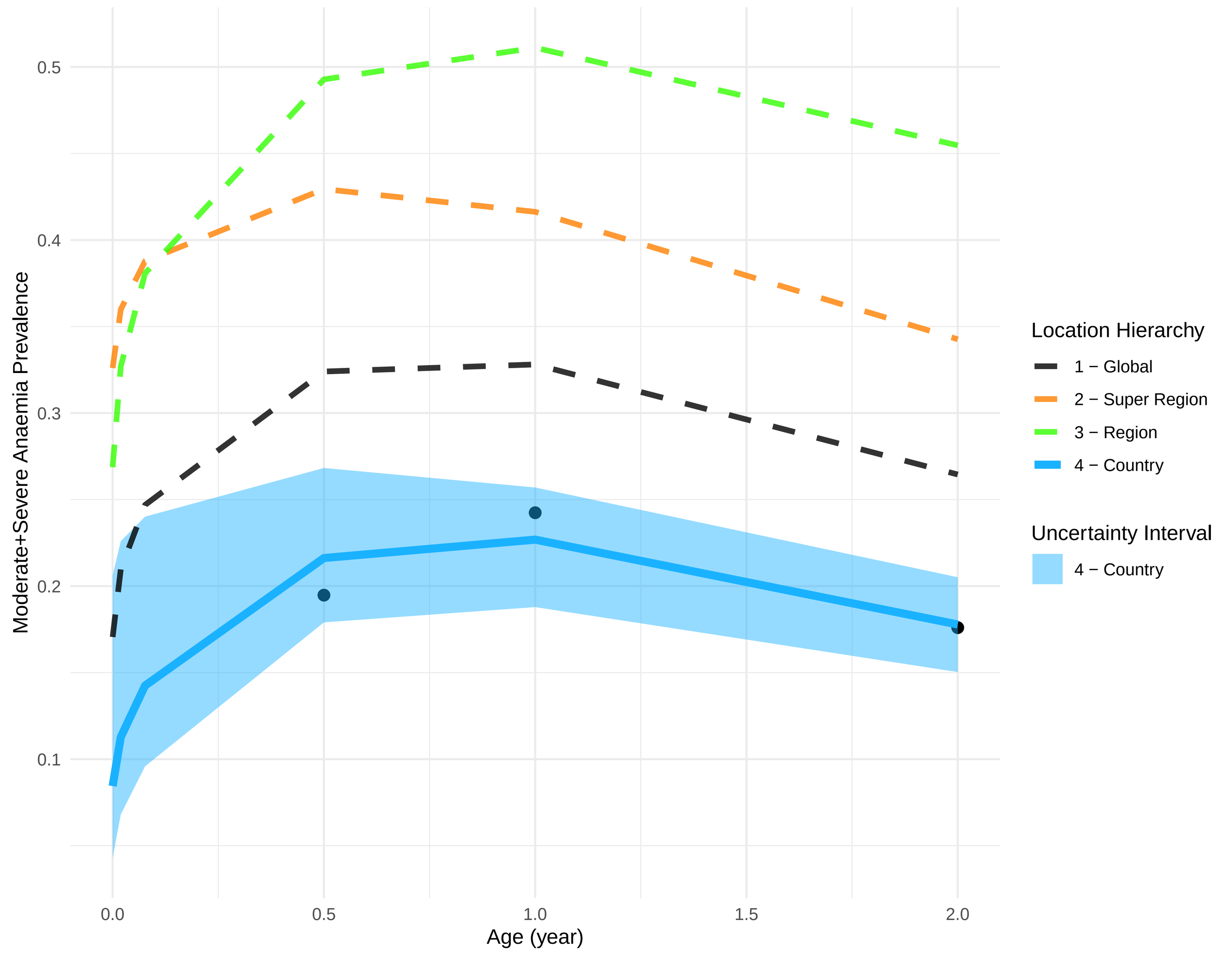

MR−BRT Age/Sex Fits − Males <2 Years Old − Moderate+Severe Anaemia Prevalence − Ghana
WHO−2001 Adjustment Method

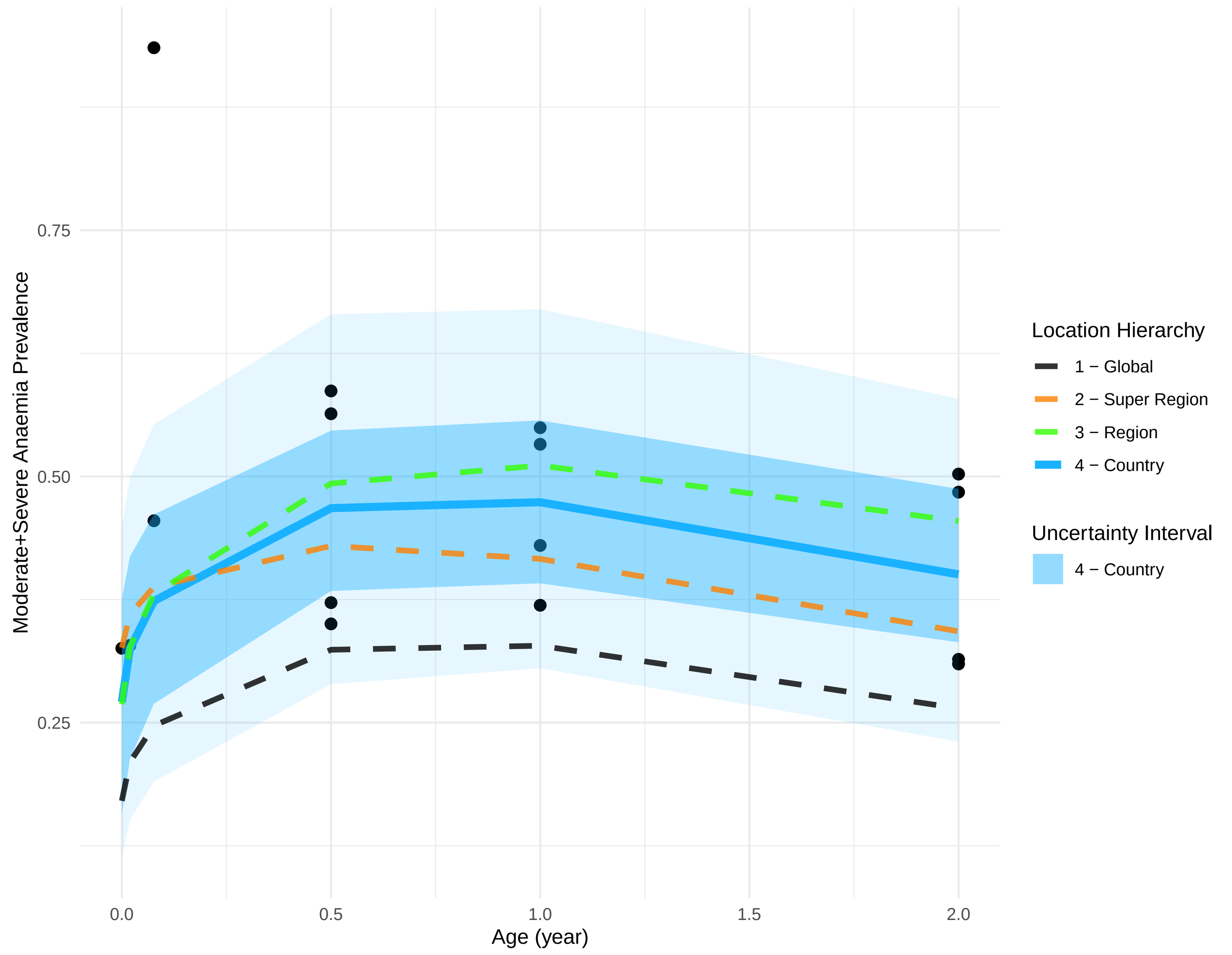

MR−BRT Age/Sex Fits − Males <2 Years Old − Moderate+Severe Anaemia Prevalence − Guinea
WHO−2001 Adjustment Method

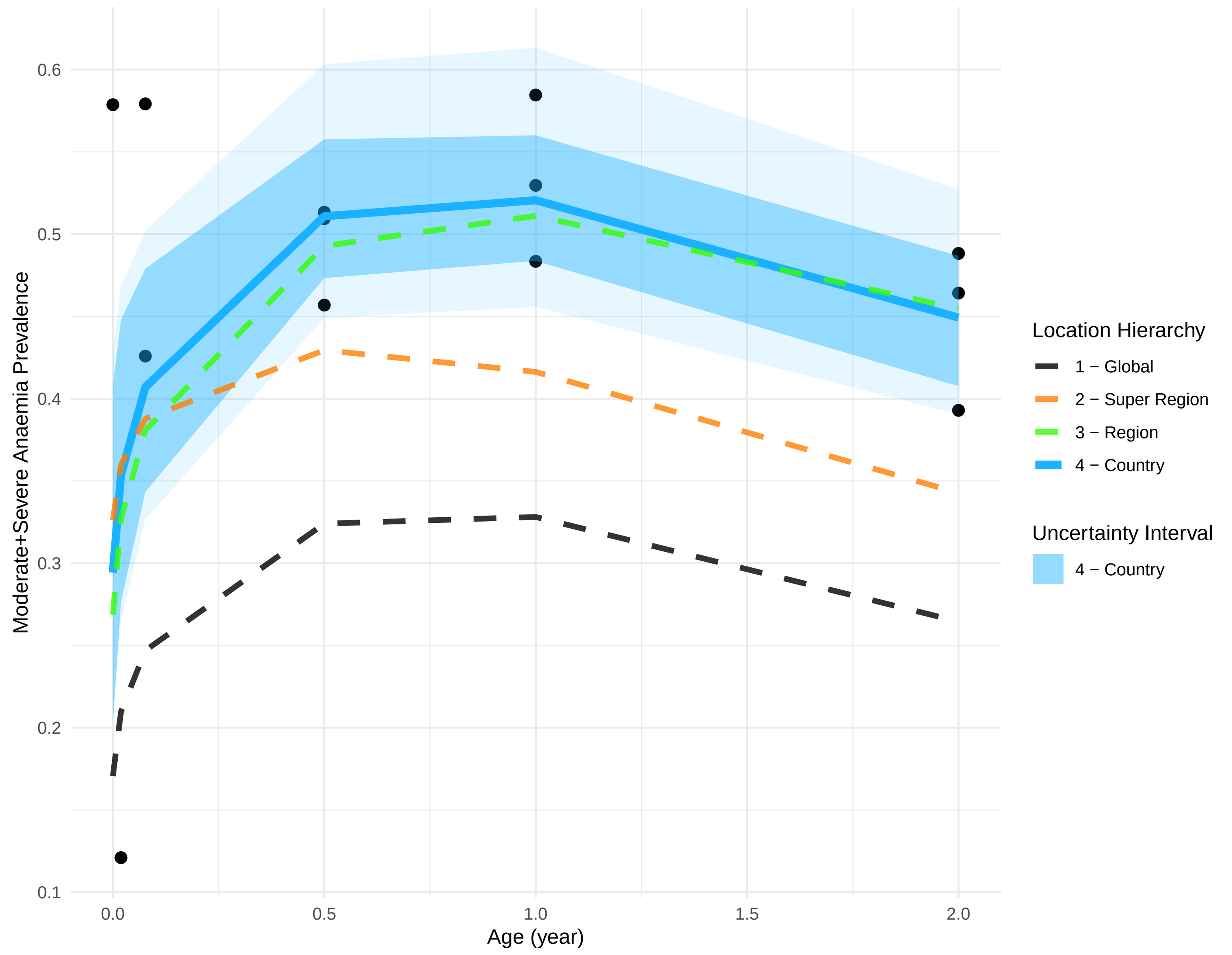

MR−BRT Age/Sex Fits − Males <2 Years Old − Moderate+Severe Anaemia Prevalence − Liberia
WHO−2001 Adjustment Method

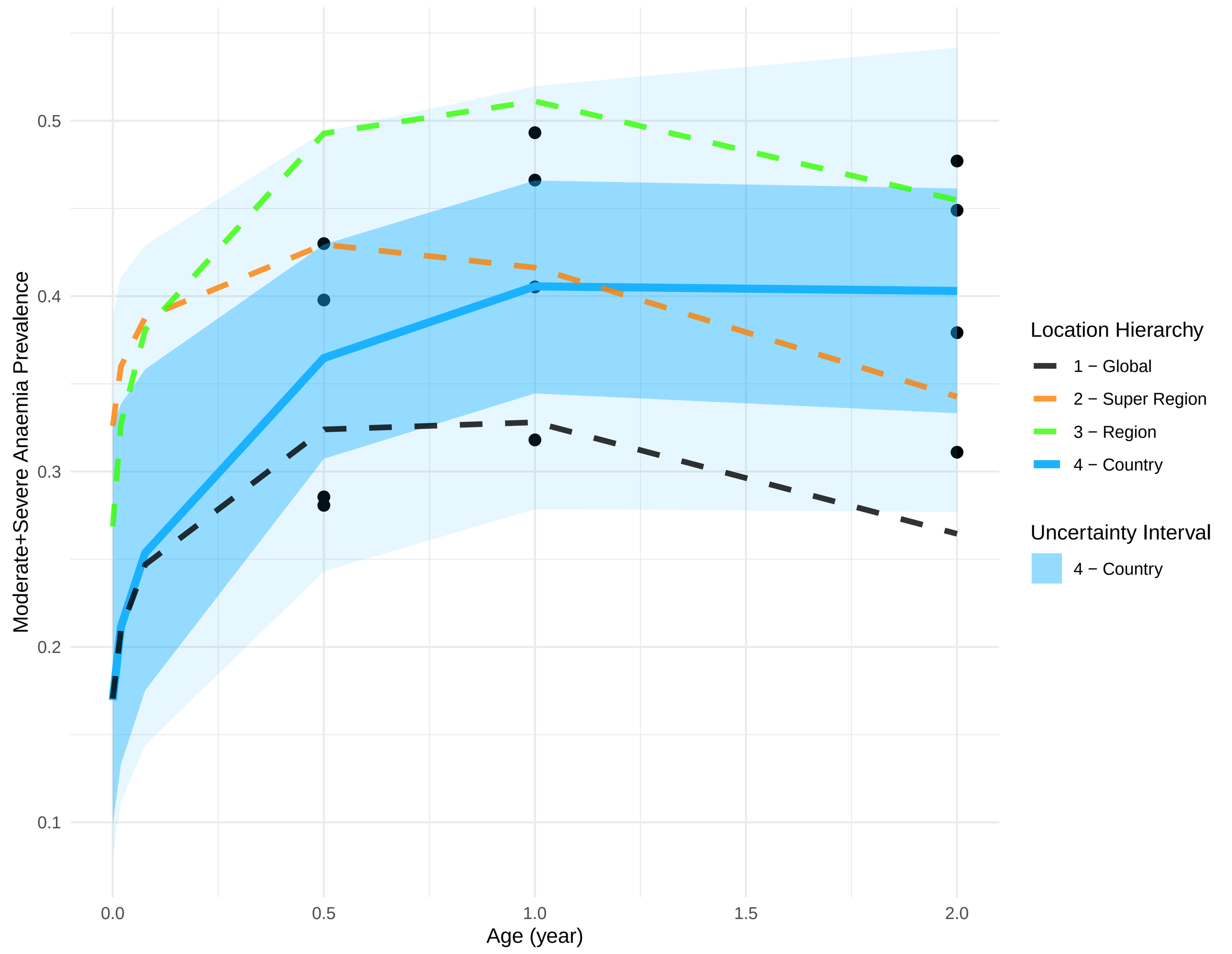

MR−BRT Age/Sex Fits − Males <2 Years Old − Moderate+Severe Anaemia Prevalence − Mali
WHO−2001 Adjustment Method

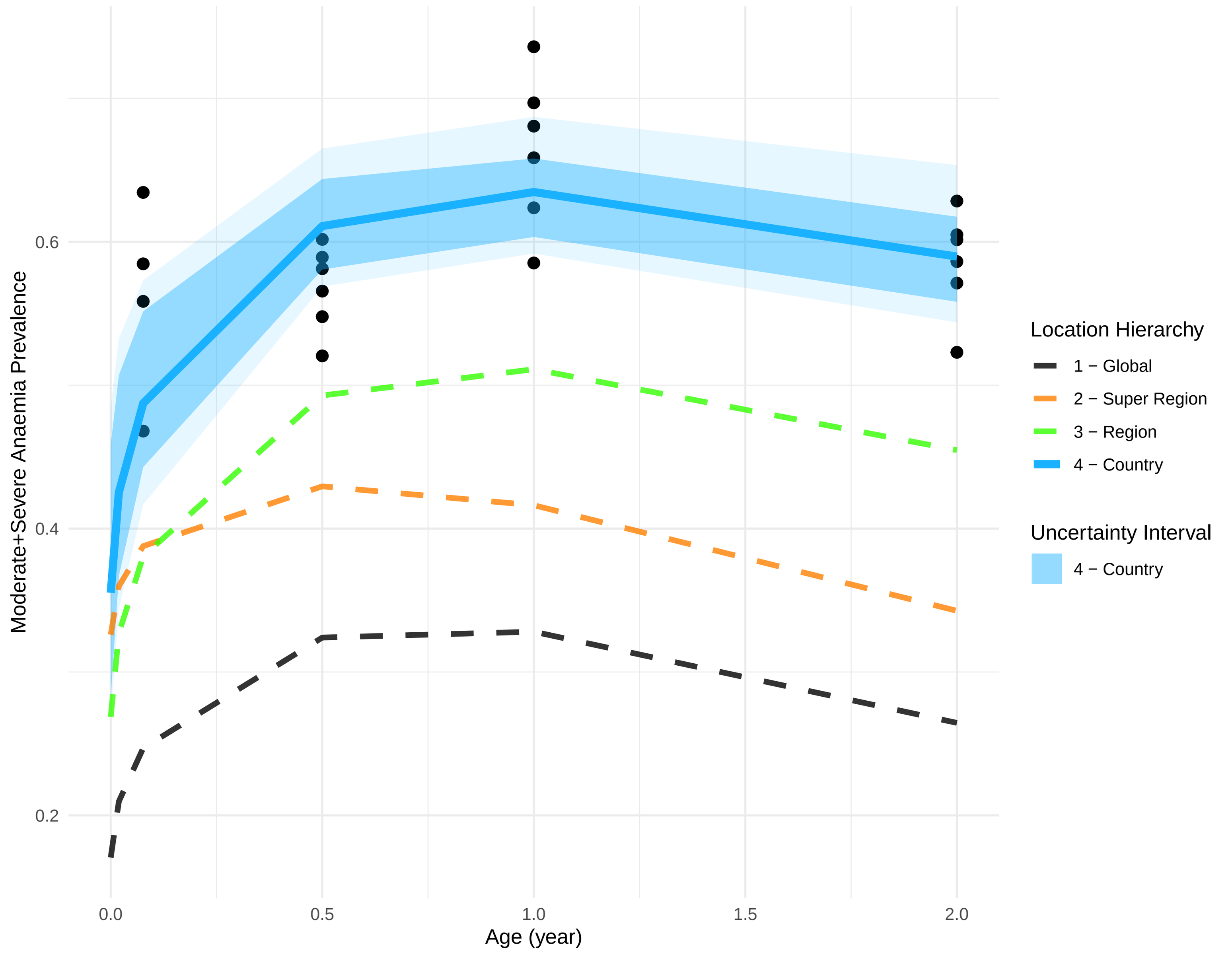

MR−BRT Age/Sex Fits − Males <2 Years Old − Moderate+Severe Anaemia Prevalence − Niger
WHO−2001 Adjustment Method

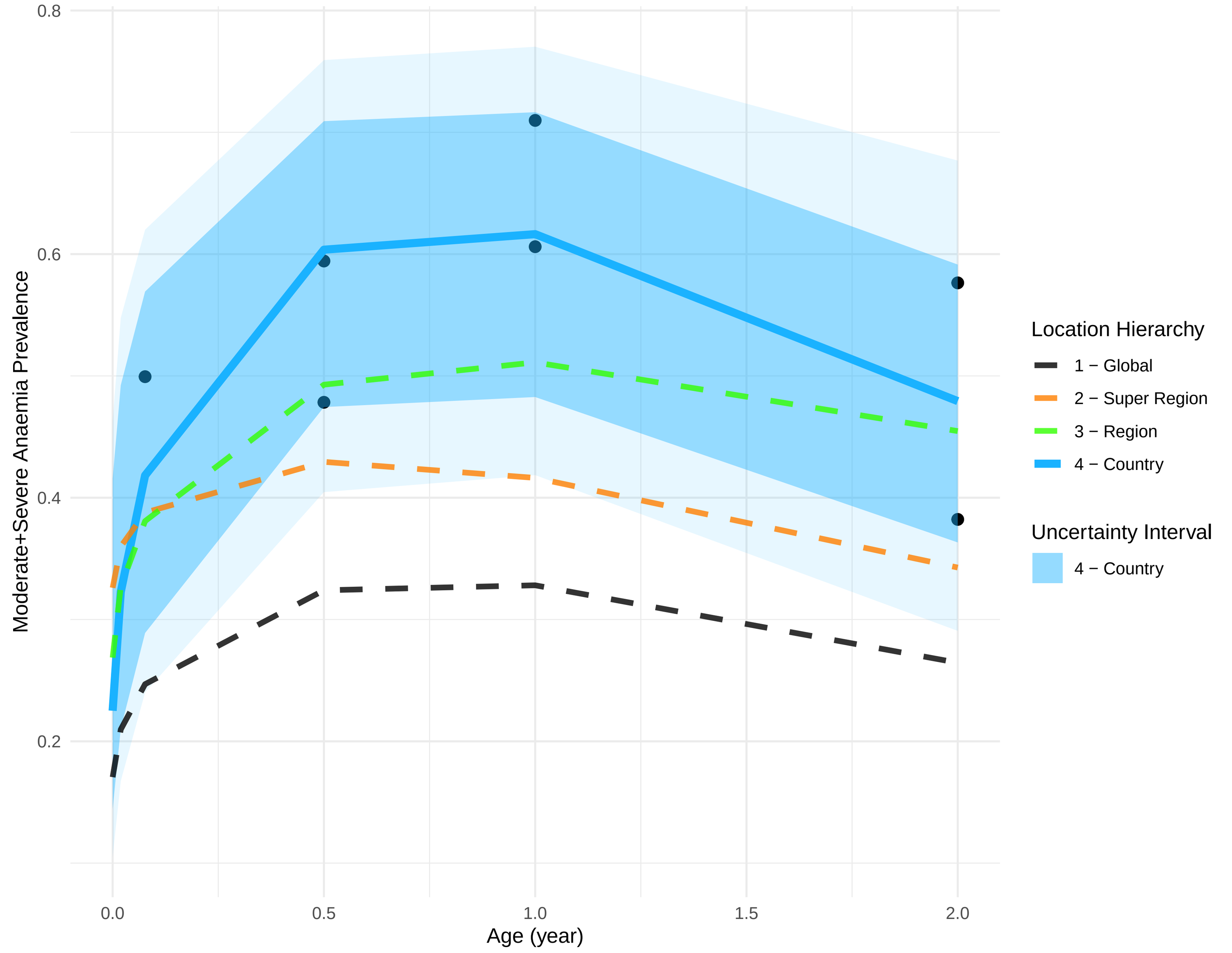

MR−BRT Age/Sex Fits − Males <2 Years Old − Moderate+Severe Anaemia Prevalence − Nigeria
WHO−2001 Adjustment Method

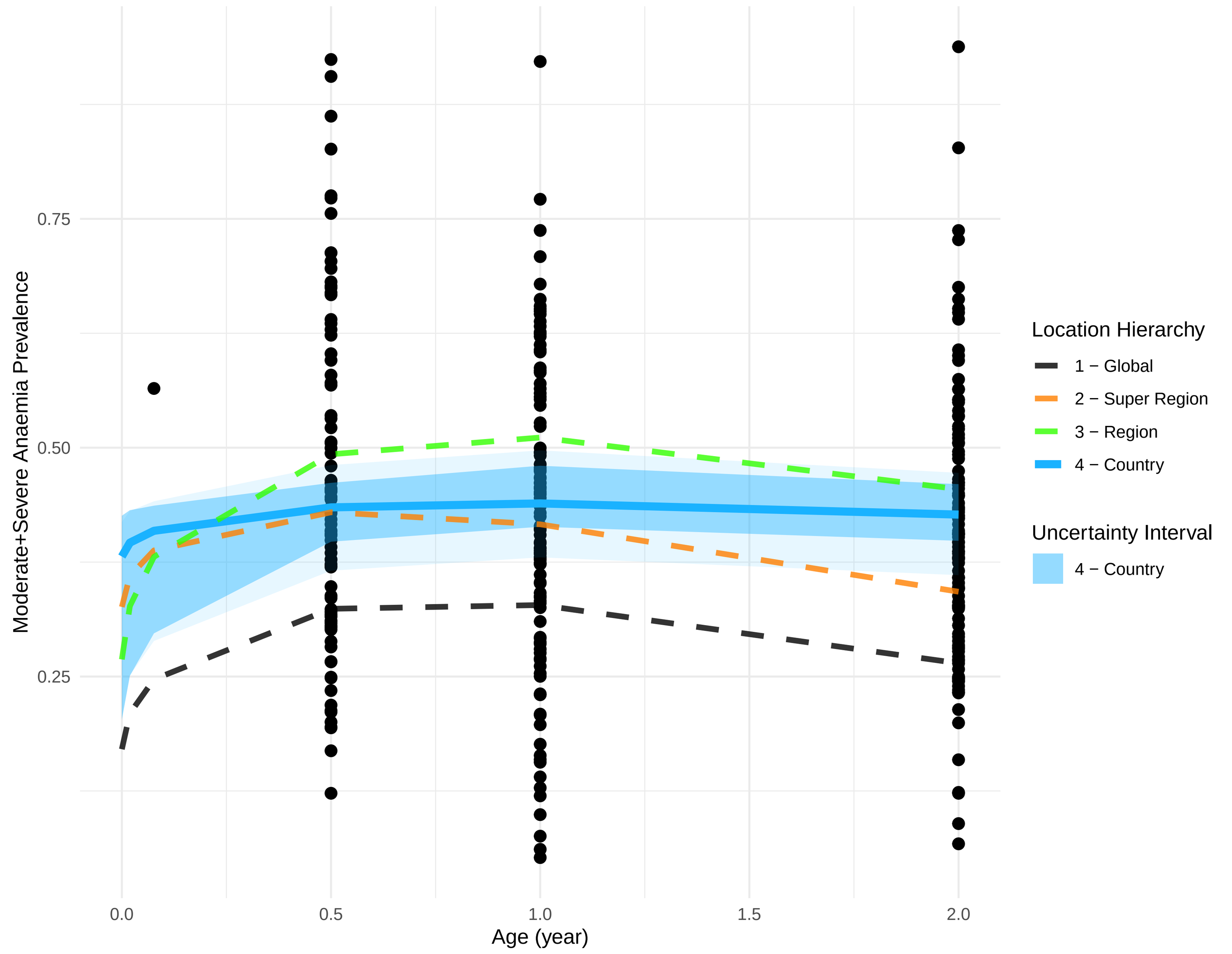

MR−BRT Age/Sex Fits − Males <2 Years Old − Moderate+Severe Anaemia Prevalence − Abia
WHO−2001 Adjustment Method

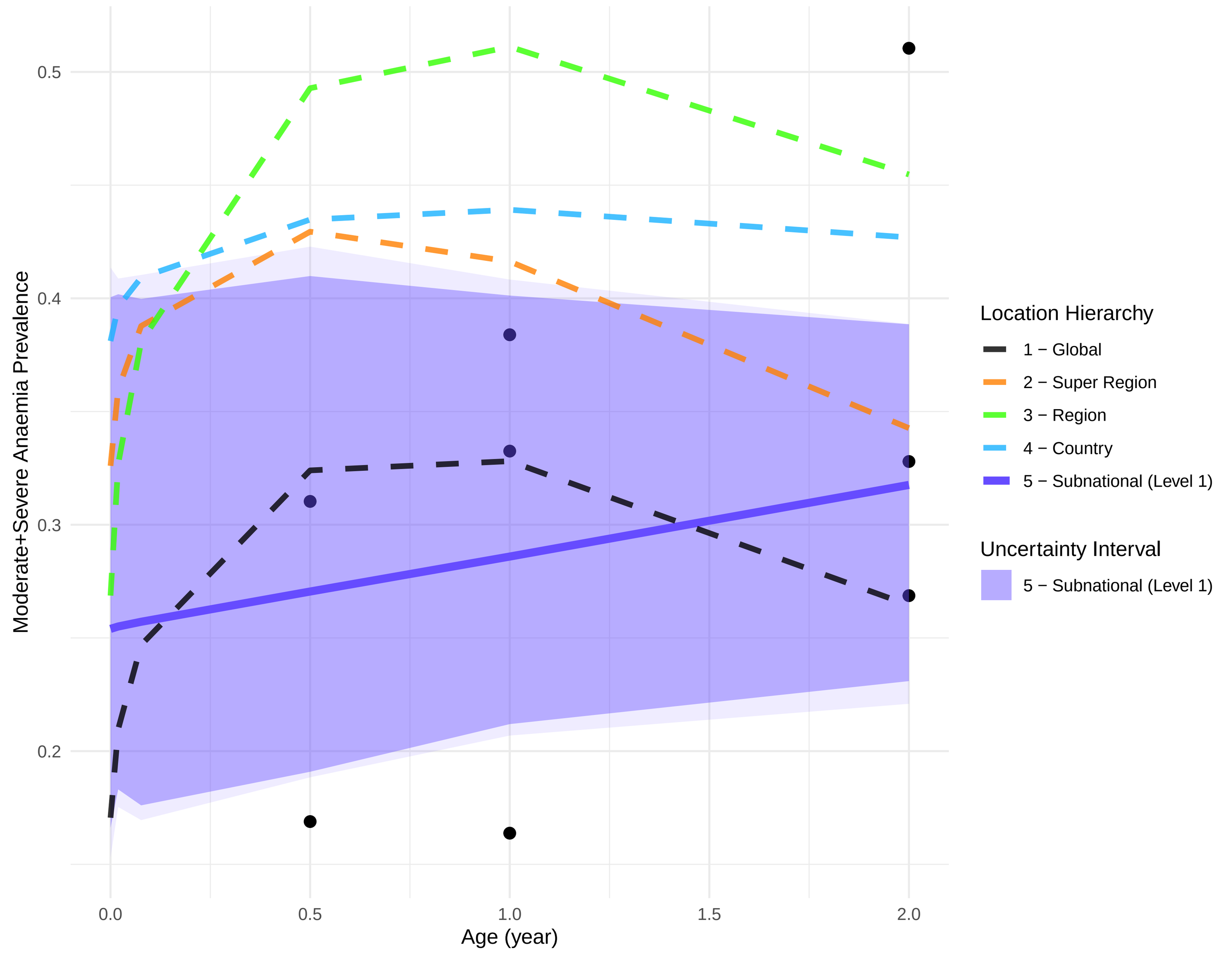

MR−BRT Age/Sex Fits − Males <2 Years Old − Moderate+Severe Anaemia Prevalence − Adamawa
WHO−2001 Adjustment Method

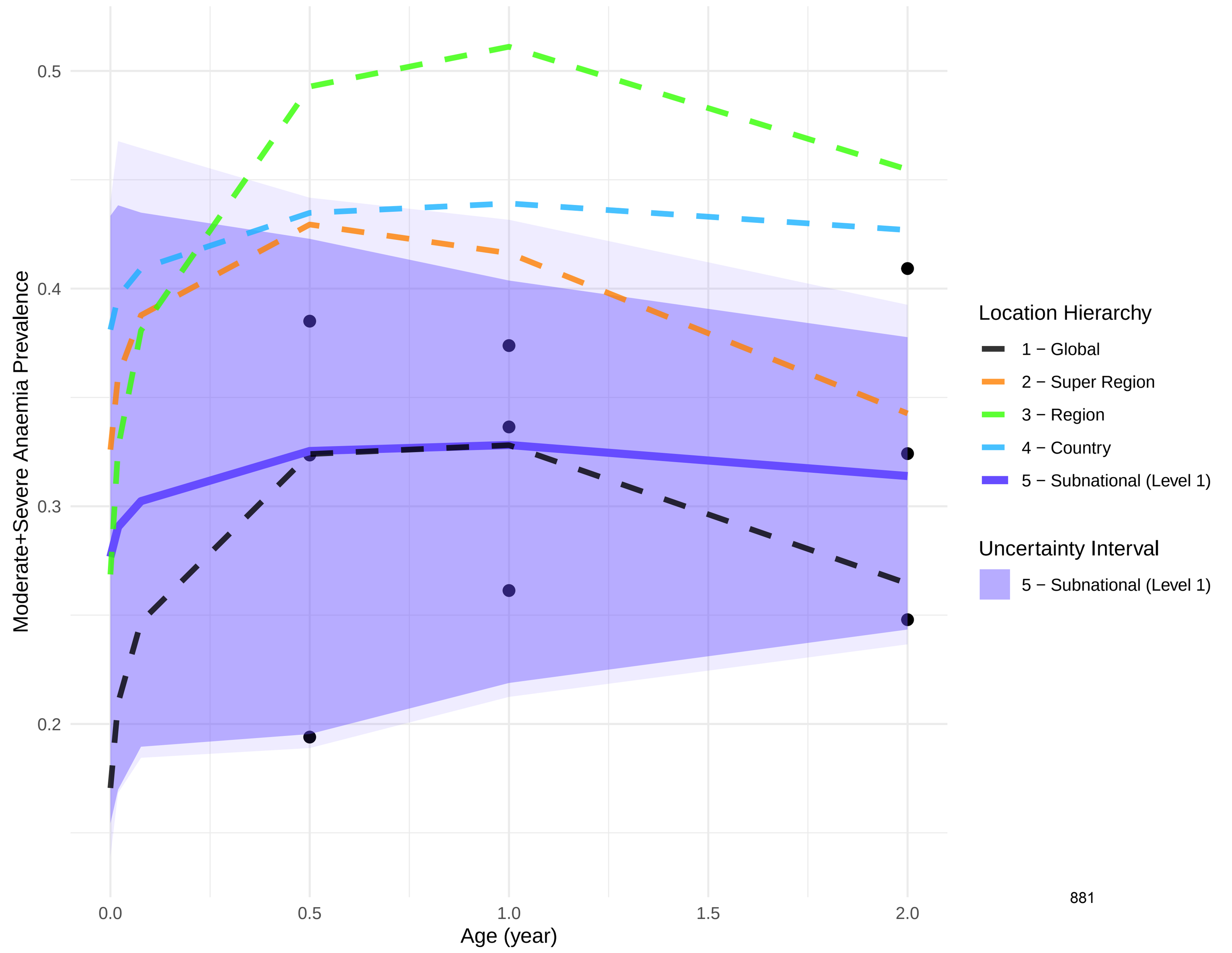

MR−BRT Age/Sex Fits − Males <2 Years Old − Moderate+Severe Anaemia Prevalence − Akwa Ibom
WHO−2001 Adjustment Method

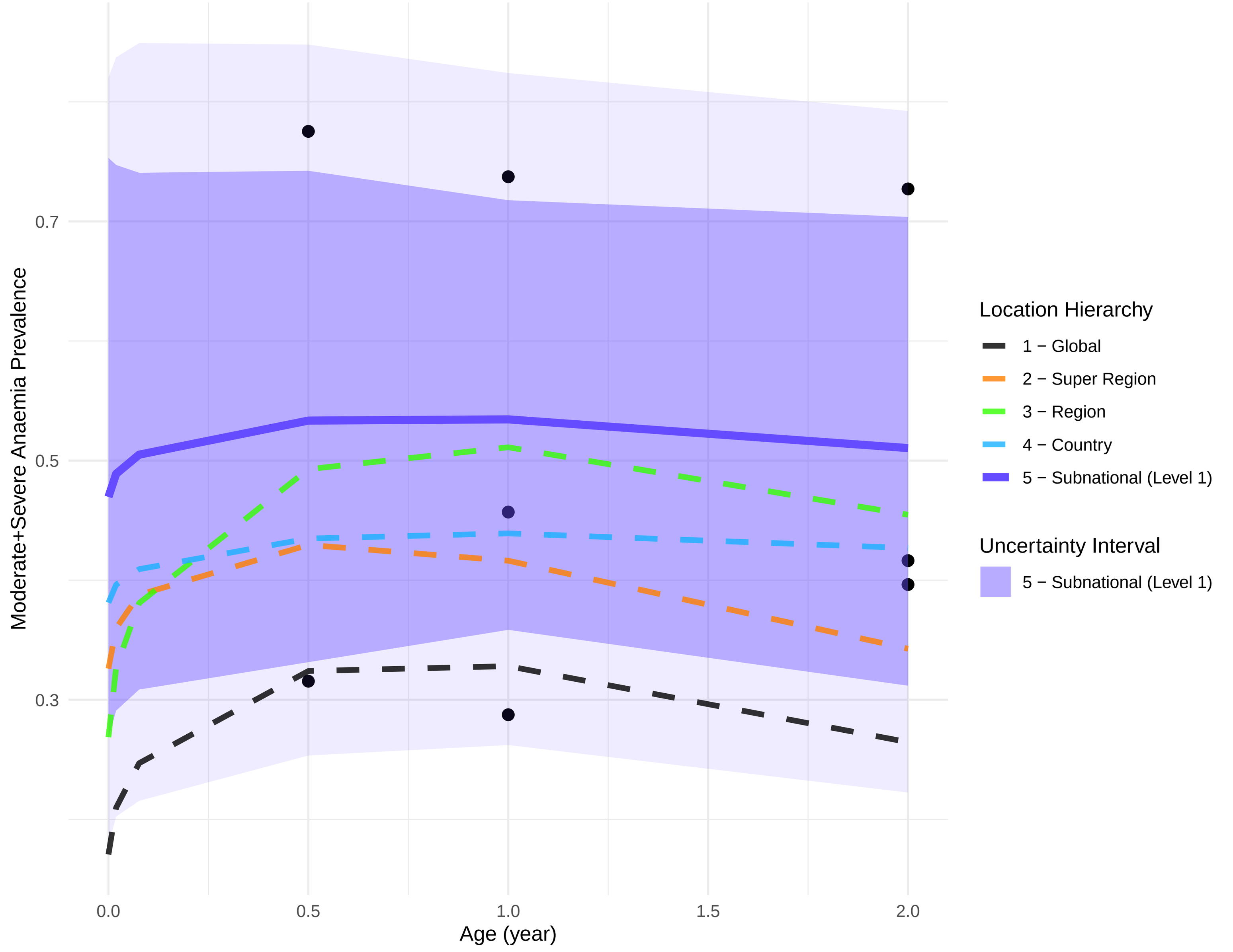

MR−BRT Age/Sex Fits − Males <2 Years Old − Moderate+Severe Anaemia Prevalence − Anambra
WHO−2001 Adjustment Method

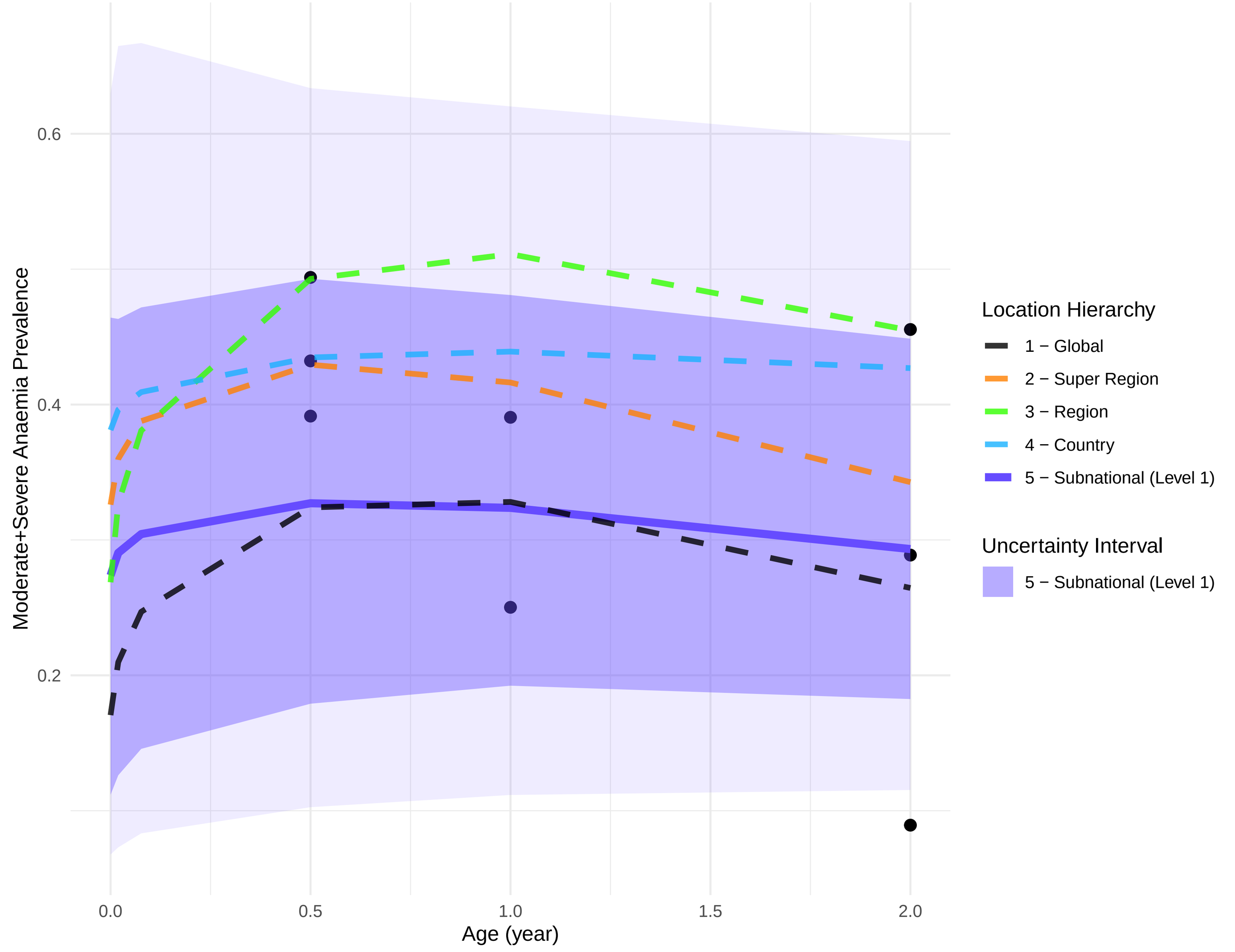

MR−BRT Age/Sex Fits − Males <2 Years Old − Moderate+Severe Anaemia Prevalence − Bauchi
WHO−2001 Adjustment Method

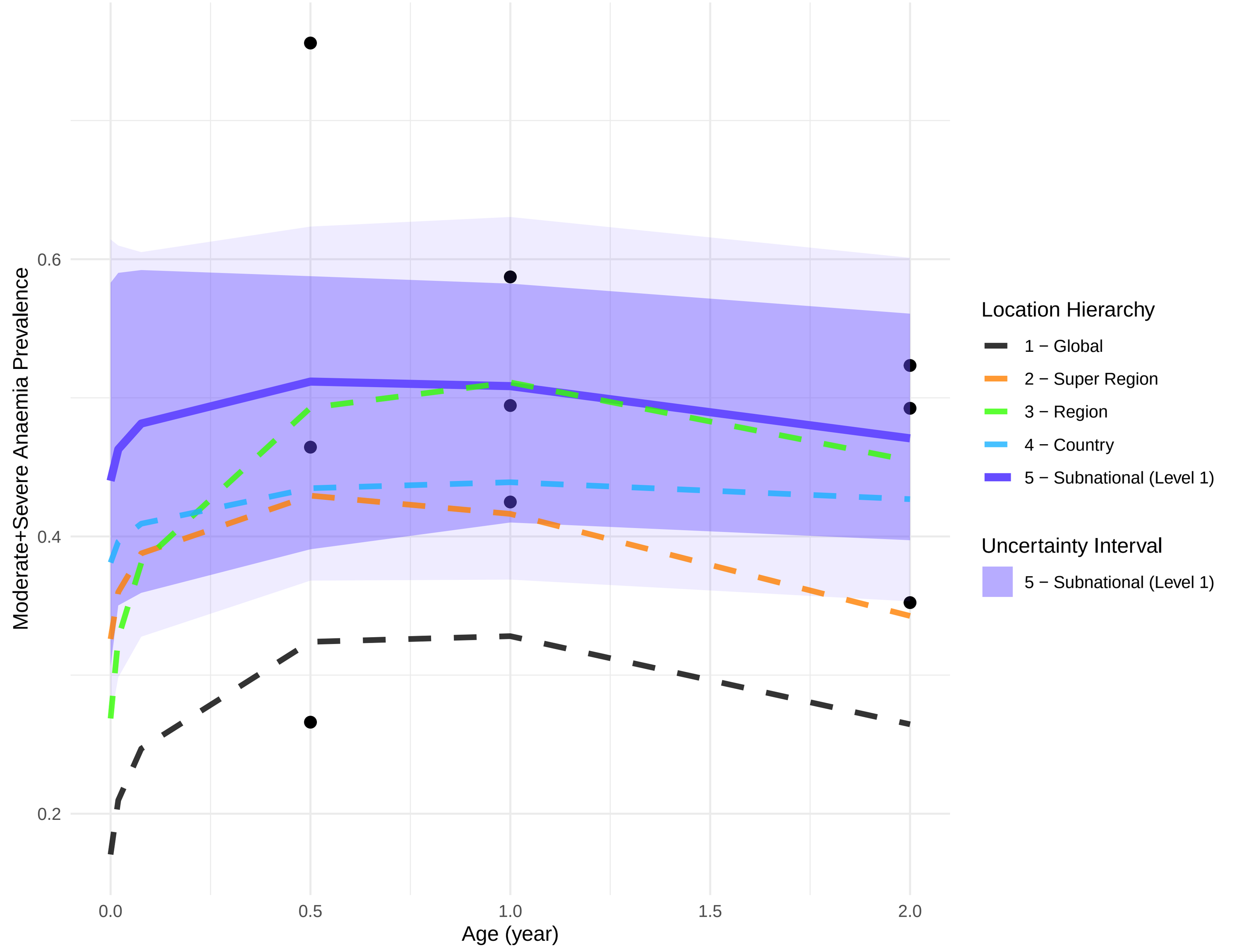

MR−BRT Age/Sex Fits − Males <2 Years Old − Moderate+Severe Anaemia Prevalence − Bayelsa
WHO−2001 Adjustment Method

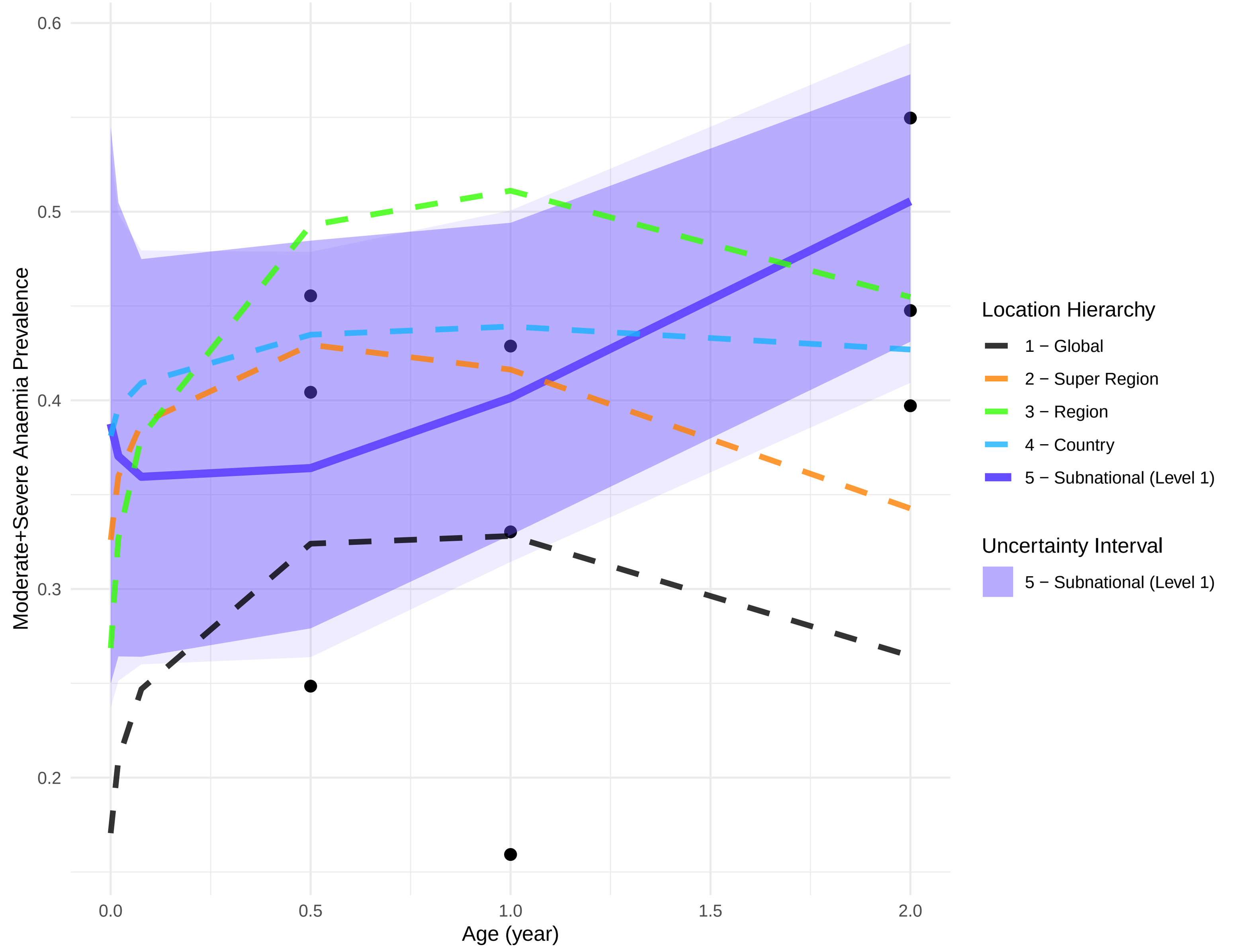

MR−BRT Age/Sex Fits − Males <2 Years Old − Moderate+Severe Anaemia Prevalence − Benue
WHO−2001 Adjustment Method

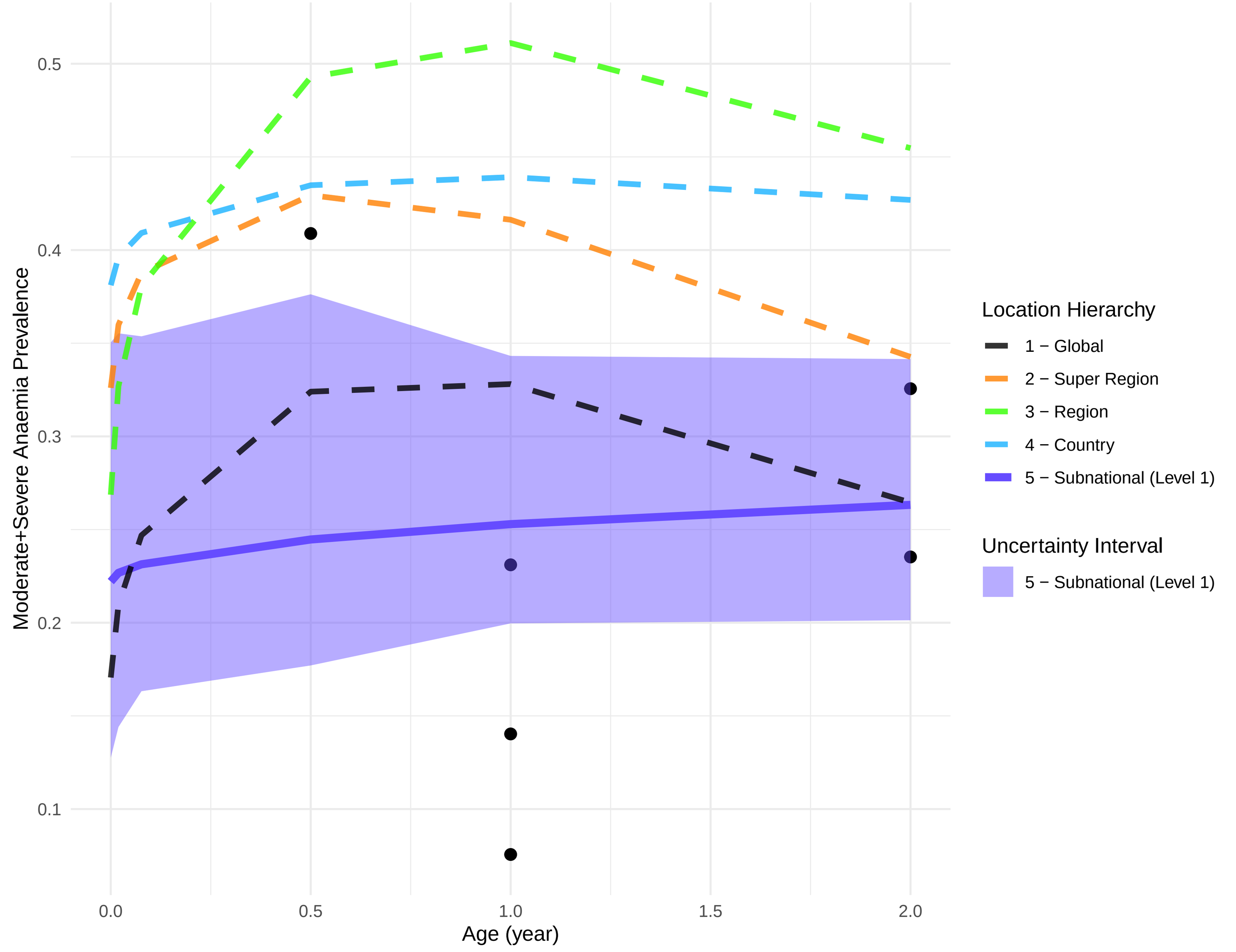

MR−BRT Age/Sex Fits − Males <2 Years Old − Moderate+Severe Anaemia Prevalence − Borno
WHO−2001 Adjustment Method

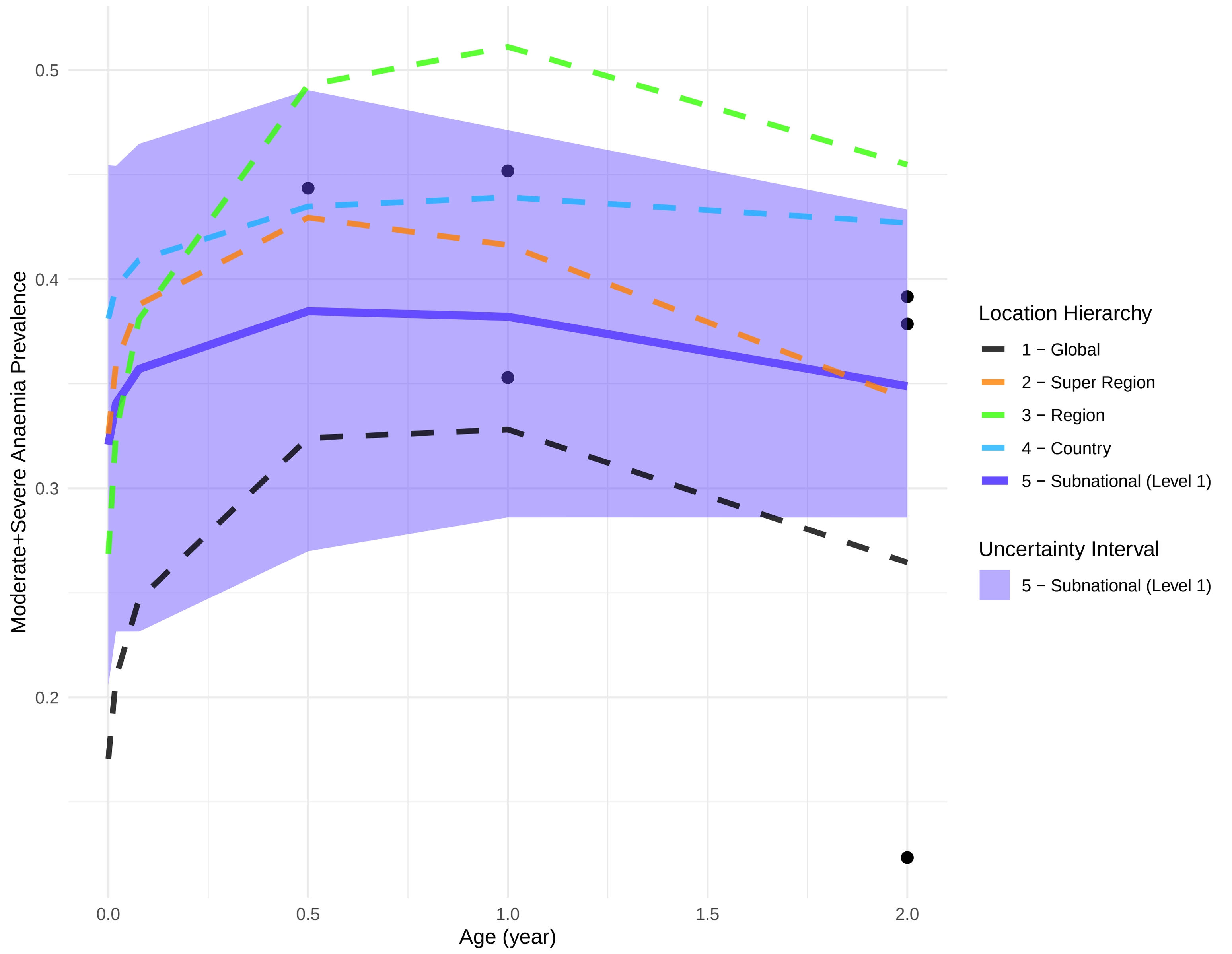

MR−BRT Age/Sex Fits − Males <2 Years Old − Moderate+Severe Anaemia Prevalence − Cross River
WHO−2001 Adjustment Method

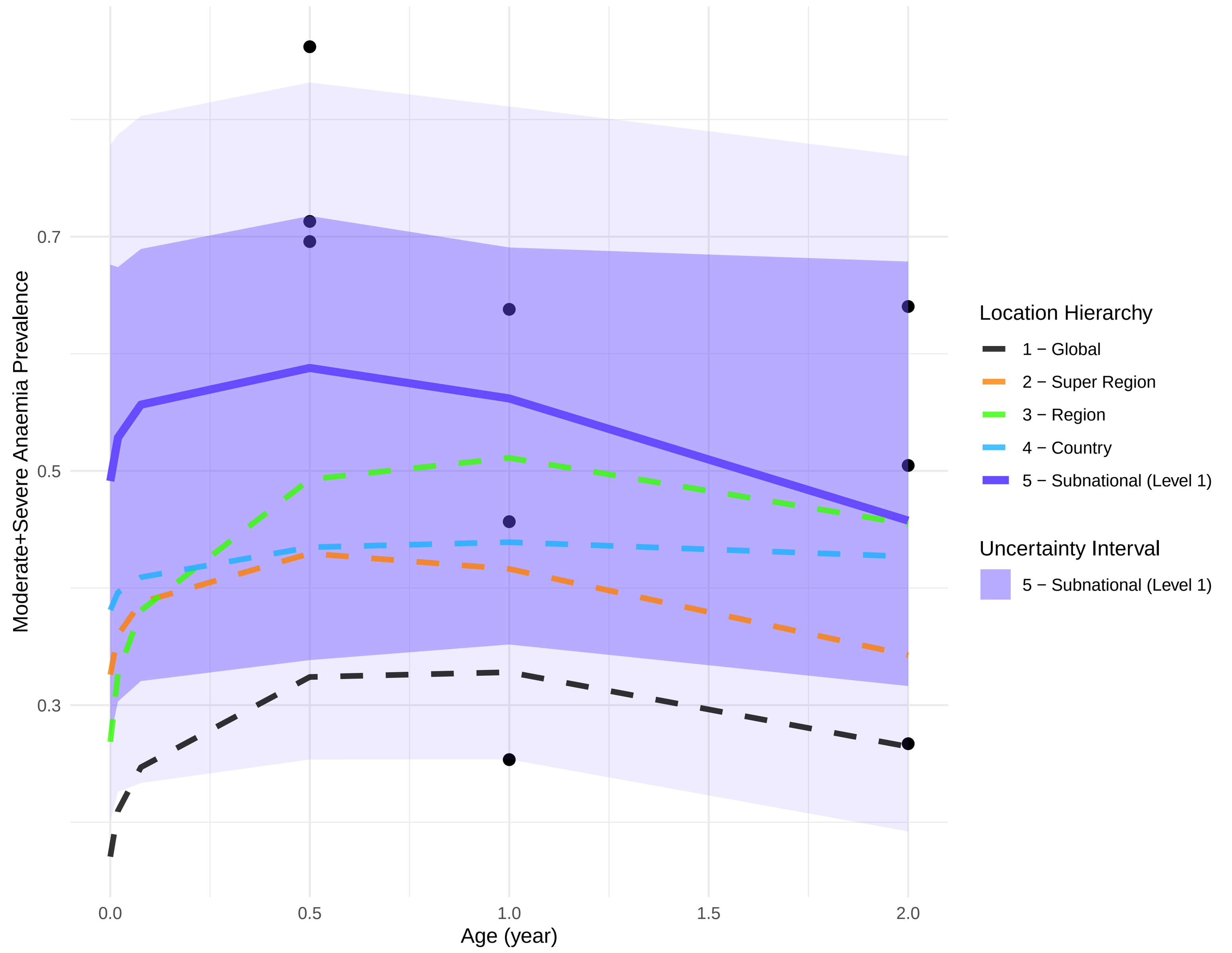

MR−BRT Age/Sex Fits − Males <2 Years Old − Moderate+Severe Anaemia Prevalence − Delta
WHO−2001 Adjustment Method

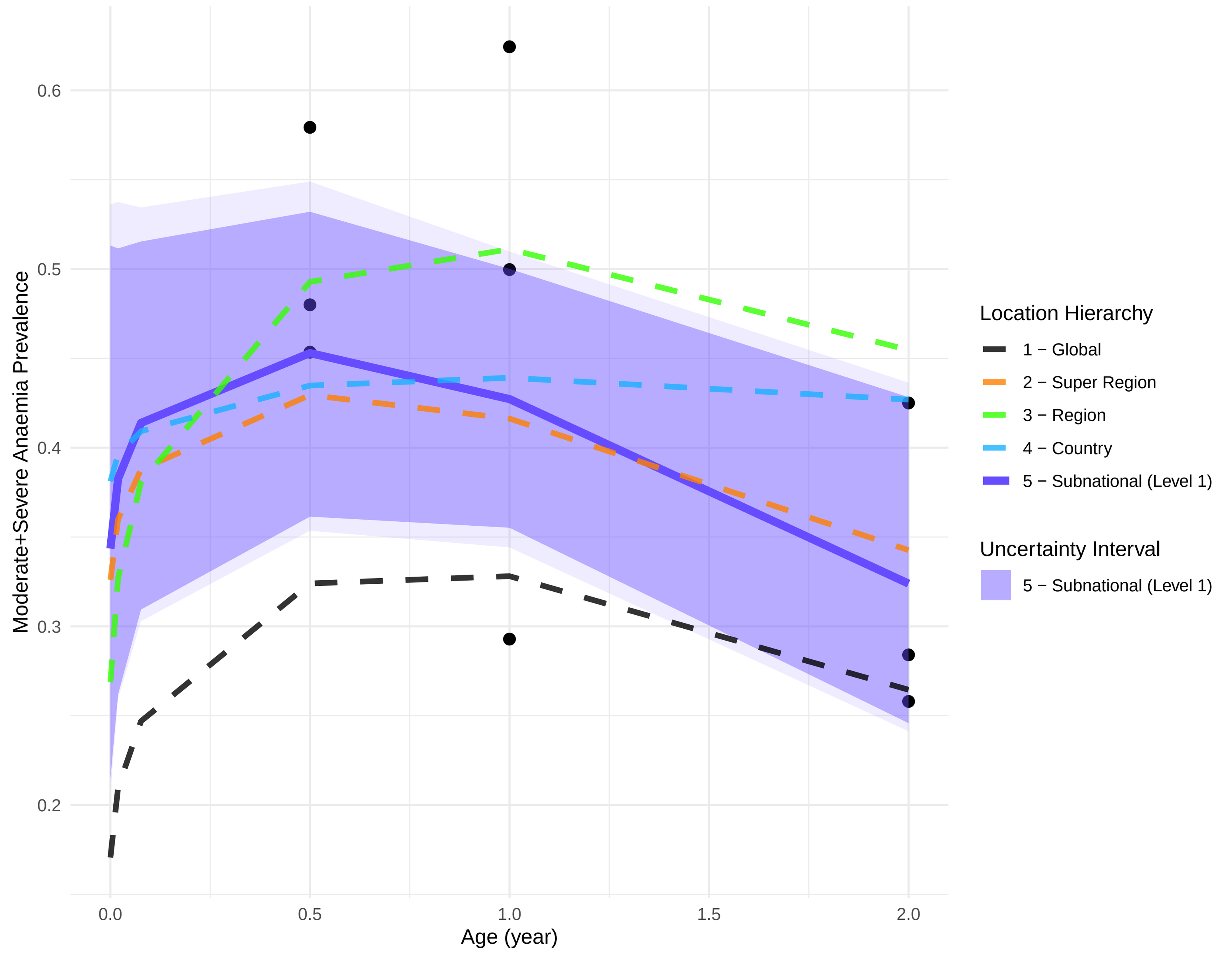

MR−BRT Age/Sex Fits − Males <2 Years Old − Moderate+Severe Anaemia Prevalence − Ebonyi
WHO−2001 Adjustment Method

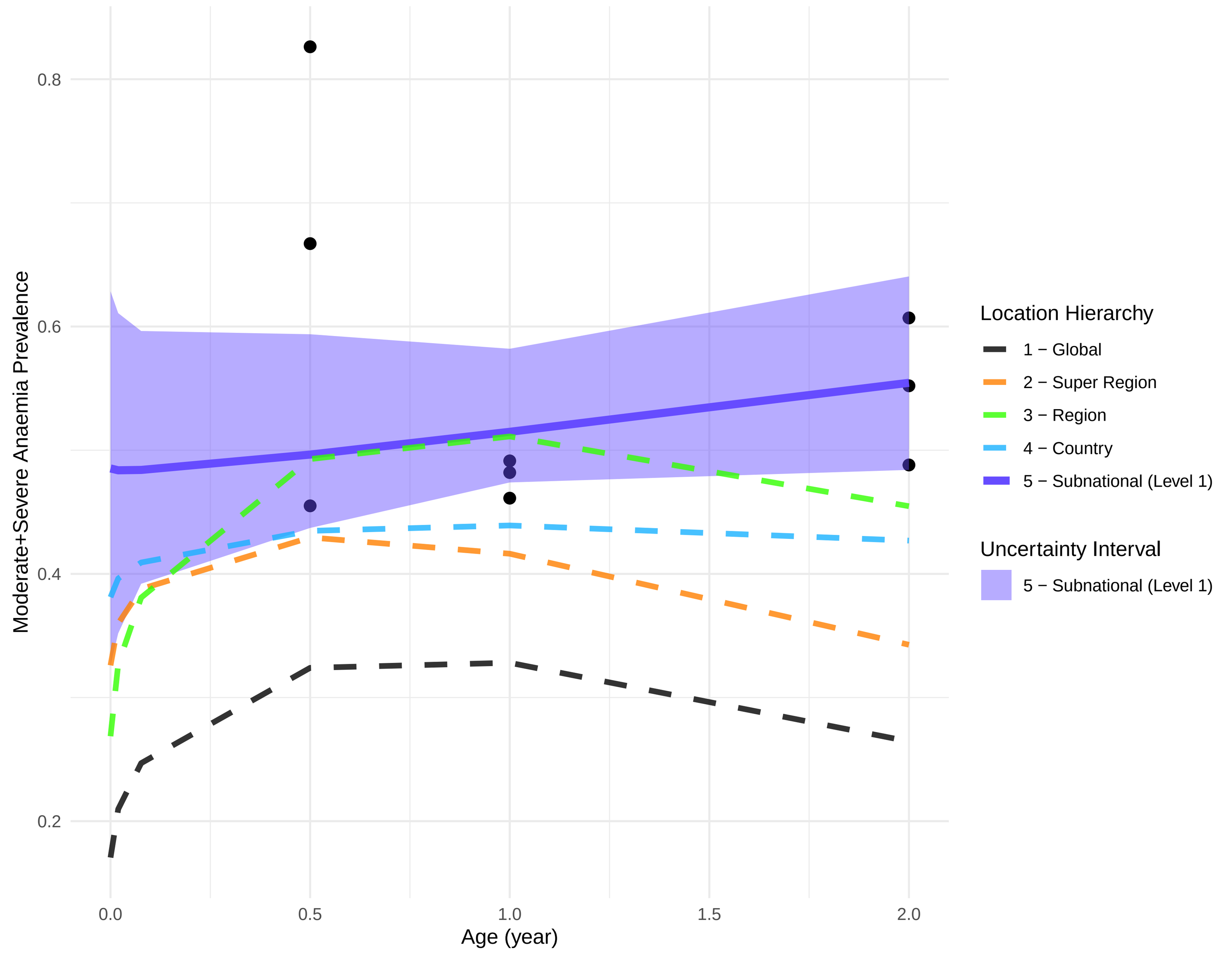

MR−BRT Age/Sex Fits − Males <2 Years Old − Moderate+Severe Anaemia Prevalence − Edo
WHO−2001 Adjustment Method

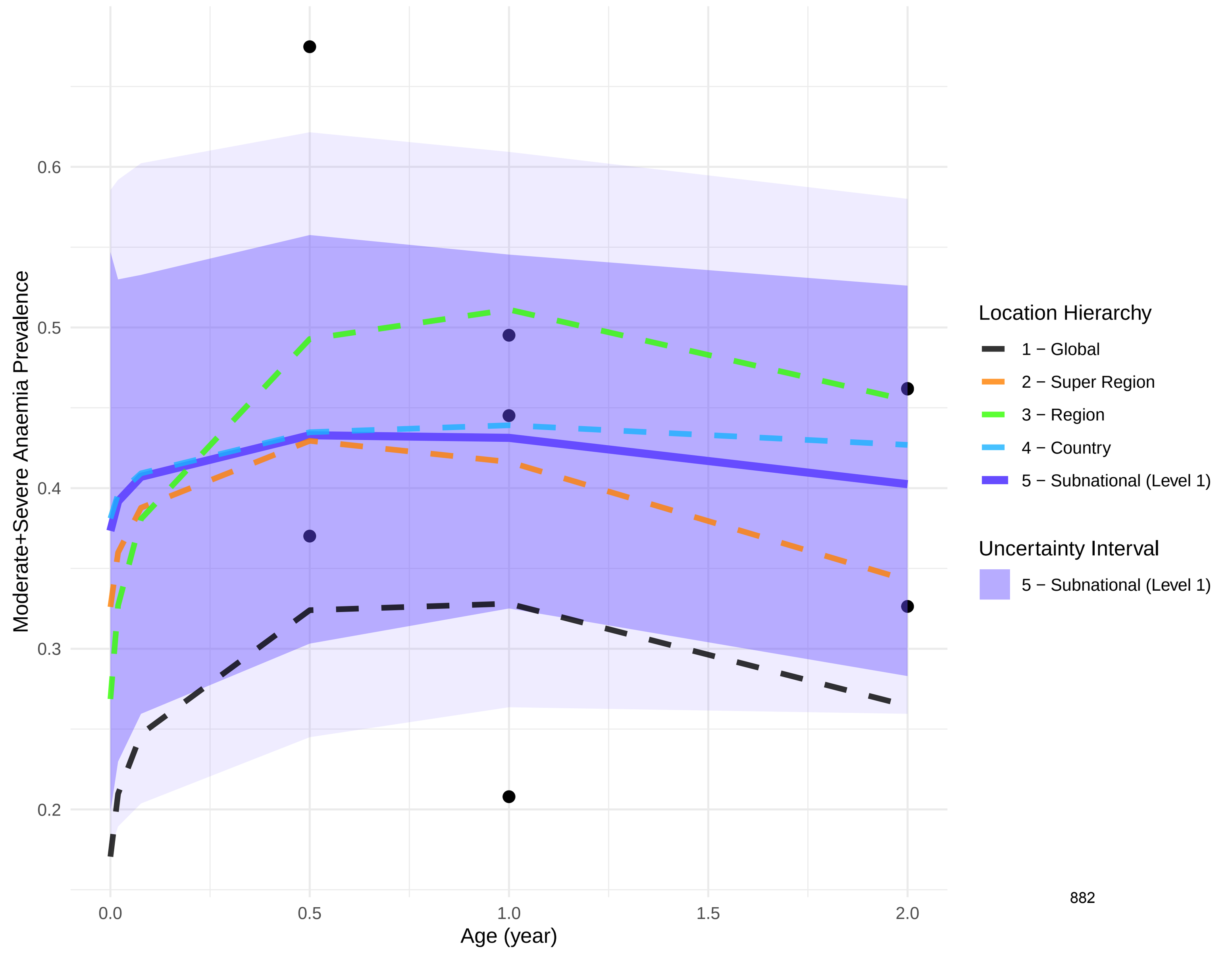

MR−BRT Age/Sex Fits − Males <2 Years Old − Moderate+Severe Anaemia Prevalence − Ekiti

WHO−2001 Adjustment Method

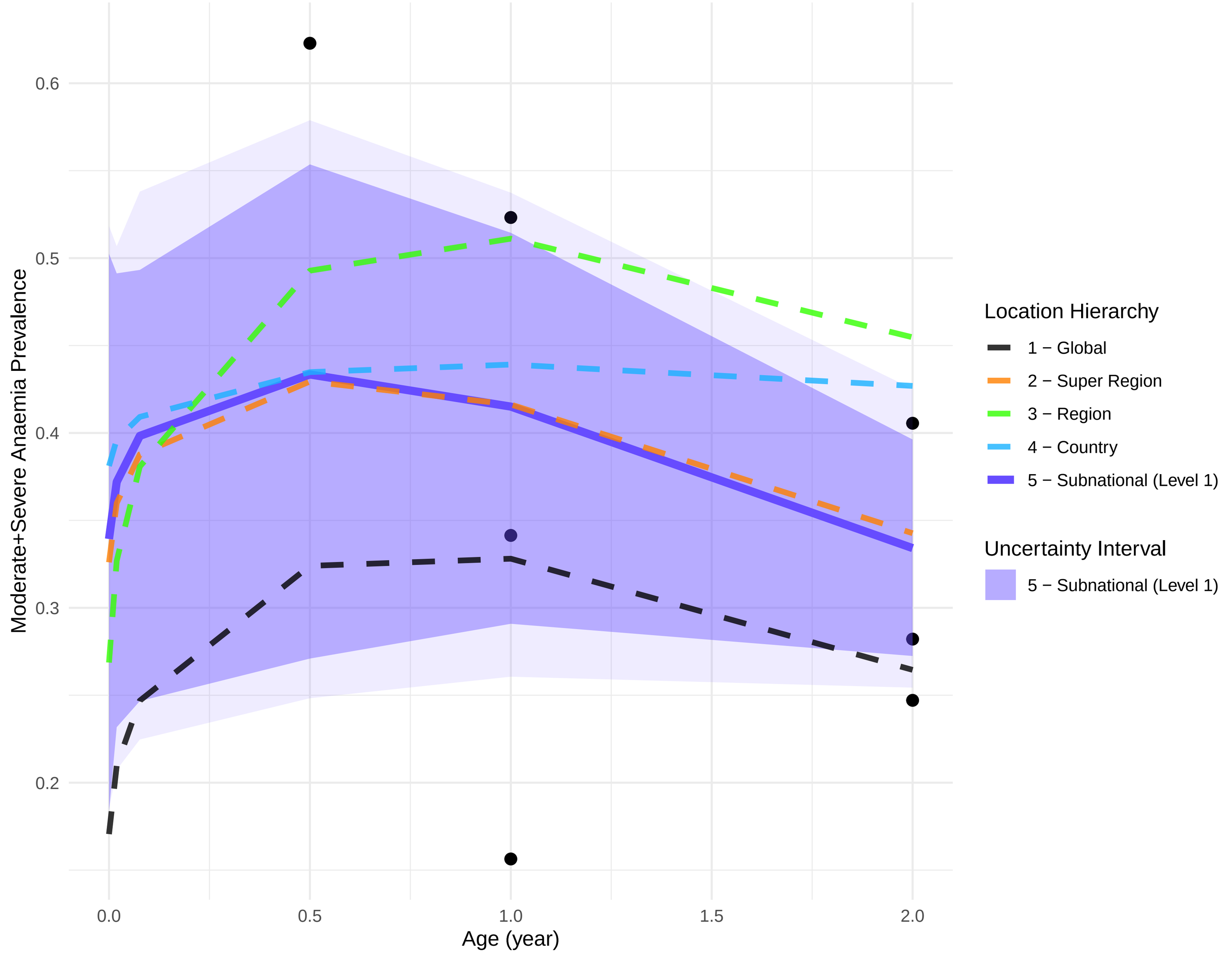

MR−BRT Age/Sex Fits − Males <2 Years Old − Moderate+Severe Anaemia Prevalence − Enugu

WHO−2001 Adjustment Method

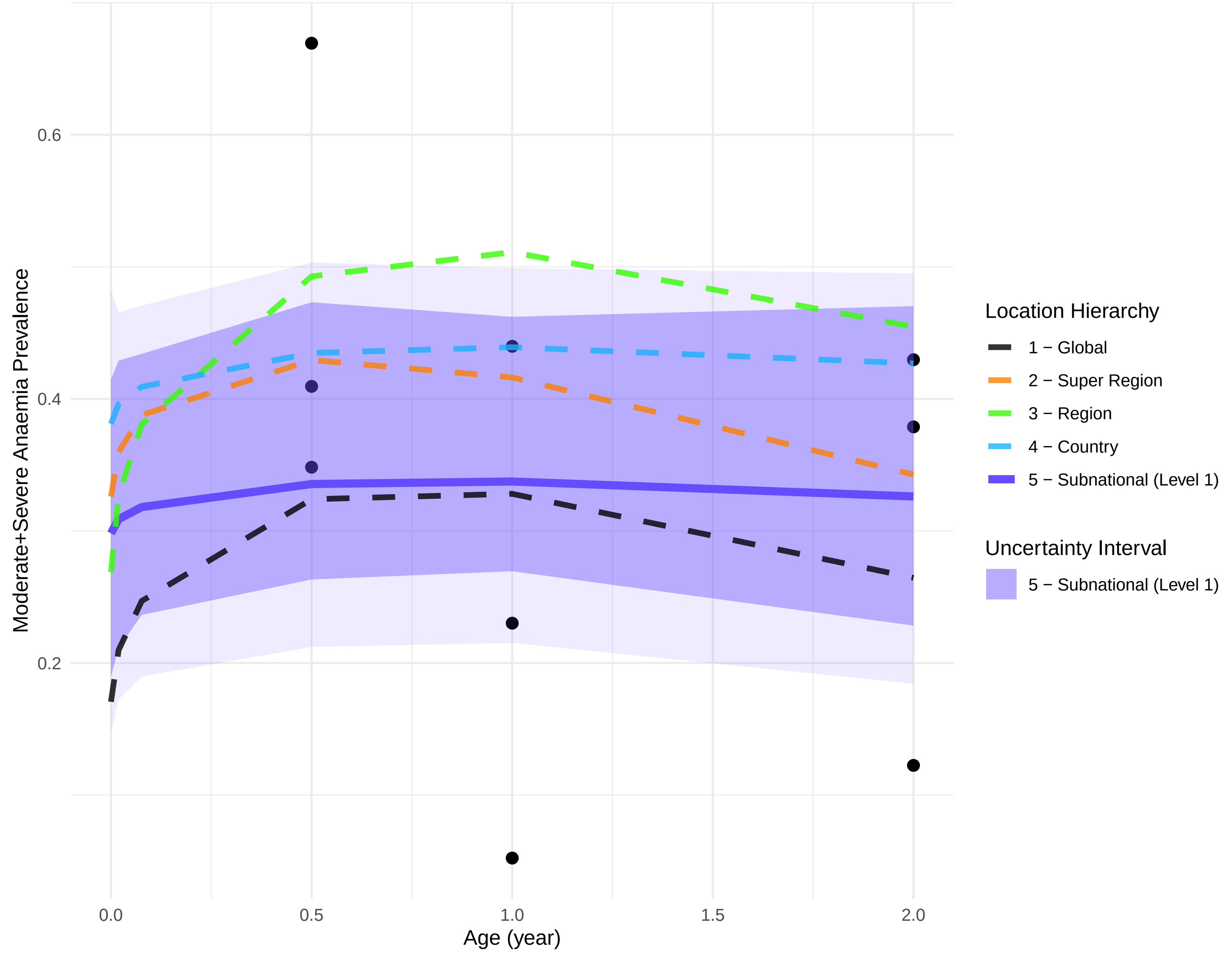

MR−BRT Age/Sex Fits − Males <2 Years Old − Moderate+Severe Anaemia Prevalence − FCT (Abuja)

WHO−2001 Adjustment Method

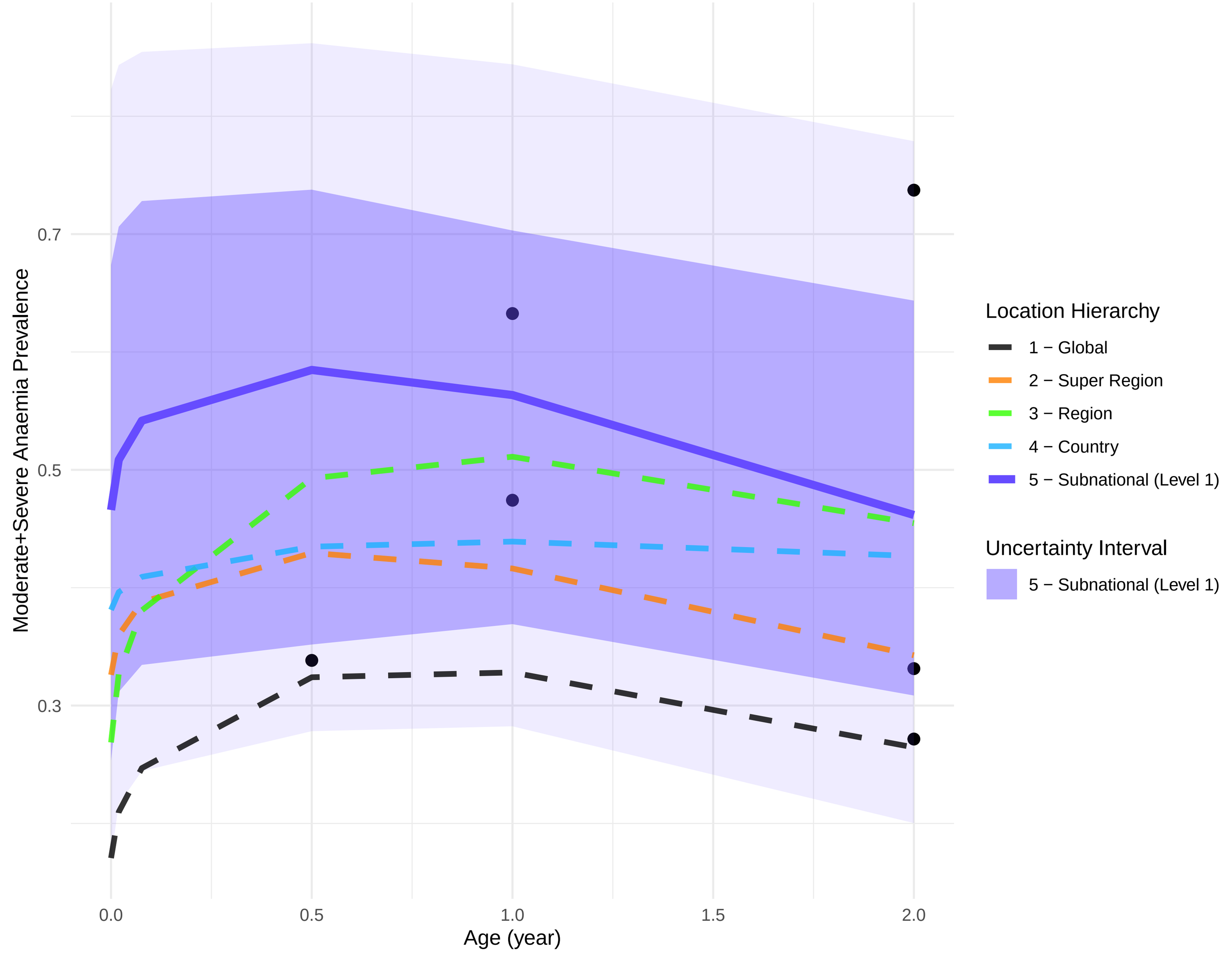

MR−BRT Age/Sex Fits − Males <2 Years Old − Moderate+Severe Anaemia Prevalence − Gombe

WHO−2001 Adjustment Method

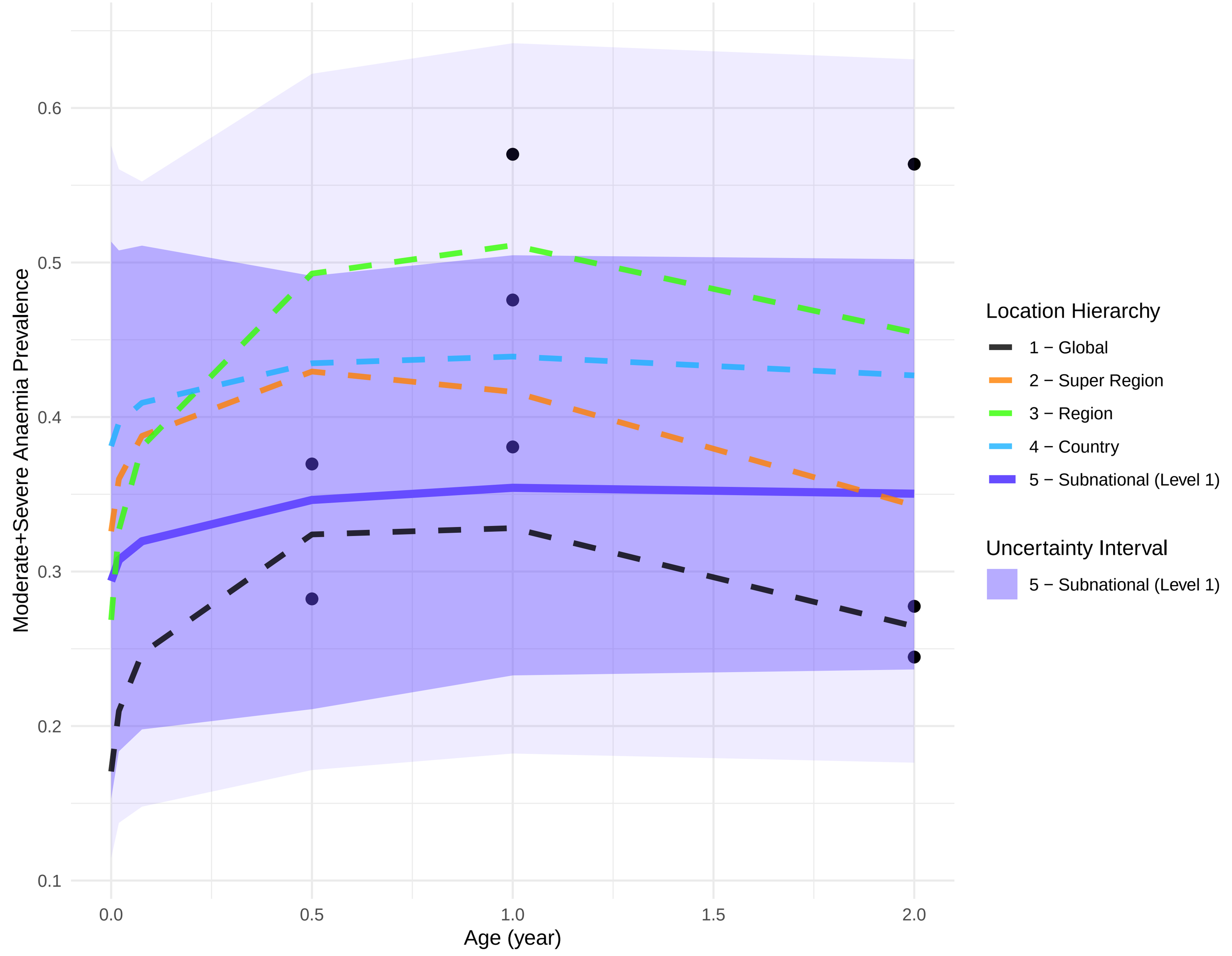

MR−BRT Age/Sex Fits − Males <2 Years Old − Moderate+Severe Anaemia Prevalence − Imo

WHO−2001 Adjustment Method

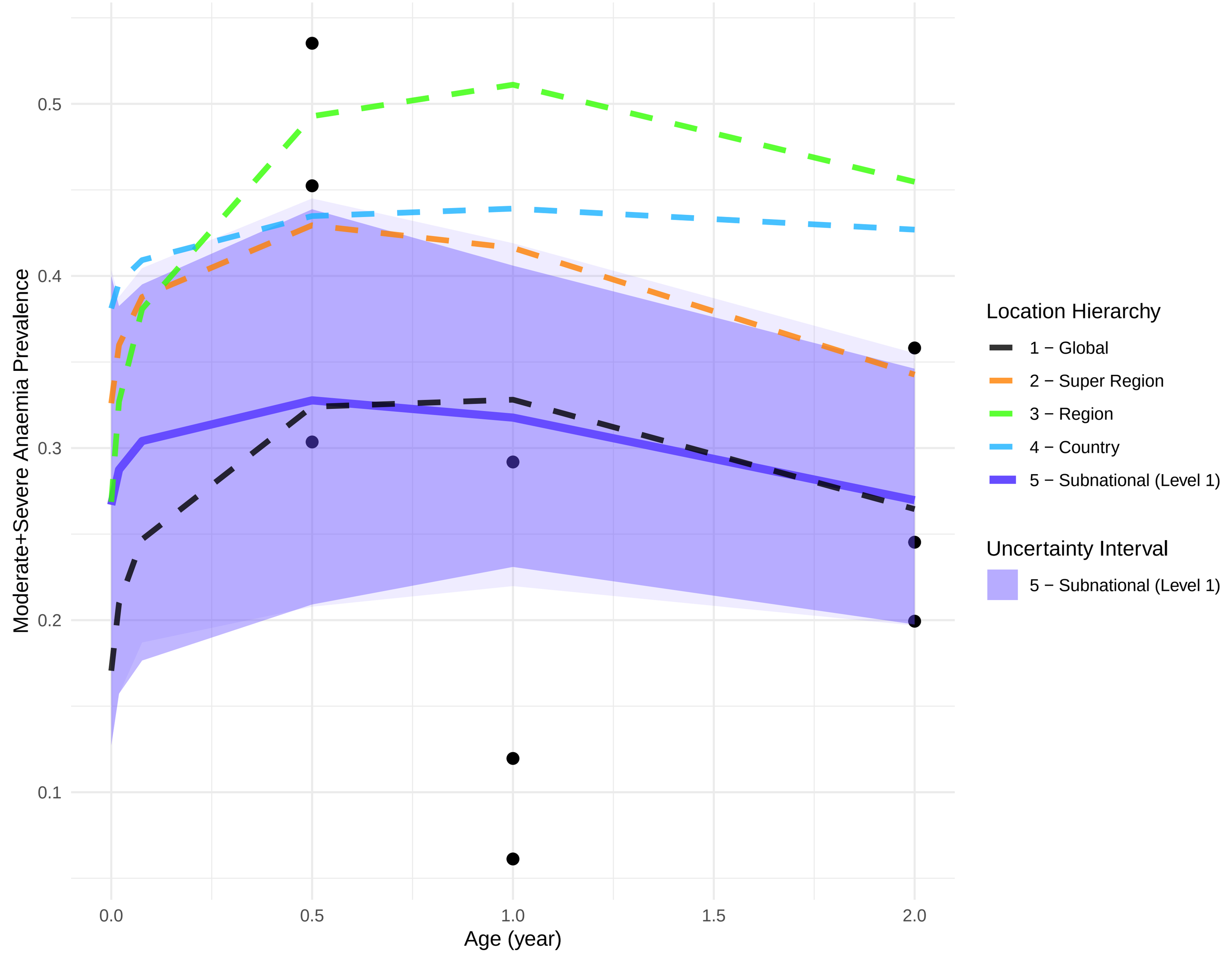

MR−BRT Age/Sex Fits − Males <2 Years Old − Moderate+Severe Anaemia Prevalence − Jigawa

WHO−2001 Adjustment Method

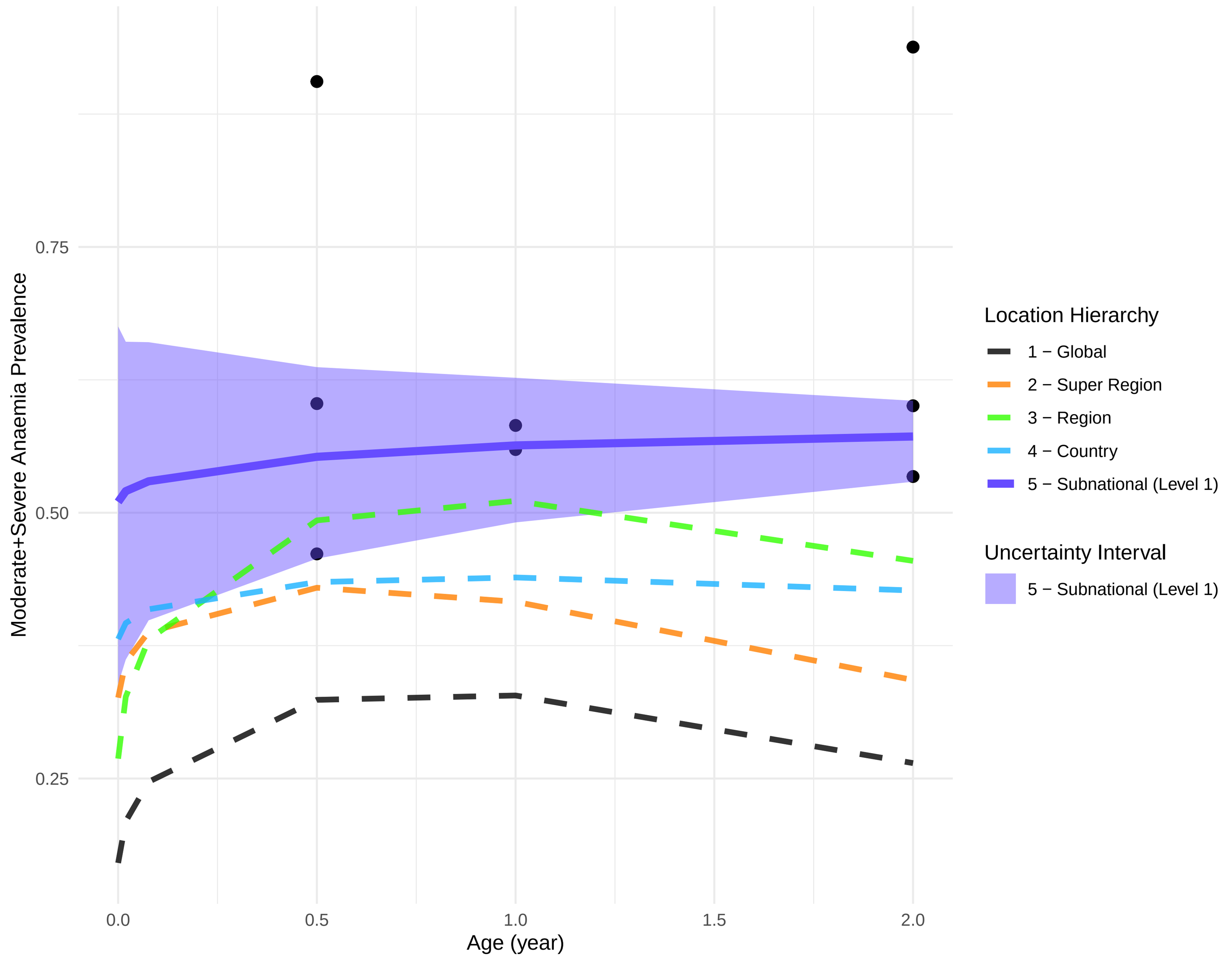

MR−BRT Age/Sex Fits − Males <2 Years Old − Moderate+Severe Anaemia Prevalence − Kaduna

WHO−2001 Adjustment Method

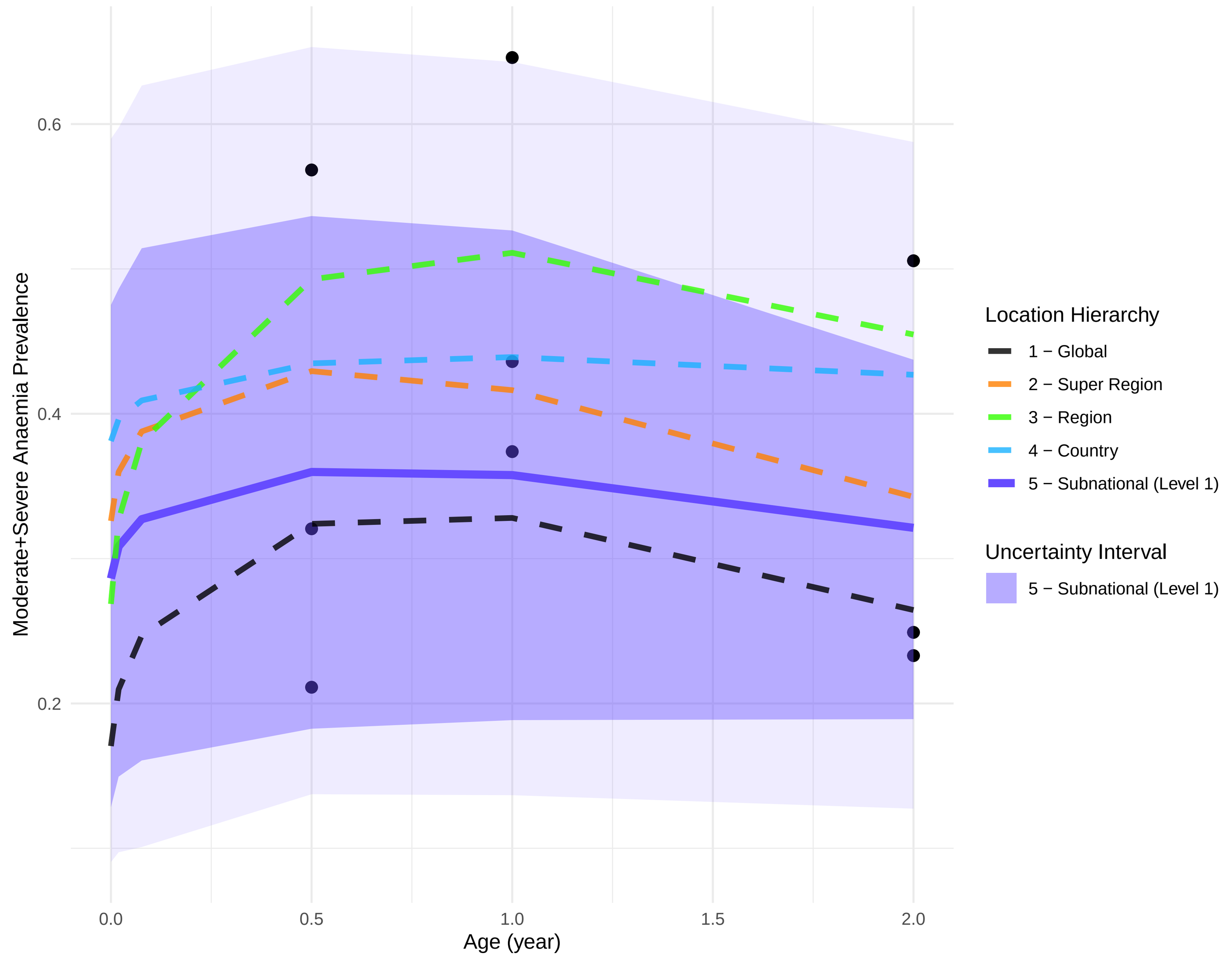

MR−BRT Age/Sex Fits − Males <2 Years Old − Moderate+Severe Anaemia Prevalence − Kano

WHO−2001 Adjustment Method

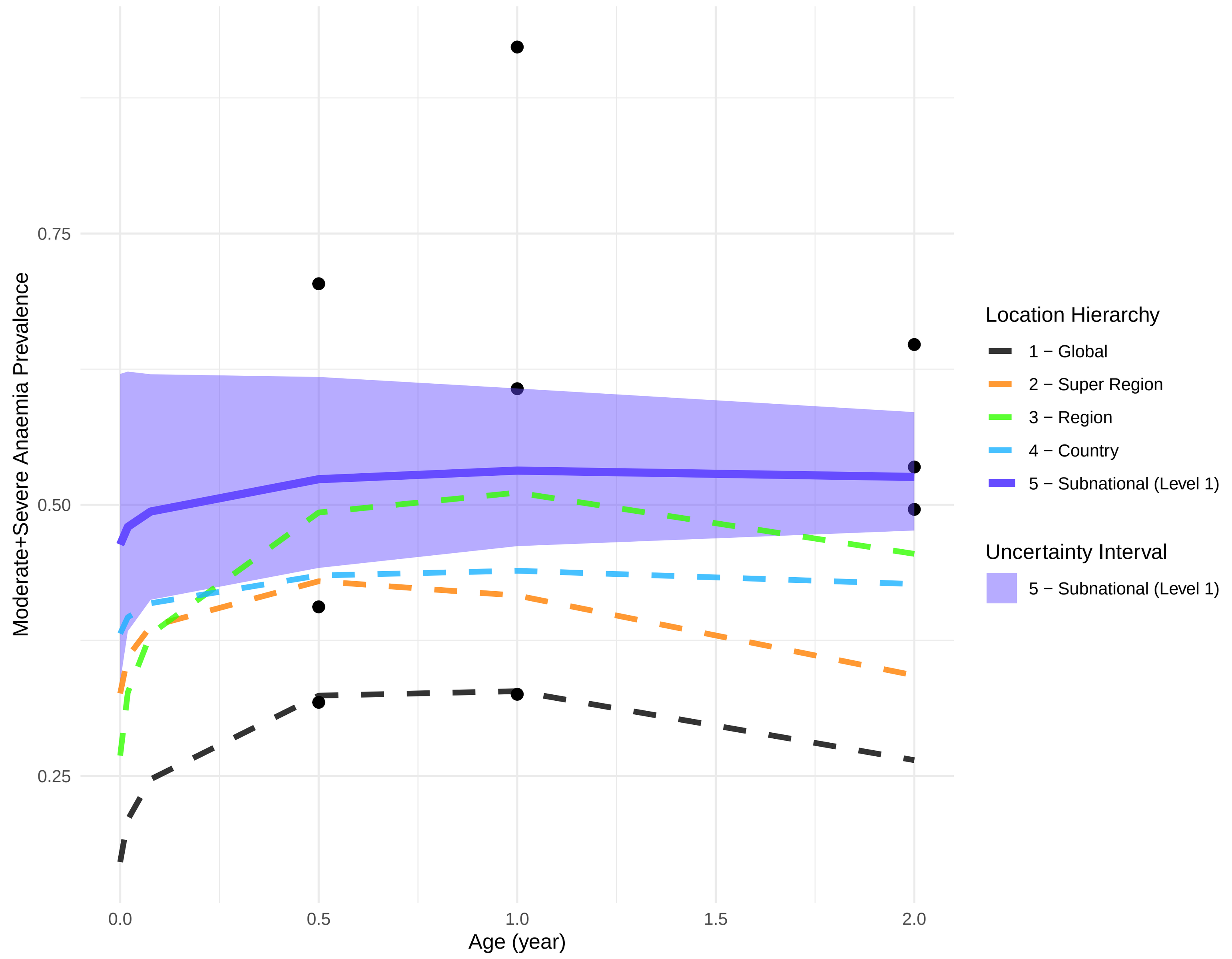

MR−BRT Age/Sex Fits − Males <2 Years Old − Moderate+Severe Anaemia Prevalence − Katsina

WHO−2001 Adjustment Method

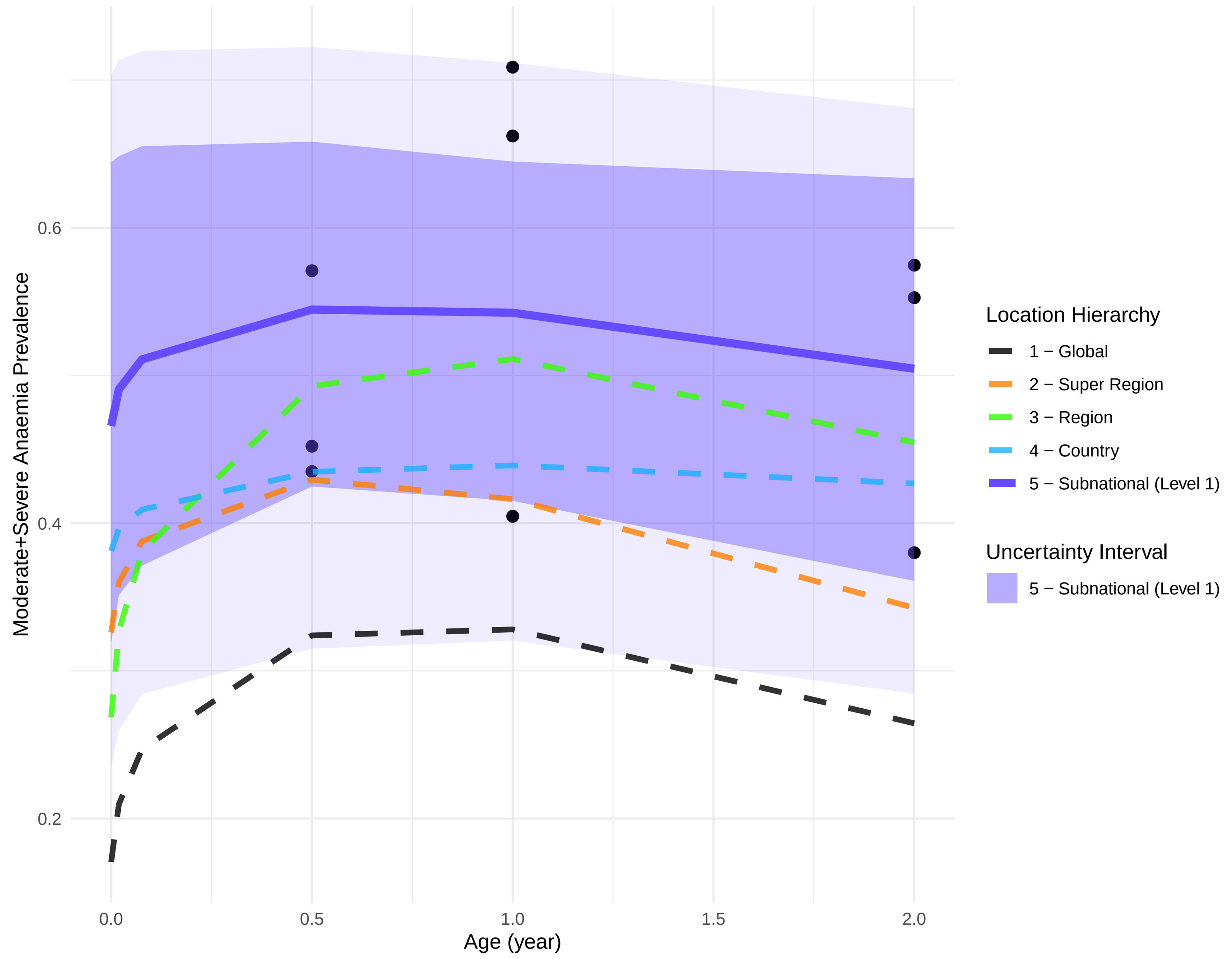

MR−BRT Age/Sex Fits − Males <2 Years Old − Moderate+Severe Anaemia Prevalence − Kebbi

WHO−2001 Adjustment Method

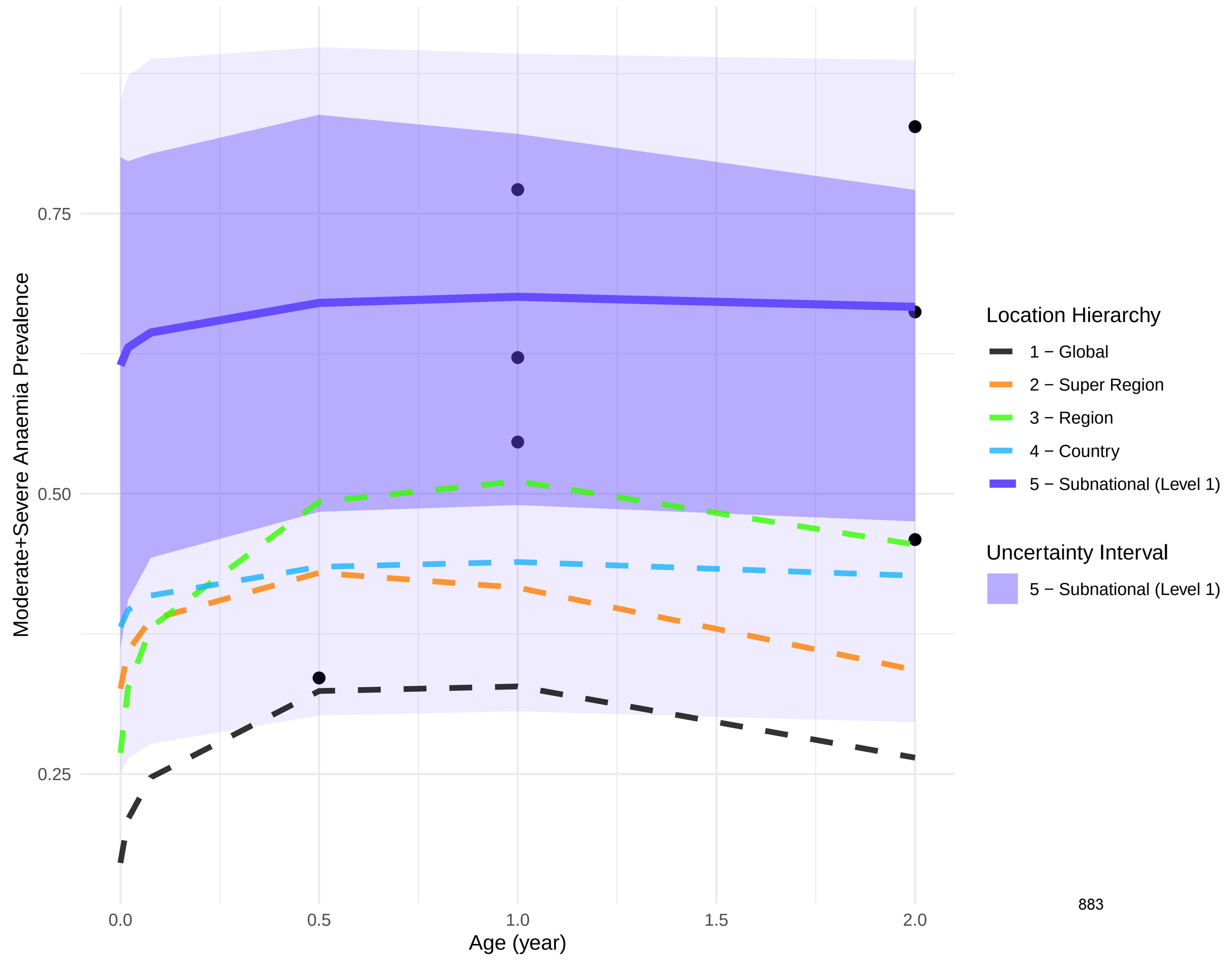

MR−BRT Age/Sex Fits − Males <2 Years Old − Moderate+Severe Anaemia Prevalence − Kogi
WHO−2001 Adjustment Method

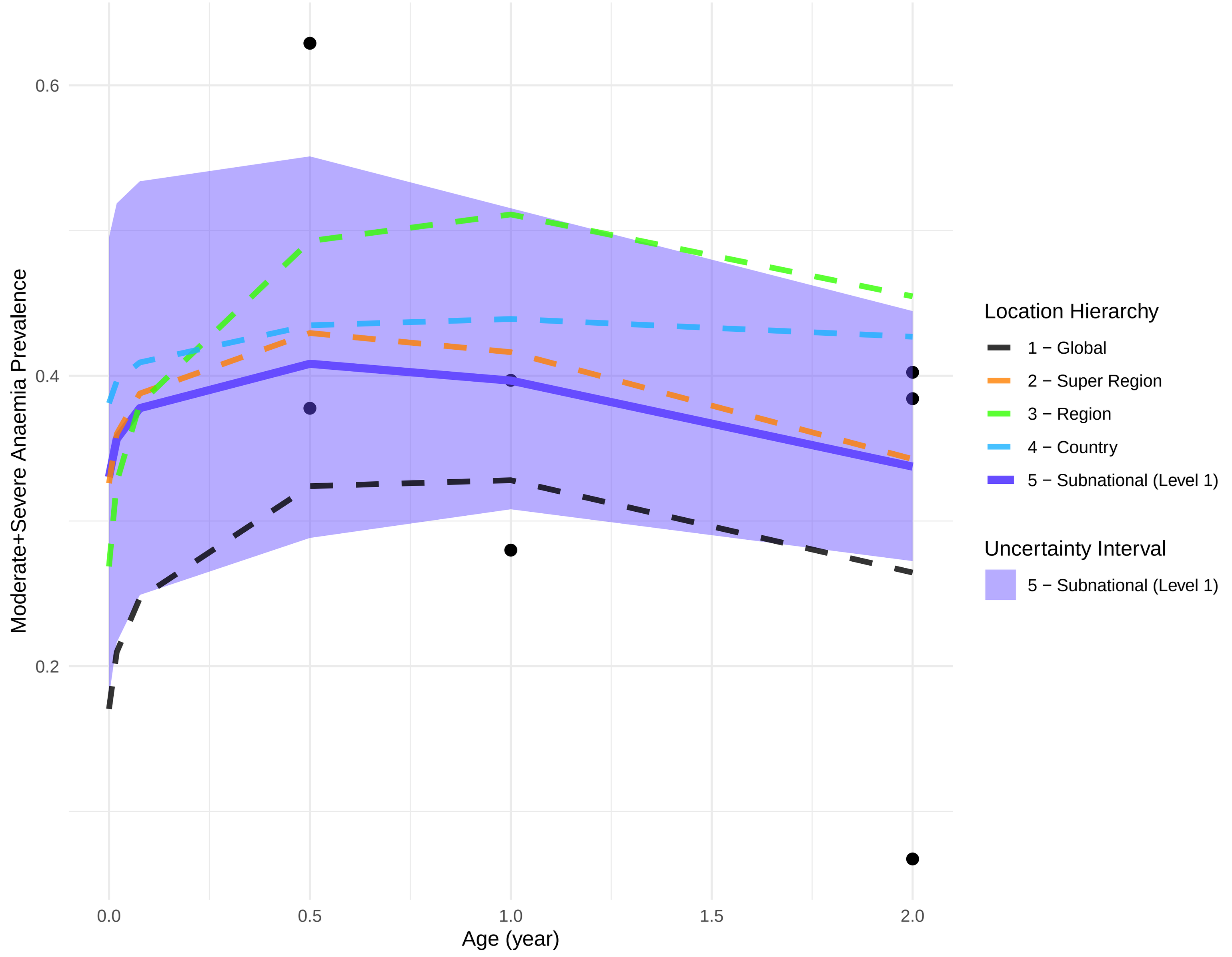

MR−BRT Age/Sex Fits − Males <2 Years Old − Moderate+Severe Anaemia Prevalence − Kwara
WHO−2001 Adjustment Method

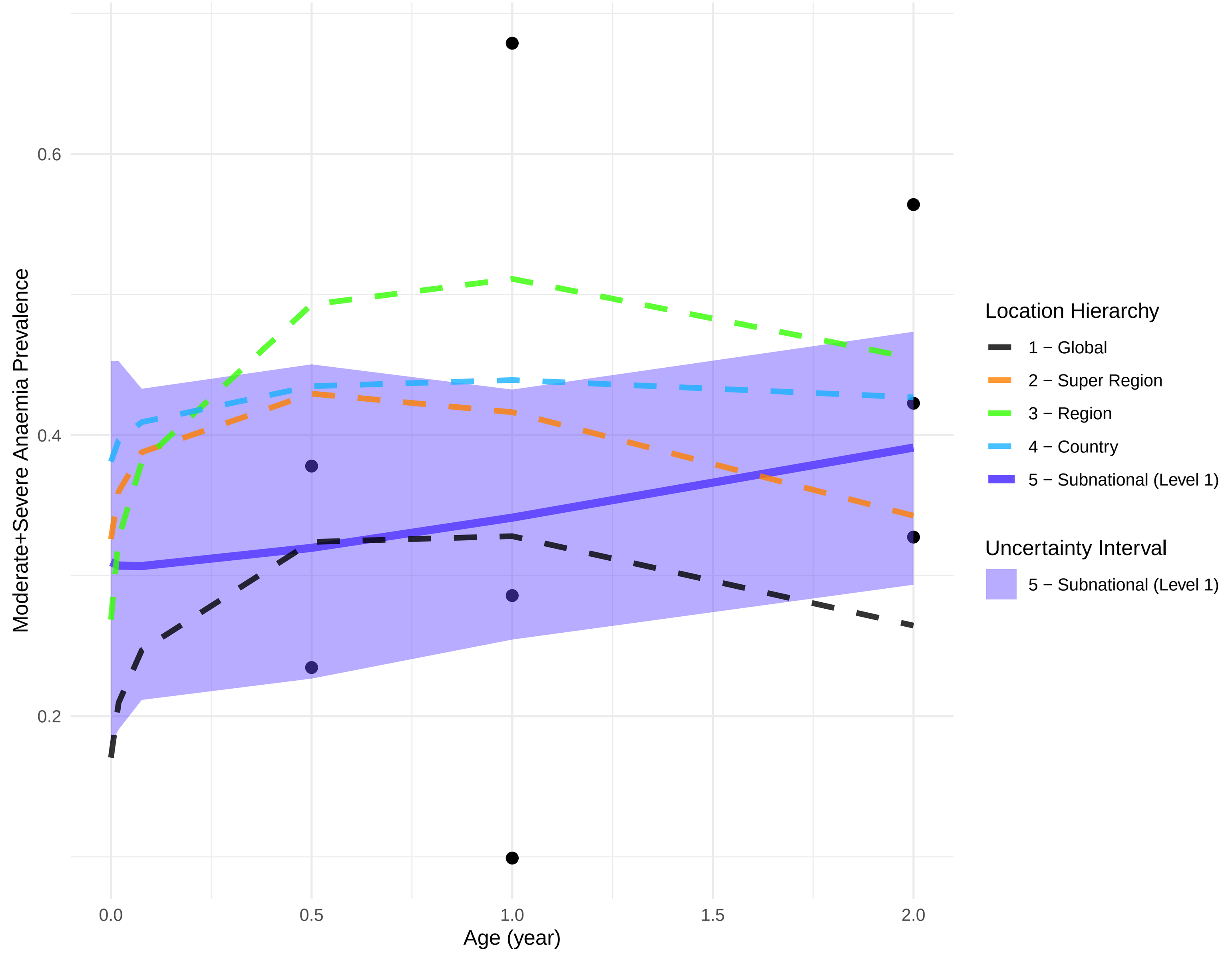

MR−BRT Age/Sex Fits − Males <2 Years Old − Moderate+Severe Anaemia Prevalence − Lagos
WHO−2001 Adjustment Method

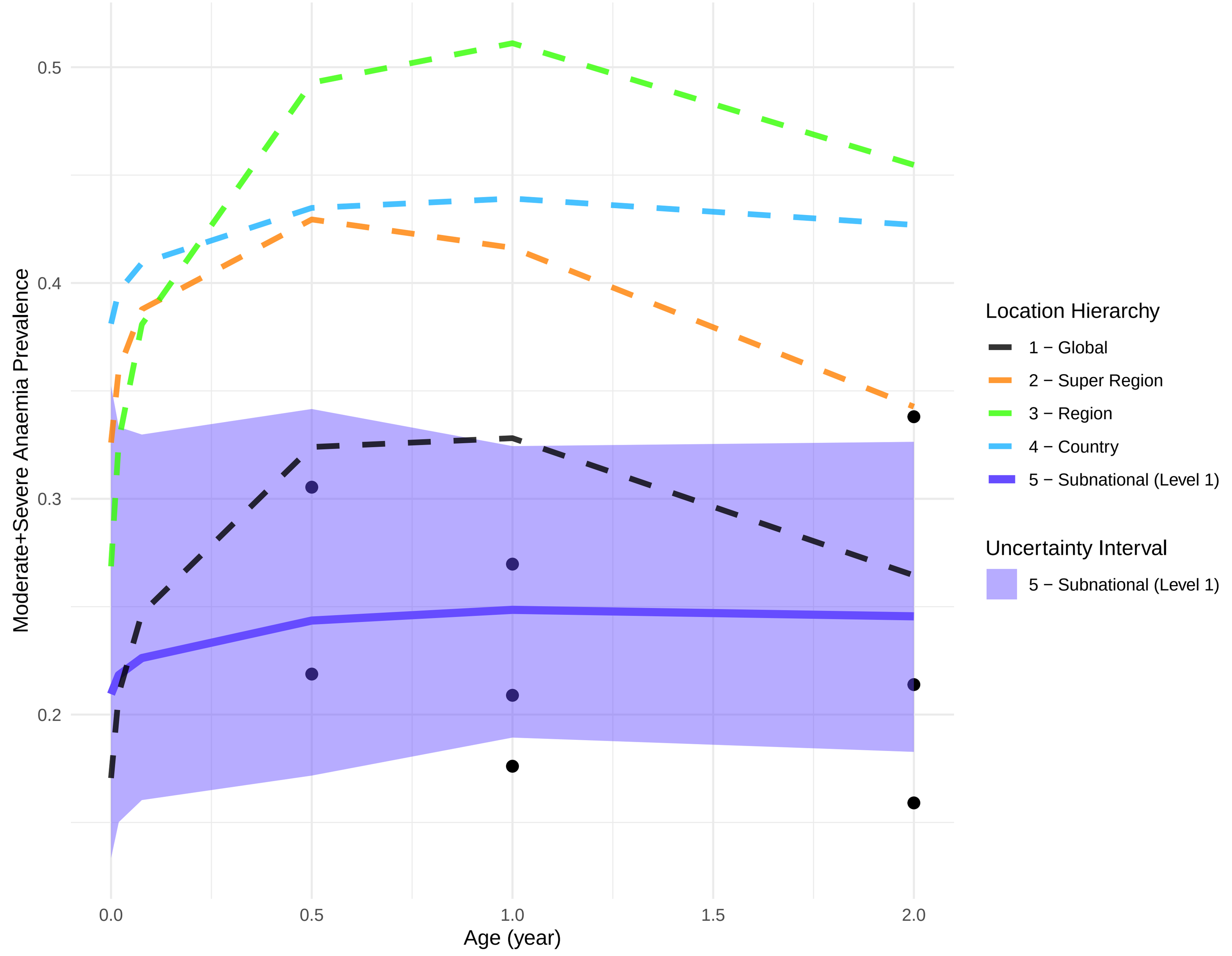

MR−BRT Age/Sex Fits − Males <2 Years Old − Moderate+Severe Anaemia Prevalence − Nasarawa
WHO−2001 Adjustment Method

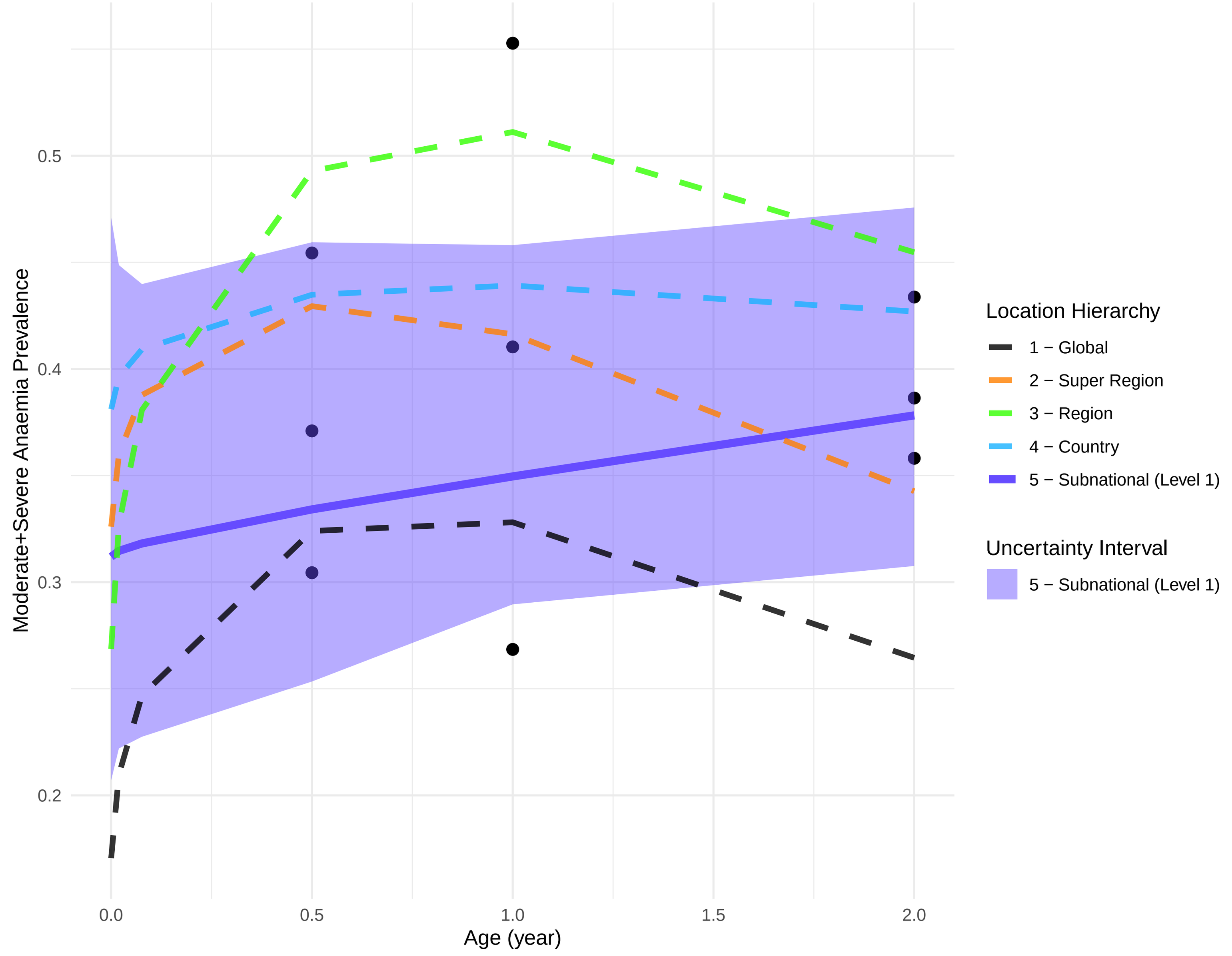

MR−BRT Age/Sex Fits − Males <2 Years Old − Moderate+Severe Anaemia Prevalence − Niger
WHO−2001 Adjustment Method

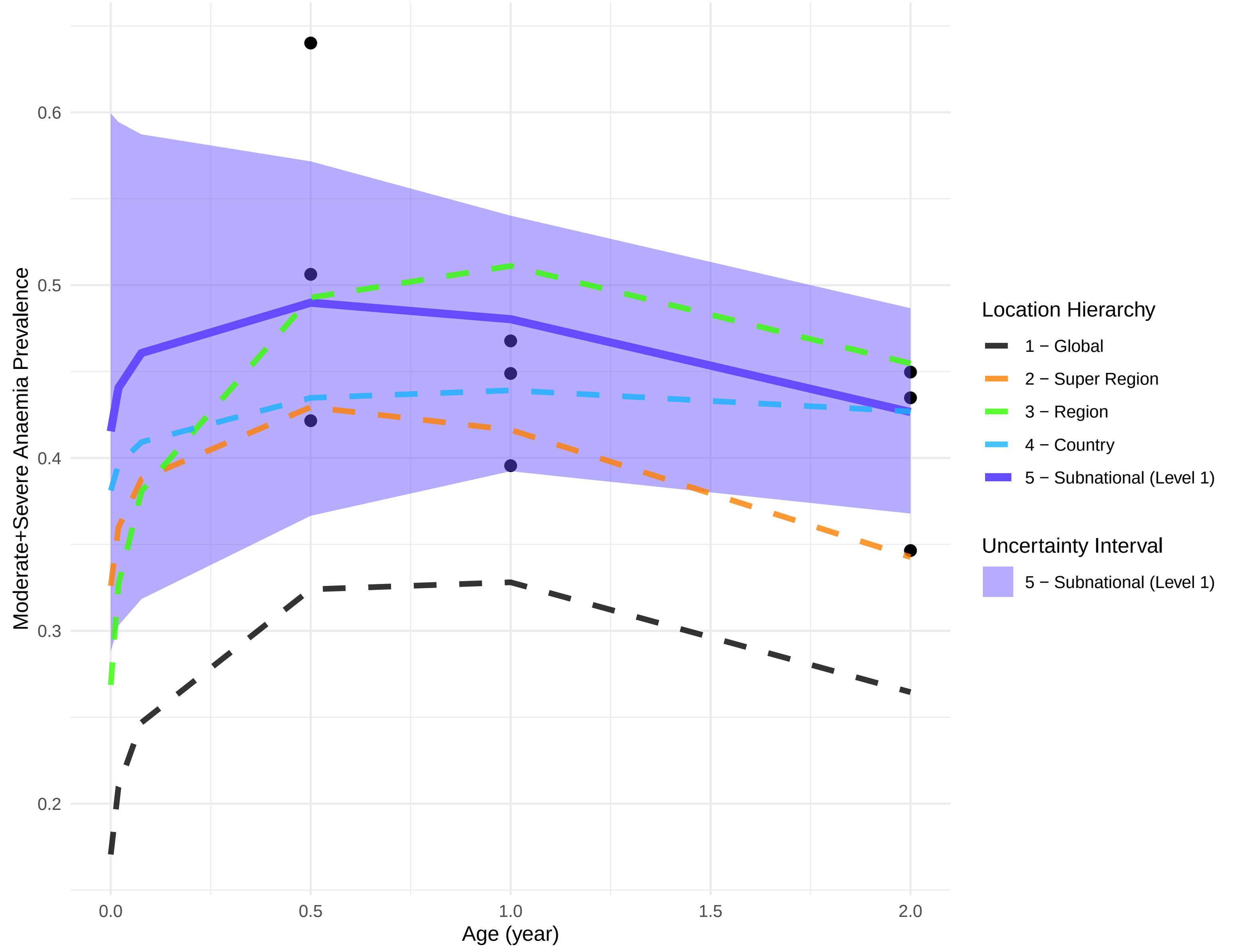

MR−BRT Age/Sex Fits − Males <2 Years Old − Moderate+Severe Anaemia Prevalence − Ogun
WHO−2001 Adjustment Method

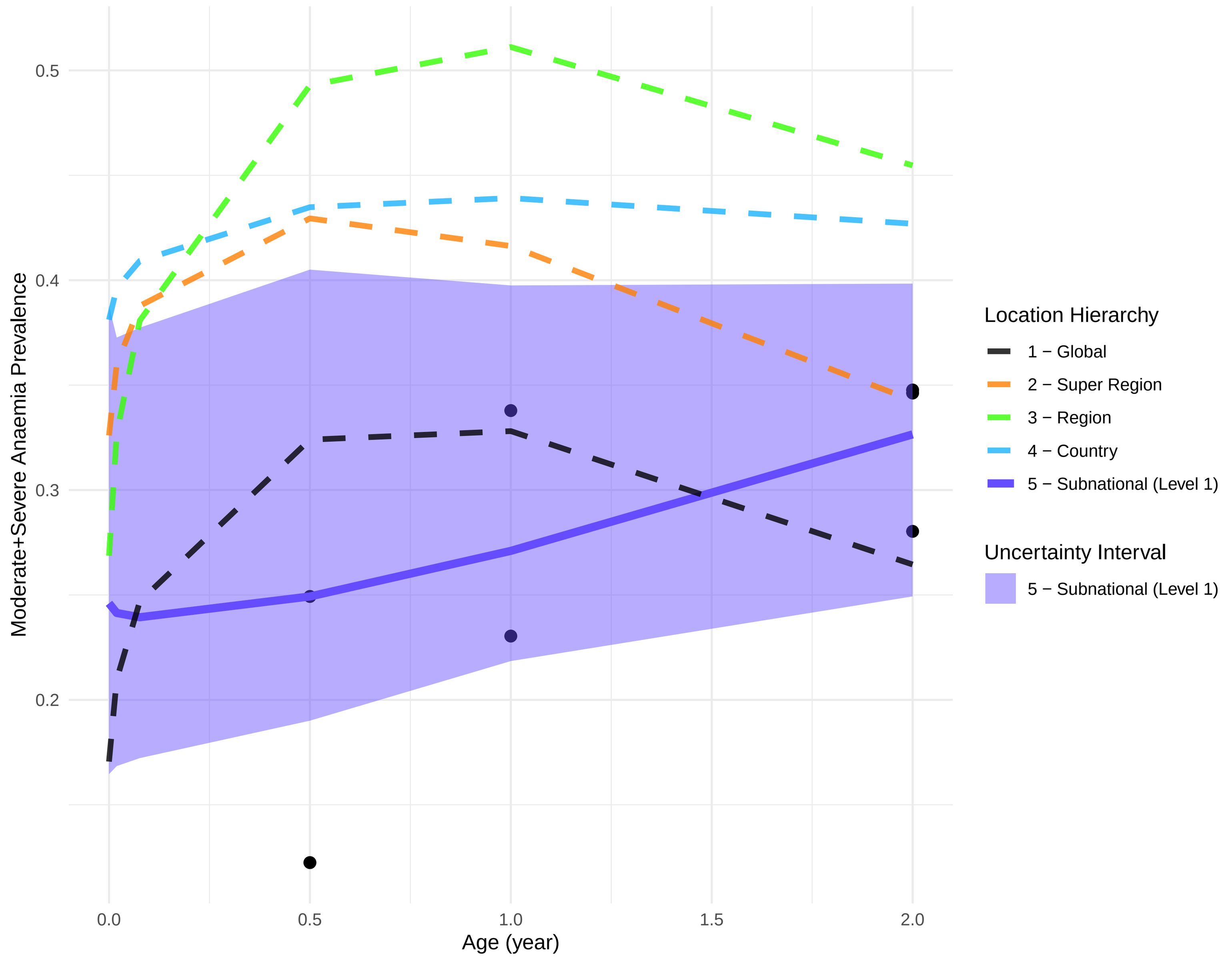

MR−BRT Age/Sex Fits − Males <2 Years Old − Moderate+Severe Anaemia Prevalence − Ondo
WHO−2001 Adjustment Method

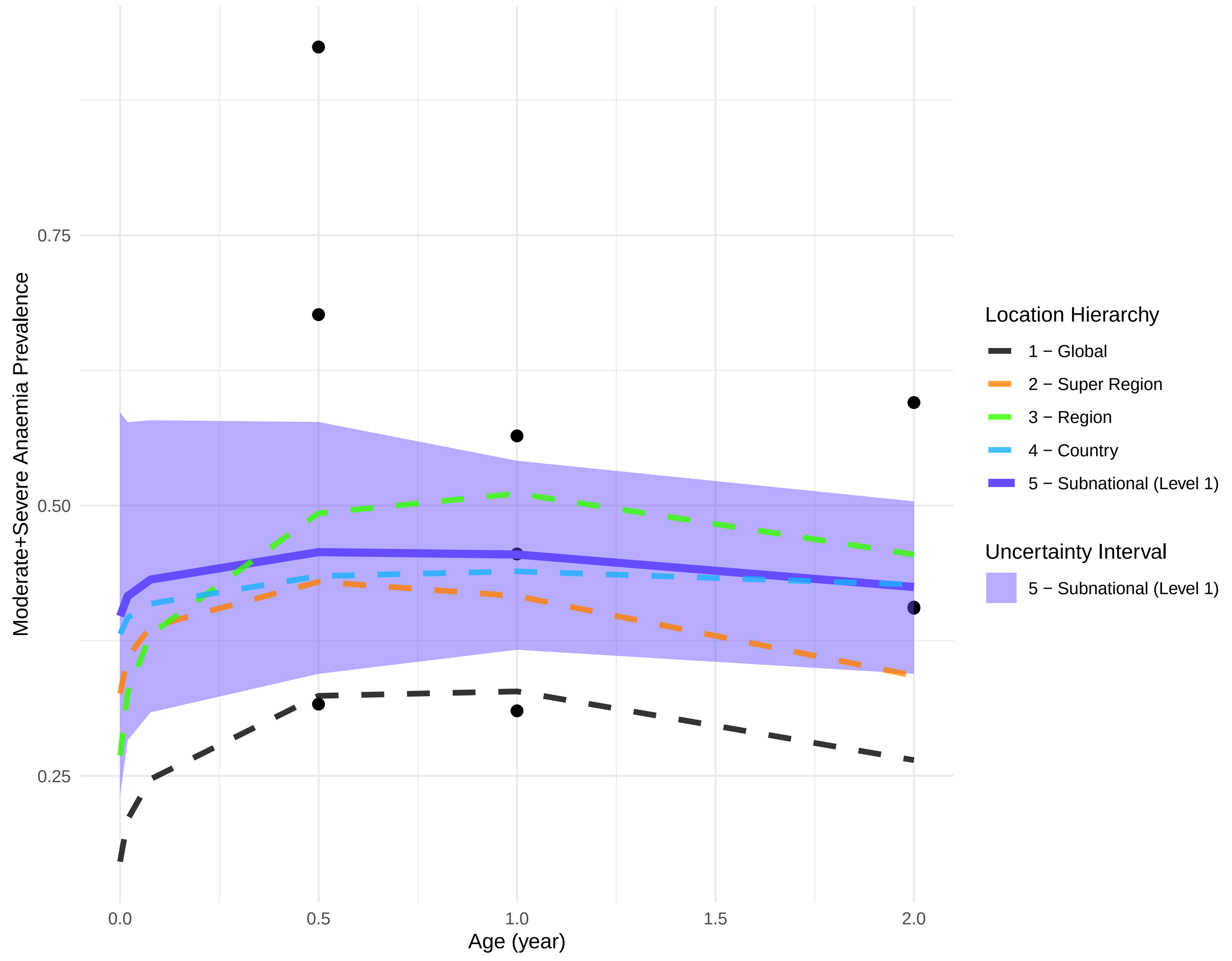

MR−BRT Age/Sex Fits − Males <2 Years Old − Moderate+Severe Anaemia Prevalence − Osun
WHO−2001 Adjustment Method

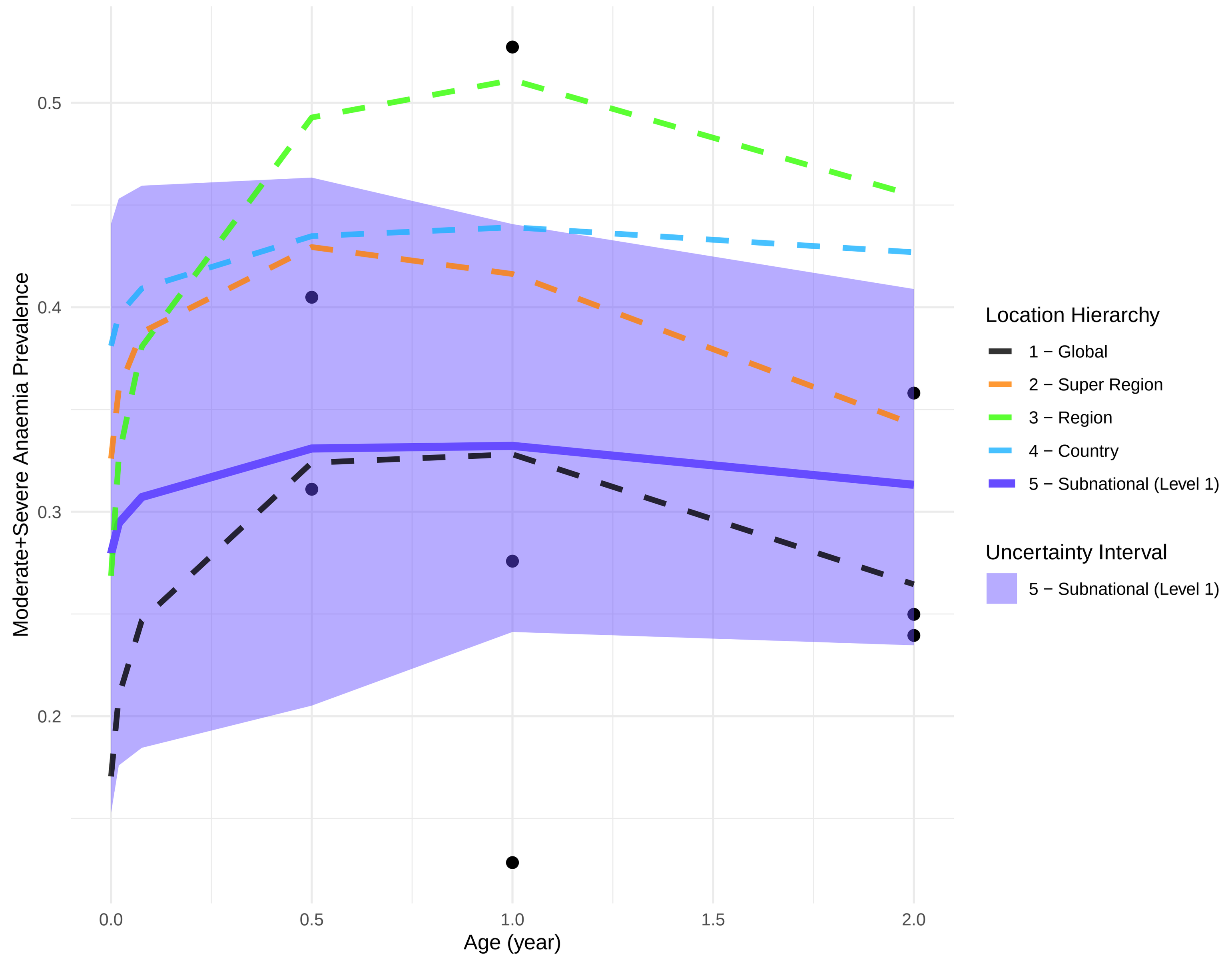

MR−BRT Age/Sex Fits − Males <2 Years Old − Moderate+Severe Anaemia Prevalence − Oyo
WHO−2001 Adjustment Method

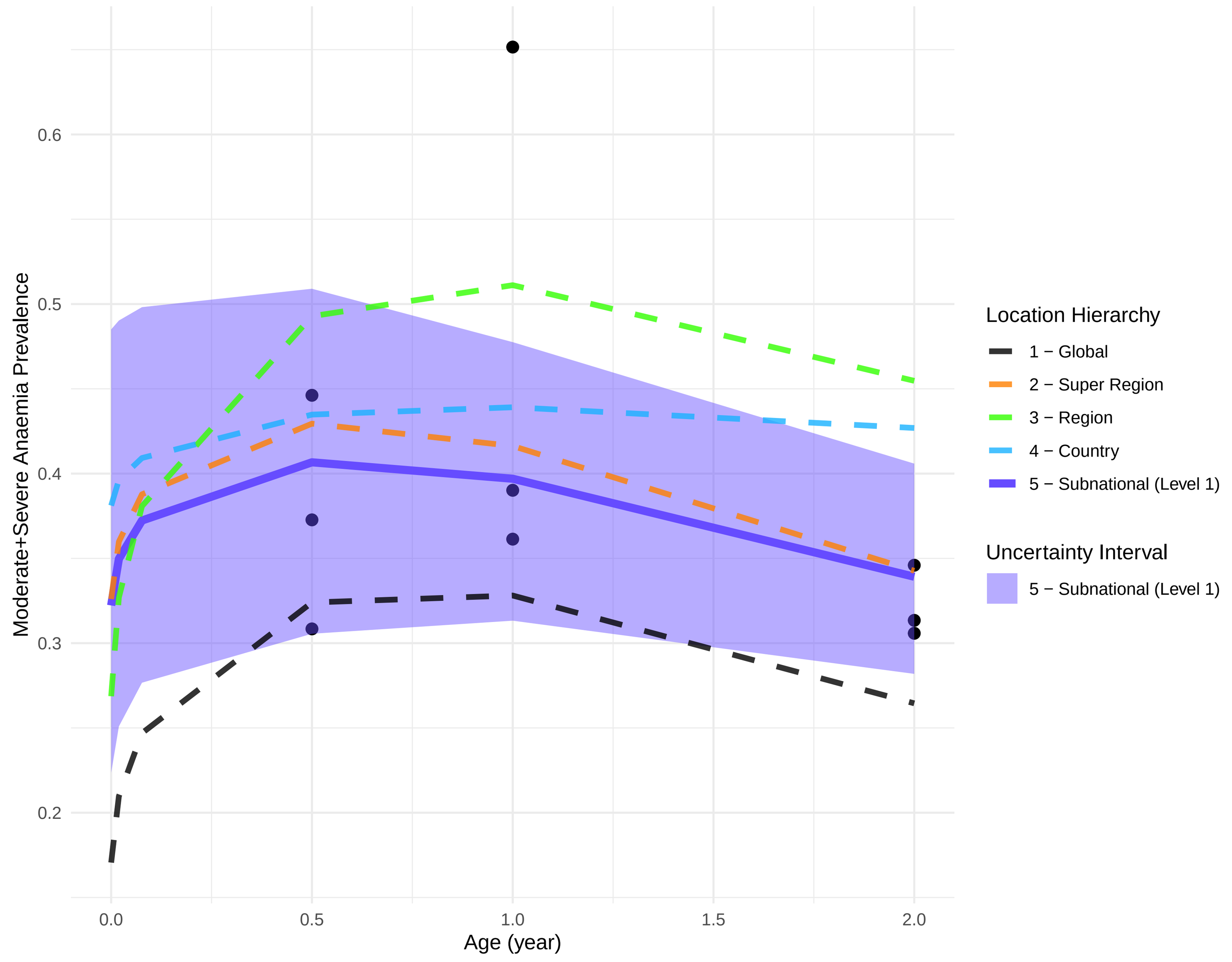

MR−BRT Age/Sex Fits − Males <2 Years Old − Moderate+Severe Anaemia Prevalence − Plateau
WHO−2001 Adjustment Method

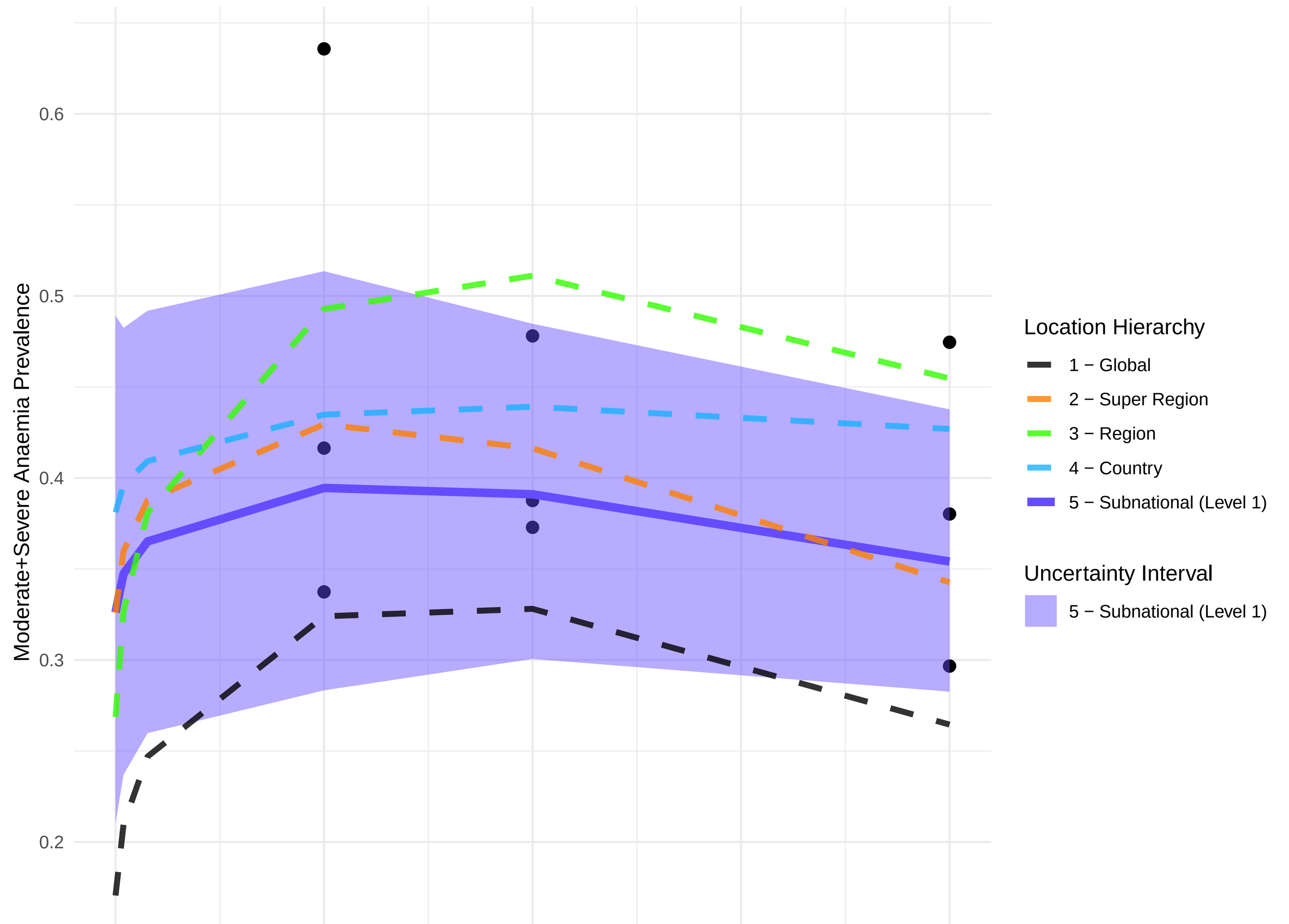

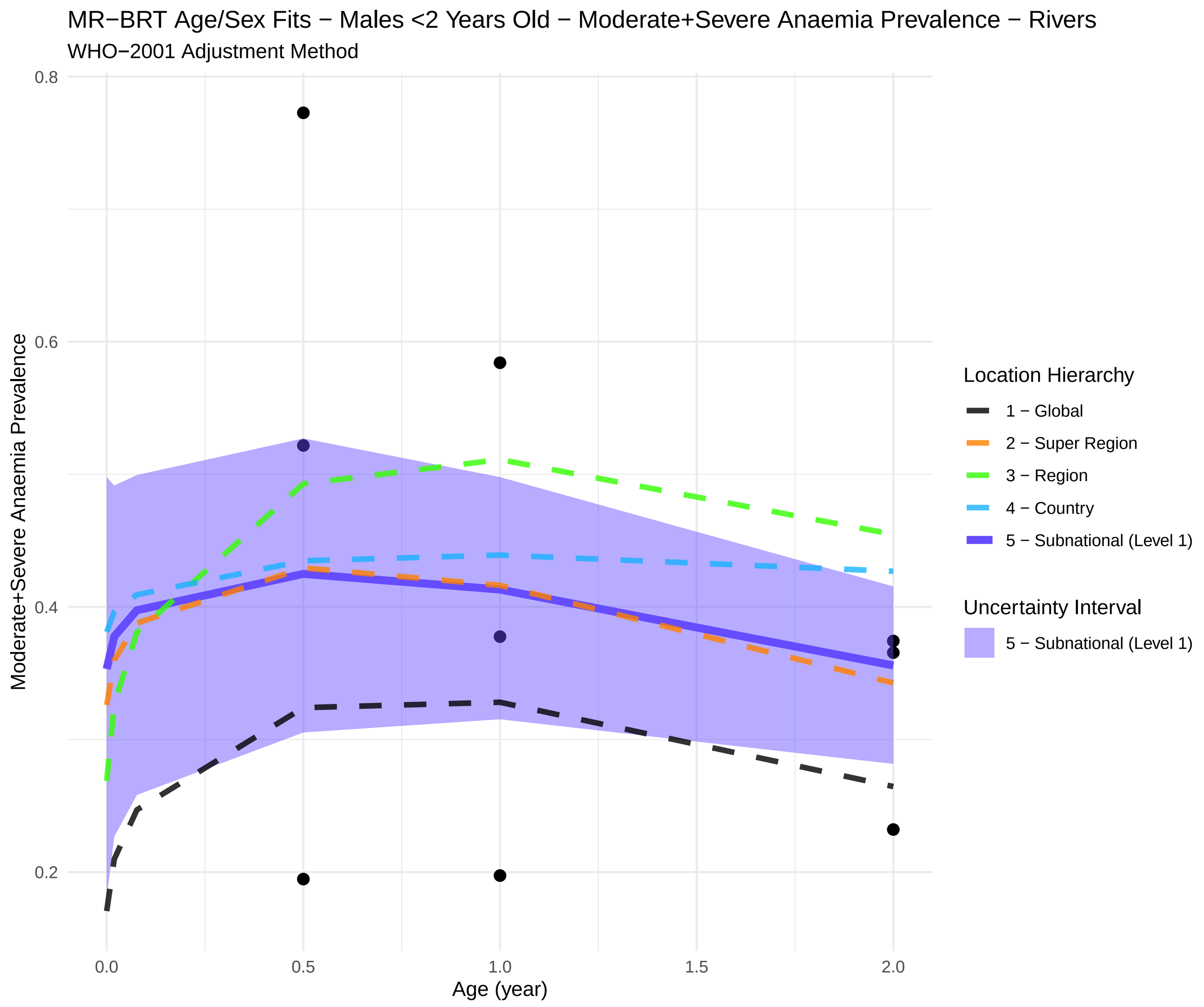
MR−BRT Age/Sex Fits − Males <2 Years Old − Moderate+Severe Anaemia Prevalence − Rivers
WHO−2001 Adjustment Method
Moderate+Severe Anaemia Prevalence
Age (year)
Location Hierarchy
1 − Global
2 − Super Region
3 − Region
4 − Country
5 − Subnational (Level 1)
Uncertainty Interval
5 − Subnational (Level 1)

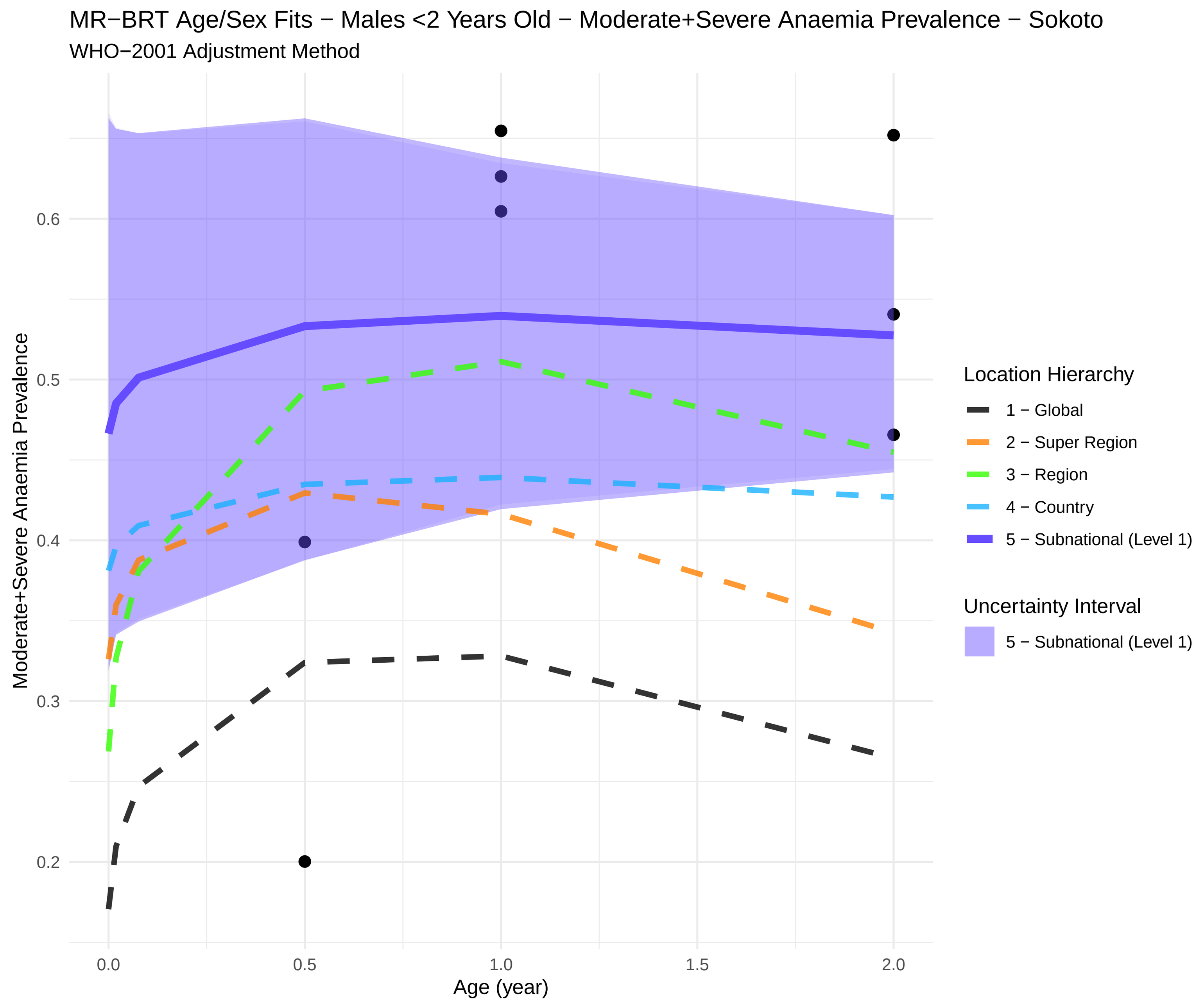
MR−BRT Age/Sex Fits − Males <2 Years Old − Moderate+Severe Anaemia Prevalence − Sokoto
WHO−2001 Adjustment Method
Moderate+Severe Anaemia Prevalence
Age (year)
Location Hierarchy
1 − Global
2 − Super Region
3 − Region
4 − Country
5 − Subnational (Level 1)
Uncertainty Interval
5 − Subnational (Level 1)

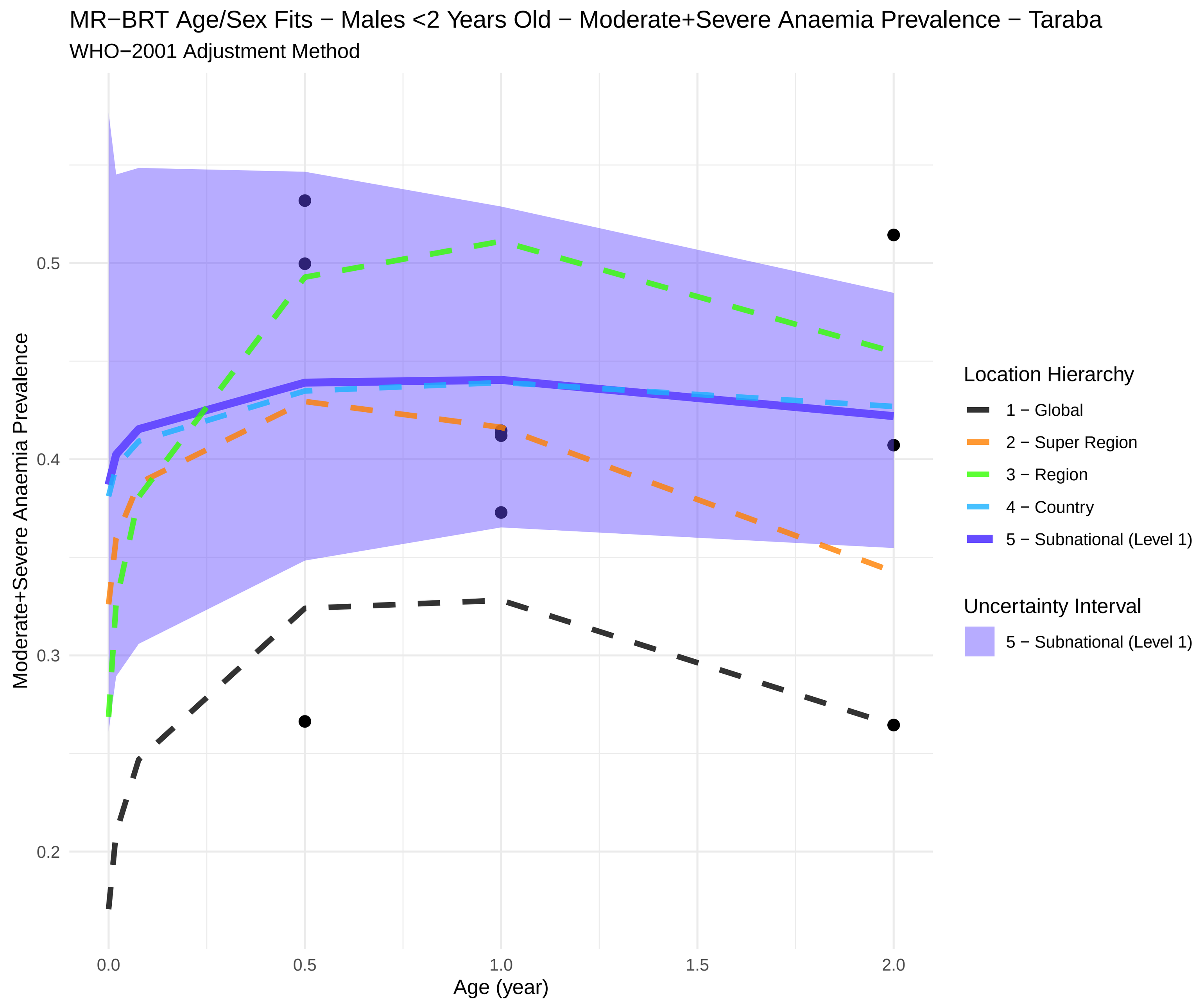
MR−BRT Age/Sex Fits − Males <2 Years Old − Moderate+Severe Anaemia Prevalence − Taraba
WHO−2001 Adjustment Method
Moderate+Severe Anaemia Prevalence
Age (year)
Location Hierarchy
1 − Global
2 − Super Region
3 − Region
4 − Country
5 − Subnational (Level 1)
Uncertainty Interval
5 − Subnational (Level 1)

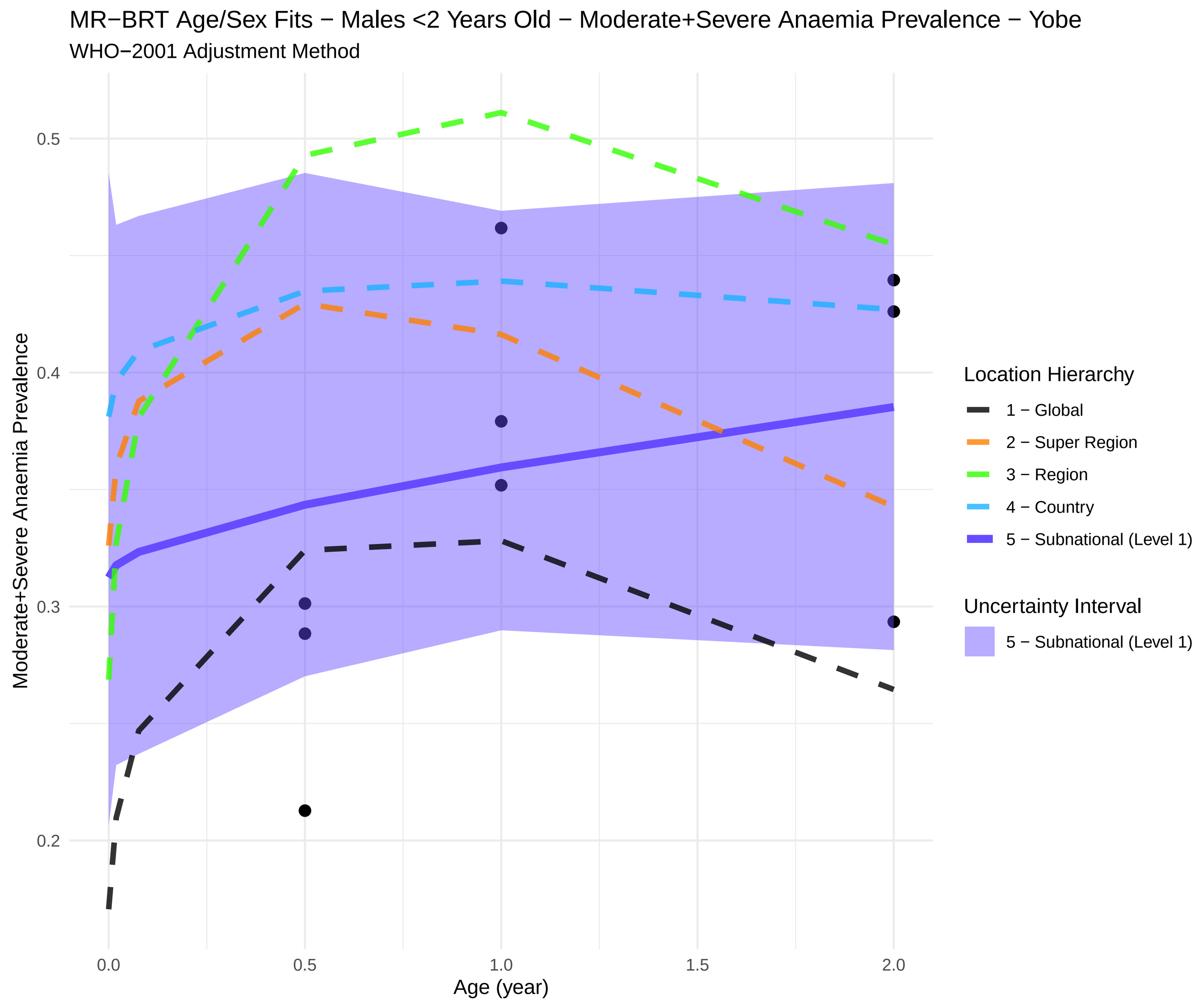
MR−BRT Age/Sex Fits − Males <2 Years Old − Moderate+Severe Anaemia Prevalence − Yobe
WHO−2001 Adjustment Method
Moderate+Severe Anaemia Prevalence
Age (year)
Location Hierarchy
1 − Global
2 − Super Region
3 − Region
4 − Country
5 − Subnational (Level 1)
Uncertainty Interval
5 − Subnational (Level 1)

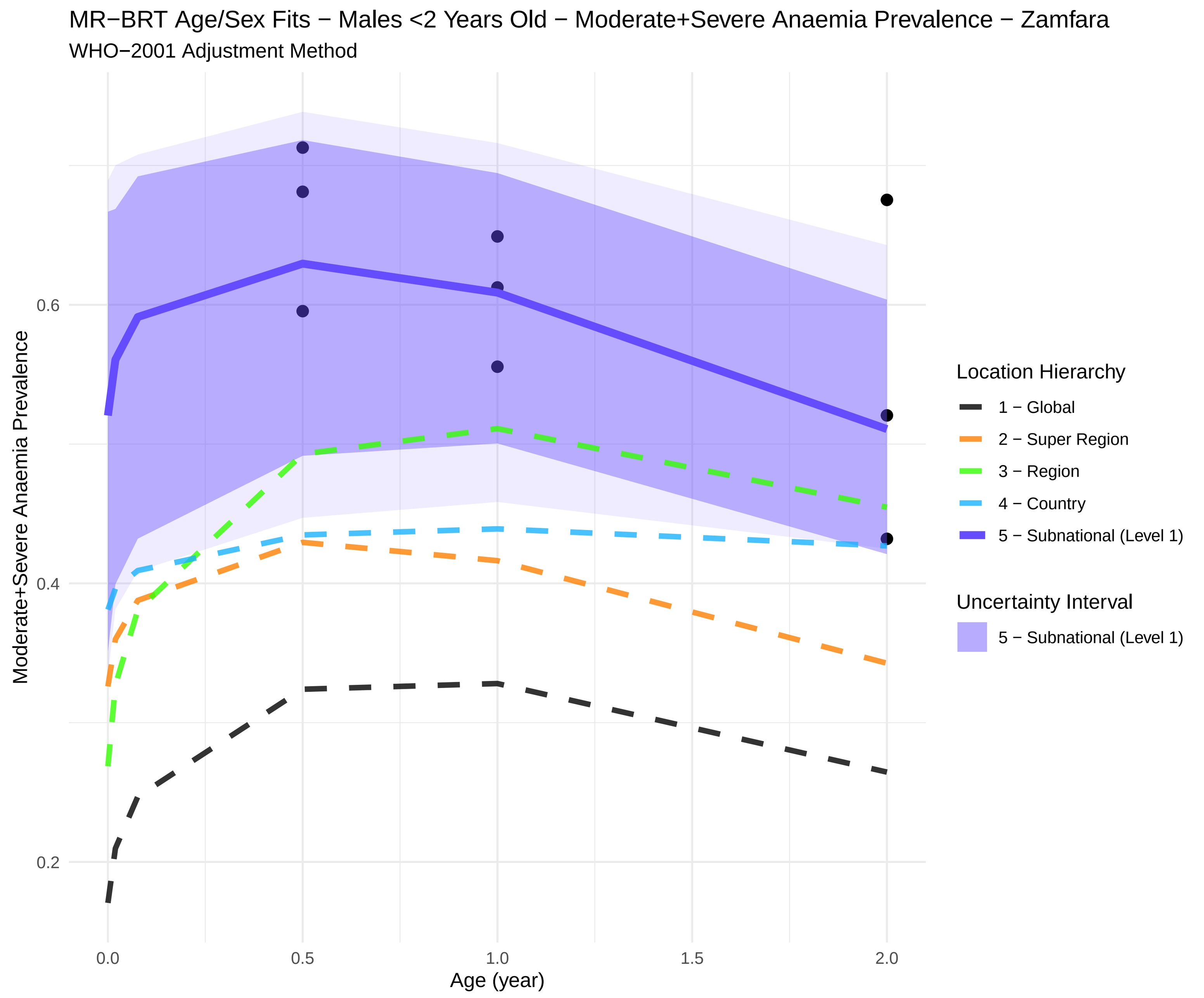
MR−BRT Age/Sex Fits − Males <2 Years Old − Moderate+Severe Anaemia Prevalence − Zamfara
WHO−2001 Adjustment Method
Moderate+Severe Anaemia Prevalence
Age (year)
Location Hierarchy
1 − Global
2 − Super Region
3 − Region
4 − Country
5 − Subnational (Level 1)
Uncertainty Interval
5 − Subnational (Level 1)

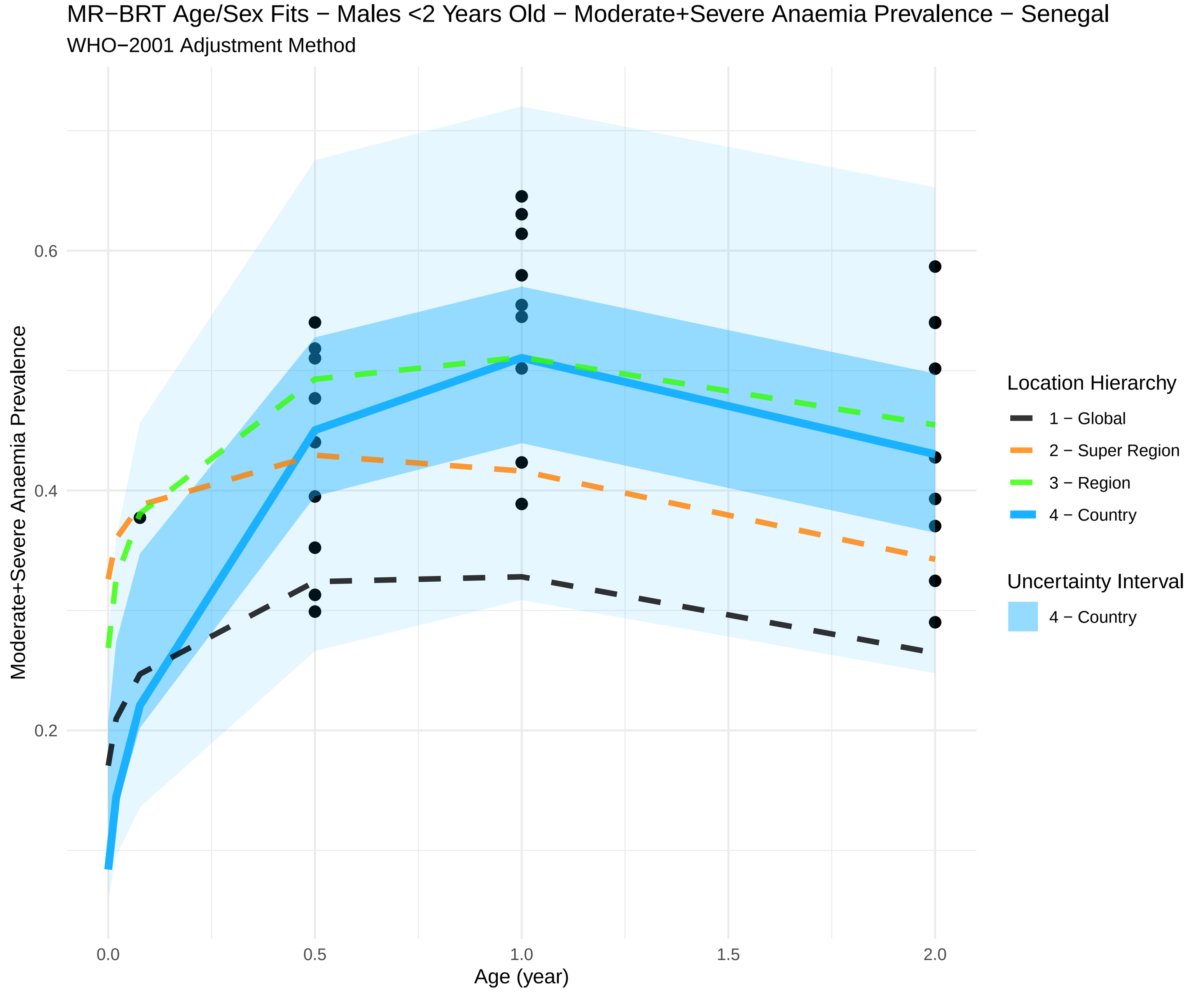
MR−BRT Age/Sex Fits − Males <2 Years Old − Moderate+Severe Anaemia Prevalence − Senegal
WHO−2001 Adjustment Method
Moderate+Severe Anaemia Prevalence
Age (year)
Location Hierarchy
1 − Global
2 − Super Region
3 − Region
4 − Country
Uncertainty Interval
4 − Country

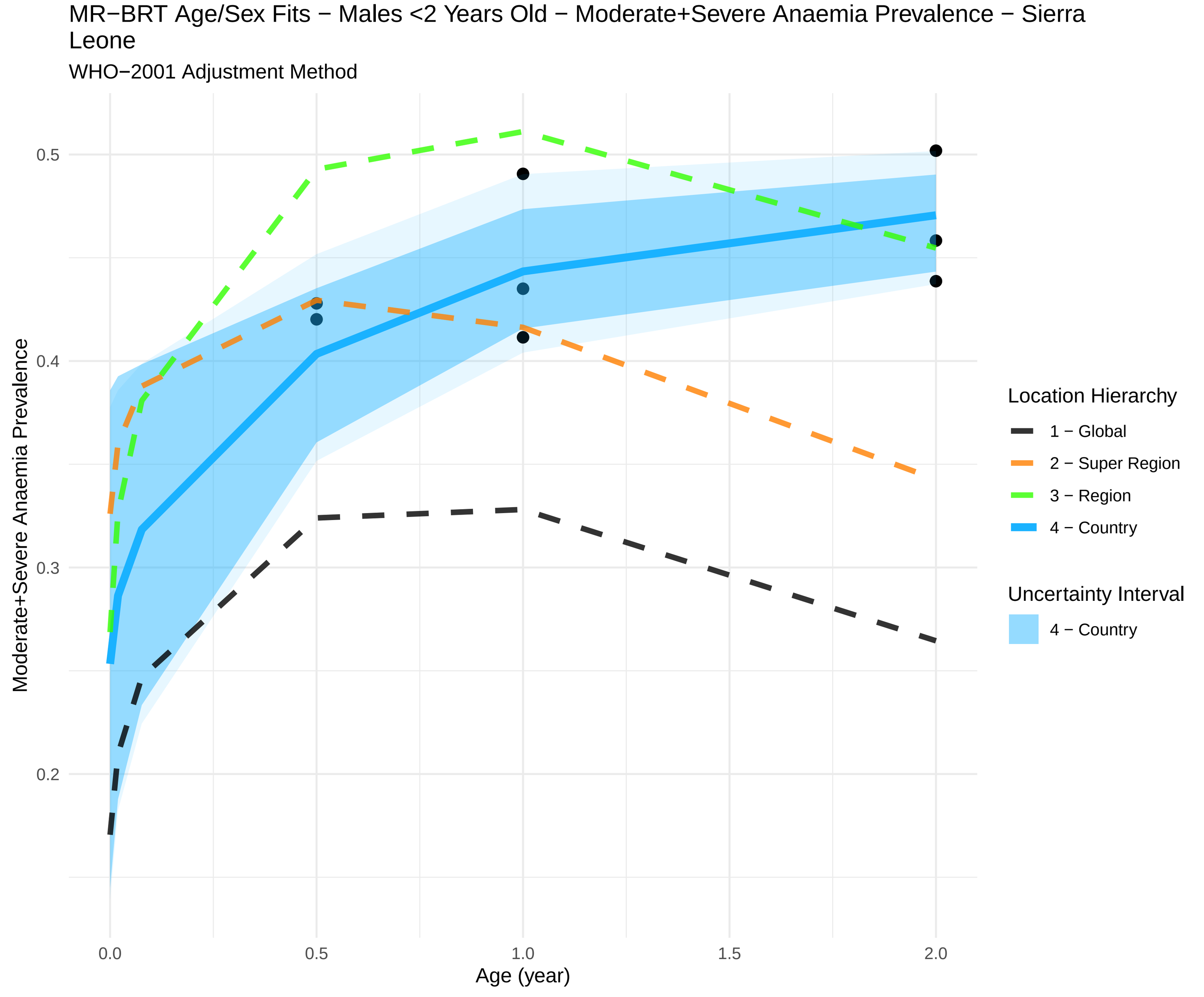
MR−BRT Age/Sex Fits − Males <2 Years Old − Moderate+Severe Anaemia Prevalence − Sierra Leone
WHO−2001 Adjustment Method
Moderate+Severe Anaemia Prevalence
Age (year)
Location Hierarchy
1 − Global
2 − Super Region
3 − Region
4 − Country
Uncertainty Interval
4 − Country

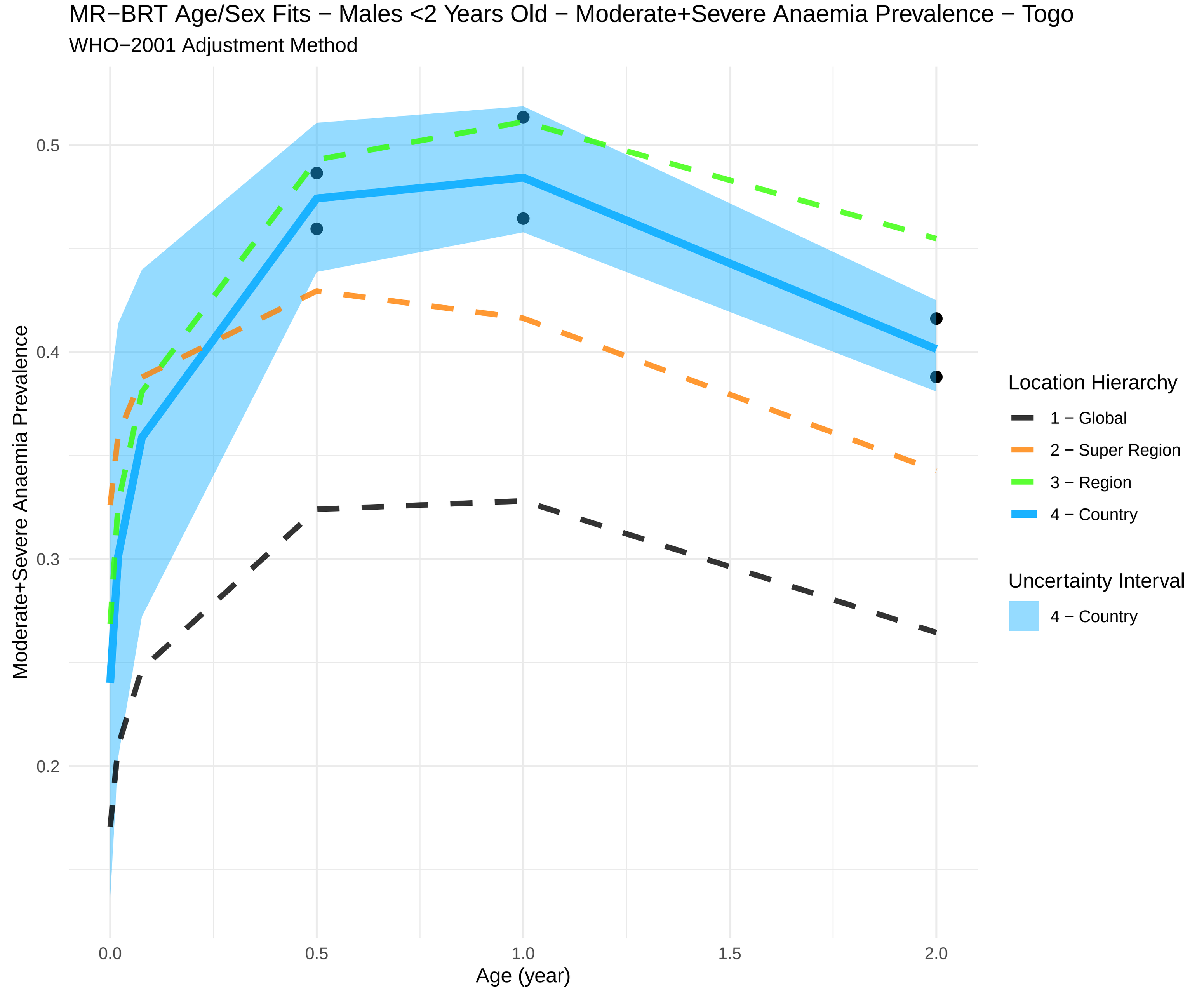
MR−BRT Age/Sex Fits − Males <2 Years Old − Moderate+Severe Anaemia Prevalence − Togo
WHO−2001 Adjustment Method
Moderate+Severe Anaemia Prevalence
Age (year)
Location Hierarchy
1 − Global
2 − Super Region
3 − Region
4 − Country
Uncertainty Interval
4 − Country

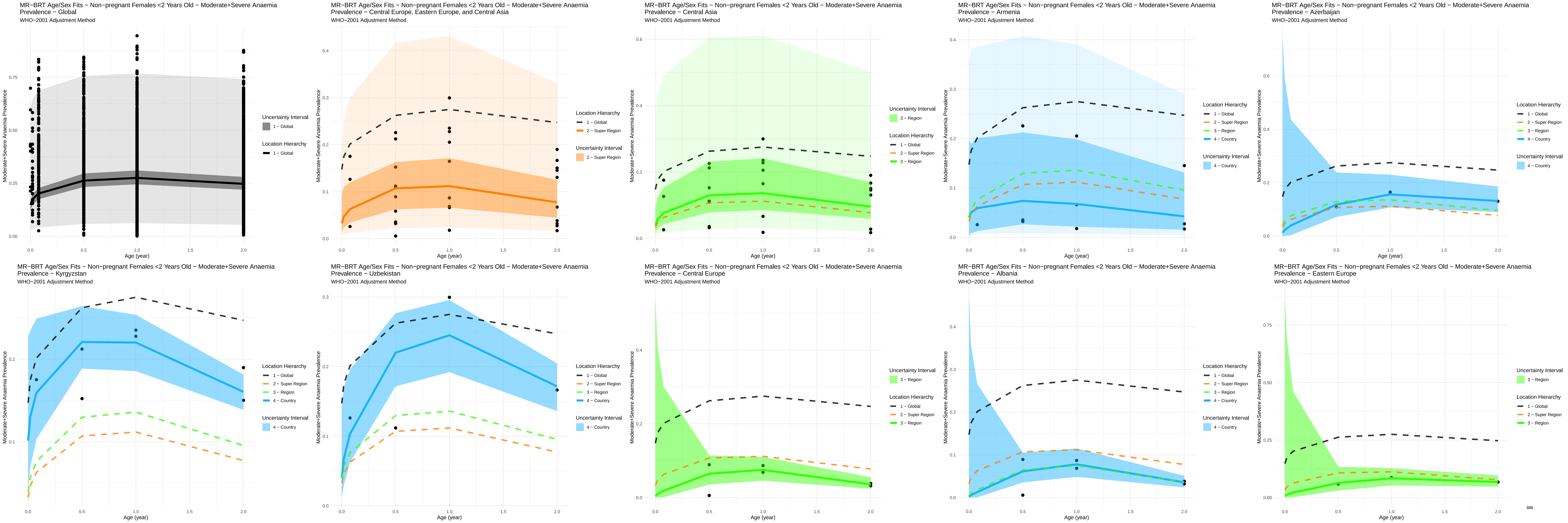
MR−BRT Age/Sex Fits − Non−pregnant Females <2 Years Old − Moderate+Severe Anaemia Prevalence − Global
WHO−2001 Adjustment Method
Uncertainty Interval
1 − Global
Location Hierarchy
1 − Global
MR−BRT Age/Sex Fits − Non−pregnant Females <2 Years Old − Moderate+Severe Anaemia Prevalence − Central Europe, Eastern Europe, and Central Asia
WHO−2001 Adjustment Method
Location Hierarchy
1 − Global
2 − Super Region
Uncertainty Interval
2 − Super Region
MR−BRT Age/Sex Fits − Non−pregnant Females <2 Years Old − Moderate+Severe Anaemia Prevalence − Central Asia
WHO−2001 Adjustment Method
Uncertainty Interval
3 − Region
Location Hierarchy
1 − Global
2 − Super Region
3 − Region
MR−BRT Age/Sex Fits − Non−pregnant Females <2 Years Old − Moderate+Severe Anaemia Prevalence − Armenia
WHO−2001 Adjustment Method
Location Hierarchy
1 − Global
2 − Super Region
3 − Region
4 − Country
Uncertainty Interval
4 − Country
MR−BRT Age/Sex Fits − Non−pregnant Females <2 Years Old − Moderate+Severe Anaemia Prevalence − Azerbaijan
WHO−2001 Adjustment Method
Location Hierarchy
1 − Global
2 − Super Region
3 − Region
4 − Country
Uncertainty Interval
4 − Country
MR−BRT Age/Sex Fits − Non−pregnant Females <2 Years Old − Moderate+Severe Anaemia Prevalence − Kyrgyzstan
WHO−2001 Adjustment Method
Location Hierarchy
1 − Global
2 − Super Region
3 − Region
4 − Country
Uncertainty Interval
4 − Country
MR−BRT Age/Sex Fits − Non−pregnant Females <2 Years Old − Moderate+Severe Anaemia Prevalence − Uzbekistan
WHO−2001 Adjustment Method
Location Hierarchy
1 − Global
2 − Super Region
3 − Region
4 − Country
Uncertainty Interval
4 − Country
MR−BRT Age/Sex Fits − Non−pregnant Females <2 Years Old − Moderate+Severe Anaemia Prevalence − Central Europe
WHO−2001 Adjustment Method
Uncertainty Interval
3 − Region
Location Hierarchy
1 − Global
2 − Super Region
3 − Region
MR−BRT Age/Sex Fits − Non−pregnant Females <2 Years Old − Moderate+Severe Anaemia Prevalence − Albania
WHO−2001 Adjustment Method
Location Hierarchy
1 − Global
2 − Super Region
3 − Region
4 − Country
Uncertainty Interval
4 − Country
MR−BRT Age/Sex Fits − Non−pregnant Females <2 Years Old − Moderate+Severe Anaemia Prevalence − Eastern Europe
WHO−2001 Adjustment Method
Uncertainty Interval
3 − Region
Location Hierarchy
1 − Global
2 − Super Region
3 − Region
Moderate+Severe Anaemia Prevalence
Age (year)

MR−BRT Age/Sex Fits − Non−pregnant Females <2 Years Old − Moderate+Severe Anaemia Prevalence − Republic of Moldova
WHO−2001 Adjustment Method

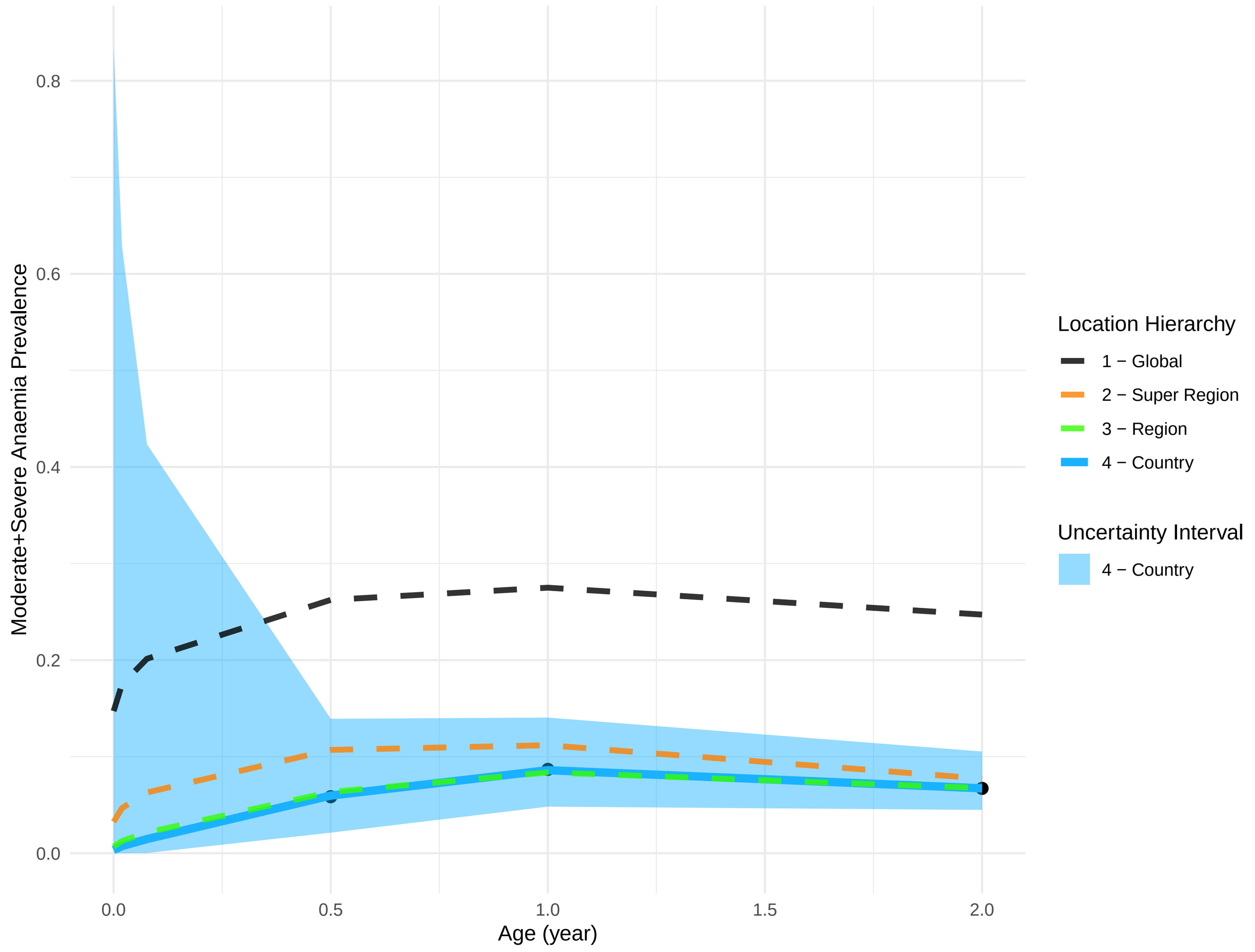

MR−BRT Age/Sex Fits − Non−pregnant Females <2 Years Old − Moderate+Severe Anaemia Prevalence − High−income
WHO−2001 Adjustment Method

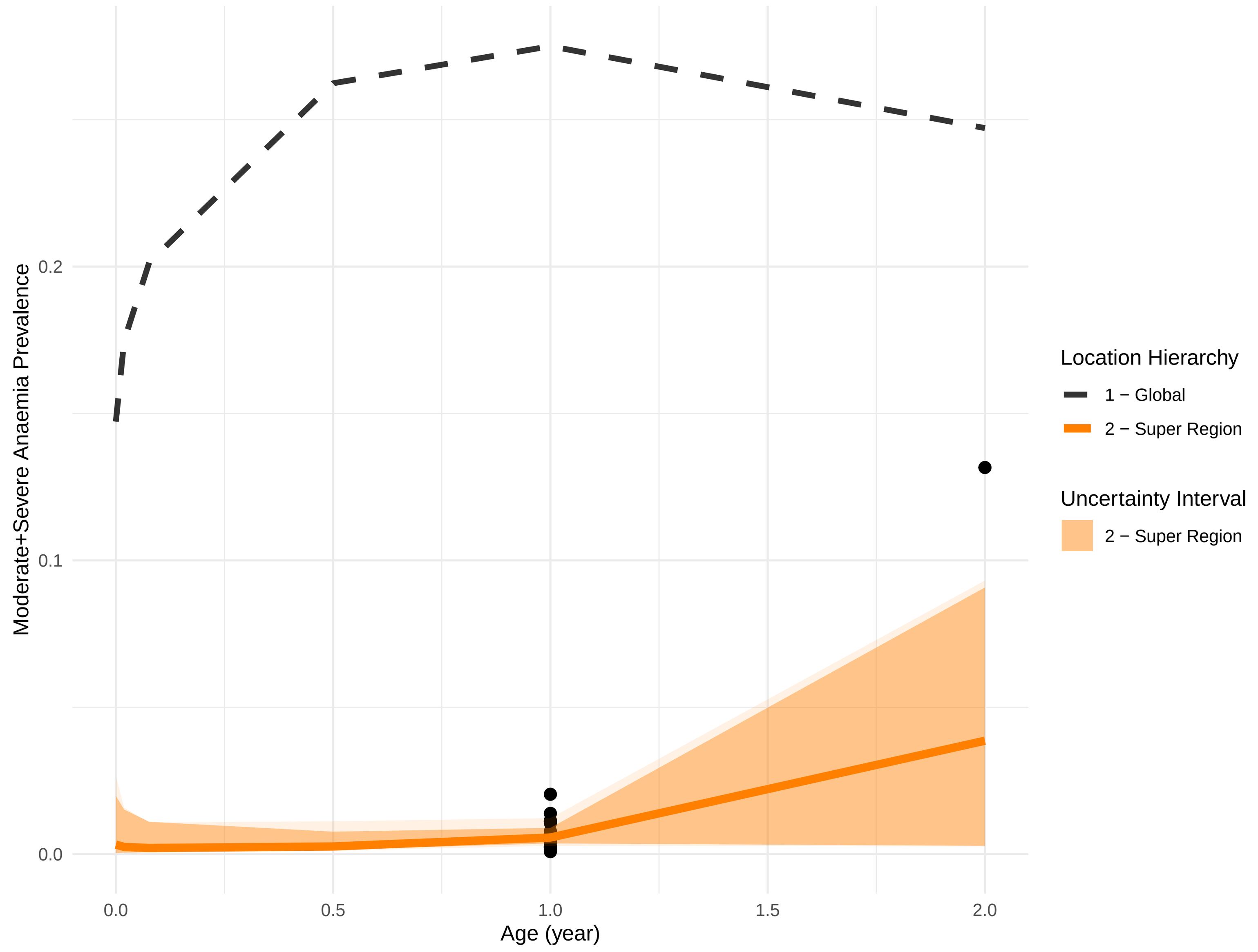

MR−BRT Age/Sex Fits − Non−pregnant Females <2 Years Old − Moderate+Severe Anaemia Prevalence − Western Europe
WHO−2001 Adjustment Method

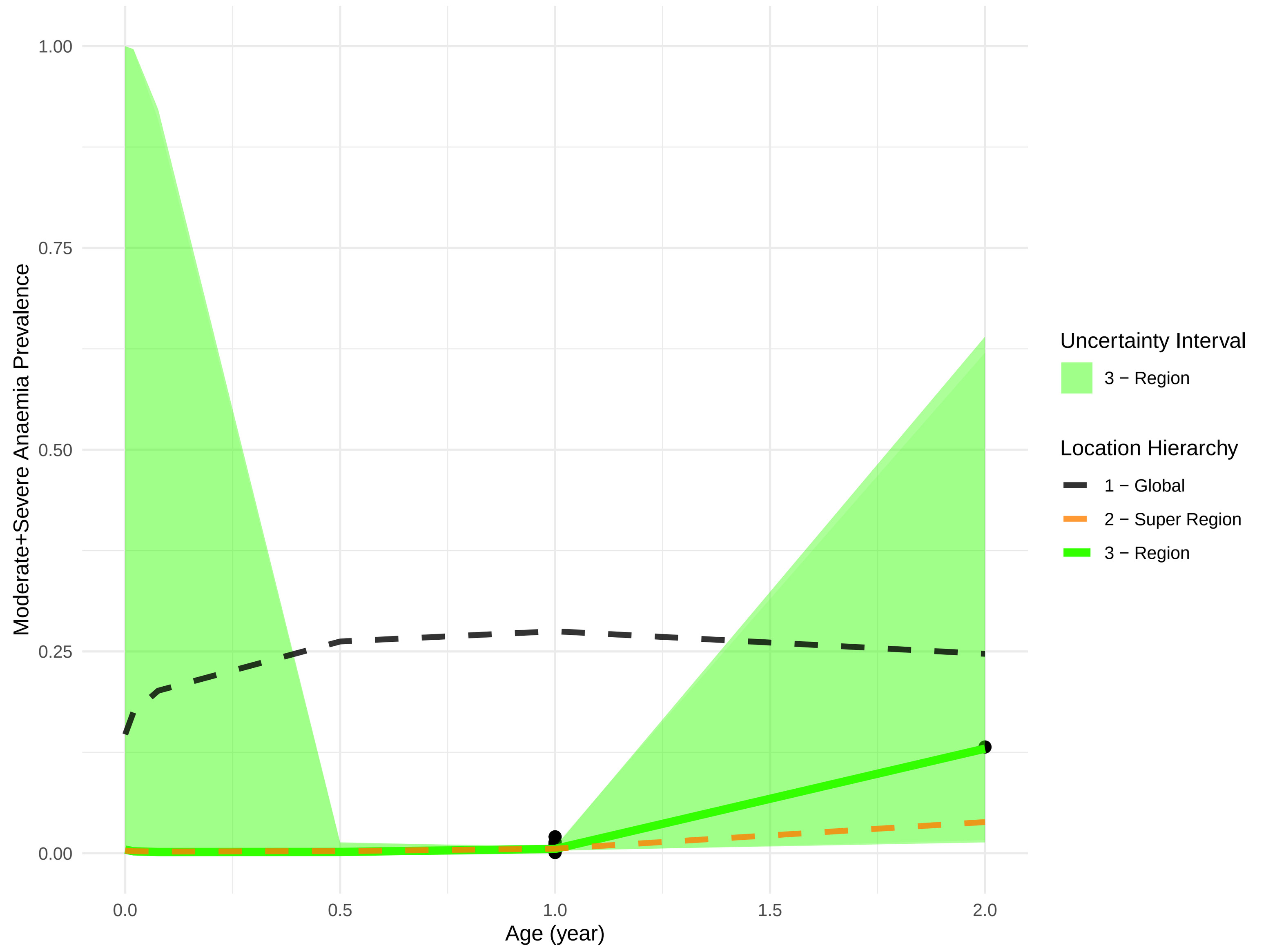

MR−BRT Age/Sex Fits − Non−pregnant Females <2 Years Old − Moderate+Severe Anaemia Prevalence − United Kingdom
WHO−2001 Adjustment Method

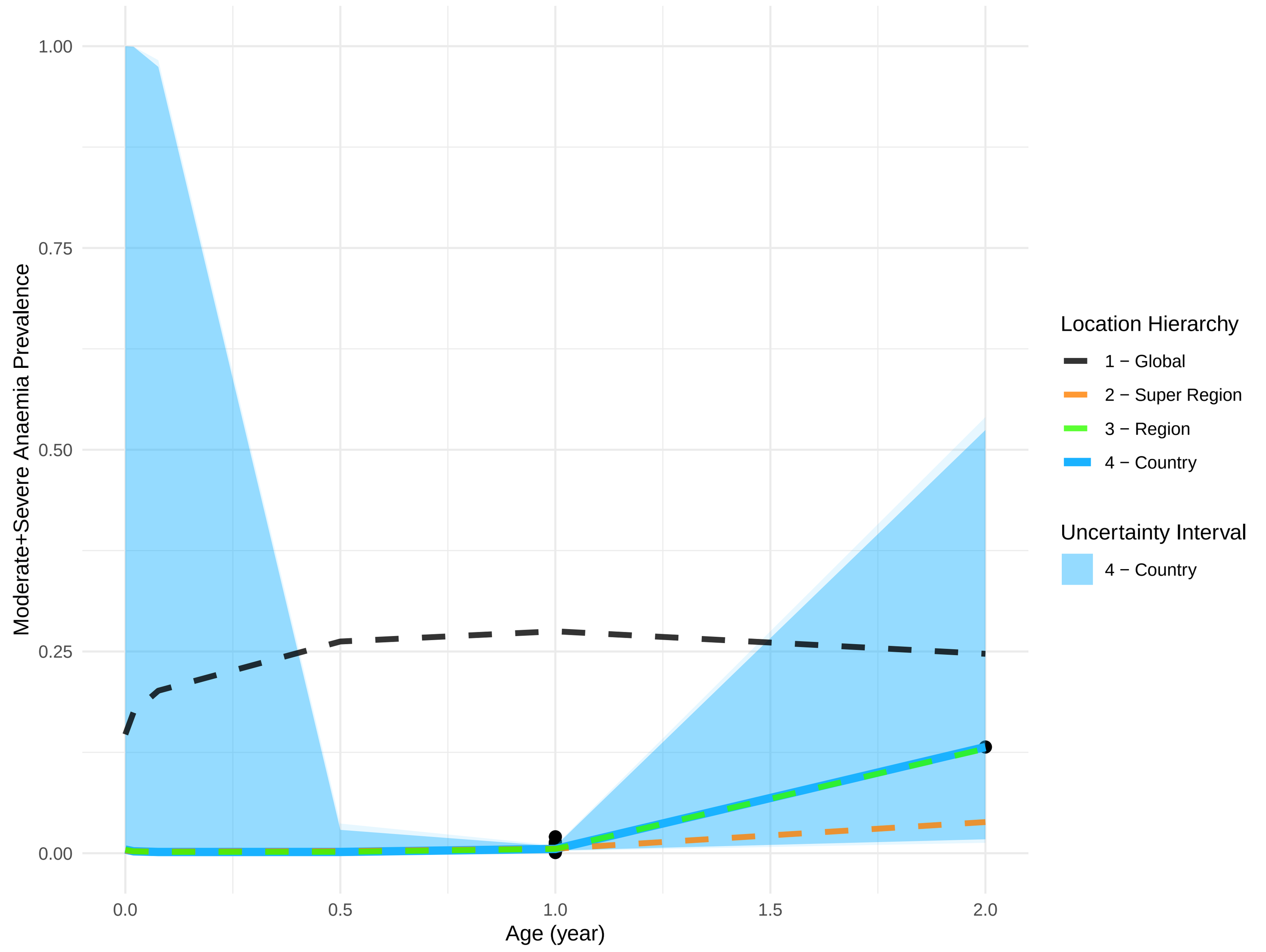

MR−BRT Age/Sex Fits − Non−pregnant Females <2 Years Old − Moderate+Severe Anaemia Prevalence − England
WHO−2001 Adjustment Method

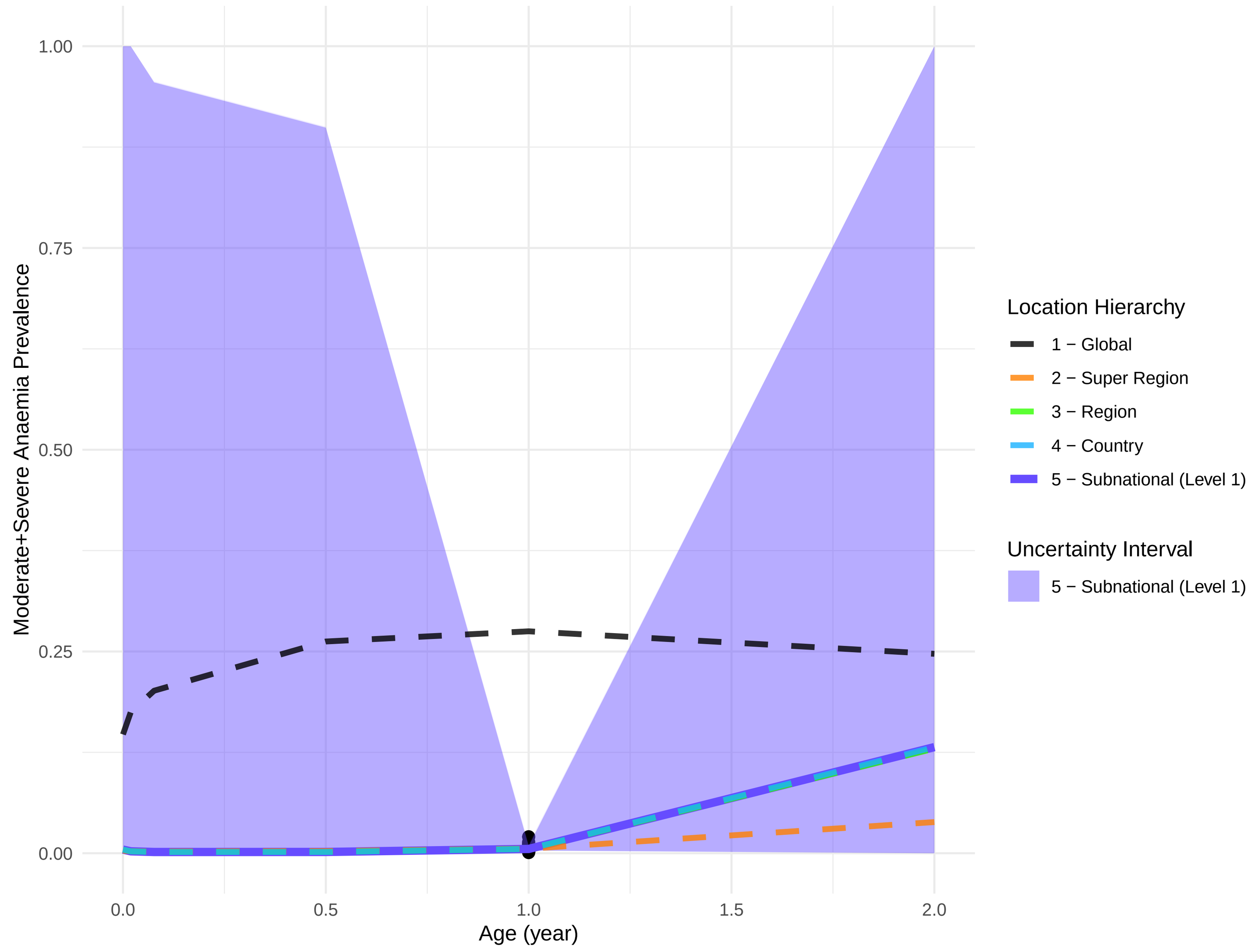

MR−BRT Age/Sex Fits − Non−pregnant Females <2 Years Old − Moderate+Severe Anaemia Prevalence − East Midlands
WHO−2001 Adjustment Method

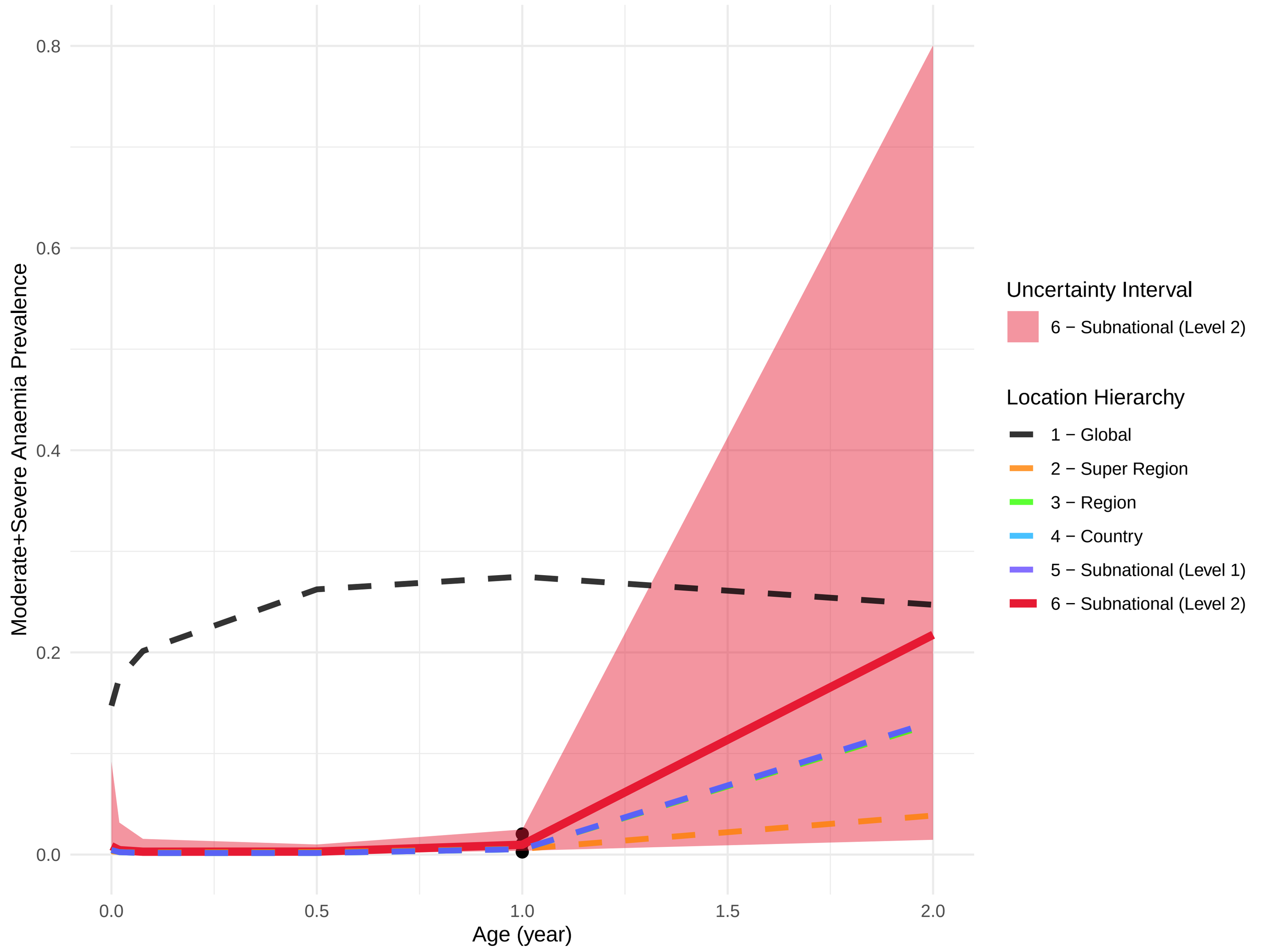

MR−BRT Age/Sex Fits − Non−pregnant Females <2 Years Old − Moderate+Severe Anaemia Prevalence − East of England
WHO−2001 Adjustment Method

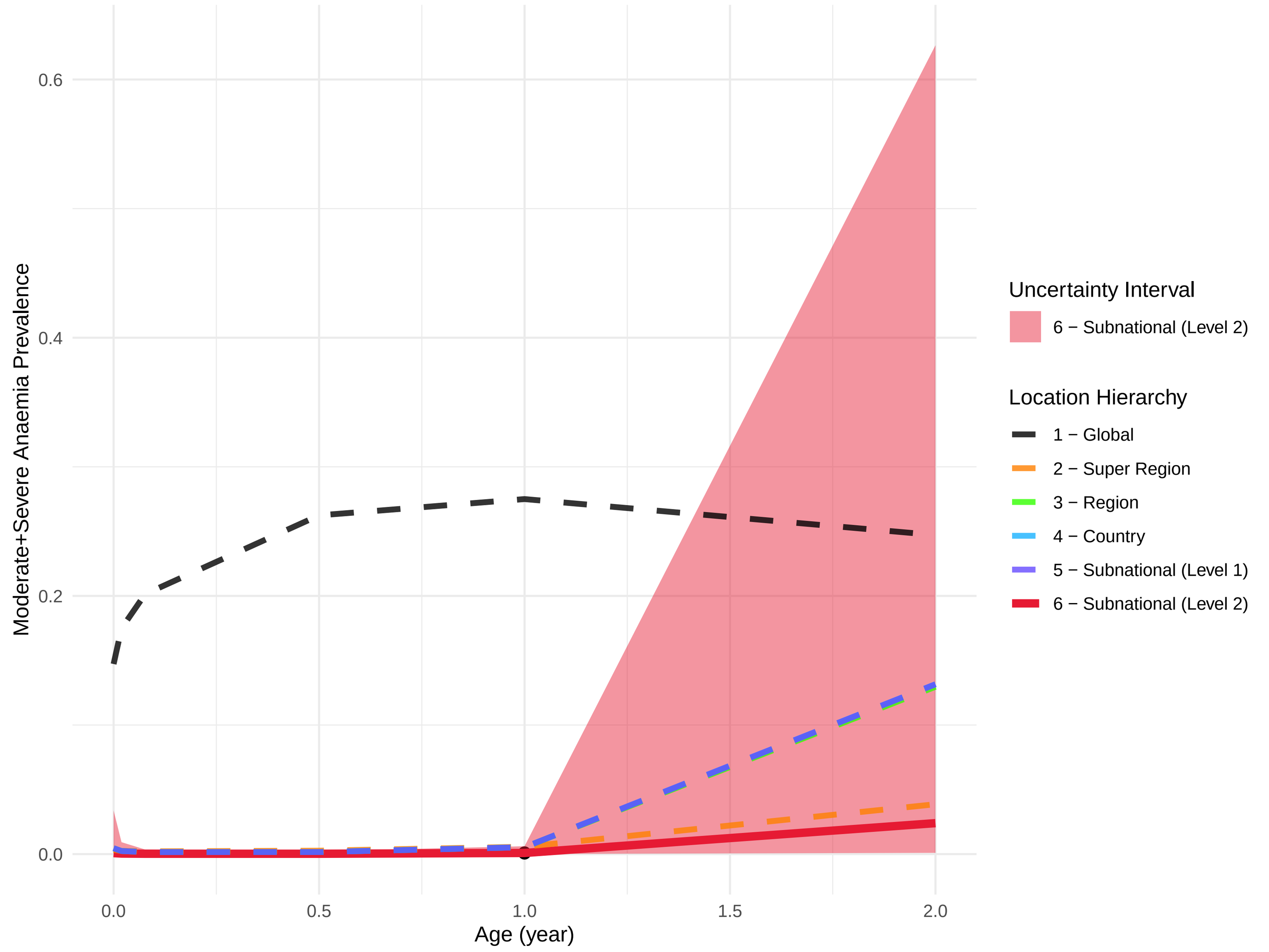

MR−BRT Age/Sex Fits − Non−pregnant Females <2 Years Old − Moderate+Severe Anaemia Prevalence − Greater London
WHO−2001 Adjustment Method

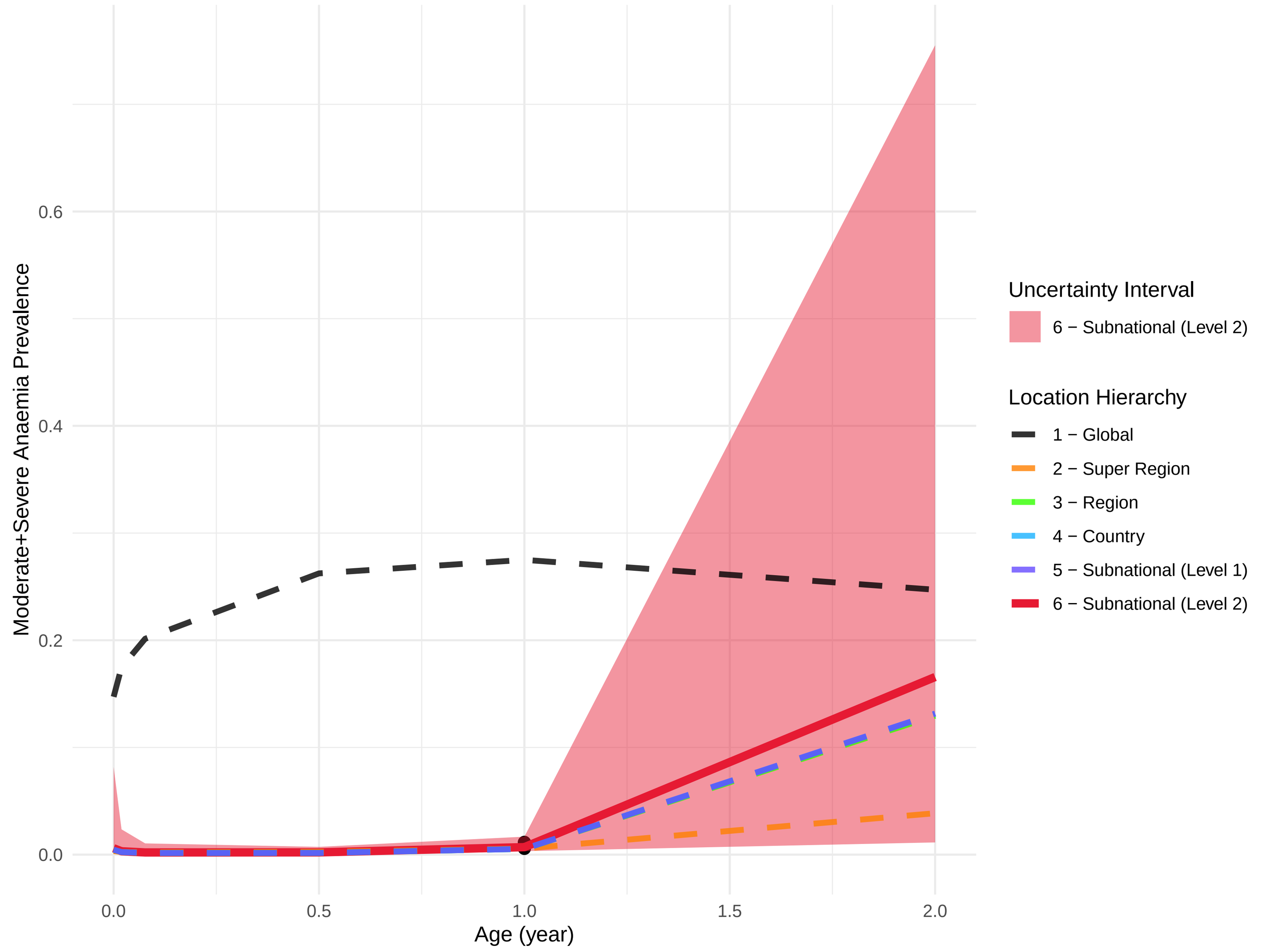

MR−BRT Age/Sex Fits − Non−pregnant Females <2 Years Old − Moderate+Severe Anaemia Prevalence − North East England
WHO−2001 Adjustment Method

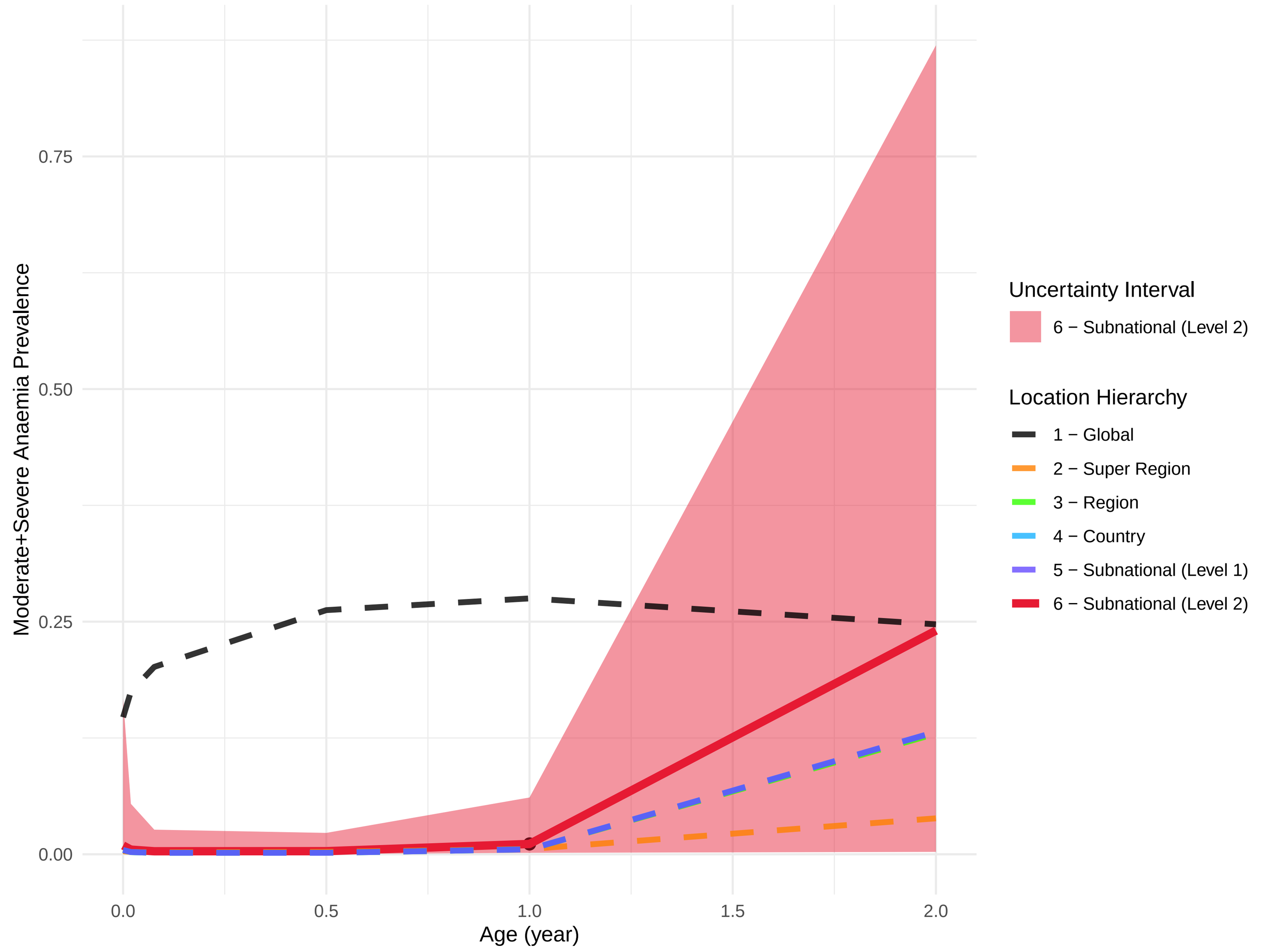

MR−BRT Age/Sex Fits − Non−pregnant Females <2 Years Old − Moderate+Severe Anaemia Prevalence − North West England
WHO−2001 Adjustment Method

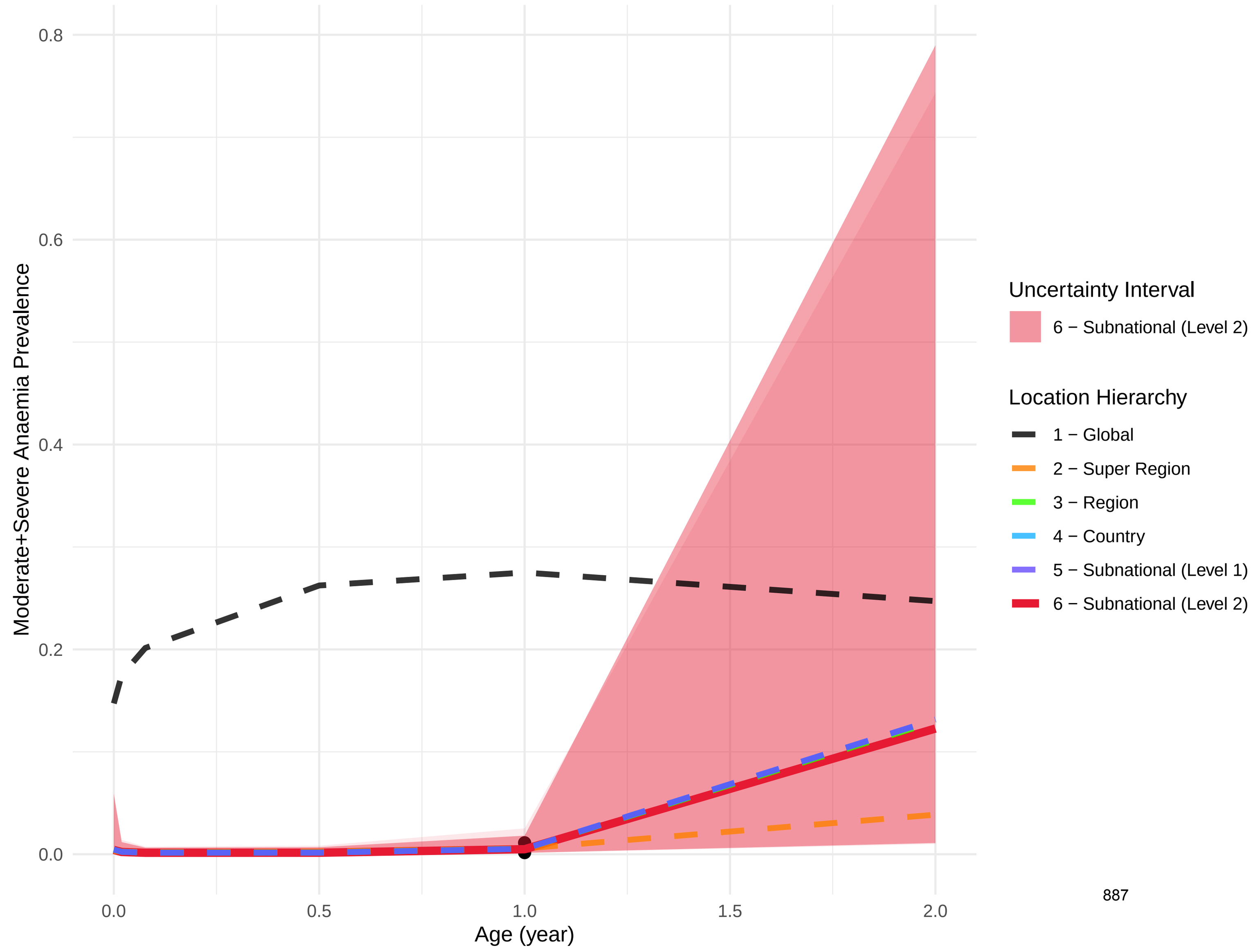

MR−BRT Age/Sex Fits − Non−pregnant Females <2 Years Old − Moderate+Severe Anaemia Prevalence − South East England
WHO−2001 Adjustment Method

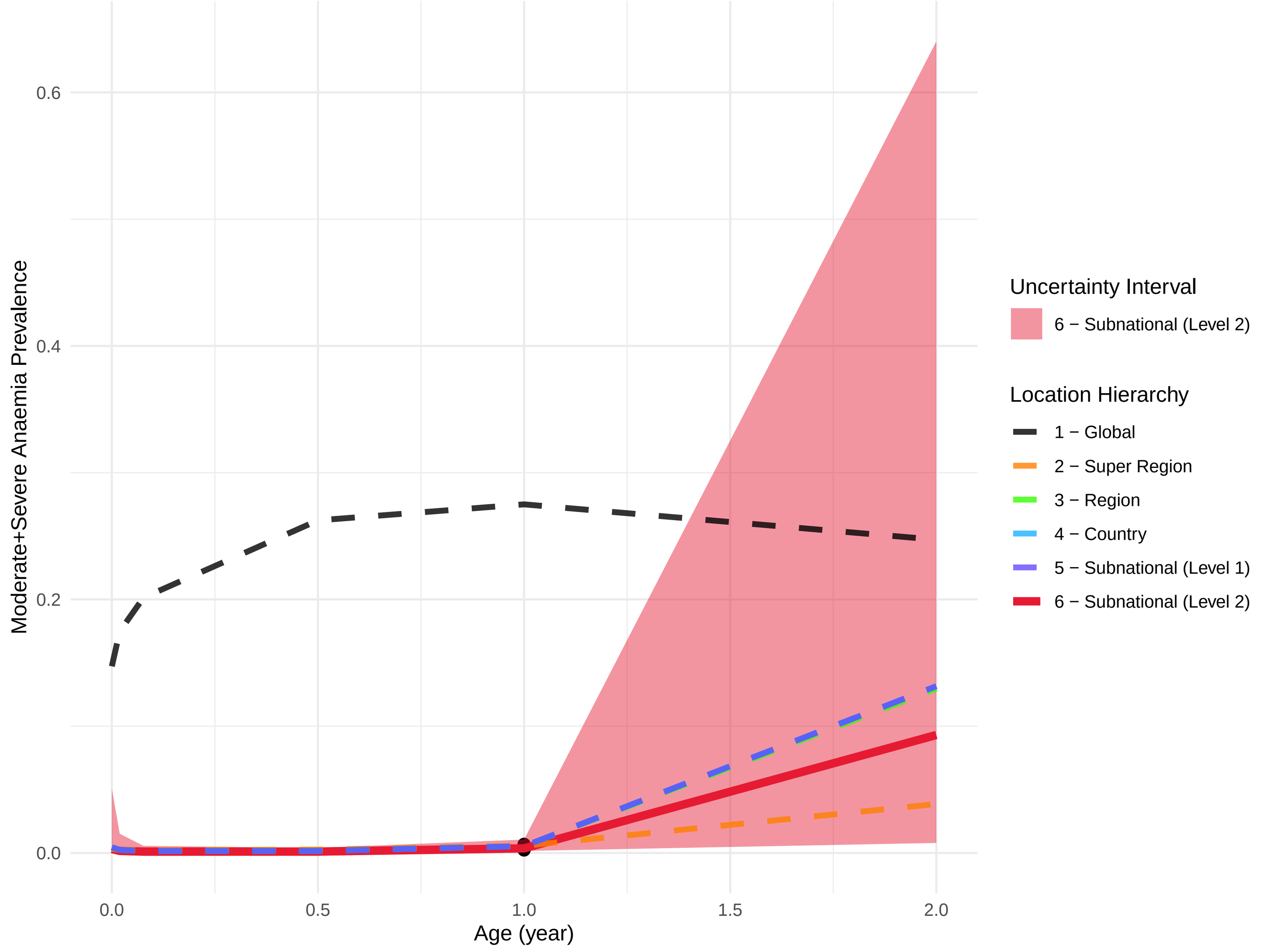

MR−BRT Age/Sex Fits − Non−pregnant Females <2 Years Old − Moderate+Severe Anaemia Prevalence − South West England
WHO−2001 Adjustment Method

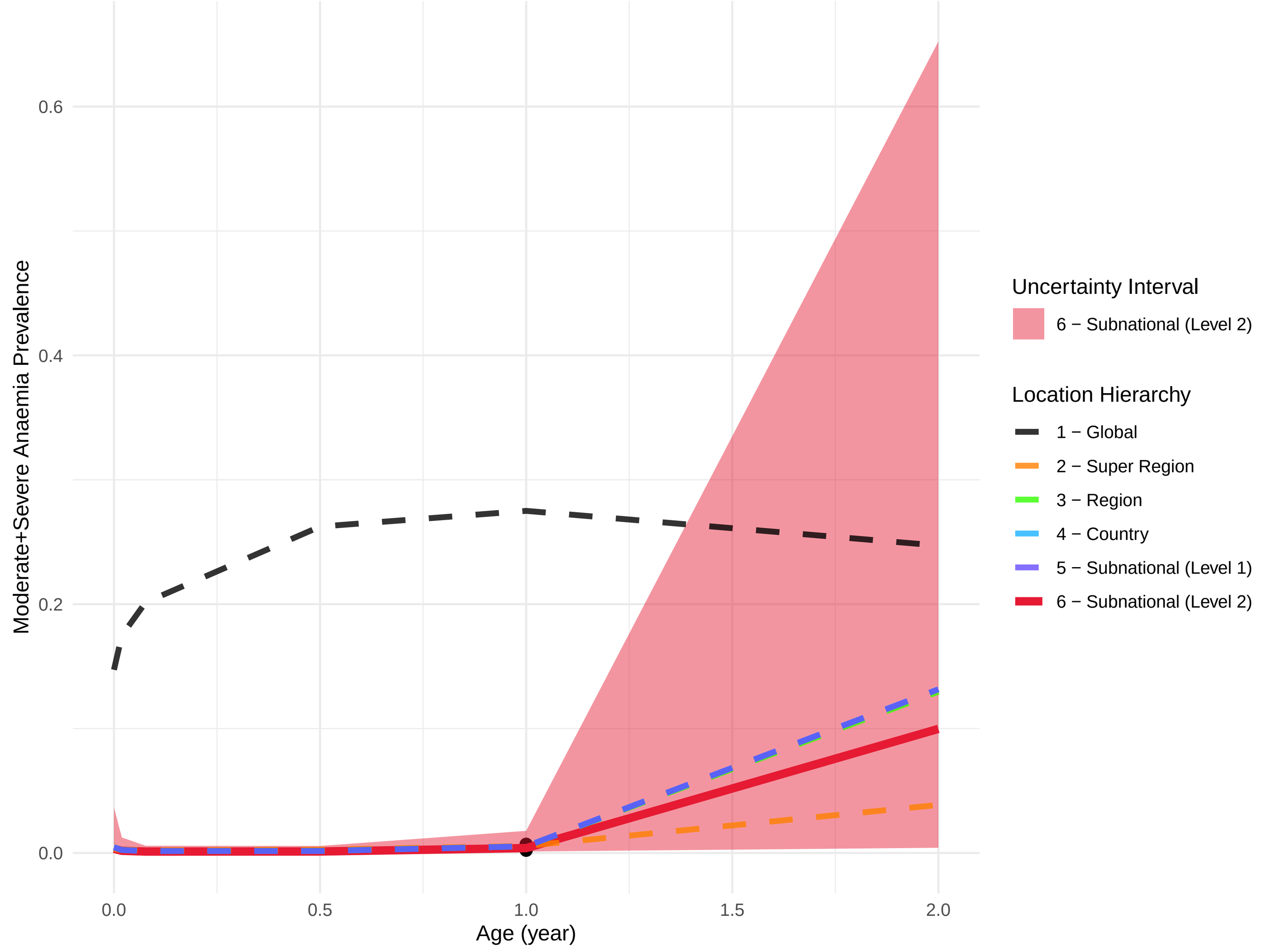

MR−BRT Age/Sex Fits − Non−pregnant Females <2 Years Old − Moderate+Severe Anaemia Prevalence − West Midlands
WHO−2001 Adjustment Method

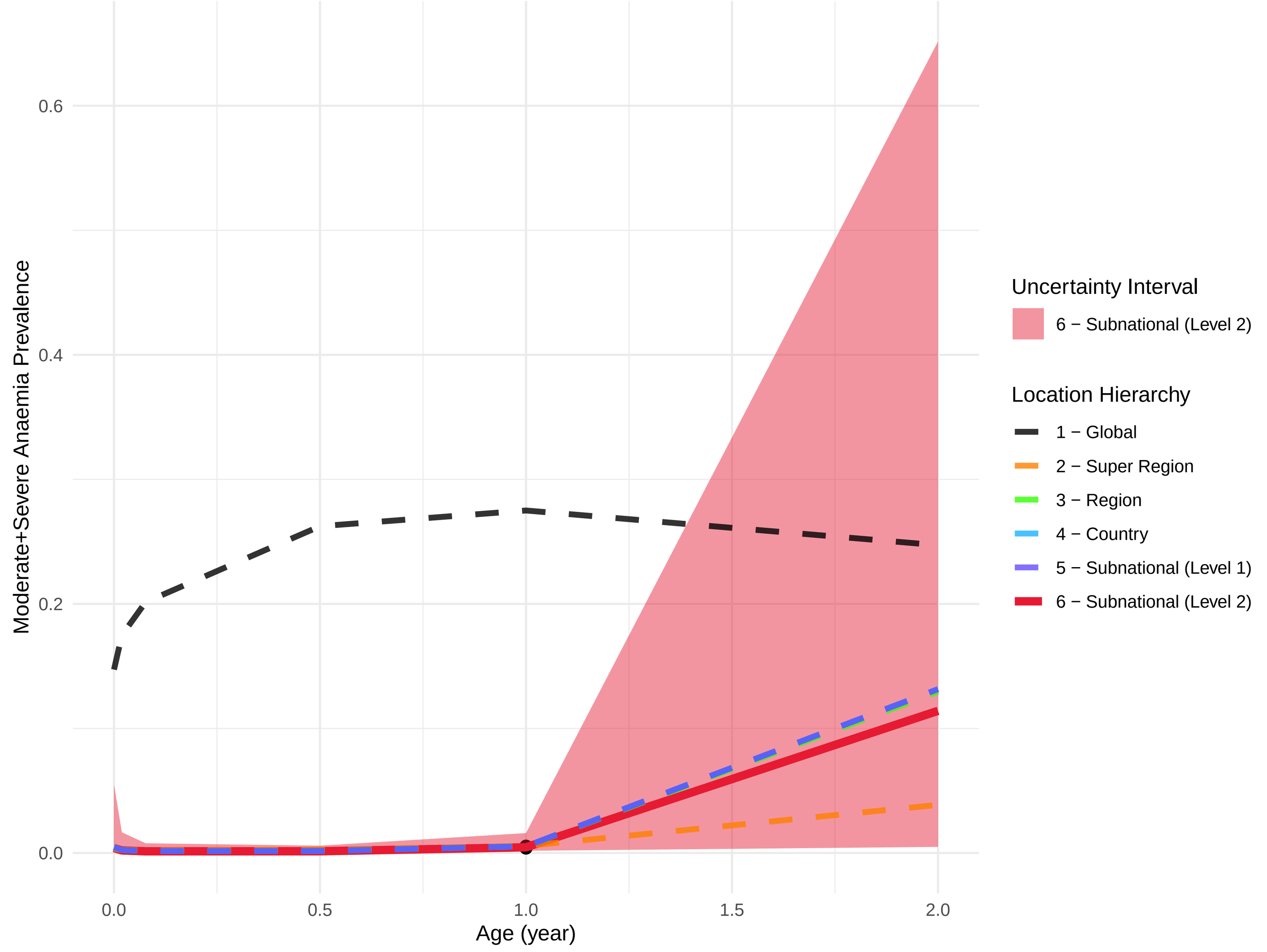

MR−BRT Age/Sex Fits − Non−pregnant Females <2 Years Old − Moderate+Severe Anaemia Prevalence − Yorkshire and the Humber
WHO−2001 Adjustment Method

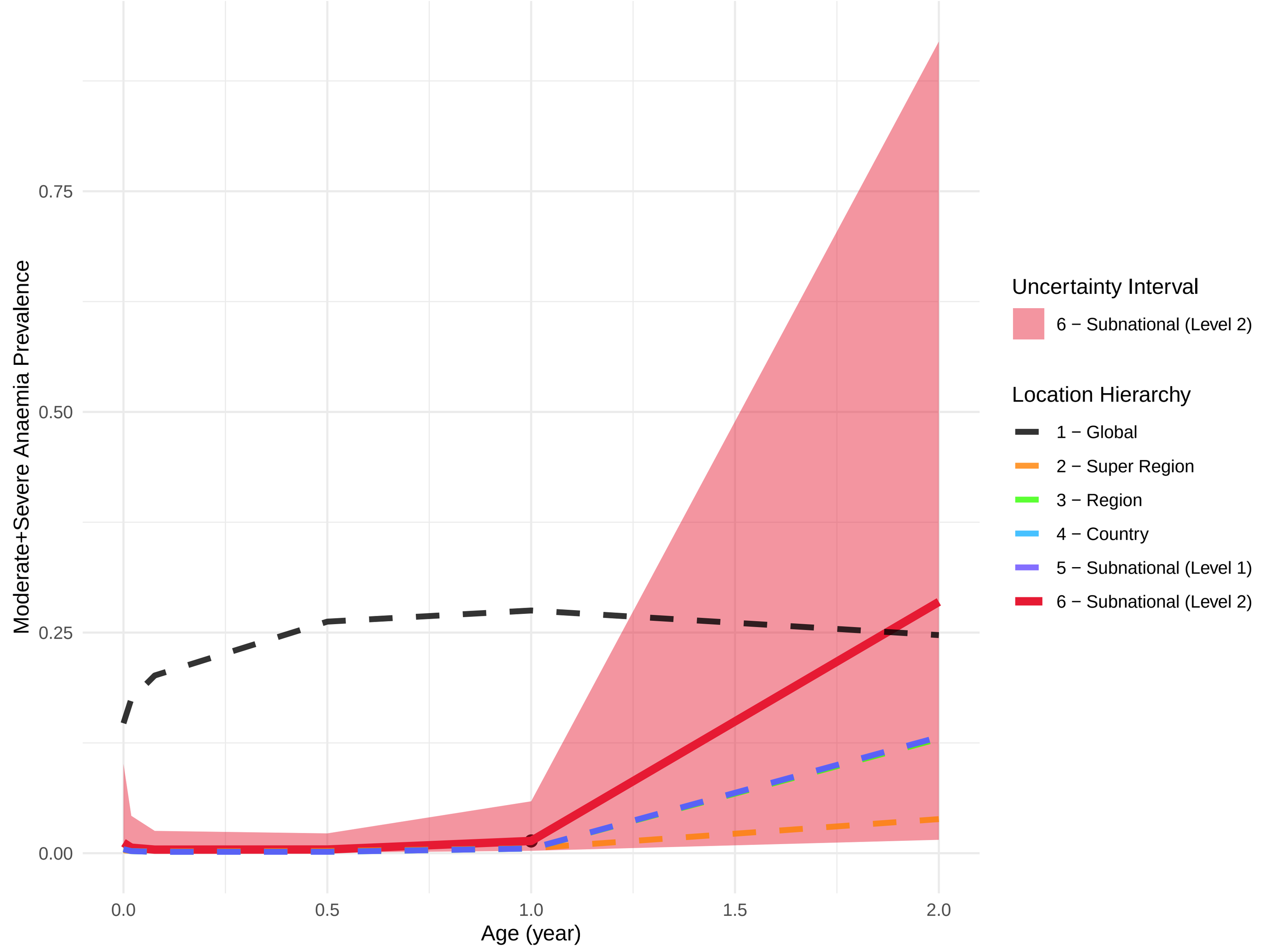

MR−BRT Age/Sex Fits − Non−pregnant Females <2 Years Old − Moderate+Severe Anaemia Prevalence − Latin America and Caribbean
WHO−2001 Adjustment Method

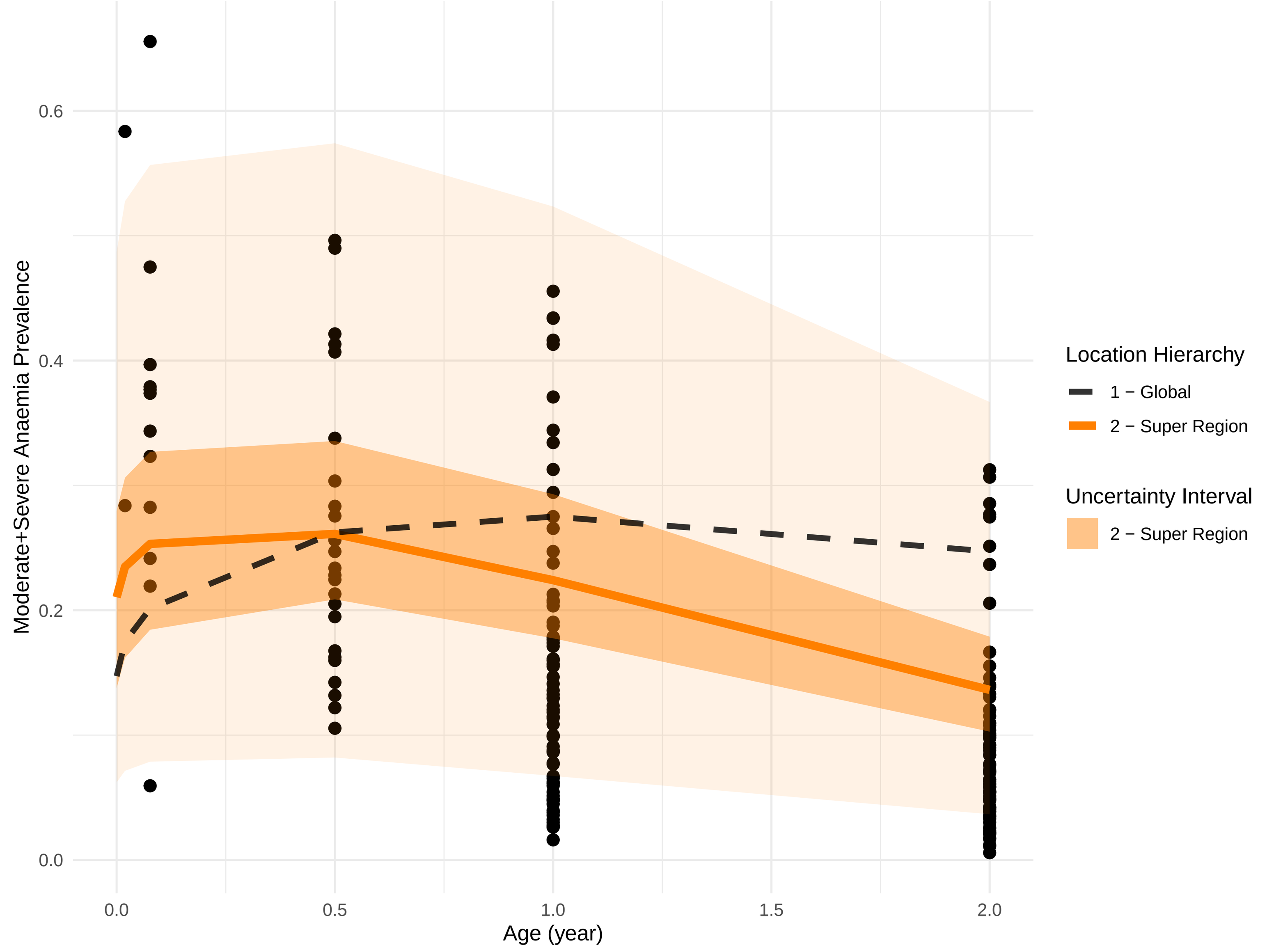

MR−BRT Age/Sex Fits − Non−pregnant Females <2 Years Old − Moderate+Severe Anaemia Prevalence − Andean Latin America
WHO−2001 Adjustment Method

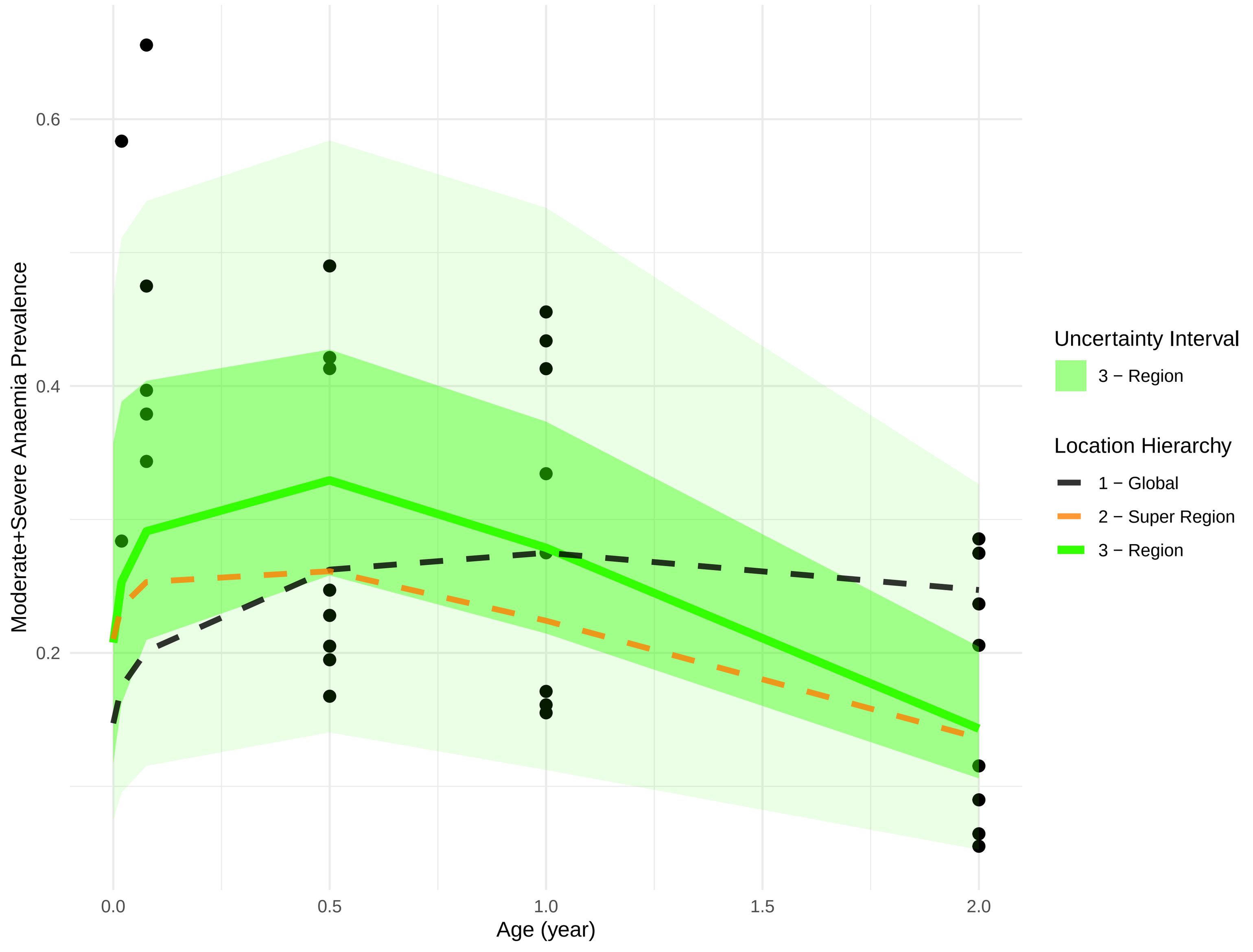

MR−BRT Age/Sex Fits − Non−pregnant Females <2 Years Old − Moderate+Severe Anaemia Prevalence − Bolivia (Plurinational State of)
WHO−2001 Adjustment Method

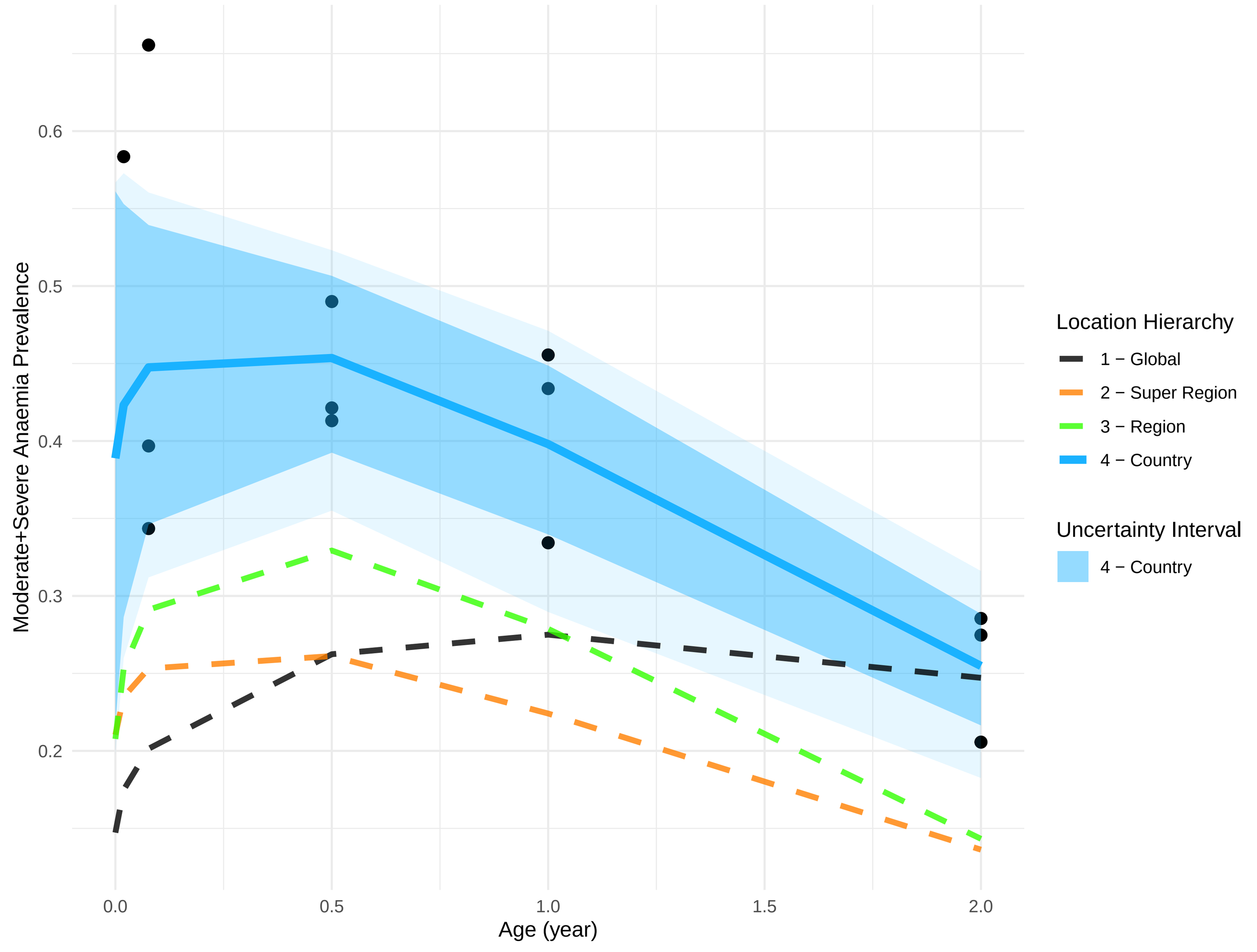

MR−BRT Age/Sex Fits − Non−pregnant Females <2 Years Old − Moderate+Severe Anaemia Prevalence − Peru
WHO−2001 Adjustment Method

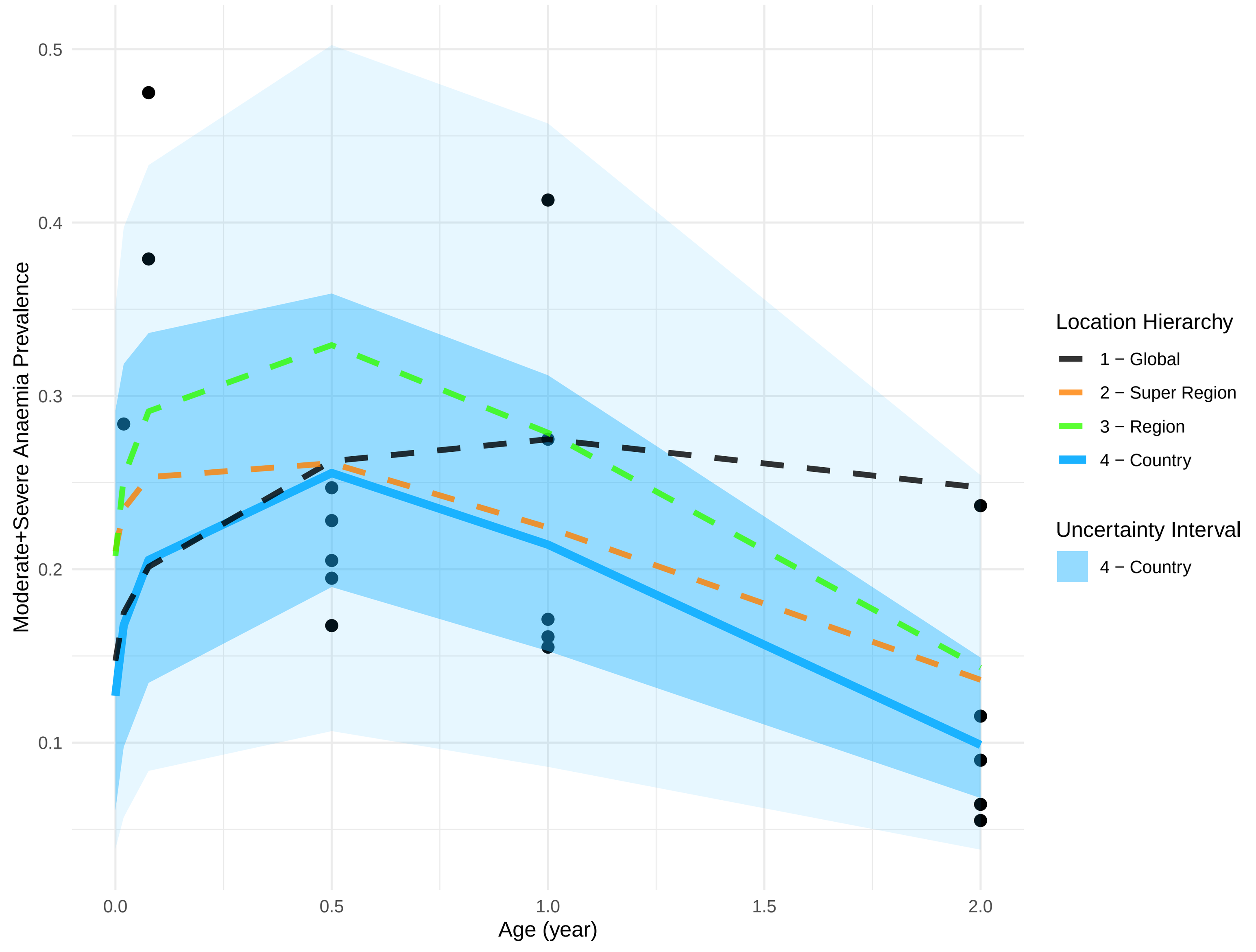

MR−BRT Age/Sex Fits − Non−pregnant Females <2 Years Old − Moderate+Severe Anaemia Prevalence − Caribbean
WHO−2001 Adjustment Method

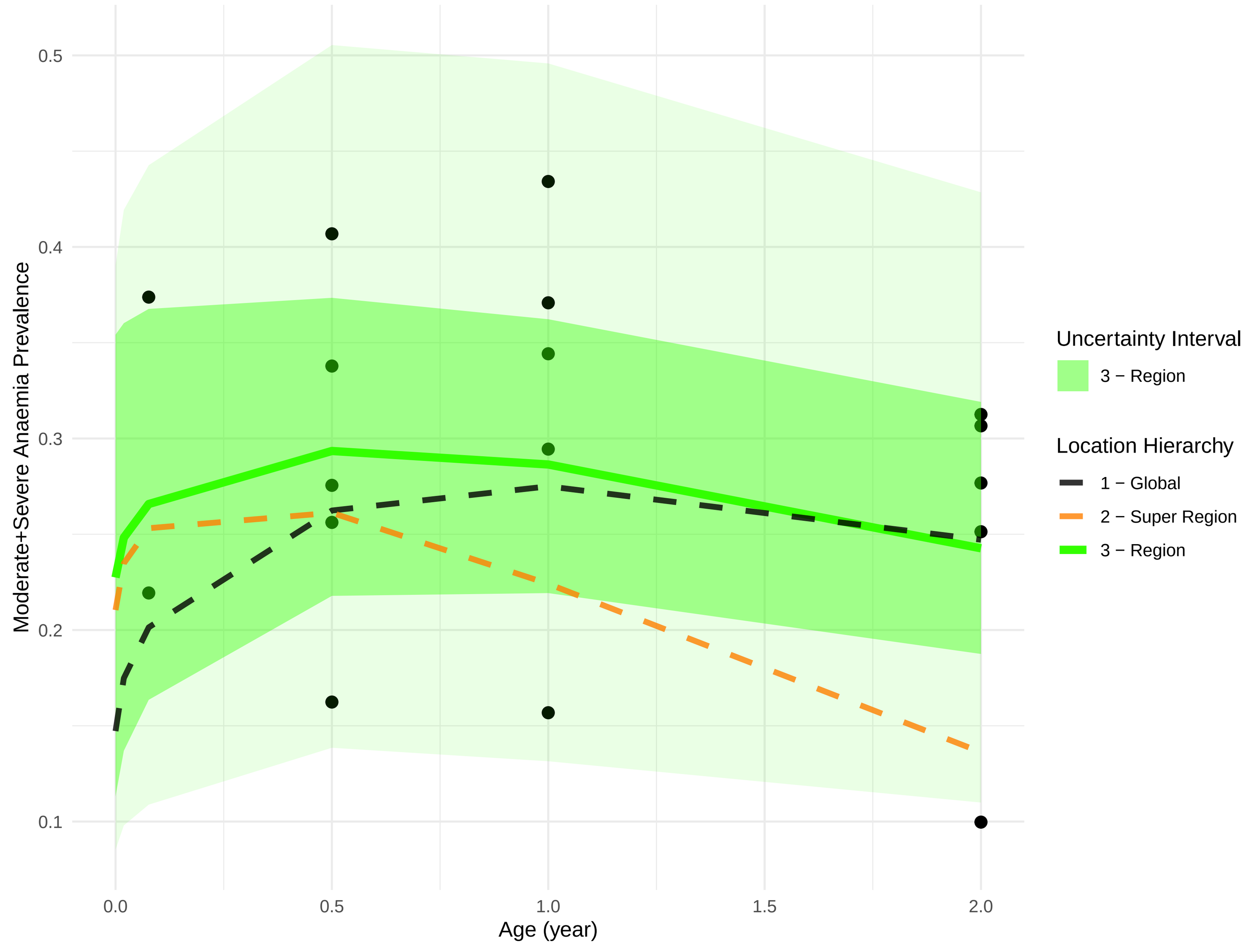

MR−BRT Age/Sex Fits − Non−pregnant Females <2 Years Old − Moderate+Severe Anaemia Prevalence − Guyana
WHO−2001 Adjustment Method

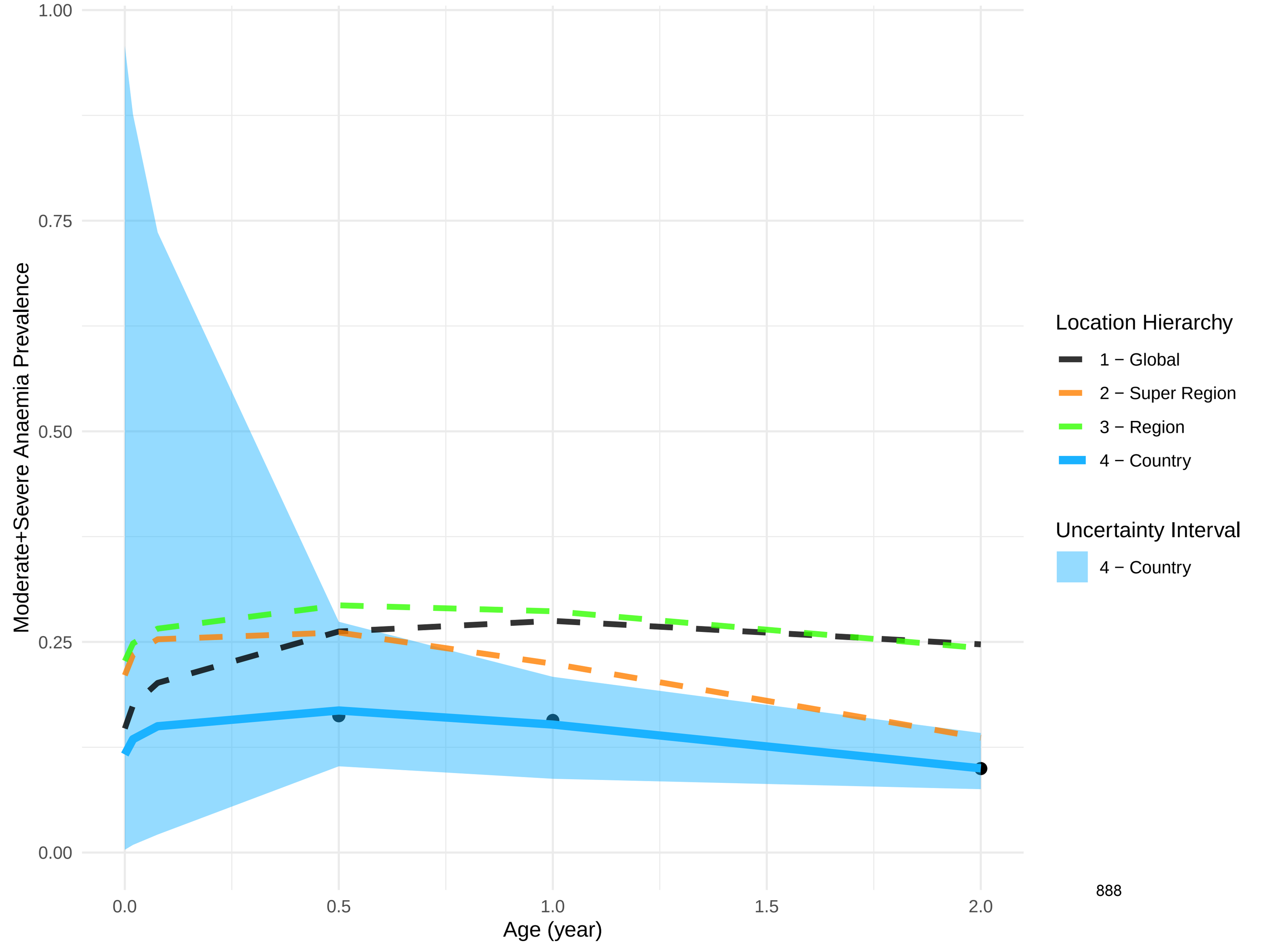

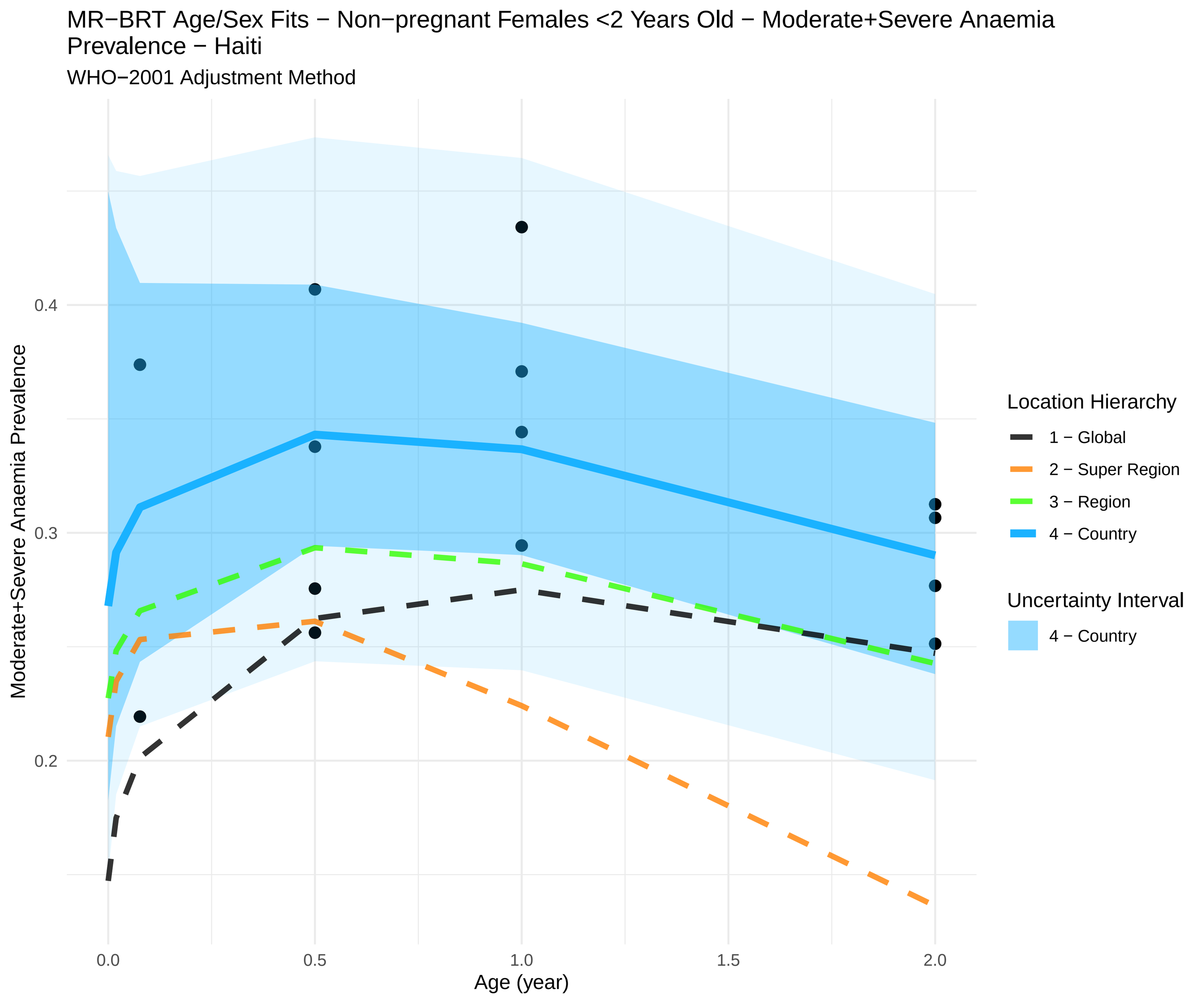
MR−BRT Age/Sex Fits − Non−pregnant Females <2 Years Old − Moderate+Severe Anaemia
Prevalence − Haiti
WHO−2001 Adjustment Method
Moderate+Severe Anaemia Prevalence
Age (year)
Location Hierarchy
1 − Global
2 − Super Region
3 − Region
4 − Country
Uncertainty Interval
4 − Country

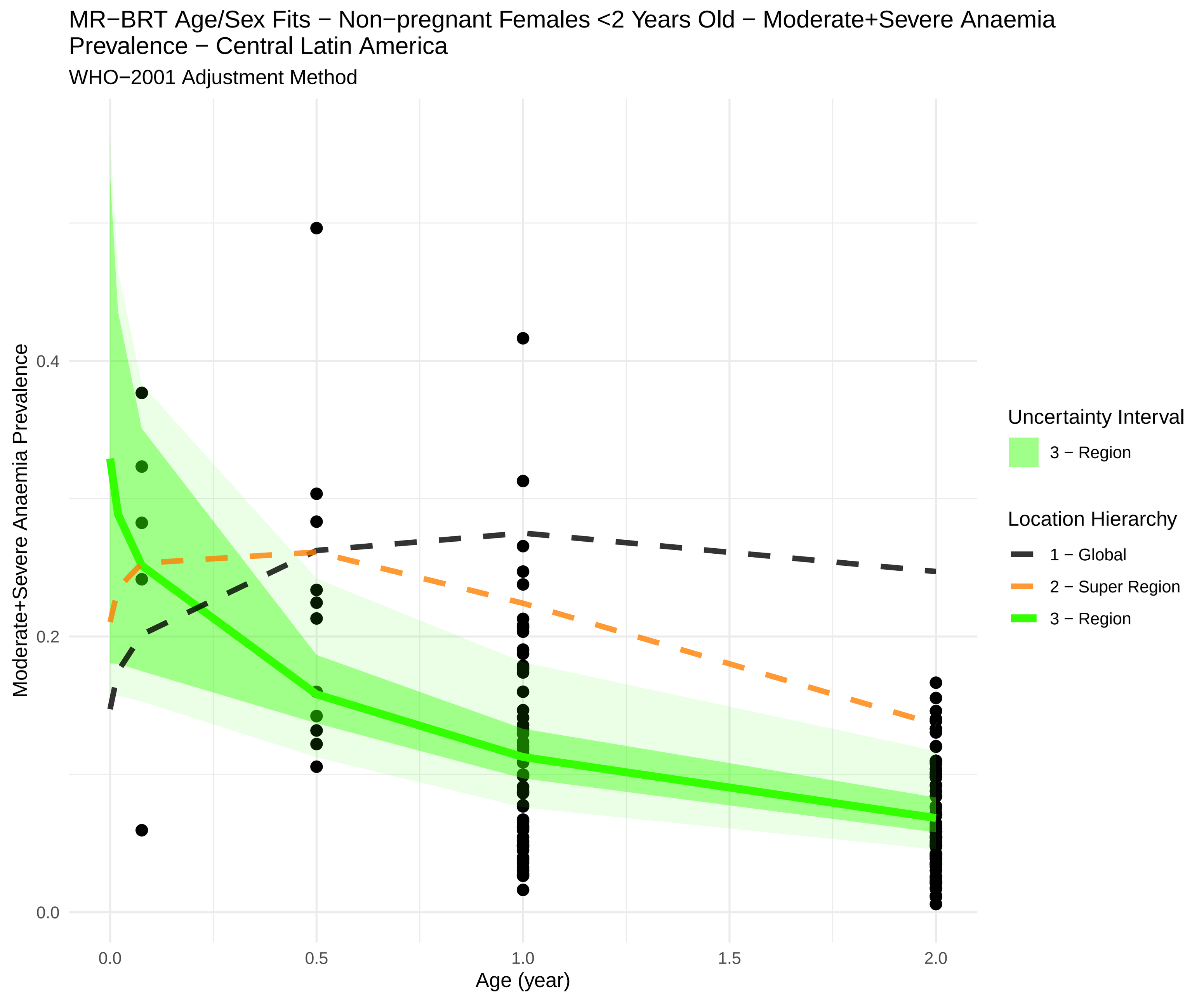
MR−BRT Age/Sex Fits − Non−pregnant Females <2 Years Old − Moderate+Severe Anaemia
Prevalence − Central Latin America
WHO−2001 Adjustment Method
Moderate+Severe Anaemia Prevalence
Age (year)
Uncertainty Interval
3 − Region
Location Hierarchy
1 − Global
2 − Super Region
3 − Region

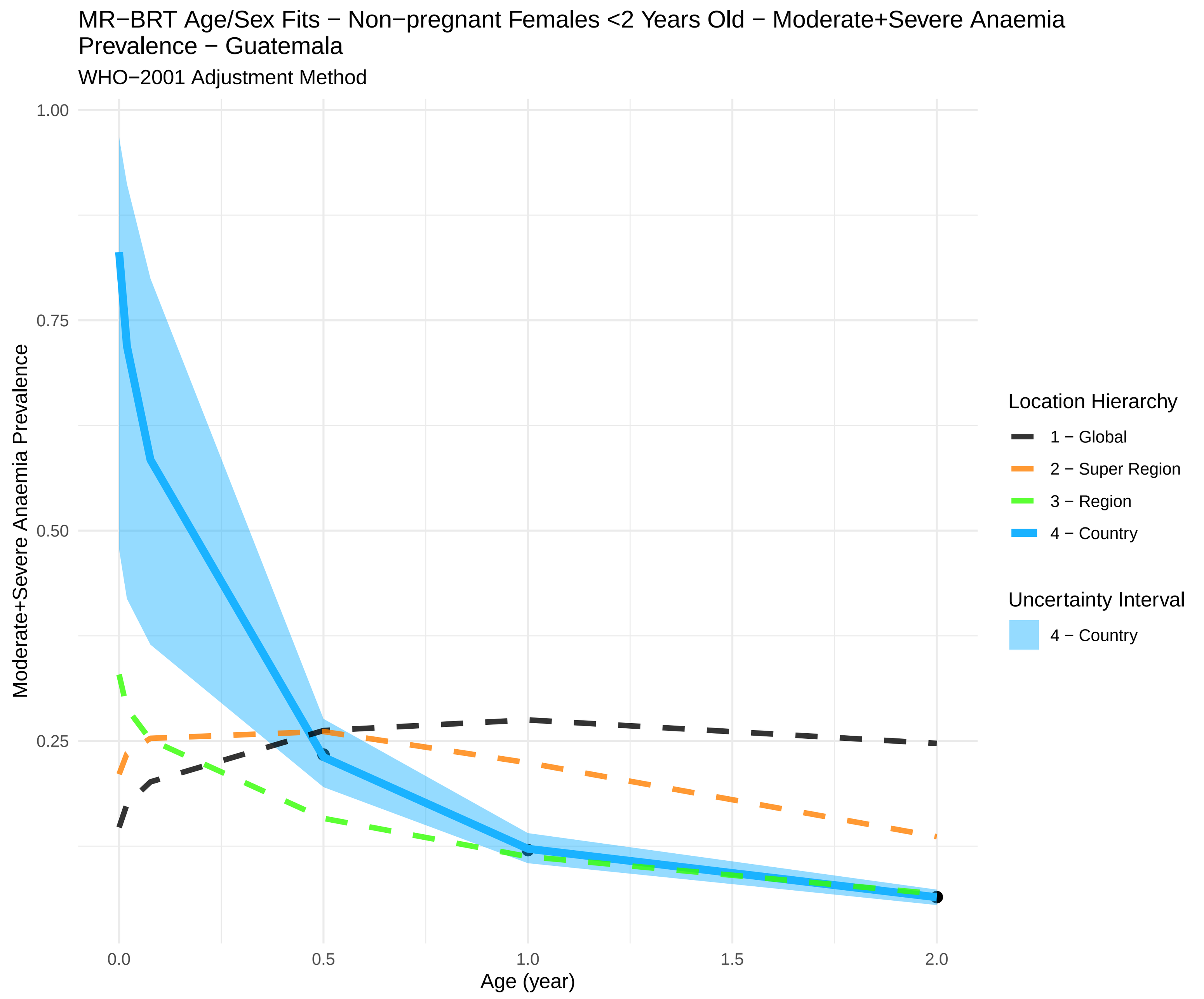
MR−BRT Age/Sex Fits − Non−pregnant Females <2 Years Old − Moderate+Severe Anaemia
Prevalence − Guatemala
WHO−2001 Adjustment Method
Moderate+Severe Anaemia Prevalence
Age (year)
Location Hierarchy
1 − Global
2 − Super Region
3 − Region
4 − Country
Uncertainty Interval
4 − Country

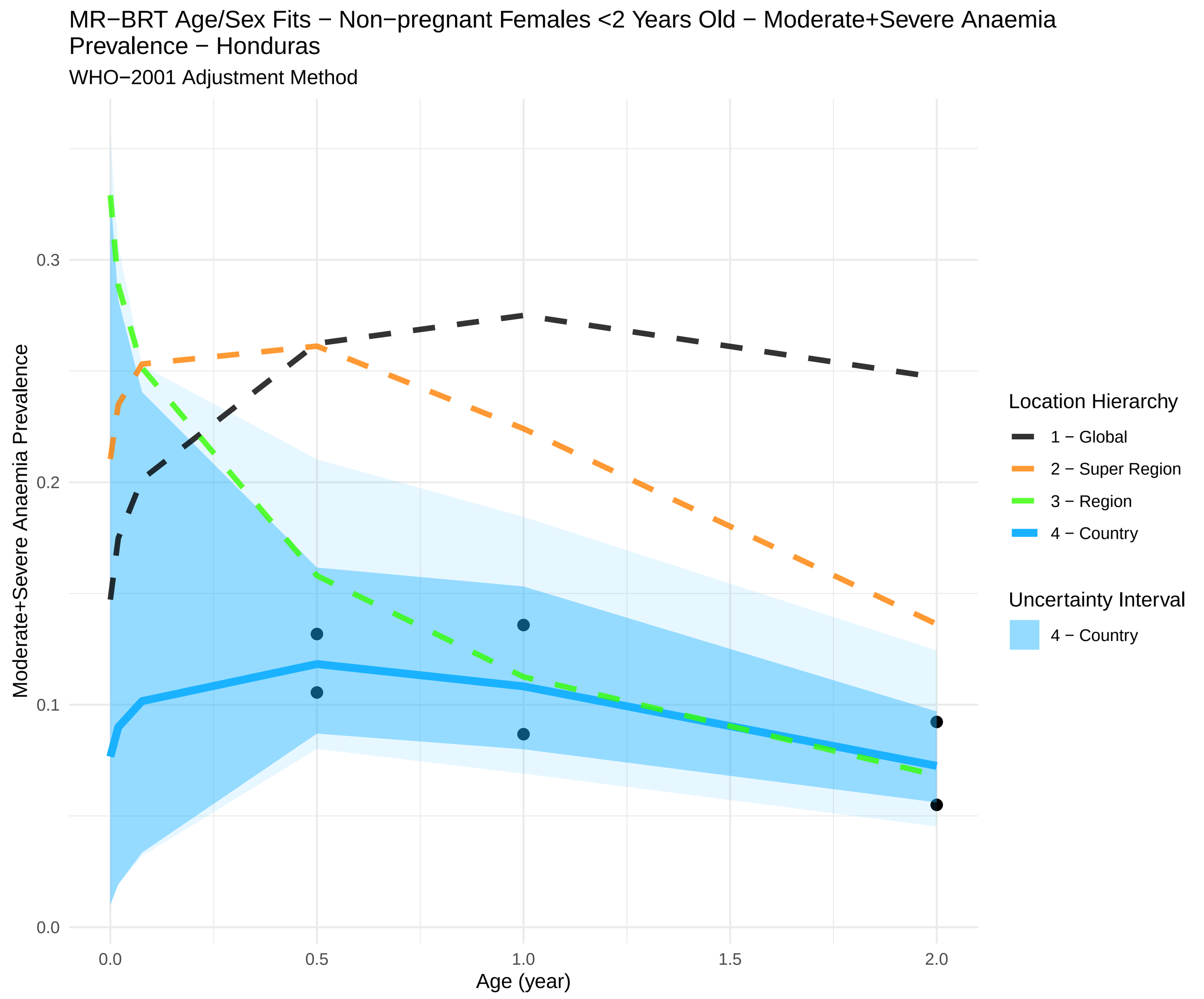
MR−BRT Age/Sex Fits − Non−pregnant Females <2 Years Old − Moderate+Severe Anaemia
Prevalence − Honduras
WHO−2001 Adjustment Method
Moderate+Severe Anaemia Prevalence
Age (year)
Location Hierarchy
1 − Global
2 − Super Region
3 − Region
4 − Country
Uncertainty Interval
4 − Country

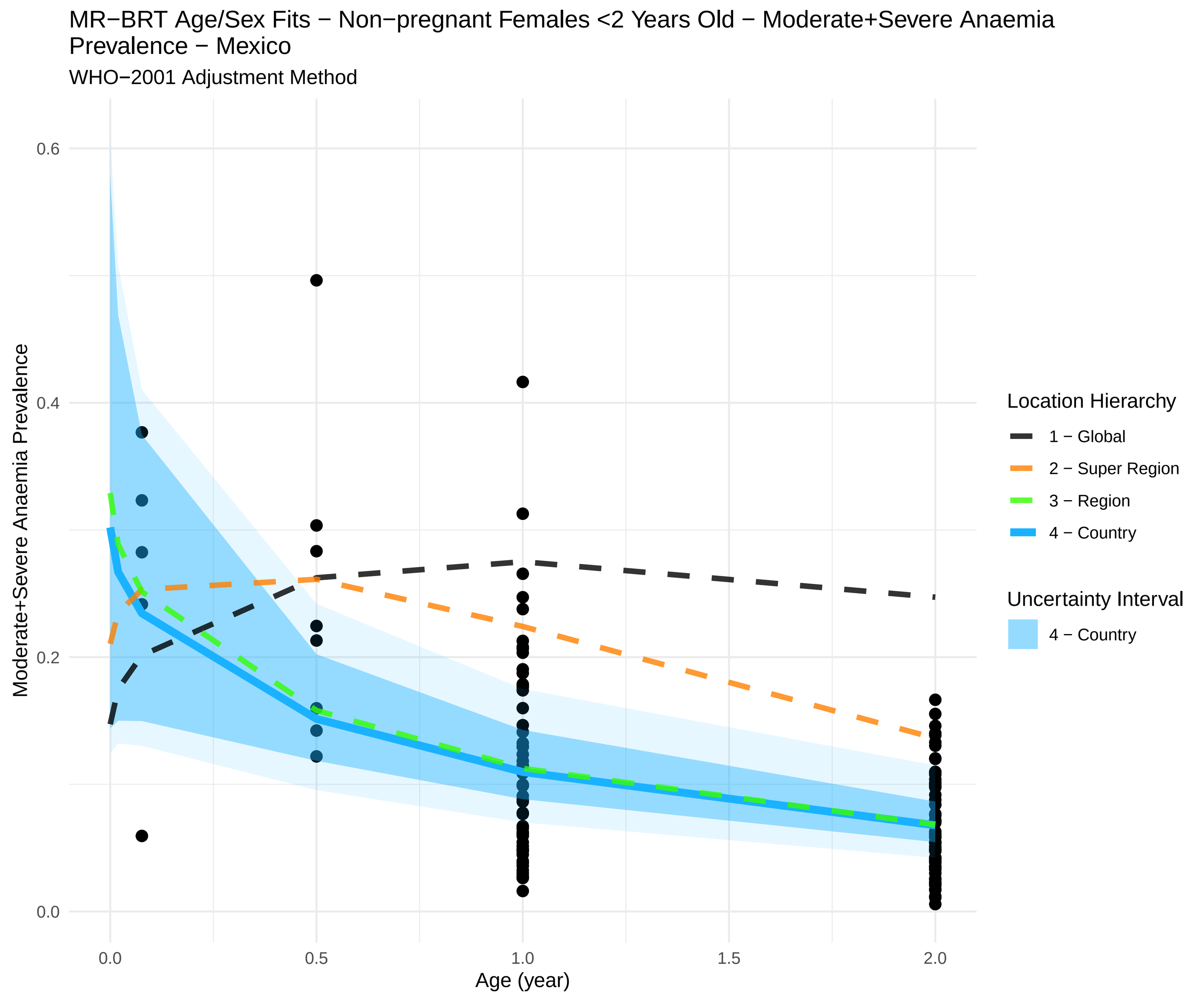
MR−BRT Age/Sex Fits − Non−pregnant Females <2 Years Old − Moderate+Severe Anaemia
Prevalence − Mexico
WHO−2001 Adjustment Method
Moderate+Severe Anaemia Prevalence
Age (year)
Location Hierarchy
1 − Global
2 − Super Region
3 − Region
4 − Country
Uncertainty Interval
4 − Country

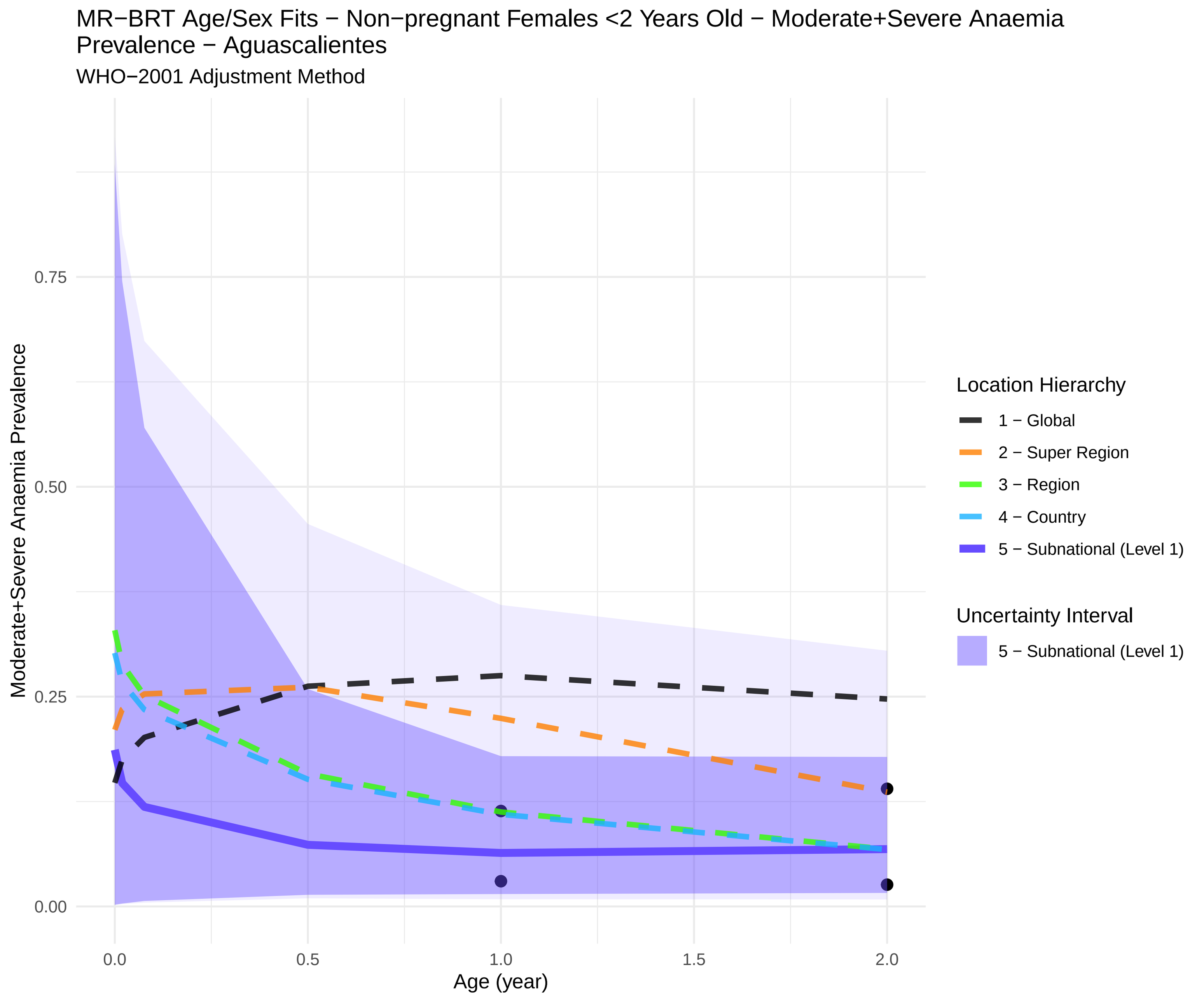
MR−BRT Age/Sex Fits − Non−pregnant Females <2 Years Old − Moderate+Severe Anaemia
Prevalence − Aguascalientes
WHO−2001 Adjustment Method
Moderate+Severe Anaemia Prevalence
Age (year)
Location Hierarchy
1 − Global
2 − Super Region
3 − Region
4 − Country
5 − Subnational (Level 1)
Uncertainty Interval
5 − Subnational (Level 1)

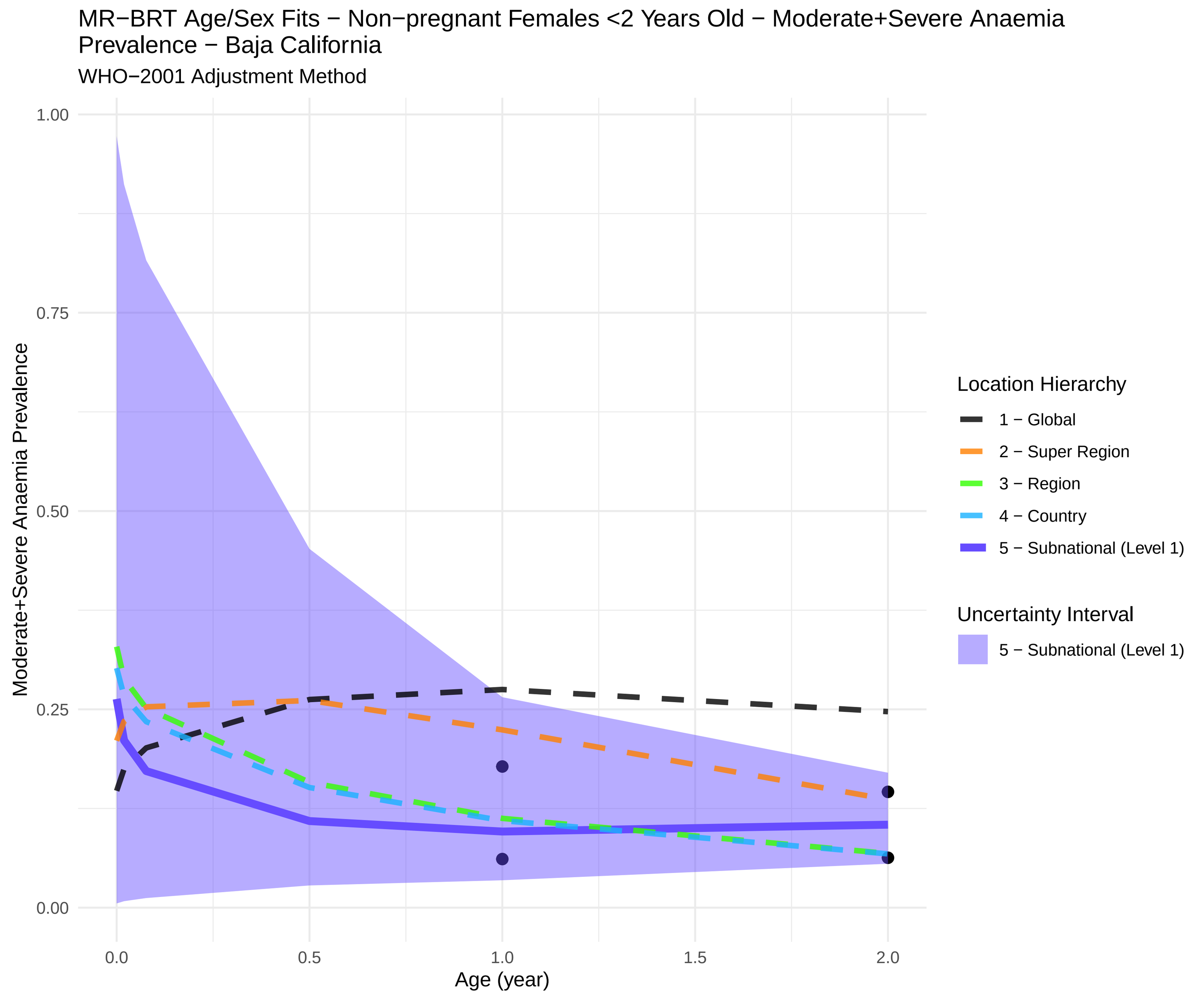
MR−BRT Age/Sex Fits − Non−pregnant Females <2 Years Old − Moderate+Severe Anaemia
Prevalence − Baja California
WHO−2001 Adjustment Method
Moderate+Severe Anaemia Prevalence
Age (year)
Location Hierarchy
1 − Global
2 − Super Region
3 − Region
4 − Country
5 − Subnational (Level 1)
Uncertainty Interval
5 − Subnational (Level 1)

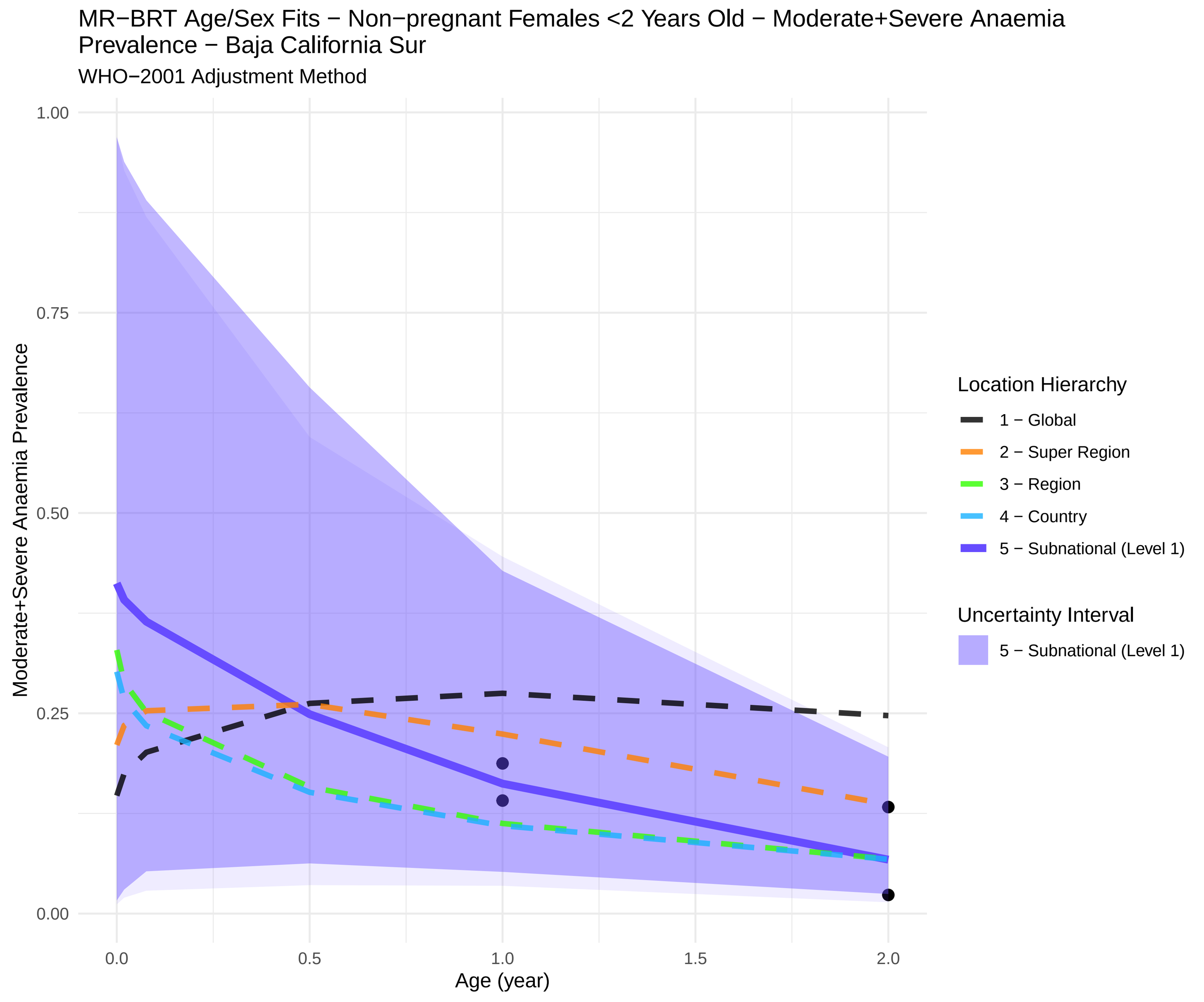
MR−BRT Age/Sex Fits − Non−pregnant Females <2 Years Old − Moderate+Severe Anaemia
Prevalence − Baja California Sur
WHO−2001 Adjustment Method
Moderate+Severe Anaemia Prevalence
Age (year)
Location Hierarchy
1 − Global
2 − Super Region
3 − Region
4 − Country
5 − Subnational (Level 1)
Uncertainty Interval
5 − Subnational (Level 1)

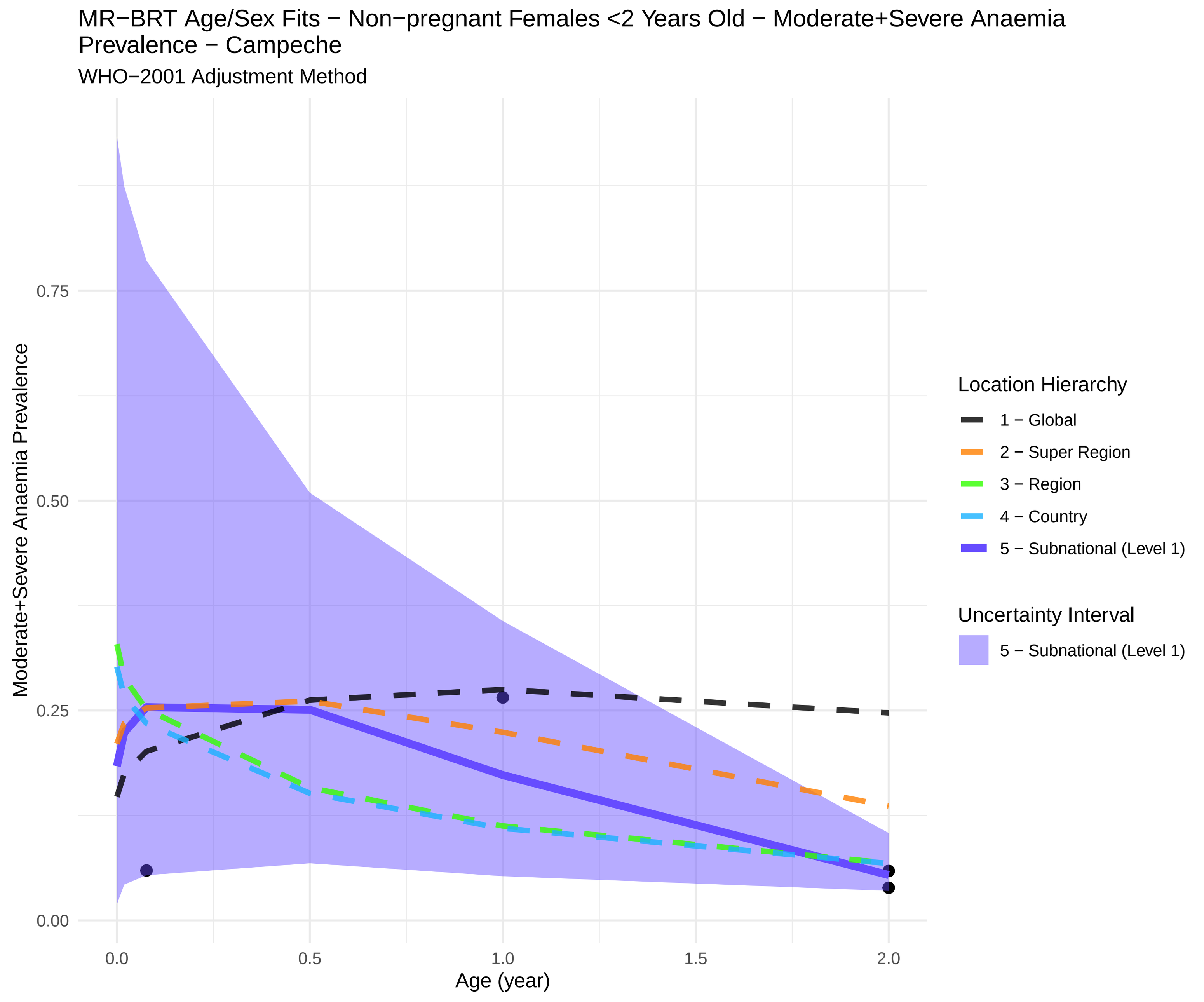
MR−BRT Age/Sex Fits − Non−pregnant Females <2 Years Old − Moderate+Severe Anaemia
Prevalence − Campeche
WHO−2001 Adjustment Method
Moderate+Severe Anaemia Prevalence
Age (year)
Location Hierarchy
1 − Global
2 − Super Region
3 − Region
4 − Country
5 − Subnational (Level 1)
Uncertainty Interval
5 − Subnational (Level 1)

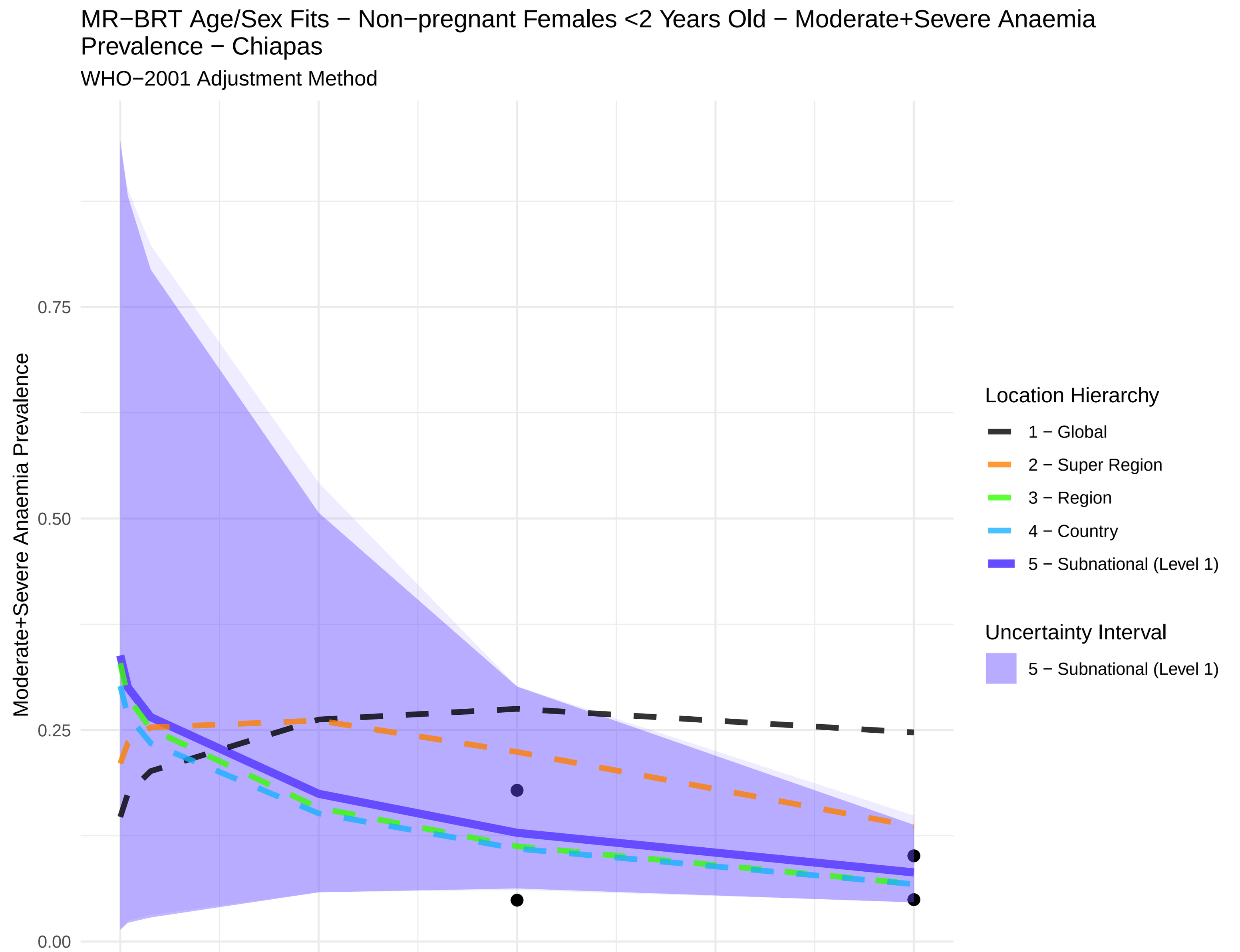
MR−BRT Age/Sex Fits − Non−pregnant Females <2 Years Old − Moderate+Severe Anaemia
Prevalence − Chiapas
WHO−2001 Adjustment Method
Moderate+Severe Anaemia Prevalence
Age (year)
Location Hierarchy
1 − Global
2 − Super Region
3 − Region
4 − Country
5 − Subnational (Level 1)
Uncertainty Interval
5 − Subnational (Level 1)

MR−BRT Age/Sex Fits − Non−pregnant Females <2 Years Old − Moderate+Severe Anaemia Prevalence − Chihuahua
WHO−2001 Adjustment Method

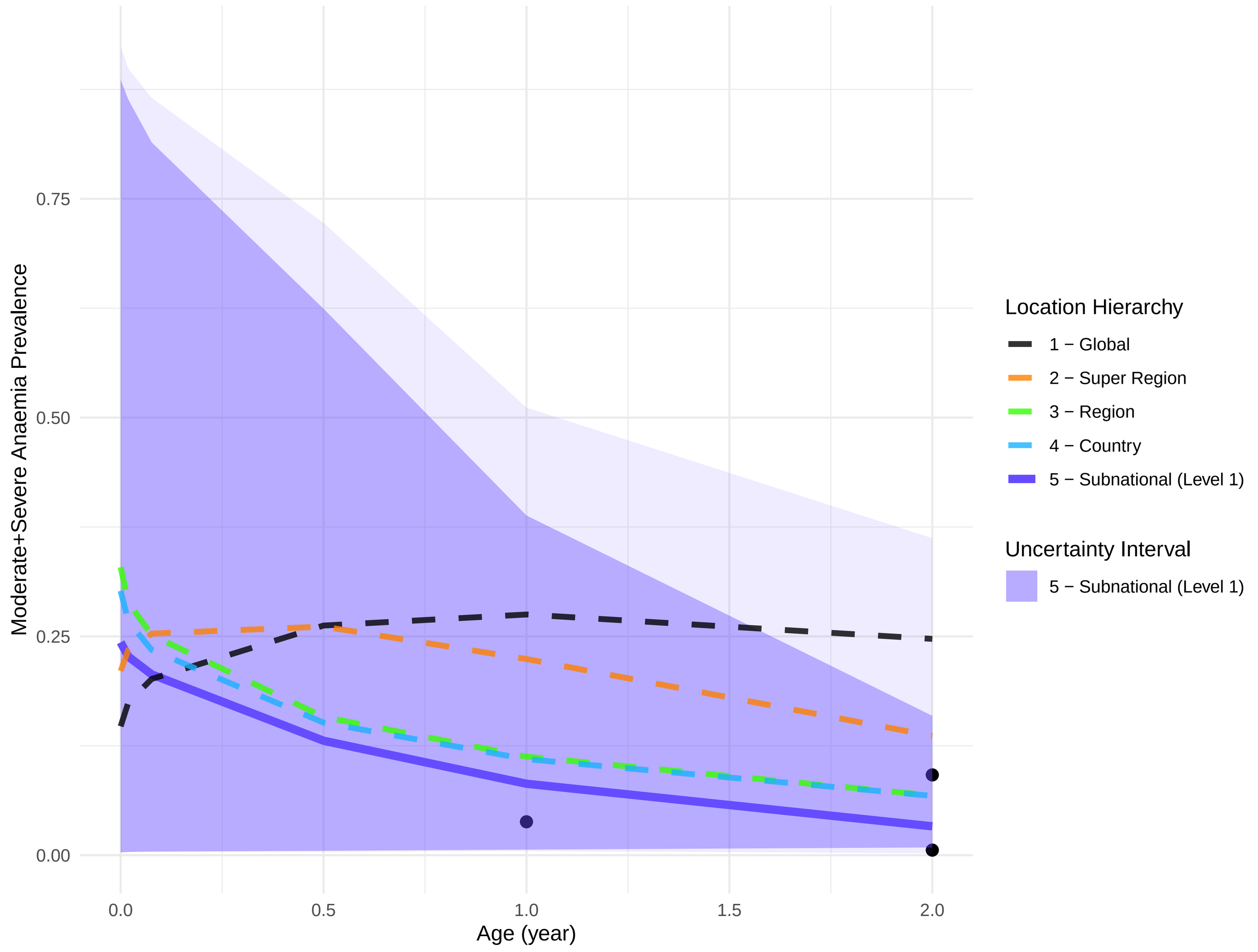

MR−BRT Age/Sex Fits − Non−pregnant Females <2 Years Old − Moderate+Severe Anaemia Prevalence − Coahuila
WHO−2001 Adjustment Method

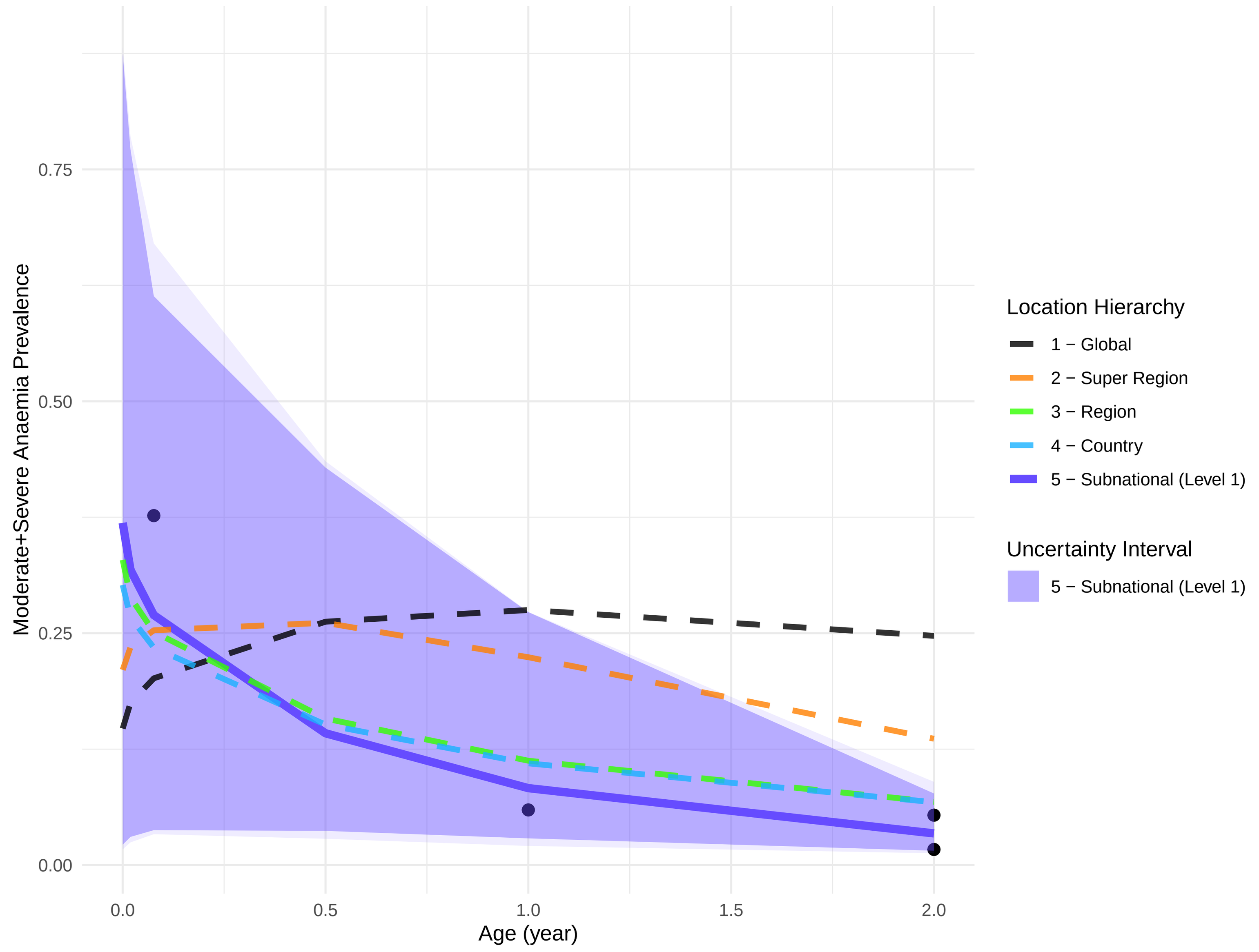

MR−BRT Age/Sex Fits − Non−pregnant Females <2 Years Old − Moderate+Severe Anaemia Prevalence − Colima
WHO−2001 Adjustment Method

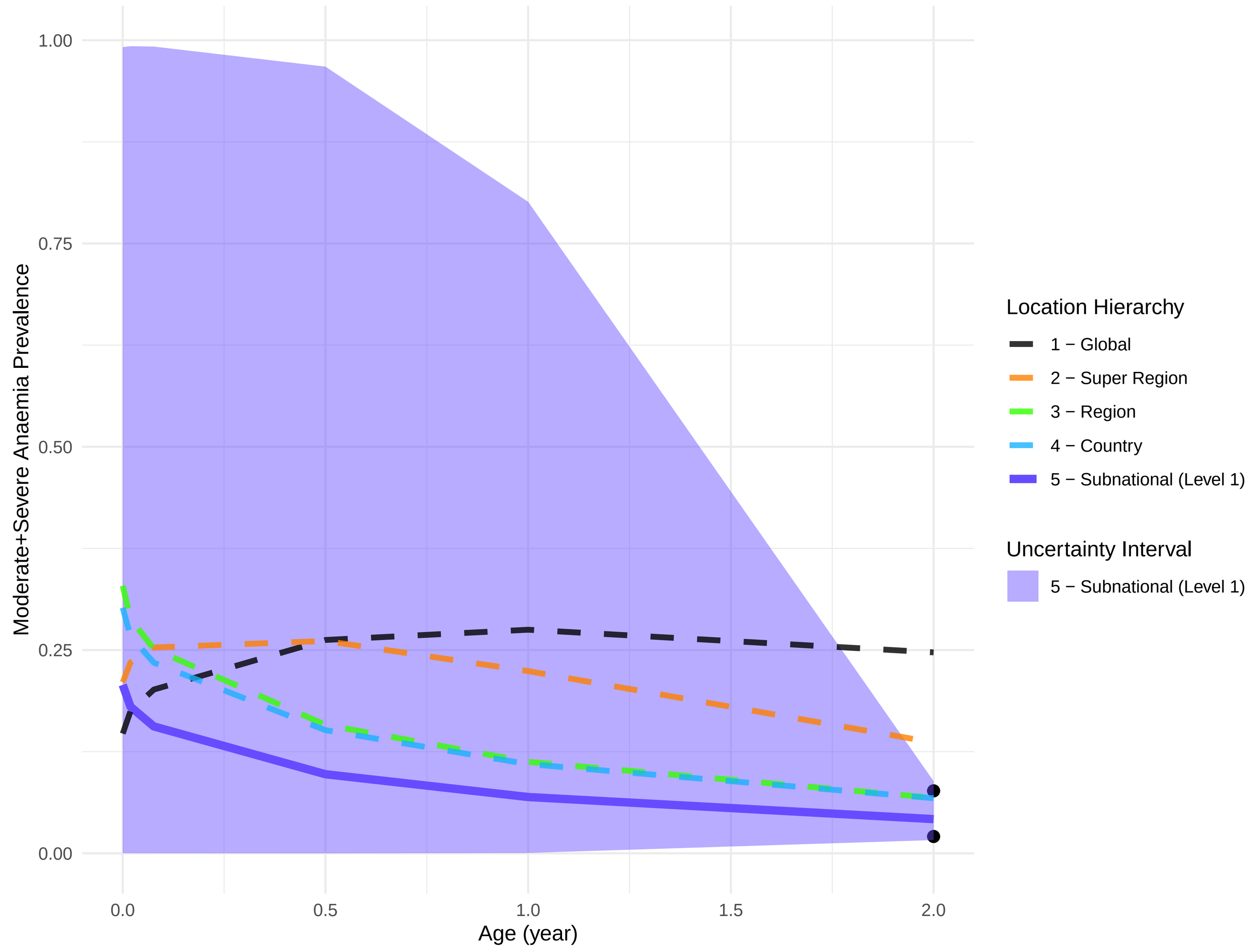

MR−BRT Age/Sex Fits − Non−pregnant Females <2 Years Old − Moderate+Severe Anaemia Prevalence − Durango
WHO−2001 Adjustment Method

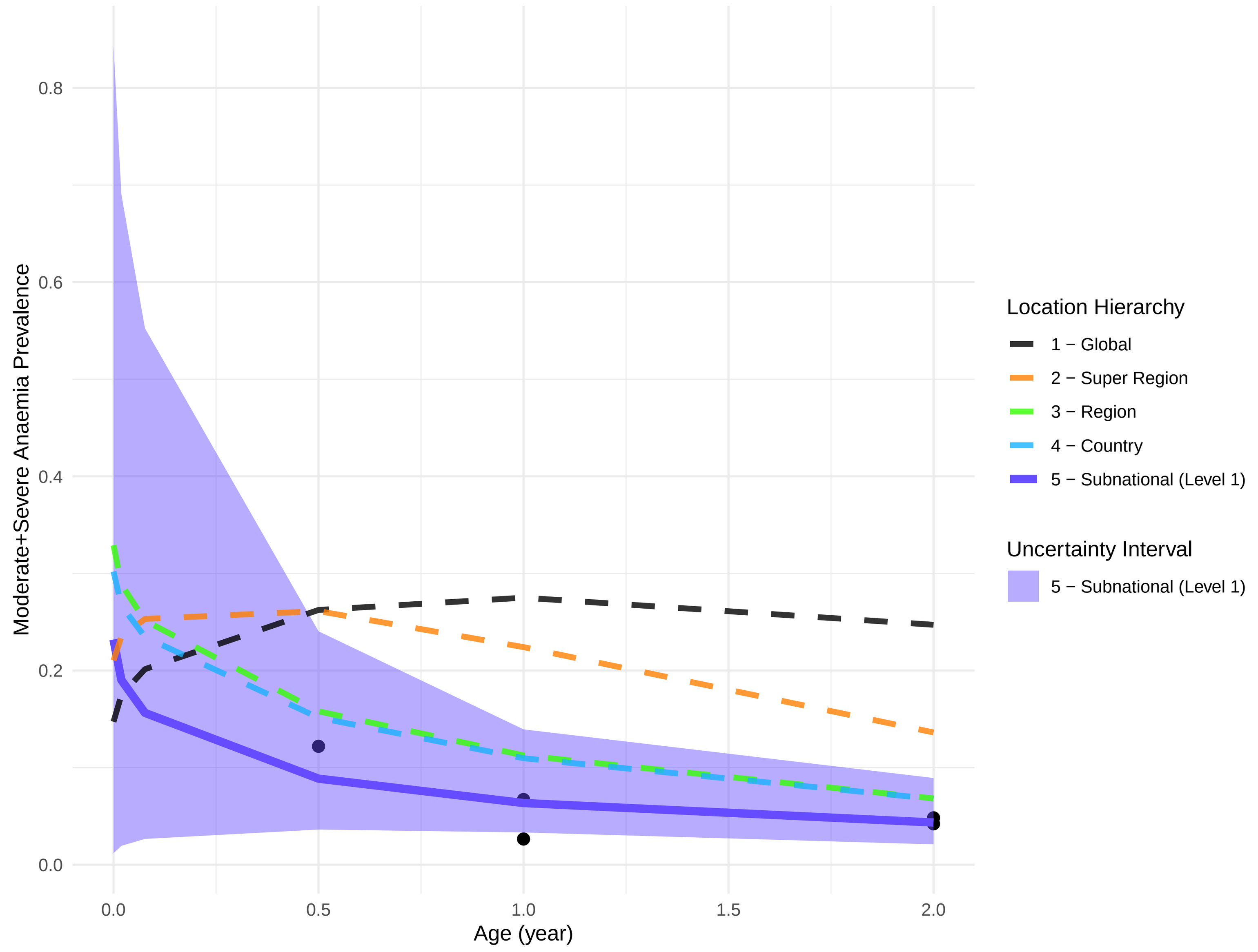

MR−BRT Age/Sex Fits − Non−pregnant Females <2 Years Old − Moderate+Severe Anaemia Prevalence − Guanajuato
WHO−2001 Adjustment Method

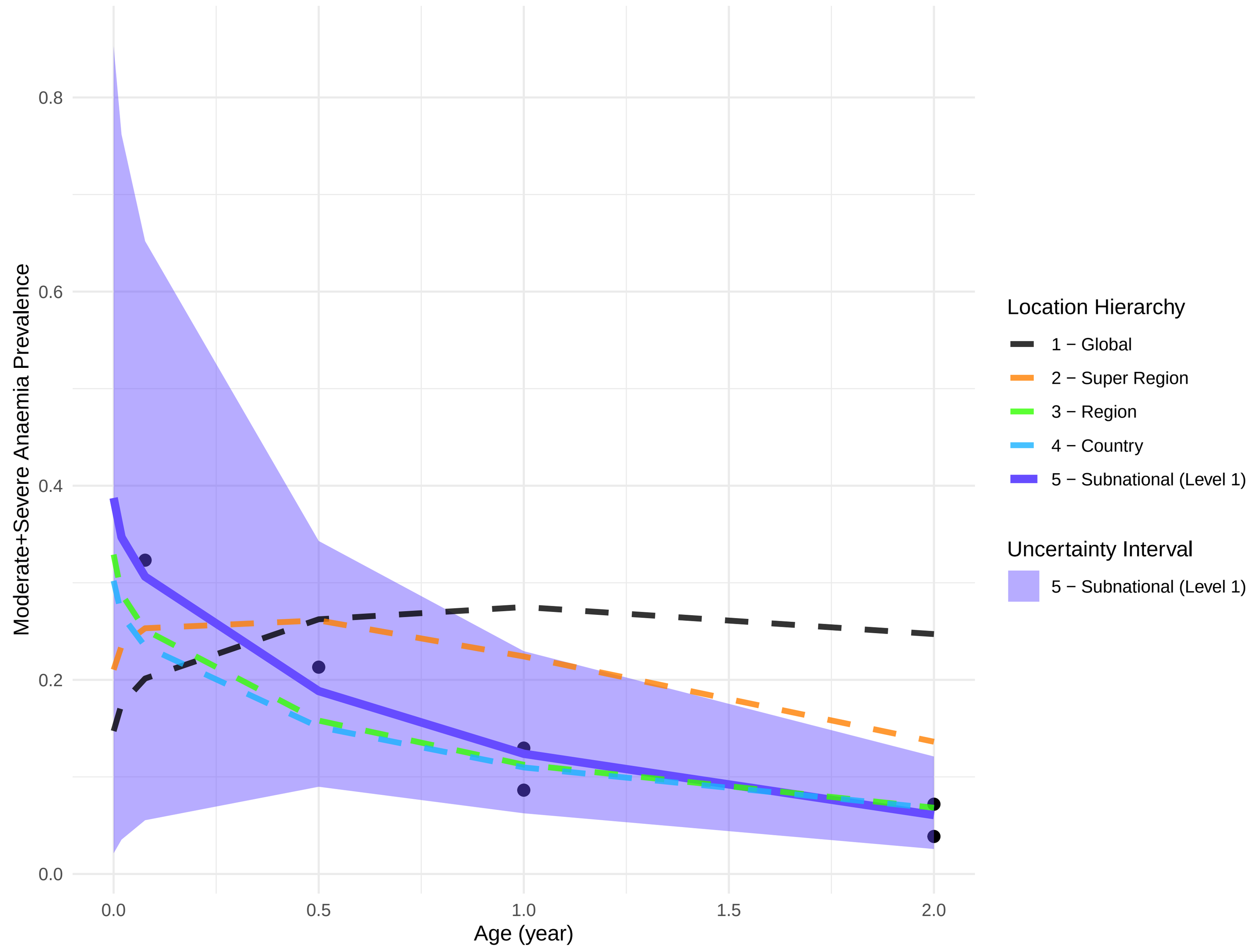

MR−BRT Age/Sex Fits − Non−pregnant Females <2 Years Old − Moderate+Severe Anaemia Prevalence − Guerrero
WHO−2001 Adjustment Method

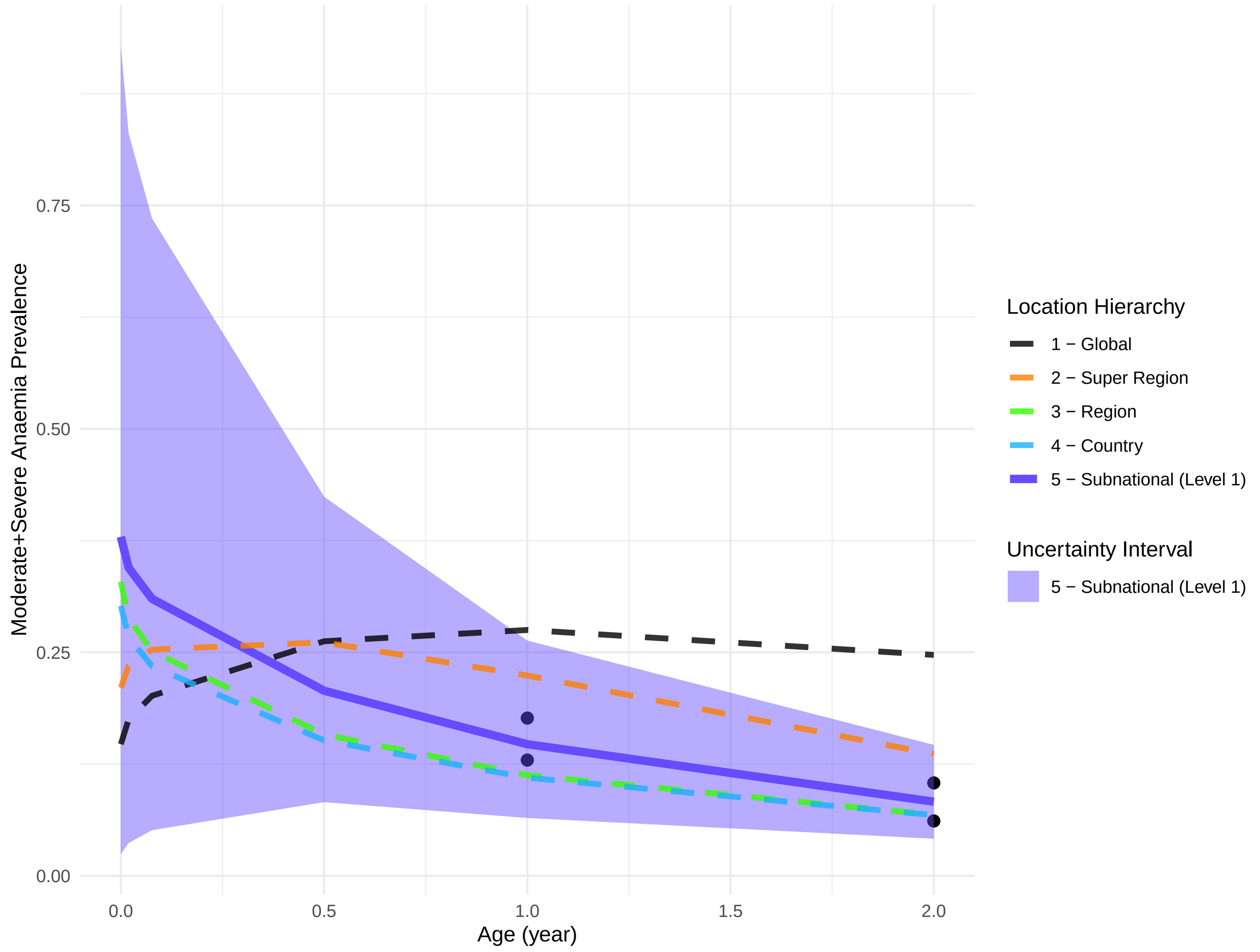

MR−BRT Age/Sex Fits − Non−pregnant Females <2 Years Old − Moderate+Severe Anaemia Prevalence − Hidalgo
WHO−2001 Adjustment Method

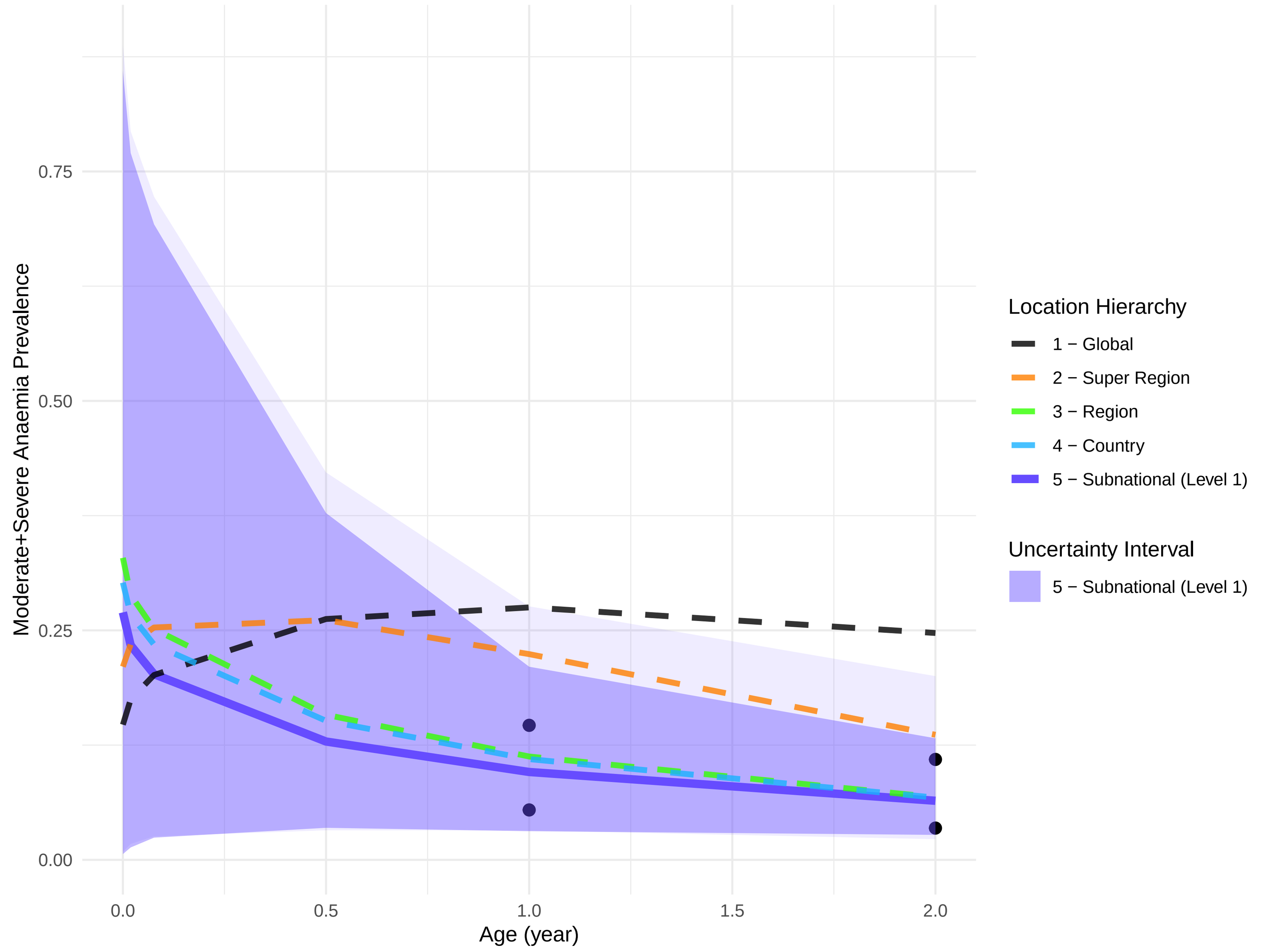

MR−BRT Age/Sex Fits − Non−pregnant Females <2 Years Old − Moderate+Severe Anaemia Prevalence − Jalisco
WHO−2001 Adjustment Method

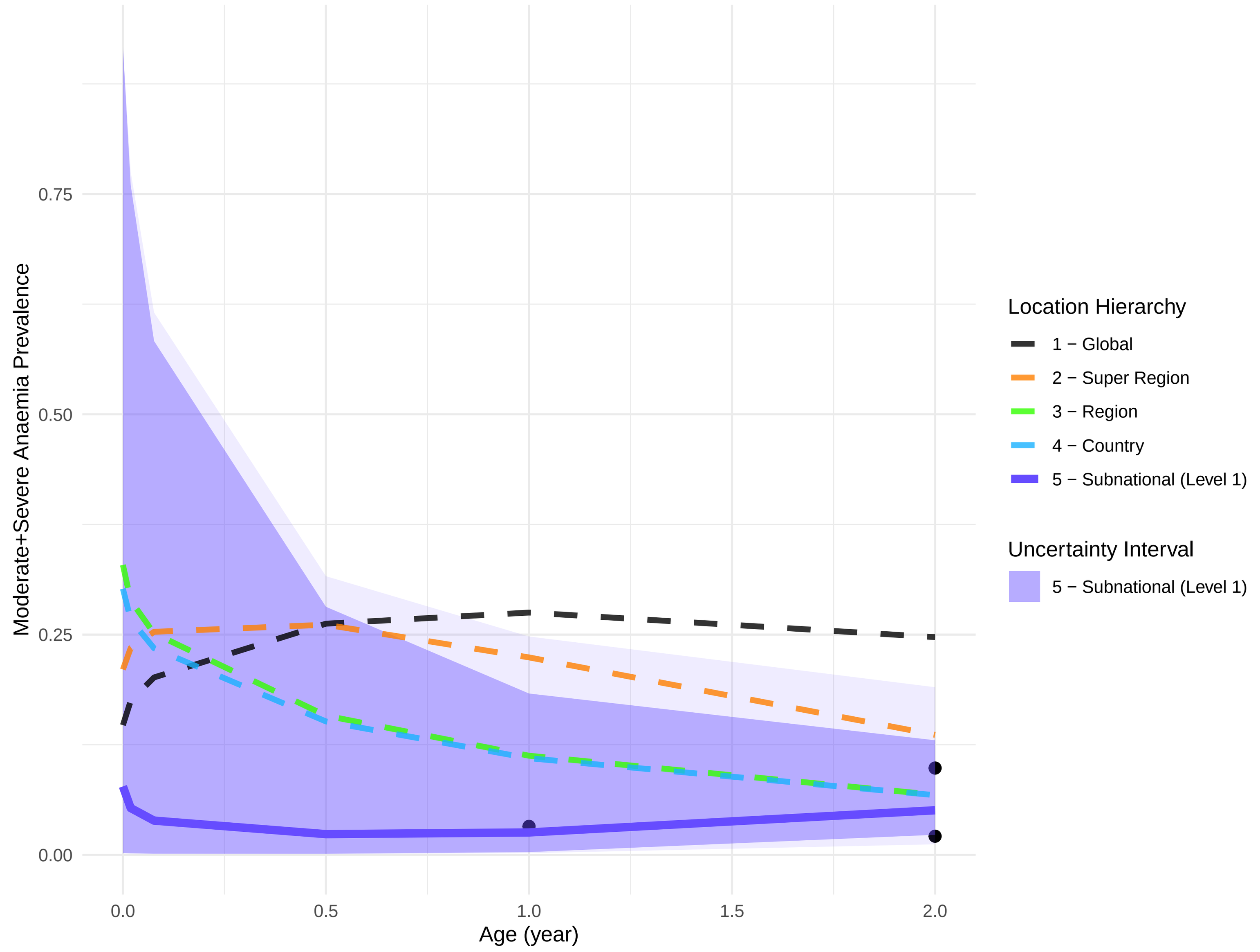

MR−BRT Age/Sex Fits − Non−pregnant Females <2 Years Old − Moderate+Severe Anaemia Prevalence − México
WHO−2001 Adjustment Method

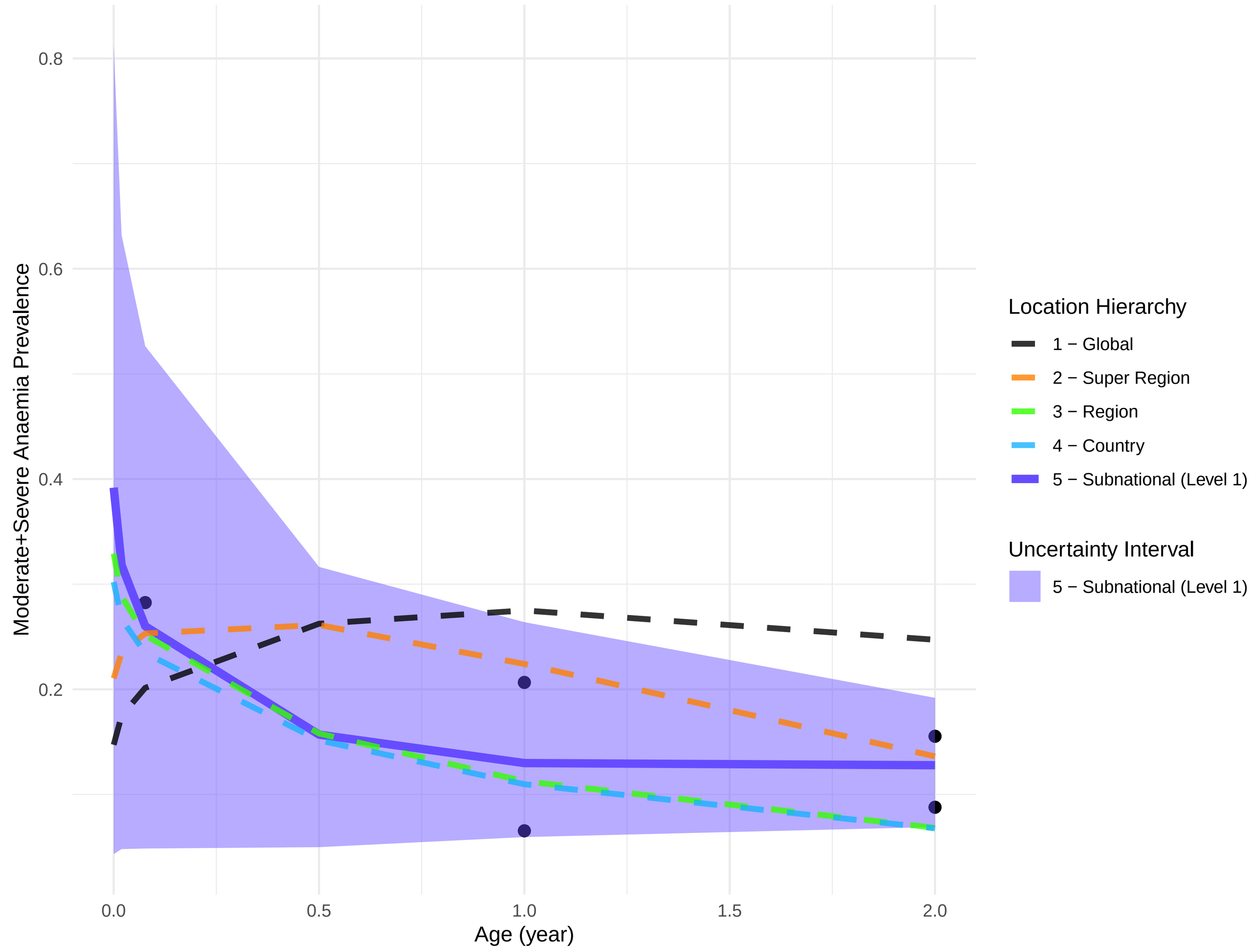

MR−BRT Age/Sex Fits − Non−pregnant Females <2 Years Old − Moderate+Severe Anaemia Prevalence − Mexico City
WHO−2001 Adjustment Method

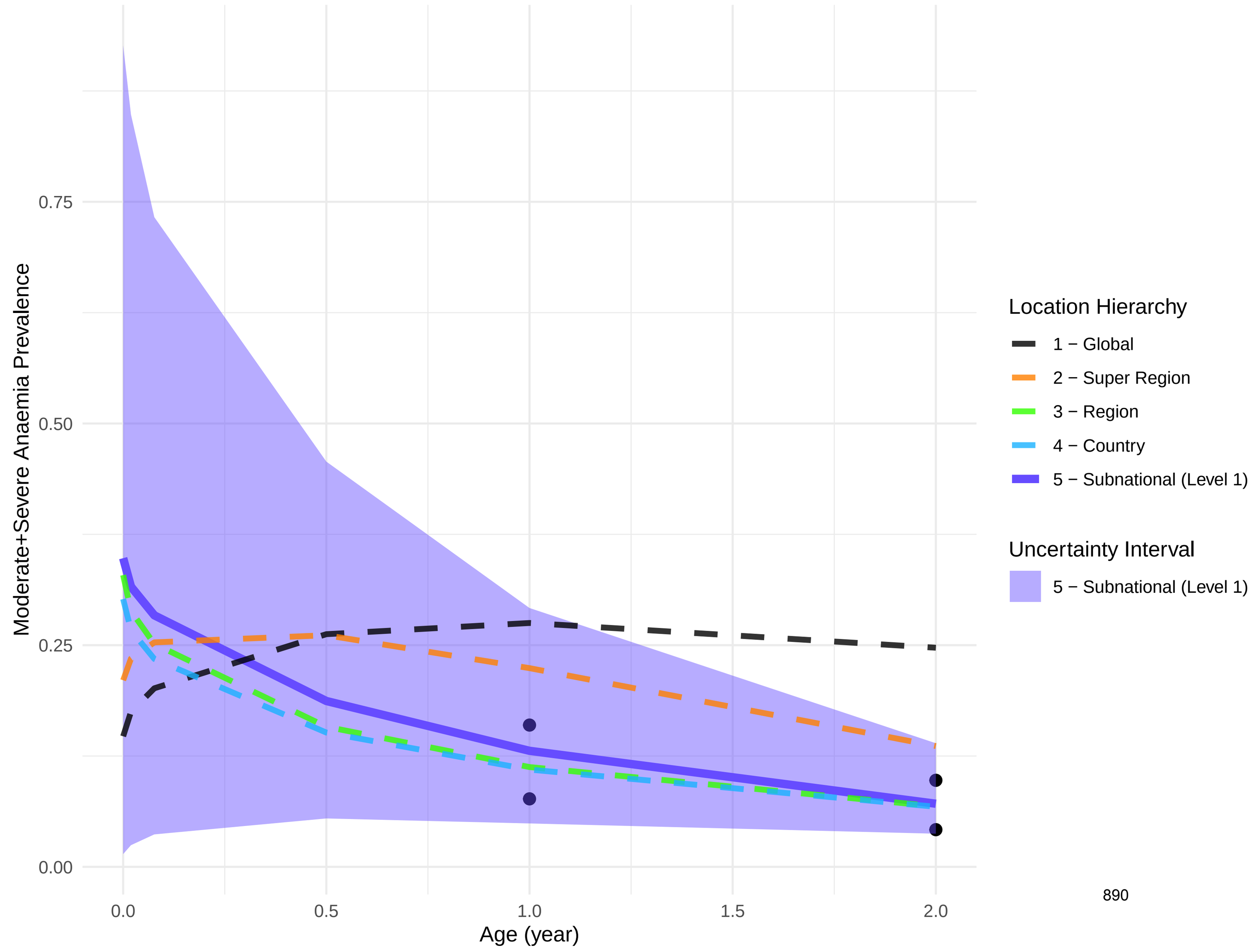

MR−BRT Age/Sex Fits − Non−pregnant Females <2 Years Old − Moderate+Severe Anaemia Prevalence − Michoacán de Ocampo
WHO−2001 Adjustment Method

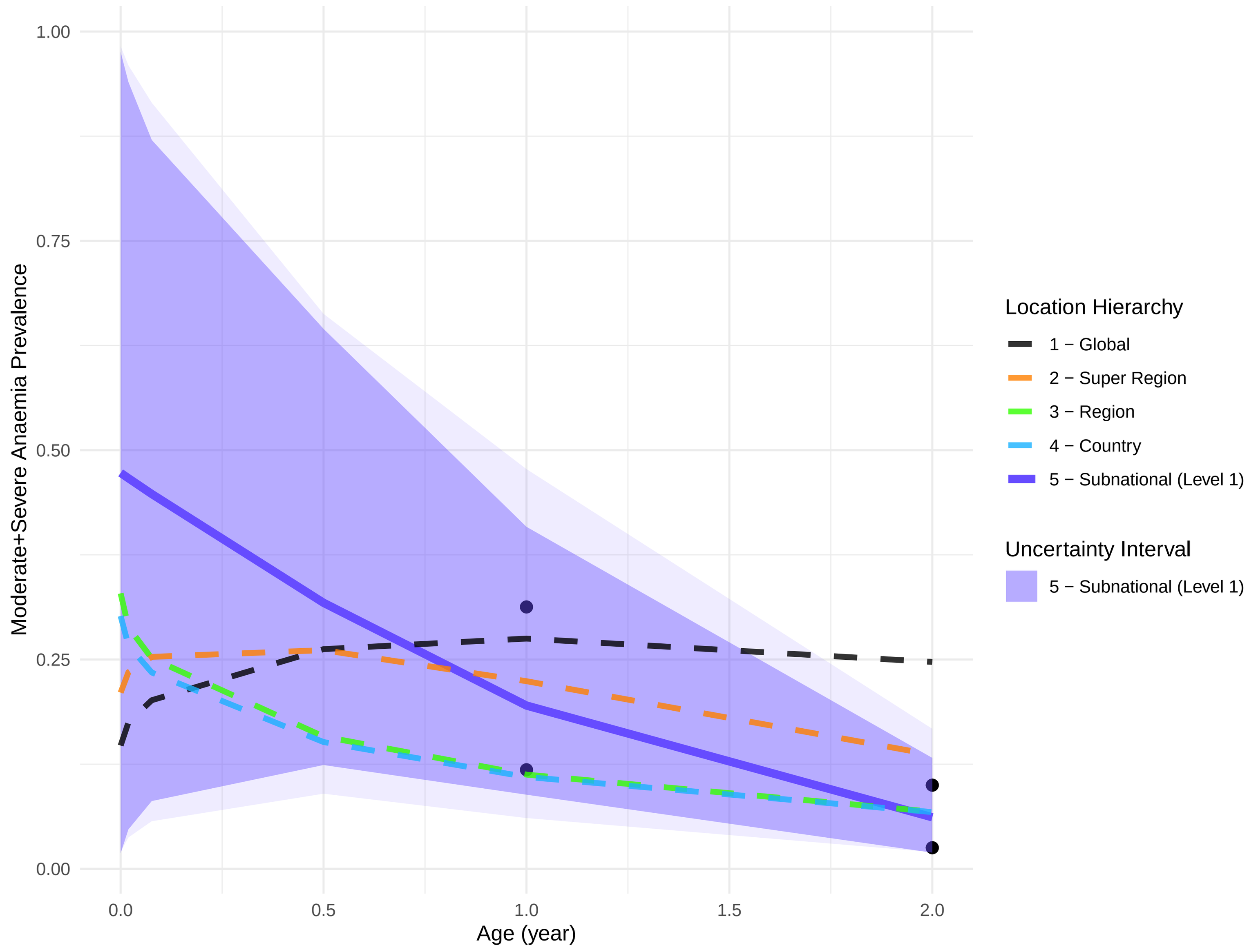

MR−BRT Age/Sex Fits − Non−pregnant Females <2 Years Old − Moderate+Severe Anaemia Prevalence − Morelos
WHO−2001 Adjustment Method

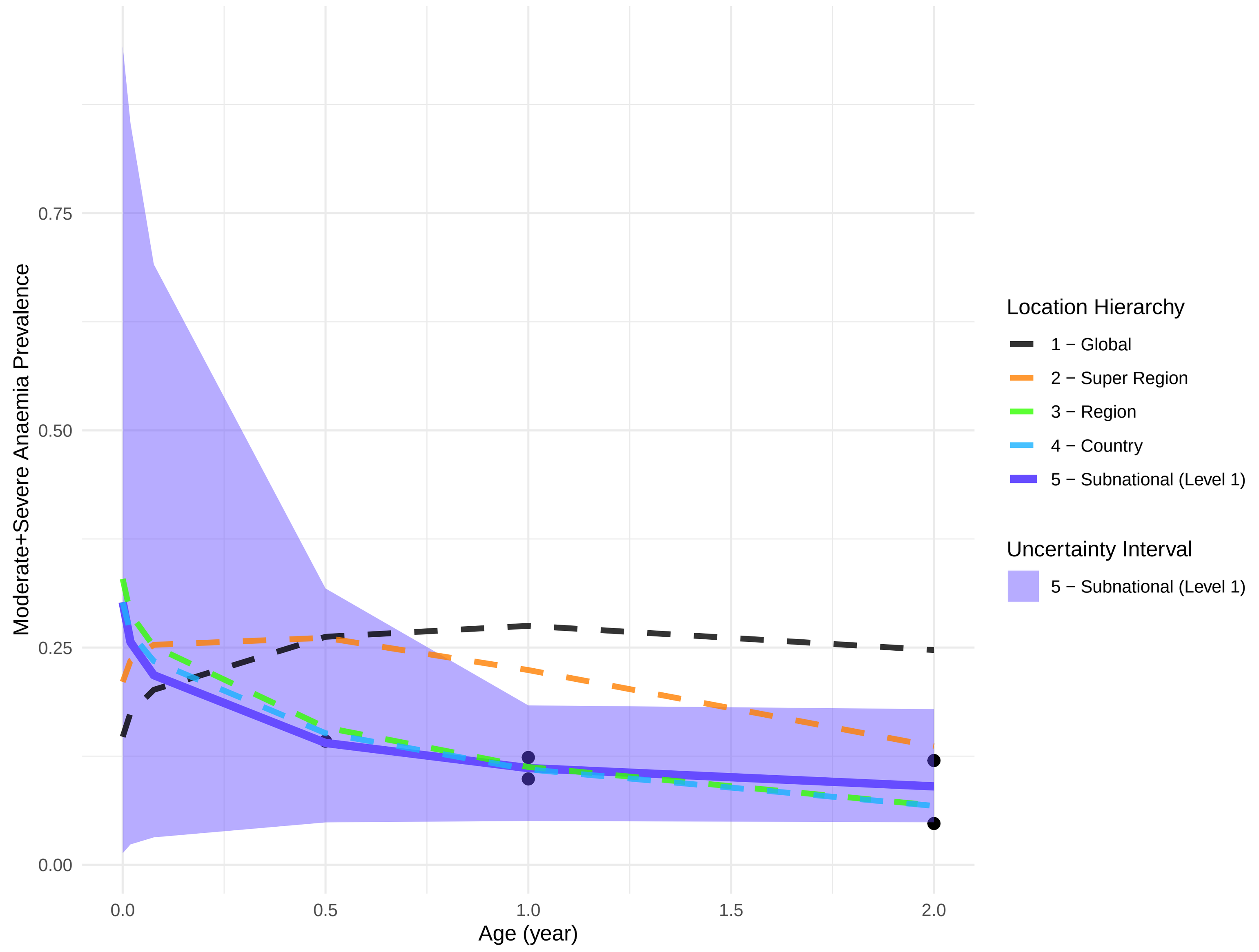

MR−BRT Age/Sex Fits − Non−pregnant Females <2 Years Old − Moderate+Severe Anaemia Prevalence − Nayarit
WHO−2001 Adjustment Method

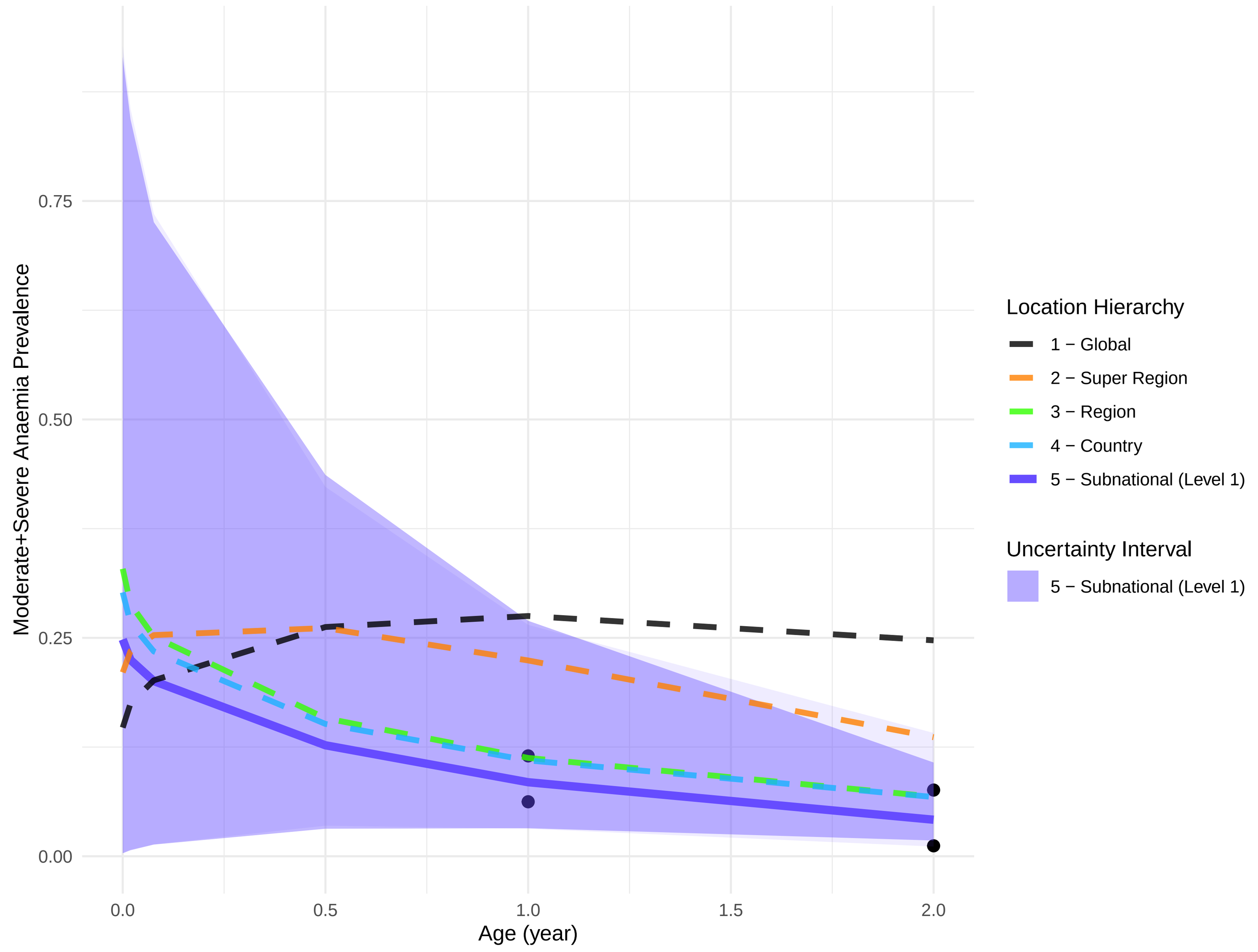

MR−BRT Age/Sex Fits − Non−pregnant Females <2 Years Old − Moderate+Severe Anaemia Prevalence − Nuevo León
WHO−2001 Adjustment Method

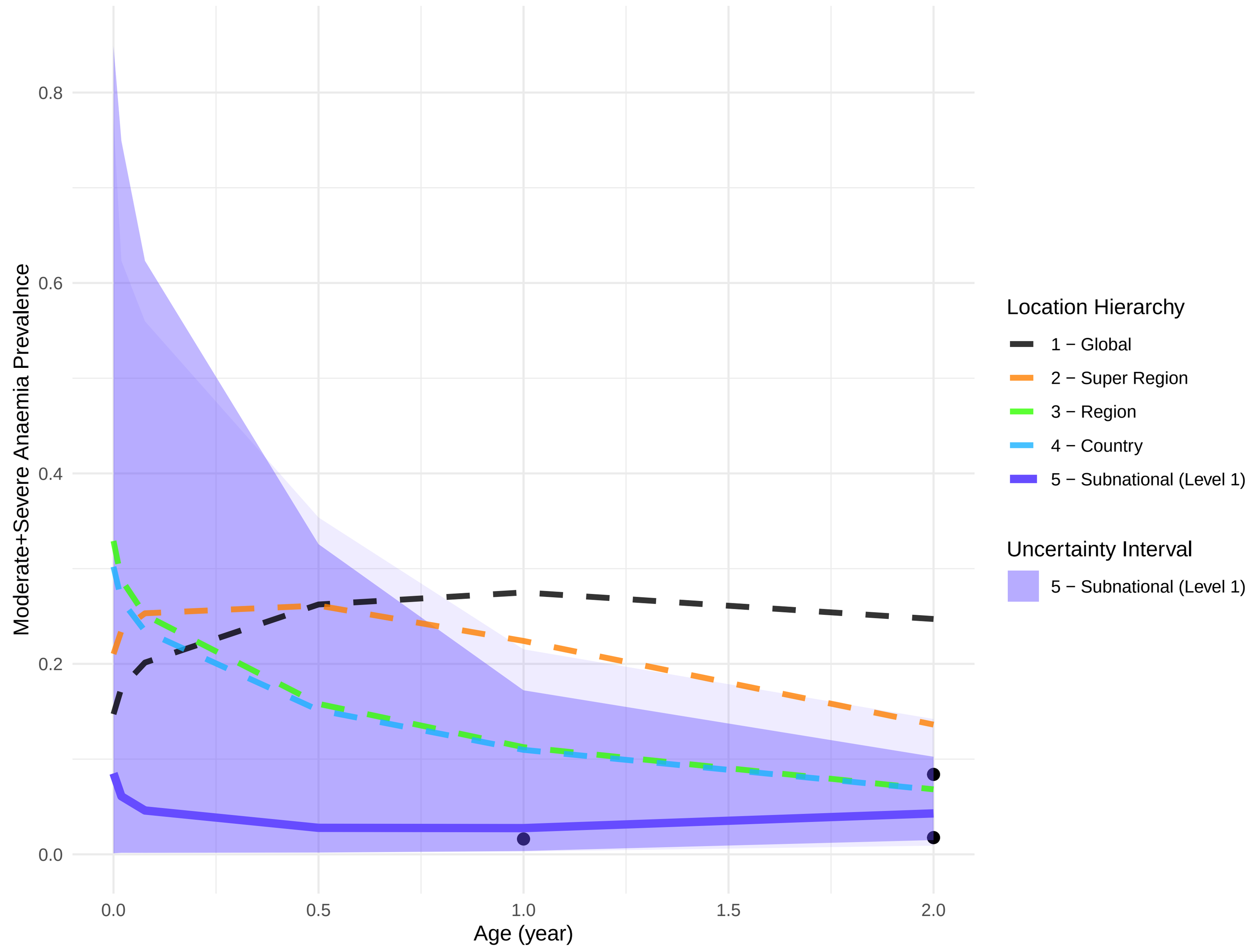

MR−BRT Age/Sex Fits − Non−pregnant Females <2 Years Old − Moderate+Severe Anaemia Prevalence − Oaxaca
WHO−2001 Adjustment Method

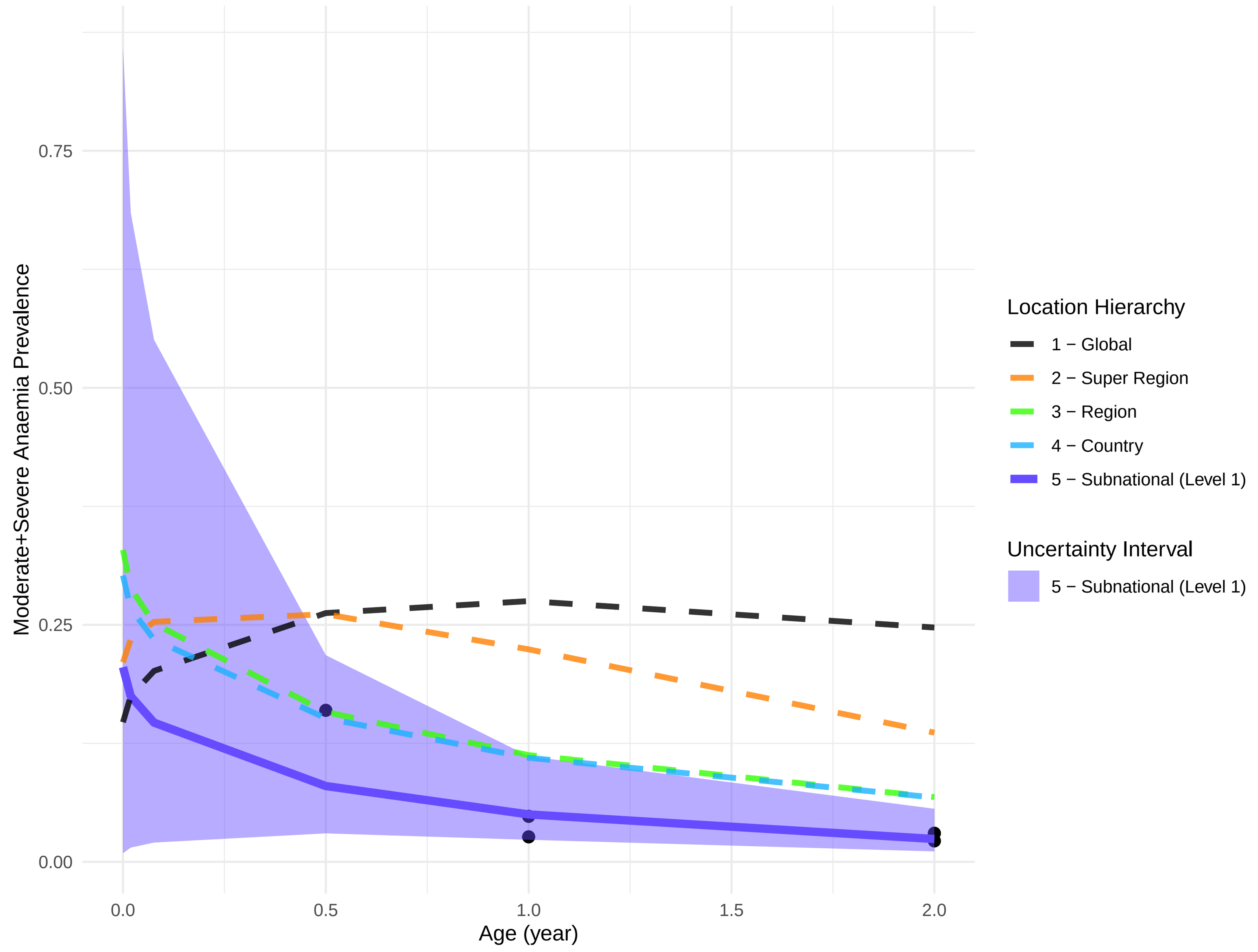

MR−BRT Age/Sex Fits − Non−pregnant Females <2 Years Old − Moderate+Severe Anaemia Prevalence − Puebla
WHO−2001 Adjustment Method

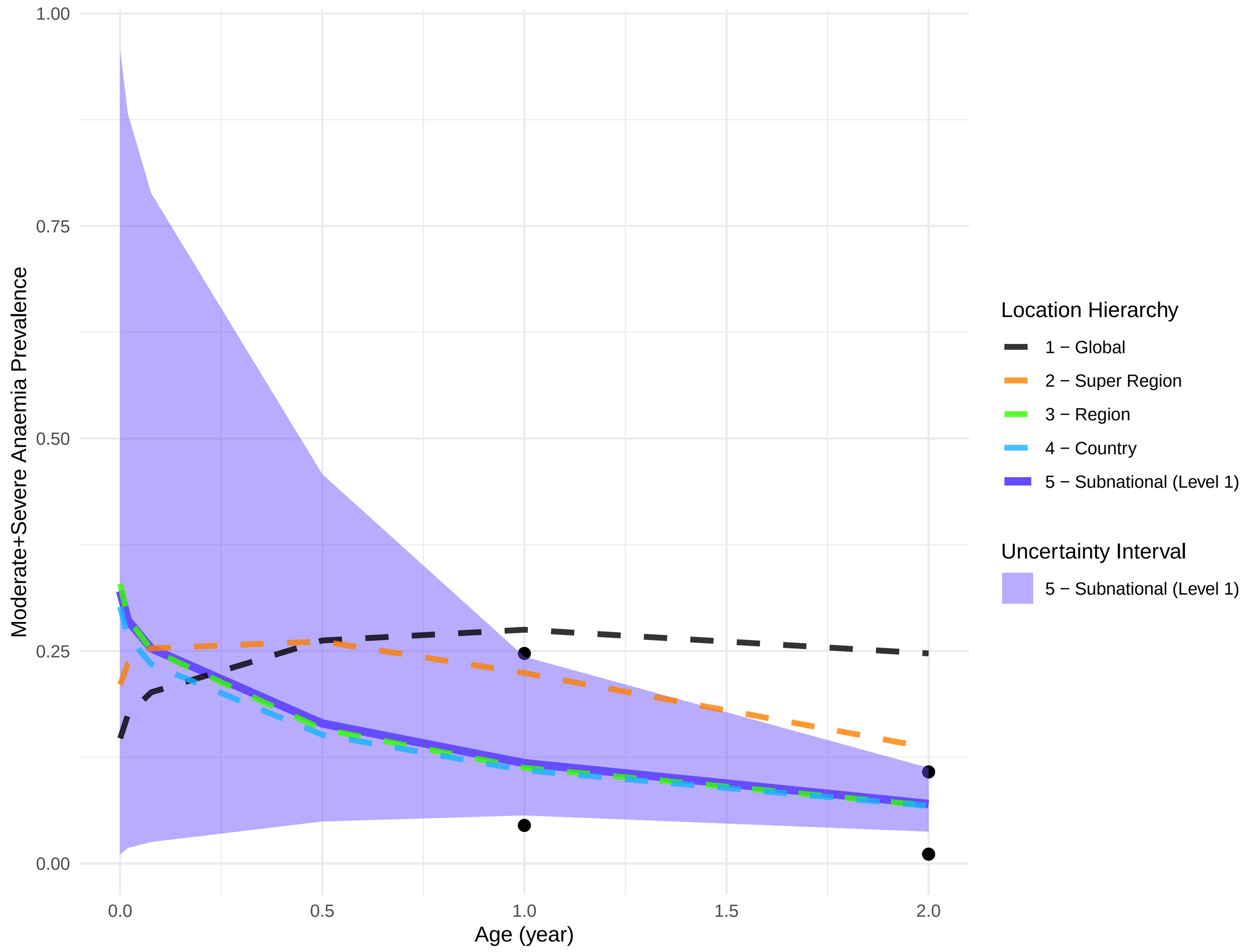

MR−BRT Age/Sex Fits − Non−pregnant Females <2 Years Old − Moderate+Severe Anaemia Prevalence − Querétaro
WHO−2001 Adjustment Method

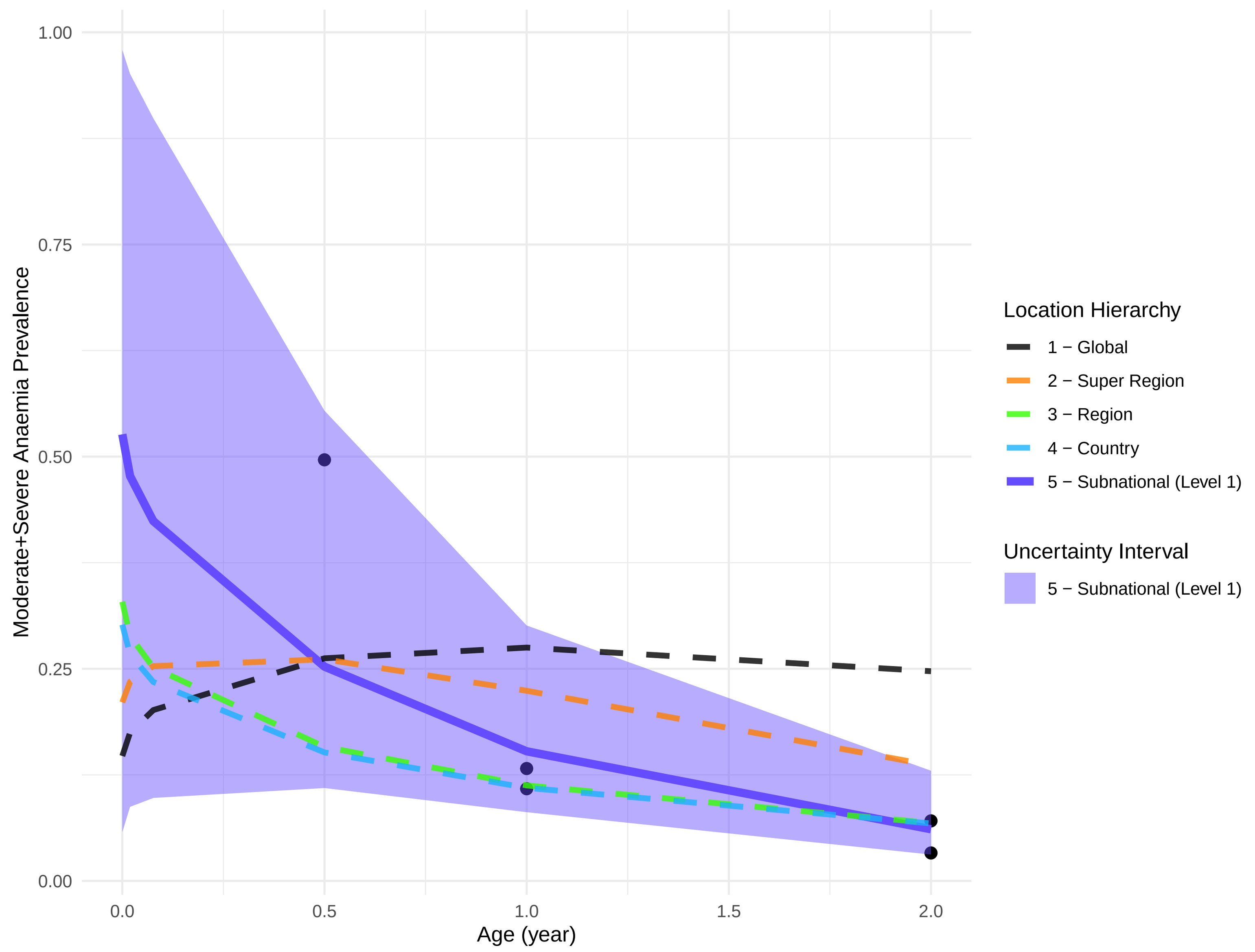

MR−BRT Age/Sex Fits − Non−pregnant Females <2 Years Old − Moderate+Severe Anaemia Prevalence − Quintana Roo
WHO−2001 Adjustment Method

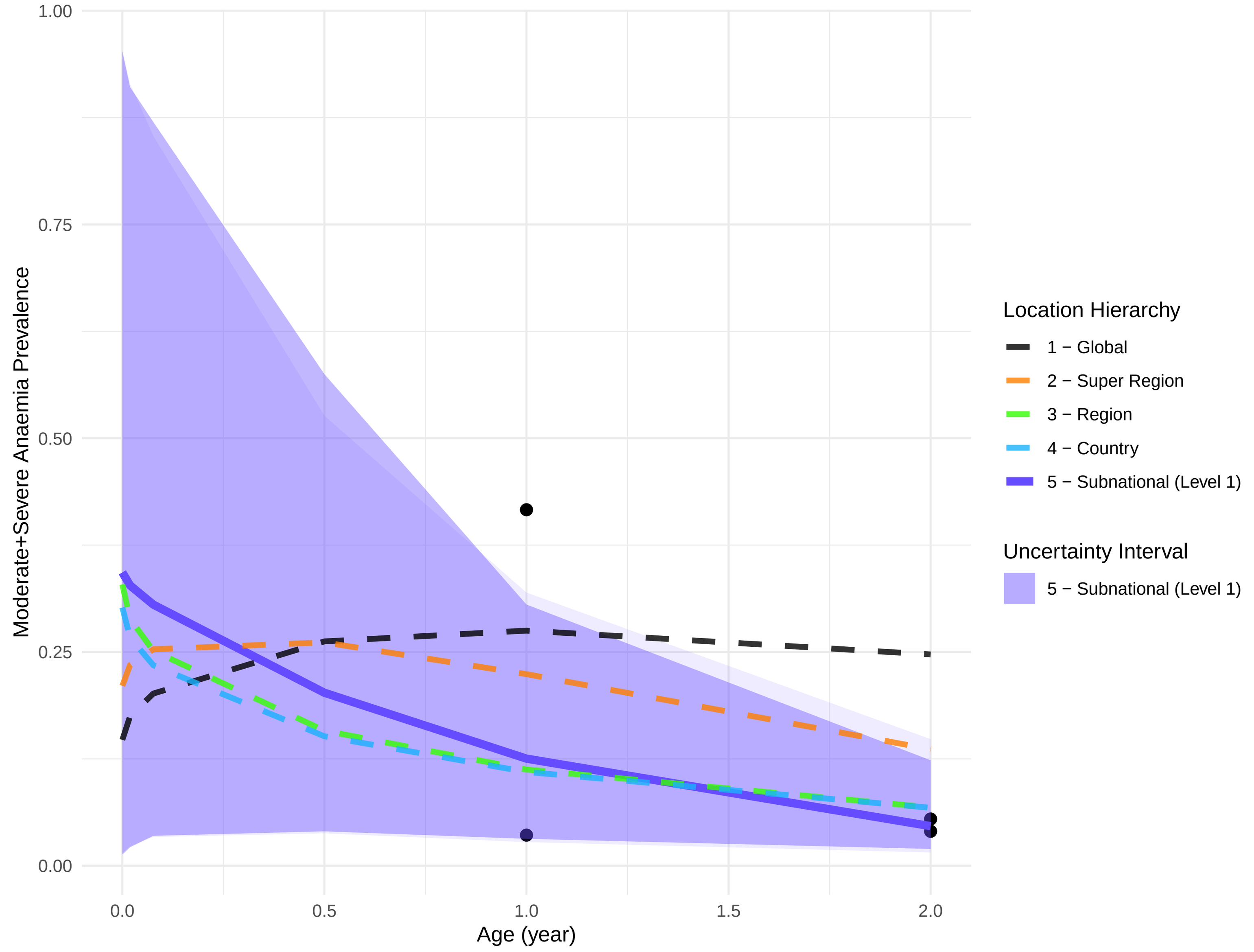

MR−BRT Age/Sex Fits − Non−pregnant Females <2 Years Old − Moderate+Severe Anaemia Prevalence − San Luis Potosí
WHO−2001 Adjustment Method

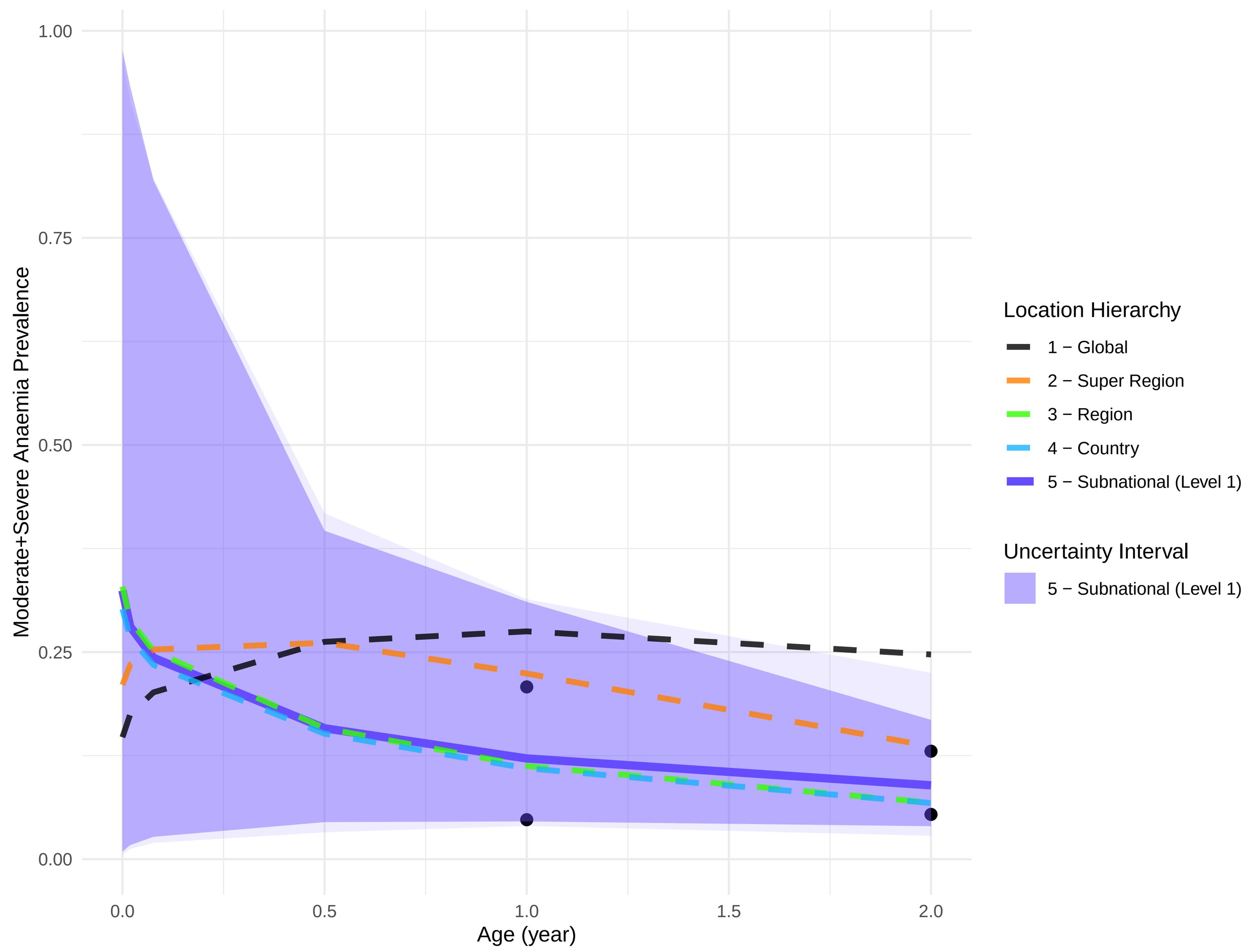

MR−BRT Age/Sex Fits − Non−pregnant Females <2 Years Old − Moderate+Severe Anaemia Prevalence − Sinaloa
WHO−2001 Adjustment Method

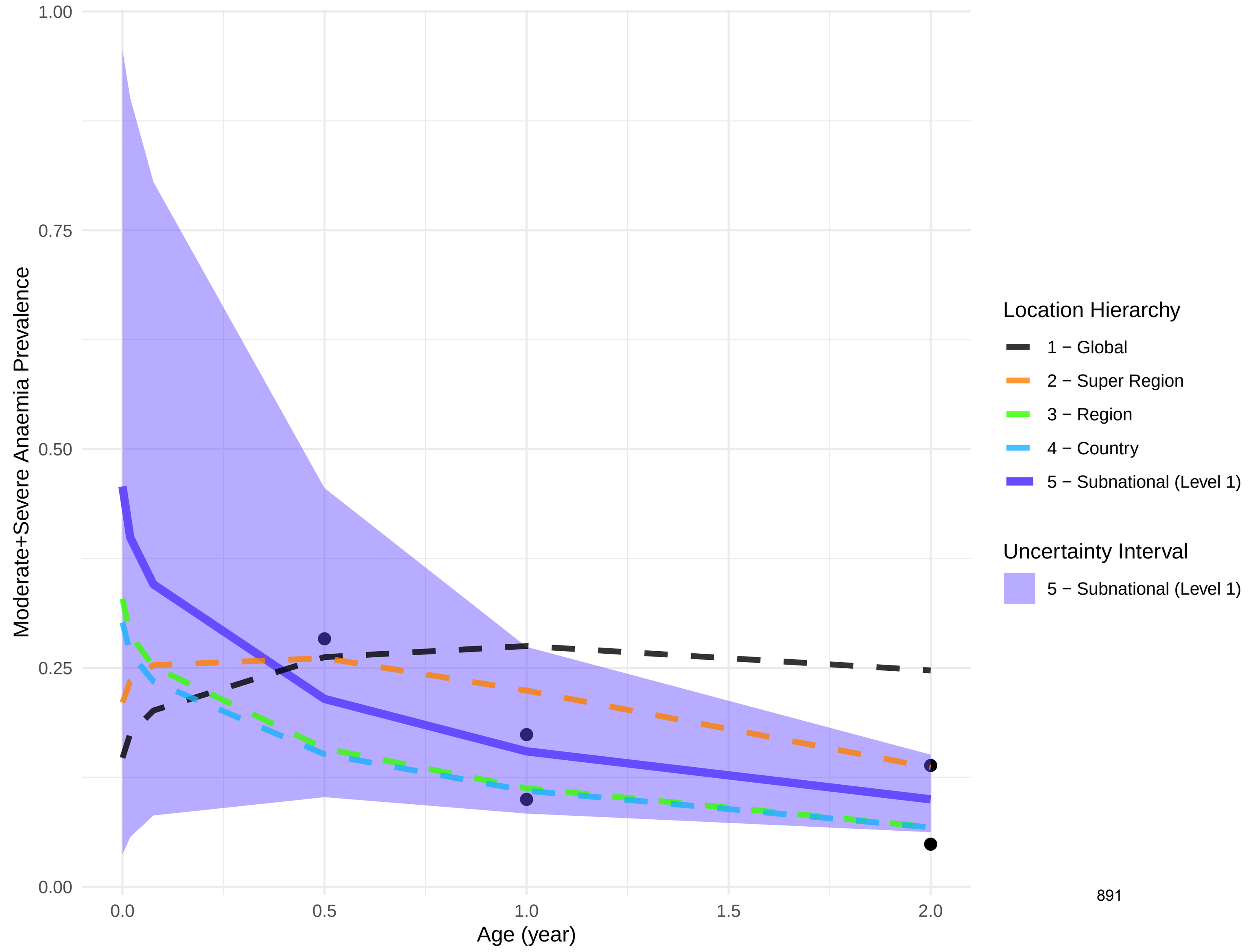

MR−BRT Age/Sex Fits − Non−pregnant Females <2 Years Old − Moderate+Severe Anaemia Prevalence − Sonora
WHO−2001 Adjustment Method

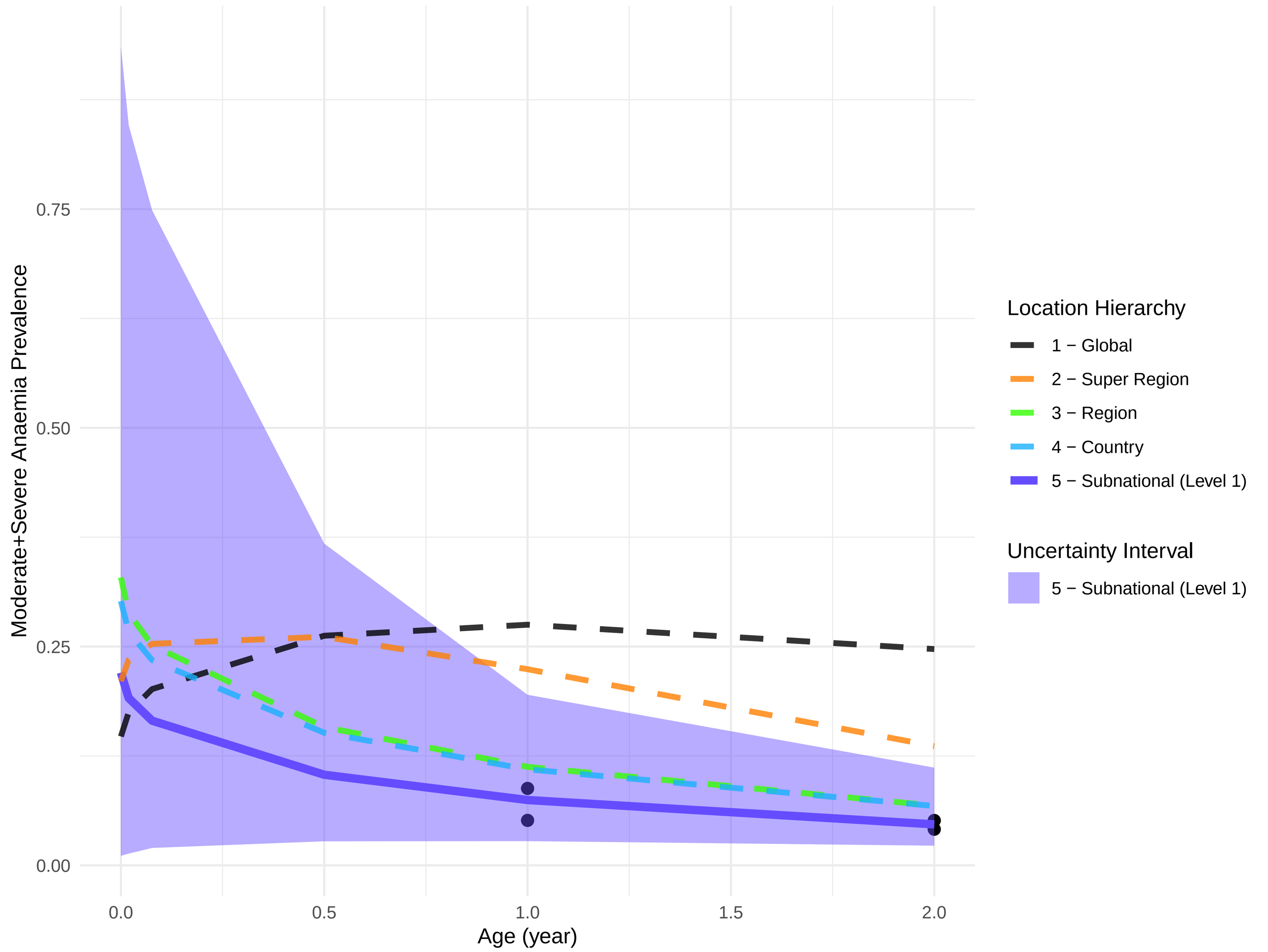

MR−BRT Age/Sex Fits − Non−pregnant Females <2 Years Old − Moderate+Severe Anaemia Prevalence − Tabasco
WHO−2001 Adjustment Method

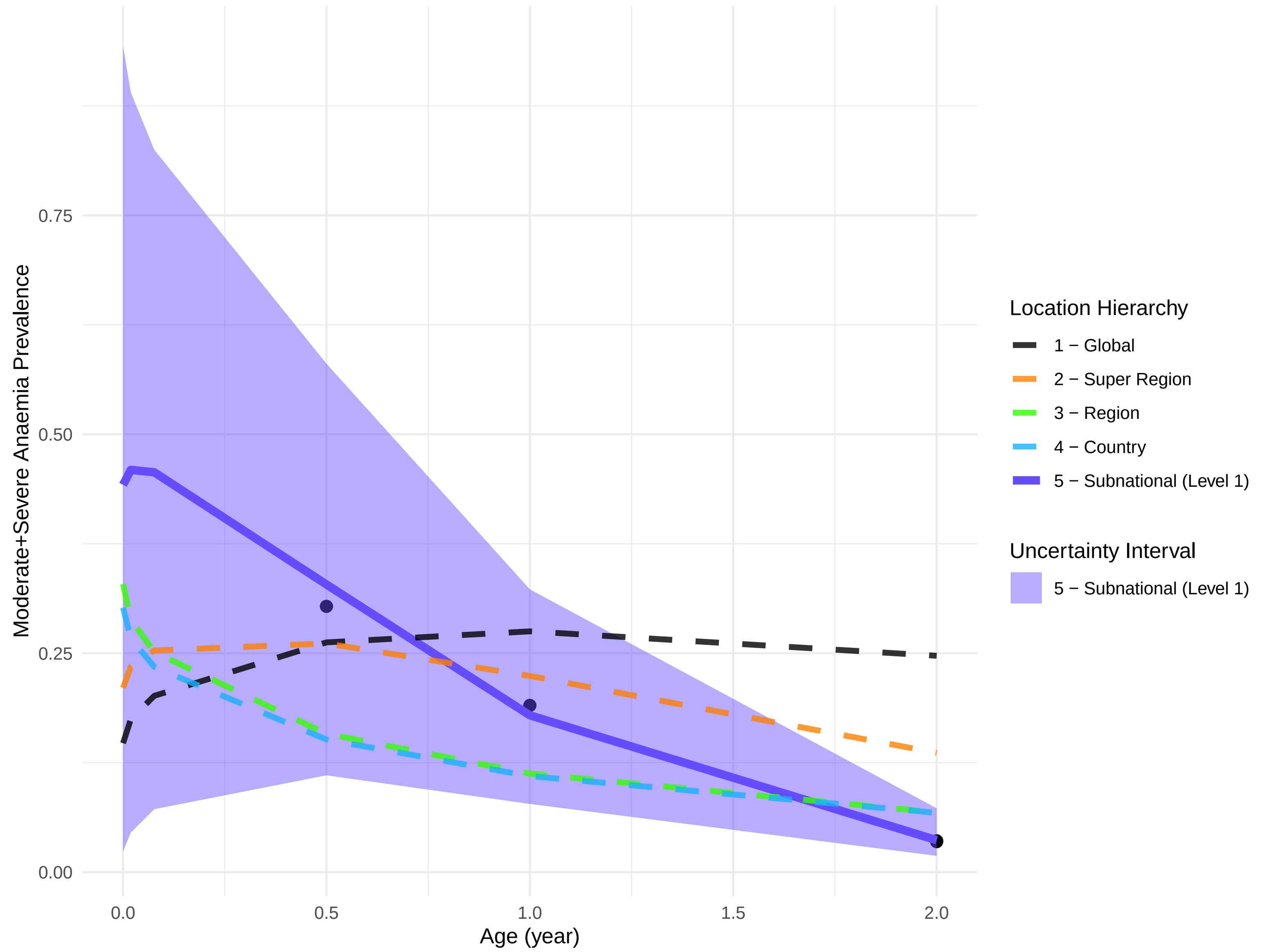

MR−BRT Age/Sex Fits − Non−pregnant Females <2 Years Old − Moderate+Severe Anaemia Prevalence − Tamaulipas
WHO−2001 Adjustment Method

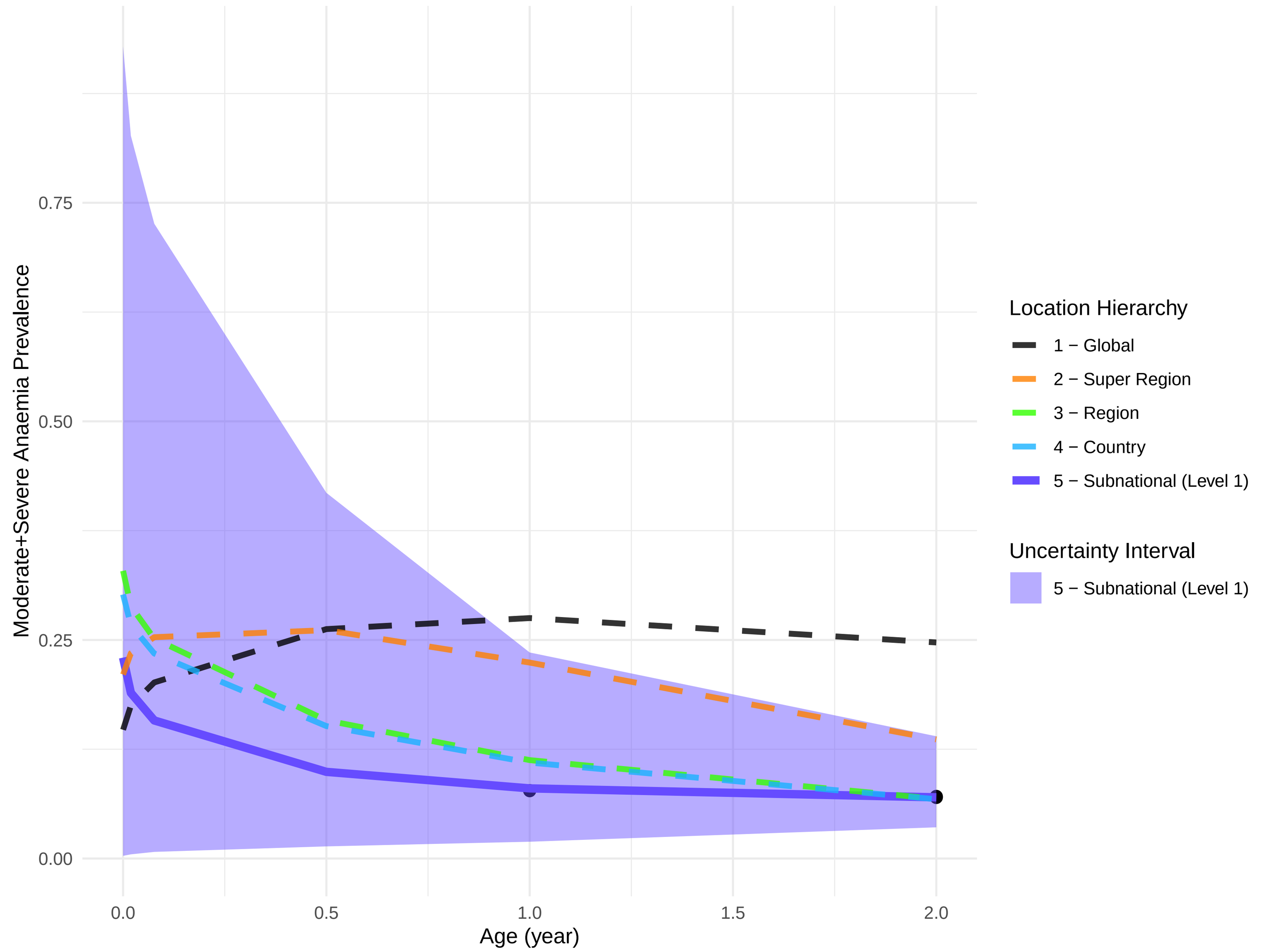

MR−BRT Age/Sex Fits − Non−pregnant Females <2 Years Old − Moderate+Severe Anaemia Prevalence − Tlaxcala
WHO−2001 Adjustment Method

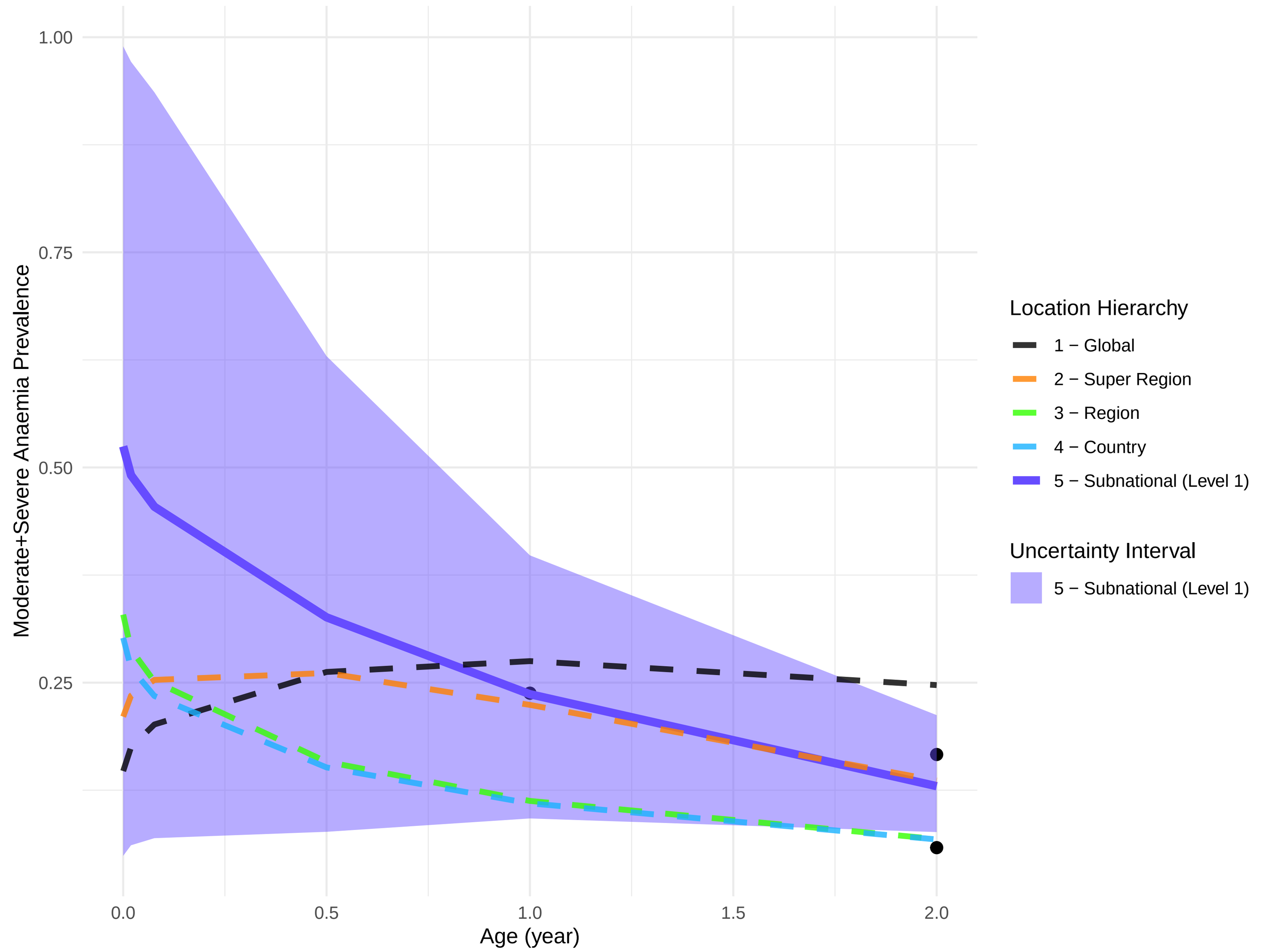

MR−BRT Age/Sex Fits − Non−pregnant Females <2 Years Old − Moderate+Severe Anaemia Prevalence − Veracruz de Ignacio de la Llave
WHO−2001 Adjustment Method

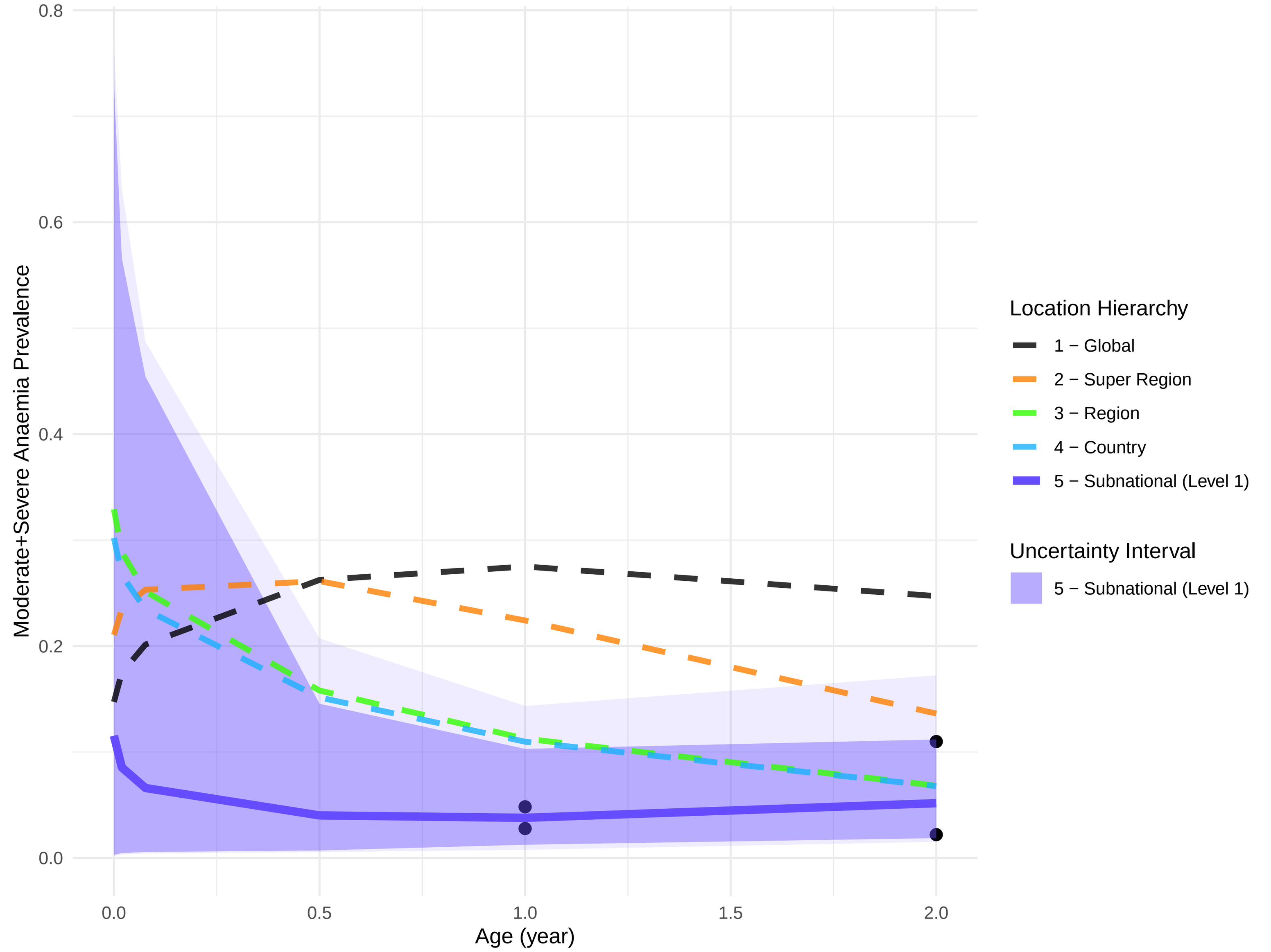

MR−BRT Age/Sex Fits − Non−pregnant Females <2 Years Old − Moderate+Severe Anaemia Prevalence − Yucatán
WHO−2001 Adjustment Method

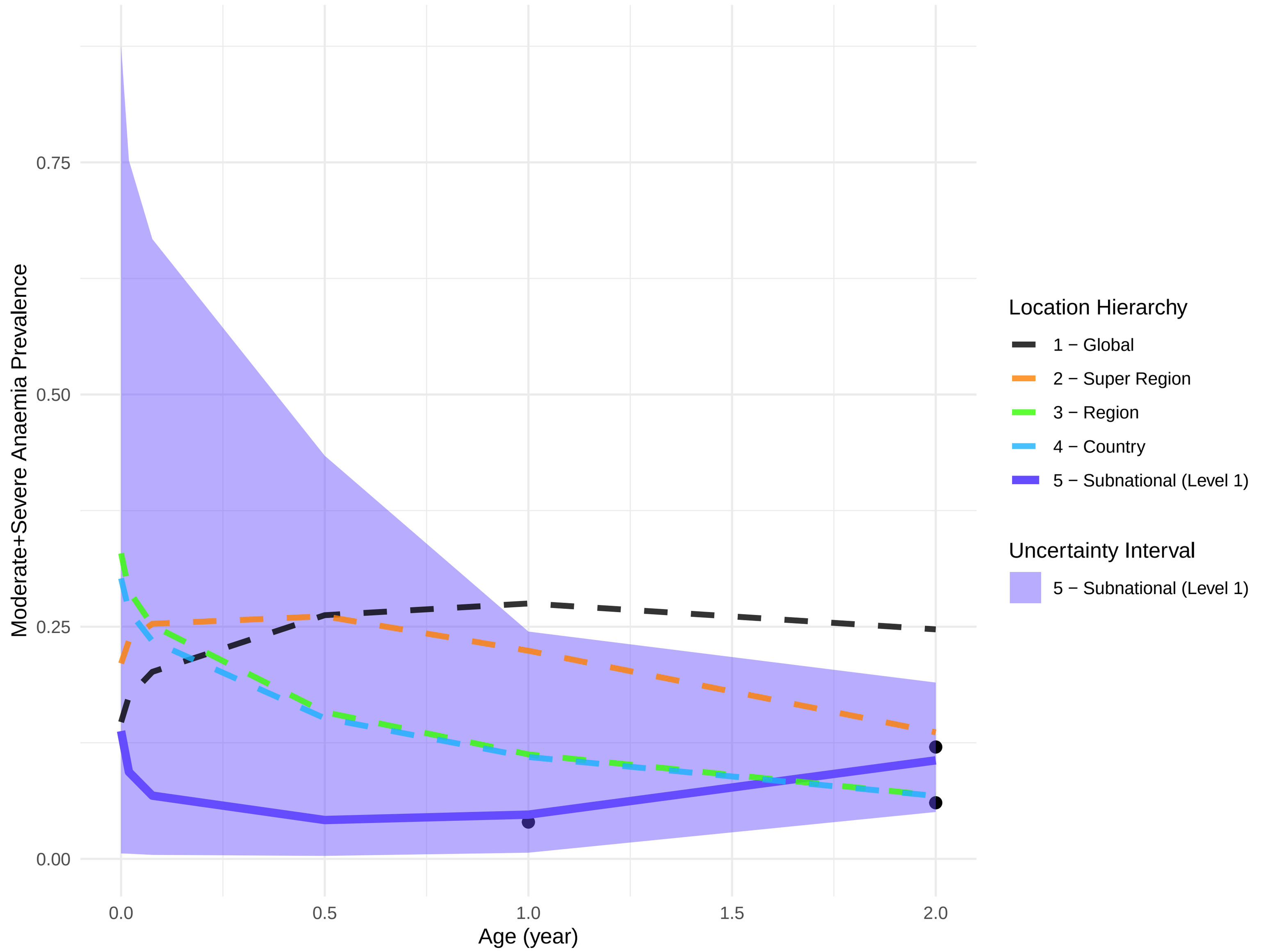

MR−BRT Age/Sex Fits − Non−pregnant Females <2 Years Old − Moderate+Severe Anaemia Prevalence − Zacatecas
WHO−2001 Adjustment Method

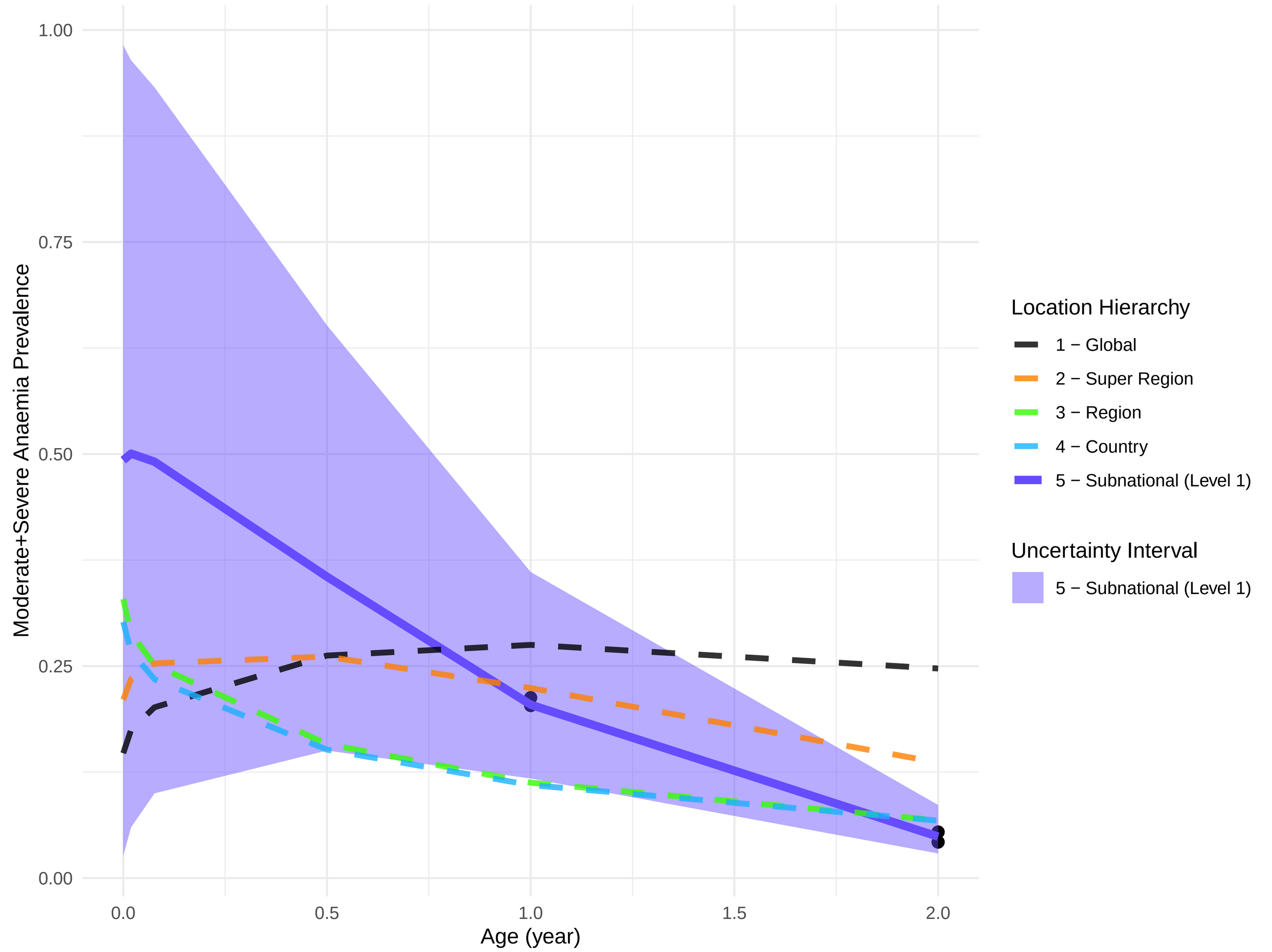

MR−BRT Age/Sex Fits − Non−pregnant Females <2 Years Old − Moderate+Severe Anaemia Prevalence − North Africa and Middle East
WHO−2001 Adjustment Method

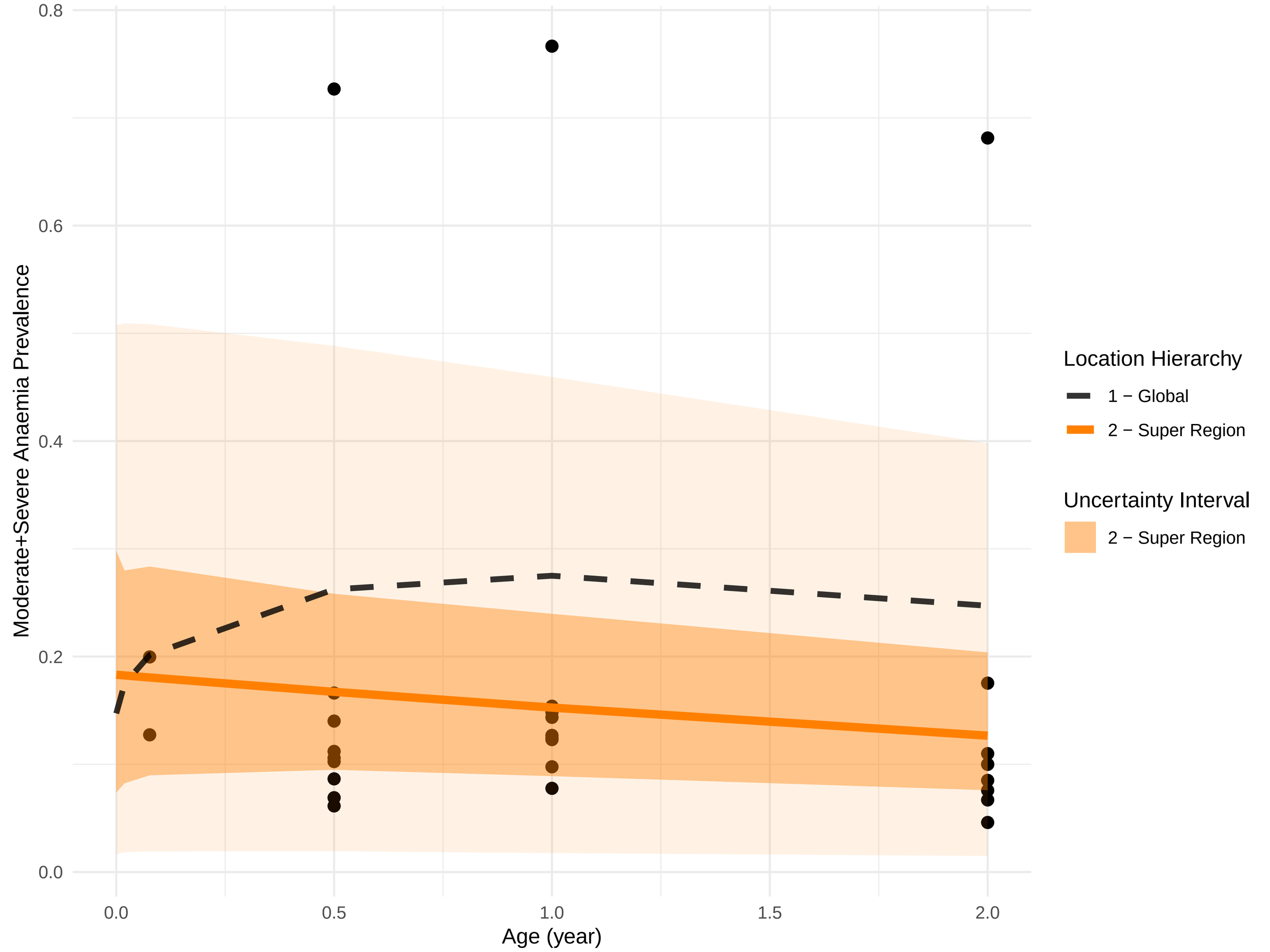

MR−BRT Age/Sex Fits − Non−pregnant Females <2 Years Old − Moderate+Severe Anaemia Prevalence − North Africa and Middle East
WHO−2001 Adjustment Method

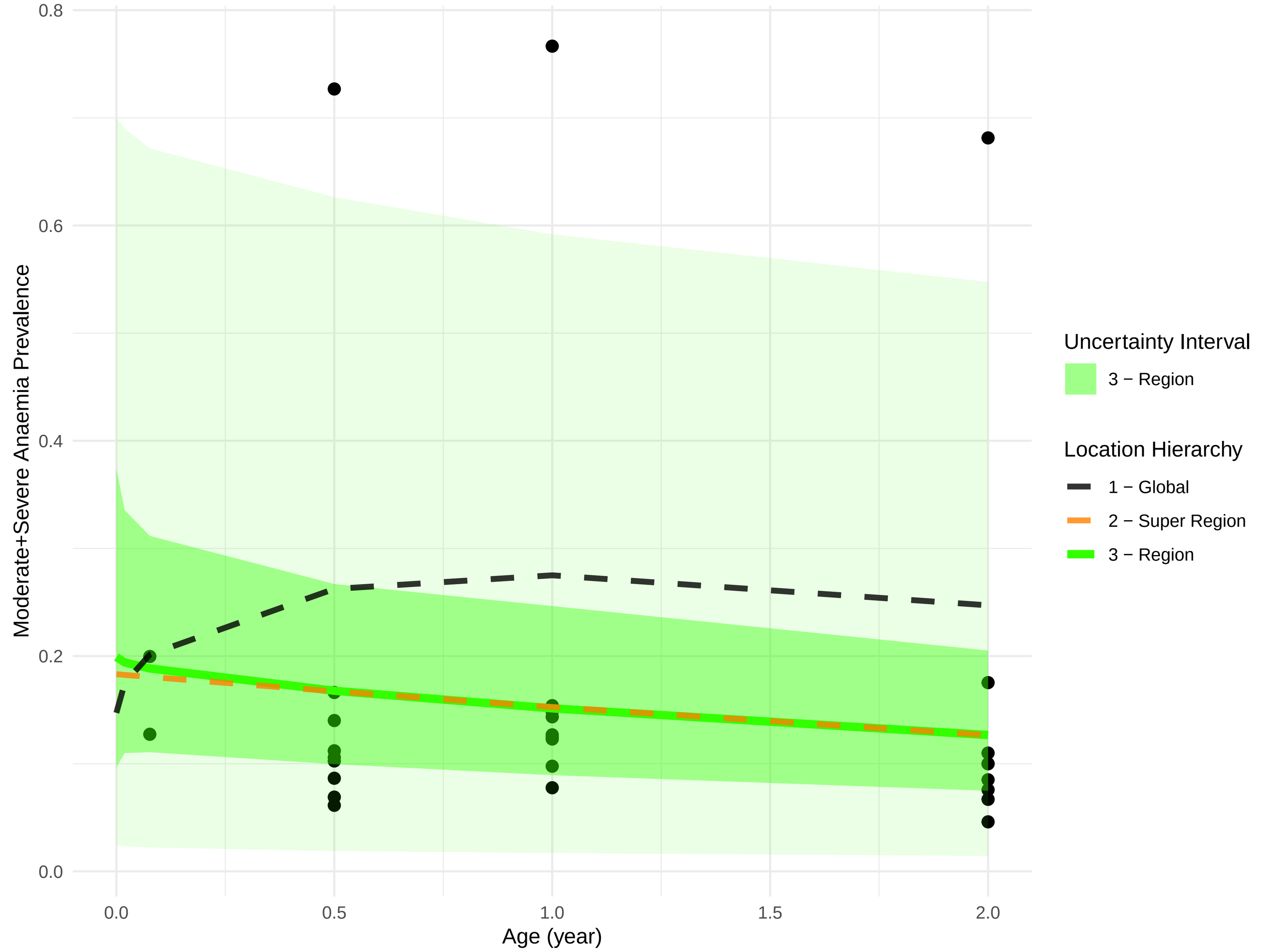

MR−BRT Age/Sex Fits − Non−pregnant Females <2 Years Old − Moderate+Severe Anaemia Prevalence − Egypt
WHO−2001 Adjustment Method

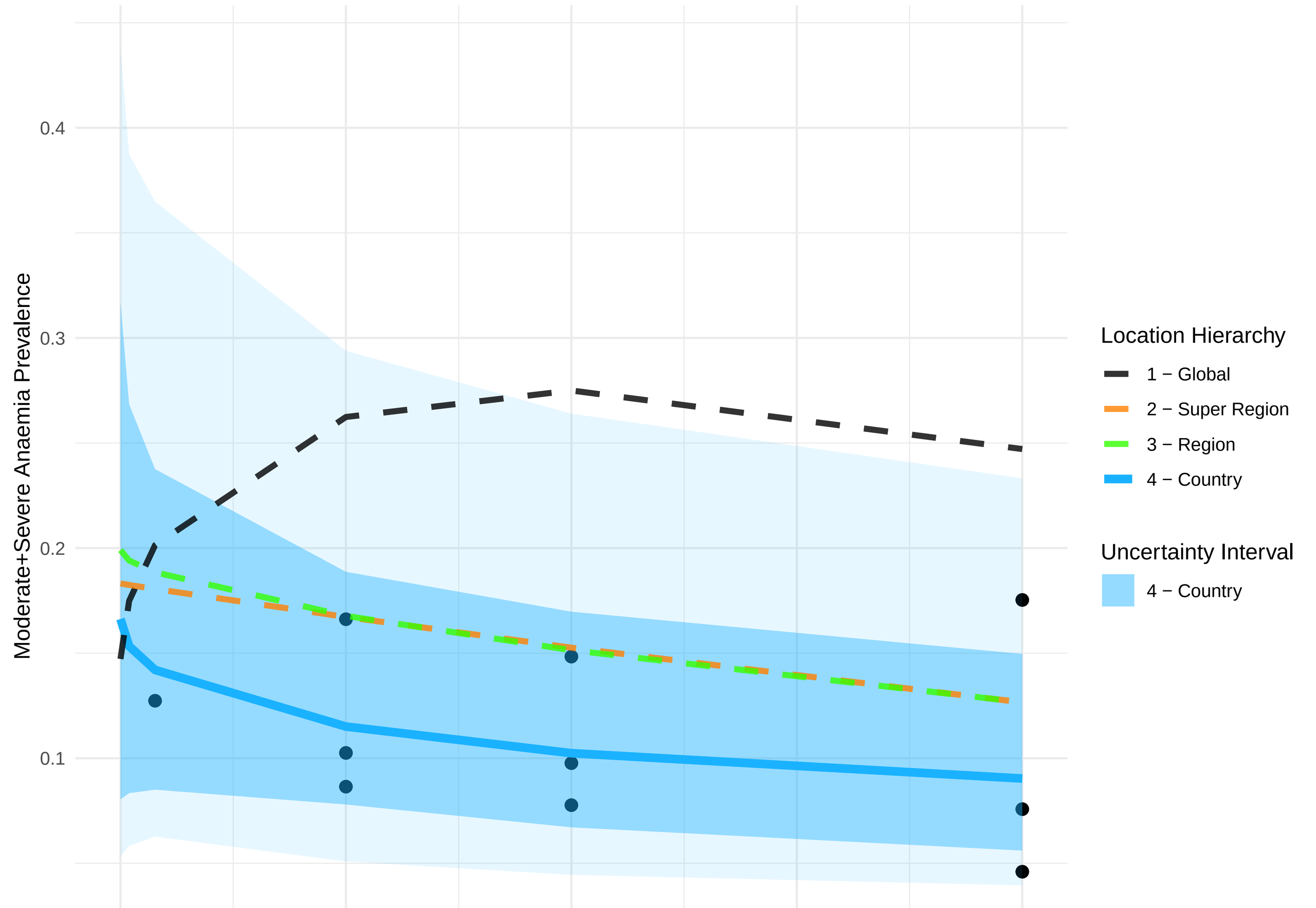

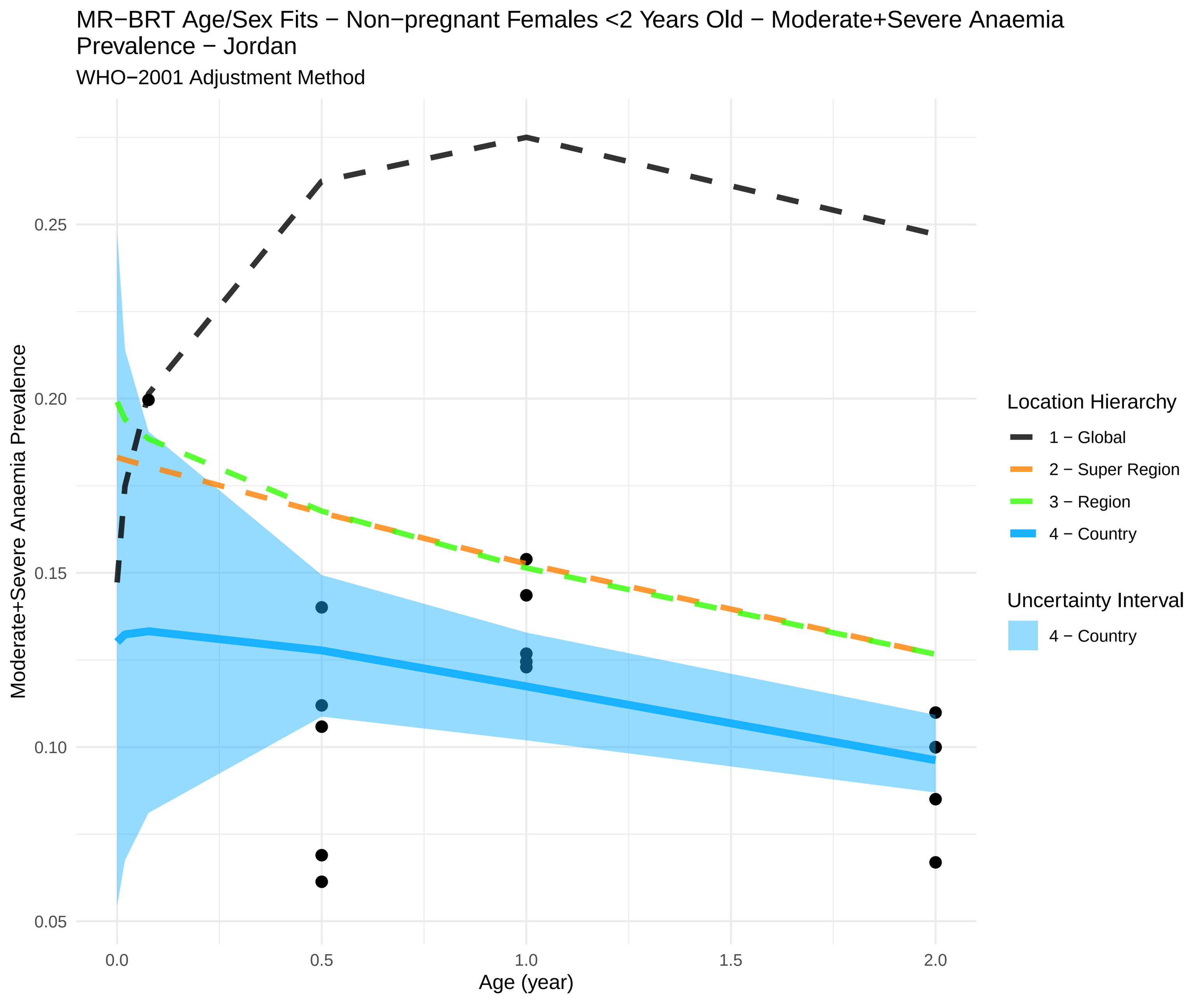
MR−BRT Age/Sex Fits − Non-pregnant Females <2 Years Old − Moderate+Severe Anaemia Prevalence − Jordan
WHO−2001 Adjustment Method
Moderate+Severe Anaemia Prevalence
Age (year)
Location Hierarchy
1 − Global
2 − Super Region
3 − Region
4 − Country
Uncertainty Interval
4 − Country

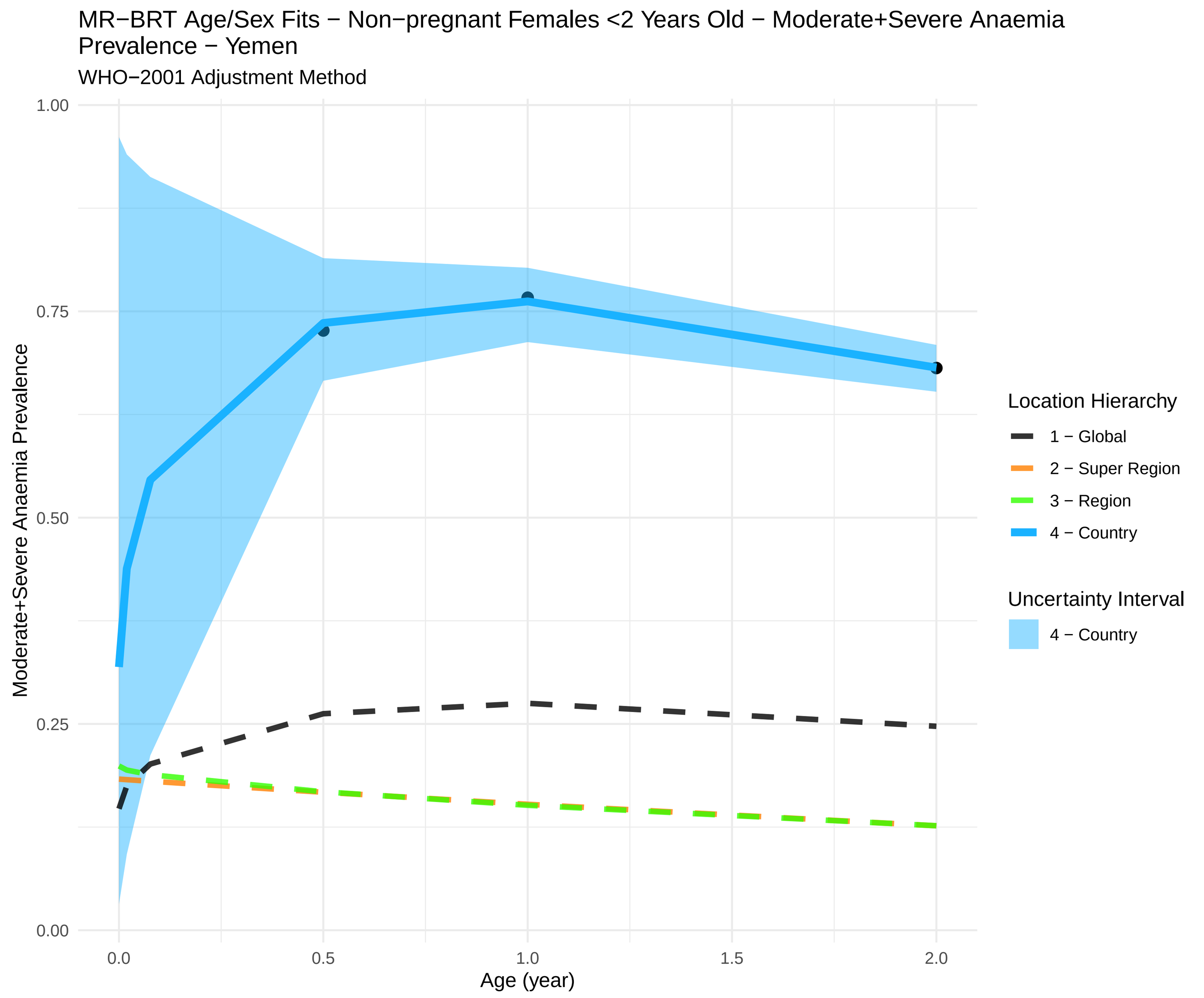
MR−BRT Age/Sex Fits − Non-pregnant Females <2 Years Old − Moderate+Severe Anaemia Prevalence − Yemen
WHO−2001 Adjustment Method
Moderate+Severe Anaemia Prevalence
Age (year)
Location Hierarchy
1 − Global
2 − Super Region
3 − Region
4 − Country
Uncertainty Interval
4 − Country

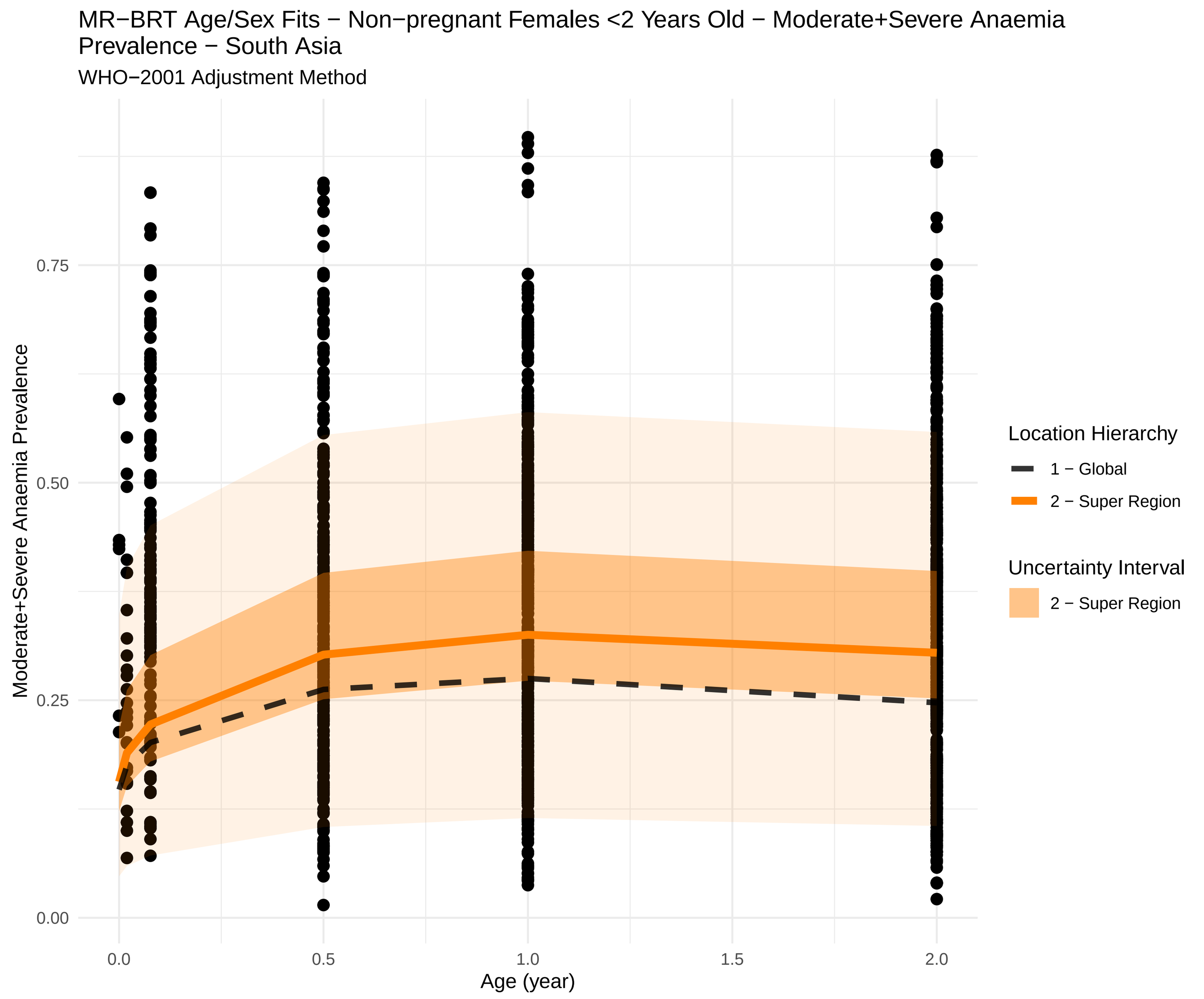
MR−BRT Age/Sex Fits − Non-pregnant Females <2 Years Old − Moderate+Severe Anaemia Prevalence − South Asia
WHO−2001 Adjustment Method
Moderate+Severe Anaemia Prevalence
Age (year)
Location Hierarchy
1 − Global
2 − Super Region
Uncertainty Interval
2 − Super Region

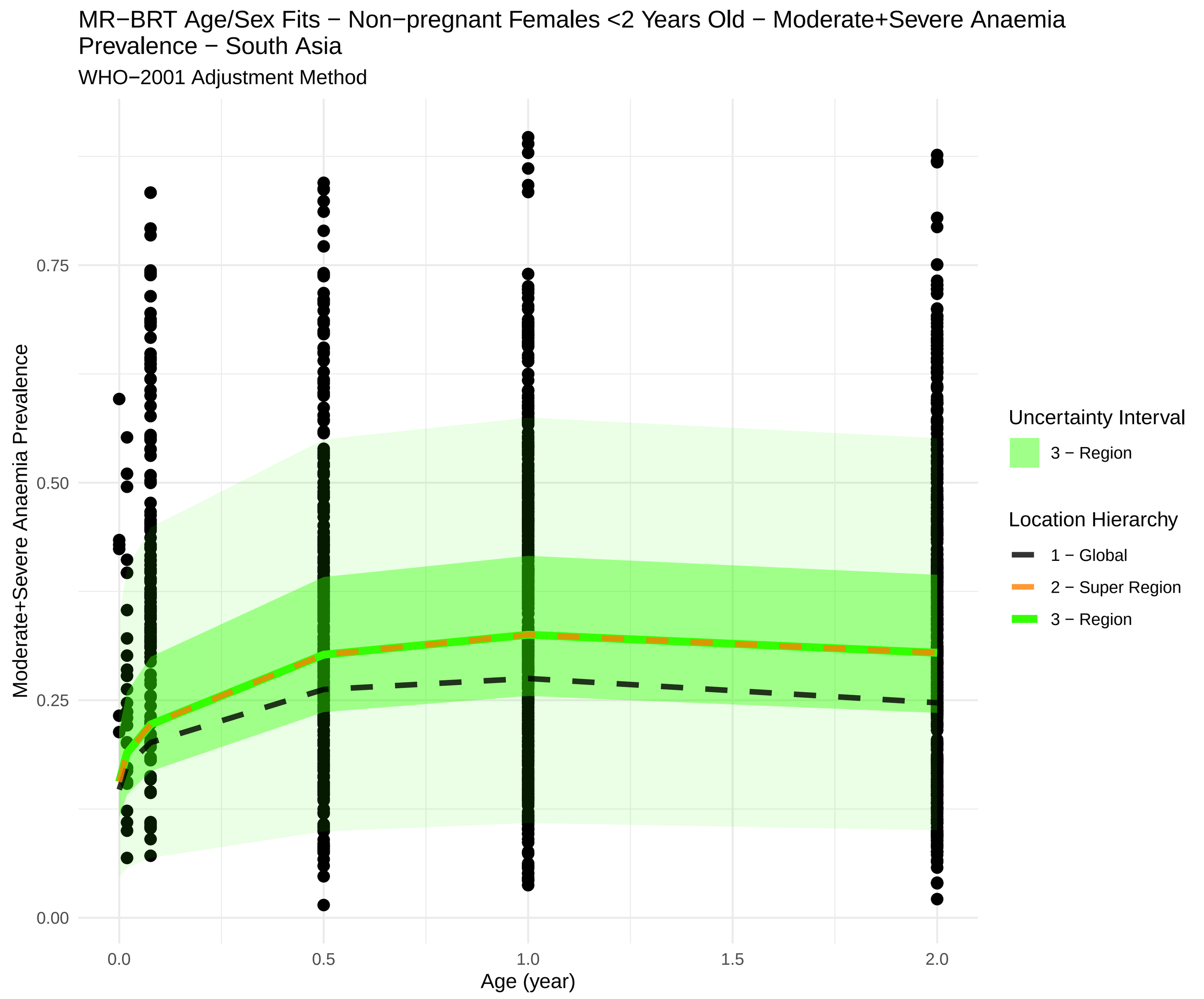
MR−BRT Age/Sex Fits − Non-pregnant Females <2 Years Old − Moderate+Severe Anaemia Prevalence − South Asia
WHO−2001 Adjustment Method
Moderate+Severe Anaemia Prevalence
Age (year)
Uncertainty Interval
3 − Region
Location Hierarchy
1 − Global
2 − Super Region
3 − Region

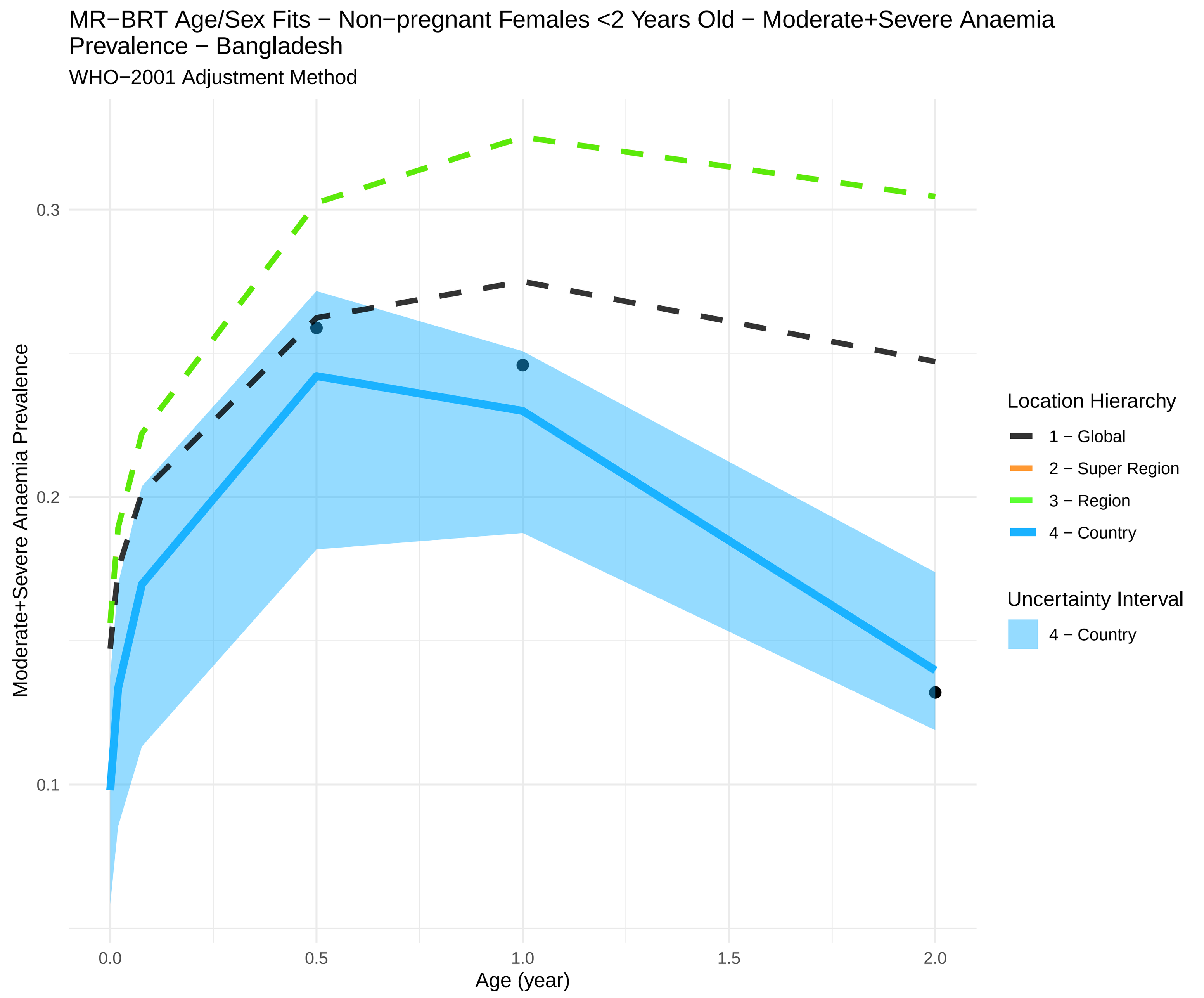
MR−BRT Age/Sex Fits − Non-pregnant Females <2 Years Old − Moderate+Severe Anaemia Prevalence − Bangladesh
WHO−2001 Adjustment Method
Moderate+Severe Anaemia Prevalence
Age (year)
Location Hierarchy
1 − Global
2 − Super Region
3 − Region
4 − Country
Uncertainty Interval
4 − Country

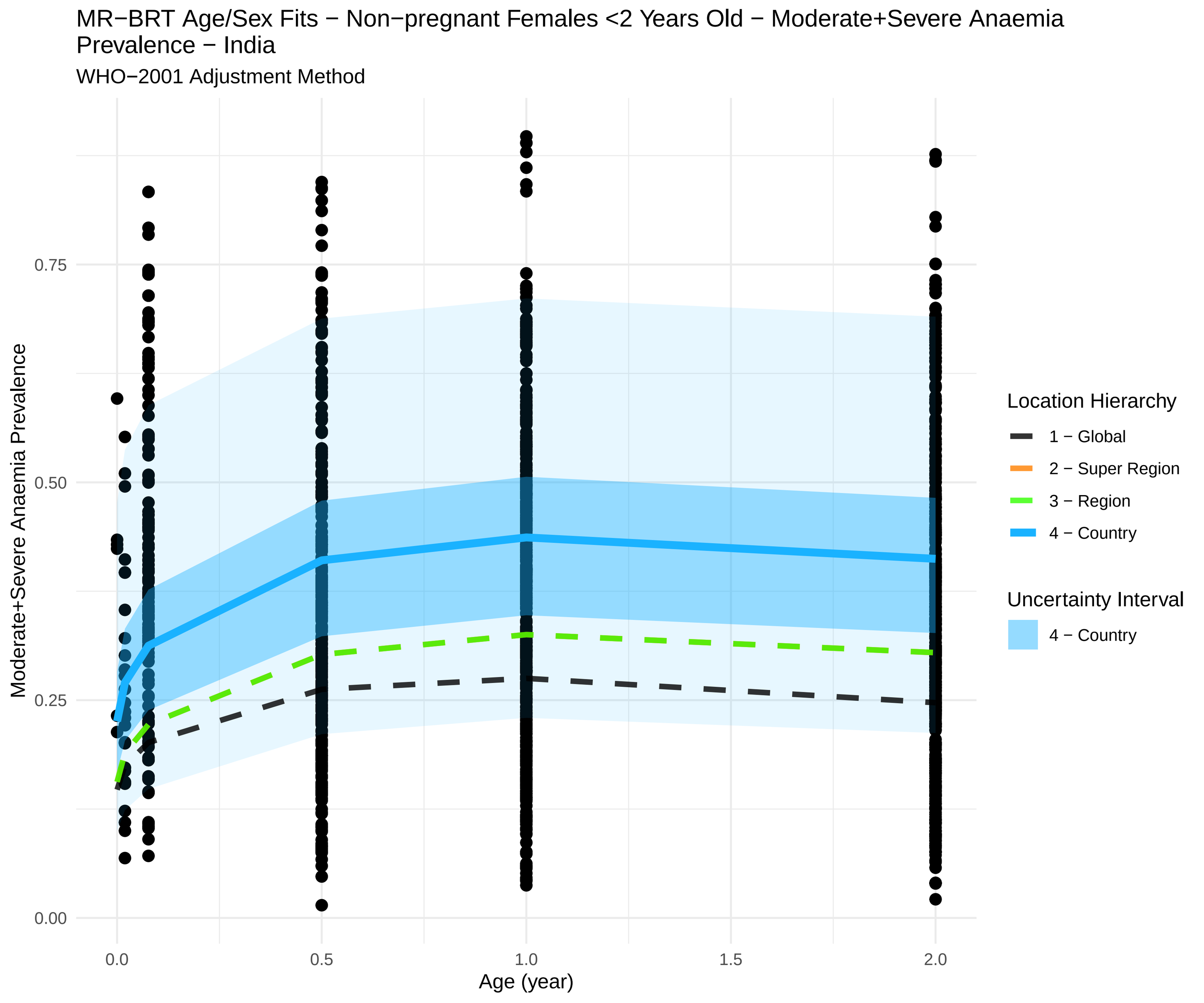
MR−BRT Age/Sex Fits − Non-pregnant Females <2 Years Old − Moderate+Severe Anaemia Prevalence − India
WHO−2001 Adjustment Method
Moderate+Severe Anaemia Prevalence
Age (year)
Location Hierarchy
1 − Global
2 − Super Region
3 − Region
4 − Country
Uncertainty Interval
4 − Country

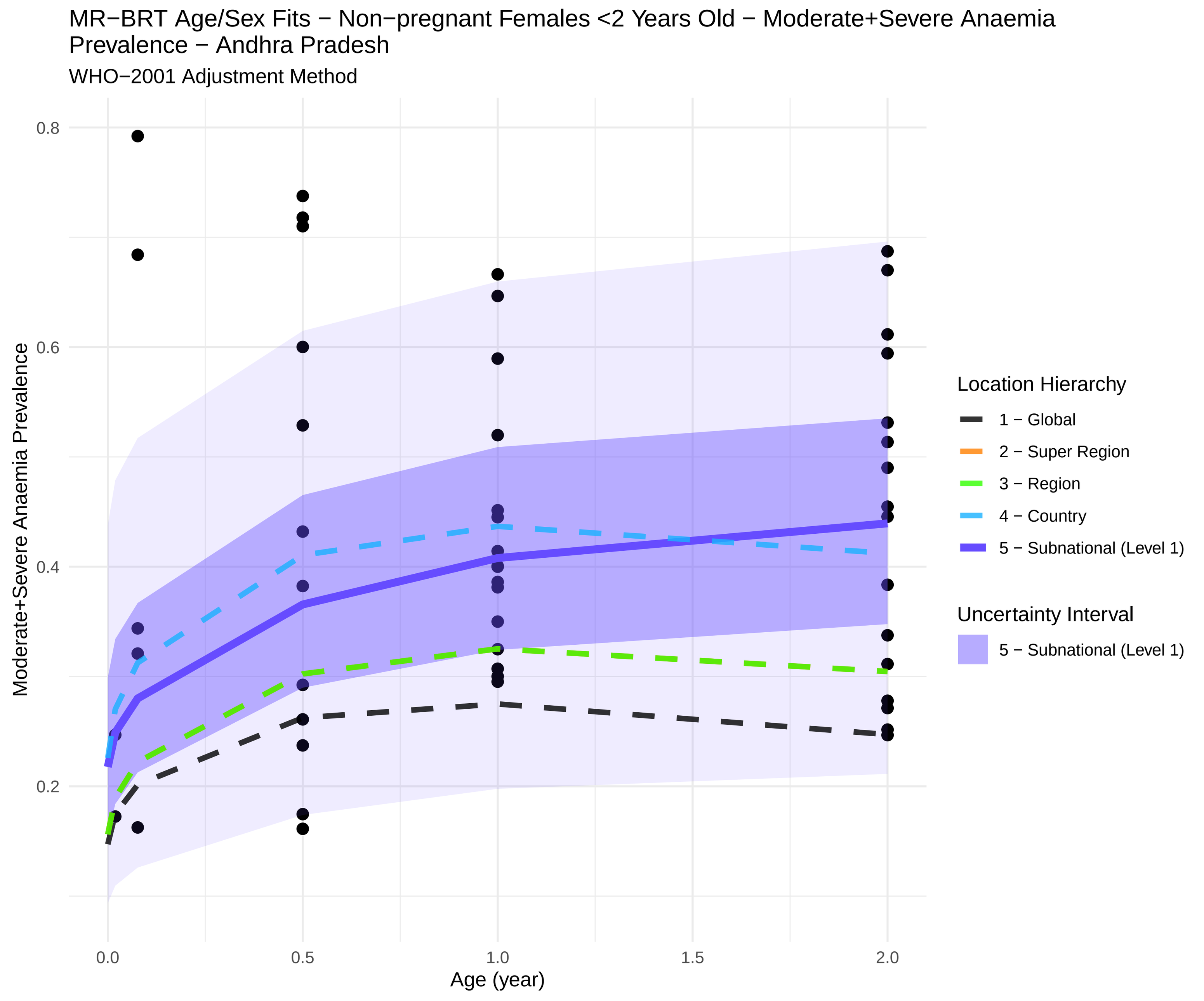
MR−BRT Age/Sex Fits − Non-pregnant Females <2 Years Old − Moderate+Severe Anaemia Prevalence − Andhra Pradesh
WHO−2001 Adjustment Method
Moderate+Severe Anaemia Prevalence
Age (year)
Location Hierarchy
1 − Global
2 − Super Region
3 − Region
4 − Country
5 − Subnational (Level 1)
Uncertainty Interval
5 − Subnational (Level 1)

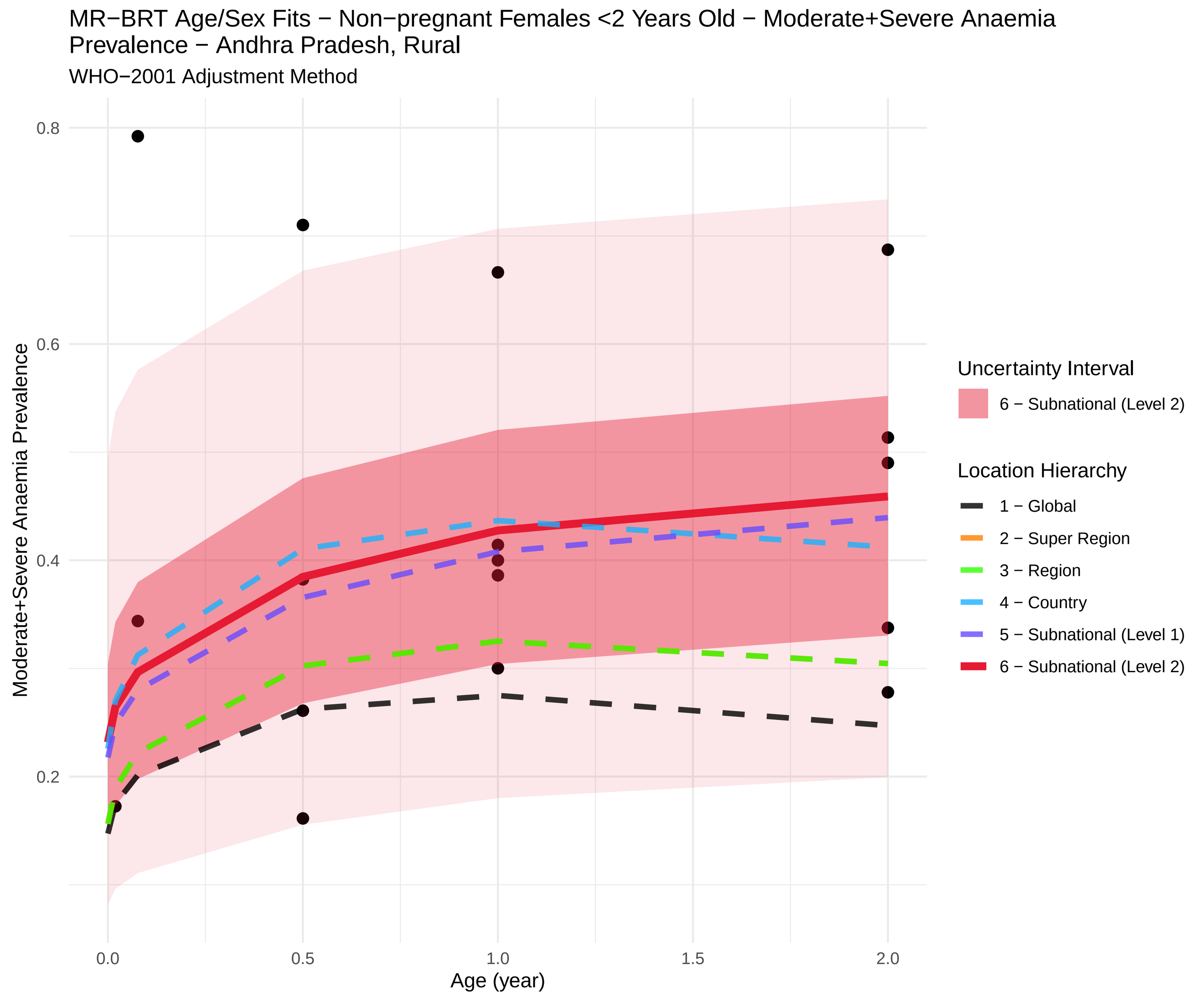
MR−BRT Age/Sex Fits − Non-pregnant Females <2 Years Old − Moderate+Severe Anaemia Prevalence − Andhra Pradesh, Rural
WHO−2001 Adjustment Method
Moderate+Severe Anaemia Prevalence
Age (year)
Uncertainty Interval
6 − Subnational (Level 2)
Location Hierarchy
1 − Global
2 − Super Region
3 − Region
4 − Country
5 − Subnational (Level 1)
6 − Subnational (Level 2)

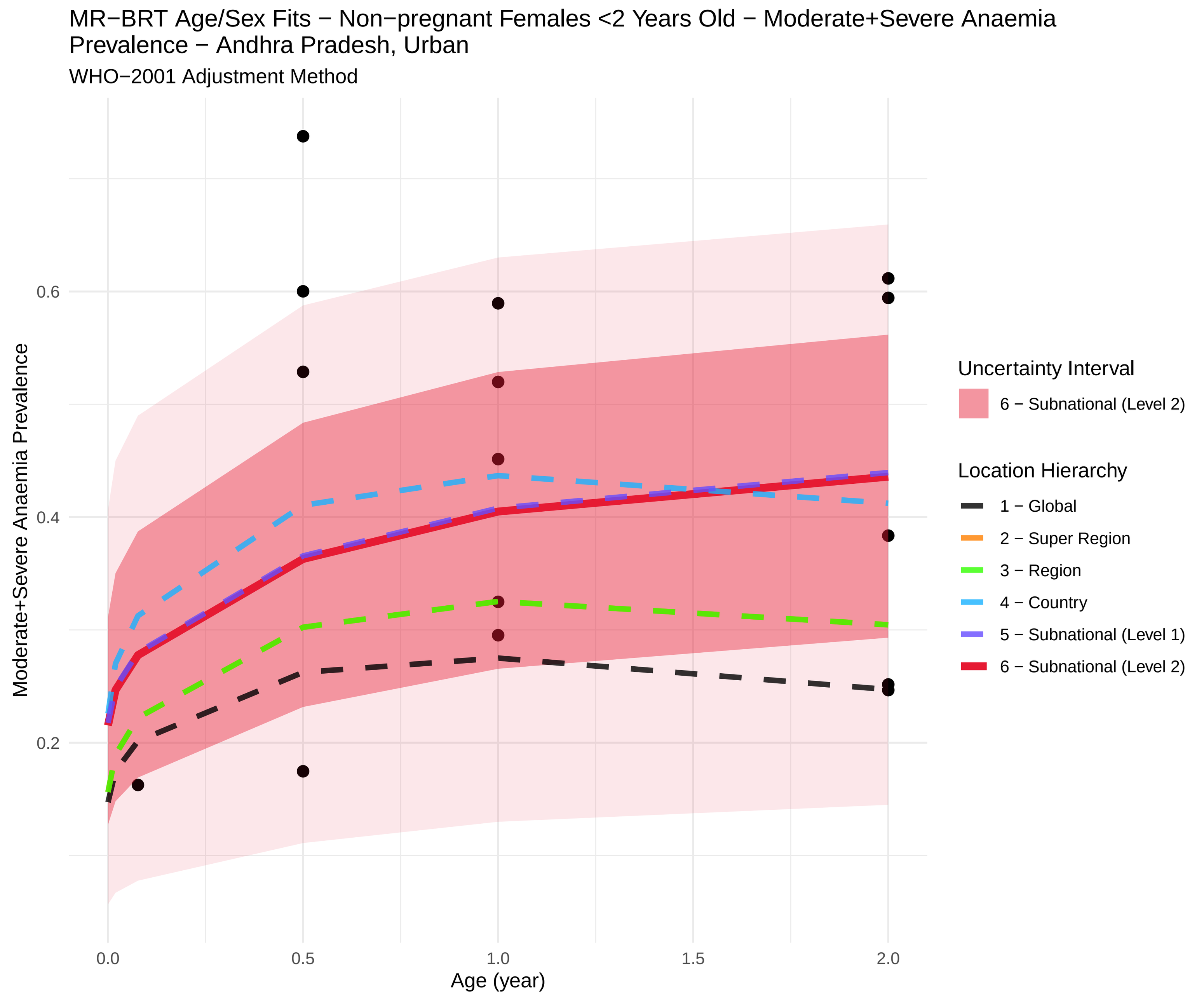
MR−BRT Age/Sex Fits − Non-pregnant Females <2 Years Old − Moderate+Severe Anaemia Prevalence − Andhra Pradesh, Urban
WHO−2001 Adjustment Method
Moderate+Severe Anaemia Prevalence
Age (year)
Uncertainty Interval
6 − Subnational (Level 2)
Location Hierarchy
1 − Global
2 − Super Region
3 − Region
4 − Country
5 − Subnational (Level 1)
6 − Subnational (Level 2)

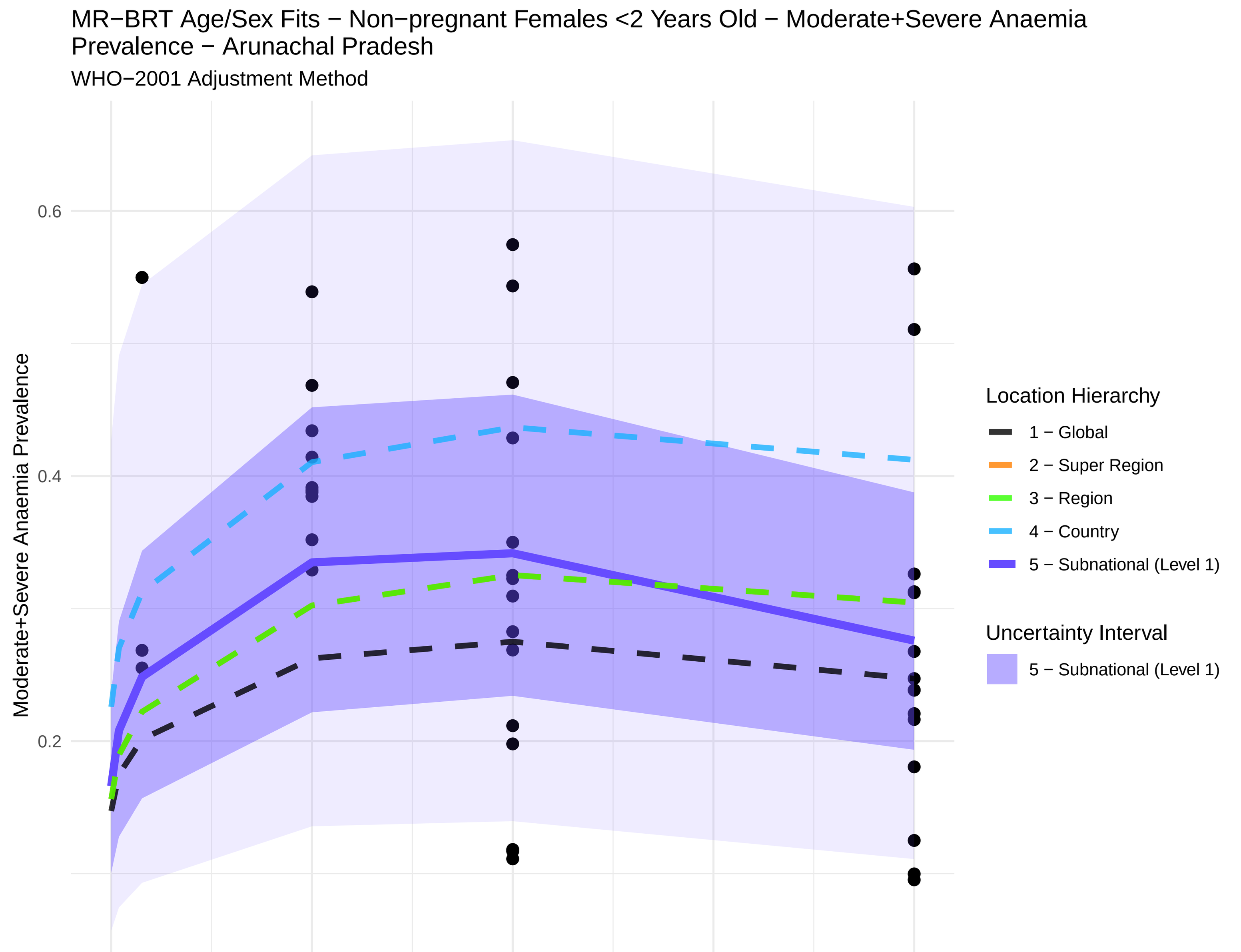
MR−BRT Age/Sex Fits − Non-pregnant Females <2 Years Old − Moderate+Severe Anaemia Prevalence − Arunachal Pradesh
WHO−2001 Adjustment Method
Moderate+Severe Anaemia Prevalence
Age (year)
Location Hierarchy
1 − Global
2 − Super Region
3 − Region
4 − Country
5 − Subnational (Level 1)
Uncertainty Interval
5 − Subnational (Level 1)

MR−BRT Age/Sex Fits − Non−pregnant Females <2 Years Old − Moderate+Severe Anaemia Prevalence − Arunachal Pradesh, Rural
WHO−2001 Adjustment Method

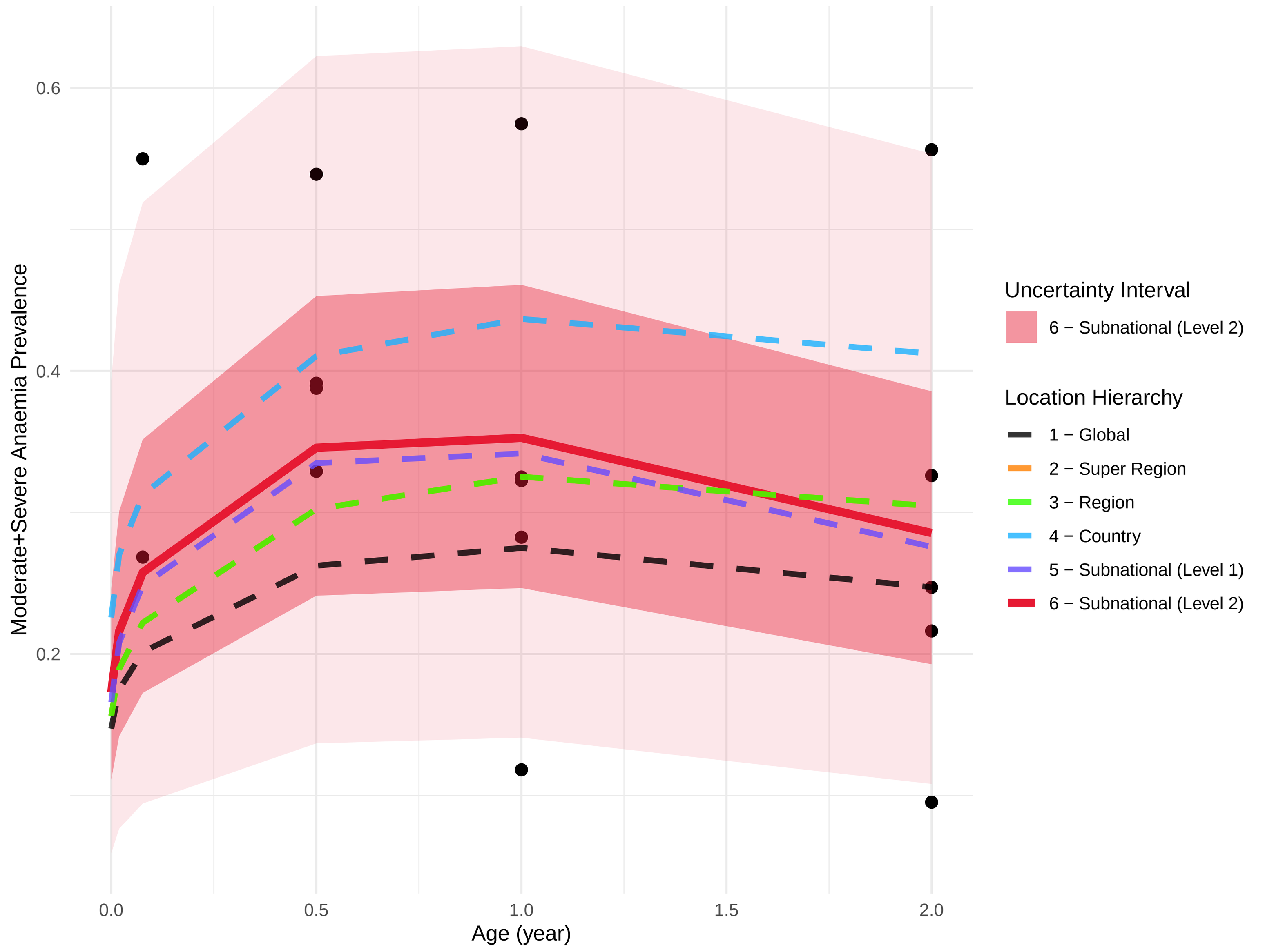

MR−BRT Age/Sex Fits − Non−pregnant Females <2 Years Old − Moderate+Severe Anaemia Prevalence − Arunachal Pradesh, Urban
WHO−2001 Adjustment Method

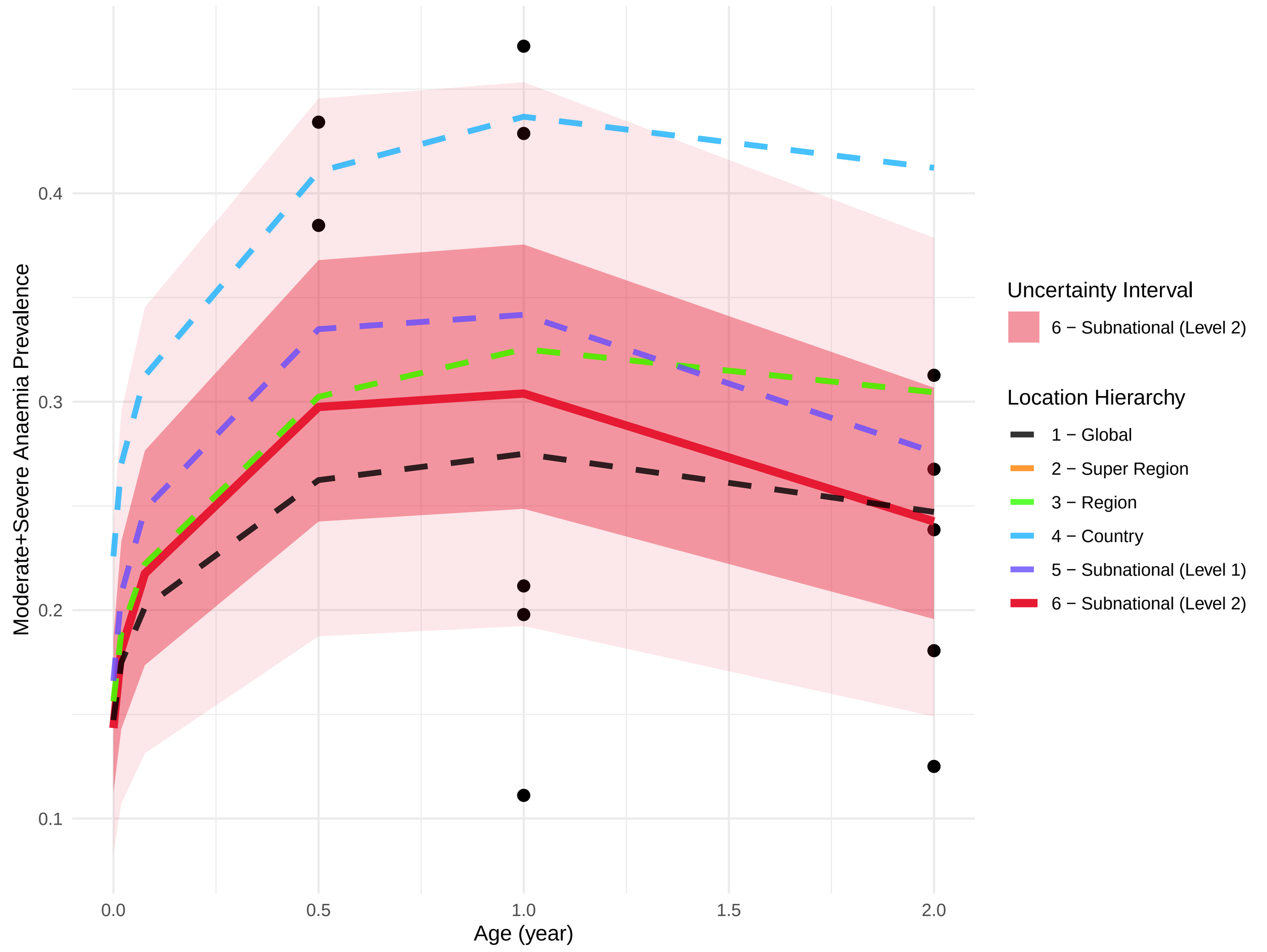

MR−BRT Age/Sex Fits − Non−pregnant Females <2 Years Old − Moderate+Severe Anaemia Prevalence − Assam
WHO−2001 Adjustment Method

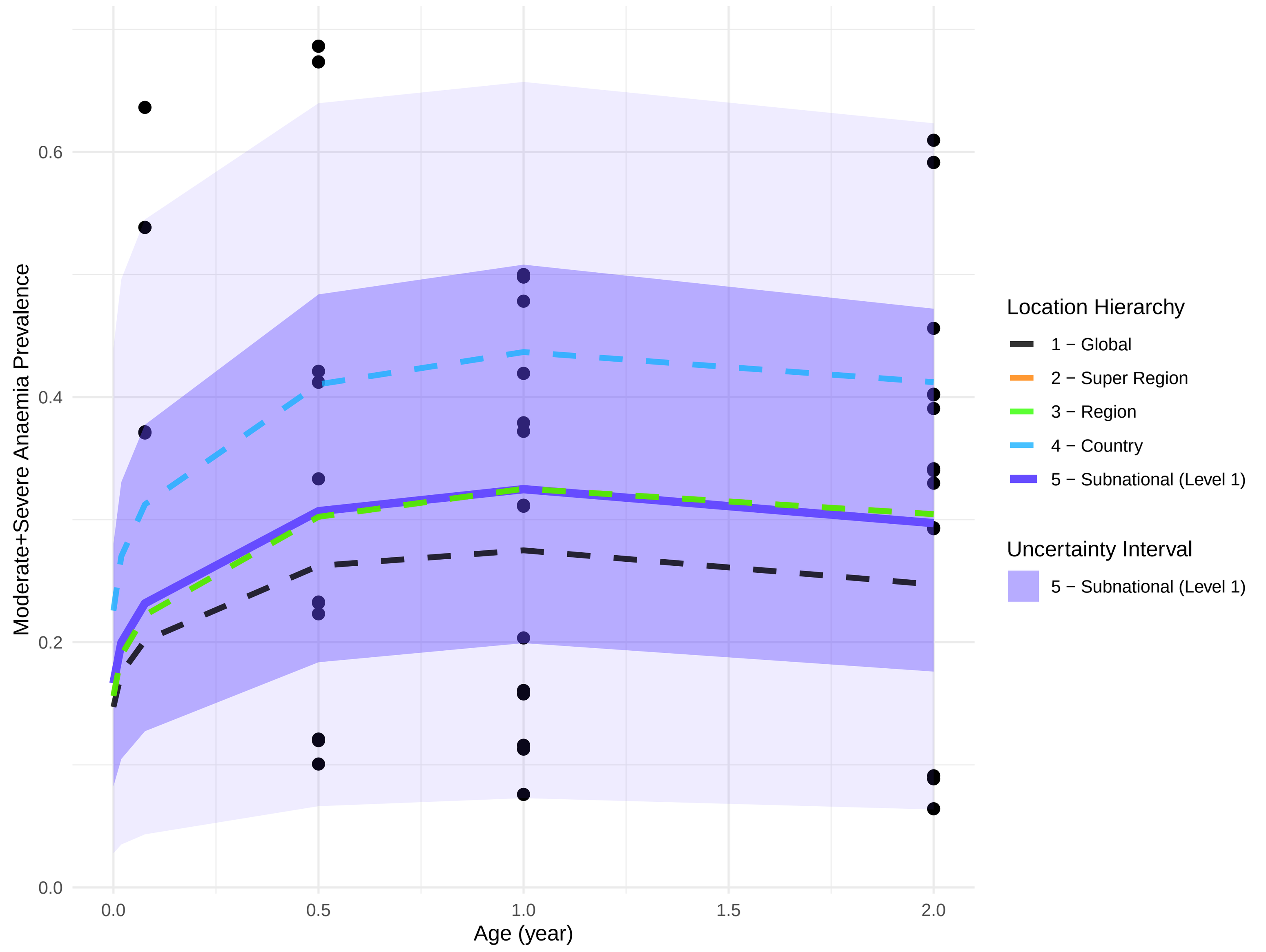

MR−BRT Age/Sex Fits − Non−pregnant Females <2 Years Old − Moderate+Severe Anaemia Prevalence − Assam, Rural
WHO−2001 Adjustment Method

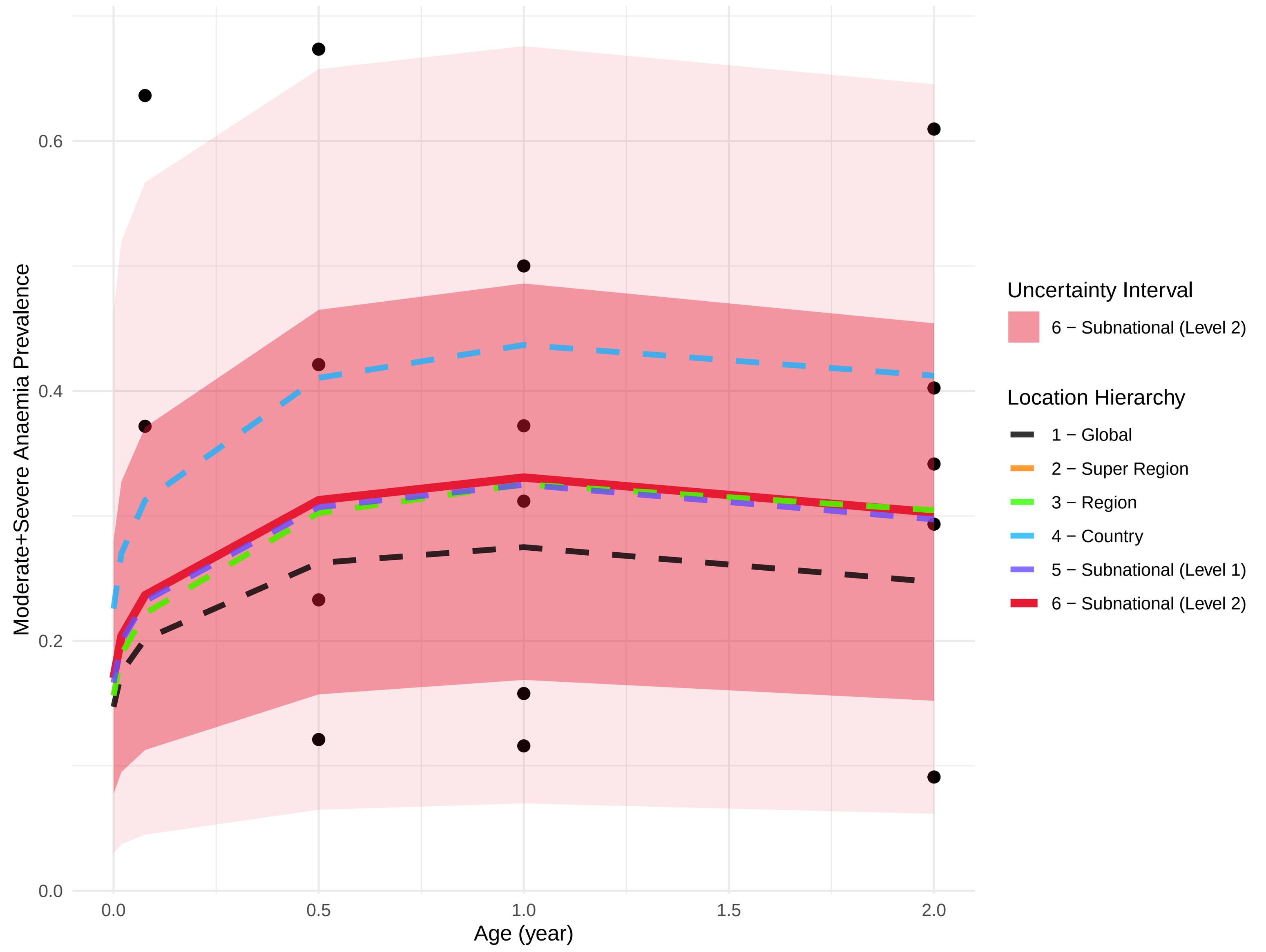

MR−BRT Age/Sex Fits − Non−pregnant Females <2 Years Old − Moderate+Severe Anaemia Prevalence − Assam, Urban
WHO−2001 Adjustment Method

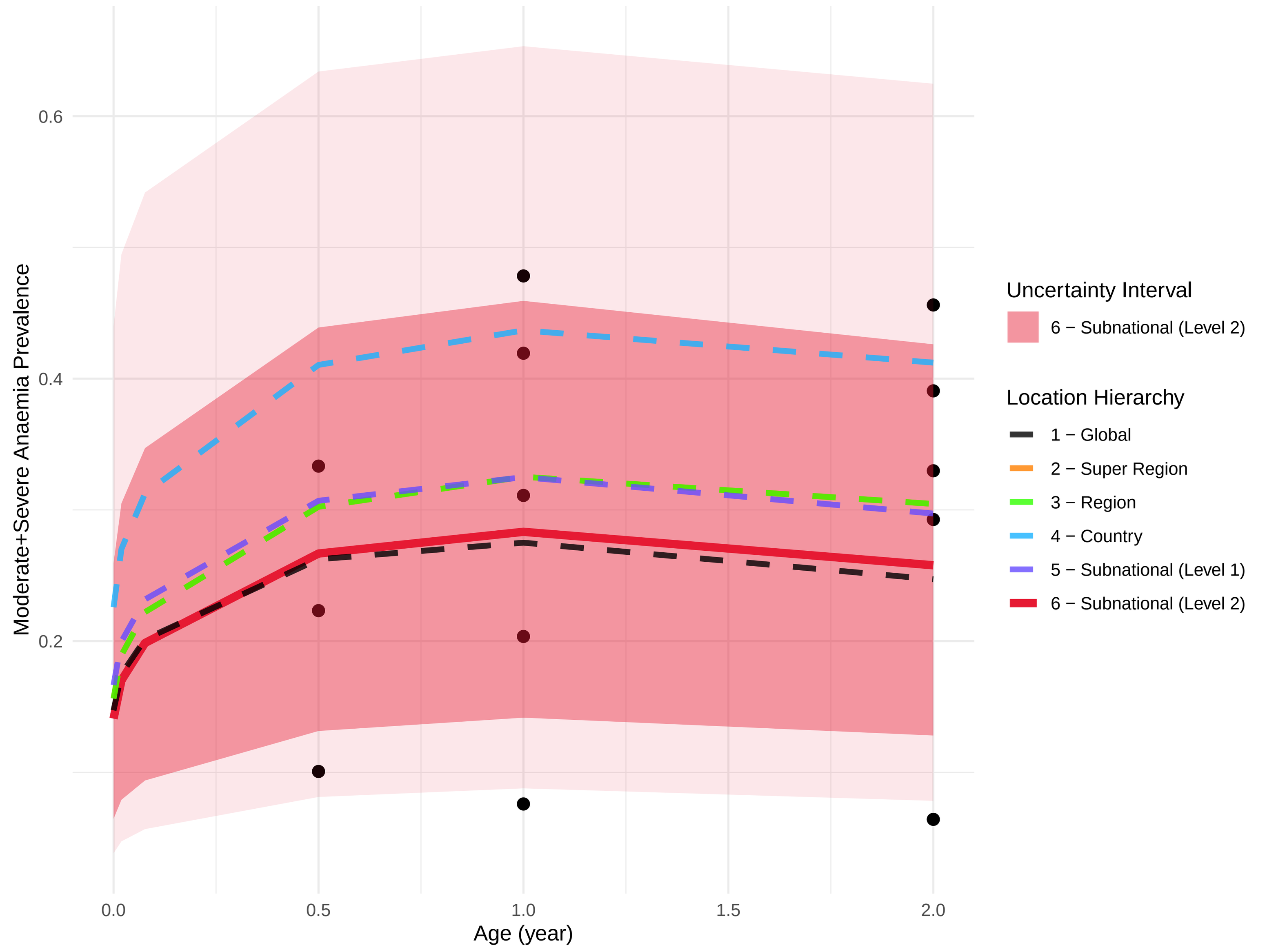

MR−BRT Age/Sex Fits − Non−pregnant Females <2 Years Old − Moderate+Severe Anaemia Prevalence − Bihar
WHO−2001 Adjustment Method

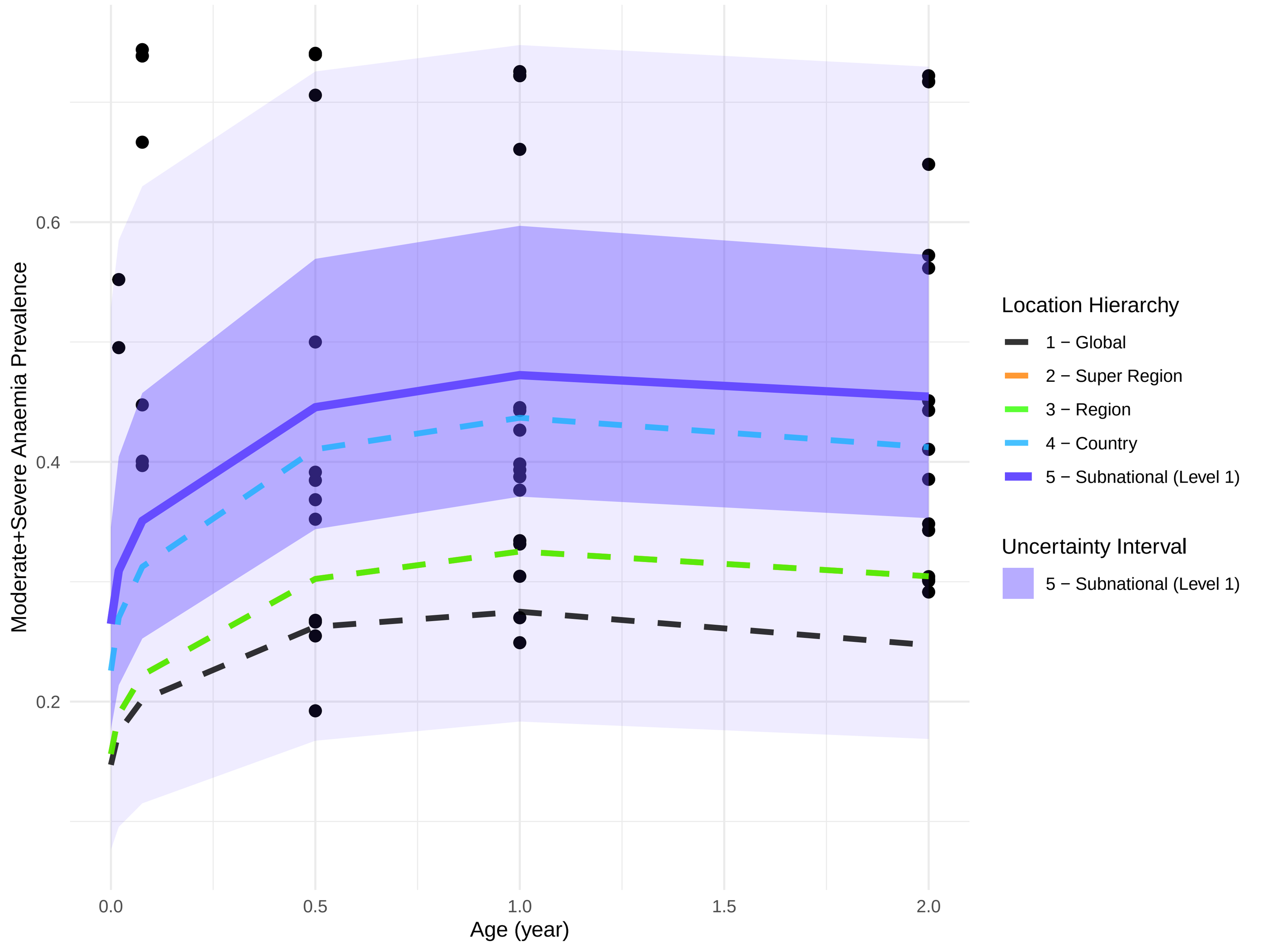

MR−BRT Age/Sex Fits − Non−pregnant Females <2 Years Old − Moderate+Severe Anaemia Prevalence − Bihar, Rural
WHO−2001 Adjustment Method

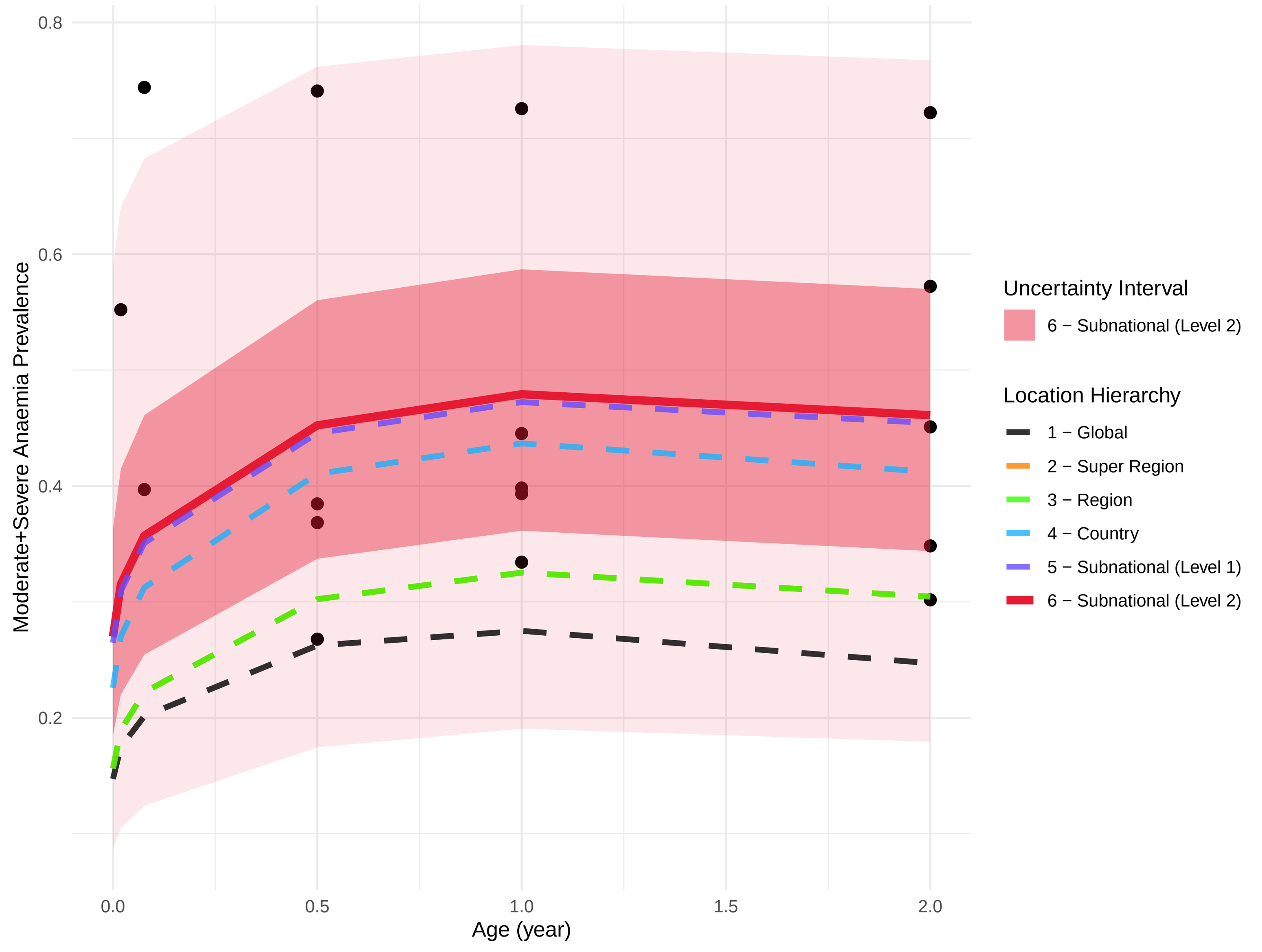

MR−BRT Age/Sex Fits − Non−pregnant Females <2 Years Old − Moderate+Severe Anaemia Prevalence − Bihar, Urban
WHO−2001 Adjustment Method

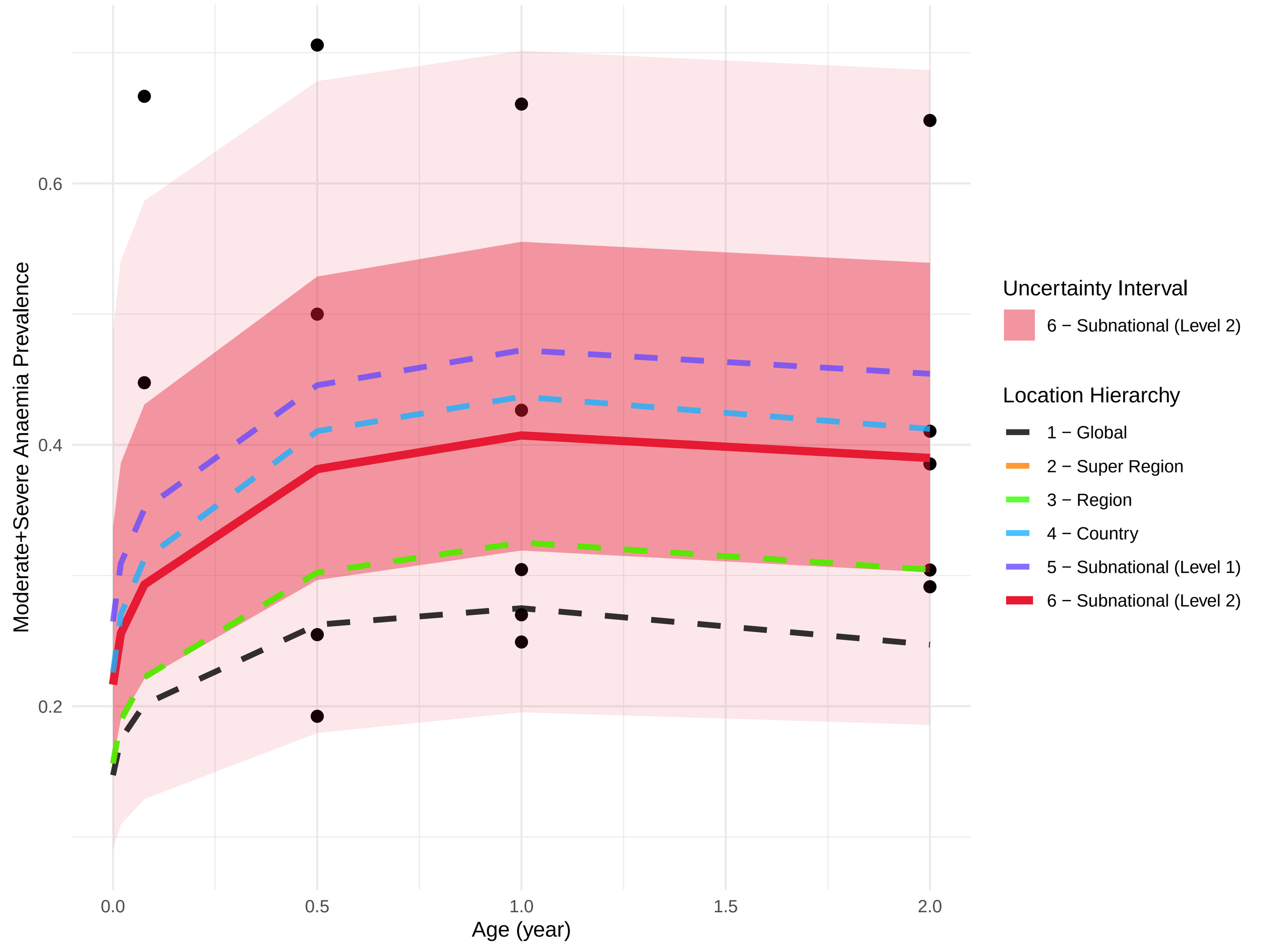

MR−BRT Age/Sex Fits − Non−pregnant Females <2 Years Old − Moderate+Severe Anaemia Prevalence − Chhattisgarh
WHO−2001 Adjustment Method

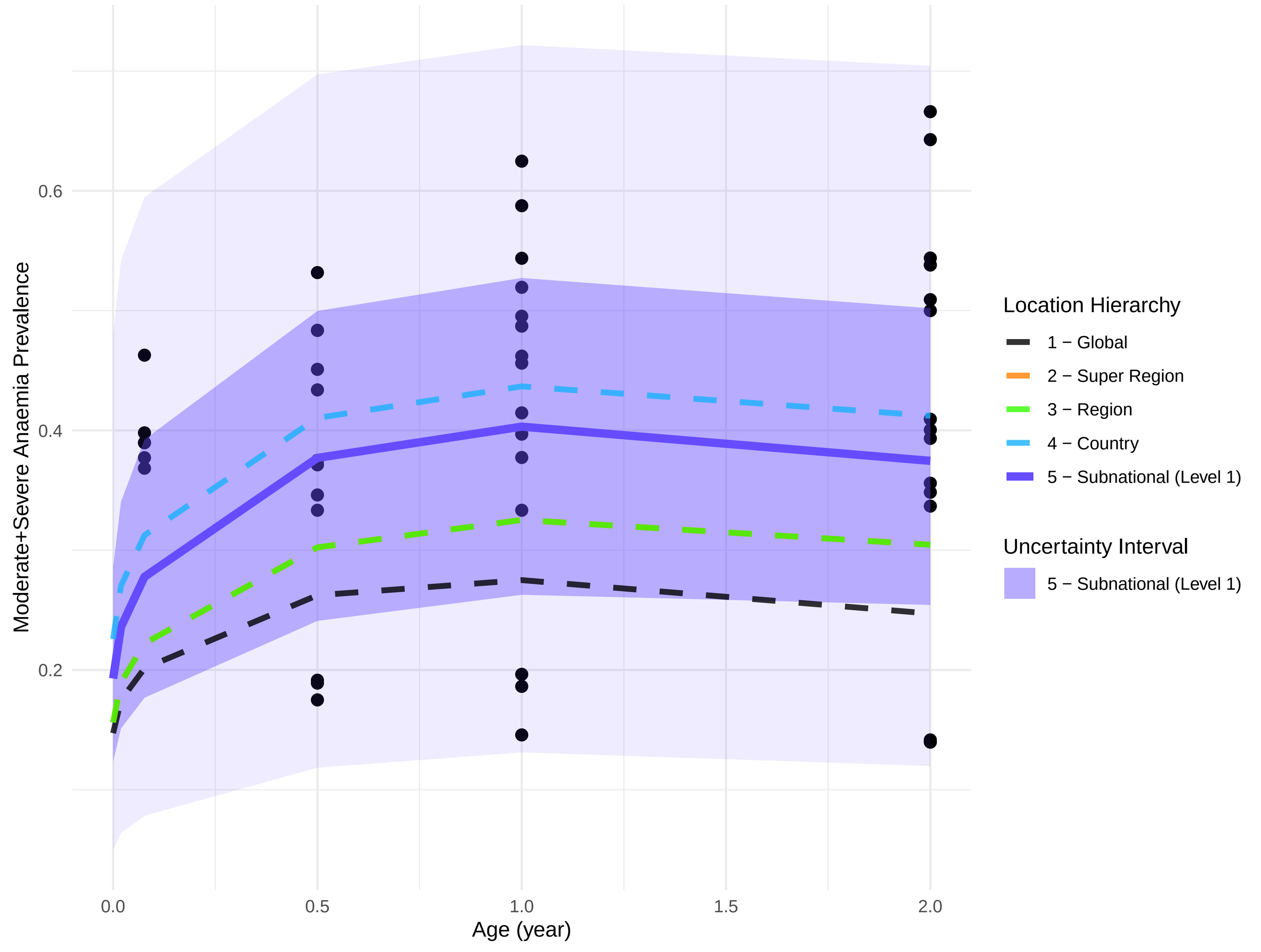

MR−BRT Age/Sex Fits − Non−pregnant Females <2 Years Old − Moderate+Severe Anaemia Prevalence − Chhattisgarh, Rural
WHO−2001 Adjustment Method

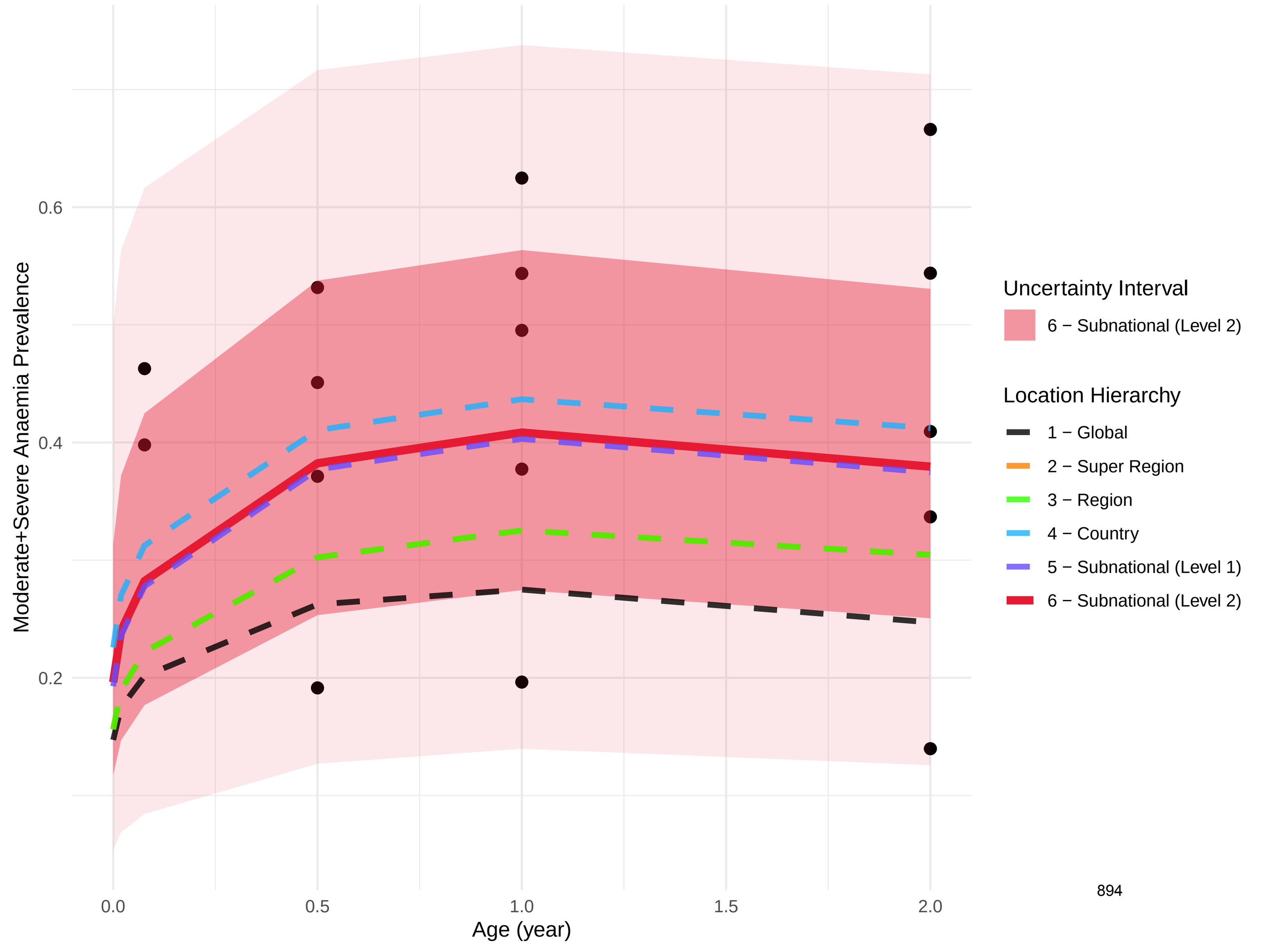

MR−BRT Age/Sex Fits − Non−pregnant Females <2 Years Old − Moderate+Severe Anaemia Prevalence − Chhattisgarh, Urban
WHO−2001 Adjustment Method

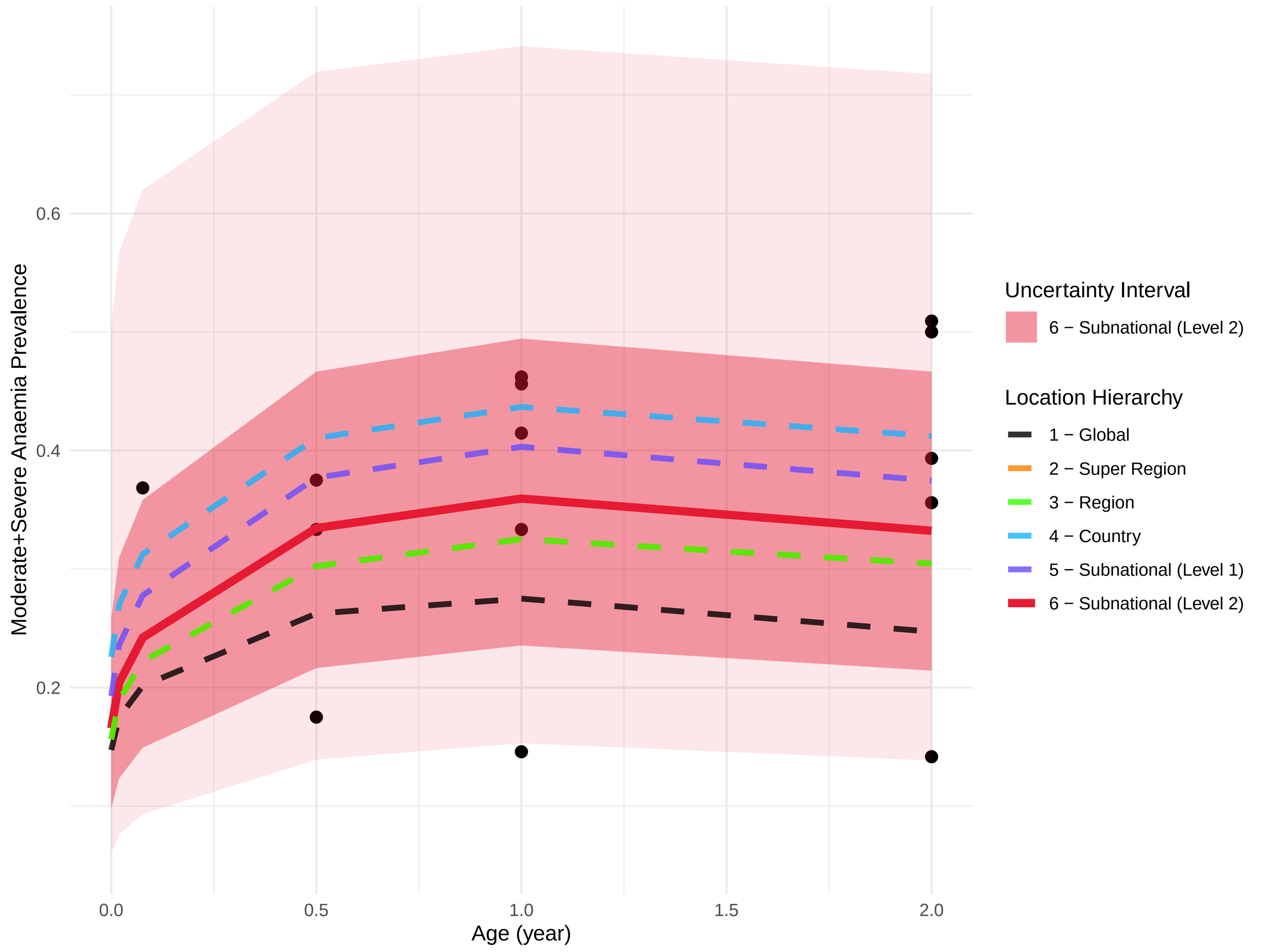

MR−BRT Age/Sex Fits − Non−pregnant Females <2 Years Old − Moderate+Severe Anaemia Prevalence − Delhi
WHO−2001 Adjustment Method

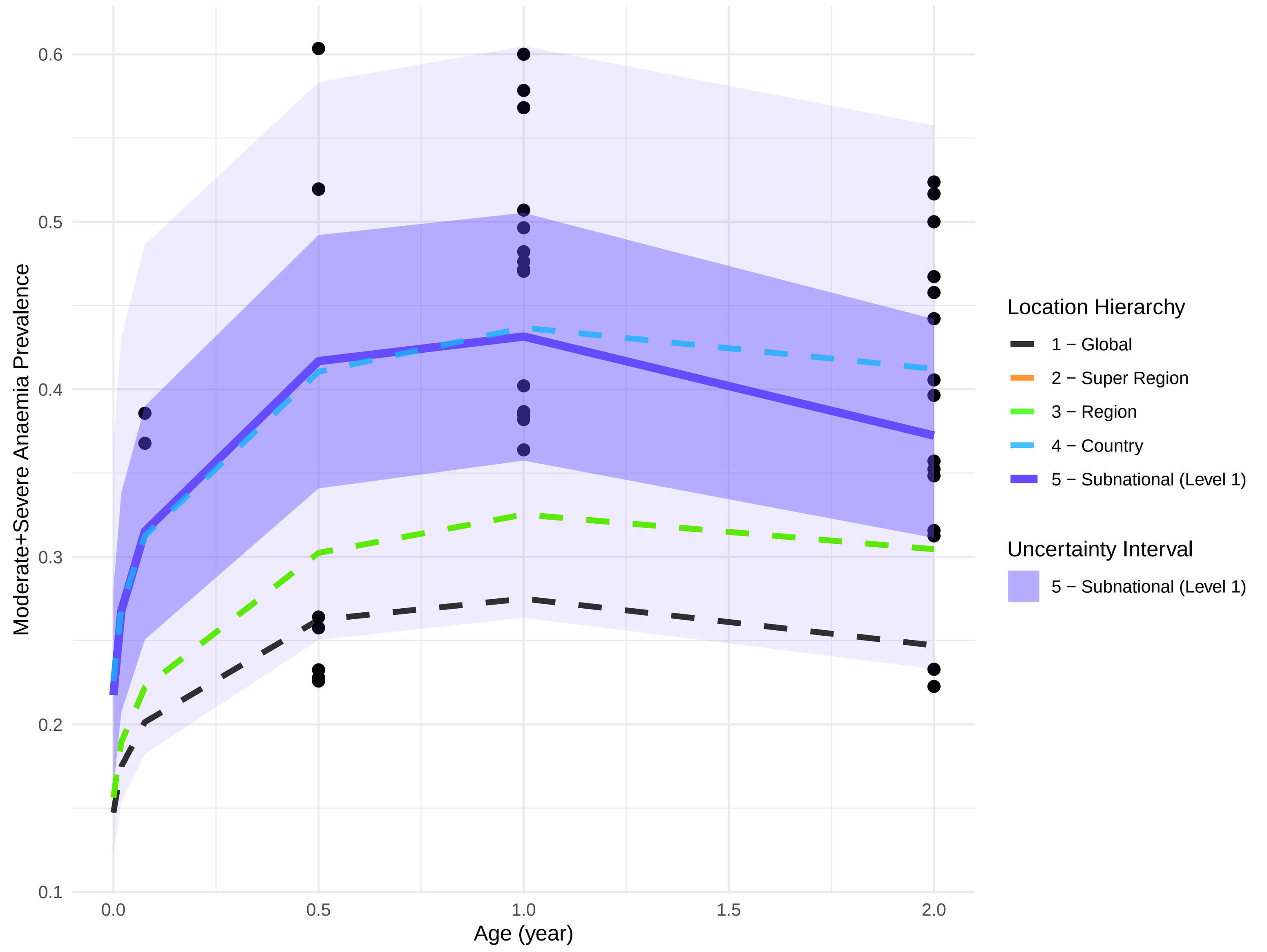

MR−BRT Age/Sex Fits − Non−pregnant Females <2 Years Old − Moderate+Severe Anaemia Prevalence − Delhi, Rural
WHO−2001 Adjustment Method

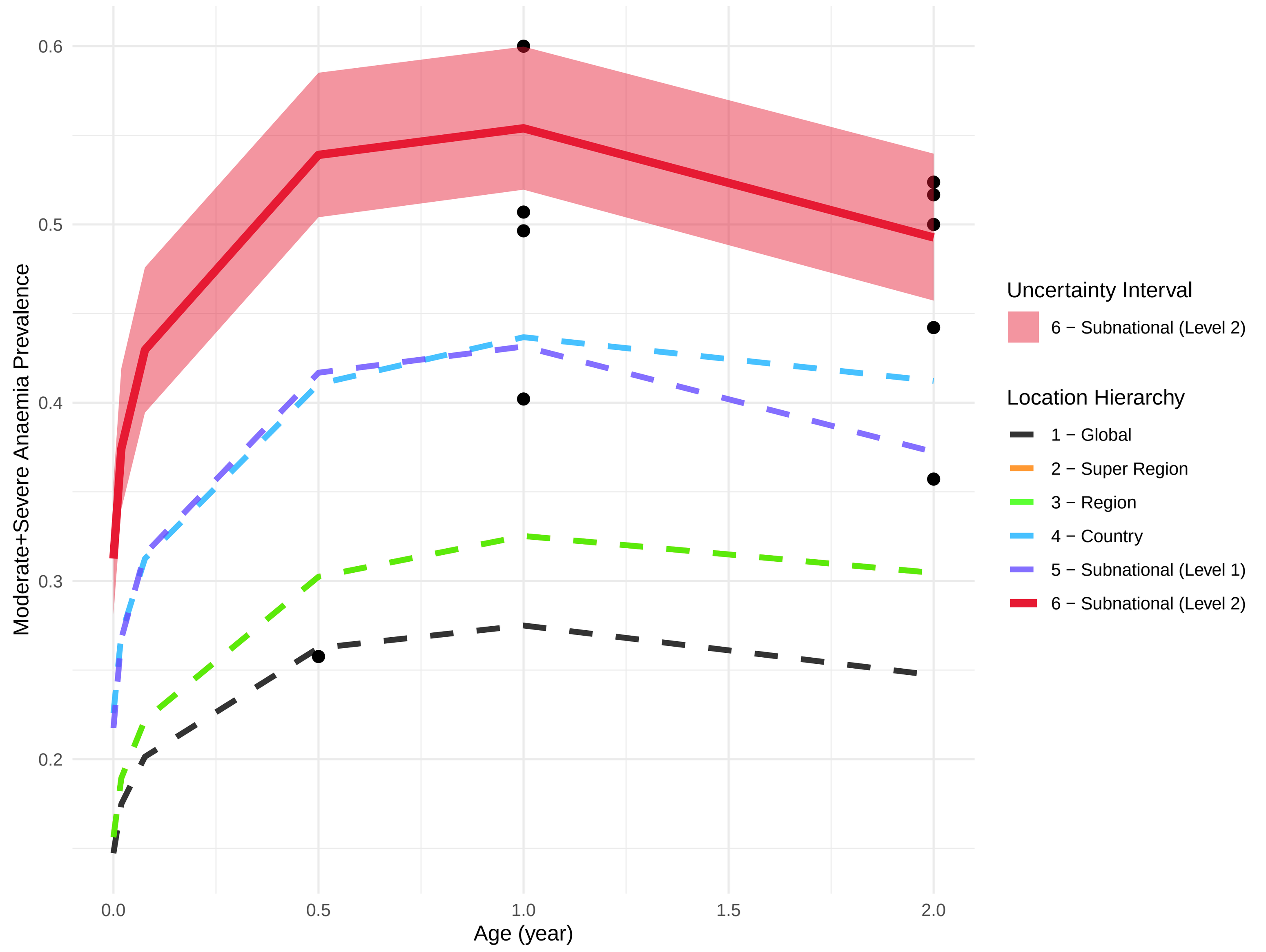

MR−BRT Age/Sex Fits − Non−pregnant Females <2 Years Old − Moderate+Severe Anaemia Prevalence − Delhi, Urban
WHO−2001 Adjustment Method

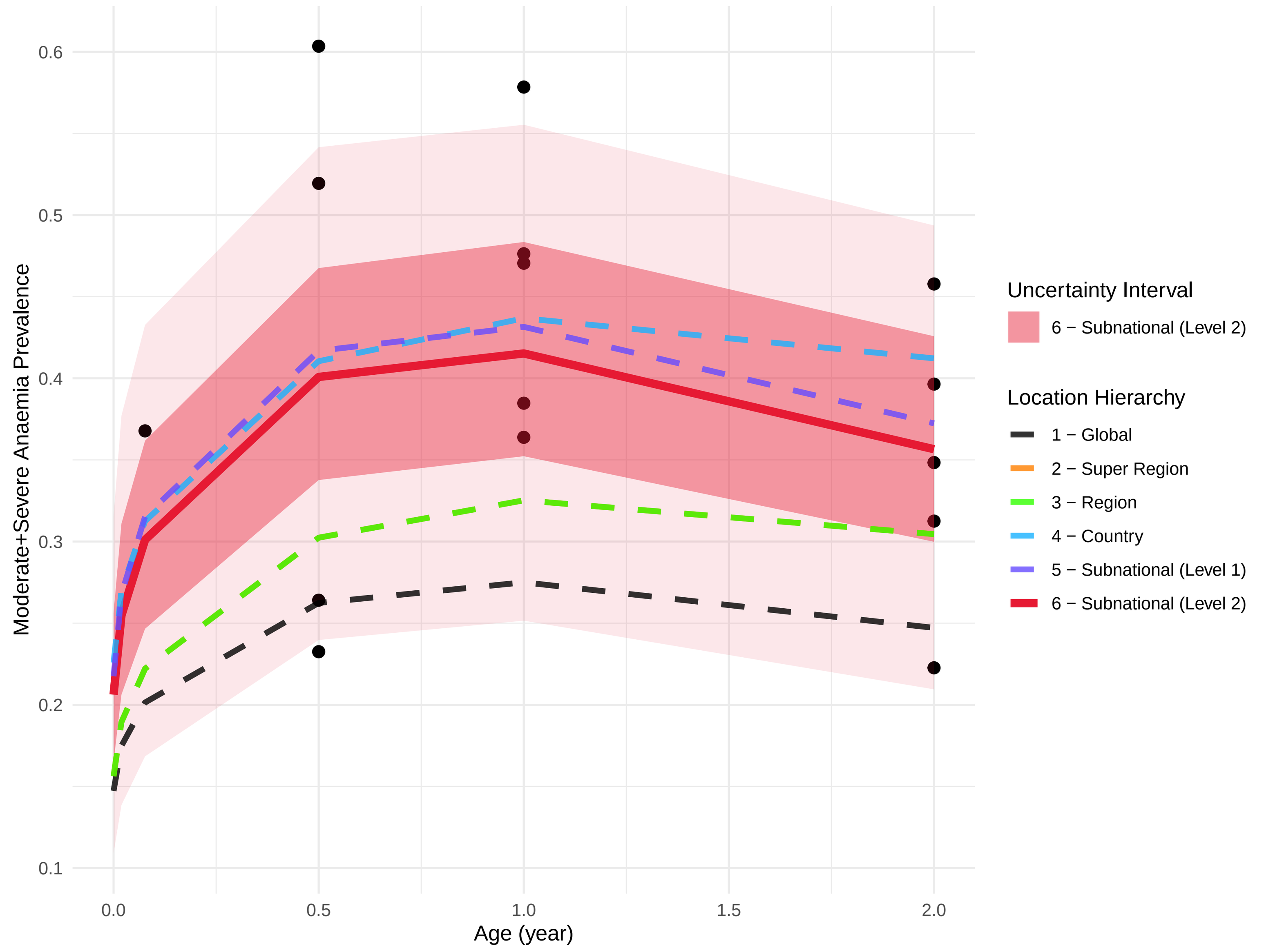

MR−BRT Age/Sex Fits − Non−pregnant Females <2 Years Old − Moderate+Severe Anaemia Prevalence − Goa
WHO−2001 Adjustment Method

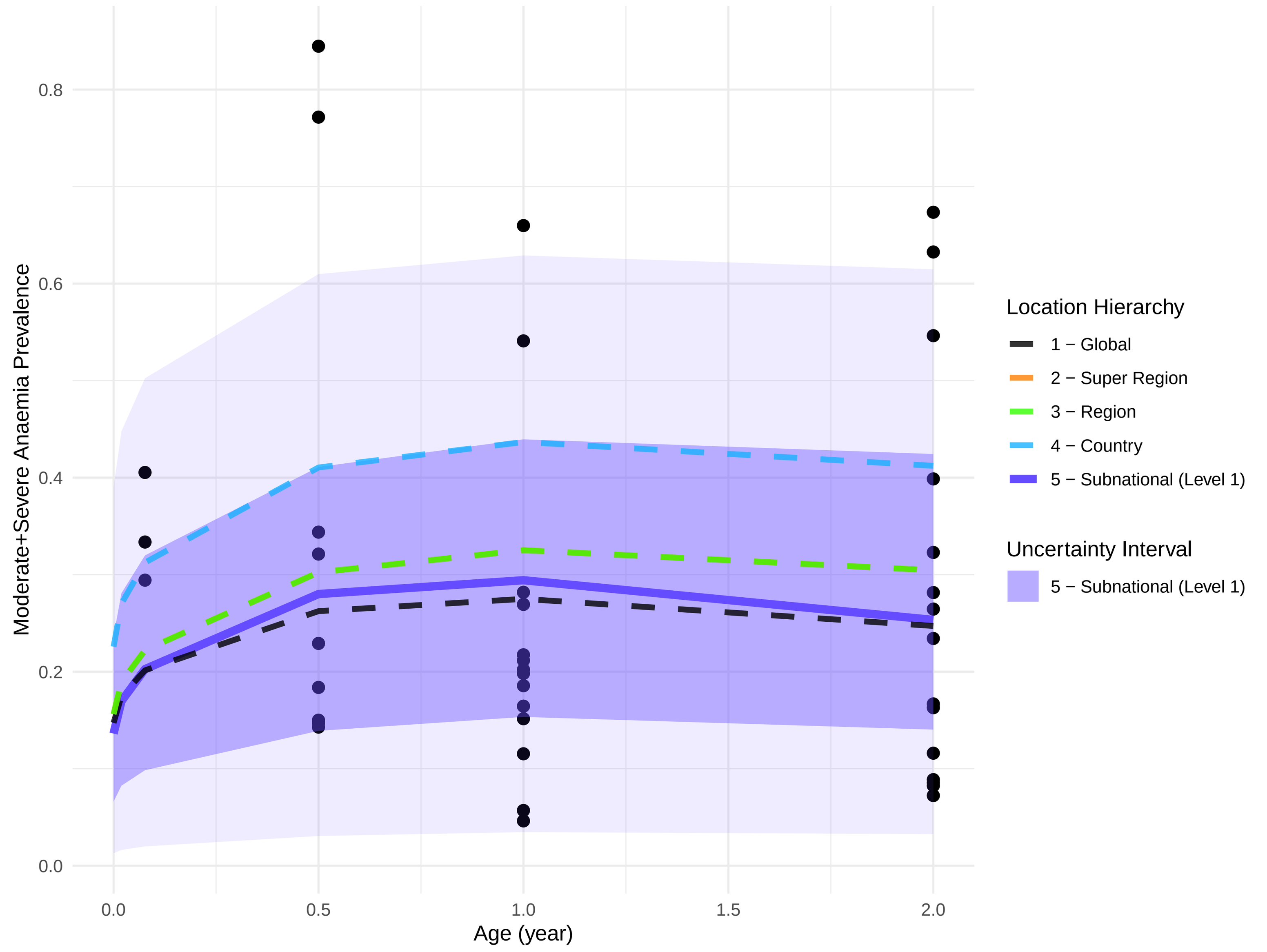

MR−BRT Age/Sex Fits − Non−pregnant Females <2 Years Old − Moderate+Severe Anaemia Prevalence − Goa, Rural
WHO−2001 Adjustment Method

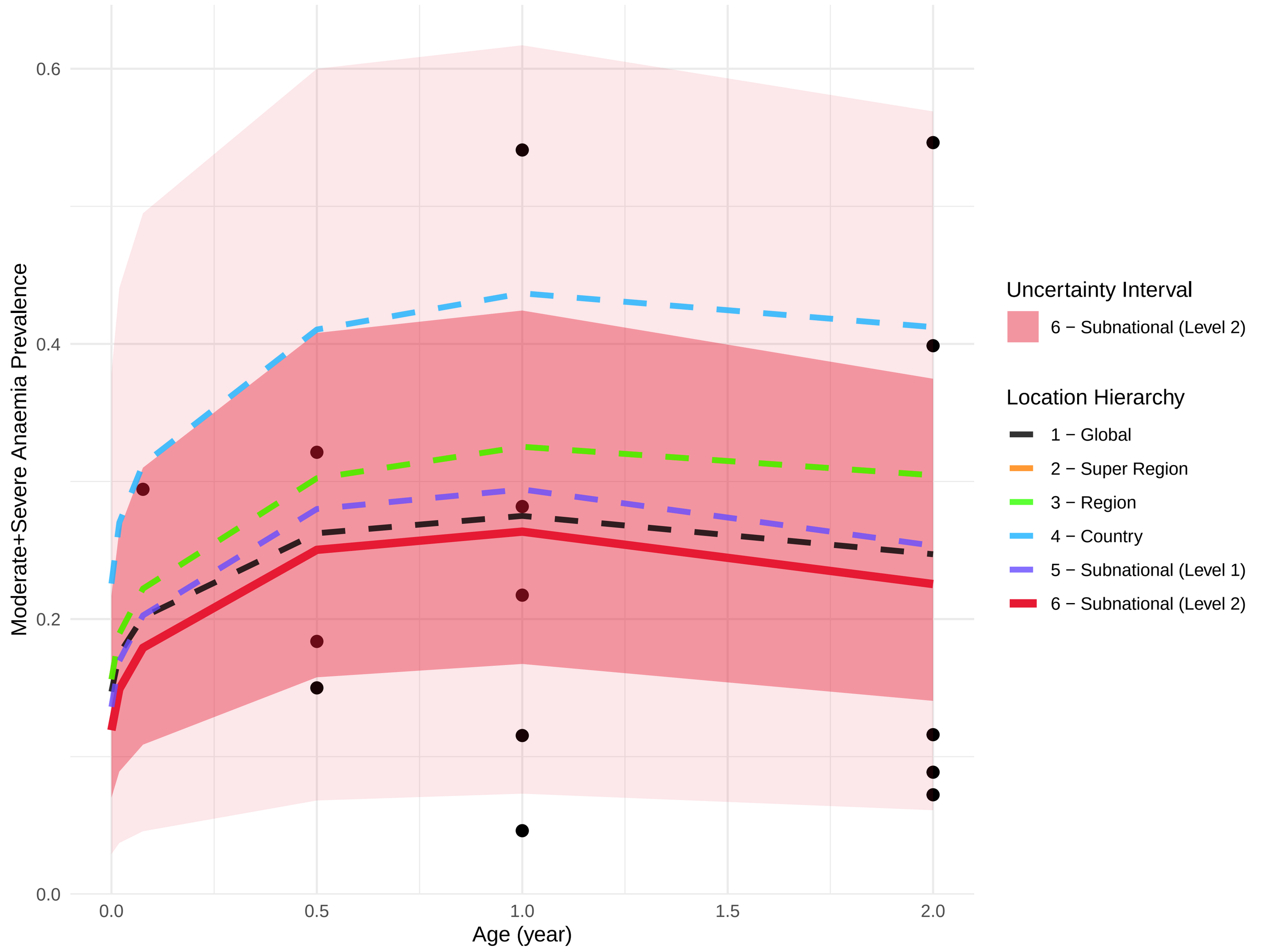

MR−BRT Age/Sex Fits − Non−pregnant Females <2 Years Old − Moderate+Severe Anaemia Prevalence − Goa, Urban
WHO−2001 Adjustment Method

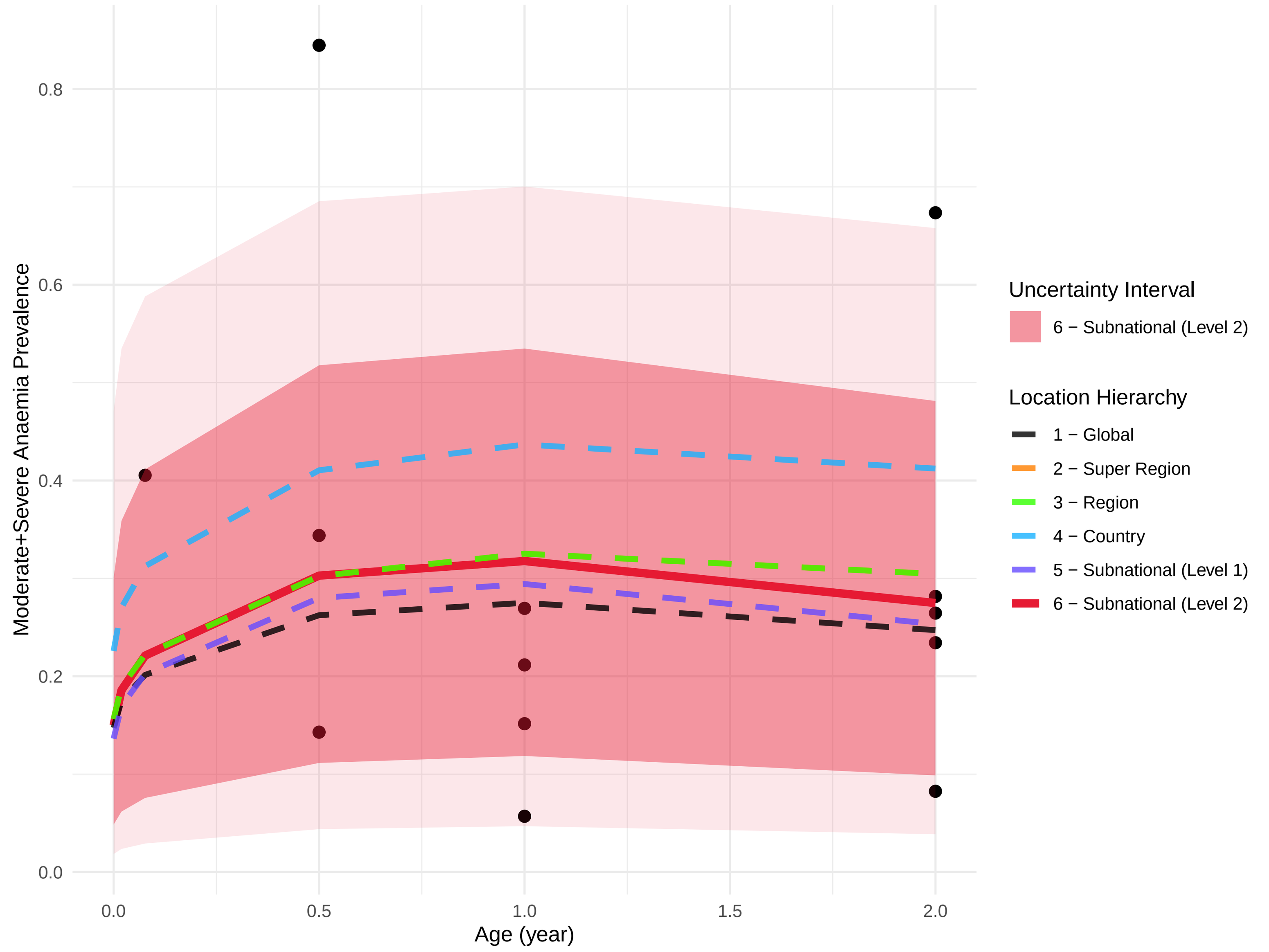

MR−BRT Age/Sex Fits − Non−pregnant Females <2 Years Old − Moderate+Severe Anaemia Prevalence − Gujarat
WHO−2001 Adjustment Method

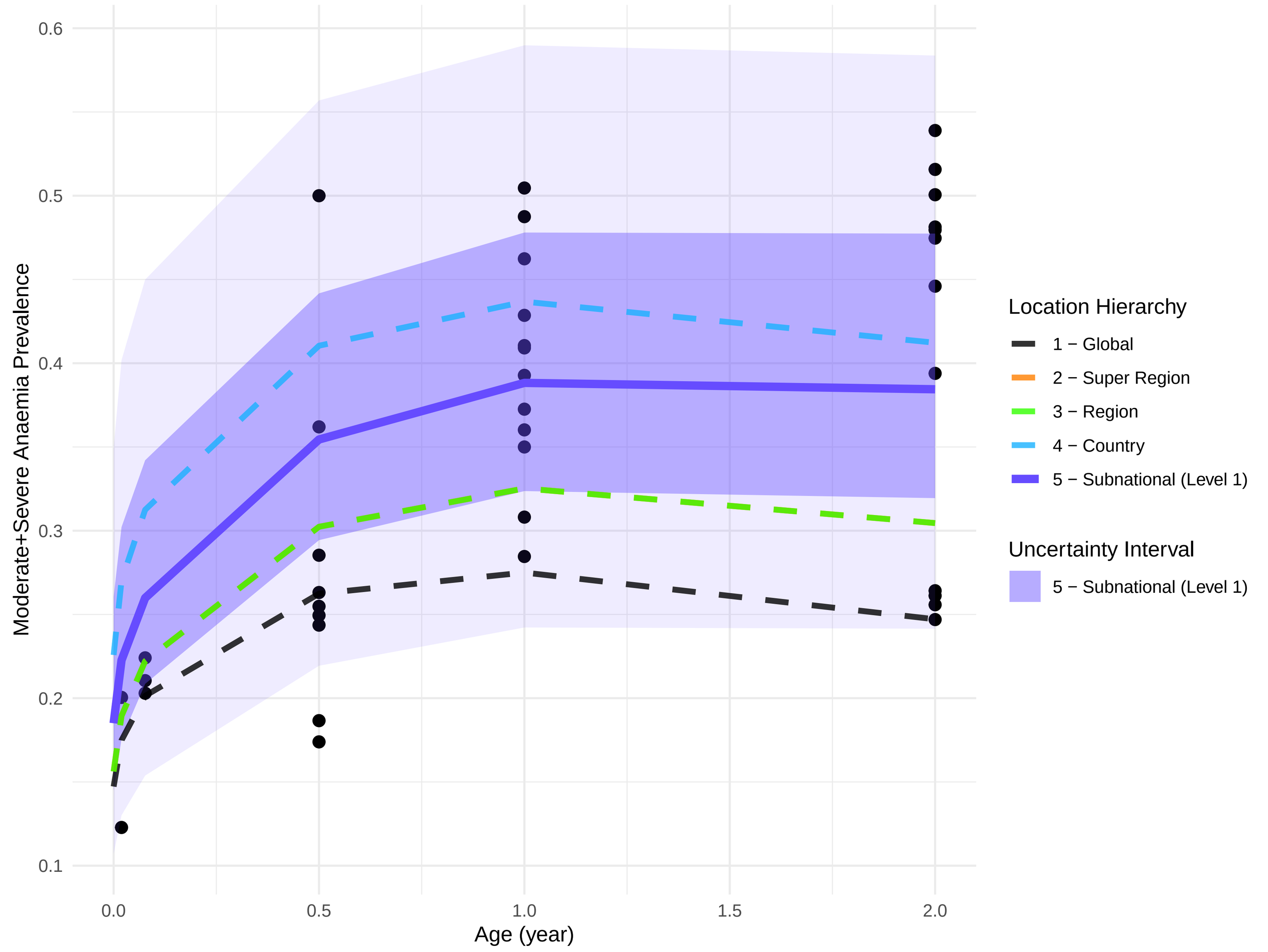

MR−BRT Age/Sex Fits − Non−pregnant Females <2 Years Old − Moderate+Severe Anaemia Prevalence − Gujarat, Rural
WHO−2001 Adjustment Method

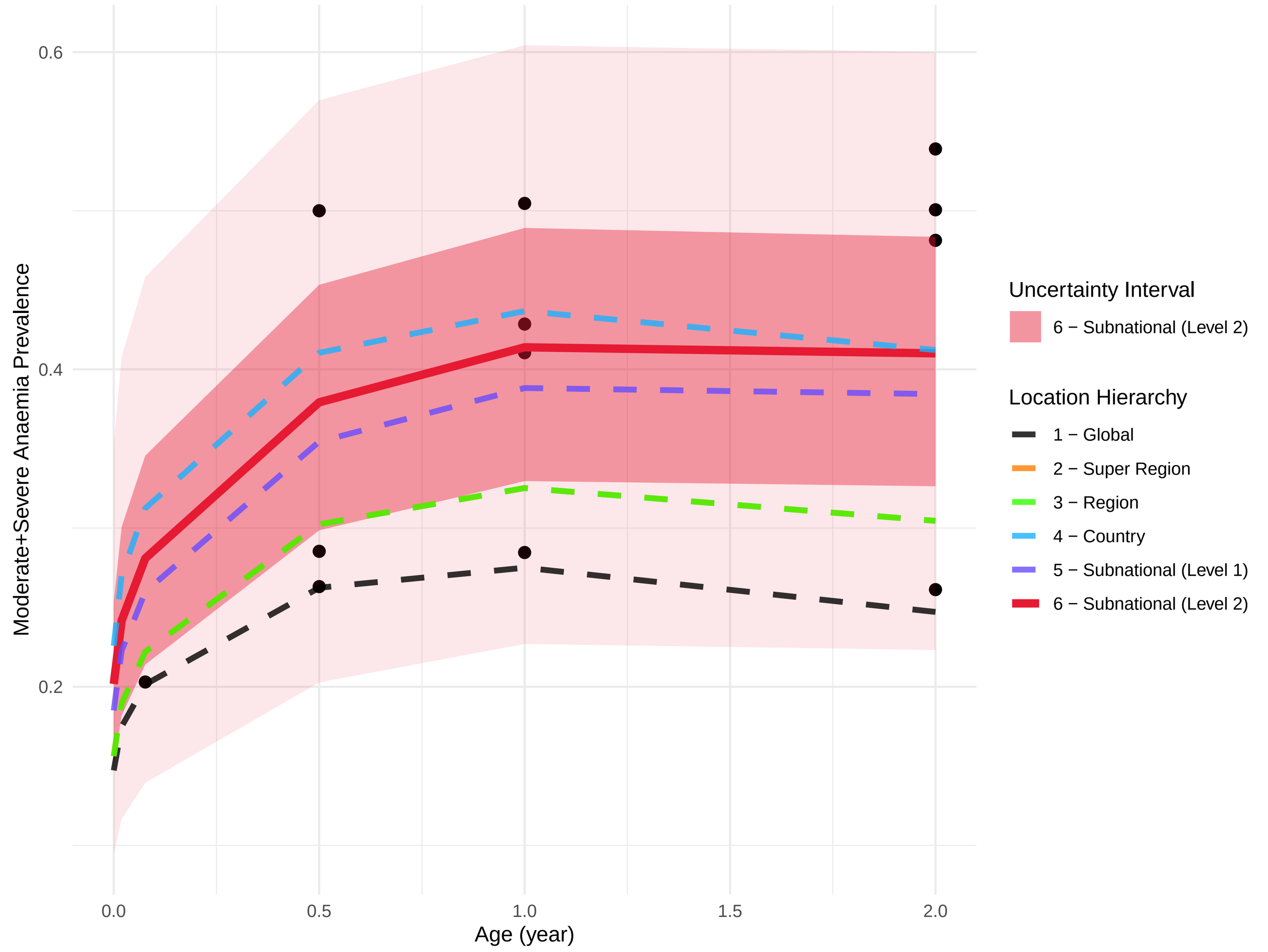

MR−BRT Age/Sex Fits − Non−pregnant Females <2 Years Old − Moderate+Severe Anaemia Prevalence − Gujarat, Urban
WHO−2001 Adjustment Method

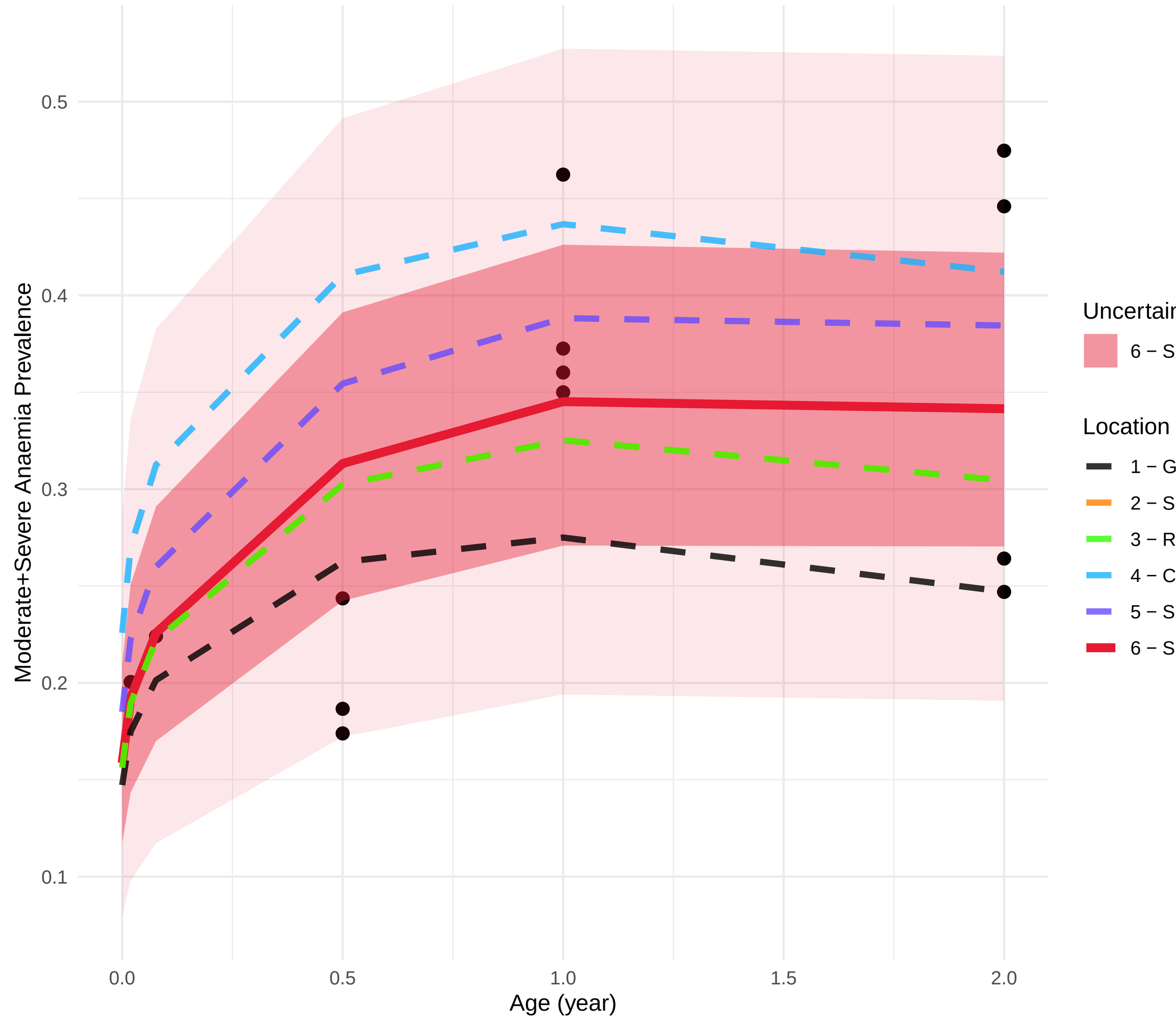

MR−BRT Age/Sex Fits − Non−pregnant Females <2 Years Old − Moderate+Severe Anaemia Prevalence − Haryana
WHO−2001 Adjustment Method

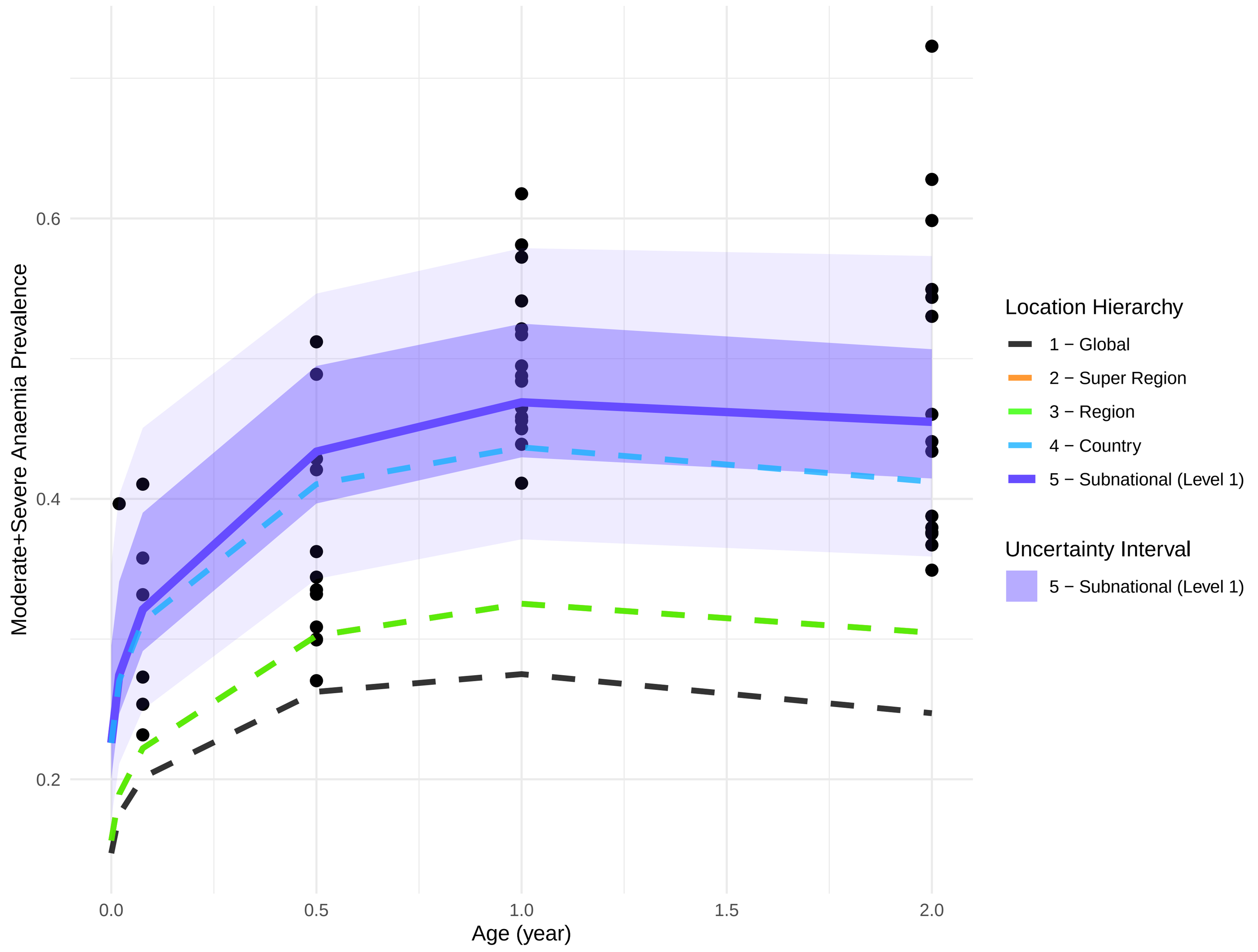

MR−BRT Age/Sex Fits − Non−pregnant Females <2 Years Old − Moderate+Severe Anaemia Prevalence − Haryana, Rural
WHO−2001 Adjustment Method

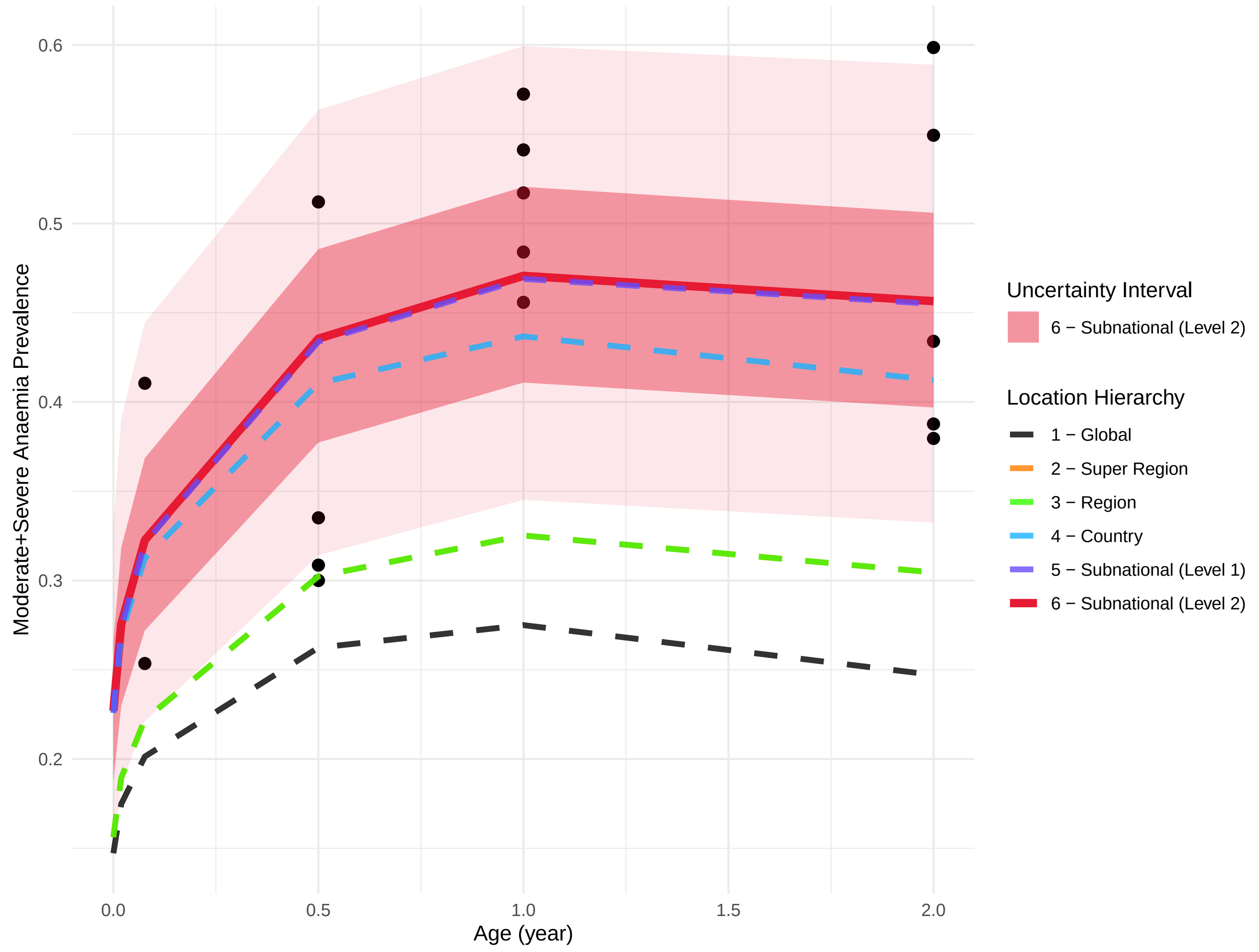

MR−BRT Age/Sex Fits − Non−pregnant Females <2 Years Old − Moderate+Severe Anaemia Prevalence − Haryana, Urban
WHO−2001 Adjustment Method

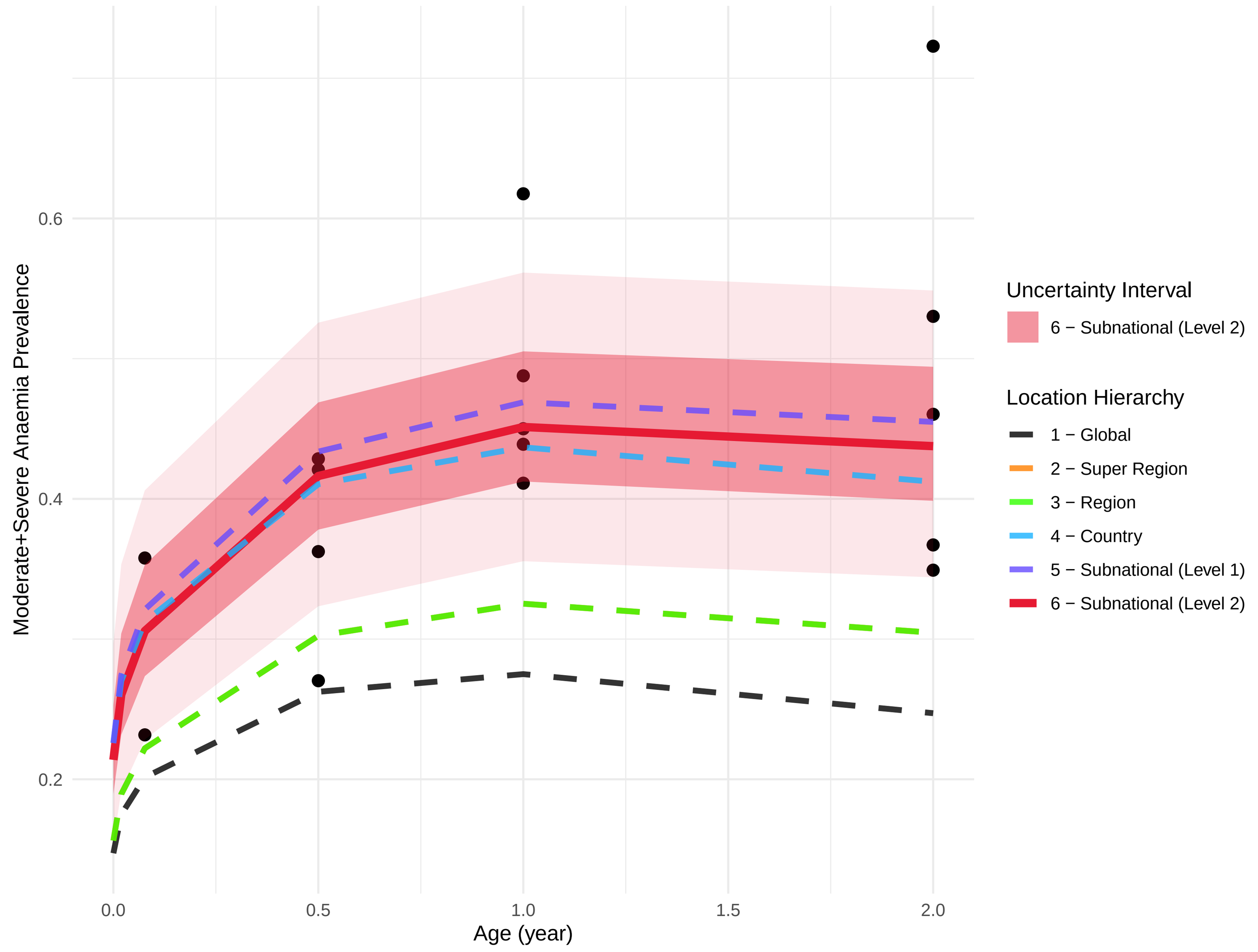

MR−BRT Age/Sex Fits − Non−pregnant Females <2 Years Old − Moderate+Severe Anaemia Prevalence − Himachal Pradesh
WHO−2001 Adjustment Method

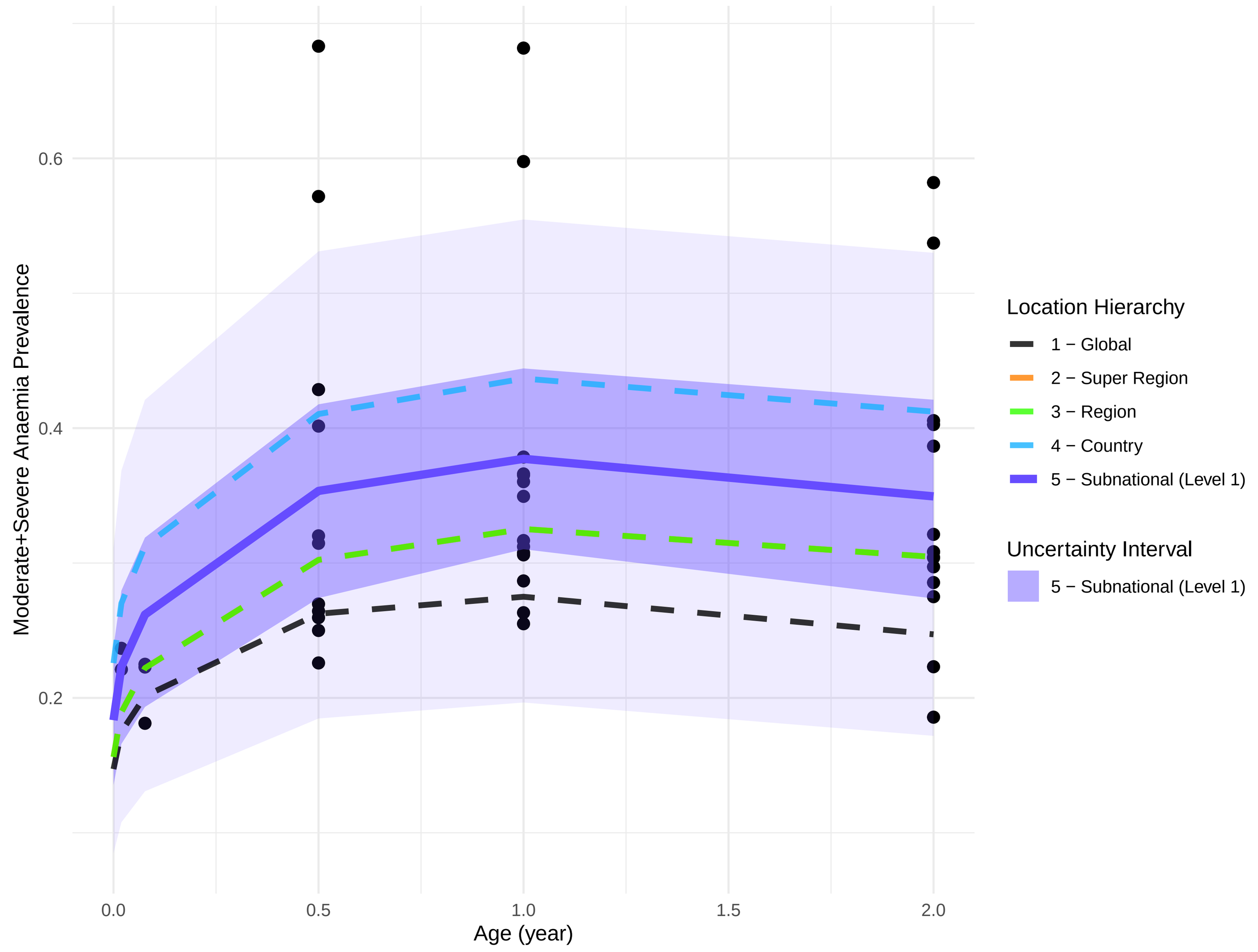

MR−BRT Age/Sex Fits − Non−pregnant Females <2 Years Old − Moderate+Severe Anaemia Prevalence − Himachal Pradesh, Rural
WHO−2001 Adjustment Method

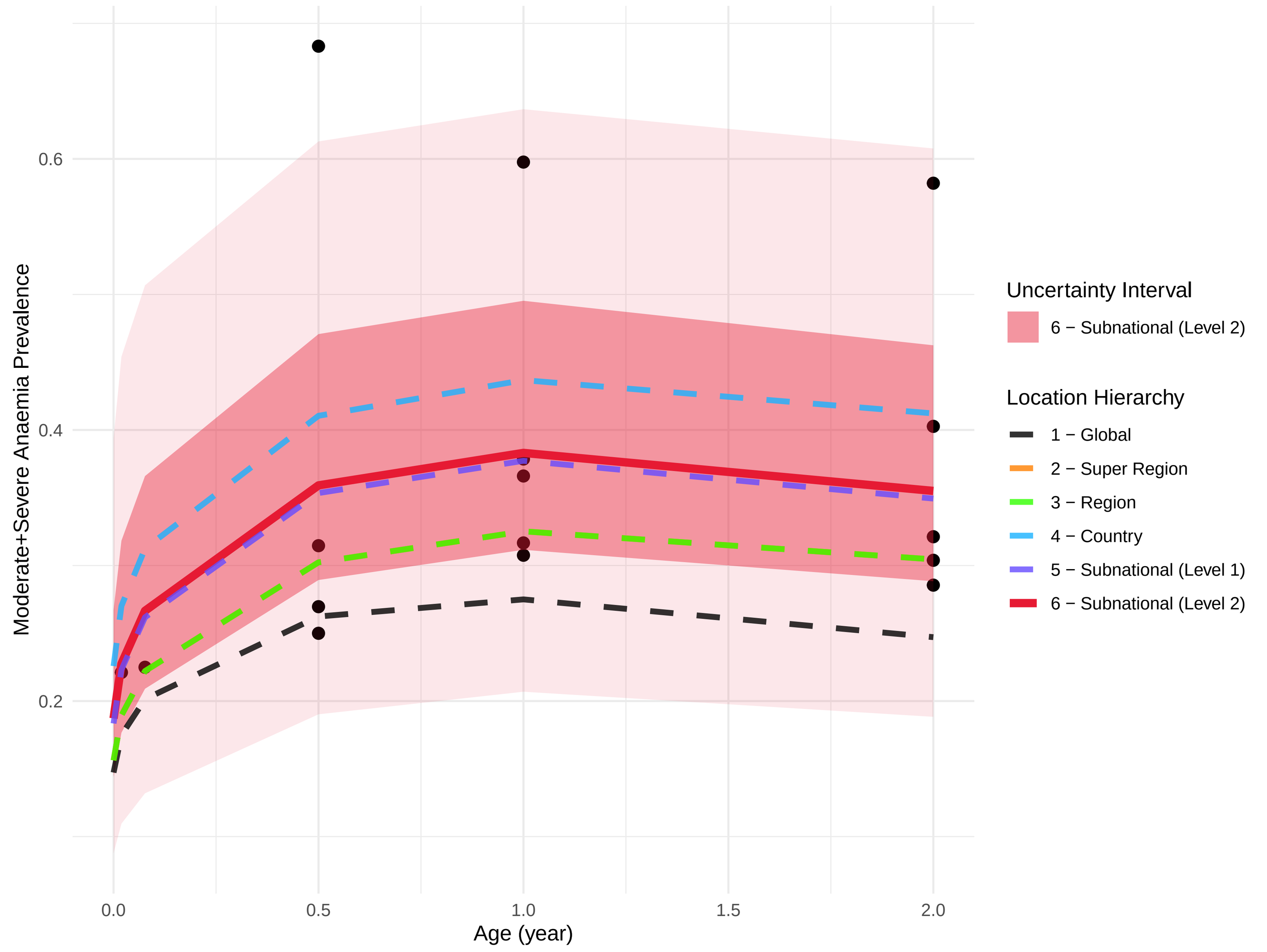

MR−BRT Age/Sex Fits − Non−pregnant Females <2 Years Old − Moderate+Severe Anaemia Prevalence − Himachal Pradesh, Urban
WHO−2001 Adjustment Method

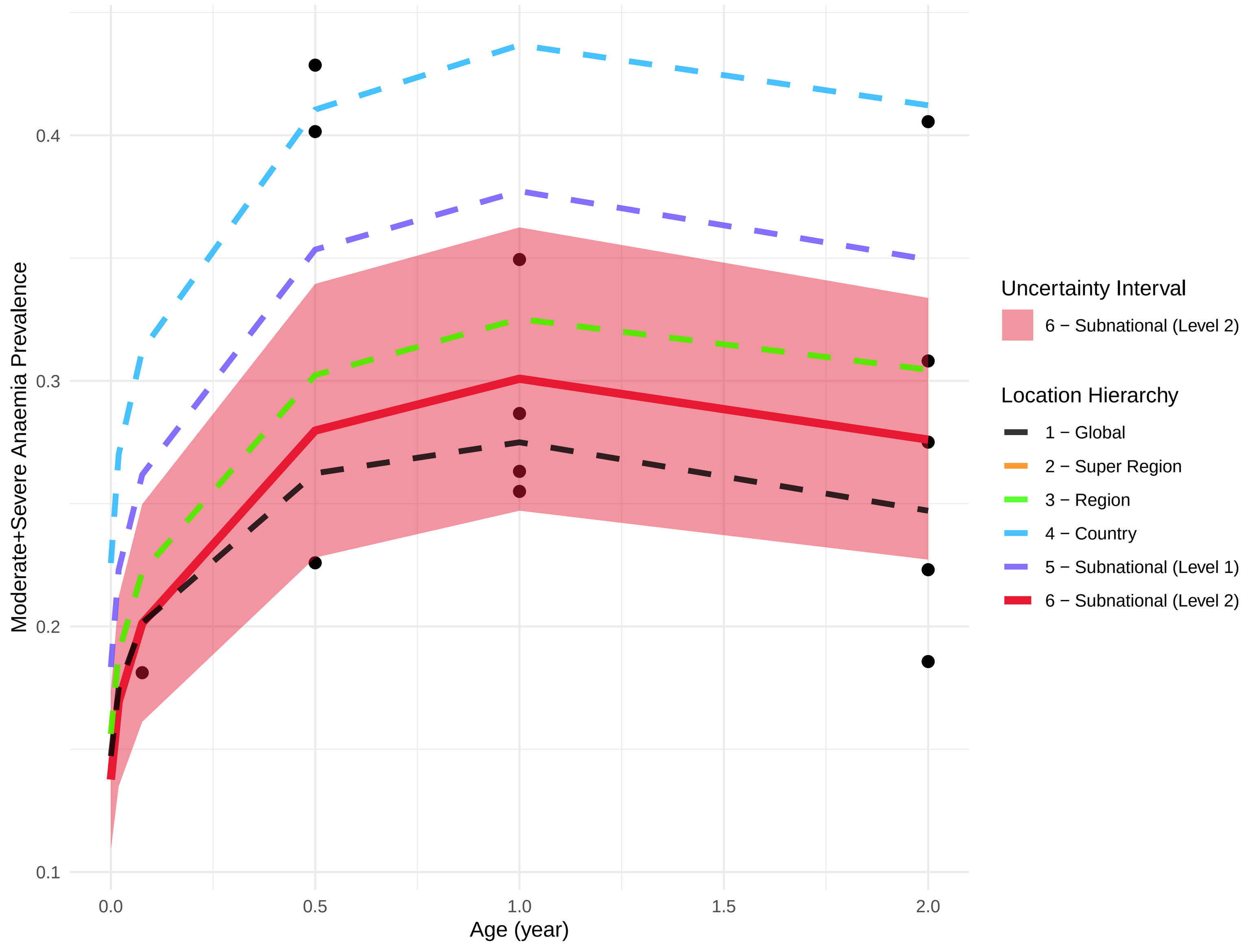

MR−BRT Age/Sex Fits − Non−pregnant Females <2 Years Old − Moderate+Severe Anaemia Prevalence − Jammu & Kashmir and Ladakh
WHO−2001 Adjustment Method

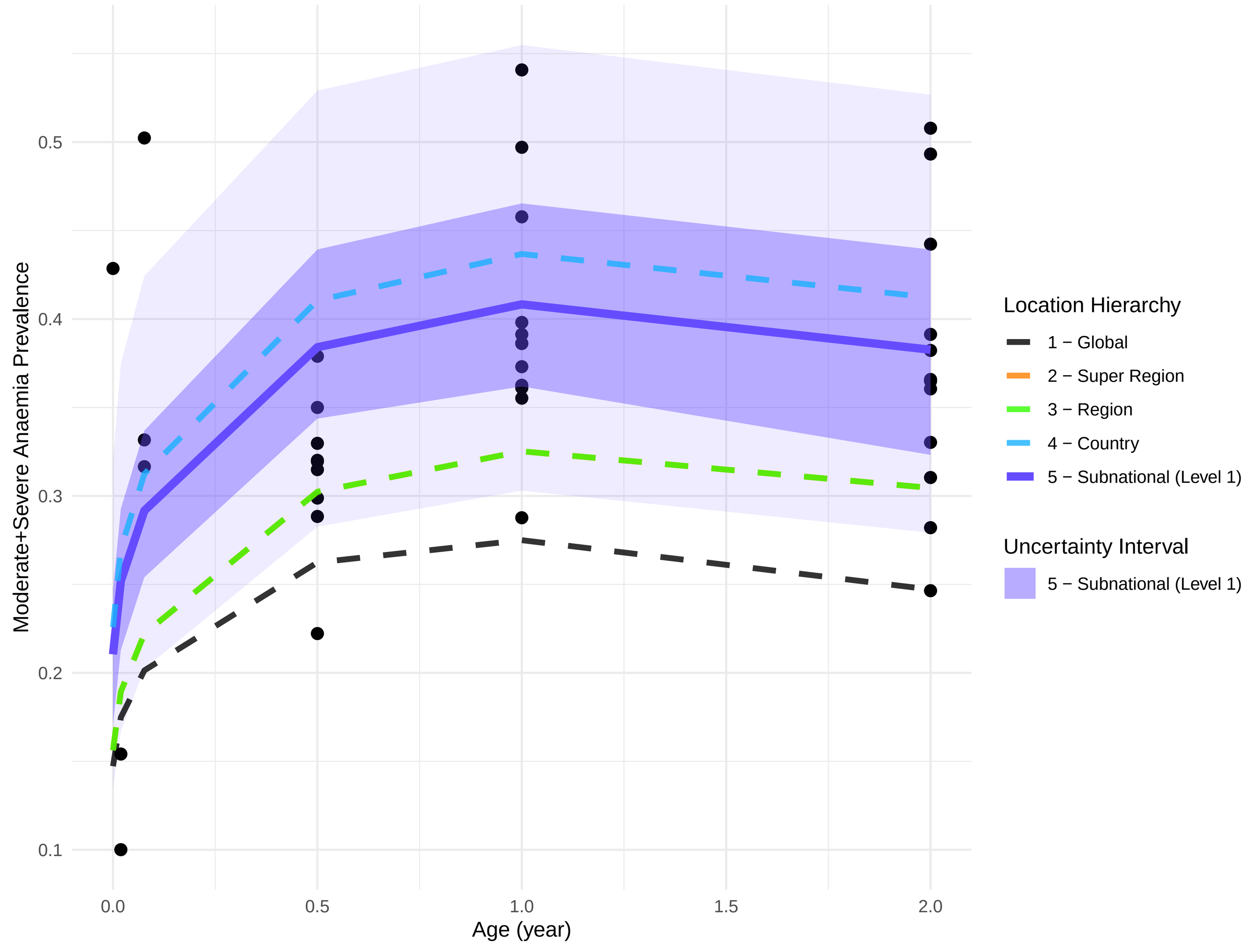

MR−BRT Age/Sex Fits − Non−pregnant Females <2 Years Old − Moderate+Severe Anaemia Prevalence − Jammu & Kashmir and Ladakh, Rural
WHO−2001 Adjustment Method

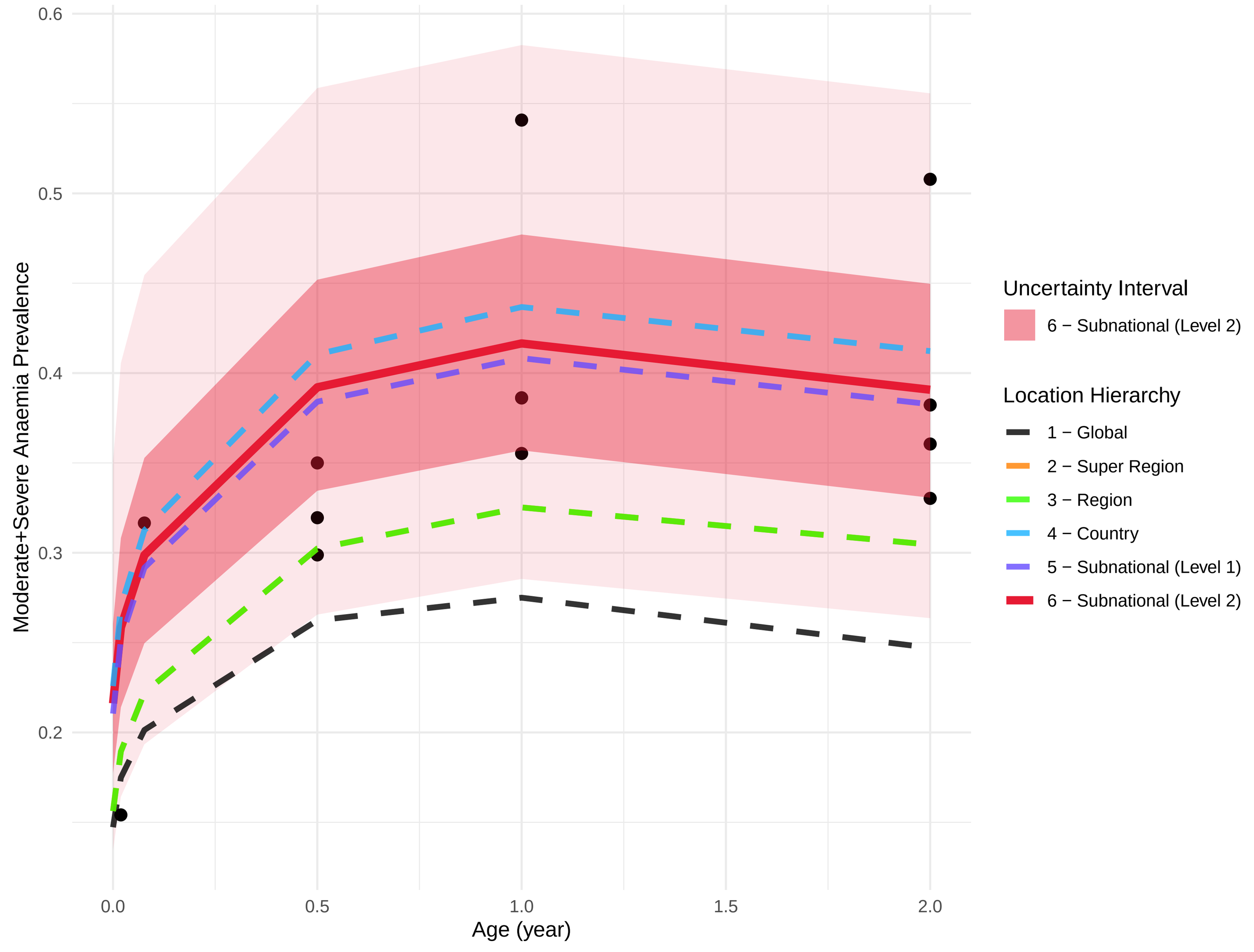

MR−BRT Age/Sex Fits − Non−pregnant Females <2 Years Old − Moderate+Severe Anaemia Prevalence − Jammu & Kashmir and Ladakh, Urban
WHO−2001 Adjustment Method

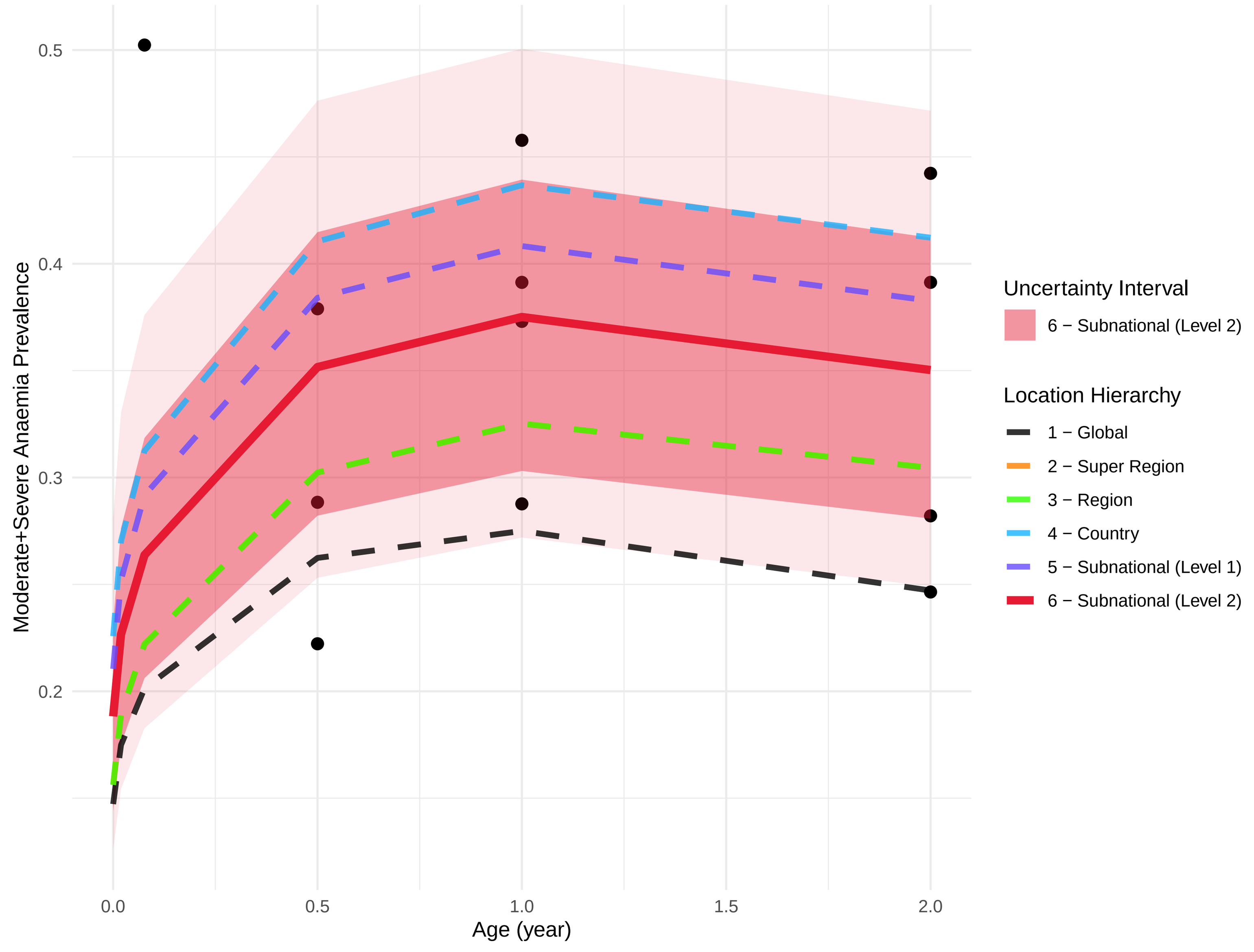

MR−BRT Age/Sex Fits − Non−pregnant Females <2 Years Old − Moderate+Severe Anaemia Prevalence − Jharkhand
WHO−2001 Adjustment Method

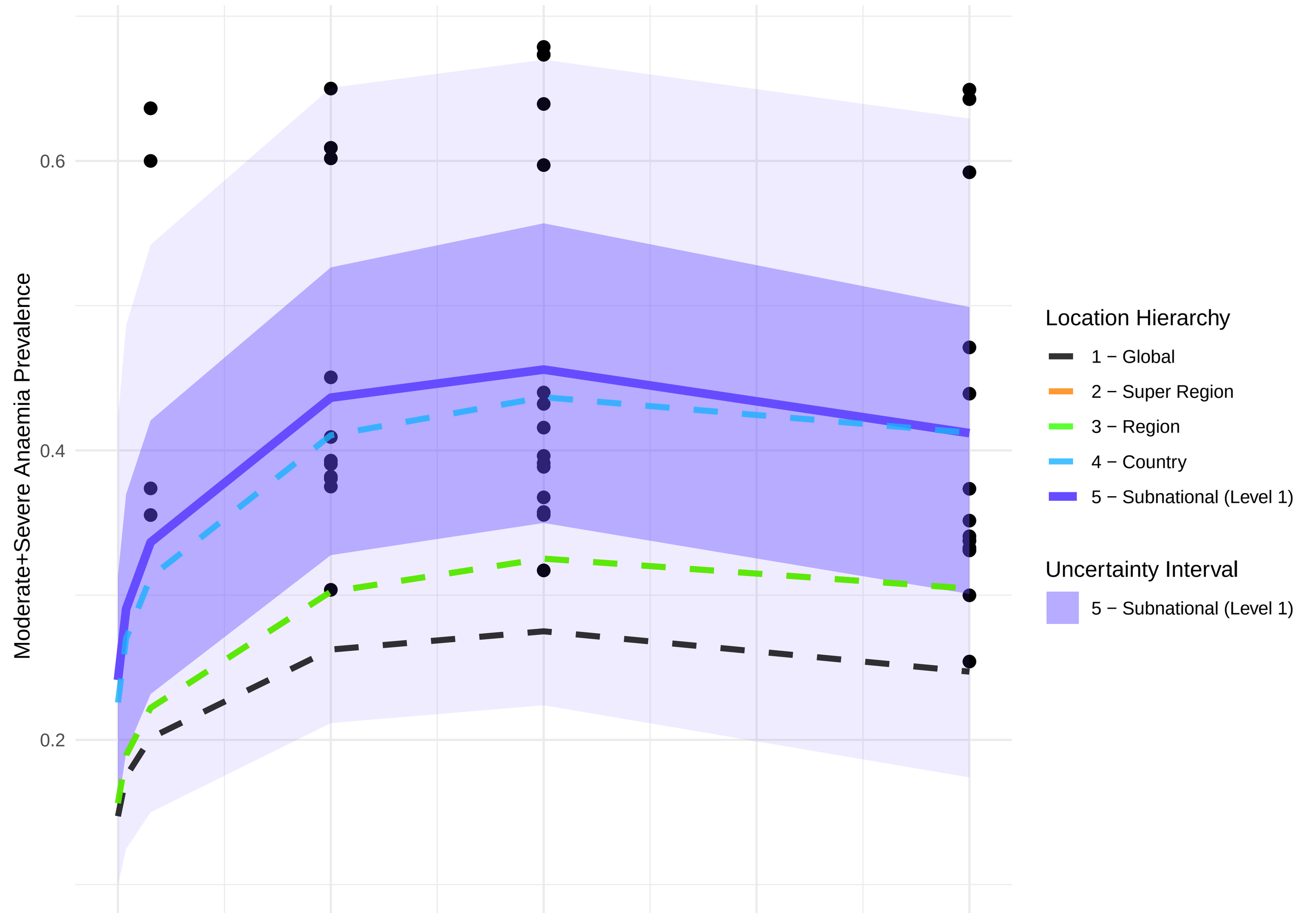

MR−BRT Age/Sex Fits − Non−pregnant Females <2 Years Old − Moderate+Severe Anaemia Prevalence − Jharkhand, Rural
WHO−2001 Adjustment Method

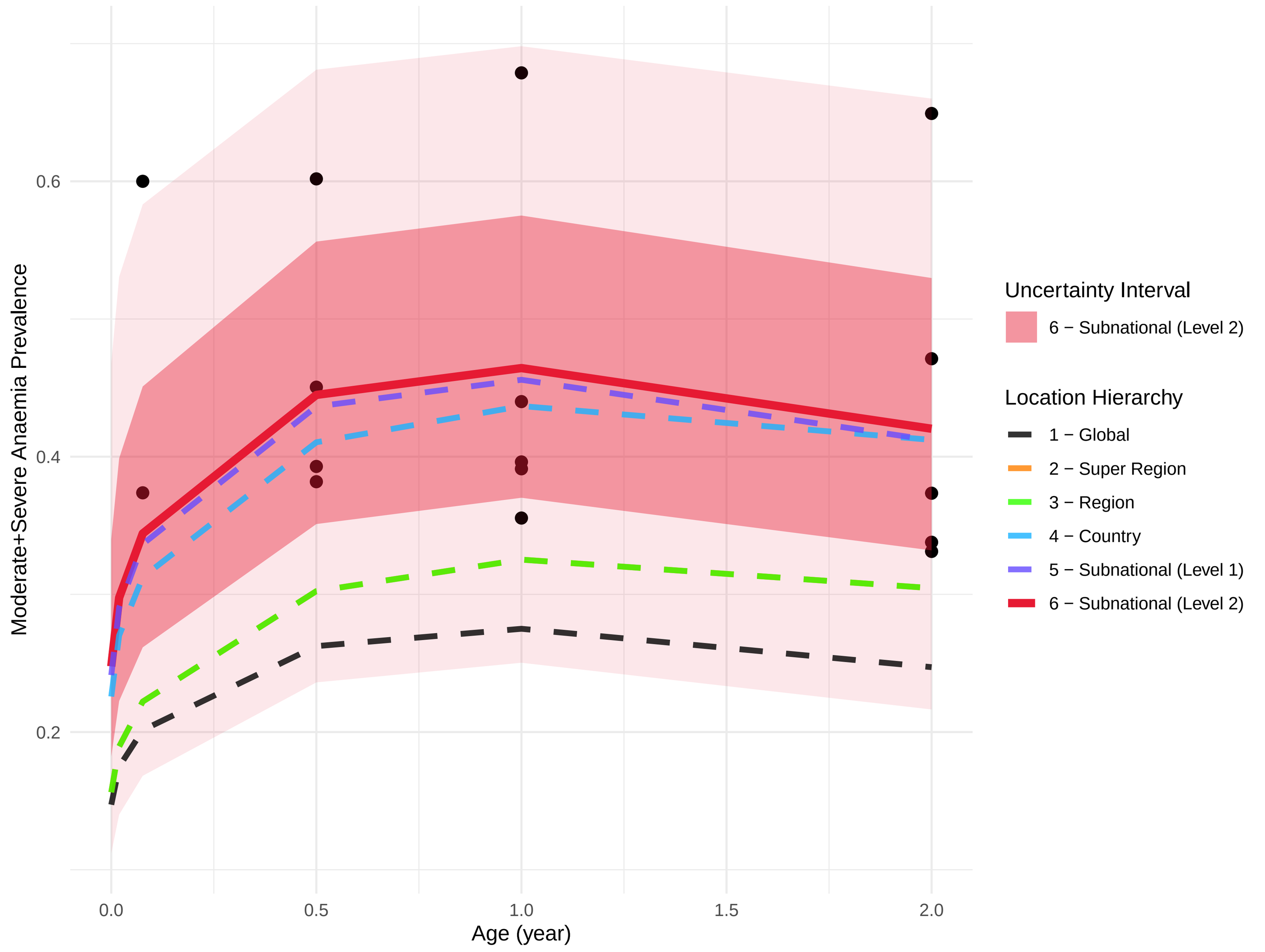

MR−BRT Age/Sex Fits − Non−pregnant Females <2 Years Old − Moderate+Severe Anaemia Prevalence − Jharkhand, Urban
WHO−2001 Adjustment Method

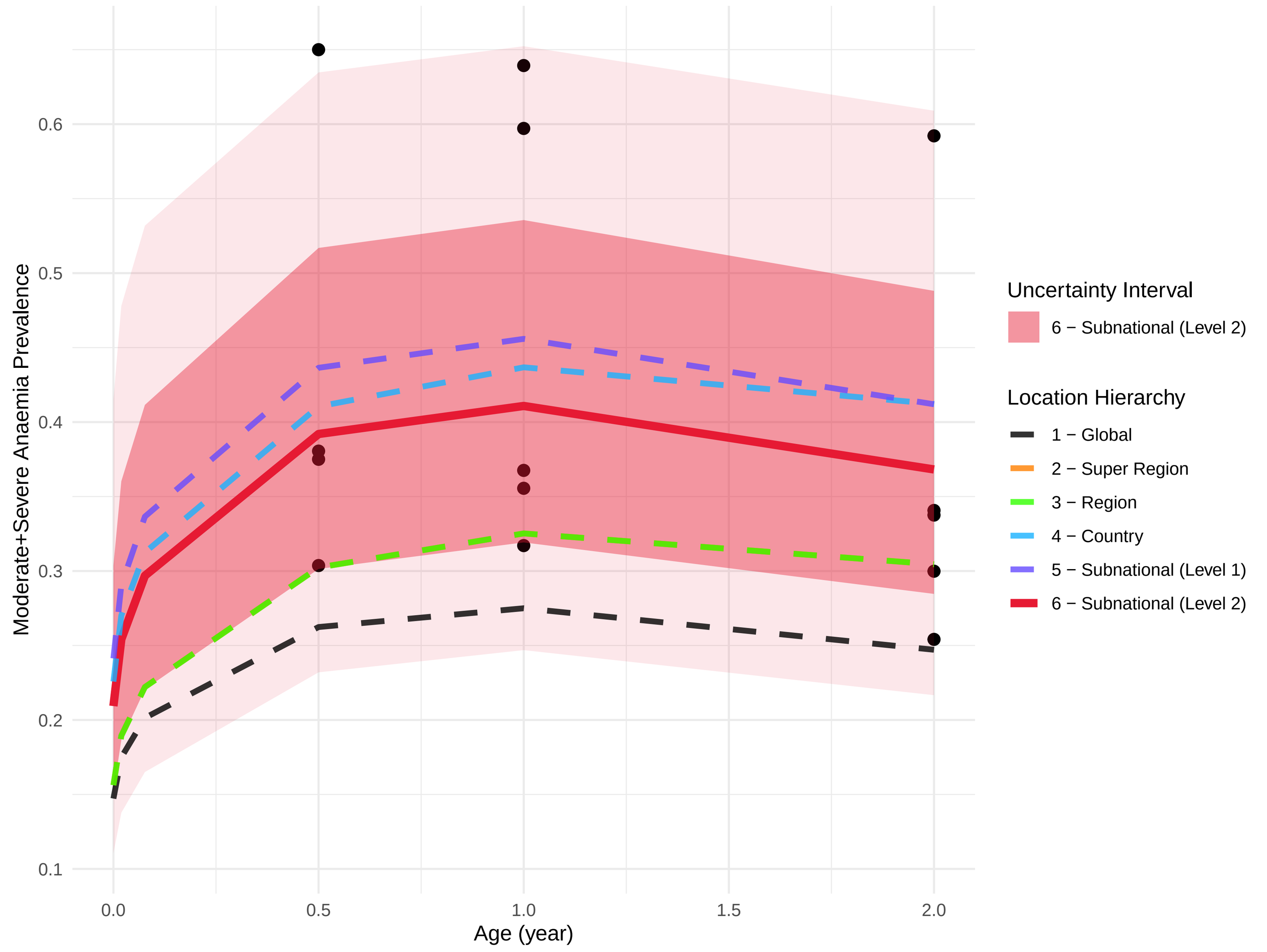

MR−BRT Age/Sex Fits − Non−pregnant Females <2 Years Old − Moderate+Severe Anaemia Prevalence − Karnataka
WHO−2001 Adjustment Method

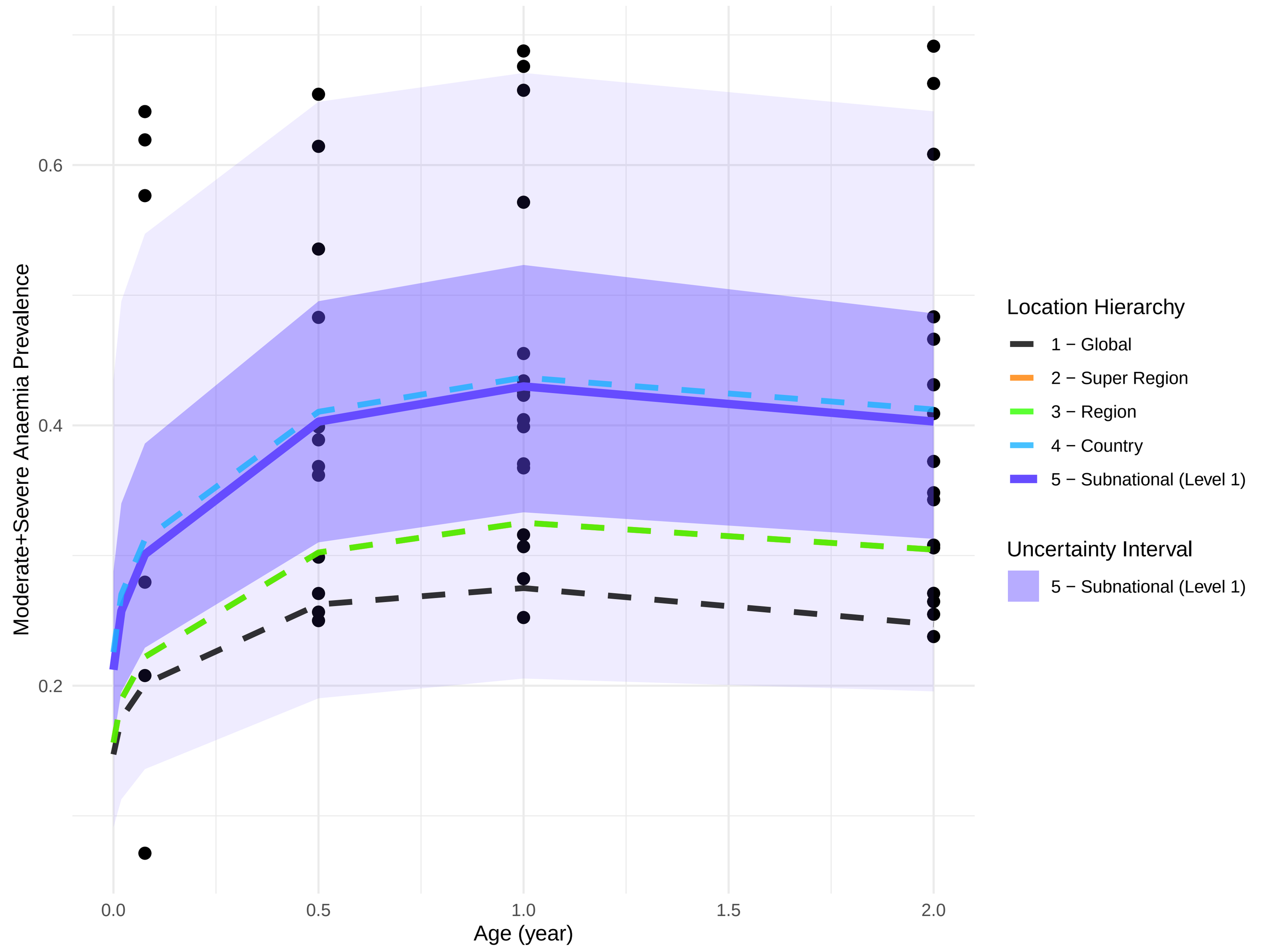

MR−BRT Age/Sex Fits − Non−pregnant Females <2 Years Old − Moderate+Severe Anaemia Prevalence − Karnataka, Rural
WHO−2001 Adjustment Method

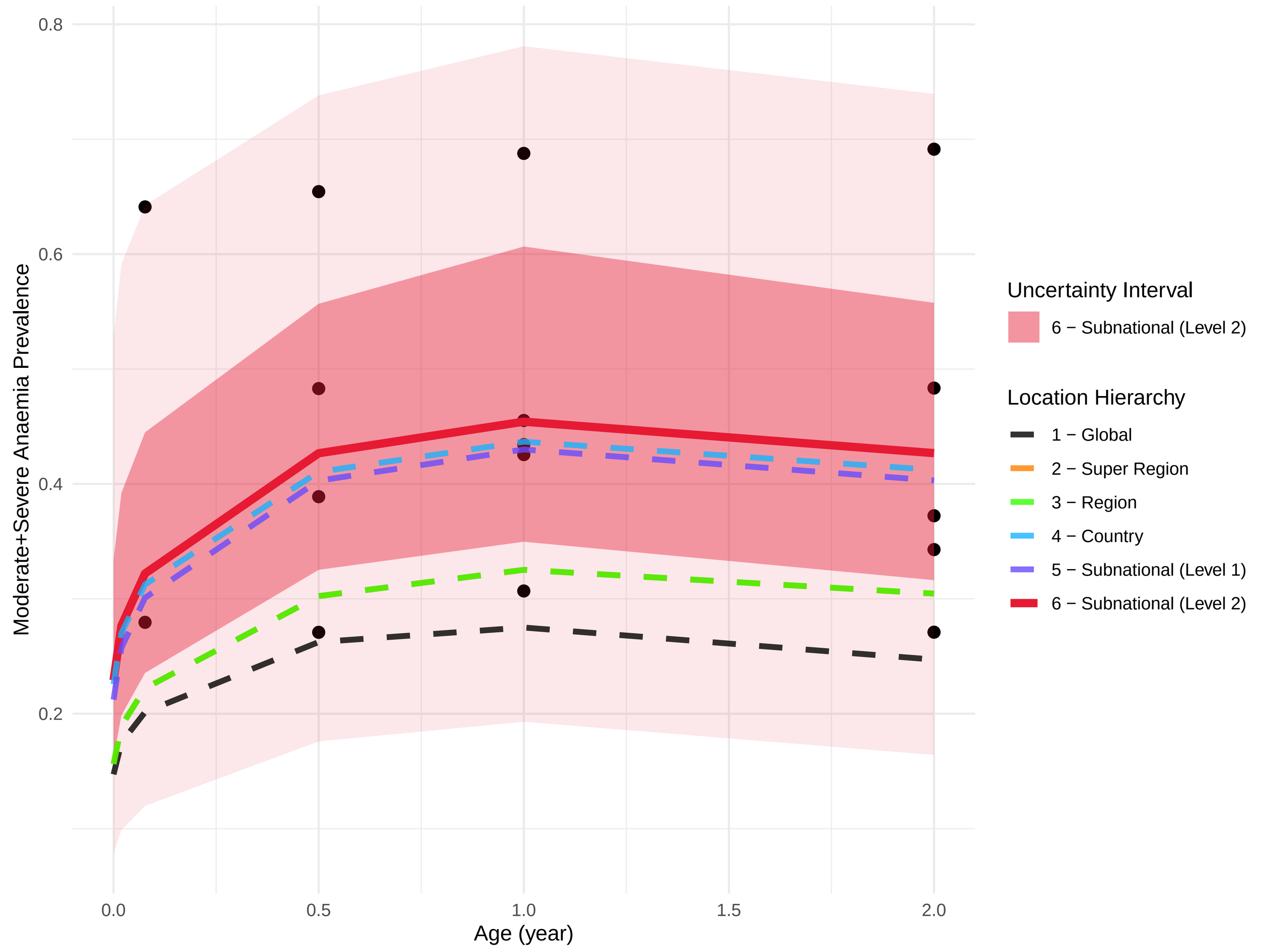

MR−BRT Age/Sex Fits − Non−pregnant Females <2 Years Old − Moderate+Severe Anaemia Prevalence − Karnataka, Urban
WHO−2001 Adjustment Method

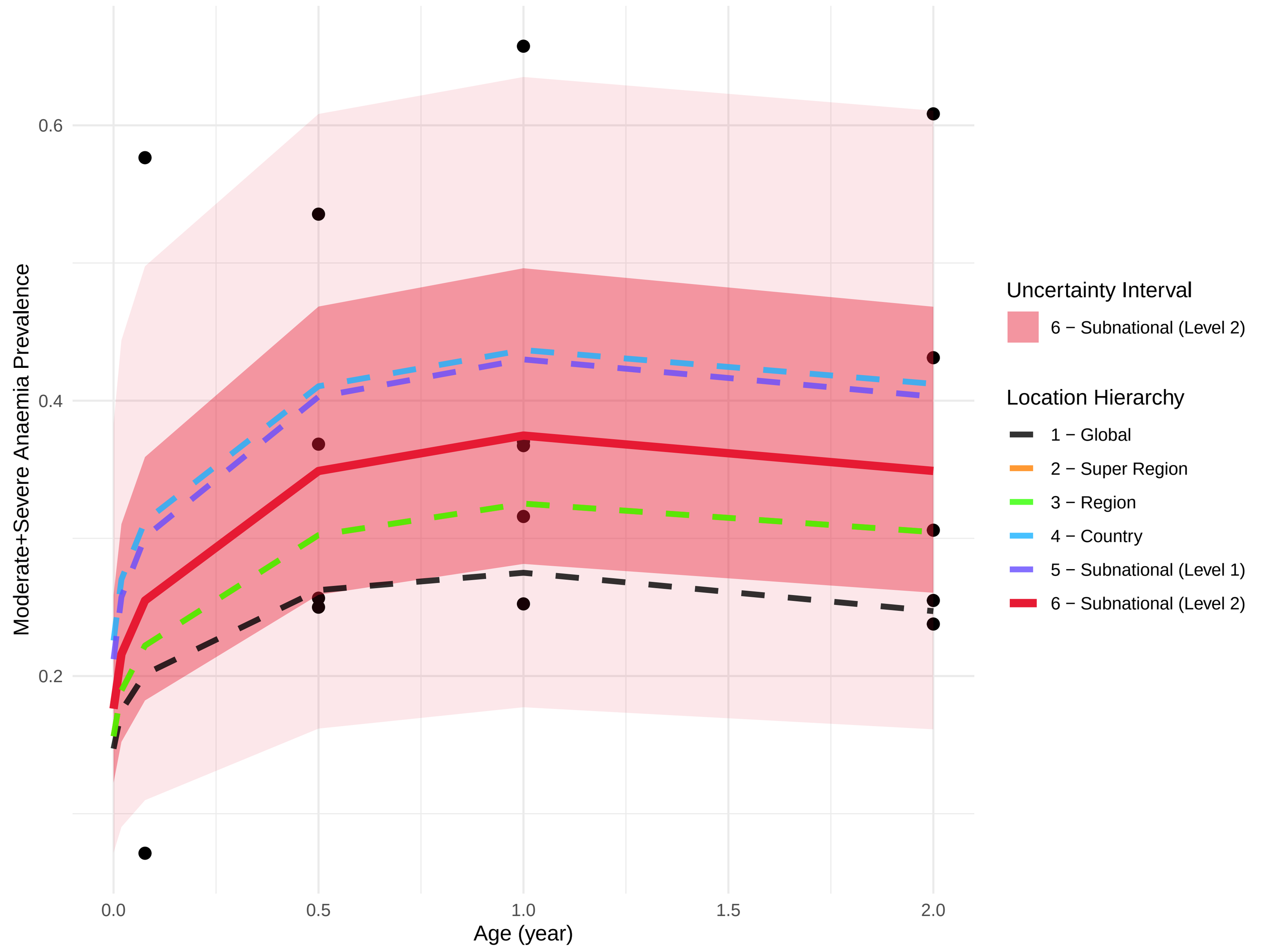

MR−BRT Age/Sex Fits − Non−pregnant Females <2 Years Old − Moderate+Severe Anaemia Prevalence − Kerala
WHO−2001 Adjustment Method

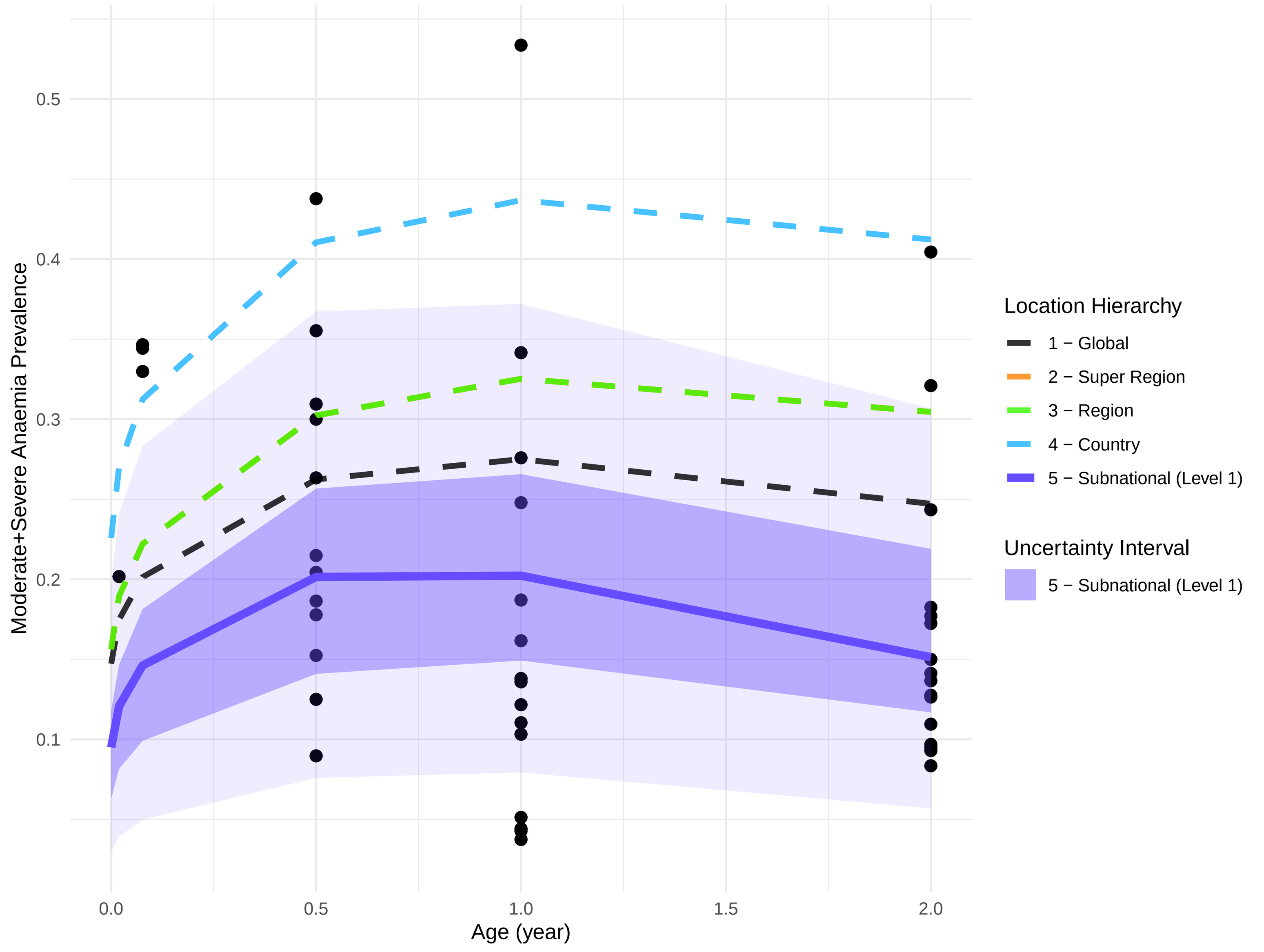

MR−BRT Age/Sex Fits − Non−pregnant Females <2 Years Old − Moderate+Severe Anaemia Prevalence − Kerala, Rural
WHO−2001 Adjustment Method

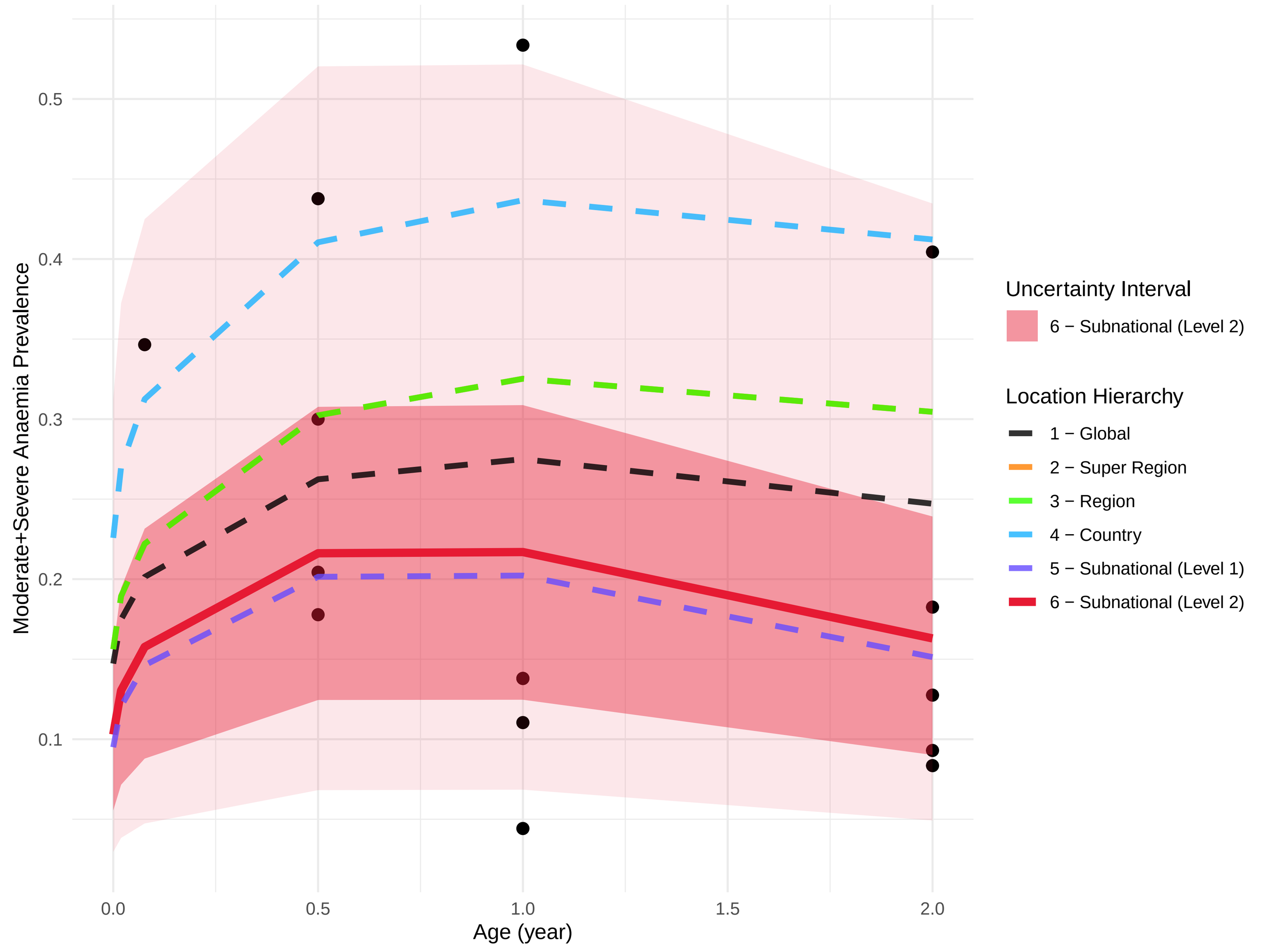

MR−BRT Age/Sex Fits − Non−pregnant Females <2 Years Old − Moderate+Severe Anaemia Prevalence − Kerala, Urban
WHO−2001 Adjustment Method

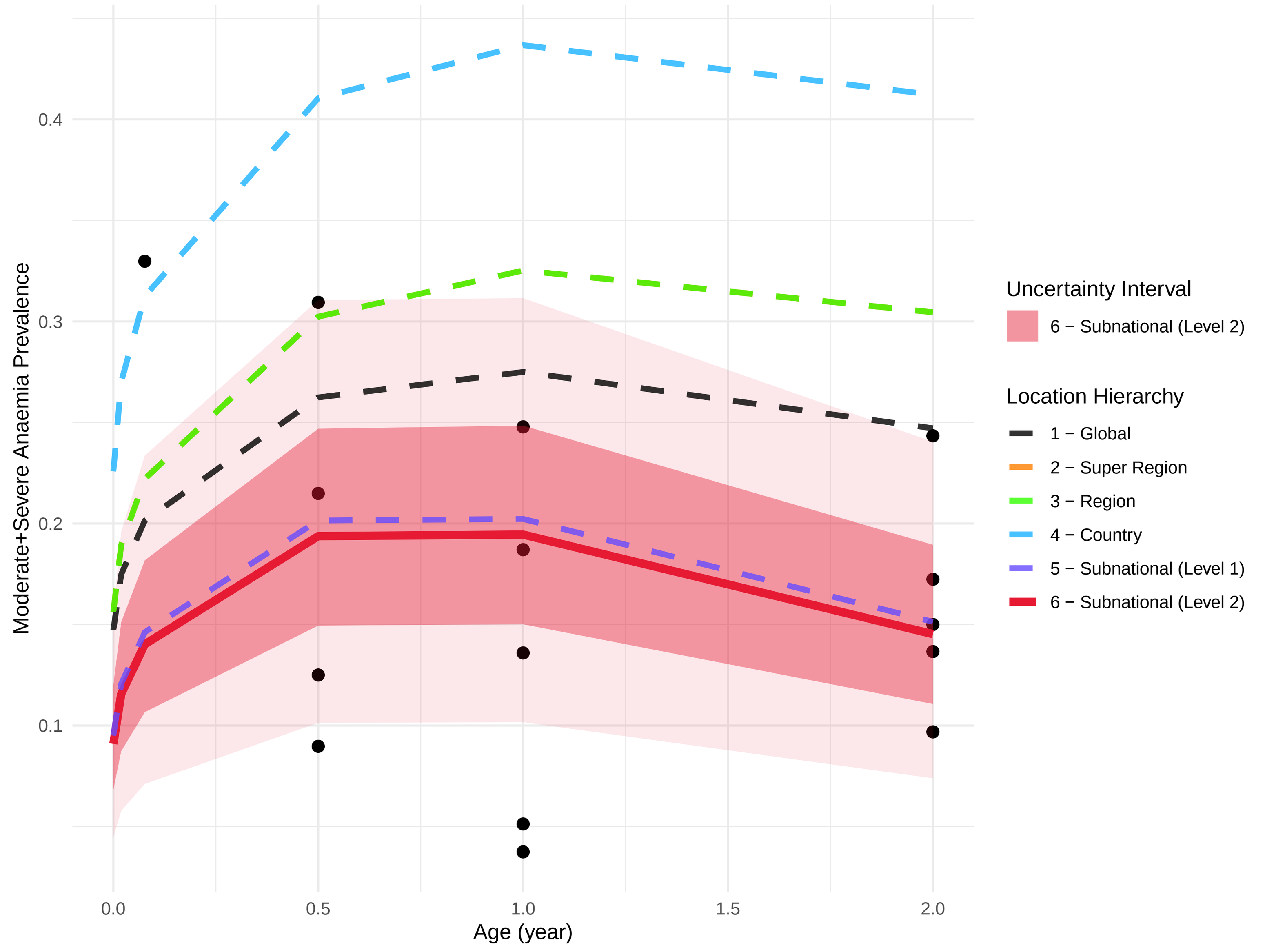

MR−BRT Age/Sex Fits − Non−pregnant Females <2 Years Old − Moderate+Severe Anaemia Prevalence − Madhya Pradesh
WHO−2001 Adjustment Method

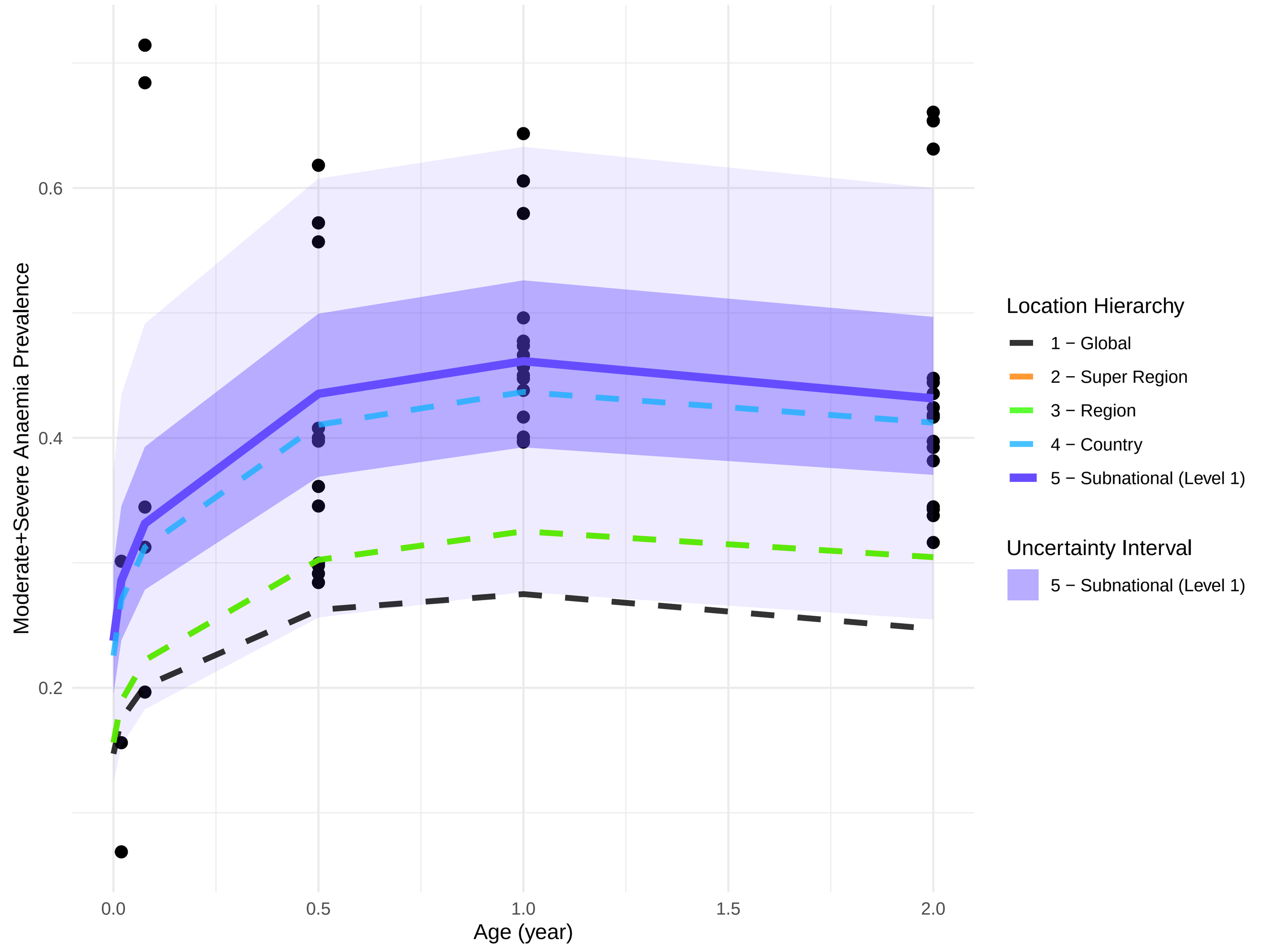

MR−BRT Age/Sex Fits − Non−pregnant Females <2 Years Old − Moderate+Severe Anaemia Prevalence − Madhya Pradesh, Rural
WHO−2001 Adjustment Method

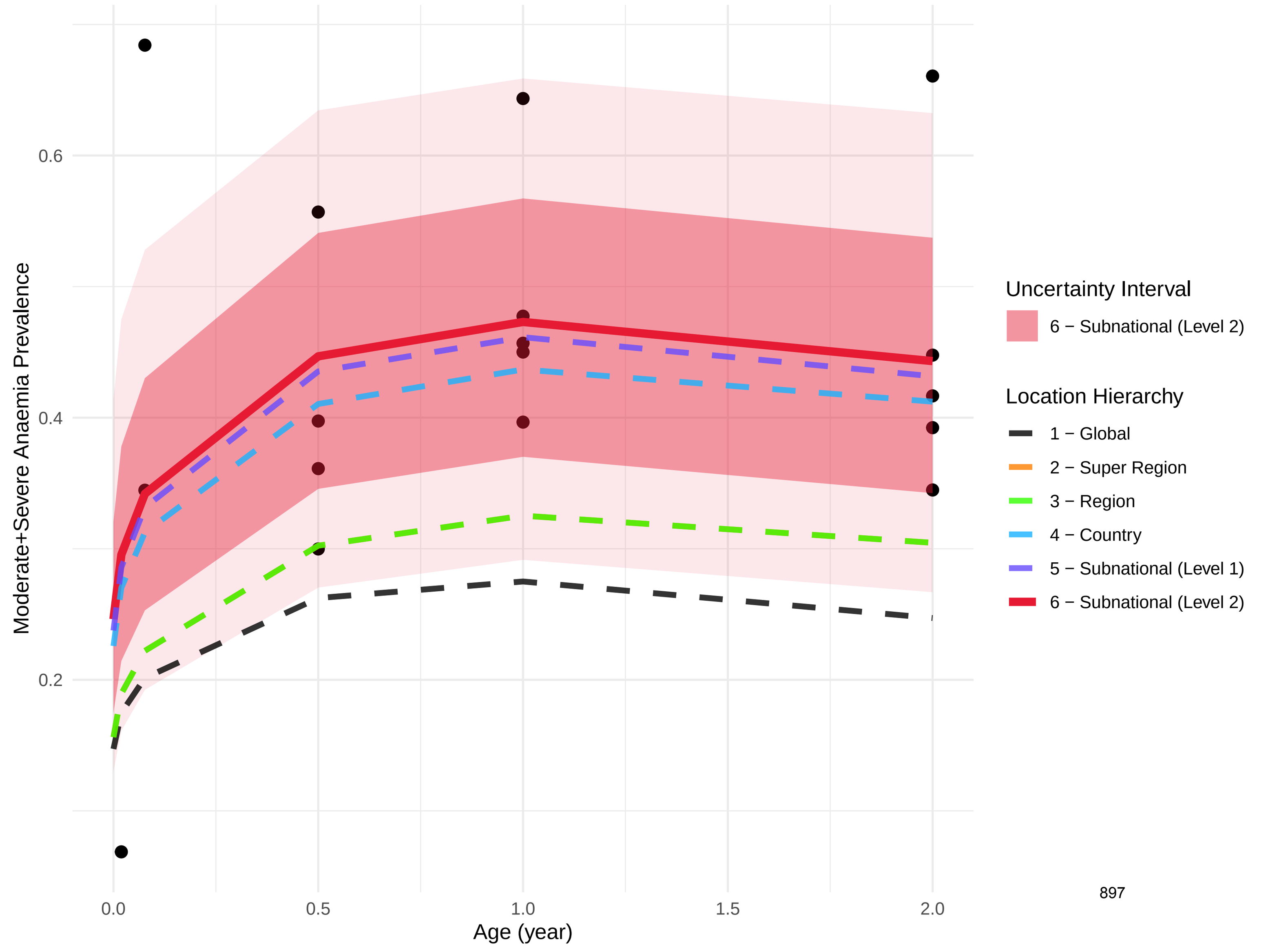

MR−BRT Age/Sex Fits − Non−pregnant Females <2 Years Old − Moderate+Severe Anaemia Prevalence − Madhya Pradesh, Urban
WHO−2001 Adjustment Method

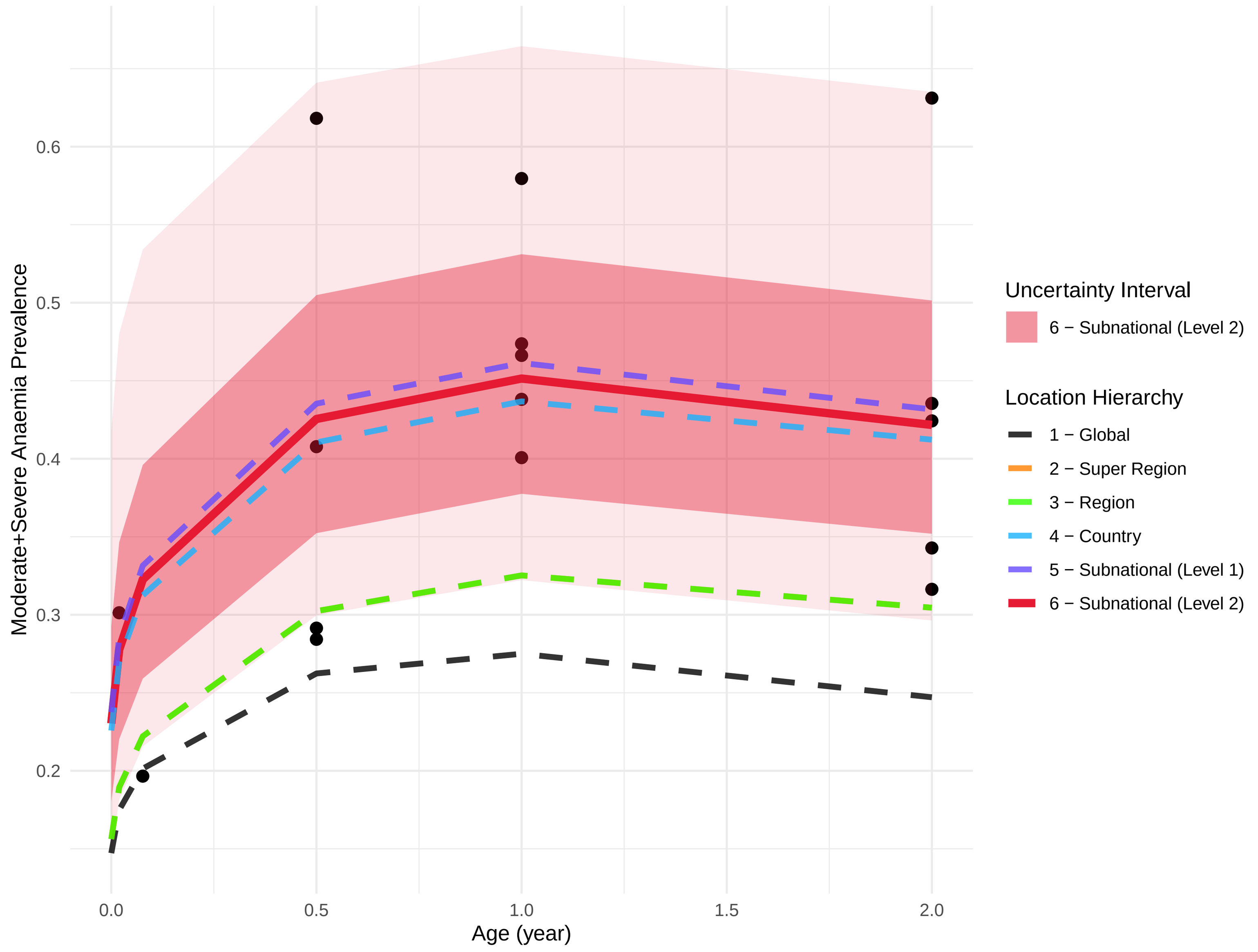

MR−BRT Age/Sex Fits − Non−pregnant Females <2 Years Old − Moderate+Severe Anaemia Prevalence − Maharashtra
WHO−2001 Adjustment Method

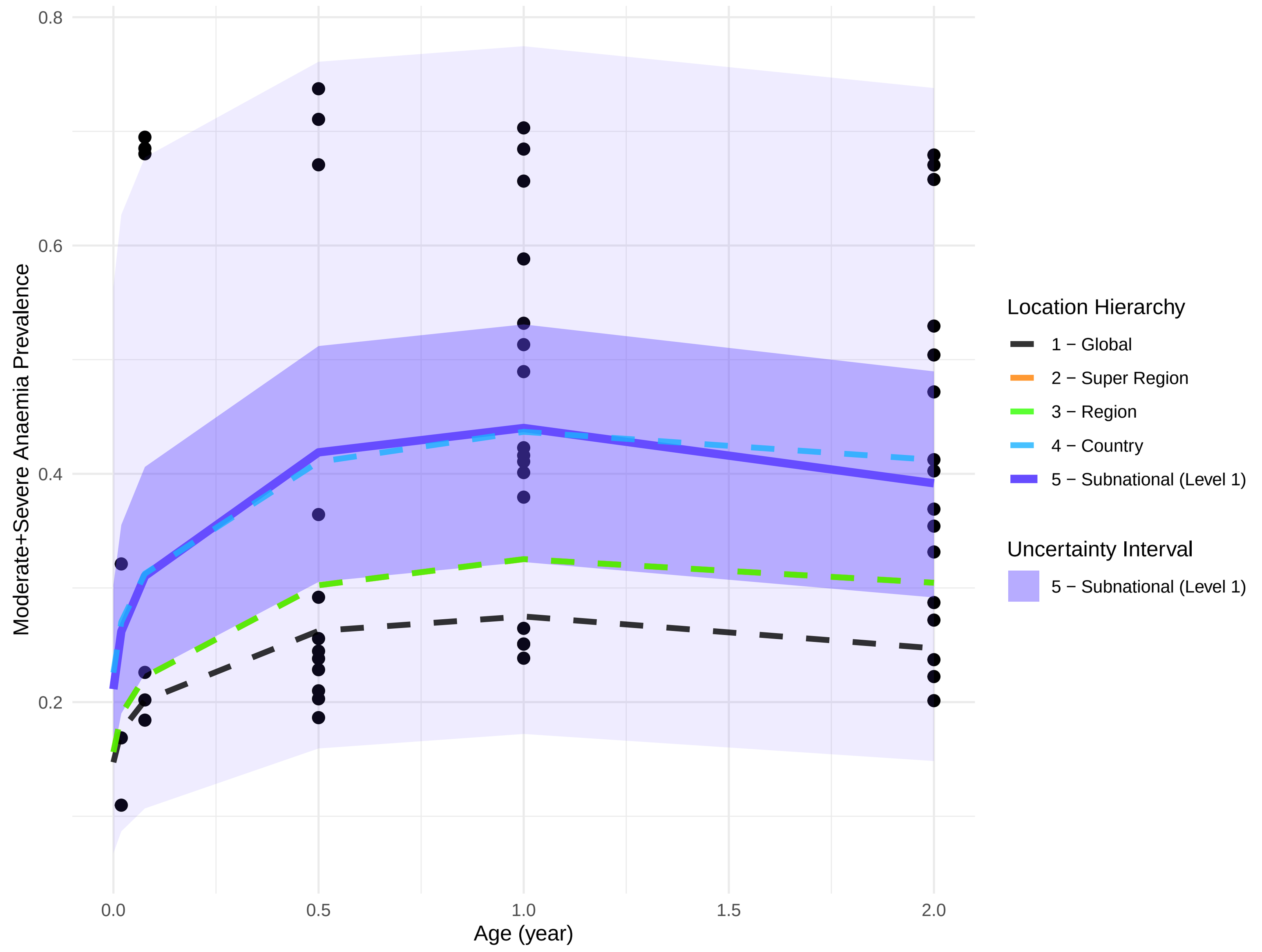

MR−BRT Age/Sex Fits − Non−pregnant Females <2 Years Old − Moderate+Severe Anaemia Prevalence − Maharashtra, Rural
WHO−2001 Adjustment Method

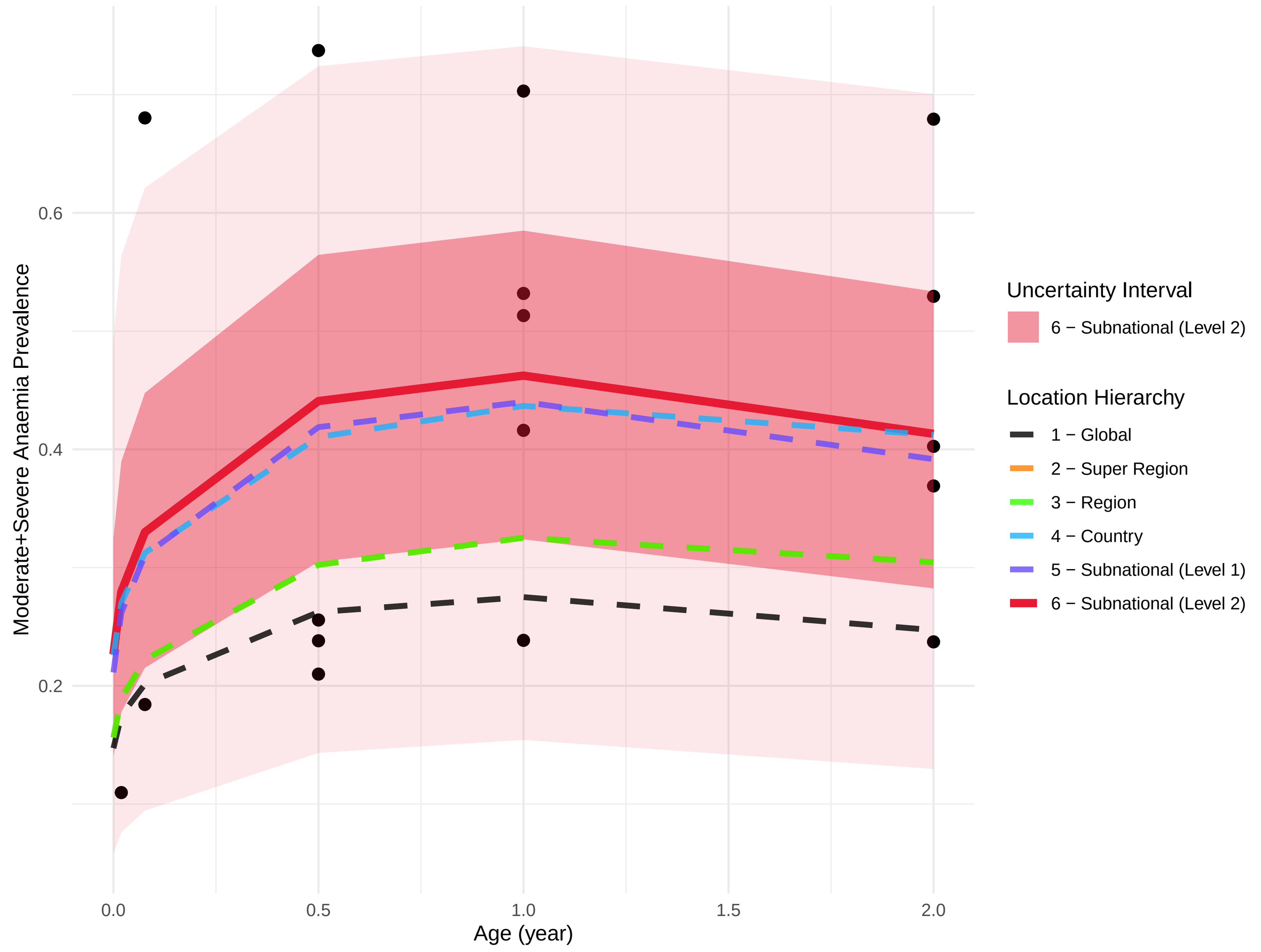

MR−BRT Age/Sex Fits − Non−pregnant Females <2 Years Old − Moderate+Severe Anaemia Prevalence − Maharashtra, Urban
WHO−2001 Adjustment Method

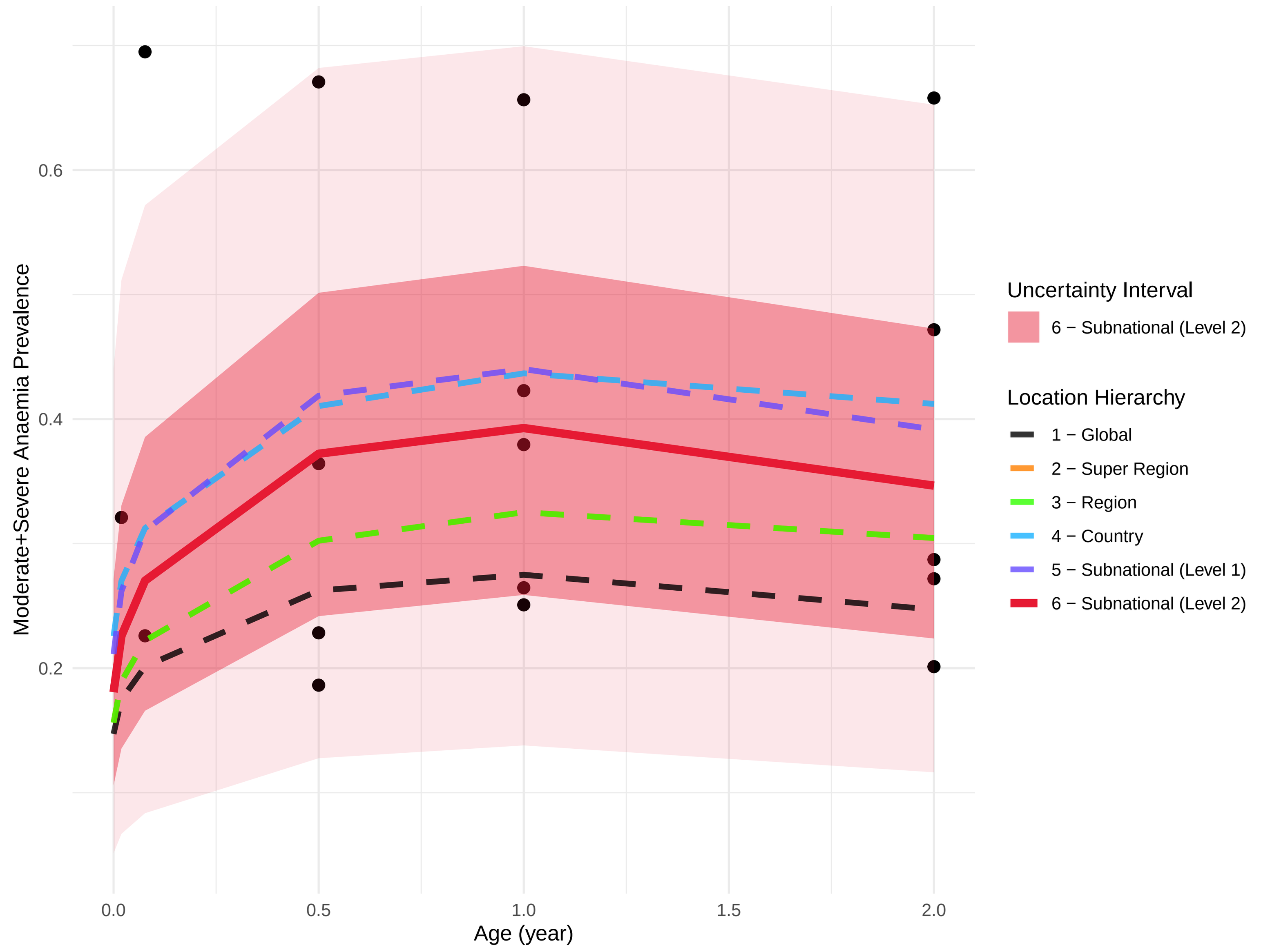

MR−BRT Age/Sex Fits − Non−pregnant Females <2 Years Old − Moderate+Severe Anaemia Prevalence − Manipur
WHO−2001 Adjustment Method

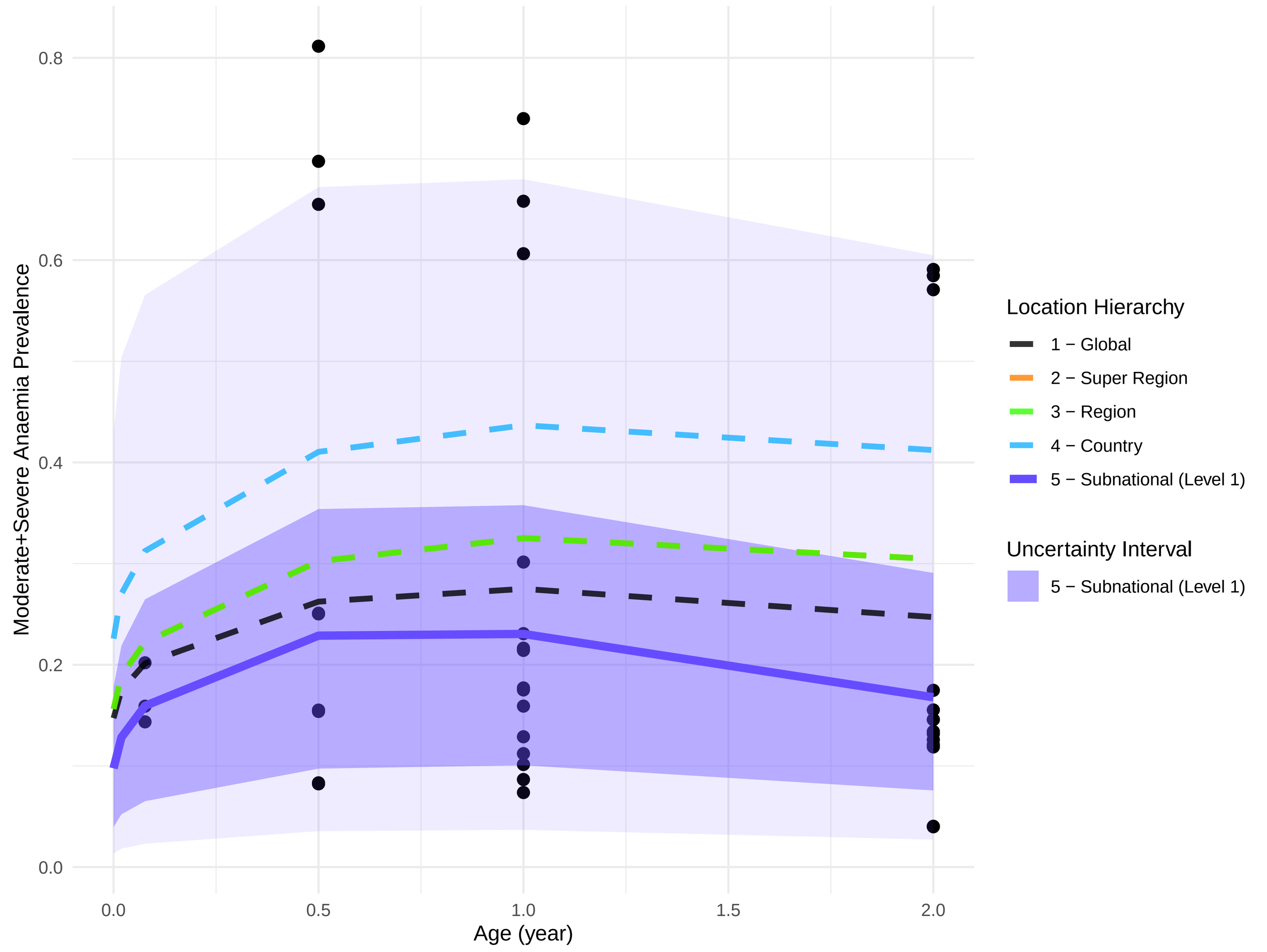

MR−BRT Age/Sex Fits − Non−pregnant Females <2 Years Old − Moderate+Severe Anaemia Prevalence − Manipur, Rural
WHO−2001 Adjustment Method

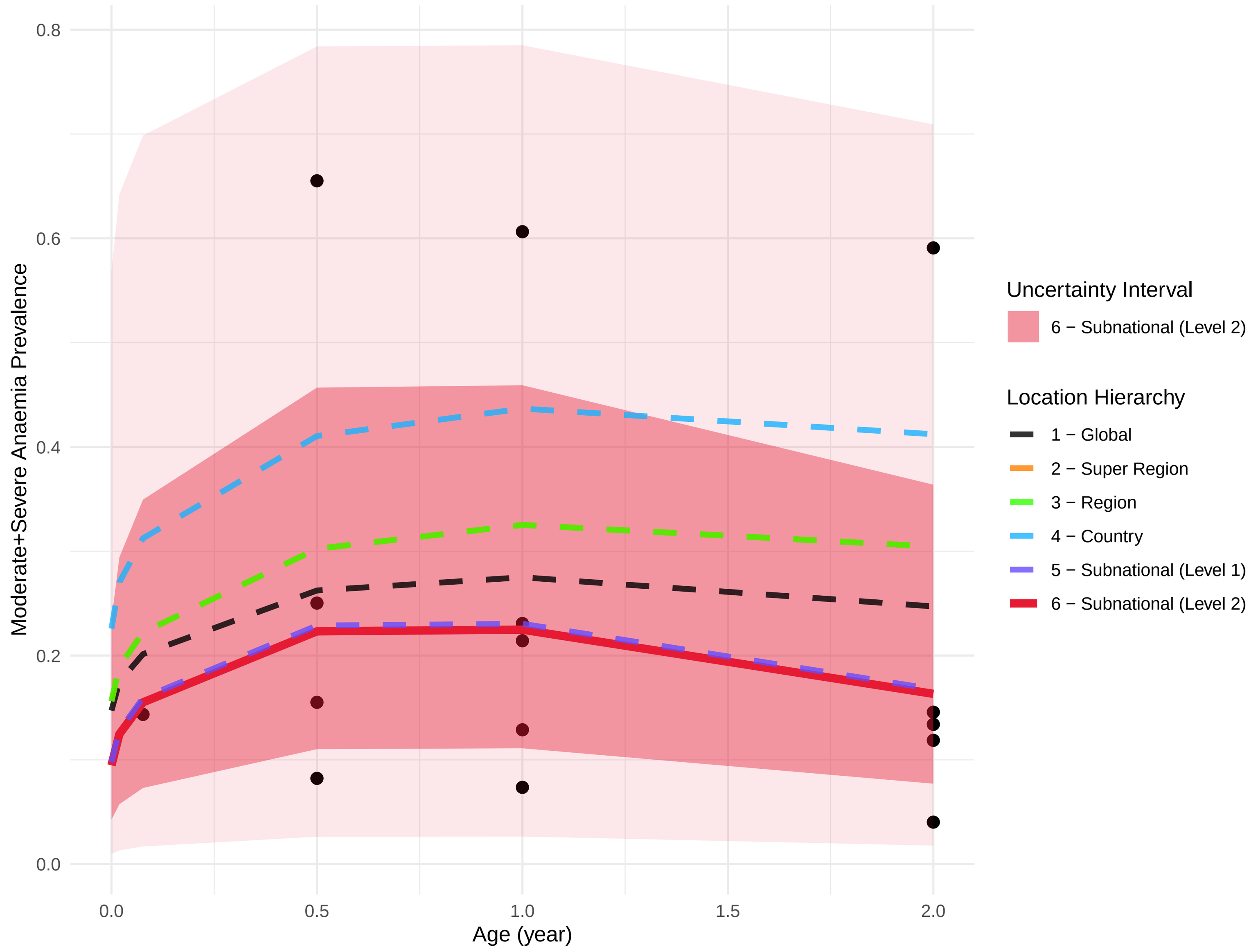

MR−BRT Age/Sex Fits − Non−pregnant Females <2 Years Old − Moderate+Severe Anaemia Prevalence − Manipur, Urban
WHO−2001 Adjustment Method

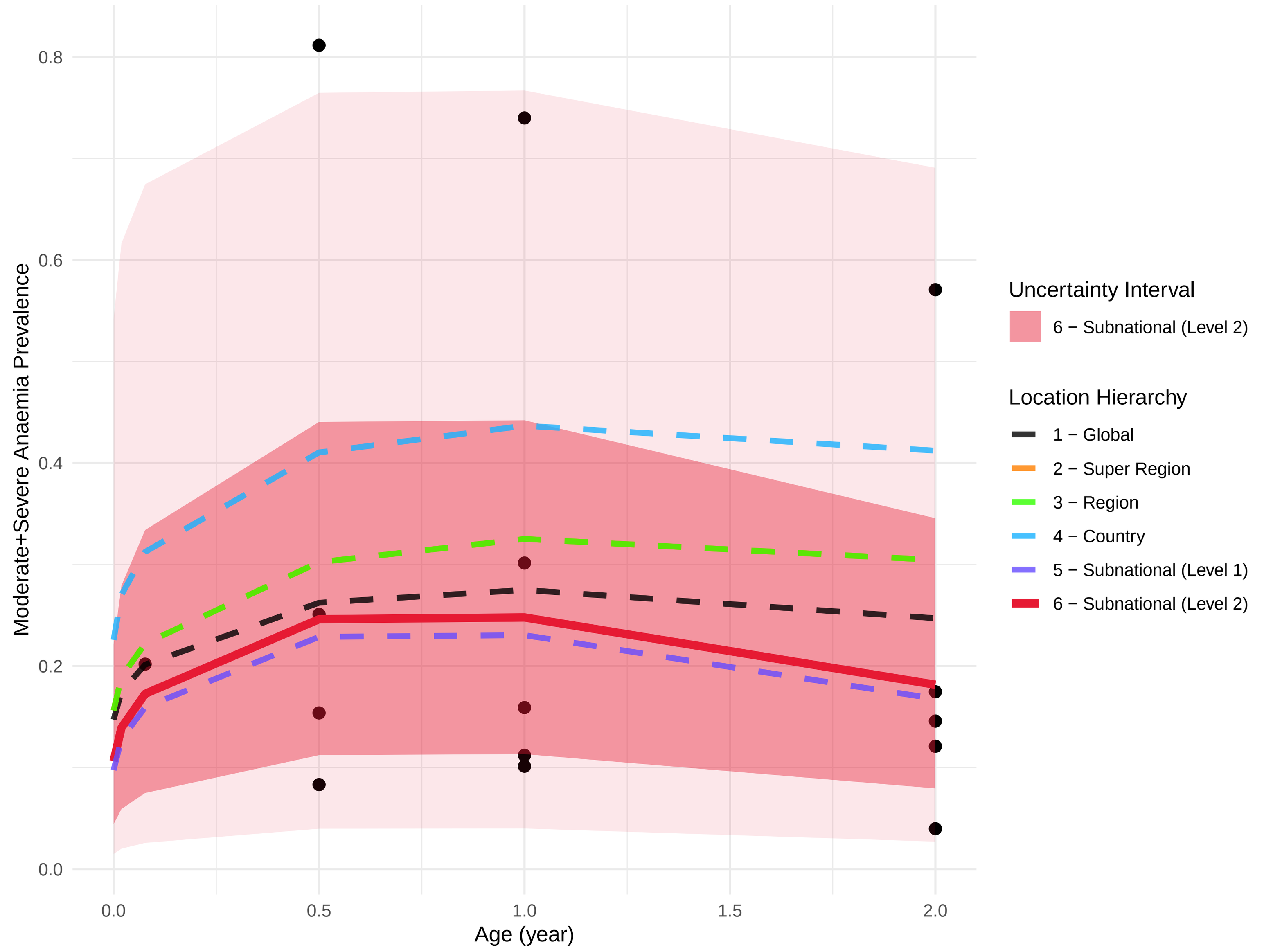

MR−BRT Age/Sex Fits − Non−pregnant Females <2 Years Old − Moderate+Severe Anaemia Prevalence − Meghalaya
WHO−2001 Adjustment Method

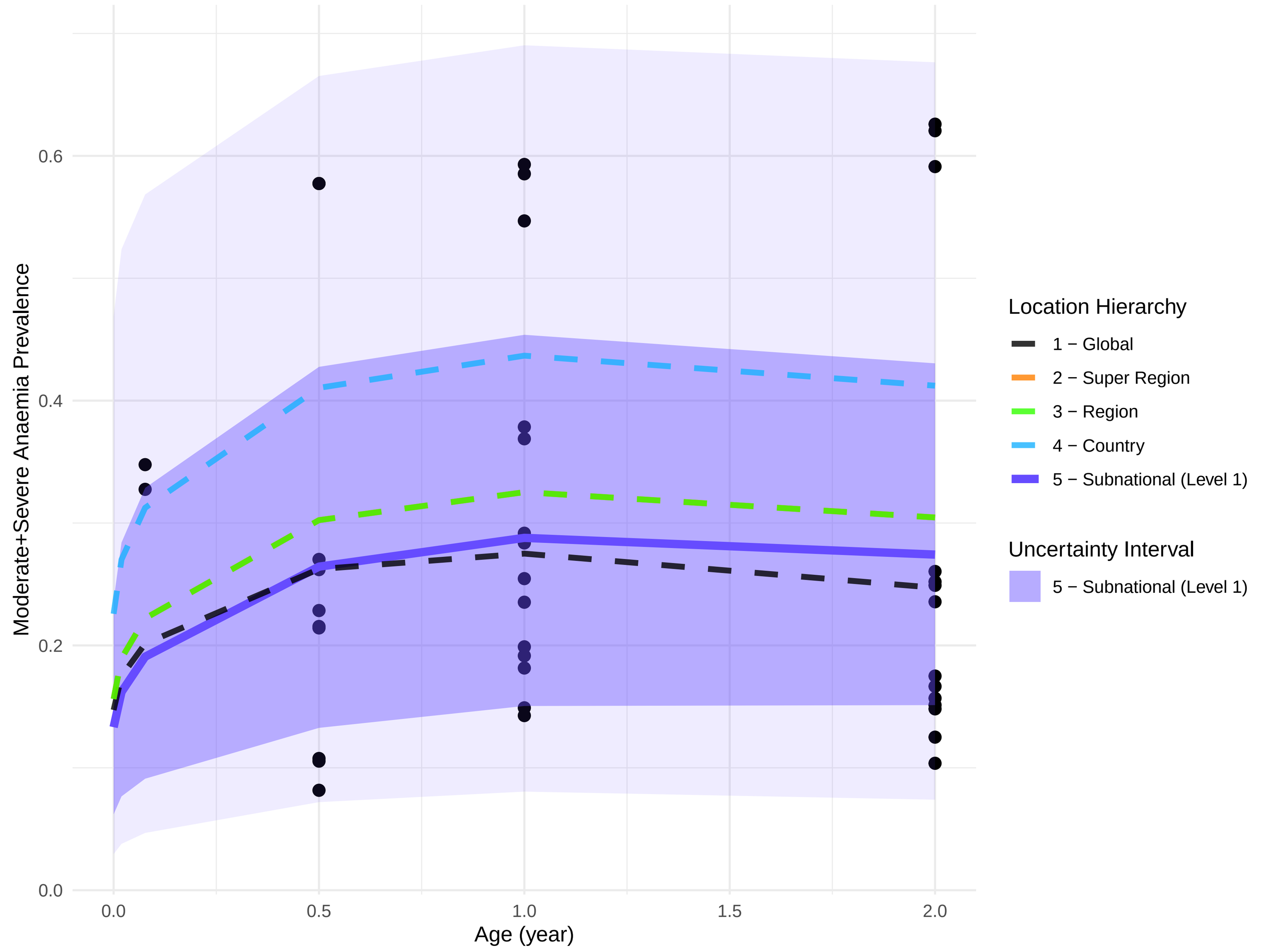

MR−BRT Age/Sex Fits − Non−pregnant Females <2 Years Old − Moderate+Severe Anaemia Prevalence − Meghalaya, Rural
WHO−2001 Adjustment Method

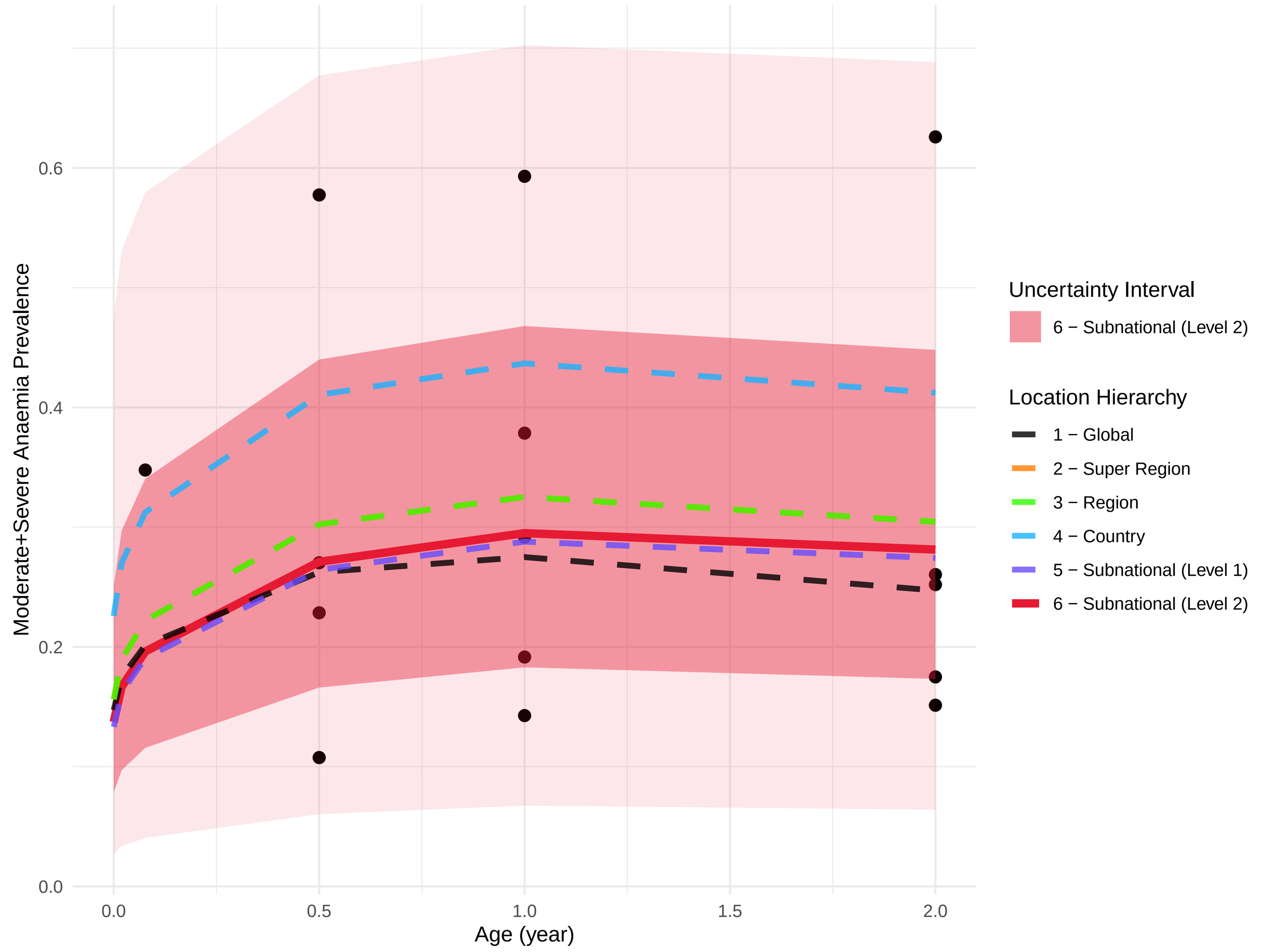

MR−BRT Age/Sex Fits − Non−pregnant Females <2 Years Old − Moderate+Severe Anaemia Prevalence − Meghalaya, Urban
WHO−2001 Adjustment Method

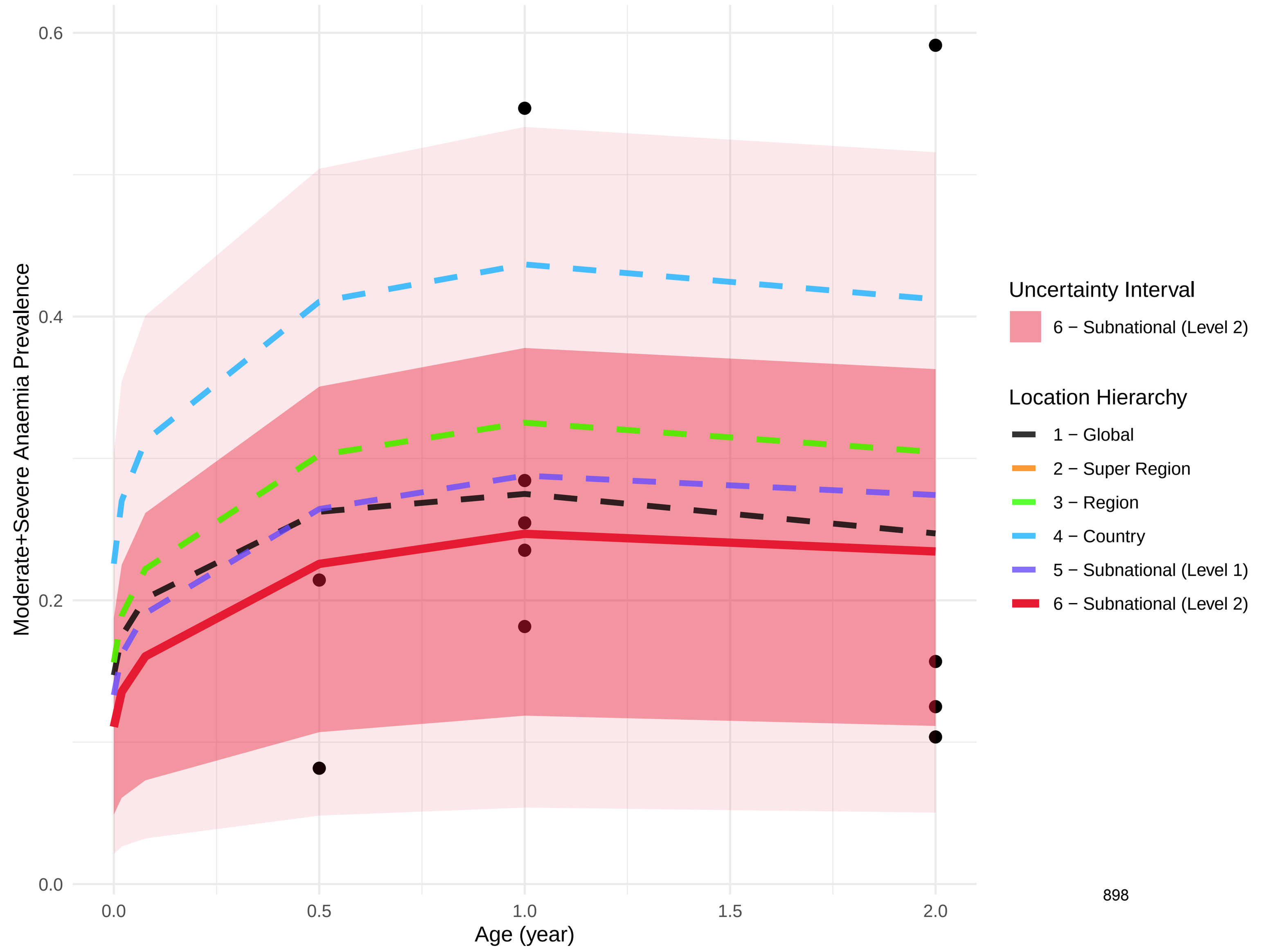

MR−BRT Age/Sex Fits − Non−pregnant Females <2 Years Old − Moderate+Severe Anaemia Prevalence − Mizoram
WHO−2001 Adjustment Method

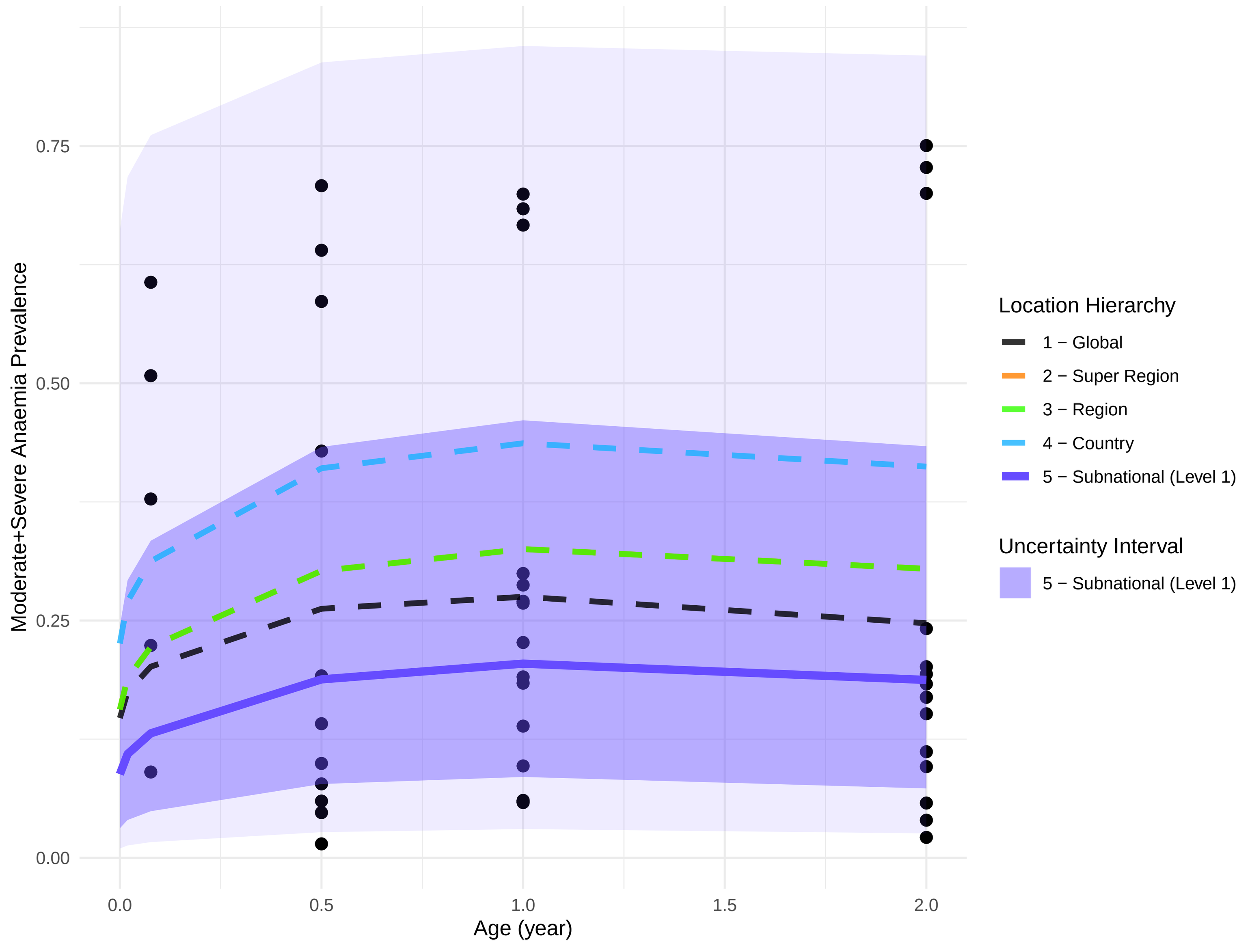

MR−BRT Age/Sex Fits − Non−pregnant Females <2 Years Old − Moderate+Severe Anaemia Prevalence − Mizoram, Rural
WHO−2001 Adjustment Method

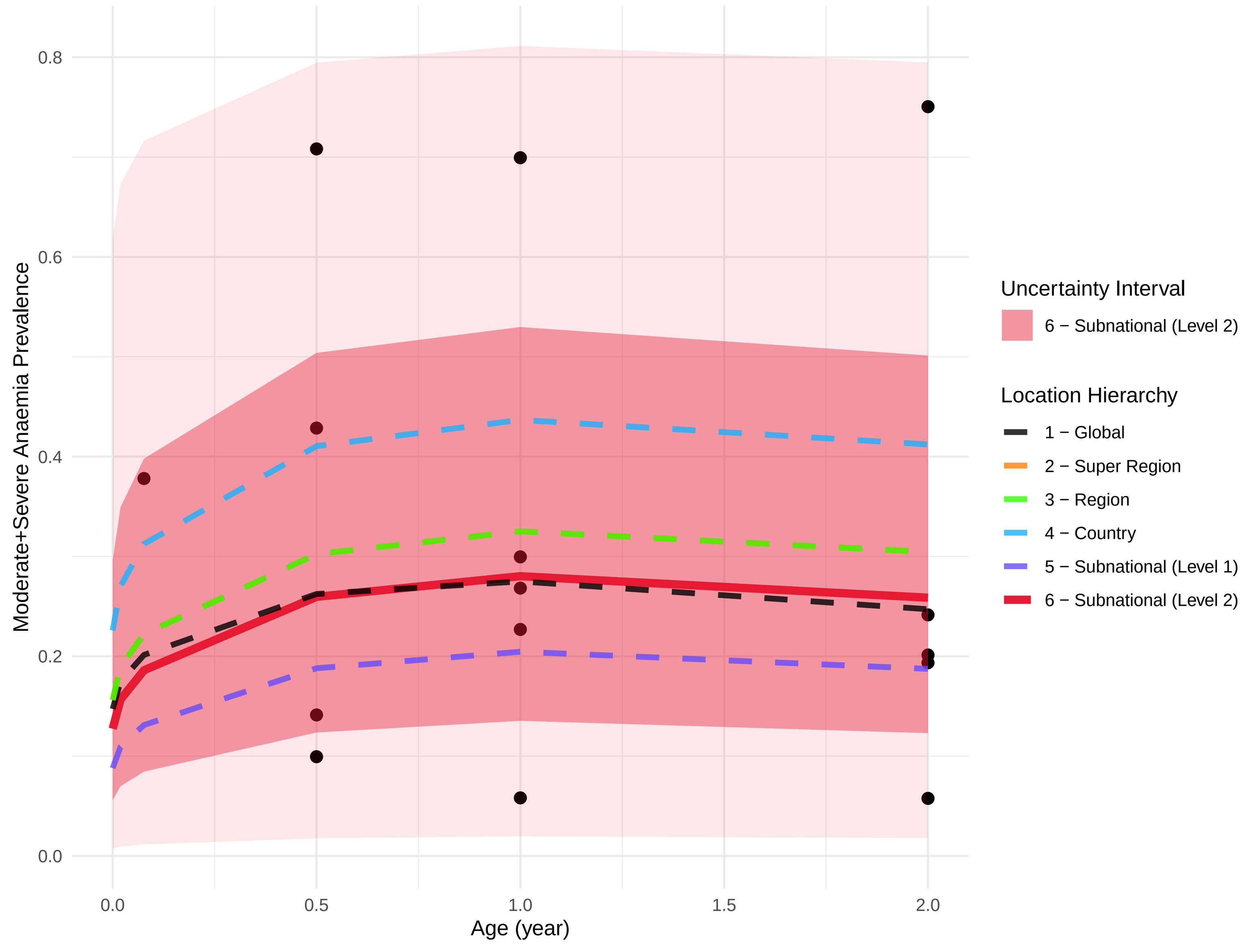

MR−BRT Age/Sex Fits − Non−pregnant Females <2 Years Old − Moderate+Severe Anaemia Prevalence − Mizoram, Urban
WHO−2001 Adjustment Method

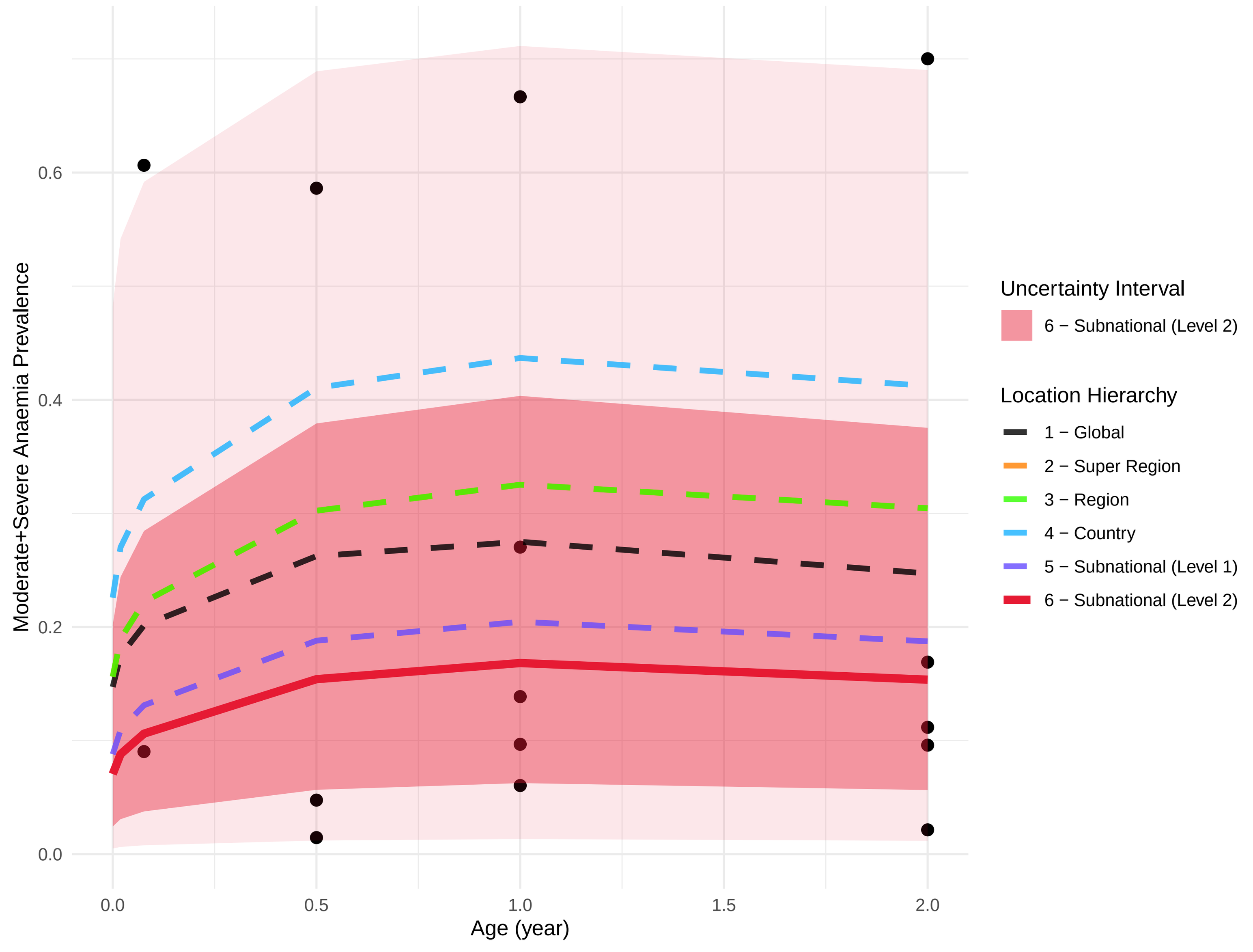

MR−BRT Age/Sex Fits − Non−pregnant Females <2 Years Old − Moderate+Severe Anaemia Prevalence − Nagaland
WHO−2001 Adjustment Method

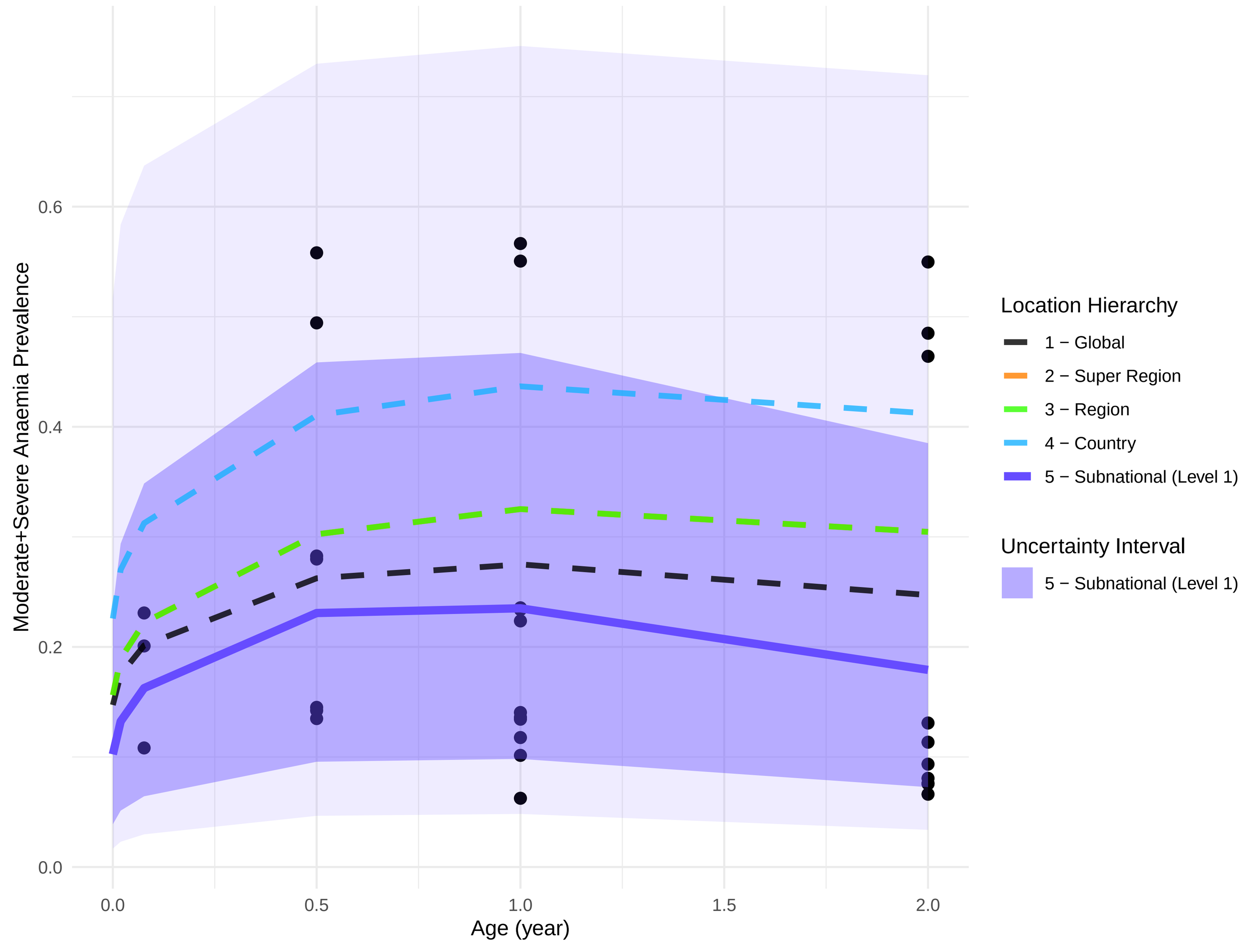

MR−BRT Age/Sex Fits − Non−pregnant Females <2 Years Old − Moderate+Severe Anaemia Prevalence − Nagaland, Rural
WHO−2001 Adjustment Method

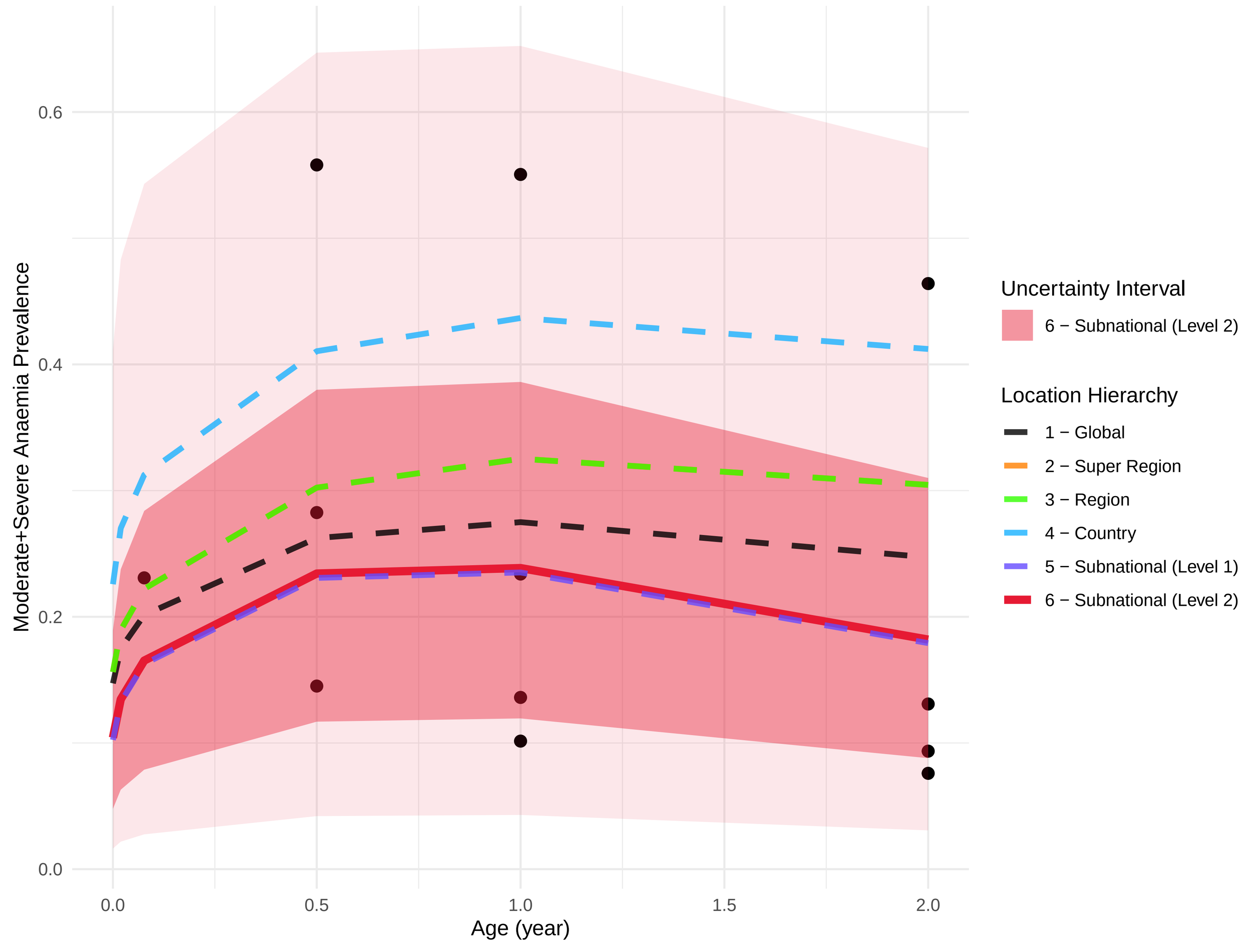

MR−BRT Age/Sex Fits − Non−pregnant Females <2 Years Old − Moderate+Severe Anaemia Prevalence − Nagaland, Urban
WHO−2001 Adjustment Method

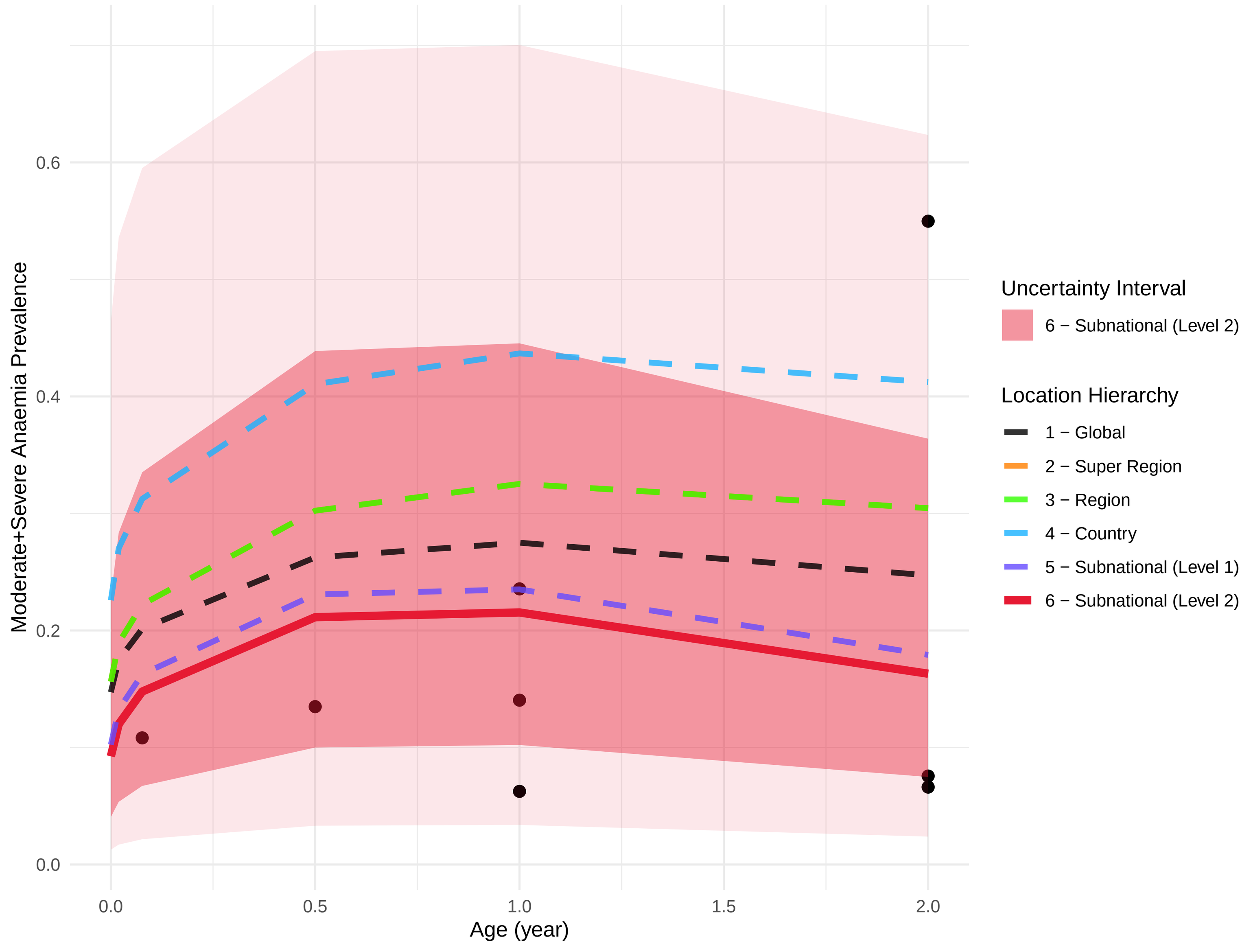

MR−BRT Age/Sex Fits − Non−pregnant Females <2 Years Old − Moderate+Severe Anaemia Prevalence − Odisha
WHO−2001 Adjustment Method

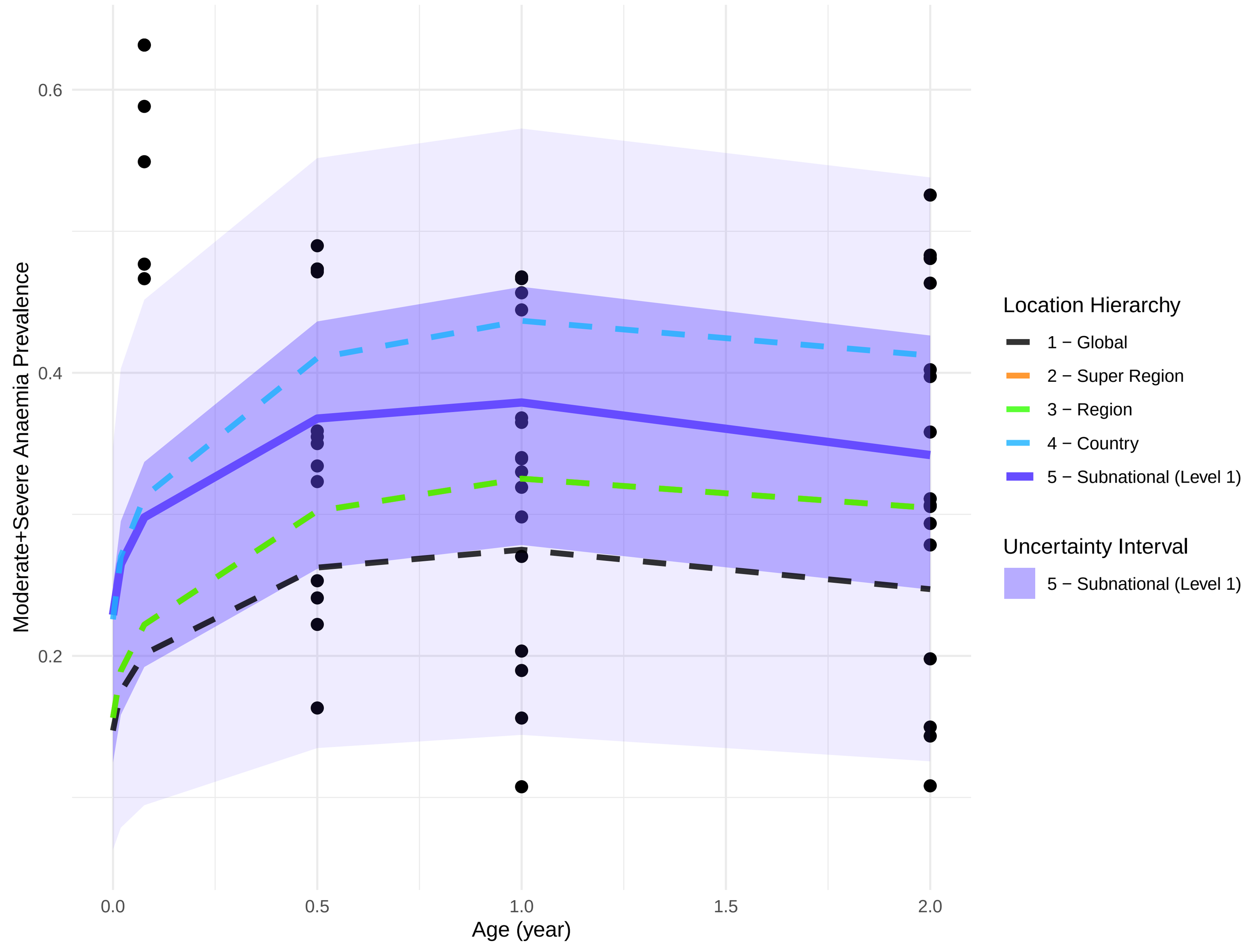

MR−BRT Age/Sex Fits − Non−pregnant Females <2 Years Old − Moderate+Severe Anaemia Prevalence − Odisha, Rural
WHO−2001 Adjustment Method

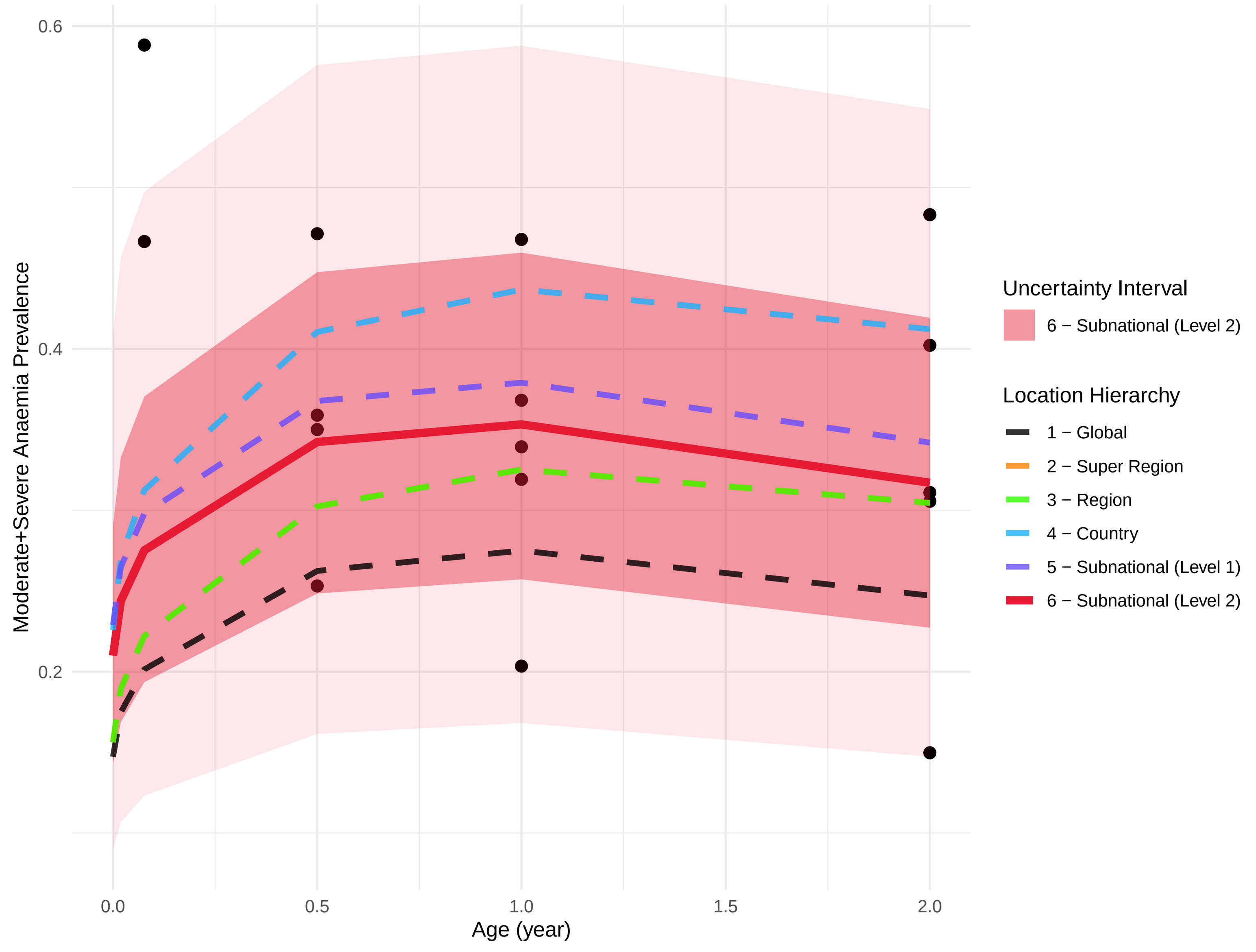

MR−BRT Age/Sex Fits − Non−pregnant Females <2 Years Old − Moderate+Severe Anaemia Prevalence − Odisha, Urban
WHO−2001 Adjustment Method

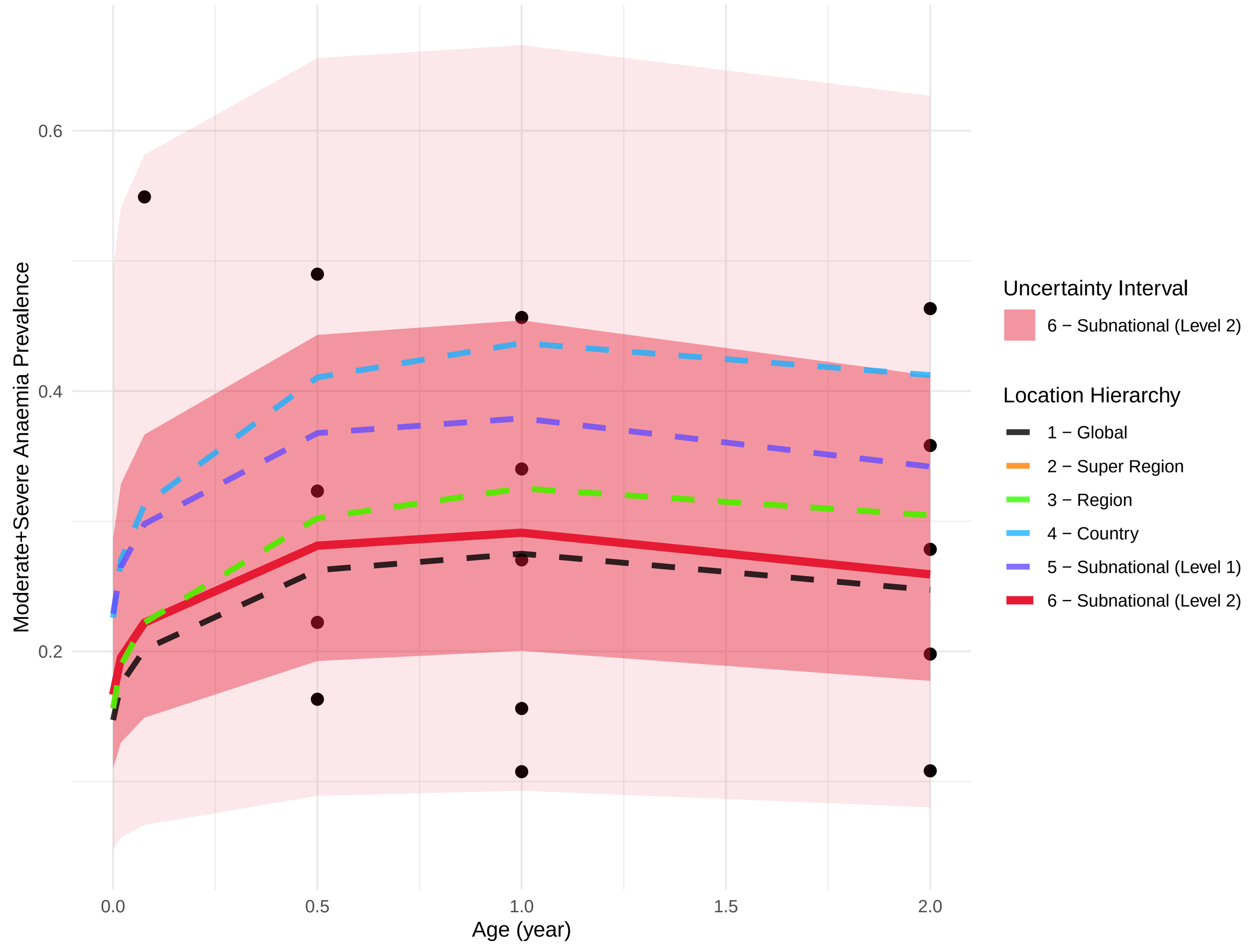

MR−BRT Age/Sex Fits − Non−pregnant Females <2 Years Old − Moderate+Severe Anaemia Prevalence − Other Union Territories
WHO−2001 Adjustment Method

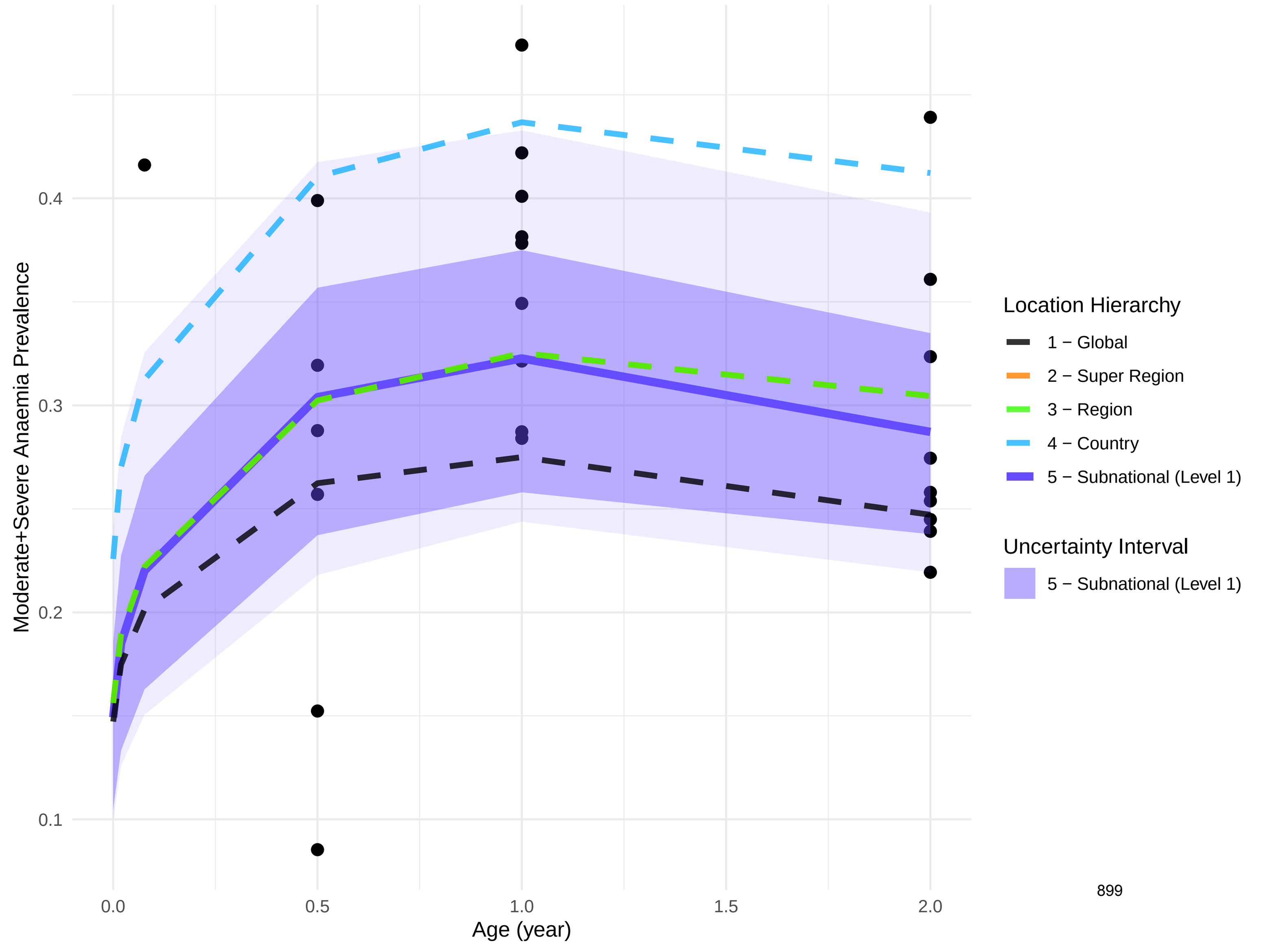

MR−BRT Age/Sex Fits − Non−pregnant Females <2 Years Old − Moderate+Severe Anaemia Prevalence − Other Union Territories, Rural
WHO−2001 Adjustment Method

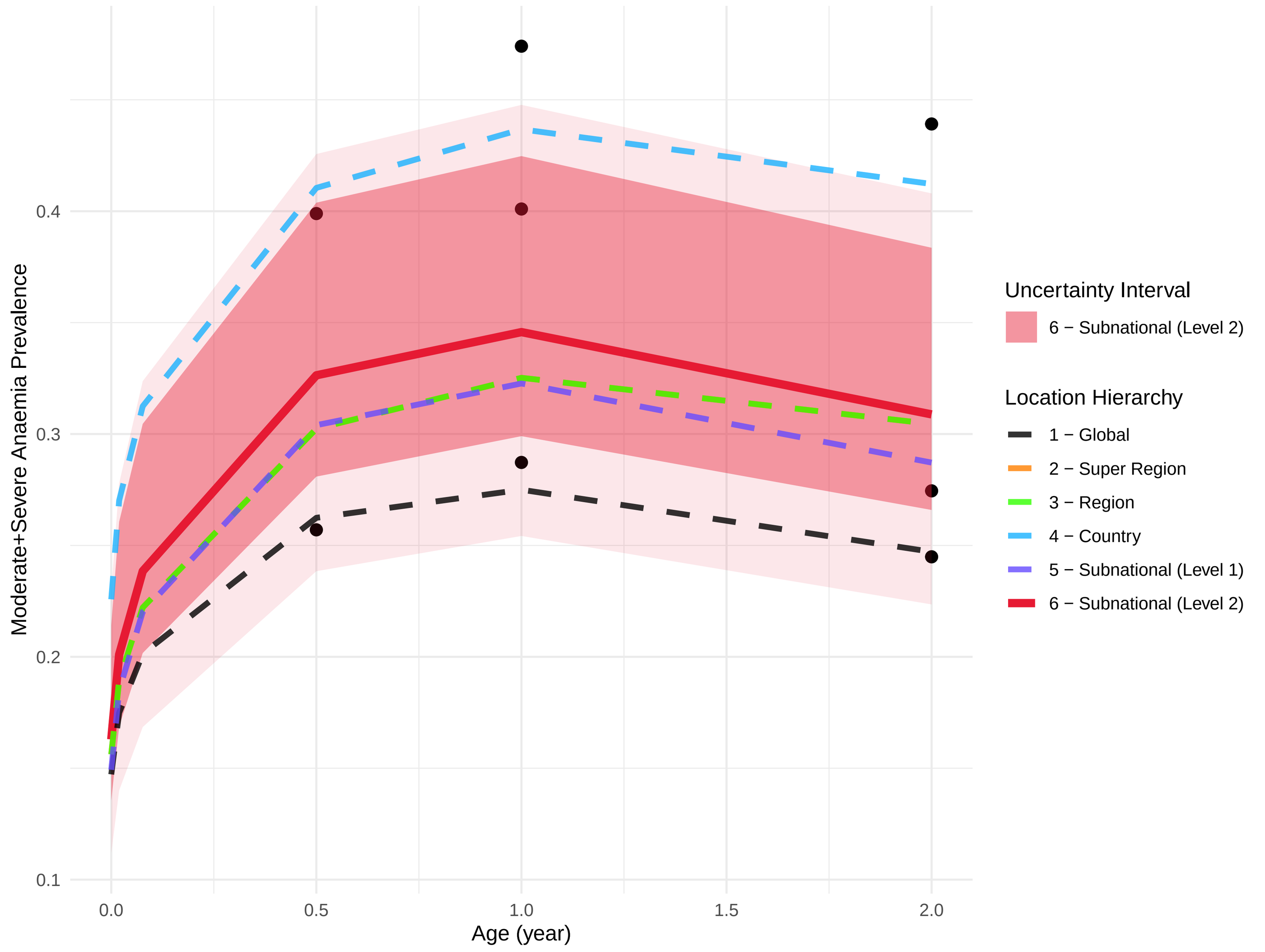

MR−BRT Age/Sex Fits − Non−pregnant Females <2 Years Old − Moderate+Severe Anaemia Prevalence − Other Union Territories, Urban
WHO−2001 Adjustment Method

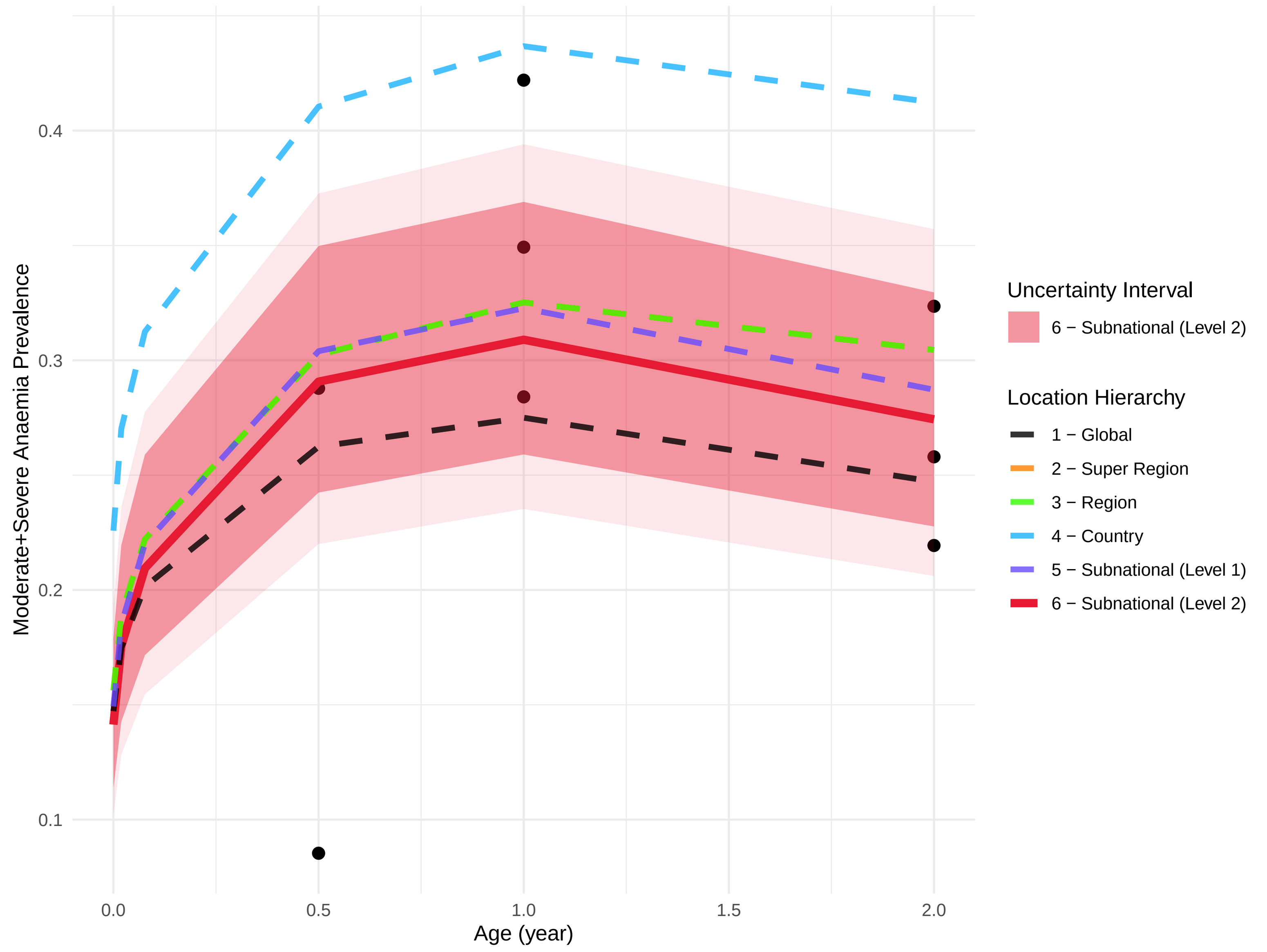

MR−BRT Age/Sex Fits − Non−pregnant Females <2 Years Old − Moderate+Severe Anaemia Prevalence − Punjab
WHO−2001 Adjustment Method

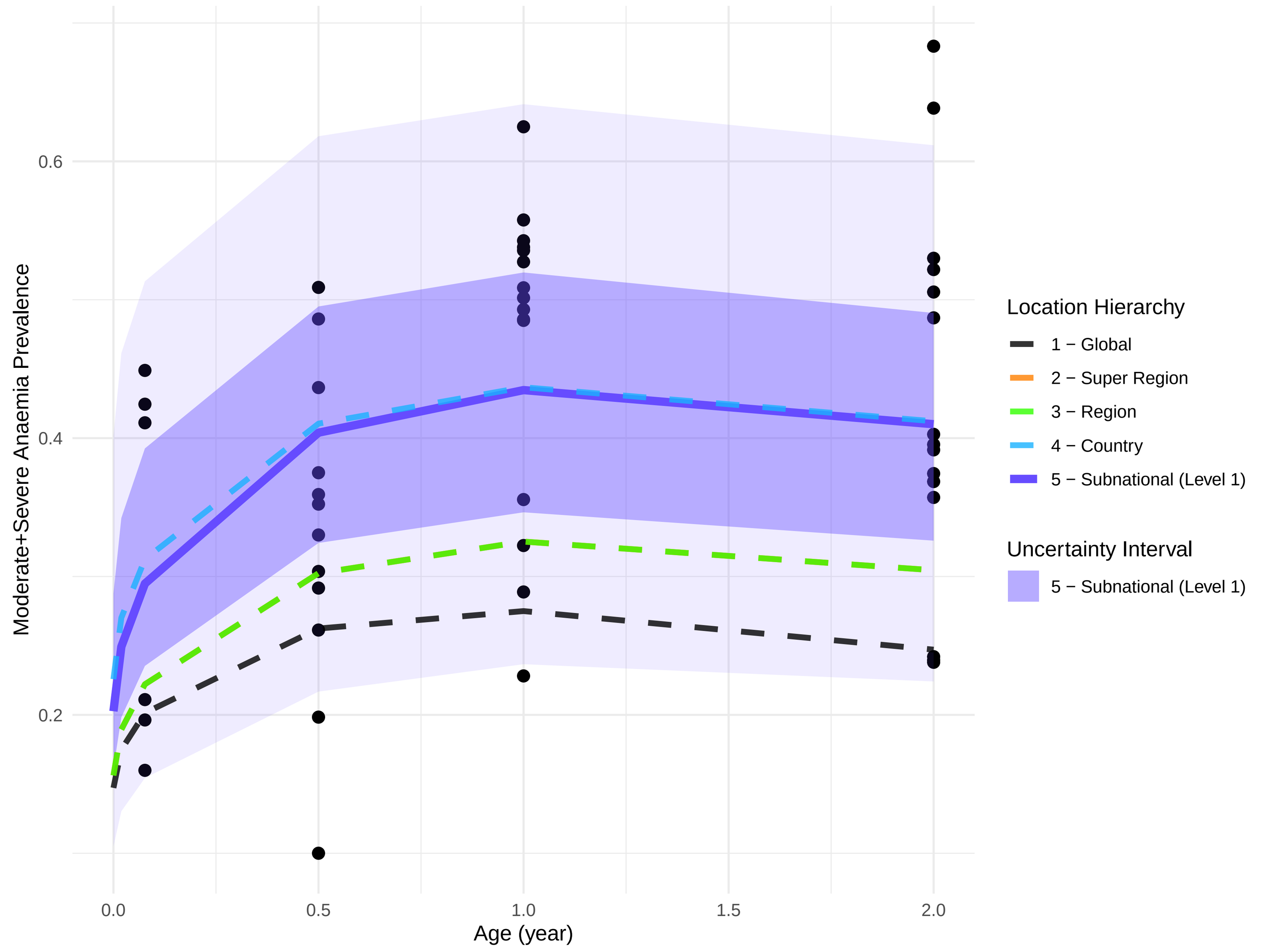

MR−BRT Age/Sex Fits − Non−pregnant Females <2 Years Old − Moderate+Severe Anaemia Prevalence − Punjab, Rural
WHO−2001 Adjustment Method

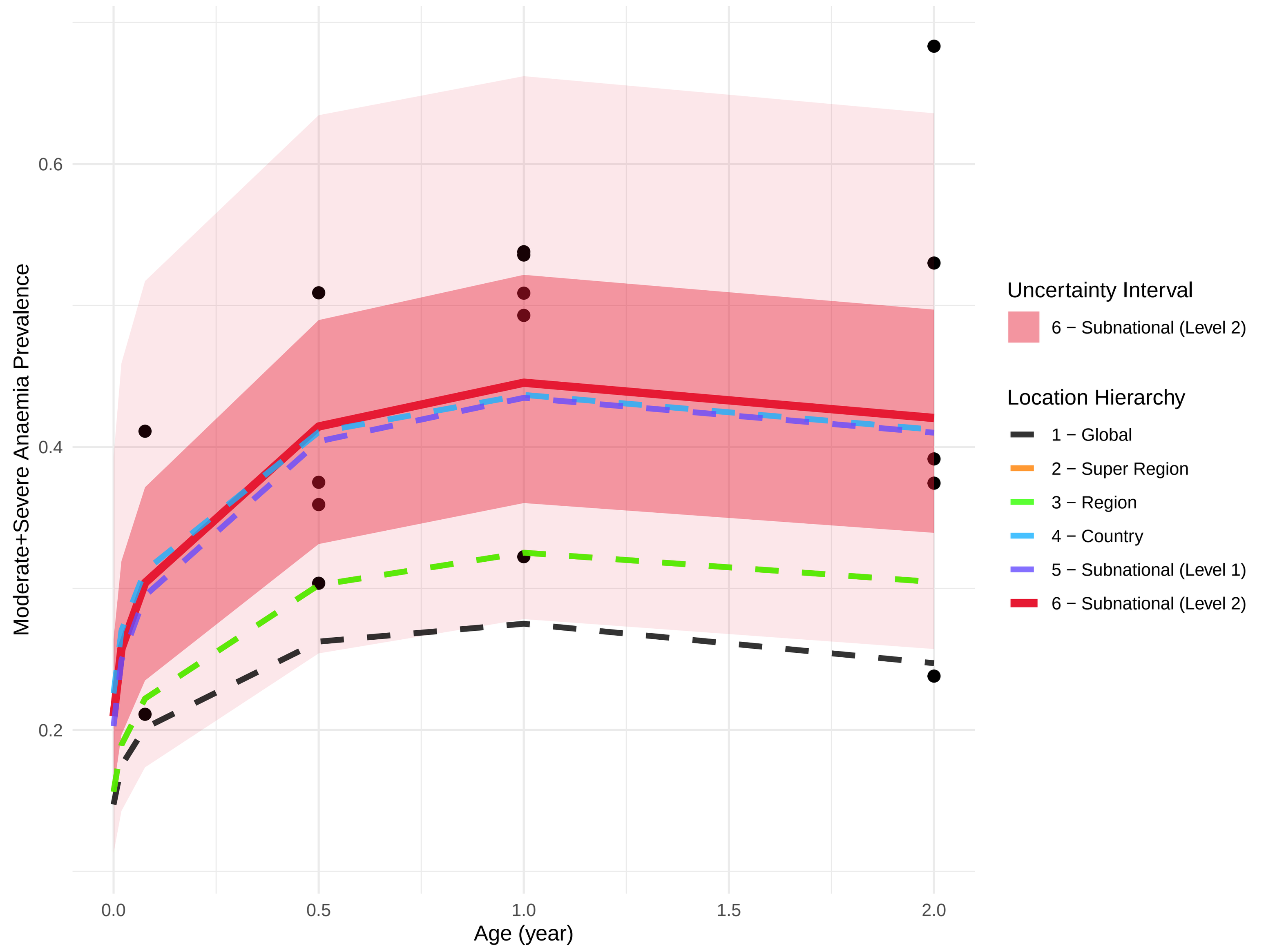

MR−BRT Age/Sex Fits − Non−pregnant Females <2 Years Old − Moderate+Severe Anaemia Prevalence − Punjab, Urban
WHO−2001 Adjustment Method

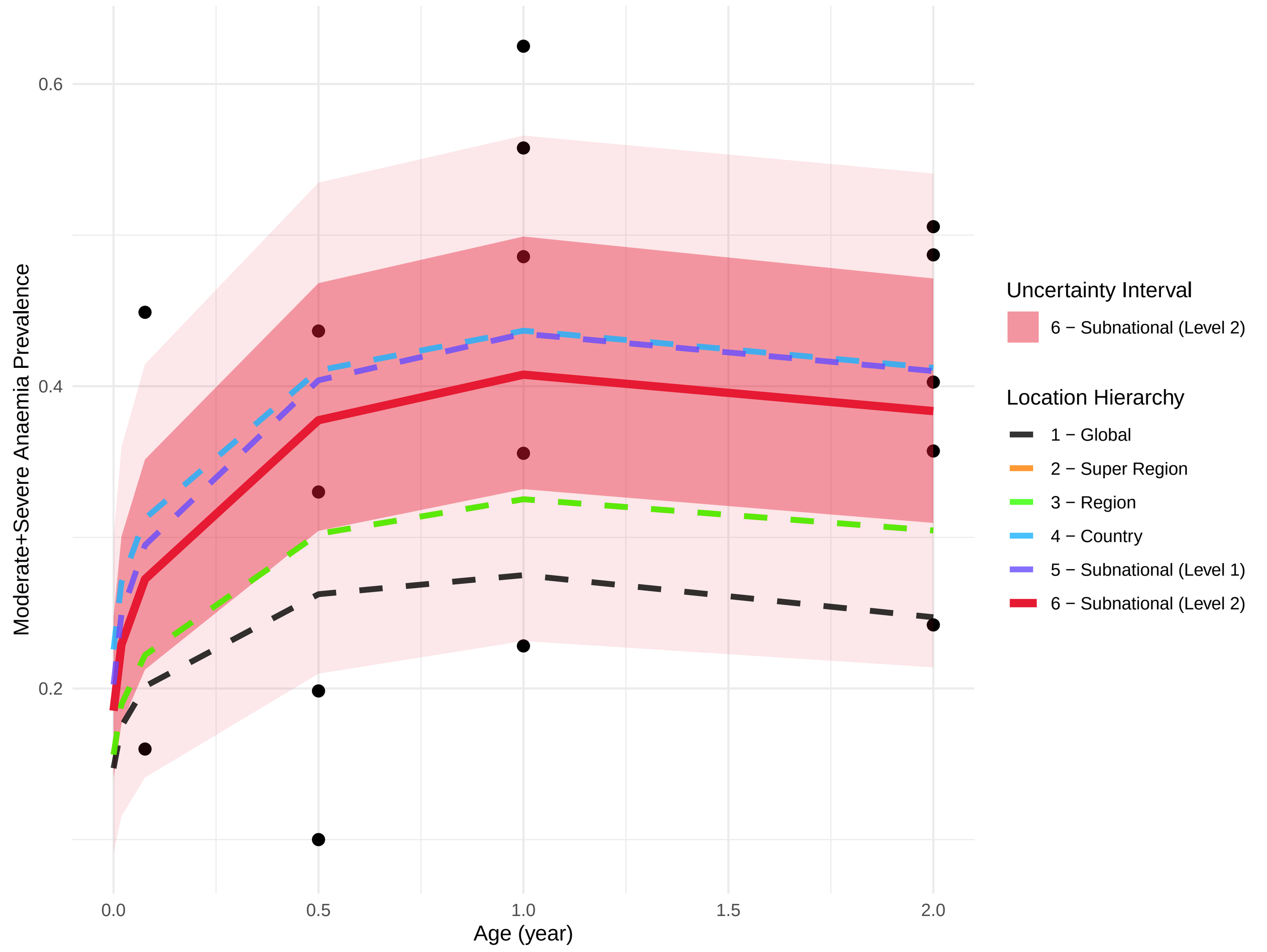

MR−BRT Age/Sex Fits − Non−pregnant Females <2 Years Old − Moderate+Severe Anaemia Prevalence − Rajasthan
WHO−2001 Adjustment Method

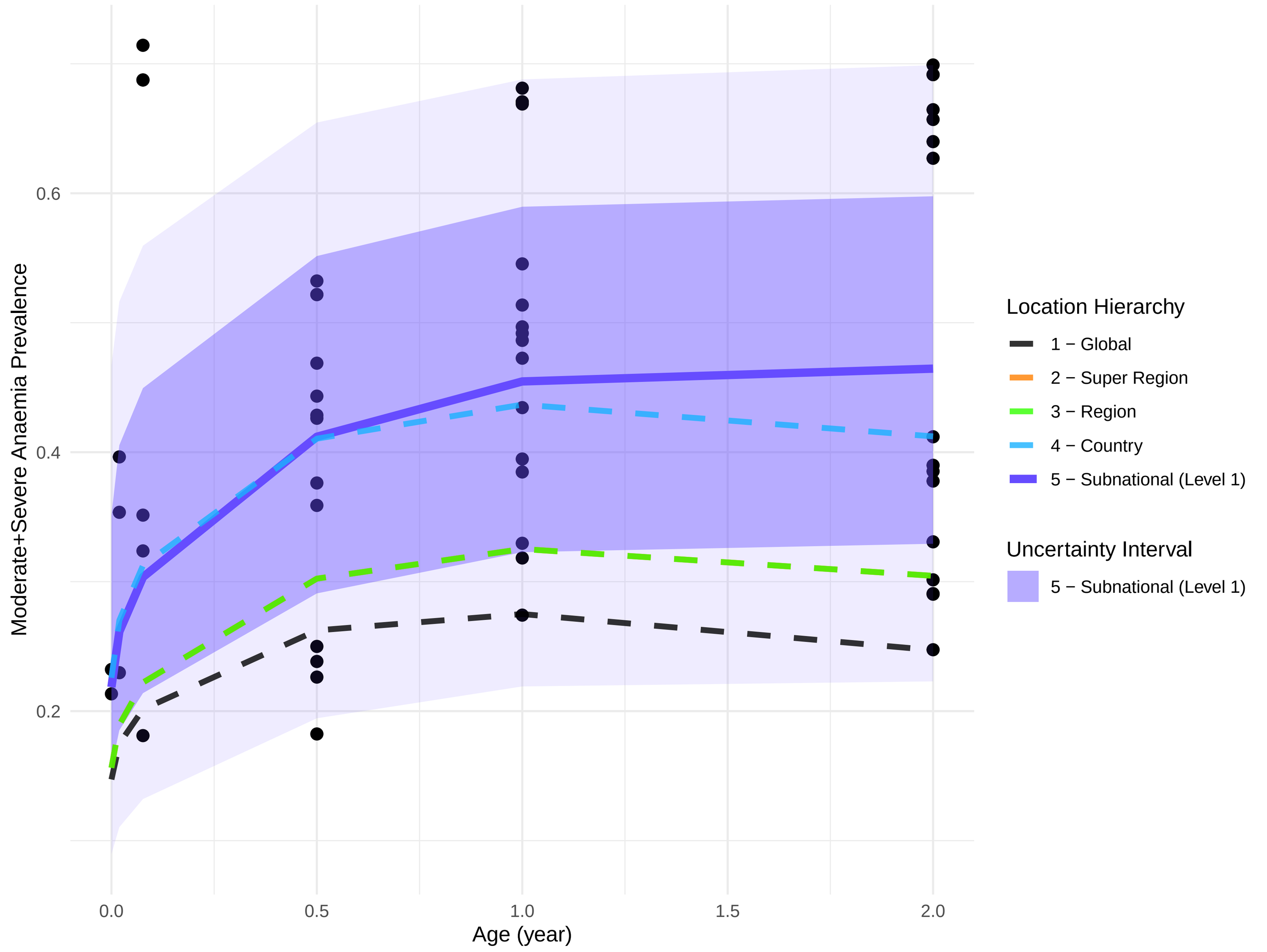

MR−BRT Age/Sex Fits − Non−pregnant Females <2 Years Old − Moderate+Severe Anaemia Prevalence − Rajasthan, Rural
WHO−2001 Adjustment Method

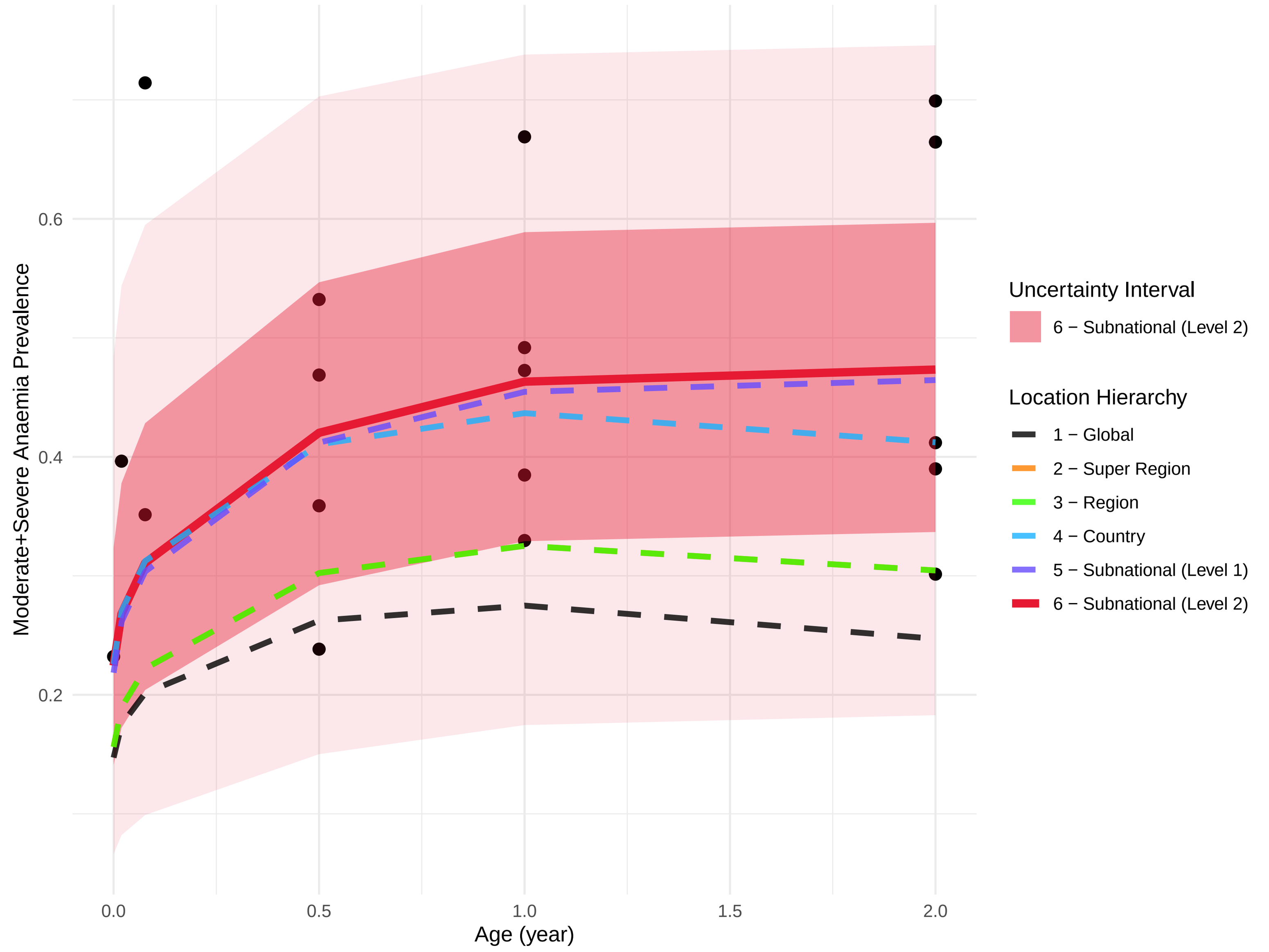

MR−BRT Age/Sex Fits − Non−pregnant Females <2 Years Old − Moderate+Severe Anaemia Prevalence − Rajasthan, Urban
WHO−2001 Adjustment Method

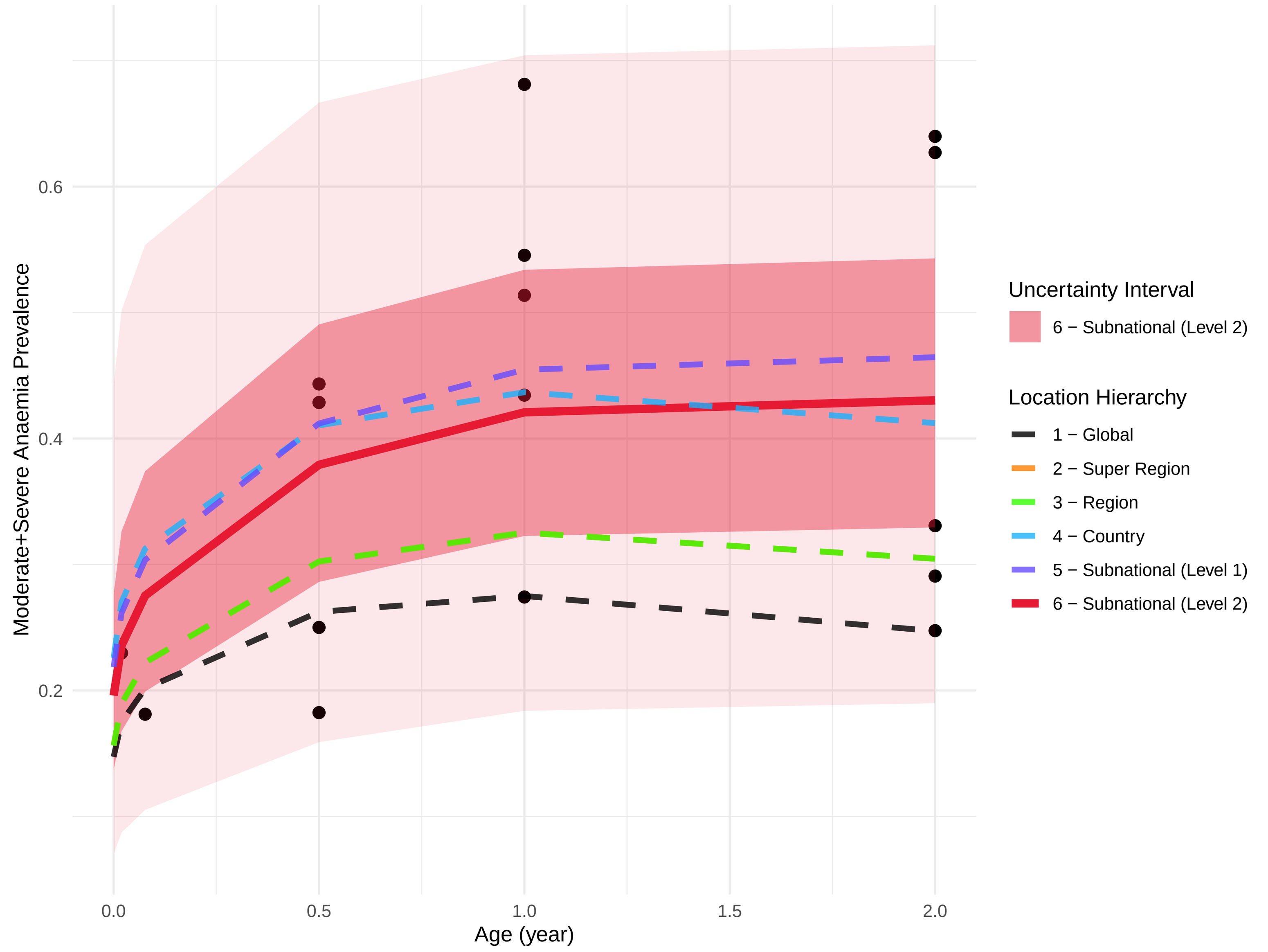

MR−BRT Age/Sex Fits − Non−pregnant Females <2 Years Old − Moderate+Severe Anaemia Prevalence − Sikkim
WHO−2001 Adjustment Method

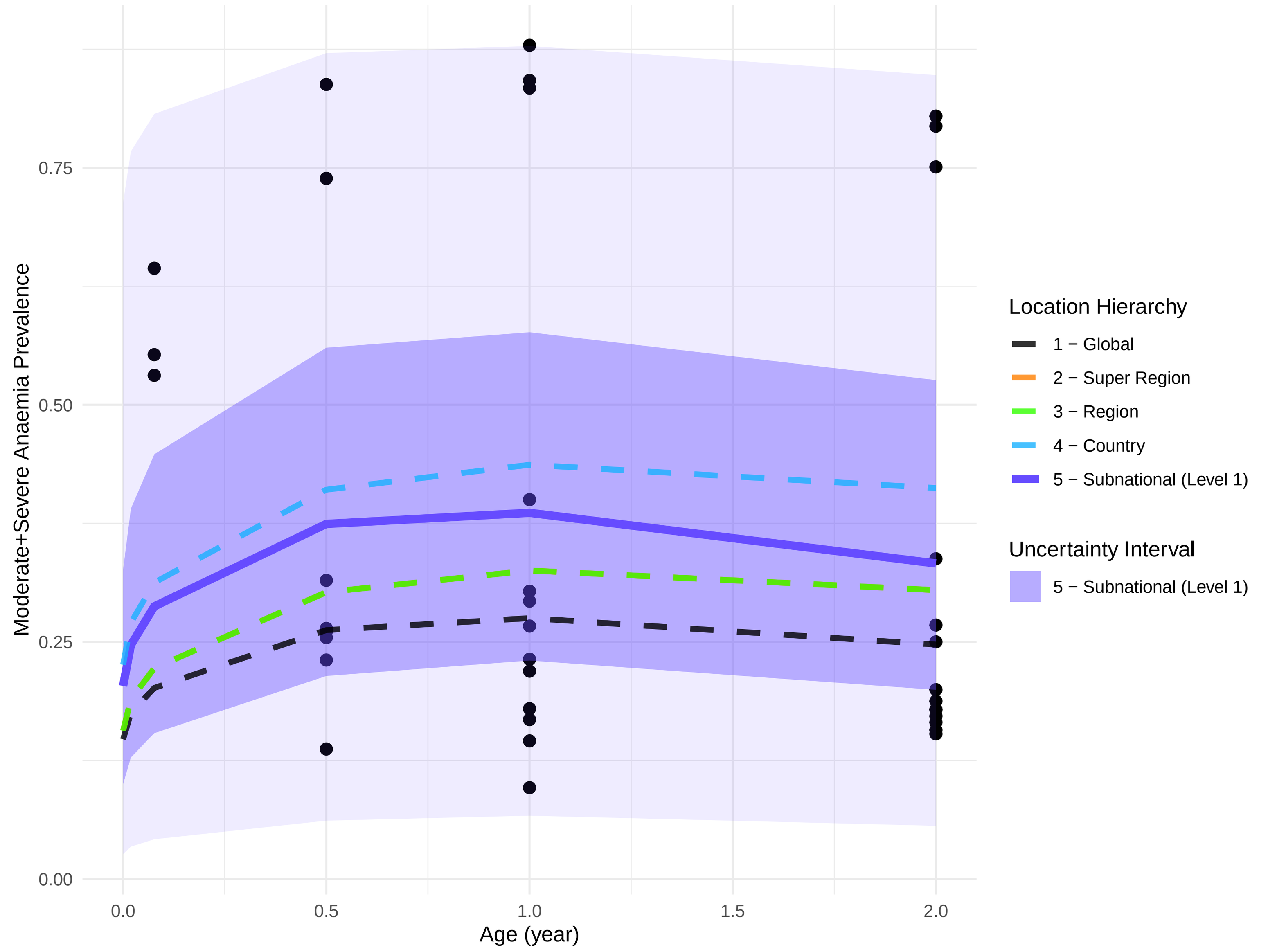

MR−BRT Age/Sex Fits − Non−pregnant Females <2 Years Old − Moderate+Severe Anaemia Prevalence − Sikkim, Rural
WHO−2001 Adjustment Method

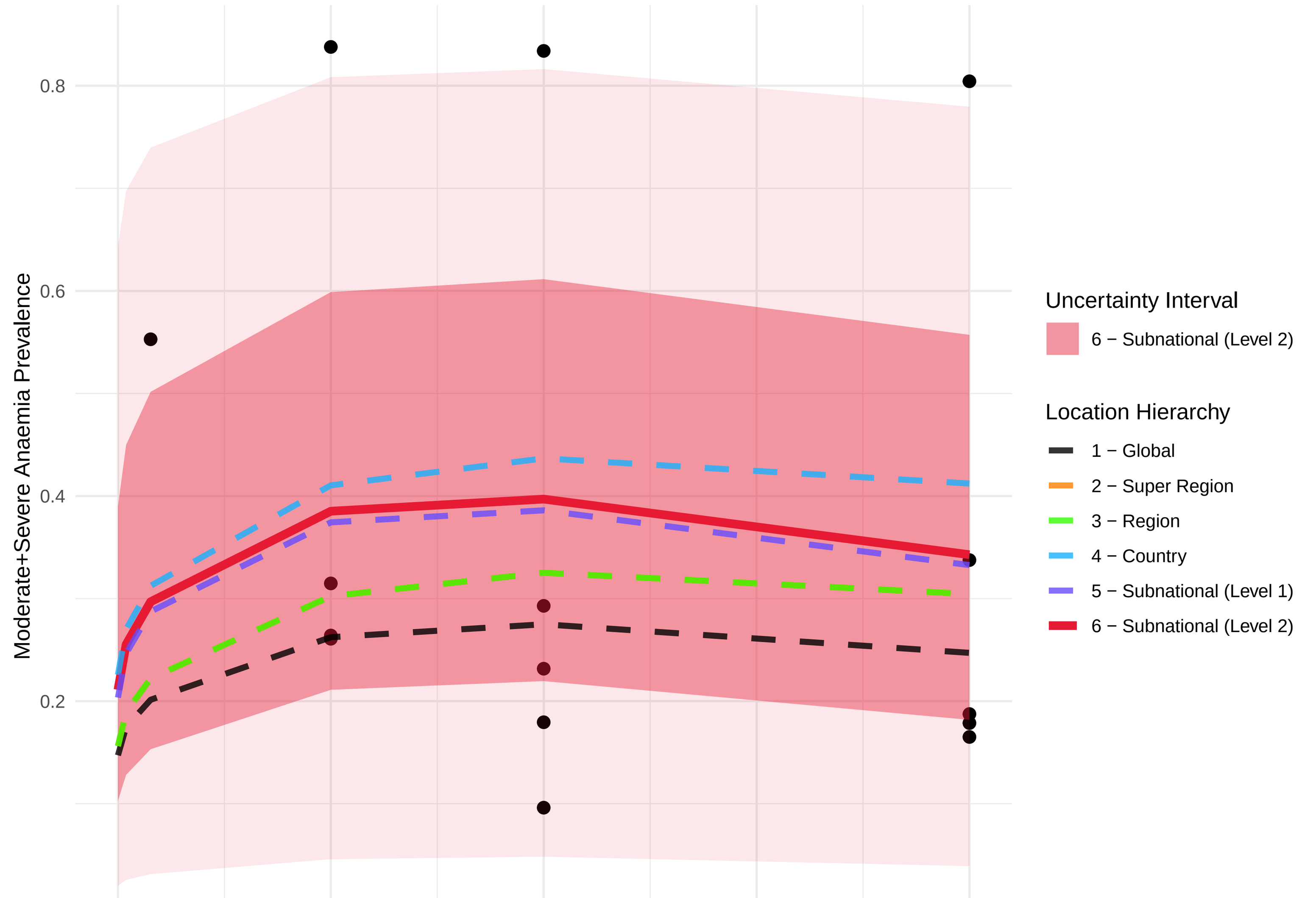

MR−BRT Age/Sex Fits − Non−pregnant Females <2 Years Old − Moderate+Severe Anaemia Prevalence − Sikkim, Urban
WHO−2001 Adjustment Method

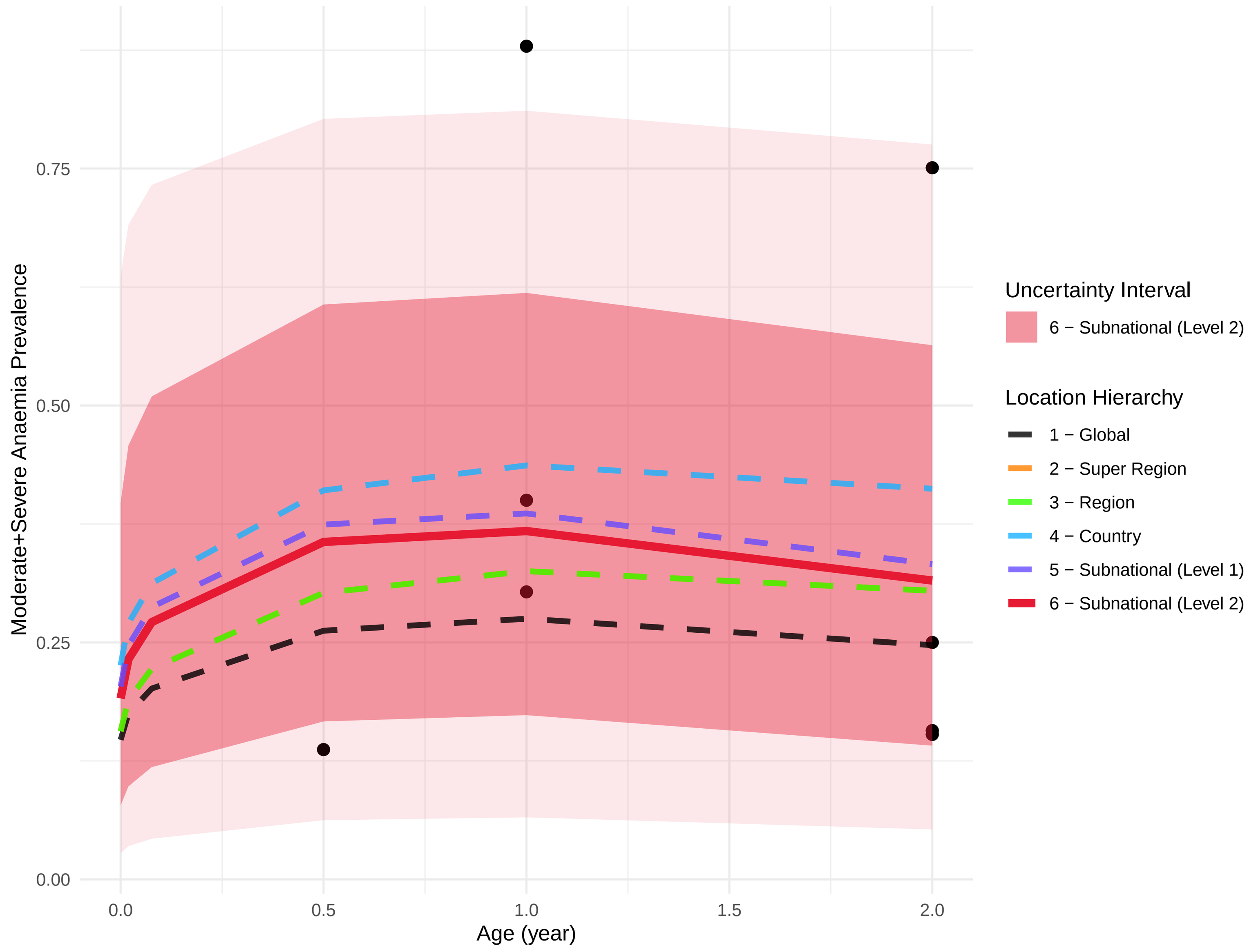

MR−BRT Age/Sex Fits − Non−pregnant Females <2 Years Old − Moderate+Severe Anaemia Prevalence − Tamil Nadu
WHO−2001 Adjustment Method

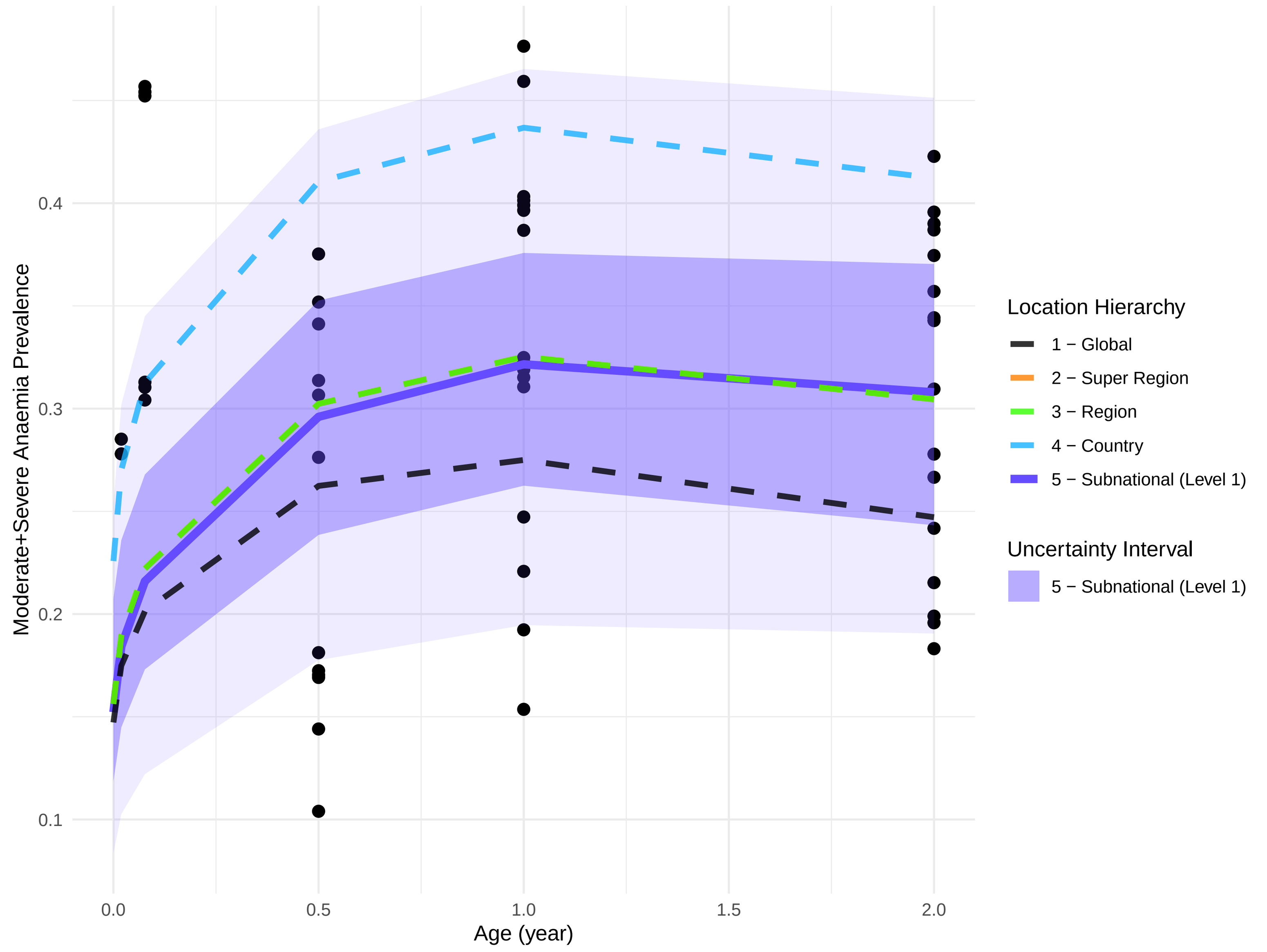

MR−BRT Age/Sex Fits − Non−pregnant Females <2 Years Old − Moderate+Severe Anaemia Prevalence − Tamil Nadu, Rural
WHO−2001 Adjustment Method

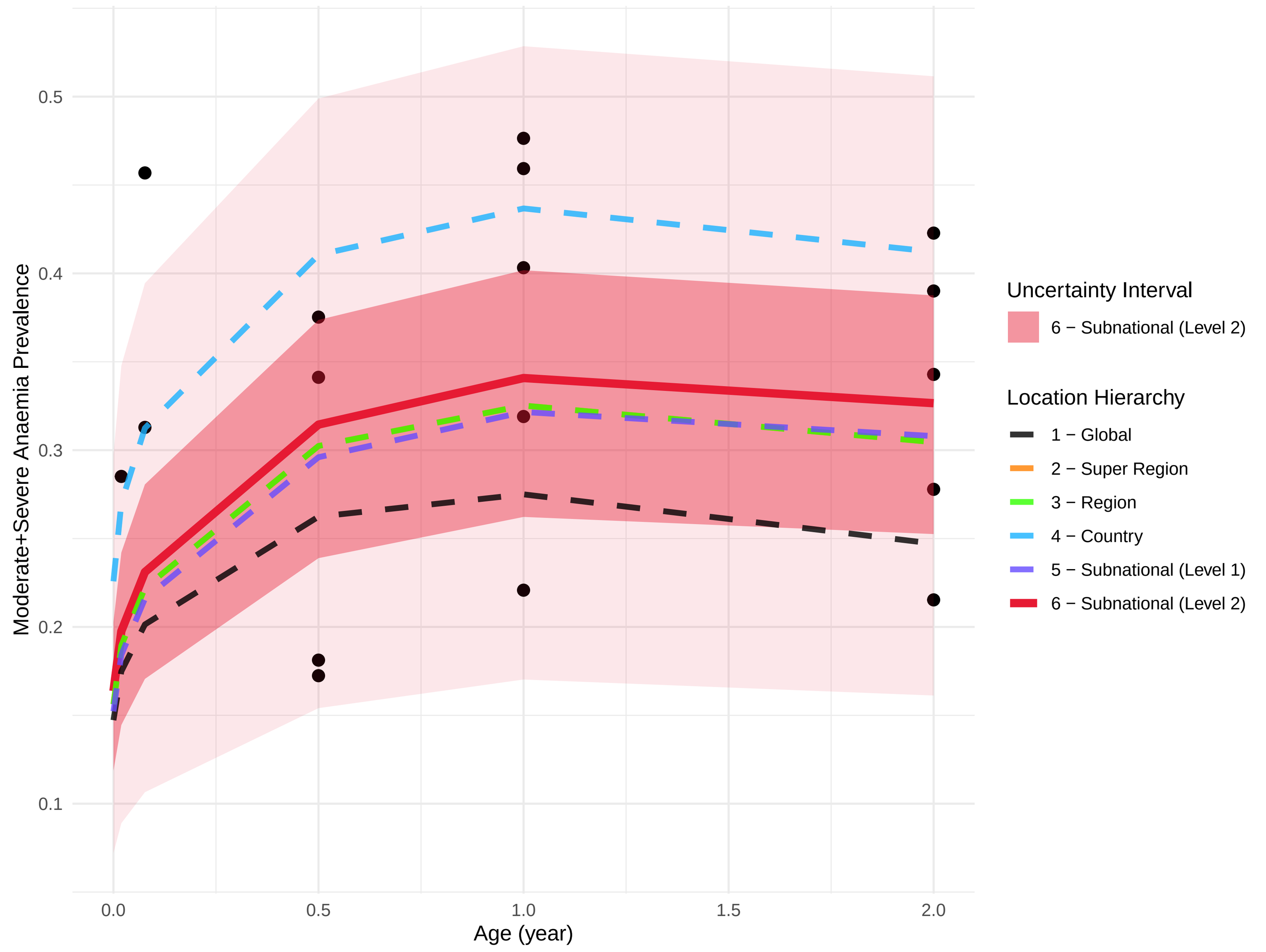

MR−BRT Age/Sex Fits − Non−pregnant Females <2 Years Old − Moderate+Severe Anaemia Prevalence − Tamil Nadu, Urban
WHO−2001 Adjustment Method

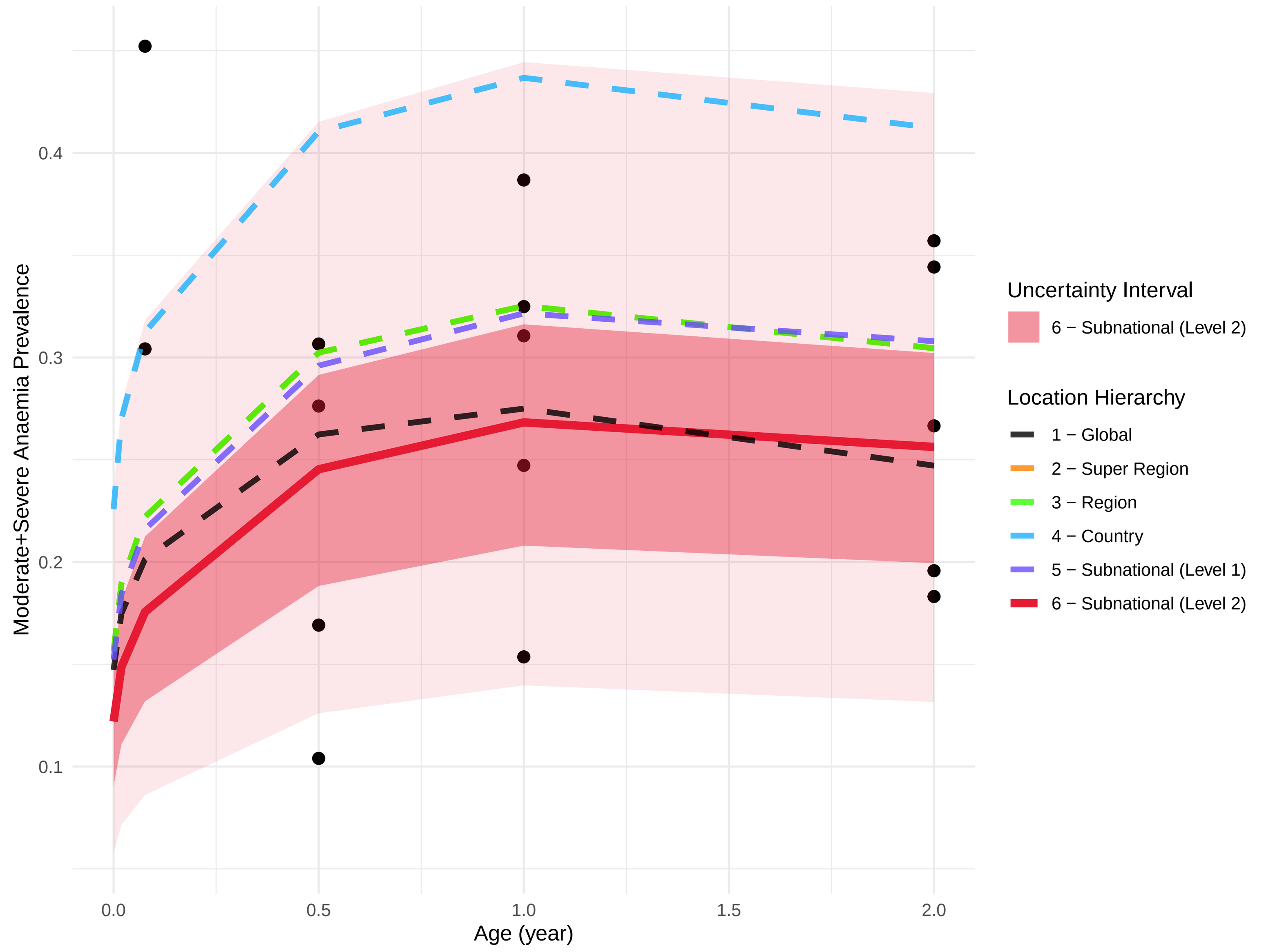

MR−BRT Age/Sex Fits − Non−pregnant Females <2 Years Old − Moderate+Severe Anaemia Prevalence − Telangana
WHO−2001 Adjustment Method

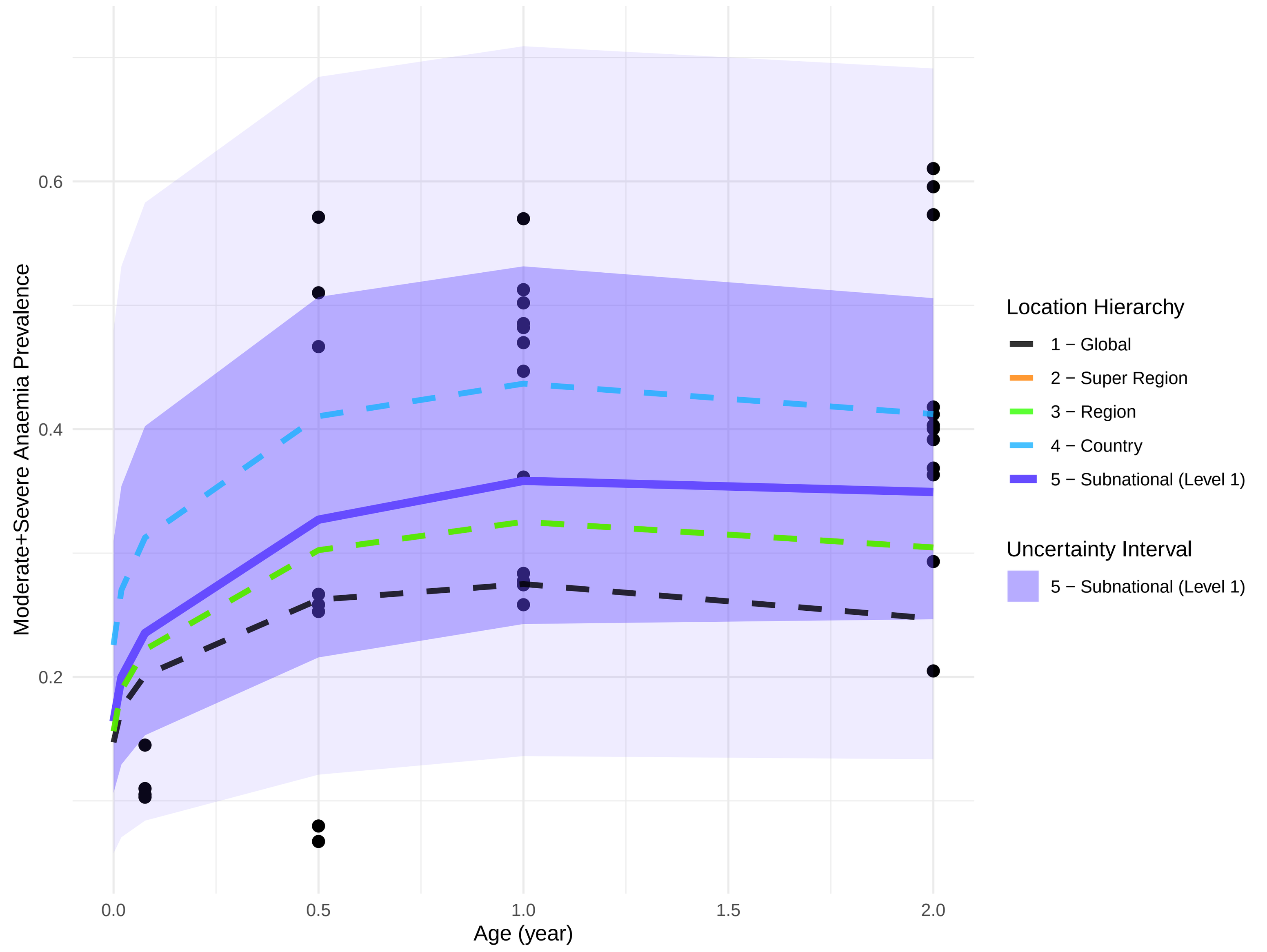

MR−BRT Age/Sex Fits − Non−pregnant Females <2 Years Old − Moderate+Severe Anaemia Prevalence − Telangana, Rural
WHO−2001 Adjustment Method

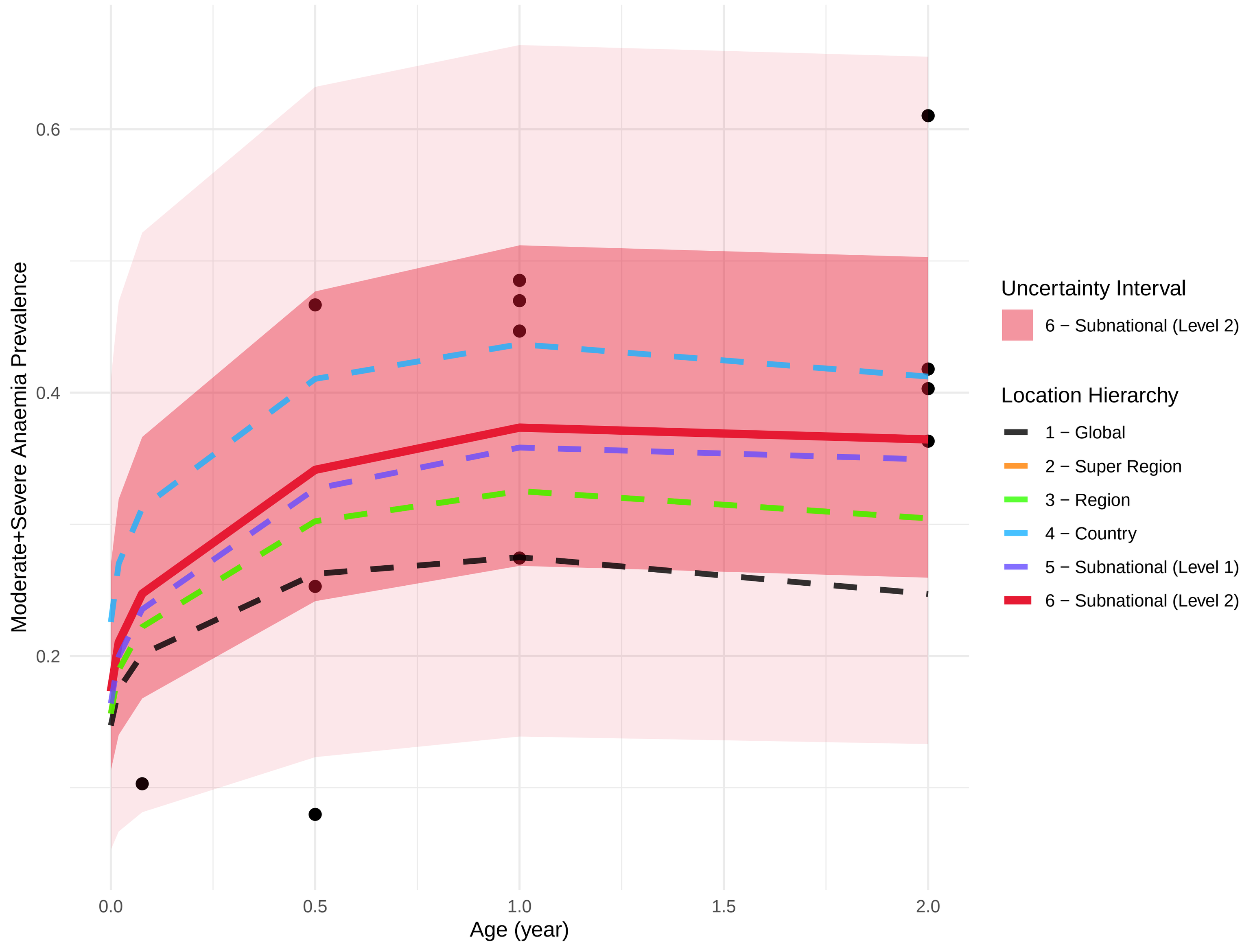

MR−BRT Age/Sex Fits − Non−pregnant Females <2 Years Old − Moderate+Severe Anaemia Prevalence − Telangana, Urban
WHO−2001 Adjustment Method

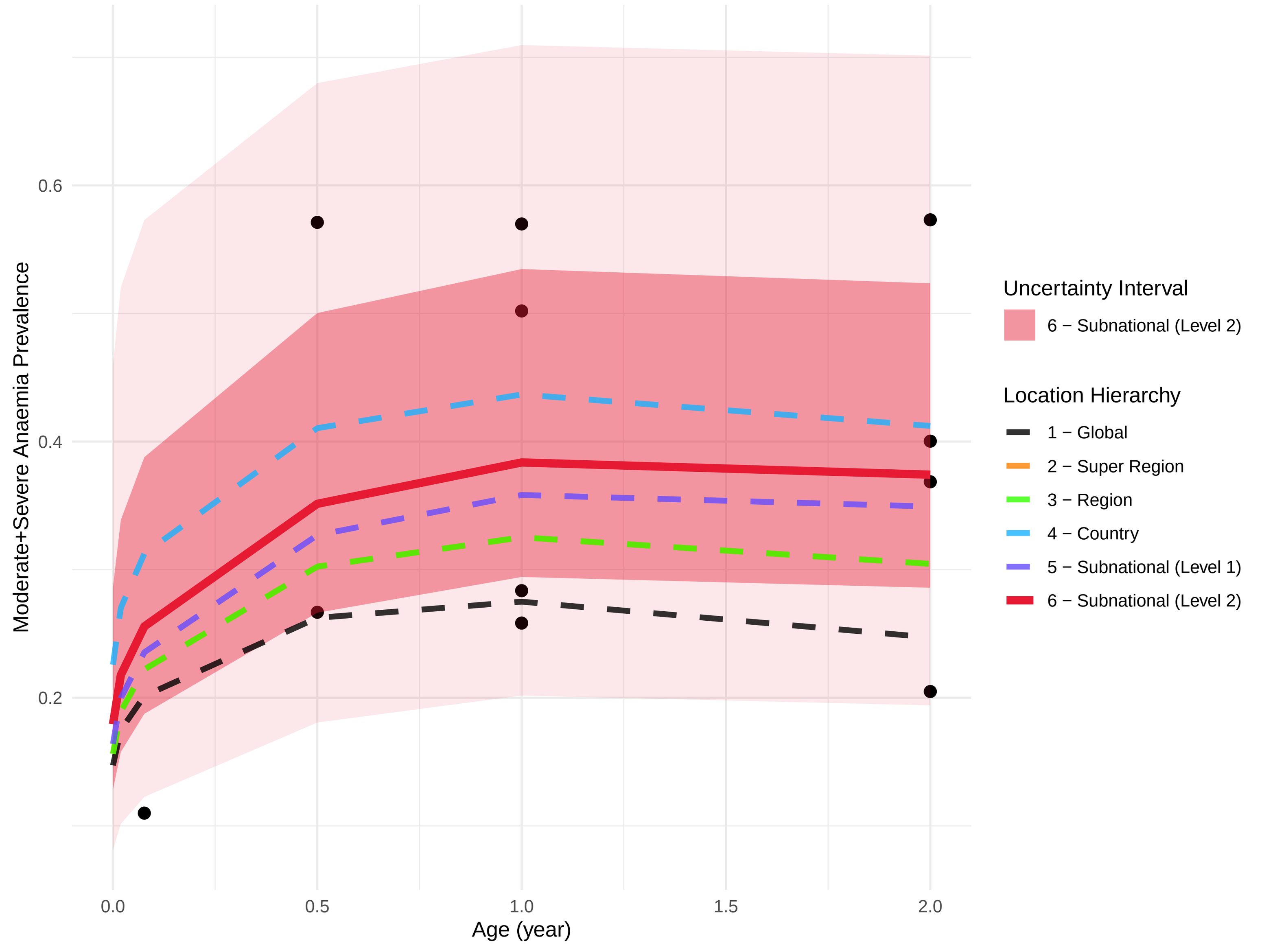

MR−BRT Age/Sex Fits − Non−pregnant Females <2 Years Old − Moderate+Severe Anaemia Prevalence − Tripura
WHO−2001 Adjustment Method

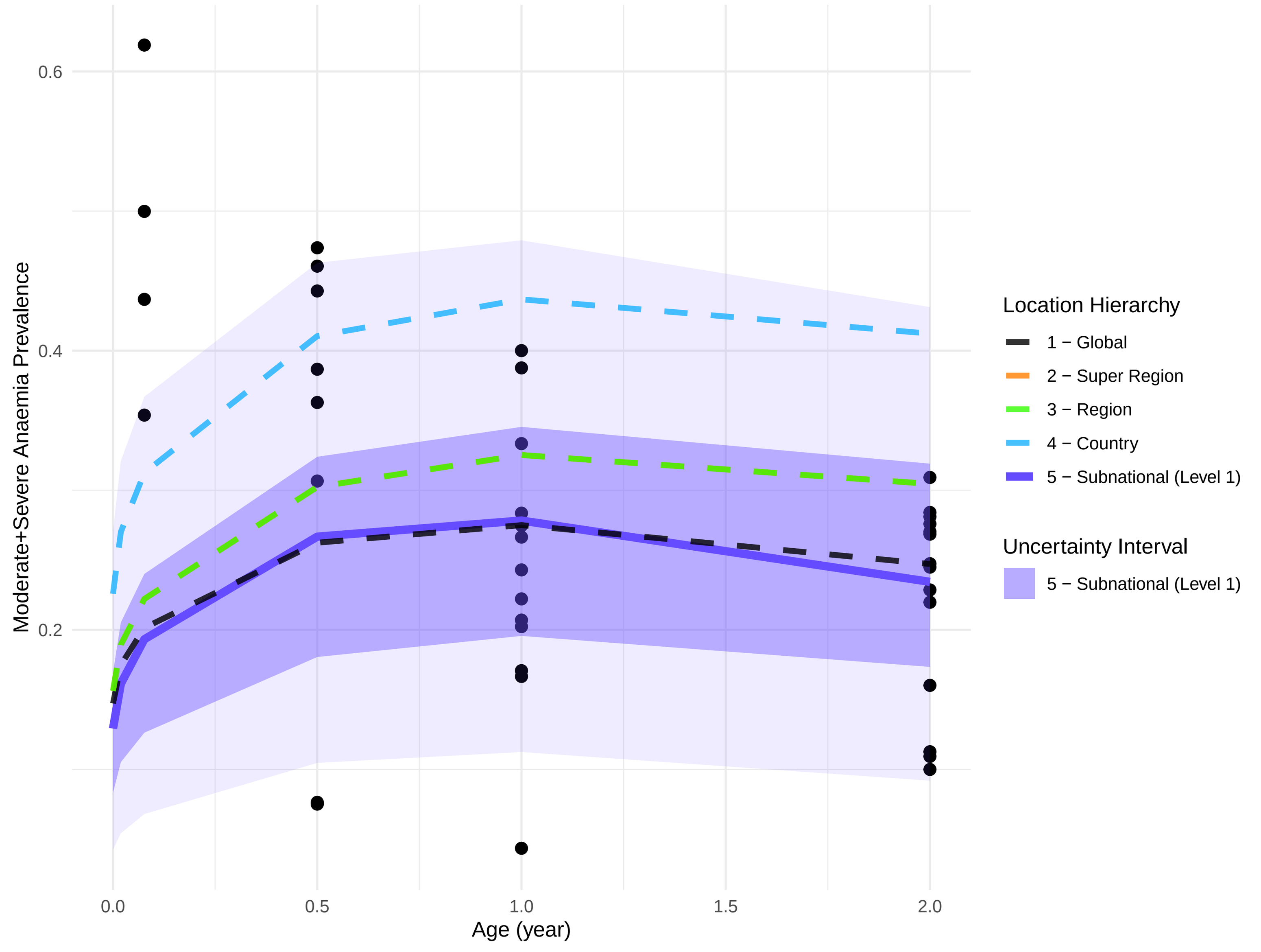

MR−BRT Age/Sex Fits − Non−pregnant Females <2 Years Old − Moderate+Severe Anaemia Prevalence − Tripura, Rural
WHO−2001 Adjustment Method

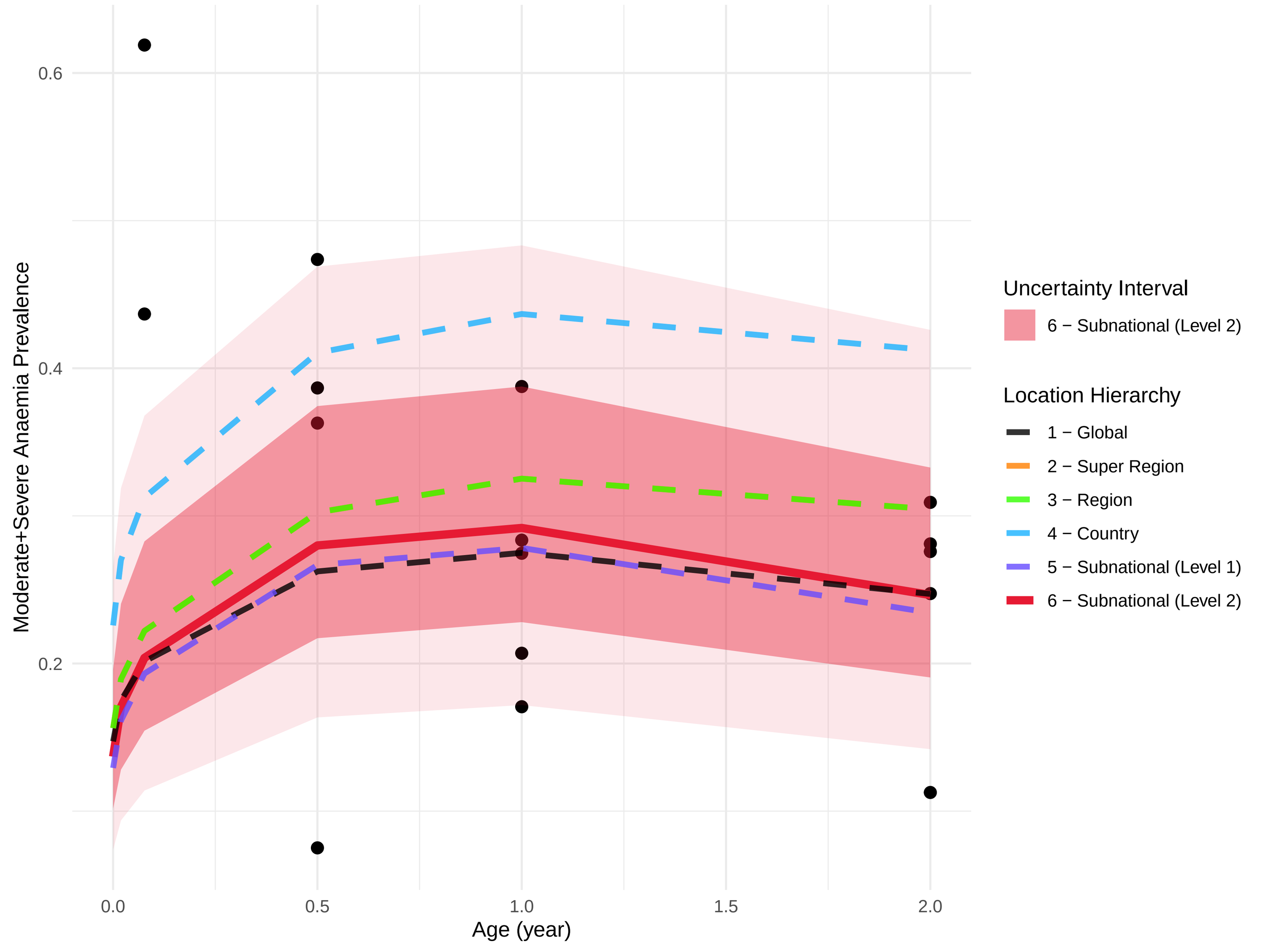

MR−BRT Age/Sex Fits − Non−pregnant Females <2 Years Old − Moderate+Severe Anaemia Prevalence − Tripura, Urban
WHO−2001 Adjustment Method

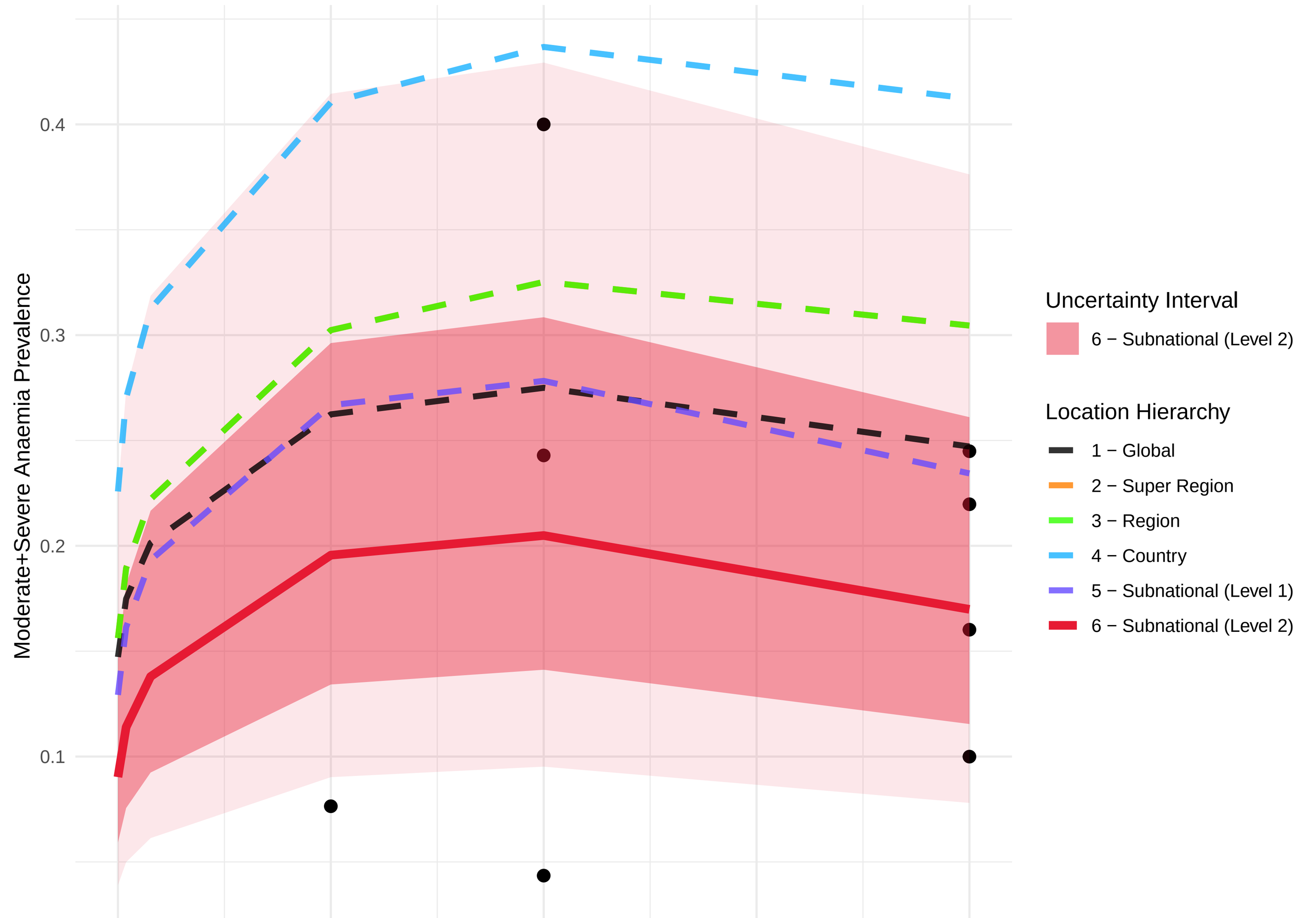

MR−BRT Age/Sex Fits − Non−pregnant Females <2 Years Old − Moderate+Severe Anaemia Prevalence − Uttar Pradesh
WHO−2001 Adjustment Method

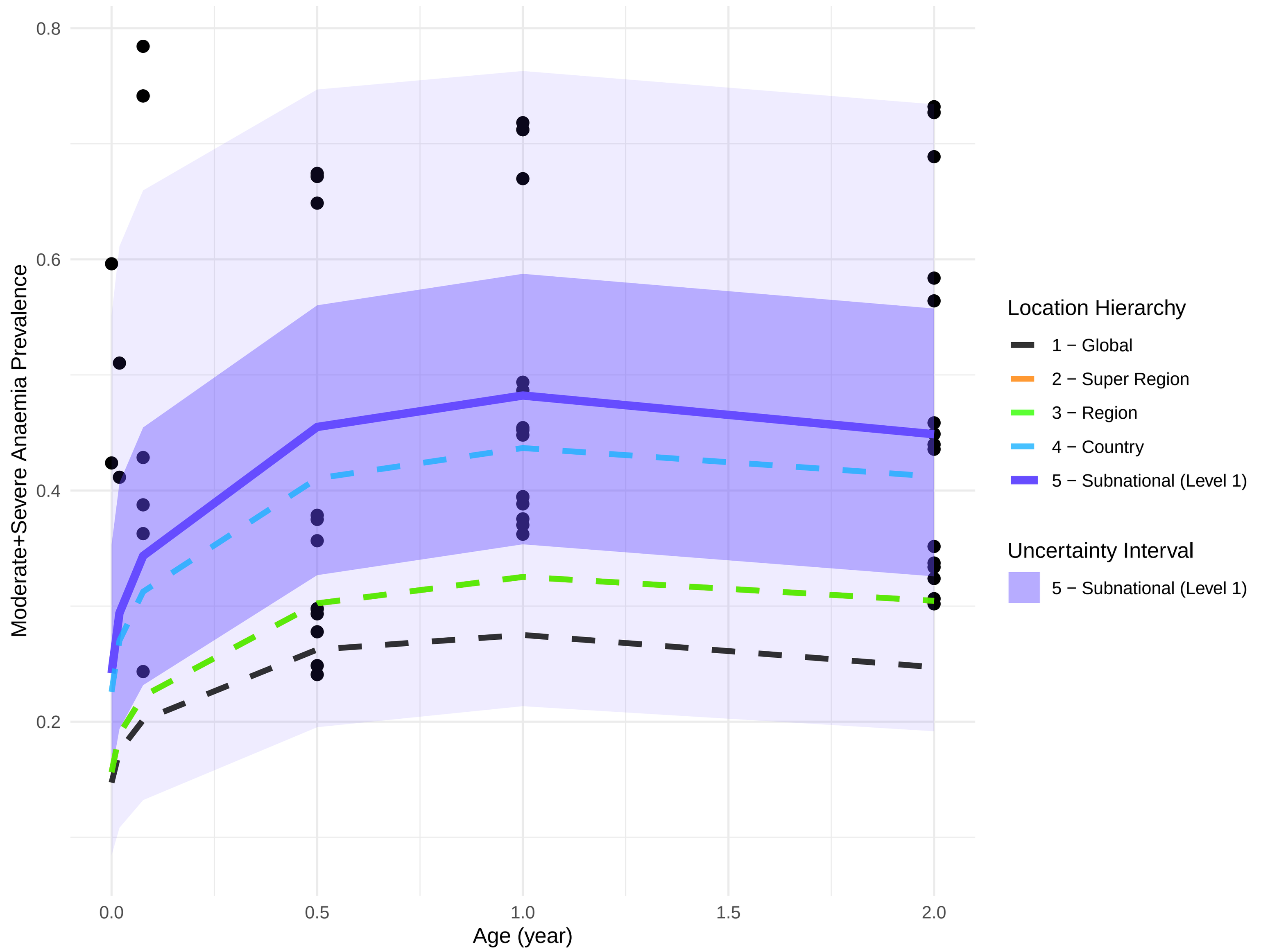

MR−BRT Age/Sex Fits − Non−pregnant Females <2 Years Old − Moderate+Severe Anaemia Prevalence − Uttar Pradesh, Rural
WHO−2001 Adjustment Method

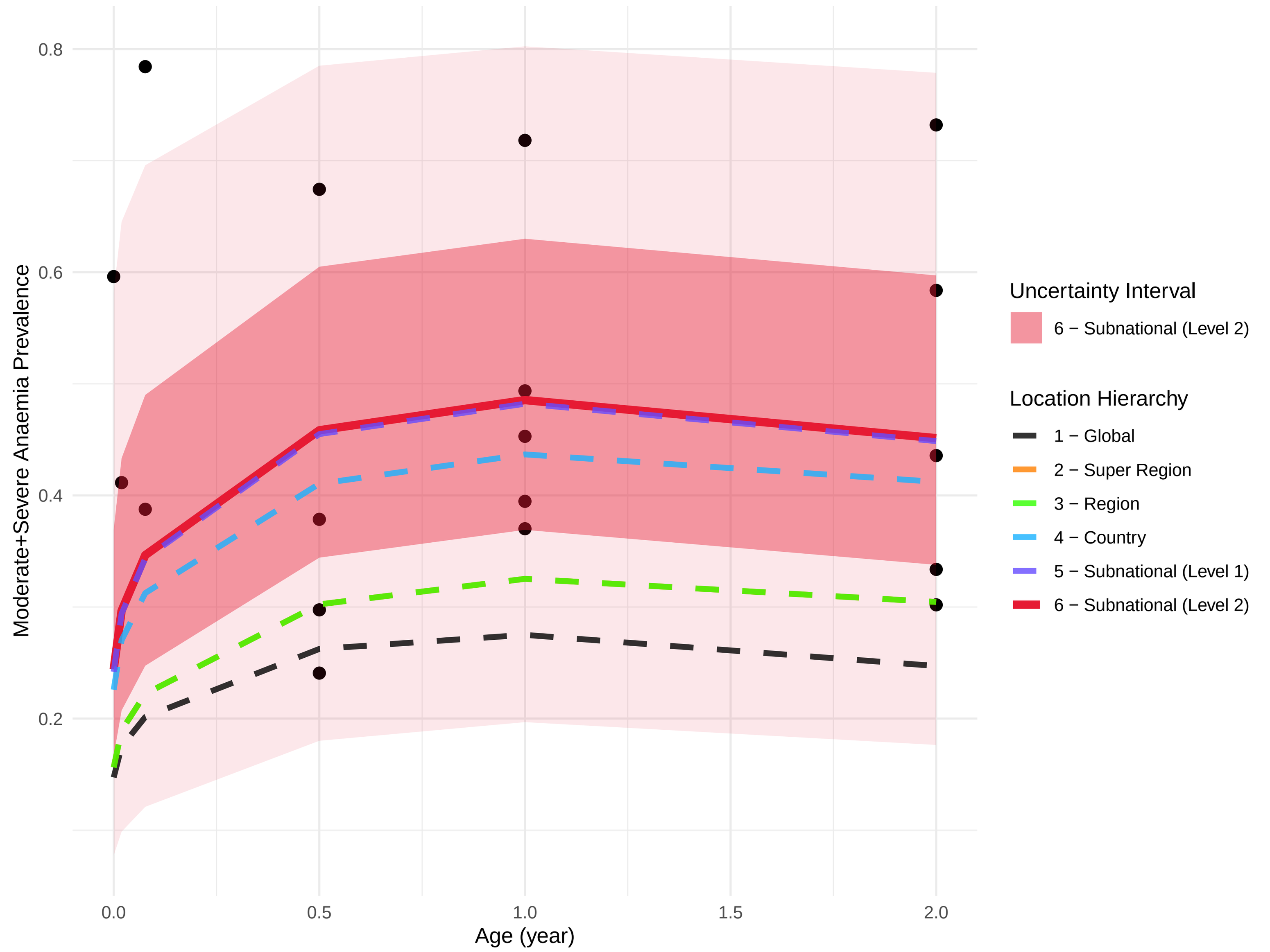

MR−BRT Age/Sex Fits − Non−pregnant Females <2 Years Old − Moderate+Severe Anaemia Prevalence − Uttar Pradesh, Urban
WHO−2001 Adjustment Method

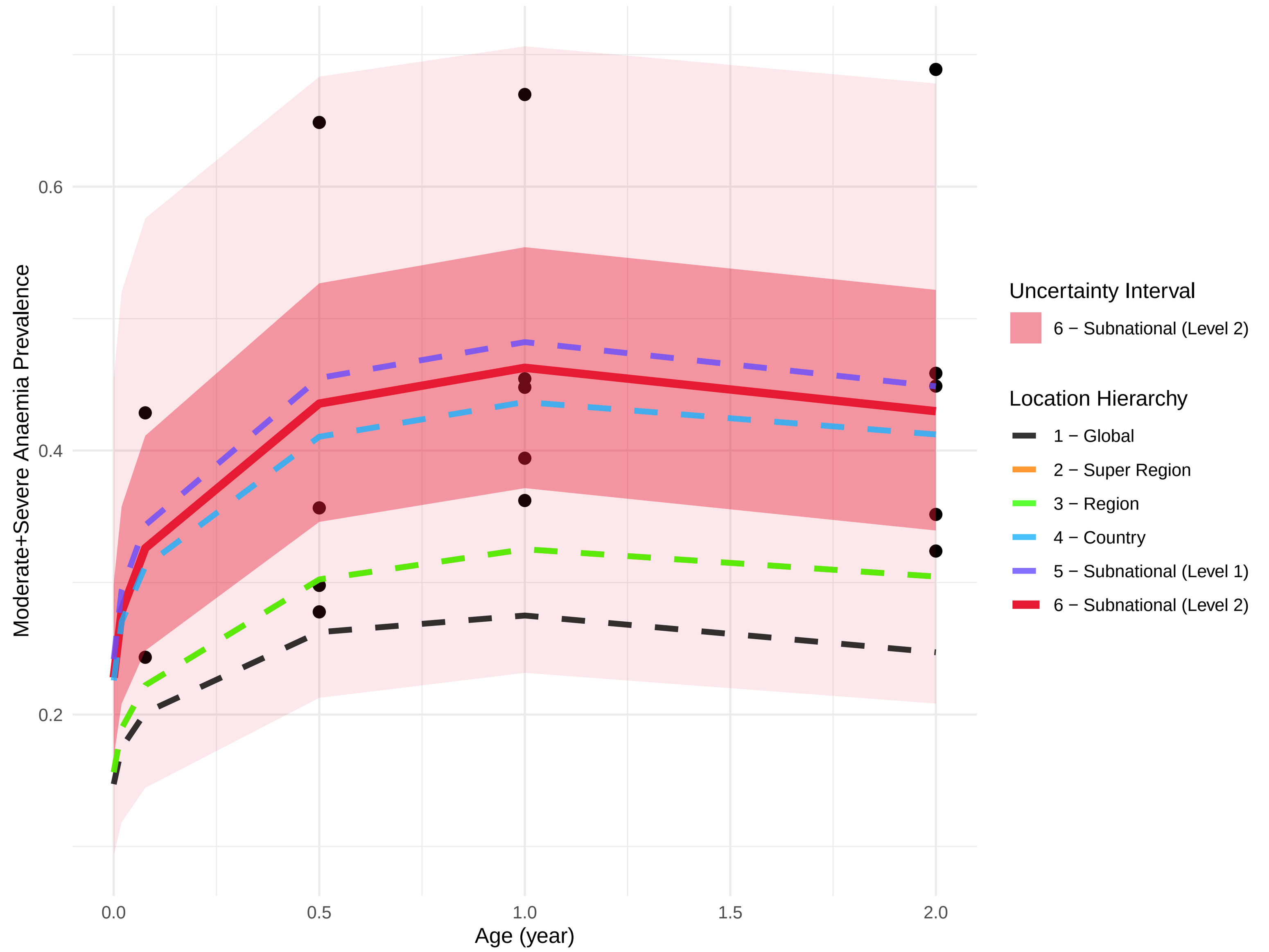

MR−BRT Age/Sex Fits − Non−pregnant Females <2 Years Old − Moderate+Severe Anaemia Prevalence − Uttarakhand
WHO−2001 Adjustment Method

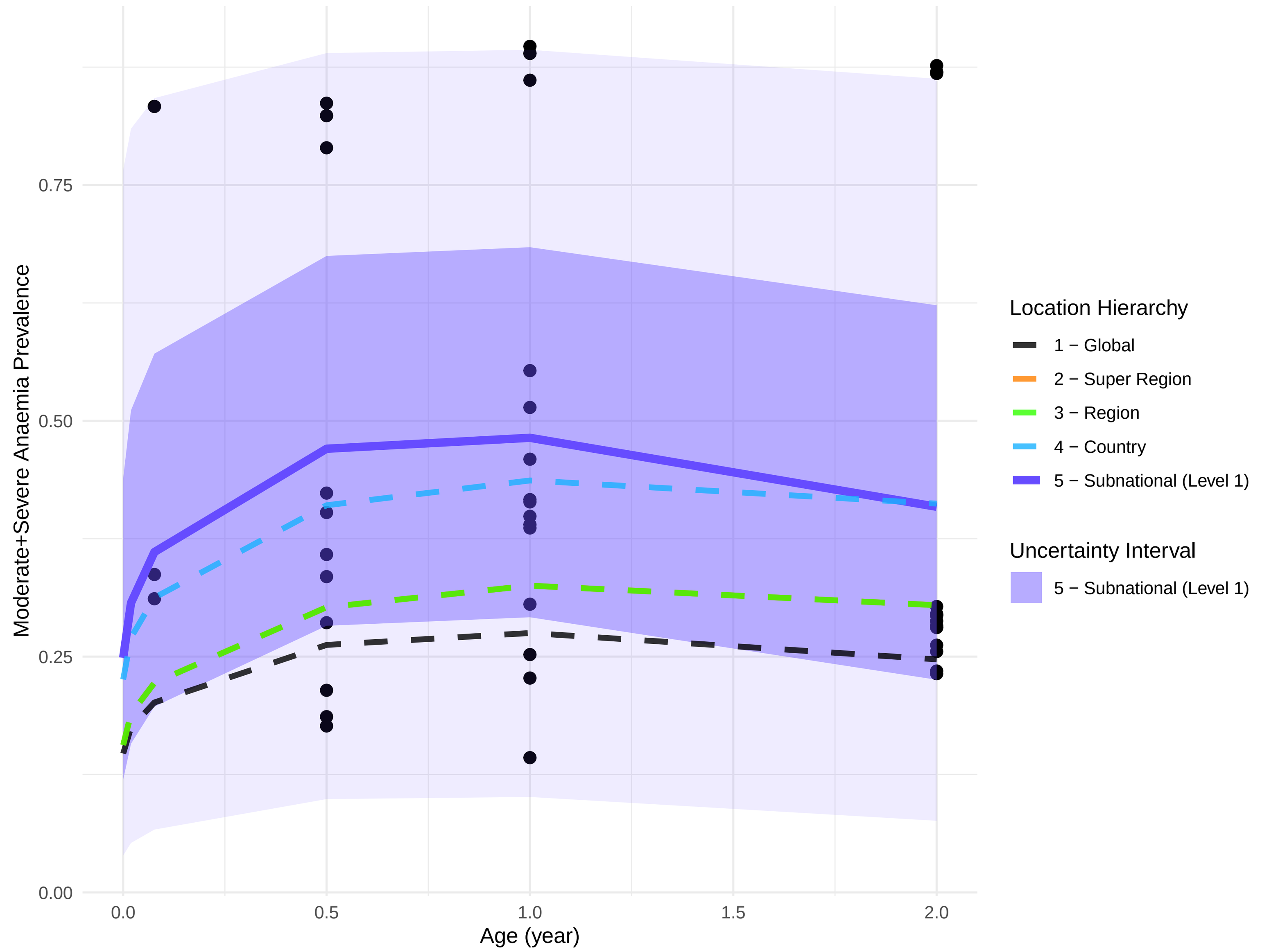

MR−BRT Age/Sex Fits − Non−pregnant Females <2 Years Old − Moderate+Severe Anaemia Prevalence − Uttarakhand, Rural
WHO−2001 Adjustment Method

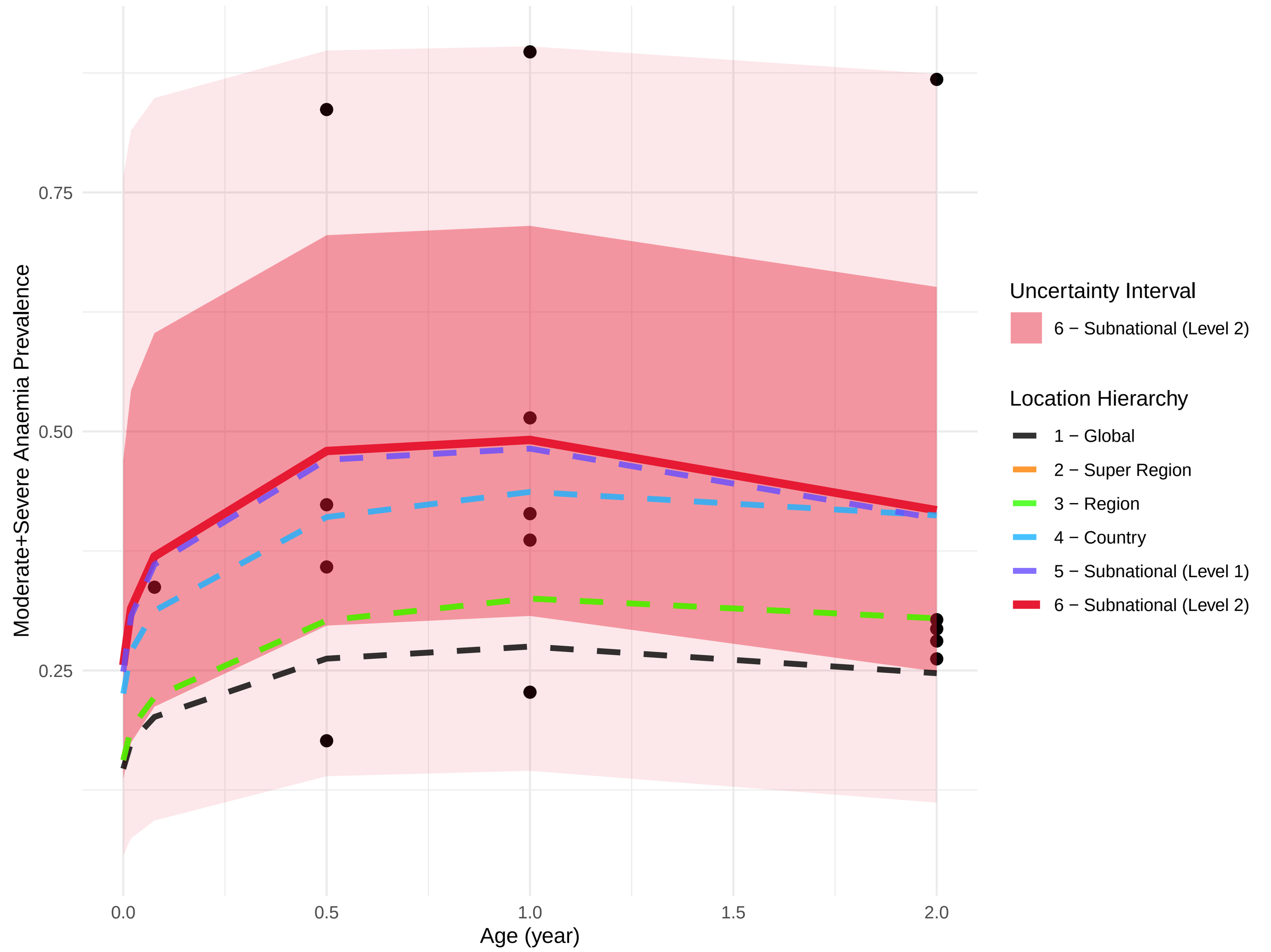

MR−BRT Age/Sex Fits − Non−pregnant Females <2 Years Old − Moderate+Severe Anaemia Prevalence − Uttarakhand, Urban
WHO−2001 Adjustment Method

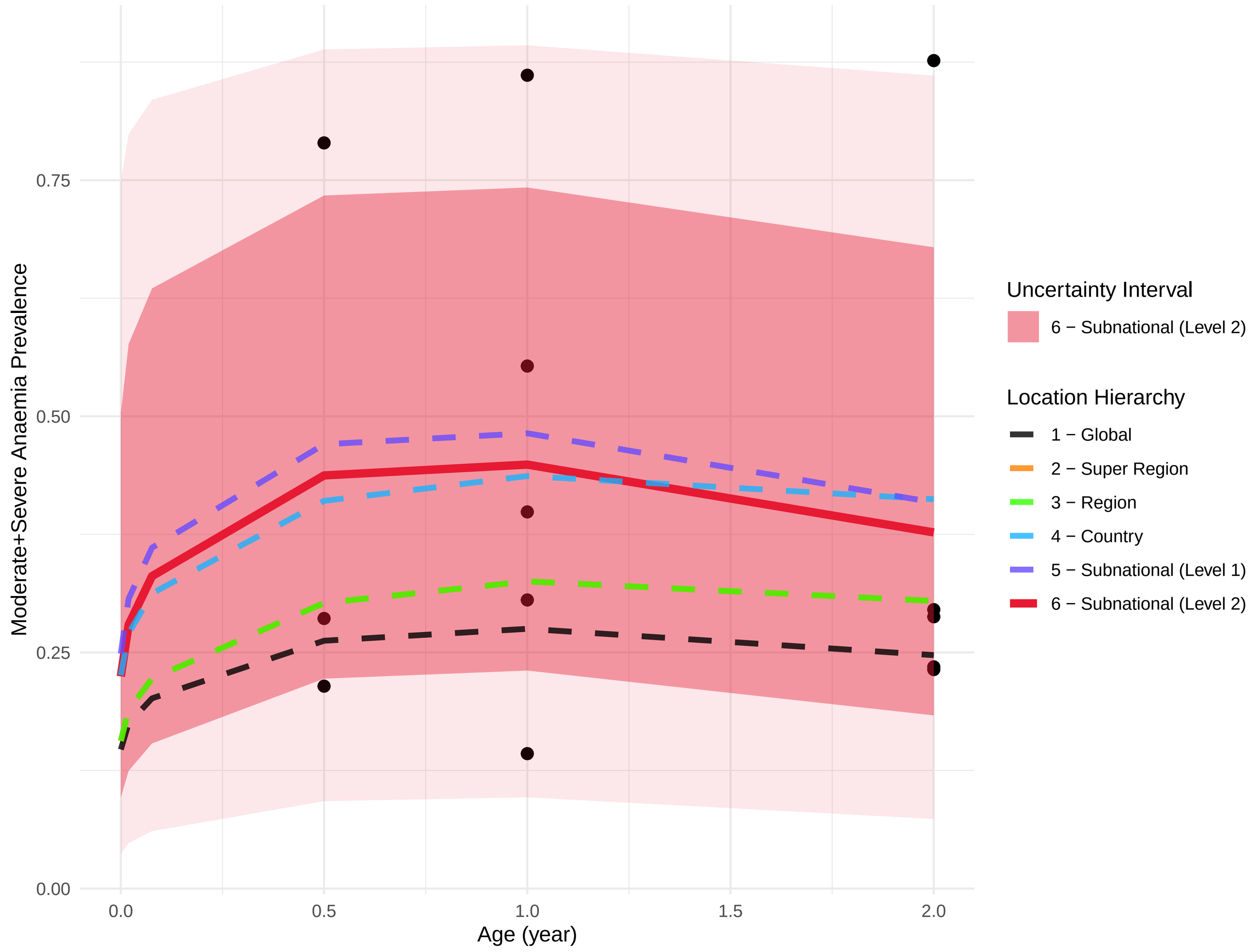

MR−BRT Age/Sex Fits − Non−pregnant Females <2 Years Old − Moderate+Severe Anaemia Prevalence − West Bengal
WHO−2001 Adjustment Method

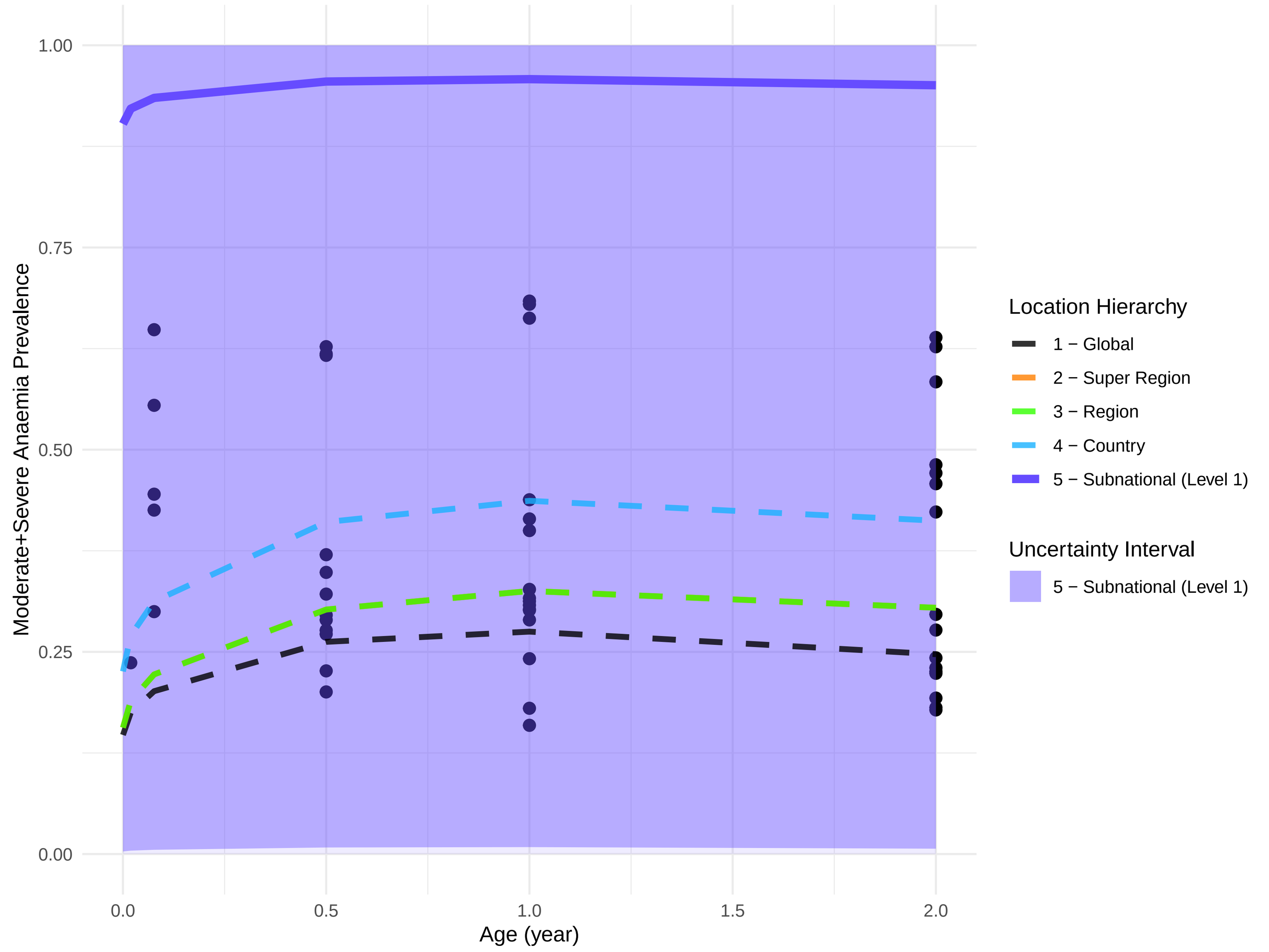

MR−BRT Age/Sex Fits − Non−pregnant Females <2 Years Old − Moderate+Severe Anaemia Prevalence − West Bengal, Rural
WHO−2001 Adjustment Method

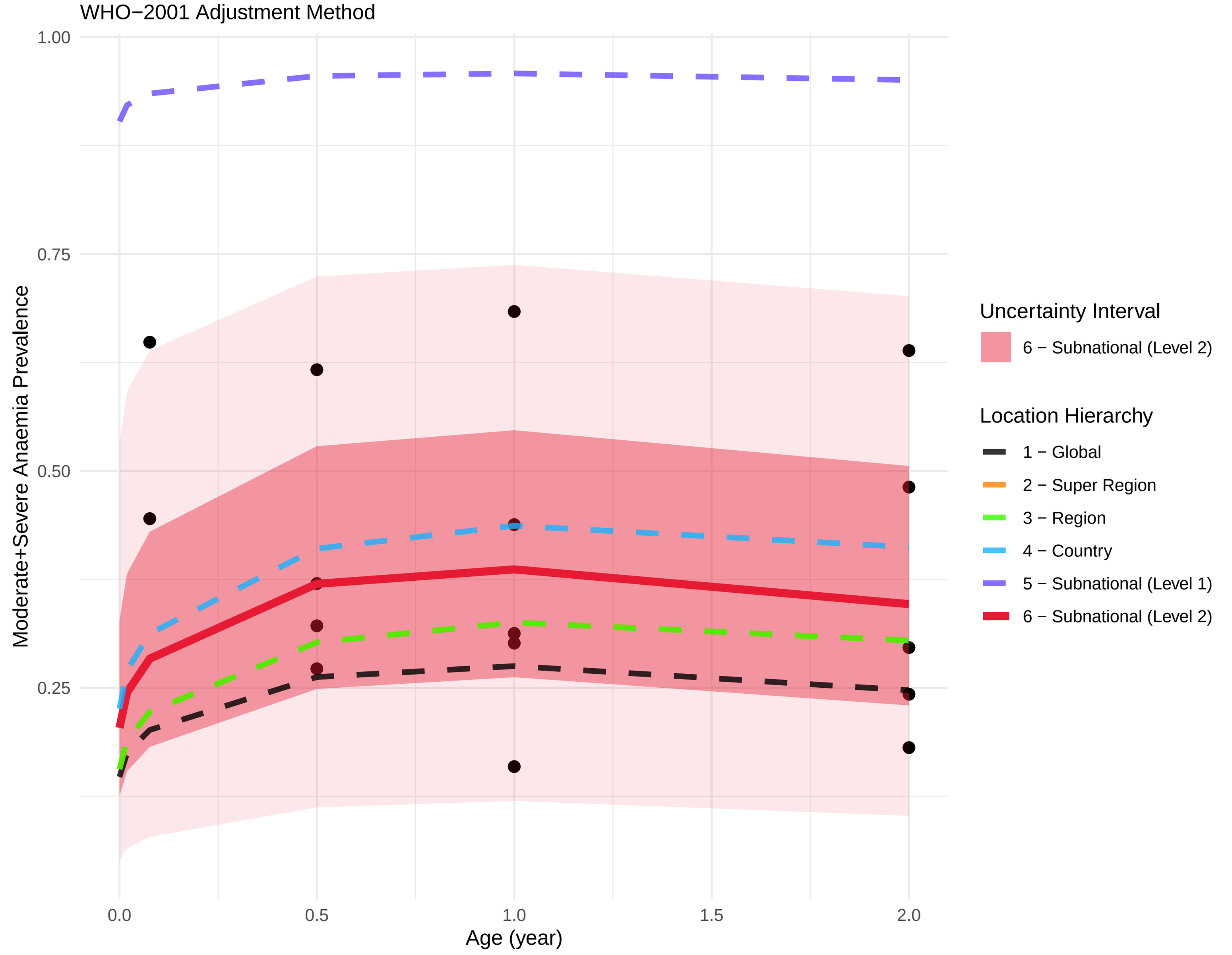

MR−BRT Age/Sex Fits − Non−pregnant Females <2 Years Old − Moderate+Severe Anaemia Prevalence − West Bengal, Urban
WHO−2001 Adjustment Method

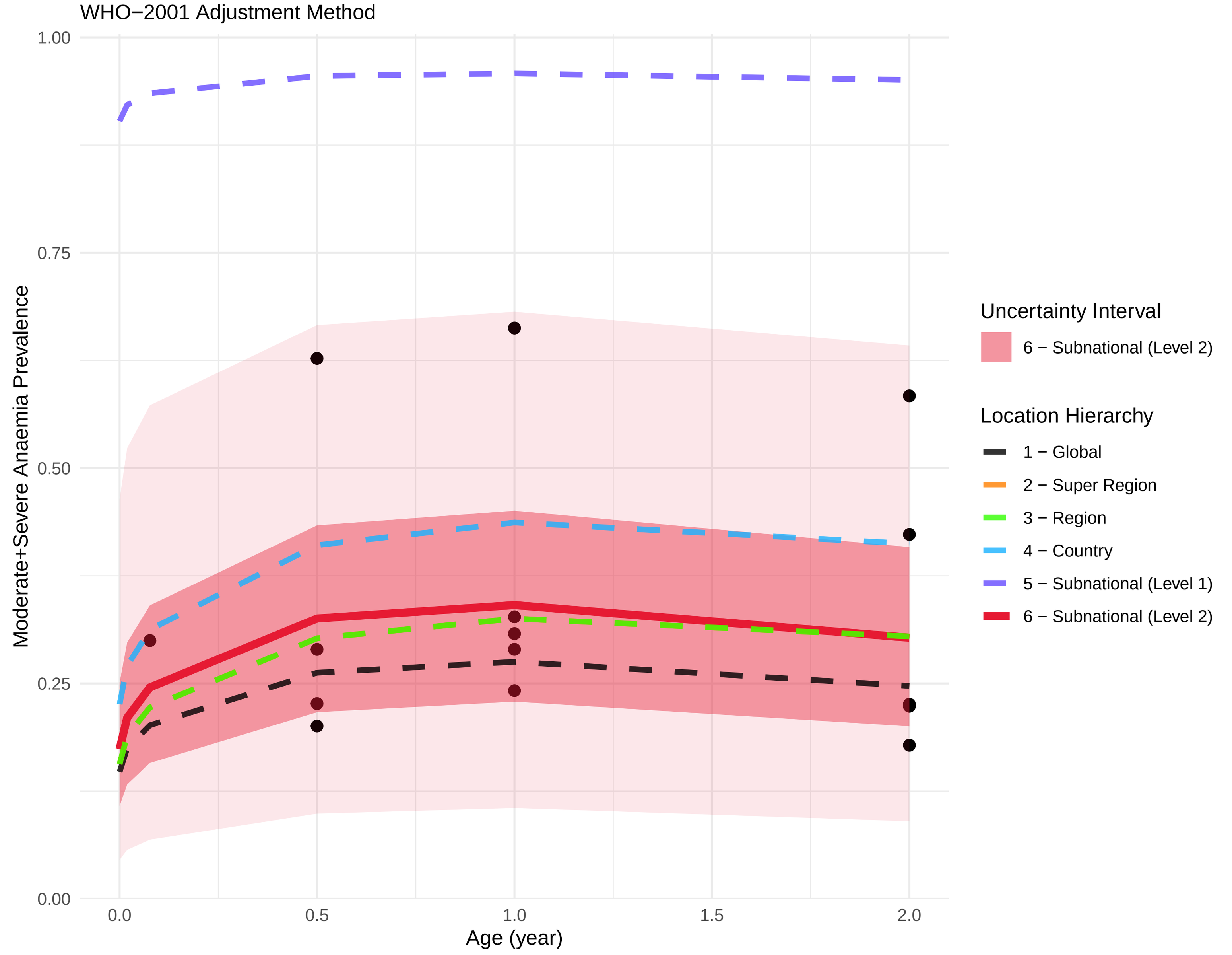

MR−BRT Age/Sex Fits − Non−pregnant Females <2 Years Old − Moderate+Severe Anaemia Prevalence − Nepal
WHO−2001 Adjustment Method

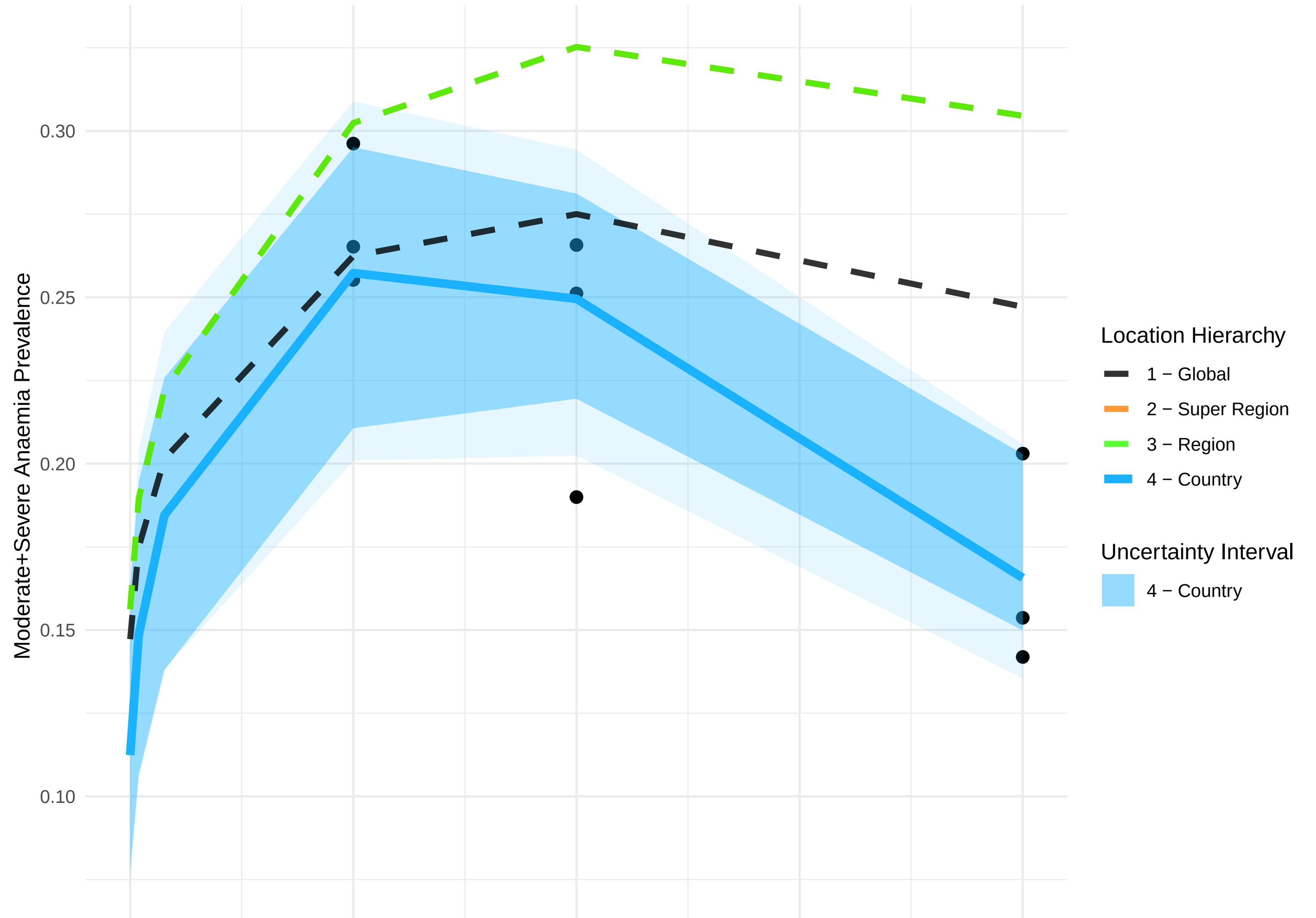

MR−BRT Age/Sex Fits − Non−pregnant Females <2 Years Old − Moderate+Severe Anaemia Prevalence − Pakistan
WHO−2001 Adjustment Method

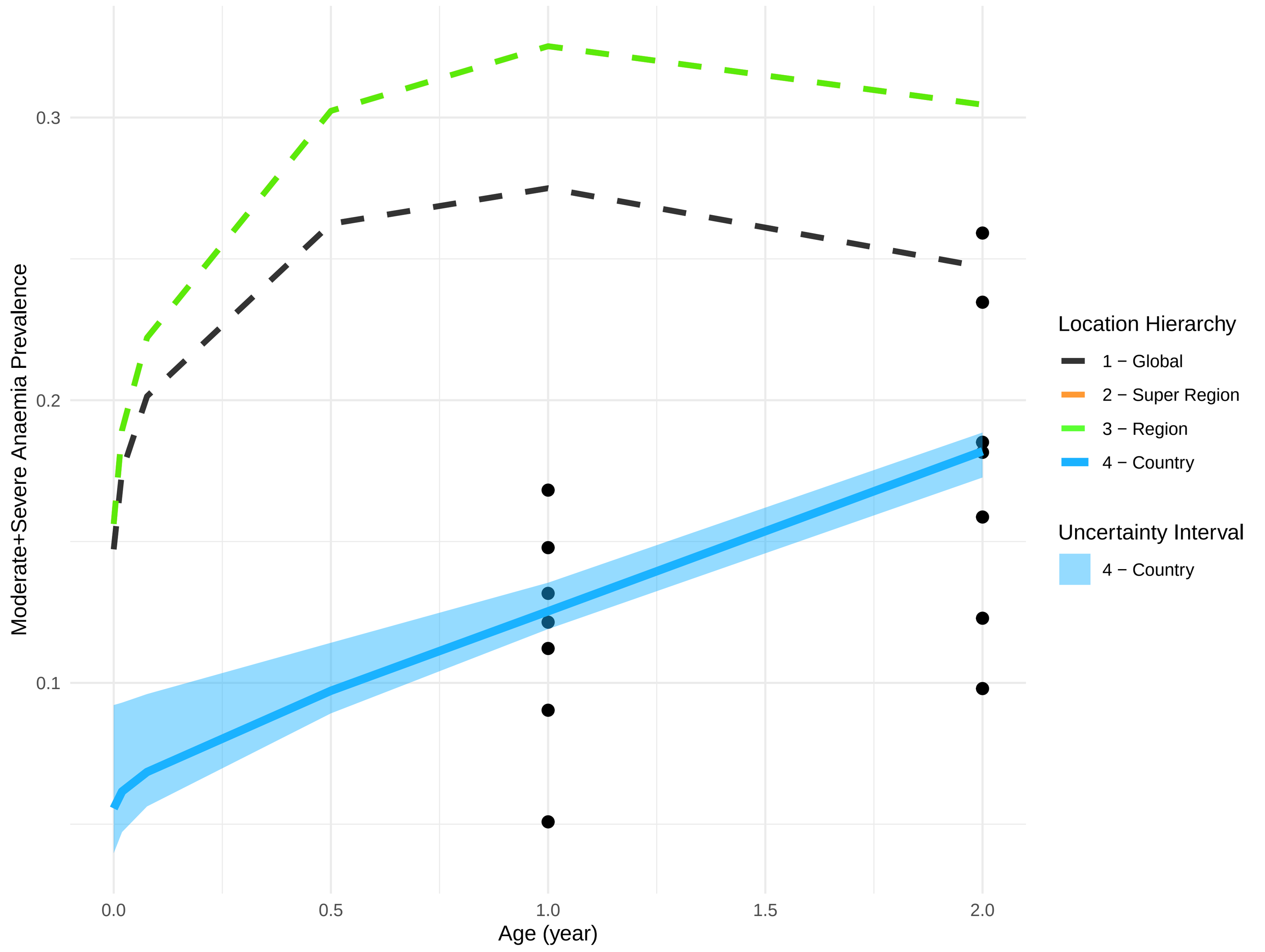

MR−BRT Age/Sex Fits − Non−pregnant Females <2 Years Old − Moderate+Severe Anaemia Prevalence − Azad Jammu & Kashmir
WHO−2001 Adjustment Method

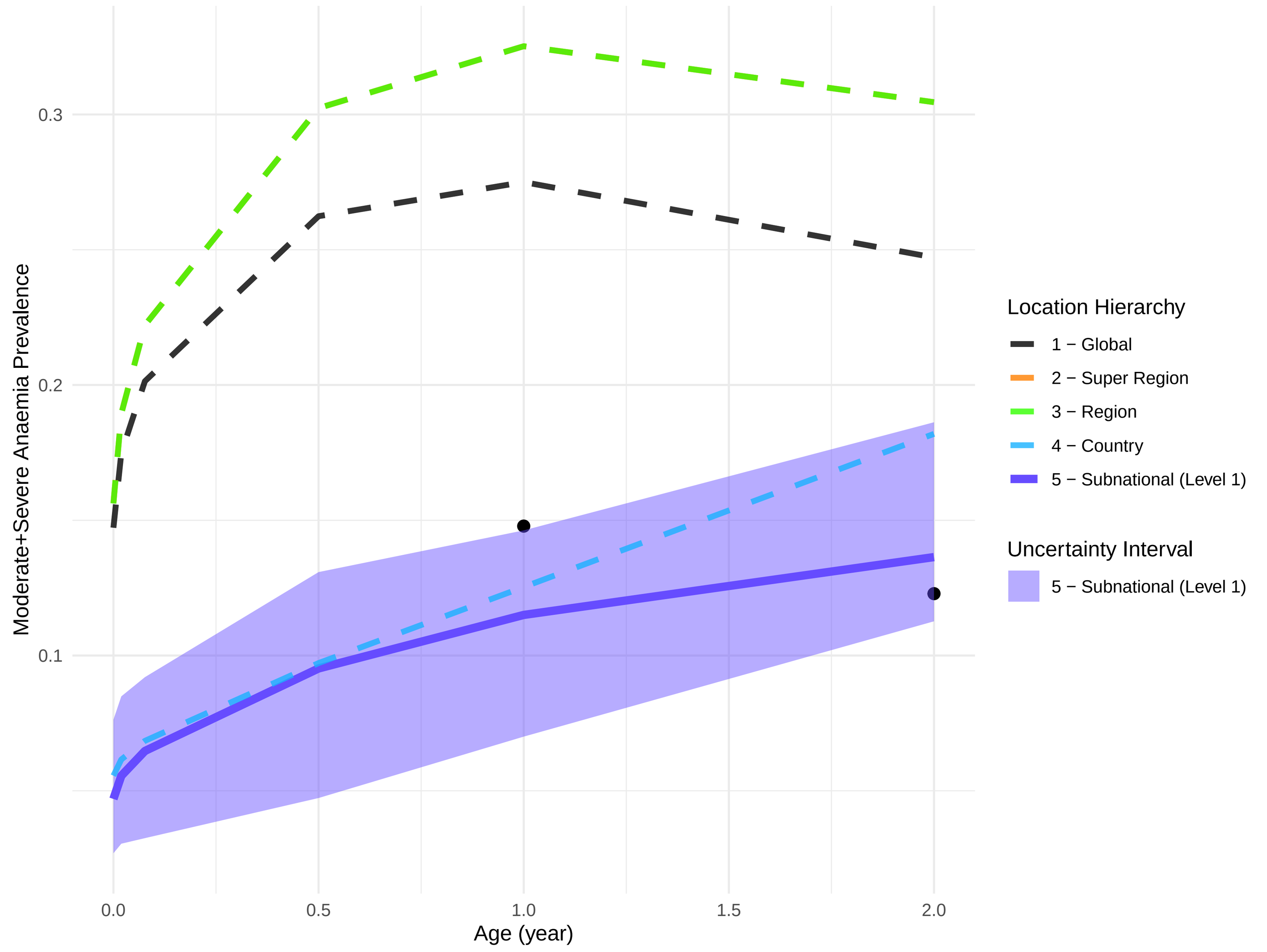

MR−BRT Age/Sex Fits − Non−pregnant Females <2 Years Old − Moderate+Severe Anaemia Prevalence − Balochistan
WHO−2001 Adjustment Method

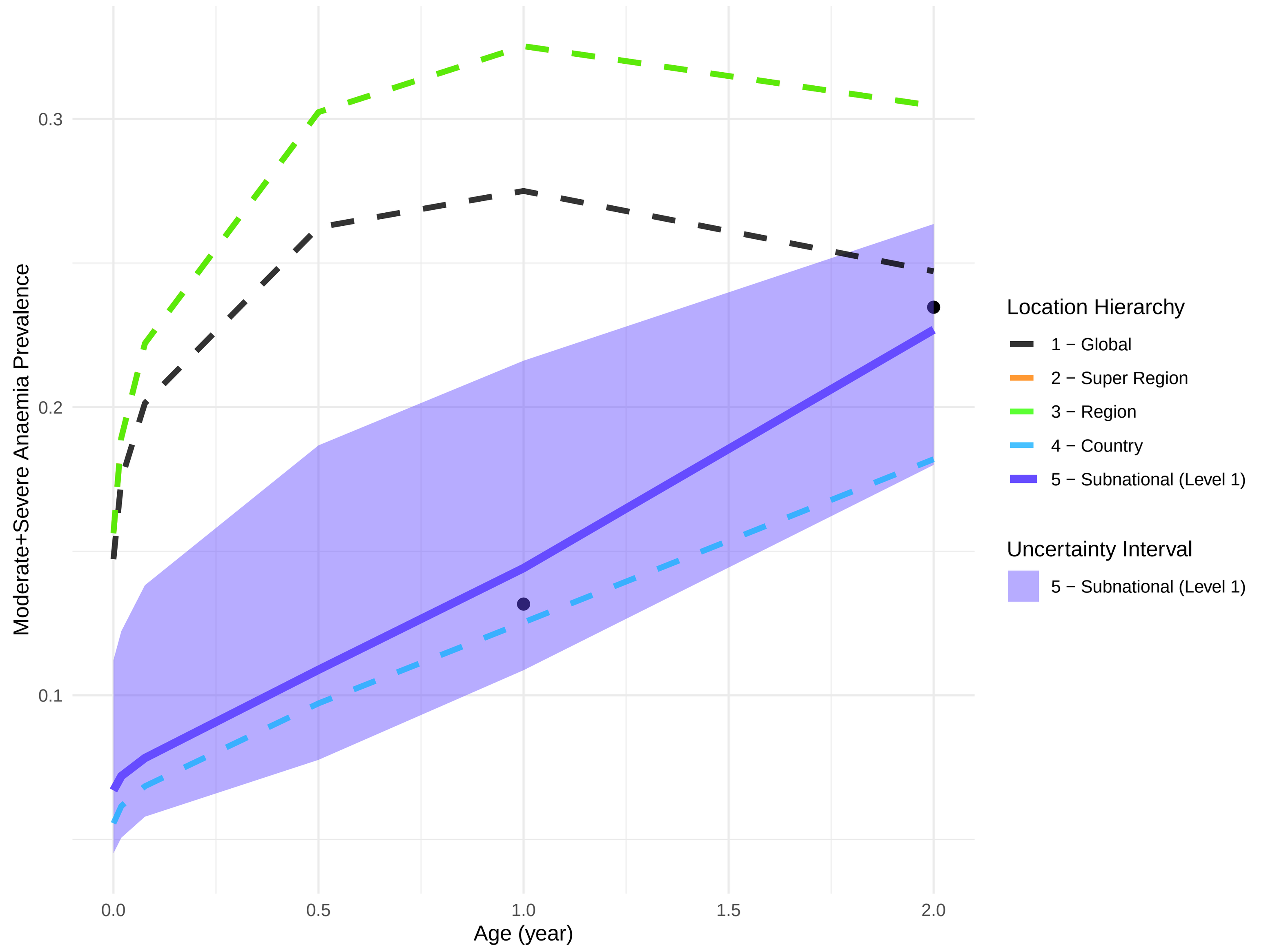

MR−BRT Age/Sex Fits − Non−pregnant Females <2 Years Old − Moderate+Severe Anaemia Prevalence − Gilgit−Baltistan
WHO−2001 Adjustment Method

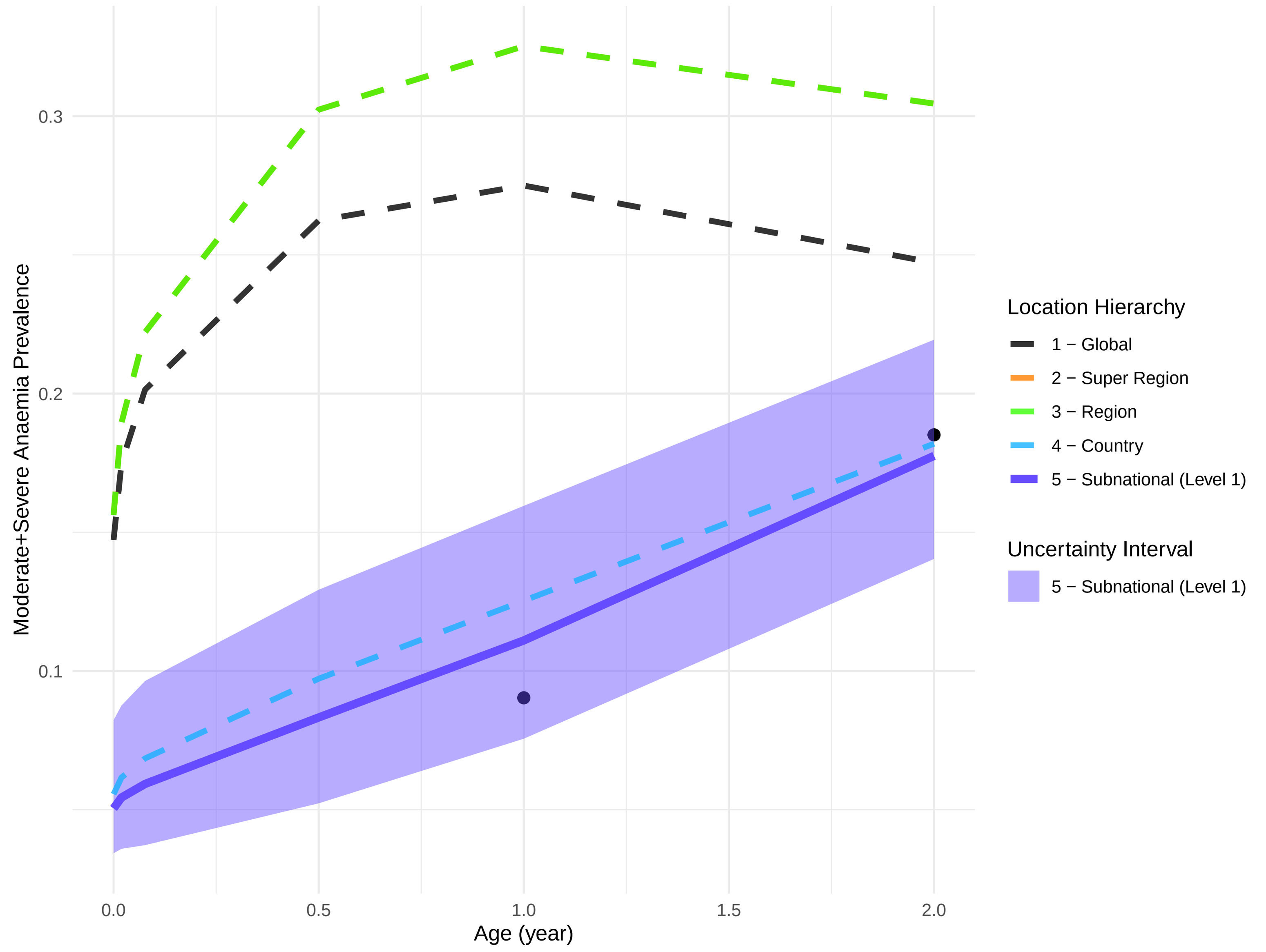

MR−BRT Age/Sex Fits − Non−pregnant Females <2 Years Old − Moderate+Severe Anaemia Prevalence − Khyber Pakhtunkhwa
WHO−2001 Adjustment Method

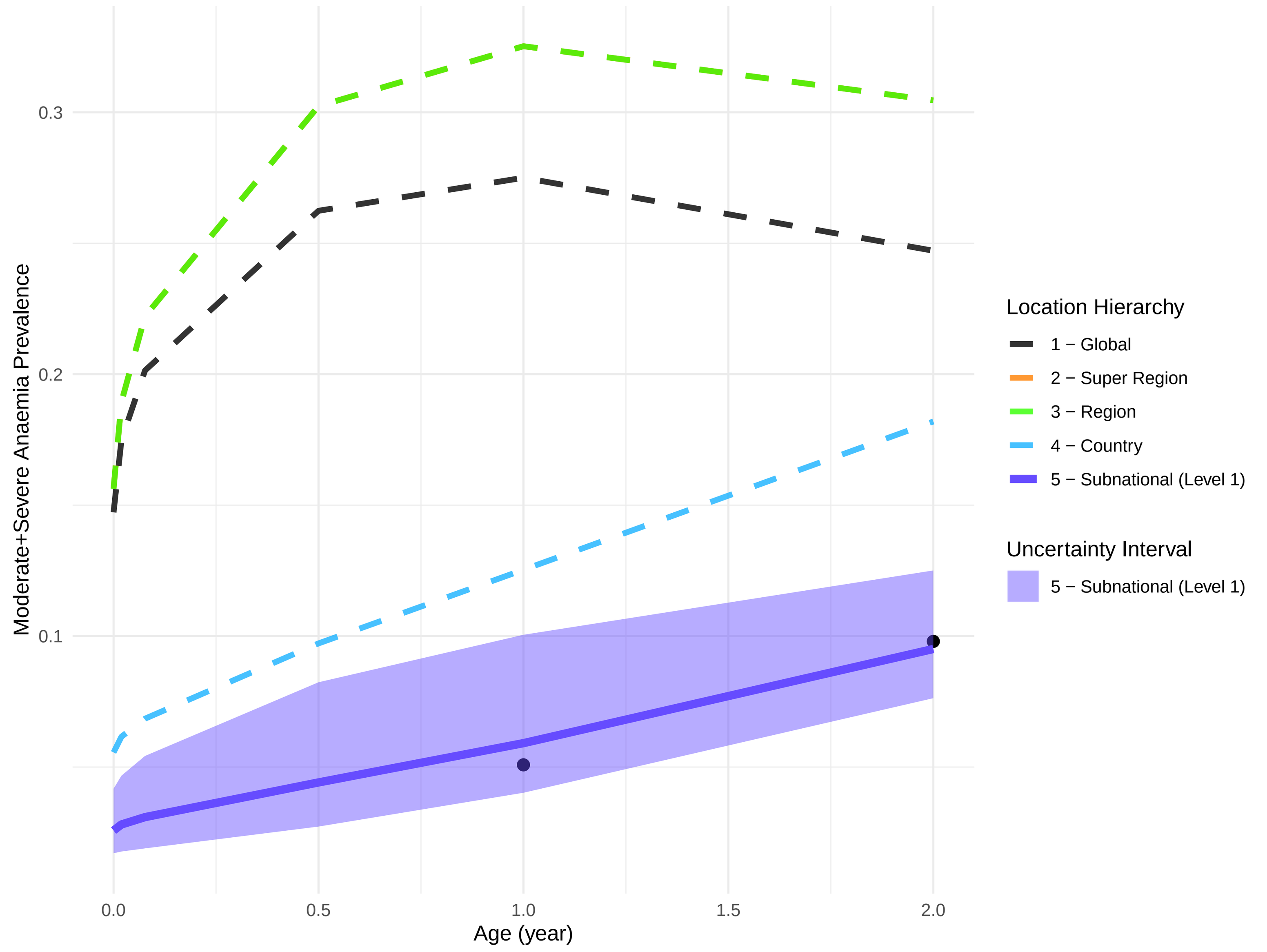

MR−BRT Age/Sex Fits − Non−pregnant Females <2 Years Old − Moderate+Severe Anaemia Prevalence − Punjab
WHO−2001 Adjustment Method

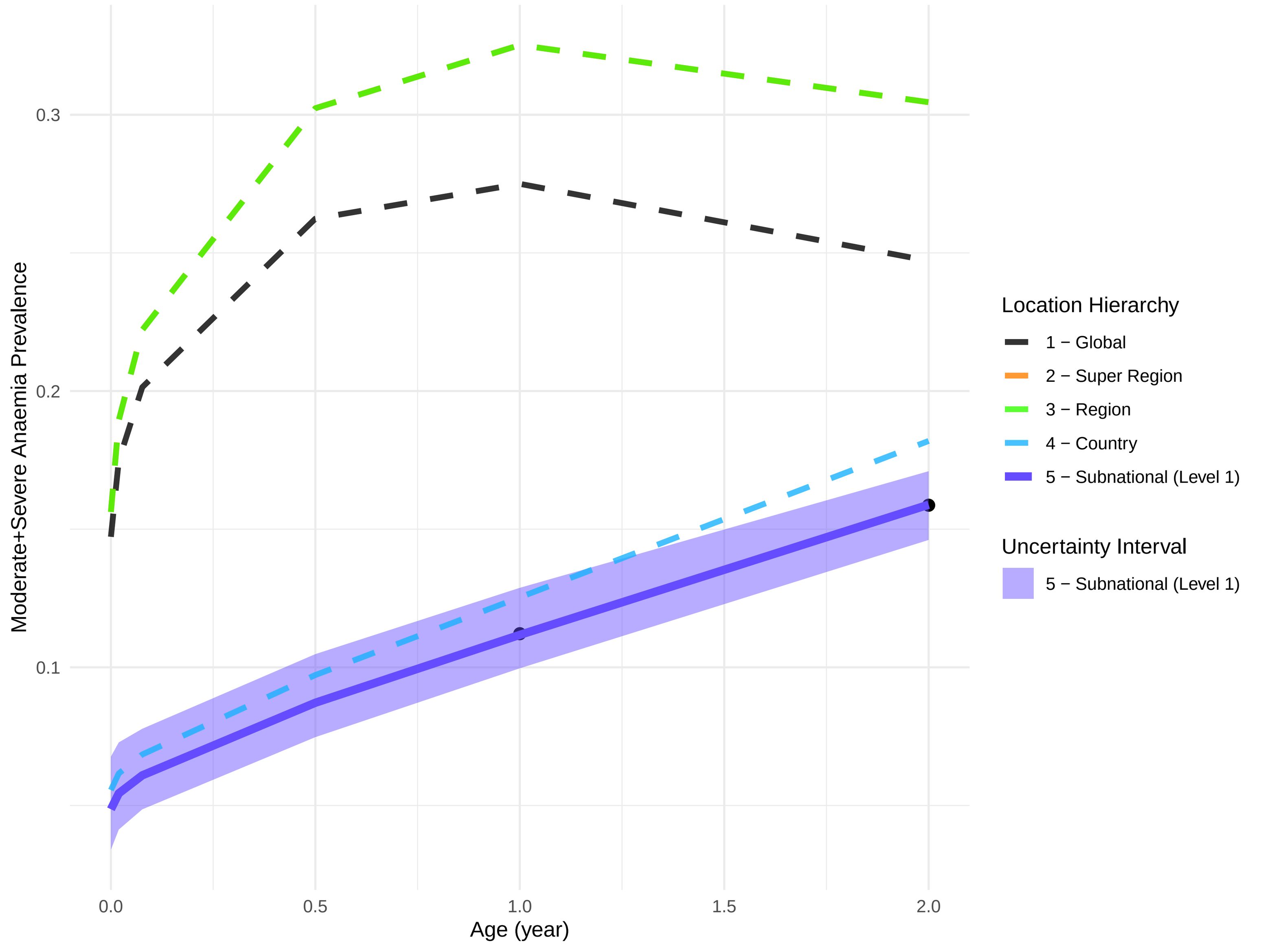

MR−BRT Age/Sex Fits − Non−pregnant Females <2 Years Old − Moderate+Severe Anaemia Prevalence − Sindh
WHO−2001 Adjustment Method

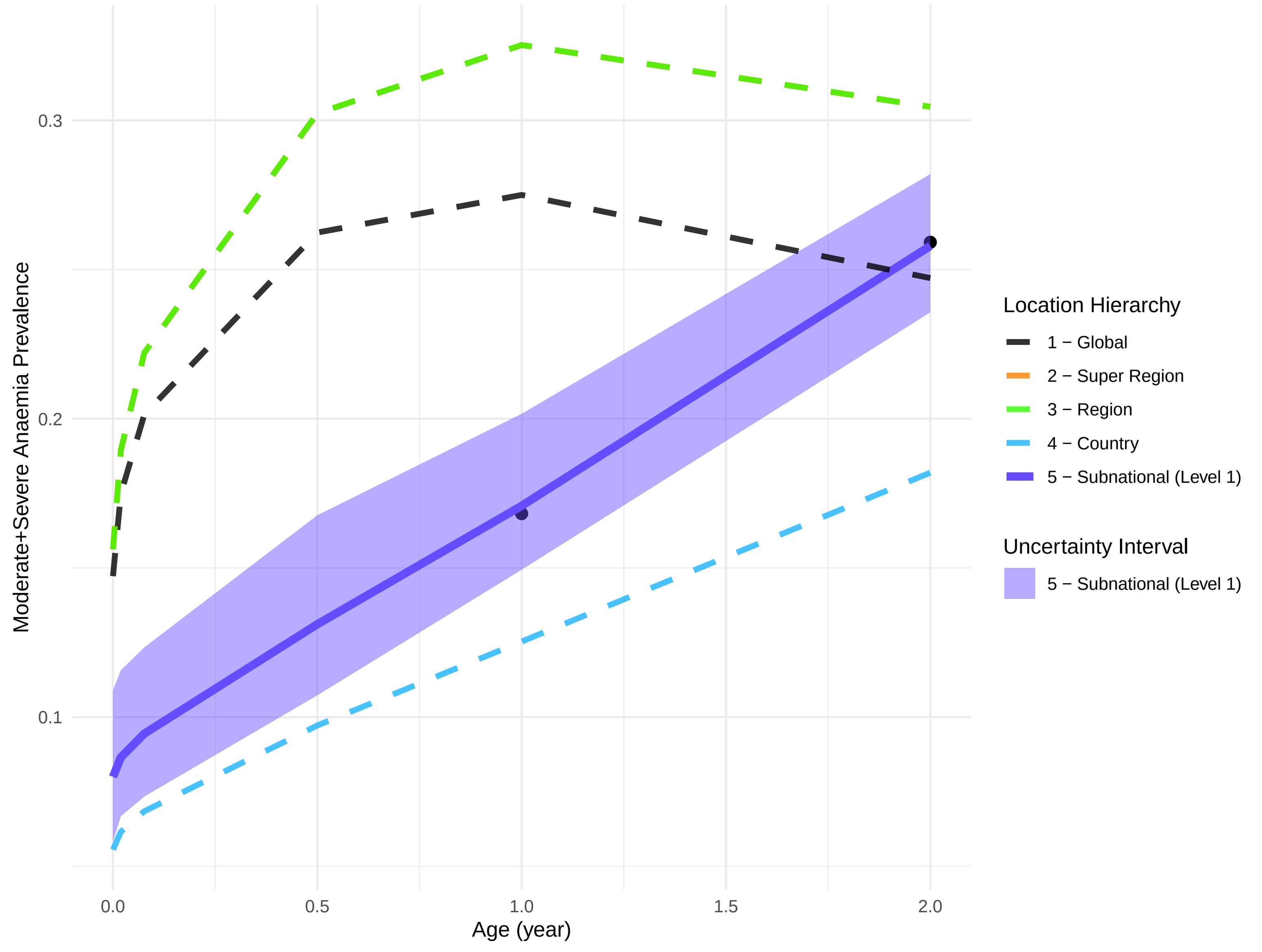

MR−BRT Age/Sex Fits − Non−pregnant Females <2 Years Old − Moderate+Severe Anaemia Prevalence − Southeast Asia, East Asia, and Oceania
WHO−2001 Adjustment Method

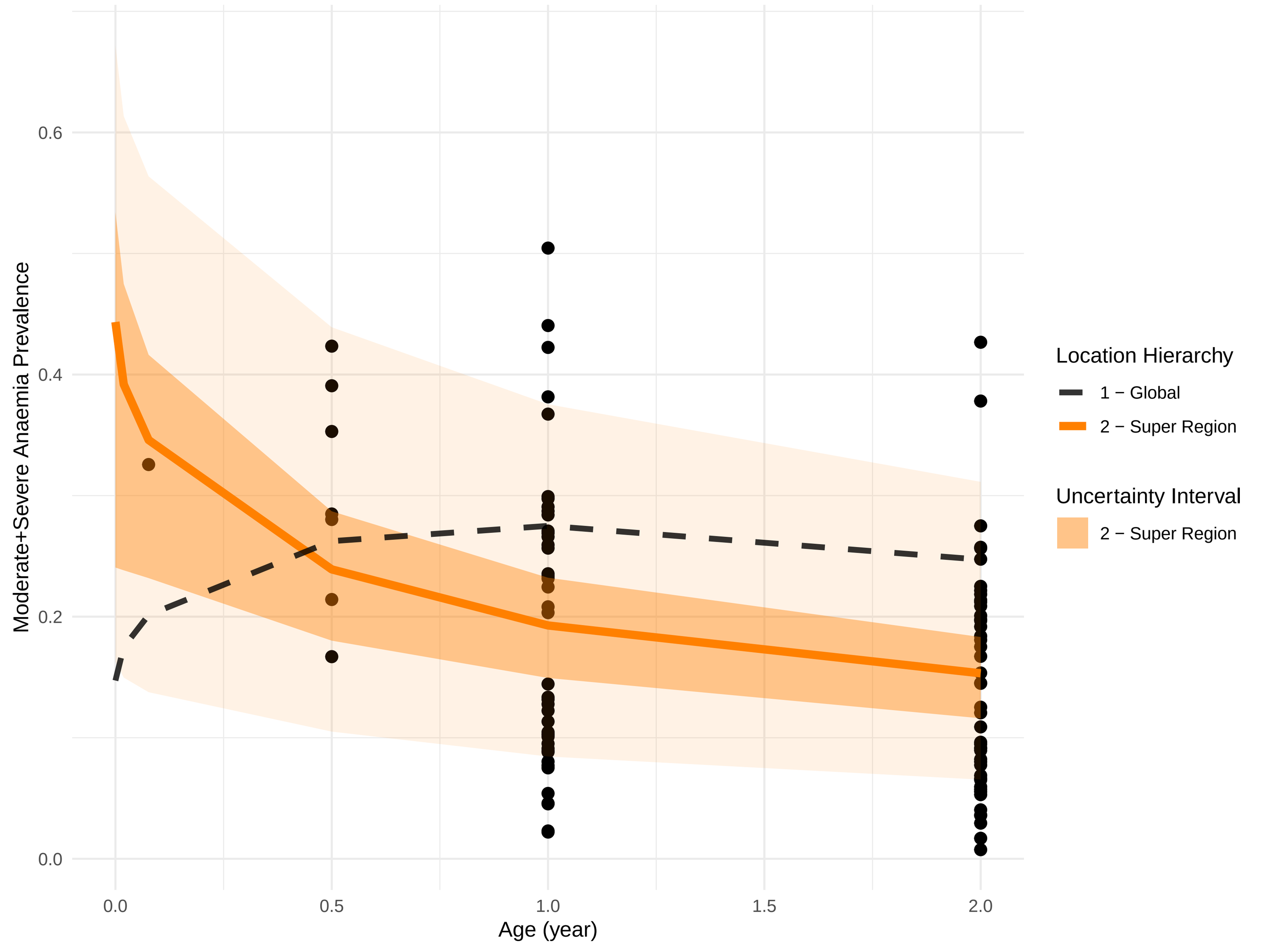

MR−BRT Age/Sex Fits − Non−pregnant Females <2 Years Old − Moderate+Severe Anaemia Prevalence − Southeast Asia
WHO−2001 Adjustment Method

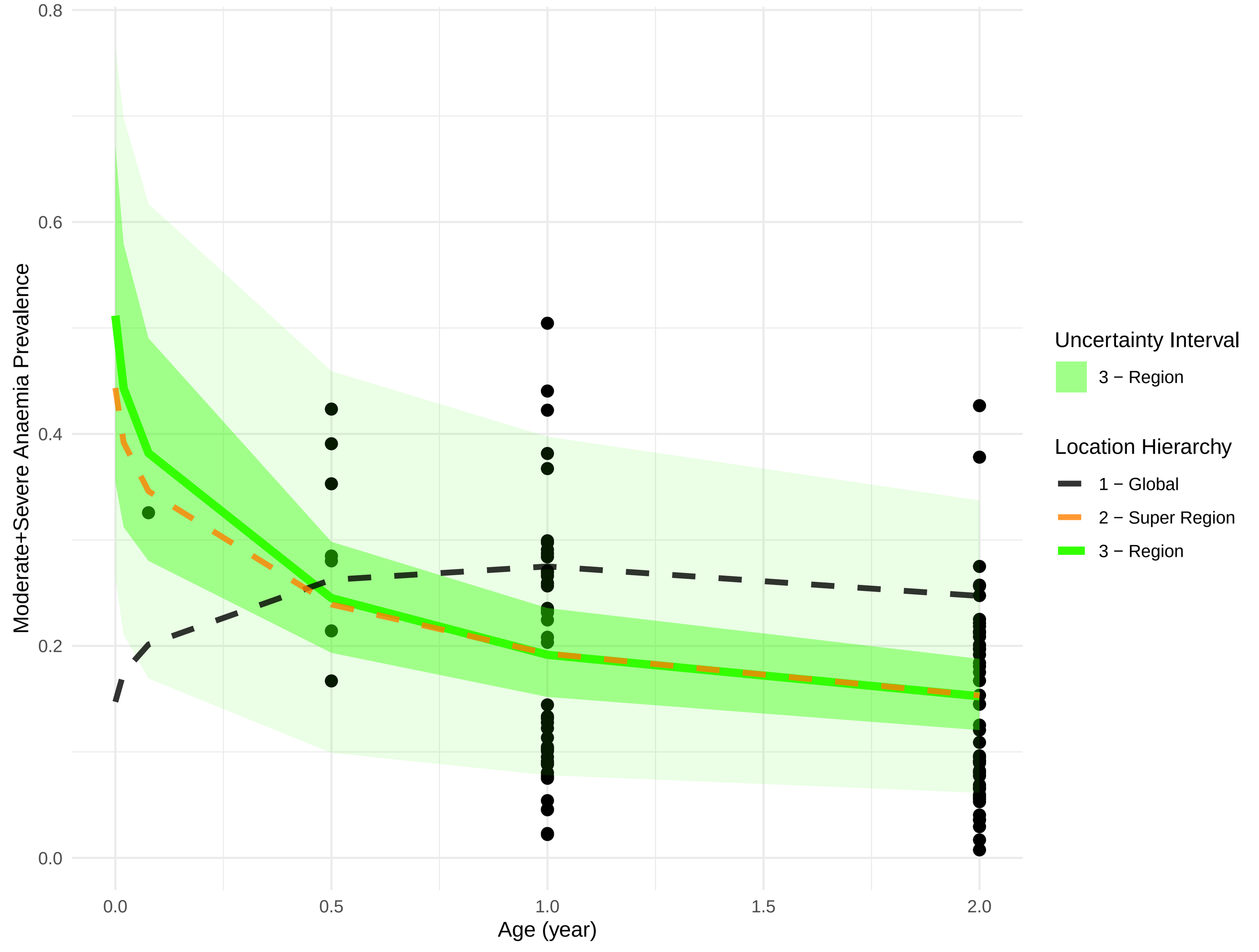

MR−BRT Age/Sex Fits − Non−pregnant Females <2 Years Old − Moderate+Severe Anaemia Prevalence − Cambodia
WHO−2001 Adjustment Method

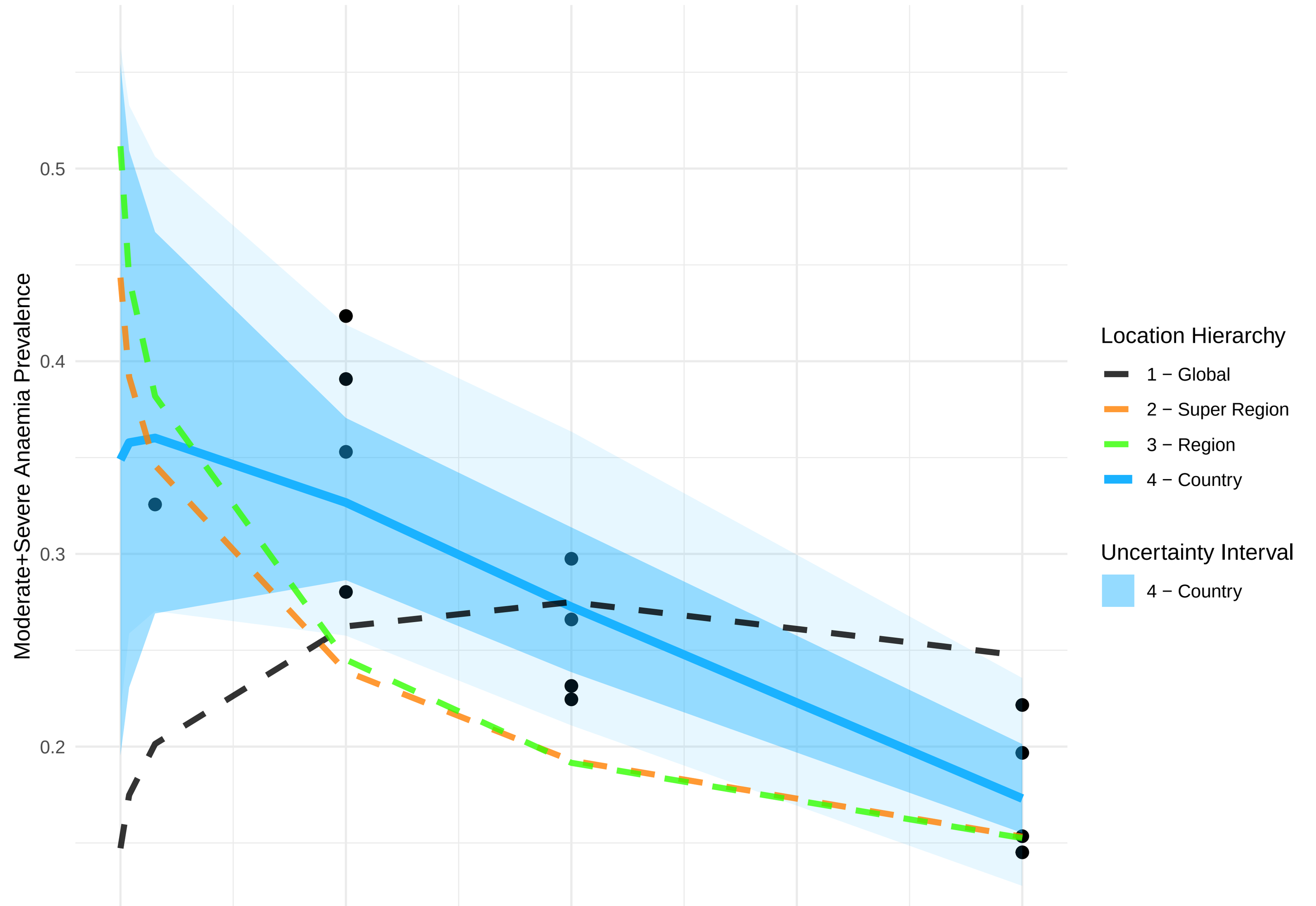

MR−BRT Age/Sex Fits − Non−pregnant Females <2 Years Old − Moderate+Severe Anaemia Prevalence − Indonesia
WHO−2001 Adjustment Method

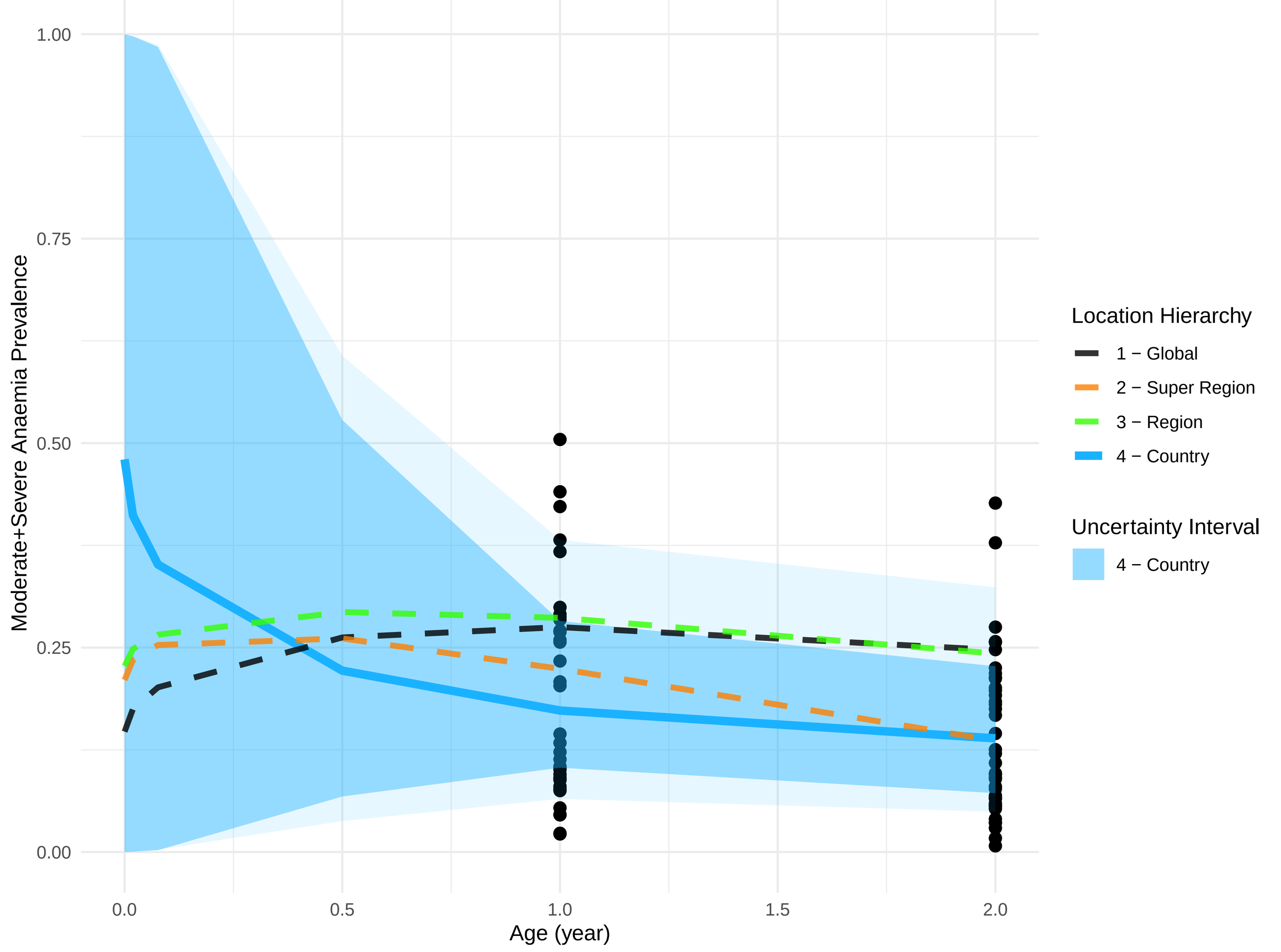

MR−BRT Age/Sex Fits − Non−pregnant Females <2 Years Old − Moderate+Severe Anaemia Prevalence − Bali
WHO−2001 Adjustment Method

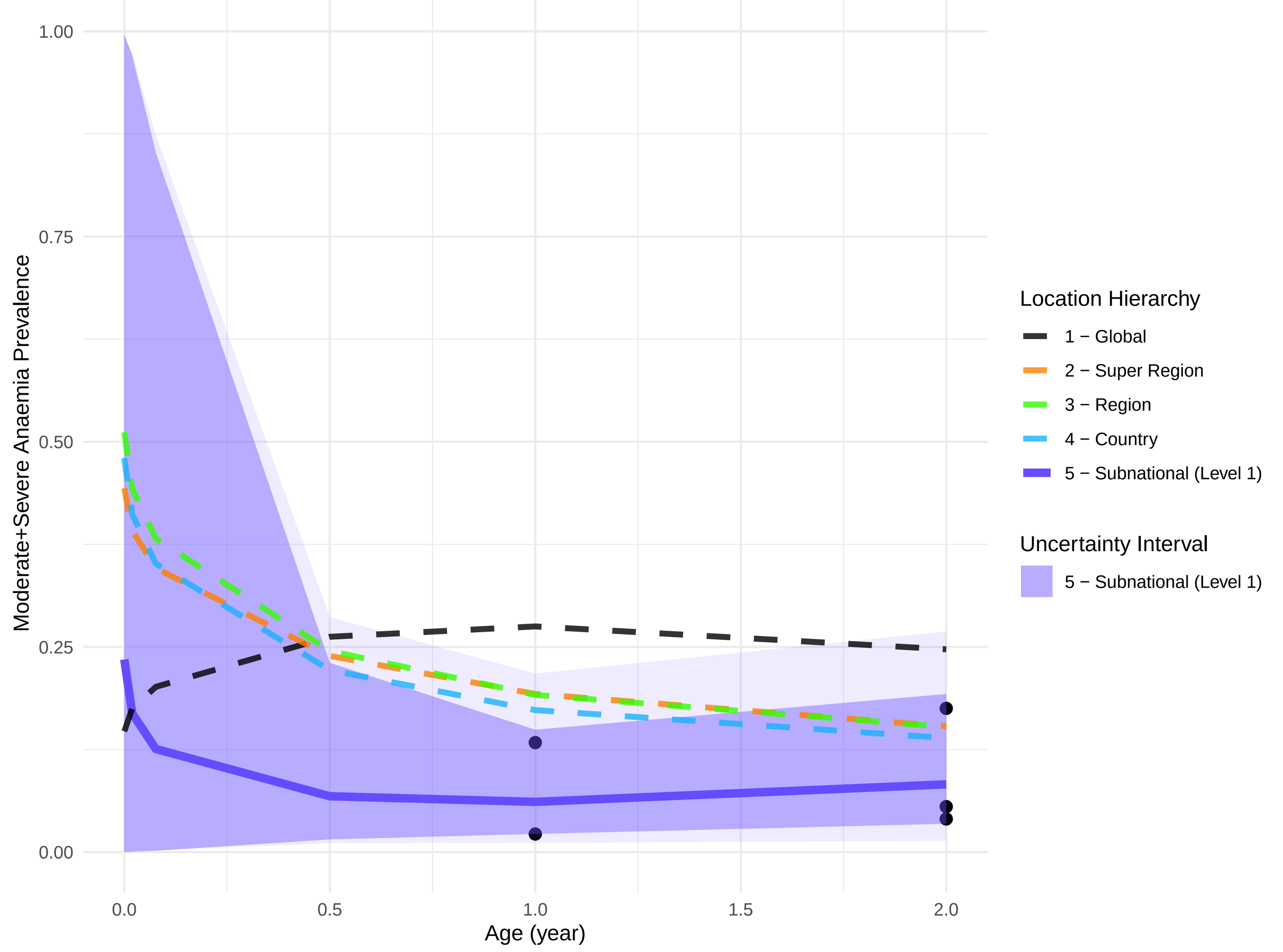

MR−BRT Age/Sex Fits − Non−pregnant Females <2 Years Old − Moderate+Severe Anaemia Prevalence − Banten
WHO−2001 Adjustment Method

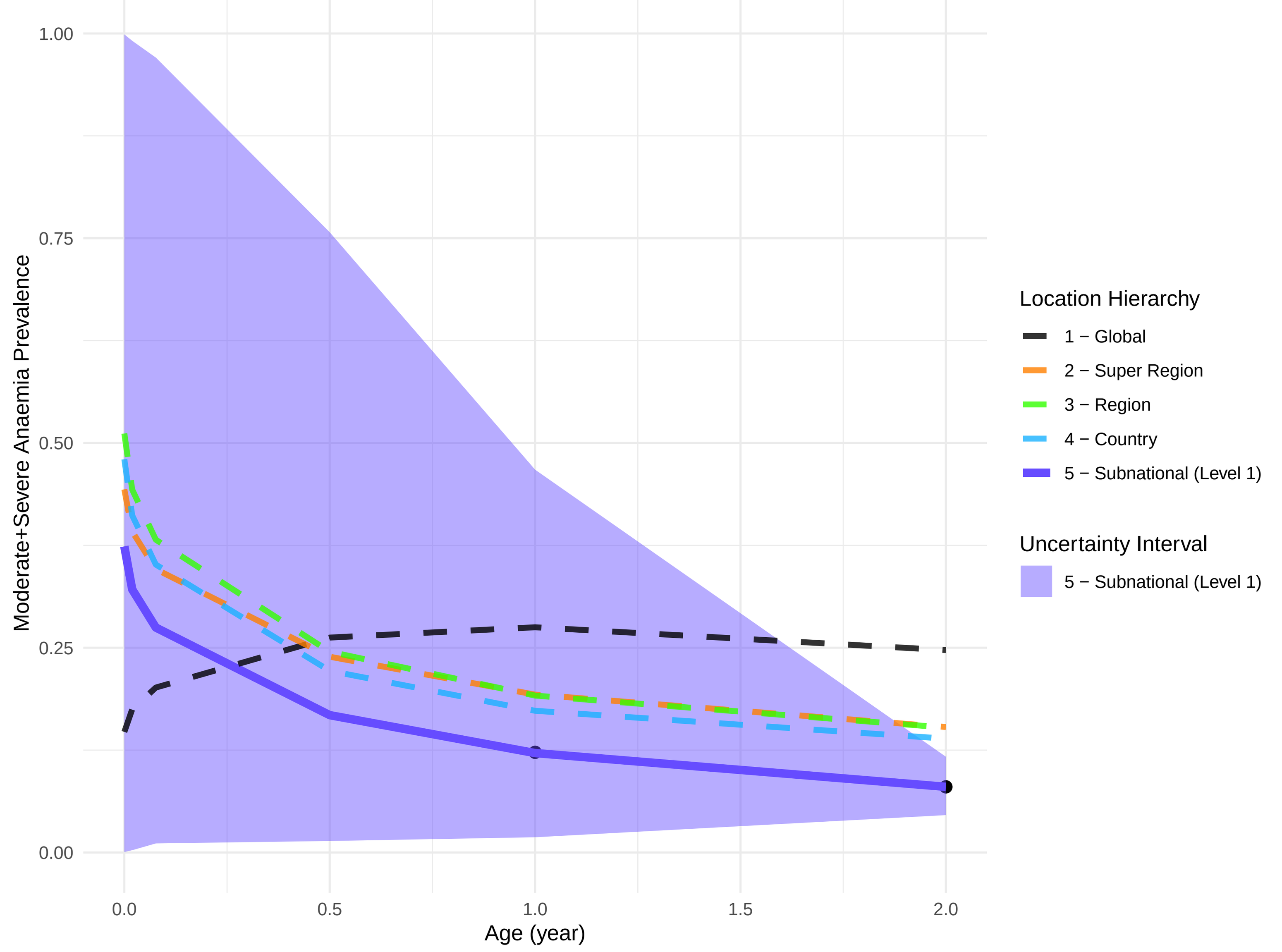

MR−BRT Age/Sex Fits − Non−pregnant Females <2 Years Old − Moderate+Severe Anaemia Prevalence − Jakarta
WHO−2001 Adjustment Method

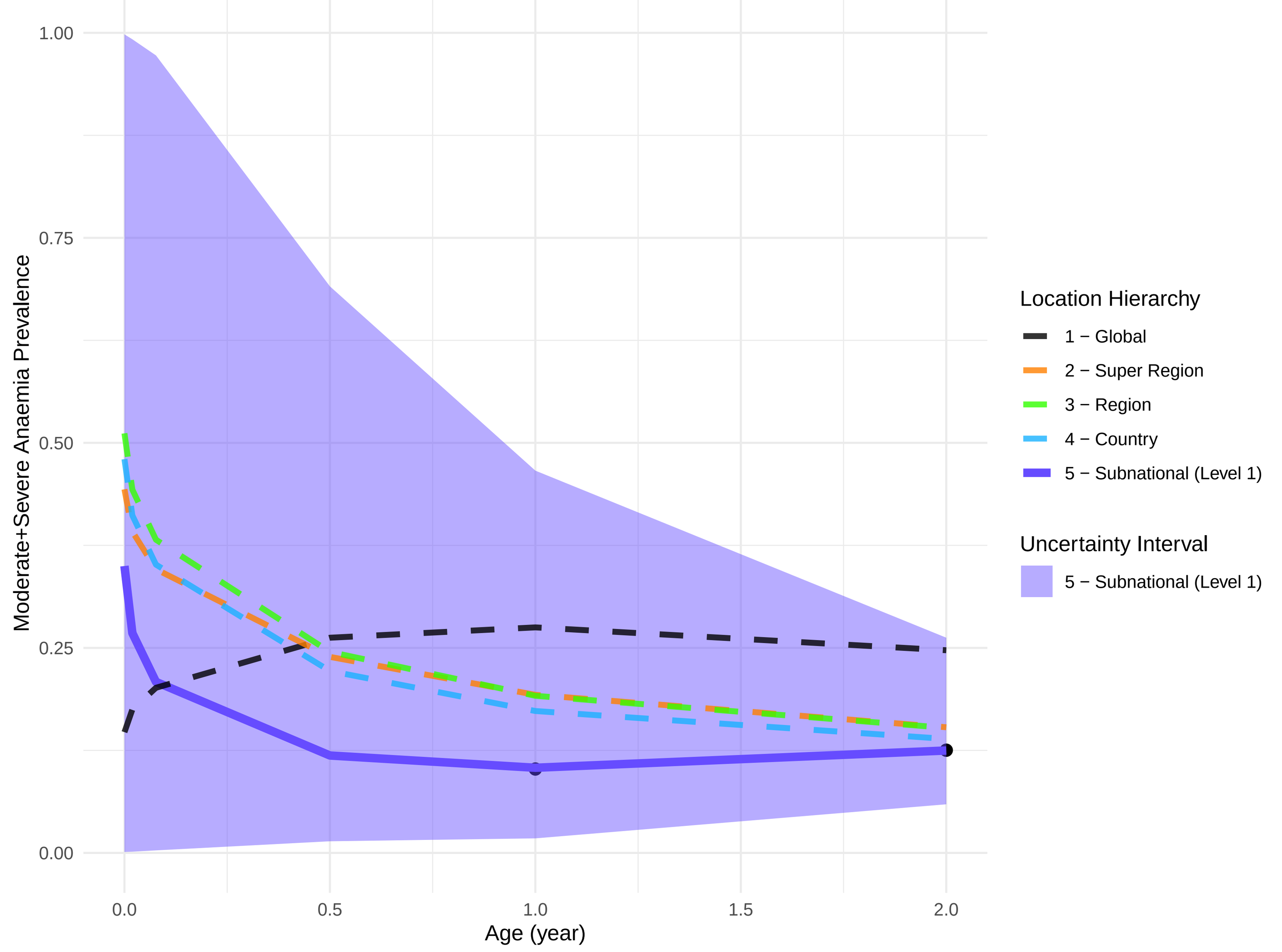

MR−BRT Age/Sex Fits − Non−pregnant Females <2 Years Old − Moderate+Severe Anaemia Prevalence − West Java
WHO−2001 Adjustment Method

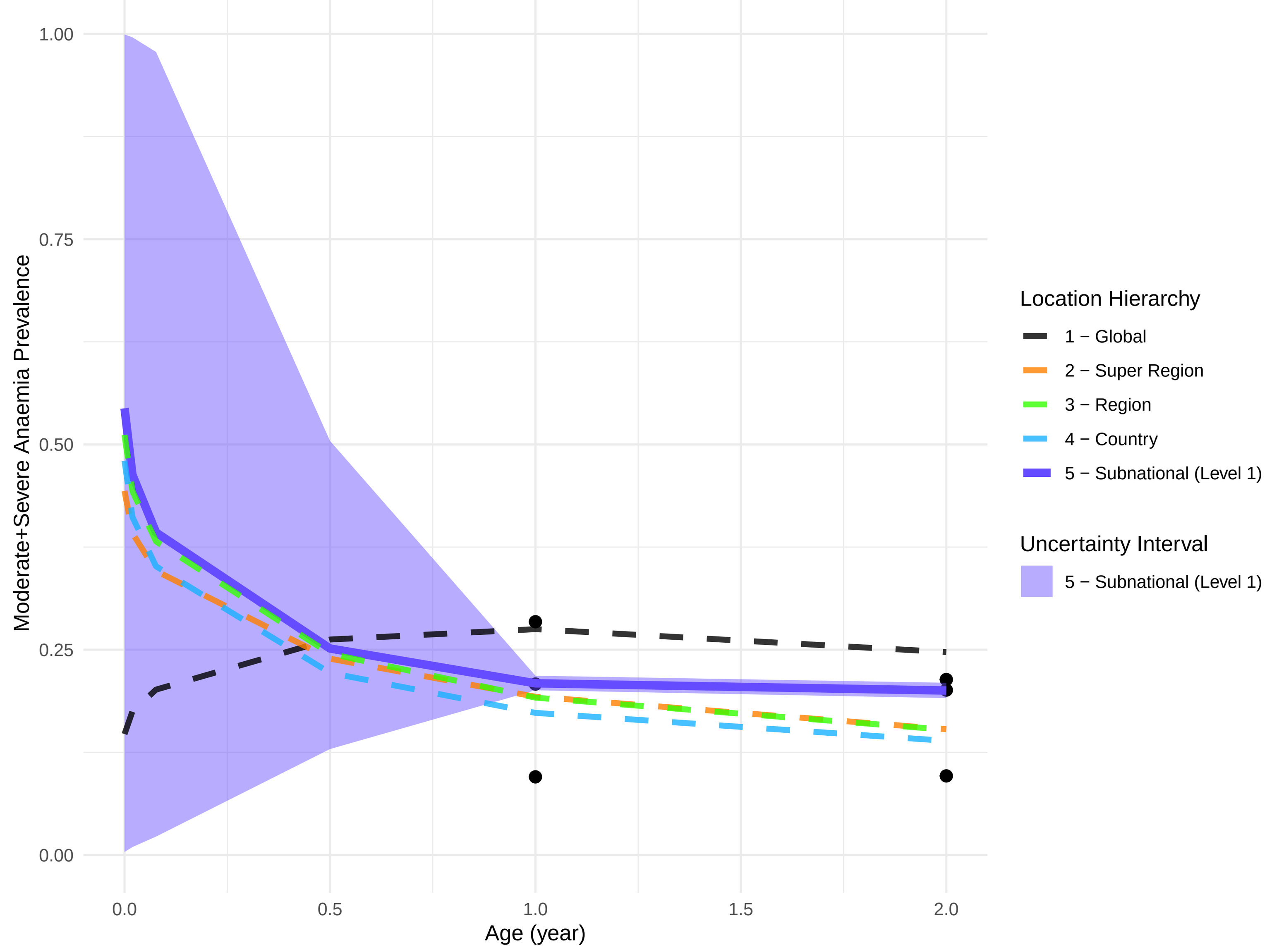

MR−BRT Age/Sex Fits − Non−pregnant Females <2 Years Old − Moderate+Severe Anaemia Prevalence − Central Java
WHO−2001 Adjustment Method

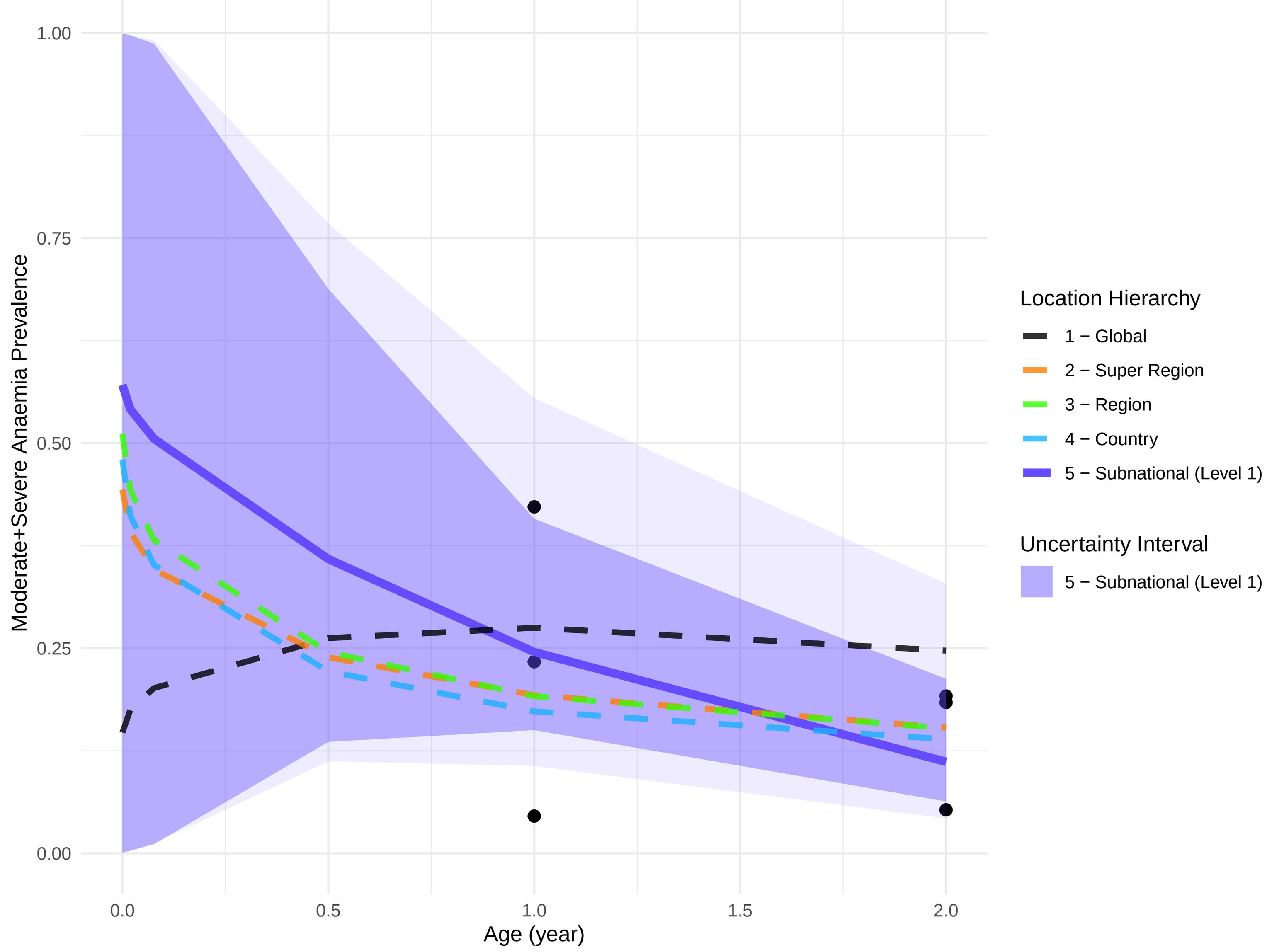

MR−BRT Age/Sex Fits − Non−pregnant Females <2 Years Old − Moderate+Severe Anaemia Prevalence − East Java
WHO−2001 Adjustment Method

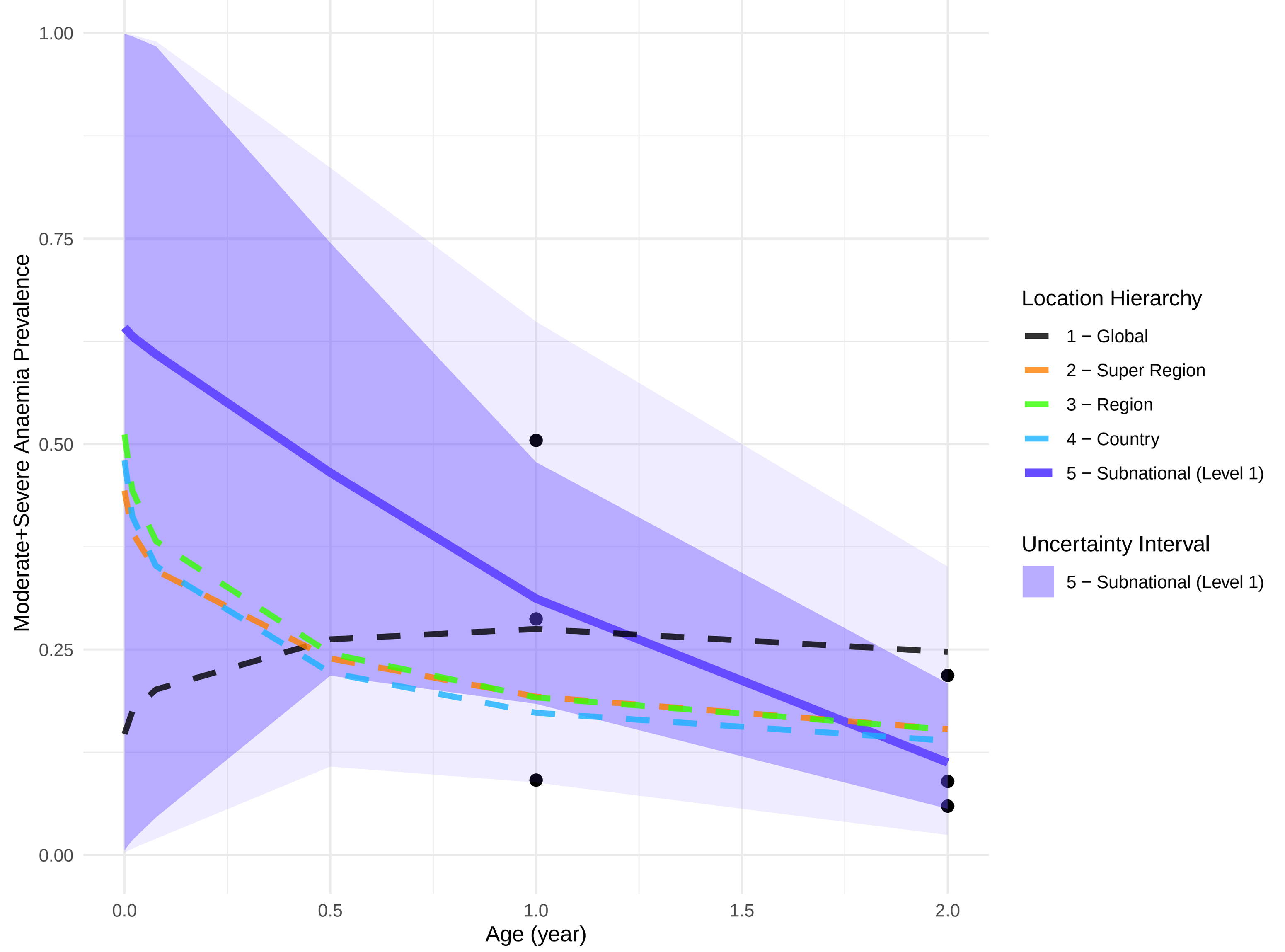

MR−BRT Age/Sex Fits − Non−pregnant Females <2 Years Old − Moderate+Severe Anaemia Prevalence − South Kalimantan
WHO−2001 Adjustment Method

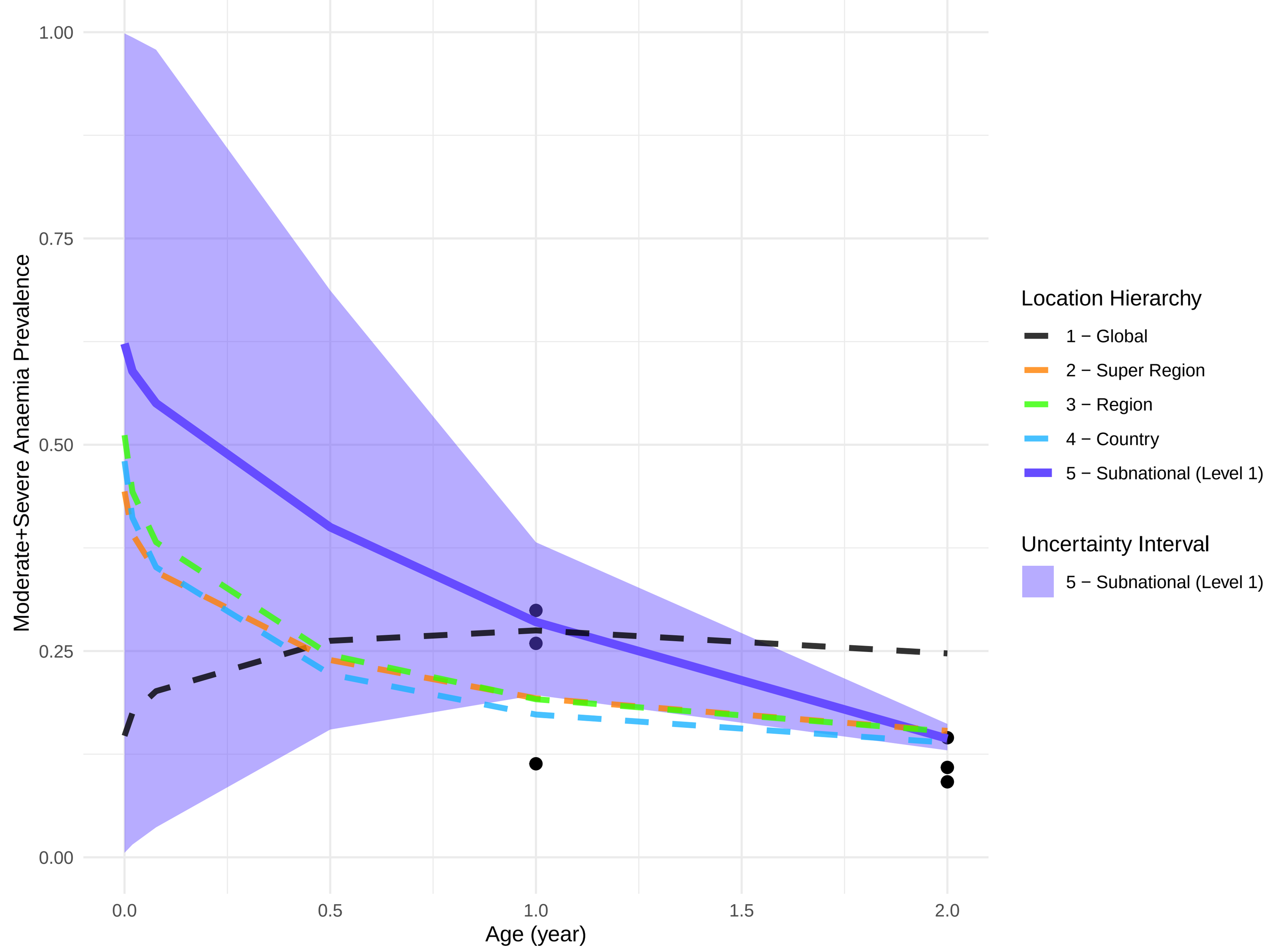

MR−BRT Age/Sex Fits − Non−pregnant Females <2 Years Old − Moderate+Severe Anaemia Prevalence − Lampung
WHO−2001 Adjustment Method

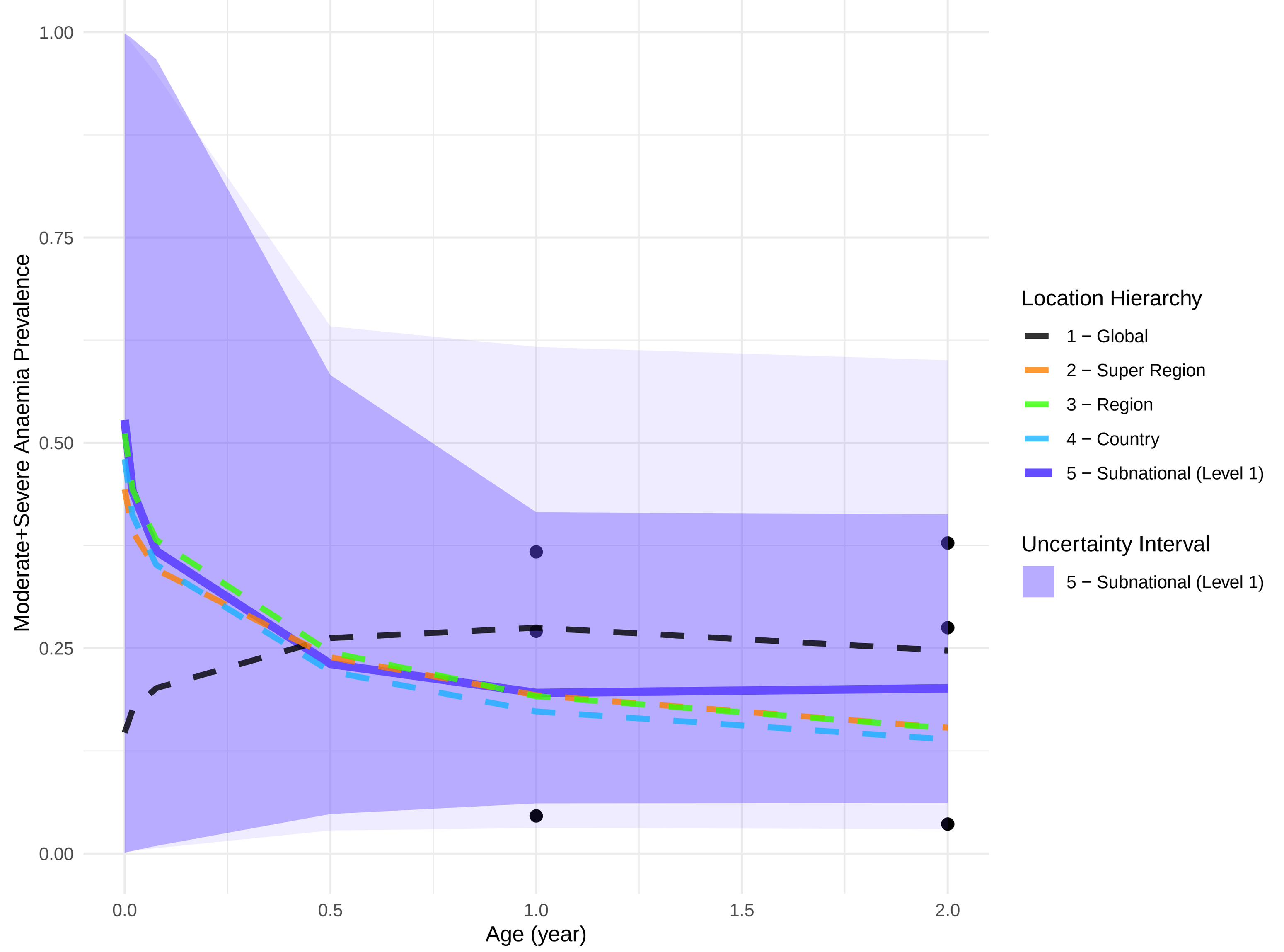

MR−BRT Age/Sex Fits − Non−pregnant Females <2 Years Old − Moderate+Severe Anaemia Prevalence − West Nusa Tenggara
WHO−2001 Adjustment Method

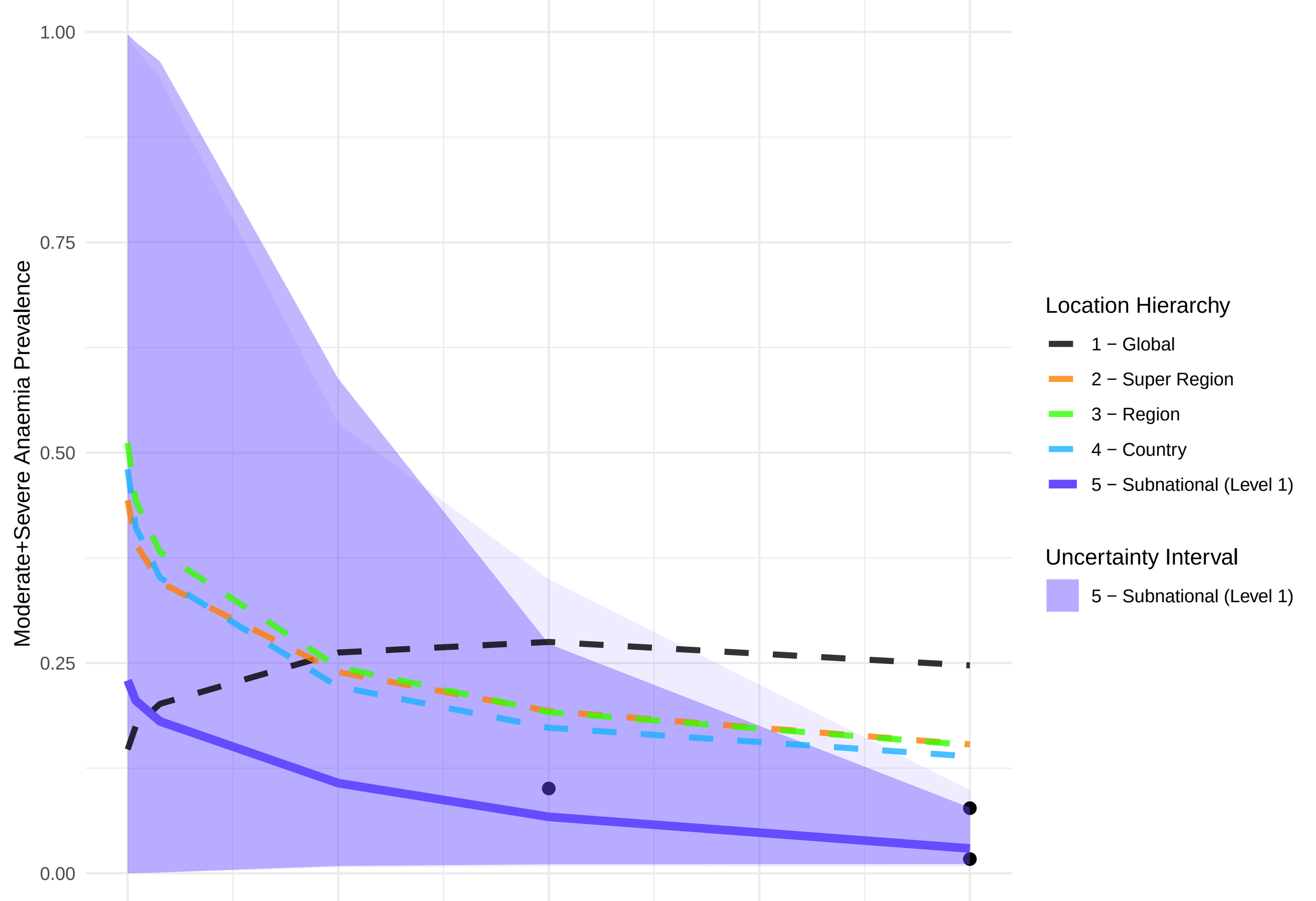

MR−BRT Age/Sex Fits − Non−pregnant Females <2 Years Old − Moderate+Severe Anaemia Prevalence − South Sulawesi
WHO−2001 Adjustment Method

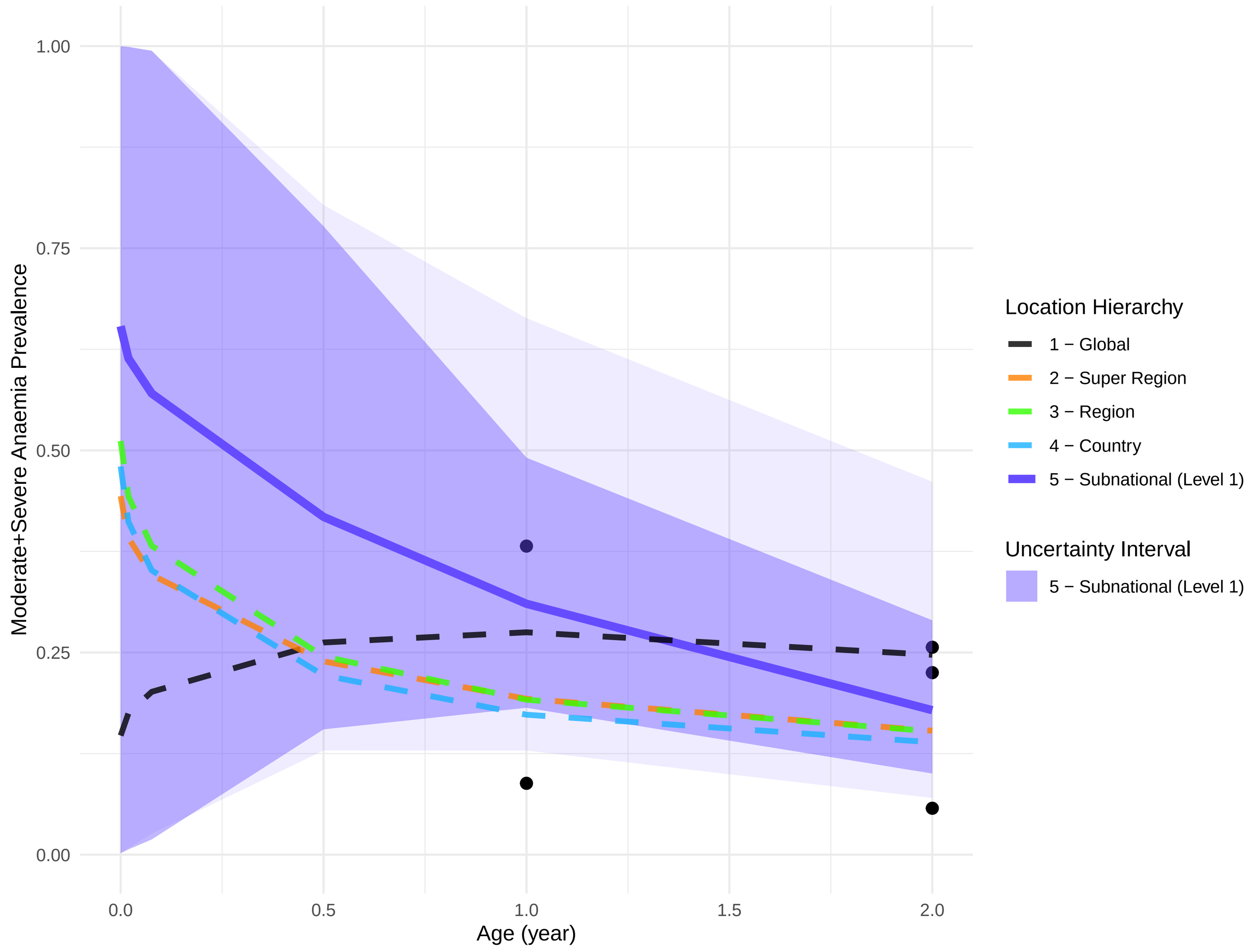

MR−BRT Age/Sex Fits − Non−pregnant Females <2 Years Old − Moderate+Severe Anaemia Prevalence − West Sumatra
WHO−2001 Adjustment Method

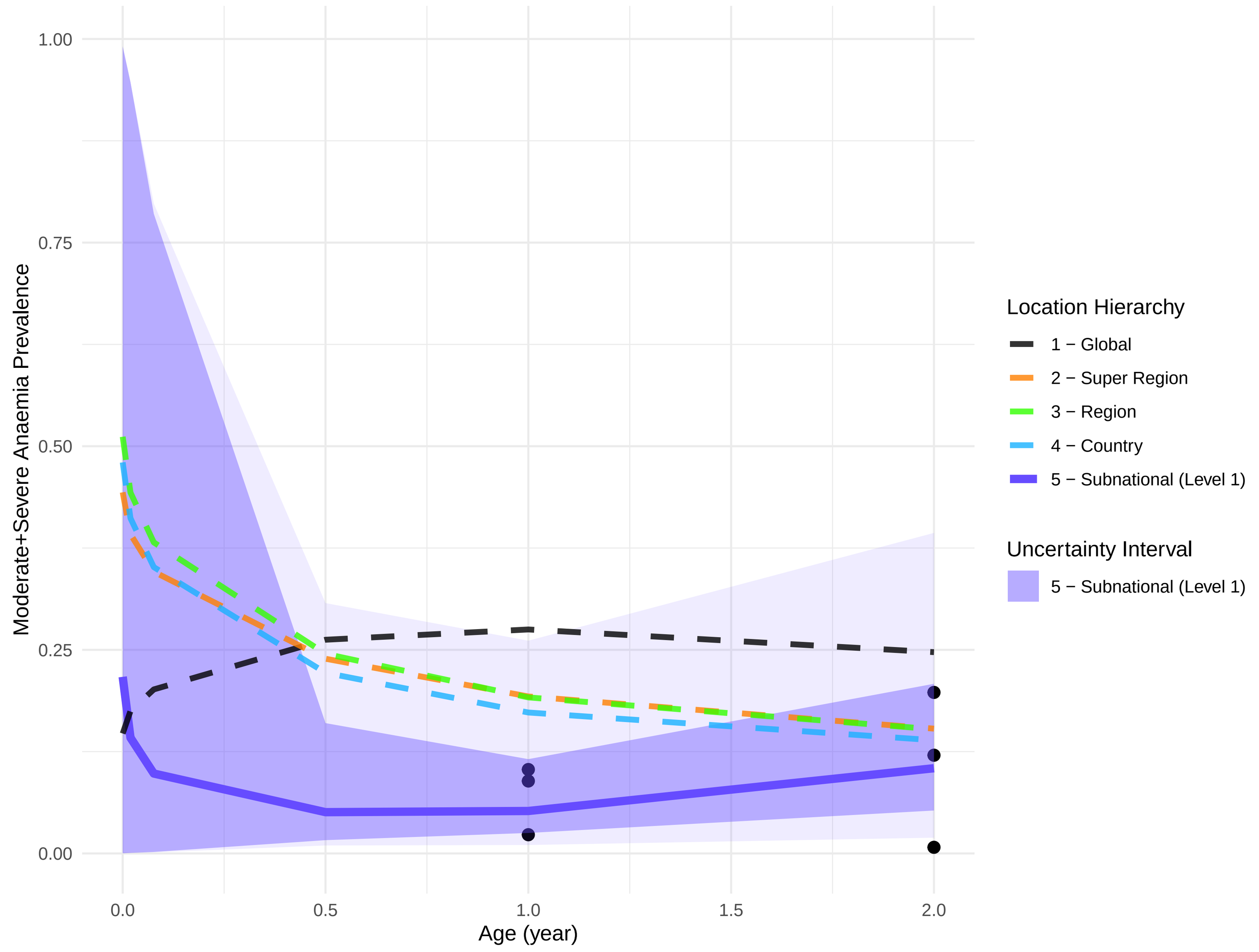

MR−BRT Age/Sex Fits − Non−pregnant Females <2 Years Old − Moderate+Severe Anaemia Prevalence − South Sumatra
WHO−2001 Adjustment Method

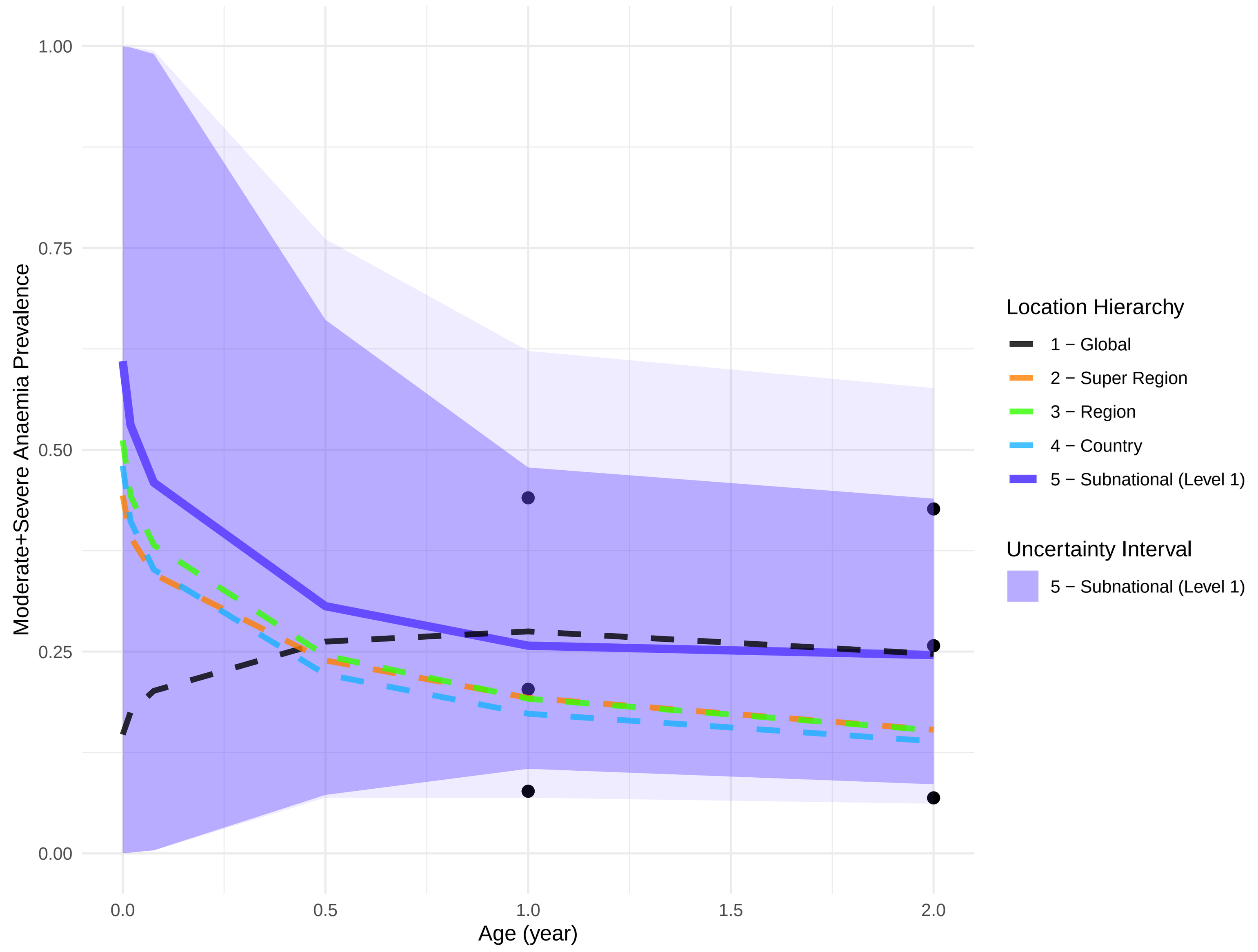

MR−BRT Age/Sex Fits − Non−pregnant Females <2 Years Old − Moderate+Severe Anaemia Prevalence − North Sumatra
WHO−2001 Adjustment Method

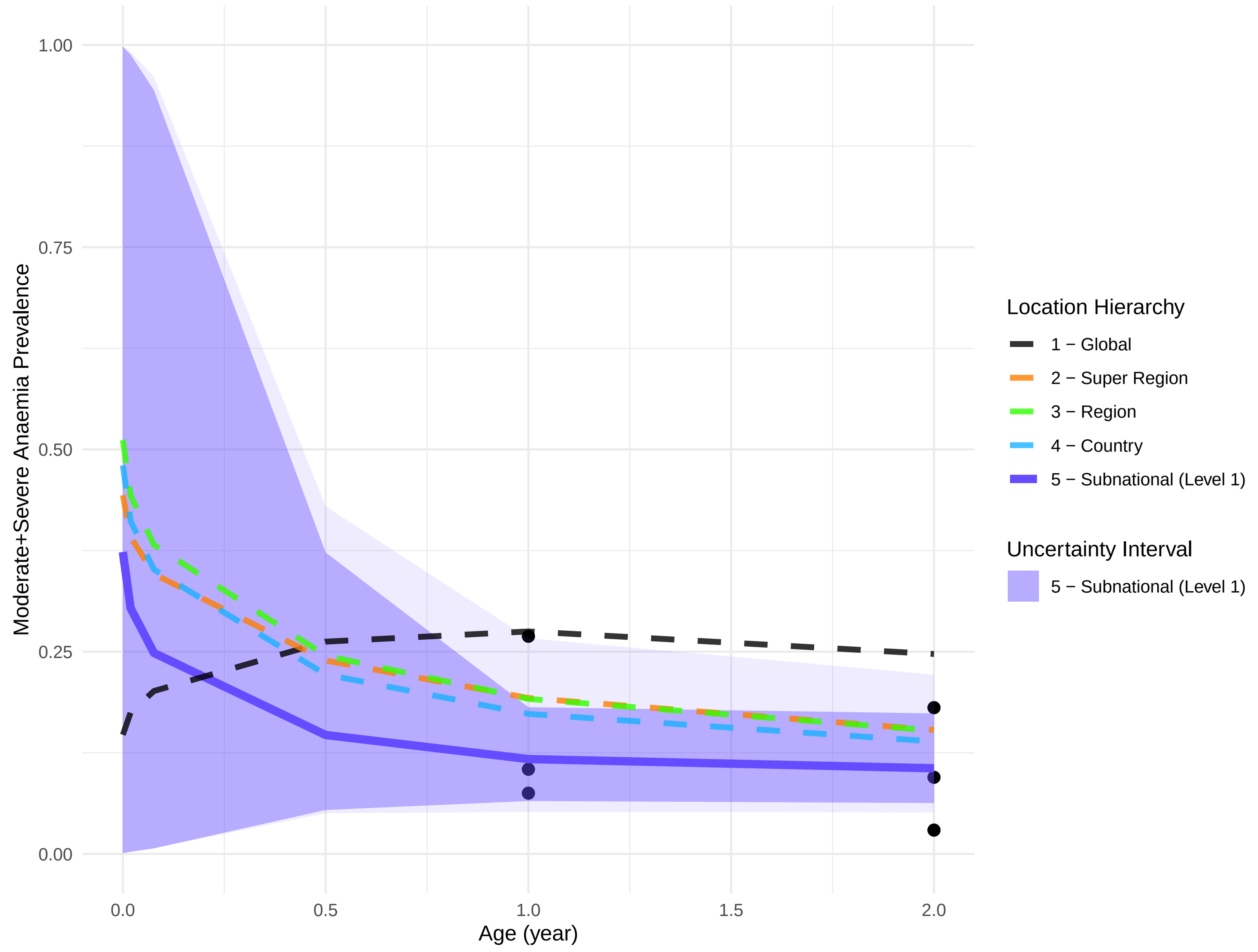

MR−BRT Age/Sex Fits − Non−pregnant Females <2 Years Old − Moderate+Severe Anaemia Prevalence − Yogyakarta
WHO−2001 Adjustment Method

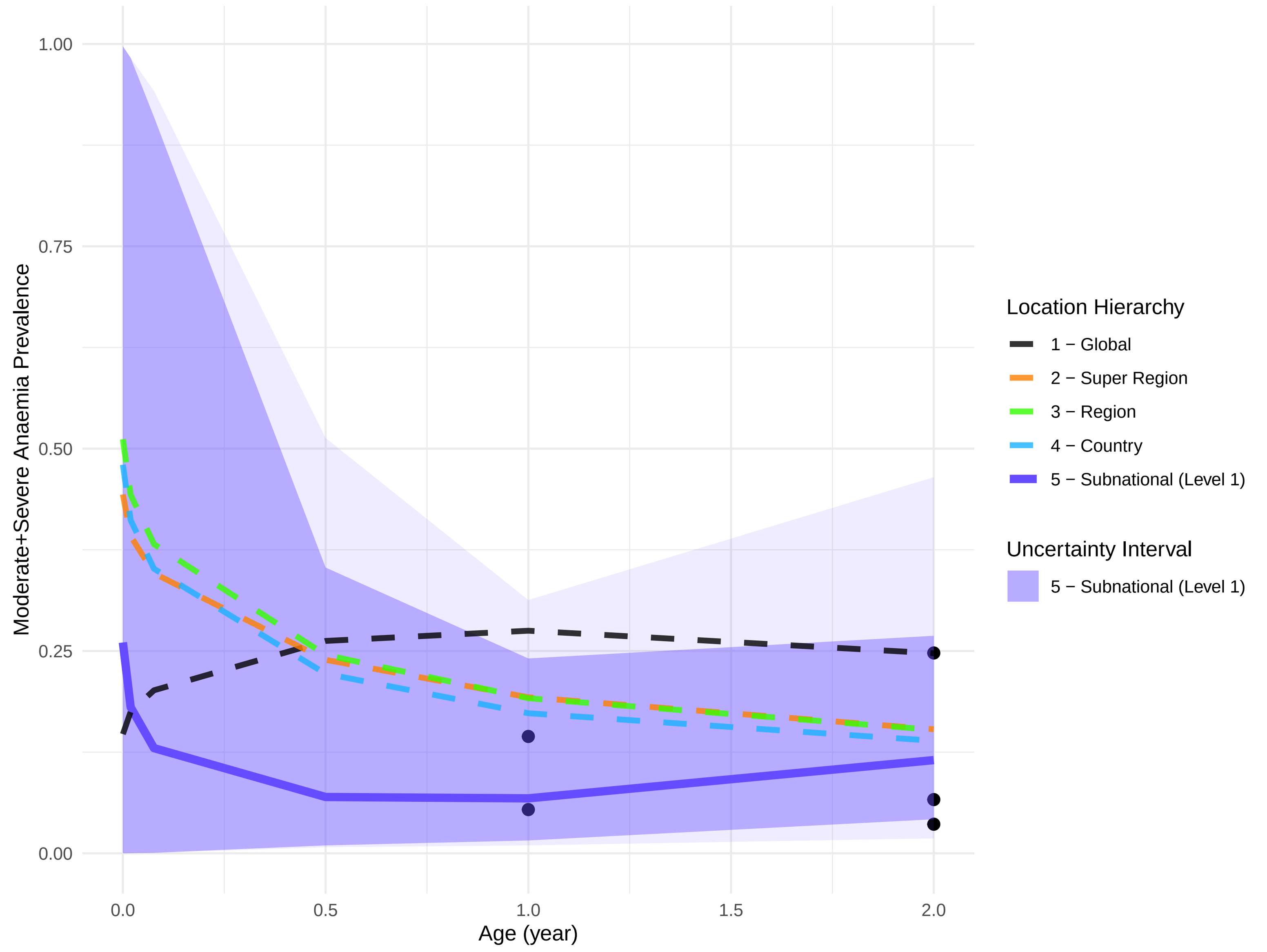

MR−BRT Age/Sex Fits − Non−pregnant Females <2 Years Old − Moderate+Severe Anaemia Prevalence − Myanmar
WHO−2001 Adjustment Method

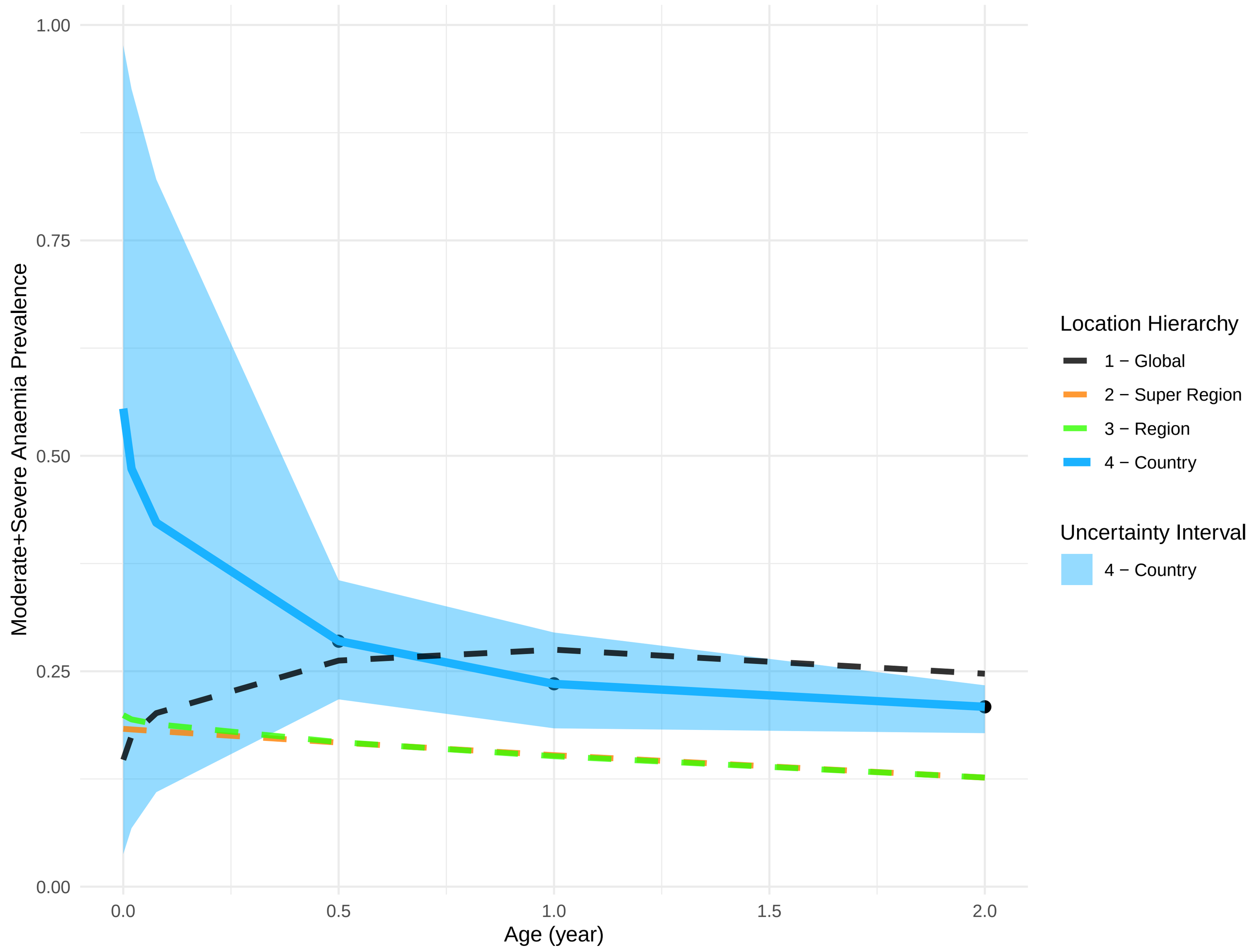

MR−BRT Age/Sex Fits − Non−pregnant Females <2 Years Old − Moderate+Severe Anaemia Prevalence − Timor−Leste
WHO−2001 Adjustment Method

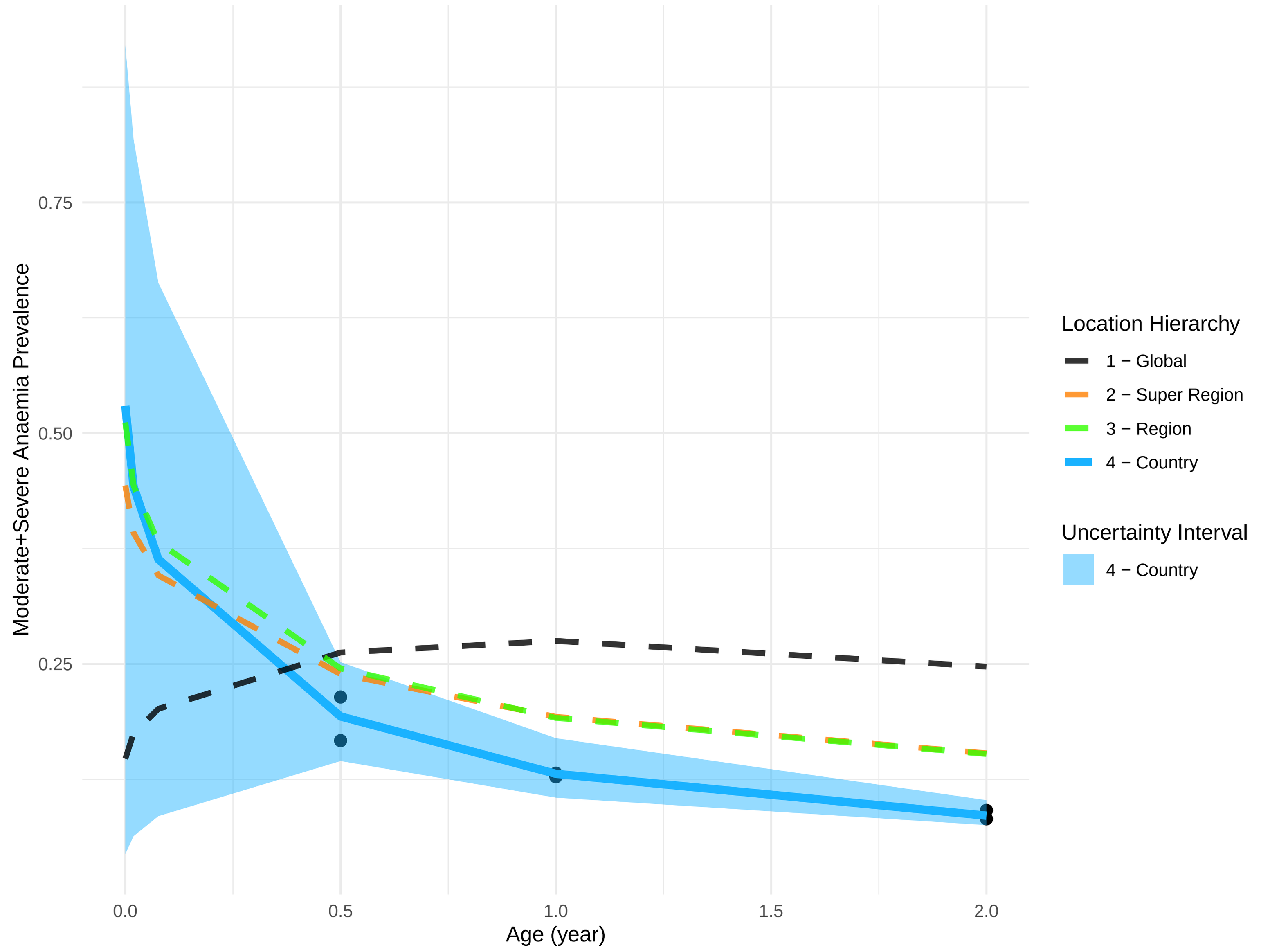

MR−BRT Age/Sex Fits − Non−pregnant Females <2 Years Old − Moderate+Severe Anaemia Prevalence − Sub−Saharan Africa
WHO−2001 Adjustment Method

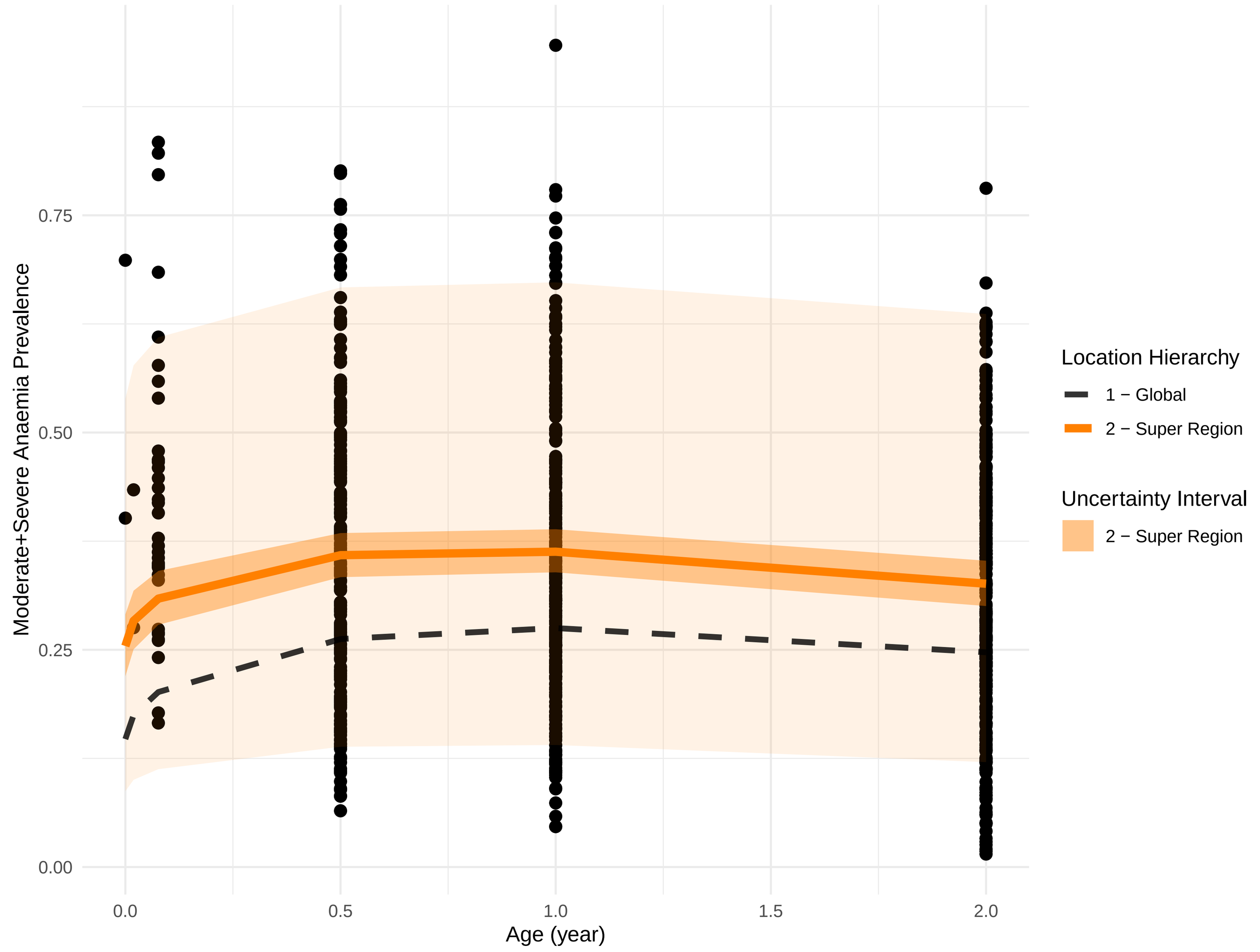

MR−BRT Age/Sex Fits − Non−pregnant Females <2 Years Old − Moderate+Severe Anaemia Prevalence − Central Sub−Saharan Africa
WHO−2001 Adjustment Method

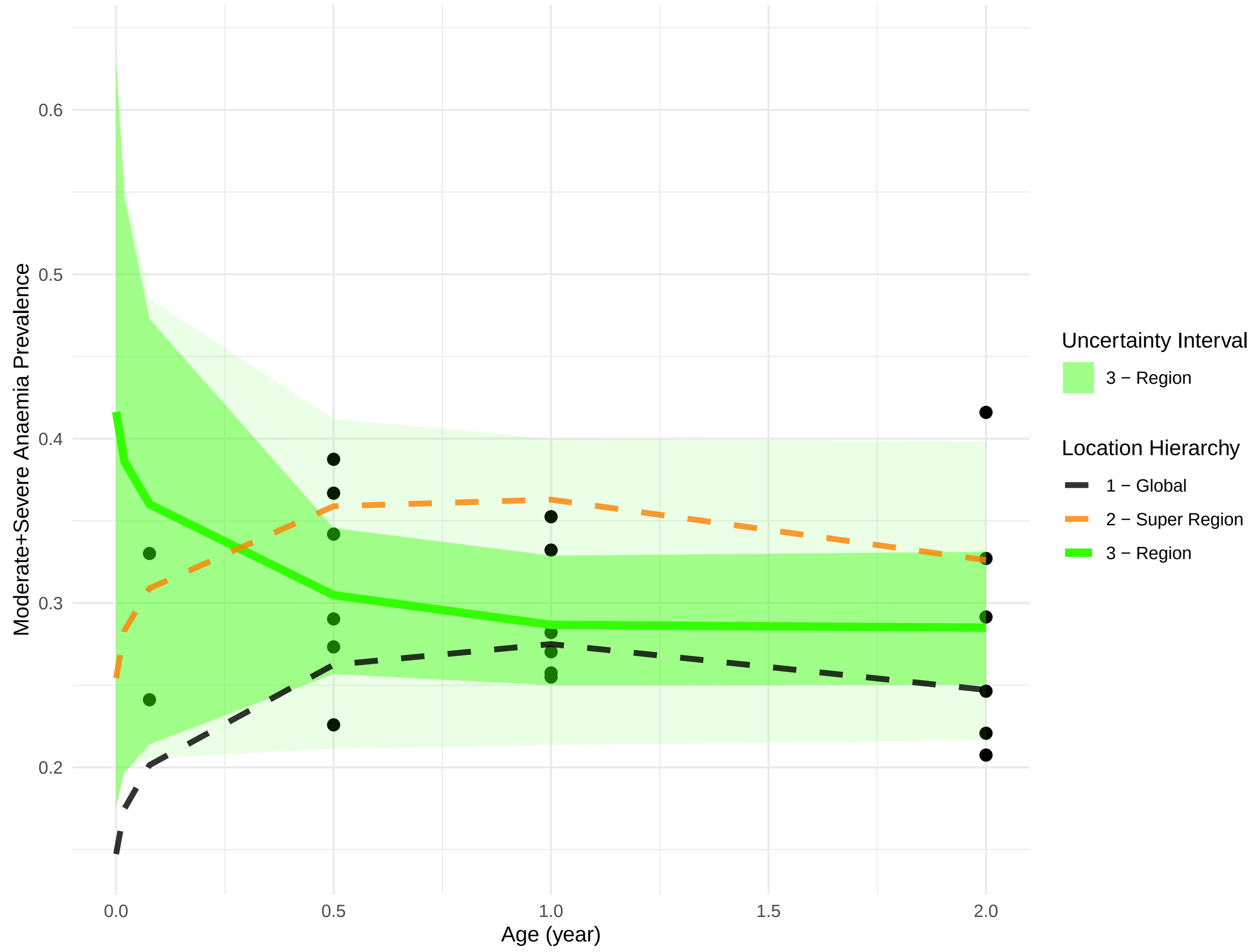

MR−BRT Age/Sex Fits − Non−pregnant Females <2 Years Old − Moderate+Severe Anaemia Prevalence − Angola
WHO−2001 Adjustment Method

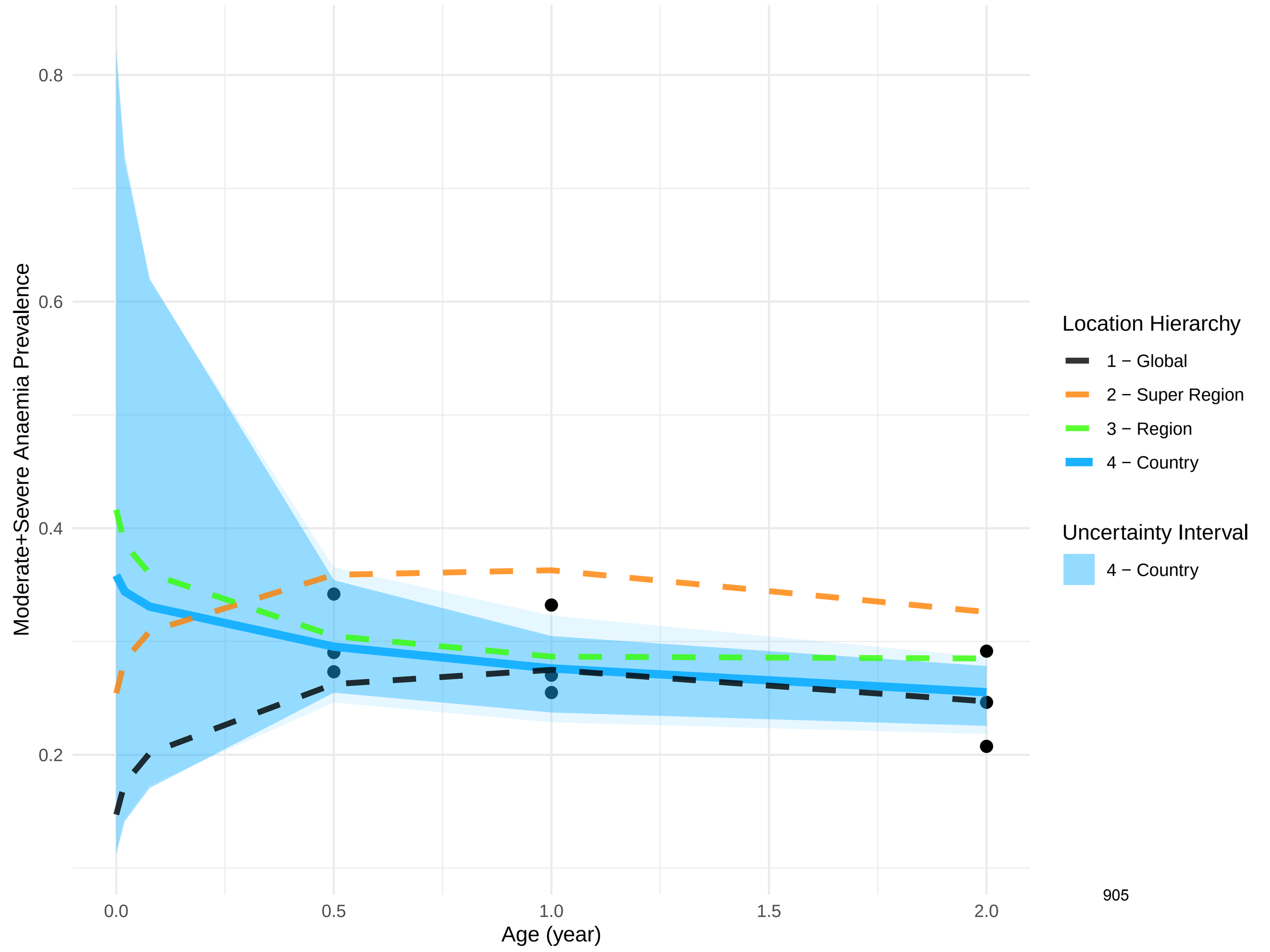

MR−BRT Age/Sex Fits − Non−pregnant Females <2 Years Old − Moderate+Severe Anaemia Prevalence − Democratic Republic of the Congo
WHO−2001 Adjustment Method

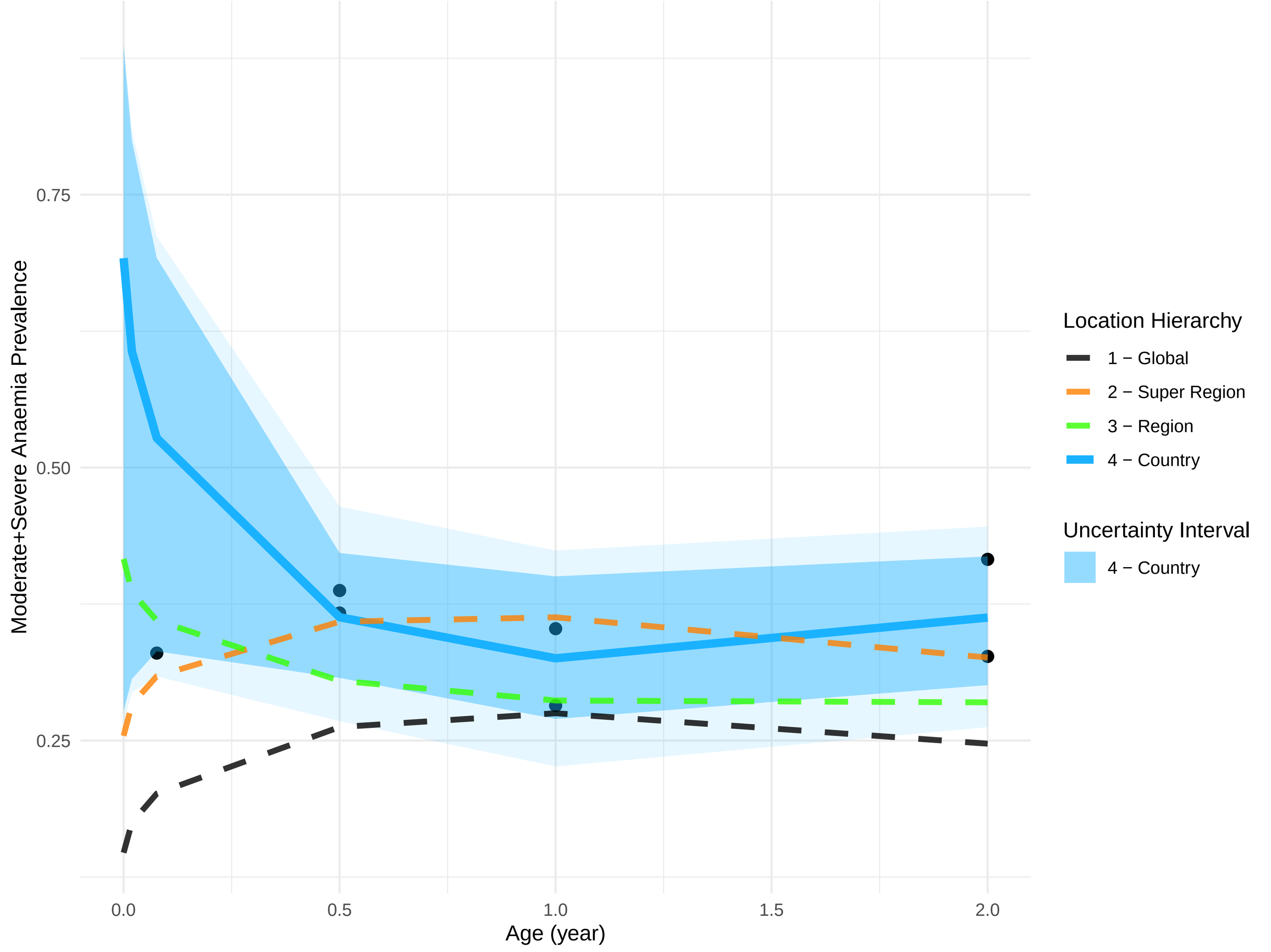

MR−BRT Age/Sex Fits − Non−pregnant Females <2 Years Old − Moderate+Severe Anaemia Prevalence − Gabon
WHO−2001 Adjustment Method

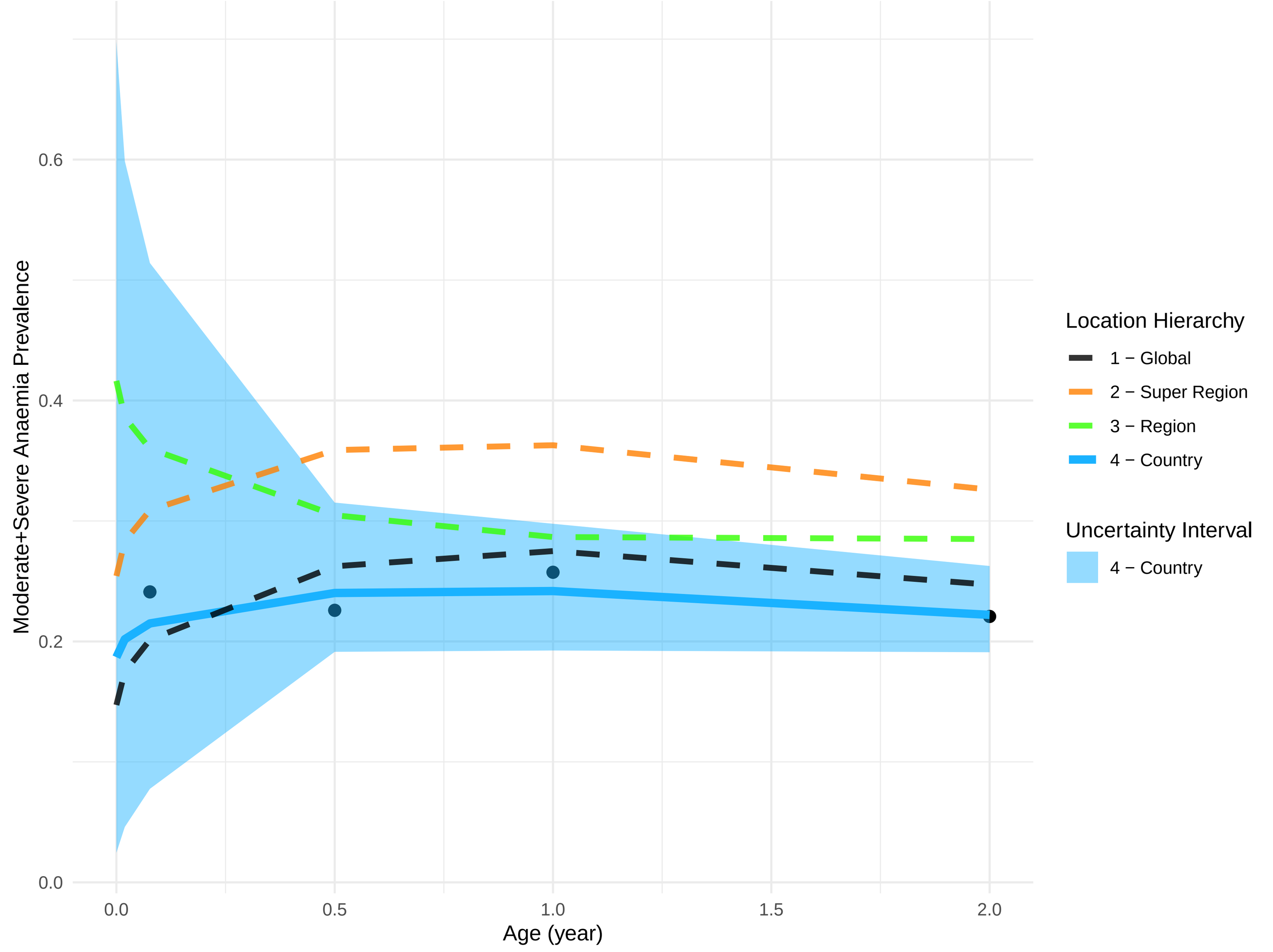

MR−BRT Age/Sex Fits − Non−pregnant Females <2 Years Old − Moderate+Severe Anaemia Prevalence − Eastern Sub−Saharan Africa
WHO−2001 Adjustment Method

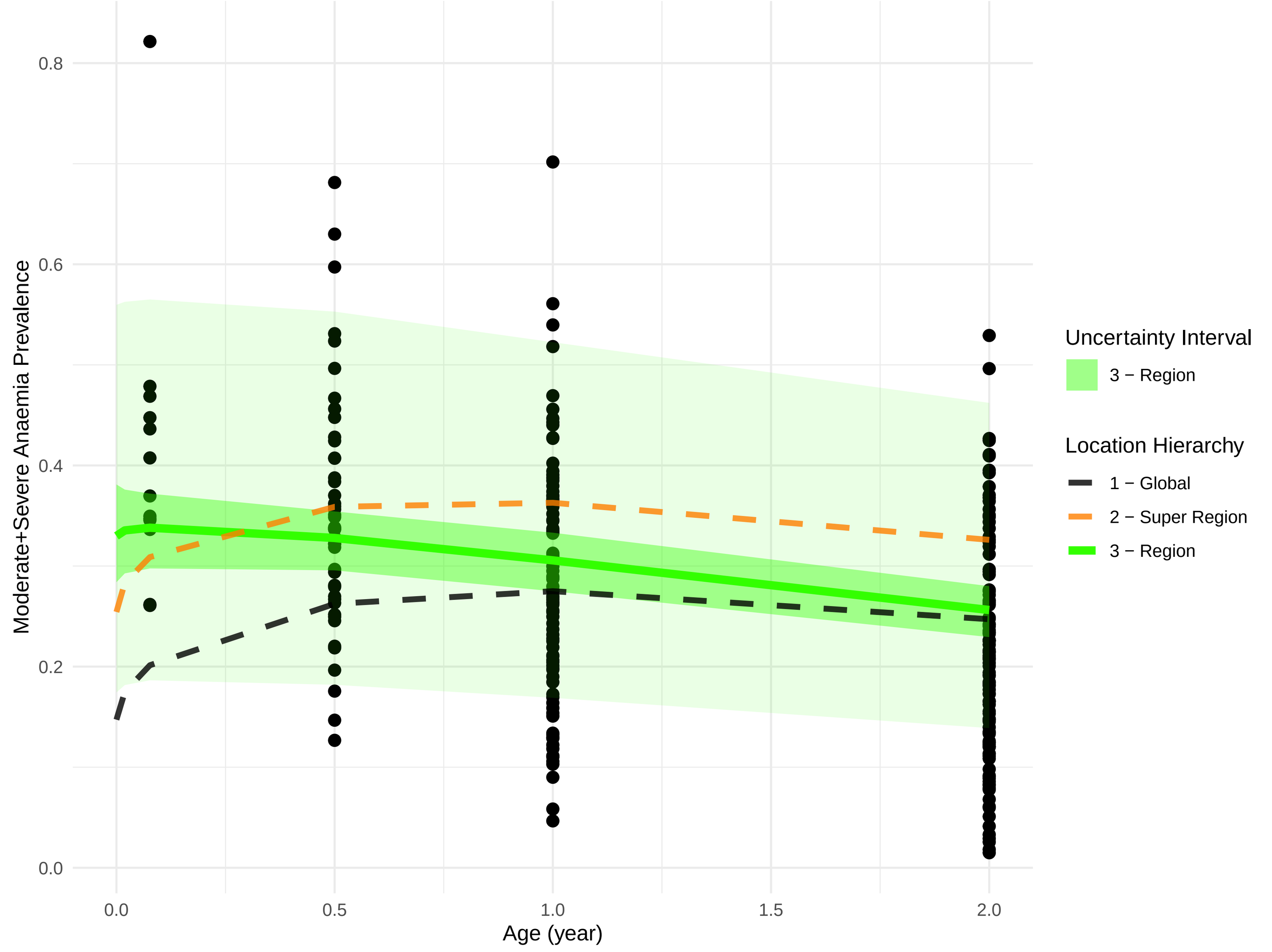

MR−BRT Age/Sex Fits − Non−pregnant Females <2 Years Old − Moderate+Severe Anaemia Prevalence − Burundi
WHO−2001 Adjustment Method

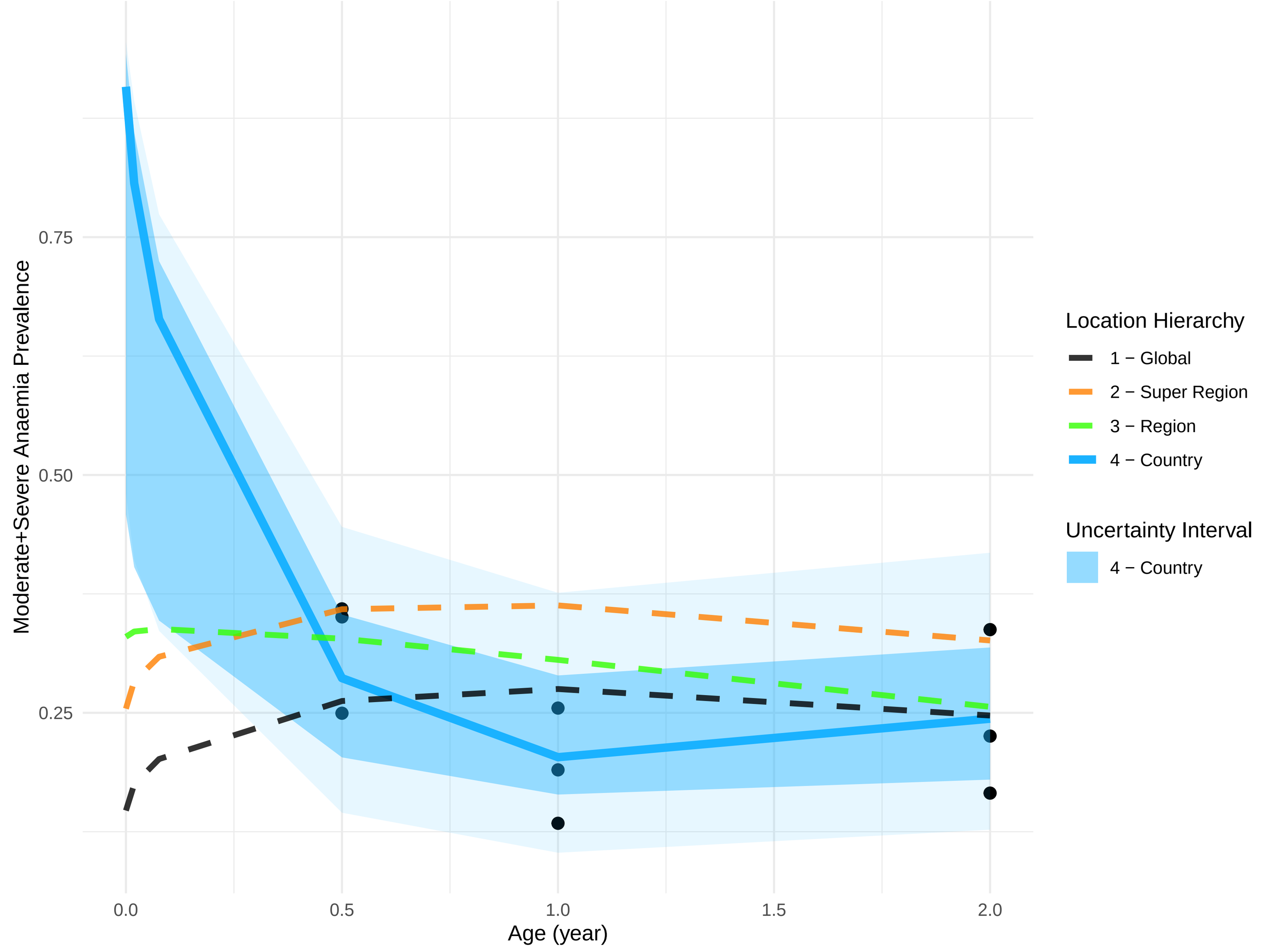

MR−BRT Age/Sex Fits − Non−pregnant Females <2 Years Old − Moderate+Severe Anaemia Prevalence − Ethiopia
WHO−2001 Adjustment Method

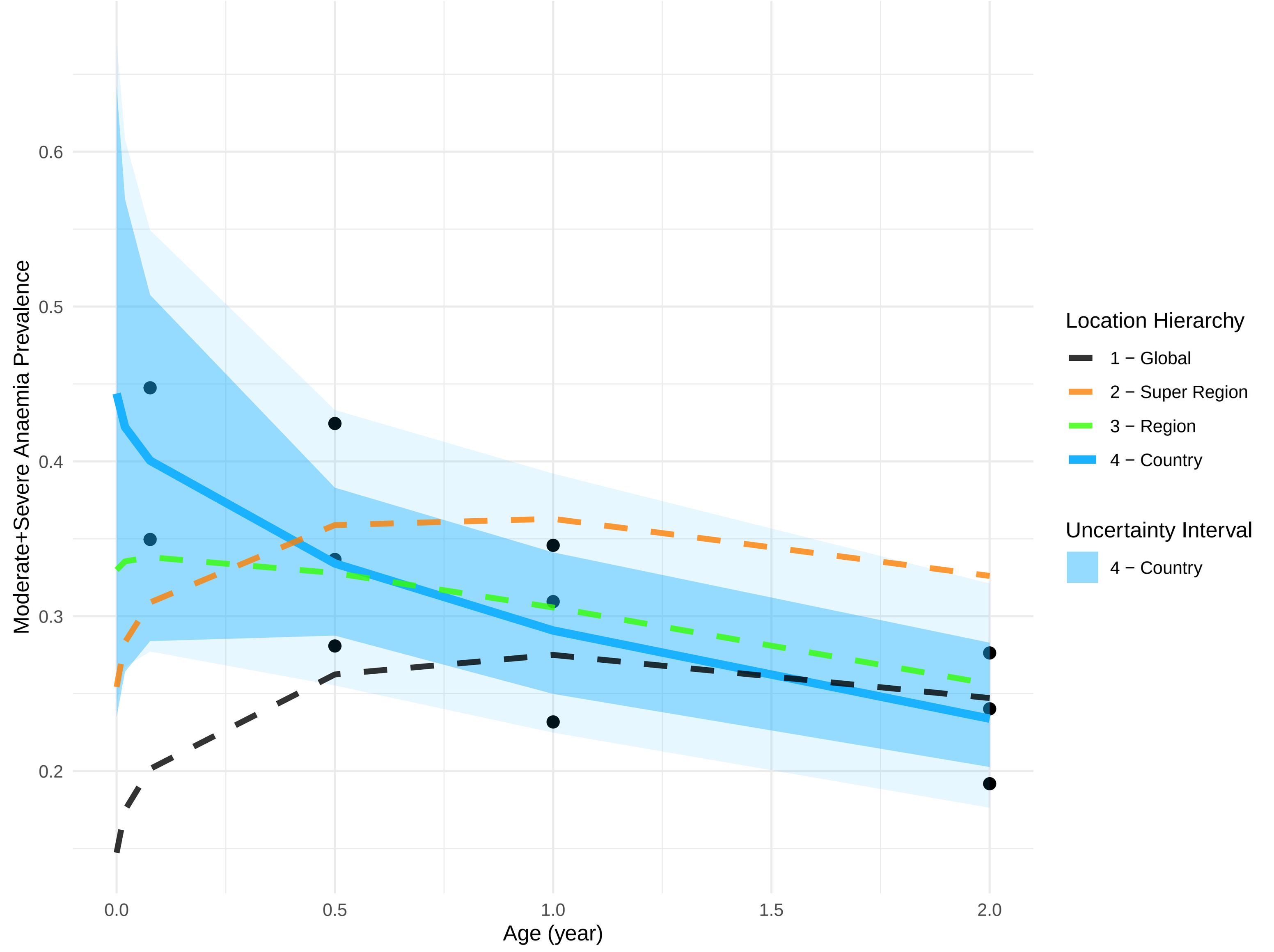

MR−BRT Age/Sex Fits − Non−pregnant Females <2 Years Old − Moderate+Severe Anaemia Prevalence − Kenya
WHO−2001 Adjustment Method

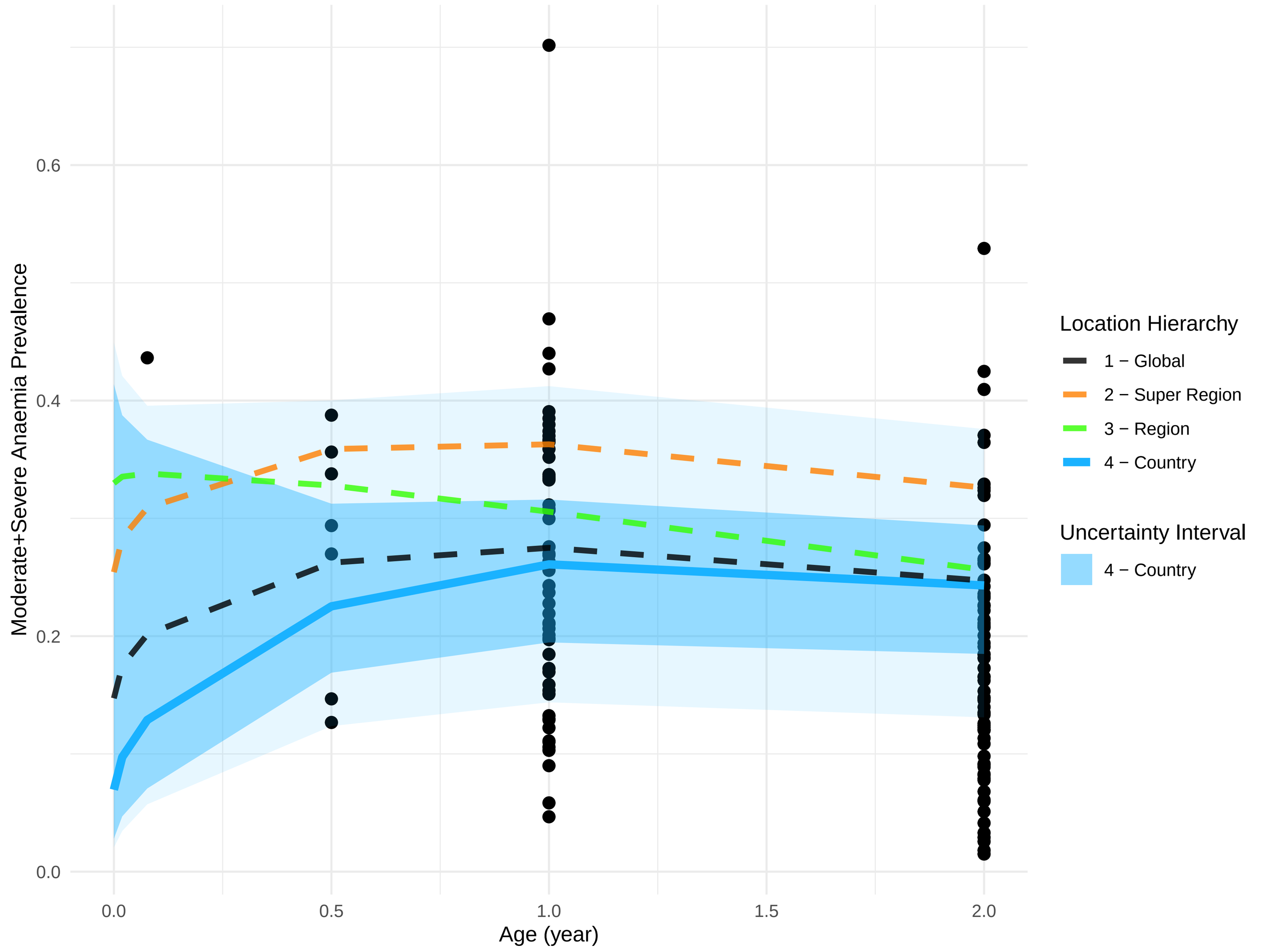

MR−BRT Age/Sex Fits − Non−pregnant Females <2 Years Old − Moderate+Severe Anaemia Prevalence − Baringo
WHO−2001 Adjustment Method

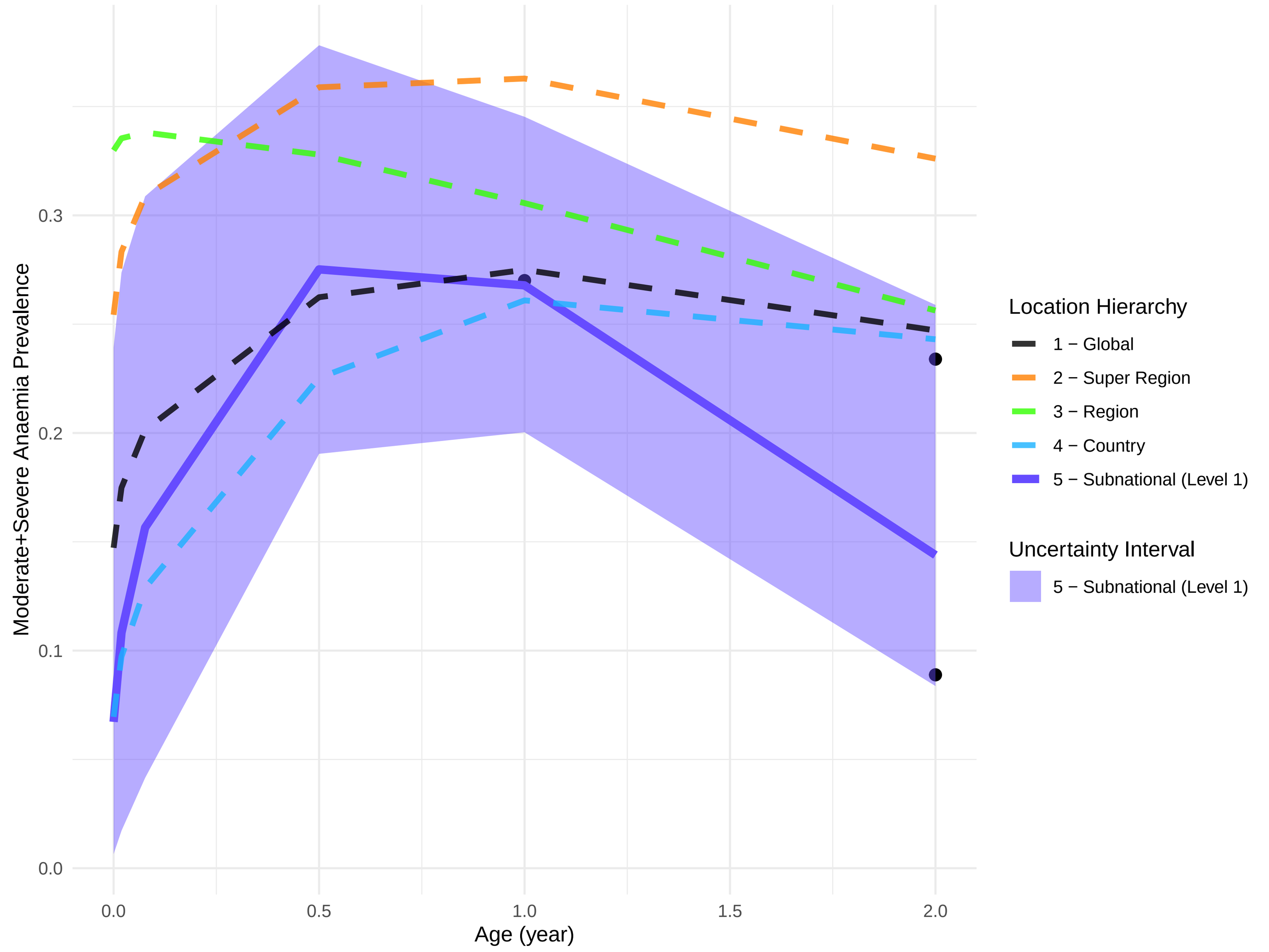

MR−BRT Age/Sex Fits − Non−pregnant Females <2 Years Old − Moderate+Severe Anaemia Prevalence − Bomet
WHO−2001 Adjustment Method

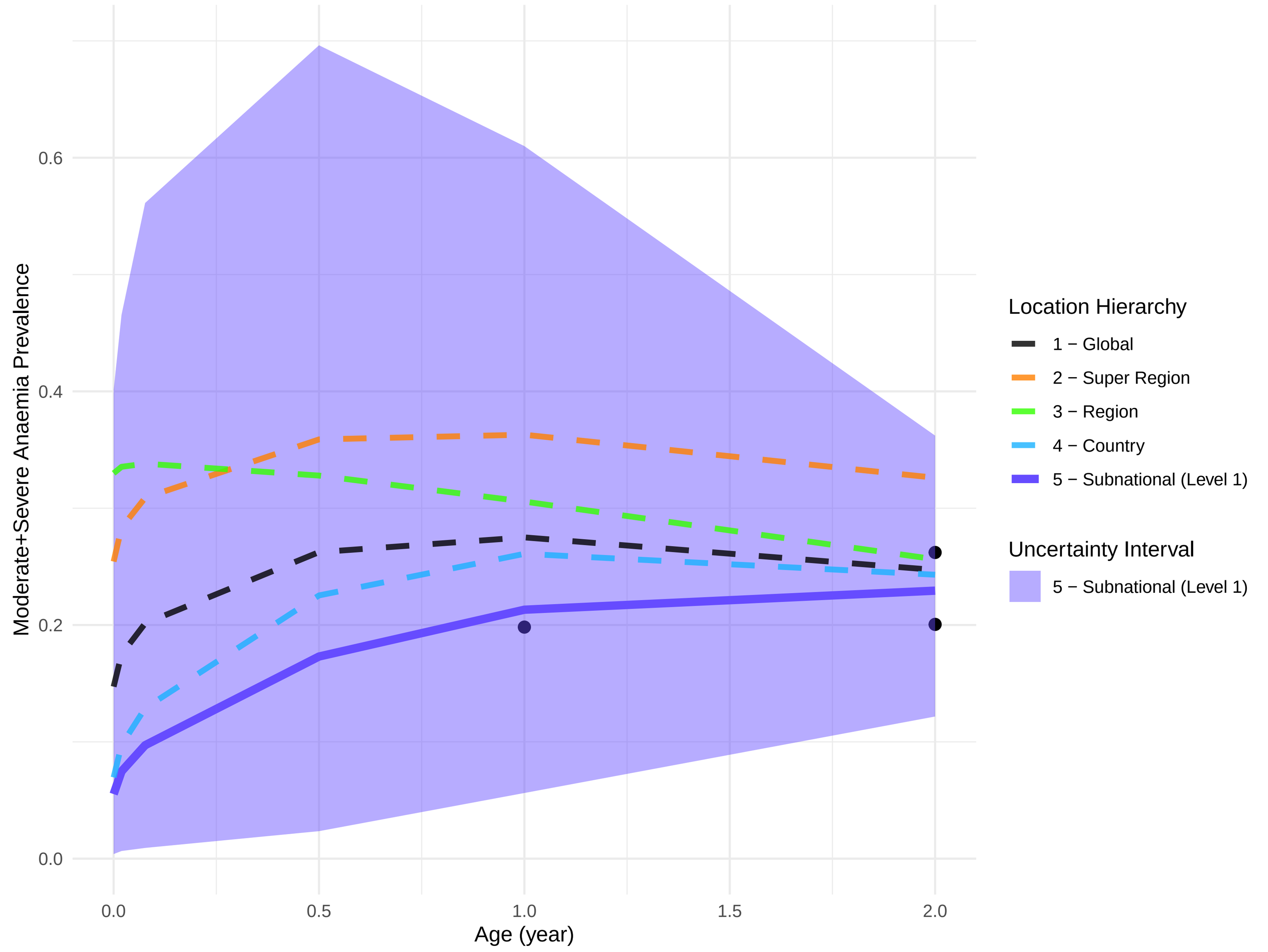

MR−BRT Age/Sex Fits − Non−pregnant Females <2 Years Old − Moderate+Severe Anaemia Prevalence − Bungoma
WHO−2001 Adjustment Method

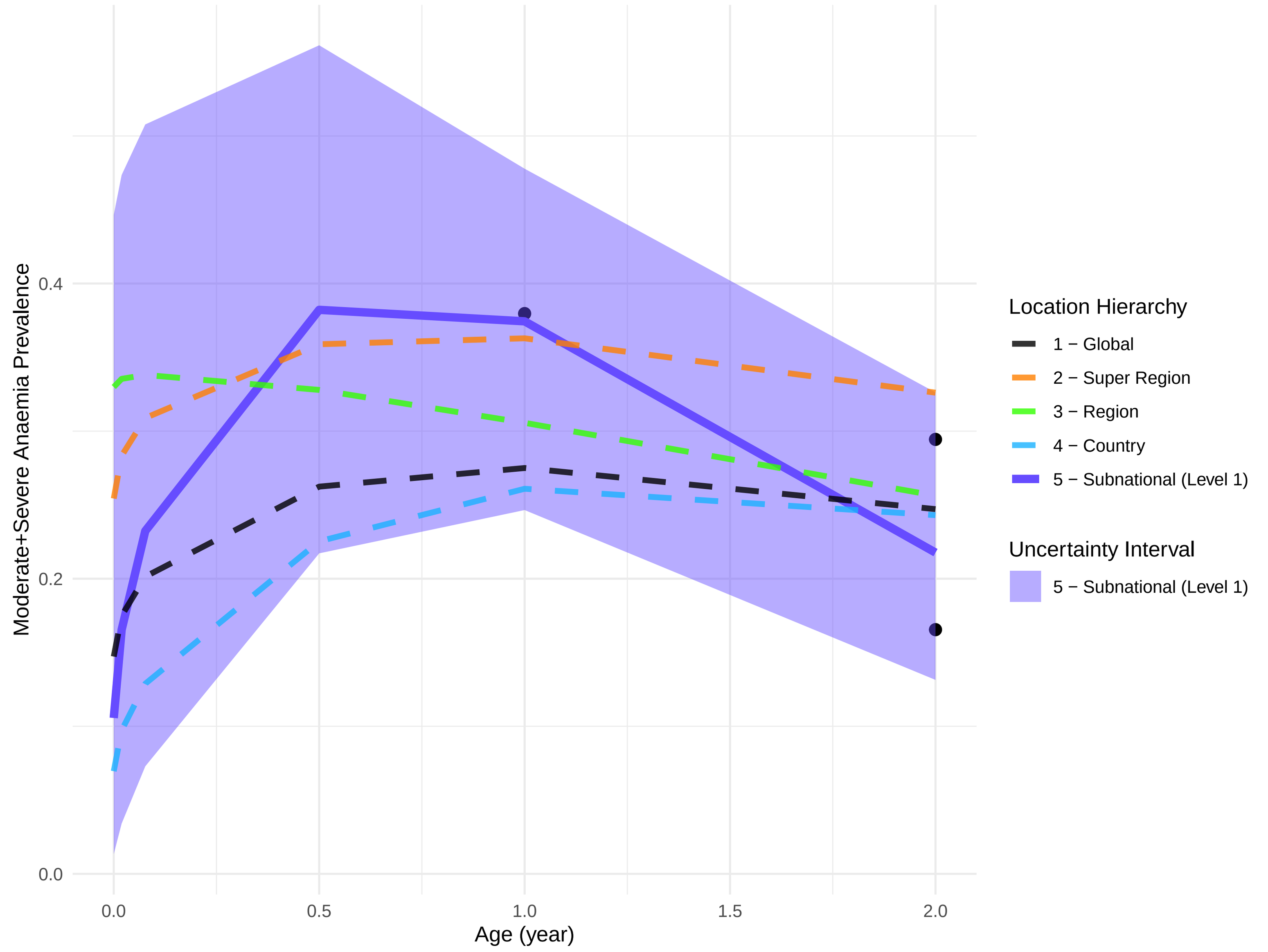

MR−BRT Age/Sex Fits − Non−pregnant Females <2 Years Old − Moderate+Severe Anaemia Prevalence − Busia
WHO−2001 Adjustment Method

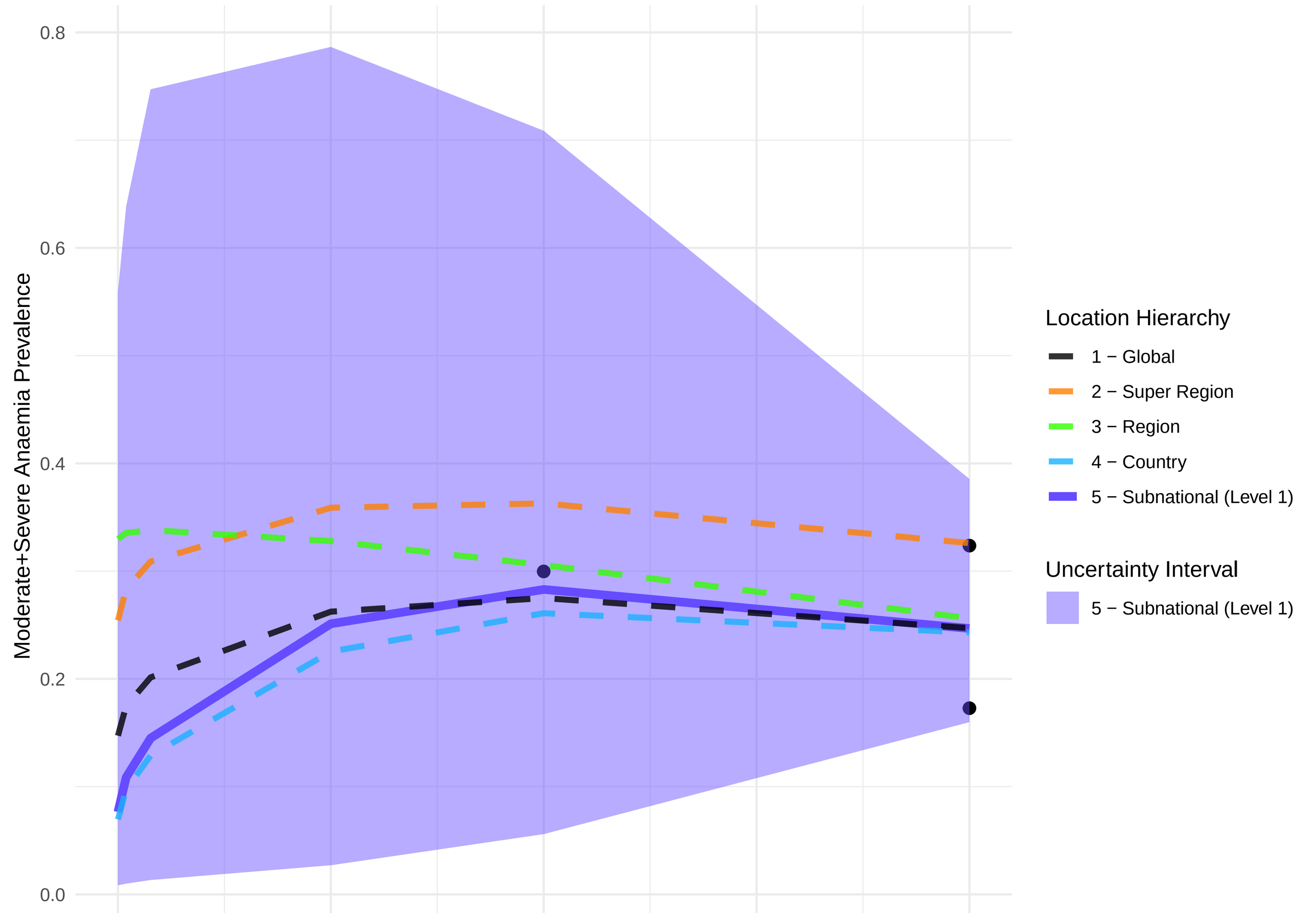

MR−BRT Age/Sex Fits − Non−pregnant Females <2 Years Old − Moderate+Severe Anaemia Prevalence − Elgeyo−Marakwet
WHO−2001 Adjustment Method

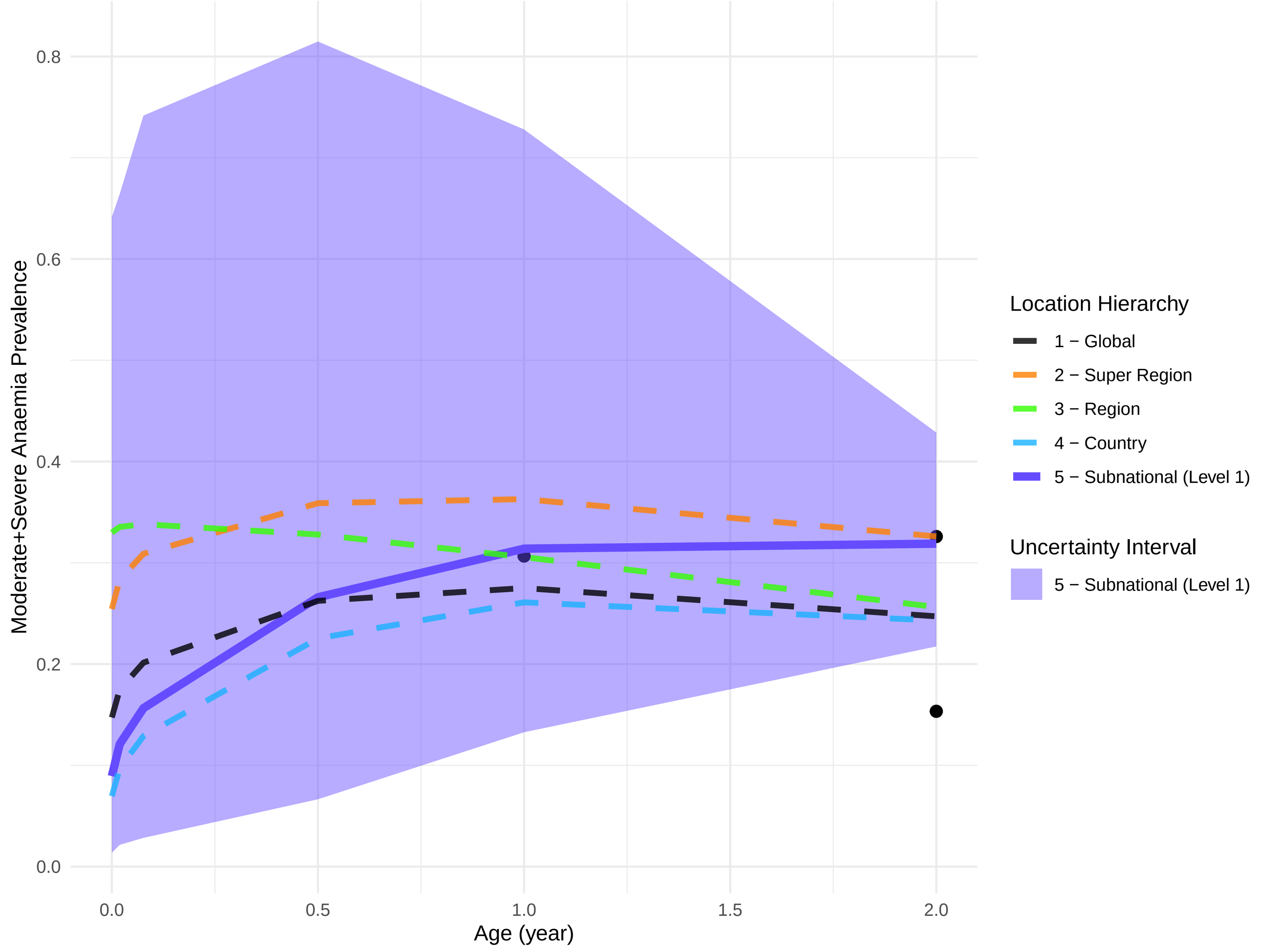

MR−BRT Age/Sex Fits − Non−pregnant Females <2 Years Old − Moderate+Severe Anaemia Prevalence − Embu
WHO−2001 Adjustment Method

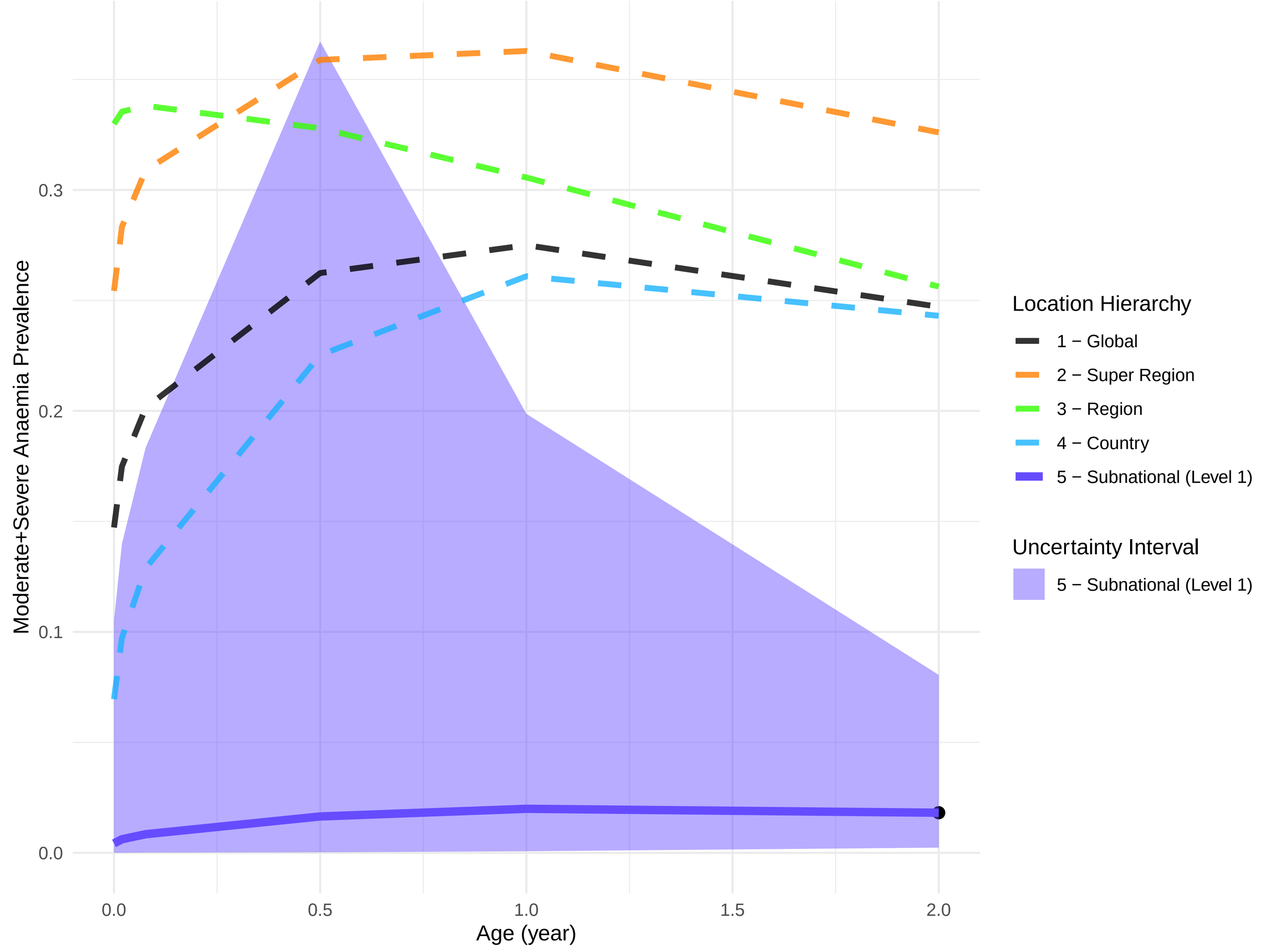

MR−BRT Age/Sex Fits − Non−pregnant Females <2 Years Old − Moderate+Severe Anaemia Prevalence − Garissa
WHO−2001 Adjustment Method

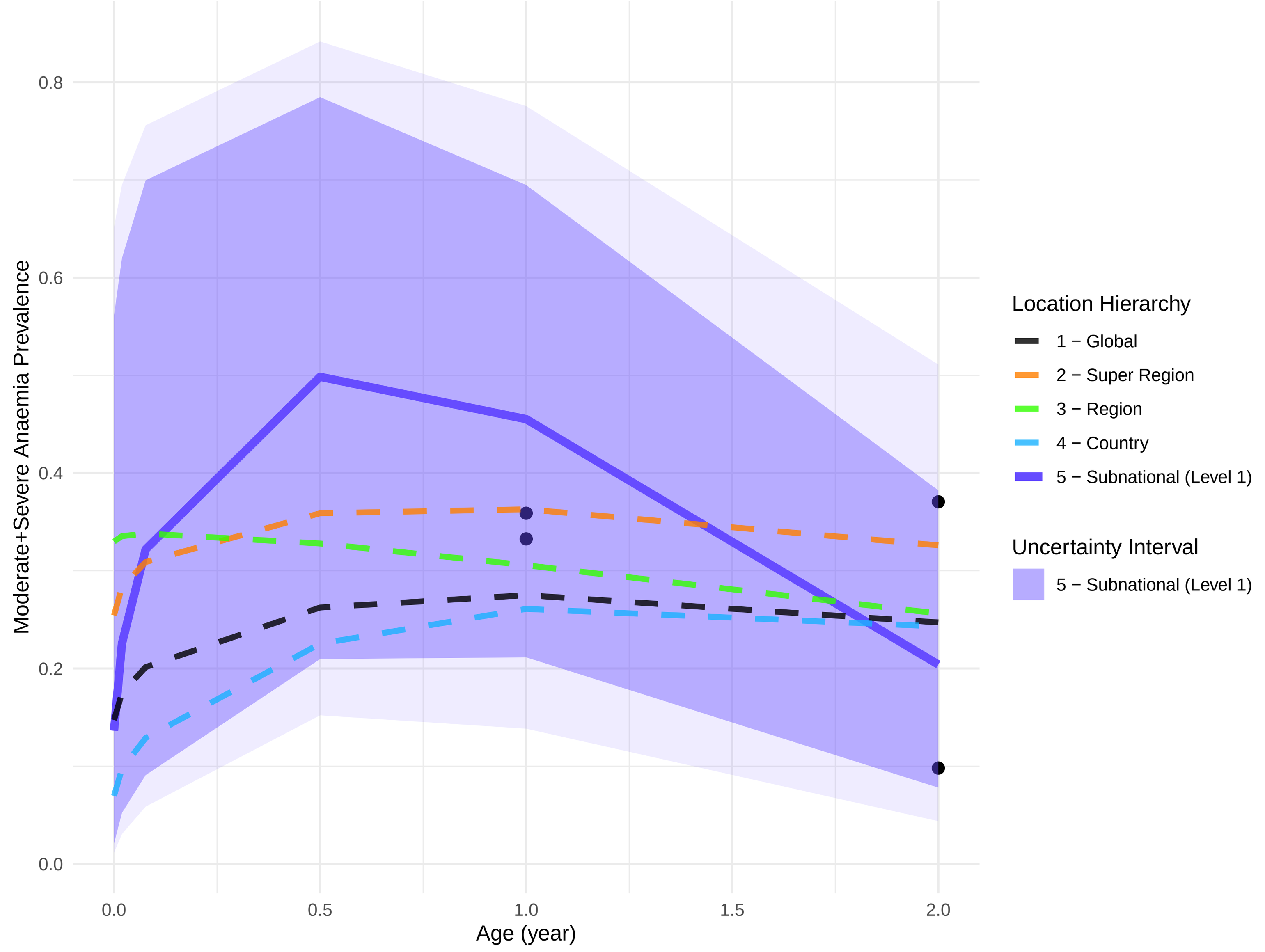

MR−BRT Age/Sex Fits − Non−pregnant Females <2 Years Old − Moderate+Severe Anaemia Prevalence − Homa Bay
WHO−2001 Adjustment Method

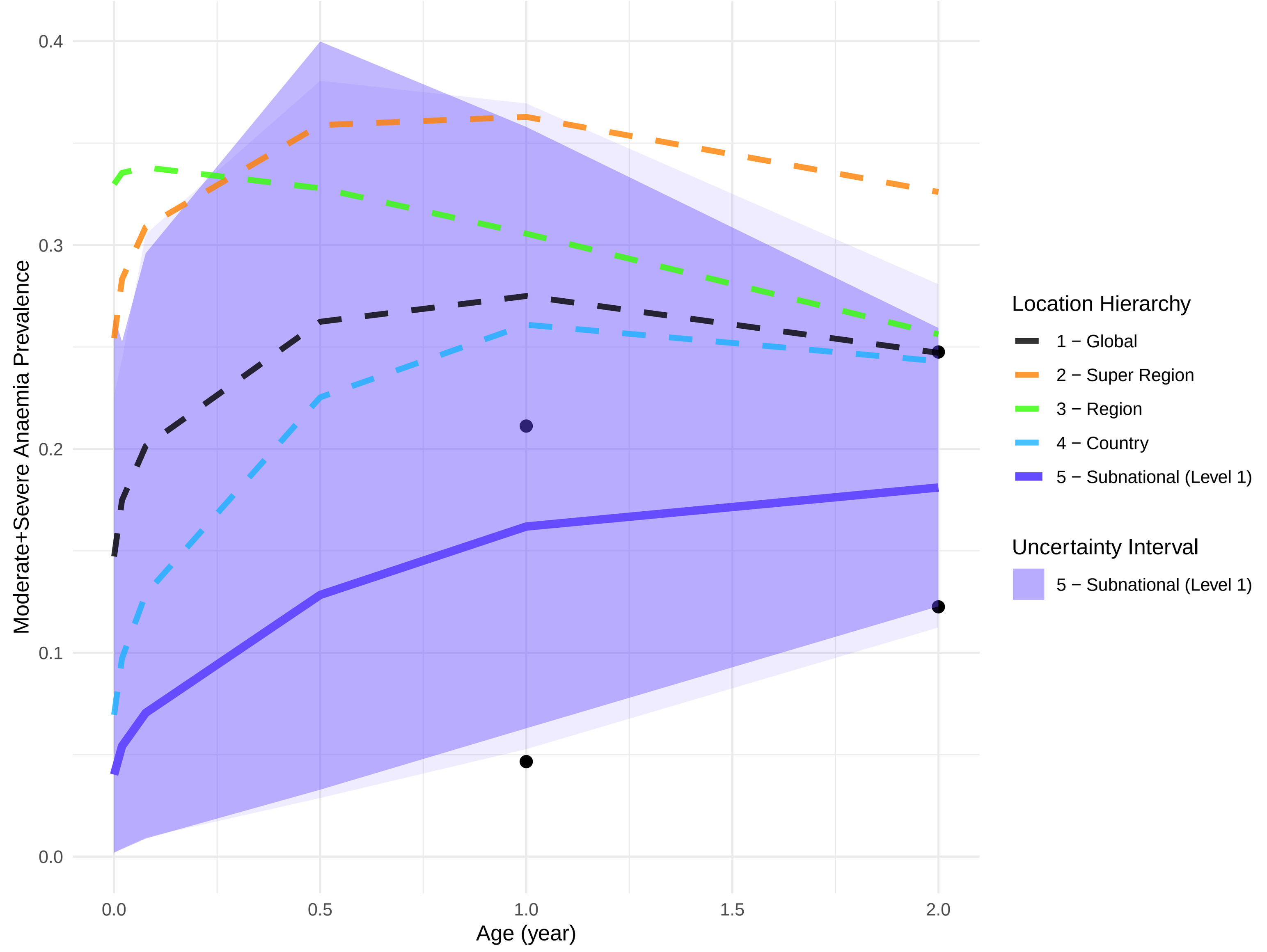

MR−BRT Age/Sex Fits − Non−pregnant Females <2 Years Old − Moderate+Severe Anaemia Prevalence − Isiolo
WHO−2001 Adjustment Method

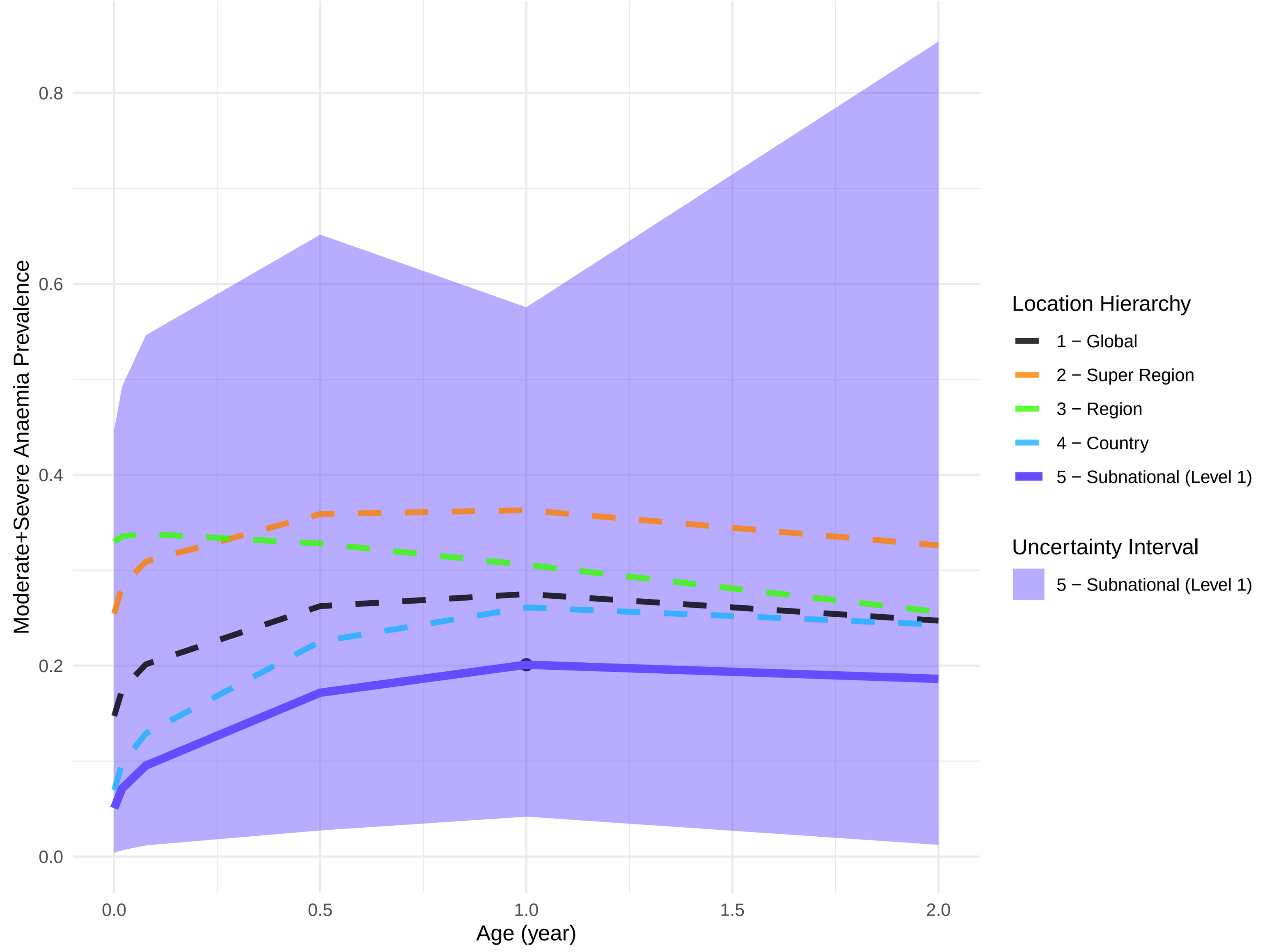

MR−BRT Age/Sex Fits − Non−pregnant Females <2 Years Old − Moderate+Severe Anaemia Prevalence − Kajiado
WHO−2001 Adjustment Method

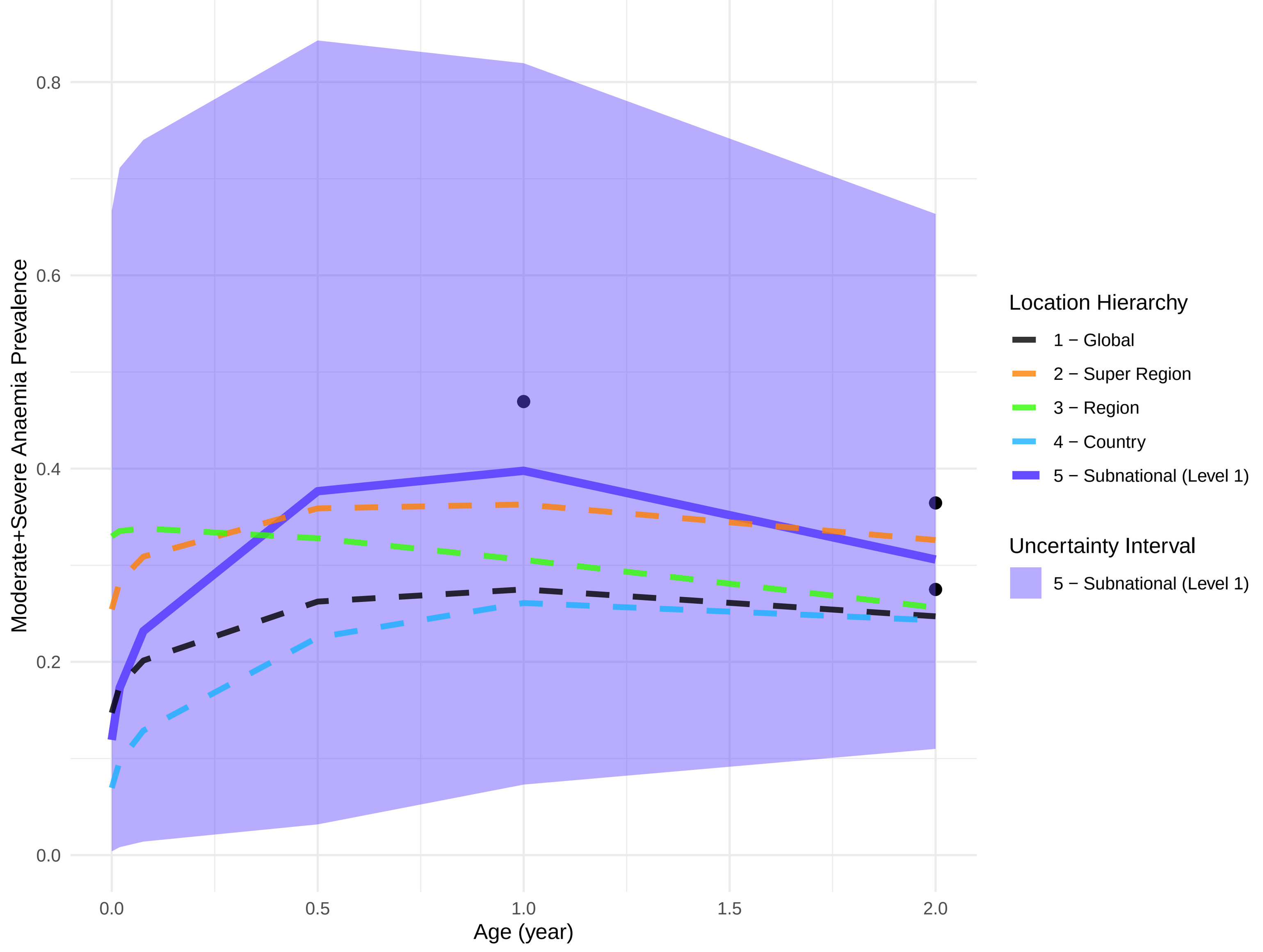

MR−BRT Age/Sex Fits − Non−pregnant Females <2 Years Old − Moderate+Severe Anaemia Prevalence − Kakamega
WHO−2001 Adjustment Method

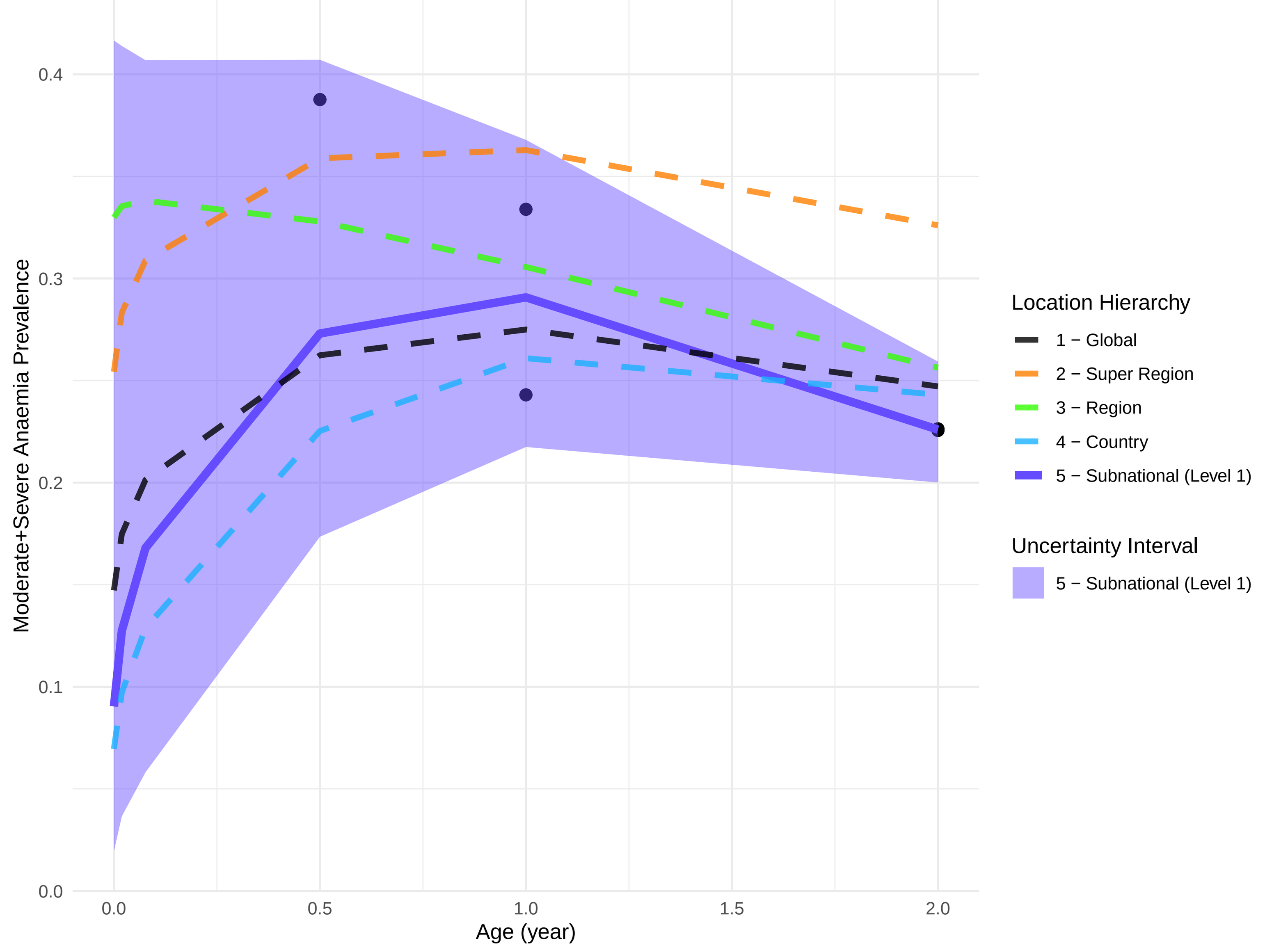

MR−BRT Age/Sex Fits − Non−pregnant Females <2 Years Old − Moderate+Severe Anaemia Prevalence − Kericho
WHO−2001 Adjustment Method

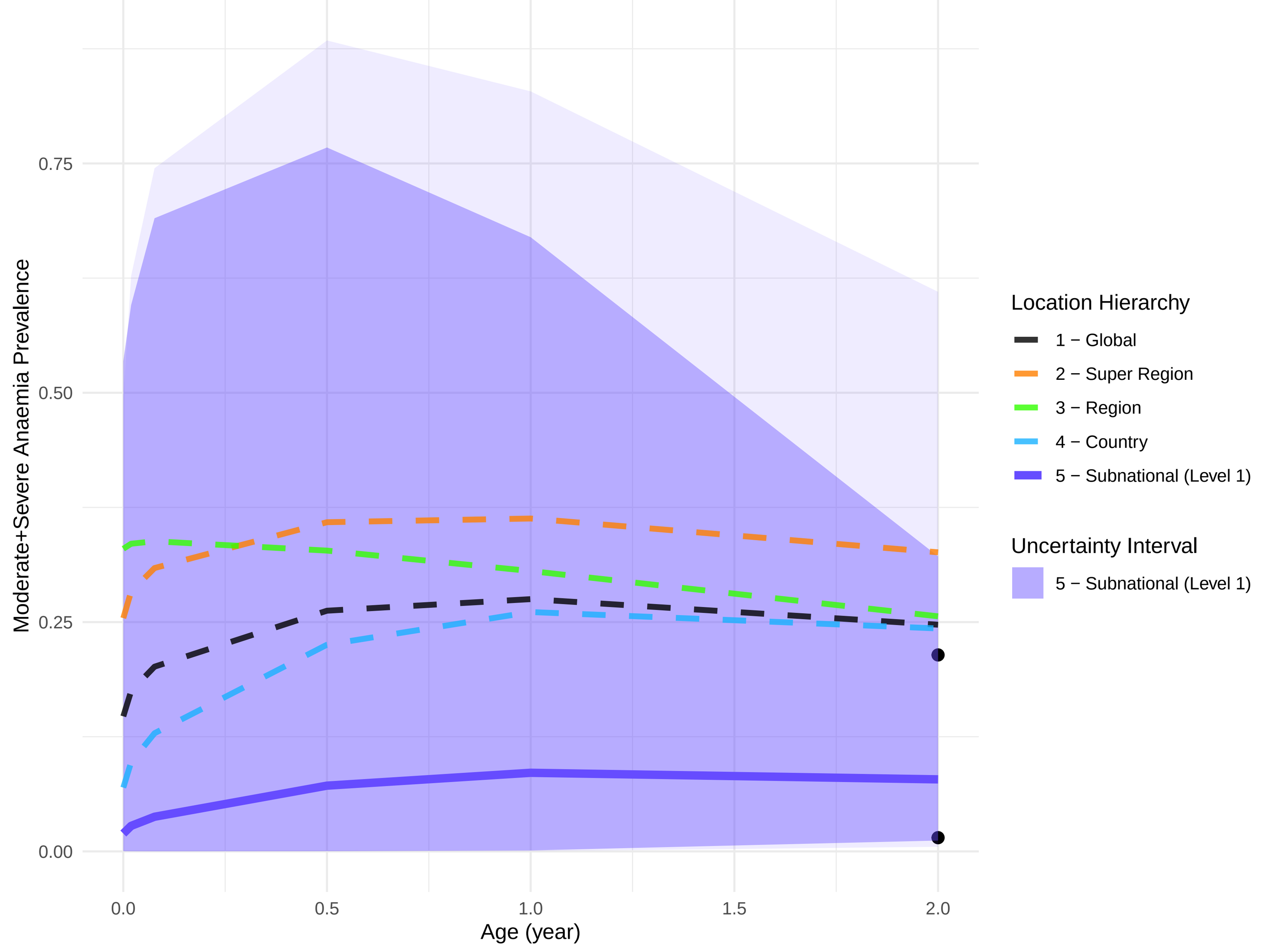

MR−BRT Age/Sex Fits − Non−pregnant Females <2 Years Old − Moderate+Severe Anaemia Prevalence − Kiambu
WHO−2001 Adjustment Method

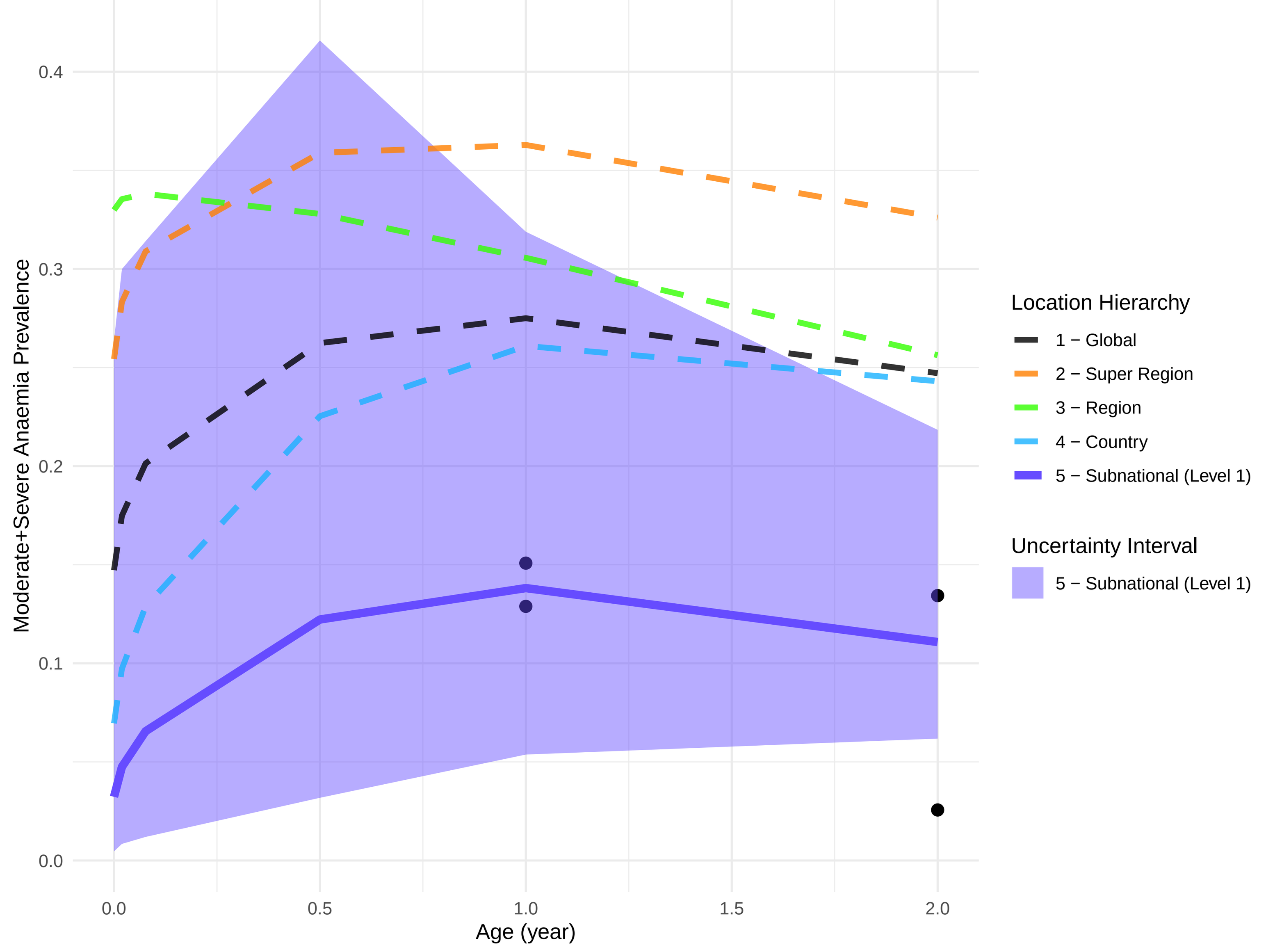

MR−BRT Age/Sex Fits − Non−pregnant Females <2 Years Old − Moderate+Severe Anaemia Prevalence − Kilifi
WHO−2001 Adjustment Method

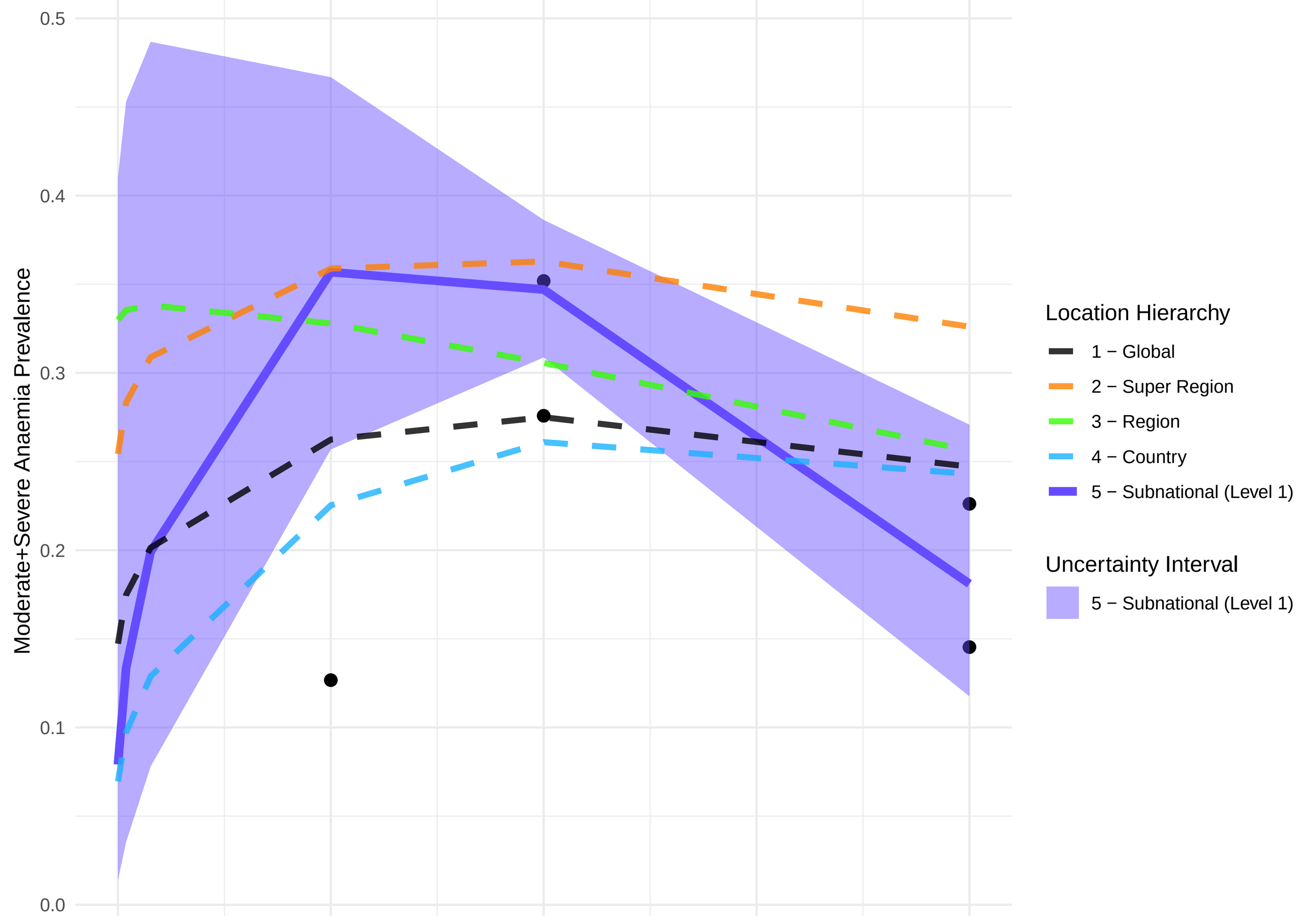

MR−BRT Age/Sex Fits − Non−pregnant Females <2 Years Old − Moderate+Severe Anaemia Prevalence − Kirinyaga
WHO−2001 Adjustment Method

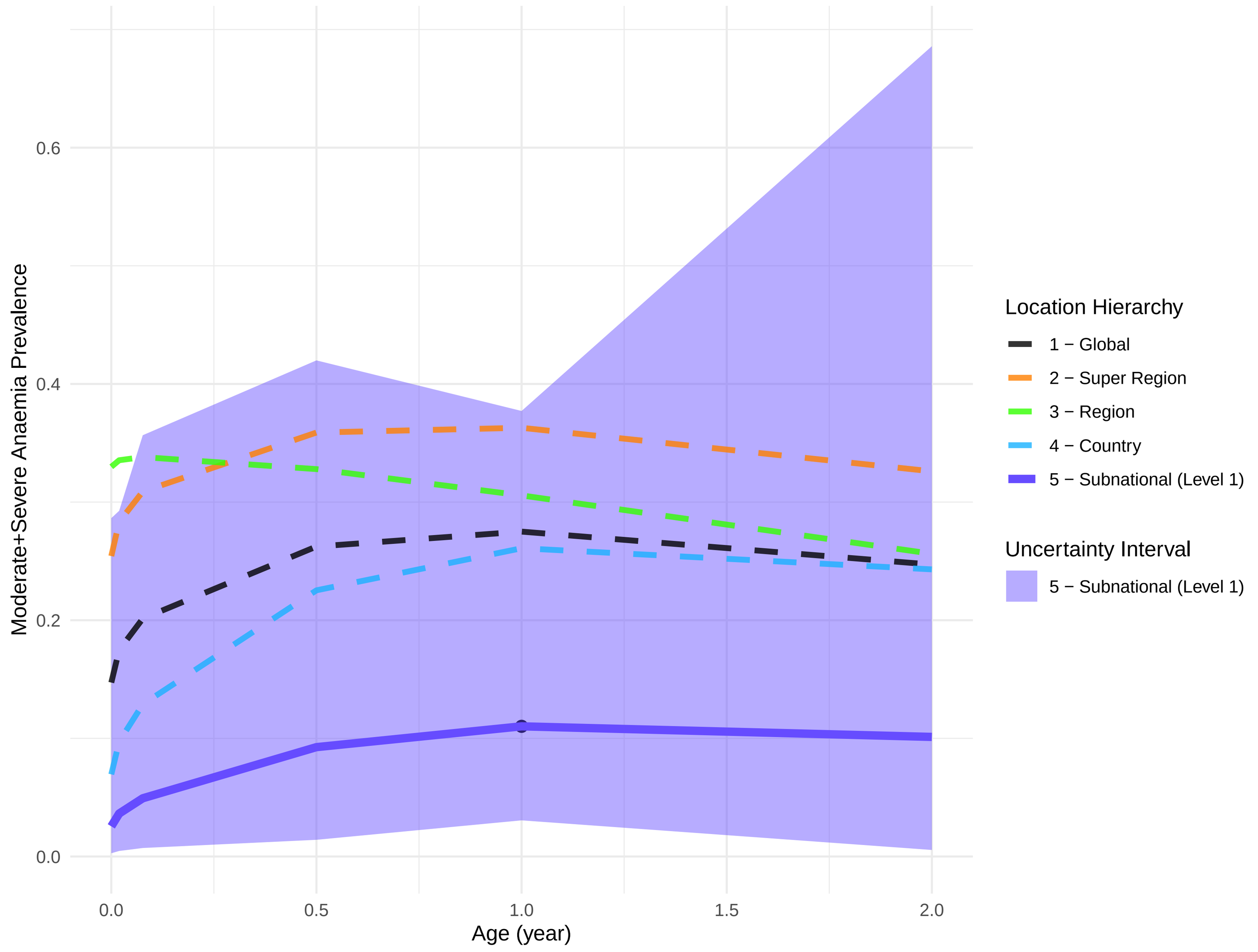

MR−BRT Age/Sex Fits − Non−pregnant Females <2 Years Old − Moderate+Severe Anaemia Prevalence − Kisii
WHO−2001 Adjustment Method

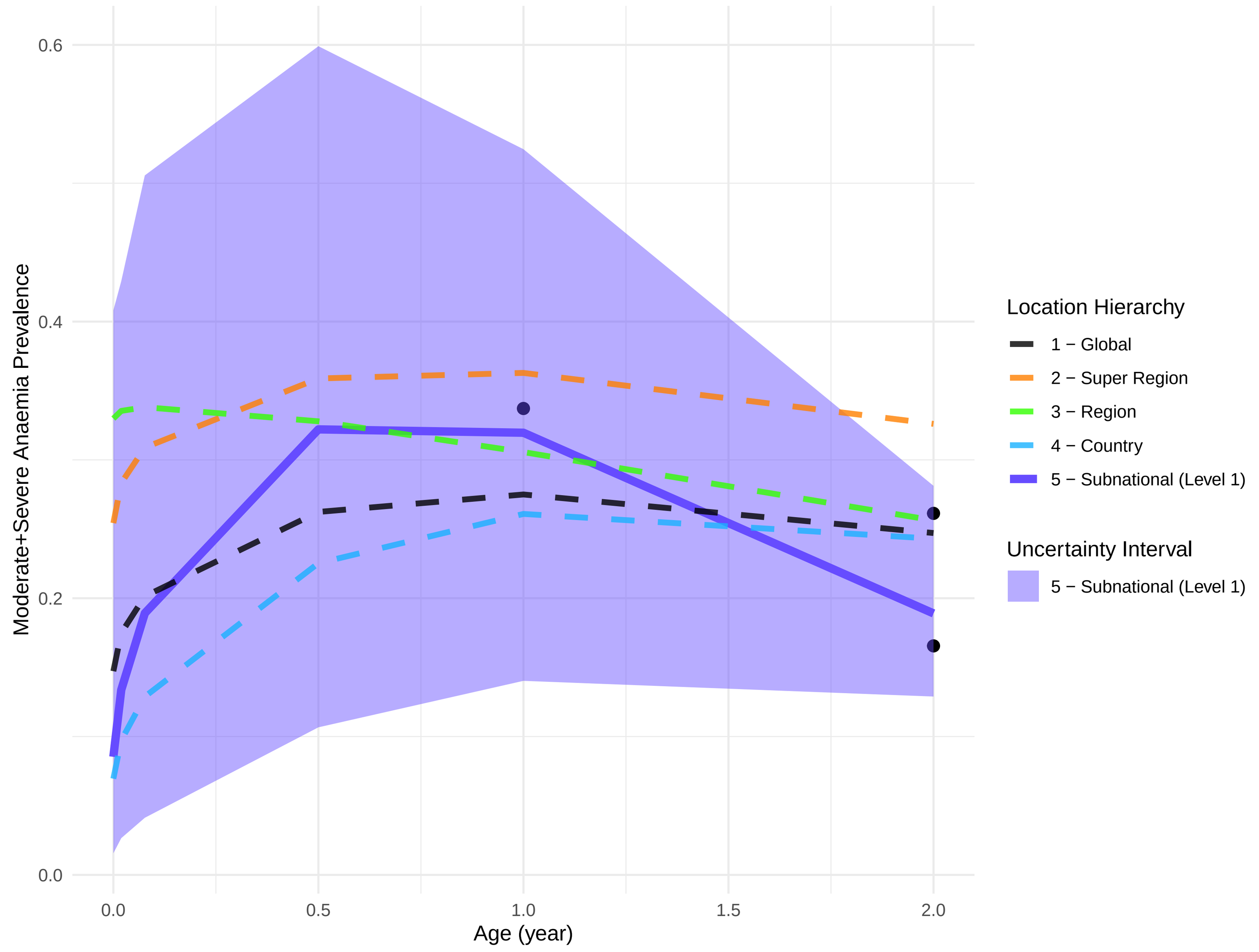

MR−BRT Age/Sex Fits − Non−pregnant Females <2 Years Old − Moderate+Severe Anaemia Prevalence − Kisumu
WHO−2001 Adjustment Method

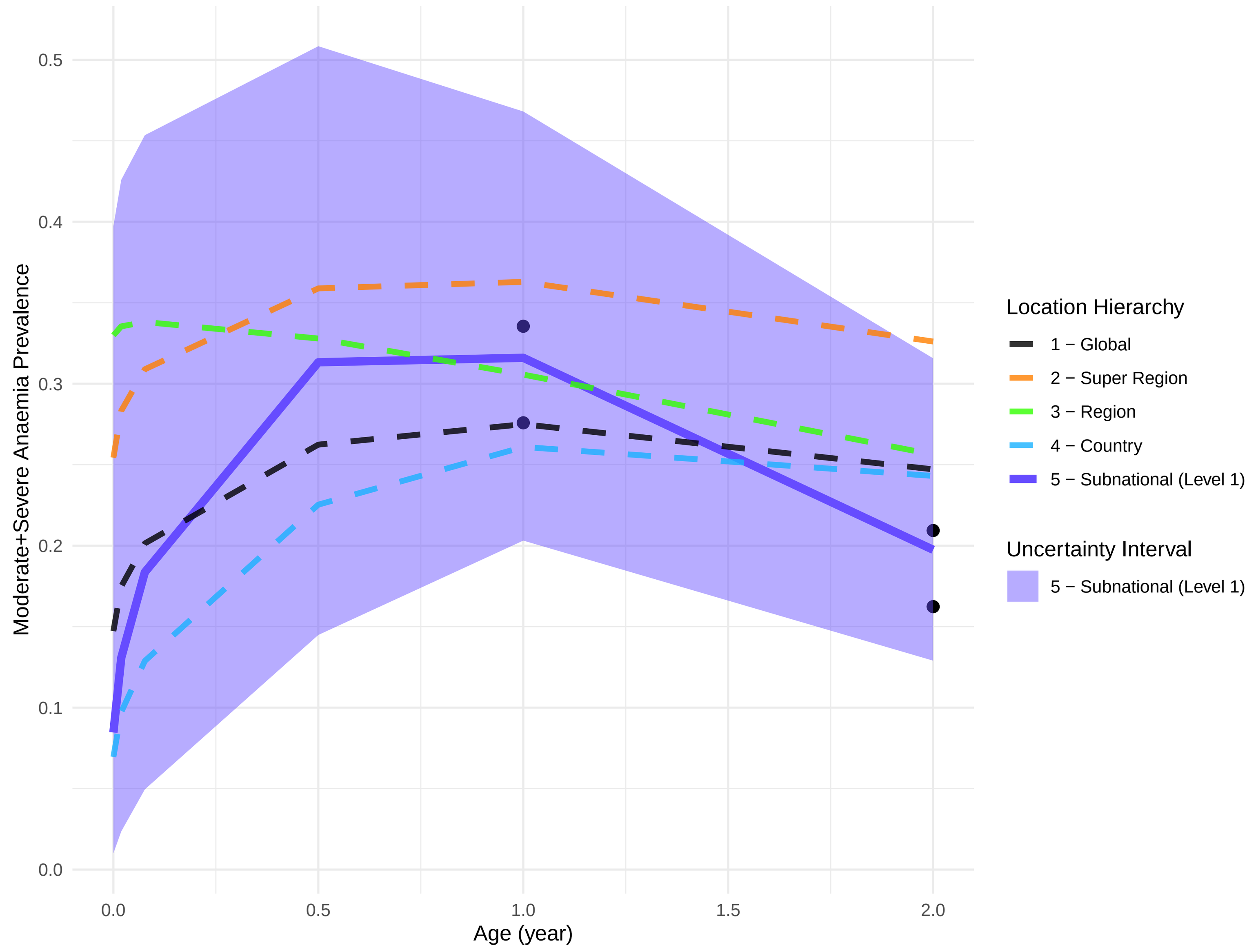

MR−BRT Age/Sex Fits − Non−pregnant Females <2 Years Old − Moderate+Severe Anaemia Prevalence − Kitui
WHO−2001 Adjustment Method

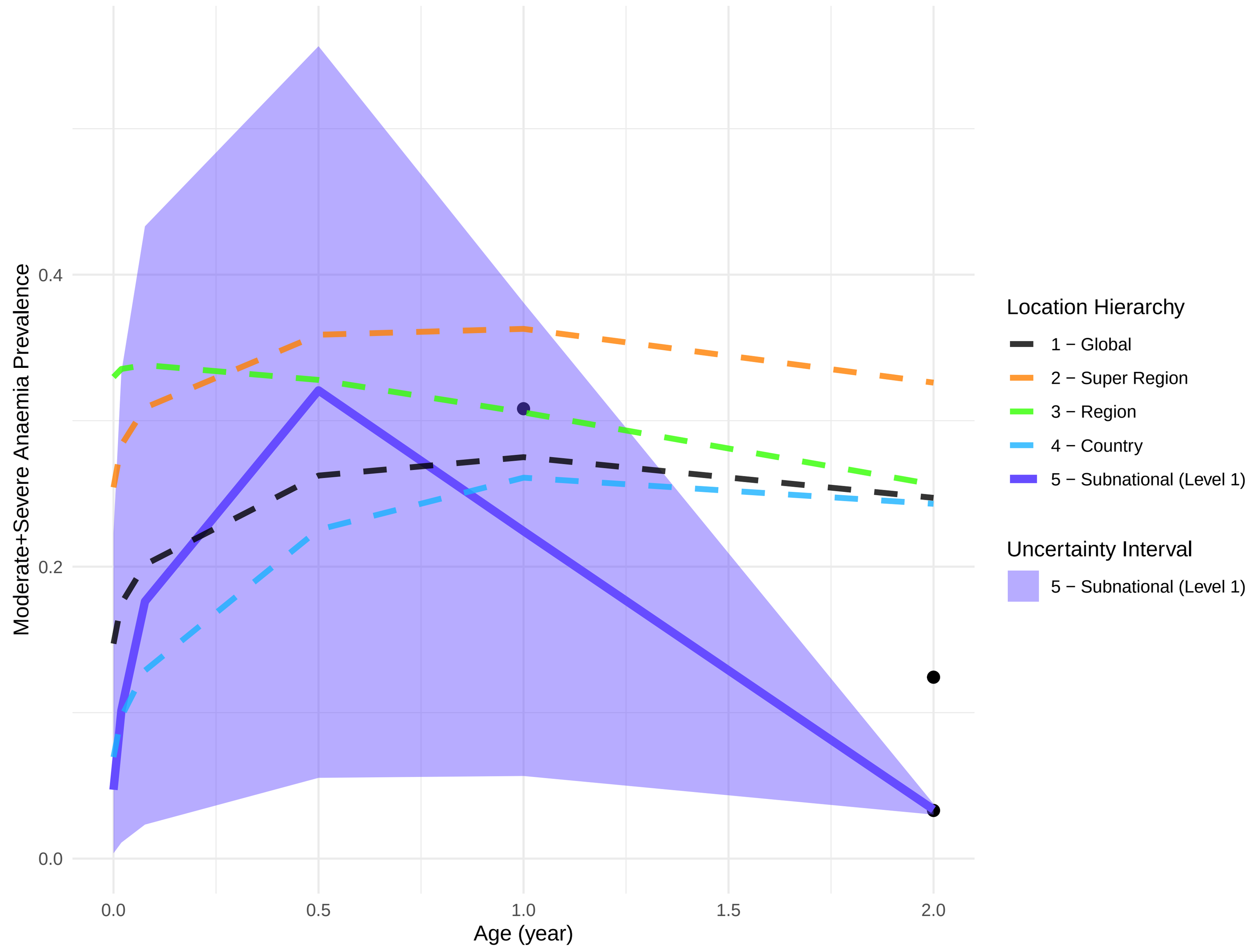

MR−BRT Age/Sex Fits − Non−pregnant Females <2 Years Old − Moderate+Severe Anaemia Prevalence − Kwale
WHO−2001 Adjustment Method

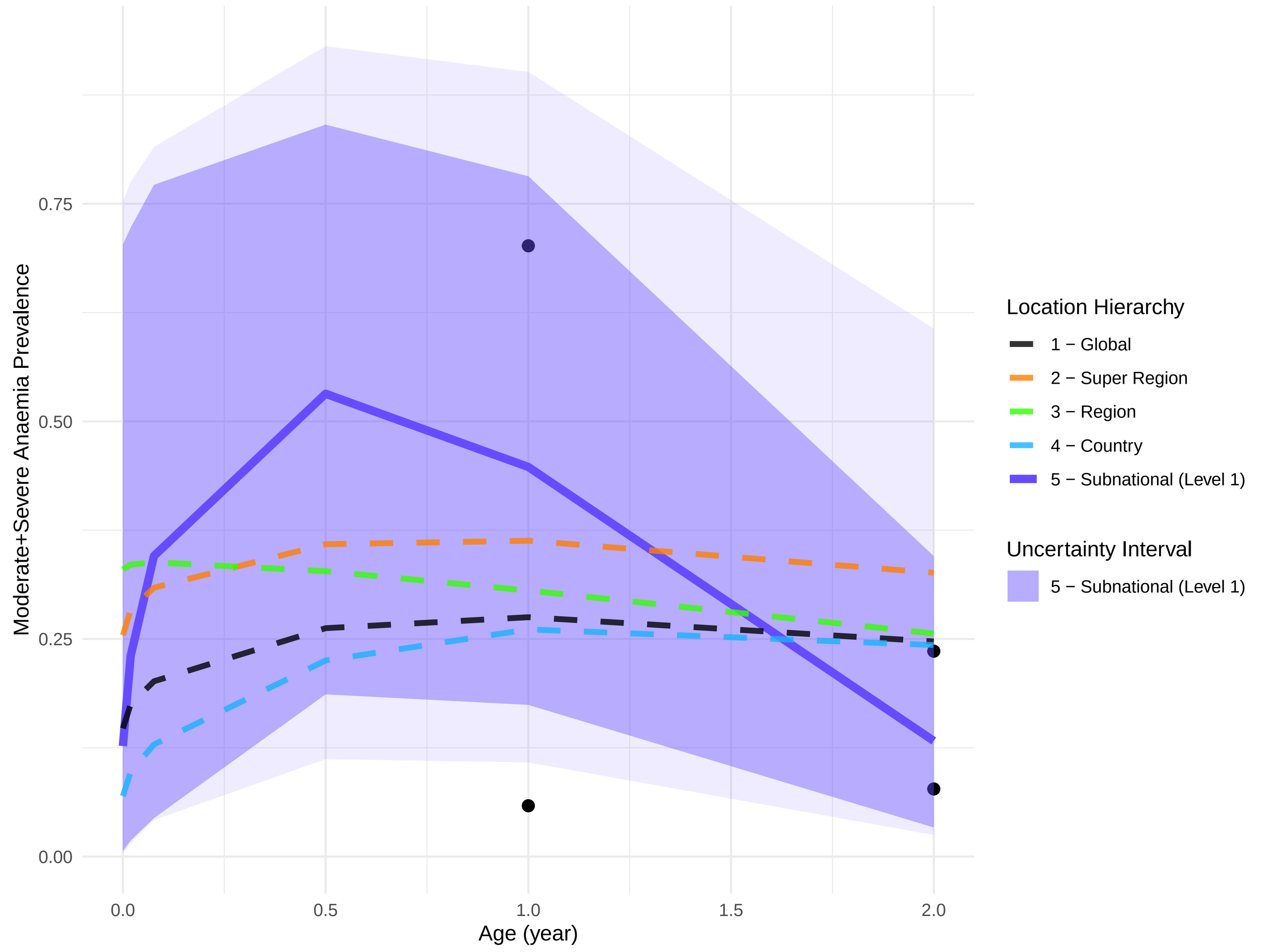

MR−BRT Age/Sex Fits − Non−pregnant Females <2 Years Old − Moderate+Severe Anaemia Prevalence − Laikipia
WHO−2001 Adjustment Method

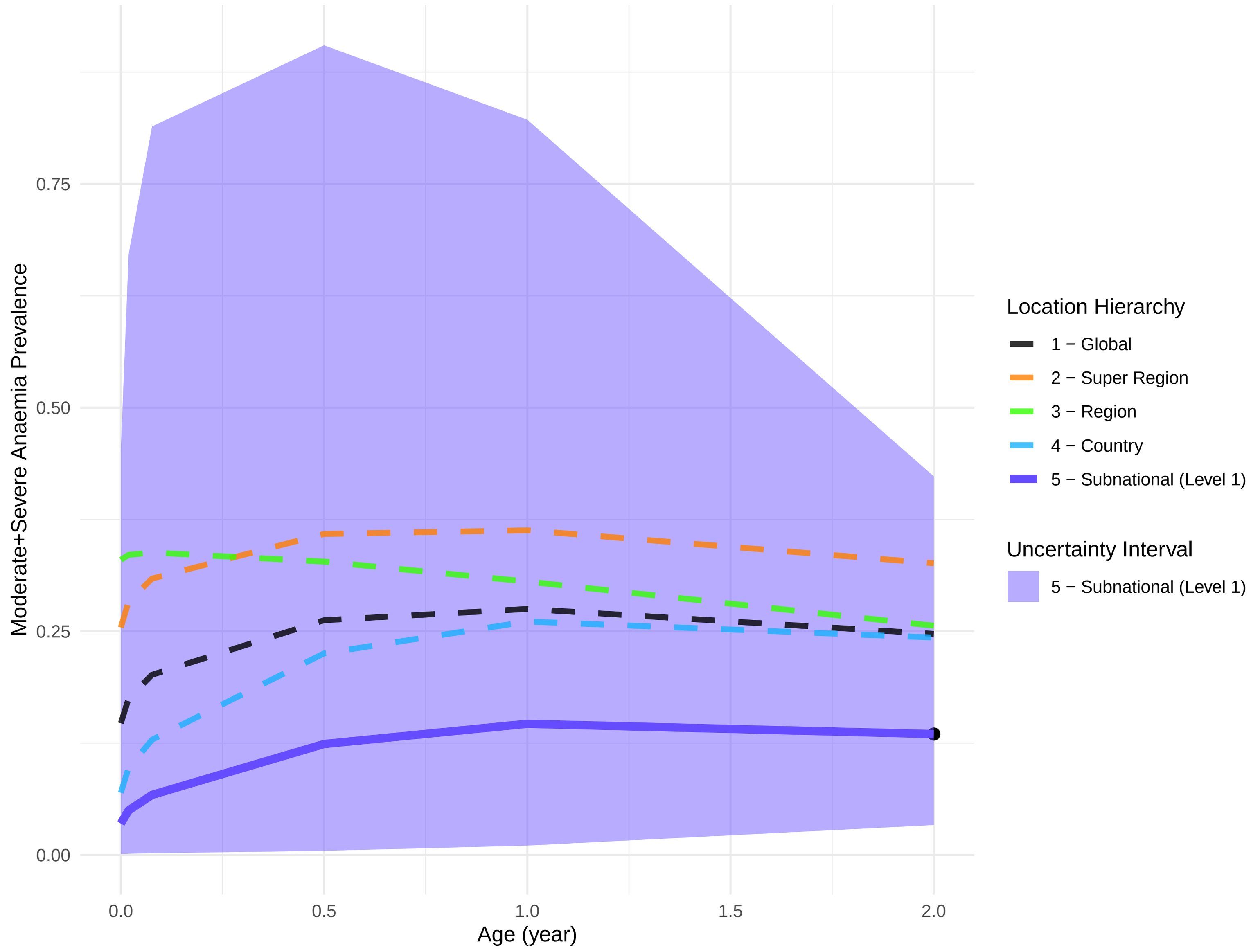

MR−BRT Age/Sex Fits − Non−pregnant Females <2 Years Old − Moderate+Severe Anaemia Prevalence − Lamu
WHO−2001 Adjustment Method

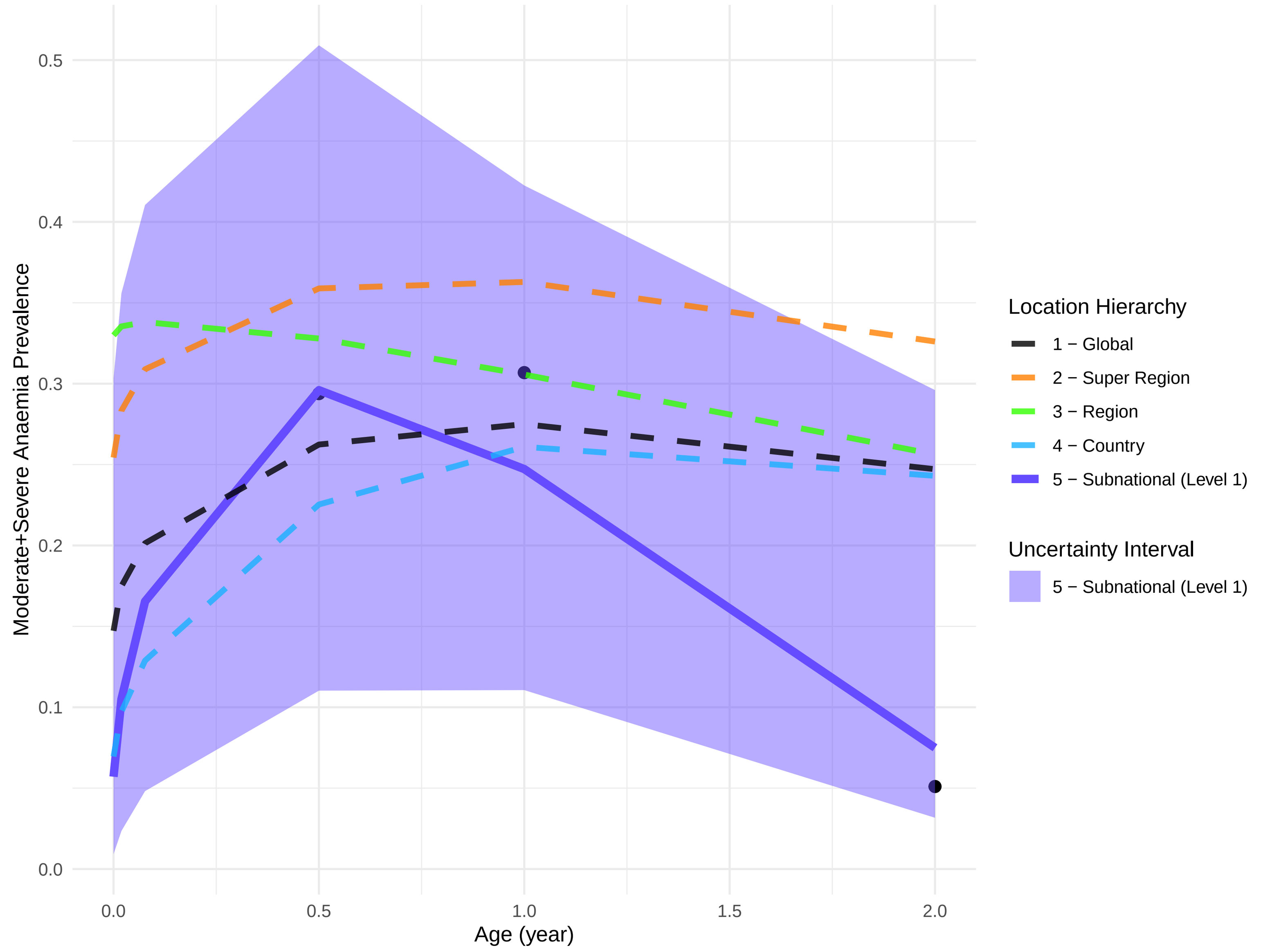

MR−BRT Age/Sex Fits − Non−pregnant Females <2 Years Old − Moderate+Severe Anaemia Prevalence − Machakos
WHO−2001 Adjustment Method

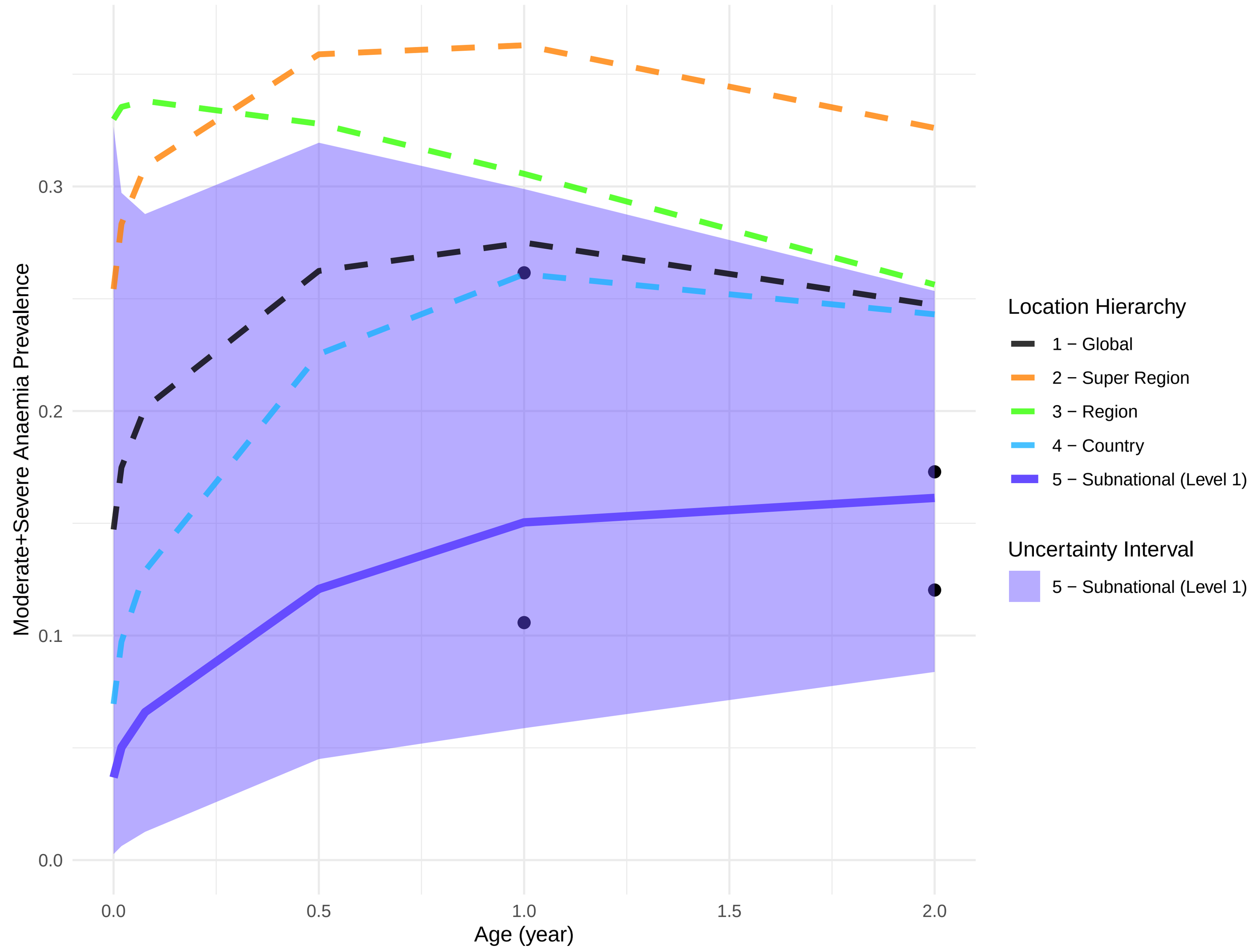

MR−BRT Age/Sex Fits − Non−pregnant Females <2 Years Old − Moderate+Severe Anaemia Prevalence − Makueni
WHO−2001 Adjustment Method

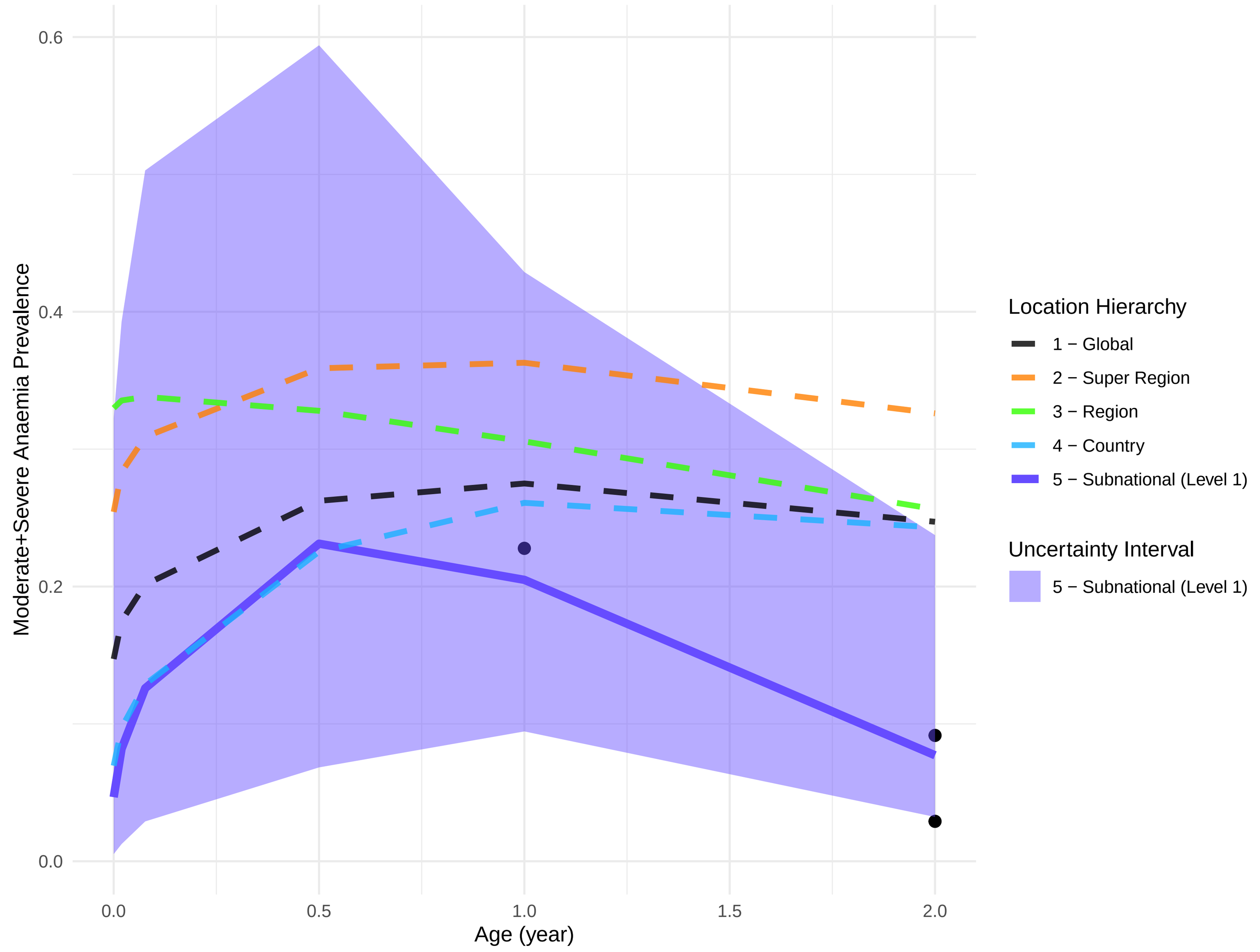

MR−BRT Age/Sex Fits − Non−pregnant Females <2 Years Old − Moderate+Severe Anaemia Prevalence − Mandera
WHO−2001 Adjustment Method

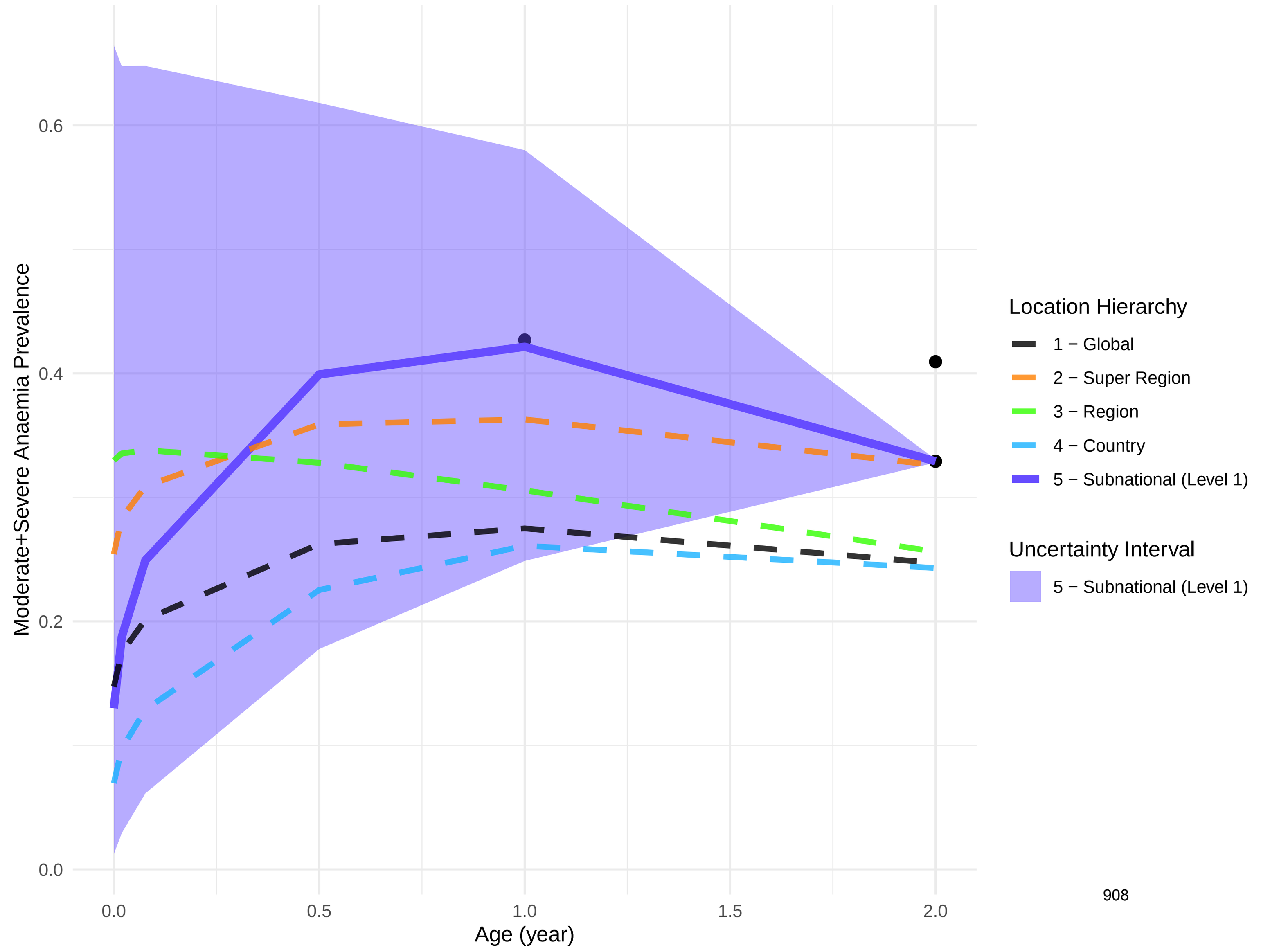

MR−BRT Age/Sex Fits − Non−pregnant Females <2 Years Old − Moderate+Severe Anaemia Prevalence − Marsabit
WHO−2001 Adjustment Method

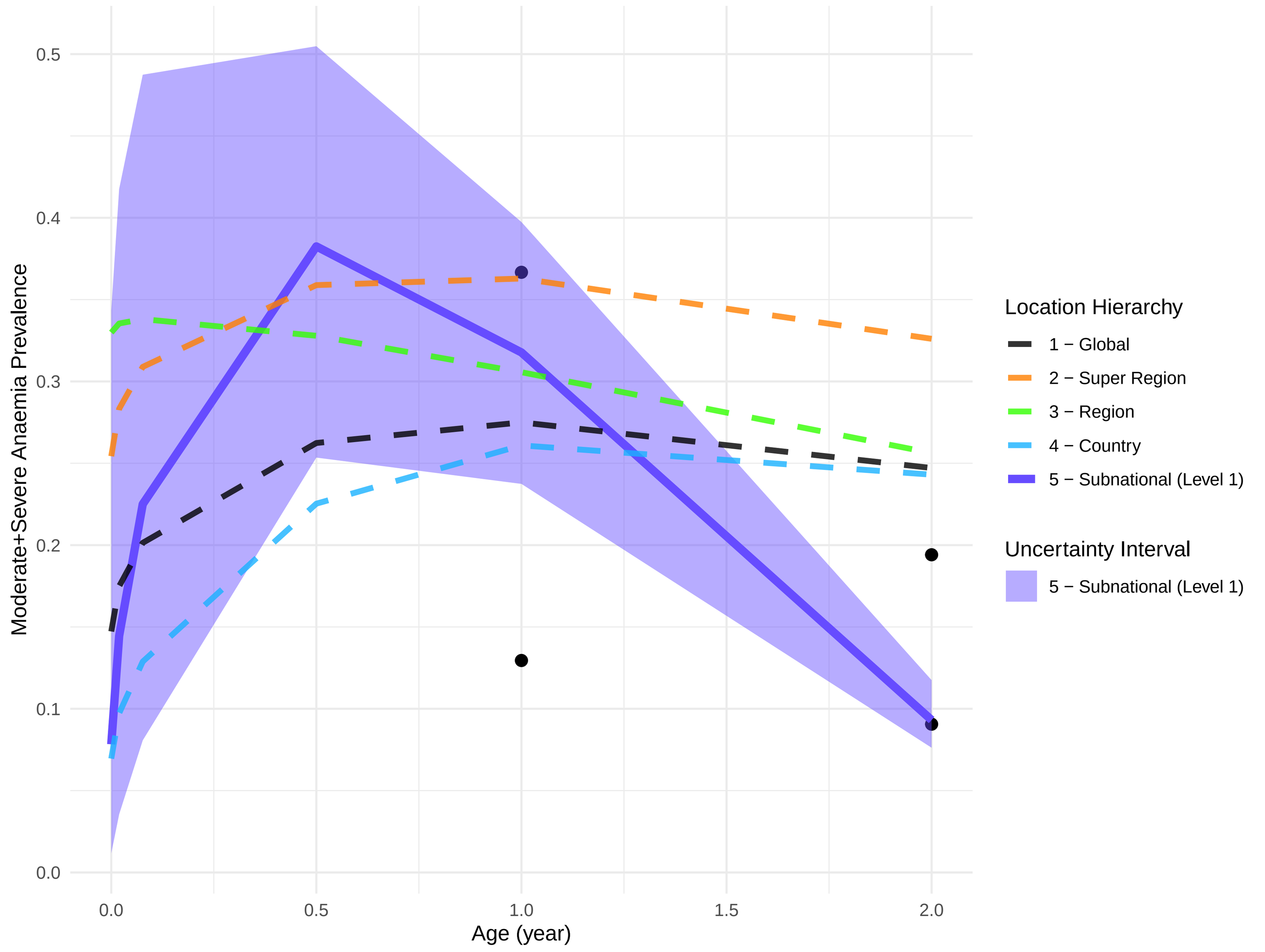

MR−BRT Age/Sex Fits − Non−pregnant Females <2 Years Old − Moderate+Severe Anaemia Prevalence − Meru
WHO−2001 Adjustment Method

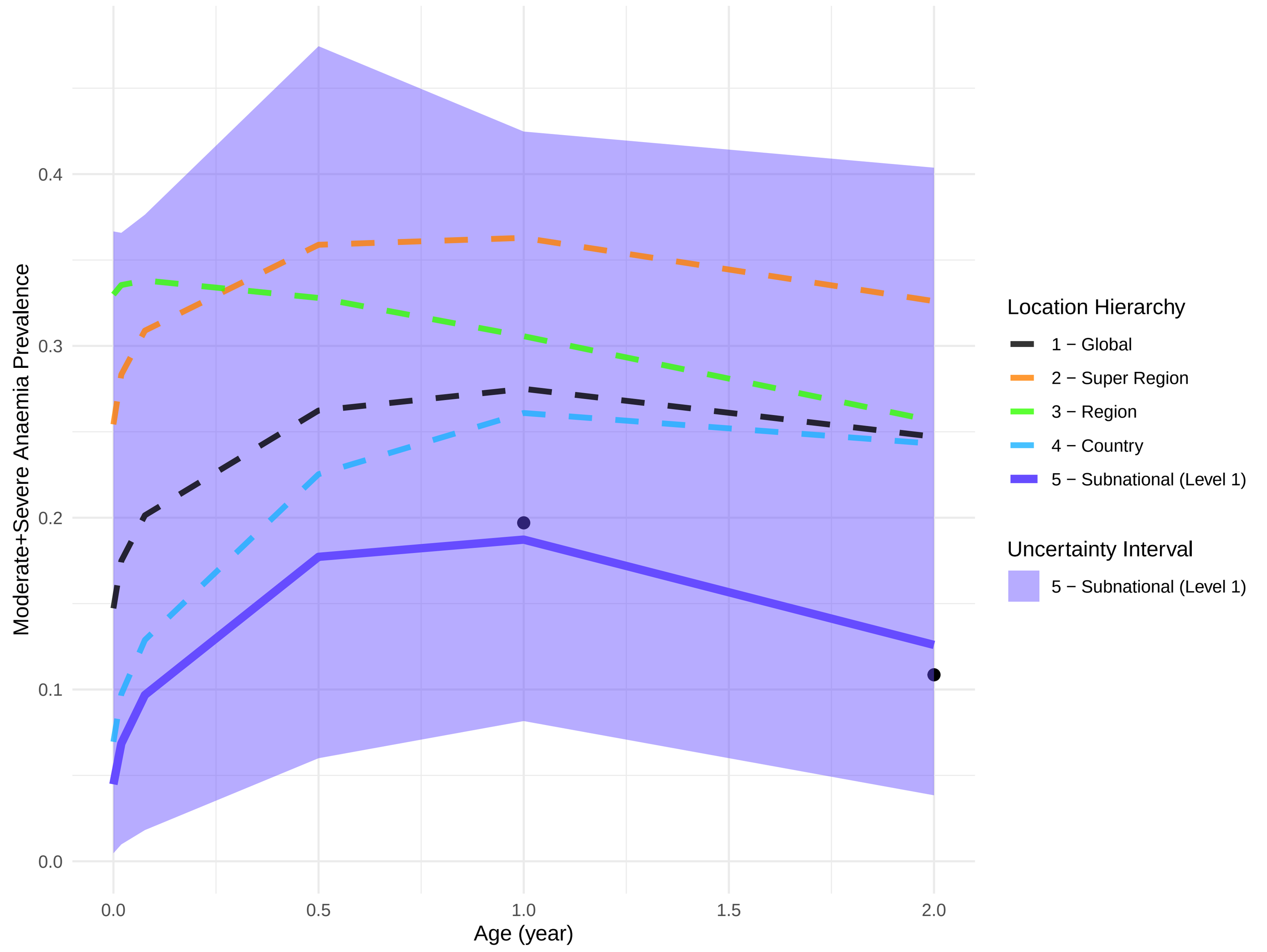

MR−BRT Age/Sex Fits − Non−pregnant Females <2 Years Old − Moderate+Severe Anaemia Prevalence − Migori
WHO−2001 Adjustment Method

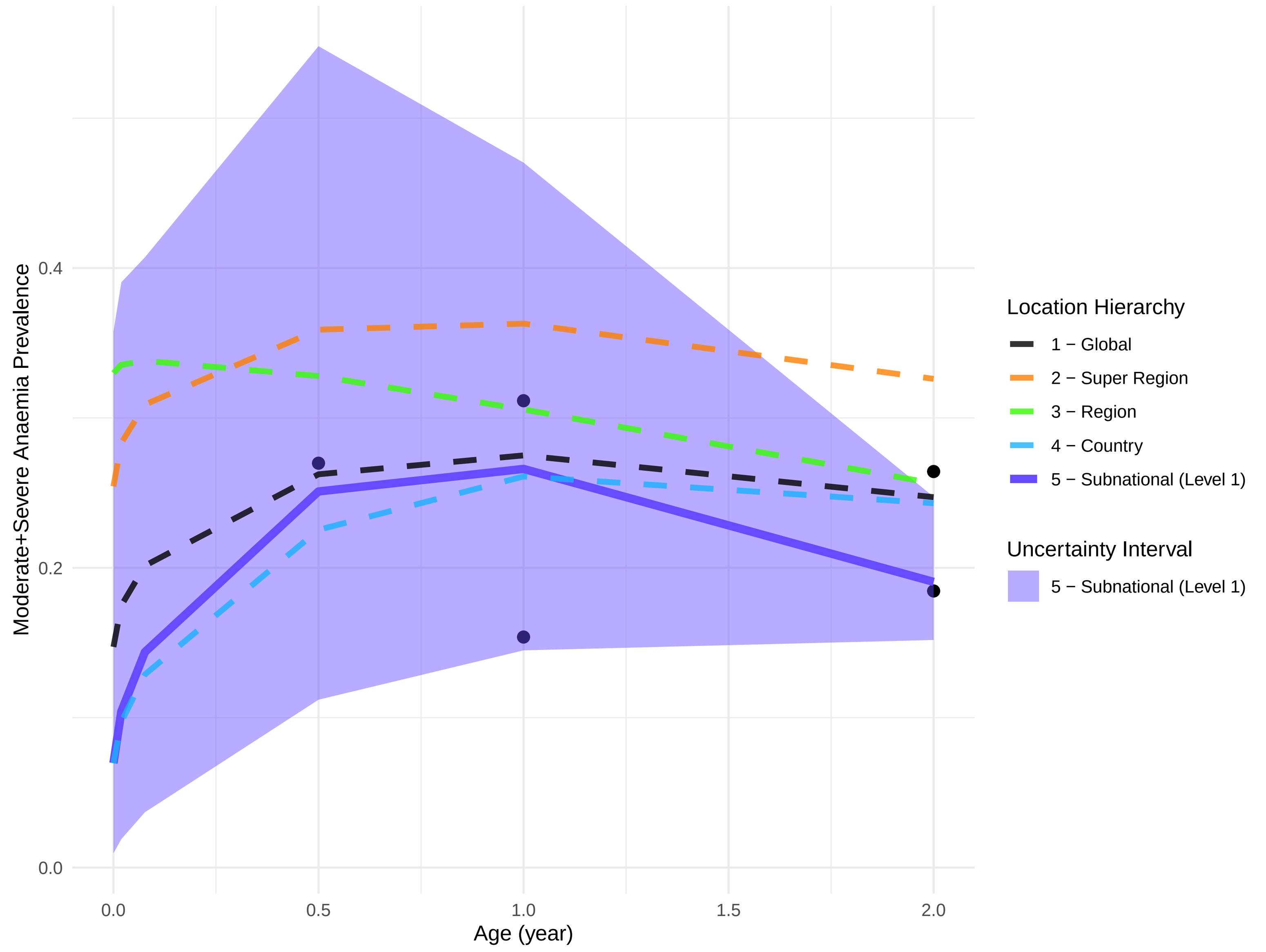

MR−BRT Age/Sex Fits − Non−pregnant Females <2 Years Old − Moderate+Severe Anaemia Prevalence − Mombasa
WHO−2001 Adjustment Method

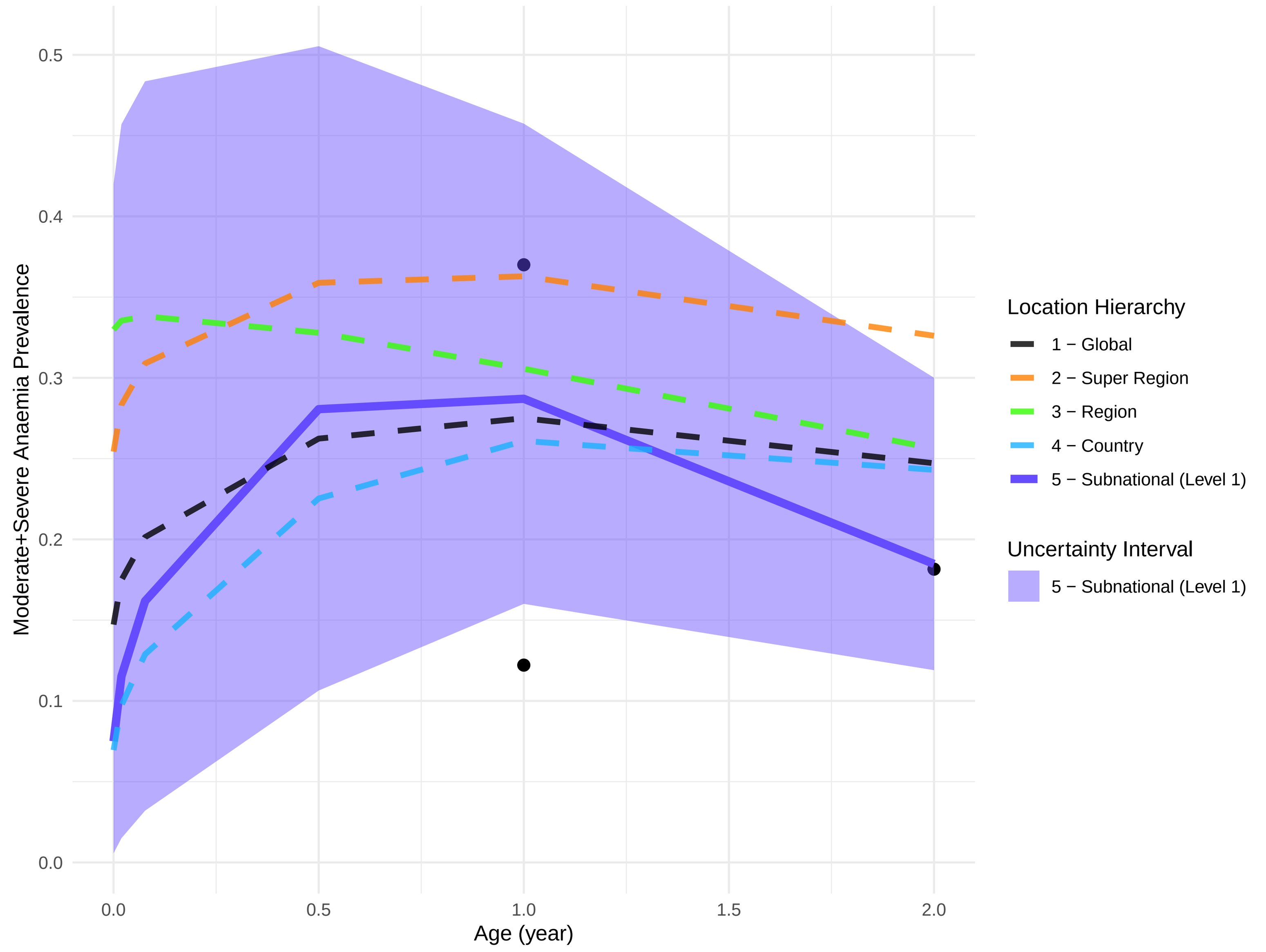

MR−BRT Age/Sex Fits − Non−pregnant Females <2 Years Old − Moderate+Severe Anaemia Prevalence − Murang'a
WHO−2001 Adjustment Method

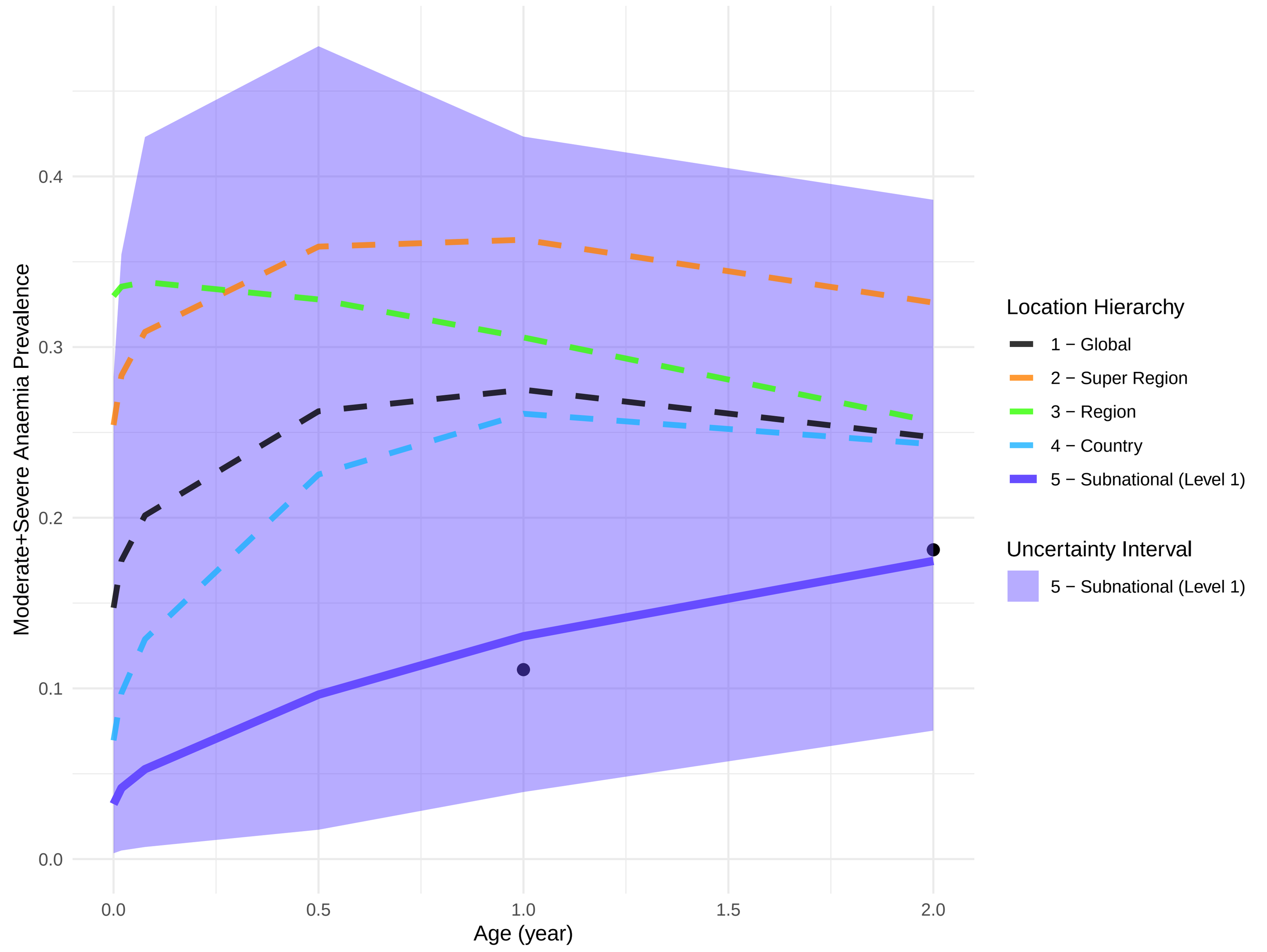

MR−BRT Age/Sex Fits − Non−pregnant Females <2 Years Old − Moderate+Severe Anaemia Prevalence − Nakuru
WHO−2001 Adjustment Method

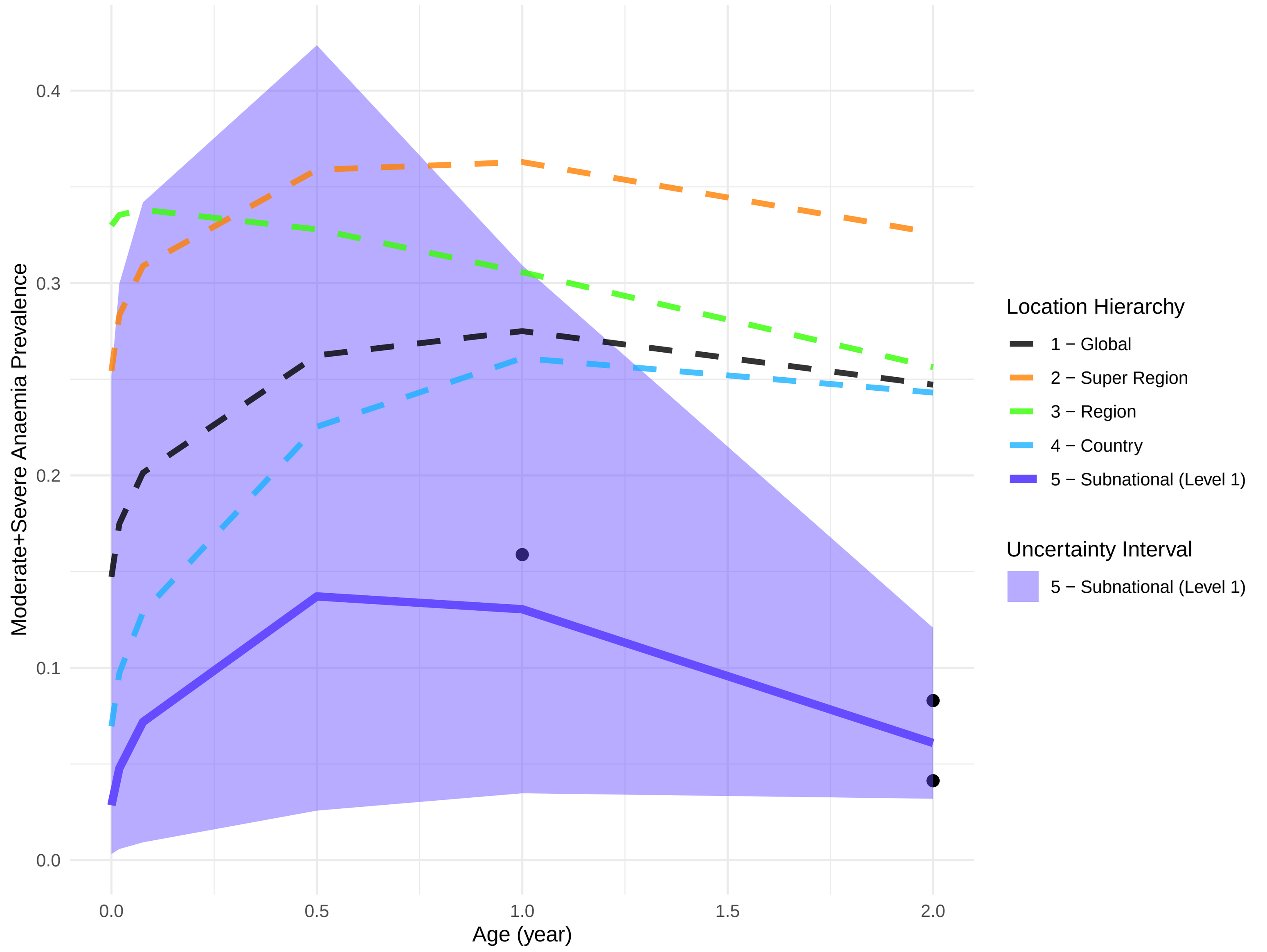

MR−BRT Age/Sex Fits − Non−pregnant Females <2 Years Old − Moderate+Severe Anaemia Prevalence − Nandi
WHO−2001 Adjustment Method

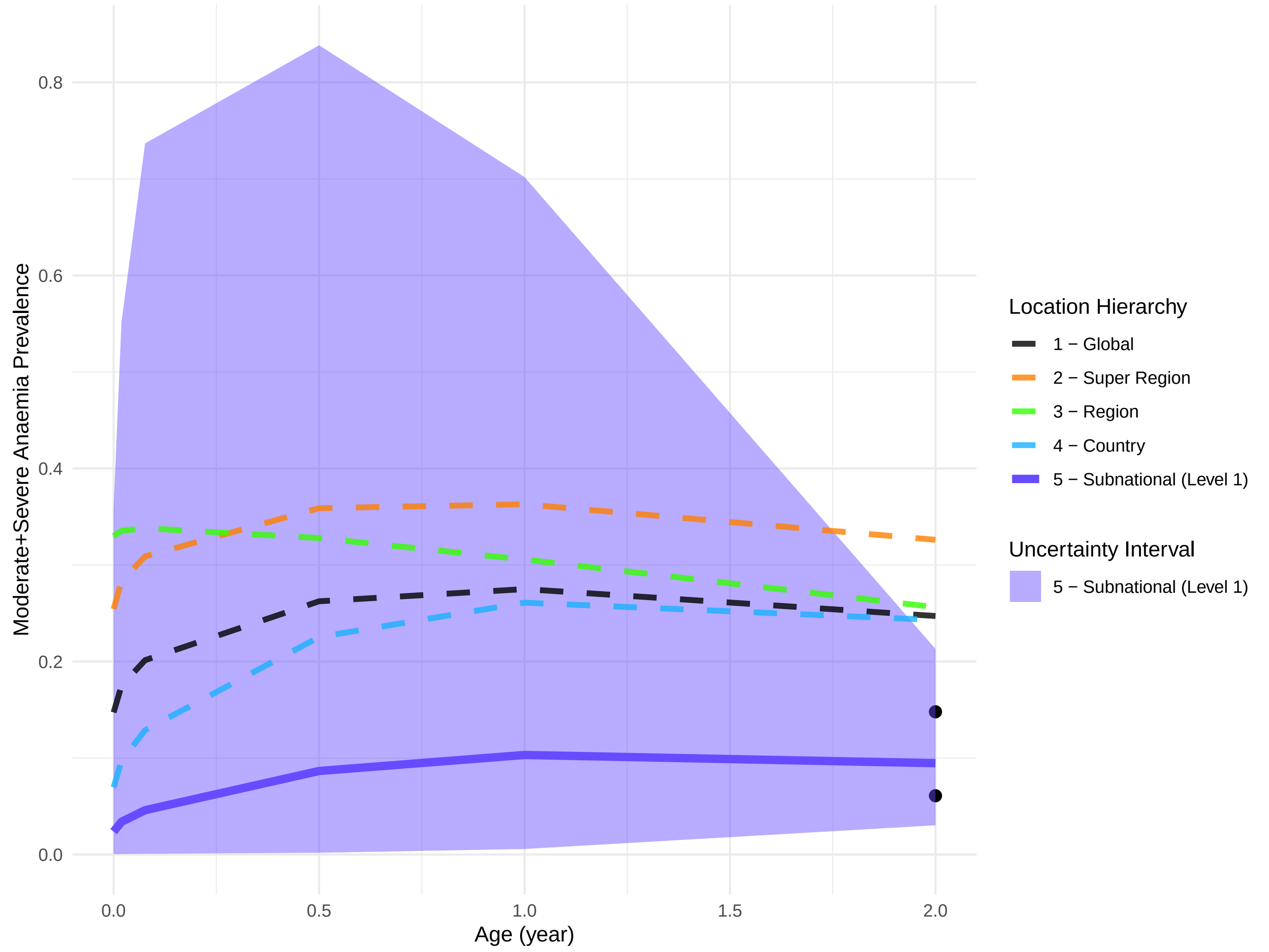

MR−BRT Age/Sex Fits − Non−pregnant Females <2 Years Old − Moderate+Severe Anaemia Prevalence − Narok
WHO−2001 Adjustment Method

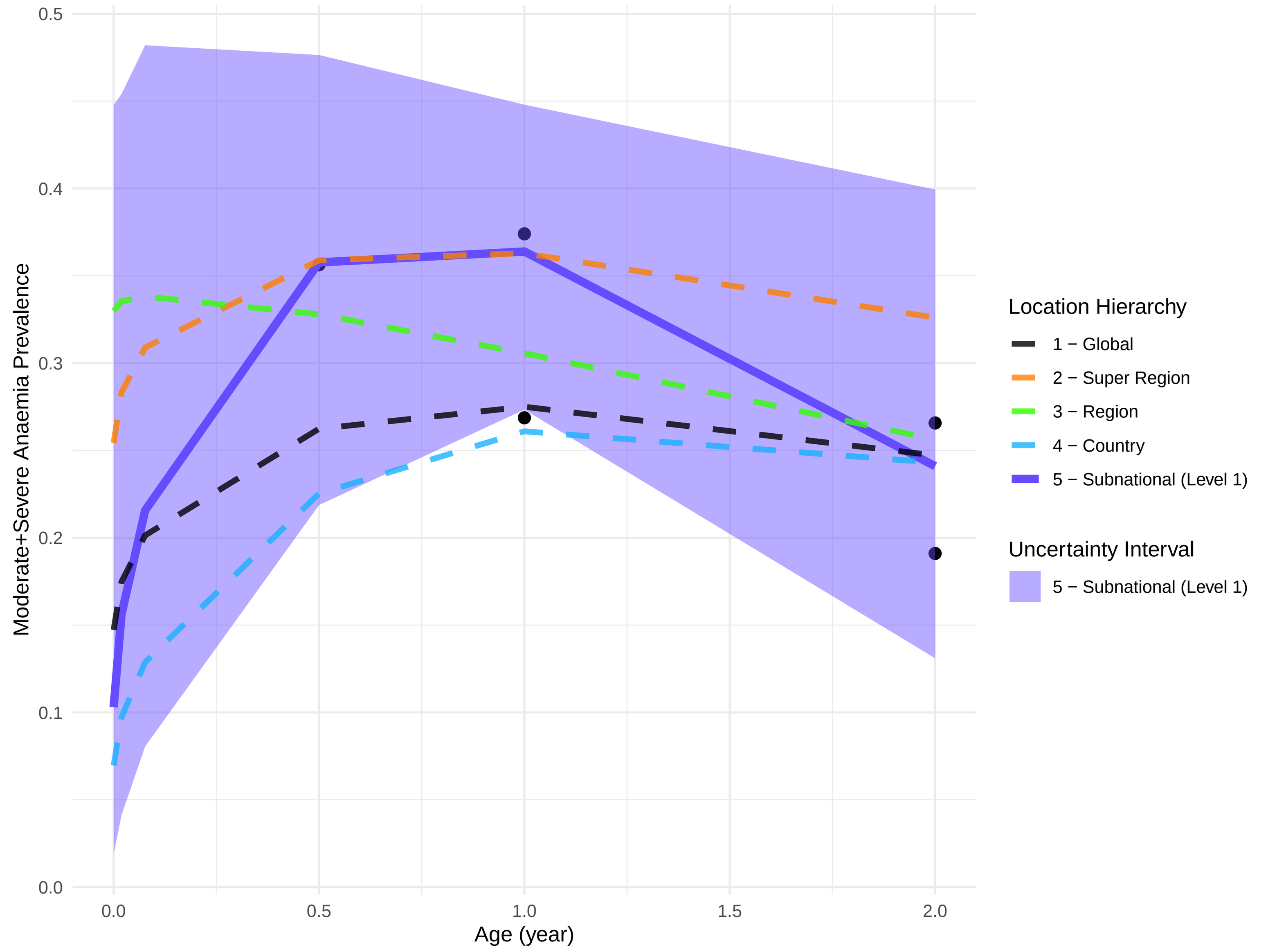

MR−BRT Age/Sex Fits − Non−pregnant Females <2 Years Old − Moderate+Severe Anaemia Prevalence − Nyamira
WHO−2001 Adjustment Method

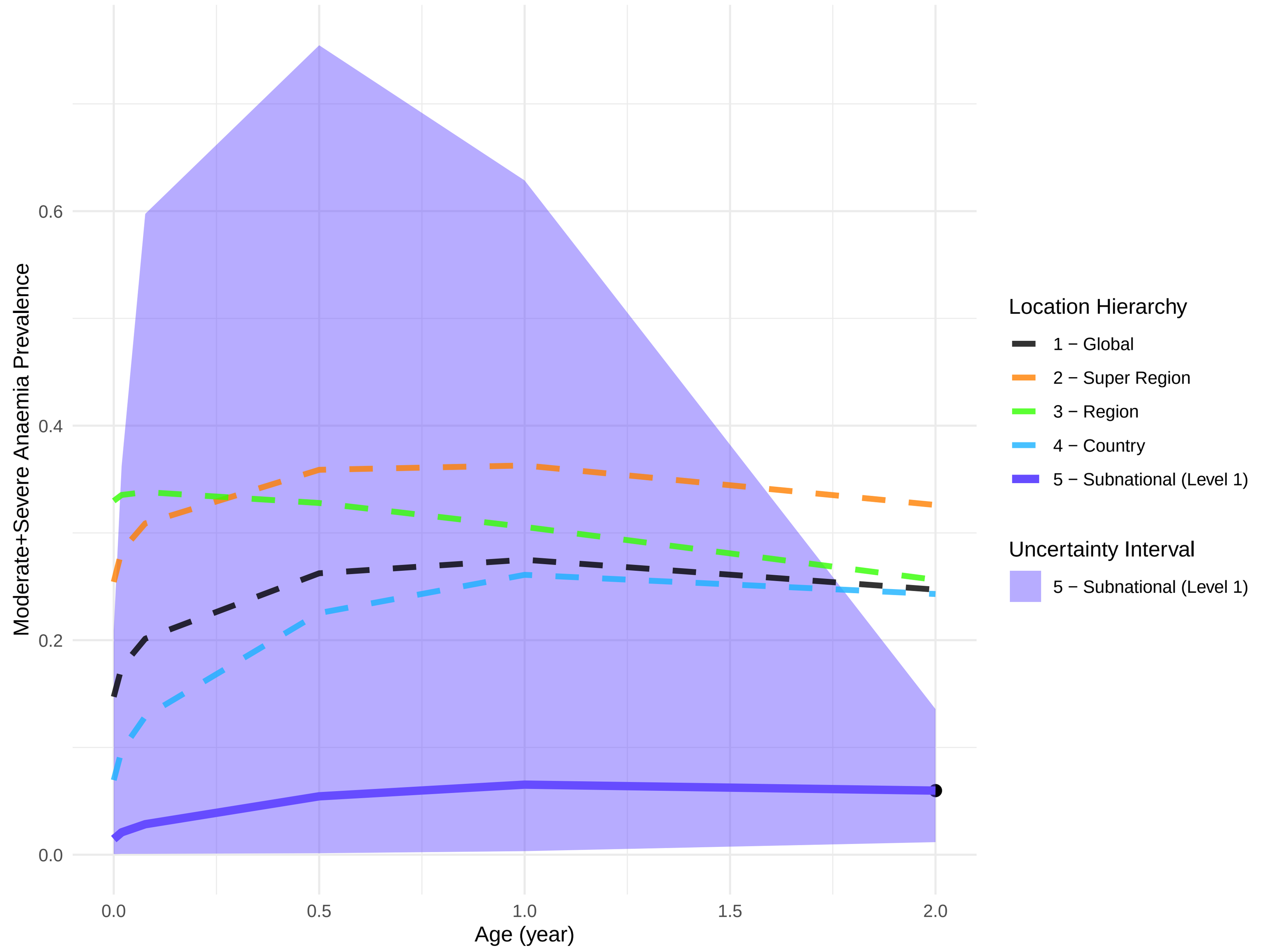

MR−BRT Age/Sex Fits − Non−pregnant Females <2 Years Old − Moderate+Severe Anaemia Prevalence − Nyandarua
WHO−2001 Adjustment Method

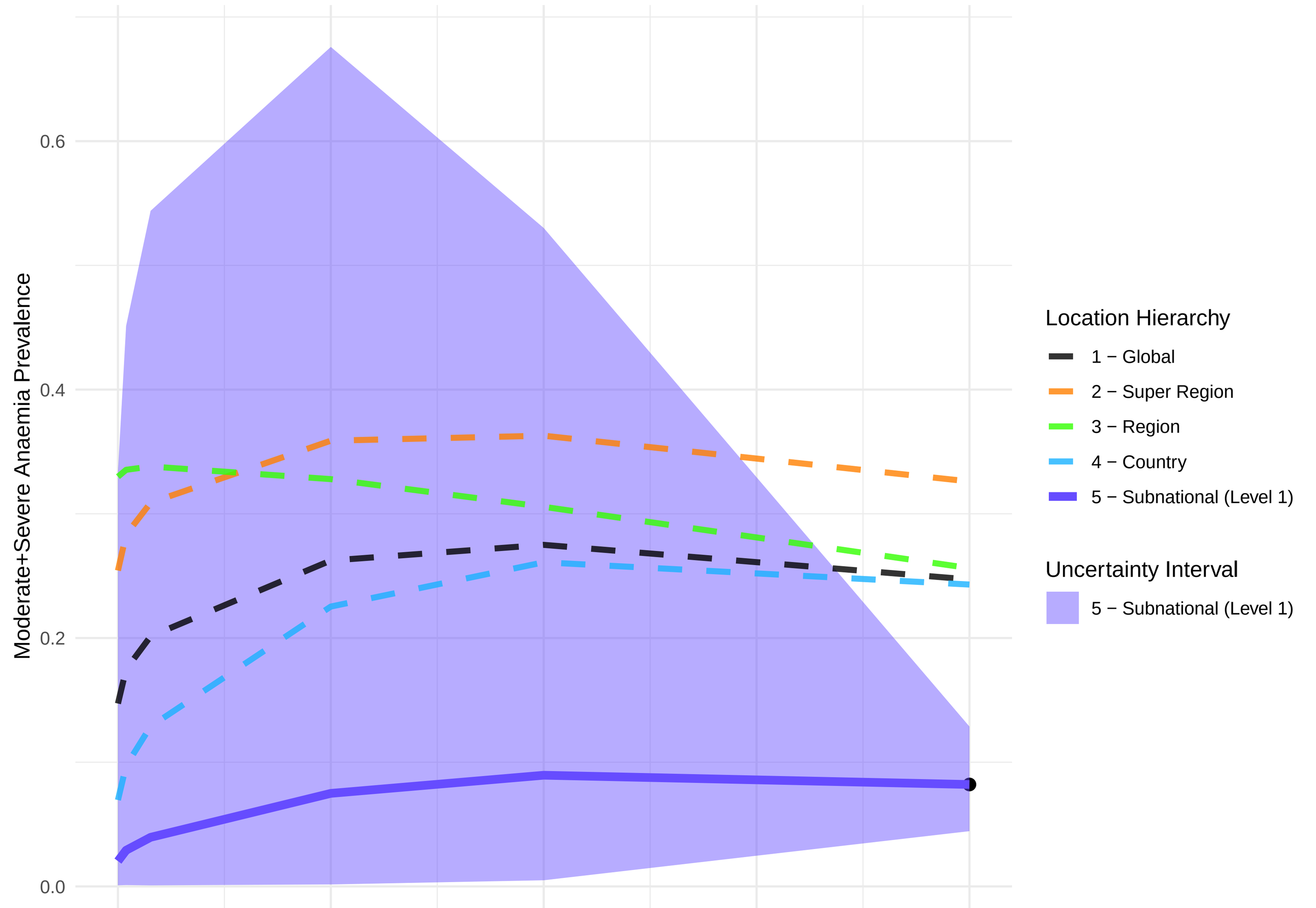

MR−BRT Age/Sex Fits − Non−pregnant Females <2 Years Old − Moderate+Severe Anaemia Prevalence − Nyeri
WHO−2001 Adjustment Method

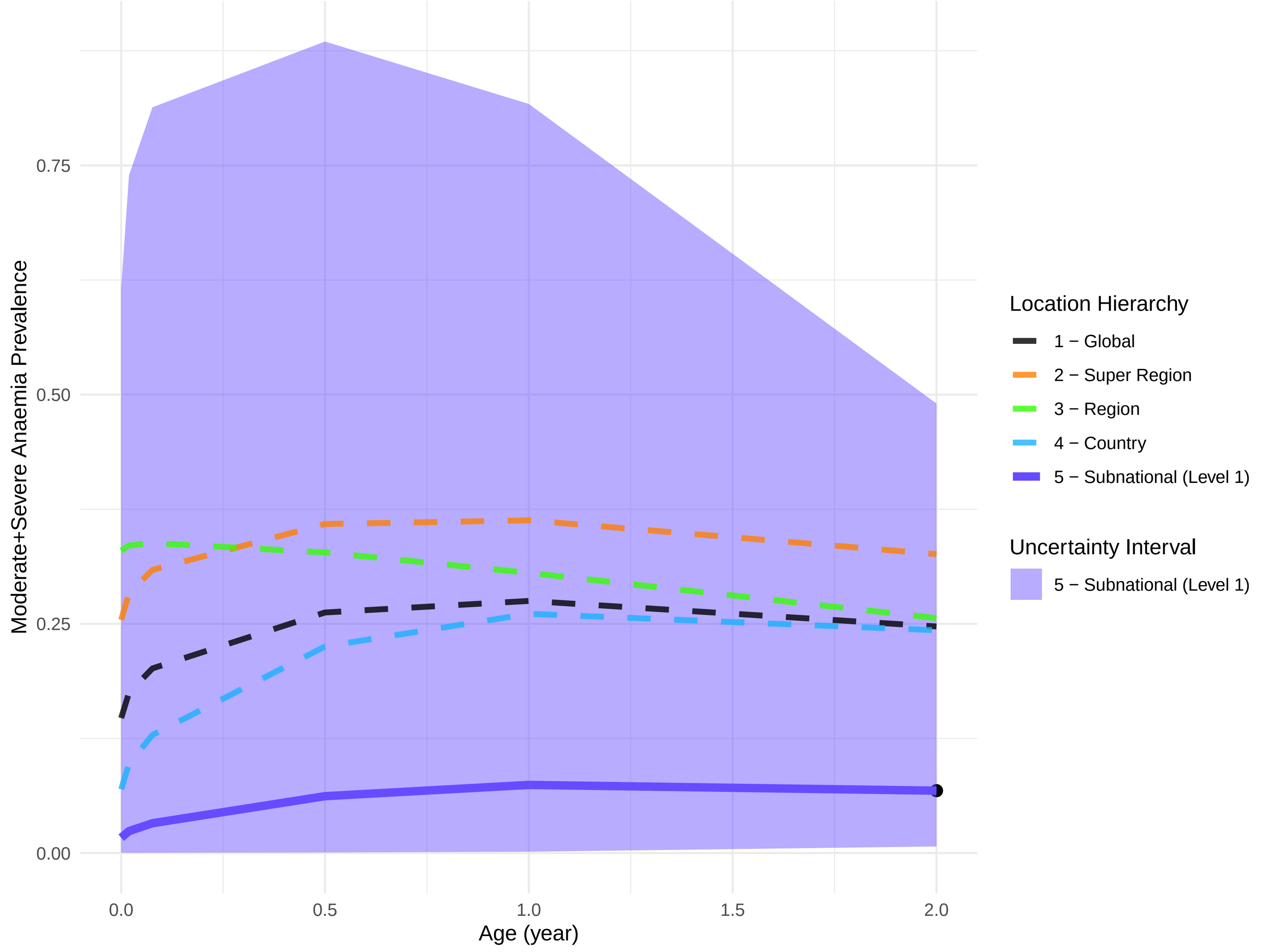

MR−BRT Age/Sex Fits − Non−pregnant Females <2 Years Old − Moderate+Severe Anaemia Prevalence − Samburu
WHO−2001 Adjustment Method

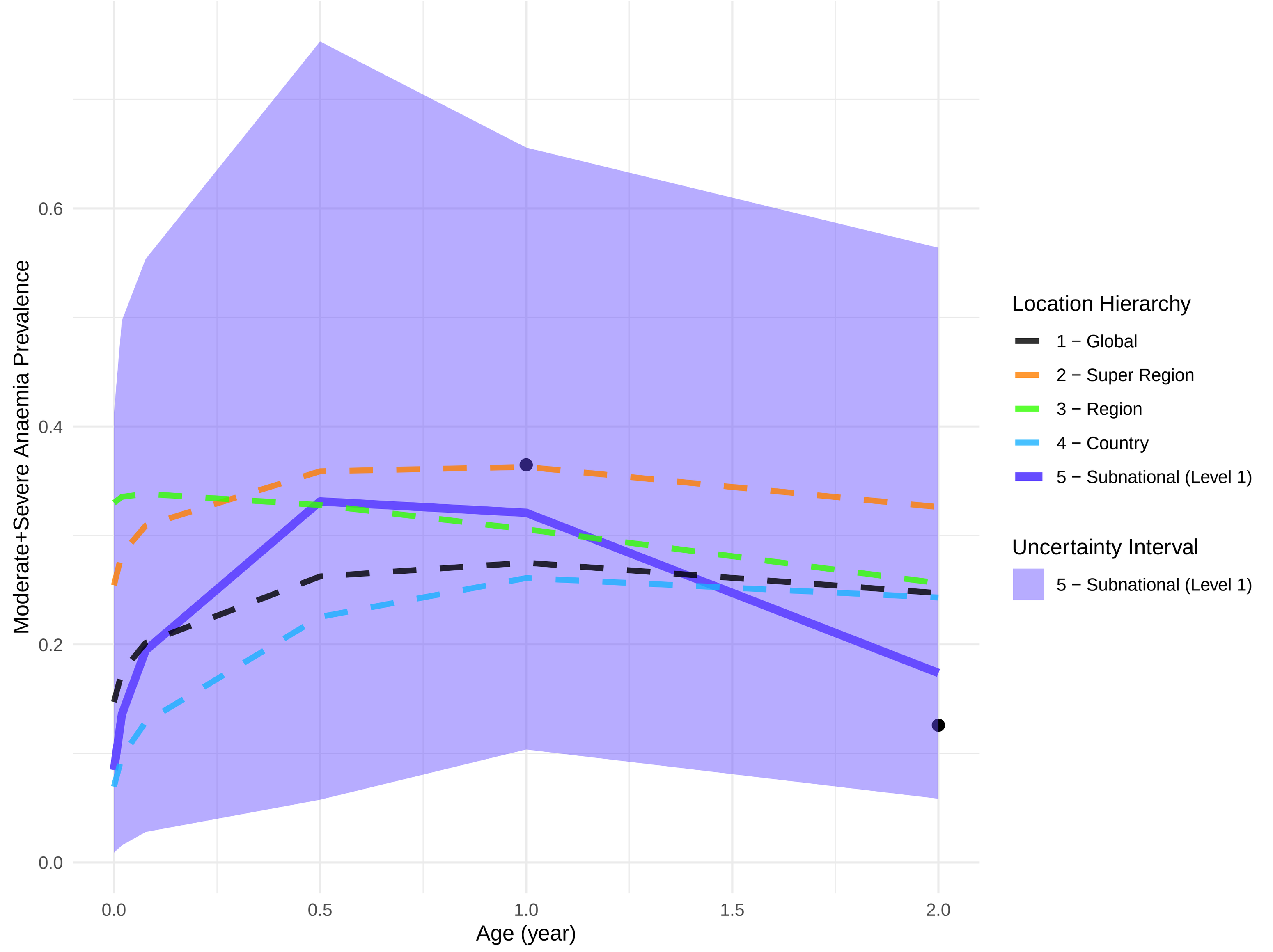

MR−BRT Age/Sex Fits − Non−pregnant Females <2 Years Old − Moderate+Severe Anaemia Prevalence − Siaya
WHO−2001 Adjustment Method

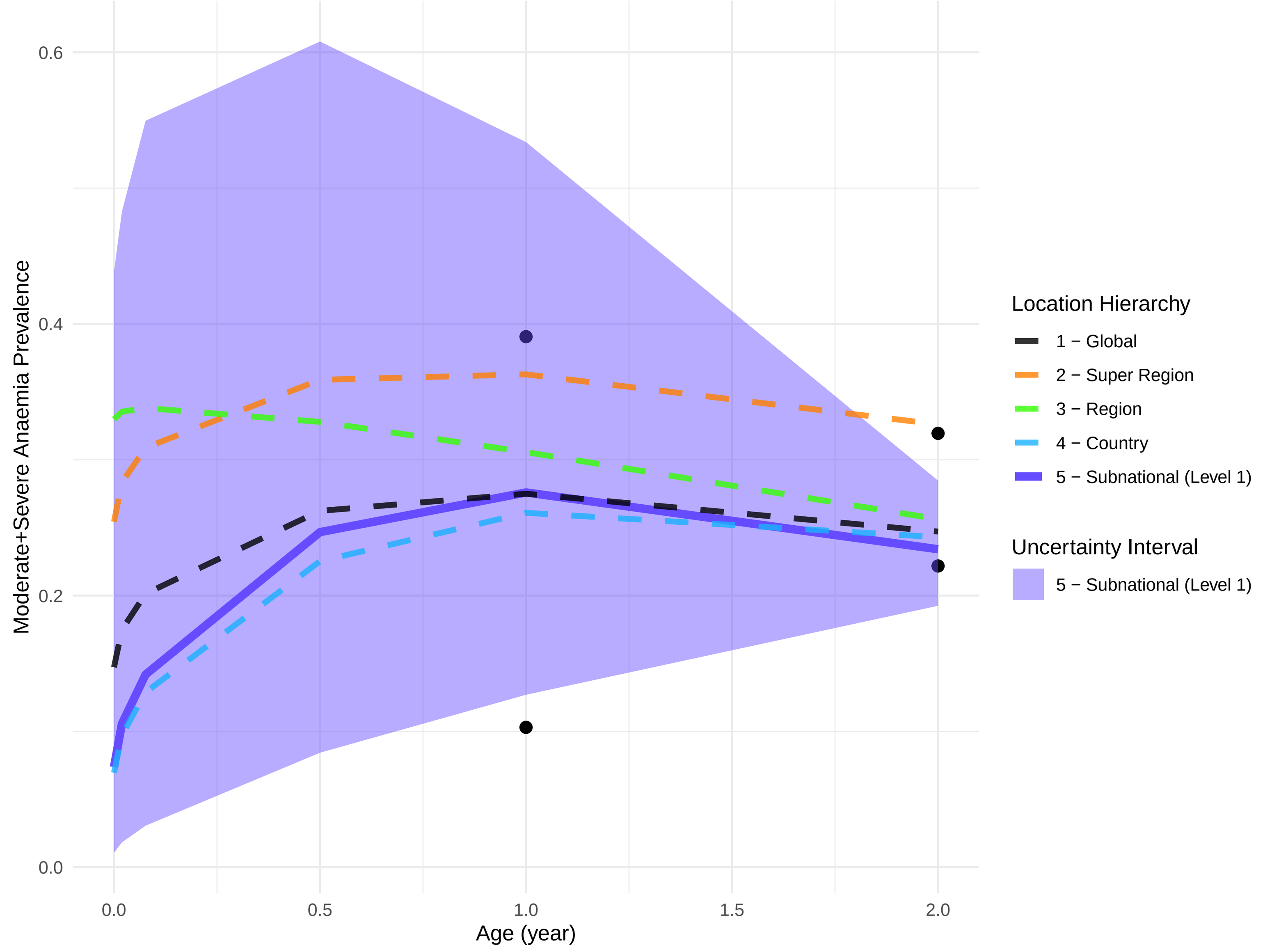

MR−BRT Age/Sex Fits − Non−pregnant Females <2 Years Old − Moderate+Severe Anaemia Prevalence − Taita Taveta
WHO−2001 Adjustment Method

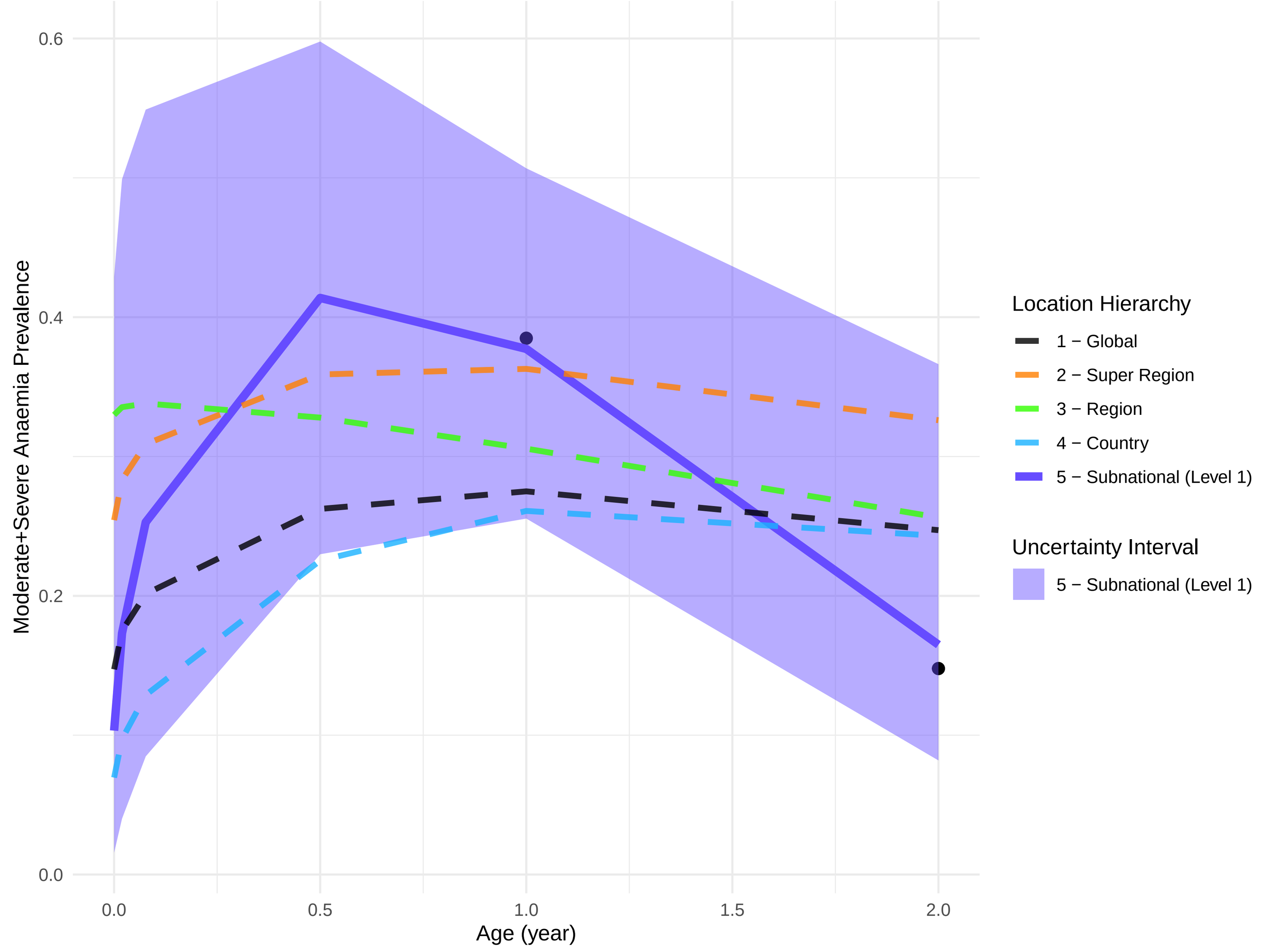

MR−BRT Age/Sex Fits − Non−pregnant Females <2 Years Old − Moderate+Severe Anaemia Prevalence − Tana River
WHO−2001 Adjustment Method

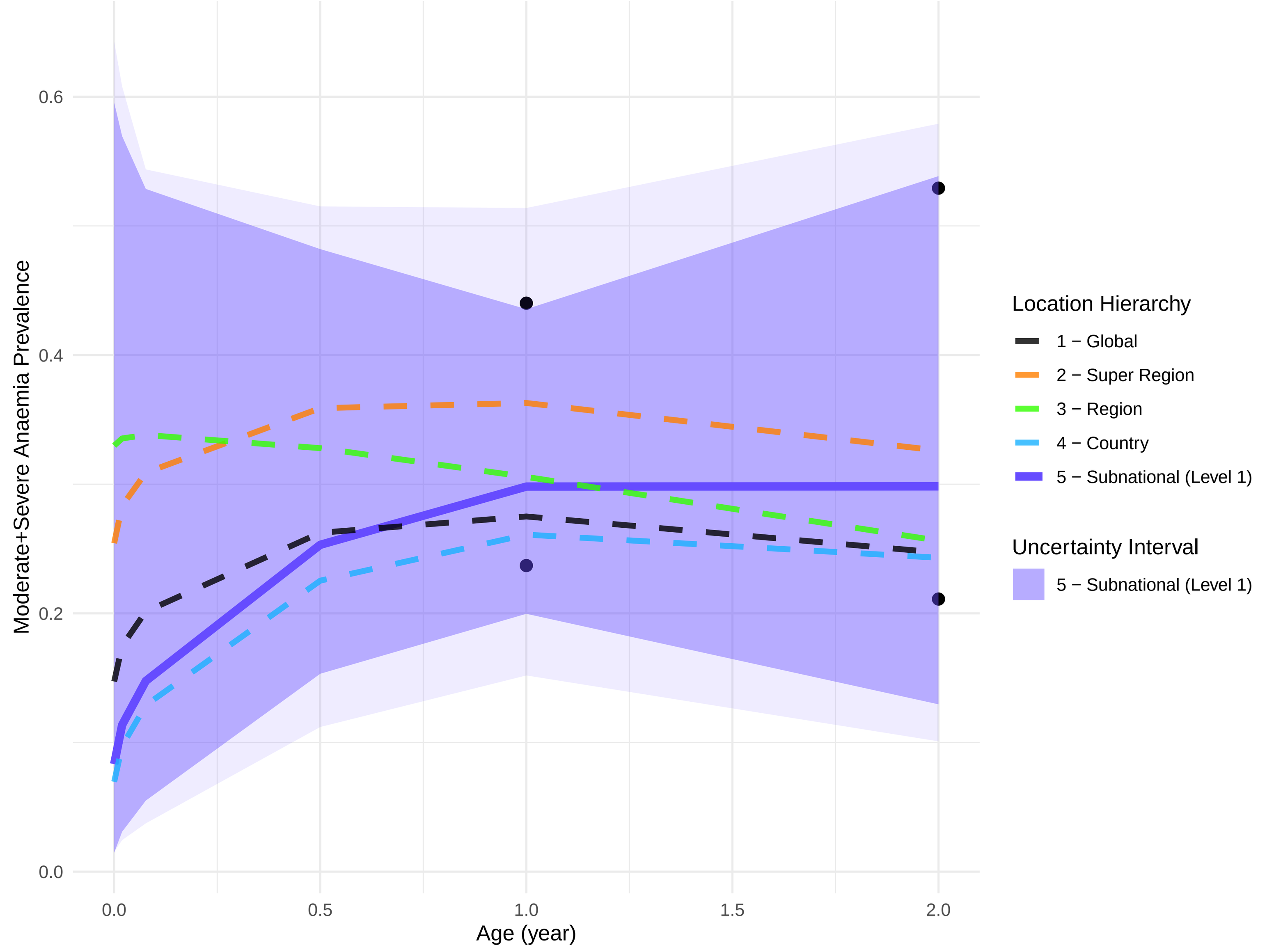

MR−BRT Age/Sex Fits − Non−pregnant Females <2 Years Old − Moderate+Severe Anaemia Prevalence − Tharaka Nithi
WHO−2001 Adjustment Method

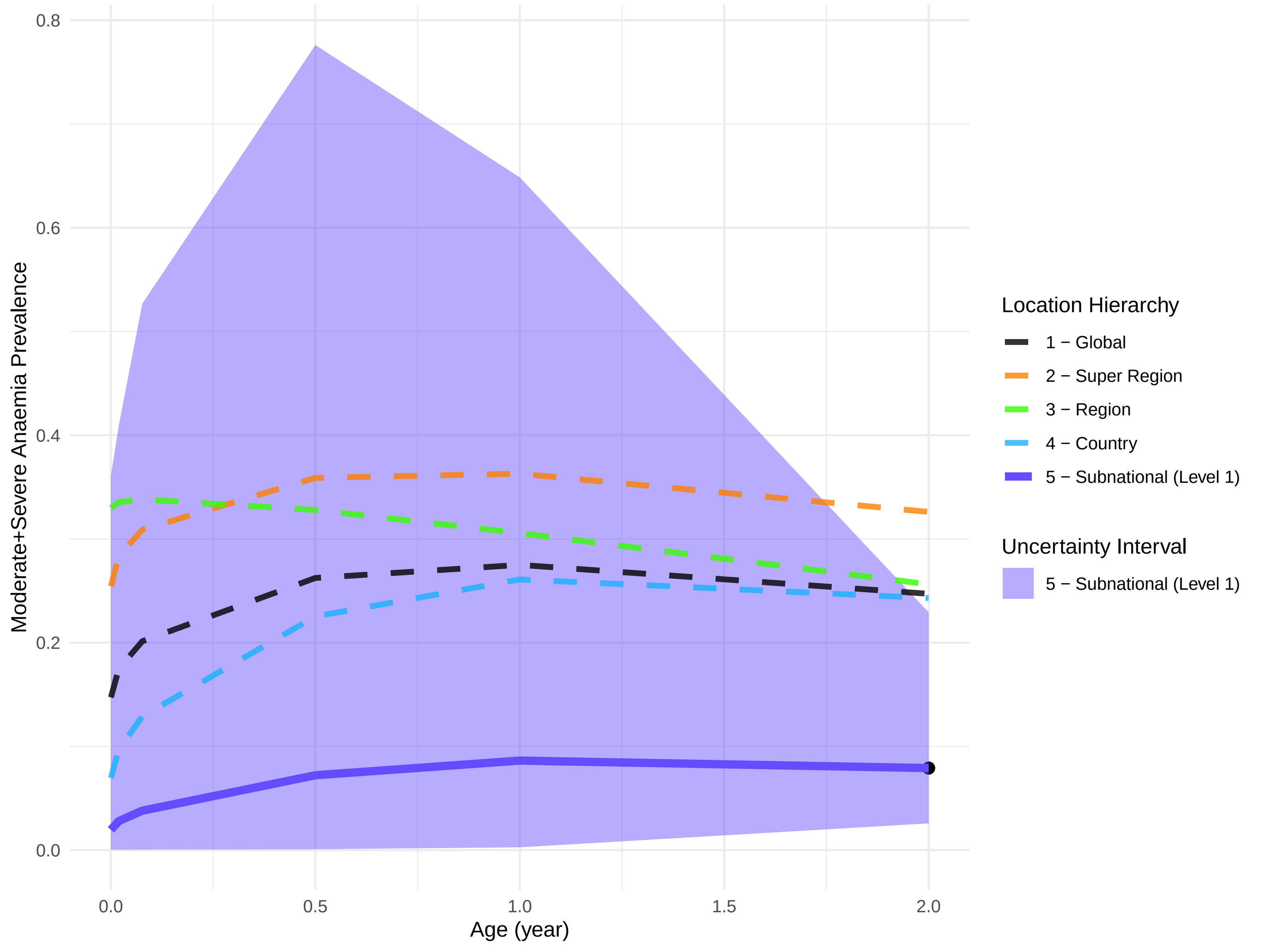

MR−BRT Age/Sex Fits − Non−pregnant Females <2 Years Old − Moderate+Severe Anaemia Prevalence − Trans Nzoia
WHO−2001 Adjustment Method

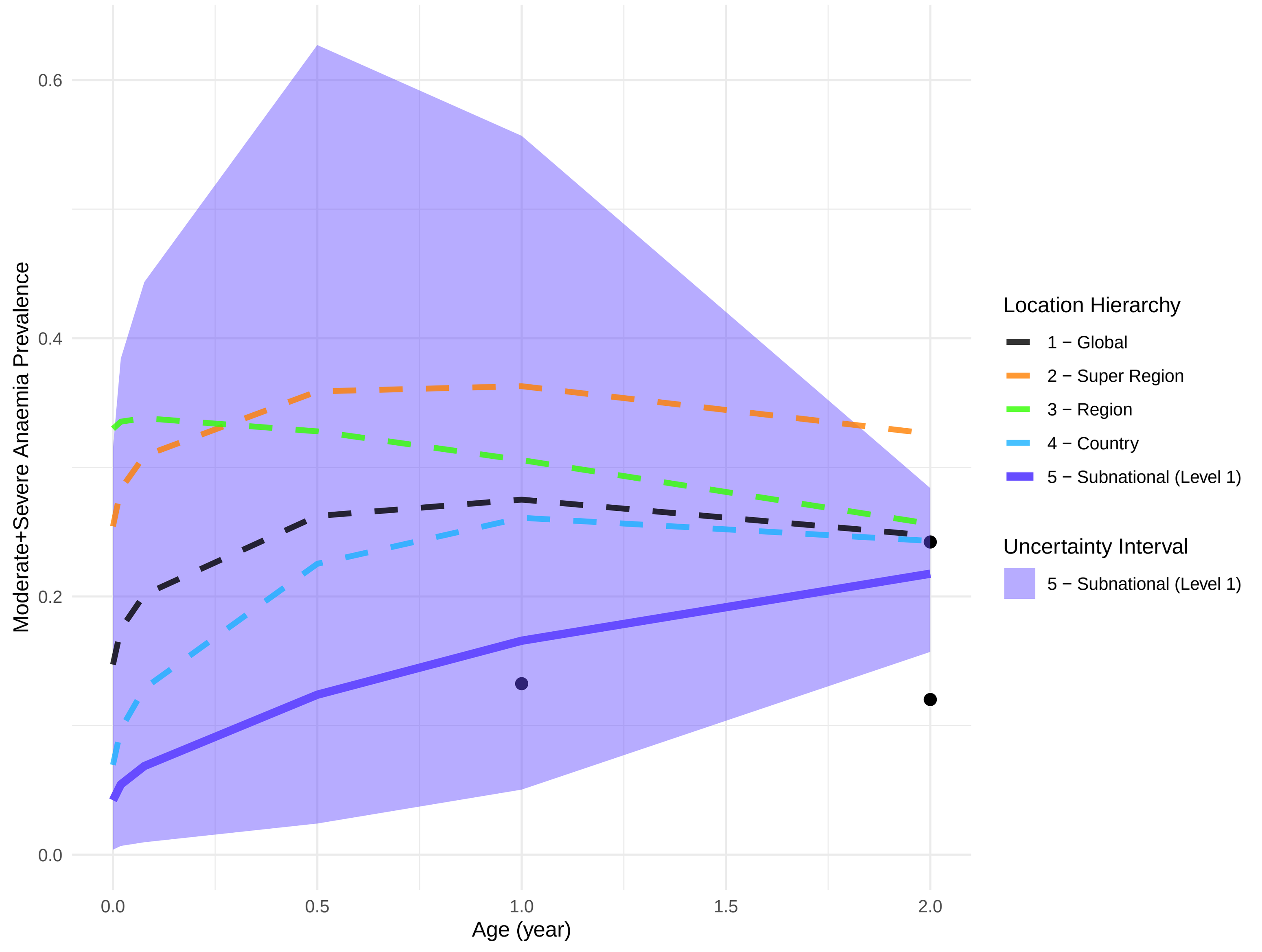

MR−BRT Age/Sex Fits − Non−pregnant Females <2 Years Old − Moderate+Severe Anaemia Prevalence − Turkana
WHO−2001 Adjustment Method

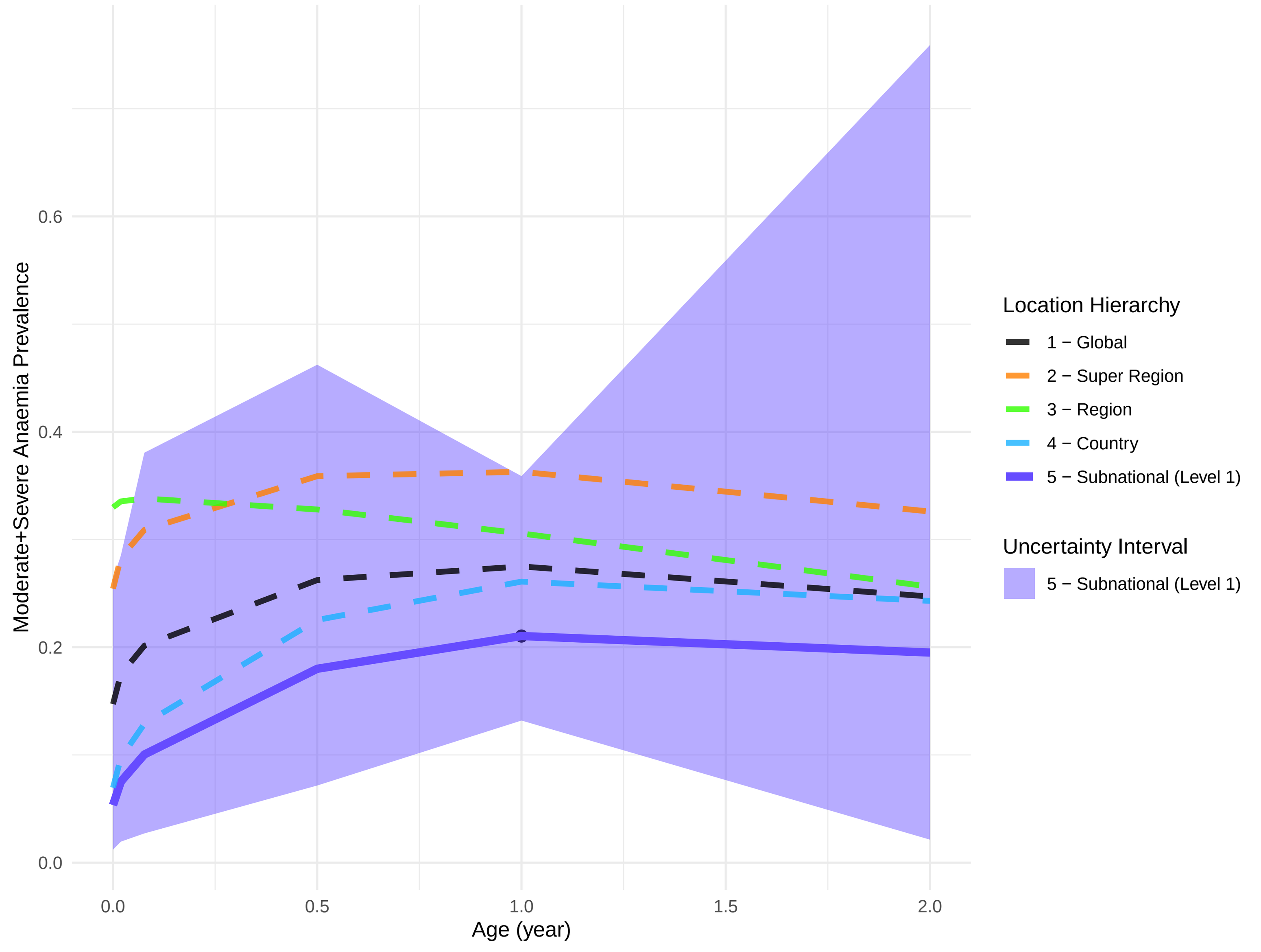

MR−BRT Age/Sex Fits − Non−pregnant Females <2 Years Old − Moderate+Severe Anaemia Prevalence − Uasin Gishu
WHO−2001 Adjustment Method

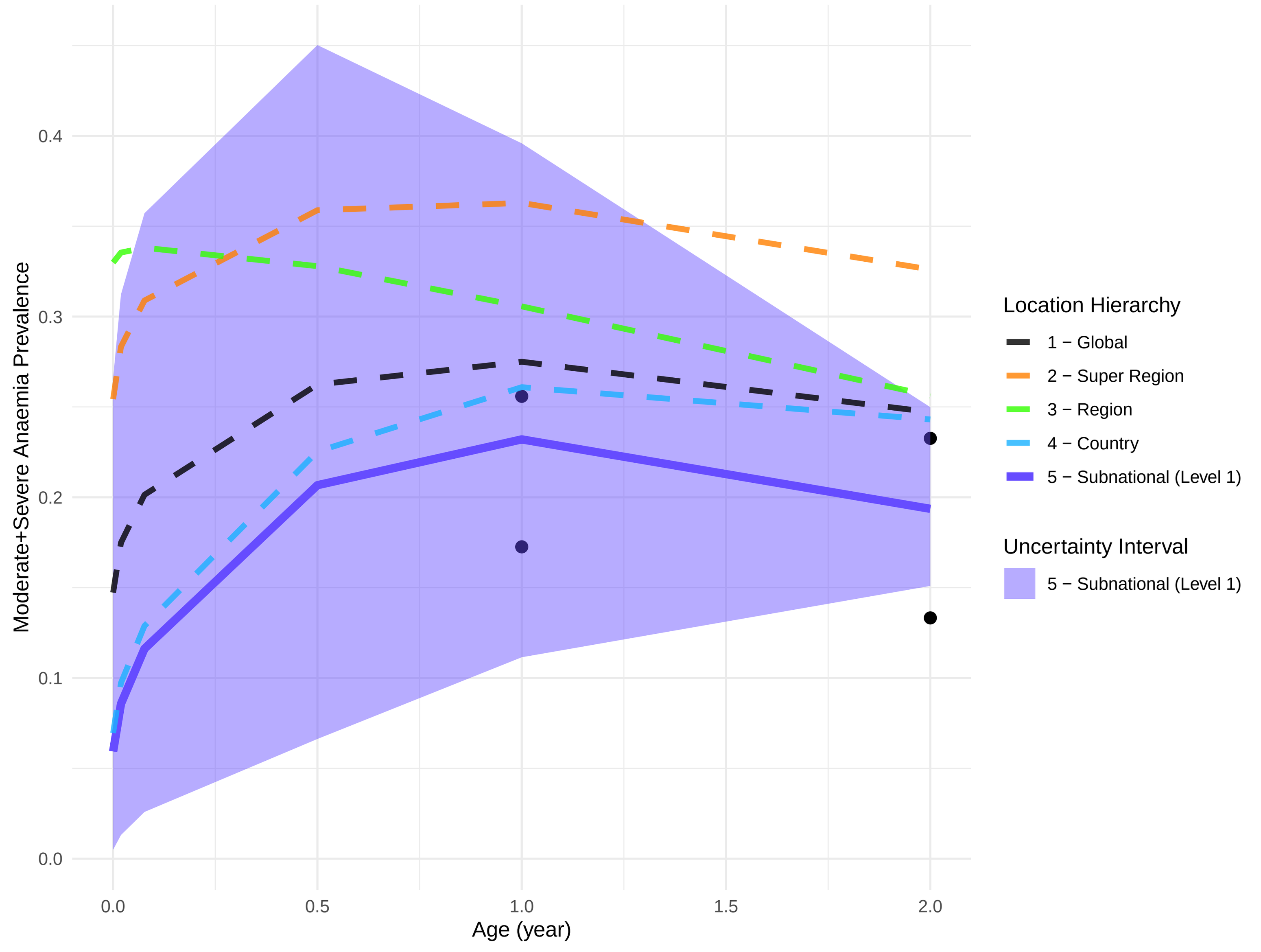

MR−BRT Age/Sex Fits − Non−pregnant Females <2 Years Old − Moderate+Severe Anaemia Prevalence − Vihiga
WHO−2001 Adjustment Method

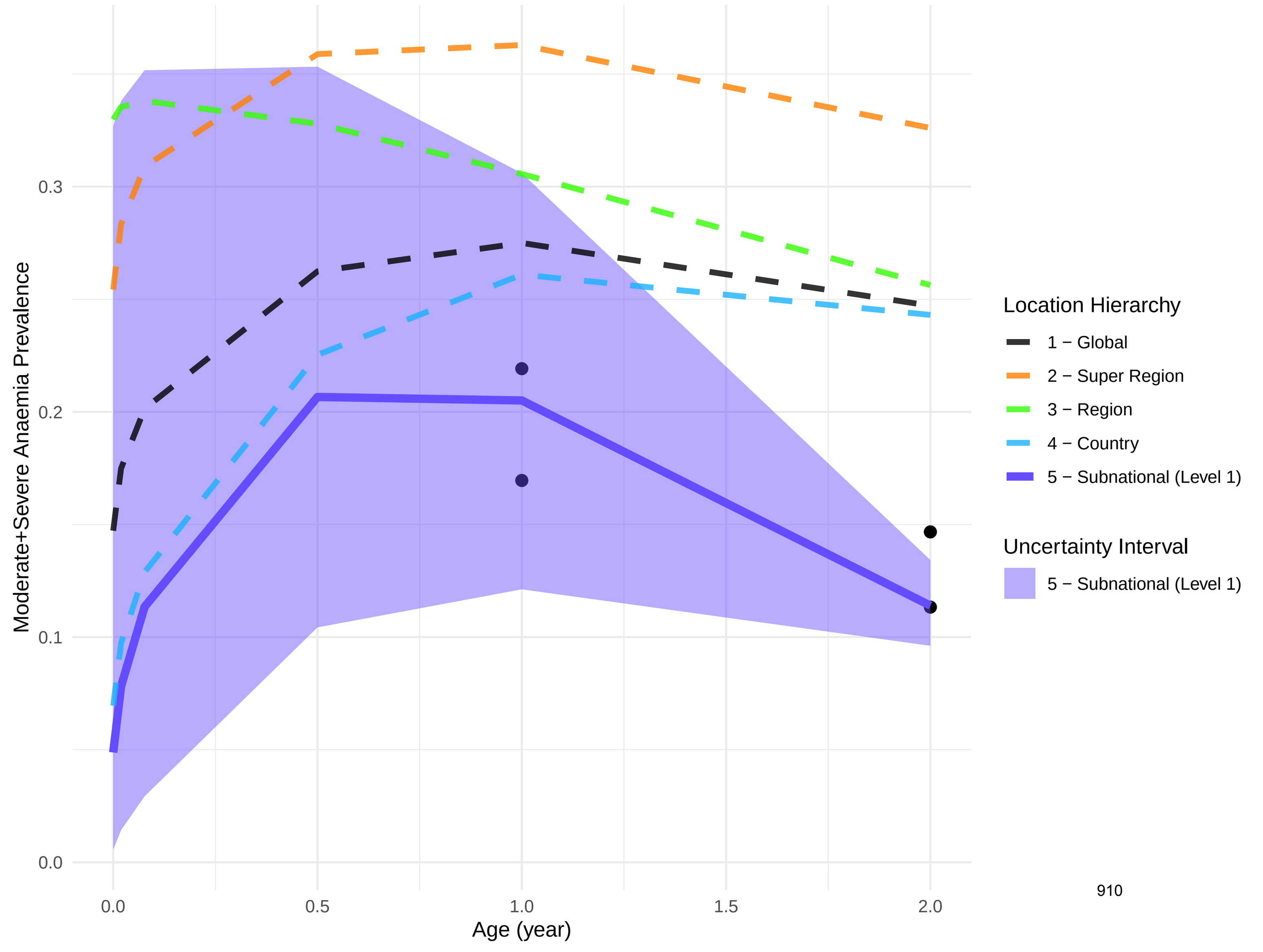

MR−BRT Age/Sex Fits − Non−pregnant Females <2 Years Old − Moderate+Severe Anaemia Prevalence − Wajir
WHO−2001 Adjustment Method

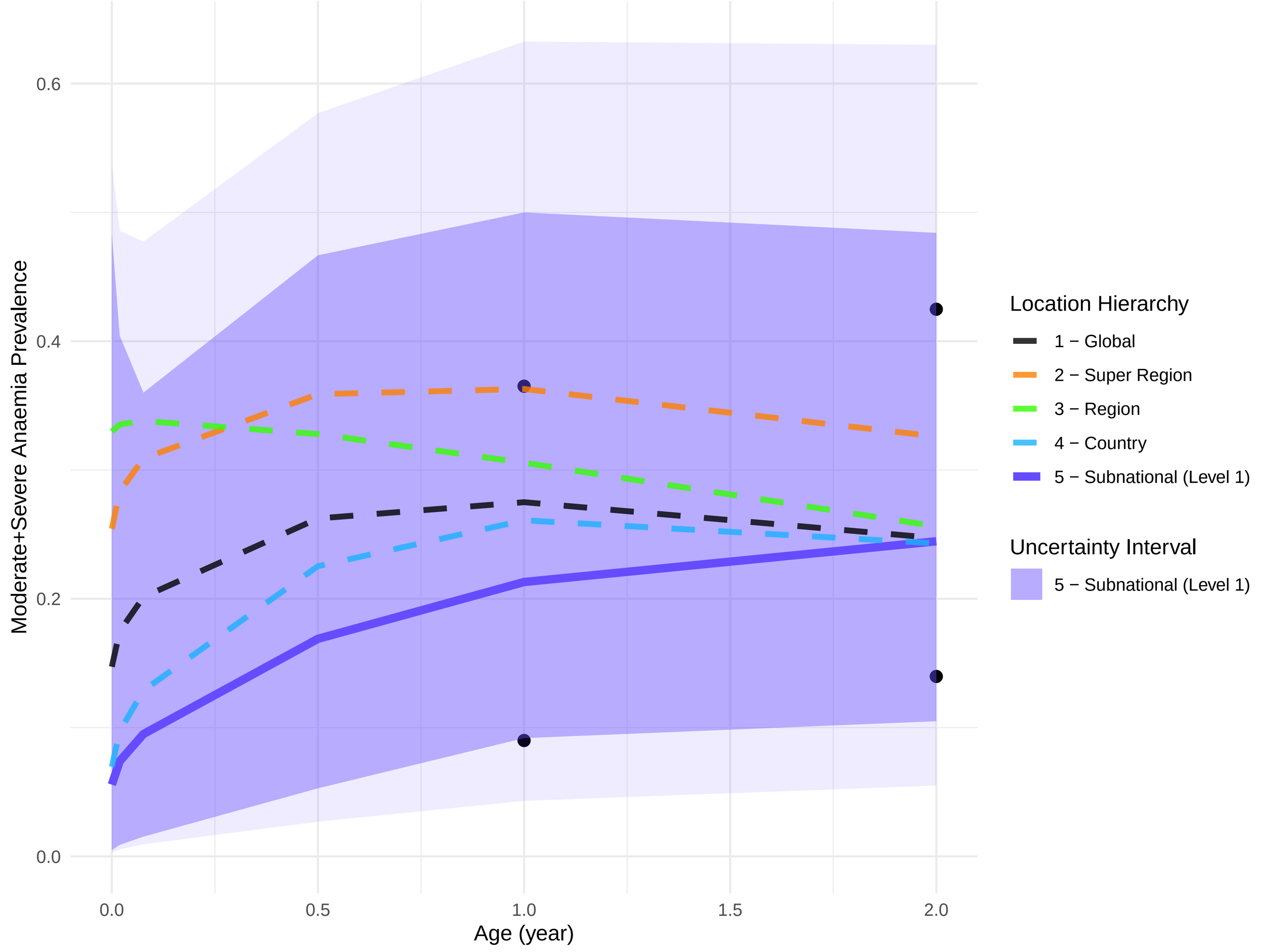

MR−BRT Age/Sex Fits − Non−pregnant Females <2 Years Old − Moderate+Severe Anaemia Prevalence − West Pokot
WHO−2001 Adjustment Method

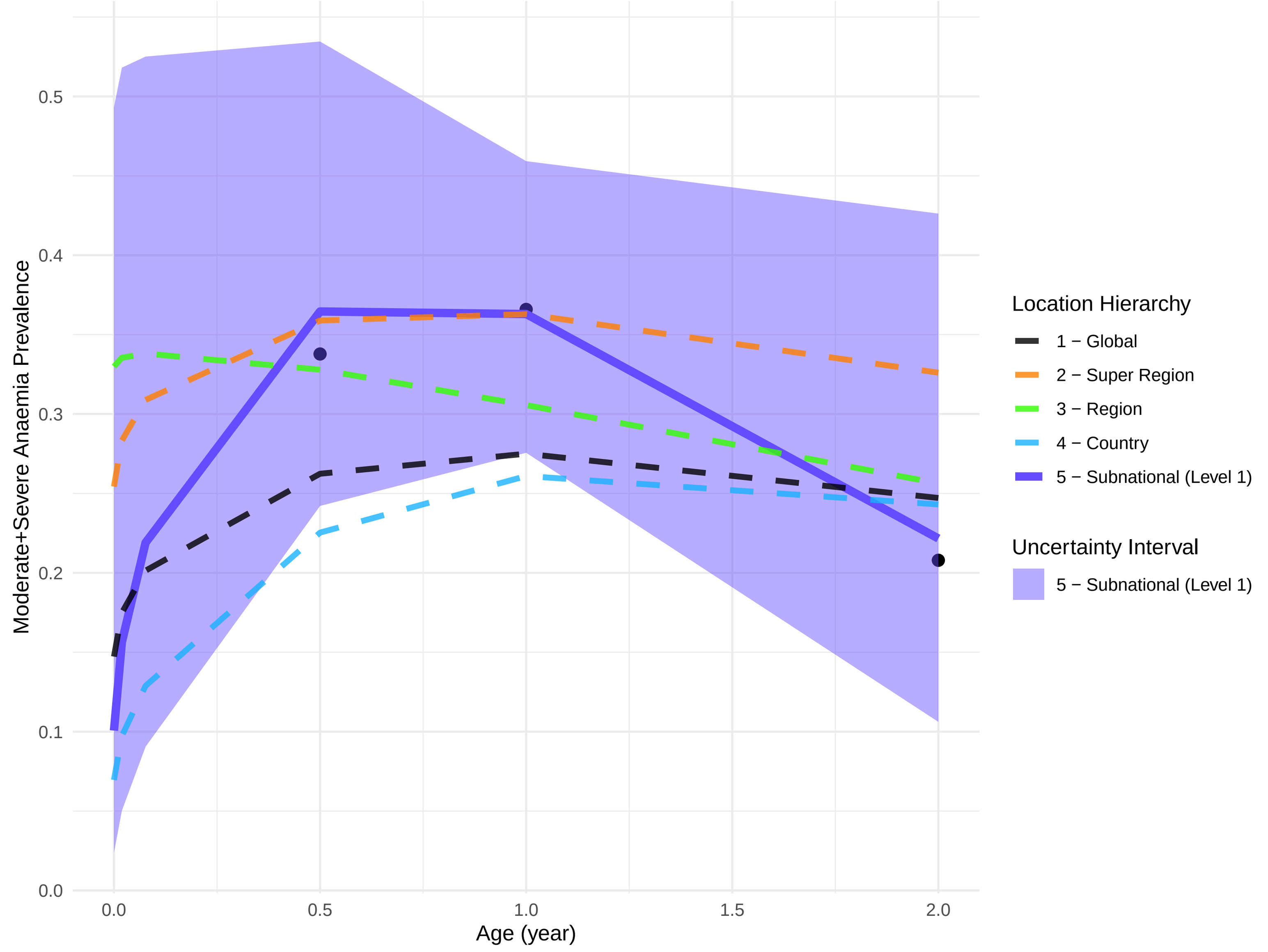

MR−BRT Age/Sex Fits − Non−pregnant Females <2 Years Old − Moderate+Severe Anaemia Prevalence − Madagascar
WHO−2001 Adjustment Method

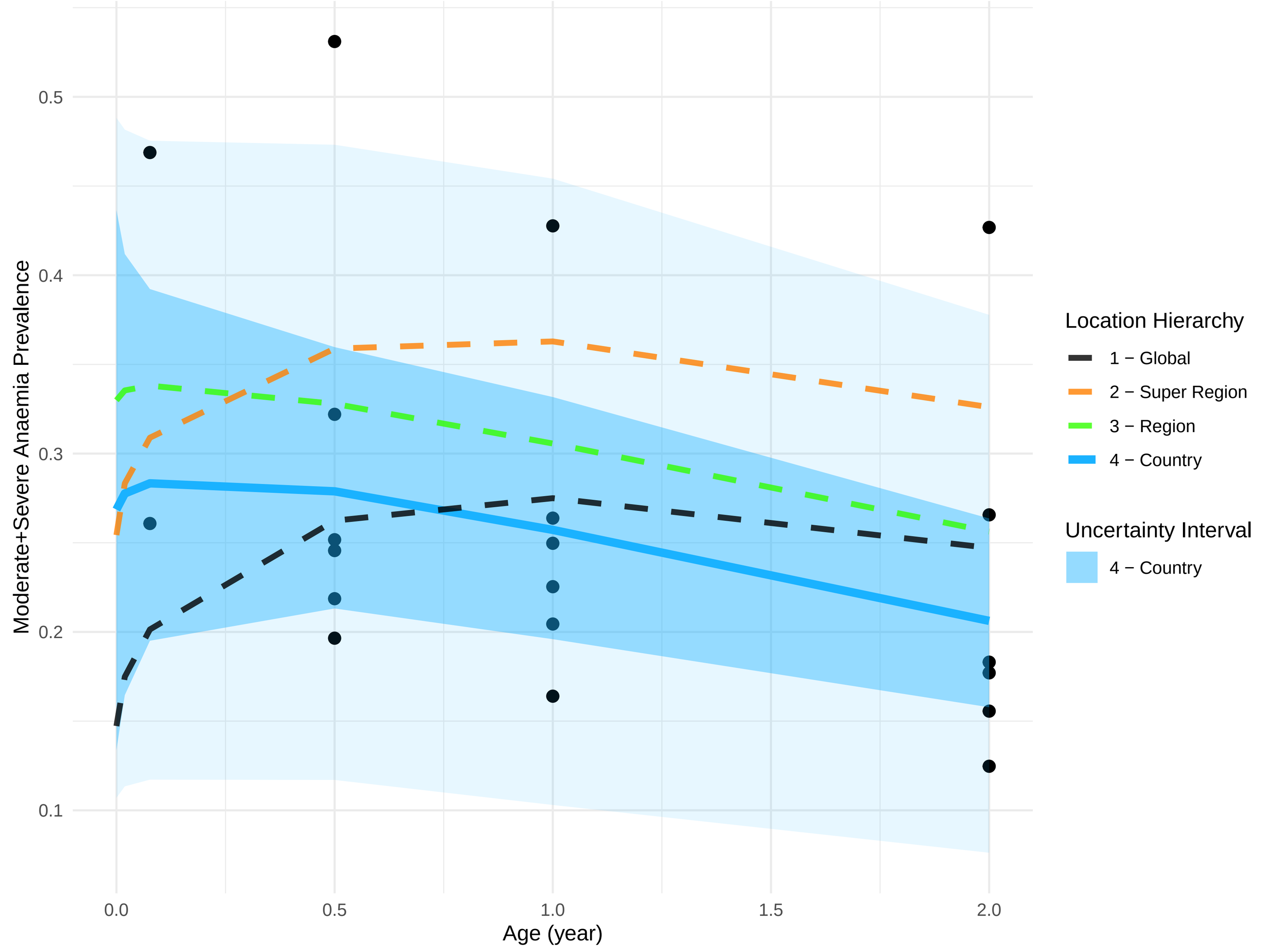

MR−BRT Age/Sex Fits − Non−pregnant Females <2 Years Old − Moderate+Severe Anaemia Prevalence − Malawi
WHO−2001 Adjustment Method

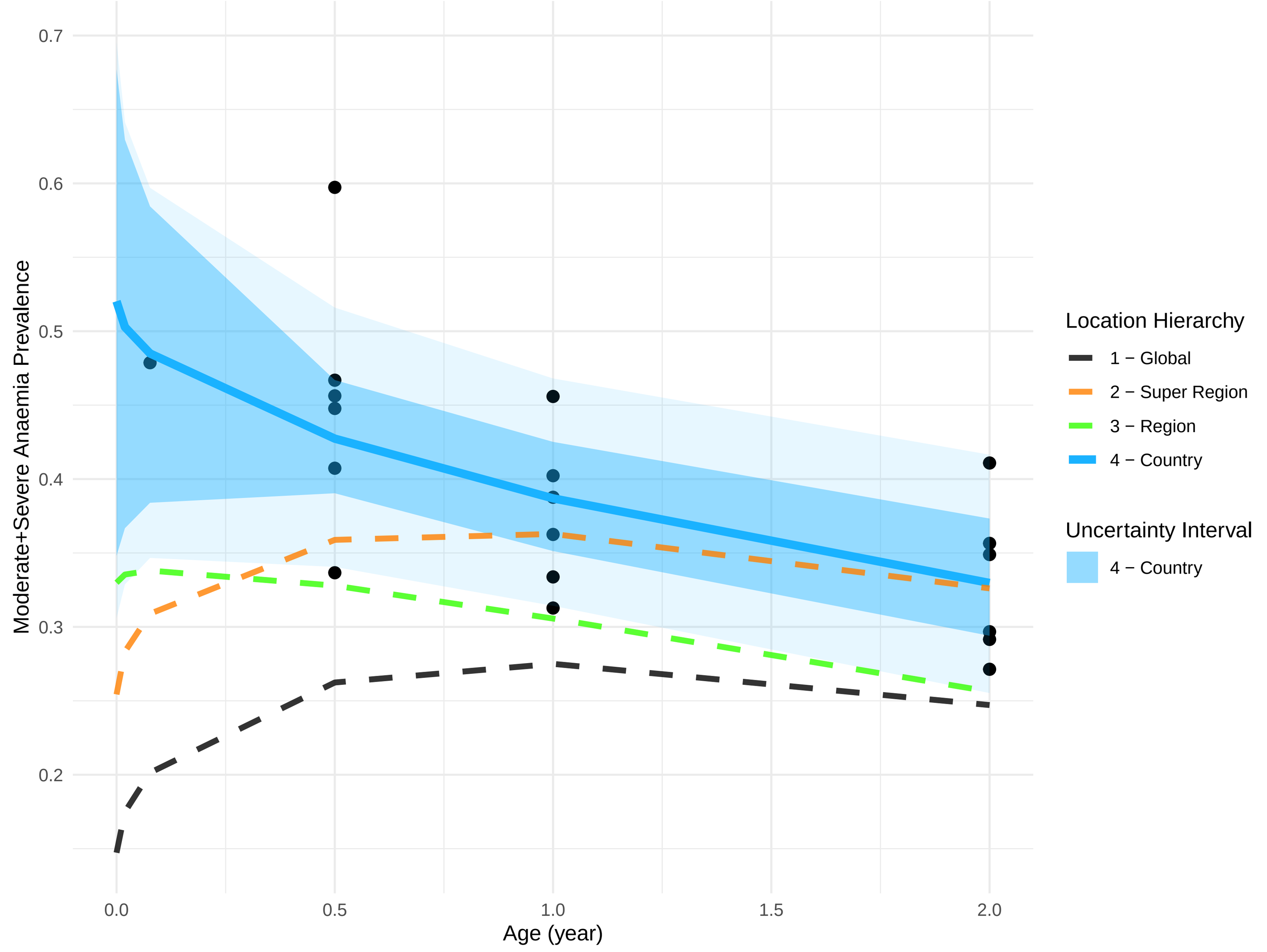

MR−BRT Age/Sex Fits − Non−pregnant Females <2 Years Old − Moderate+Severe Anaemia Prevalence − Mozambique
WHO−2001 Adjustment Method

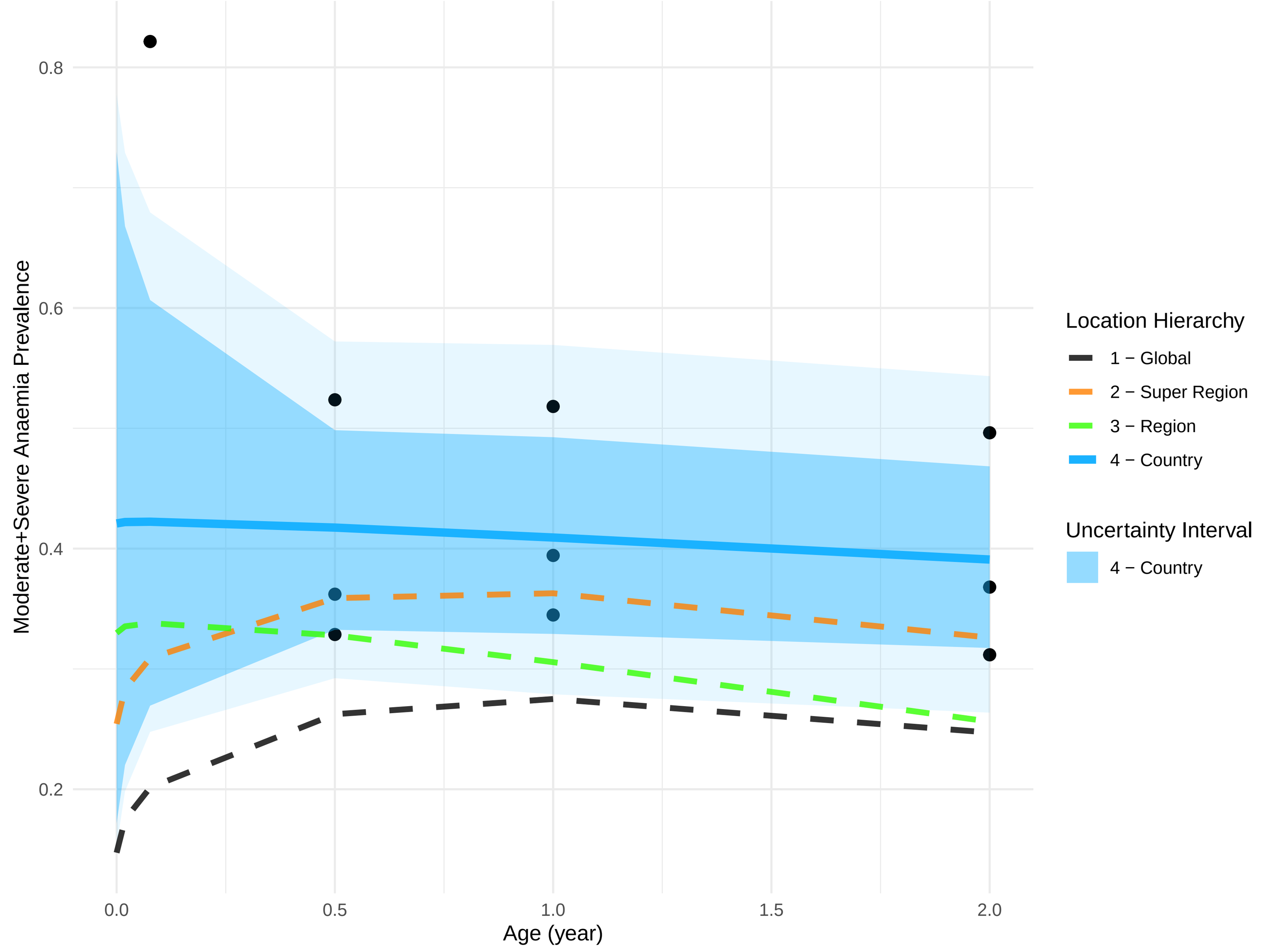

MR−BRT Age/Sex Fits − Non−pregnant Females <2 Years Old − Moderate+Severe Anaemia Prevalence − Rwanda
WHO−2001 Adjustment Method

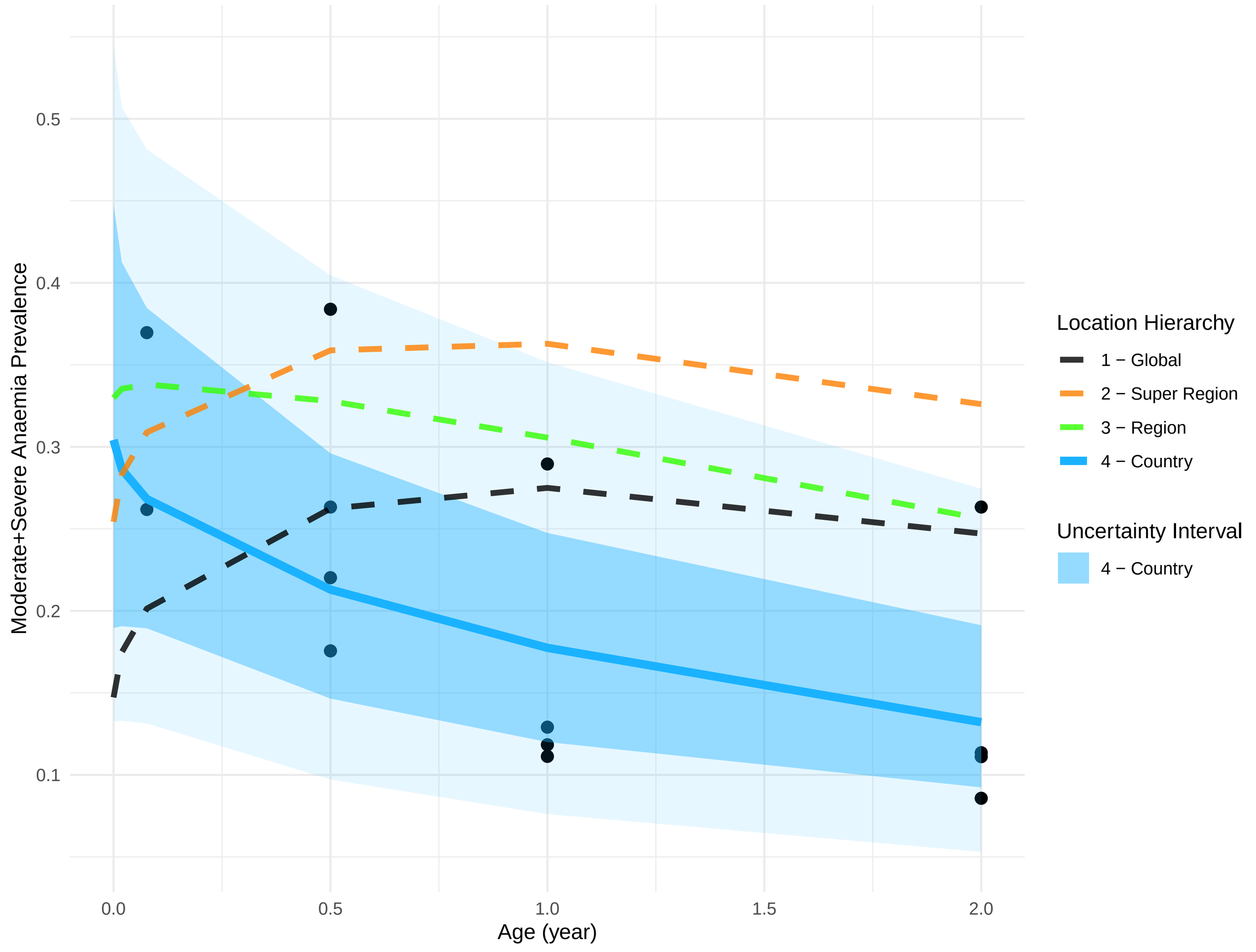

MR−BRT Age/Sex Fits − Non−pregnant Females <2 Years Old − Moderate+Severe Anaemia Prevalence − Uganda
WHO−2001 Adjustment Method

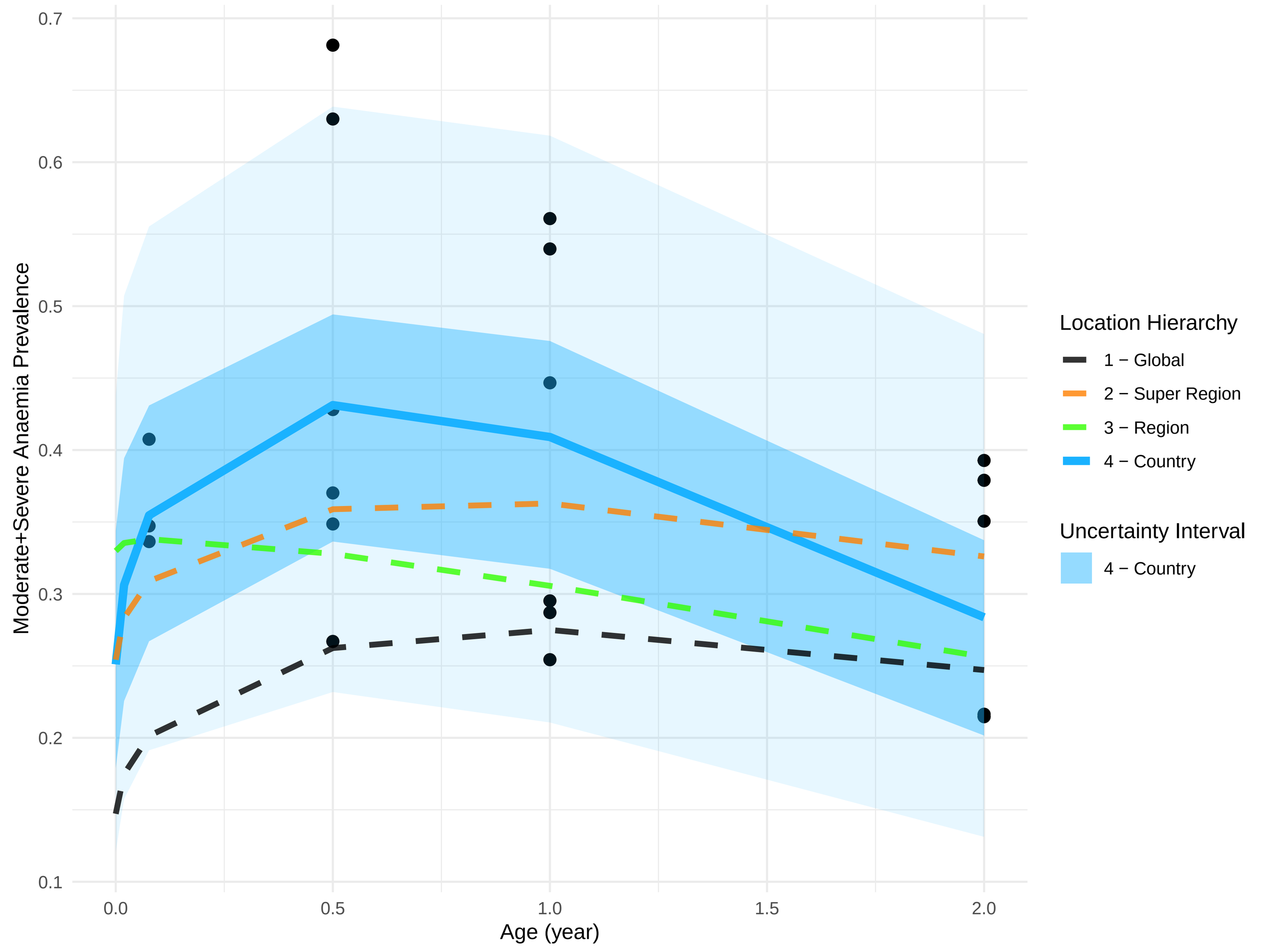

MR−BRT Age/Sex Fits − Non−pregnant Females <2 Years Old − Moderate+Severe Anaemia Prevalence − United Republic of Tanzania
WHO−2001 Adjustment Method

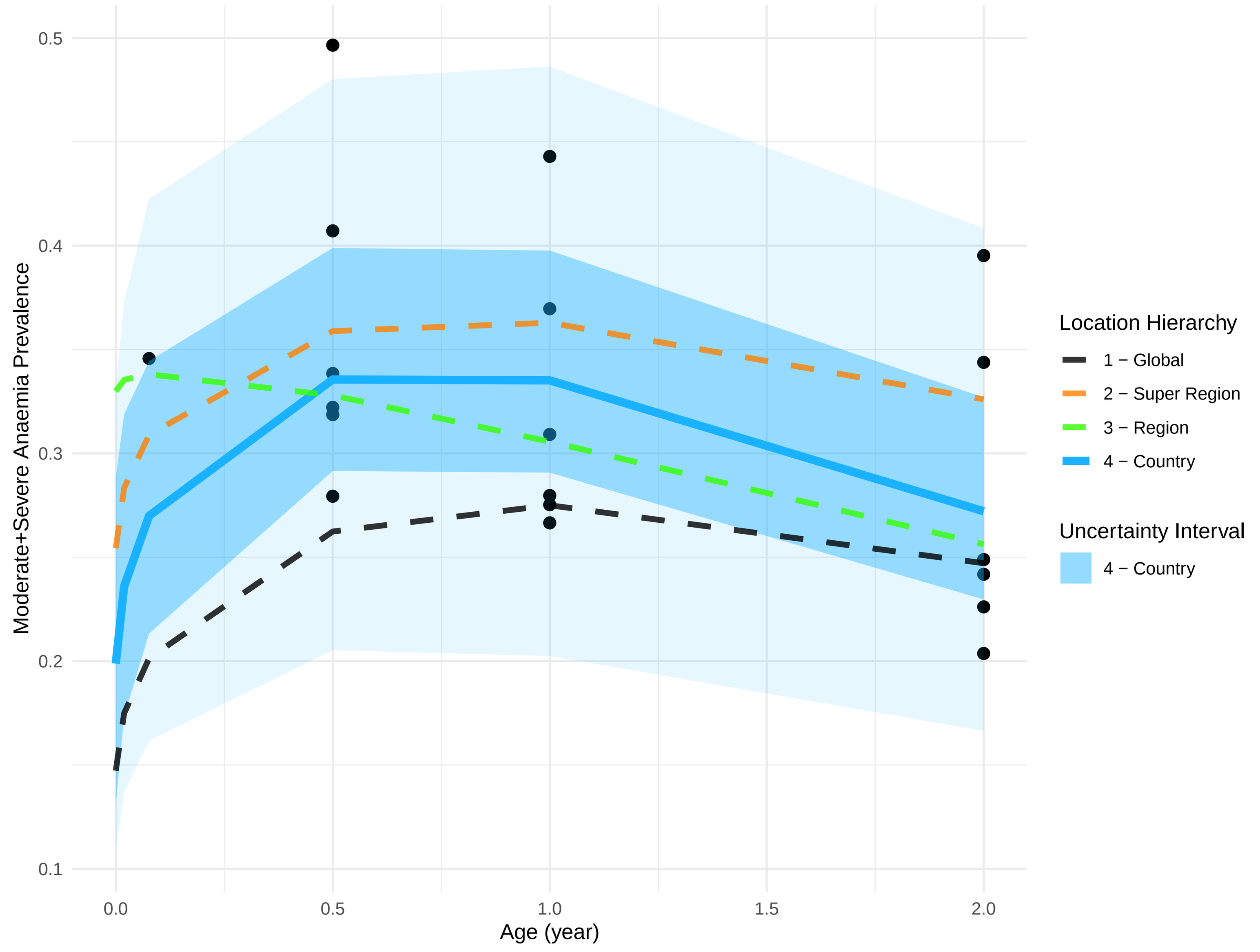

MR−BRT Age/Sex Fits − Non−pregnant Females <2 Years Old − Moderate+Severe Anaemia Prevalence − Zambia
WHO−2001 Adjustment Method

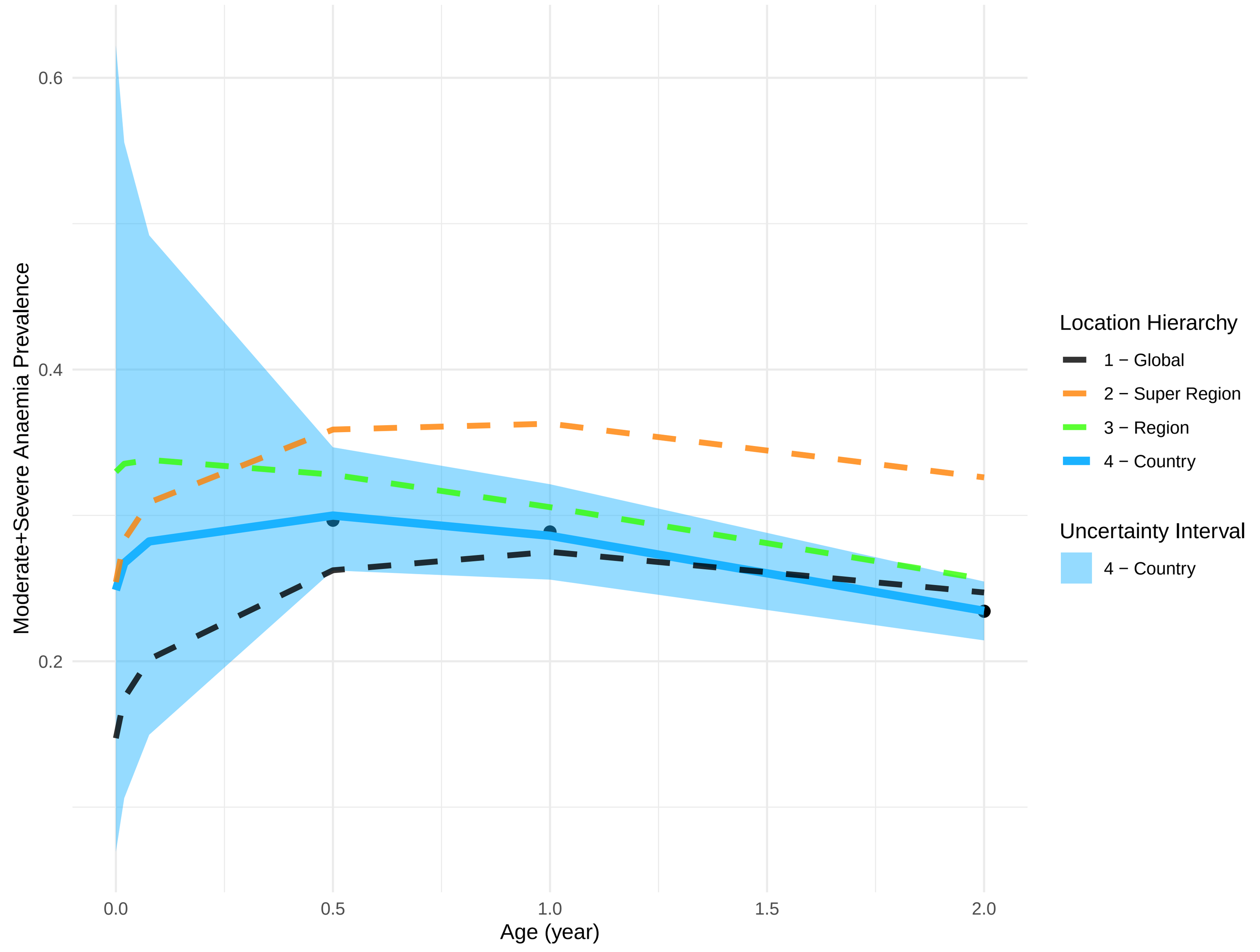

MR−BRT Age/Sex Fits − Non−pregnant Females <2 Years Old − Moderate+Severe Anaemia Prevalence − Southern Sub−Saharan Africa
WHO−2001 Adjustment Method

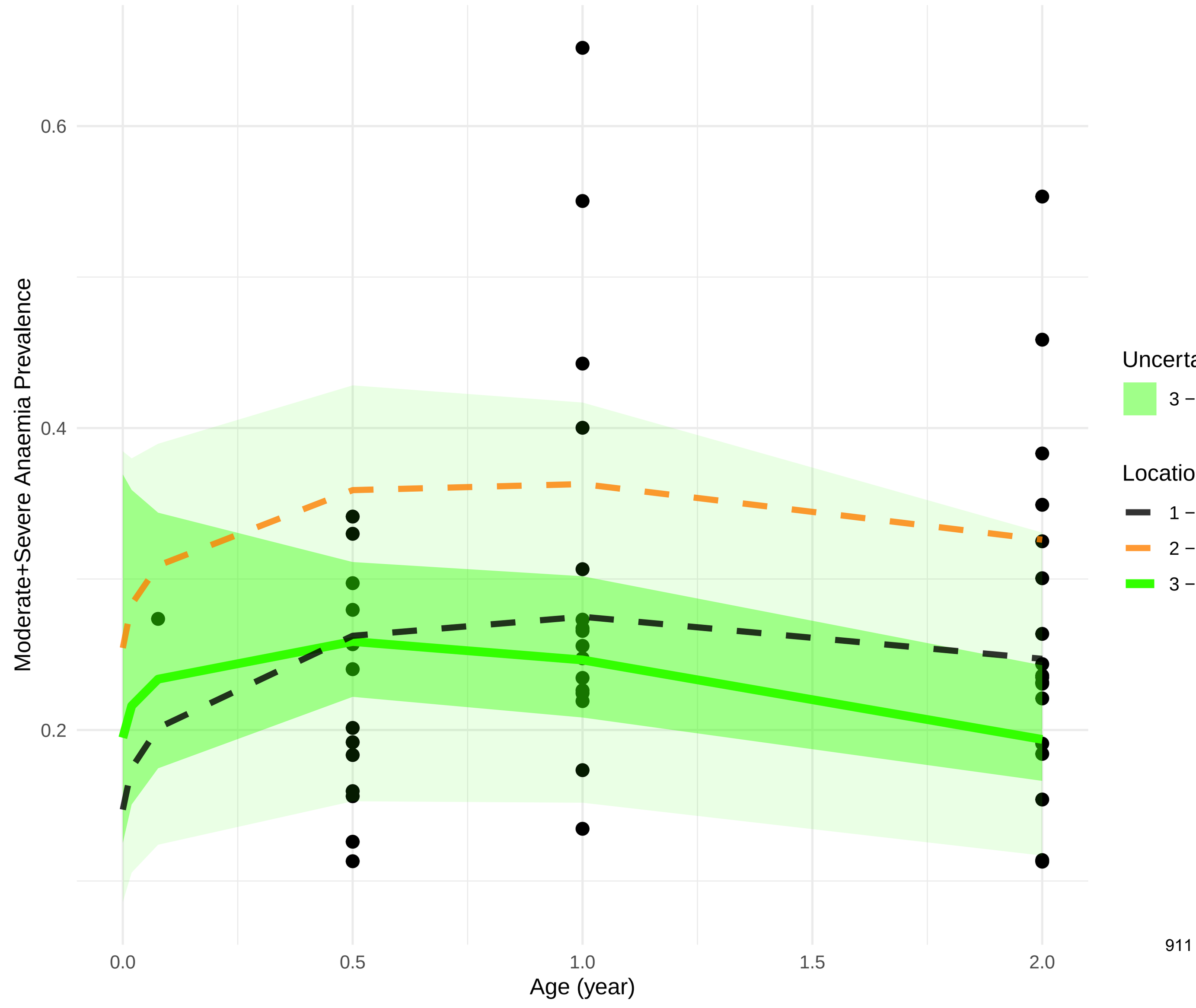

MR−BRT Age/Sex Fits − Non−pregnant Females <2 Years Old − Moderate+Severe Anaemia Prevalence − Eswatini
WHO−2001 Adjustment Method

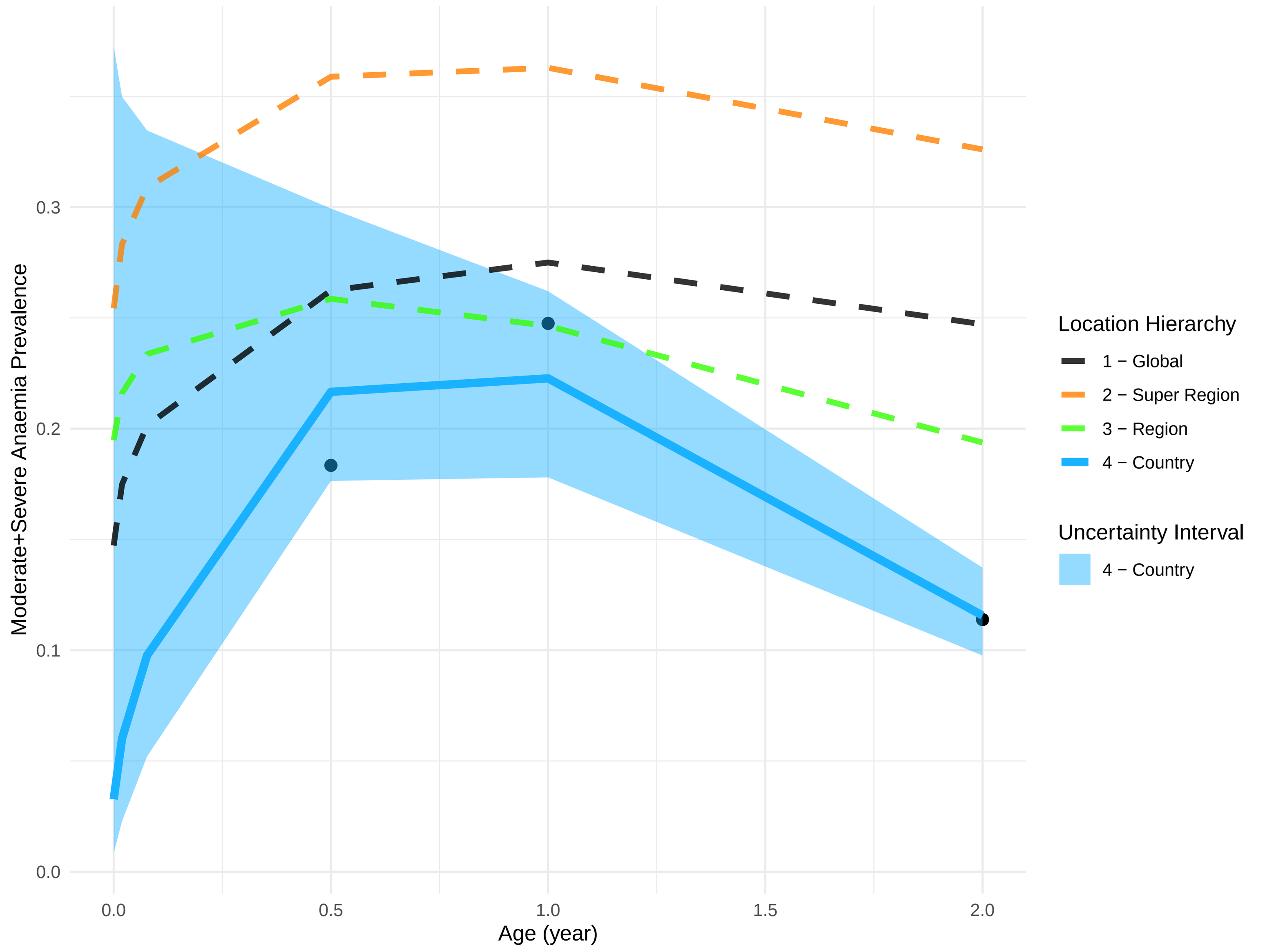

MR−BRT Age/Sex Fits − Non−pregnant Females <2 Years Old − Moderate+Severe Anaemia Prevalence − Lesotho
WHO−2001 Adjustment Method

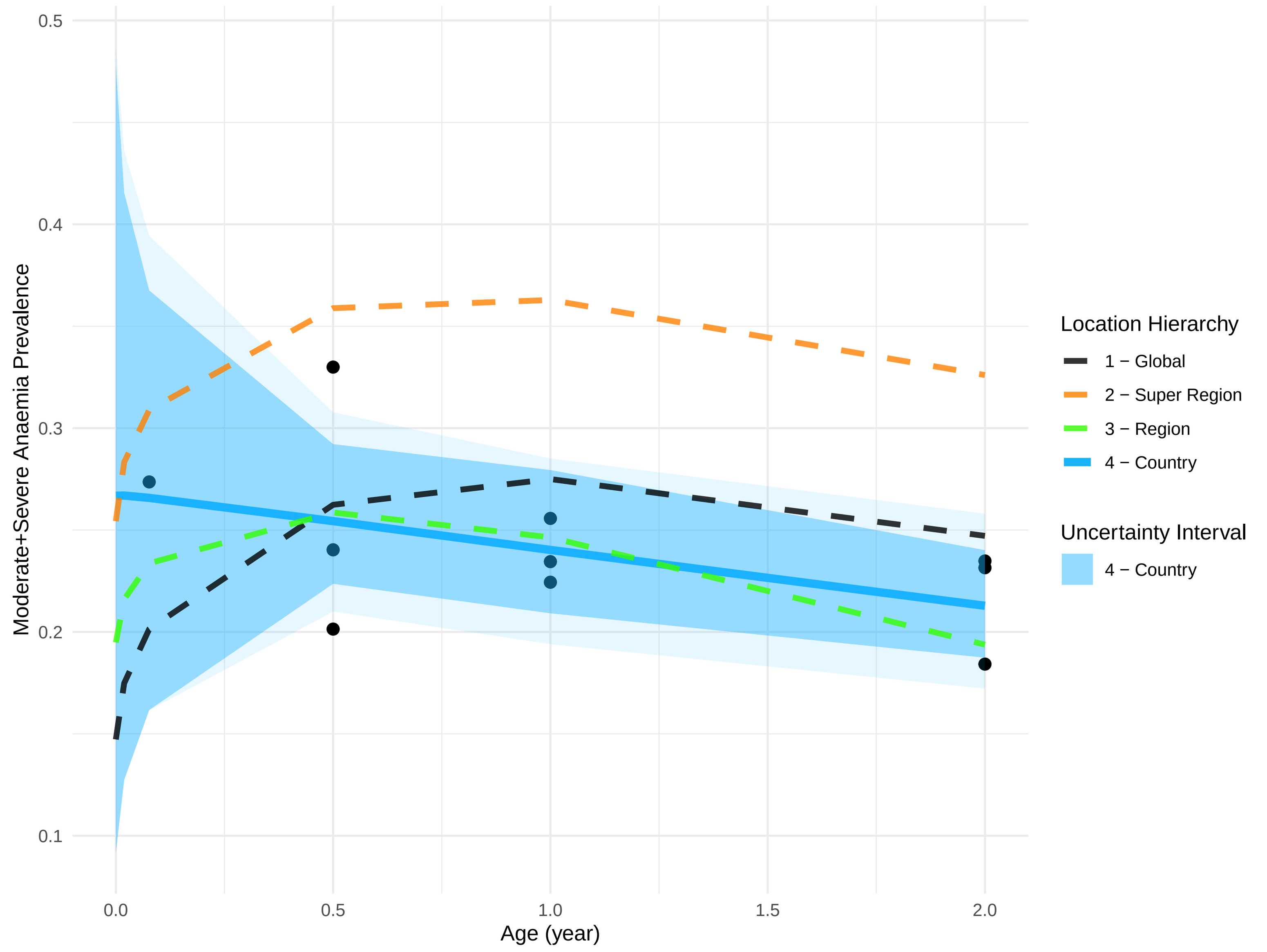

MR−BRT Age/Sex Fits − Non−pregnant Females <2 Years Old − Moderate+Severe Anaemia Prevalence − Namibia
WHO−2001 Adjustment Method

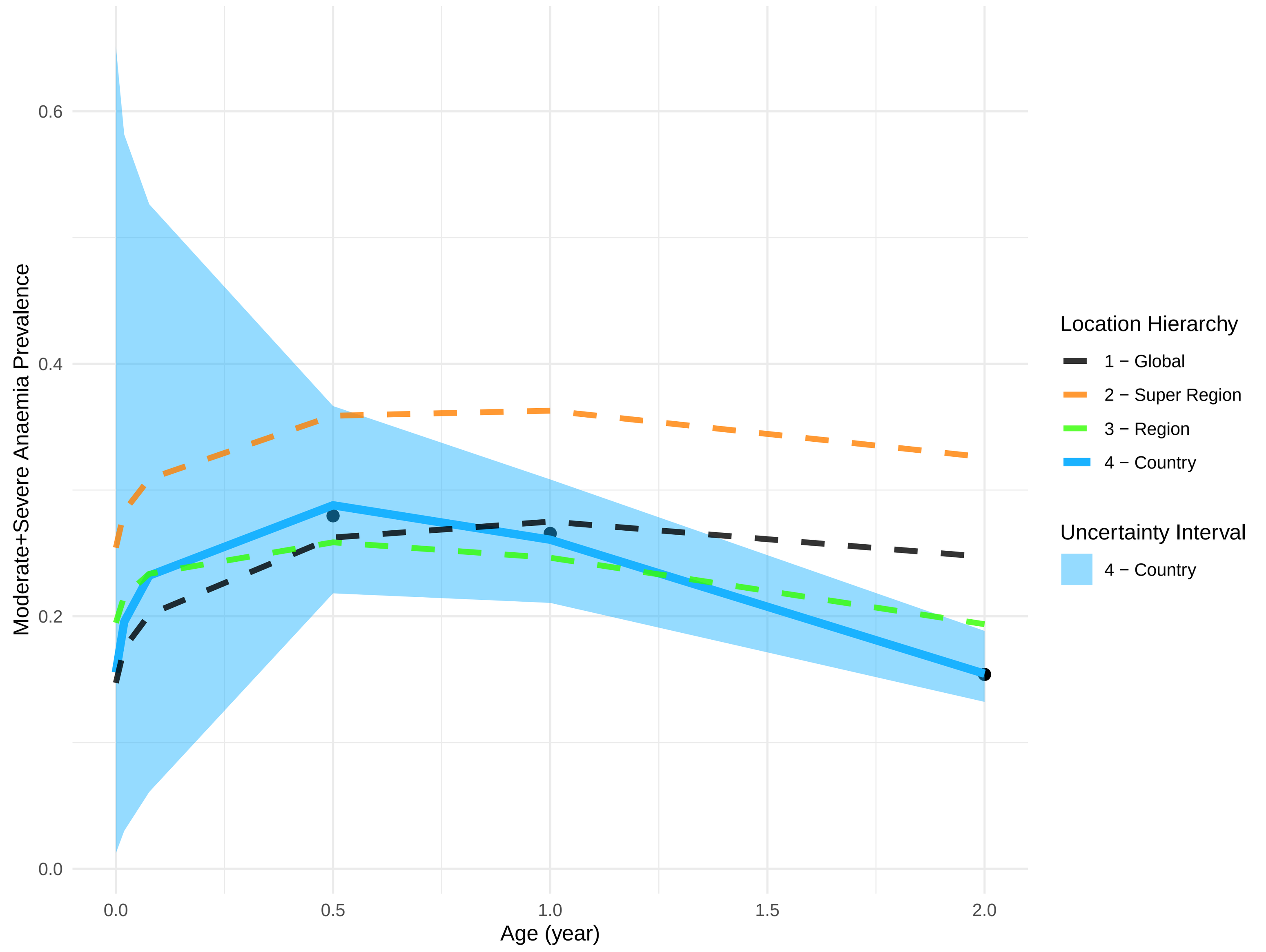

MR−BRT Age/Sex Fits − Non−pregnant Females <2 Years Old − Moderate+Severe Anaemia Prevalence − South Africa
WHO−2001 Adjustment Method

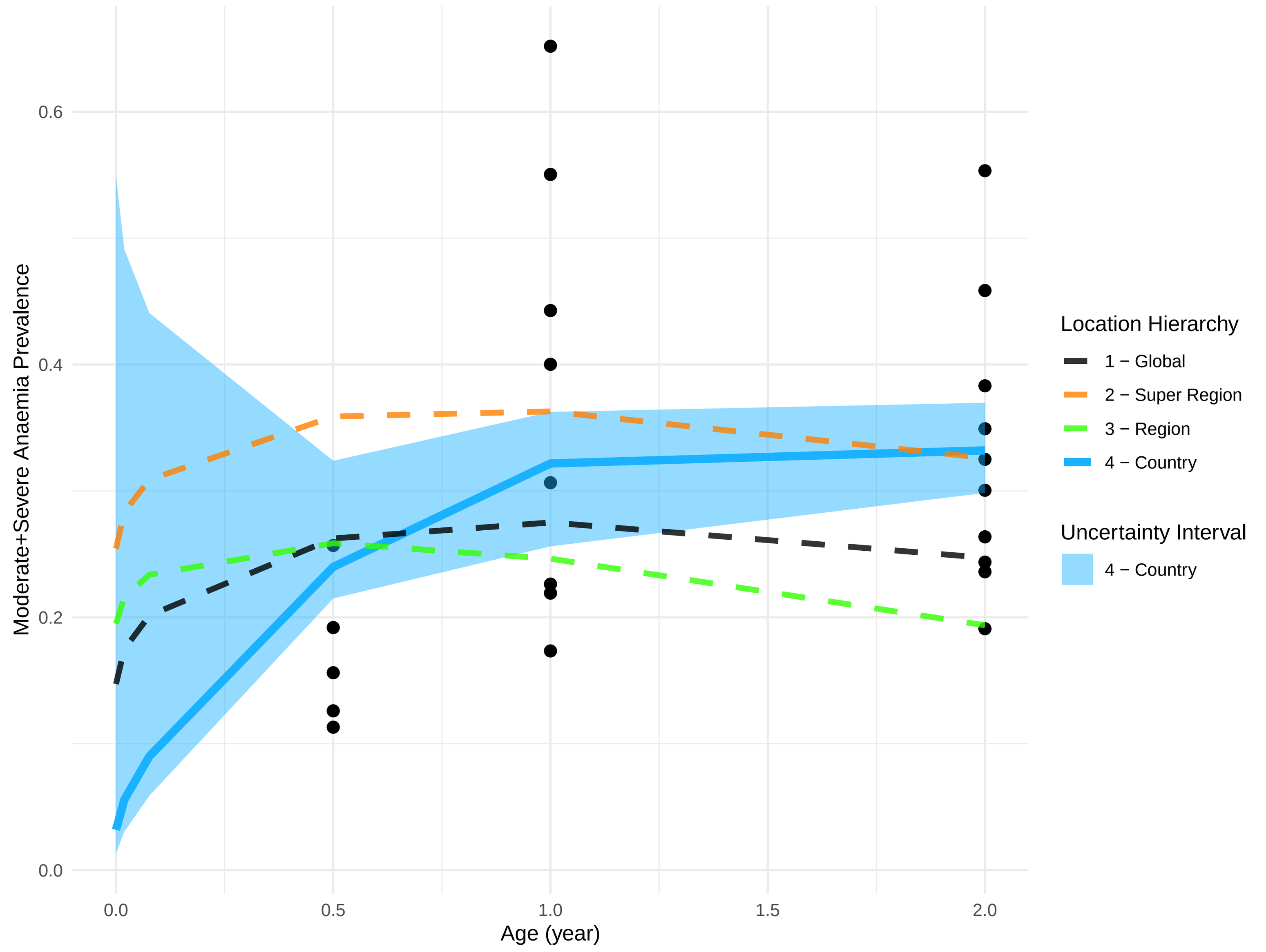

MR−BRT Age/Sex Fits − Non−pregnant Females <2 Years Old − Moderate+Severe Anaemia Prevalence − Eastern Cape
WHO−2001 Adjustment Method

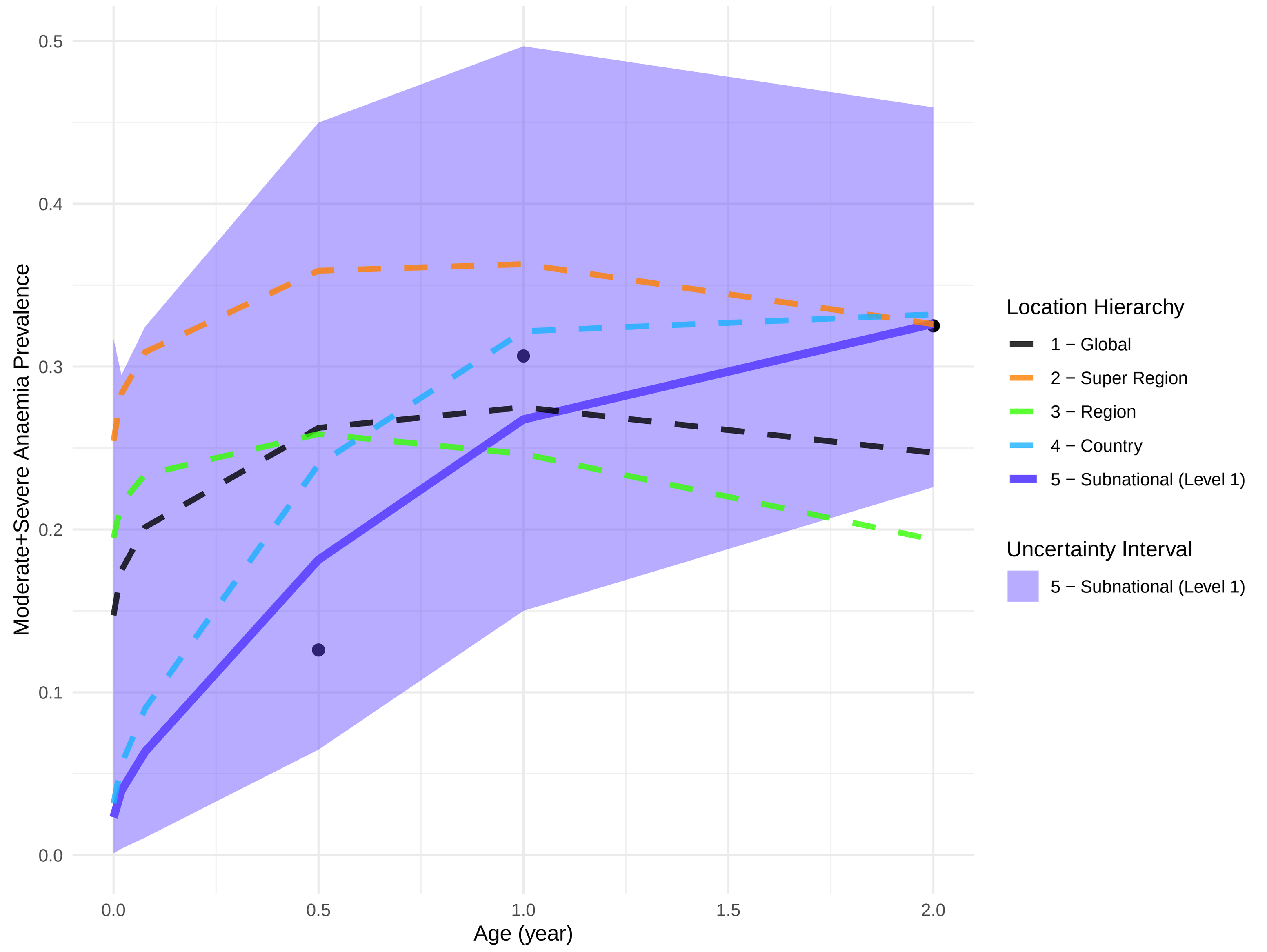

MR−BRT Age/Sex Fits − Non−pregnant Females <2 Years Old − Moderate+Severe Anaemia Prevalence − Free State
WHO−2001 Adjustment Method

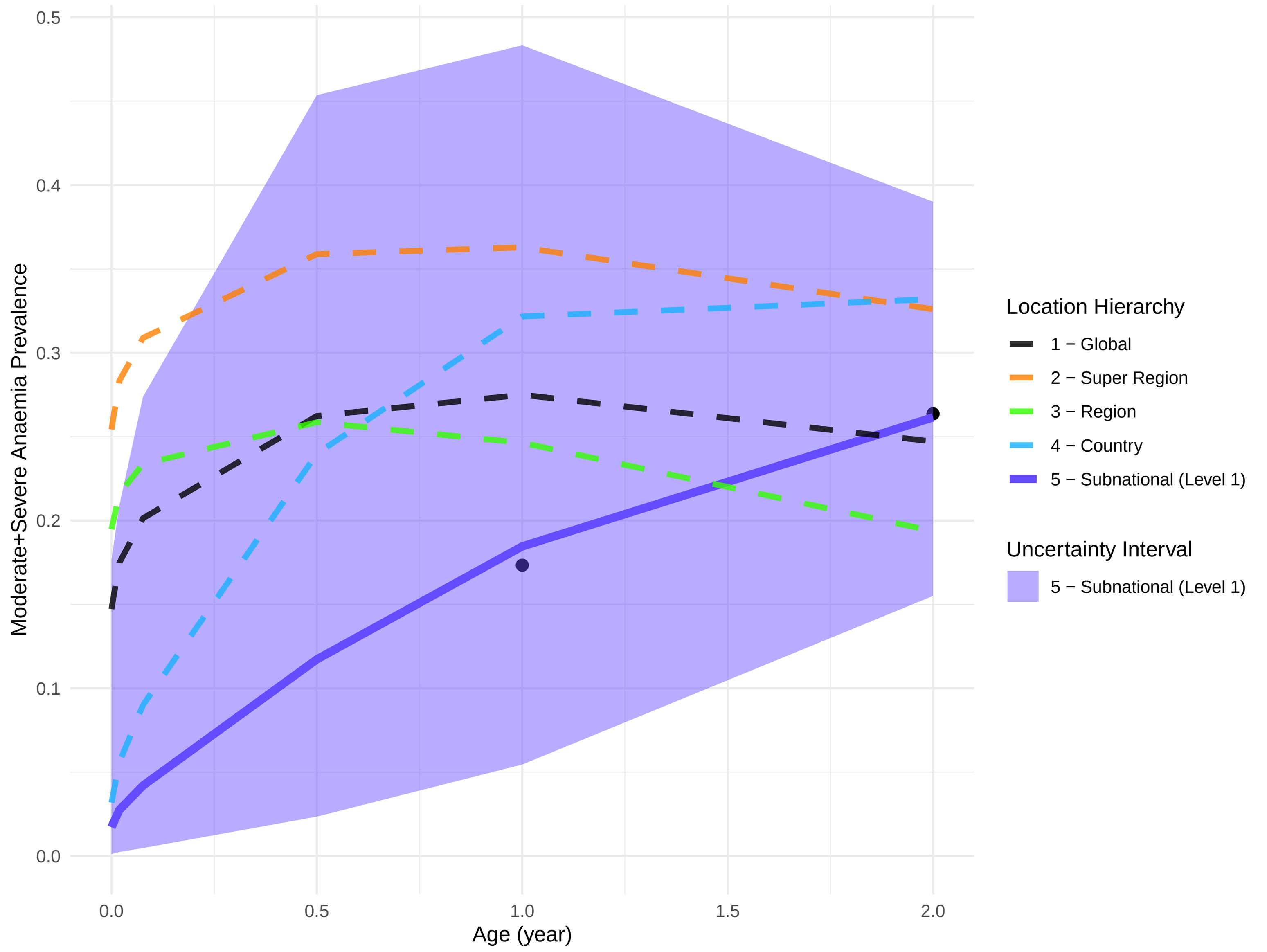

MR−BRT Age/Sex Fits − Non−pregnant Females <2 Years Old − Moderate+Severe Anaemia Prevalence − Gauteng
WHO−2001 Adjustment Method

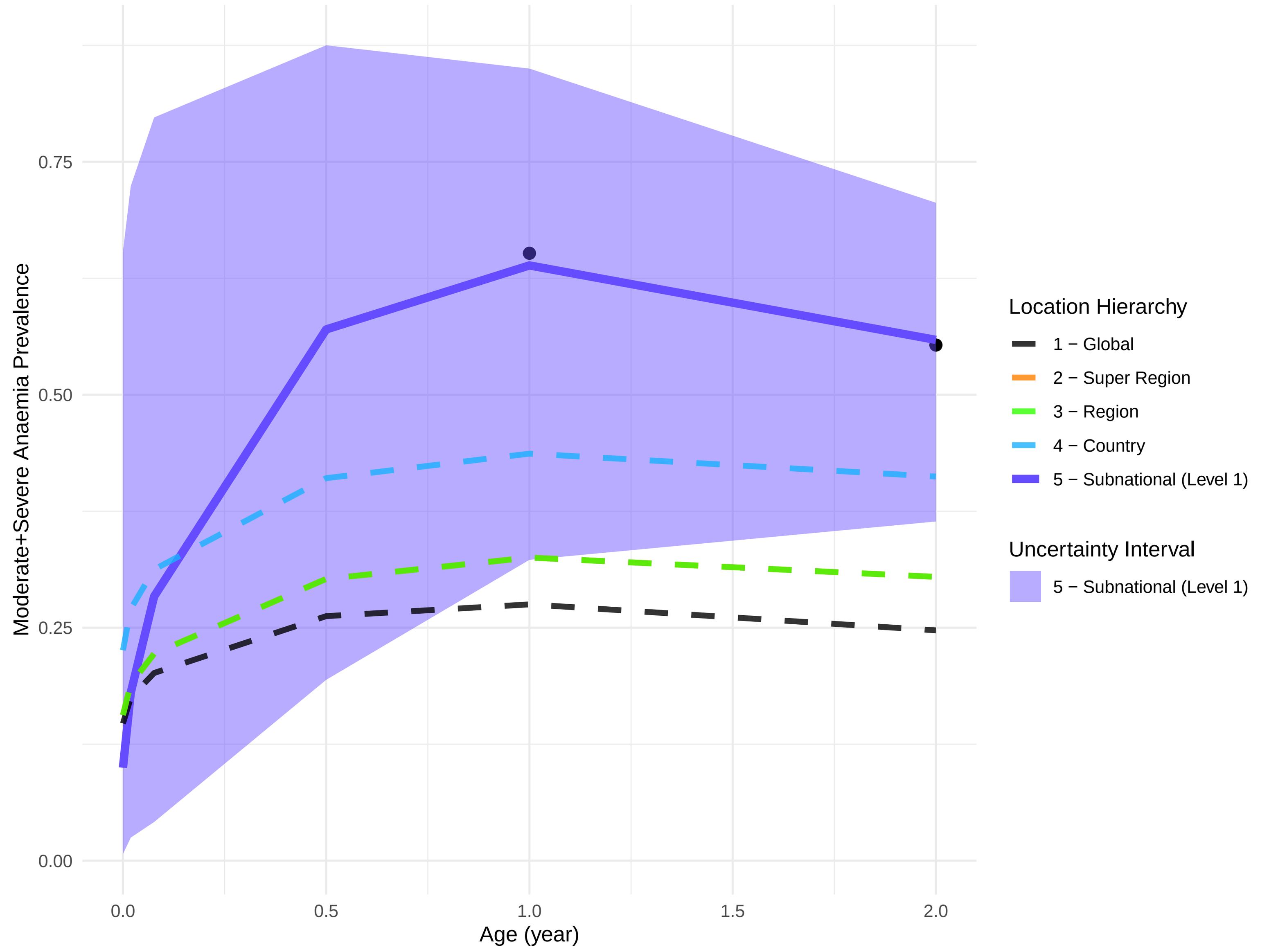

MR−BRT Age/Sex Fits − Non−pregnant Females <2 Years Old − Moderate+Severe Anaemia Prevalence − KwaZulu−Natal
WHO−2001 Adjustment Method

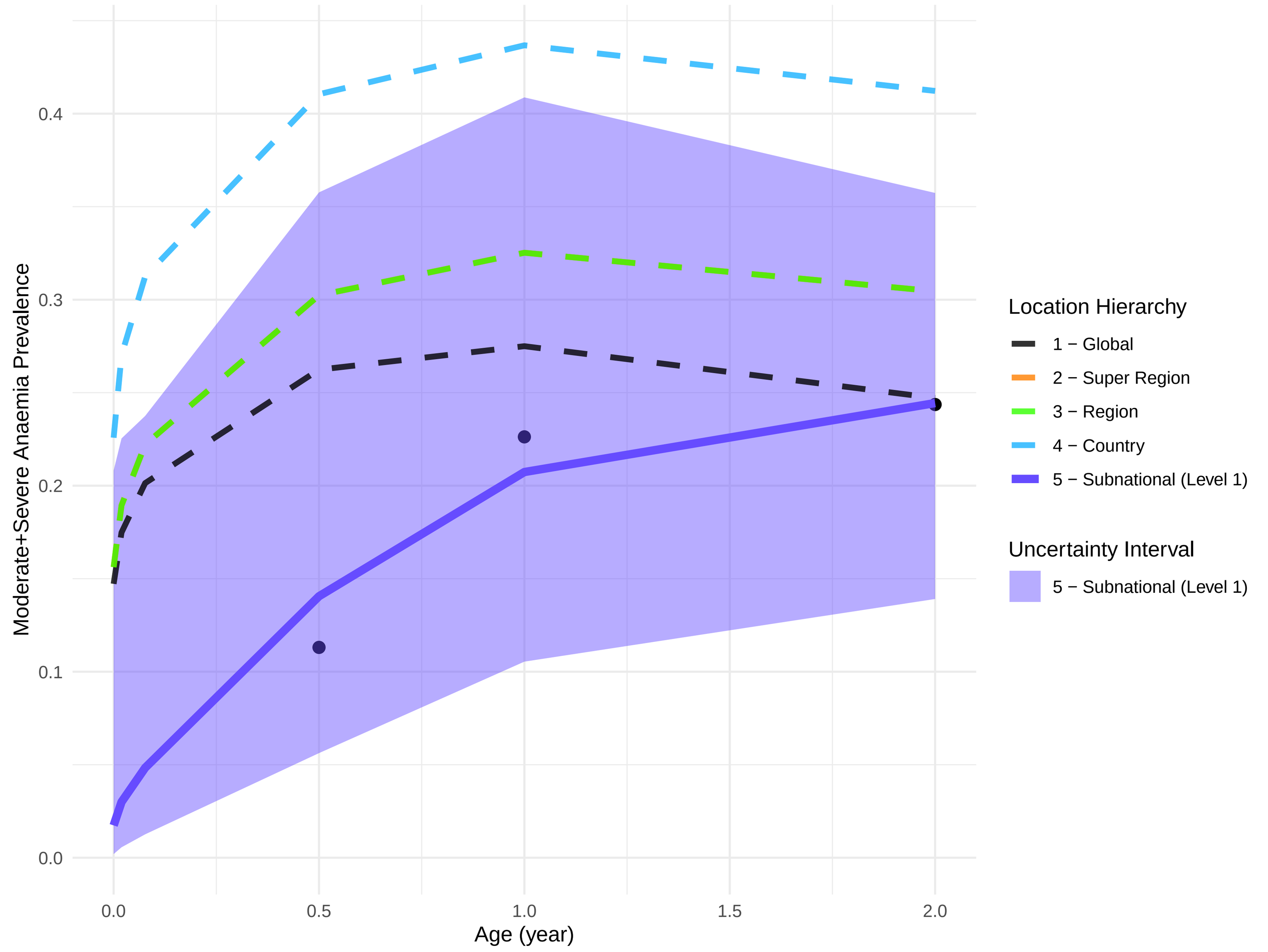

MR−BRT Age/Sex Fits − Non−pregnant Females <2 Years Old − Moderate+Severe Anaemia Prevalence − Limpopo
WHO−2001 Adjustment Method

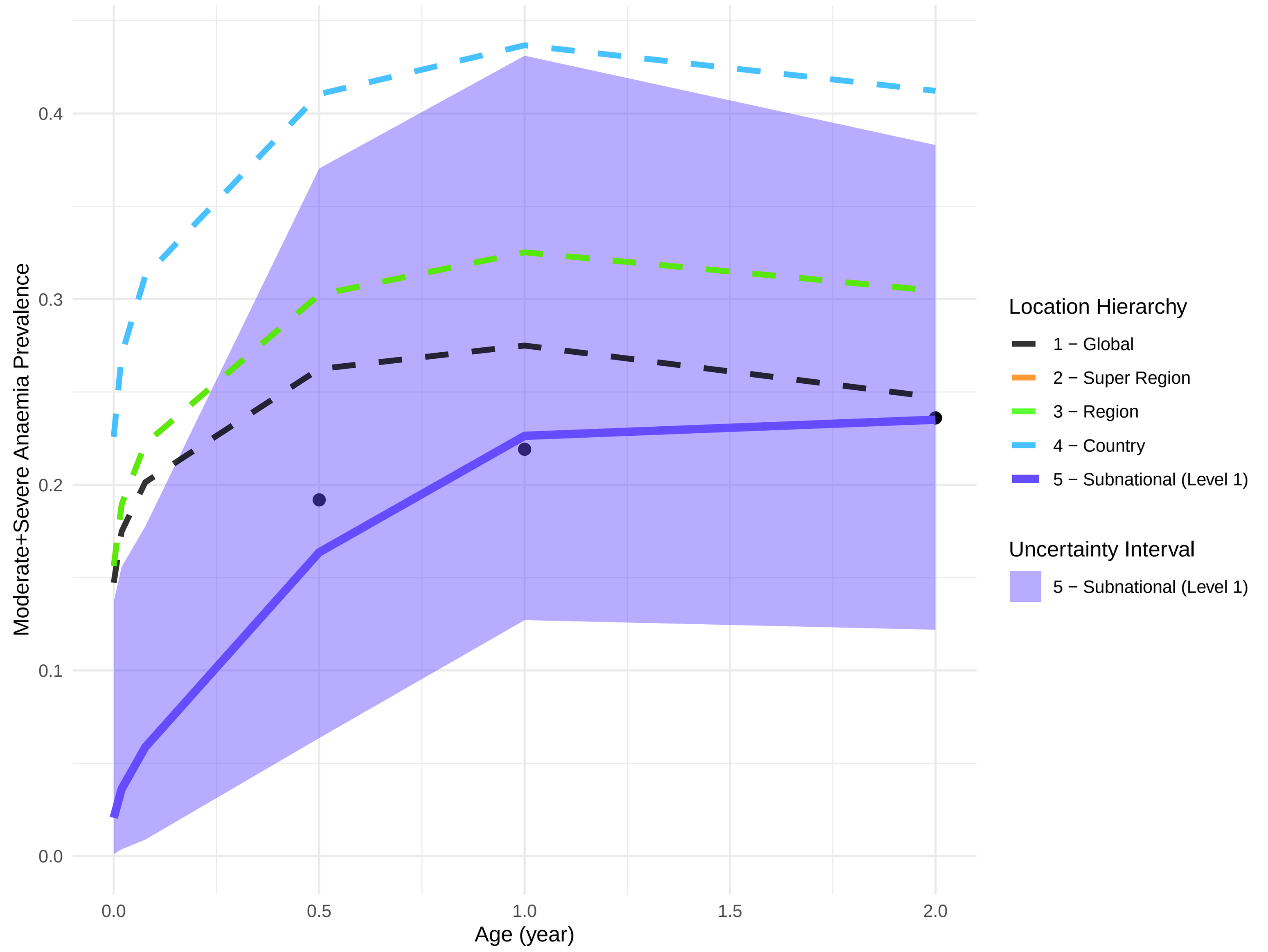

MR−BRT Age/Sex Fits − Non−pregnant Females <2 Years Old − Moderate+Severe Anaemia Prevalence − Mpumalanga
WHO−2001 Adjustment Method

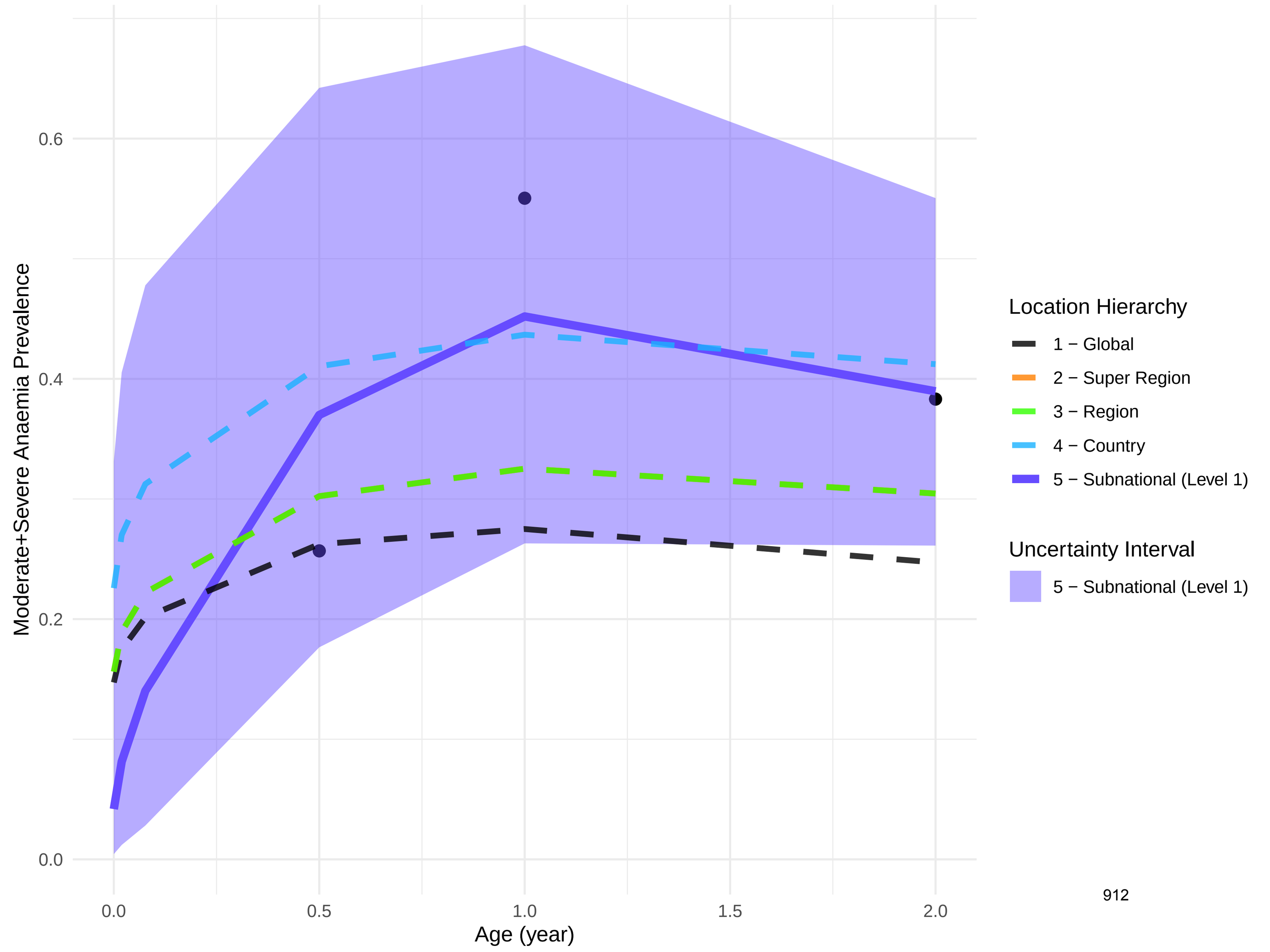

MR−BRT Age/Sex Fits − Non−pregnant Females <2 Years Old − Moderate+Severe Anaemia Prevalence − North−West
WHO−2001 Adjustment Method

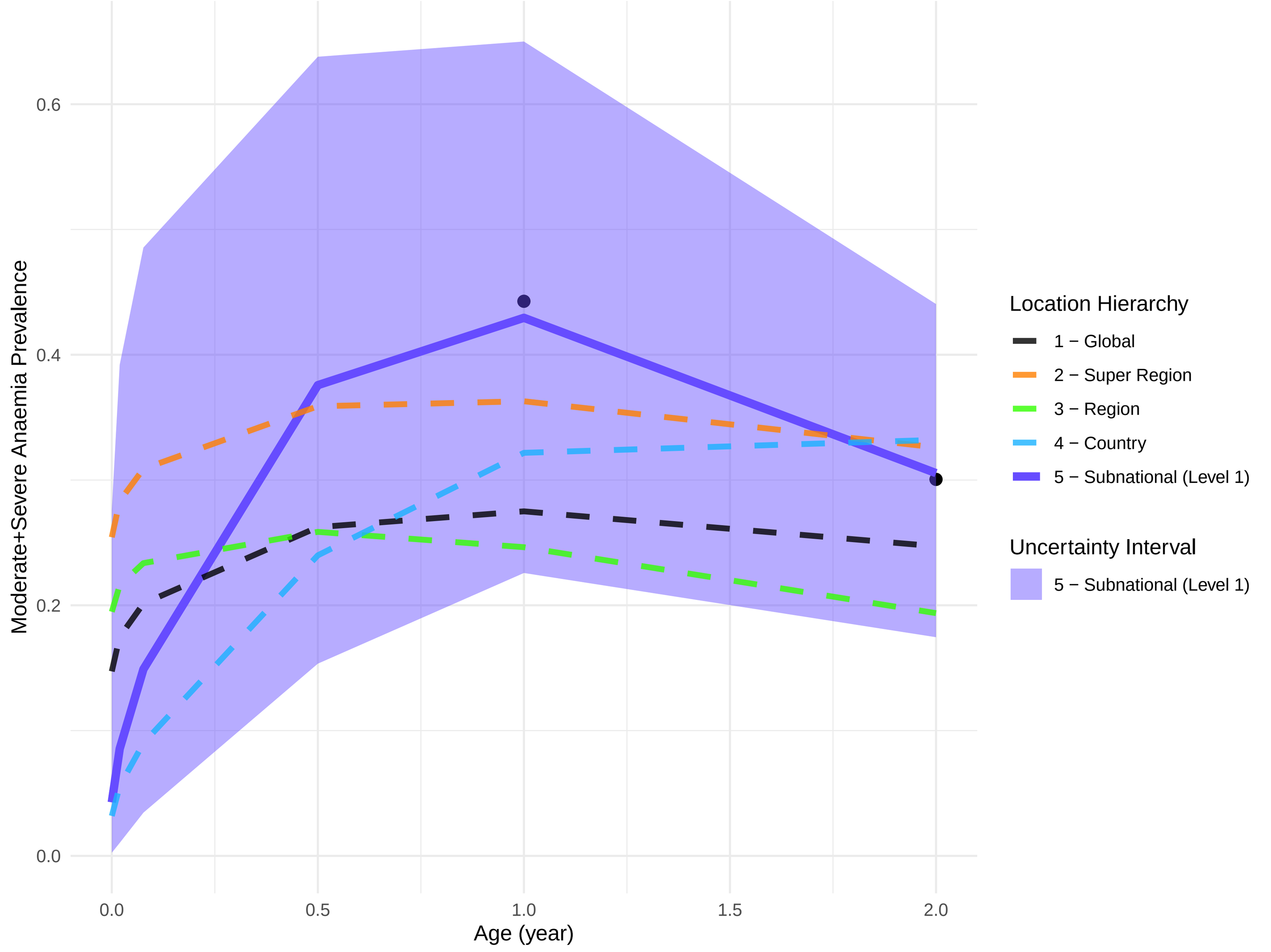

MR−BRT Age/Sex Fits − Non−pregnant Females <2 Years Old − Moderate+Severe Anaemia Prevalence − Northern Cape
WHO−2001 Adjustment Method

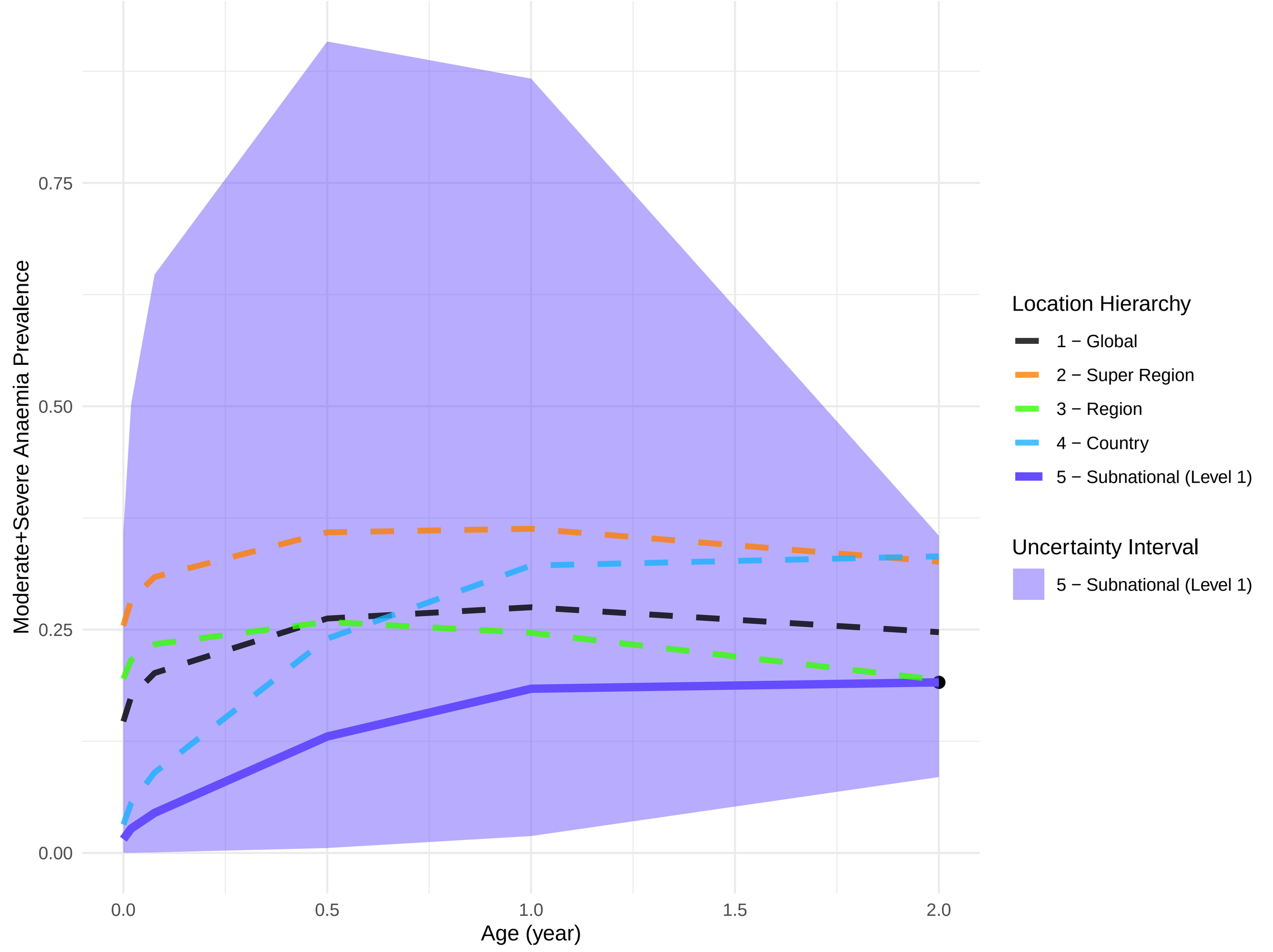

MR−BRT Age/Sex Fits − Non−pregnant Females <2 Years Old − Moderate+Severe Anaemia Prevalence − Western Cape
WHO−2001 Adjustment Method

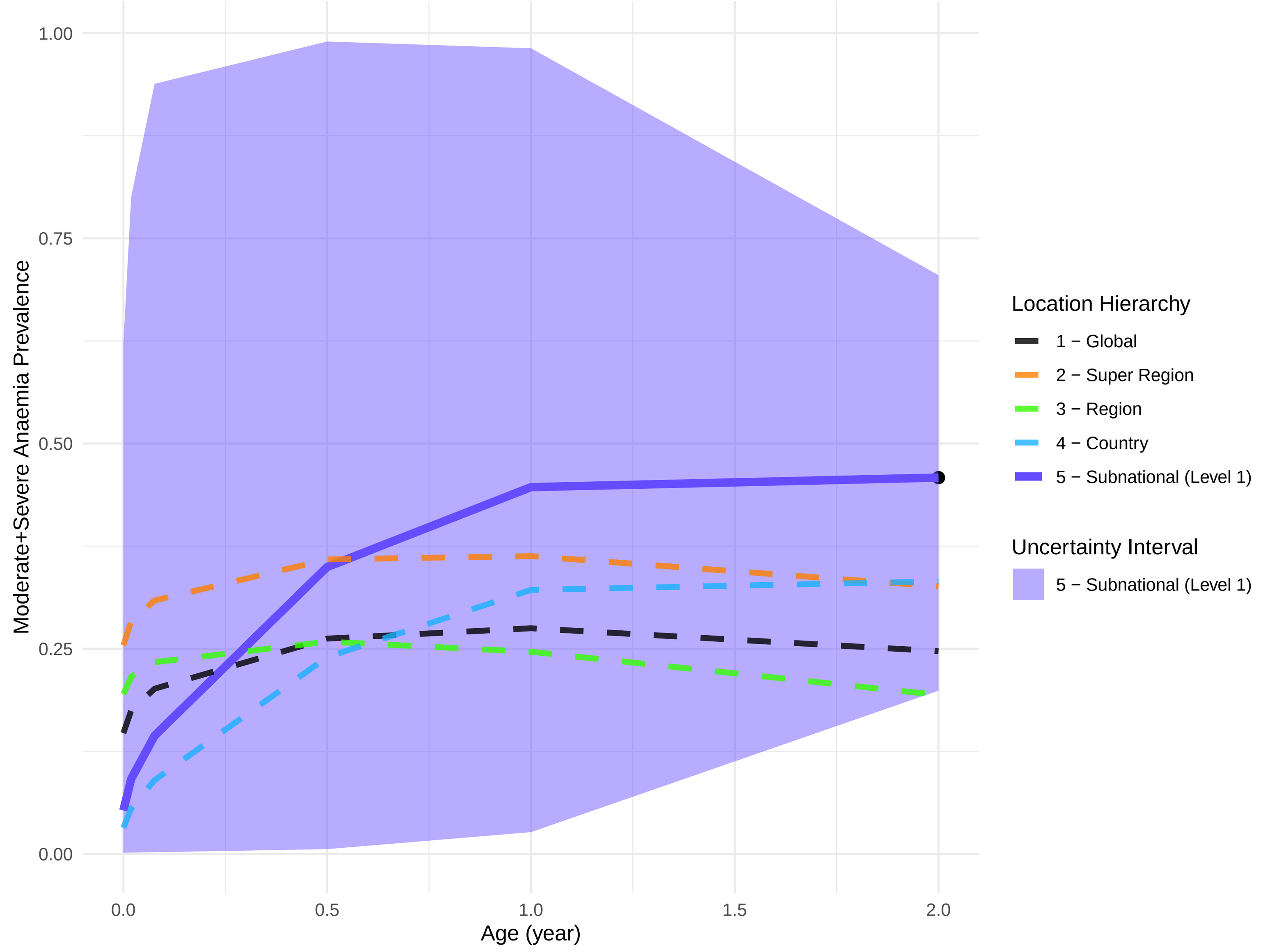

MR−BRT Age/Sex Fits − Non−pregnant Females <2 Years Old − Moderate+Severe Anaemia Prevalence − Zimbabwe
WHO−2001 Adjustment Method

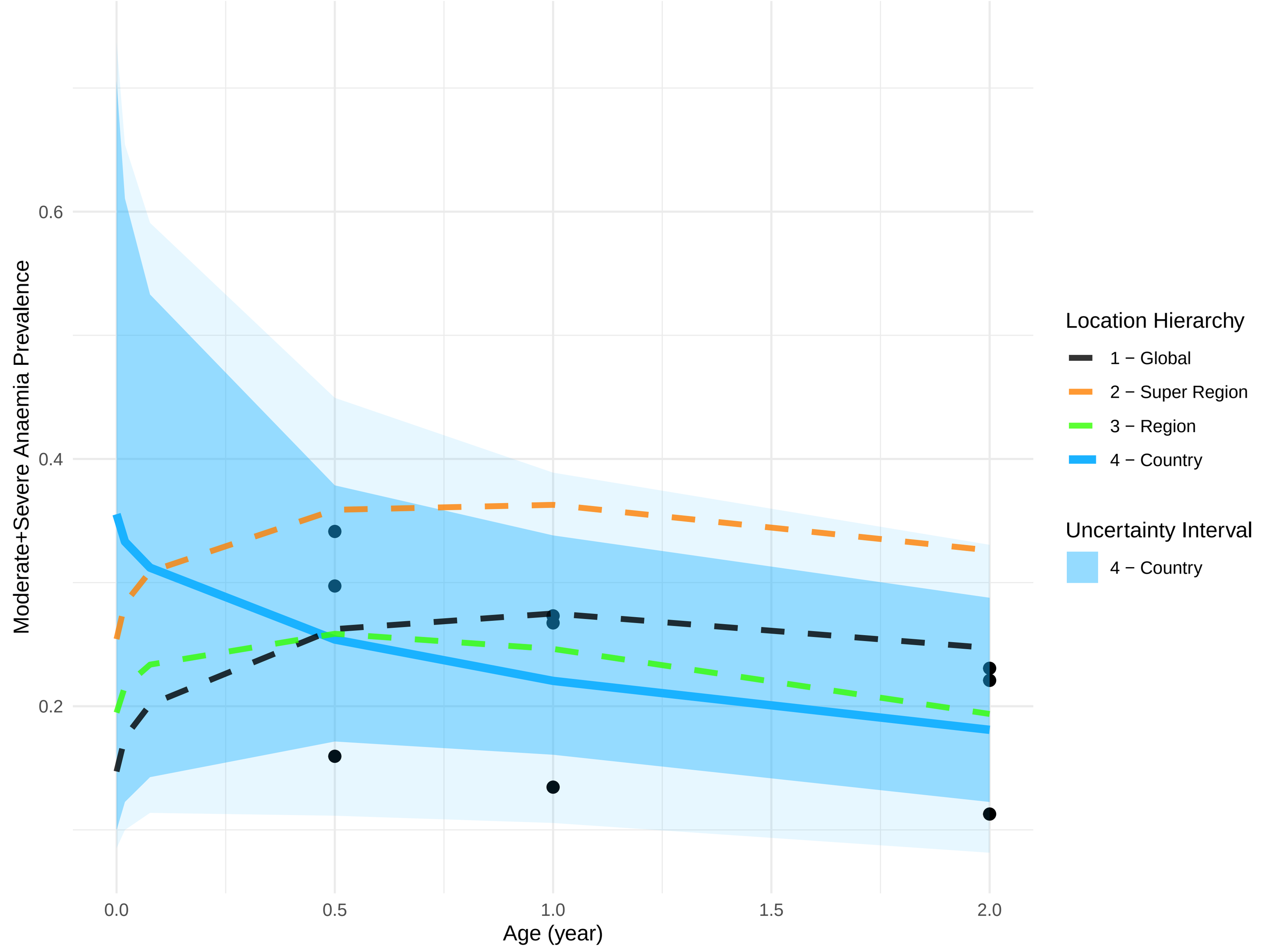

MR−BRT Age/Sex Fits − Non−pregnant Females <2 Years Old − Moderate+Severe Anaemia Prevalence − Western Sub-Saharan Africa
WHO−2001 Adjustment Method

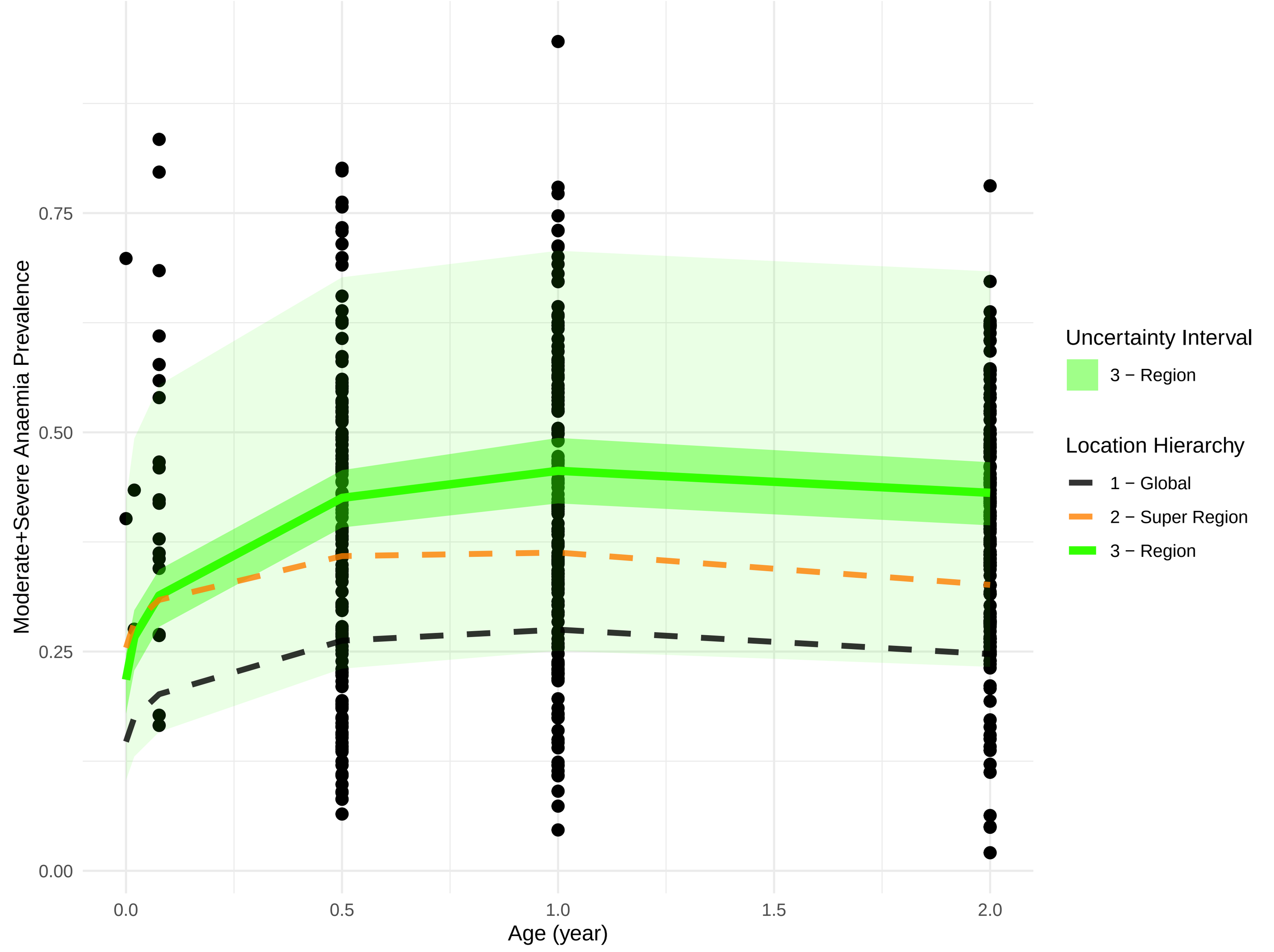

MR−BRT Age/Sex Fits − Non−pregnant Females <2 Years Old − Moderate+Severe Anaemia Prevalence − Benin
WHO−2001 Adjustment Method

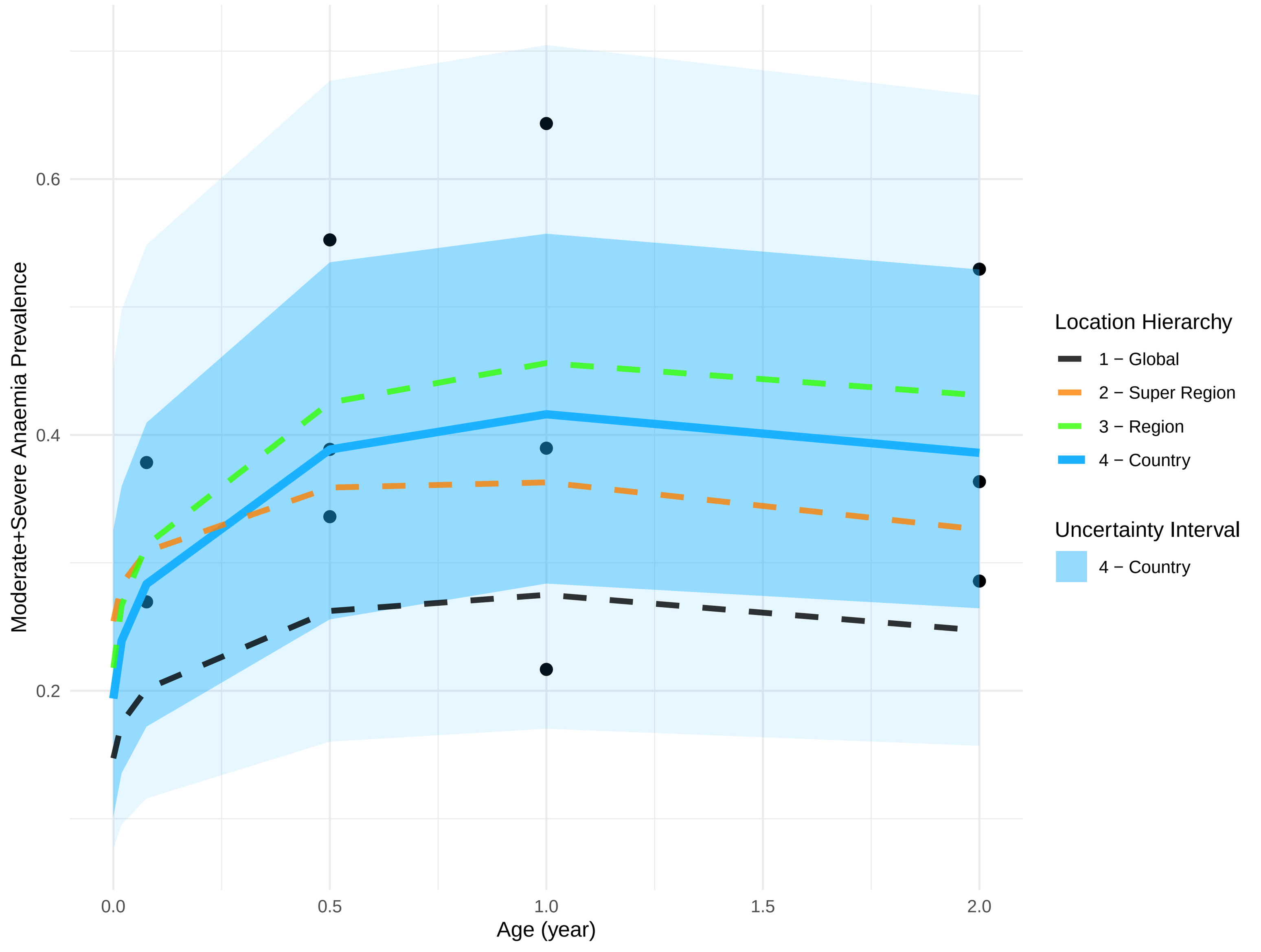

MR−BRT Age/Sex Fits − Non−pregnant Females <2 Years Old − Moderate+Severe Anaemia Prevalence − Burkina Faso
WHO−2001 Adjustment Method

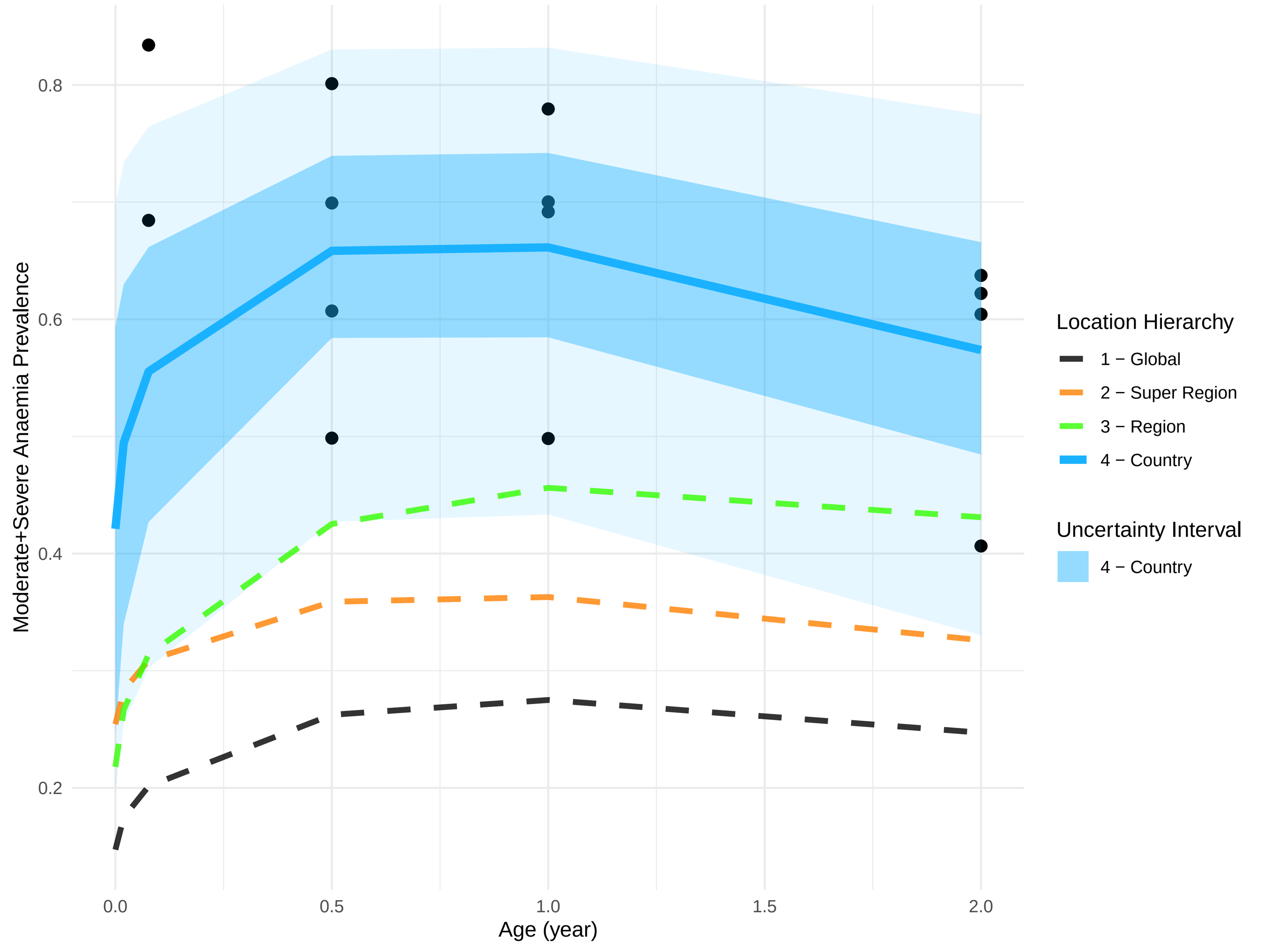

MR−BRT Age/Sex Fits − Non−pregnant Females <2 Years Old − Moderate+Severe Anaemia Prevalence − Cameroon
WHO−2001 Adjustment Method

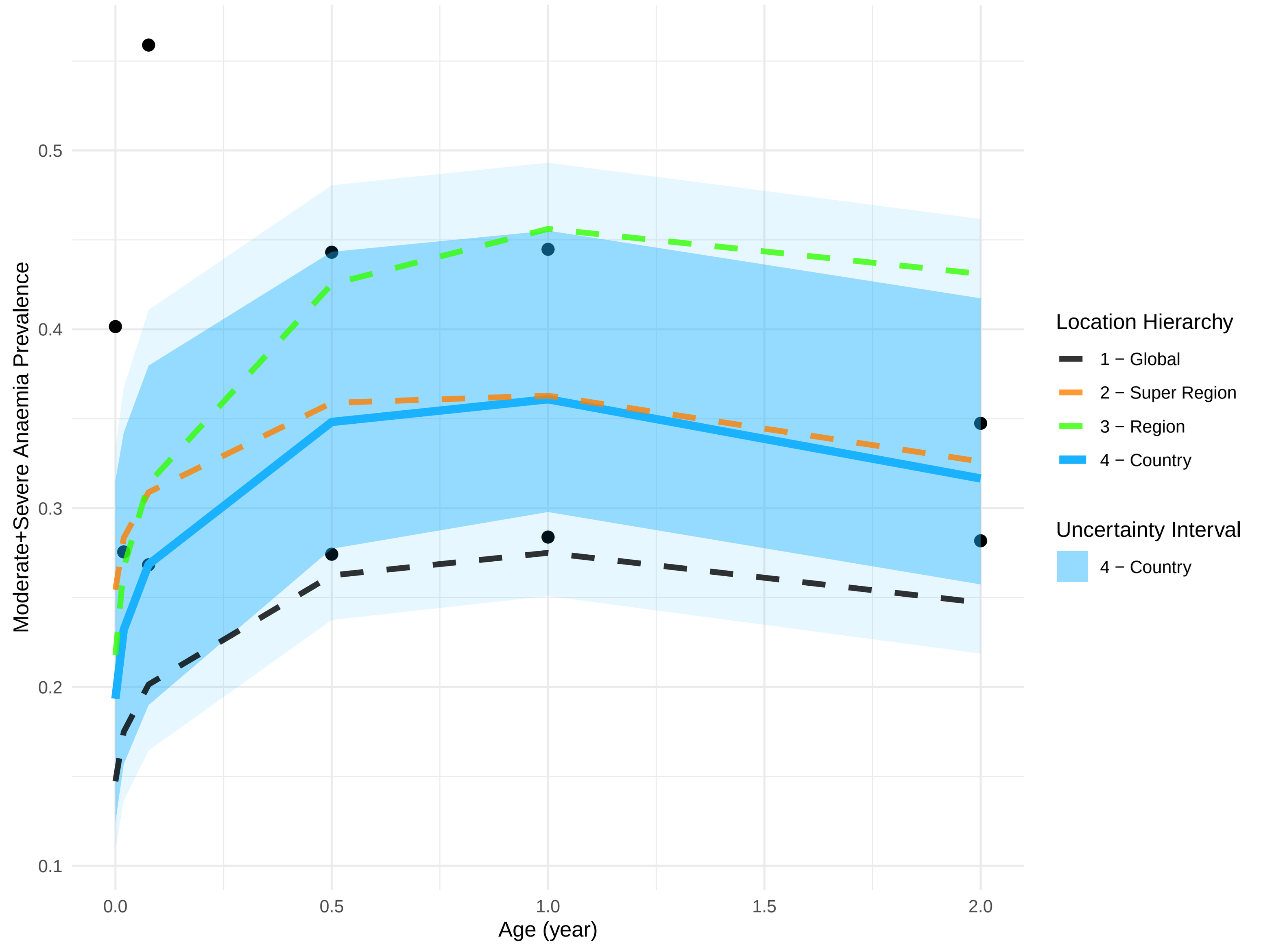

MR−BRT Age/Sex Fits − Non−pregnant Females <2 Years Old − Moderate+Severe Anaemia Prevalence − Côte d'Ivoire
WHO−2001 Adjustment Method

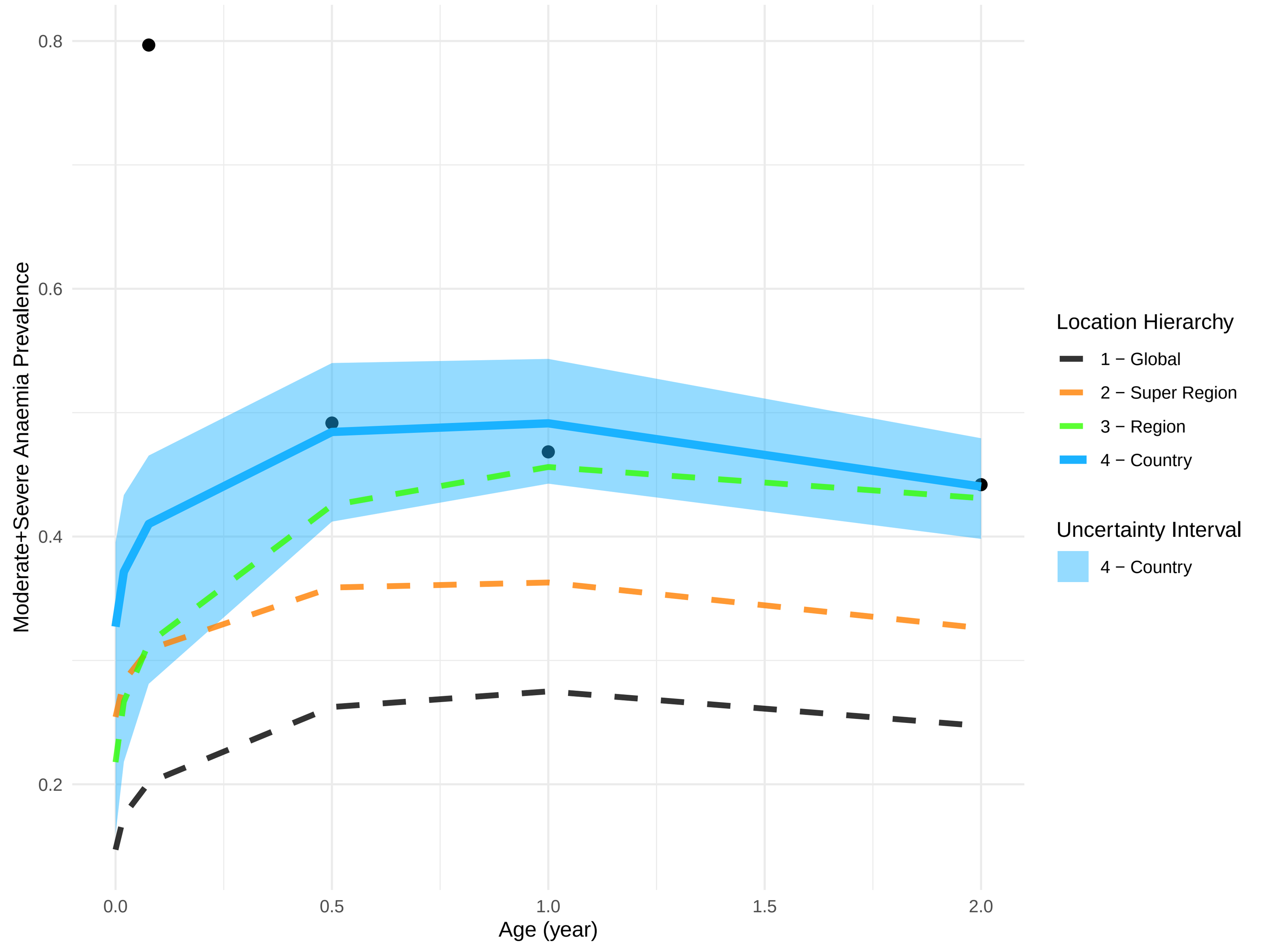

MR−BRT Age/Sex Fits − Non−pregnant Females <2 Years Old − Moderate+Severe Anaemia Prevalence − Gambia
WHO−2001 Adjustment Method

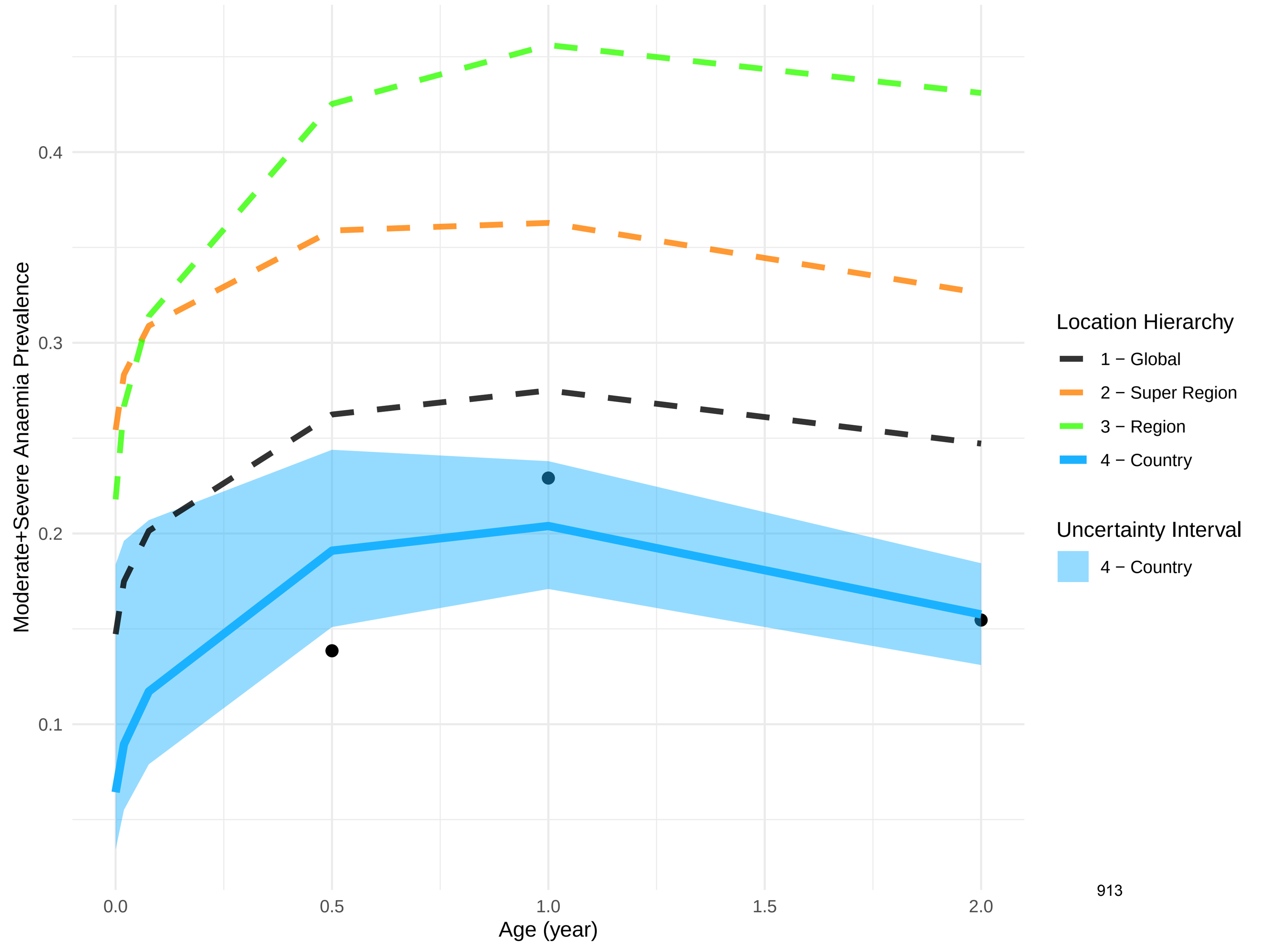

MR−BRT Age/Sex Fits − Non−pregnant Females <2 Years Old − Moderate+Severe Anaemia Prevalence − Ghana
WHO−2001 Adjustment Method

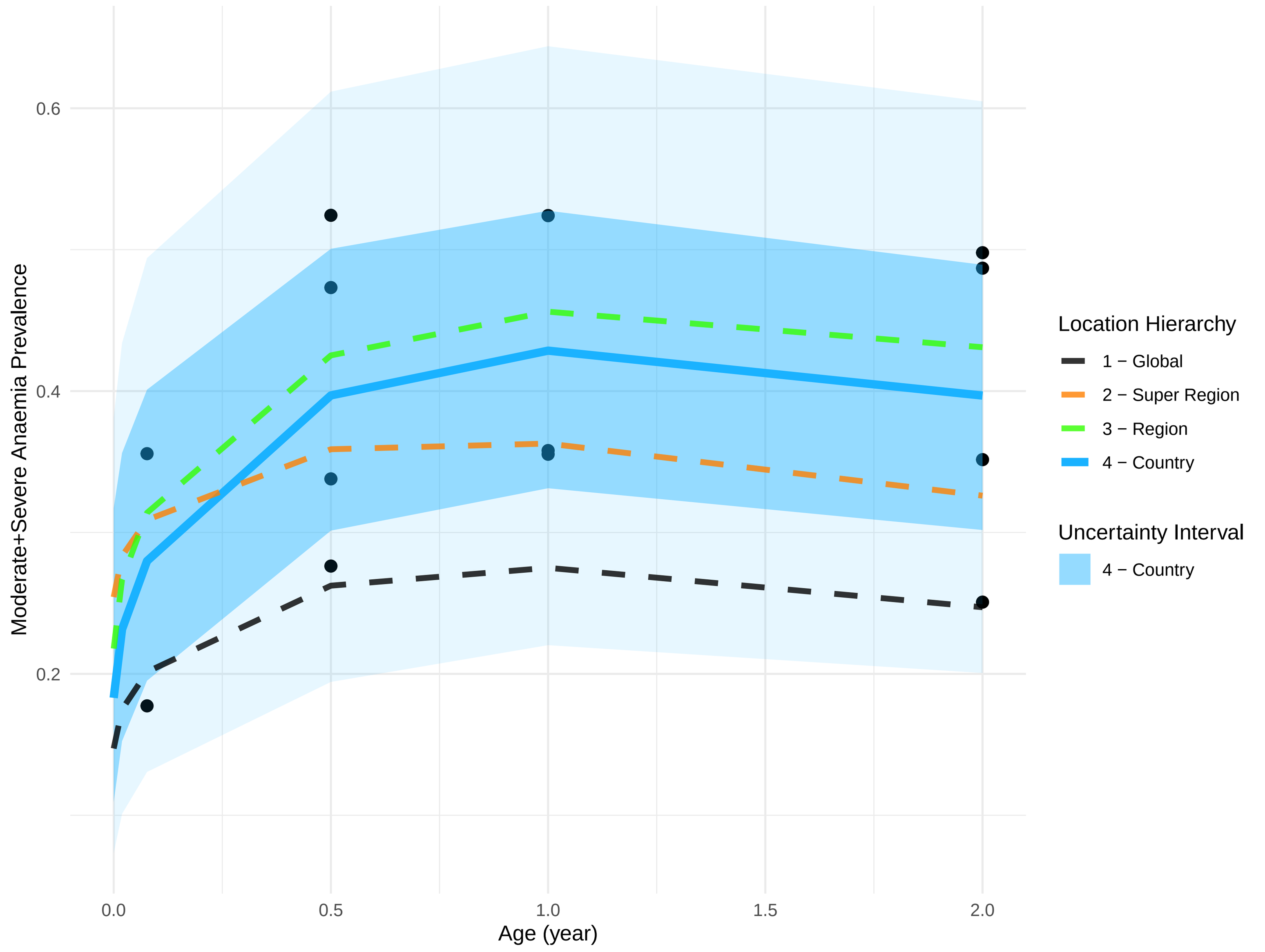

MR−BRT Age/Sex Fits − Non−pregnant Females <2 Years Old − Moderate+Severe Anaemia Prevalence − Guinea
WHO−2001 Adjustment Method

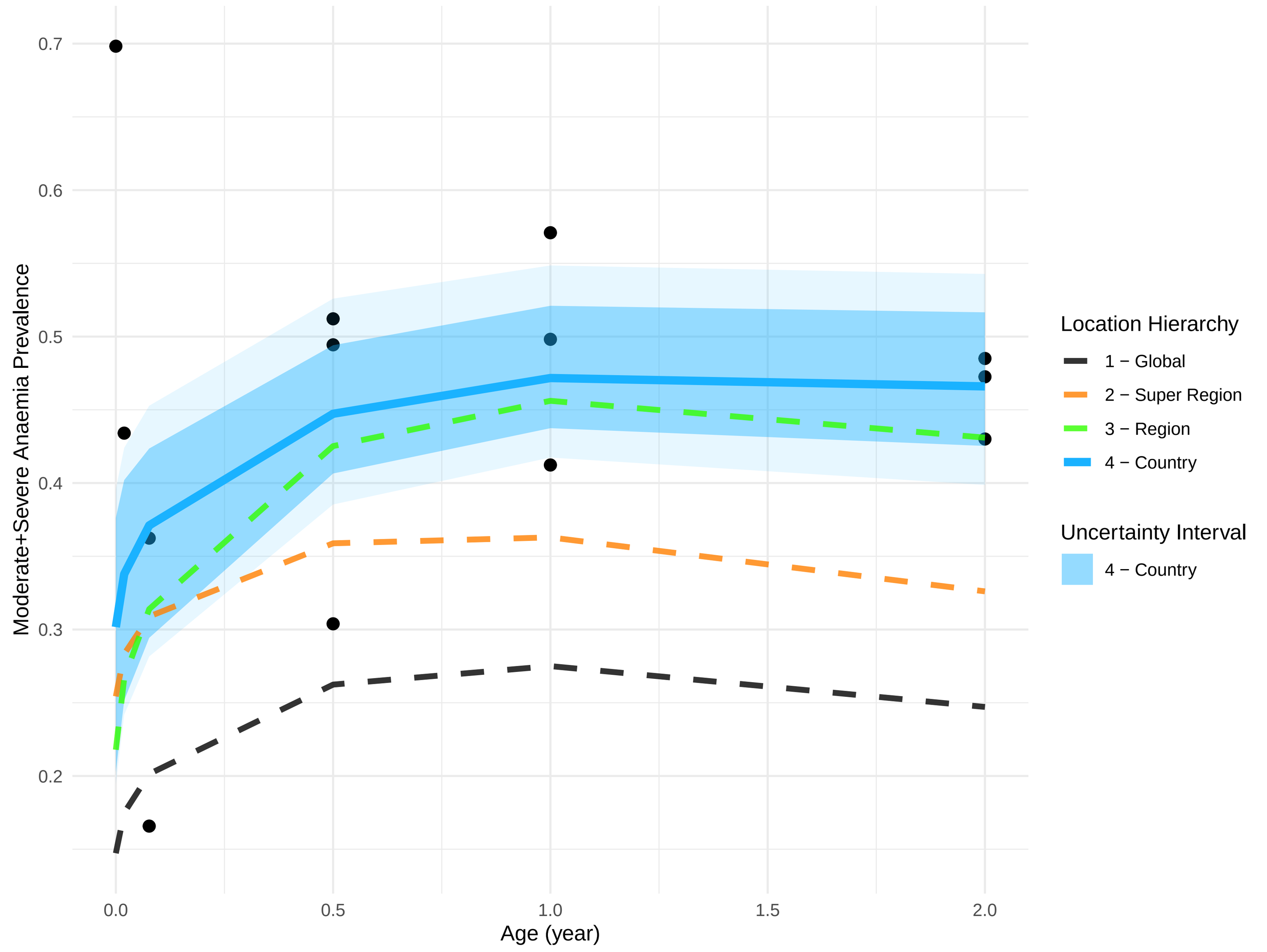

MR−BRT Age/Sex Fits − Non−pregnant Females <2 Years Old − Moderate+Severe Anaemia Prevalence − Liberia
WHO−2001 Adjustment Method

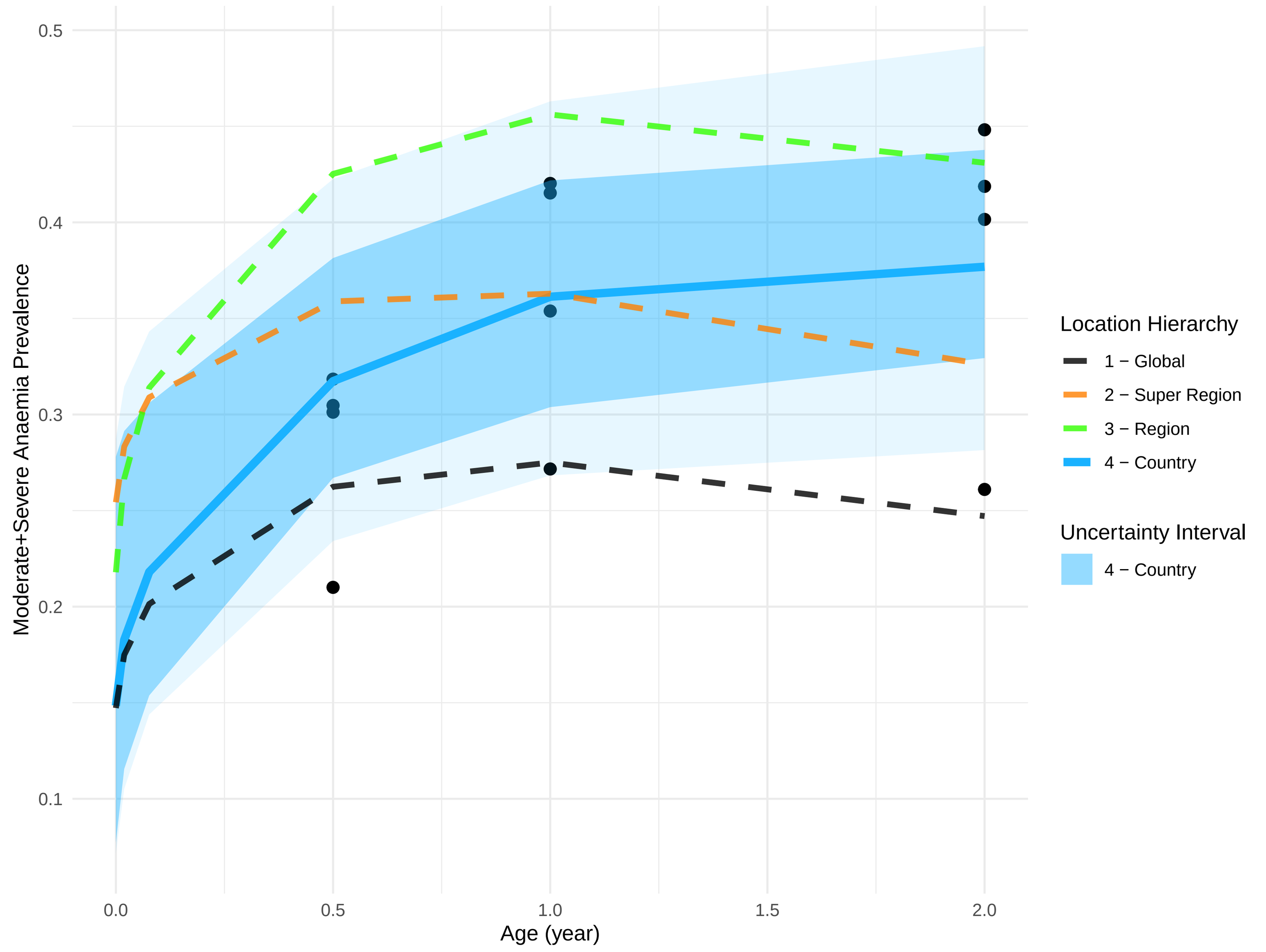

MR−BRT Age/Sex Fits − Non−pregnant Females <2 Years Old − Moderate+Severe Anaemia Prevalence − Mali
WHO−2001 Adjustment Method

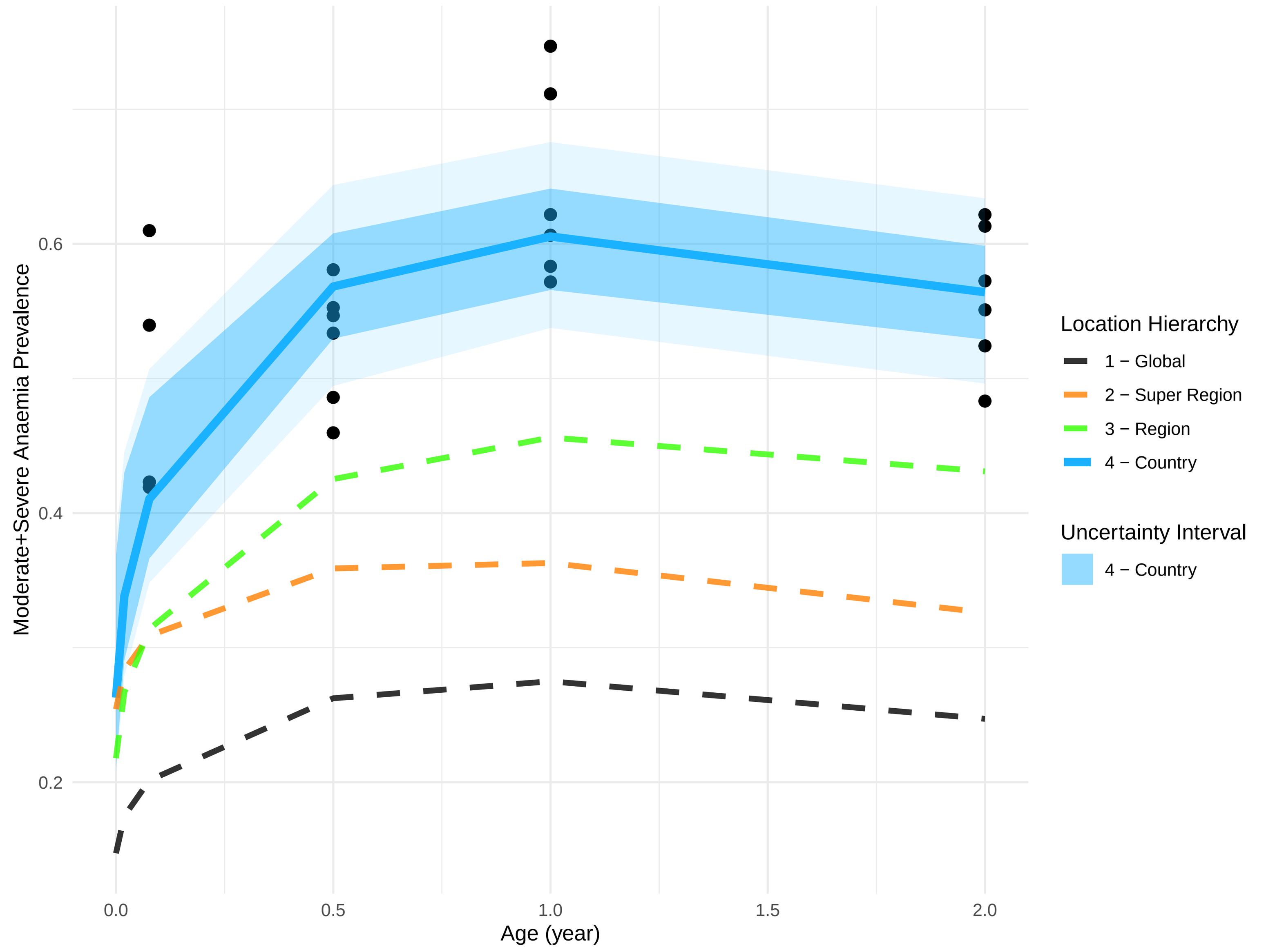

MR−BRT Age/Sex Fits − Non−pregnant Females <2 Years Old − Moderate+Severe Anaemia Prevalence − Niger
WHO−2001 Adjustment Method

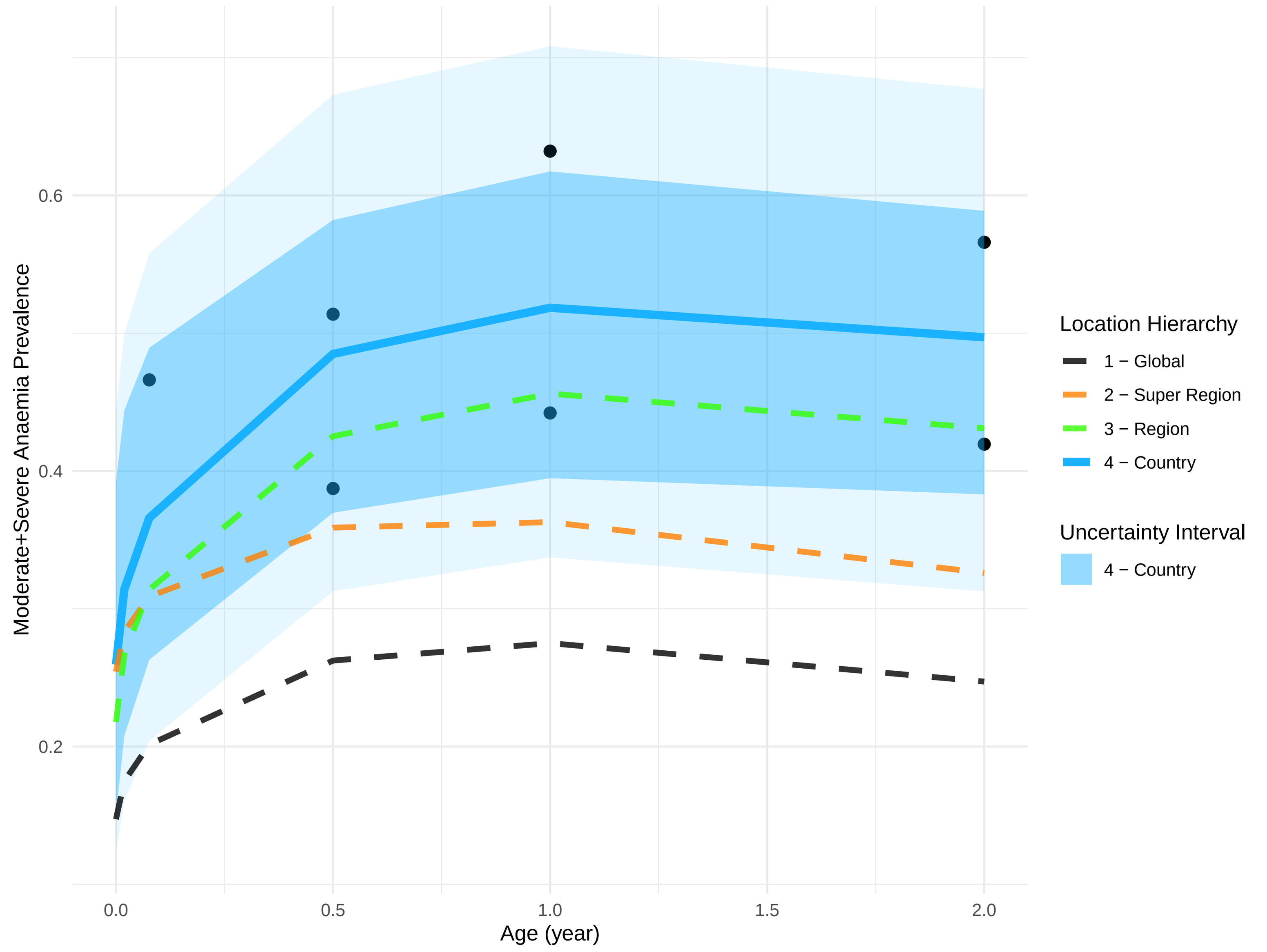

MR−BRT Age/Sex Fits − Non−pregnant Females <2 Years Old − Moderate+Severe Anaemia Prevalence − Nigeria
WHO−2001 Adjustment Method

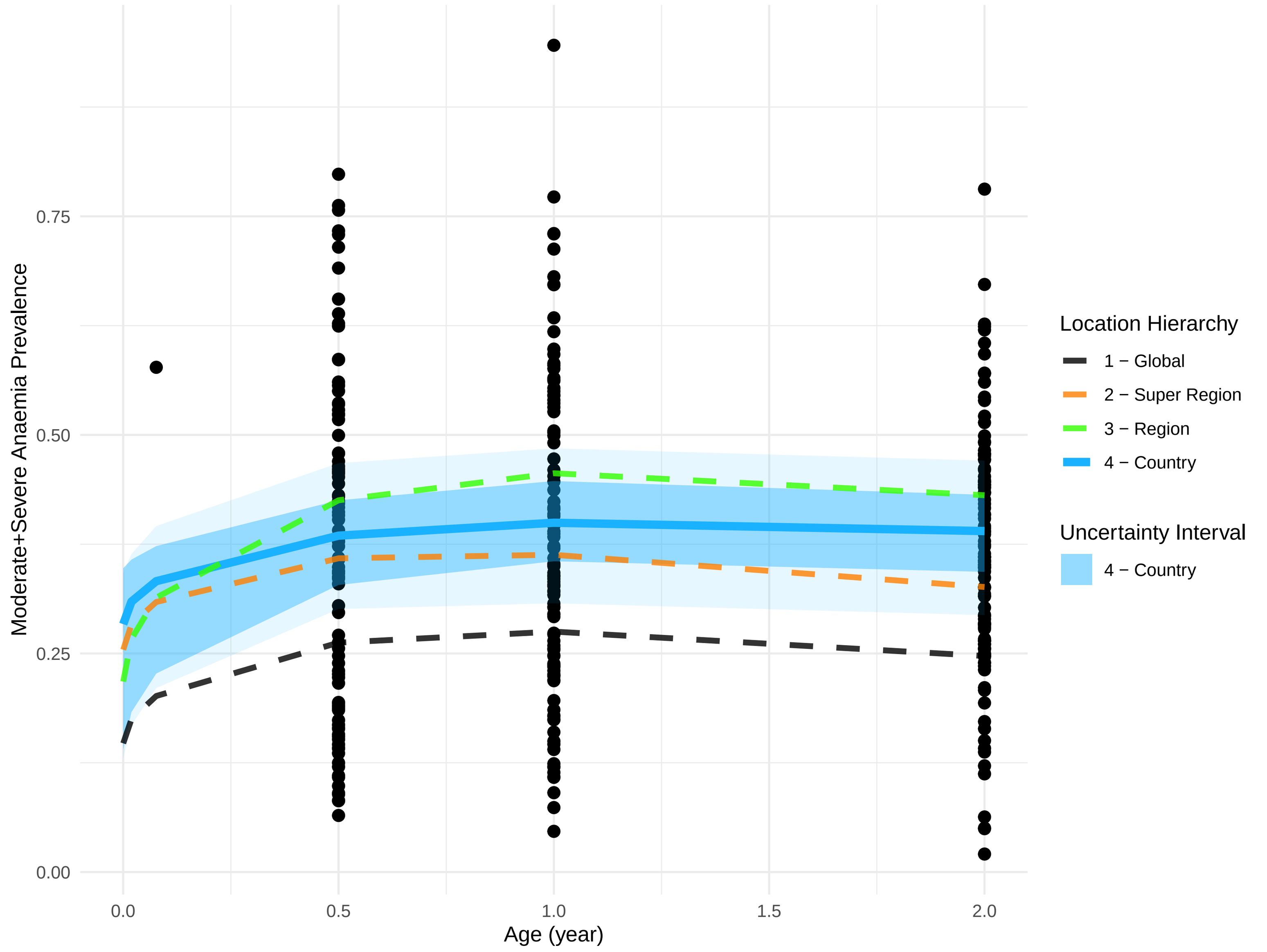

MR−BRT Age/Sex Fits − Non−pregnant Females <2 Years Old − Moderate+Severe Anaemia Prevalence − Abia
WHO−2001 Adjustment Method

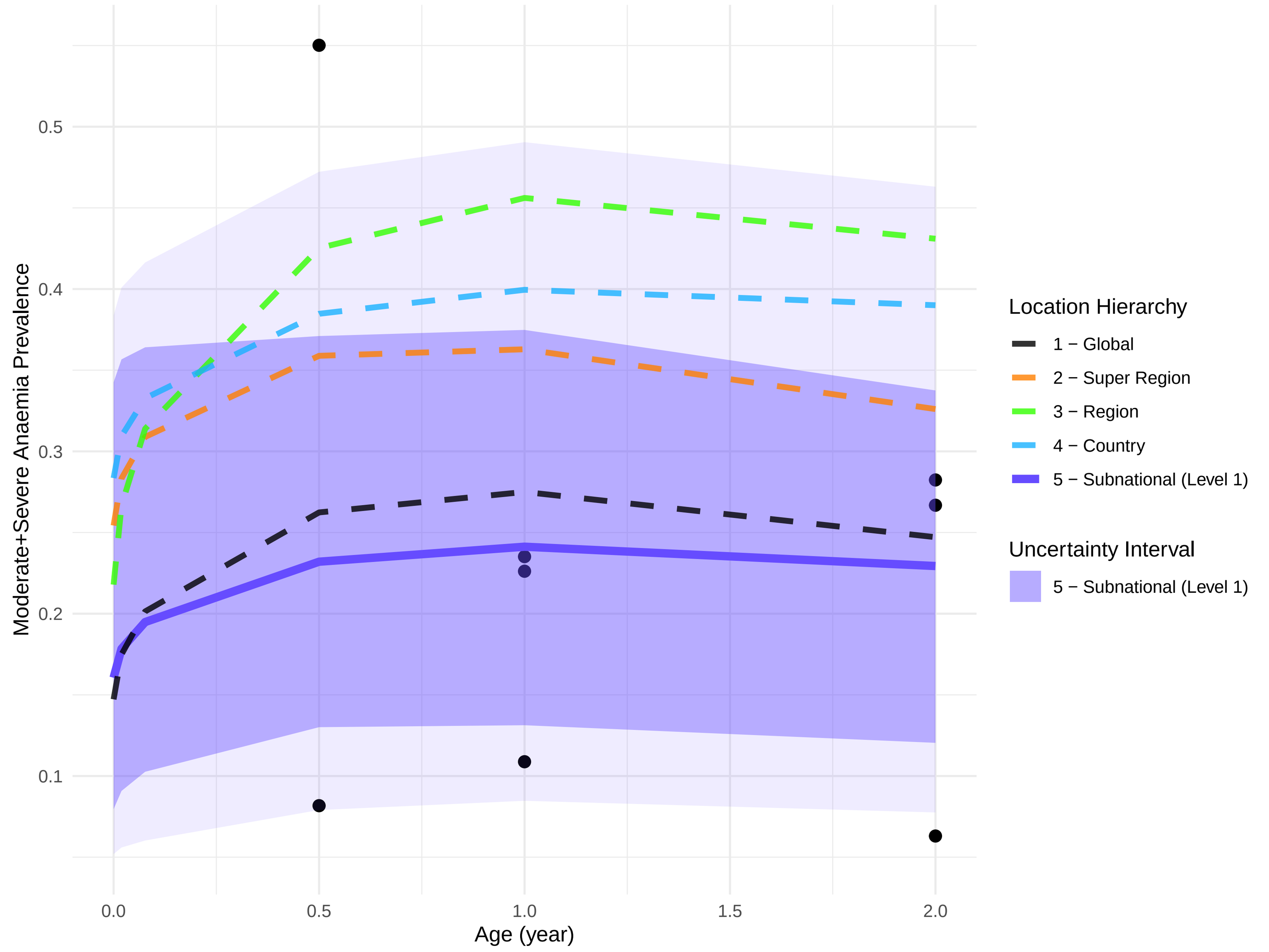

MR−BRT Age/Sex Fits − Non−pregnant Females <2 Years Old − Moderate+Severe Anaemia Prevalence − Adamawa
WHO−2001 Adjustment Method

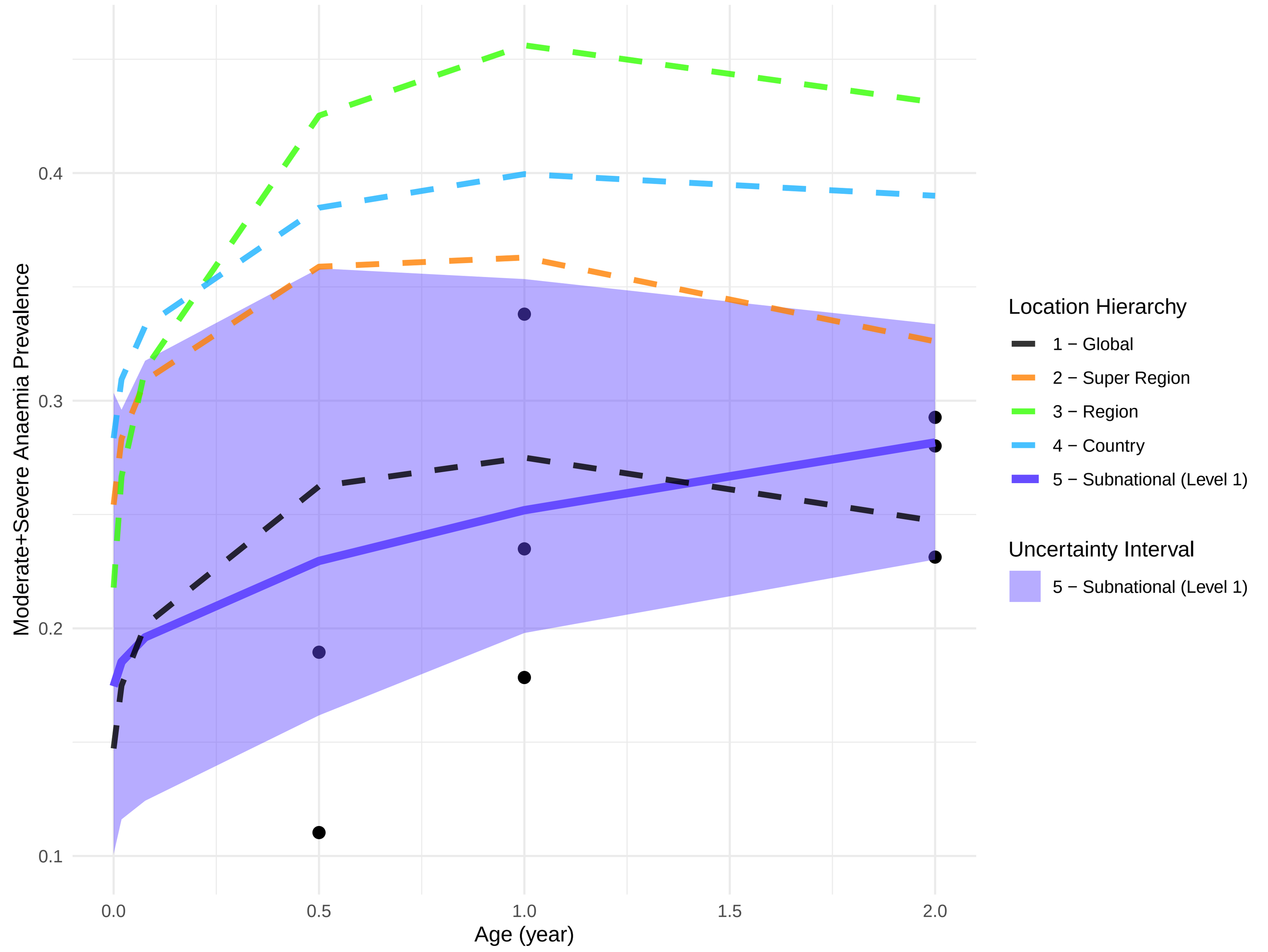

MR−BRT Age/Sex Fits − Non−pregnant Females <2 Years Old − Moderate+Severe Anaemia Prevalence − Akwa Ibom
WHO−2001 Adjustment Method

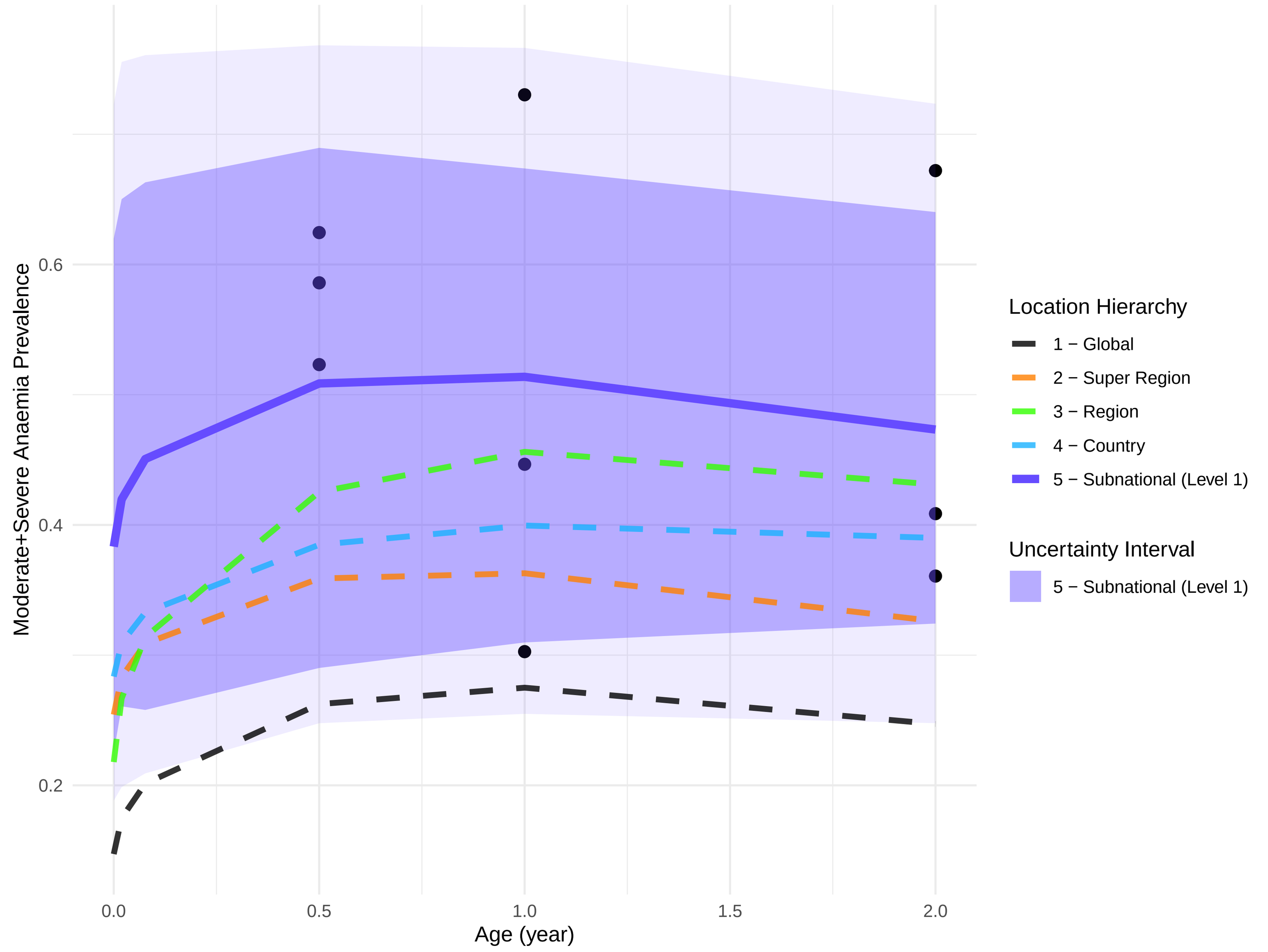

MR−BRT Age/Sex Fits − Non−pregnant Females <2 Years Old − Moderate+Severe Anaemia Prevalence − Anambra
WHO−2001 Adjustment Method

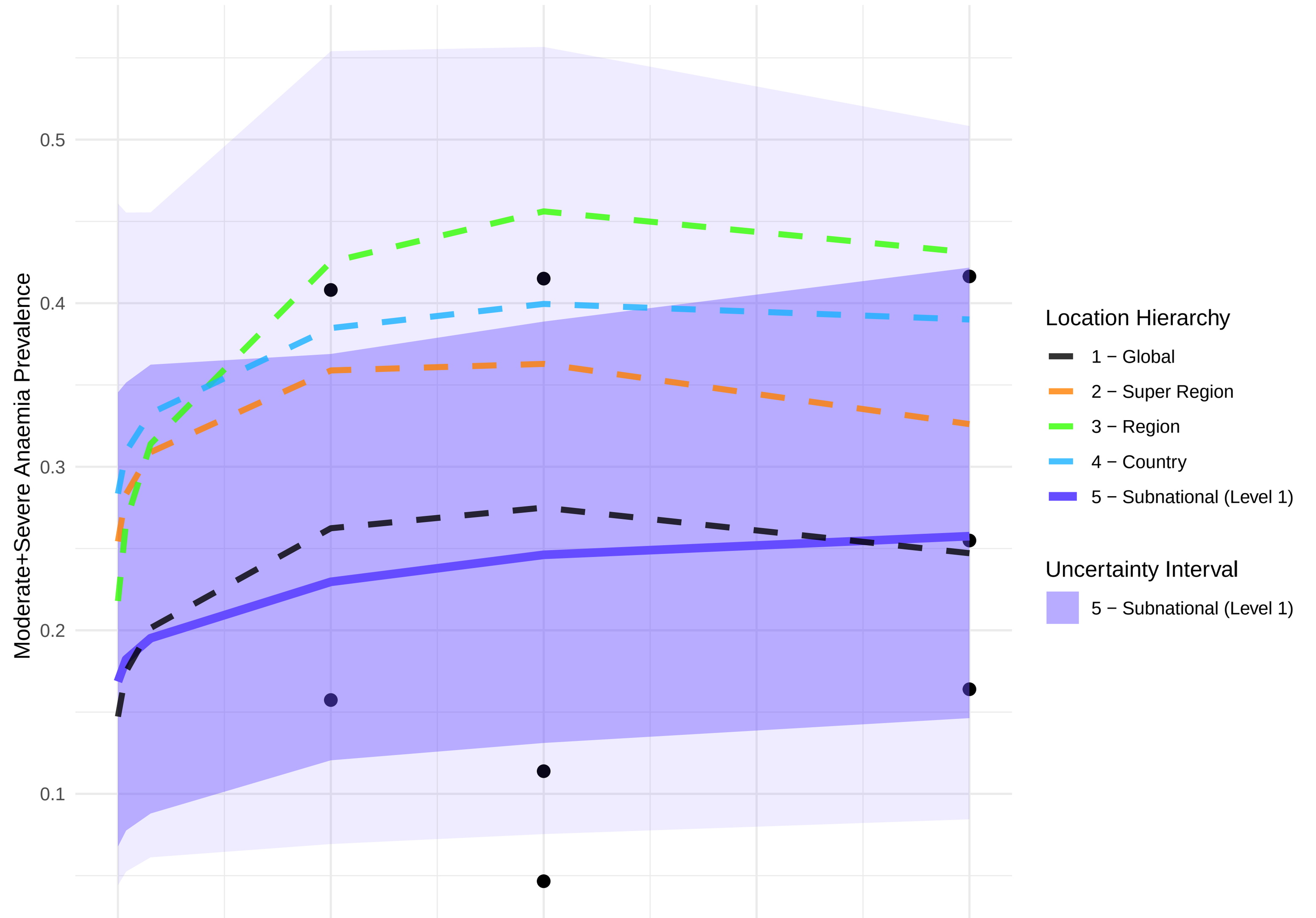

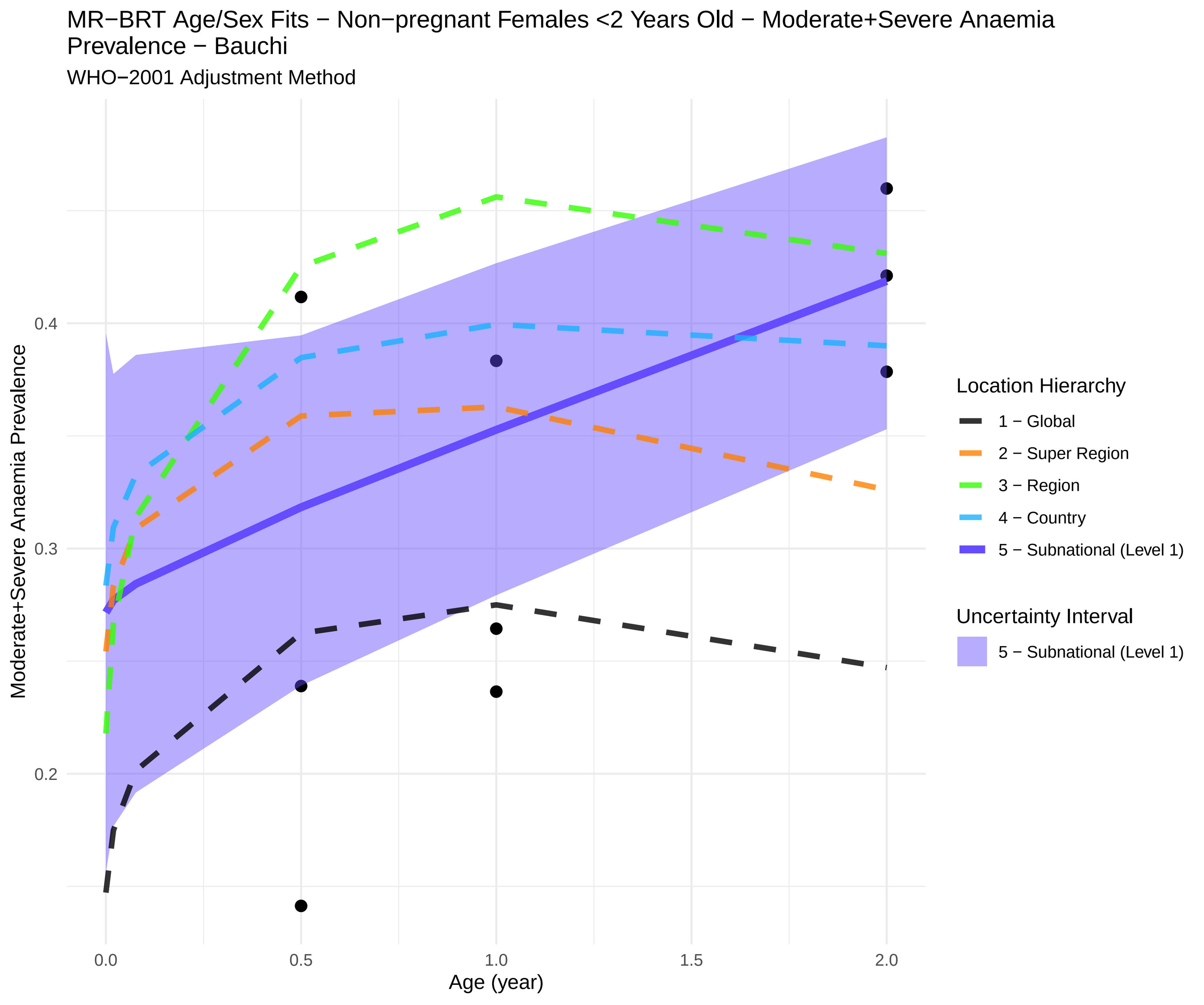
MR−BRT Age/Sex Fits − Non−pregnant Females <2 Years Old − Moderate+Severe Anaemia
Prevalence − Bauchi
WHO−2001 Adjustment Method
Moderate+Severe Anaemia Prevalence
Age (year)
Location Hierarchy
1 − Global
2 − Super Region
3 − Region
4 − Country
5 − Subnational (Level 1)
Uncertainty Interval
5 − Subnational (Level 1)

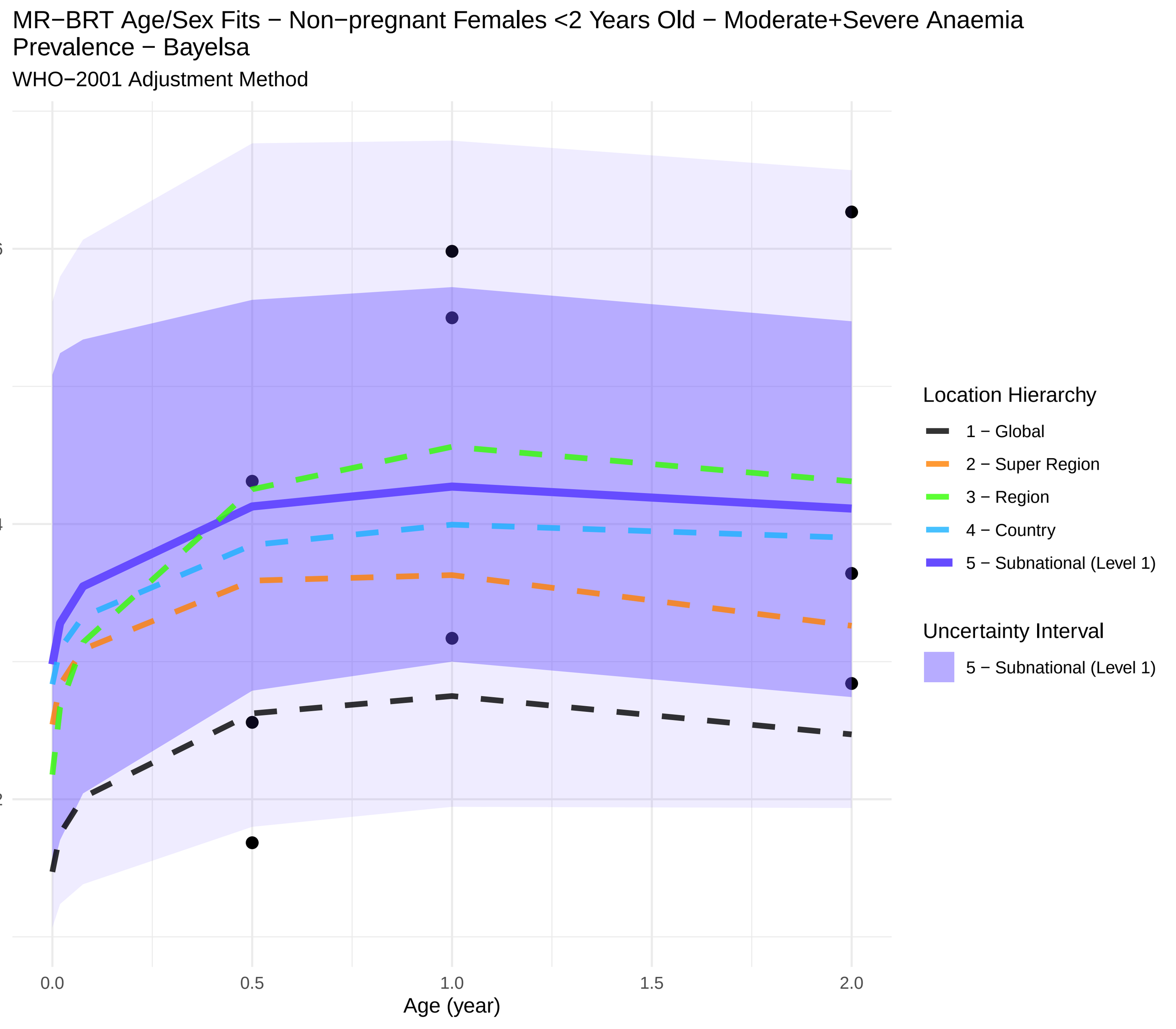
MR−BRT Age/Sex Fits − Non−pregnant Females <2 Years Old − Moderate+Severe Anaemia
Prevalence − Bayelsa
WHO−2001 Adjustment Method
Moderate+Severe Anaemia Prevalence
Age (year)
Location Hierarchy
1 − Global
2 − Super Region
3 − Region
4 − Country
5 − Subnational (Level 1)
Uncertainty Interval
5 − Subnational (Level 1)

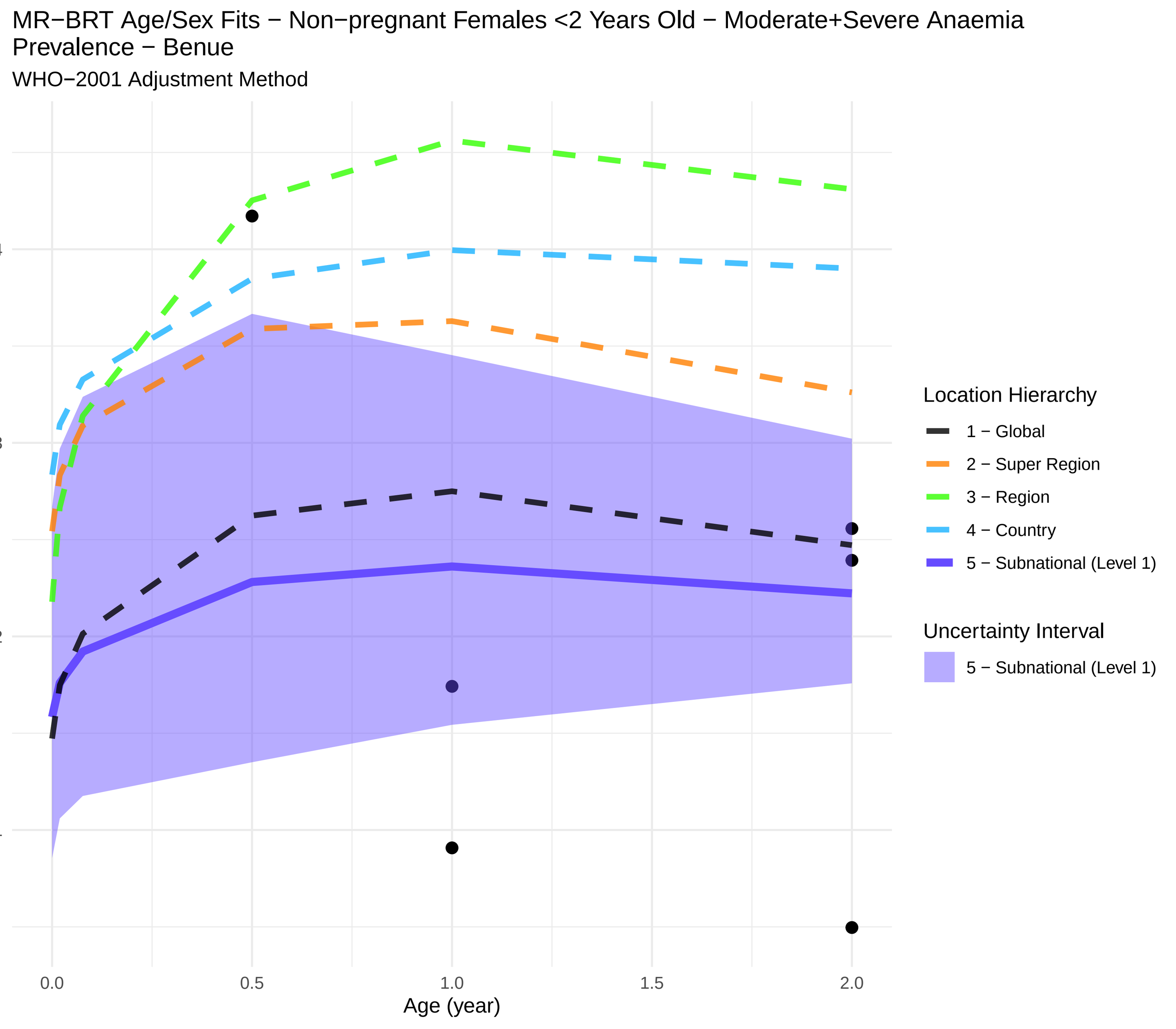
MR−BRT Age/Sex Fits − Non−pregnant Females <2 Years Old − Moderate+Severe Anaemia
Prevalence − Benue
WHO−2001 Adjustment Method
Moderate+Severe Anaemia Prevalence
Age (year)
Location Hierarchy
1 − Global
2 − Super Region
3 − Region
4 − Country
5 − Subnational (Level 1)
Uncertainty Interval
5 − Subnational (Level 1)

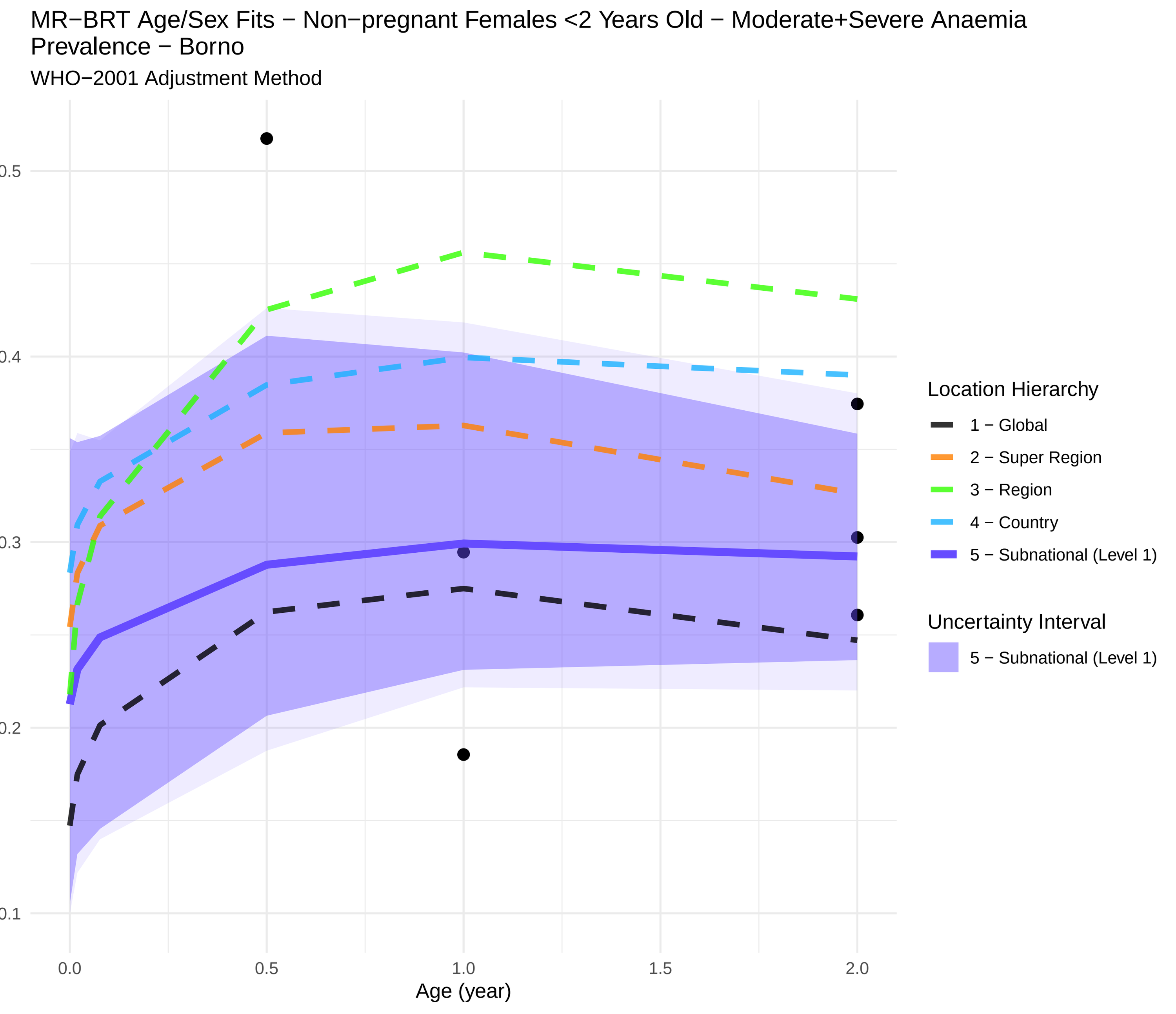
MR−BRT Age/Sex Fits − Non−pregnant Females <2 Years Old − Moderate+Severe Anaemia
Prevalence − Borno
WHO−2001 Adjustment Method
Moderate+Severe Anaemia Prevalence
Age (year)
Location Hierarchy
1 − Global
2 − Super Region
3 − Region
4 − Country
5 − Subnational (Level 1)
Uncertainty Interval
5 − Subnational (Level 1)

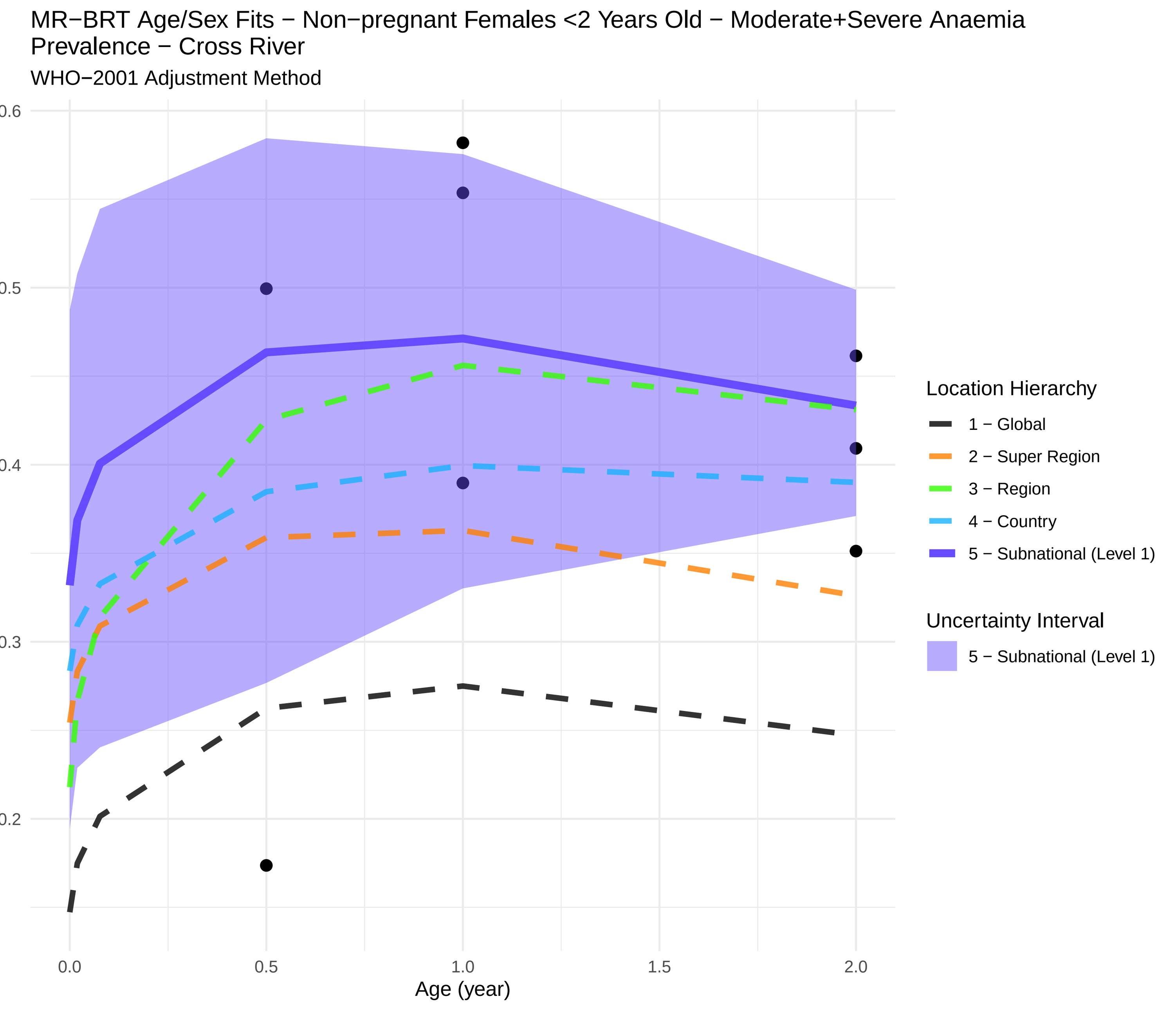
MR−BRT Age/Sex Fits − Non−pregnant Females <2 Years Old − Moderate+Severe Anaemia
Prevalence − Cross River
WHO−2001 Adjustment Method
Moderate+Severe Anaemia Prevalence
Age (year)
Location Hierarchy
1 − Global
2 − Super Region
3 − Region
4 − Country
5 − Subnational (Level 1)
Uncertainty Interval
5 − Subnational (Level 1)

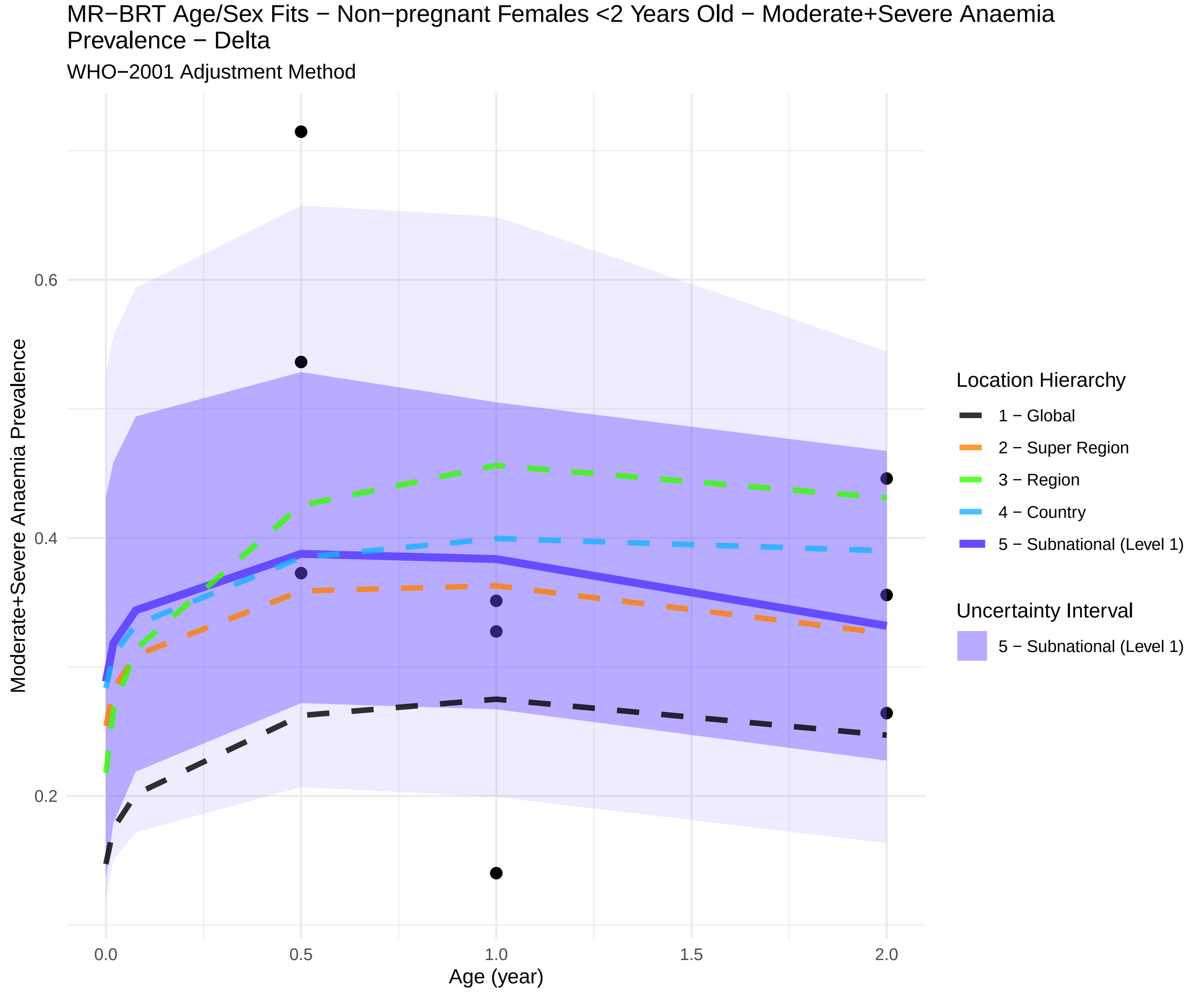
MR−BRT Age/Sex Fits − Non−pregnant Females <2 Years Old − Moderate+Severe Anaemia
Prevalence − Delta
WHO−2001 Adjustment Method
Moderate+Severe Anaemia Prevalence
Age (year)
Location Hierarchy
1 − Global
2 − Super Region
3 − Region
4 − Country
5 − Subnational (Level 1)
Uncertainty Interval
5 − Subnational (Level 1)

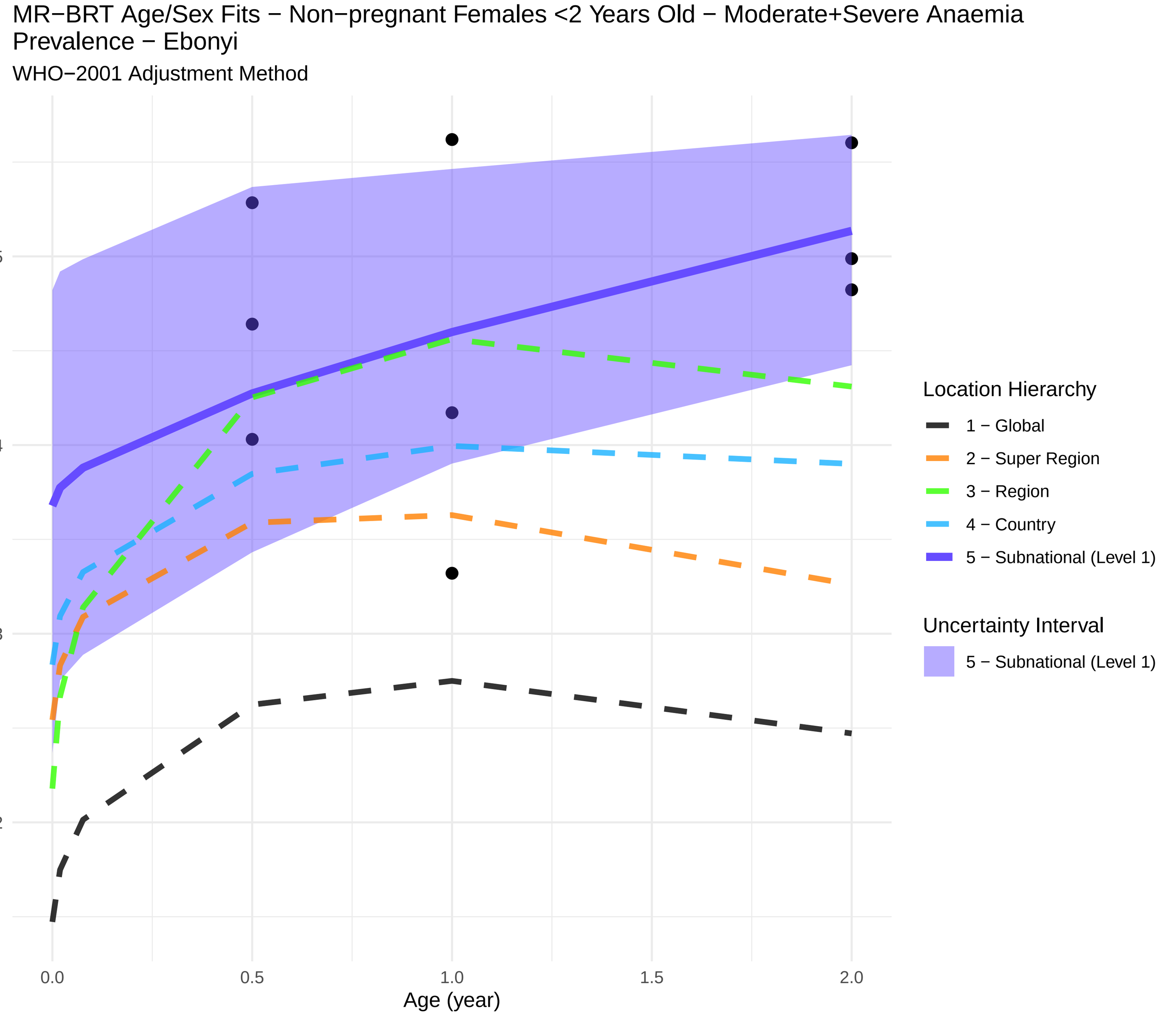
MR−BRT Age/Sex Fits − Non−pregnant Females <2 Years Old − Moderate+Severe Anaemia
Prevalence − Ebonyi
WHO−2001 Adjustment Method
Moderate+Severe Anaemia Prevalence
Age (year)
Location Hierarchy
1 − Global
2 − Super Region
3 − Region
4 − Country
5 − Subnational (Level 1)
Uncertainty Interval
5 − Subnational (Level 1)

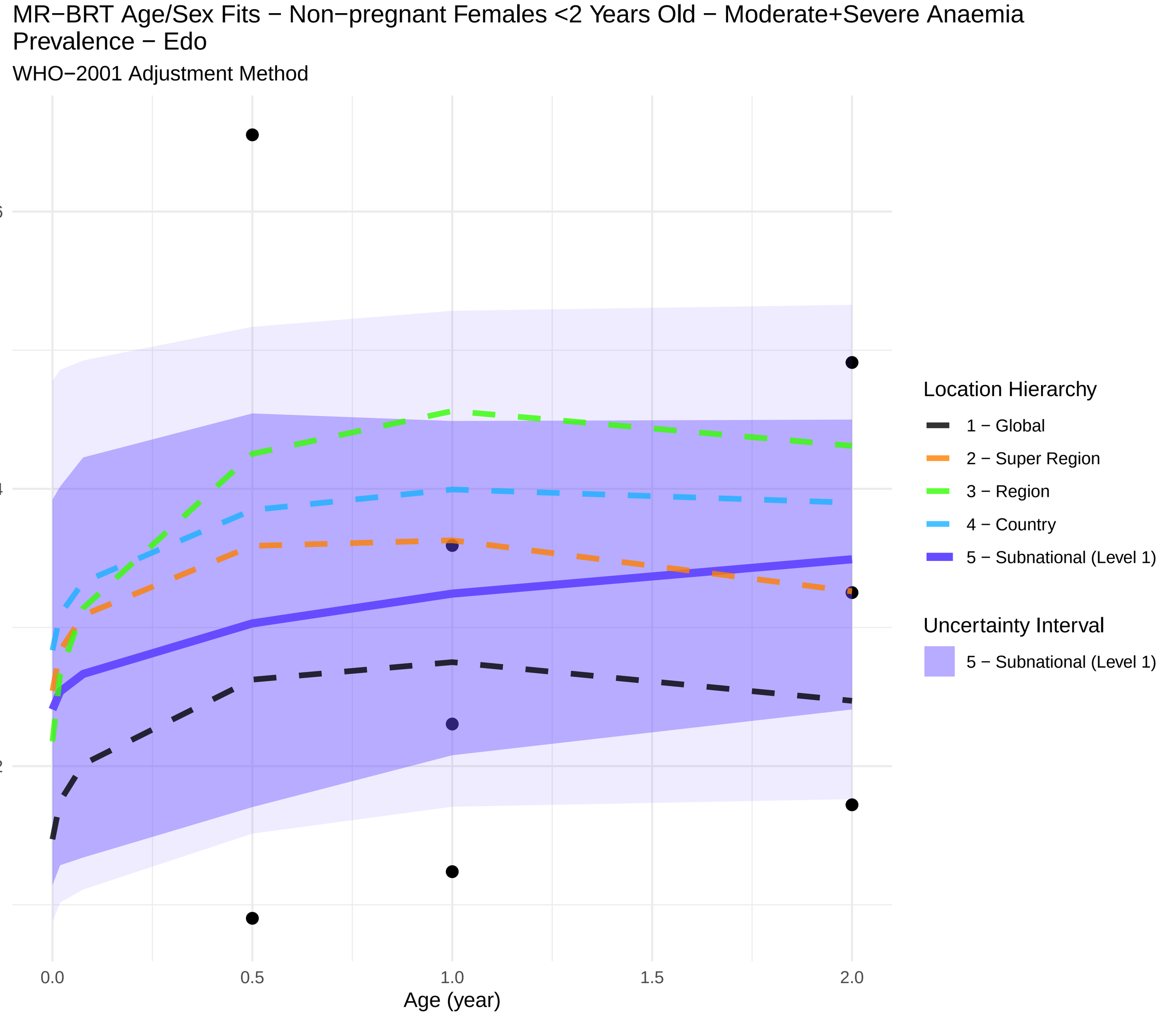
MR−BRT Age/Sex Fits − Non−pregnant Females <2 Years Old − Moderate+Severe Anaemia
Prevalence − Edo
WHO−2001 Adjustment Method
Moderate+Severe Anaemia Prevalence
Age (year)
Location Hierarchy
1 − Global
2 − Super Region
3 − Region
4 − Country
5 − Subnational (Level 1)
Uncertainty Interval
5 − Subnational (Level 1)

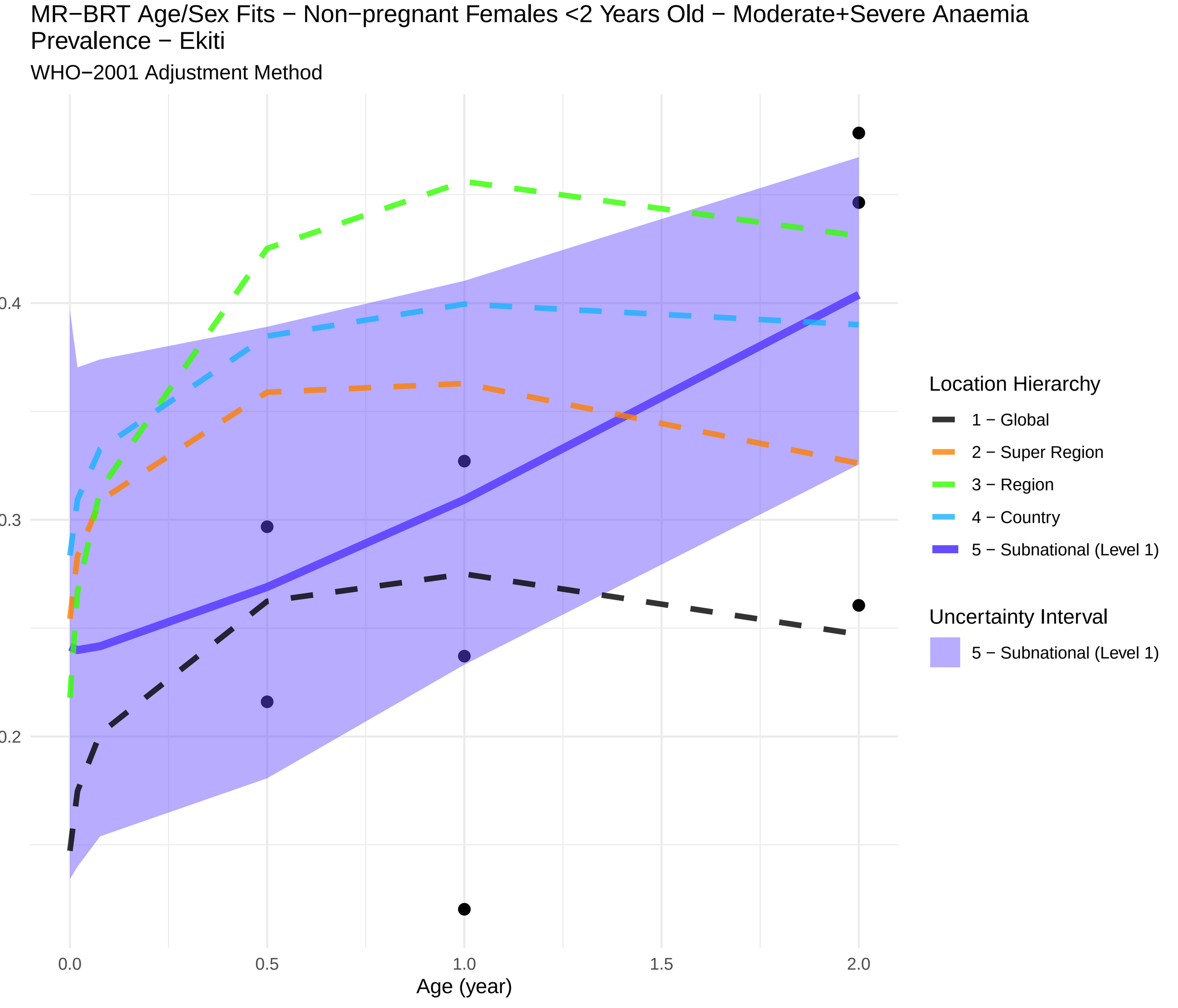
MR−BRT Age/Sex Fits − Non−pregnant Females <2 Years Old − Moderate+Severe Anaemia
Prevalence − Ekiti
WHO−2001 Adjustment Method
Moderate+Severe Anaemia Prevalence
Age (year)
Location Hierarchy
1 − Global
2 − Super Region
3 − Region
4 − Country
5 − Subnational (Level 1)
Uncertainty Interval
5 − Subnational (Level 1)

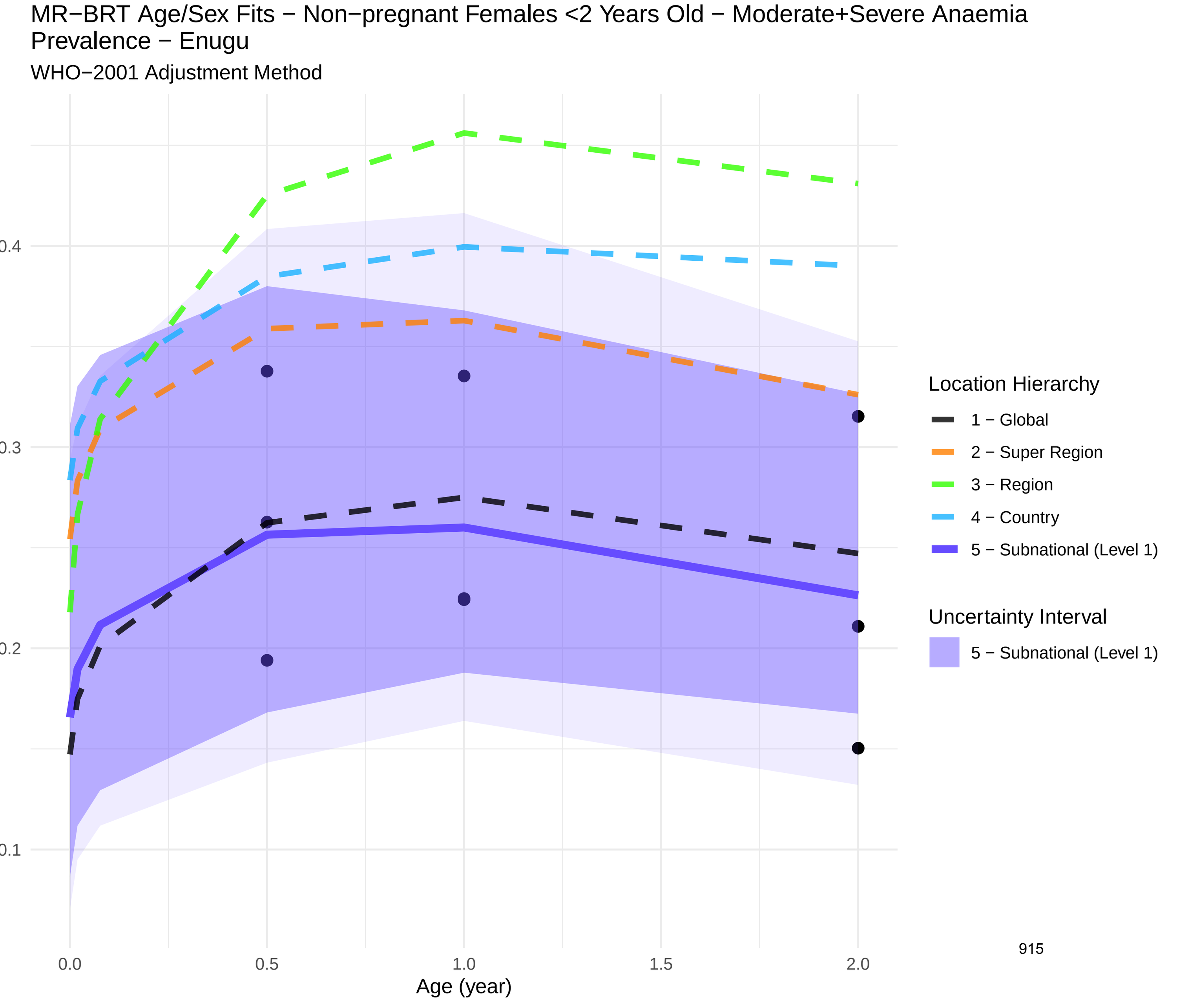
MR−BRT Age/Sex Fits − Non−pregnant Females <2 Years Old − Moderate+Severe Anaemia
Prevalence − Enugu
WHO−2001 Adjustment Method
Moderate+Severe Anaemia Prevalence
Age (year)
Location Hierarchy
1 − Global
2 − Super Region
3 − Region
4 − Country
5 − Subnational (Level 1)
Uncertainty Interval
5 − Subnational (Level 1)

MR−BRT Age/Sex Fits − Non−pregnant Females <2 Years Old − Moderate+Severe Anaemia Prevalence − FCT (Abuja)
WHO−2001 Adjustment Method

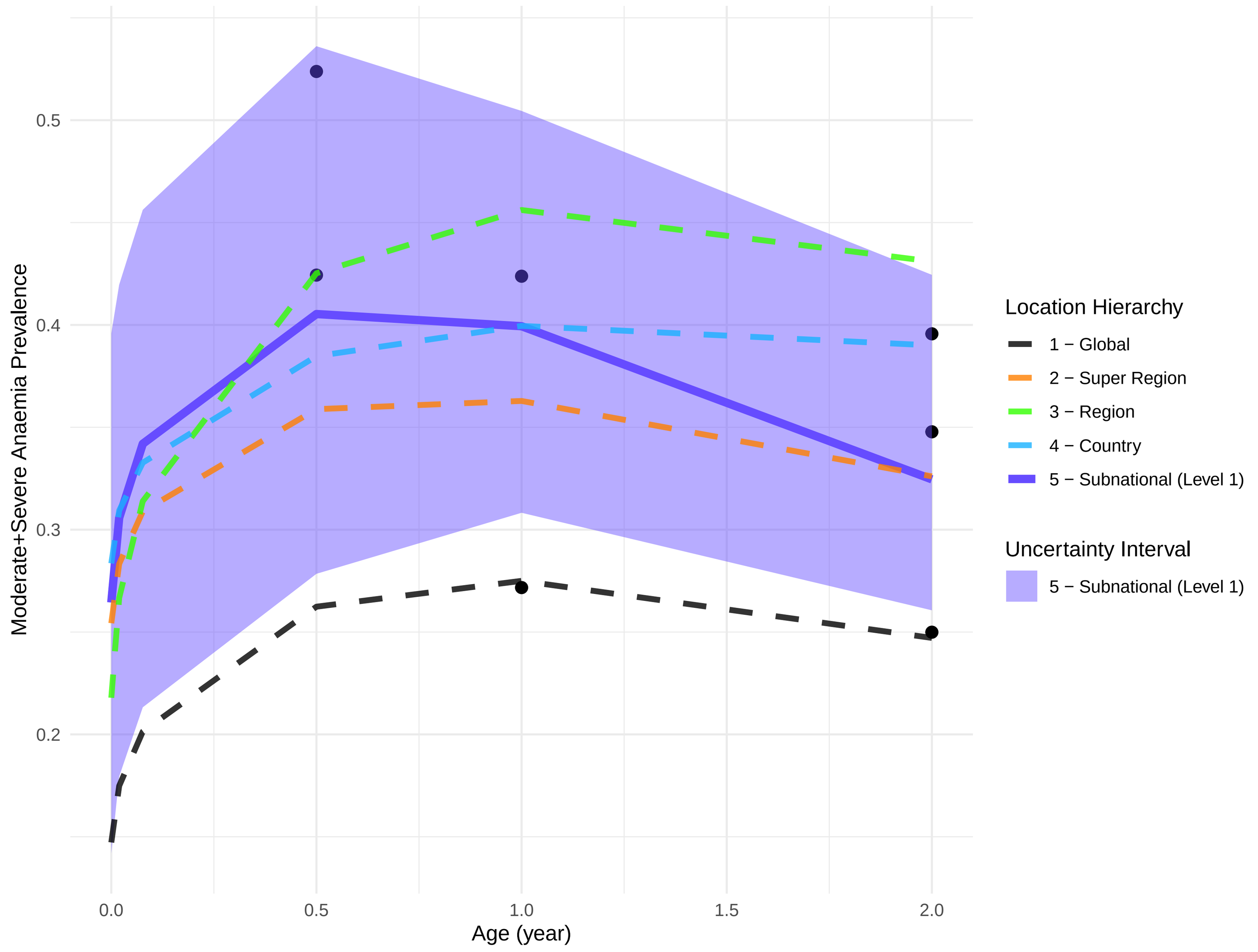

MR−BRT Age/Sex Fits − Non−pregnant Females <2 Years Old − Moderate+Severe Anaemia Prevalence − Gombe
WHO−2001 Adjustment Method

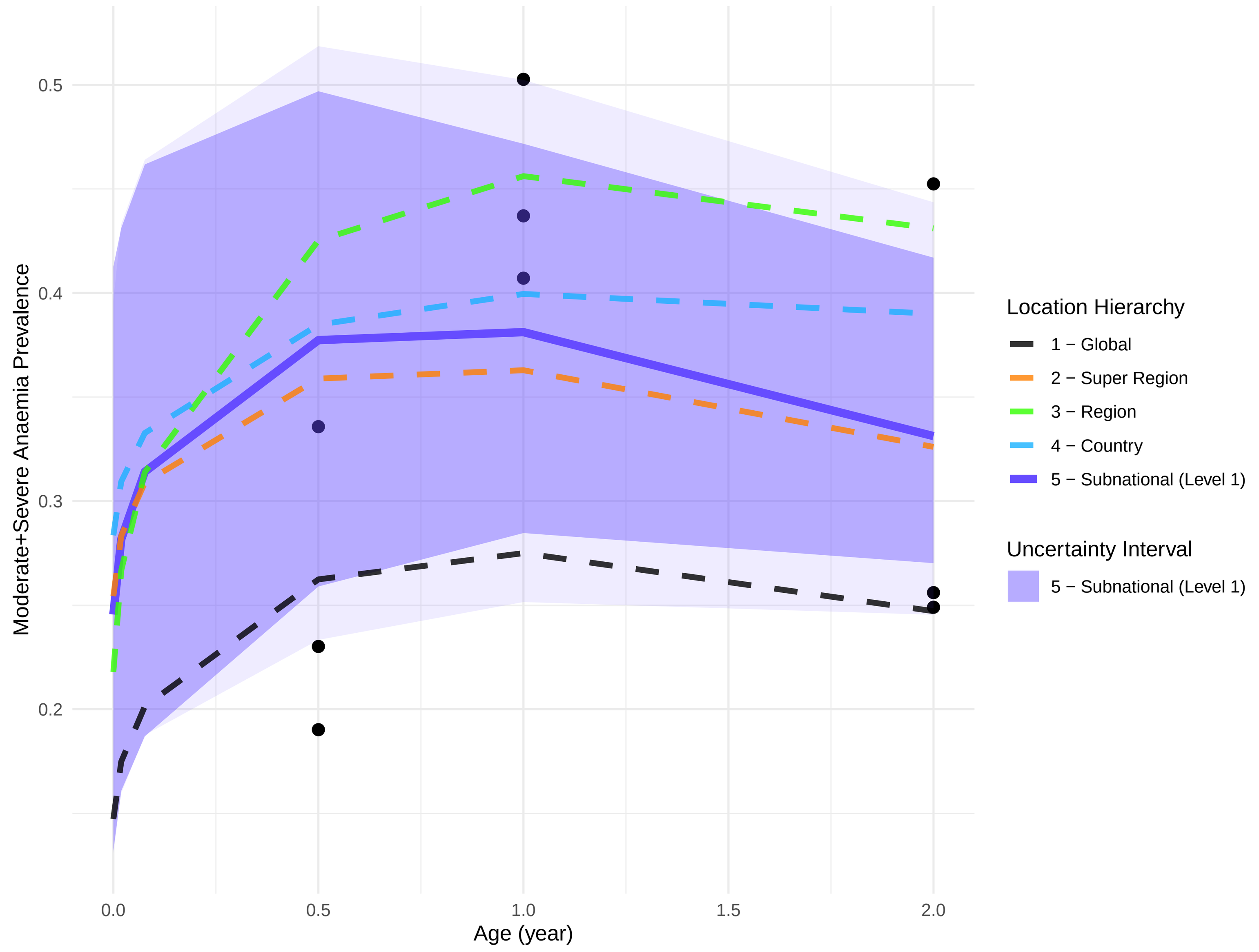

MR−BRT Age/Sex Fits − Non−pregnant Females <2 Years Old − Moderate+Severe Anaemia Prevalence − Imo
WHO−2001 Adjustment Method

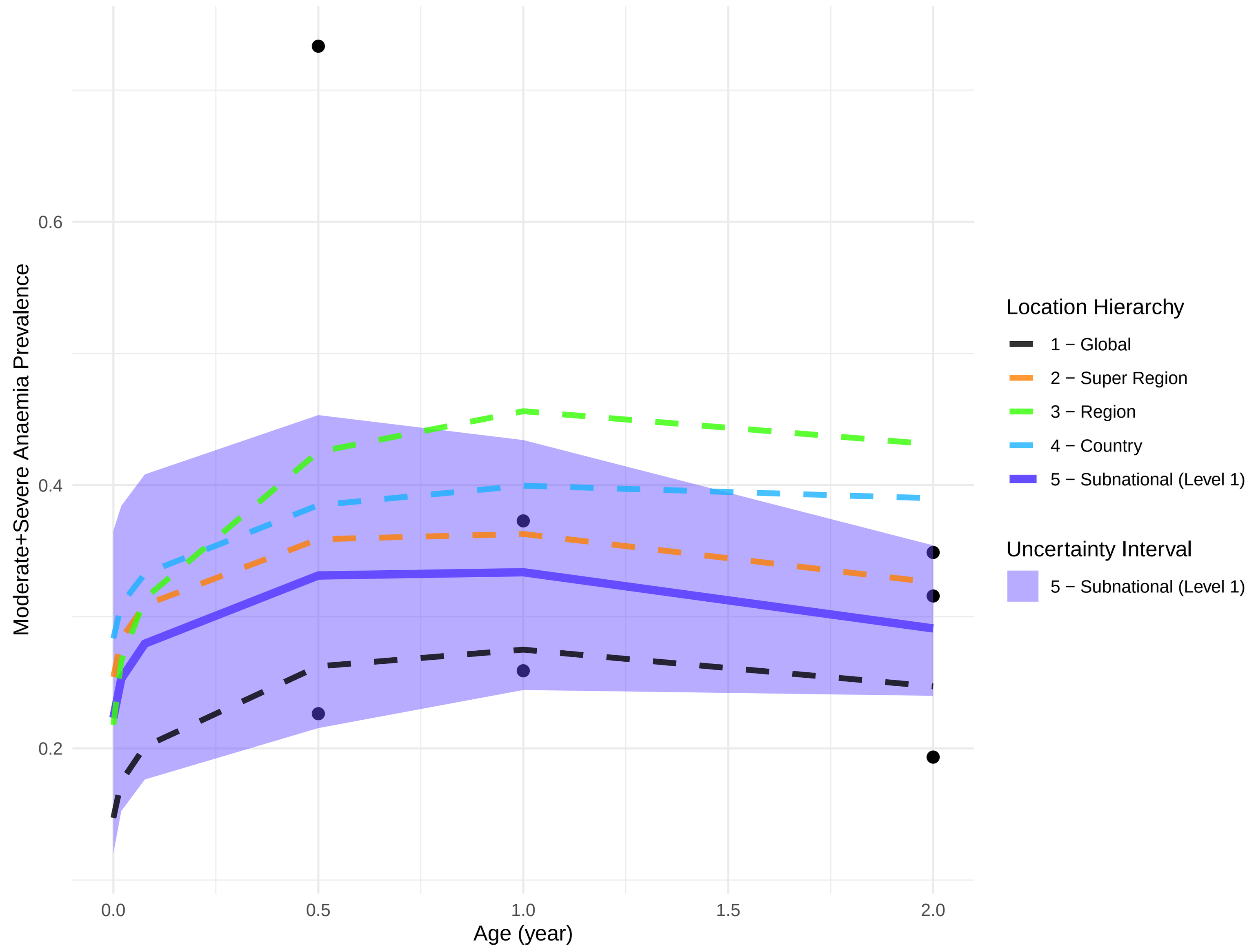

MR−BRT Age/Sex Fits − Non−pregnant Females <2 Years Old − Moderate+Severe Anaemia Prevalence − Jigawa
WHO−2001 Adjustment Method

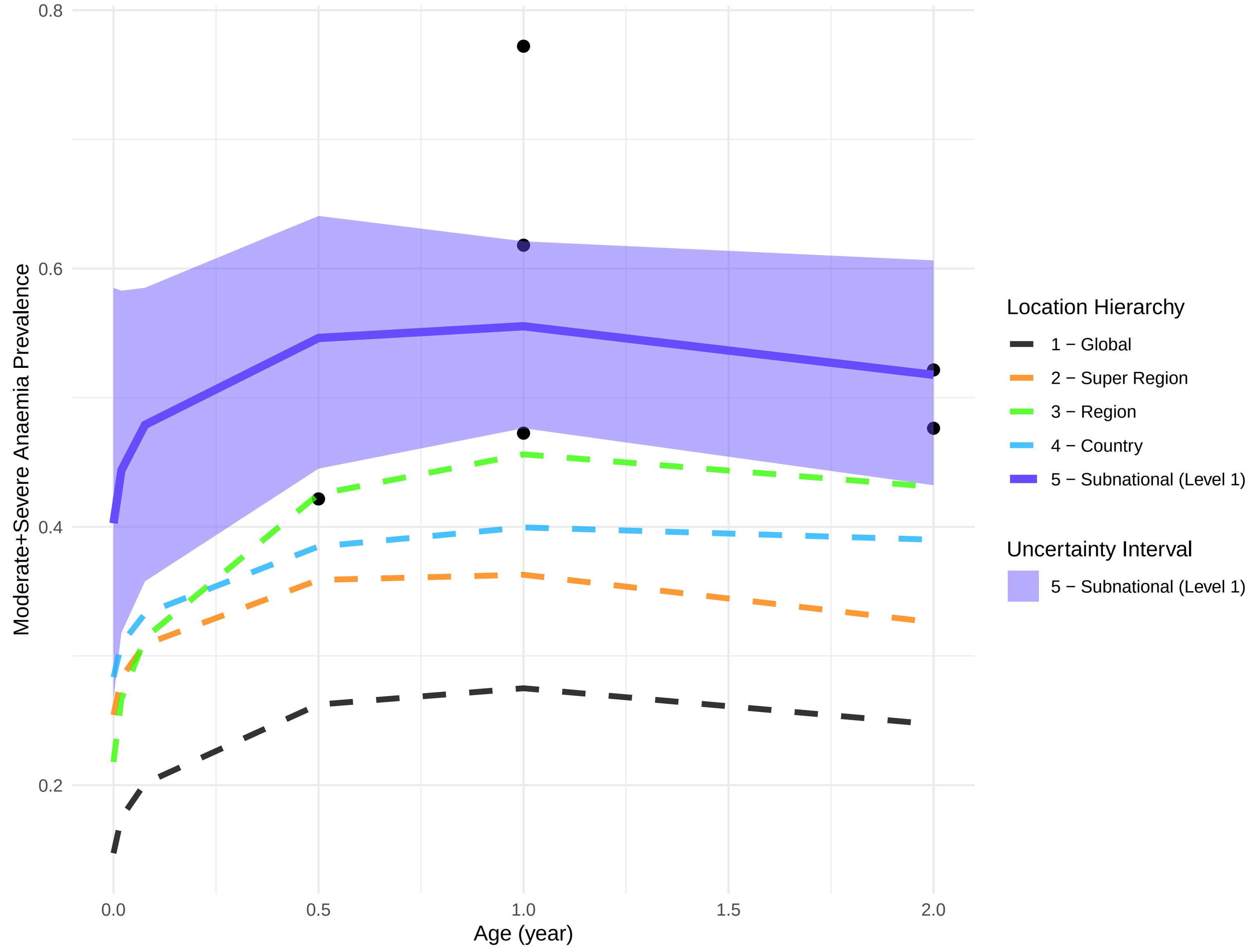

MR−BRT Age/Sex Fits − Non−pregnant Females <2 Years Old − Moderate+Severe Anaemia Prevalence − Kaduna
WHO−2001 Adjustment Method

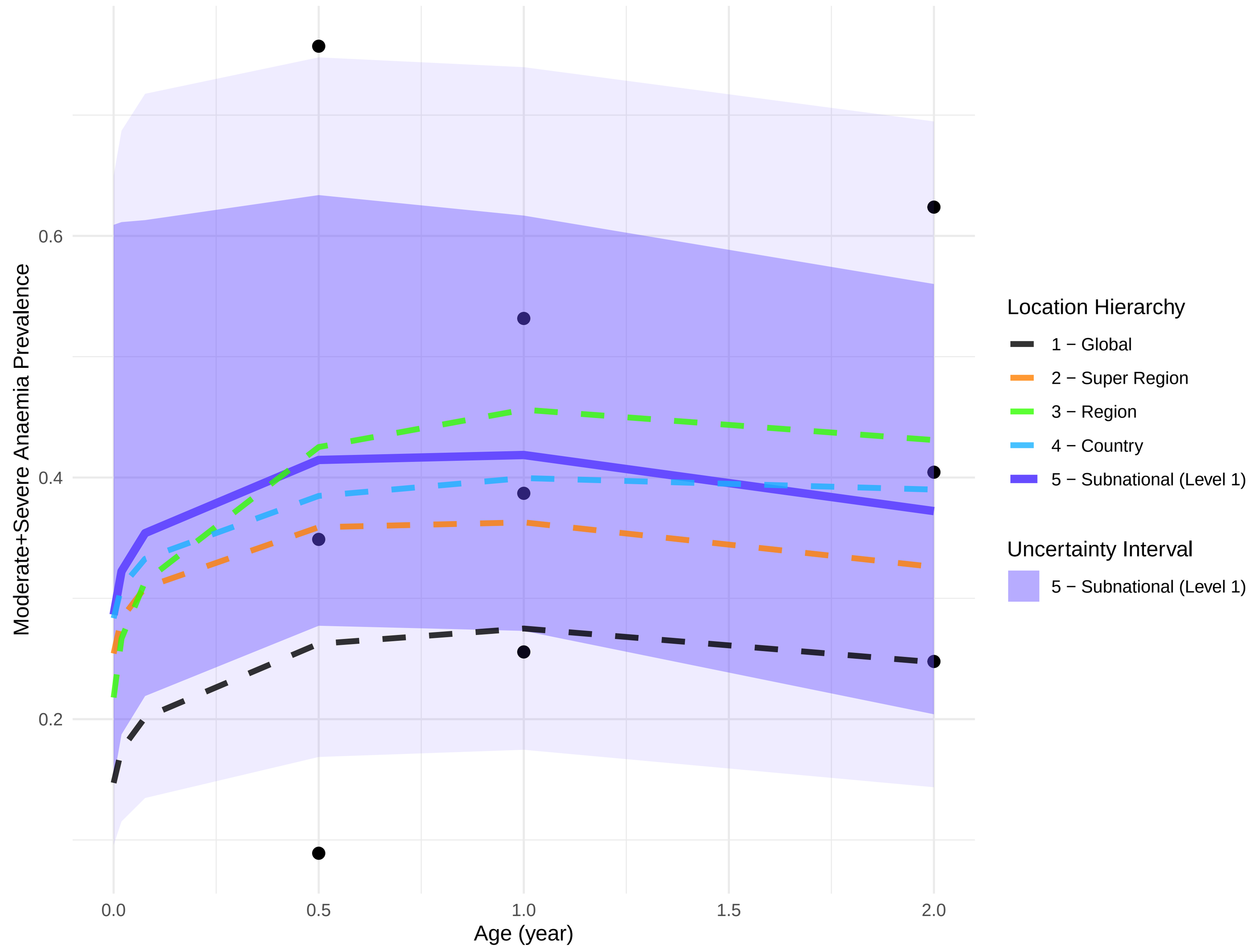

MR−BRT Age/Sex Fits − Non−pregnant Females <2 Years Old − Moderate+Severe Anaemia Prevalence − Kano
WHO−2001 Adjustment Method

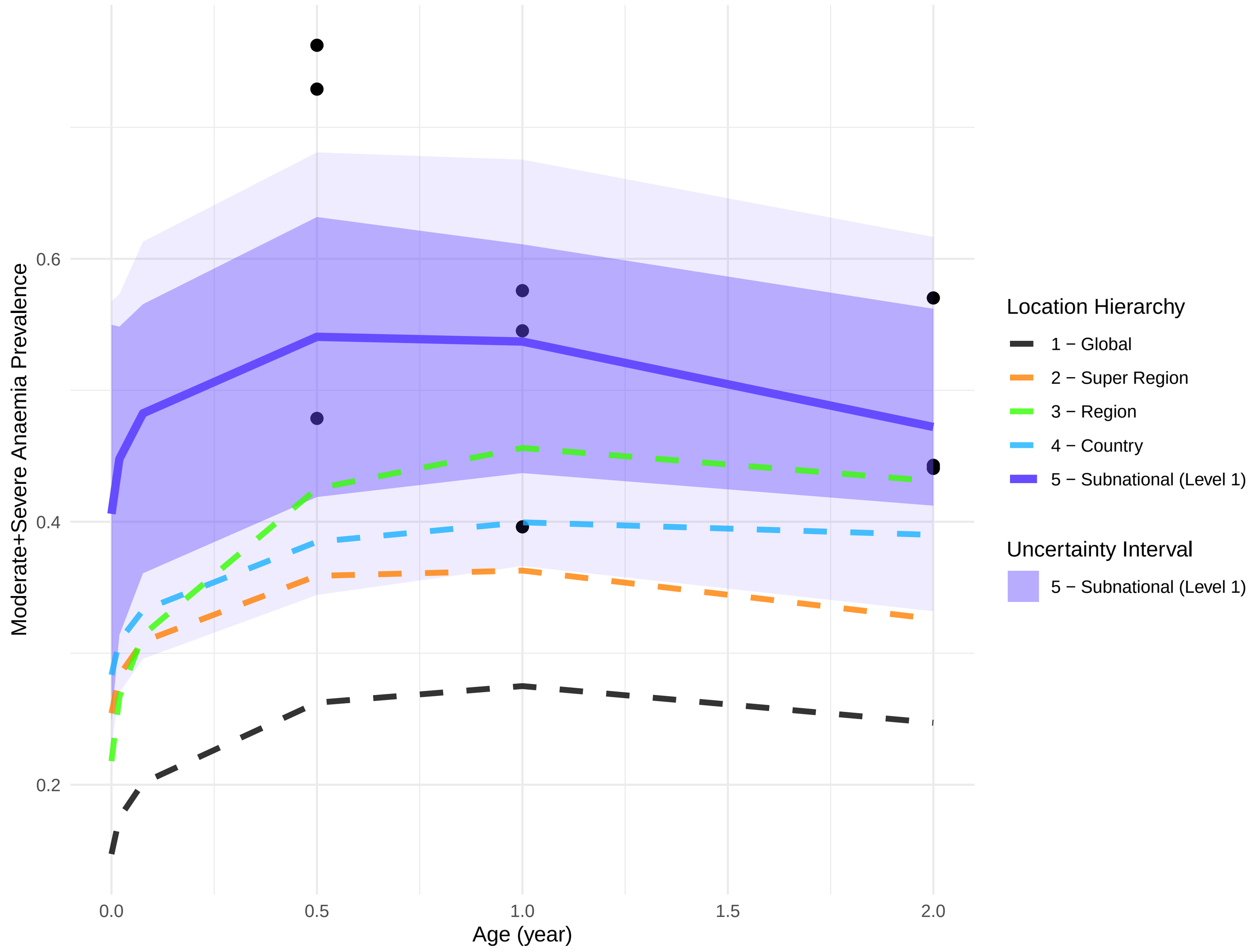

MR−BRT Age/Sex Fits − Non−pregnant Females <2 Years Old − Moderate+Severe Anaemia Prevalence − Katsina
WHO−2001 Adjustment Method

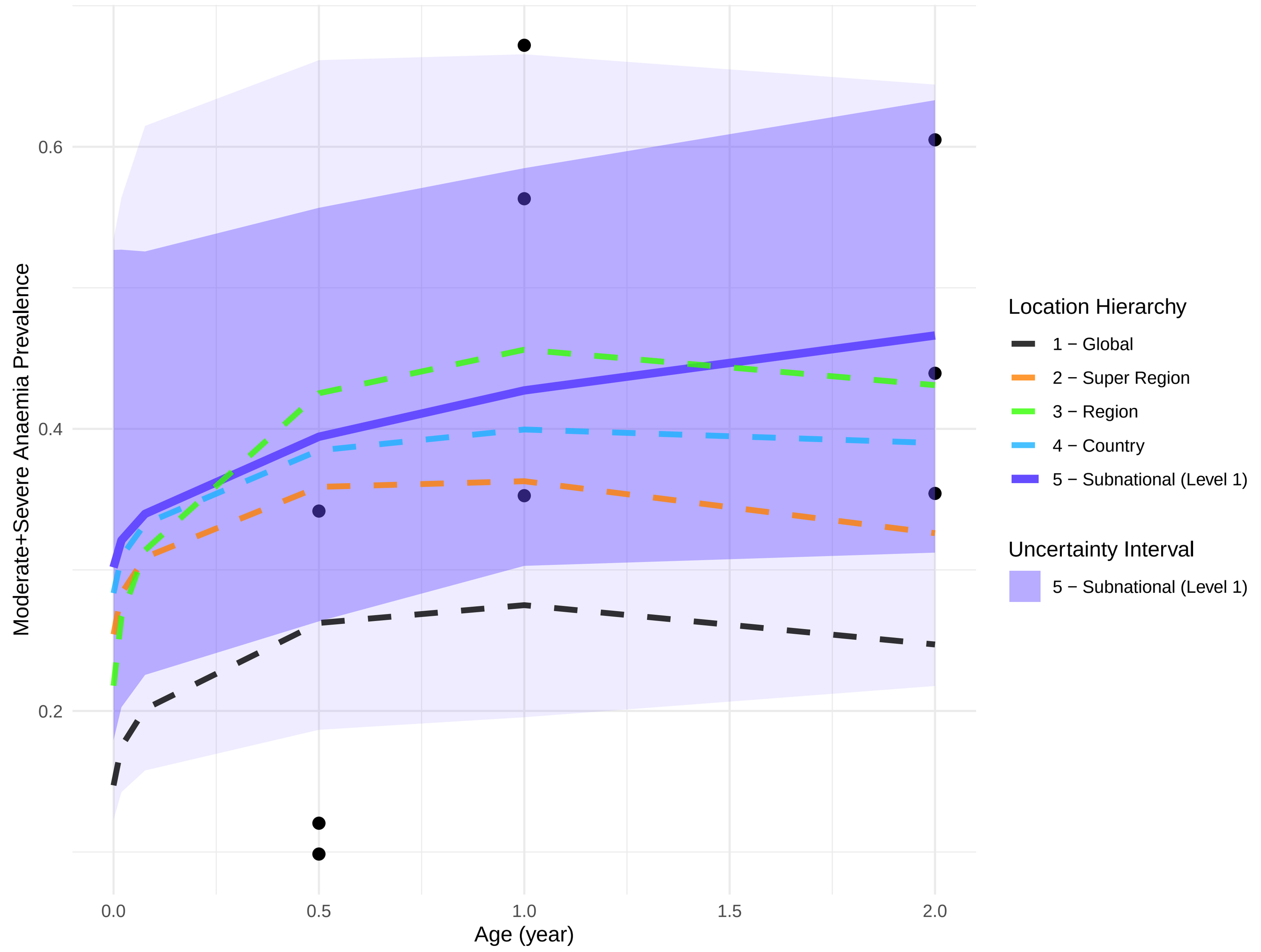

MR−BRT Age/Sex Fits − Non−pregnant Females <2 Years Old − Moderate+Severe Anaemia Prevalence − Kebbi
WHO−2001 Adjustment Method

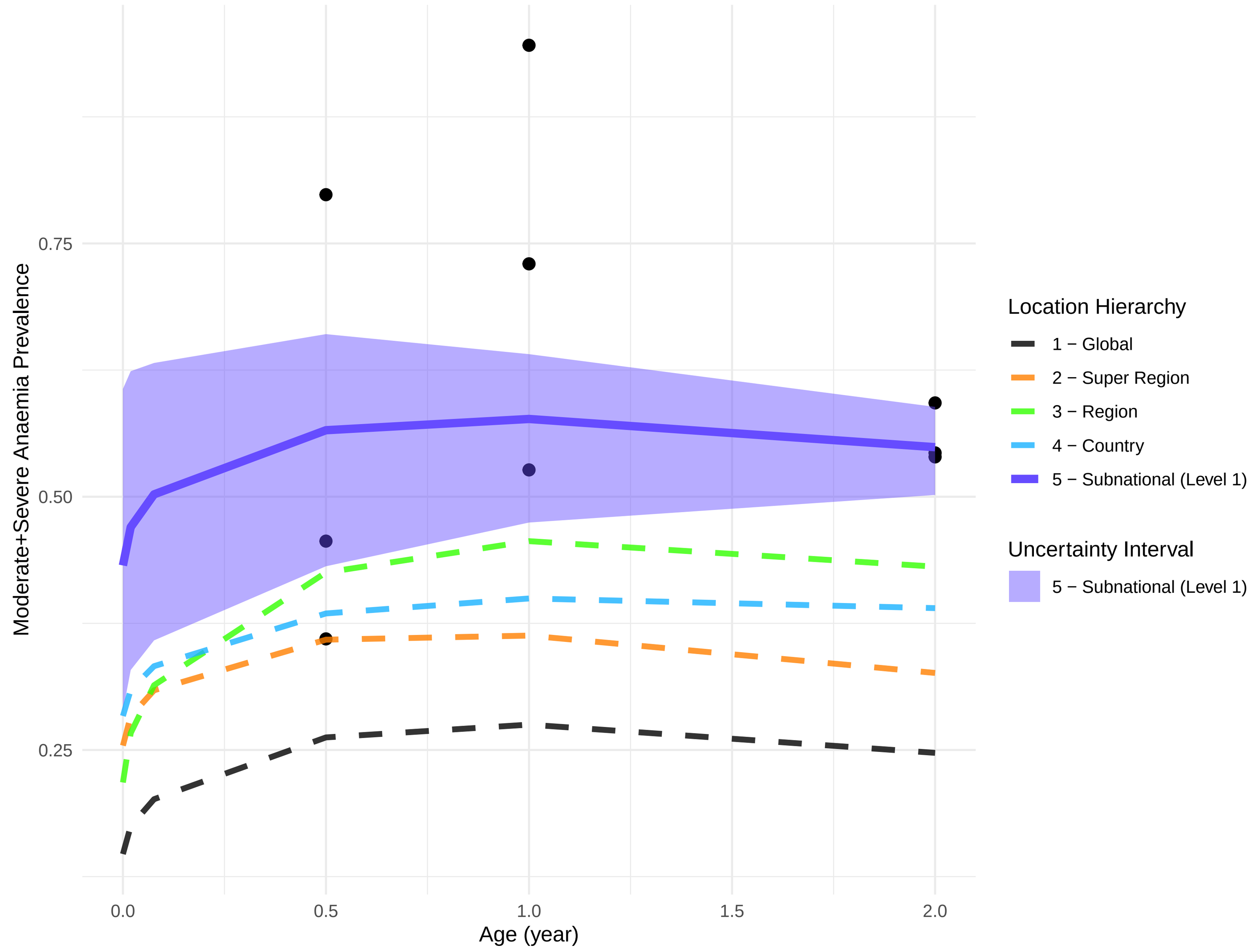

MR−BRT Age/Sex Fits − Non−pregnant Females <2 Years Old − Moderate+Severe Anaemia Prevalence − Kogi
WHO−2001 Adjustment Method

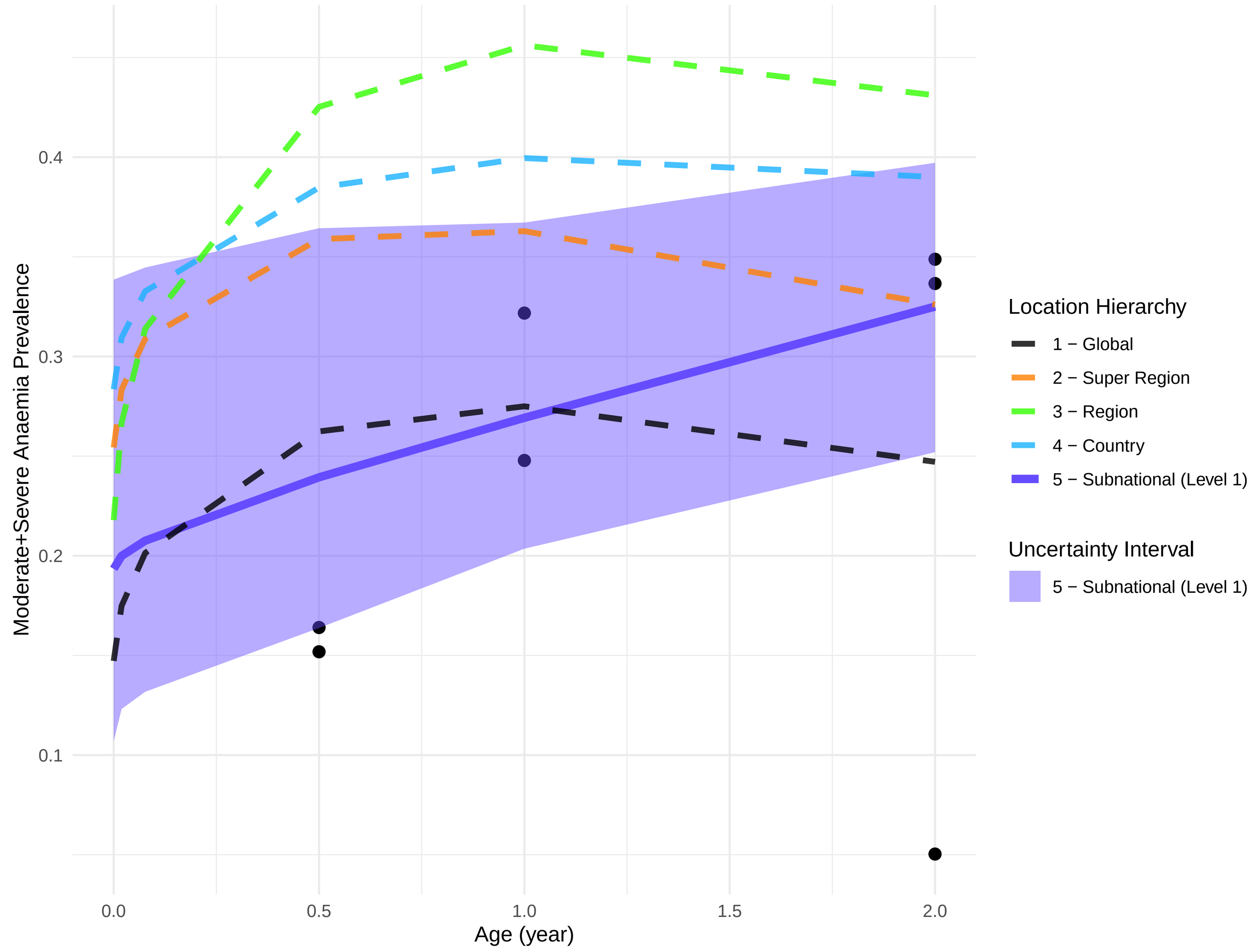

MR−BRT Age/Sex Fits − Non−pregnant Females <2 Years Old − Moderate+Severe Anaemia Prevalence − Kwara
WHO−2001 Adjustment Method

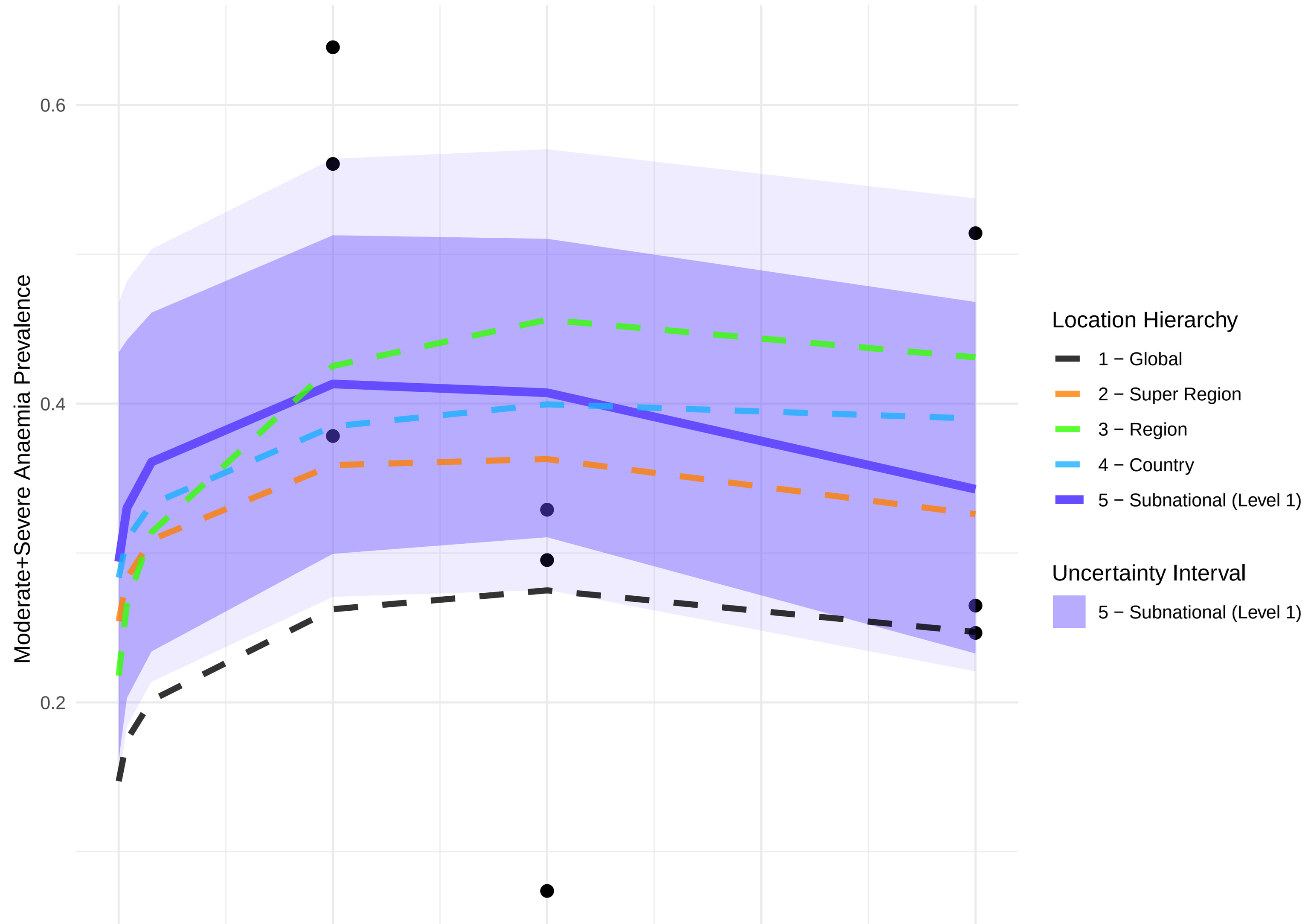

MR−BRT Age/Sex Fits − Non−pregnant Females <2 Years Old − Moderate+Severe Anaemia Prevalence − Lagos
WHO−2001 Adjustment Method

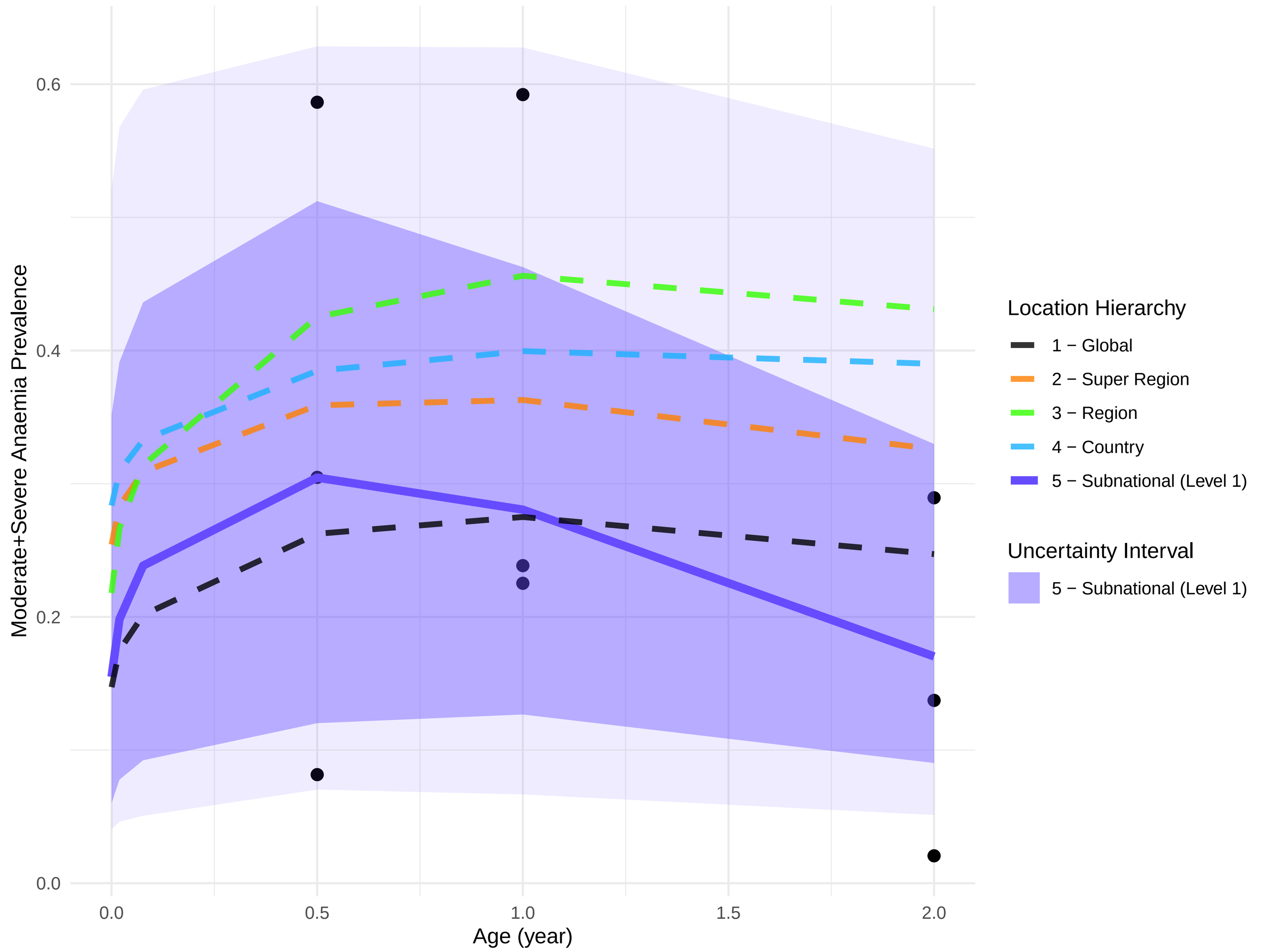

MR−BRT Age/Sex Fits − Non−pregnant Females <2 Years Old − Moderate+Severe Anaemia Prevalence − Nasarawa
WHO−2001 Adjustment Method

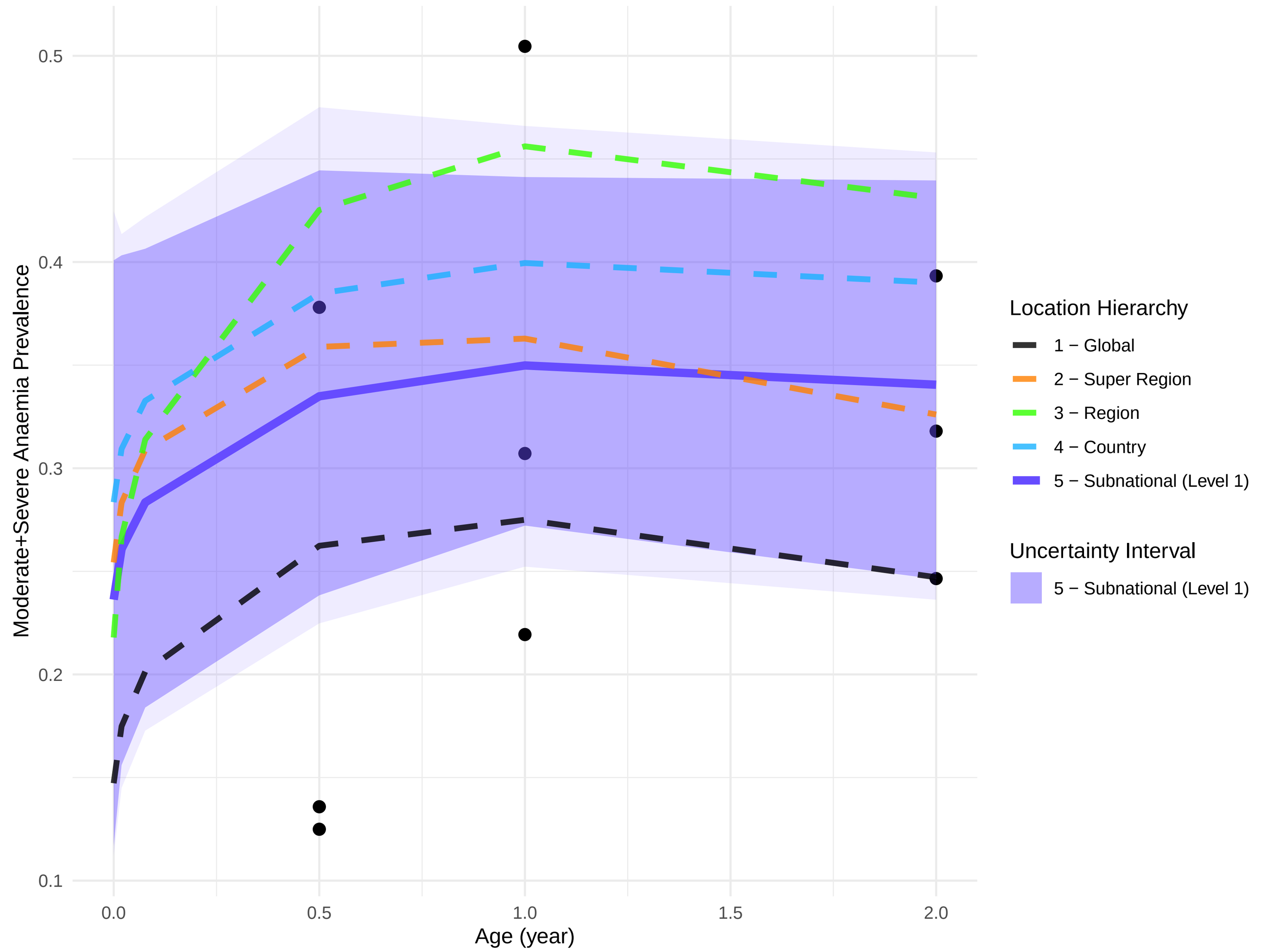

MR−BRT Age/Sex Fits − Non−pregnant Females <2 Years Old − Moderate+Severe Anaemia Prevalence − Niger
WHO−2001 Adjustment Method

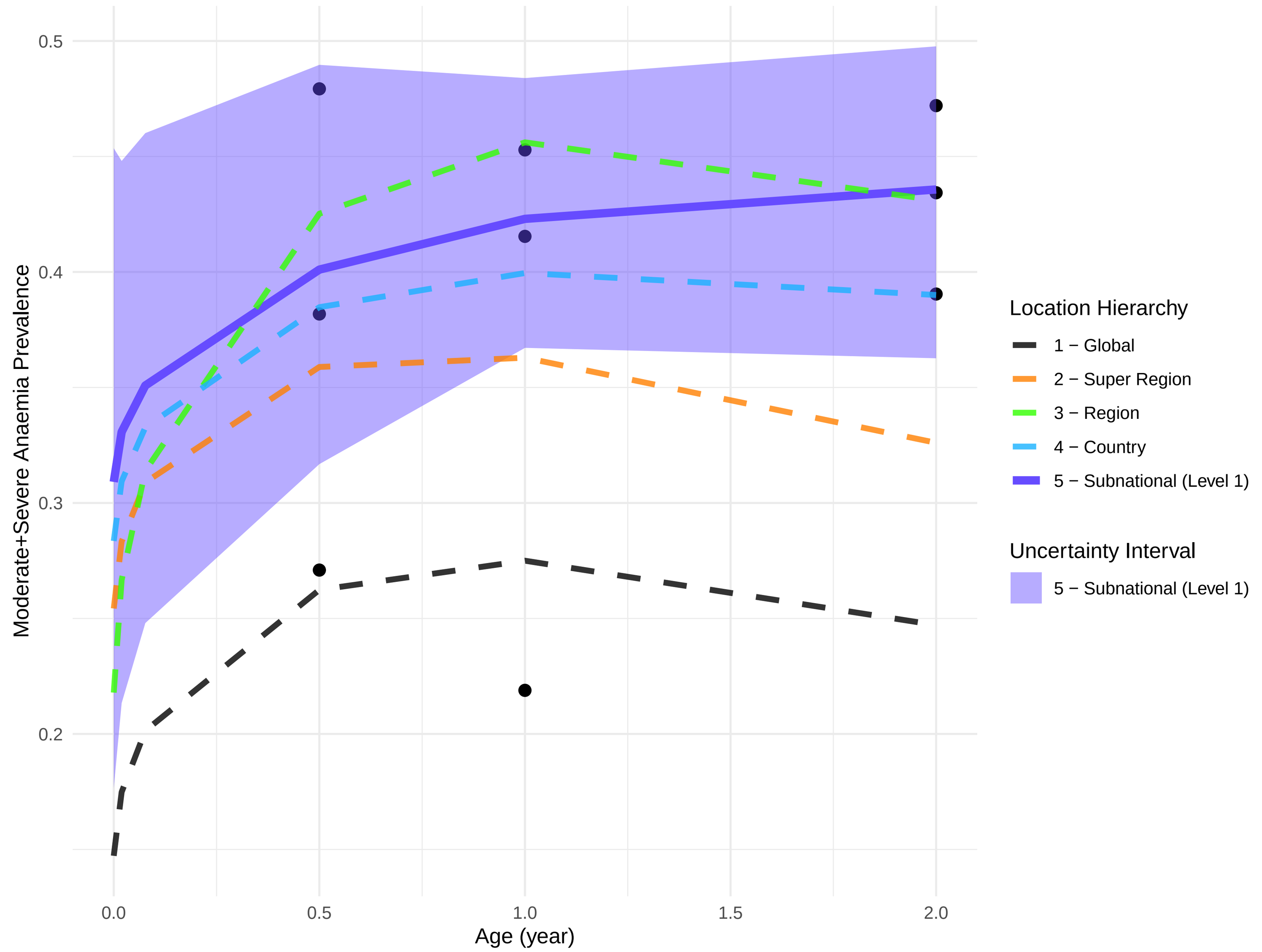

MR−BRT Age/Sex Fits − Non−pregnant Females <2 Years Old − Moderate+Severe Anaemia Prevalence − Ogun
WHO−2001 Adjustment Method

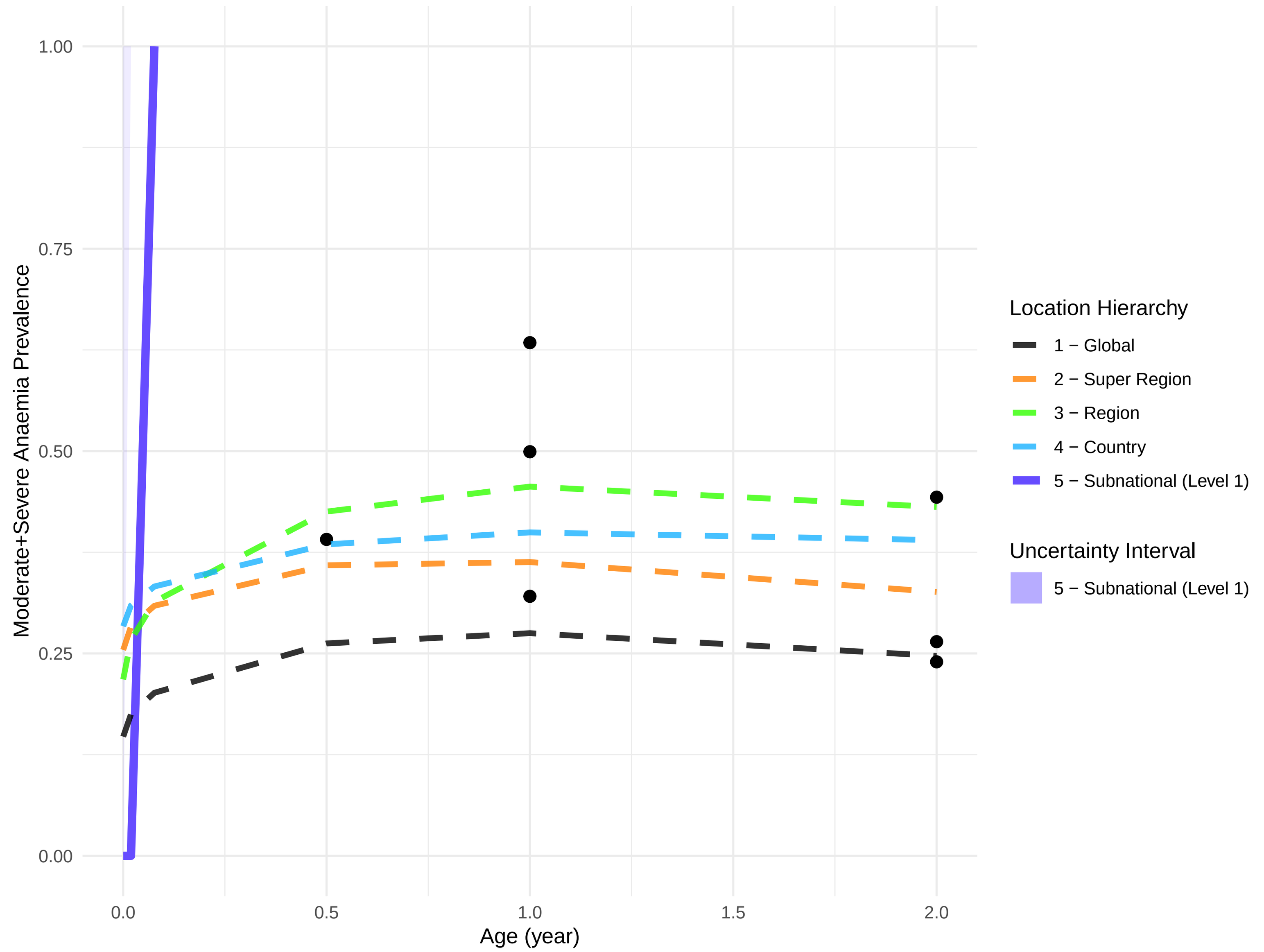

MR−BRT Age/Sex Fits − Non−pregnant Females <2 Years Old − Moderate+Severe Anaemia Prevalence − Ondo
WHO−2001 Adjustment Method

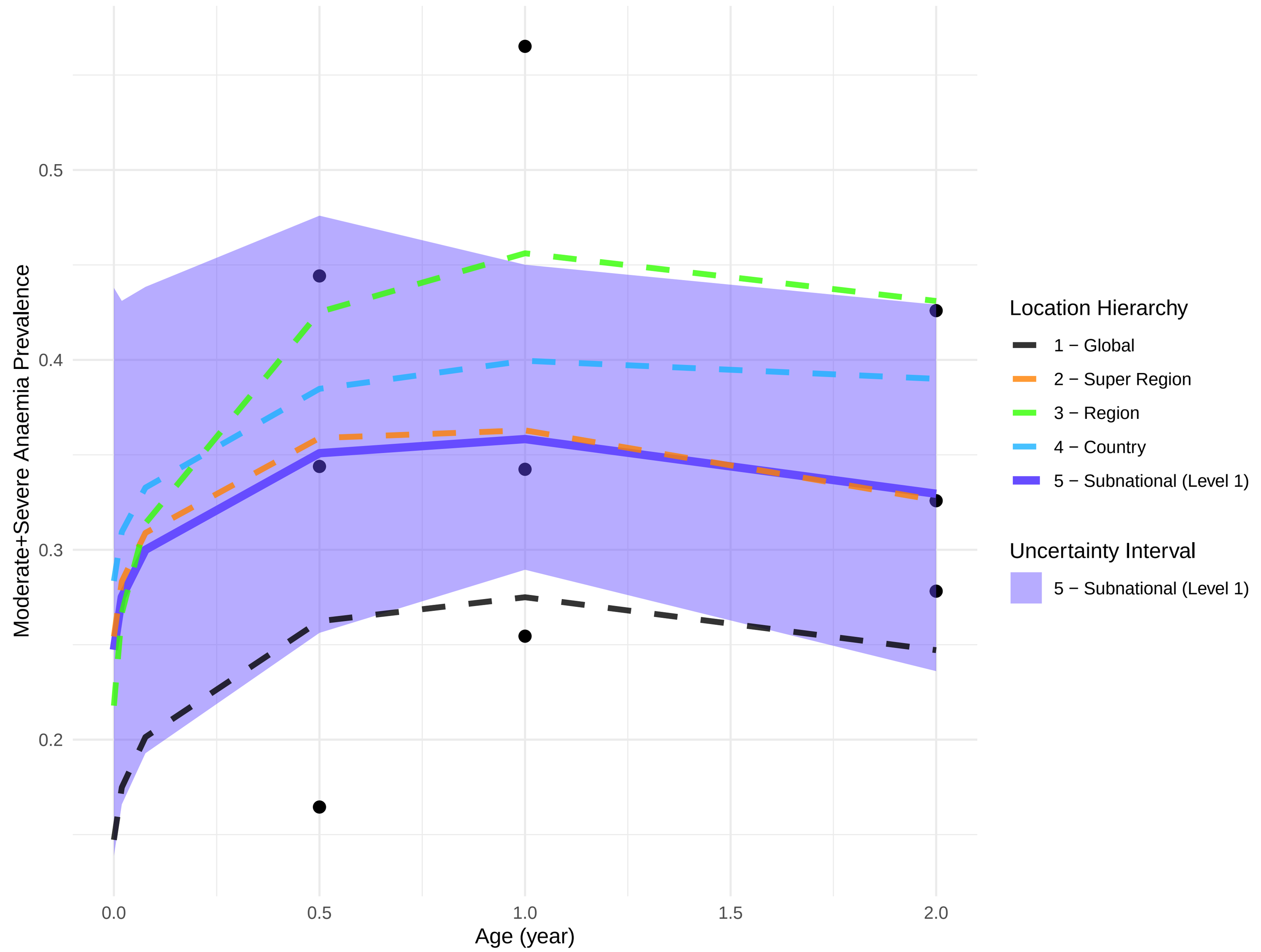

MR−BRT Age/Sex Fits − Non−pregnant Females <2 Years Old − Moderate+Severe Anaemia Prevalence − Osun
WHO−2001 Adjustment Method

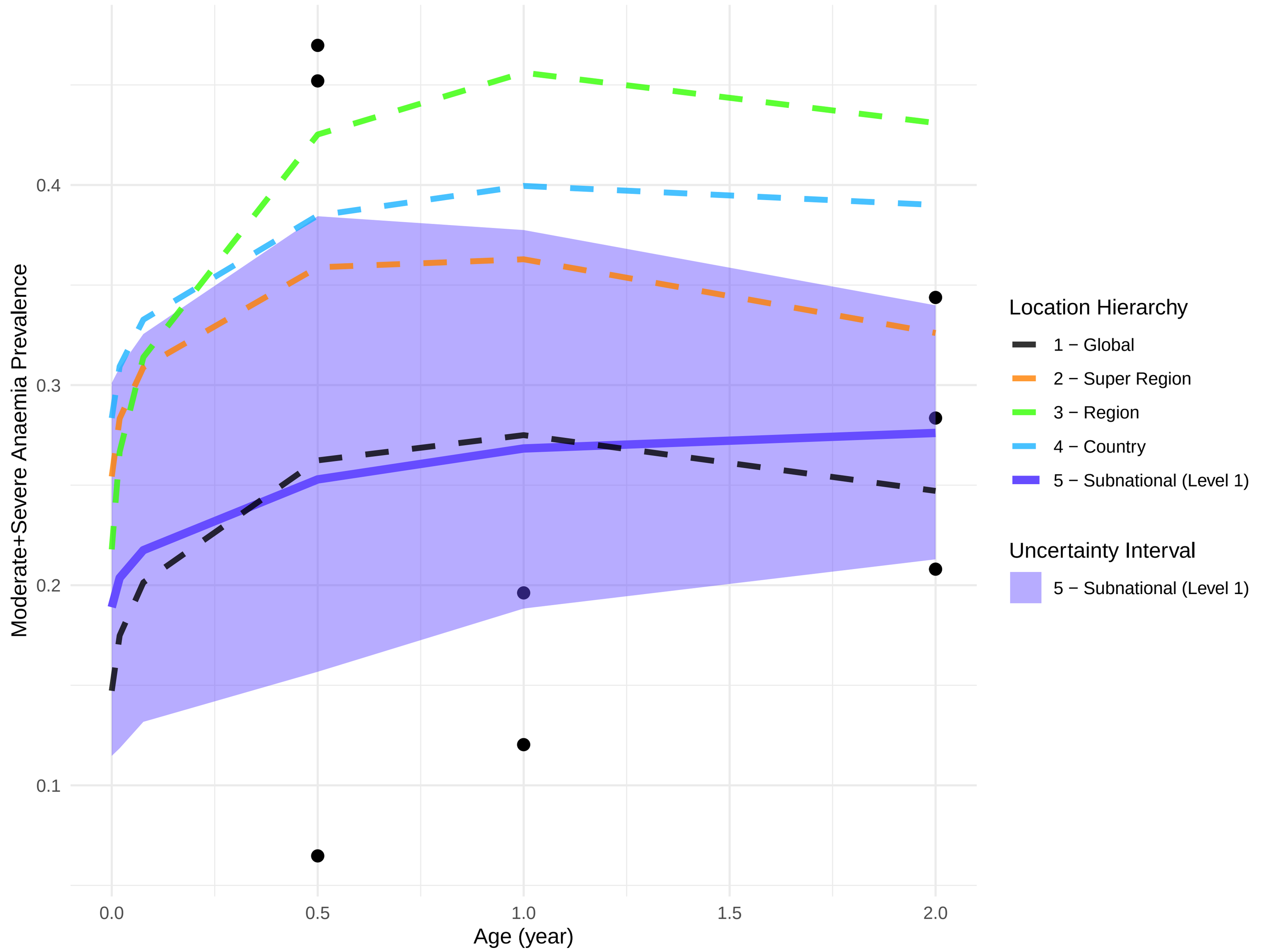

MR−BRT Age/Sex Fits − Non−pregnant Females <2 Years Old − Moderate+Severe Anaemia Prevalence − Oyo
WHO−2001 Adjustment Method

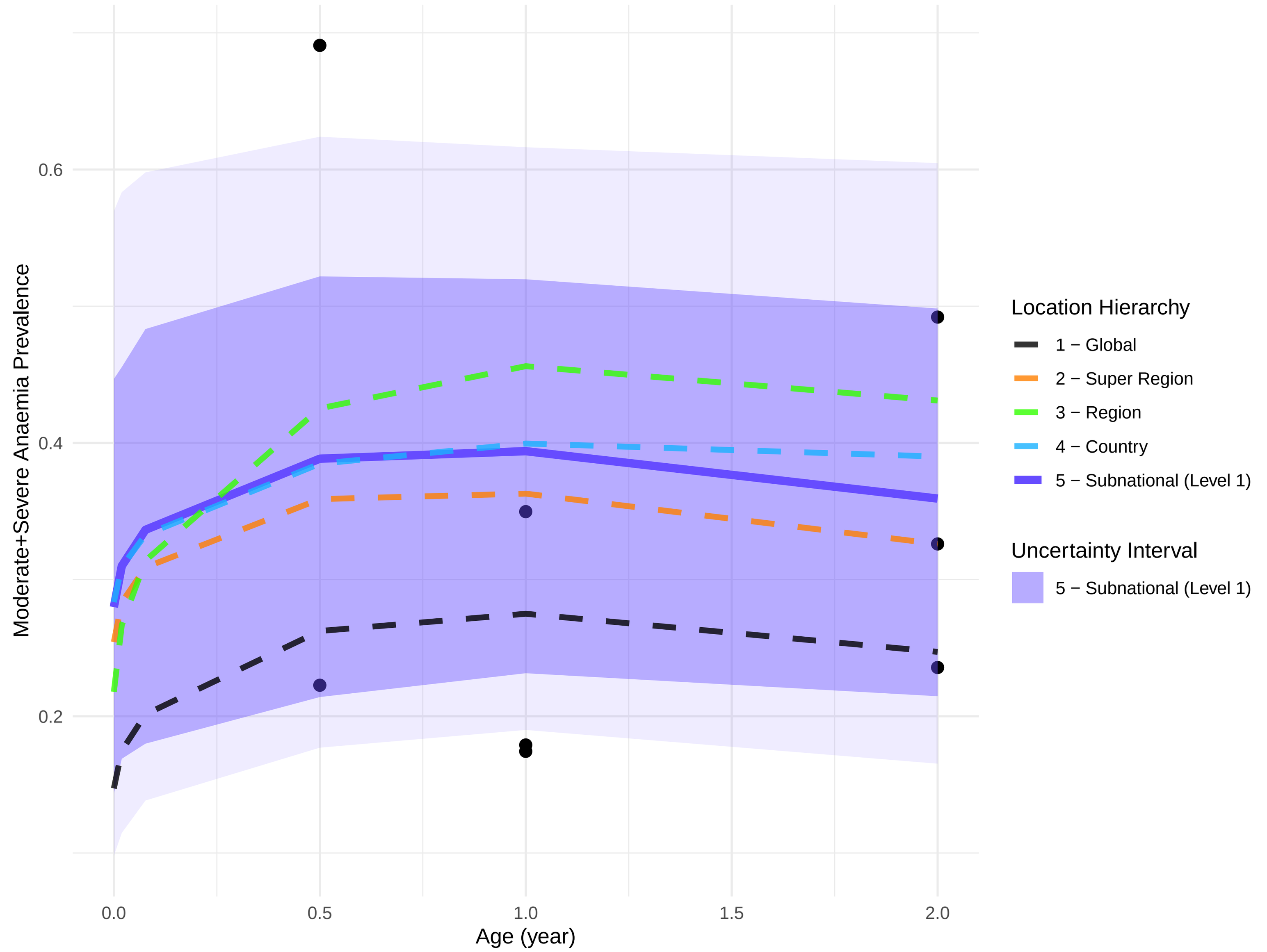

MR−BRT Age/Sex Fits − Non−pregnant Females <2 Years Old − Moderate+Severe Anaemia Prevalence − Plateau
WHO−2001 Adjustment Method

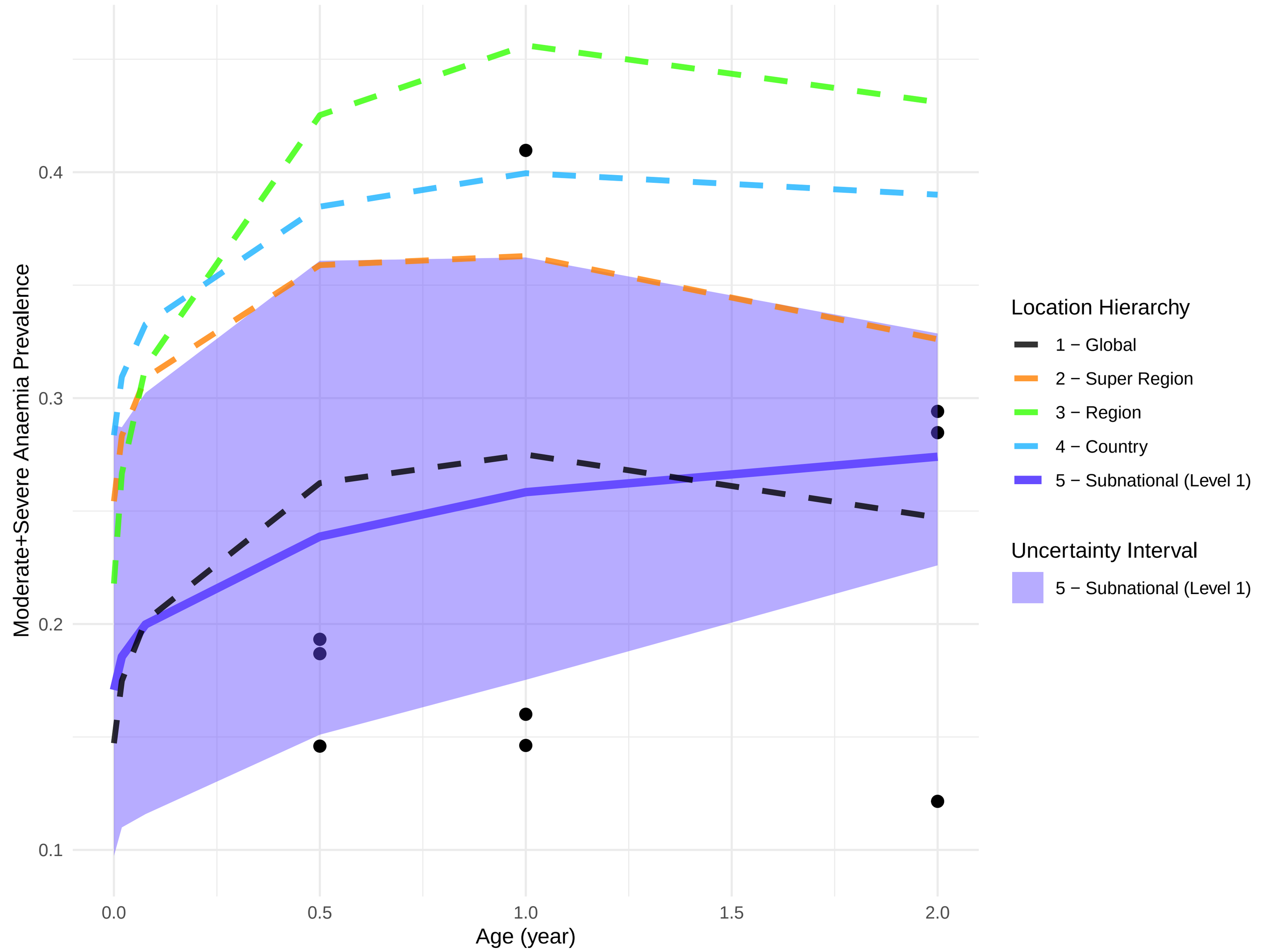

MR−BRT Age/Sex Fits − Non−pregnant Females <2 Years Old − Moderate+Severe Anaemia Prevalence − Rivers
WHO−2001 Adjustment Method

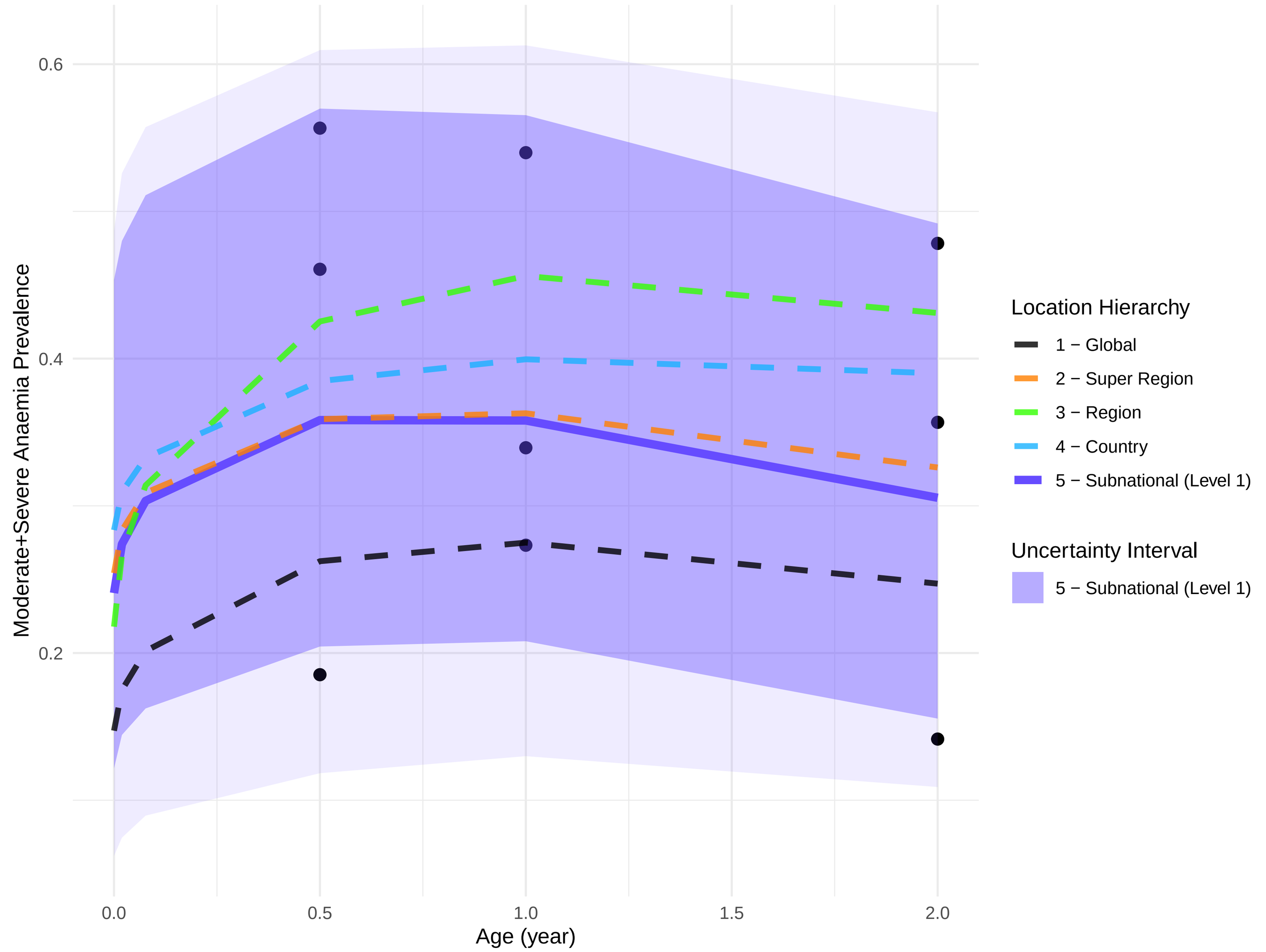

MR−BRT Age/Sex Fits − Non−pregnant Females <2 Years Old − Moderate+Severe Anaemia Prevalence − Sokoto
WHO−2001 Adjustment Method

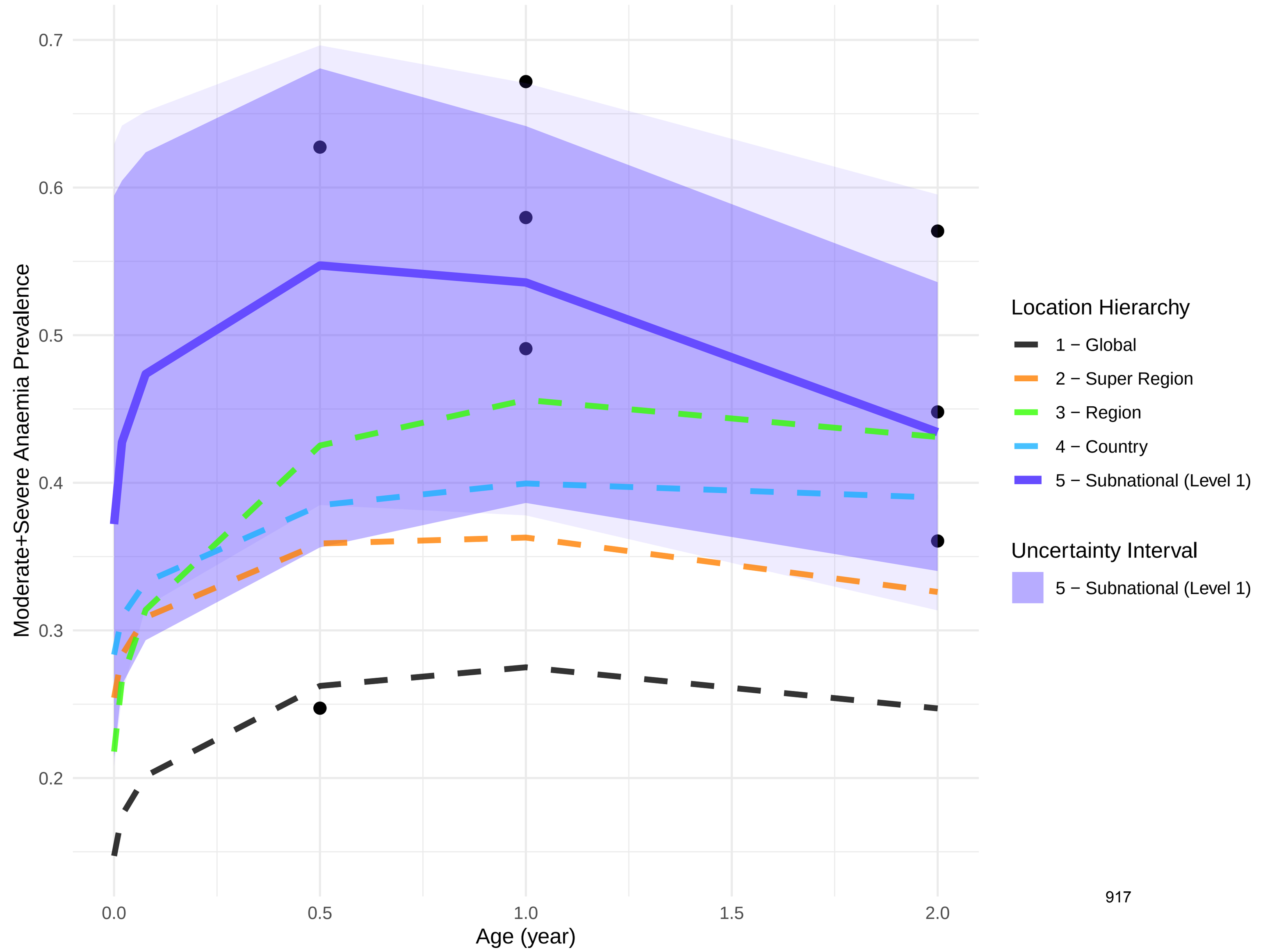

MR−BRT Age/Sex Fits − Non−pregnant Females <2 Years Old − Moderate+Severe Anaemia Prevalence − Taraba
WHO−2001 Adjustment Method

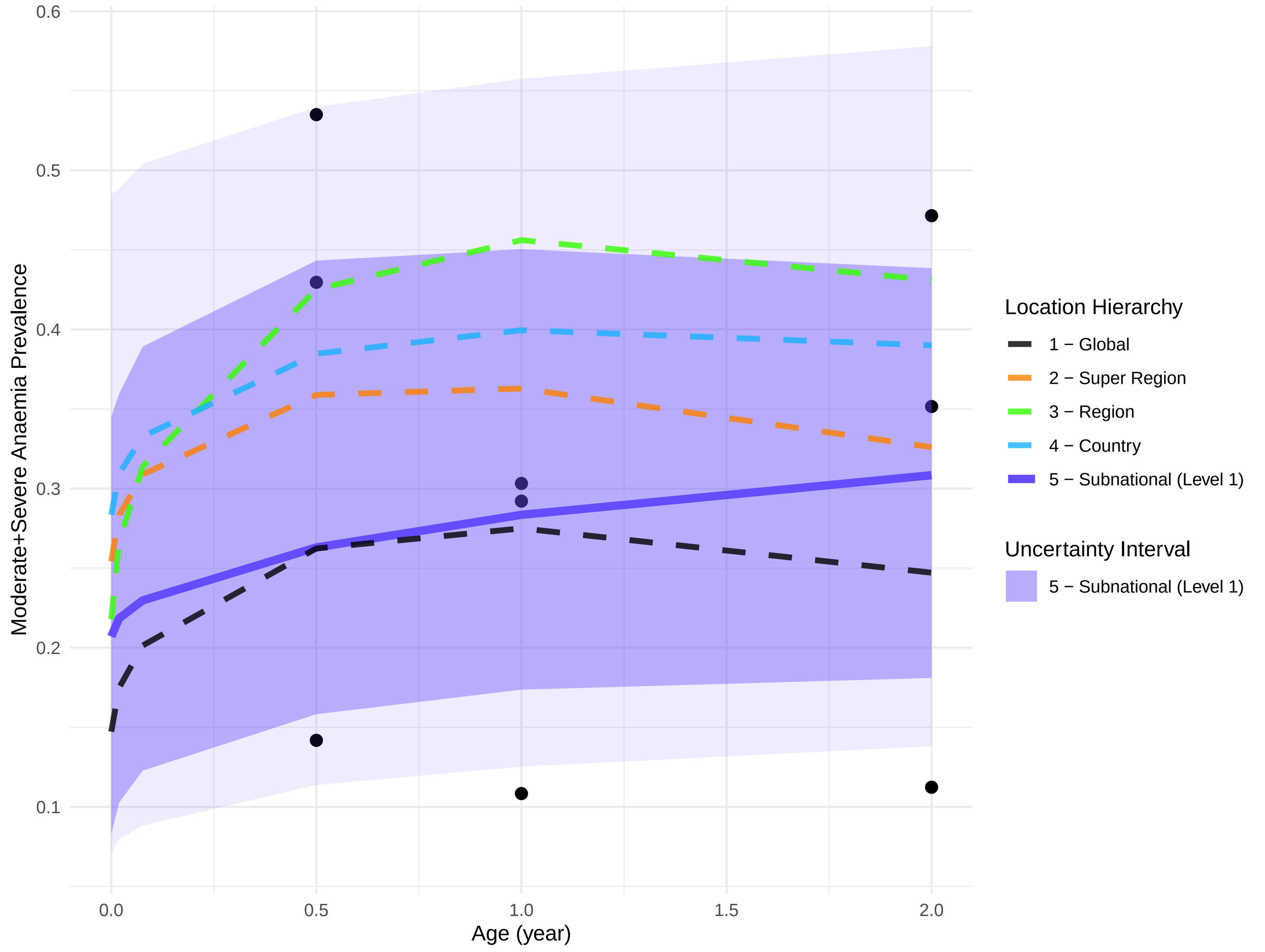

MR−BRT Age/Sex Fits − Non−pregnant Females <2 Years Old − Moderate+Severe Anaemia Prevalence − Yobe
WHO−2001 Adjustment Method

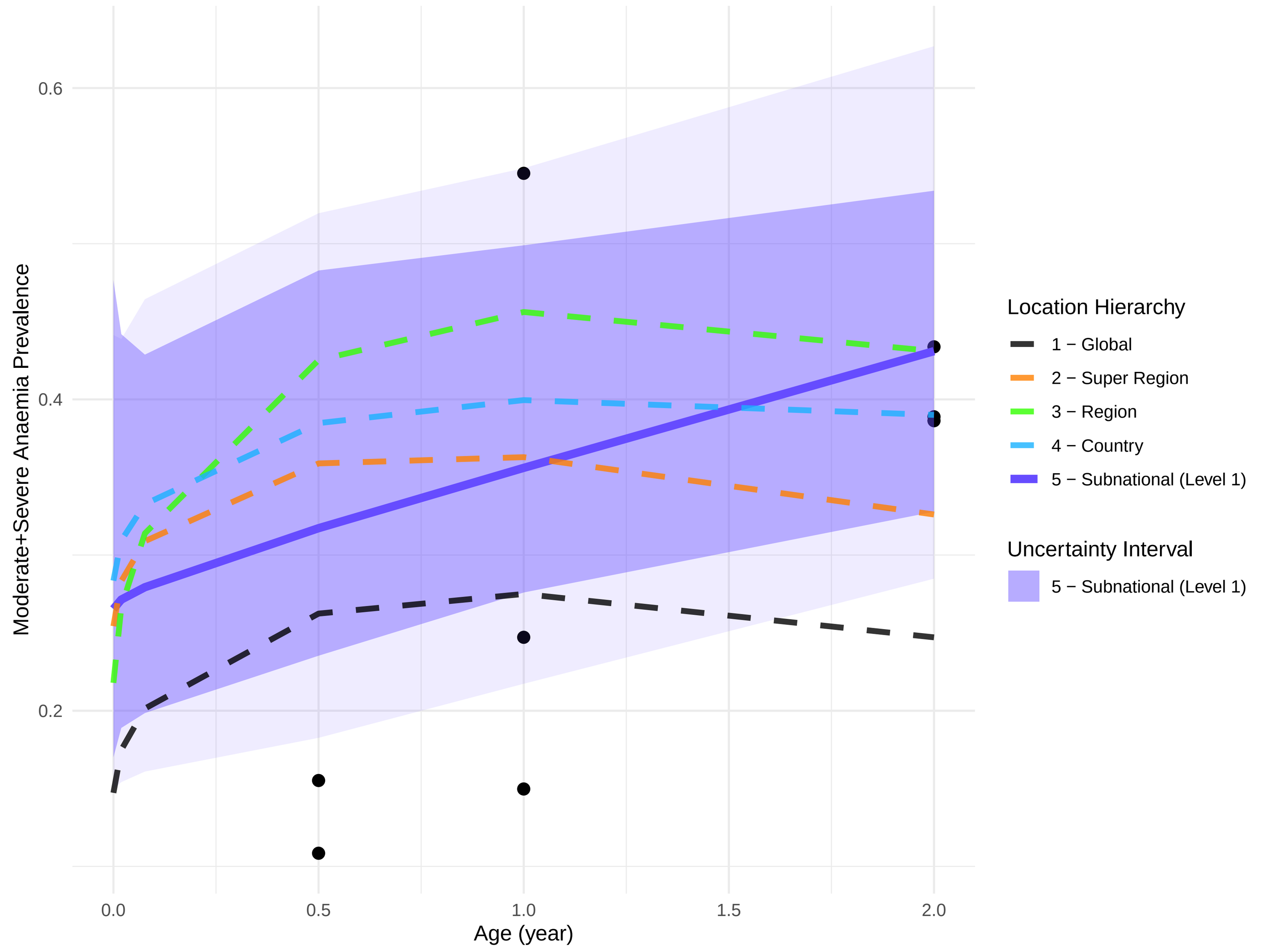

MR−BRT Age/Sex Fits − Non−pregnant Females <2 Years Old − Moderate+Severe Anaemia Prevalence − Zamfara
WHO−2001 Adjustment Method

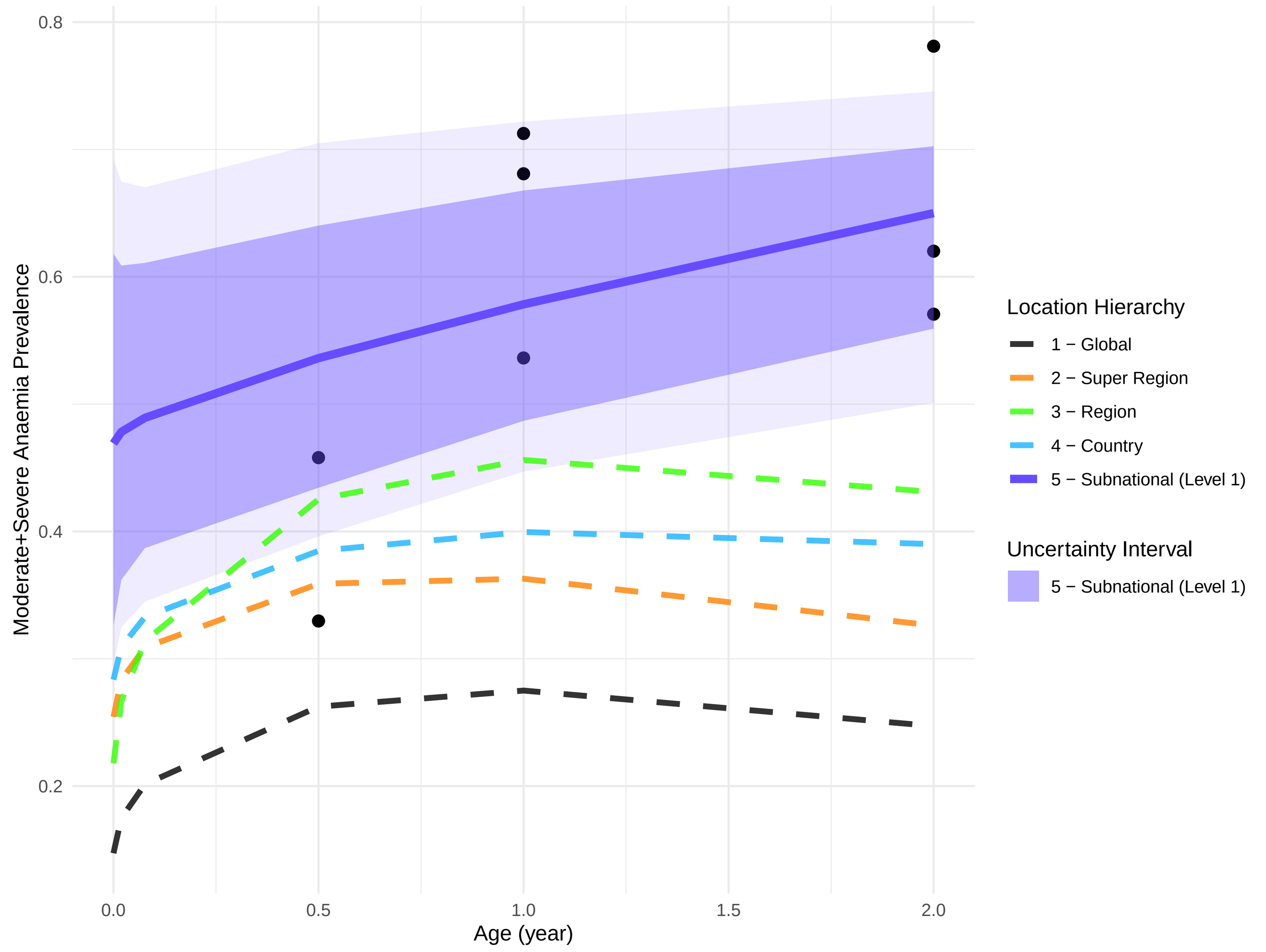

MR−BRT Age/Sex Fits − Non−pregnant Females <2 Years Old − Moderate+Severe Anaemia Prevalence − Senegal
WHO−2001 Adjustment Method

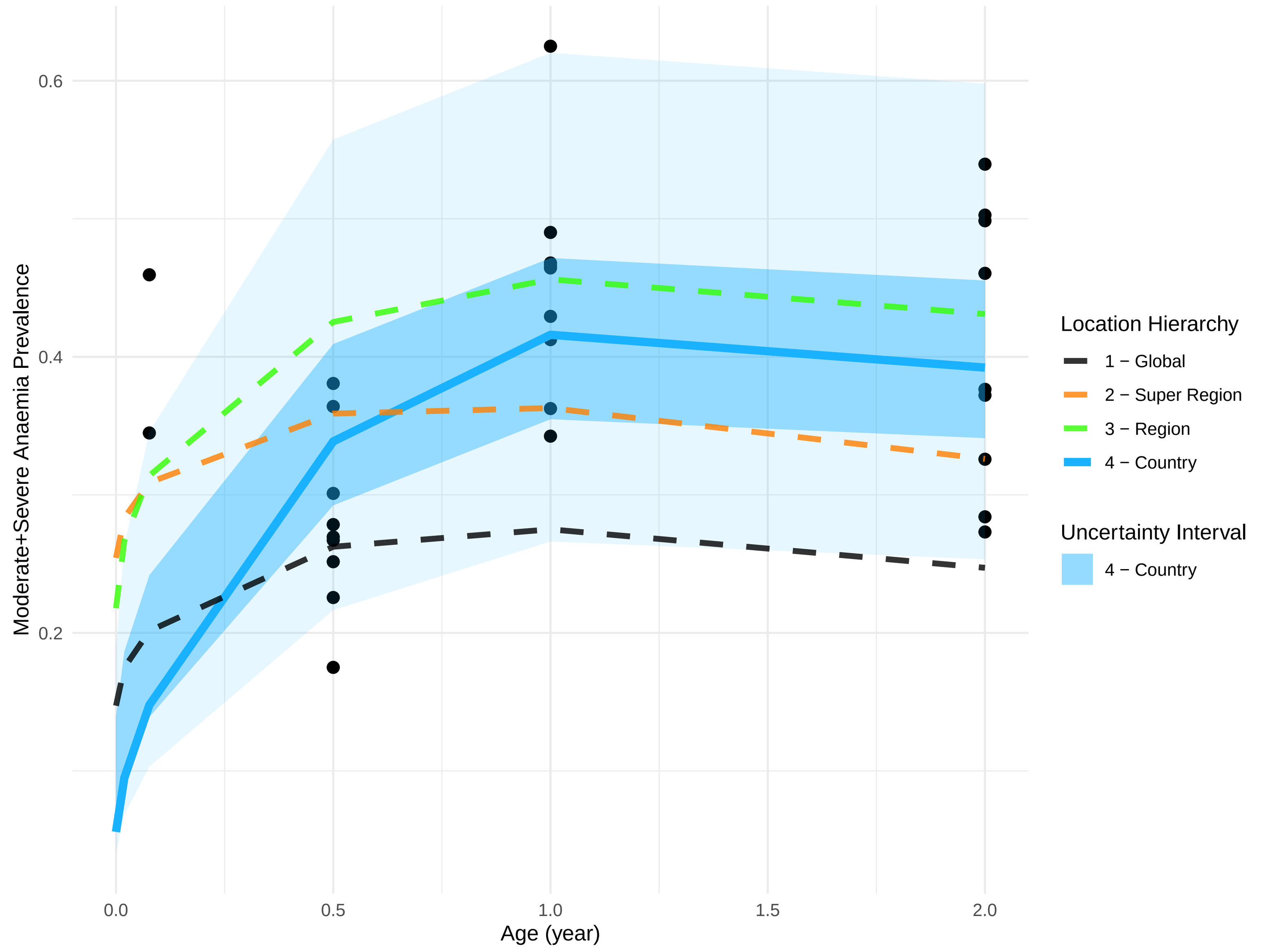

MR−BRT Age/Sex Fits − Non−pregnant Females <2 Years Old − Moderate+Severe Anaemia Prevalence − Sierra Leone
WHO−2001 Adjustment Method

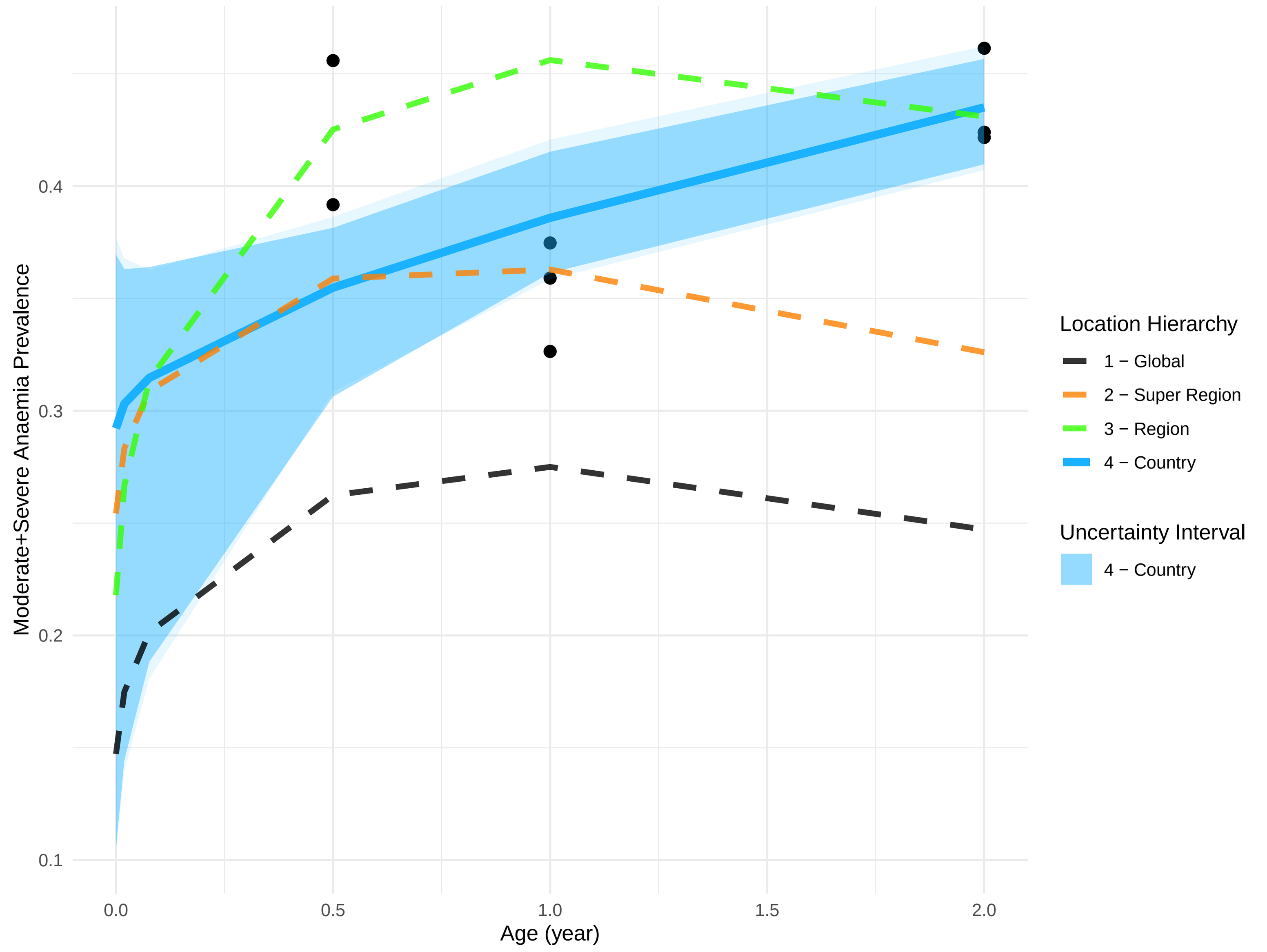

MR−BRT Age/Sex Fits − Non−pregnant Females <2 Years Old − Moderate+Severe Anaemia Prevalence − Togo
WHO−2001 Adjustment Method

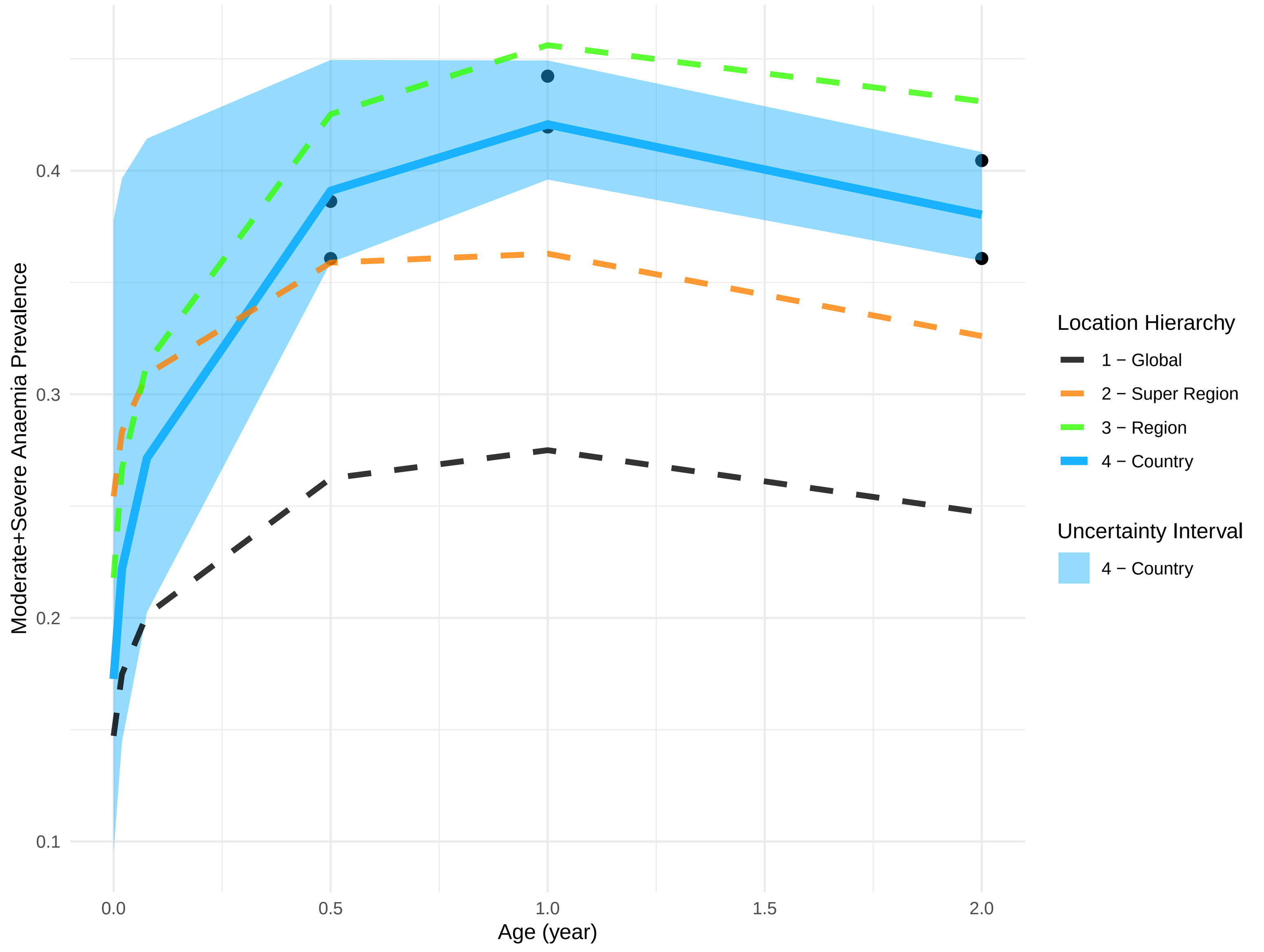

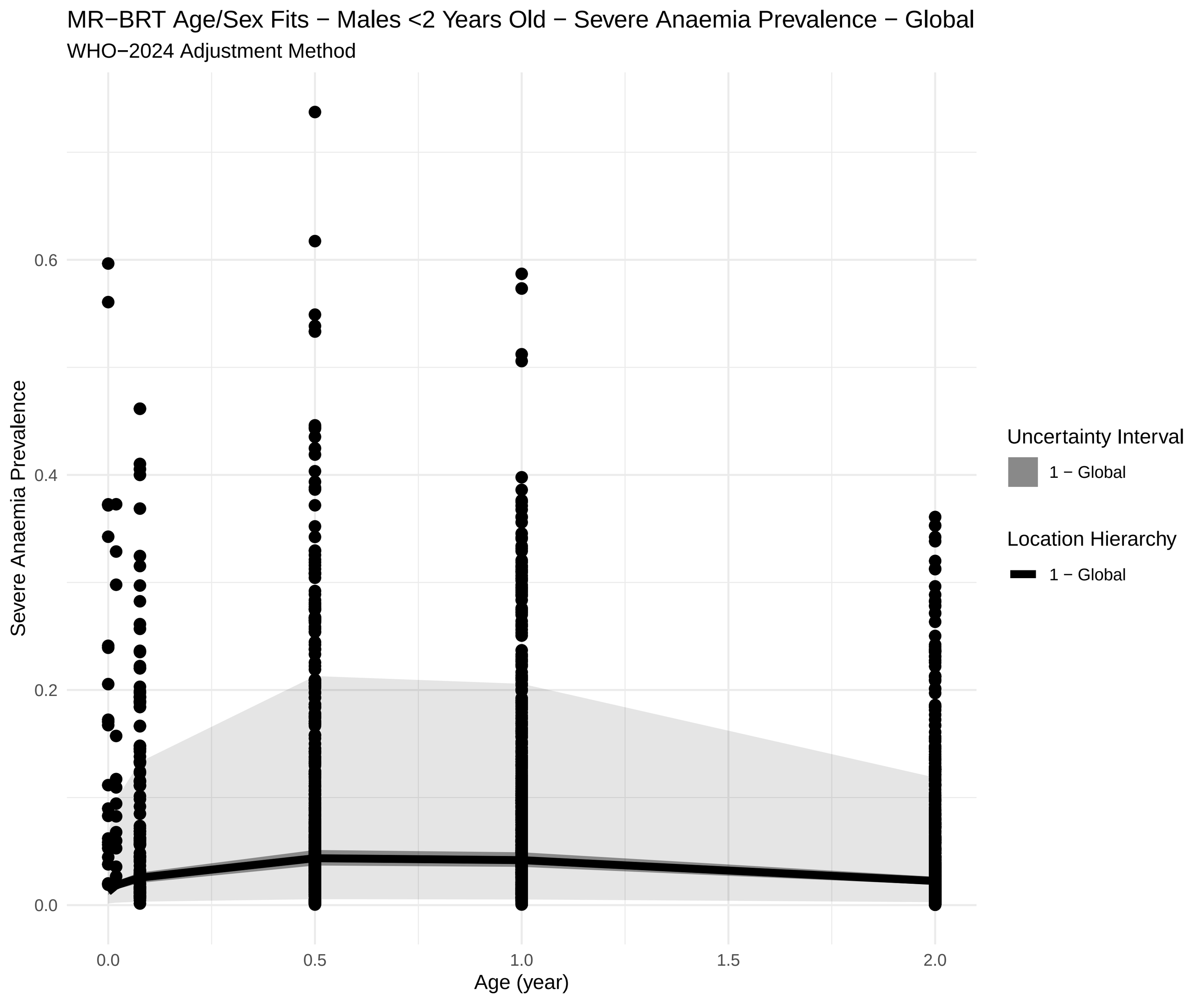
MR−BRT Age/Sex Fits − Males <2 Years Old − Severe Anaemia Prevalence − Global
WHO−2024 Adjustment Method
Severe Anaemia Prevalence
Age (year)
Uncertainty Interval
1 − Global
Location Hierarchy
1 − Global

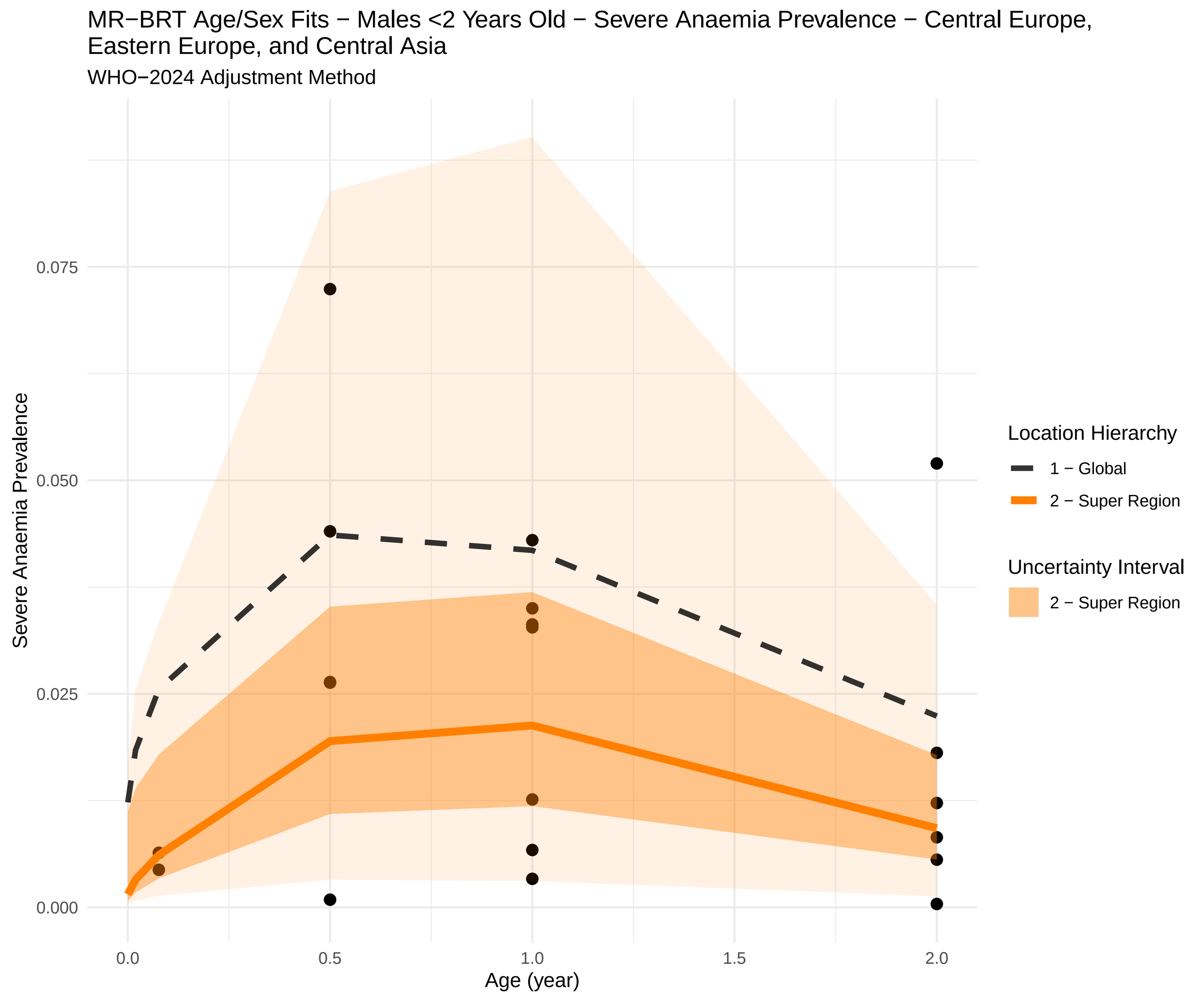
MR−BRT Age/Sex Fits − Males <2 Years Old − Severe Anaemia Prevalence − Central Europe, Eastern Europe, and Central Asia
WHO−2024 Adjustment Method
Severe Anaemia Prevalence
Age (year)
Location Hierarchy
1 − Global
2 − Super Region
Uncertainty Interval
2 − Super Region

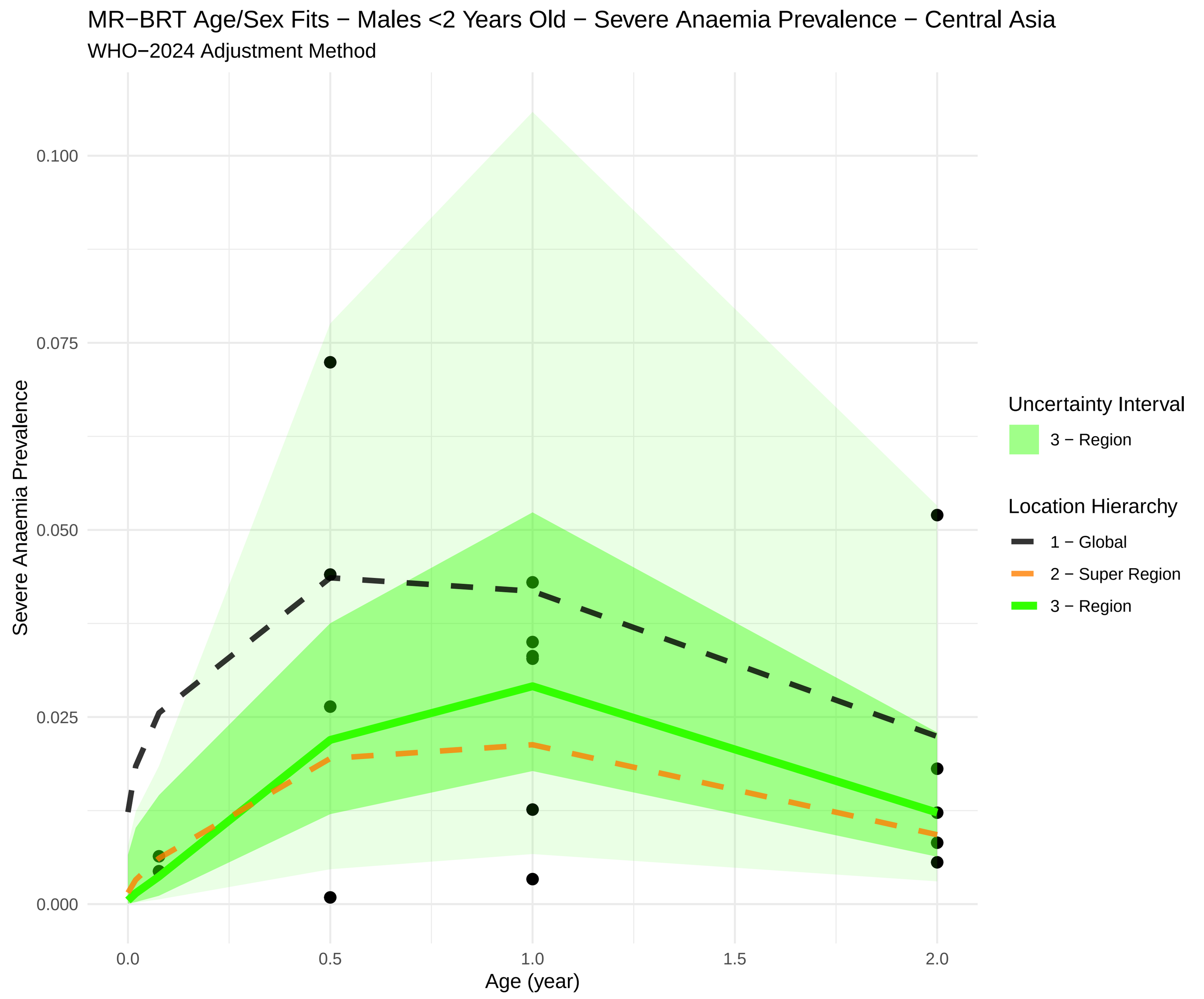
MR−BRT Age/Sex Fits − Males <2 Years Old − Severe Anaemia Prevalence − Central Asia
WHO−2024 Adjustment Method
Severe Anaemia Prevalence
Age (year)
Uncertainty Interval
3 − Region
Location Hierarchy
1 − Global
2 − Super Region
3 − Region

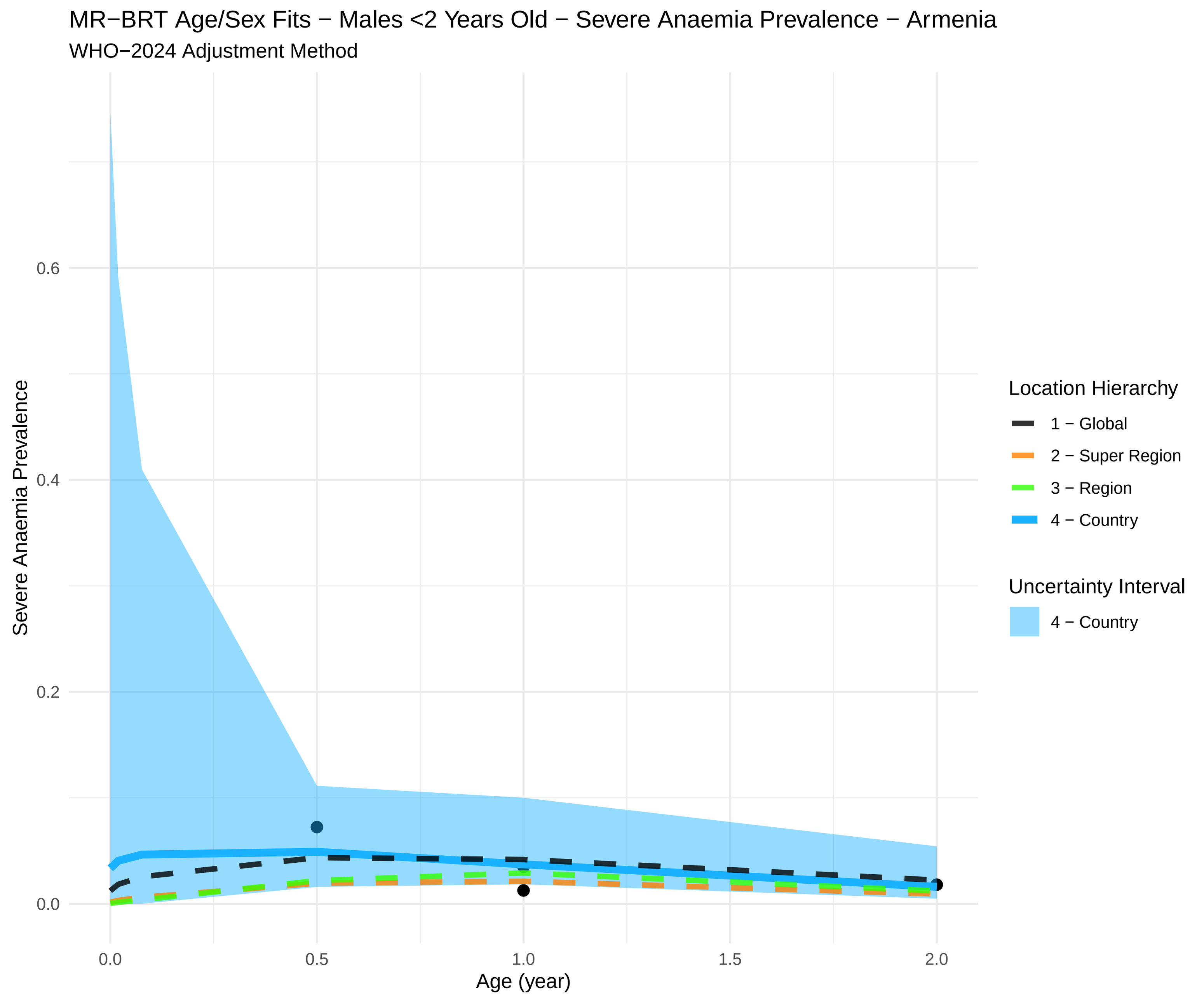
MR−BRT Age/Sex Fits − Males <2 Years Old − Severe Anaemia Prevalence − Armenia
WHO−2024 Adjustment Method
Severe Anaemia Prevalence
Age (year)
Location Hierarchy
1 − Global
2 − Super Region
3 − Region
4 − Country
Uncertainty Interval
4 − Country

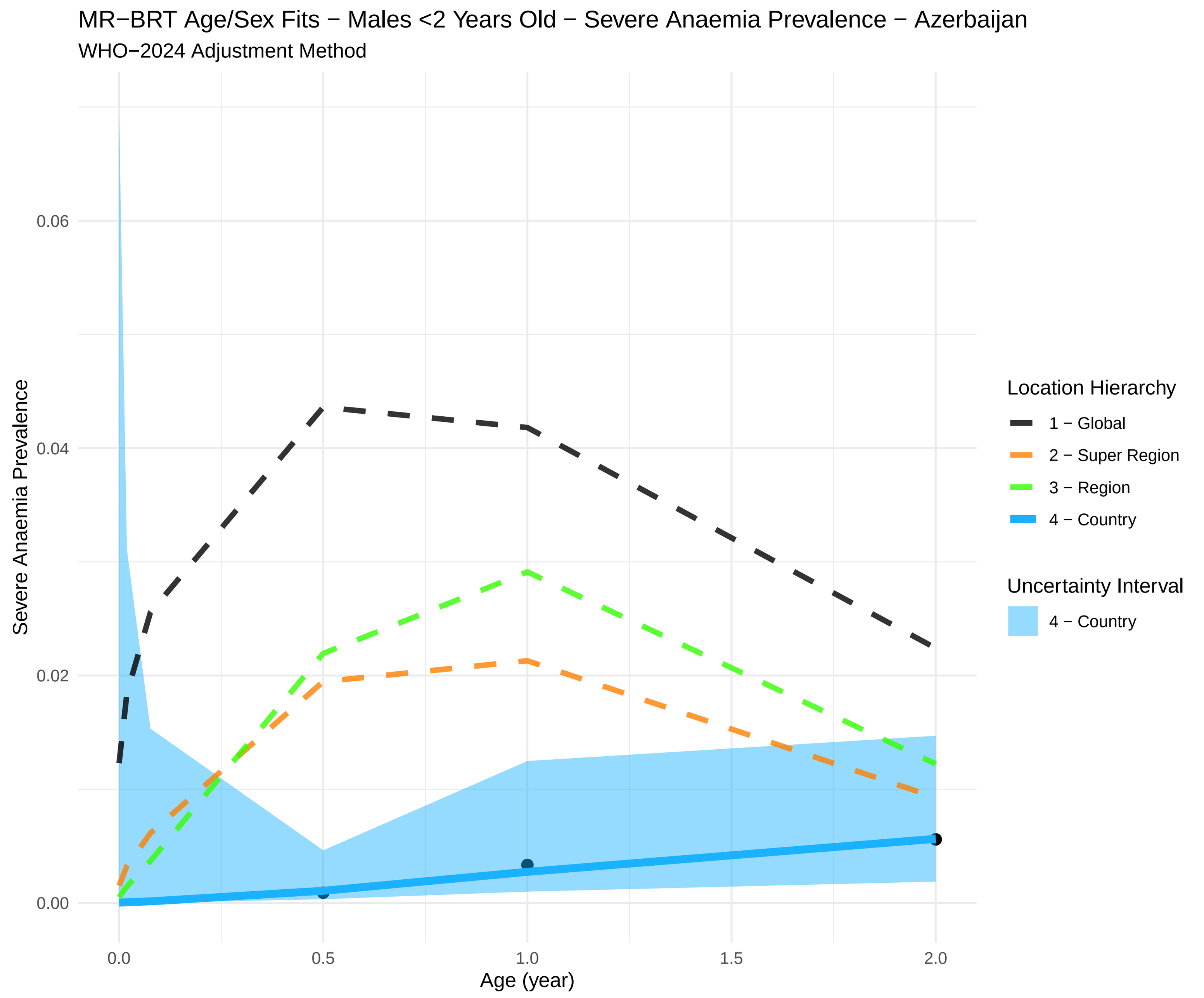
MR−BRT Age/Sex Fits − Males <2 Years Old − Severe Anaemia Prevalence − Azerbaijan
WHO−2024 Adjustment Method
Severe Anaemia Prevalence
Age (year)
Location Hierarchy
1 − Global
2 − Super Region
3 − Region
4 − Country
Uncertainty Interval
4 − Country

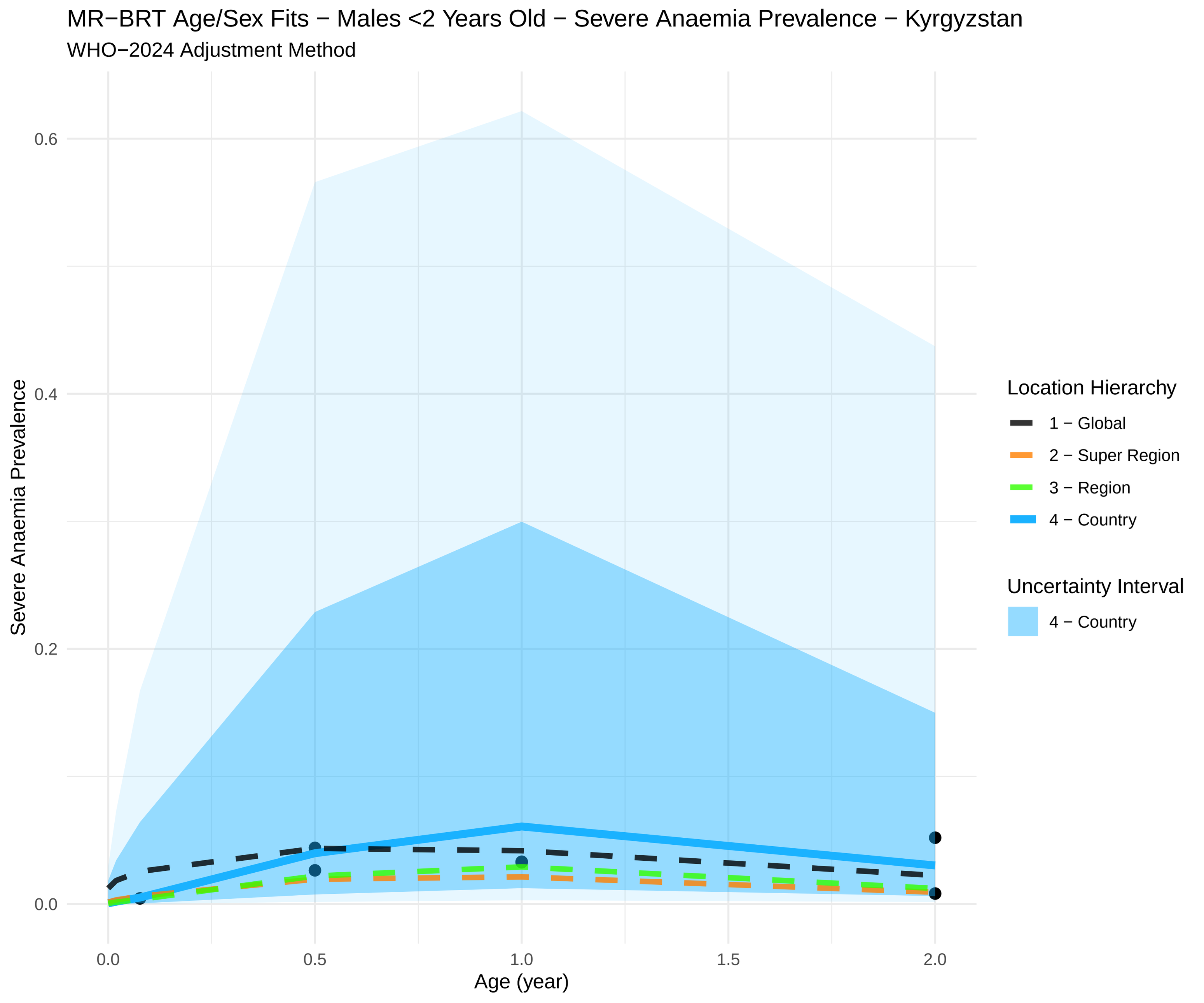
MR−BRT Age/Sex Fits − Males <2 Years Old − Severe Anaemia Prevalence − Kyrgyzstan
WHO−2024 Adjustment Method
Severe Anaemia Prevalence
Age (year)
Location Hierarchy
1 − Global
2 − Super Region
3 − Region
4 − Country
Uncertainty Interval
4 − Country

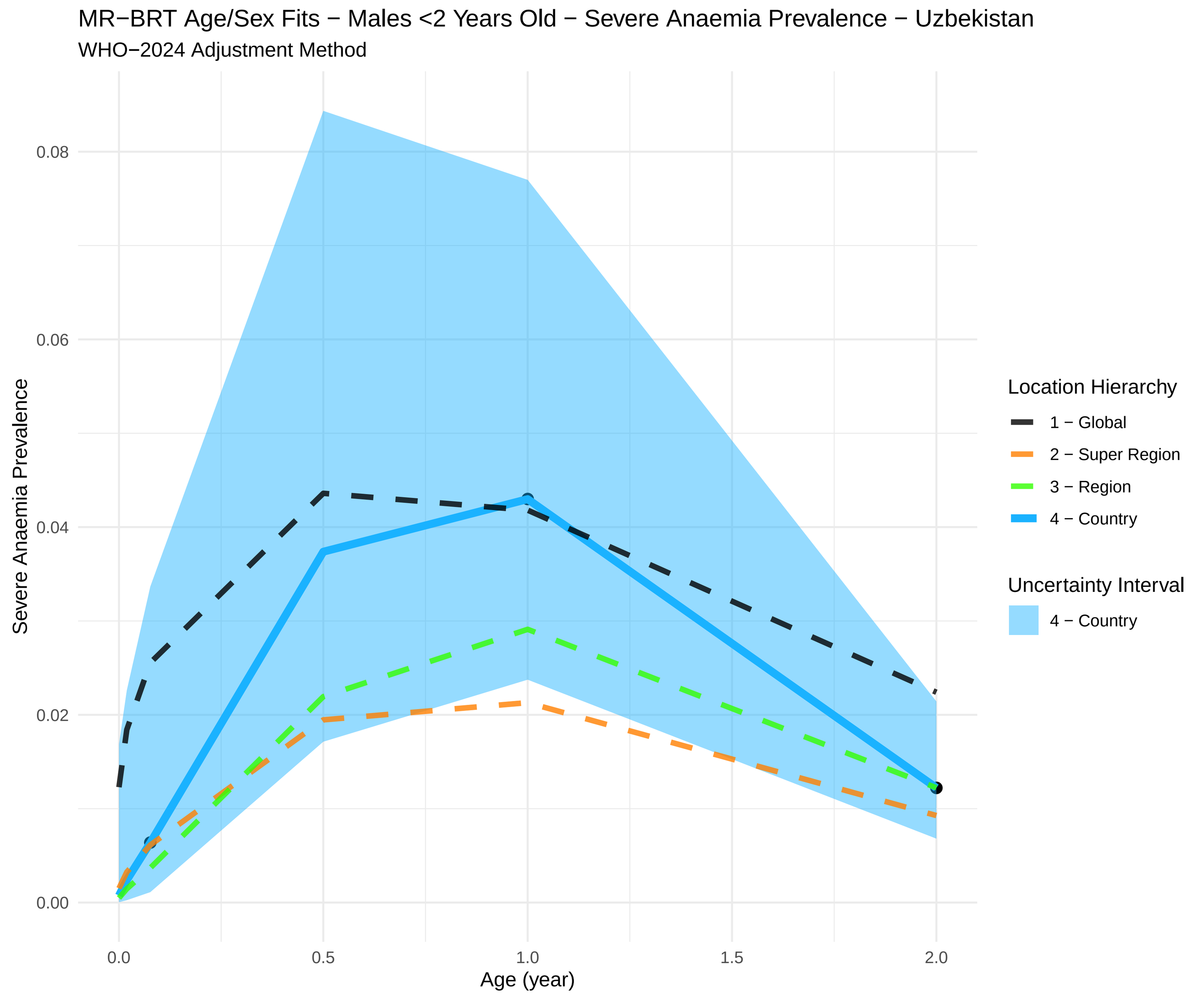
MR−BRT Age/Sex Fits − Males <2 Years Old − Severe Anaemia Prevalence − Uzbekistan
WHO−2024 Adjustment Method
Severe Anaemia Prevalence
Age (year)
Location Hierarchy
1 − Global
2 − Super Region
3 − Region
4 − Country
Uncertainty Interval
4 − Country

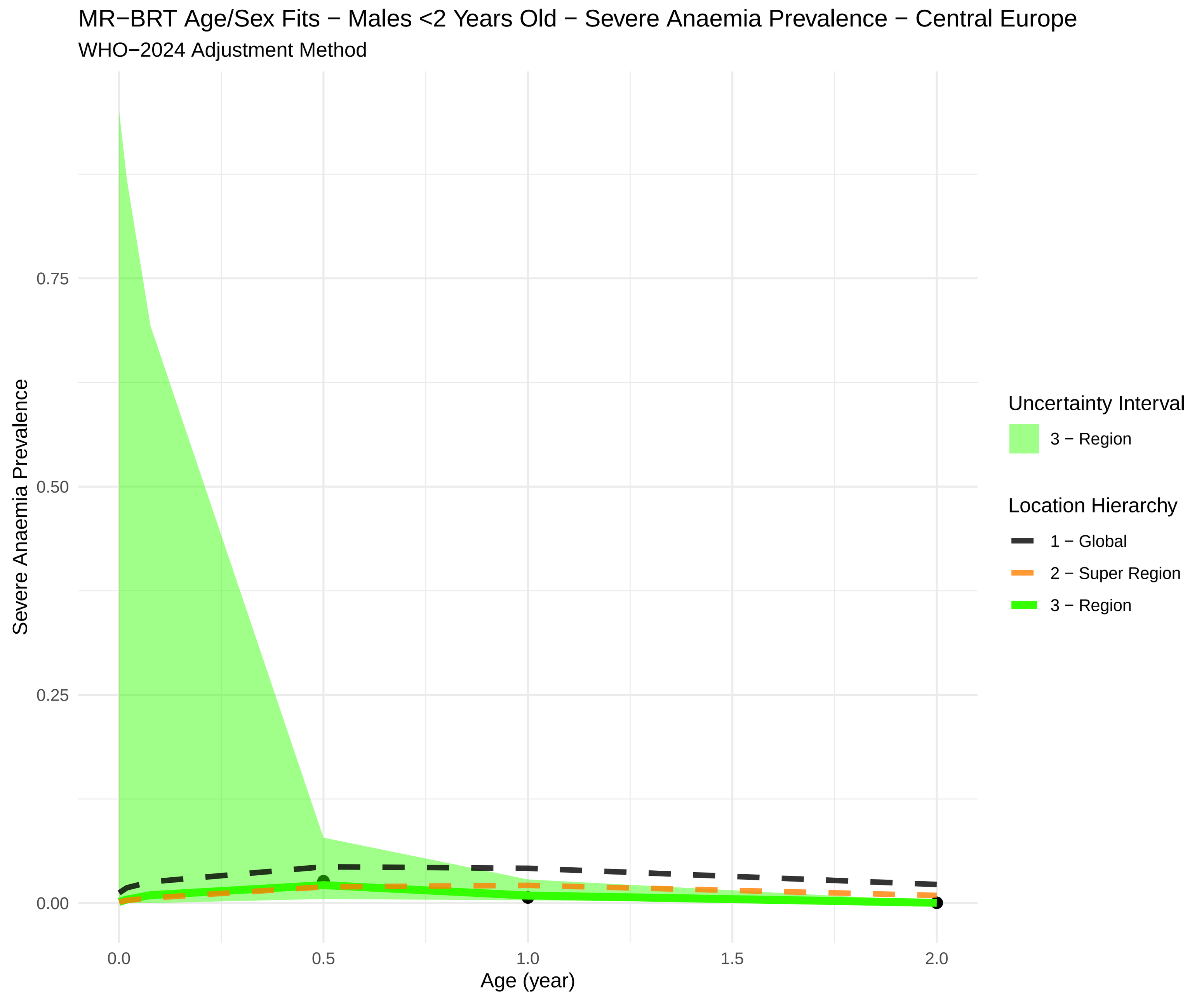
MR−BRT Age/Sex Fits − Males <2 Years Old − Severe Anaemia Prevalence − Central Europe
WHO−2024 Adjustment Method
Severe Anaemia Prevalence
Age (year)
Uncertainty Interval
3 − Region
Location Hierarchy
1 − Global
2 − Super Region
3 − Region

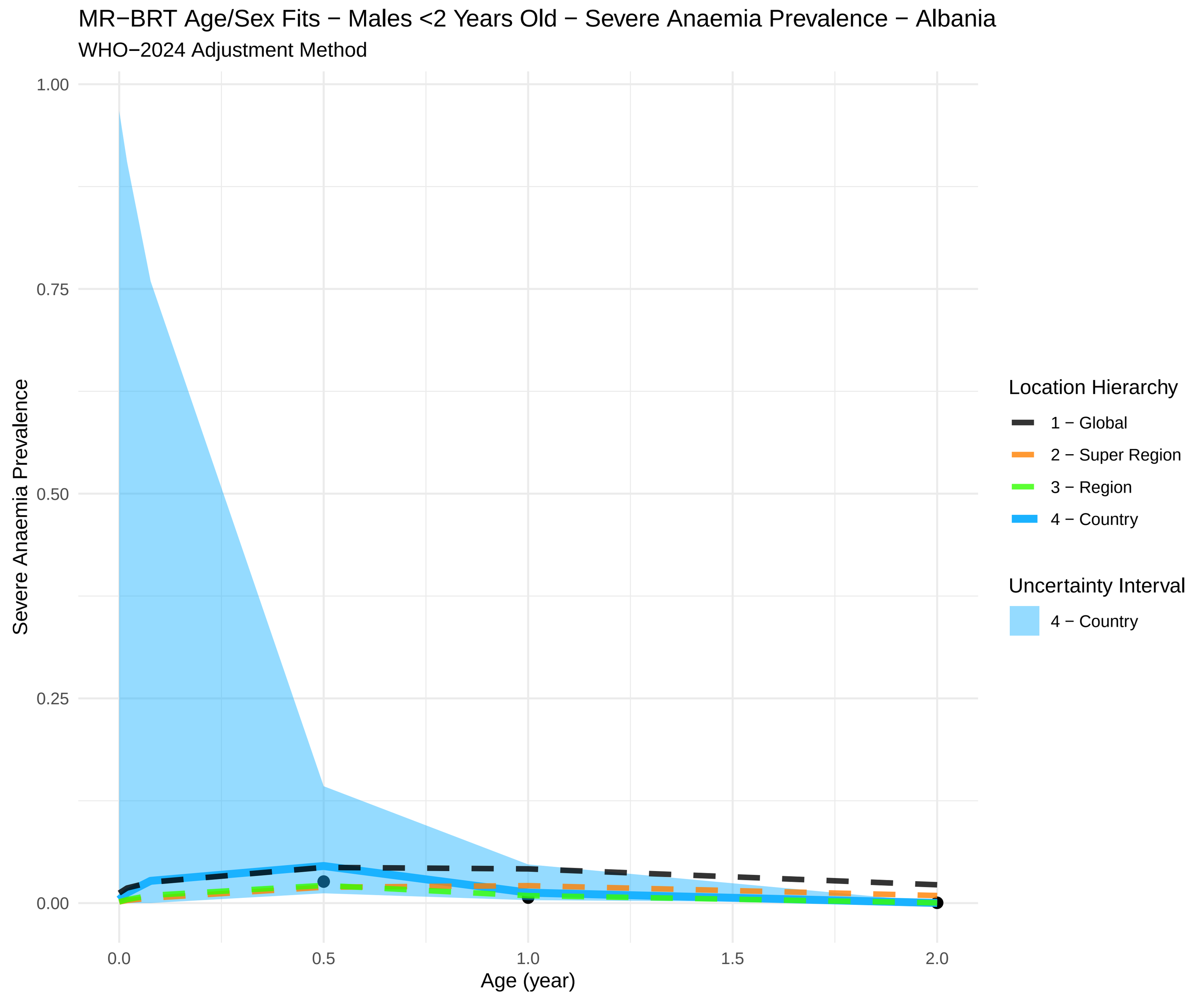
MR−BRT Age/Sex Fits − Males <2 Years Old − Severe Anaemia Prevalence − Albania
WHO−2024 Adjustment Method
Severe Anaemia Prevalence
Age (year)
Location Hierarchy
1 − Global
2 − Super Region
3 − Region
4 − Country
Uncertainty Interval
4 − Country

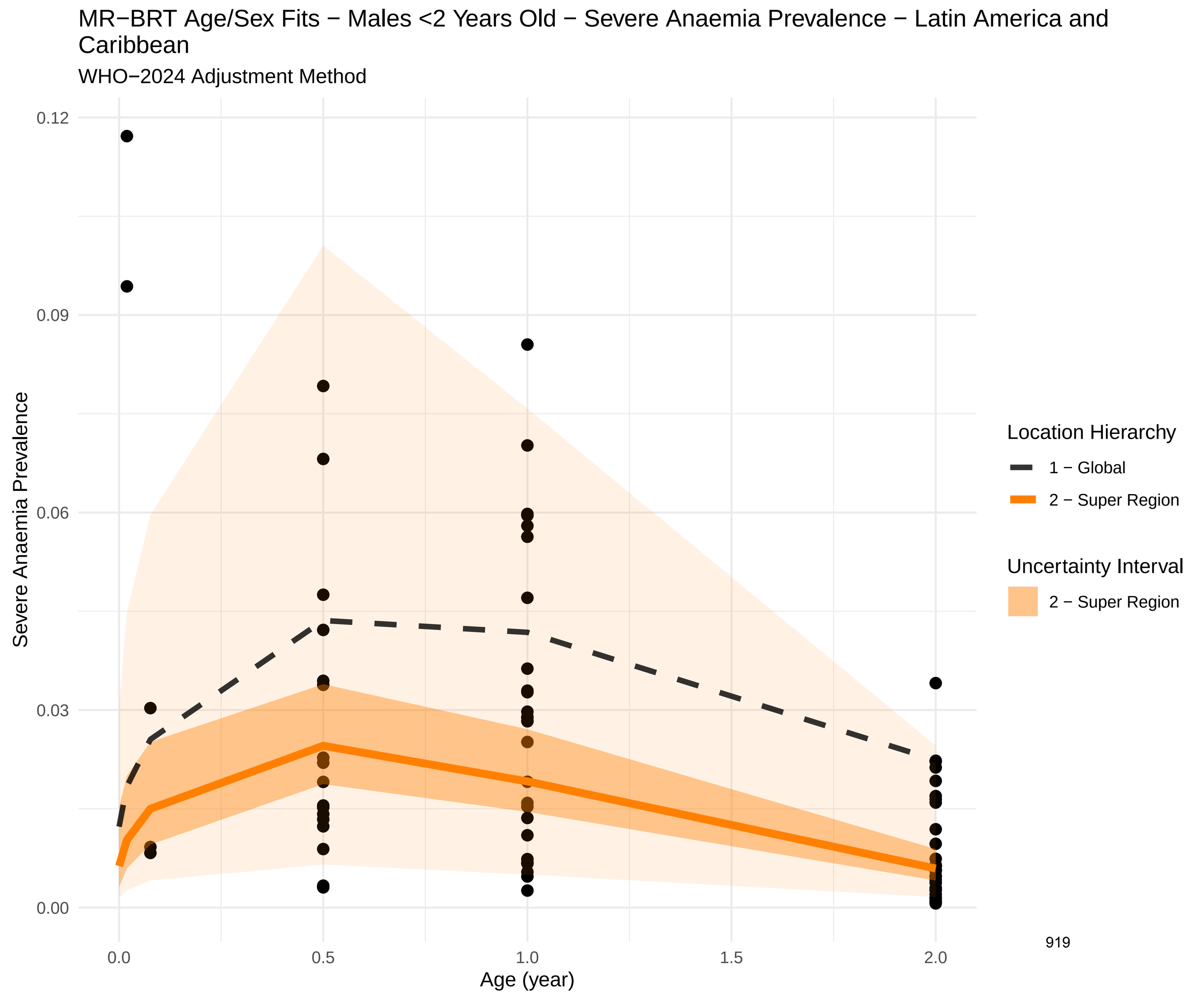
MR−BRT Age/Sex Fits − Males <2 Years Old − Severe Anaemia Prevalence − Latin America and Caribbean
WHO−2024 Adjustment Method
Severe Anaemia Prevalence
Age (year)
Location Hierarchy
1 − Global
2 − Super Region
Uncertainty Interval
2 − Super Region

MR−BRT Age/Sex Fits − Males <2 Years Old − Severe Anaemia Prevalence − Andean Latin America
WHO−2024 Adjustment Method

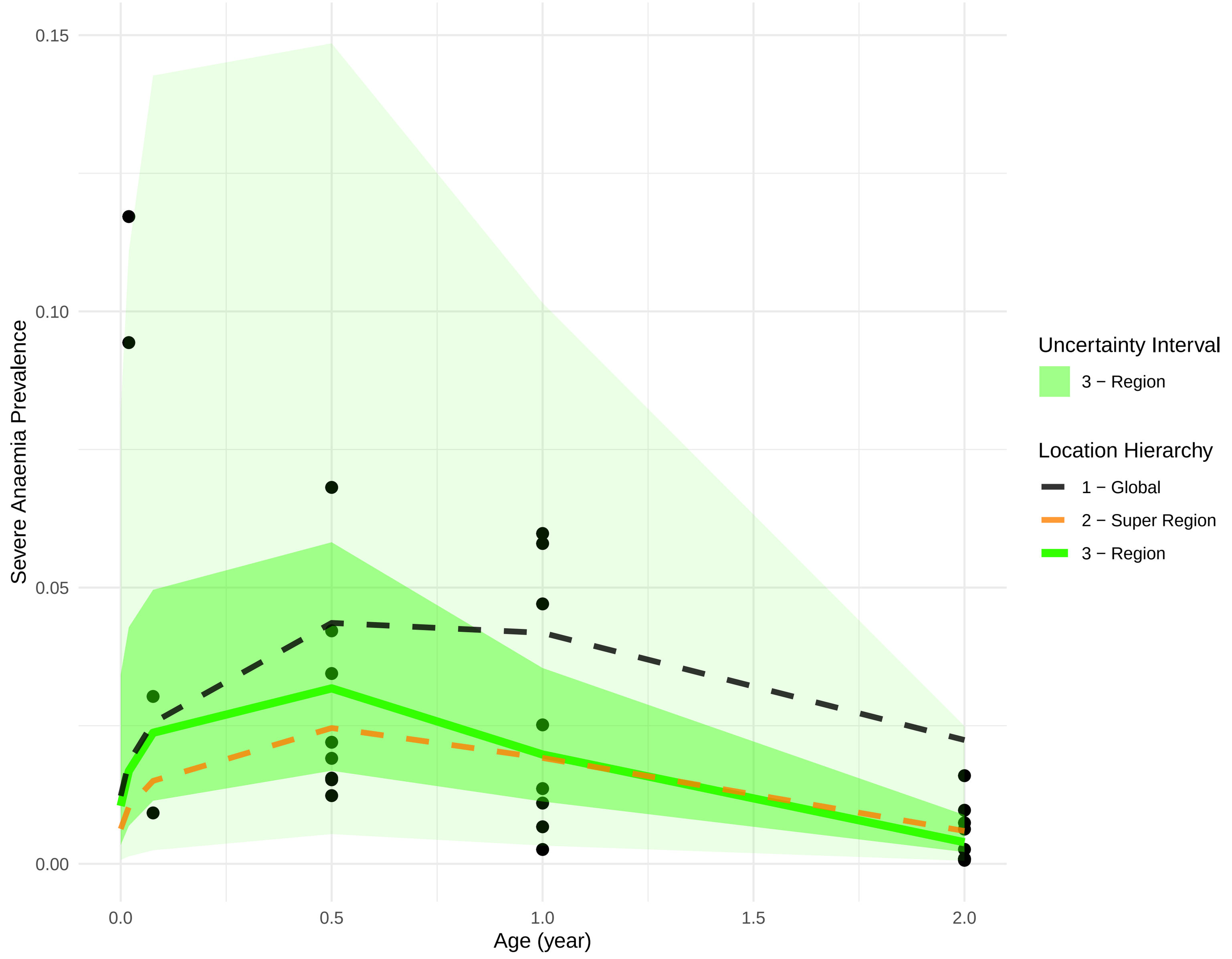

MR−BRT Age/Sex Fits − Males <2 Years Old − Severe Anaemia Prevalence − Bolivia (Plurinational State of)
WHO−2024 Adjustment Method

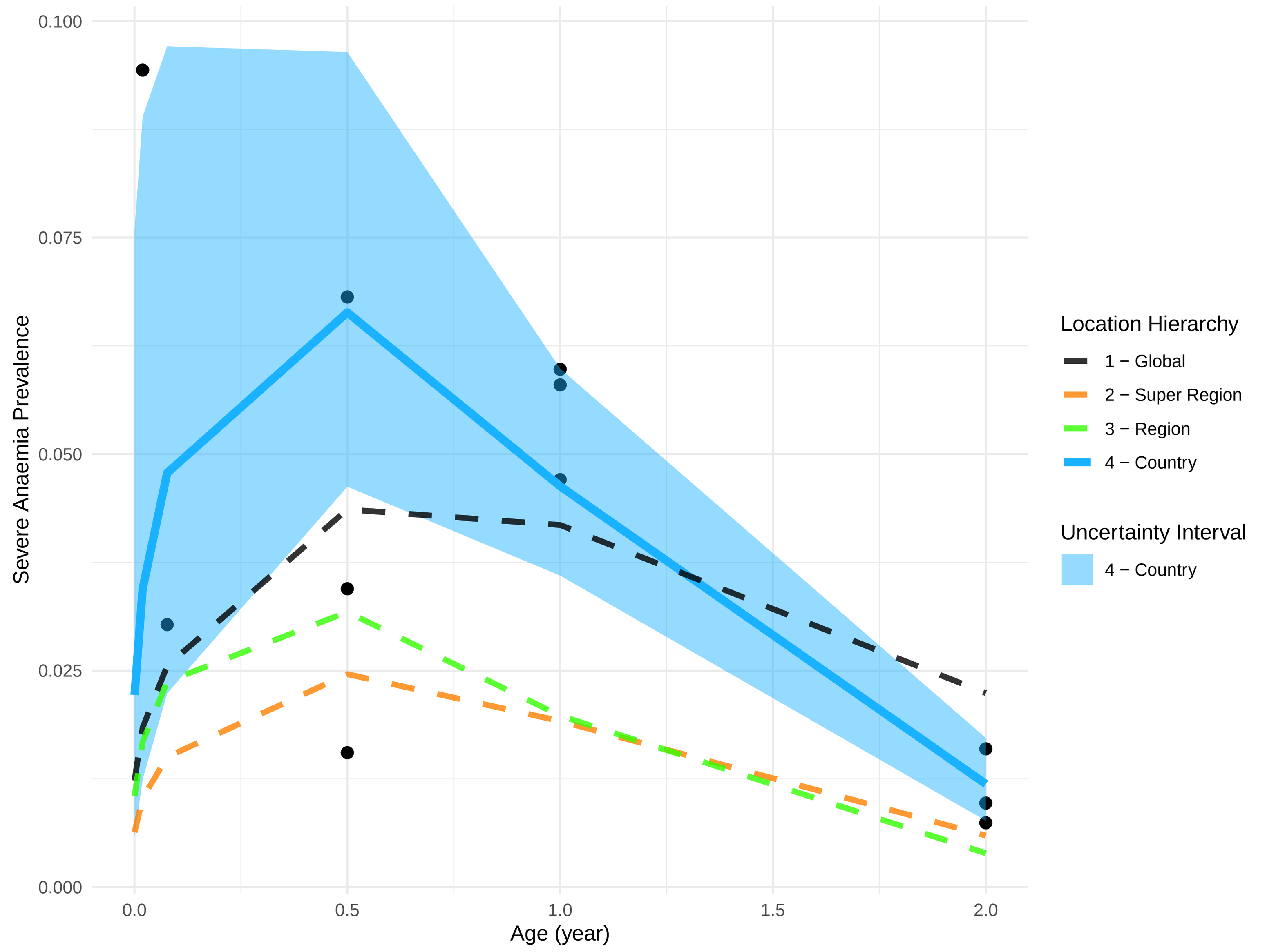

MR−BRT Age/Sex Fits − Males <2 Years Old − Severe Anaemia Prevalence − Peru
WHO−2024 Adjustment Method

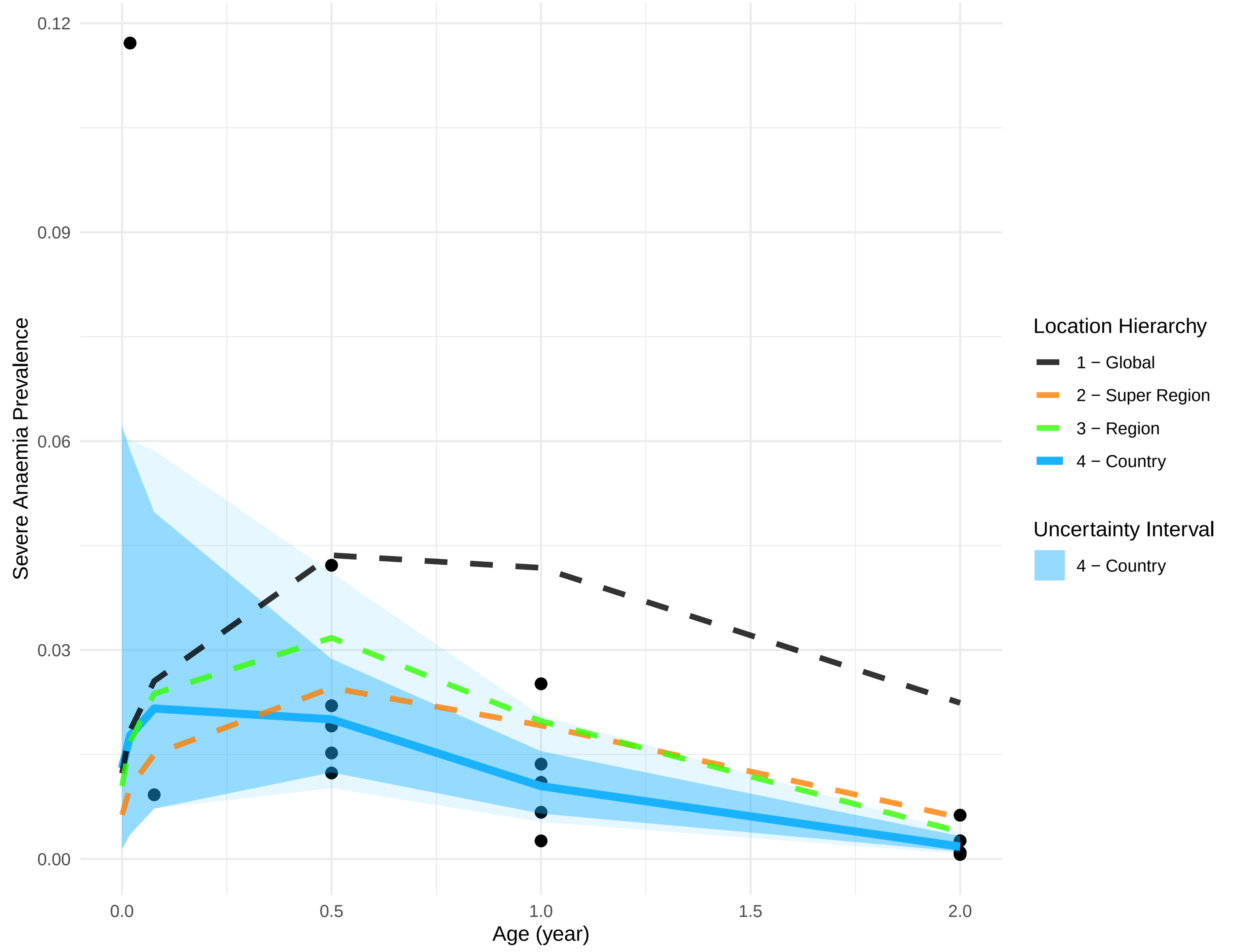

MR−BRT Age/Sex Fits − Males <2 Years Old − Severe Anaemia Prevalence − Caribbean
WHO−2024 Adjustment Method

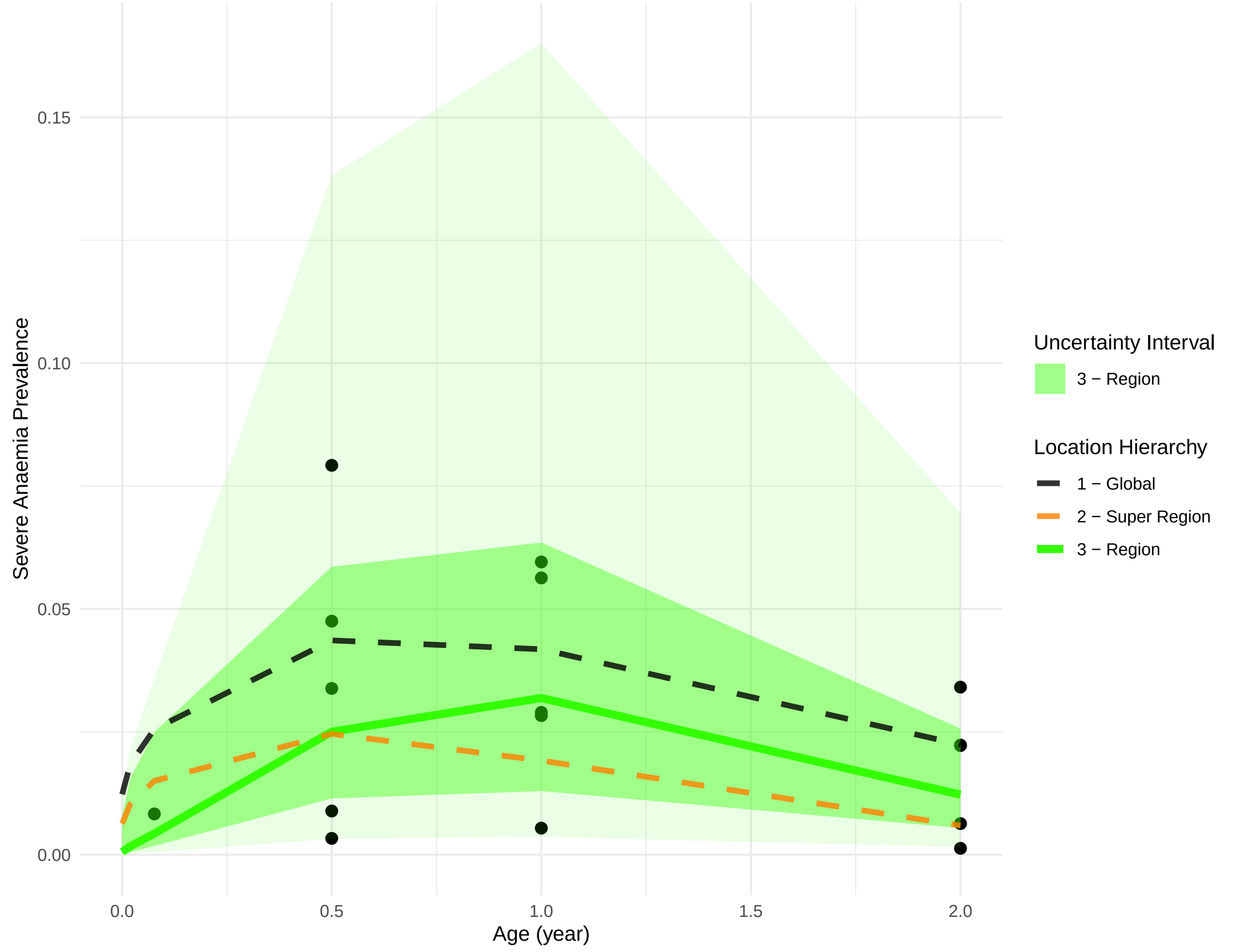

MR−BRT Age/Sex Fits − Males <2 Years Old − Severe Anaemia Prevalence − Guyana
WHO−2024 Adjustment Method

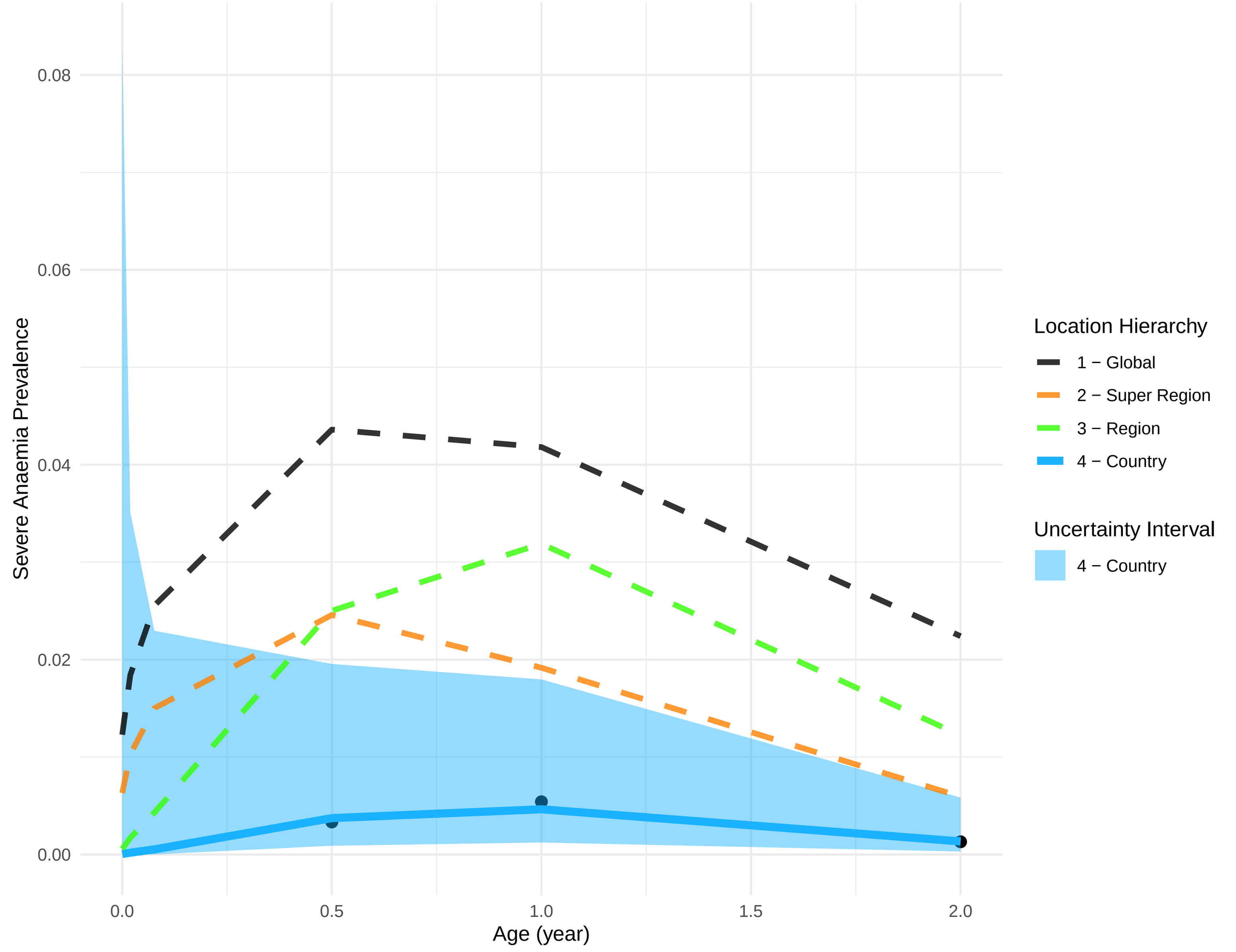

MR−BRT Age/Sex Fits − Males <2 Years Old − Severe Anaemia Prevalence − Haiti
WHO−2024 Adjustment Method

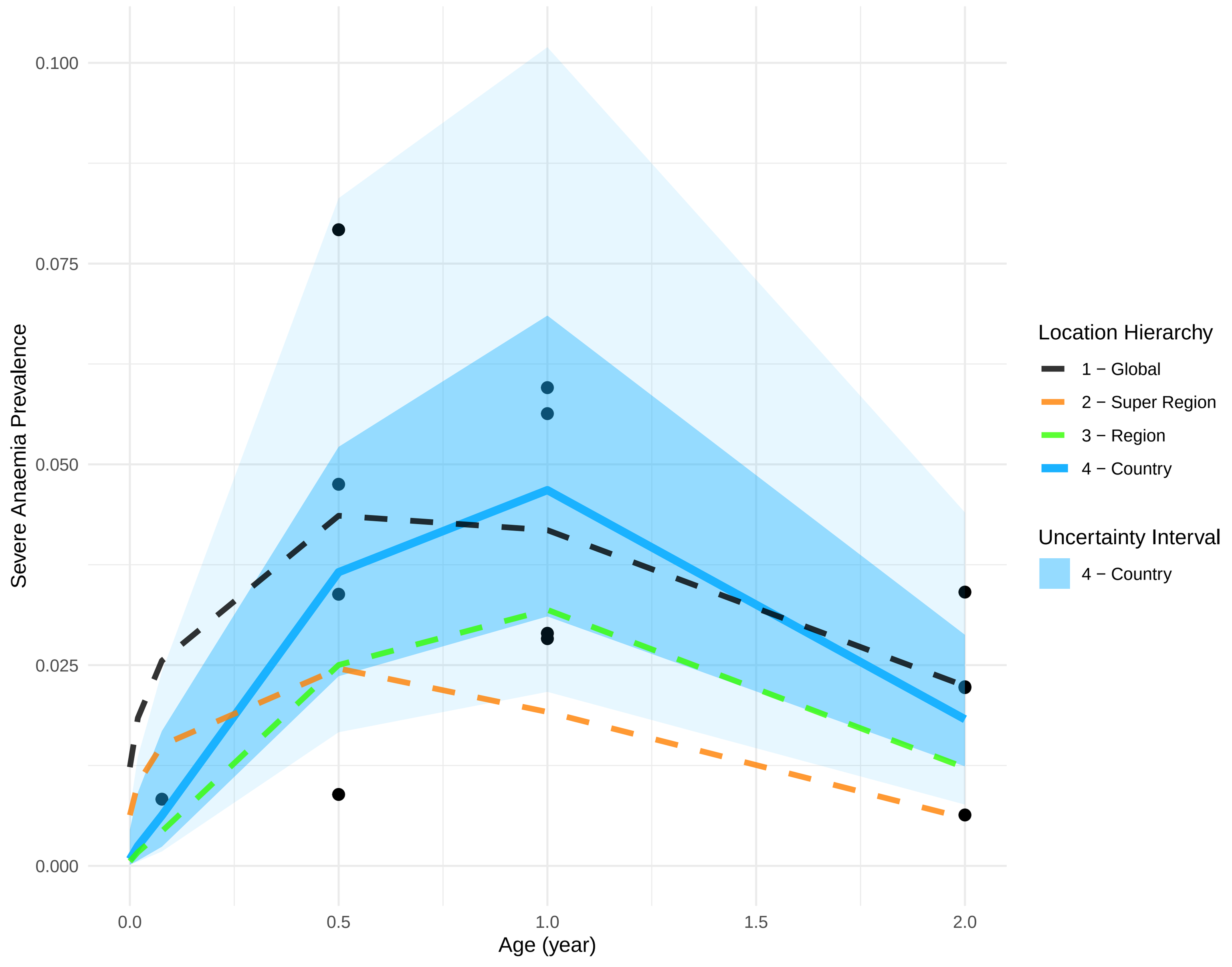

MR−BRT Age/Sex Fits − Males <2 Years Old − Severe Anaemia Prevalence − Central Latin America
WHO−2024 Adjustment Method

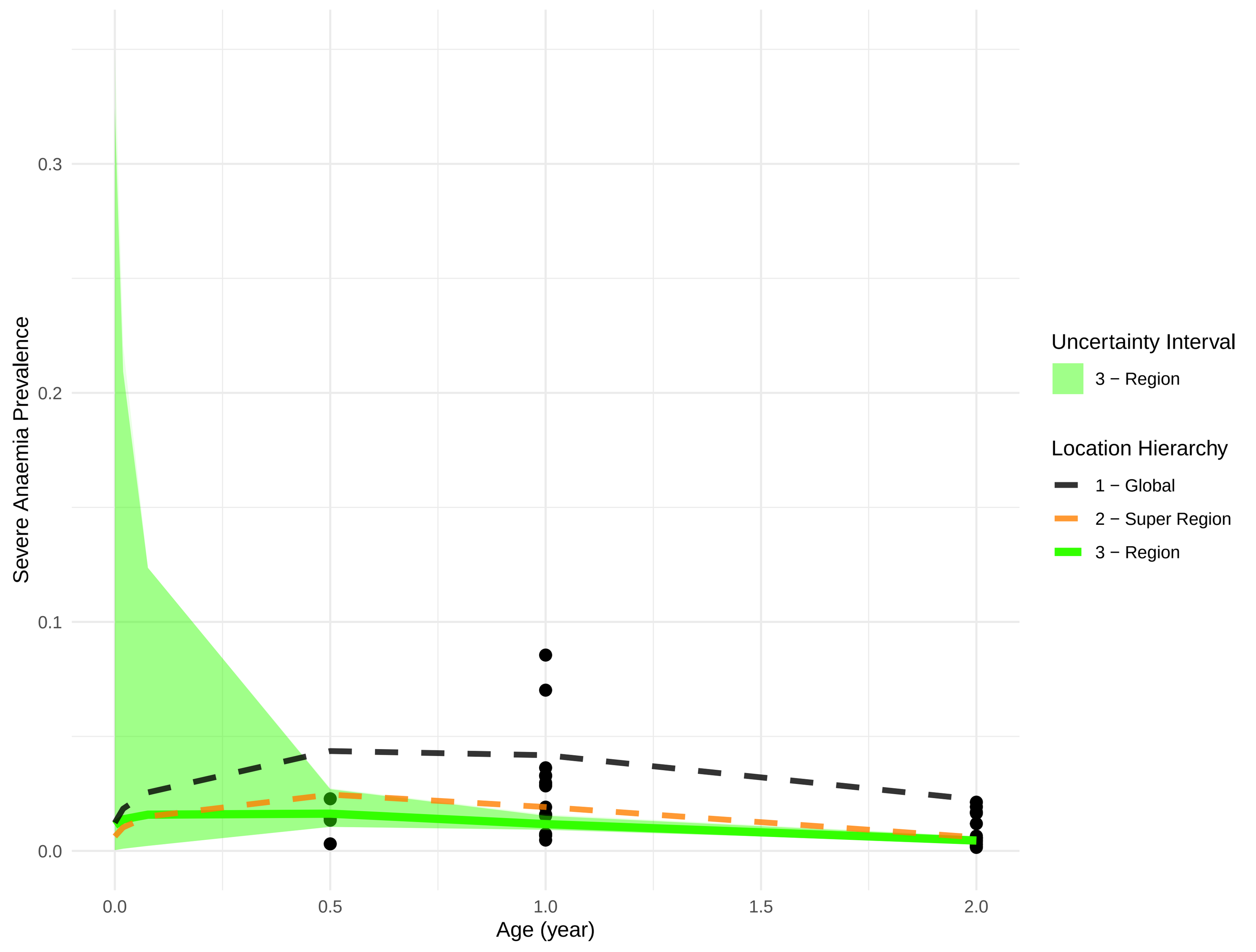

MR−BRT Age/Sex Fits − Males <2 Years Old − Severe Anaemia Prevalence − Guatemala
WHO−2024 Adjustment Method

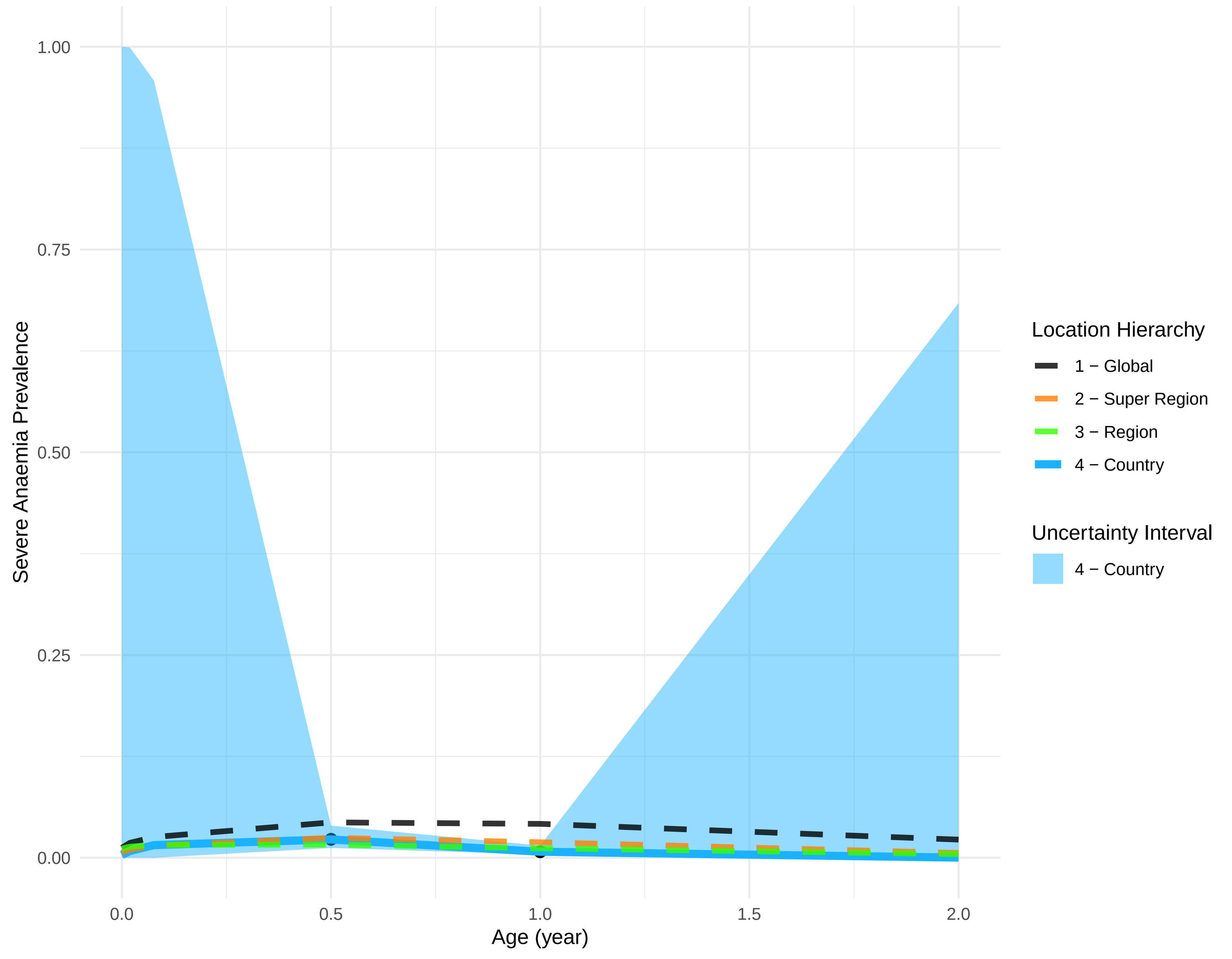

MR−BRT Age/Sex Fits − Males <2 Years Old − Severe Anaemia Prevalence − Honduras
WHO−2024 Adjustment Method

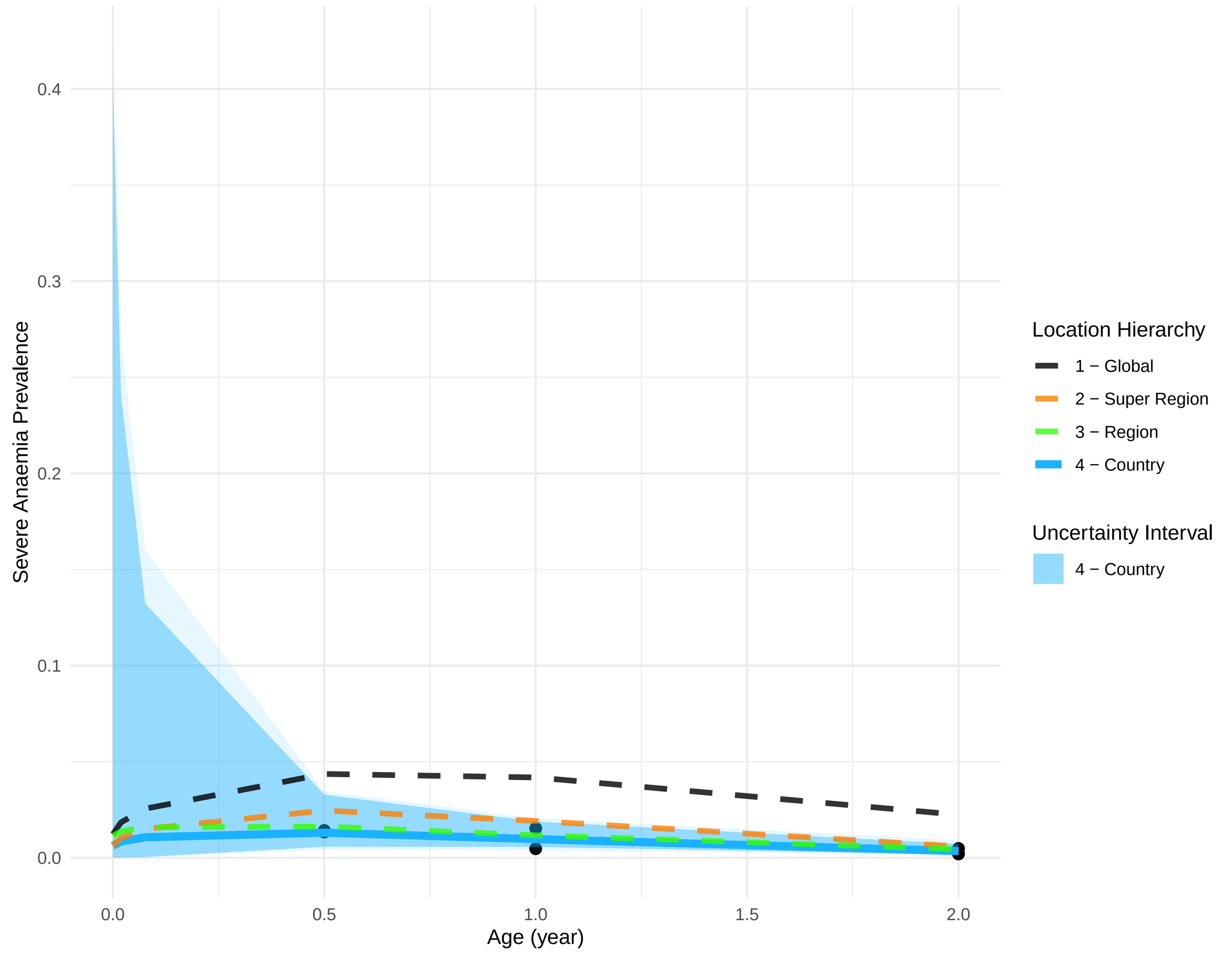

MR−BRT Age/Sex Fits − Males <2 Years Old − Severe Anaemia Prevalence − Mexico
WHO−2024 Adjustment Method

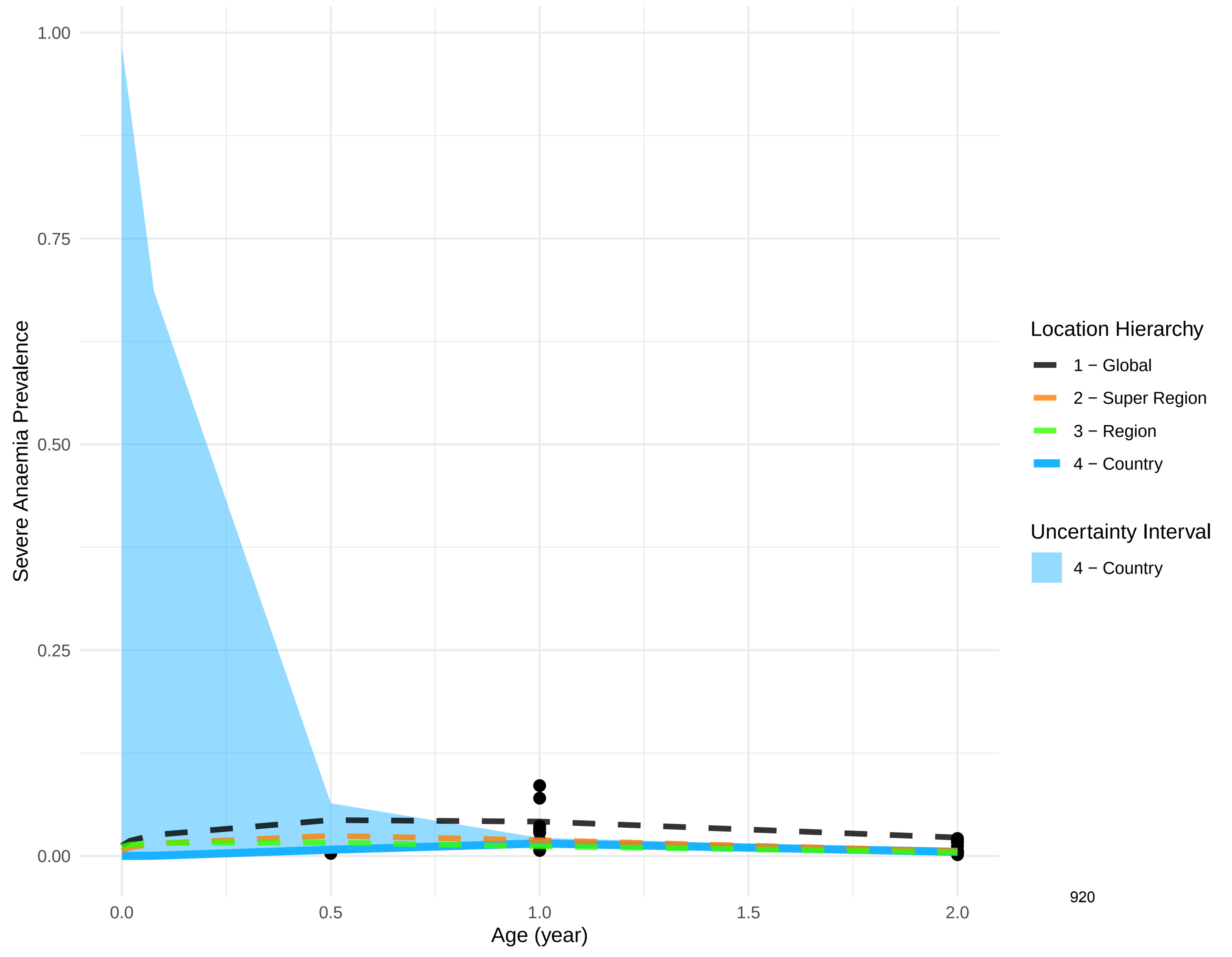

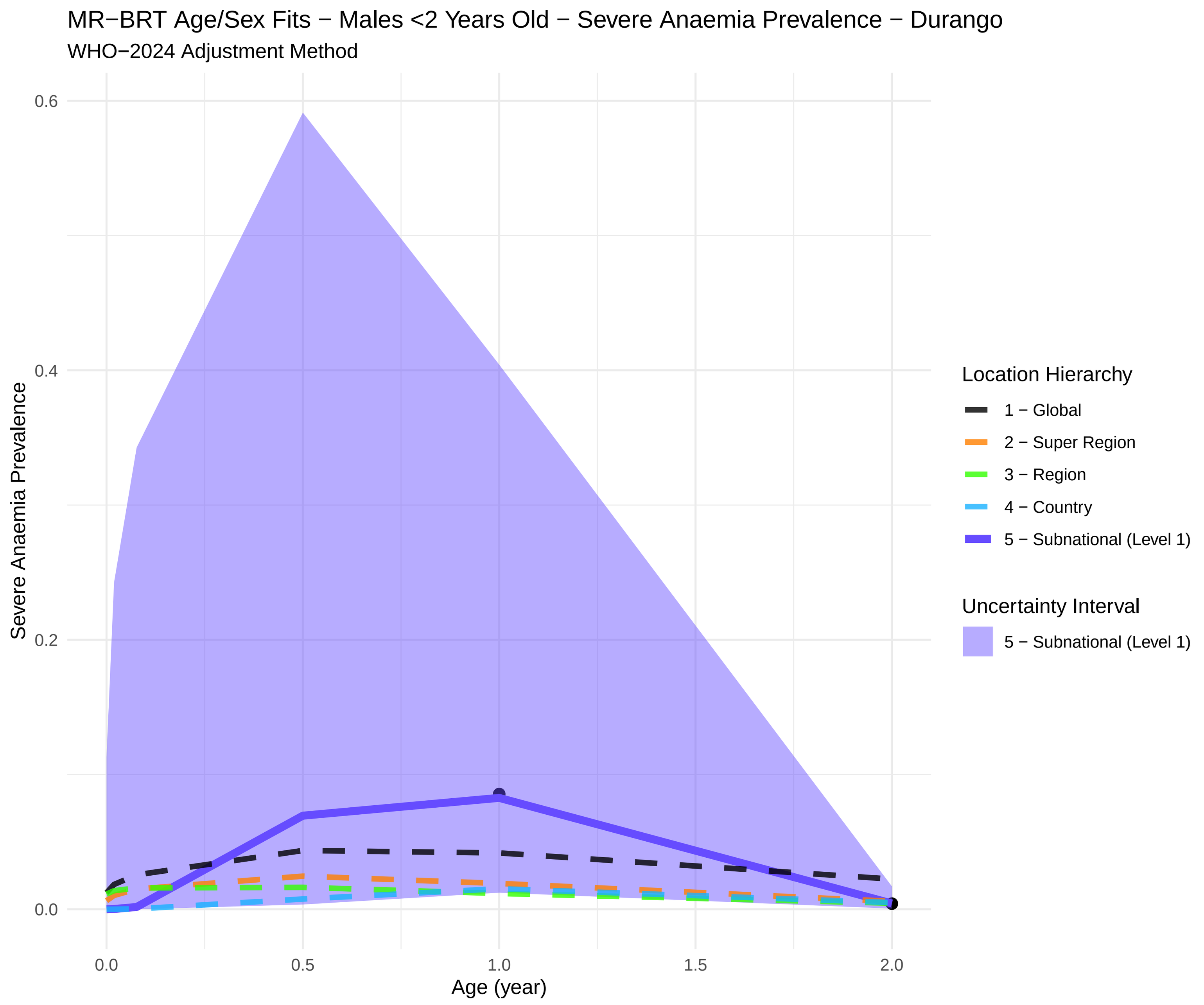
MR−BRT Age/Sex Fits − Males <2 Years Old − Severe Anaemia Prevalence − Durango
WHO−2024 Adjustment Method
Severe Anaemia Prevalence
Age (year)
Location Hierarchy
1 − Global
2 − Super Region
3 − Region
4 − Country
5 − Subnational (Level 1)
Uncertainty Interval
5 − Subnational (Level 1)

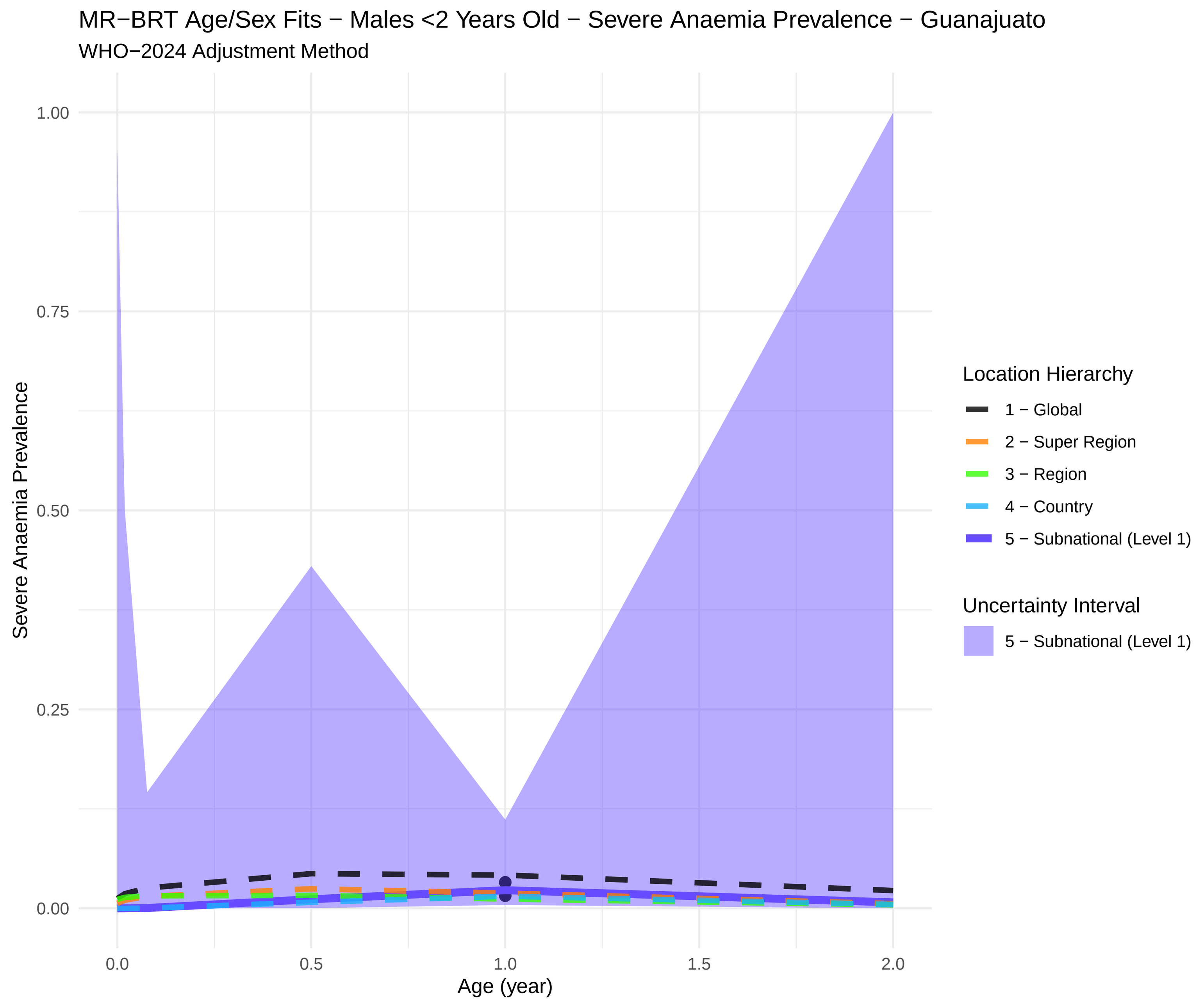
MR−BRT Age/Sex Fits − Males <2 Years Old − Severe Anaemia Prevalence − Guanajuato
WHO−2024 Adjustment Method
Severe Anaemia Prevalence
Age (year)
Location Hierarchy
1 − Global
2 − Super Region
3 − Region
4 − Country
5 − Subnational (Level 1)
Uncertainty Interval
5 − Subnational (Level 1)

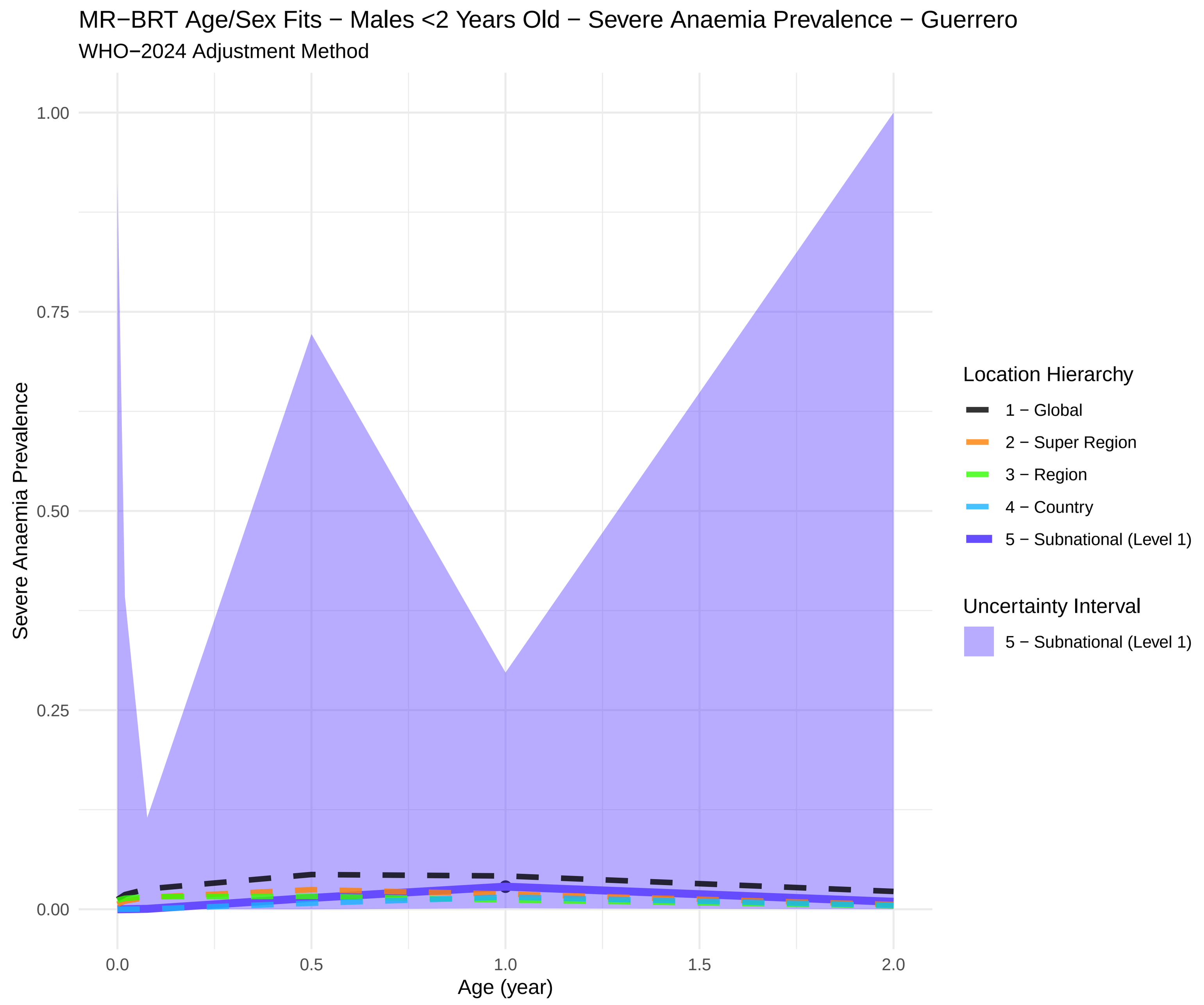
MR−BRT Age/Sex Fits − Males <2 Years Old − Severe Anaemia Prevalence − Guerrero
WHO−2024 Adjustment Method
Severe Anaemia Prevalence
Age (year)
Location Hierarchy
1 − Global
2 − Super Region
3 − Region
4 − Country
5 − Subnational (Level 1)
Uncertainty Interval
5 − Subnational (Level 1)

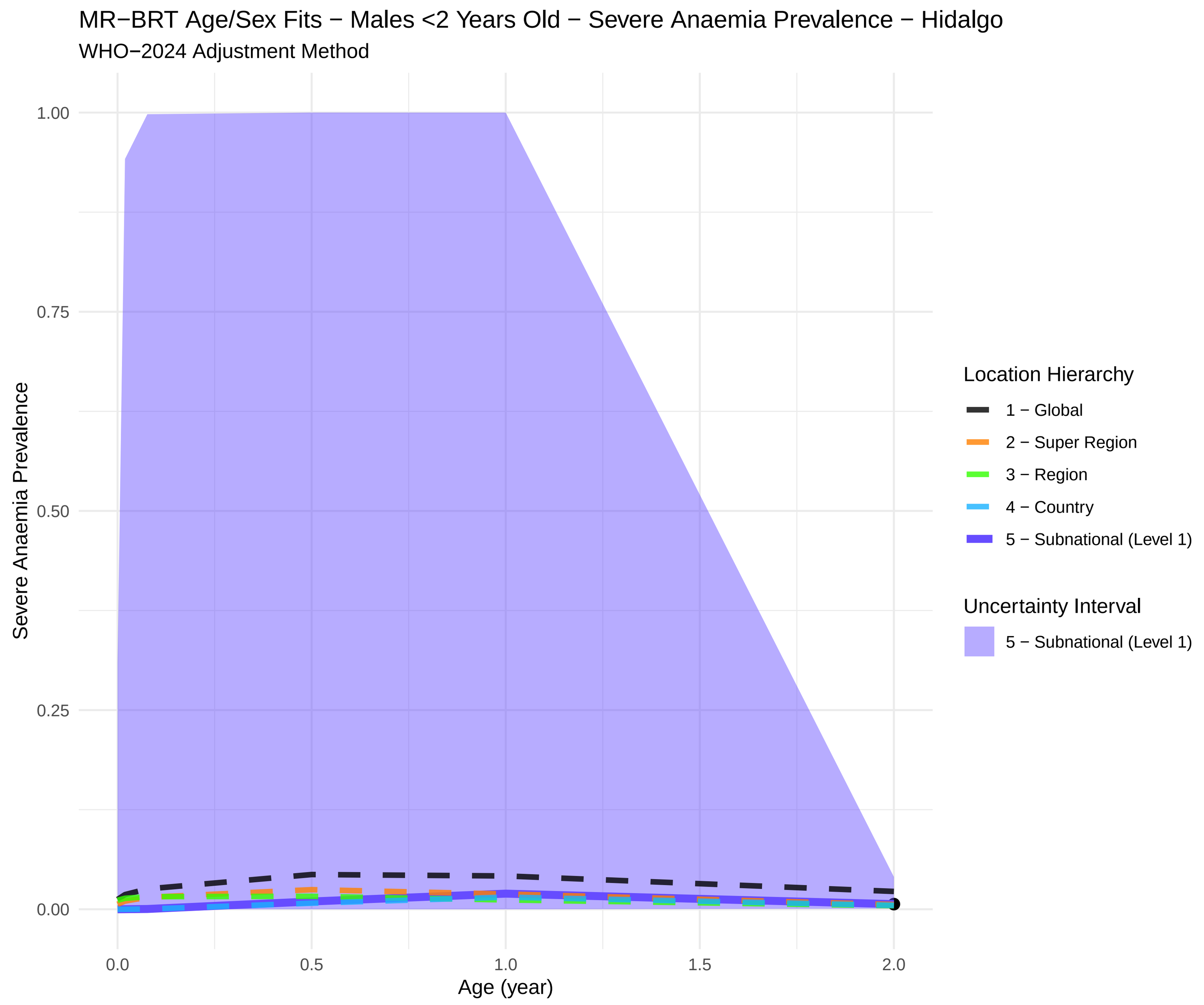
MR−BRT Age/Sex Fits − Males <2 Years Old − Severe Anaemia Prevalence − Hidalgo
WHO−2024 Adjustment Method
Severe Anaemia Prevalence
Age (year)
Location Hierarchy
1 − Global
2 − Super Region
3 − Region
4 − Country
5 − Subnational (Level 1)
Uncertainty Interval
5 − Subnational (Level 1)

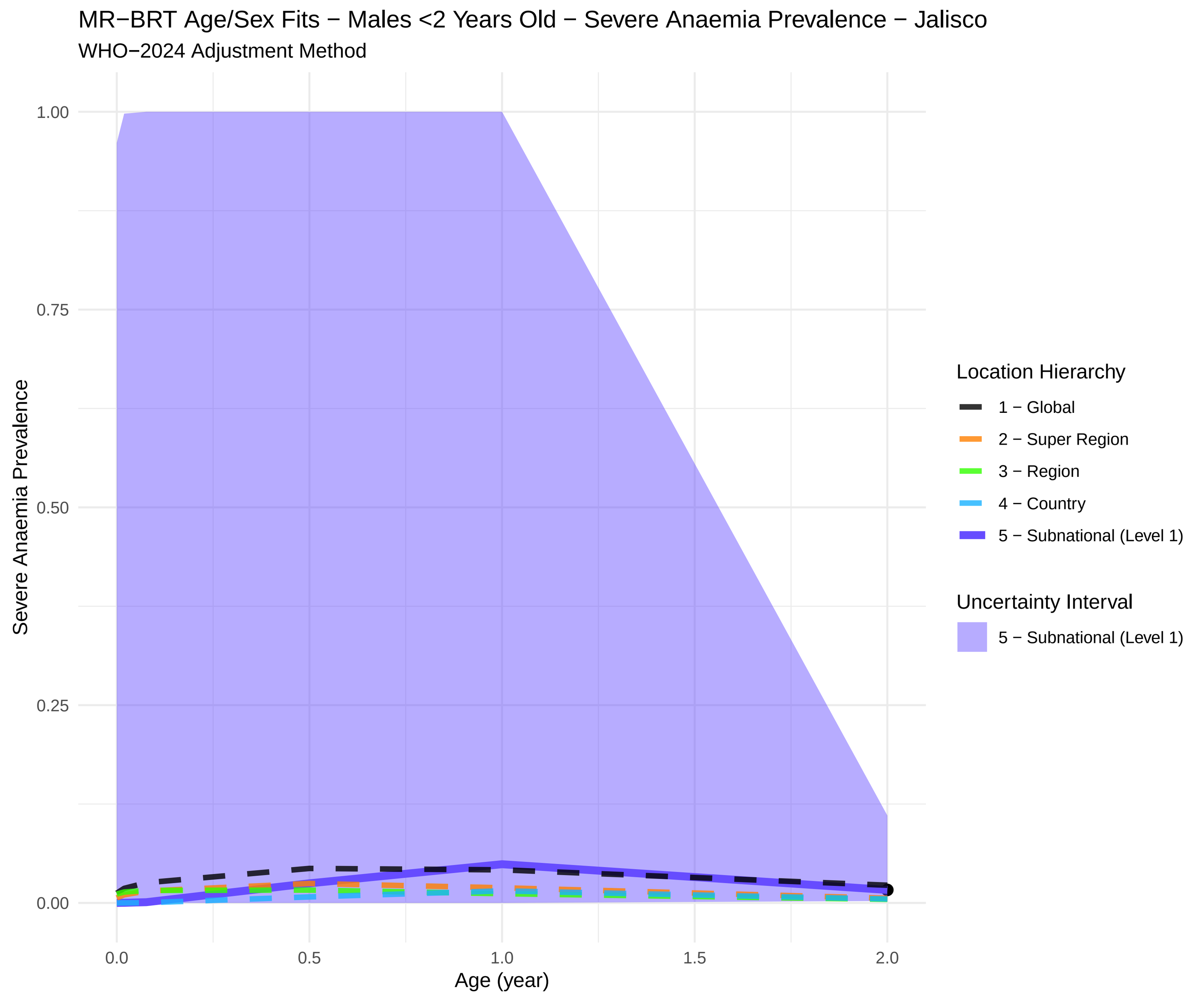
MR−BRT Age/Sex Fits − Males <2 Years Old − Severe Anaemia Prevalence − Jalisco
WHO−2024 Adjustment Method
Severe Anaemia Prevalence
Age (year)
Location Hierarchy
1 − Global
2 − Super Region
3 − Region
4 − Country
5 − Subnational (Level 1)
Uncertainty Interval
5 − Subnational (Level 1)

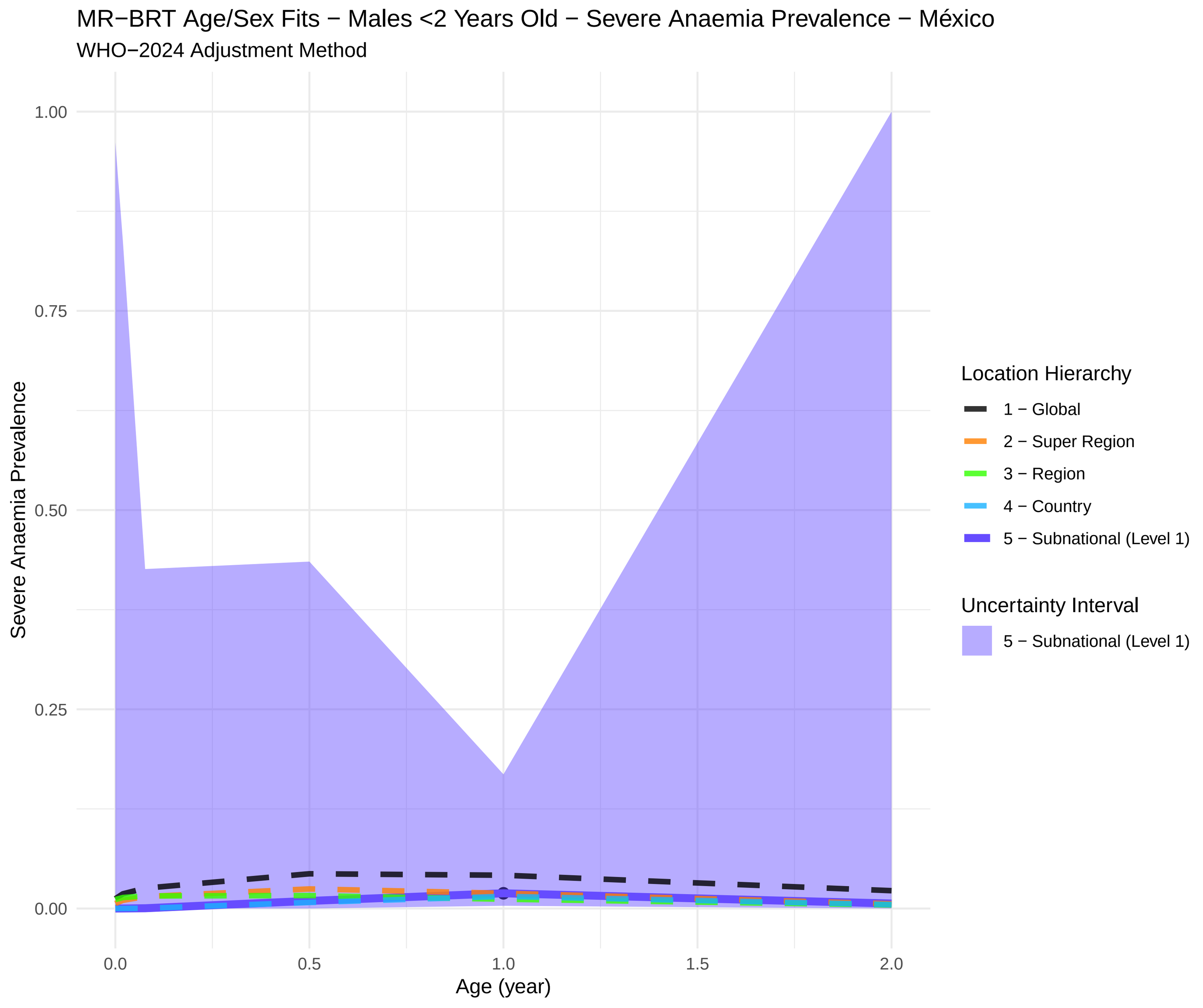
MR−BRT Age/Sex Fits − Males <2 Years Old − Severe Anaemia Prevalence − México
WHO−2024 Adjustment Method
Severe Anaemia Prevalence
Age (year)
Location Hierarchy
1 − Global
2 − Super Region
3 − Region
4 − Country
5 − Subnational (Level 1)
Uncertainty Interval
5 − Subnational (Level 1)

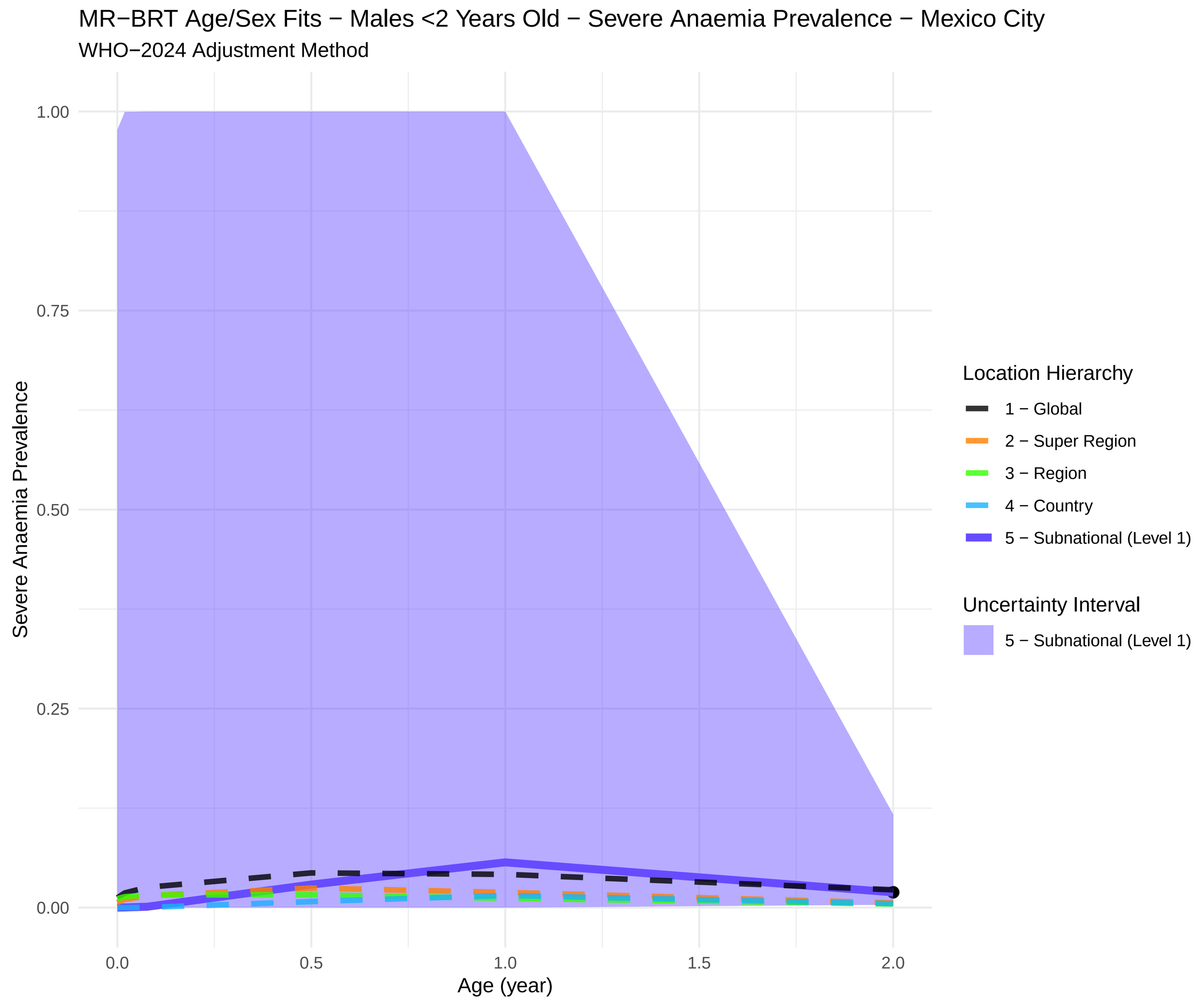
MR−BRT Age/Sex Fits − Males <2 Years Old − Severe Anaemia Prevalence − Mexico City
WHO−2024 Adjustment Method
Severe Anaemia Prevalence
Age (year)
Location Hierarchy
1 − Global
2 − Super Region
3 − Region
4 − Country
5 − Subnational (Level 1)
Uncertainty Interval
5 − Subnational (Level 1)

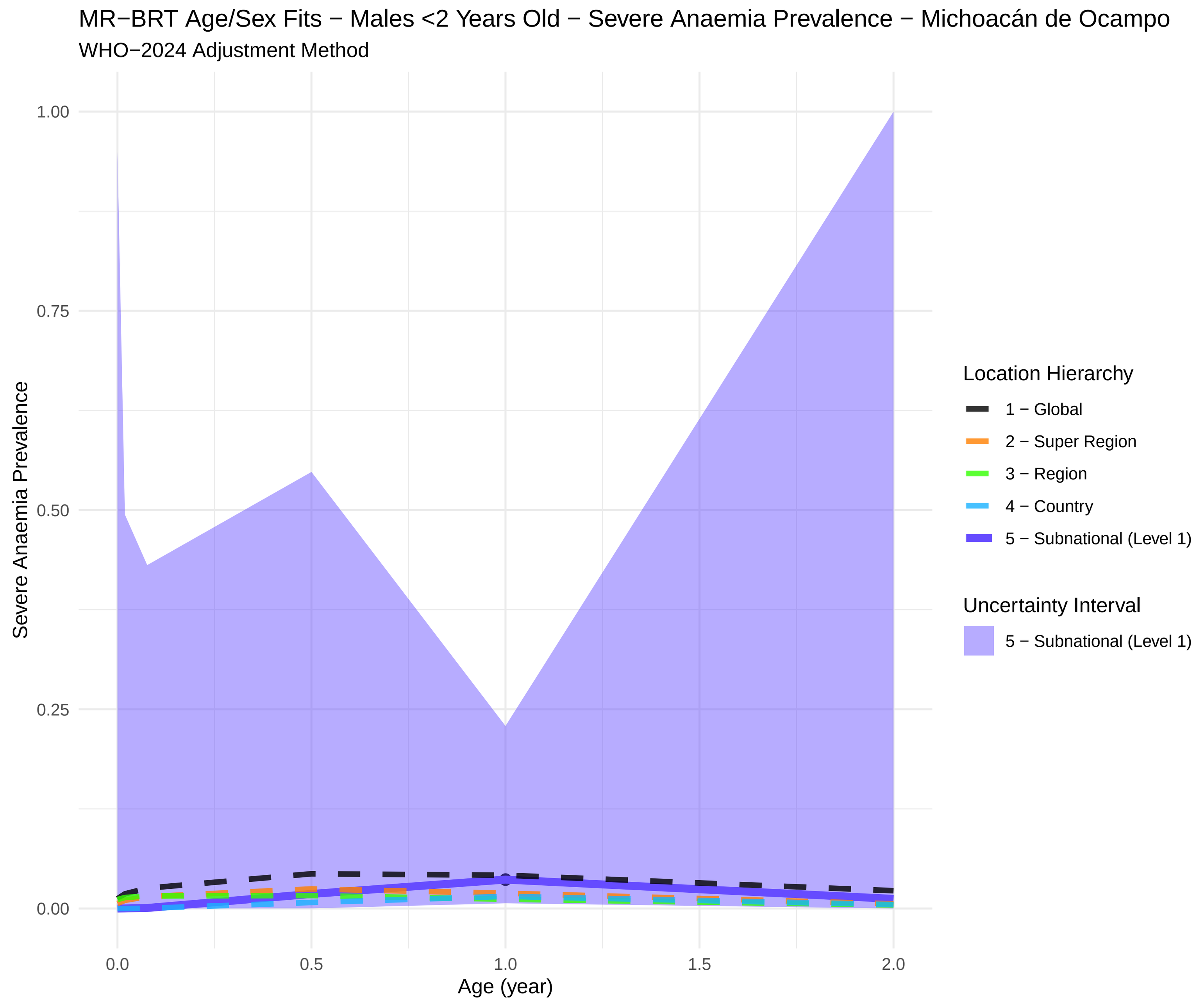
MR−BRT Age/Sex Fits − Males <2 Years Old − Severe Anaemia Prevalence − Michoacán de Ocampo
WHO−2024 Adjustment Method
Severe Anaemia Prevalence
Age (year)
Location Hierarchy
1 − Global
2 − Super Region
3 − Region
4 − Country
5 − Subnational (Level 1)
Uncertainty Interval
5 − Subnational (Level 1)

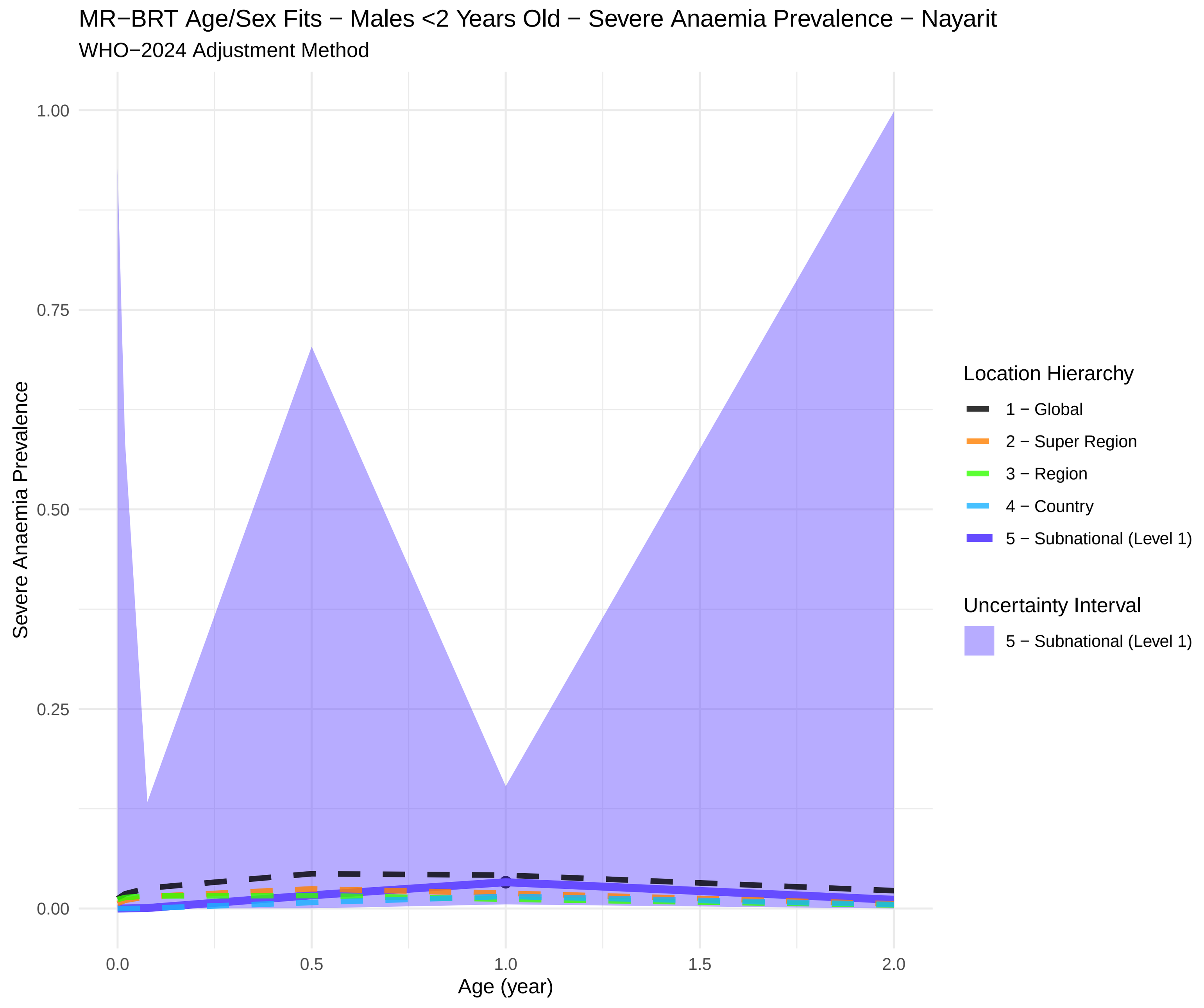
MR−BRT Age/Sex Fits − Males <2 Years Old − Severe Anaemia Prevalence − Nayarit
WHO−2024 Adjustment Method
Severe Anaemia Prevalence
Age (year)
Location Hierarchy
1 − Global
2 − Super Region
3 − Region
4 − Country
5 − Subnational (Level 1)
Uncertainty Interval
5 − Subnational (Level 1)

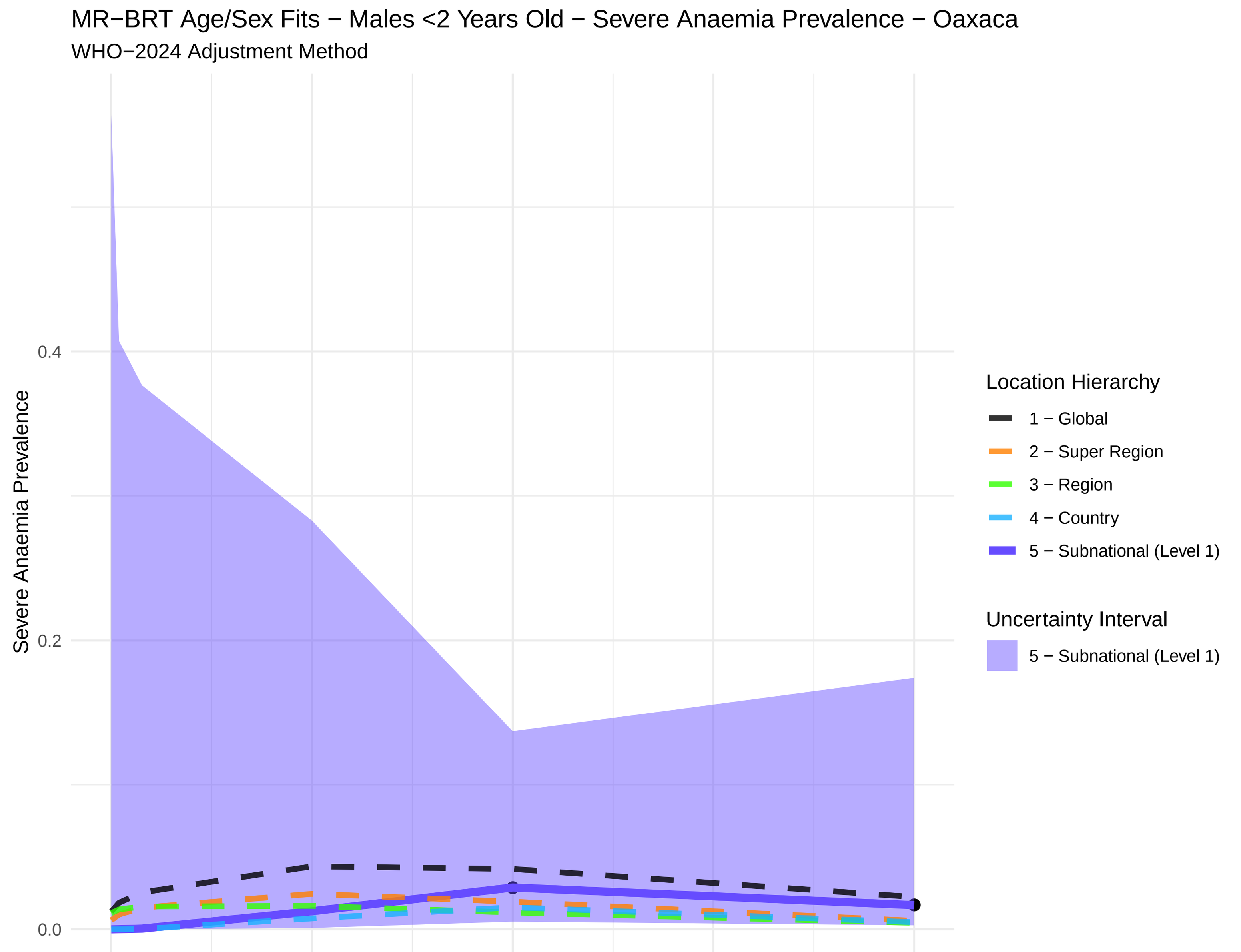
MR−BRT Age/Sex Fits − Males <2 Years Old − Severe Anaemia Prevalence − Oaxaca
WHO−2024 Adjustment Method
Severe Anaemia Prevalence
Age (year)
Location Hierarchy
1 − Global
2 − Super Region
3 − Region
4 − Country
5 − Subnational (Level 1)
Uncertainty Interval
5 − Subnational (Level 1)

MR−BRT Age/Sex Fits − Males <2 Years Old − Severe Anaemia Prevalence − Puebla
WHO−2024 Adjustment Method

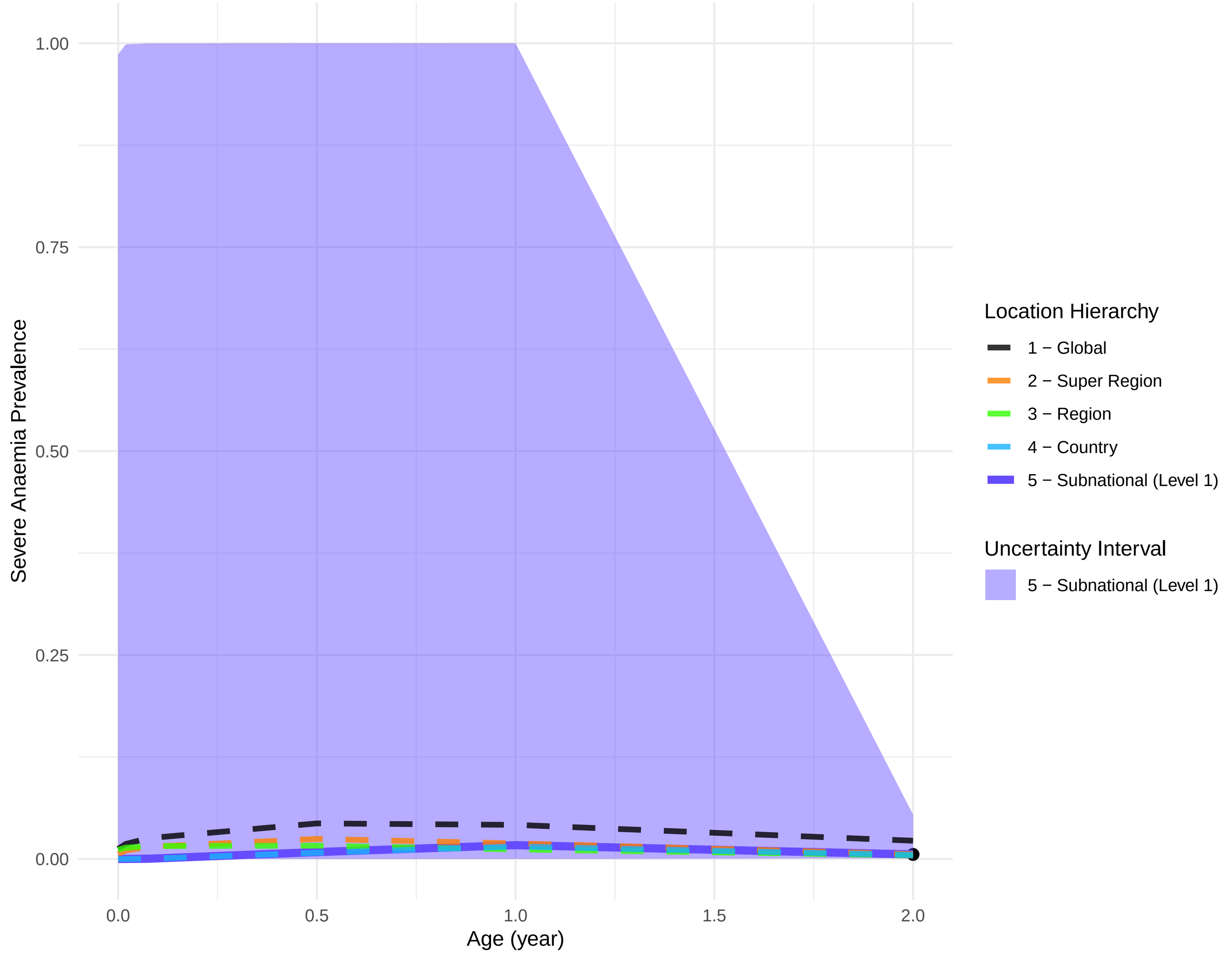

MR−BRT Age/Sex Fits − Males <2 Years Old − Severe Anaemia Prevalence − Querétaro
WHO−2024 Adjustment Method

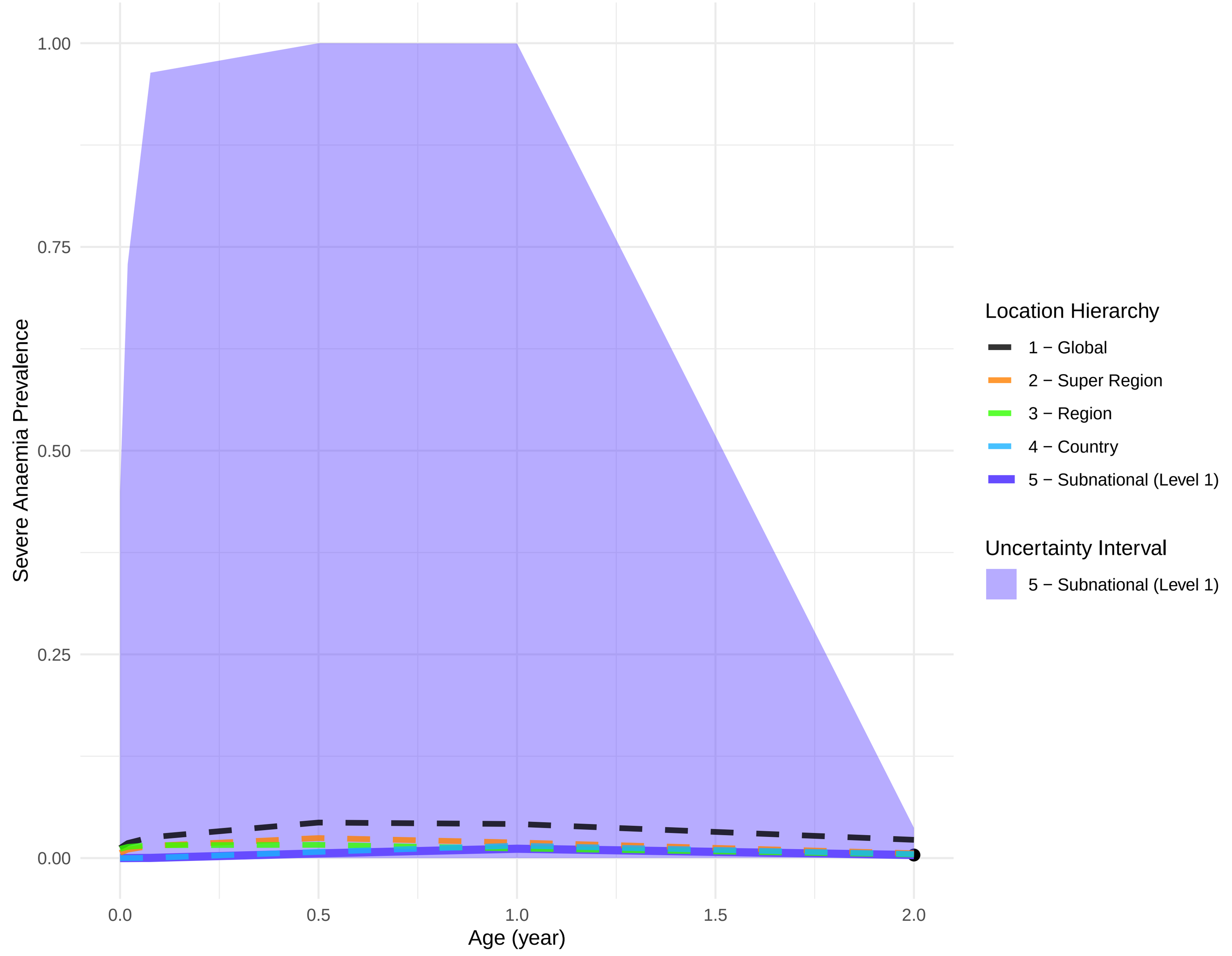

MR−BRT Age/Sex Fits − Males <2 Years Old − Severe Anaemia Prevalence − Quintana Roo
WHO−2024 Adjustment Method

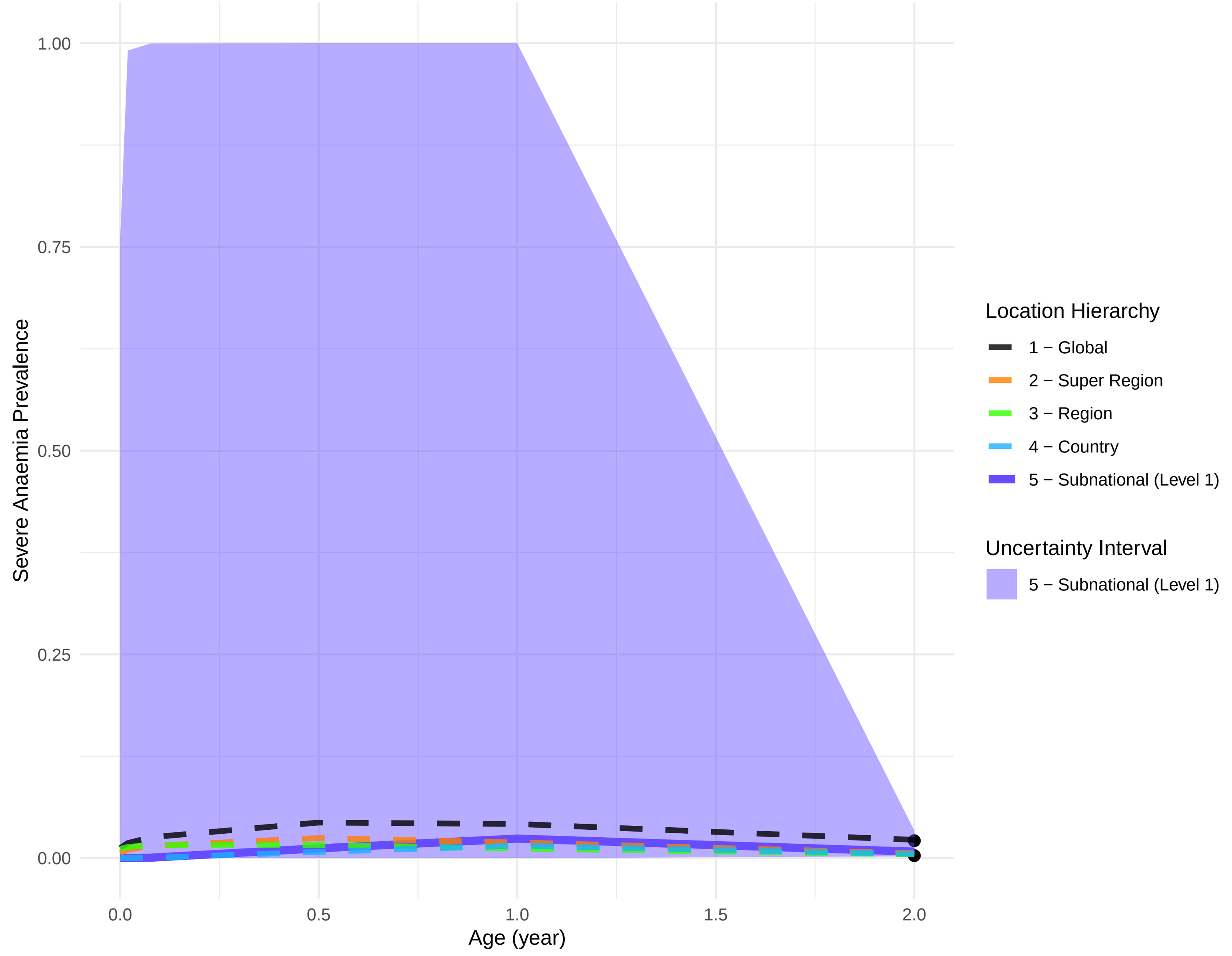

MR−BRT Age/Sex Fits − Males <2 Years Old − Severe Anaemia Prevalence − San Luis Potosí
WHO−2024 Adjustment Method

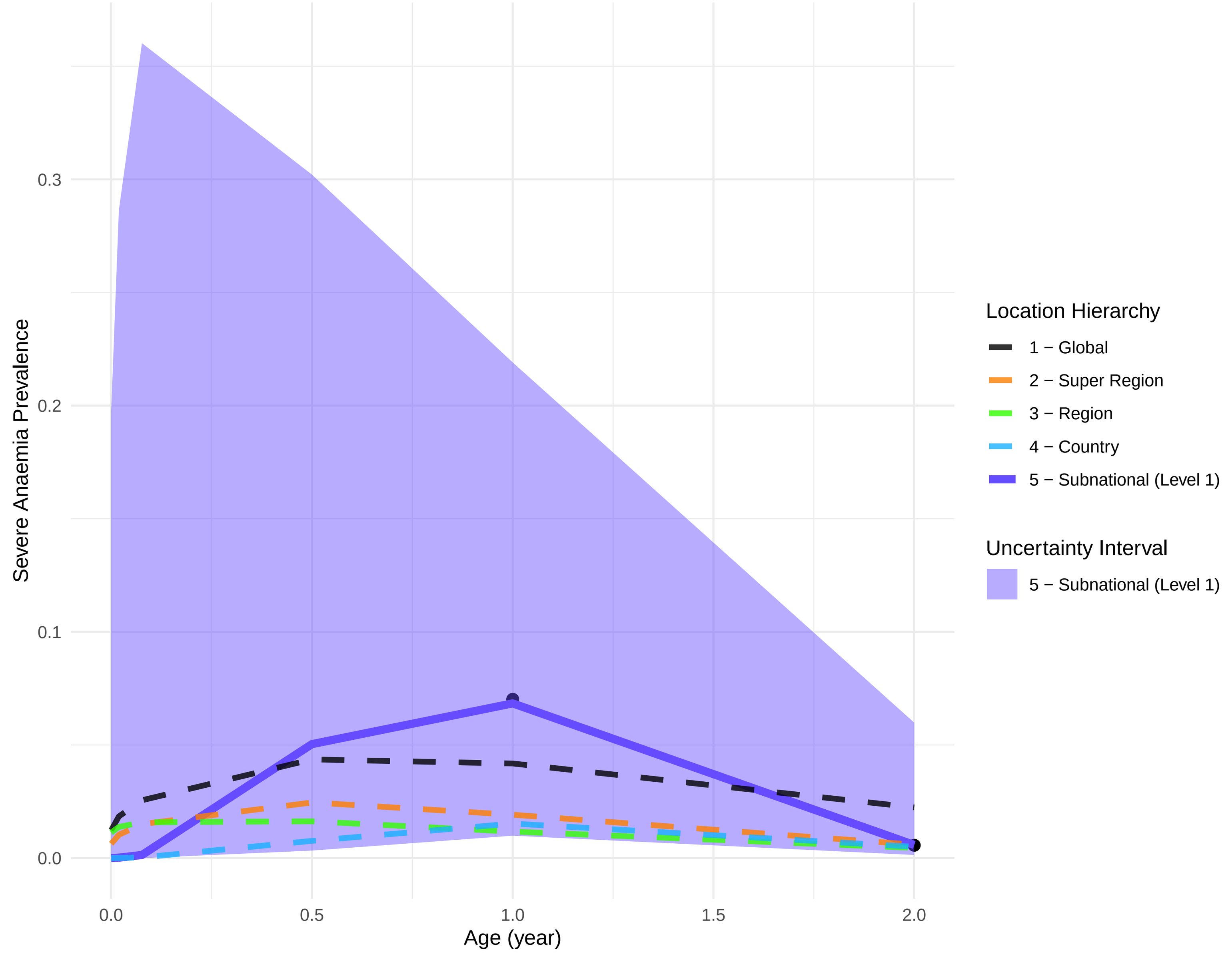

MR−BRT Age/Sex Fits − Males <2 Years Old − Severe Anaemia Prevalence − Sinaloa
WHO−2024 Adjustment Method

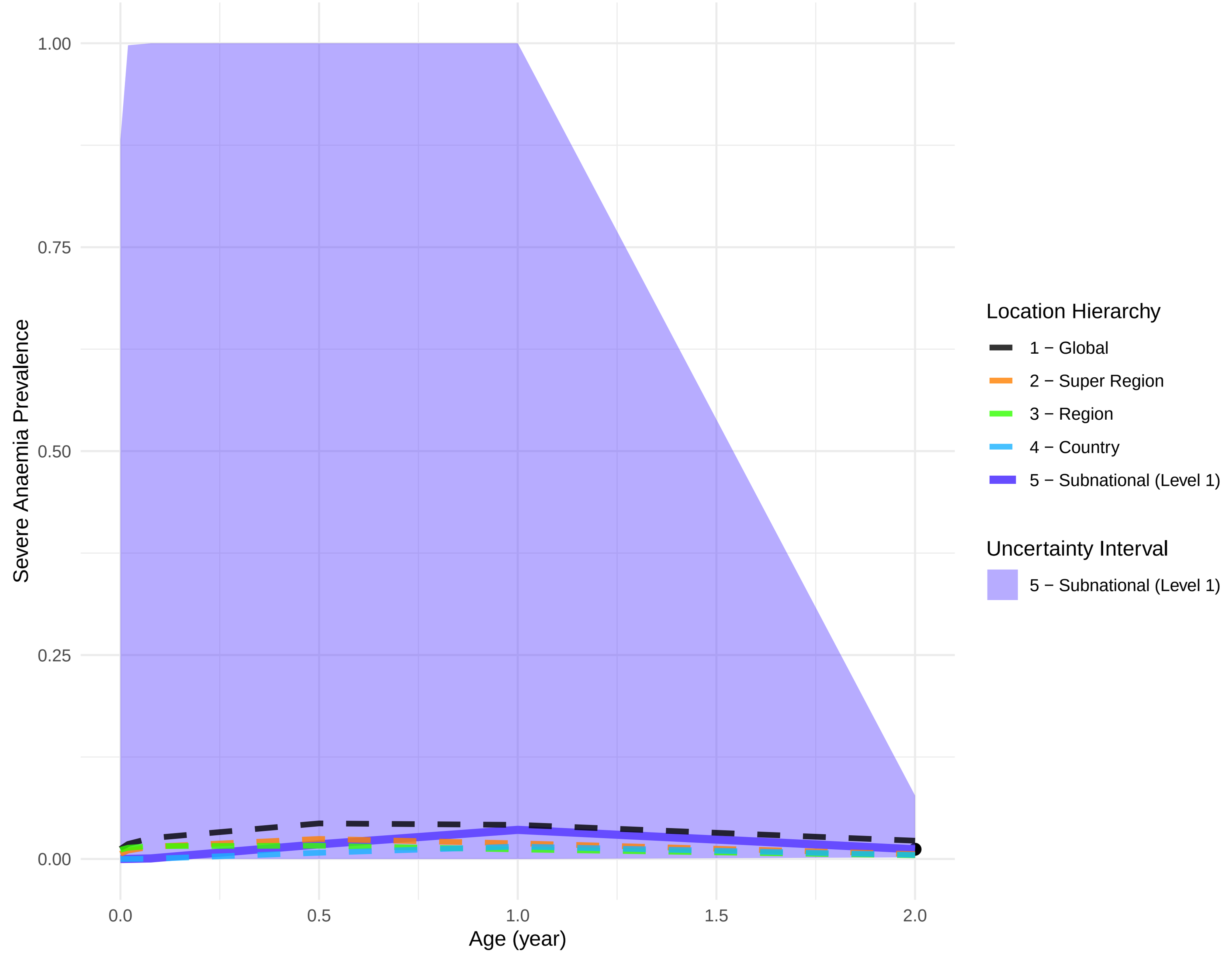

MR−BRT Age/Sex Fits − Males <2 Years Old − Severe Anaemia Prevalence − Tlaxcala
WHO−2024 Adjustment Method

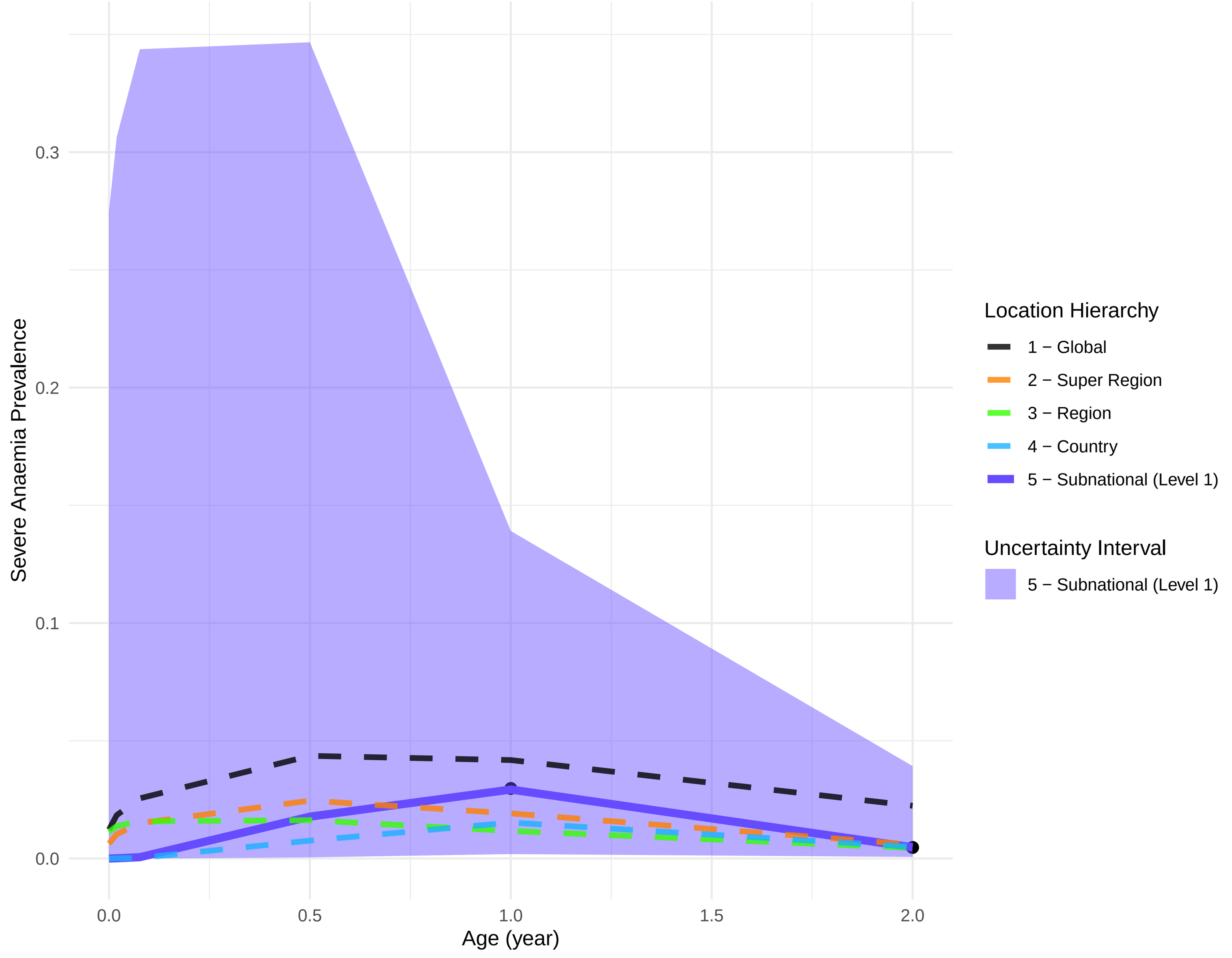

MR−BRT Age/Sex Fits − Males <2 Years Old − Severe Anaemia Prevalence − Veracruz de Ignacio de la Llave
WHO−2024 Adjustment Method

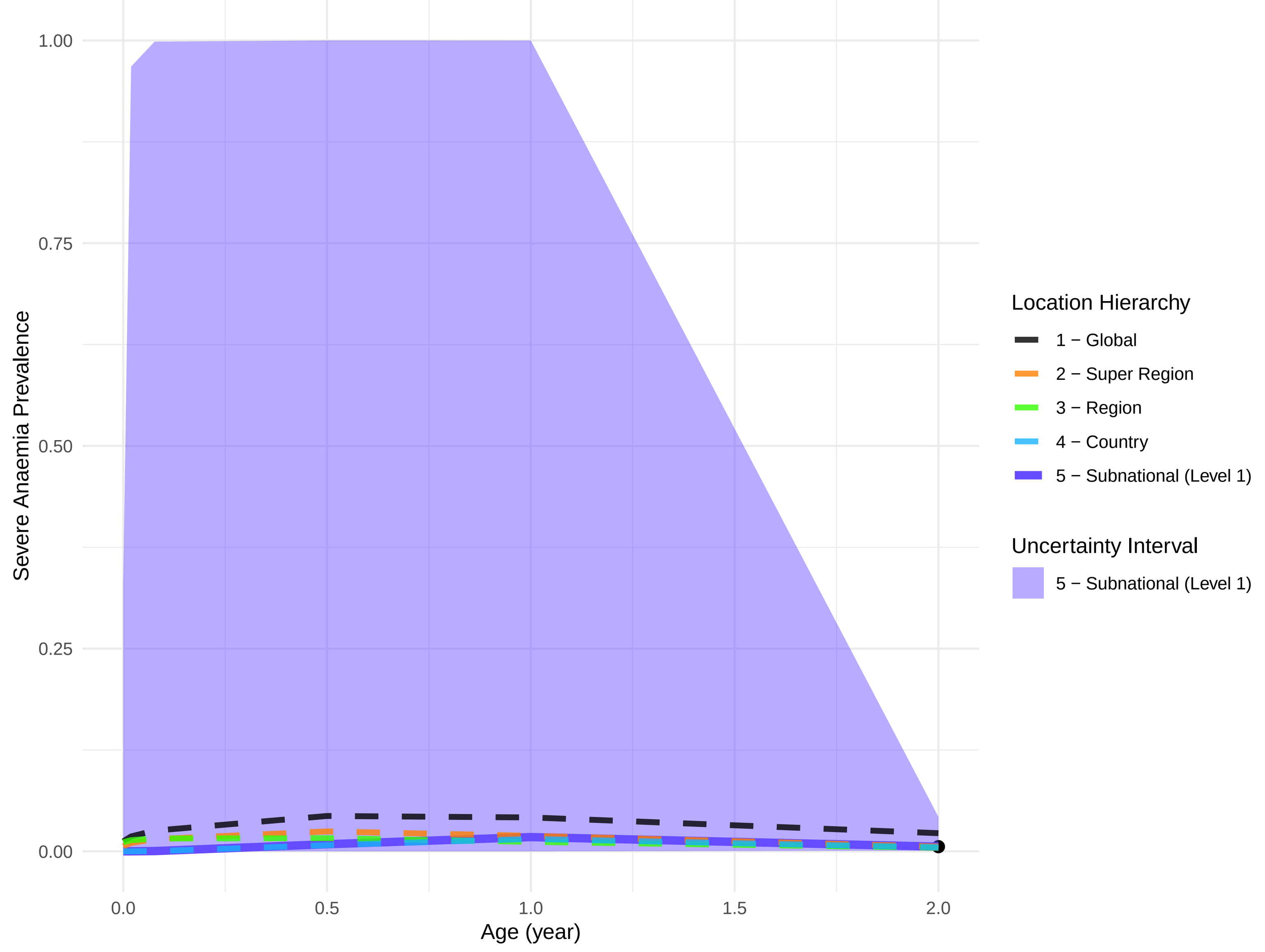

MR−BRT Age/Sex Fits − Males <2 Years Old − Severe Anaemia Prevalence − North Africa and Middle East
WHO−2024 Adjustment Method

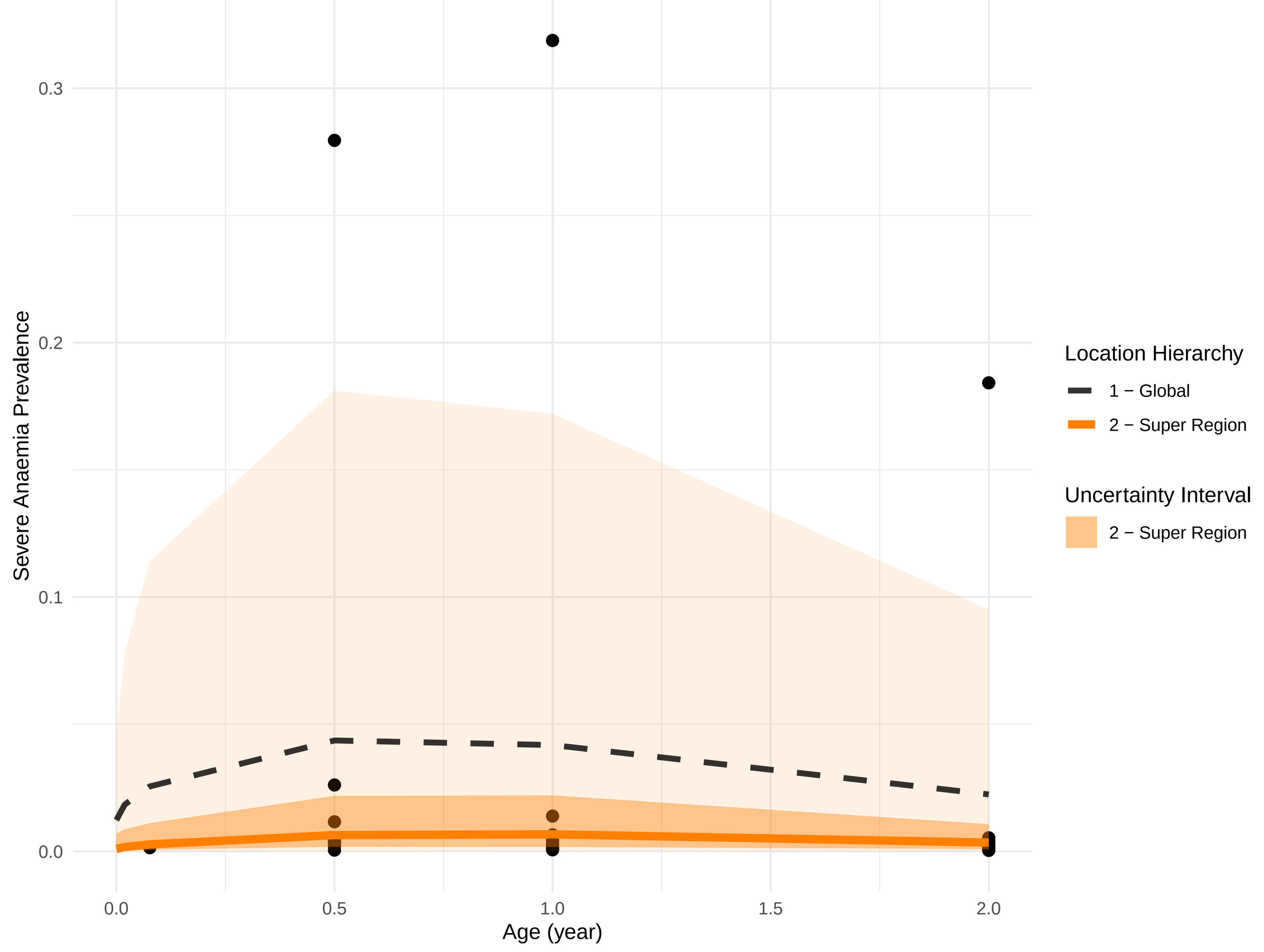

MR−BRT Age/Sex Fits − Males <2 Years Old − Severe Anaemia Prevalence − North Africa and Middle East
WHO−2024 Adjustment Method

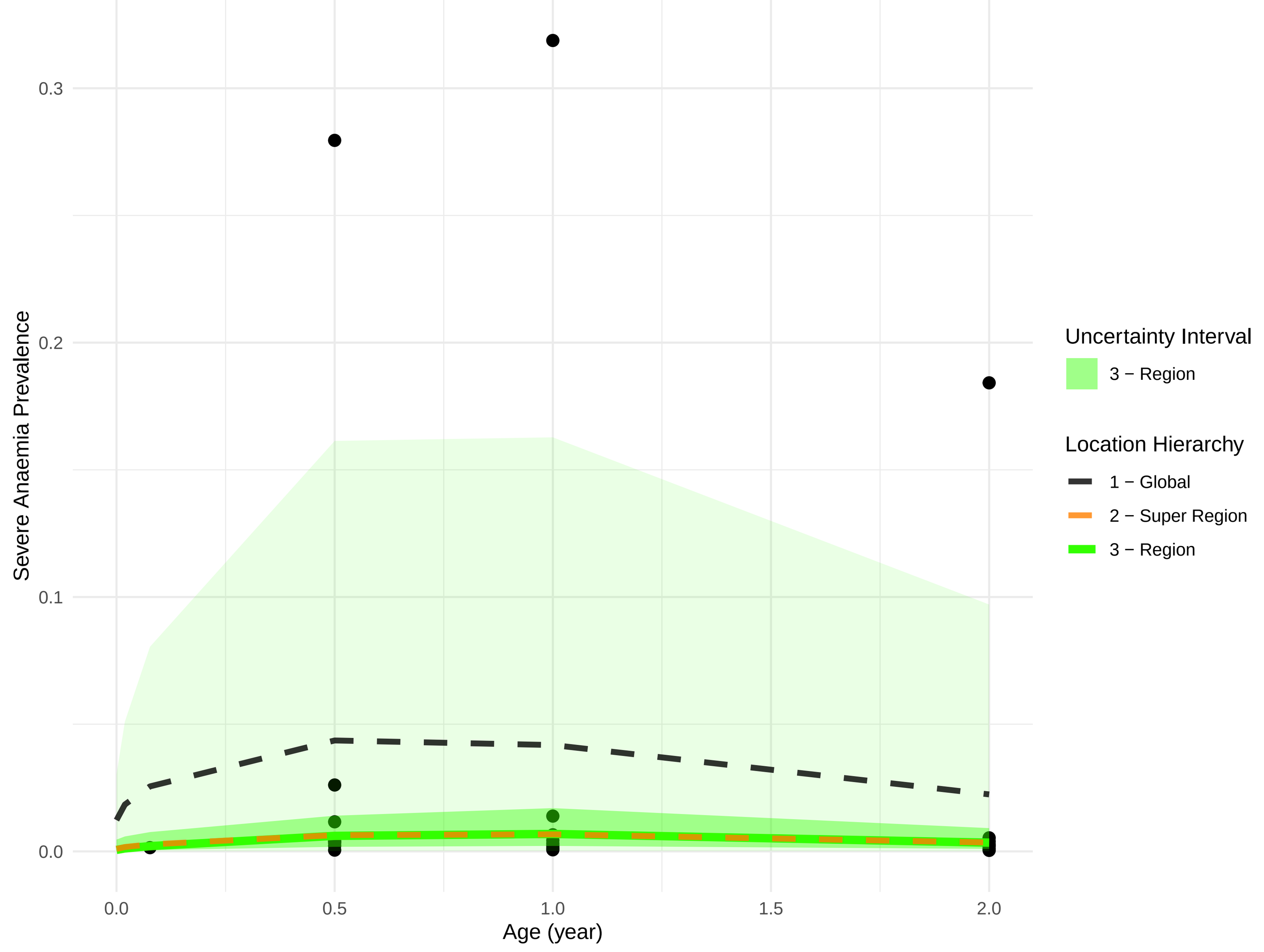

MR−BRT Age/Sex Fits − Males <2 Years Old − Severe Anaemia Prevalence − Egypt
WHO−2024 Adjustment Method

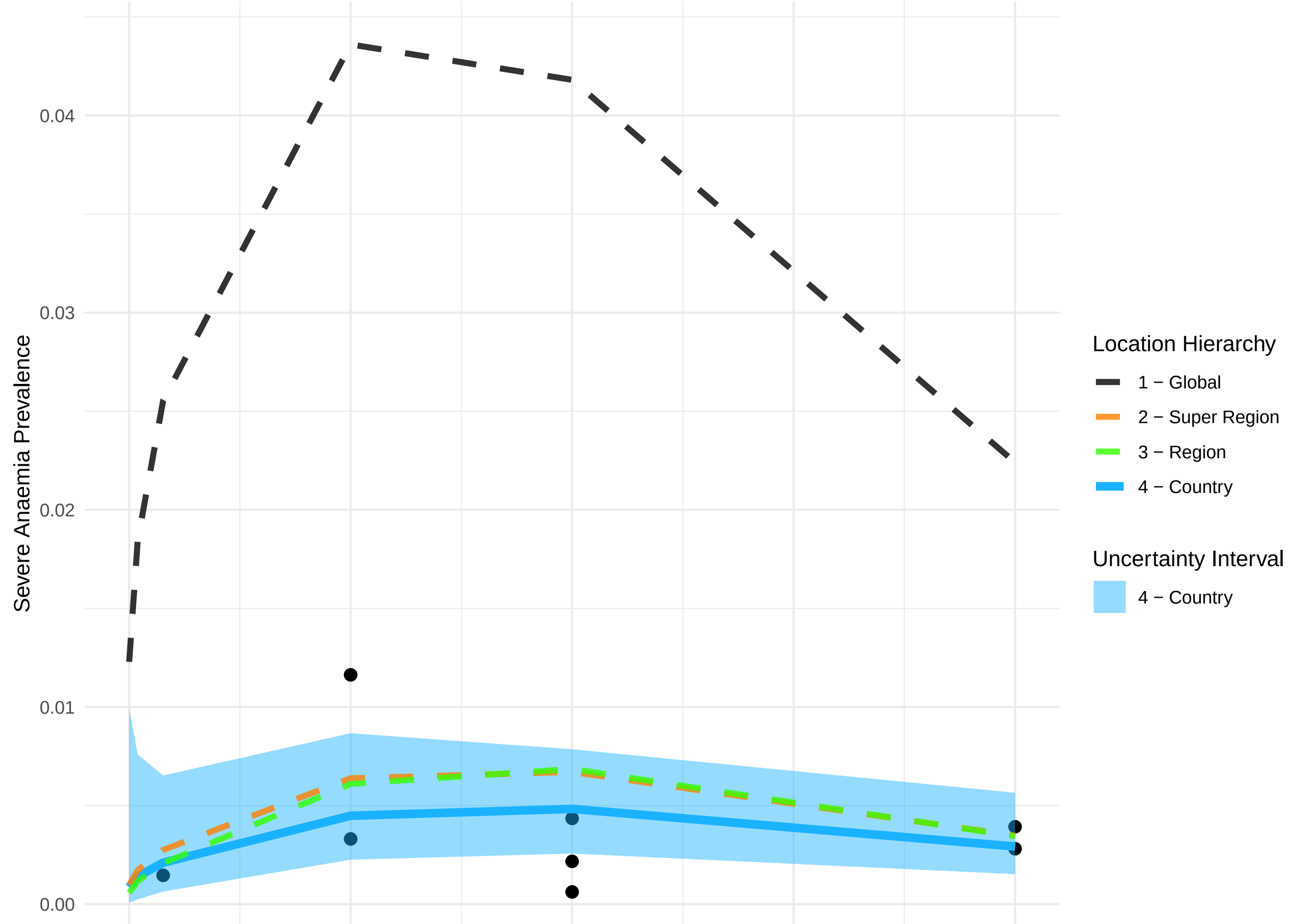

MR−BRT Age/Sex Fits − Males <2 Years Old − Severe Anaemia Prevalence − Jordan
WHO−2024 Adjustment Method

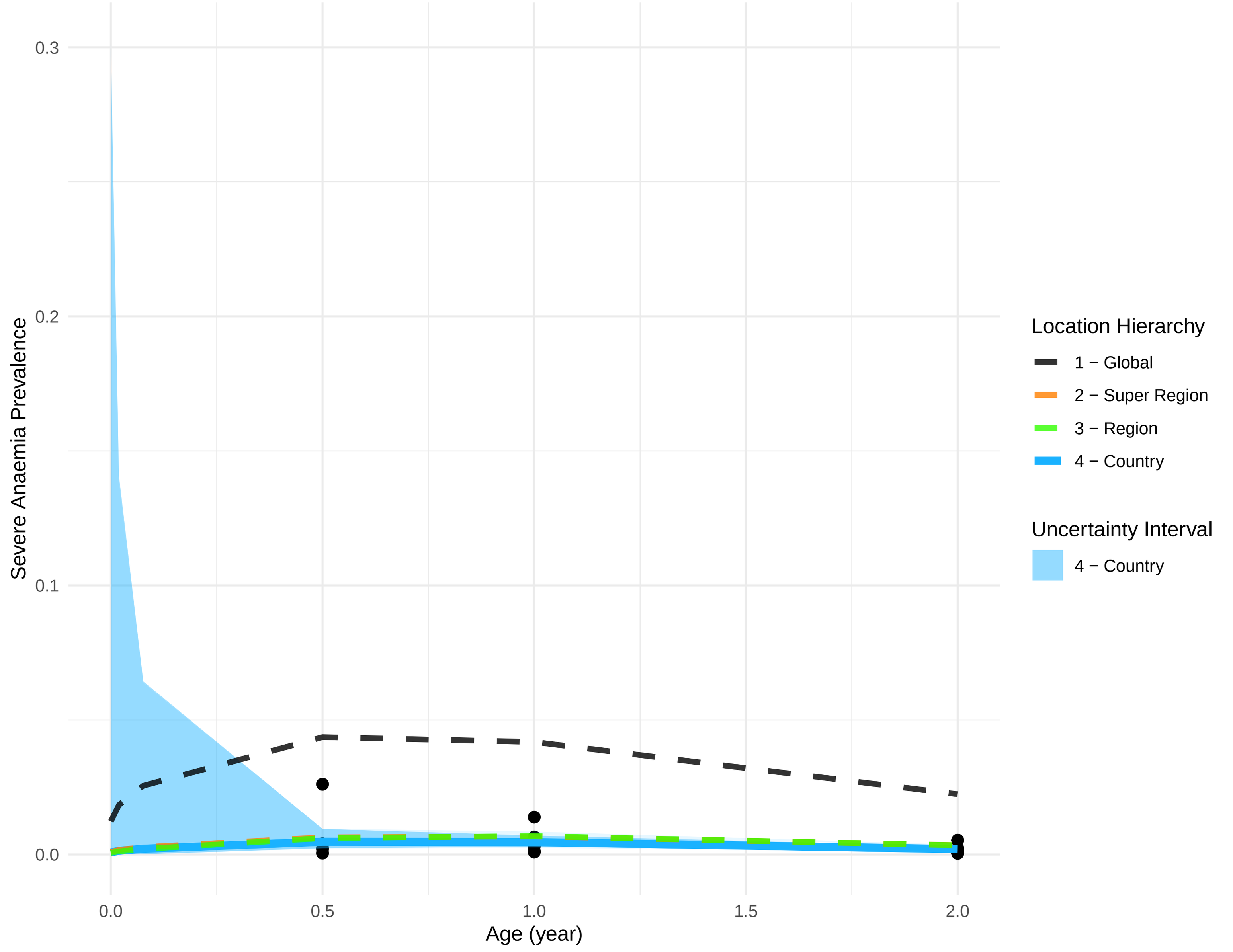

MR−BRT Age/Sex Fits − Males <2 Years Old − Severe Anaemia Prevalence − Yemen
WHO−2024 Adjustment Method

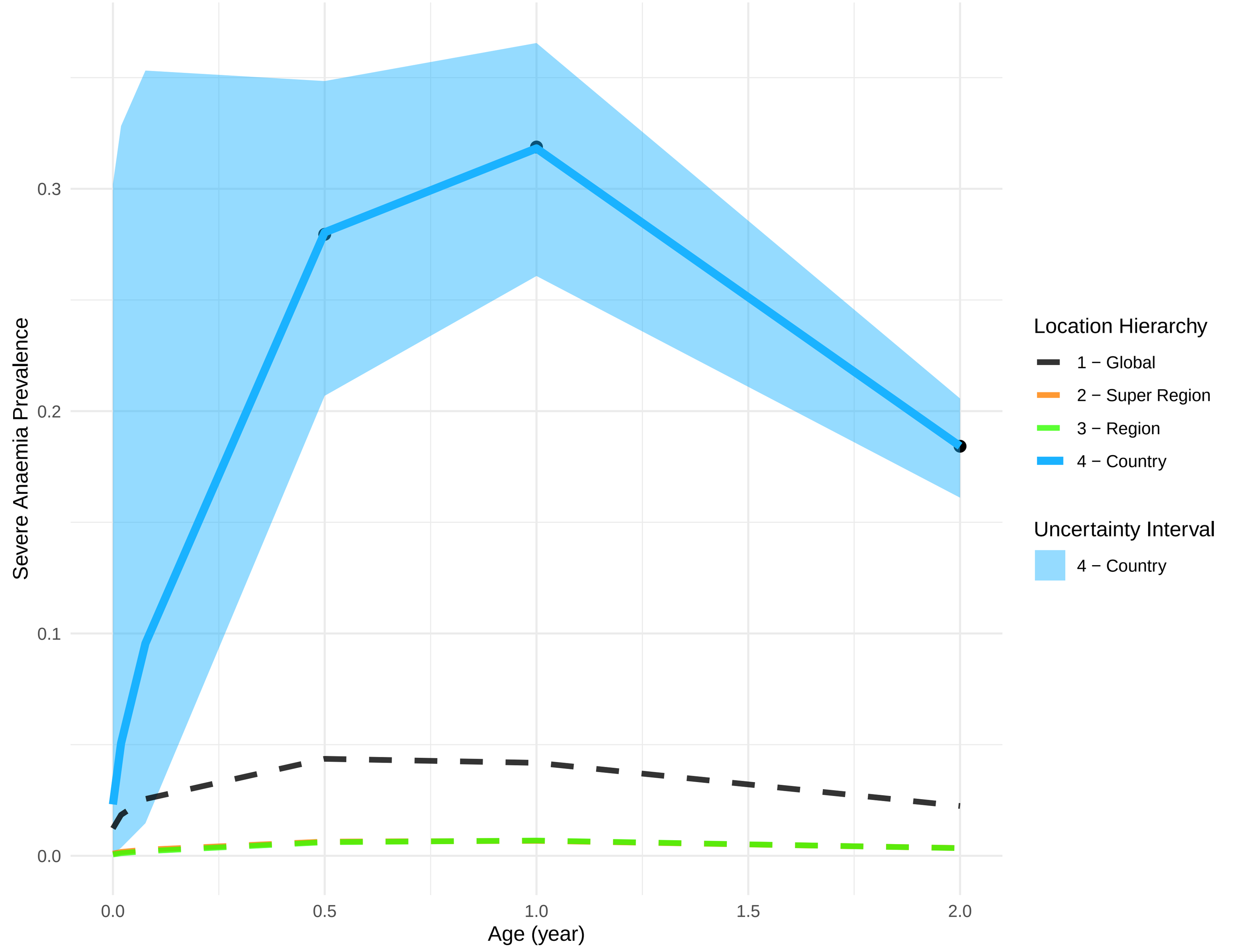

MR−BRT Age/Sex Fits − Males <2 Years Old − Severe Anaemia Prevalence − South Asia
WHO−2024 Adjustment Method

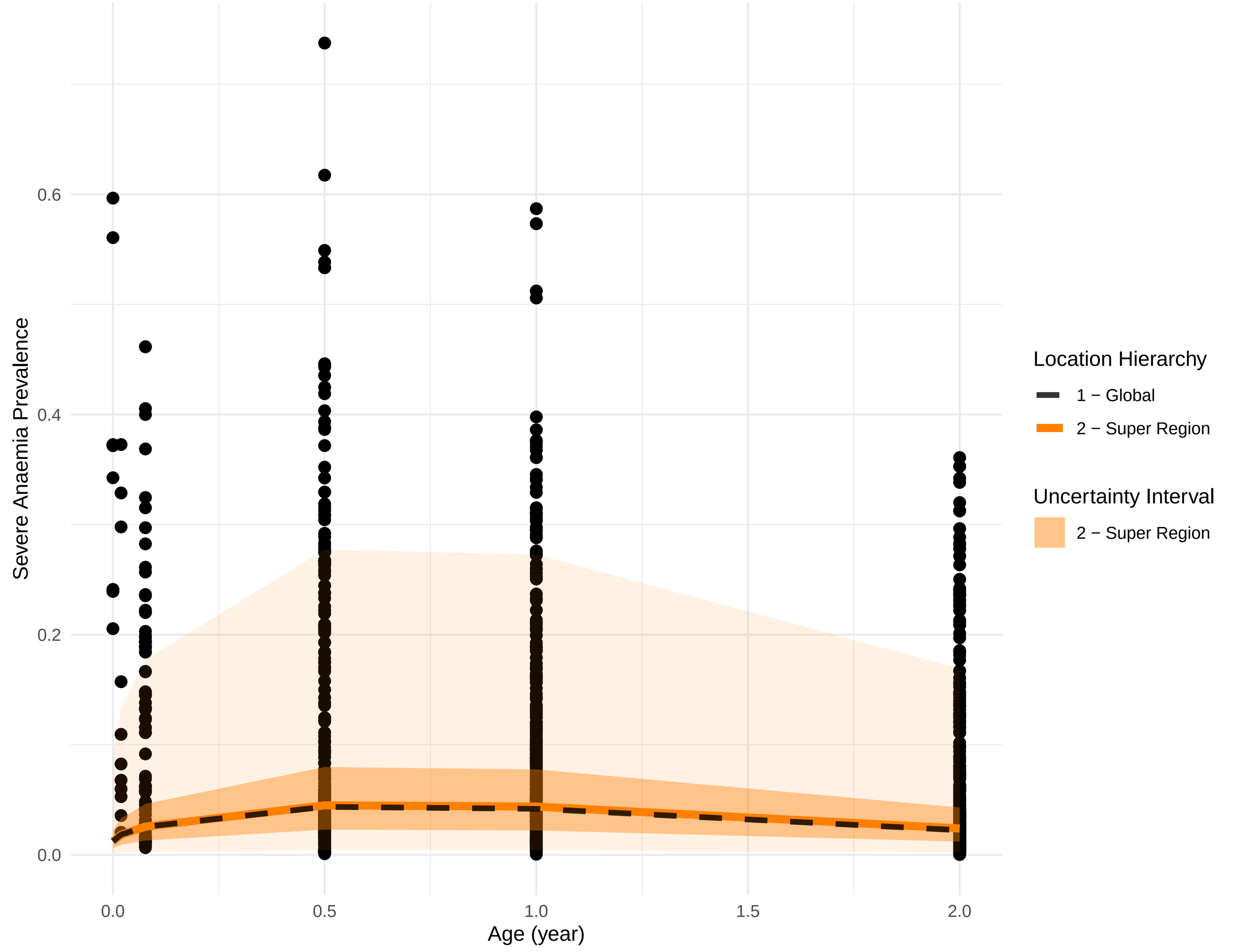

MR−BRT Age/Sex Fits − Males <2 Years Old − Severe Anaemia Prevalence − South Asia
WHO−2024 Adjustment Method

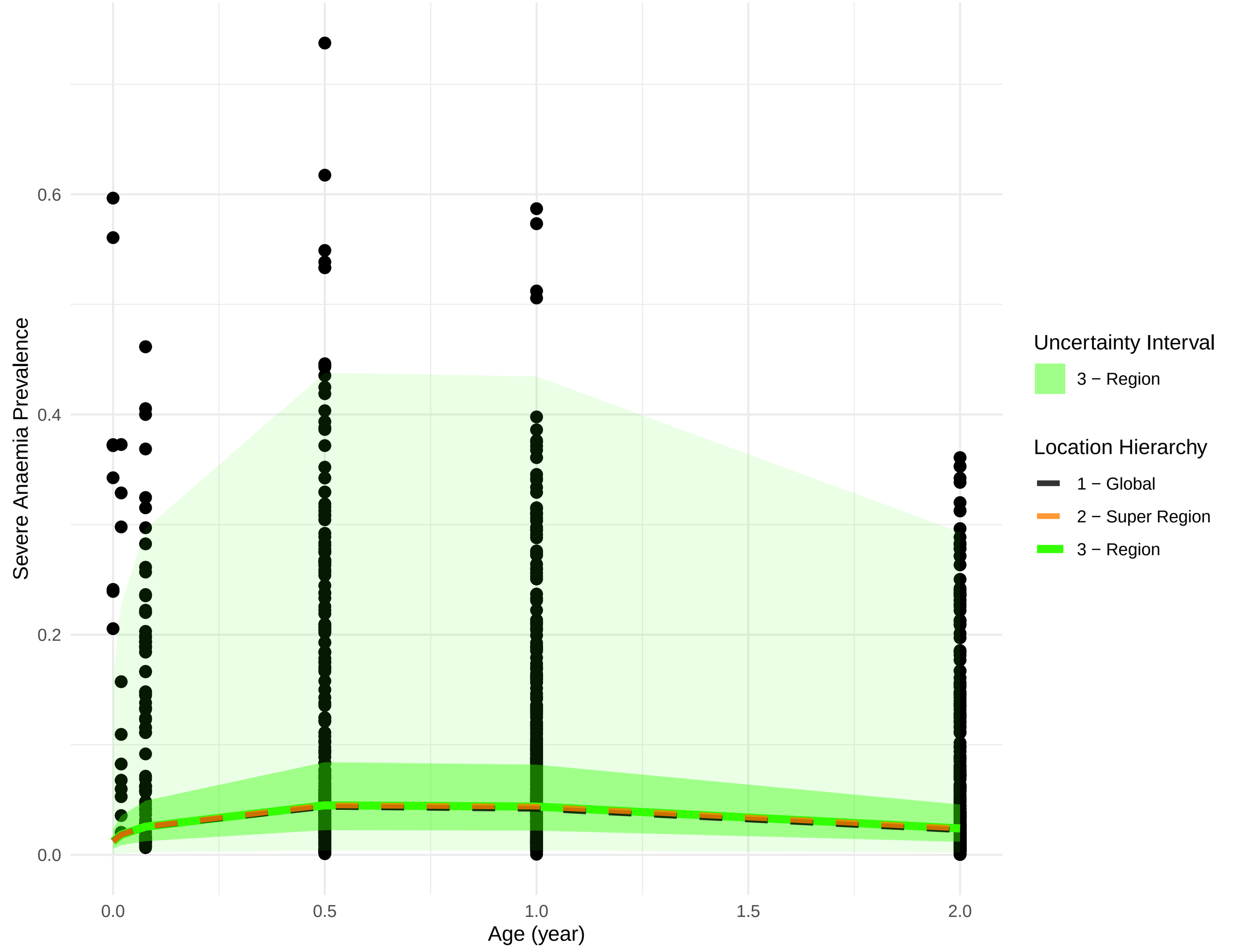

MR−BRT Age/Sex Fits − Males <2 Years Old − Severe Anaemia Prevalence − Bangladesh
WHO−2024 Adjustment Method

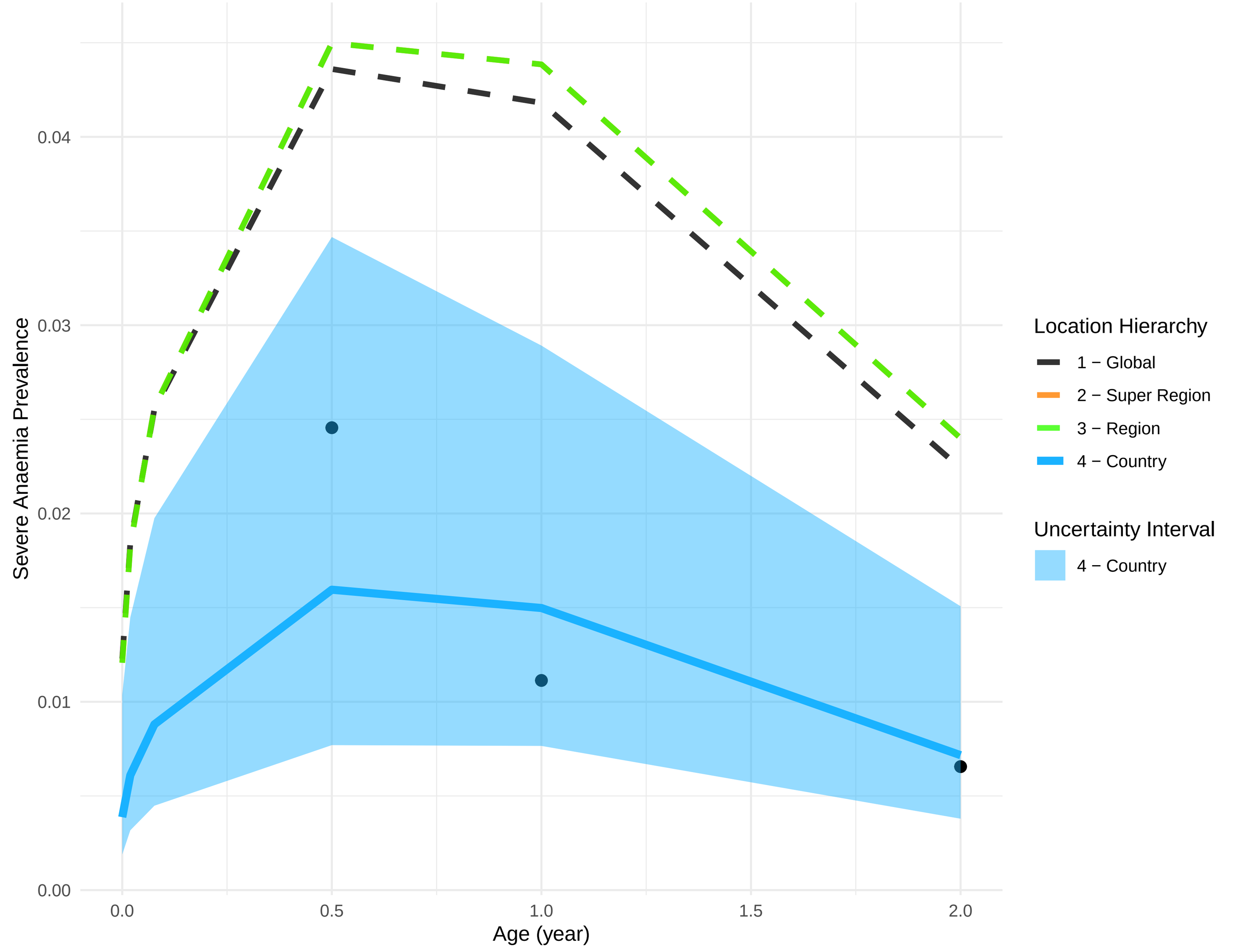

MR−BRT Age/Sex Fits − Males <2 Years Old − Severe Anaemia Prevalence − India
WHO−2024 Adjustment Method

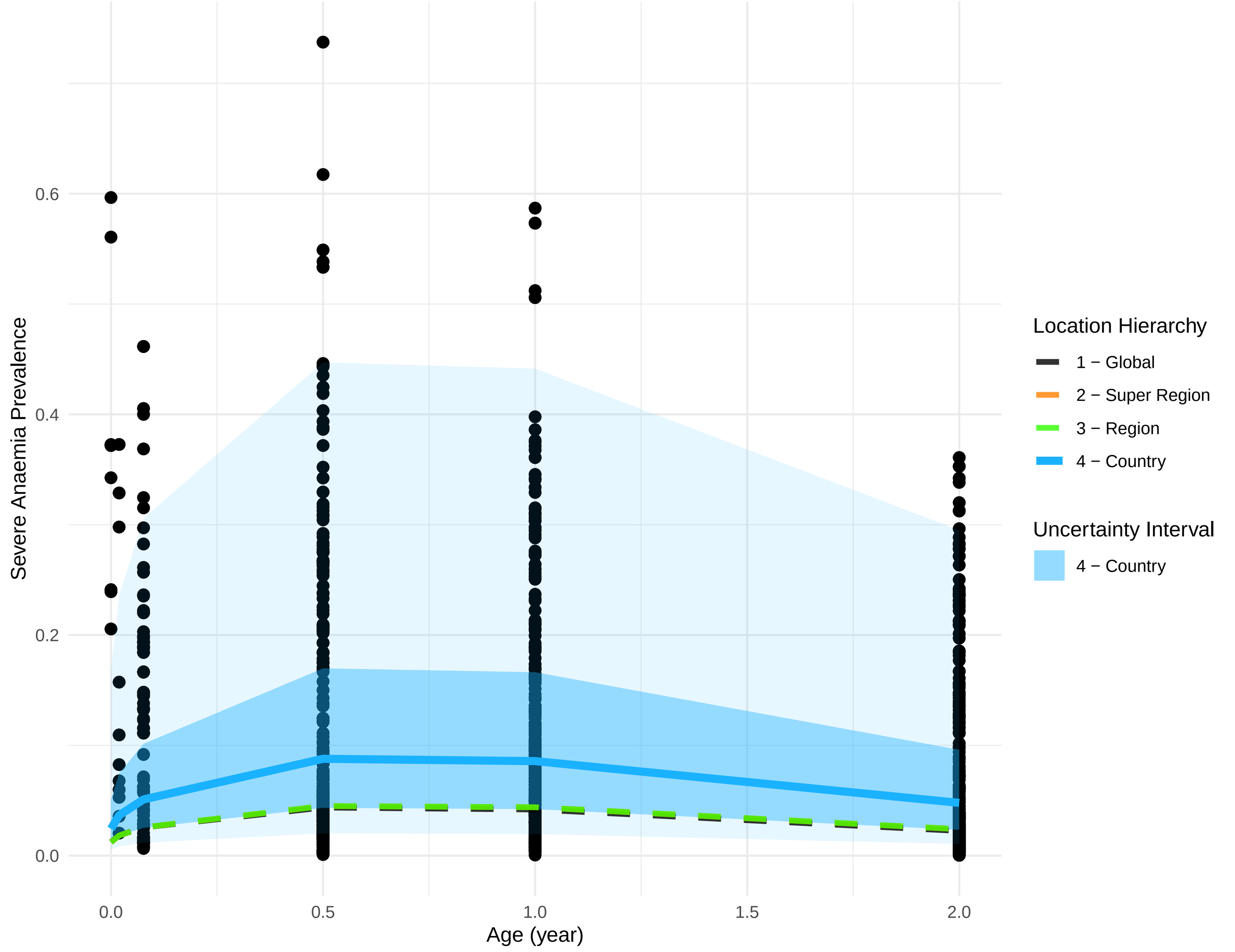

MR−BRT Age/Sex Fits − Males <2 Years Old − Severe Anaemia Prevalence − Andhra Pradesh
WHO−2024 Adjustment Method

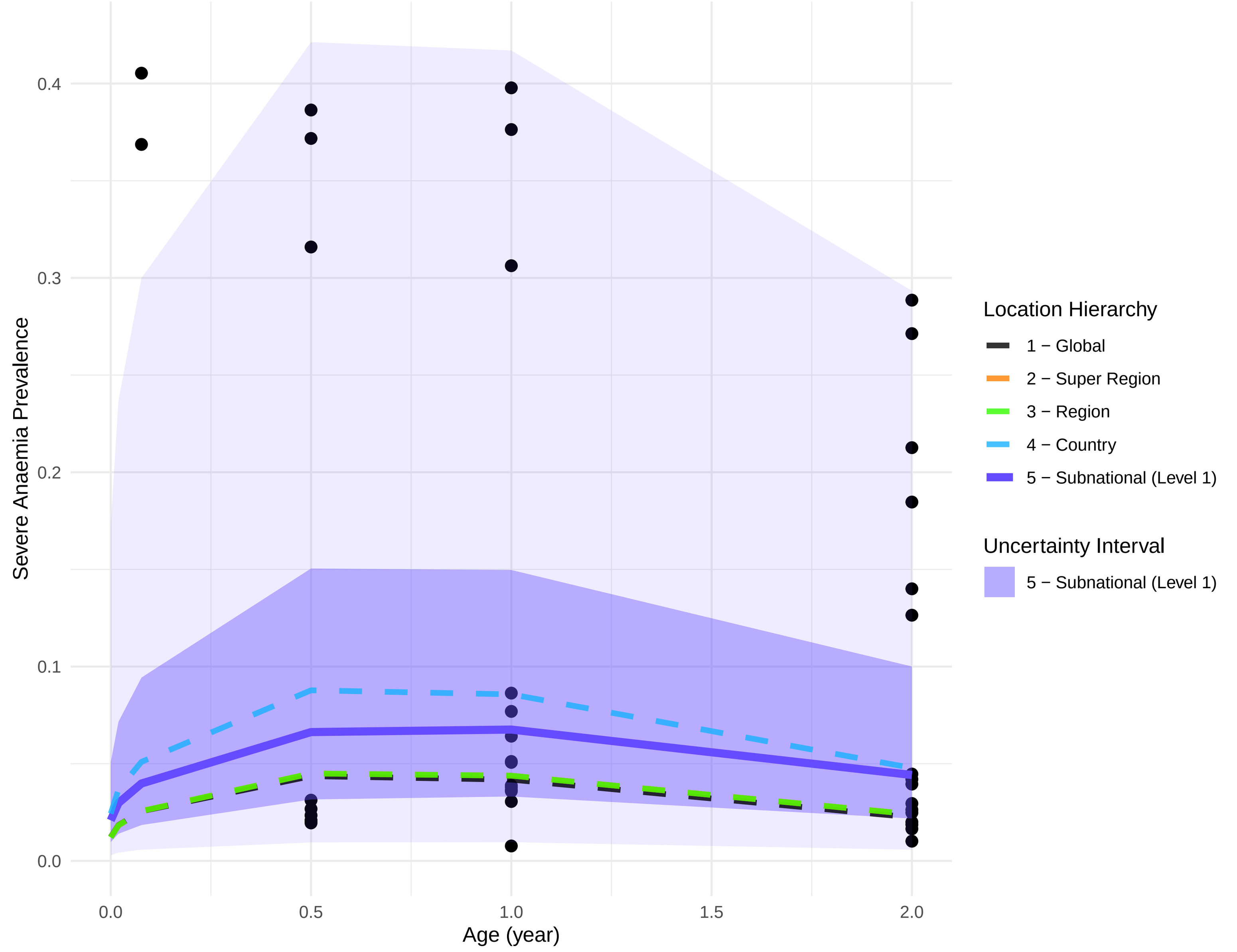

MR−BRT Age/Sex Fits − Males <2 Years Old − Severe Anaemia Prevalence − Andhra Pradesh, Rural
WHO−2024 Adjustment Method

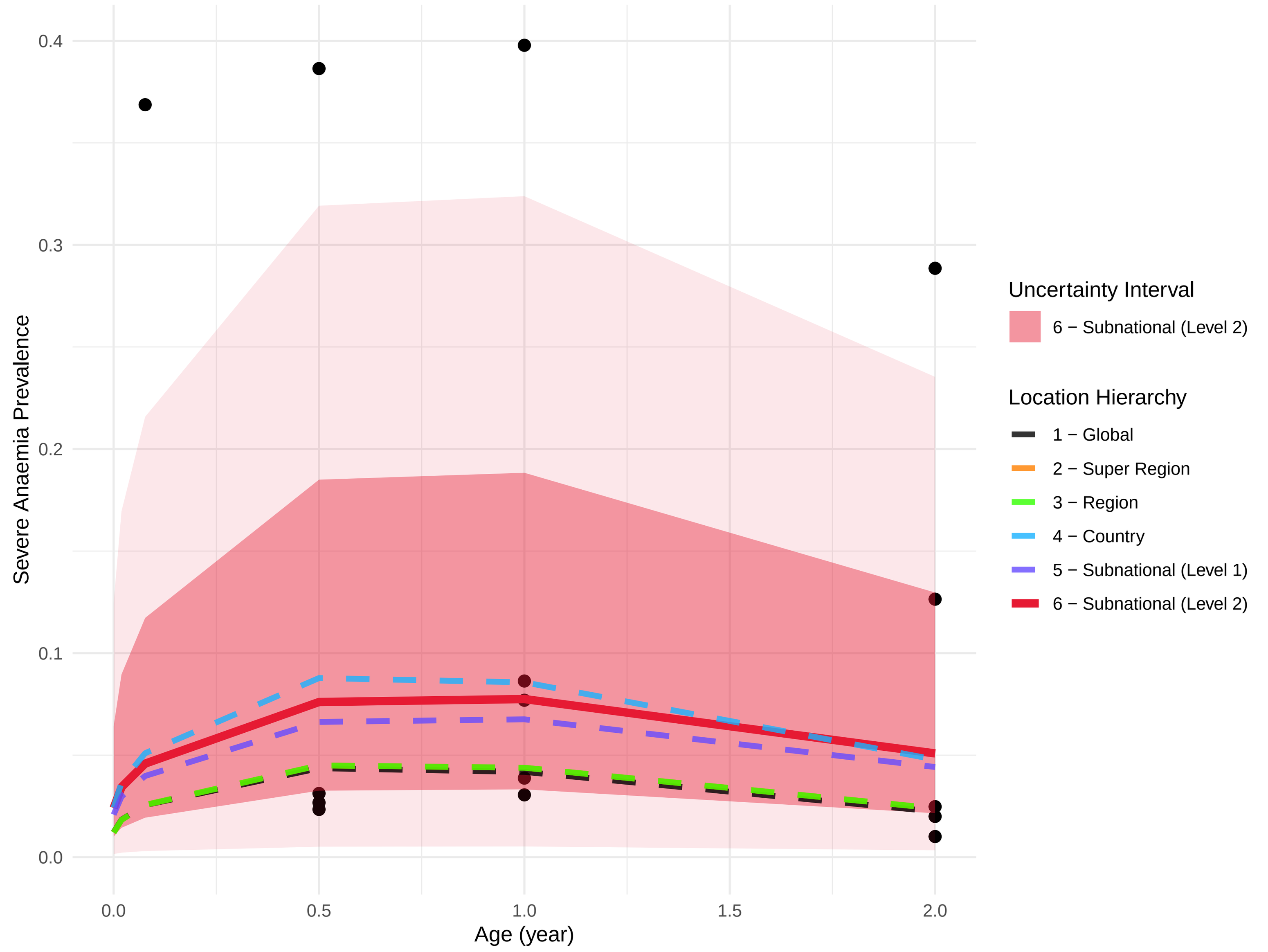

MR−BRT Age/Sex Fits − Males <2 Years Old − Severe Anaemia Prevalence − Andhra Pradesh, Urban
WHO−2024 Adjustment Method

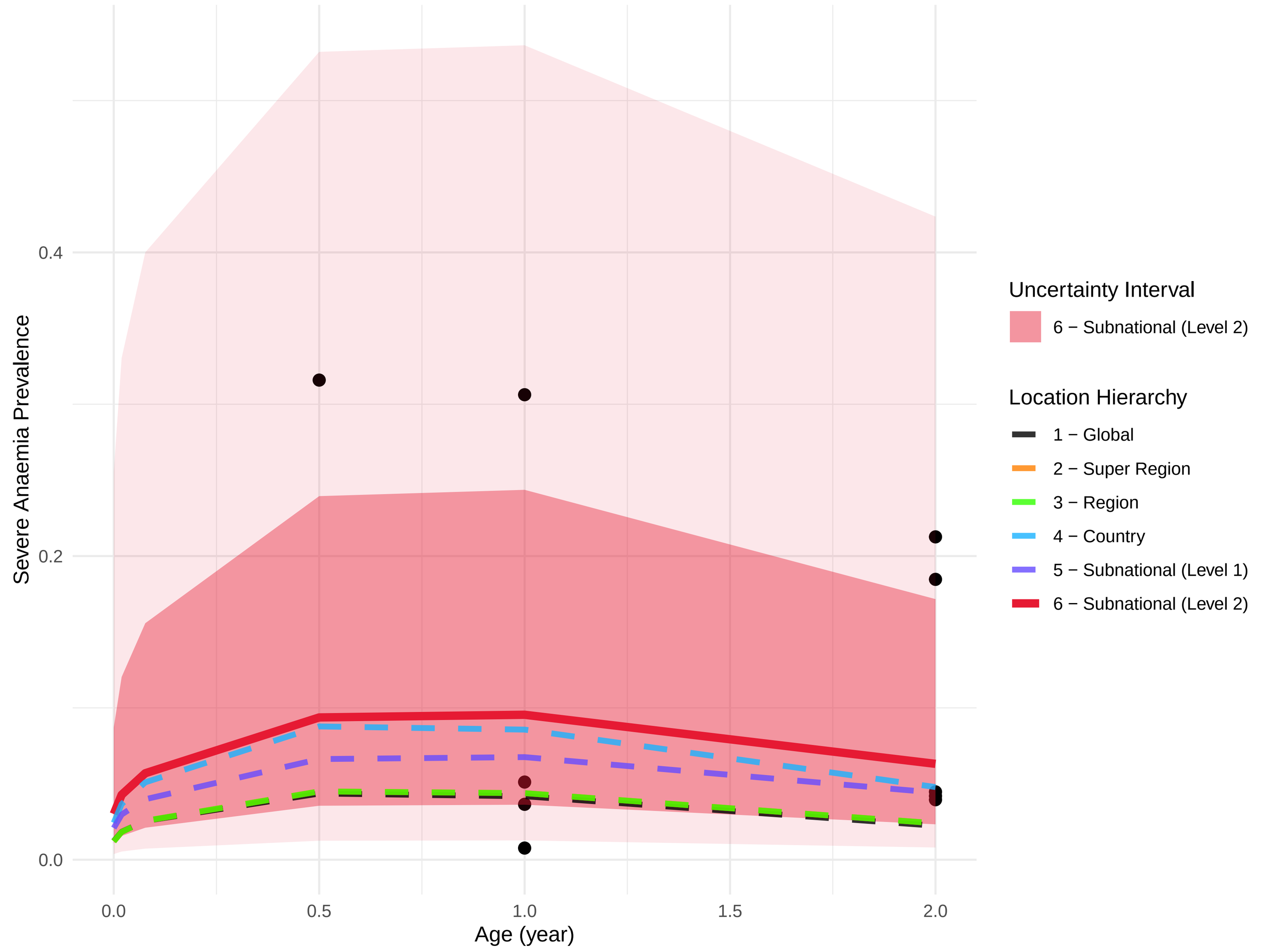

MR−BRT Age/Sex Fits − Males <2 Years Old − Severe Anaemia Prevalence − Arunachal Pradesh
WHO−2024 Adjustment Method

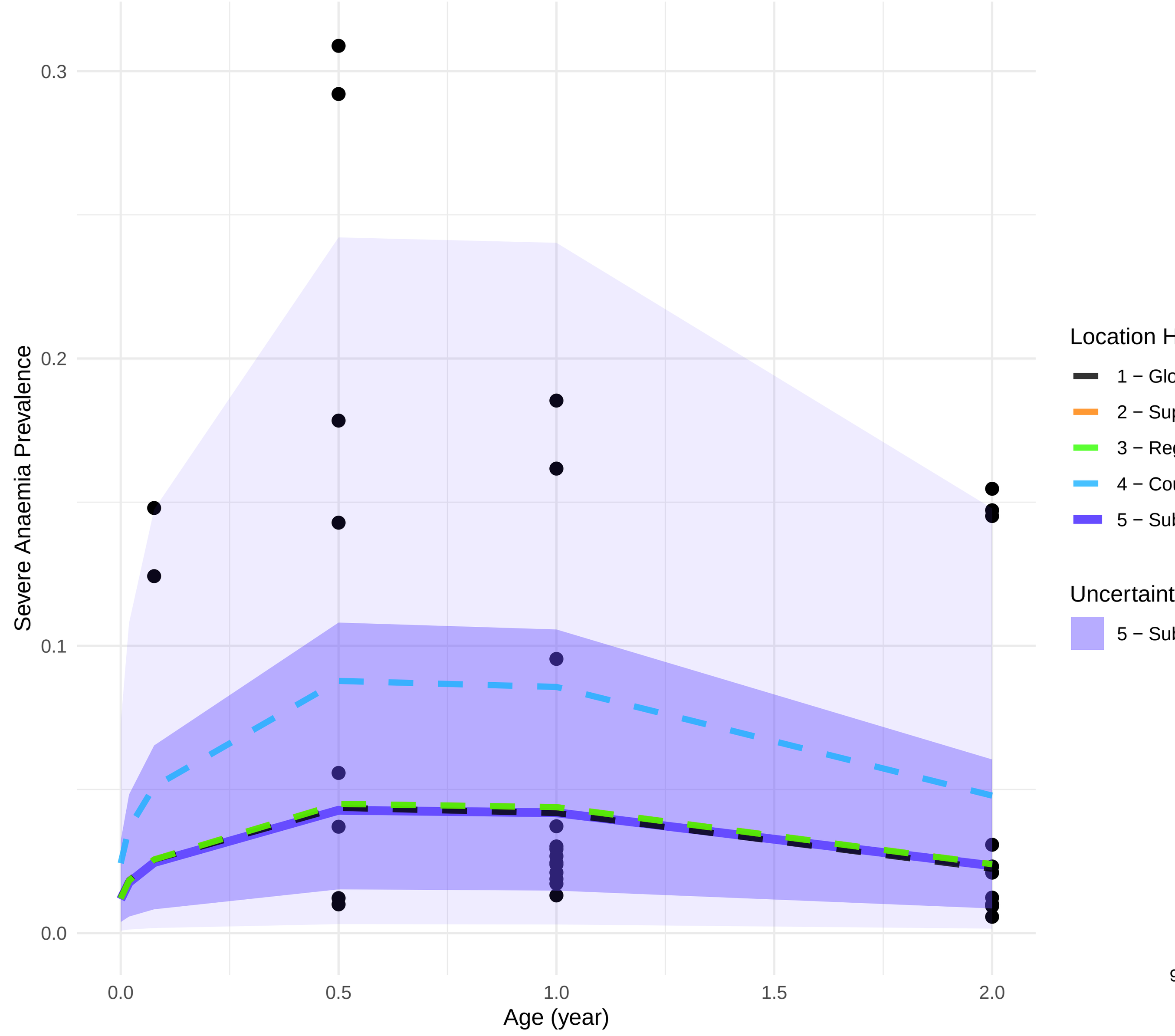

MR−BRT Age/Sex Fits − Males <2 Years Old − Severe Anaemia Prevalence − Arunachal Pradesh, Rural
WHO−2024 Adjustment Method

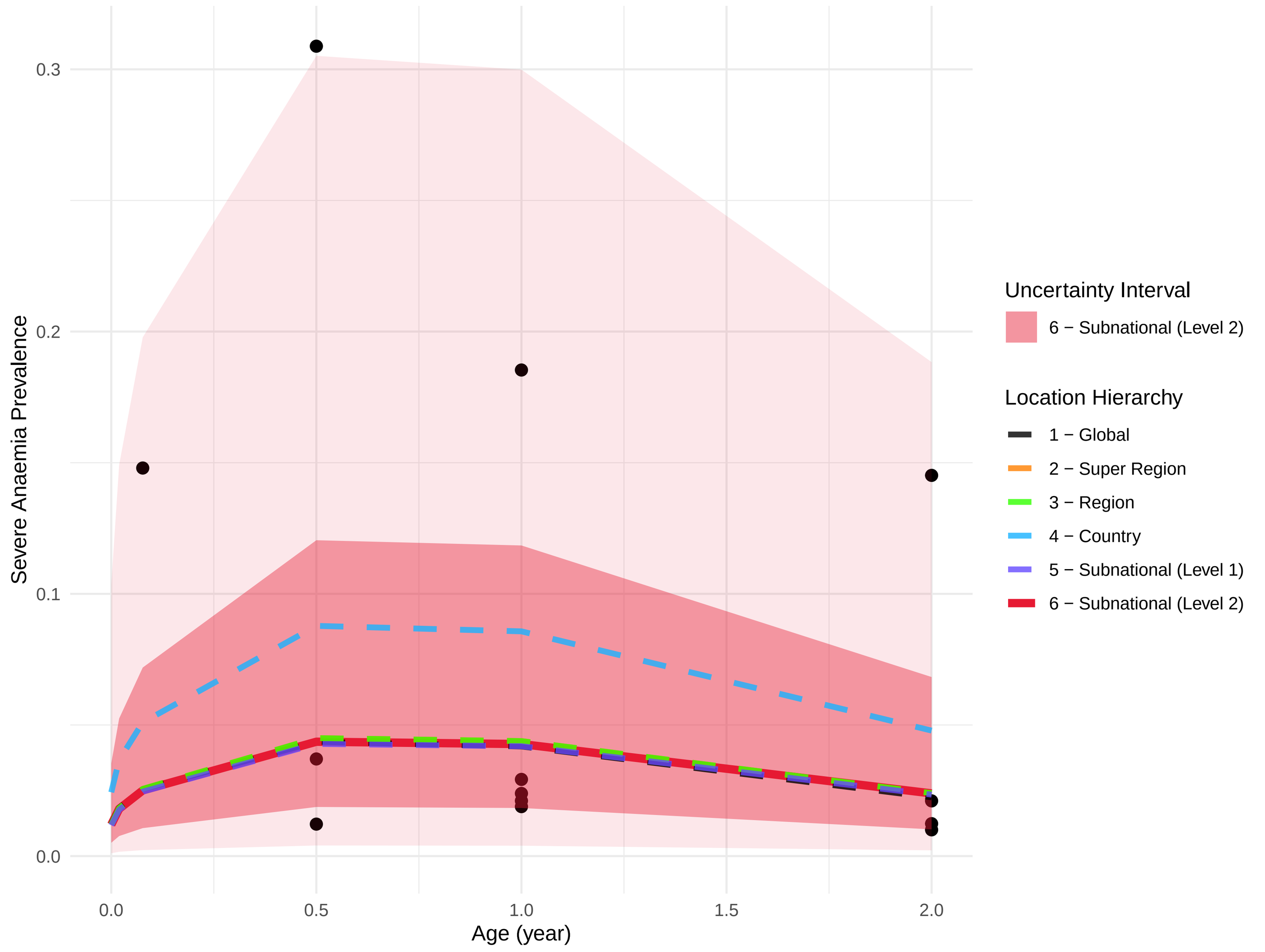

MR−BRT Age/Sex Fits − Males <2 Years Old − Severe Anaemia Prevalence − Arunachal Pradesh, Urban
WHO−2024 Adjustment Method

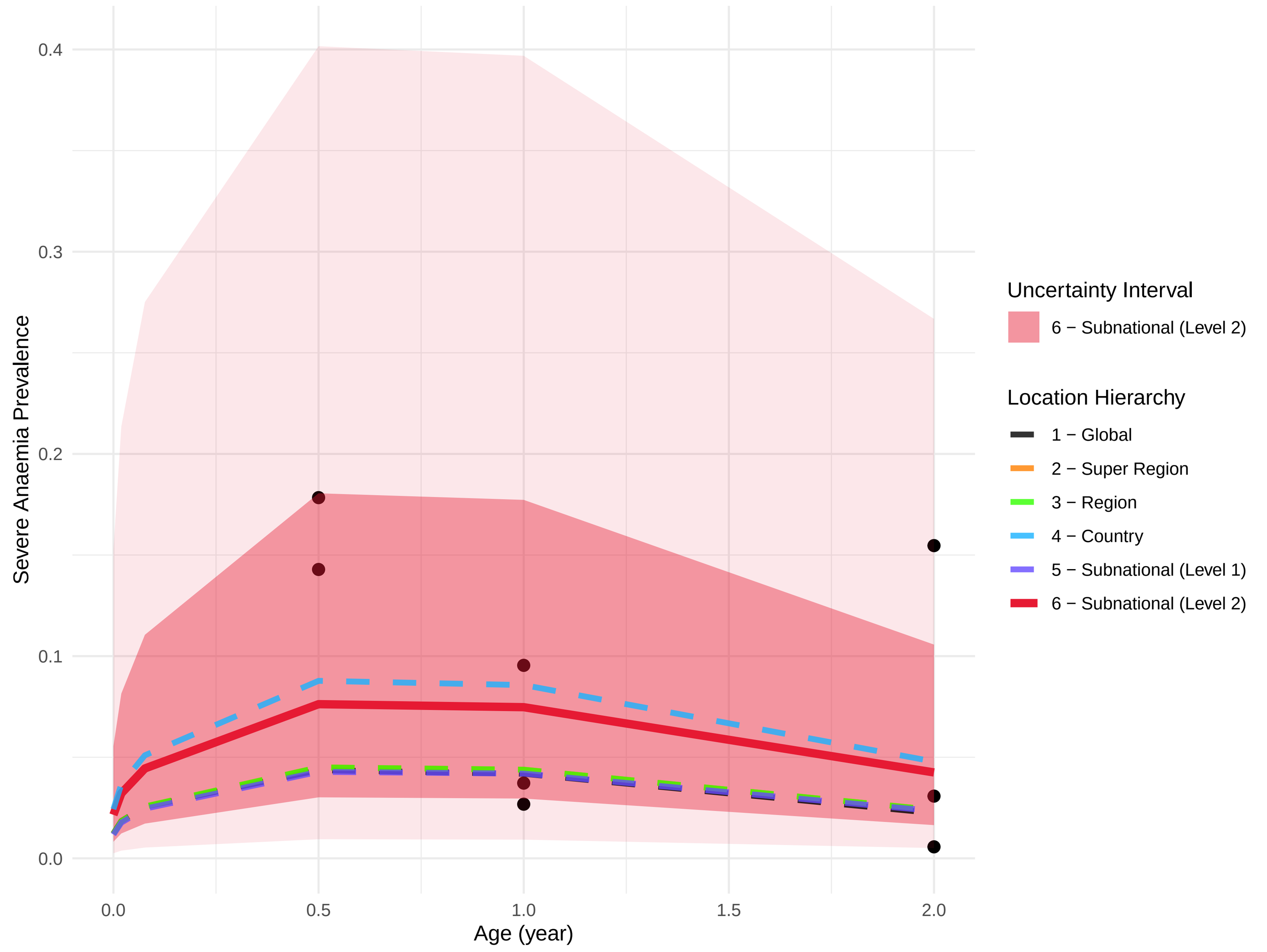

MR−BRT Age/Sex Fits − Males <2 Years Old − Severe Anaemia Prevalence − Assam
WHO−2024 Adjustment Method

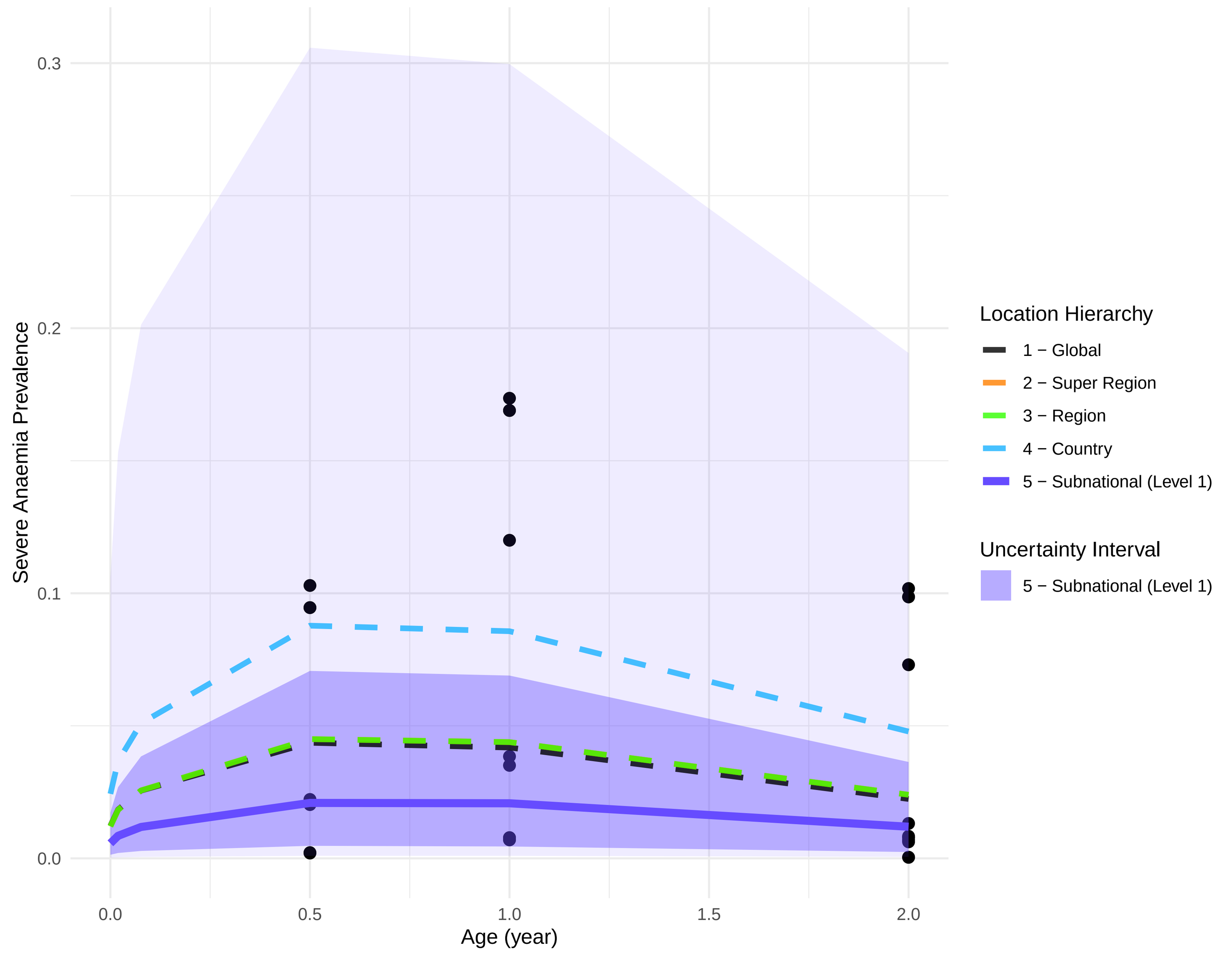

MR−BRT Age/Sex Fits − Males <2 Years Old − Severe Anaemia Prevalence − Assam, Rural
WHO−2024 Adjustment Method

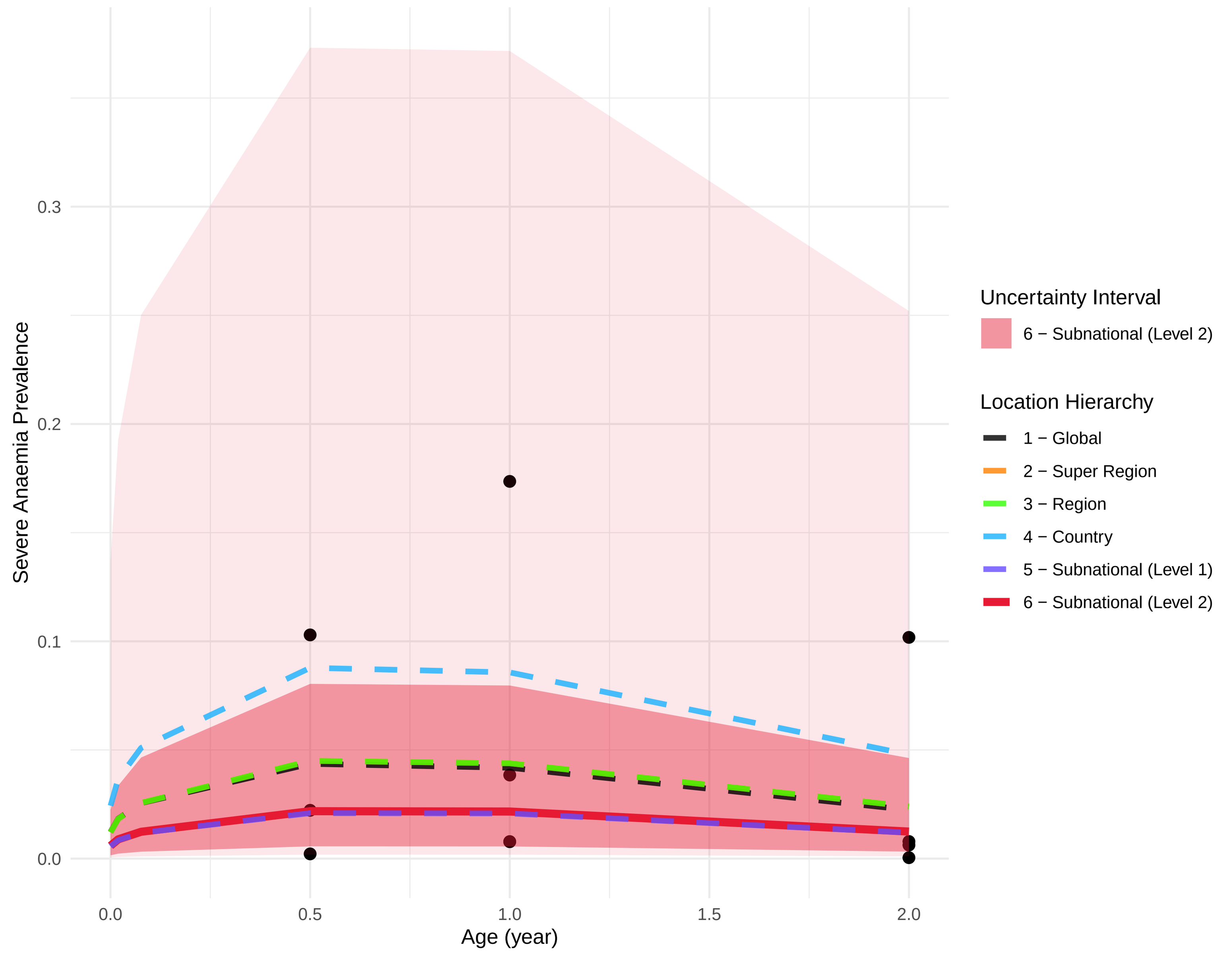

MR−BRT Age/Sex Fits − Males <2 Years Old − Severe Anaemia Prevalence − Assam, Urban
WHO−2024 Adjustment Method

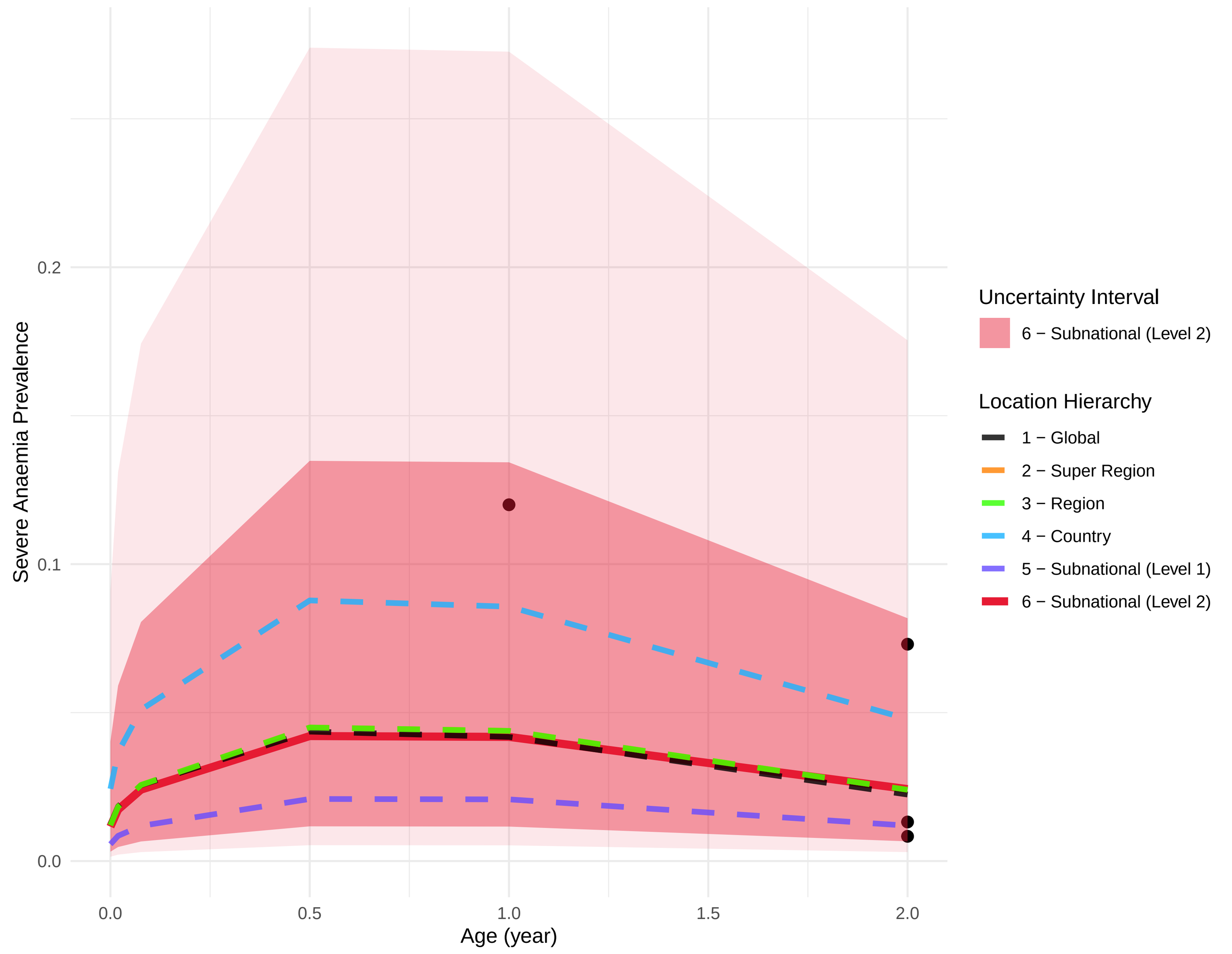

MR−BRT Age/Sex Fits − Males <2 Years Old − Severe Anaemia Prevalence − Bihar
WHO−2024 Adjustment Method

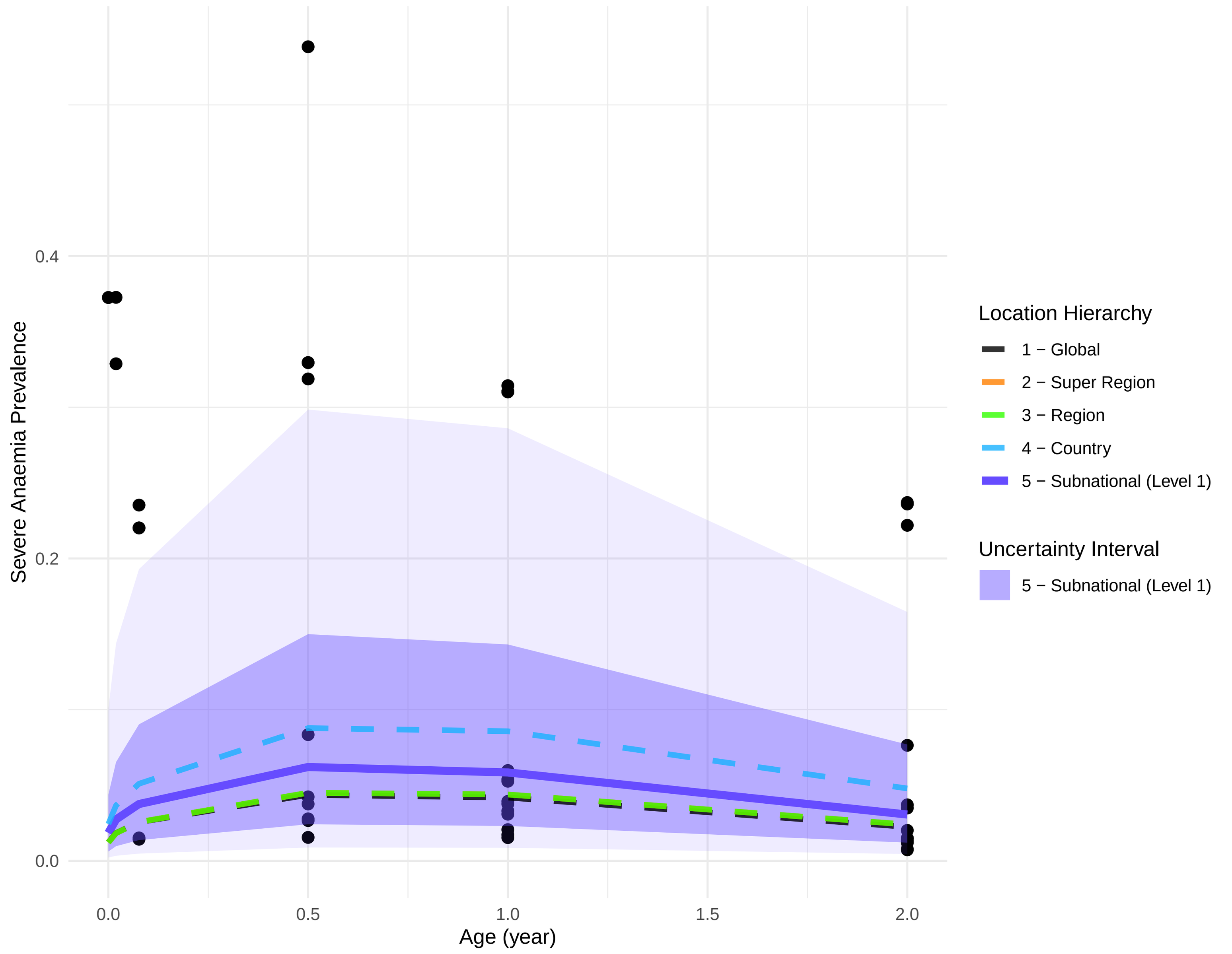

MR−BRT Age/Sex Fits − Males <2 Years Old − Severe Anaemia Prevalence − Bihar, Rural
WHO−2024 Adjustment Method

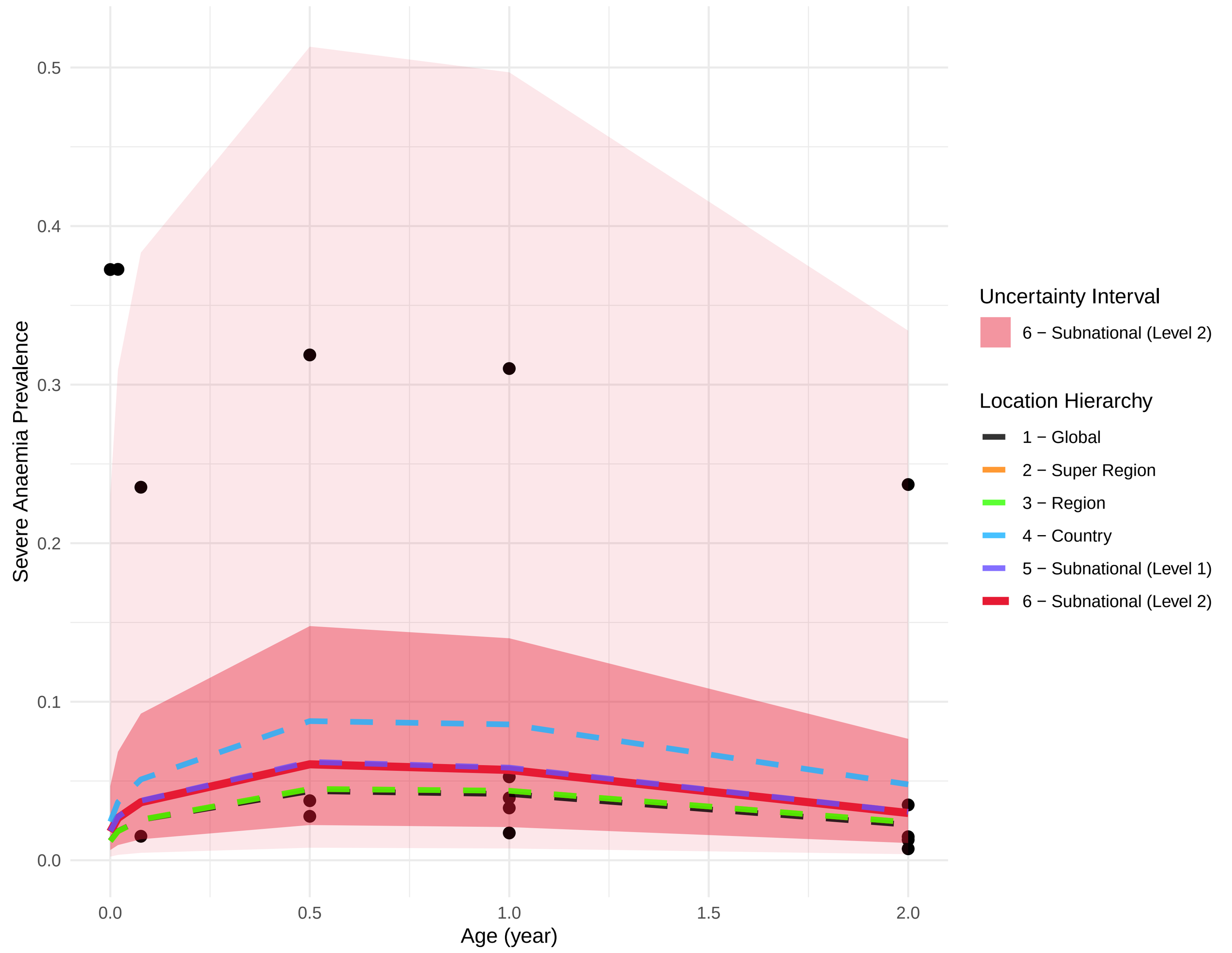

MR−BRT Age/Sex Fits − Males <2 Years Old − Severe Anaemia Prevalence − Bihar, Urban
WHO−2024 Adjustment Method

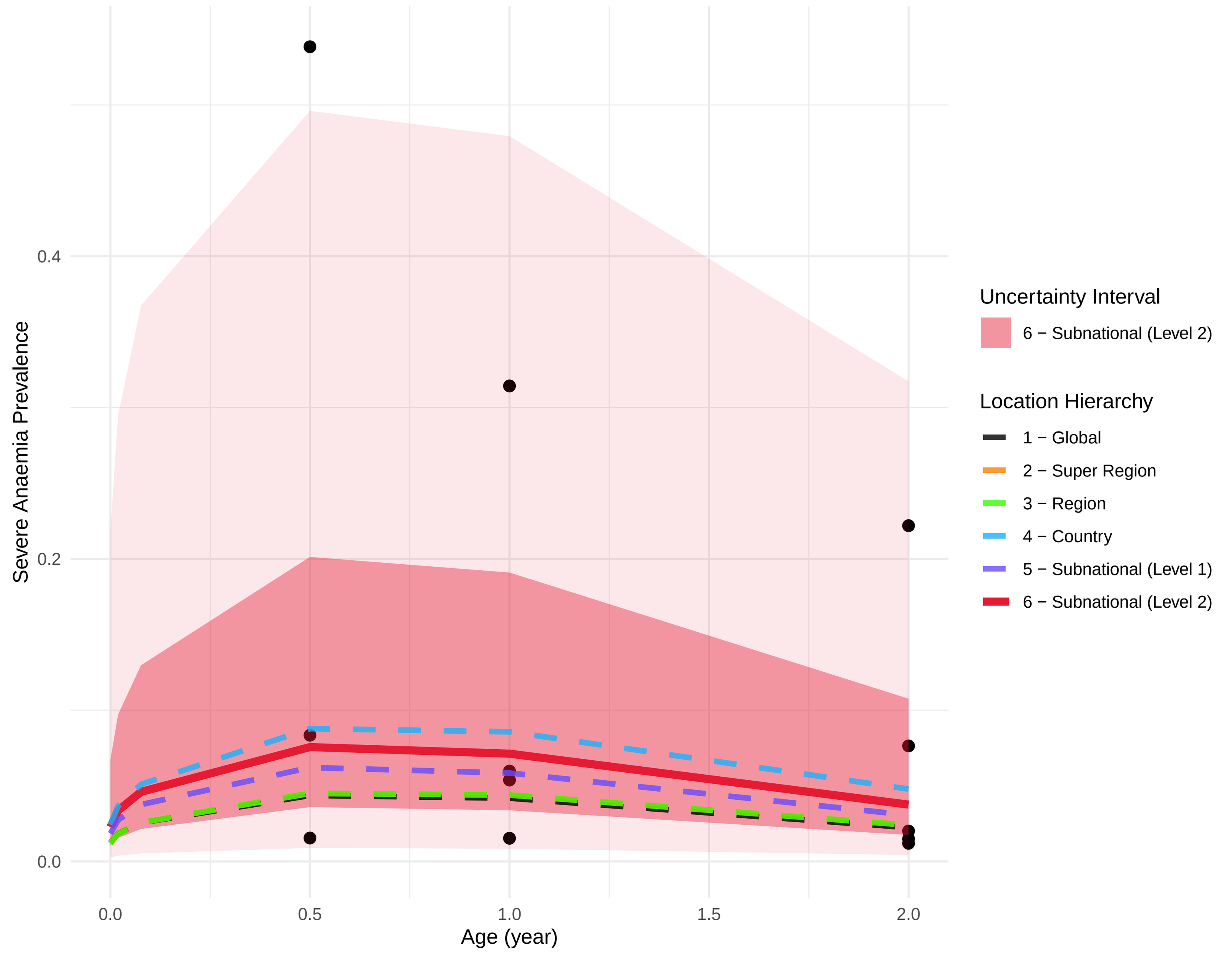

MR−BRT Age/Sex Fits − Males <2 Years Old − Severe Anaemia Prevalence − Chhattisgarh
WHO−2024 Adjustment Method

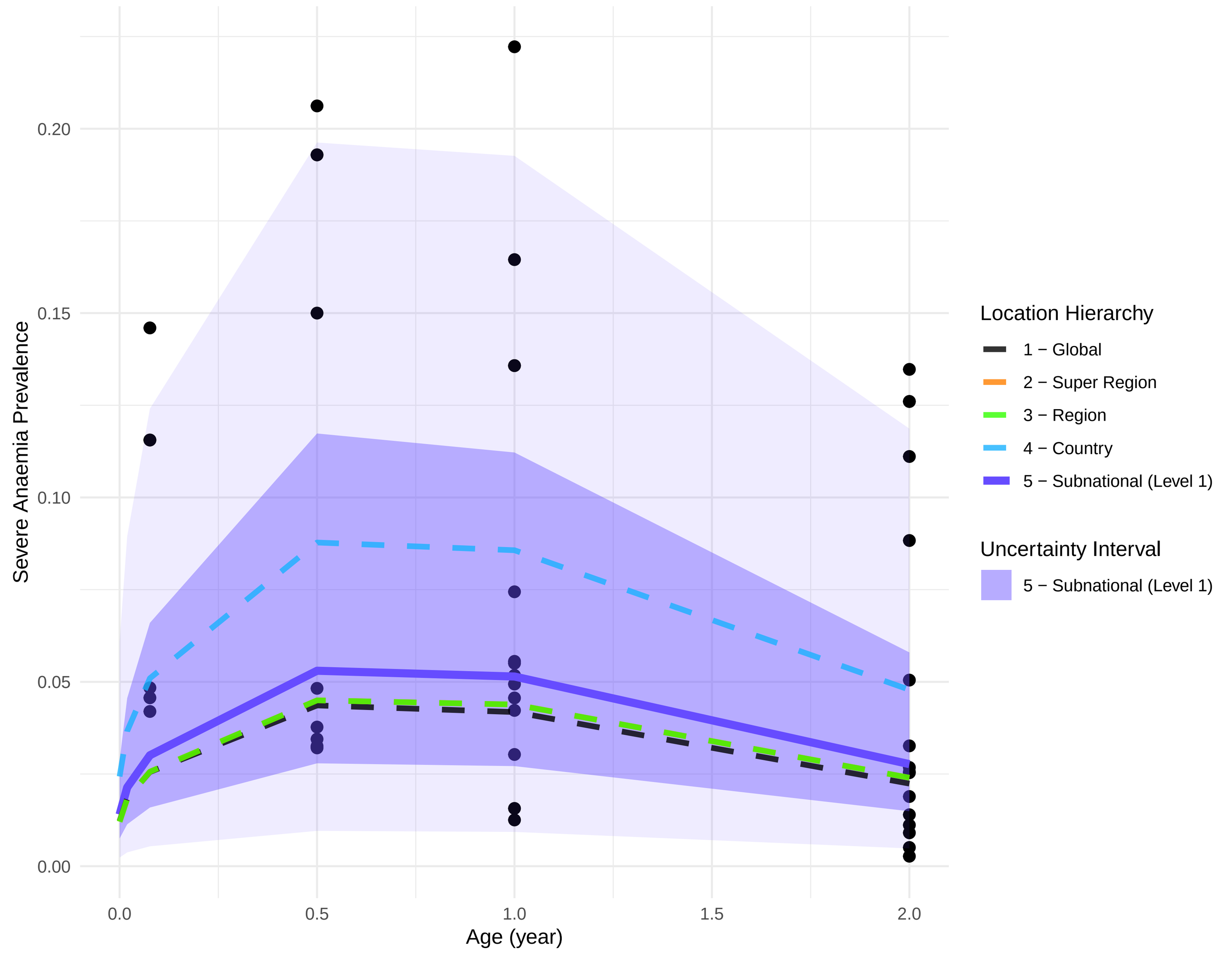

MR−BRT Age/Sex Fits − Males <2 Years Old − Severe Anaemia Prevalence − Chhattisgarh, Rural
WHO−2024 Adjustment Method

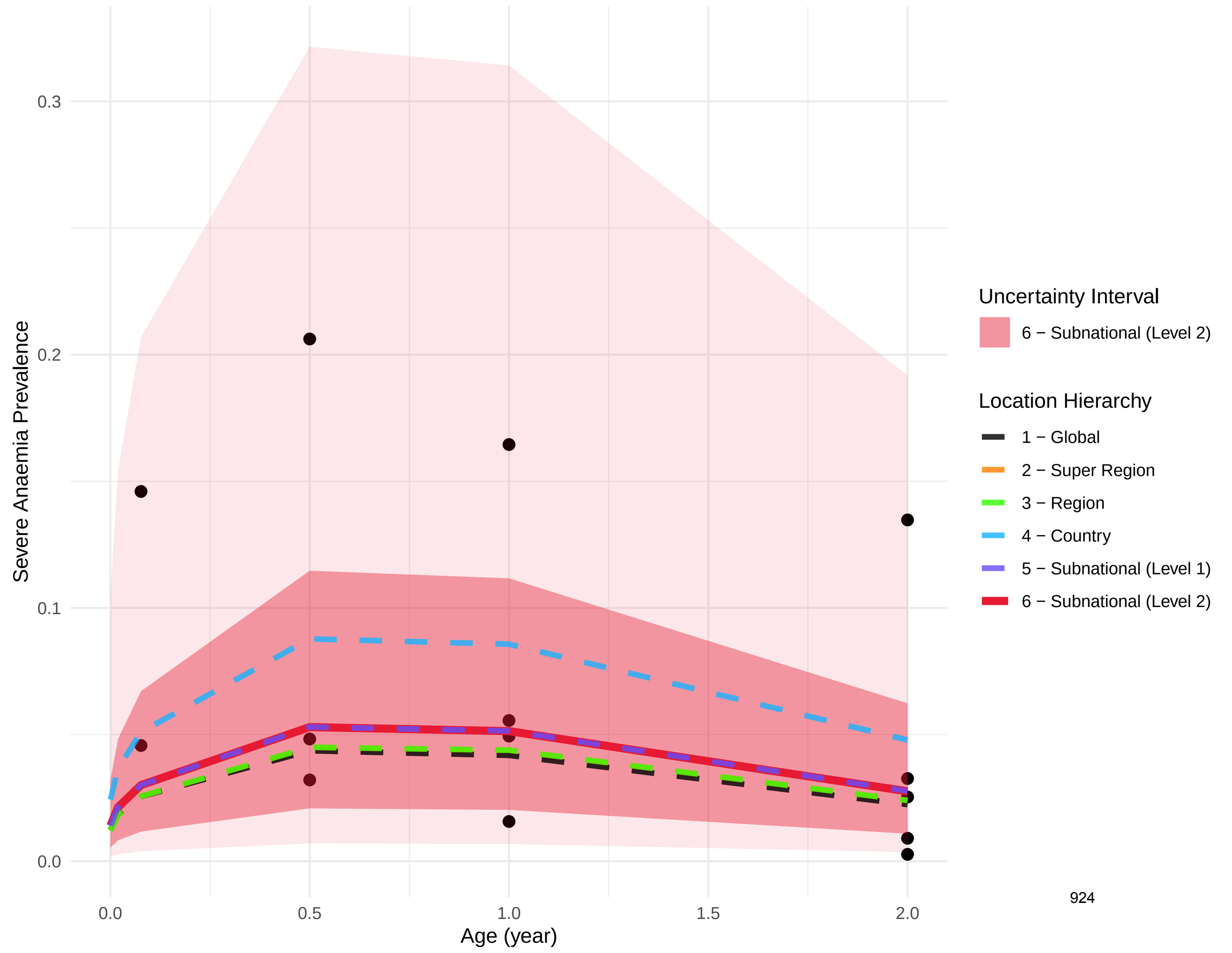

MR−BRT Age/Sex Fits − Males <2 Years Old − Severe Anaemia Prevalence − Chhattisgarh, Urban
WHO−2024 Adjustment Method

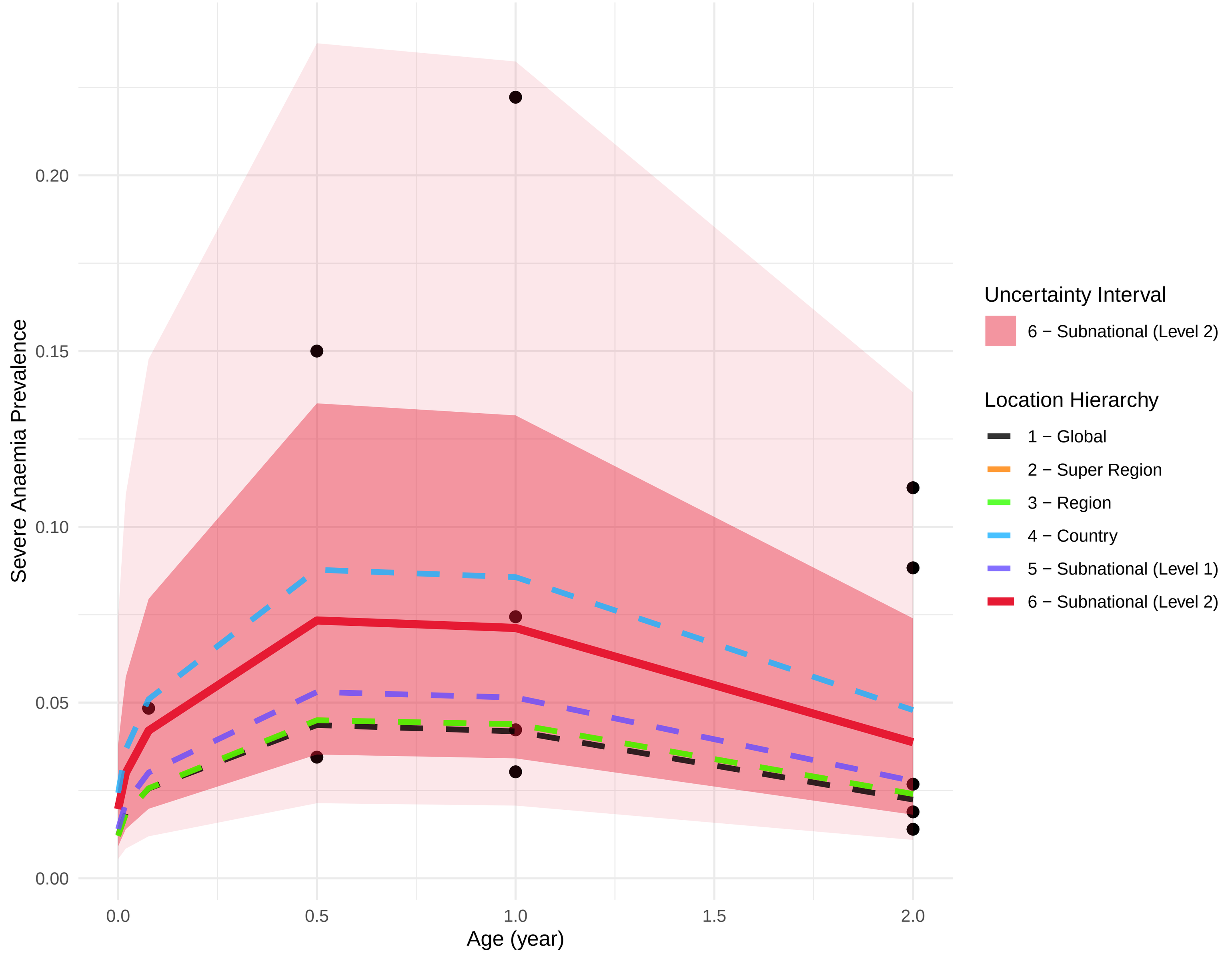

MR−BRT Age/Sex Fits − Males <2 Years Old − Severe Anaemia Prevalence − Delhi
WHO−2024 Adjustment Method

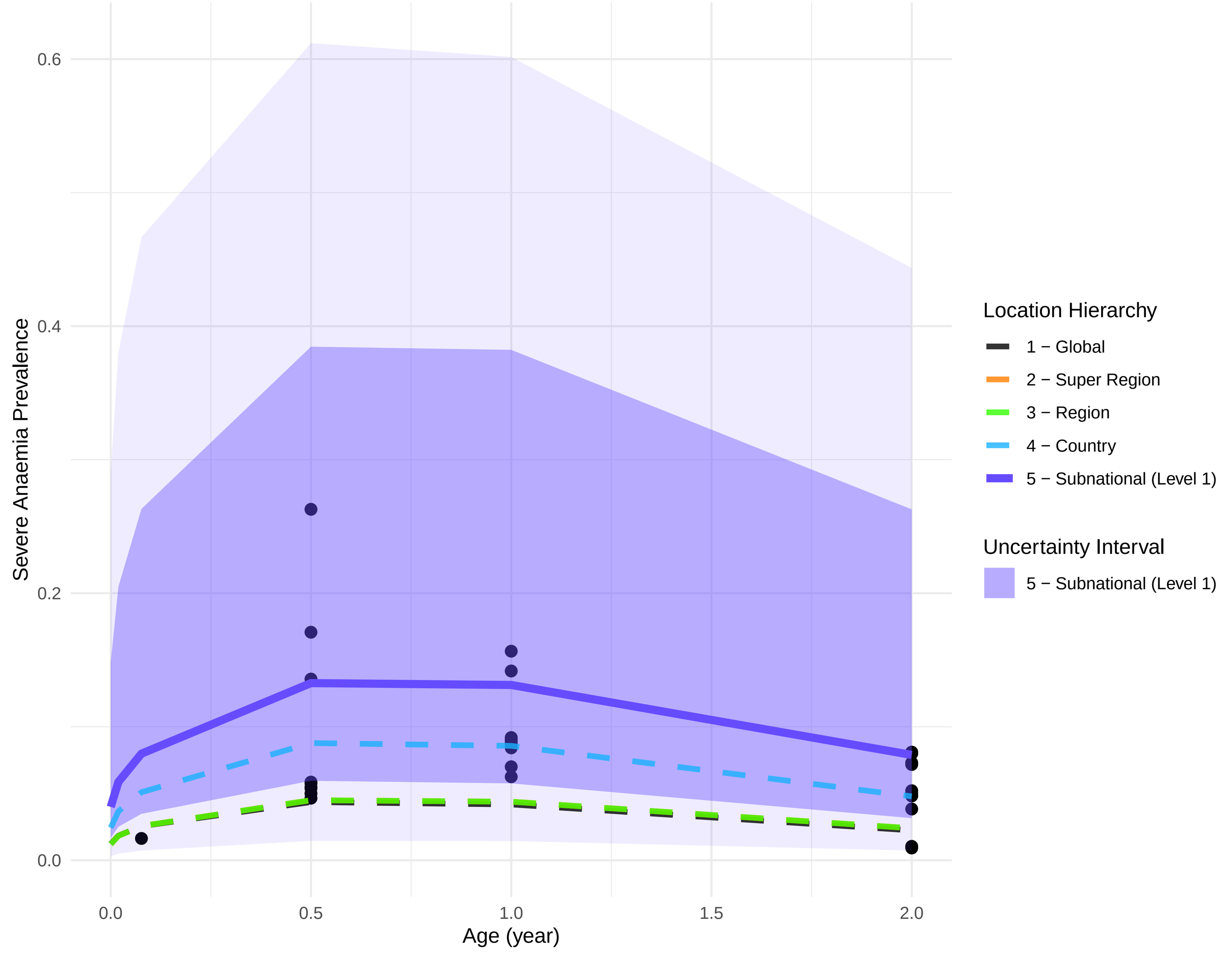

MR−BRT Age/Sex Fits − Males <2 Years Old − Severe Anaemia Prevalence − Delhi, Rural
WHO−2024 Adjustment Method

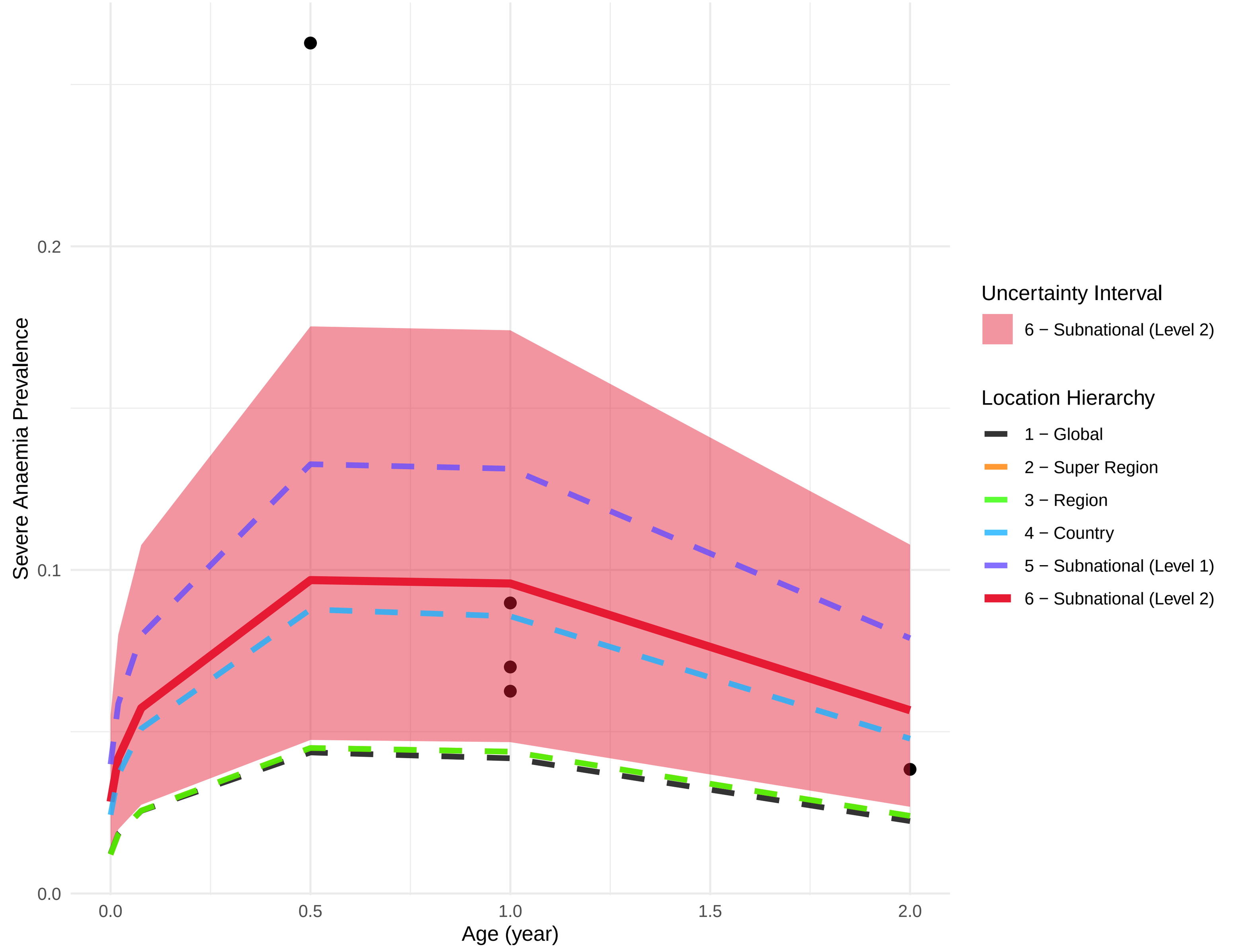

MR−BRT Age/Sex Fits − Males <2 Years Old − Severe Anaemia Prevalence − Delhi, Urban
WHO−2024 Adjustment Method

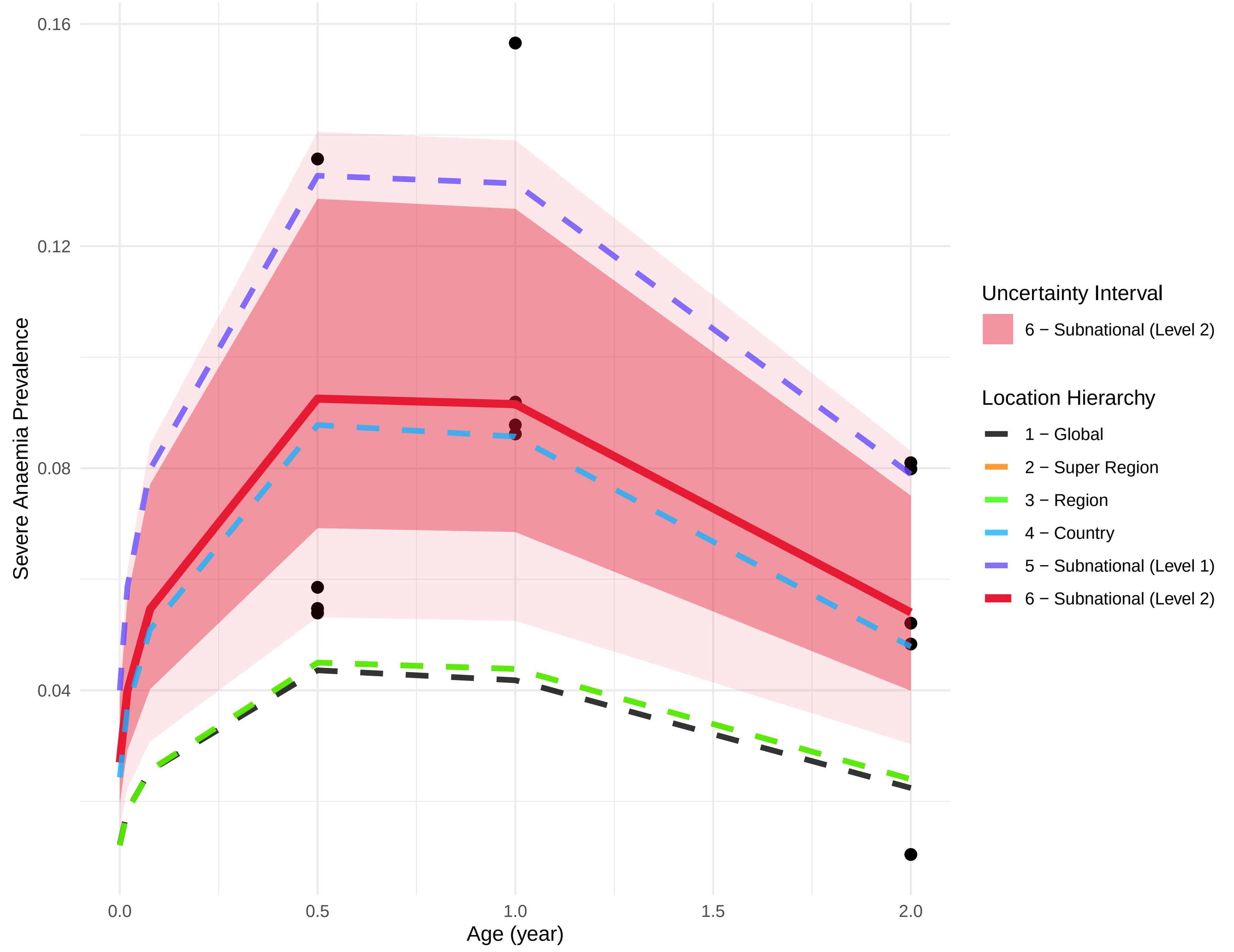

MR−BRT Age/Sex Fits − Males <2 Years Old − Severe Anaemia Prevalence − Goa
WHO−2024 Adjustment Method

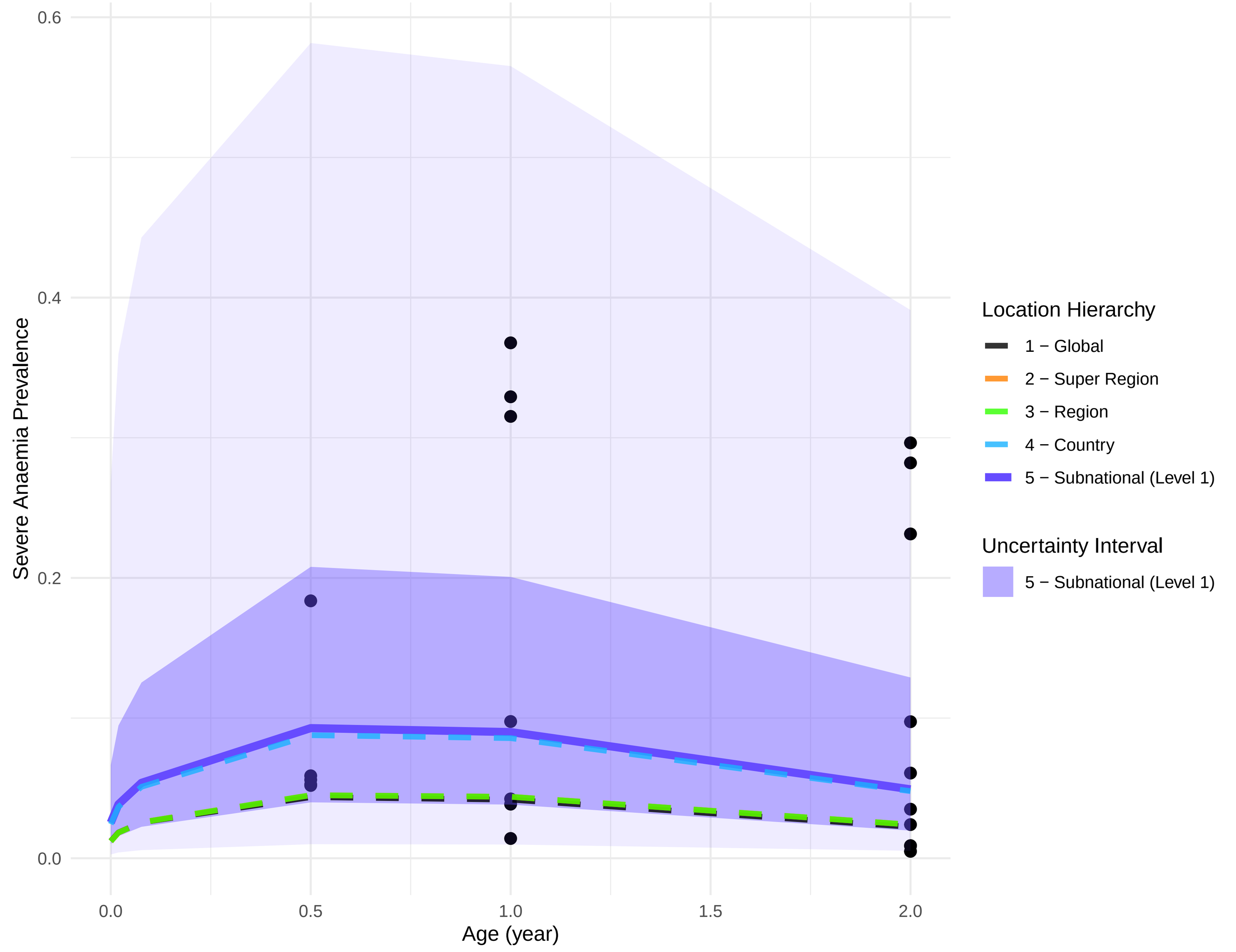

MR−BRT Age/Sex Fits − Males <2 Years Old − Severe Anaemia Prevalence − Goa, Rural
WHO−2024 Adjustment Method

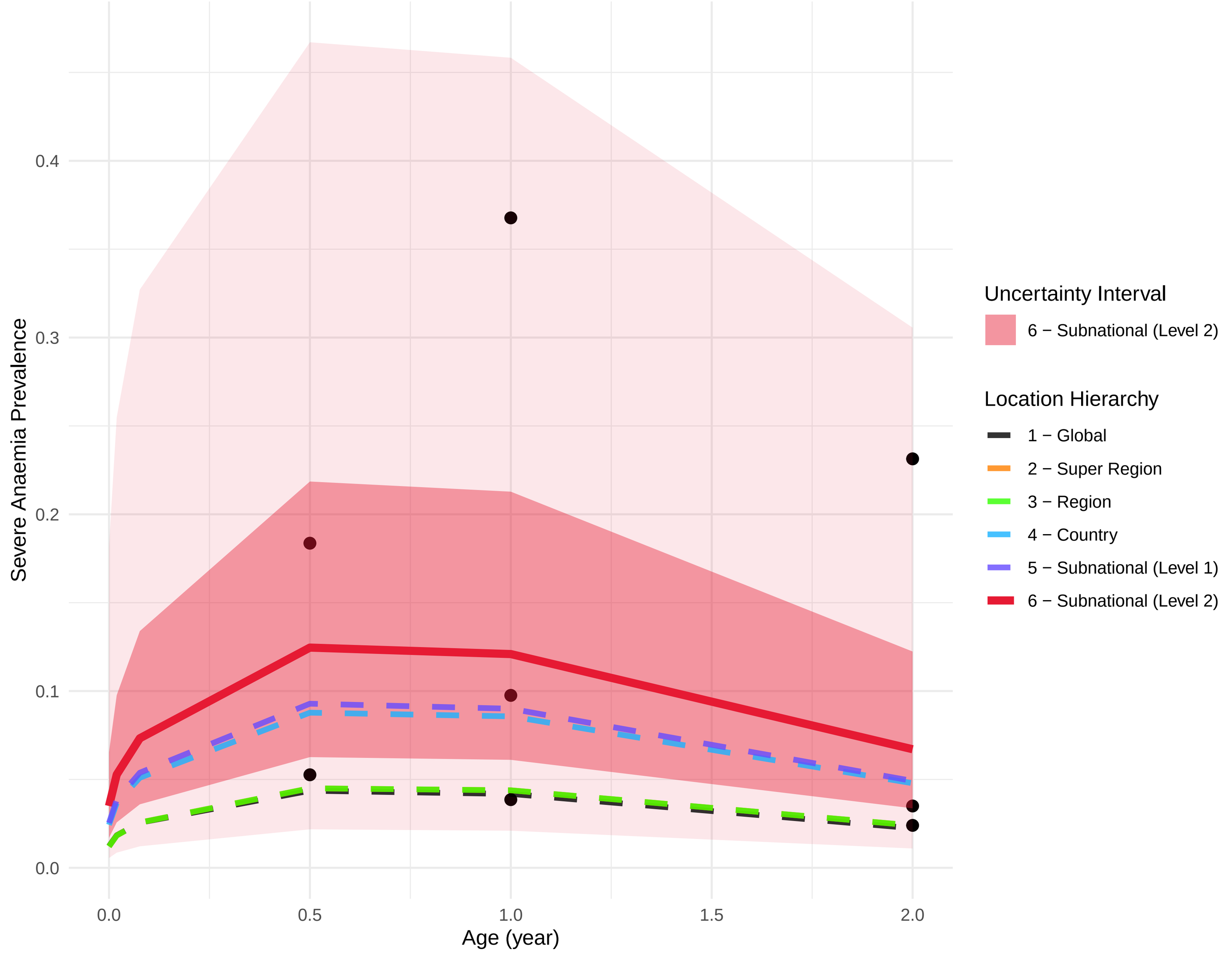

MR−BRT Age/Sex Fits − Males <2 Years Old − Severe Anaemia Prevalence − Goa, Urban
WHO−2024 Adjustment Method

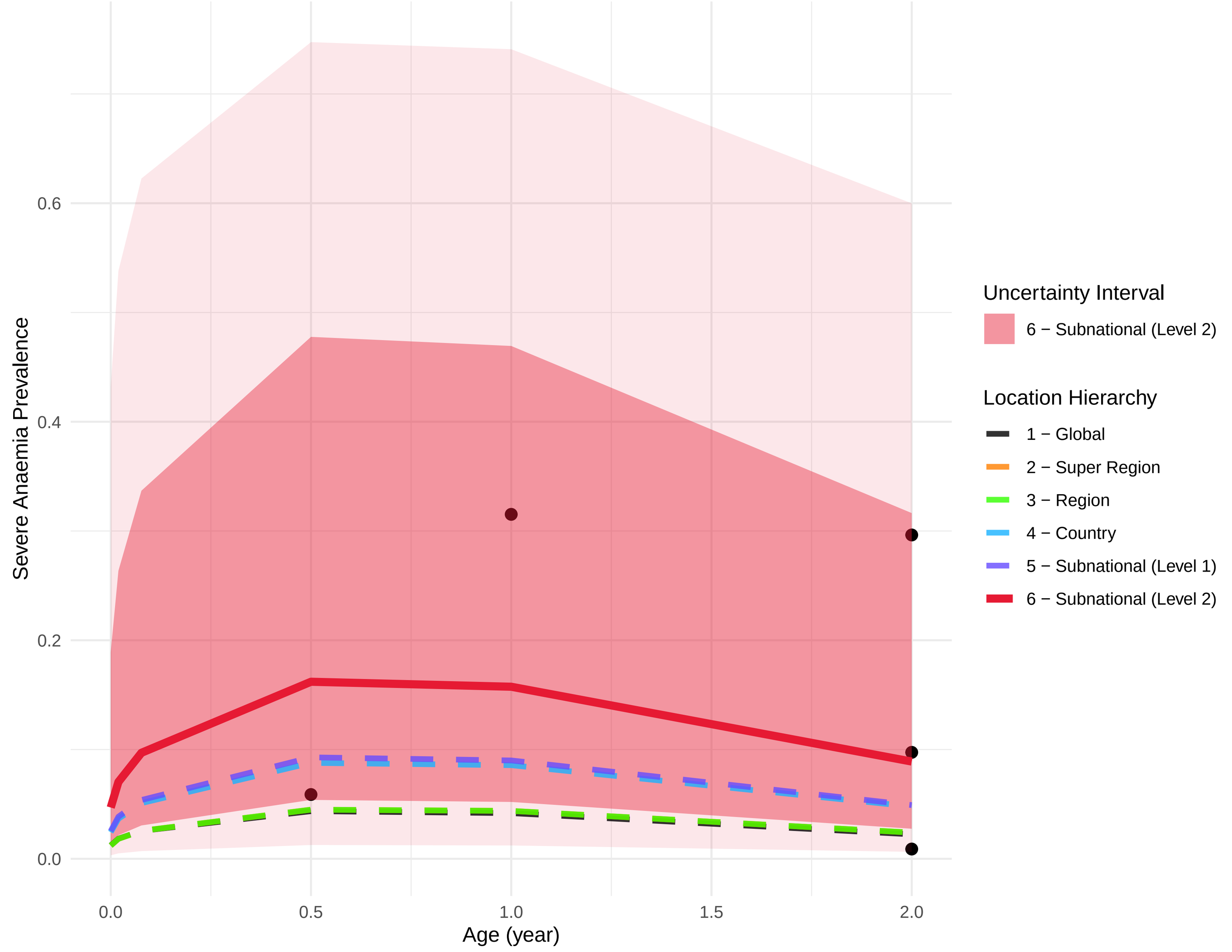

MR−BRT Age/Sex Fits − Males <2 Years Old − Severe Anaemia Prevalence − Gujarat
WHO−2024 Adjustment Method

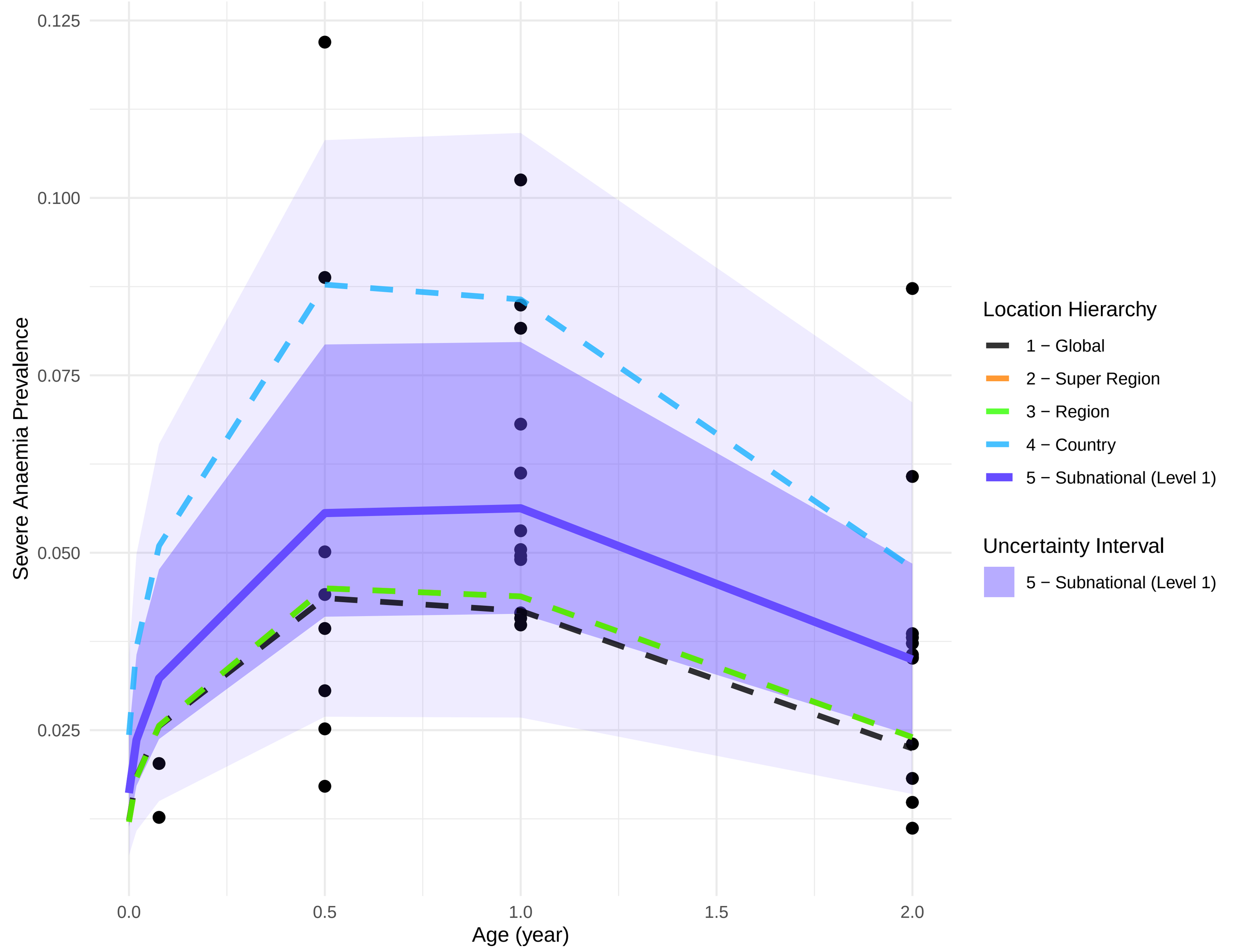

MR−BRT Age/Sex Fits − Males <2 Years Old − Severe Anaemia Prevalence − Gujarat, Rural
WHO−2024 Adjustment Method

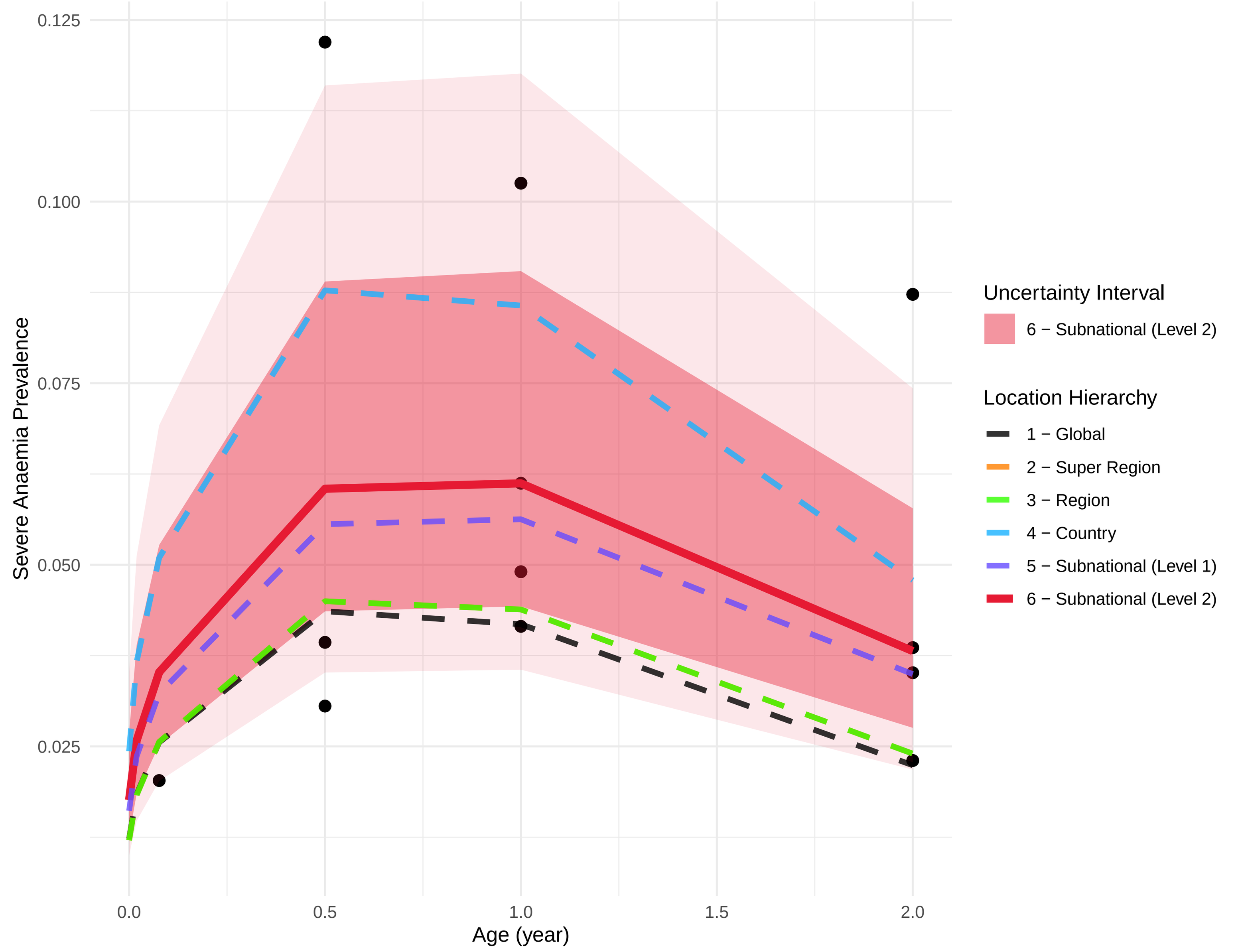

MR−BRT Age/Sex Fits − Males <2 Years Old − Severe Anaemia Prevalence − Gujarat, Urban
WHO−2024 Adjustment Method

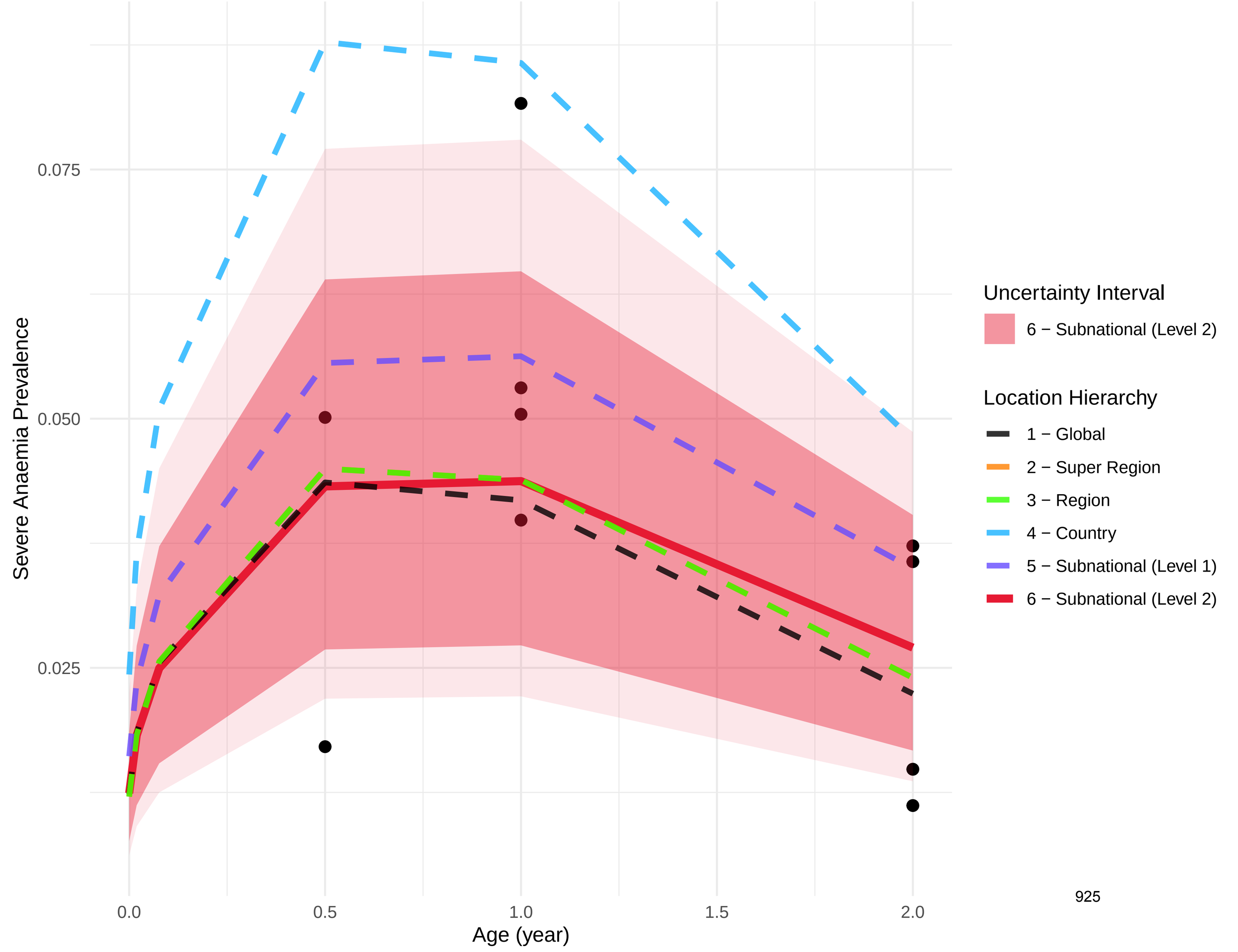

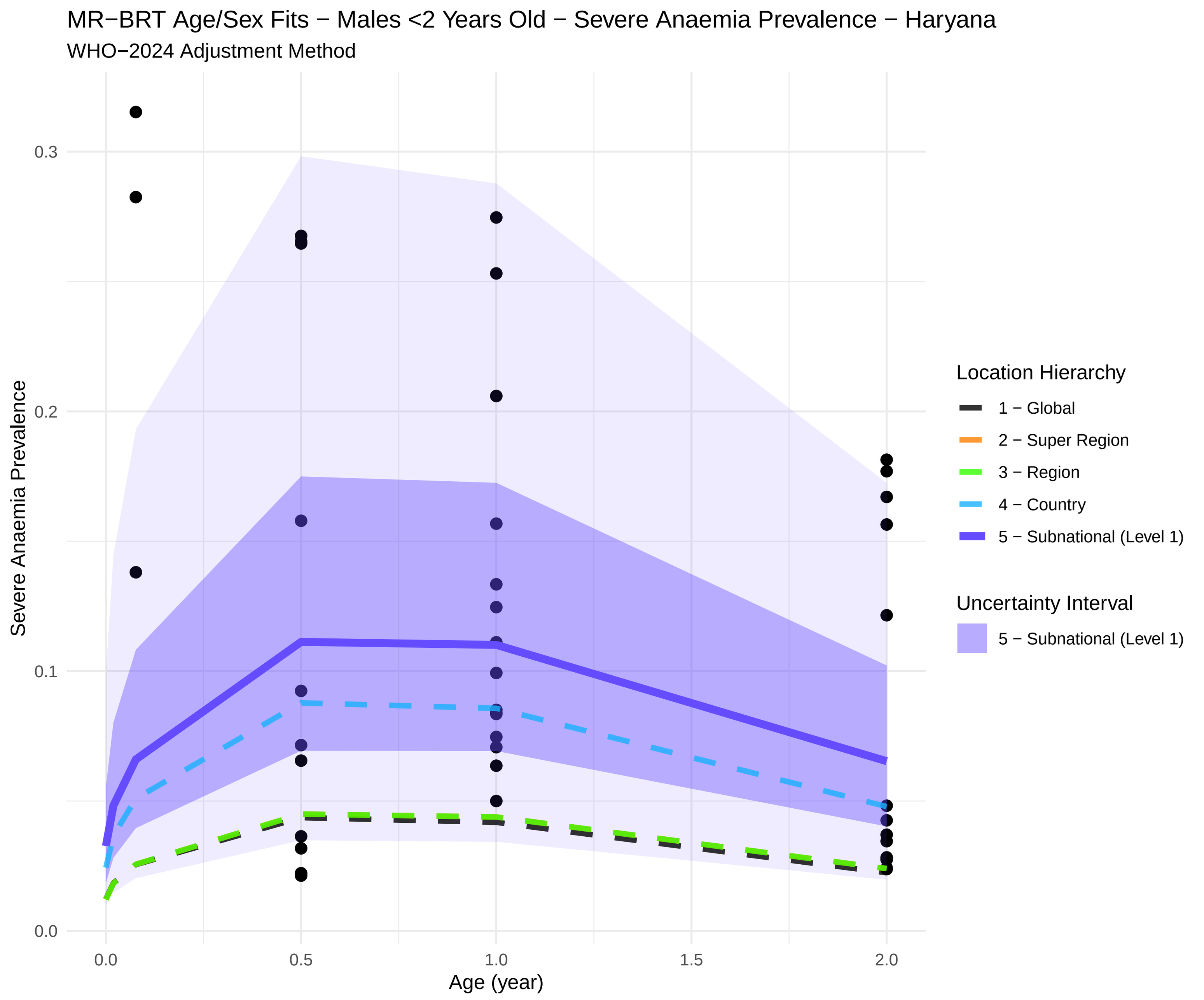
MR−BRT Age/Sex Fits − Males <2 Years Old − Severe Anaemia Prevalence − Haryana
WHO−2024 Adjustment Method
Severe Anaemia Prevalence
Age (year)
Location Hierarchy
1 − Global
2 − Super Region
3 − Region
4 − Country
5 − Subnational (Level 1)
Uncertainty Interval
5 − Subnational (Level 1)

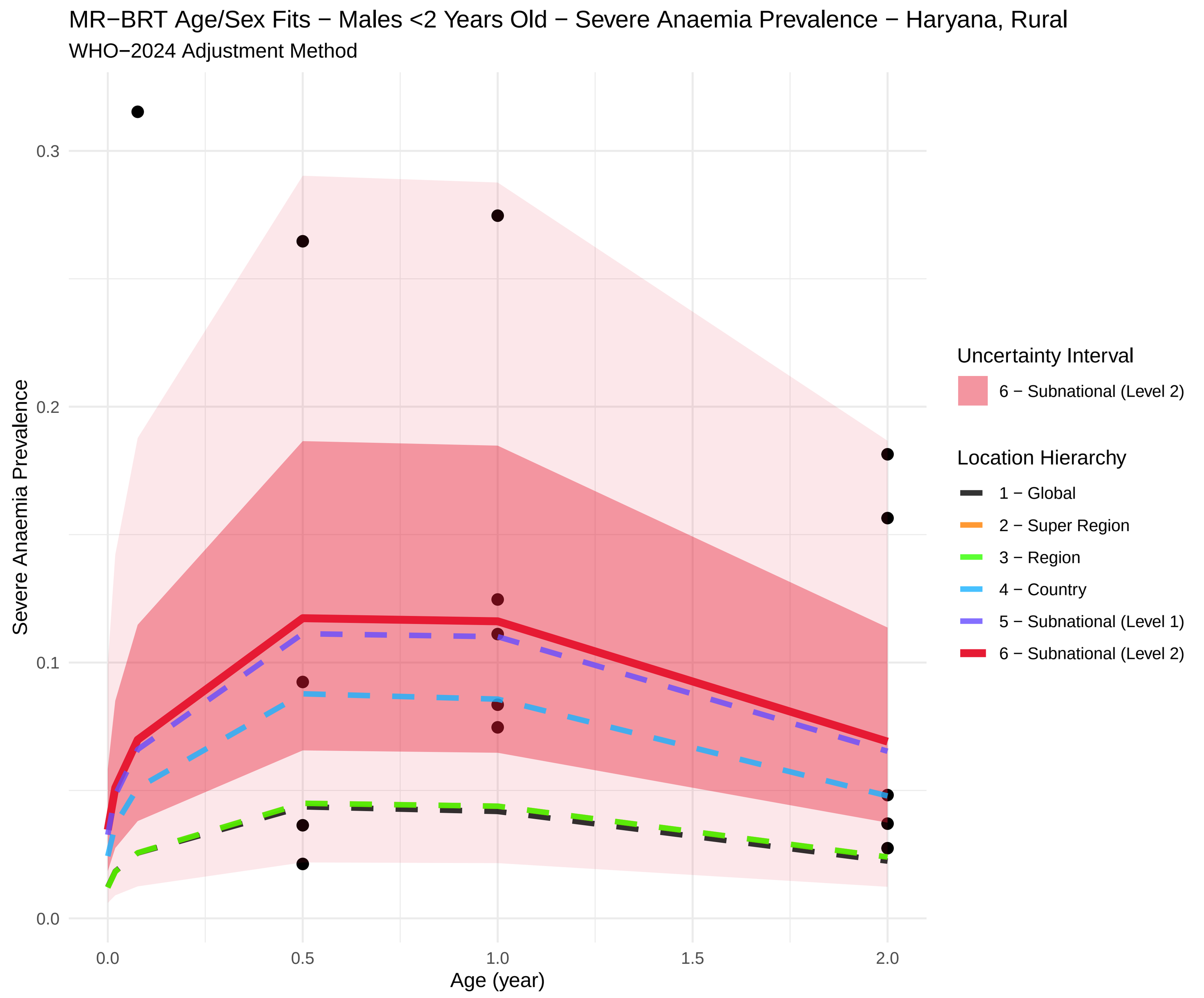
MR−BRT Age/Sex Fits − Males <2 Years Old − Severe Anaemia Prevalence − Haryana, Rural
WHO−2024 Adjustment Method
Severe Anaemia Prevalence
Age (year)
Uncertainty Interval
6 − Subnational (Level 2)
Location Hierarchy
1 − Global
2 − Super Region
3 − Region
4 − Country
5 − Subnational (Level 1)
6 − Subnational (Level 2)

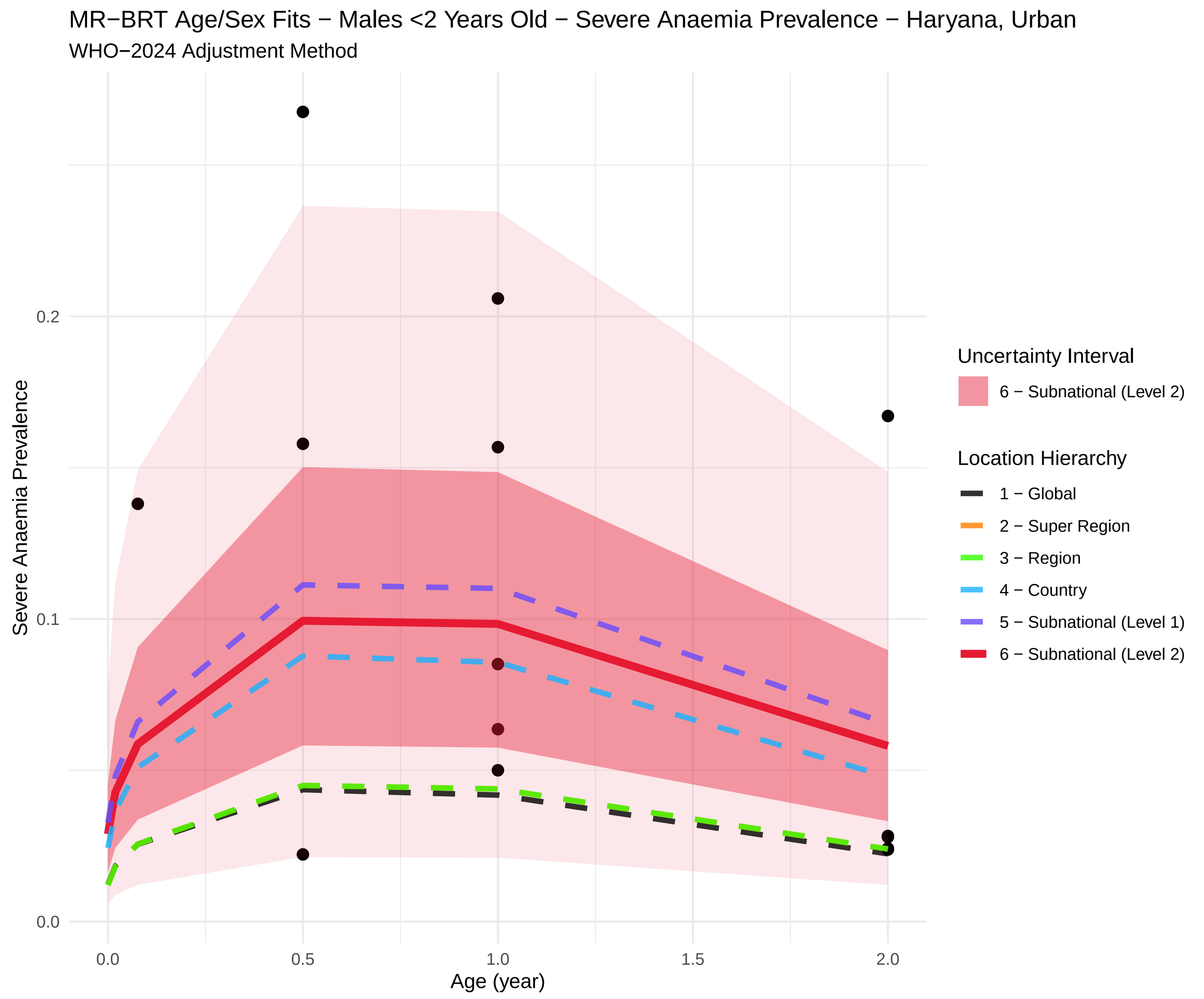
MR−BRT Age/Sex Fits − Males <2 Years Old − Severe Anaemia Prevalence − Haryana, Urban
WHO−2024 Adjustment Method
Severe Anaemia Prevalence
Age (year)
Uncertainty Interval
6 − Subnational (Level 2)
Location Hierarchy
1 − Global
2 − Super Region
3 − Region
4 − Country
5 − Subnational (Level 1)
6 − Subnational (Level 2)

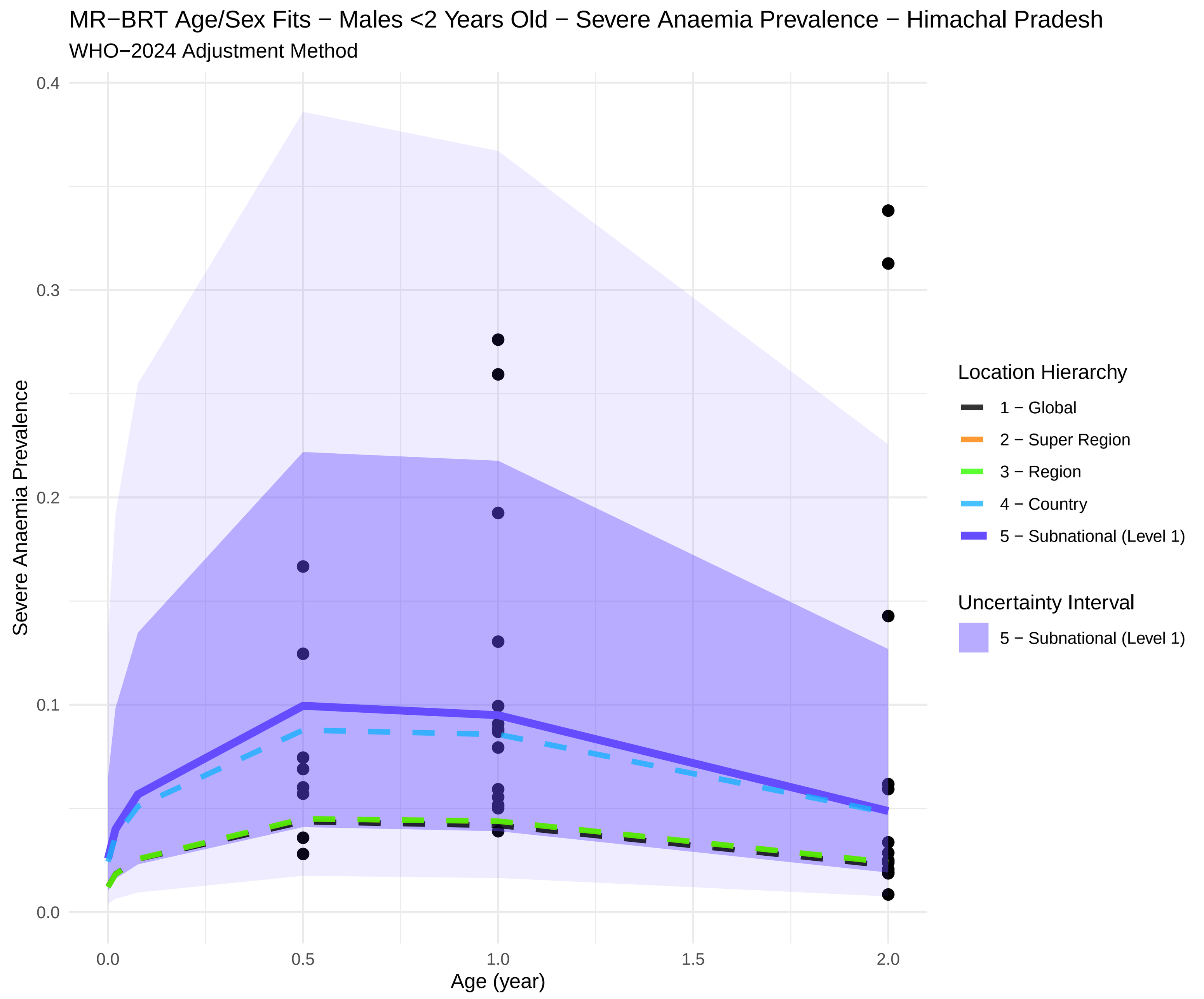
MR−BRT Age/Sex Fits − Males <2 Years Old − Severe Anaemia Prevalence − Himachal Pradesh
WHO−2024 Adjustment Method
Severe Anaemia Prevalence
Age (year)
Location Hierarchy
1 − Global
2 − Super Region
3 − Region
4 − Country
5 − Subnational (Level 1)
Uncertainty Interval
5 − Subnational (Level 1)

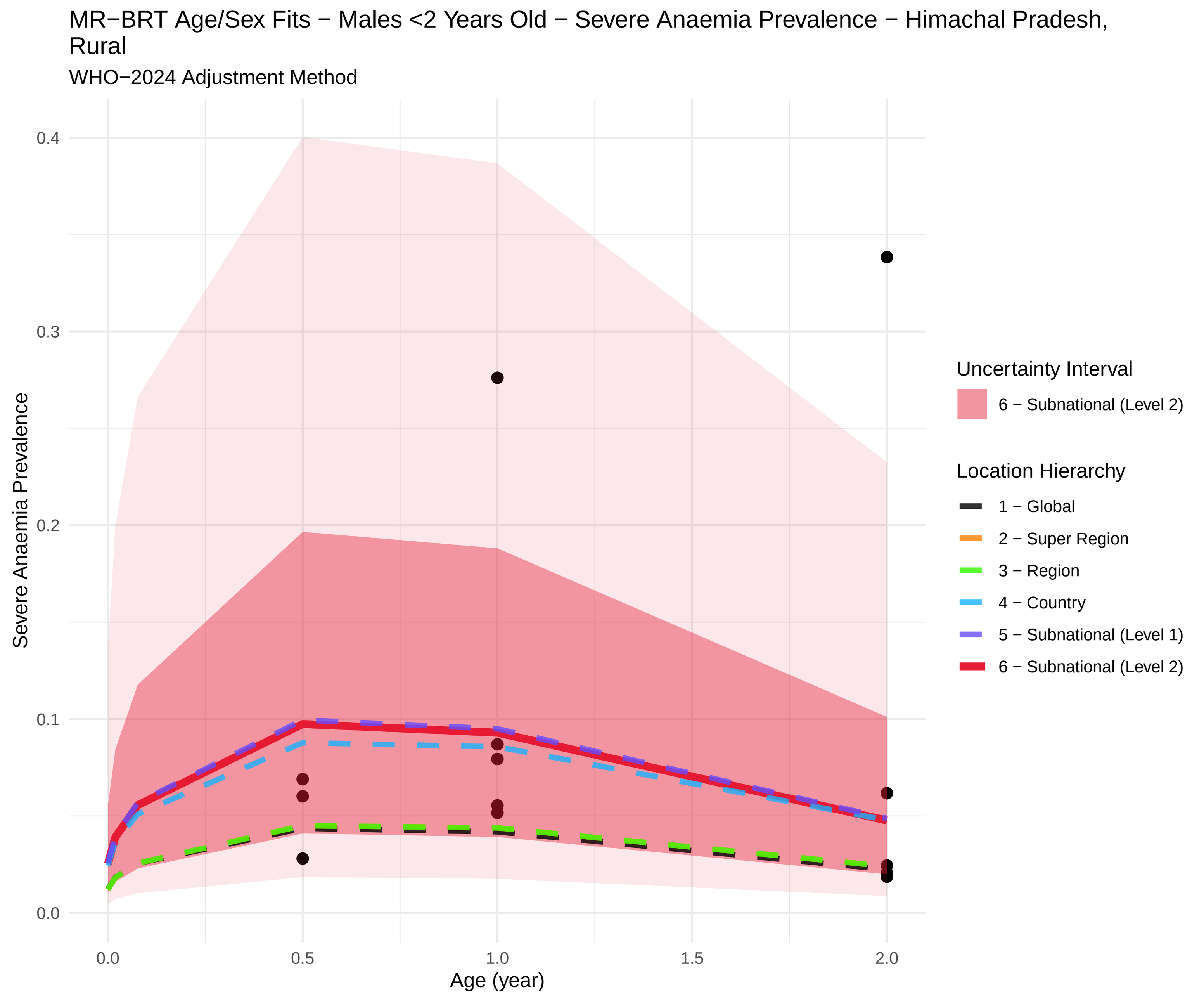
MR−BRT Age/Sex Fits − Males <2 Years Old − Severe Anaemia Prevalence − Himachal Pradesh, Rural
WHO−2024 Adjustment Method
Severe Anaemia Prevalence
Age (year)
Uncertainty Interval
6 − Subnational (Level 2)
Location Hierarchy
1 − Global
2 − Super Region
3 − Region
4 − Country
5 − Subnational (Level 1)
6 − Subnational (Level 2)

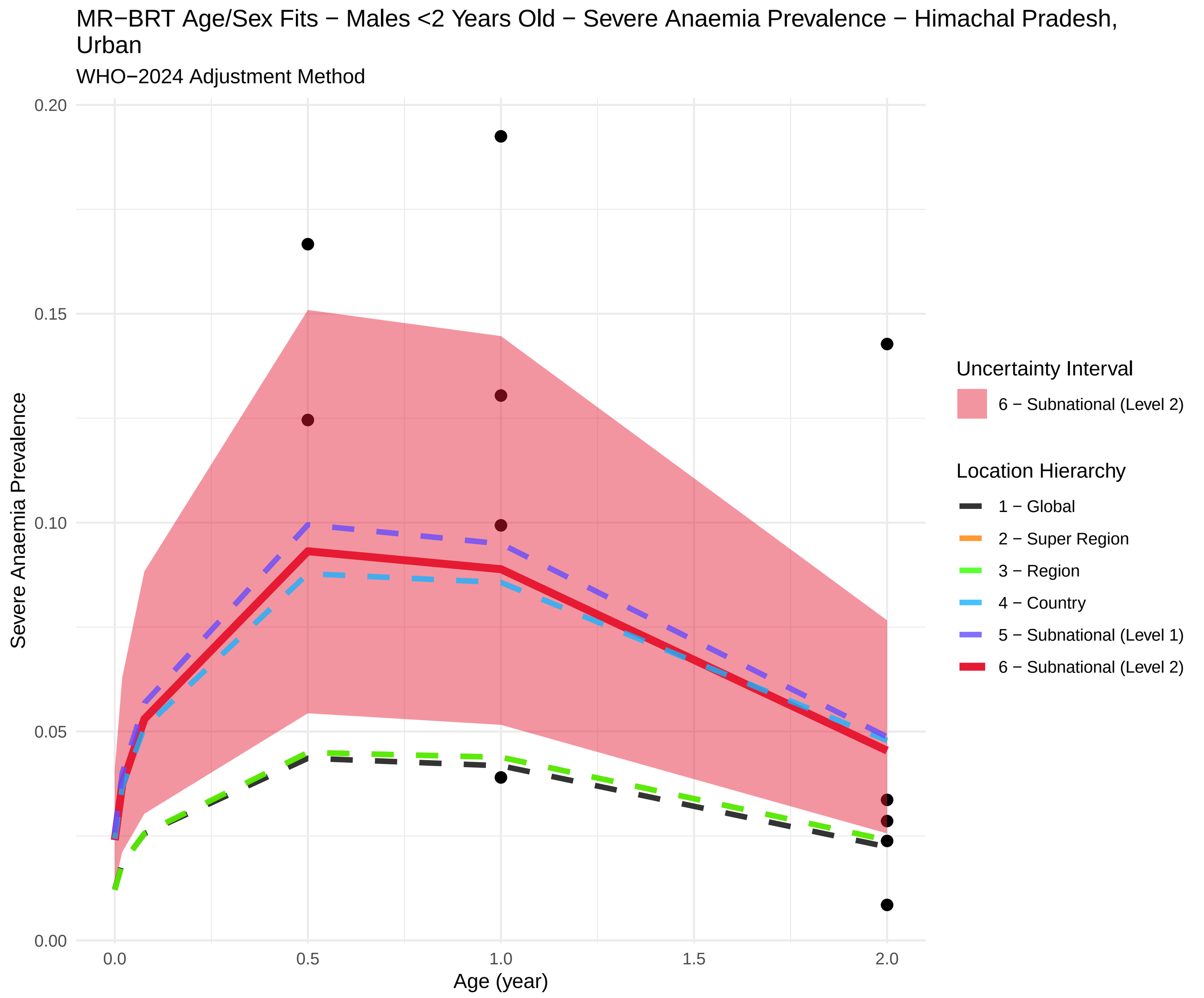
MR−BRT Age/Sex Fits − Males <2 Years Old − Severe Anaemia Prevalence − Himachal Pradesh, Urban
WHO−2024 Adjustment Method
Severe Anaemia Prevalence
Age (year)
Uncertainty Interval
6 − Subnational (Level 2)
Location Hierarchy
1 − Global
2 − Super Region
3 − Region
4 − Country
5 − Subnational (Level 1)
6 − Subnational (Level 2)

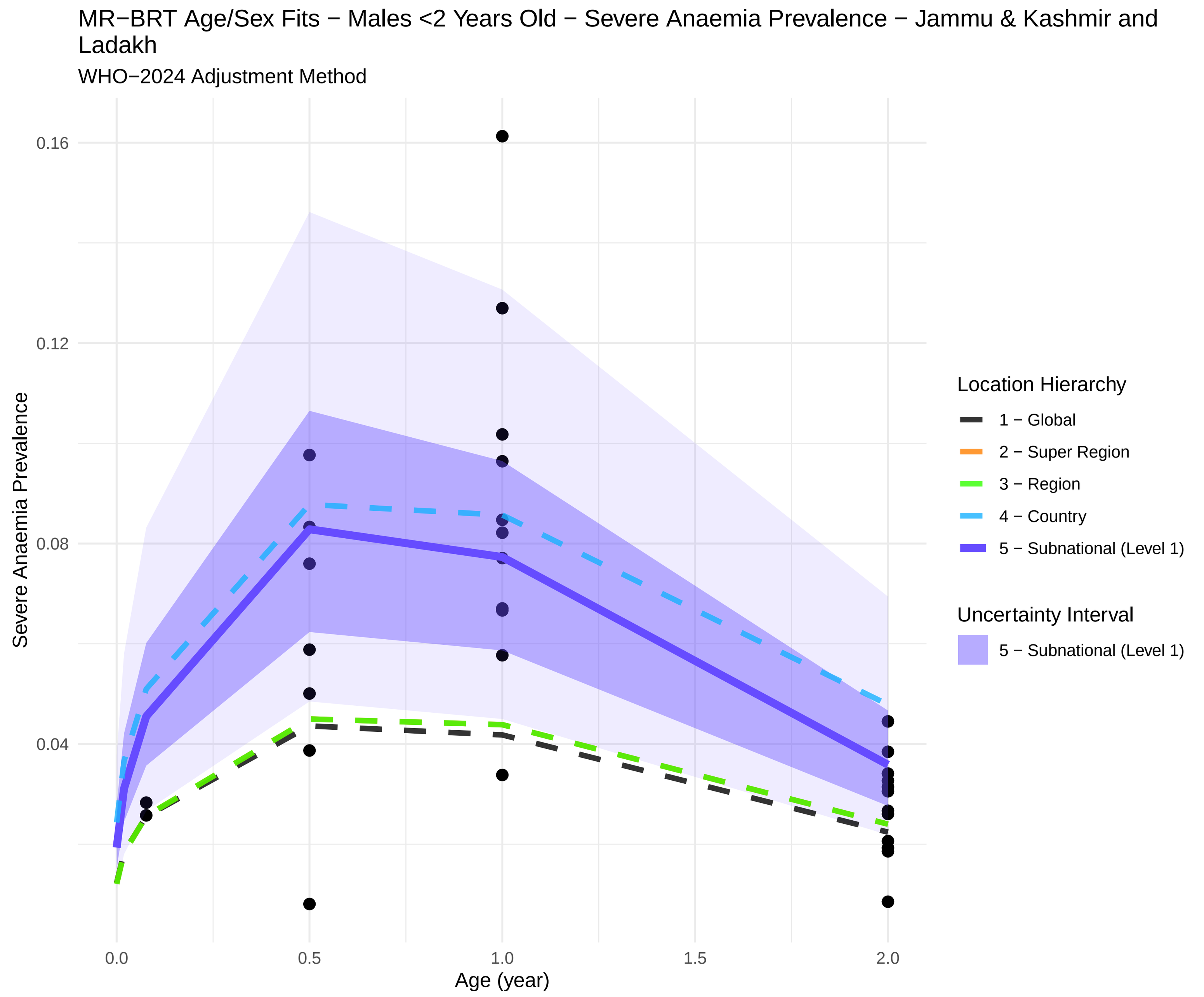
MR−BRT Age/Sex Fits − Males <2 Years Old − Severe Anaemia Prevalence − Jammu & Kashmir and Ladakh
WHO−2024 Adjustment Method
Severe Anaemia Prevalence
Age (year)
Location Hierarchy
1 − Global
2 − Super Region
3 − Region
4 − Country
5 − Subnational (Level 1)
Uncertainty Interval
5 − Subnational (Level 1)

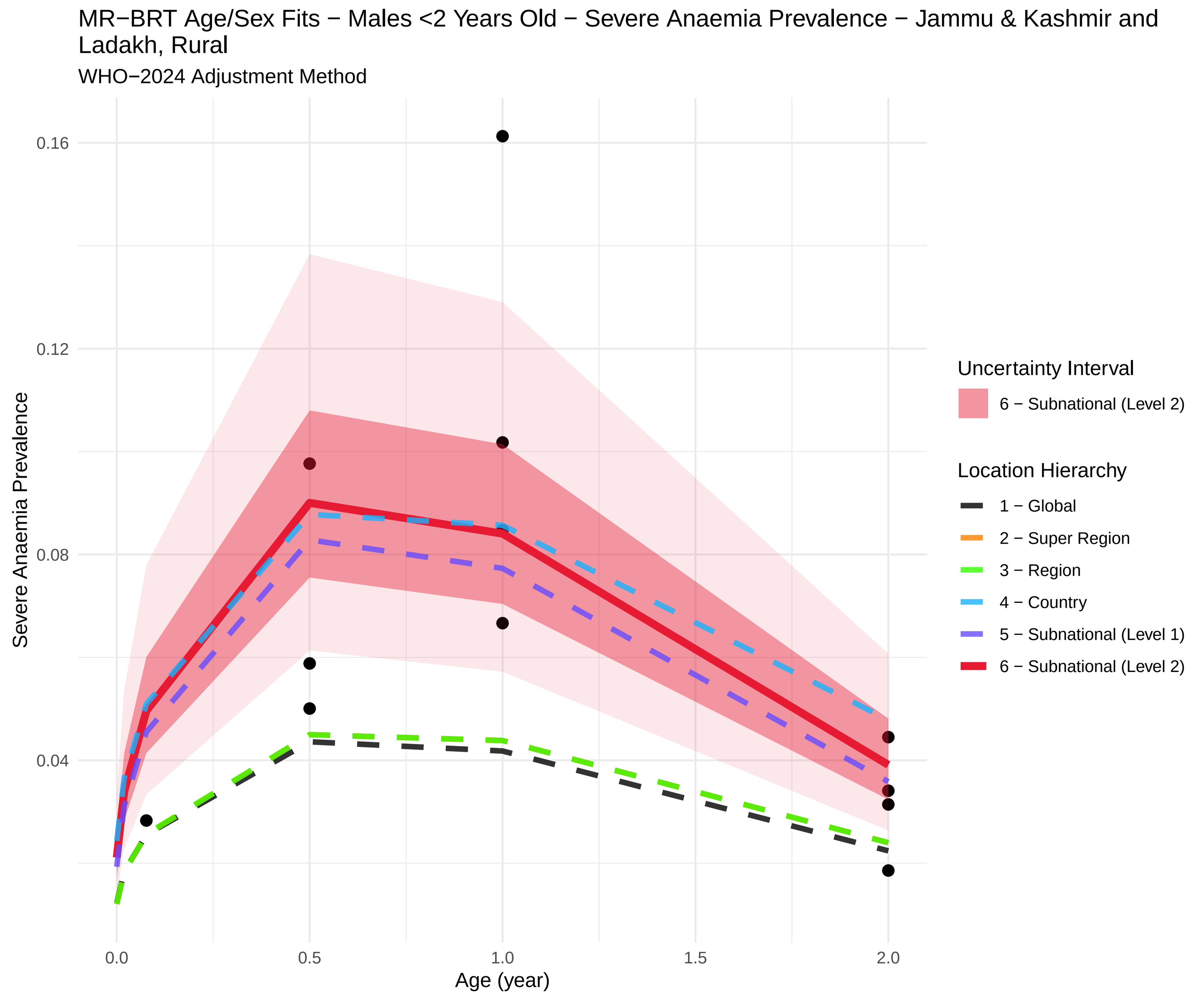
MR−BRT Age/Sex Fits − Males <2 Years Old − Severe Anaemia Prevalence − Jammu & Kashmir and Ladakh, Rural
WHO−2024 Adjustment Method
Severe Anaemia Prevalence
Age (year)
Uncertainty Interval
6 − Subnational (Level 2)
Location Hierarchy
1 − Global
2 − Super Region
3 − Region
4 − Country
5 − Subnational (Level 1)
6 − Subnational (Level 2)

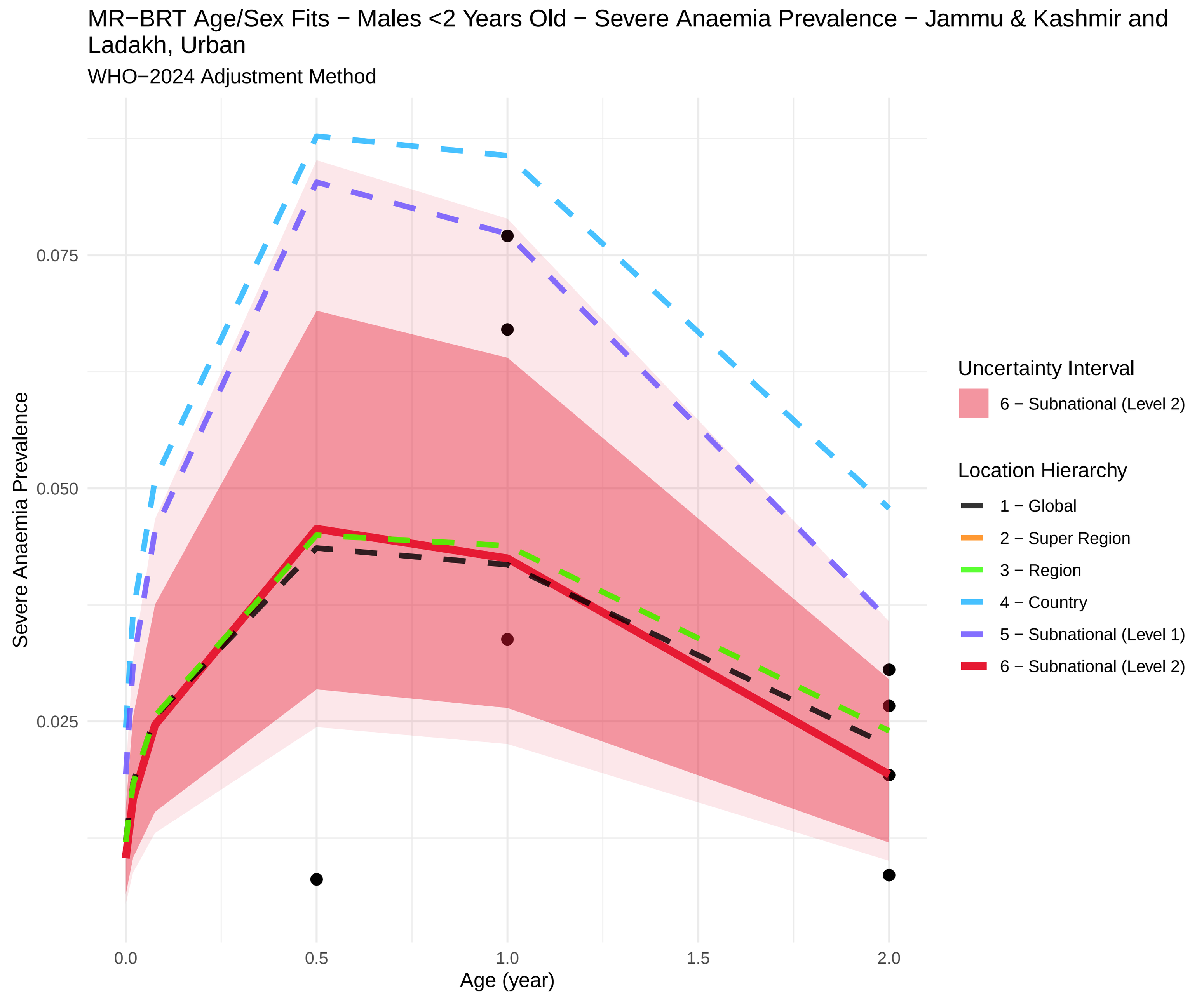
MR−BRT Age/Sex Fits − Males <2 Years Old − Severe Anaemia Prevalence − Jammu & Kashmir and Ladakh, Urban
WHO−2024 Adjustment Method
Severe Anaemia Prevalence
Age (year)
Uncertainty Interval
6 − Subnational (Level 2)
Location Hierarchy
1 − Global
2 − Super Region
3 − Region
4 − Country
5 − Subnational (Level 1)
6 − Subnational (Level 2)

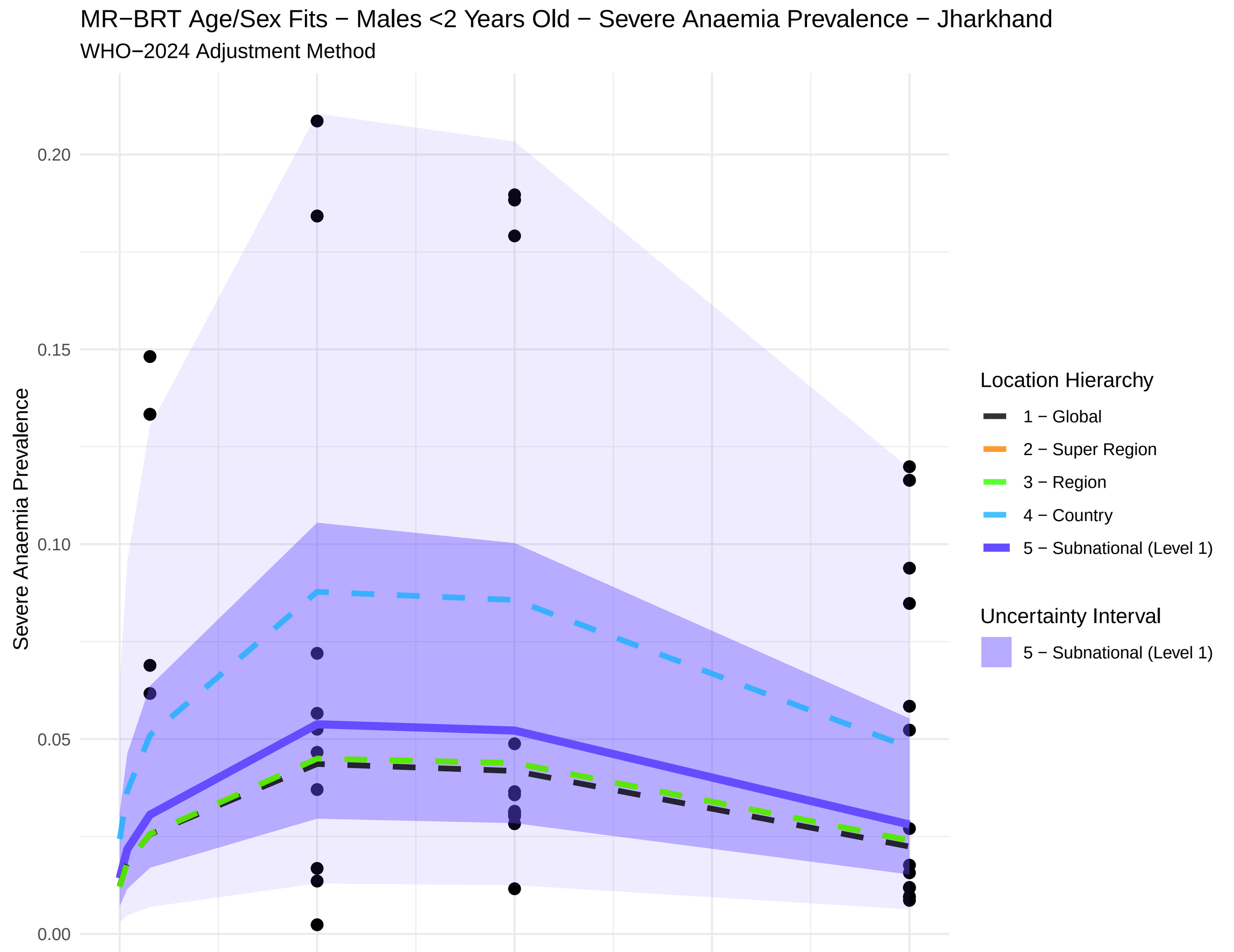
MR−BRT Age/Sex Fits − Males <2 Years Old − Severe Anaemia Prevalence − Jharkhand
WHO−2024 Adjustment Method
Severe Anaemia Prevalence
Age (year)
Location Hierarchy
1 − Global
2 − Super Region
3 − Region
4 − Country
5 − Subnational (Level 1)
Uncertainty Interval
5 − Subnational (Level 1)

MR−BRT Age/Sex Fits − Males <2 Years Old − Severe Anaemia Prevalence − Jharkhand, Rural
WHO−2024 Adjustment Method

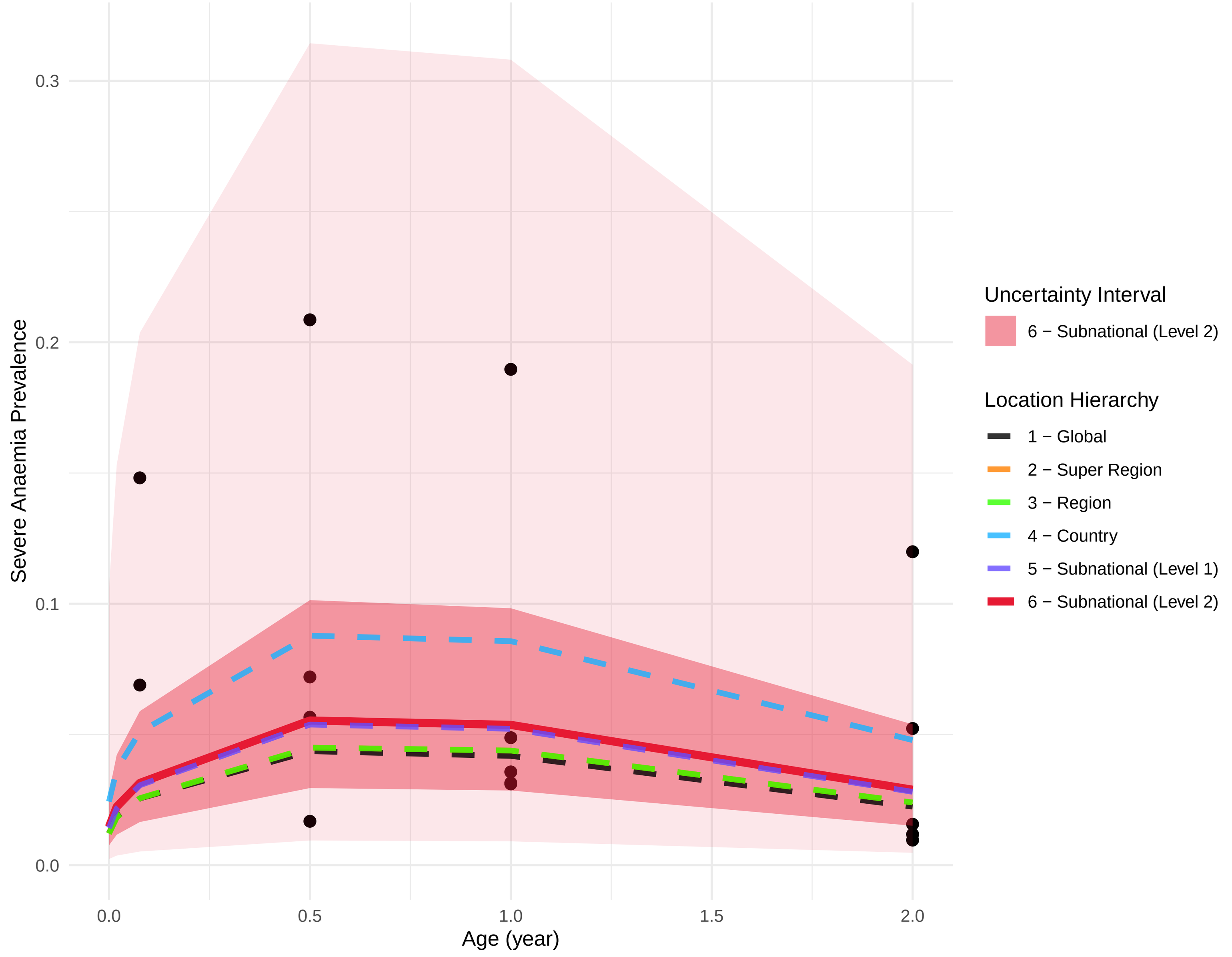

MR−BRT Age/Sex Fits − Males <2 Years Old − Severe Anaemia Prevalence − Jharkhand, Urban
WHO−2024 Adjustment Method

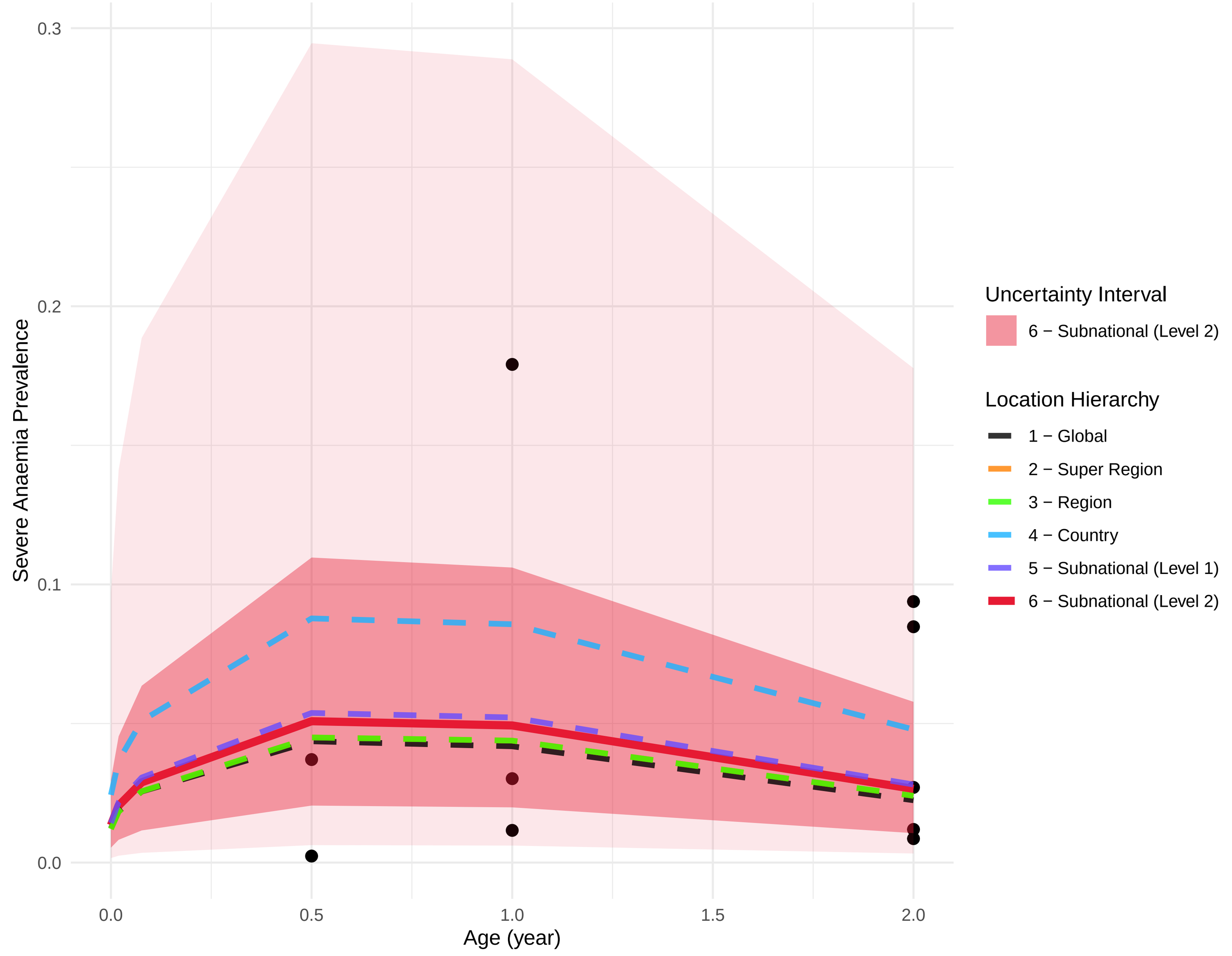

MR−BRT Age/Sex Fits − Males <2 Years Old − Severe Anaemia Prevalence − Karnataka
WHO−2024 Adjustment Method

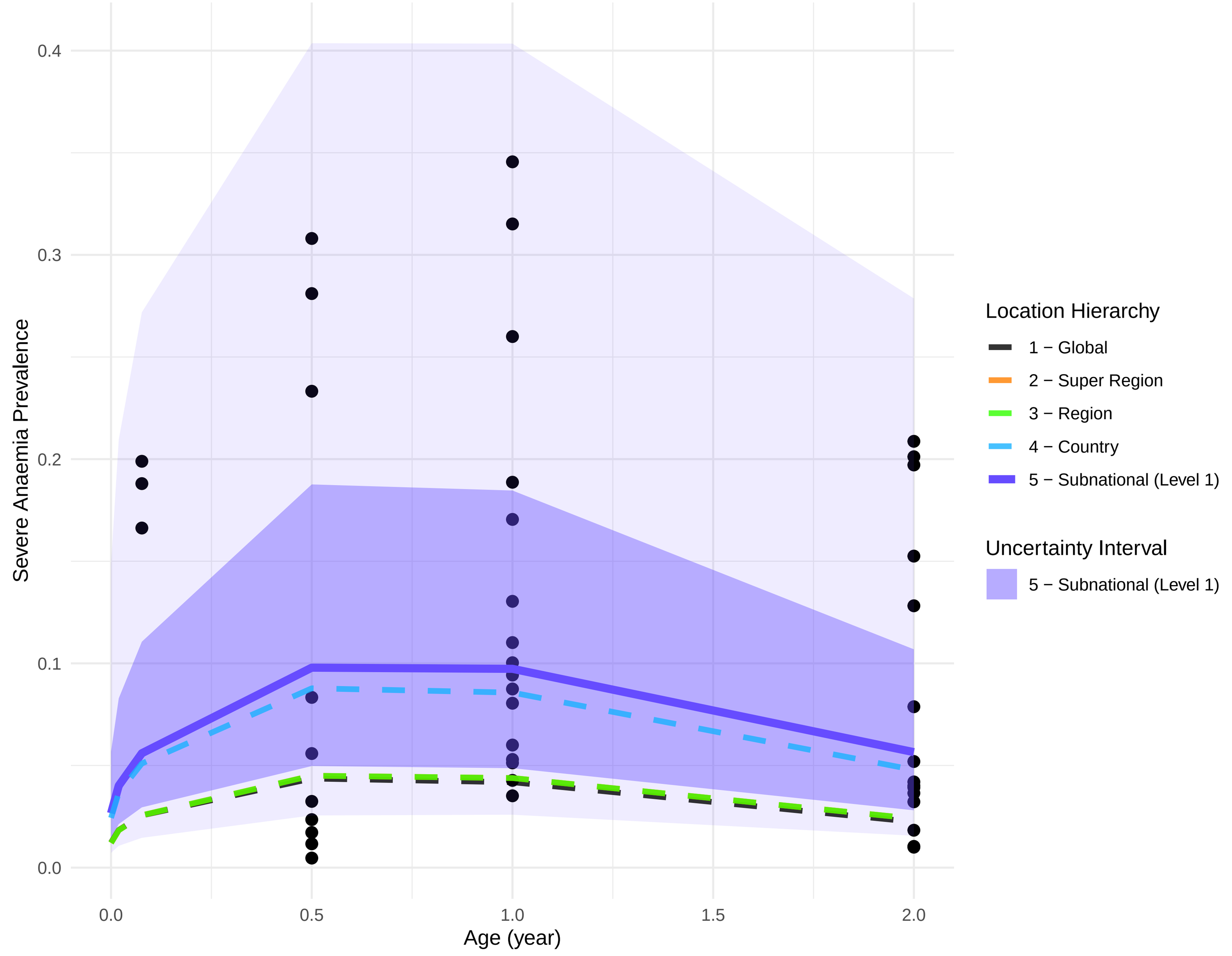

MR−BRT Age/Sex Fits − Males <2 Years Old − Severe Anaemia Prevalence − Karnataka, Rural
WHO−2024 Adjustment Method

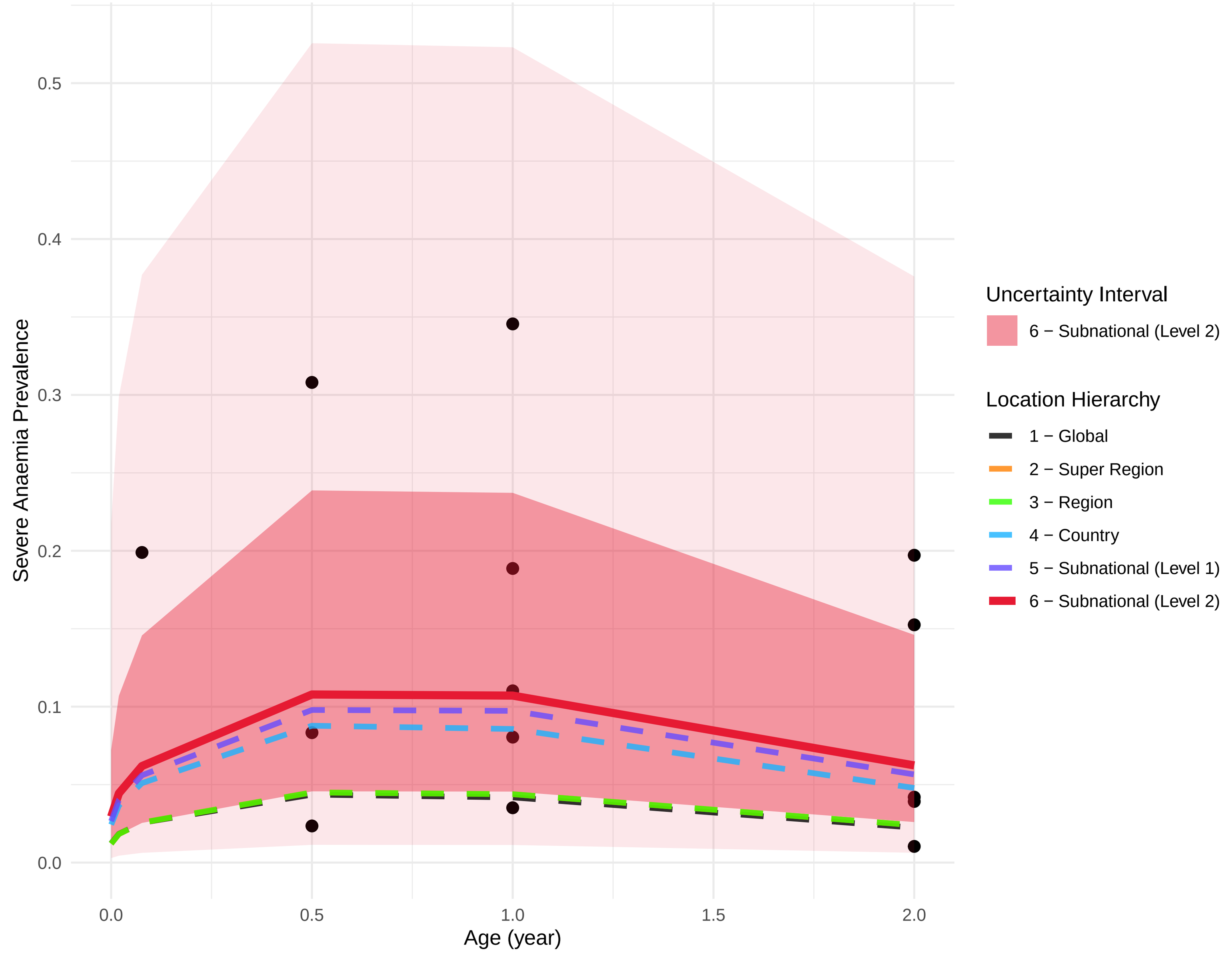

MR−BRT Age/Sex Fits − Males <2 Years Old − Severe Anaemia Prevalence − Karnataka, Urban
WHO−2024 Adjustment Method

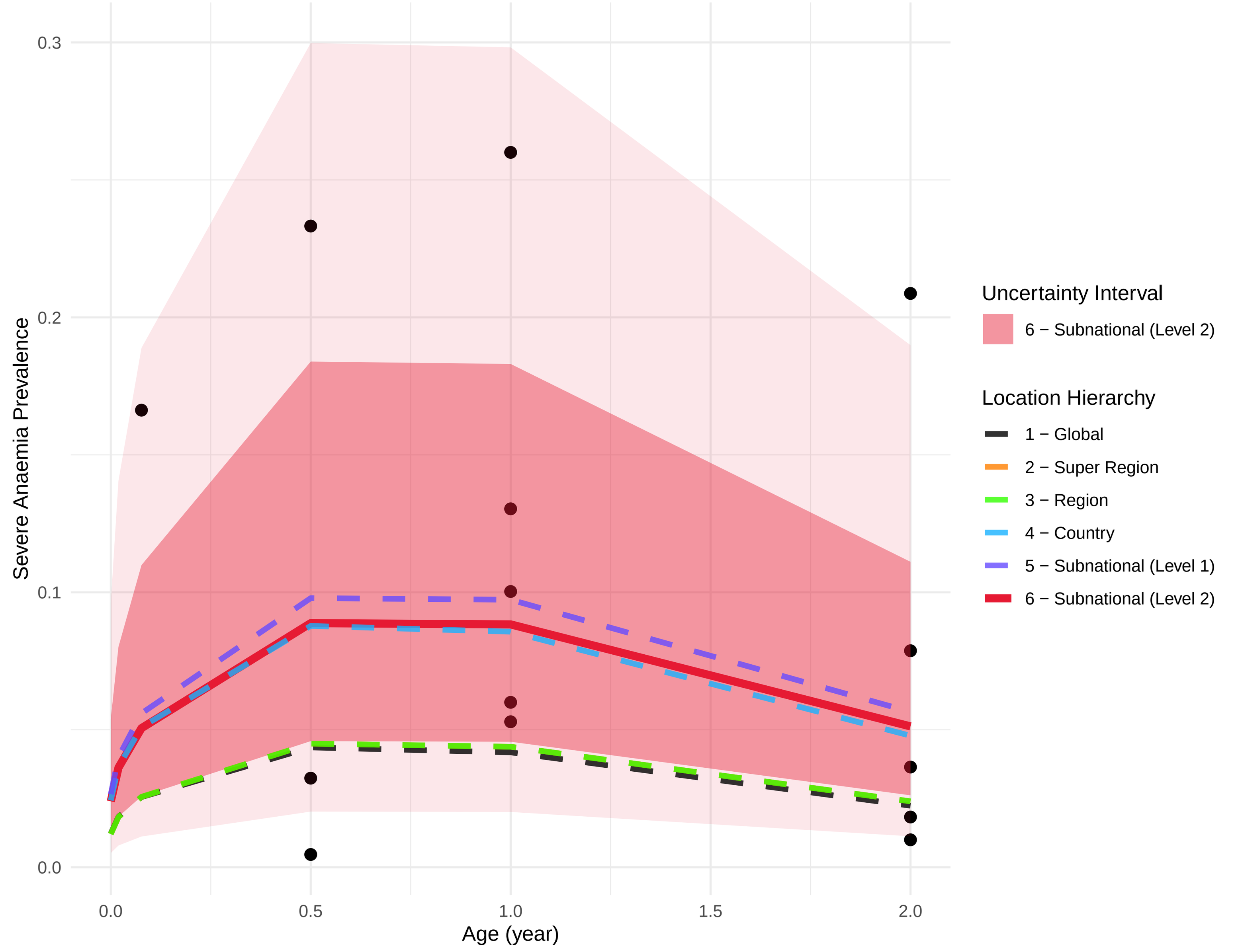

MR−BRT Age/Sex Fits − Males <2 Years Old − Severe Anaemia Prevalence − Kerala
WHO−2024 Adjustment Method

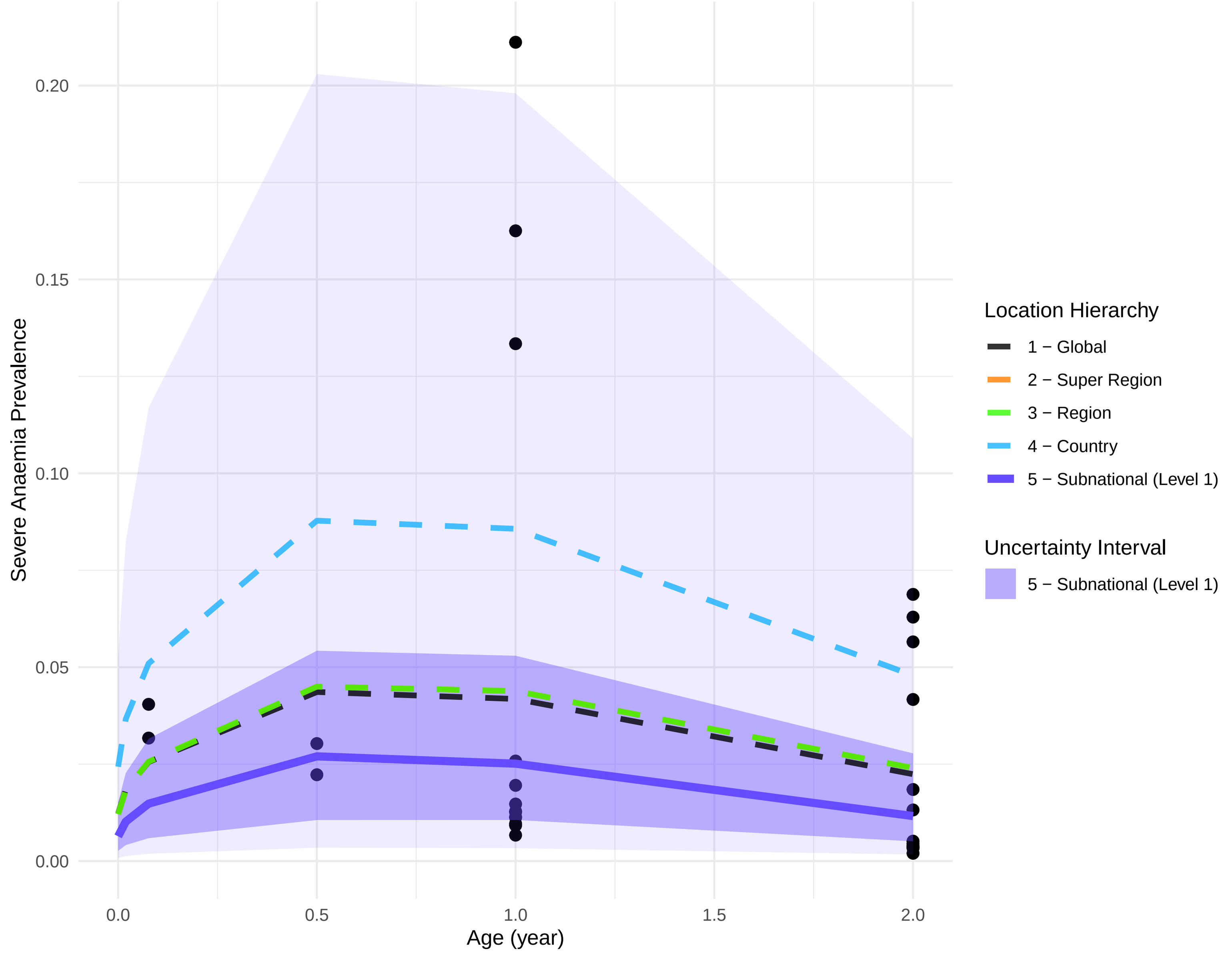

MR−BRT Age/Sex Fits − Males <2 Years Old − Severe Anaemia Prevalence − Kerala, Rural
WHO−2024 Adjustment Method

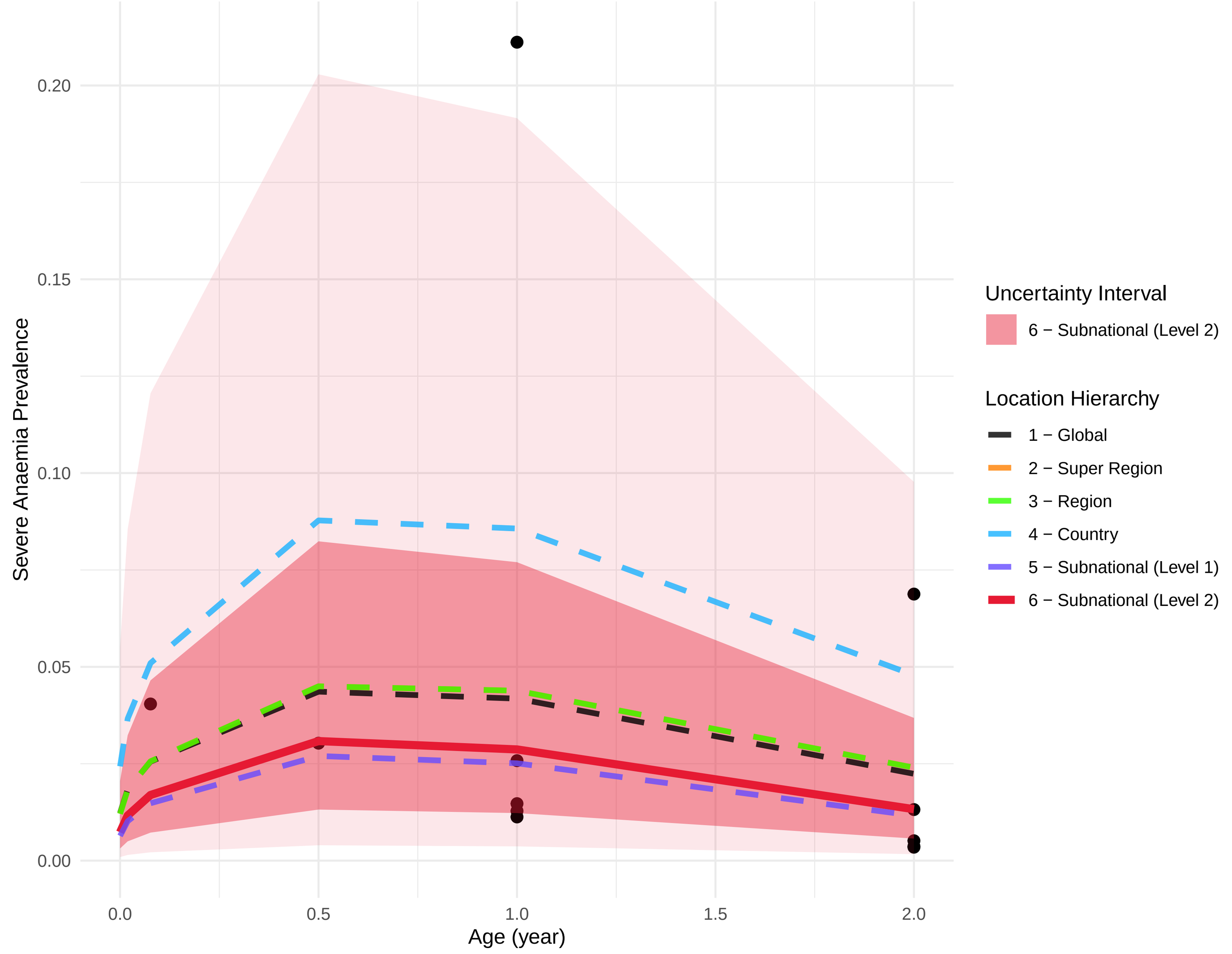

MR−BRT Age/Sex Fits − Males <2 Years Old − Severe Anaemia Prevalence − Kerala, Urban
WHO−2024 Adjustment Method

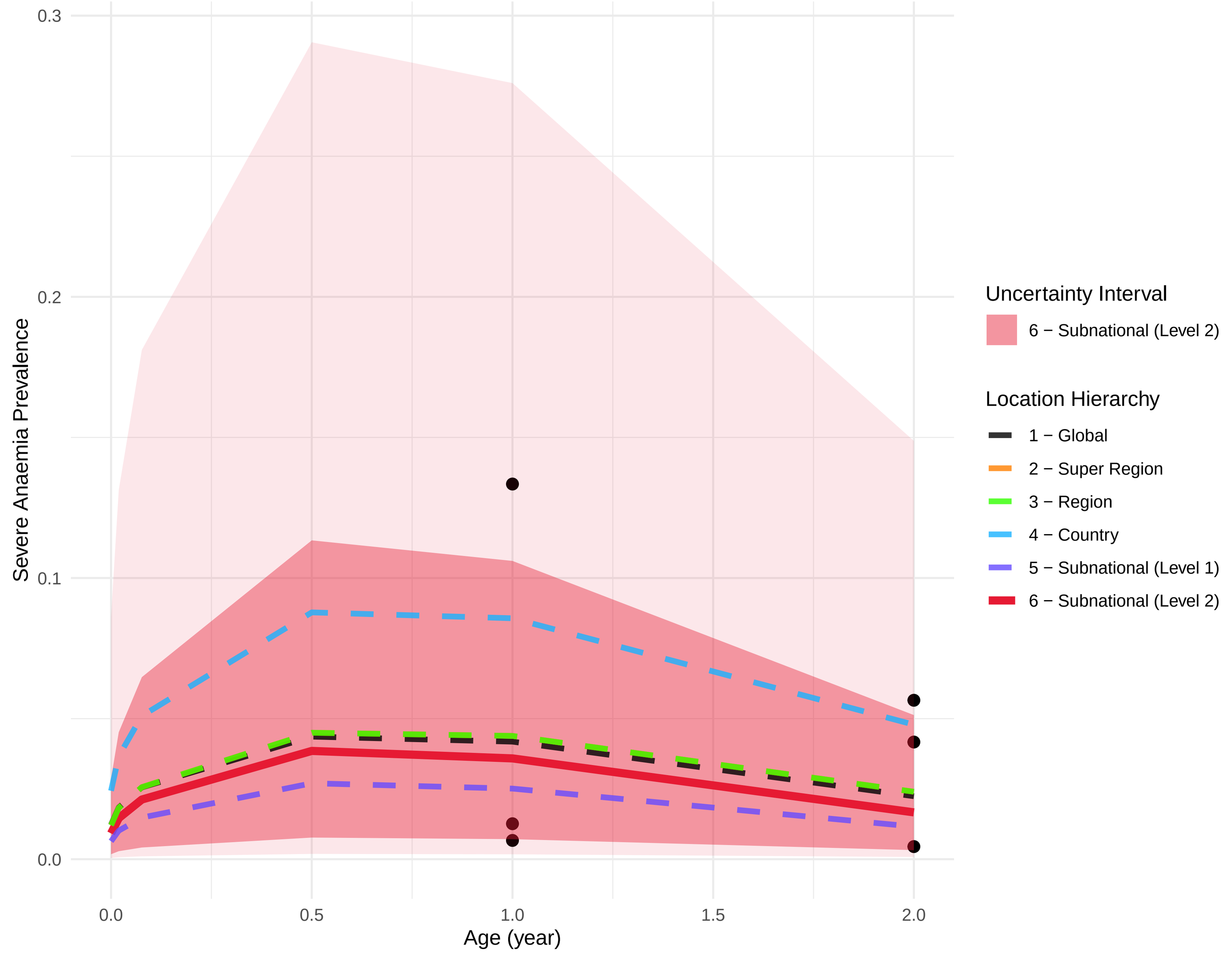

MR−BRT Age/Sex Fits − Males <2 Years Old − Severe Anaemia Prevalence − Madhya Pradesh
WHO−2024 Adjustment Method

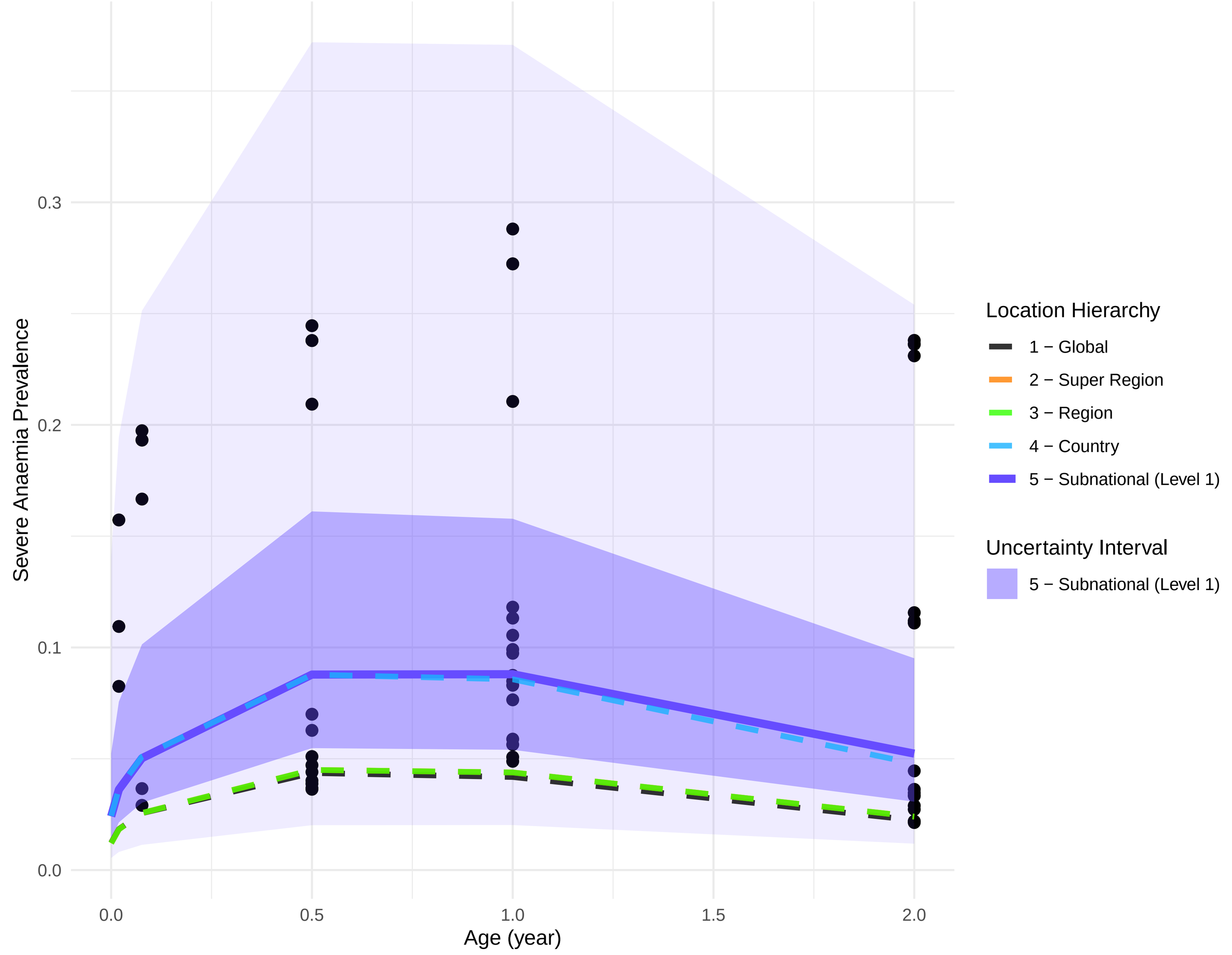

MR−BRT Age/Sex Fits − Males <2 Years Old − Severe Anaemia Prevalence − Madhya Pradesh, Rural
WHO−2024 Adjustment Method

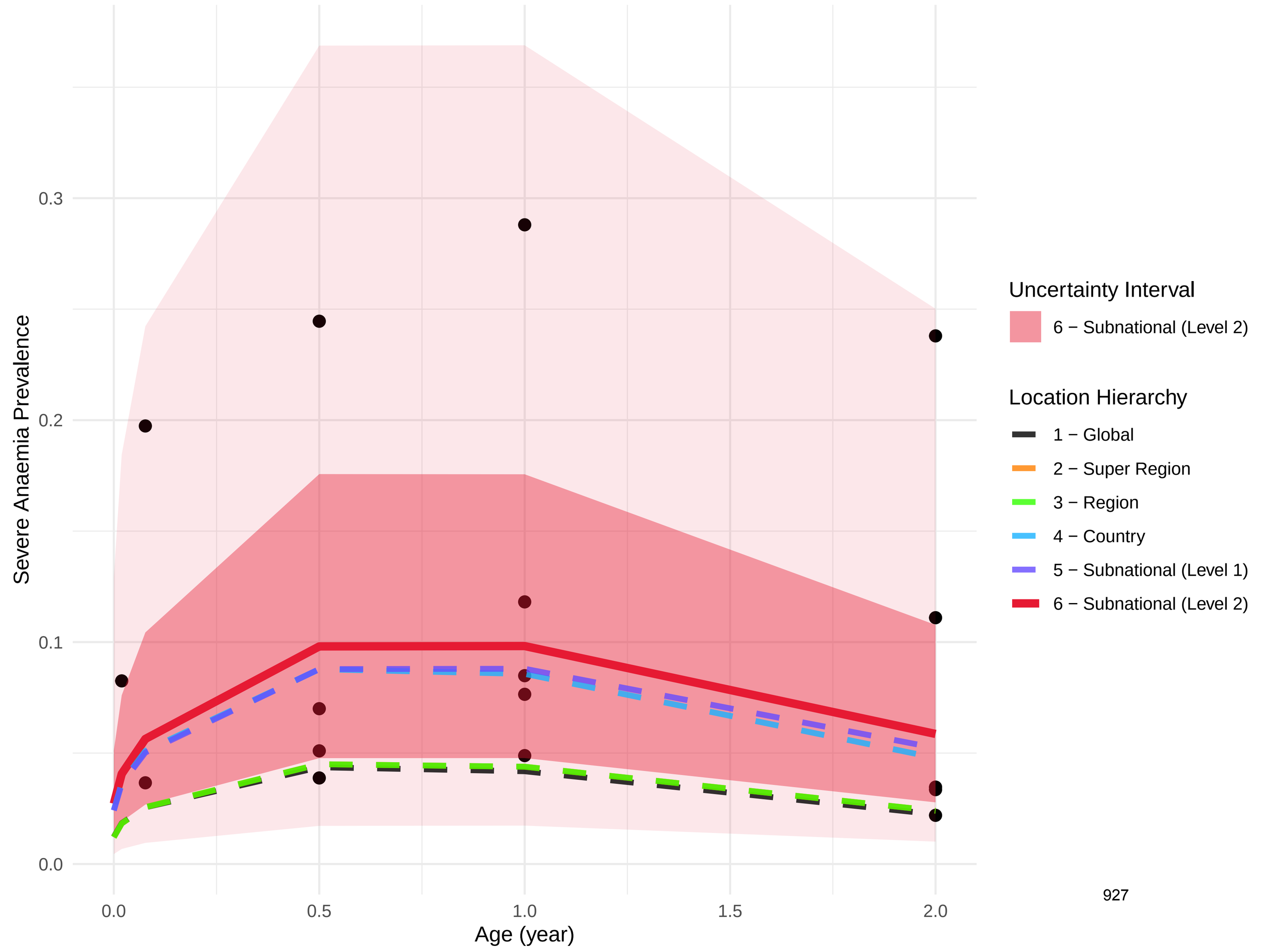

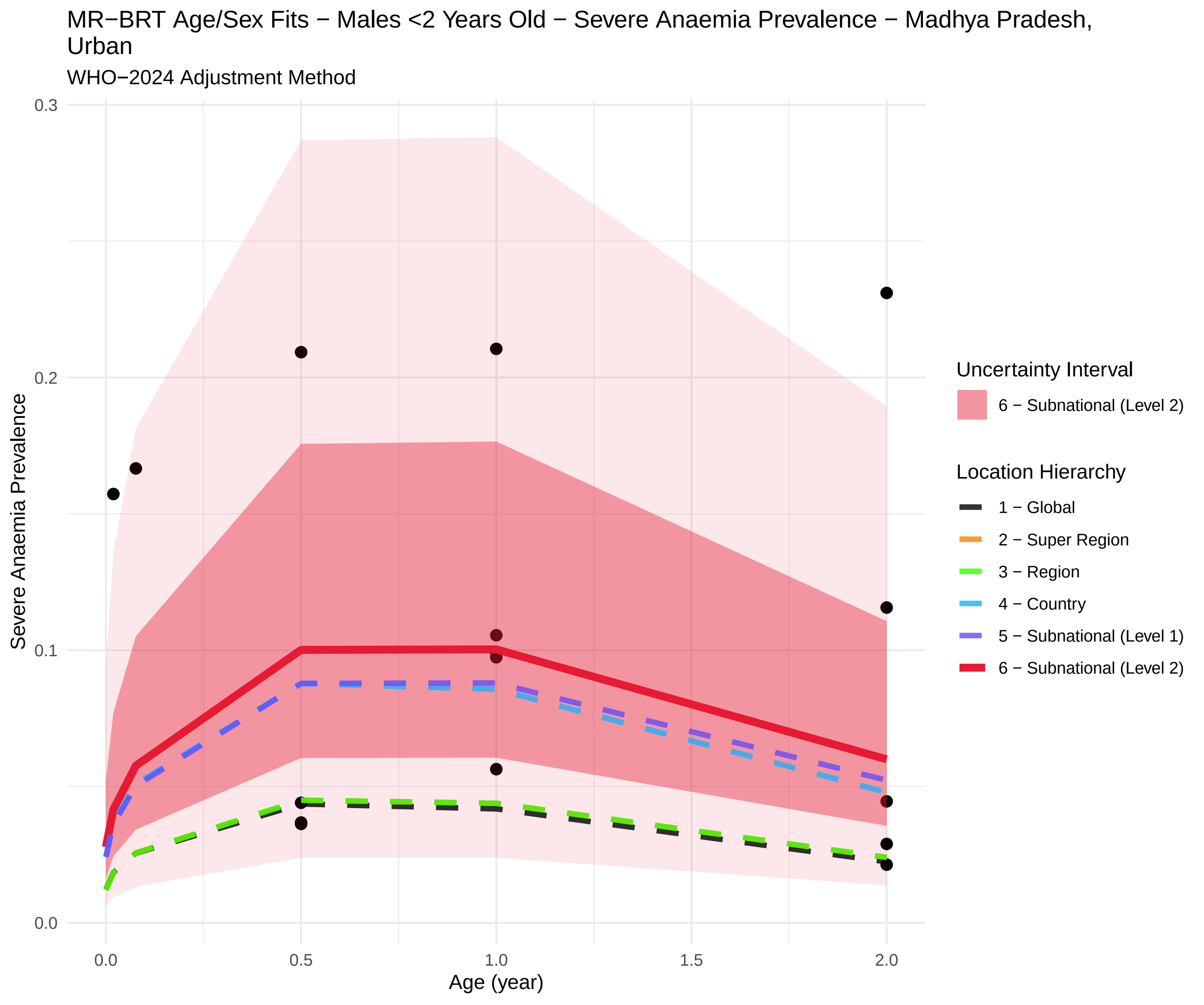
MR−BRT Age/Sex Fits − Males <2 Years Old − Severe Anaemia Prevalence − Madhya Pradesh, Urban
WHO−2024 Adjustment Method
Severe Anaemia Prevalence
Age (year)
Uncertainty Interval
6 − Subnational (Level 2)
Location Hierarchy
1 − Global
2 − Super Region
3 − Region
4 − Country
5 − Subnational (Level 1)
6 − Subnational (Level 2)

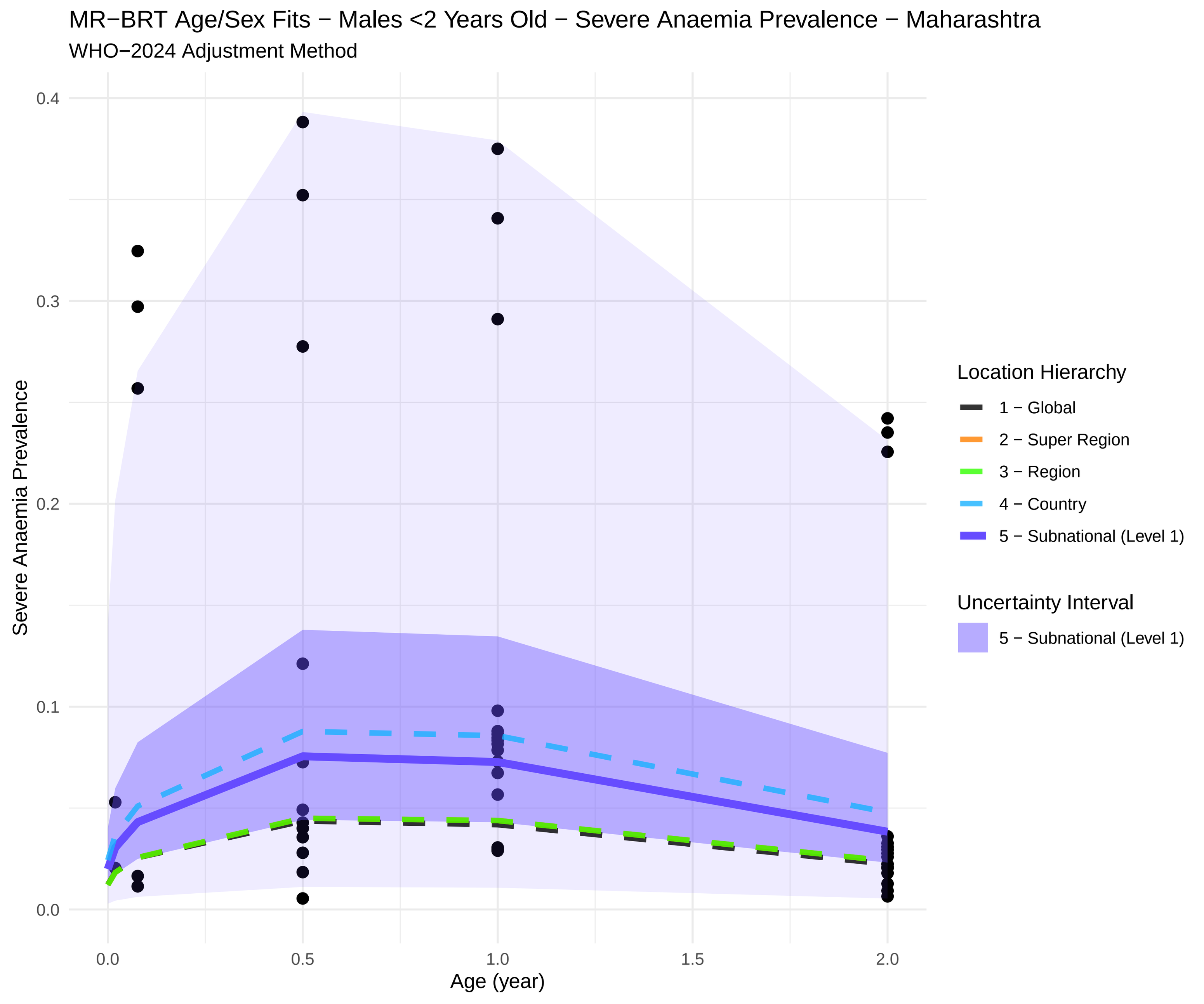
MR−BRT Age/Sex Fits − Males <2 Years Old − Severe Anaemia Prevalence − Maharashtra
WHO−2024 Adjustment Method
Severe Anaemia Prevalence
Age (year)
Location Hierarchy
1 − Global
2 − Super Region
3 − Region
4 − Country
5 − Subnational (Level 1)
Uncertainty Interval
5 − Subnational (Level 1)

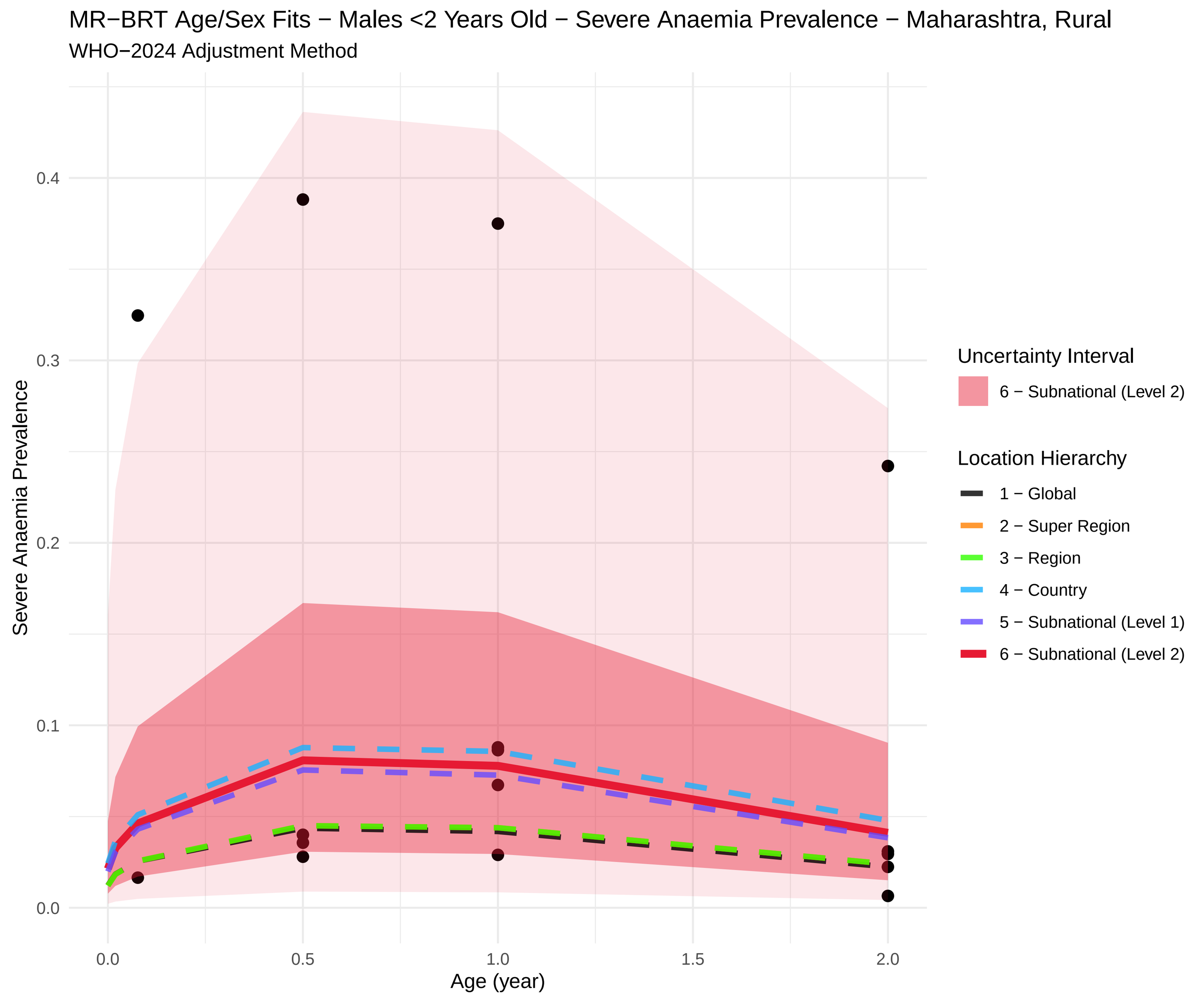
MR−BRT Age/Sex Fits − Males <2 Years Old − Severe Anaemia Prevalence − Maharashtra, Rural
WHO−2024 Adjustment Method
Severe Anaemia Prevalence
Age (year)
Uncertainty Interval
6 − Subnational (Level 2)
Location Hierarchy
1 − Global
2 − Super Region
3 − Region
4 − Country
5 − Subnational (Level 1)
6 − Subnational (Level 2)

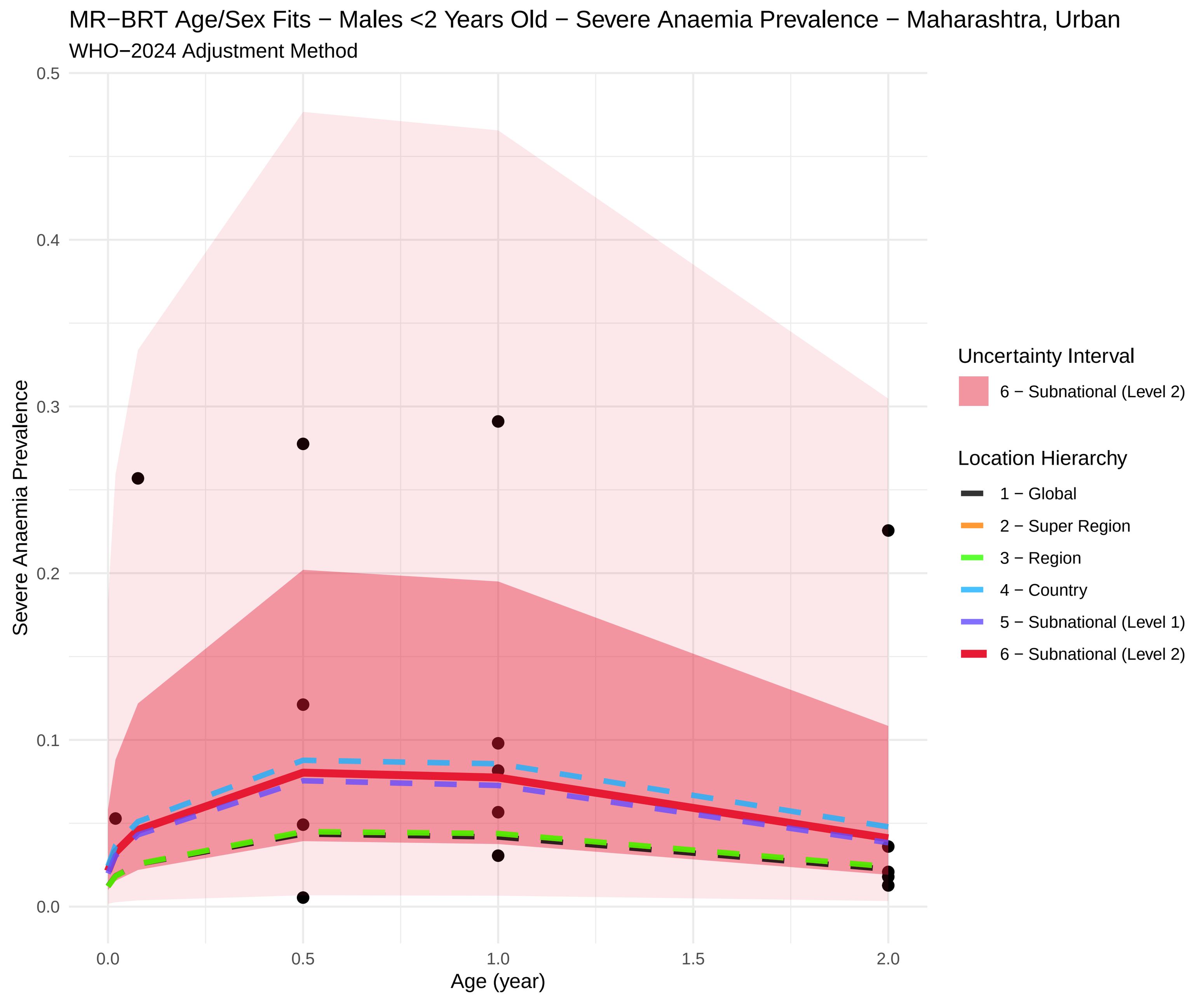
MR−BRT Age/Sex Fits − Males <2 Years Old − Severe Anaemia Prevalence − Maharashtra, Urban
WHO−2024 Adjustment Method
Severe Anaemia Prevalence
Age (year)
Uncertainty Interval
6 − Subnational (Level 2)
Location Hierarchy
1 − Global
2 − Super Region
3 − Region
4 − Country
5 − Subnational (Level 1)
6 − Subnational (Level 2)

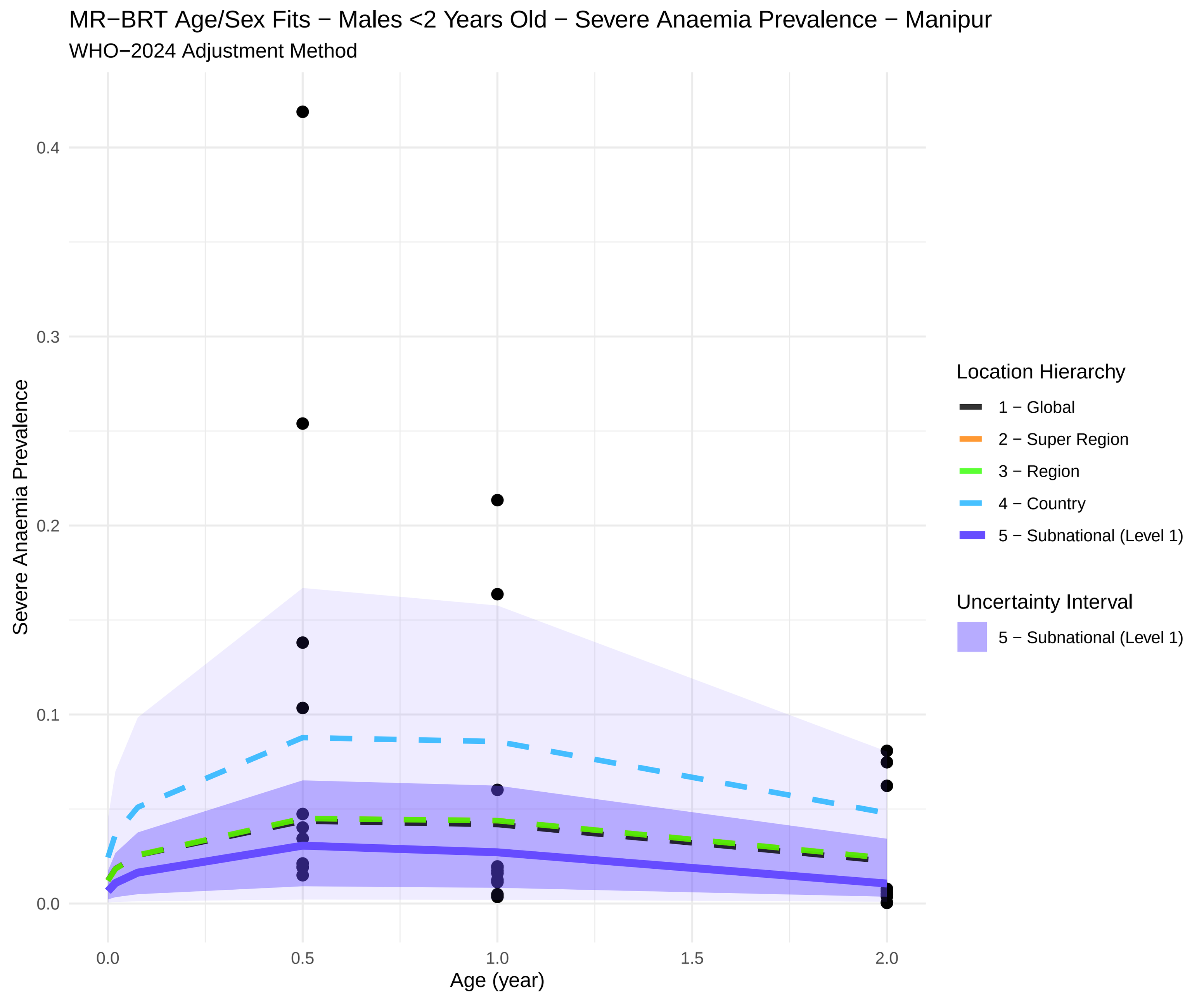
MR−BRT Age/Sex Fits − Males <2 Years Old − Severe Anaemia Prevalence − Manipur
WHO−2024 Adjustment Method
Severe Anaemia Prevalence
Age (year)
Location Hierarchy
1 − Global
2 − Super Region
3 − Region
4 − Country
5 − Subnational (Level 1)
Uncertainty Interval
5 − Subnational (Level 1)

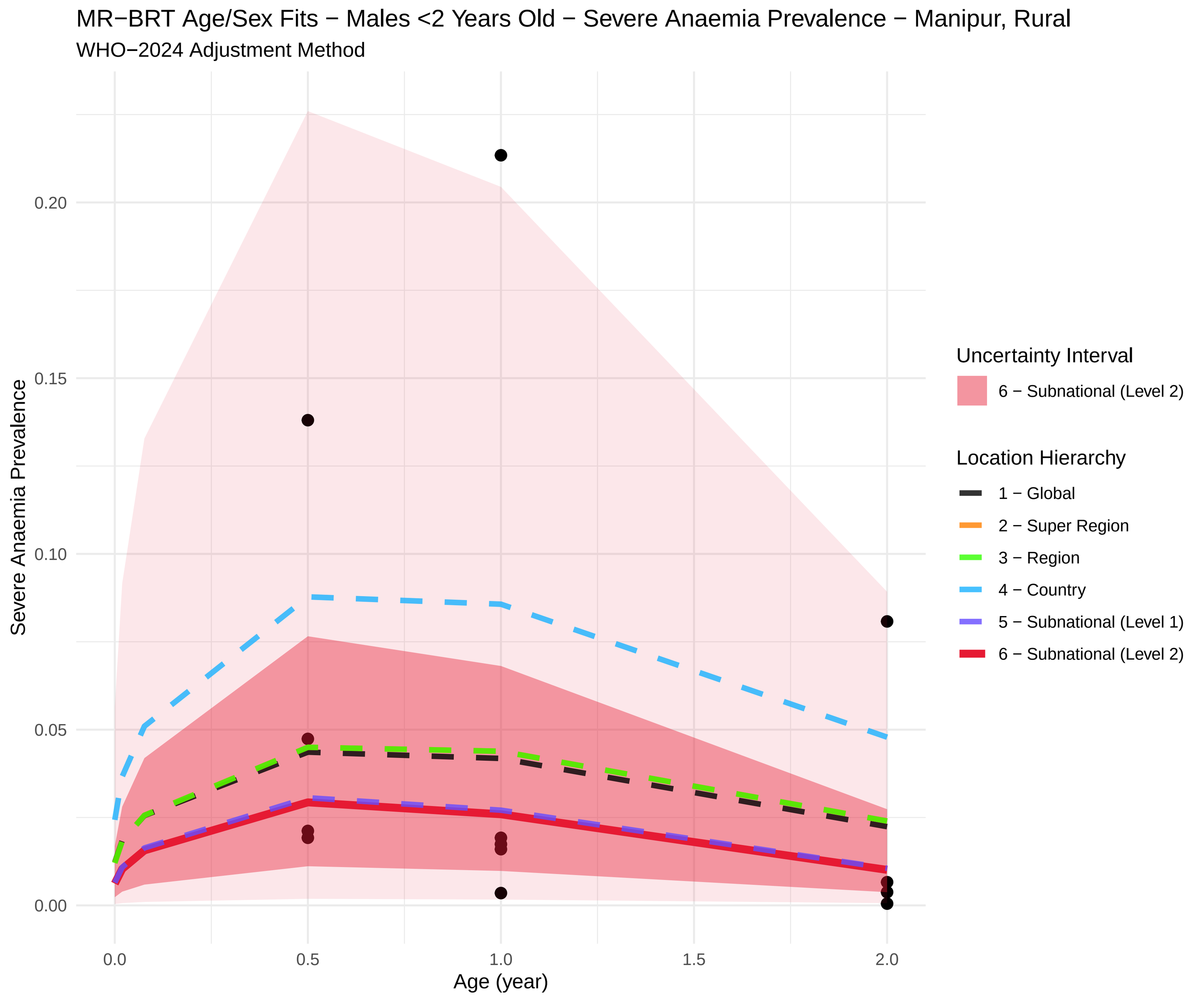
MR−BRT Age/Sex Fits − Males <2 Years Old − Severe Anaemia Prevalence − Manipur, Rural
WHO−2024 Adjustment Method
Severe Anaemia Prevalence
Age (year)
Uncertainty Interval
6 − Subnational (Level 2)
Location Hierarchy
1 − Global
2 − Super Region
3 − Region
4 − Country
5 − Subnational (Level 1)
6 − Subnational (Level 2)

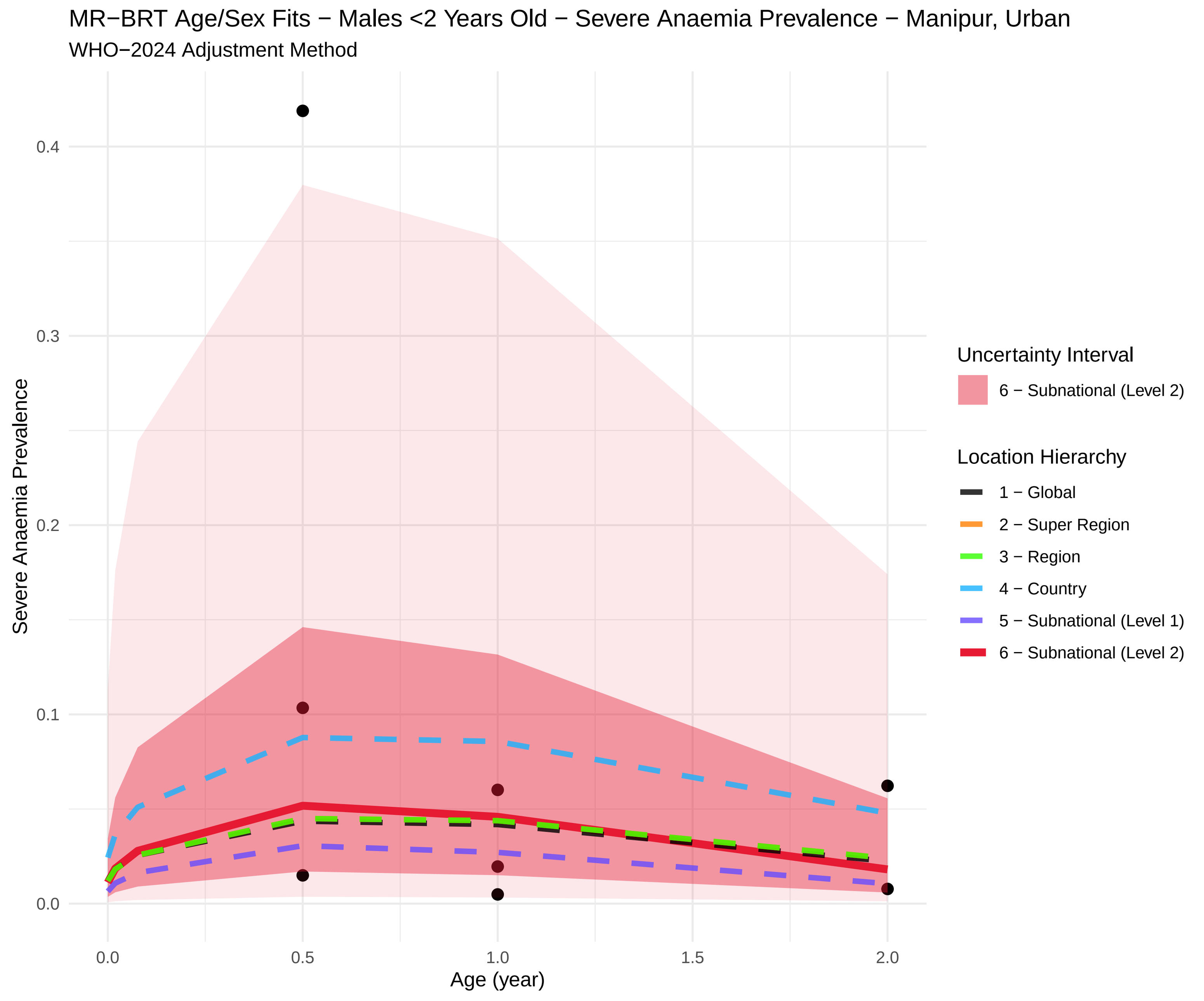
MR−BRT Age/Sex Fits − Males <2 Years Old − Severe Anaemia Prevalence − Manipur, Urban
WHO−2024 Adjustment Method
Severe Anaemia Prevalence
Age (year)
Uncertainty Interval
6 − Subnational (Level 2)
Location Hierarchy
1 − Global
2 − Super Region
3 − Region
4 − Country
5 − Subnational (Level 1)
6 − Subnational (Level 2)

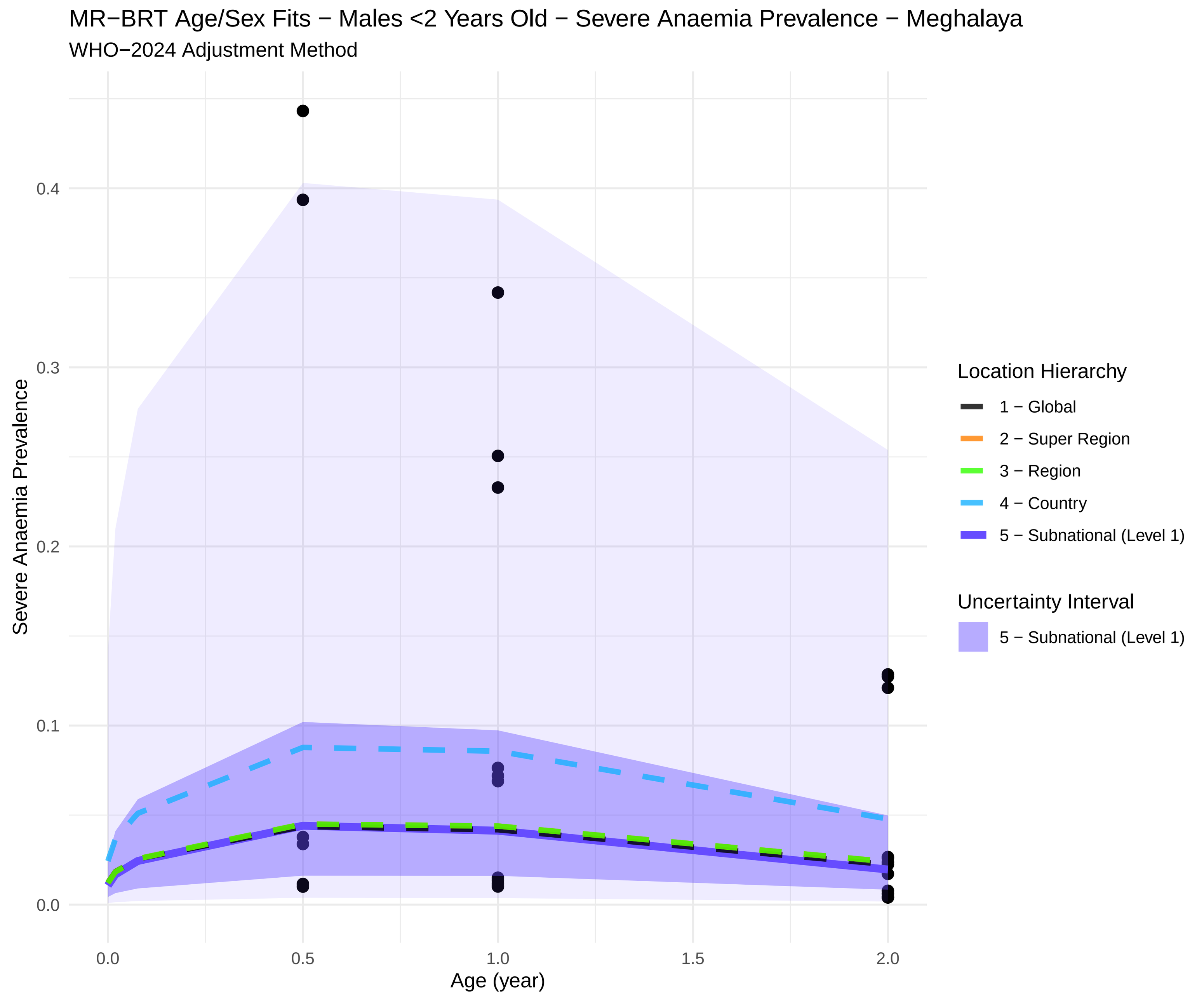
MR−BRT Age/Sex Fits − Males <2 Years Old − Severe Anaemia Prevalence − Meghalaya
WHO−2024 Adjustment Method
Severe Anaemia Prevalence
Age (year)
Location Hierarchy
1 − Global
2 − Super Region
3 − Region
4 − Country
5 − Subnational (Level 1)
Uncertainty Interval
5 − Subnational (Level 1)

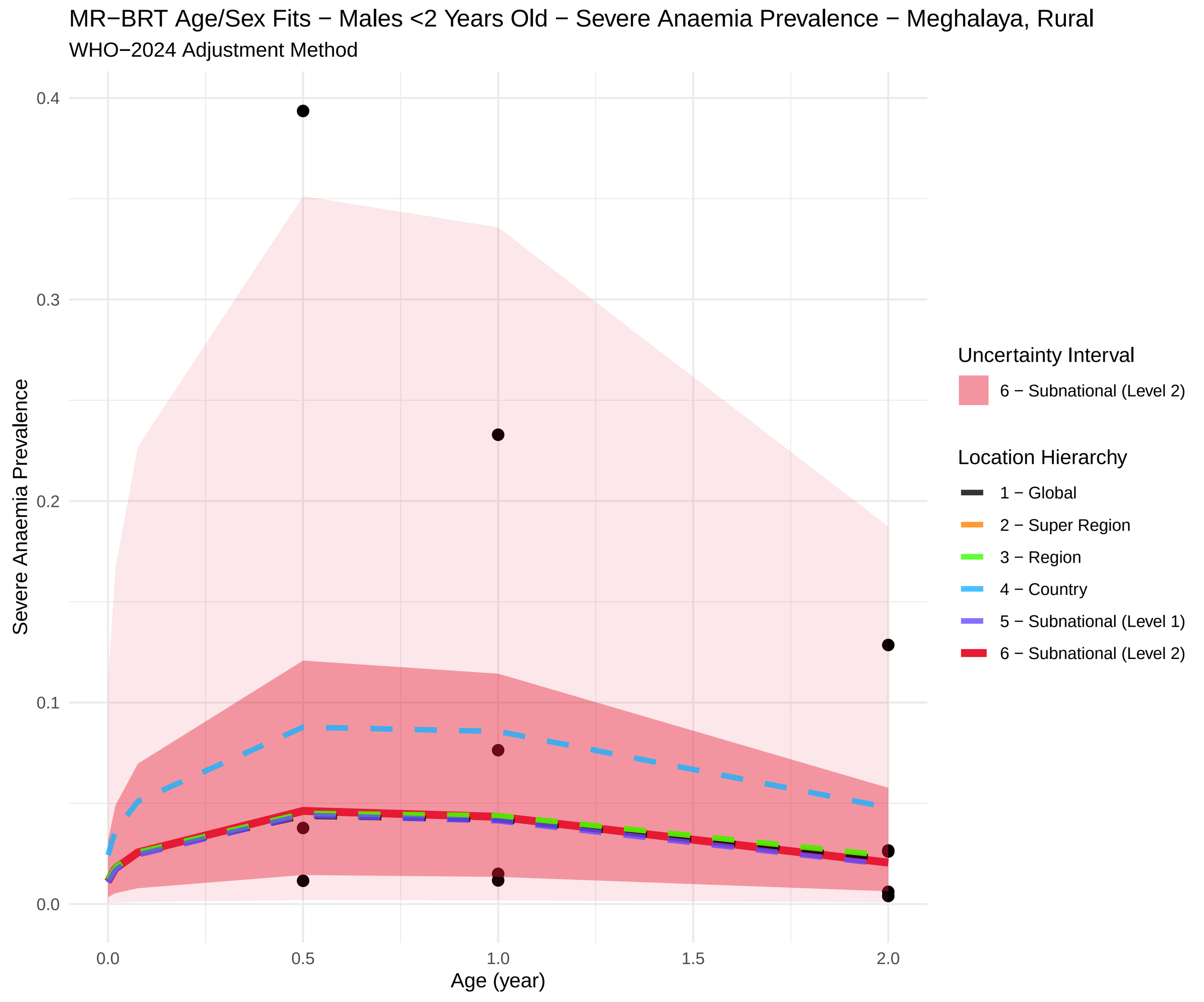
MR−BRT Age/Sex Fits − Males <2 Years Old − Severe Anaemia Prevalence − Meghalaya, Rural
WHO−2024 Adjustment Method
Severe Anaemia Prevalence
Age (year)
Uncertainty Interval
6 − Subnational (Level 2)
Location Hierarchy
1 − Global
2 − Super Region
3 − Region
4 − Country
5 − Subnational (Level 1)
6 − Subnational (Level 2)

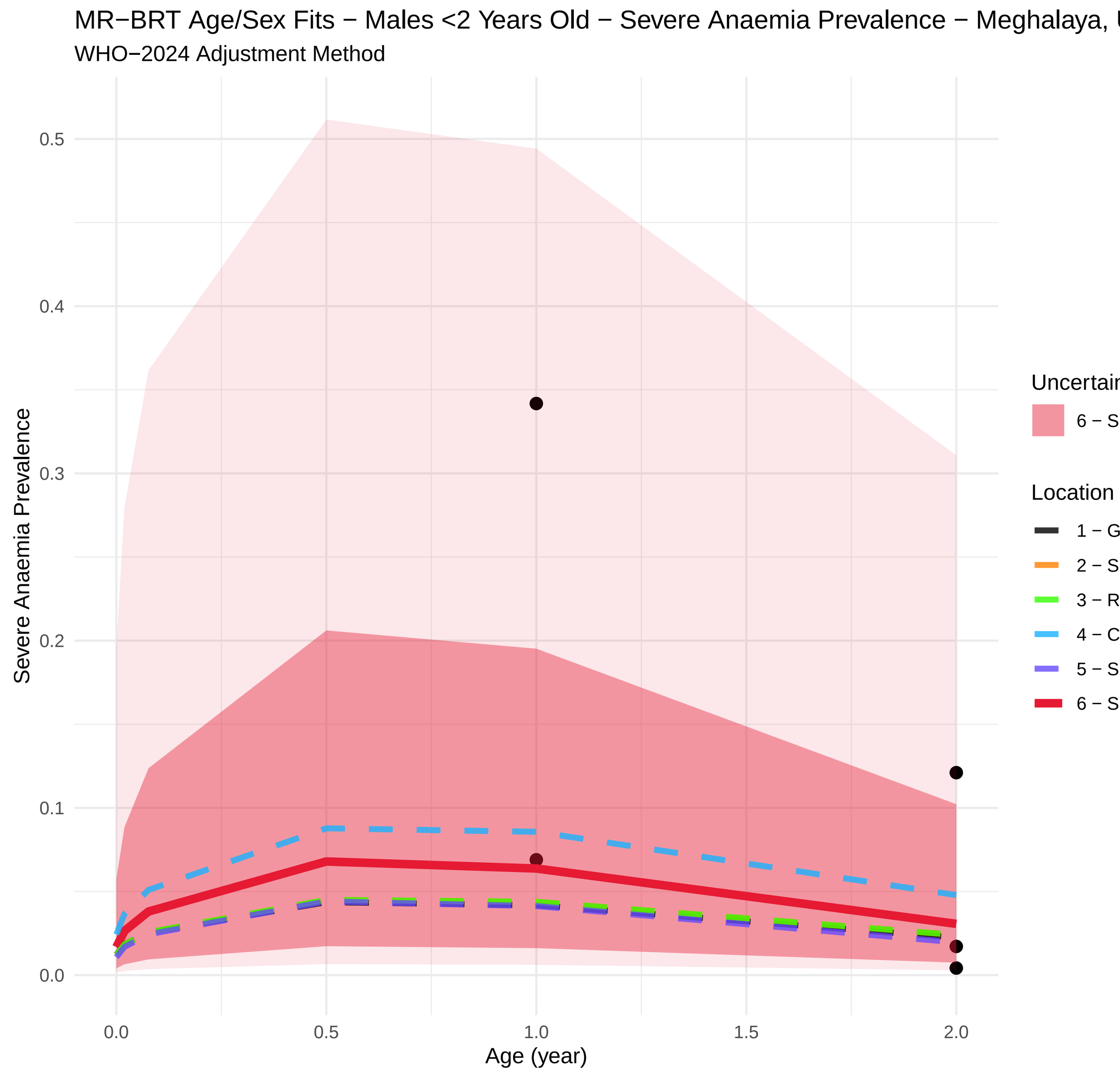
MR−BRT Age/Sex Fits − Males <2 Years Old − Severe Anaemia Prevalence − Meghalaya, Urban
WHO−2024 Adjustment Method
Severe Anaemia Prevalence
Age (year)
Uncertainty Interval
6 − Subnational (Level 2)
Location Hierarchy
1 − Global
2 − Super Region
3 − Region
4 − Country
5 − Subnational (Level 1)
6 − Subnational (Level 2)

MR−BRT Age/Sex Fits − Males <2 Years Old − Severe Anaemia Prevalence − Mizoram
WHO−2024 Adjustment Method

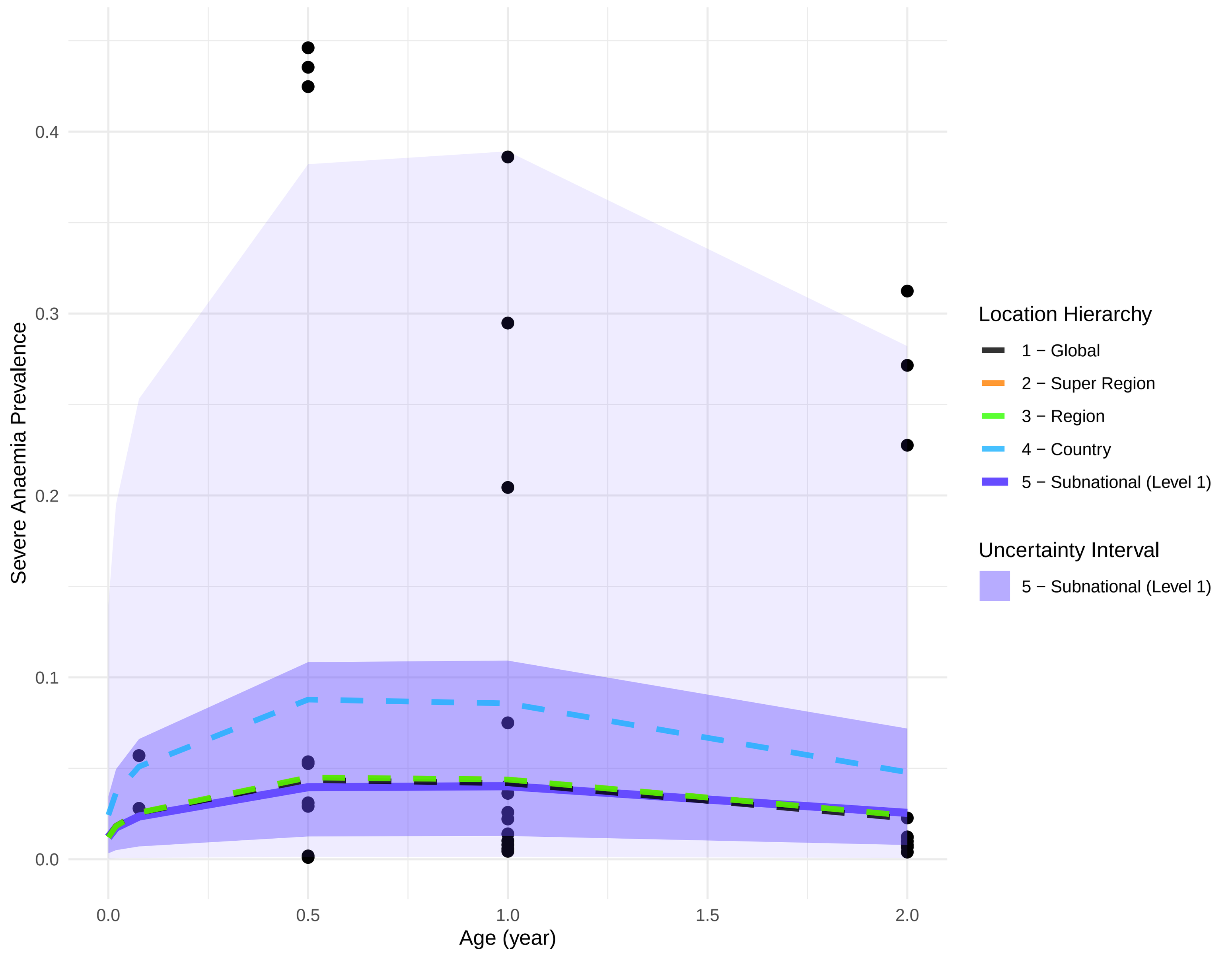

MR−BRT Age/Sex Fits − Males <2 Years Old − Severe Anaemia Prevalence − Mizoram, Rural
WHO−2024 Adjustment Method

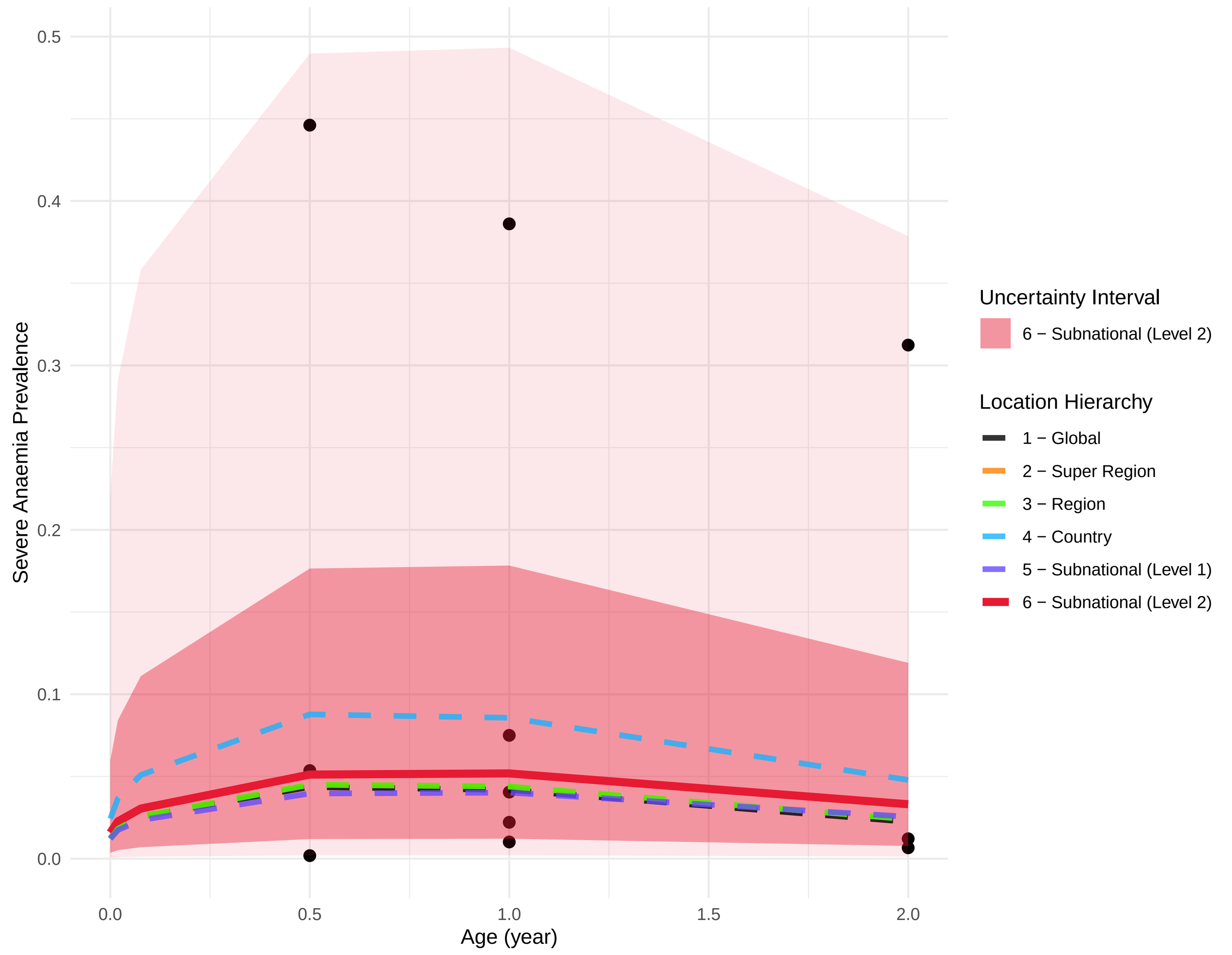

MR−BRT Age/Sex Fits − Males <2 Years Old − Severe Anaemia Prevalence − Mizoram, Urban
WHO−2024 Adjustment Method

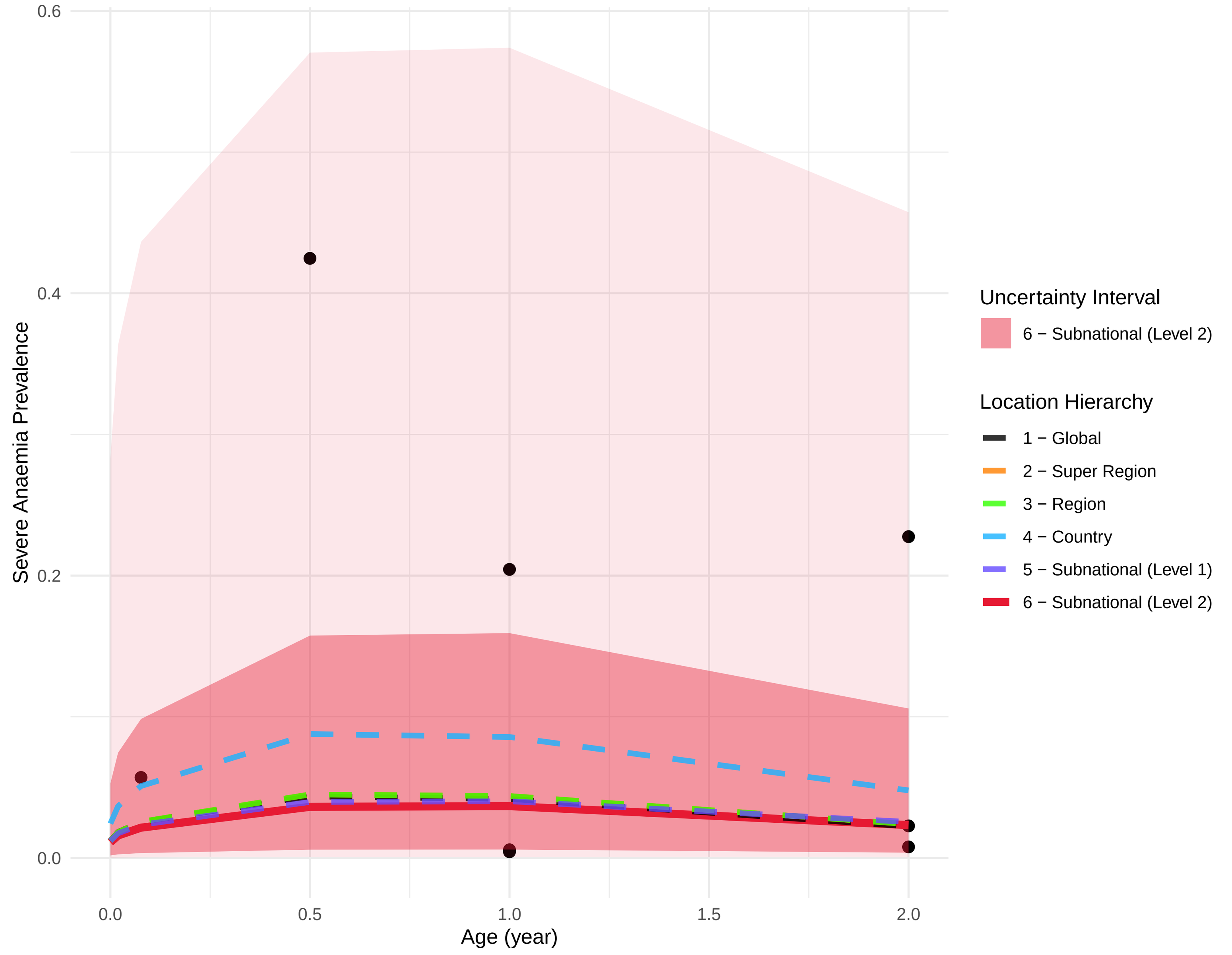

MR−BRT Age/Sex Fits − Males <2 Years Old − Severe Anaemia Prevalence − Nagaland
WHO−2024 Adjustment Method

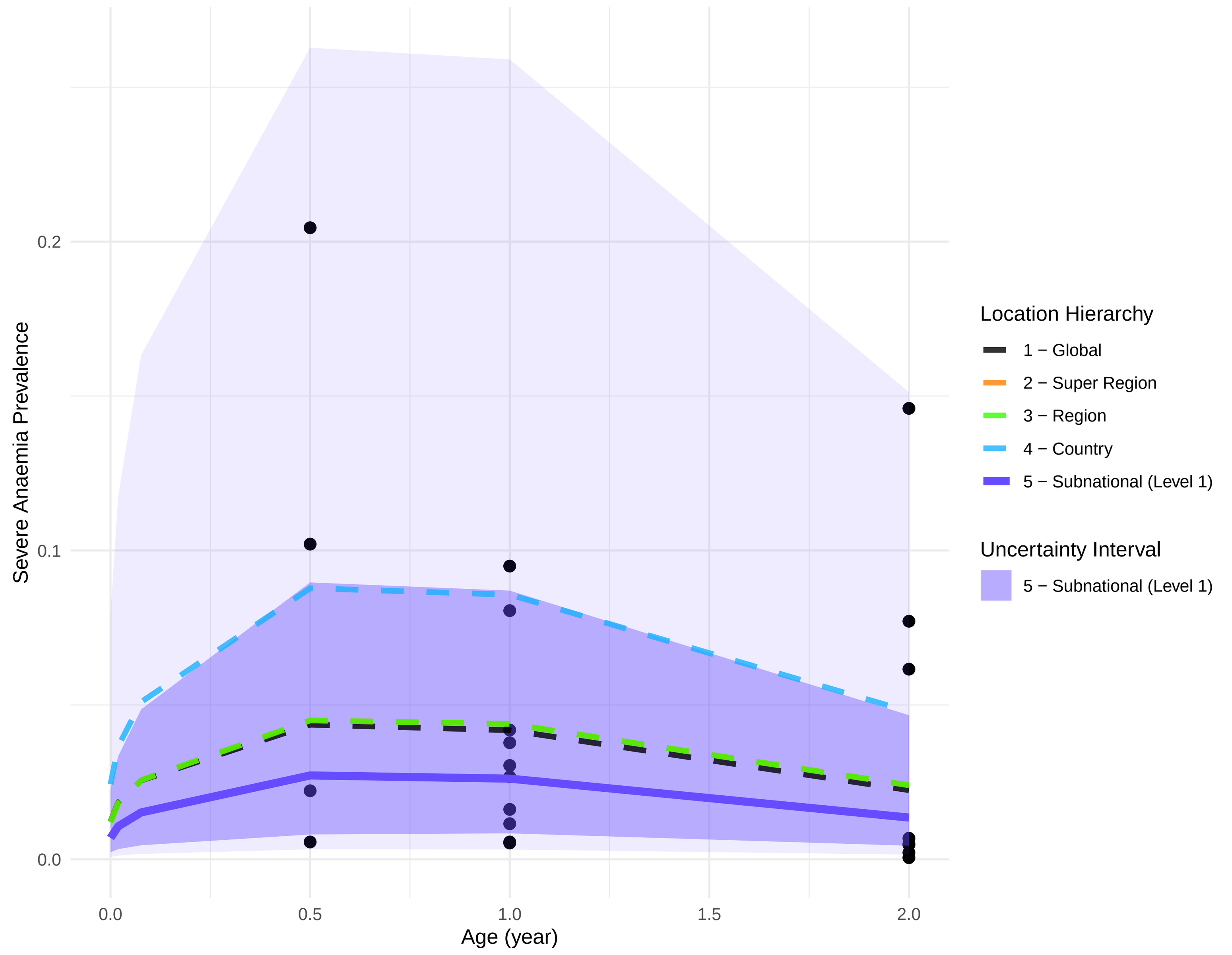

MR−BRT Age/Sex Fits − Males <2 Years Old − Severe Anaemia Prevalence − Nagaland, Rural
WHO−2024 Adjustment Method

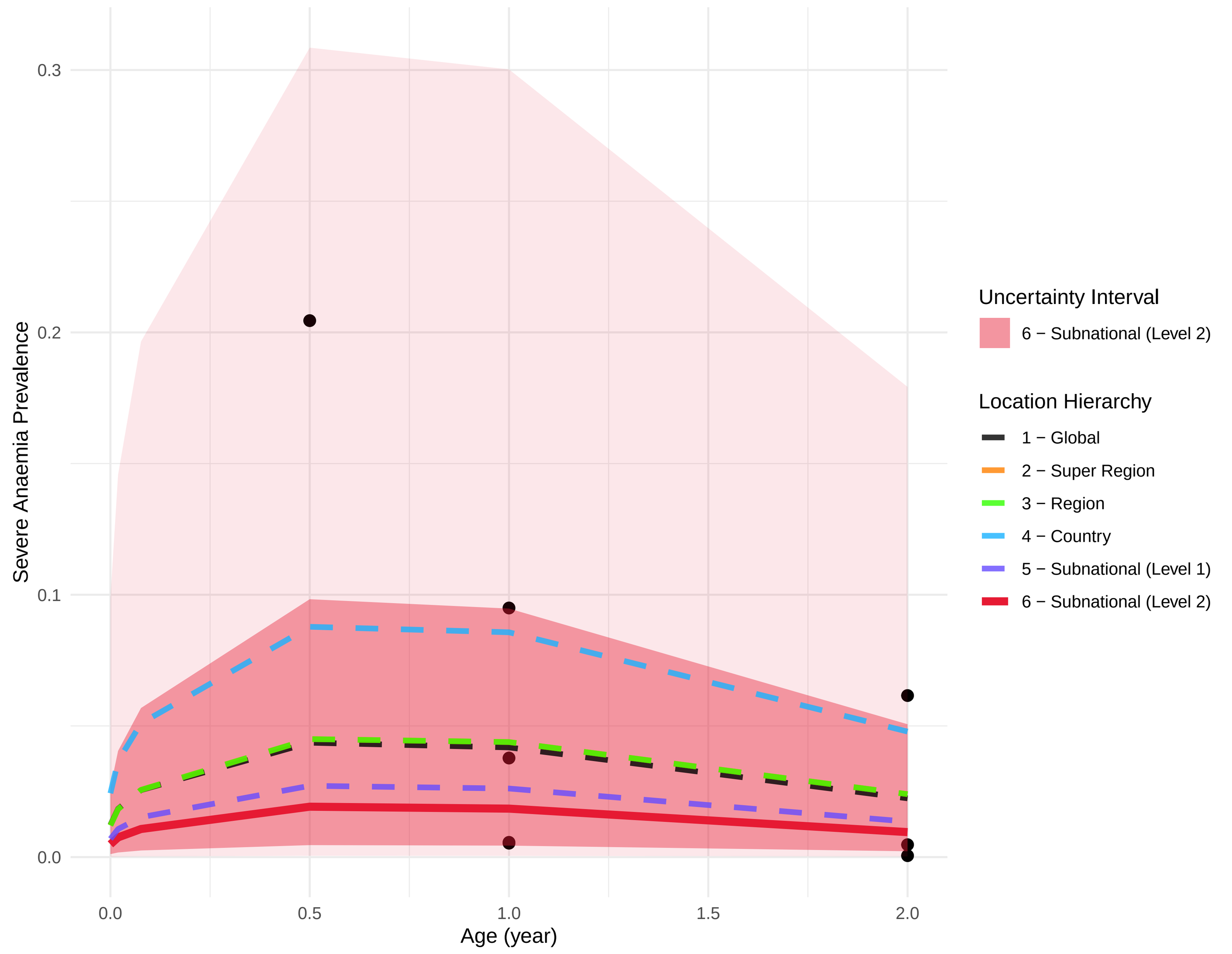

MR−BRT Age/Sex Fits − Males <2 Years Old − Severe Anaemia Prevalence − Nagaland, Urban
WHO−2024 Adjustment Method

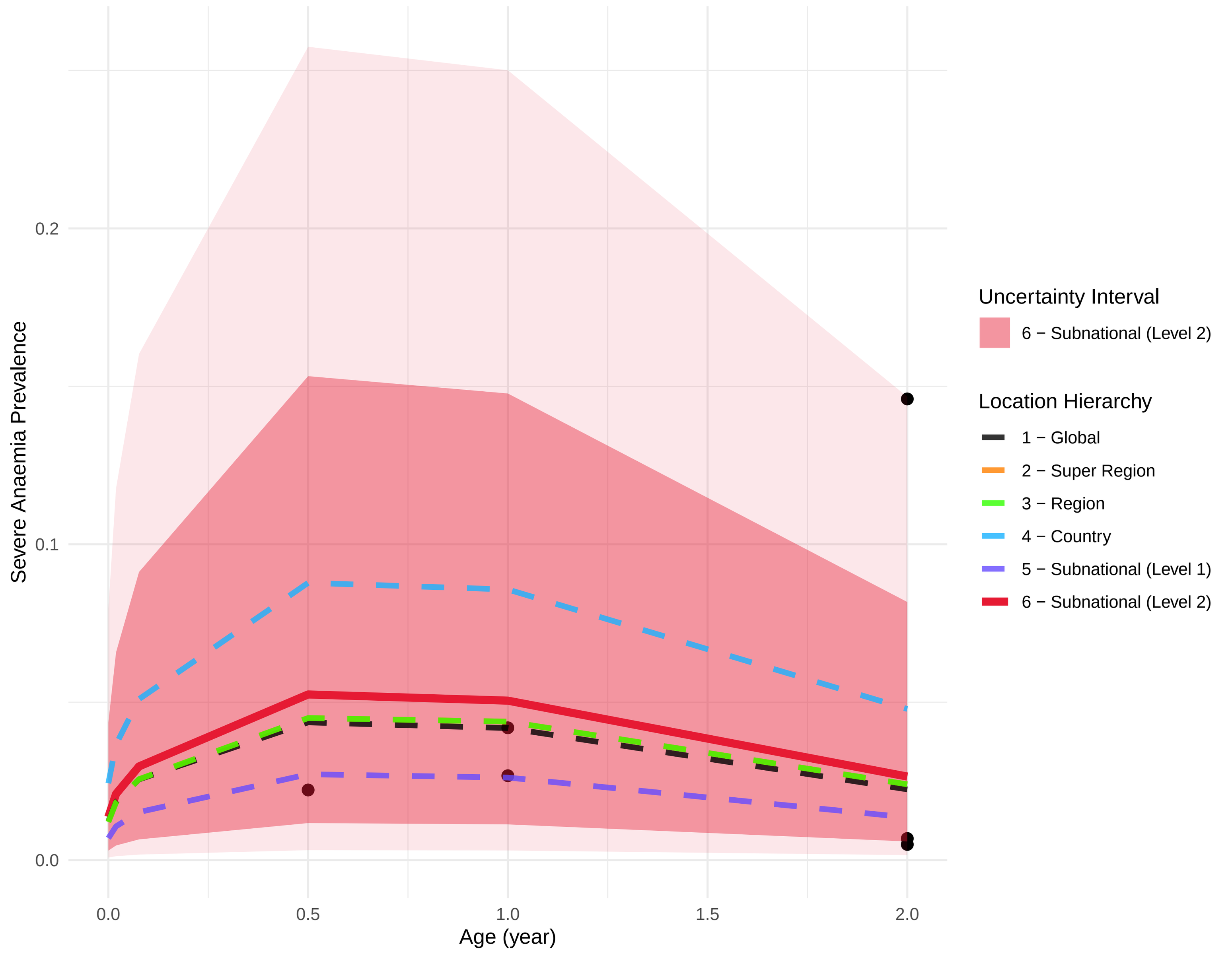

MR−BRT Age/Sex Fits − Males <2 Years Old − Severe Anaemia Prevalence − Odisha
WHO−2024 Adjustment Method

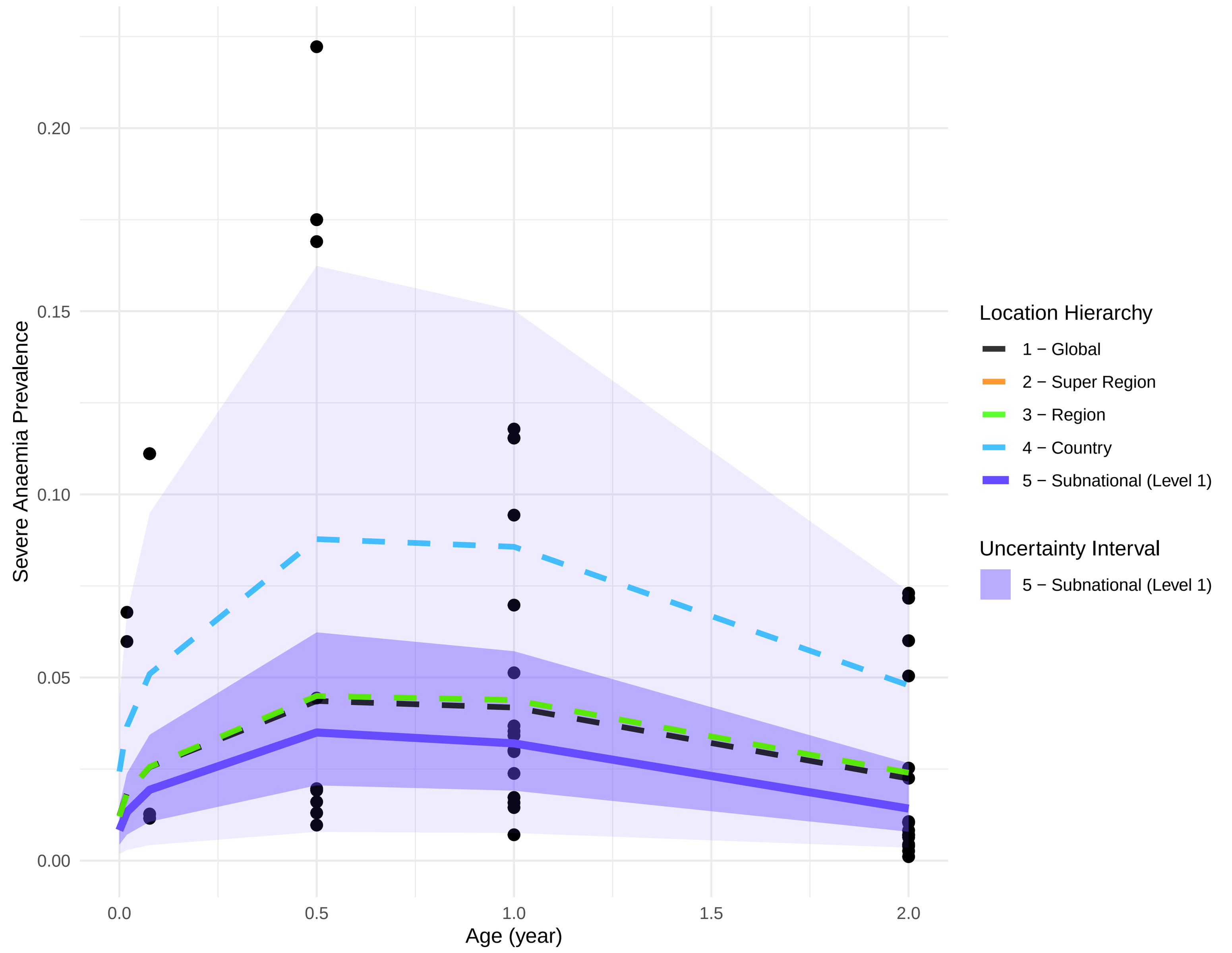

MR−BRT Age/Sex Fits − Males <2 Years Old − Severe Anaemia Prevalence − Odisha, Rural
WHO−2024 Adjustment Method

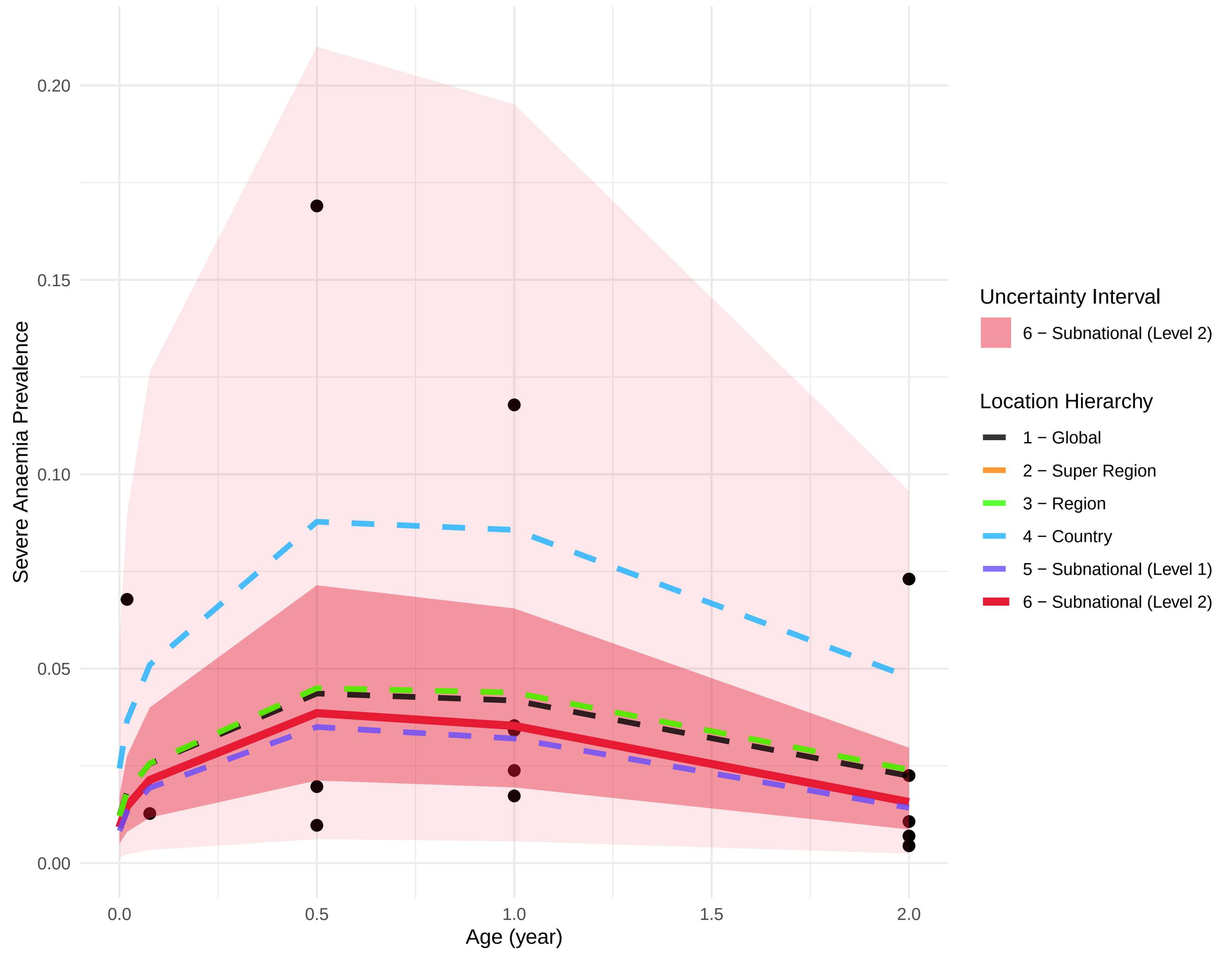

MR−BRT Age/Sex Fits − Males <2 Years Old − Severe Anaemia Prevalence − Odisha, Urban
WHO−2024 Adjustment Method

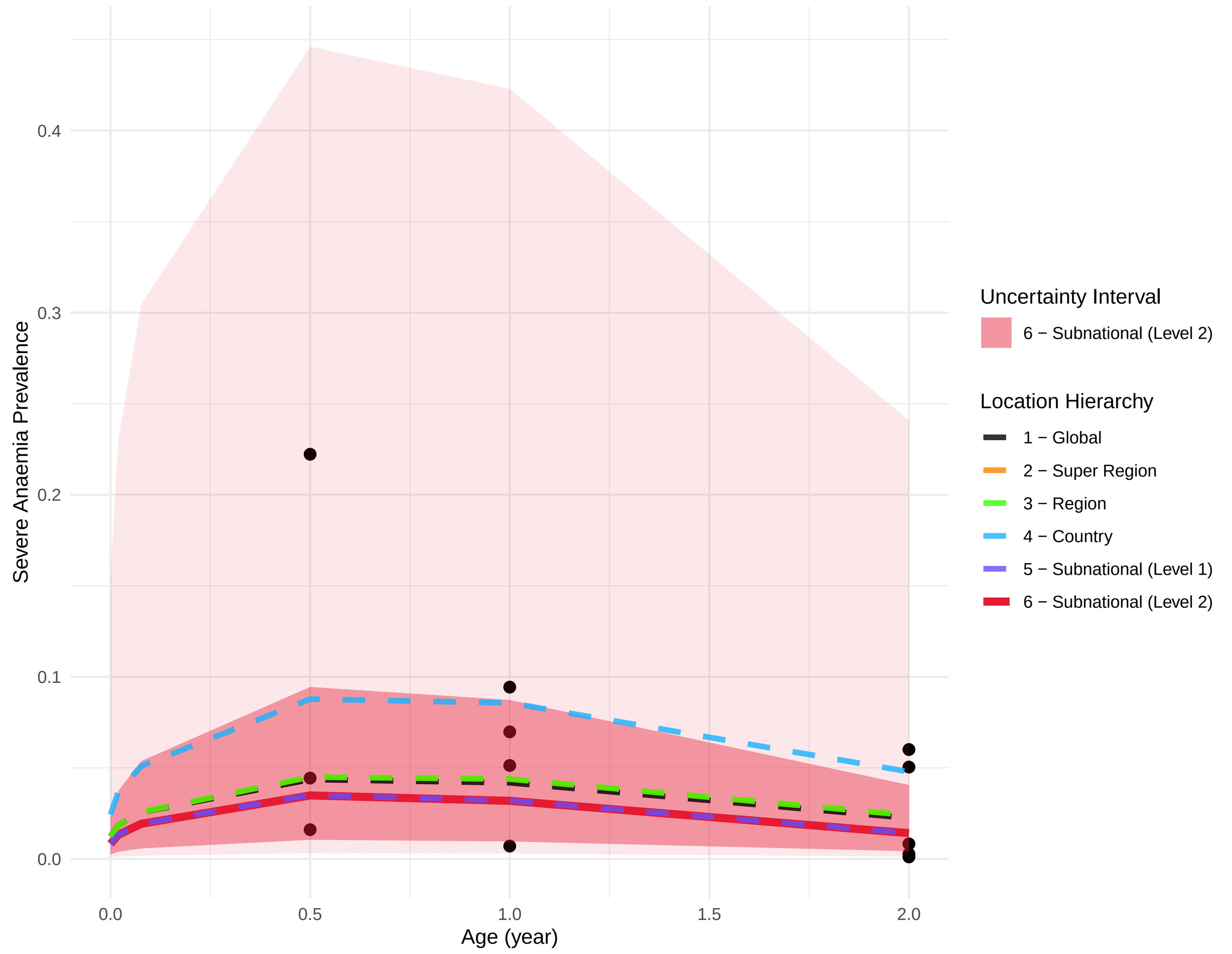

MR−BRT Age/Sex Fits − Males <2 Years Old − Severe Anaemia Prevalence − Other Union Territories
WHO−2024 Adjustment Method

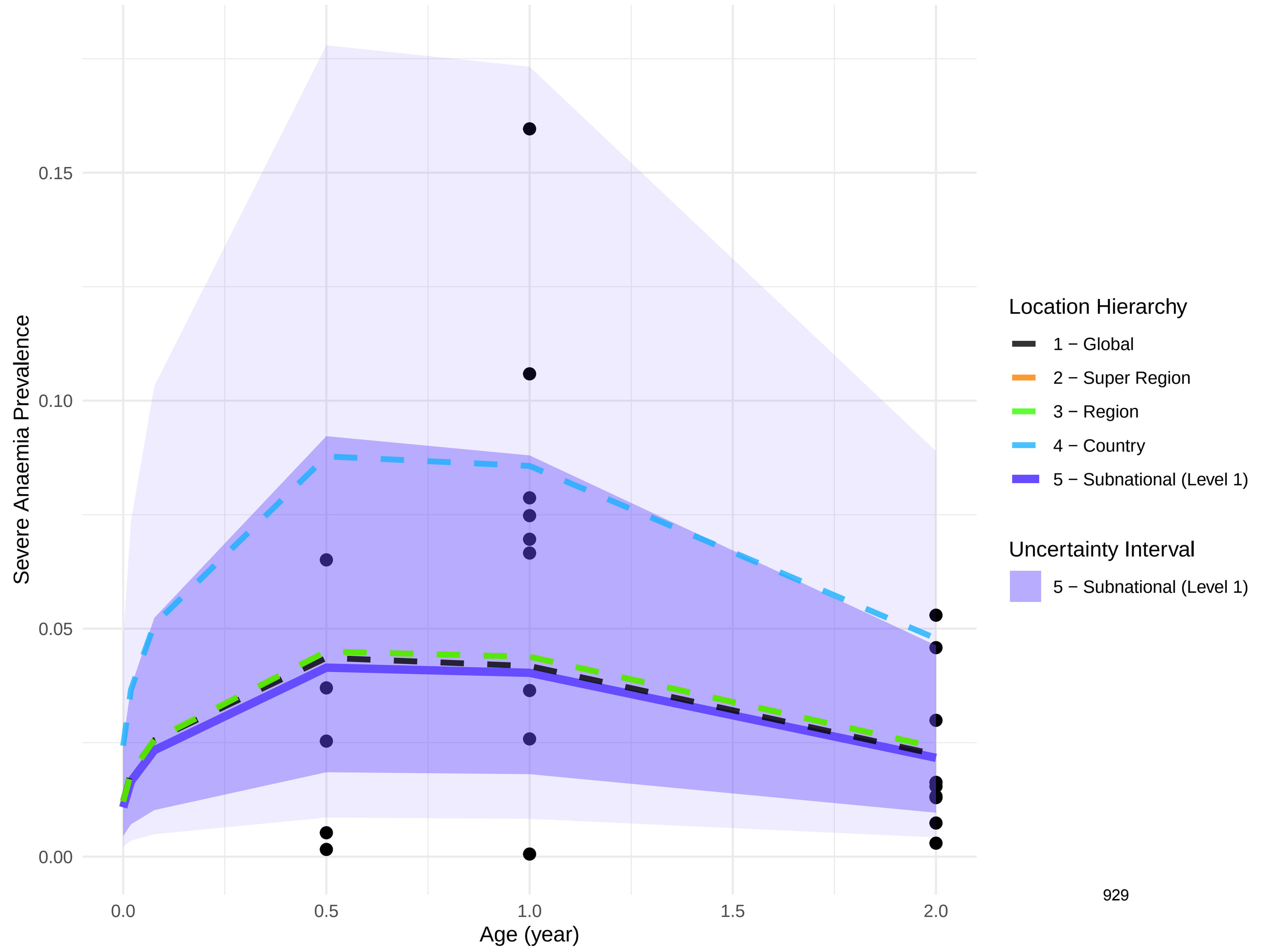

MR−BRT Age/Sex Fits − Males <2 Years Old − Severe Anaemia Prevalence − Other Union Territories, Rural
WHO−2024 Adjustment Method

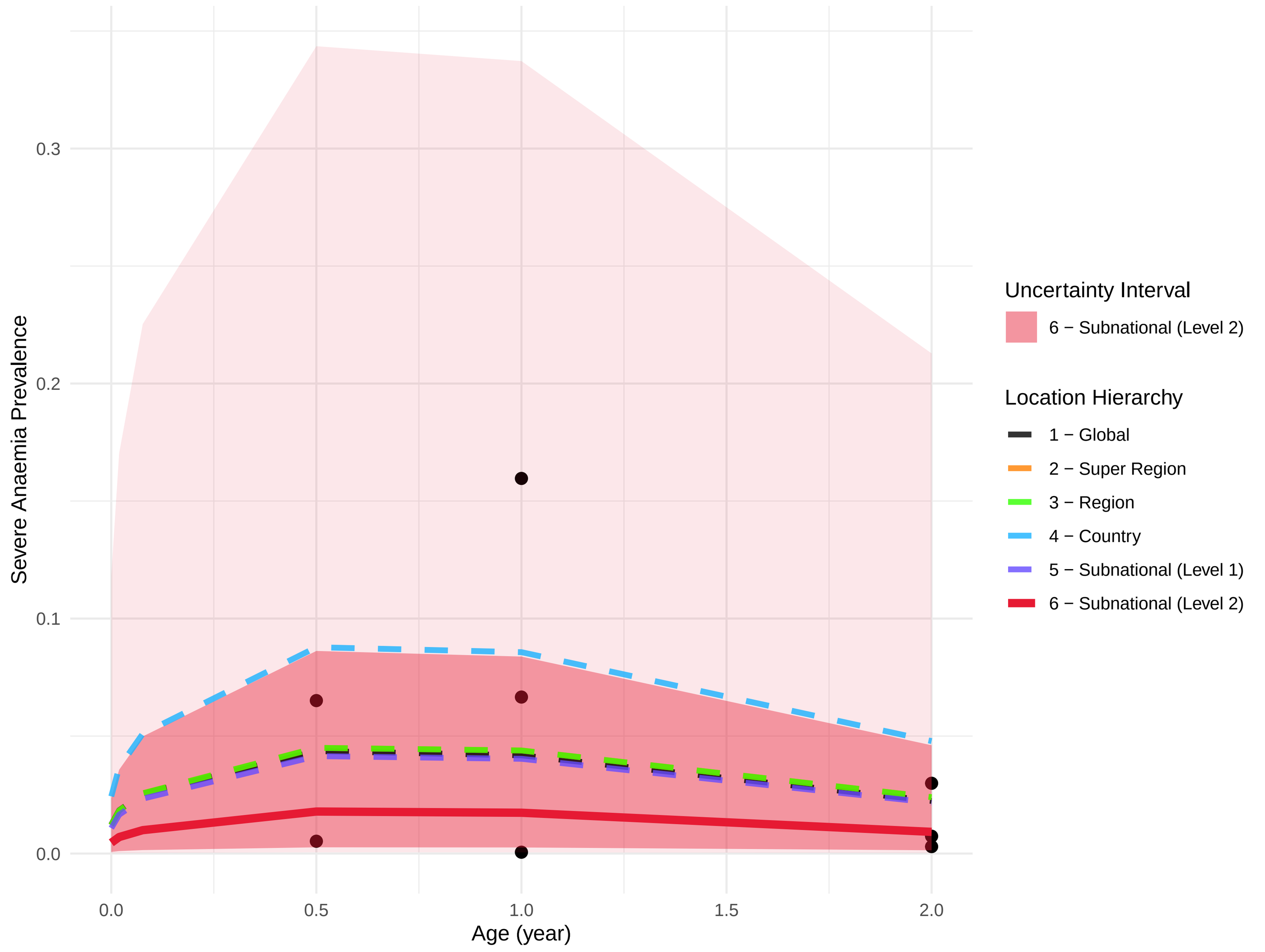

MR−BRT Age/Sex Fits − Males <2 Years Old − Severe Anaemia Prevalence − Other Union Territories, Urban
WHO−2024 Adjustment Method

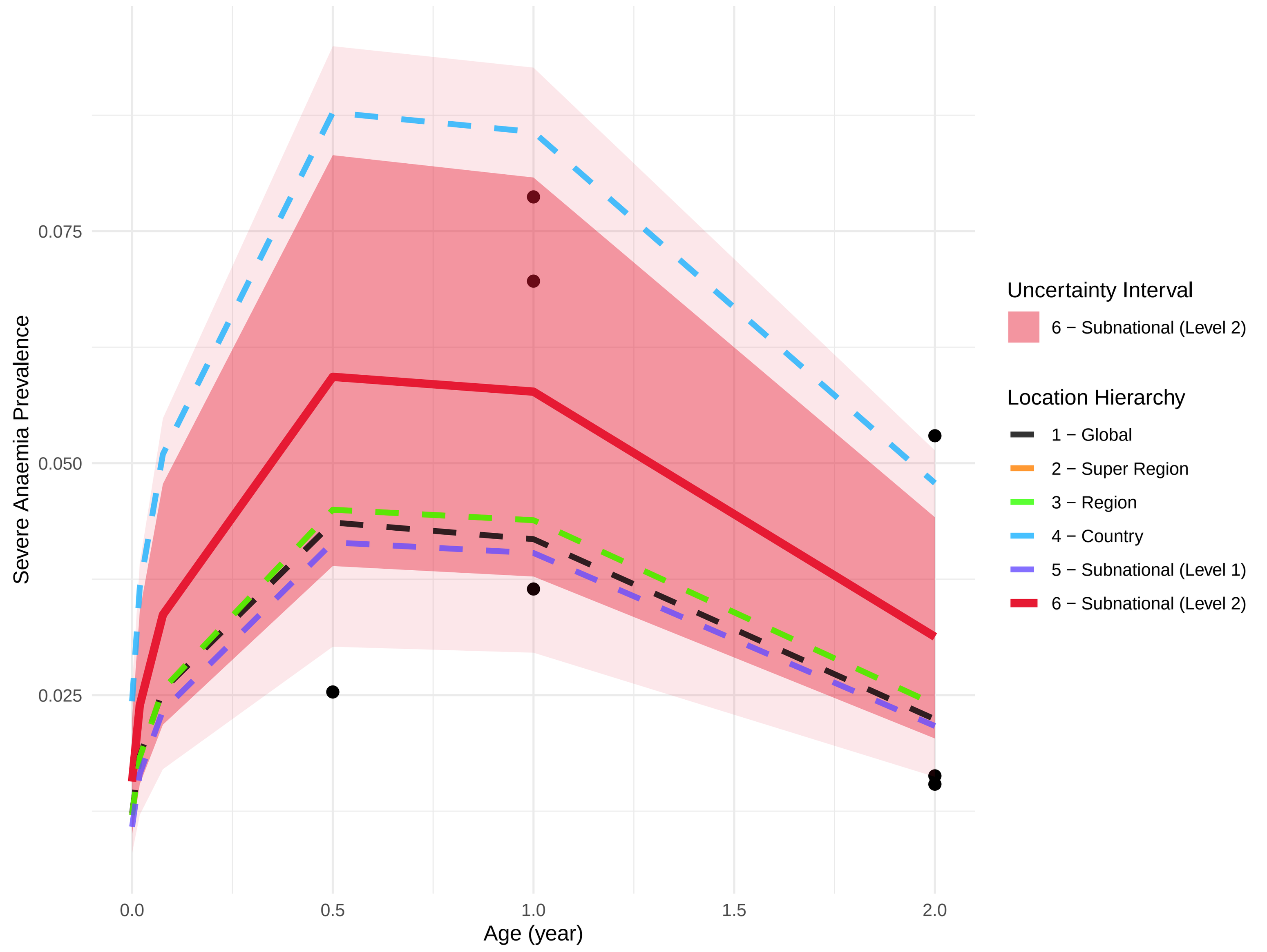

MR−BRT Age/Sex Fits − Males <2 Years Old − Severe Anaemia Prevalence − Punjab
WHO−2024 Adjustment Method

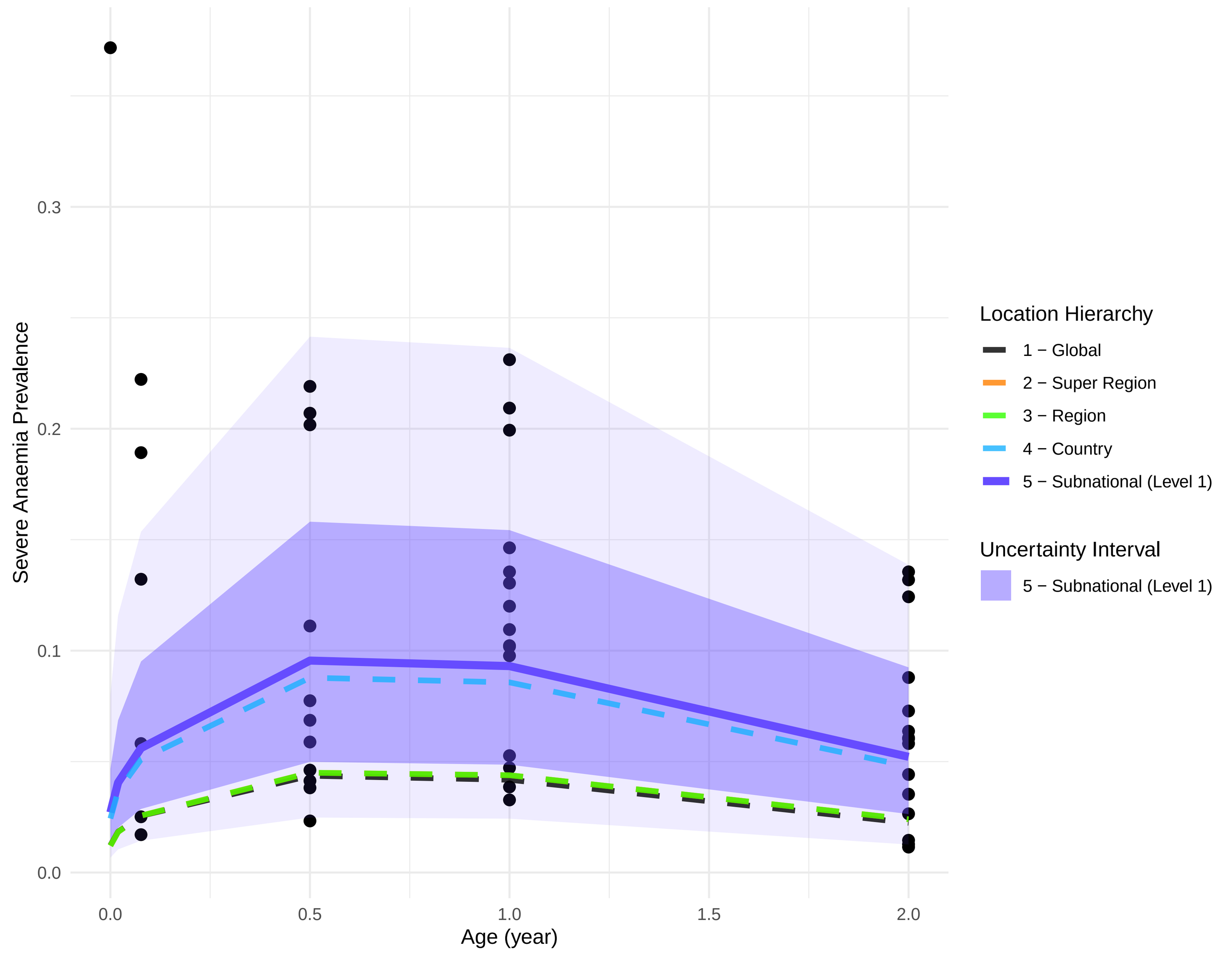

MR−BRT Age/Sex Fits − Males <2 Years Old − Severe Anaemia Prevalence − Punjab, Rural
WHO−2024 Adjustment Method

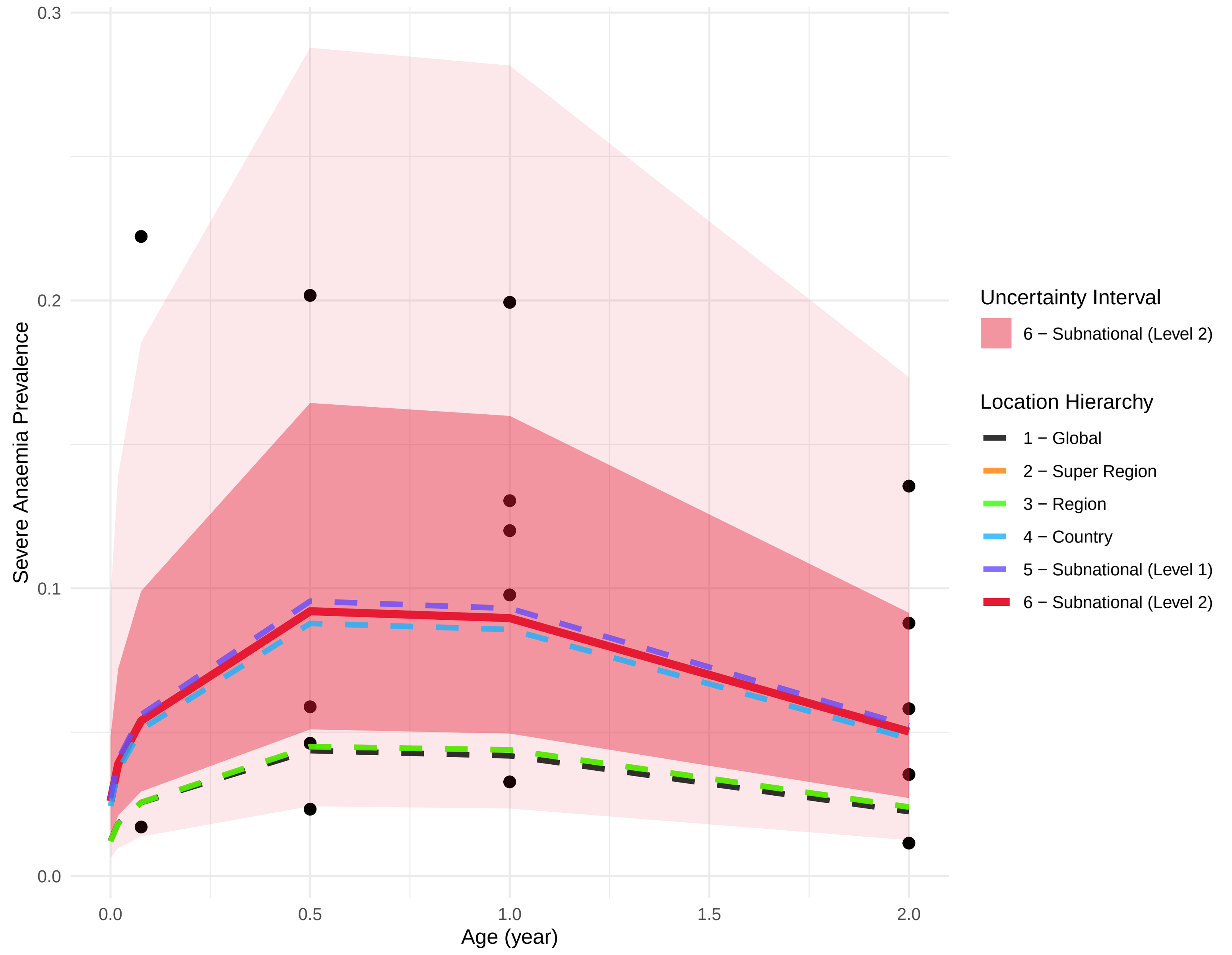

MR−BRT Age/Sex Fits − Males <2 Years Old − Severe Anaemia Prevalence − Punjab, Urban
WHO−2024 Adjustment Method

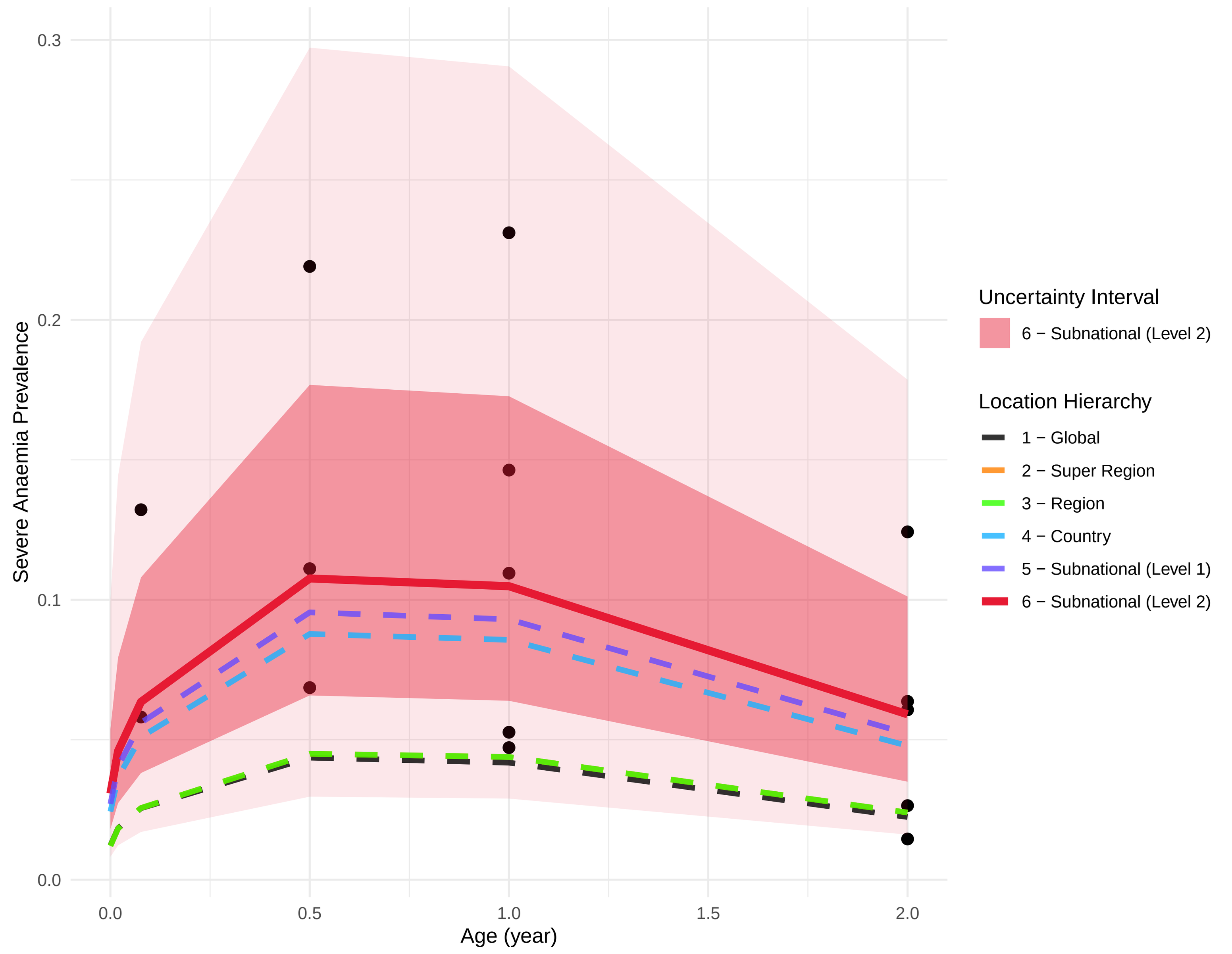

MR−BRT Age/Sex Fits − Males <2 Years Old − Severe Anaemia Prevalence − Rajasthan
WHO−2024 Adjustment Method

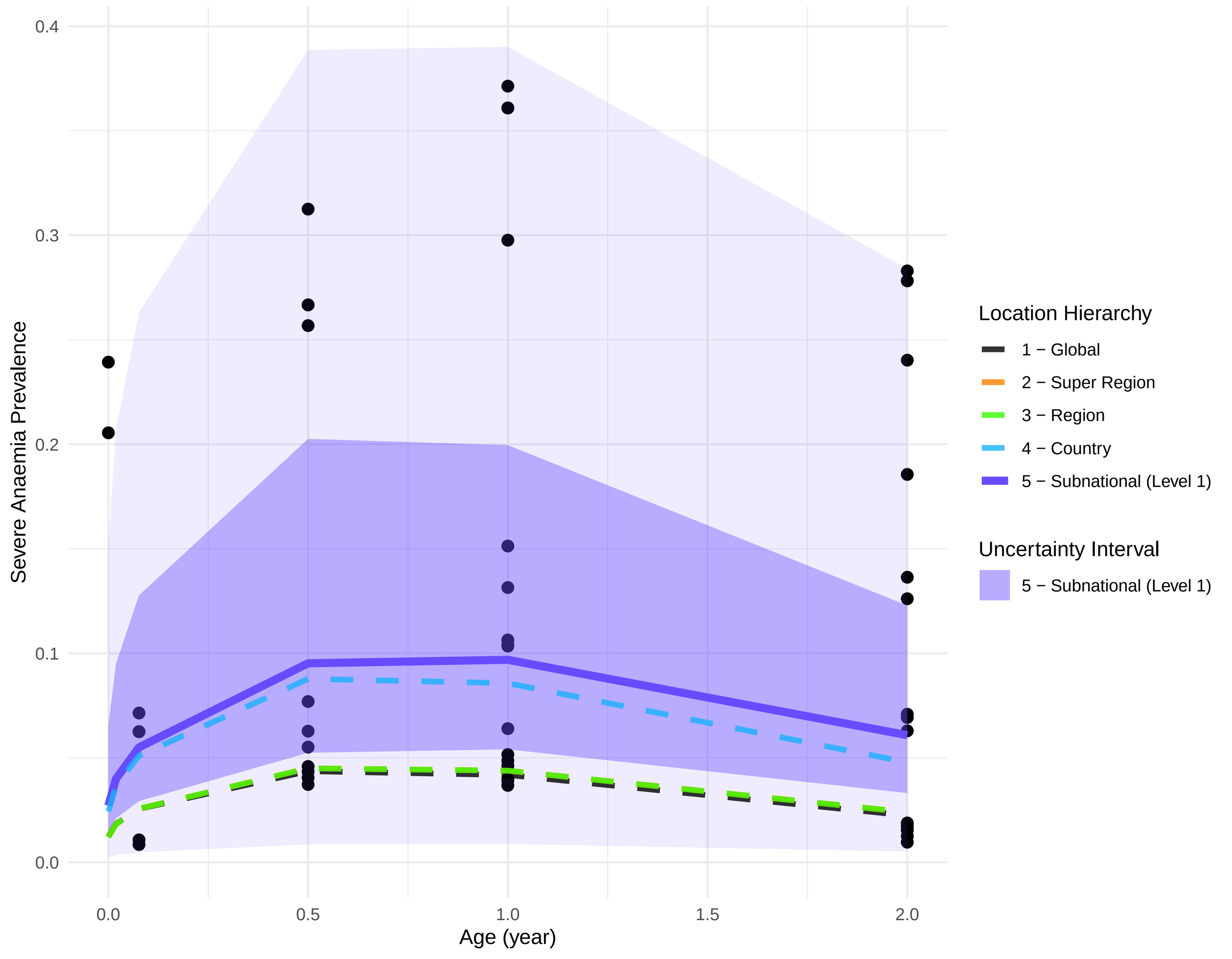

MR−BRT Age/Sex Fits − Males <2 Years Old − Severe Anaemia Prevalence − Rajasthan, Rural
WHO−2024 Adjustment Method

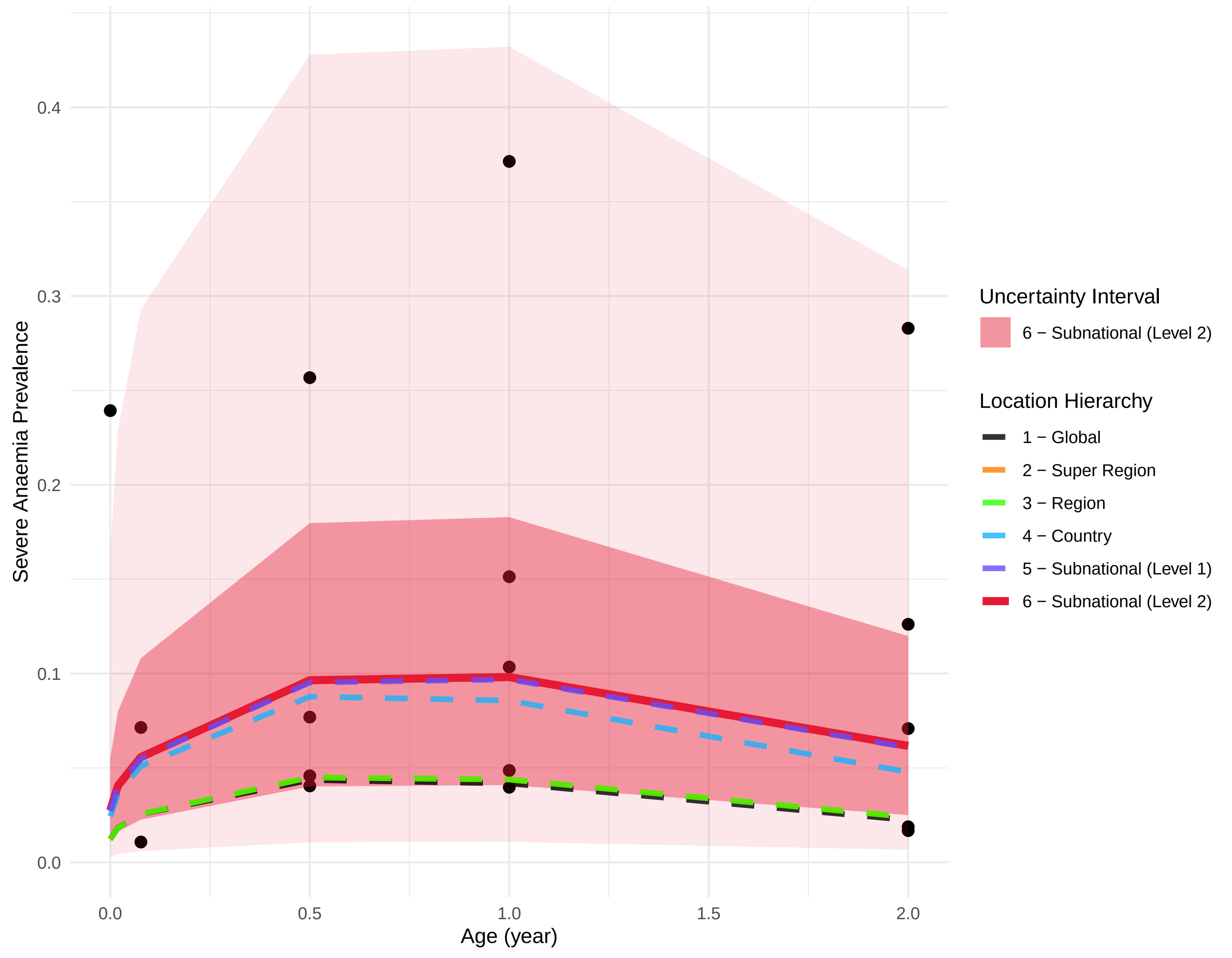

MR−BRT Age/Sex Fits − Males <2 Years Old − Severe Anaemia Prevalence − Rajasthan, Urban
WHO−2024 Adjustment Method

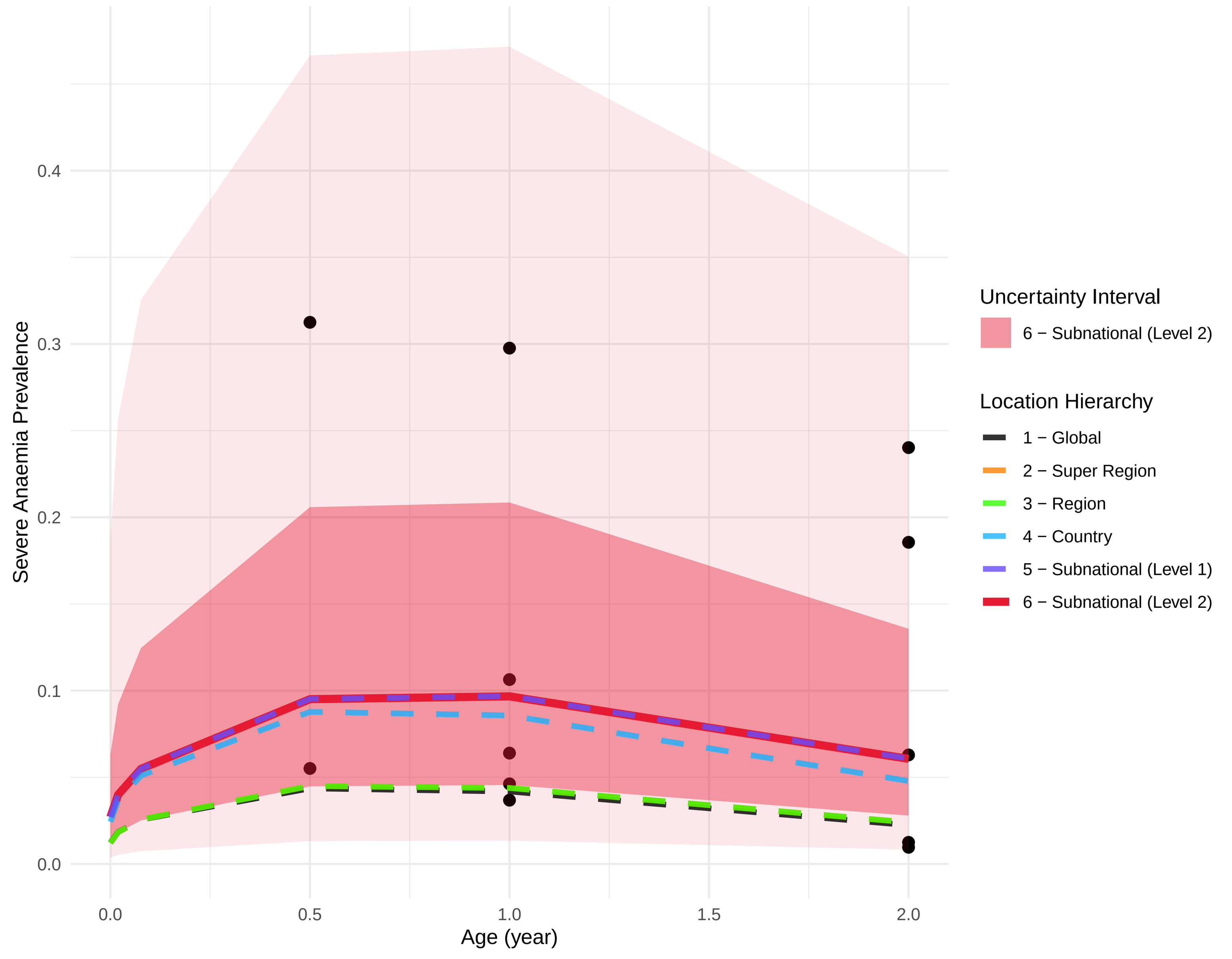

MR−BRT Age/Sex Fits − Males <2 Years Old − Severe Anaemia Prevalence − Sikkim
WHO−2024 Adjustment Method

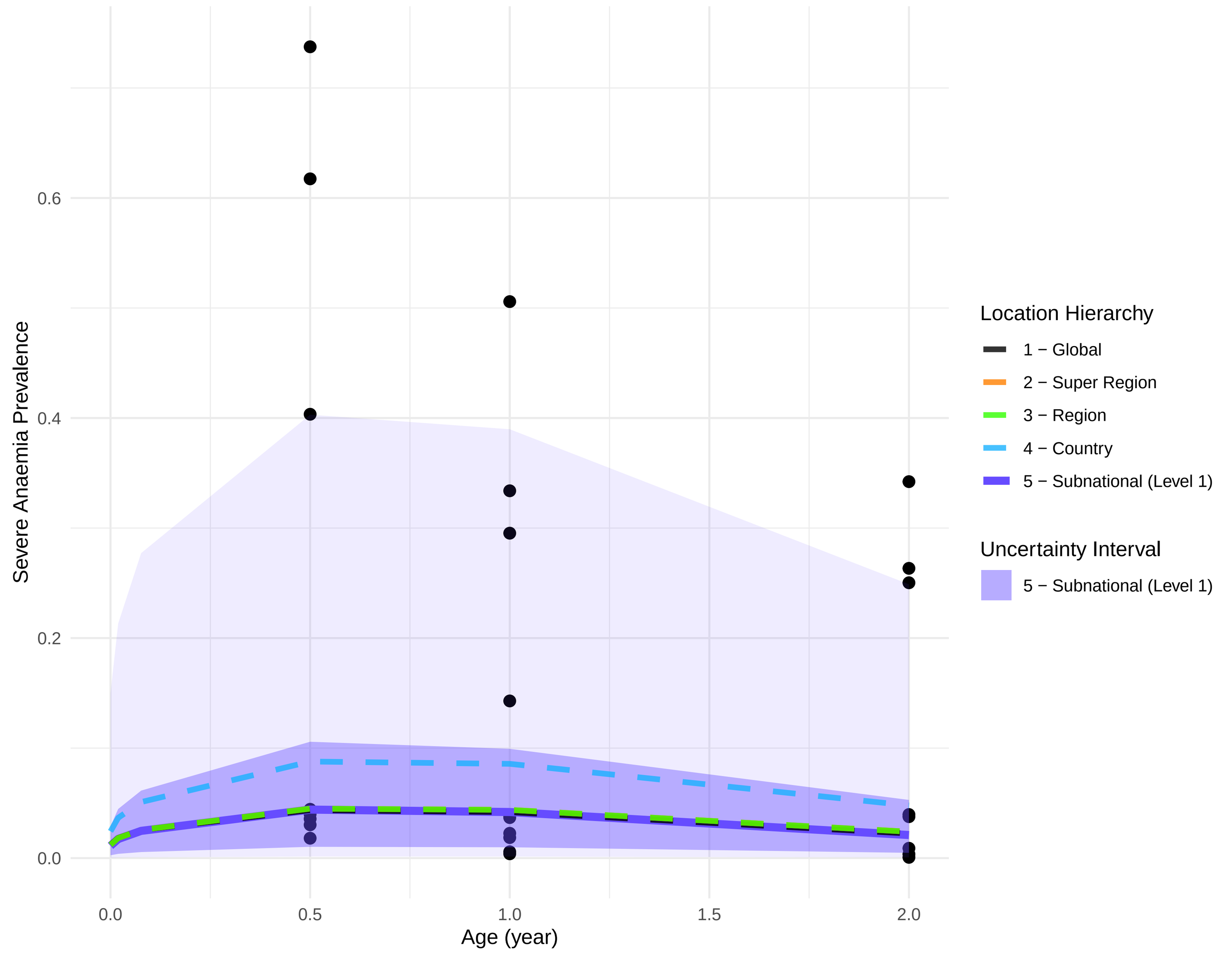

MR−BRT Age/Sex Fits − Males <2 Years Old − Severe Anaemia Prevalence − Sikkim, Rural
WHO−2024 Adjustment Method

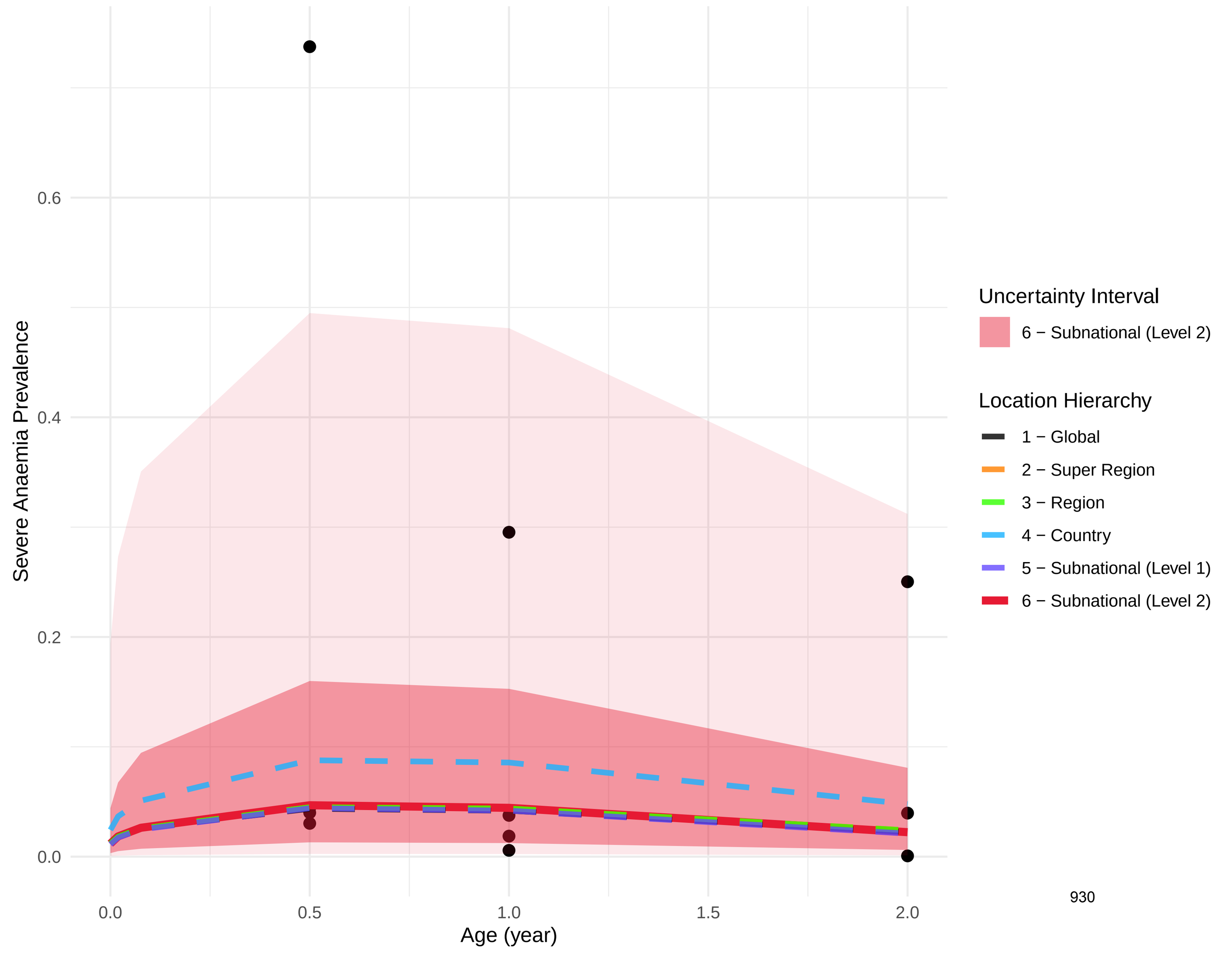

MR−BRT Age/Sex Fits − Males <2 Years Old − Severe Anaemia Prevalence − Sikkim, Urban
WHO−2024 Adjustment Method

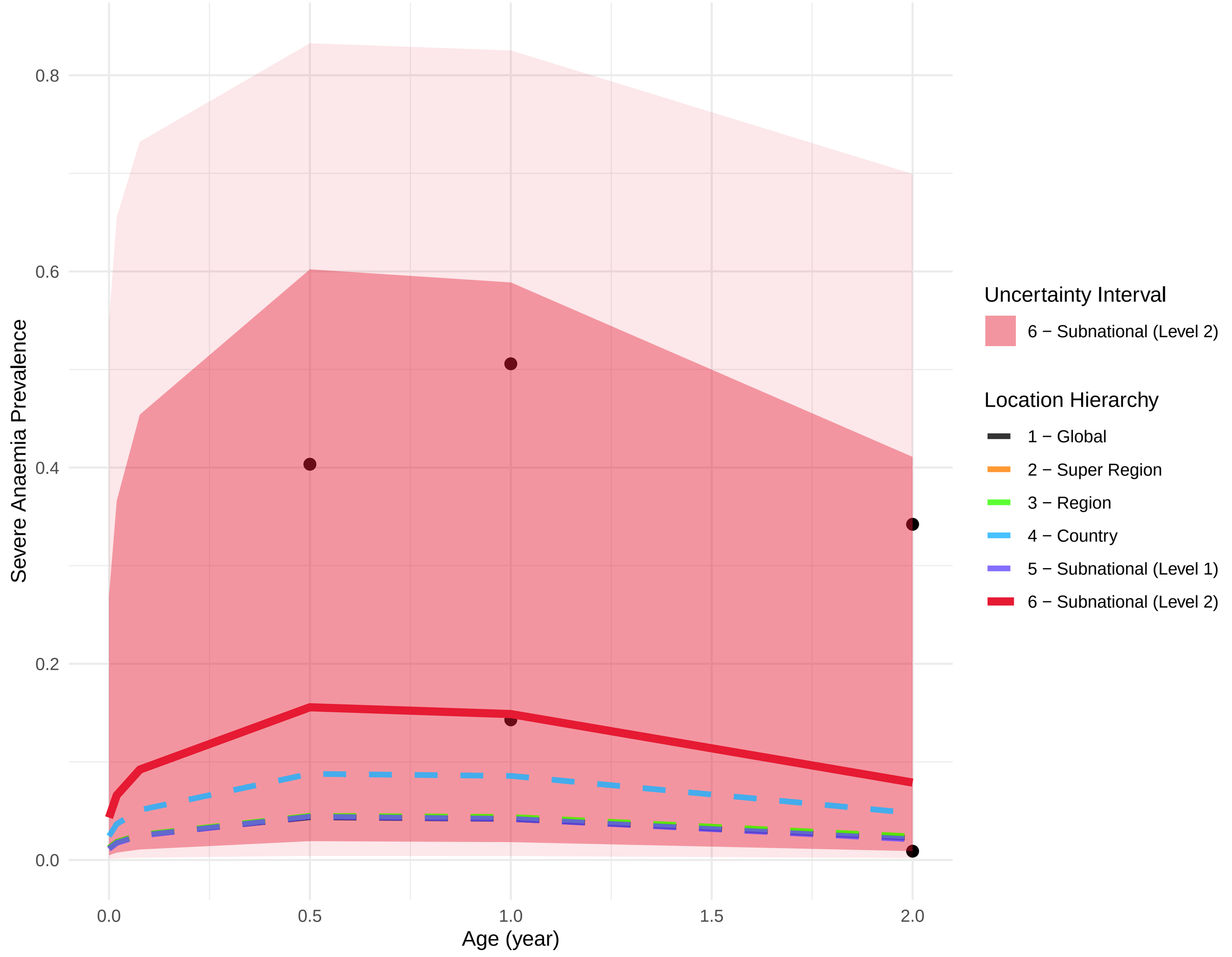

MR−BRT Age/Sex Fits − Males <2 Years Old − Severe Anaemia Prevalence − Tamil Nadu
WHO−2024 Adjustment Method

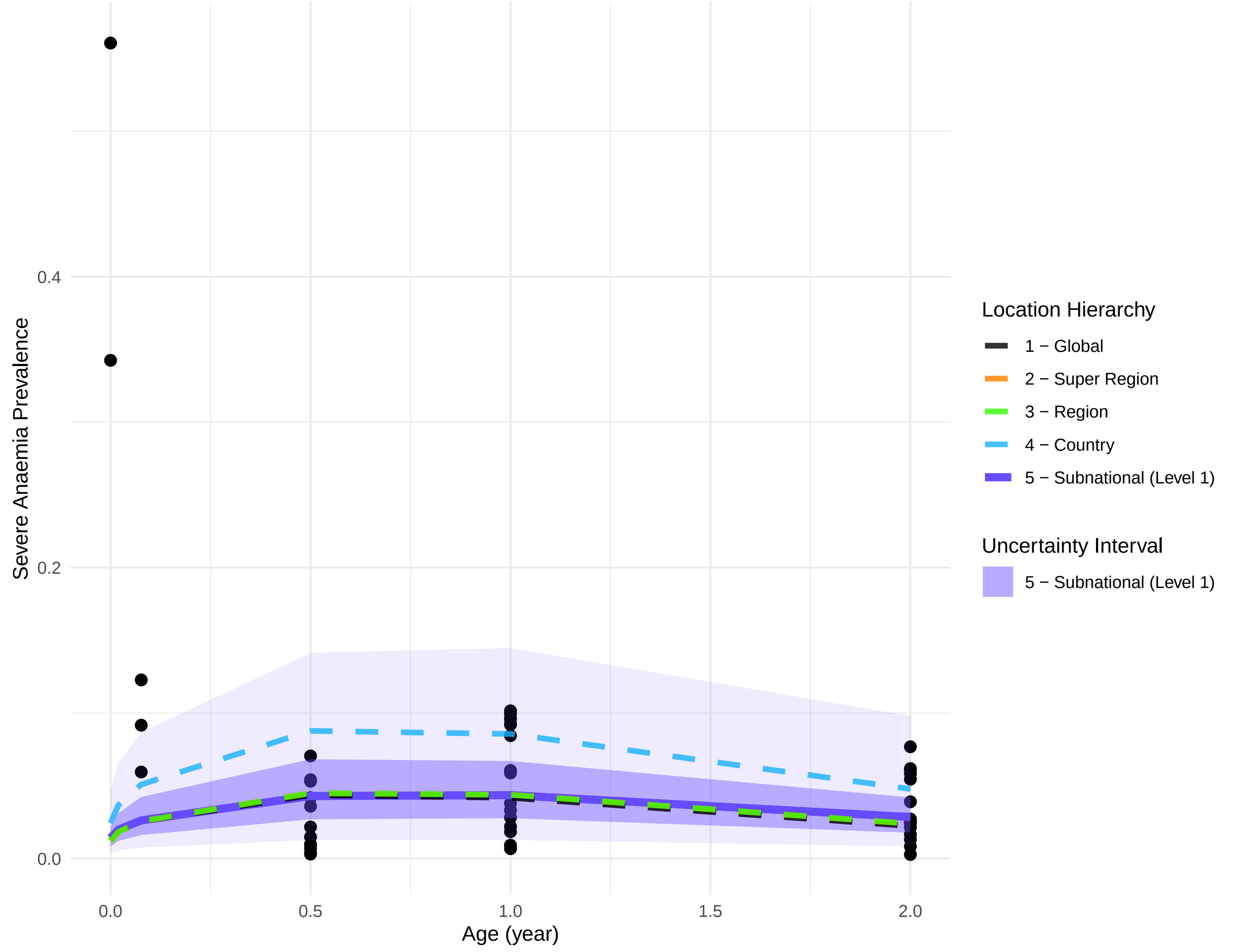

MR−BRT Age/Sex Fits − Males <2 Years Old − Severe Anaemia Prevalence − Tamil Nadu, Rural
WHO−2024 Adjustment Method

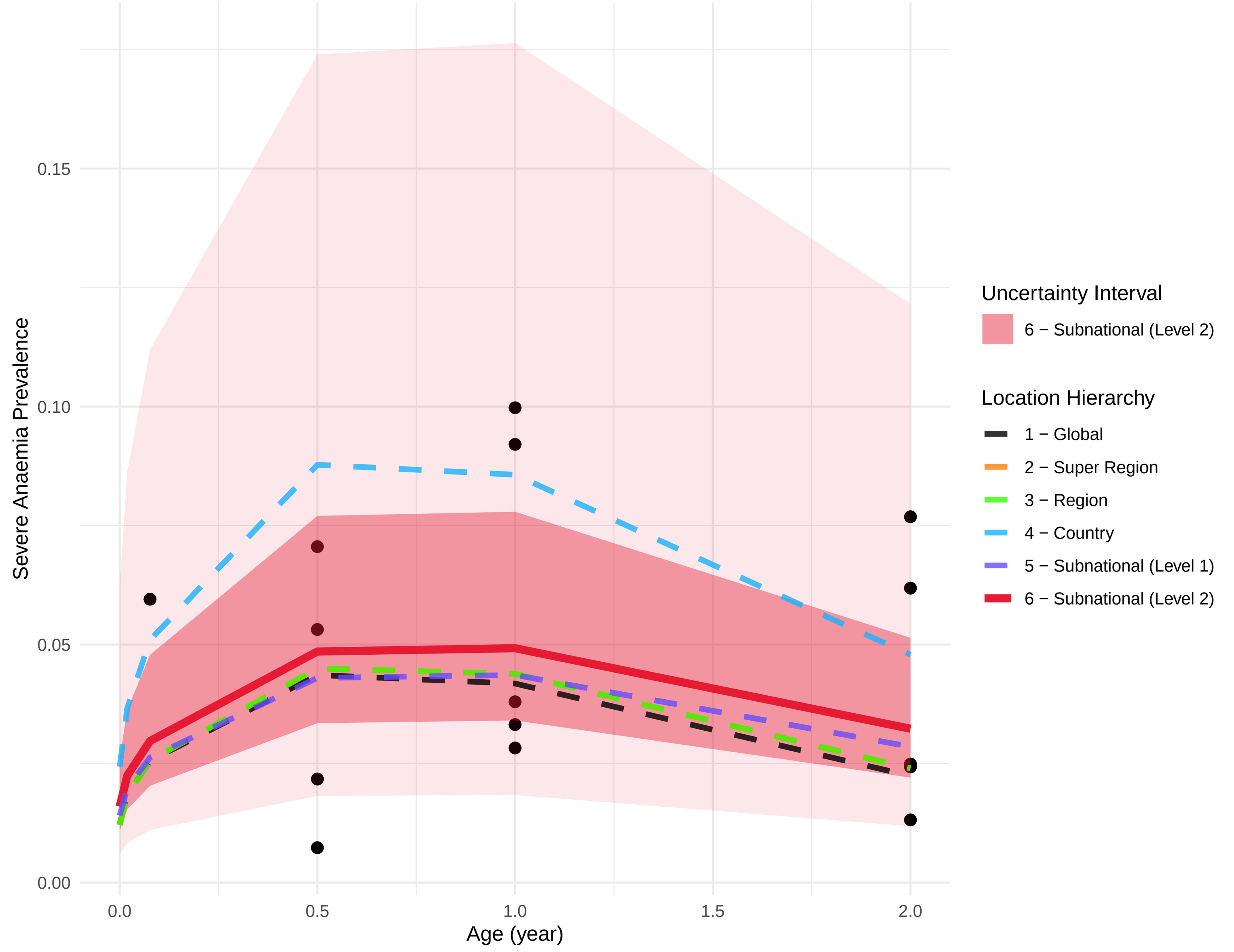

MR−BRT Age/Sex Fits − Males <2 Years Old − Severe Anaemia Prevalence − Tamil Nadu, Urban
WHO−2024 Adjustment Method

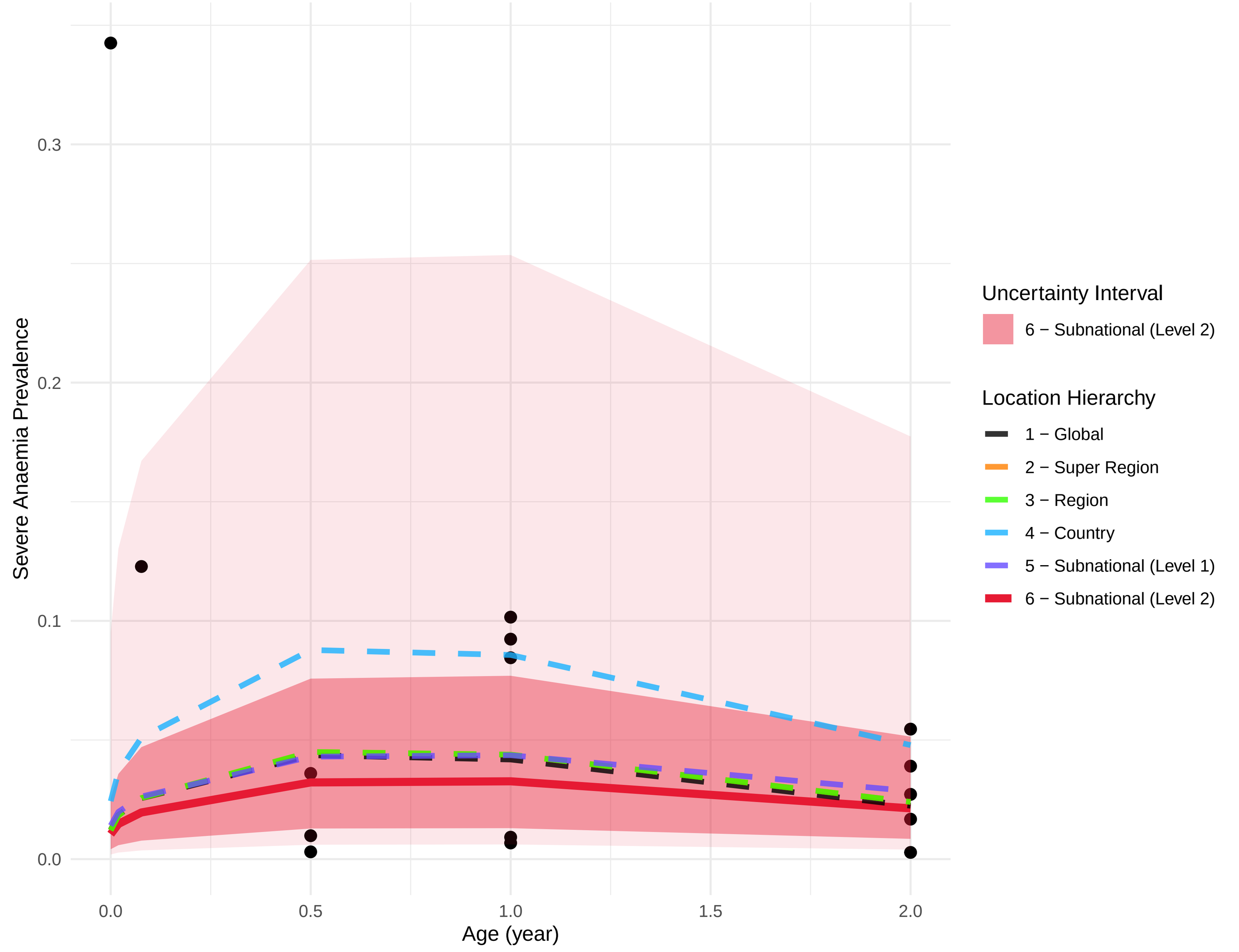

MR−BRT Age/Sex Fits − Males <2 Years Old − Severe Anaemia Prevalence − Telangana
WHO−2024 Adjustment Method

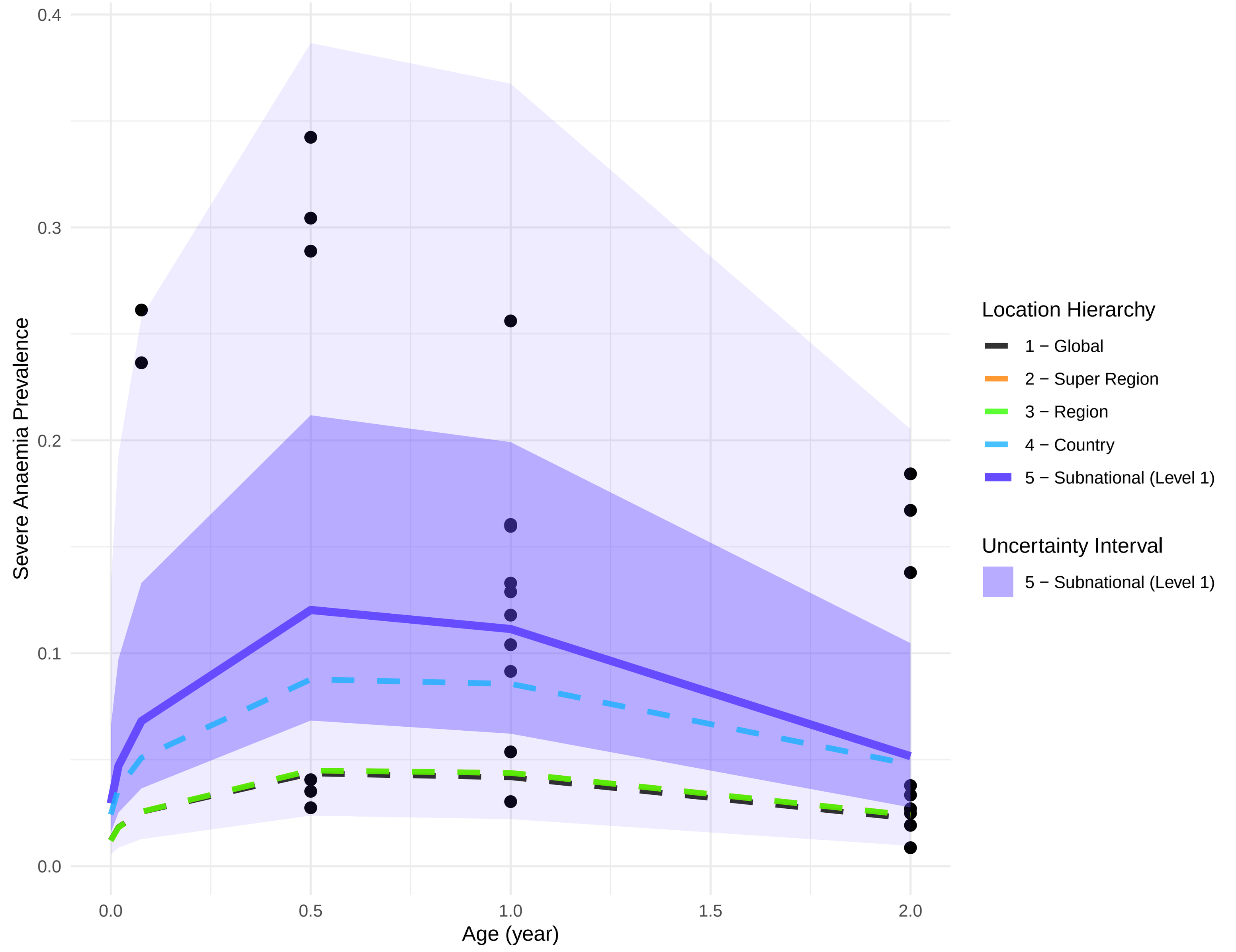

MR−BRT Age/Sex Fits − Males <2 Years Old − Severe Anaemia Prevalence − Telangana, Rural
WHO−2024 Adjustment Method

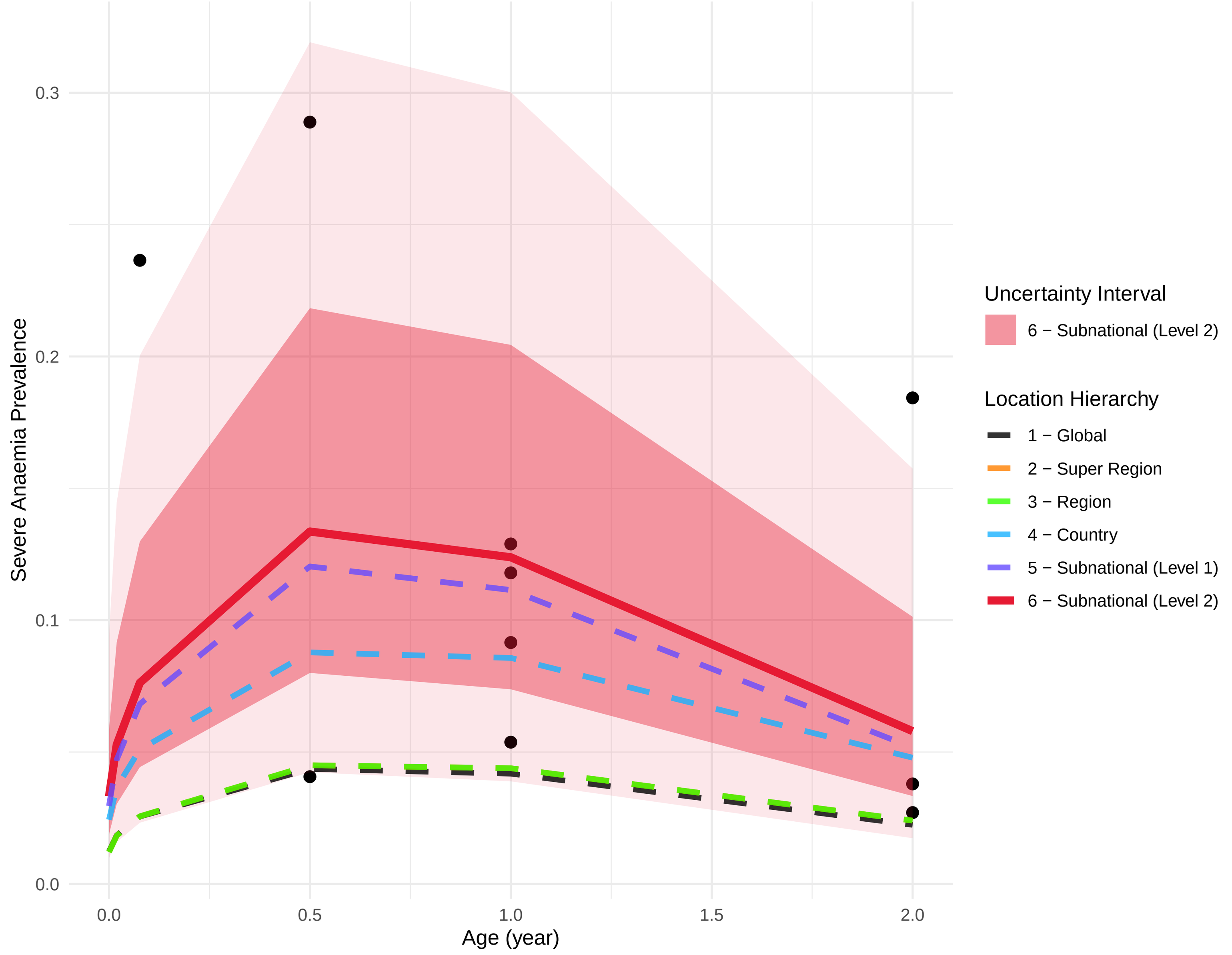

MR−BRT Age/Sex Fits − Males <2 Years Old − Severe Anaemia Prevalence − Telangana, Urban
WHO−2024 Adjustment Method

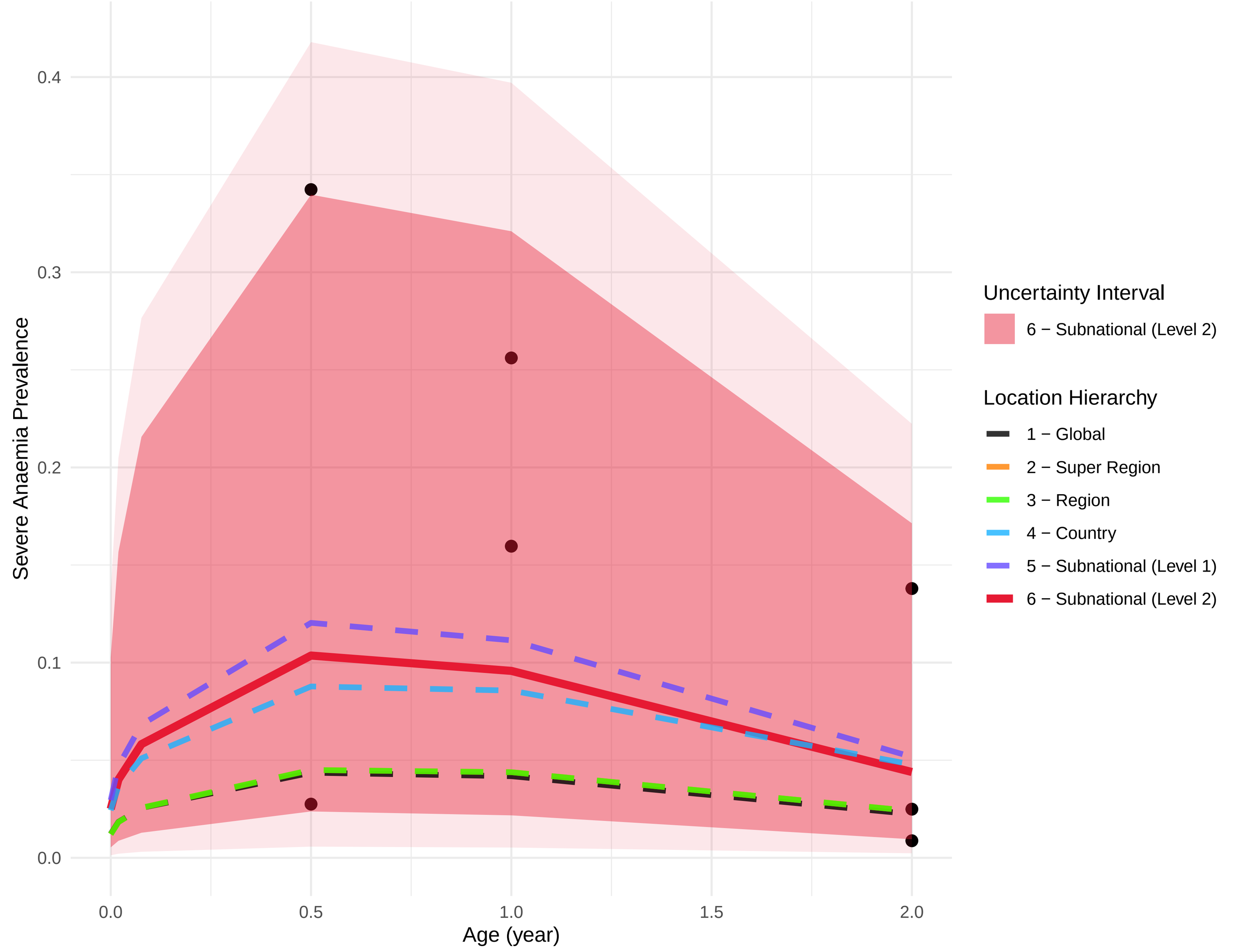

MR−BRT Age/Sex Fits − Males <2 Years Old − Severe Anaemia Prevalence − Tripura
WHO−2024 Adjustment Method

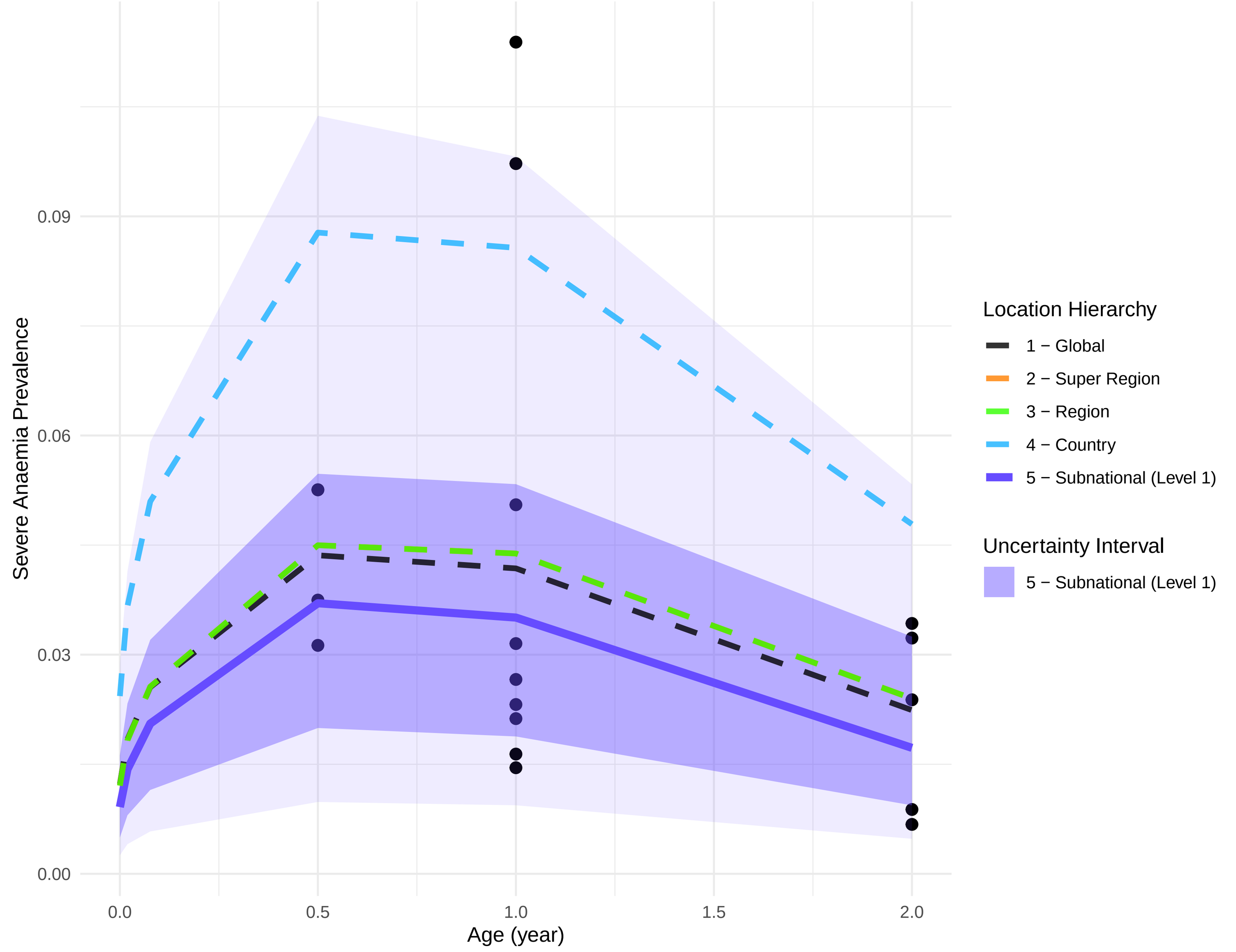

MR−BRT Age/Sex Fits − Males <2 Years Old − Severe Anaemia Prevalence − Tripura, Rural
WHO−2024 Adjustment Method

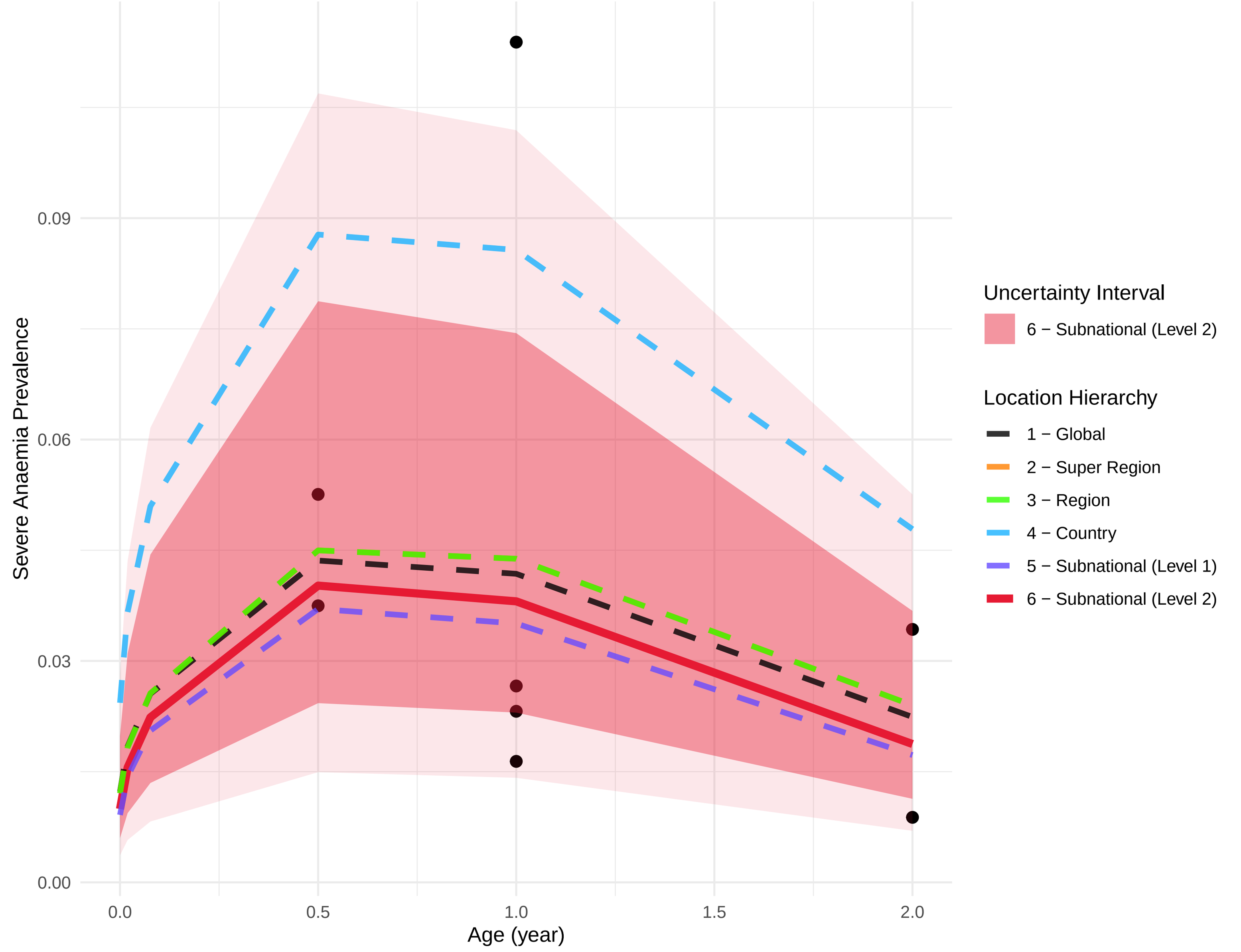

MR−BRT Age/Sex Fits − Males <2 Years Old − Severe Anaemia Prevalence − Tripura, Urban
WHO−2024 Adjustment Method

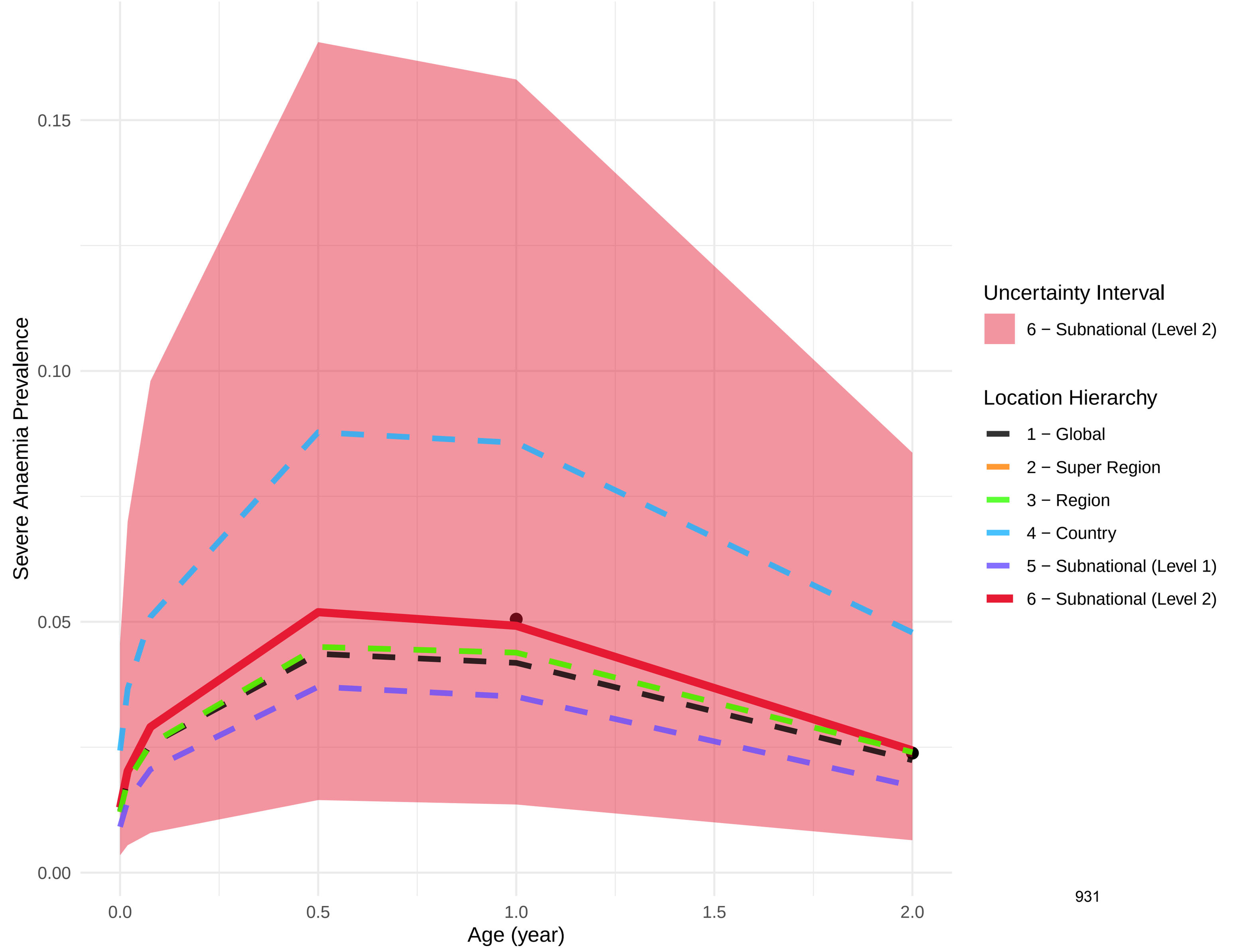

MR−BRT Age/Sex Fits − Males <2 Years Old − Severe Anaemia Prevalence − Uttar Pradesh
WHO−2024 Adjustment Method

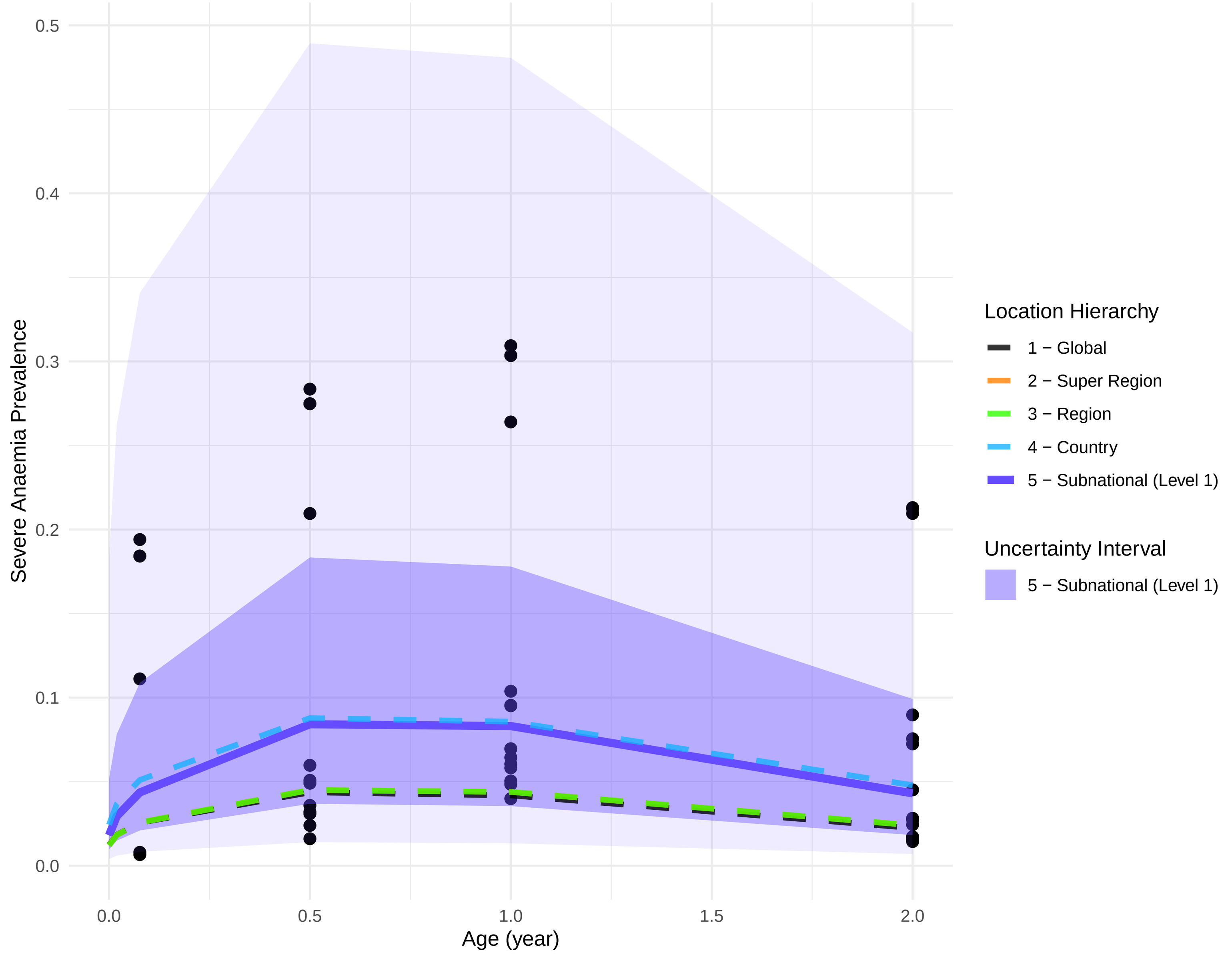

MR−BRT Age/Sex Fits − Males <2 Years Old − Severe Anaemia Prevalence − Uttar Pradesh, Rural
WHO−2024 Adjustment Method

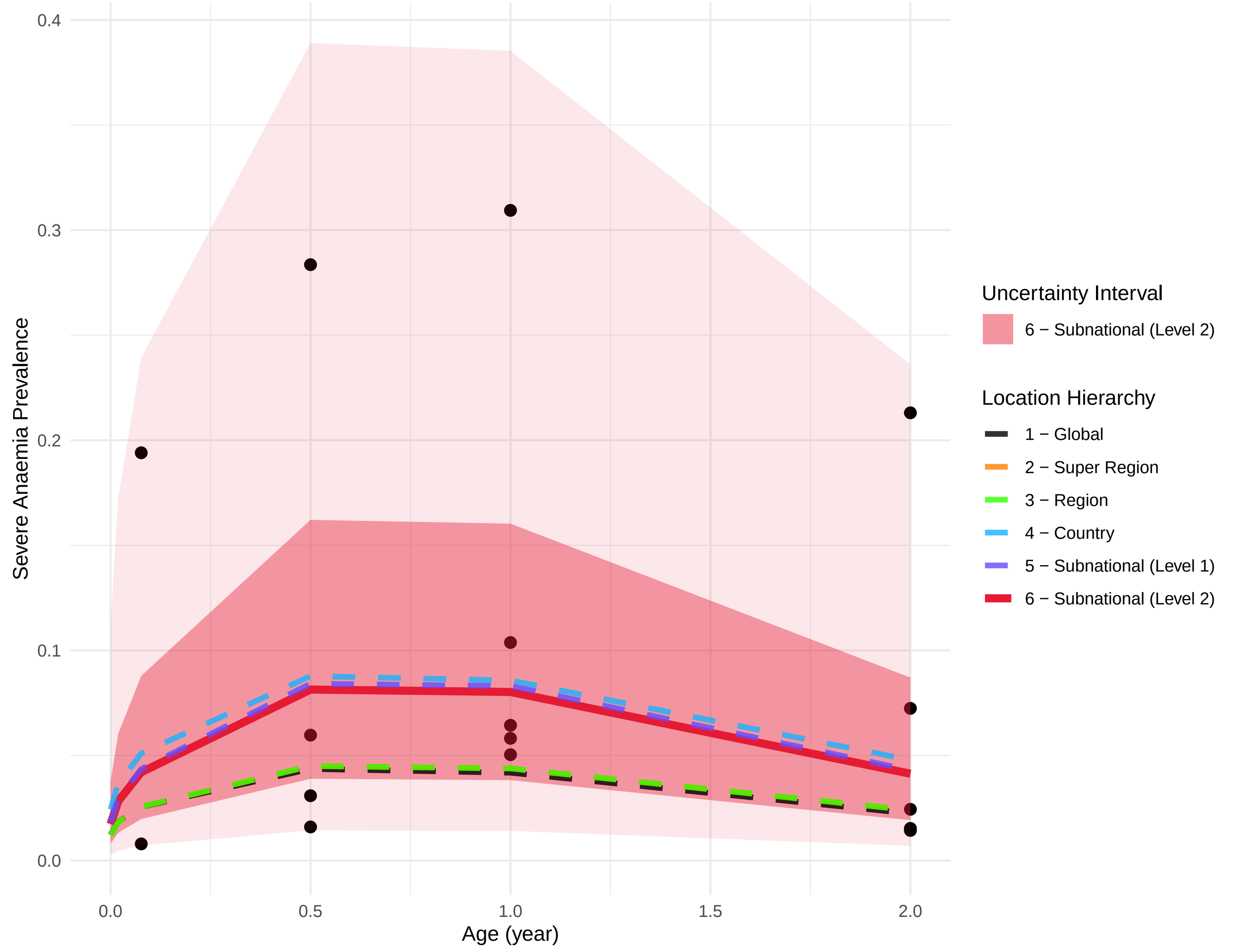

MR−BRT Age/Sex Fits − Males <2 Years Old − Severe Anaemia Prevalence − Uttar Pradesh, Urban
WHO−2024 Adjustment Method

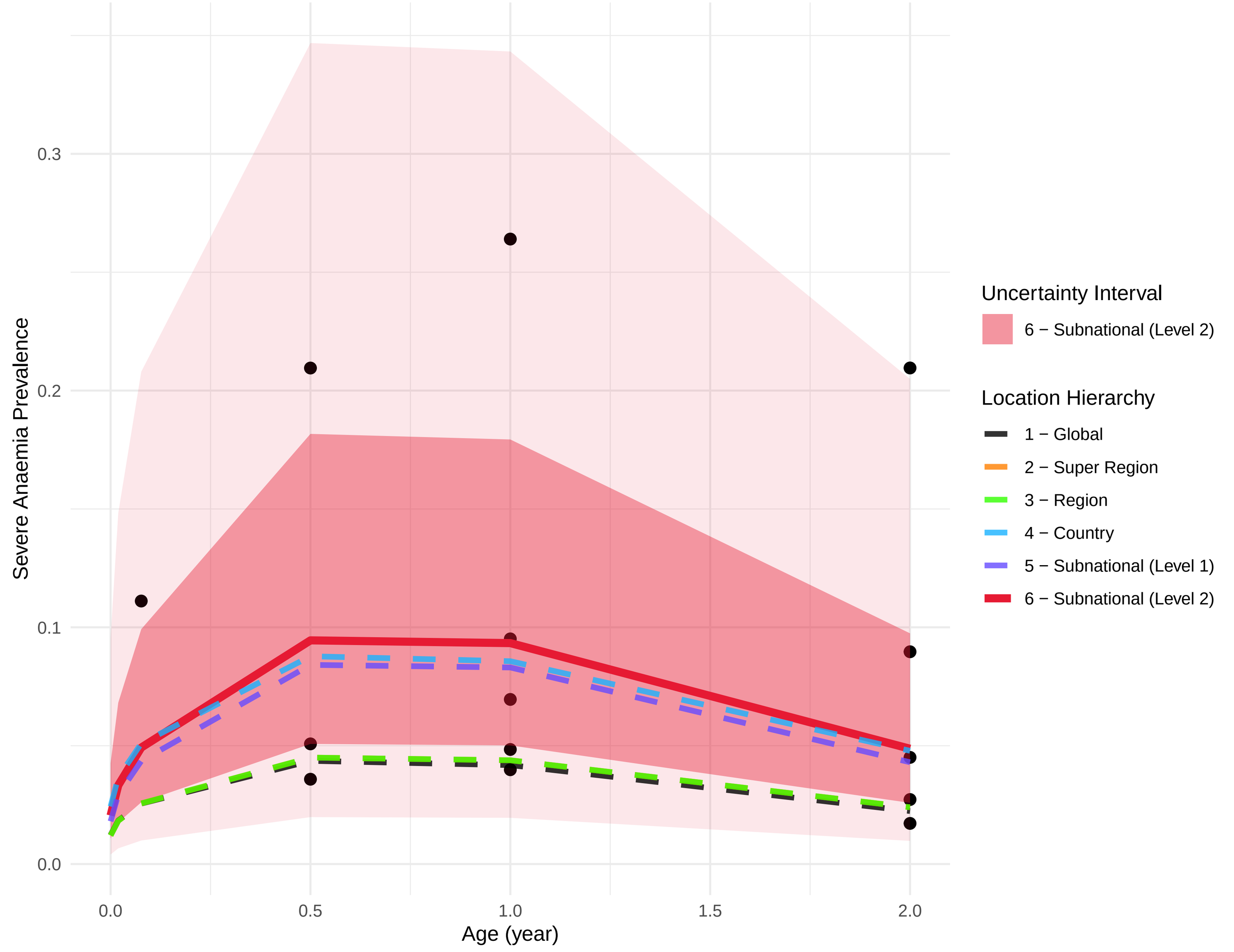

MR−BRT Age/Sex Fits − Males <2 Years Old − Severe Anaemia Prevalence − Uttarakhand
WHO−2024 Adjustment Method

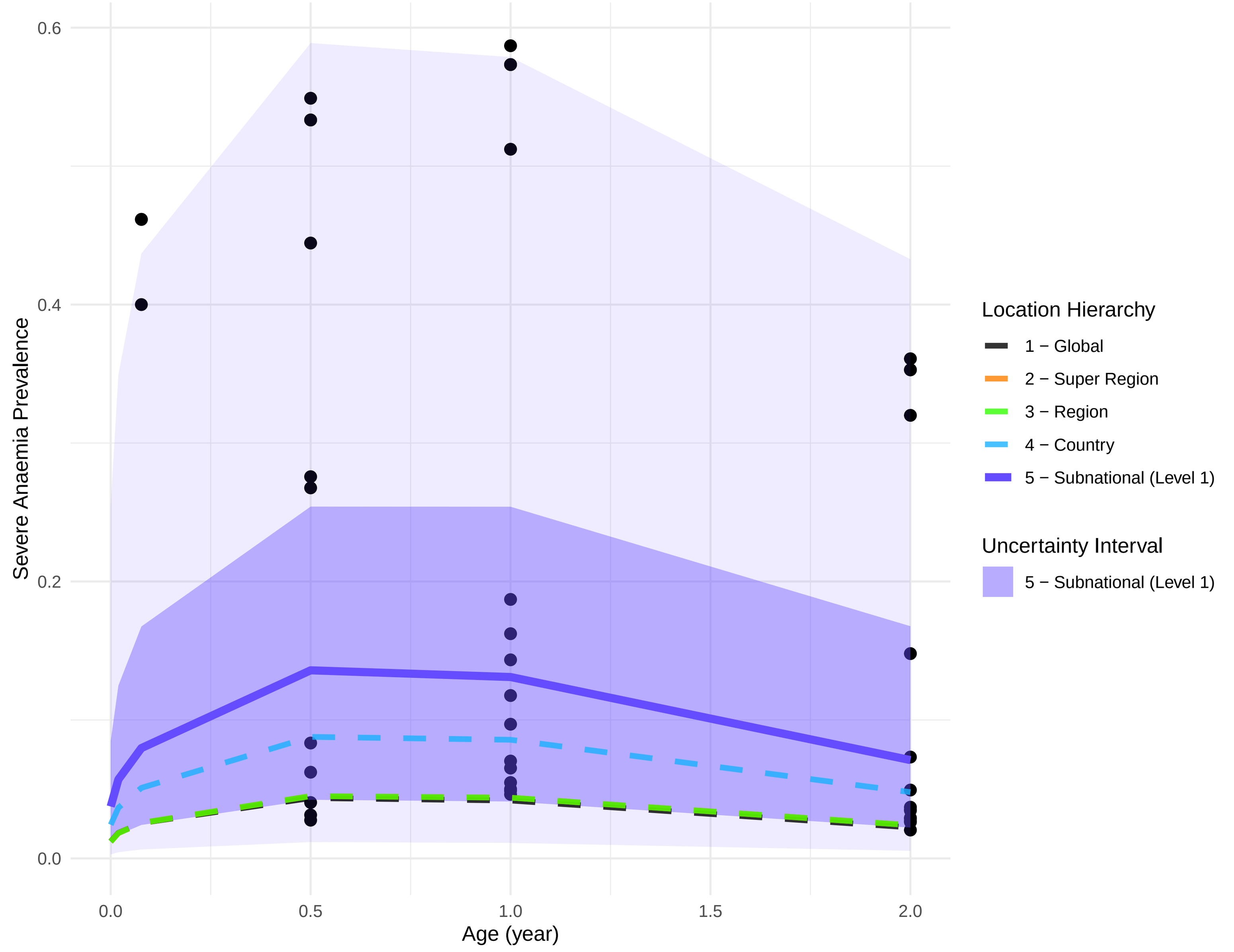

MR−BRT Age/Sex Fits − Males <2 Years Old − Severe Anaemia Prevalence − Uttarakhand, Rural
WHO−2024 Adjustment Method

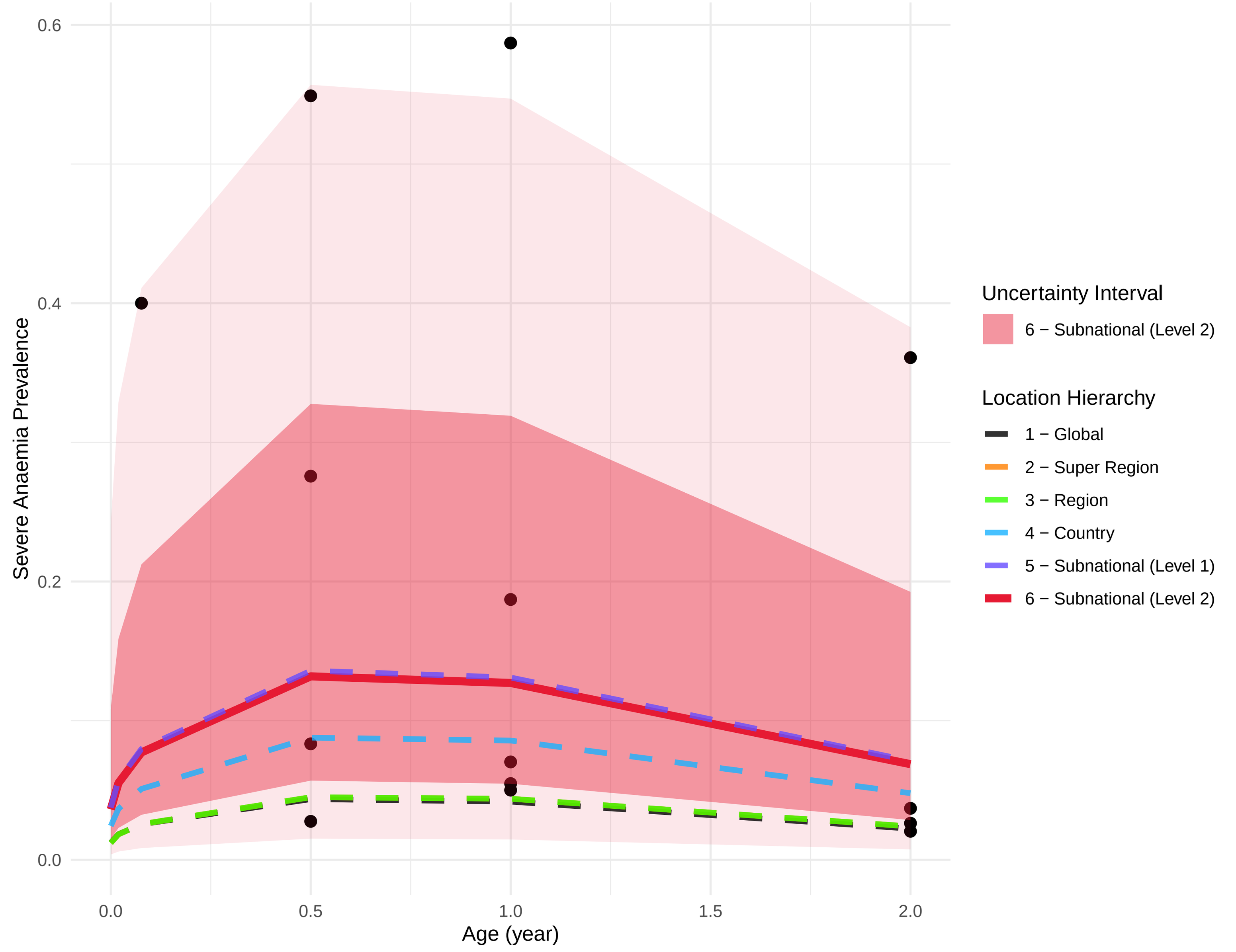

MR−BRT Age/Sex Fits − Males <2 Years Old − Severe Anaemia Prevalence − Uttarakhand, Urban
WHO−2024 Adjustment Method

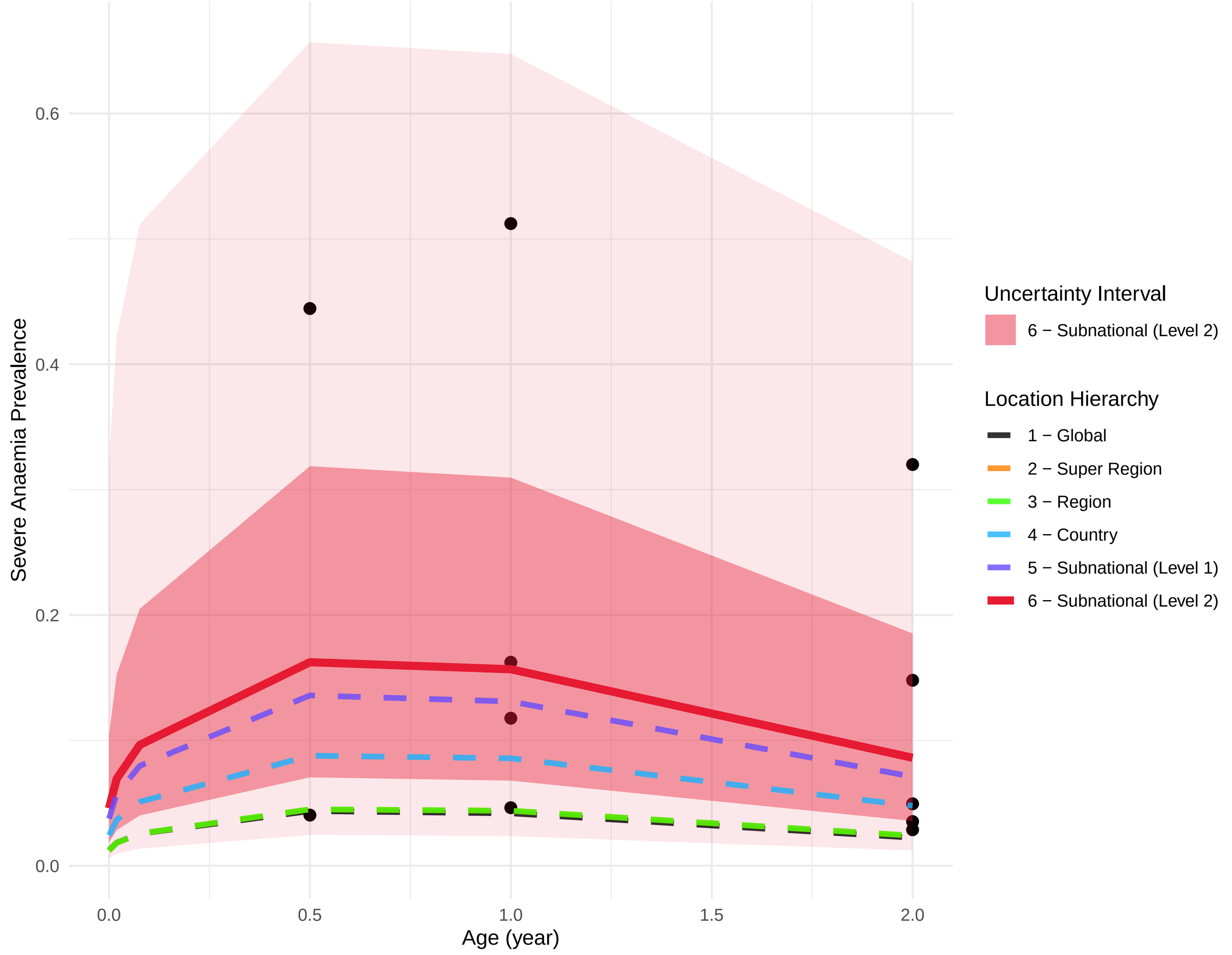

MR−BRT Age/Sex Fits − Males <2 Years Old − Severe Anaemia Prevalence − West Bengal
WHO−2024 Adjustment Method

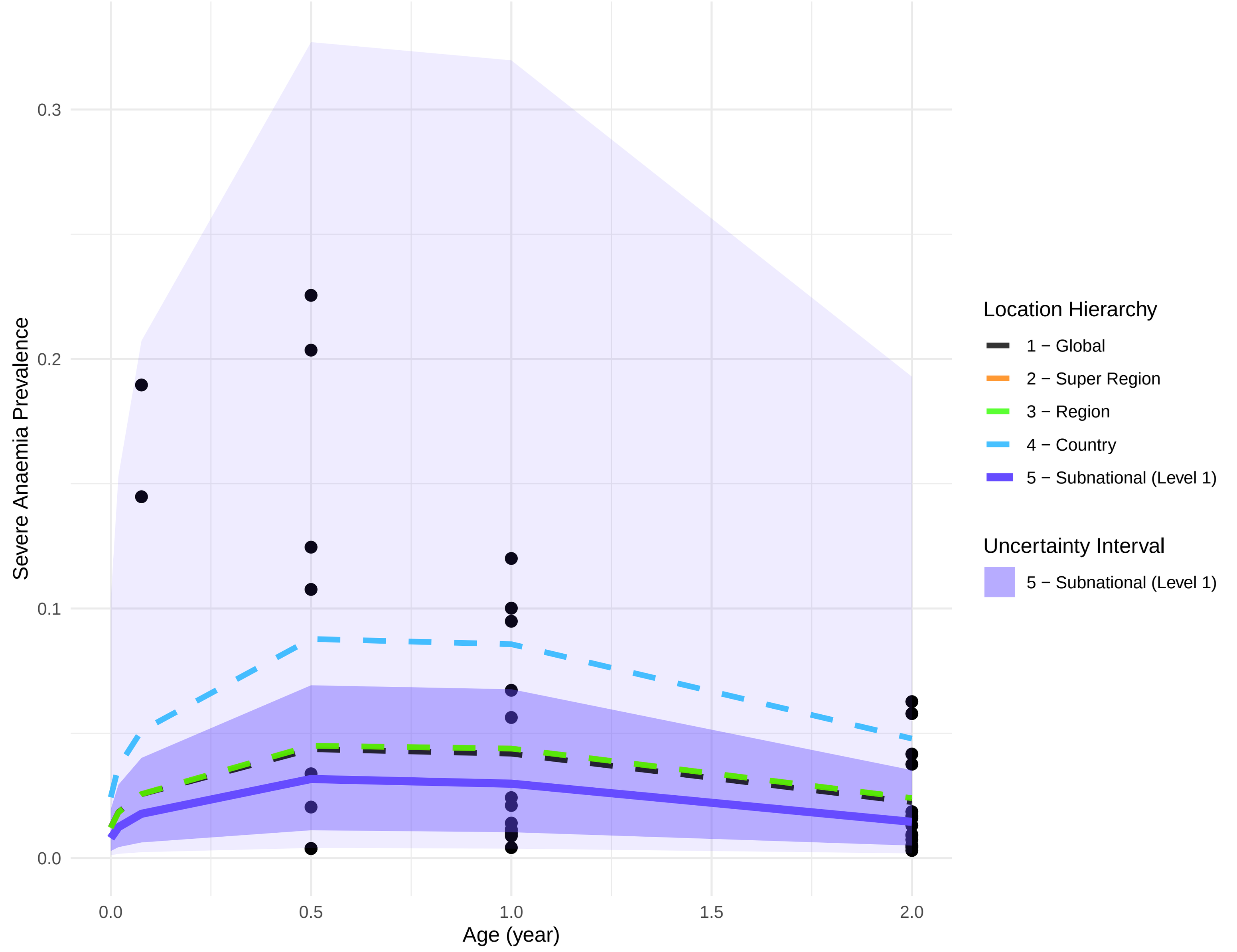

MR−BRT Age/Sex Fits − Males <2 Years Old − Severe Anaemia Prevalence − West Bengal, Rural
WHO−2024 Adjustment Method

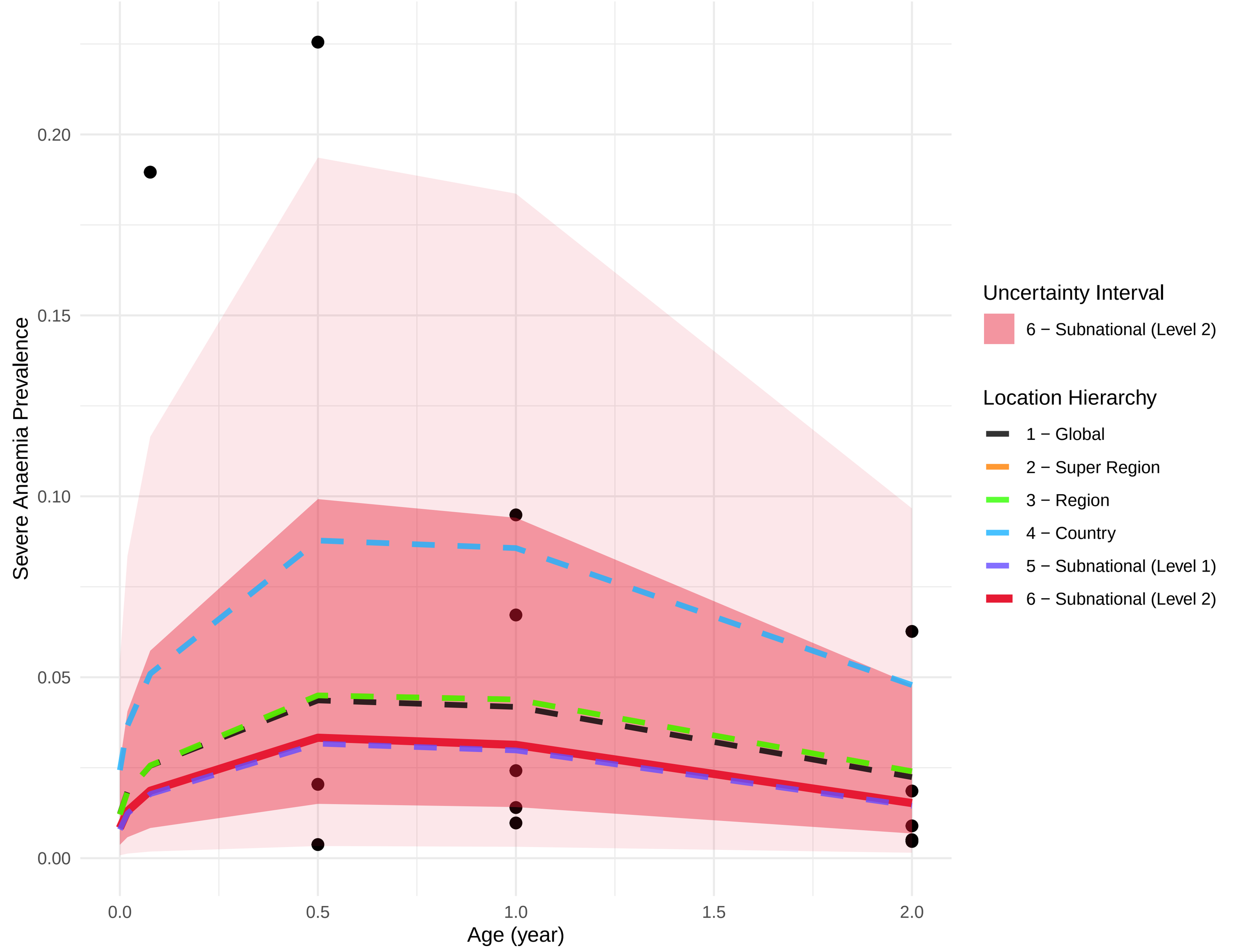

MR−BRT Age/Sex Fits − Males <2 Years Old − Severe Anaemia Prevalence − West Bengal, Urban
WHO−2024 Adjustment Method

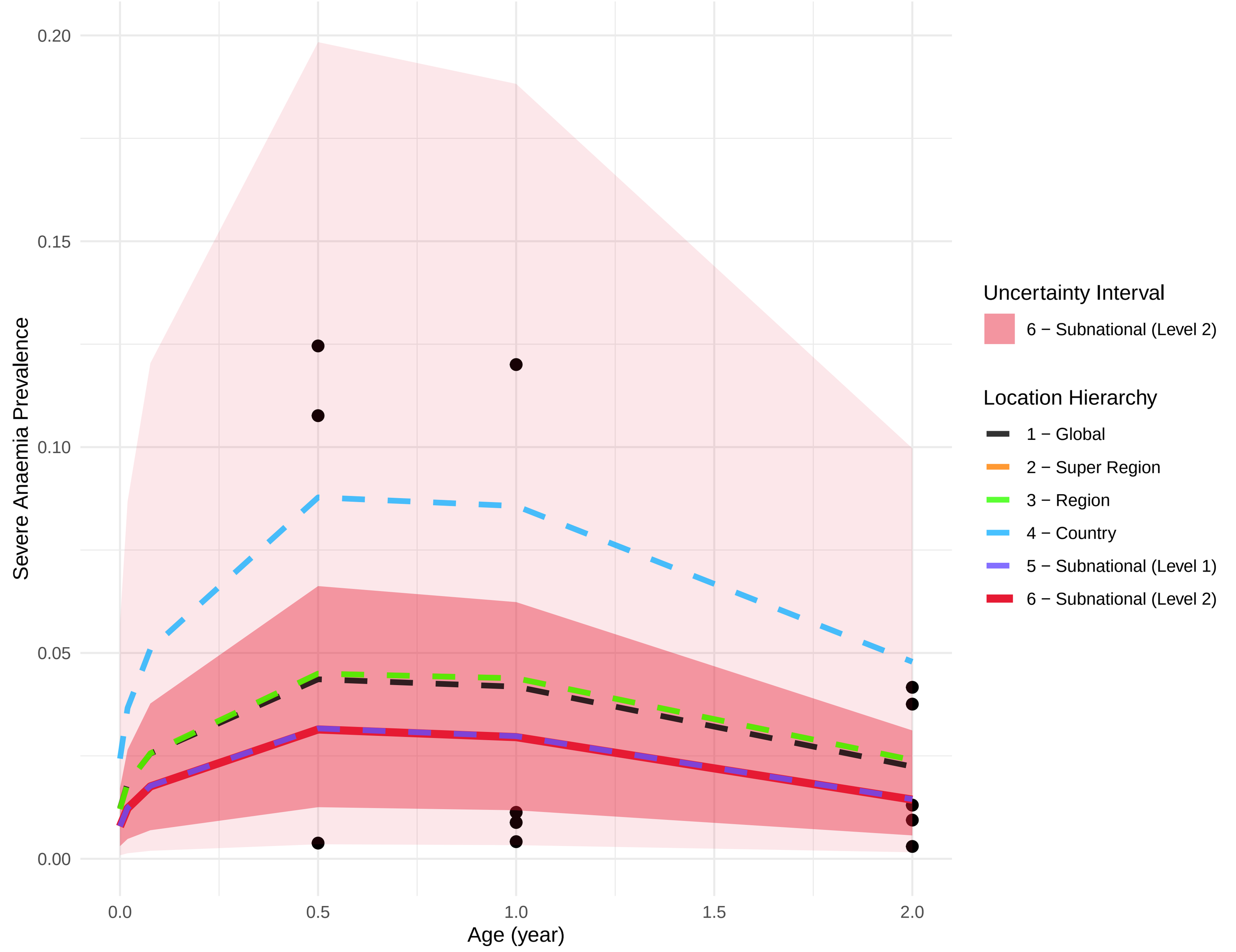

MR−BRT Age/Sex Fits − Males <2 Years Old − Severe Anaemia Prevalence − Nepal
WHO−2024 Adjustment Method

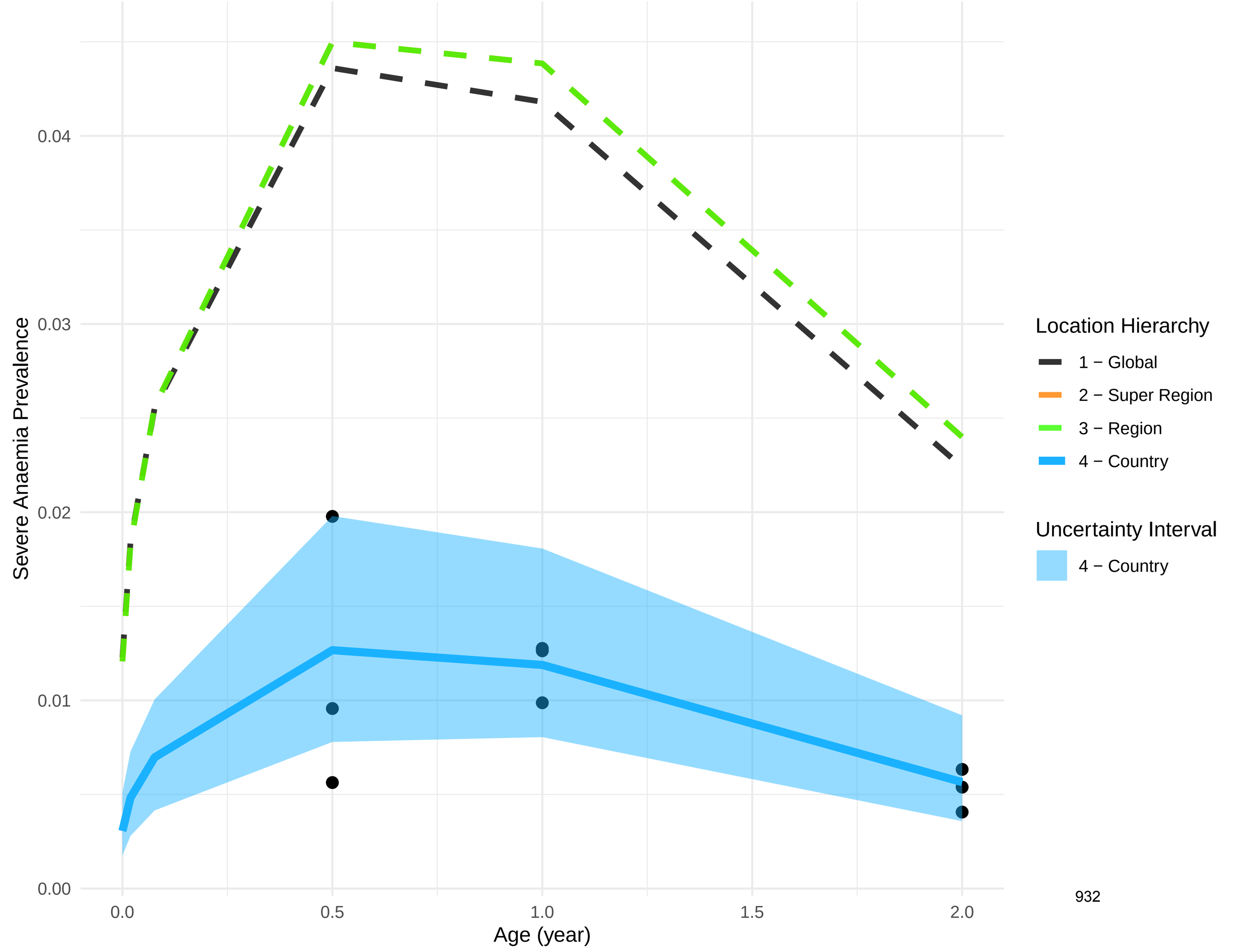

MR−BRT Age/Sex Fits − Males <2 Years Old − Severe Anaemia Prevalence − Southeast Asia, East Asia, and Oceania
WHO−2024 Adjustment Method

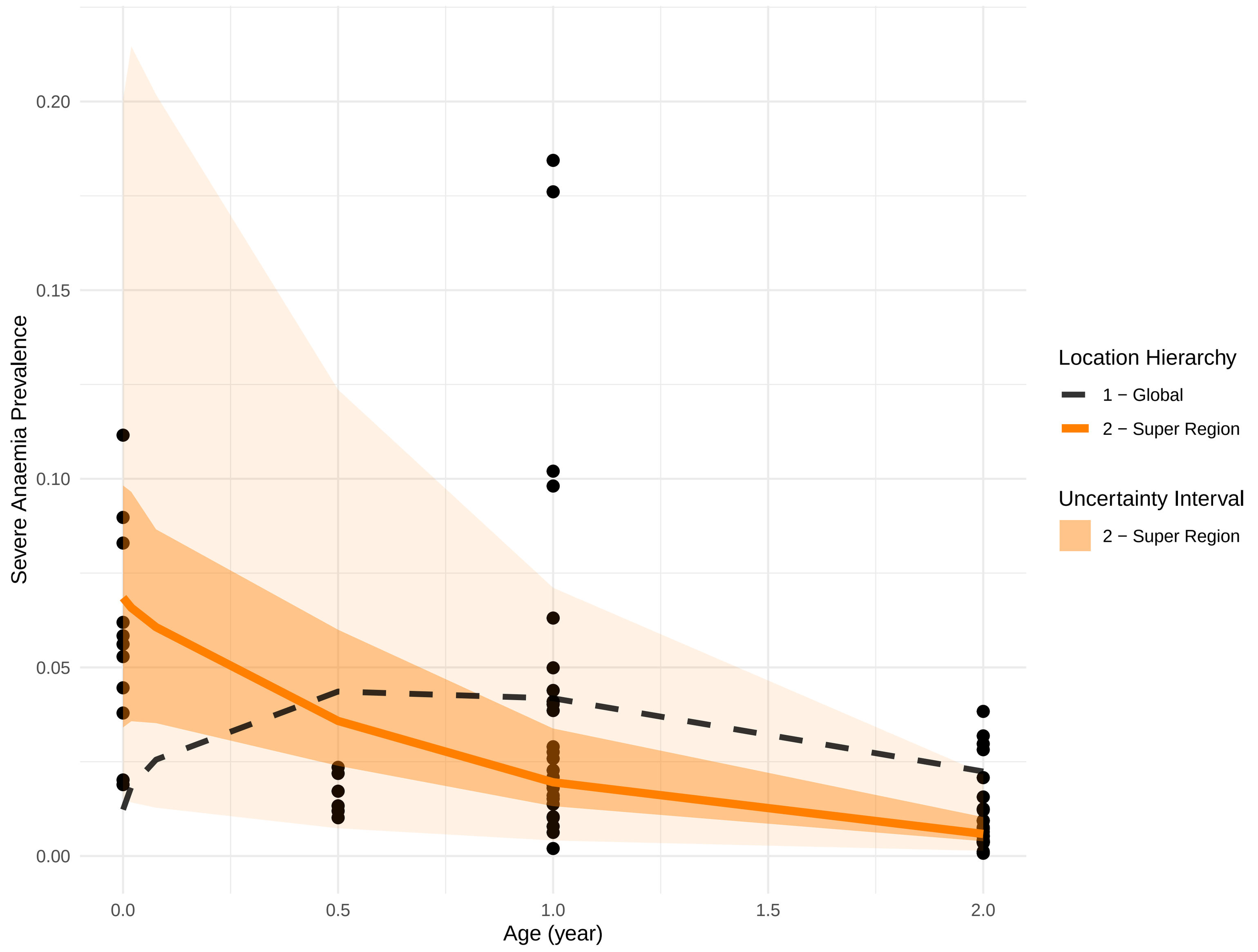

MR−BRT Age/Sex Fits − Males <2 Years Old − Severe Anaemia Prevalence − Southeast Asia
WHO−2024 Adjustment Method

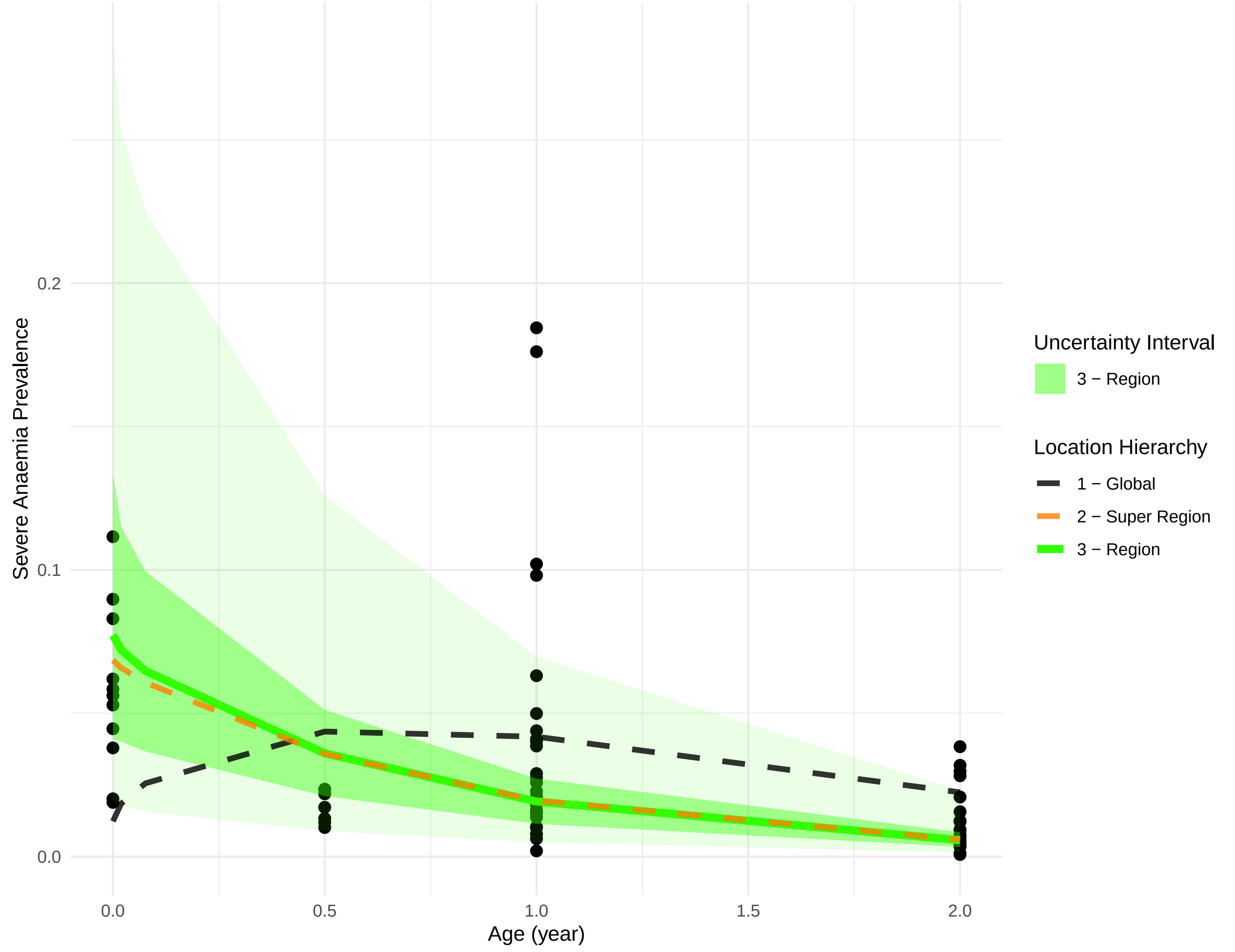

MR−BRT Age/Sex Fits − Males <2 Years Old − Severe Anaemia Prevalence − Cambodia
WHO−2024 Adjustment Method

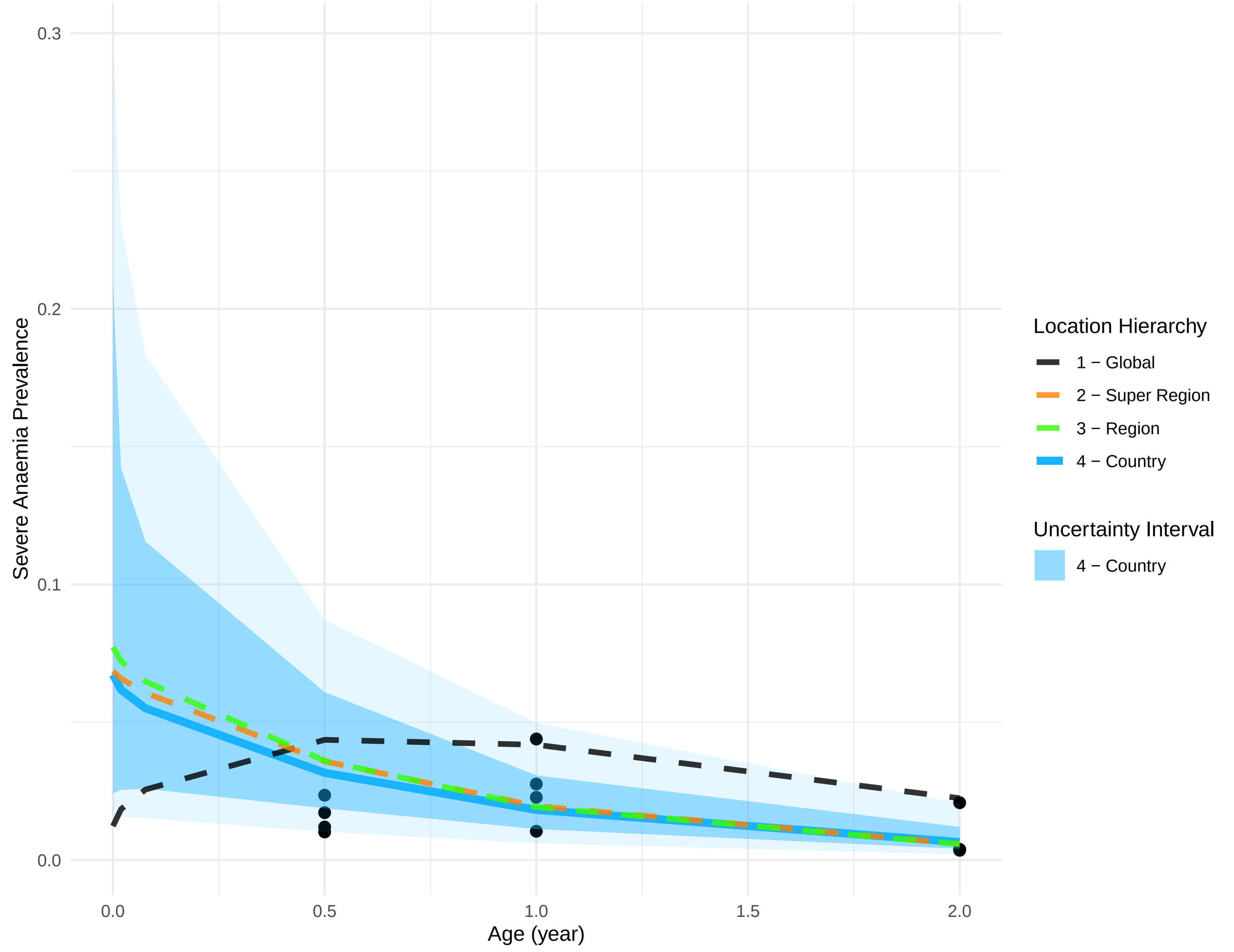

MR−BRT Age/Sex Fits − Males <2 Years Old − Severe Anaemia Prevalence − Indonesia
WHO−2024 Adjustment Method

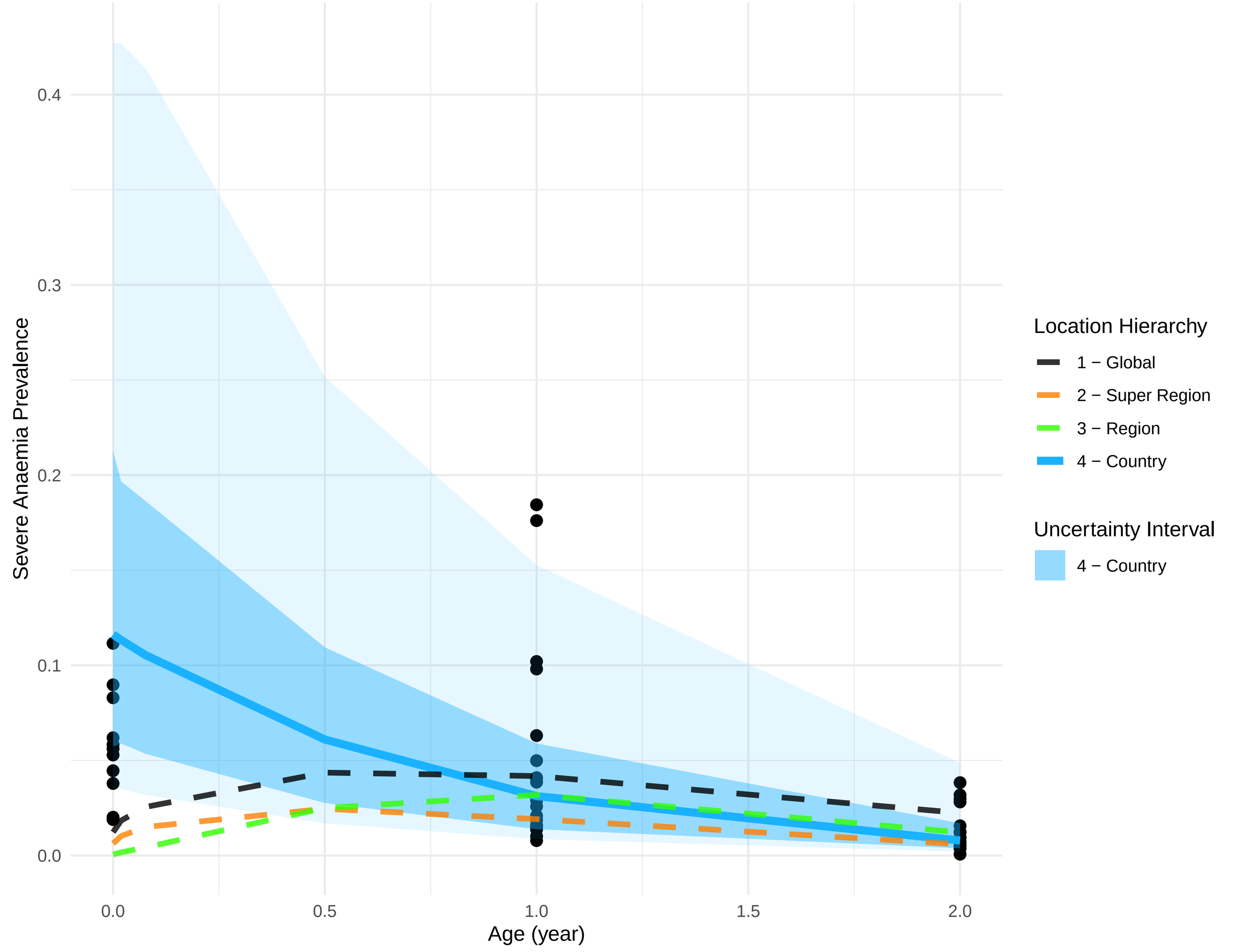

MR−BRT Age/Sex Fits − Males <2 Years Old − Severe Anaemia Prevalence − Bali
WHO−2024 Adjustment Method

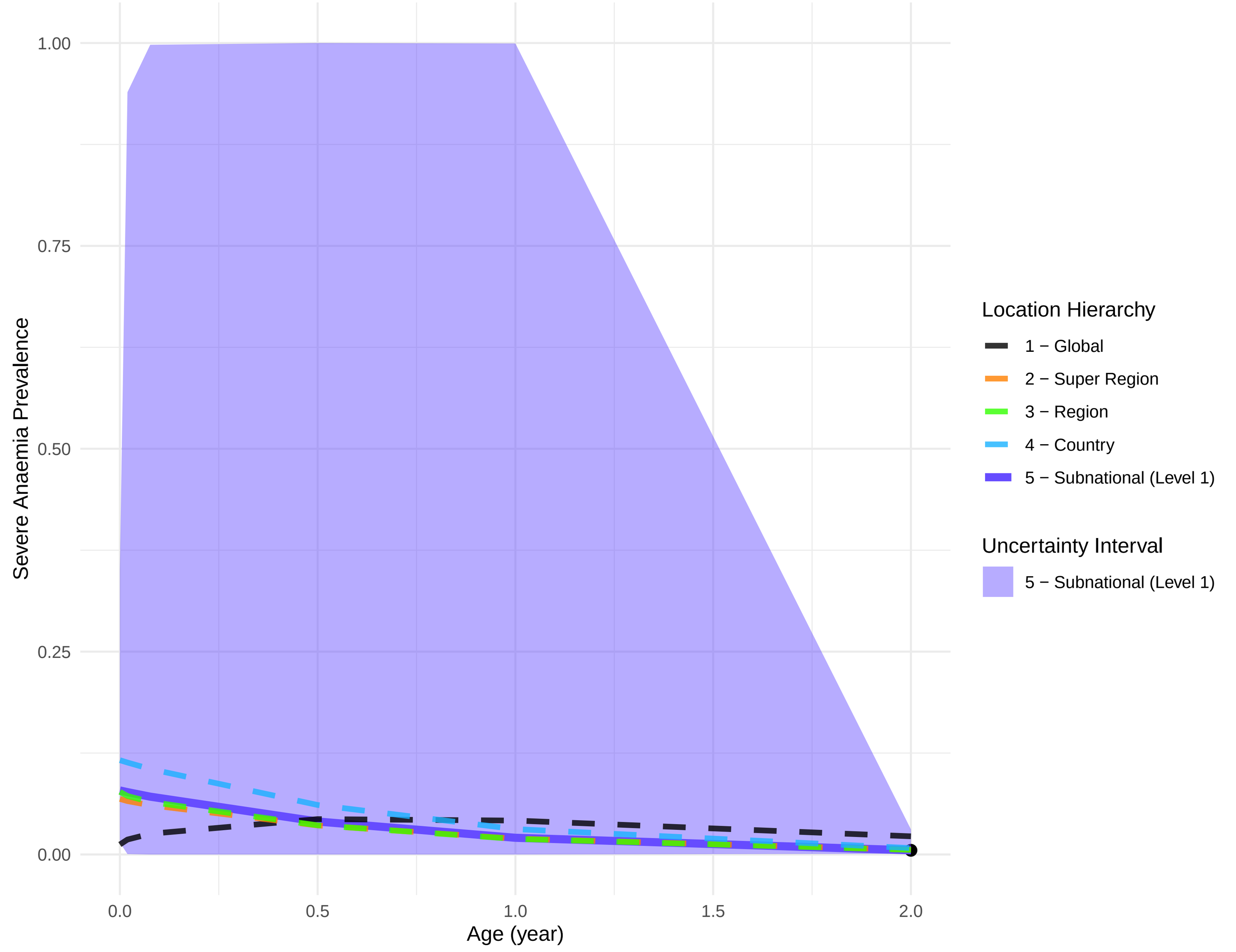

MR−BRT Age/Sex Fits − Males <2 Years Old − Severe Anaemia Prevalence − Banten
WHO−2024 Adjustment Method

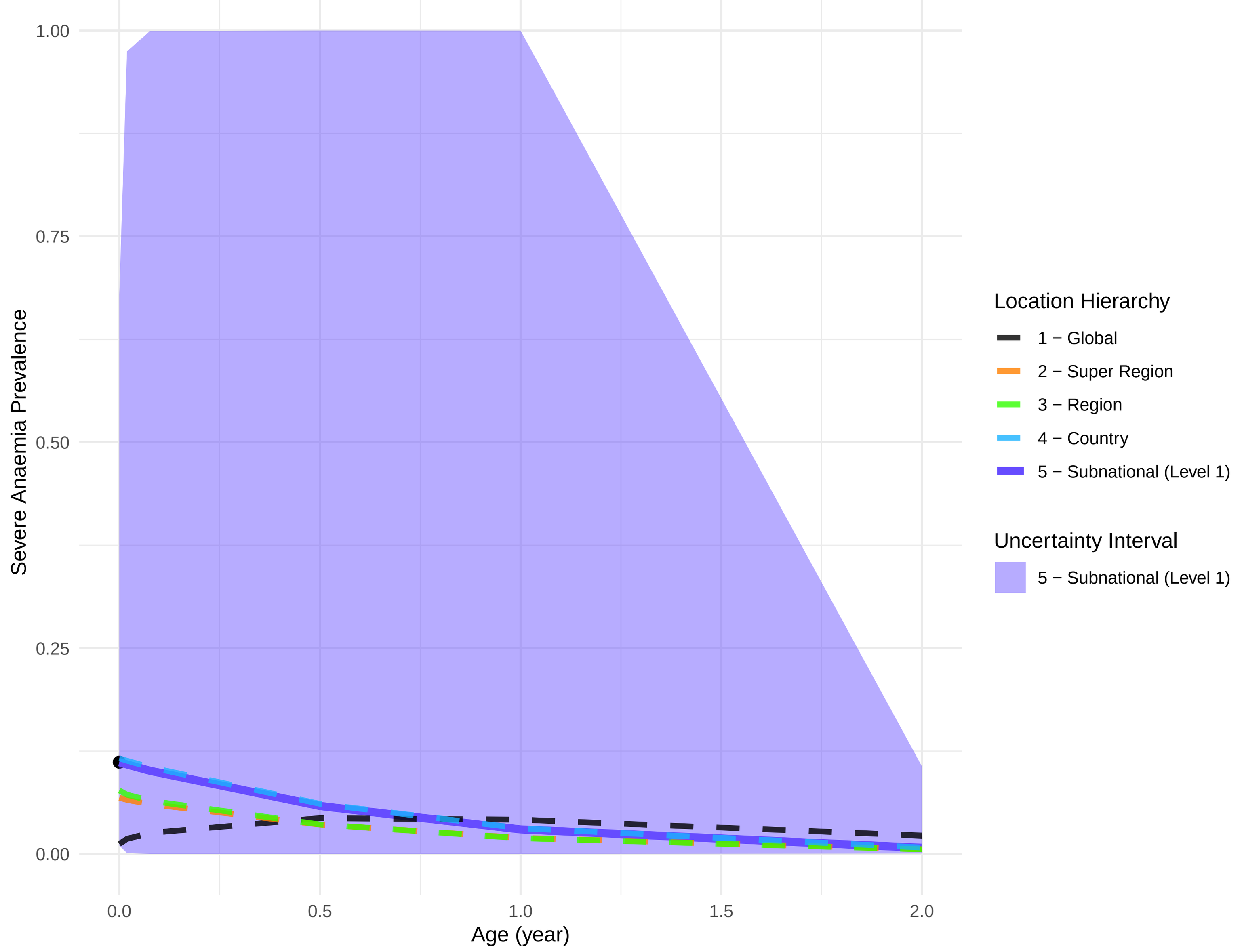

MR−BRT Age/Sex Fits − Males <2 Years Old − Severe Anaemia Prevalence − West Java
WHO−2024 Adjustment Method

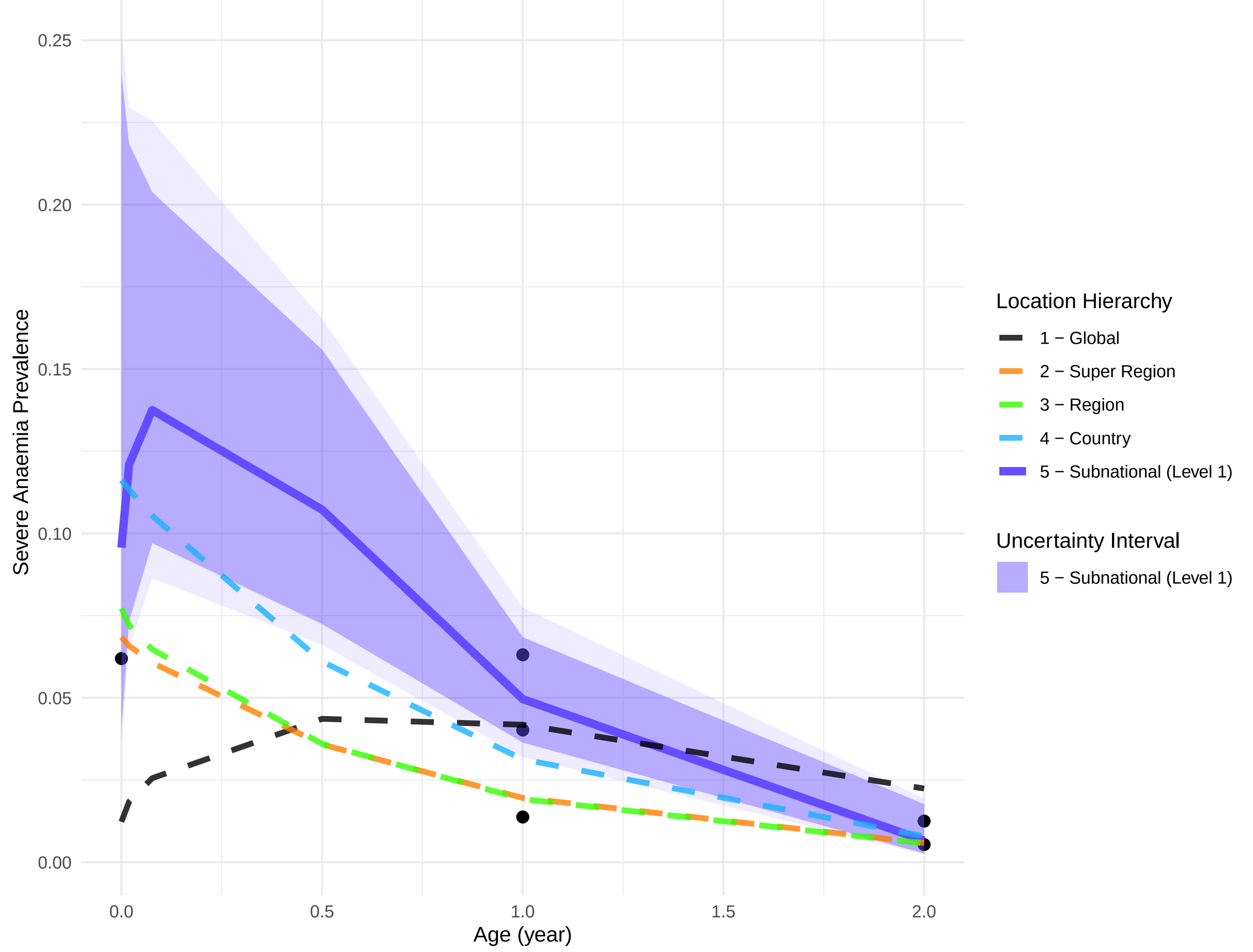

MR−BRT Age/Sex Fits − Males <2 Years Old − Severe Anaemia Prevalence − Central Java
WHO−2024 Adjustment Method

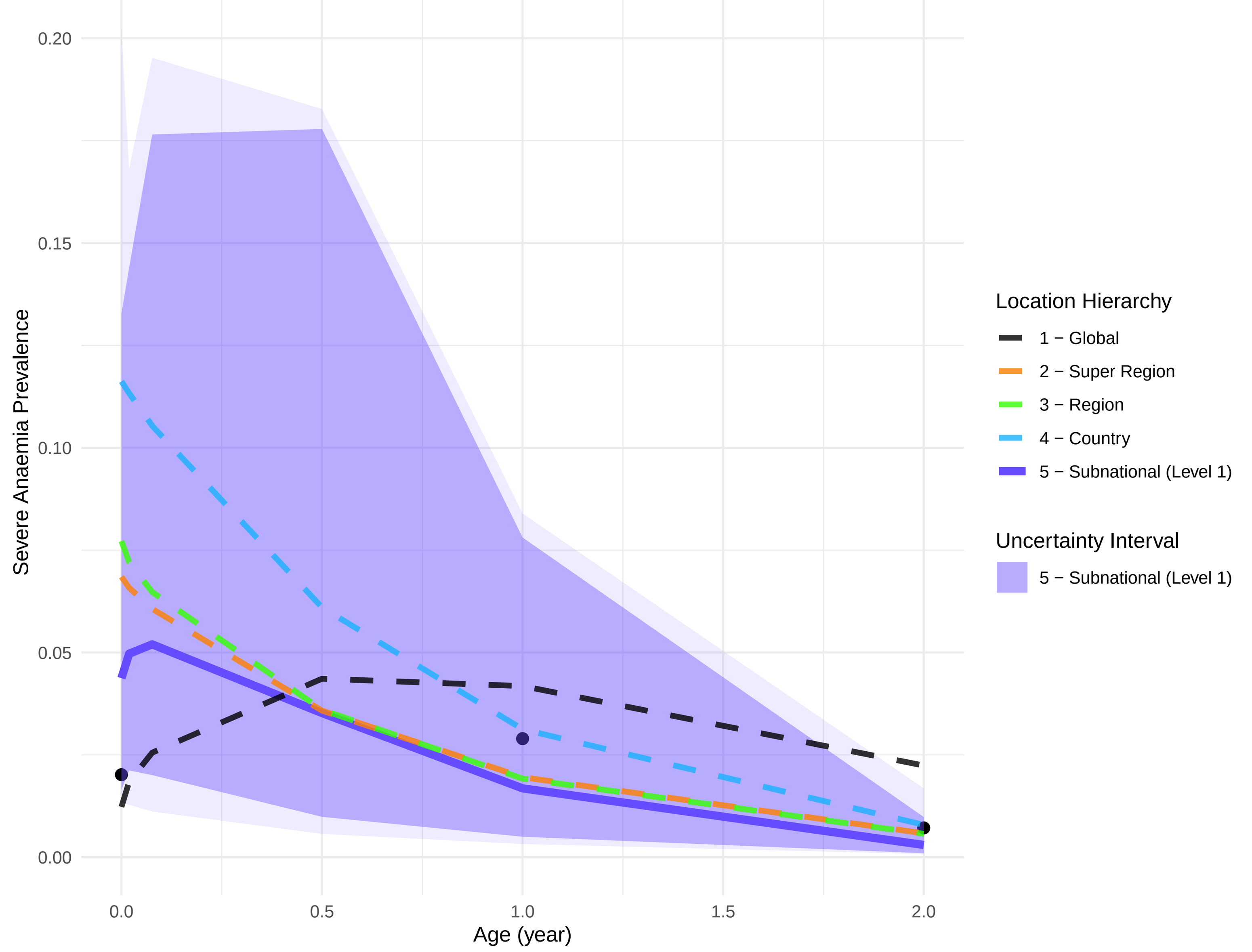

MR−BRT Age/Sex Fits − Males <2 Years Old − Severe Anaemia Prevalence − East Java
WHO−2024 Adjustment Method

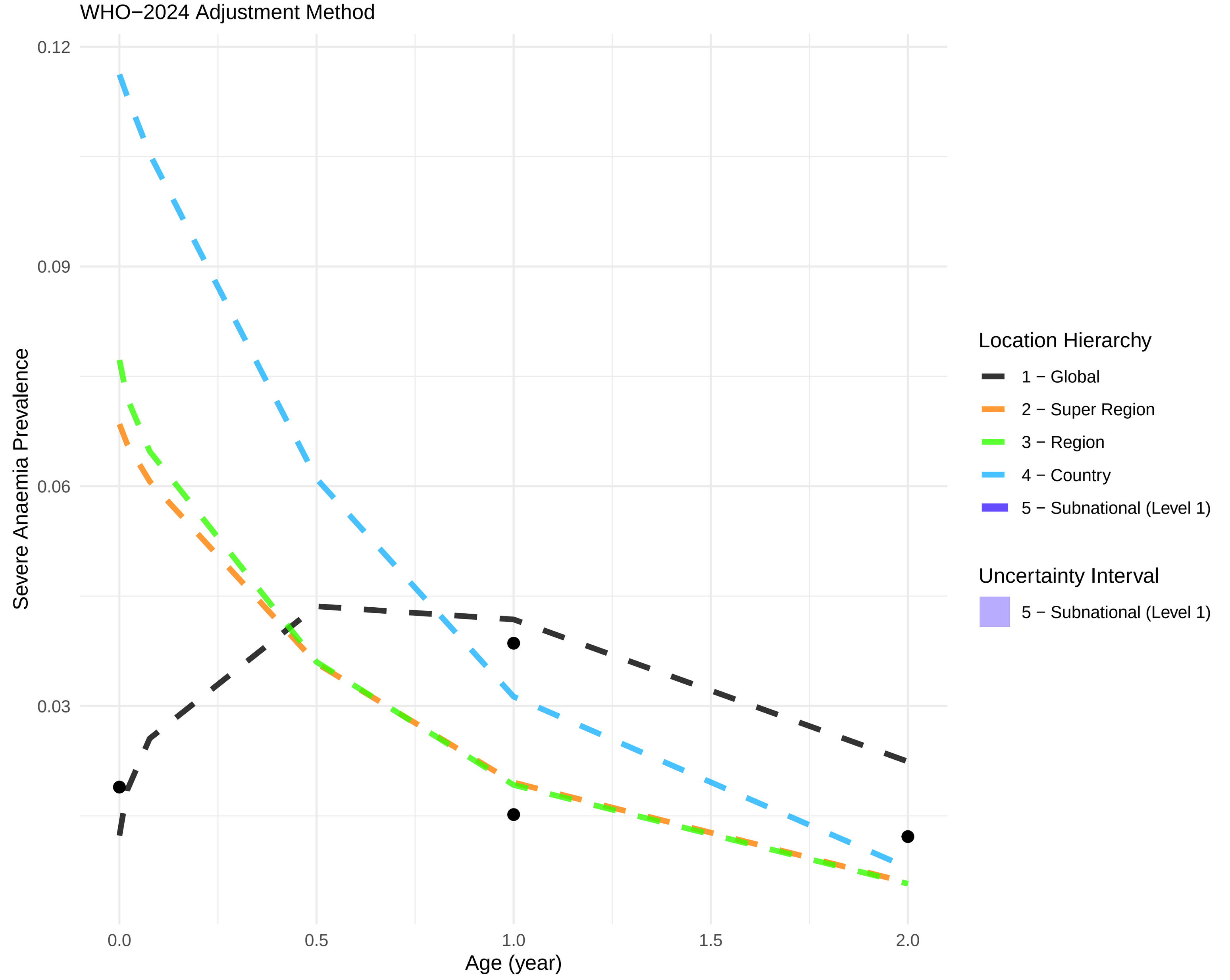

MR−BRT Age/Sex Fits − Males <2 Years Old − Severe Anaemia Prevalence − South Kalimantan
WHO−2024 Adjustment Method

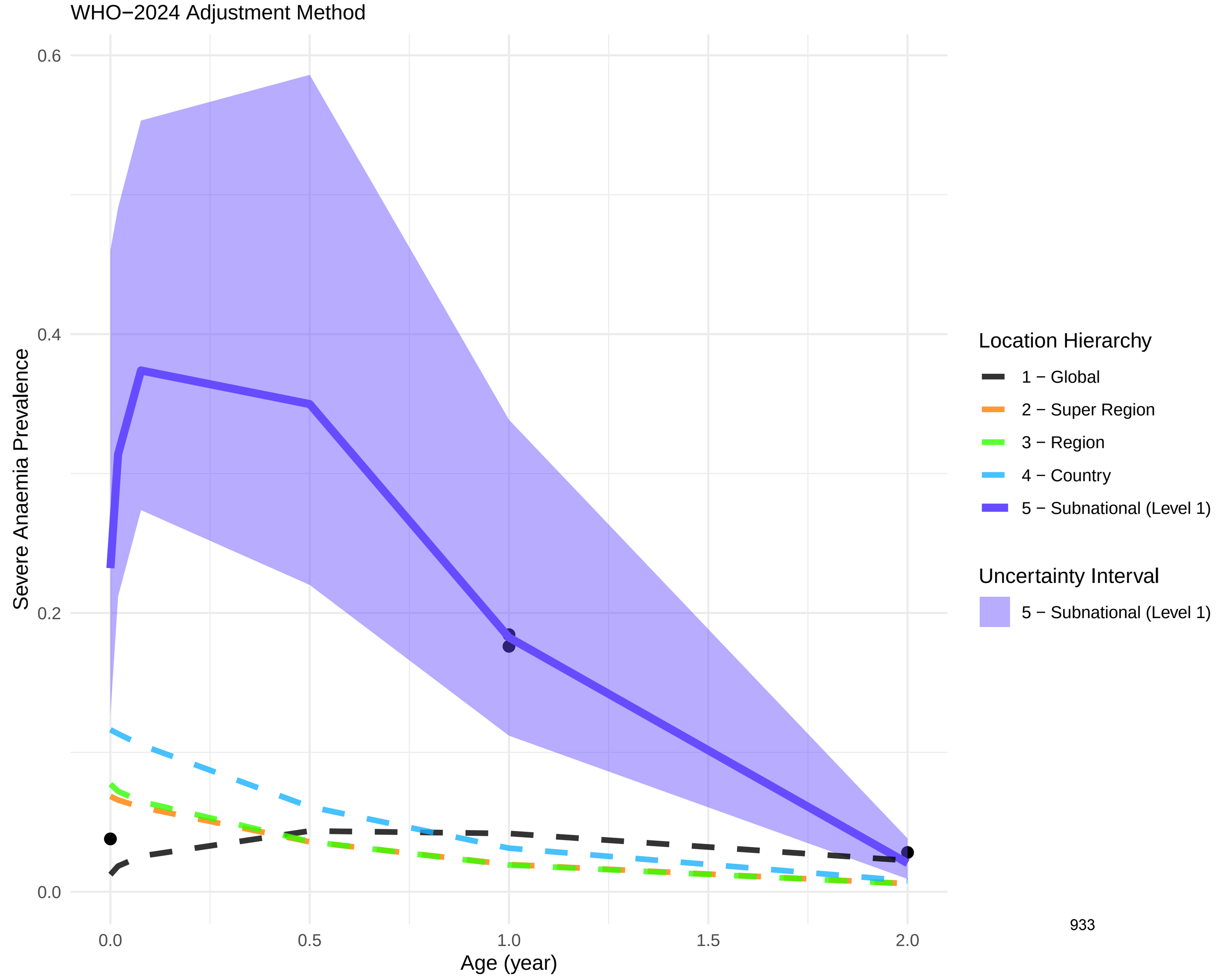

MR−BRT Age/Sex Fits − Males <2 Years Old − Severe Anaemia Prevalence − Lampung
WHO−2024 Adjustment Method

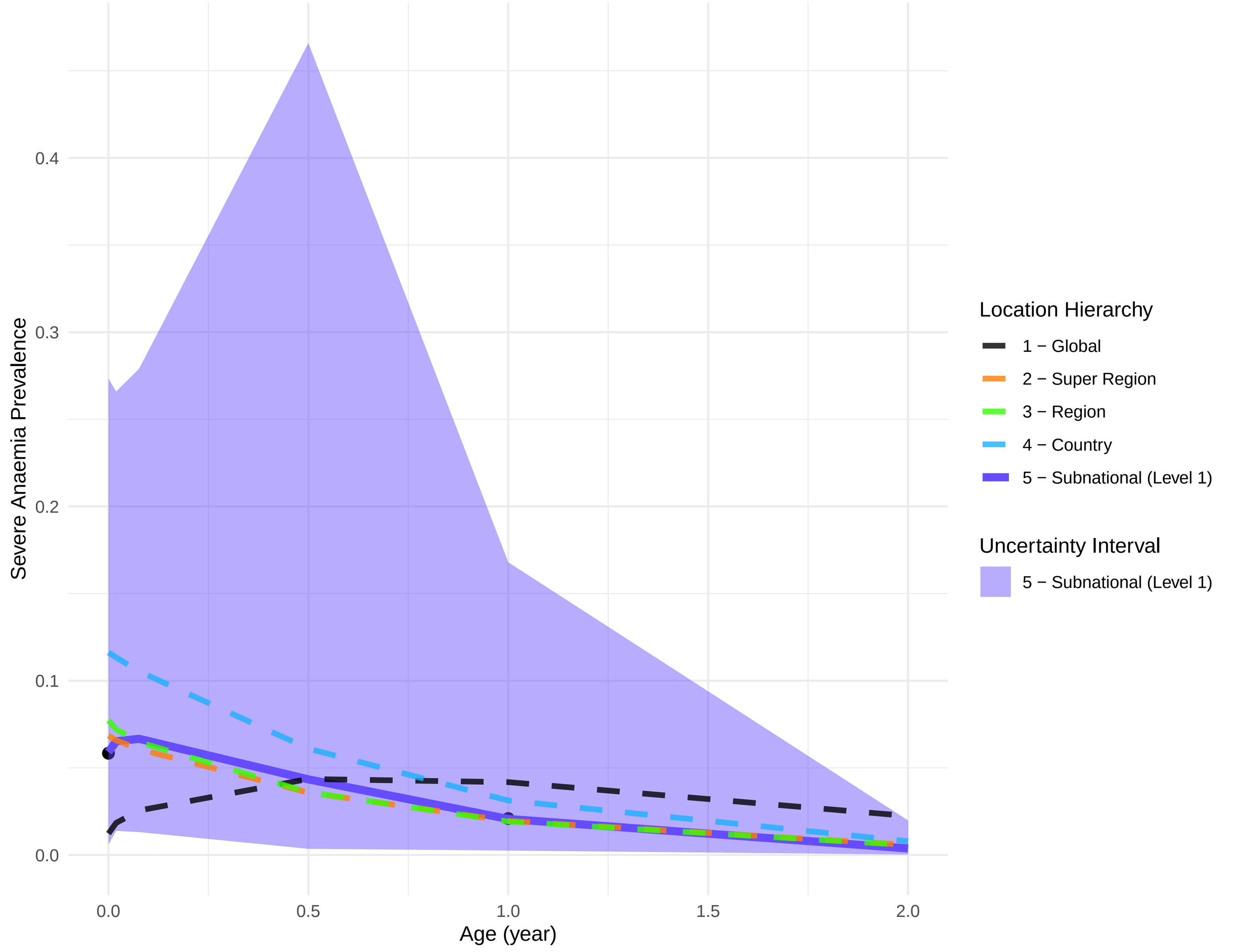

MR−BRT Age/Sex Fits − Males <2 Years Old − Severe Anaemia Prevalence − West Nusa Tenggara
WHO−2024 Adjustment Method

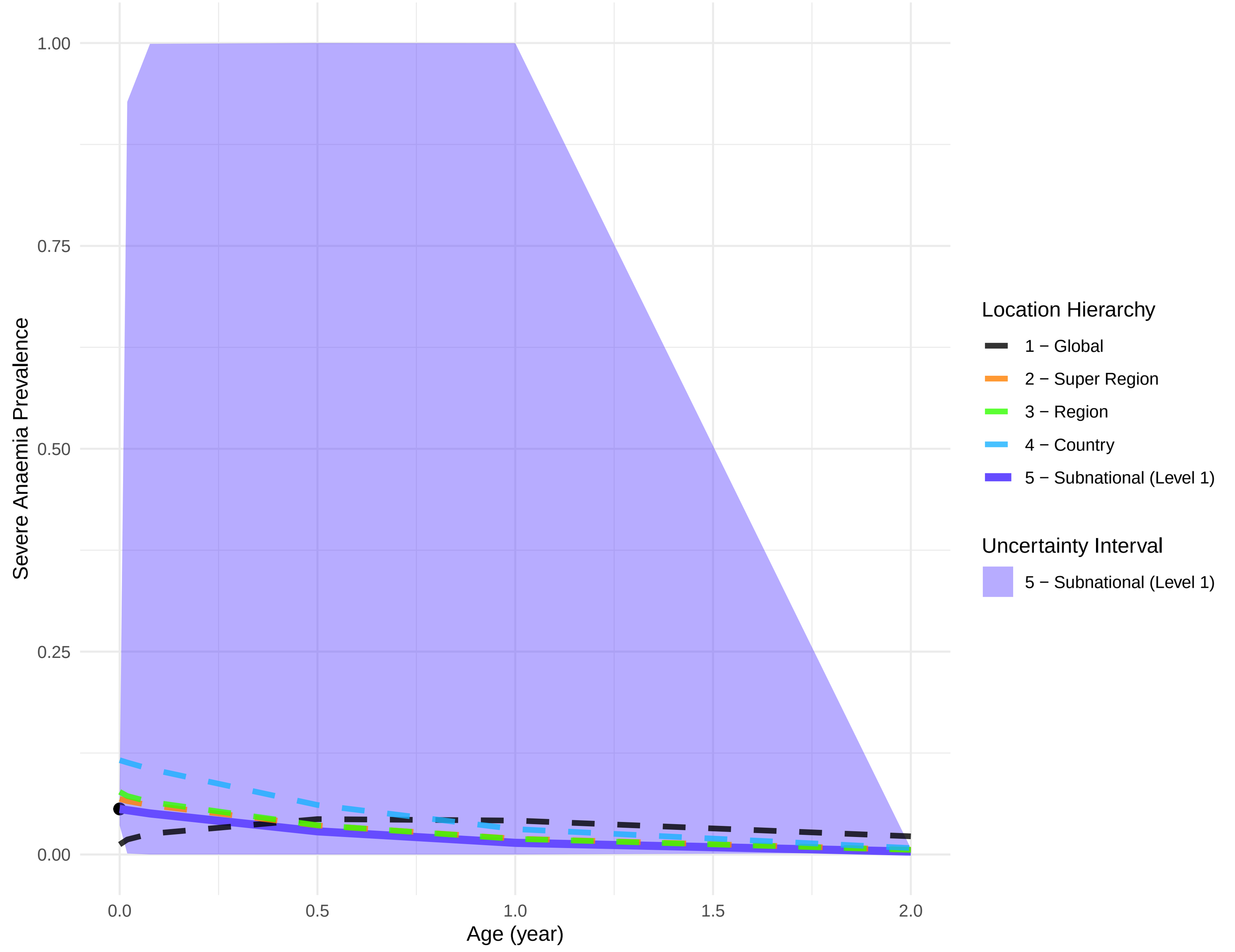

MR−BRT Age/Sex Fits − Males <2 Years Old − Severe Anaemia Prevalence − South Sulawesi
WHO−2024 Adjustment Method

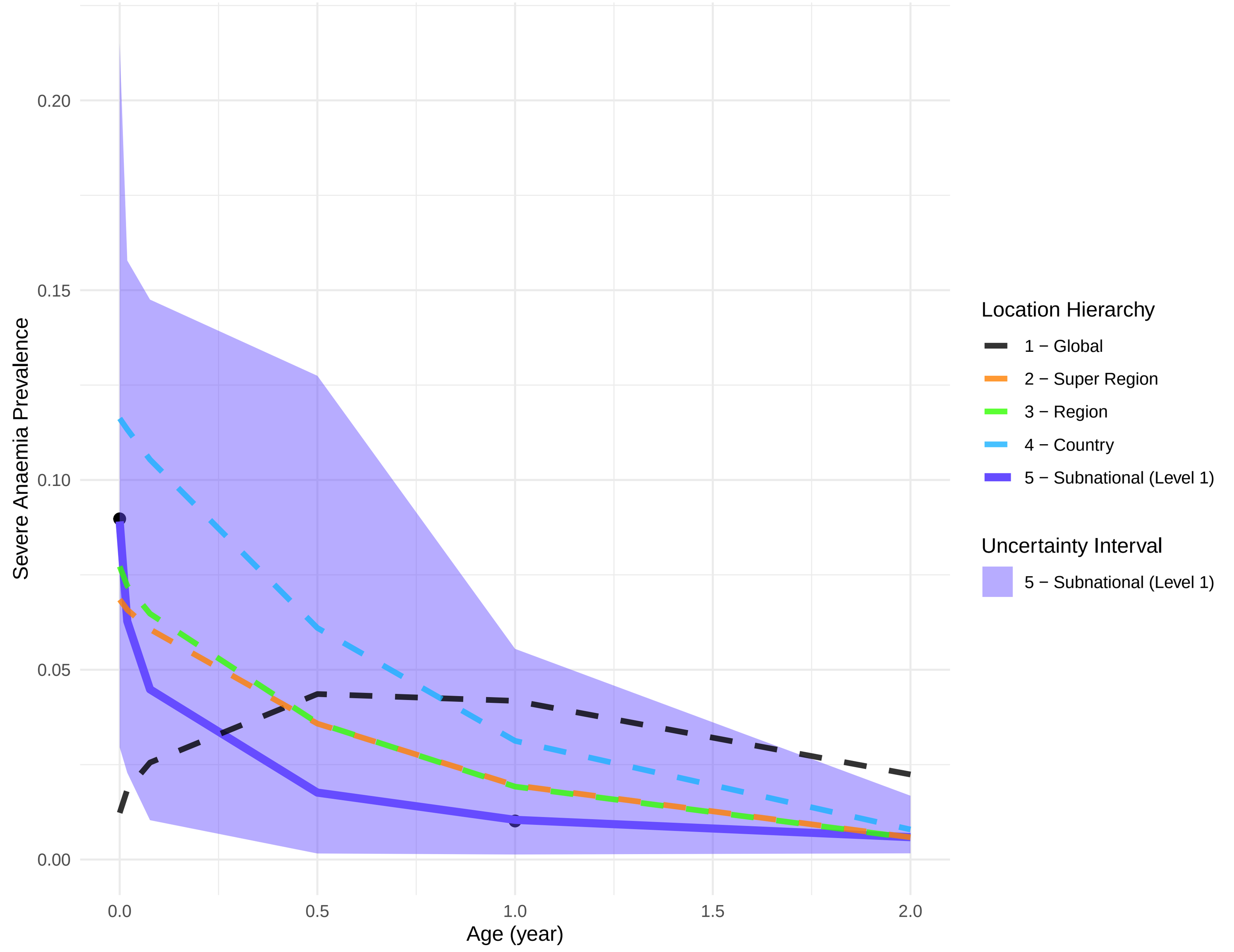

MR−BRT Age/Sex Fits − Males <2 Years Old − Severe Anaemia Prevalence − West Sumatra
WHO−2024 Adjustment Method

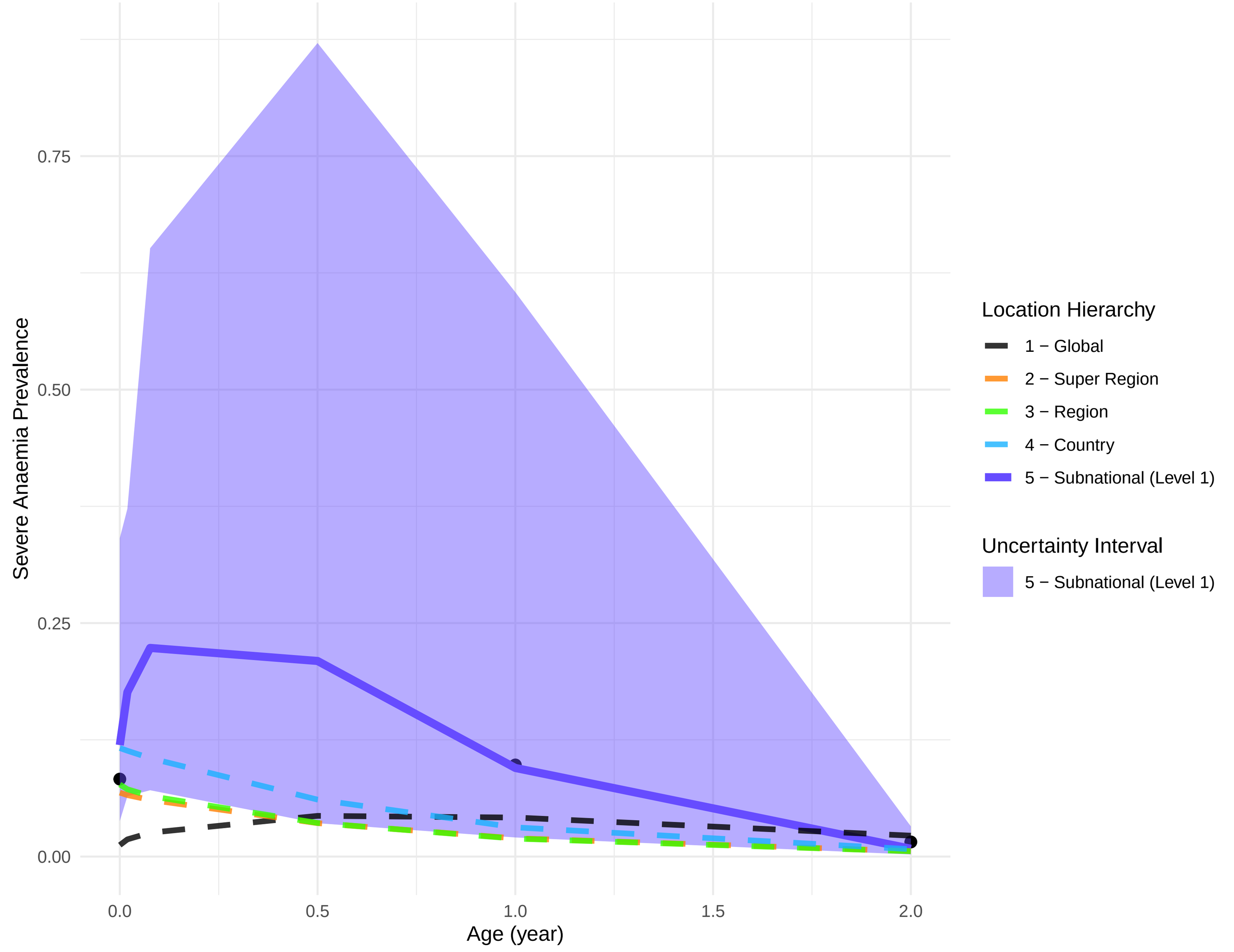

MR−BRT Age/Sex Fits − Males <2 Years Old − Severe Anaemia Prevalence − South Sumatra
WHO−2024 Adjustment Method

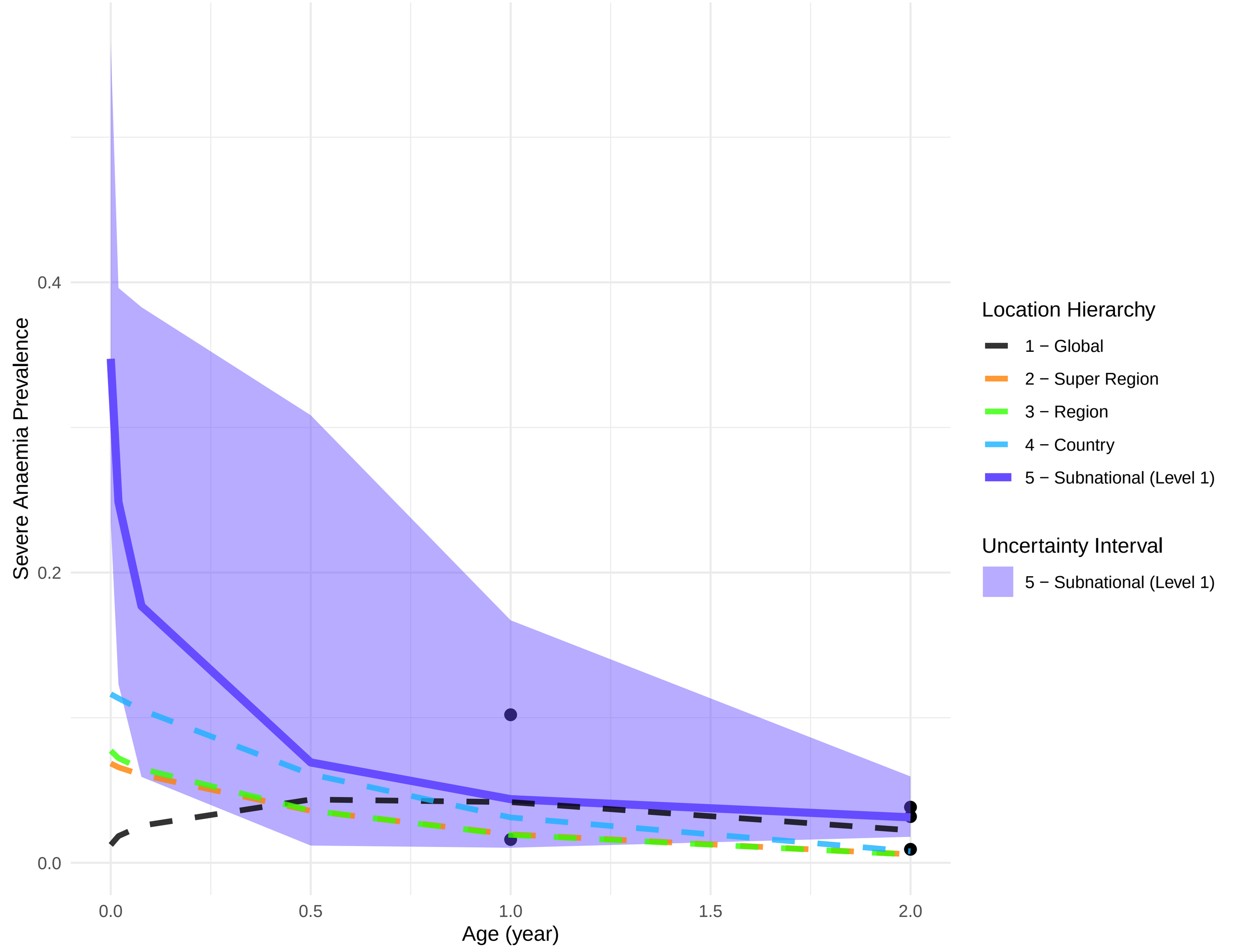

MR−BRT Age/Sex Fits − Males <2 Years Old − Severe Anaemia Prevalence − North Sumatra
WHO−2024 Adjustment Method

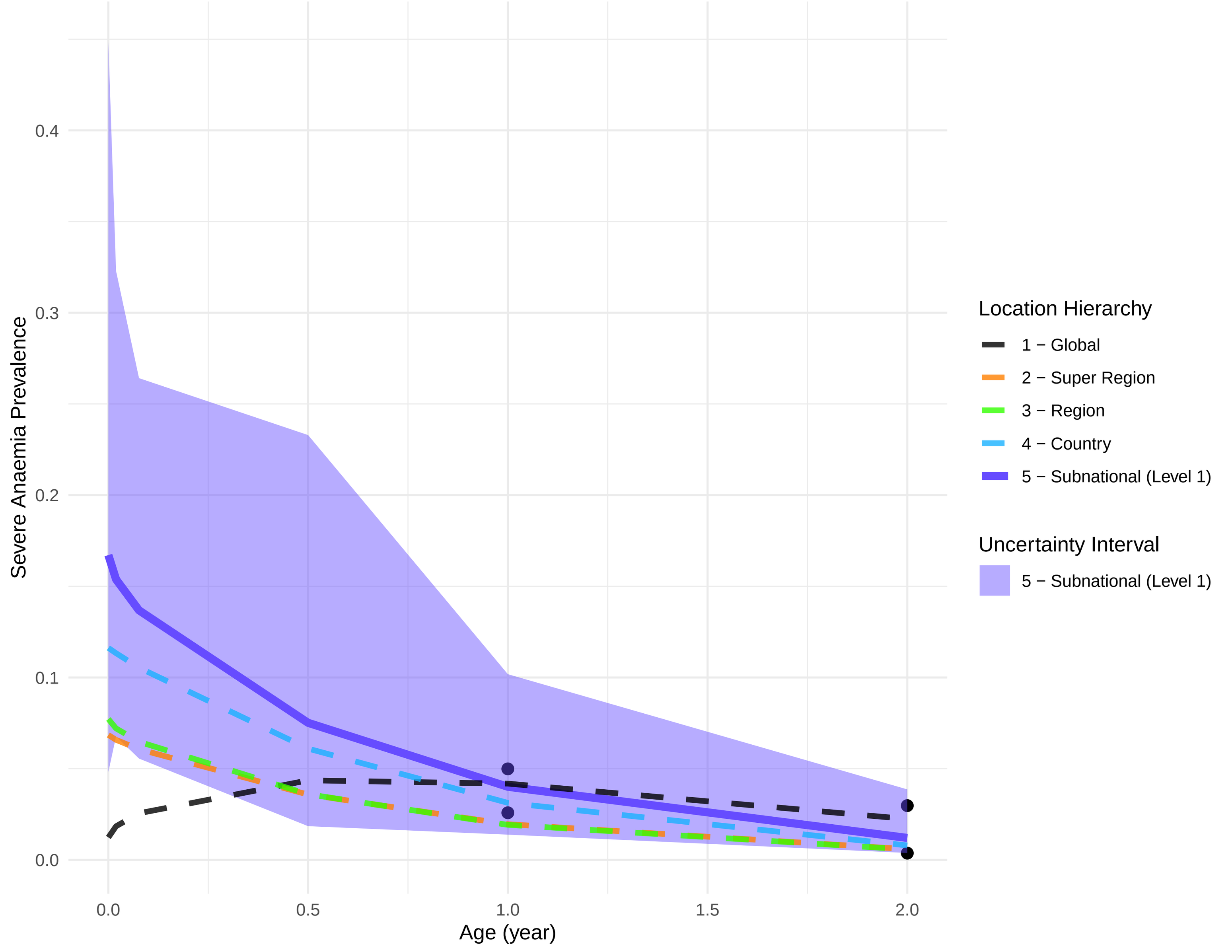

MR−BRT Age/Sex Fits − Males <2 Years Old − Severe Anaemia Prevalence − Yogyakarta
WHO−2024 Adjustment Method

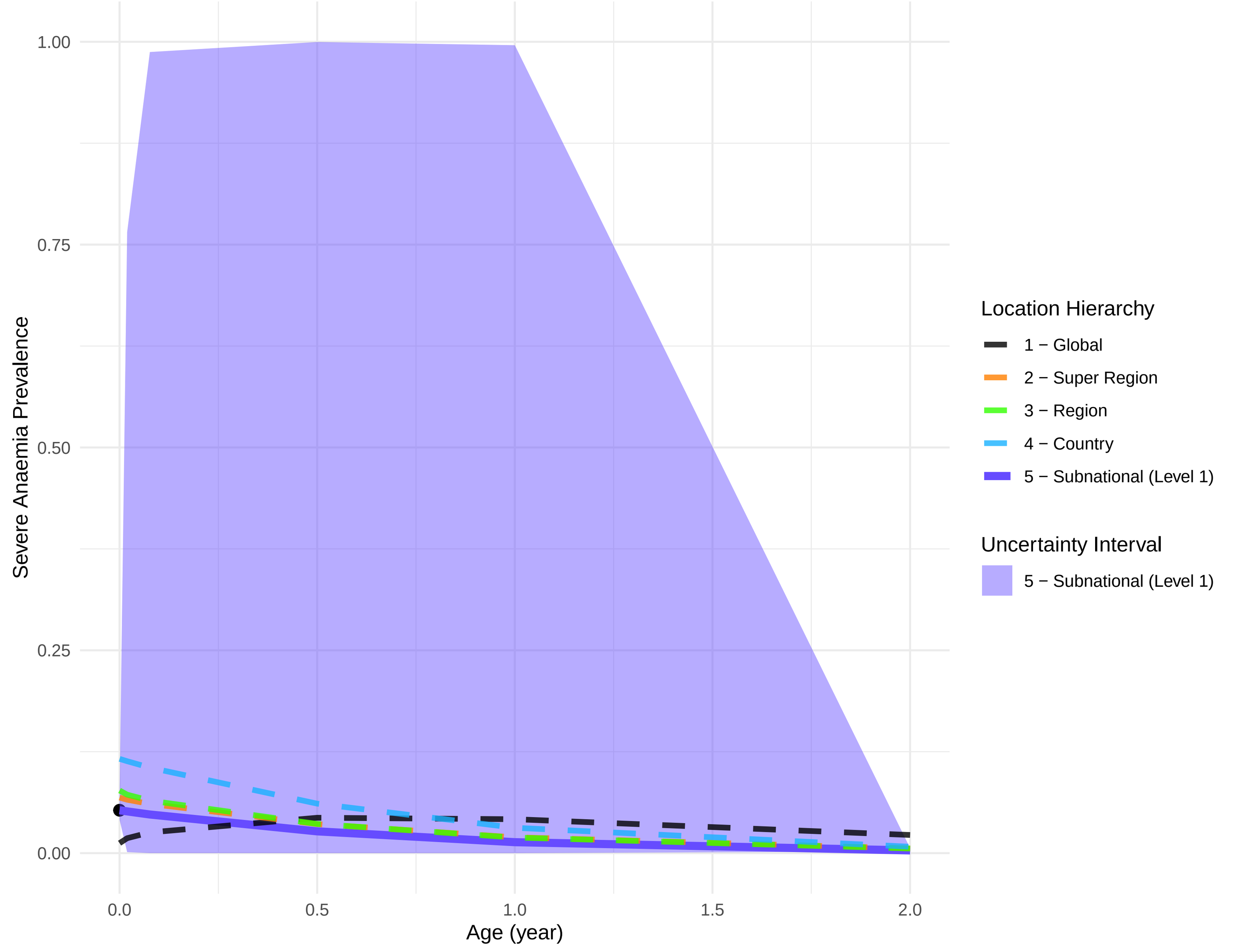

MR−BRT Age/Sex Fits − Males <2 Years Old − Severe Anaemia Prevalence − Myanmar
WHO−2024 Adjustment Method

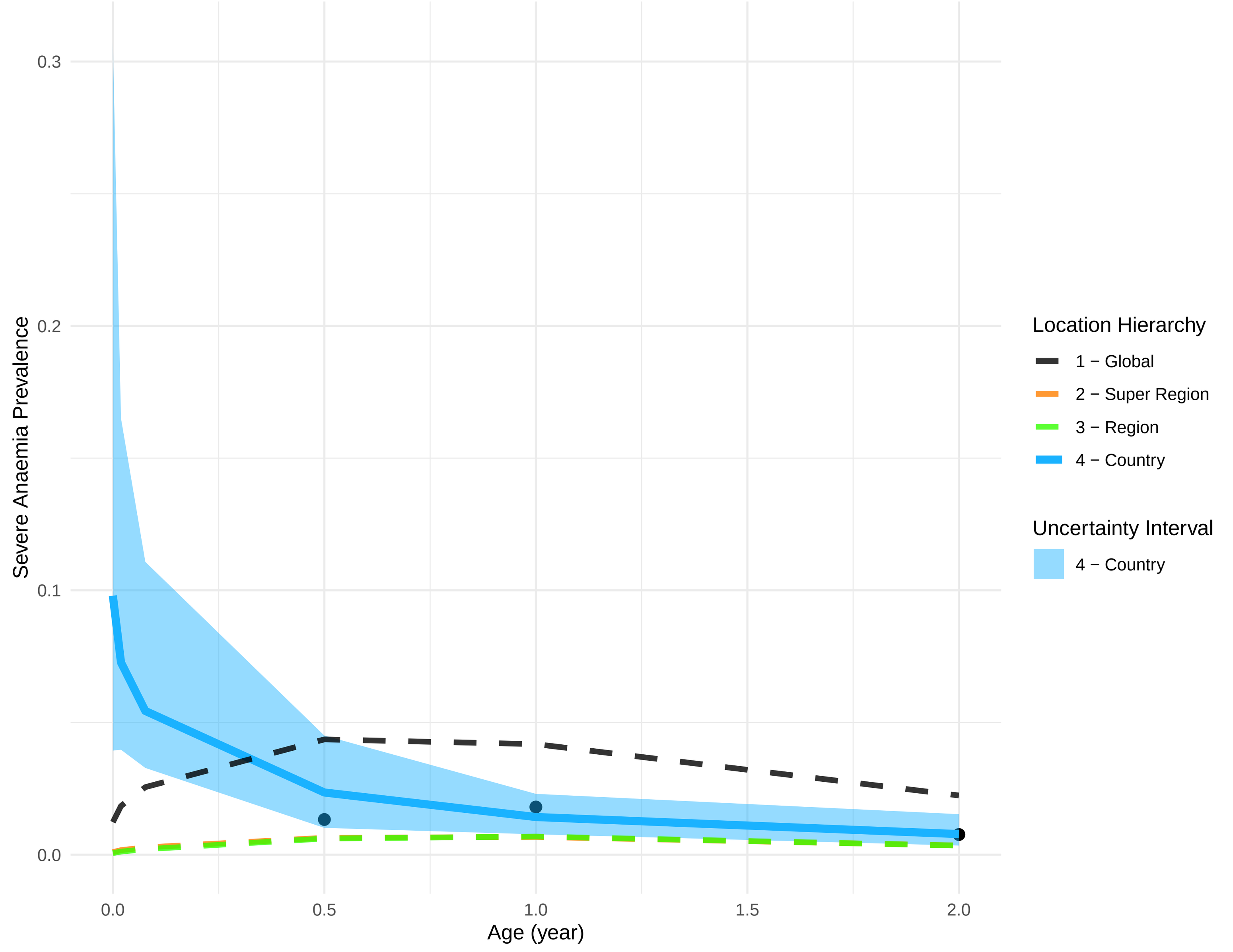

MR−BRT Age/Sex Fits − Males <2 Years Old − Severe Anaemia Prevalence − Timor−Leste
WHO−2024 Adjustment Method

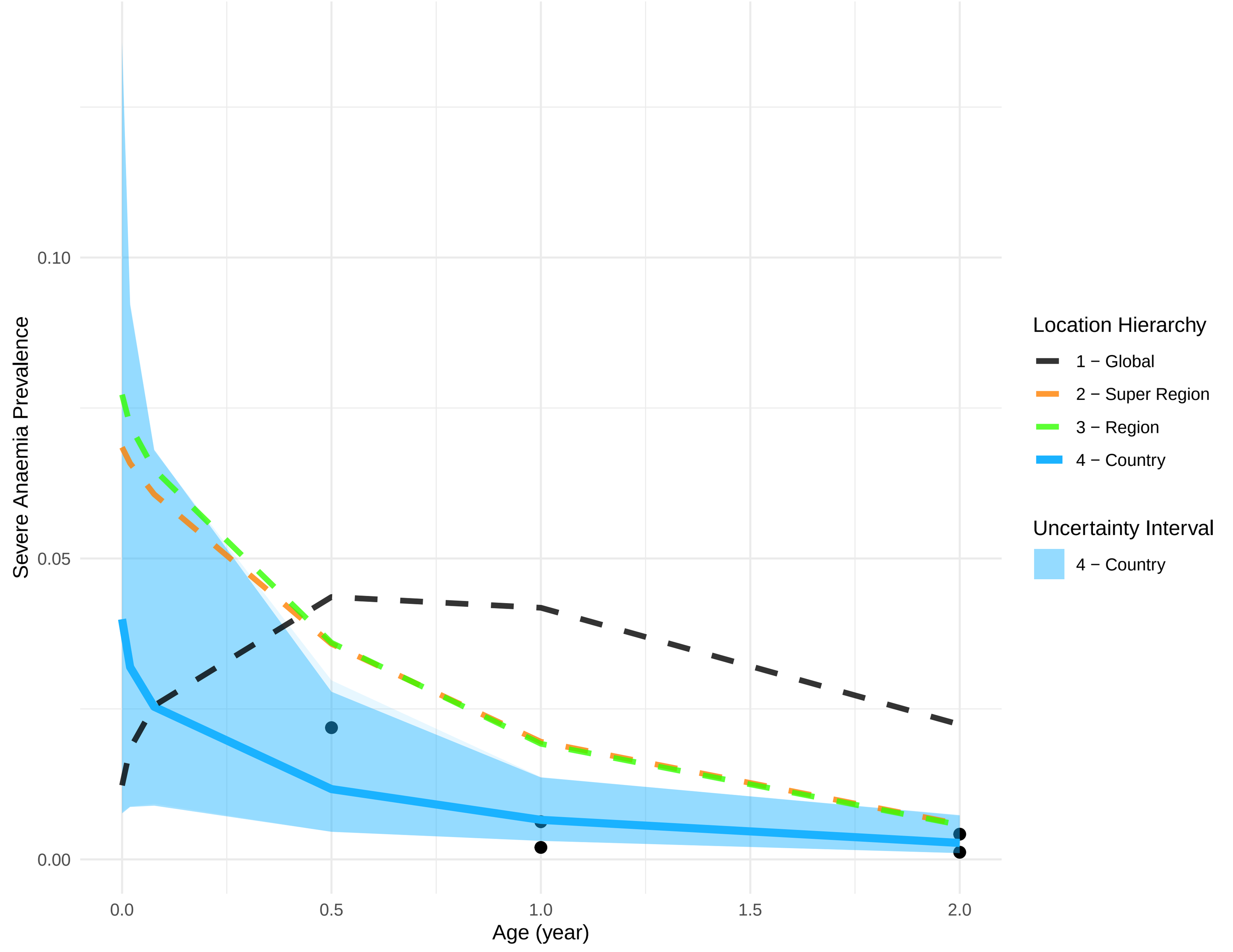

MR−BRT Age/Sex Fits − Males <2 Years Old − Severe Anaemia Prevalence − Sub−Saharan Africa
WHO−2024 Adjustment Method

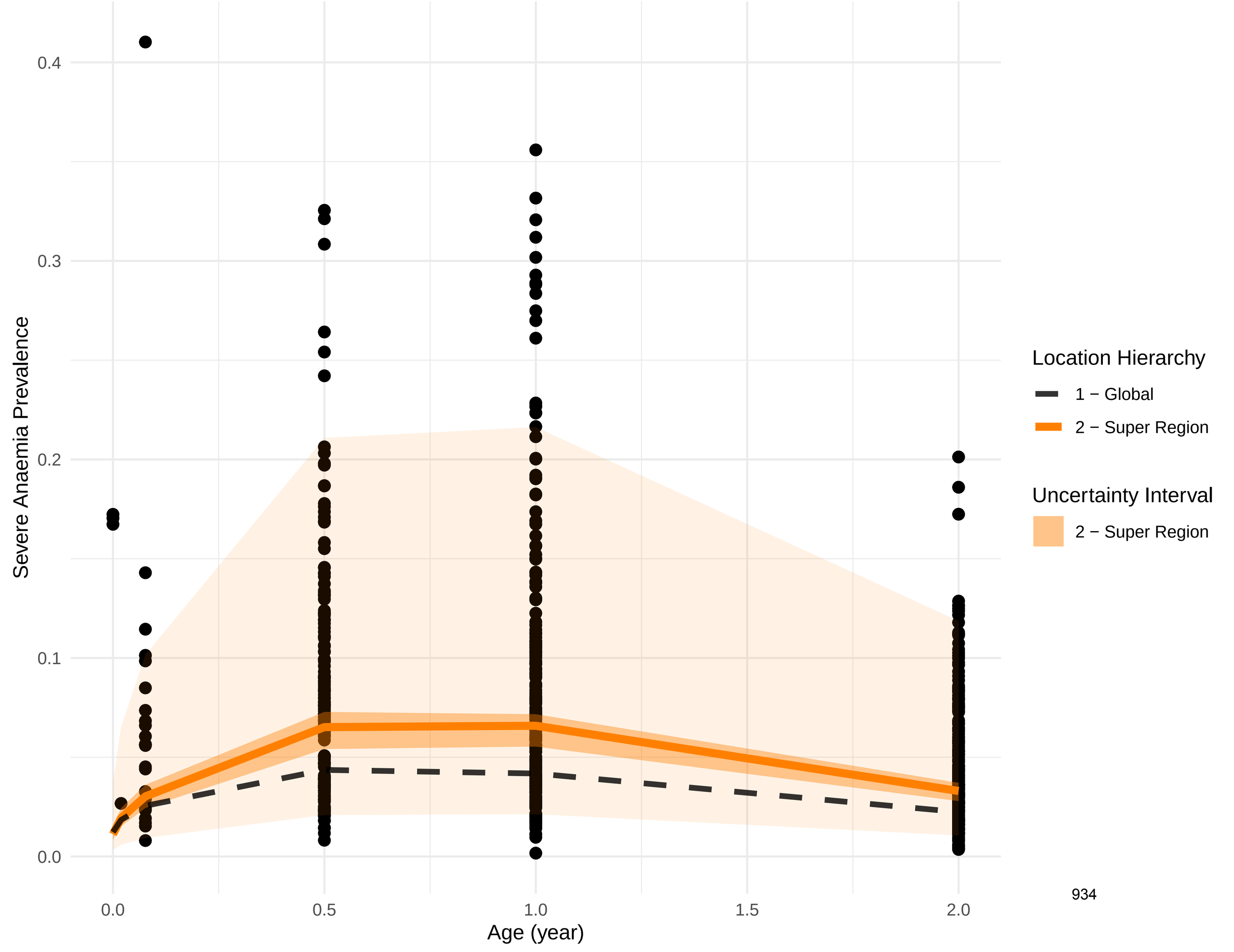

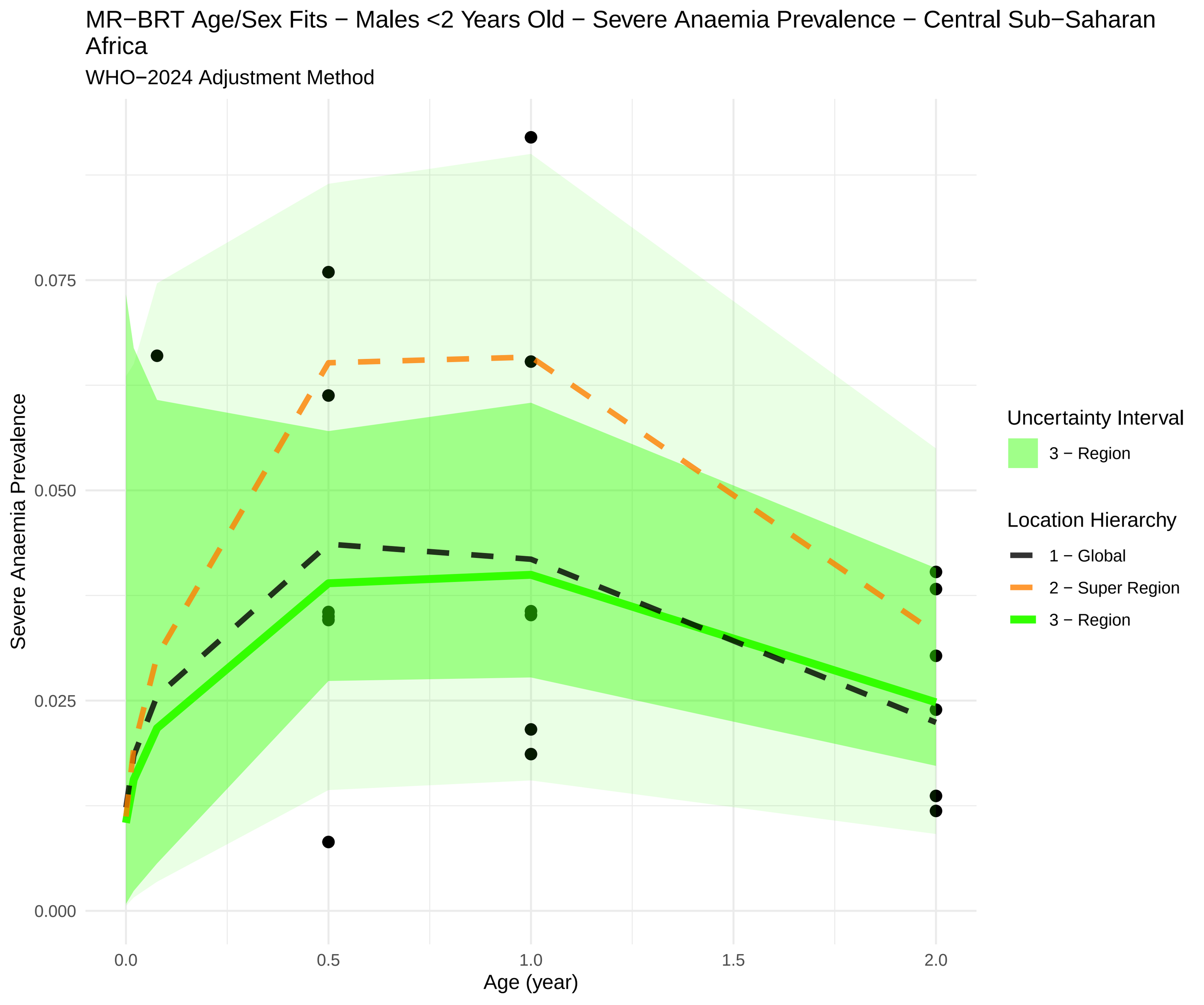
MR−BRT Age/Sex Fits − Males <2 Years Old − Severe Anaemia Prevalence − Central Sub−Saharan Africa
WHO−2024 Adjustment Method
Severe Anaemia Prevalence
Age (year)
Uncertainty Interval
3 − Region
Location Hierarchy
1 − Global
2 − Super Region
3 − Region

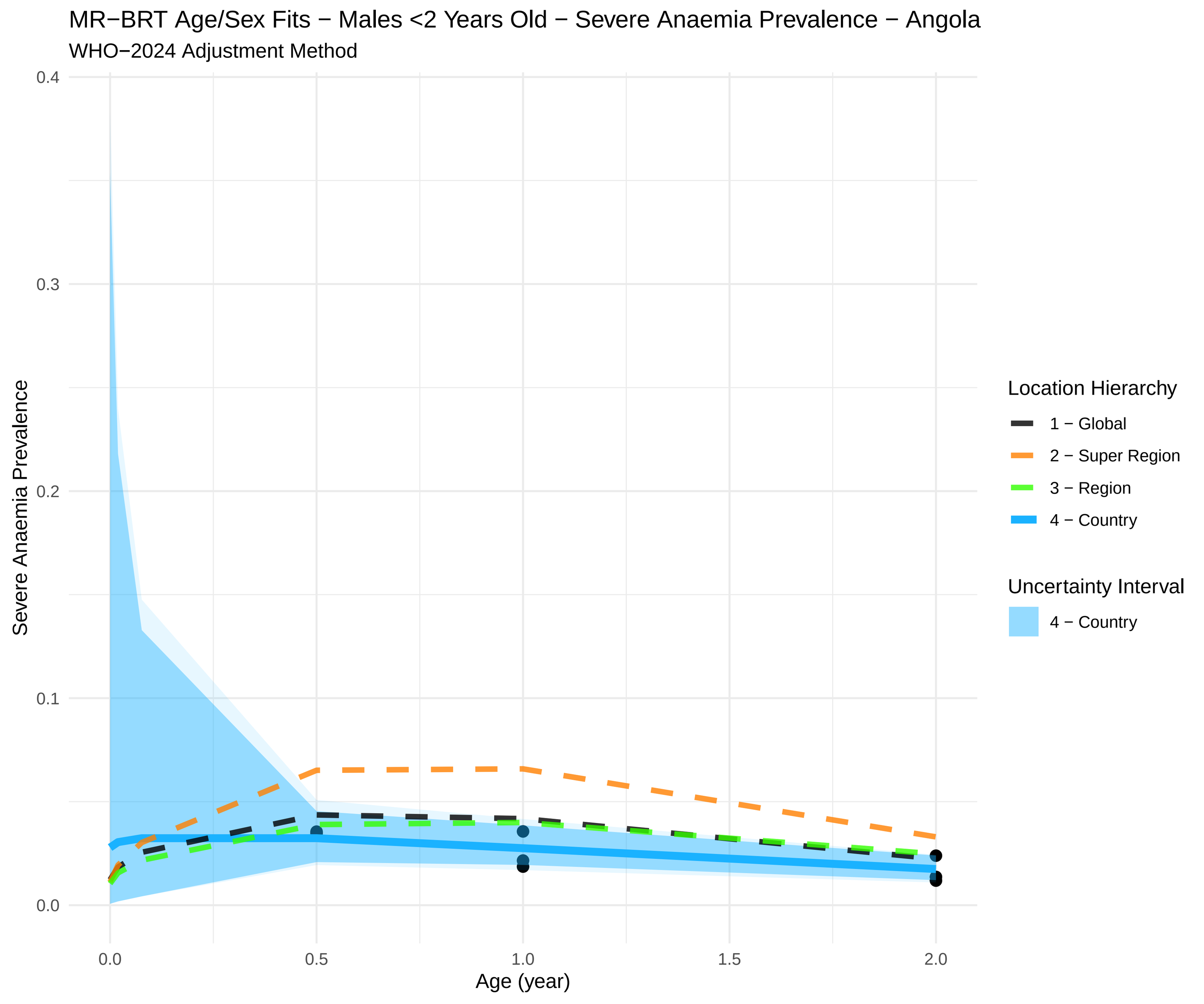
MR−BRT Age/Sex Fits − Males <2 Years Old − Severe Anaemia Prevalence − Angola
WHO−2024 Adjustment Method
Severe Anaemia Prevalence
Age (year)
Location Hierarchy
1 − Global
2 − Super Region
3 − Region
4 − Country
Uncertainty Interval
4 − Country

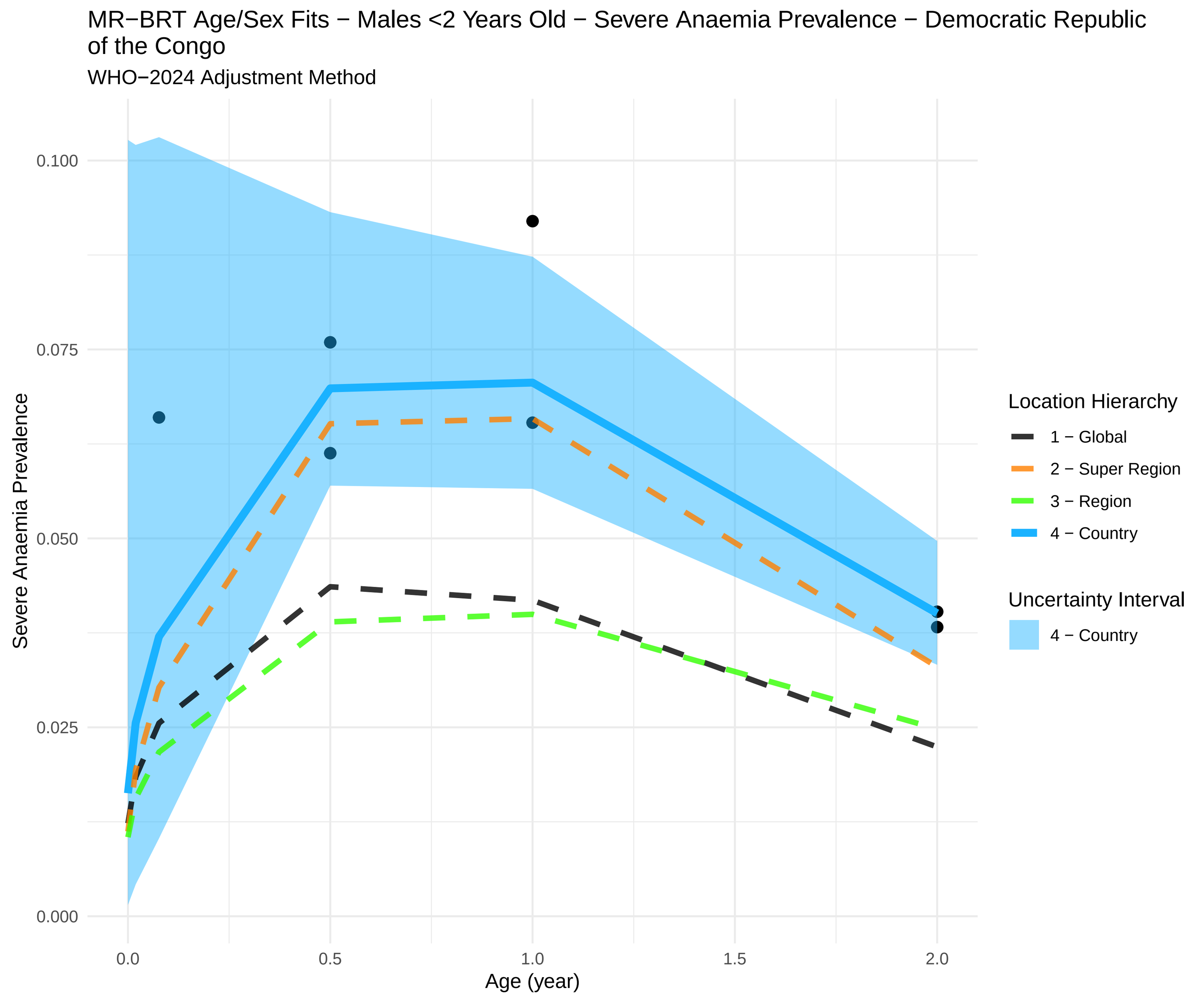
MR−BRT Age/Sex Fits − Males <2 Years Old − Severe Anaemia Prevalence − Democratic Republic of the Congo
WHO−2024 Adjustment Method
Severe Anaemia Prevalence
Age (year)
Location Hierarchy
1 − Global
2 − Super Region
3 − Region
4 − Country
Uncertainty Interval
4 − Country

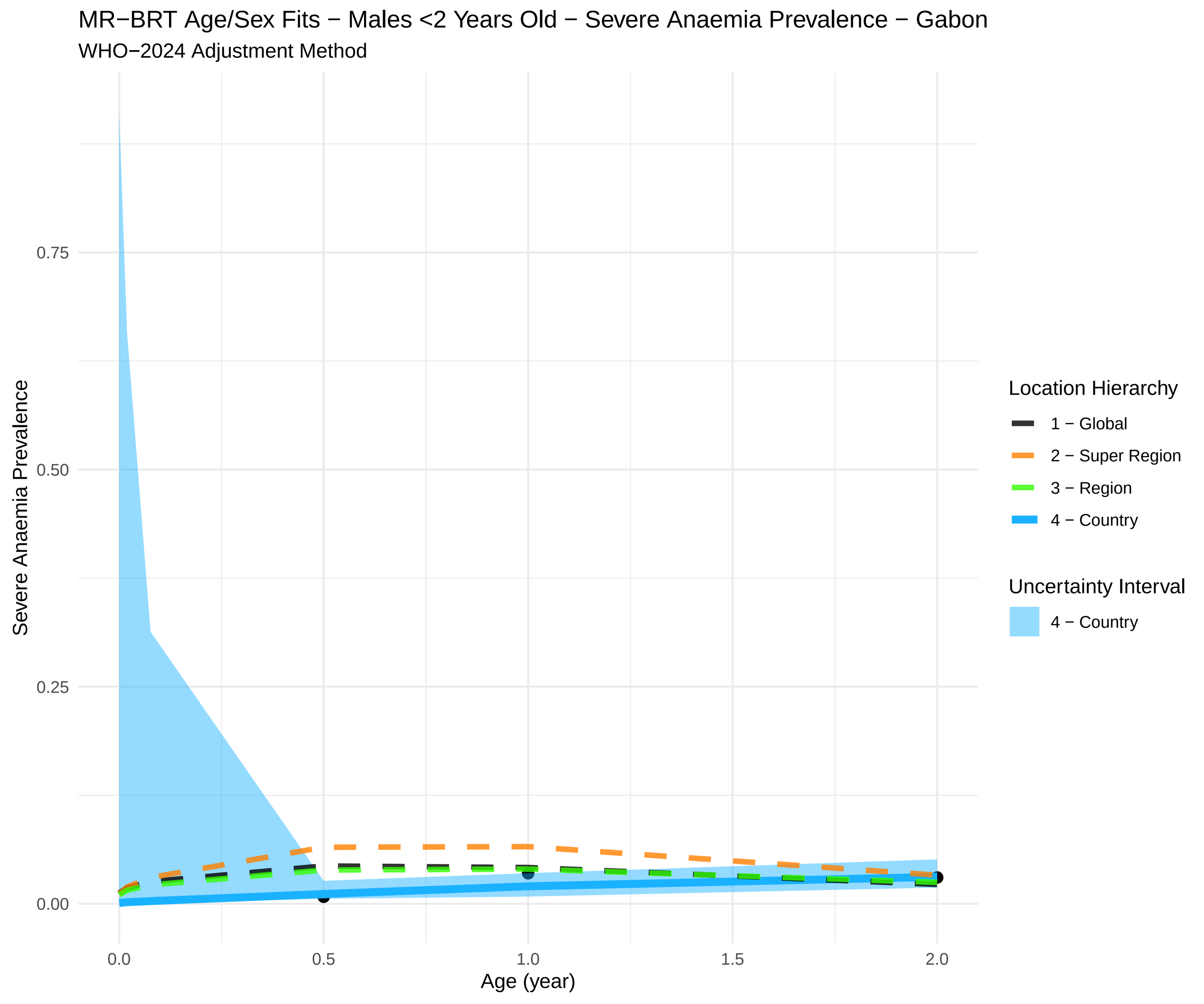
MR−BRT Age/Sex Fits − Males <2 Years Old − Severe Anaemia Prevalence − Gabon
WHO−2024 Adjustment Method
Severe Anaemia Prevalence
Age (year)
Location Hierarchy
1 − Global
2 − Super Region
3 − Region
4 − Country
Uncertainty Interval
4 − Country

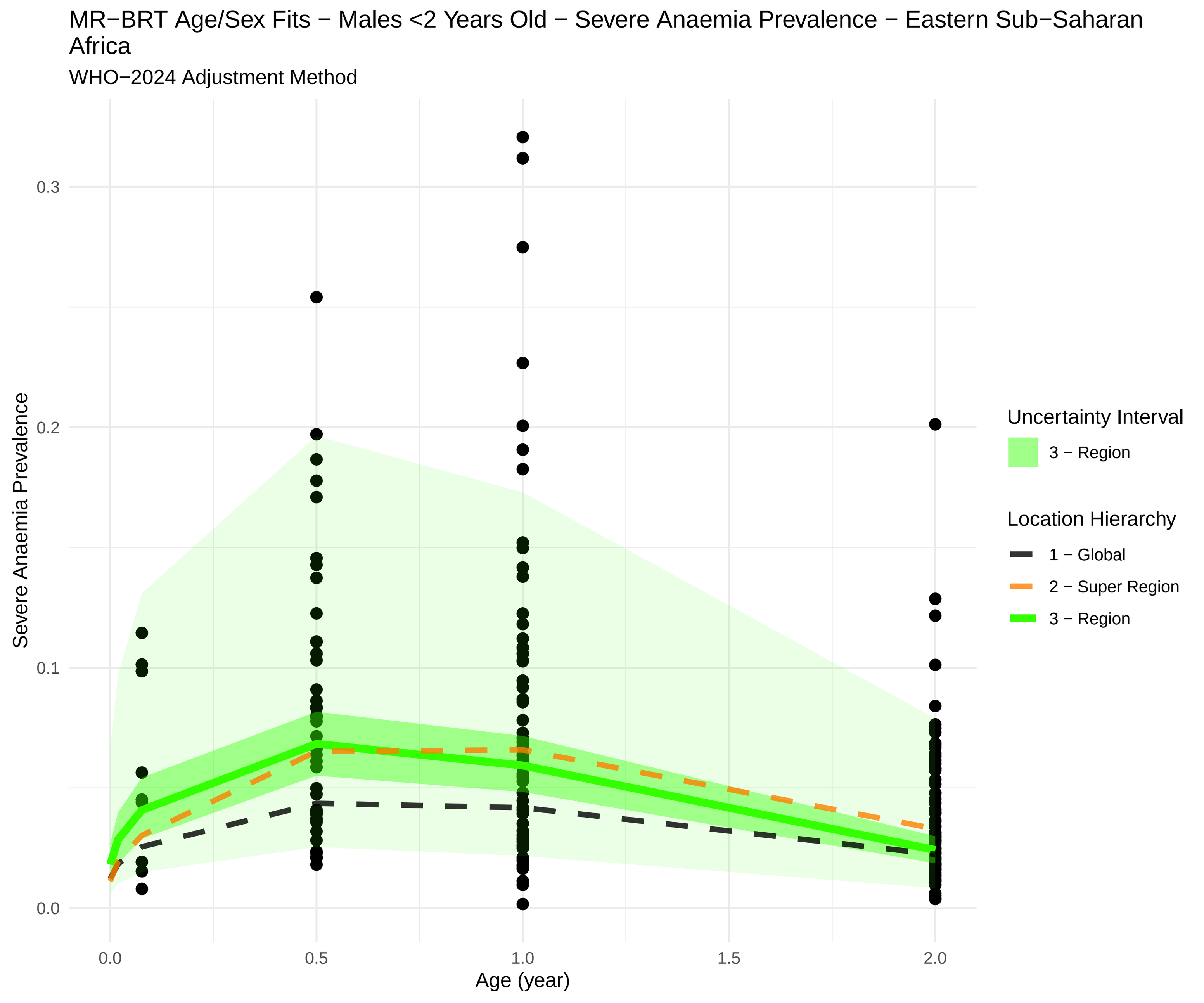
MR−BRT Age/Sex Fits − Males <2 Years Old − Severe Anaemia Prevalence − Eastern Sub−Saharan Africa
WHO−2024 Adjustment Method
Severe Anaemia Prevalence
Age (year)
Uncertainty Interval
3 − Region
Location Hierarchy
1 − Global
2 − Super Region
3 − Region

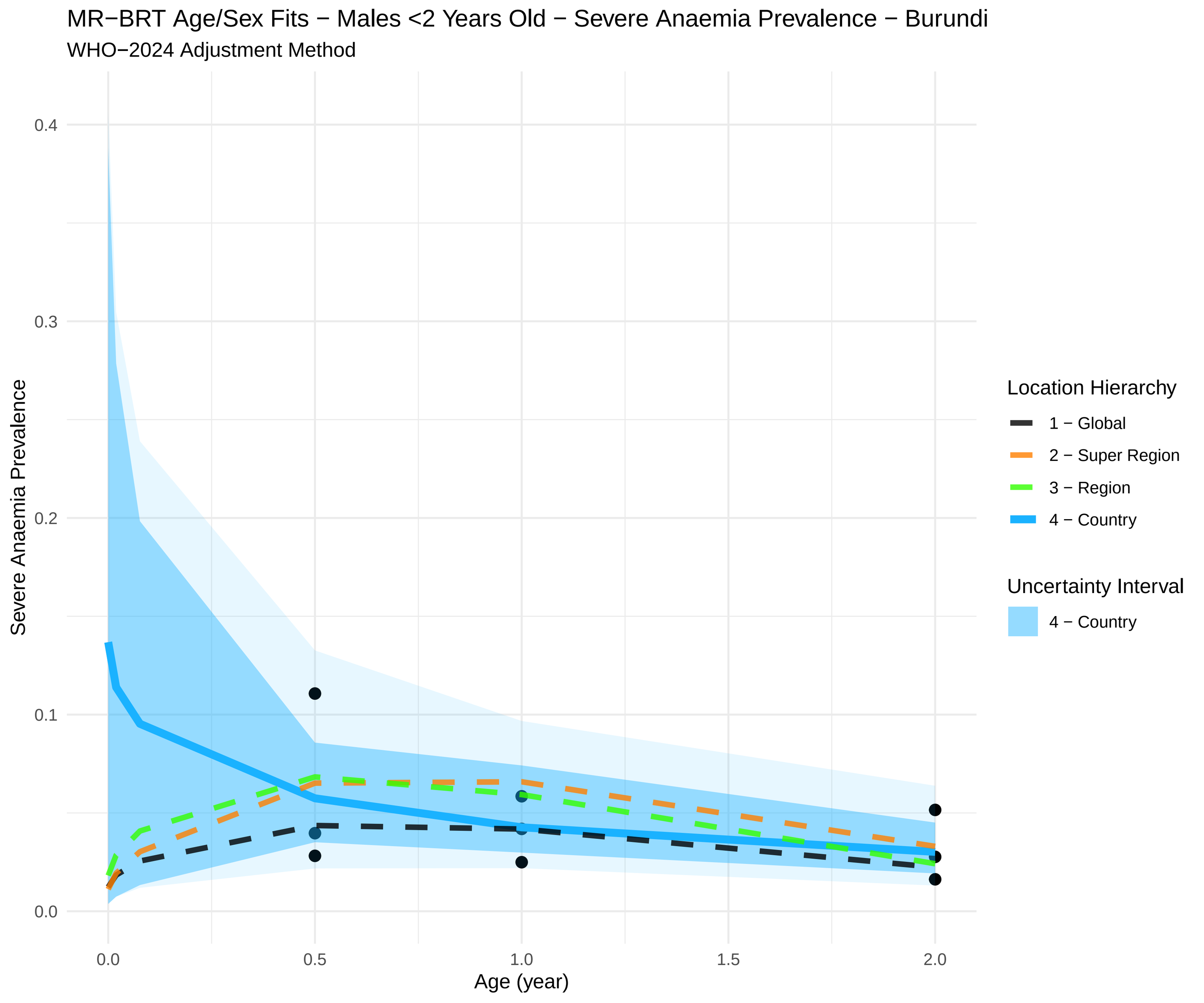
MR−BRT Age/Sex Fits − Males <2 Years Old − Severe Anaemia Prevalence − Burundi
WHO−2024 Adjustment Method
Severe Anaemia Prevalence
Age (year)
Location Hierarchy
1 − Global
2 − Super Region
3 − Region
4 − Country
Uncertainty Interval
4 − Country

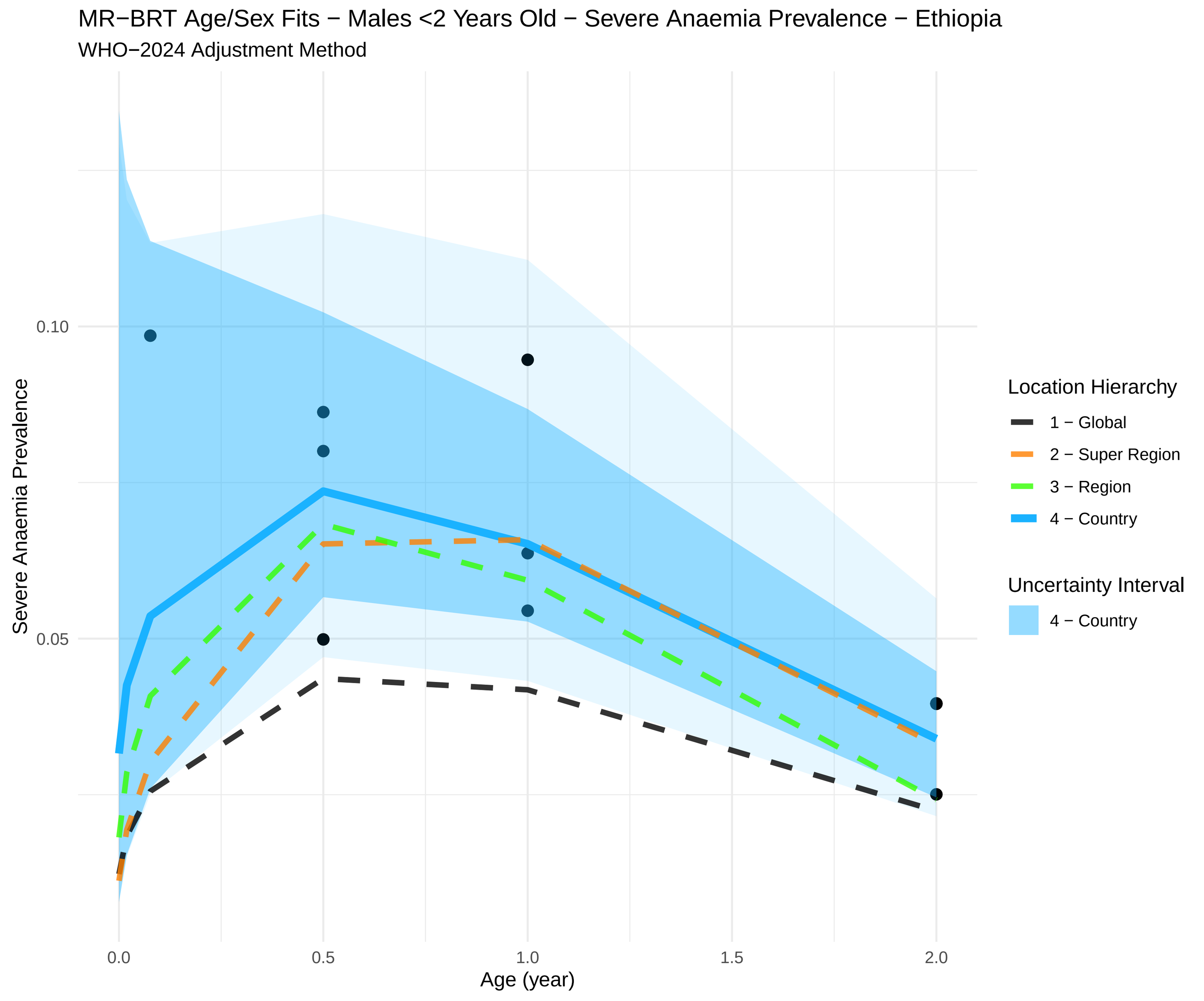
MR−BRT Age/Sex Fits − Males <2 Years Old − Severe Anaemia Prevalence − Ethiopia
WHO−2024 Adjustment Method
Severe Anaemia Prevalence
Age (year)
Location Hierarchy
1 − Global
2 − Super Region
3 − Region
4 − Country
Uncertainty Interval
4 − Country

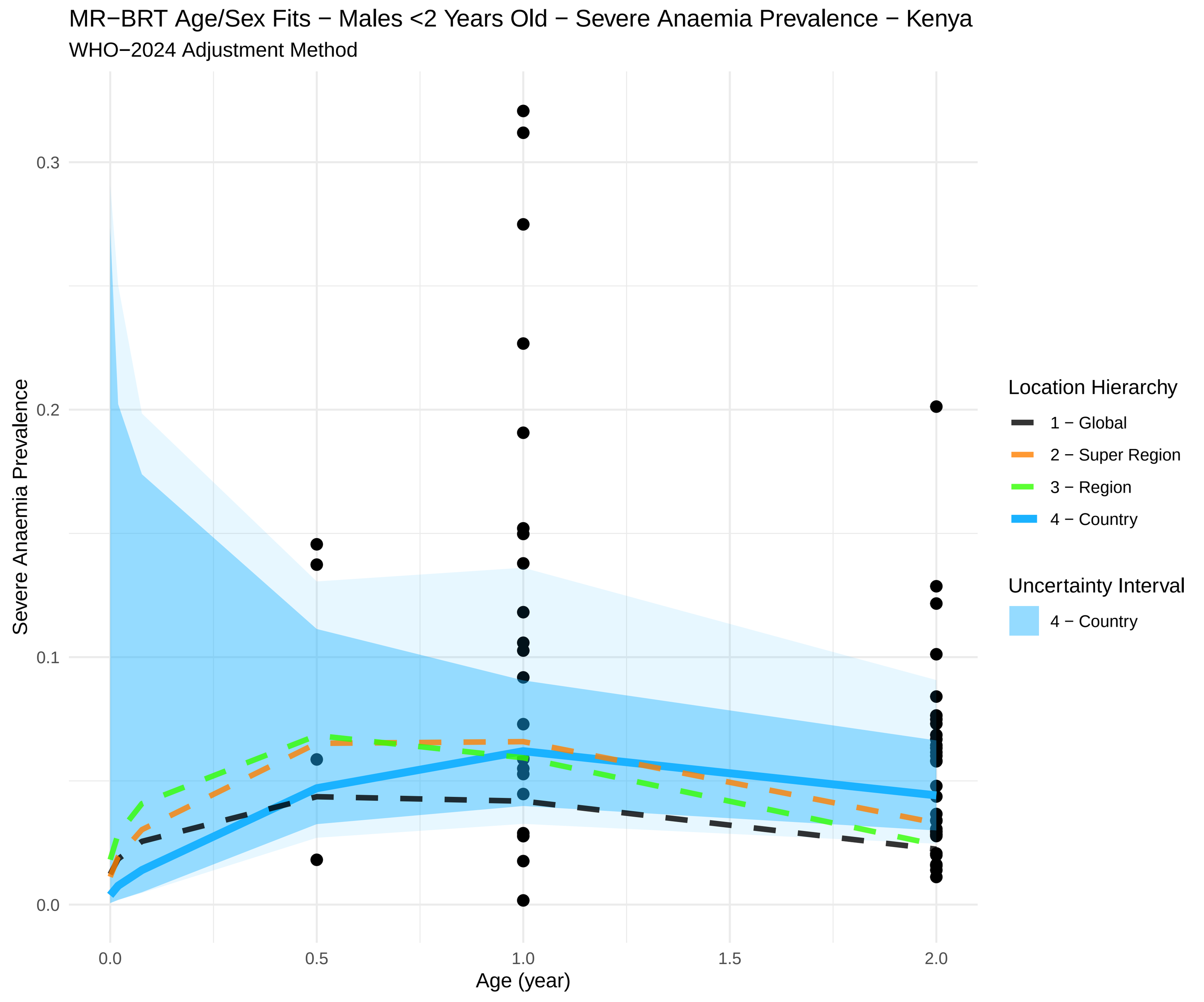
MR−BRT Age/Sex Fits − Males <2 Years Old − Severe Anaemia Prevalence − Kenya
WHO−2024 Adjustment Method
Severe Anaemia Prevalence
Age (year)
Location Hierarchy
1 − Global
2 − Super Region
3 − Region
4 − Country
Uncertainty Interval
4 − Country

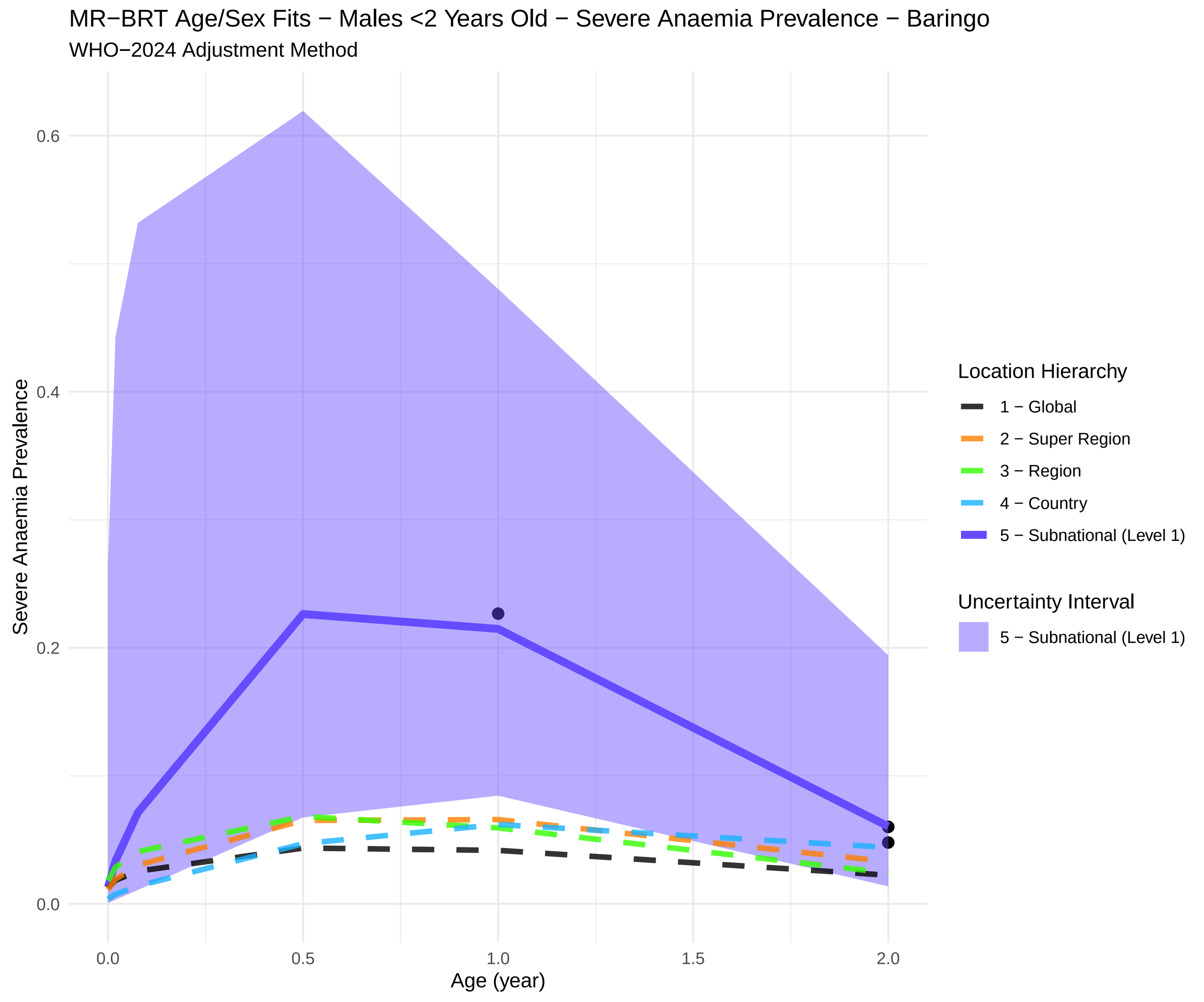
MR−BRT Age/Sex Fits − Males <2 Years Old − Severe Anaemia Prevalence − Baringo
WHO−2024 Adjustment Method
Severe Anaemia Prevalence
Age (year)
Location Hierarchy
1 − Global
2 − Super Region
3 − Region
4 − Country
5 − Subnational (Level 1)
Uncertainty Interval
5 − Subnational (Level 1)

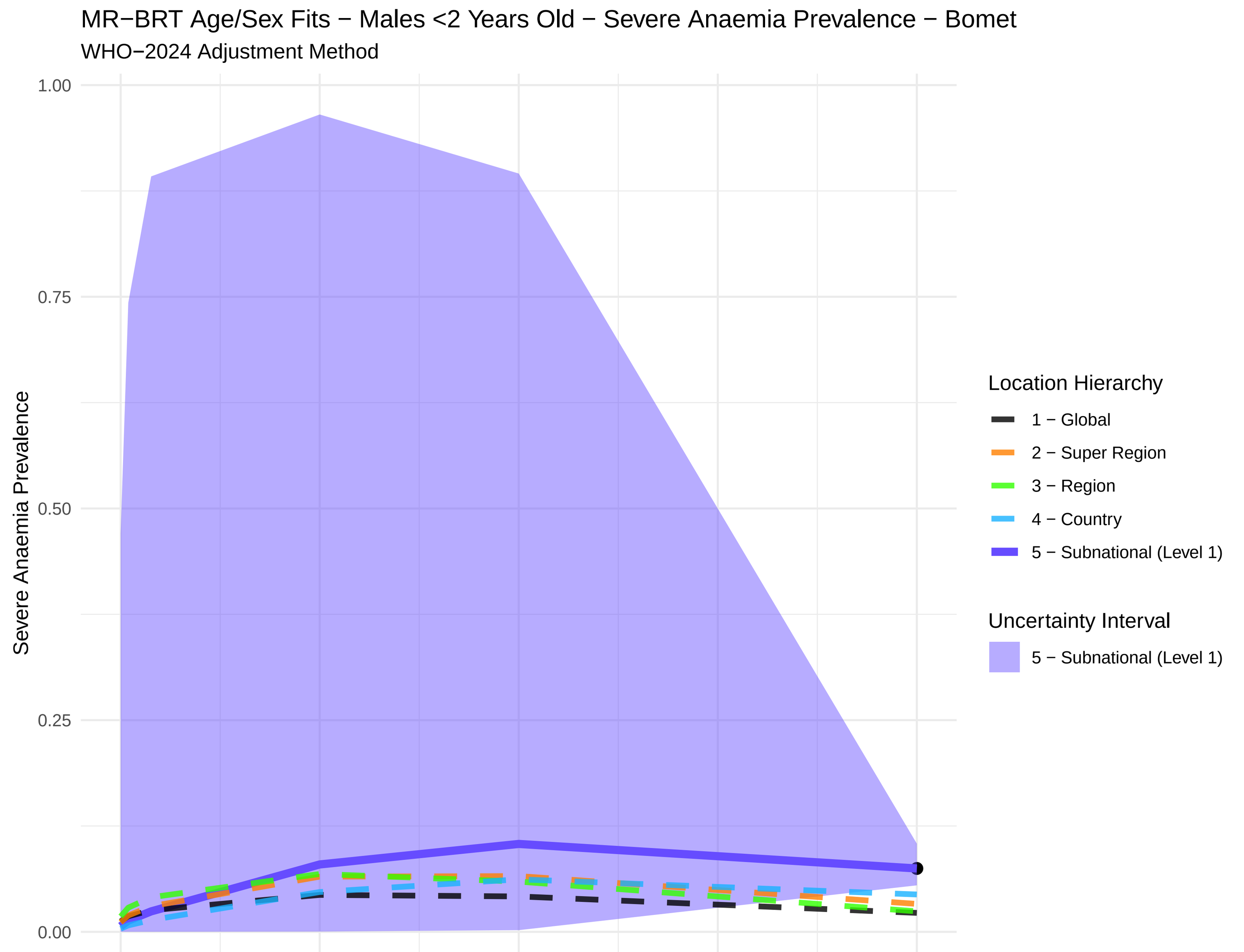
MR−BRT Age/Sex Fits − Males <2 Years Old − Severe Anaemia Prevalence − Bomet
WHO−2024 Adjustment Method
Severe Anaemia Prevalence
Age (year)
Location Hierarchy
1 − Global
2 − Super Region
3 − Region
4 − Country
5 − Subnational (Level 1)
Uncertainty Interval
5 − Subnational (Level 1)

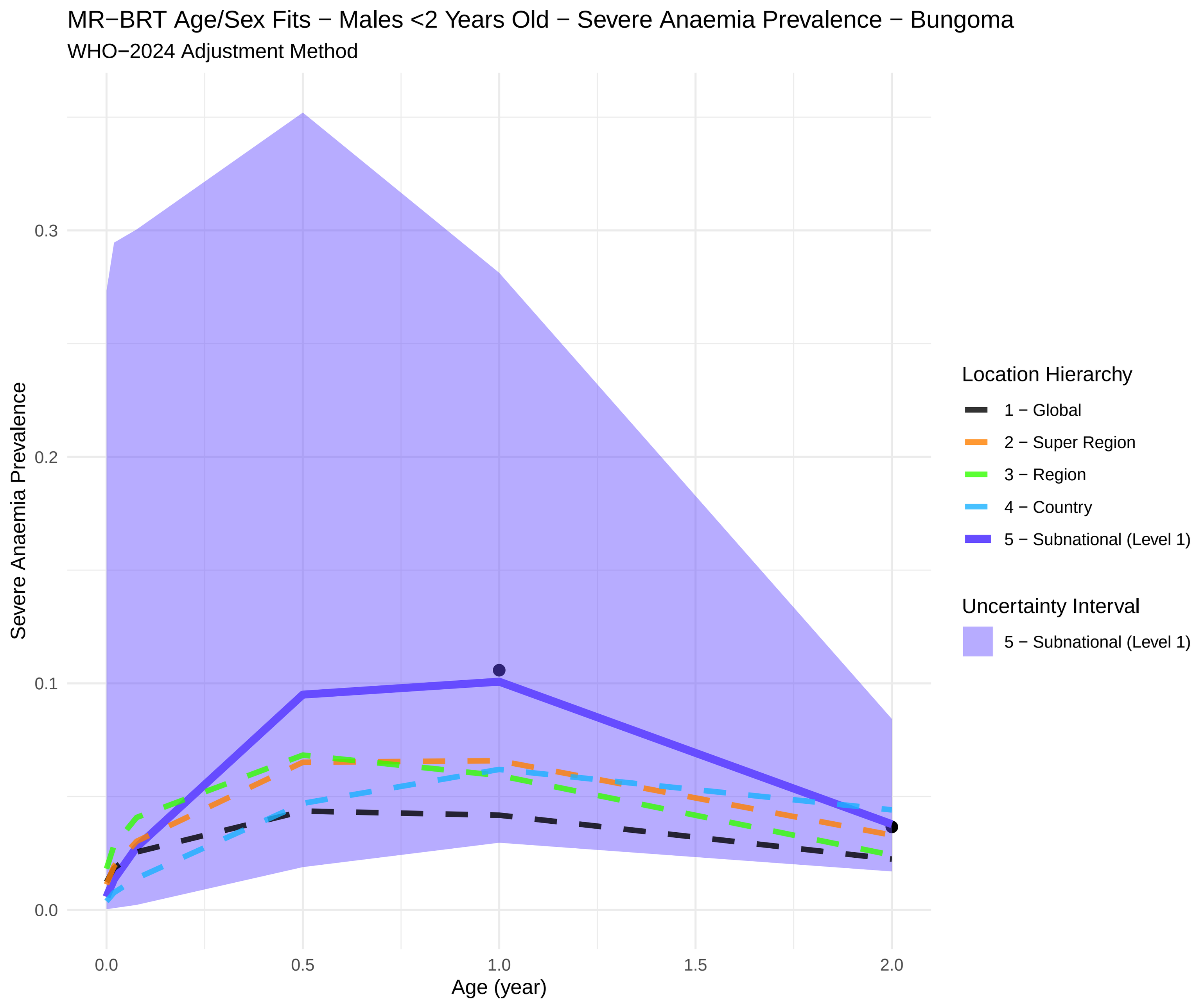
MR−BRT Age/Sex Fits − Males <2 Years Old − Severe Anaemia Prevalence − Bungoma
WHO−2024 Adjustment Method
Severe Anaemia Prevalence
Age (year)
Location Hierarchy
1 − Global
2 − Super Region
3 − Region
4 − Country
5 − Subnational (Level 1)
Uncertainty Interval
5 − Subnational (Level 1)

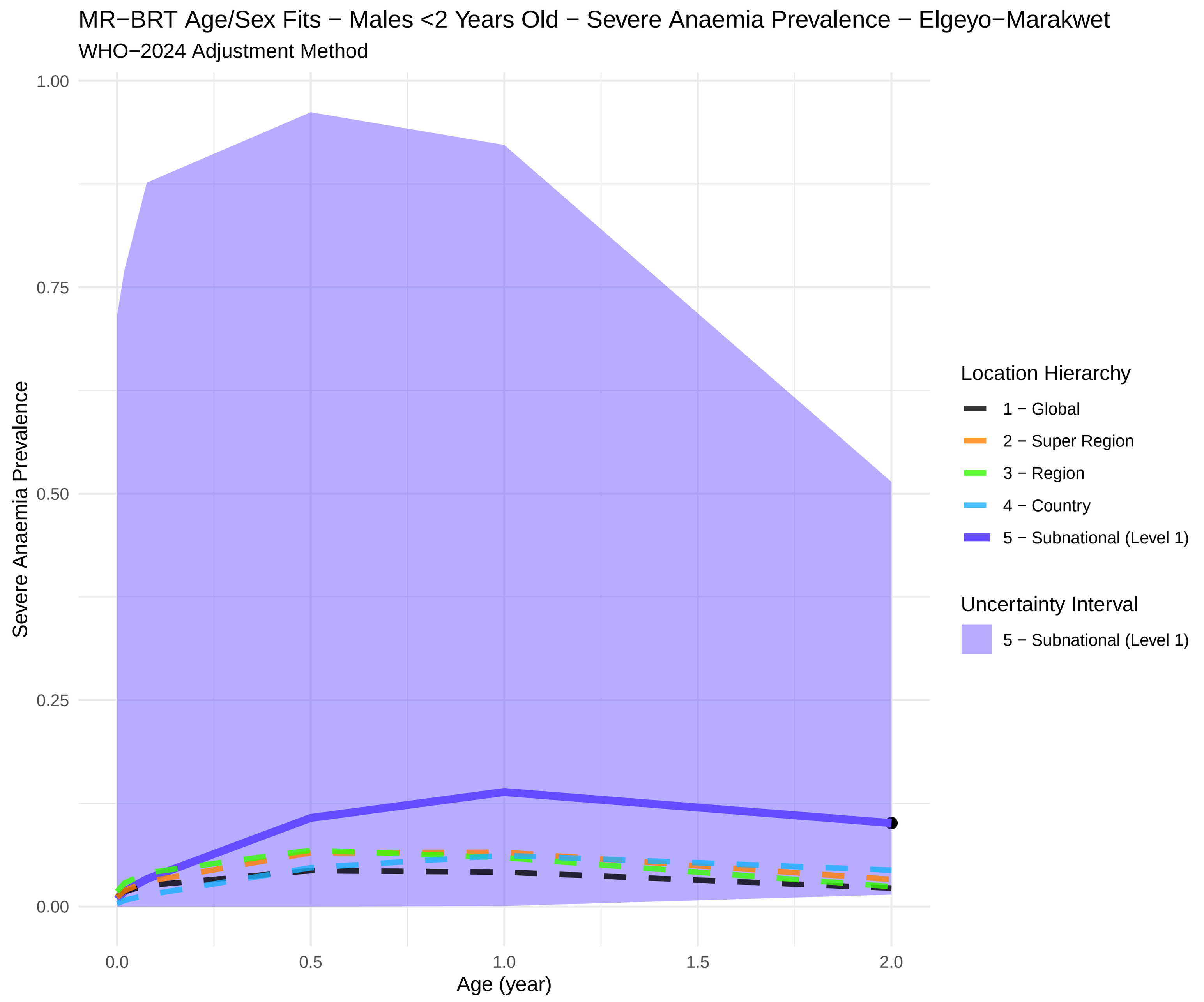
MR−BRT Age/Sex Fits − Males <2 Years Old − Severe Anaemia Prevalence − Elgeyo−Marakwet
WHO−2024 Adjustment Method
Severe Anaemia Prevalence
Age (year)
Location Hierarchy
1 − Global
2 − Super Region
3 − Region
4 − Country
5 − Subnational (Level 1)
Uncertainty Interval
5 − Subnational (Level 1)

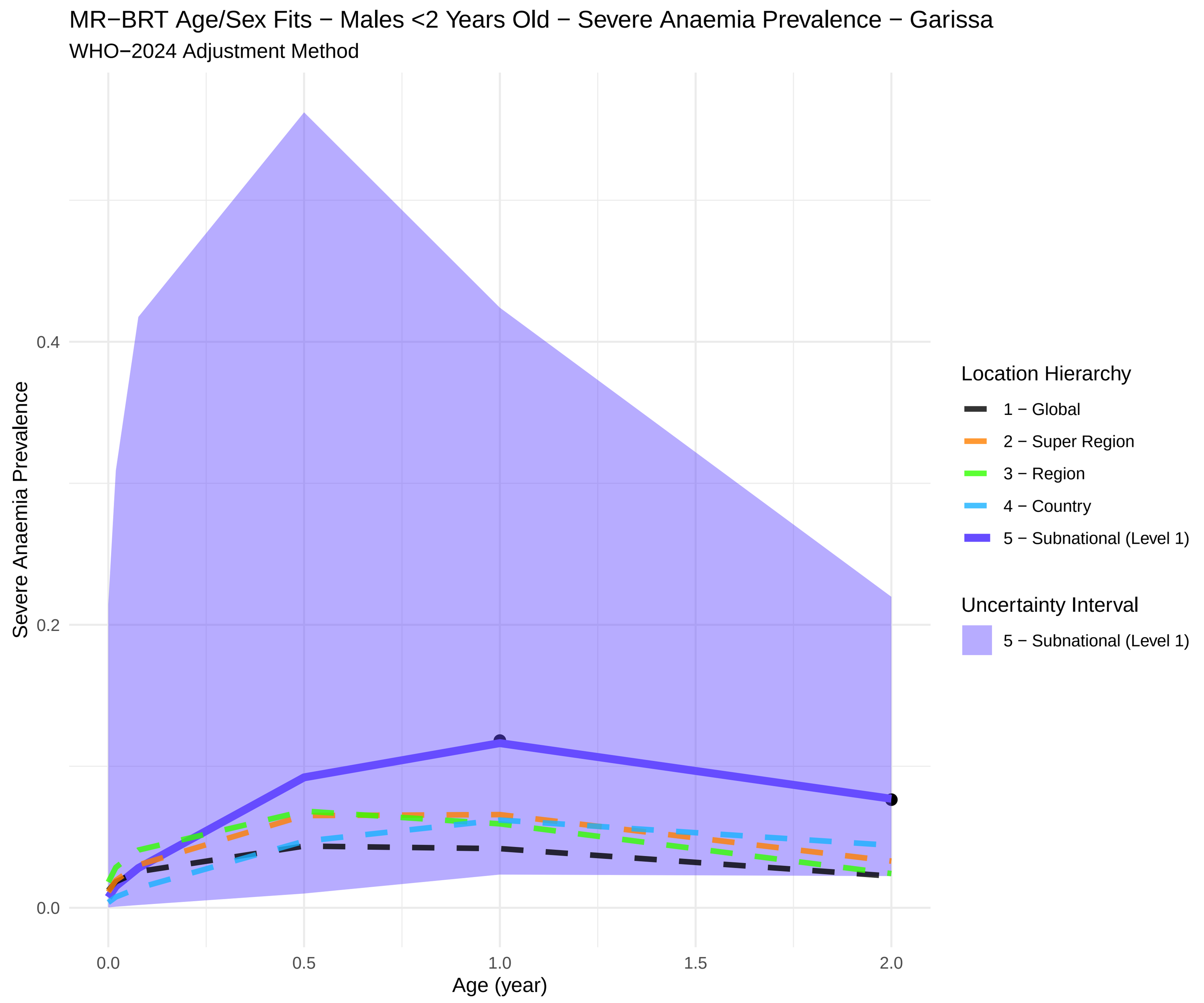
MR−BRT Age/Sex Fits − Males <2 Years Old − Severe Anaemia Prevalence − Garissa
WHO−2024 Adjustment Method
Severe Anaemia Prevalence
Age (year)
Location Hierarchy
1 − Global
2 − Super Region
3 − Region
4 − Country
5 − Subnational (Level 1)
Uncertainty Interval
5 − Subnational (Level 1)

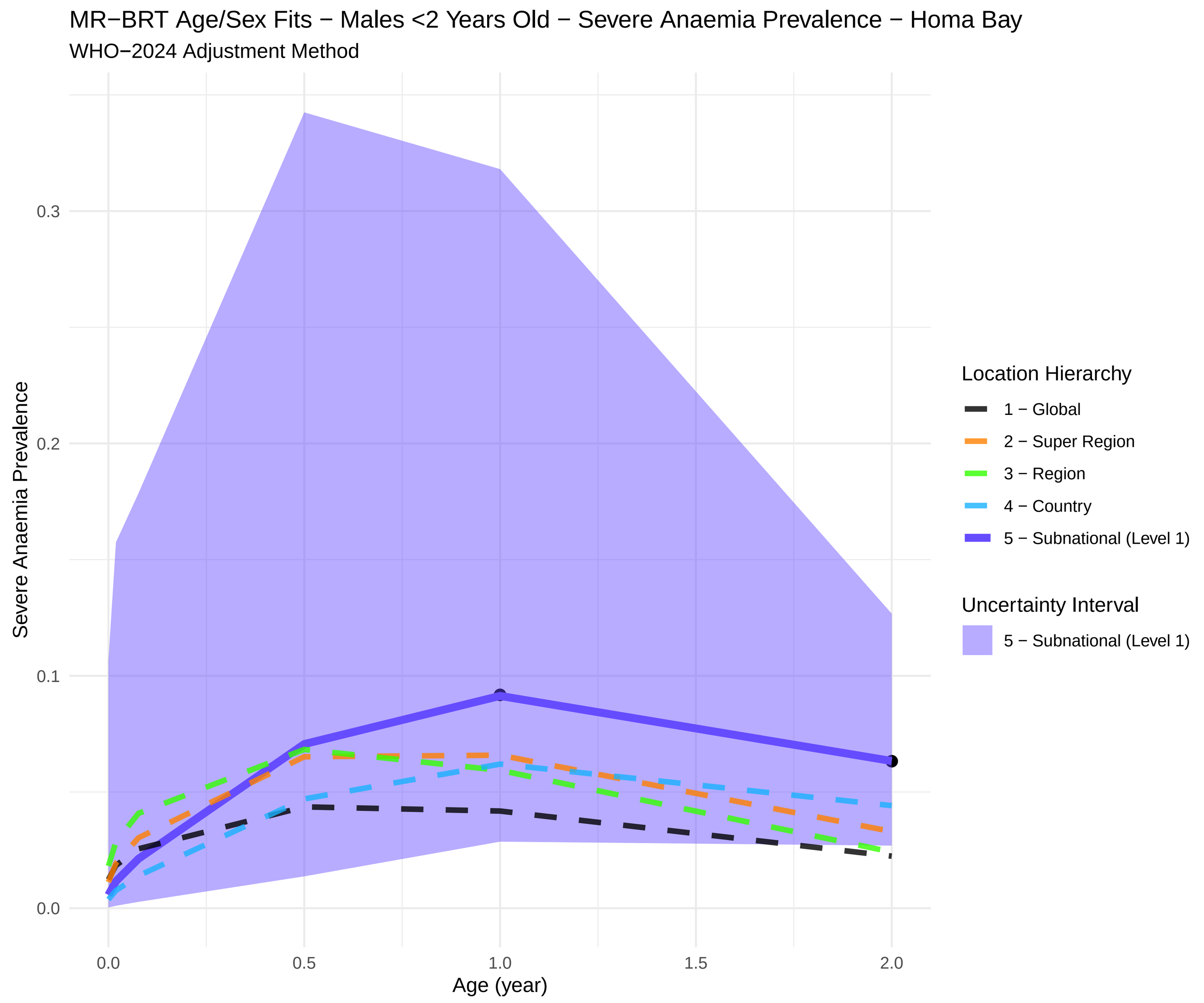
MR−BRT Age/Sex Fits − Males <2 Years Old − Severe Anaemia Prevalence − Homa Bay
WHO−2024 Adjustment Method
Severe Anaemia Prevalence
Age (year)
Location Hierarchy
1 − Global
2 − Super Region
3 − Region
4 − Country
5 − Subnational (Level 1)
Uncertainty Interval
5 − Subnational (Level 1)

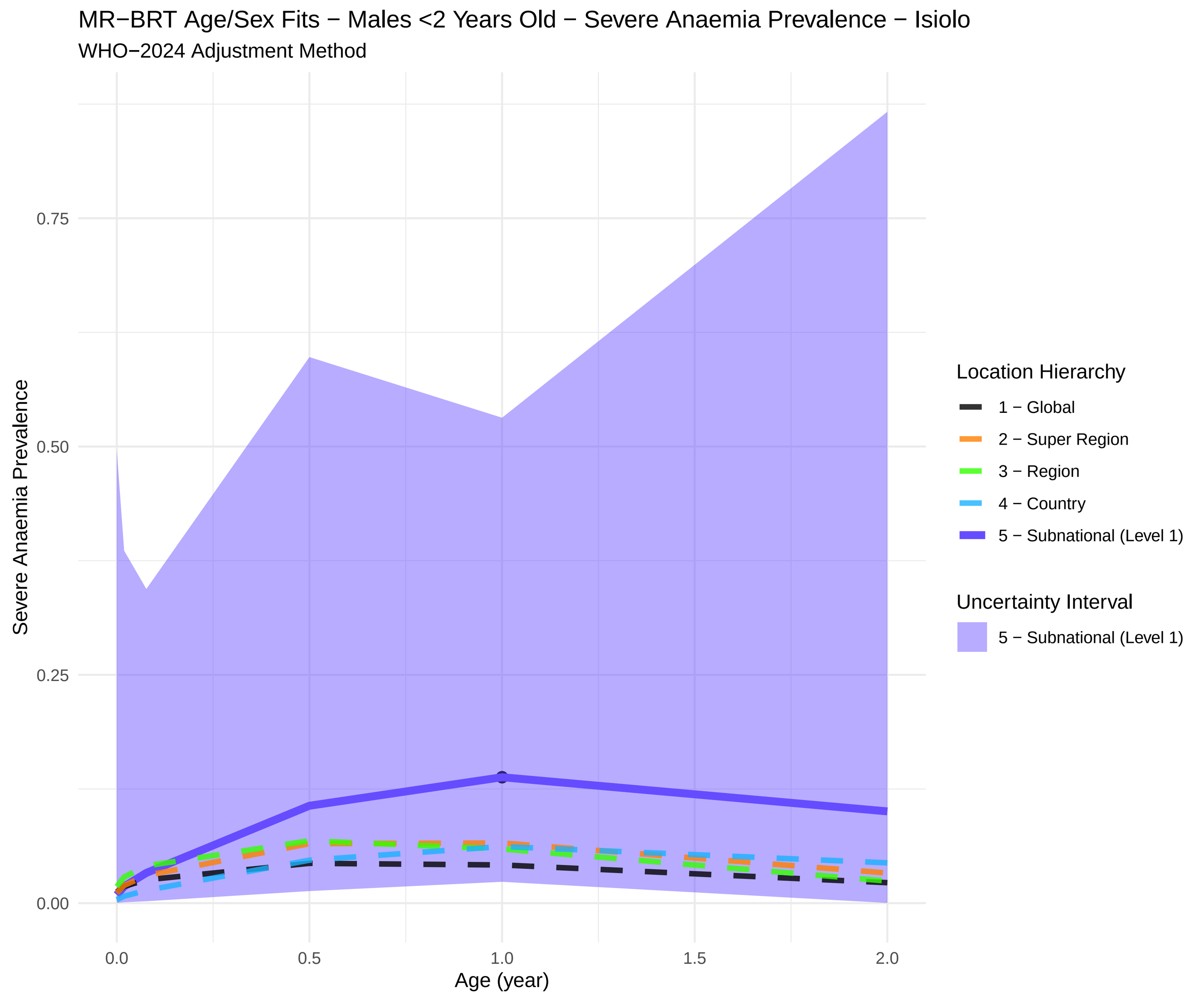
MR−BRT Age/Sex Fits − Males <2 Years Old − Severe Anaemia Prevalence − Isiolo
WHO−2024 Adjustment Method
Severe Anaemia Prevalence
Age (year)
Location Hierarchy
1 − Global
2 − Super Region
3 − Region
4 − Country
5 − Subnational (Level 1)
Uncertainty Interval
5 − Subnational (Level 1)

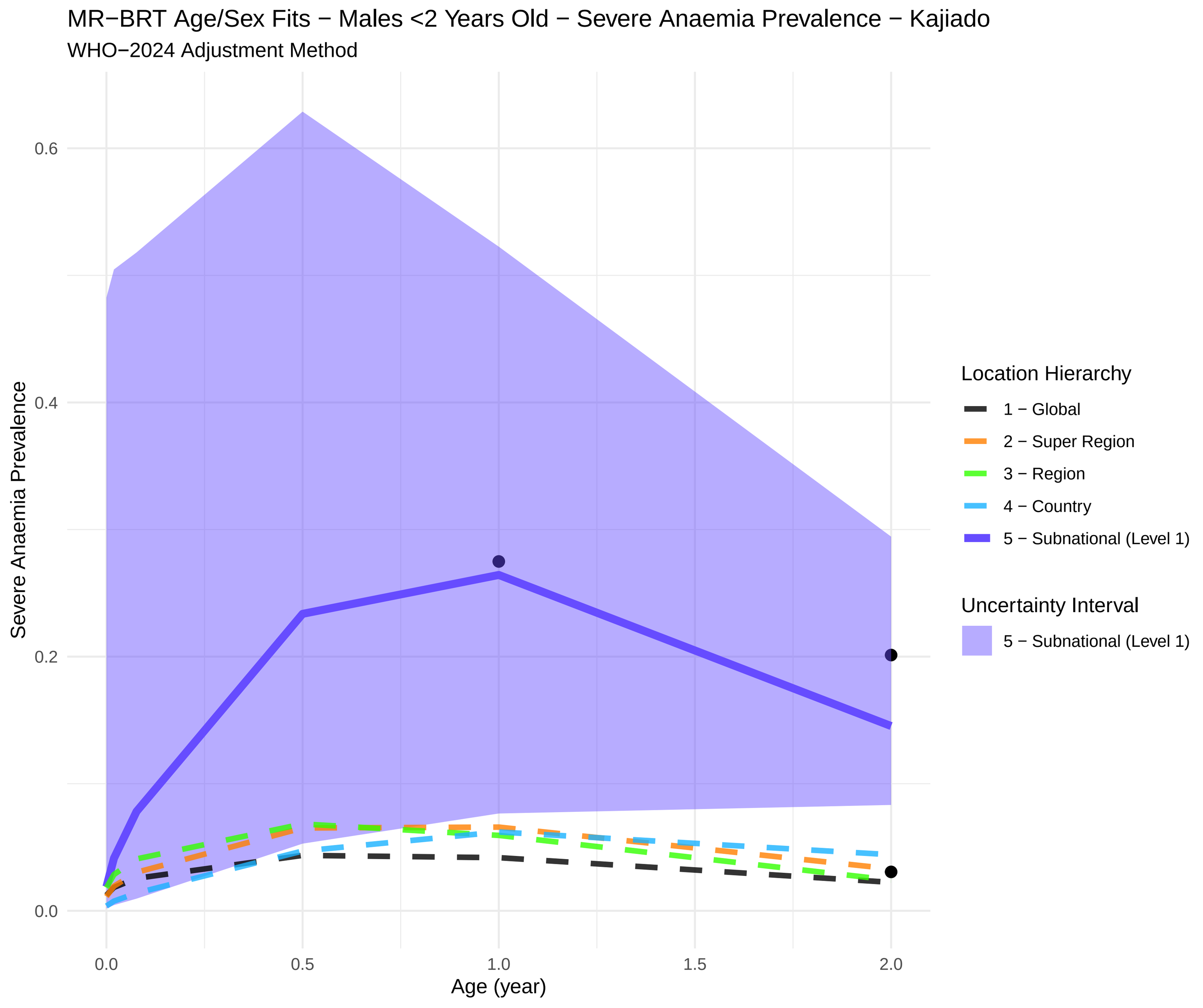
MR−BRT Age/Sex Fits − Males <2 Years Old − Severe Anaemia Prevalence − Kajiado
WHO−2024 Adjustment Method
Severe Anaemia Prevalence
Age (year)
Location Hierarchy
1 − Global
2 − Super Region
3 − Region
4 − Country
5 − Subnational (Level 1)
Uncertainty Interval
5 − Subnational (Level 1)

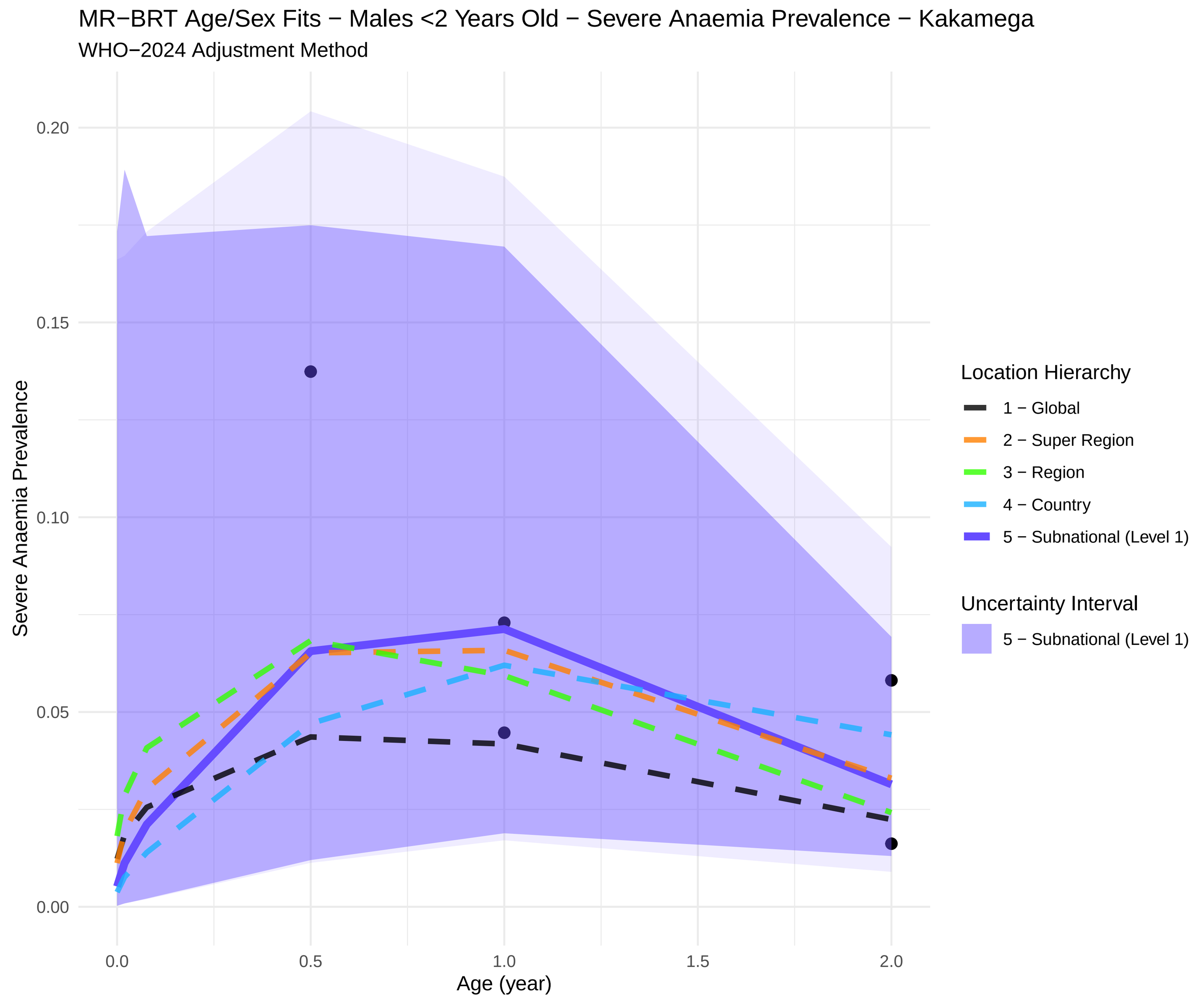
MR−BRT Age/Sex Fits − Males <2 Years Old − Severe Anaemia Prevalence − Kakamega
WHO−2024 Adjustment Method
Severe Anaemia Prevalence
Age (year)
Location Hierarchy
1 − Global
2 − Super Region
3 − Region
4 − Country
5 − Subnational (Level 1)
Uncertainty Interval
5 − Subnational (Level 1)

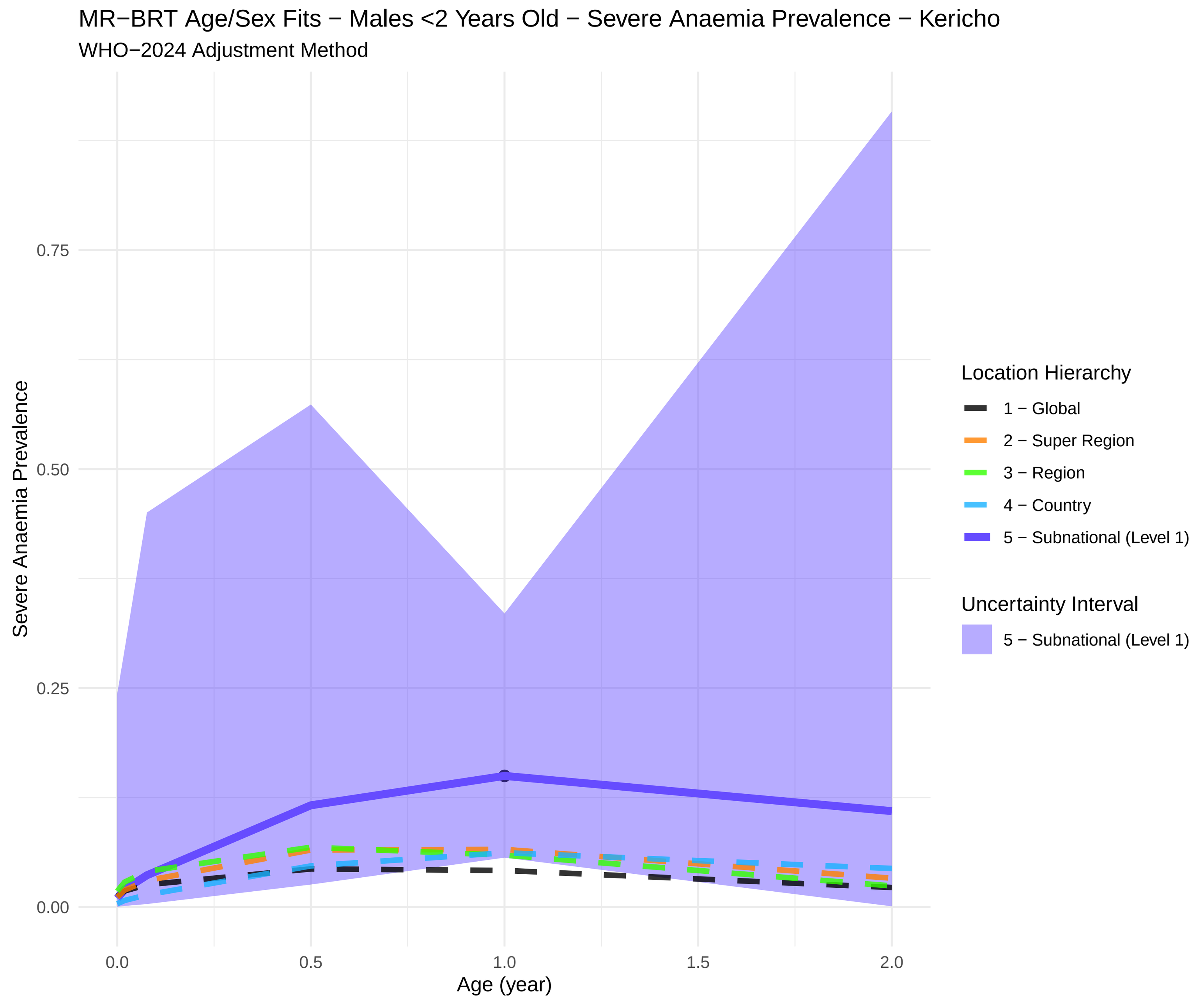
MR−BRT Age/Sex Fits − Males <2 Years Old − Severe Anaemia Prevalence − Kericho
WHO−2024 Adjustment Method
Severe Anaemia Prevalence
Age (year)
Location Hierarchy
1 − Global
2 − Super Region
3 − Region
4 − Country
5 − Subnational (Level 1)
Uncertainty Interval
5 − Subnational (Level 1)

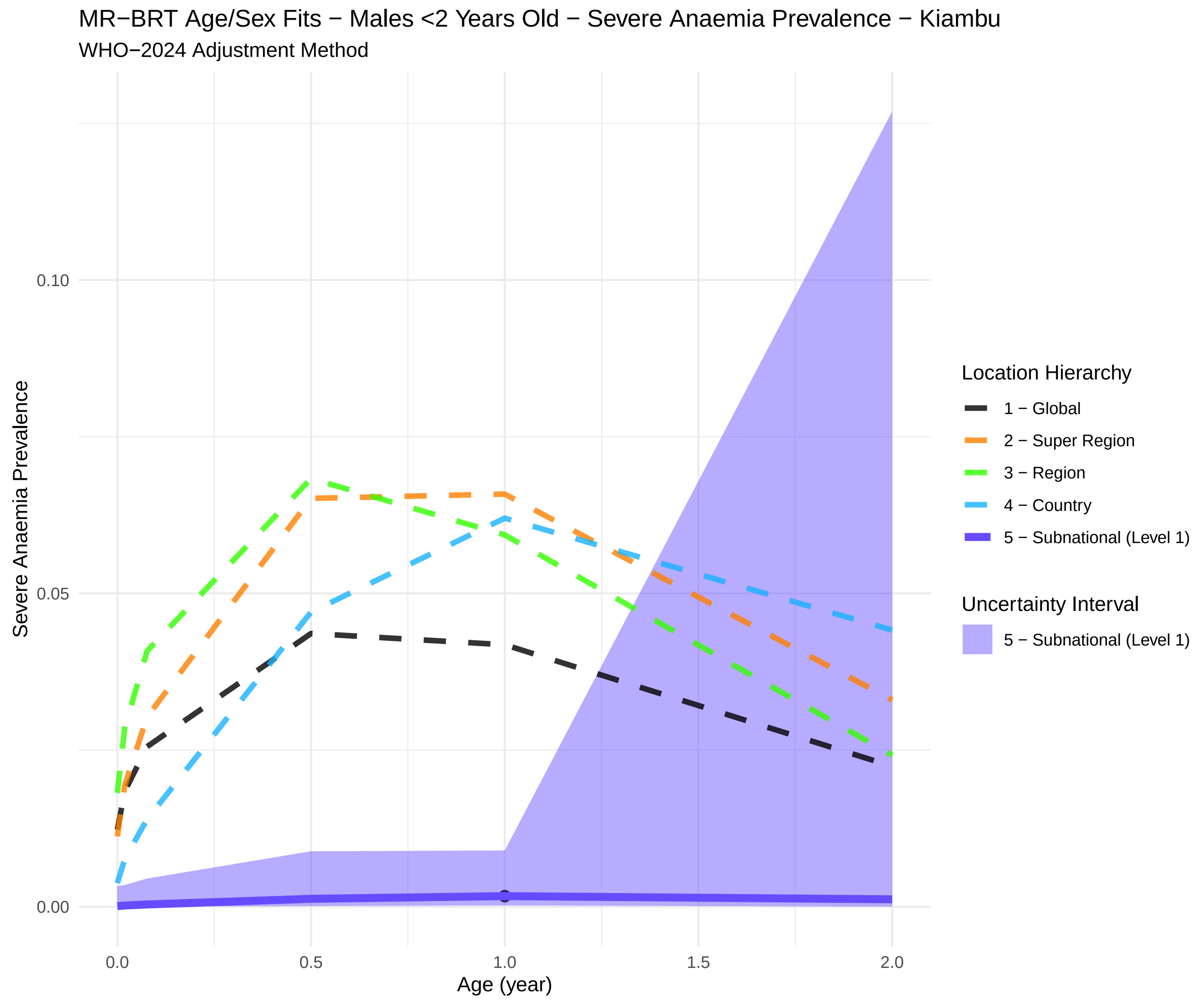
MR−BRT Age/Sex Fits − Males <2 Years Old − Severe Anaemia Prevalence − Kiambu
WHO−2024 Adjustment Method
Severe Anaemia Prevalence
Age (year)
Location Hierarchy
1 − Global
2 − Super Region
3 − Region
4 − Country
5 − Subnational (Level 1)
Uncertainty Interval
5 − Subnational (Level 1)

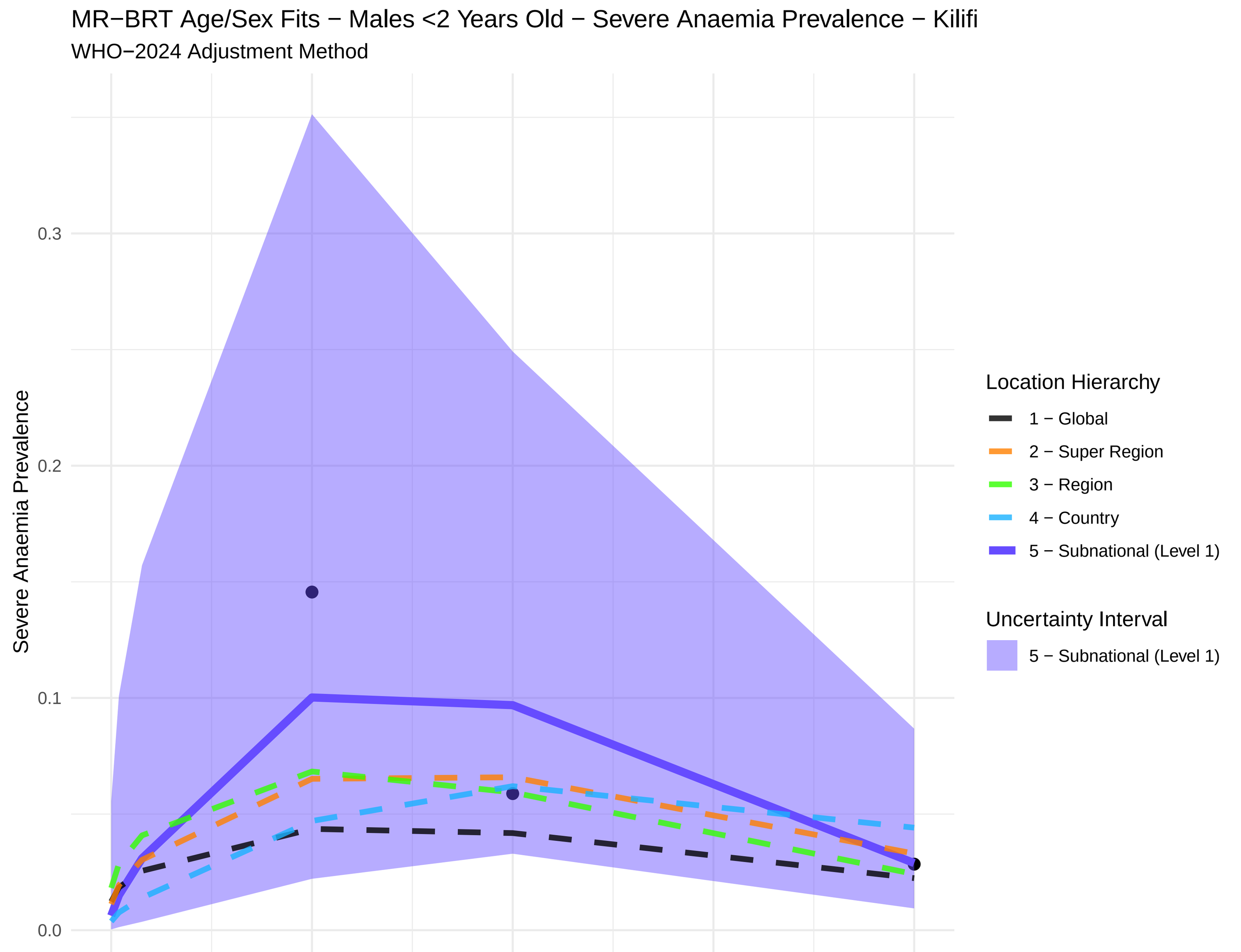
MR−BRT Age/Sex Fits − Males <2 Years Old − Severe Anaemia Prevalence − Kilifi
WHO−2024 Adjustment Method
Severe Anaemia Prevalence
Age (year)
Location Hierarchy
1 − Global
2 − Super Region
3 − Region
4 − Country
5 − Subnational (Level 1)
Uncertainty Interval
5 − Subnational (Level 1)

MR−BRT Age/Sex Fits − Males <2 Years Old − Severe Anaemia Prevalence − Kisumu
WHO−2024 Adjustment Method

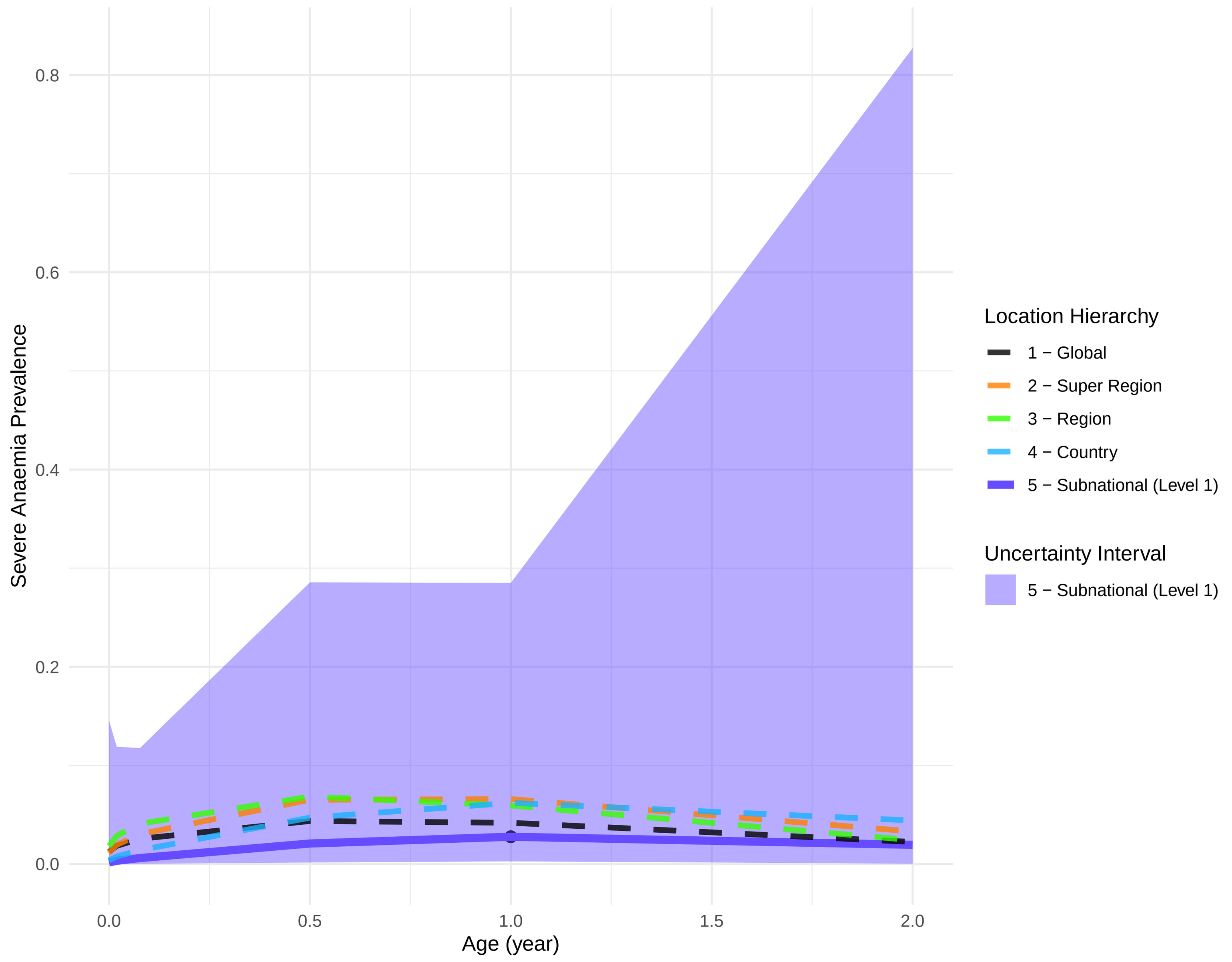

MR−BRT Age/Sex Fits − Males <2 Years Old − Severe Anaemia Prevalence − Kitui
WHO−2024 Adjustment Method

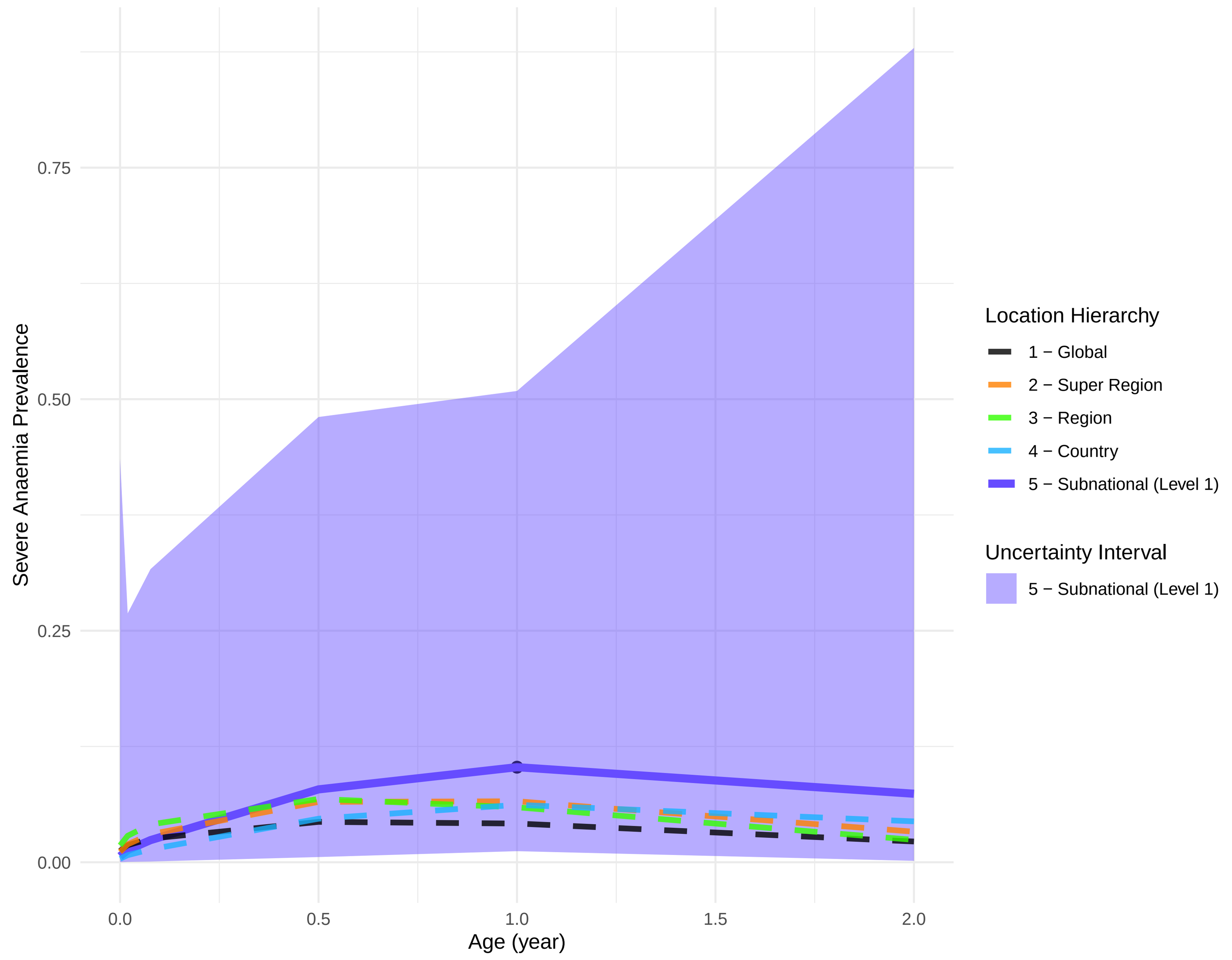

MR−BRT Age/Sex Fits − Males <2 Years Old − Severe Anaemia Prevalence − Kwale
WHO−2024 Adjustment Method

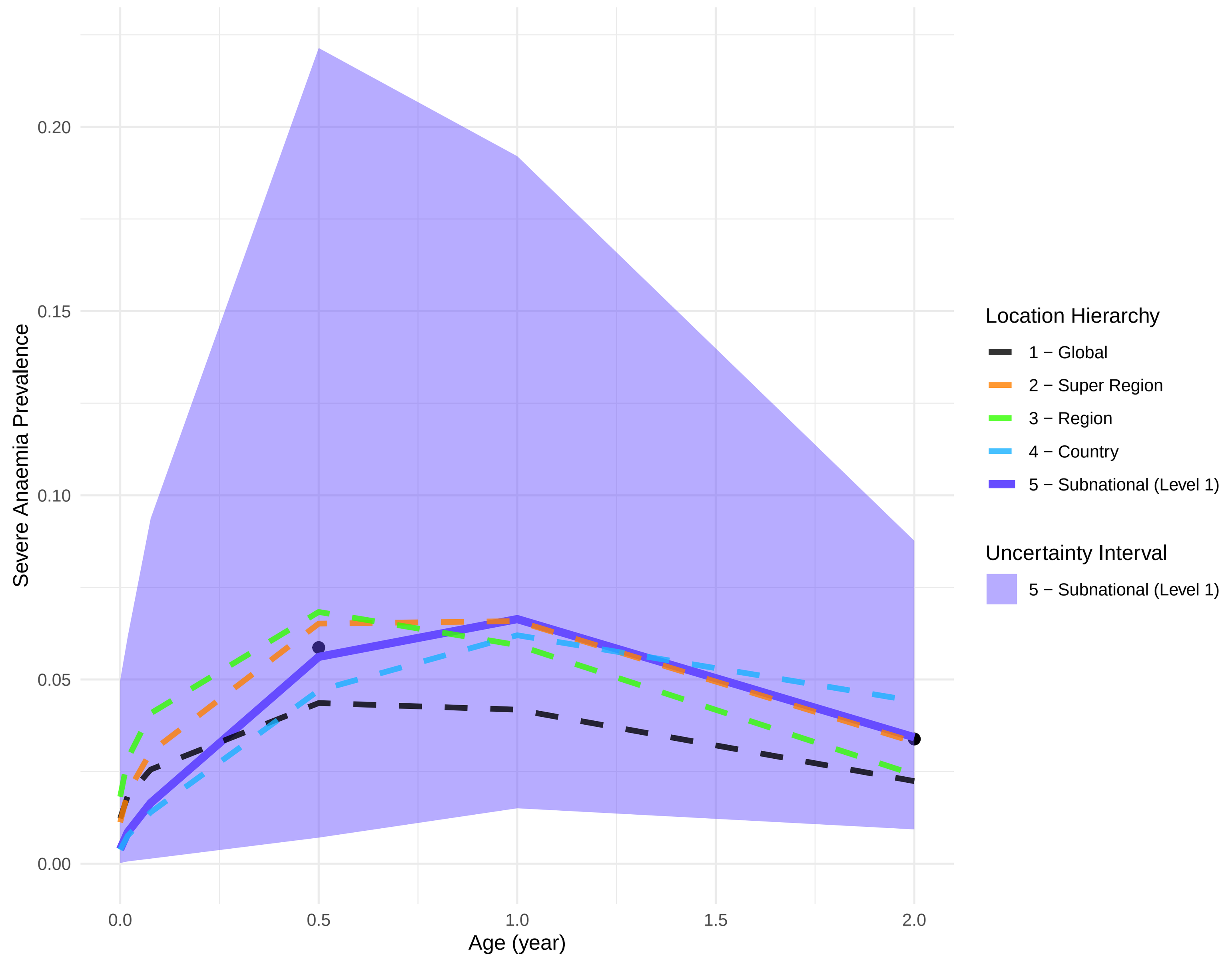

MR−BRT Age/Sex Fits − Males <2 Years Old − Severe Anaemia Prevalence − Machakos
WHO−2024 Adjustment Method

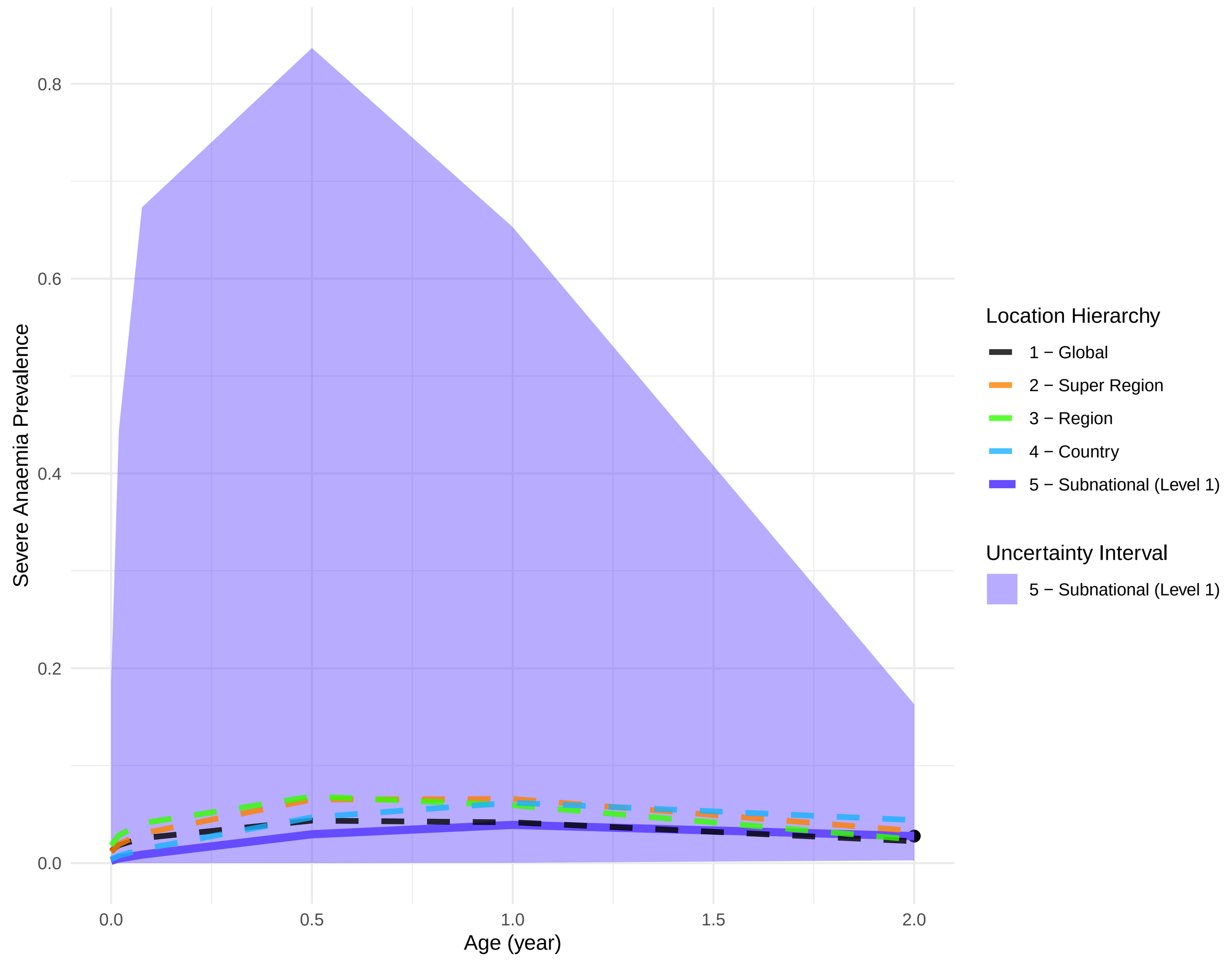

MR−BRT Age/Sex Fits − Males <2 Years Old − Severe Anaemia Prevalence − Mandera
WHO−2024 Adjustment Method

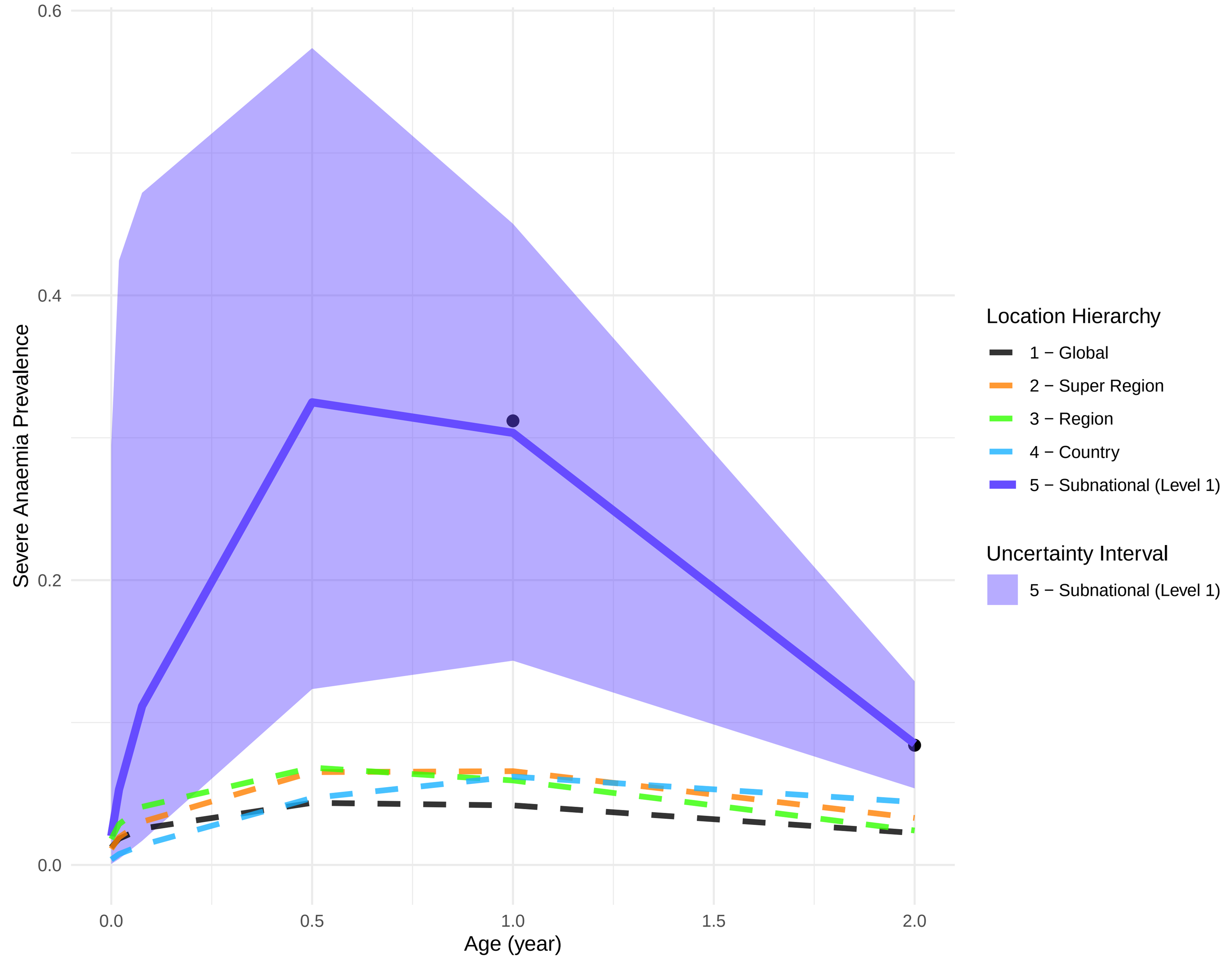

MR−BRT Age/Sex Fits − Males <2 Years Old − Severe Anaemia Prevalence − Meru
WHO−2024 Adjustment Method

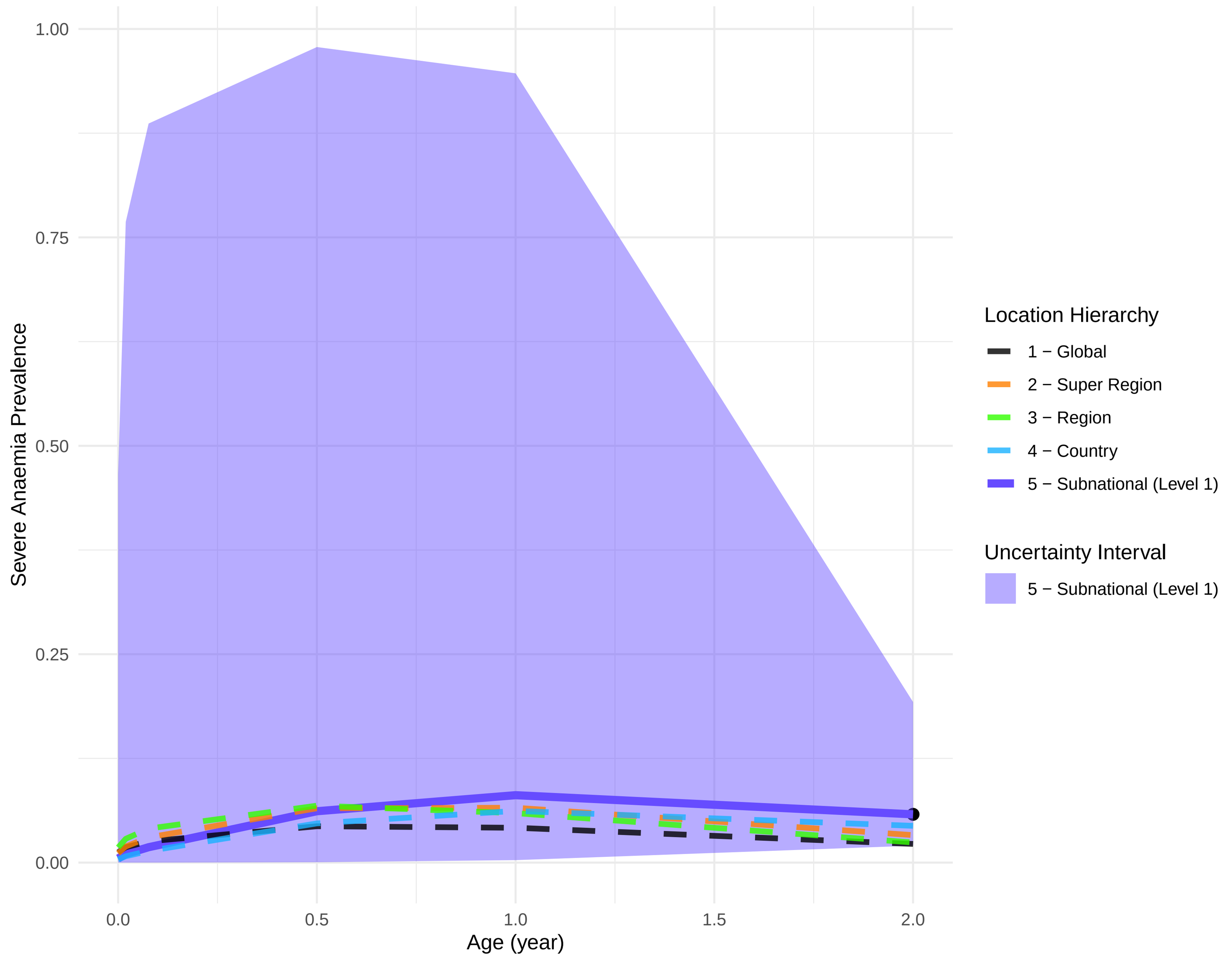

MR−BRT Age/Sex Fits − Males <2 Years Old − Severe Anaemia Prevalence − Migori
WHO−2024 Adjustment Method

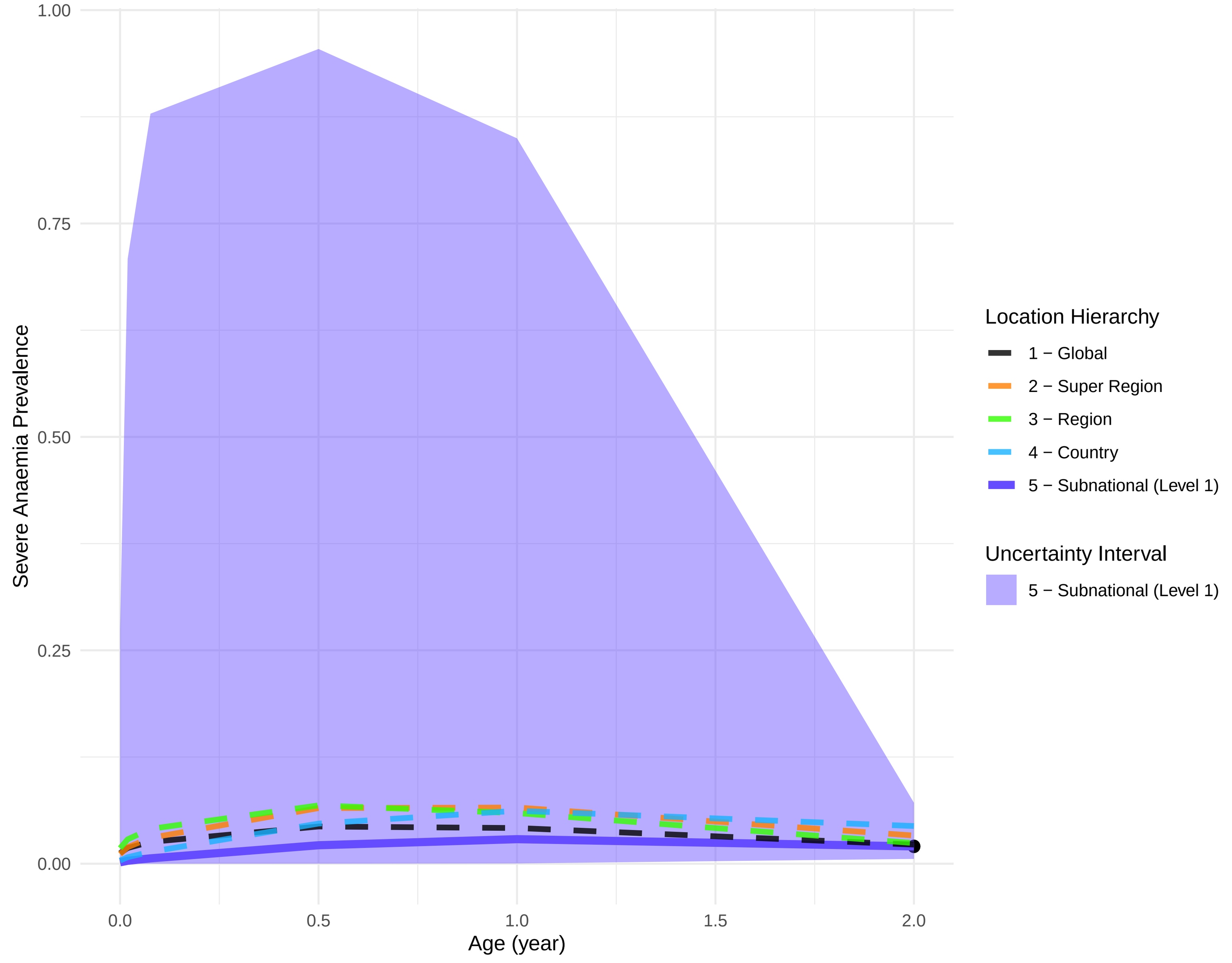

MR−BRT Age/Sex Fits − Males <2 Years Old − Severe Anaemia Prevalence − Murang'a
WHO−2024 Adjustment Method

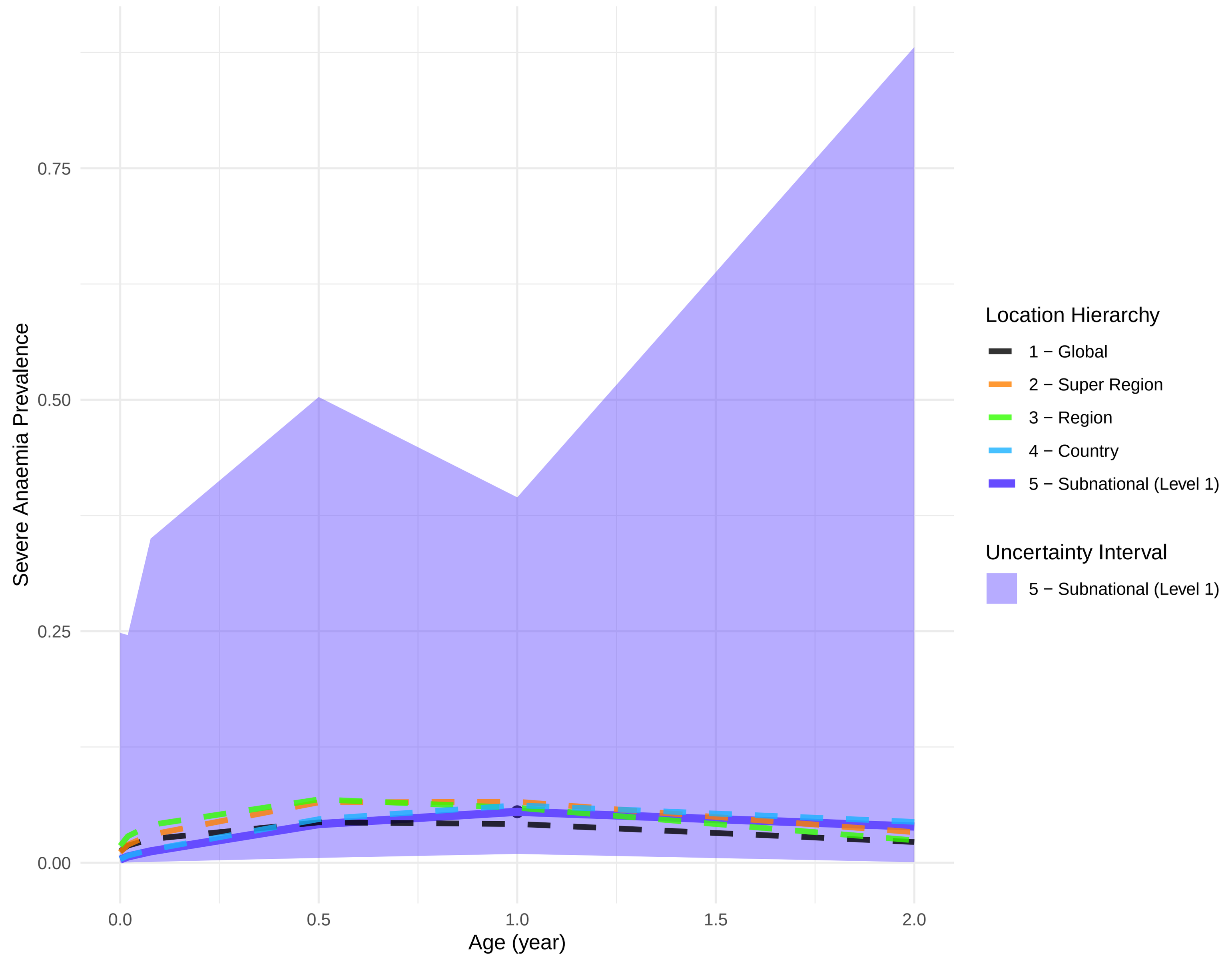

MR−BRT Age/Sex Fits − Males <2 Years Old − Severe Anaemia Prevalence − Nakuru
WHO−2024 Adjustment Method

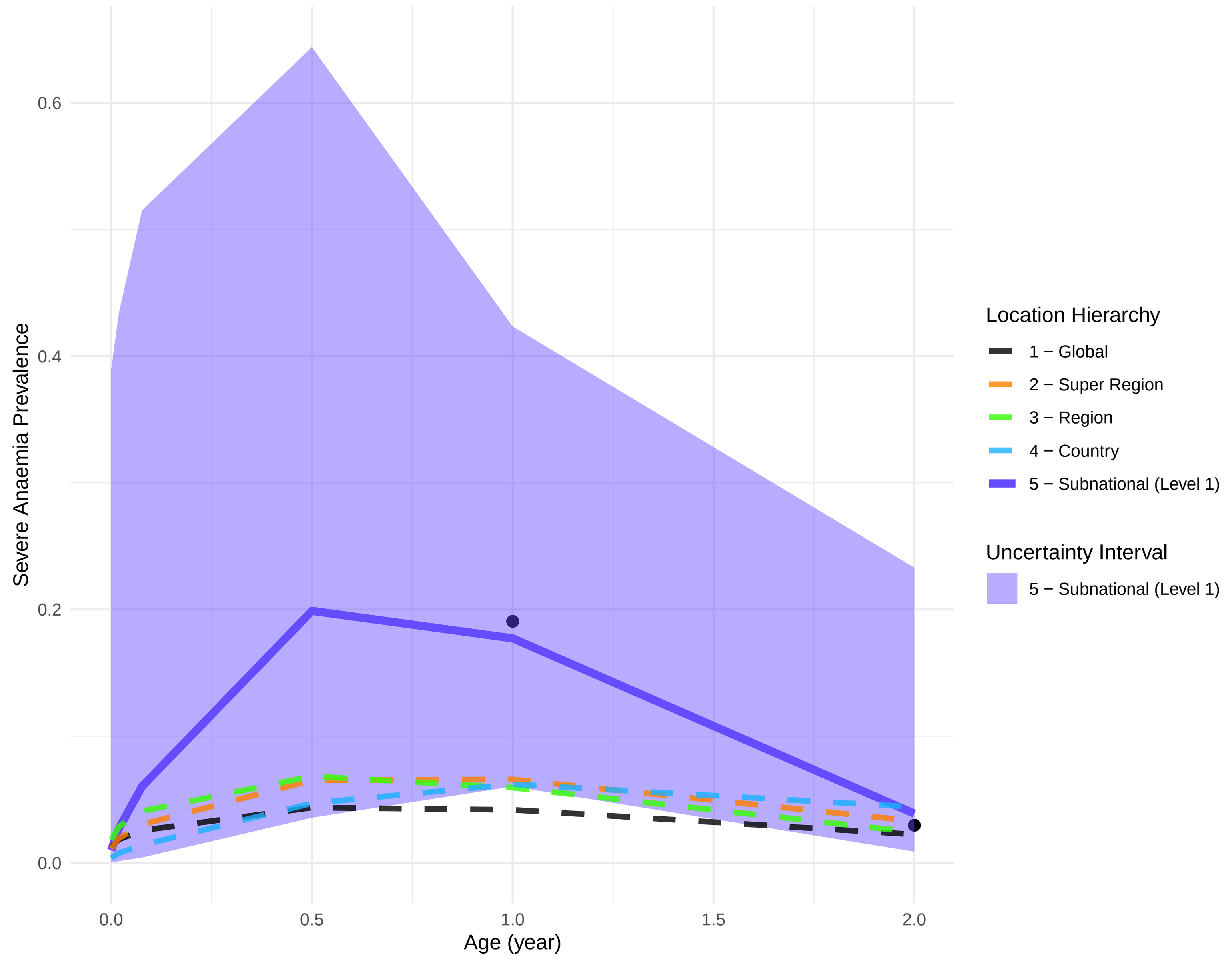

MR−BRT Age/Sex Fits − Males <2 Years Old − Severe Anaemia Prevalence − Narok
WHO−2024 Adjustment Method

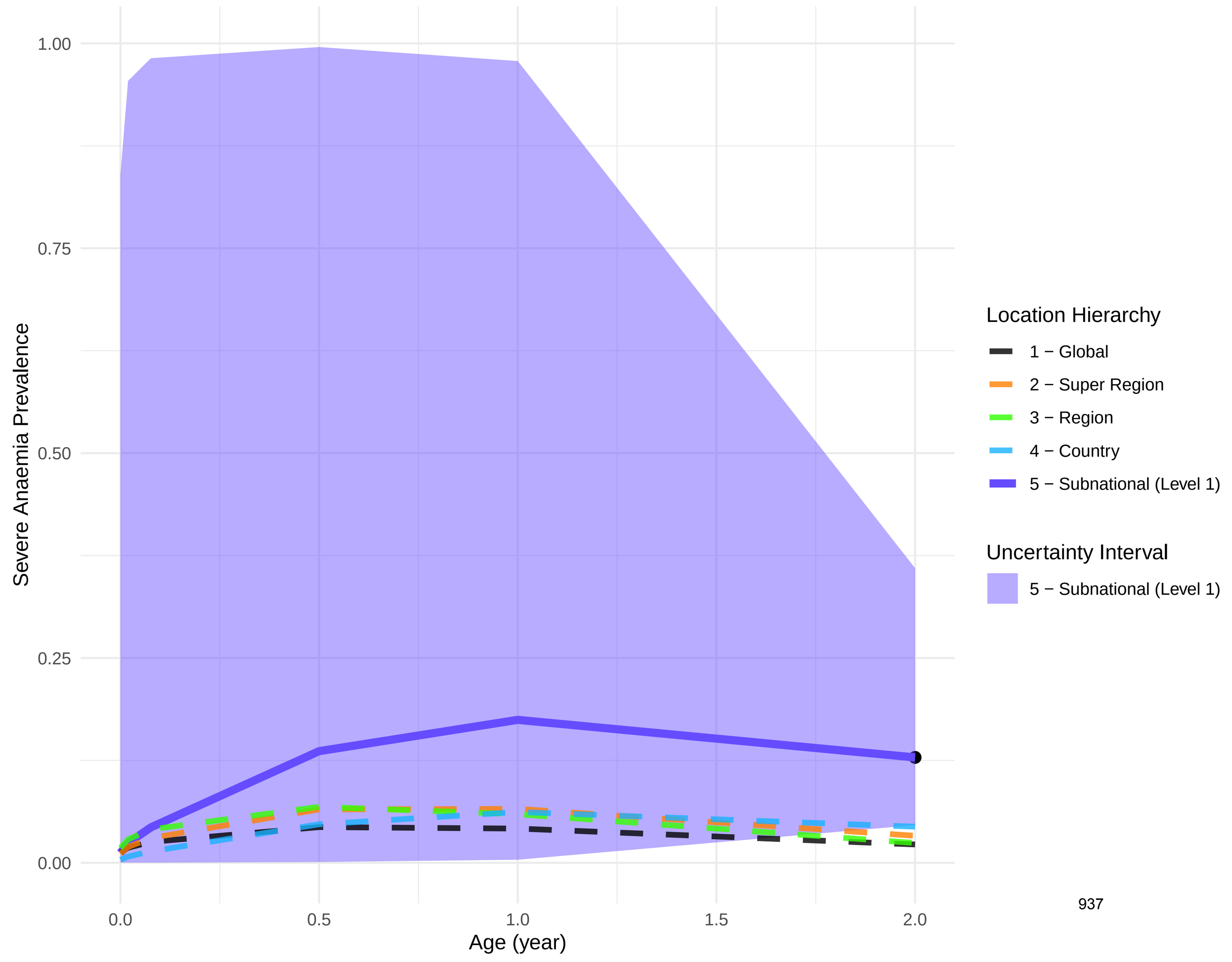

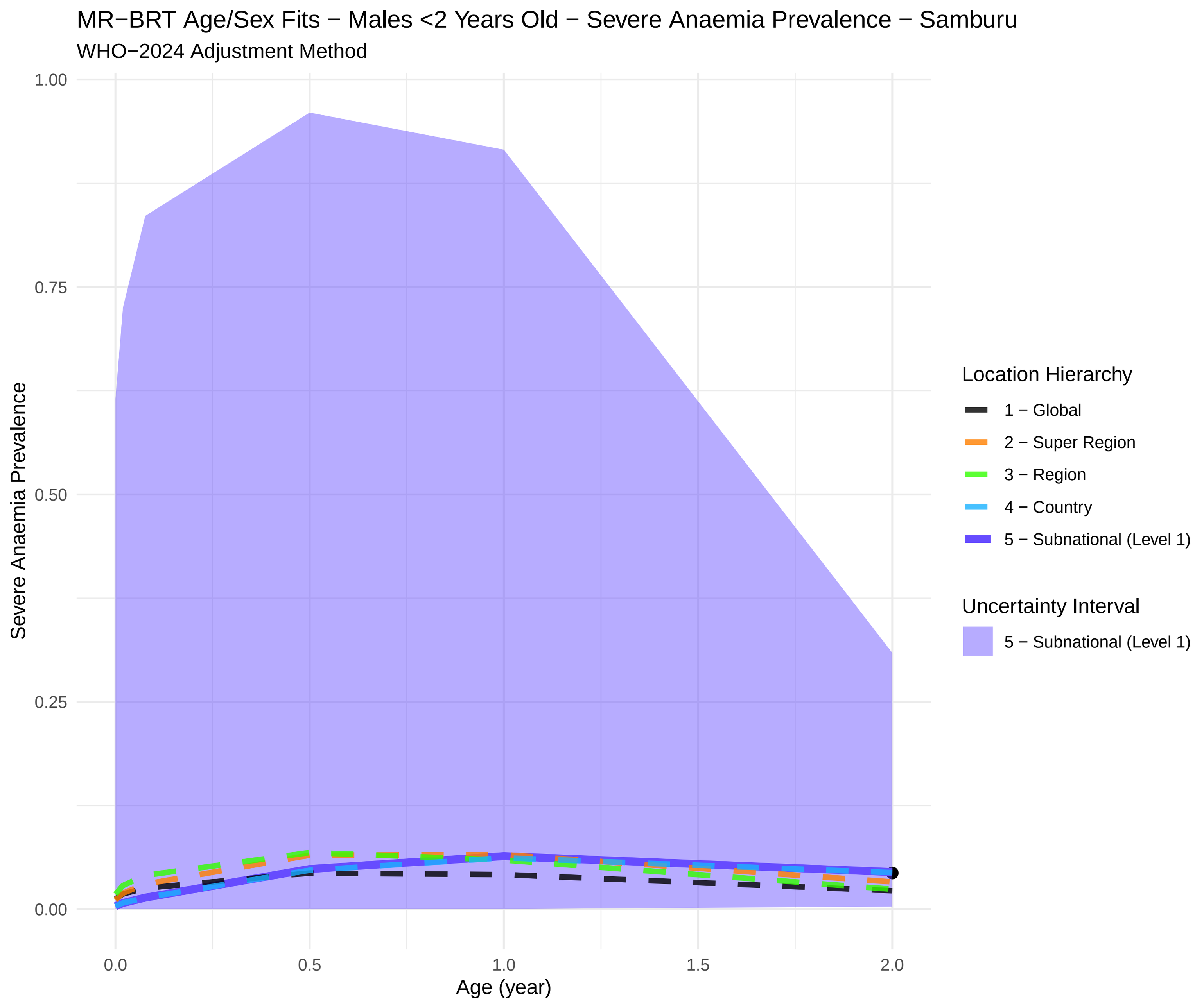
MR−BRT Age/Sex Fits − Males <2 Years Old − Severe Anaemia Prevalence − Samburu
WHO−2024 Adjustment Method
Severe Anaemia Prevalence
Age (year)
Location Hierarchy
1 − Global
2 − Super Region
3 − Region
4 − Country
5 − Subnational (Level 1)
Uncertainty Interval
5 − Subnational (Level 1)

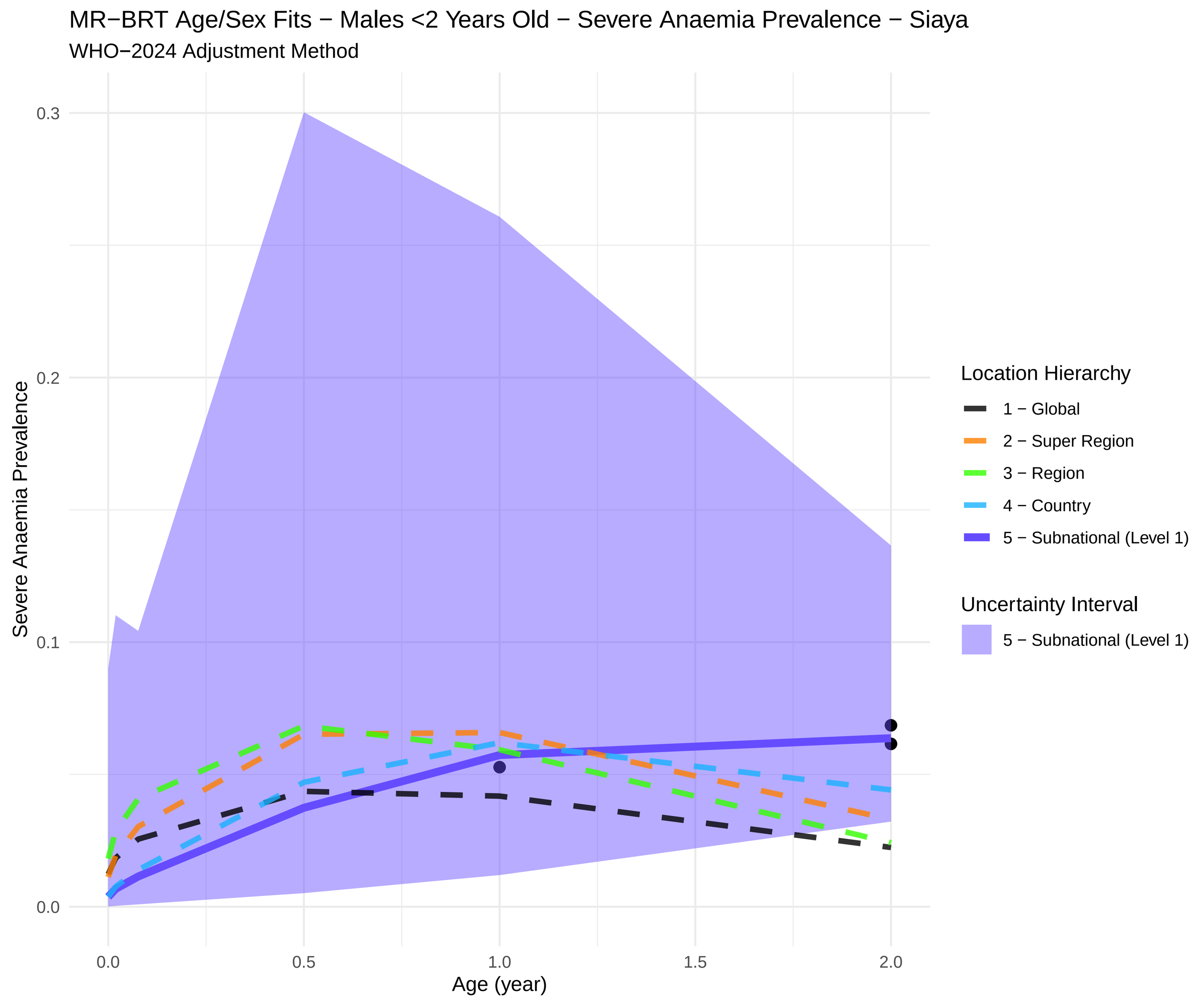
MR−BRT Age/Sex Fits − Males <2 Years Old − Severe Anaemia Prevalence − Siaya
WHO−2024 Adjustment Method
Severe Anaemia Prevalence
Age (year)
Location Hierarchy
1 − Global
2 − Super Region
3 − Region
4 − Country
5 − Subnational (Level 1)
Uncertainty Interval
5 − Subnational (Level 1)

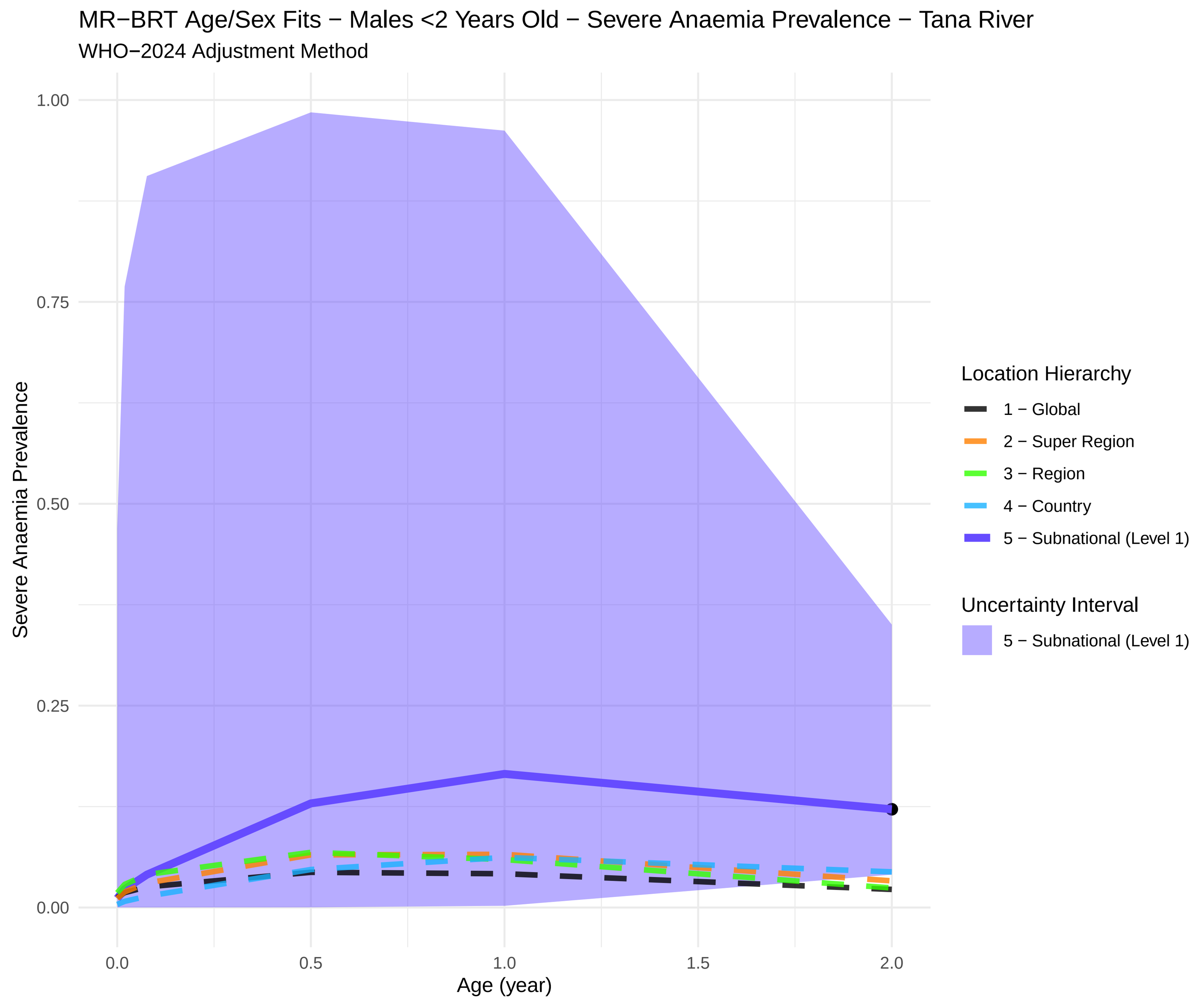
MR−BRT Age/Sex Fits − Males <2 Years Old − Severe Anaemia Prevalence − Tana River
WHO−2024 Adjustment Method
Severe Anaemia Prevalence
Age (year)
Location Hierarchy
1 − Global
2 − Super Region
3 − Region
4 − Country
5 − Subnational (Level 1)
Uncertainty Interval
5 − Subnational (Level 1)

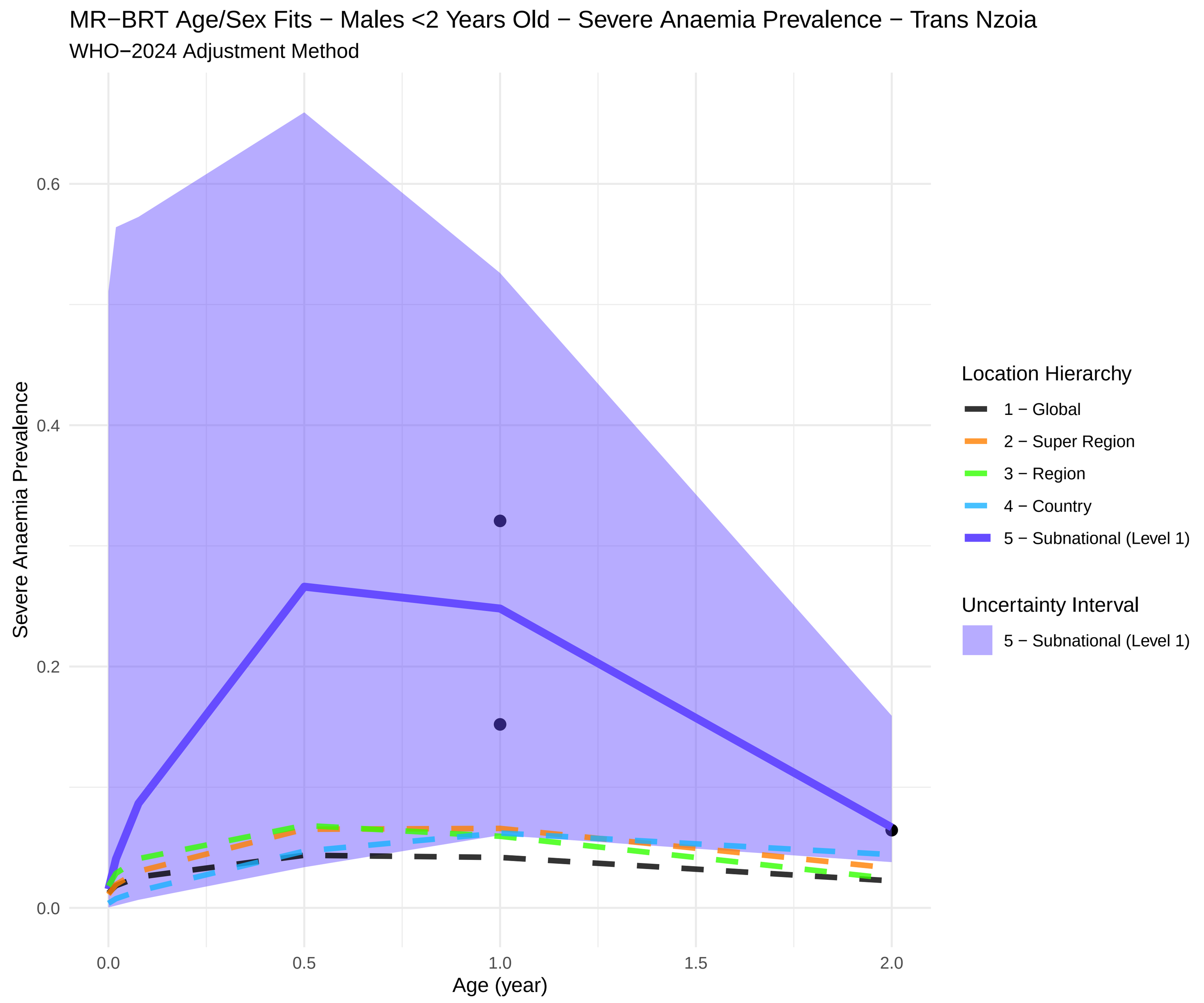
MR−BRT Age/Sex Fits − Males <2 Years Old − Severe Anaemia Prevalence − Trans Nzoia
WHO−2024 Adjustment Method
Severe Anaemia Prevalence
Age (year)
Location Hierarchy
1 − Global
2 − Super Region
3 − Region
4 − Country
5 − Subnational (Level 1)
Uncertainty Interval
5 − Subnational (Level 1)

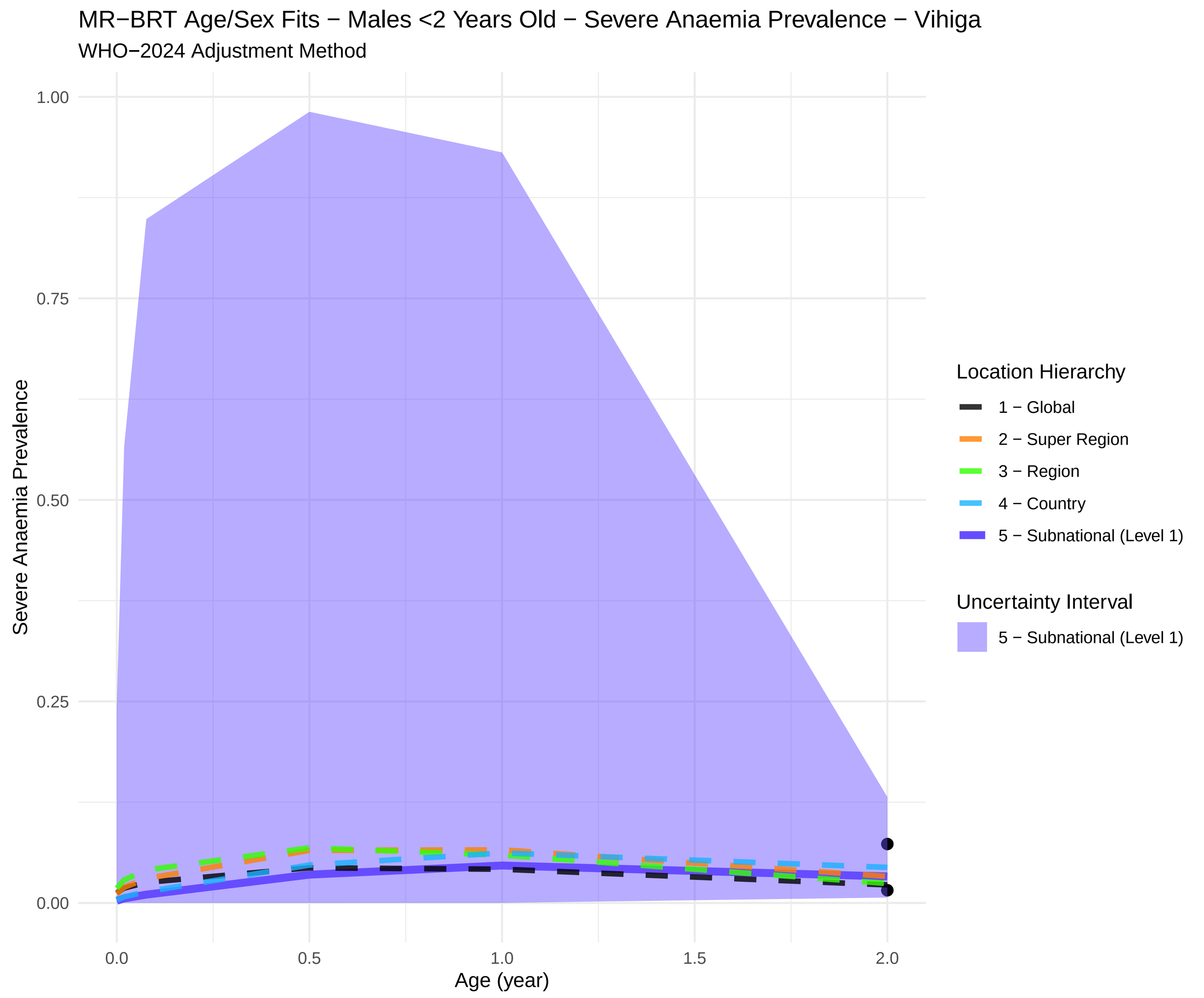
MR−BRT Age/Sex Fits − Males <2 Years Old − Severe Anaemia Prevalence − Vihiga
WHO−2024 Adjustment Method
Severe Anaemia Prevalence
Age (year)
Location Hierarchy
1 − Global
2 − Super Region
3 − Region
4 − Country
5 − Subnational (Level 1)
Uncertainty Interval
5 − Subnational (Level 1)

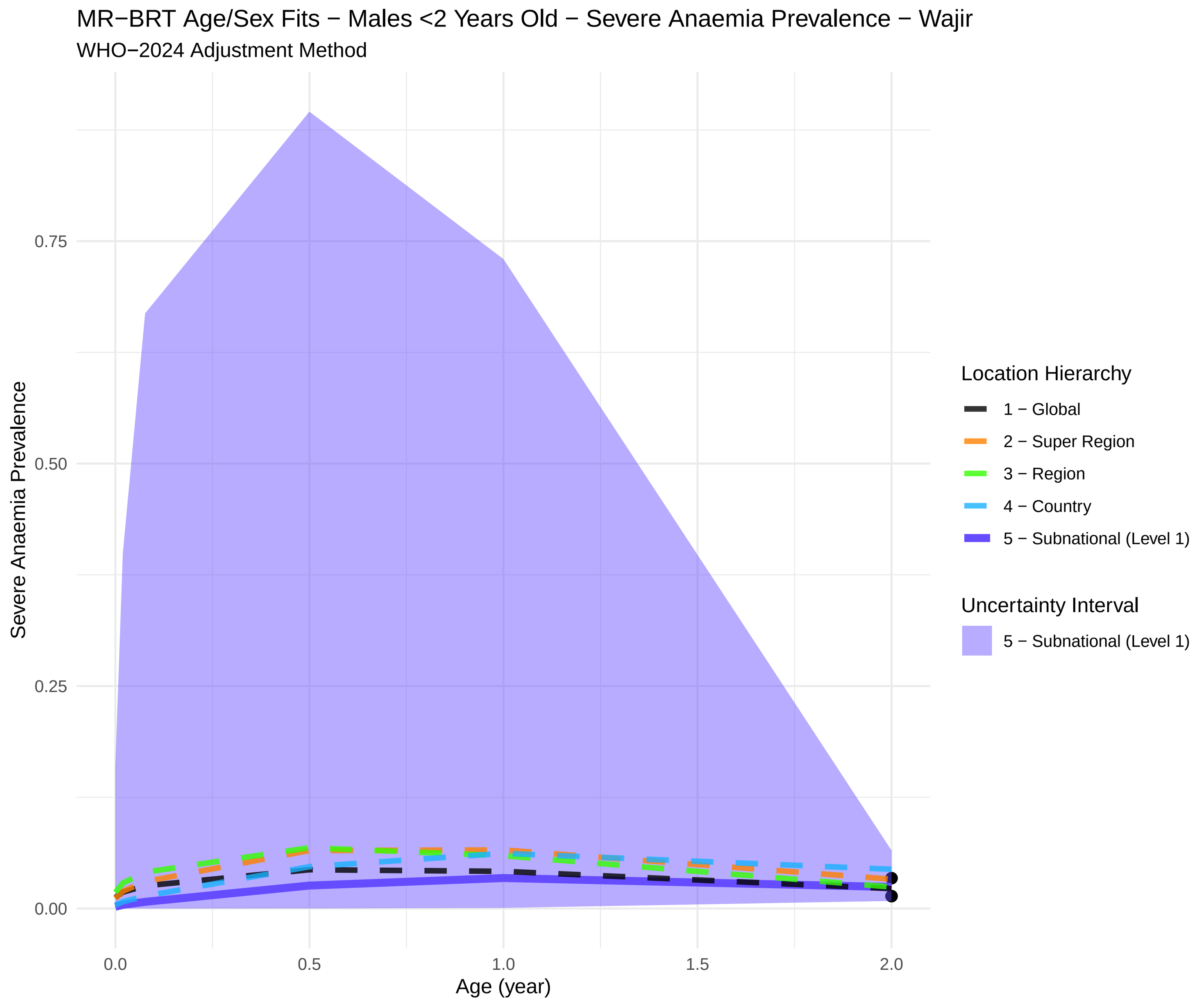
MR−BRT Age/Sex Fits − Males <2 Years Old − Severe Anaemia Prevalence − Wajir
WHO−2024 Adjustment Method
Severe Anaemia Prevalence
Age (year)
Location Hierarchy
1 − Global
2 − Super Region
3 − Region
4 − Country
5 − Subnational (Level 1)
Uncertainty Interval
5 − Subnational (Level 1)

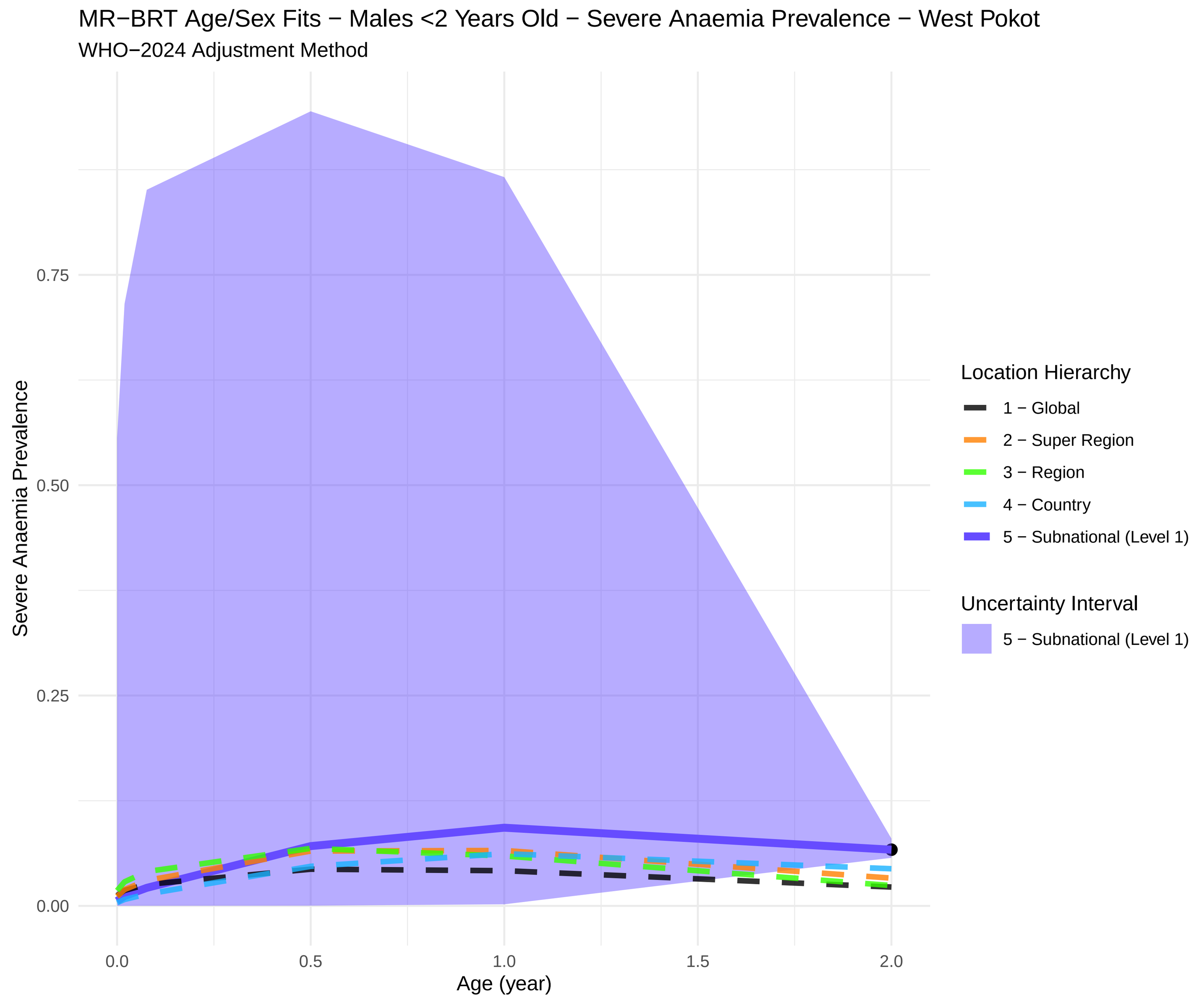
MR−BRT Age/Sex Fits − Males <2 Years Old − Severe Anaemia Prevalence − West Pokot
WHO−2024 Adjustment Method
Severe Anaemia Prevalence
Age (year)
Location Hierarchy
1 − Global
2 − Super Region
3 − Region
4 − Country
5 − Subnational (Level 1)
Uncertainty Interval
5 − Subnational (Level 1)

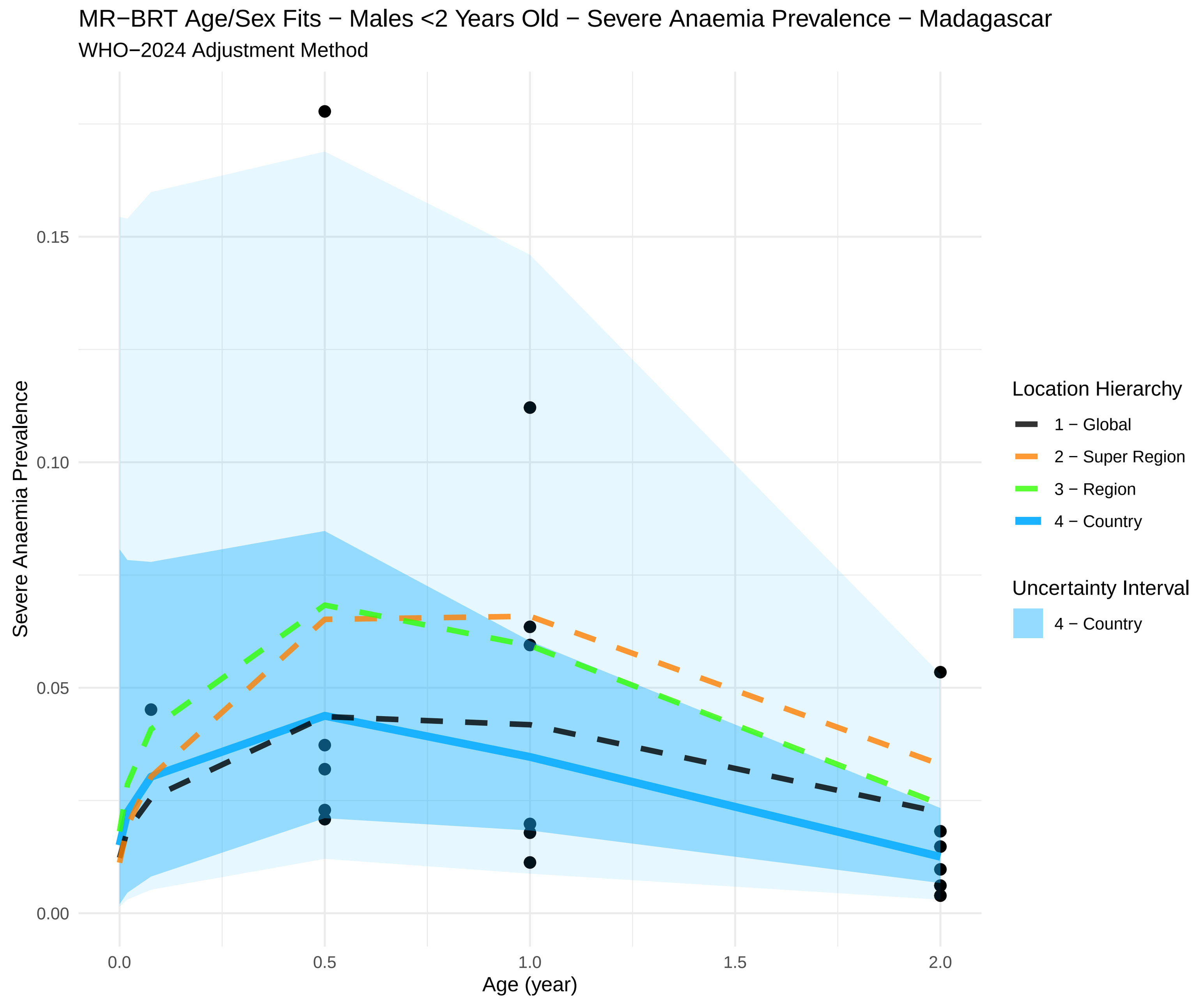
MR−BRT Age/Sex Fits − Males <2 Years Old − Severe Anaemia Prevalence − Madagascar
WHO−2024 Adjustment Method
Severe Anaemia Prevalence
Age (year)
Location Hierarchy
1 − Global
2 − Super Region
3 − Region
4 − Country
Uncertainty Interval
4 − Country

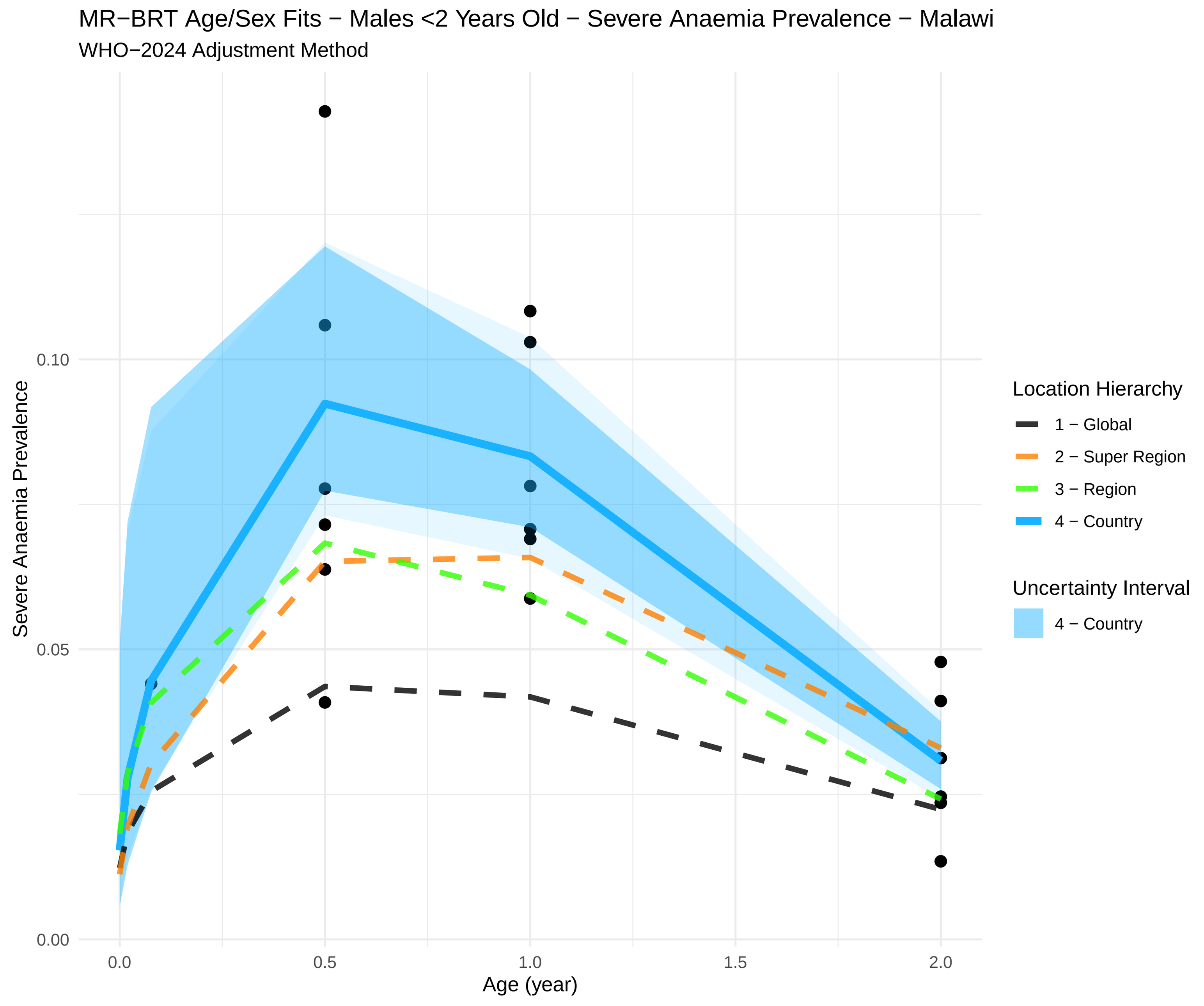
MR−BRT Age/Sex Fits − Males <2 Years Old − Severe Anaemia Prevalence − Malawi
WHO−2024 Adjustment Method
Severe Anaemia Prevalence
Age (year)
Location Hierarchy
1 − Global
2 − Super Region
3 − Region
4 − Country
Uncertainty Interval
4 − Country

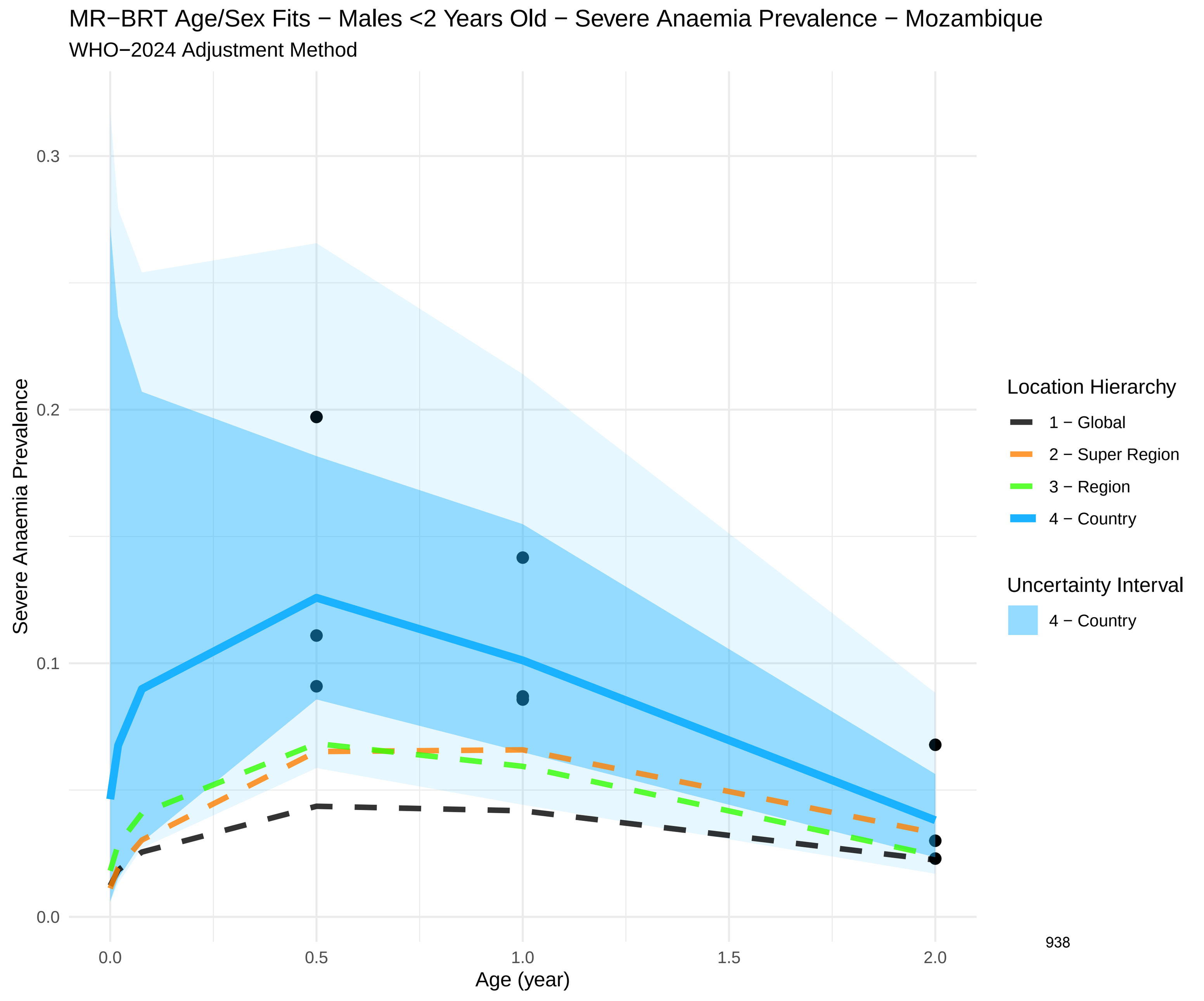
MR−BRT Age/Sex Fits − Males <2 Years Old − Severe Anaemia Prevalence − Mozambique
WHO−2024 Adjustment Method
Severe Anaemia Prevalence
Age (year)
Location Hierarchy
1 − Global
2 − Super Region
3 − Region
4 − Country
Uncertainty Interval
4 − Country

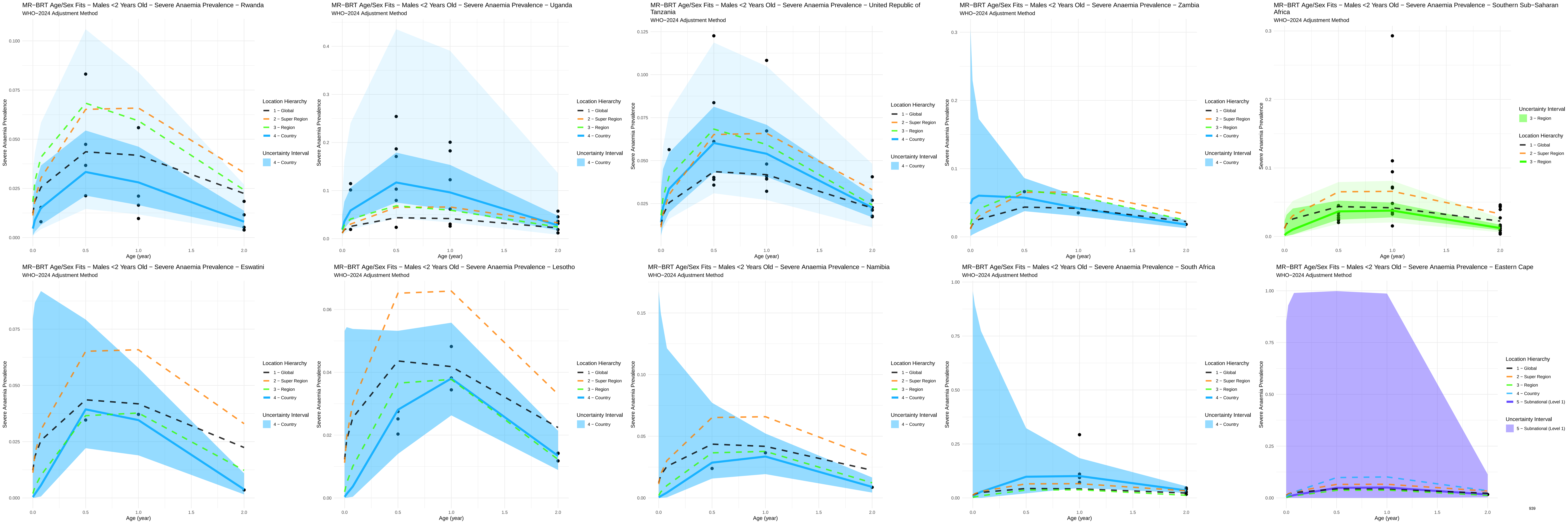
MR−BRT Age/Sex Fits − Males <2 Years Old − Severe Anaemia Prevalence − Rwanda
WHO−2024 Adjustment Method
MR−BRT Age/Sex Fits − Males <2 Years Old − Severe Anaemia Prevalence − Uganda
WHO−2024 Adjustment Method
MR−BRT Age/Sex Fits − Males <2 Years Old − Severe Anaemia Prevalence − United Republic of Tanzania
WHO−2024 Adjustment Method
MR−BRT Age/Sex Fits − Males <2 Years Old − Severe Anaemia Prevalence − Zambia
WHO−2024 Adjustment Method
MR−BRT Age/Sex Fits − Males <2 Years Old − Severe Anaemia Prevalence − Southern Sub−Saharan Africa
WHO−2024 Adjustment Method
MR−BRT Age/Sex Fits − Males <2 Years Old − Severe Anaemia Prevalence − Eswatini
WHO−2024 Adjustment Method
MR−BRT Age/Sex Fits − Males <2 Years Old − Severe Anaemia Prevalence − Lesotho
WHO−2024 Adjustment Method
MR−BRT Age/Sex Fits − Males <2 Years Old − Severe Anaemia Prevalence − Namibia
WHO−2024 Adjustment Method
MR−BRT Age/Sex Fits − Males <2 Years Old − Severe Anaemia Prevalence − South Africa
WHO−2024 Adjustment Method
MR−BRT Age/Sex Fits − Males <2 Years Old − Severe Anaemia Prevalence − Eastern Cape
WHO−2024 Adjustment Method
Severe Anaemia Prevalence
Age (year)
Location Hierarchy
1 − Global
2 − Super Region
3 − Region
4 − Country
5 − Subnational (Level 1)
Uncertainty Interval
3 − Region
4 − Country
5 − Subnational (Level 1)

MR−BRT Age/Sex Fits − Males <2 Years Old − Severe Anaemia Prevalence − Free State

WHO−2024 Adjustment Method

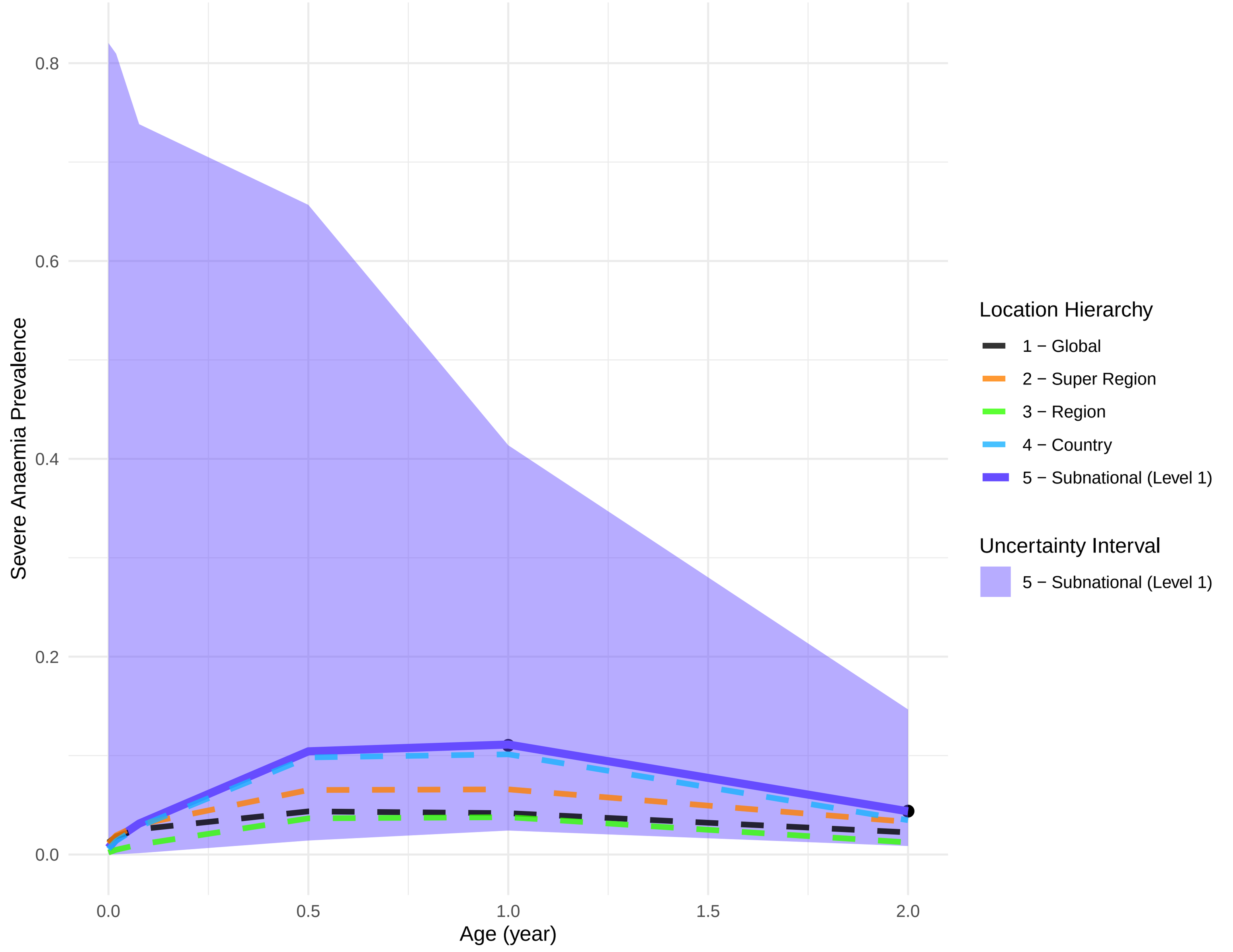

MR−BRT Age/Sex Fits − Males <2 Years Old − Severe Anaemia Prevalence − Gauteng

WHO−2024 Adjustment Method

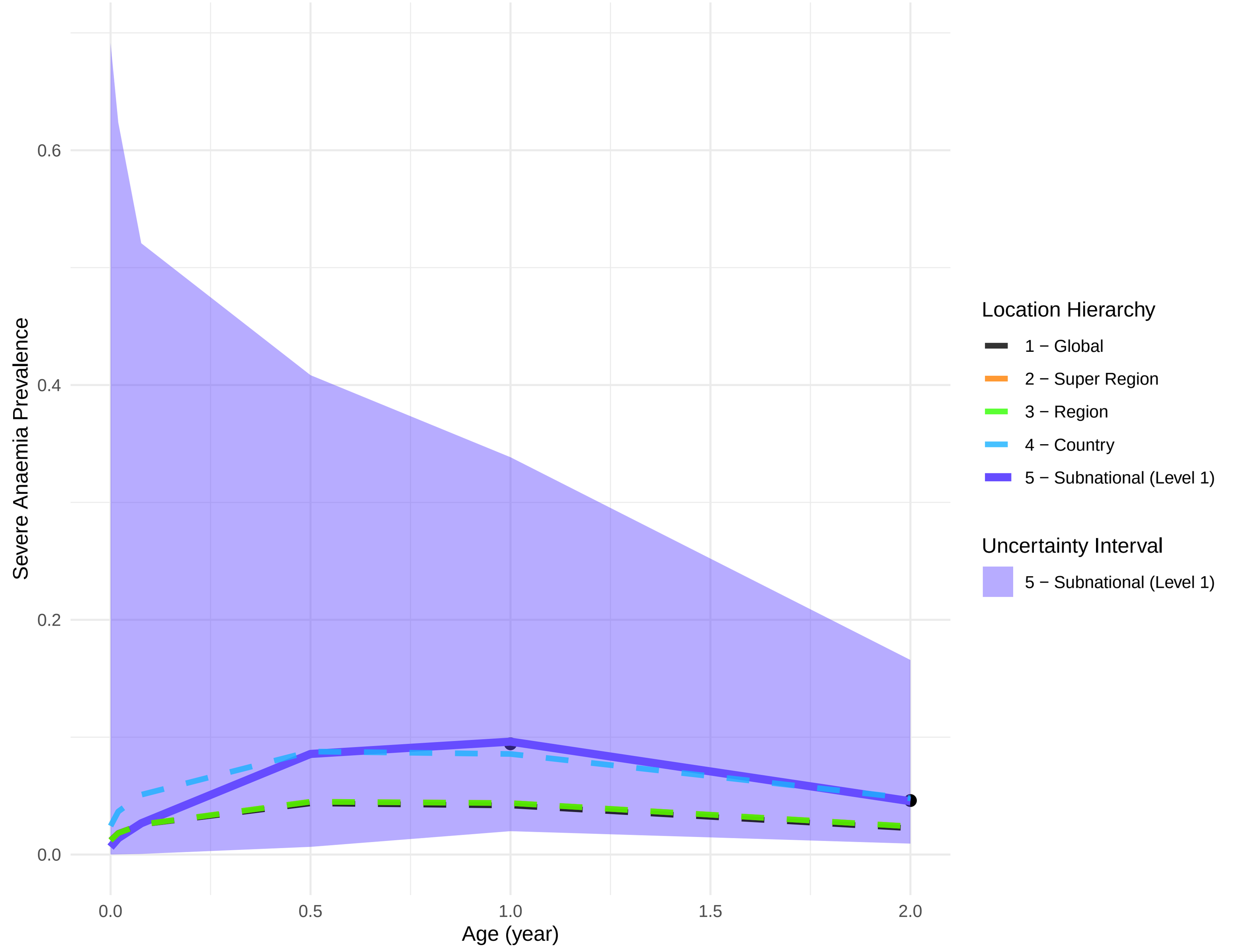

MR−BRT Age/Sex Fits − Males <2 Years Old − Severe Anaemia Prevalence − Limpopo

WHO−2024 Adjustment Method

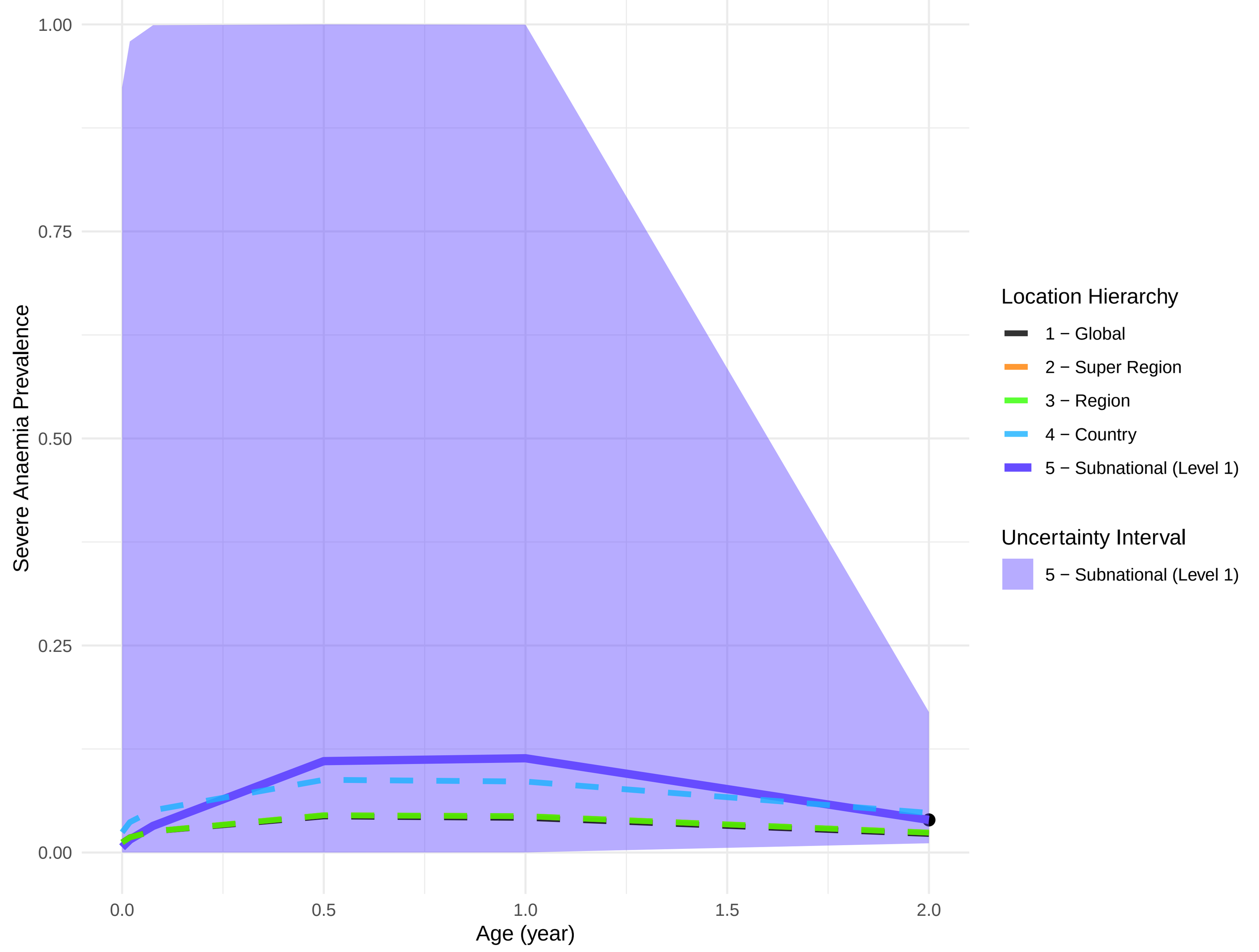

MR−BRT Age/Sex Fits − Males <2 Years Old − Severe Anaemia Prevalence − Mpumalanga

WHO−2024 Adjustment Method

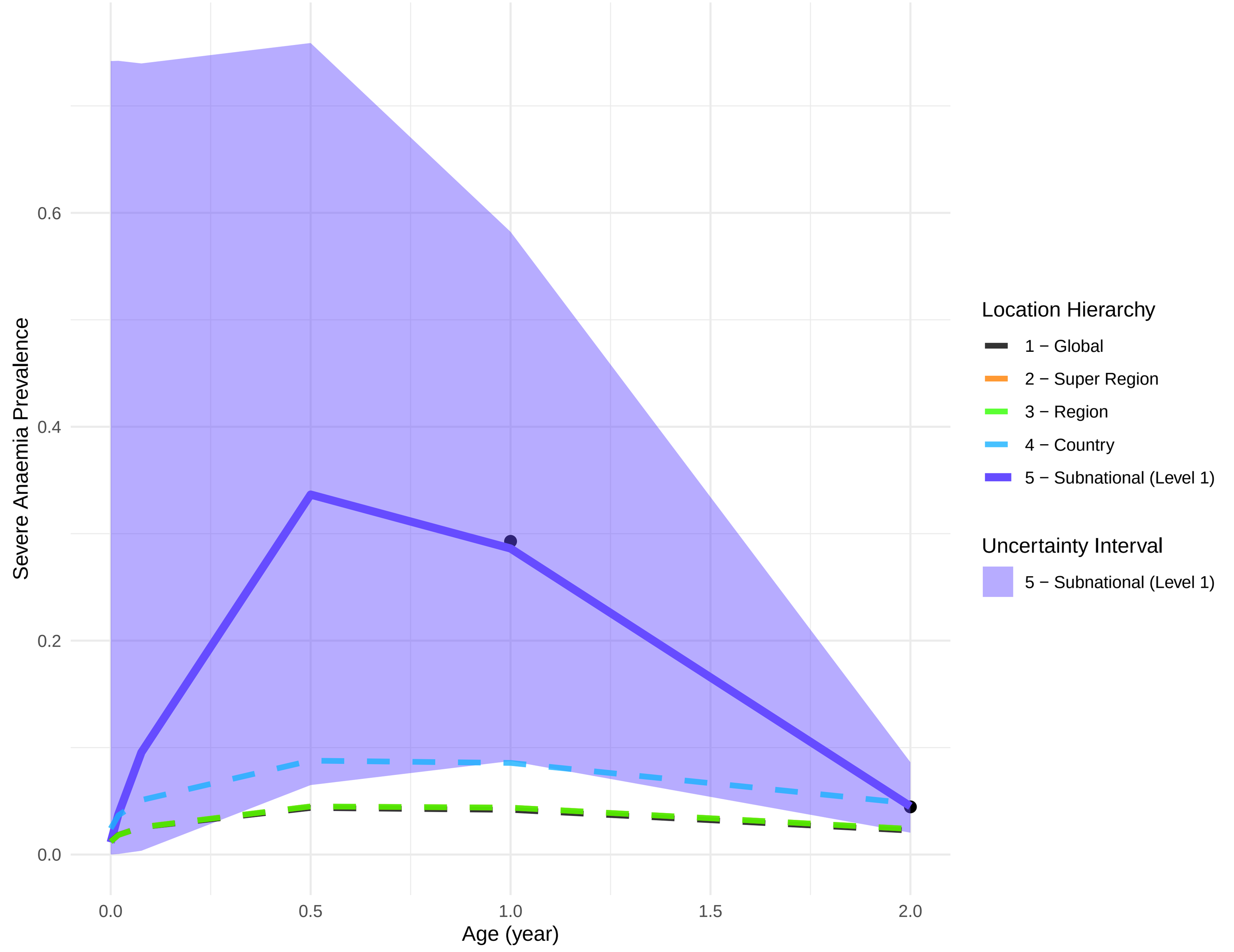

MR−BRT Age/Sex Fits − Males <2 Years Old − Severe Anaemia Prevalence − North−West

WHO−2024 Adjustment Method

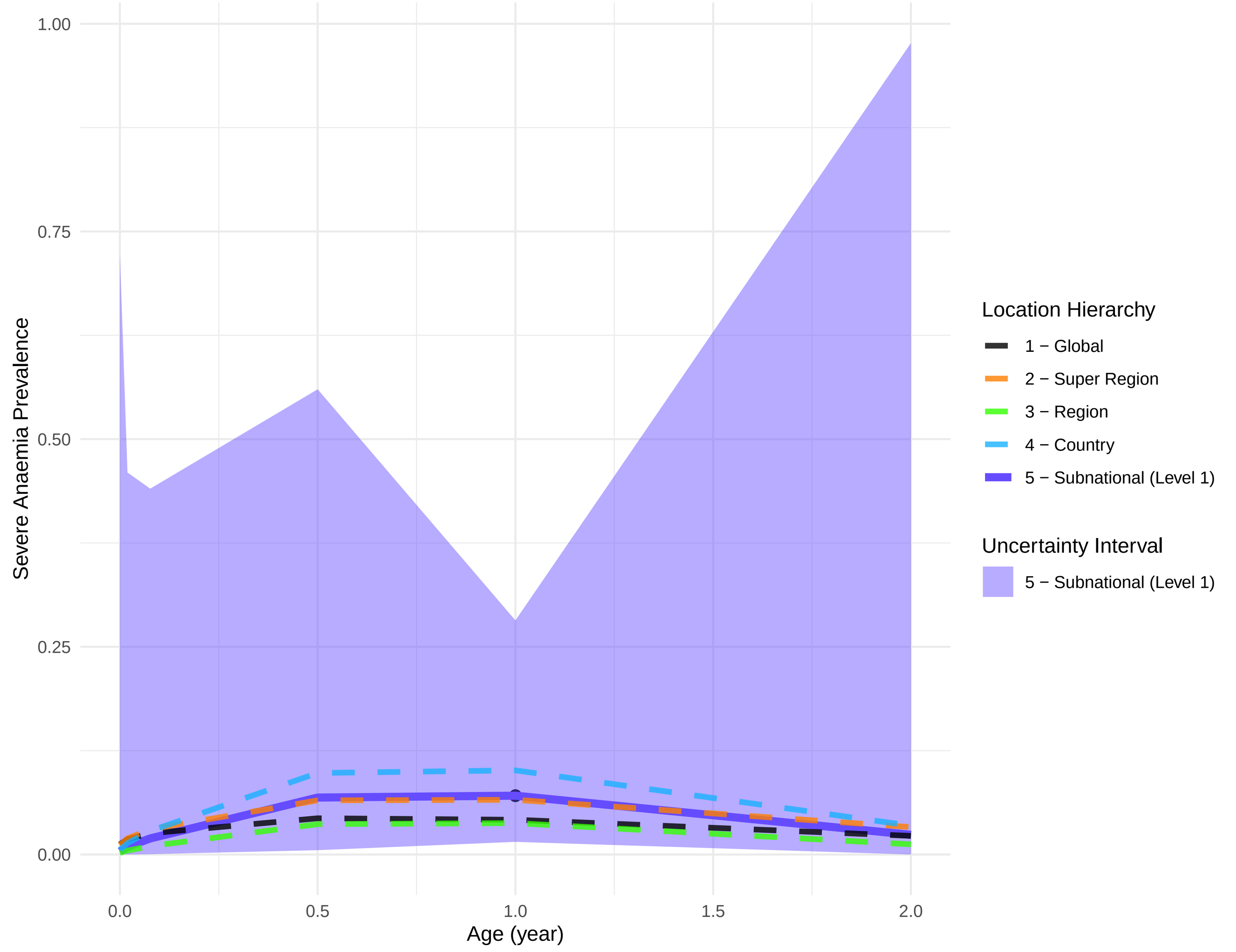

MR−BRT Age/Sex Fits − Males <2 Years Old − Severe Anaemia Prevalence − Northern Cape

WHO−2024 Adjustment Method

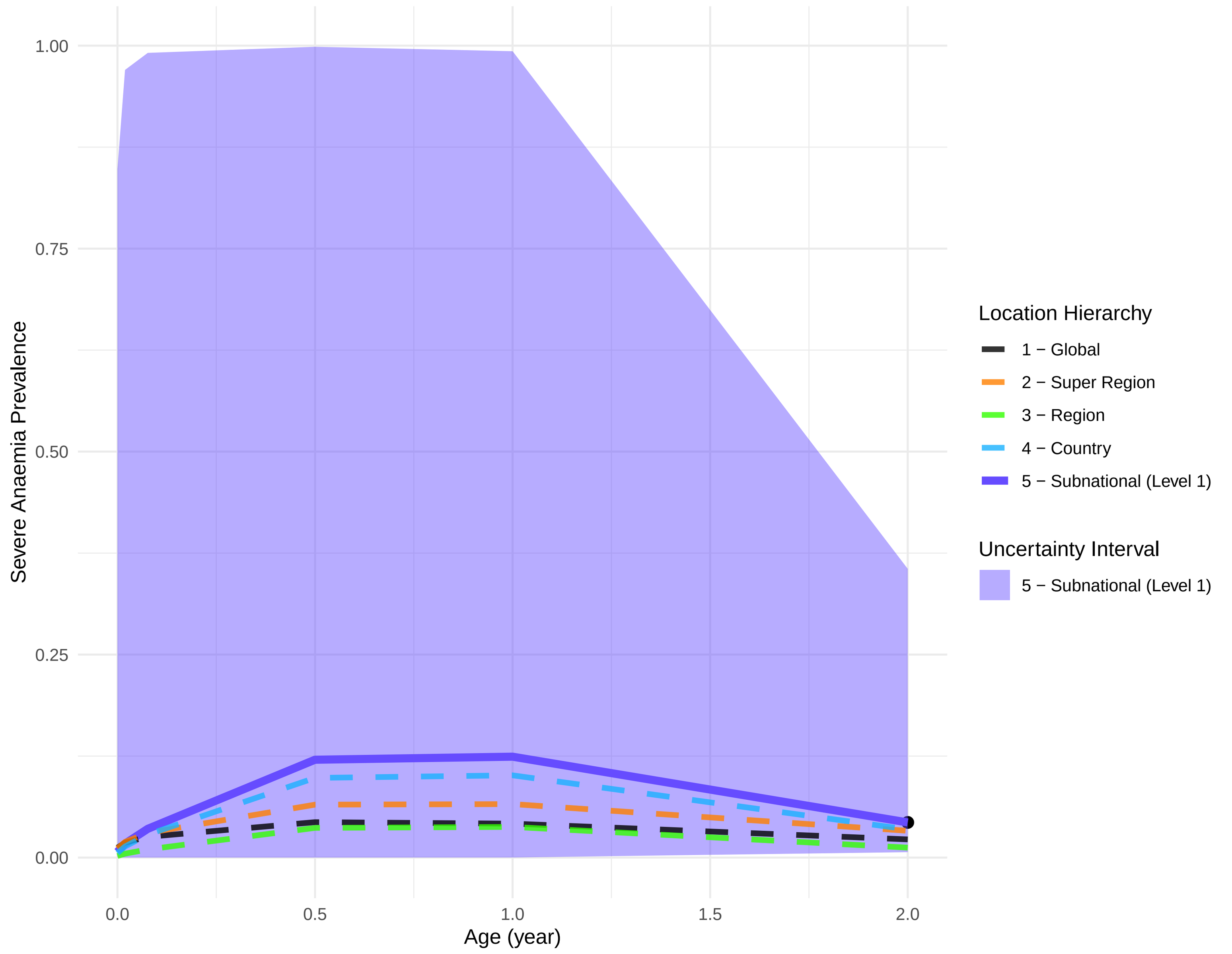

MR−BRT Age/Sex Fits − Males <2 Years Old − Severe Anaemia Prevalence − Zimbabwe

WHO−2024 Adjustment Method

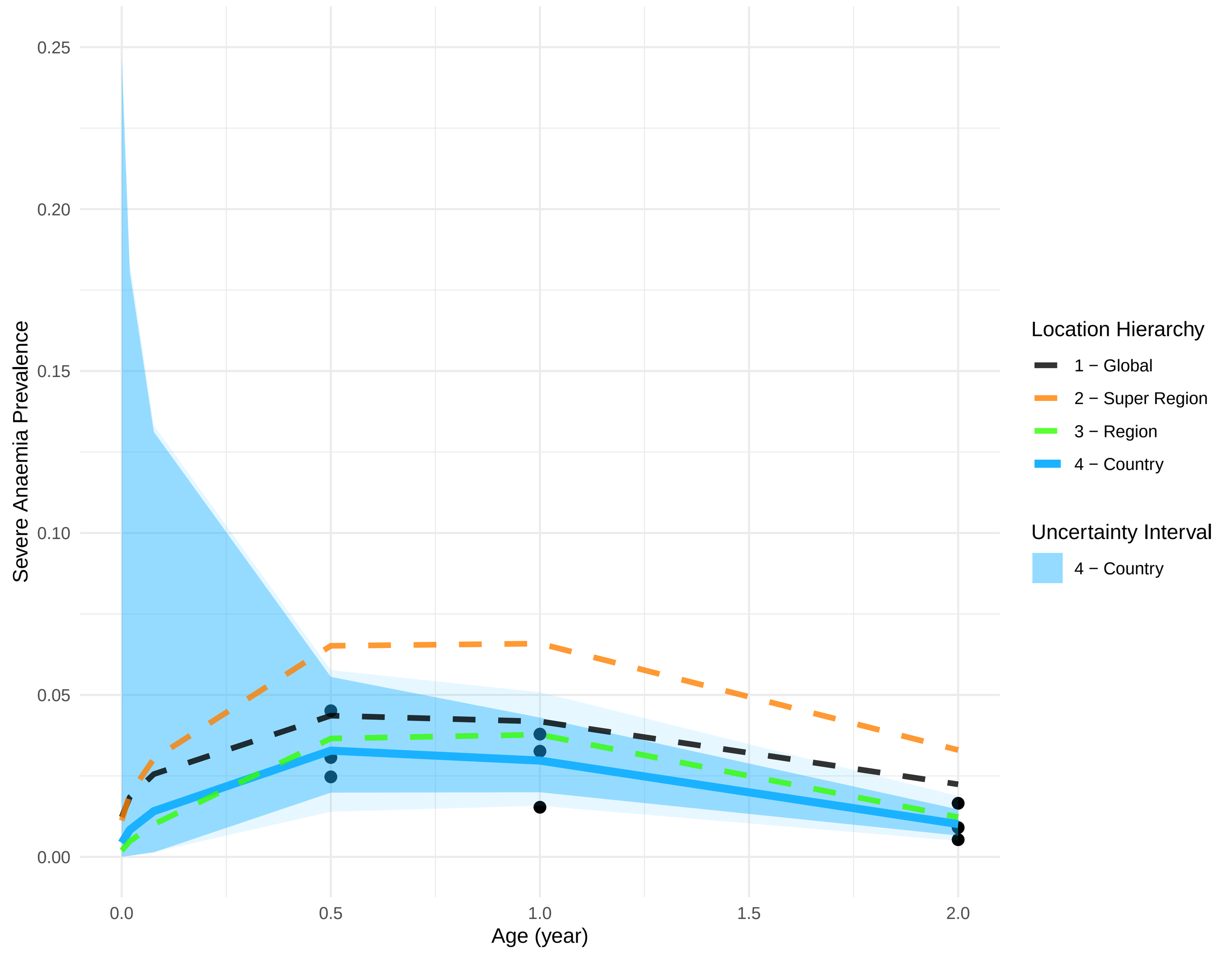

MR−BRT Age/Sex Fits − Males <2 Years Old − Severe Anaemia Prevalence − Western Sub−Saharan Africa

WHO−2024 Adjustment Method

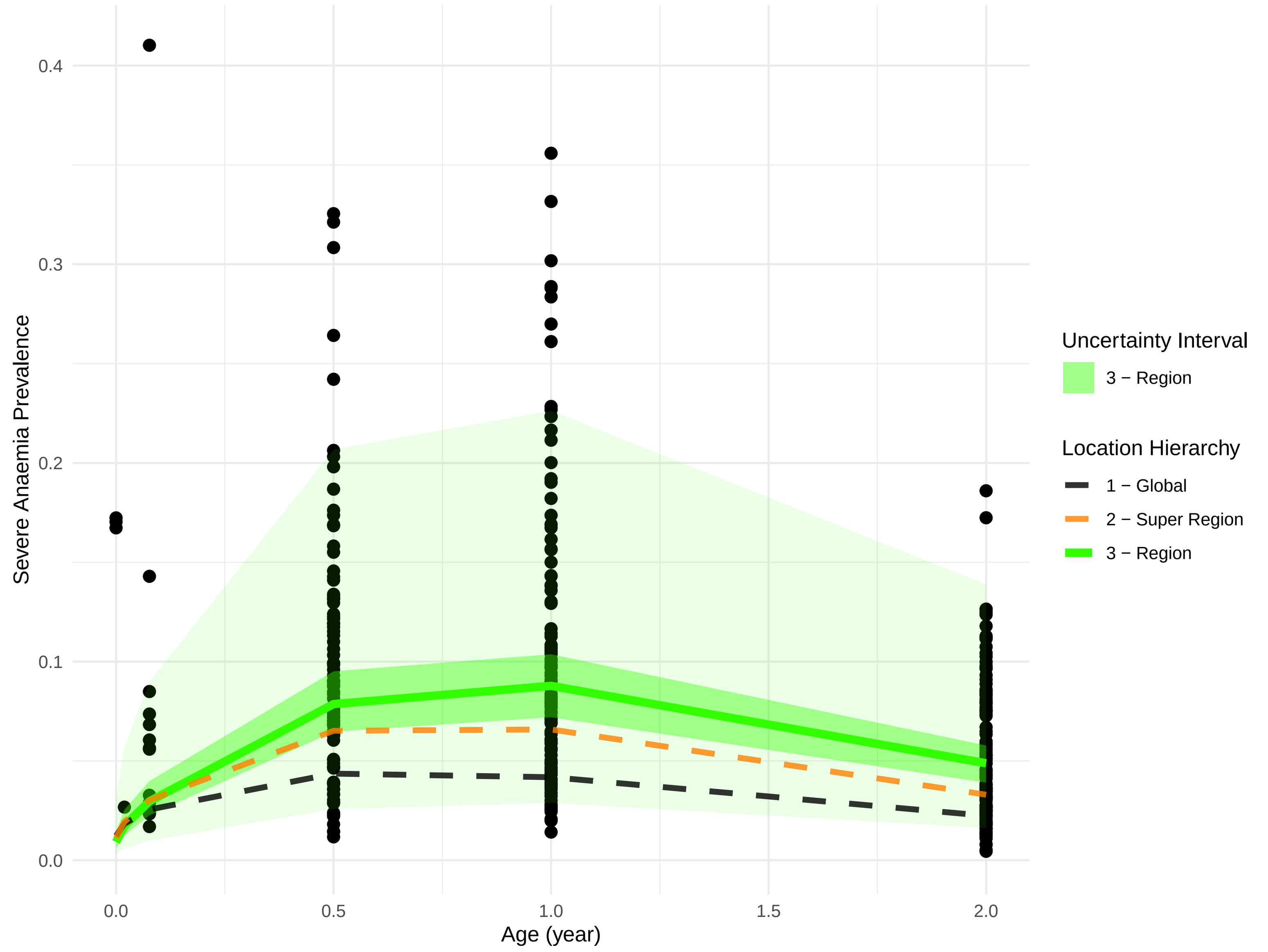

MR−BRT Age/Sex Fits − Males <2 Years Old − Severe Anaemia Prevalence − Benin

WHO−2024 Adjustment Method

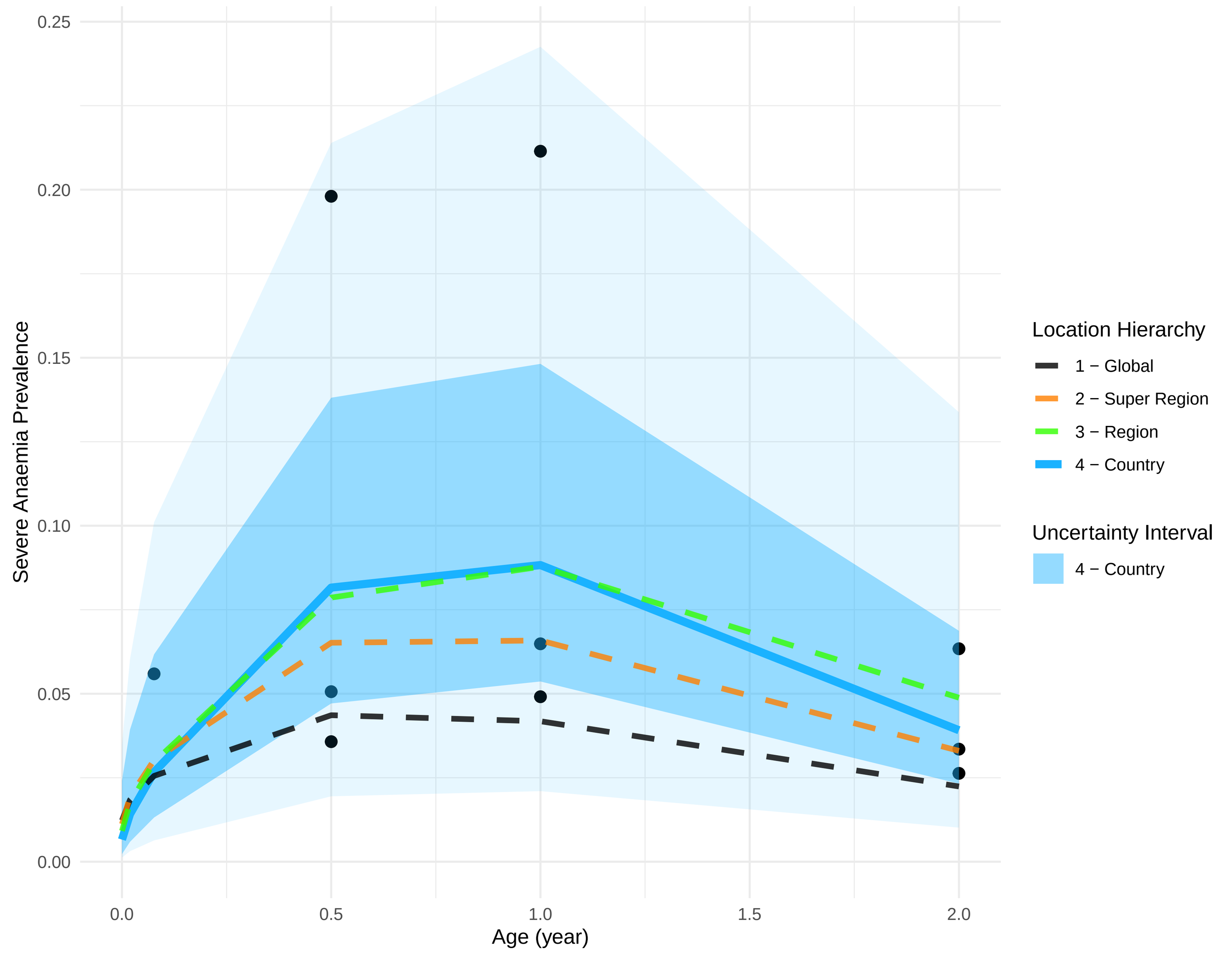

MR−BRT Age/Sex Fits − Males <2 Years Old − Severe Anaemia Prevalence − Burkina Faso

WHO−2024 Adjustment Method

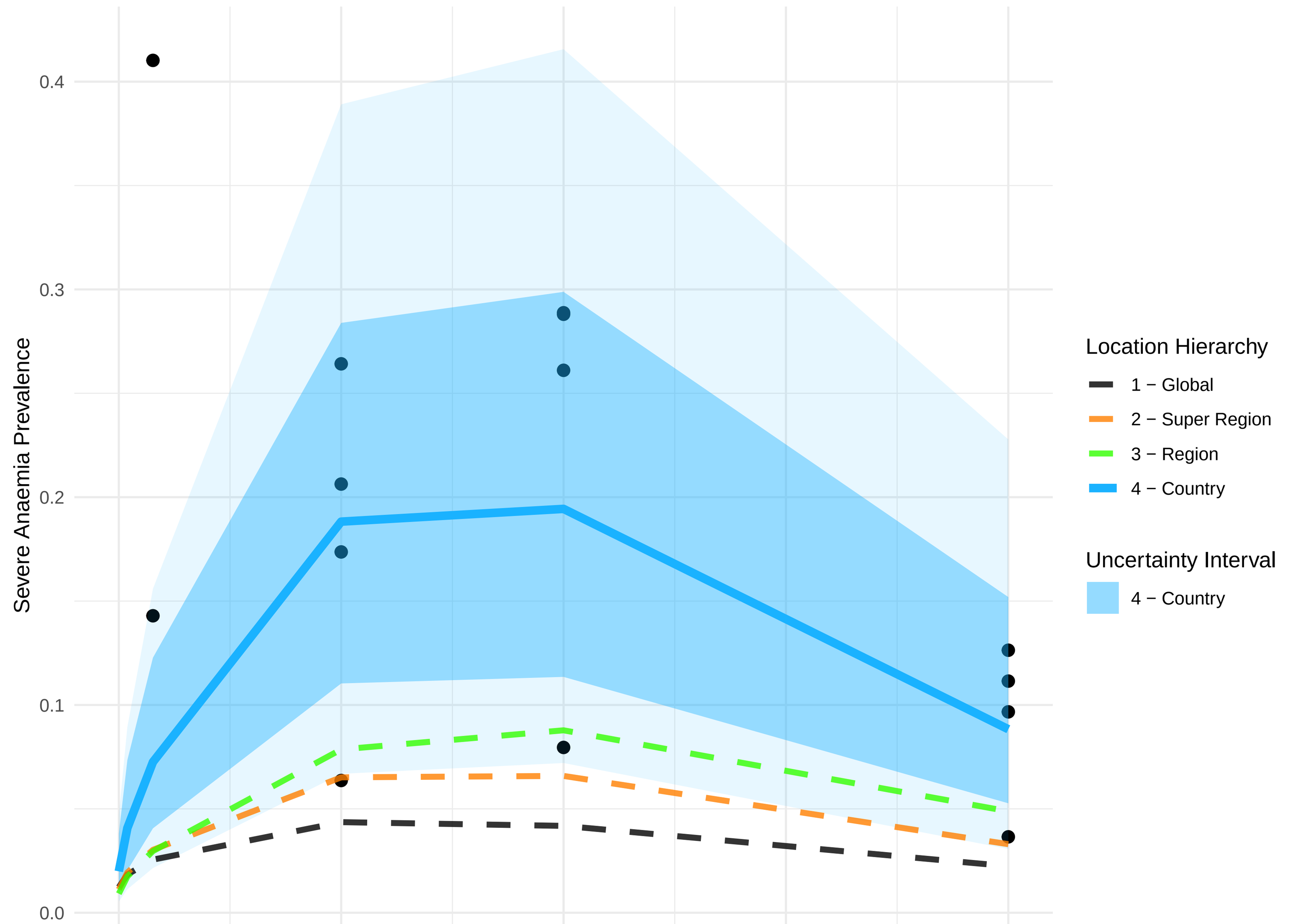

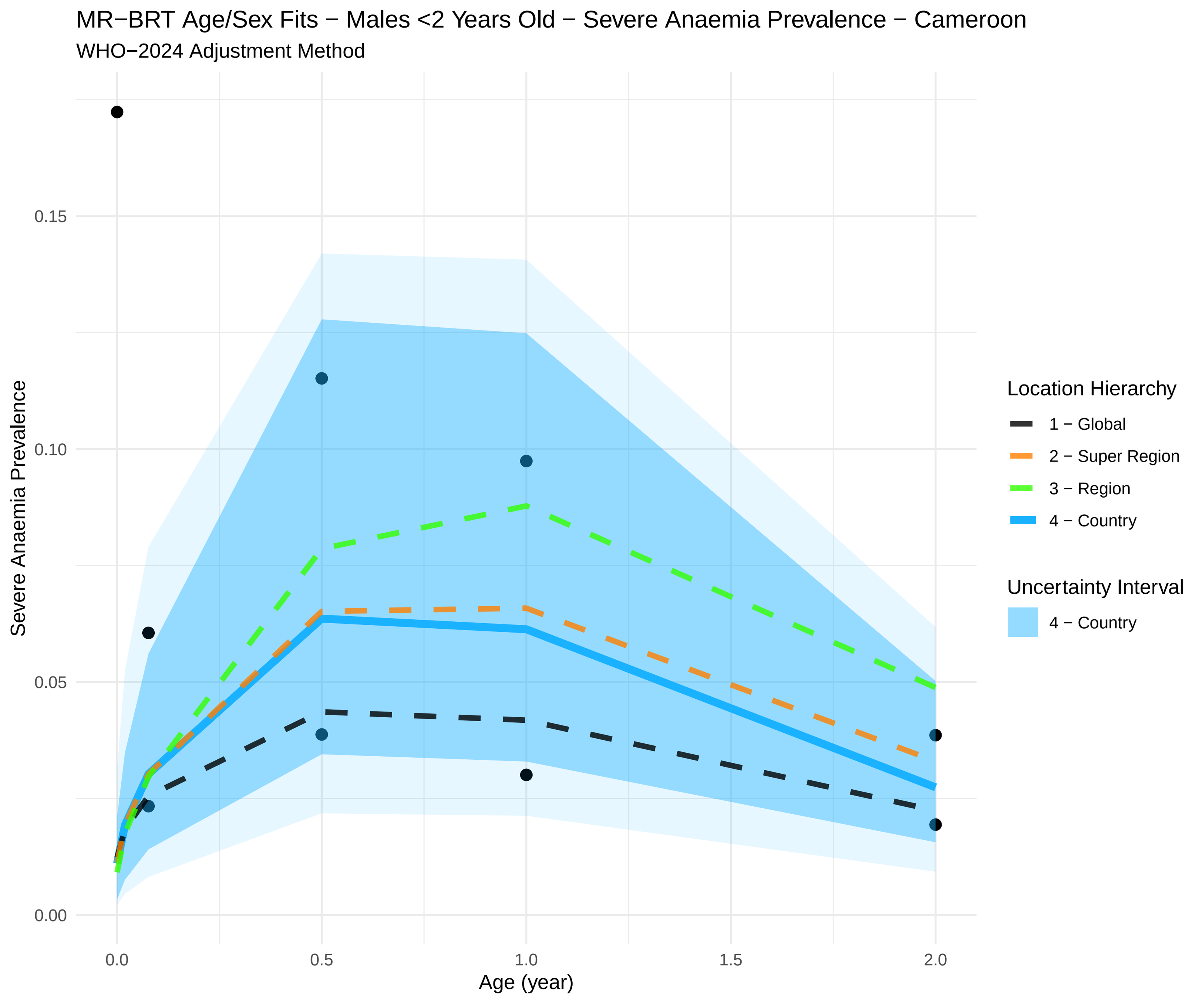
MR−BRT Age/Sex Fits − Males <2 Years Old − Severe Anaemia Prevalence − Cameroon
WHO−2024 Adjustment Method
Severe Anaemia Prevalence
Age (year)
Location Hierarchy
1 − Global
2 − Super Region
3 − Region
4 − Country
Uncertainty Interval
4 − Country

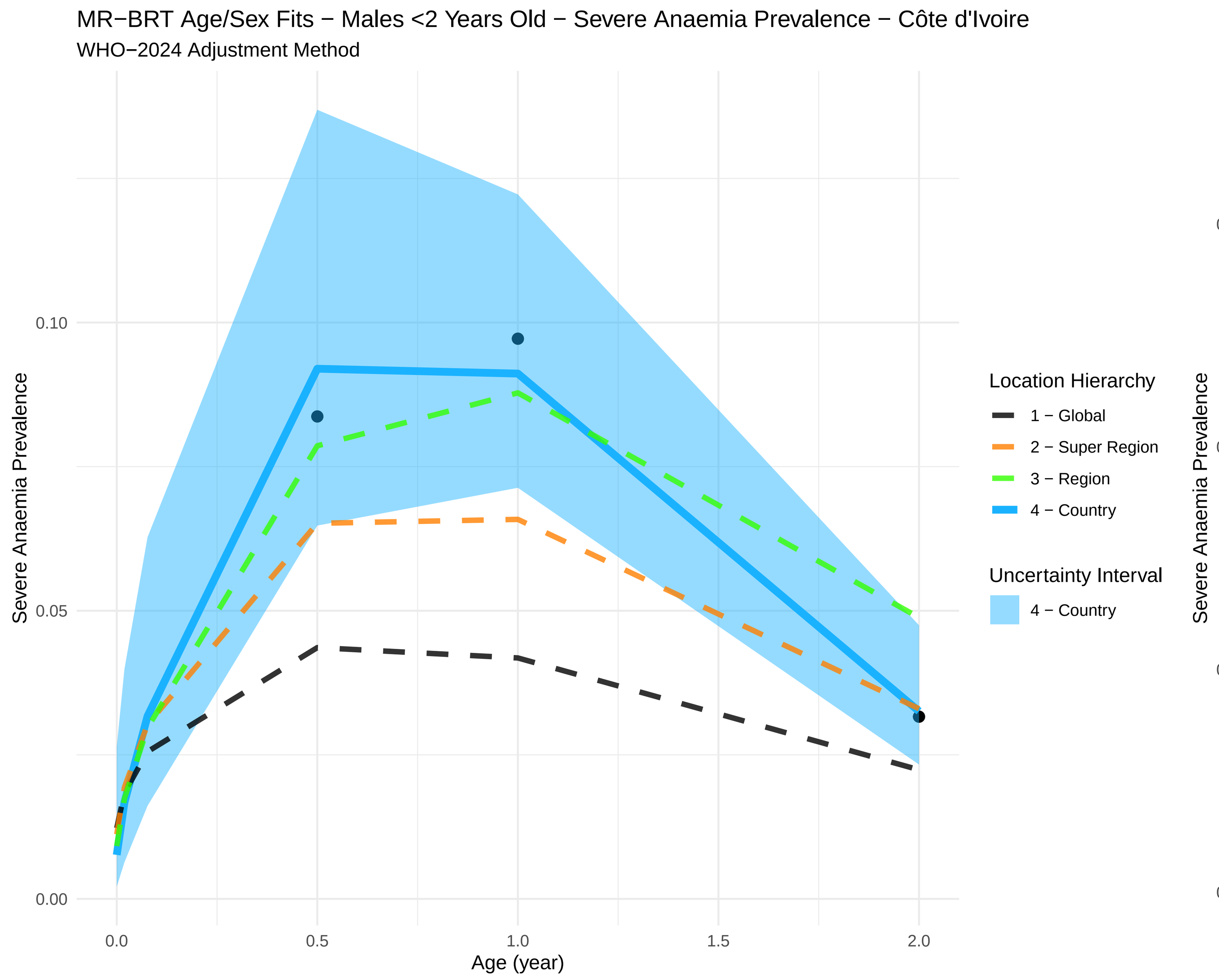
MR−BRT Age/Sex Fits − Males <2 Years Old − Severe Anaemia Prevalence − Côte d'Ivoire
WHO−2024 Adjustment Method
Severe Anaemia Prevalence
Age (year)
Location Hierarchy
1 − Global
2 − Super Region
3 − Region
4 − Country
Uncertainty Interval
4 − Country

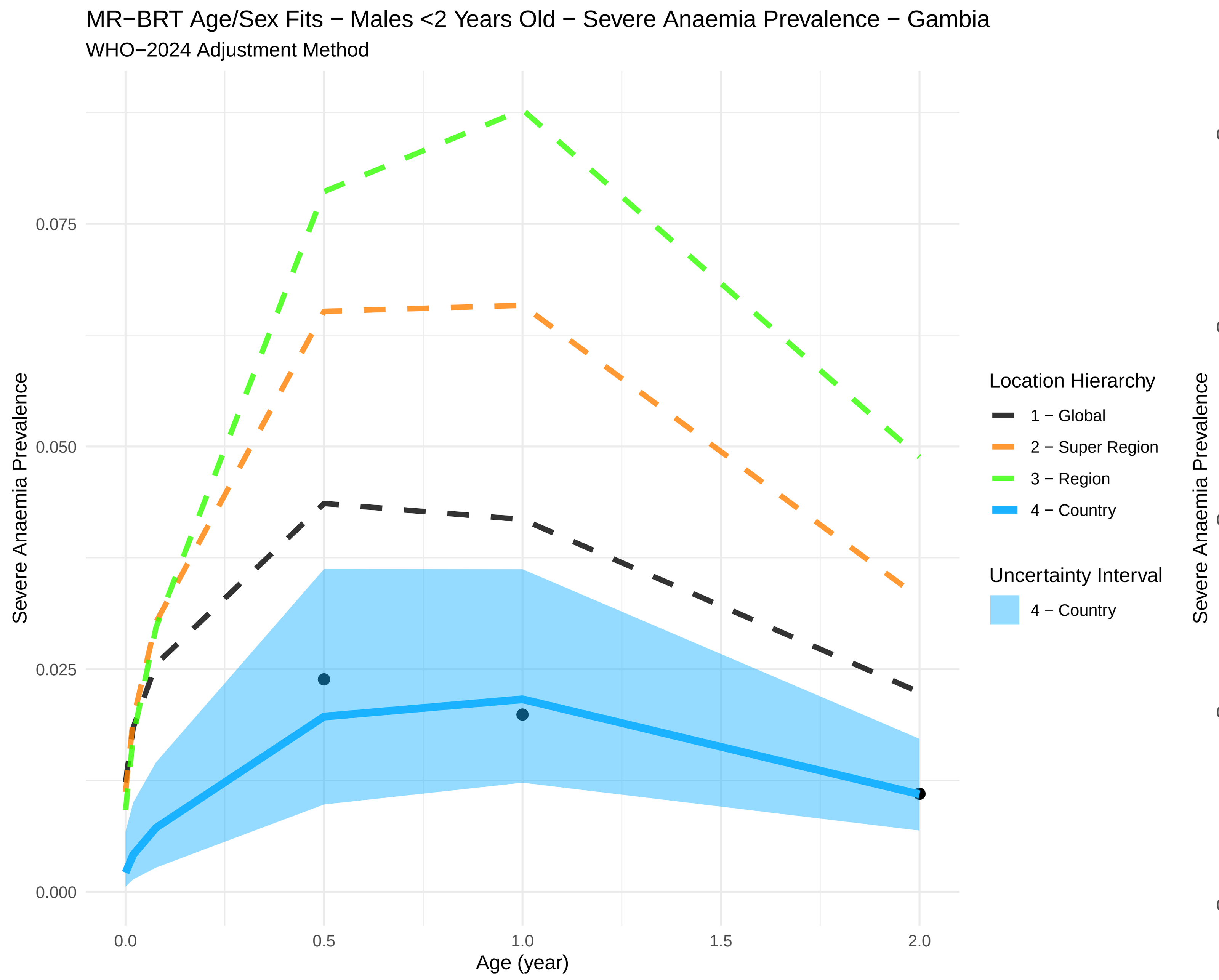
MR−BRT Age/Sex Fits − Males <2 Years Old − Severe Anaemia Prevalence − Gambia
WHO−2024 Adjustment Method
Severe Anaemia Prevalence
Age (year)
Location Hierarchy
1 − Global
2 − Super Region
3 − Region
4 − Country
Uncertainty Interval
4 − Country

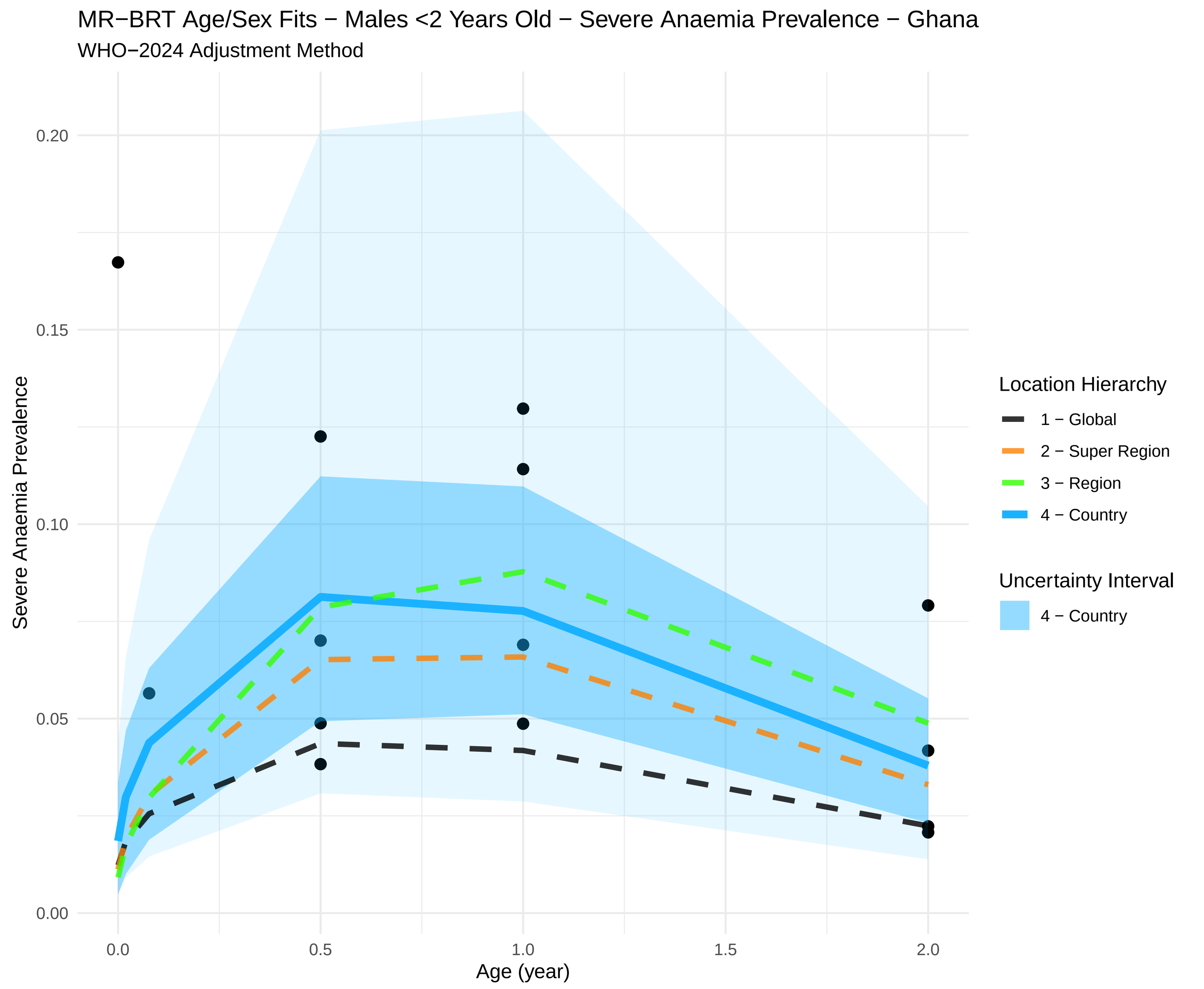
MR−BRT Age/Sex Fits − Males <2 Years Old − Severe Anaemia Prevalence − Ghana
WHO−2024 Adjustment Method
Severe Anaemia Prevalence
Age (year)
Location Hierarchy
1 − Global
2 − Super Region
3 − Region
4 − Country
Uncertainty Interval
4 − Country

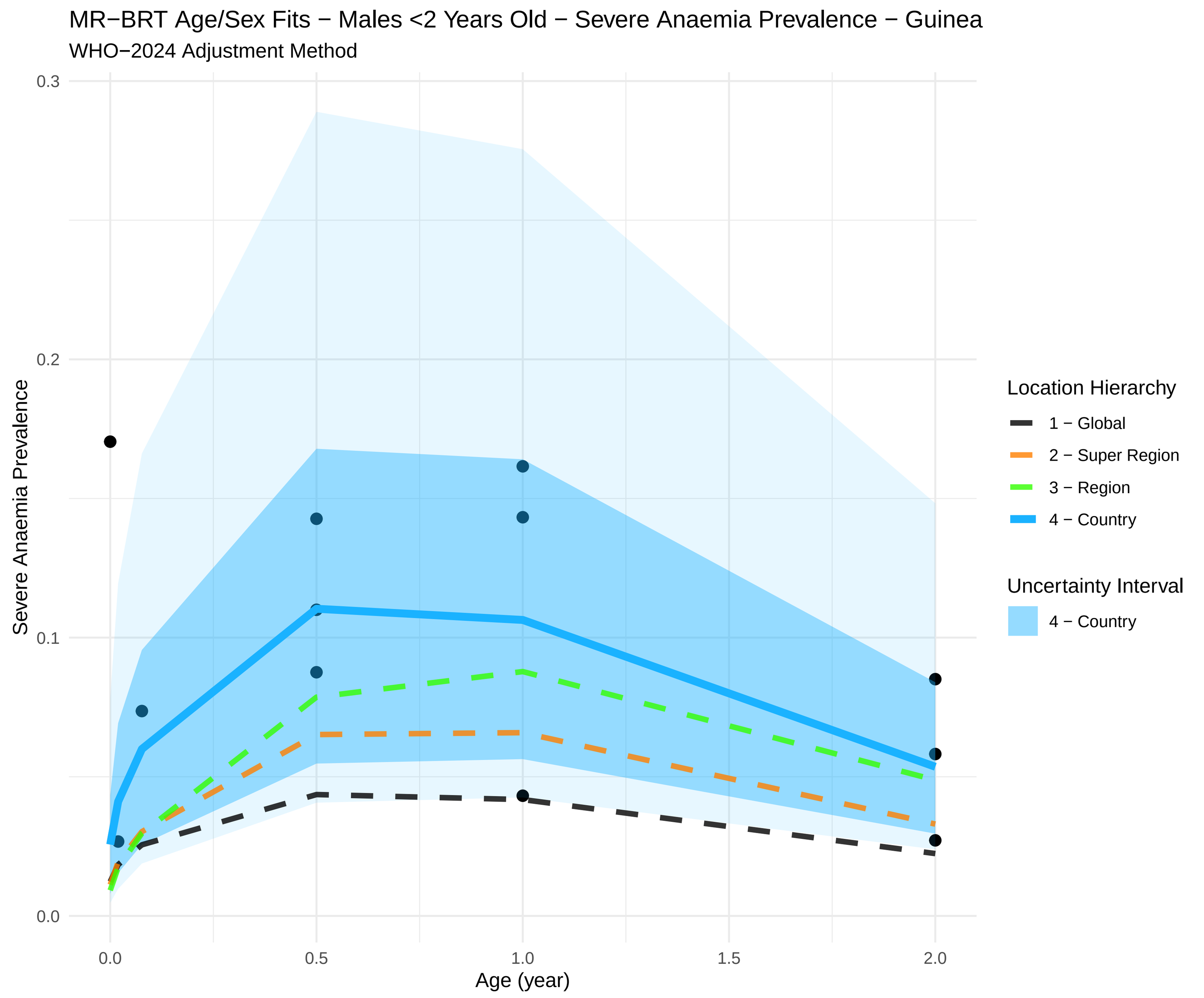
MR−BRT Age/Sex Fits − Males <2 Years Old − Severe Anaemia Prevalence − Guinea
WHO−2024 Adjustment Method
Severe Anaemia Prevalence
Age (year)
Location Hierarchy
1 − Global
2 − Super Region
3 − Region
4 − Country
Uncertainty Interval
4 − Country

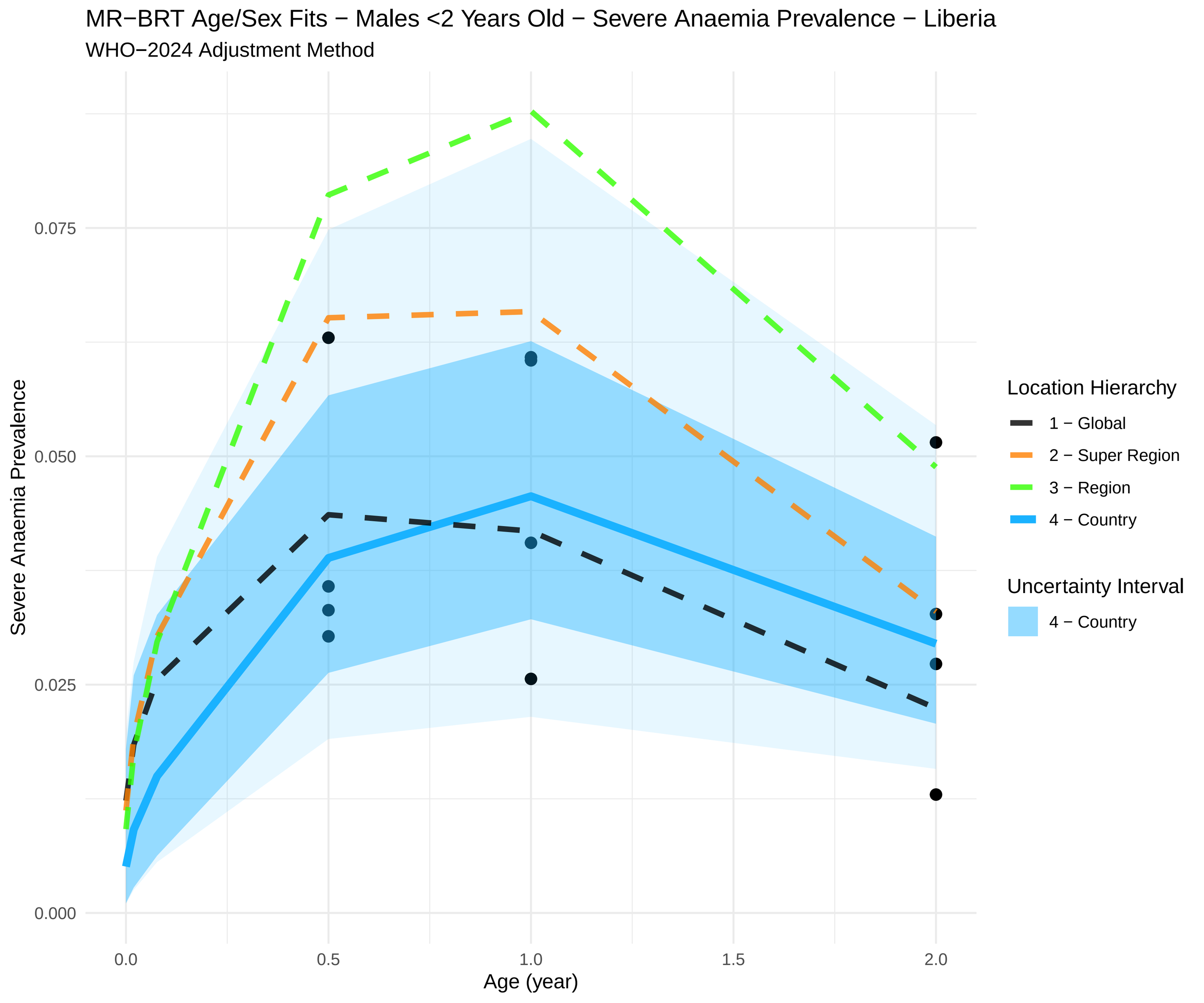
MR−BRT Age/Sex Fits − Males <2 Years Old − Severe Anaemia Prevalence − Liberia
WHO−2024 Adjustment Method
Severe Anaemia Prevalence
Age (year)
Location Hierarchy
1 − Global
2 − Super Region
3 − Region
4 − Country
Uncertainty Interval
4 − Country

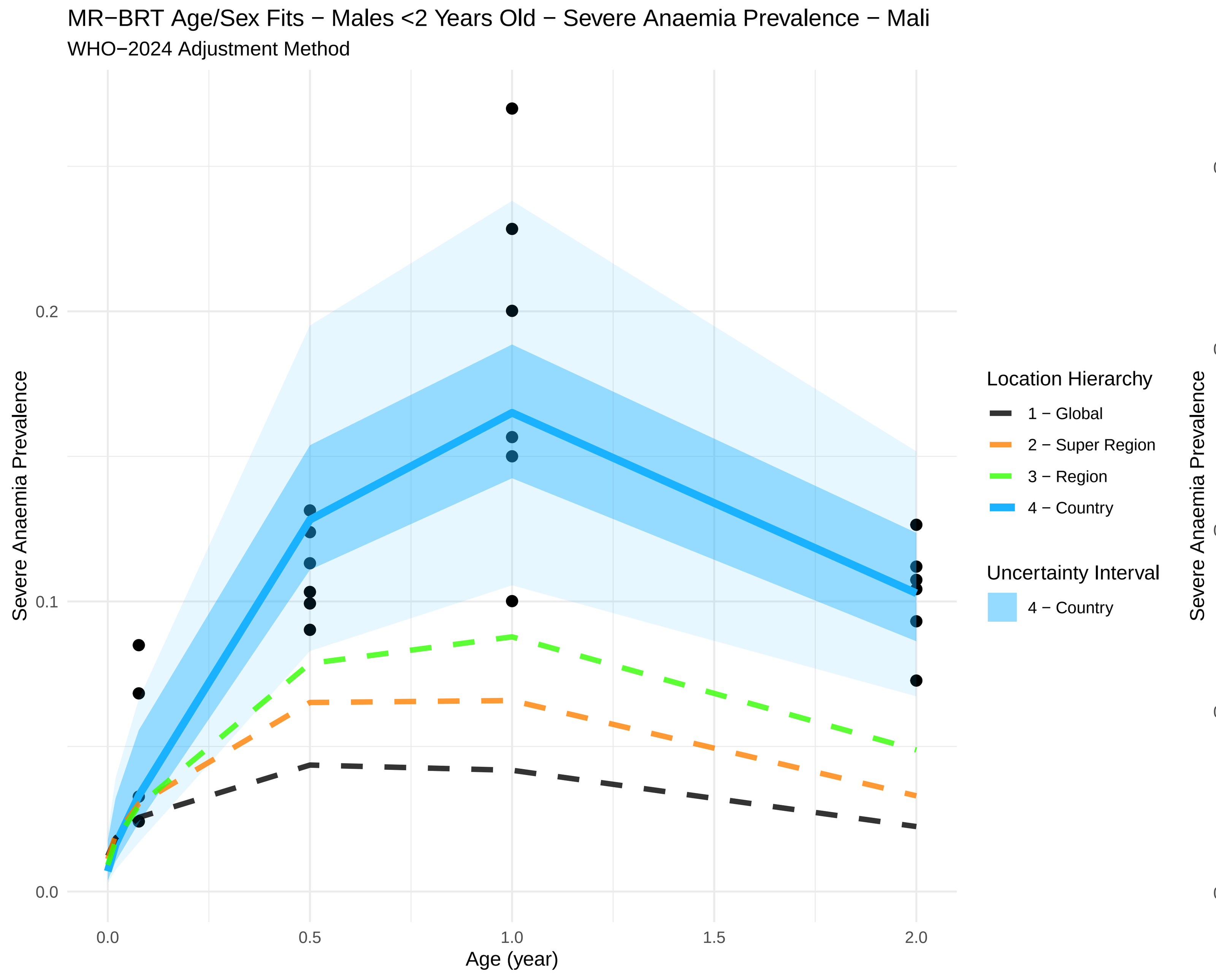
MR−BRT Age/Sex Fits − Males <2 Years Old − Severe Anaemia Prevalence − Mali
WHO−2024 Adjustment Method
Severe Anaemia Prevalence
Age (year)
Location Hierarchy
1 − Global
2 − Super Region
3 − Region
4 − Country
Uncertainty Interval
4 − Country

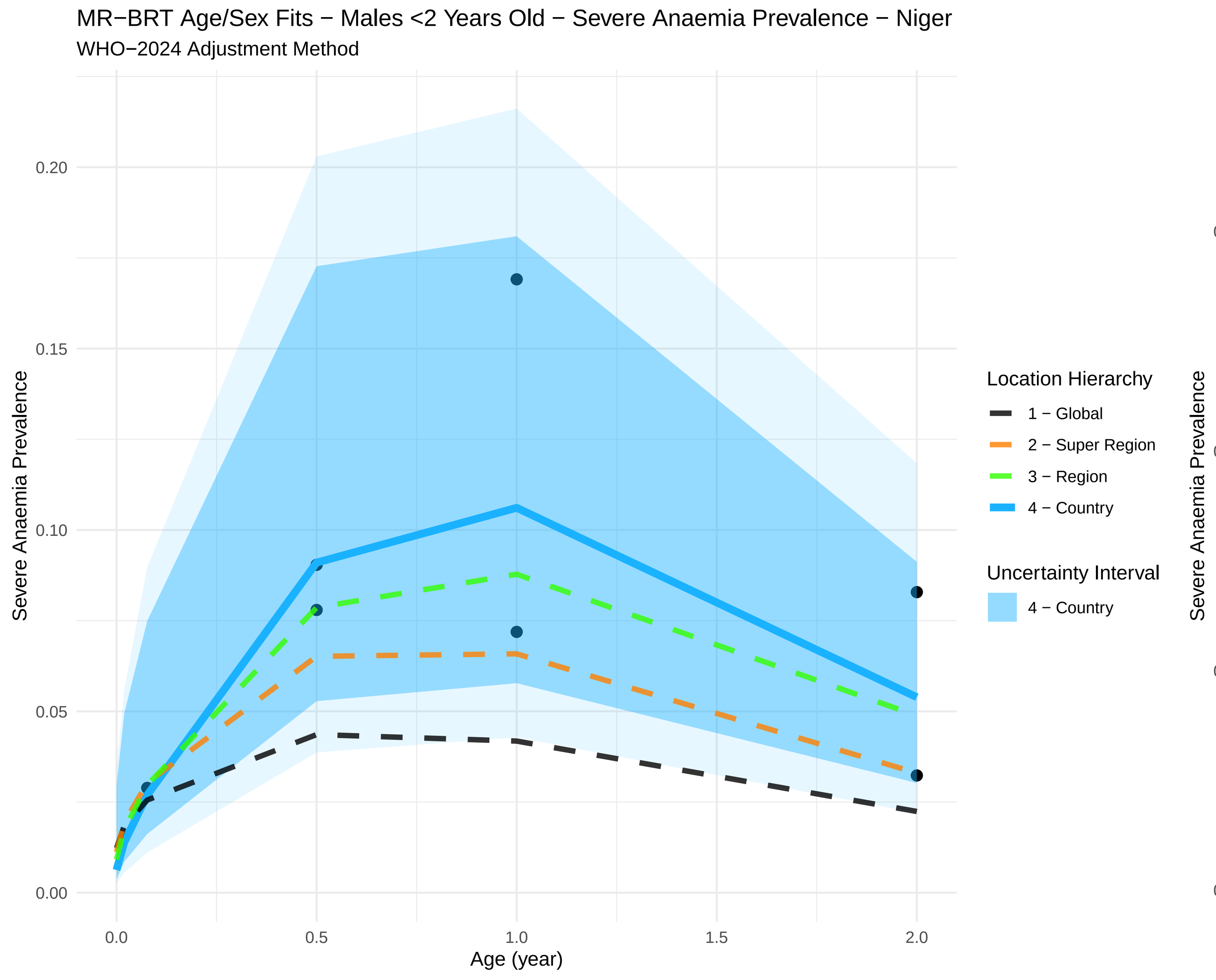
MR−BRT Age/Sex Fits − Males <2 Years Old − Severe Anaemia Prevalence − Niger
WHO−2024 Adjustment Method
Severe Anaemia Prevalence
Age (year)
Location Hierarchy
1 − Global
2 − Super Region
3 − Region
4 − Country
Uncertainty Interval
4 − Country

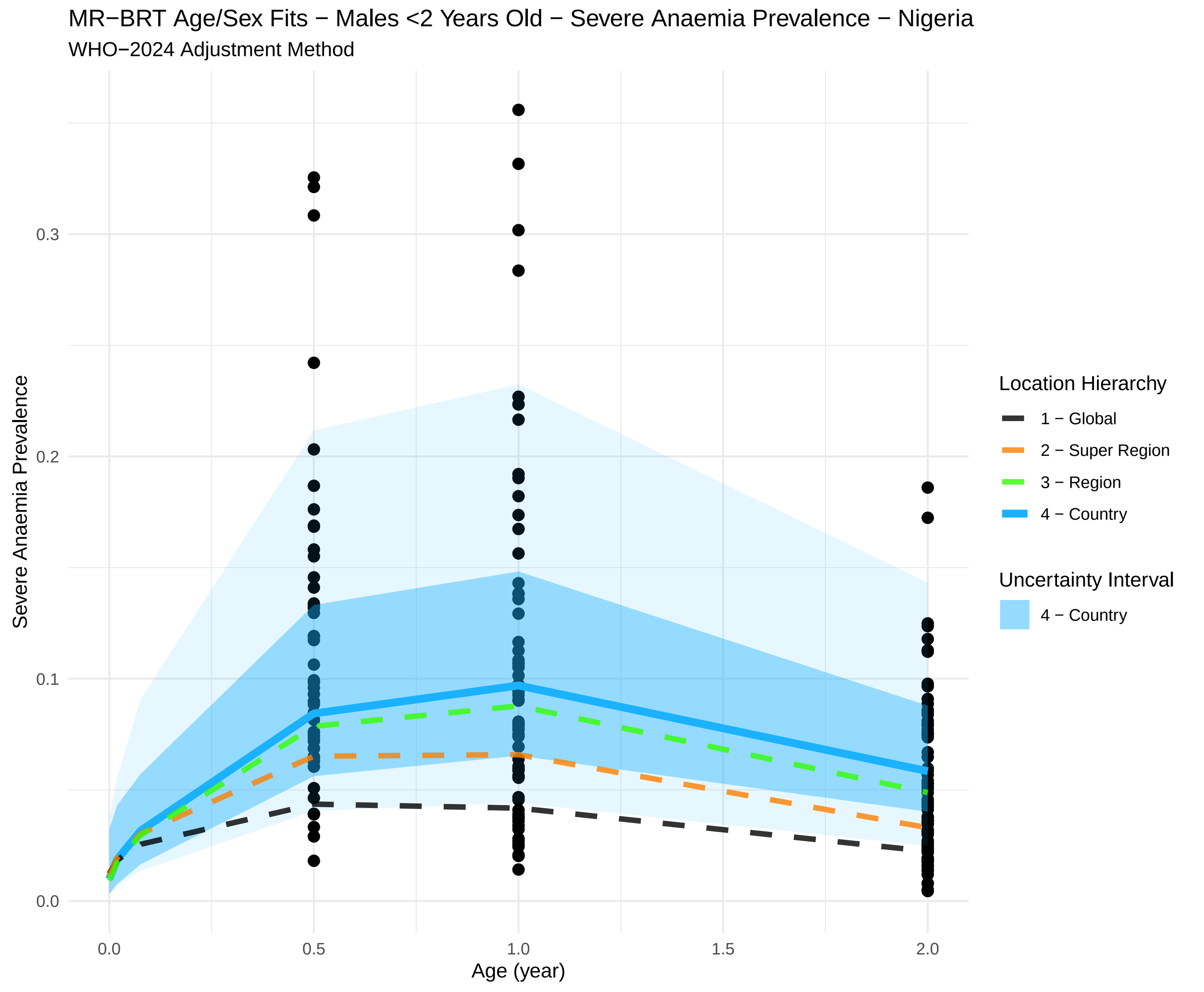
MR−BRT Age/Sex Fits − Males <2 Years Old − Severe Anaemia Prevalence − Nigeria
WHO−2024 Adjustment Method
Severe Anaemia Prevalence
Age (year)
Location Hierarchy
1 − Global
2 − Super Region
3 − Region
4 − Country
Uncertainty Interval
4 − Country

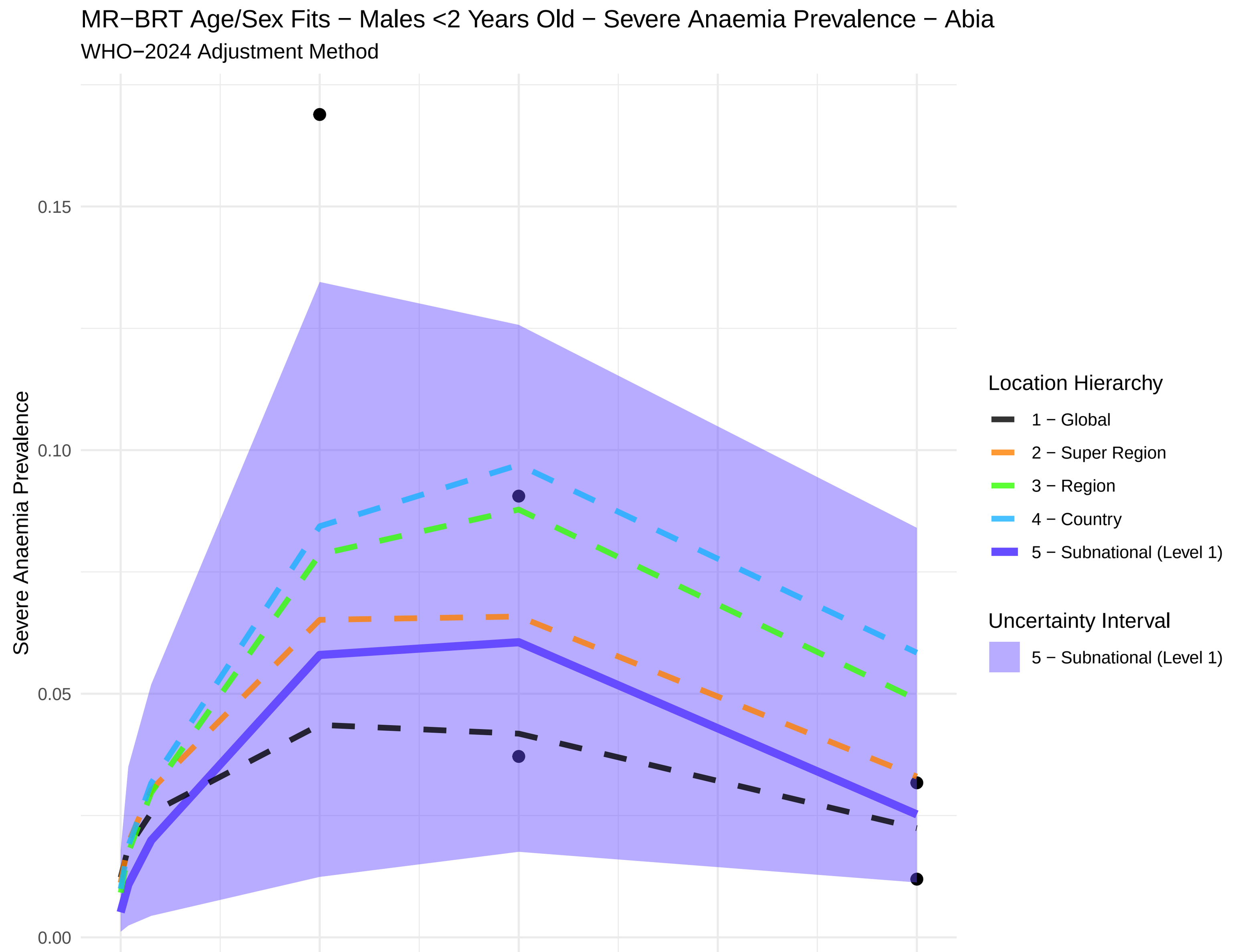
MR−BRT Age/Sex Fits − Males <2 Years Old − Severe Anaemia Prevalence − Abia
WHO−2024 Adjustment Method
Severe Anaemia Prevalence
Age (year)
Location Hierarchy
1 − Global
2 − Super Region
3 − Region
4 − Country
5 − Subnational (Level 1)
Uncertainty Interval
5 − Subnational (Level 1)

MR−BRT Age/Sex Fits − Males <2 Years Old − Severe Anaemia Prevalence − Adamawa
WHO−2024 Adjustment Method

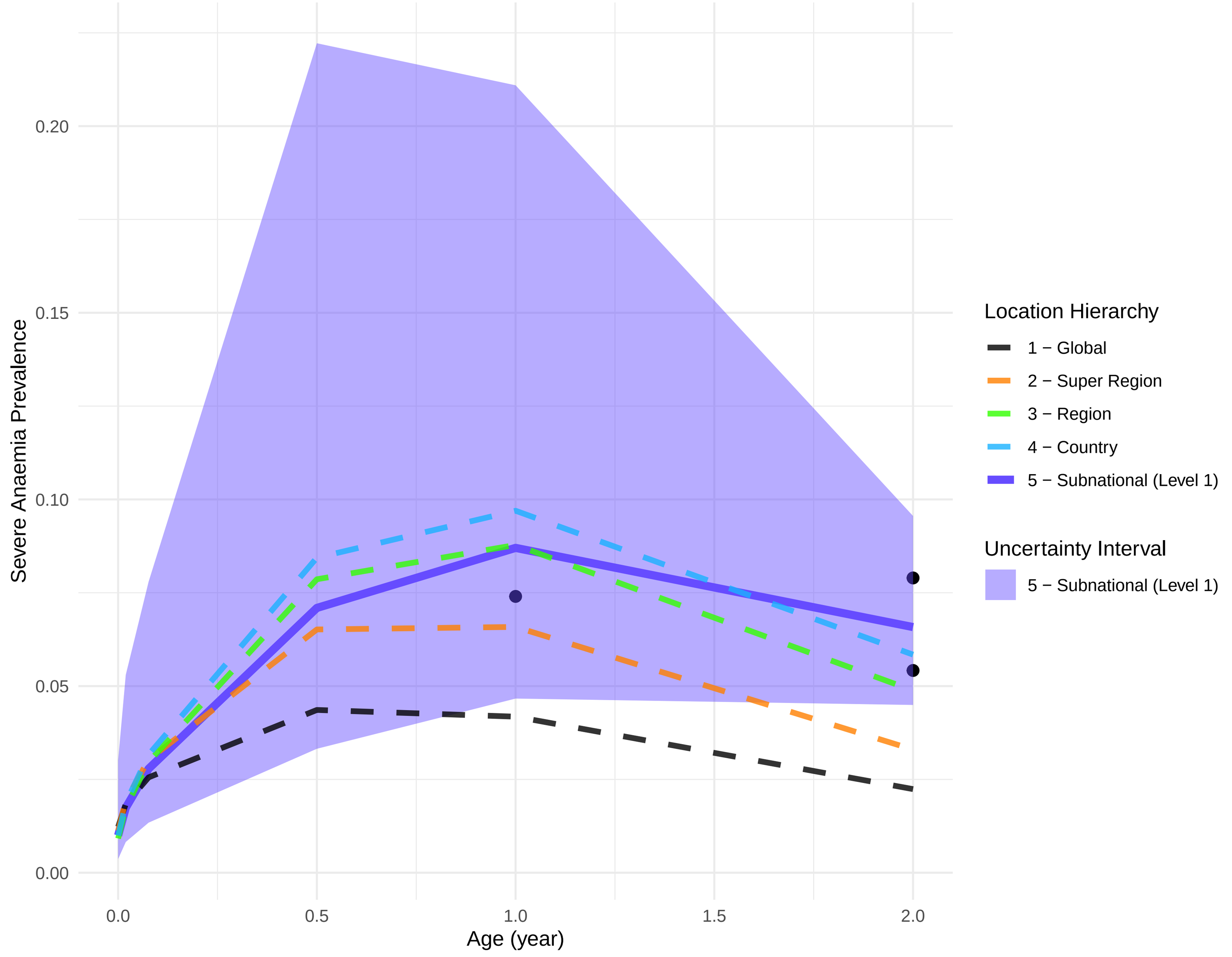

MR−BRT Age/Sex Fits − Males <2 Years Old − Severe Anaemia Prevalence − Akwa Ibom
WHO−2024 Adjustment Method

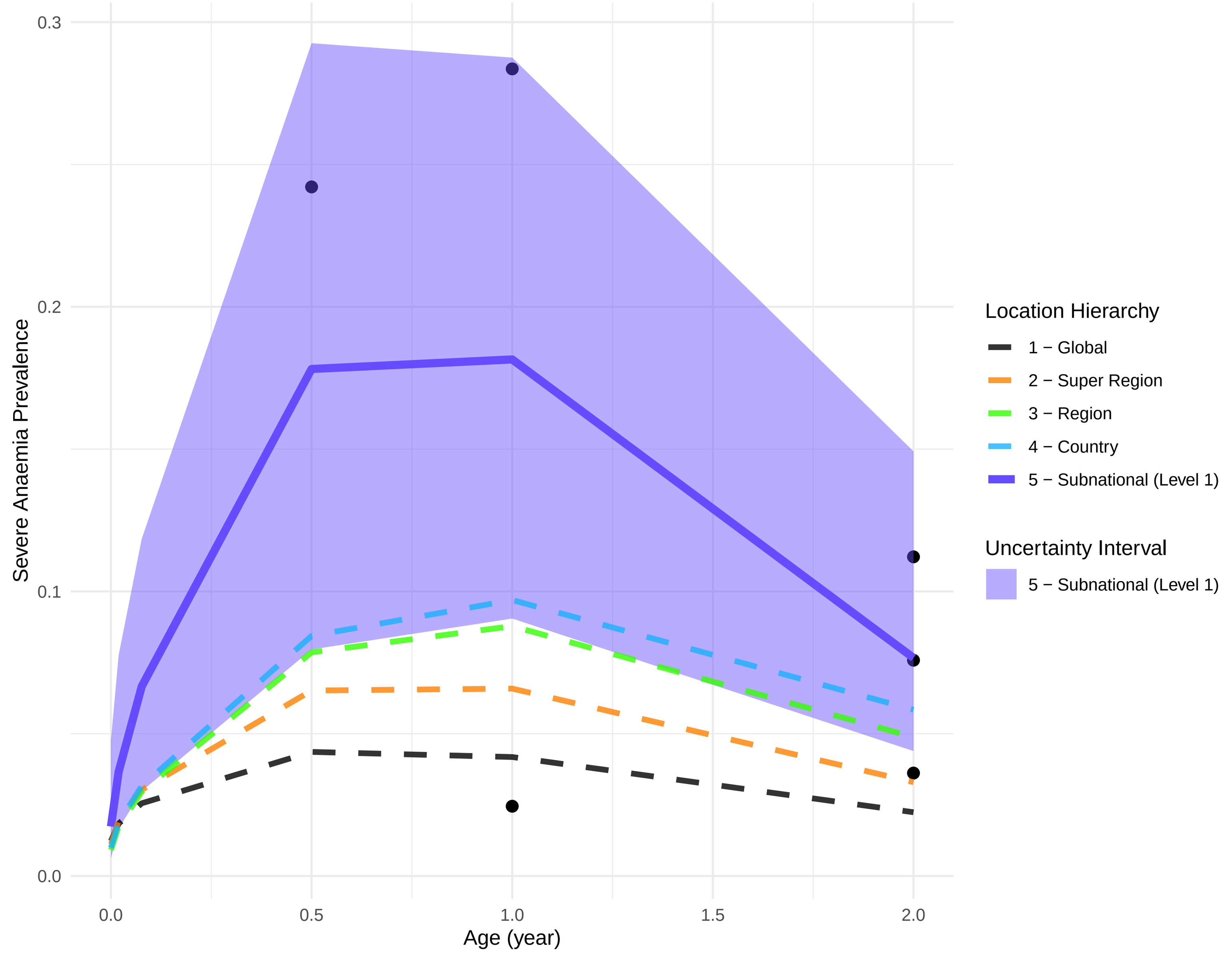

MR−BRT Age/Sex Fits − Males <2 Years Old − Severe Anaemia Prevalence − Anambra
WHO−2024 Adjustment Method

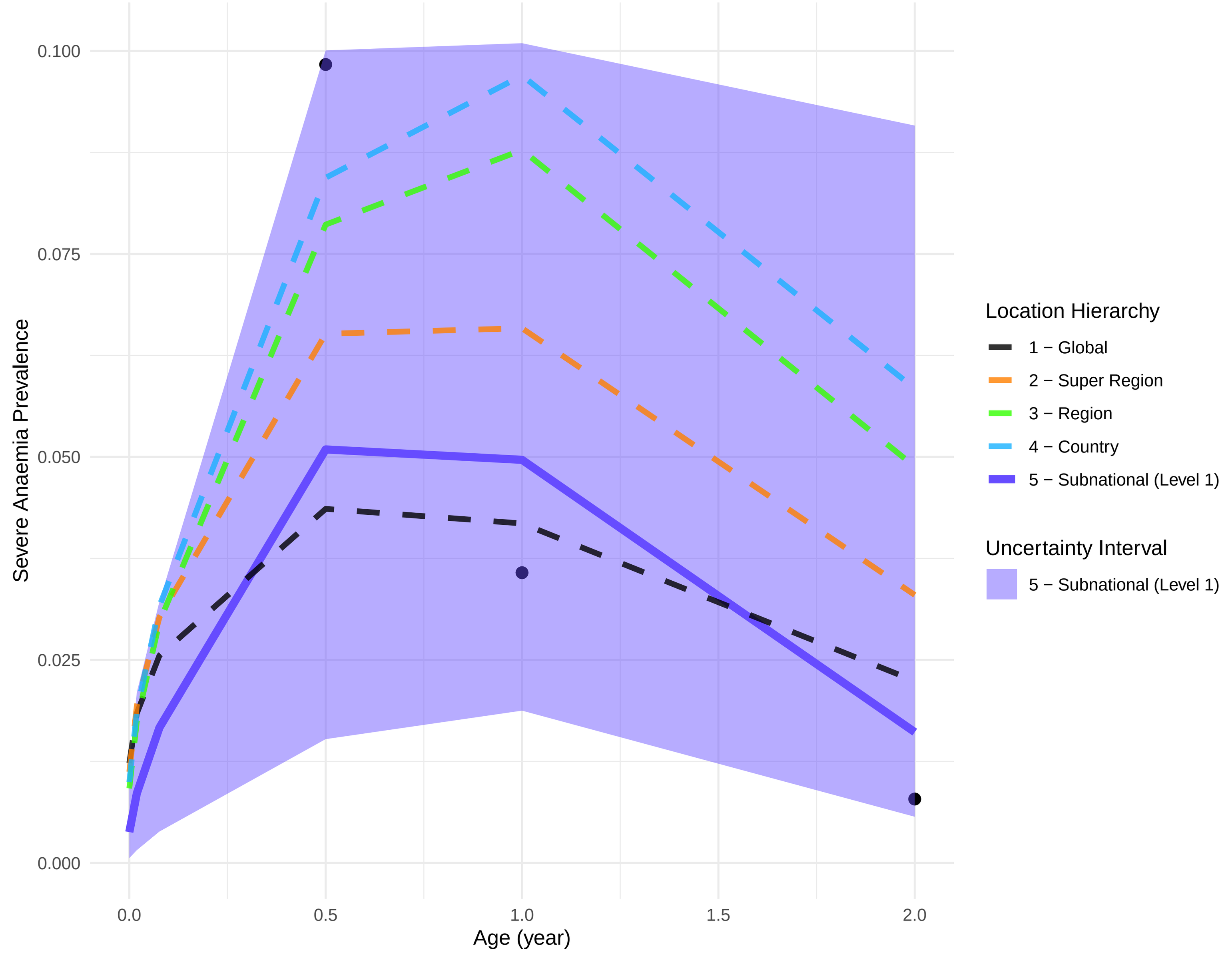

MR−BRT Age/Sex Fits − Males <2 Years Old − Severe Anaemia Prevalence − Bauchi
WHO−2024 Adjustment Method

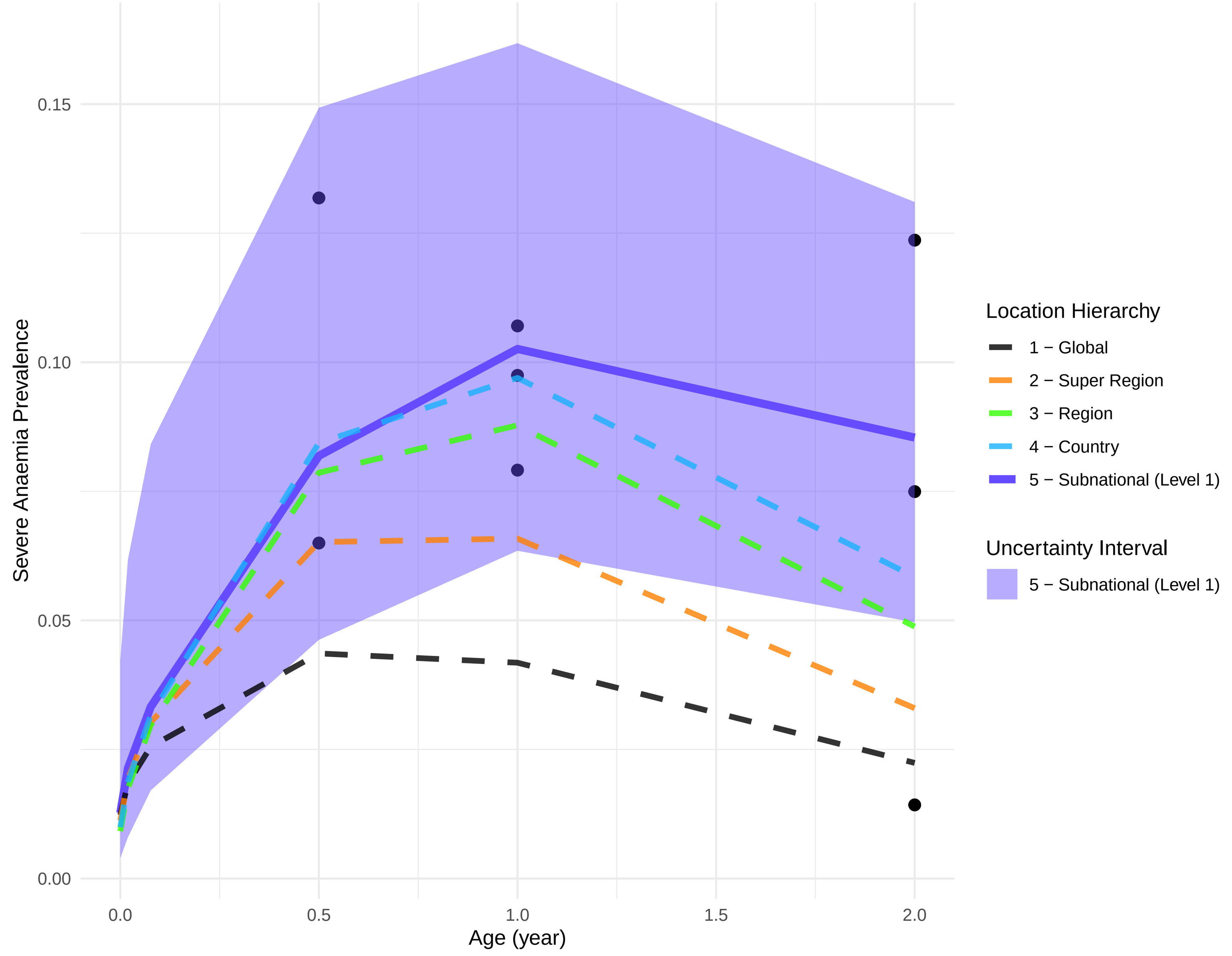

MR−BRT Age/Sex Fits − Males <2 Years Old − Severe Anaemia Prevalence − Bayelsa
WHO−2024 Adjustment Method

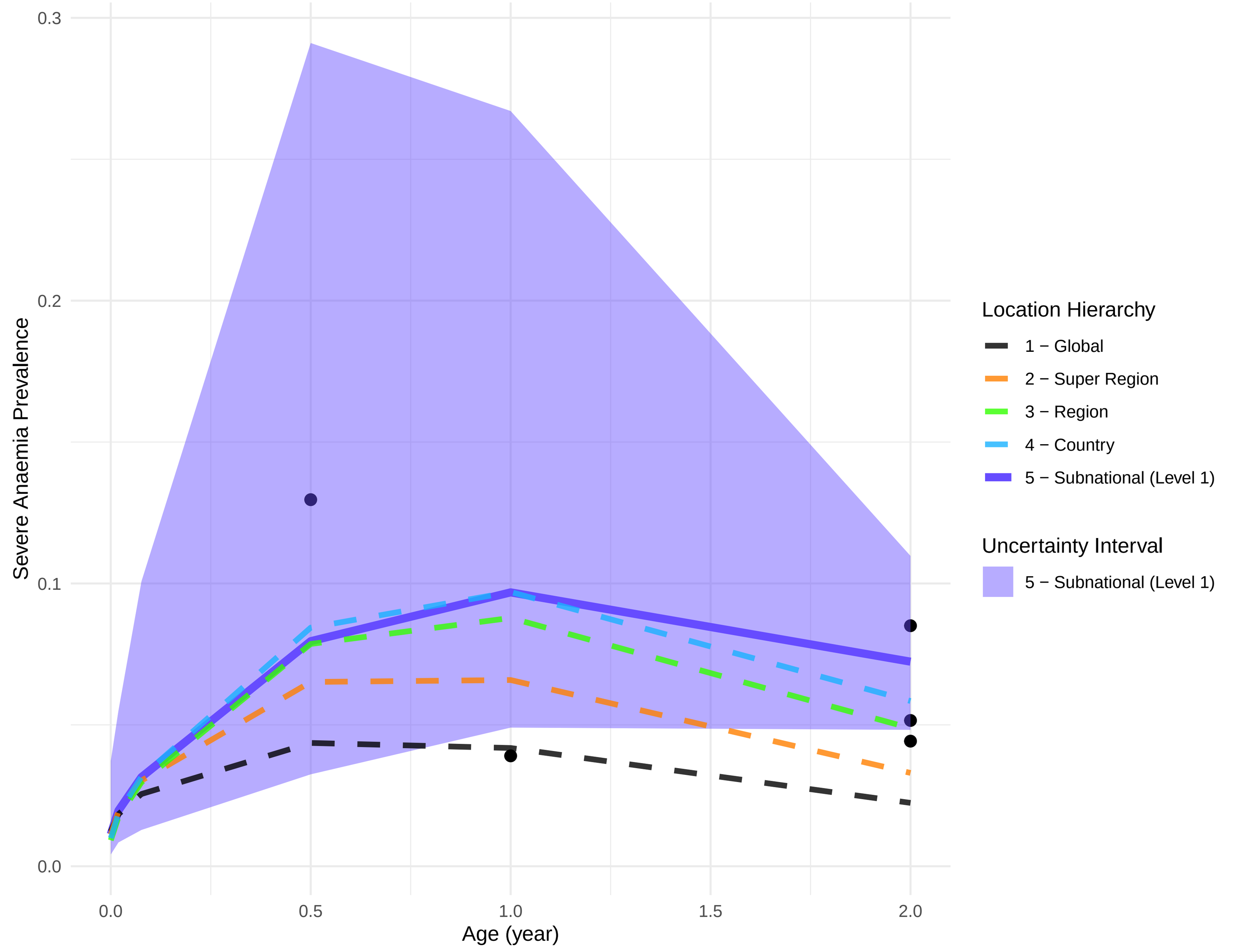

MR−BRT Age/Sex Fits − Males <2 Years Old − Severe Anaemia Prevalence − Benue
WHO−2024 Adjustment Method

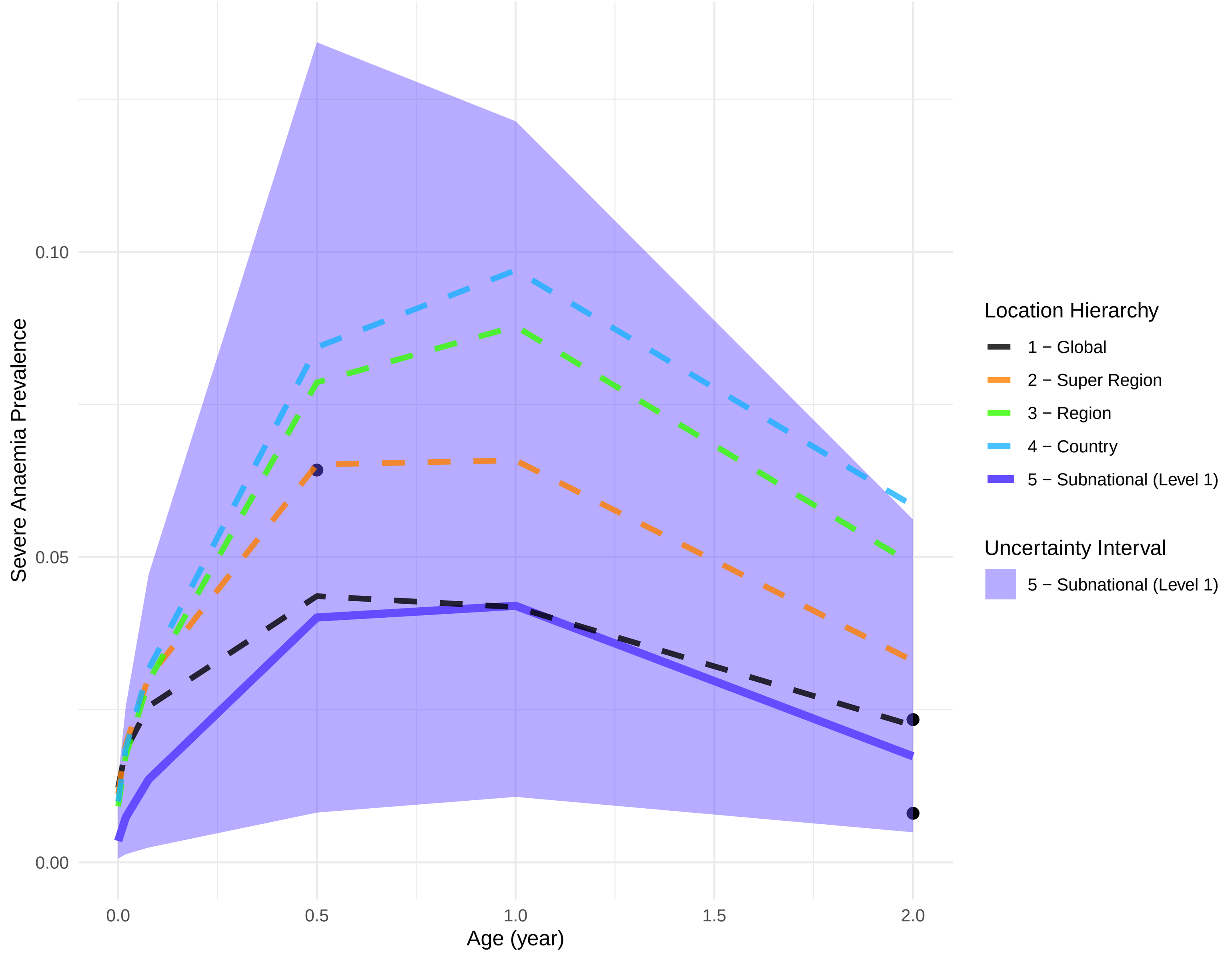

MR−BRT Age/Sex Fits − Males <2 Years Old − Severe Anaemia Prevalence − Borno
WHO−2024 Adjustment Method

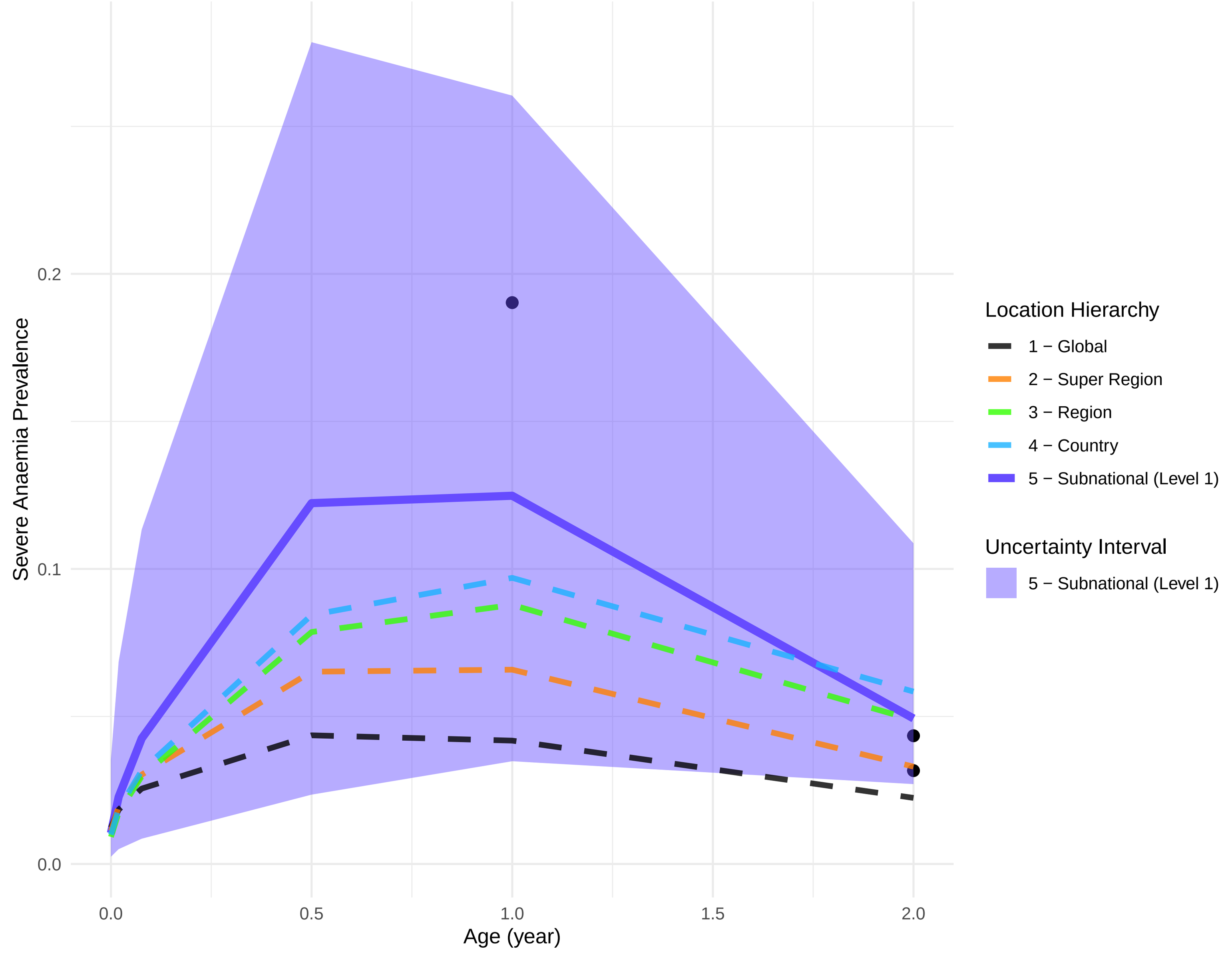

MR−BRT Age/Sex Fits − Males <2 Years Old − Severe Anaemia Prevalence − Cross River
WHO−2024 Adjustment Method

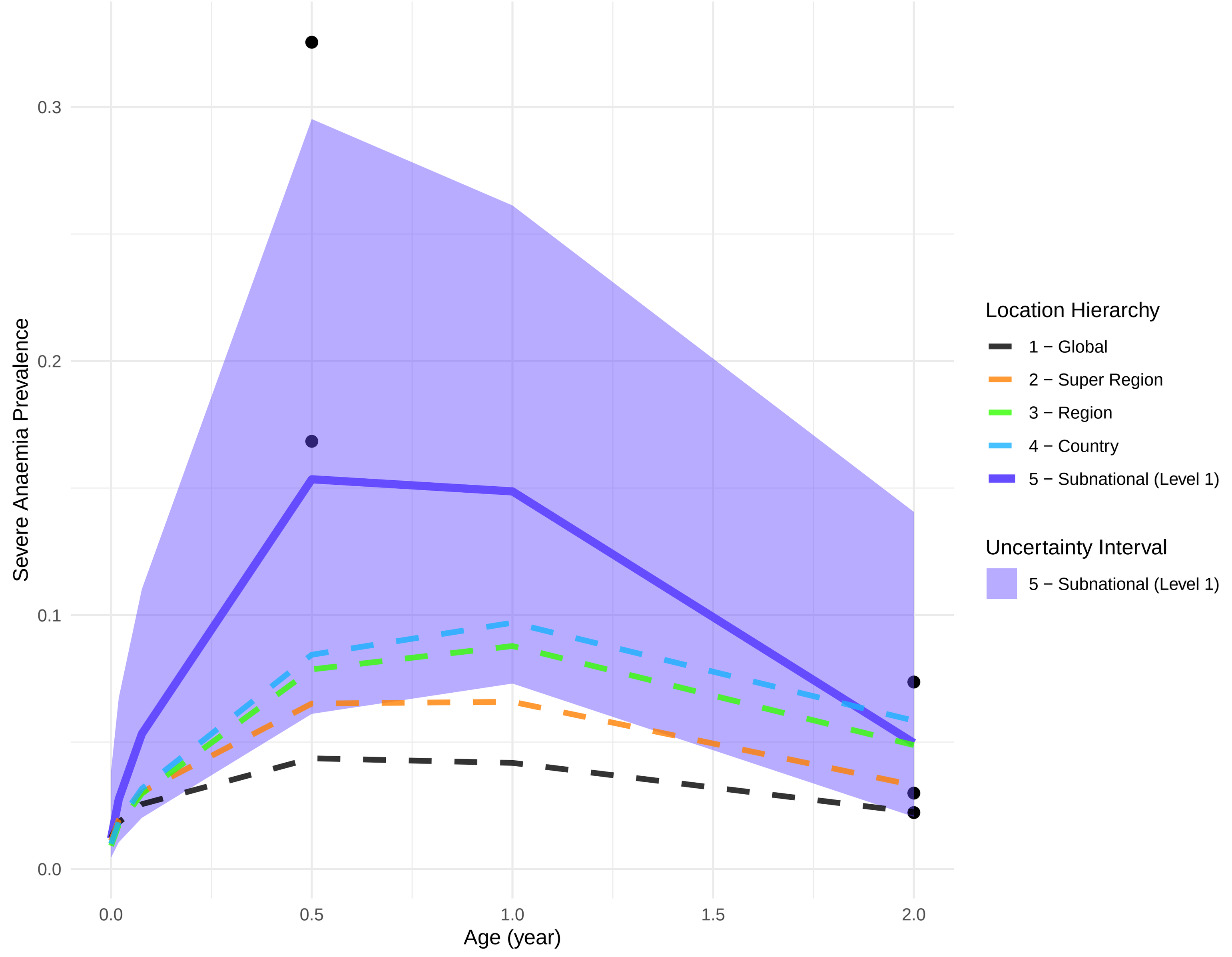

MR−BRT Age/Sex Fits − Males <2 Years Old − Severe Anaemia Prevalence − Delta
WHO−2024 Adjustment Method

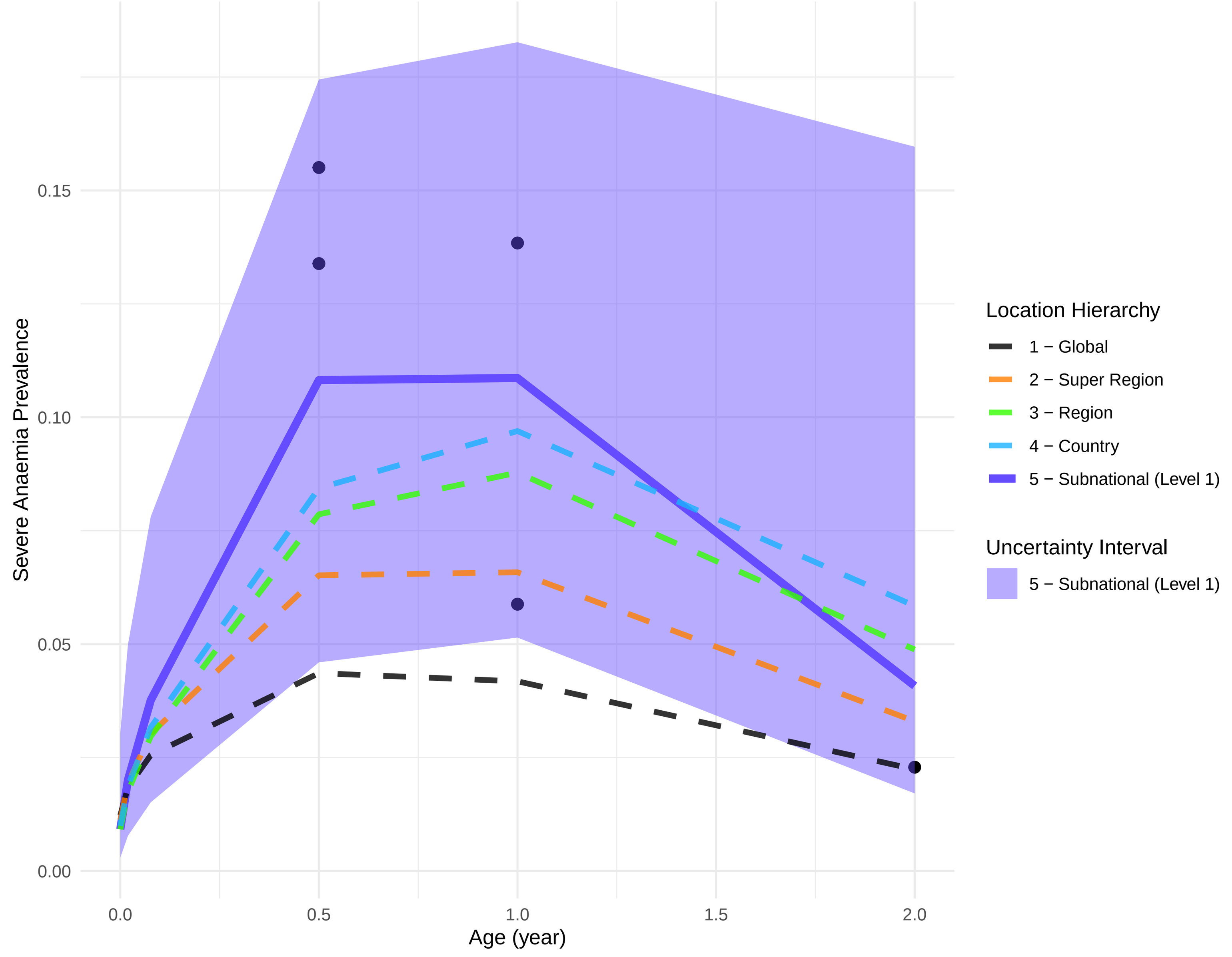

MR−BRT Age/Sex Fits − Males <2 Years Old − Severe Anaemia Prevalence − Ebonyi
WHO−2024 Adjustment Method

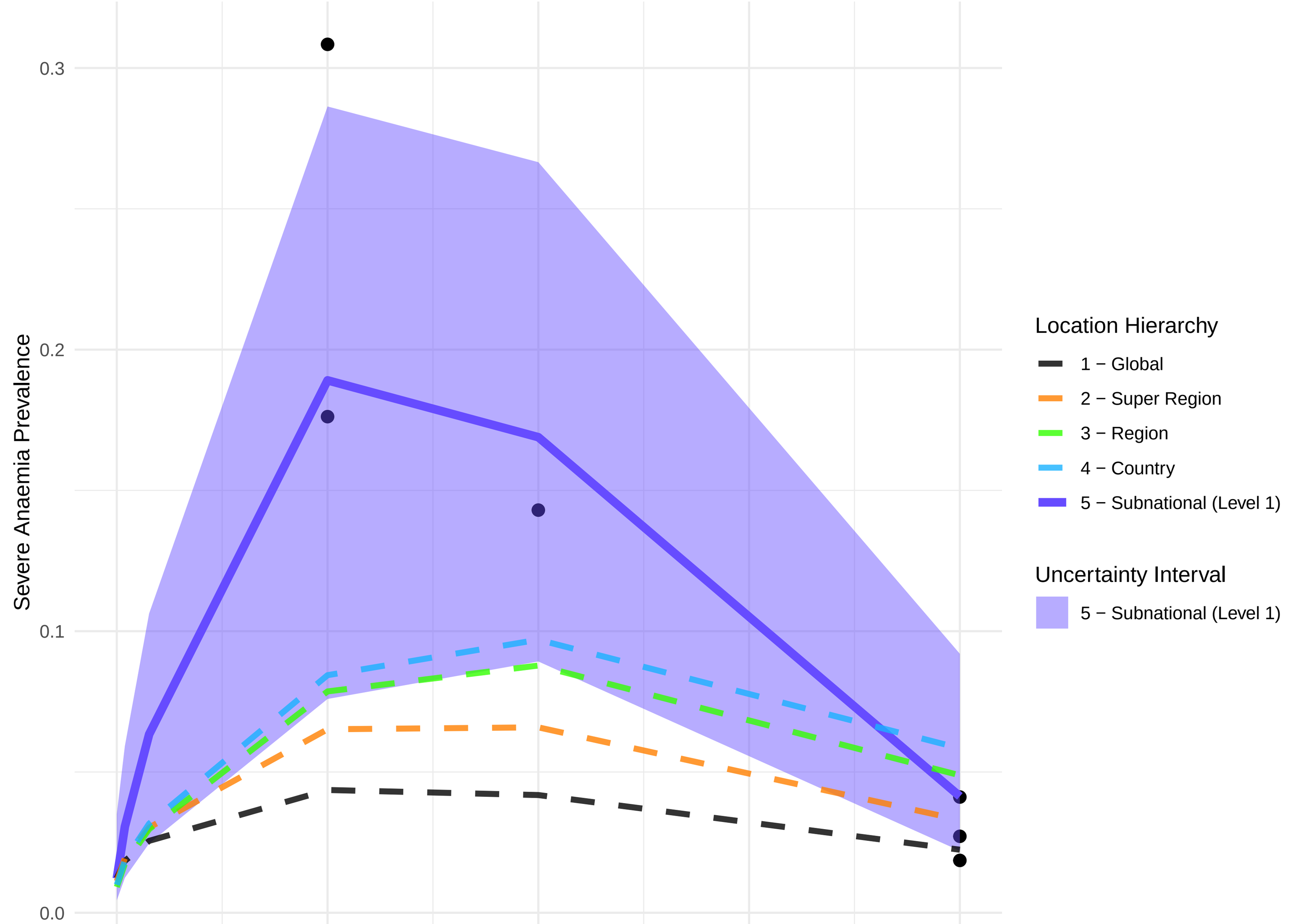

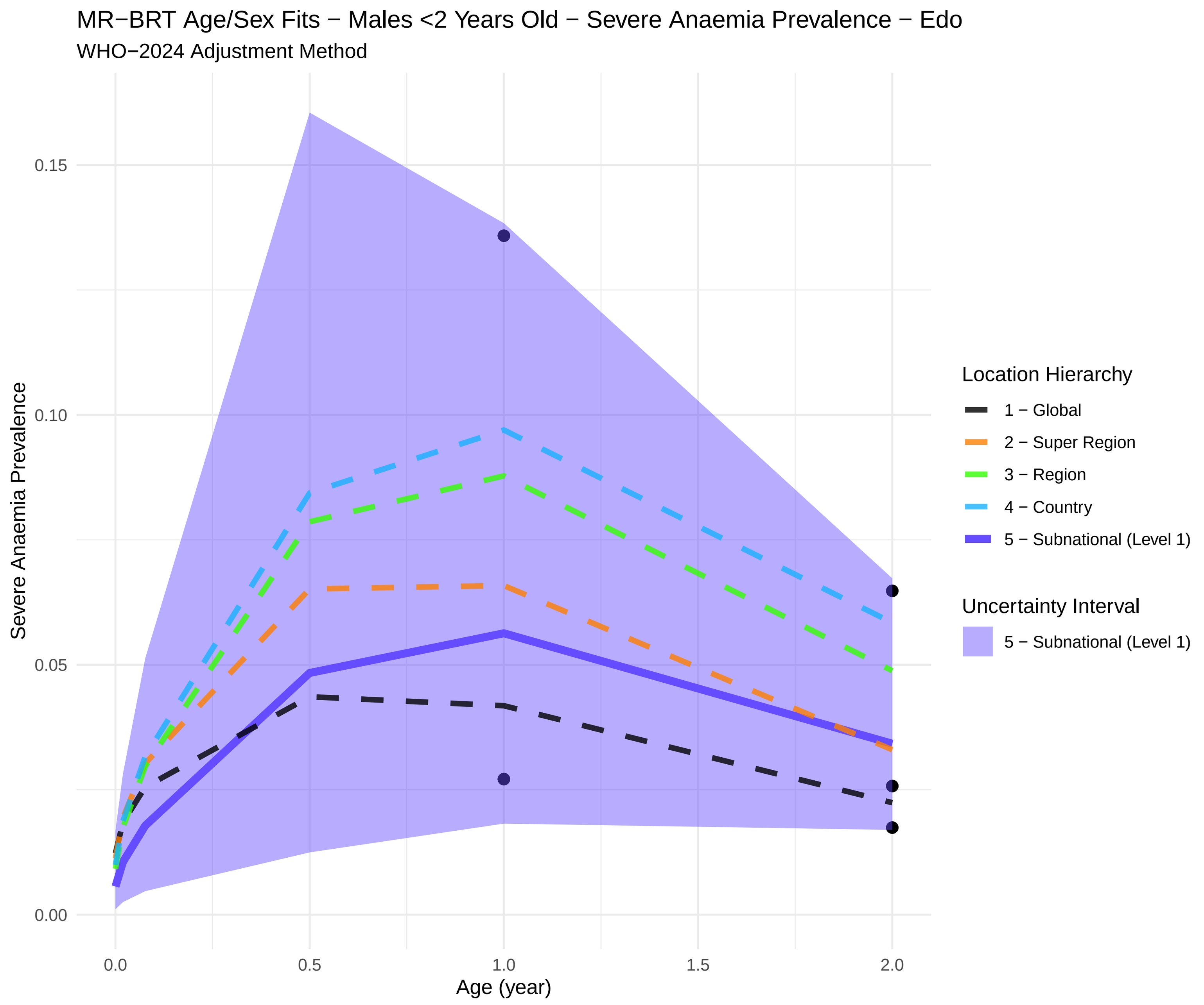

MR−BRT Age/Sex Fits − Males <2 Years Old − Severe Anaemia Prevalence − Edo
WHO−2024 Adjustment Method
Severe Anaemia Prevalence
Age (year)
Location Hierarchy
1 − Global
2 − Super Region
3 − Region
4 − Country
5 − Subnational (Level 1)
Uncertainty Interval
5 − Subnational (Level 1)

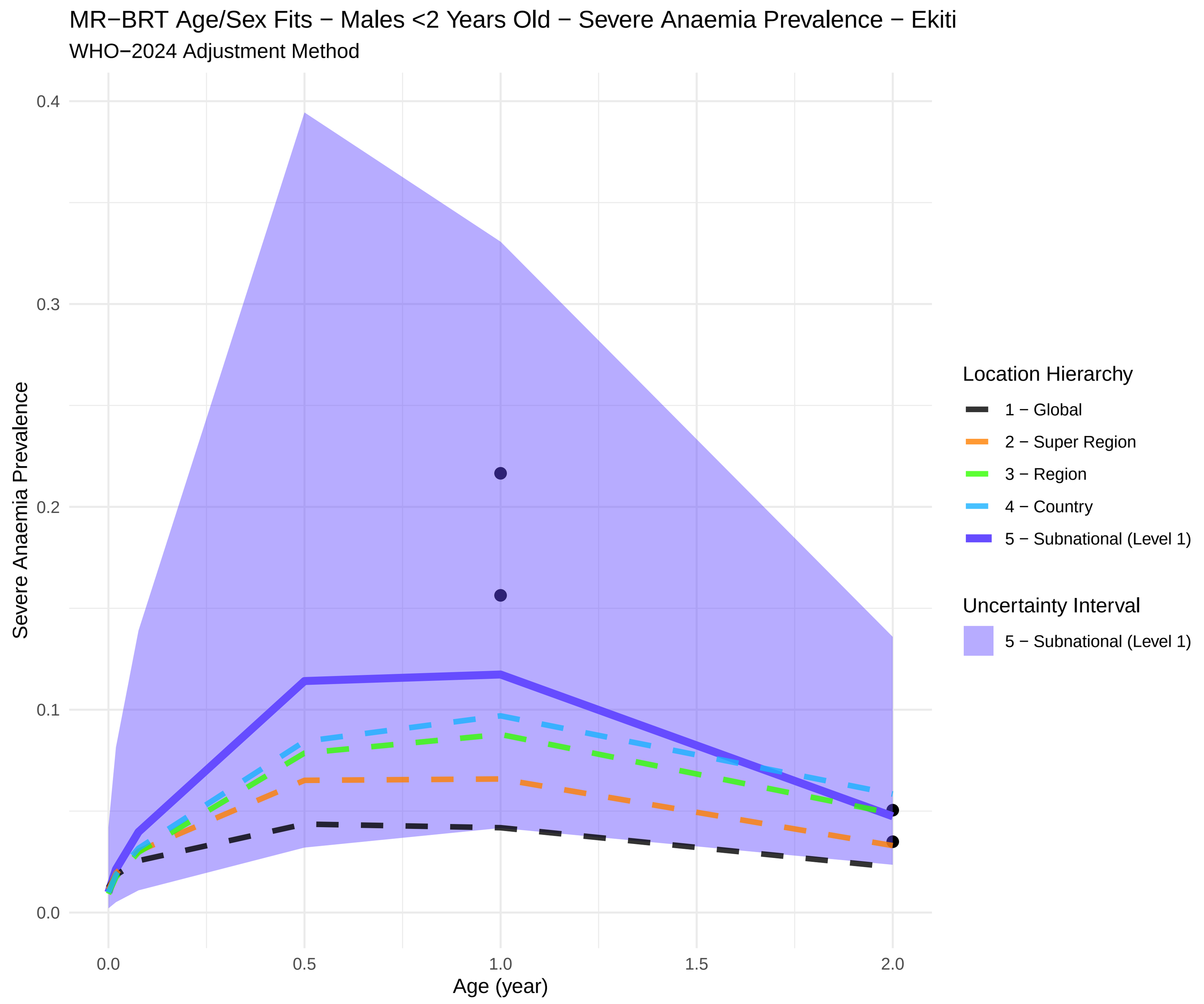

MR−BRT Age/Sex Fits − Males <2 Years Old − Severe Anaemia Prevalence − Ekiti
WHO−2024 Adjustment Method
Severe Anaemia Prevalence
Age (year)
Location Hierarchy
1 − Global
2 − Super Region
3 − Region
4 − Country
5 − Subnational (Level 1)
Uncertainty Interval
5 − Subnational (Level 1)

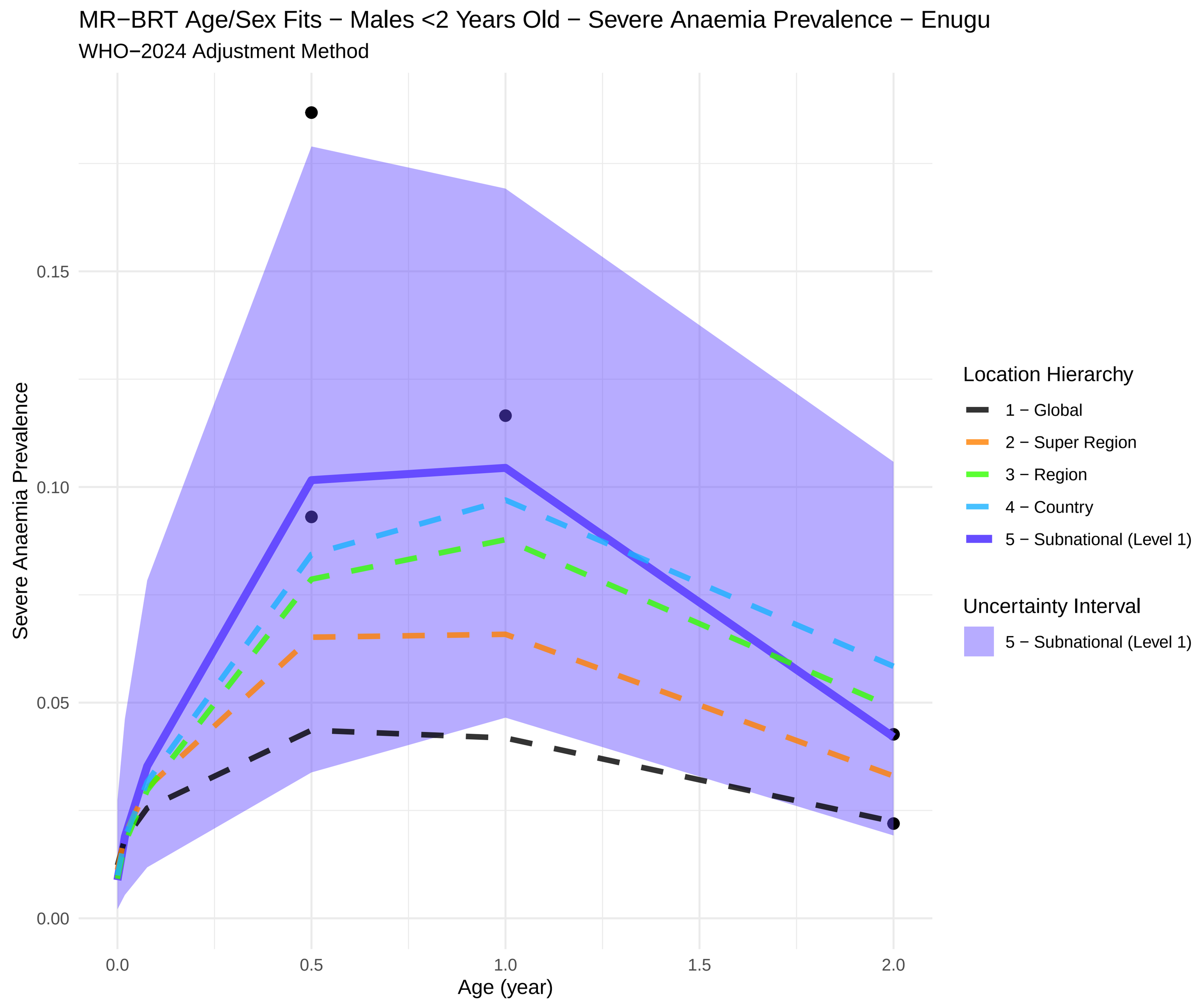

MR−BRT Age/Sex Fits − Males <2 Years Old − Severe Anaemia Prevalence − Enugu
WHO−2024 Adjustment Method
Severe Anaemia Prevalence
Age (year)
Location Hierarchy
1 − Global
2 − Super Region
3 − Region
4 − Country
5 − Subnational (Level 1)
Uncertainty Interval
5 − Subnational (Level 1)

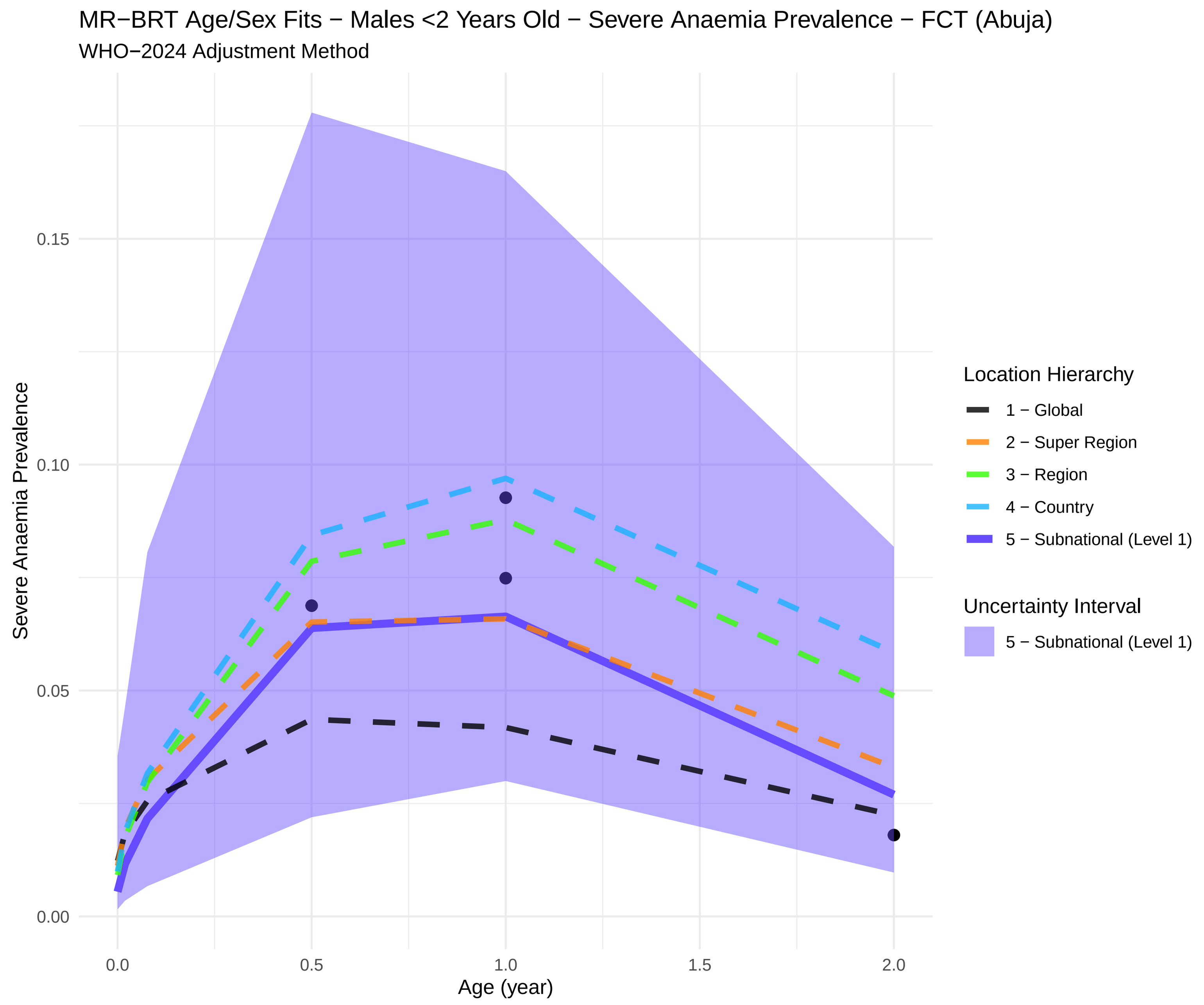

MR−BRT Age/Sex Fits − Males <2 Years Old − Severe Anaemia Prevalence − FCT (Abuja)
WHO−2024 Adjustment Method
Severe Anaemia Prevalence
Age (year)
Location Hierarchy
1 − Global
2 − Super Region
3 − Region
4 − Country
5 − Subnational (Level 1)
Uncertainty Interval
5 − Subnational (Level 1)

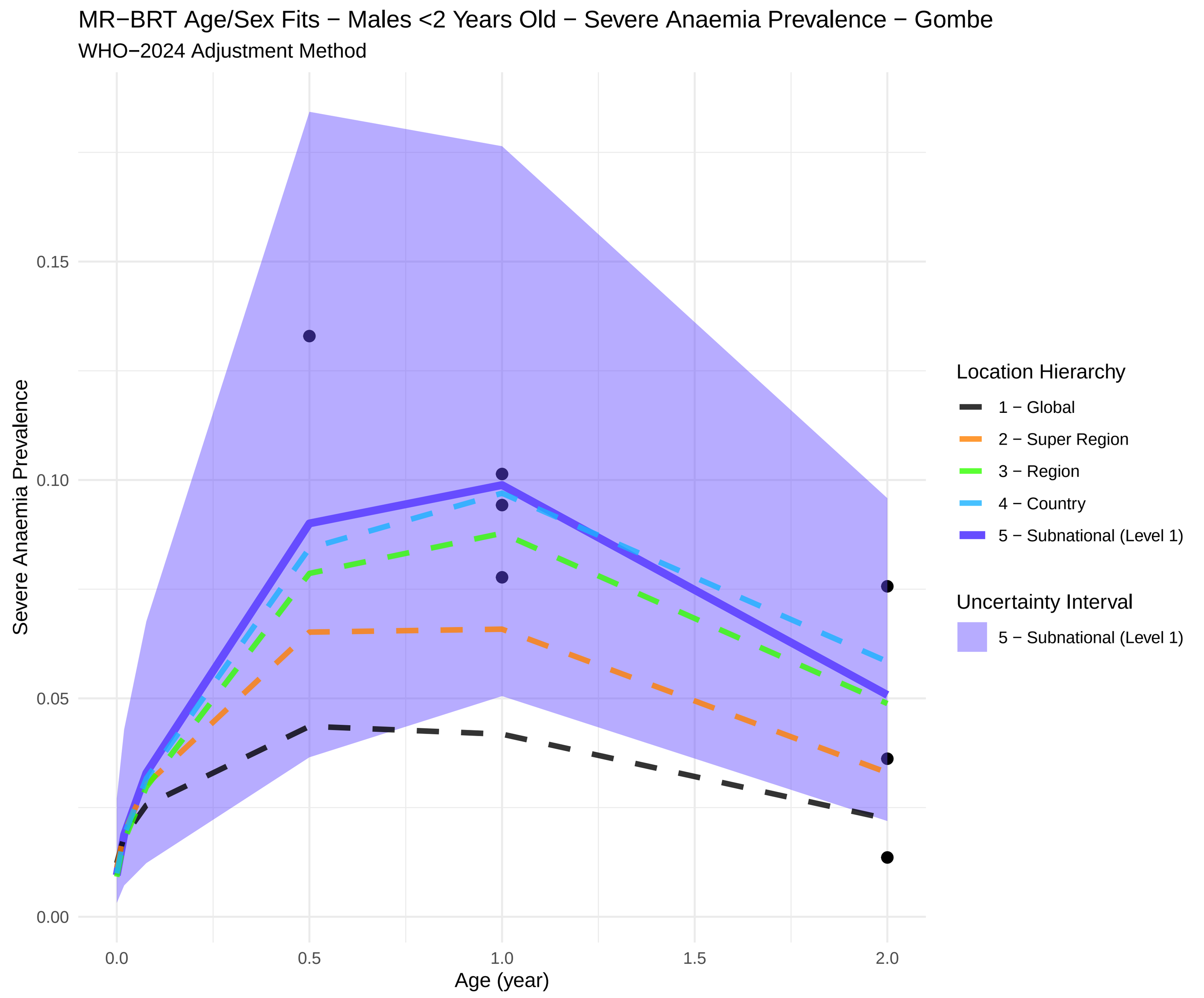

MR−BRT Age/Sex Fits − Males <2 Years Old − Severe Anaemia Prevalence − Gombe
WHO−2024 Adjustment Method
Severe Anaemia Prevalence
Age (year)
Location Hierarchy
1 − Global
2 − Super Region
3 − Region
4 − Country
5 − Subnational (Level 1)
Uncertainty Interval
5 − Subnational (Level 1)

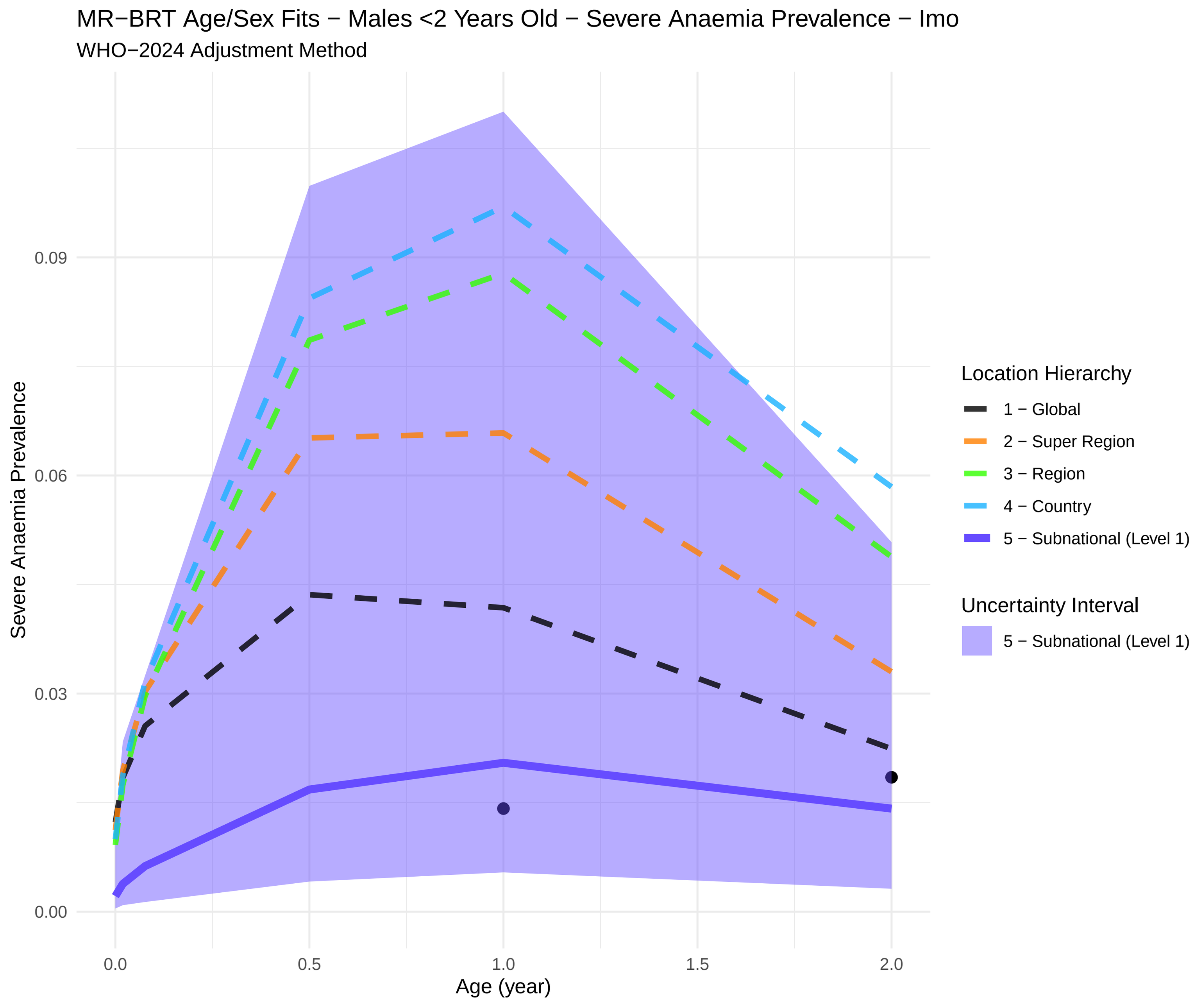

MR−BRT Age/Sex Fits − Males <2 Years Old − Severe Anaemia Prevalence − Imo
WHO−2024 Adjustment Method
Severe Anaemia Prevalence
Age (year)
Location Hierarchy
1 − Global
2 − Super Region
3 − Region
4 − Country
5 − Subnational (Level 1)
Uncertainty Interval
5 − Subnational (Level 1)

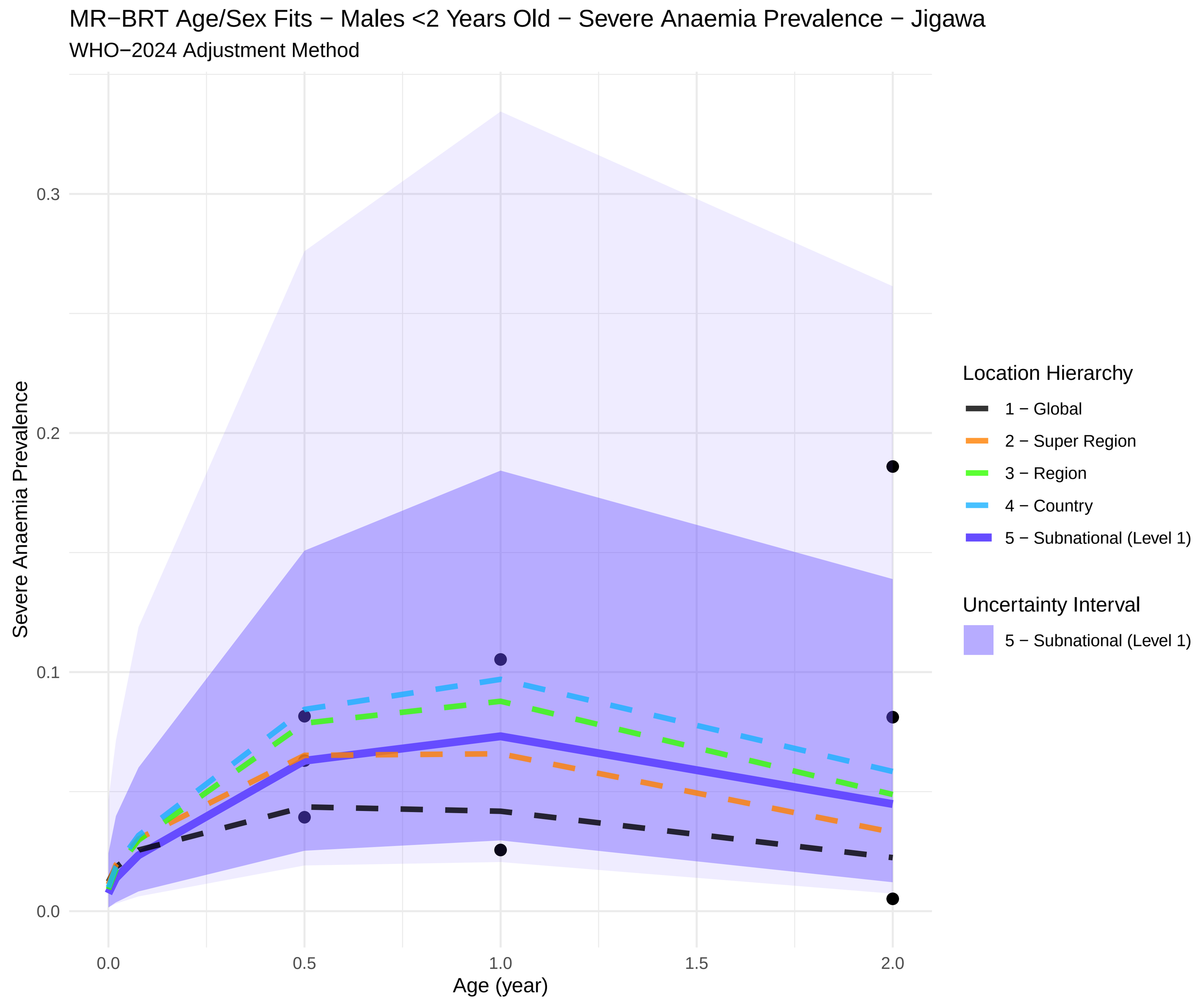

MR−BRT Age/Sex Fits − Males <2 Years Old − Severe Anaemia Prevalence − Jigawa
WHO−2024 Adjustment Method
Severe Anaemia Prevalence
Age (year)
Location Hierarchy
1 − Global
2 − Super Region
3 − Region
4 − Country
5 − Subnational (Level 1)
Uncertainty Interval
5 − Subnational (Level 1)

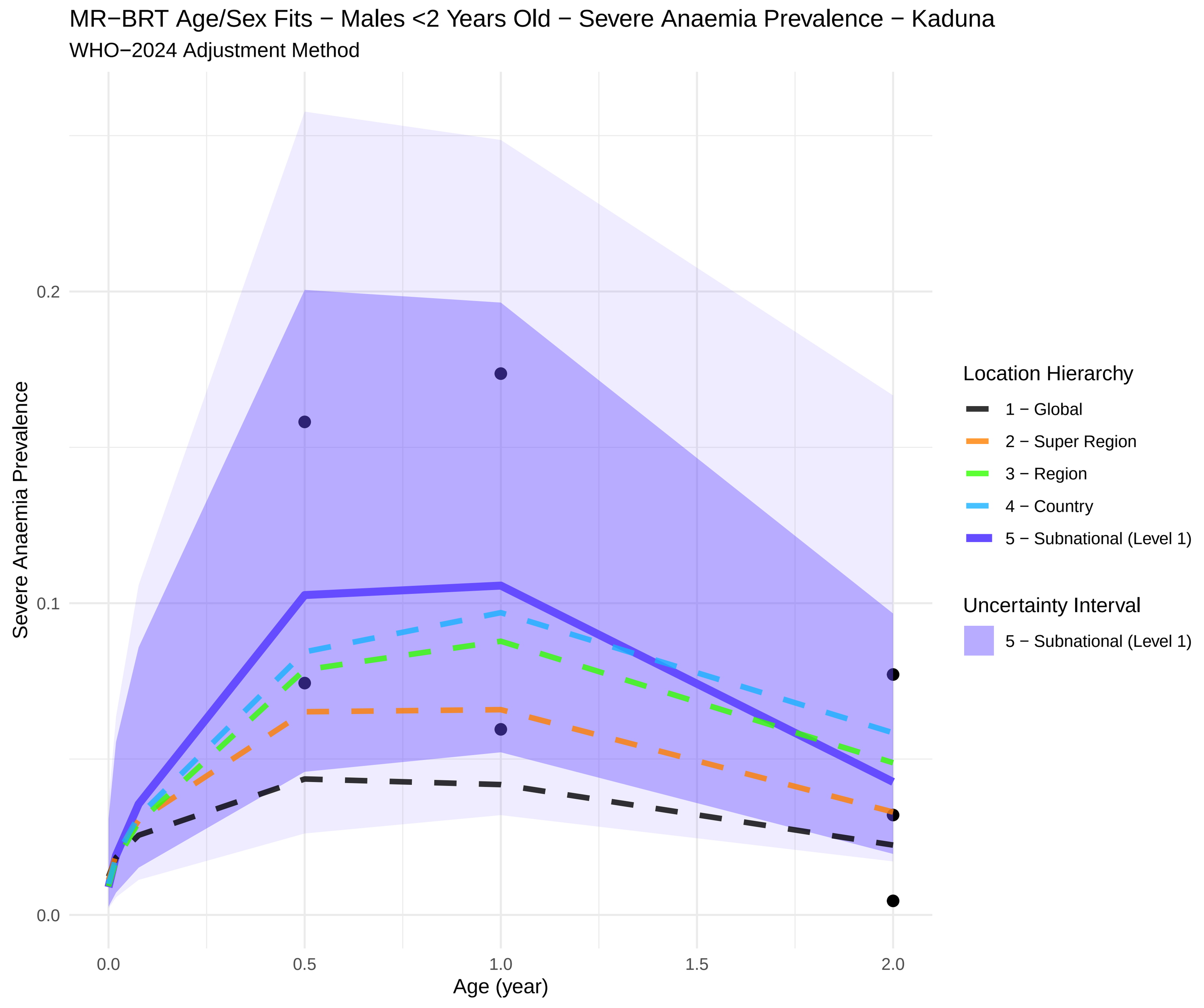

MR−BRT Age/Sex Fits − Males <2 Years Old − Severe Anaemia Prevalence − Kaduna
WHO−2024 Adjustment Method
Severe Anaemia Prevalence
Age (year)
Location Hierarchy
1 − Global
2 − Super Region
3 − Region
4 − Country
5 − Subnational (Level 1)
Uncertainty Interval
5 − Subnational (Level 1)

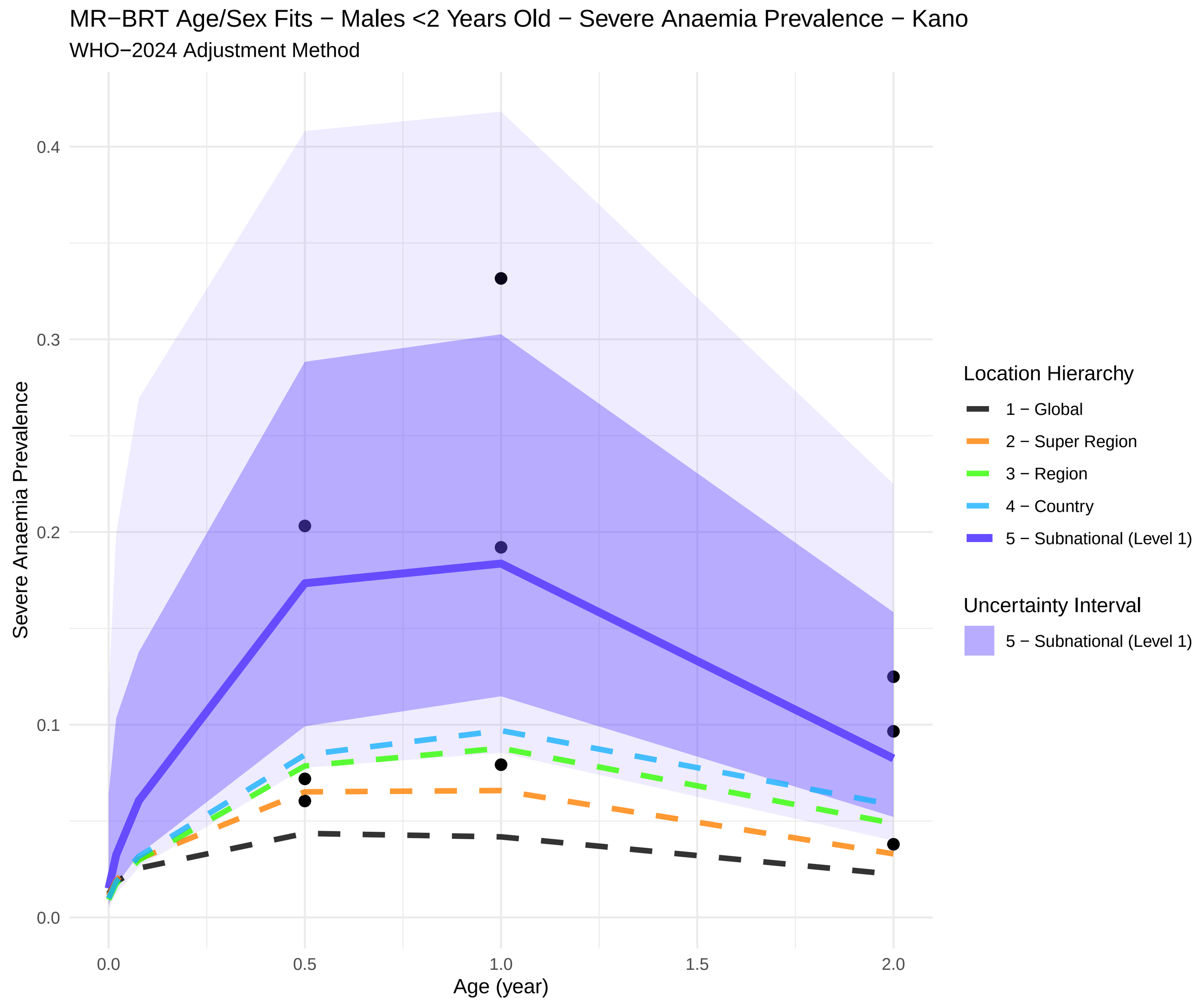

MR−BRT Age/Sex Fits − Males <2 Years Old − Severe Anaemia Prevalence − Kano
WHO−2024 Adjustment Method
Severe Anaemia Prevalence
Age (year)
Location Hierarchy
1 − Global
2 − Super Region
3 − Region
4 − Country
5 − Subnational (Level 1)
Uncertainty Interval
5 − Subnational (Level 1)

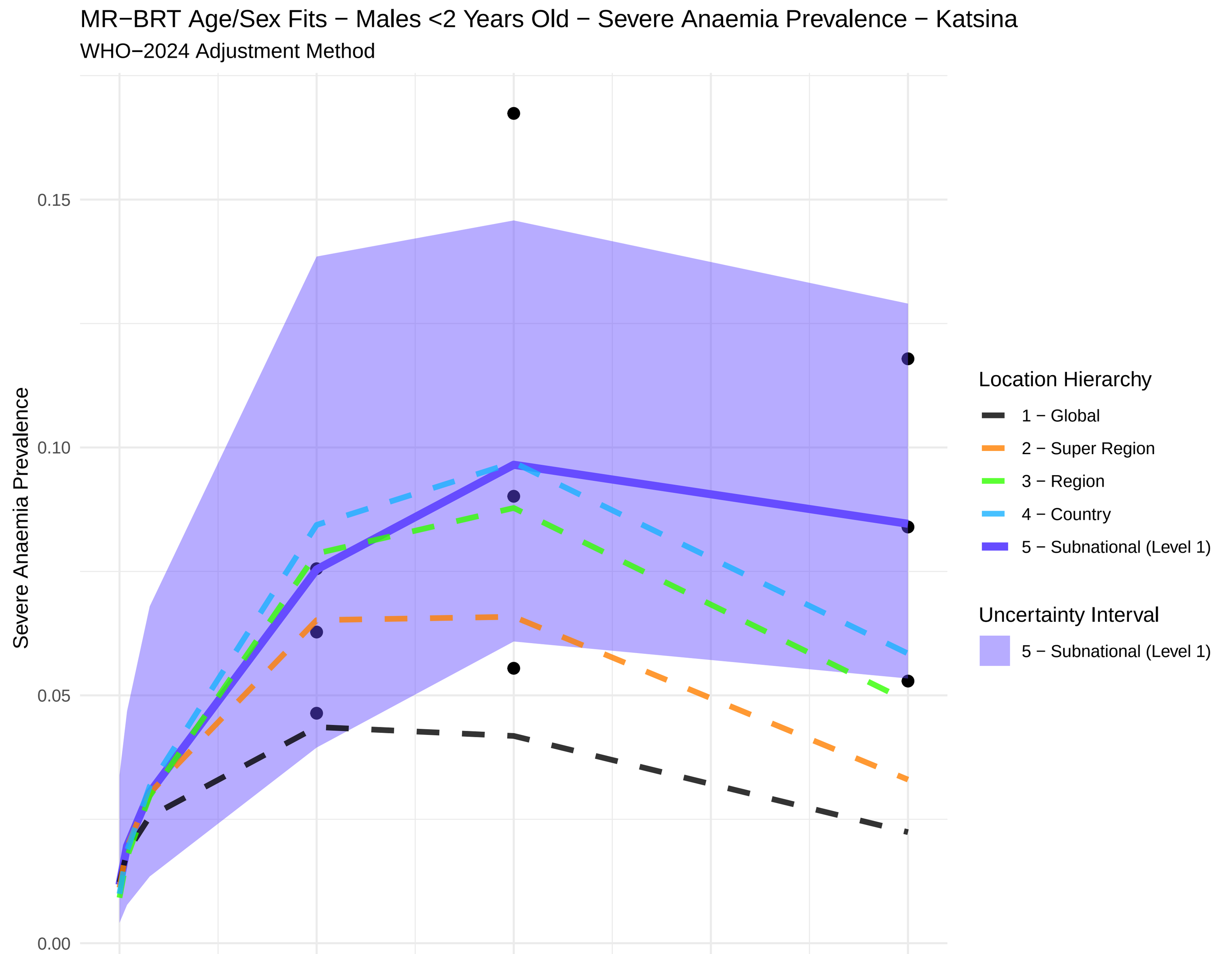

MR−BRT Age/Sex Fits − Males <2 Years Old − Severe Anaemia Prevalence − Katsina
WHO−2024 Adjustment Method
Severe Anaemia Prevalence
Age (year)
Location Hierarchy
1 − Global
2 − Super Region
3 − Region
4 − Country
5 − Subnational (Level 1)
Uncertainty Interval
5 − Subnational (Level 1)

MR−BRT Age/Sex Fits − Males <2 Years Old − Severe Anaemia Prevalence − Kebbi
WHO−2024 Adjustment Method

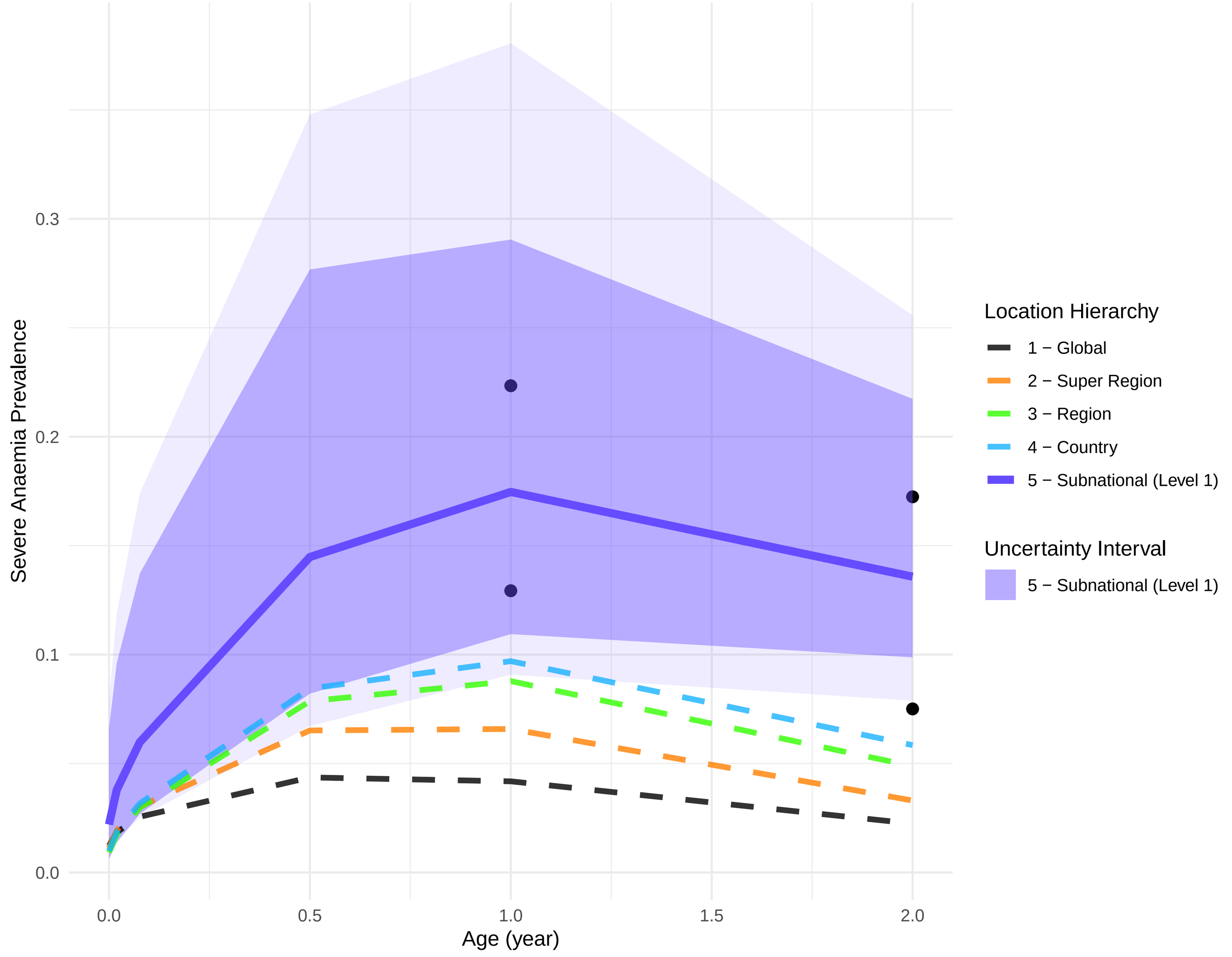

MR−BRT Age/Sex Fits − Males <2 Years Old − Severe Anaemia Prevalence − Kogi
WHO−2024 Adjustment Method

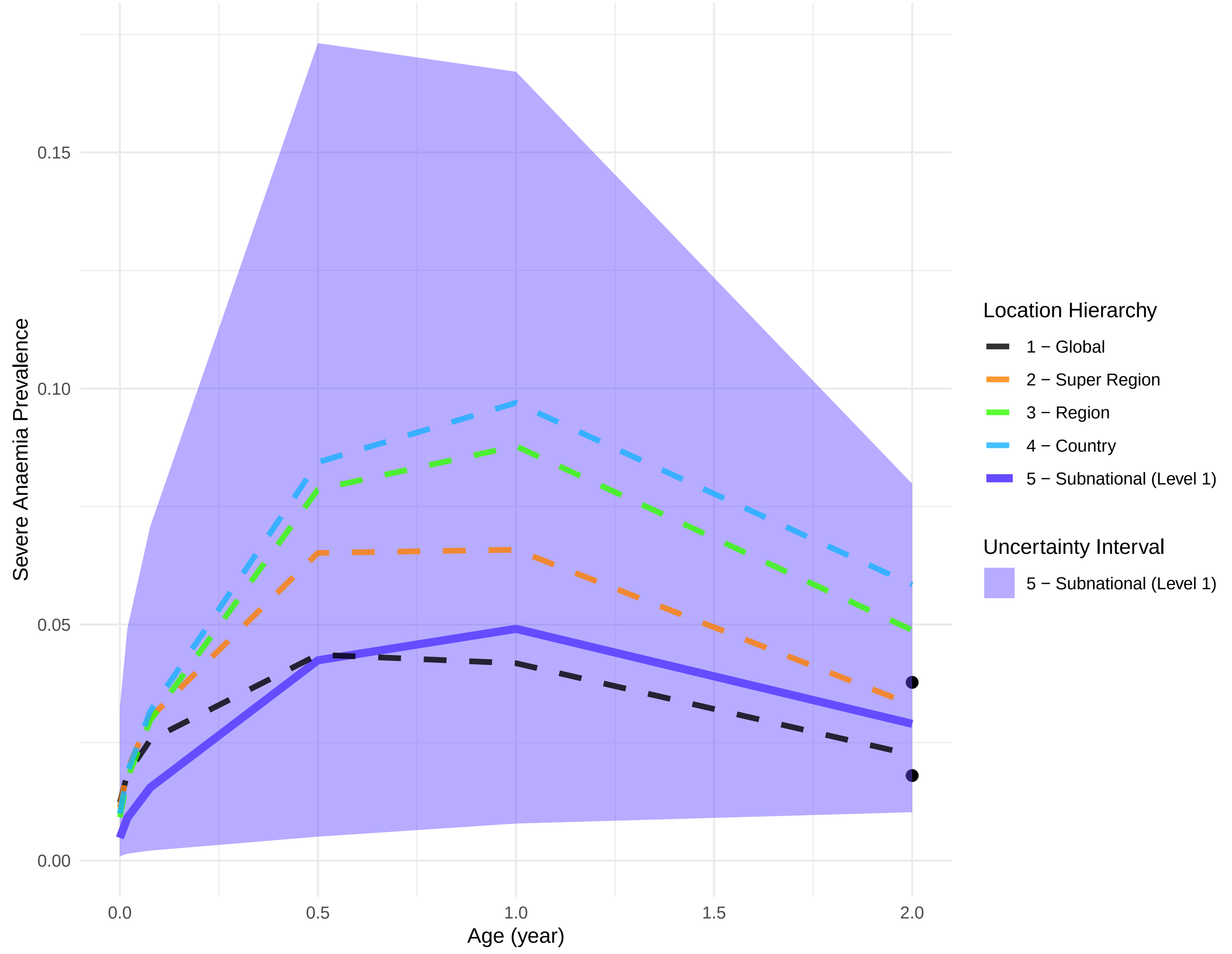

MR−BRT Age/Sex Fits − Males <2 Years Old − Severe Anaemia Prevalence − Kwara
WHO−2024 Adjustment Method

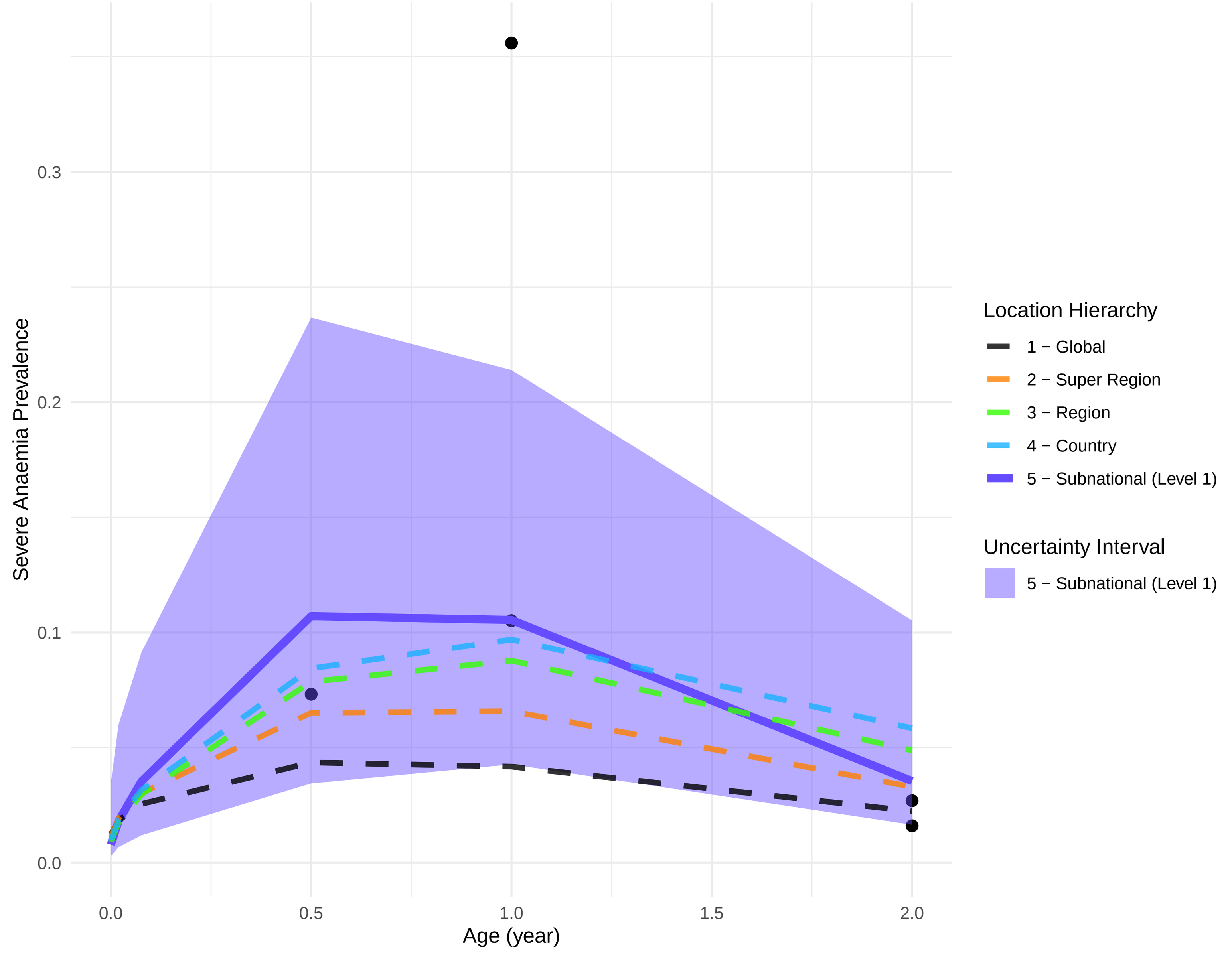

MR−BRT Age/Sex Fits − Males <2 Years Old − Severe Anaemia Prevalence − Lagos
WHO−2024 Adjustment Method

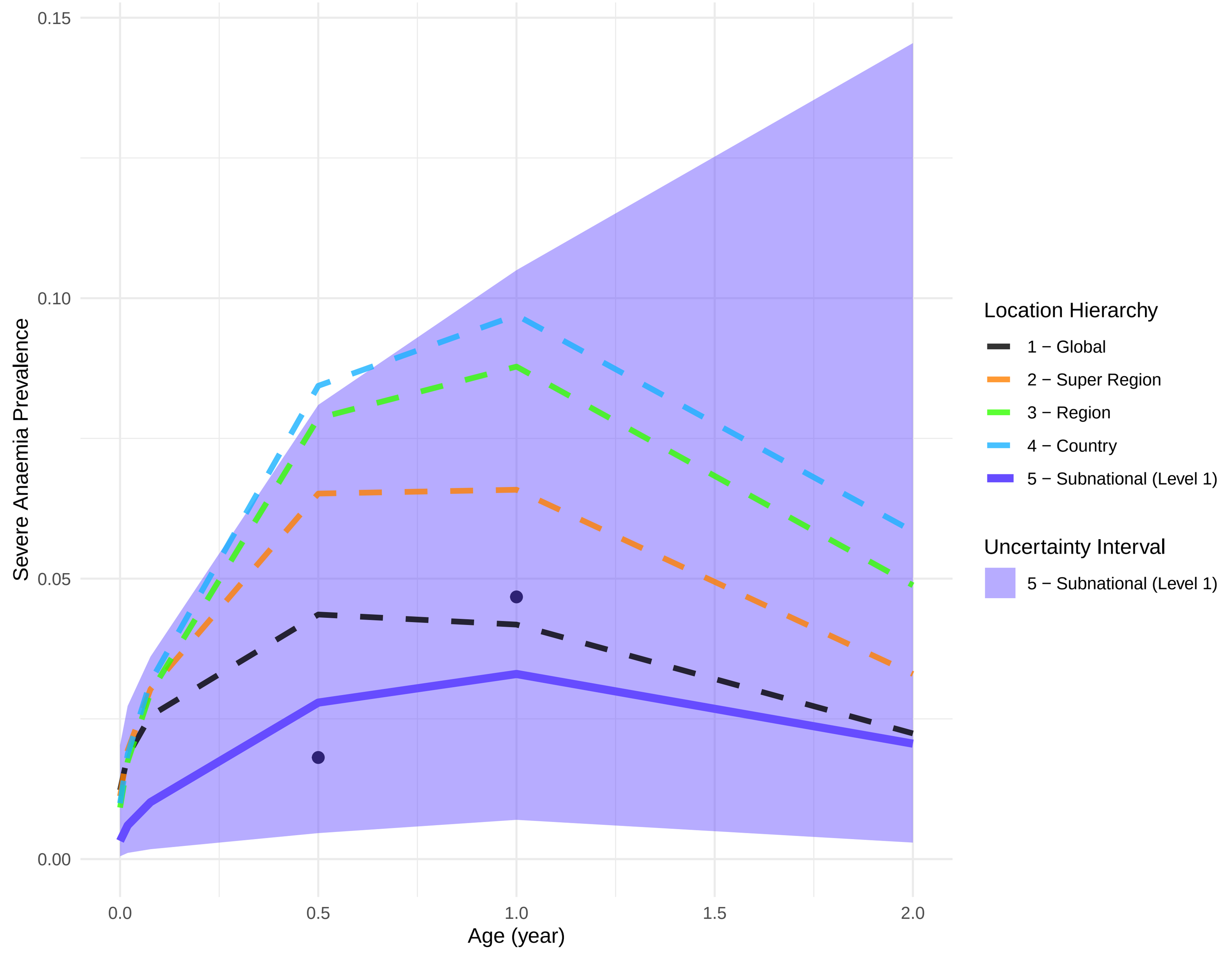

MR−BRT Age/Sex Fits − Males <2 Years Old − Severe Anaemia Prevalence − Nasarawa
WHO−2024 Adjustment Method

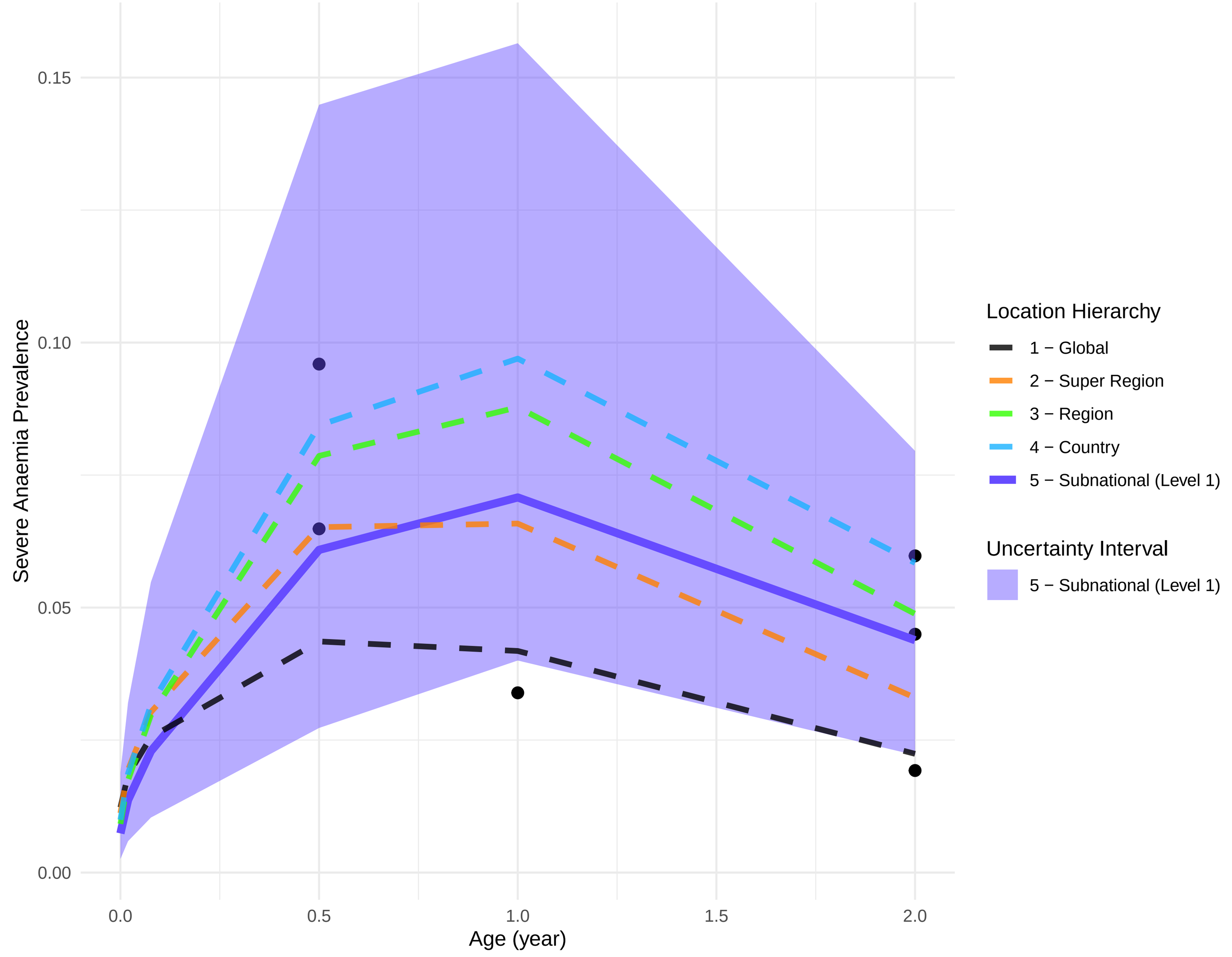

MR−BRT Age/Sex Fits − Males <2 Years Old − Severe Anaemia Prevalence − Niger
WHO−2024 Adjustment Method

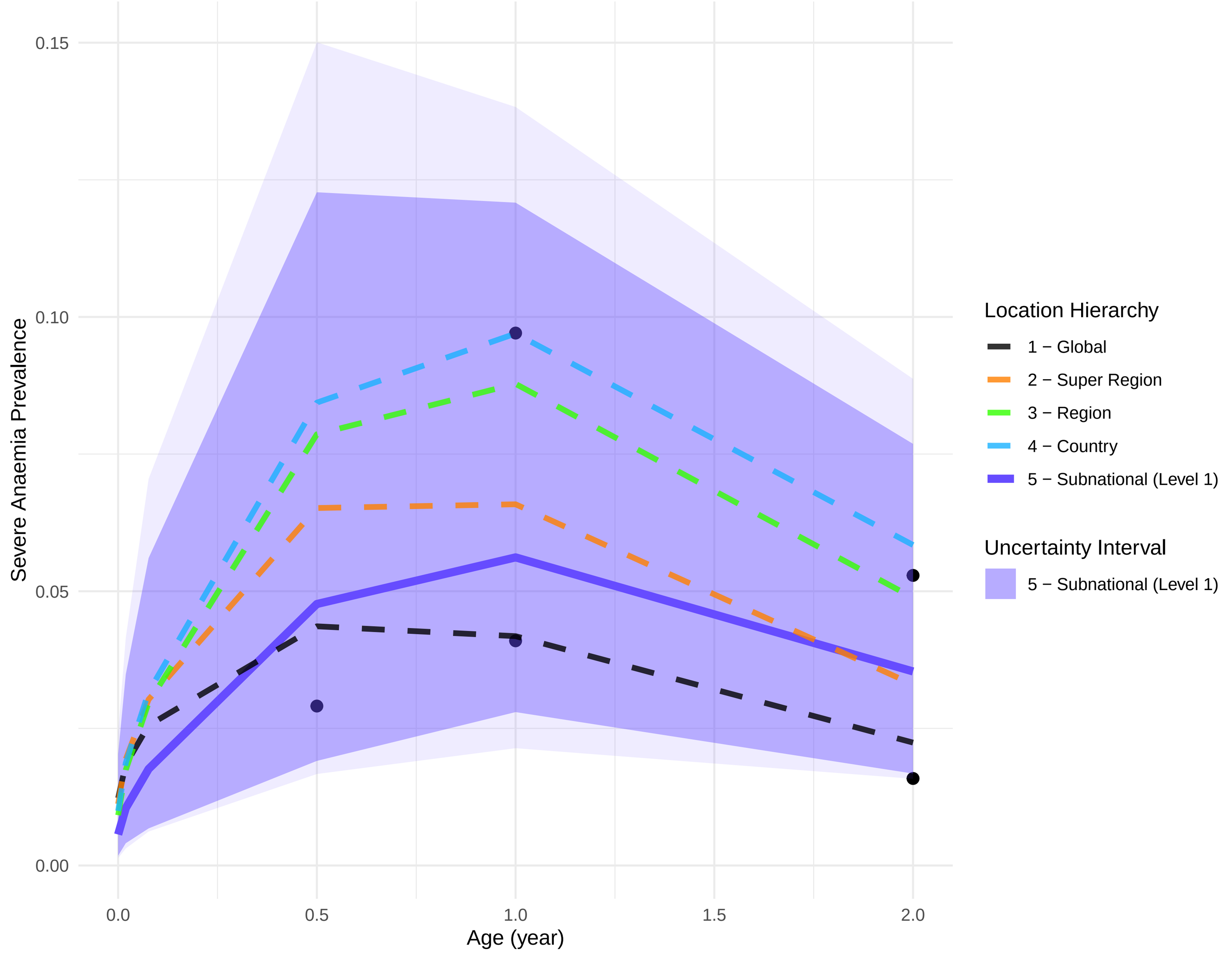

MR−BRT Age/Sex Fits − Males <2 Years Old − Severe Anaemia Prevalence − Ogun
WHO−2024 Adjustment Method

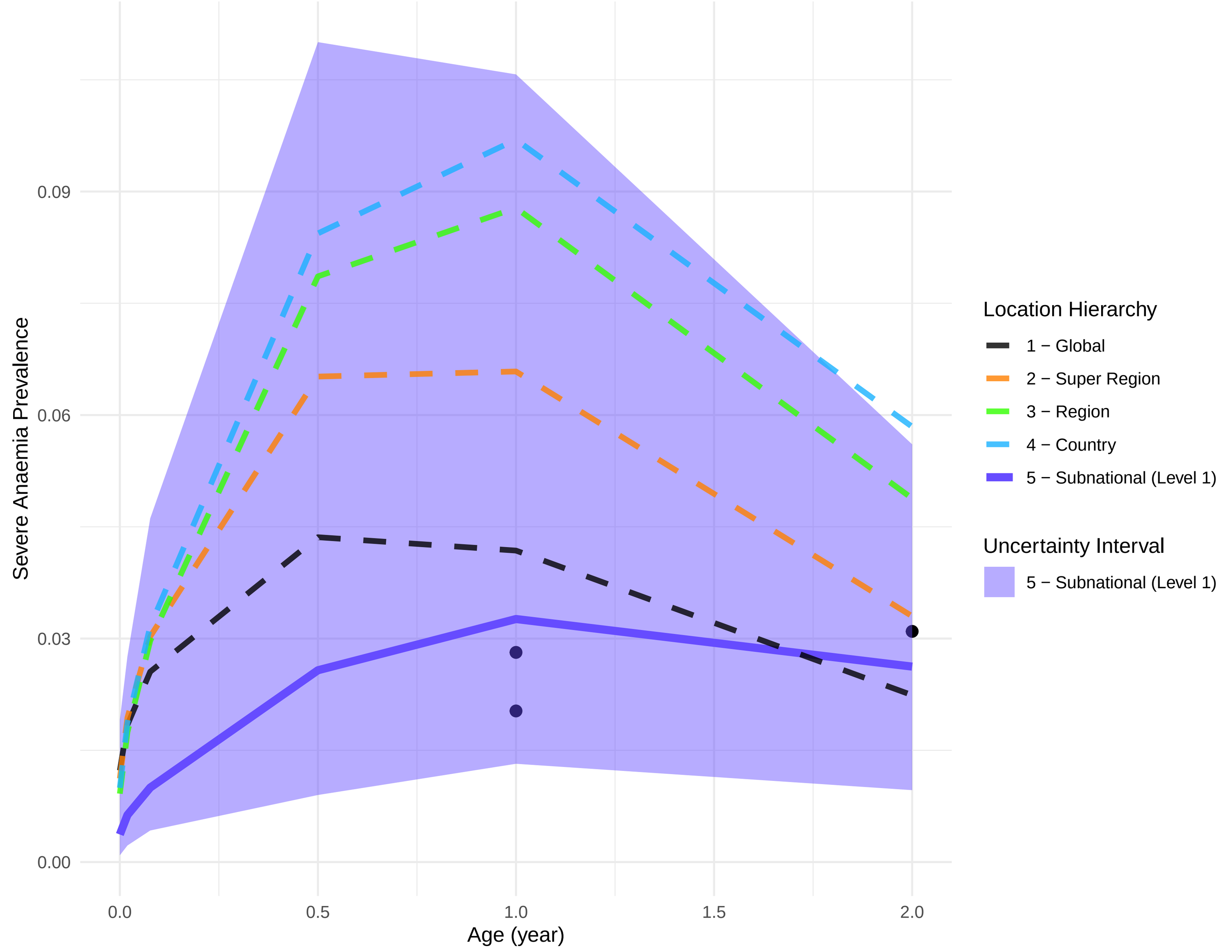

MR−BRT Age/Sex Fits − Males <2 Years Old − Severe Anaemia Prevalence − Ondo
WHO−2024 Adjustment Method

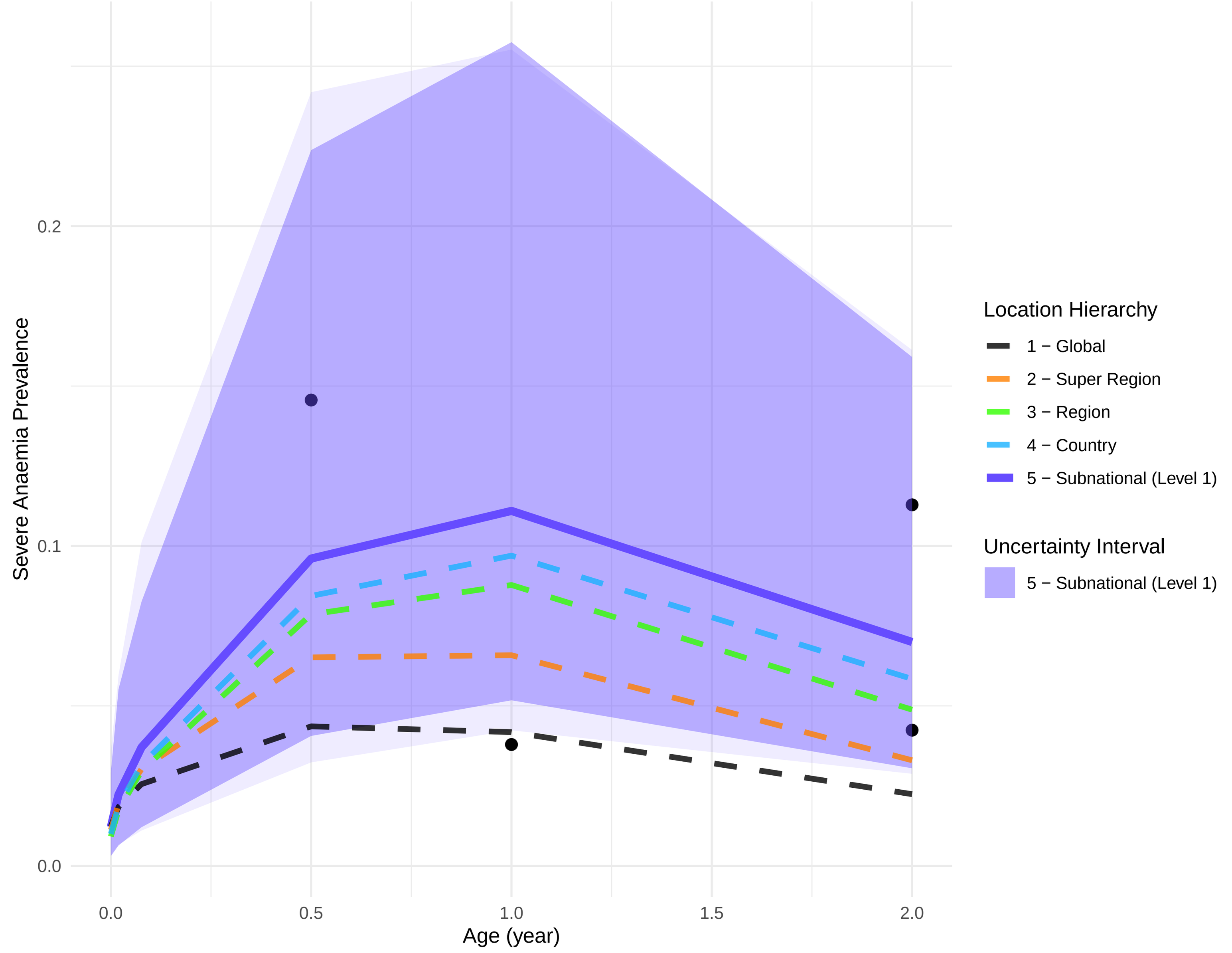

MR−BRT Age/Sex Fits − Males <2 Years Old − Severe Anaemia Prevalence − Osun
WHO−2024 Adjustment Method

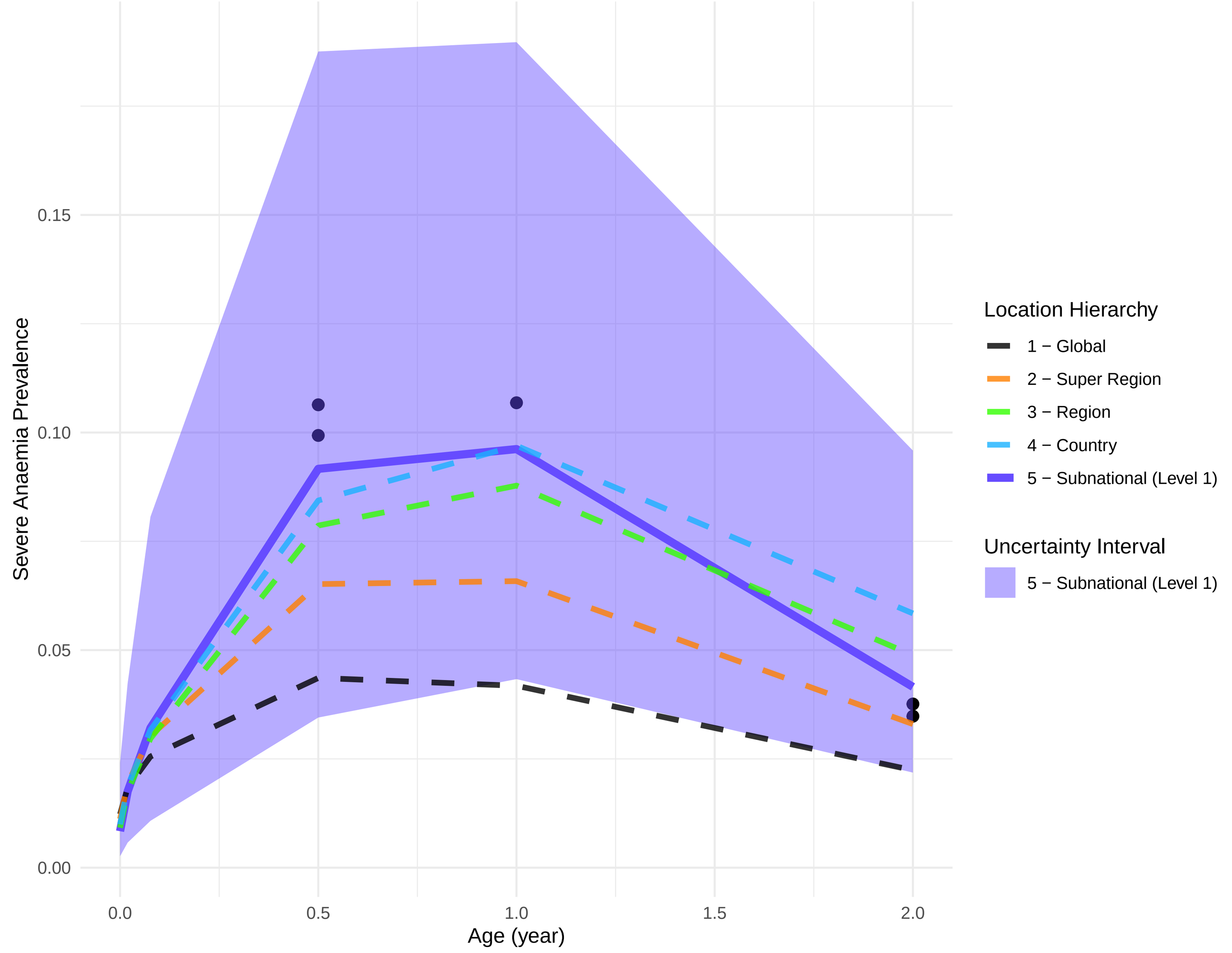

MR−BRT Age/Sex Fits − Males <2 Years Old − Severe Anaemia Prevalence − Oyo
WHO−2024 Adjustment Method

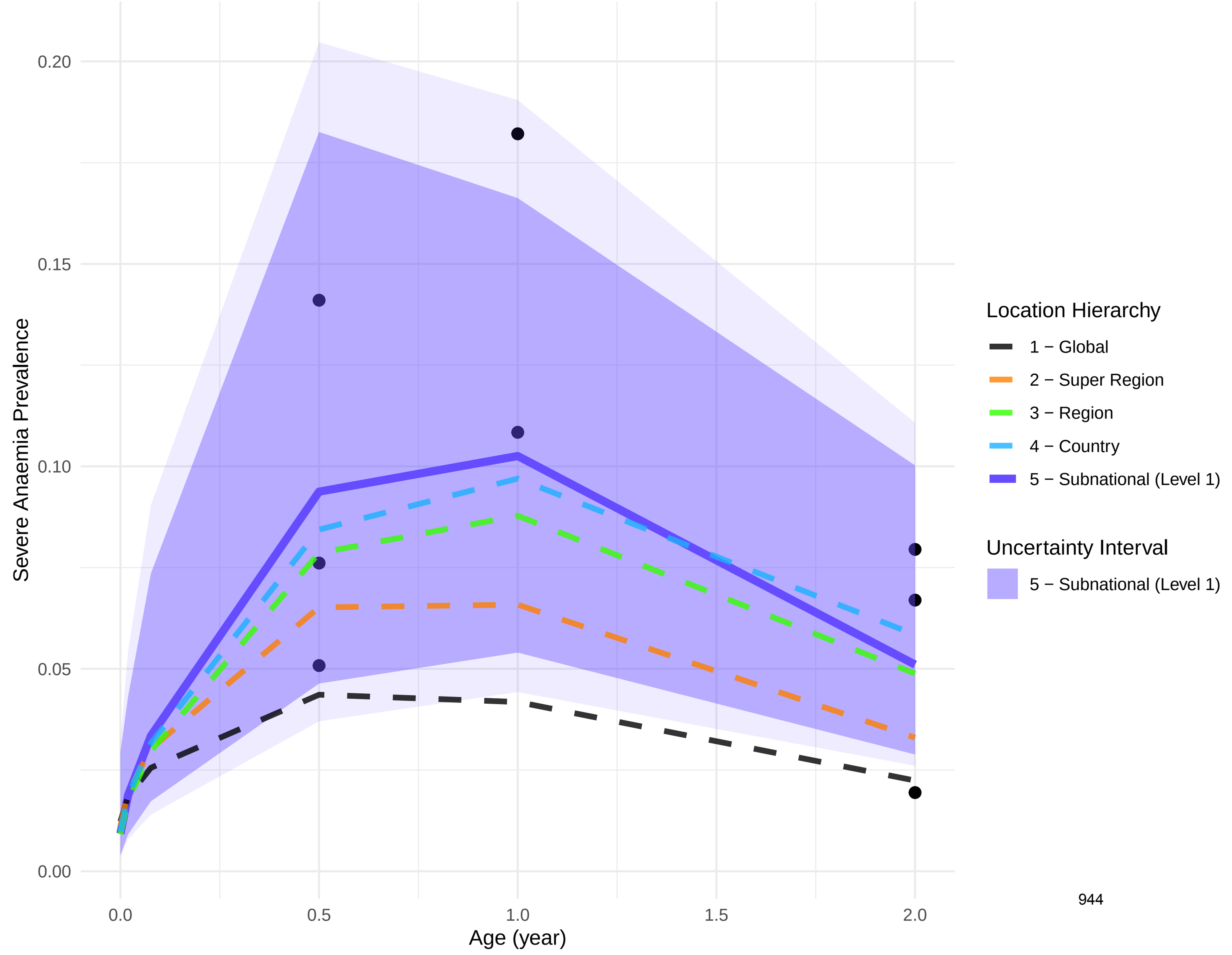

MR−BRT Age/Sex Fits − Males <2 Years Old − Severe Anaemia Prevalence − Plateau
WHO−2024 Adjustment Method

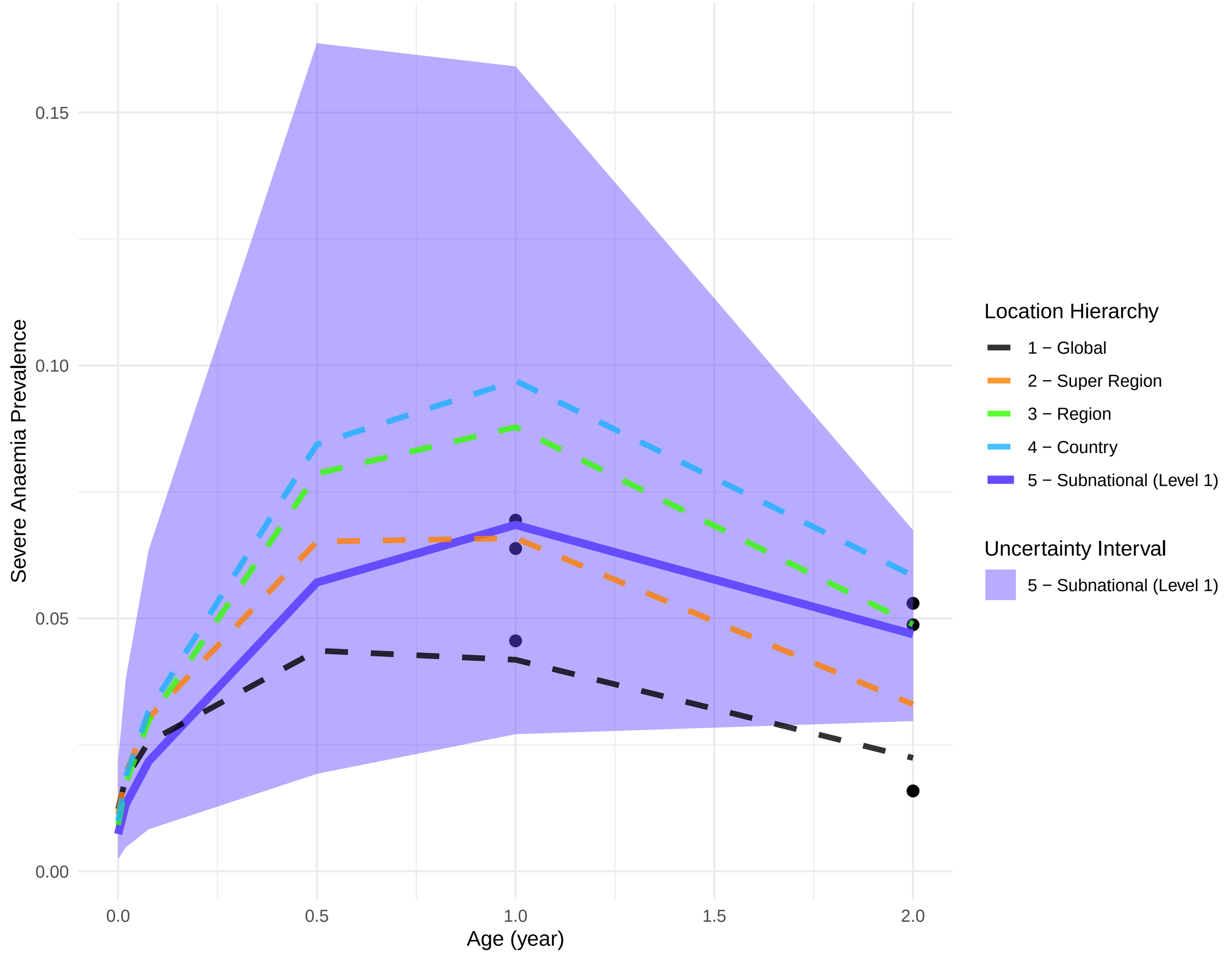

MR−BRT Age/Sex Fits − Males <2 Years Old − Severe Anaemia Prevalence − Rivers
WHO−2024 Adjustment Method

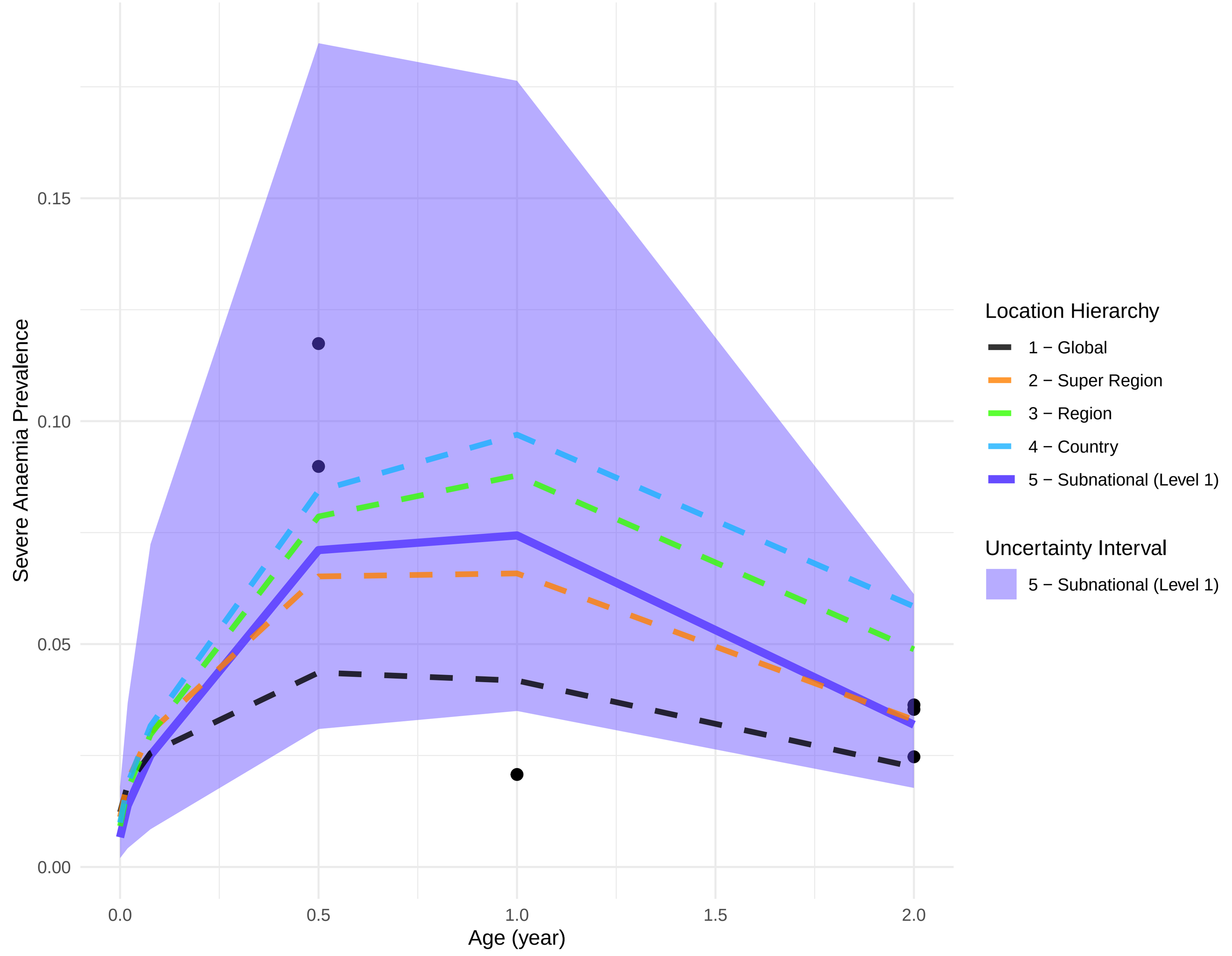

MR−BRT Age/Sex Fits − Males <2 Years Old − Severe Anaemia Prevalence − Sokoto
WHO−2024 Adjustment Method

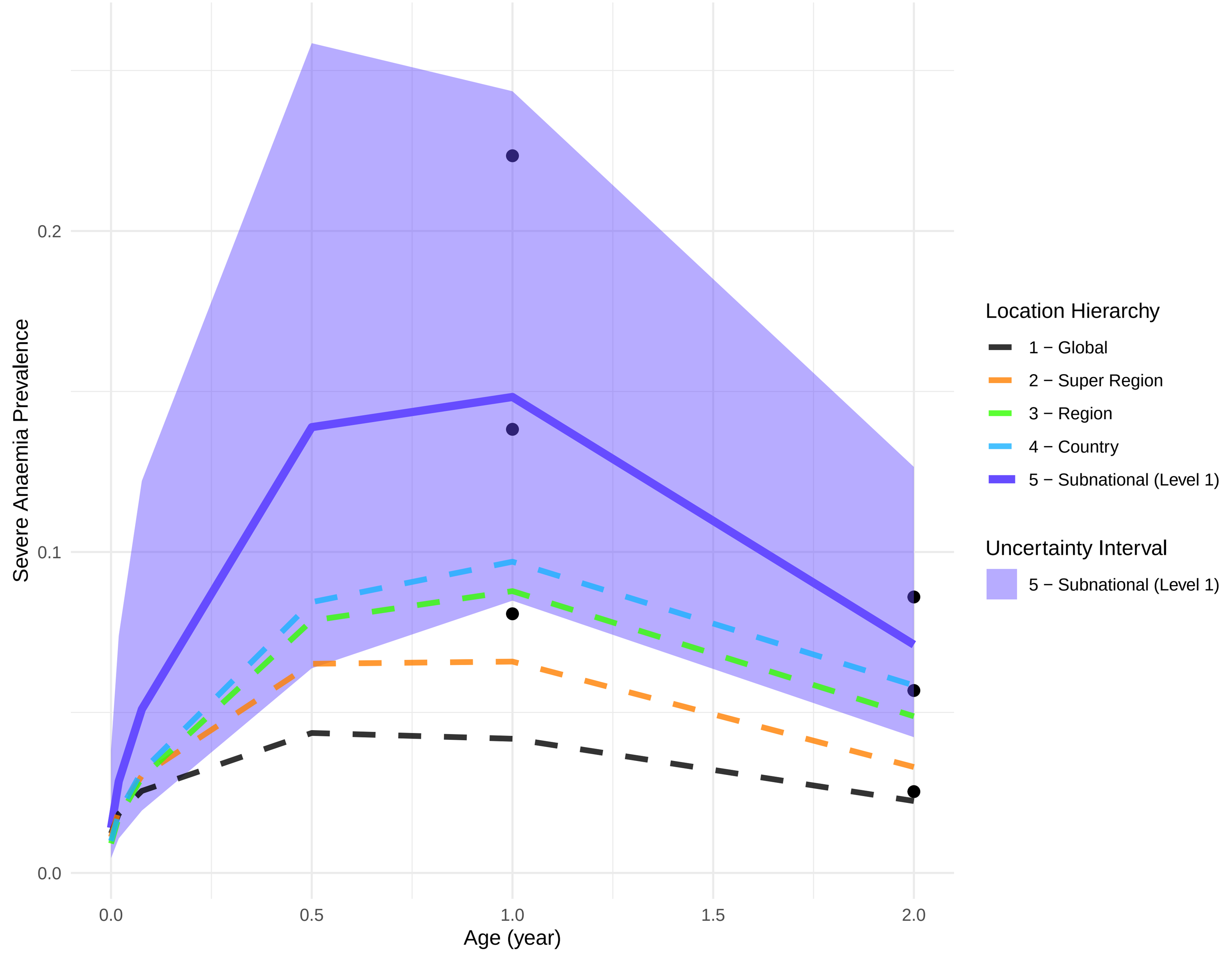

MR−BRT Age/Sex Fits − Males <2 Years Old − Severe Anaemia Prevalence − Taraba
WHO−2024 Adjustment Method

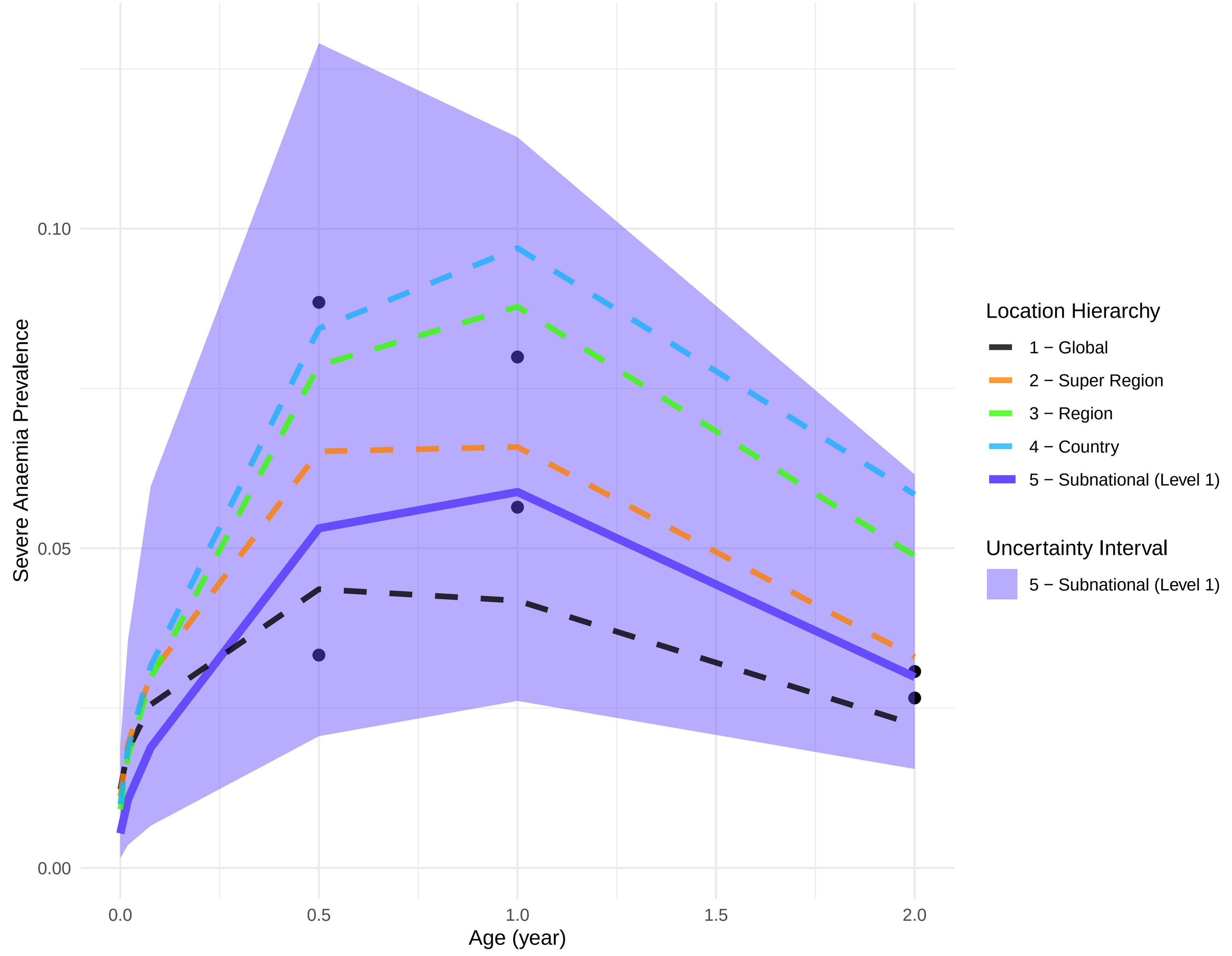

MR−BRT Age/Sex Fits − Males <2 Years Old − Severe Anaemia Prevalence − Yobe
WHO−2024 Adjustment Method

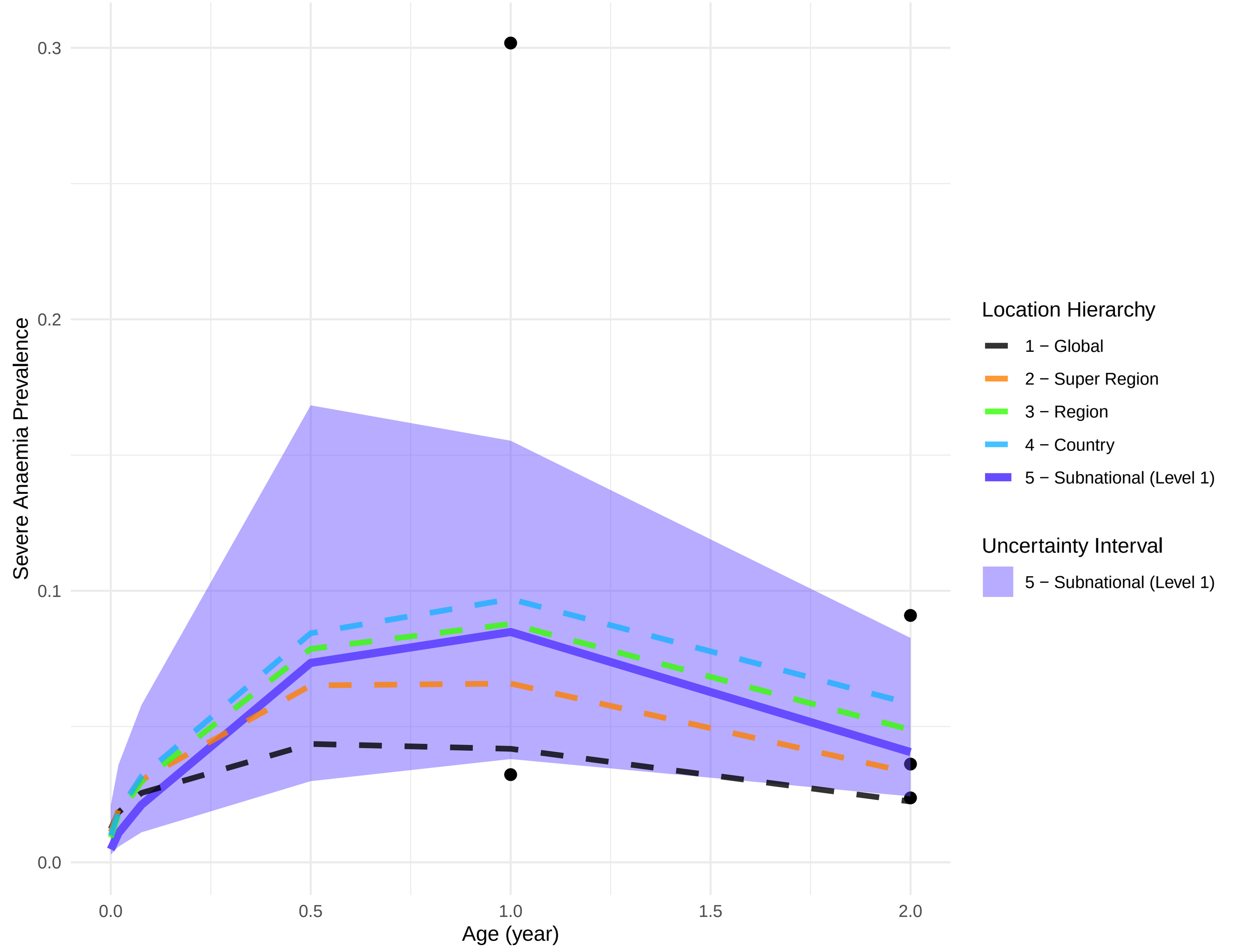

MR−BRT Age/Sex Fits − Males <2 Years Old − Severe Anaemia Prevalence − Zamfara
WHO−2024 Adjustment Method

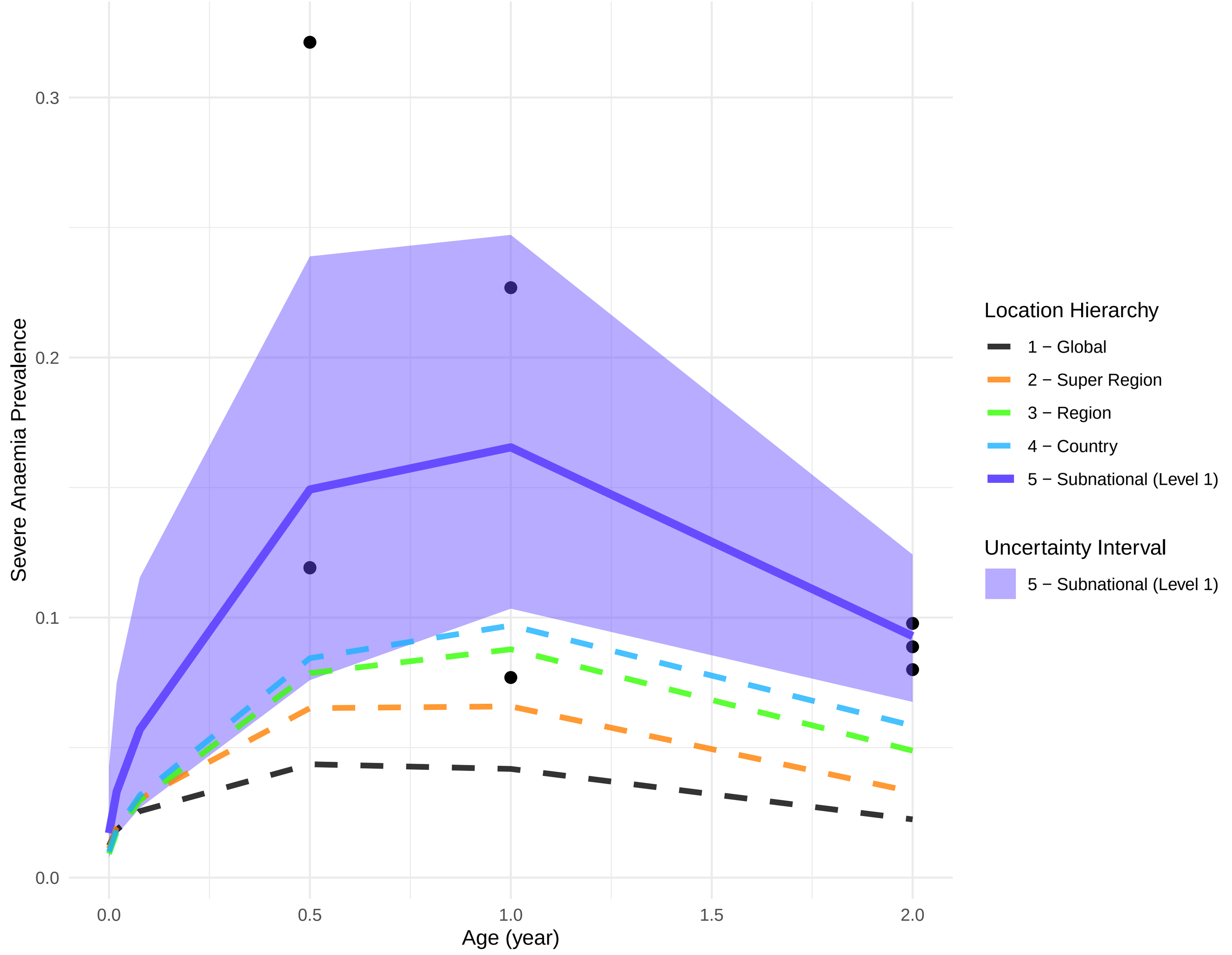

MR−BRT Age/Sex Fits − Males <2 Years Old − Severe Anaemia Prevalence − Senegal
WHO−2024 Adjustment Method

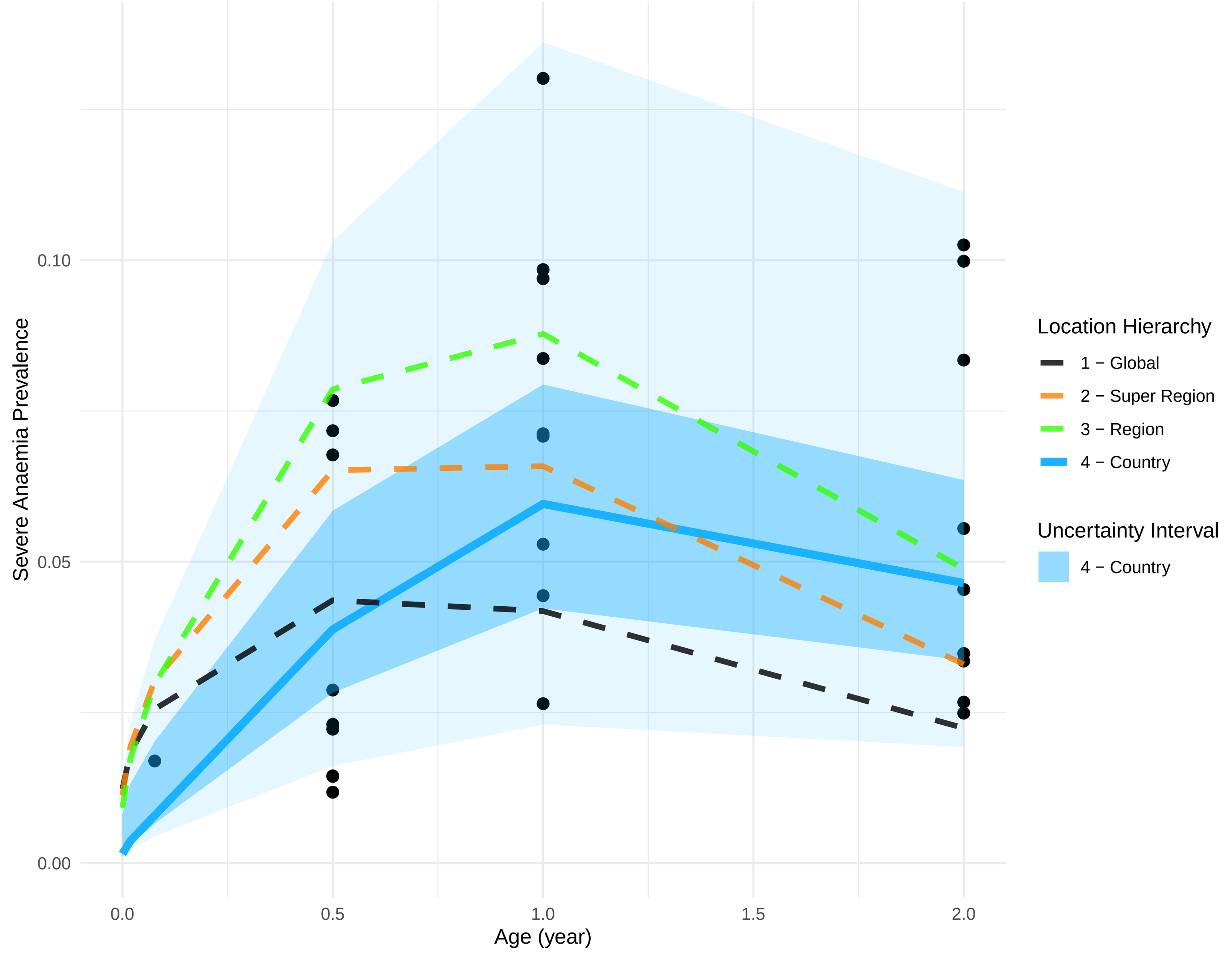

MR−BRT Age/Sex Fits − Males <2 Years Old − Severe Anaemia Prevalence − Sierra Leone
WHO−2024 Adjustment Method

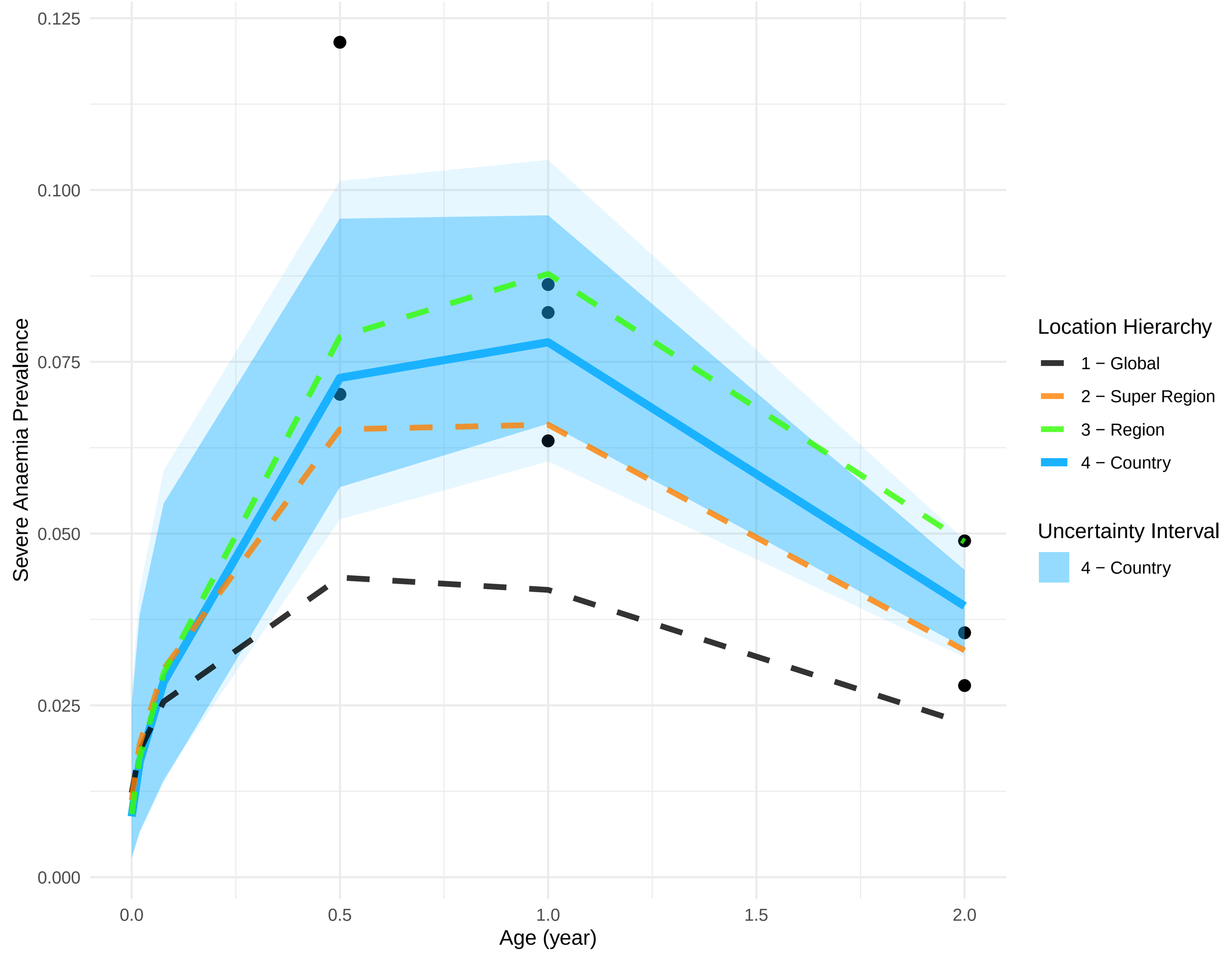

MR−BRT Age/Sex Fits − Males <2 Years Old − Severe Anaemia Prevalence − Togo
WHO−2024 Adjustment Method

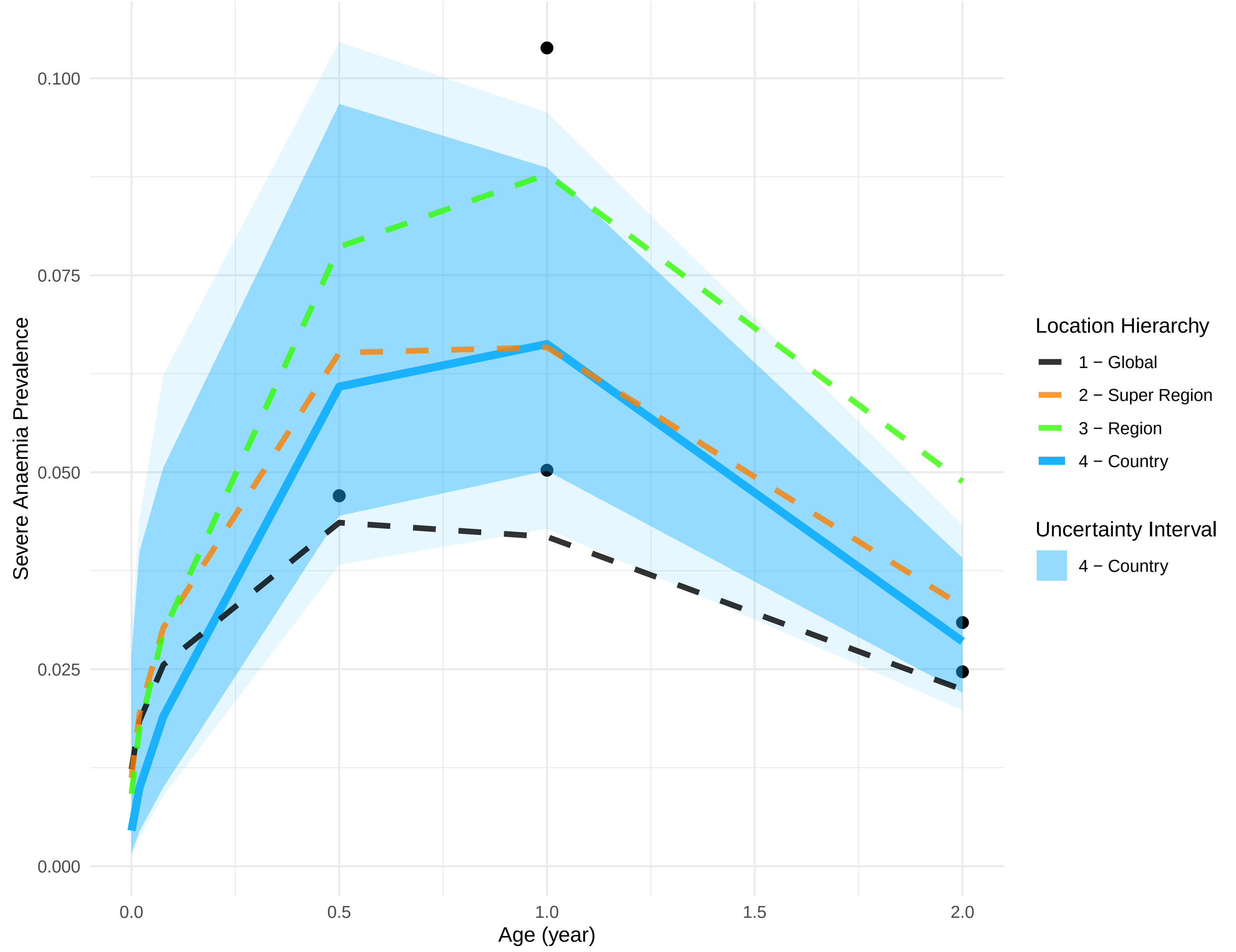

MR−BRT Age/Sex Fits − Non−pregnant Females <2 Years Old − Severe Anaemia Prevalence − Global
WHO−2024 Adjustment Method

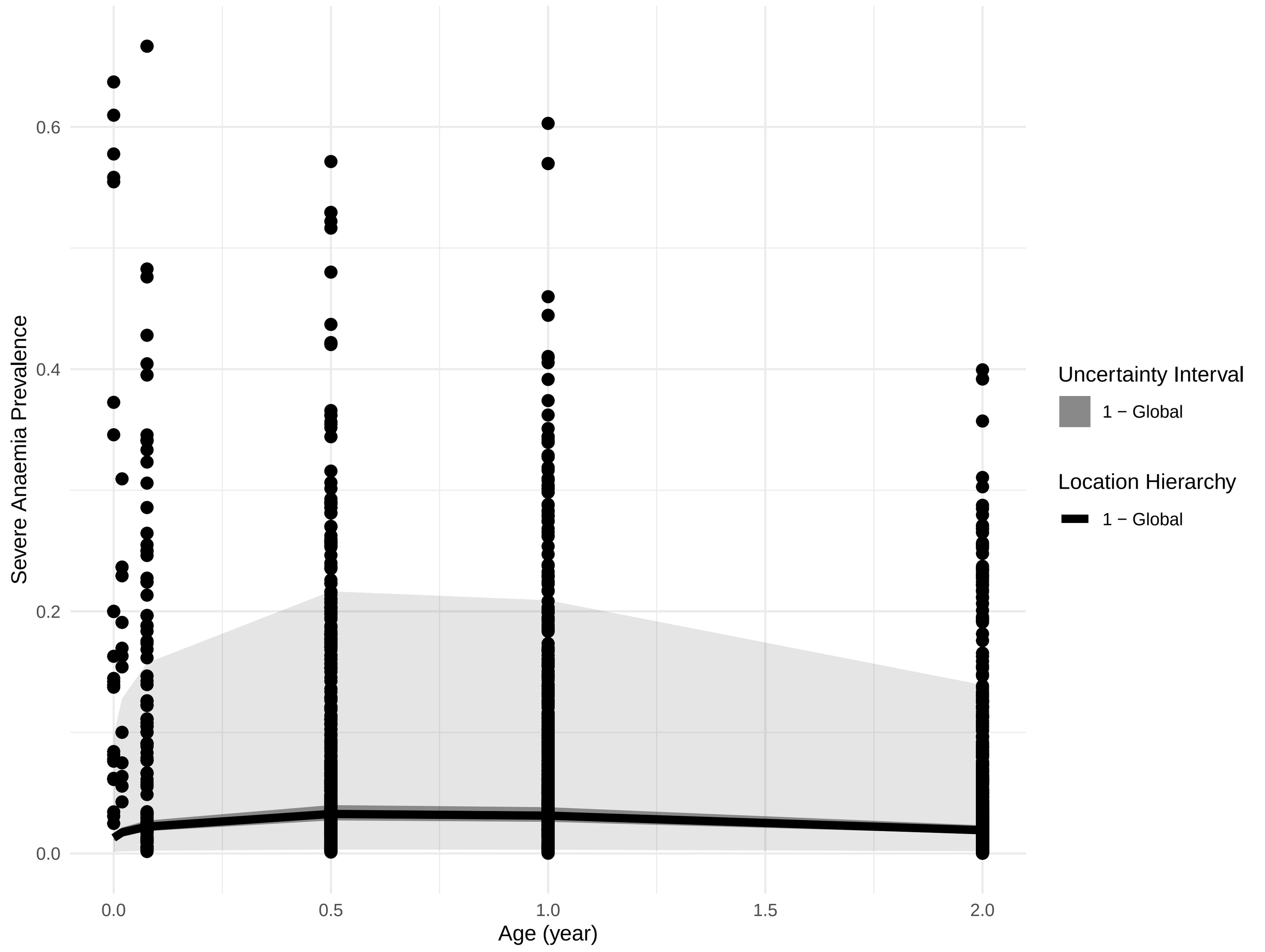

MR−BRT Age/Sex Fits − Non−pregnant Females <2 Years Old − Severe Anaemia Prevalence − Central Europe, Eastern Europe, and Central Asia
WHO−2024 Adjustment Method

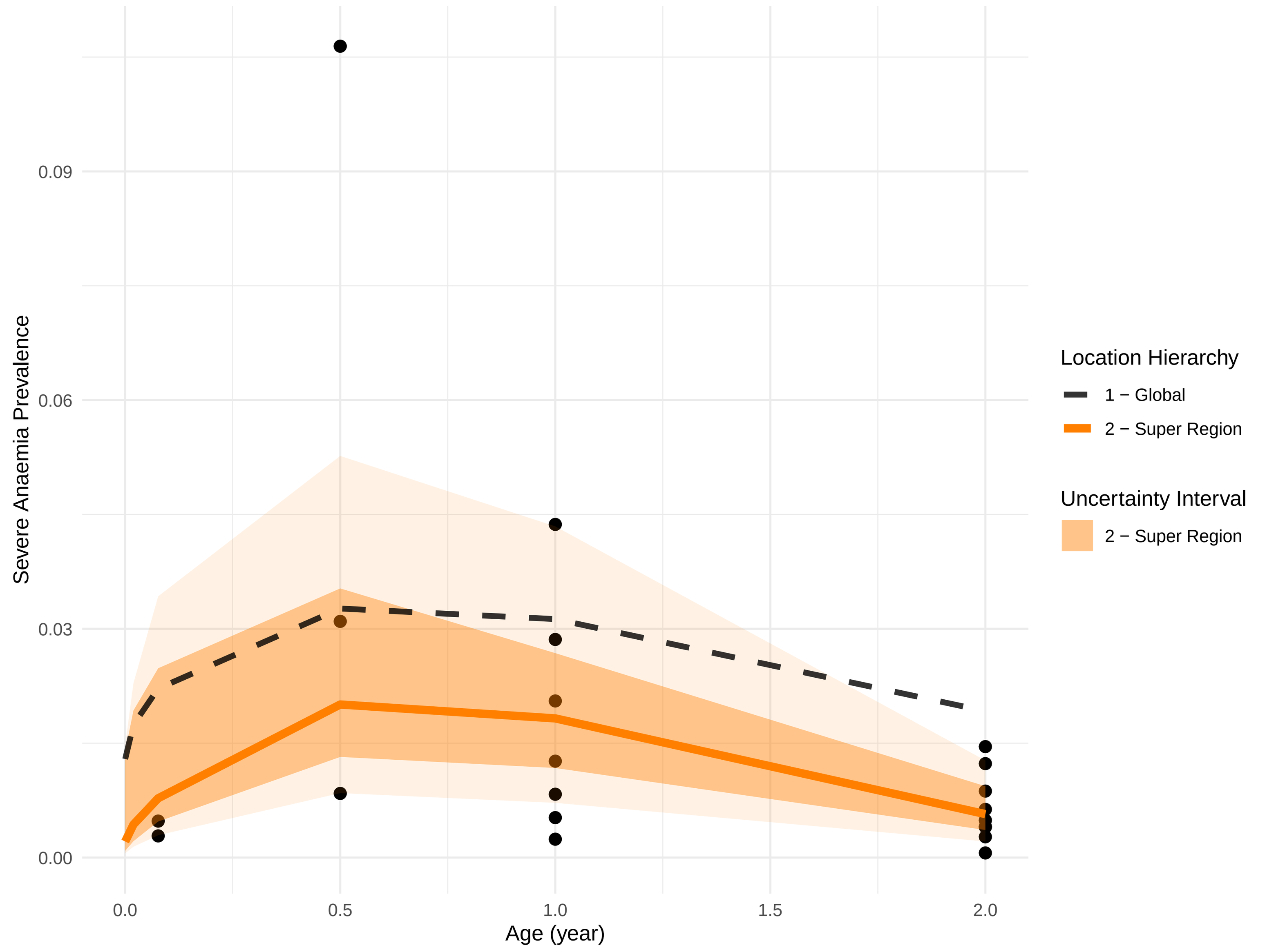

MR−BRT Age/Sex Fits − Non−pregnant Females <2 Years Old − Severe Anaemia Prevalence − Central Asia
WHO−2024 Adjustment Method

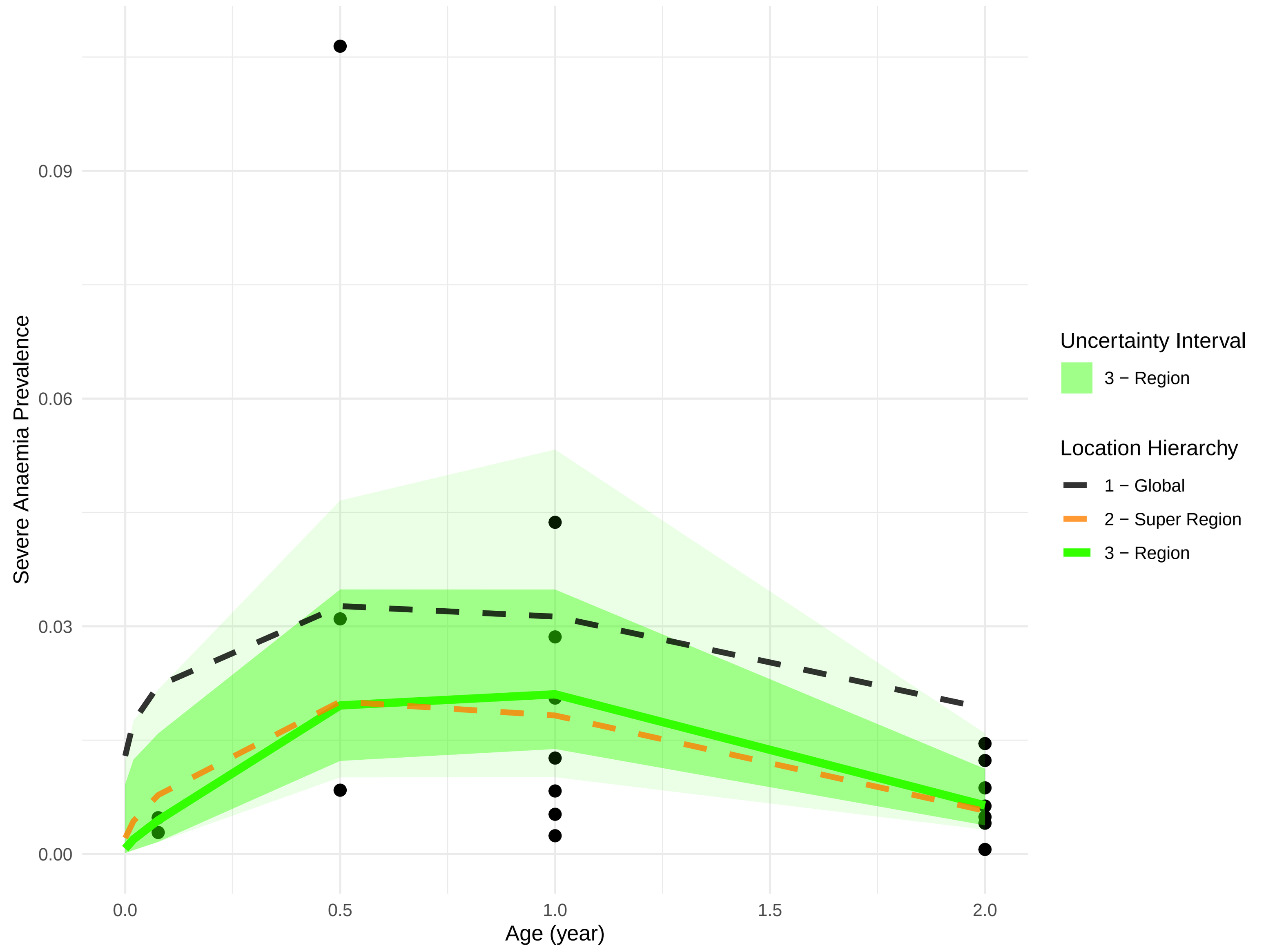

MR−BRT Age/Sex Fits − Non−pregnant Females <2 Years Old − Severe Anaemia Prevalence − Armenia
WHO−2024 Adjustment Method

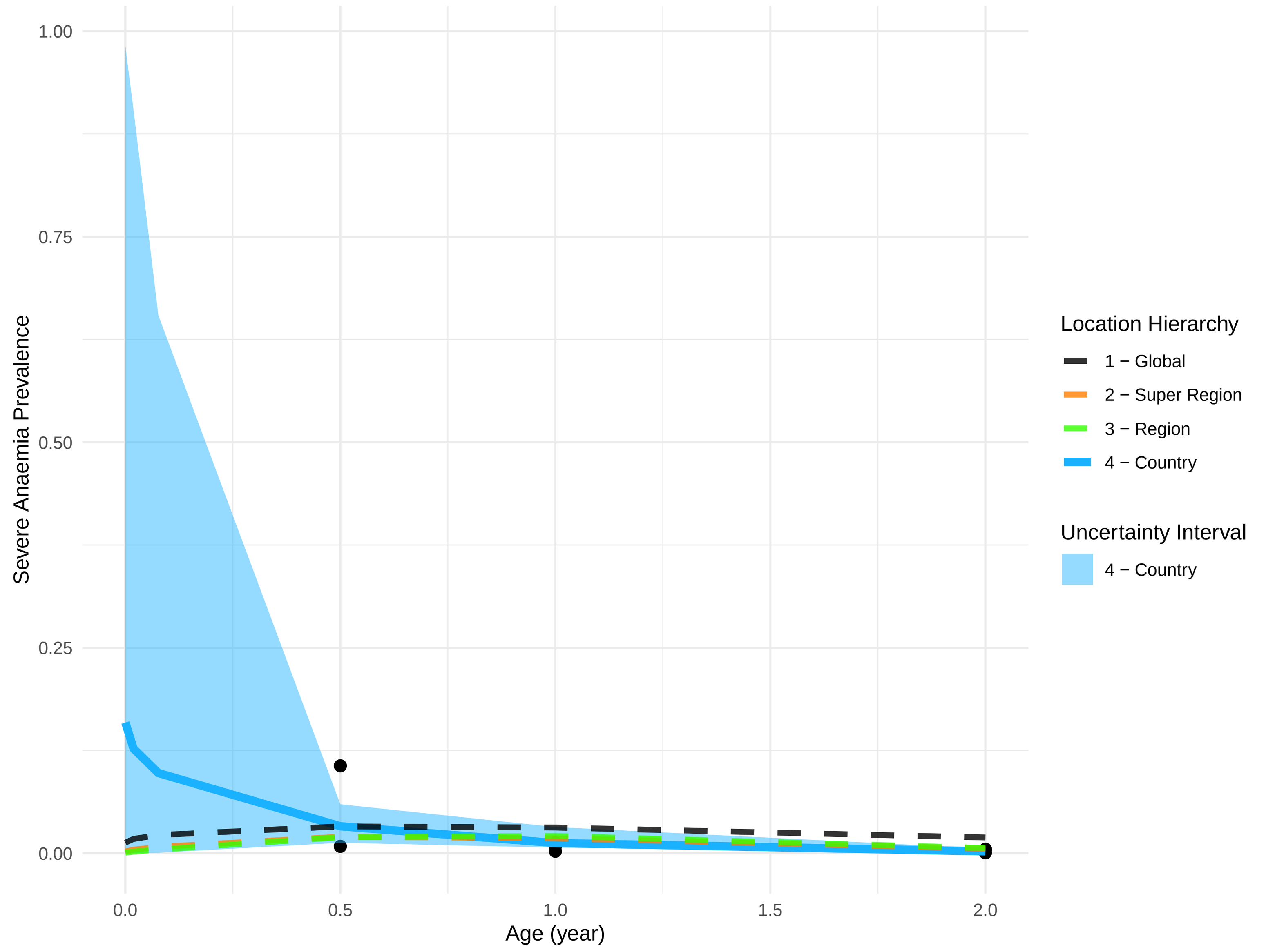

MR−BRT Age/Sex Fits − Non−pregnant Females <2 Years Old − Severe Anaemia Prevalence − Azerbaijan
WHO−2024 Adjustment Method

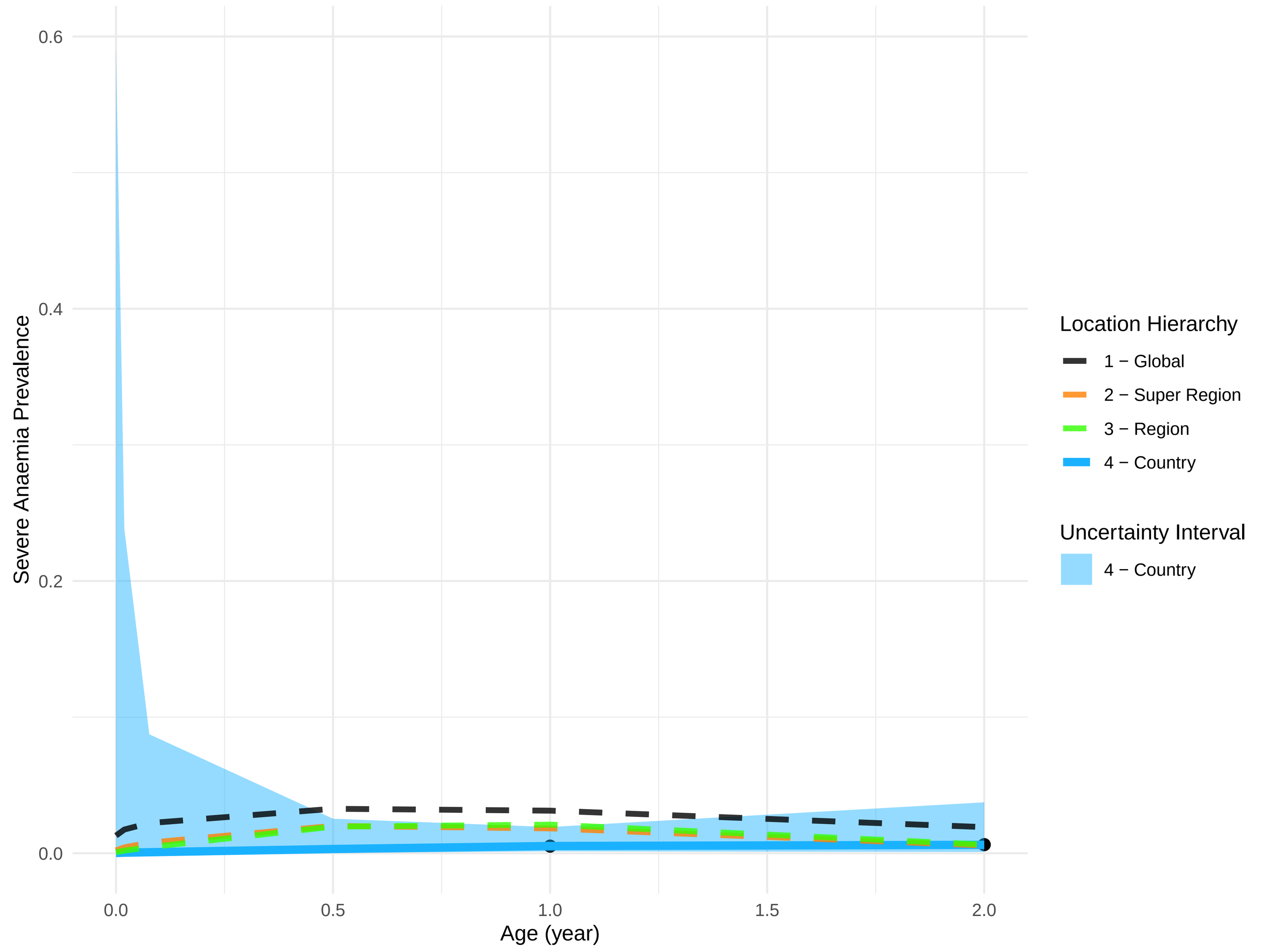

MR−BRT Age/Sex Fits − Non−pregnant Females <2 Years Old − Severe Anaemia Prevalence − Kyrgyzstan
WHO−2024 Adjustment Method

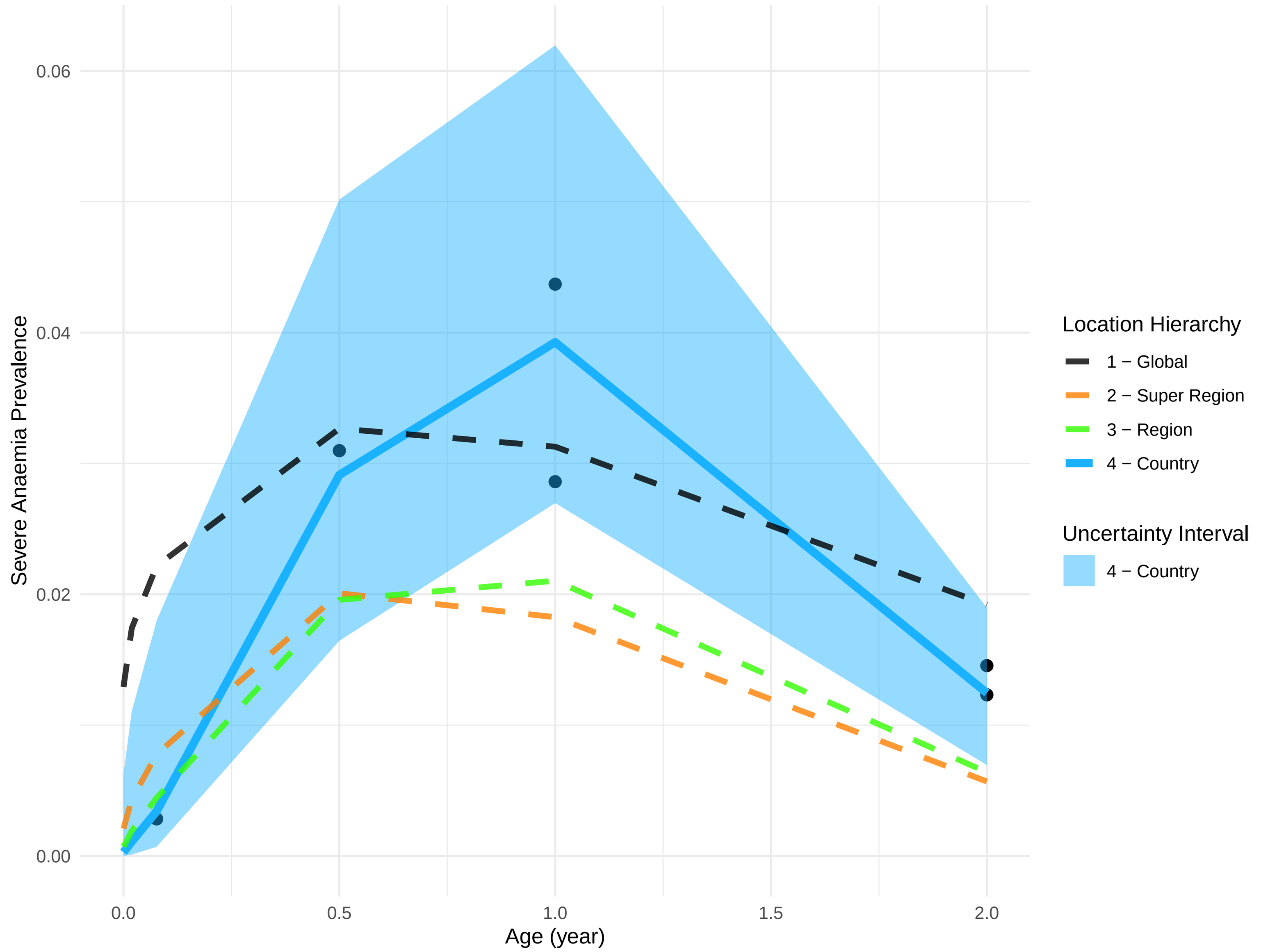

MR−BRT Age/Sex Fits − Non−pregnant Females <2 Years Old − Severe Anaemia Prevalence − Uzbekistan
WHO−2024 Adjustment Method

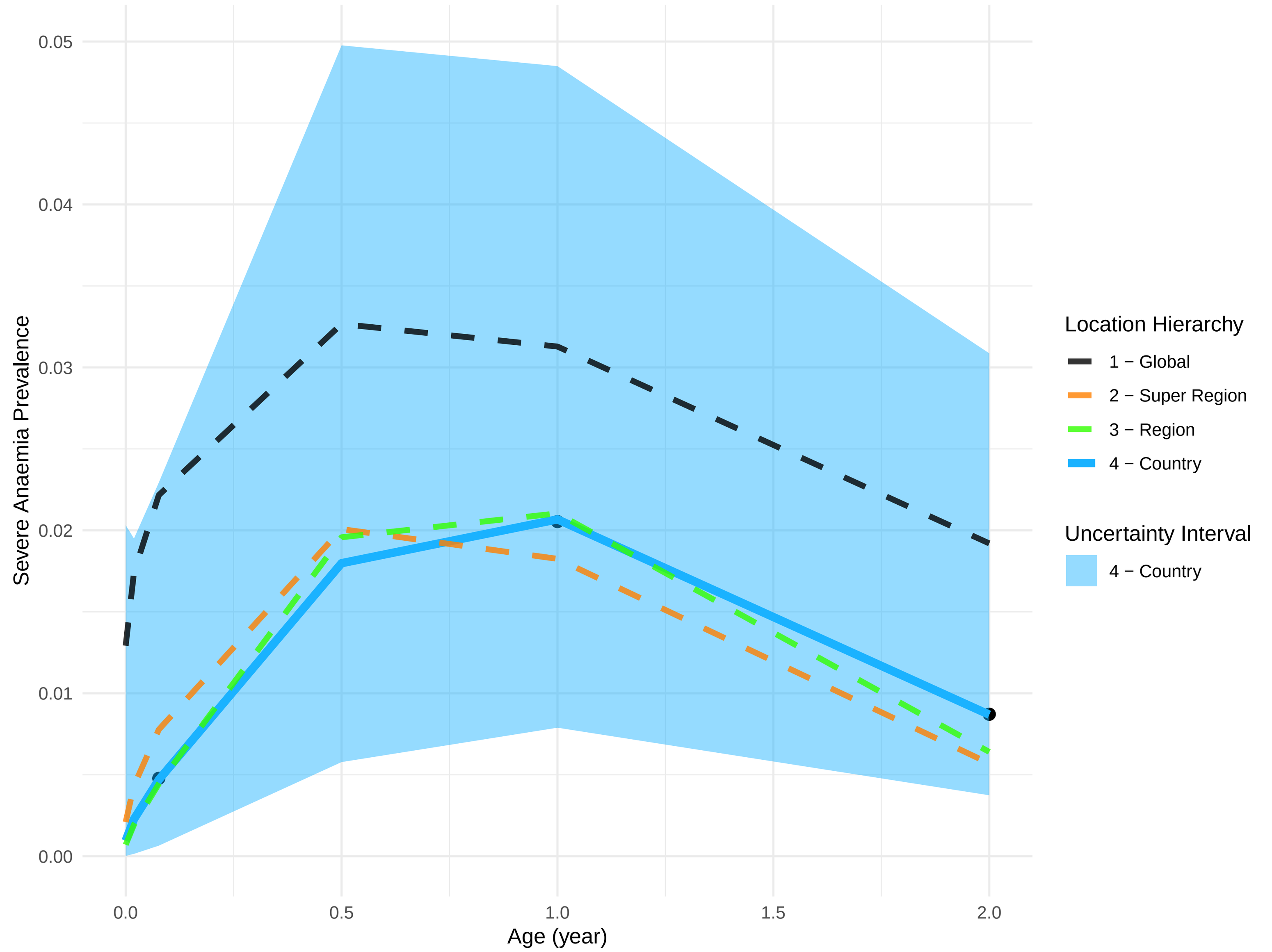

MR−BRT Age/Sex Fits − Non−pregnant Females <2 Years Old − Severe Anaemia Prevalence − Central Europe
WHO−2024 Adjustment Method

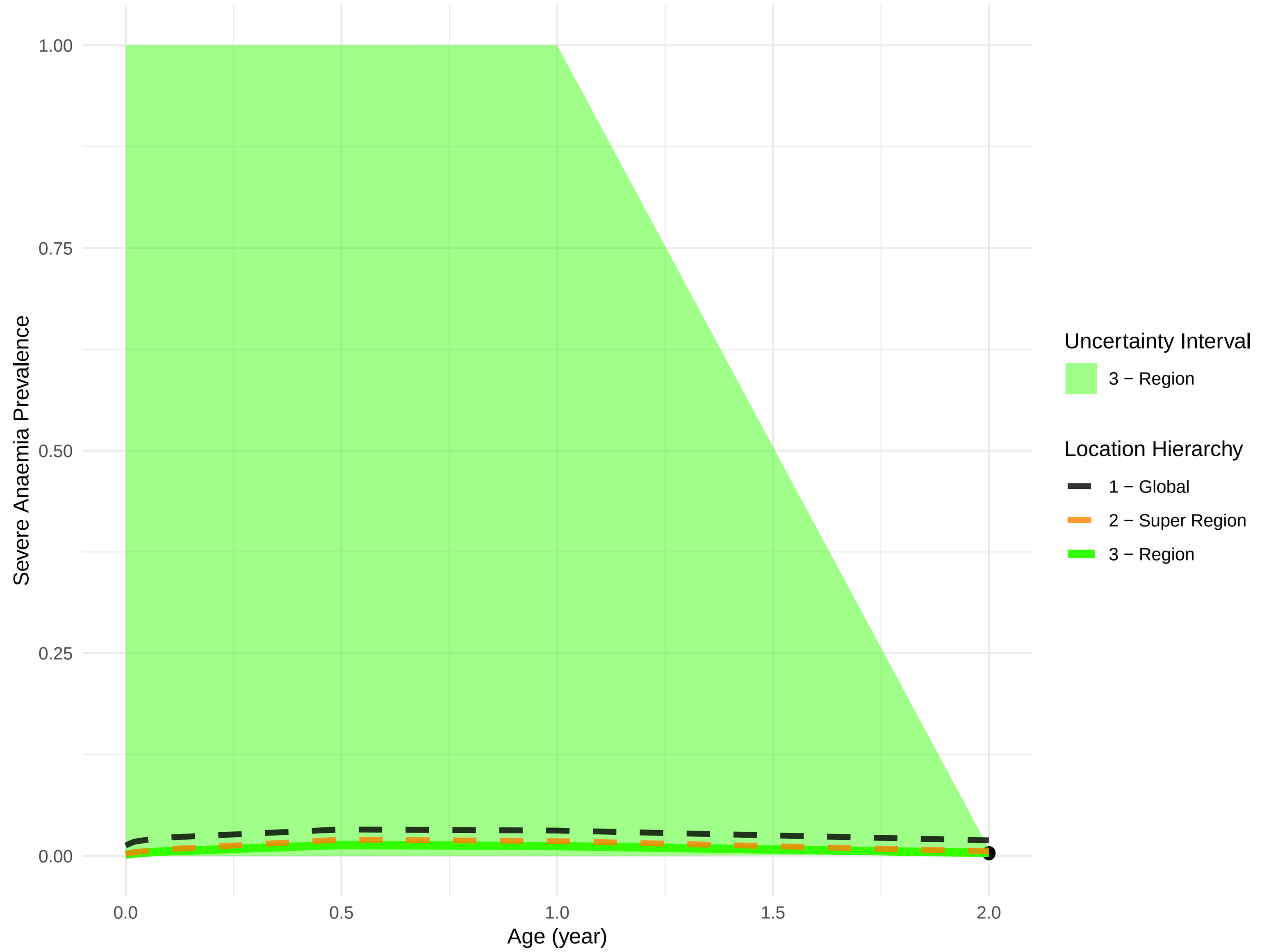

MR−BRT Age/Sex Fits − Non−pregnant Females <2 Years Old − Severe Anaemia Prevalence − Albania
WHO−2024 Adjustment Method

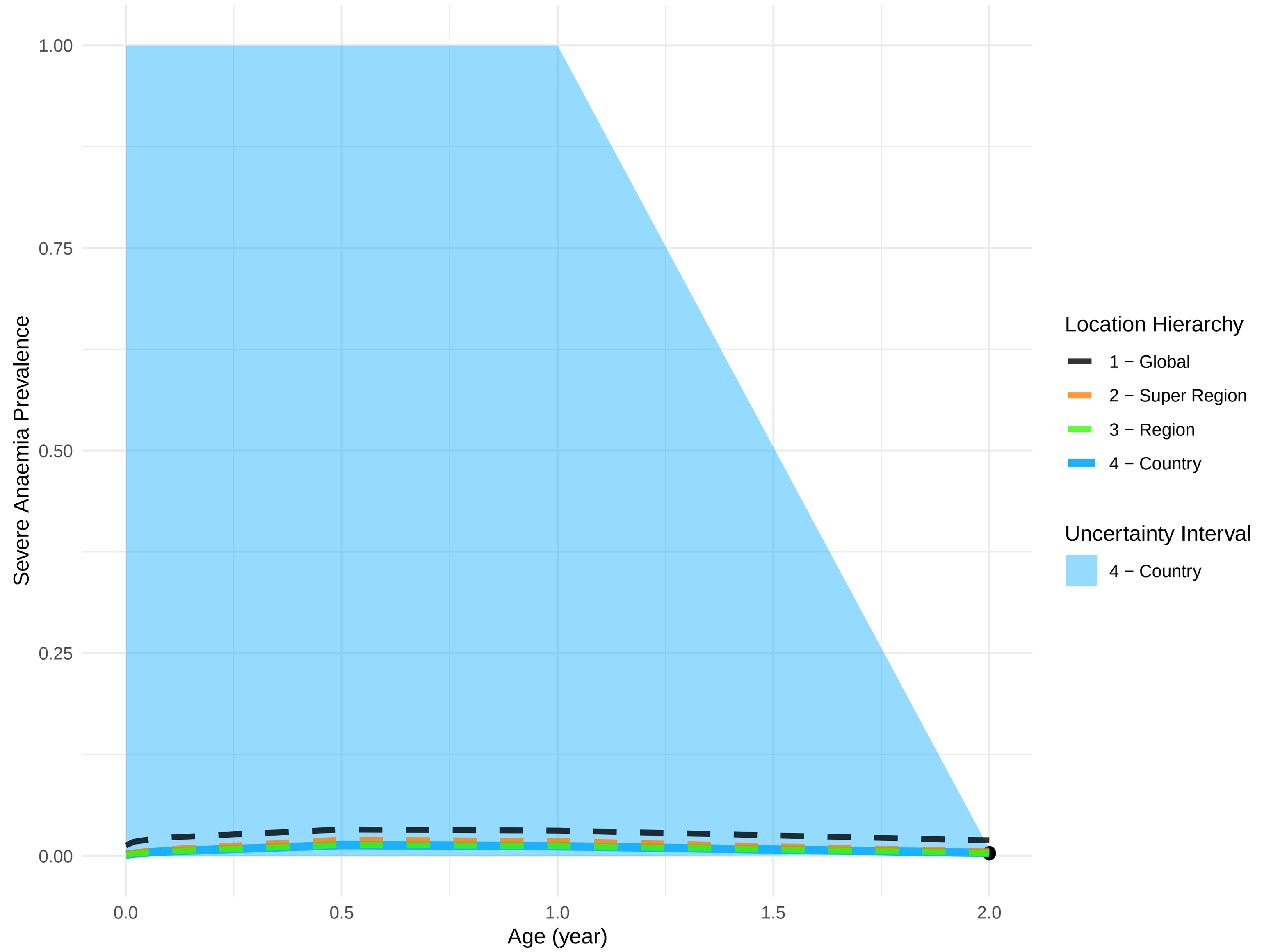

MR−BRT Age/Sex Fits − Non−pregnant Females <2 Years Old − Severe Anaemia Prevalence − High−income
WHO−2024 Adjustment Method

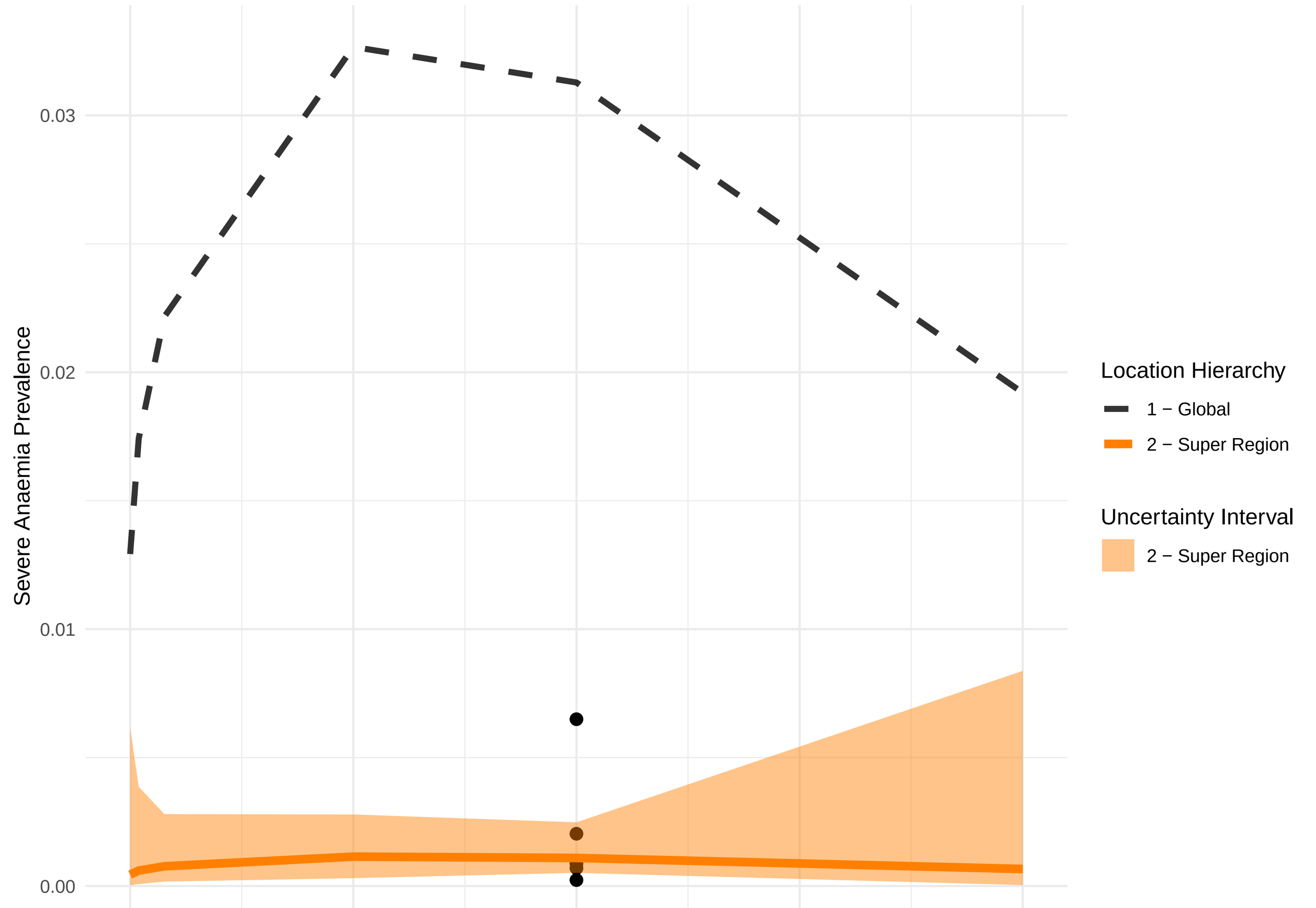

MR−BRT Age/Sex Fits − Non−pregnant Females <2 Years Old − Severe Anaemia Prevalence − Western Europe
WHO−2024 Adjustment Method

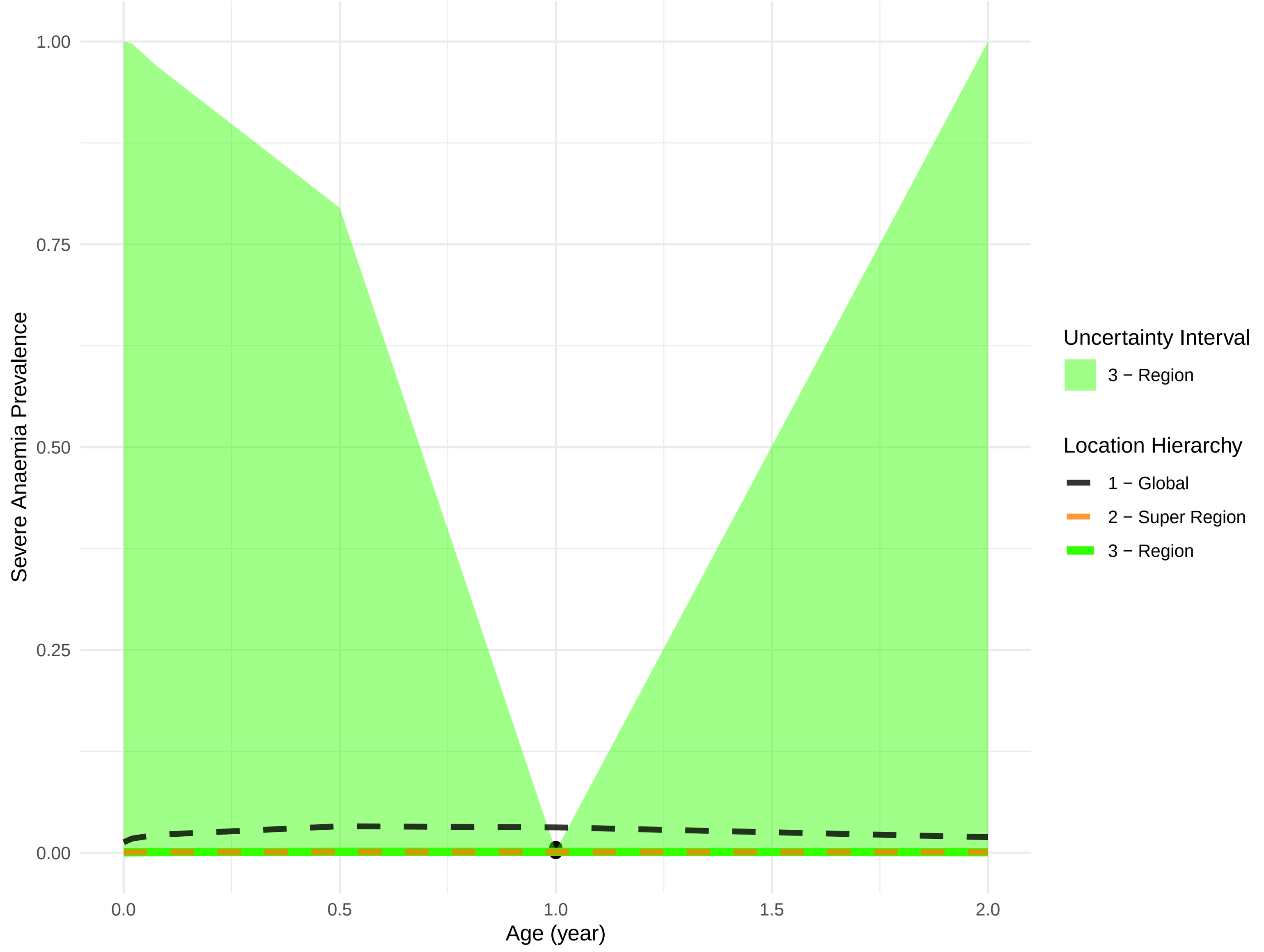

MR−BRT Age/Sex Fits − Non−pregnant Females <2 Years Old − Severe Anaemia Prevalence − United Kingdom
WHO−2024 Adjustment Method

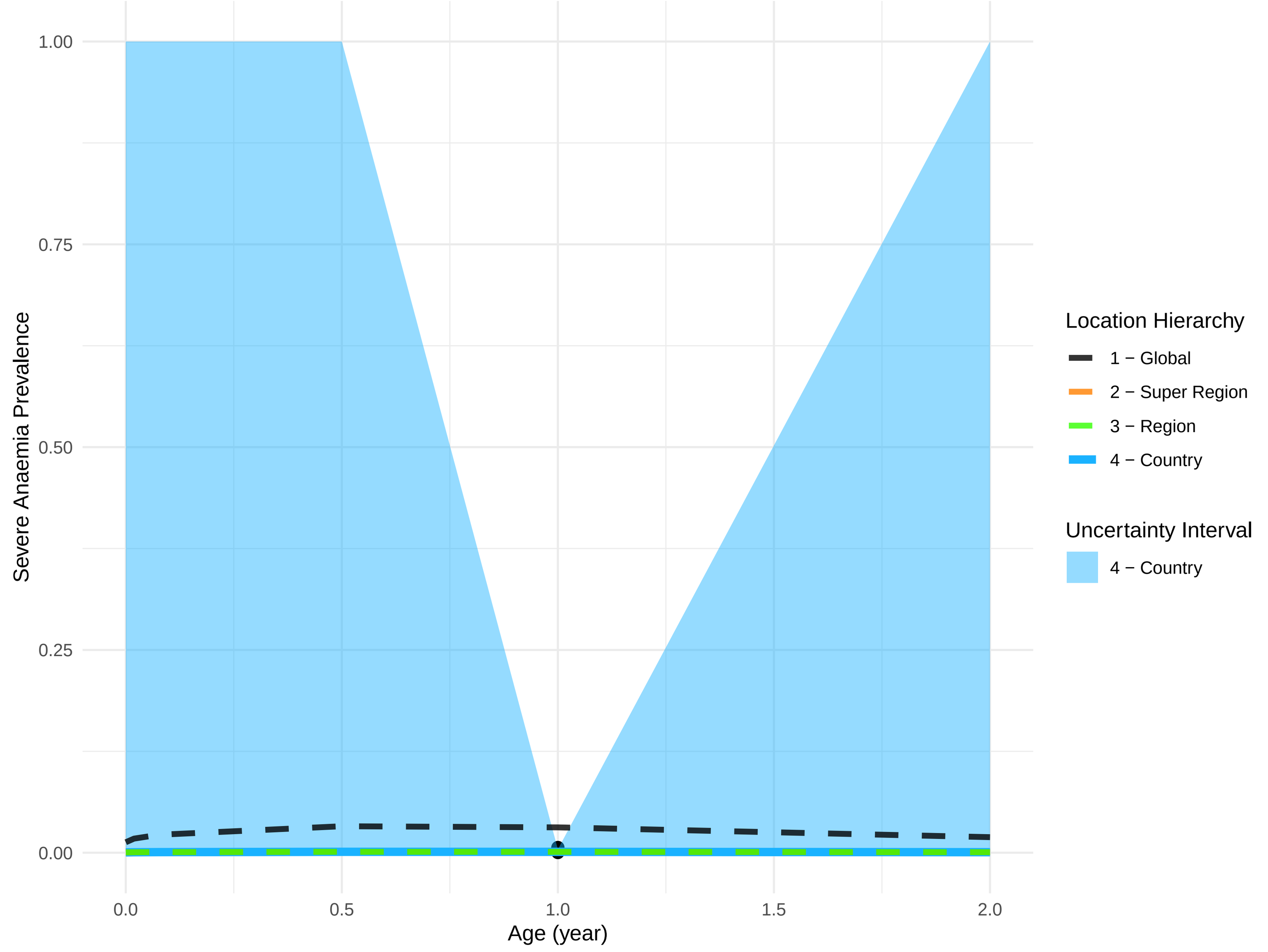

MR−BRT Age/Sex Fits − Non−pregnant Females <2 Years Old − Severe Anaemia Prevalence − England
WHO−2024 Adjustment Method

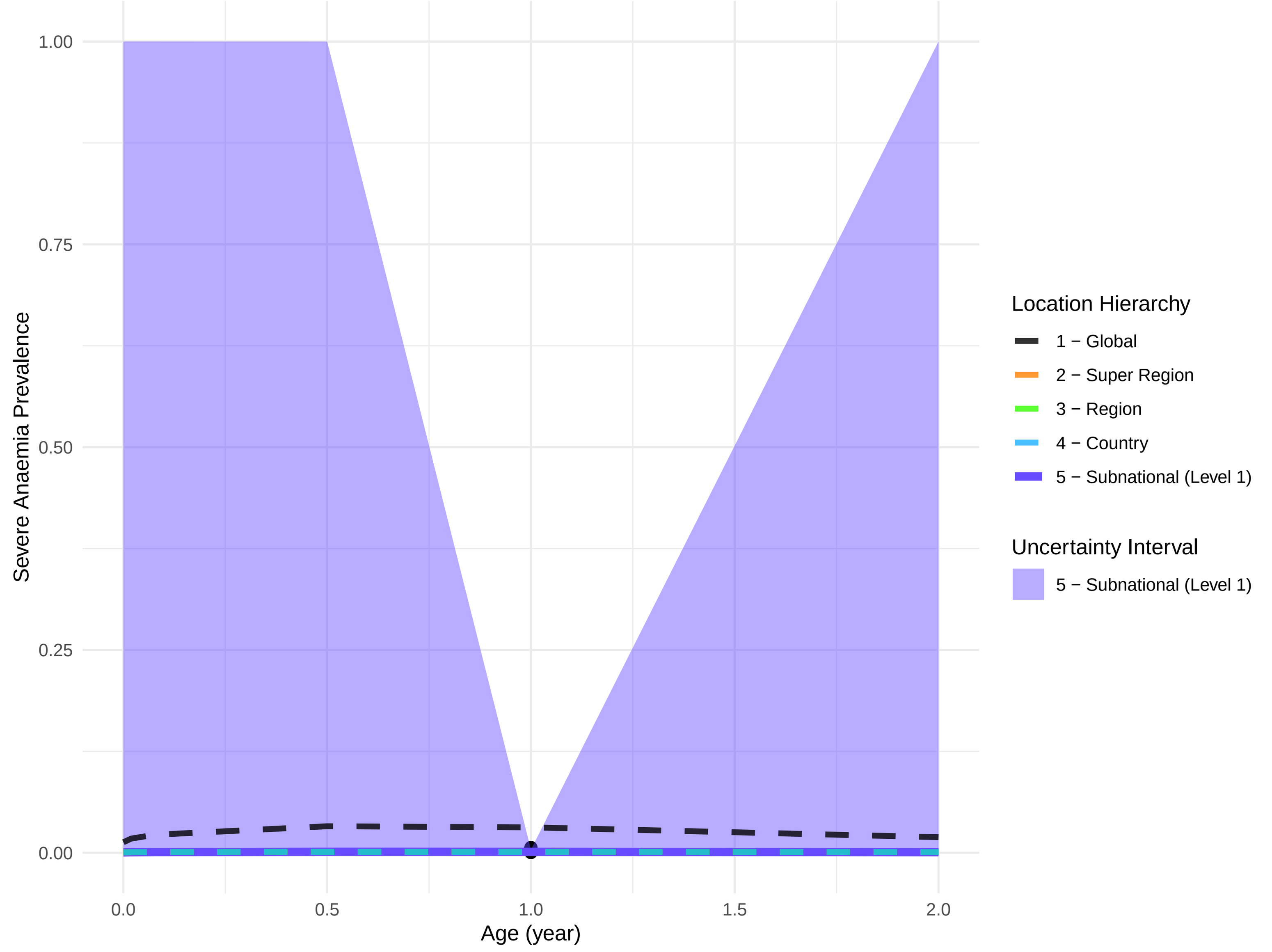

MR−BRT Age/Sex Fits − Non−pregnant Females <2 Years Old − Severe Anaemia Prevalence − East Midlands
WHO−2024 Adjustment Method

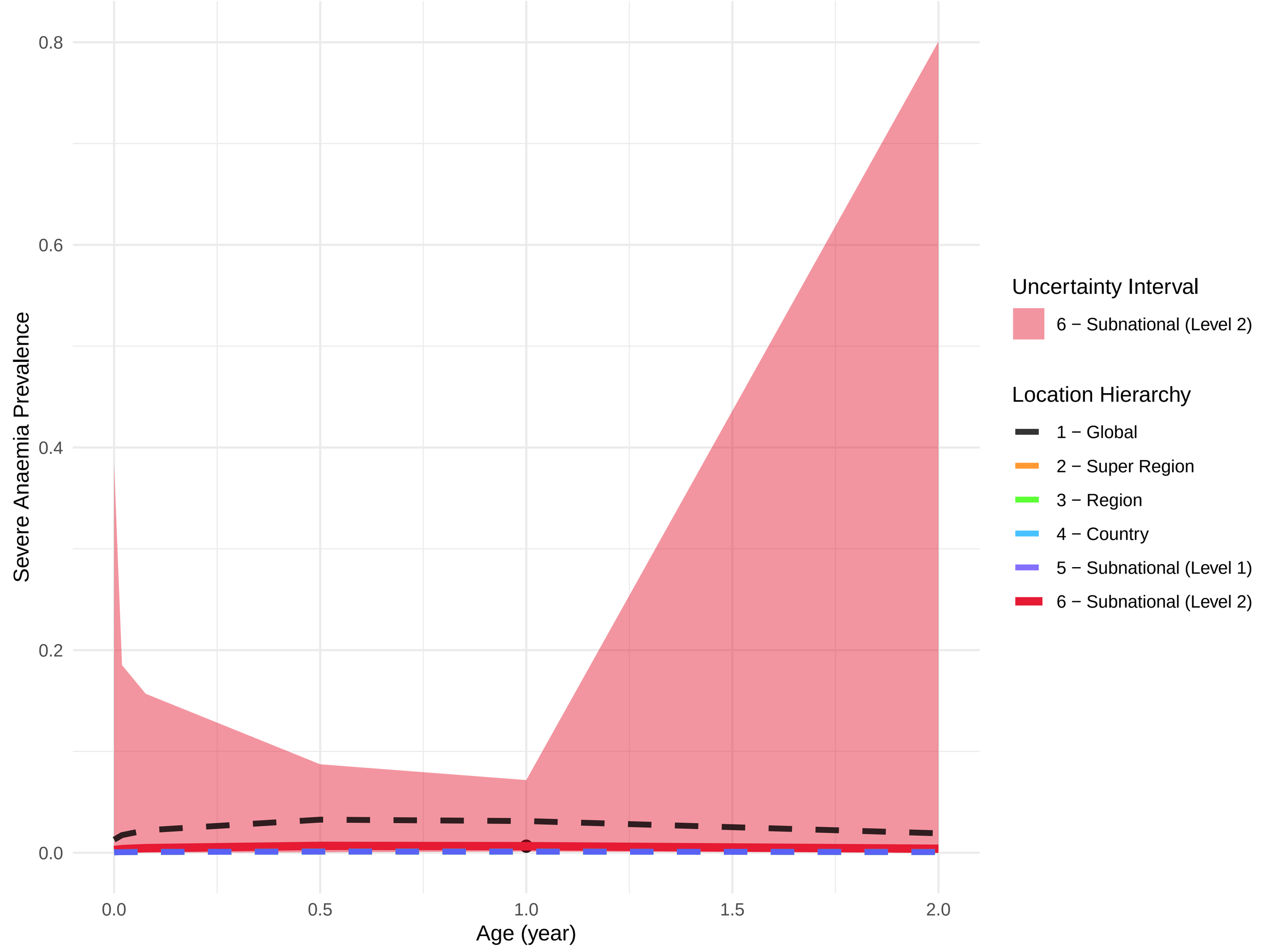

MR−BRT Age/Sex Fits − Non−pregnant Females <2 Years Old − Severe Anaemia Prevalence − East of England
WHO−2024 Adjustment Method

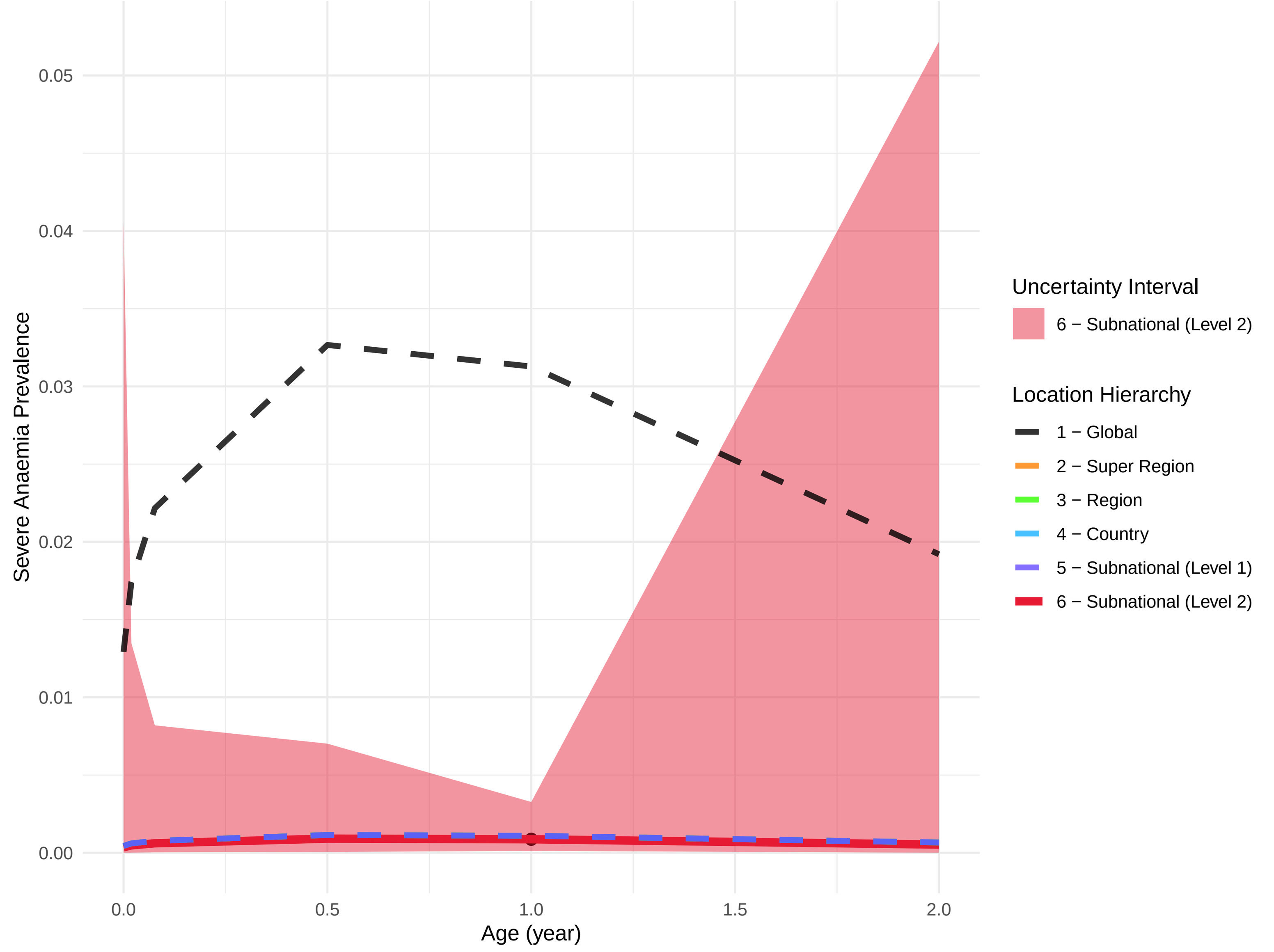

MR−BRT Age/Sex Fits − Non−pregnant Females <2 Years Old − Severe Anaemia Prevalence − West Midlands
WHO−2024 Adjustment Method

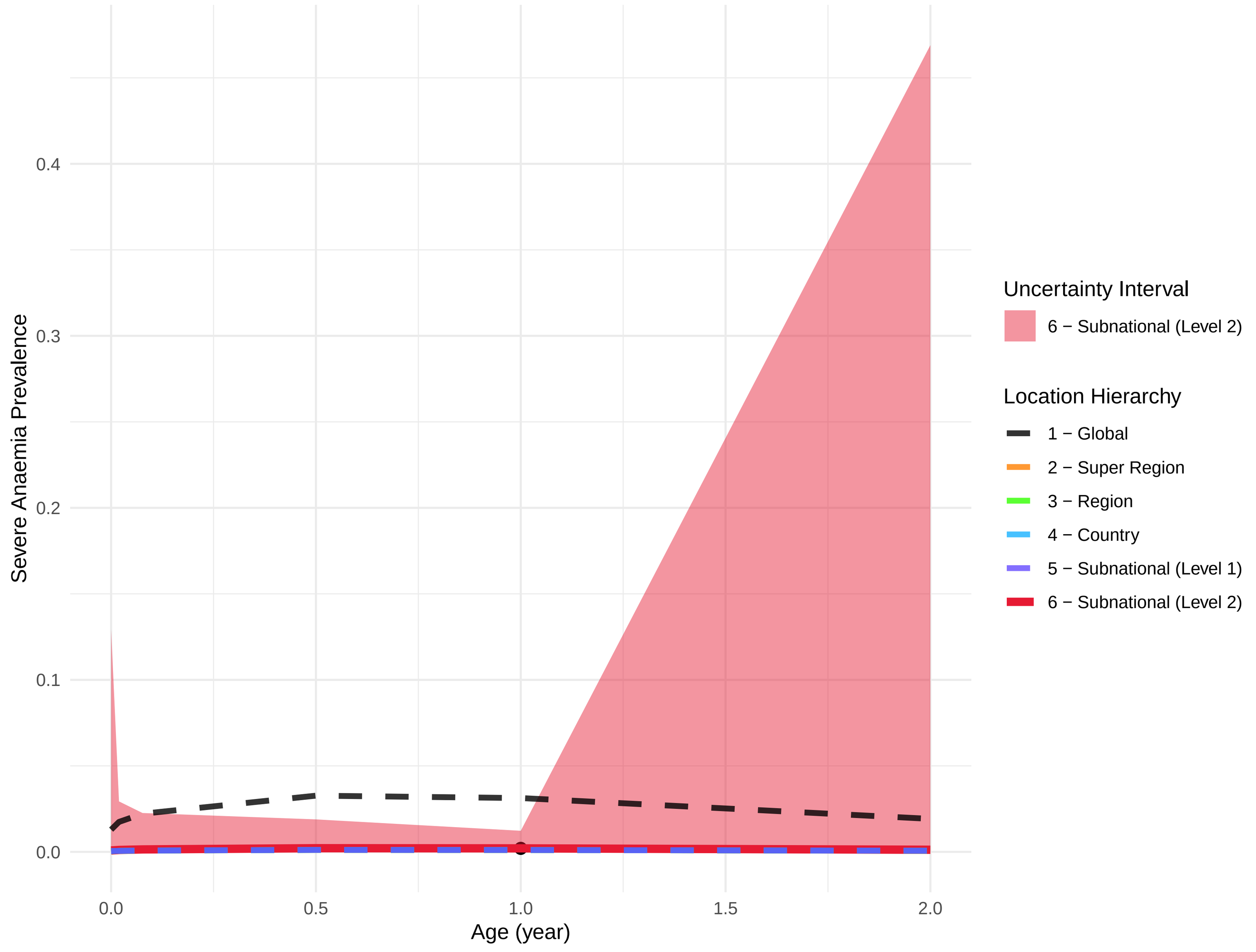

MR−BRT Age/Sex Fits − Non−pregnant Females <2 Years Old − Severe Anaemia Prevalence − Latin America and Caribbean
WHO−2024 Adjustment Method

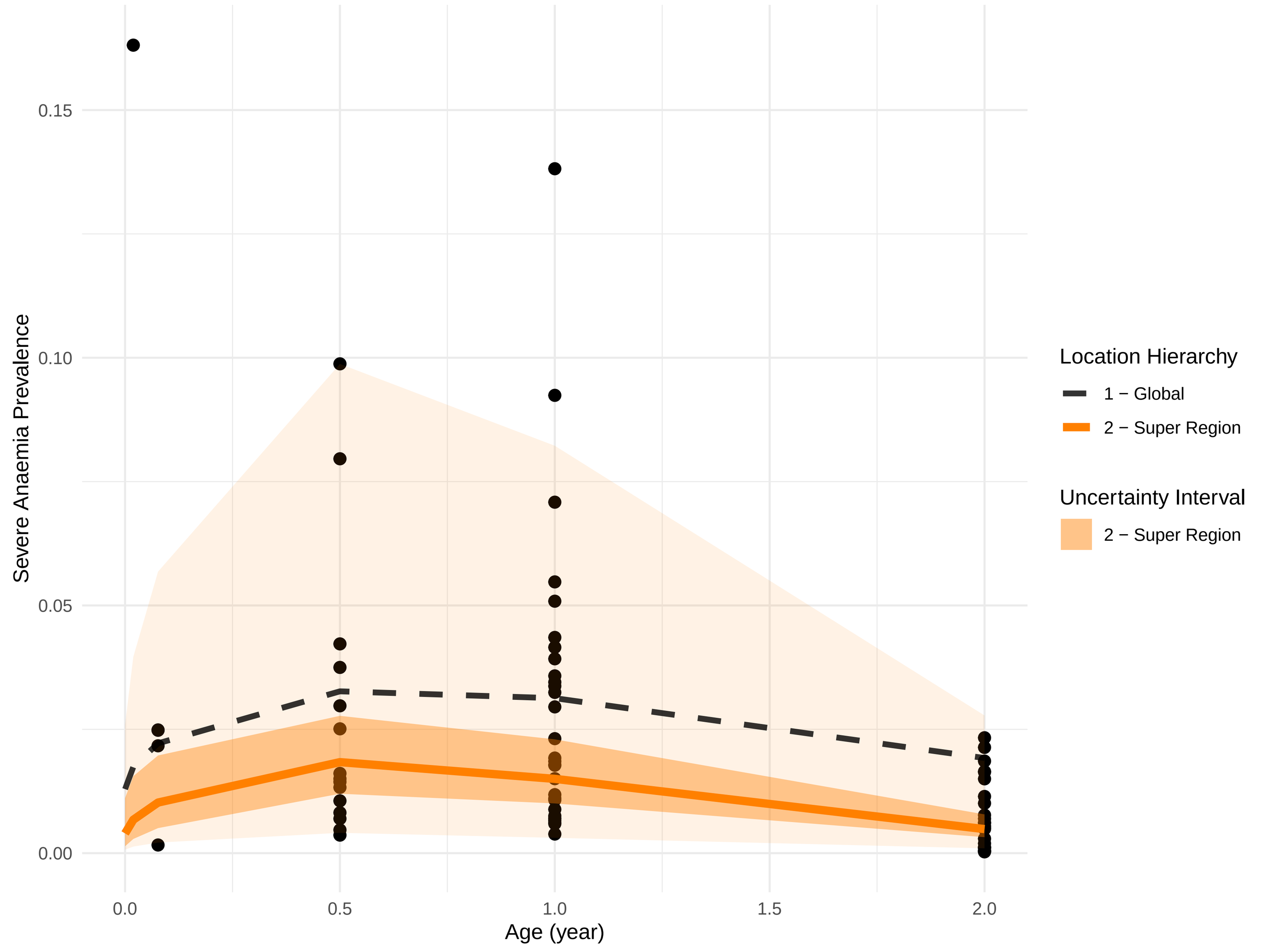

MR−BRT Age/Sex Fits − Non−pregnant Females <2 Years Old − Severe Anaemia Prevalence − Andean Latin America
WHO−2024 Adjustment Method

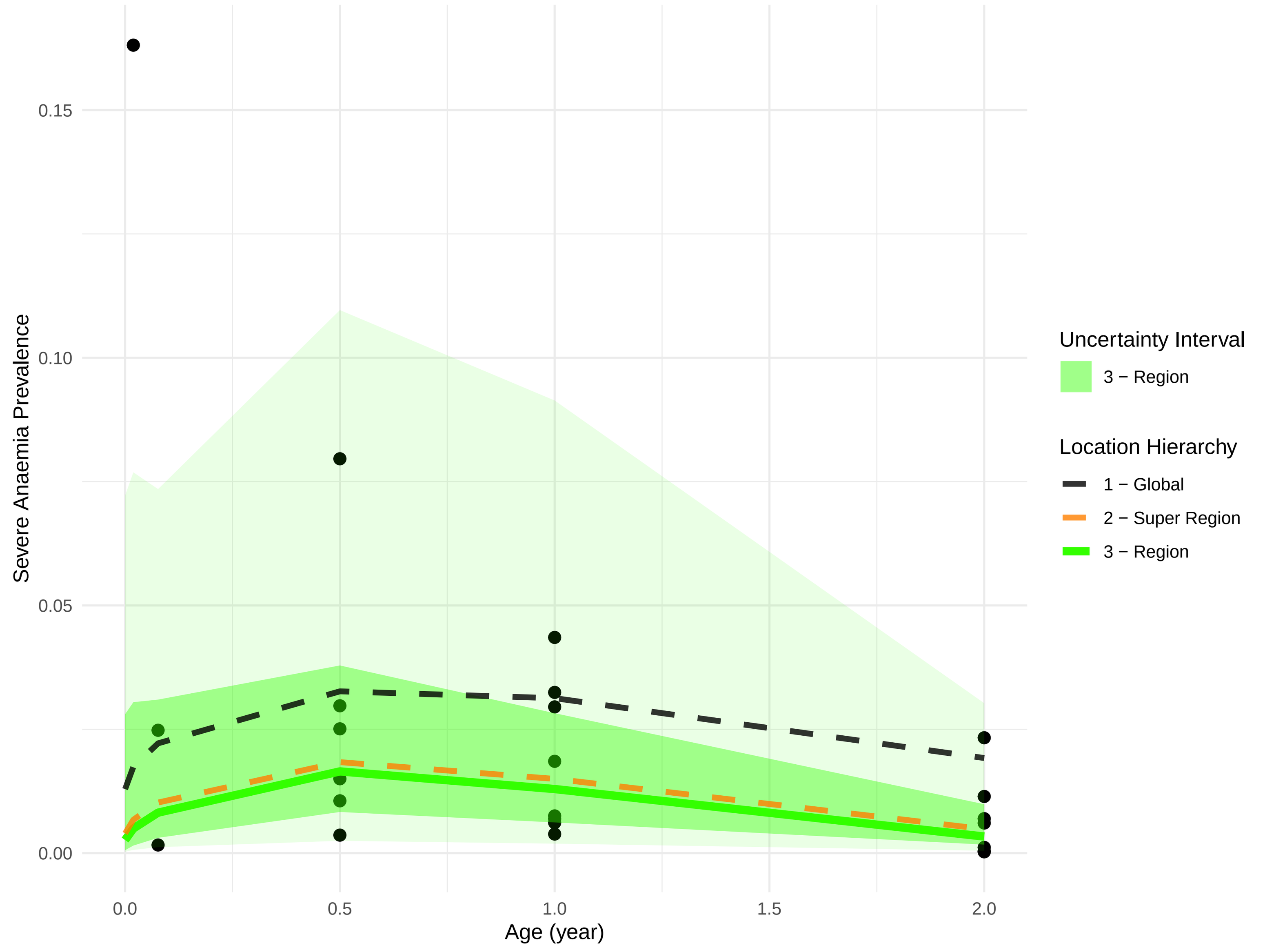

MR−BRT Age/Sex Fits − Non−pregnant Females <2 Years Old − Severe Anaemia Prevalence − Bolivia (Plurinational State of)
WHO−2024 Adjustment Method

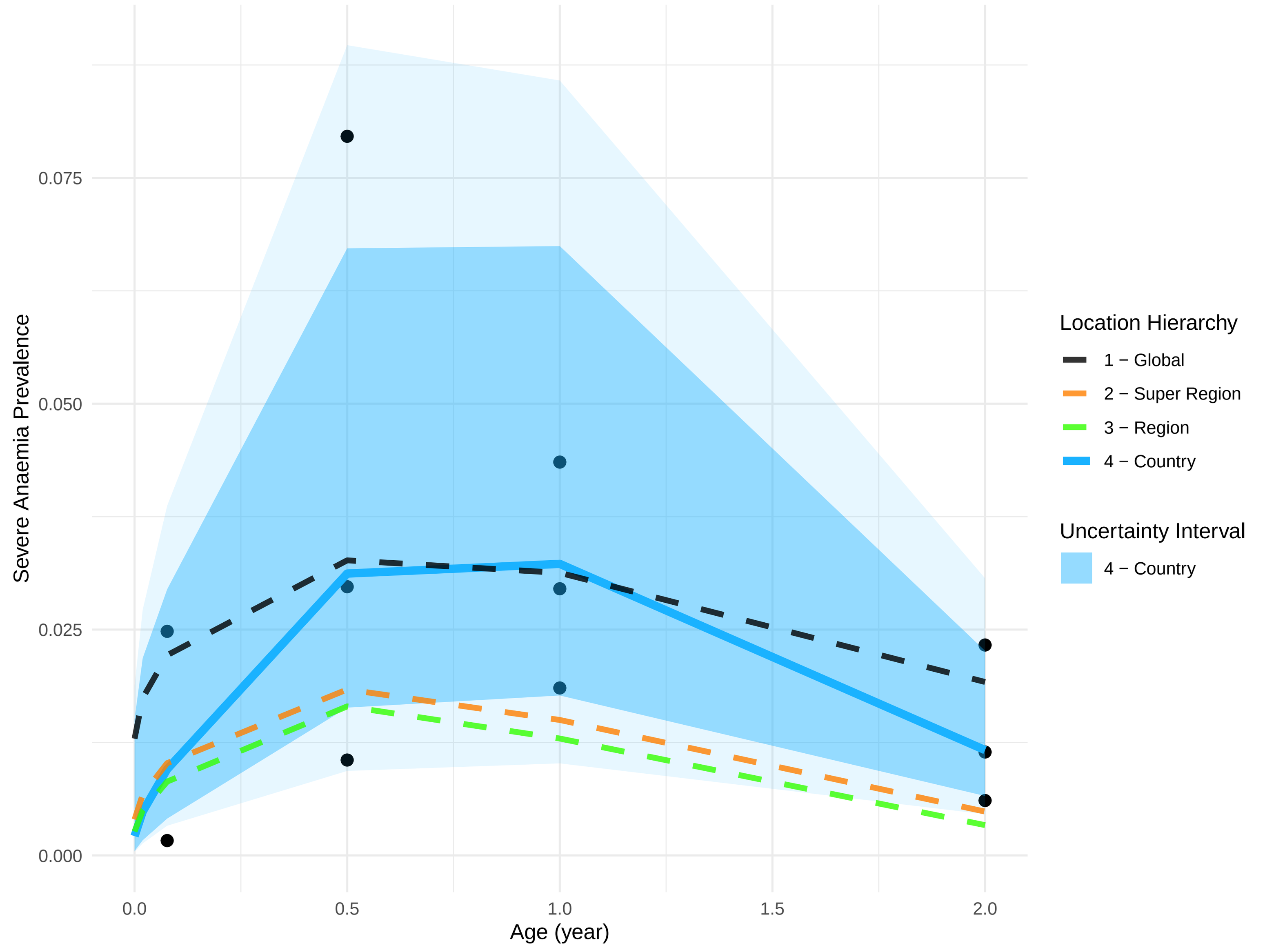

MR−BRT Age/Sex Fits − Non−pregnant Females <2 Years Old − Severe Anaemia Prevalence − Peru
WHO−2024 Adjustment Method

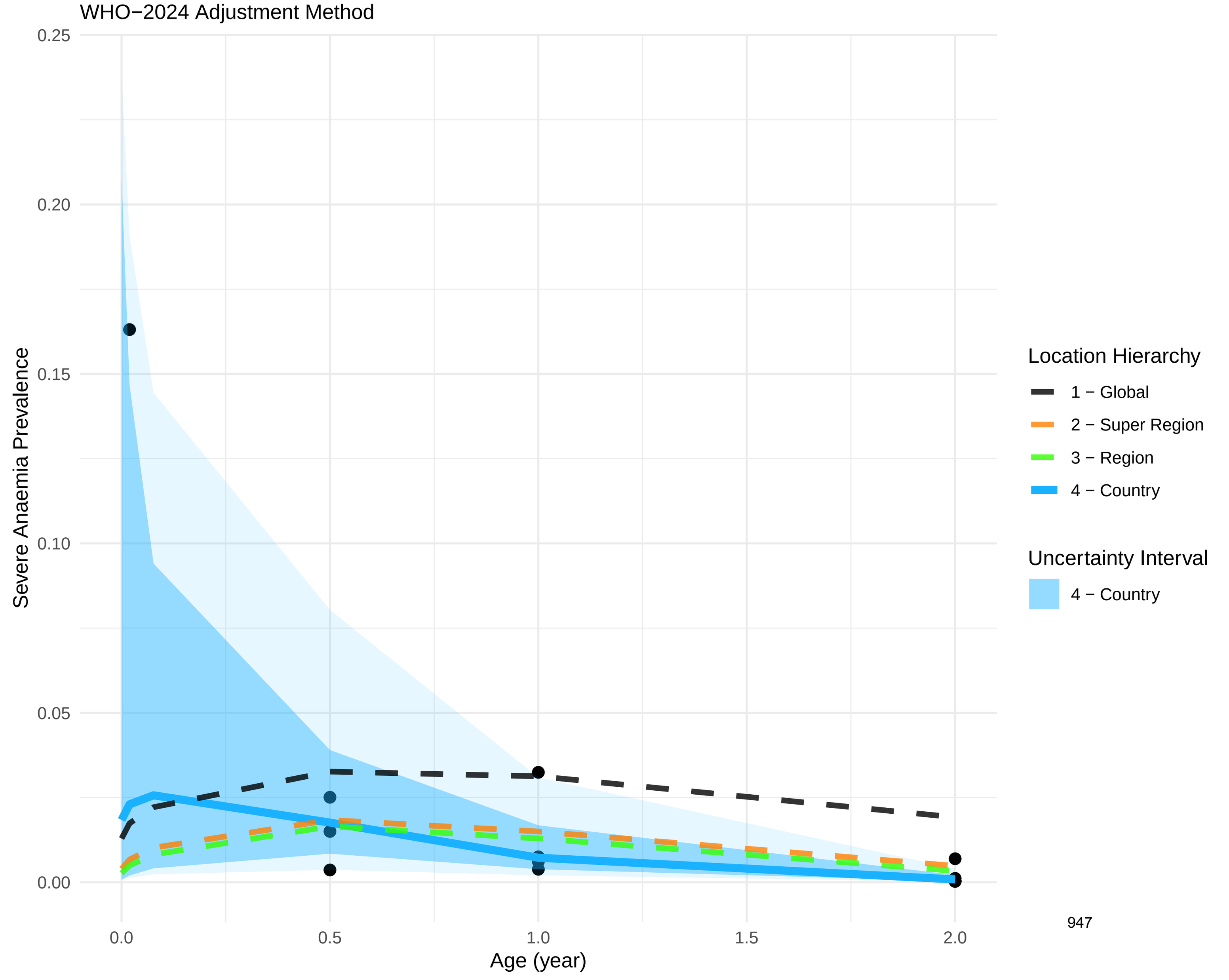

MR−BRT Age/Sex Fits − Non−pregnant Females <2 Years Old − Severe Anaemia Prevalence − Caribbean
WHO−2024 Adjustment Method

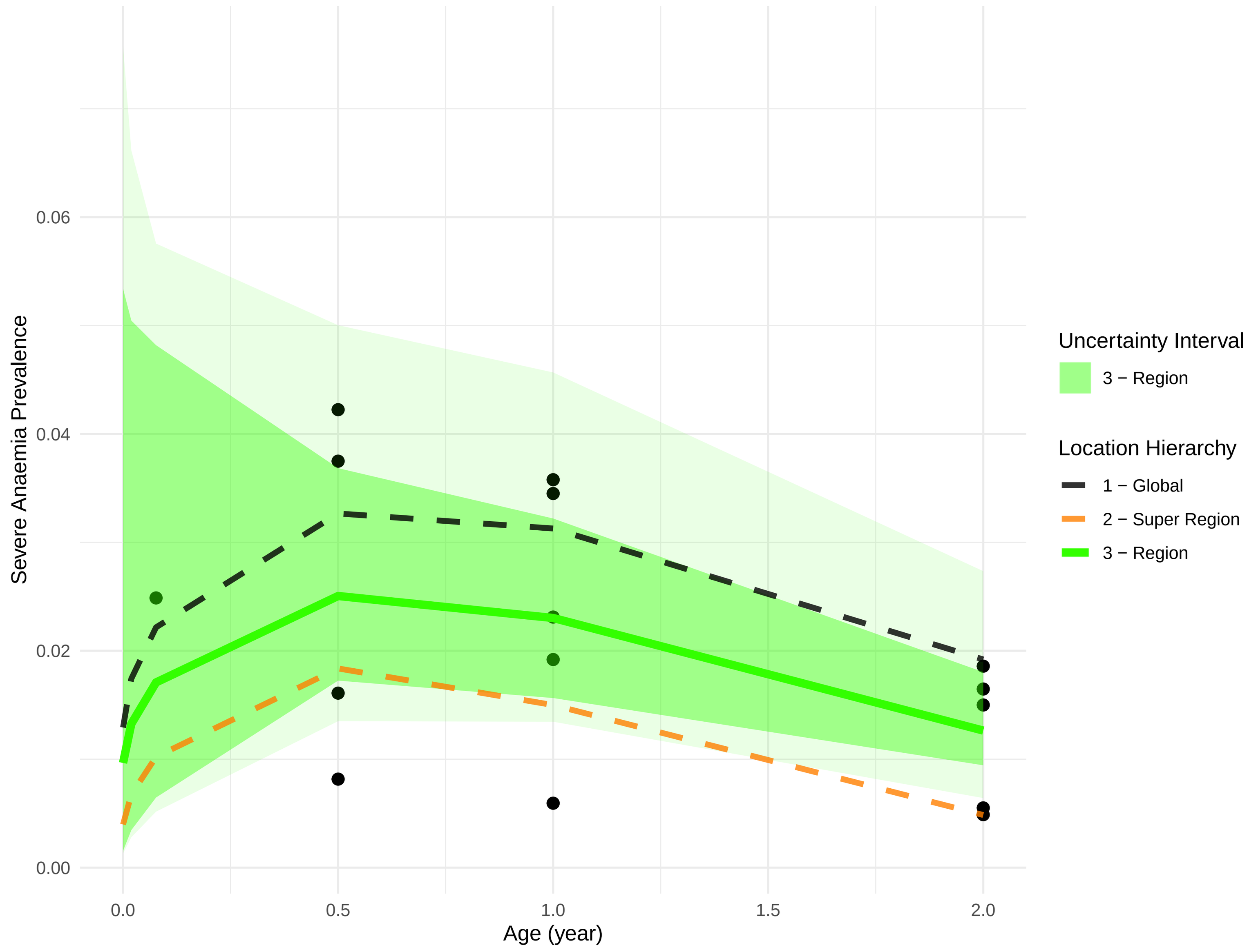

MR−BRT Age/Sex Fits − Non−pregnant Females <2 Years Old − Severe Anaemia Prevalence − Guyana
WHO−2024 Adjustment Method

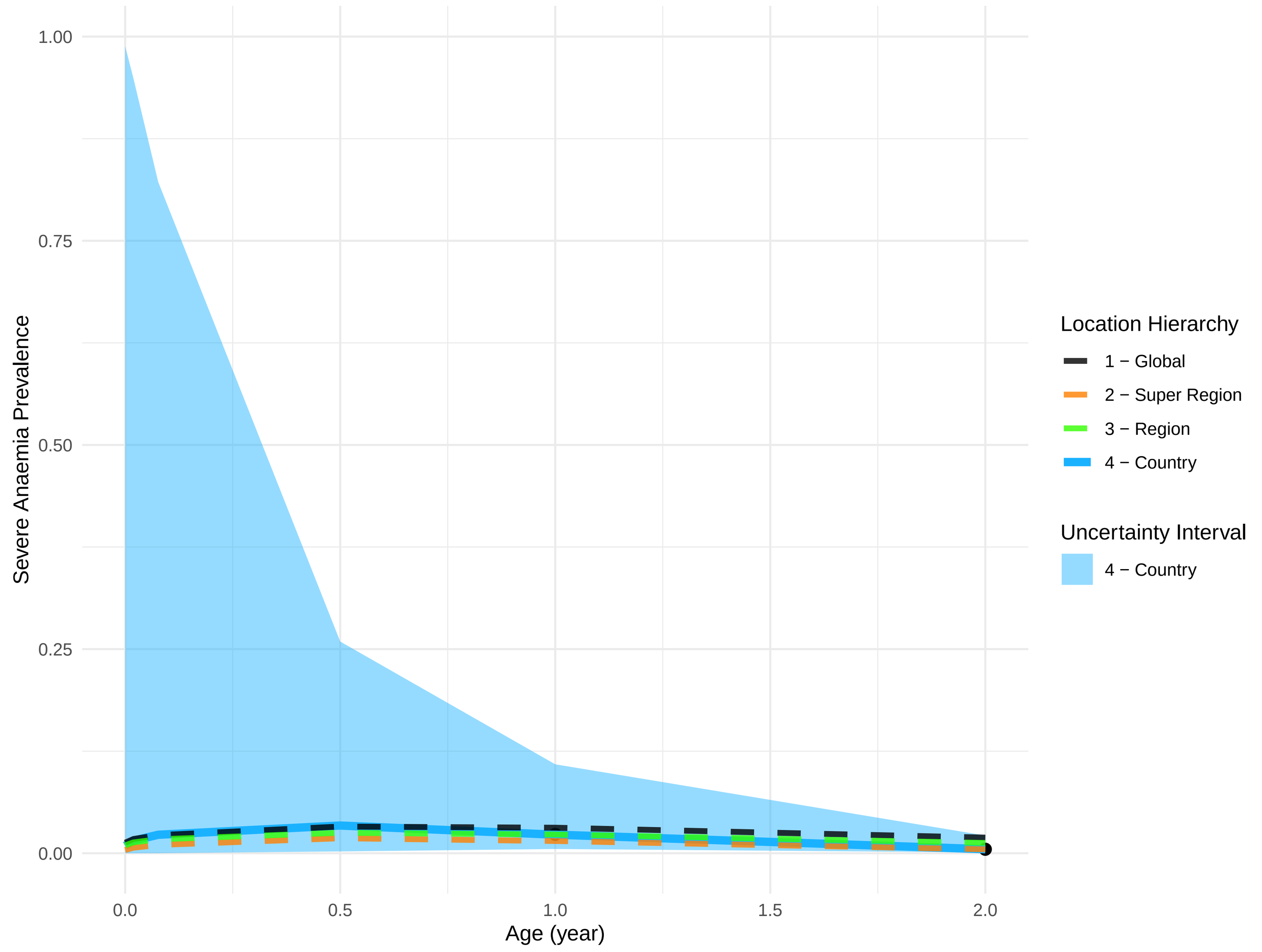

MR−BRT Age/Sex Fits − Non−pregnant Females <2 Years Old − Severe Anaemia Prevalence − Haiti
WHO−2024 Adjustment Method

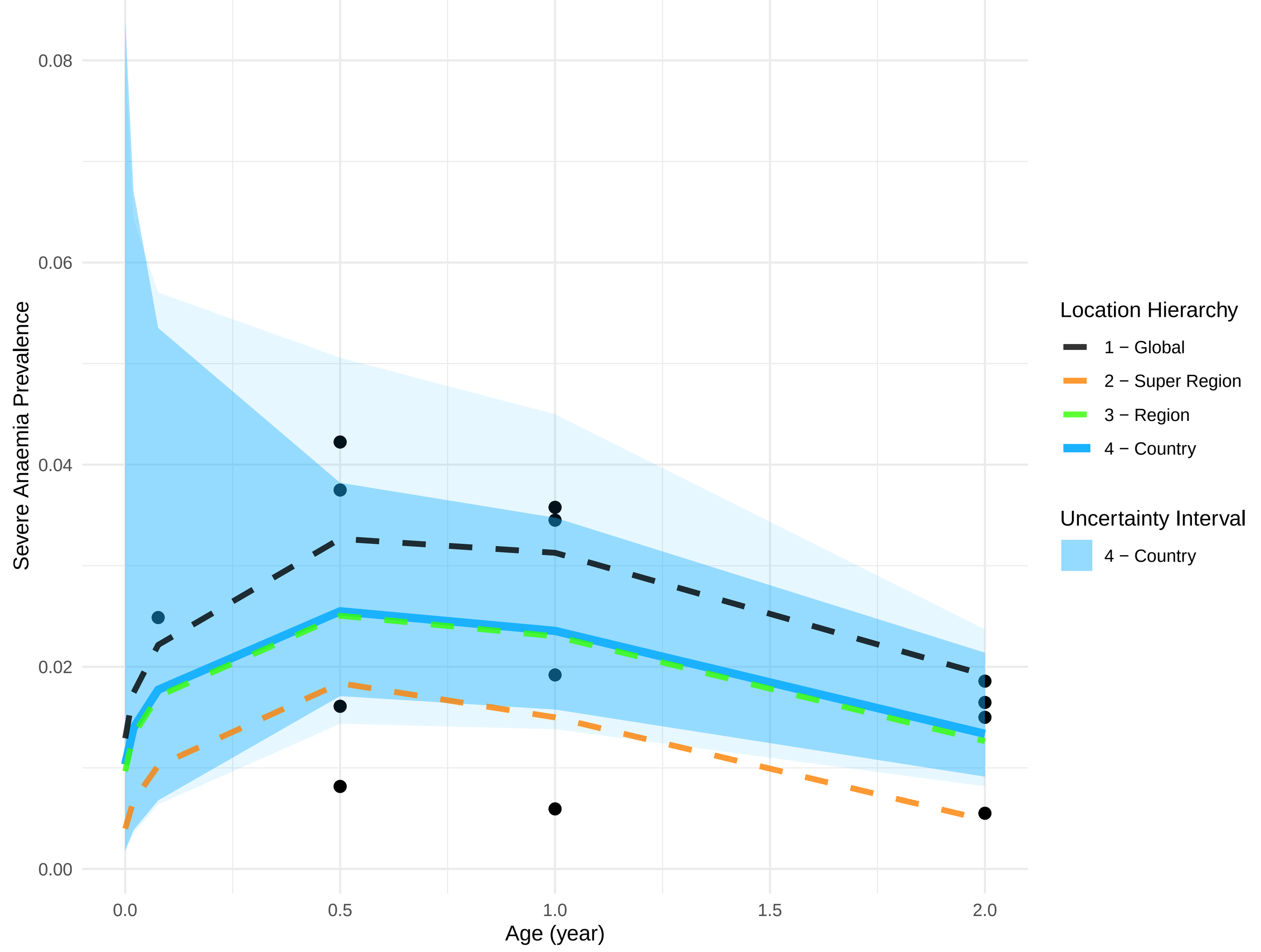

MR−BRT Age/Sex Fits − Non−pregnant Females <2 Years Old − Severe Anaemia Prevalence − Central Latin America
WHO−2024 Adjustment Method

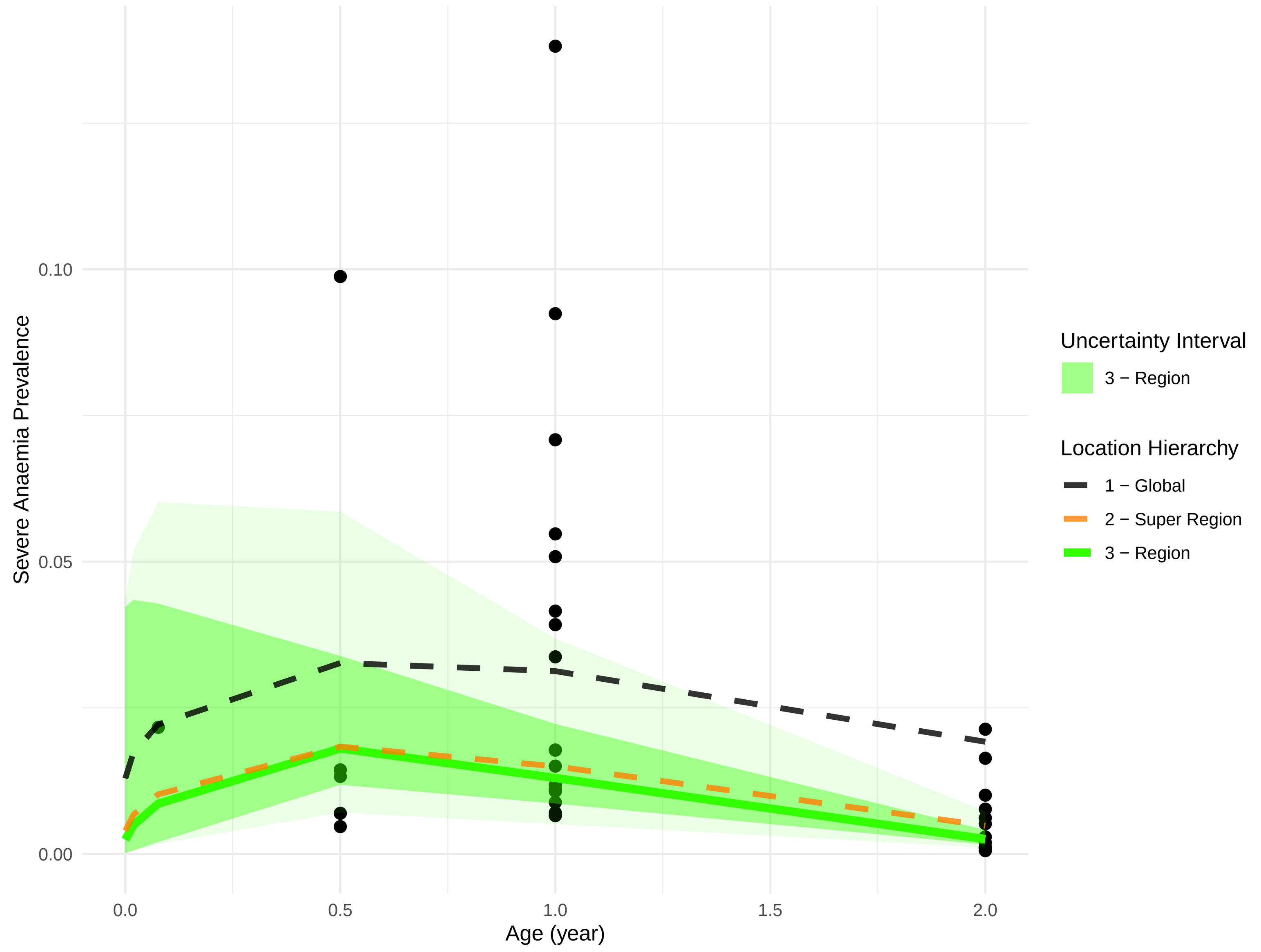

MR−BRT Age/Sex Fits − Non−pregnant Females <2 Years Old − Severe Anaemia Prevalence − Guatemala
WHO−2024 Adjustment Method

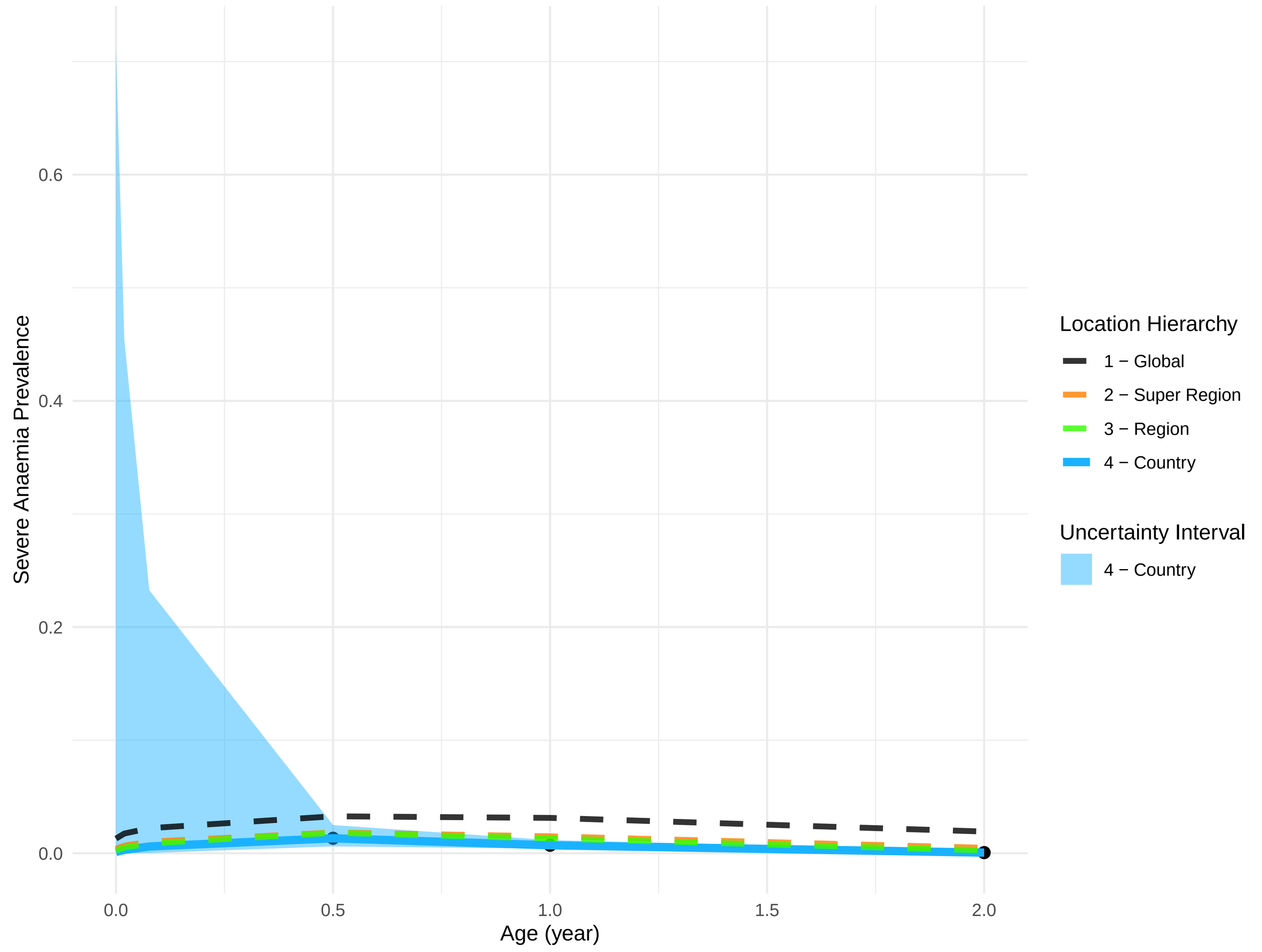

MR−BRT Age/Sex Fits − Non−pregnant Females <2 Years Old − Severe Anaemia Prevalence − Honduras
WHO−2024 Adjustment Method

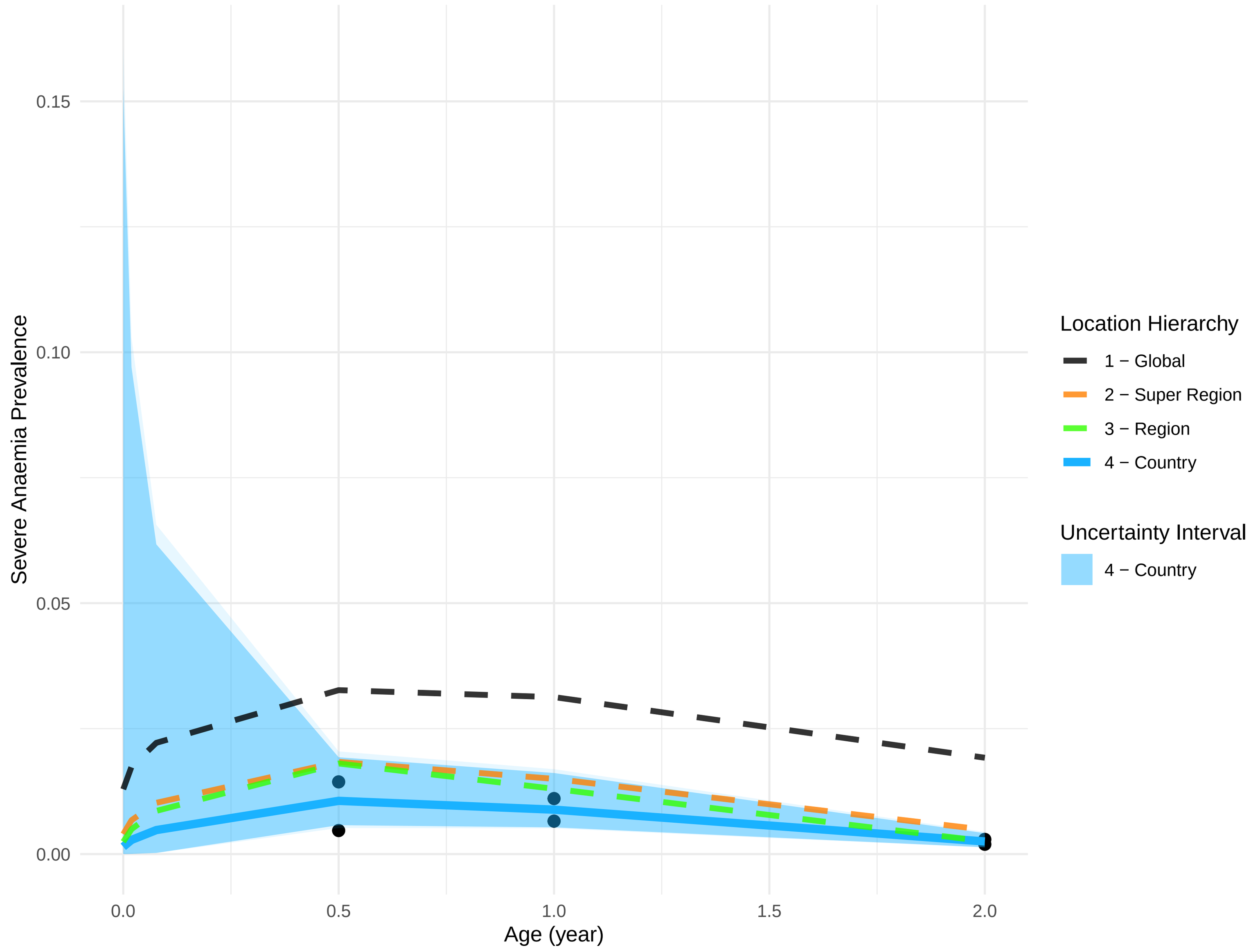

MR−BRT Age/Sex Fits − Non−pregnant Females <2 Years Old − Severe Anaemia Prevalence − Mexico
WHO−2024 Adjustment Method

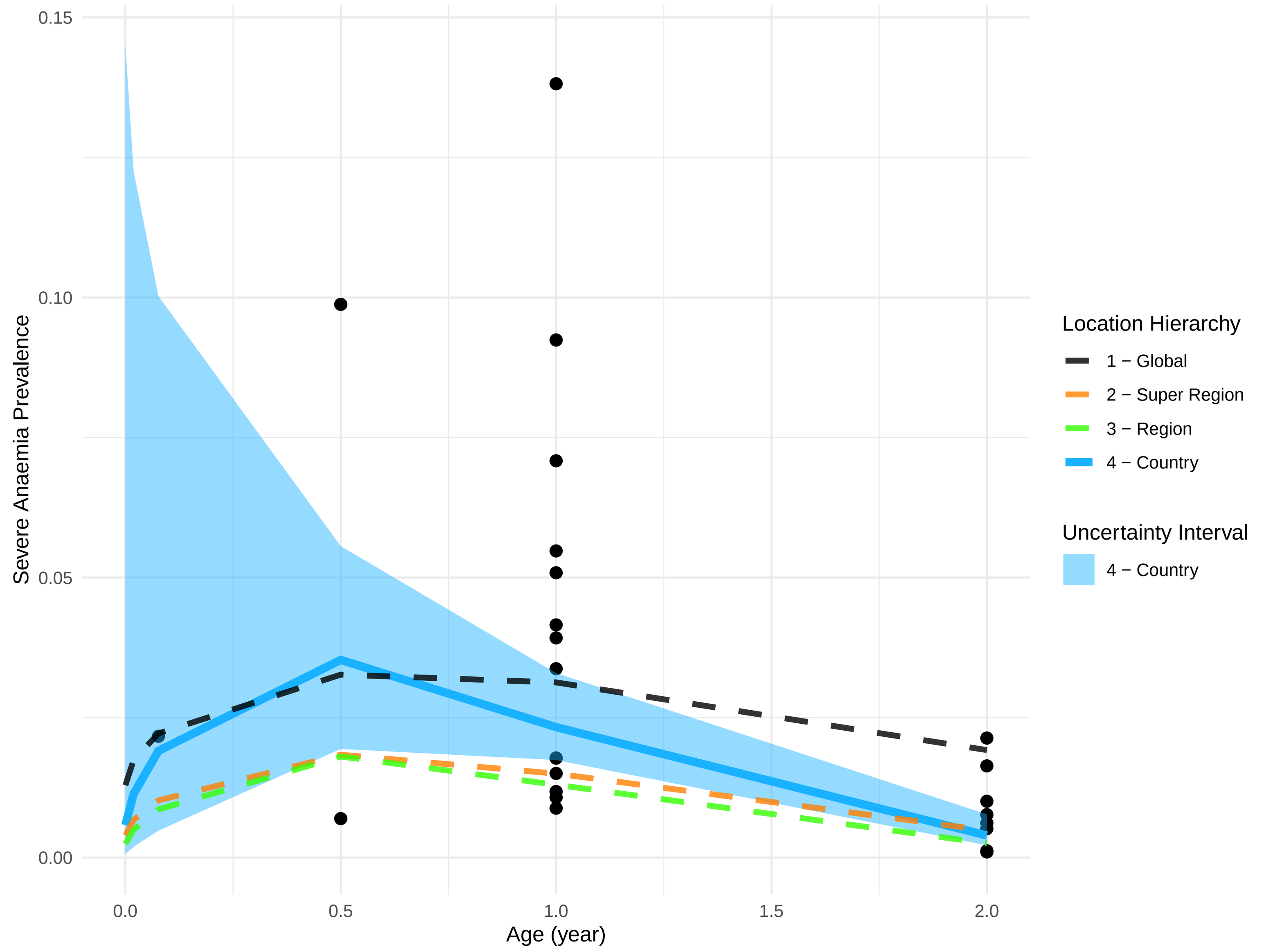

MR−BRT Age/Sex Fits − Non−pregnant Females <2 Years Old − Severe Anaemia Prevalence − Baja California
WHO−2024 Adjustment Method

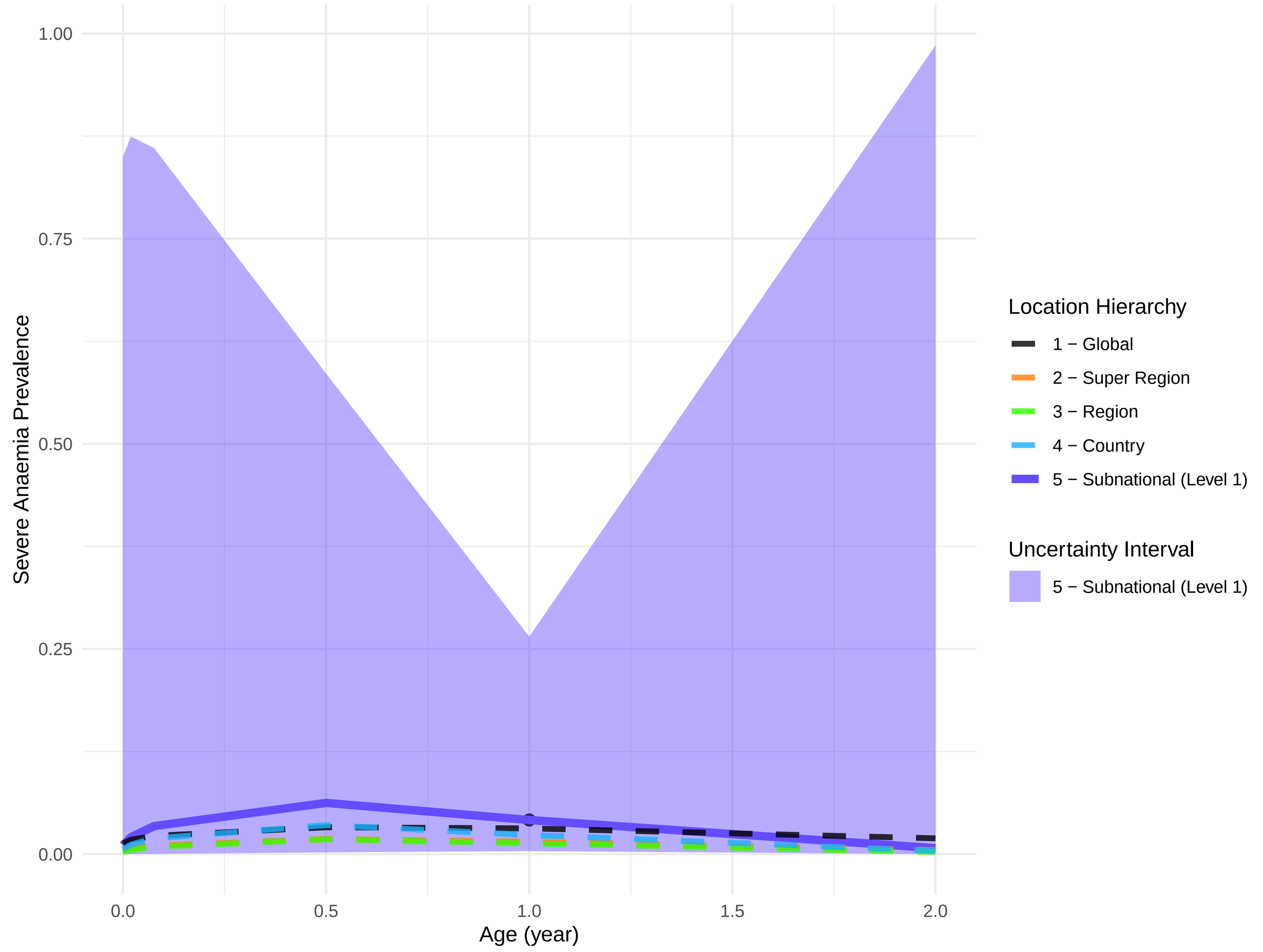

MR−BRT Age/Sex Fits − Non−pregnant Females <2 Years Old − Severe Anaemia Prevalence − Baja California Sur
WHO−2024 Adjustment Method

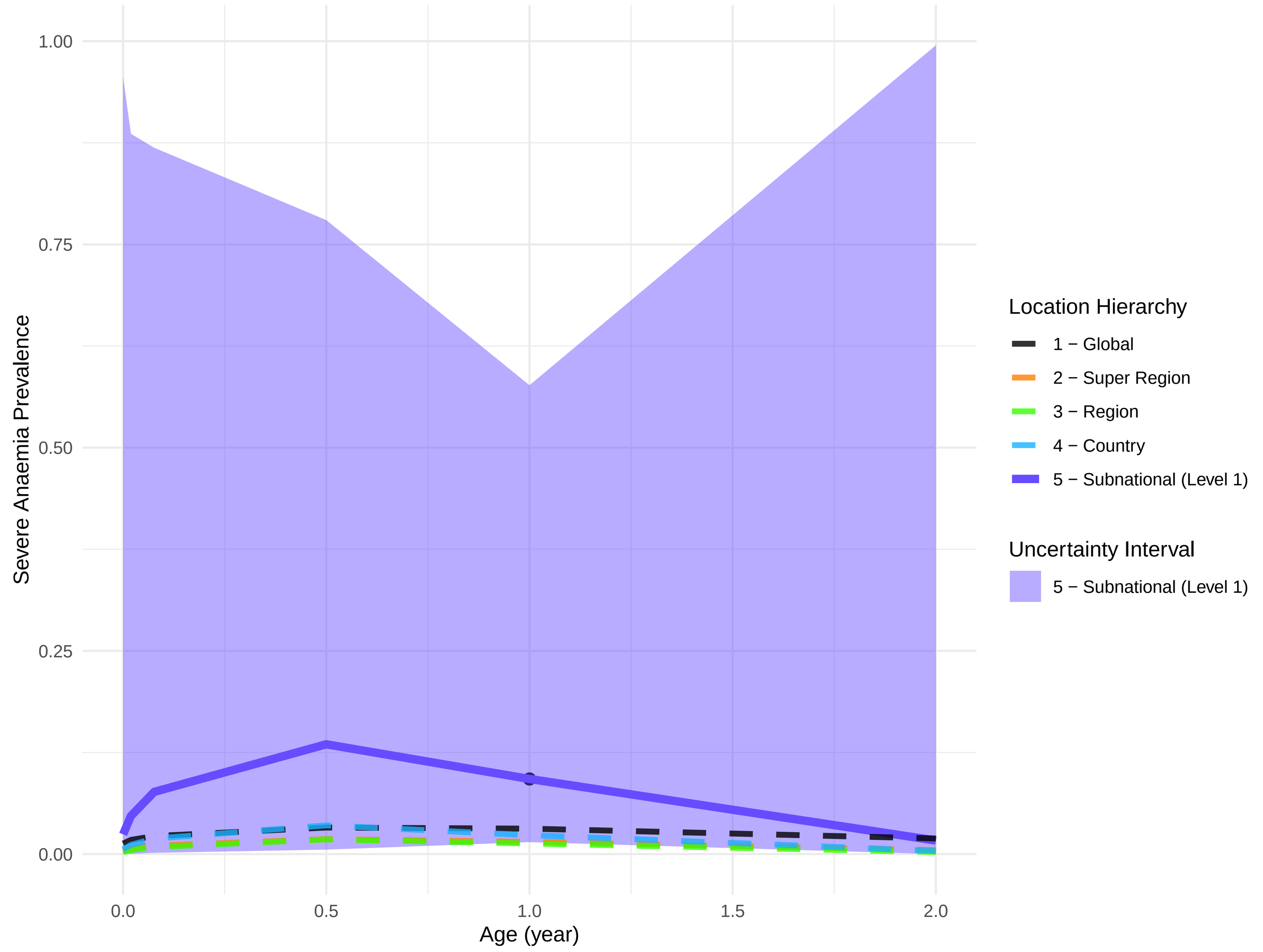

MR−BRT Age/Sex Fits − Non−pregnant Females <2 Years Old − Severe Anaemia Prevalence − Guanajuato
WHO−2024 Adjustment Method

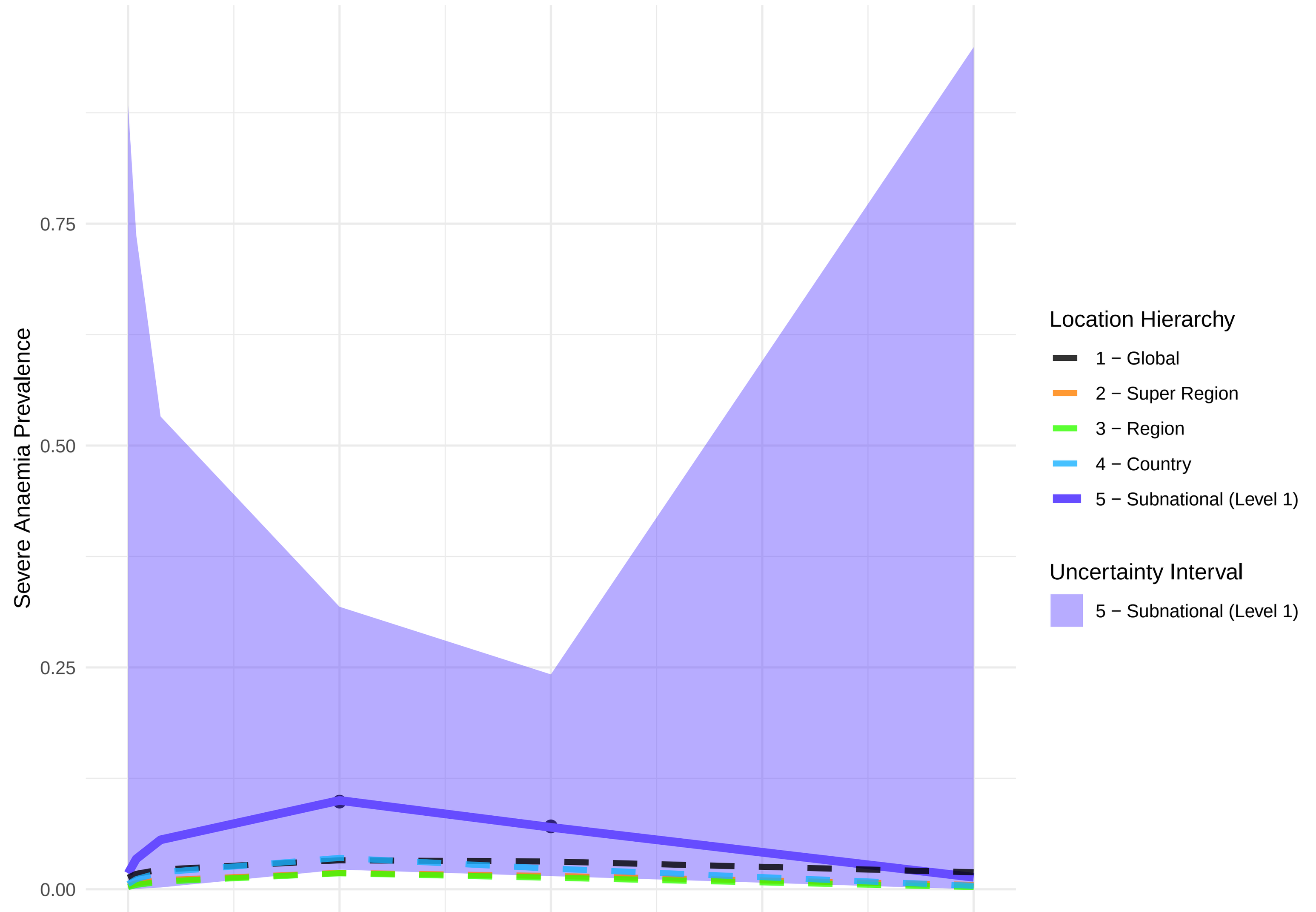

MR−BRT Age/Sex Fits − Non−pregnant Females <2 Years Old − Severe Anaemia Prevalence − Guerrero
WHO−2024 Adjustment Method

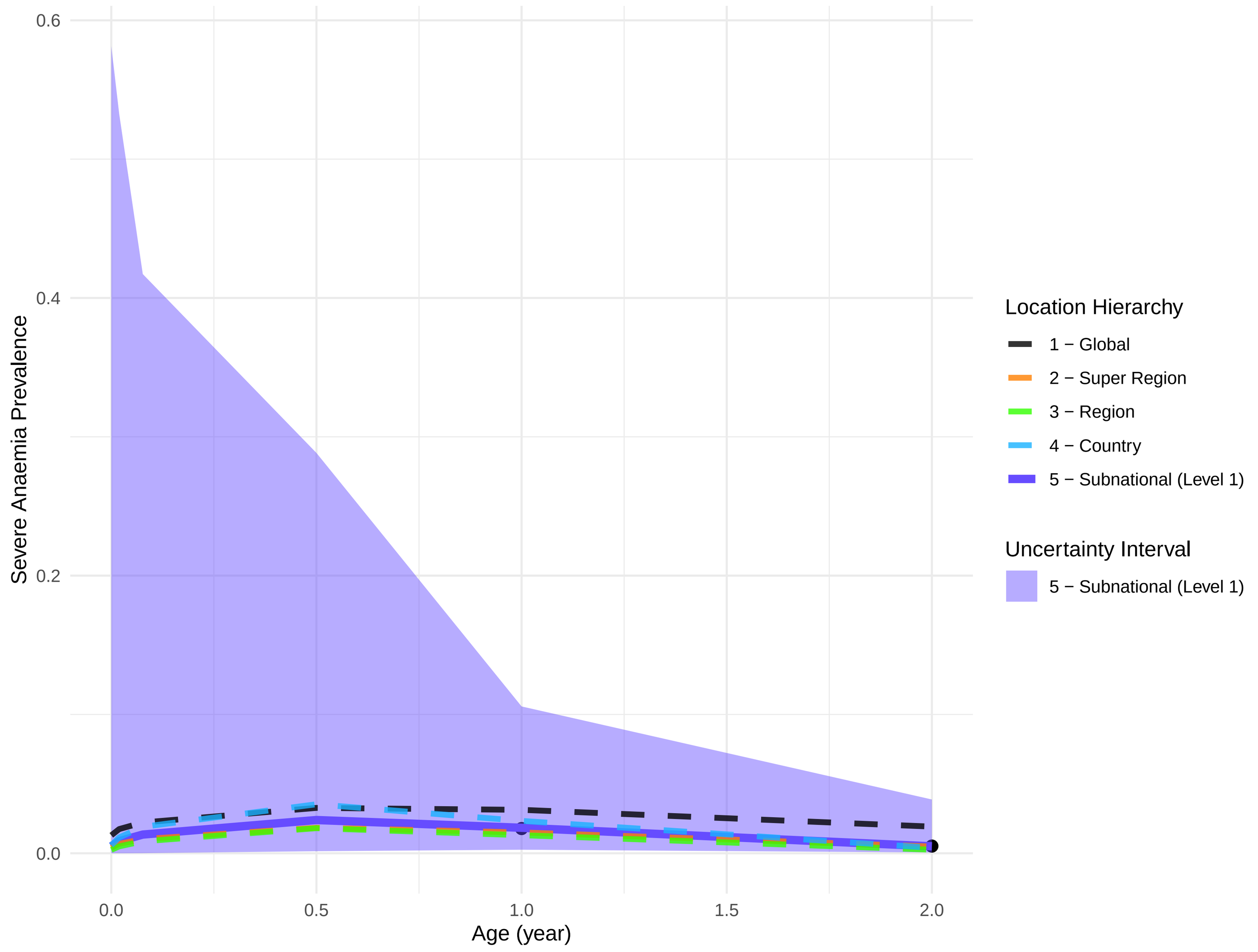

MR−BRT Age/Sex Fits − Non−pregnant Females <2 Years Old − Severe Anaemia Prevalence − Jalisco
WHO−2024 Adjustment Method

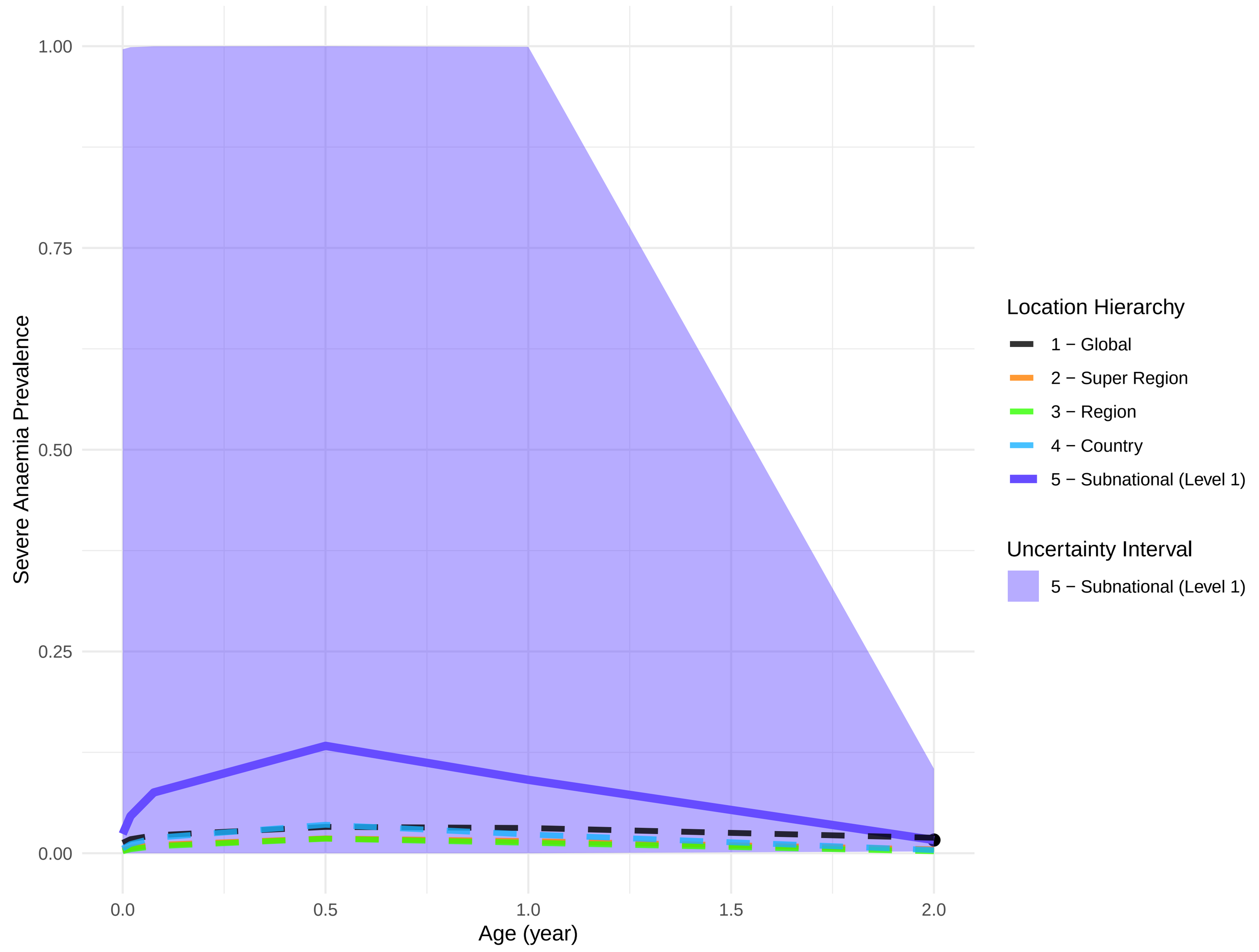

MR−BRT Age/Sex Fits − Non−pregnant Females <2 Years Old − Severe Anaemia Prevalence − México
WHO−2024 Adjustment Method

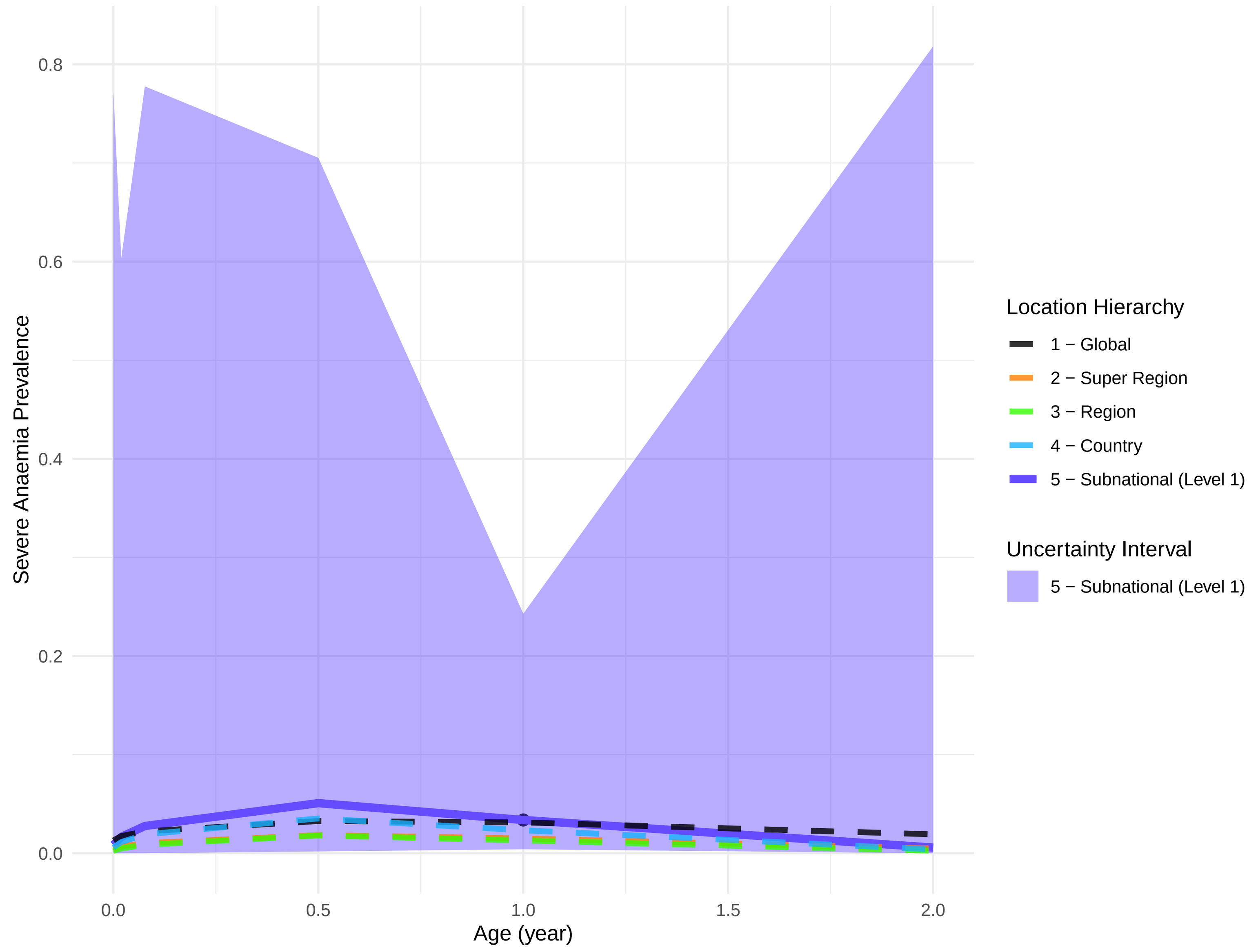

MR−BRT Age/Sex Fits − Non−pregnant Females <2 Years Old − Severe Anaemia Prevalence − Mexico City
WHO−2024 Adjustment Method

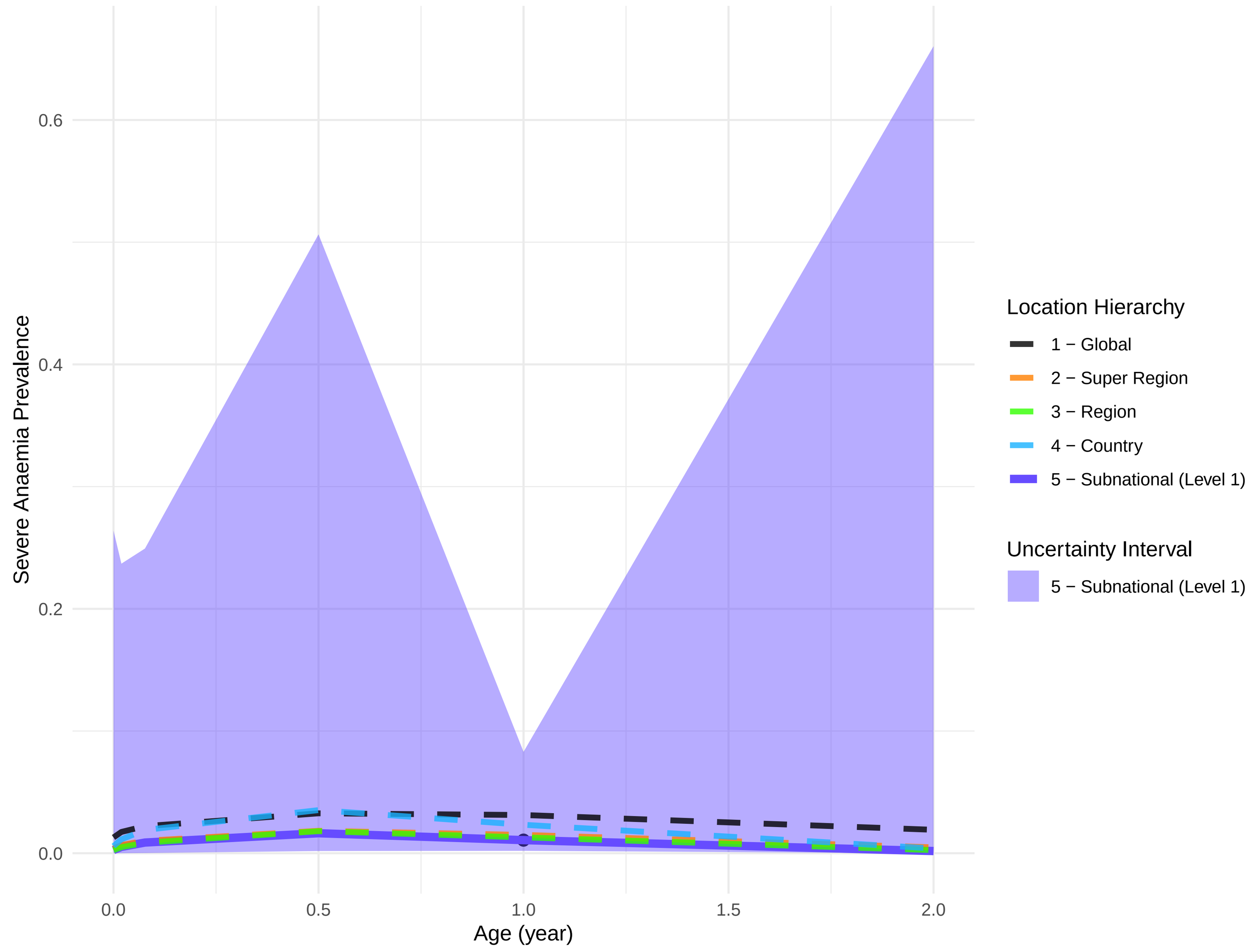

MR−BRT Age/Sex Fits − Non−pregnant Females <2 Years Old − Severe Anaemia Prevalence − Michoacán de Ocampo
WHO−2024 Adjustment Method

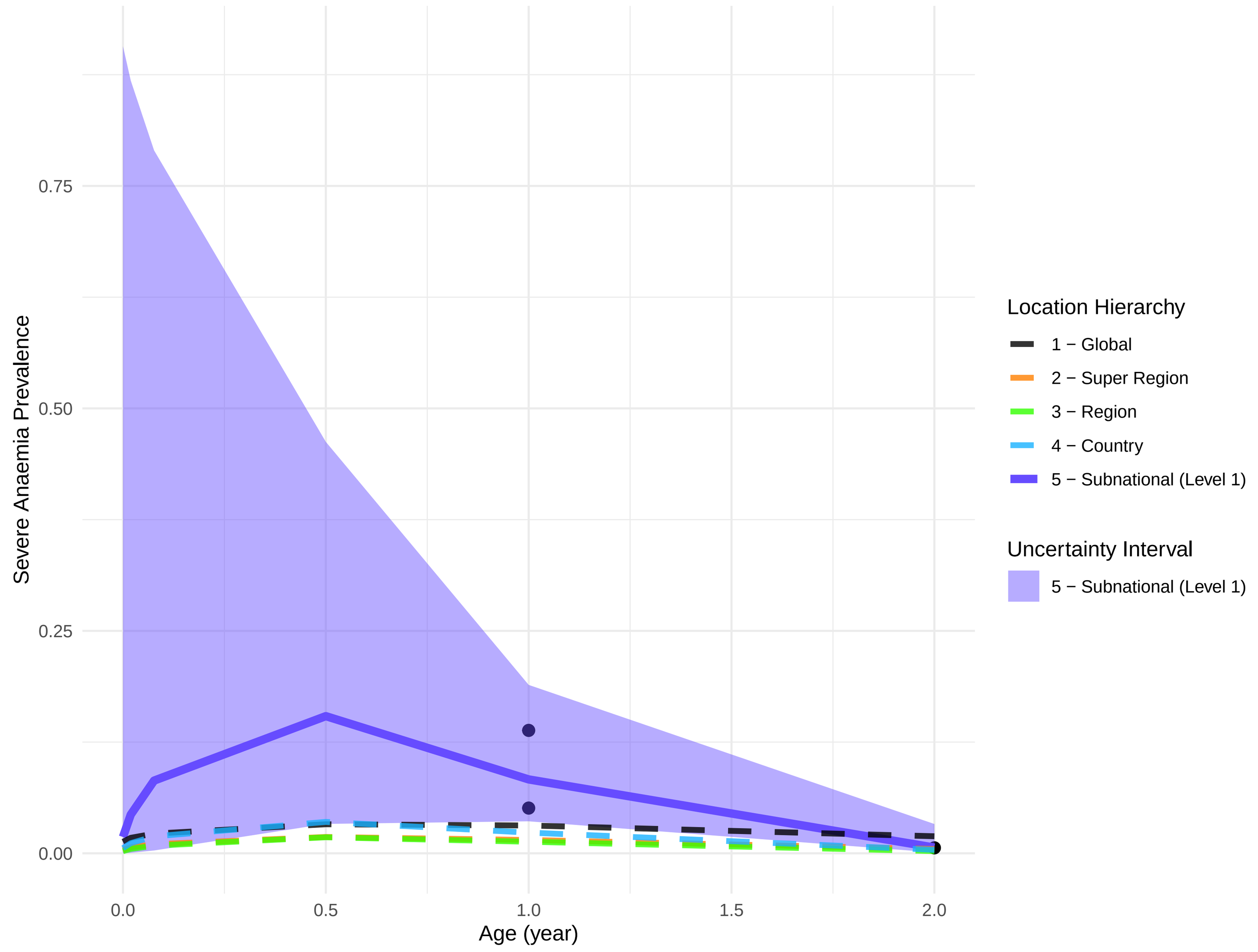

MR−BRT Age/Sex Fits − Non−pregnant Females <2 Years Old − Severe Anaemia Prevalence − Querétaro
WHO−2024 Adjustment Method

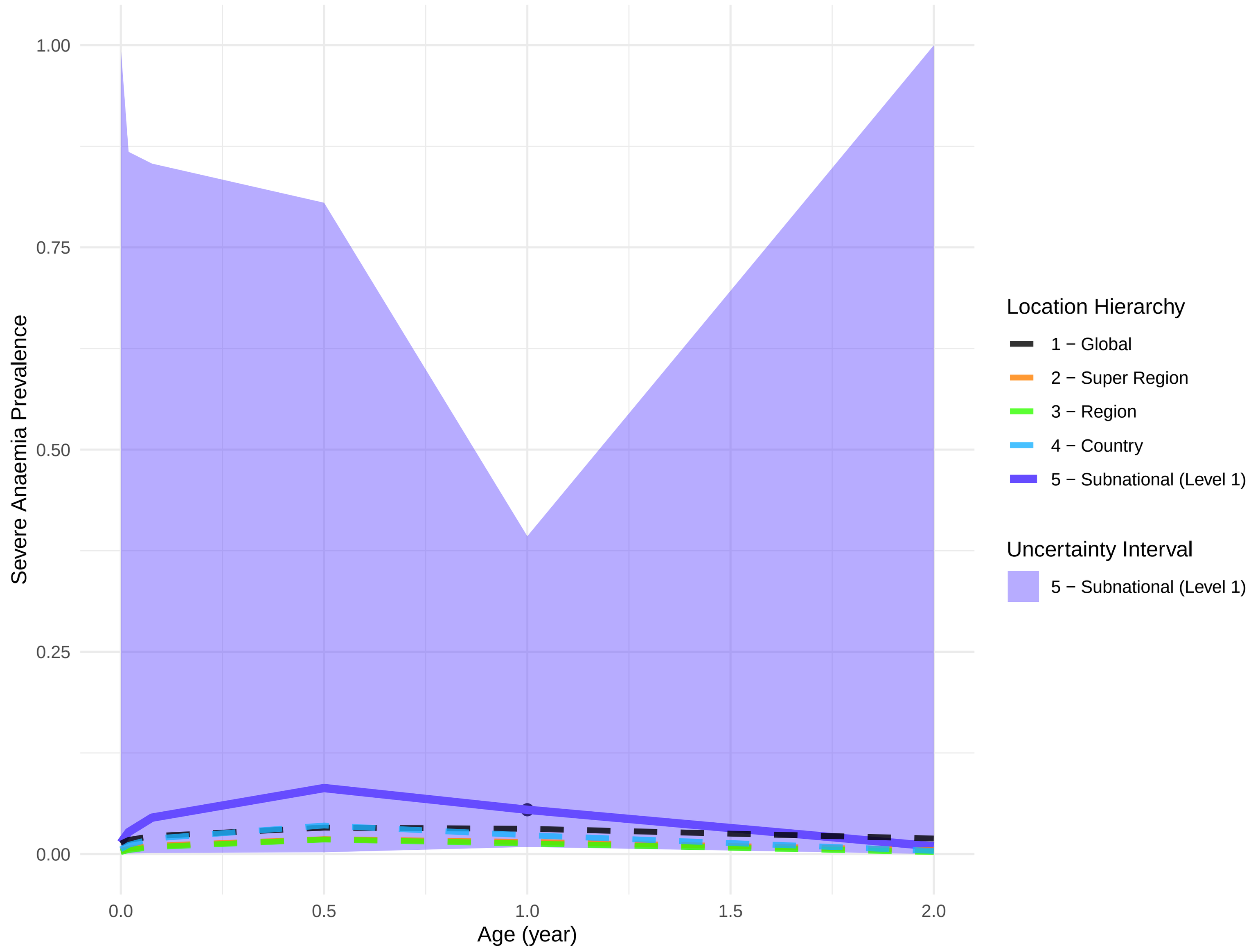

MR−BRT Age/Sex Fits − Non−pregnant Females <2 Years Old − Severe Anaemia Prevalence − San Luis Potosí
WHO−2024 Adjustment Method

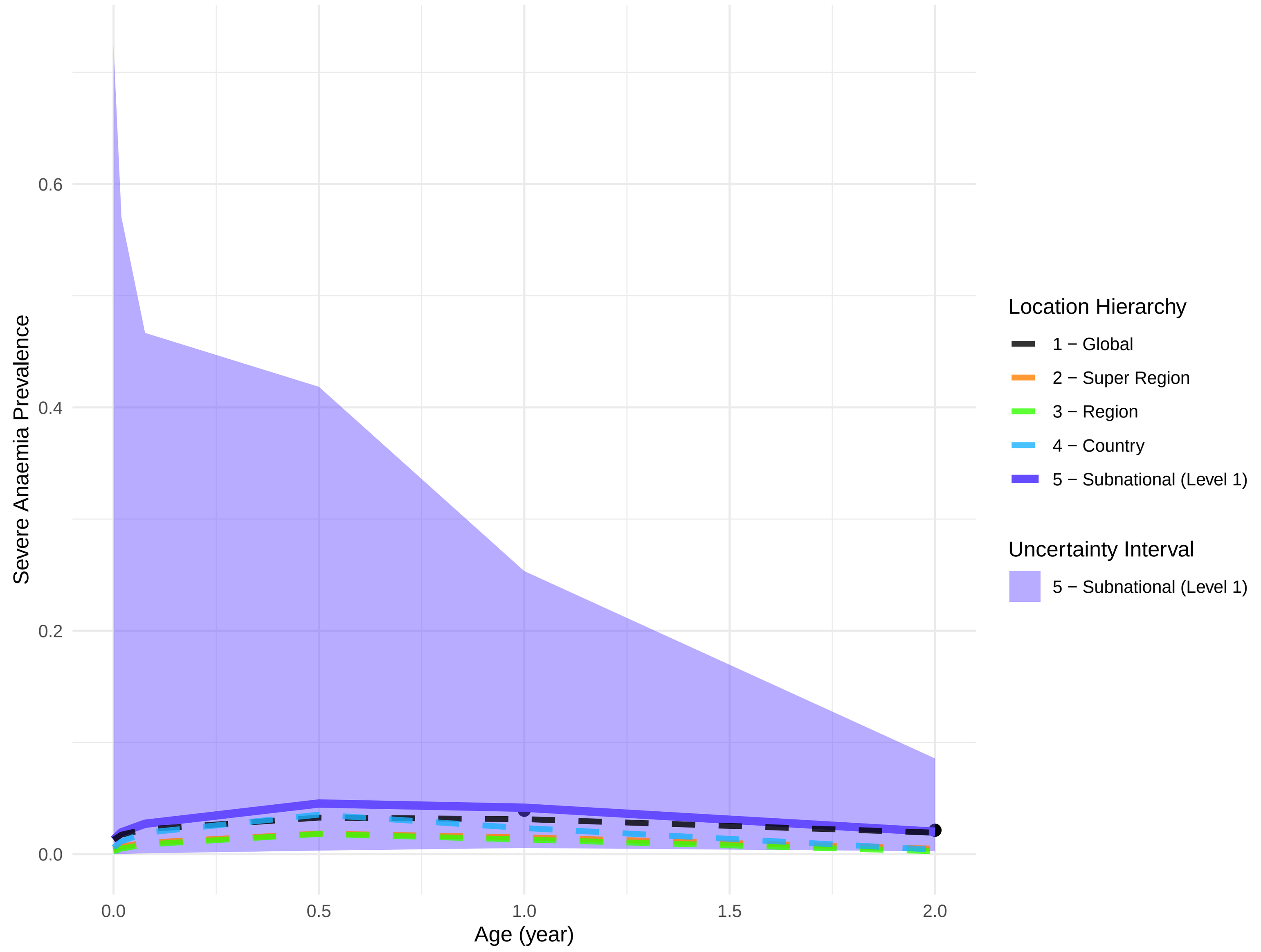

MR−BRT Age/Sex Fits − Non−pregnant Females <2 Years Old − Severe Anaemia Prevalence − Sinaloa
WHO−2024 Adjustment Method

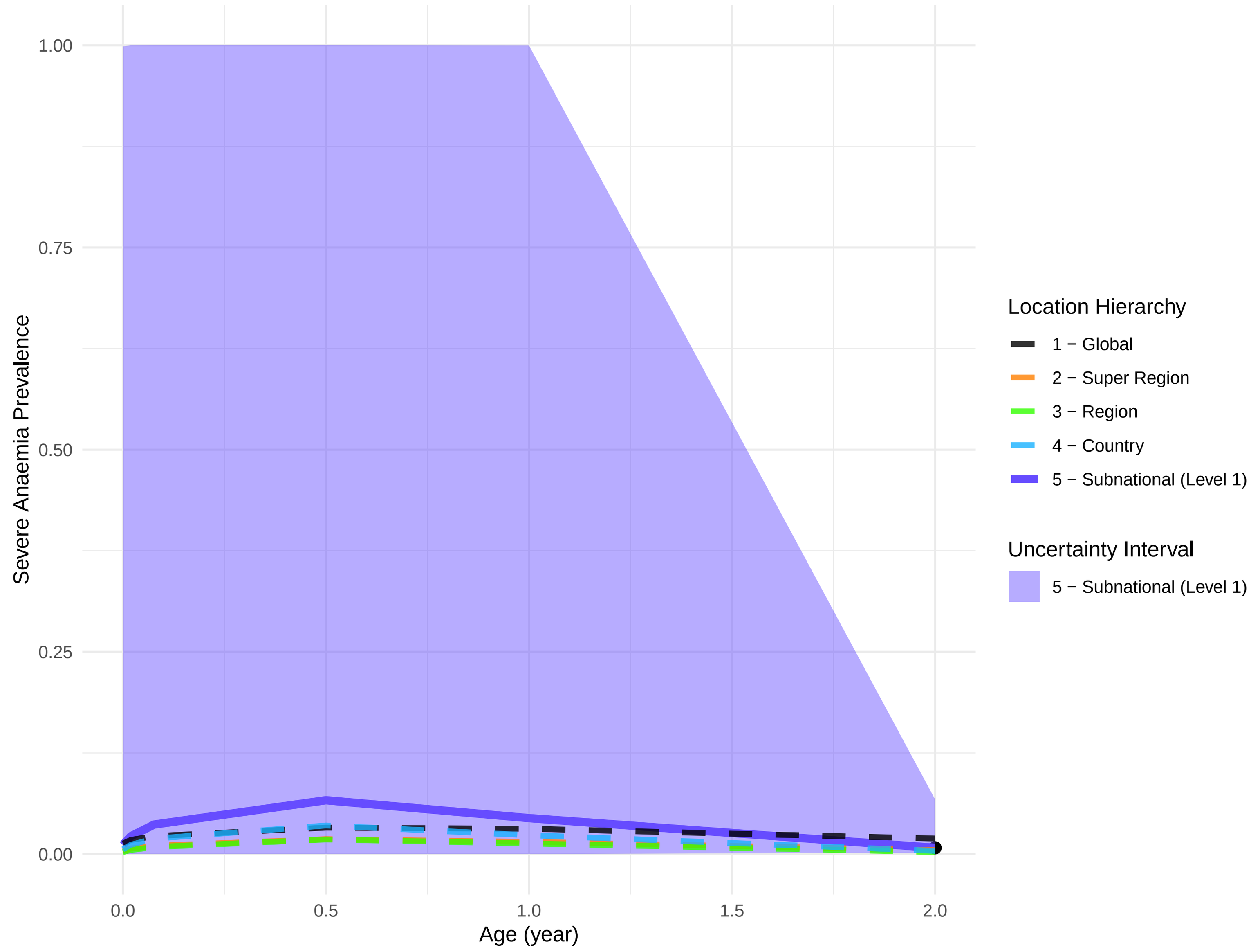

MR−BRT Age/Sex Fits − Non−pregnant Females <2 Years Old − Severe Anaemia Prevalence − Sonora
WHO−2024 Adjustment Method

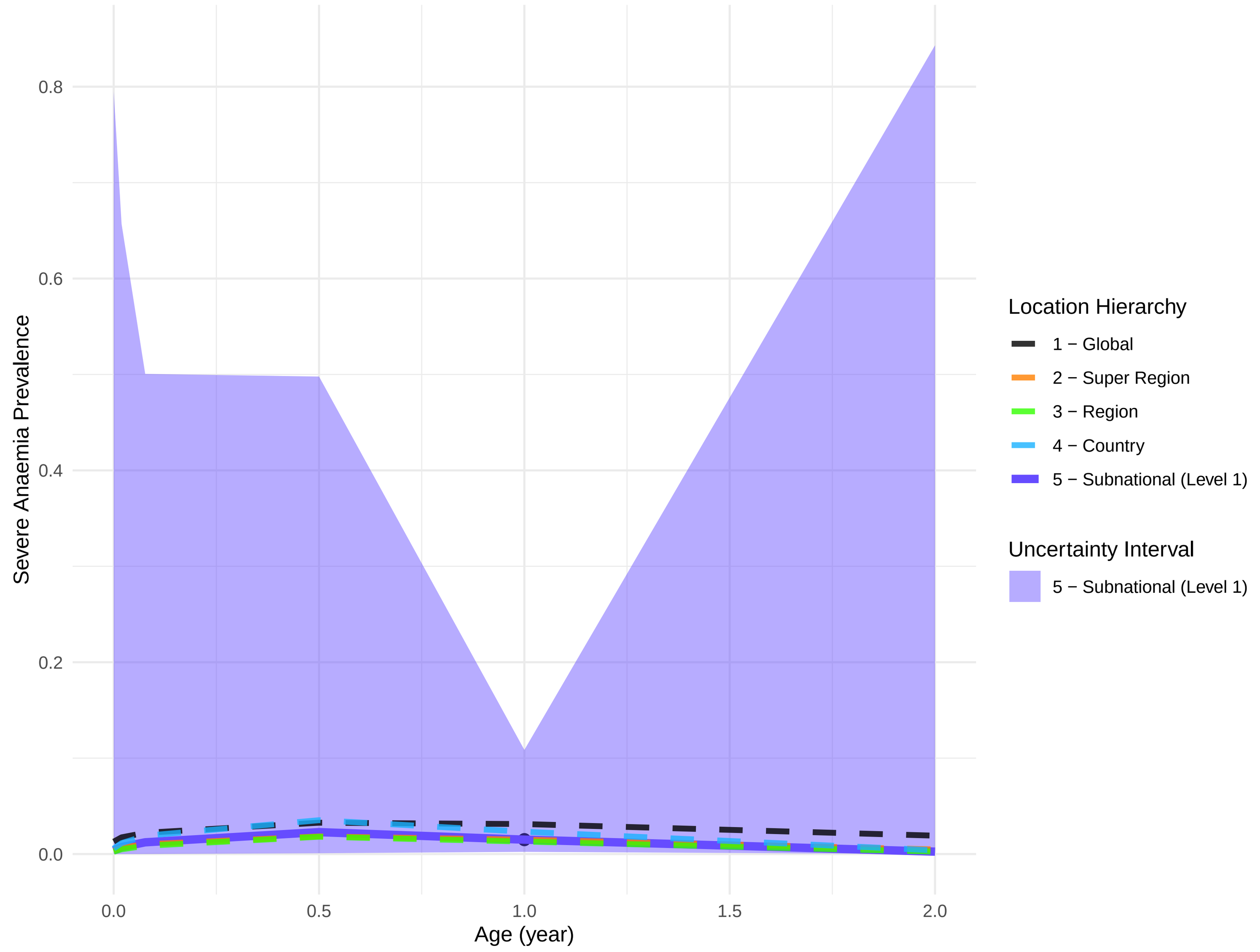

MR−BRT Age/Sex Fits − Non−pregnant Females <2 Years Old − Severe Anaemia Prevalence − Tlaxcala
WHO−2024 Adjustment Method

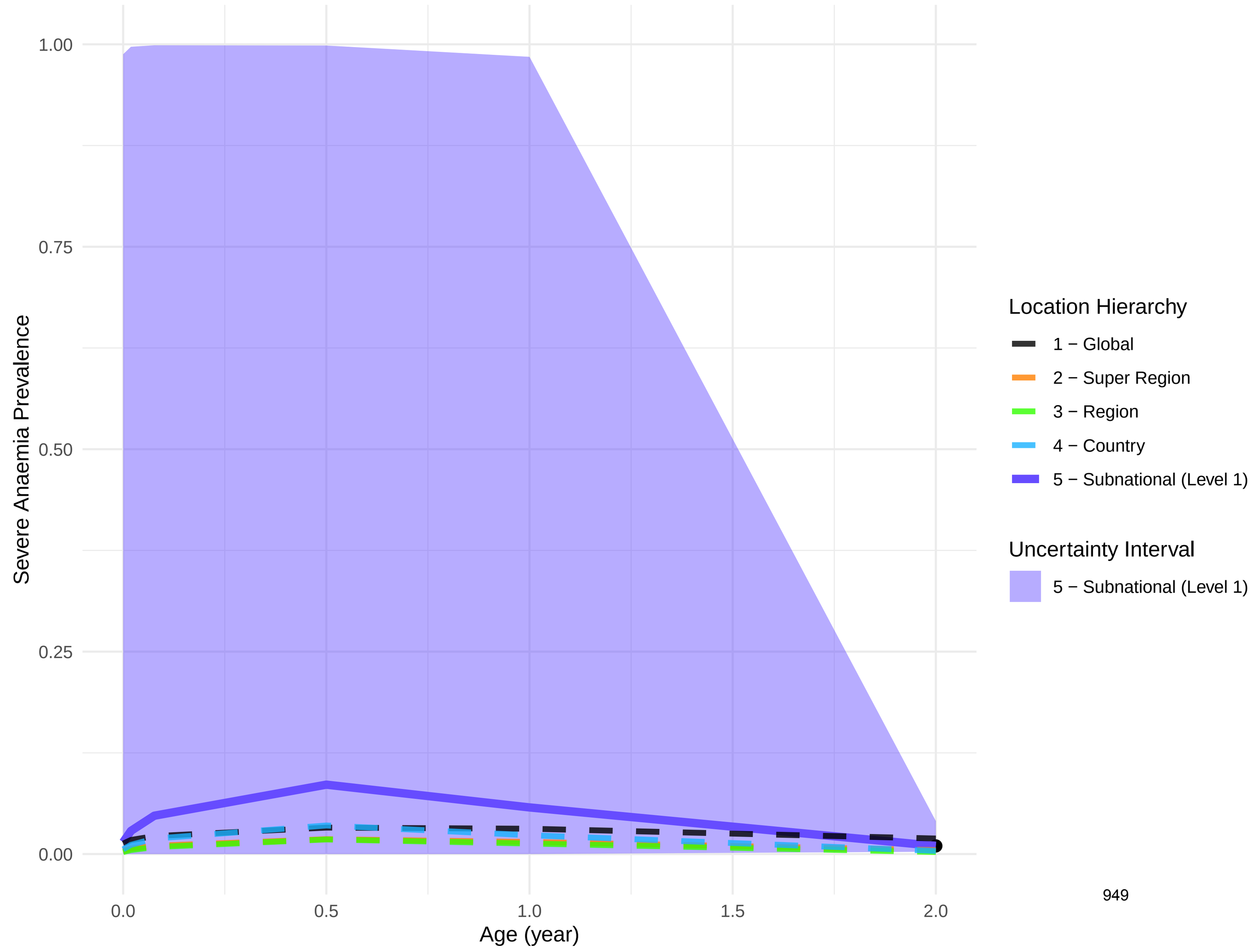

MR−BRT Age/Sex Fits − Non−pregnant Females <2 Years Old − Severe Anaemia Prevalence − Veracruz de Ignacio de la Llave
WHO−2024 Adjustment Method

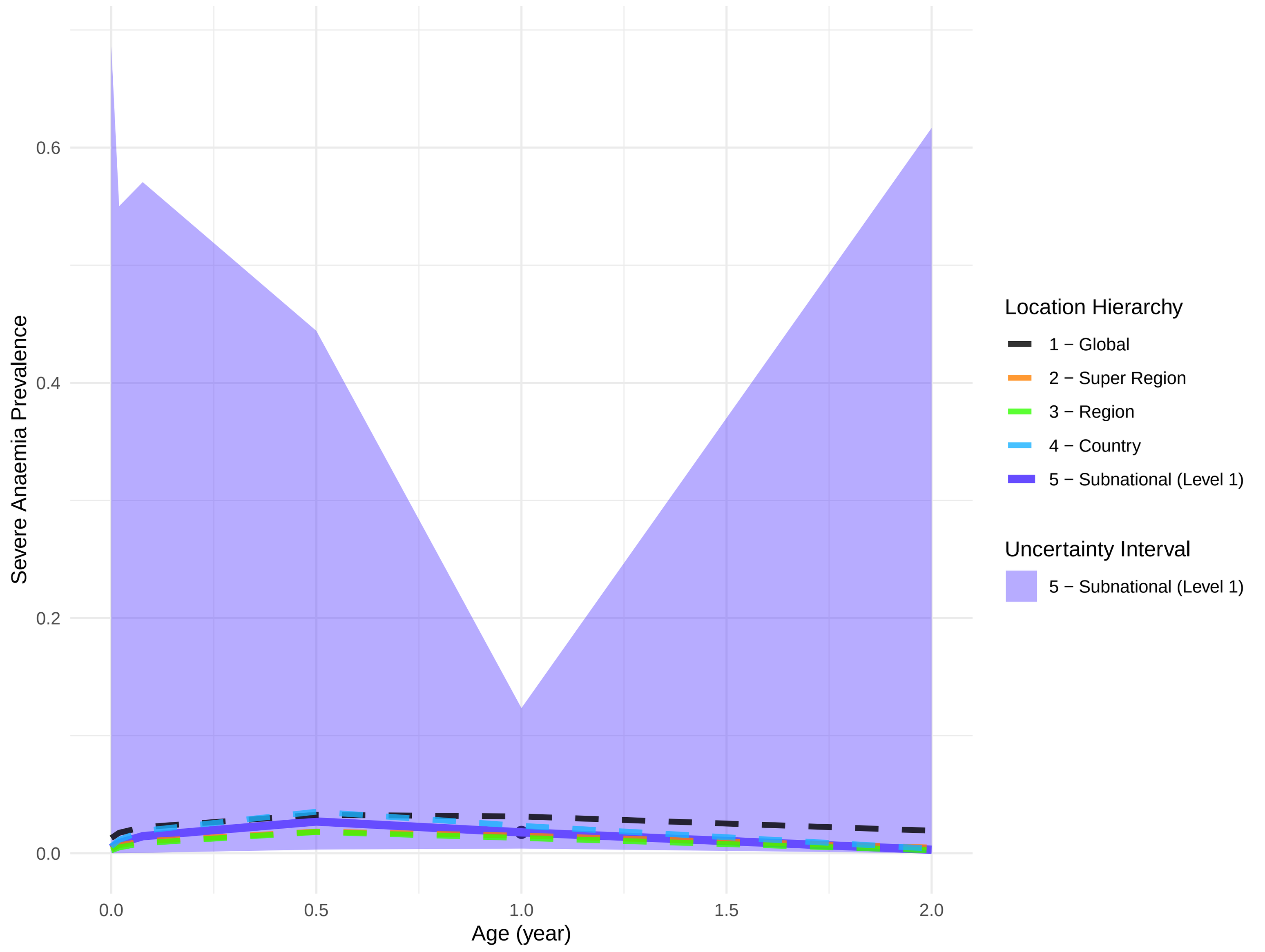

MR−BRT Age/Sex Fits − Non−pregnant Females <2 Years Old − Severe Anaemia Prevalence − North Africa and Middle East
WHO−2024 Adjustment Method

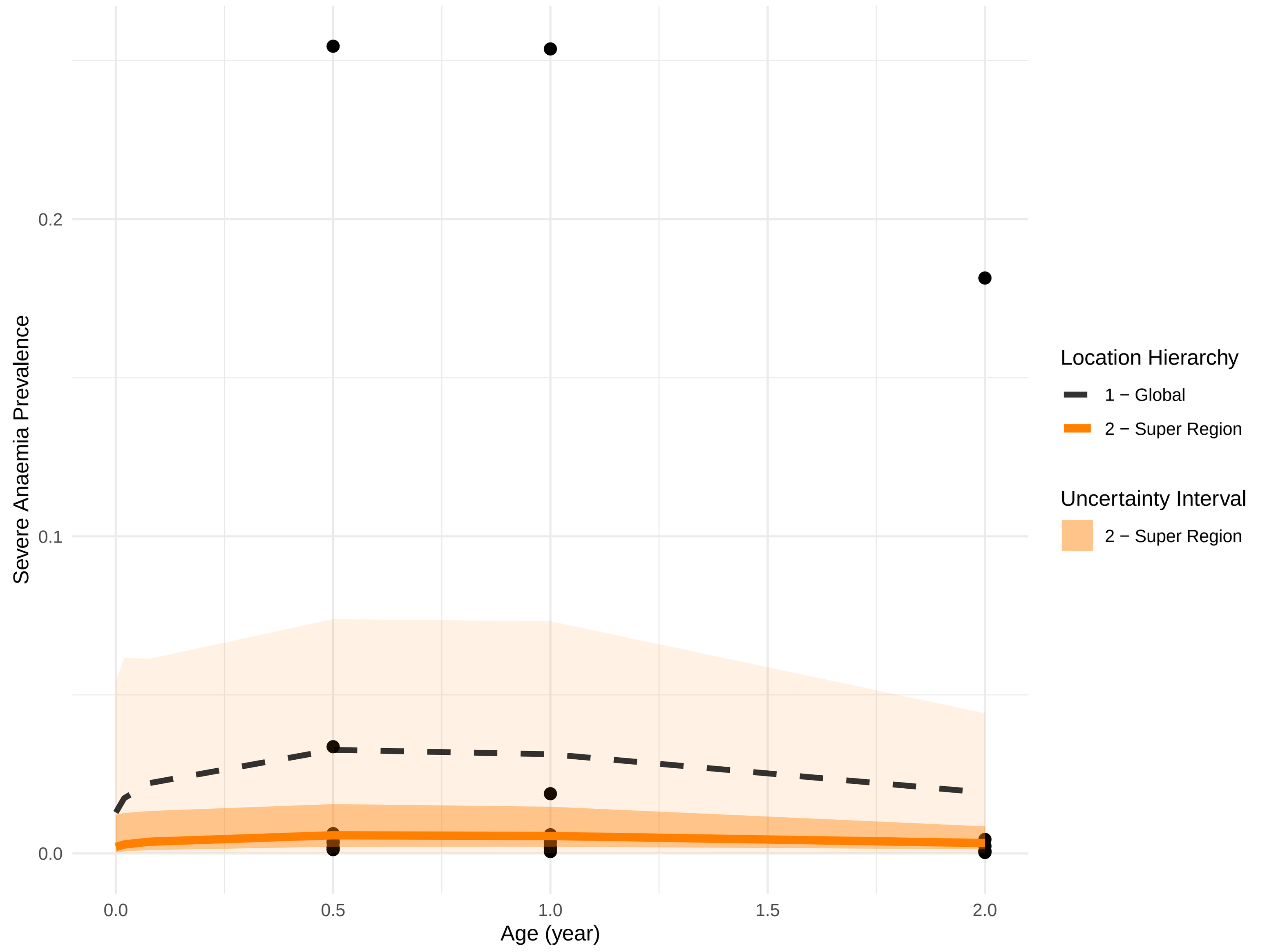

MR−BRT Age/Sex Fits − Non−pregnant Females <2 Years Old − Severe Anaemia Prevalence − North Africa and Middle East
WHO−2024 Adjustment Method

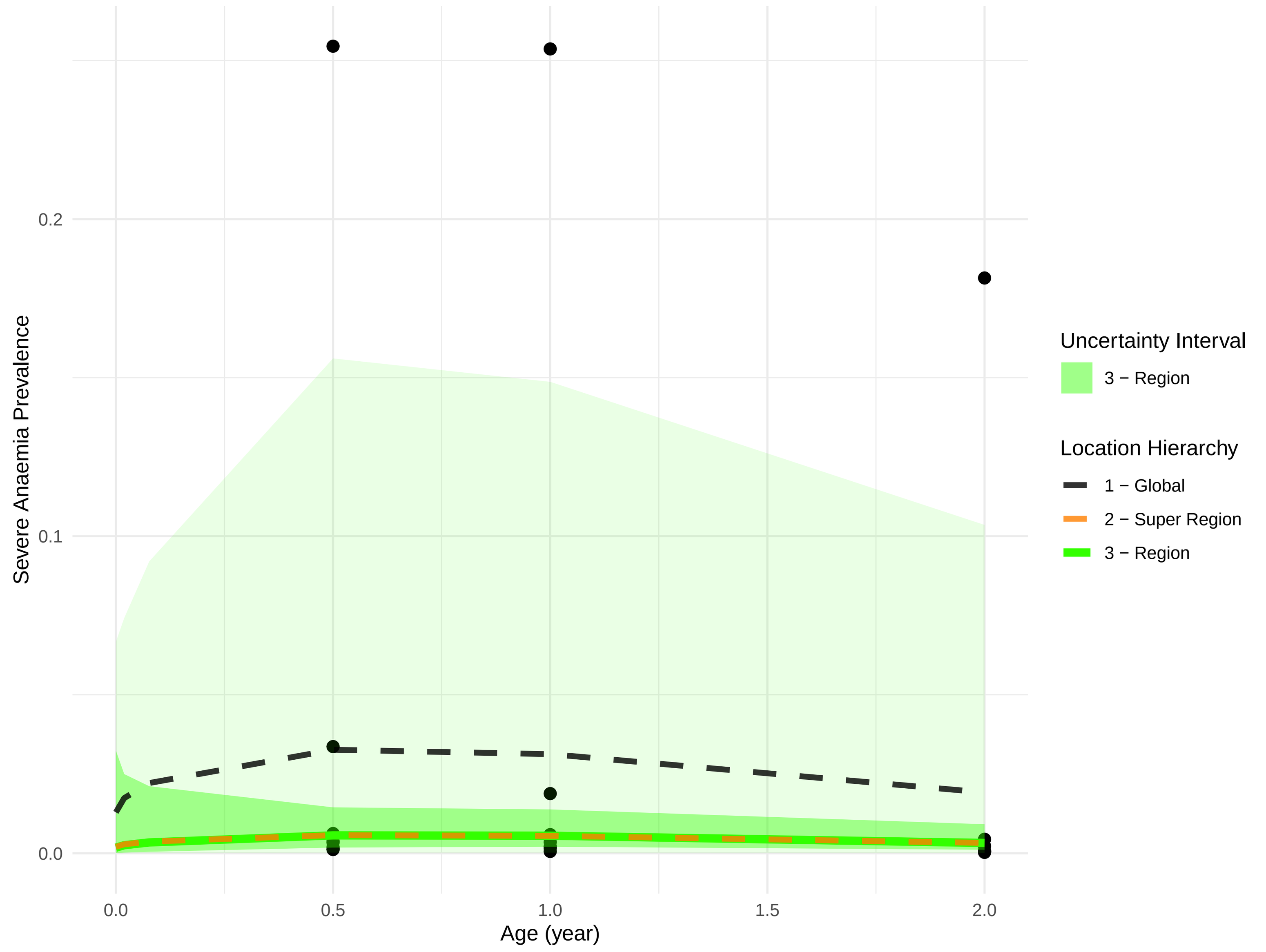

MR−BRT Age/Sex Fits − Non−pregnant Females <2 Years Old − Severe Anaemia Prevalence − Egypt
WHO−2024 Adjustment Method

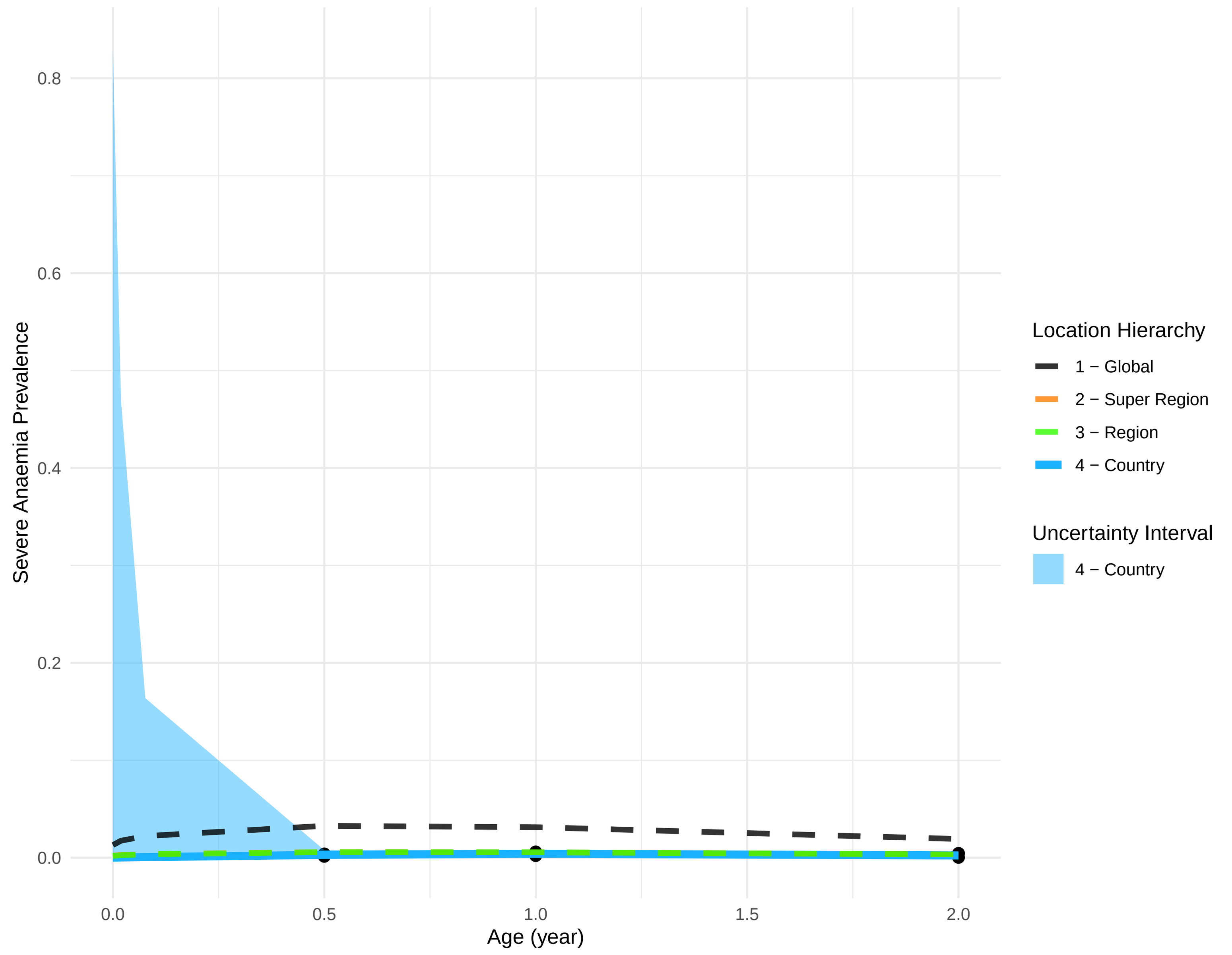

MR−BRT Age/Sex Fits − Non−pregnant Females <2 Years Old − Severe Anaemia Prevalence − Jordan
WHO−2024 Adjustment Method

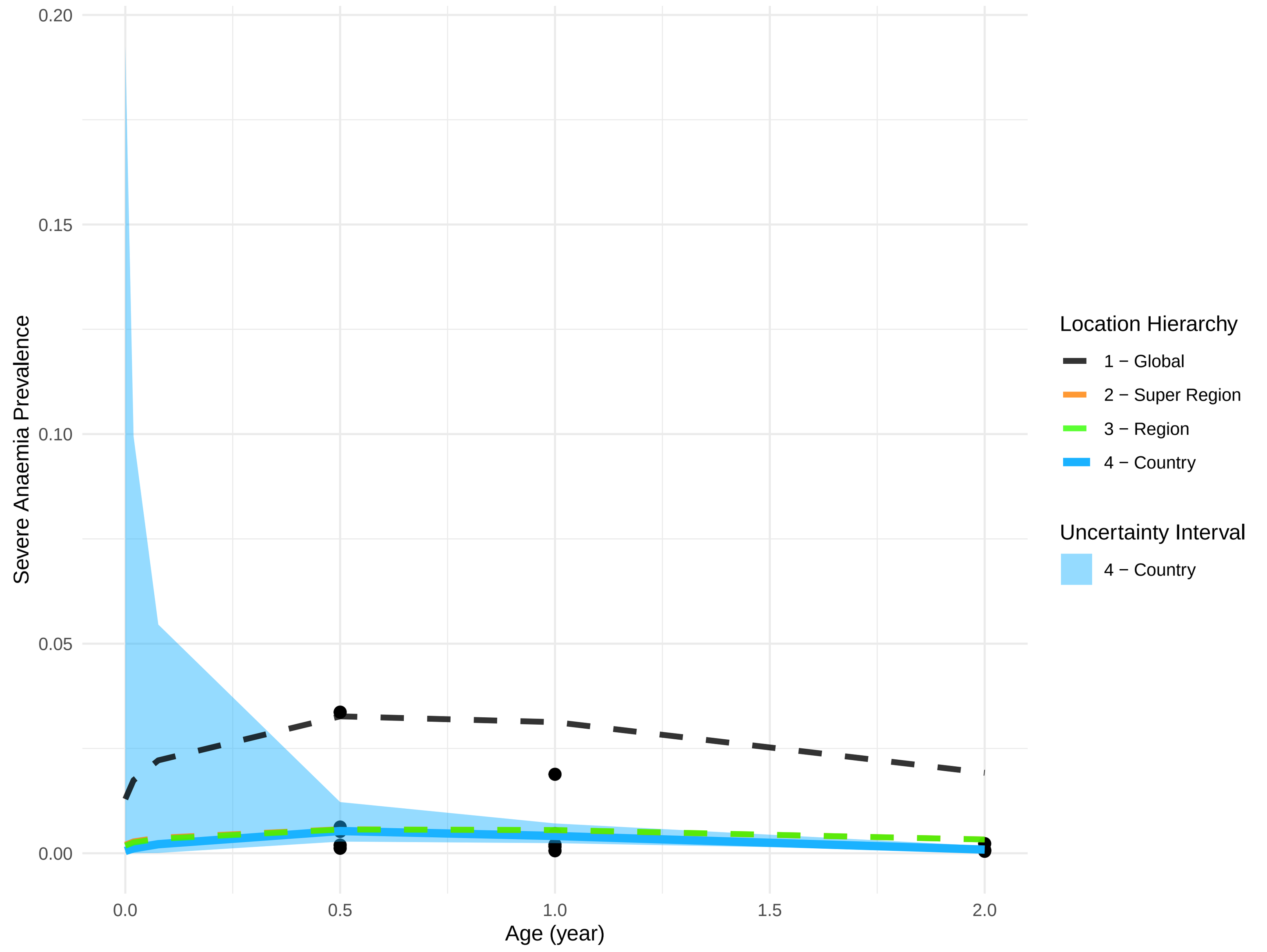

MR−BRT Age/Sex Fits − Non−pregnant Females <2 Years Old − Severe Anaemia Prevalence − Yemen
WHO−2024 Adjustment Method

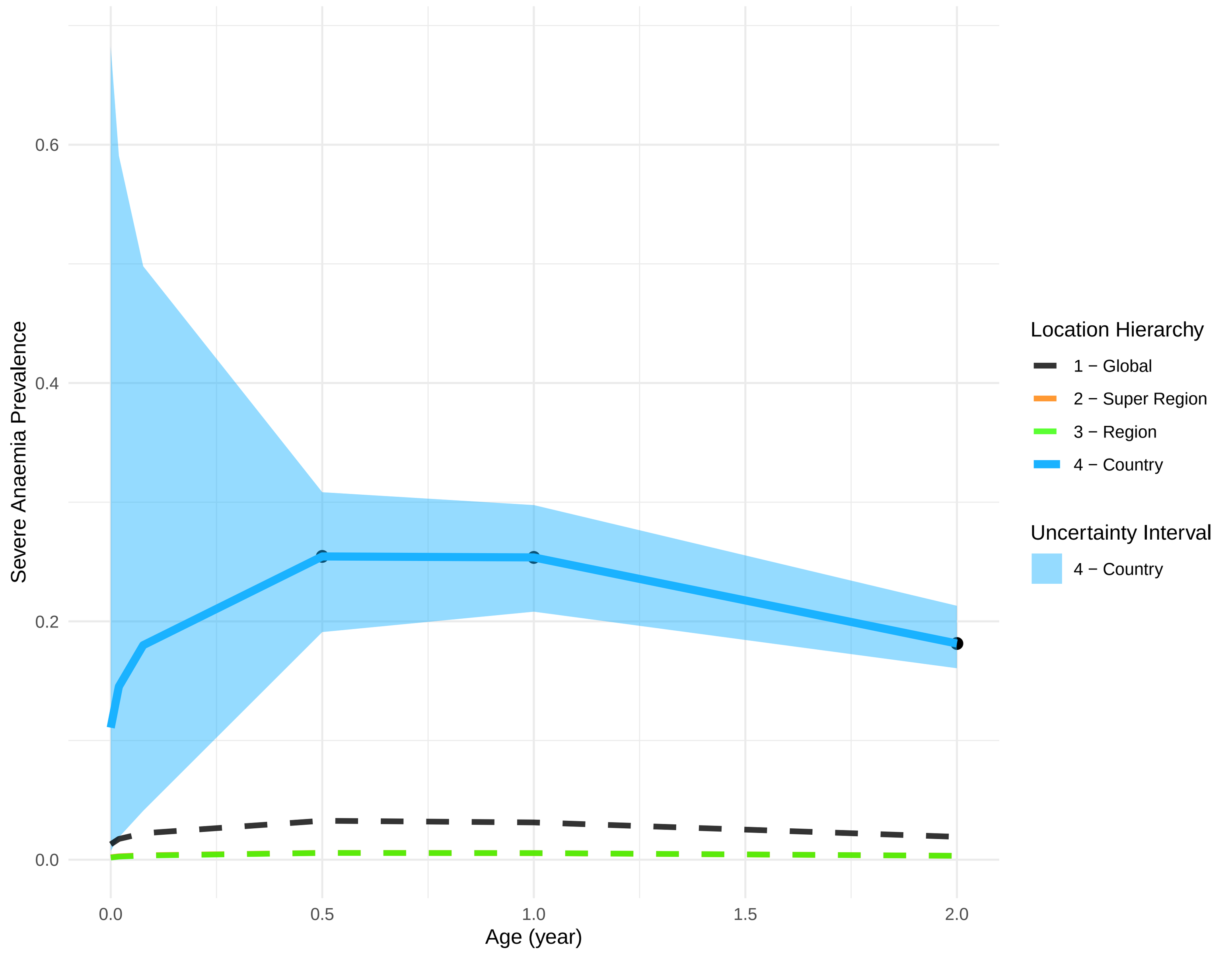

MR−BRT Age/Sex Fits − Non−pregnant Females <2 Years Old − Severe Anaemia Prevalence − South Asia
WHO−2024 Adjustment Method

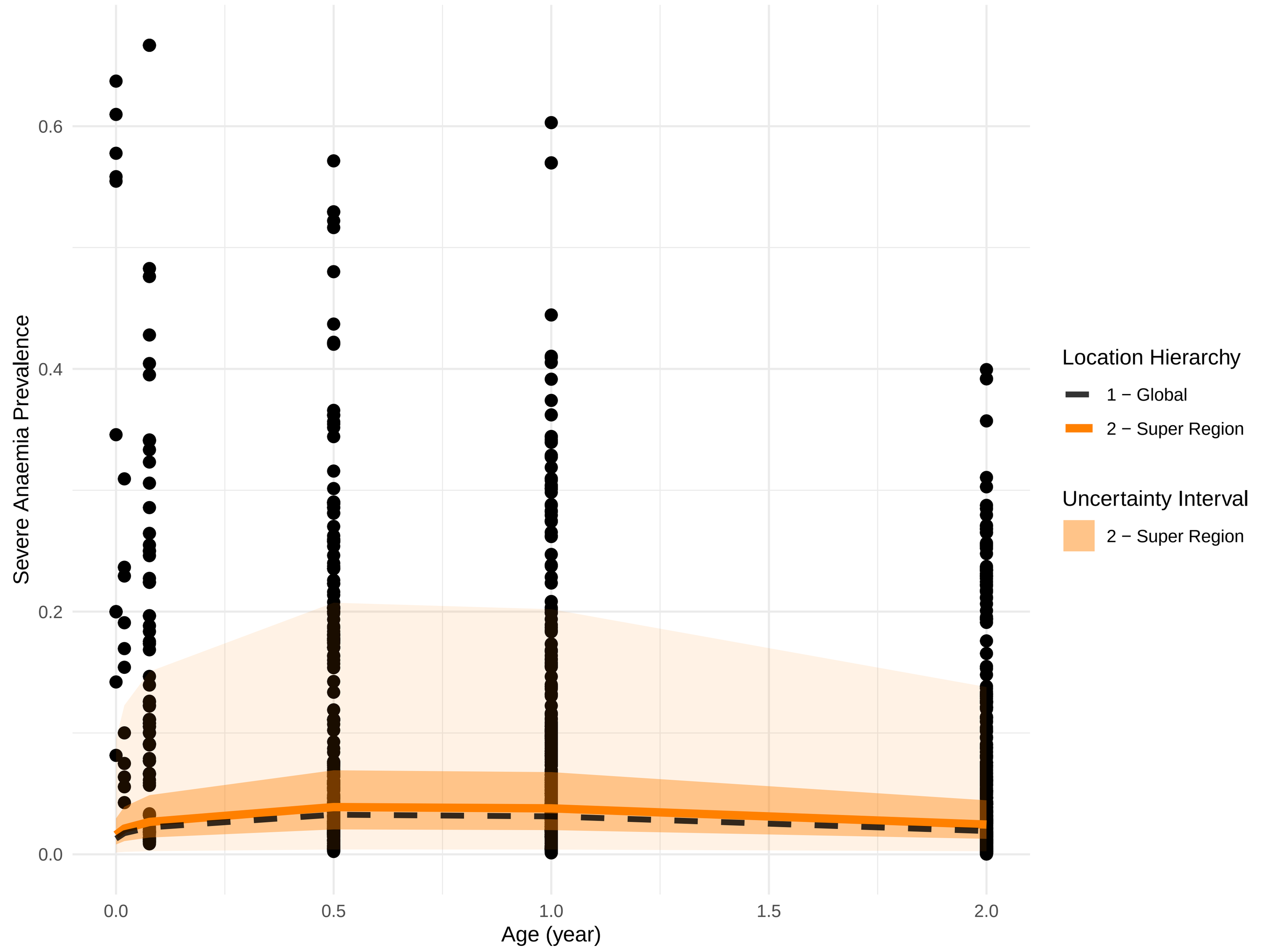

MR−BRT Age/Sex Fits − Non−pregnant Females <2 Years Old − Severe Anaemia Prevalence − South Asia
WHO−2024 Adjustment Method

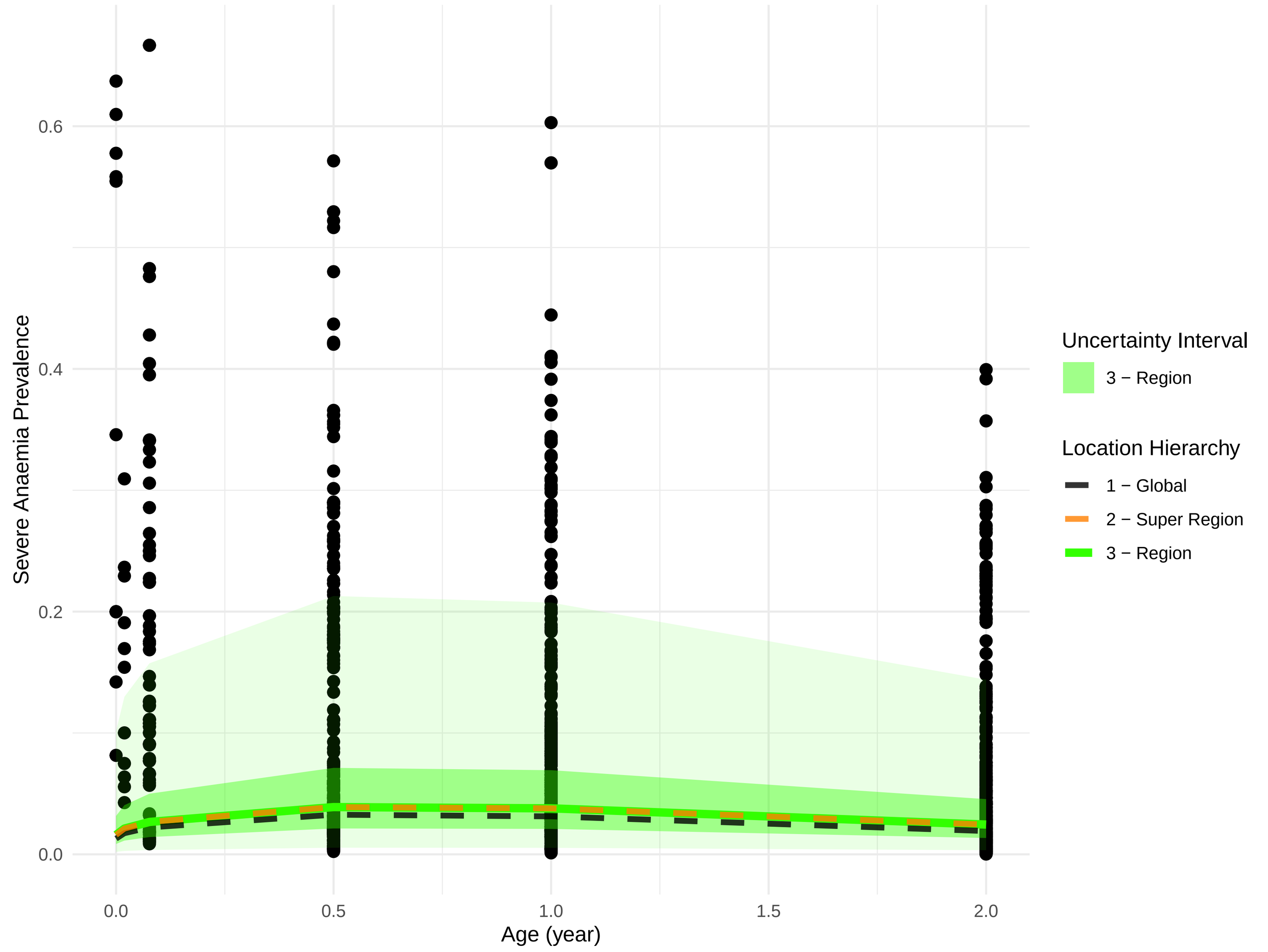

MR−BRT Age/Sex Fits − Non−pregnant Females <2 Years Old − Severe Anaemia Prevalence − Bangladesh
WHO−2024 Adjustment Method

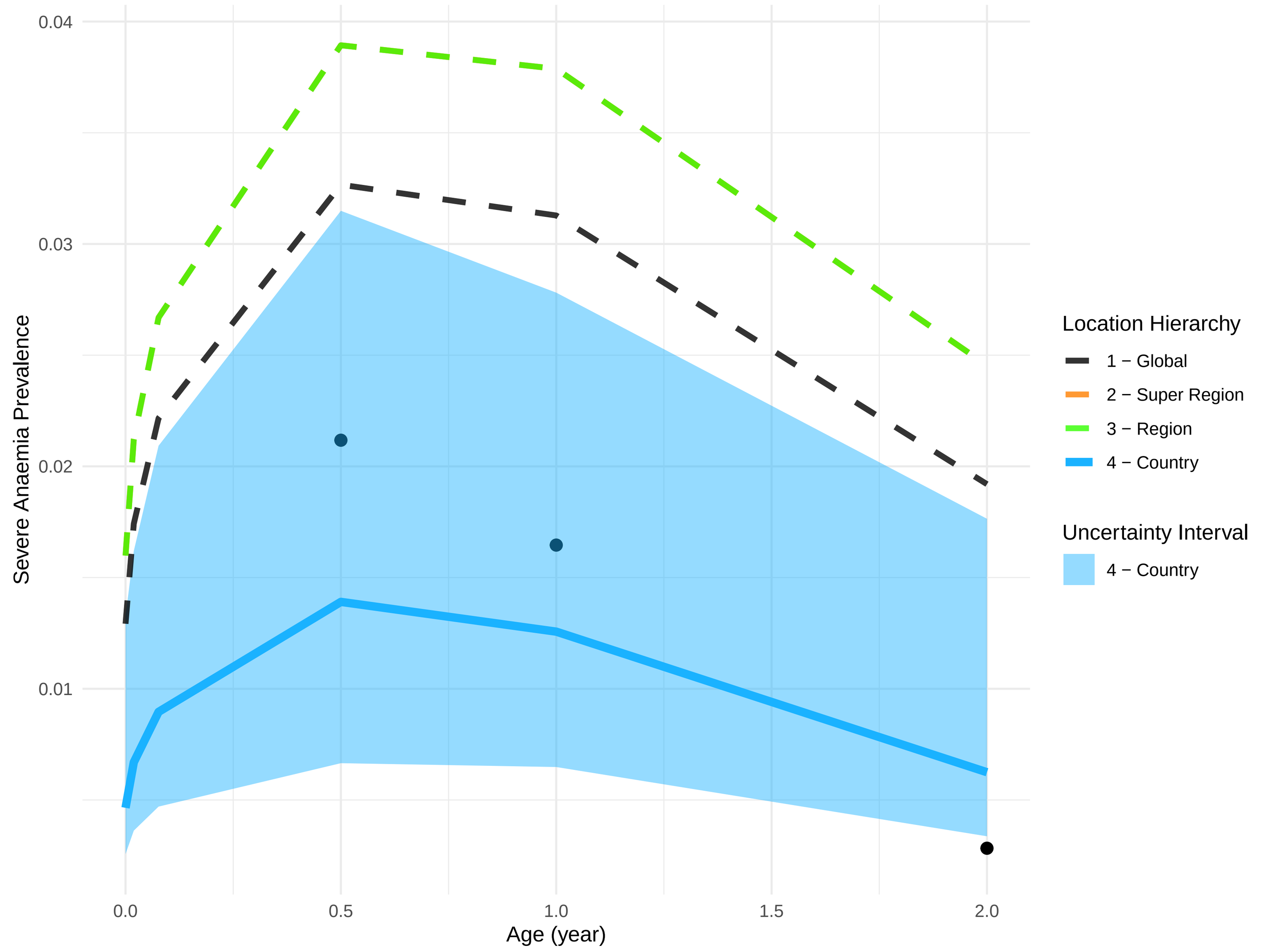

MR−BRT Age/Sex Fits − Non−pregnant Females <2 Years Old − Severe Anaemia Prevalence − India
WHO−2024 Adjustment Method

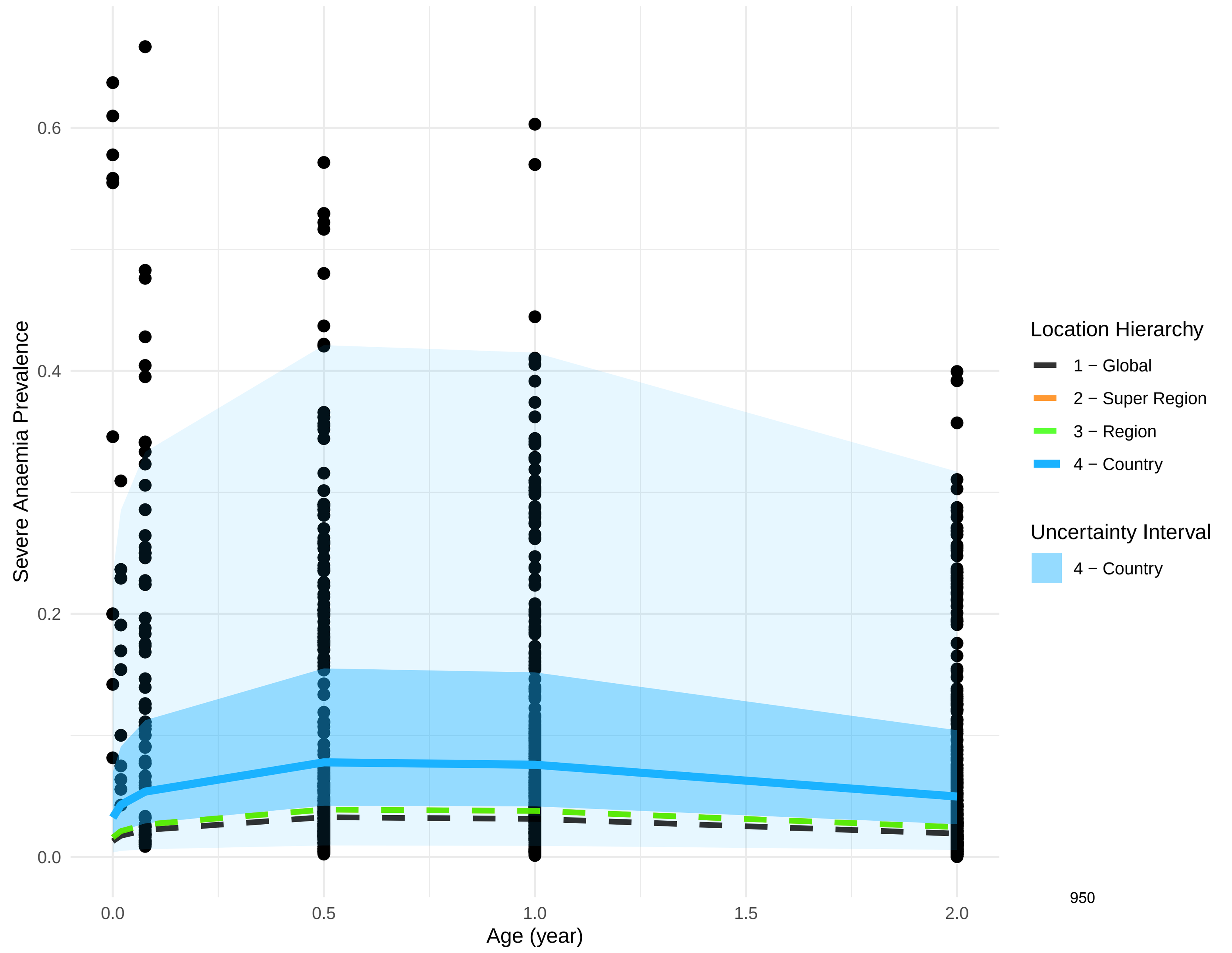

MR−BRT Age/Sex Fits − Non−pregnant Females <2 Years Old − Severe Anaemia Prevalence − Andhra Pradesh
WHO−2024 Adjustment Method

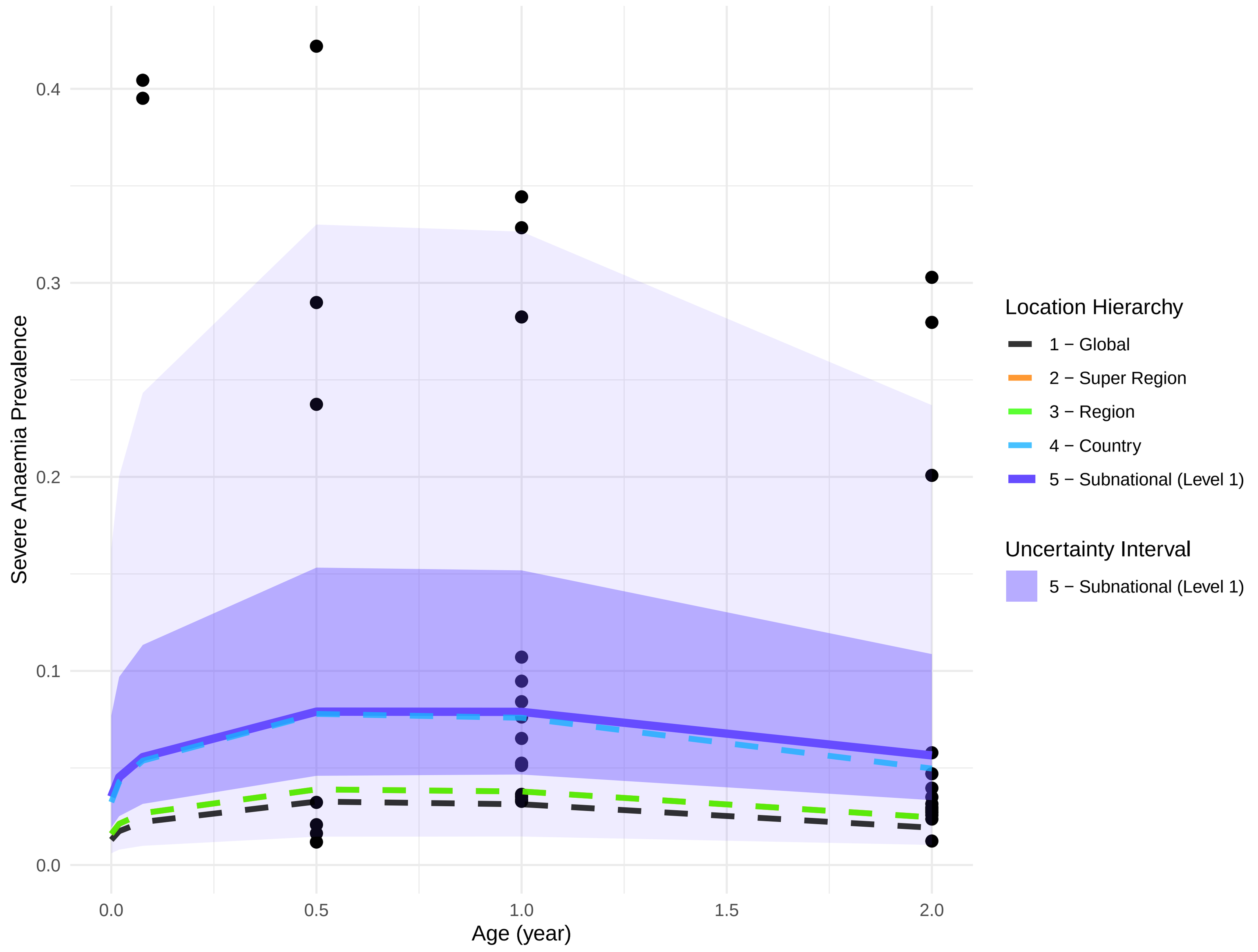

MR−BRT Age/Sex Fits − Non−pregnant Females <2 Years Old − Severe Anaemia Prevalence − Andhra Pradesh, Rural
WHO−2024 Adjustment Method

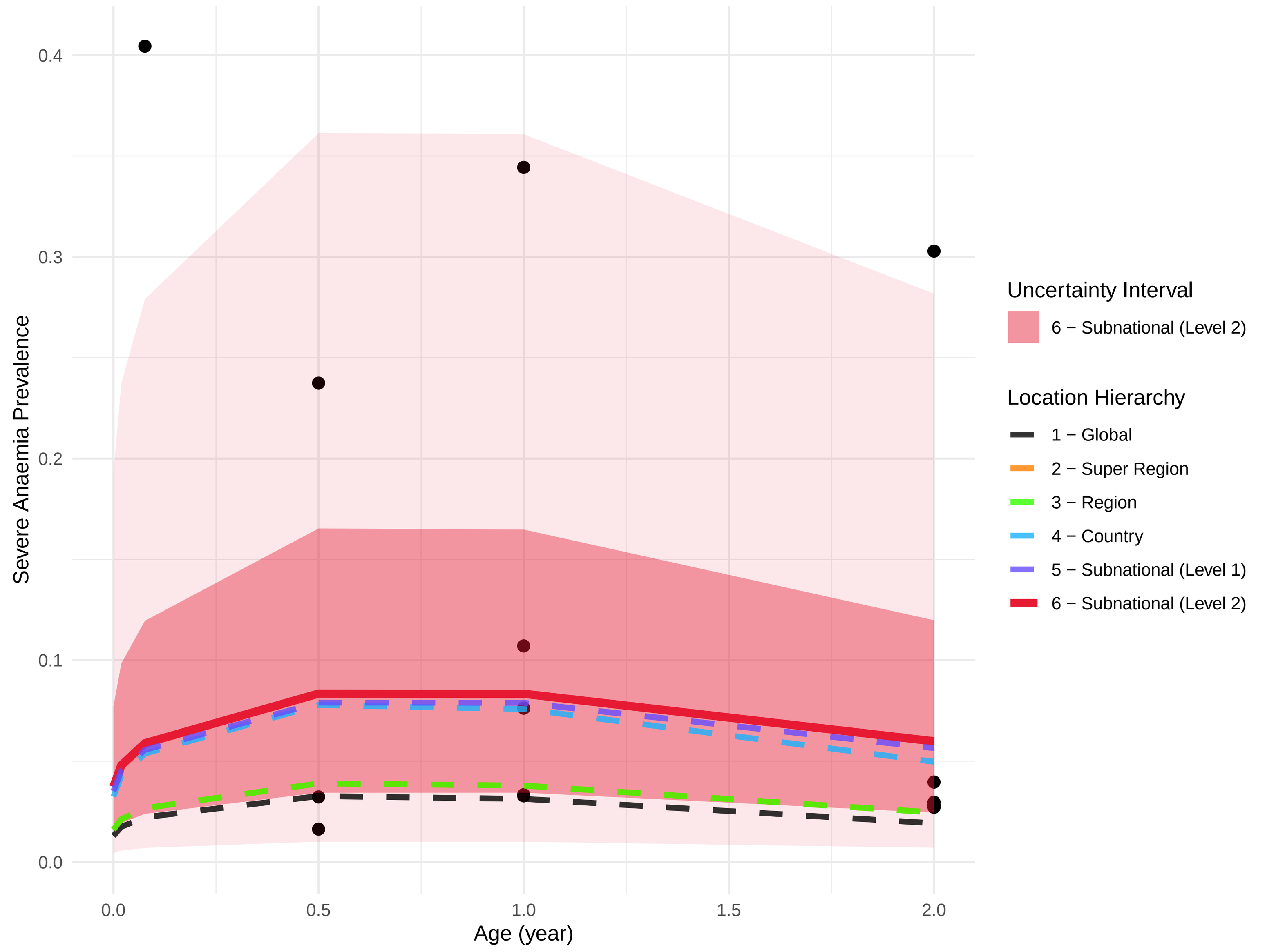

MR−BRT Age/Sex Fits − Non−pregnant Females <2 Years Old − Severe Anaemia Prevalence − Andhra Pradesh, Urban
WHO−2024 Adjustment Method

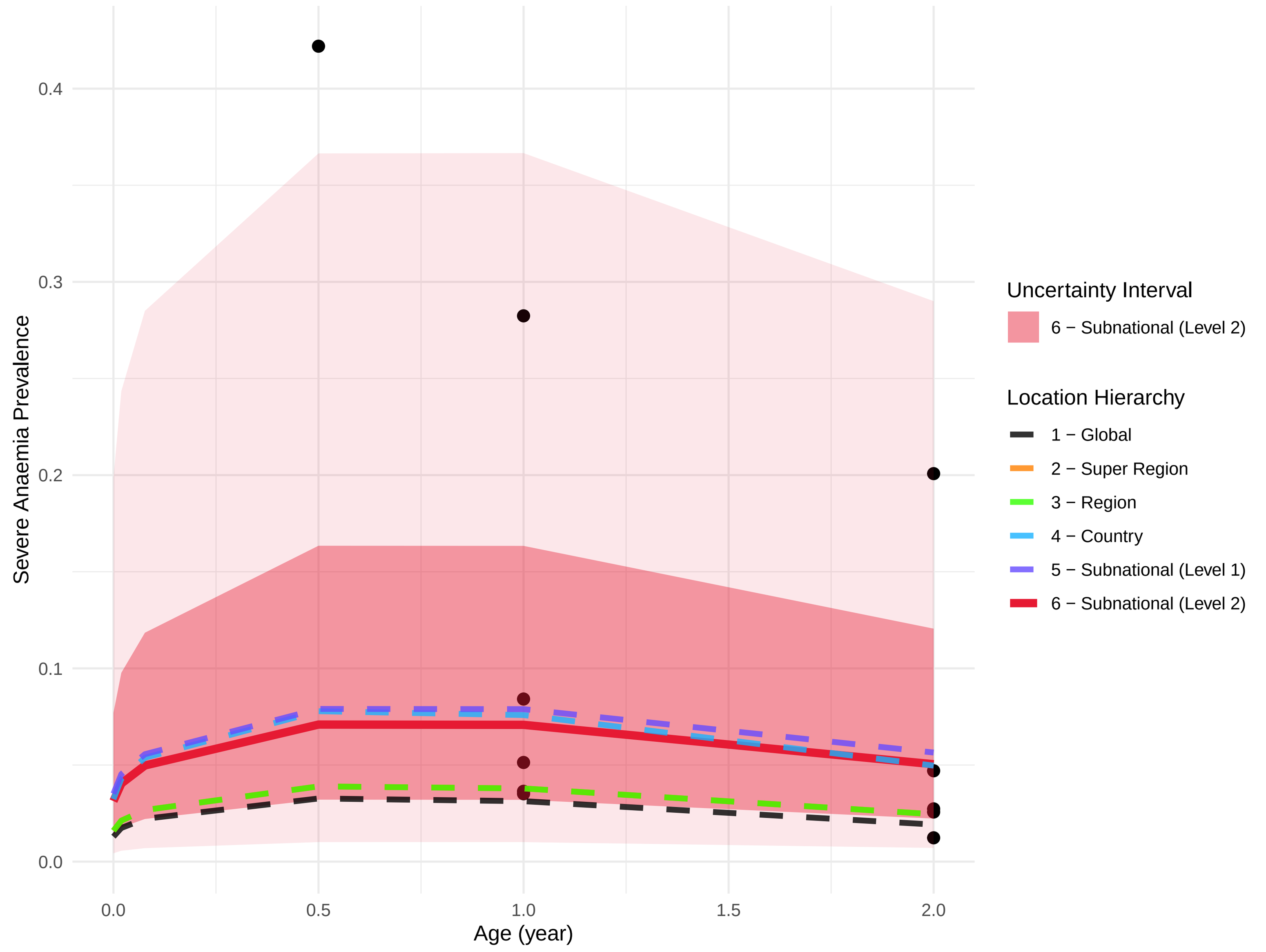

MR−BRT Age/Sex Fits − Non−pregnant Females <2 Years Old − Severe Anaemia Prevalence − Arunachal Pradesh
WHO−2024 Adjustment Method

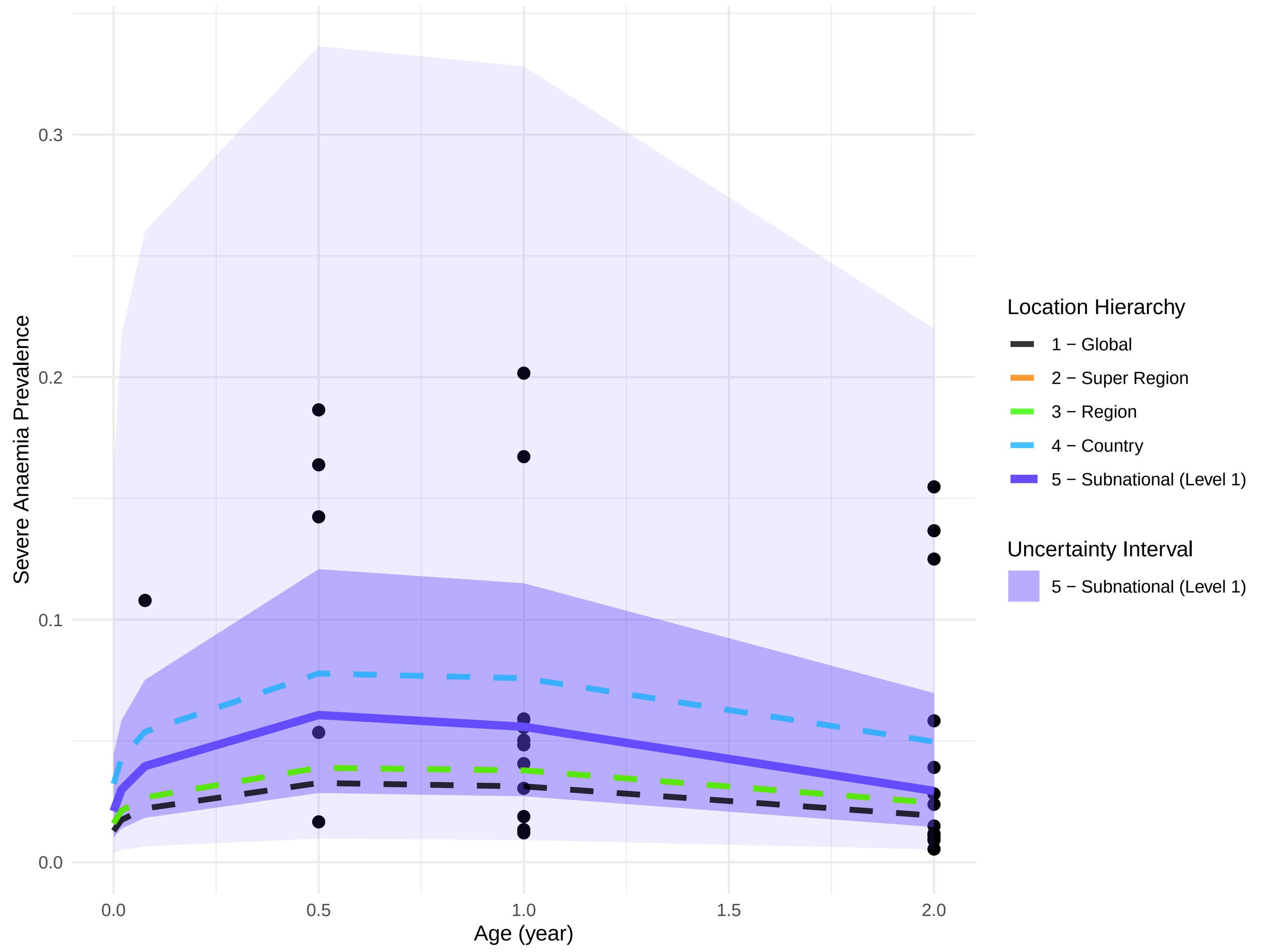

MR−BRT Age/Sex Fits − Non−pregnant Females <2 Years Old − Severe Anaemia Prevalence − Arunachal Pradesh, Rural
WHO−2024 Adjustment Method

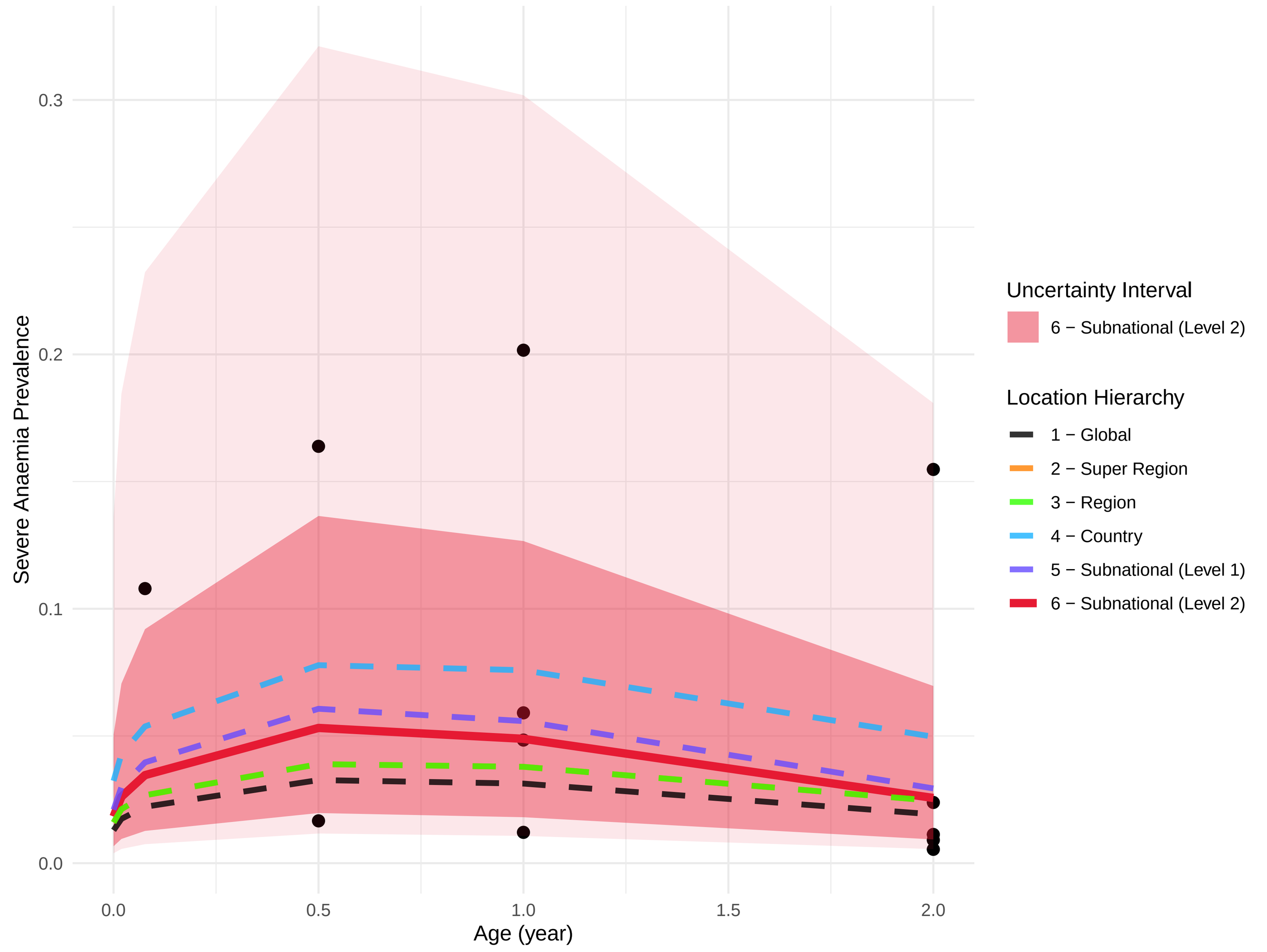

MR−BRT Age/Sex Fits − Non−pregnant Females <2 Years Old − Severe Anaemia Prevalence − Arunachal Pradesh, Urban
WHO−2024 Adjustment Method

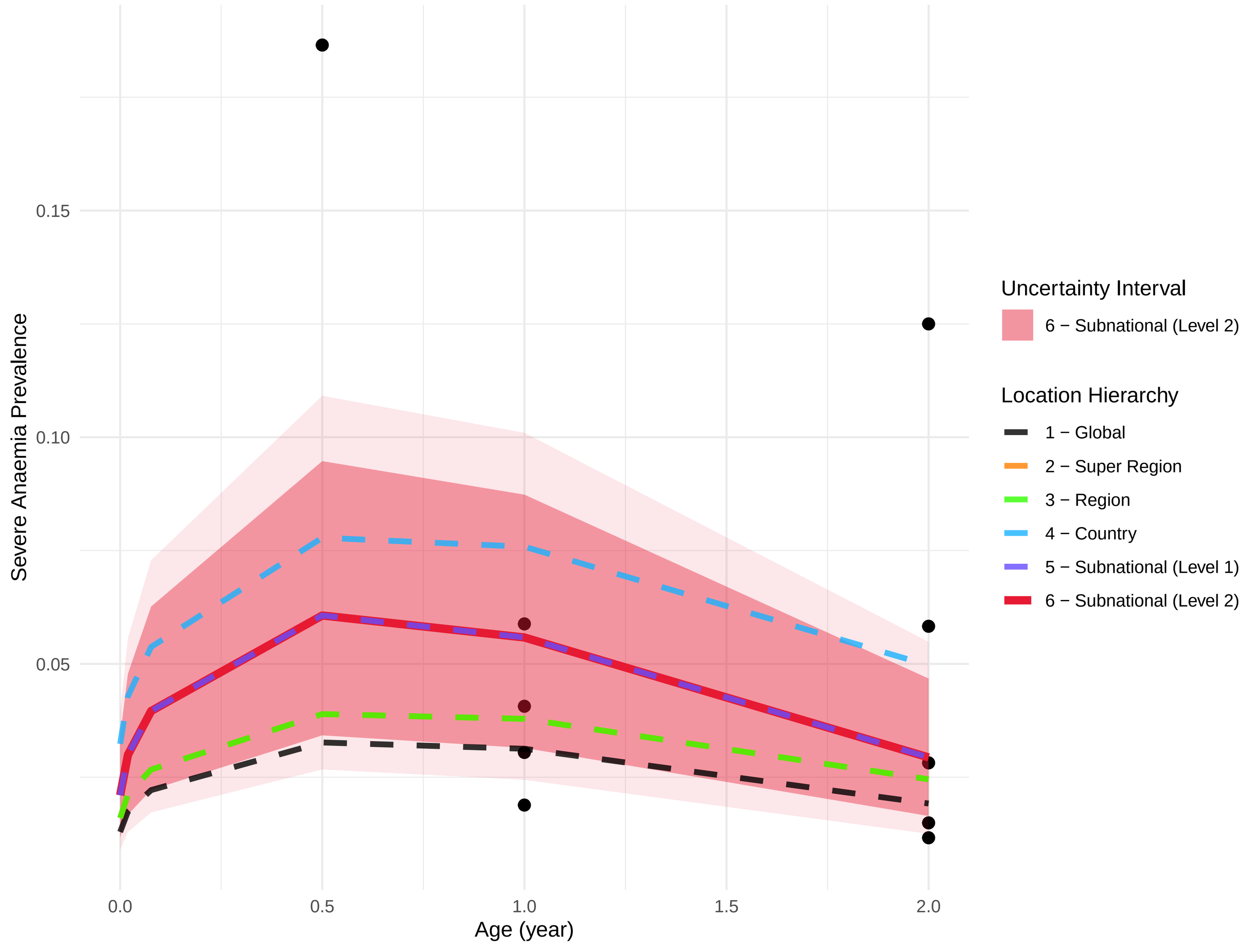

MR−BRT Age/Sex Fits − Non−pregnant Females <2 Years Old − Severe Anaemia Prevalence − Assam
WHO−2024 Adjustment Method

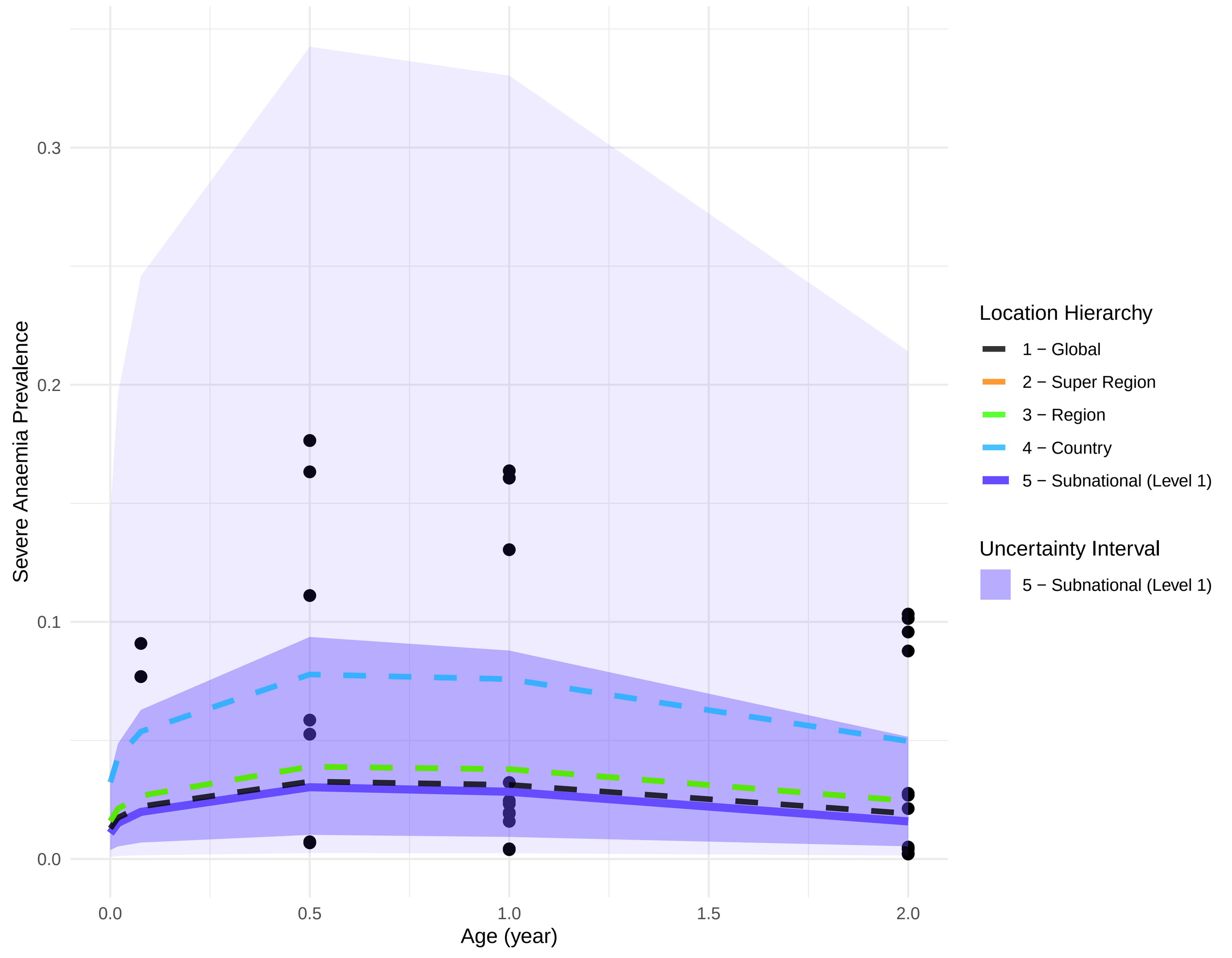

MR−BRT Age/Sex Fits − Non−pregnant Females <2 Years Old − Severe Anaemia Prevalence − Assam, Rural
WHO−2024 Adjustment Method

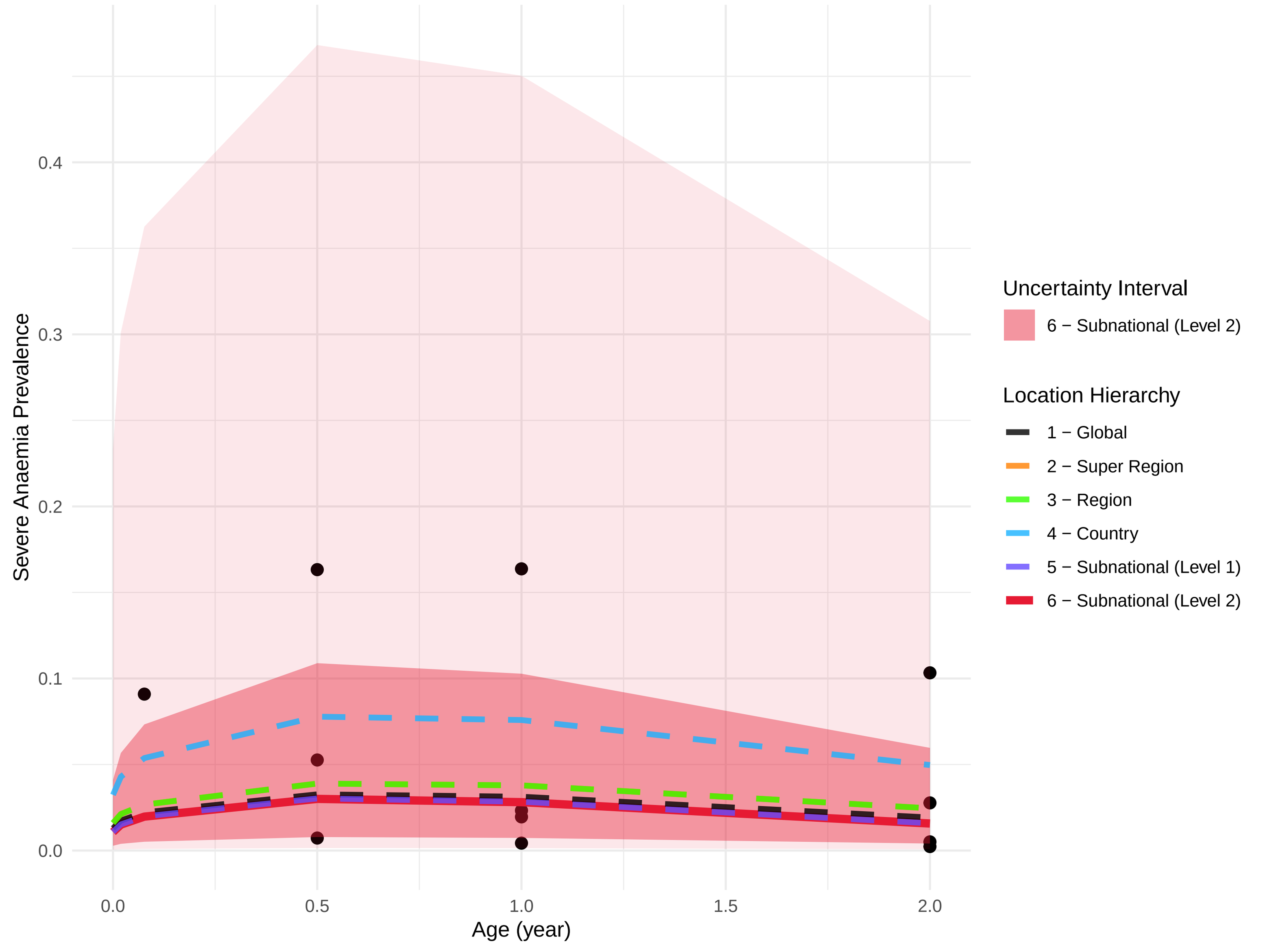

MR−BRT Age/Sex Fits − Non−pregnant Females <2 Years Old − Severe Anaemia Prevalence − Assam, Urban
WHO−2024 Adjustment Method

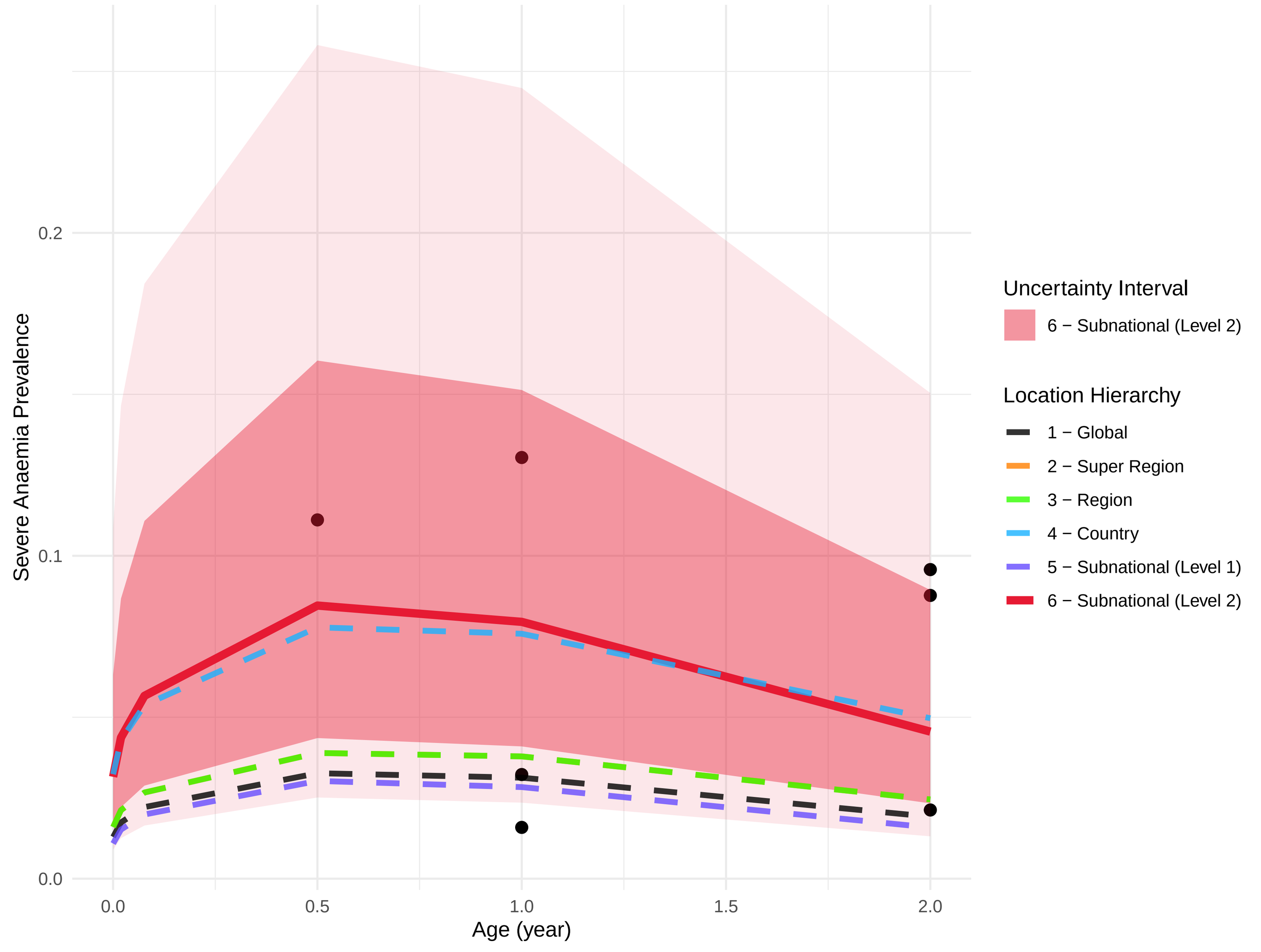

MR−BRT Age/Sex Fits − Non−pregnant Females <2 Years Old − Severe Anaemia Prevalence − Bihar
WHO−2024 Adjustment Method

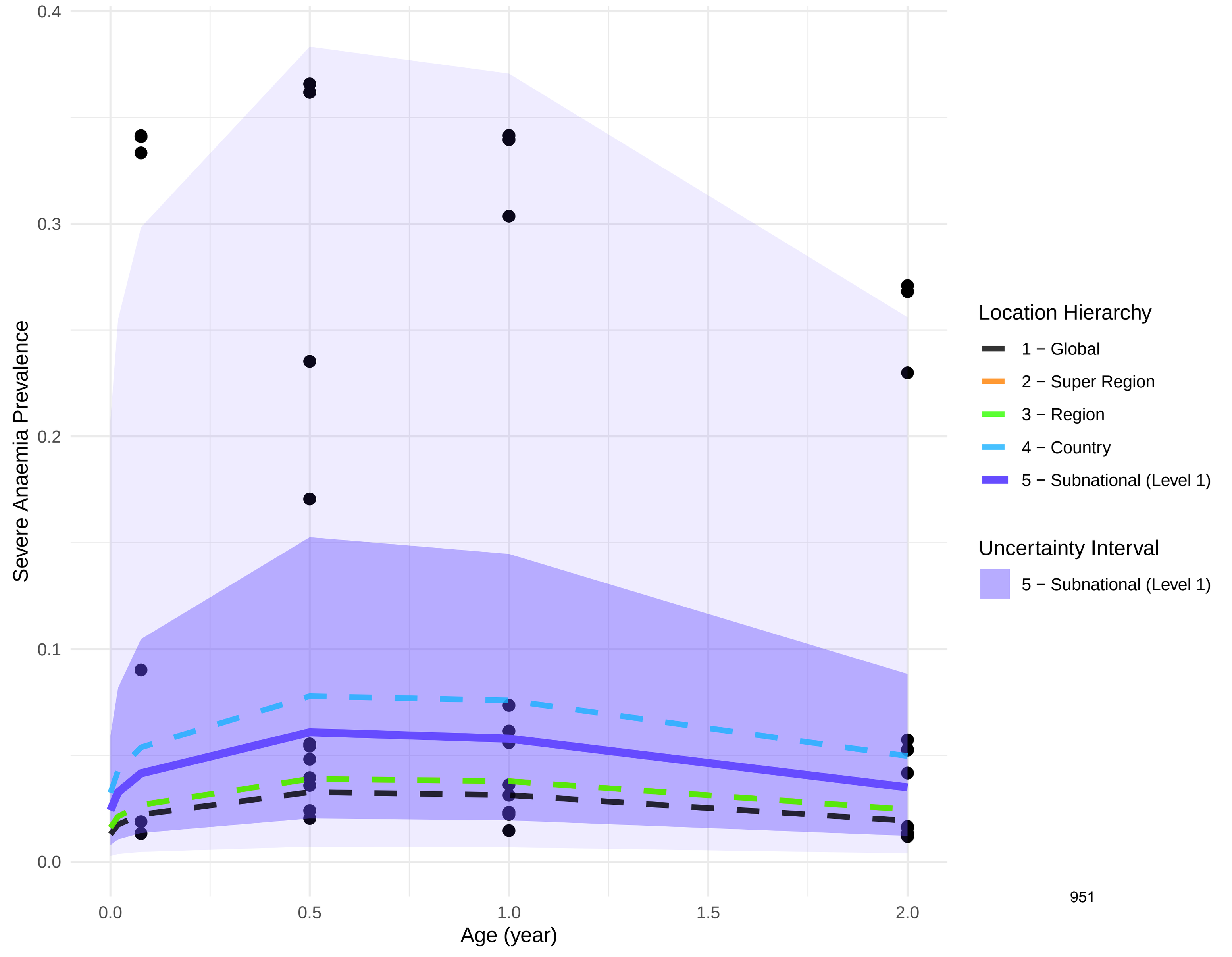

MR−BRT Age/Sex Fits − Non−pregnant Females <2 Years Old − Severe Anaemia Prevalence − Bihar, Rural
WHO−2024 Adjustment Method

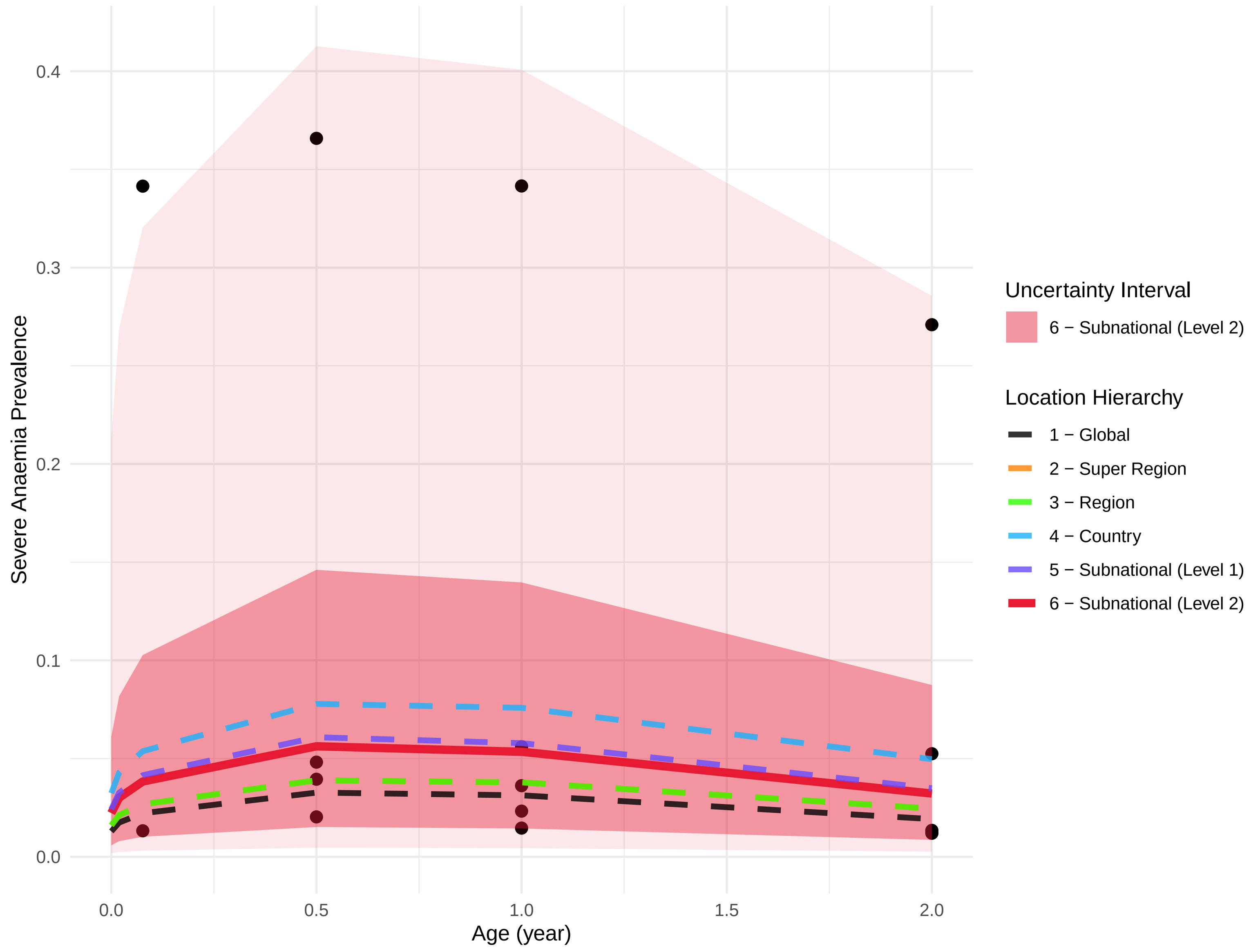

MR−BRT Age/Sex Fits − Non−pregnant Females <2 Years Old − Severe Anaemia Prevalence − Bihar, Urban
WHO−2024 Adjustment Method

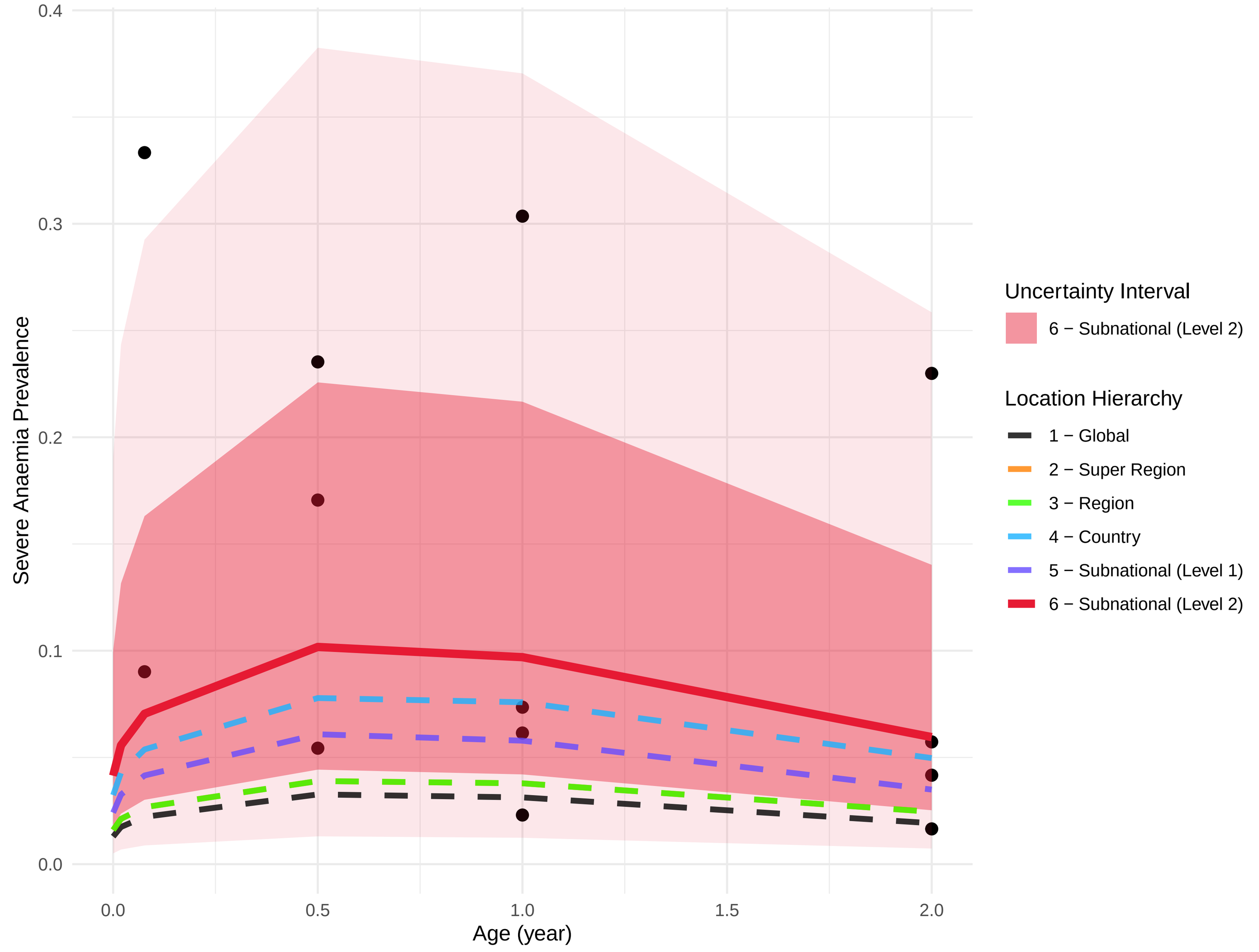

MR−BRT Age/Sex Fits − Non−pregnant Females <2 Years Old − Severe Anaemia Prevalence − Chhattisgarh
WHO−2024 Adjustment Method

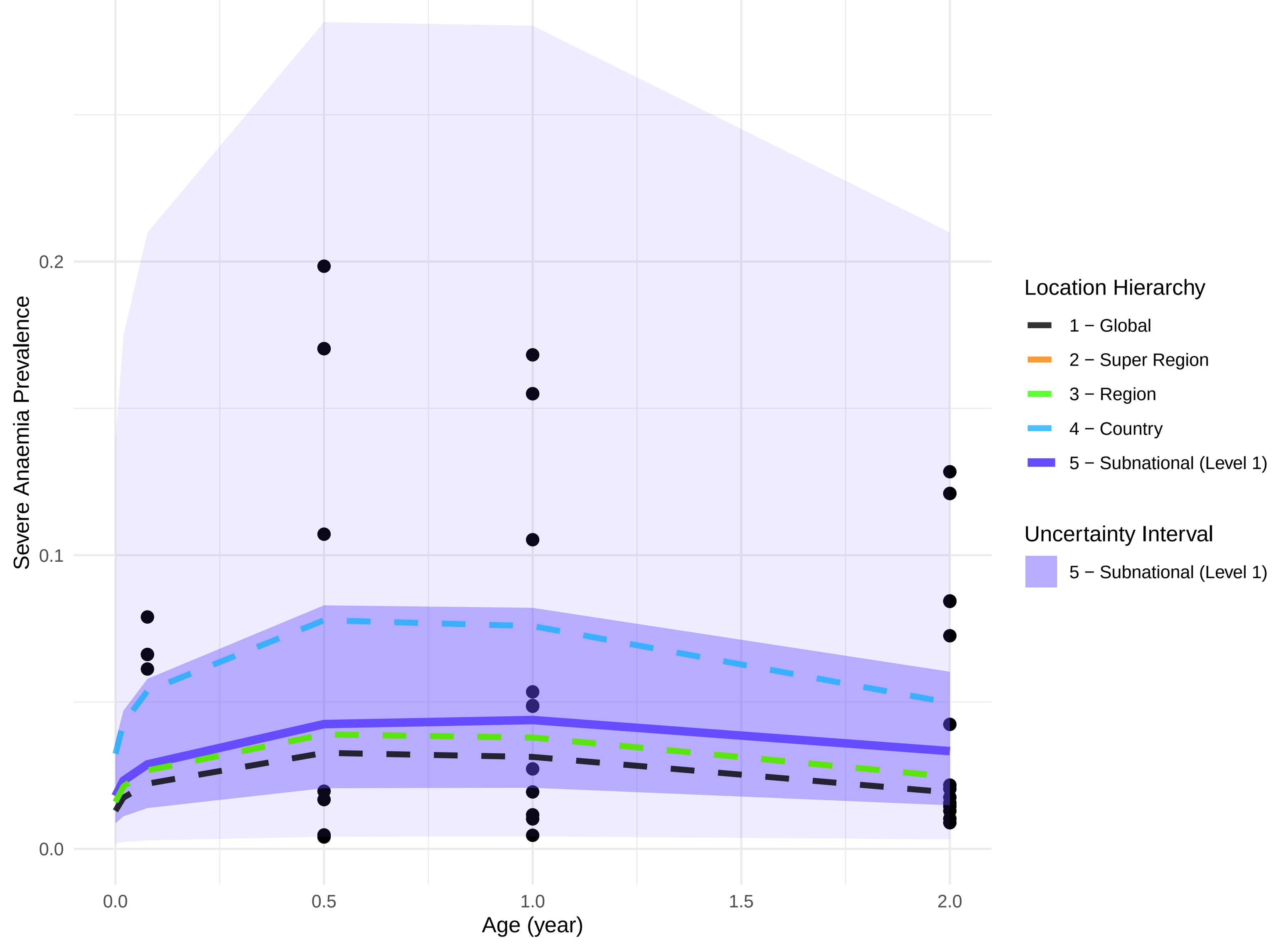

MR−BRT Age/Sex Fits − Non−pregnant Females <2 Years Old − Severe Anaemia Prevalence − Chhattisgarh, Rural
WHO−2024 Adjustment Method

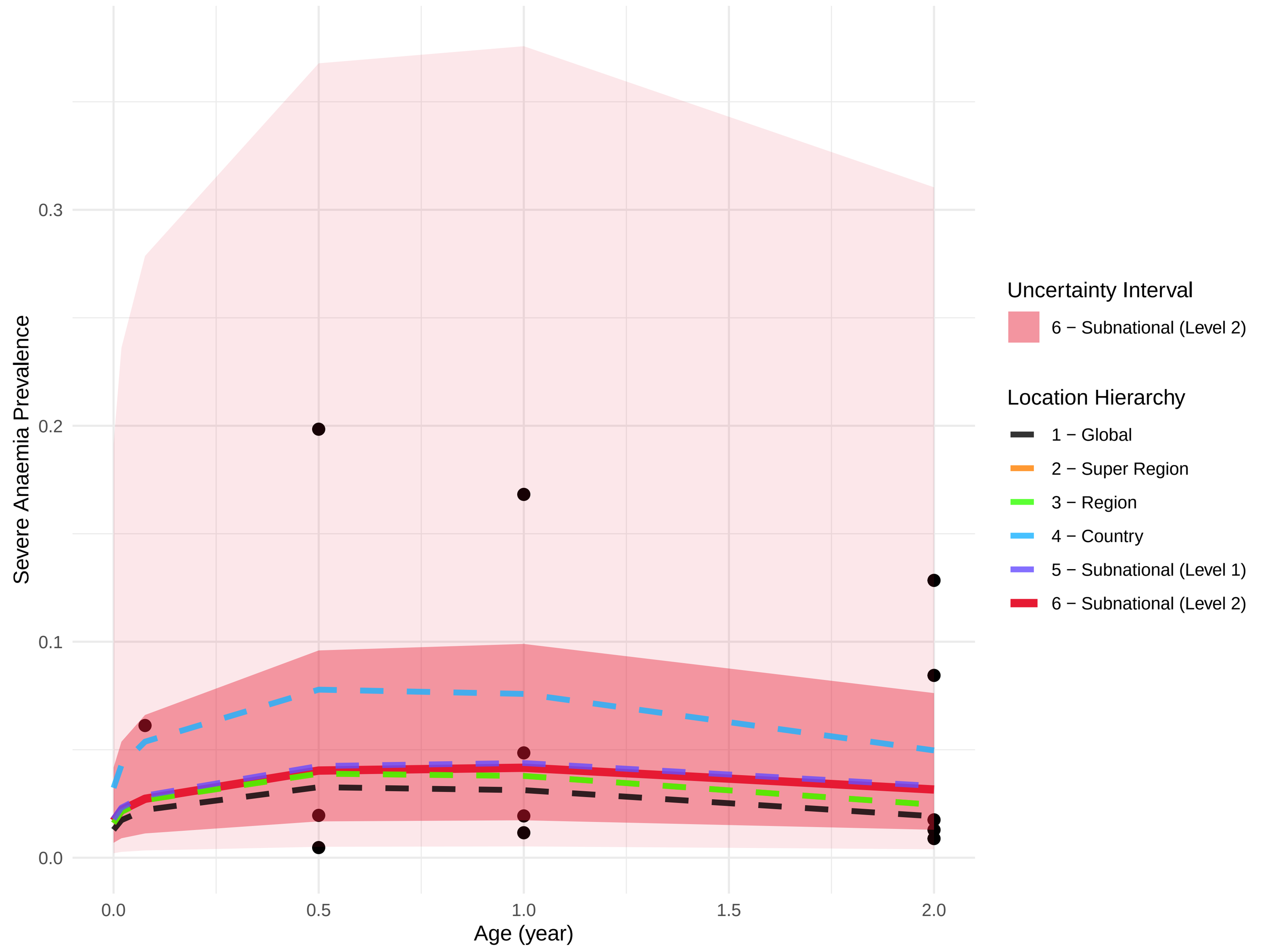

MR−BRT Age/Sex Fits − Non−pregnant Females <2 Years Old − Severe Anaemia Prevalence − Chhattisgarh, Urban
WHO−2024 Adjustment Method

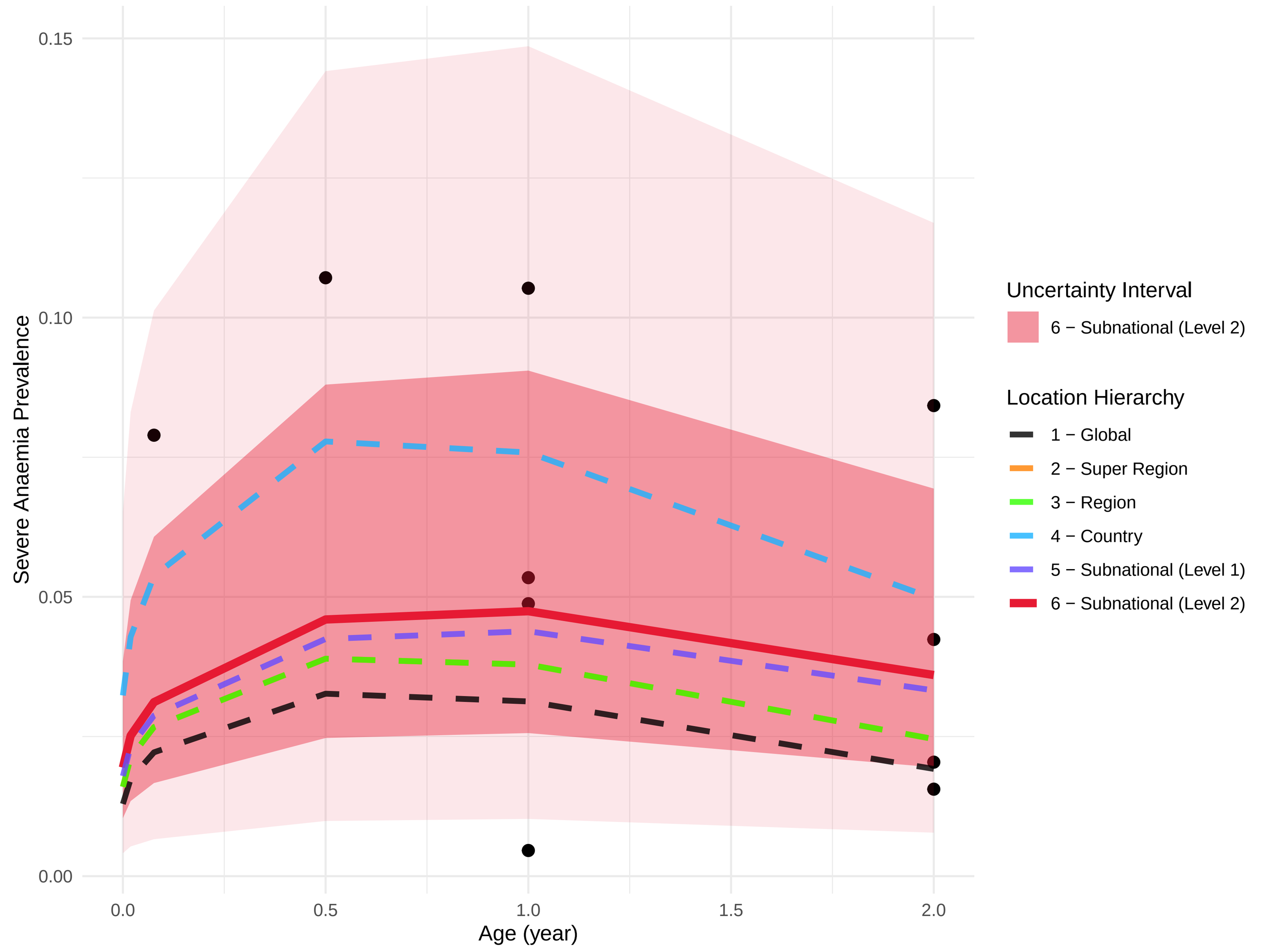

MR−BRT Age/Sex Fits − Non−pregnant Females <2 Years Old − Severe Anaemia Prevalence − Delhi
WHO−2024 Adjustment Method

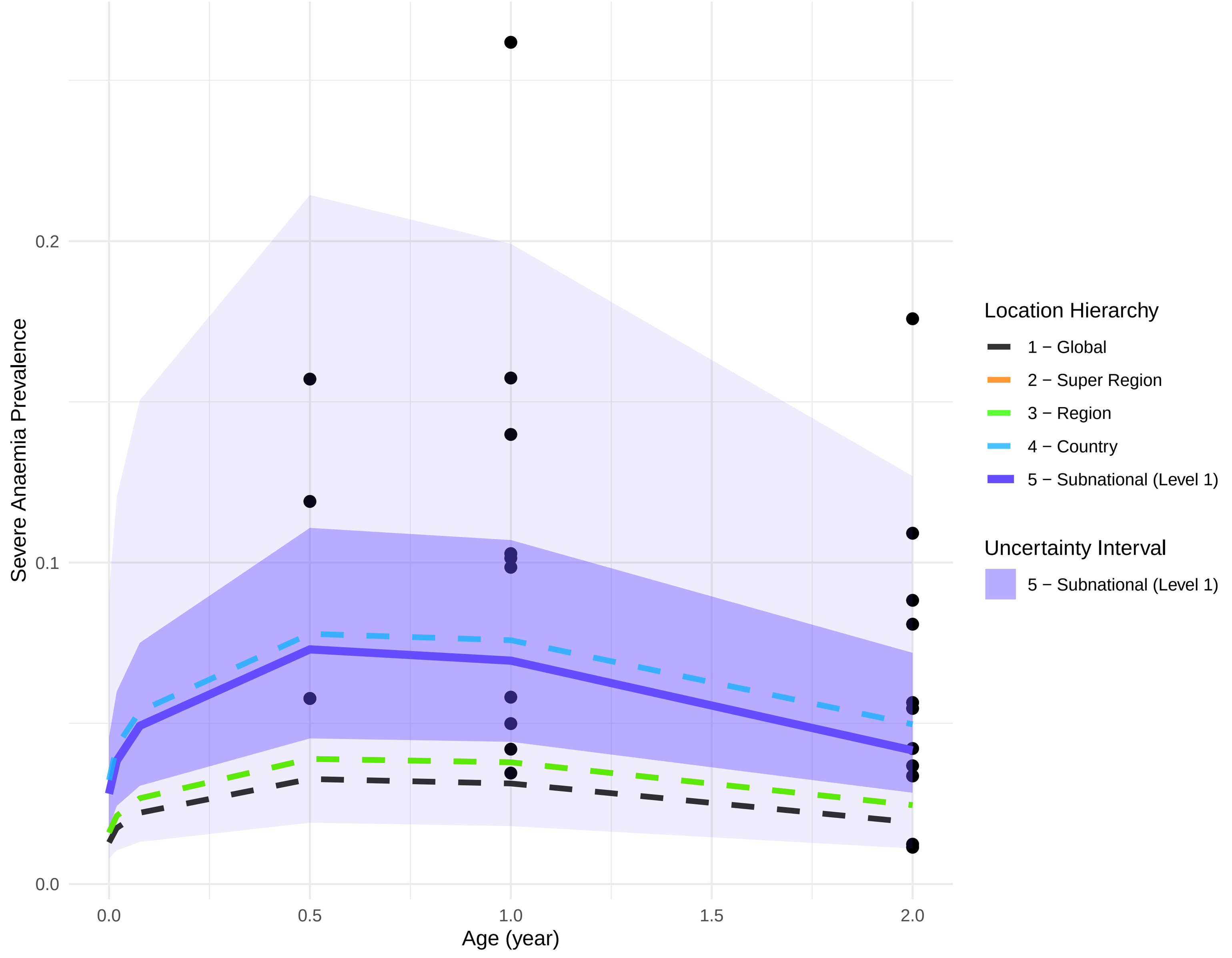

MR−BRT Age/Sex Fits − Non−pregnant Females <2 Years Old − Severe Anaemia Prevalence − Delhi, Rural
WHO−2024 Adjustment Method

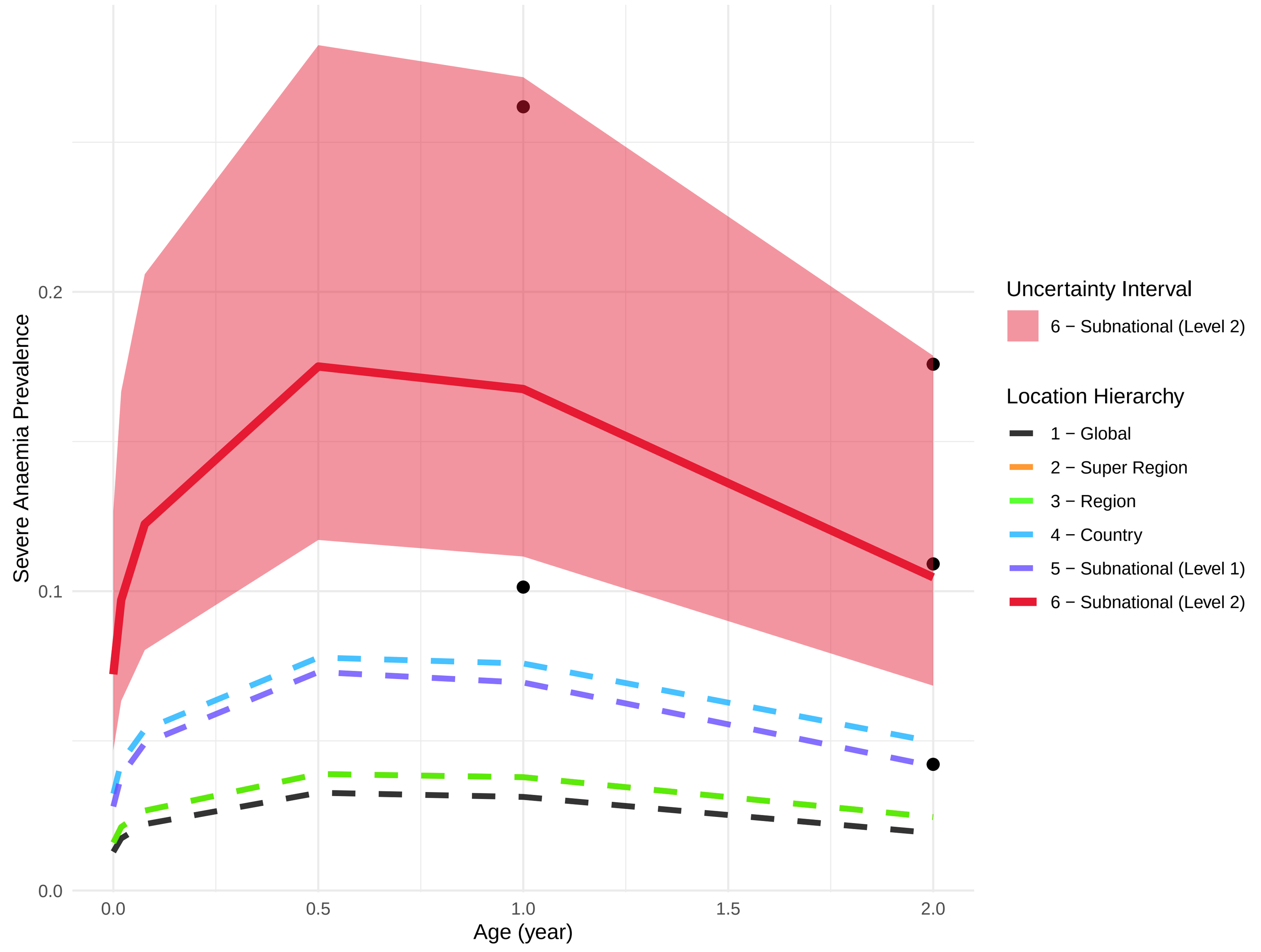

MR−BRT Age/Sex Fits − Non−pregnant Females <2 Years Old − Severe Anaemia Prevalence − Delhi, Urban
WHO−2024 Adjustment Method

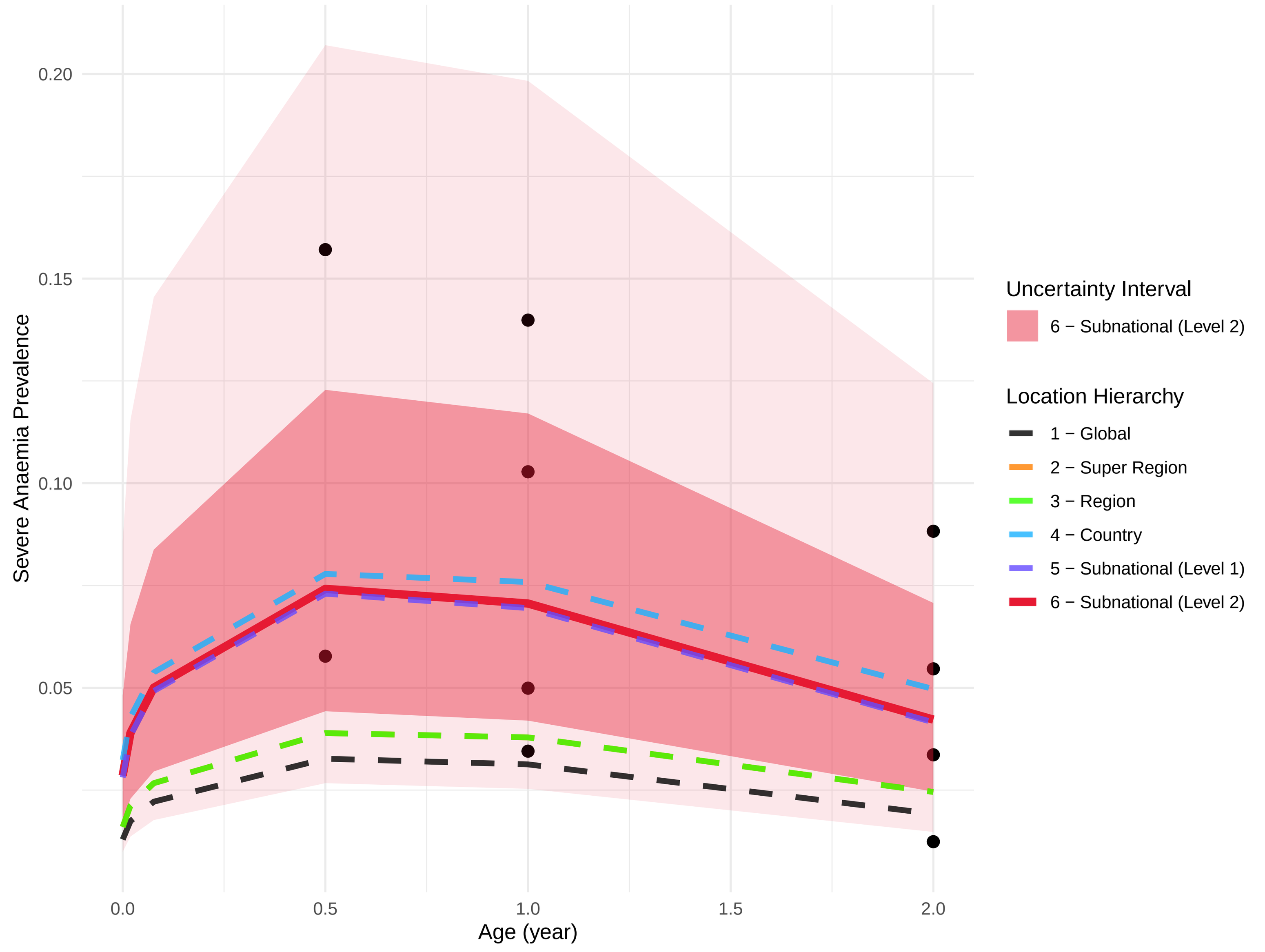

MR−BRT Age/Sex Fits − Non−pregnant Females <2 Years Old − Severe Anaemia Prevalence − Goa
WHO−2024 Adjustment Method

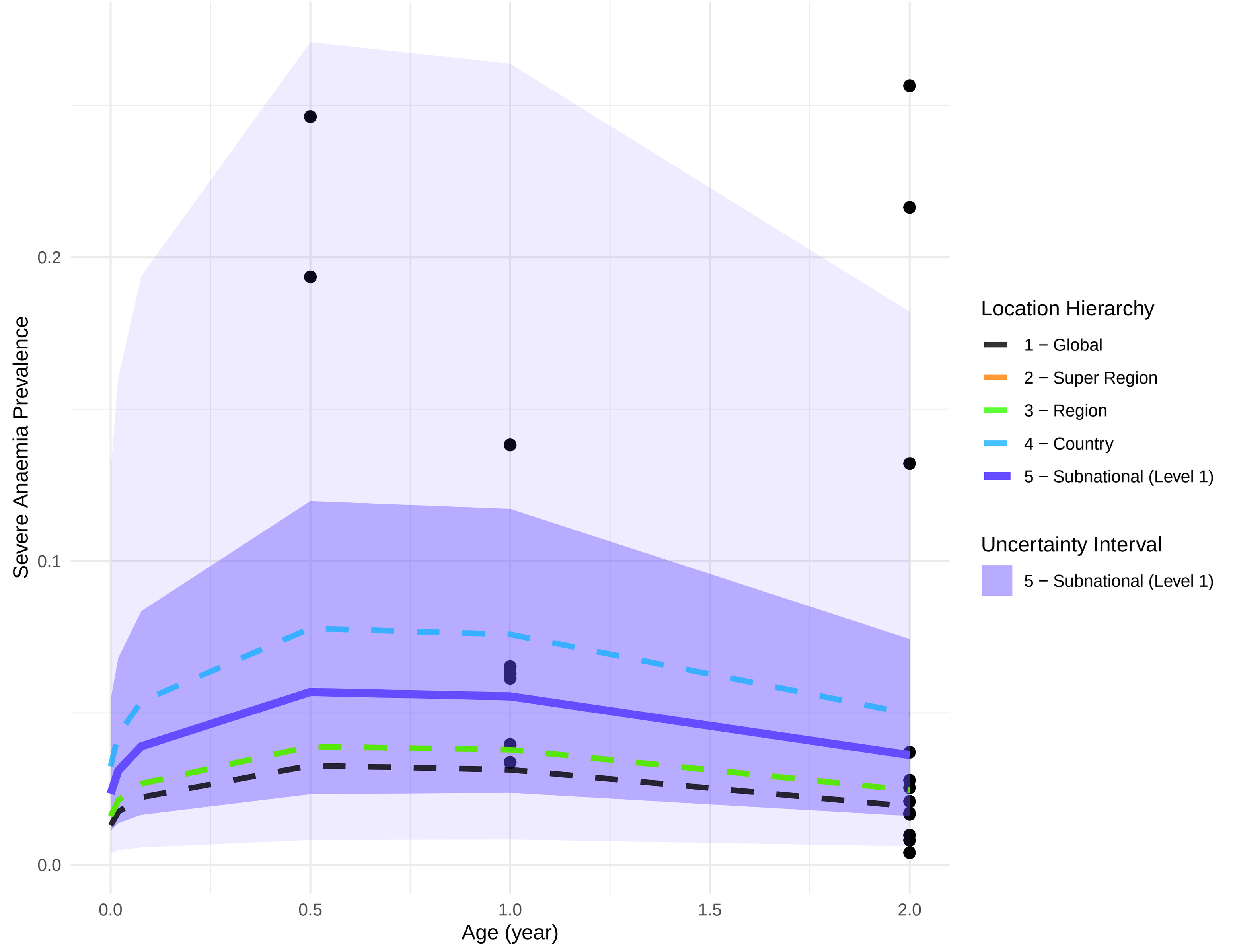

MR−BRT Age/Sex Fits − Non−pregnant Females <2 Years Old − Severe Anaemia Prevalence − Goa, Rural
WHO−2024 Adjustment Method

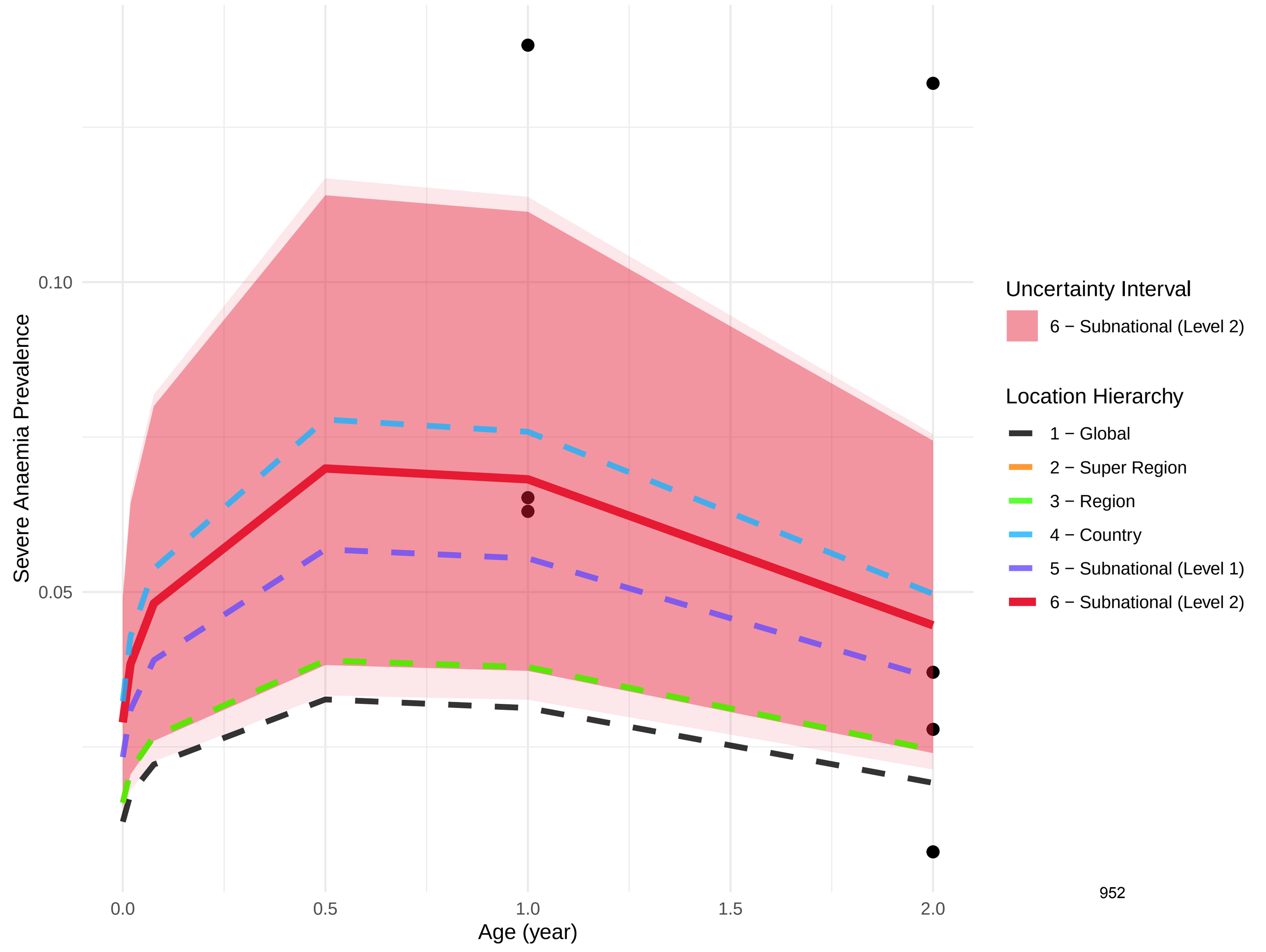

MR−BRT Age/Sex Fits − Non−pregnant Females <2 Years Old − Severe Anaemia Prevalence − Goa, Urban
WHO−2024 Adjustment Method

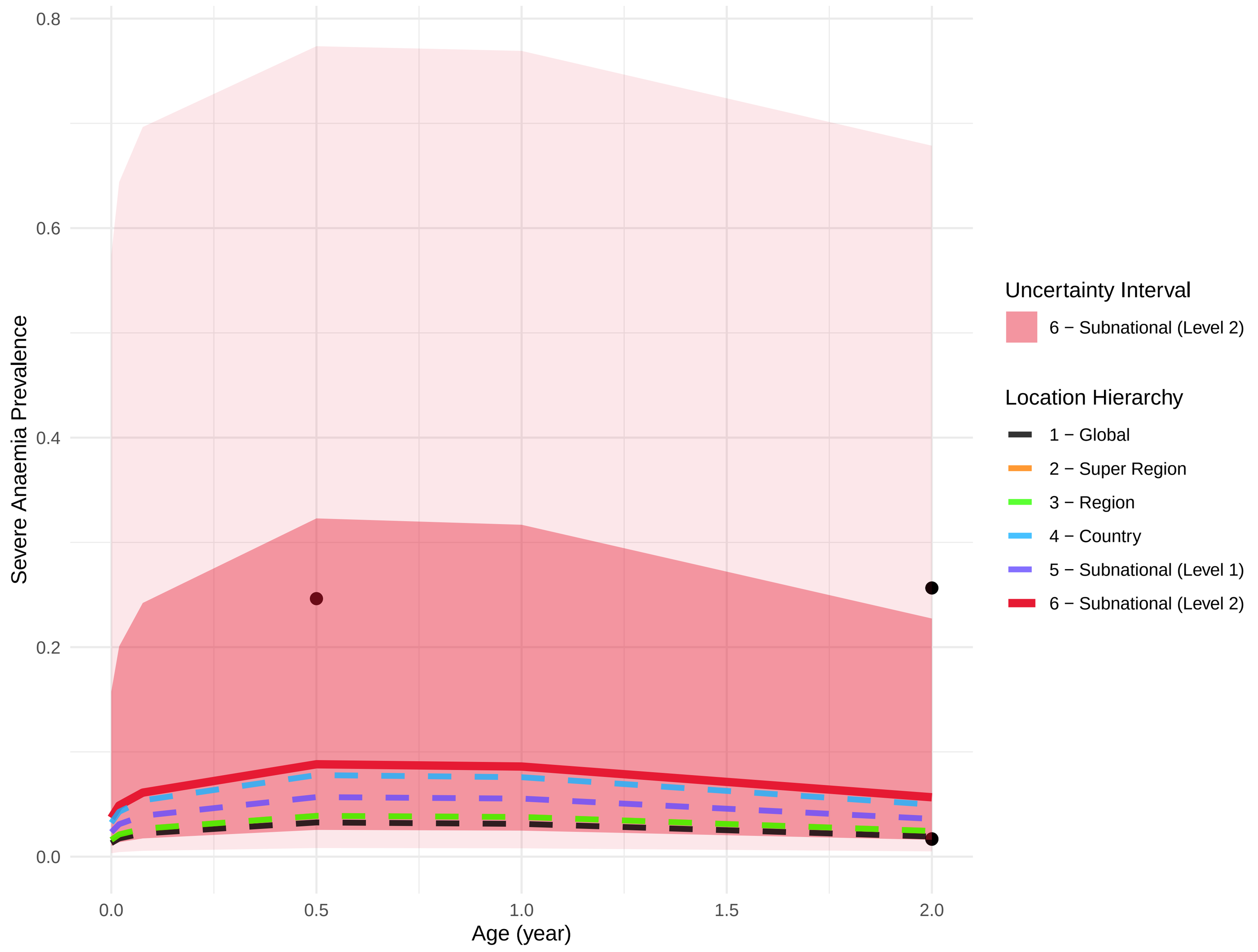

MR−BRT Age/Sex Fits − Non−pregnant Females <2 Years Old − Severe Anaemia Prevalence − Gujarat
WHO−2024 Adjustment Method

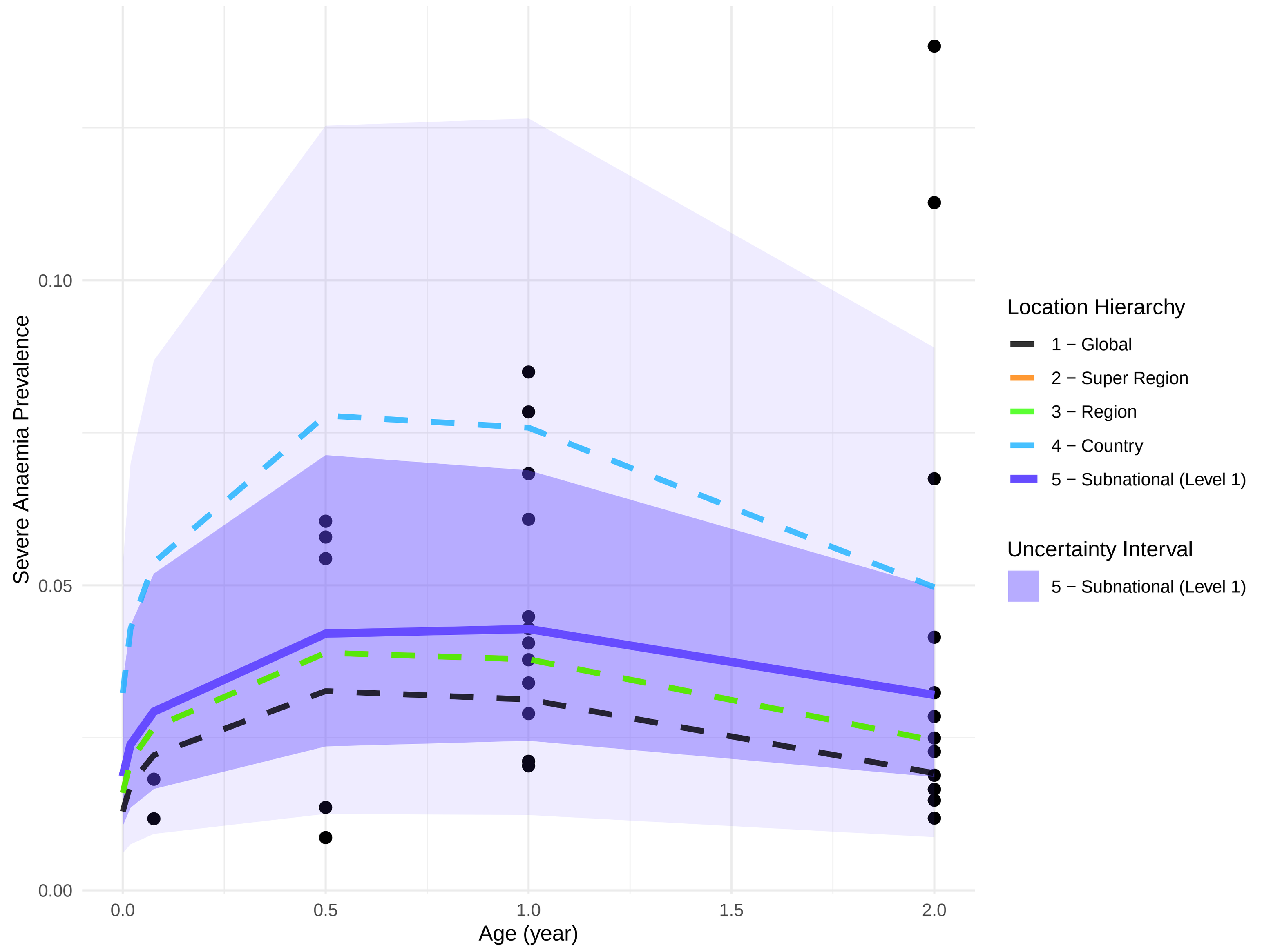

MR−BRT Age/Sex Fits − Non−pregnant Females <2 Years Old − Severe Anaemia Prevalence − Gujarat, Rural
WHO−2024 Adjustment Method

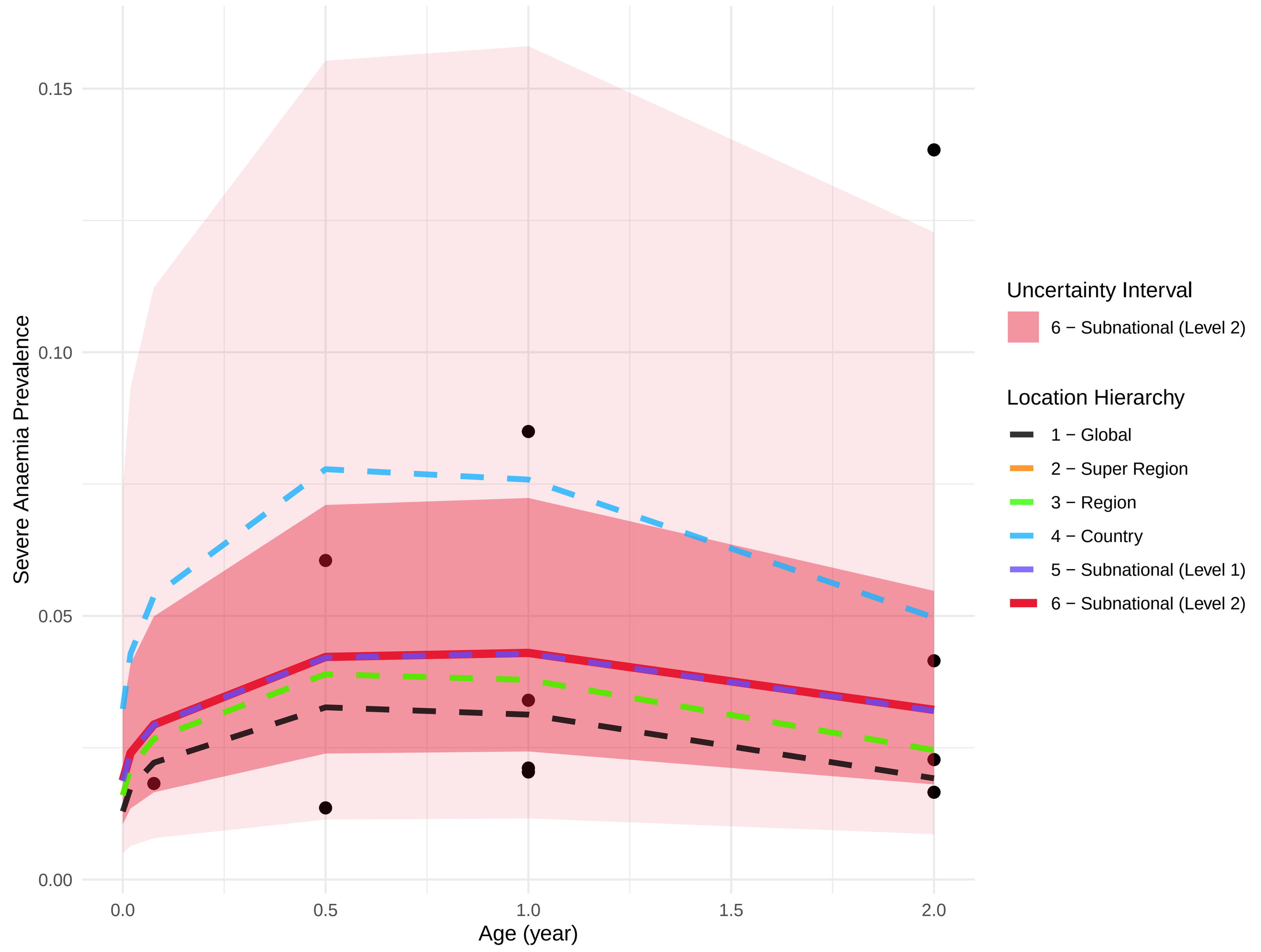

MR−BRT Age/Sex Fits − Non−pregnant Females <2 Years Old − Severe Anaemia Prevalence − Gujarat, Urban
WHO−2024 Adjustment Method

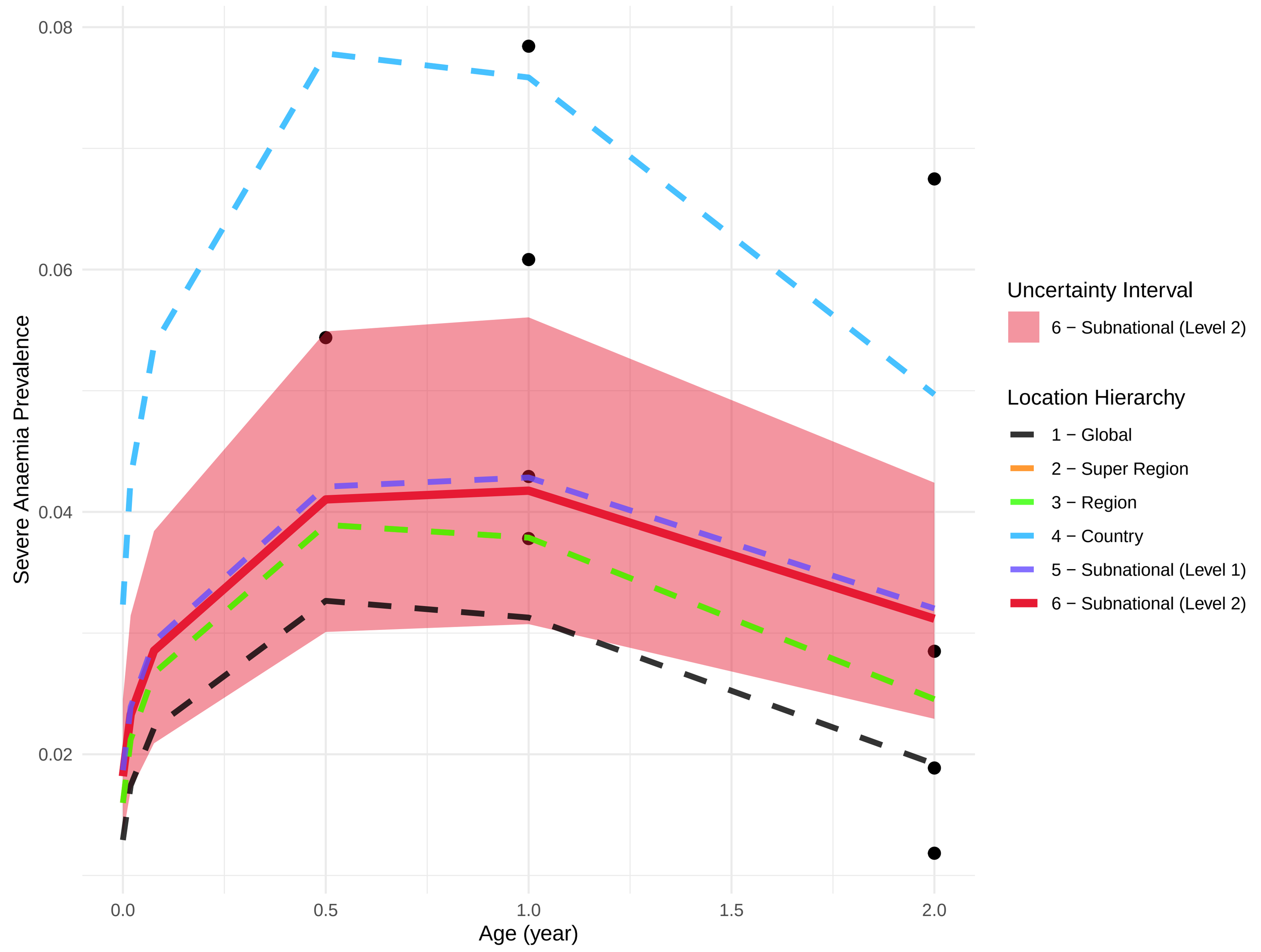

MR−BRT Age/Sex Fits − Non−pregnant Females <2 Years Old − Severe Anaemia Prevalence − Haryana
WHO−2024 Adjustment Method

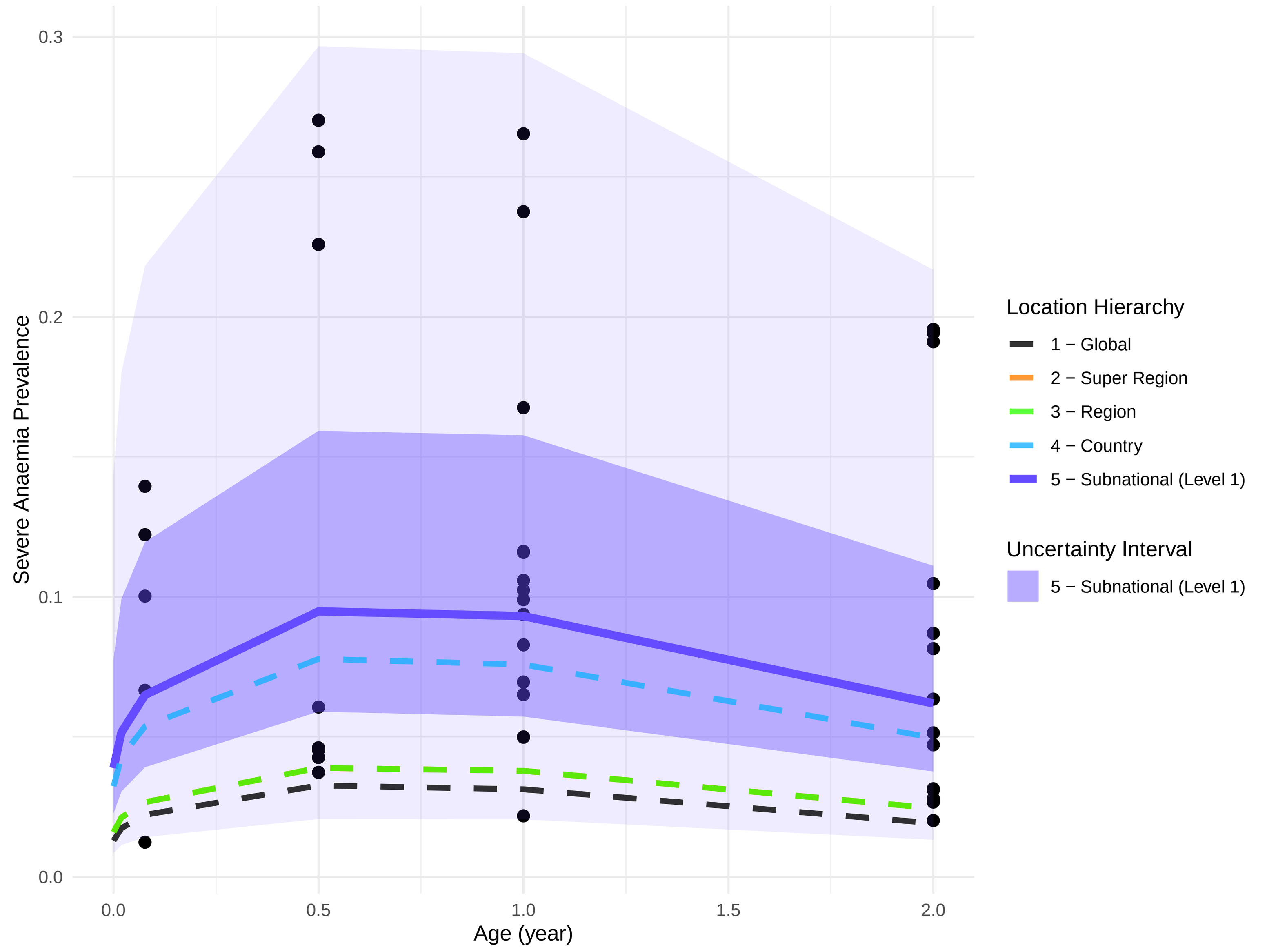

MR−BRT Age/Sex Fits − Non−pregnant Females <2 Years Old − Severe Anaemia Prevalence − Haryana, Rural
WHO−2024 Adjustment Method

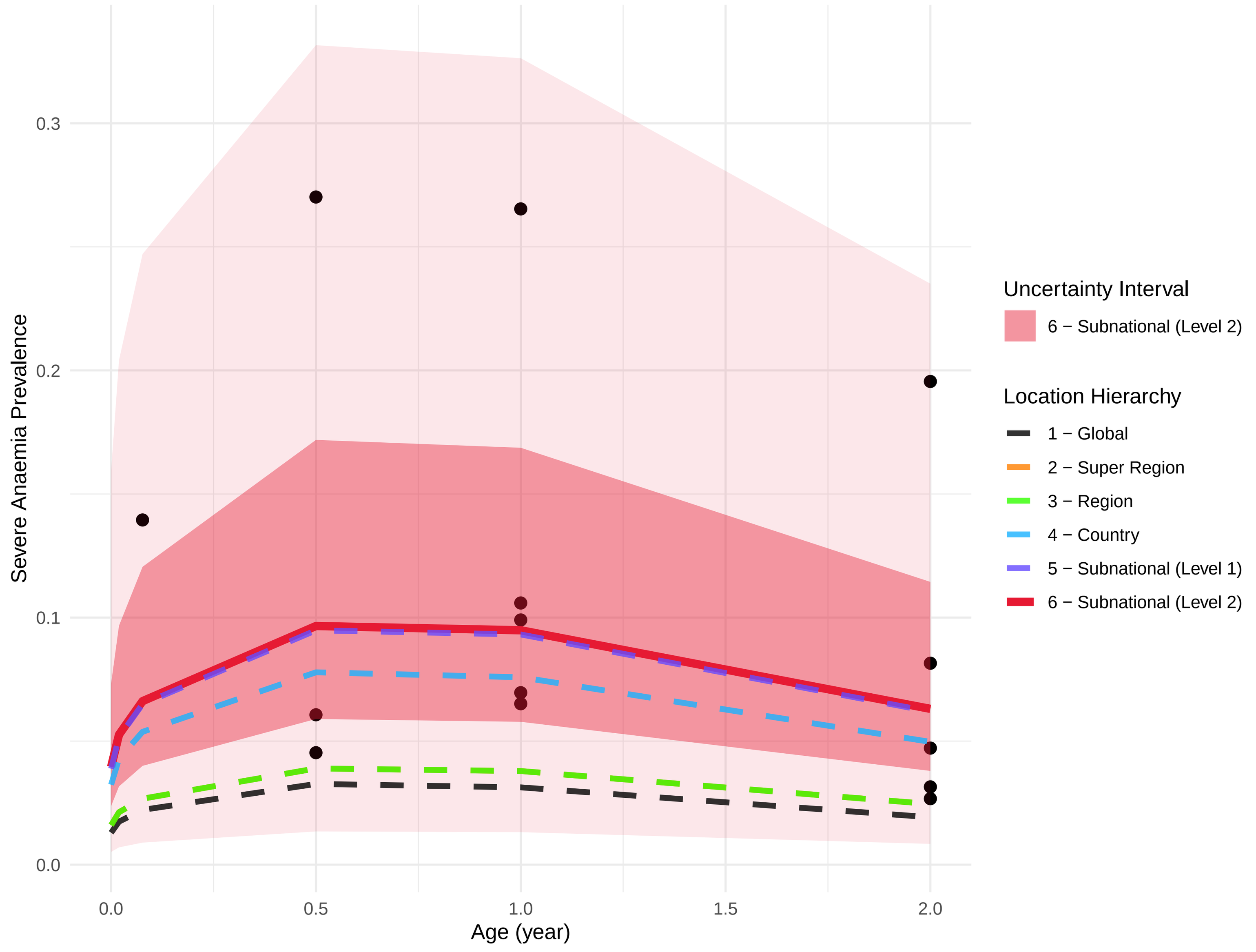

MR−BRT Age/Sex Fits − Non−pregnant Females <2 Years Old − Severe Anaemia Prevalence − Haryana, Urban
WHO−2024 Adjustment Method

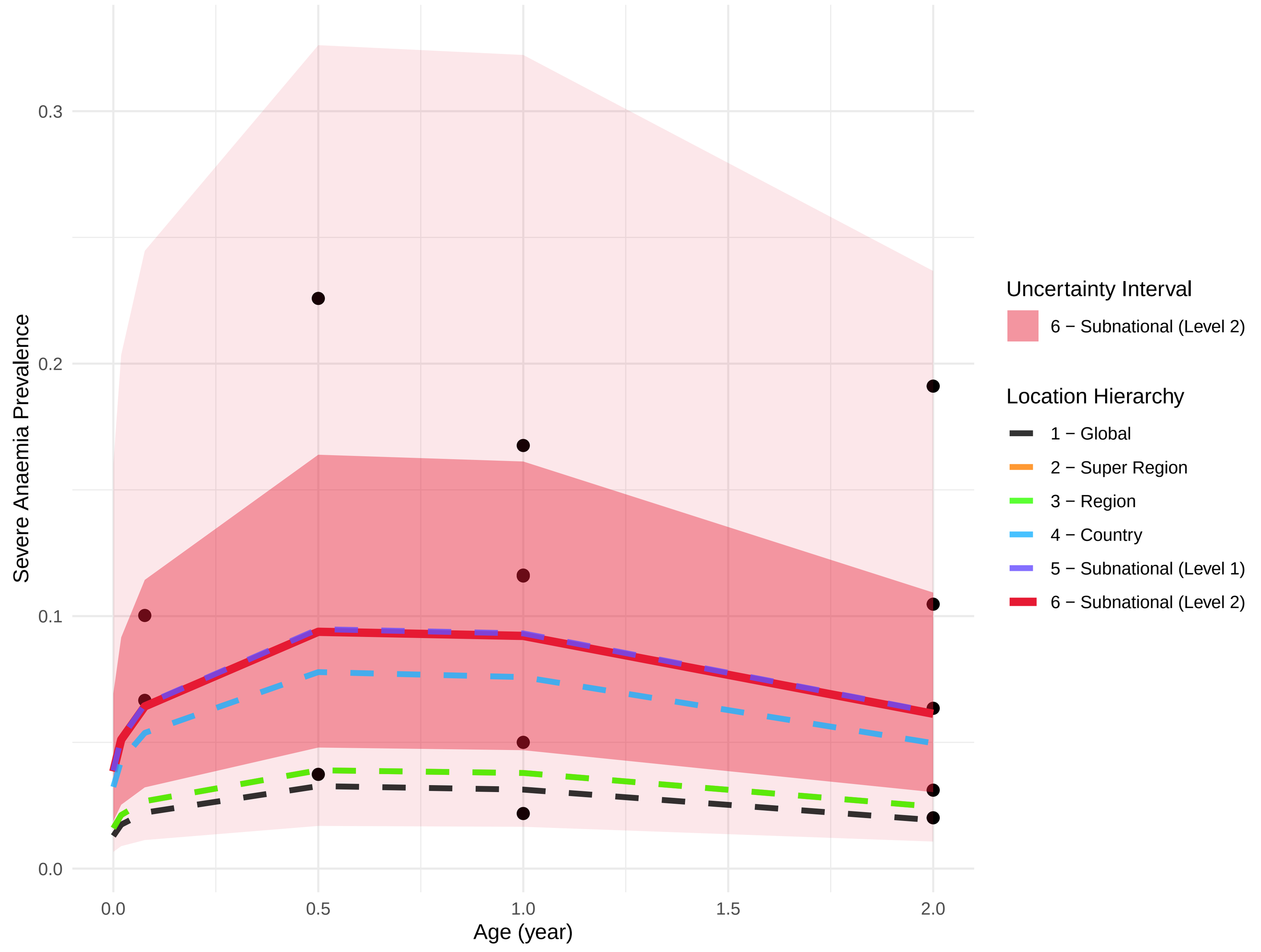

MR−BRT Age/Sex Fits − Non−pregnant Females <2 Years Old − Severe Anaemia Prevalence − Himachal Pradesh
WHO−2024 Adjustment Method

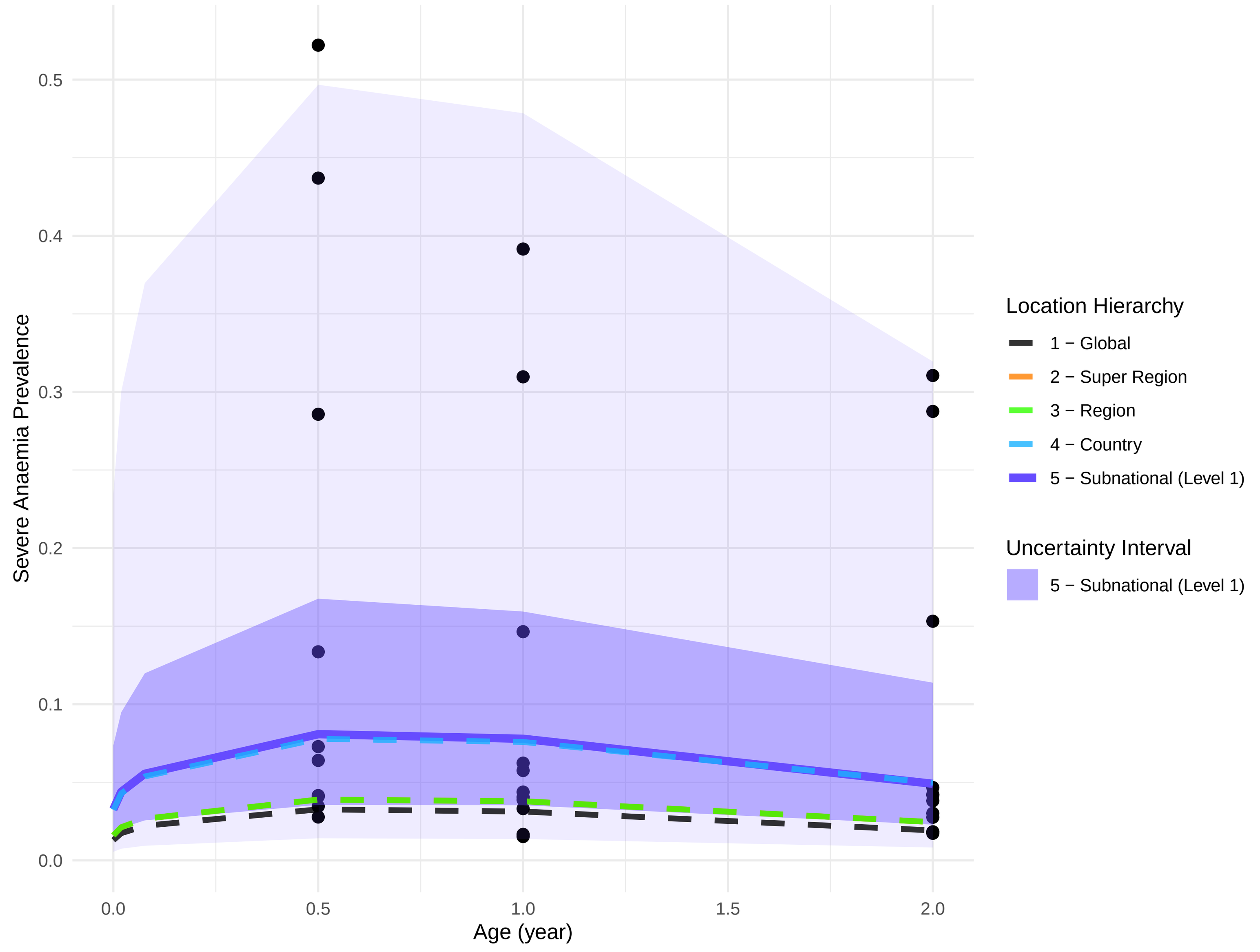

MR−BRT Age/Sex Fits − Non−pregnant Females <2 Years Old − Severe Anaemia Prevalence − Himachal Pradesh, Rural
WHO−2024 Adjustment Method

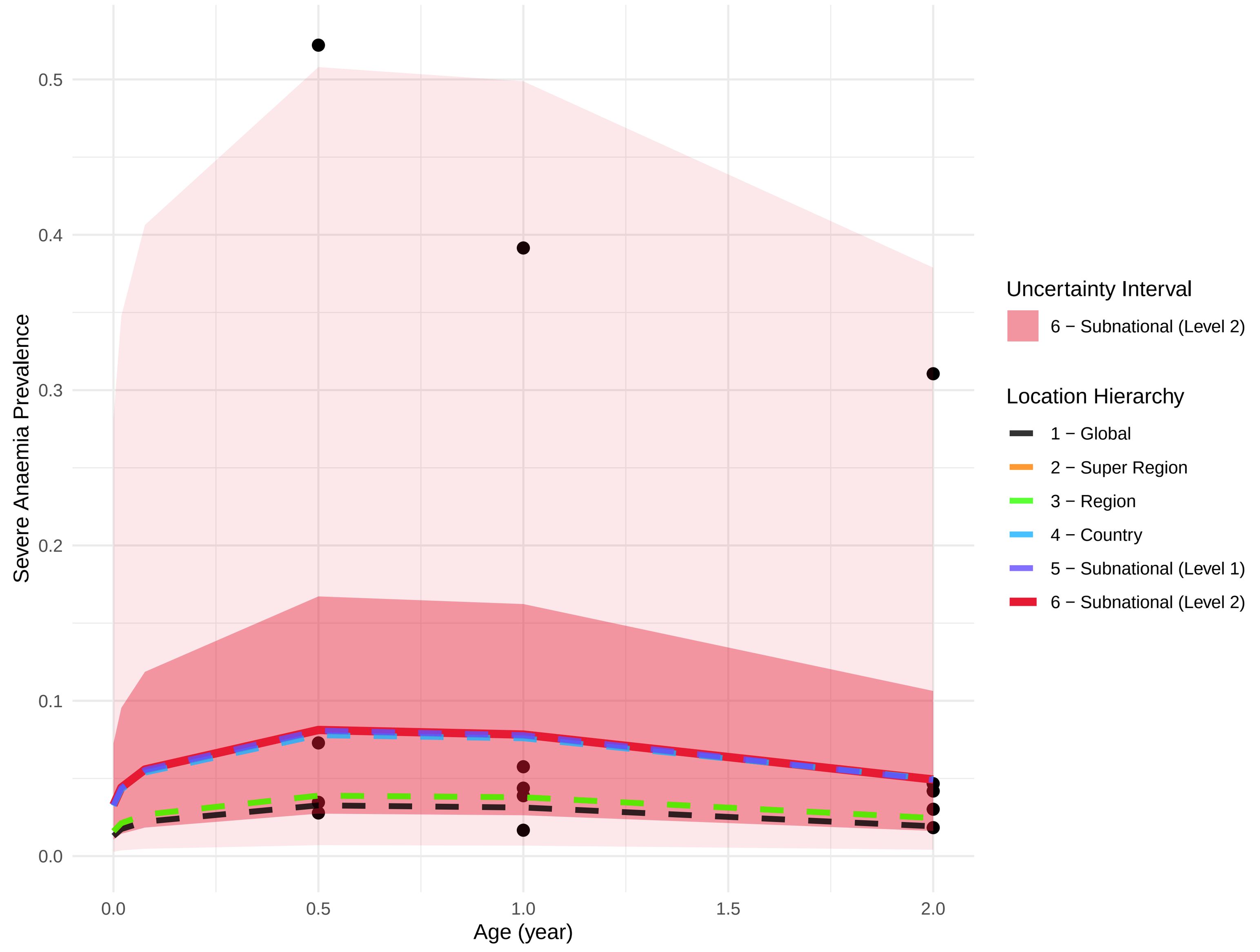

MR−BRT Age/Sex Fits − Non−pregnant Females <2 Years Old − Severe Anaemia Prevalence − Himachal Pradesh, Urban
WHO−2024 Adjustment Method

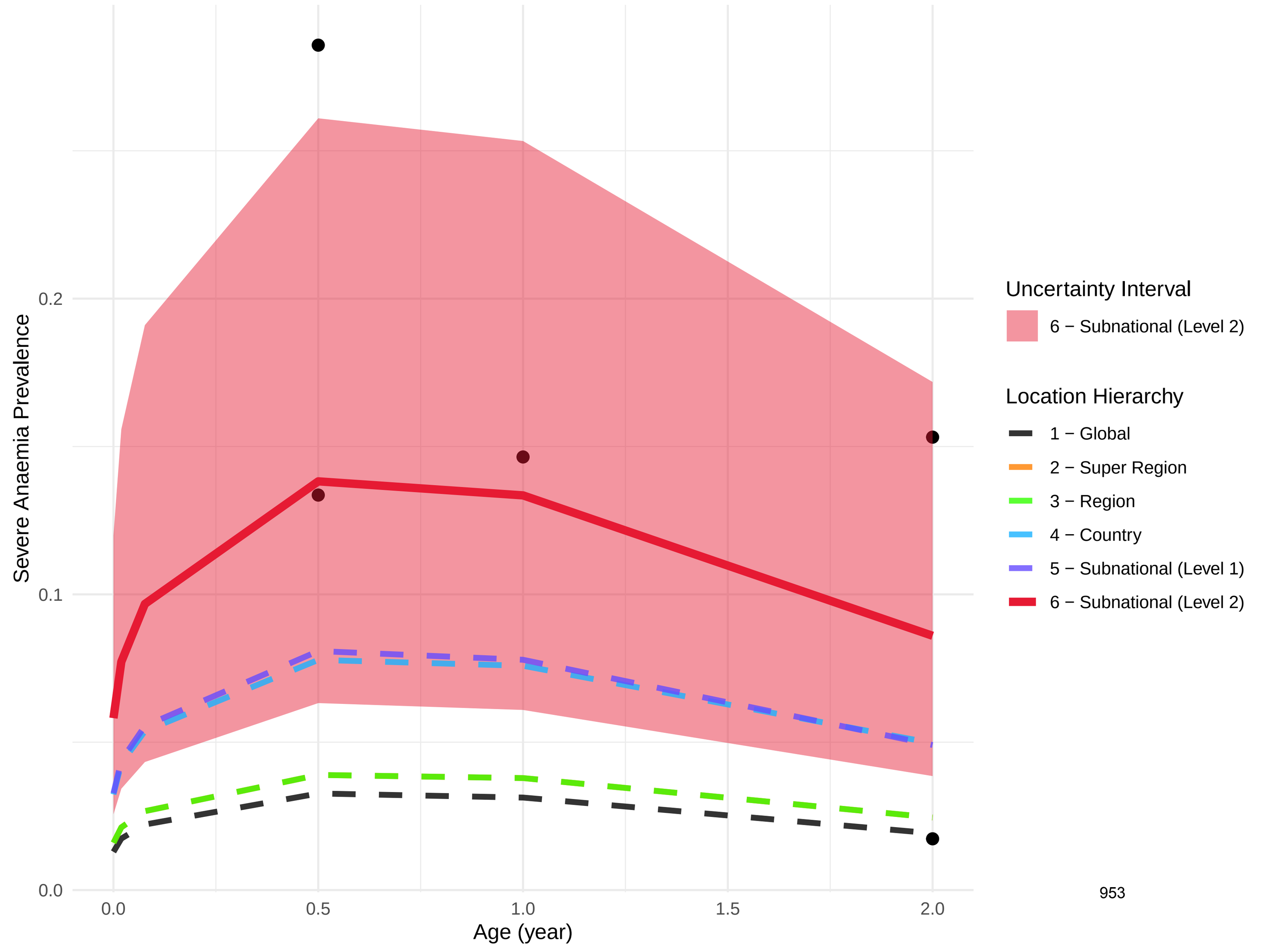

MR−BRT Age/Sex Fits − Non−pregnant Females <2 Years Old − Severe Anaemia Prevalence − Jammu & Kashmir and Ladakh
WHO−2024 Adjustment Method

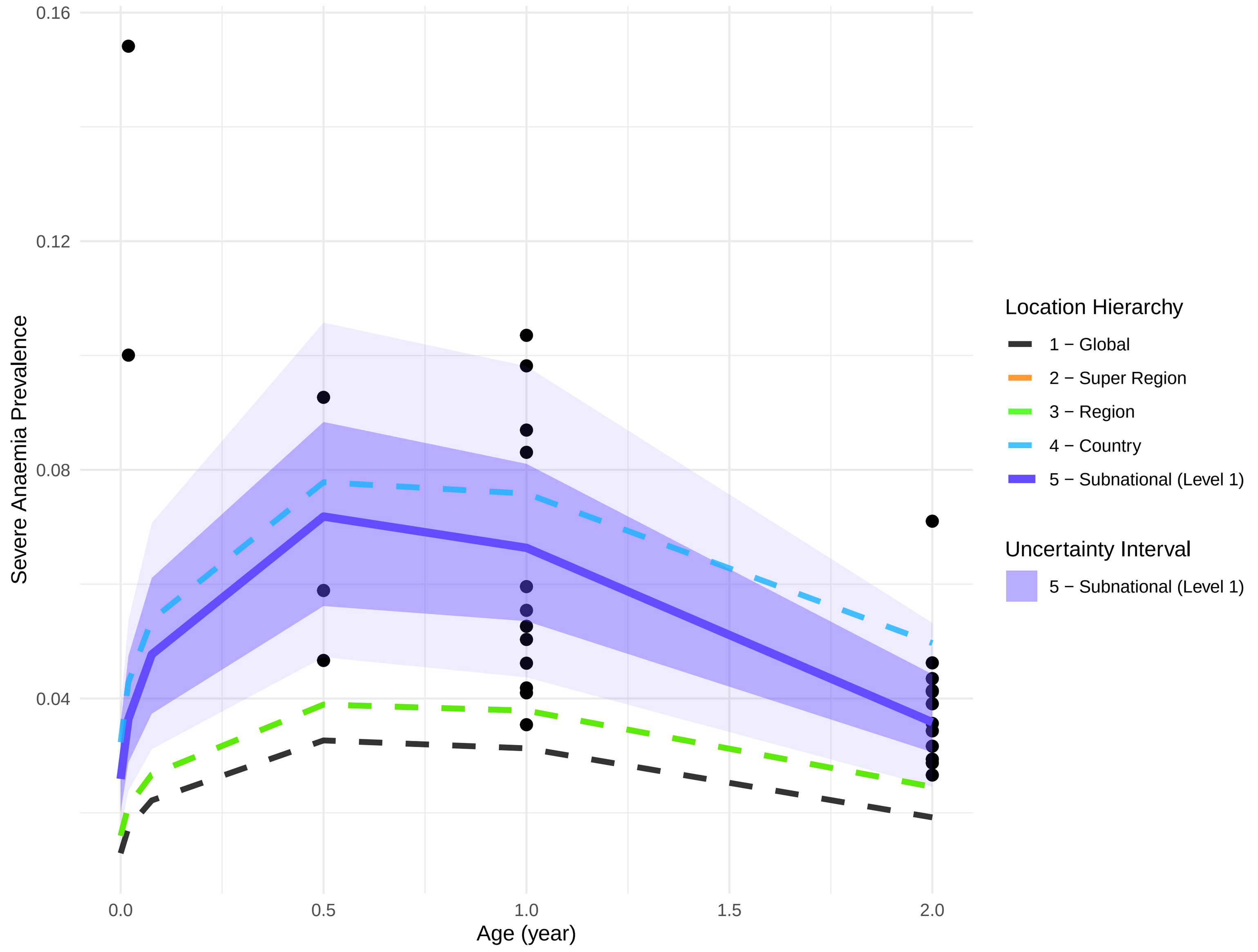

MR−BRT Age/Sex Fits − Non−pregnant Females <2 Years Old − Severe Anaemia Prevalence − Jammu & Kashmir and Ladakh, Rural
WHO−2024 Adjustment Method

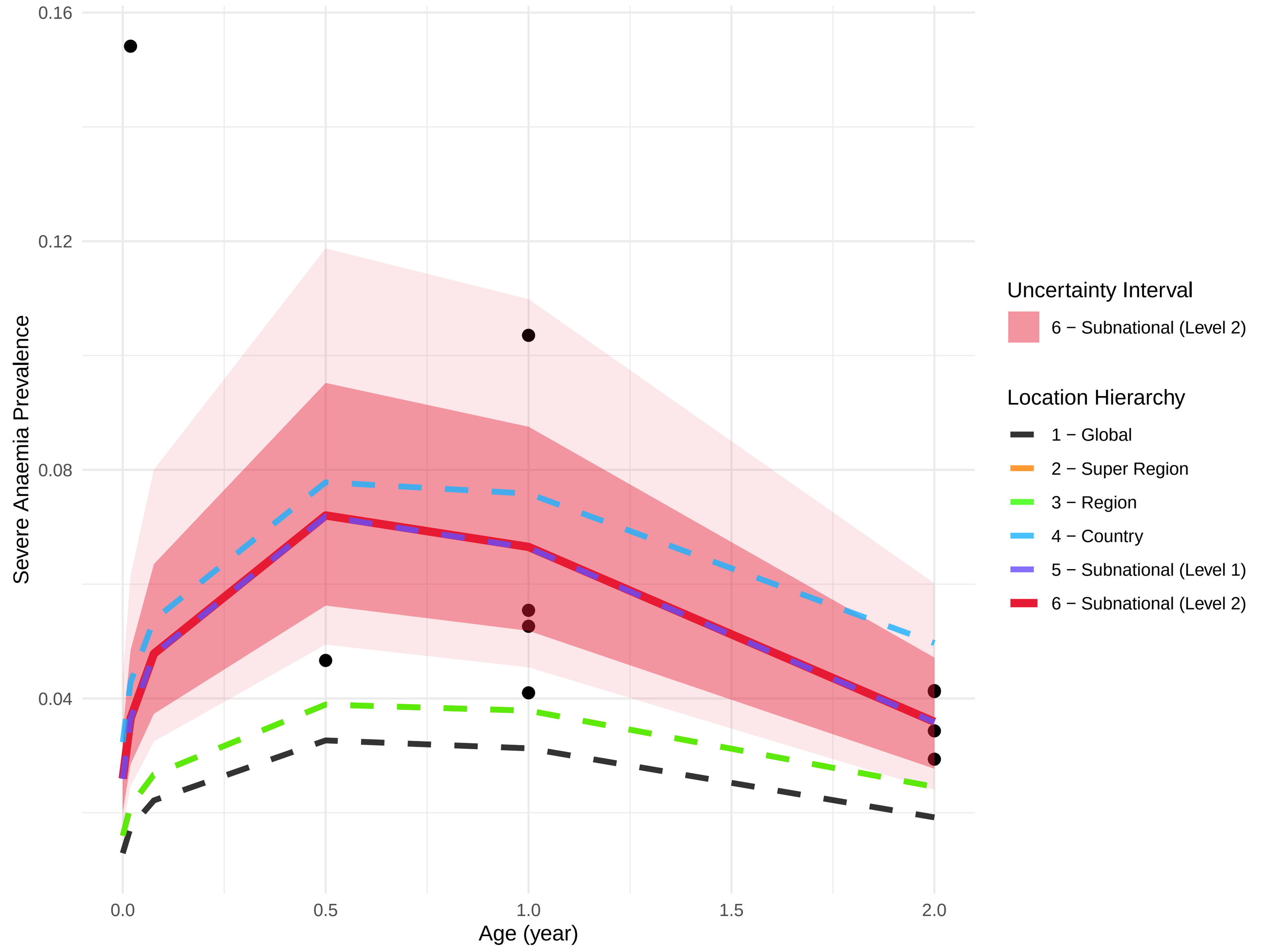

MR−BRT Age/Sex Fits − Non−pregnant Females <2 Years Old − Severe Anaemia Prevalence − Jammu & Kashmir and Ladakh, Urban
WHO−2024 Adjustment Method

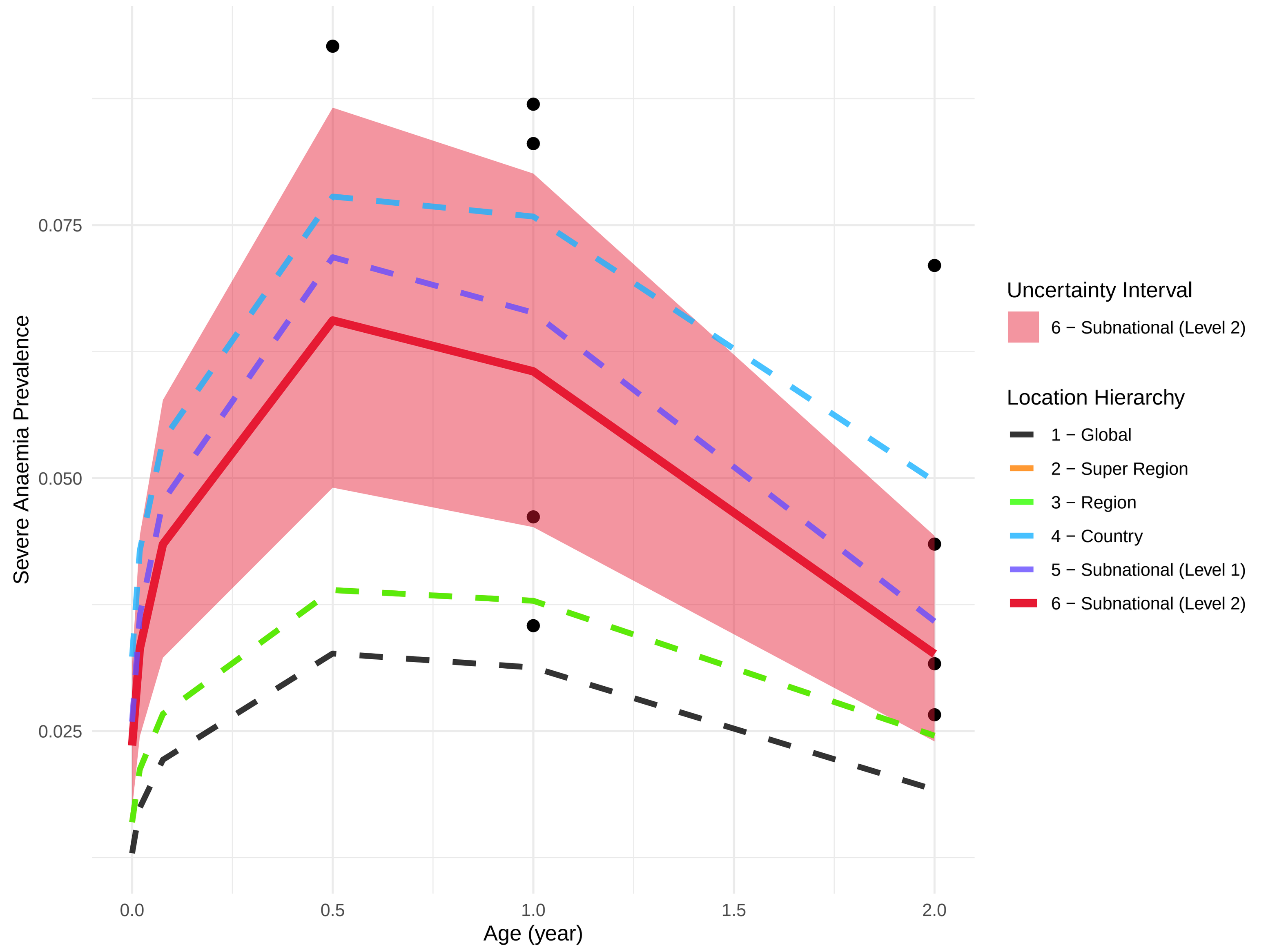

MR−BRT Age/Sex Fits − Non−pregnant Females <2 Years Old − Severe Anaemia Prevalence − Jharkhand
WHO−2024 Adjustment Method

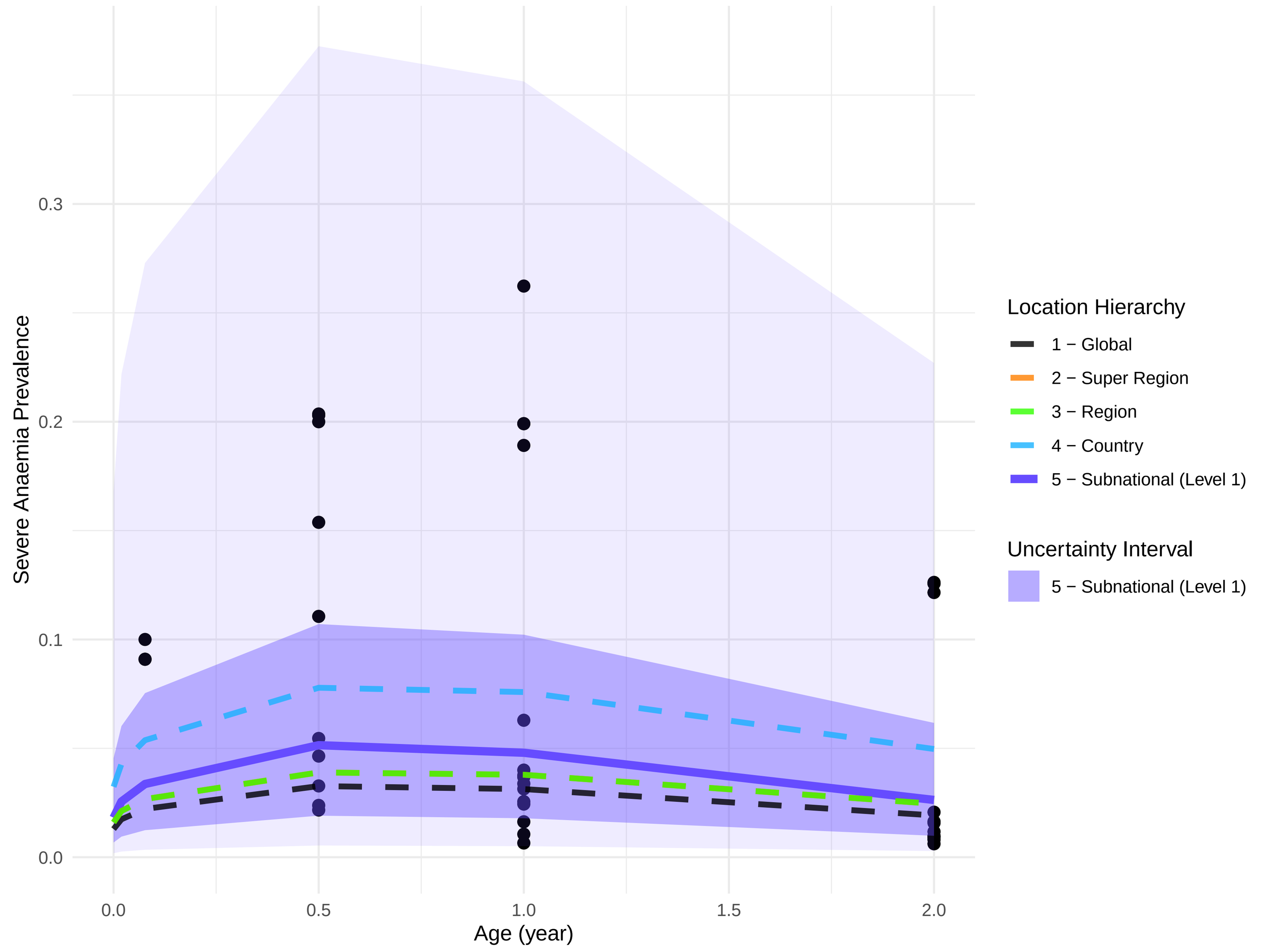

MR−BRT Age/Sex Fits − Non−pregnant Females <2 Years Old − Severe Anaemia Prevalence − Jharkhand, Rural
WHO−2024 Adjustment Method

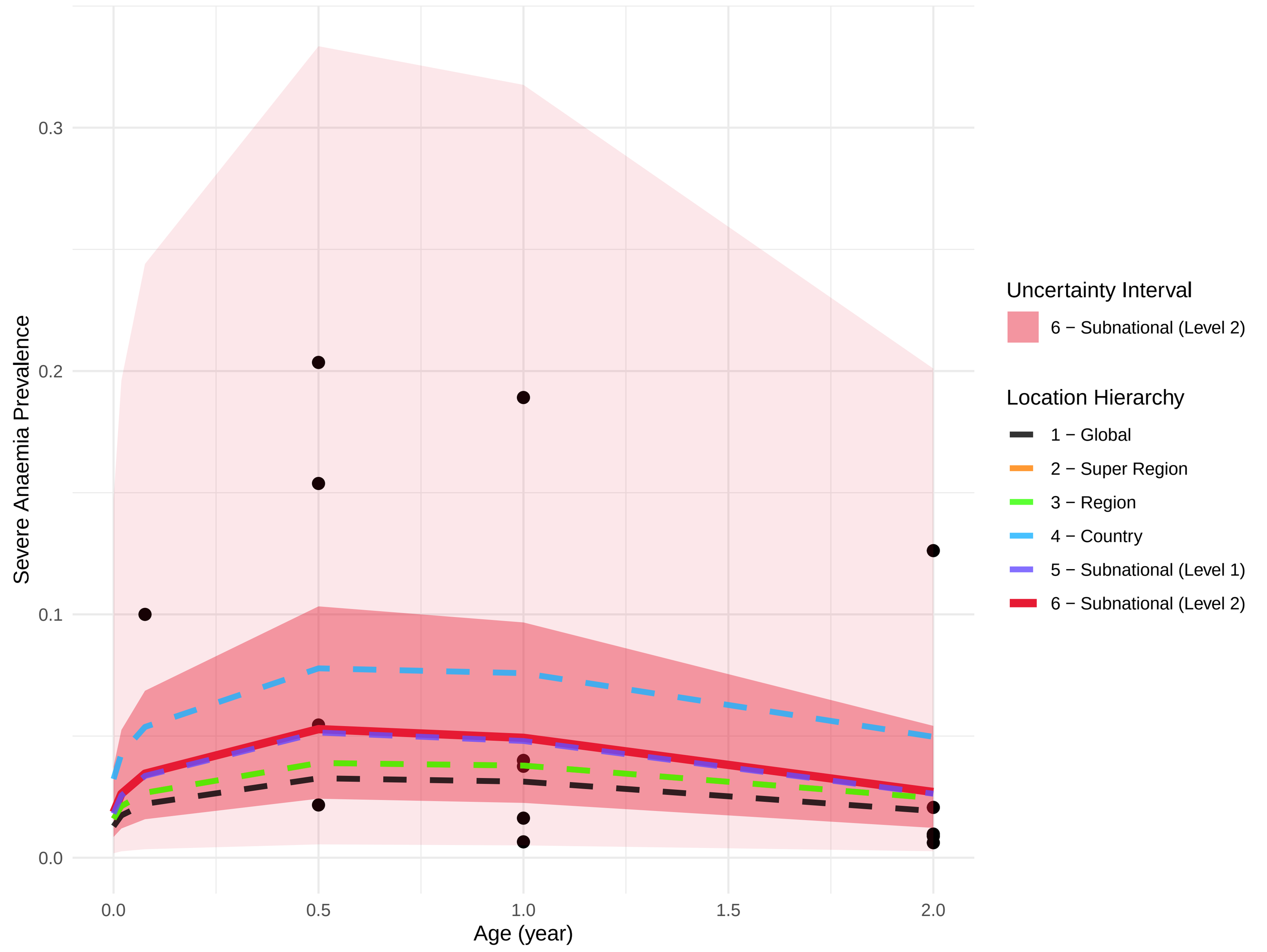

MR−BRT Age/Sex Fits − Non−pregnant Females <2 Years Old − Severe Anaemia Prevalence − Jharkhand, Urban
WHO−2024 Adjustment Method

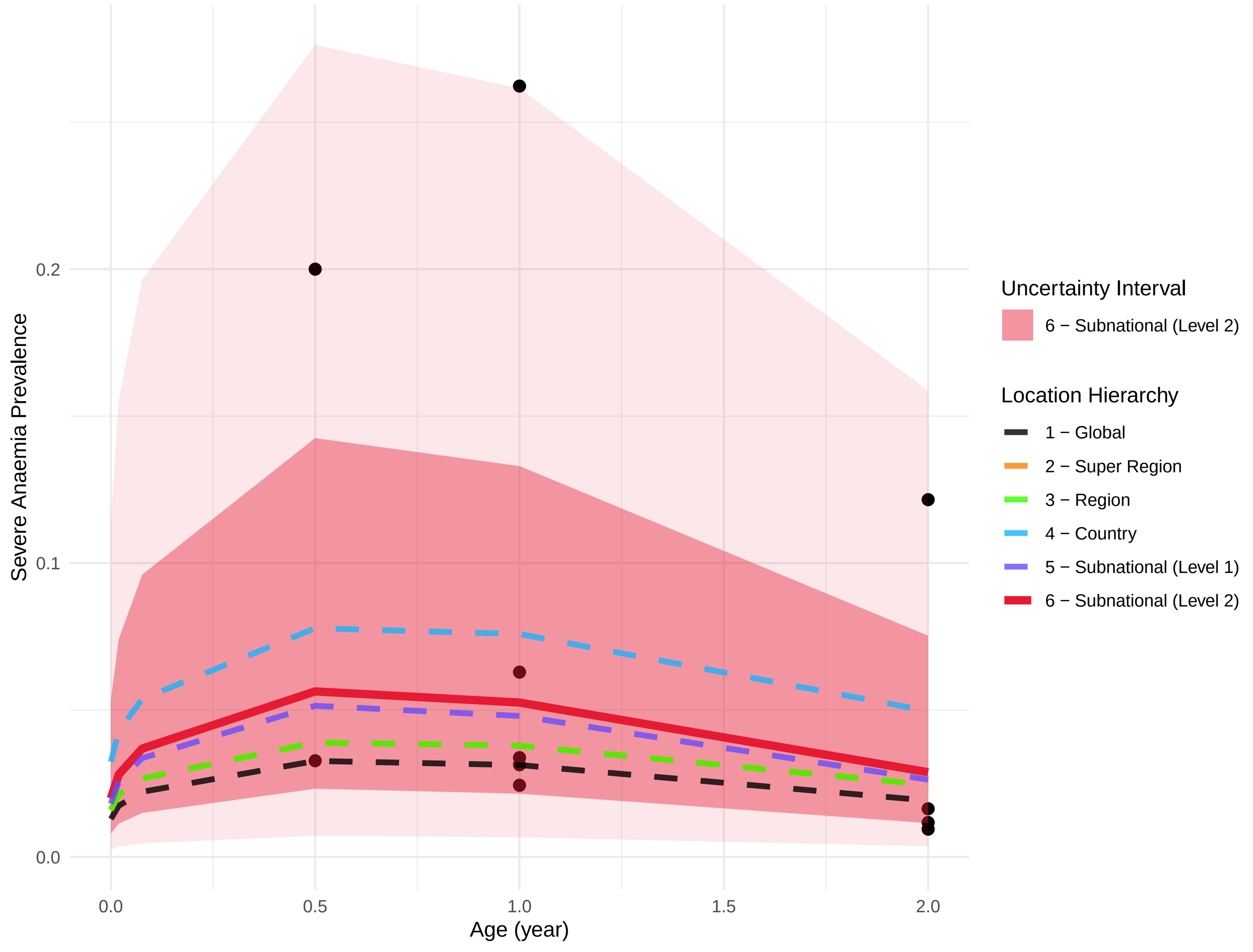

MR−BRT Age/Sex Fits − Non−pregnant Females <2 Years Old − Severe Anaemia Prevalence − Karnataka
WHO−2024 Adjustment Method

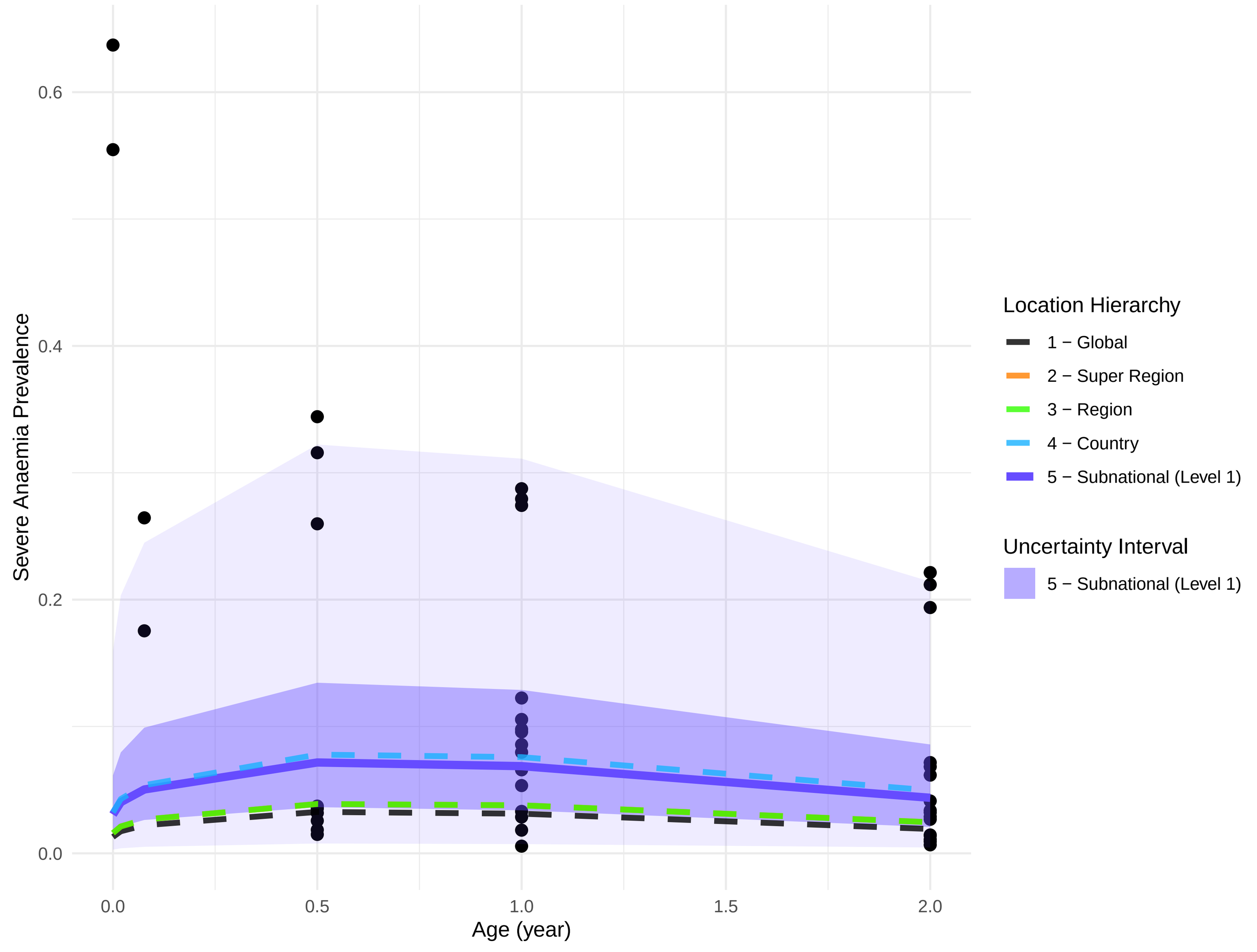

MR−BRT Age/Sex Fits − Non−pregnant Females <2 Years Old − Severe Anaemia Prevalence − Karnataka, Rural
WHO−2024 Adjustment Method

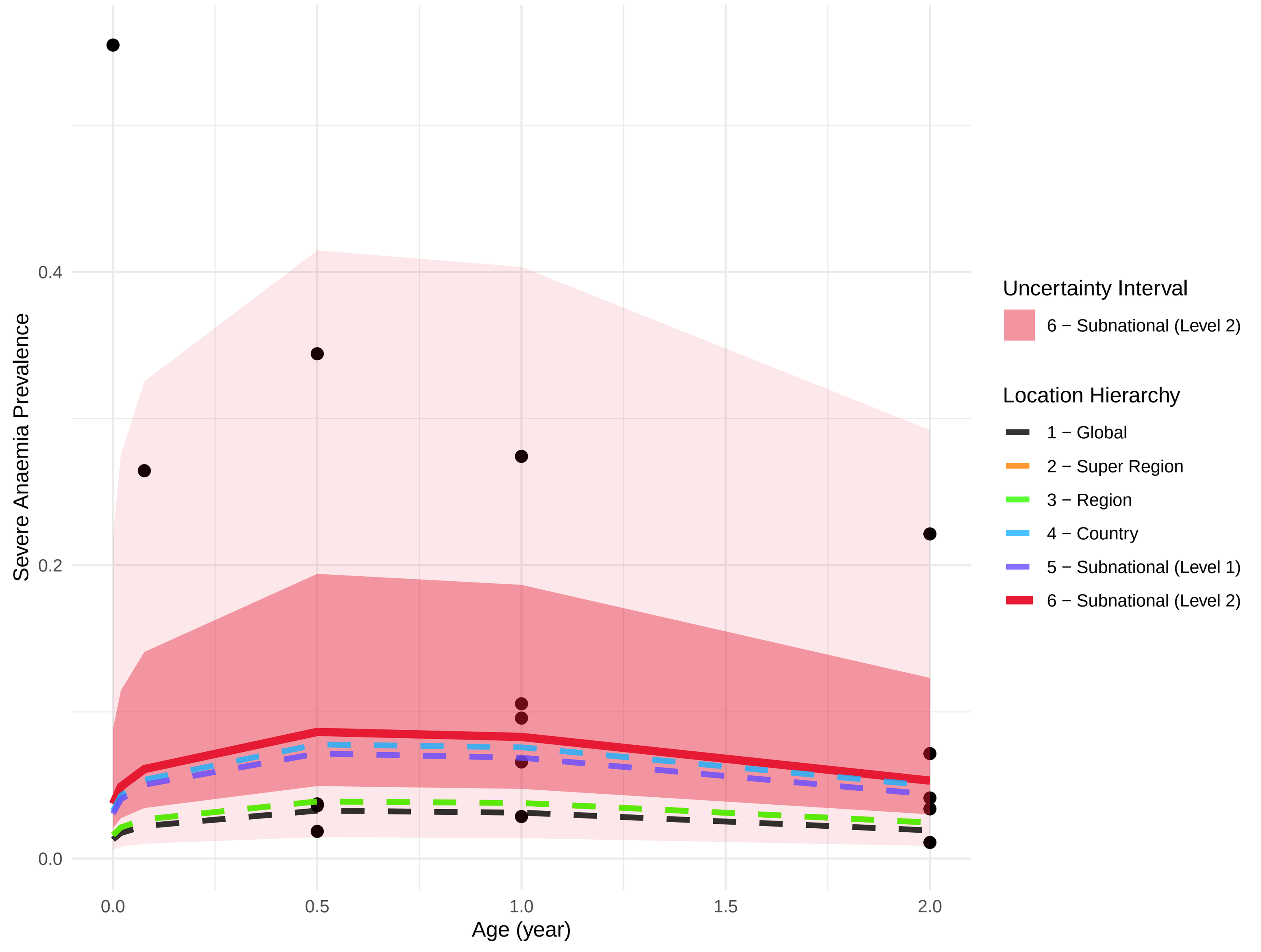

MR−BRT Age/Sex Fits − Non−pregnant Females <2 Years Old − Severe Anaemia Prevalence − Karnataka, Urban
WHO−2024 Adjustment Method

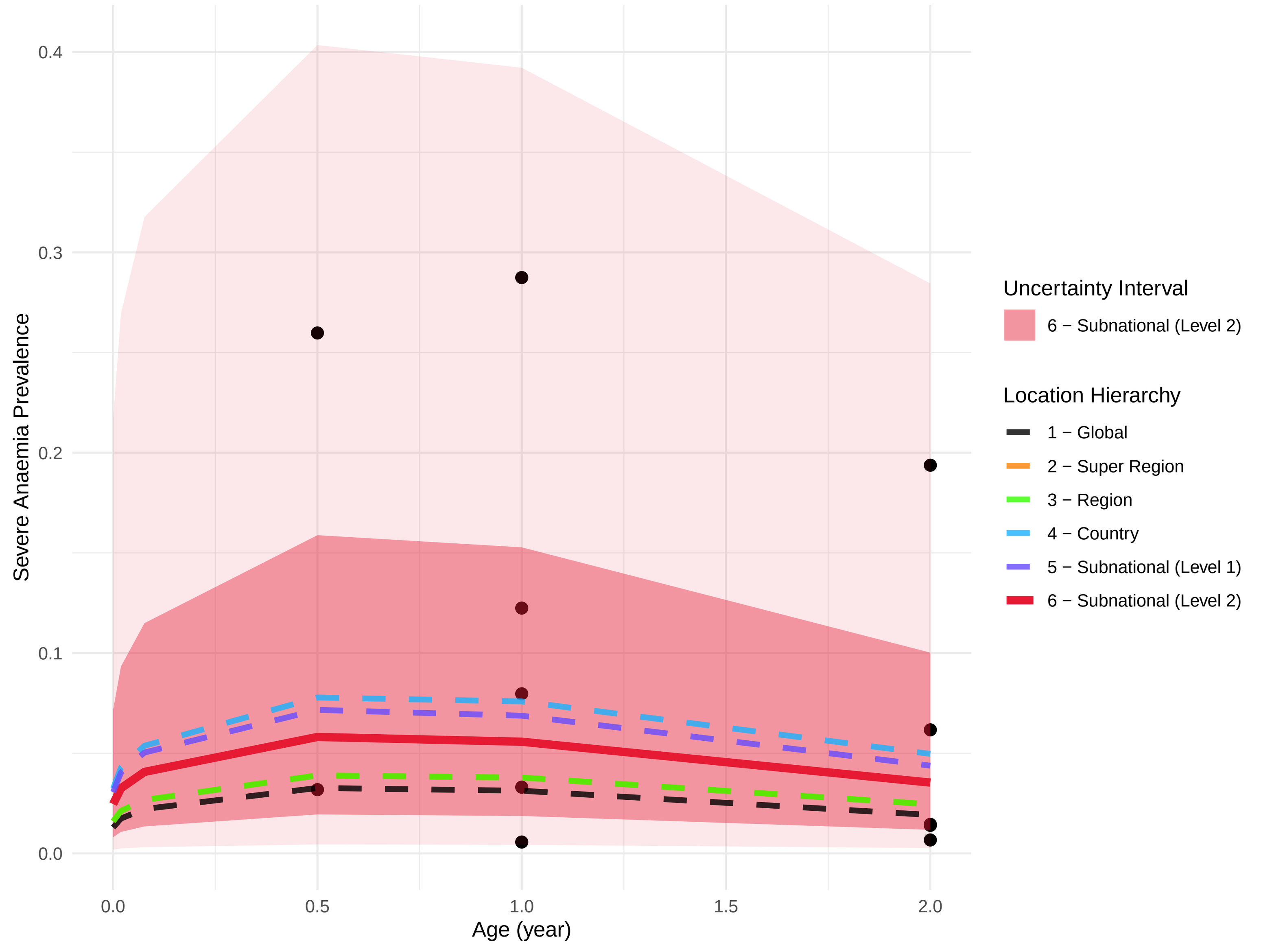

MR−BRT Age/Sex Fits − Non−pregnant Females <2 Years Old − Severe Anaemia Prevalence − Kerala
WHO−2024 Adjustment Method

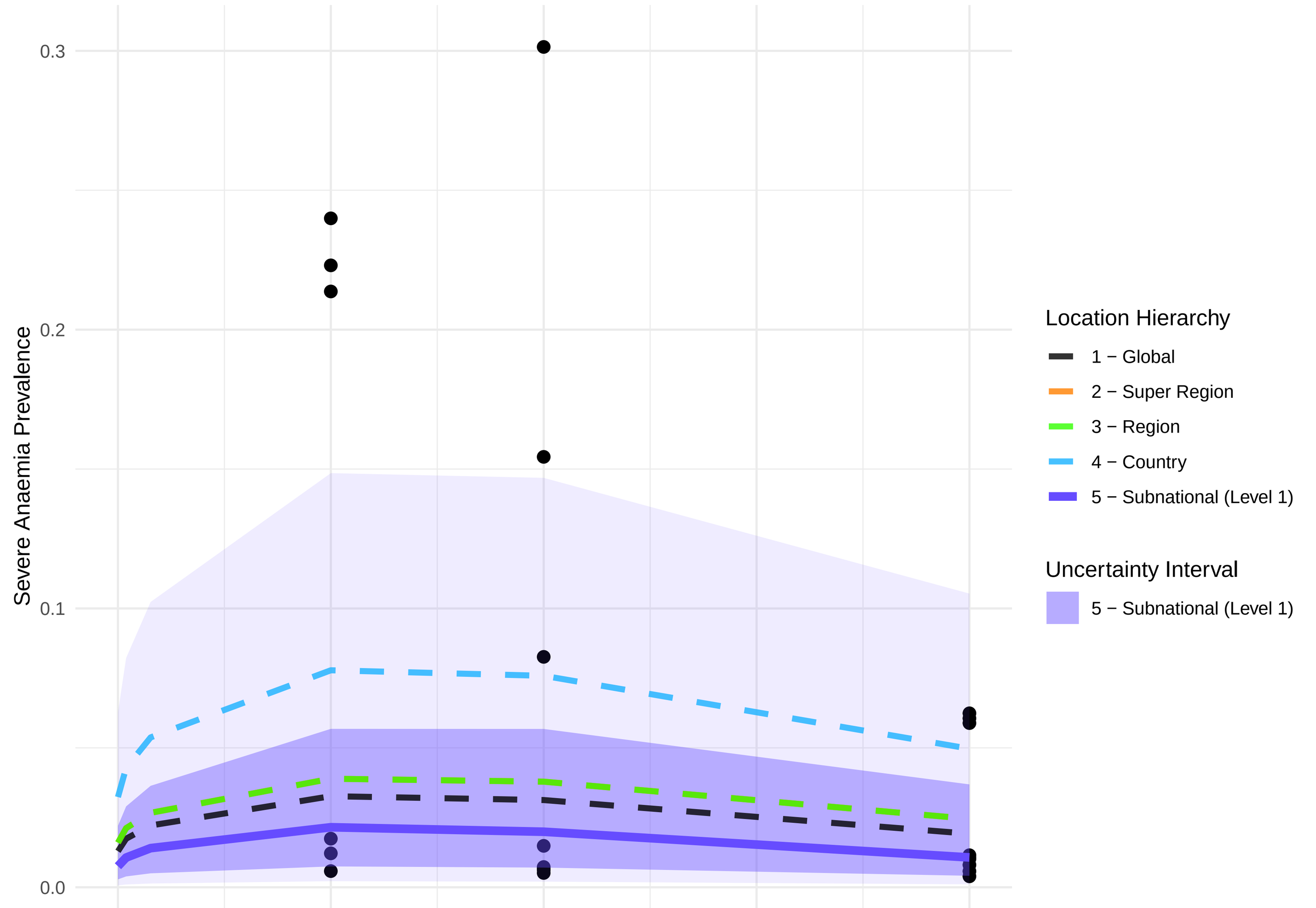

MR−BRT Age/Sex Fits − Non−pregnant Females <2 Years Old − Severe Anaemia Prevalence − Kerala, Rural
WHO−2024 Adjustment Method

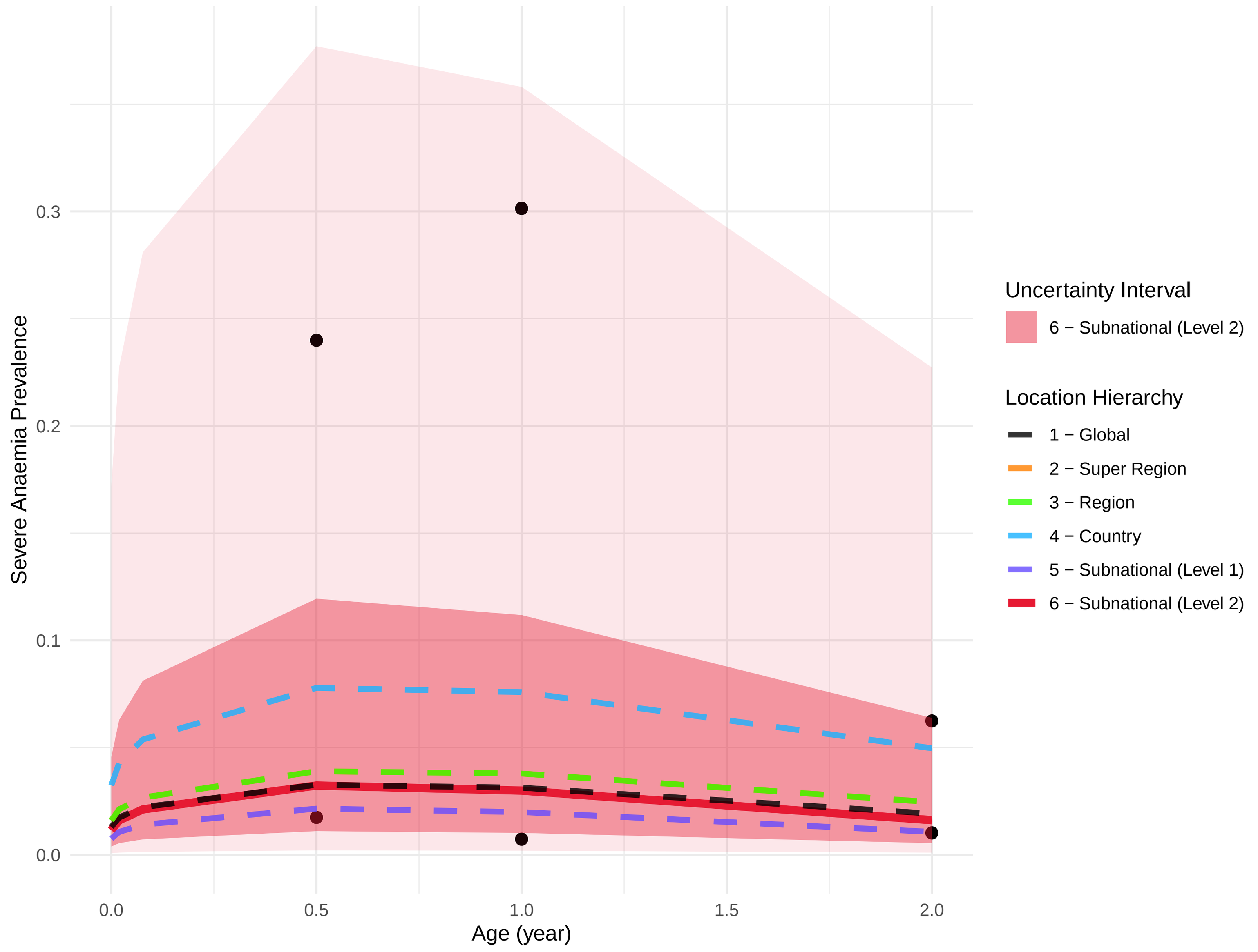

MR−BRT Age/Sex Fits − Non−pregnant Females <2 Years Old − Severe Anaemia Prevalence − Kerala, Urban
WHO−2024 Adjustment Method

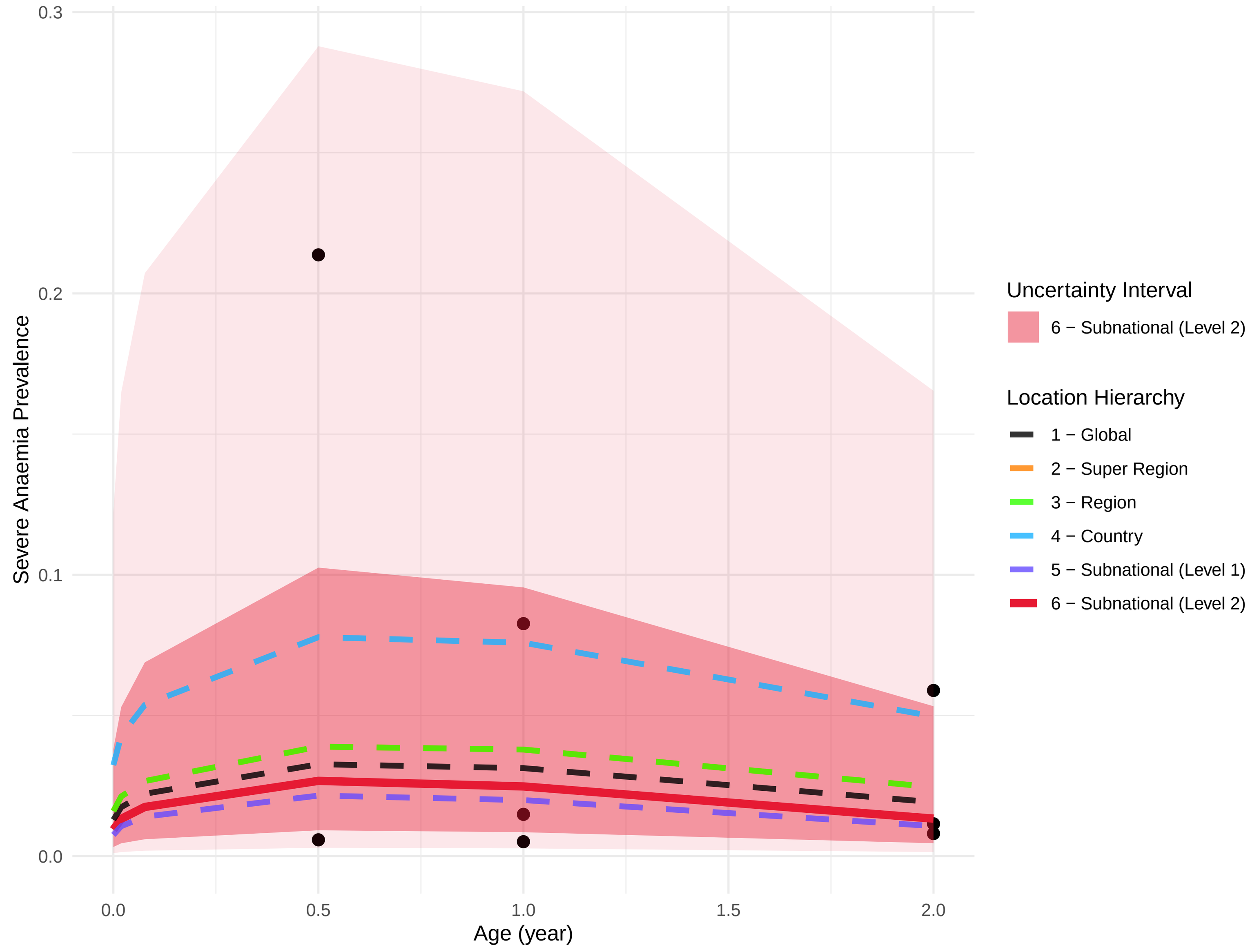

MR−BRT Age/Sex Fits − Non−pregnant Females <2 Years Old − Severe Anaemia Prevalence − Madhya Pradesh
WHO−2024 Adjustment Method

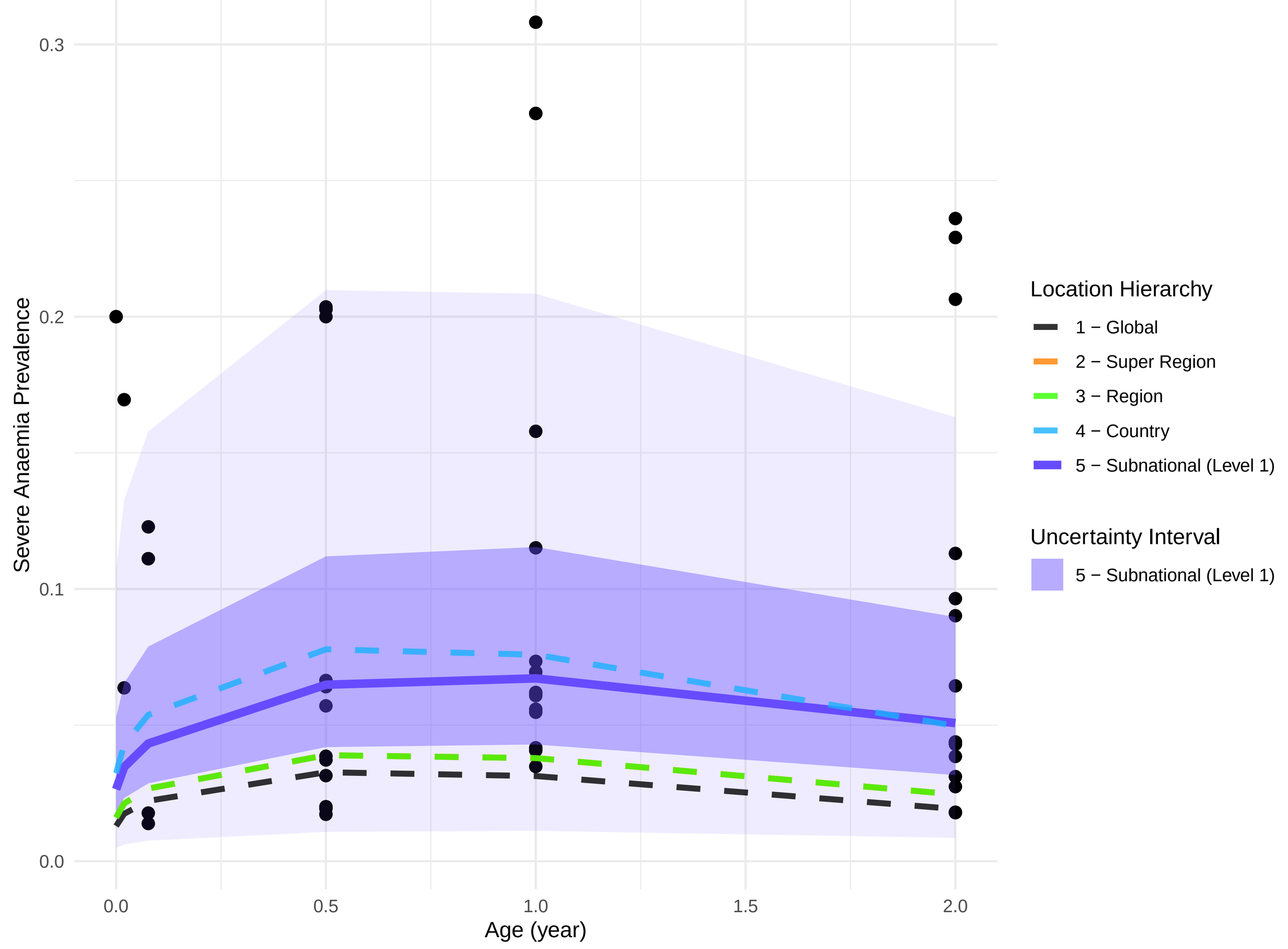

MR−BRT Age/Sex Fits − Non−pregnant Females <2 Years Old − Severe Anaemia Prevalence − Madhya Pradesh, Rural
WHO−2024 Adjustment Method

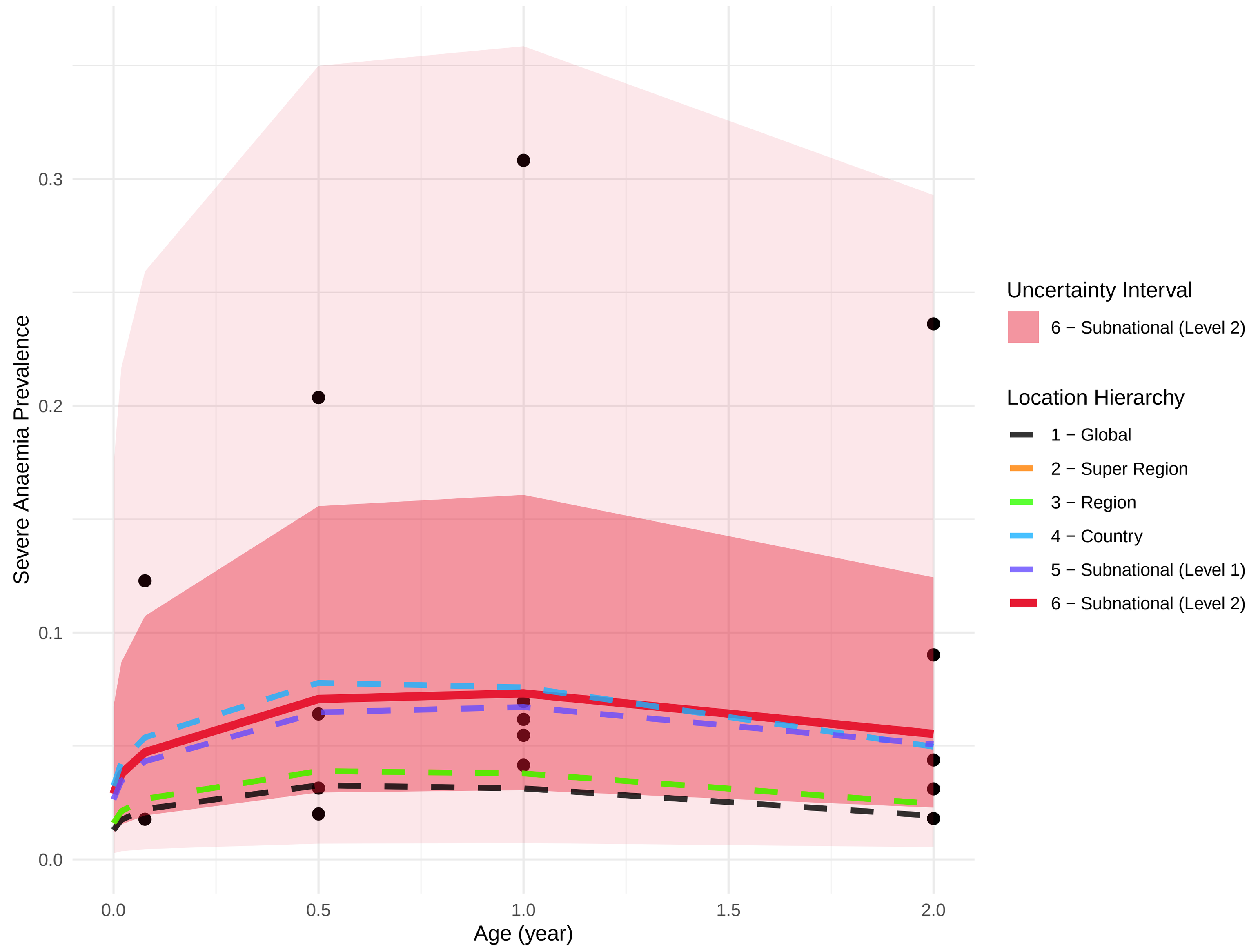

MR−BRT Age/Sex Fits − Non−pregnant Females <2 Years Old − Severe Anaemia Prevalence − Madhya Pradesh, Urban
WHO−2024 Adjustment Method

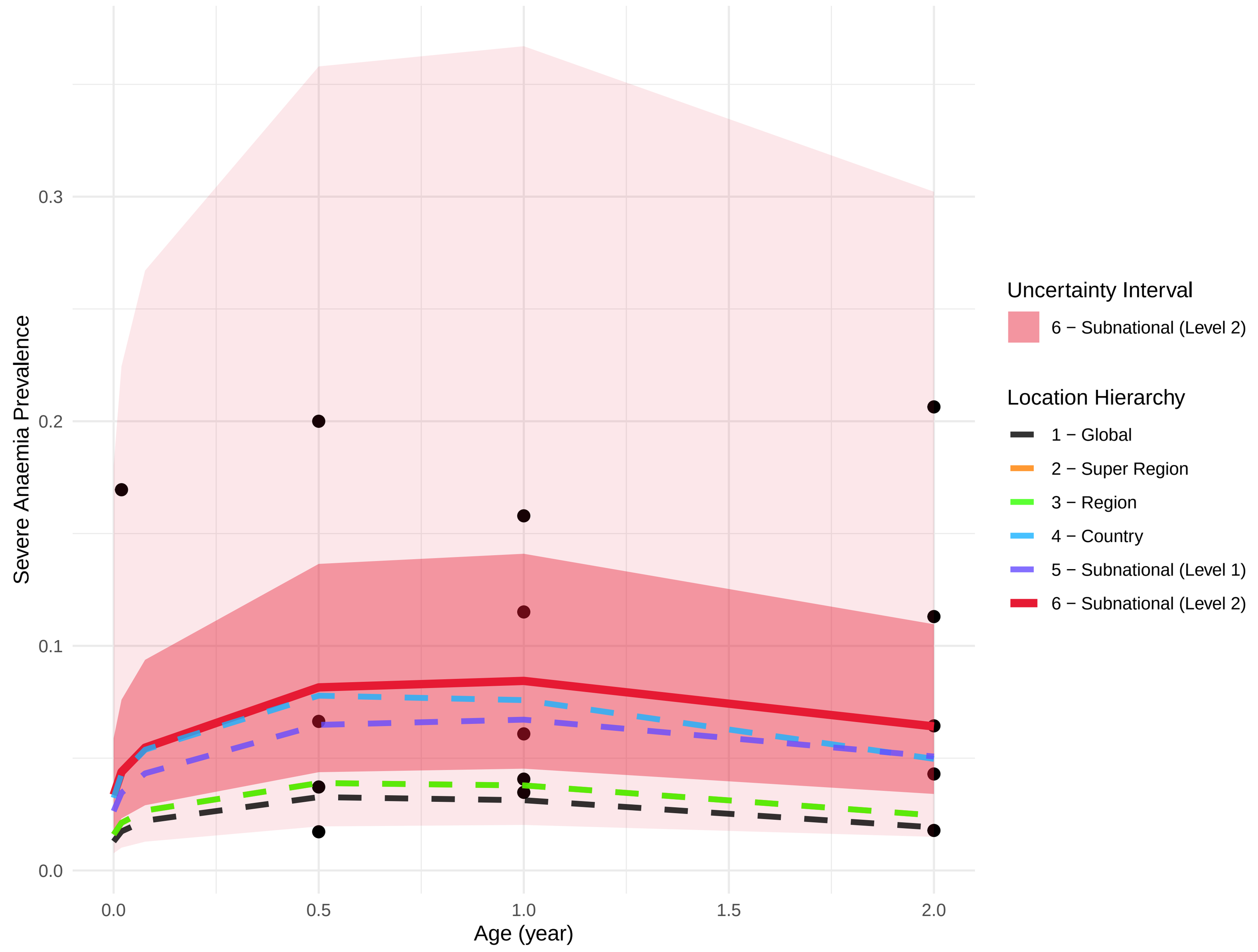

MR−BRT Age/Sex Fits − Non−pregnant Females <2 Years Old − Severe Anaemia Prevalence − Maharashtra
WHO−2024 Adjustment Method

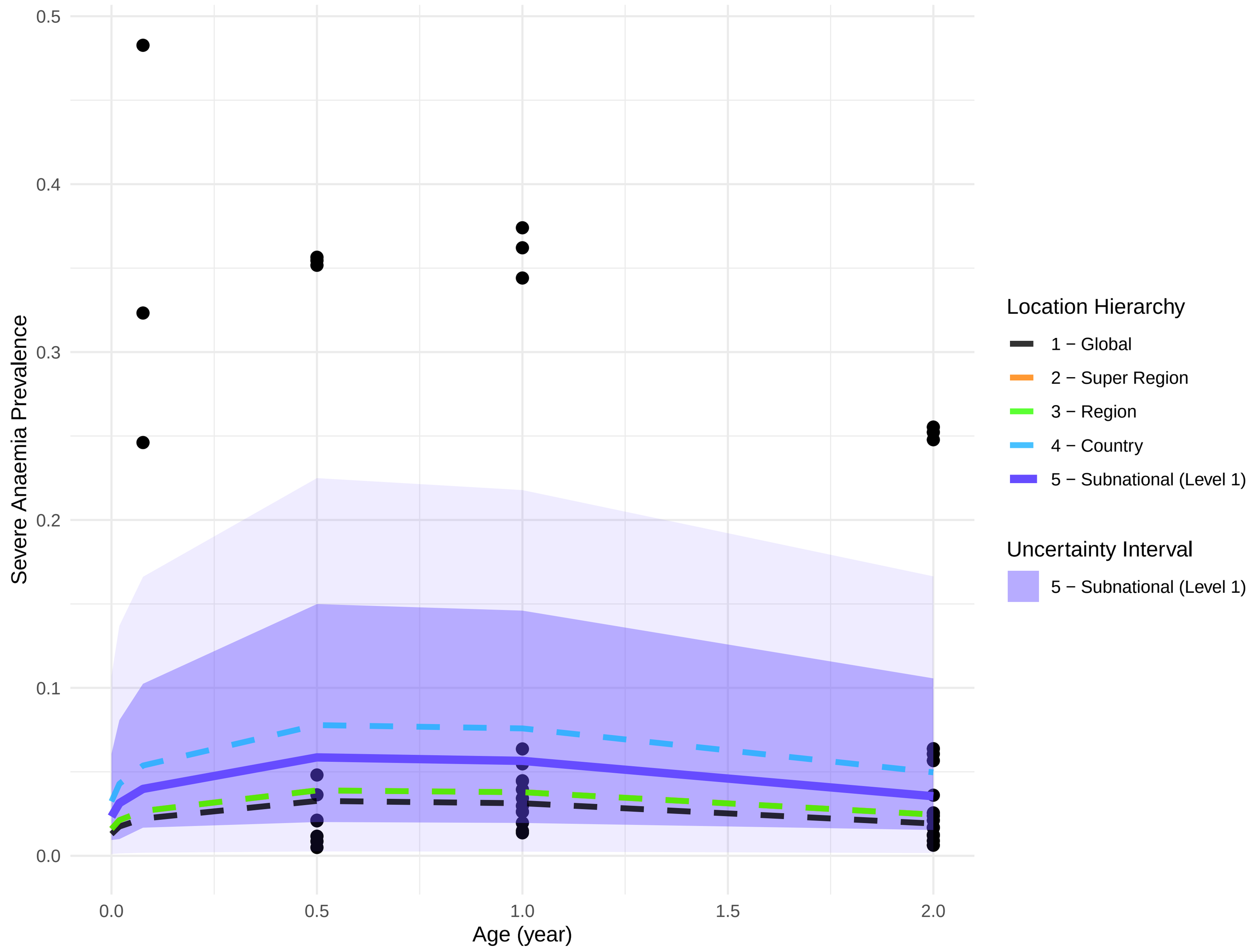

MR−BRT Age/Sex Fits − Non−pregnant Females <2 Years Old − Severe Anaemia Prevalence − Maharashtra, Rural
WHO−2024 Adjustment Method

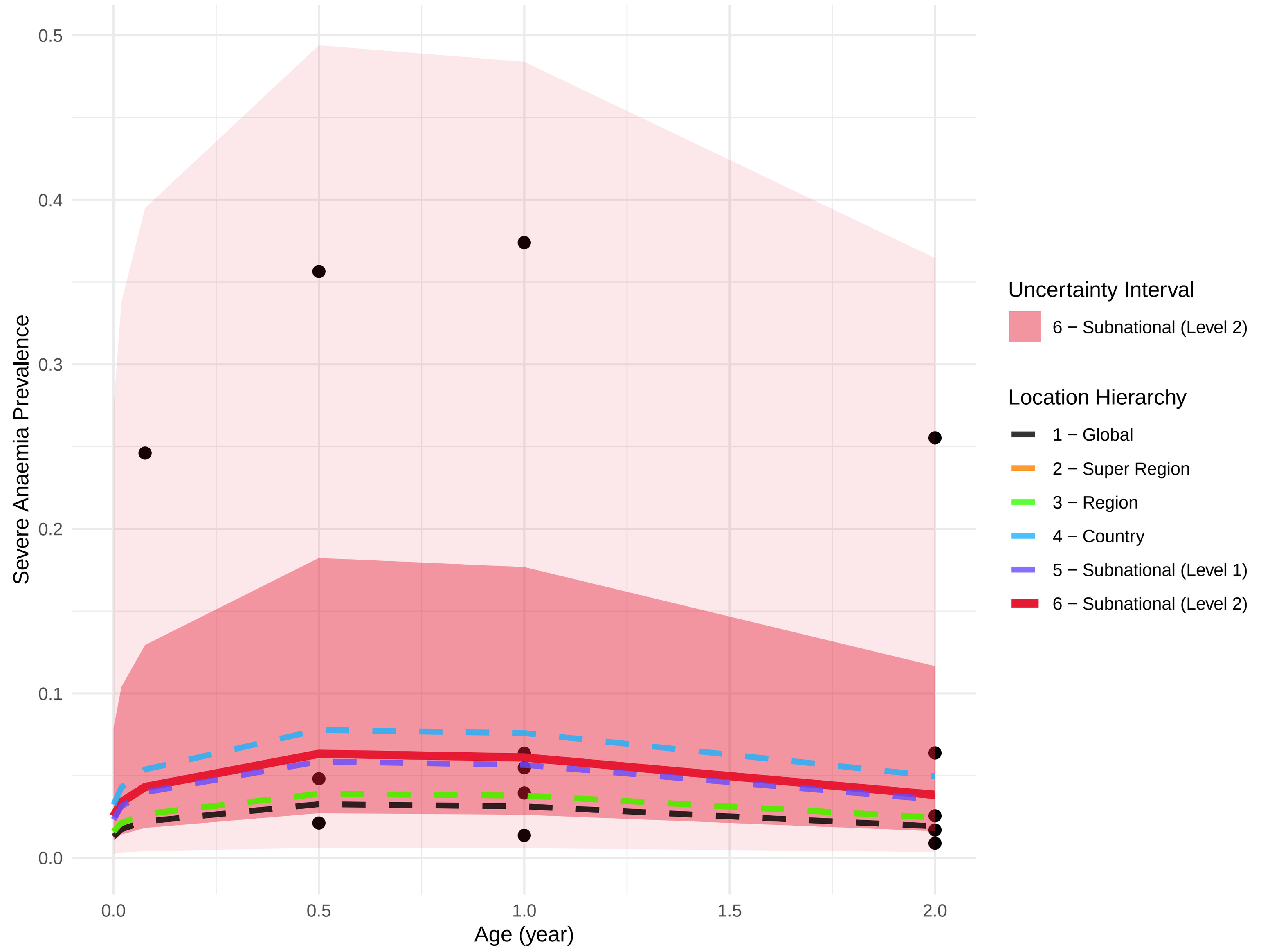

MR−BRT Age/Sex Fits − Non−pregnant Females <2 Years Old − Severe Anaemia Prevalence − Maharashtra, Urban
WHO−2024 Adjustment Method

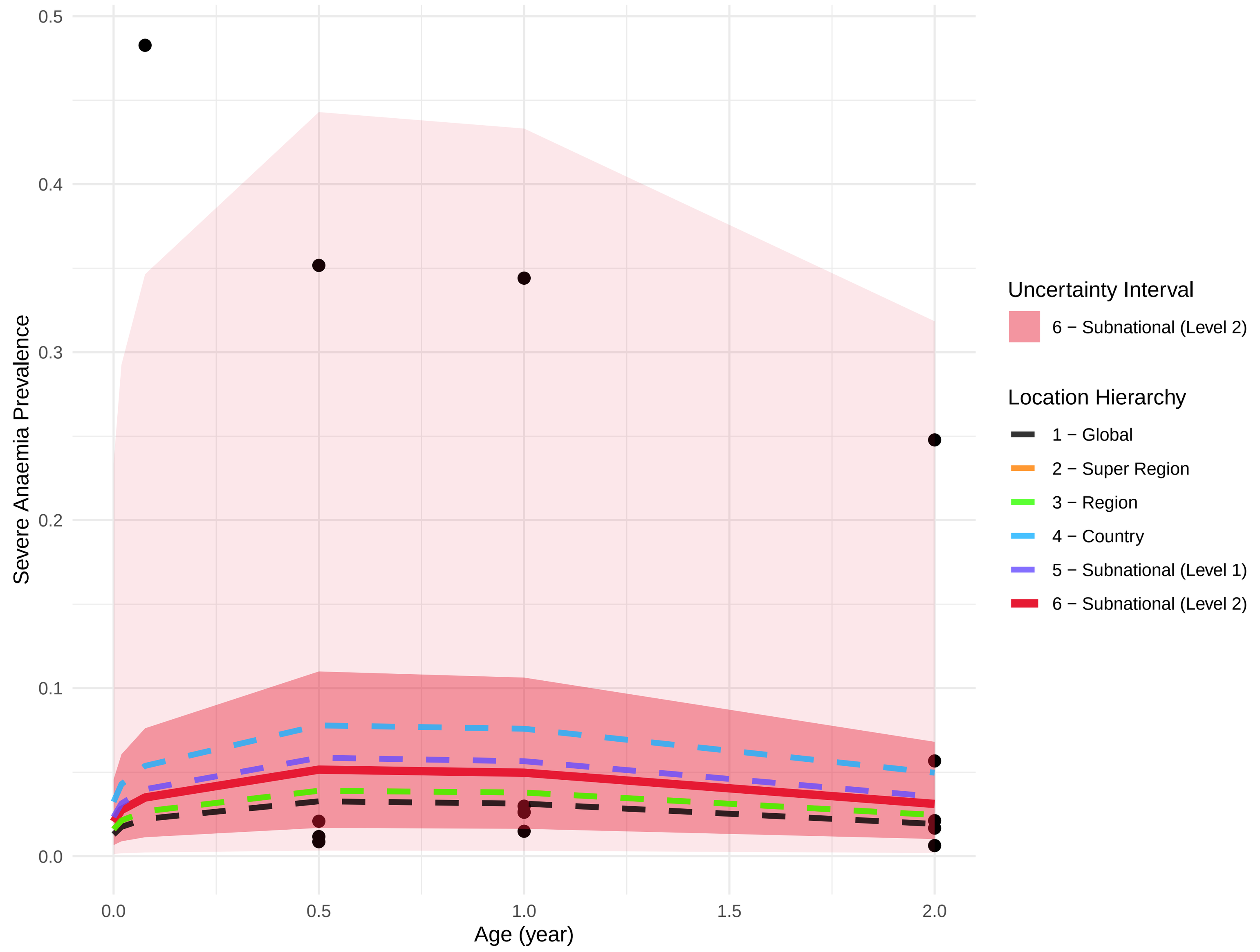

MR−BRT Age/Sex Fits − Non−pregnant Females <2 Years Old − Severe Anaemia Prevalence − Manipur
WHO−2024 Adjustment Method

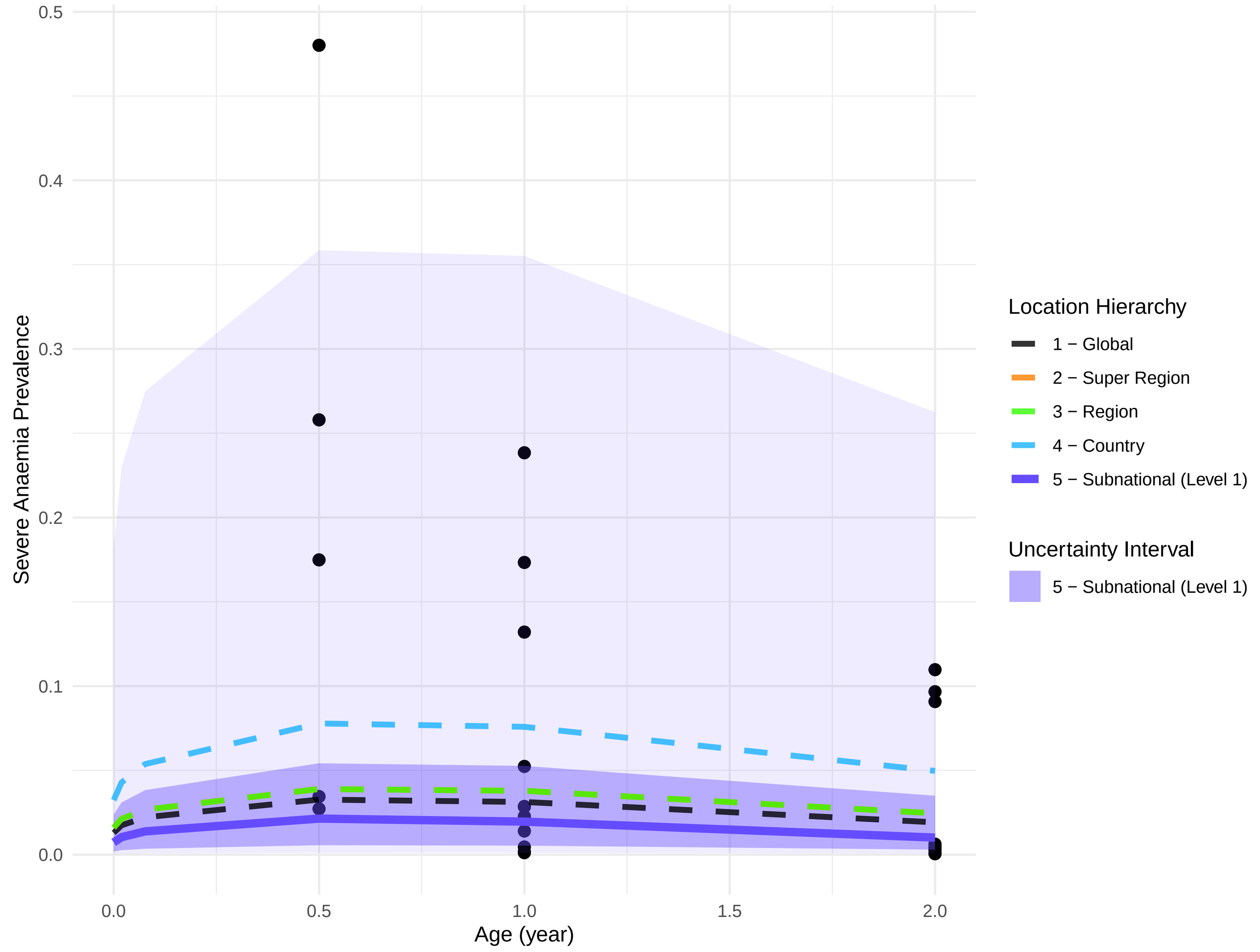

MR−BRT Age/Sex Fits − Non−pregnant Females <2 Years Old − Severe Anaemia Prevalence − Manipur, Rural
WHO−2024 Adjustment Method

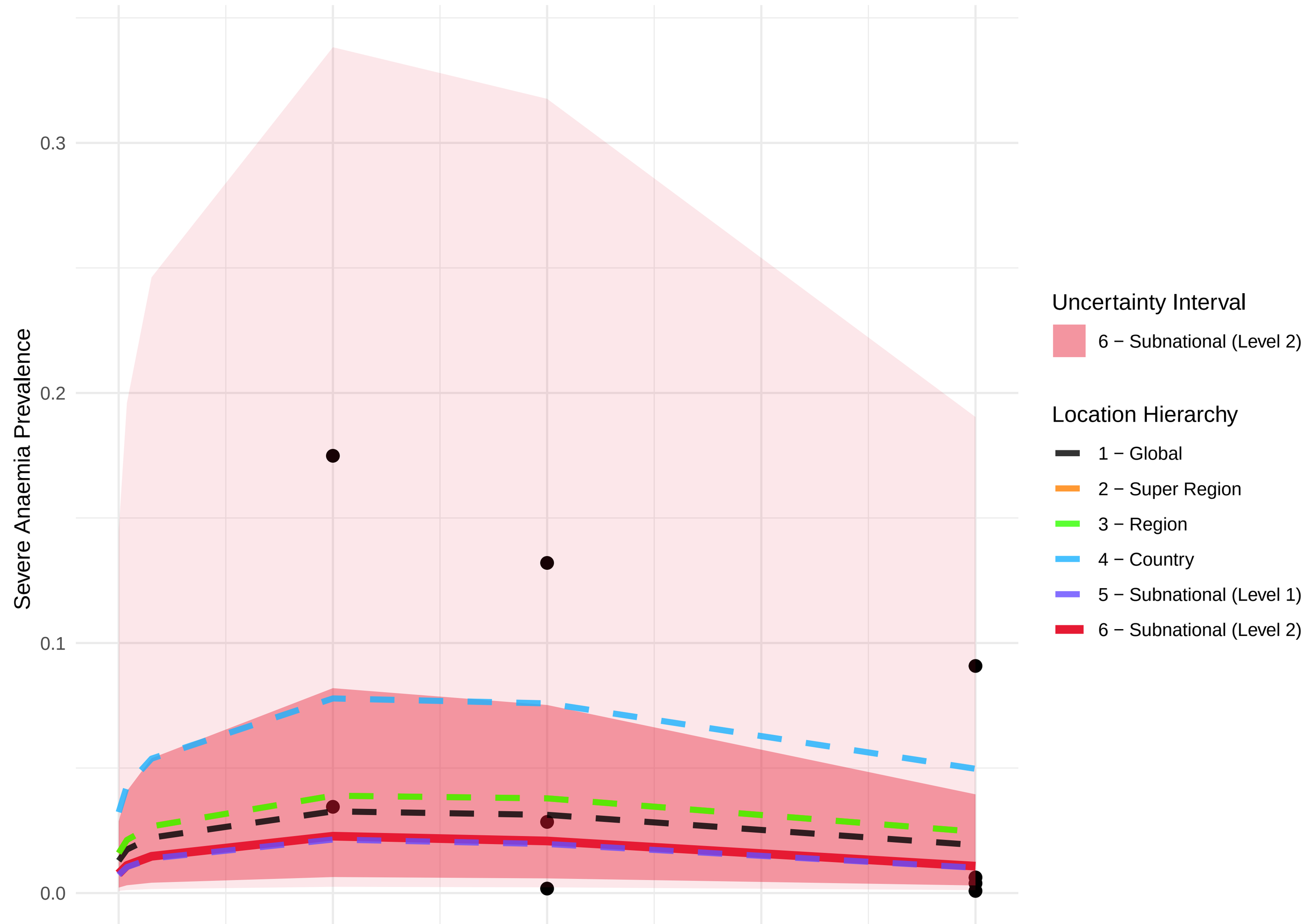

MR−BRT Age/Sex Fits − Non−pregnant Females <2 Years Old − Severe Anaemia Prevalence − Manipur, Urban
WHO−2024 Adjustment Method

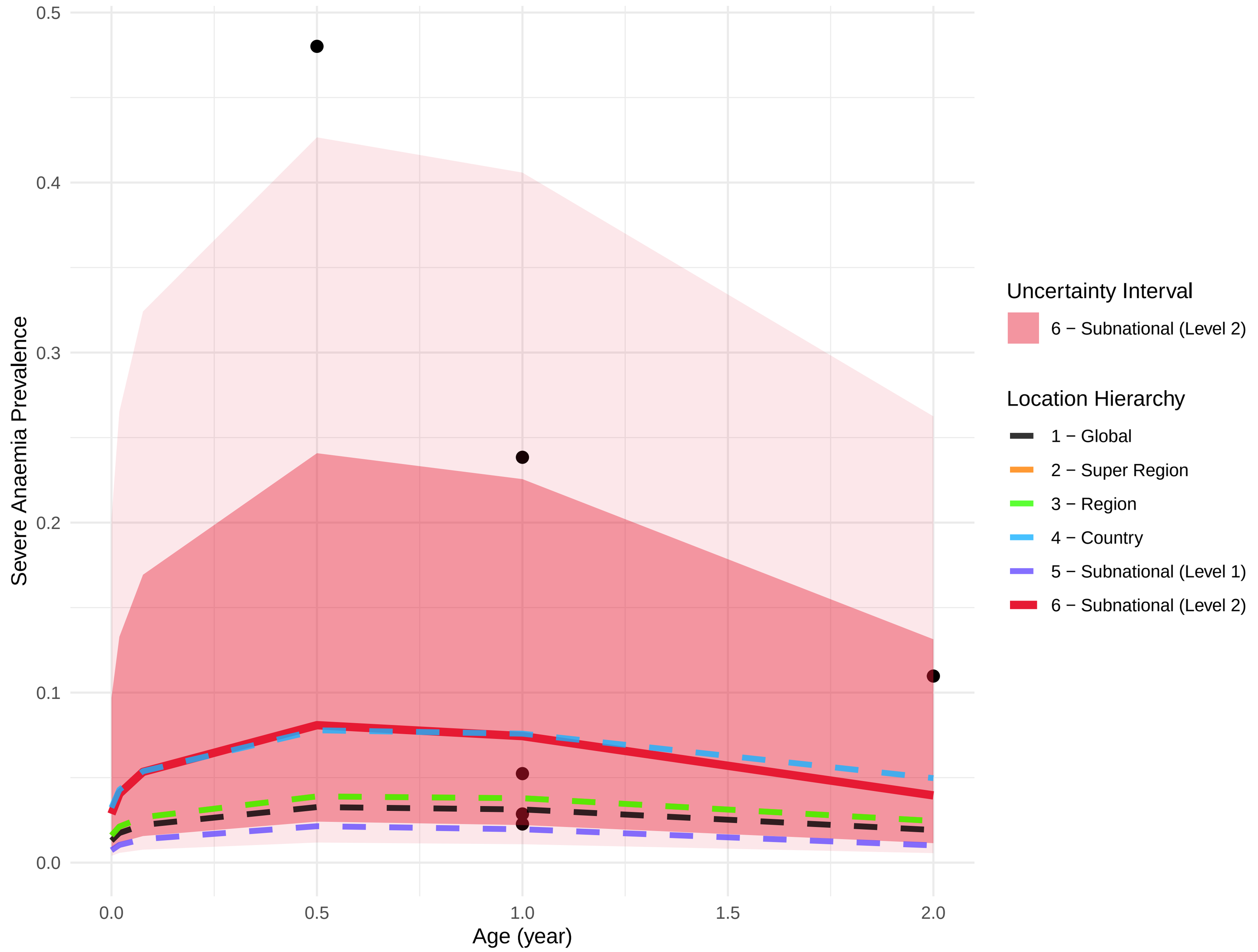

MR−BRT Age/Sex Fits − Non−pregnant Females <2 Years Old − Severe Anaemia Prevalence − Meghalaya
WHO−2024 Adjustment Method

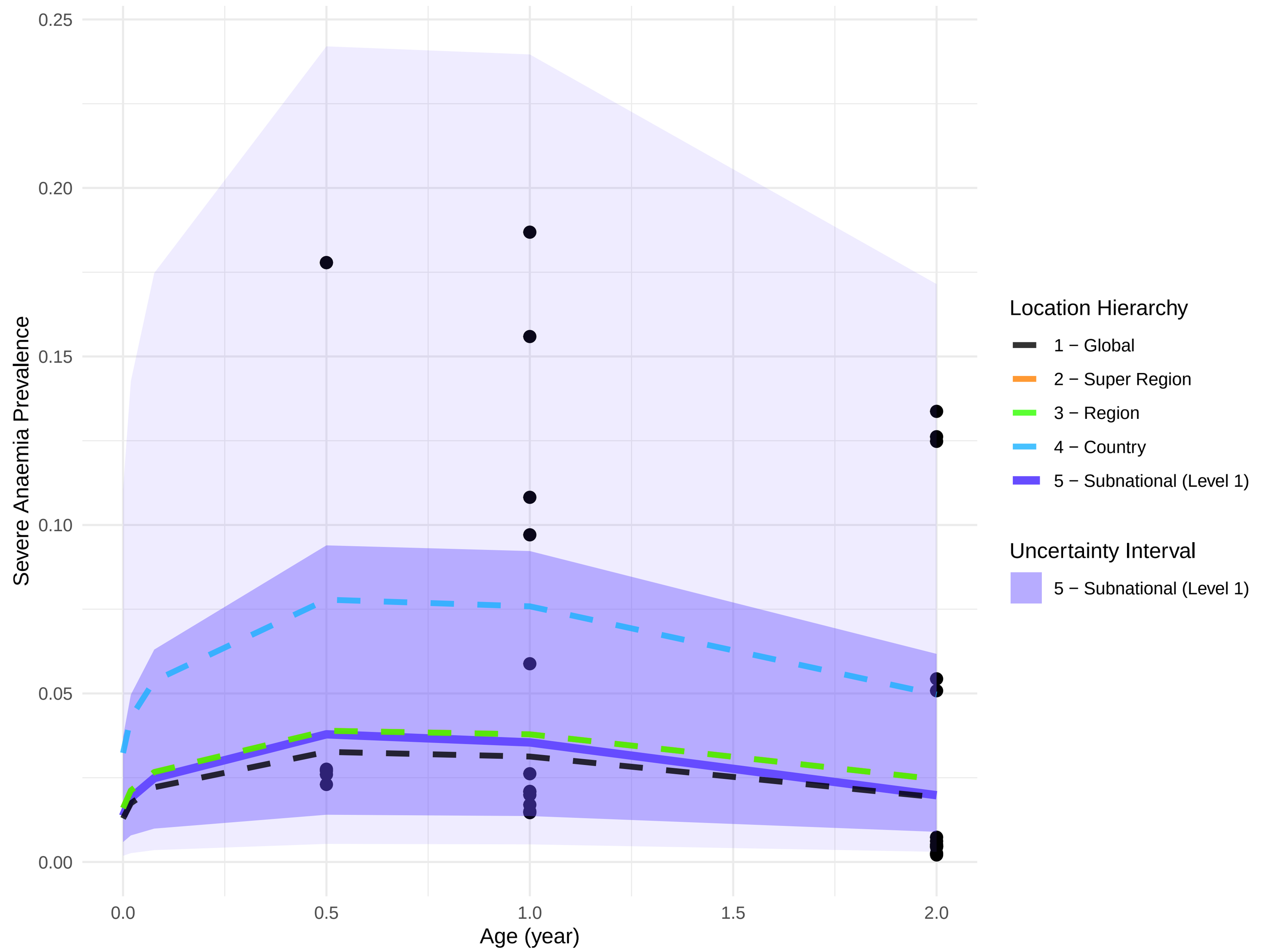

MR−BRT Age/Sex Fits − Non−pregnant Females <2 Years Old − Severe Anaemia Prevalence − Meghalaya, Rural
WHO−2024 Adjustment Method

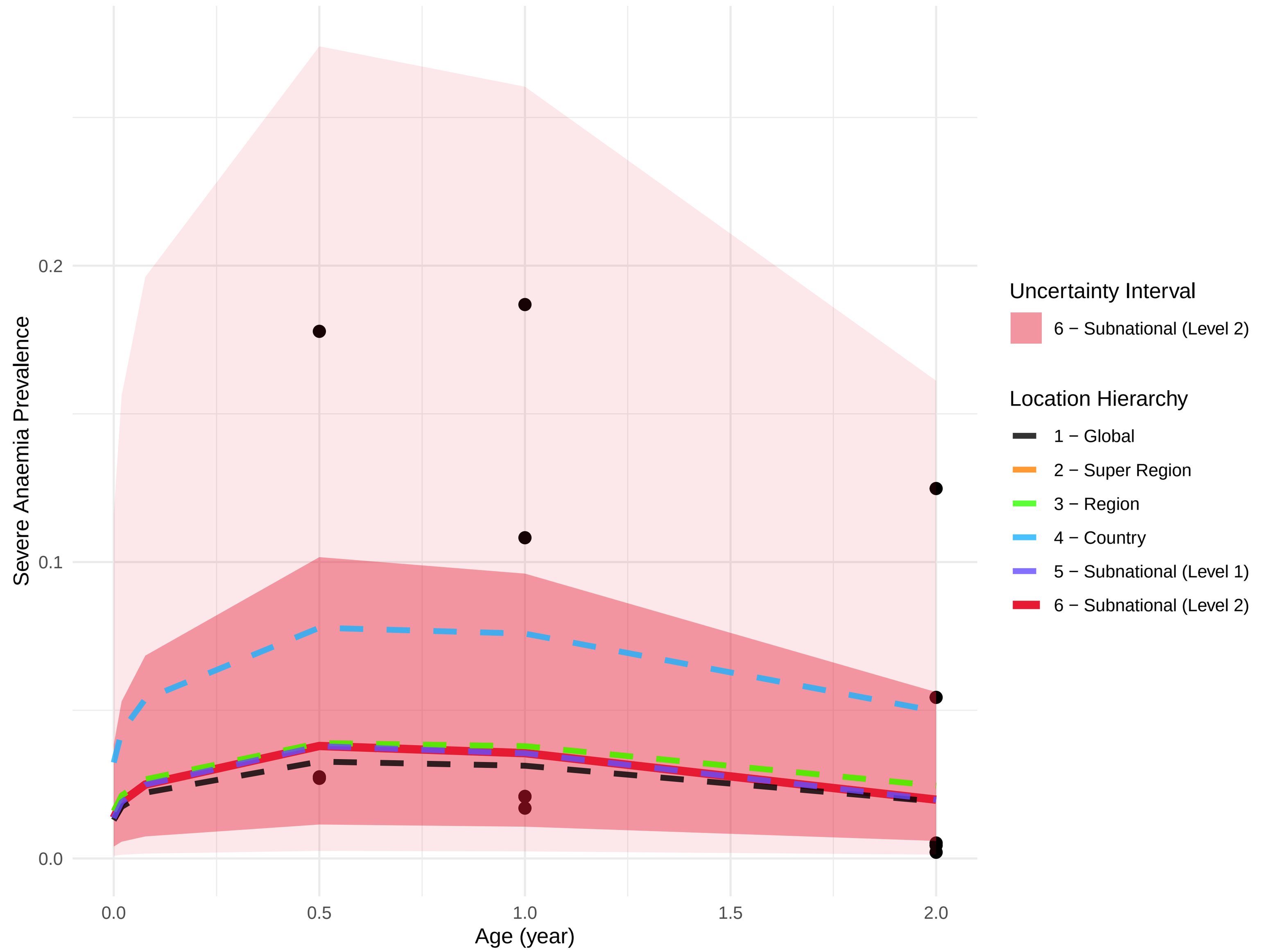

MR−BRT Age/Sex Fits − Non−pregnant Females <2 Years Old − Severe Anaemia Prevalence − Meghalaya, Urban
WHO−2024 Adjustment Method

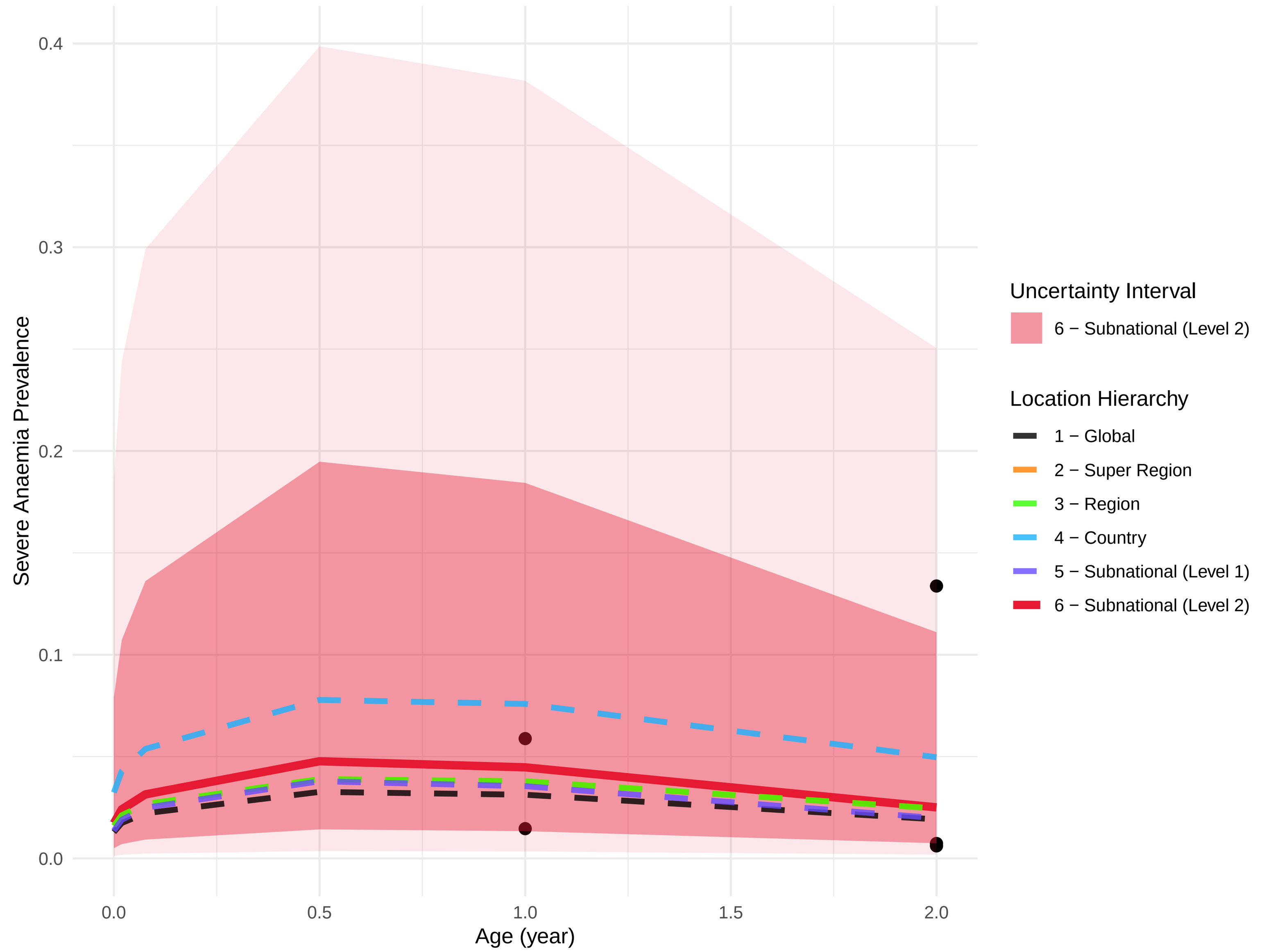

MR−BRT Age/Sex Fits − Non−pregnant Females <2 Years Old − Severe Anaemia Prevalence − Mizoram
WHO−2024 Adjustment Method

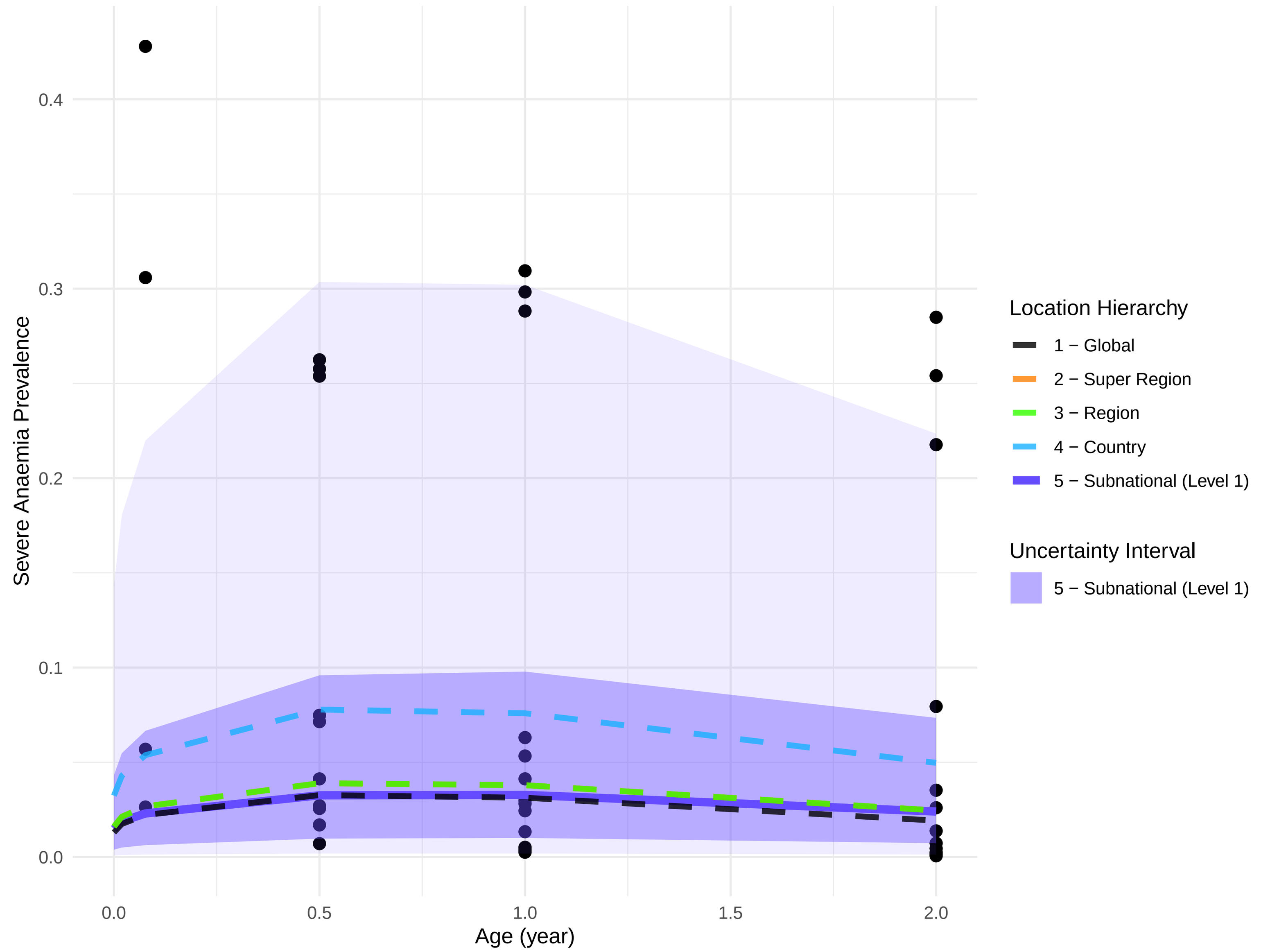

MR−BRT Age/Sex Fits − Non−pregnant Females <2 Years Old − Severe Anaemia Prevalence − Mizoram, Rural
WHO−2024 Adjustment Method

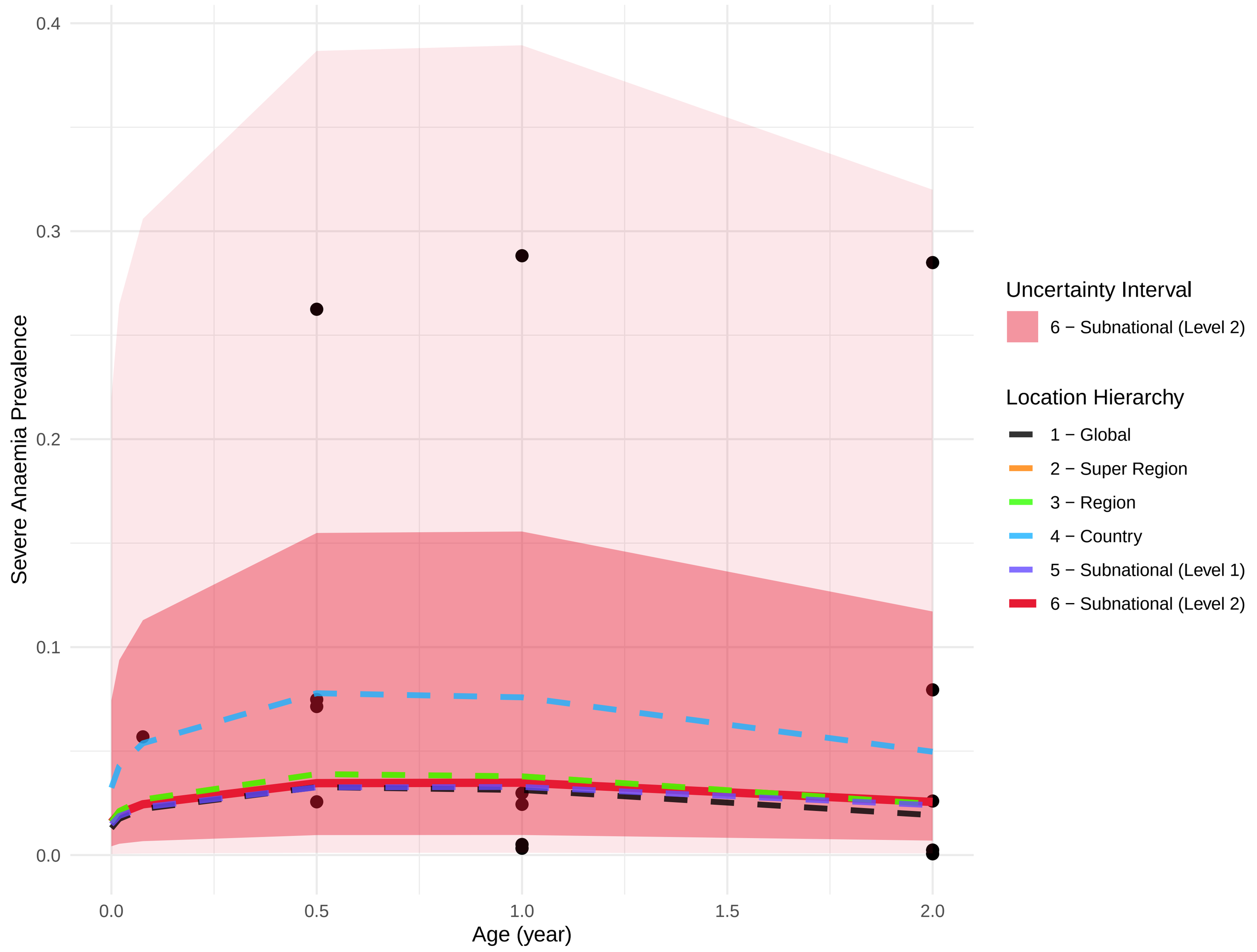

MR−BRT Age/Sex Fits − Non−pregnant Females <2 Years Old − Severe Anaemia Prevalence − Mizoram, Urban
WHO−2024 Adjustment Method

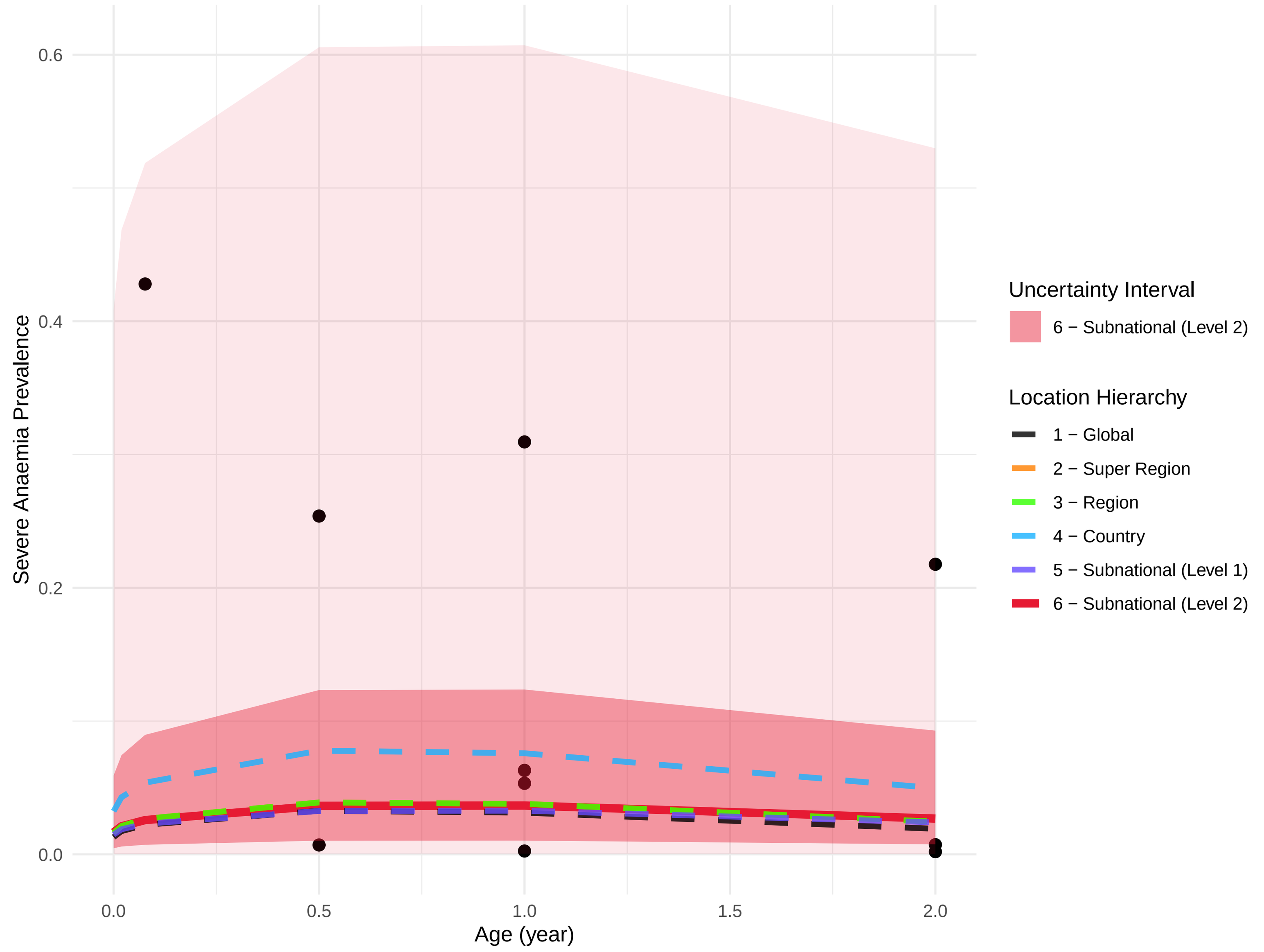

MR−BRT Age/Sex Fits − Non−pregnant Females <2 Years Old − Severe Anaemia Prevalence − Nagaland
WHO−2024 Adjustment Method

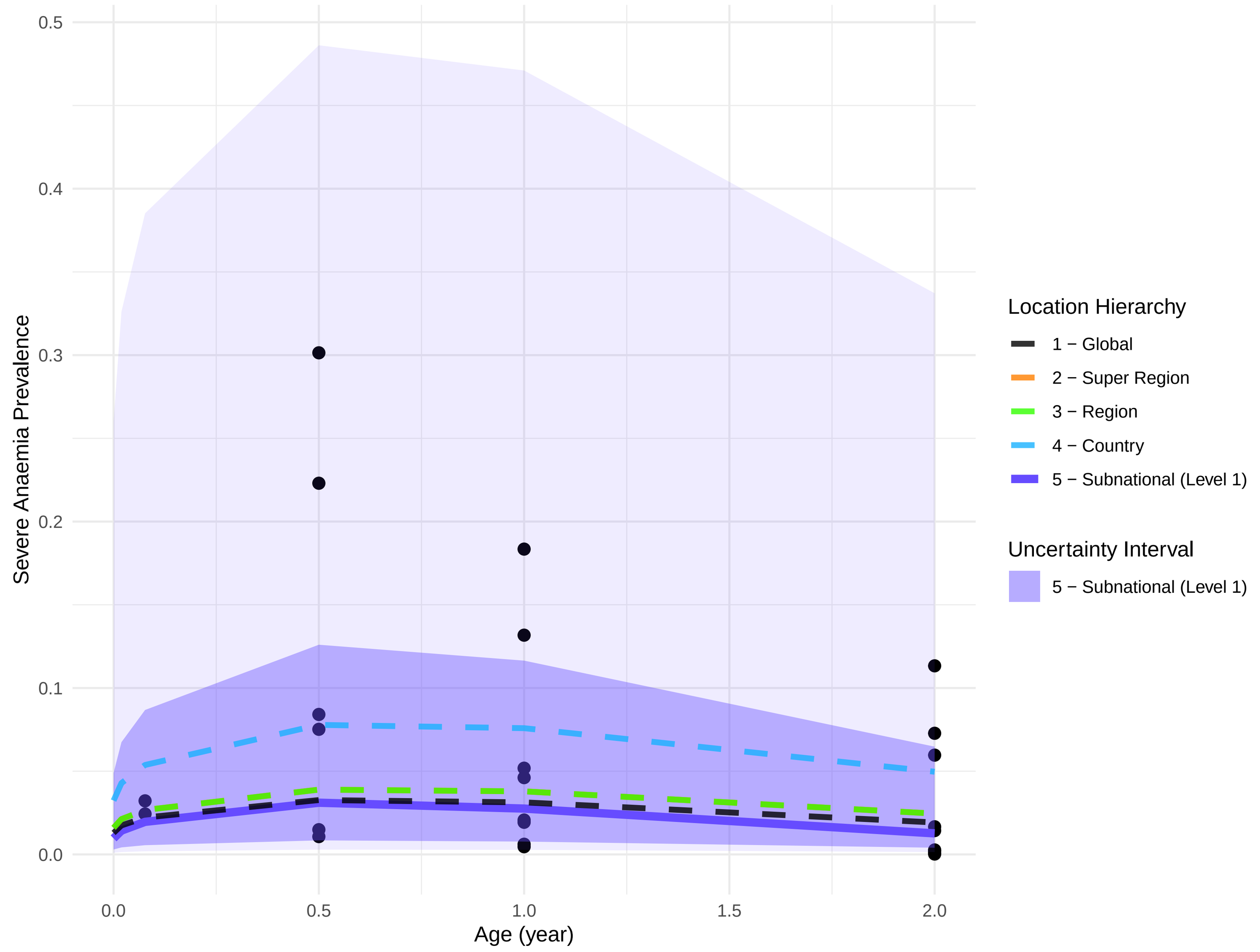

MR−BRT Age/Sex Fits − Non−pregnant Females <2 Years Old − Severe Anaemia Prevalence − Nagaland, Rural
WHO−2024 Adjustment Method

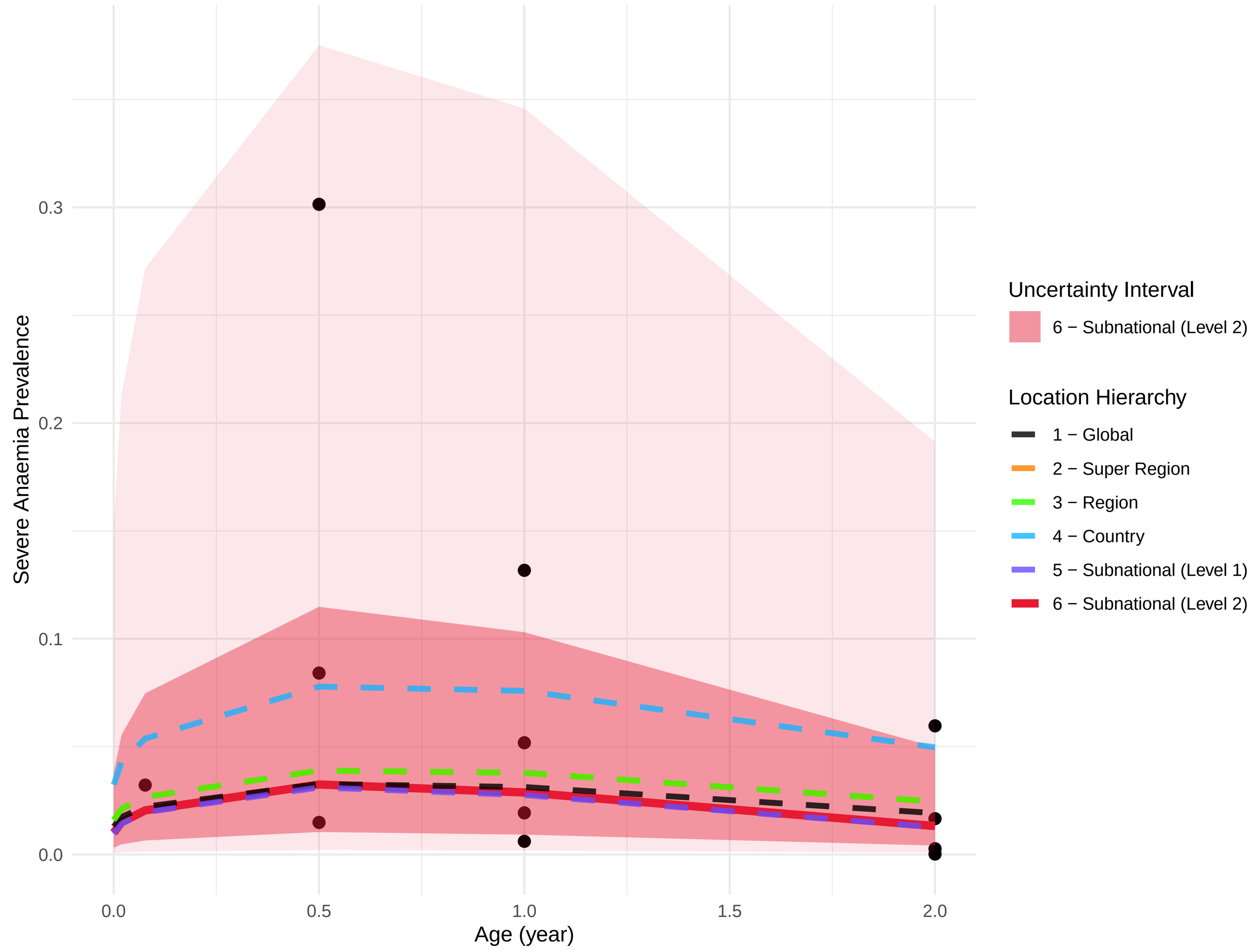

MR−BRT Age/Sex Fits − Non−pregnant Females <2 Years Old − Severe Anaemia Prevalence − Nagaland, Urban
WHO−2024 Adjustment Method

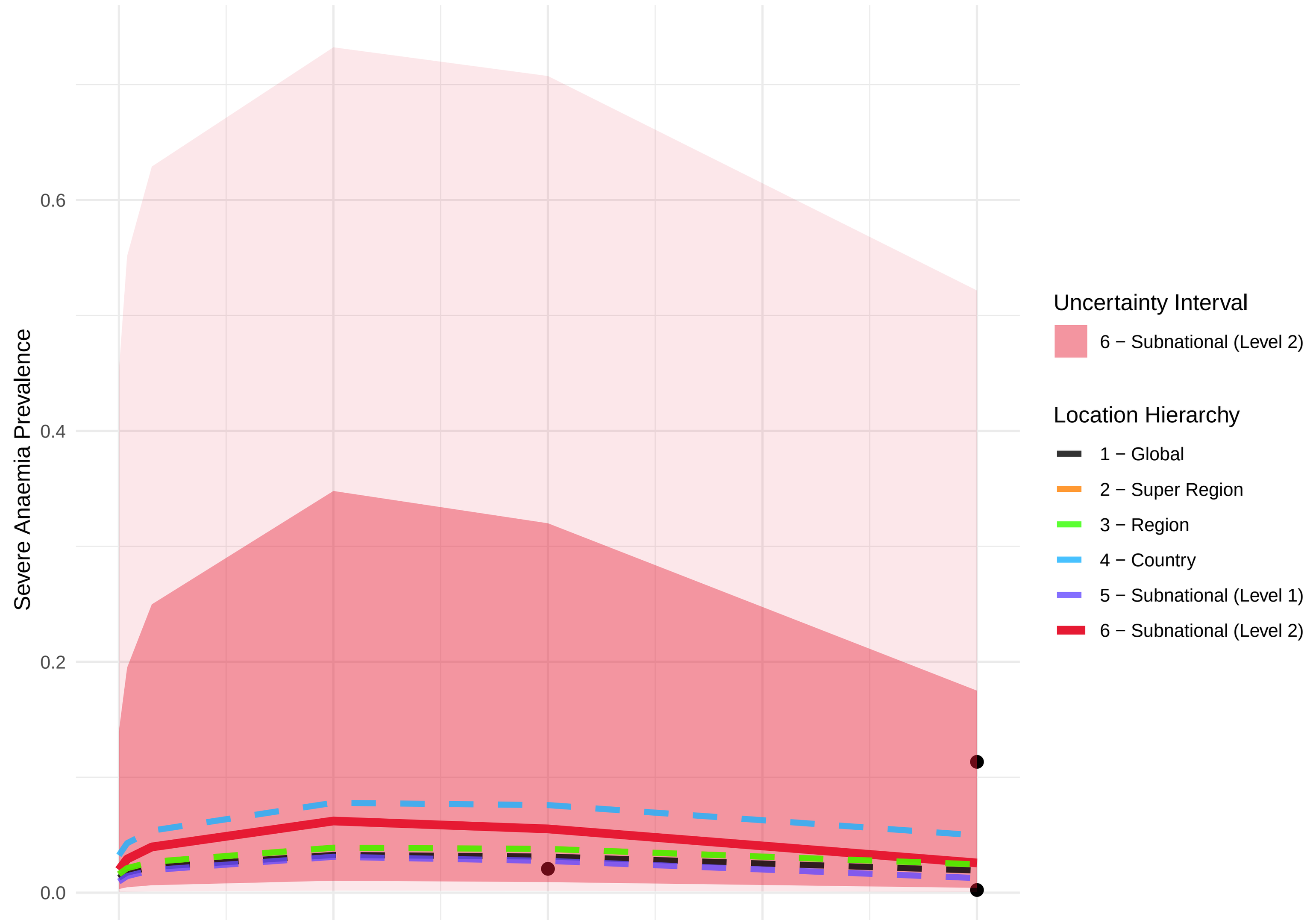

MR−BRT Age/Sex Fits − Non−pregnant Females <2 Years Old − Severe Anaemia Prevalence − Odisha
WHO−2024 Adjustment Method

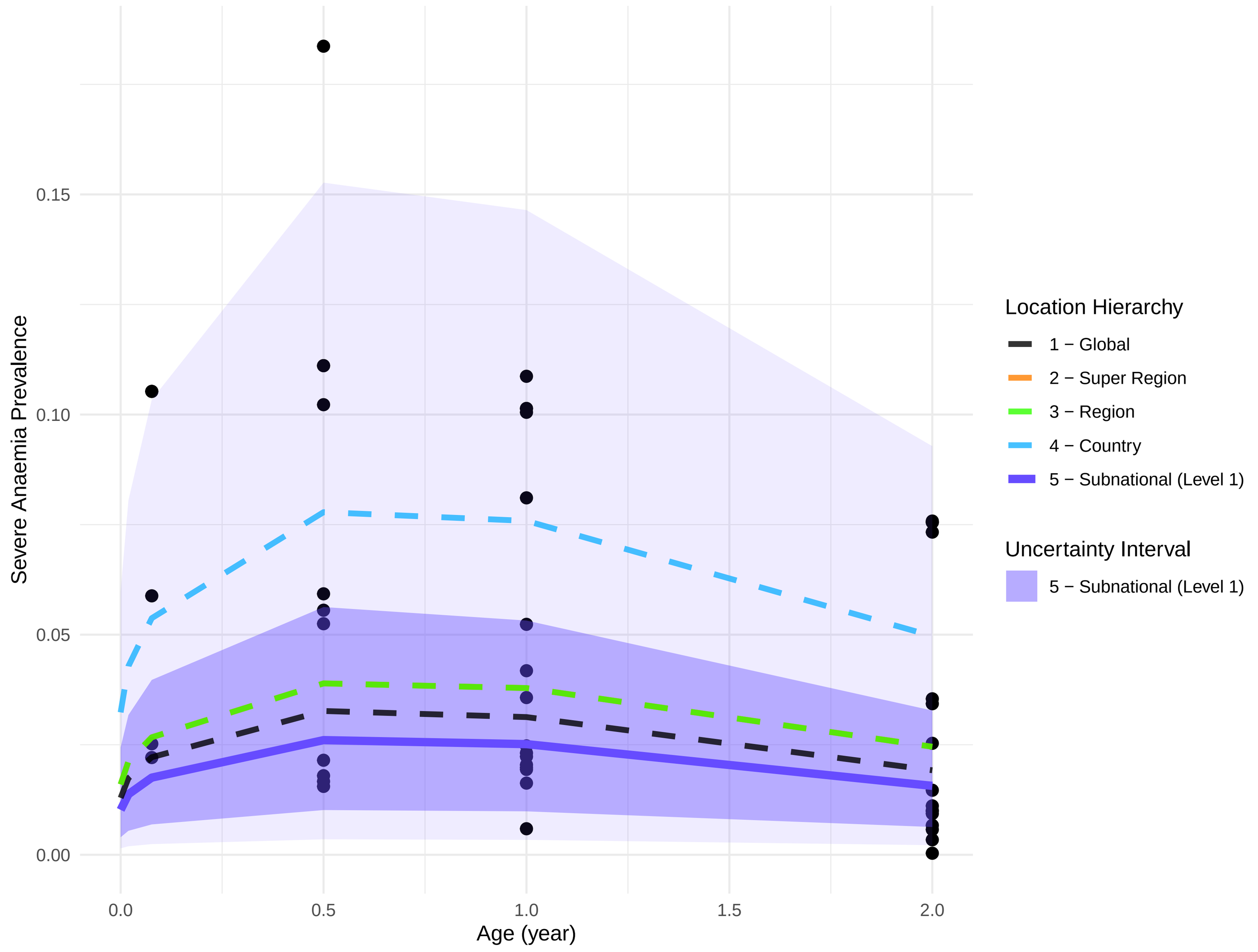

MR−BRT Age/Sex Fits − Non−pregnant Females <2 Years Old − Severe Anaemia Prevalence − Odisha, Rural
WHO−2024 Adjustment Method

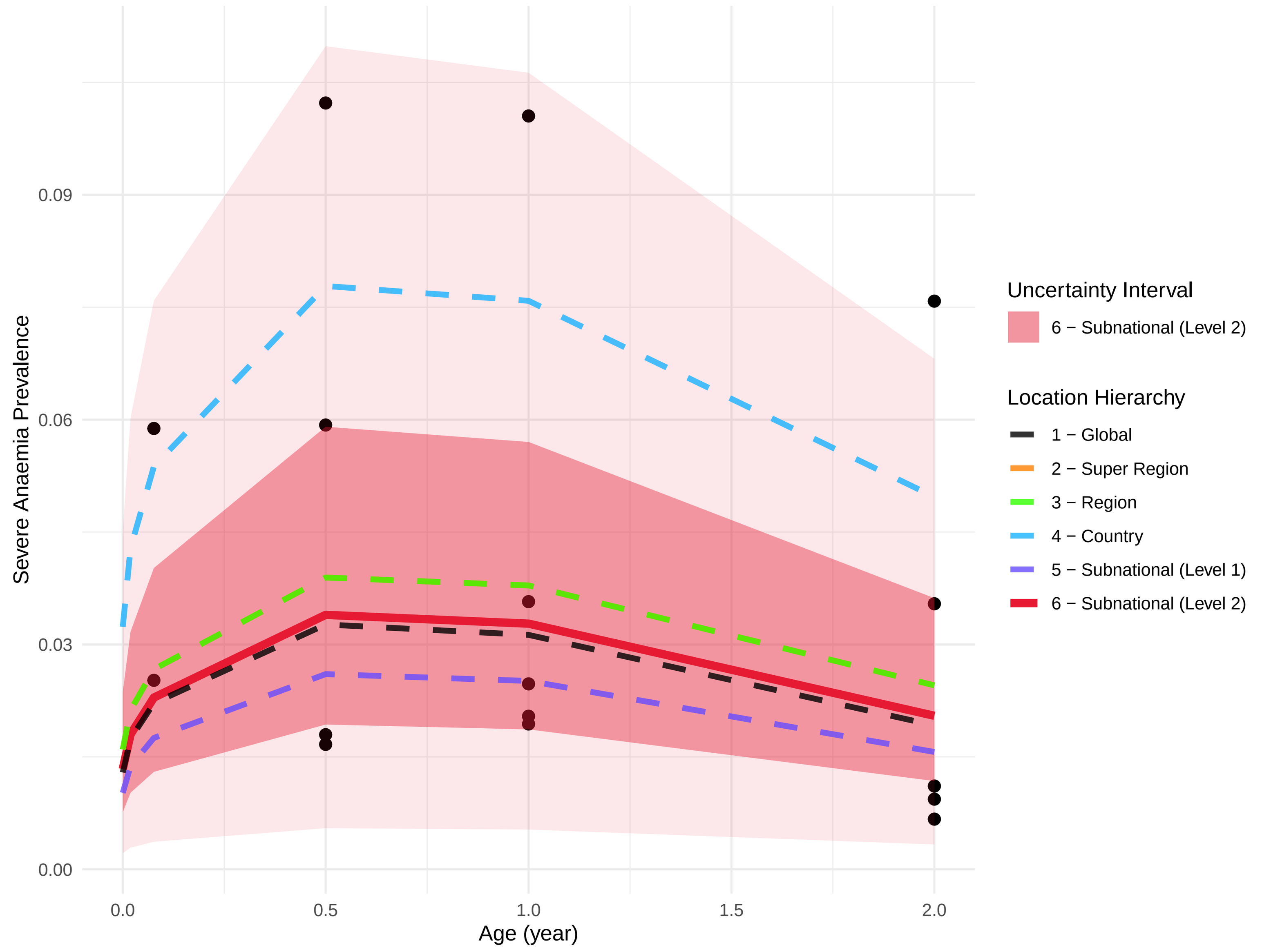

MR−BRT Age/Sex Fits − Non−pregnant Females <2 Years Old − Severe Anaemia Prevalence − Odisha, Urban
WHO−2024 Adjustment Method

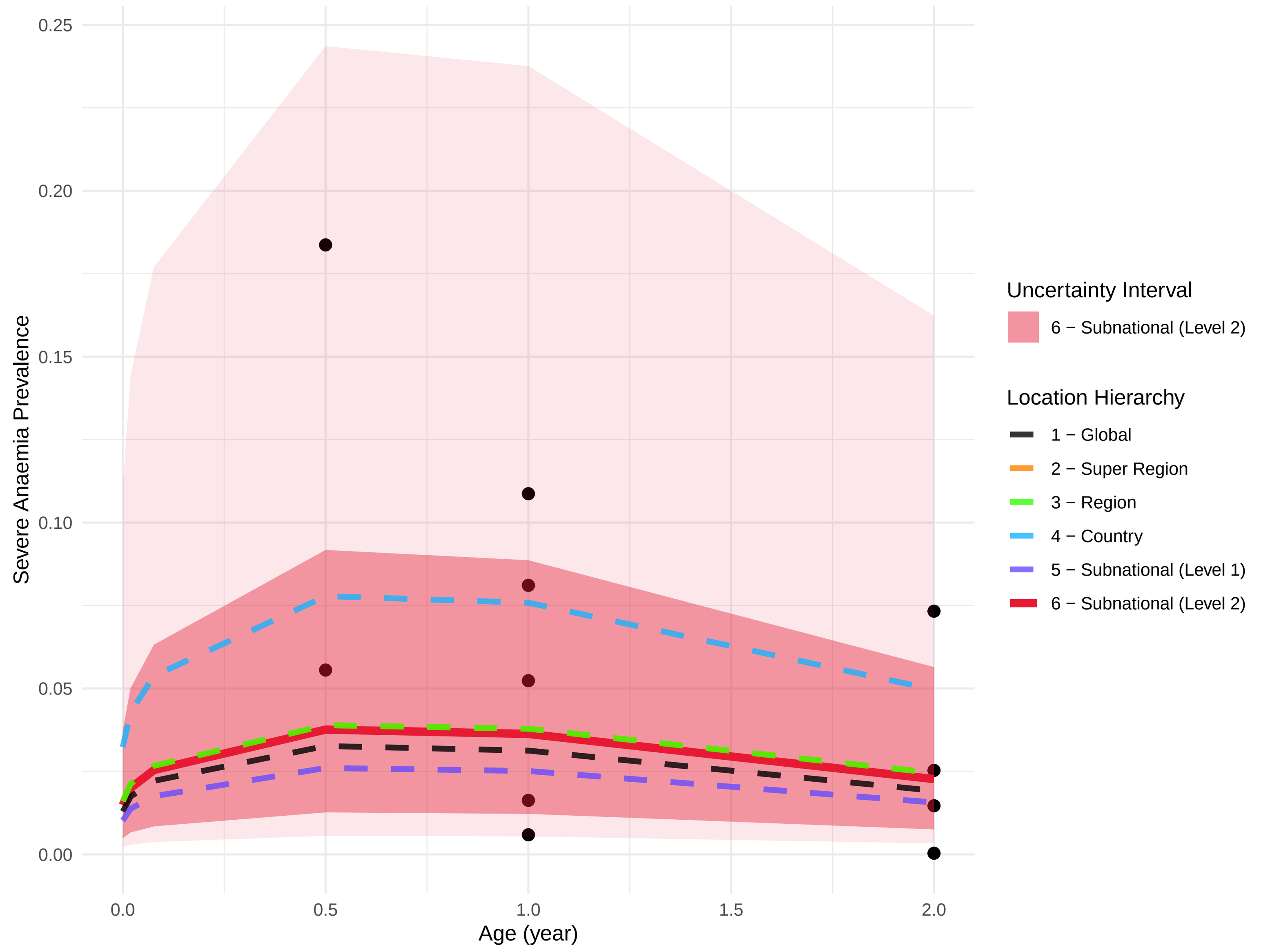

MR−BRT Age/Sex Fits − Non−pregnant Females <2 Years Old − Severe Anaemia Prevalence − Other Union Territories
WHO−2024 Adjustment Method

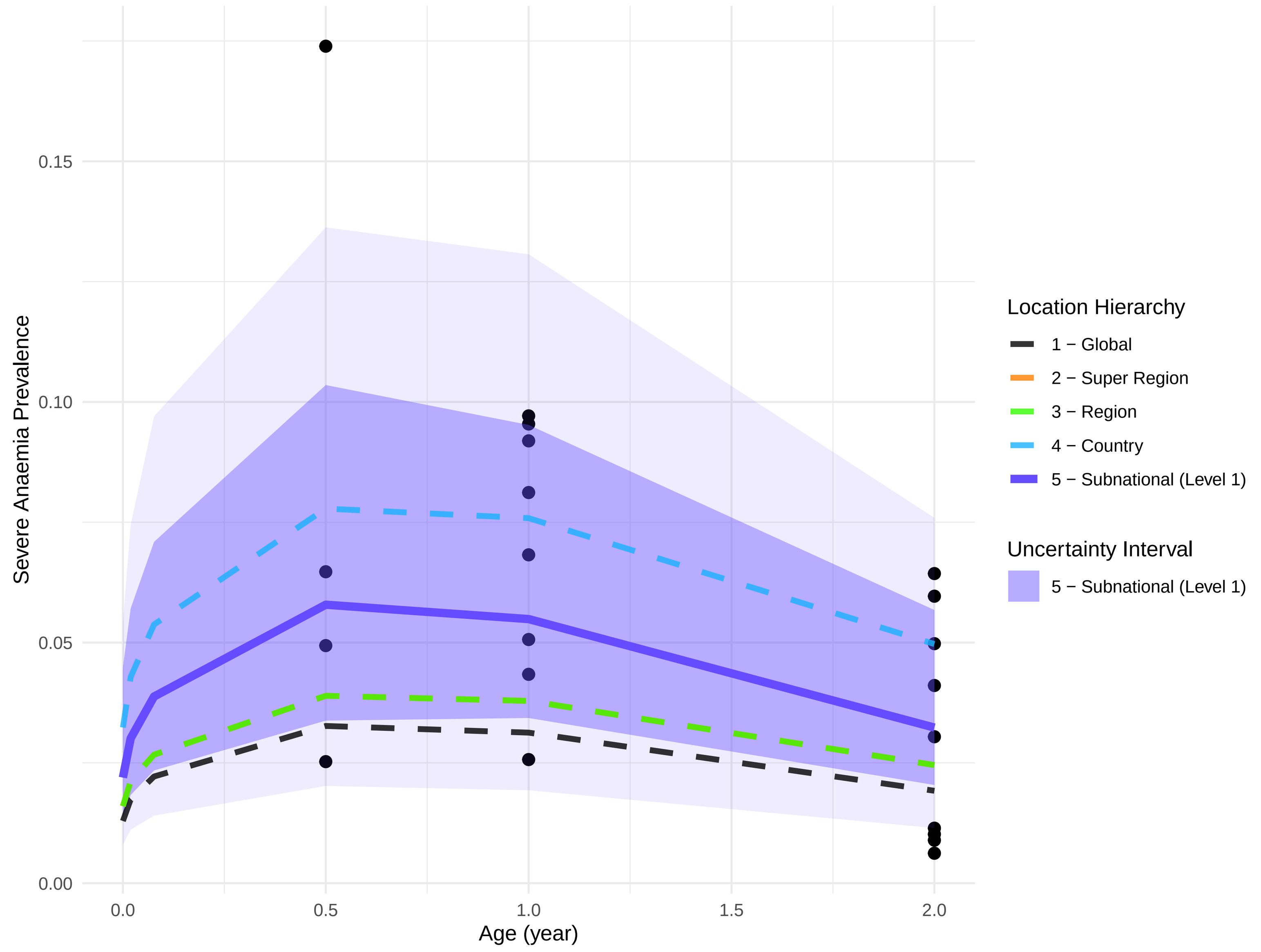

MR−BRT Age/Sex Fits − Non−pregnant Females <2 Years Old − Severe Anaemia Prevalence − Other Union Territories, Rural
WHO−2024 Adjustment Method

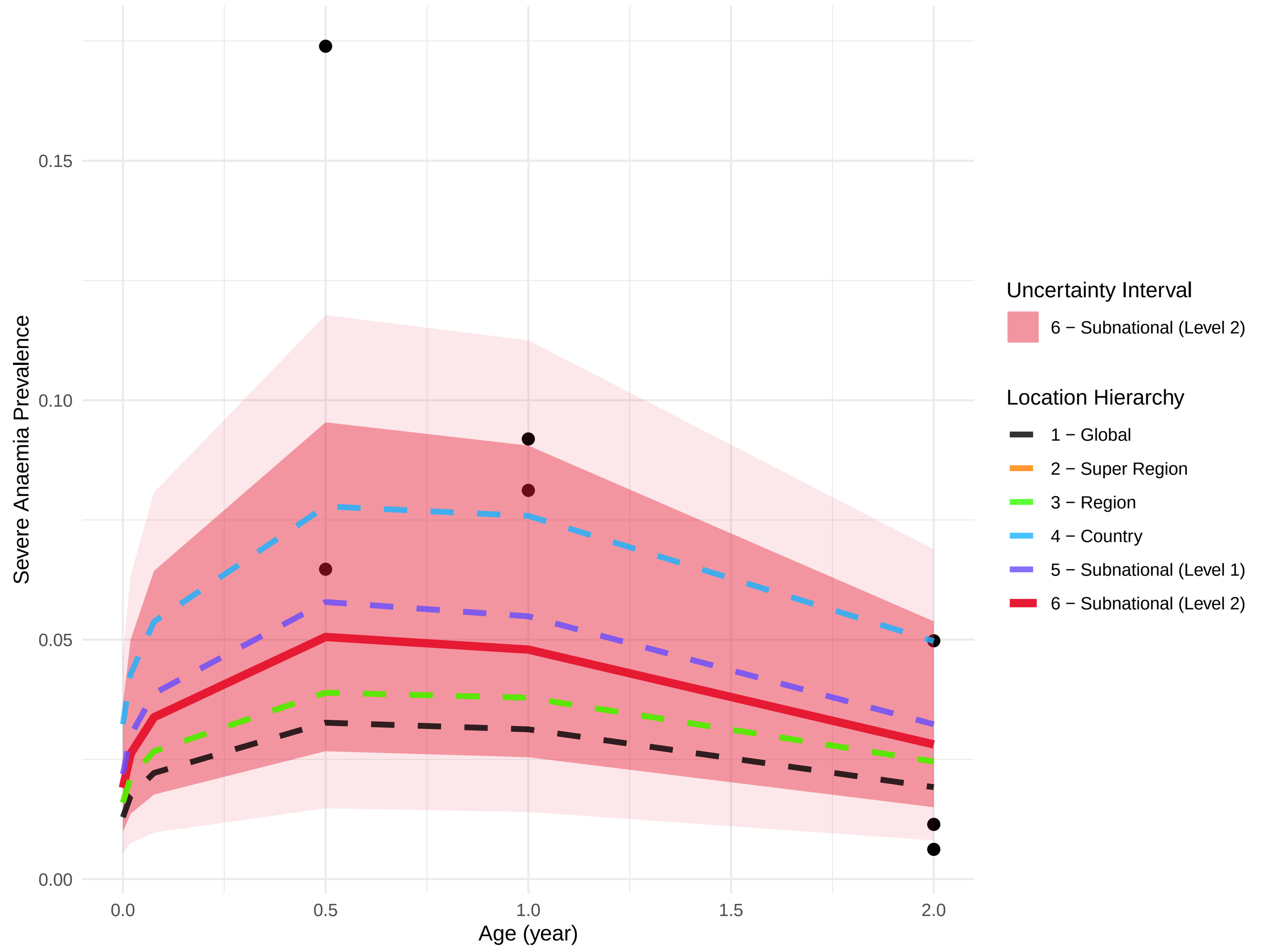

MR−BRT Age/Sex Fits − Non−pregnant Females <2 Years Old − Severe Anaemia Prevalence − Other Union Territories, Urban
WHO−2024 Adjustment Method

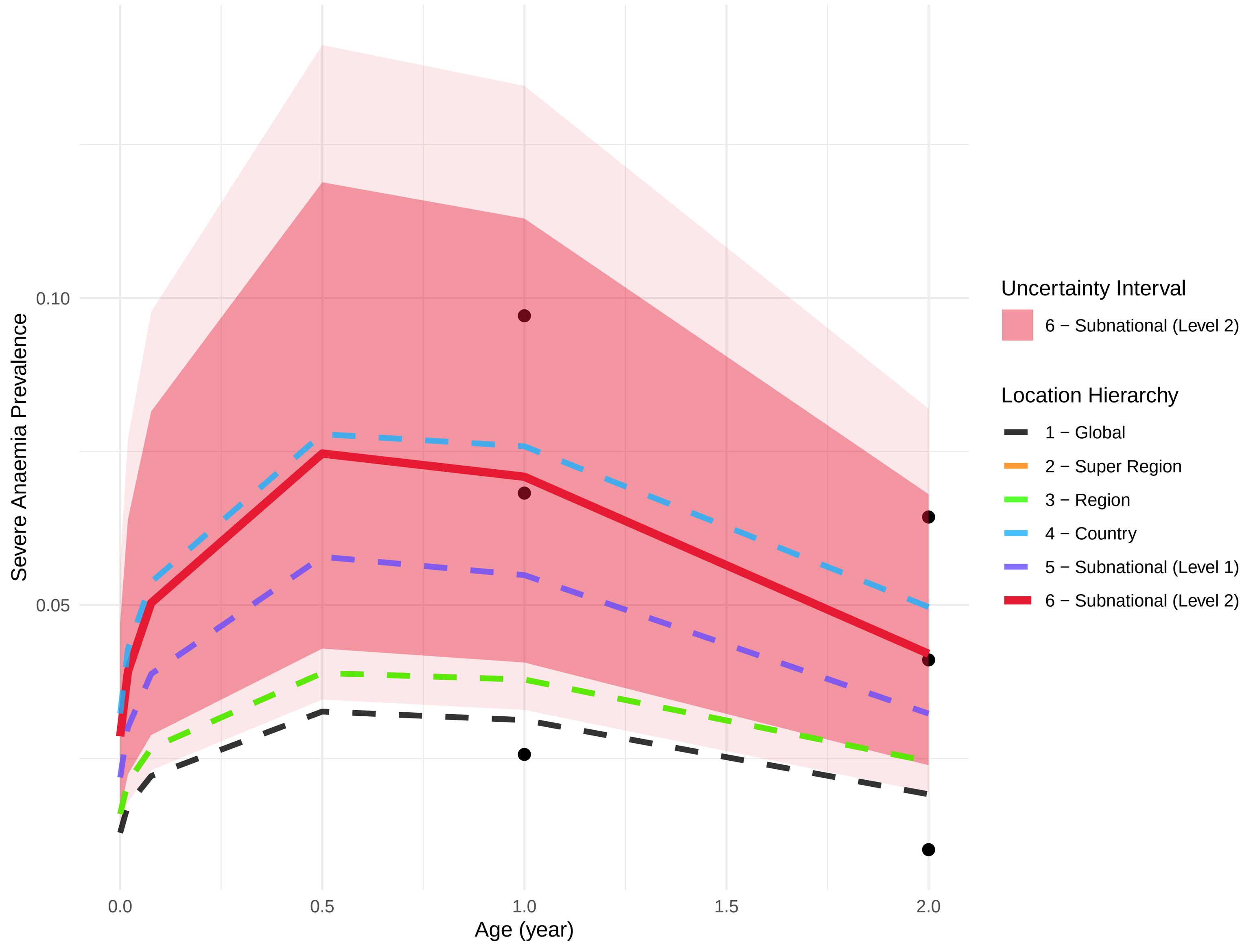

MR−BRT Age/Sex Fits − Non−pregnant Females <2 Years Old − Severe Anaemia Prevalence − Punjab
WHO−2024 Adjustment Method

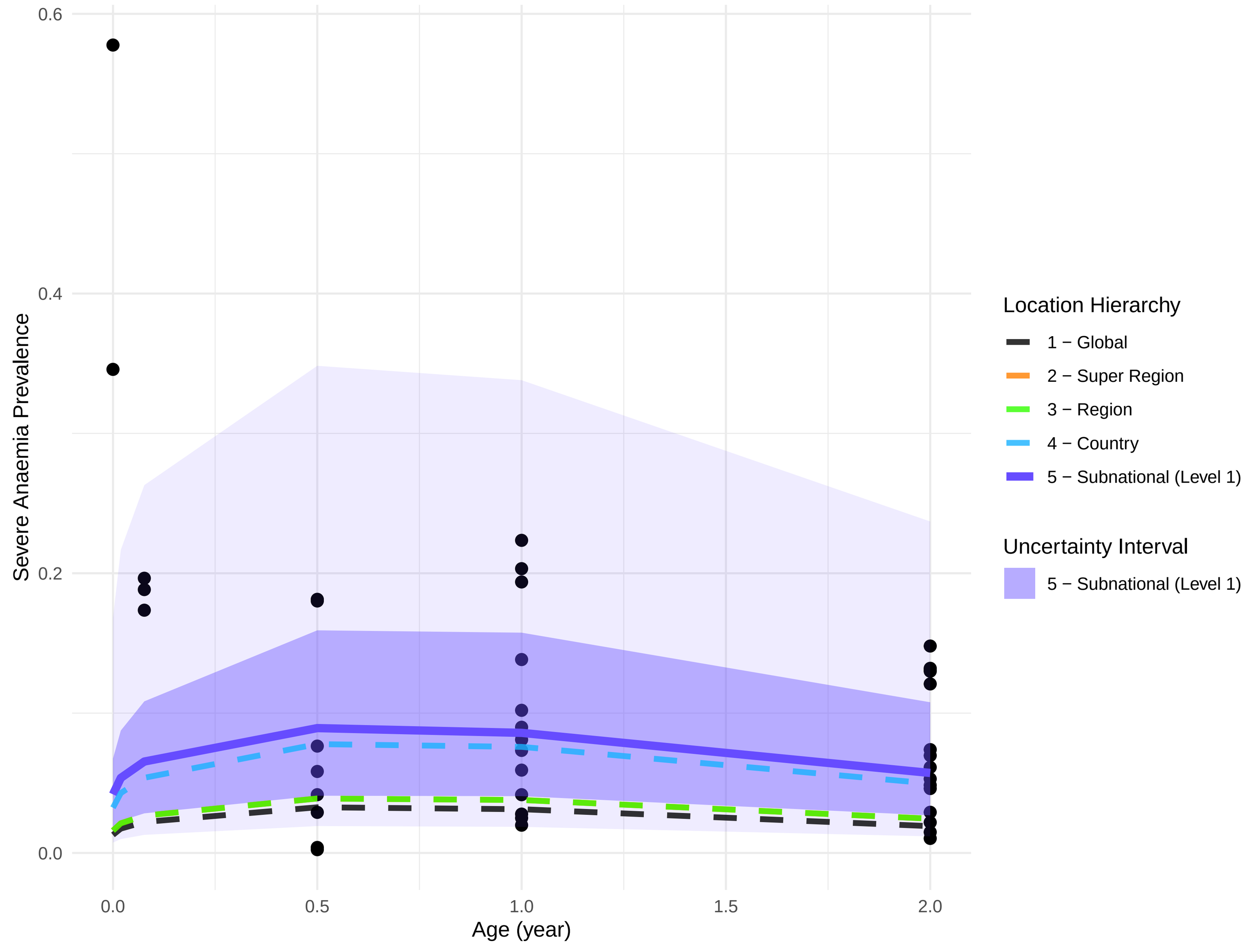

MR−BRT Age/Sex Fits − Non−pregnant Females <2 Years Old − Severe Anaemia Prevalence − Punjab, Rural
WHO−2024 Adjustment Method

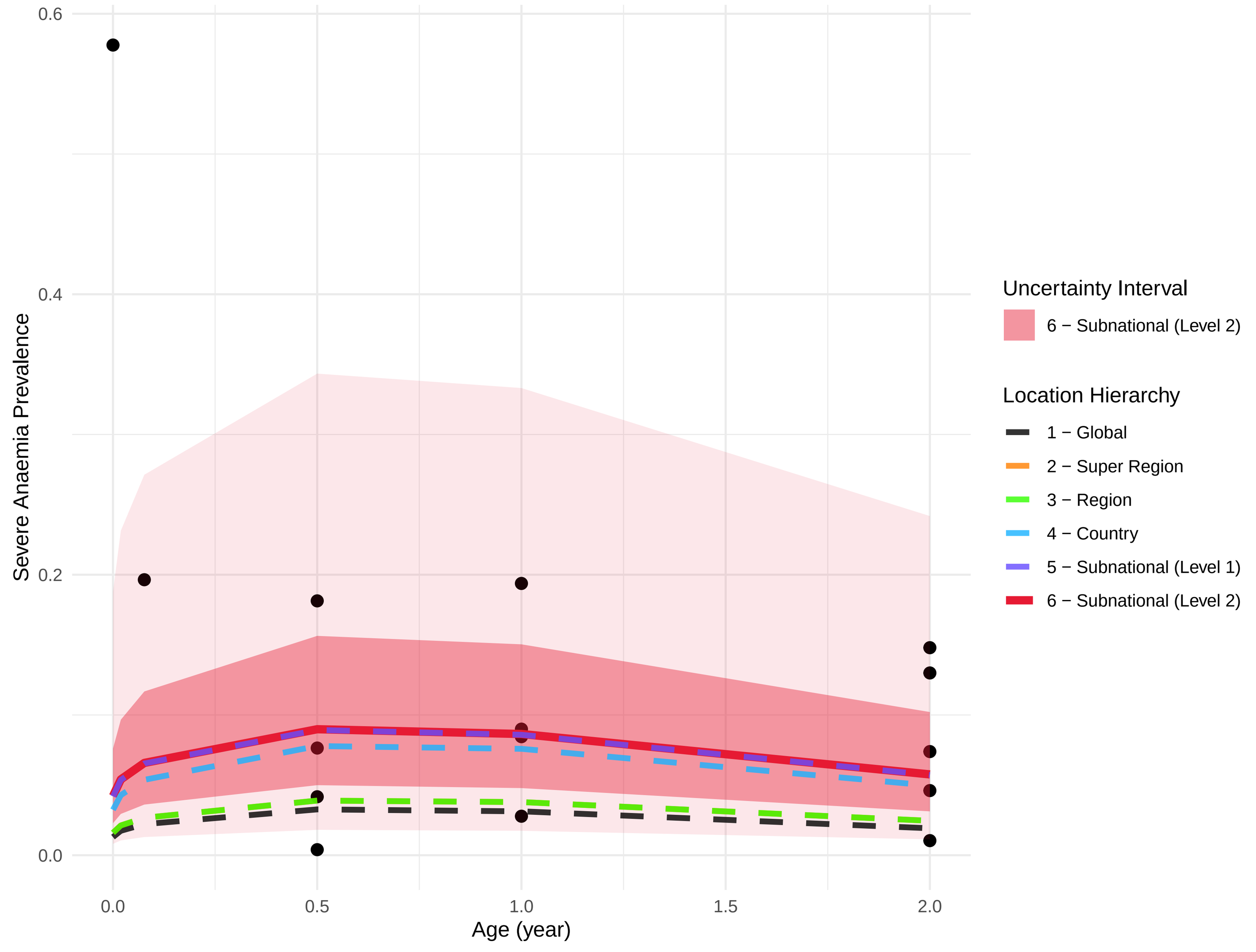

MR−BRT Age/Sex Fits − Non−pregnant Females <2 Years Old − Severe Anaemia Prevalence − Punjab, Urban
WHO−2024 Adjustment Method

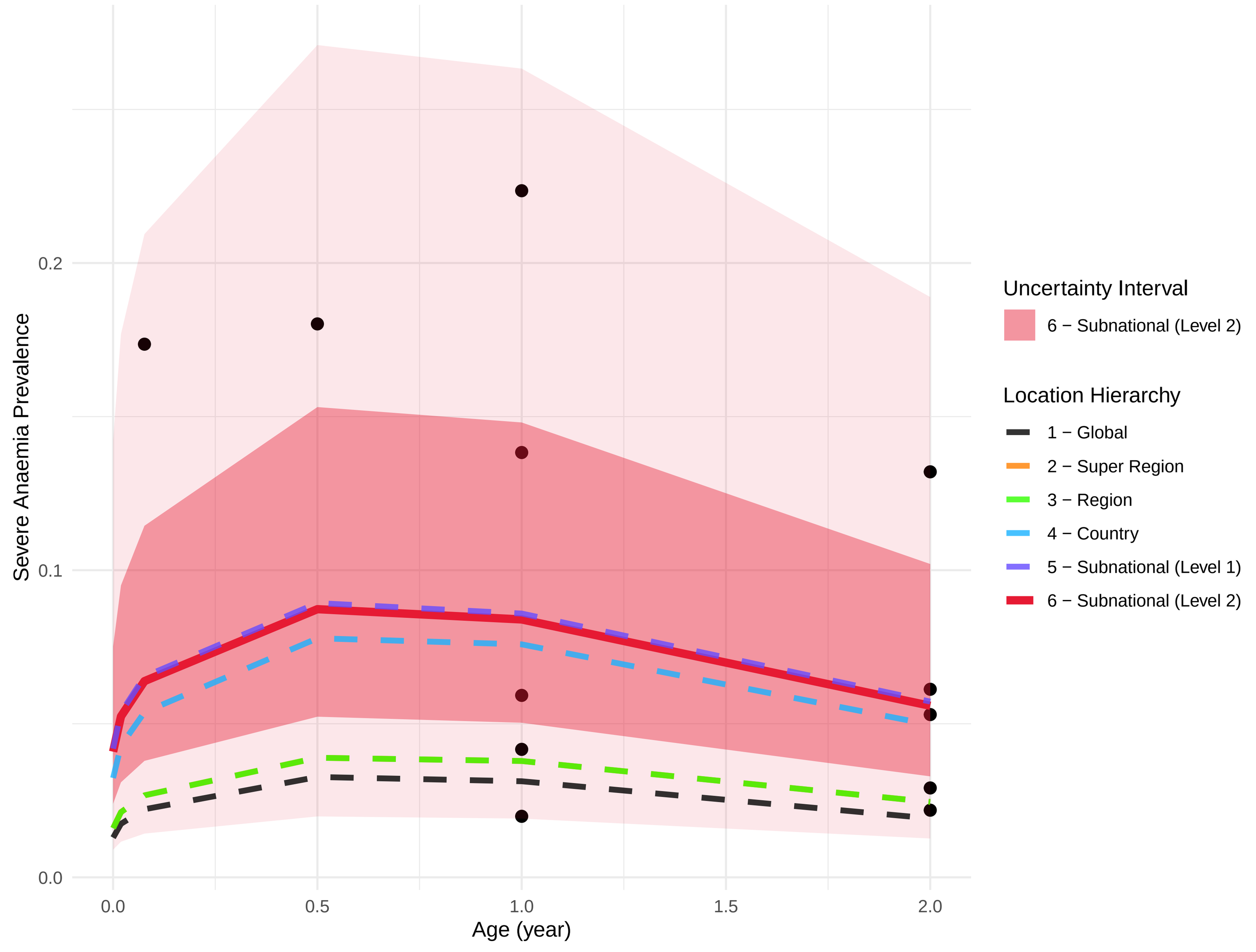

MR−BRT Age/Sex Fits − Non−pregnant Females <2 Years Old − Severe Anaemia Prevalence − Rajasthan
WHO−2024 Adjustment Method

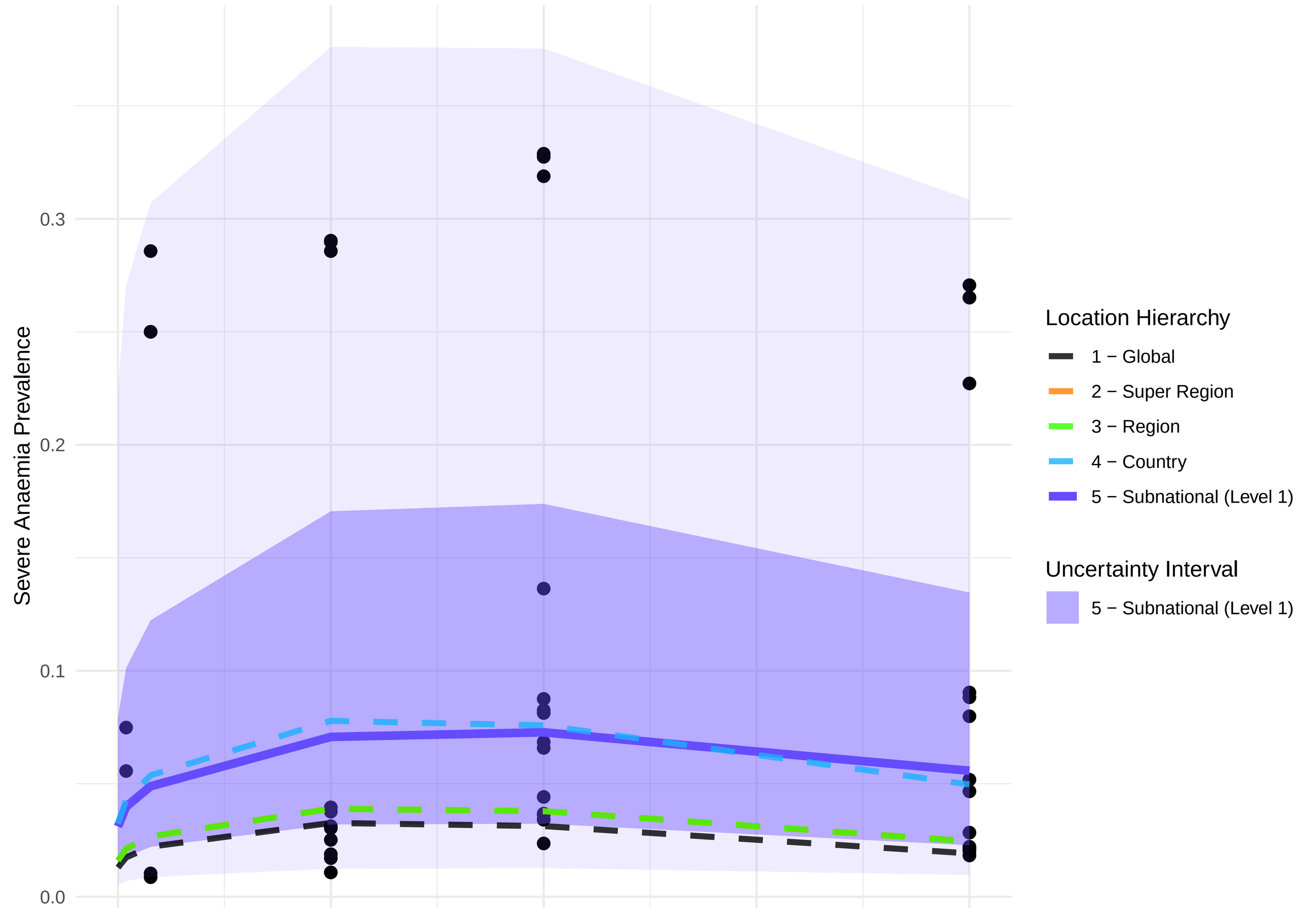

MR−BRT Age/Sex Fits − Non−pregnant Females <2 Years Old − Severe Anaemia Prevalence − Rajasthan, Rural
WHO−2024 Adjustment Method

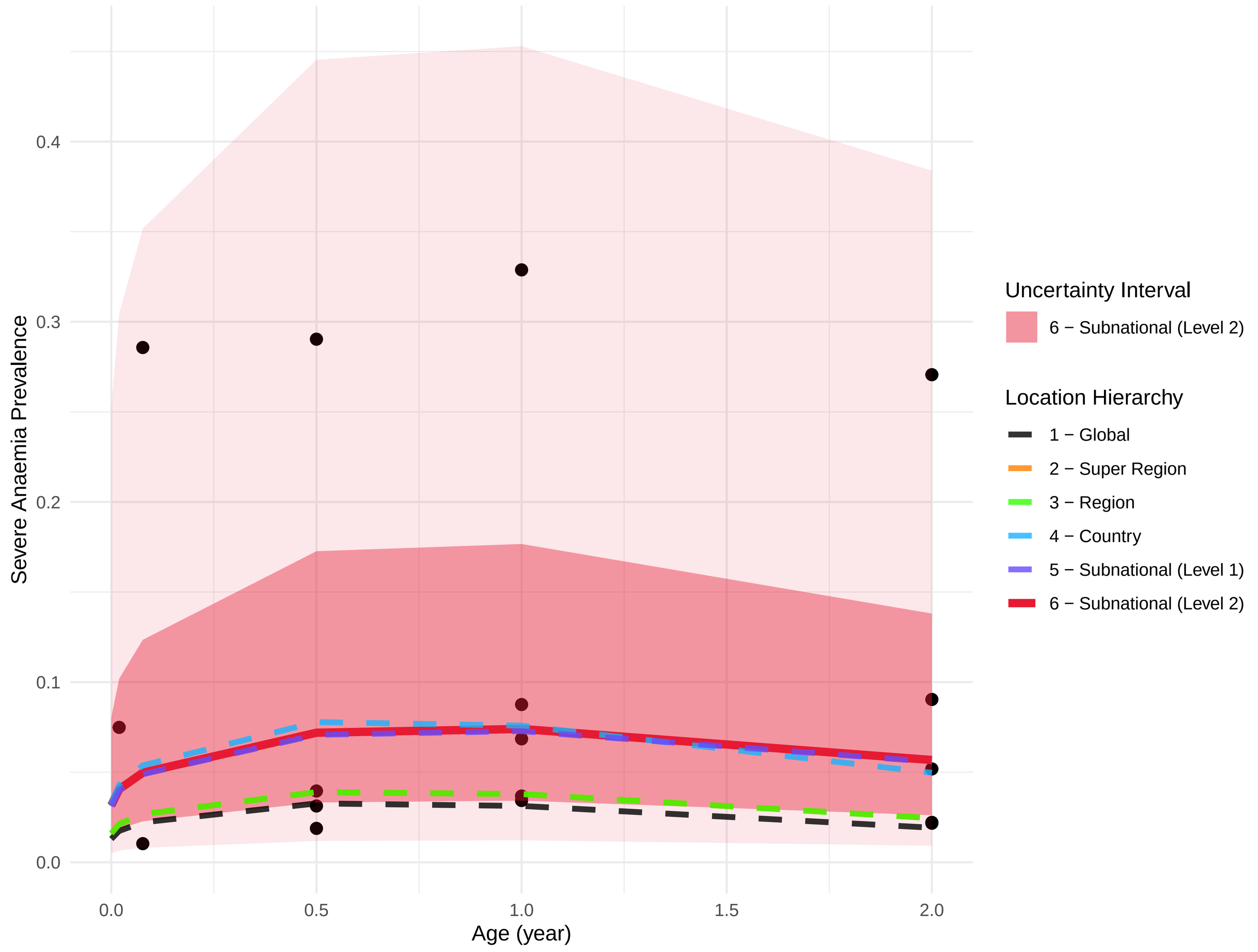

MR−BRT Age/Sex Fits − Non−pregnant Females <2 Years Old − Severe Anaemia Prevalence − Rajasthan, Urban
WHO−2024 Adjustment Method

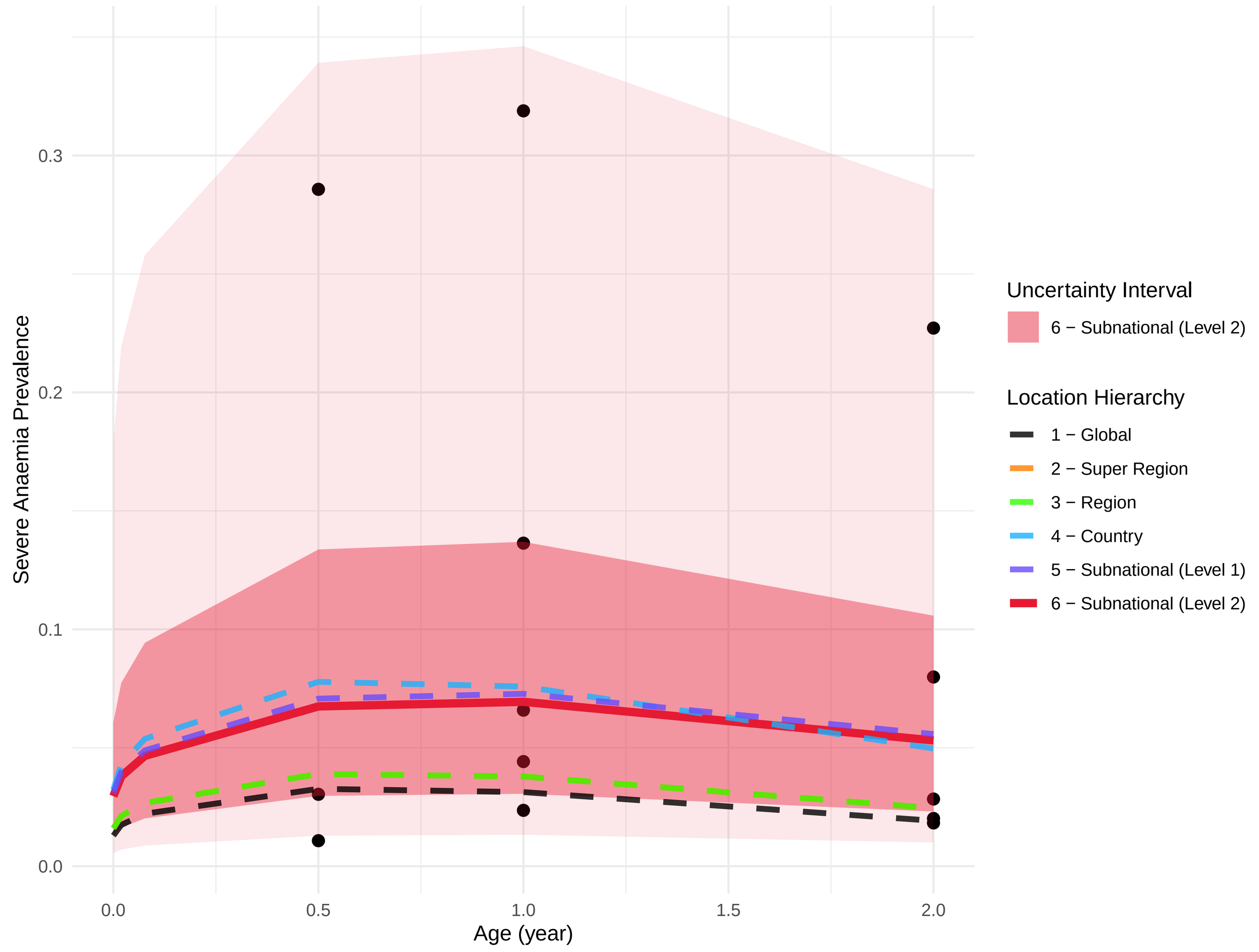

MR−BRT Age/Sex Fits − Non−pregnant Females <2 Years Old − Severe Anaemia Prevalence − Sikkim
WHO−2024 Adjustment Method

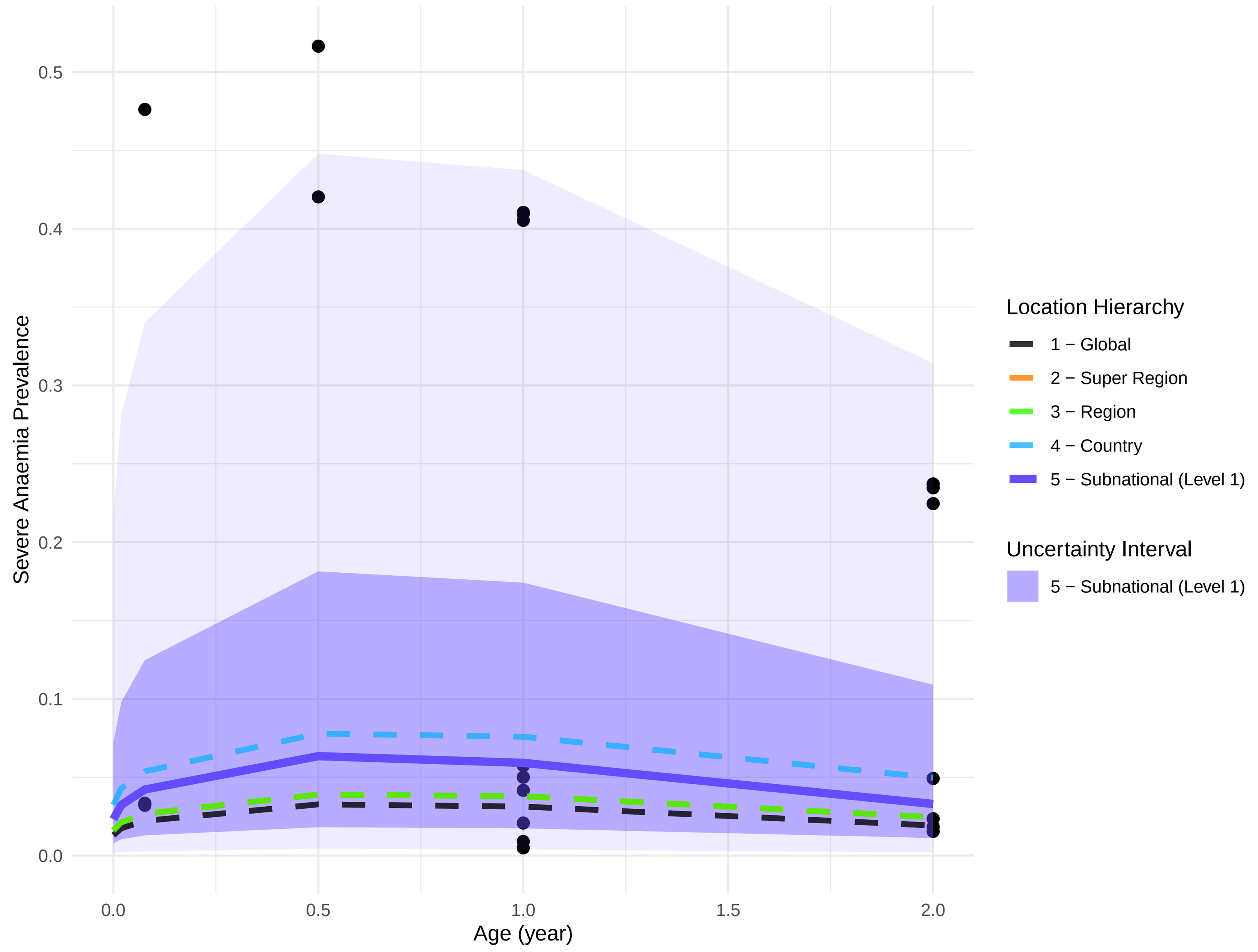

MR−BRT Age/Sex Fits − Non−pregnant Females <2 Years Old − Severe Anaemia Prevalence − Sikkim, Rural
WHO−2024 Adjustment Method

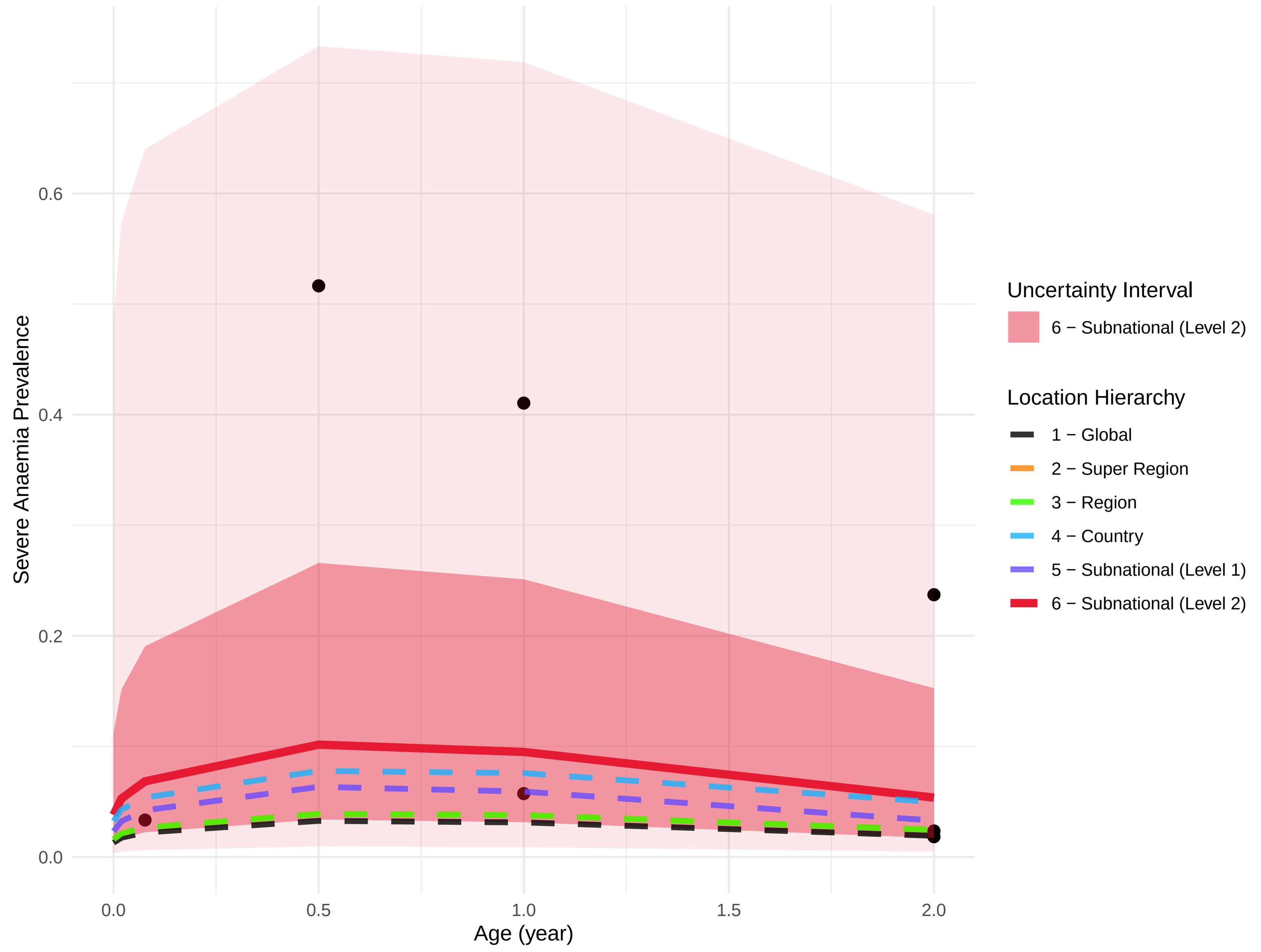

MR−BRT Age/Sex Fits − Non−pregnant Females <2 Years Old − Severe Anaemia Prevalence − Sikkim, Urban
WHO−2024 Adjustment Method

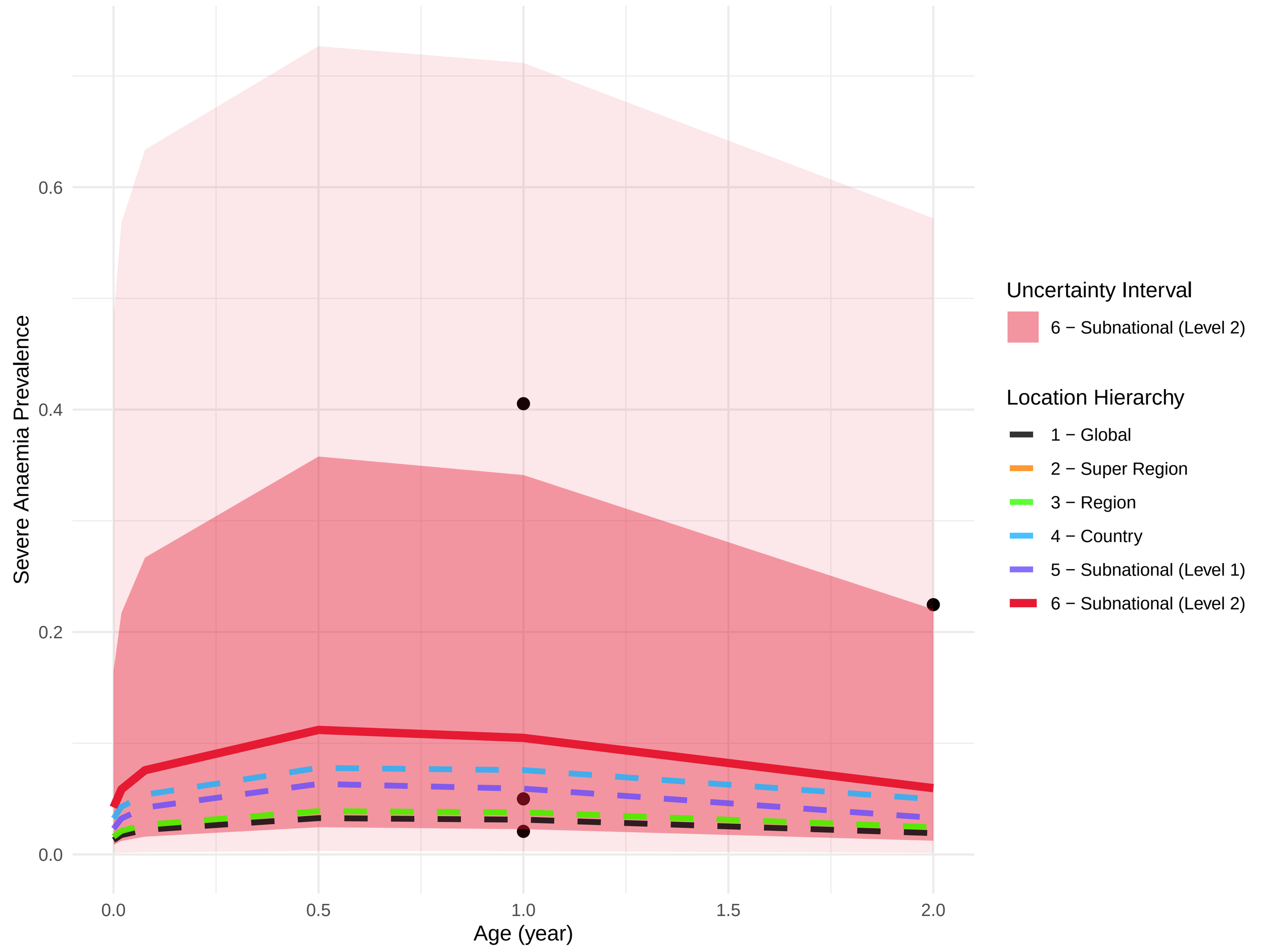

MR−BRT Age/Sex Fits − Non−pregnant Females <2 Years Old − Severe Anaemia Prevalence − Tamil Nadu
WHO−2024 Adjustment Method

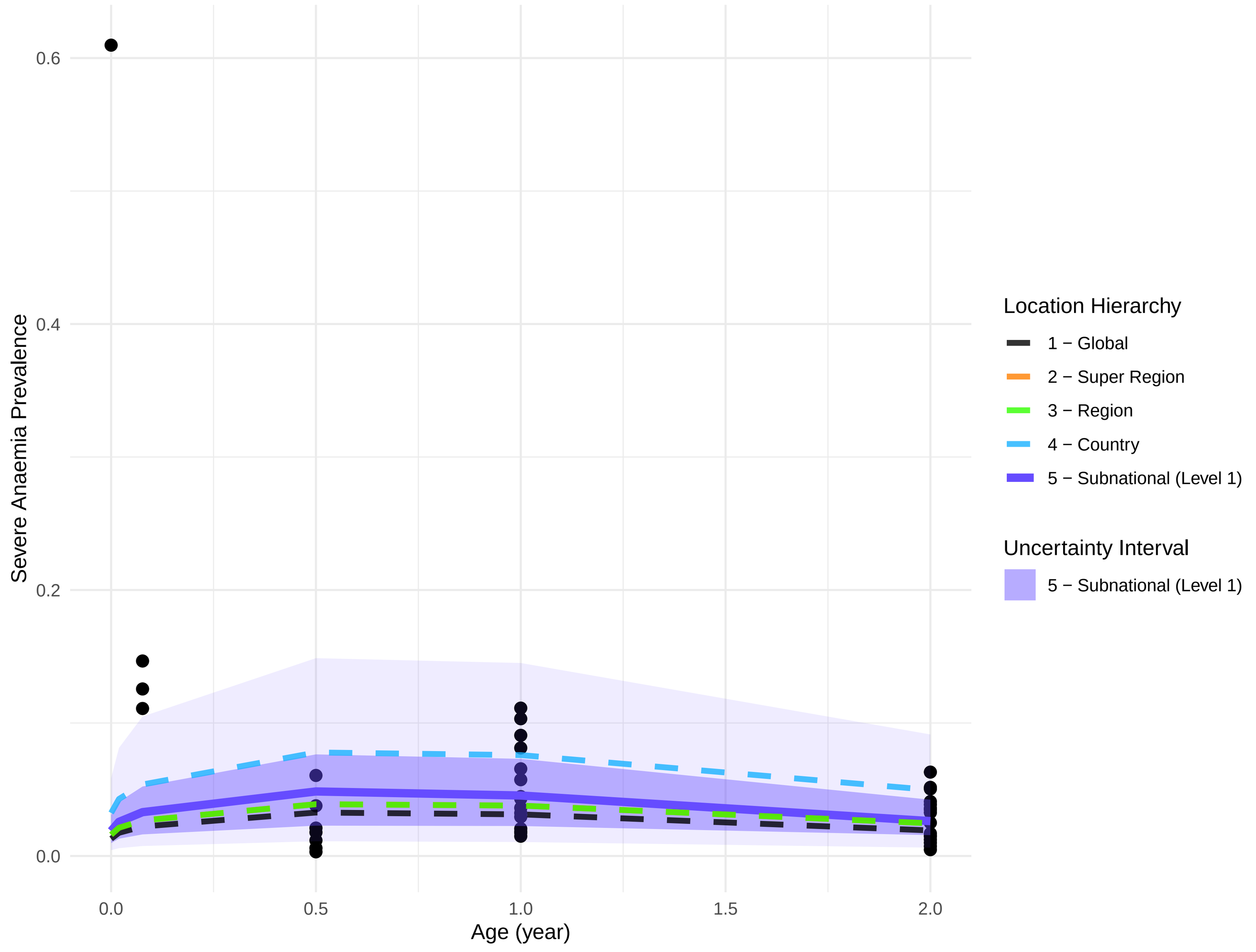

MR−BRT Age/Sex Fits − Non−pregnant Females <2 Years Old − Severe Anaemia Prevalence − Tamil Nadu, Rural
WHO−2024 Adjustment Method

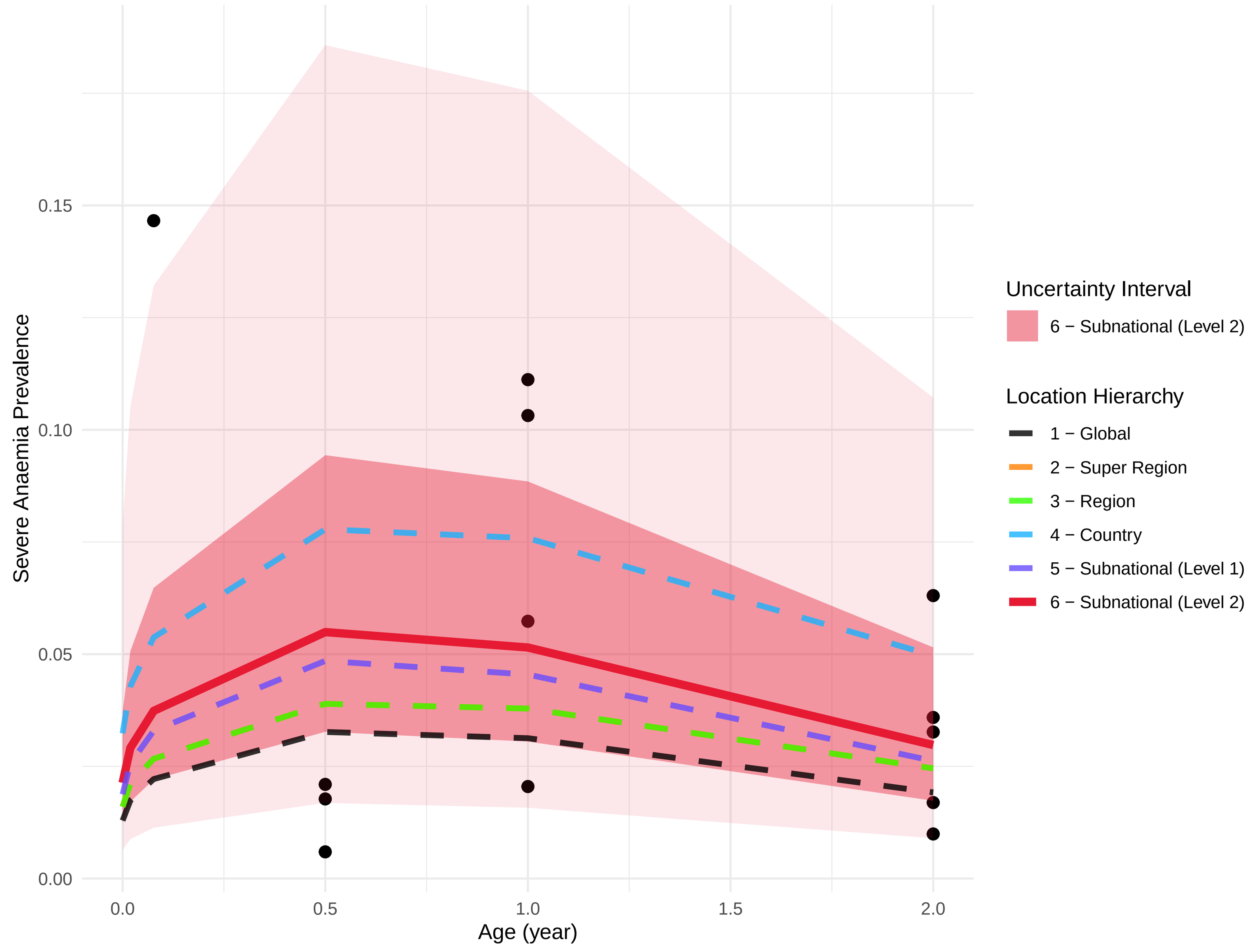

MR−BRT Age/Sex Fits − Non−pregnant Females <2 Years Old − Severe Anaemia Prevalence − Tamil Nadu, Urban
WHO−2024 Adjustment Method

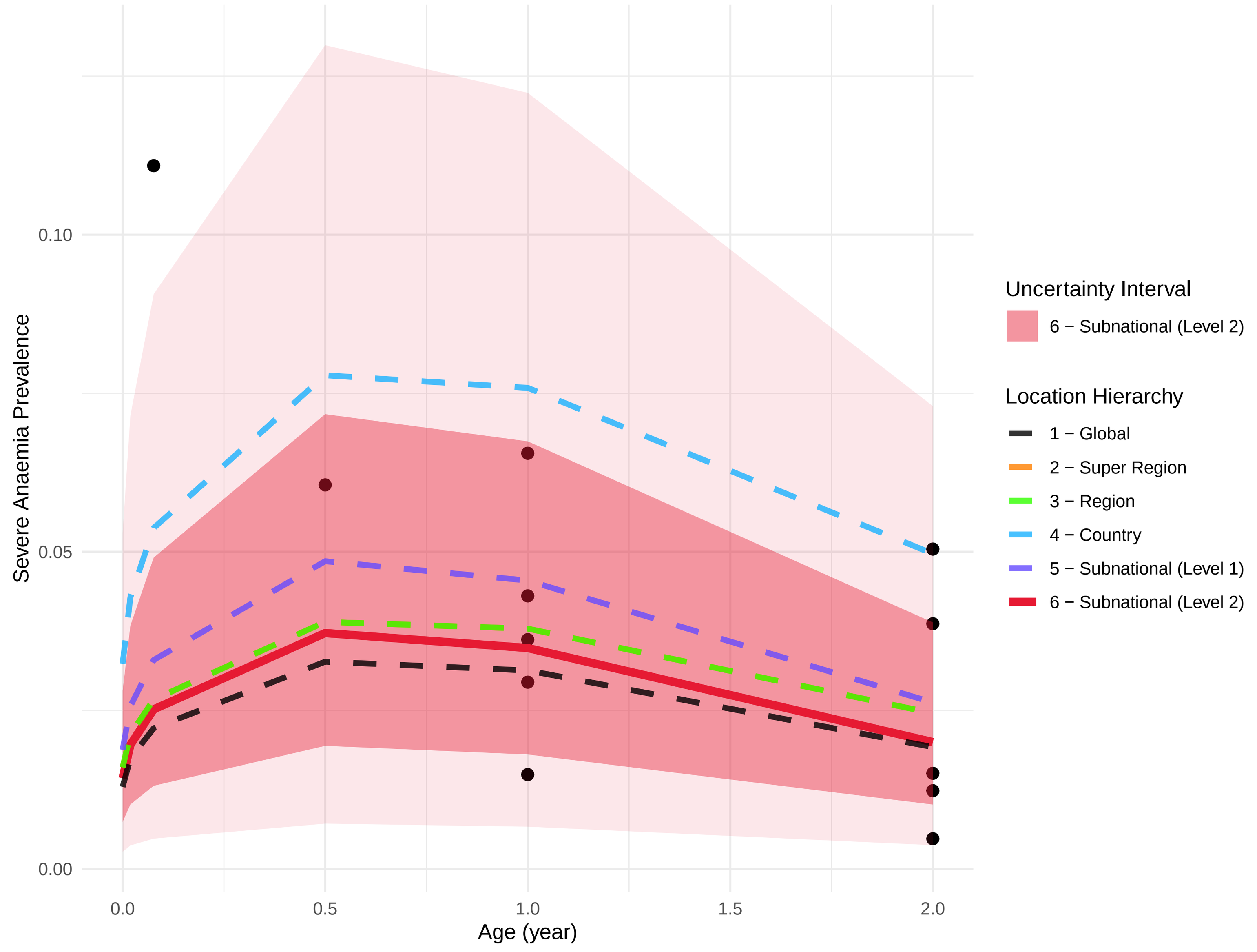

MR−BRT Age/Sex Fits − Non−pregnant Females <2 Years Old − Severe Anaemia Prevalence − Telangana
WHO−2024 Adjustment Method

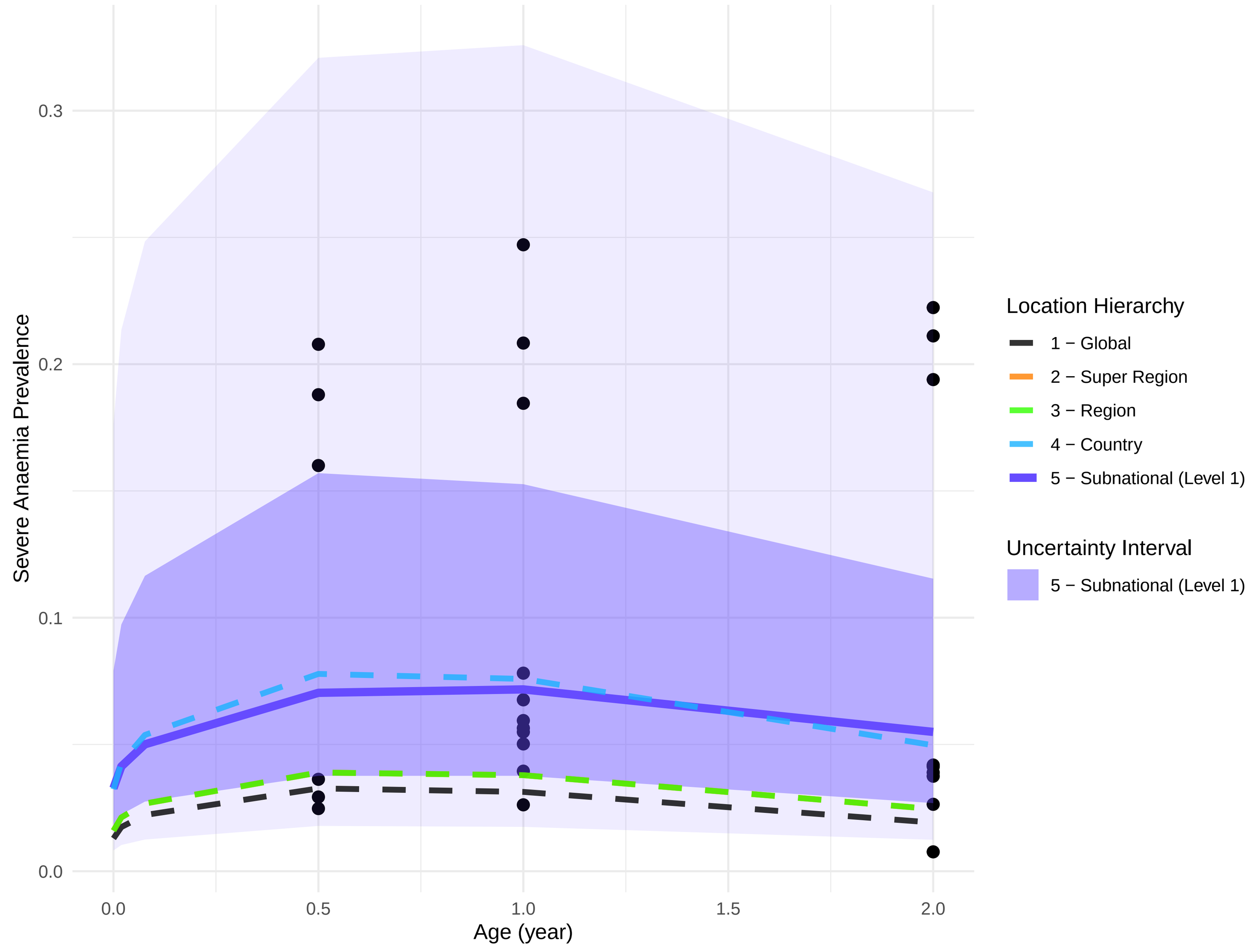

MR−BRT Age/Sex Fits − Non−pregnant Females <2 Years Old − Severe Anaemia Prevalence − Telangana, Rural
WHO−2024 Adjustment Method

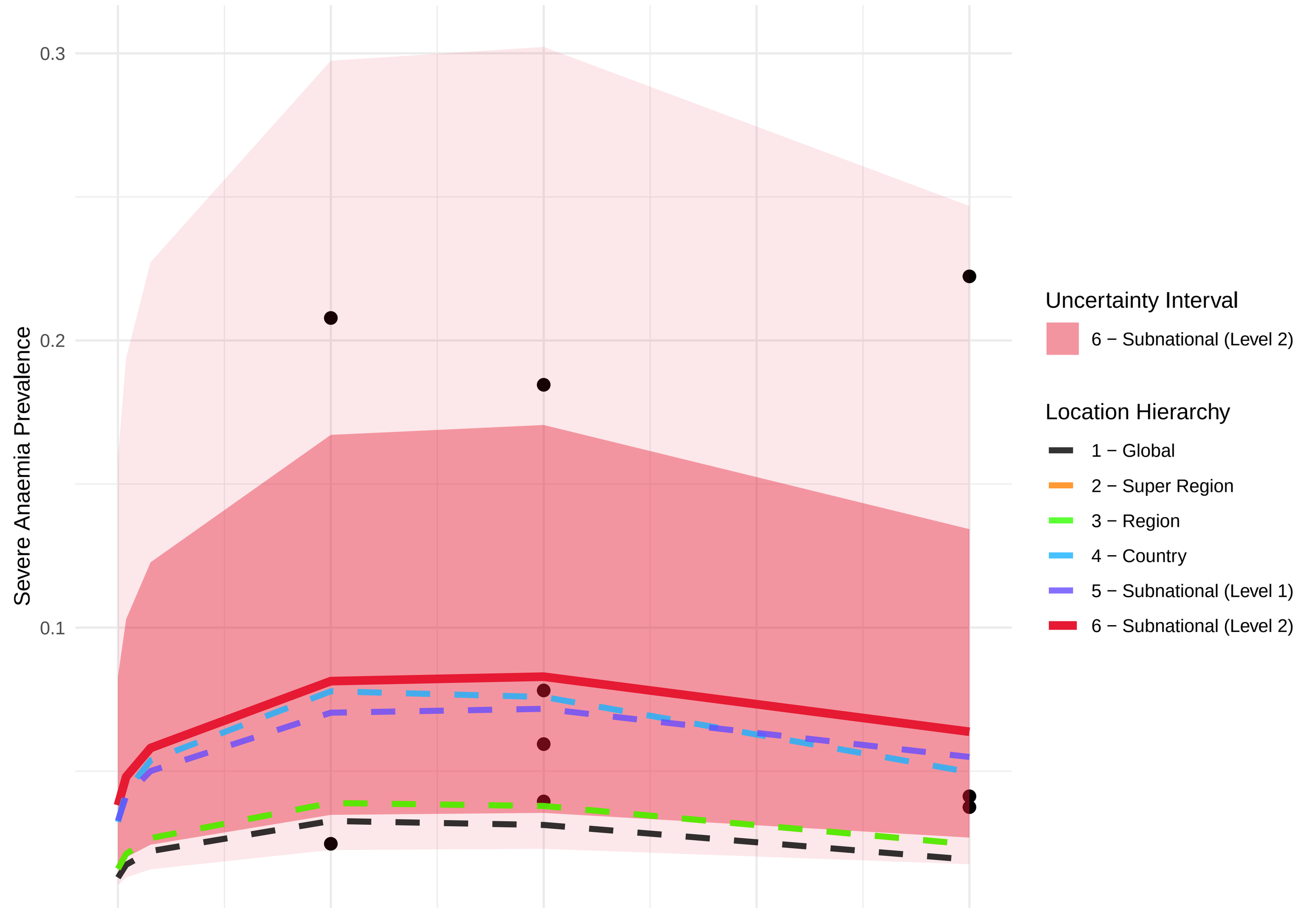

MR−BRT Age/Sex Fits − Non-pregnant Females <2 Years Old − Severe Anaemia Prevalence − Telangana, Urban
WHO−2024 Adjustment Method

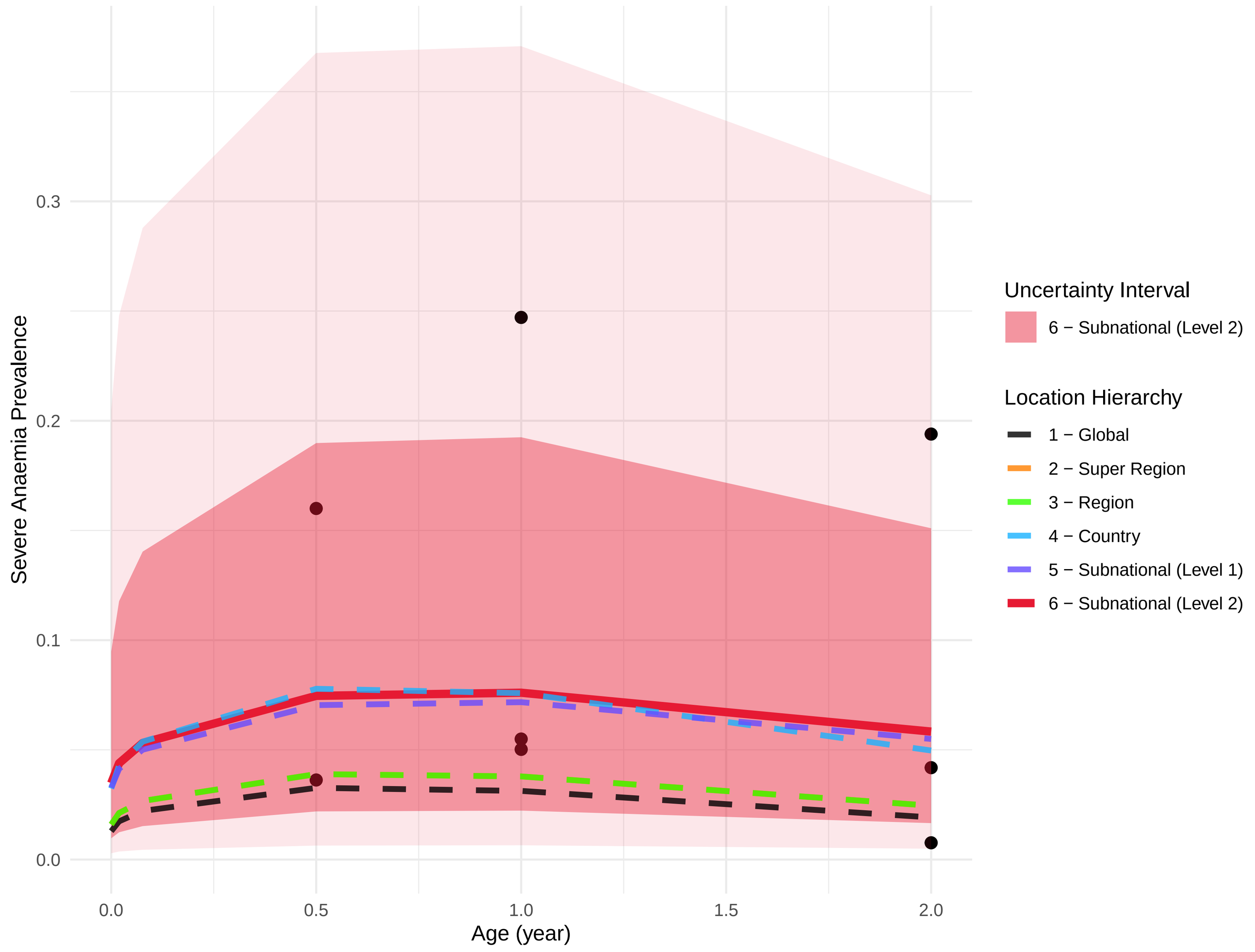

MR−BRT Age/Sex Fits − Non-pregnant Females <2 Years Old − Severe Anaemia Prevalence − Tripura
WHO−2024 Adjustment Method

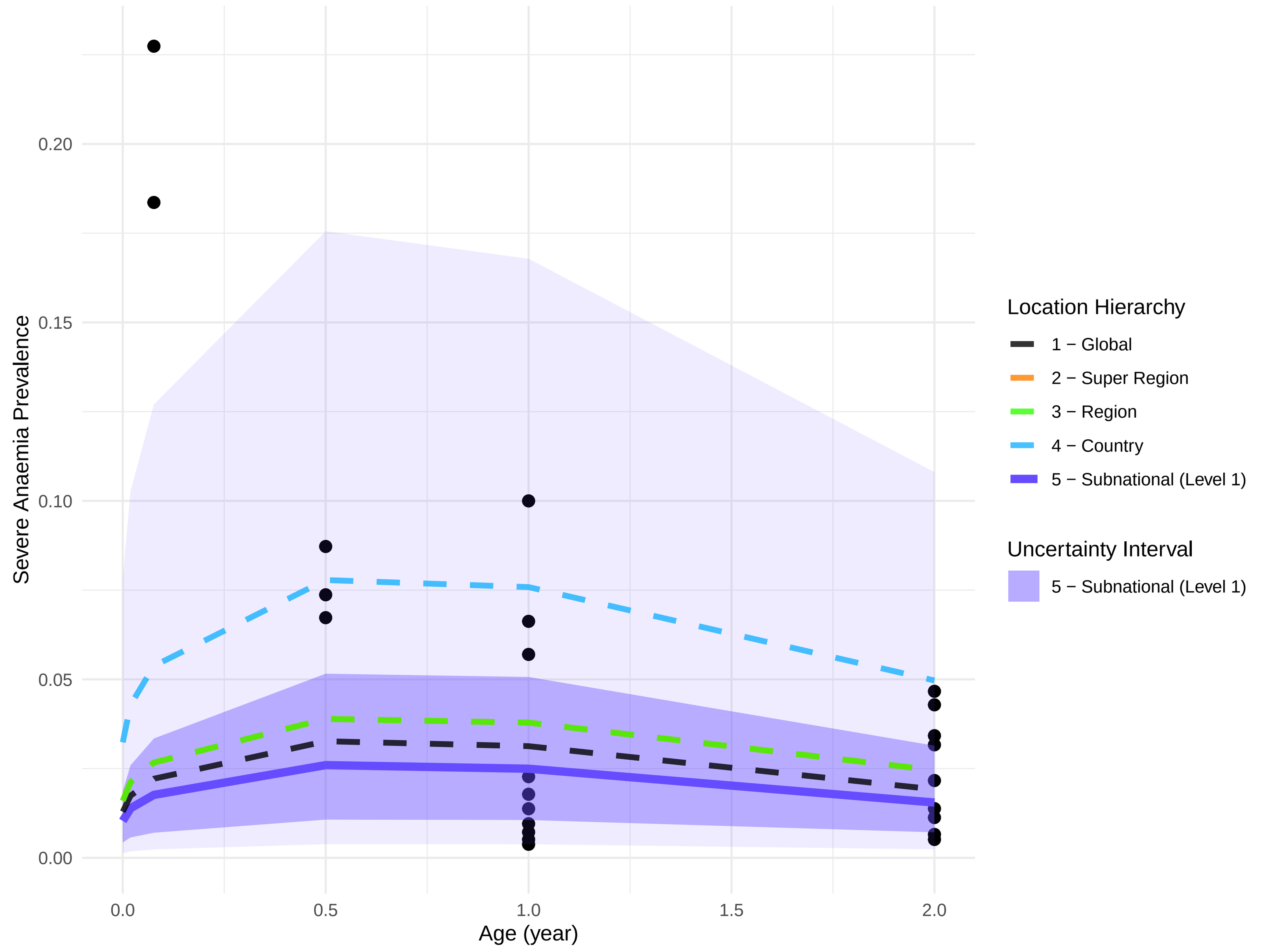

MR−BRT Age/Sex Fits − Non-pregnant Females <2 Years Old − Severe Anaemia Prevalence − Tripura, Rural
WHO−2024 Adjustment Method

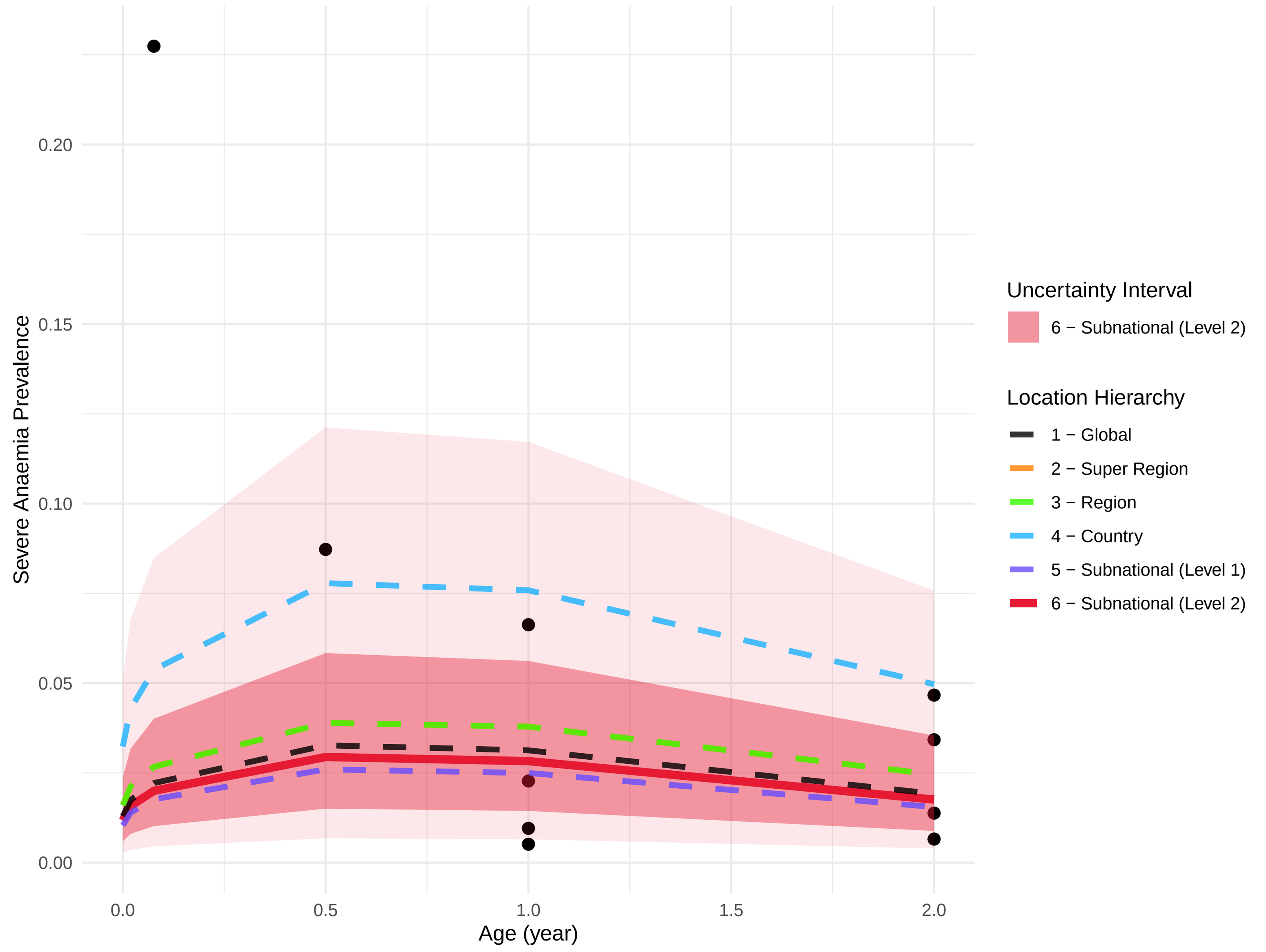

MR−BRT Age/Sex Fits − Non-pregnant Females <2 Years Old − Severe Anaemia Prevalence − Tripura, Urban
WHO−2024 Adjustment Method

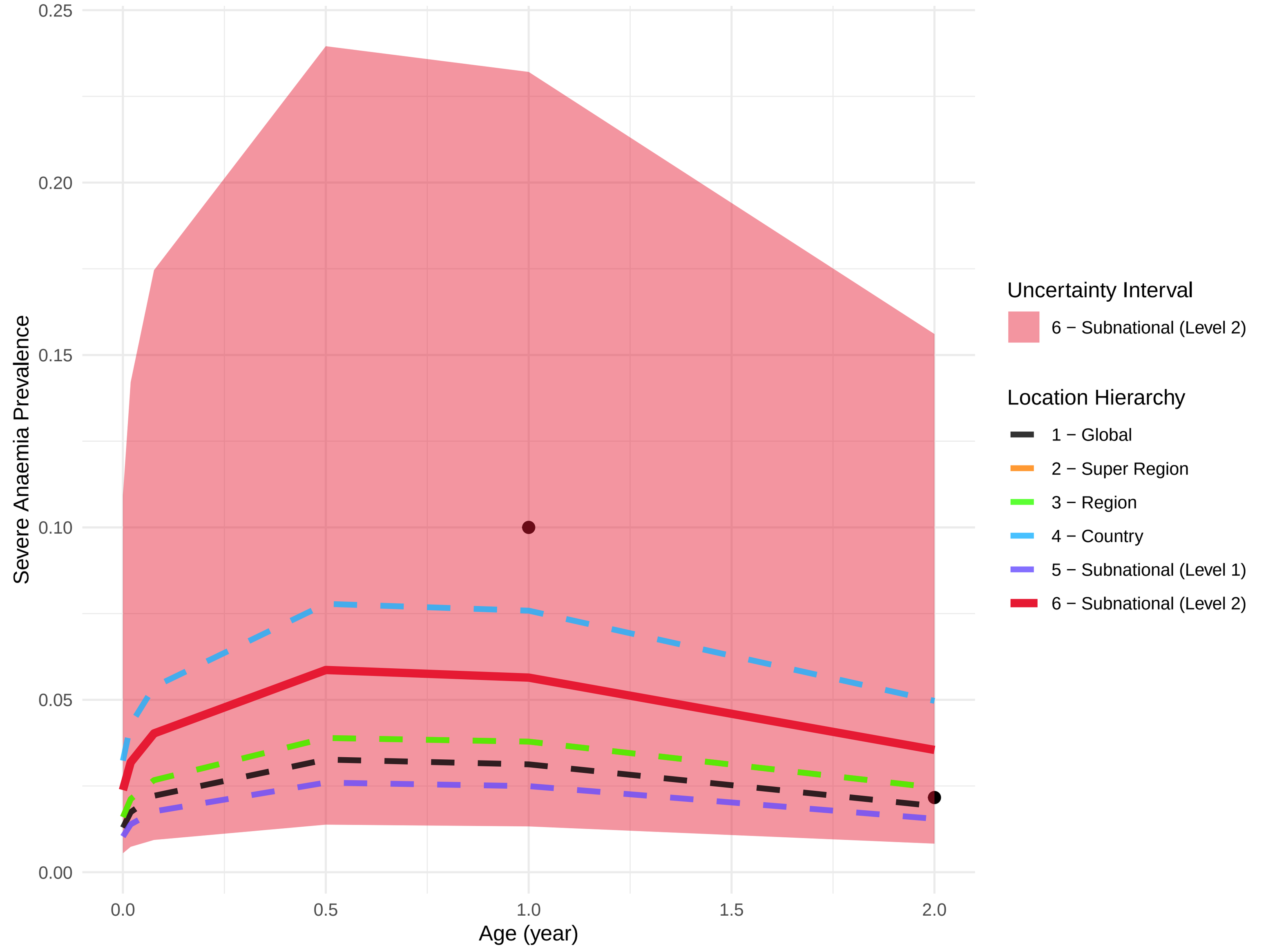

MR−BRT Age/Sex Fits − Non-pregnant Females <2 Years Old − Severe Anaemia Prevalence − Uttar Pradesh
WHO−2024 Adjustment Method

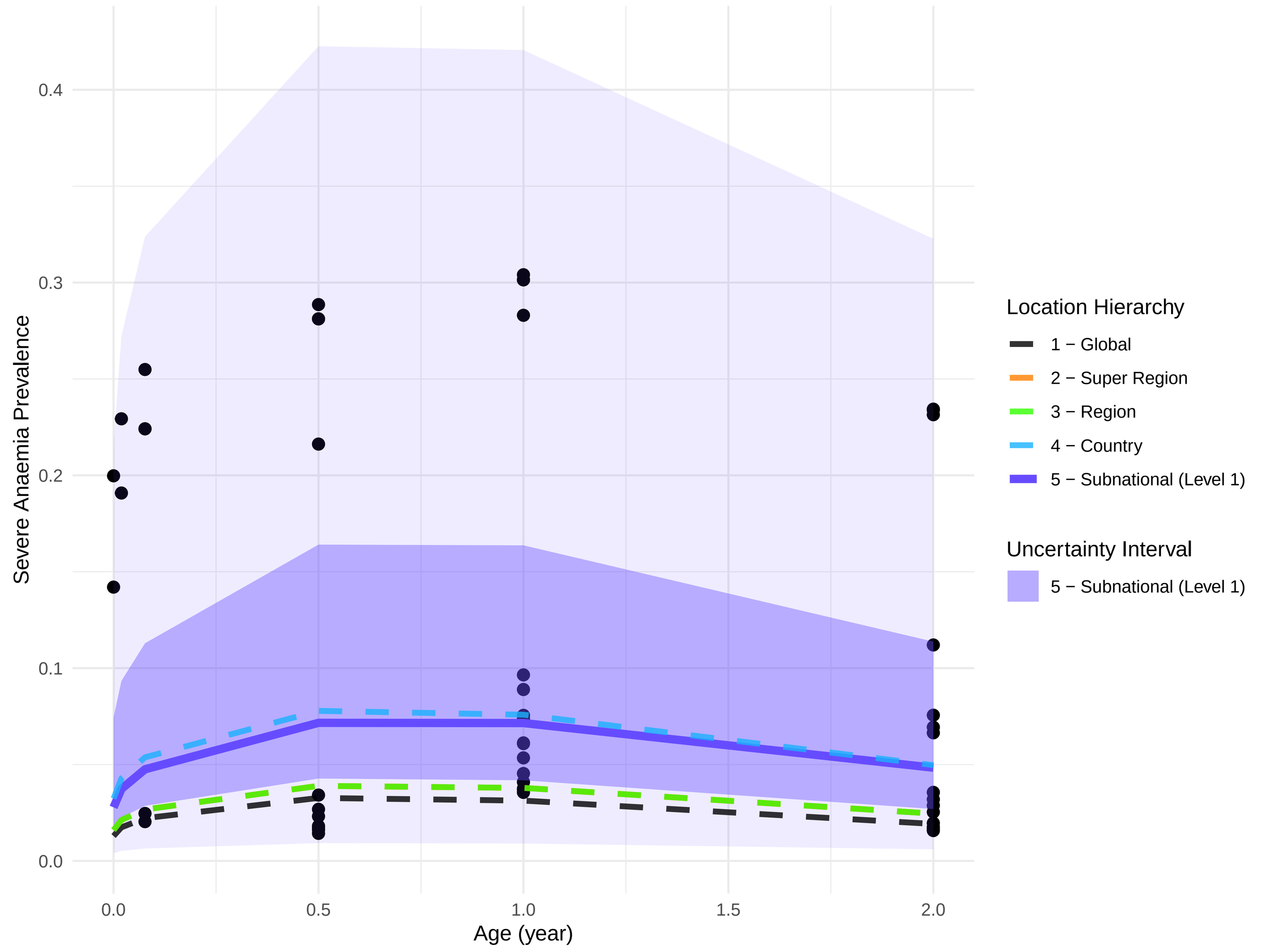

MR−BRT Age/Sex Fits − Non-pregnant Females <2 Years Old − Severe Anaemia Prevalence − Uttar Pradesh, Rural
WHO−2024 Adjustment Method

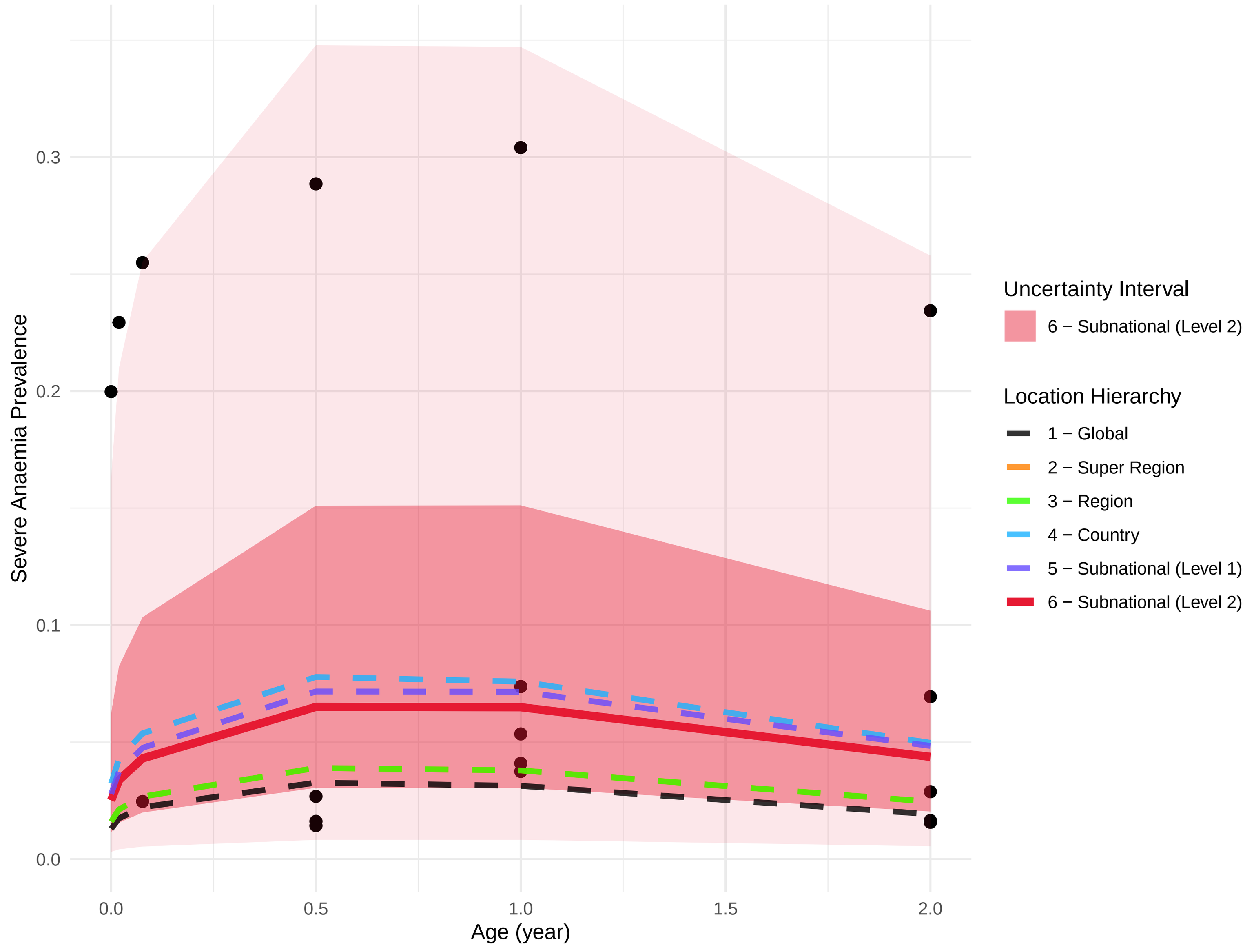

MR−BRT Age/Sex Fits − Non-pregnant Females <2 Years Old − Severe Anaemia Prevalence − Uttar Pradesh, Urban
WHO−2024 Adjustment Method

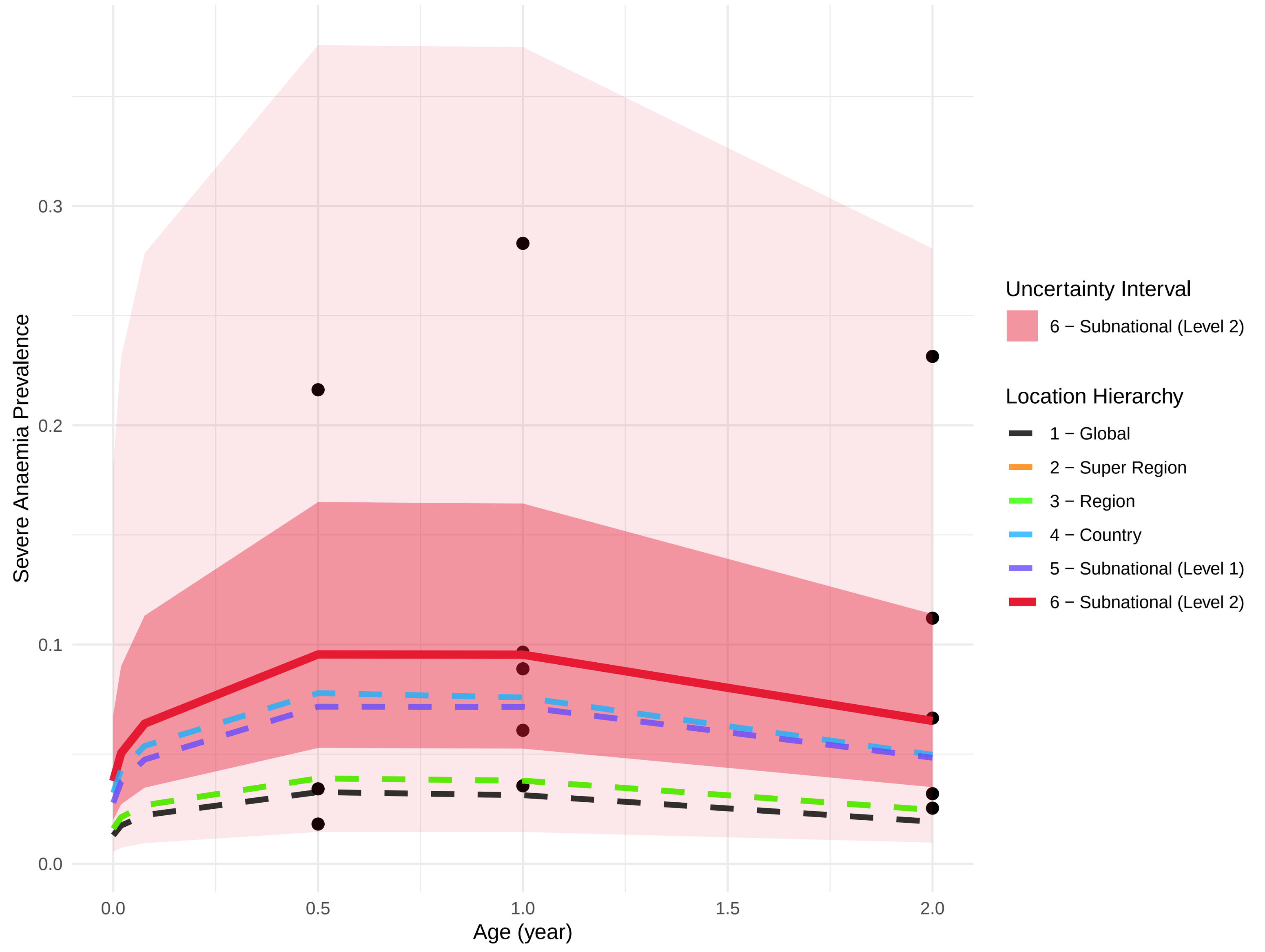

MR−BRT Age/Sex Fits − Non-pregnant Females <2 Years Old − Severe Anaemia Prevalence − Uttarakhand
WHO−2024 Adjustment Method

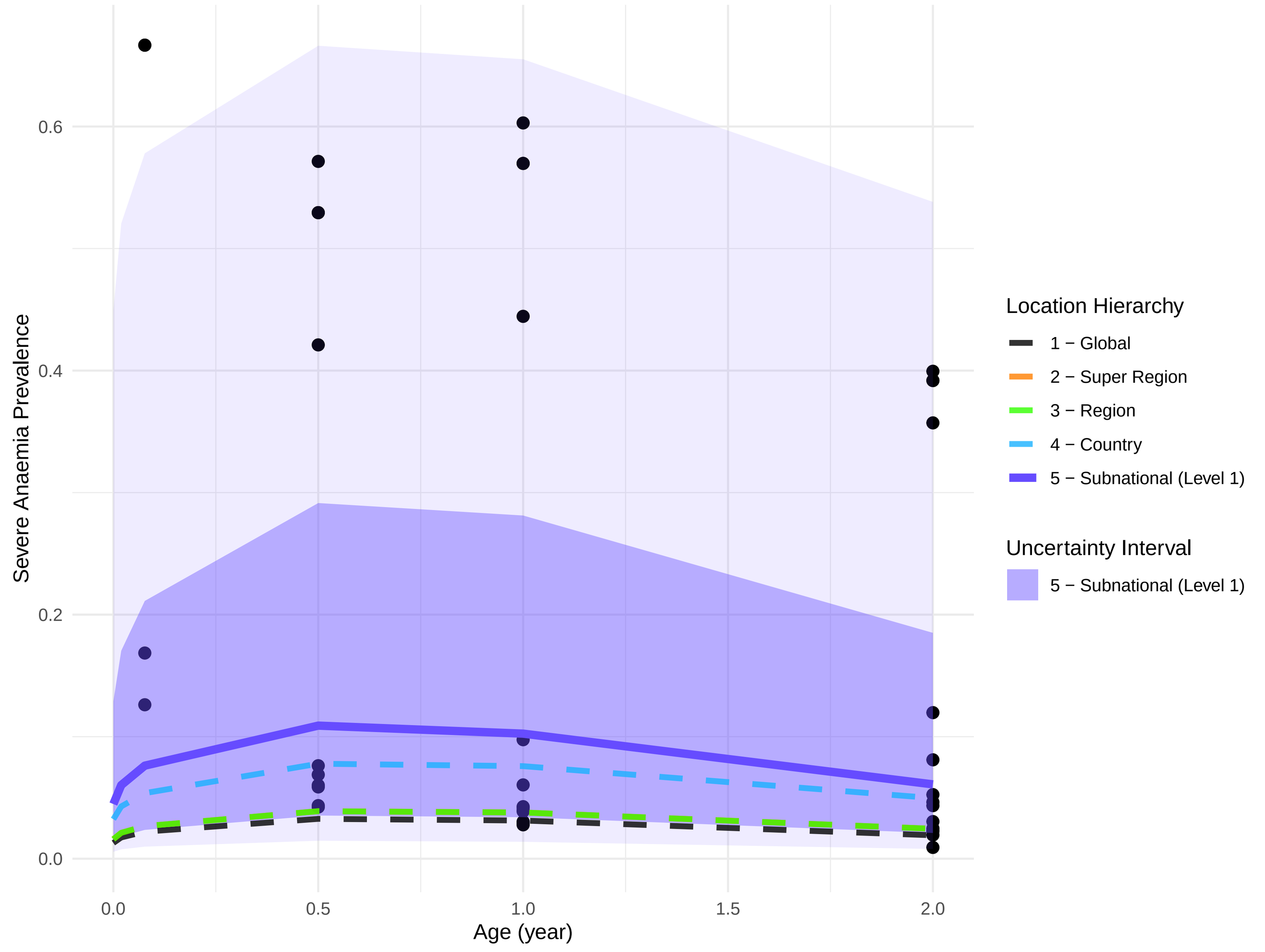

MR−BRT Age/Sex Fits − Non-pregnant Females <2 Years Old − Severe Anaemia Prevalence − Uttarakhand, Rural
WHO−2024 Adjustment Method

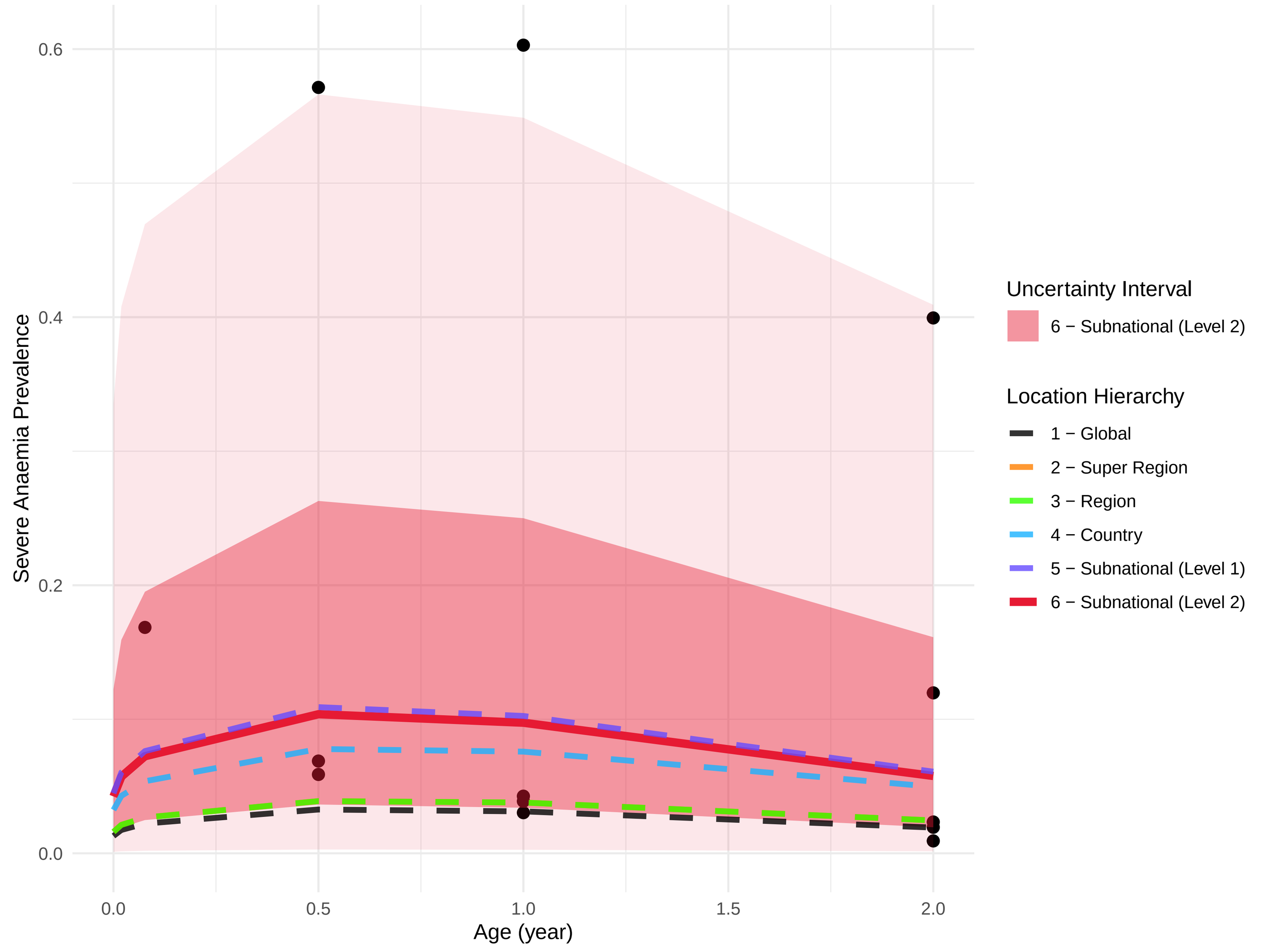

MR−BRT Age/Sex Fits − Non-pregnant Females <2 Years Old − Severe Anaemia Prevalence − Uttarakhand, Urban
WHO−2024 Adjustment Method

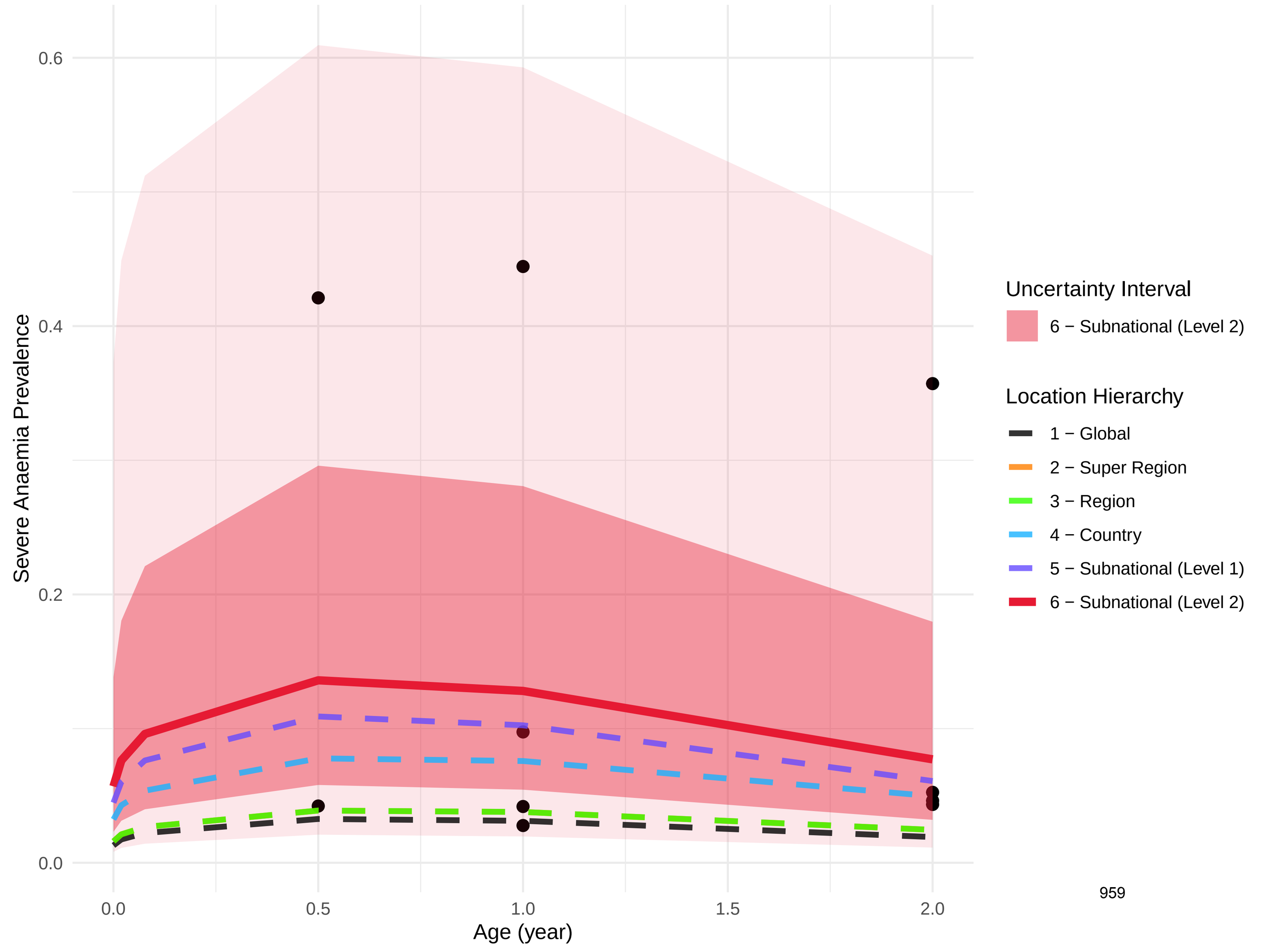

MR−BRT Age/Sex Fits − Non−pregnant Females <2 Years Old − Severe Anaemia Prevalence − West Bengal
WHO−2024 Adjustment Method

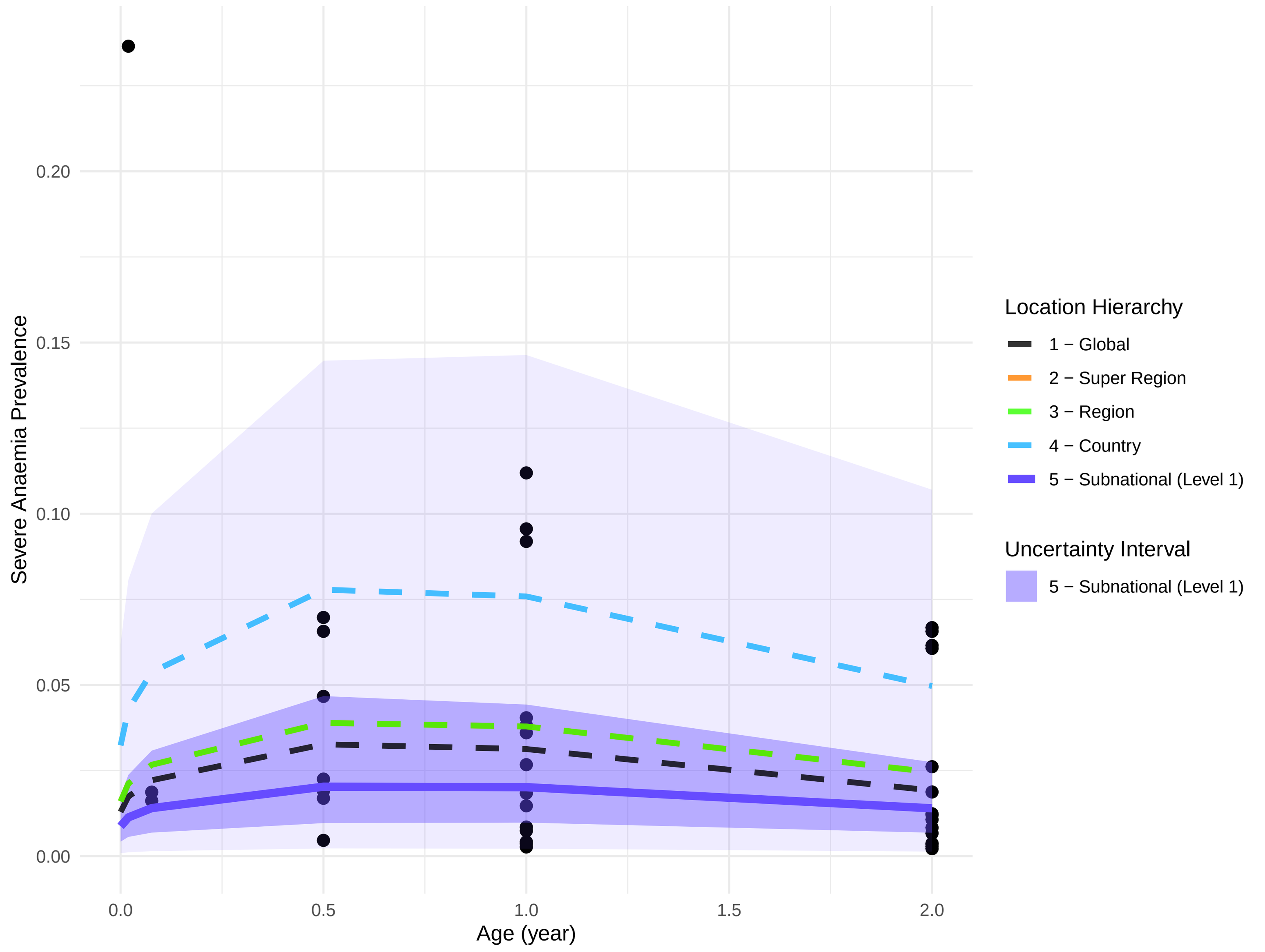

MR−BRT Age/Sex Fits − Non−pregnant Females <2 Years Old − Severe Anaemia Prevalence − West Bengal, Rural
WHO−2024 Adjustment Method

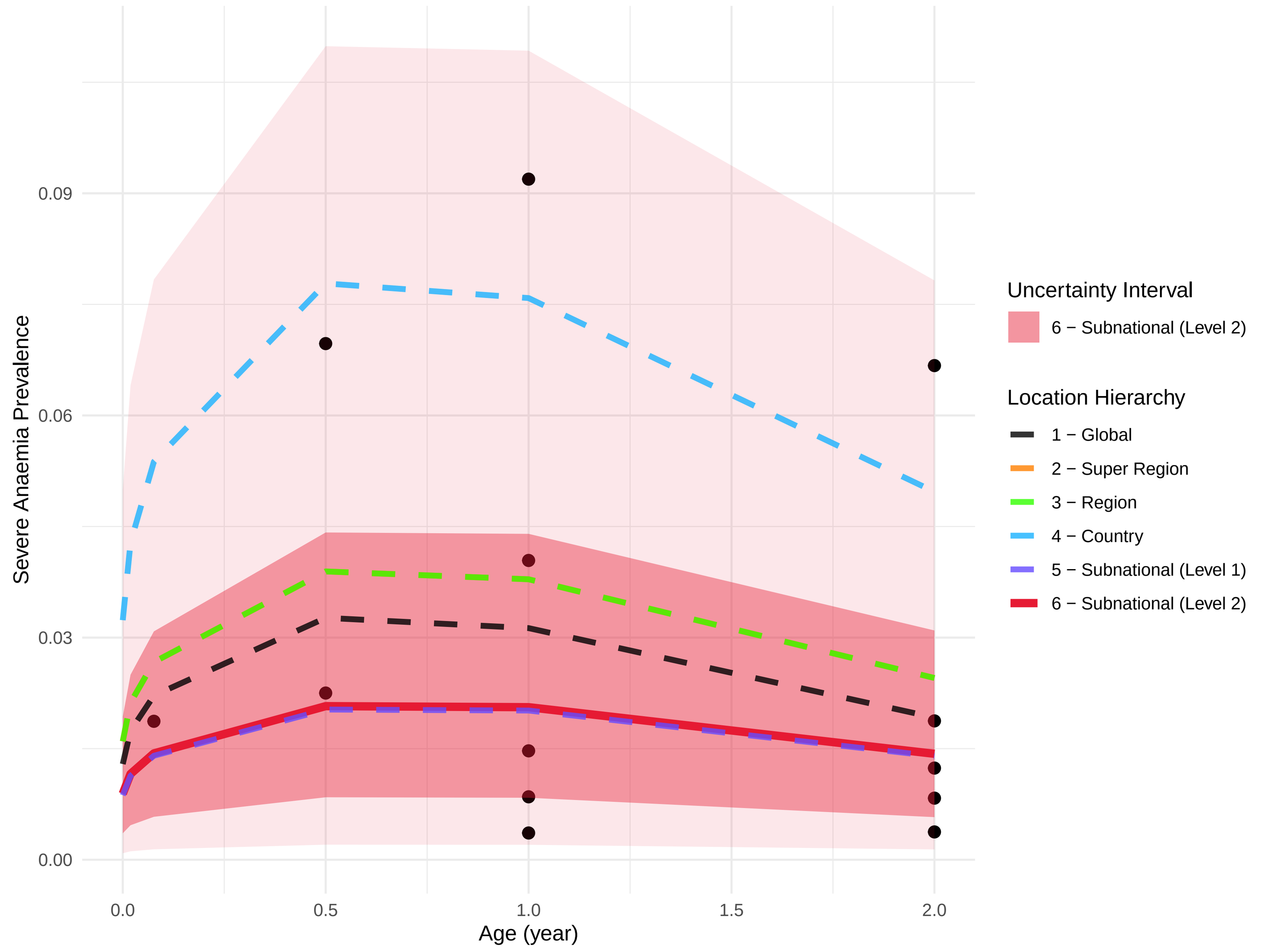

MR−BRT Age/Sex Fits − Non−pregnant Females <2 Years Old − Severe Anaemia Prevalence − West Bengal, Urban
WHO−2024 Adjustment Method

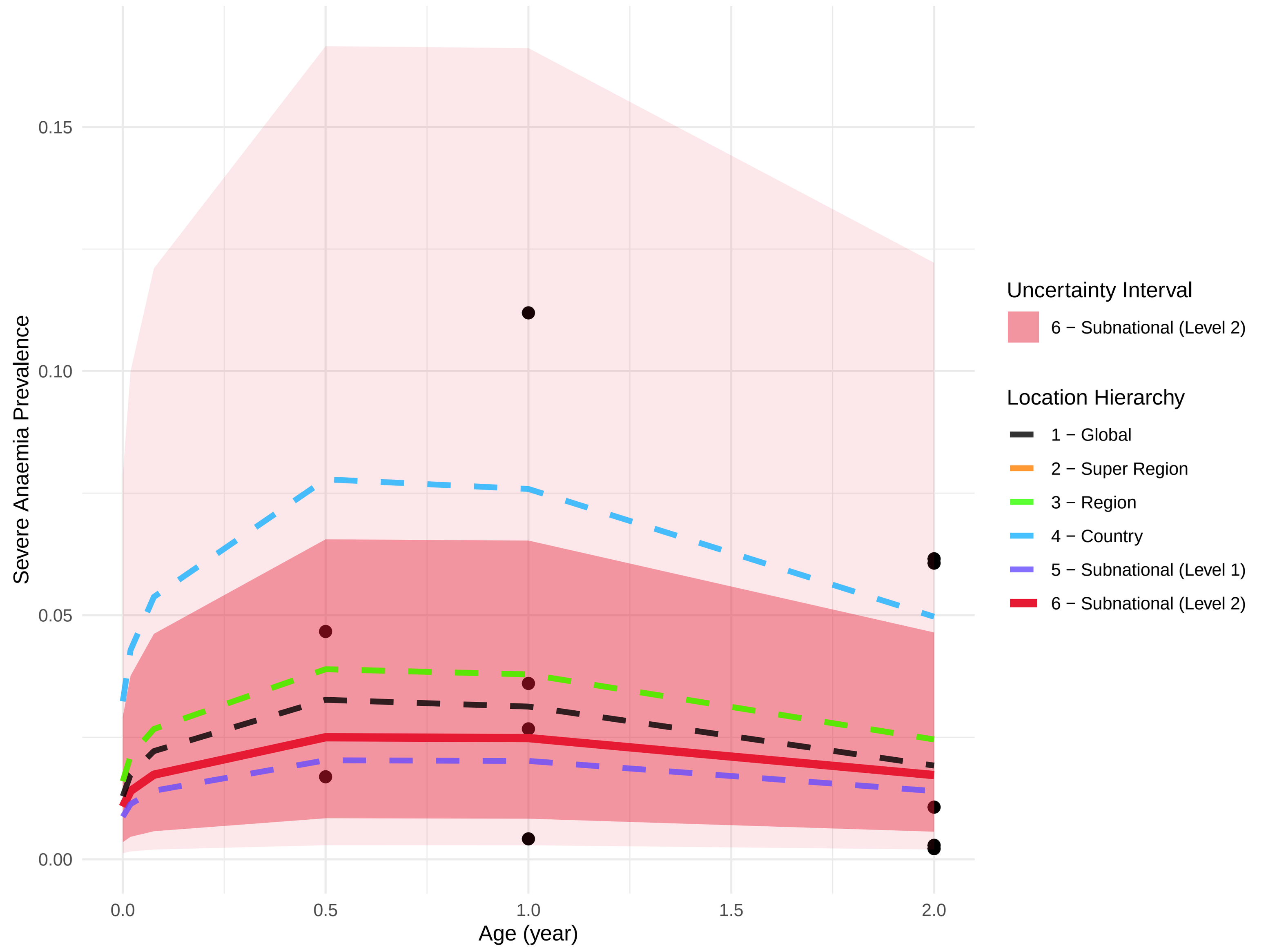

MR−BRT Age/Sex Fits − Non−pregnant Females <2 Years Old − Severe Anaemia Prevalence − Nepal
WHO−2024 Adjustment Method

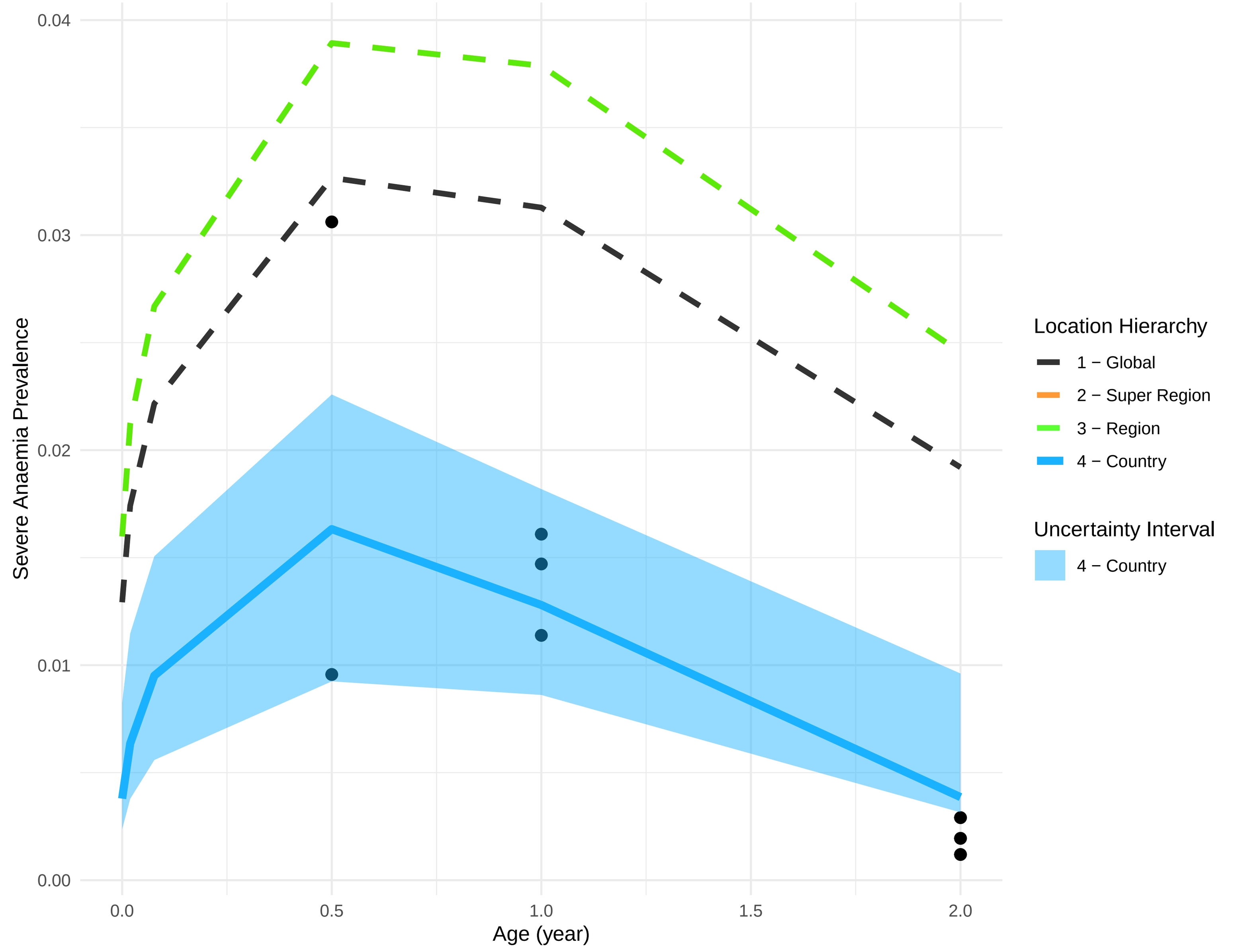

MR−BRT Age/Sex Fits − Non−pregnant Females <2 Years Old − Severe Anaemia Prevalence − Pakistan
WHO−2024 Adjustment Method

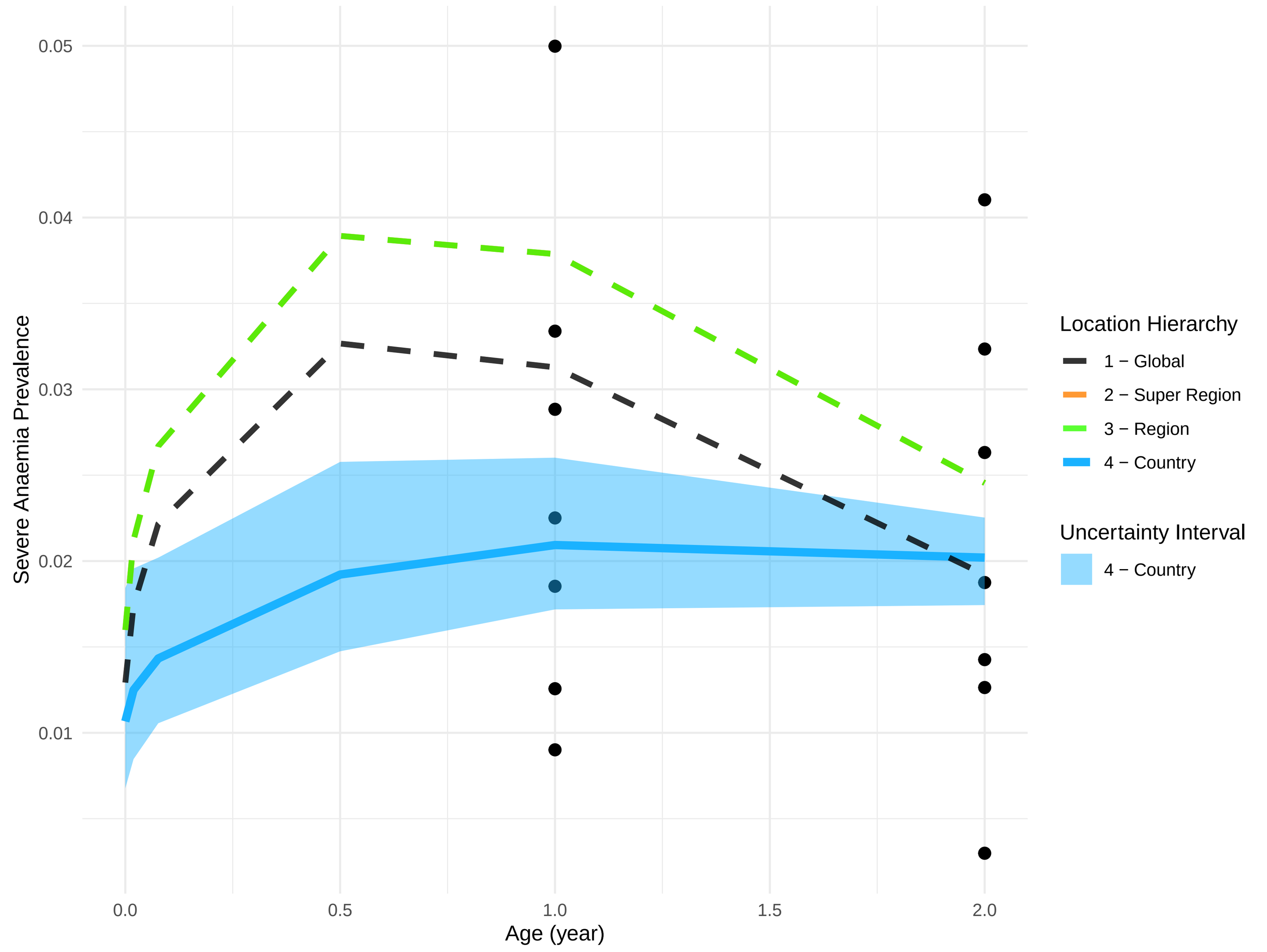

MR−BRT Age/Sex Fits − Non−pregnant Females <2 Years Old − Severe Anaemia Prevalence − Azad Jammu & Kashmir
WHO−2024 Adjustment Method

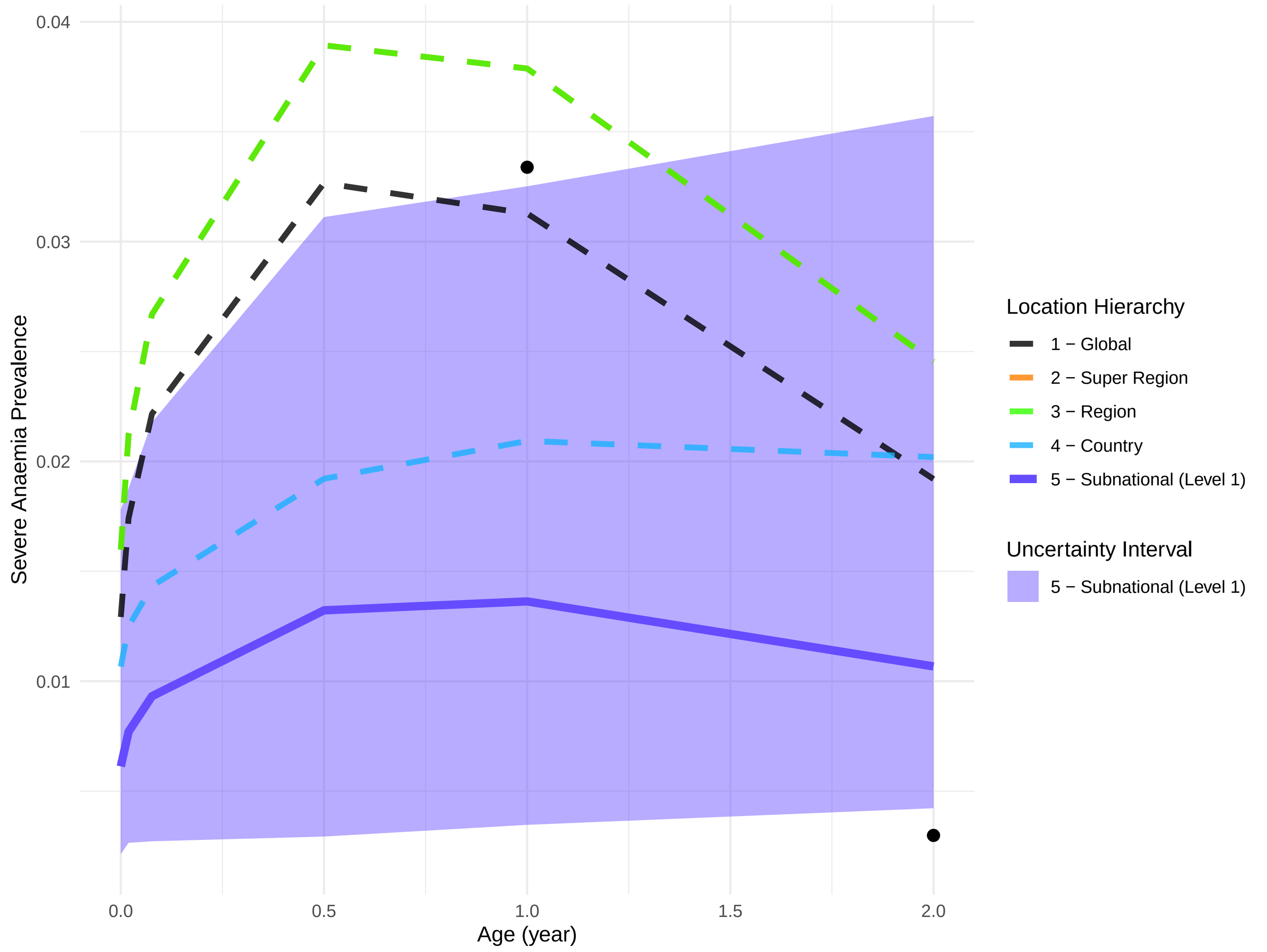

MR−BRT Age/Sex Fits − Non−pregnant Females <2 Years Old − Severe Anaemia Prevalence − Balochistan
WHO−2024 Adjustment Method

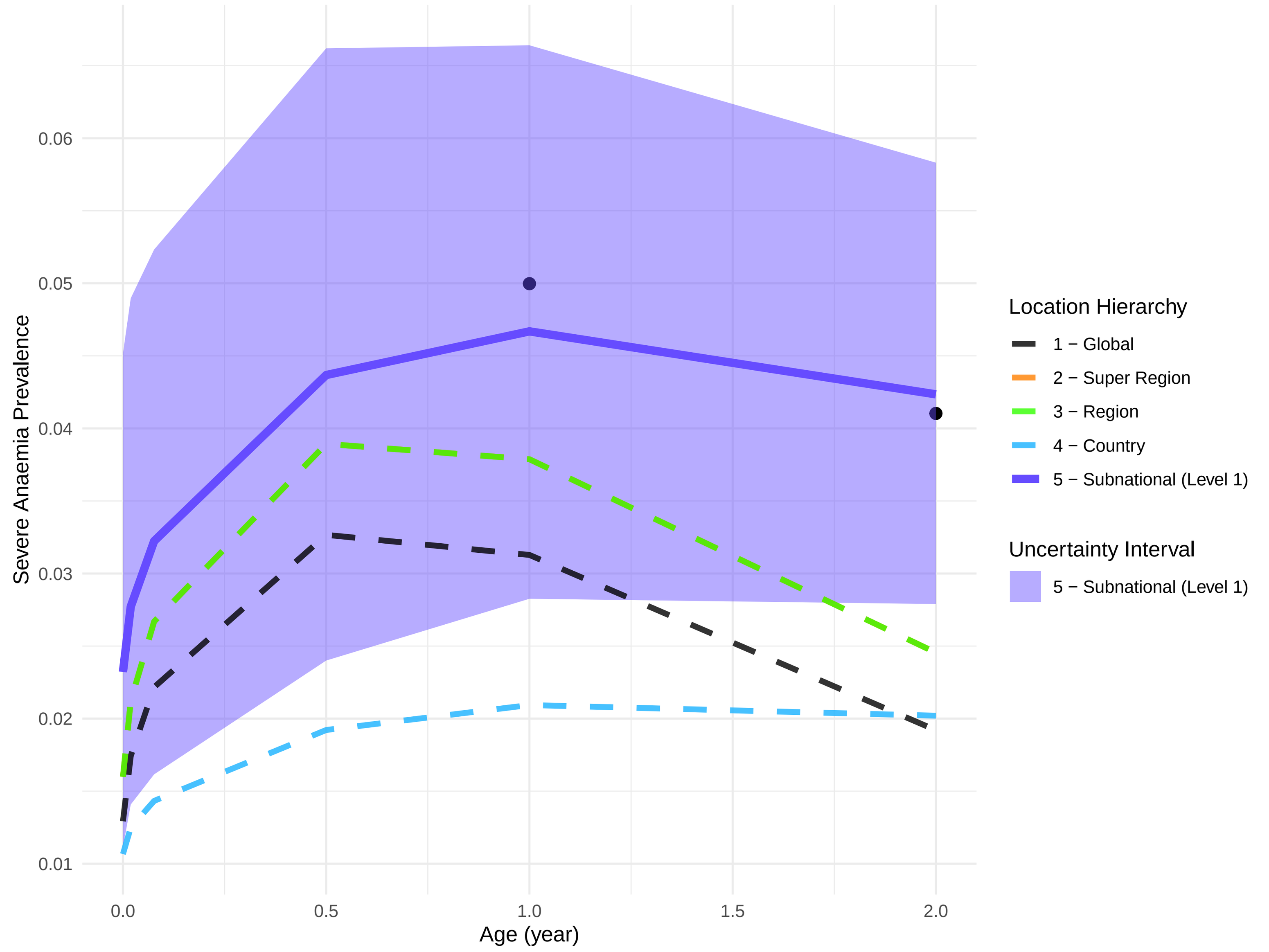

MR−BRT Age/Sex Fits − Non−pregnant Females <2 Years Old − Severe Anaemia Prevalence − Gilgit−Baltistan
WHO−2024 Adjustment Method

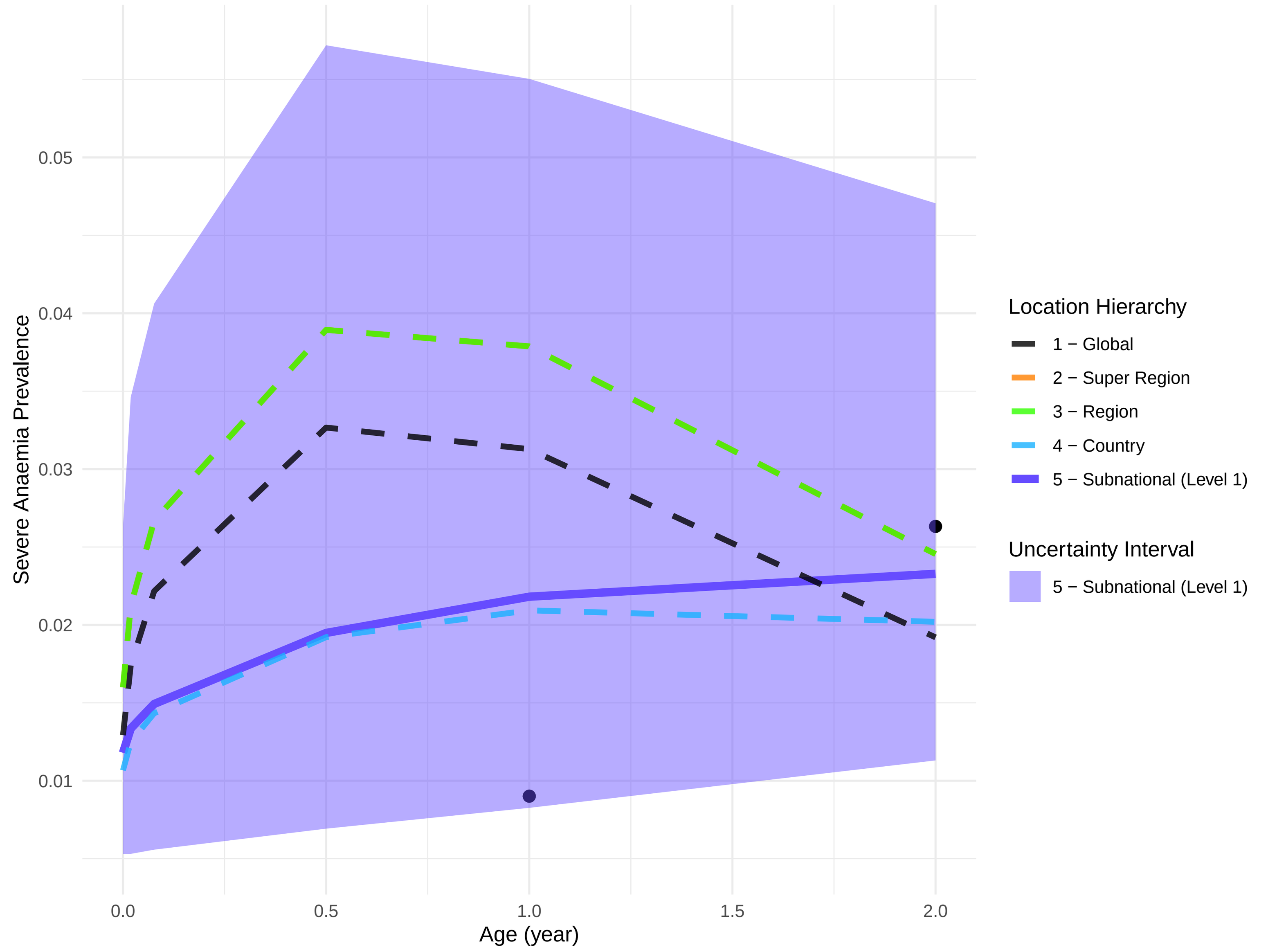

MR−BRT Age/Sex Fits − Non−pregnant Females <2 Years Old − Severe Anaemia Prevalence − Khyber Pakhtunkhwa
WHO−2024 Adjustment Method

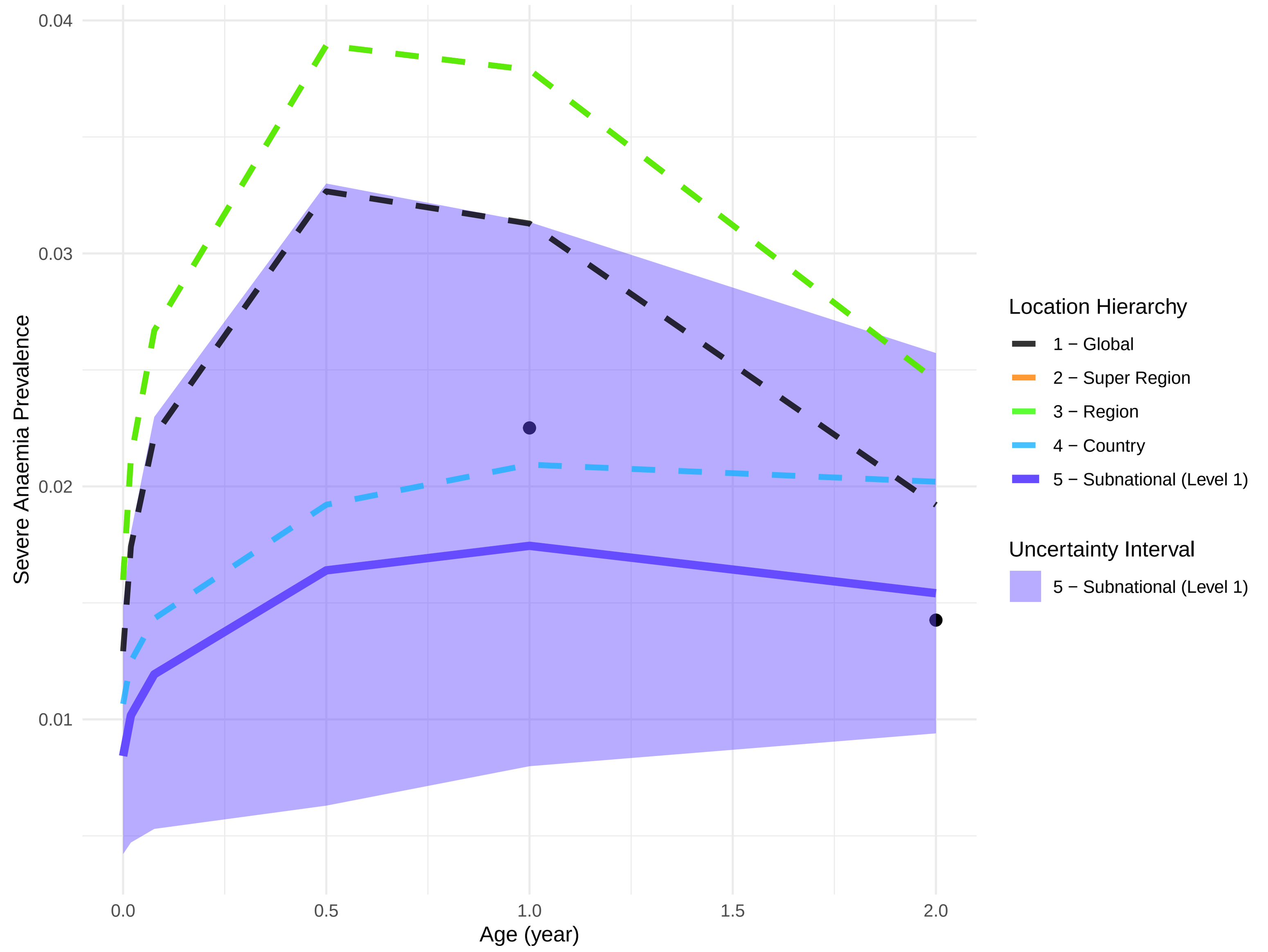

MR−BRT Age/Sex Fits − Non−pregnant Females <2 Years Old − Severe Anaemia Prevalence − Punjab
WHO−2024 Adjustment Method

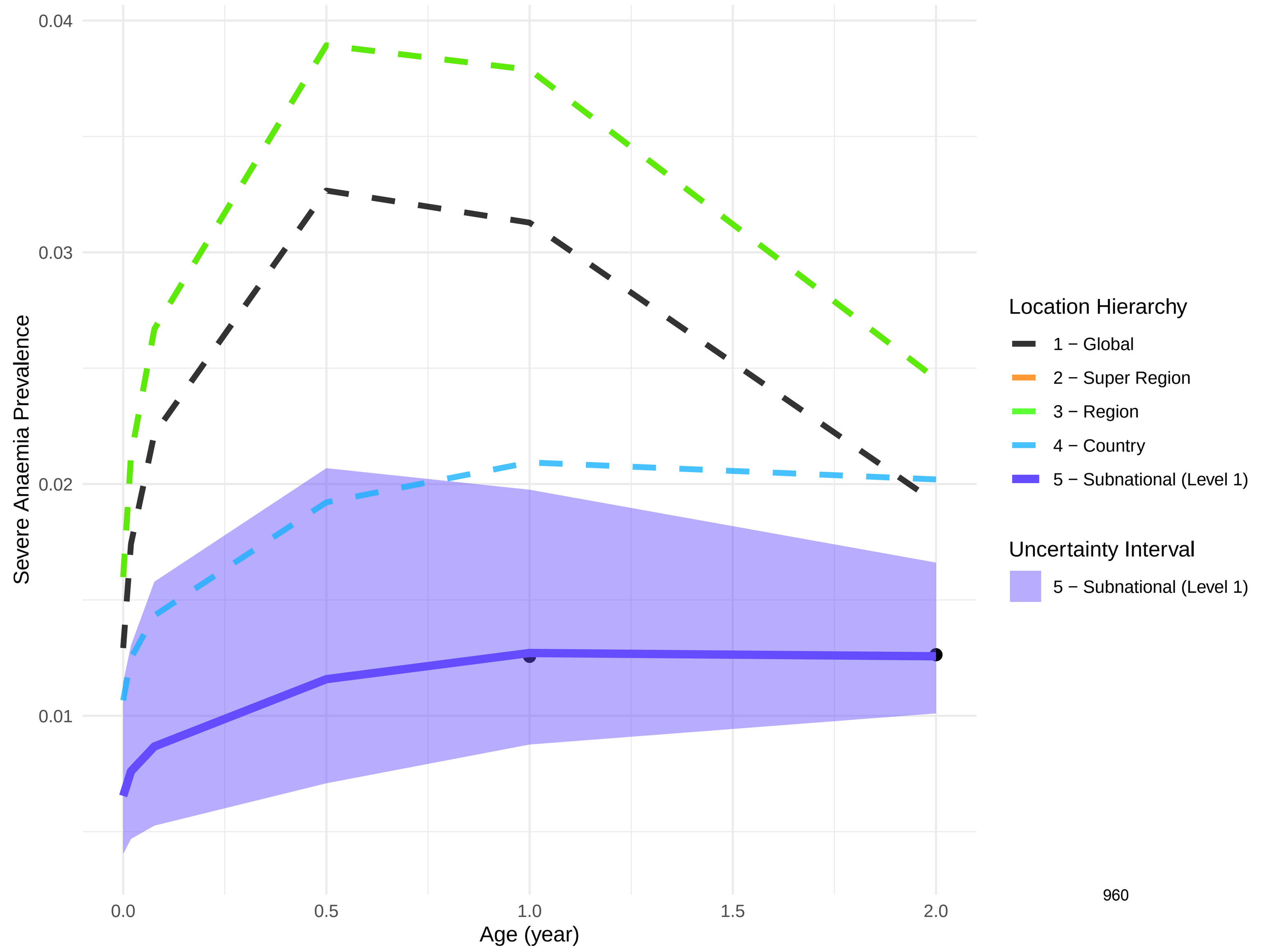

MR−BRT Age/Sex Fits − Non−pregnant Females <2 Years Old − Severe Anaemia Prevalence − Sindh
WHO−2024 Adjustment Method

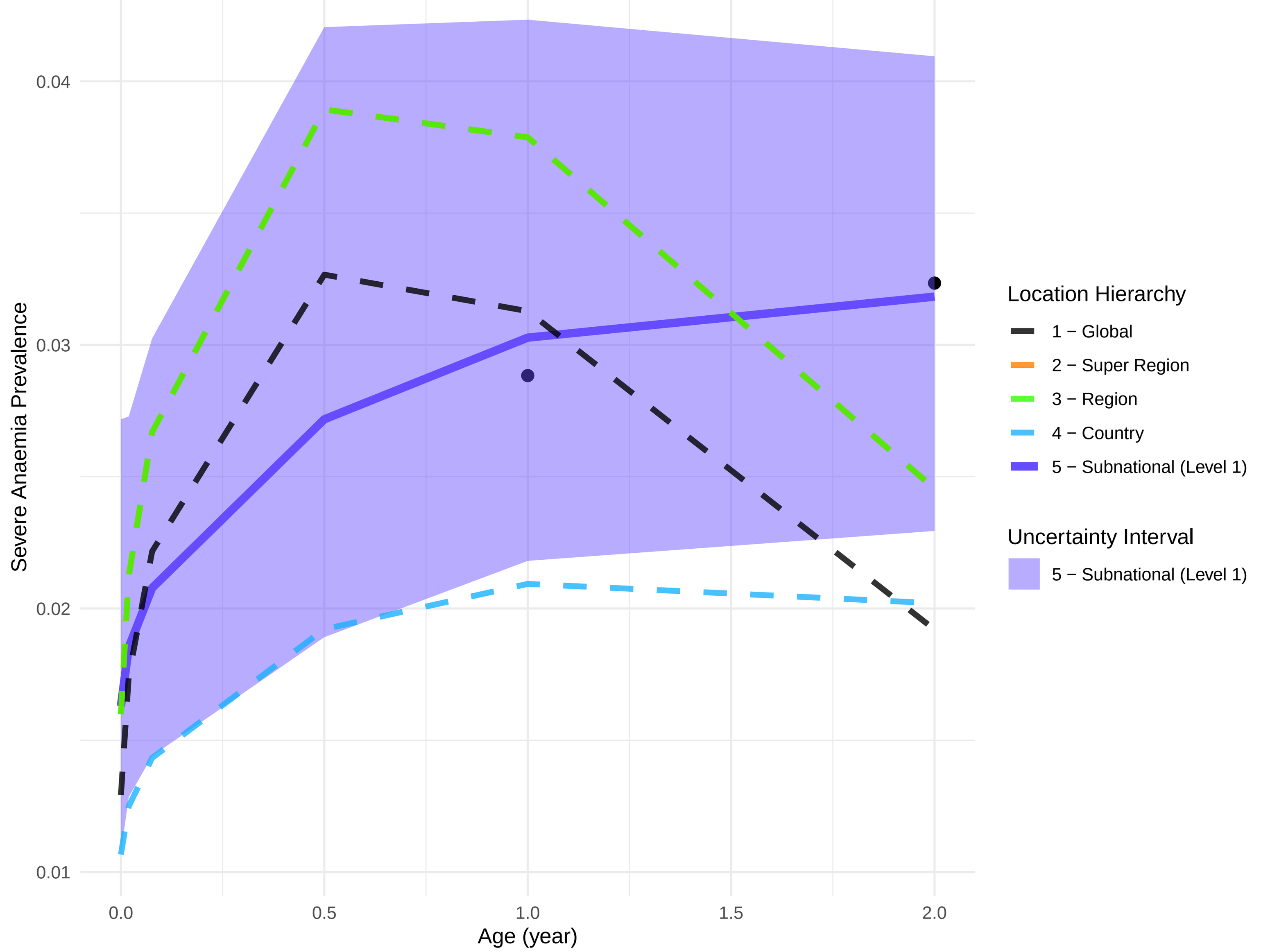

MR−BRT Age/Sex Fits − Non−pregnant Females <2 Years Old − Severe Anaemia Prevalence − Southeast Asia, East Asia, and Oceania
WHO−2024 Adjustment Method

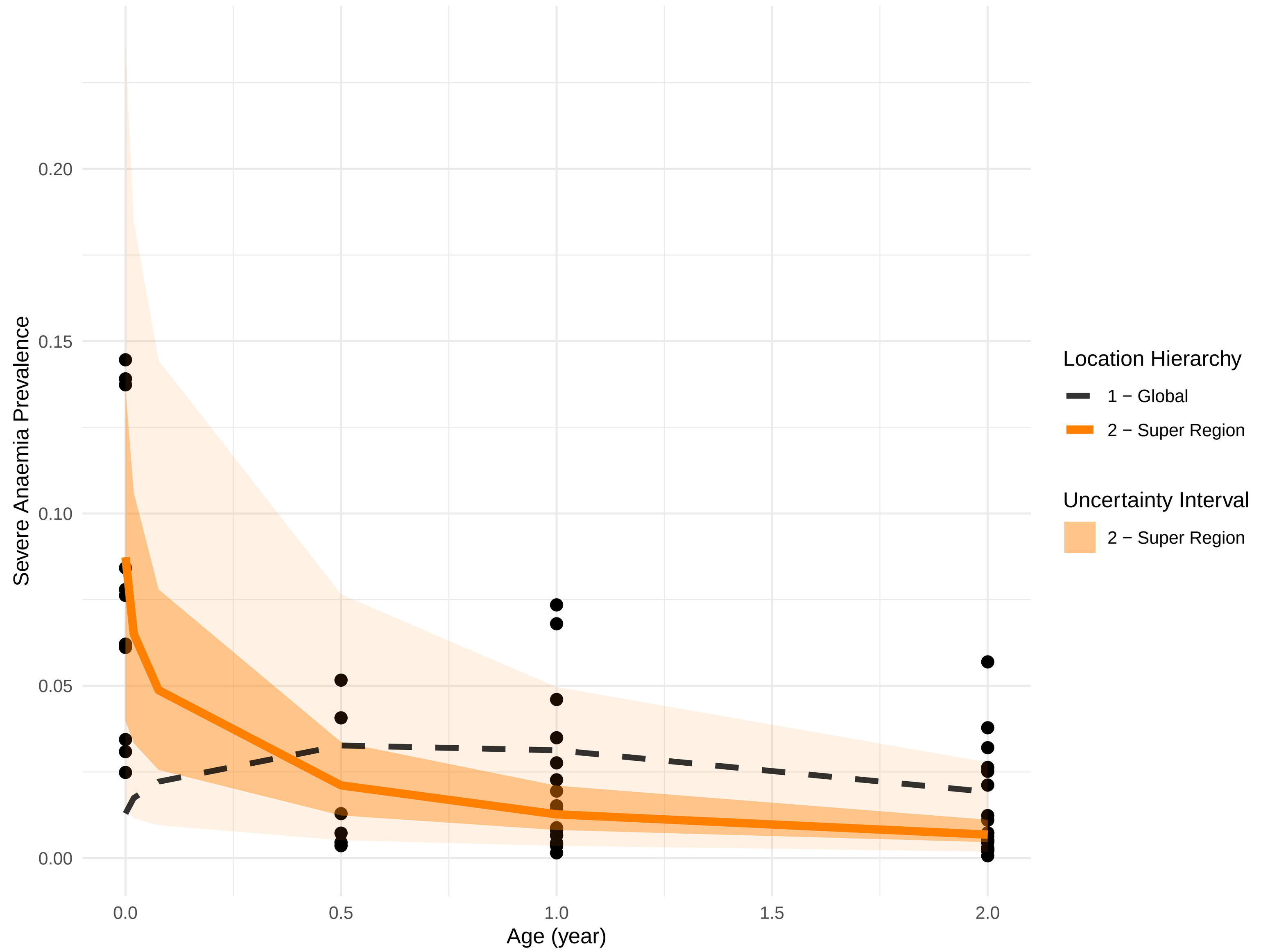

MR−BRT Age/Sex Fits − Non−pregnant Females <2 Years Old − Severe Anaemia Prevalence − Southeast Asia
WHO−2024 Adjustment Method

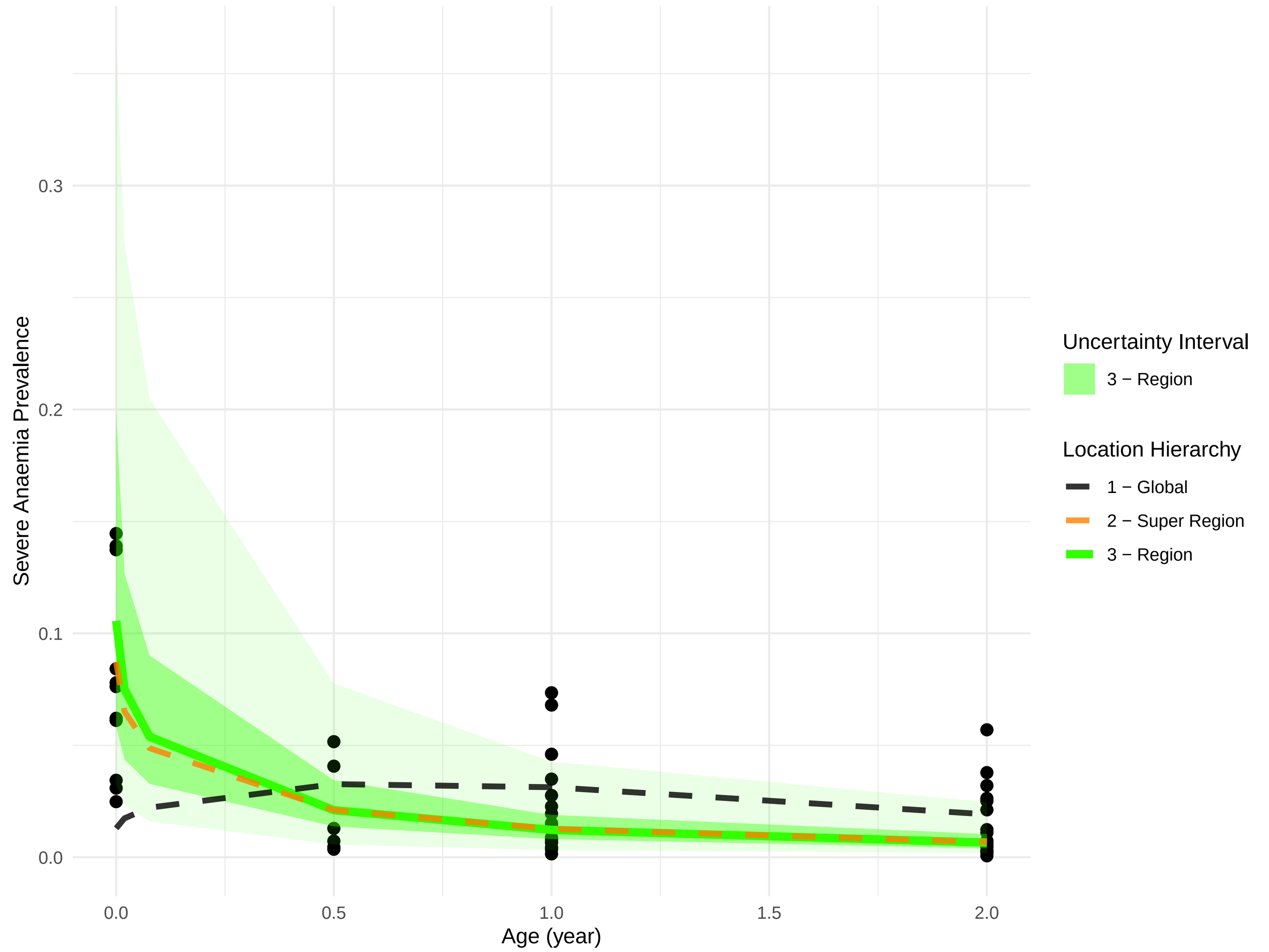

MR−BRT Age/Sex Fits − Non−pregnant Females <2 Years Old − Severe Anaemia Prevalence − Cambodia
WHO−2024 Adjustment Method

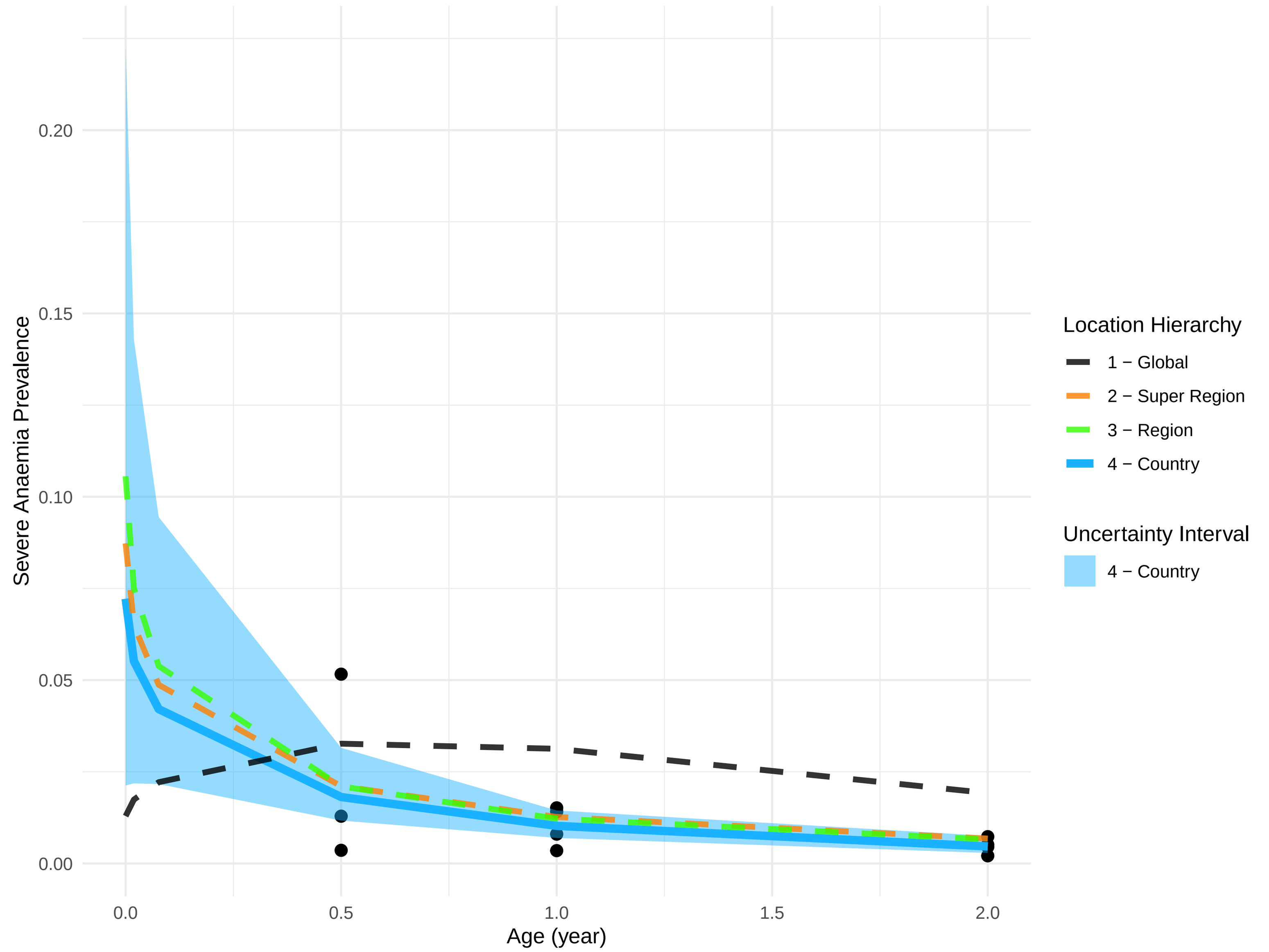

MR−BRT Age/Sex Fits − Non−pregnant Females <2 Years Old − Severe Anaemia Prevalence − Indonesia
WHO−2024 Adjustment Method

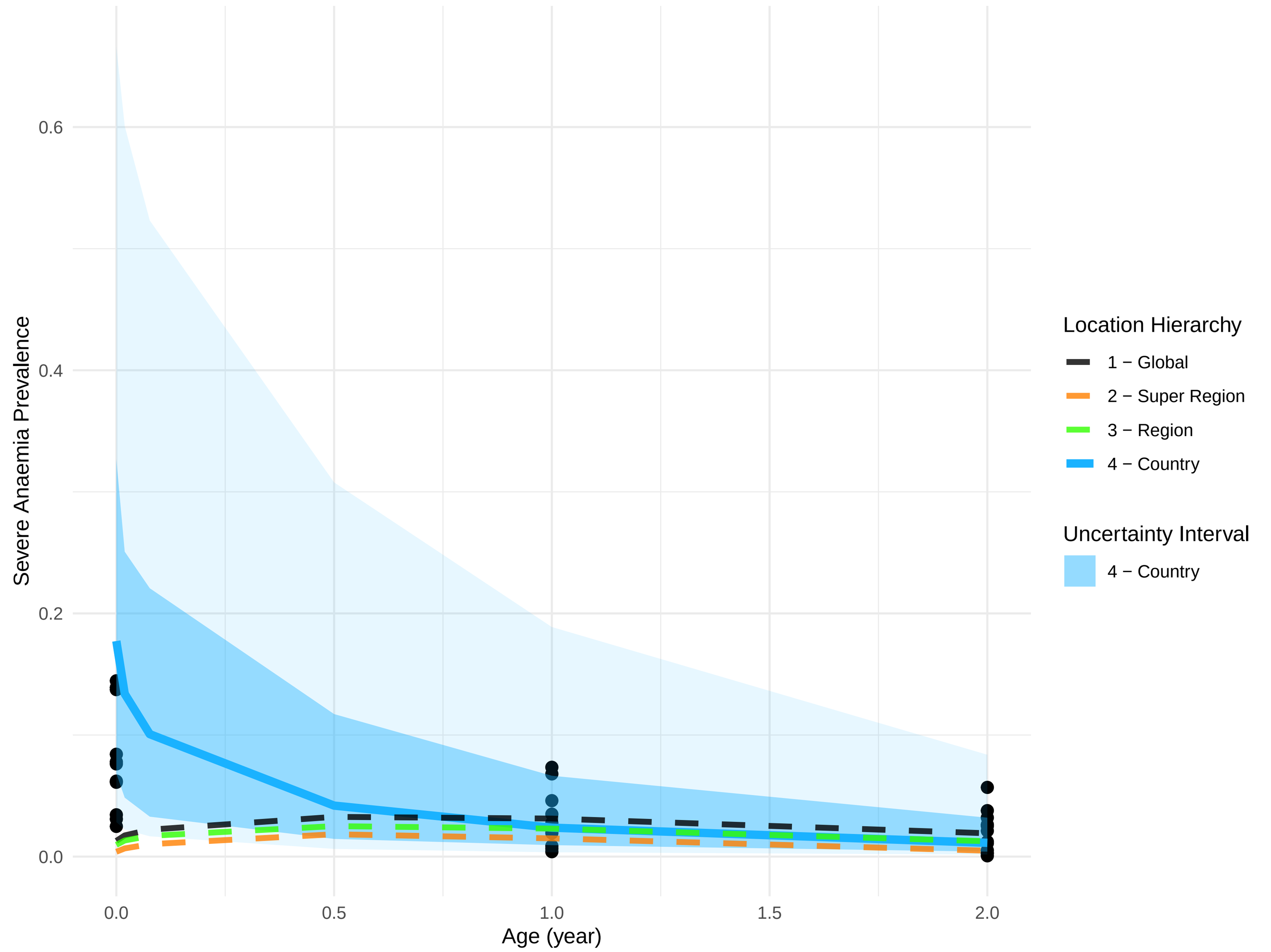

MR−BRT Age/Sex Fits − Non−pregnant Females <2 Years Old − Severe Anaemia Prevalence − Banten
WHO−2024 Adjustment Method

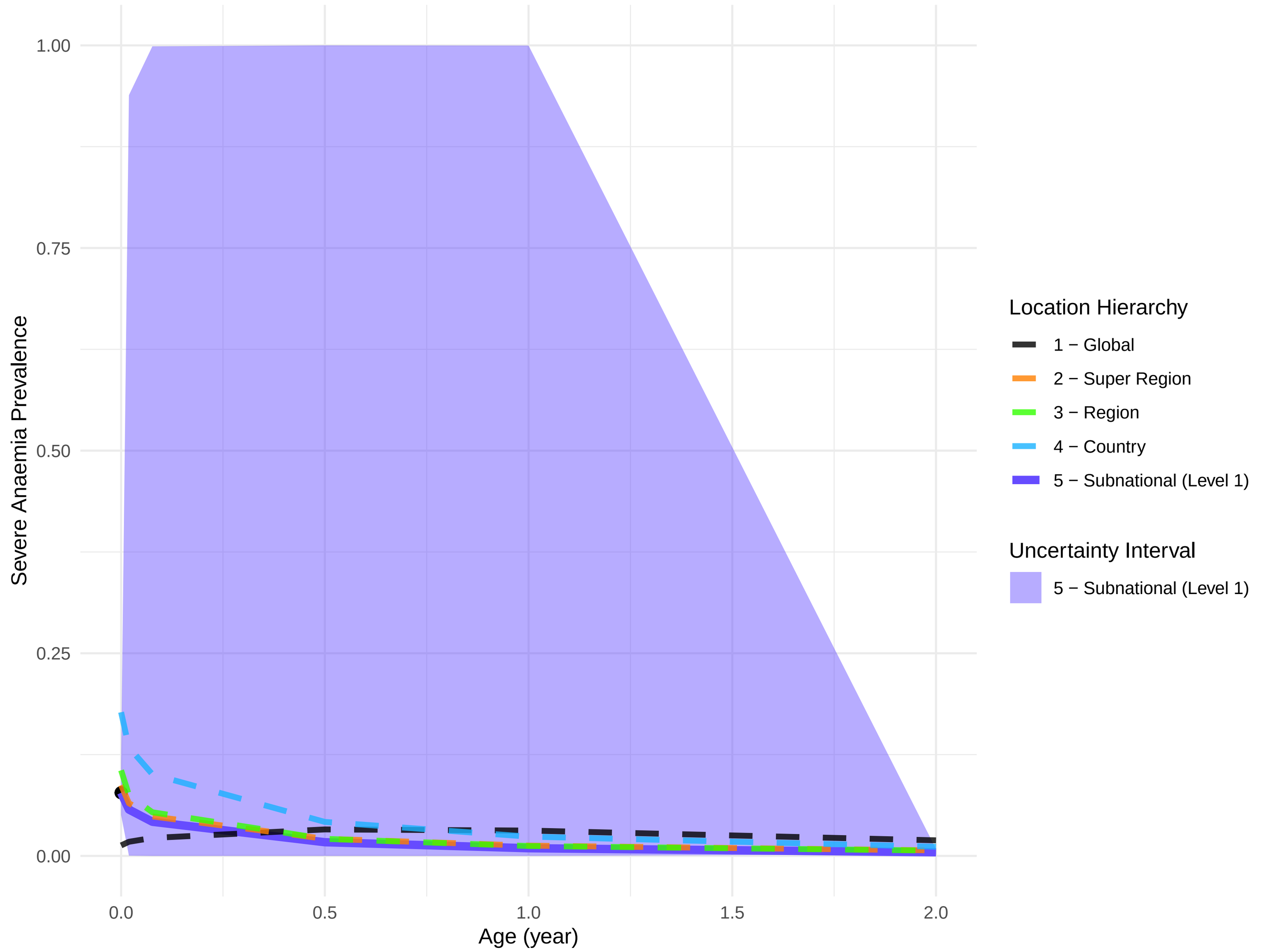

MR−BRT Age/Sex Fits − Non−pregnant Females <2 Years Old − Severe Anaemia Prevalence − West Java
WHO−2024 Adjustment Method

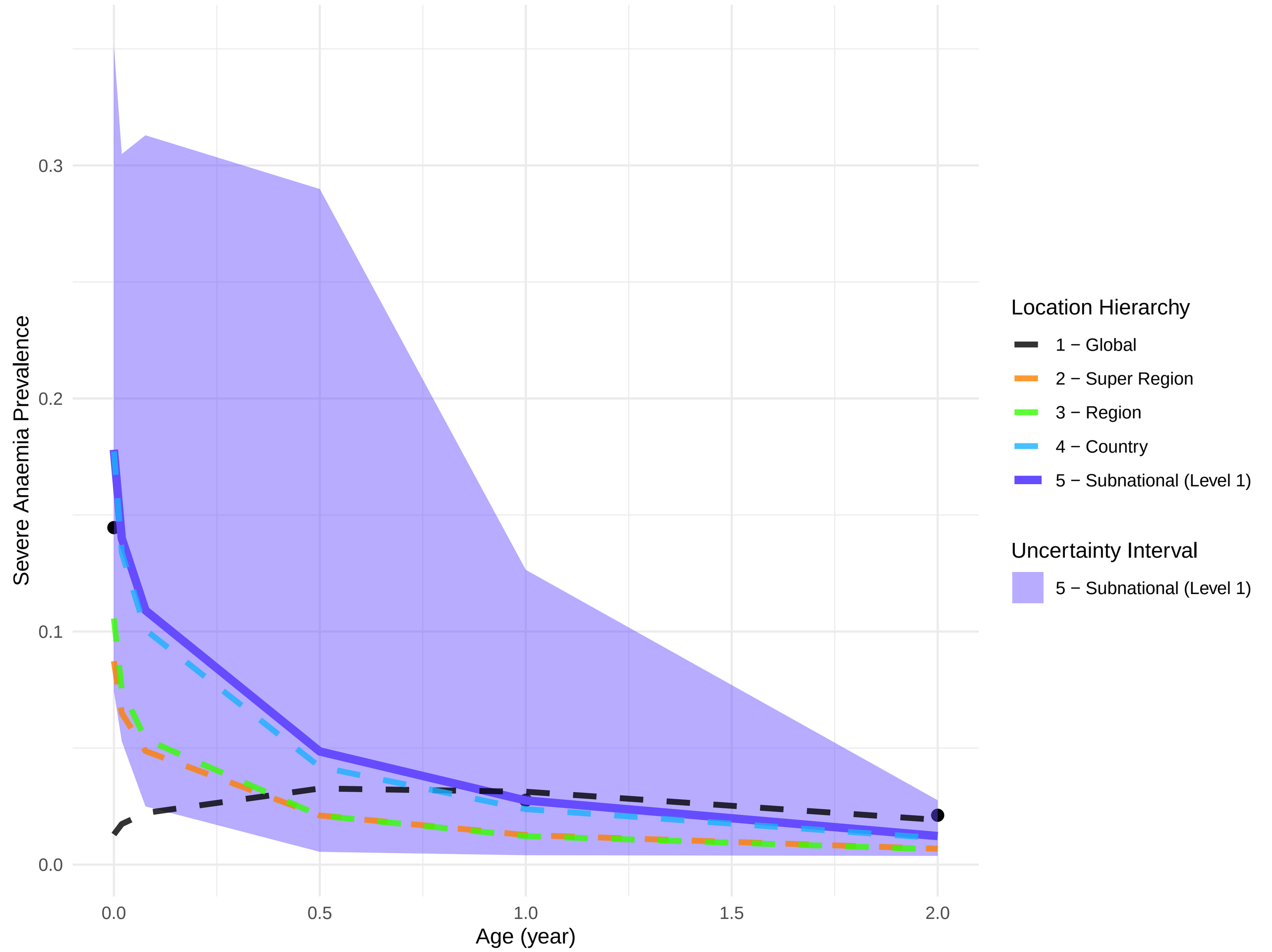

MR−BRT Age/Sex Fits − Non−pregnant Females <2 Years Old − Severe Anaemia Prevalence − Central Java
WHO−2024 Adjustment Method

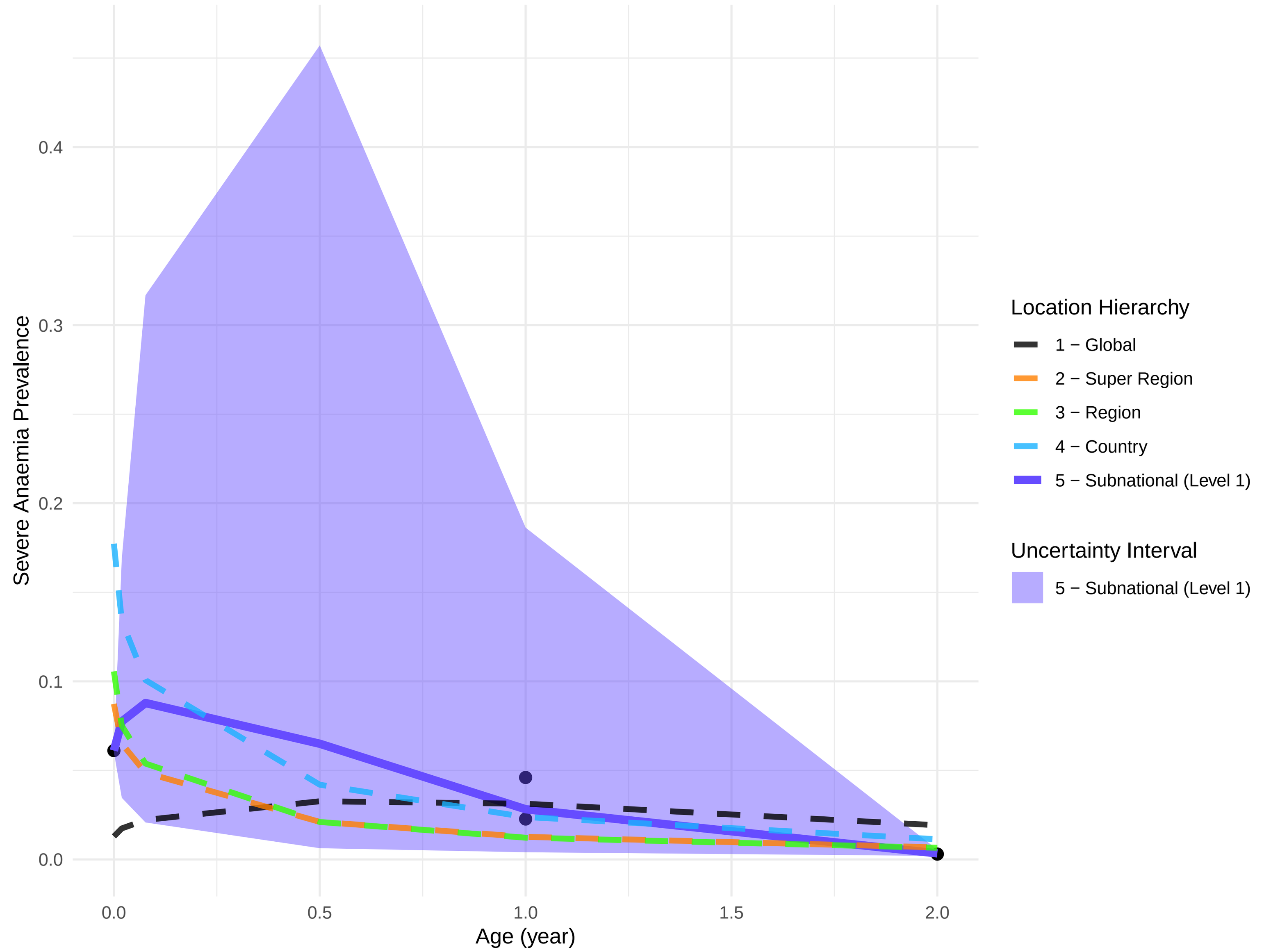

MR−BRT Age/Sex Fits − Non−pregnant Females <2 Years Old − Severe Anaemia Prevalence − East Java
WHO−2024 Adjustment Method

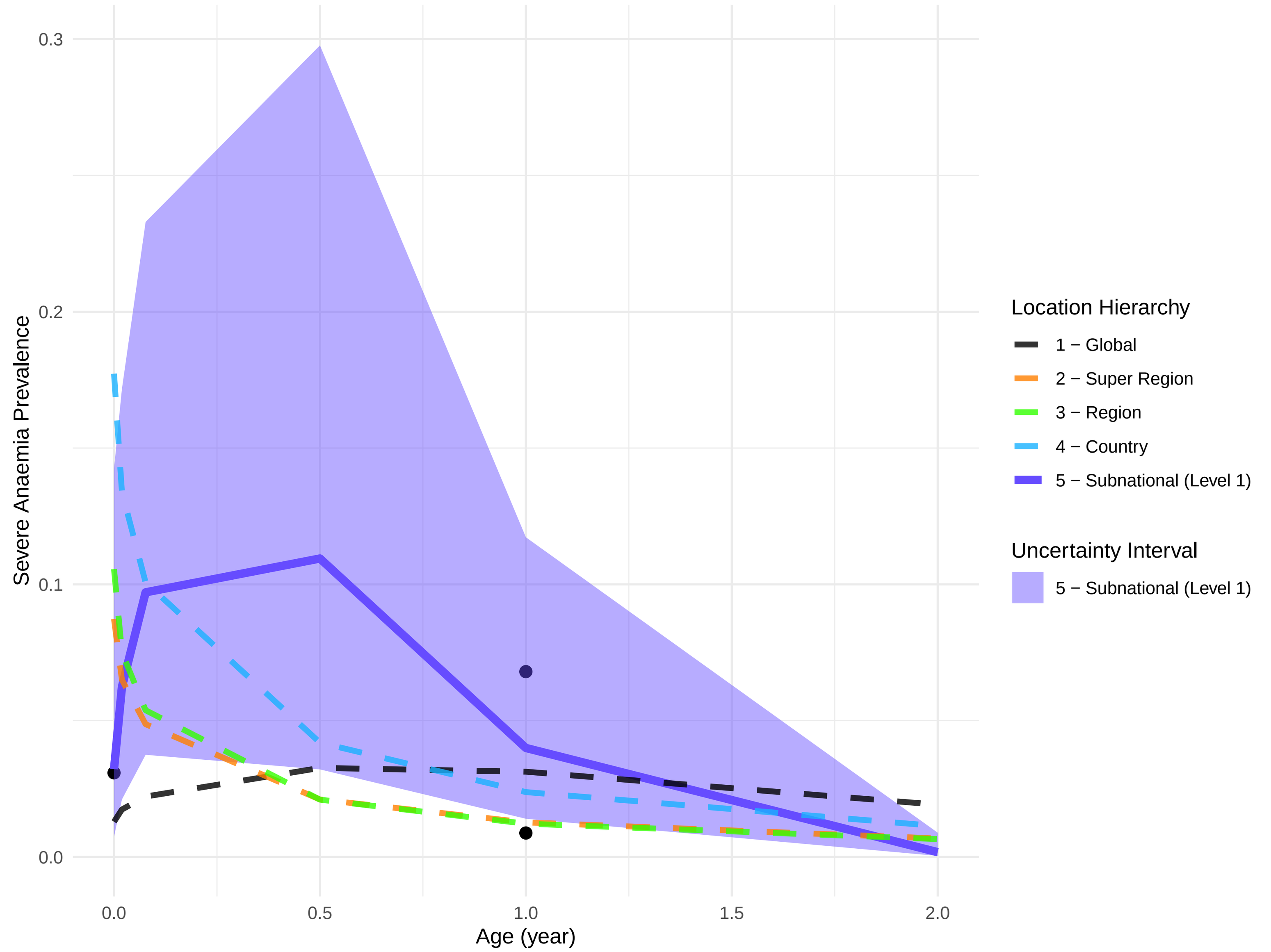

MR−BRT Age/Sex Fits − Non−pregnant Females <2 Years Old − Severe Anaemia Prevalence − South Kalimantan
WHO−2024 Adjustment Method

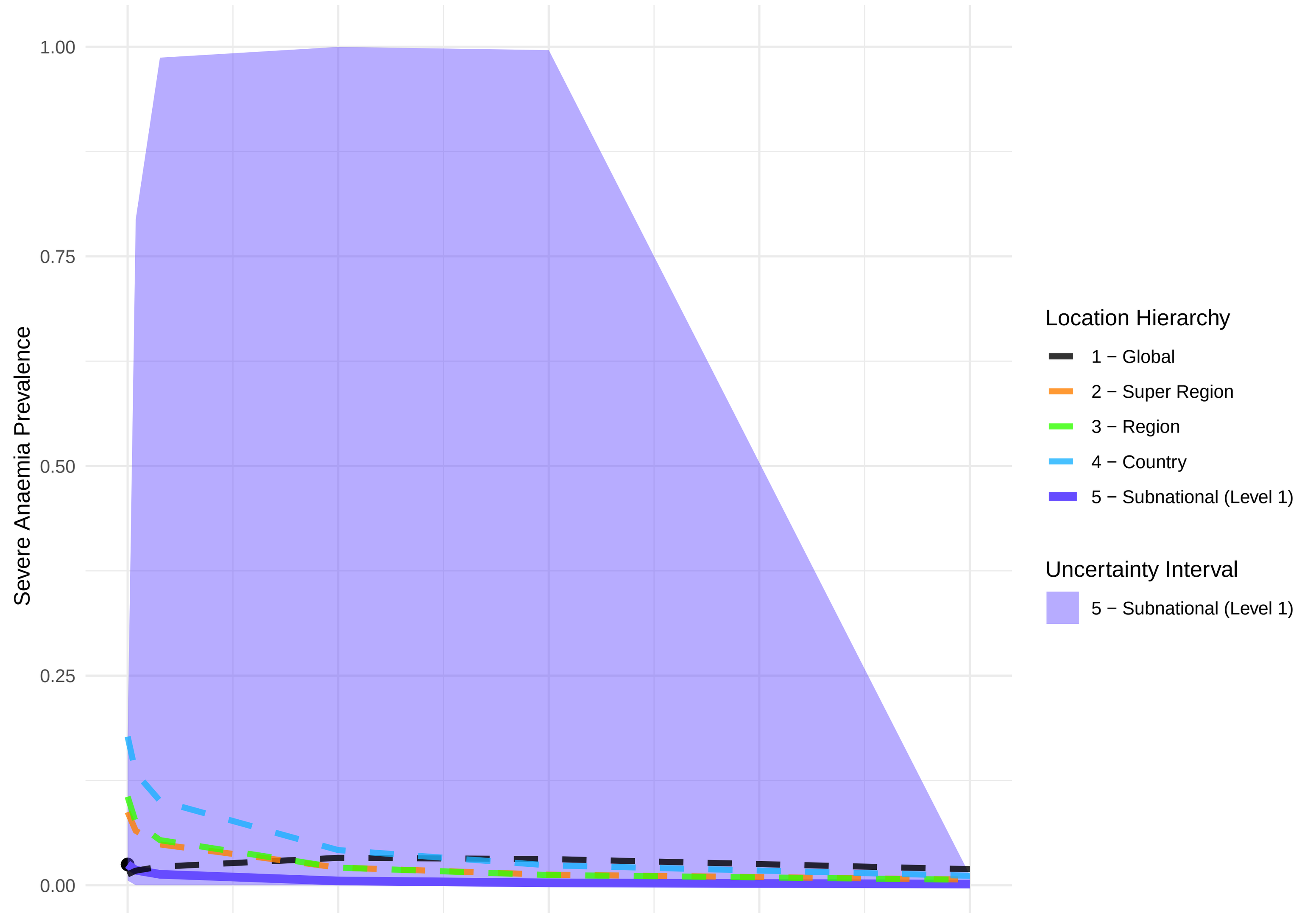

MR−BRT Age/Sex Fits − Non−pregnant Females <2 Years Old − Severe Anaemia Prevalence − Lampung
WHO−2024 Adjustment Method

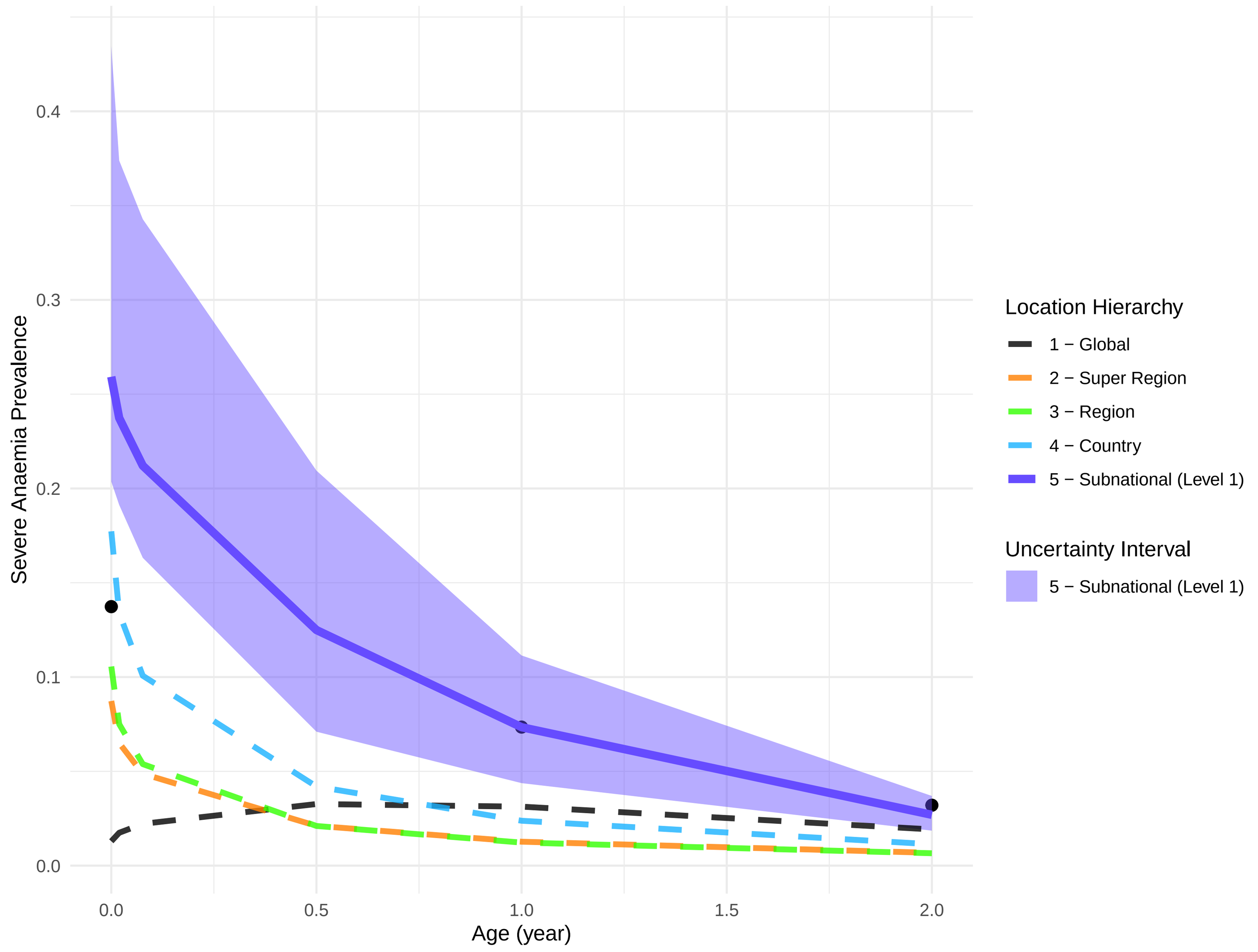

MR−BRT Age/Sex Fits − Non−pregnant Females <2 Years Old − Severe Anaemia Prevalence − West Nusa Tenggara
WHO−2024 Adjustment Method

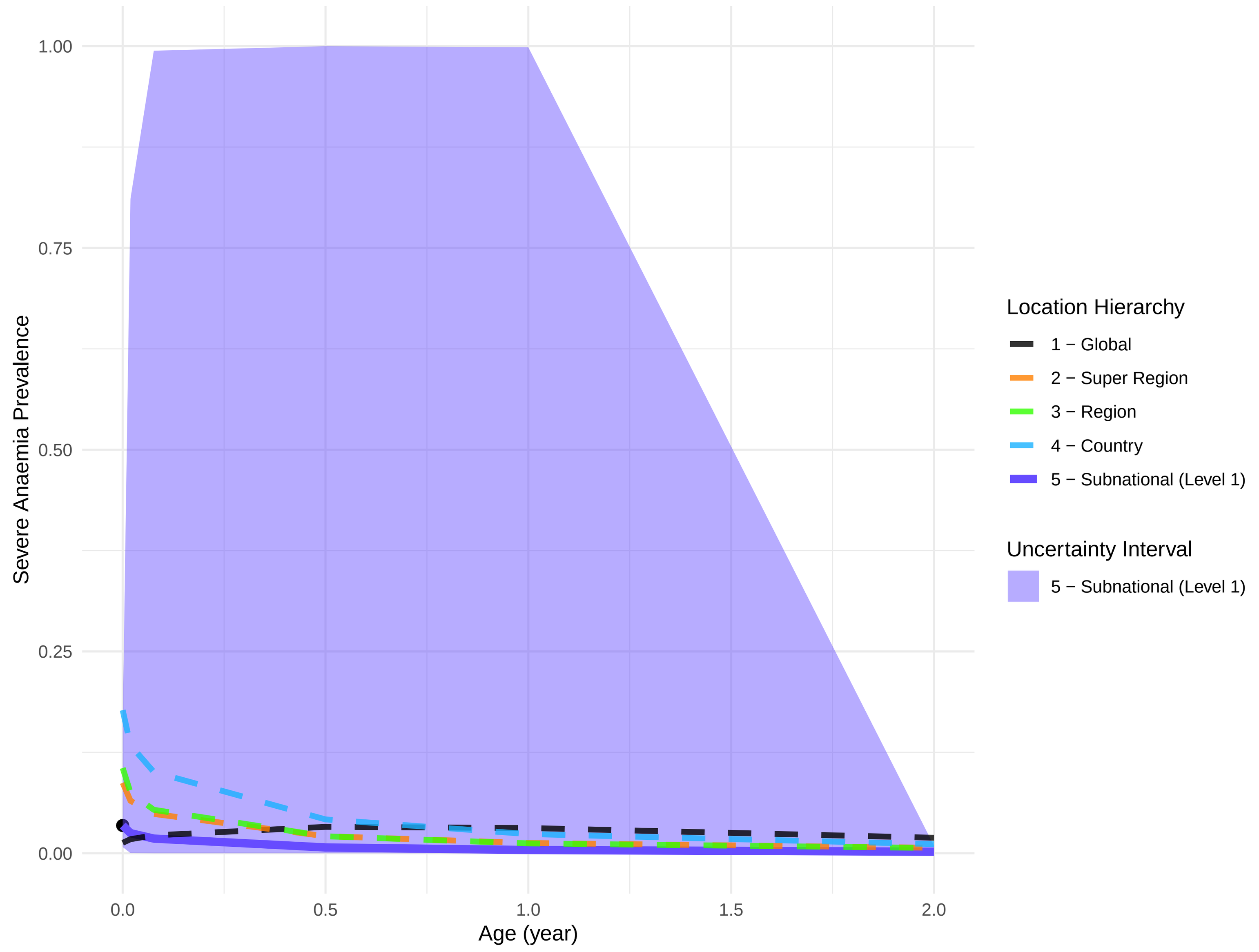

MR−BRT Age/Sex Fits − Non−pregnant Females <2 Years Old − Severe Anaemia Prevalence − South Sulawesi
WHO−2024 Adjustment Method

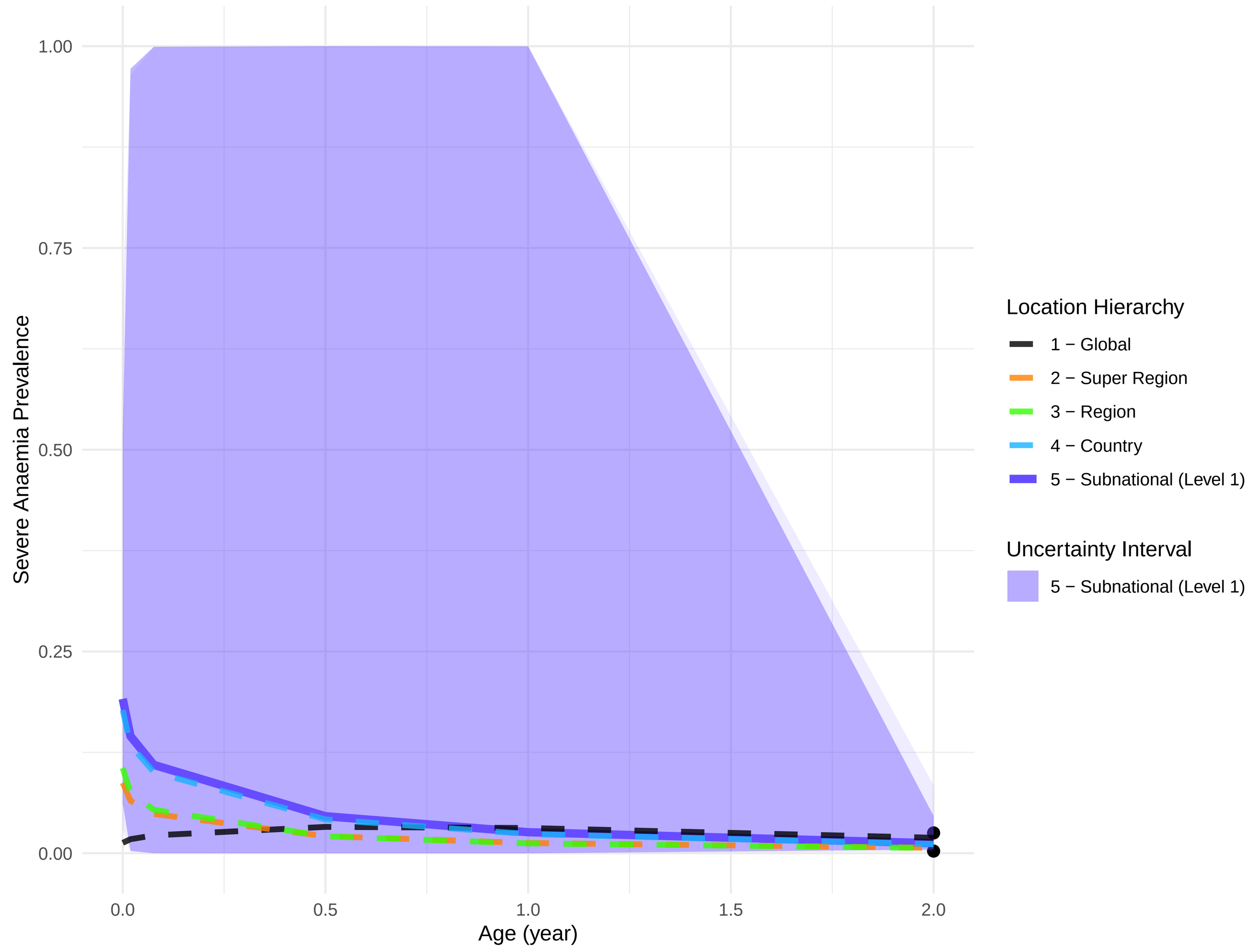

MR−BRT Age/Sex Fits − Non−pregnant Females <2 Years Old − Severe Anaemia Prevalence − West Sumatra
WHO−2024 Adjustment Method

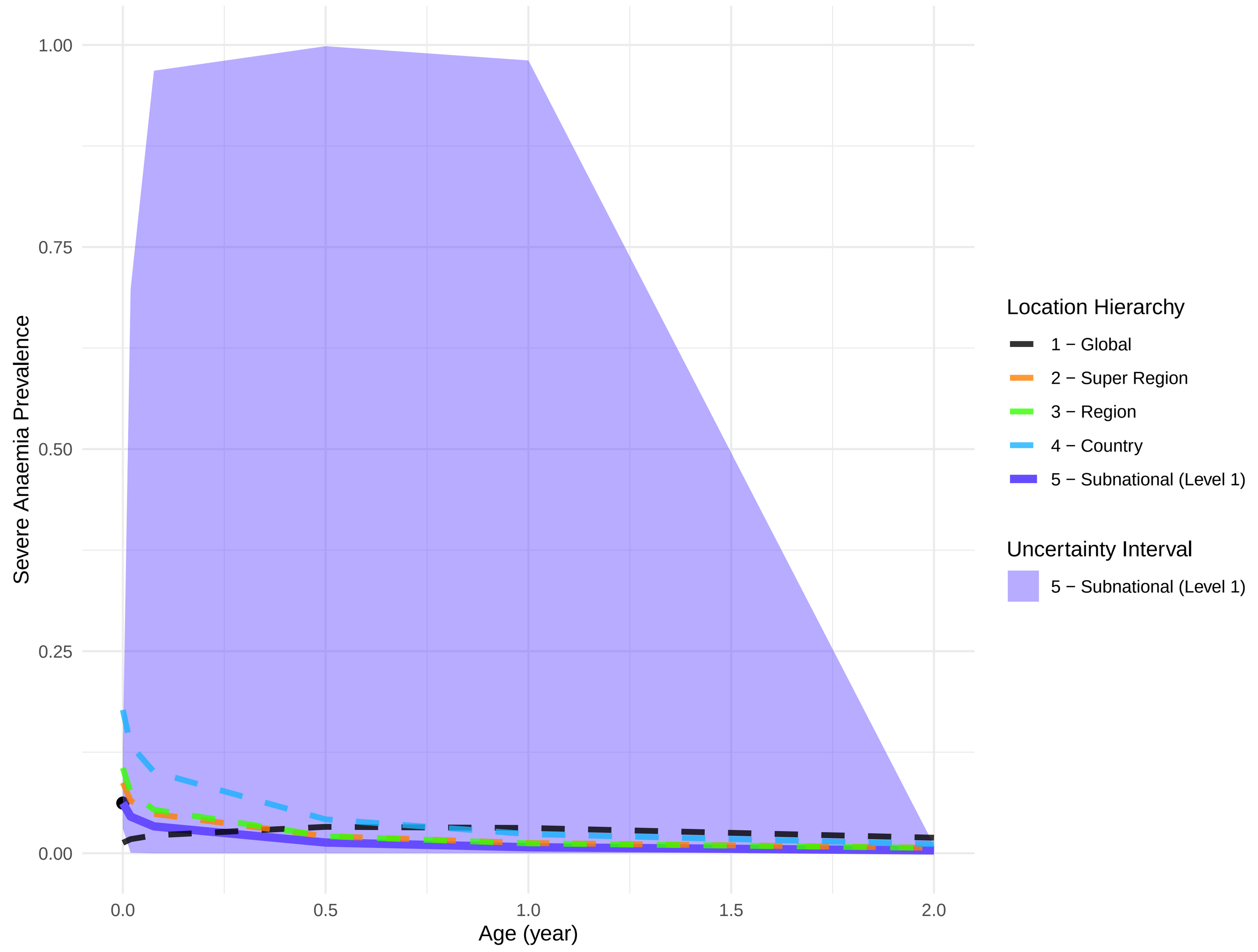

MR−BRT Age/Sex Fits − Non−pregnant Females <2 Years Old − Severe Anaemia Prevalence − South Sumatra
WHO−2024 Adjustment Method

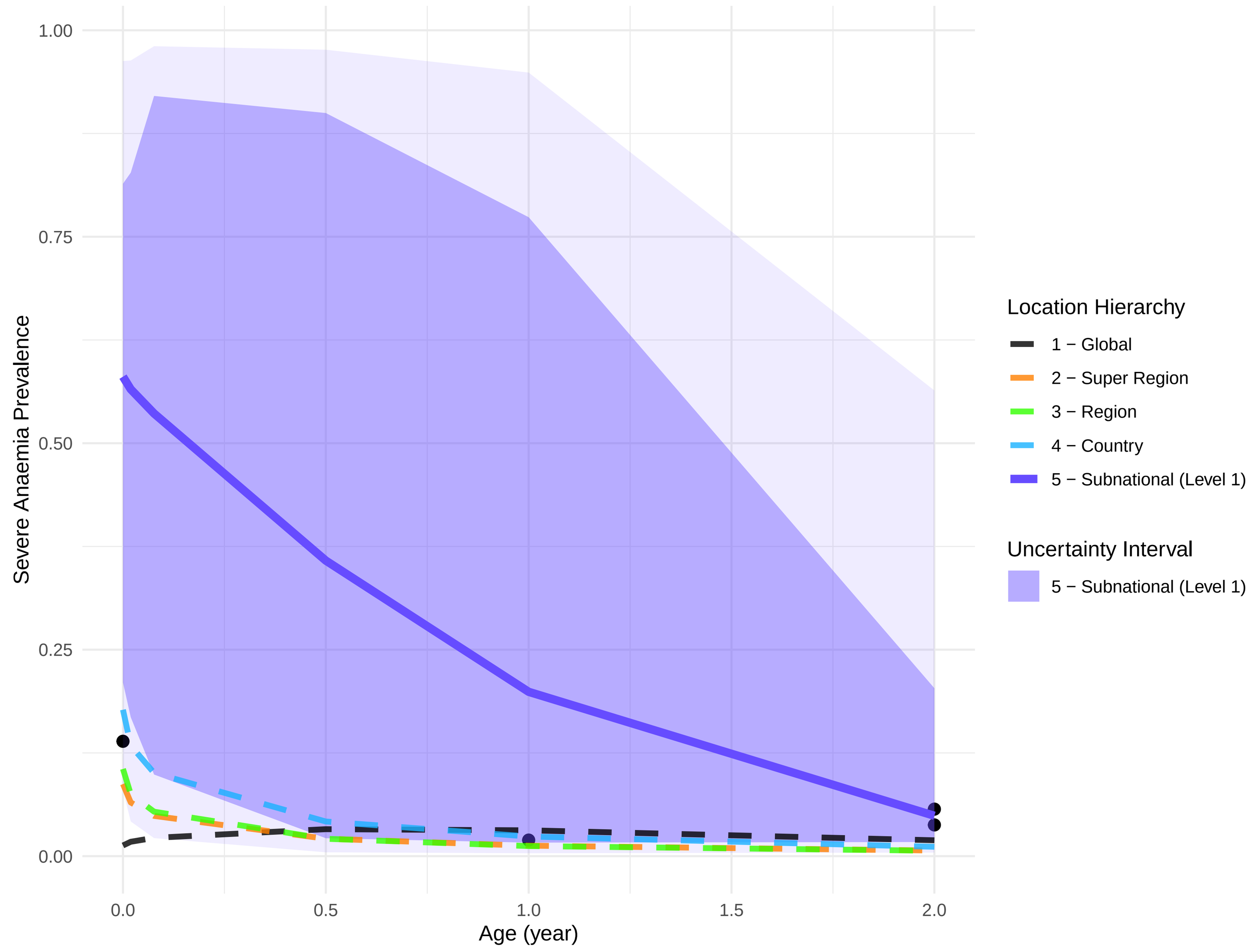

MR−BRT Age/Sex Fits − Non−pregnant Females <2 Years Old − Severe Anaemia Prevalence − North Sumatra
WHO−2024 Adjustment Method

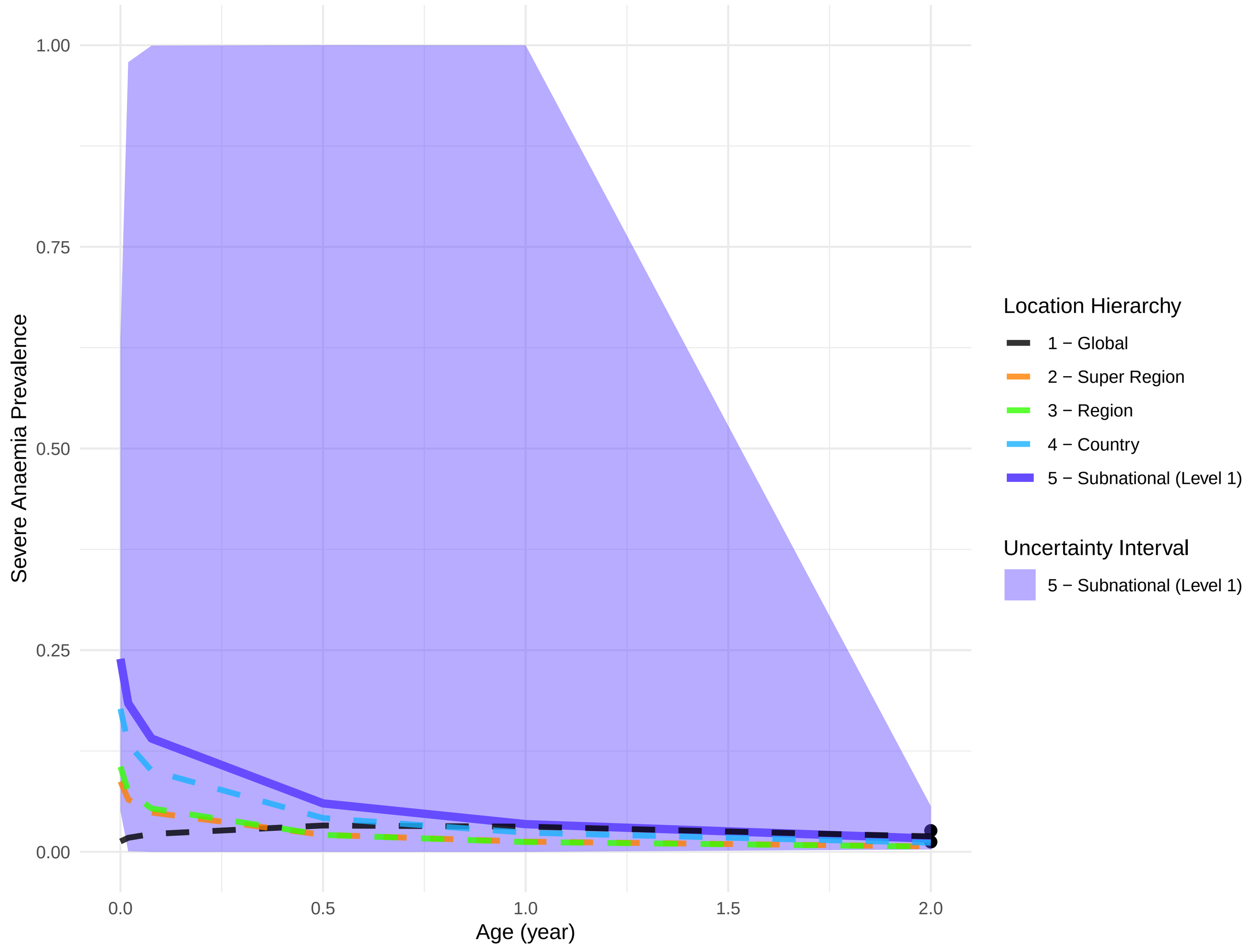

MR−BRT Age/Sex Fits − Non−pregnant Females <2 Years Old − Severe Anaemia Prevalence − Yogyakarta
WHO−2024 Adjustment Method

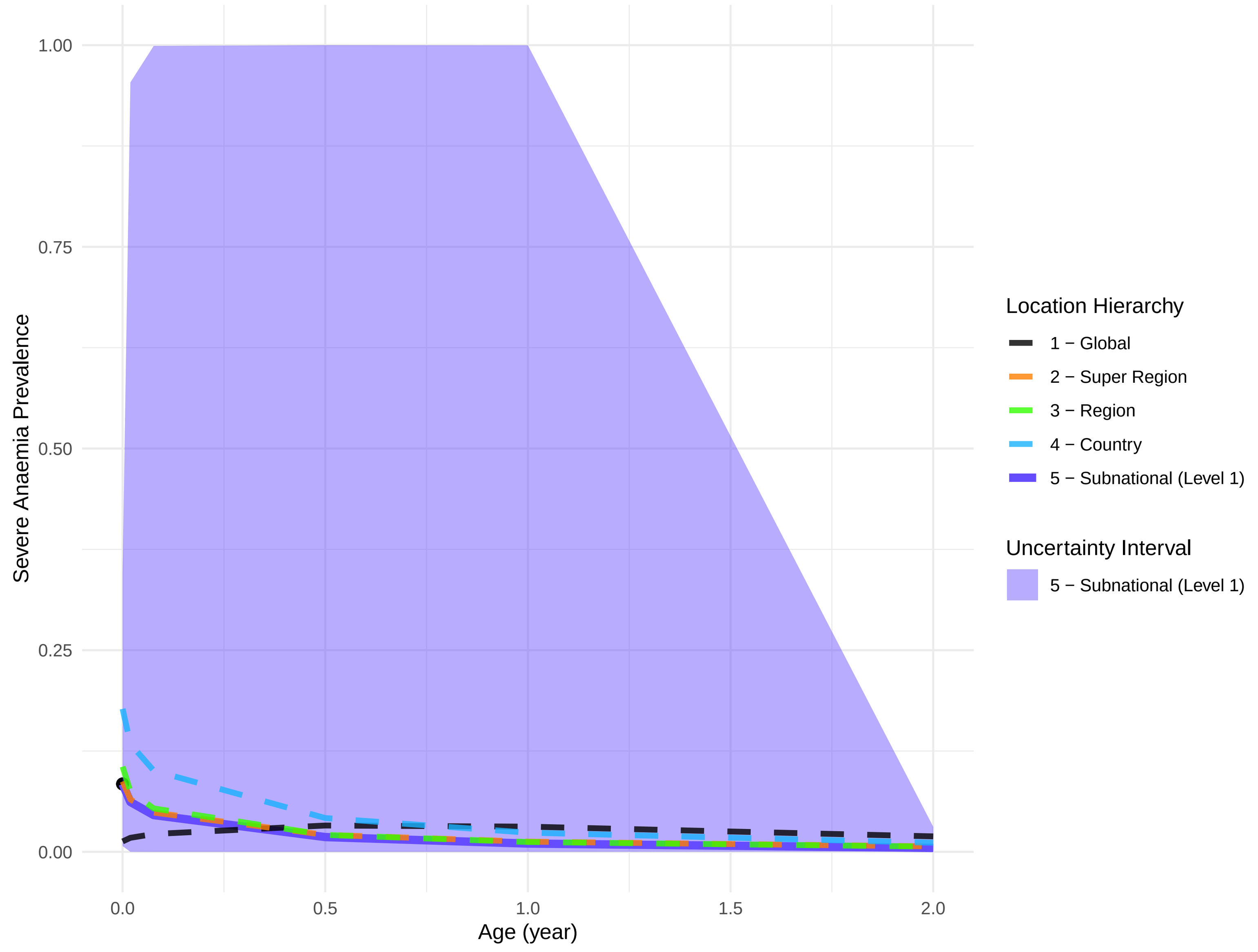

MR−BRT Age/Sex Fits − Non−pregnant Females <2 Years Old − Severe Anaemia Prevalence − Myanmar
WHO−2024 Adjustment Method

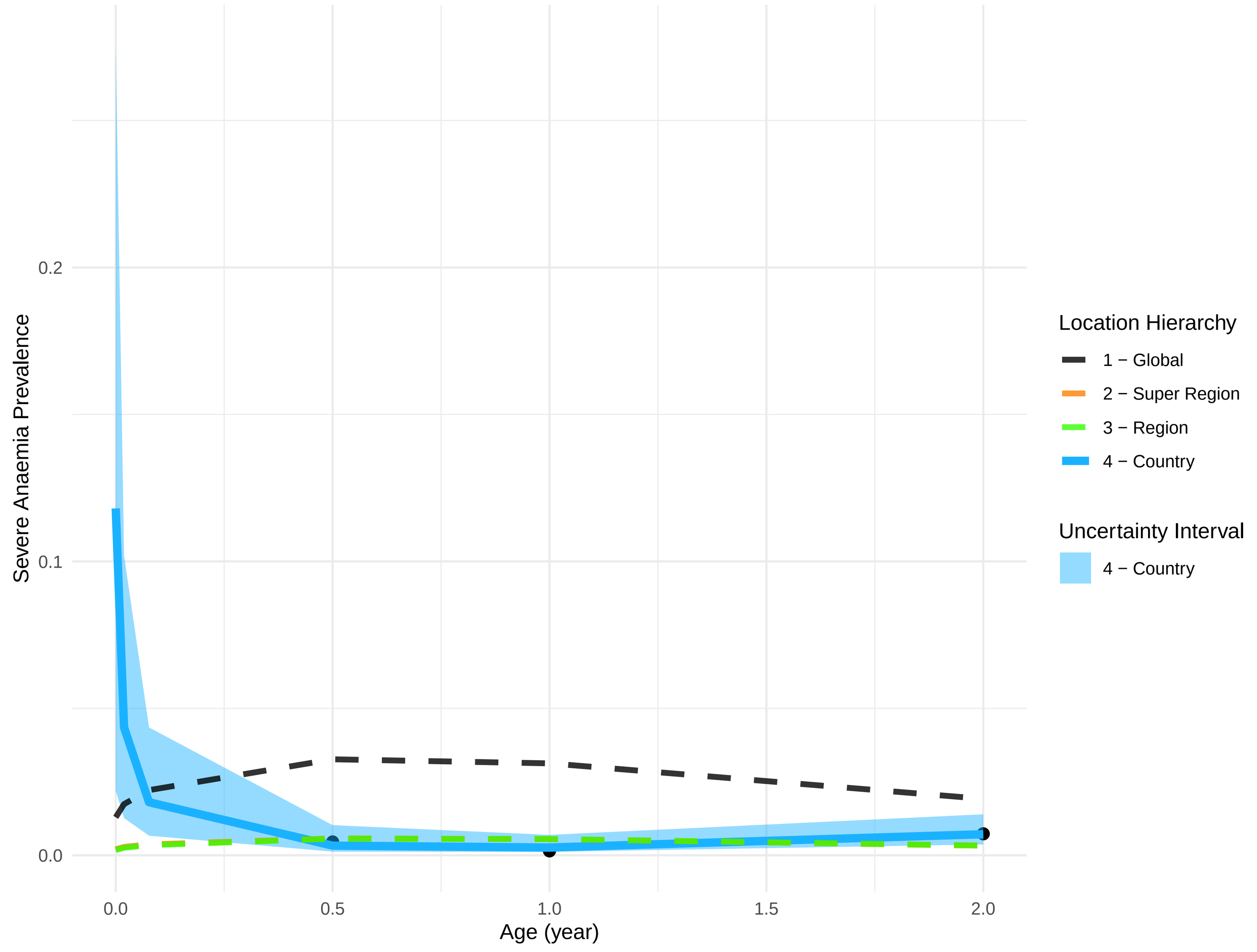

MR−BRT Age/Sex Fits − Non−pregnant Females <2 Years Old − Severe Anaemia Prevalence − Timor−Leste
WHO−2024 Adjustment Method

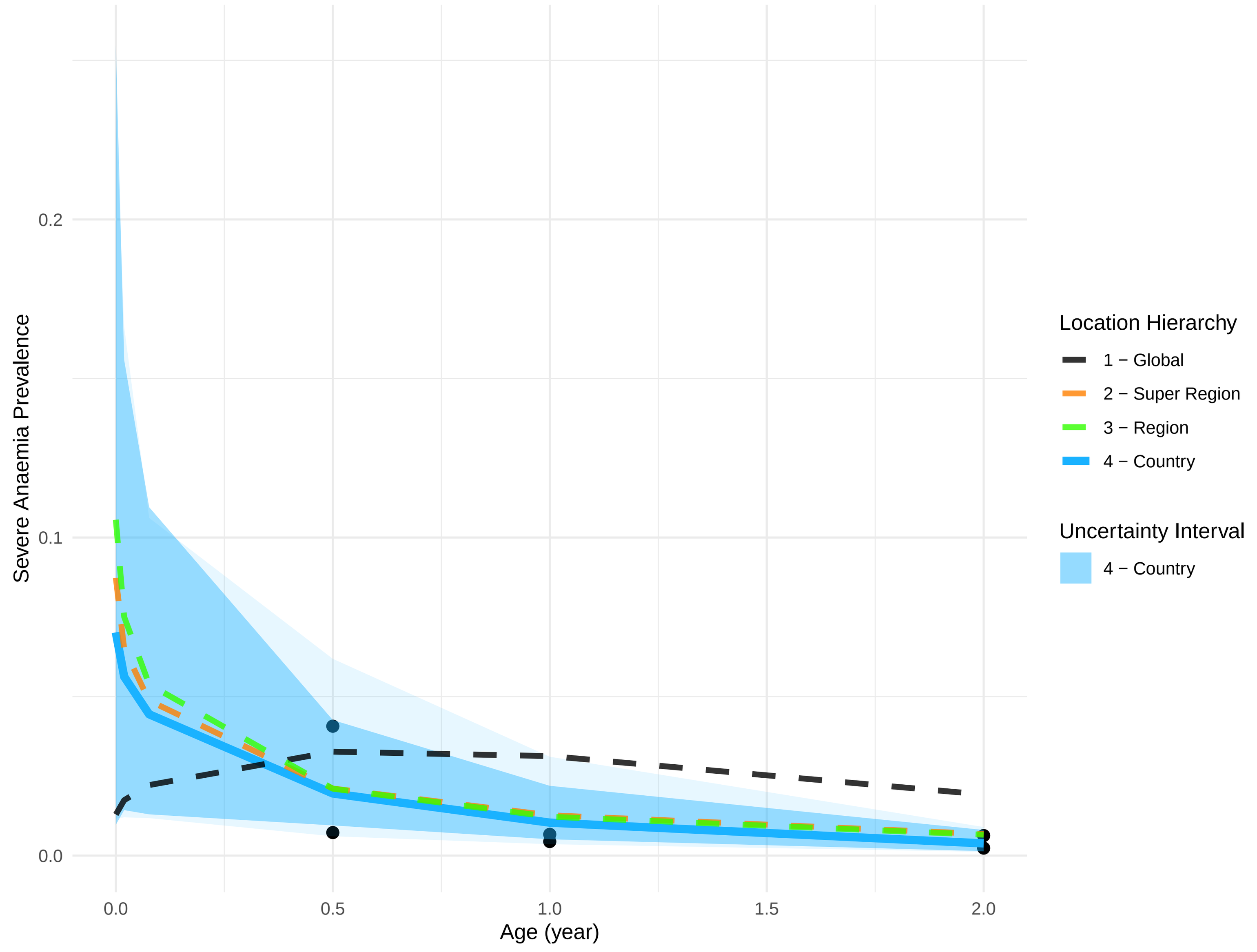

MR−BRT Age/Sex Fits − Non−pregnant Females <2 Years Old − Severe Anaemia Prevalence − Sub−Saharan Africa
WHO−2024 Adjustment Method

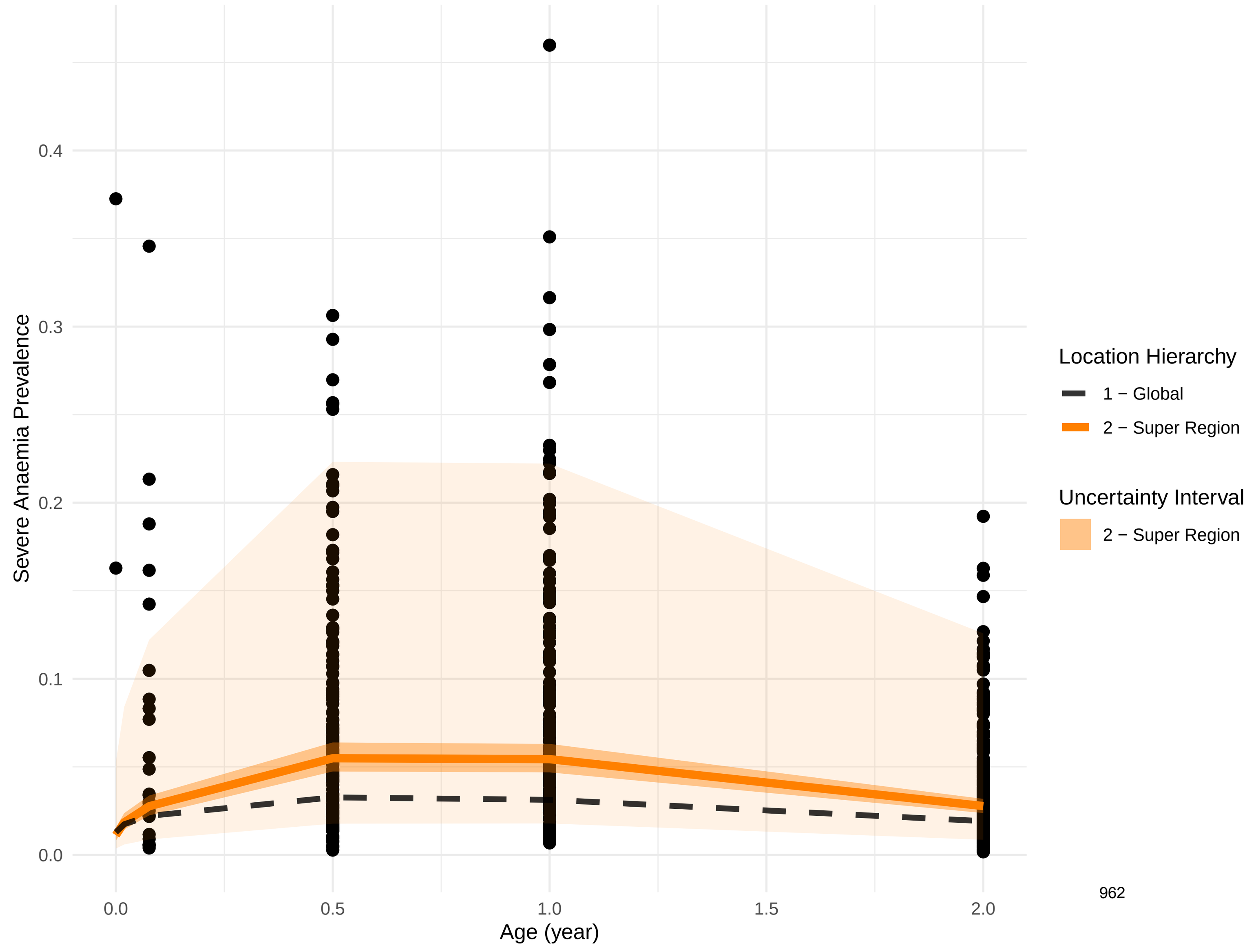

MR−BRT Age/Sex Fits − Non−pregnant Females <2 Years Old − Severe Anaemia Prevalence − Central Sub−Saharan Africa
WHO−2024 Adjustment Method

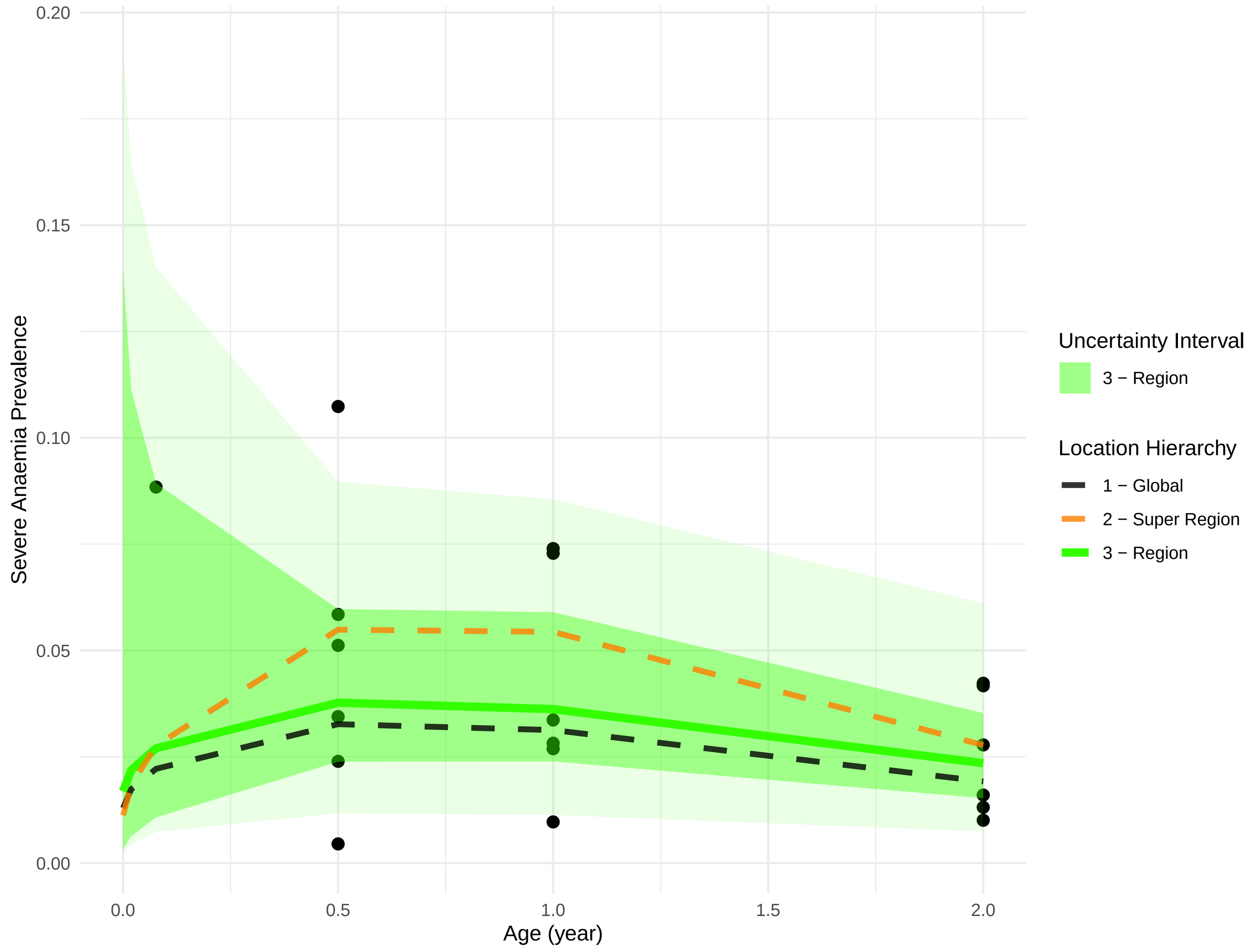

MR−BRT Age/Sex Fits − Non−pregnant Females <2 Years Old − Severe Anaemia Prevalence − Angola
WHO−2024 Adjustment Method

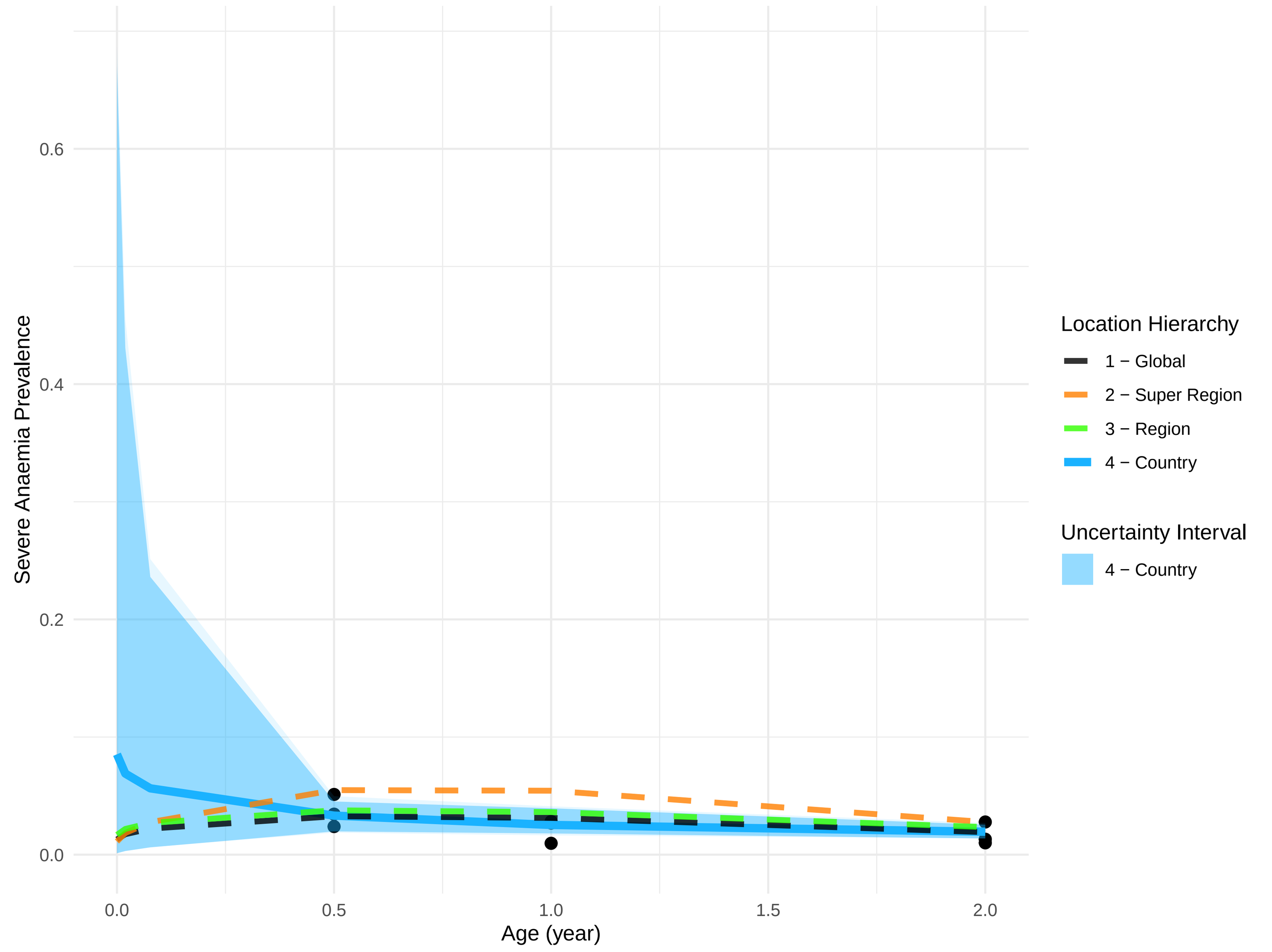

MR−BRT Age/Sex Fits − Non−pregnant Females <2 Years Old − Severe Anaemia Prevalence − Democratic Republic of the Congo
WHO−2024 Adjustment Method

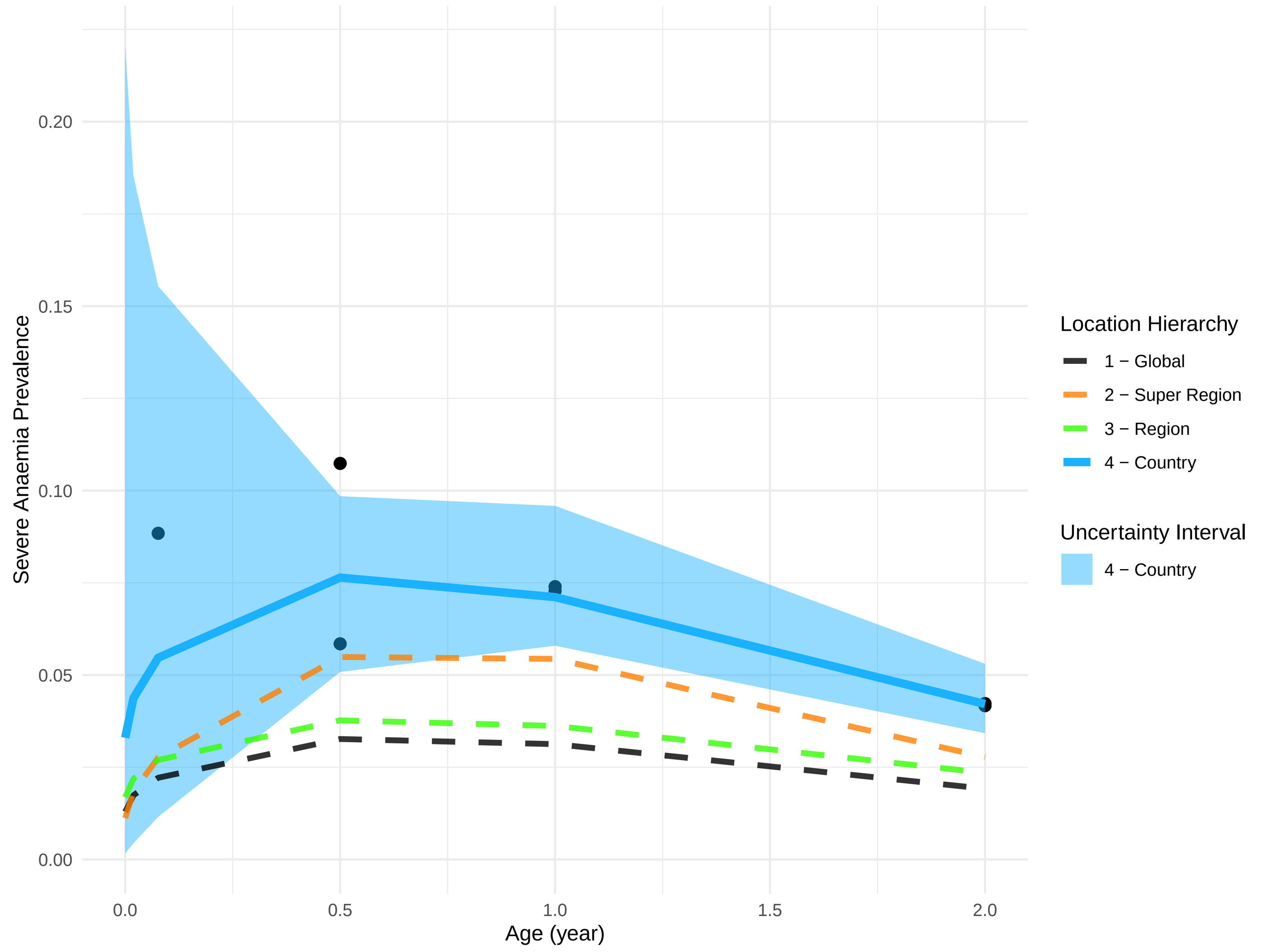

MR−BRT Age/Sex Fits − Non−pregnant Females <2 Years Old − Severe Anaemia Prevalence − Gabon
WHO−2024 Adjustment Method

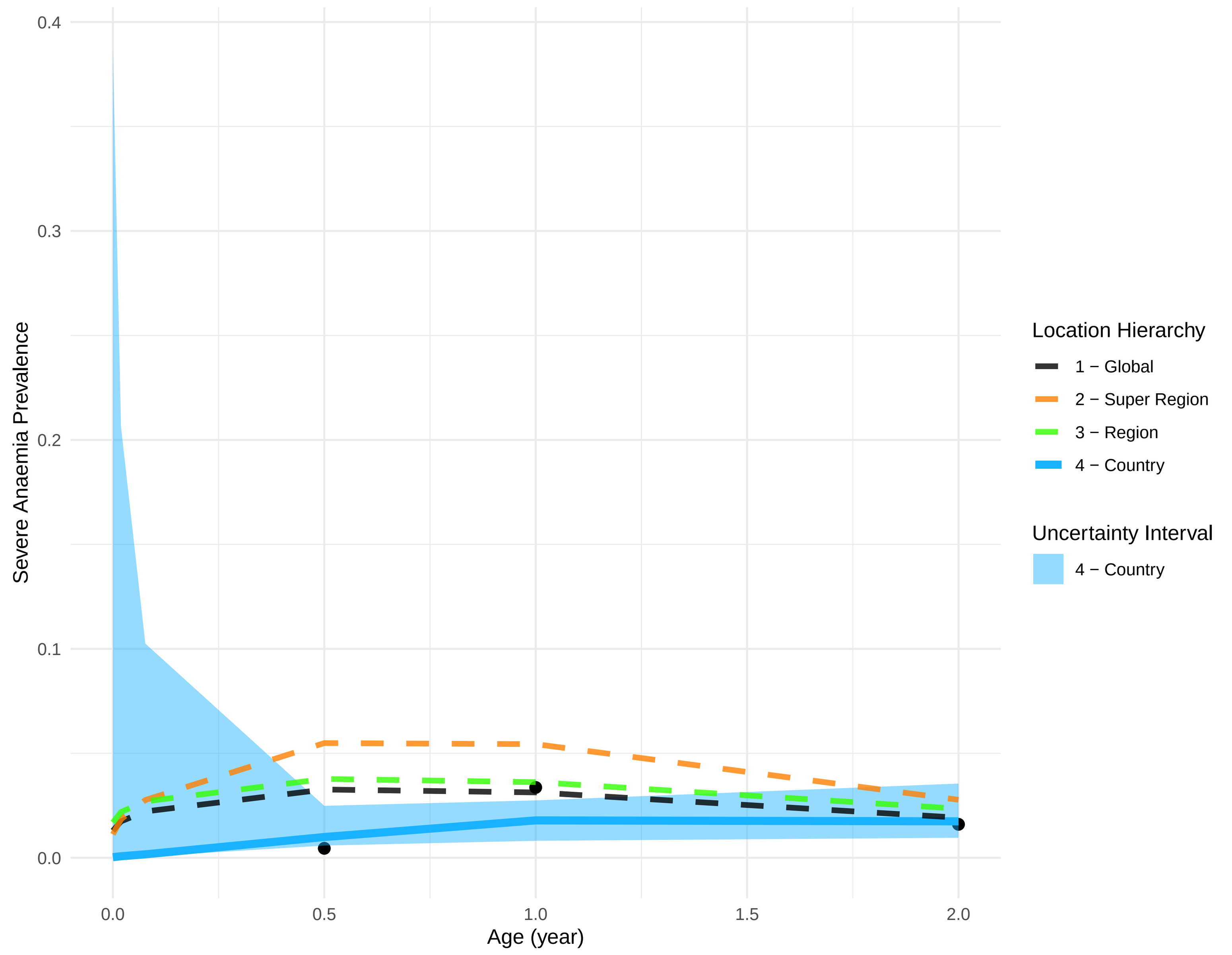

MR−BRT Age/Sex Fits − Non−pregnant Females <2 Years Old − Severe Anaemia Prevalence − Eastern Sub−Saharan Africa
WHO−2024 Adjustment Method

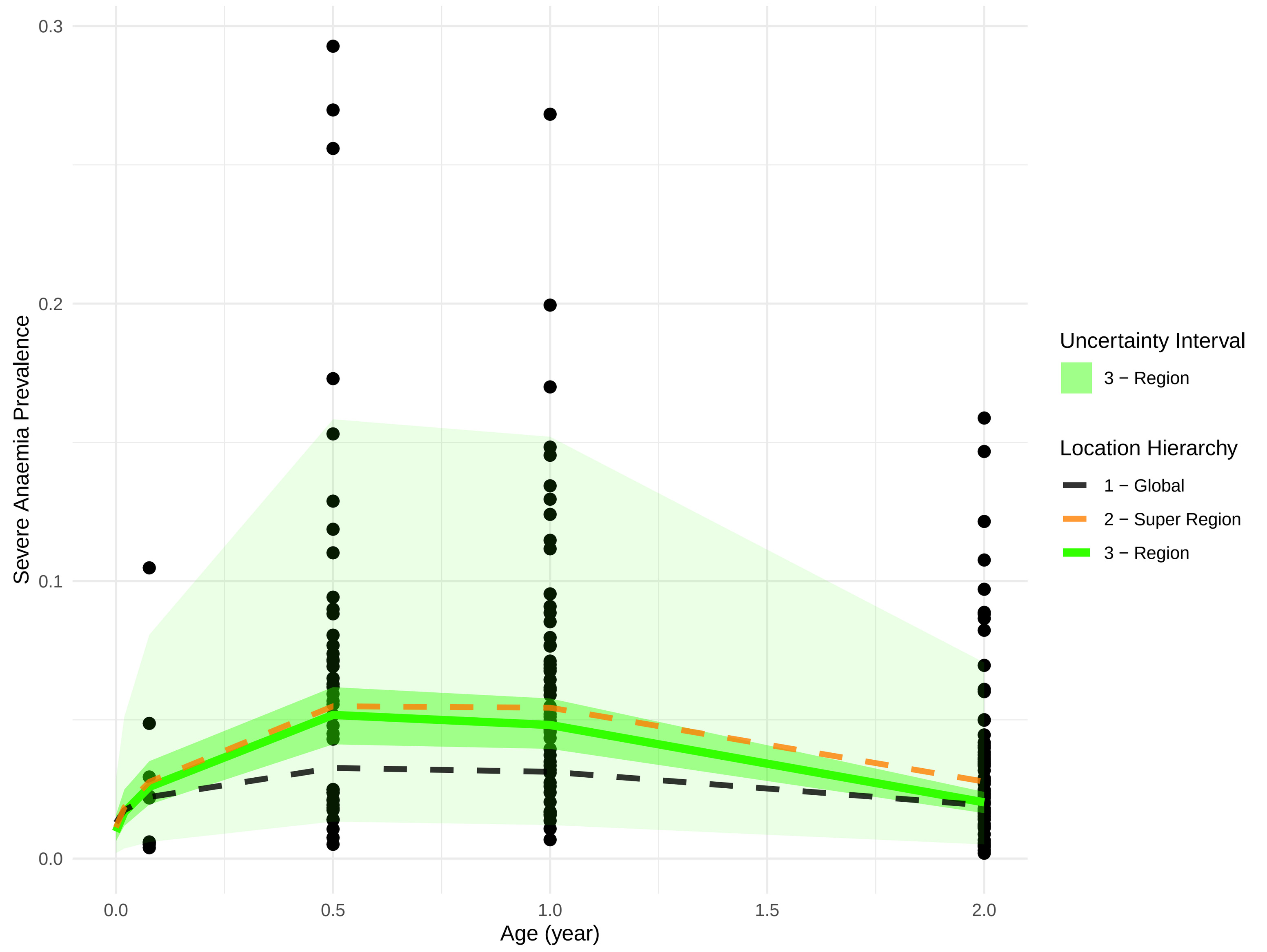

MR−BRT Age/Sex Fits − Non−pregnant Females <2 Years Old − Severe Anaemia Prevalence − Burundi
WHO−2024 Adjustment Method

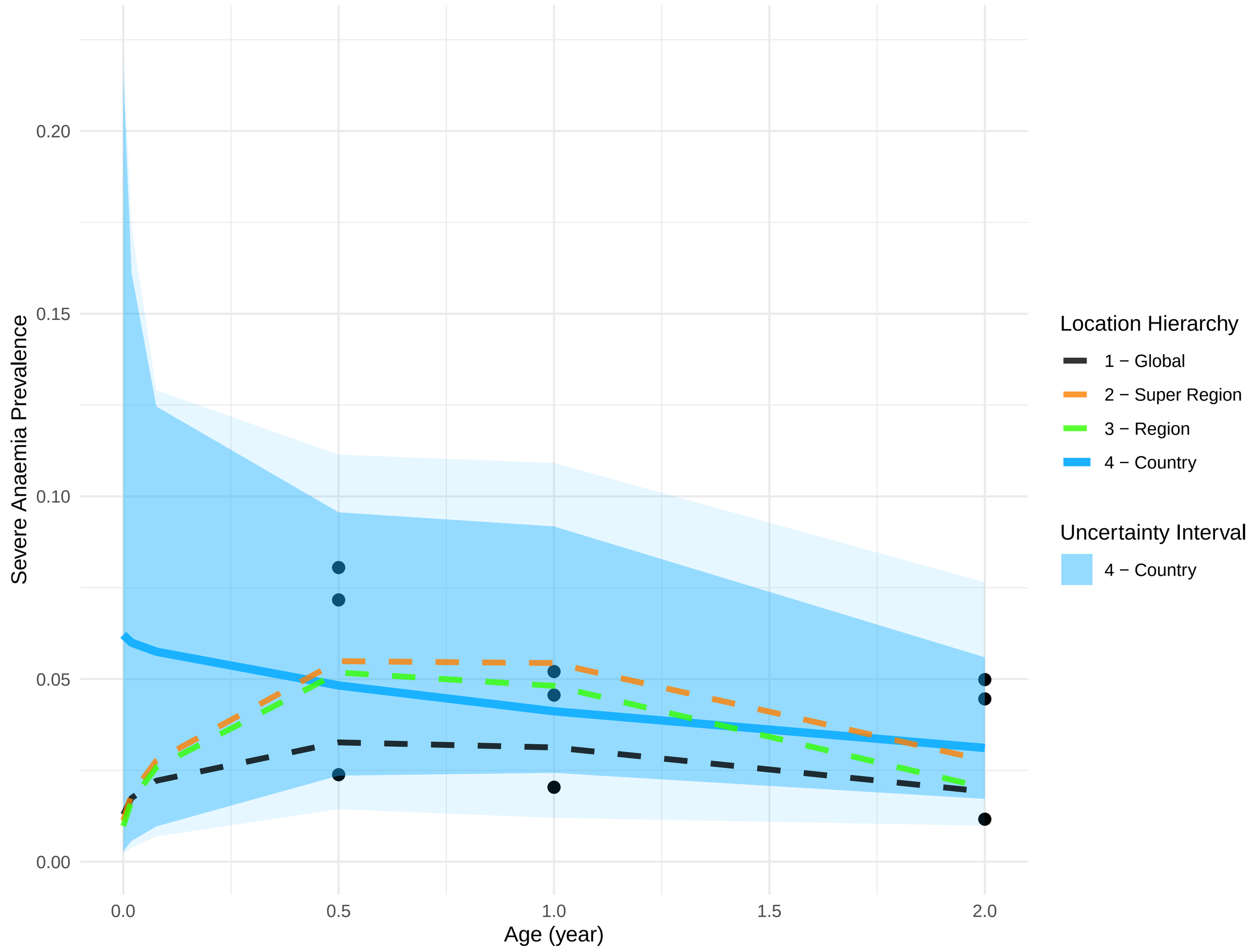

MR−BRT Age/Sex Fits − Non−pregnant Females <2 Years Old − Severe Anaemia Prevalence − Ethiopia
WHO−2024 Adjustment Method

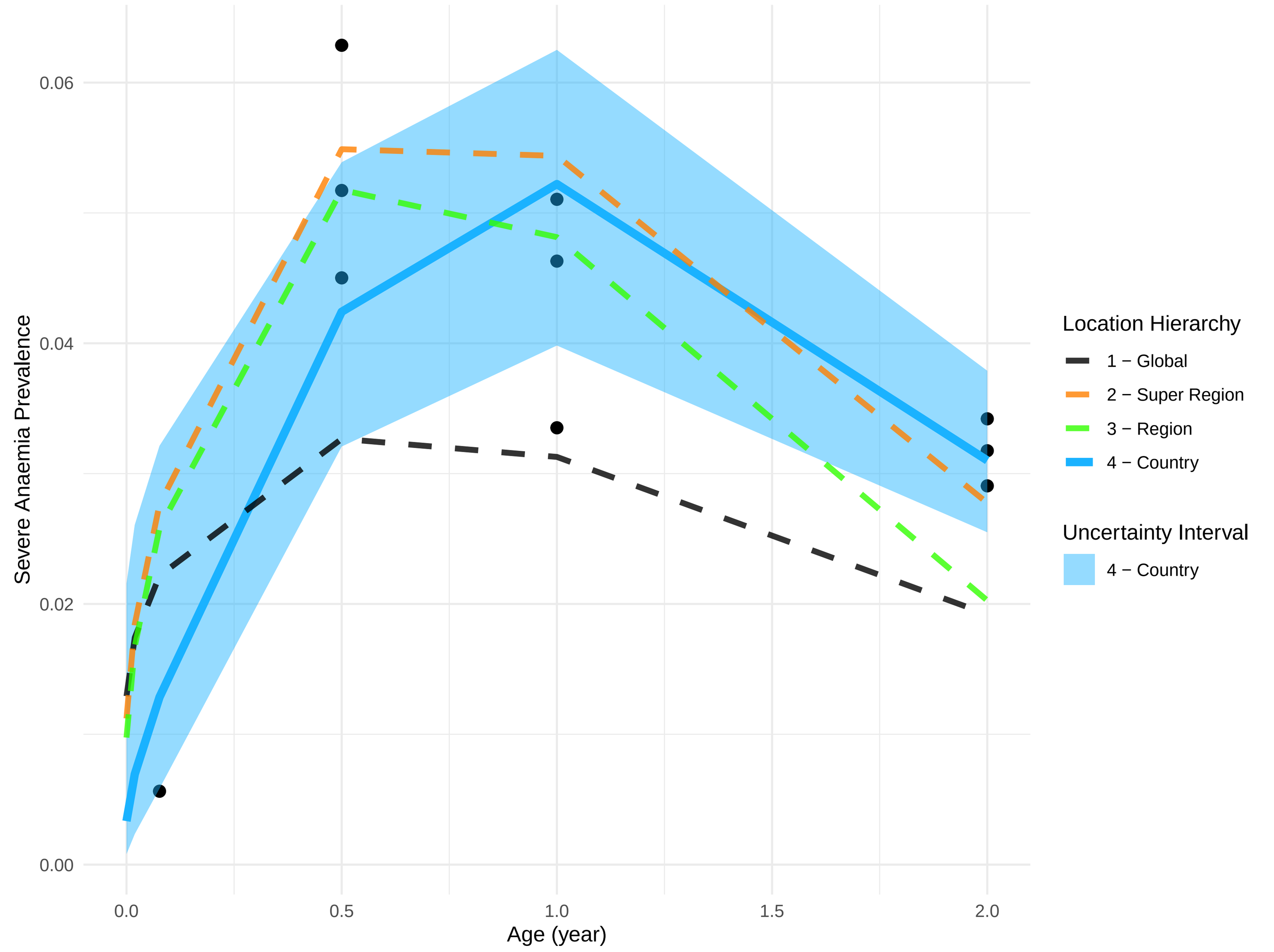

MR−BRT Age/Sex Fits − Non−pregnant Females <2 Years Old − Severe Anaemia Prevalence − Kenya
WHO−2024 Adjustment Method

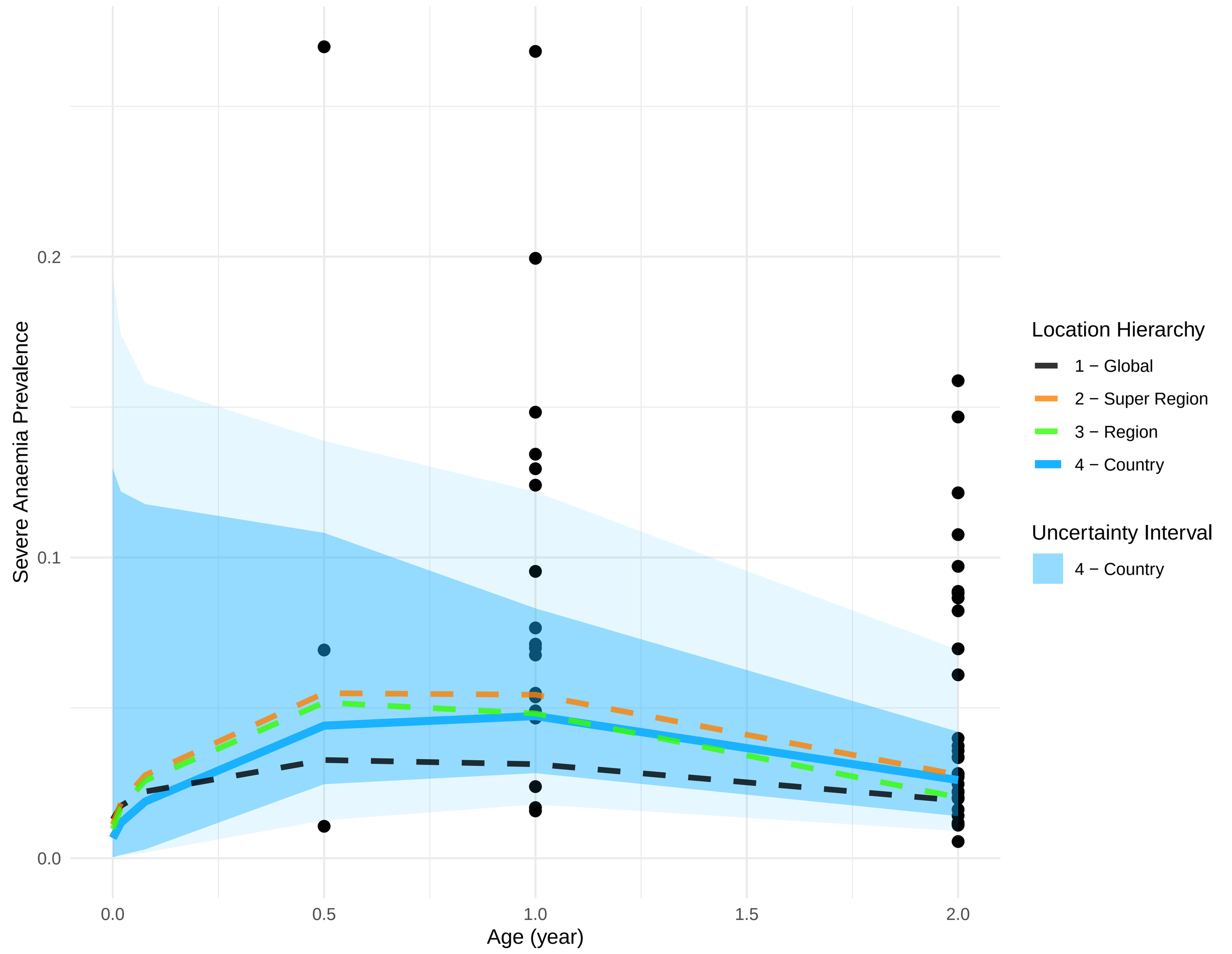

MR−BRT Age/Sex Fits − Non−pregnant Females <2 Years Old − Severe Anaemia Prevalence − Bungoma
WHO−2024 Adjustment Method

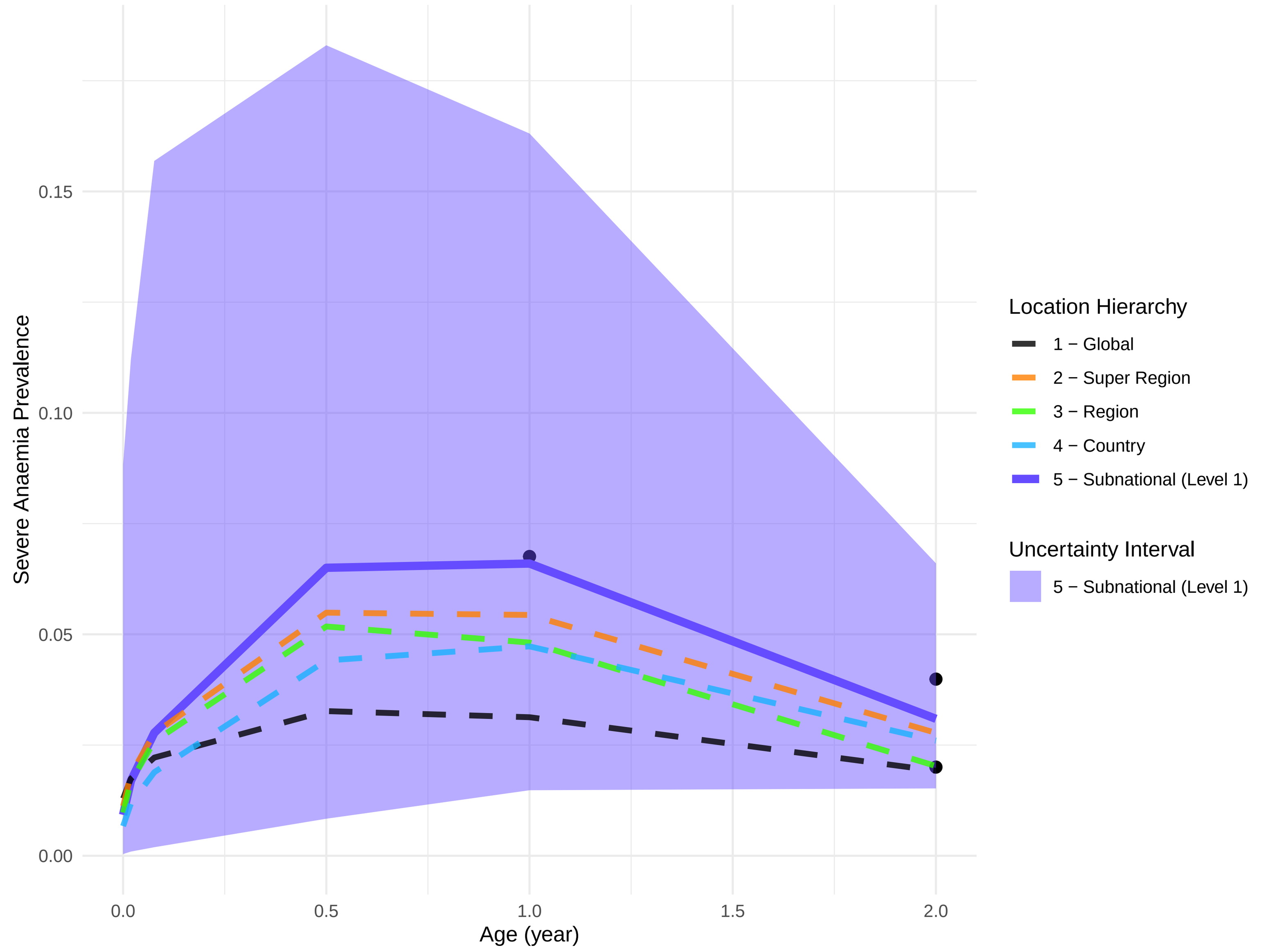

MR−BRT Age/Sex Fits − Non−pregnant Females <2 Years Old − Severe Anaemia Prevalence − Busia
WHO−2024 Adjustment Method

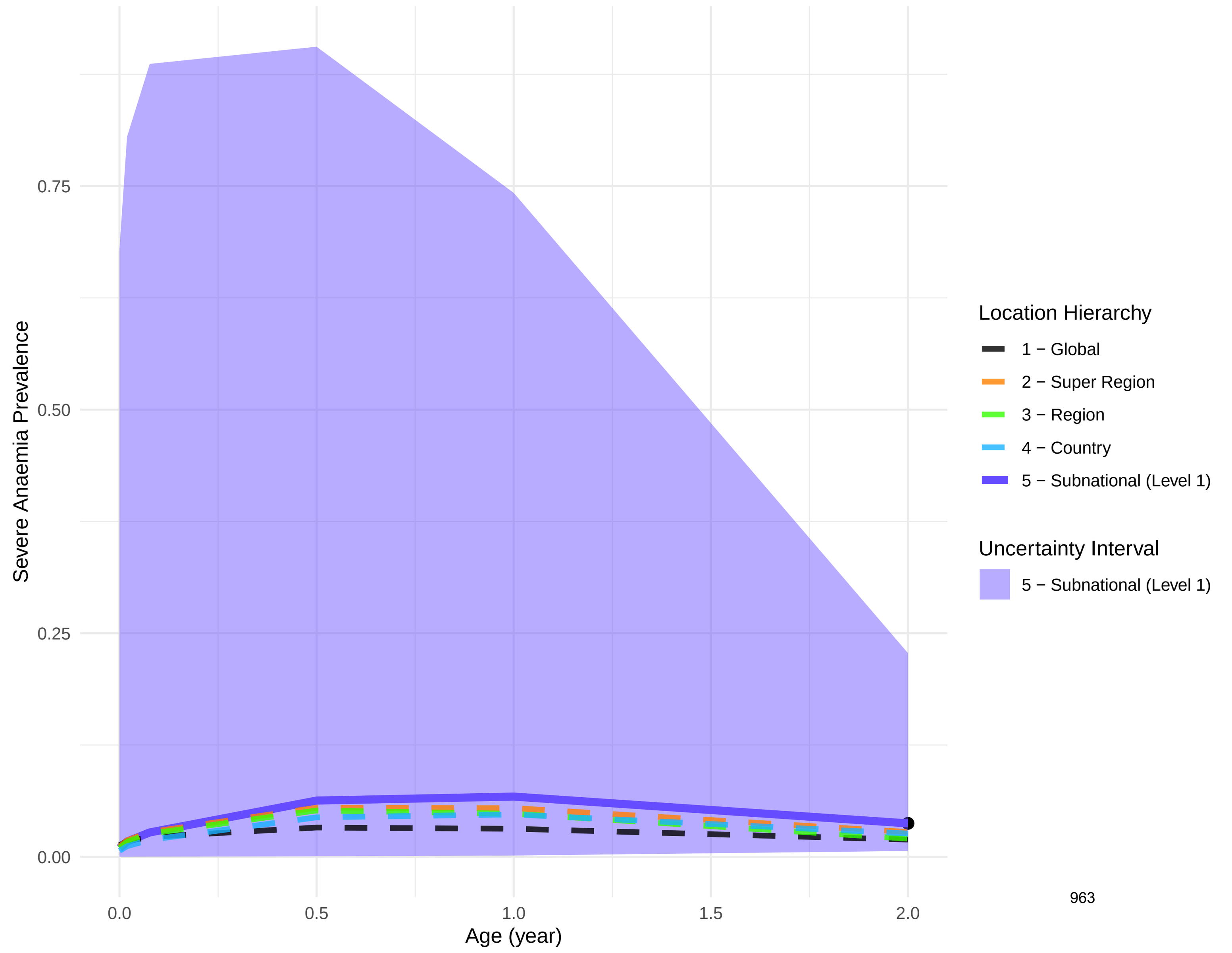

MR−BRT Age/Sex Fits − Non−pregnant Females <2 Years Old − Severe Anaemia Prevalence − Elgeyo−Marakwet
WHO−2024 Adjustment Method

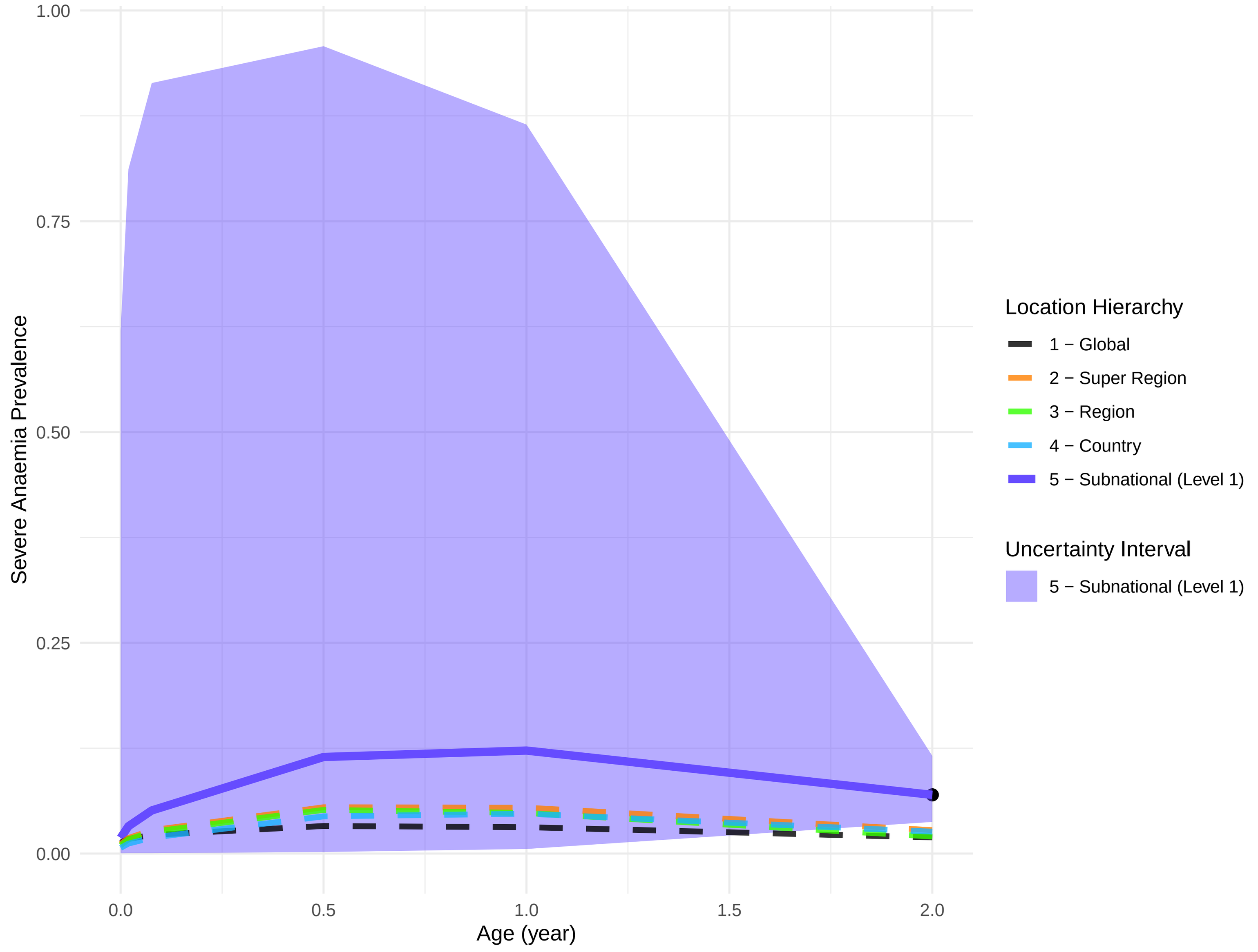

MR−BRT Age/Sex Fits − Non−pregnant Females <2 Years Old − Severe Anaemia Prevalence − Garissa
WHO−2024 Adjustment Method

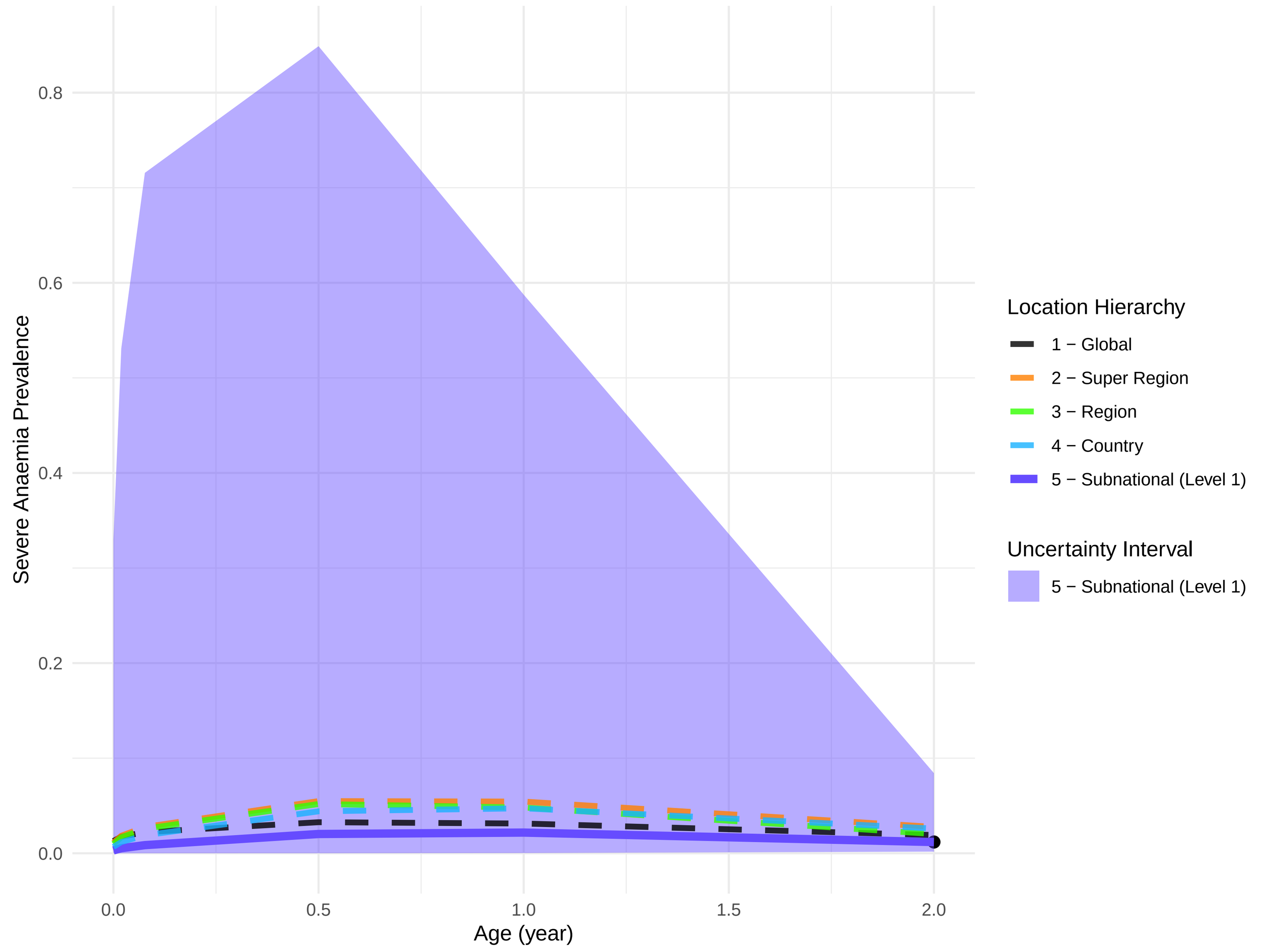

MR−BRT Age/Sex Fits − Non−pregnant Females <2 Years Old − Severe Anaemia Prevalence − Homa Bay
WHO−2024 Adjustment Method

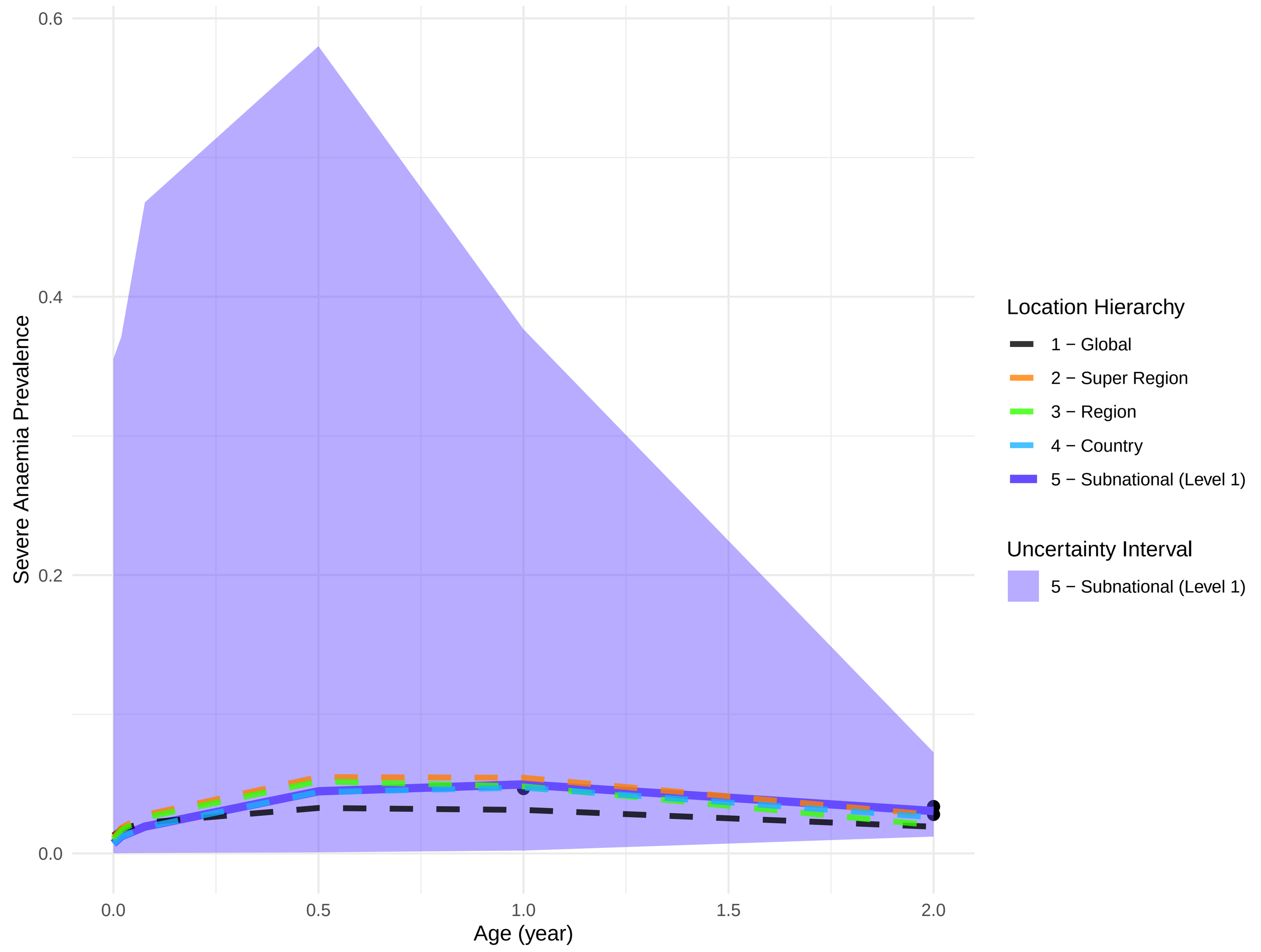

MR−BRT Age/Sex Fits − Non−pregnant Females <2 Years Old − Severe Anaemia Prevalence − Kajiado
WHO−2024 Adjustment Method

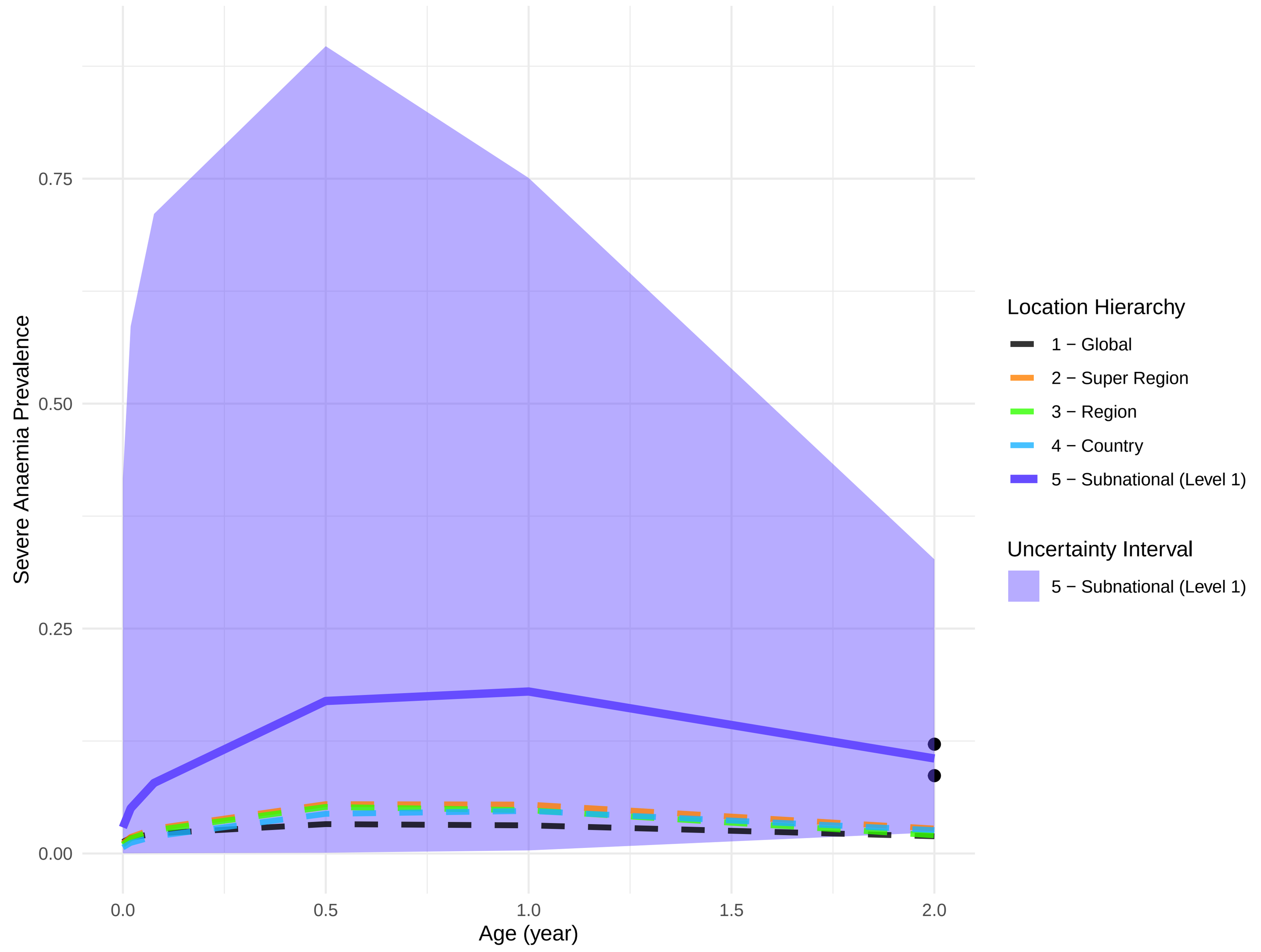

MR−BRT Age/Sex Fits − Non−pregnant Females <2 Years Old − Severe Anaemia Prevalence − Kakamega
WHO−2024 Adjustment Method

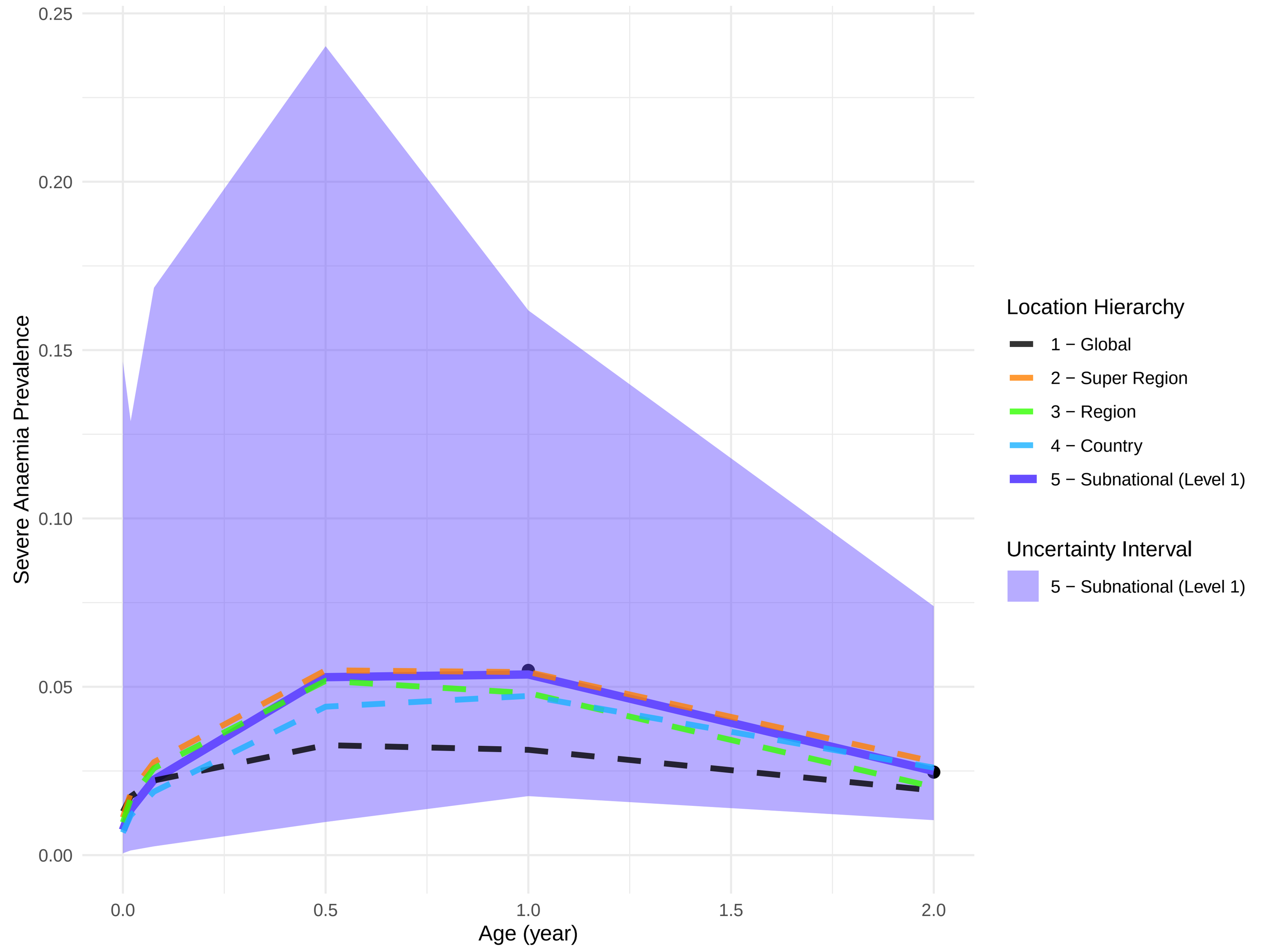

MR−BRT Age/Sex Fits − Non−pregnant Females <2 Years Old − Severe Anaemia Prevalence − Kericho
WHO−2024 Adjustment Method

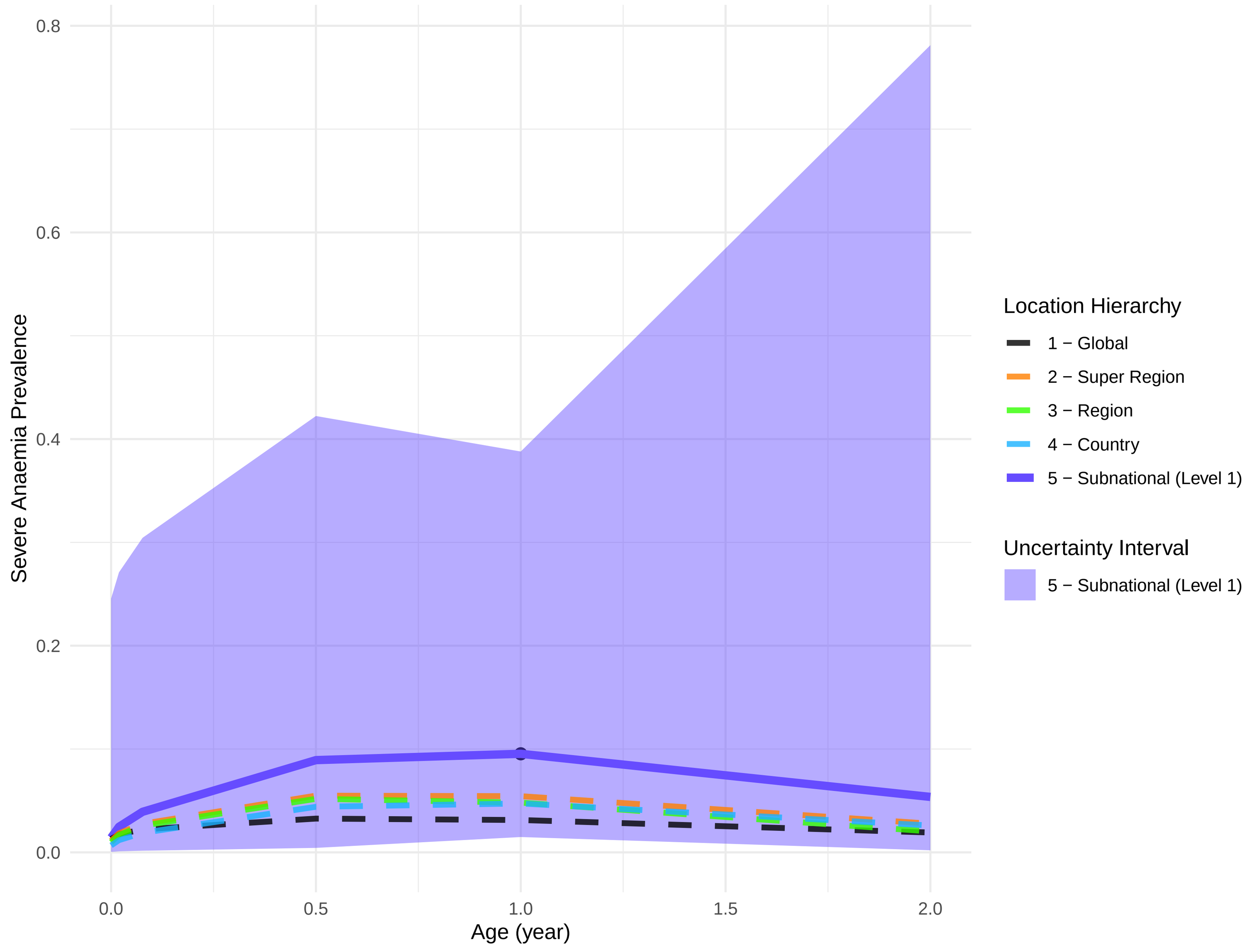

MR−BRT Age/Sex Fits − Non−pregnant Females <2 Years Old − Severe Anaemia Prevalence − Kiambu
WHO−2024 Adjustment Method

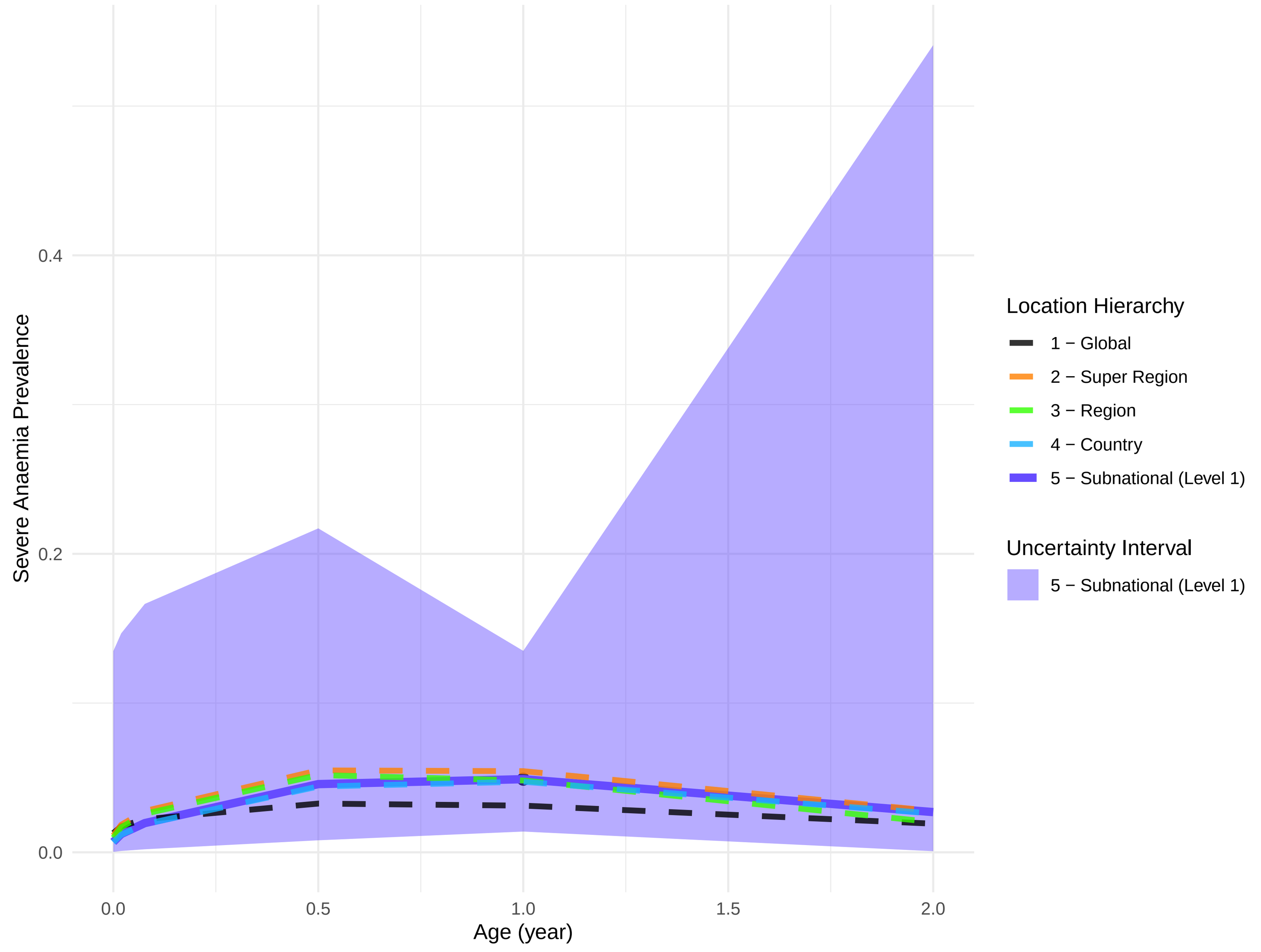

MR−BRT Age/Sex Fits − Non−pregnant Females <2 Years Old − Severe Anaemia Prevalence − Kilifi
WHO−2024 Adjustment Method

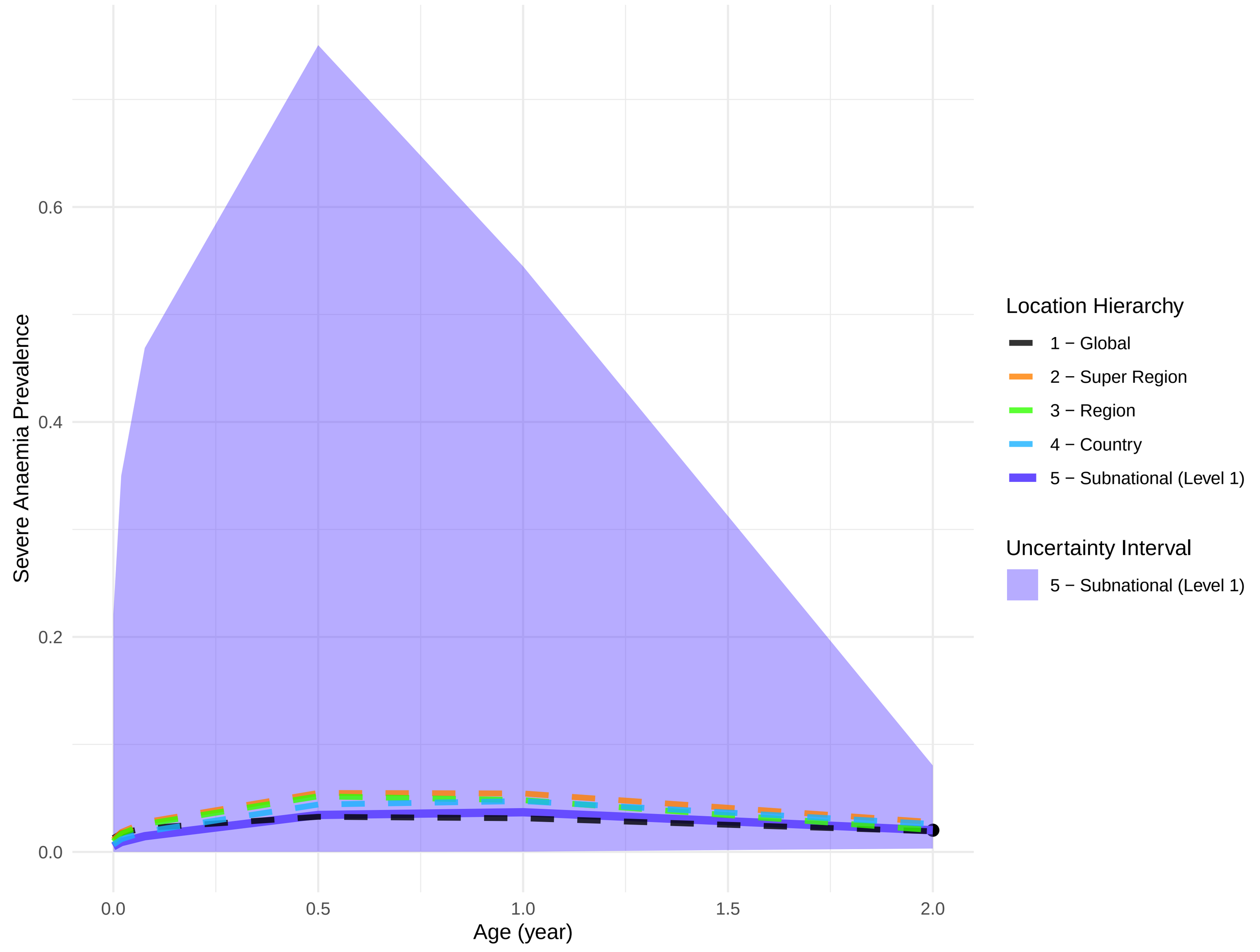

MR−BRT Age/Sex Fits − Non−pregnant Females <2 Years Old − Severe Anaemia Prevalence − Kisii
WHO−2024 Adjustment Method

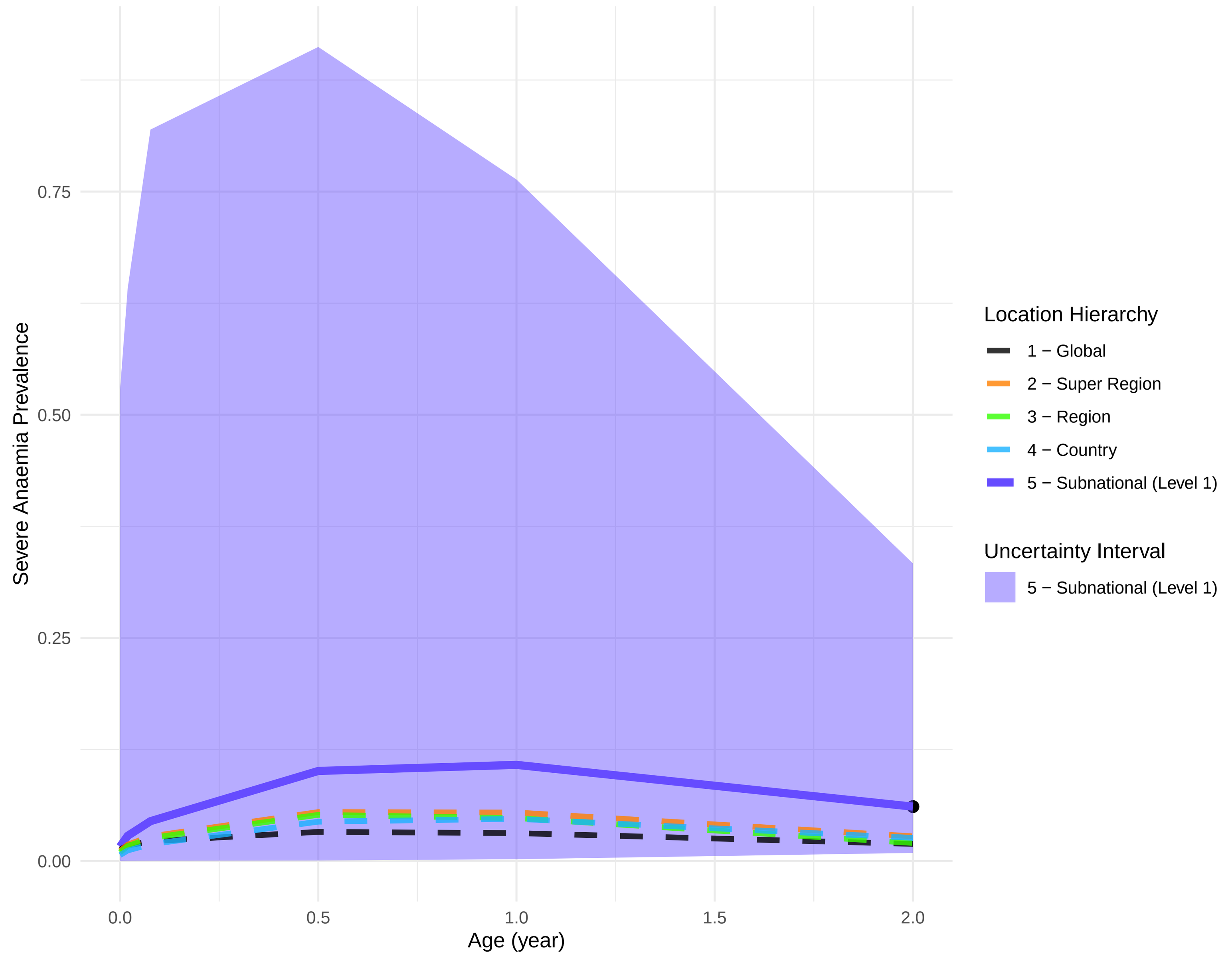

MR−BRT Age/Sex Fits − Non−pregnant Females <2 Years Old − Severe Anaemia Prevalence − Kisumu
WHO−2024 Adjustment Method

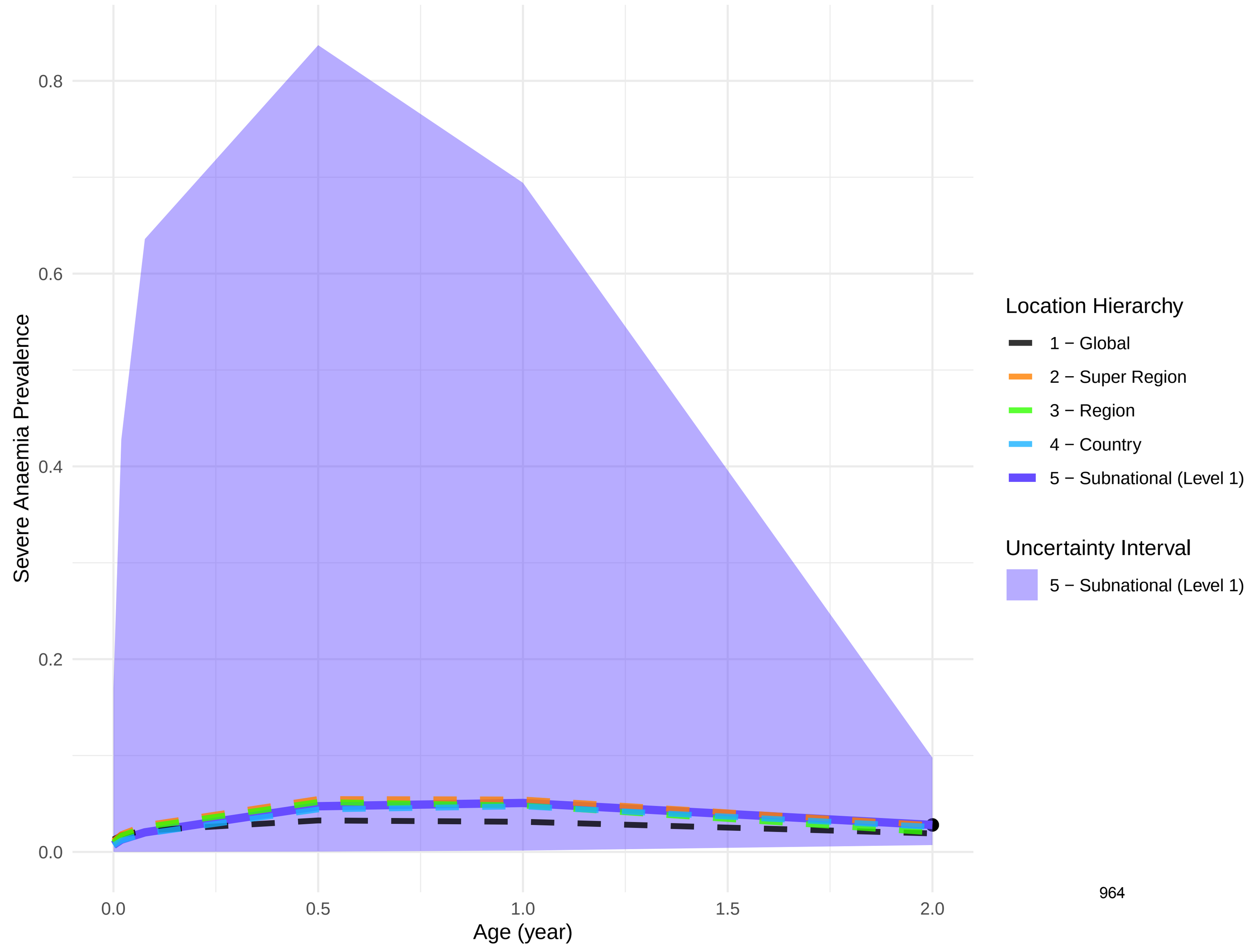

MR−BRT Age/Sex Fits − Non−pregnant Females <2 Years Old − Severe Anaemia Prevalence − Kwale
WHO−2024 Adjustment Method

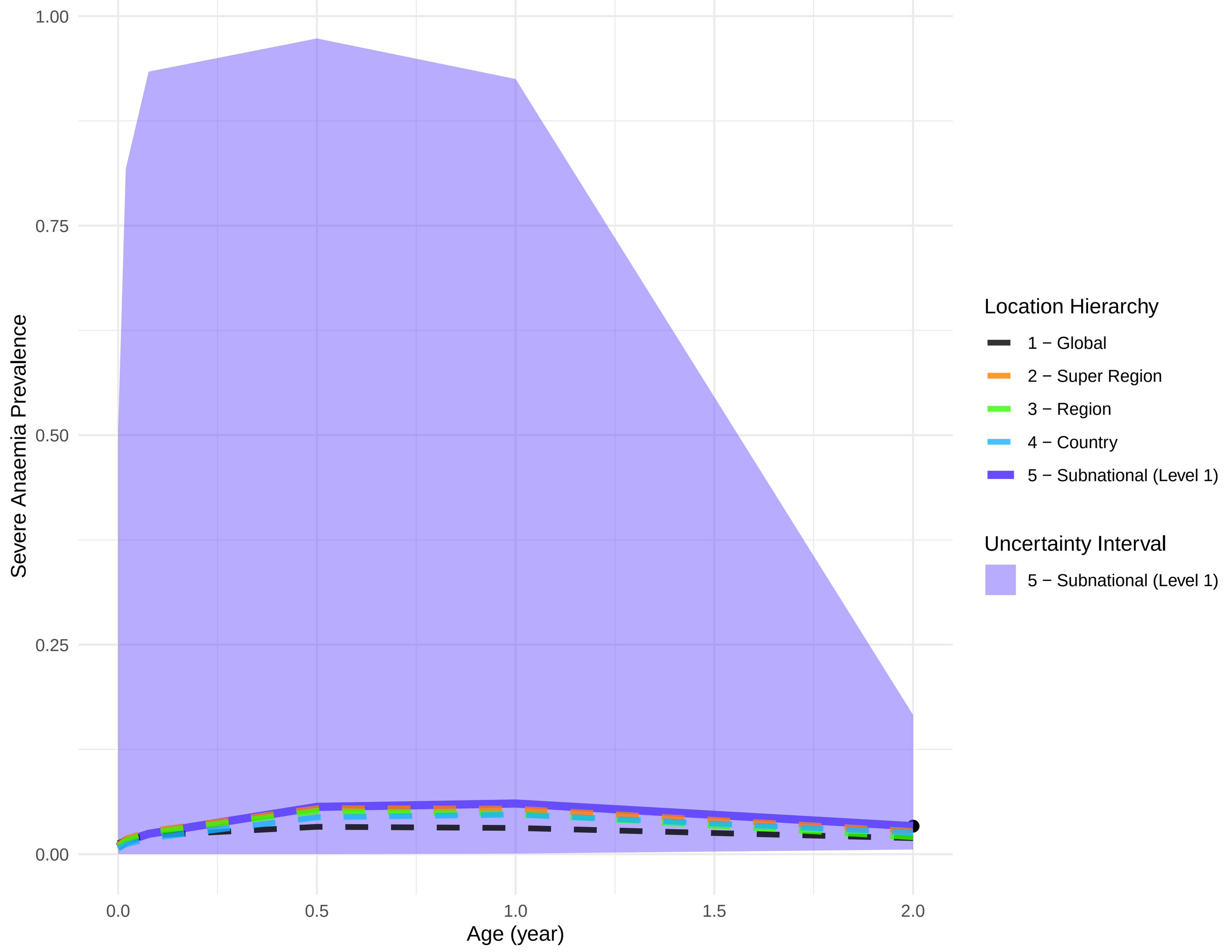

MR−BRT Age/Sex Fits − Non−pregnant Females <2 Years Old − Severe Anaemia Prevalence − Lamu
WHO−2024 Adjustment Method

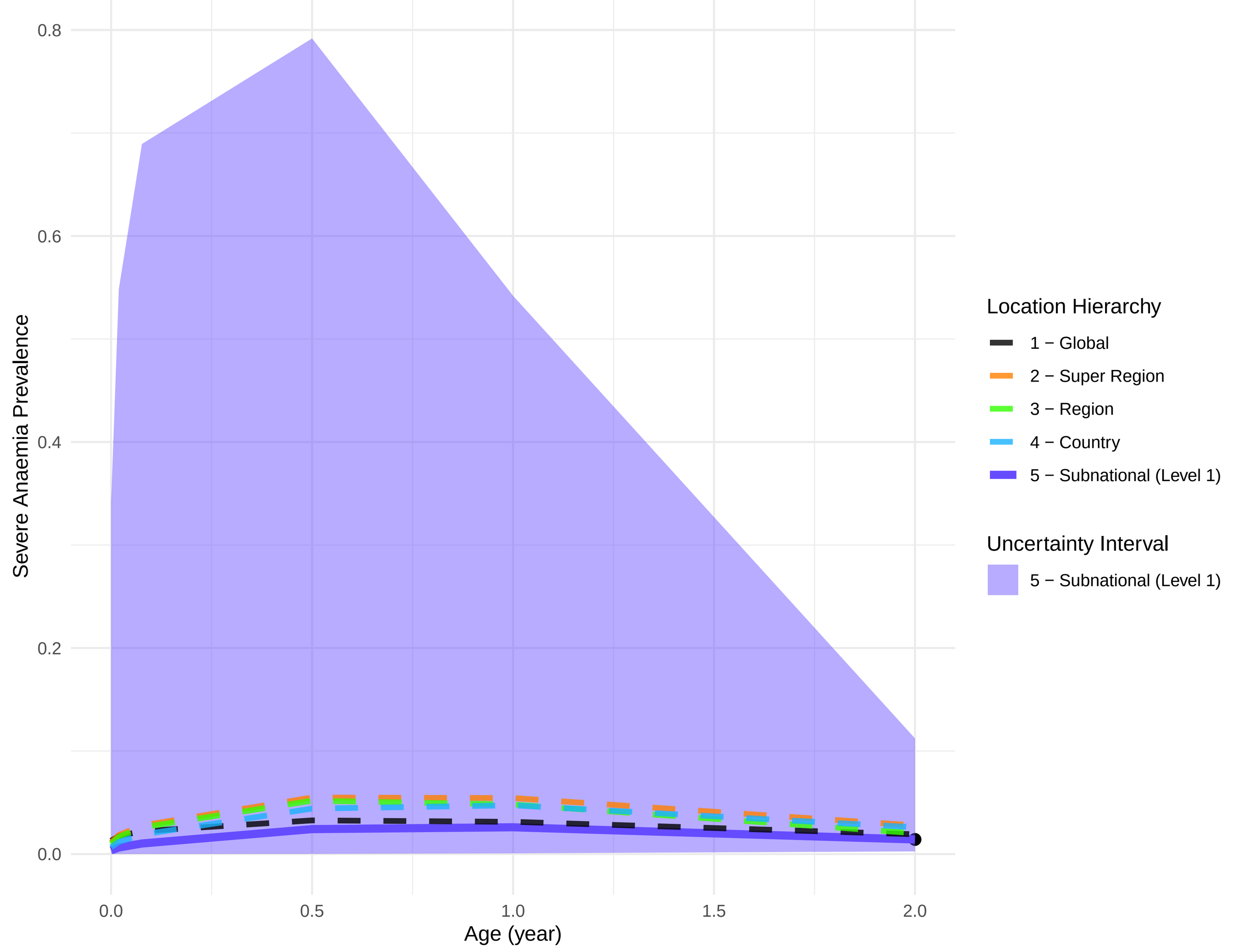

MR−BRT Age/Sex Fits − Non−pregnant Females <2 Years Old − Severe Anaemia Prevalence − Machakos
WHO−2024 Adjustment Method

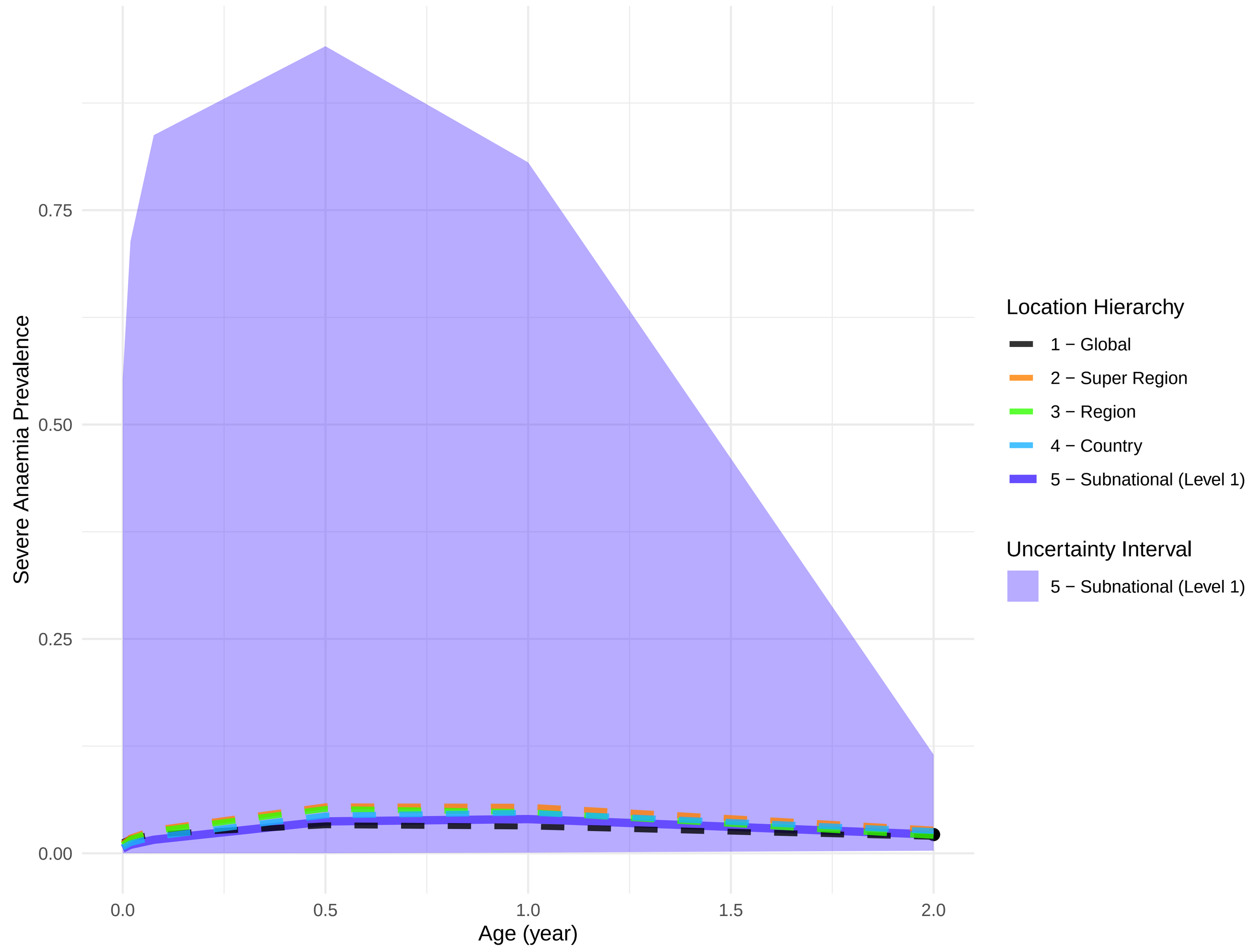

MR−BRT Age/Sex Fits − Non−pregnant Females <2 Years Old − Severe Anaemia Prevalence − Mandera
WHO−2024 Adjustment Method

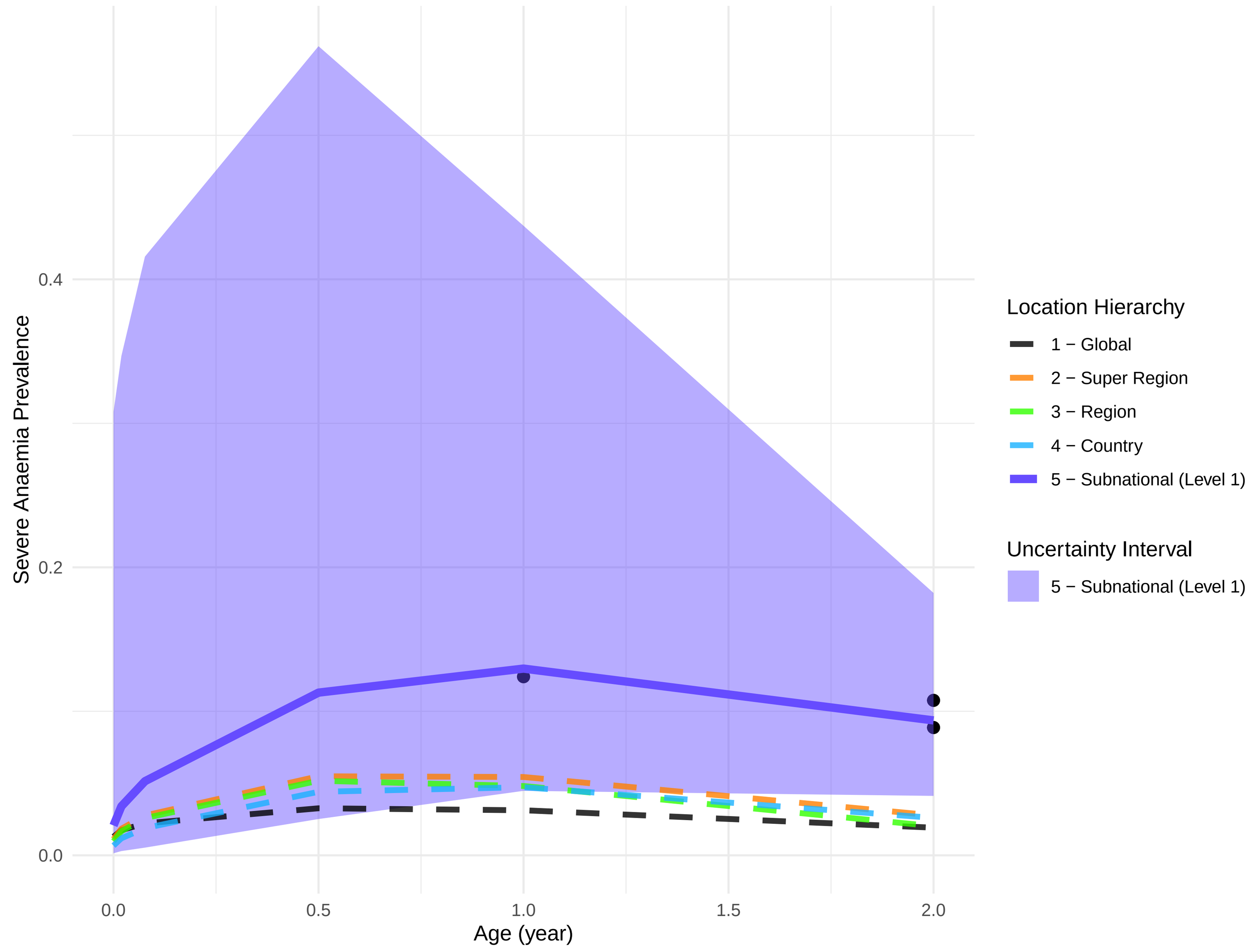

MR−BRT Age/Sex Fits − Non−pregnant Females <2 Years Old − Severe Anaemia Prevalence − Marsabit
WHO−2024 Adjustment Method

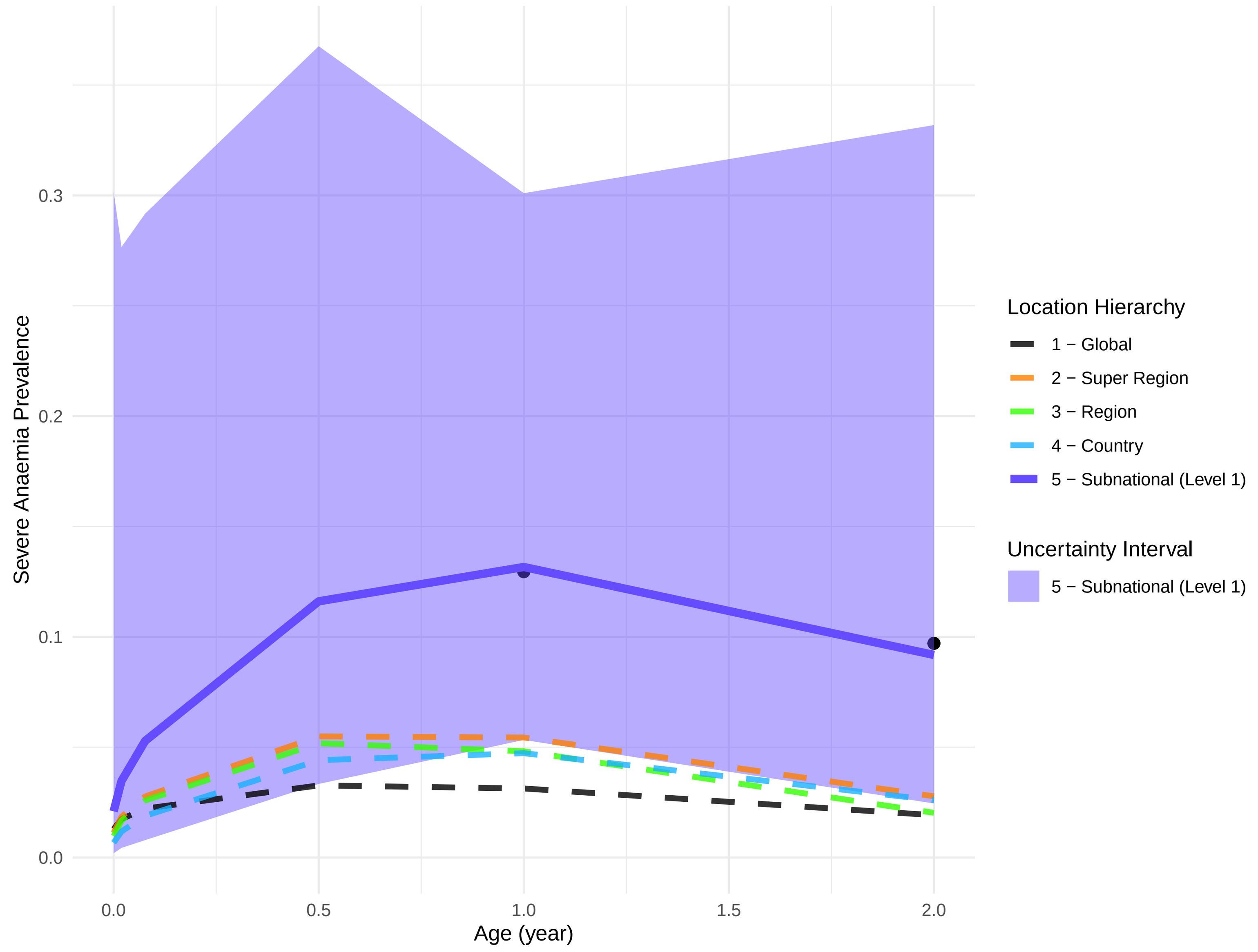

MR−BRT Age/Sex Fits − Non−pregnant Females <2 Years Old − Severe Anaemia Prevalence − Migori
WHO−2024 Adjustment Method

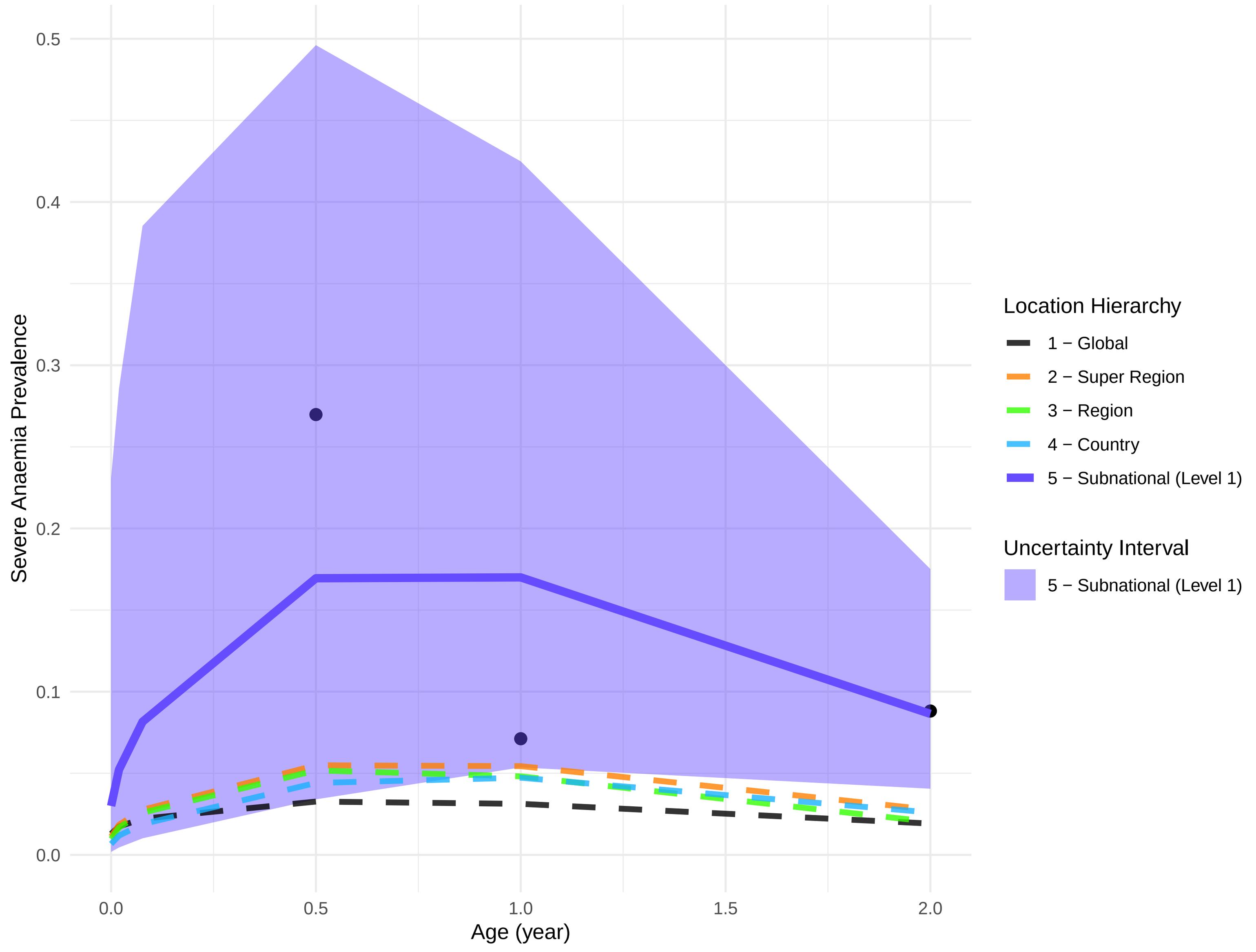

MR−BRT Age/Sex Fits − Non−pregnant Females <2 Years Old − Severe Anaemia Prevalence − Mombasa
WHO−2024 Adjustment Method

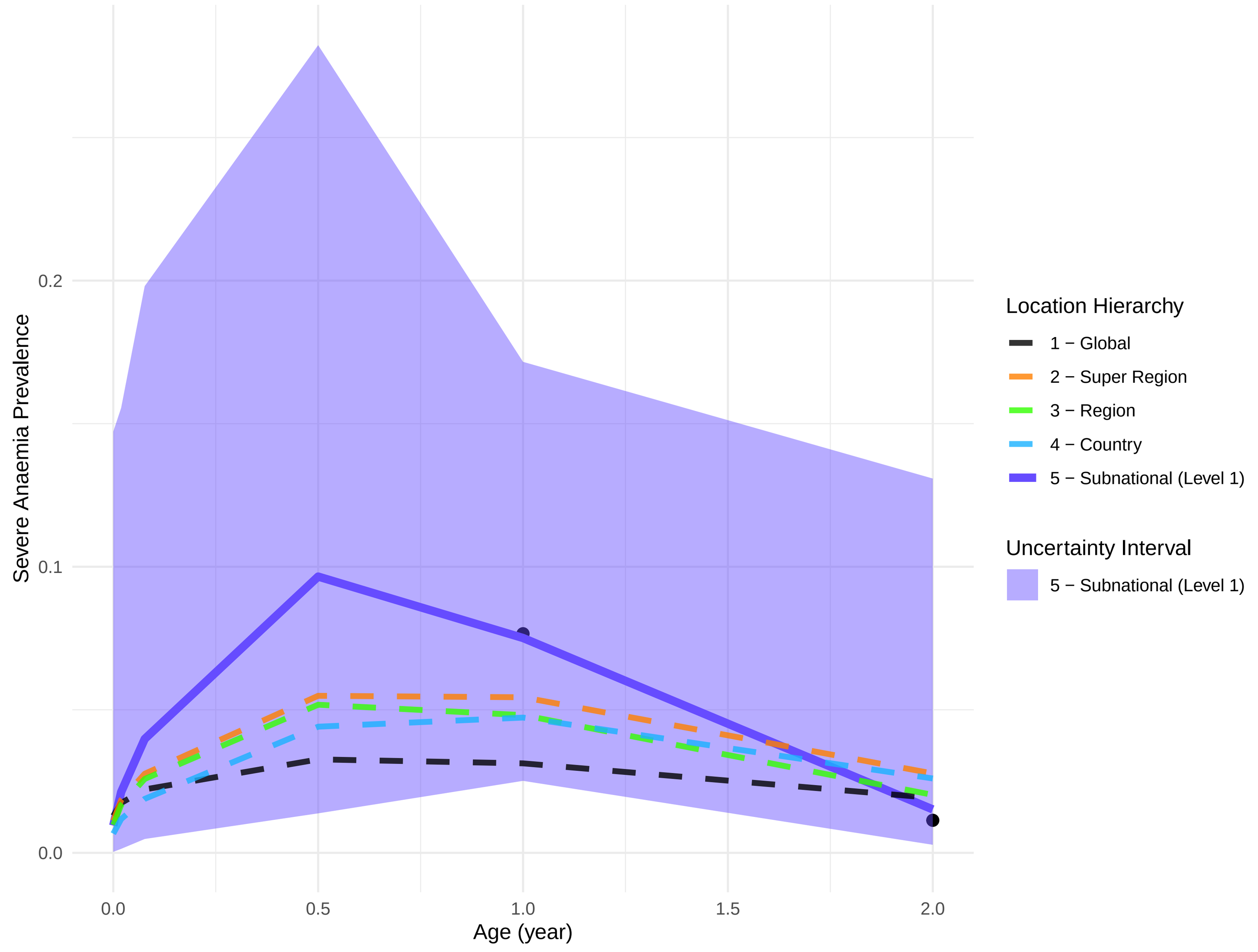

MR−BRT Age/Sex Fits − Non−pregnant Females <2 Years Old − Severe Anaemia Prevalence − Narok
WHO−2024 Adjustment Method

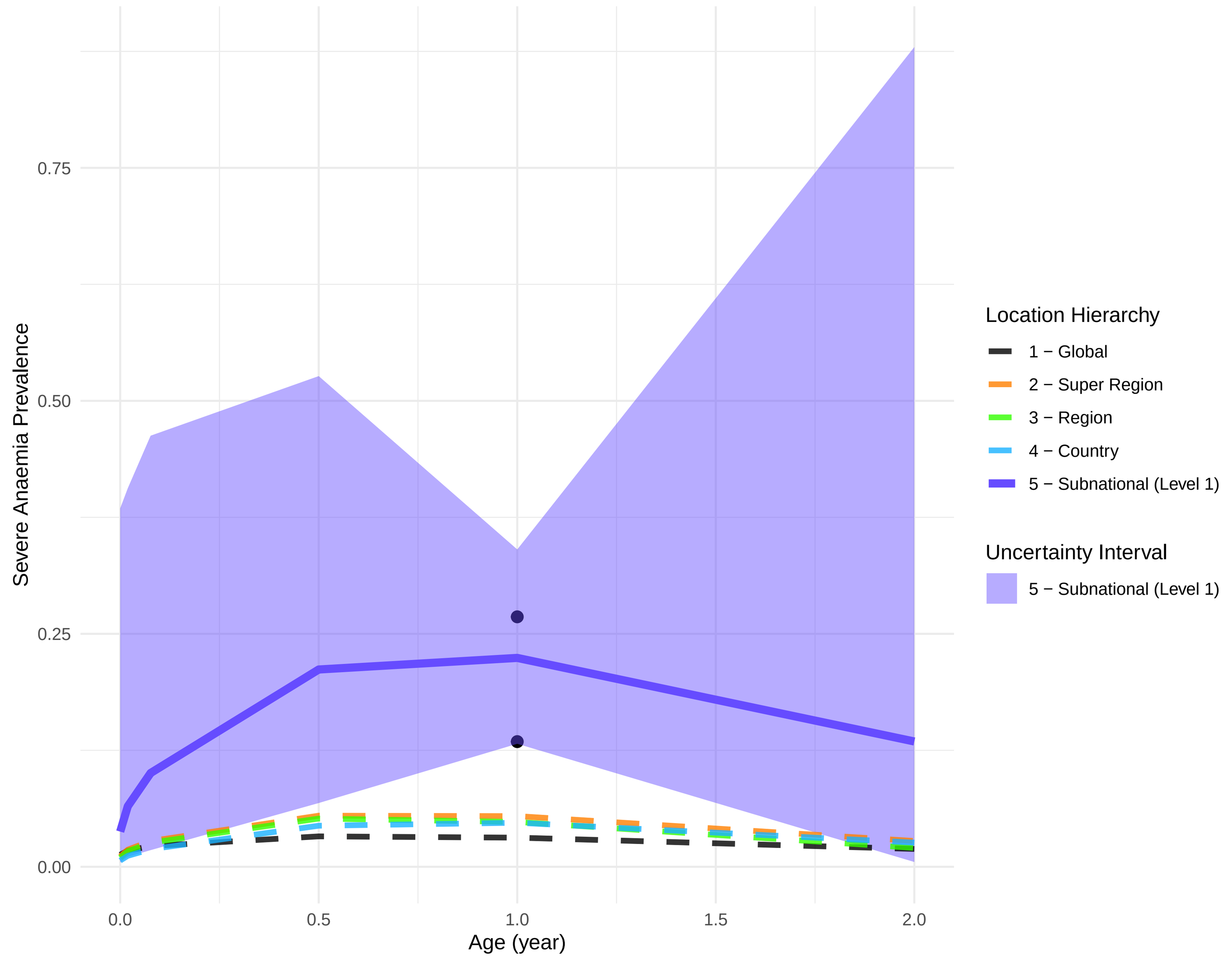

MR−BRT Age/Sex Fits − Non−pregnant Females <2 Years Old − Severe Anaemia Prevalence − Siaya
WHO−2024 Adjustment Method

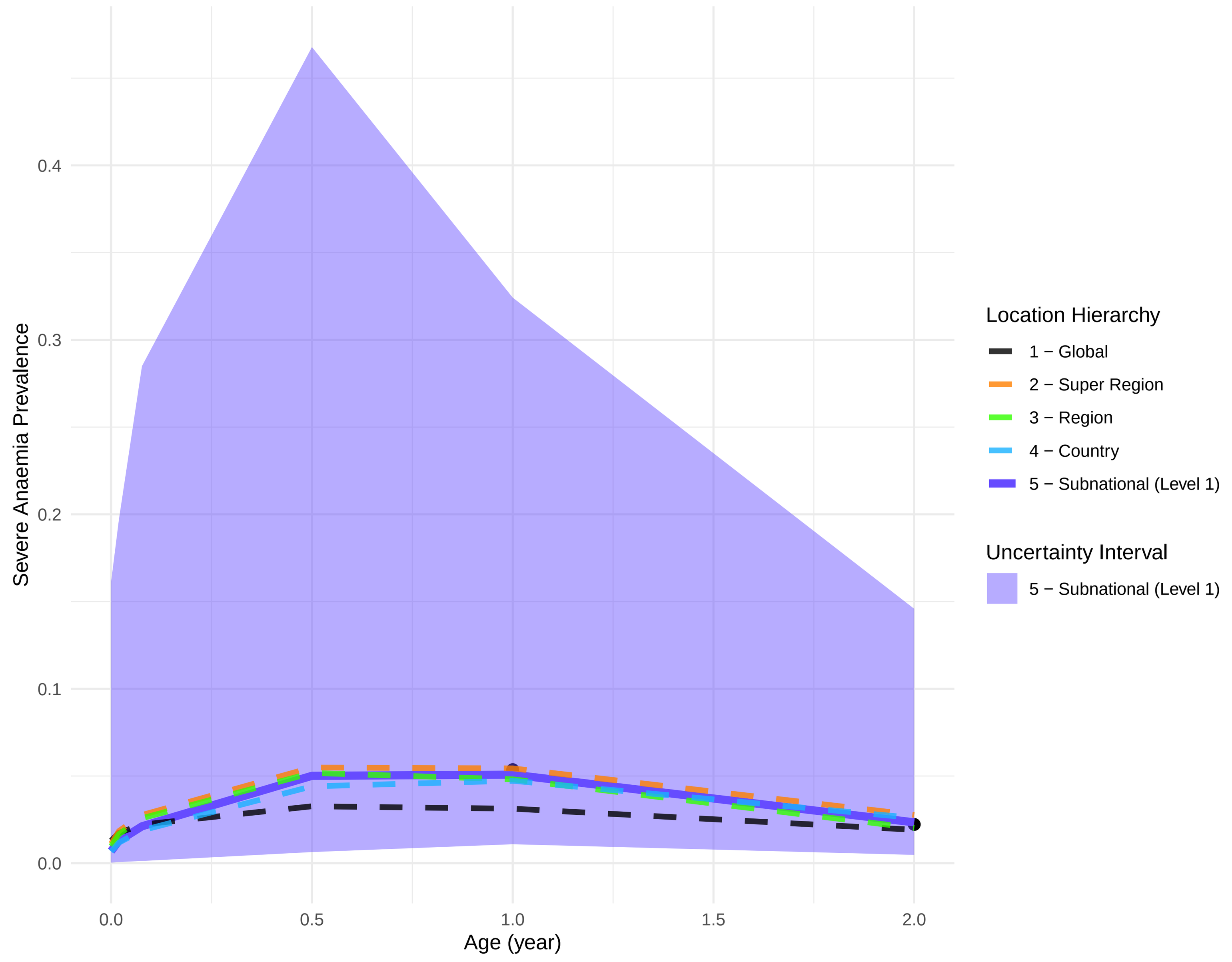

MR−BRT Age/Sex Fits − Non−pregnant Females <2 Years Old − Severe Anaemia Prevalence − Tana River
WHO−2024 Adjustment Method

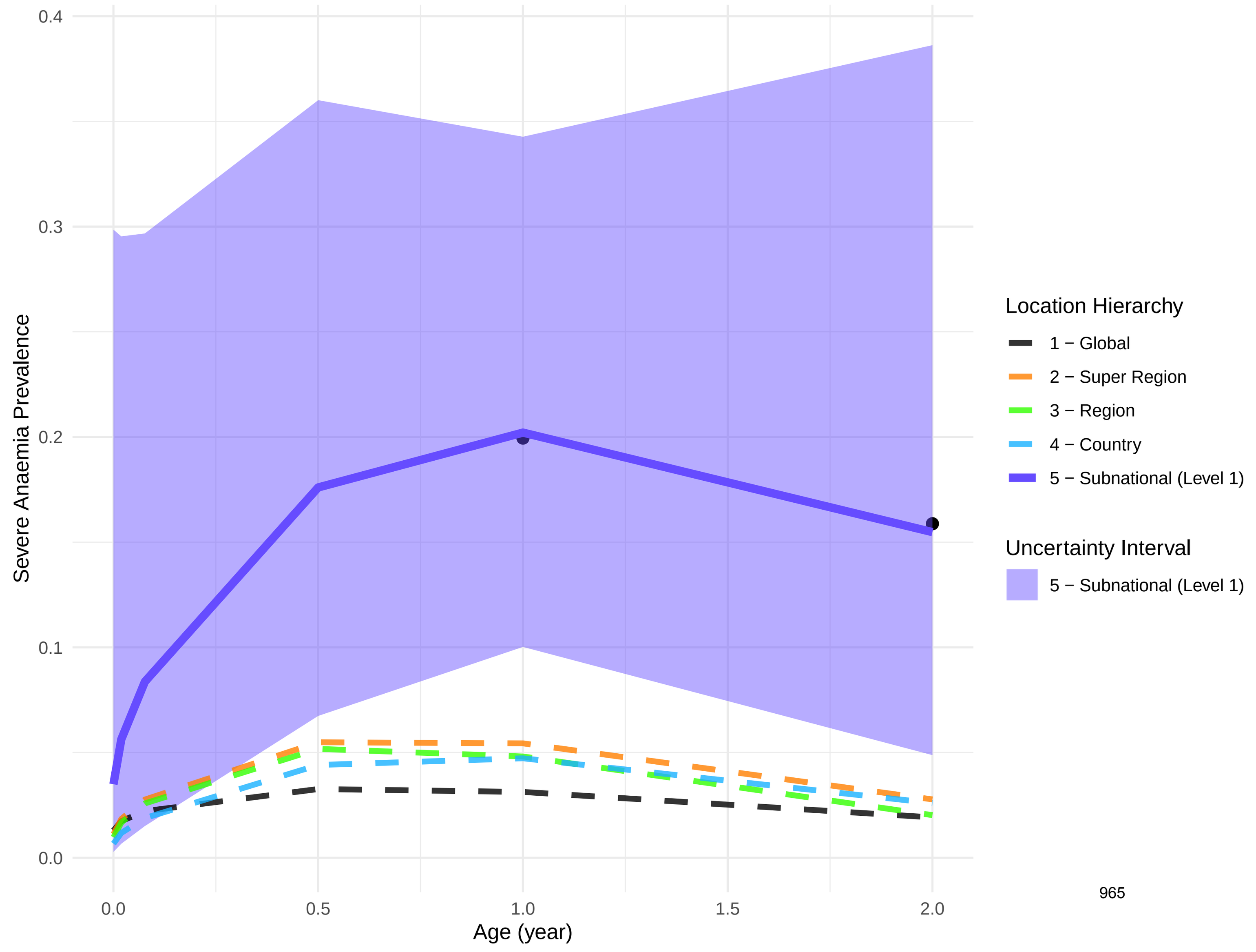

MR−BRT Age/Sex Fits − Non−pregnant Females <2 Years Old − Severe Anaemia Prevalence − Vihiga
WHO−2024 Adjustment Method

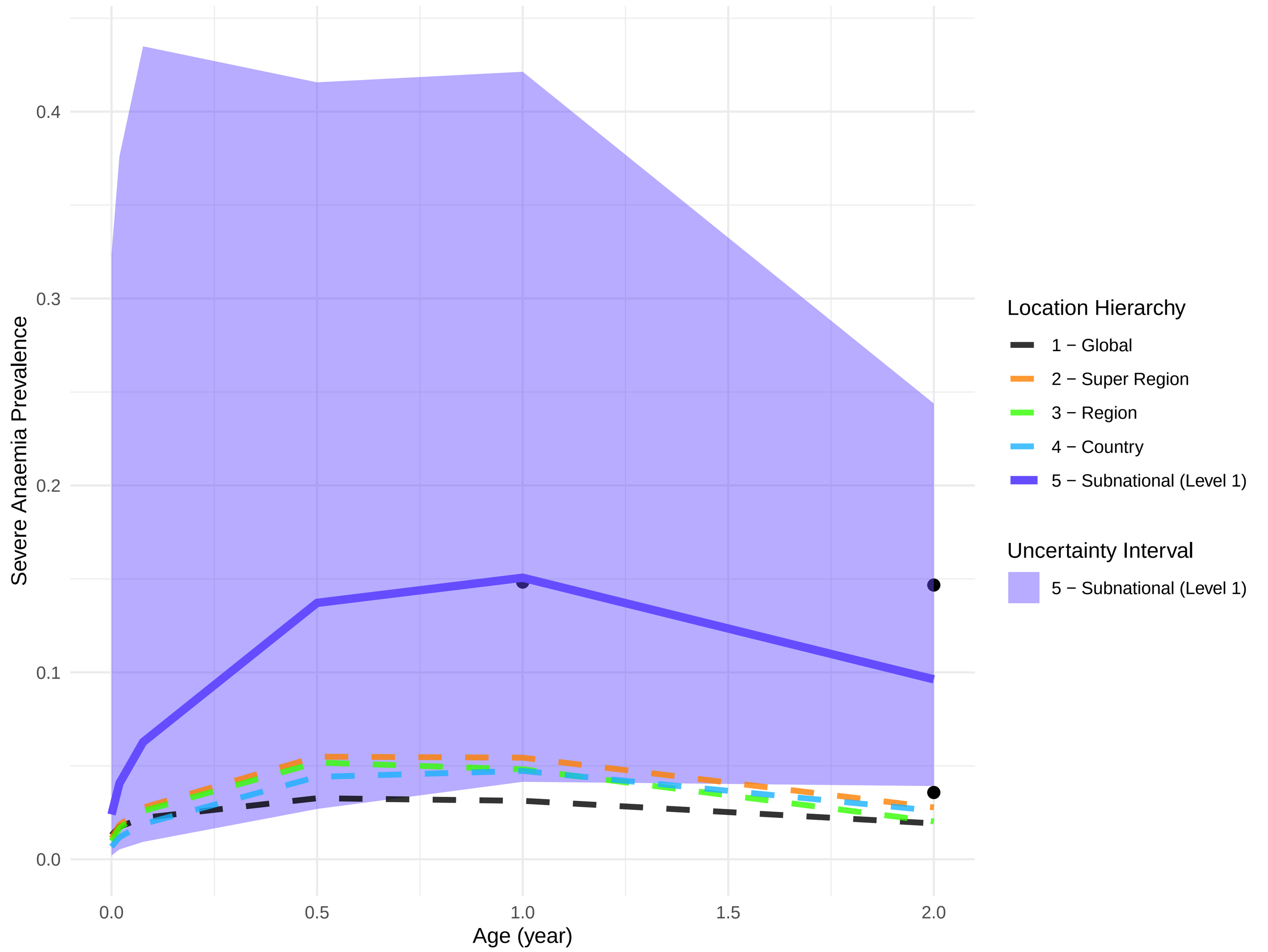

MR−BRT Age/Sex Fits − Non−pregnant Females <2 Years Old − Severe Anaemia Prevalence − Wajir
WHO−2024 Adjustment Method

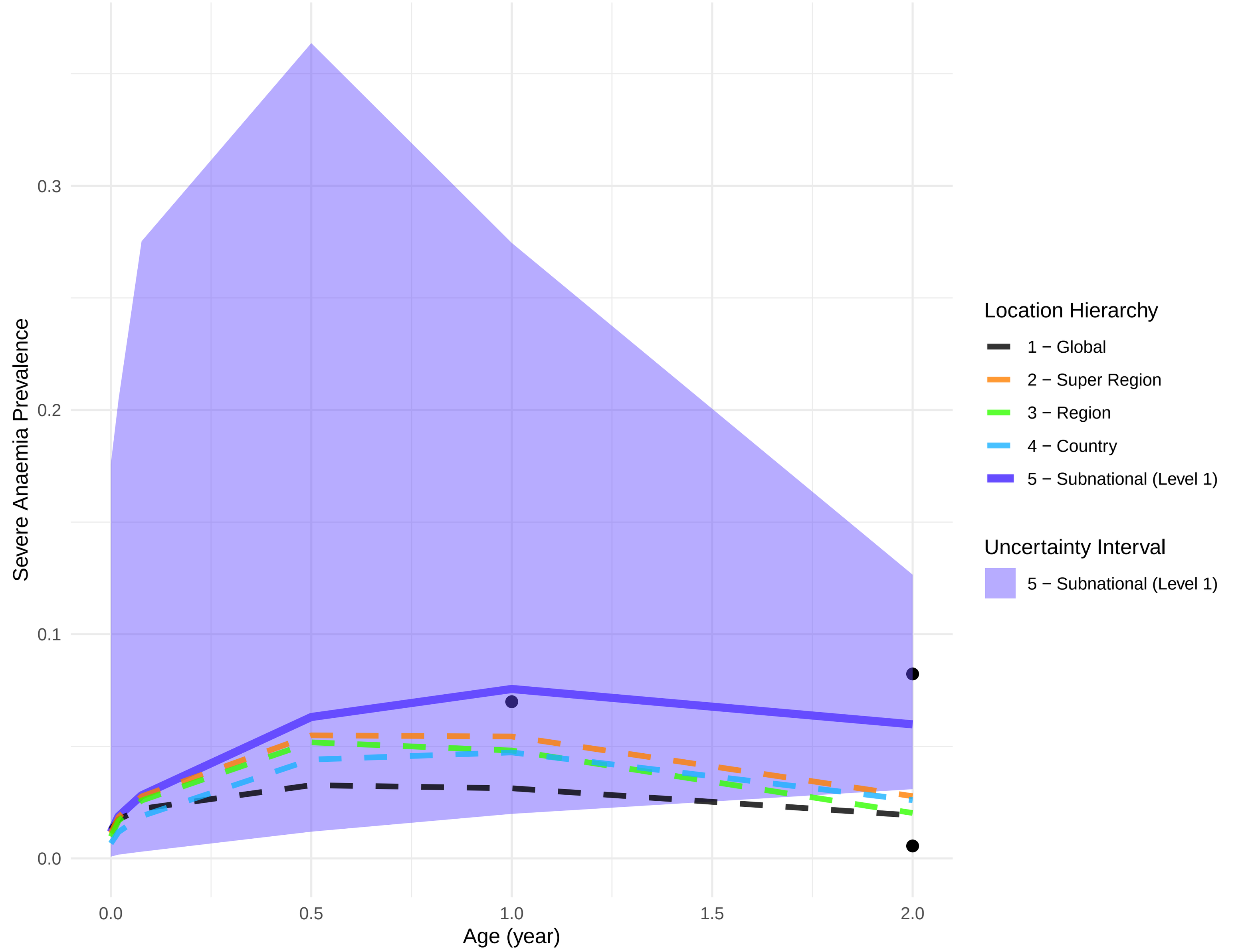

MR−BRT Age/Sex Fits − Non−pregnant Females <2 Years Old − Severe Anaemia Prevalence − West Pokot
WHO−2024 Adjustment Method

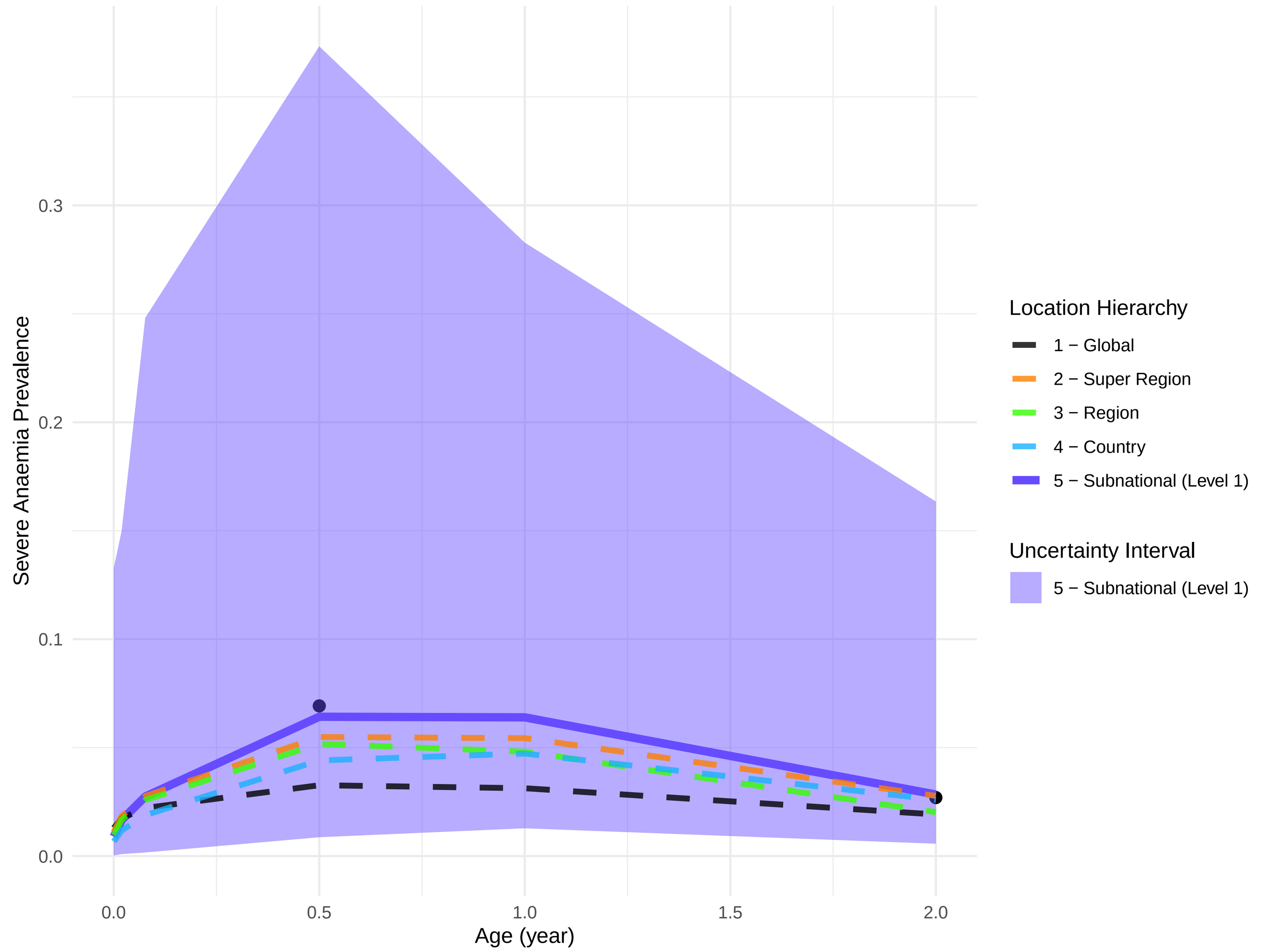

MR−BRT Age/Sex Fits − Non−pregnant Females <2 Years Old − Severe Anaemia Prevalence − Madagascar
WHO−2024 Adjustment Method

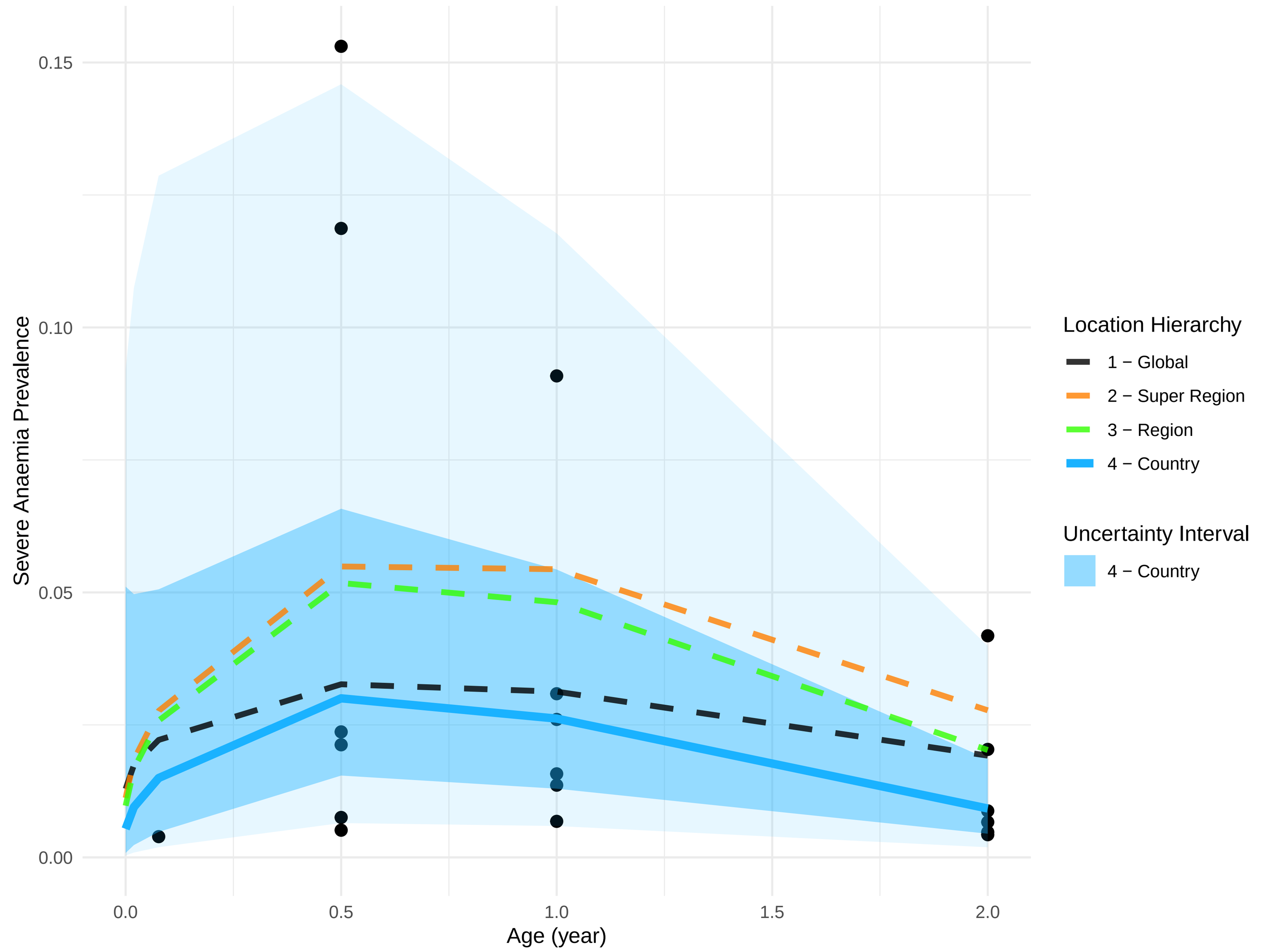

MR−BRT Age/Sex Fits − Non−pregnant Females <2 Years Old − Severe Anaemia Prevalence − Malawi
WHO−2024 Adjustment Method

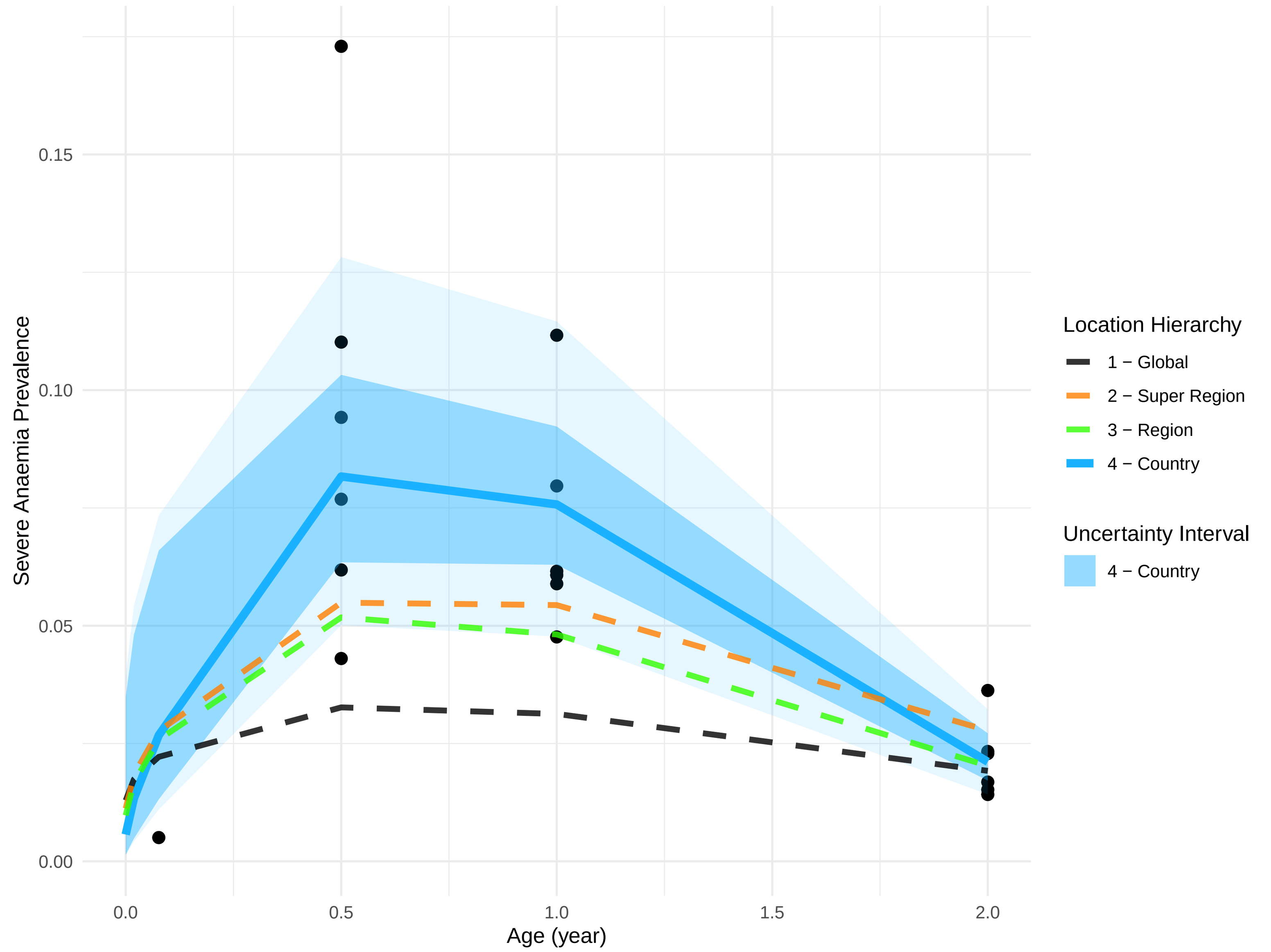

MR−BRT Age/Sex Fits − Non−pregnant Females <2 Years Old − Severe Anaemia Prevalence − Mozambique
WHO−2024 Adjustment Method

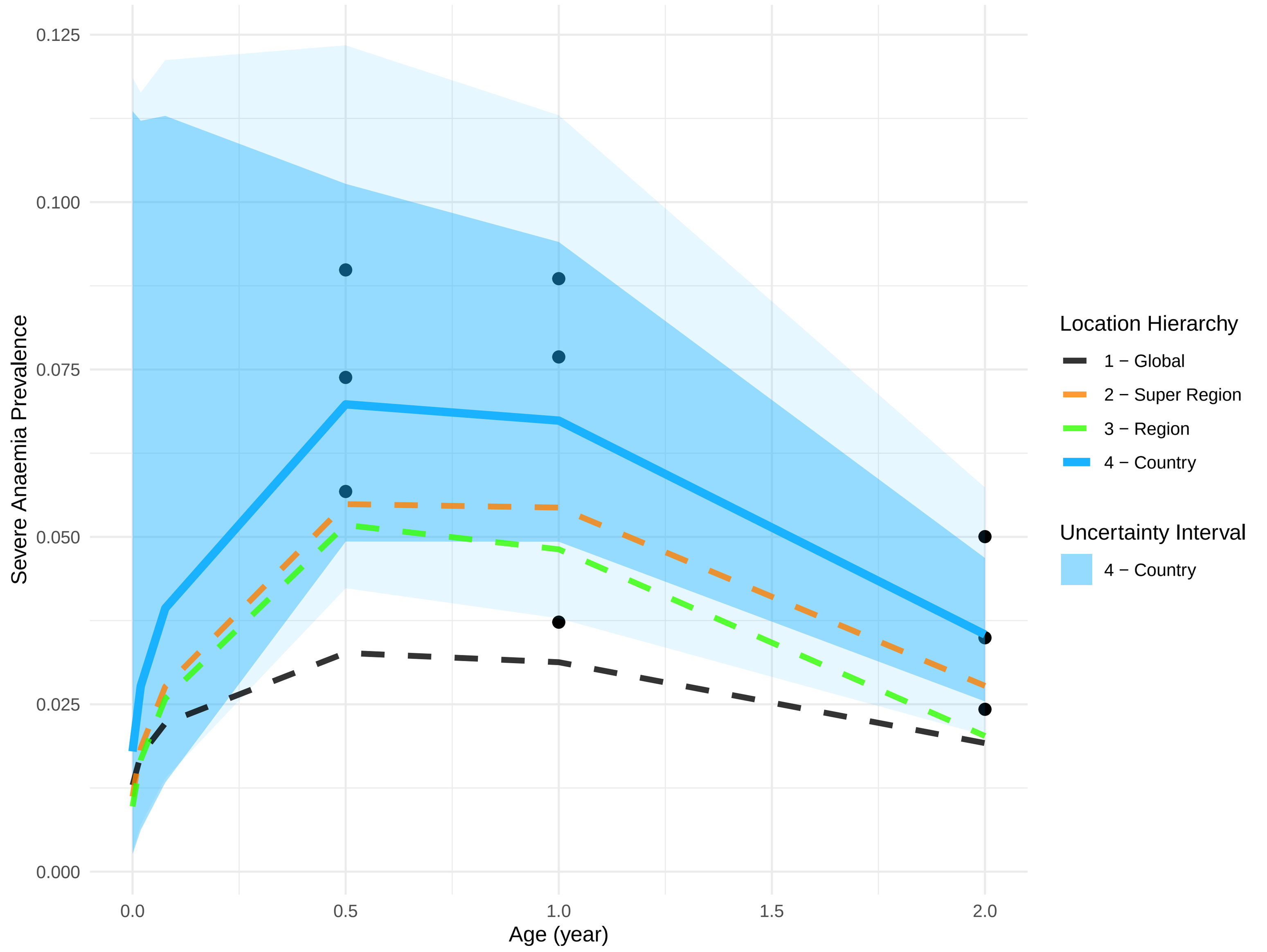

MR−BRT Age/Sex Fits − Non−pregnant Females <2 Years Old − Severe Anaemia Prevalence − Rwanda
WHO−2024 Adjustment Method

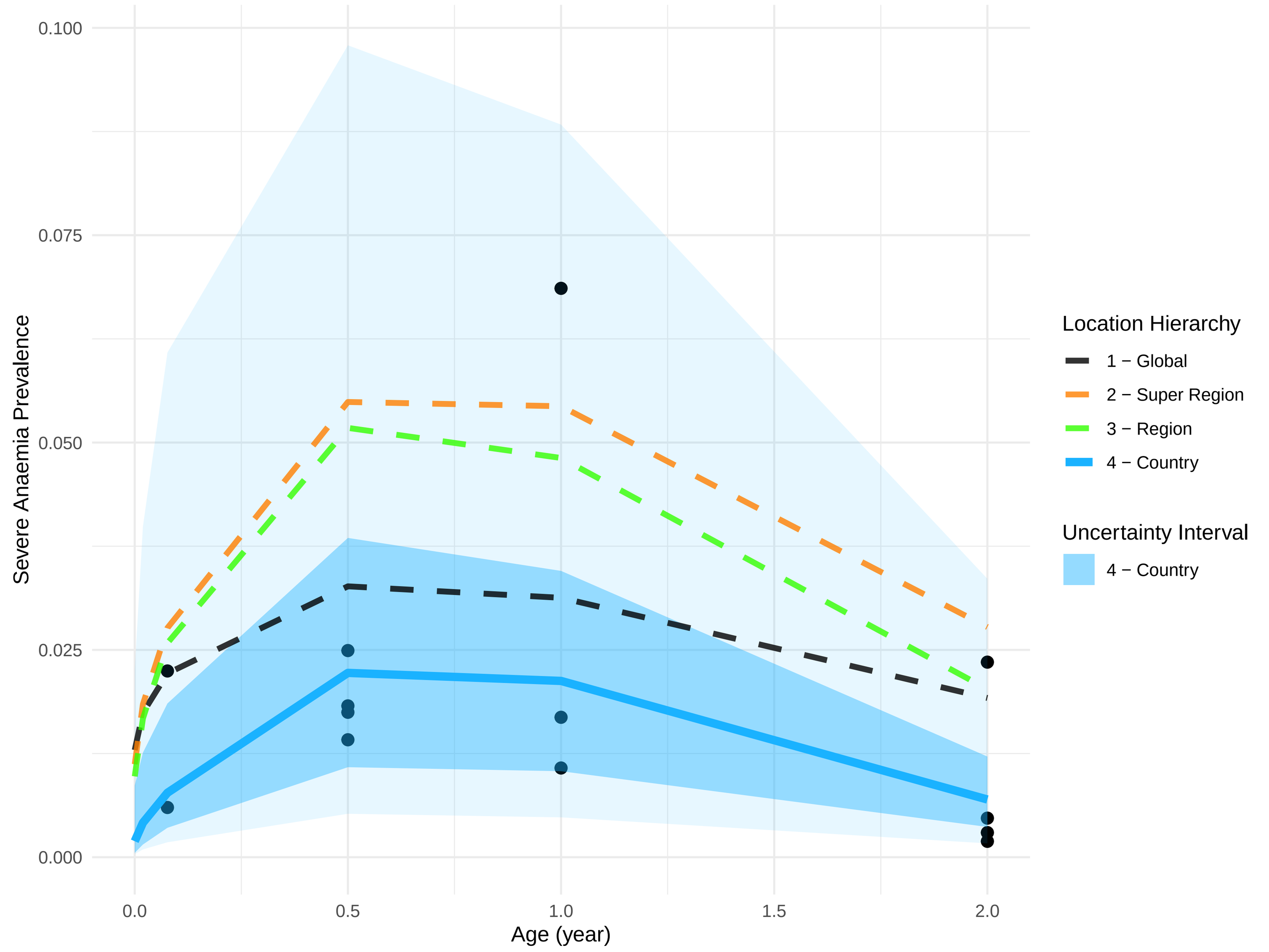

MR−BRT Age/Sex Fits − Non−pregnant Females <2 Years Old − Severe Anaemia Prevalence − Uganda
WHO−2024 Adjustment Method

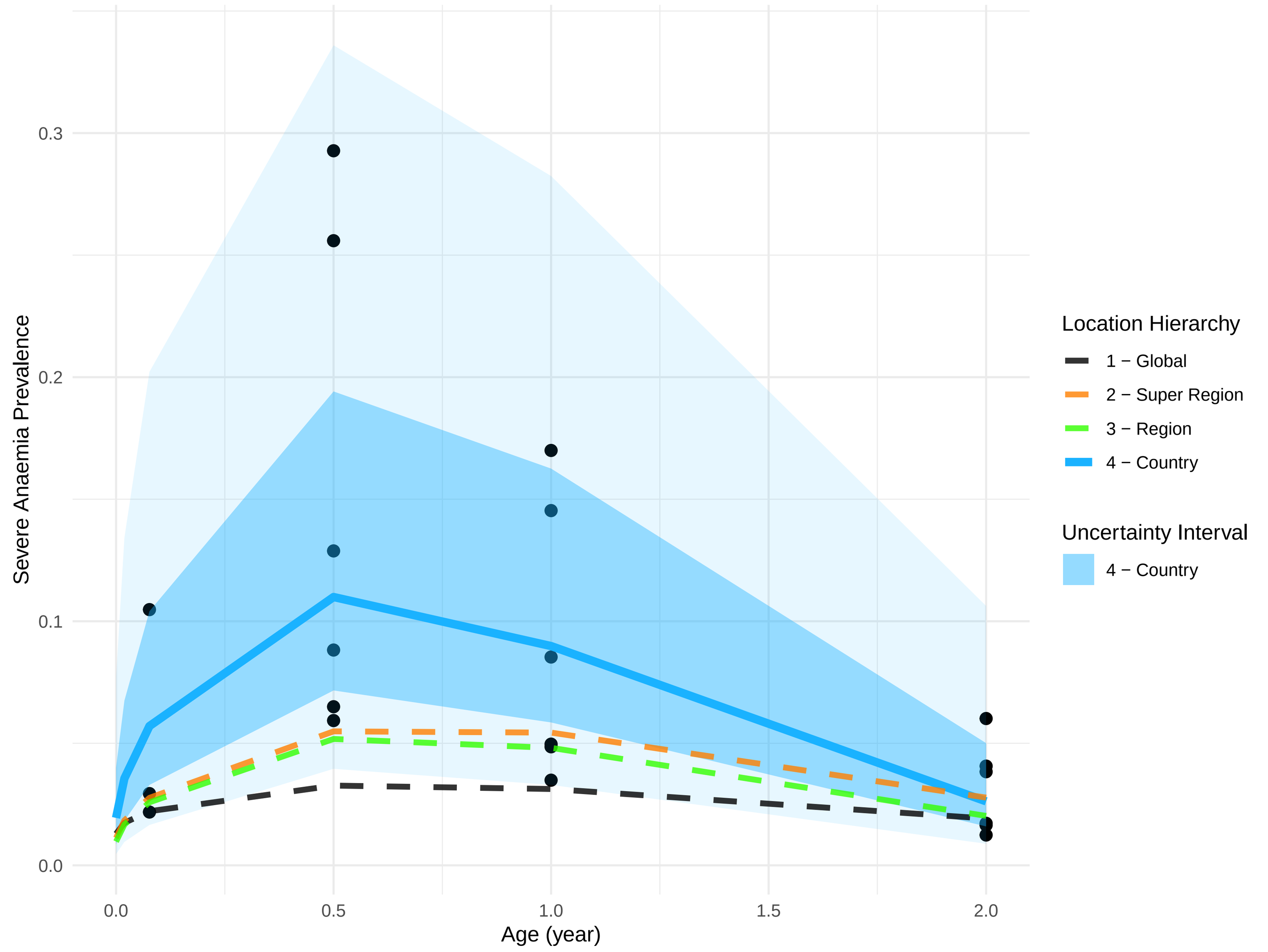

MR−BRT Age/Sex Fits − Non−pregnant Females <2 Years Old − Severe Anaemia Prevalence − United Republic of Tanzania
WHO−2024 Adjustment Method

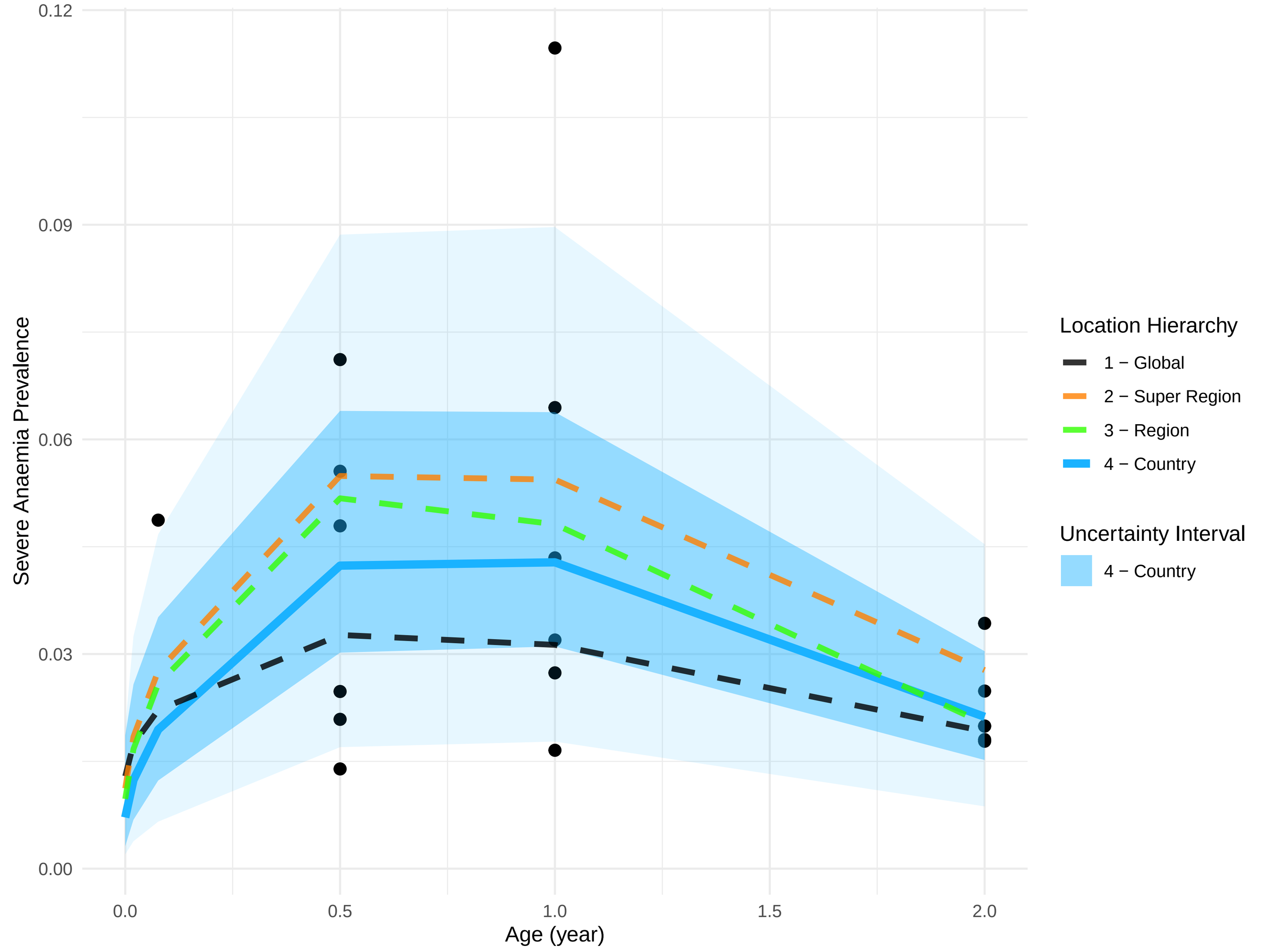

MR−BRT Age/Sex Fits − Non−pregnant Females <2 Years Old − Severe Anaemia Prevalence − Zambia
WHO−2024 Adjustment Method

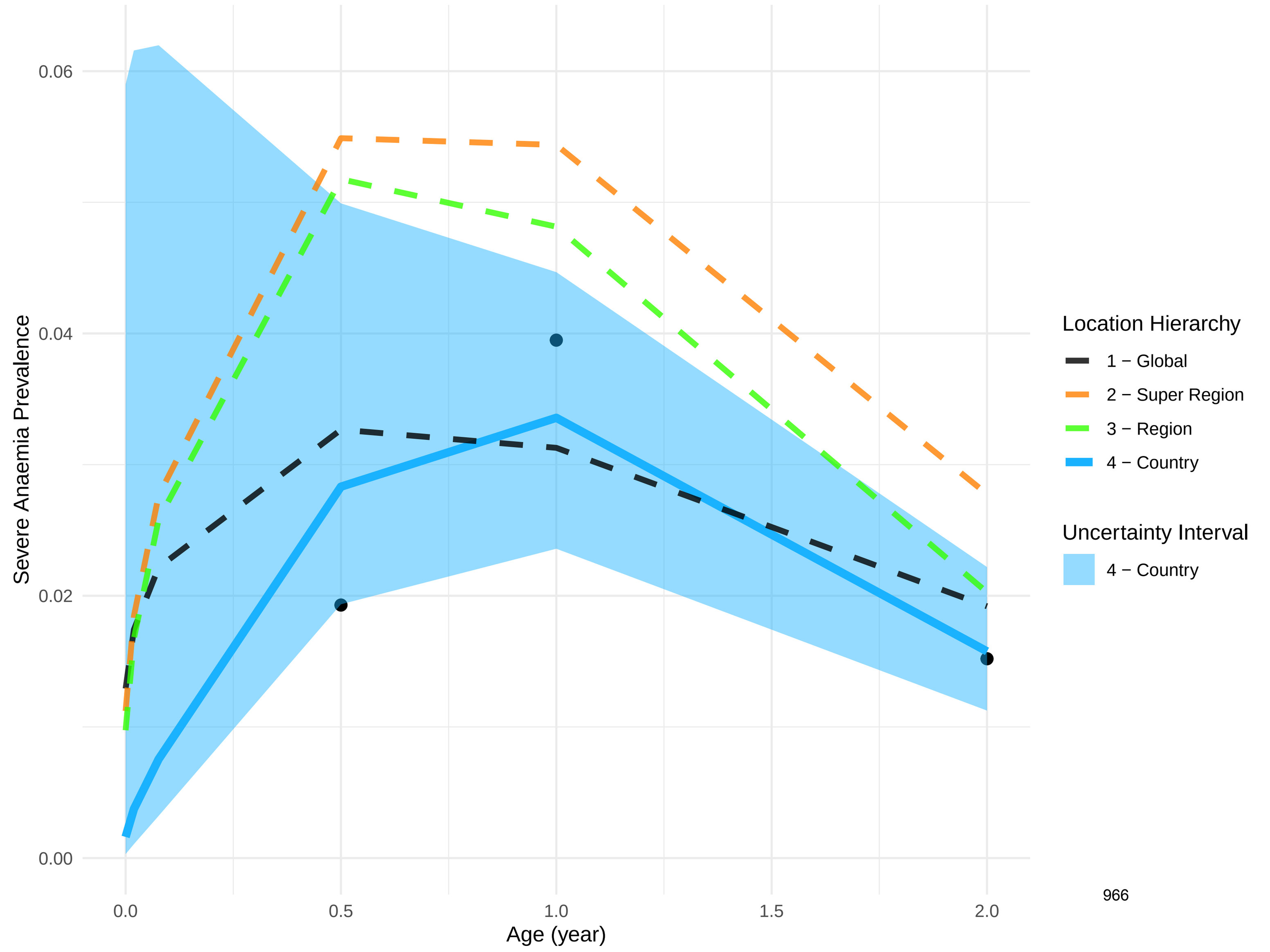

MR−BRT Age/Sex Fits − Non-pregnant Females <2 Years Old − Severe Anaemia Prevalence − Southern Sub−Saharan Africa
WHO−2024 Adjustment Method

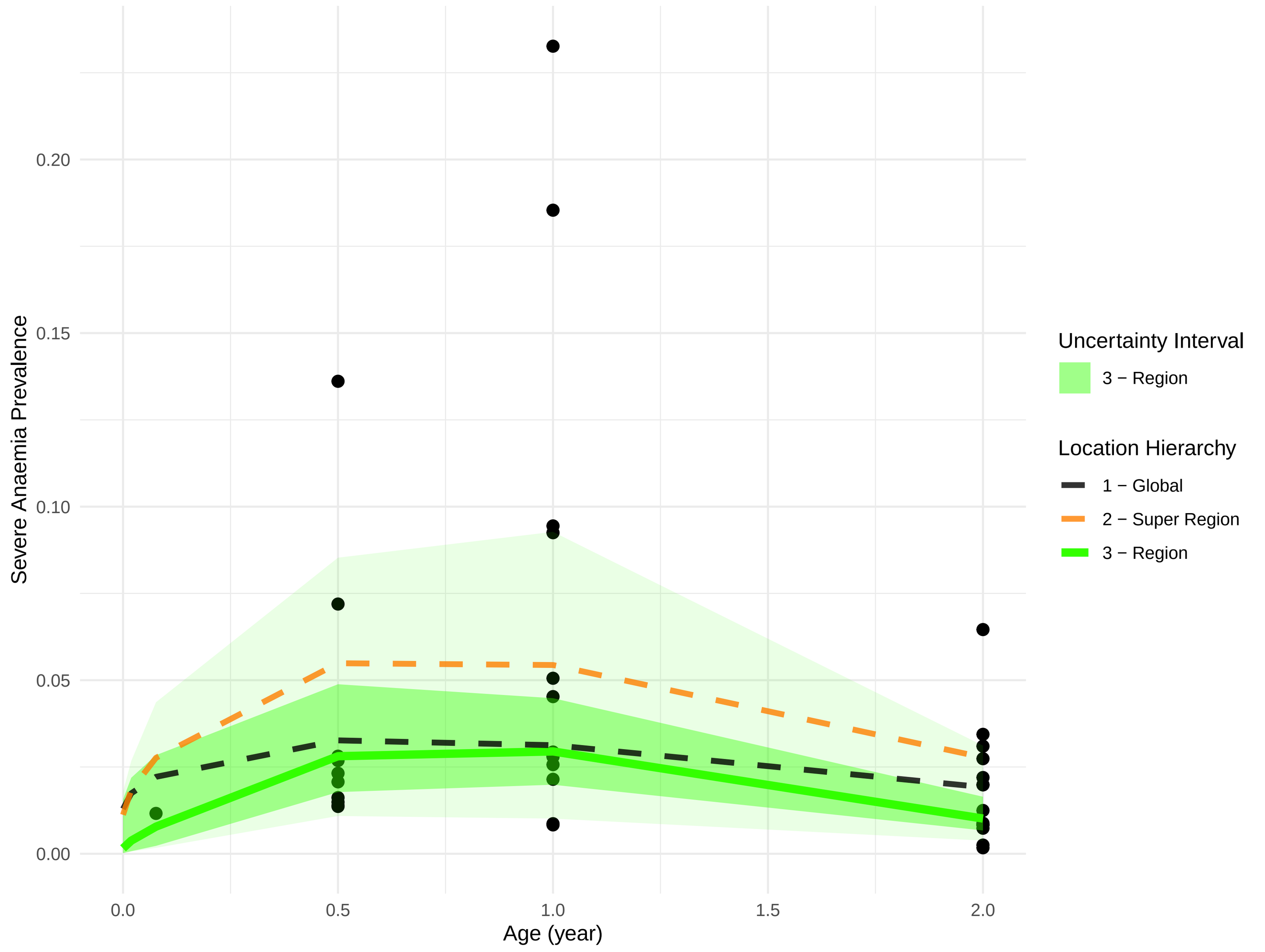

MR−BRT Age/Sex Fits − Non-pregnant Females <2 Years Old − Severe Anaemia Prevalence − Eswatini
WHO−2024 Adjustment Method

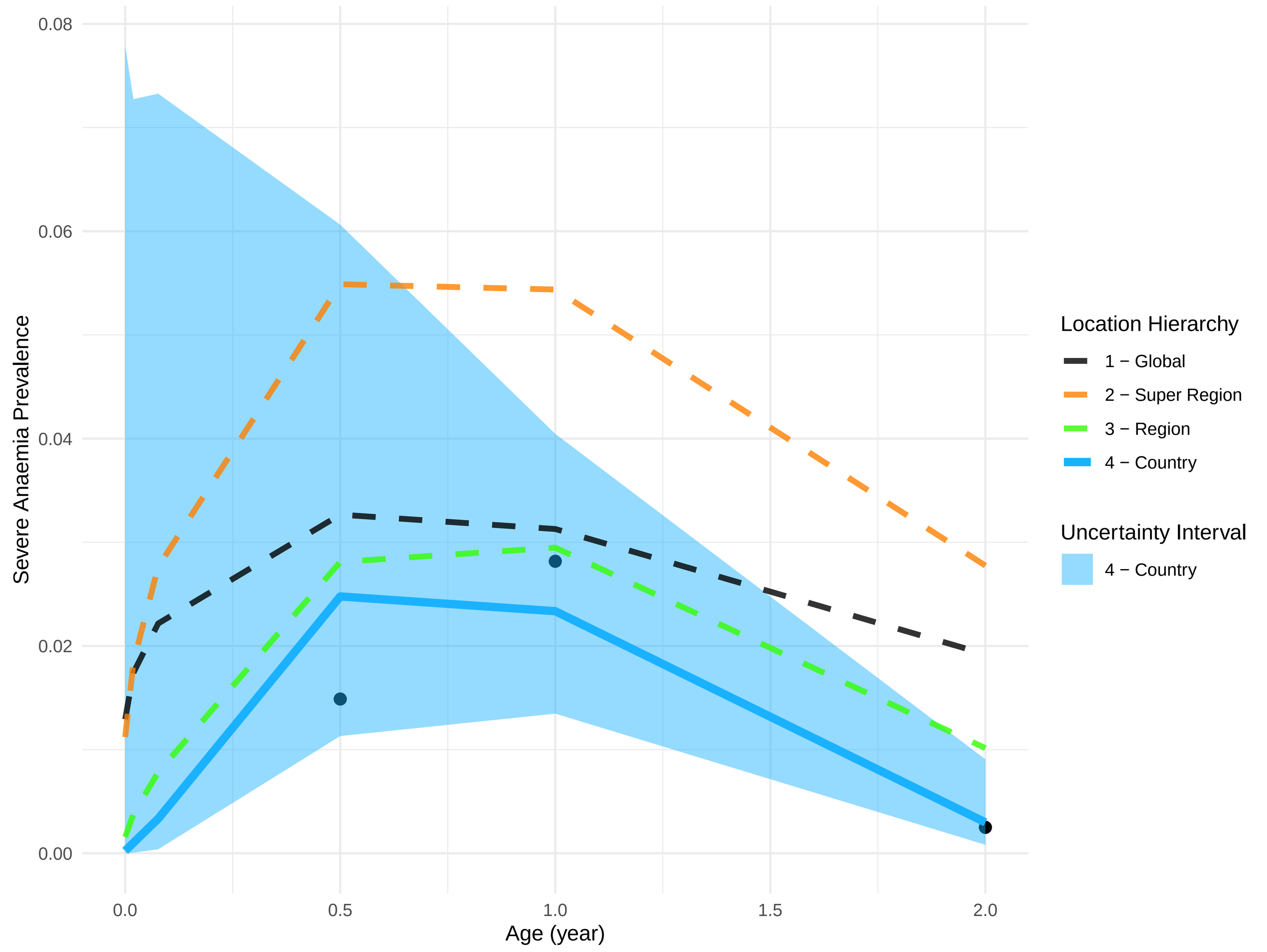

MR−BRT Age/Sex Fits − Non-pregnant Females <2 Years Old − Severe Anaemia Prevalence − Lesotho
WHO−2024 Adjustment Method

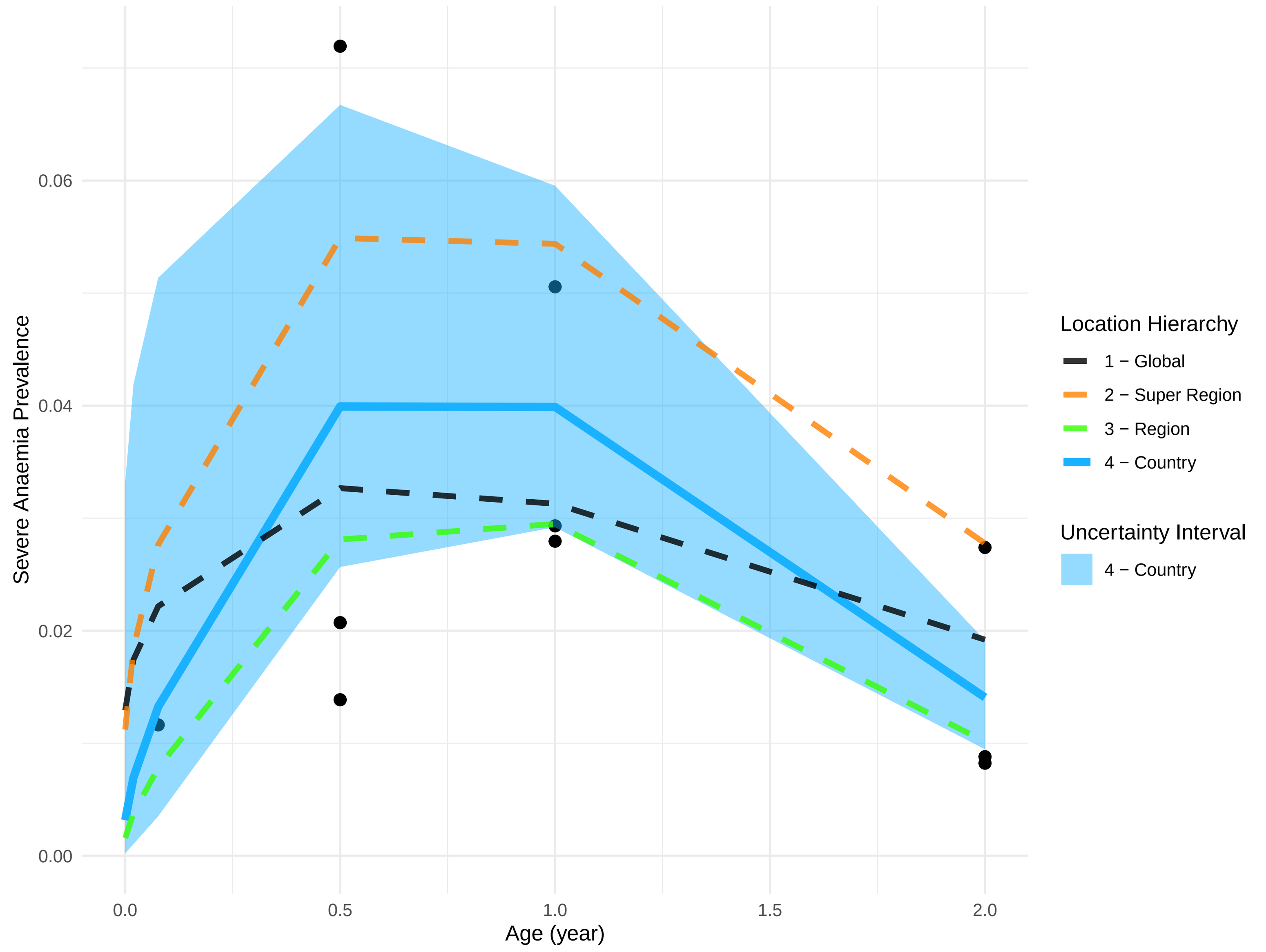

MR−BRT Age/Sex Fits − Non-pregnant Females <2 Years Old − Severe Anaemia Prevalence − Namibia
WHO−2024 Adjustment Method

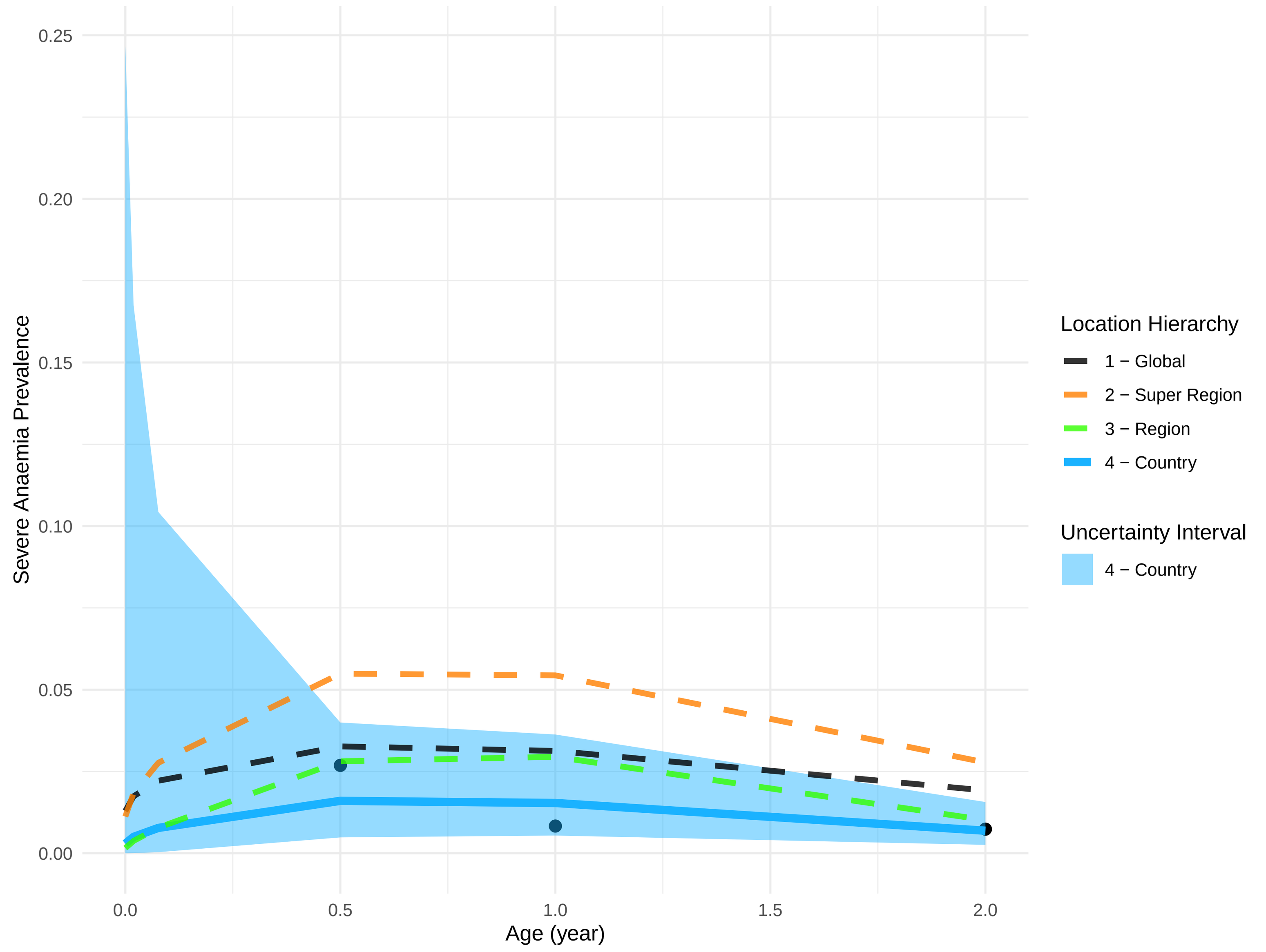

MR−BRT Age/Sex Fits − Non-pregnant Females <2 Years Old − Severe Anaemia Prevalence − South Africa
WHO−2024 Adjustment Method

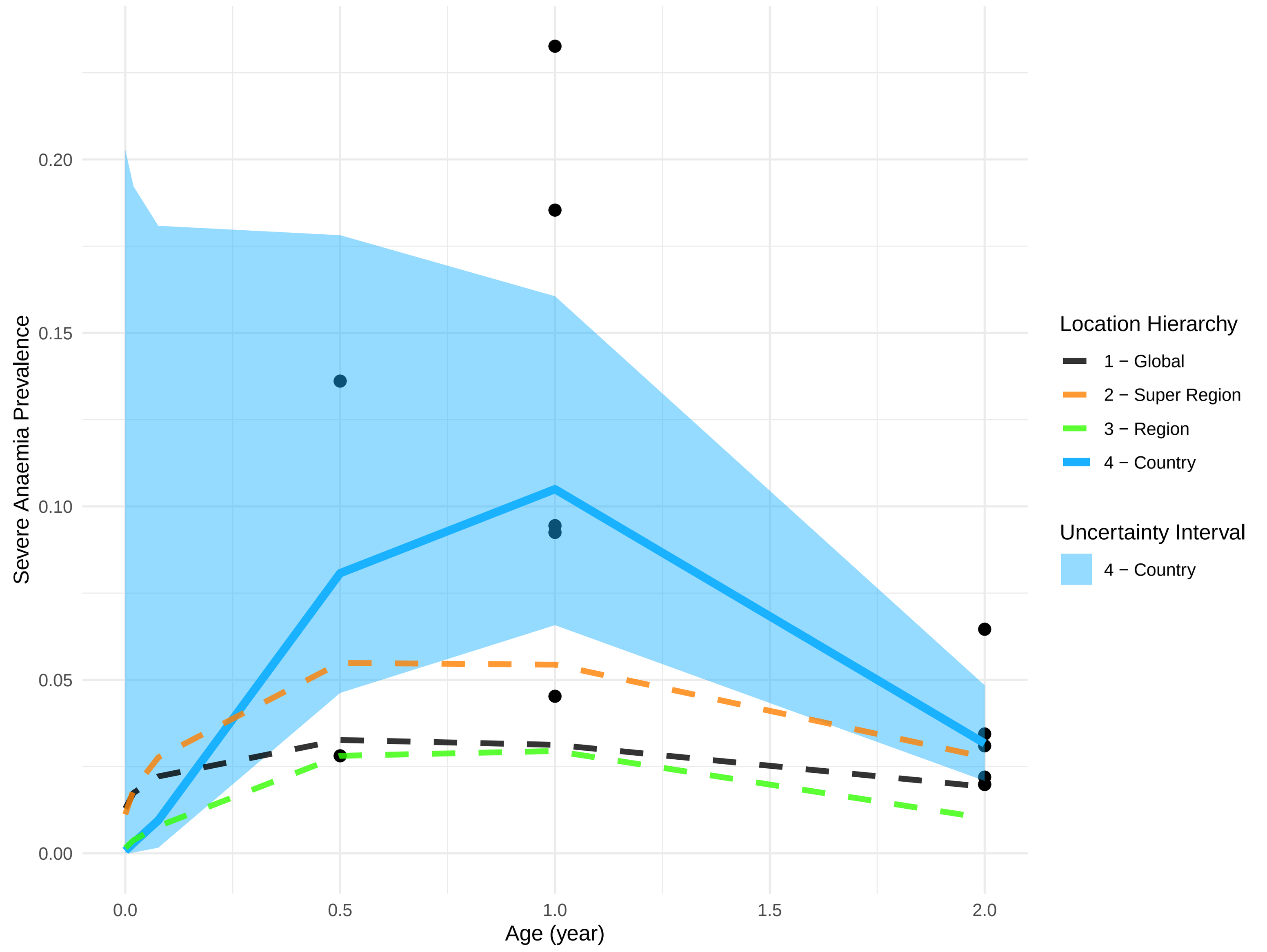

MR−BRT Age/Sex Fits − Non-pregnant Females <2 Years Old − Severe Anaemia Prevalence − Free State
WHO−2024 Adjustment Method

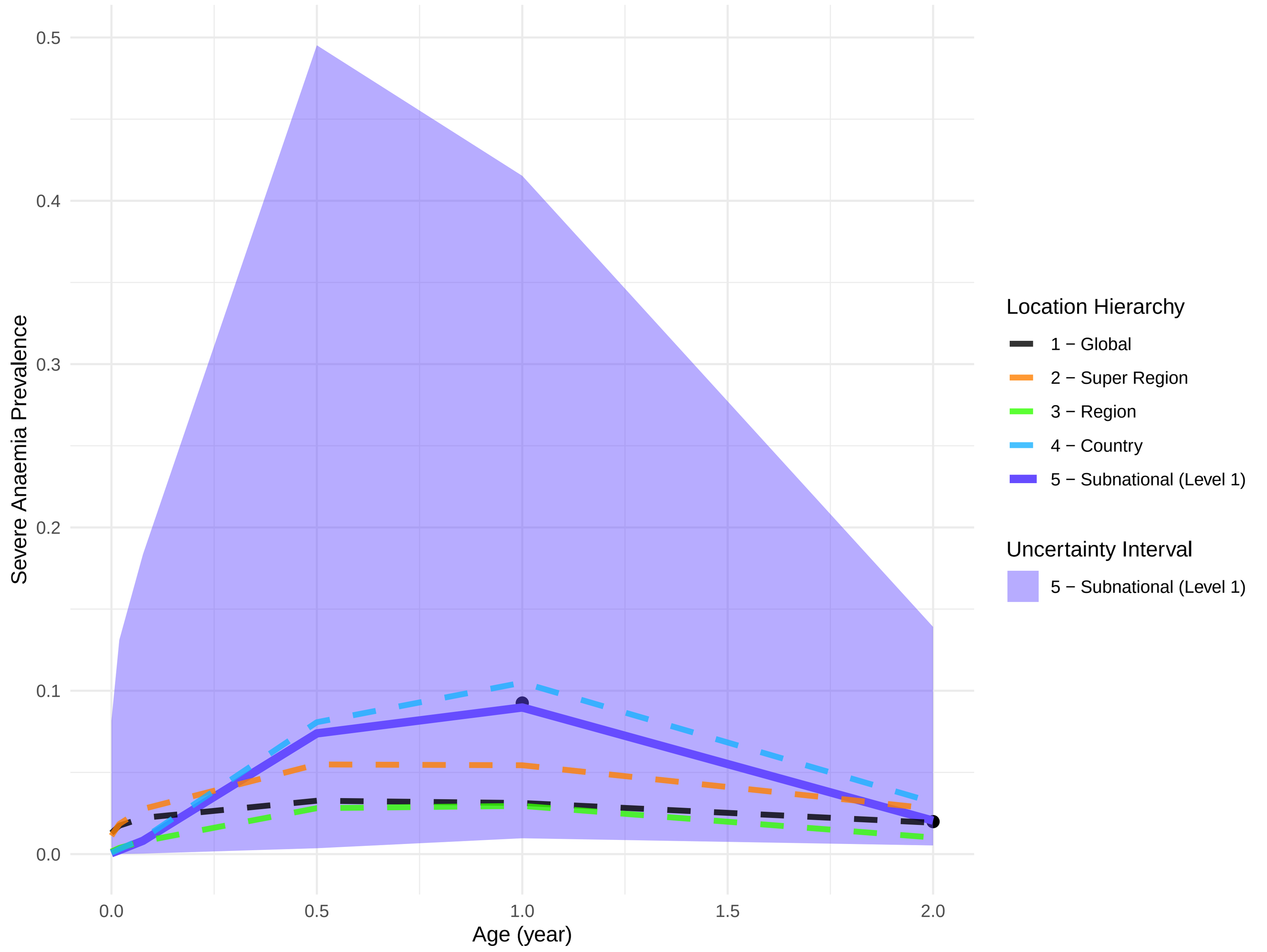

MR−BRT Age/Sex Fits − Non-pregnant Females <2 Years Old − Severe Anaemia Prevalence − Gauteng
WHO−2024 Adjustment Method

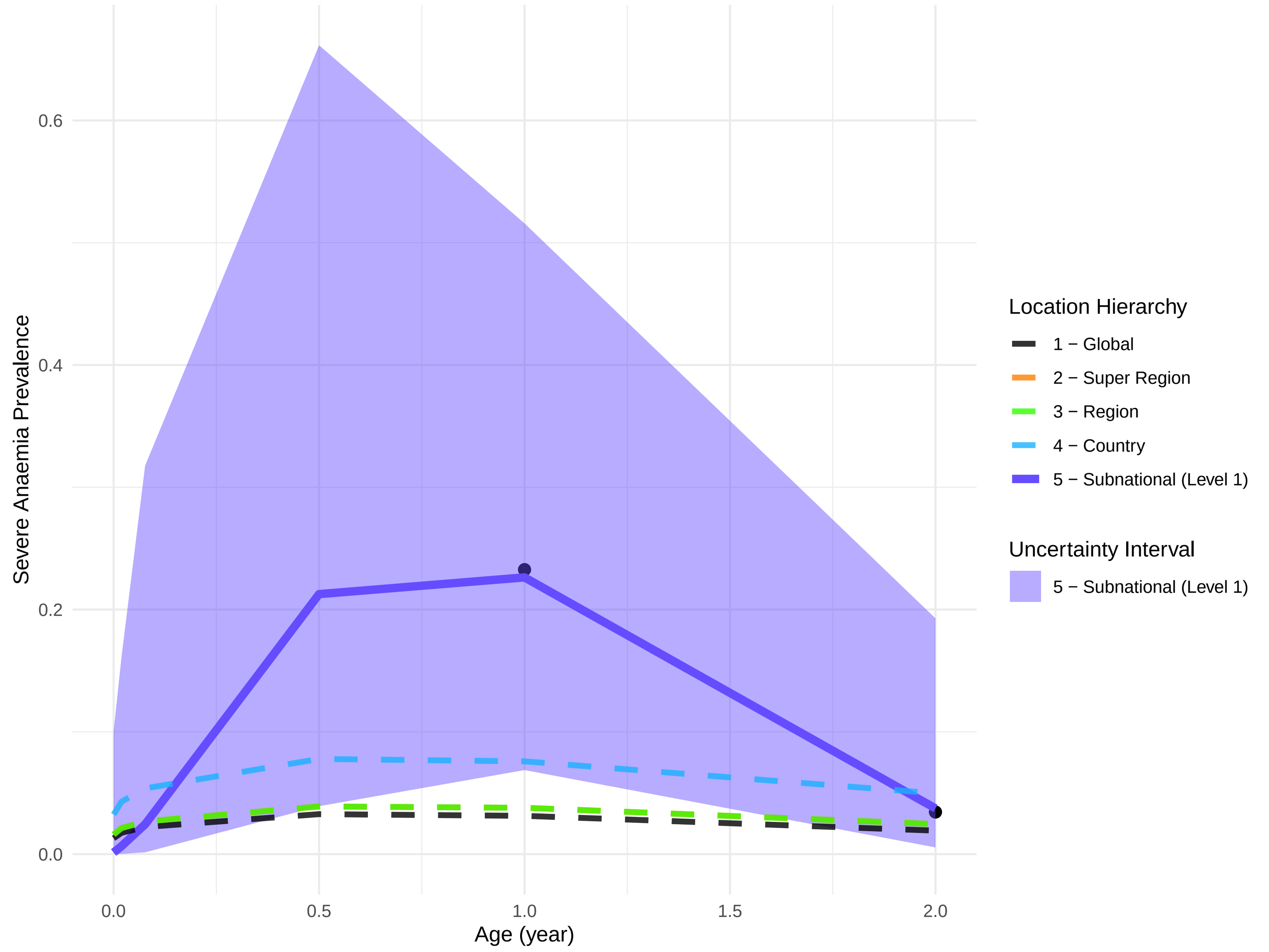

MR−BRT Age/Sex Fits − Non-pregnant Females <2 Years Old − Severe Anaemia Prevalence − KwaZulu−Natal
WHO−2024 Adjustment Method

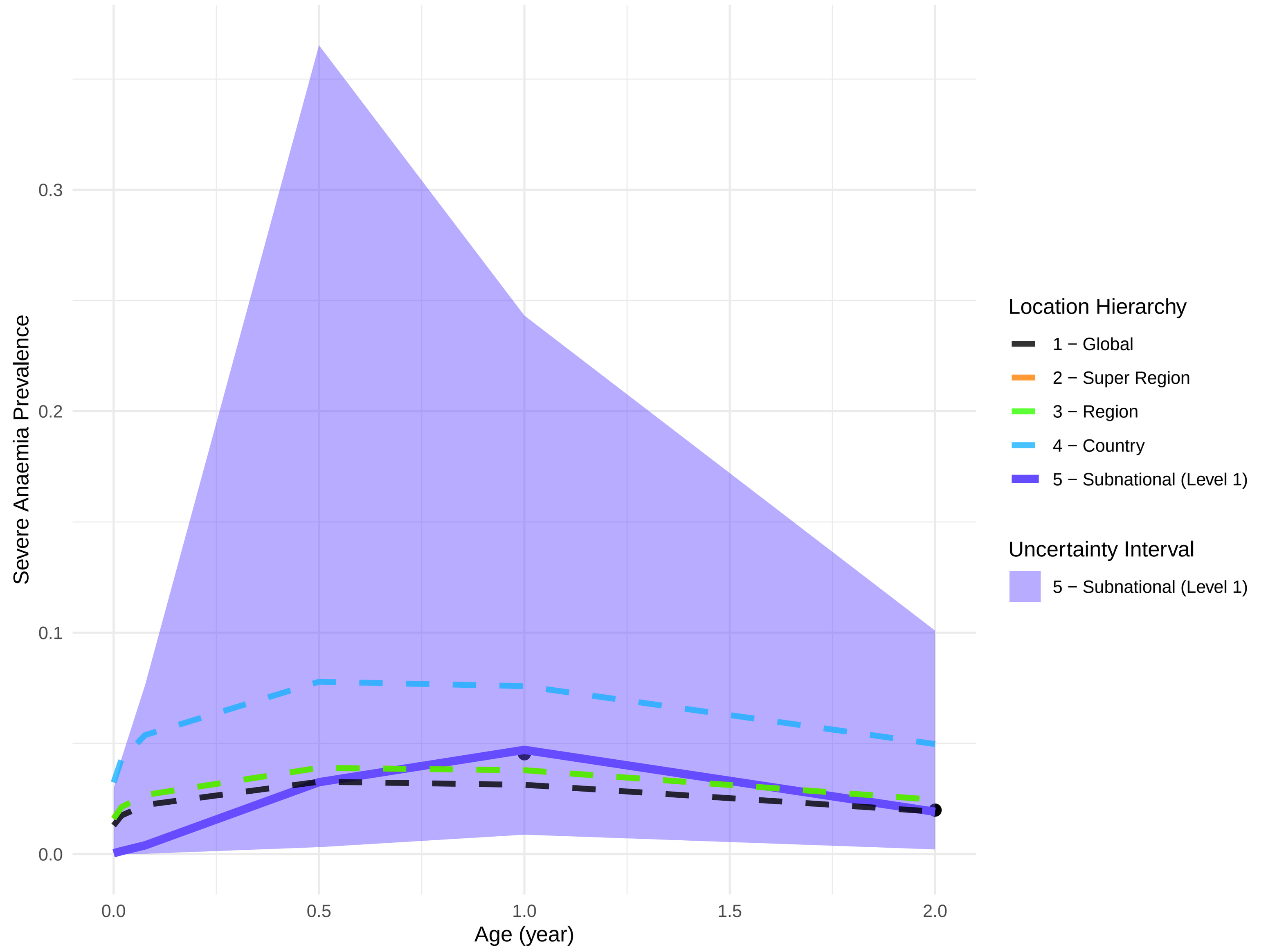

MR−BRT Age/Sex Fits − Non-pregnant Females <2 Years Old − Severe Anaemia Prevalence − Mpumalanga
WHO−2024 Adjustment Method

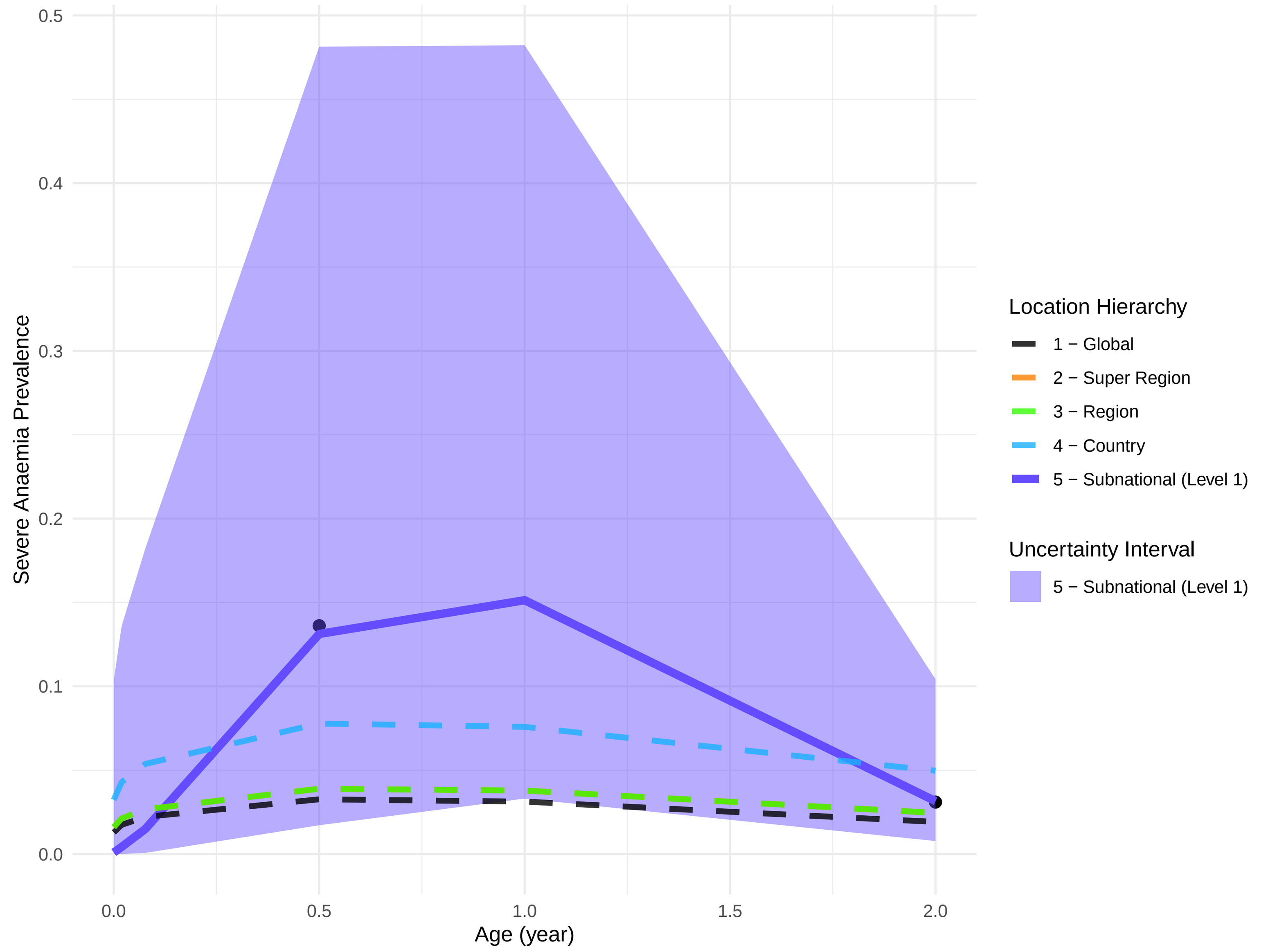

MR−BRT Age/Sex Fits − Non-pregnant Females <2 Years Old − Severe Anaemia Prevalence − North−West
WHO−2024 Adjustment Method

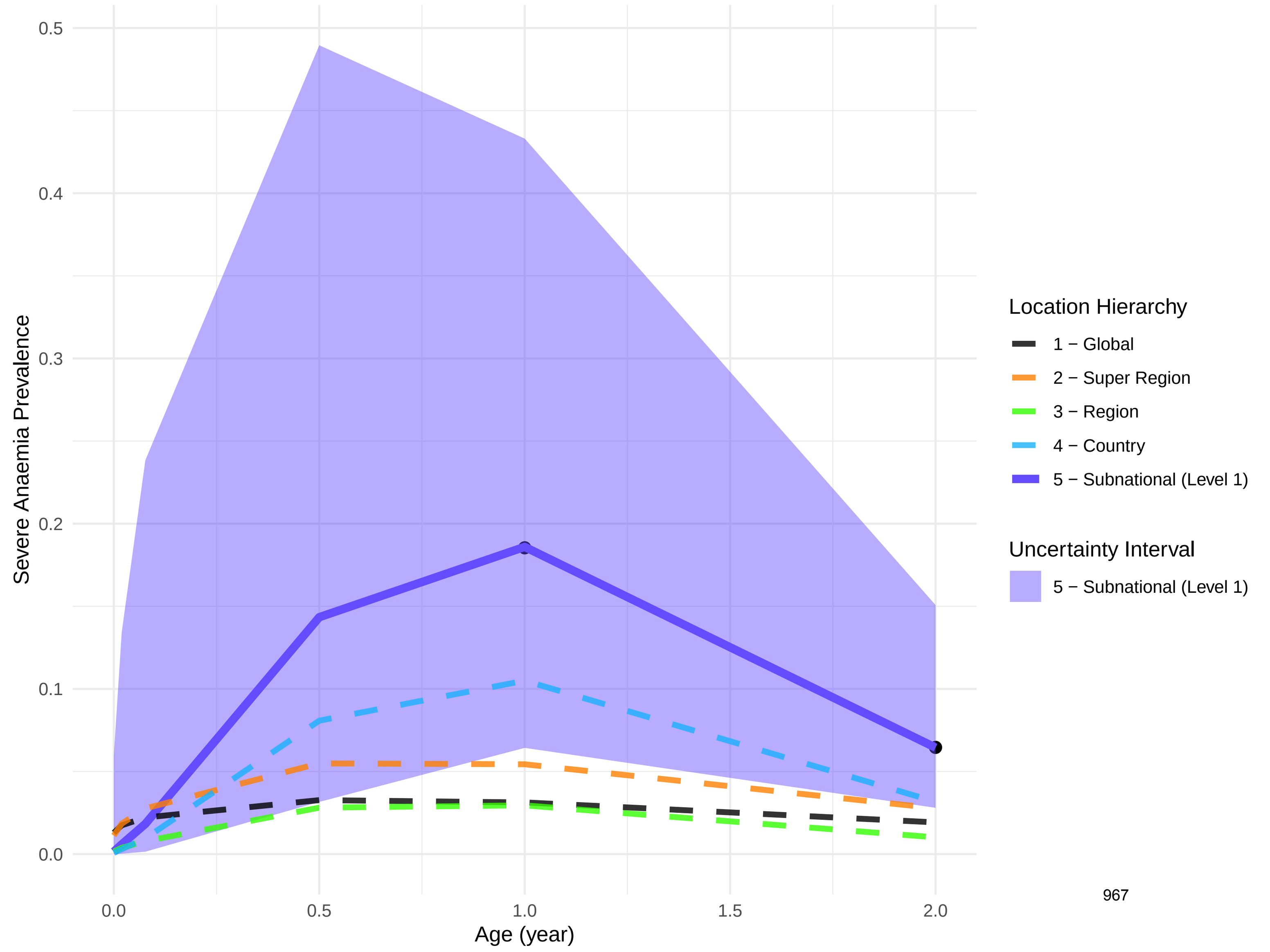

MR−BRT Age/Sex Fits − Non−pregnant Females <2 Years Old − Severe Anaemia Prevalence − Zimbabwe
WHO−2024 Adjustment Method

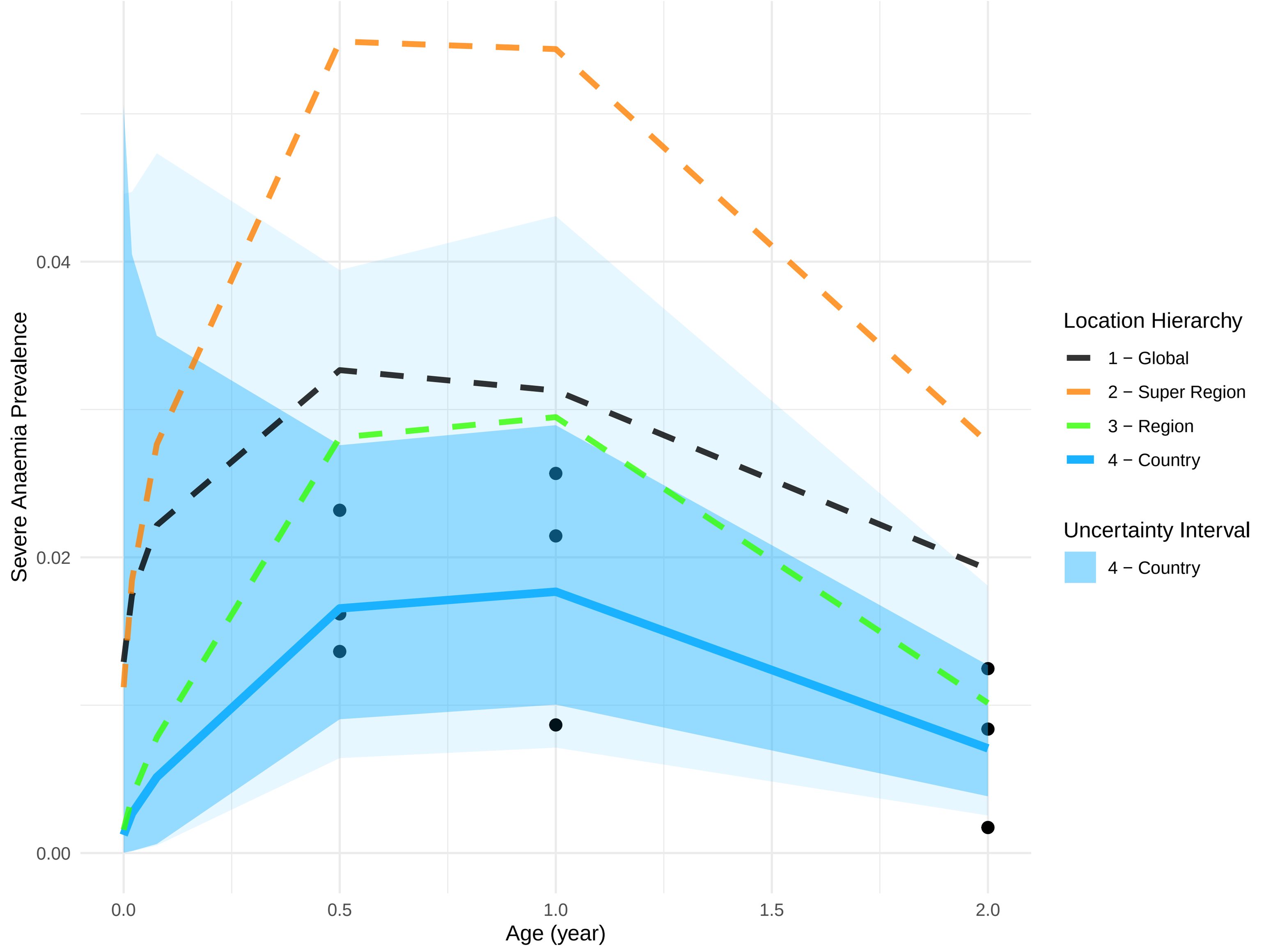

MR−BRT Age/Sex Fits − Non−pregnant Females <2 Years Old − Severe Anaemia Prevalence − Western Sub−Saharan Africa
WHO−2024 Adjustment Method

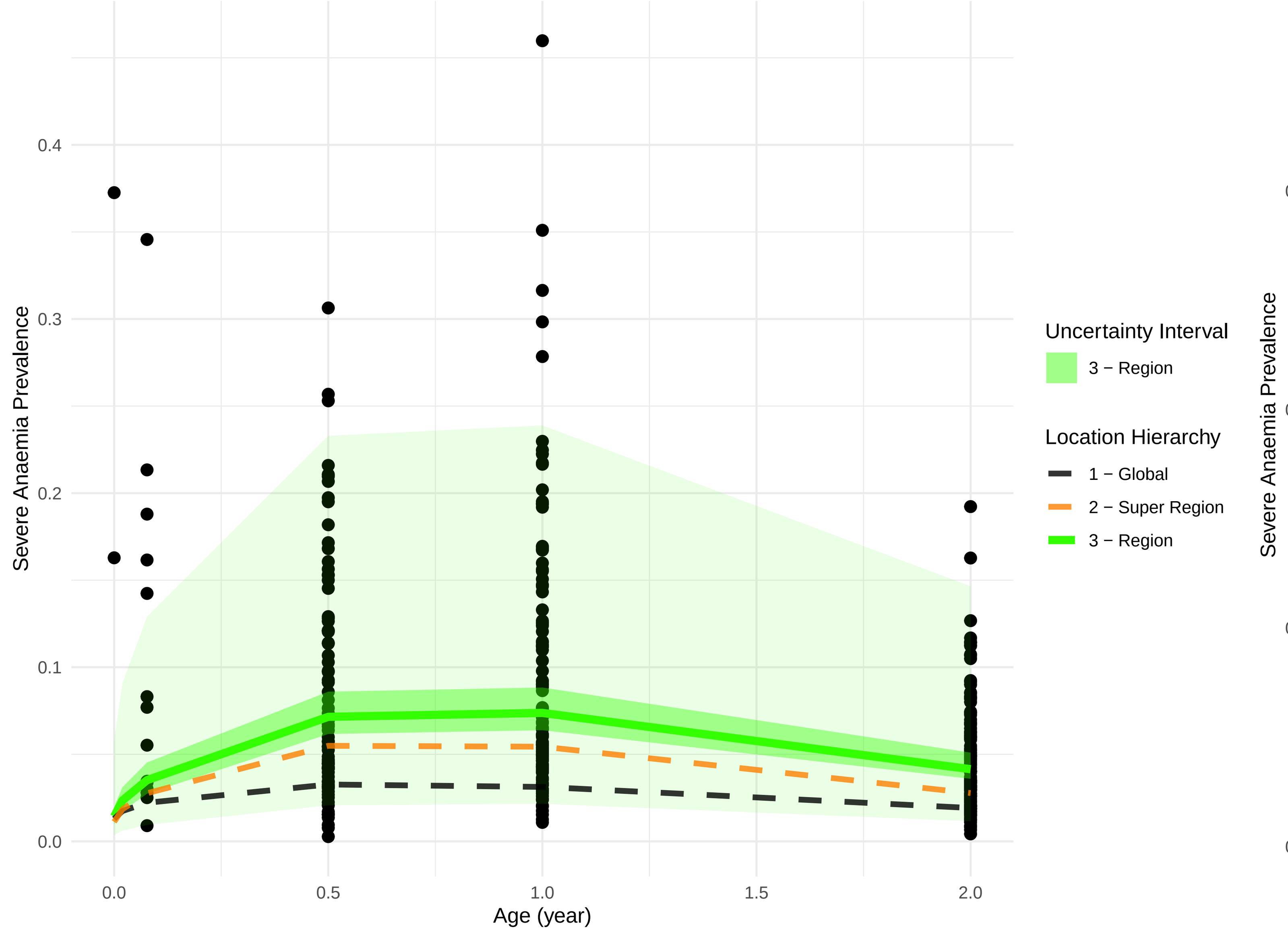

MR−BRT Age/Sex Fits − Non−pregnant Females <2 Years Old − Severe Anaemia Prevalence − Benin
WHO−2024 Adjustment Method

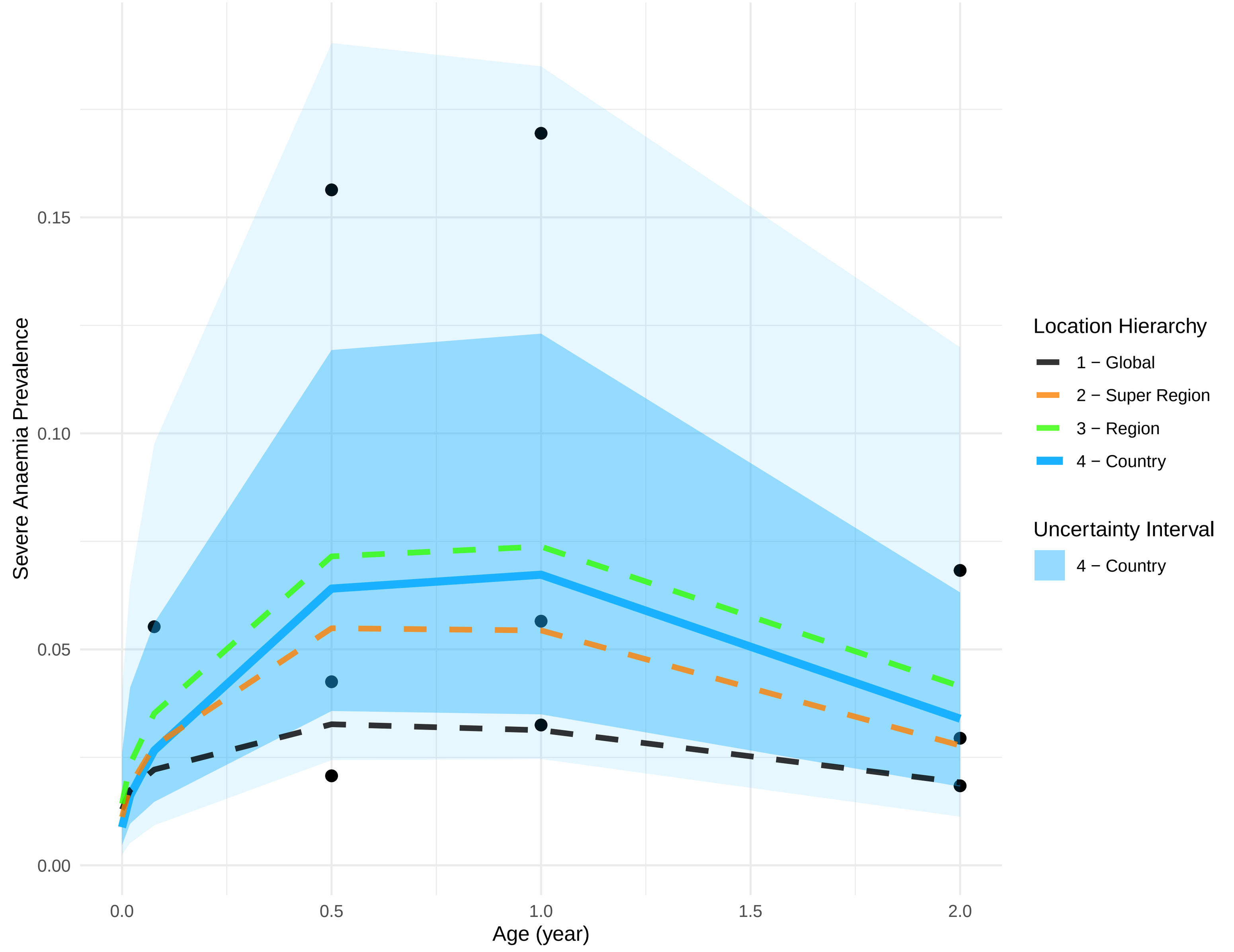

MR−BRT Age/Sex Fits − Non−pregnant Females <2 Years Old − Severe Anaemia Prevalence − Burkina Faso
WHO−2024 Adjustment Method

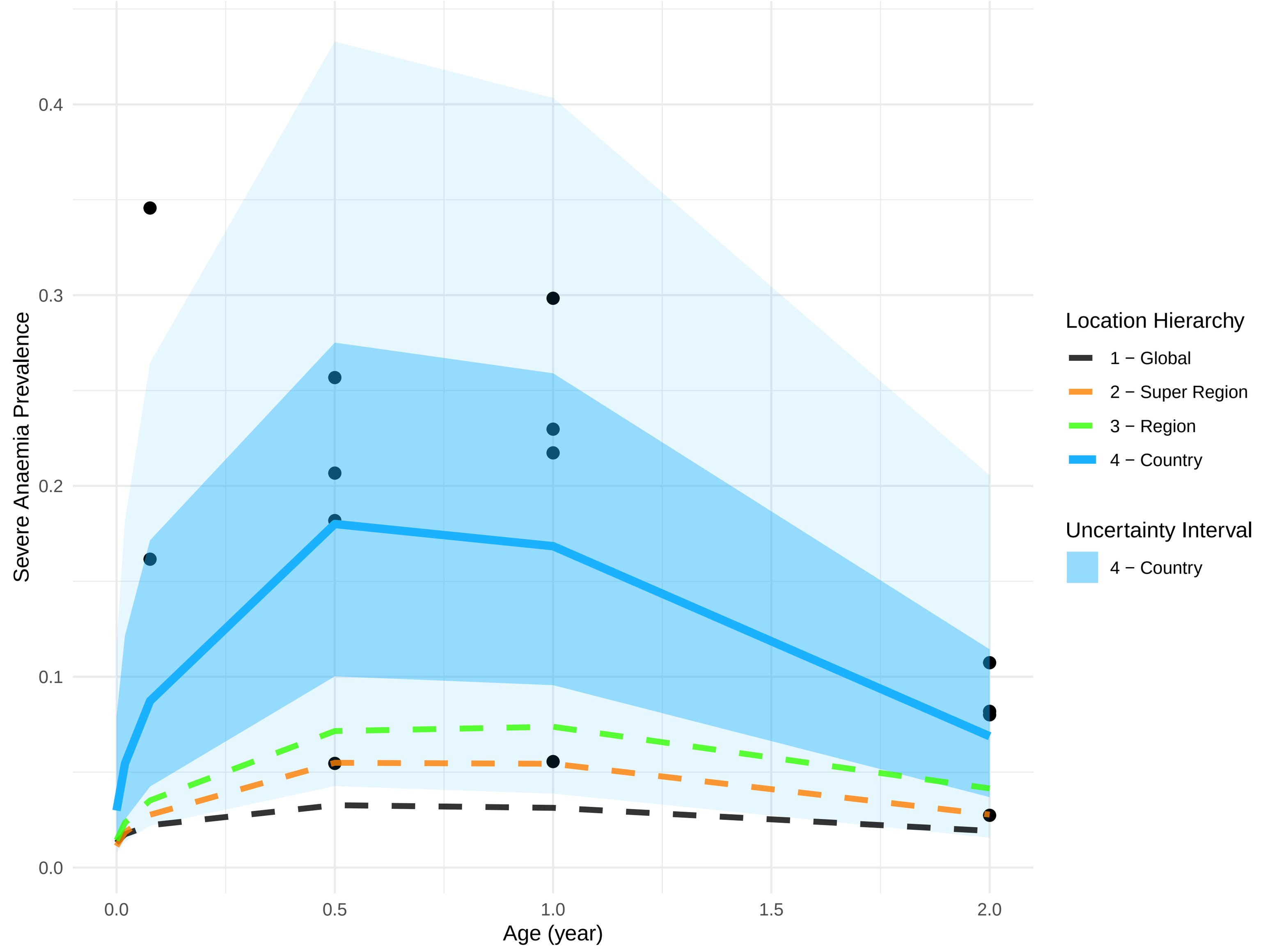

MR−BRT Age/Sex Fits − Non−pregnant Females <2 Years Old − Severe Anaemia Prevalence − Cameroon
WHO−2024 Adjustment Method

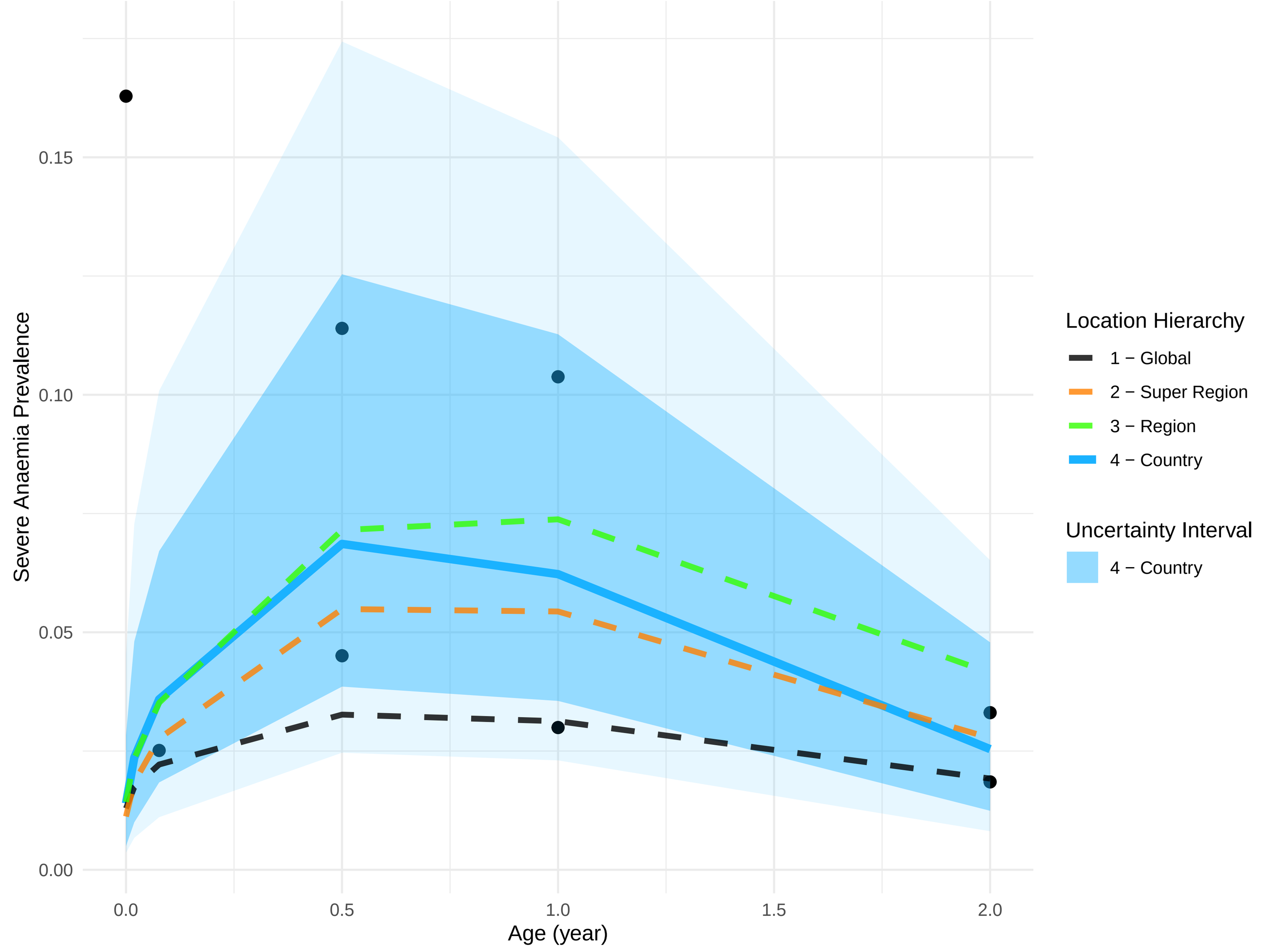

MR−BRT Age/Sex Fits − Non−pregnant Females <2 Years Old − Severe Anaemia Prevalence − Côte d'Ivoire
WHO−2024 Adjustment Method

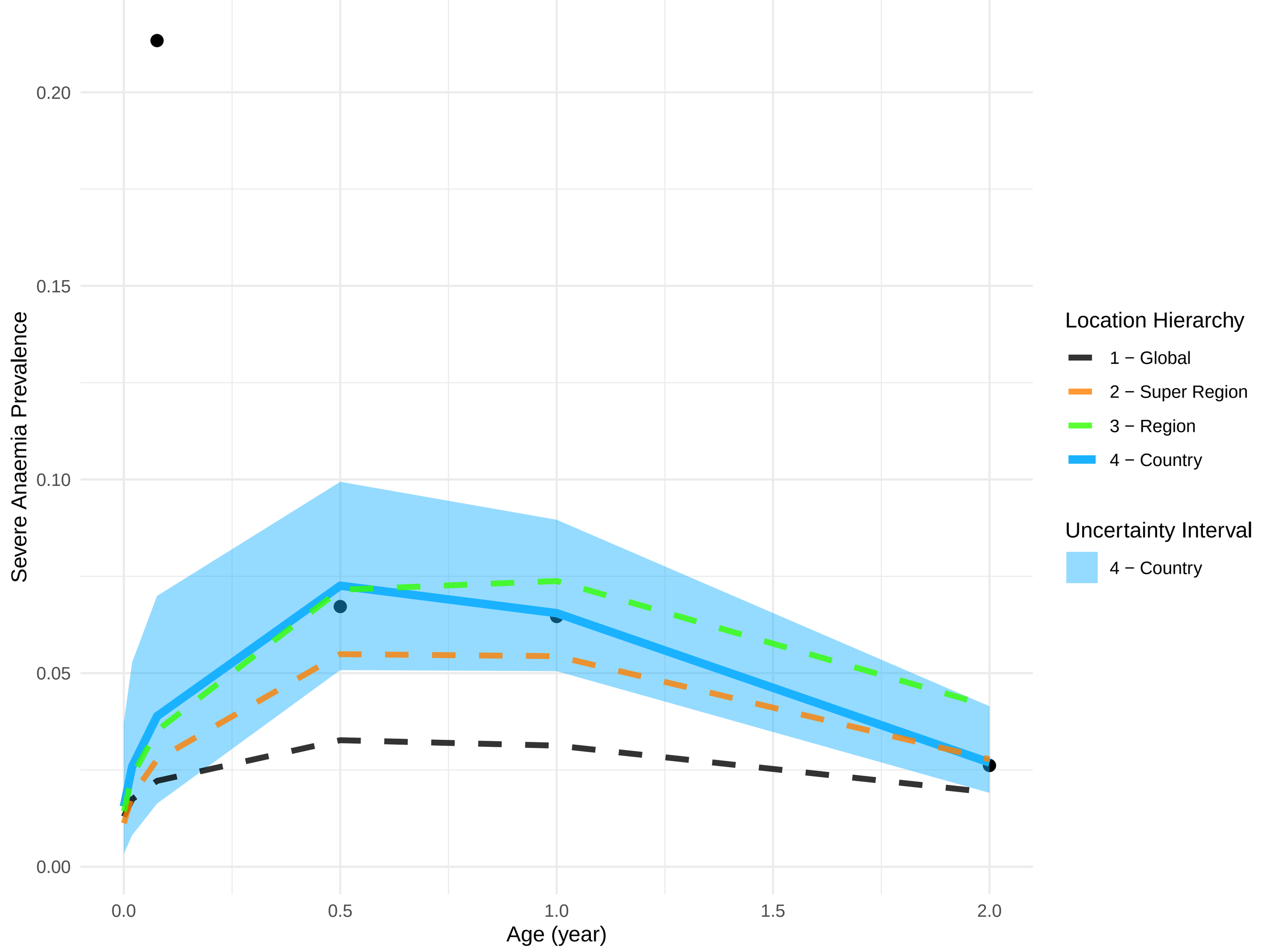

MR−BRT Age/Sex Fits − Non−pregnant Females <2 Years Old − Severe Anaemia Prevalence − Gambia
WHO−2024 Adjustment Method

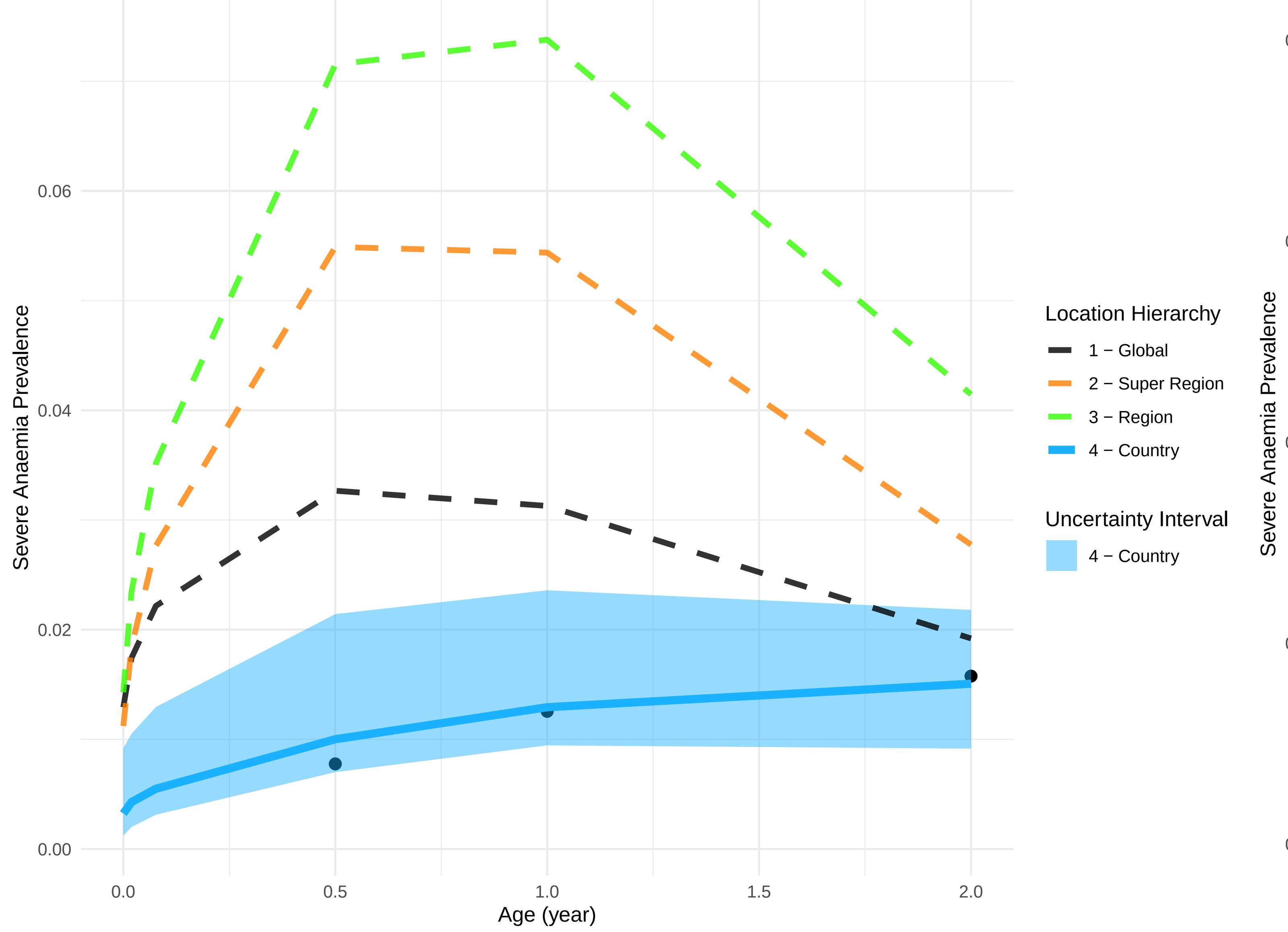

MR−BRT Age/Sex Fits − Non−pregnant Females <2 Years Old − Severe Anaemia Prevalence − Ghana
WHO−2024 Adjustment Method

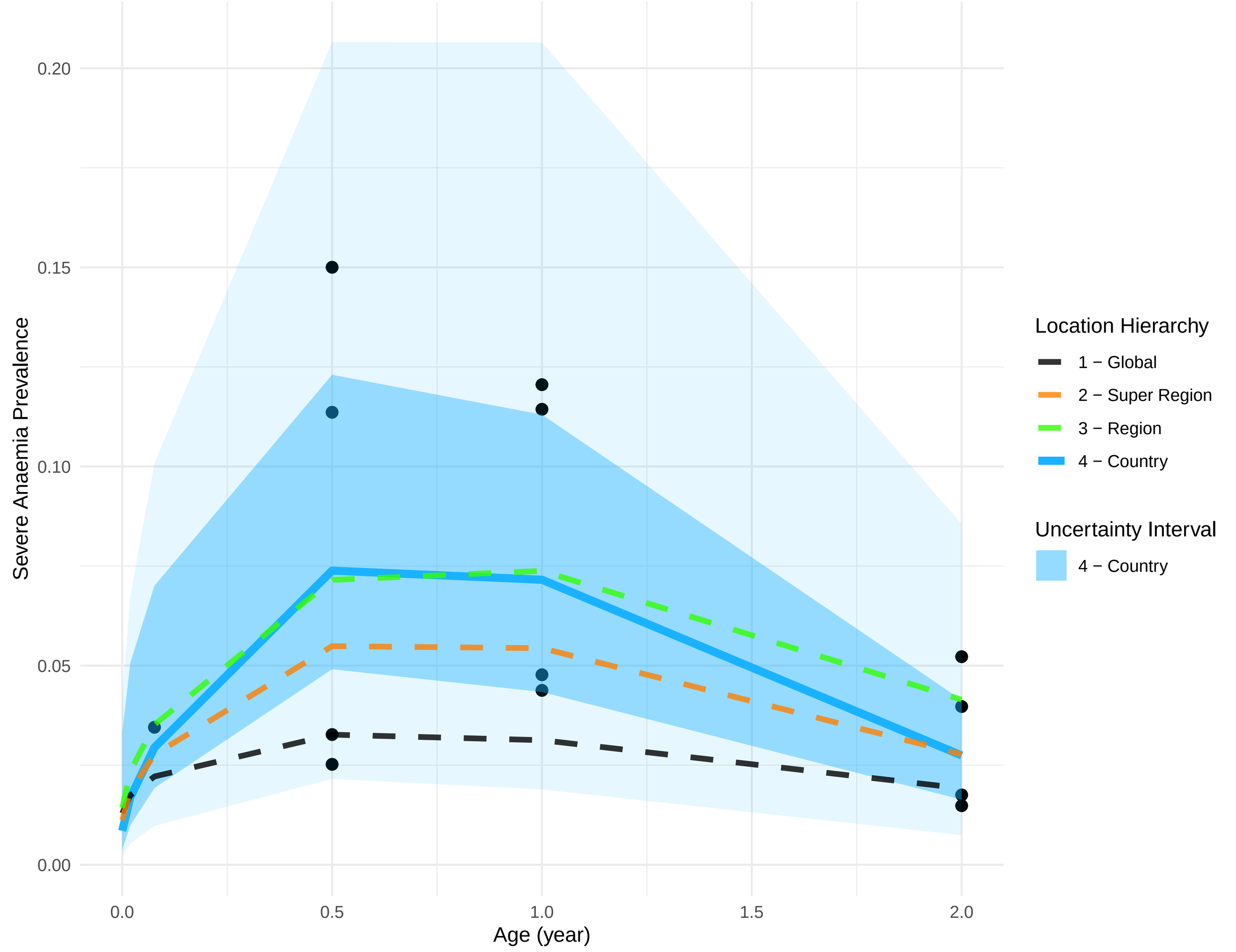

MR−BRT Age/Sex Fits − Non−pregnant Females <2 Years Old − Severe Anaemia Prevalence − Guinea
WHO−2024 Adjustment Method

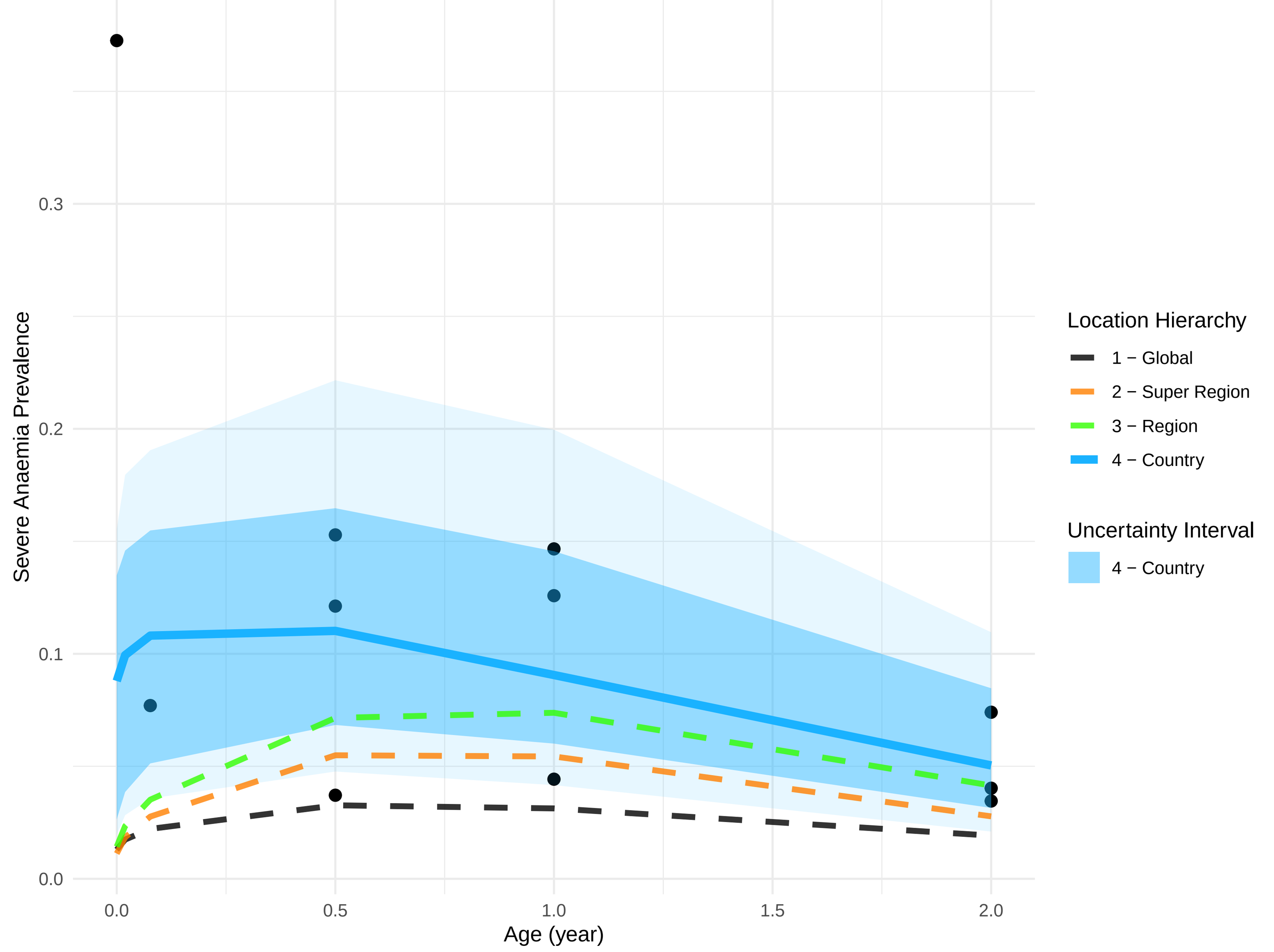

MR−BRT Age/Sex Fits − Non−pregnant Females <2 Years Old − Severe Anaemia Prevalence − Liberia
WHO−2024 Adjustment Method

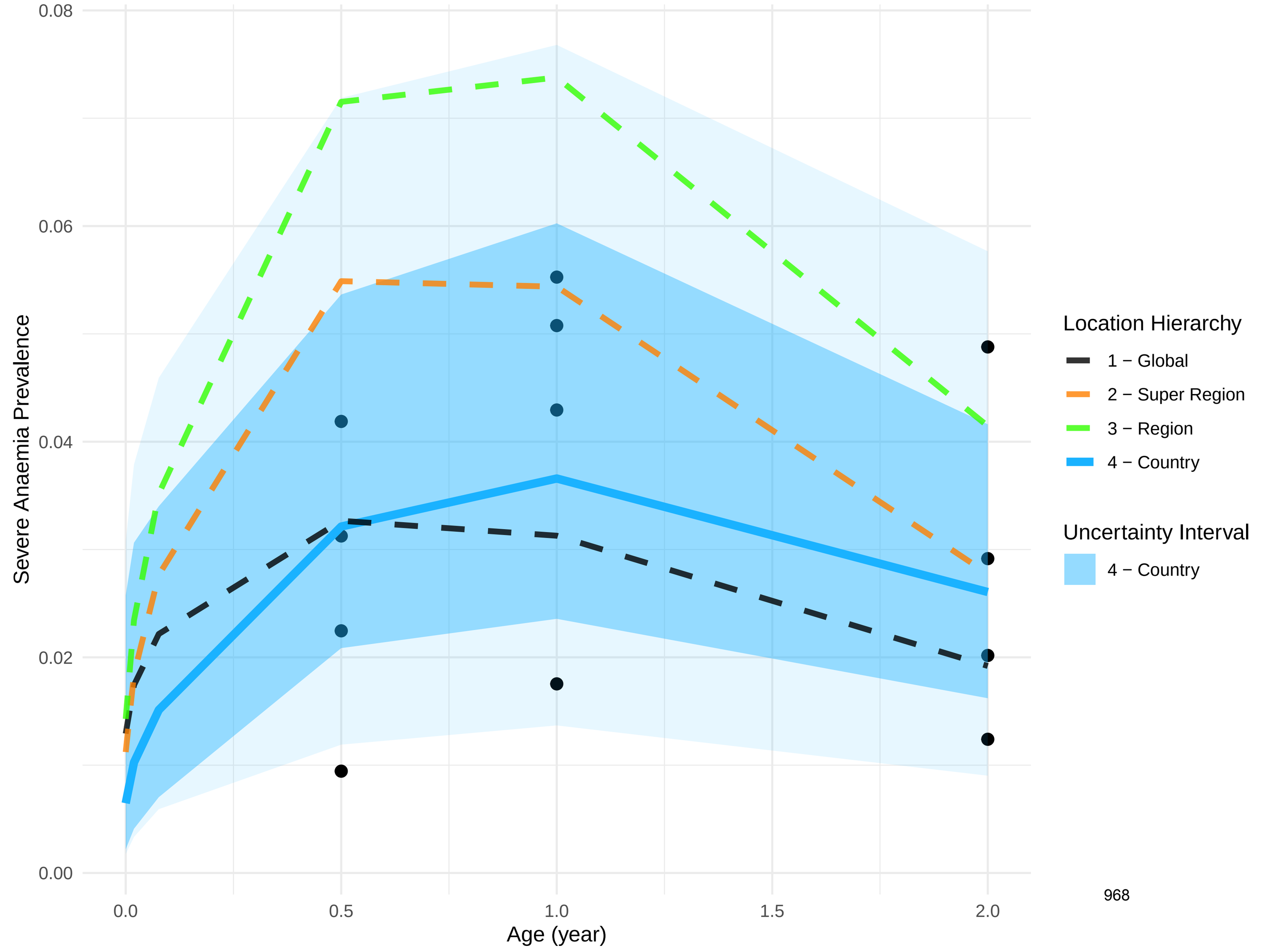

MR−BRT Age/Sex Fits − Non−pregnant Females <2 Years Old − Severe Anaemia Prevalence − Mali
WHO−2024 Adjustment Method

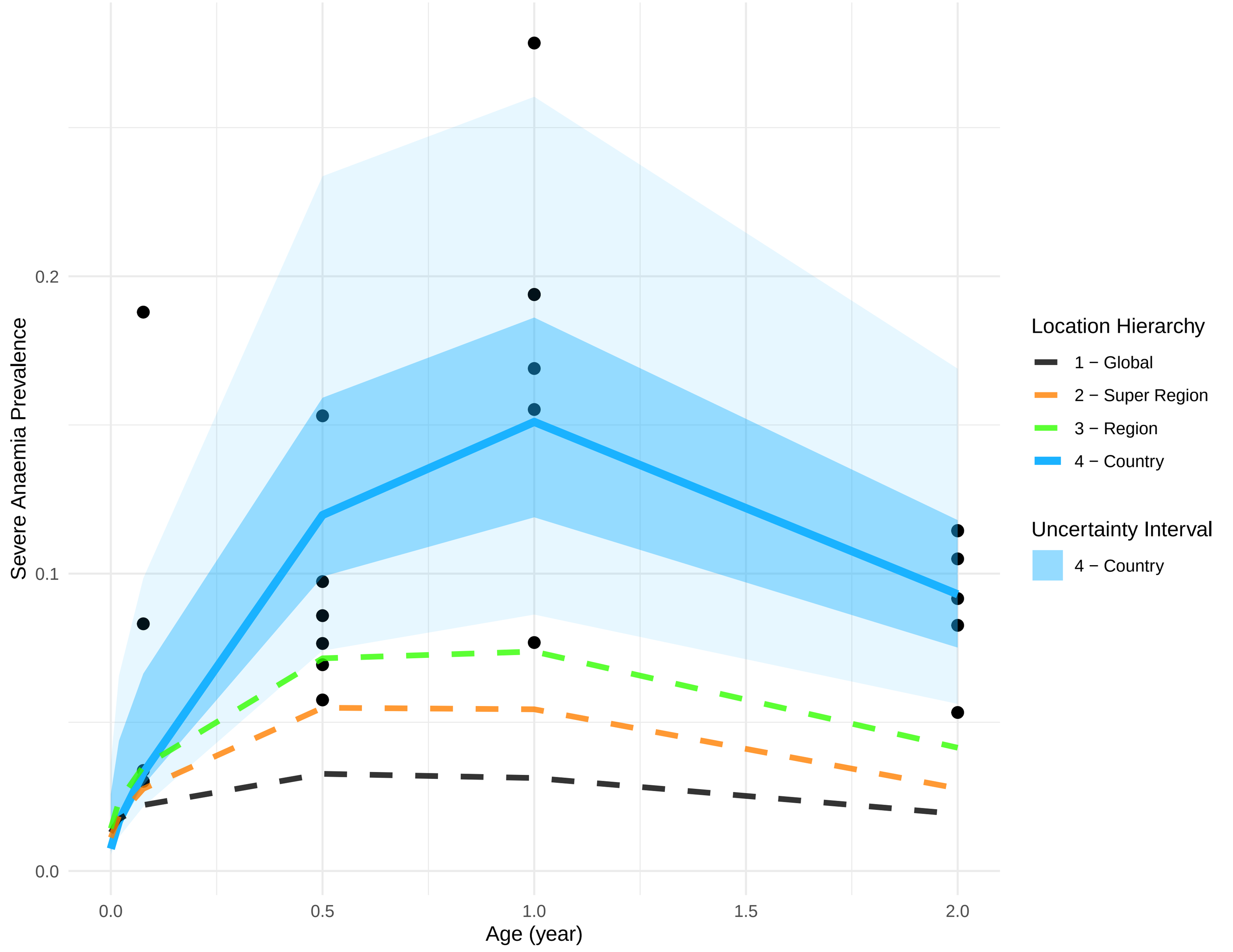

MR−BRT Age/Sex Fits − Non−pregnant Females <2 Years Old − Severe Anaemia Prevalence − Niger
WHO−2024 Adjustment Method

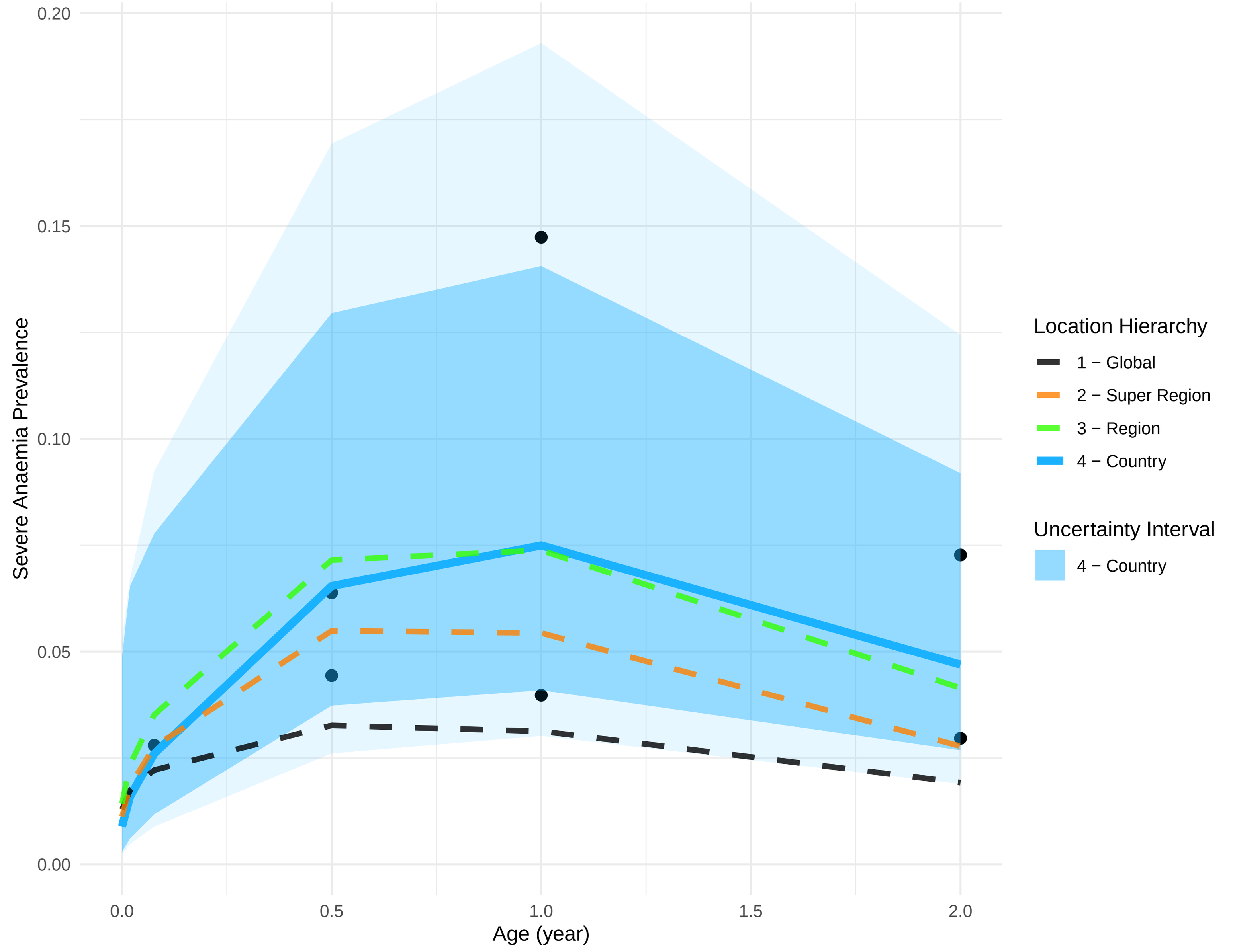

MR−BRT Age/Sex Fits − Non−pregnant Females <2 Years Old − Severe Anaemia Prevalence − Nigeria
WHO−2024 Adjustment Method

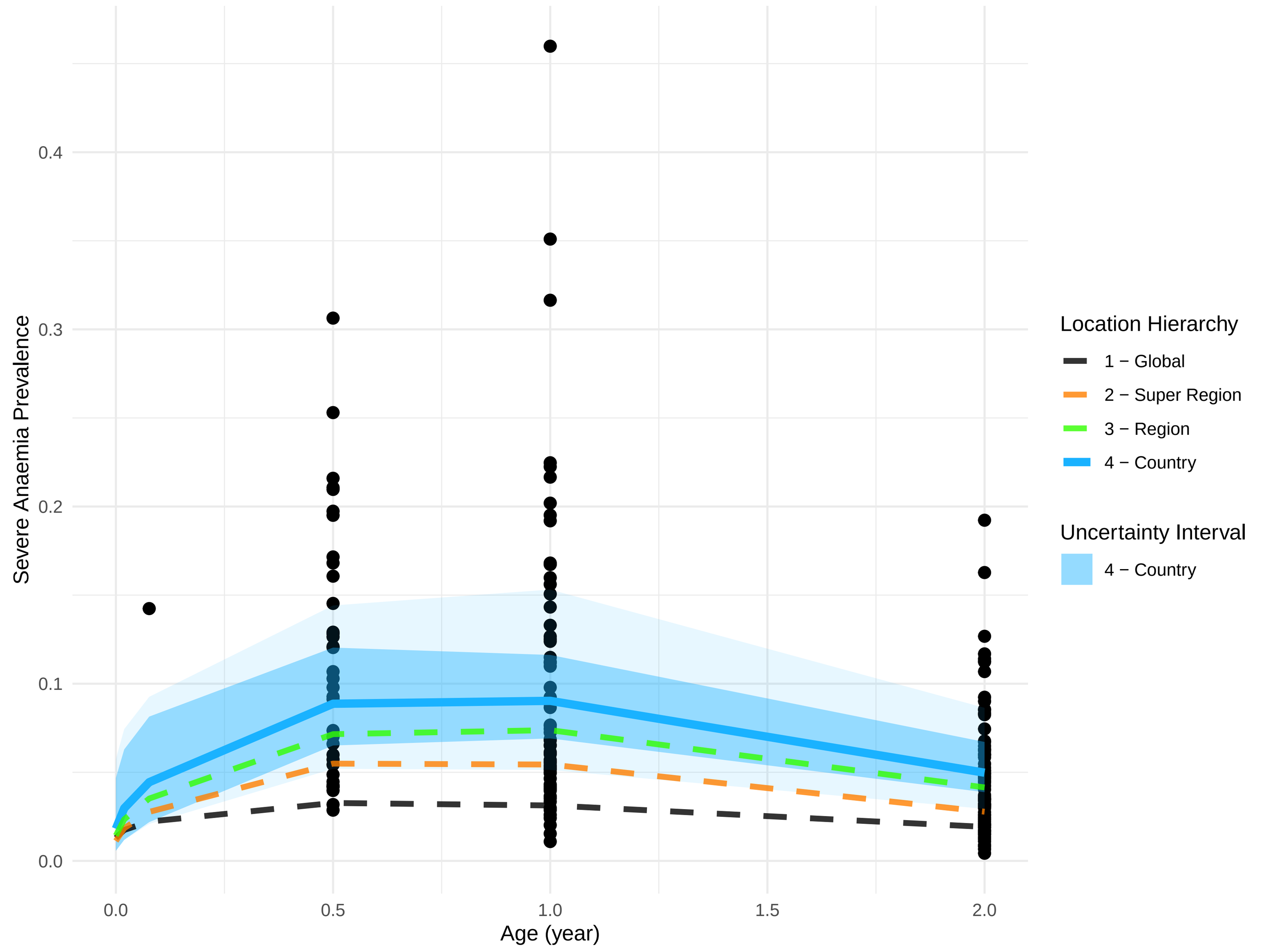

MR−BRT Age/Sex Fits − Non−pregnant Females <2 Years Old − Severe Anaemia Prevalence − Abia
WHO−2024 Adjustment Method

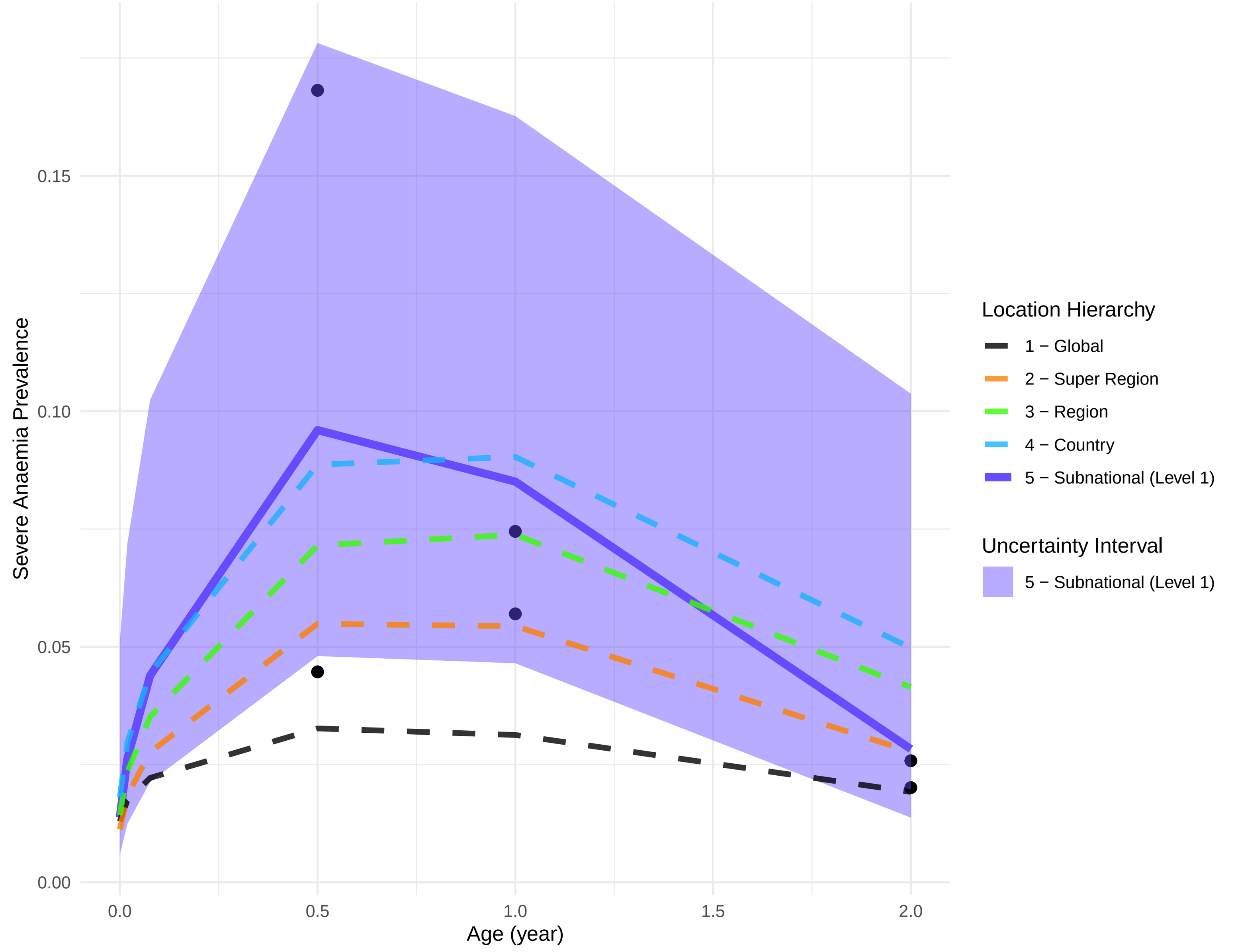

MR−BRT Age/Sex Fits − Non−pregnant Females <2 Years Old − Severe Anaemia Prevalence − Adamawa
WHO−2024 Adjustment Method

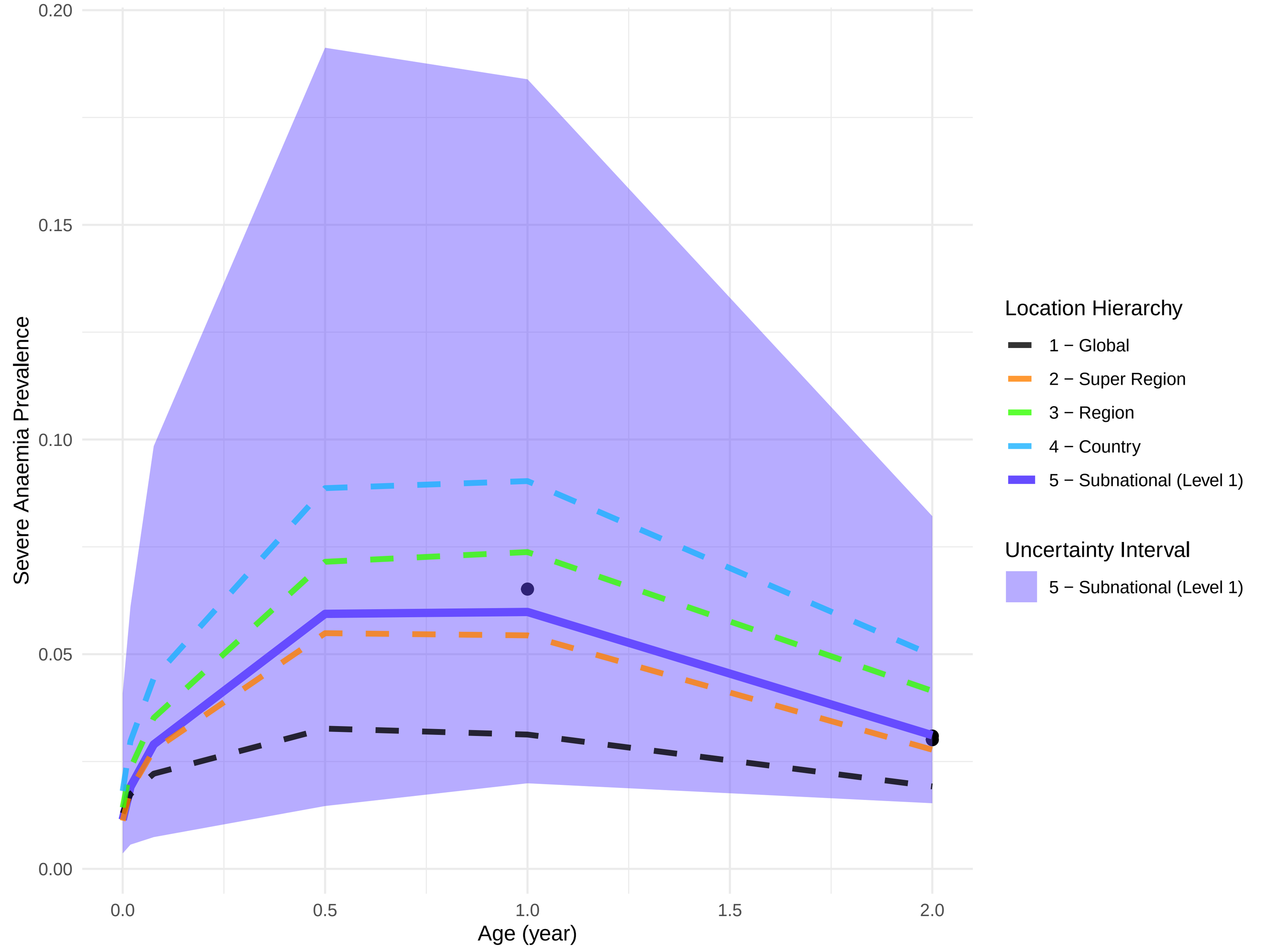

MR−BRT Age/Sex Fits − Non−pregnant Females <2 Years Old − Severe Anaemia Prevalence − Akwa Ibom
WHO−2024 Adjustment Method

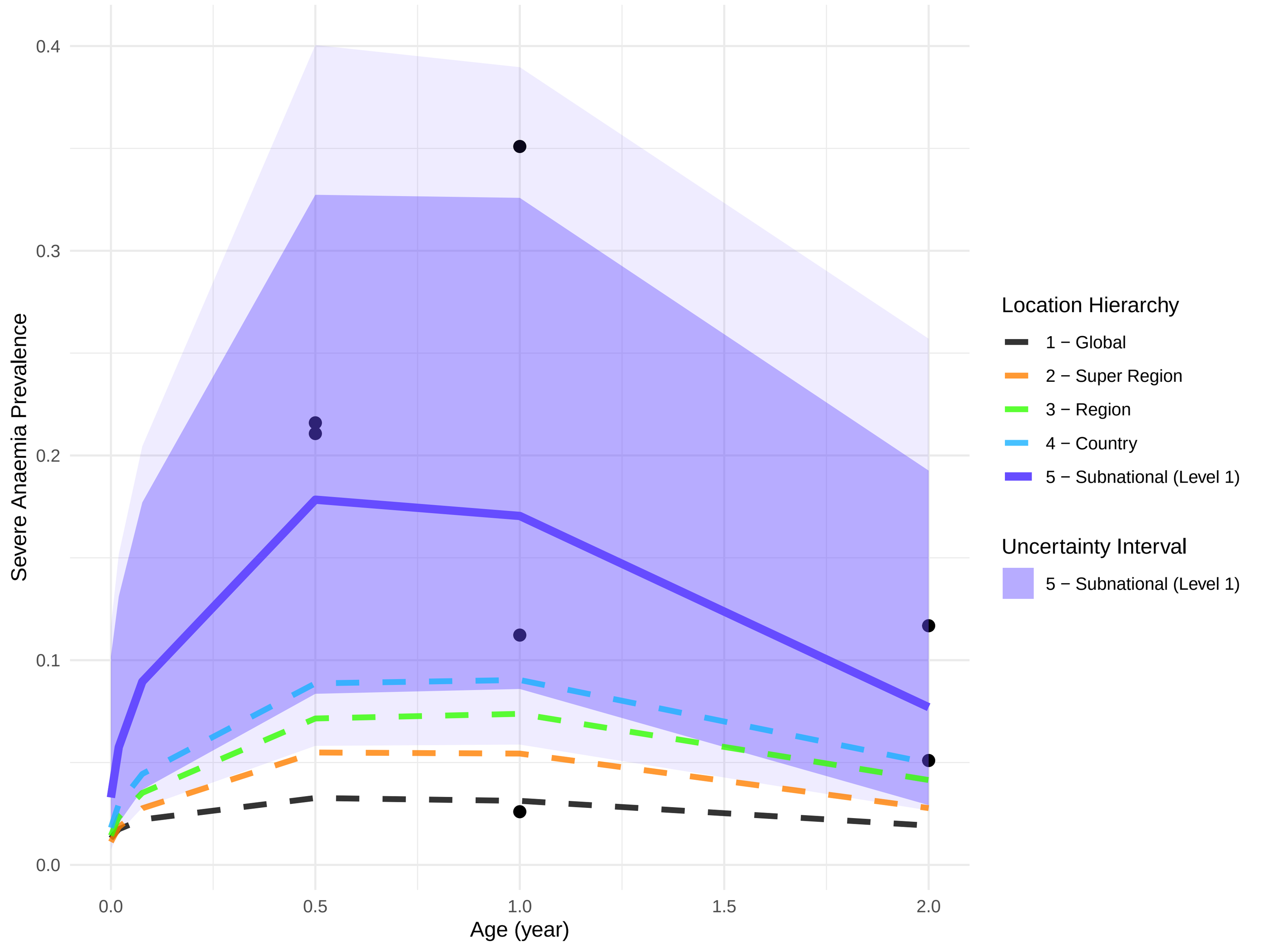

MR−BRT Age/Sex Fits − Non−pregnant Females <2 Years Old − Severe Anaemia Prevalence − Anambra
WHO−2024 Adjustment Method

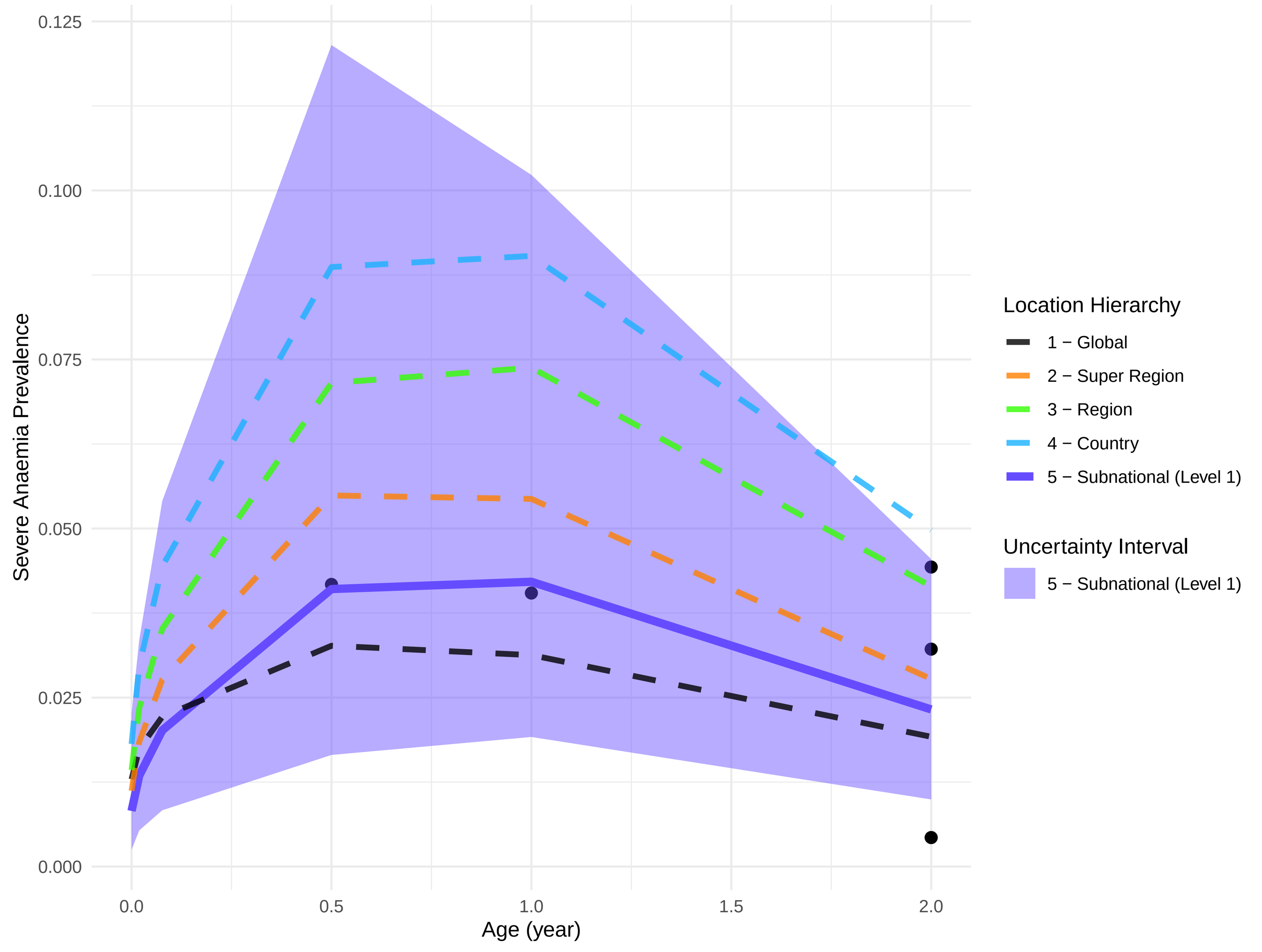

MR−BRT Age/Sex Fits − Non−pregnant Females <2 Years Old − Severe Anaemia Prevalence − Bauchi
WHO−2024 Adjustment Method

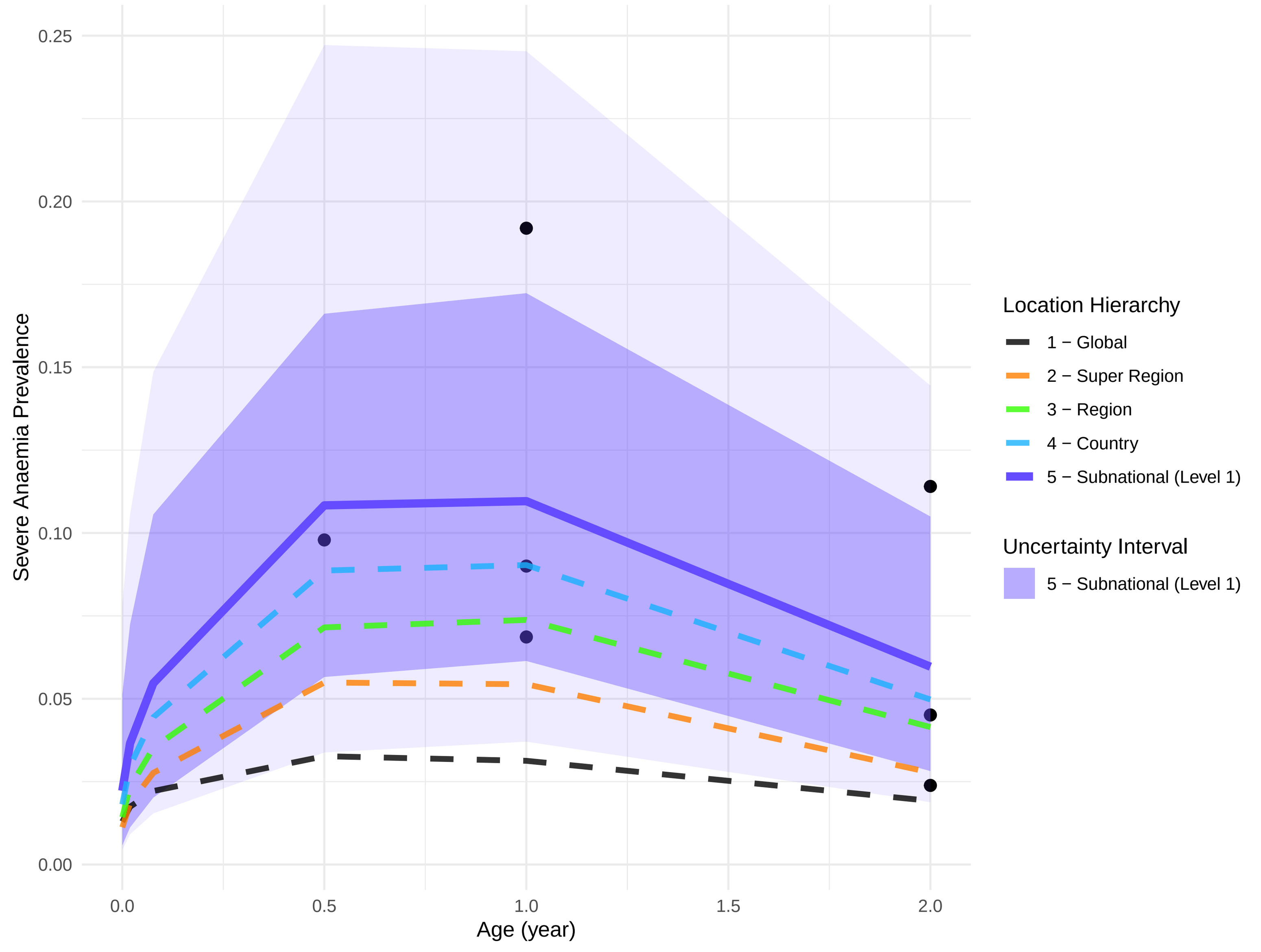

MR−BRT Age/Sex Fits − Non−pregnant Females <2 Years Old − Severe Anaemia Prevalence − Bayelsa
WHO−2024 Adjustment Method

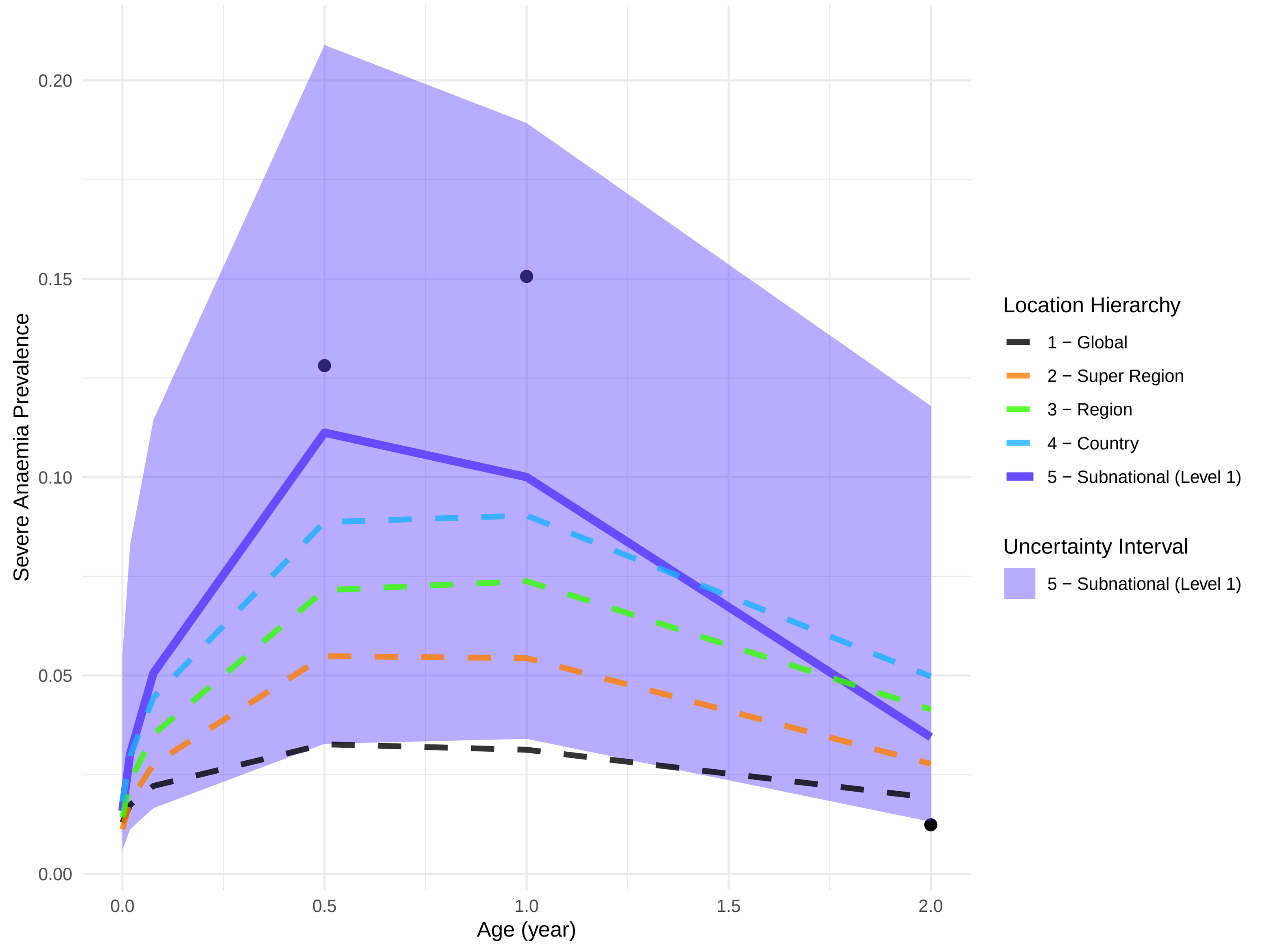

MR−BRT Age/Sex Fits − Non−pregnant Females <2 Years Old − Severe Anaemia Prevalence − Benue
WHO−2024 Adjustment Method

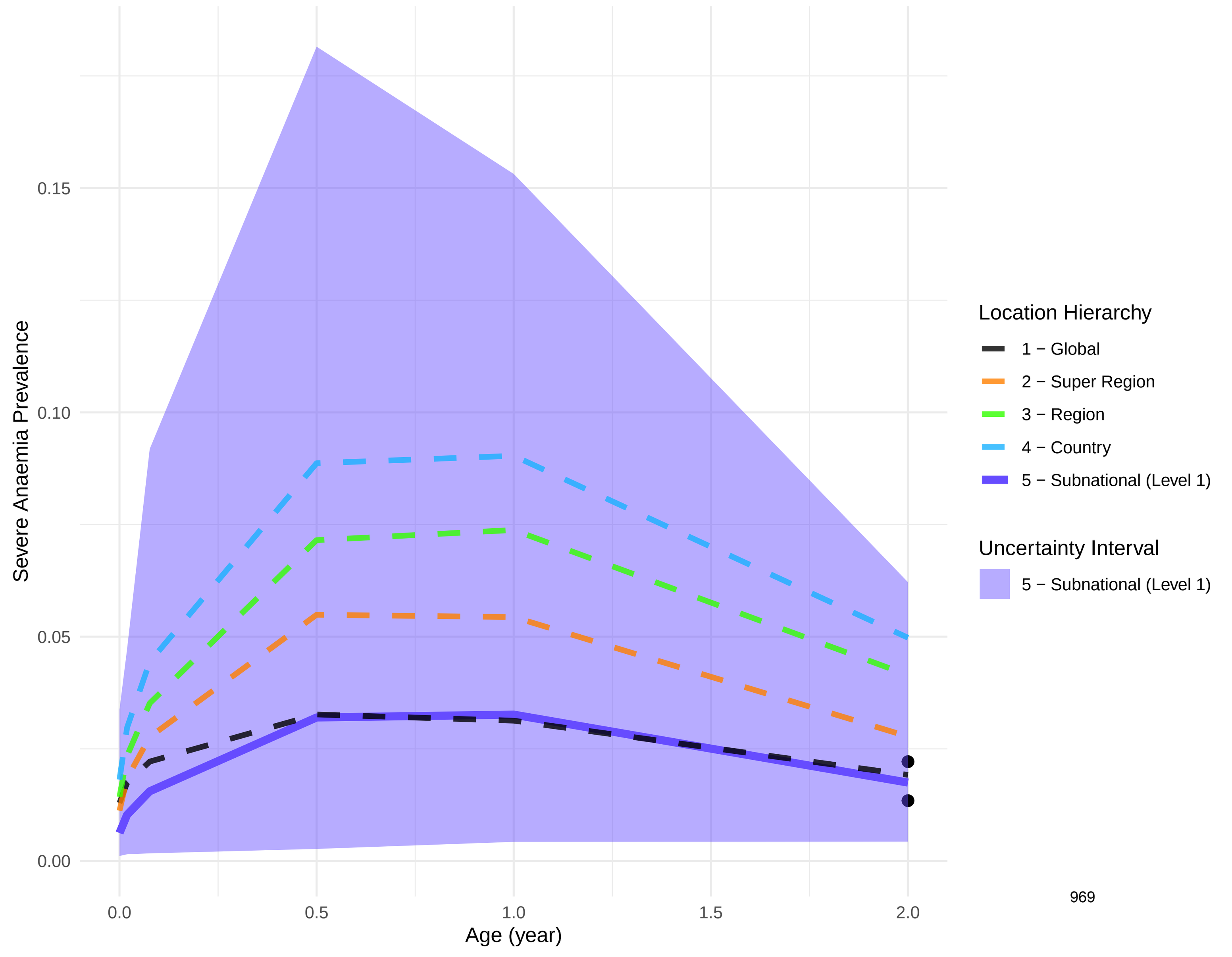

MR−BRT Age/Sex Fits − Non−pregnant Females <2 Years Old − Severe Anaemia Prevalence − Borno
WHO−2024 Adjustment Method

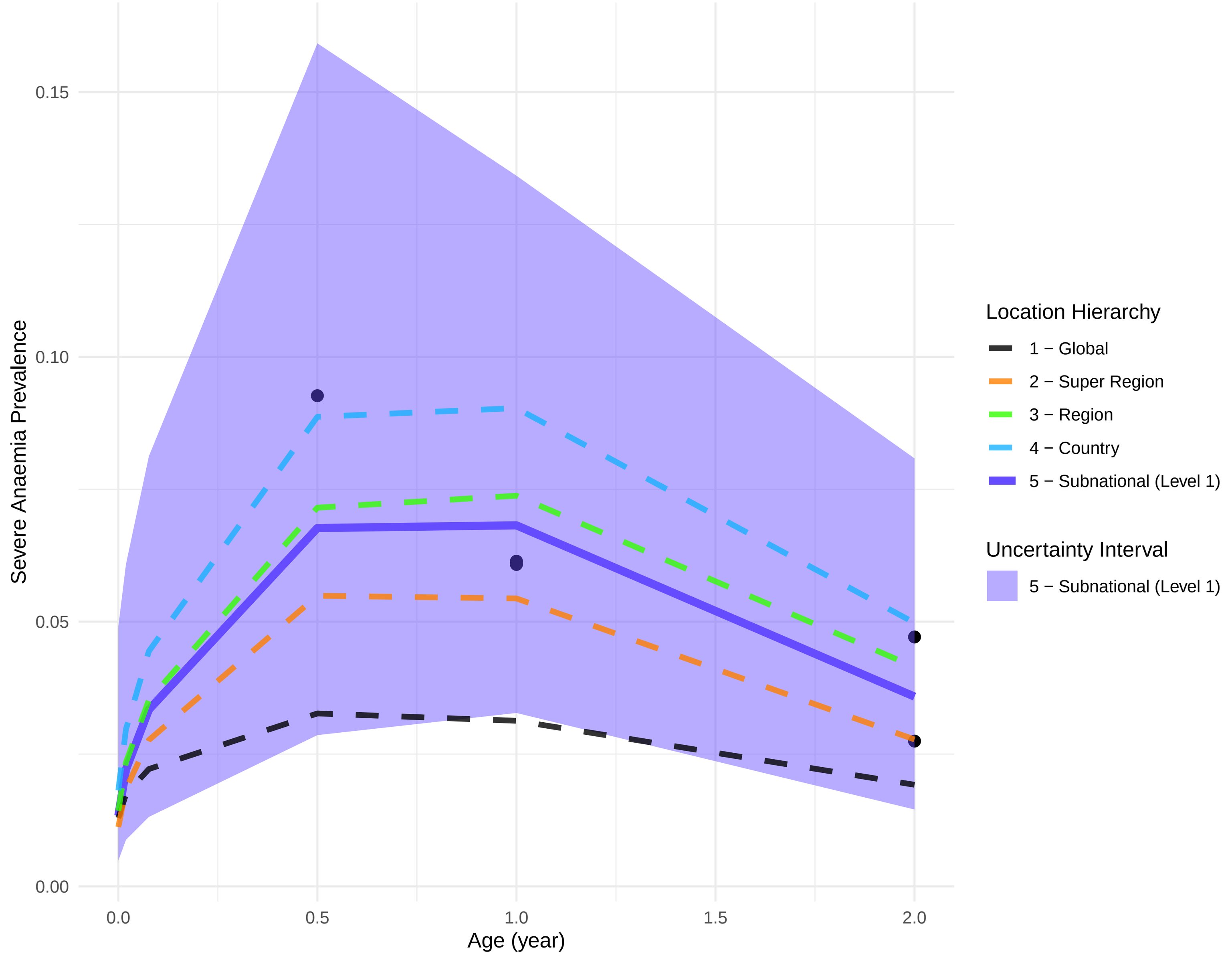

MR−BRT Age/Sex Fits − Non−pregnant Females <2 Years Old − Severe Anaemia Prevalence − Cross River
WHO−2024 Adjustment Method

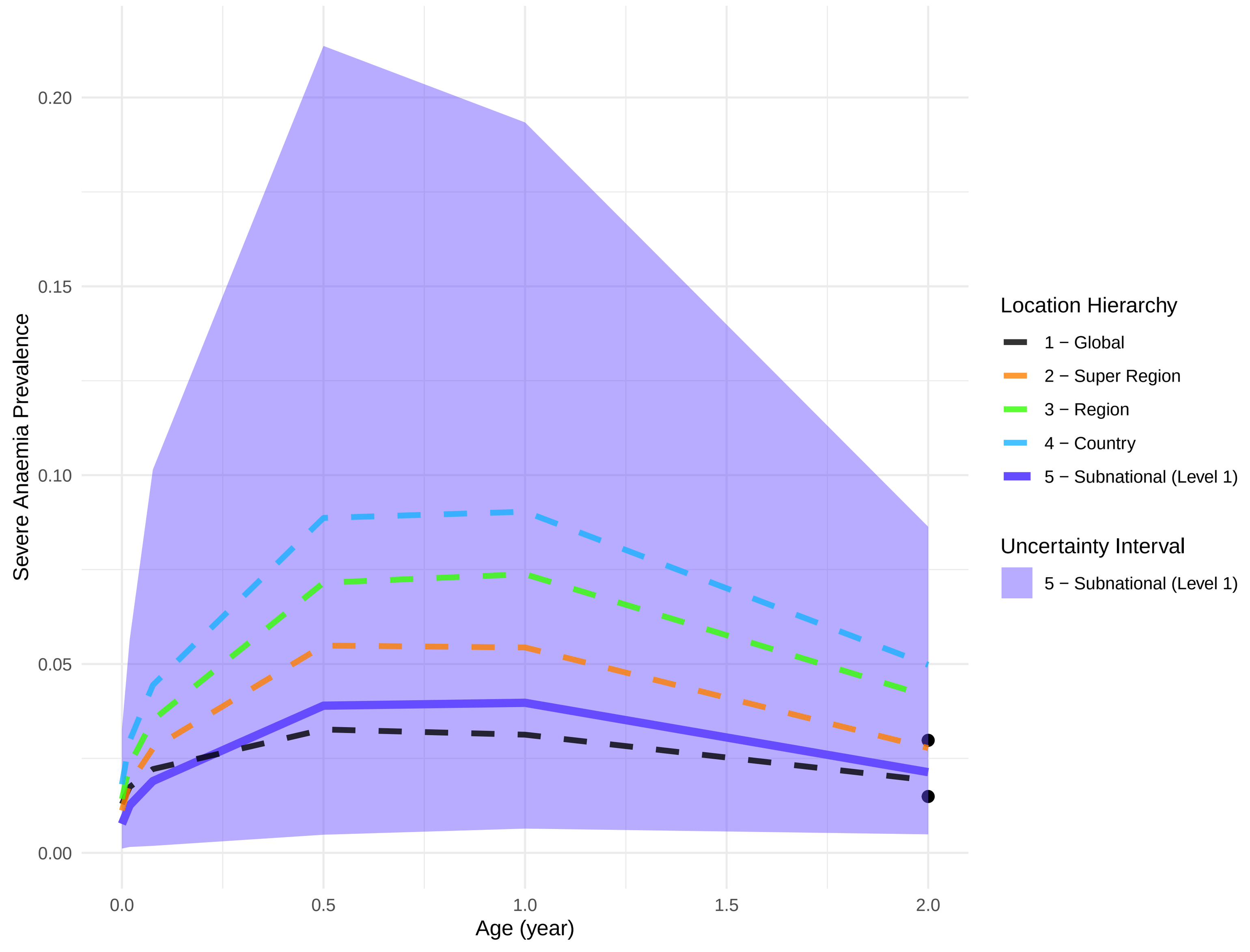

MR−BRT Age/Sex Fits − Non−pregnant Females <2 Years Old − Severe Anaemia Prevalence − Delta
WHO−2024 Adjustment Method

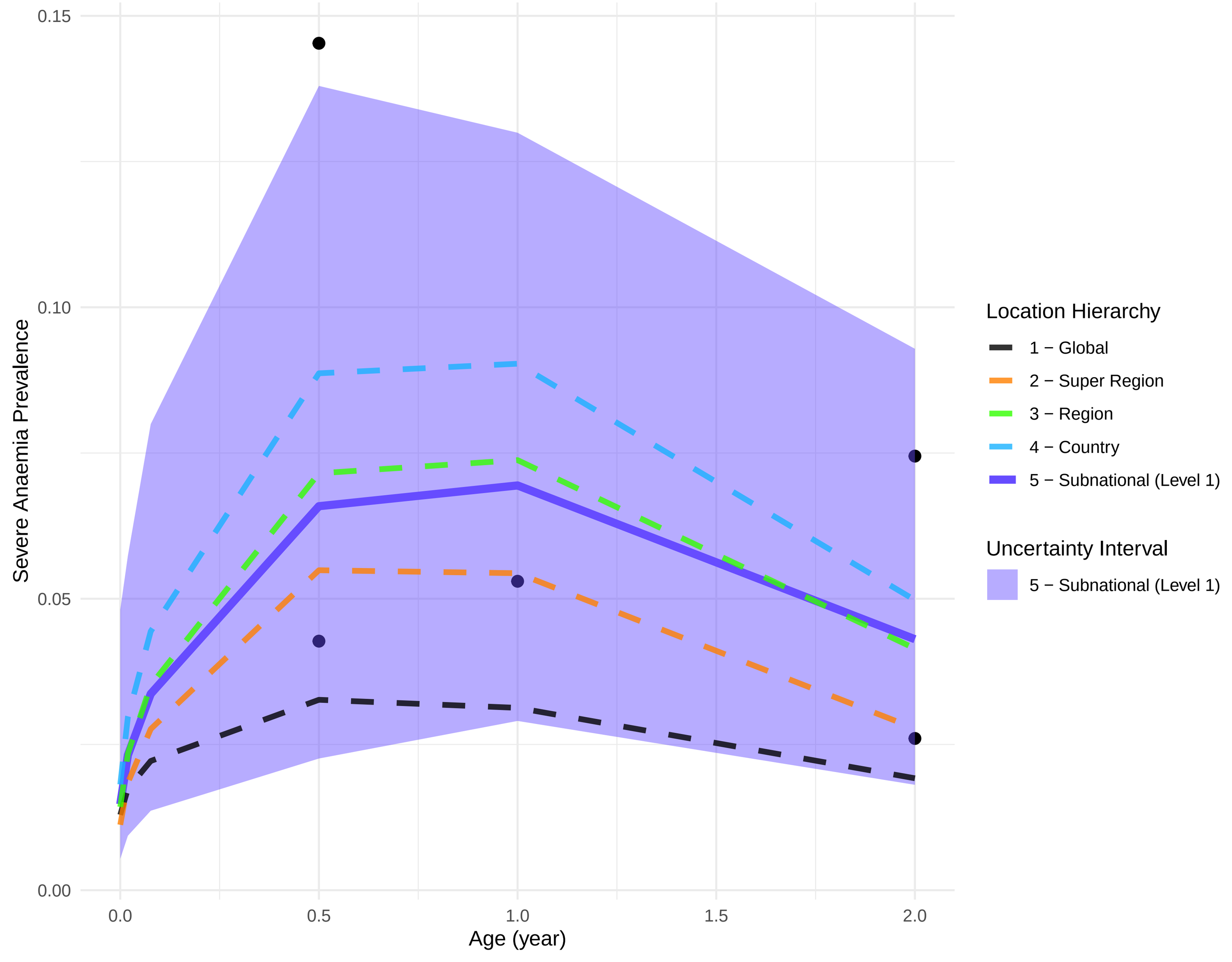

MR−BRT Age/Sex Fits − Non−pregnant Females <2 Years Old − Severe Anaemia Prevalence − Ebonyi
WHO−2024 Adjustment Method

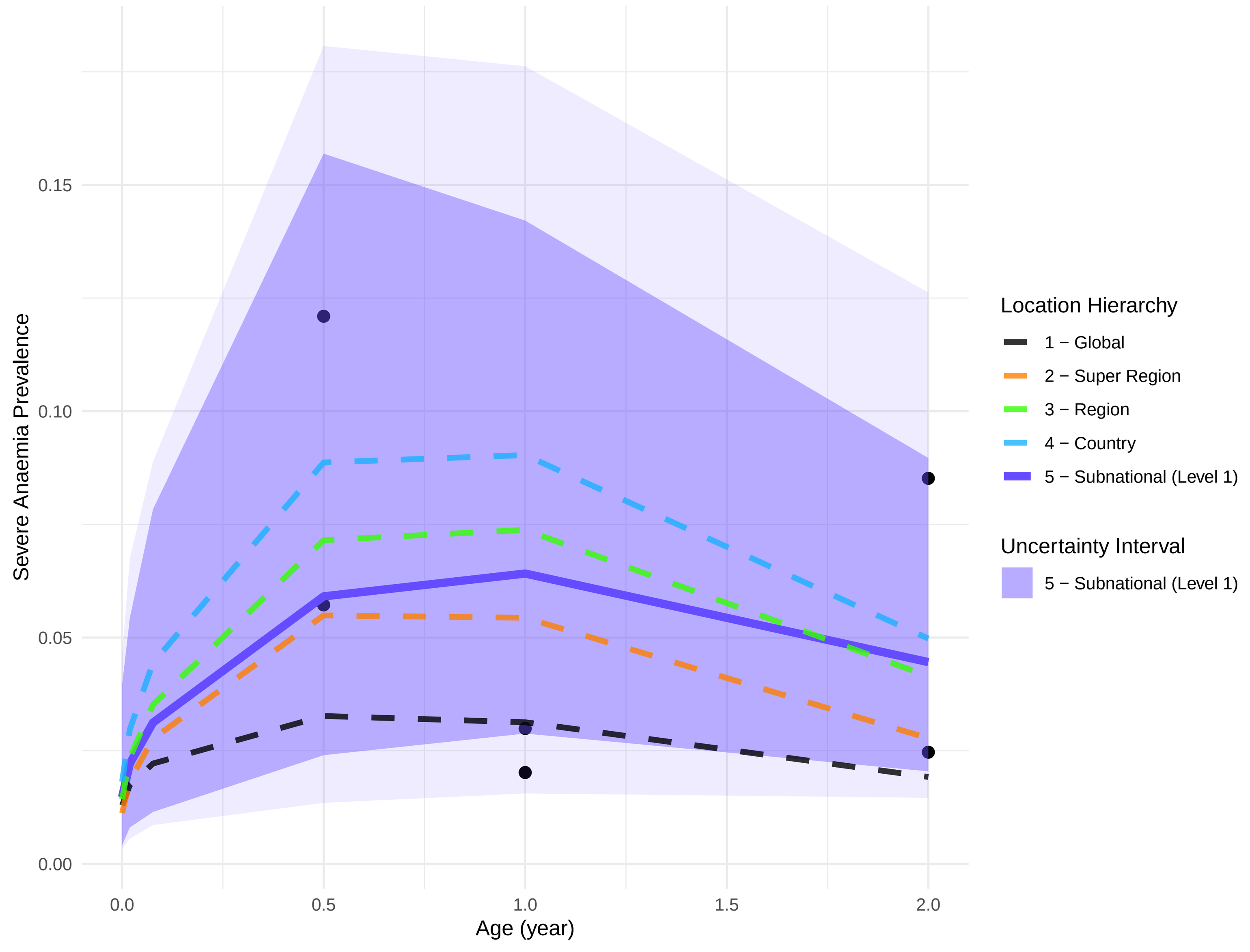

MR−BRT Age/Sex Fits − Non−pregnant Females <2 Years Old − Severe Anaemia Prevalence − Edo
WHO−2024 Adjustment Method

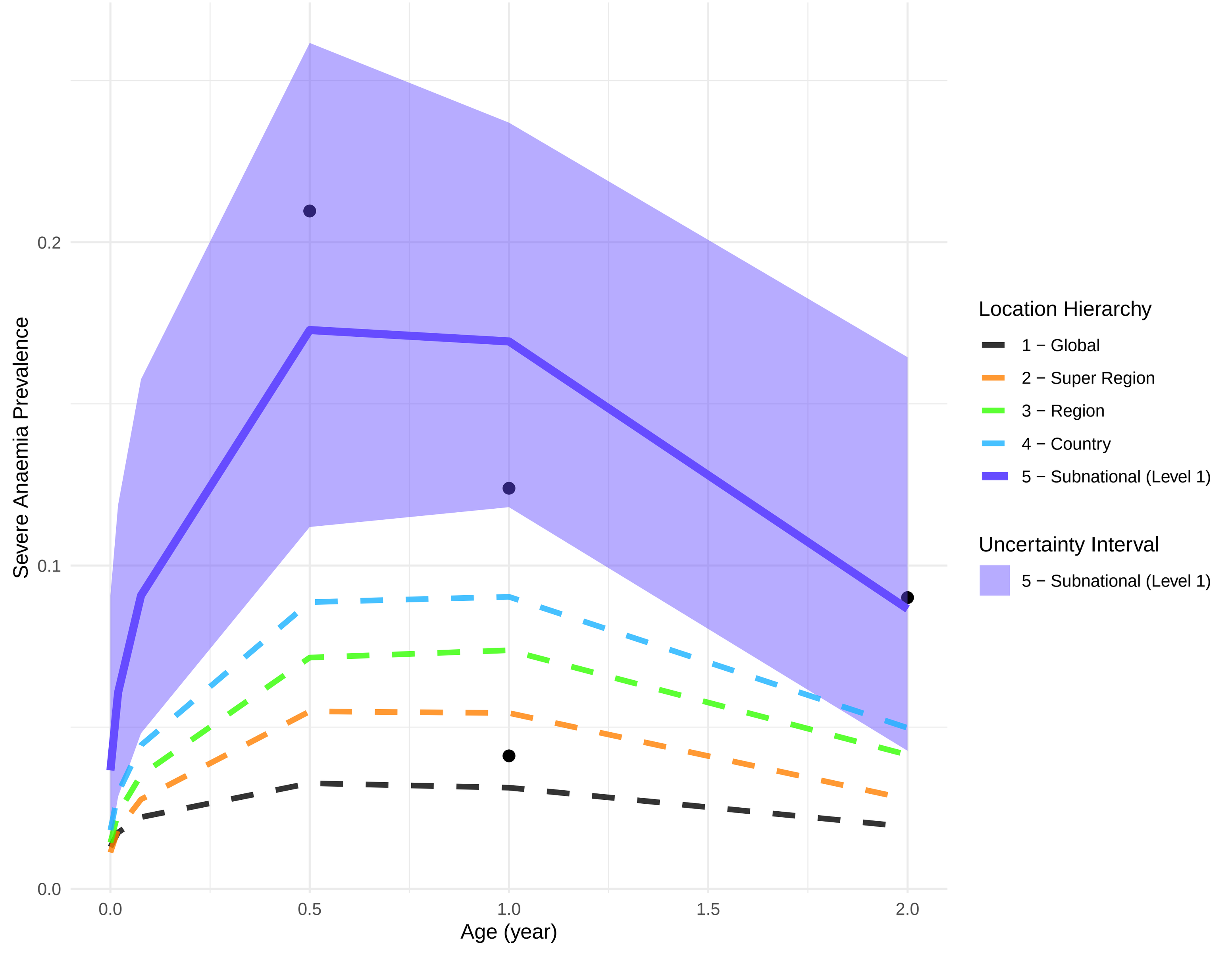

MR−BRT Age/Sex Fits − Non−pregnant Females <2 Years Old − Severe Anaemia Prevalence − Ekiti
WHO−2024 Adjustment Method

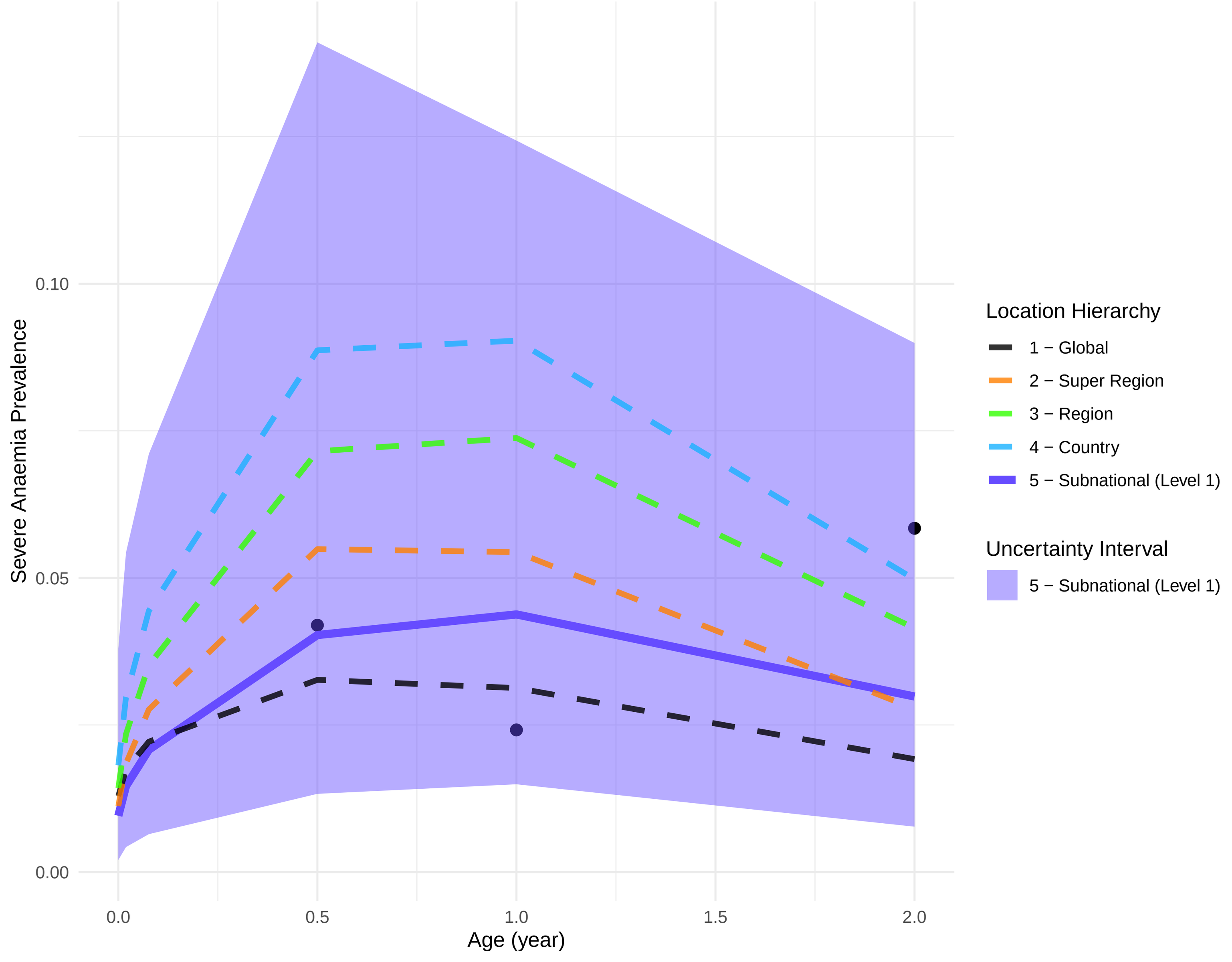

MR−BRT Age/Sex Fits − Non−pregnant Females <2 Years Old − Severe Anaemia Prevalence − Enugu
WHO−2024 Adjustment Method

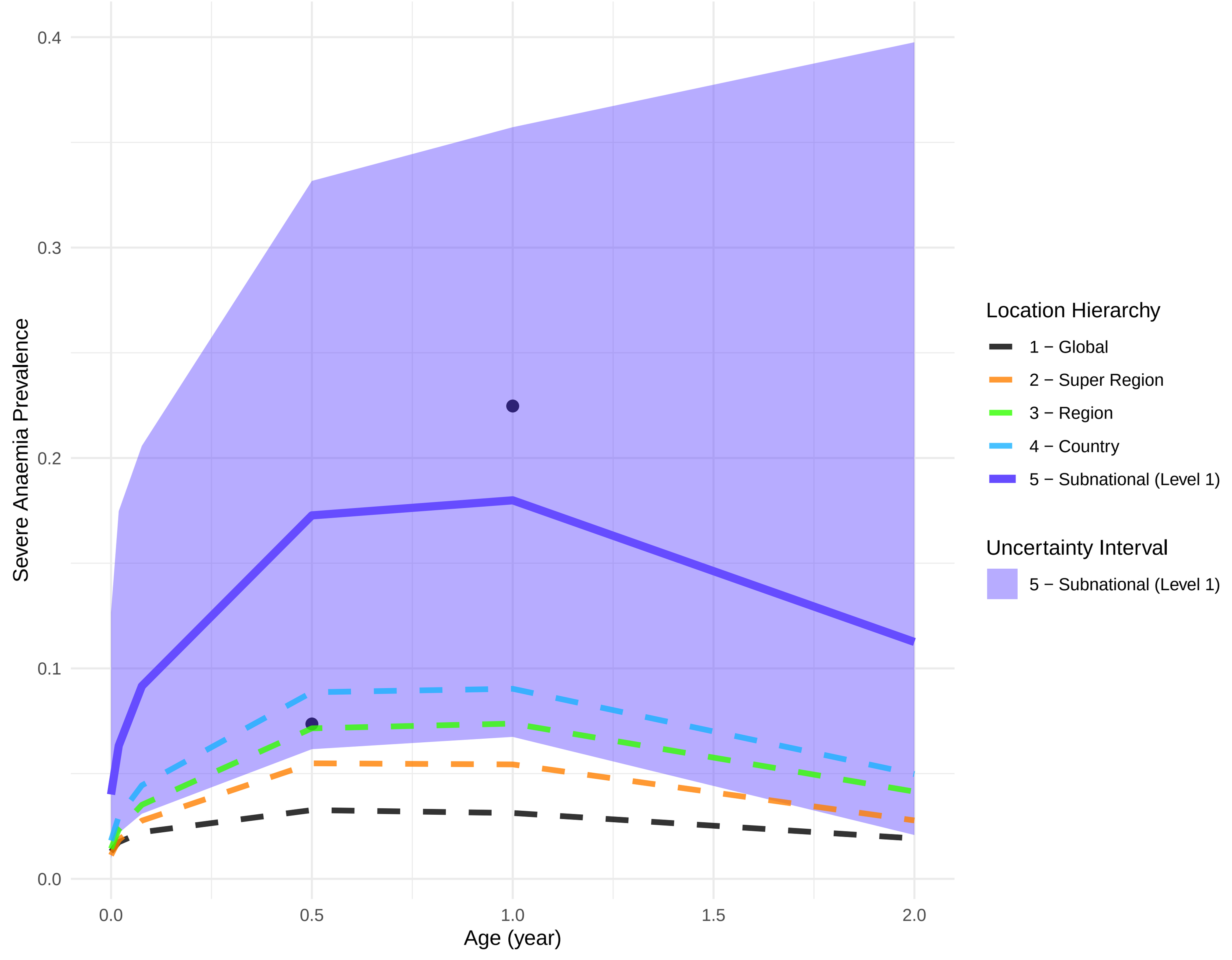

MR−BRT Age/Sex Fits − Non−pregnant Females <2 Years Old − Severe Anaemia Prevalence − FCT (Abuja)
WHO−2024 Adjustment Method

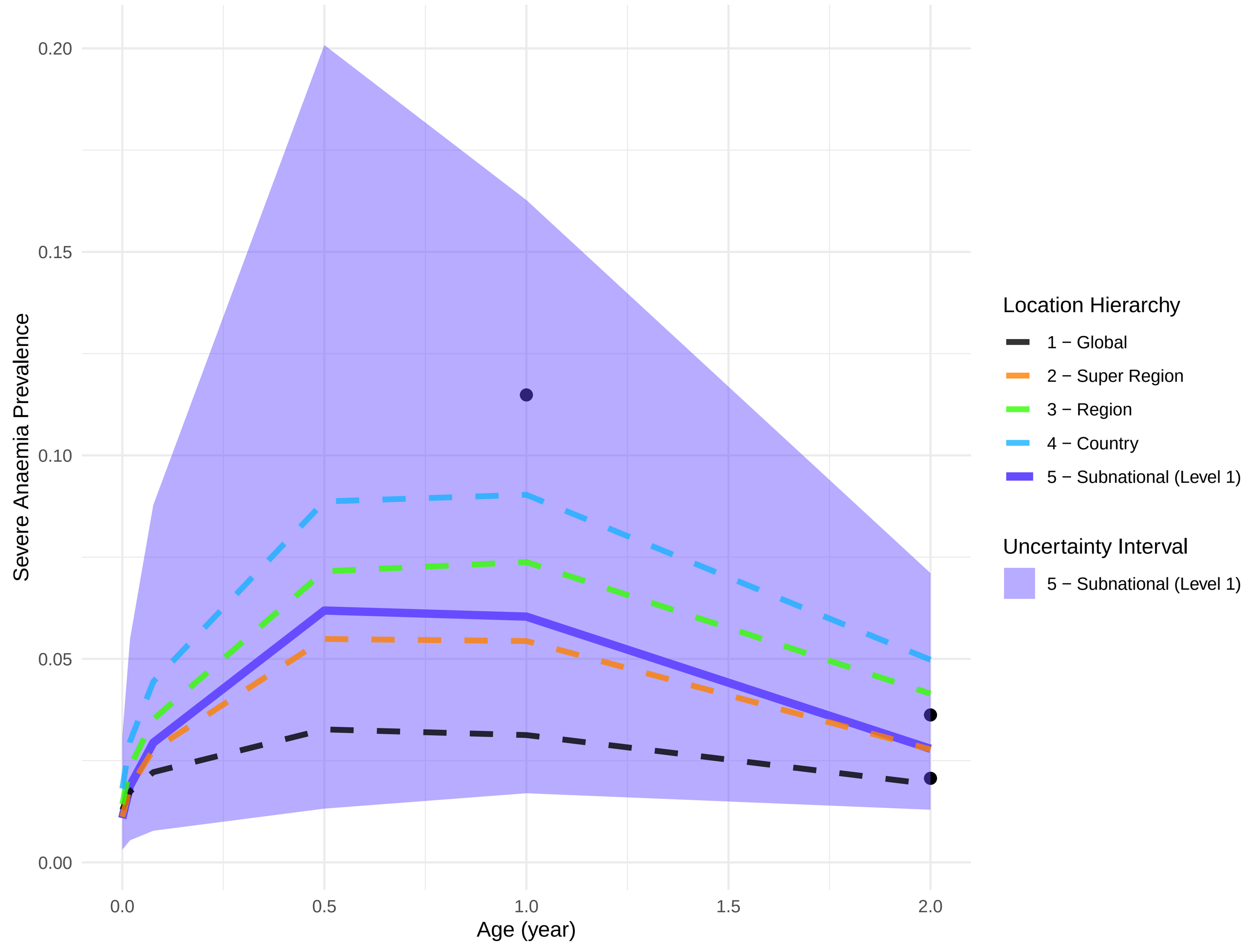

MR−BRT Age/Sex Fits − Non−pregnant Females <2 Years Old − Severe Anaemia Prevalence − Gombe
WHO−2024 Adjustment Method

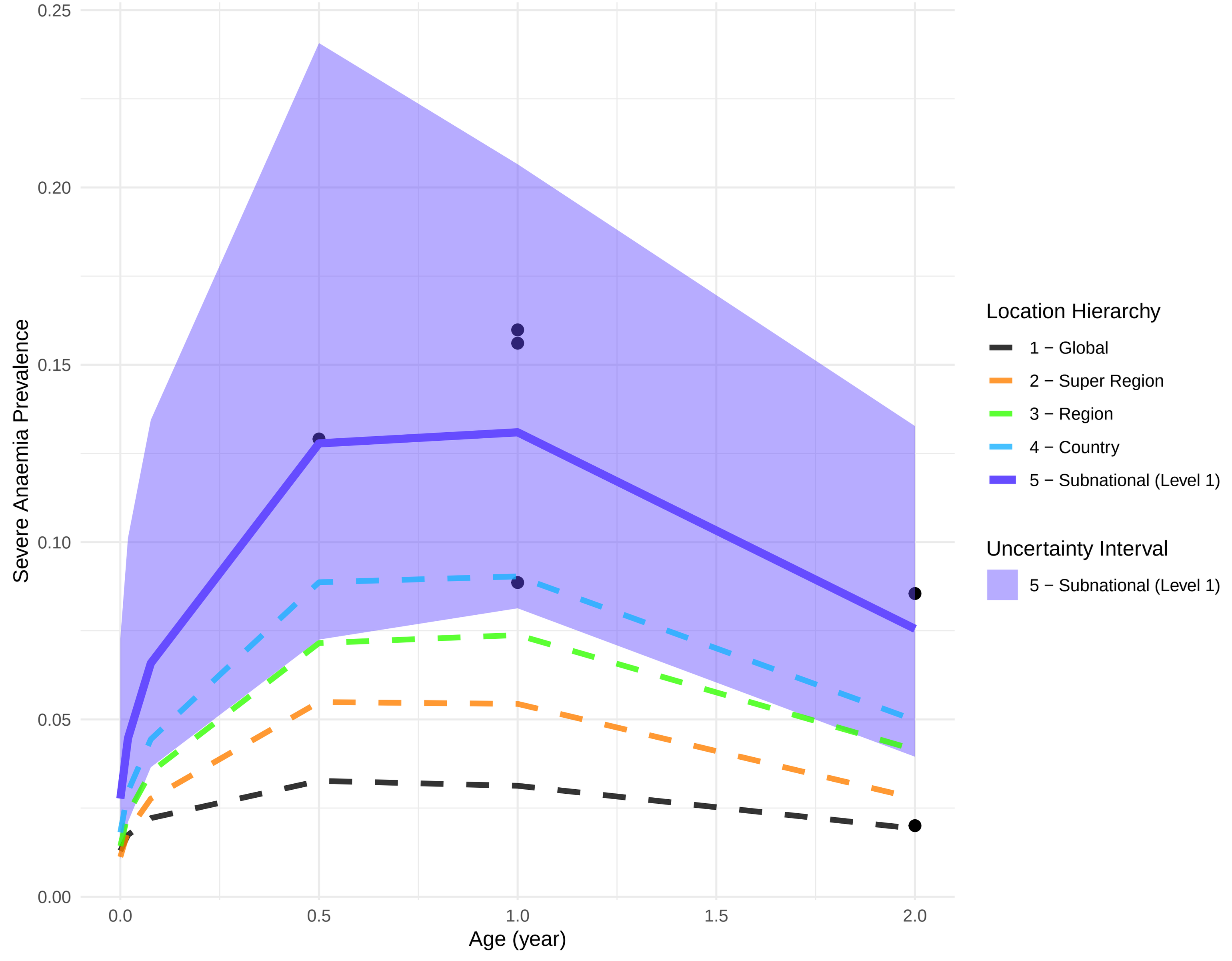

MR−BRT Age/Sex Fits − Non−pregnant Females <2 Years Old − Severe Anaemia Prevalence − Imo
WHO−2024 Adjustment Method

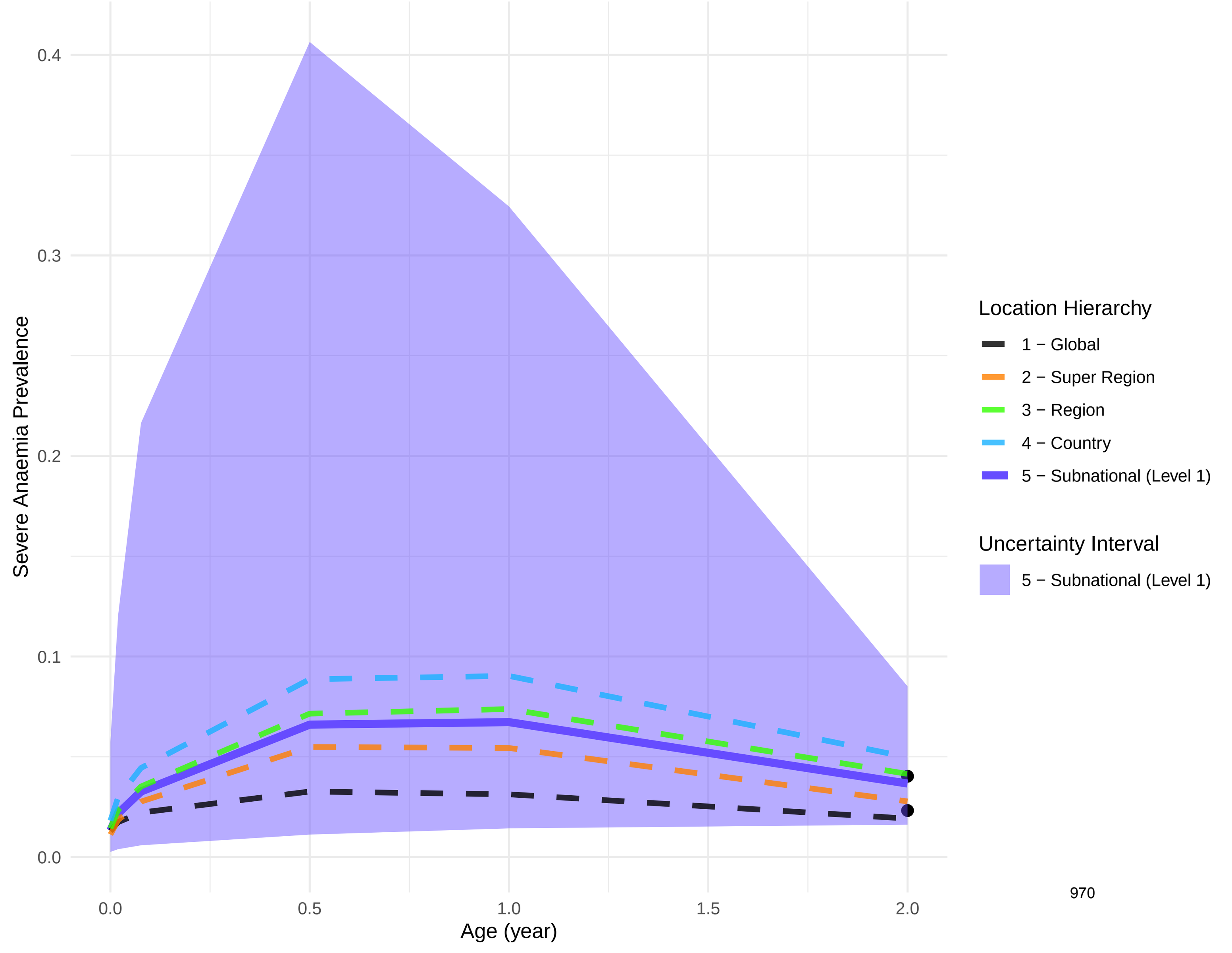

MR−BRT Age/Sex Fits − Non−pregnant Females <2 Years Old − Severe Anaemia Prevalence − Jigawa
WHO−2024 Adjustment Method

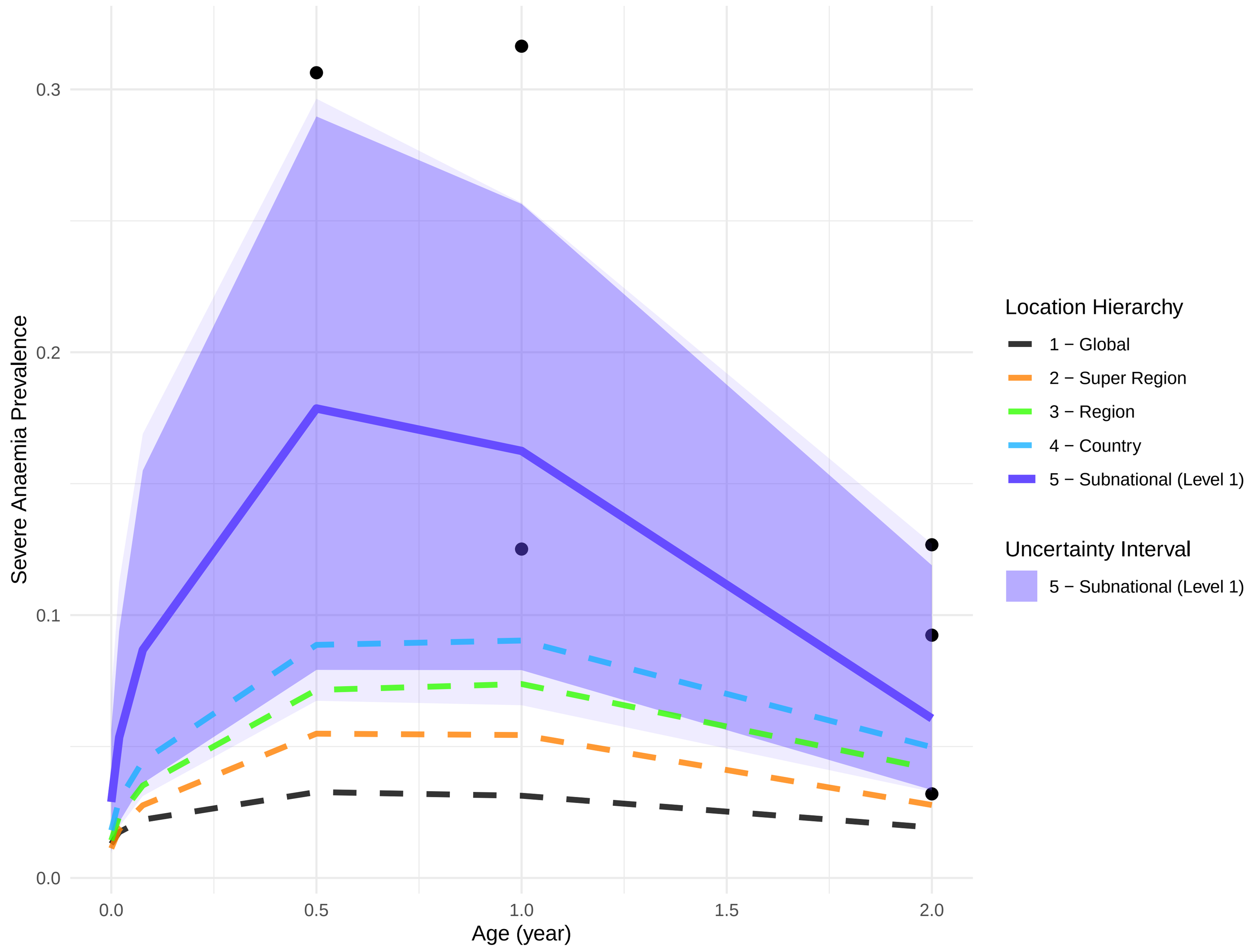

MR−BRT Age/Sex Fits − Non−pregnant Females <2 Years Old − Severe Anaemia Prevalence − Kaduna
WHO−2024 Adjustment Method

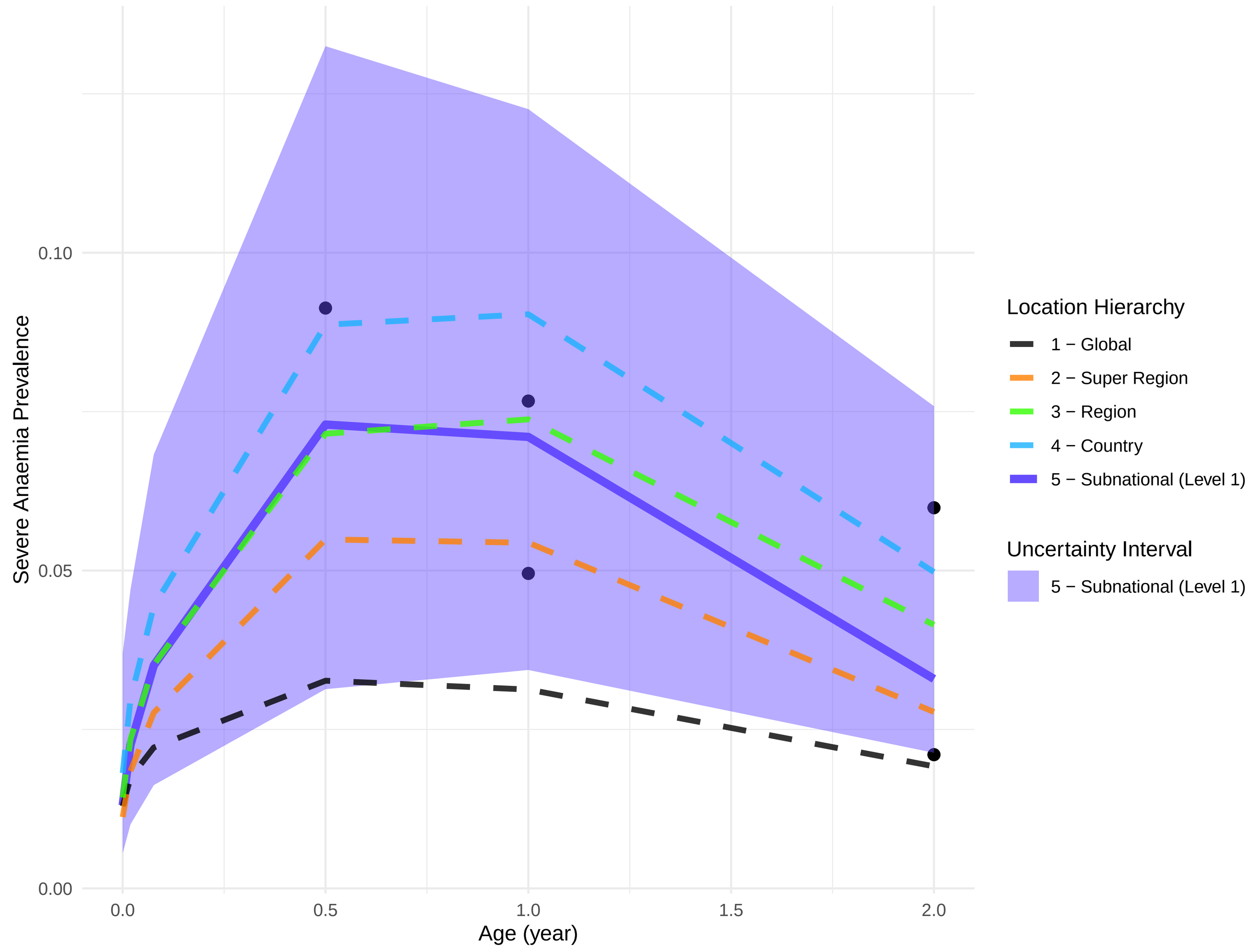

MR−BRT Age/Sex Fits − Non−pregnant Females <2 Years Old − Severe Anaemia Prevalence − Kano
WHO−2024 Adjustment Method

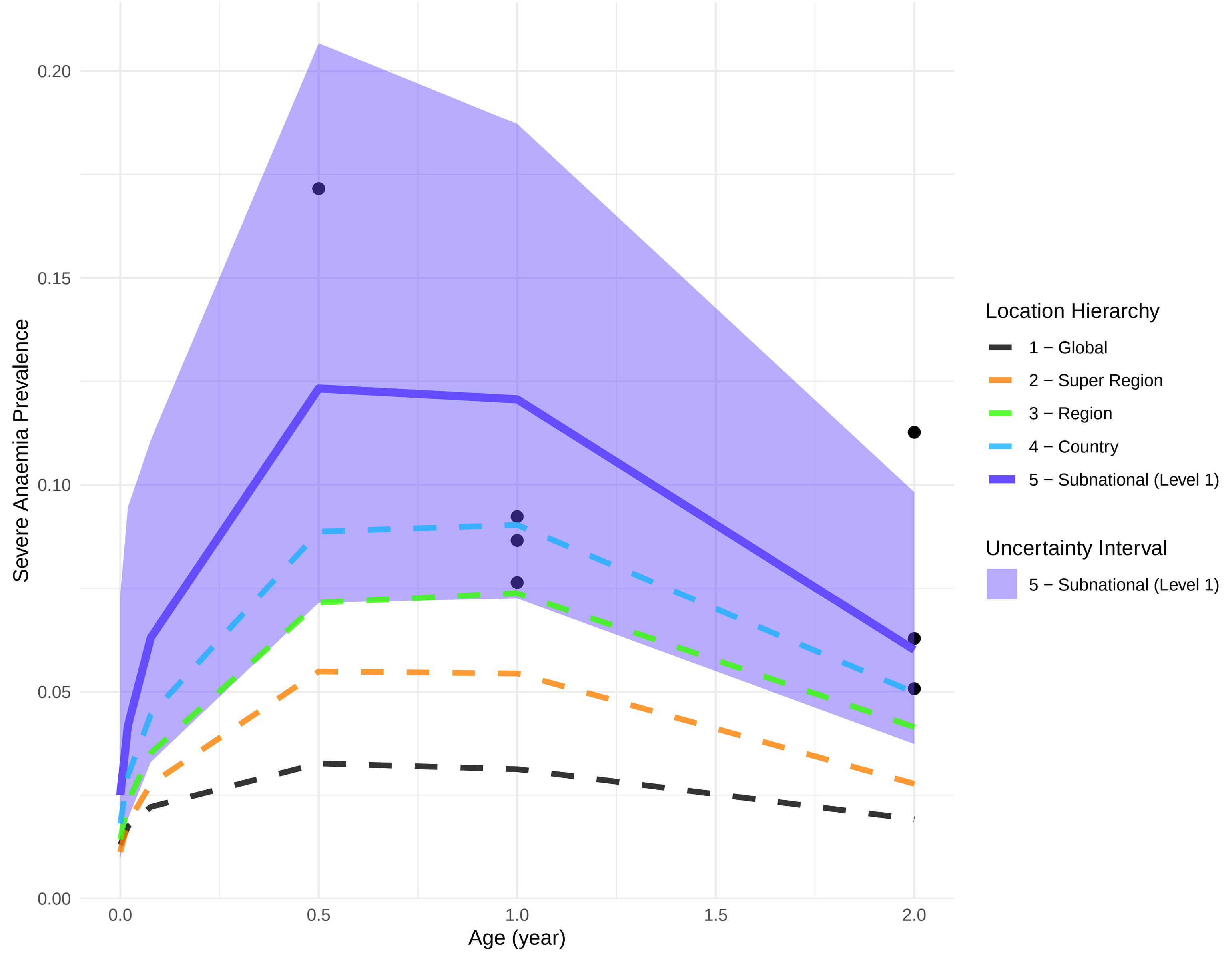

MR−BRT Age/Sex Fits − Non−pregnant Females <2 Years Old − Severe Anaemia Prevalence − Katsina
WHO−2024 Adjustment Method

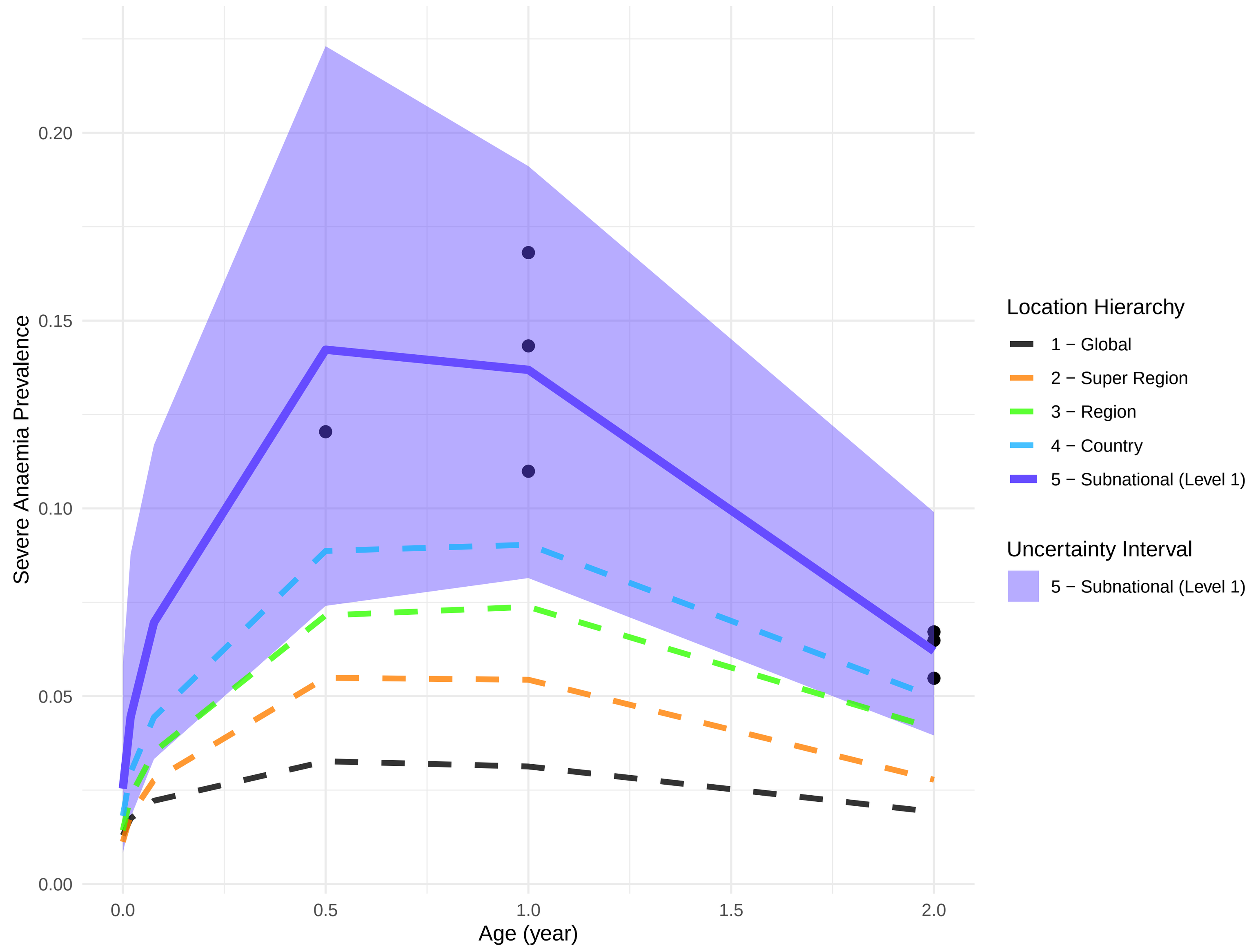

MR−BRT Age/Sex Fits − Non−pregnant Females <2 Years Old − Severe Anaemia Prevalence − Kebbi
WHO−2024 Adjustment Method

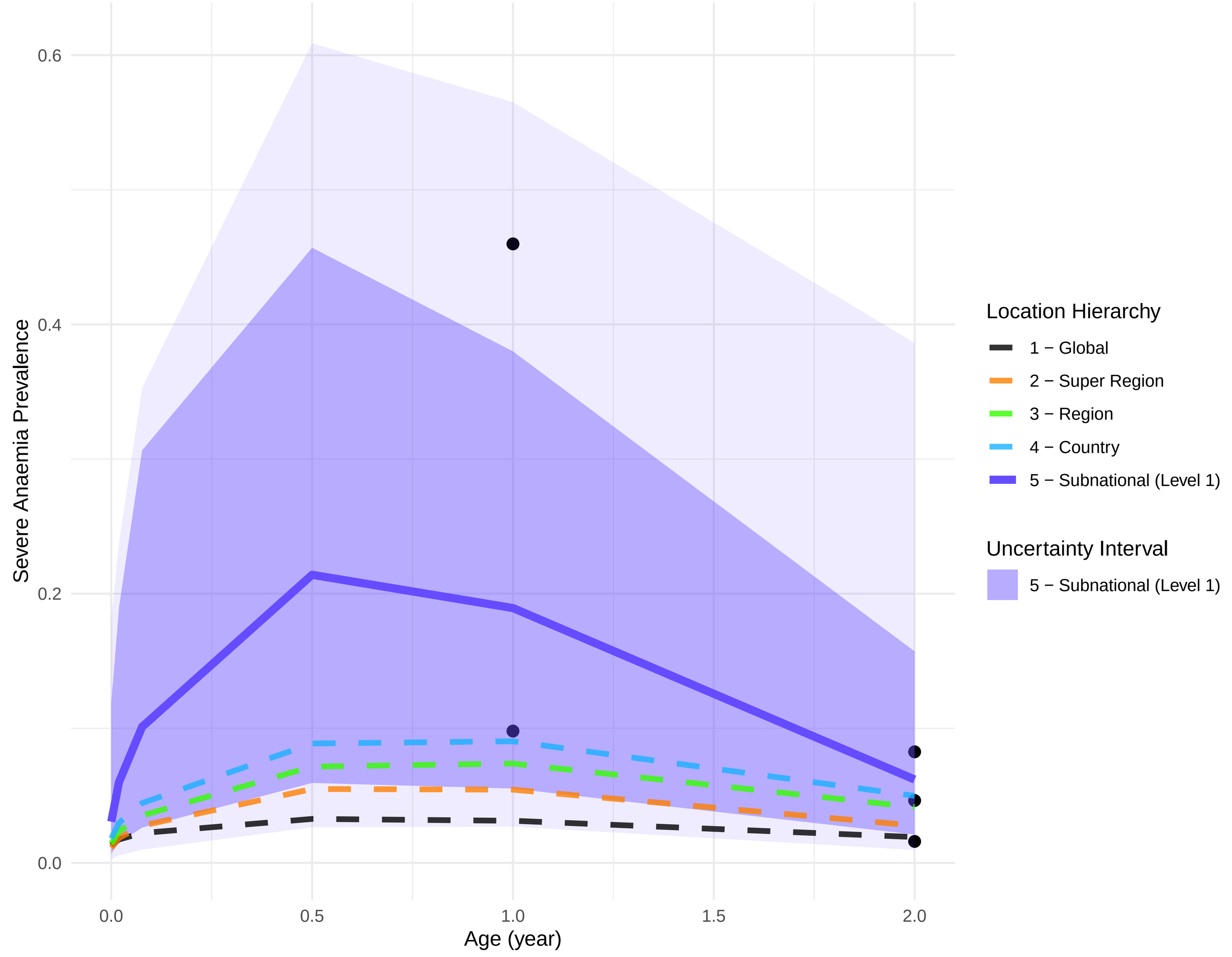

MR−BRT Age/Sex Fits − Non−pregnant Females <2 Years Old − Severe Anaemia Prevalence − Kogi
WHO−2024 Adjustment Method

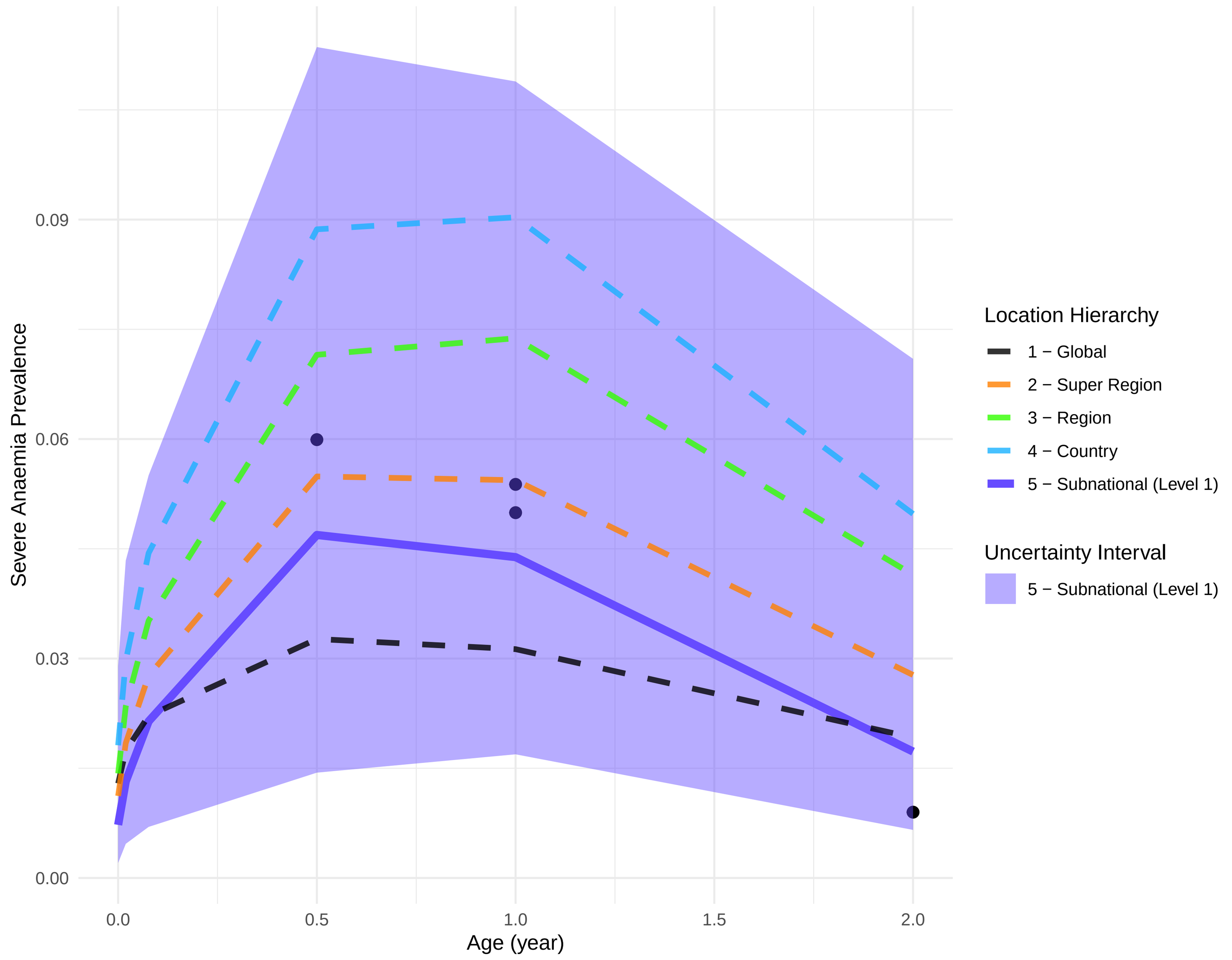

MR−BRT Age/Sex Fits − Non−pregnant Females <2 Years Old − Severe Anaemia Prevalence − Kwara
WHO−2024 Adjustment Method

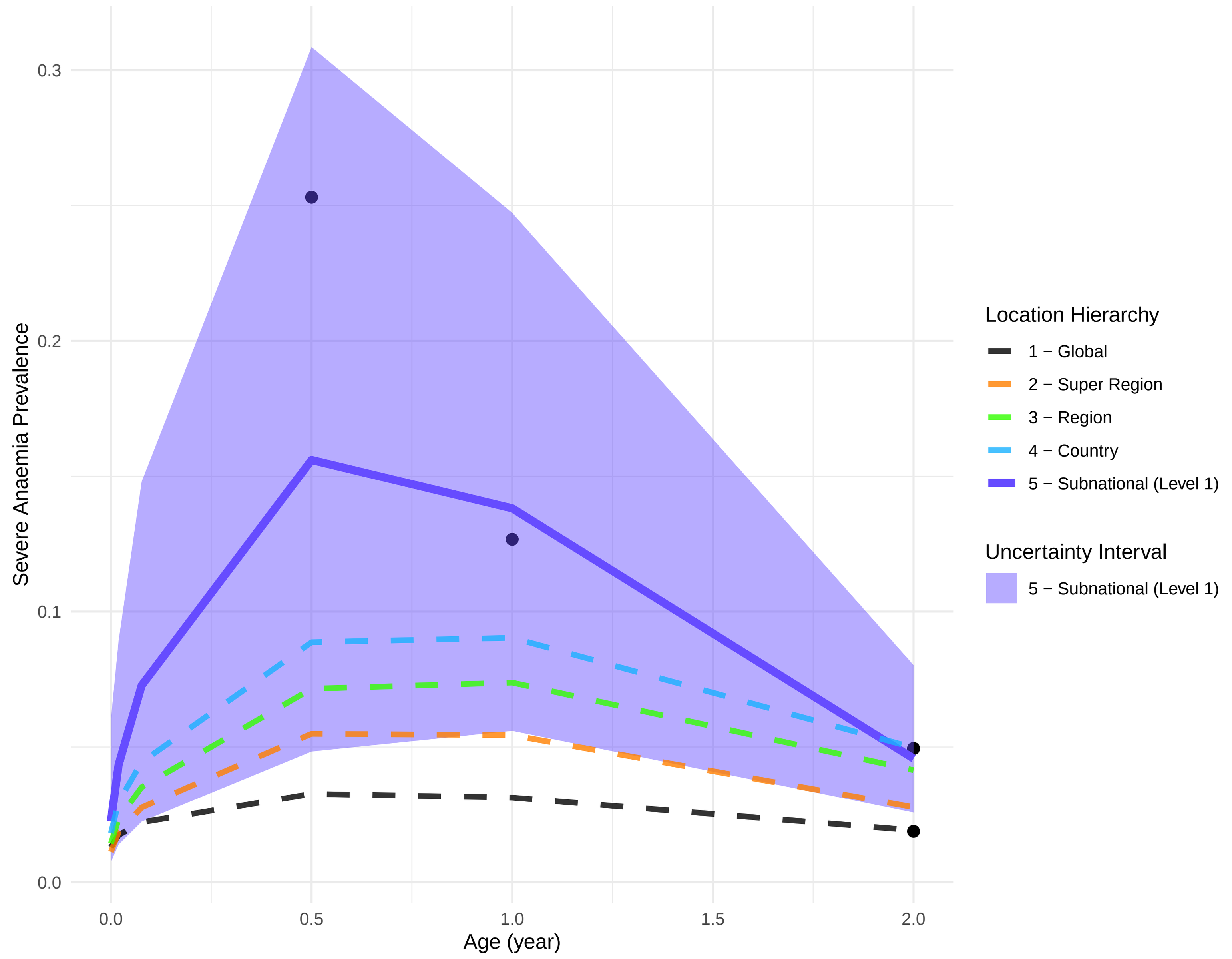

MR−BRT Age/Sex Fits − Non−pregnant Females <2 Years Old − Severe Anaemia Prevalence − Lagos
WHO−2024 Adjustment Method

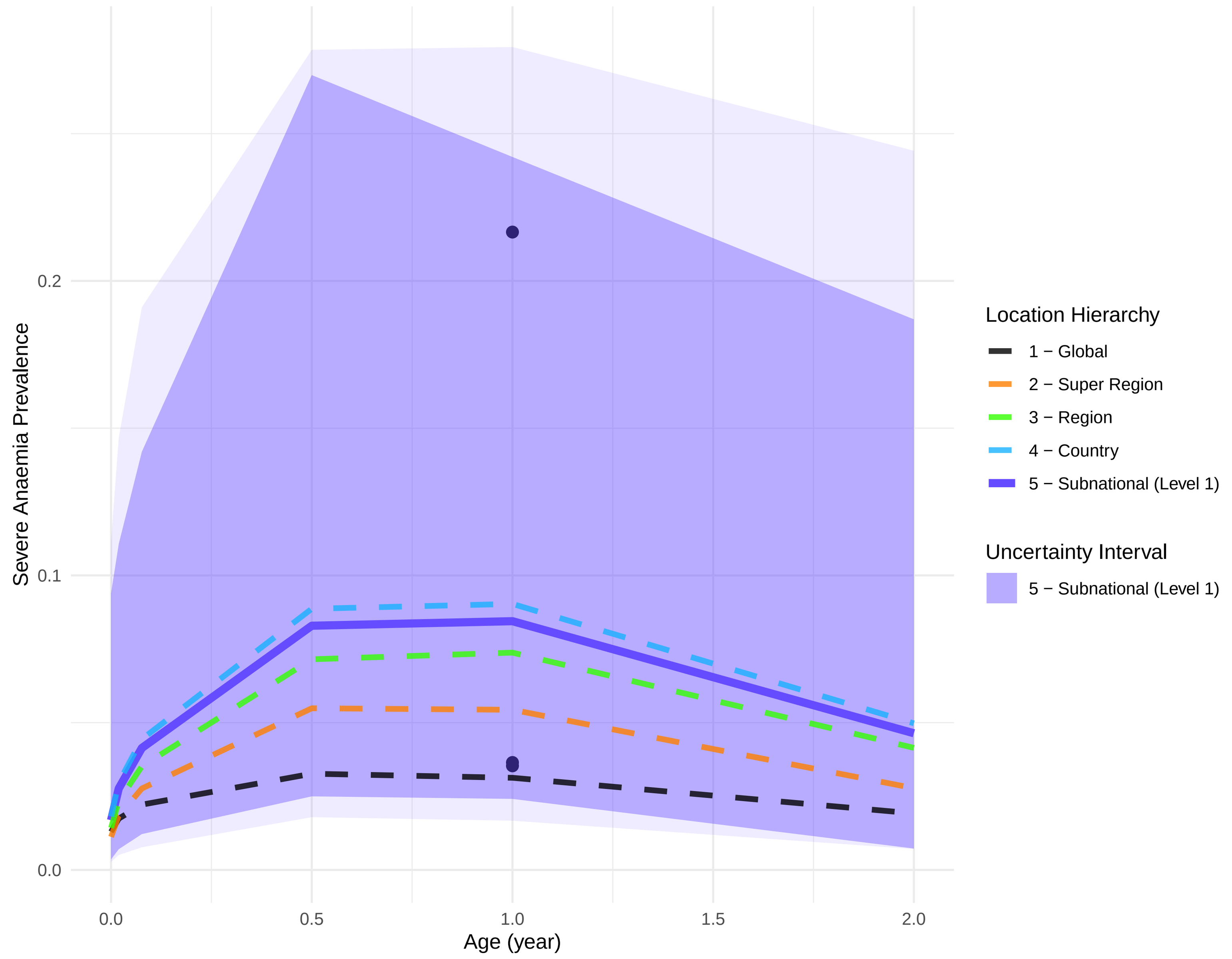

MR−BRT Age/Sex Fits − Non−pregnant Females <2 Years Old − Severe Anaemia Prevalence − Nasarawa
WHO−2024 Adjustment Method

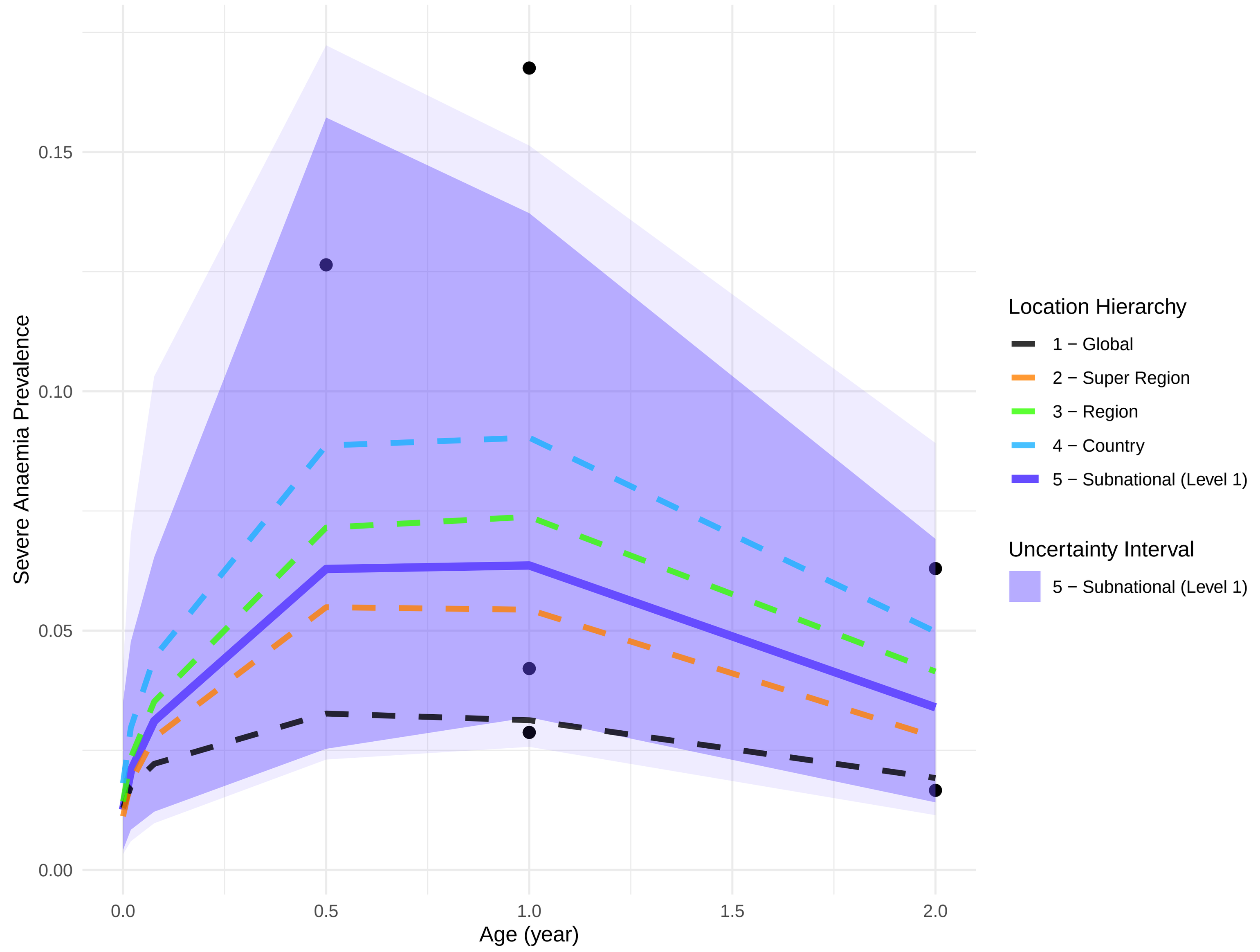

MR−BRT Age/Sex Fits − Non−pregnant Females <2 Years Old − Severe Anaemia Prevalence − Niger
WHO−2024 Adjustment Method

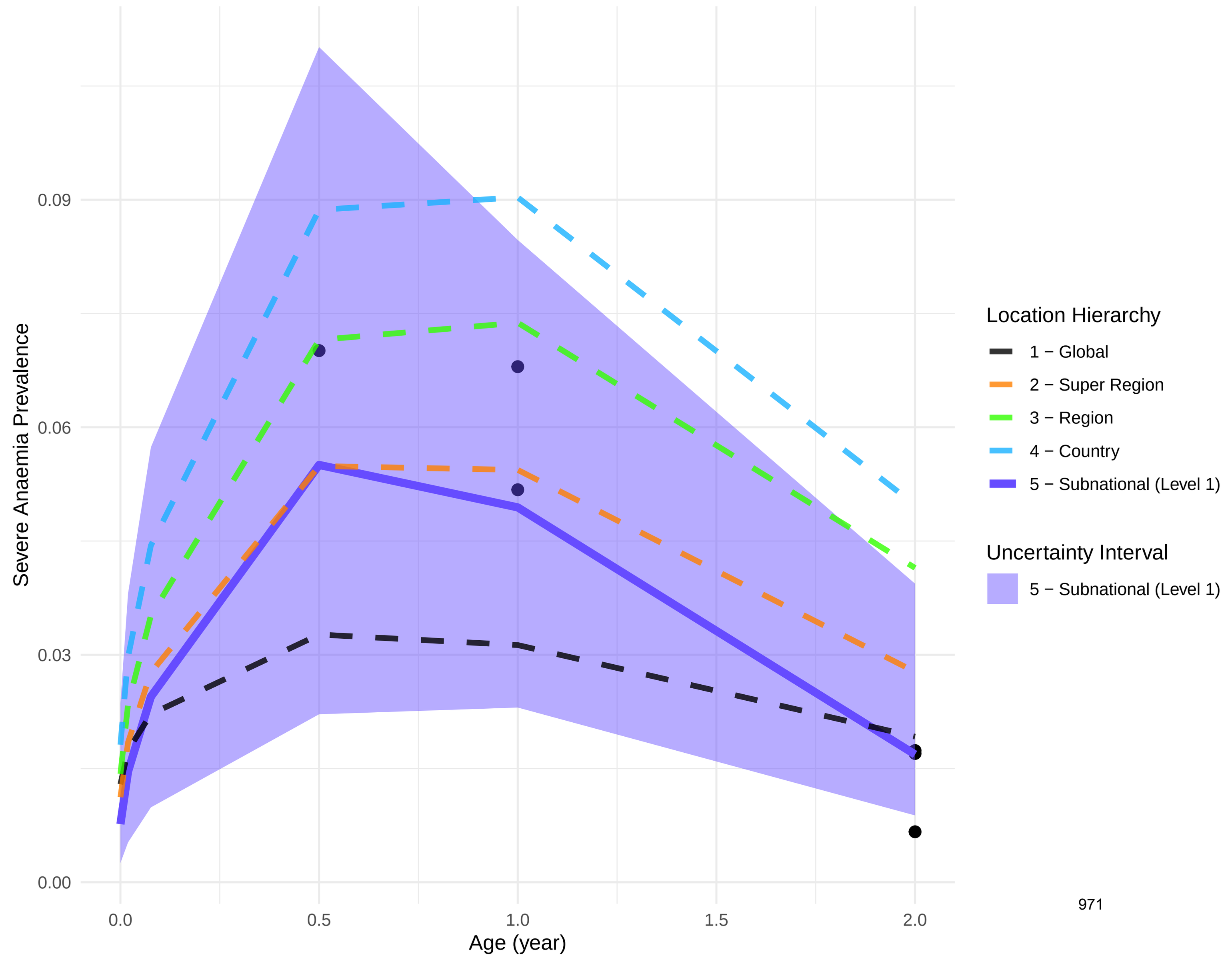

MR−BRT Age/Sex Fits − Non-pregnant Females <2 Years Old − Severe Anaemia Prevalence − Ogun
WHO−2024 Adjustment Method

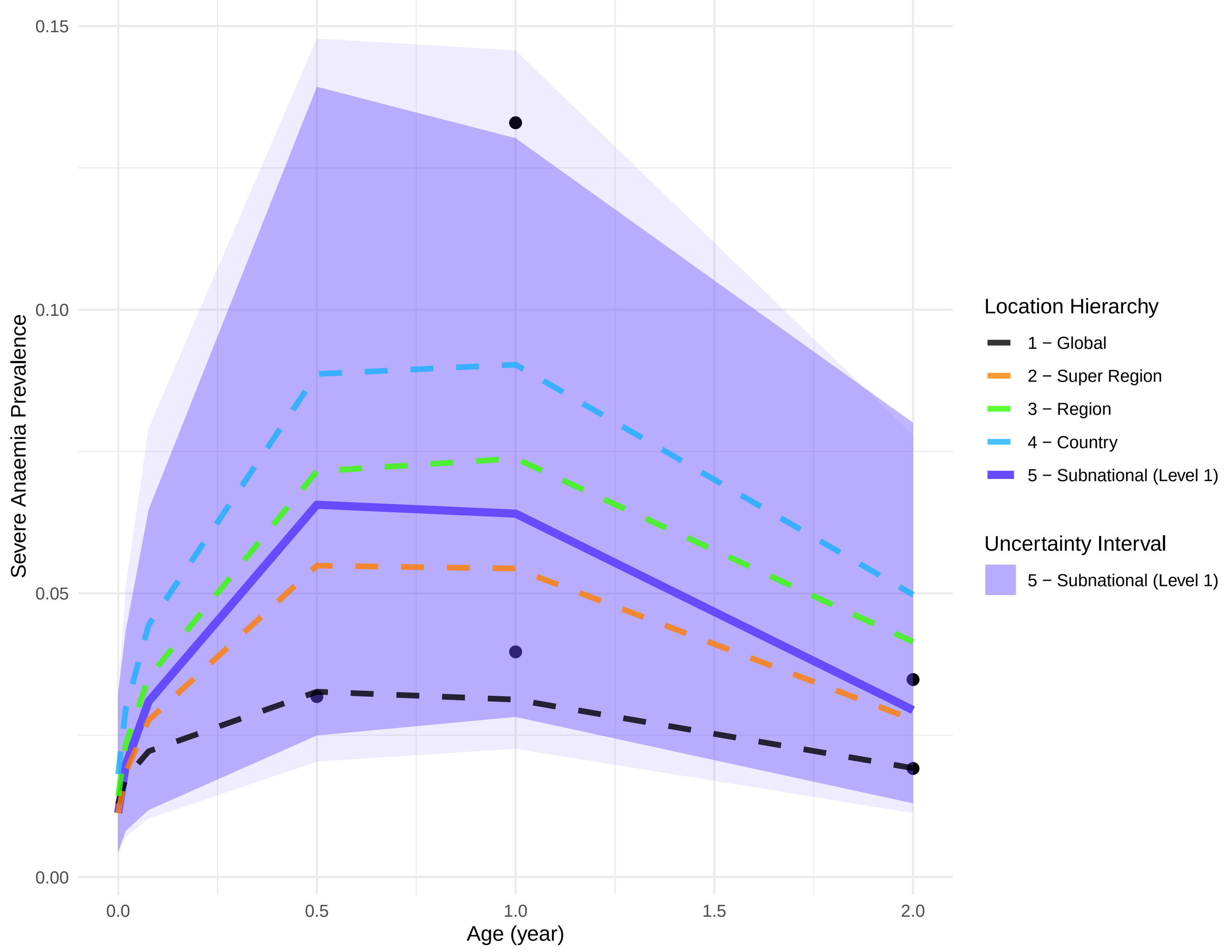

MR−BRT Age/Sex Fits − Non-pregnant Females <2 Years Old − Severe Anaemia Prevalence − Ondo
WHO−2024 Adjustment Method

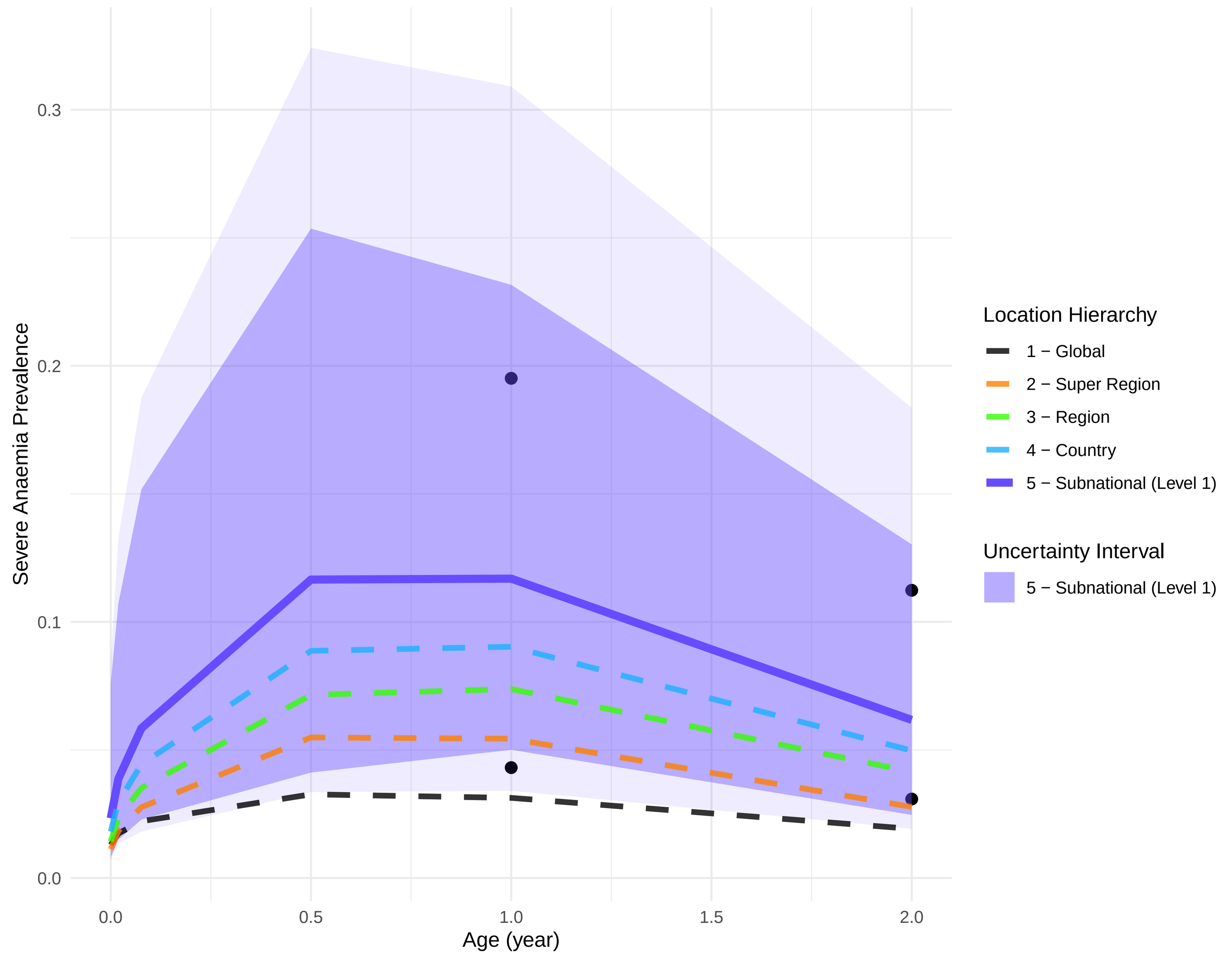

MR−BRT Age/Sex Fits − Non-pregnant Females <2 Years Old − Severe Anaemia Prevalence − Osun
WHO−2024 Adjustment Method

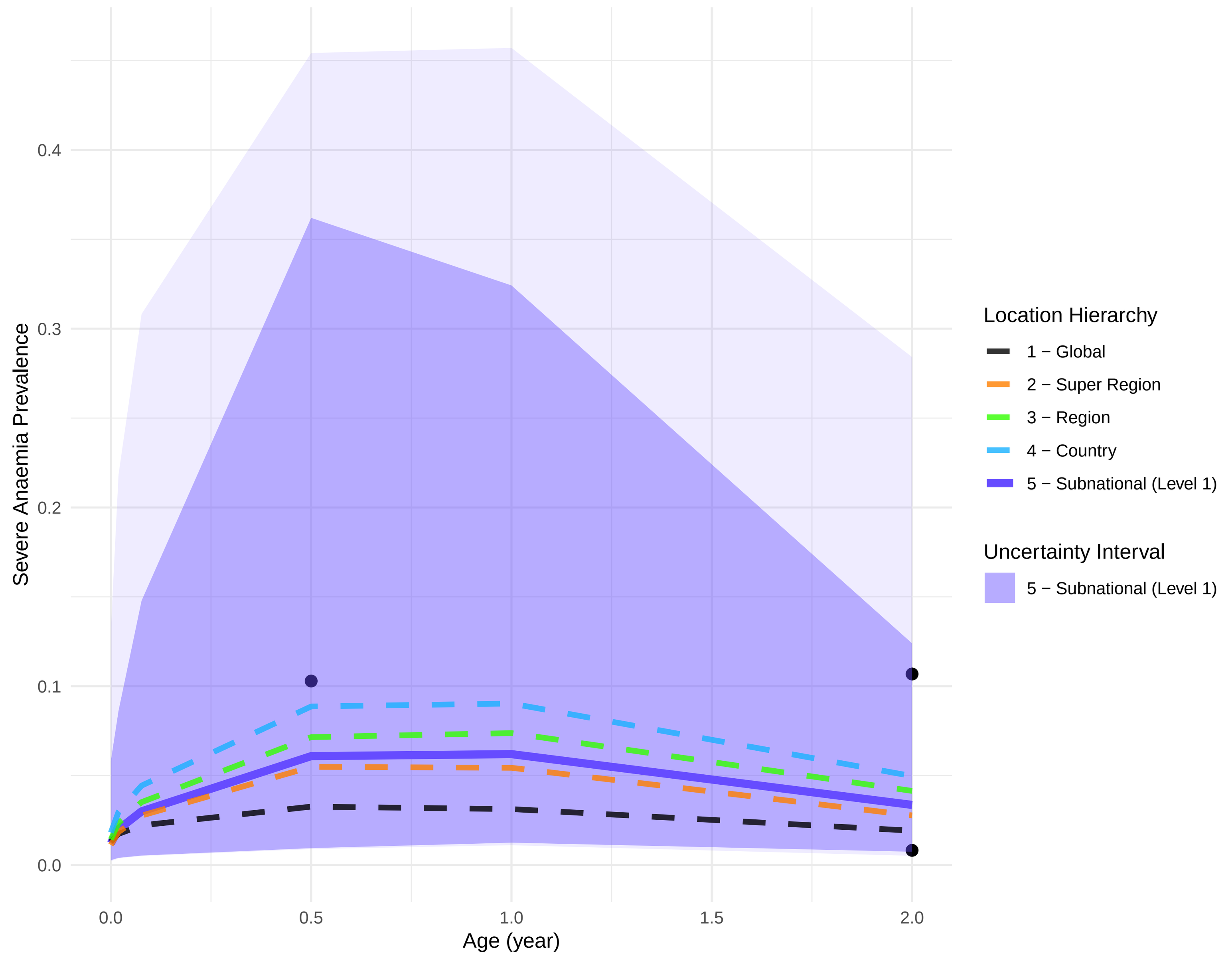

MR−BRT Age/Sex Fits − Non-pregnant Females <2 Years Old − Severe Anaemia Prevalence − Oyo
WHO−2024 Adjustment Method

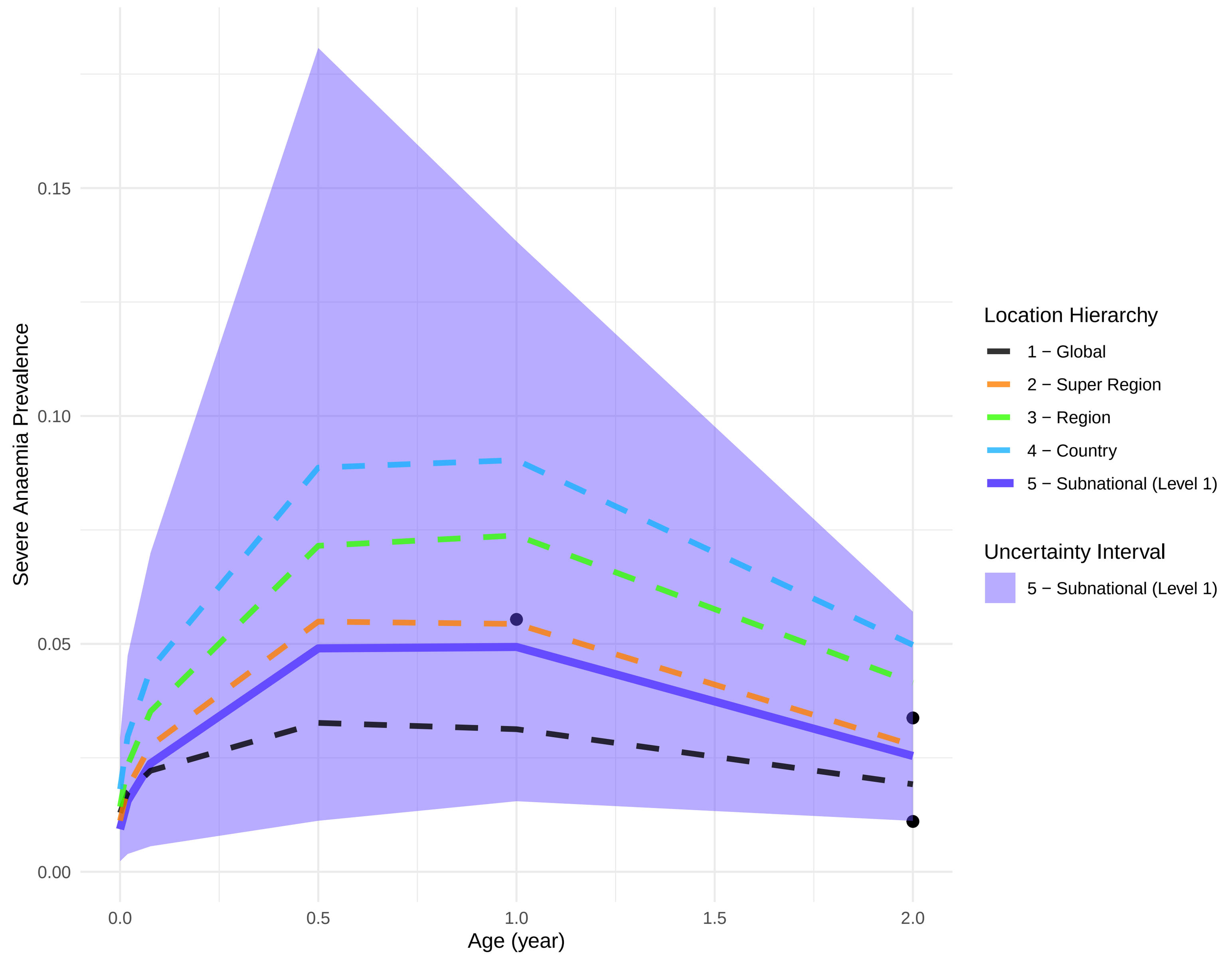

MR−BRT Age/Sex Fits − Non-pregnant Females <2 Years Old − Severe Anaemia Prevalence − Plateau
WHO−2024 Adjustment Method

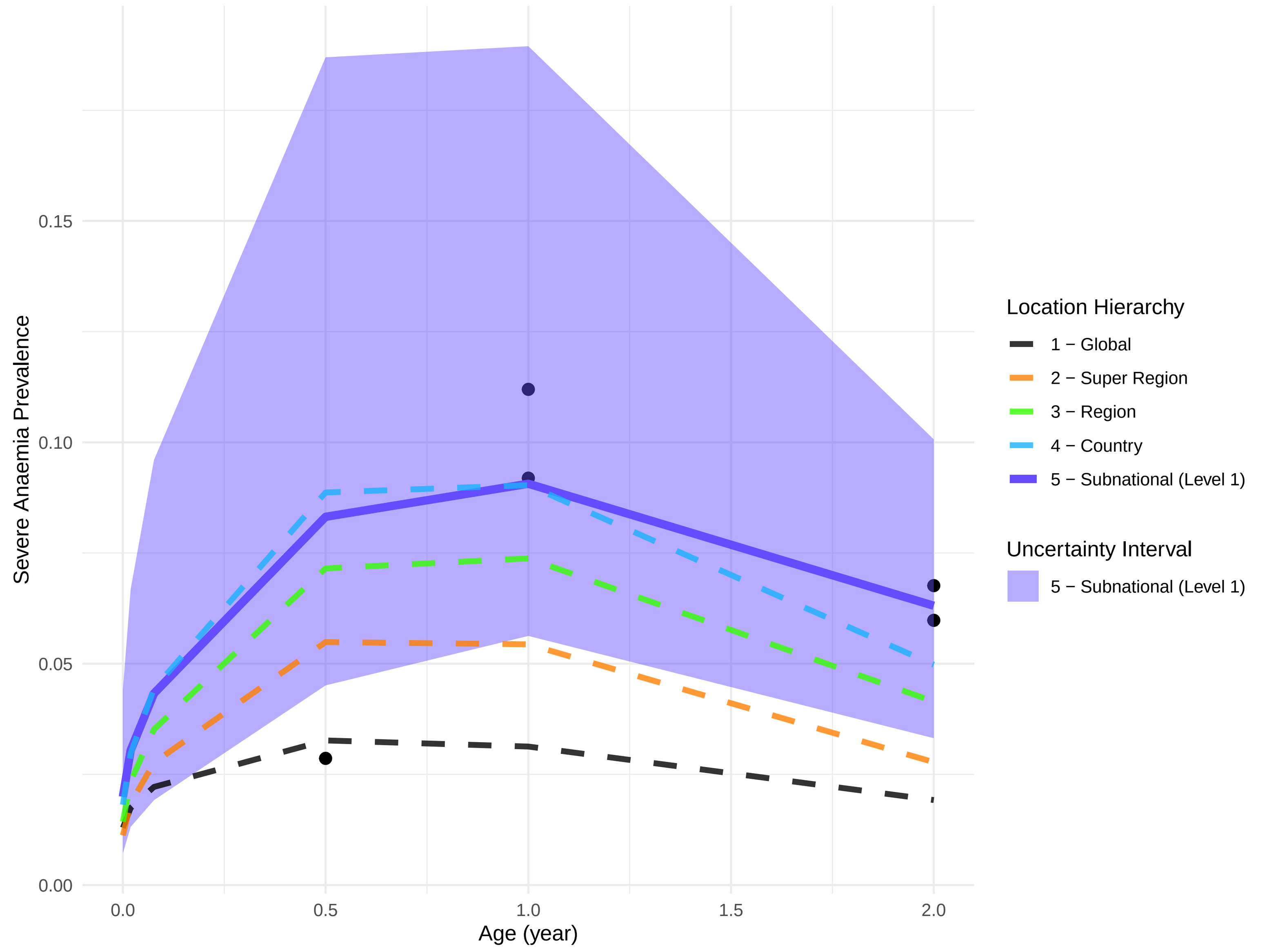

MR−BRT Age/Sex Fits − Non-pregnant Females <2 Years Old − Severe Anaemia Prevalence − Rivers
WHO−2024 Adjustment Method

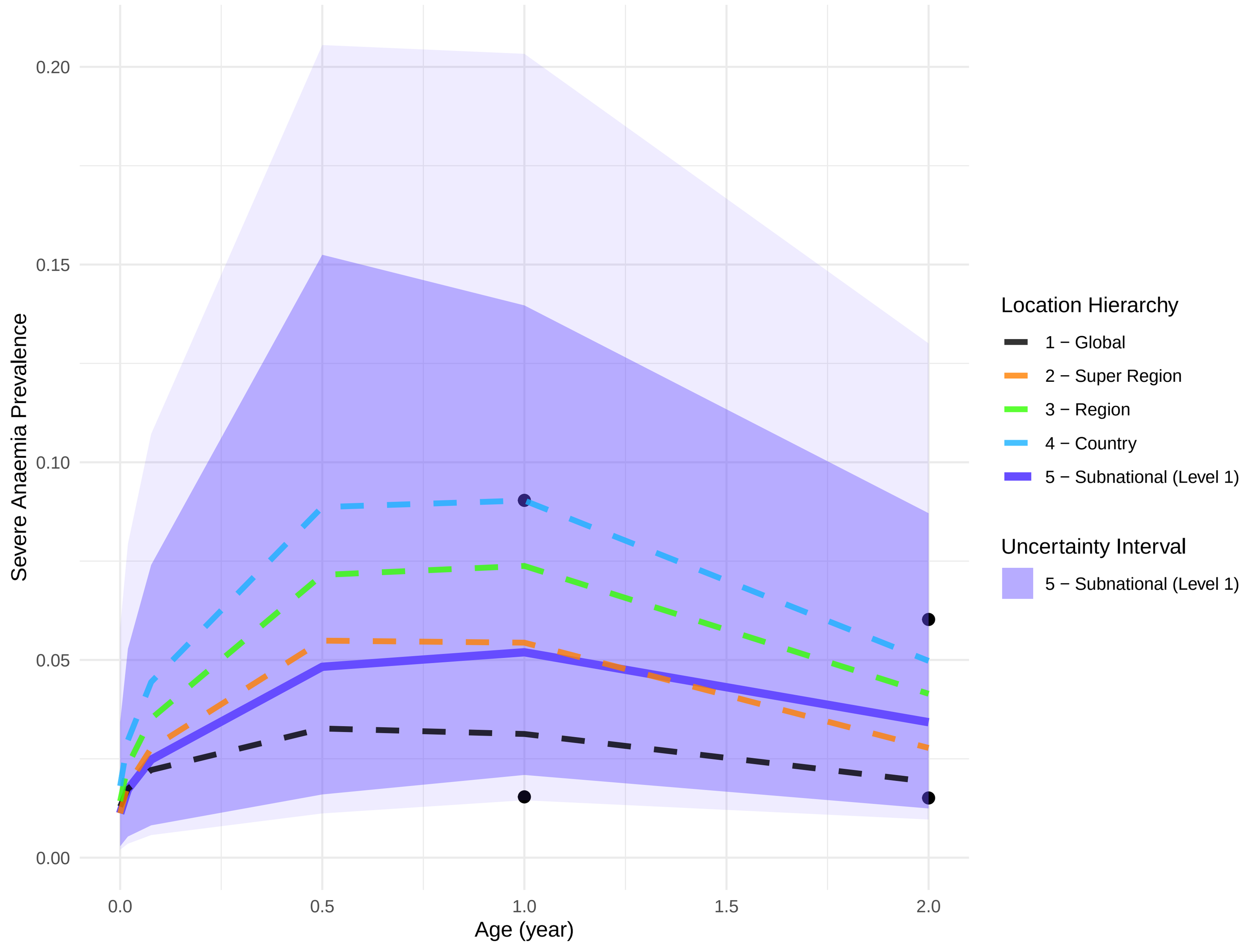

MR−BRT Age/Sex Fits − Non-pregnant Females <2 Years Old − Severe Anaemia Prevalence − Sokoto
WHO−2024 Adjustment Method

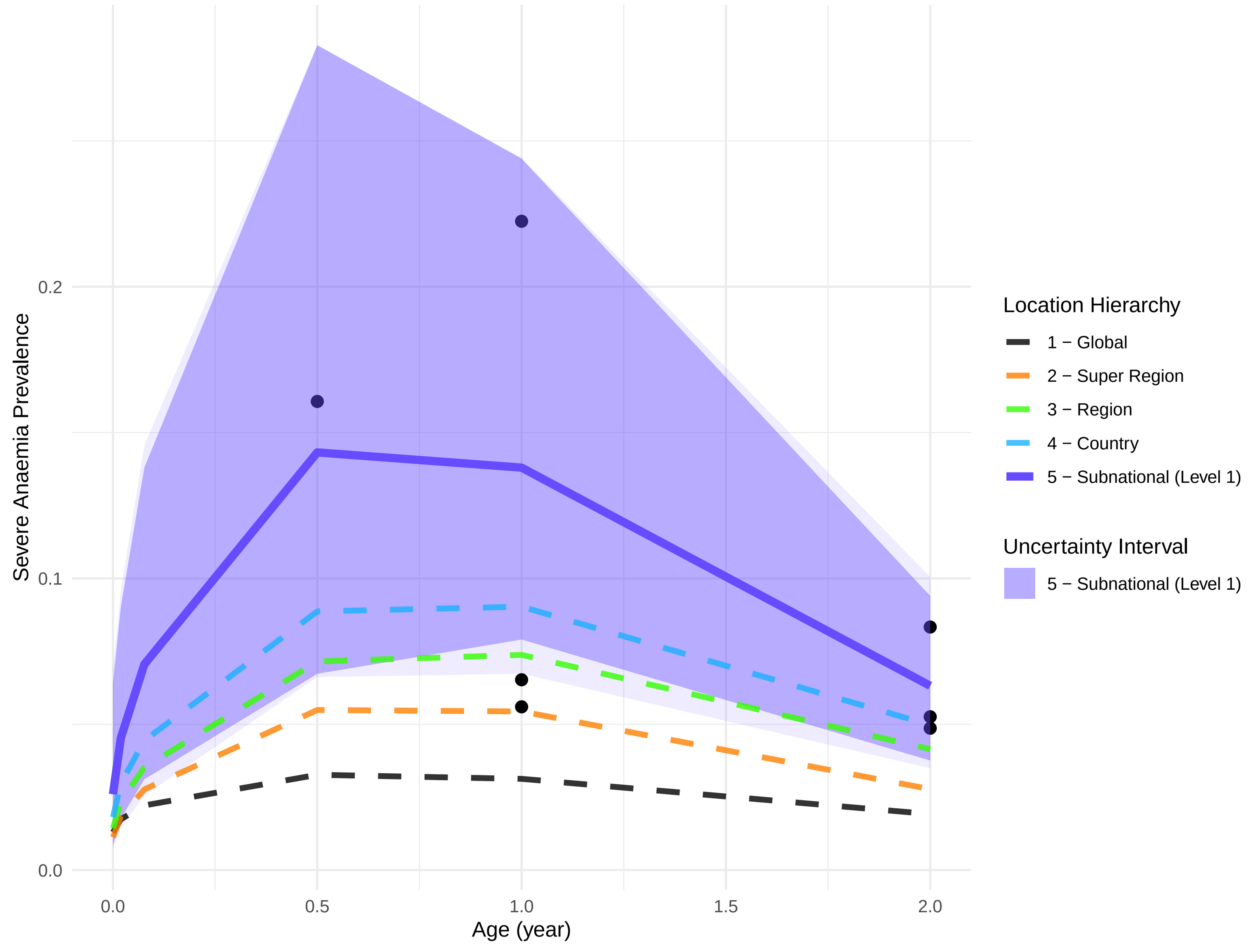

MR−BRT Age/Sex Fits − Non-pregnant Females <2 Years Old − Severe Anaemia Prevalence − Taraba
WHO−2024 Adjustment Method

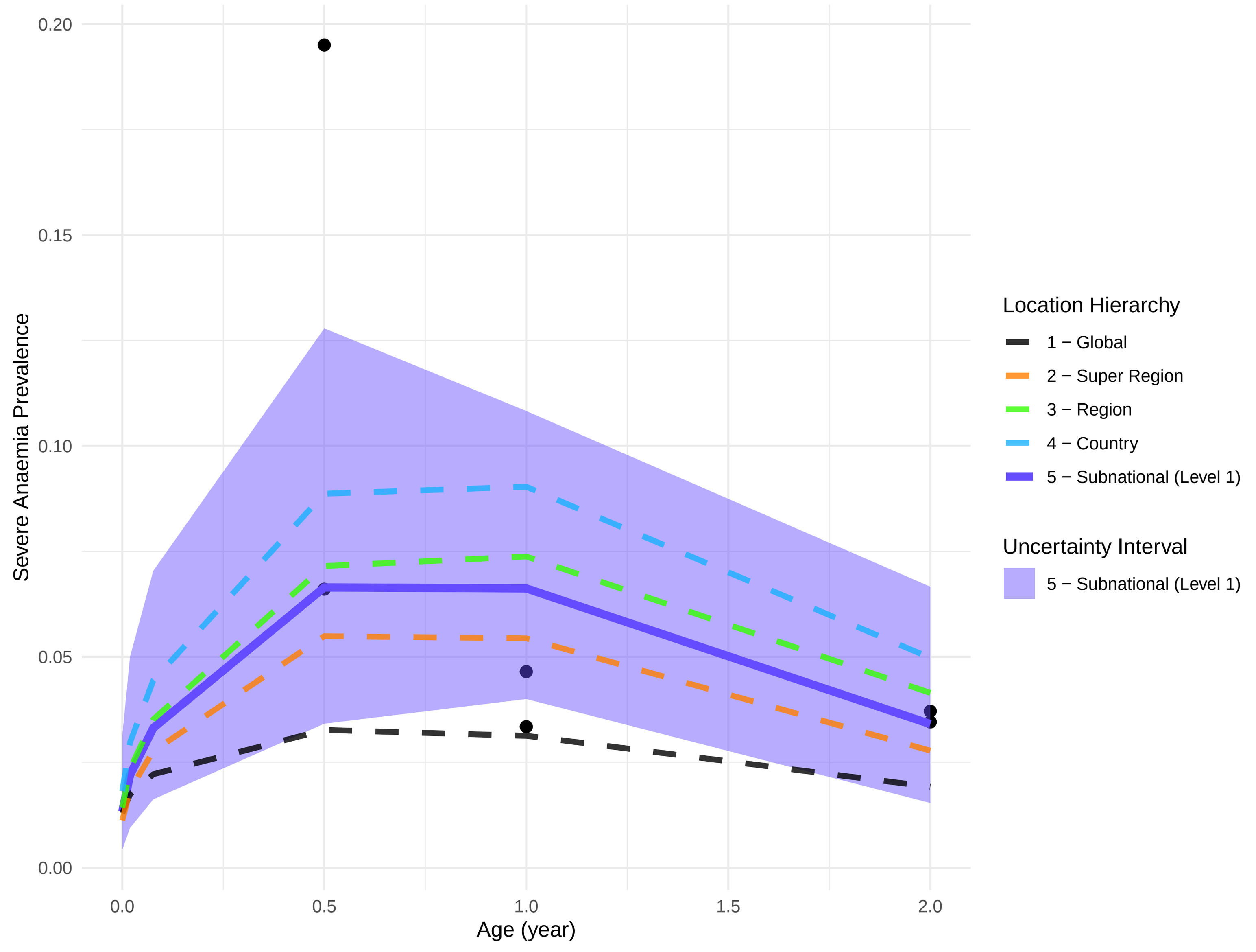

MR−BRT Age/Sex Fits − Non-pregnant Females <2 Years Old − Severe Anaemia Prevalence − Yobe
WHO−2024 Adjustment Method

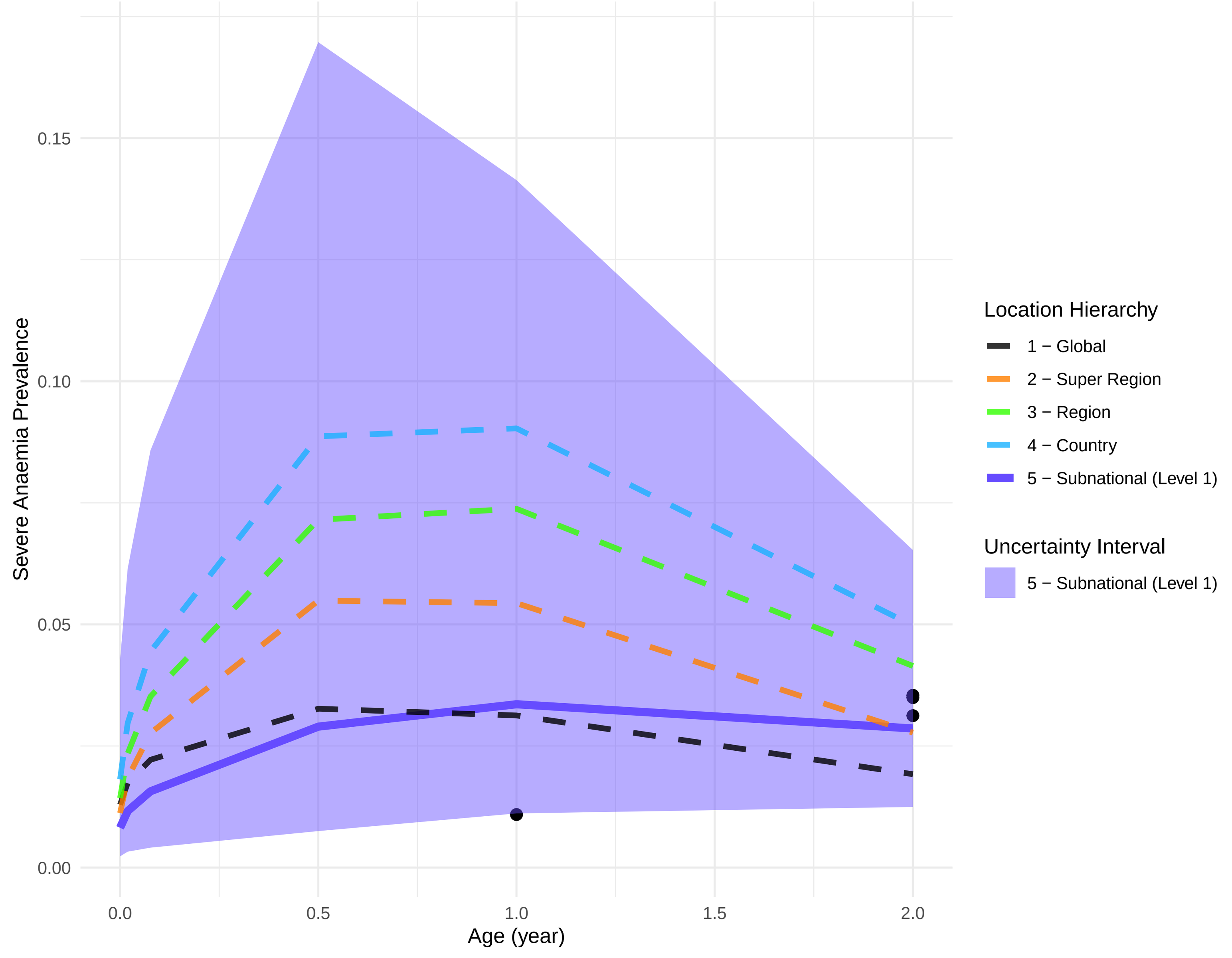

MR−BRT Age/Sex Fits − Non-pregnant Females <2 Years Old − Severe Anaemia Prevalence − Zamfara
WHO−2024 Adjustment Method

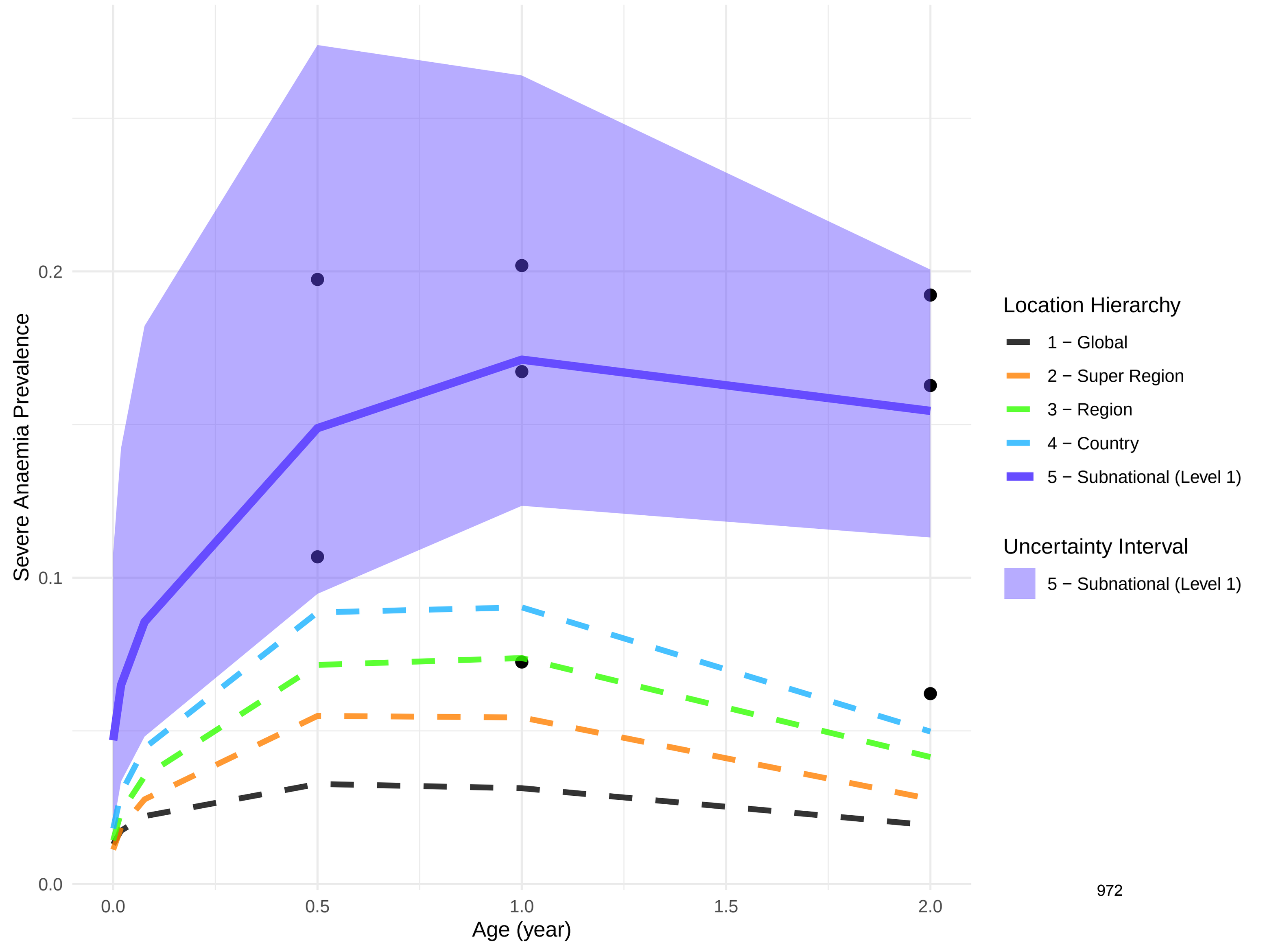

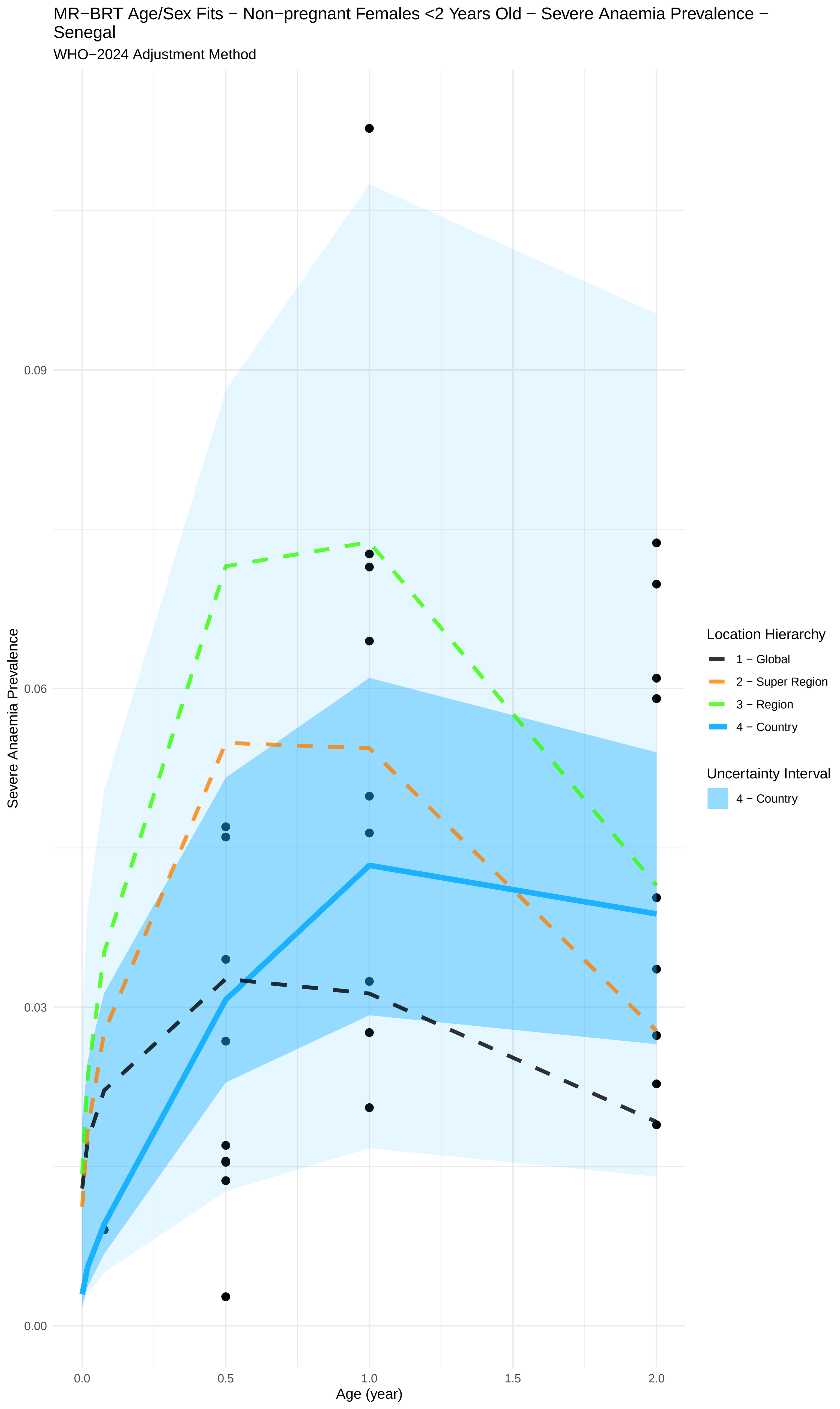

MR−BRT Age/Sex Fits − Non−pregnant Females <2 Years Old − Severe Anaemia Prevalence − Senegal
WHO−2024 Adjustment Method
Severe Anaemia Prevalence
Age (year)
Location Hierarchy
1 − Global
2 − Super Region
3 − Region
4 − Country
Uncertainty Interval
4 − Country

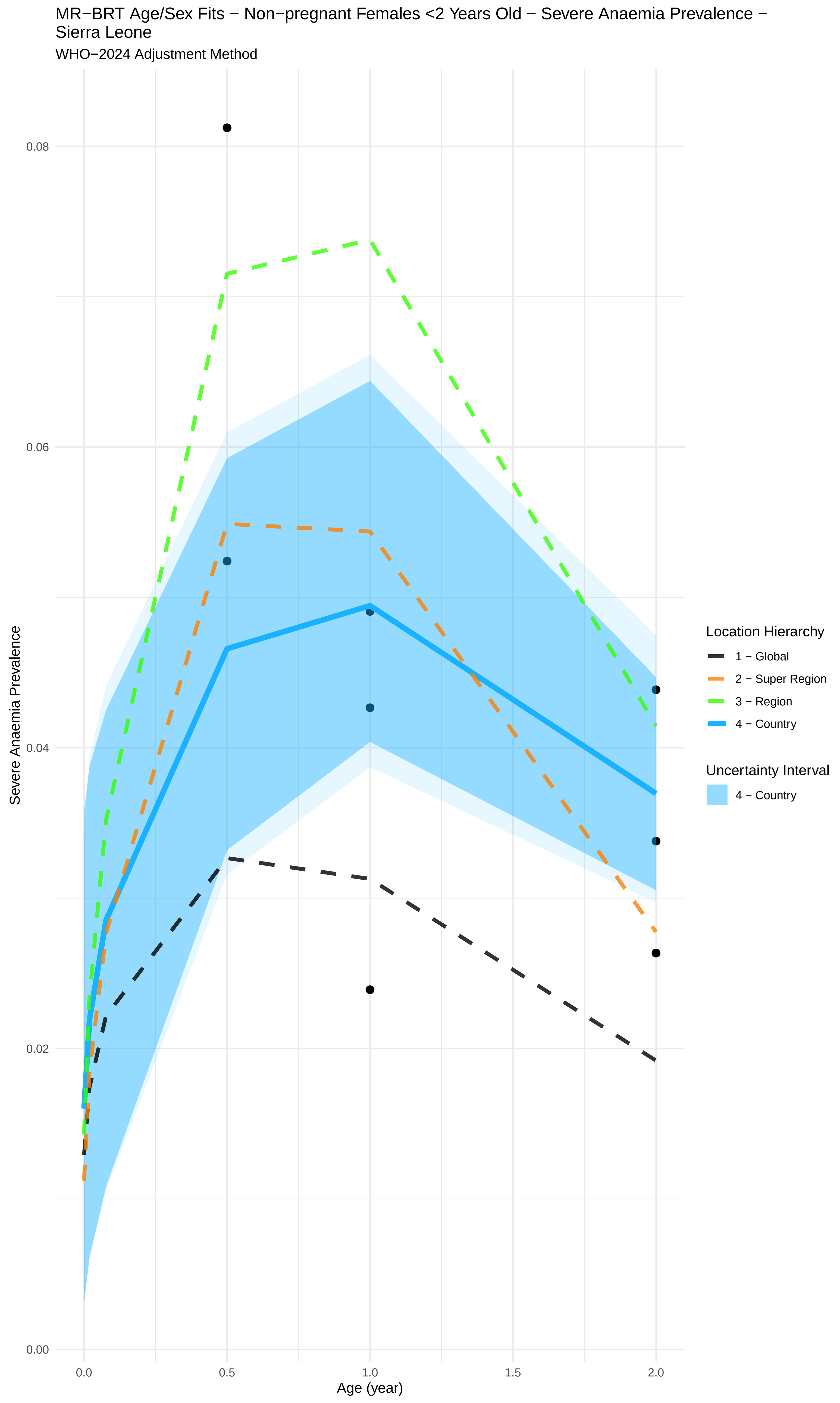

MR−BRT Age/Sex Fits − Non−pregnant Females <2 Years Old − Severe Anaemia Prevalence − Sierra Leone
WHO−2024 Adjustment Method
Severe Anaemia Prevalence
Age (year)
Location Hierarchy
1 − Global
2 − Super Region
3 − Region
4 − Country
Uncertainty Interval
4 − Country

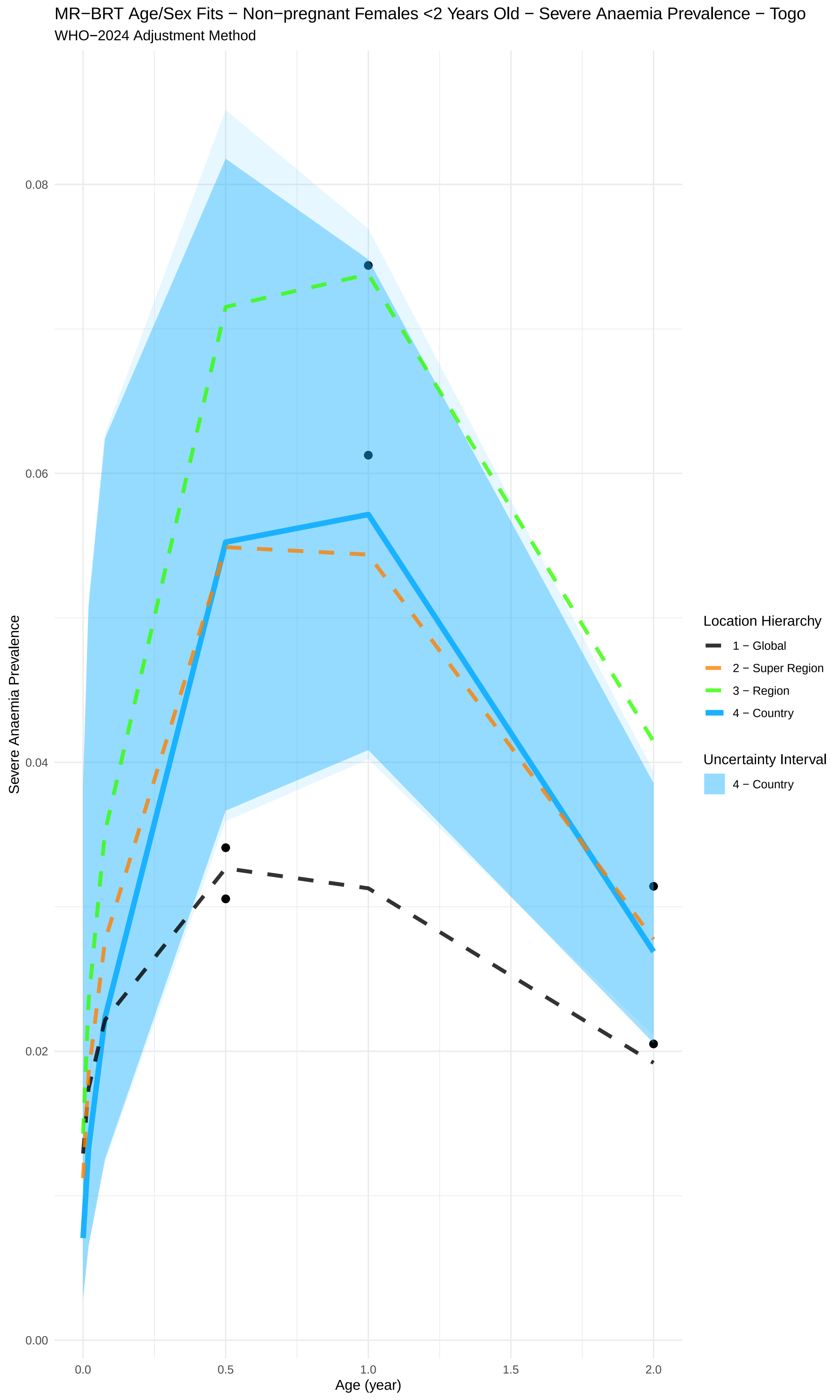

MR−BRT Age/Sex Fits − Non−pregnant Females <2 Years Old − Severe Anaemia Prevalence − Togo
WHO−2024 Adjustment Method
Severe Anaemia Prevalence
Age (year)
Location Hierarchy
1 − Global
2 − Super Region
3 − Region
4 − Country
Uncertainty Interval
4 − Country

MR−BRT Age/Sex Fits − Males <2 Years Old − Severe Anaemia Prevalence − Global
WHO−2001 Adjustment Method

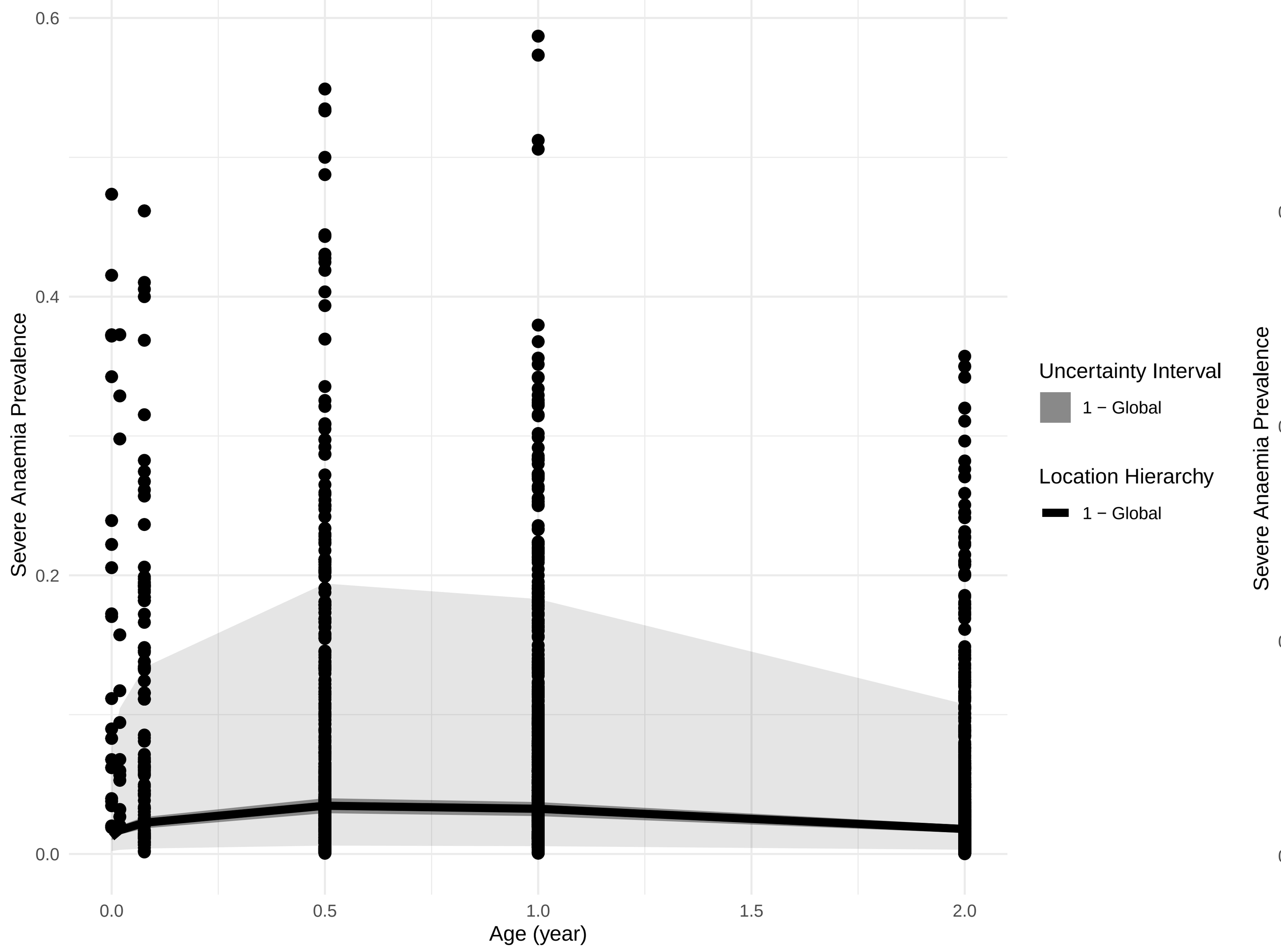

MR−BRT Age/Sex Fits − Males <2 Years Old − Severe Anaemia Prevalence − Central Europe, Eastern Europe, and Central Asia
WHO−2001 Adjustment Method

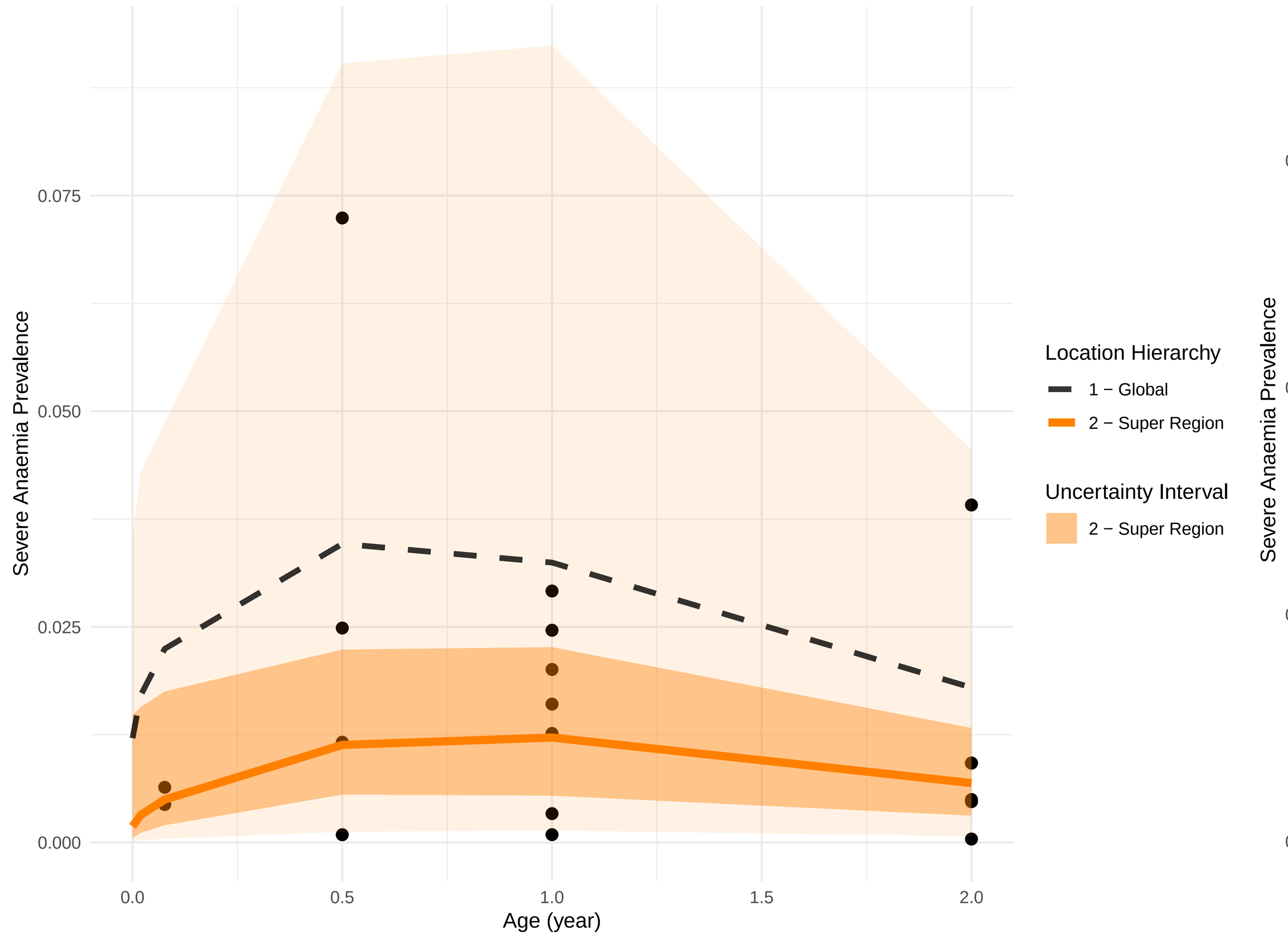

MR−BRT Age/Sex Fits − Males <2 Years Old − Severe Anaemia Prevalence − Central Asia
WHO−2001 Adjustment Method

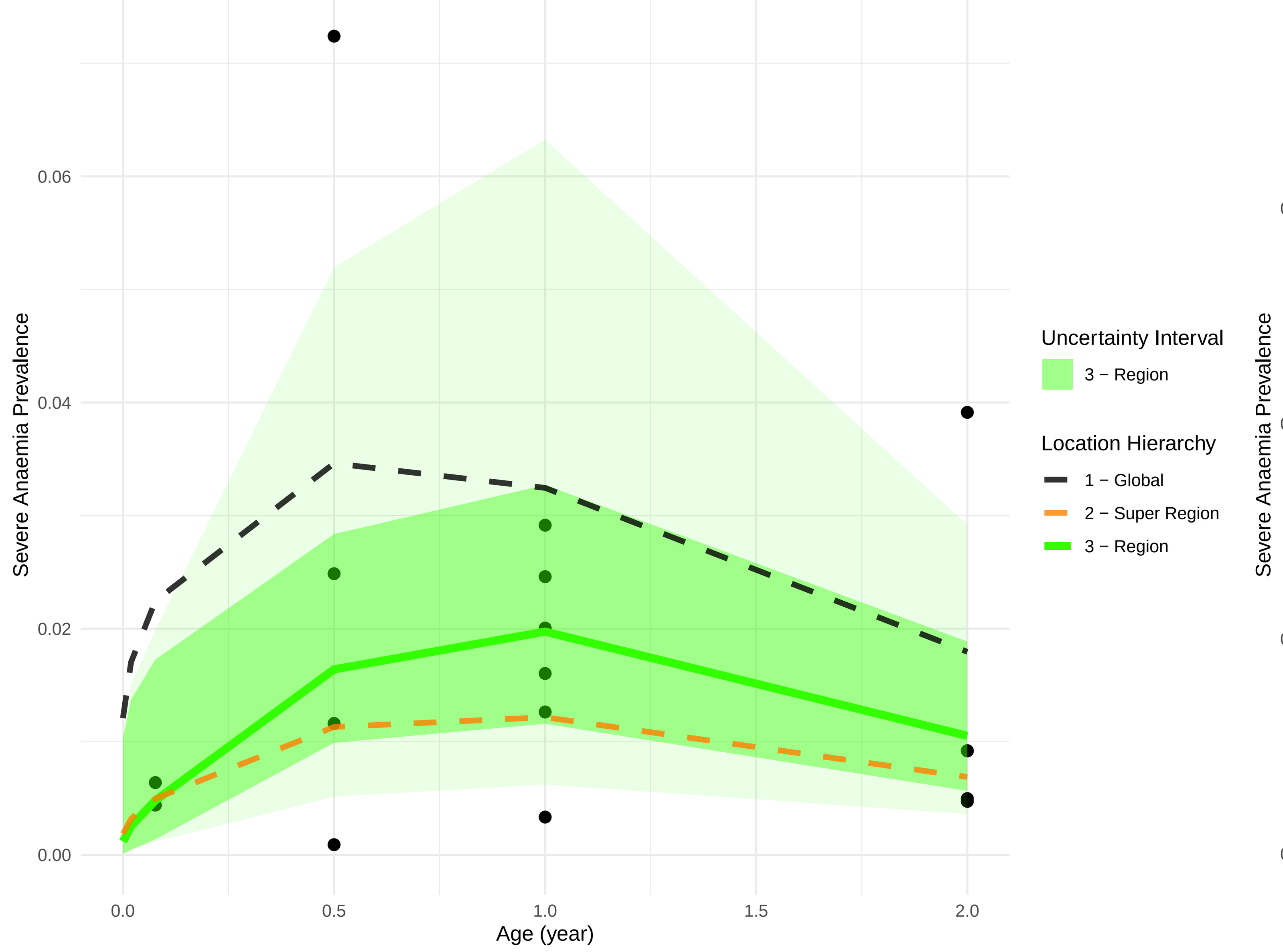

MR−BRT Age/Sex Fits − Males <2 Years Old − Severe Anaemia Prevalence − Armenia
WHO−2001 Adjustment Method

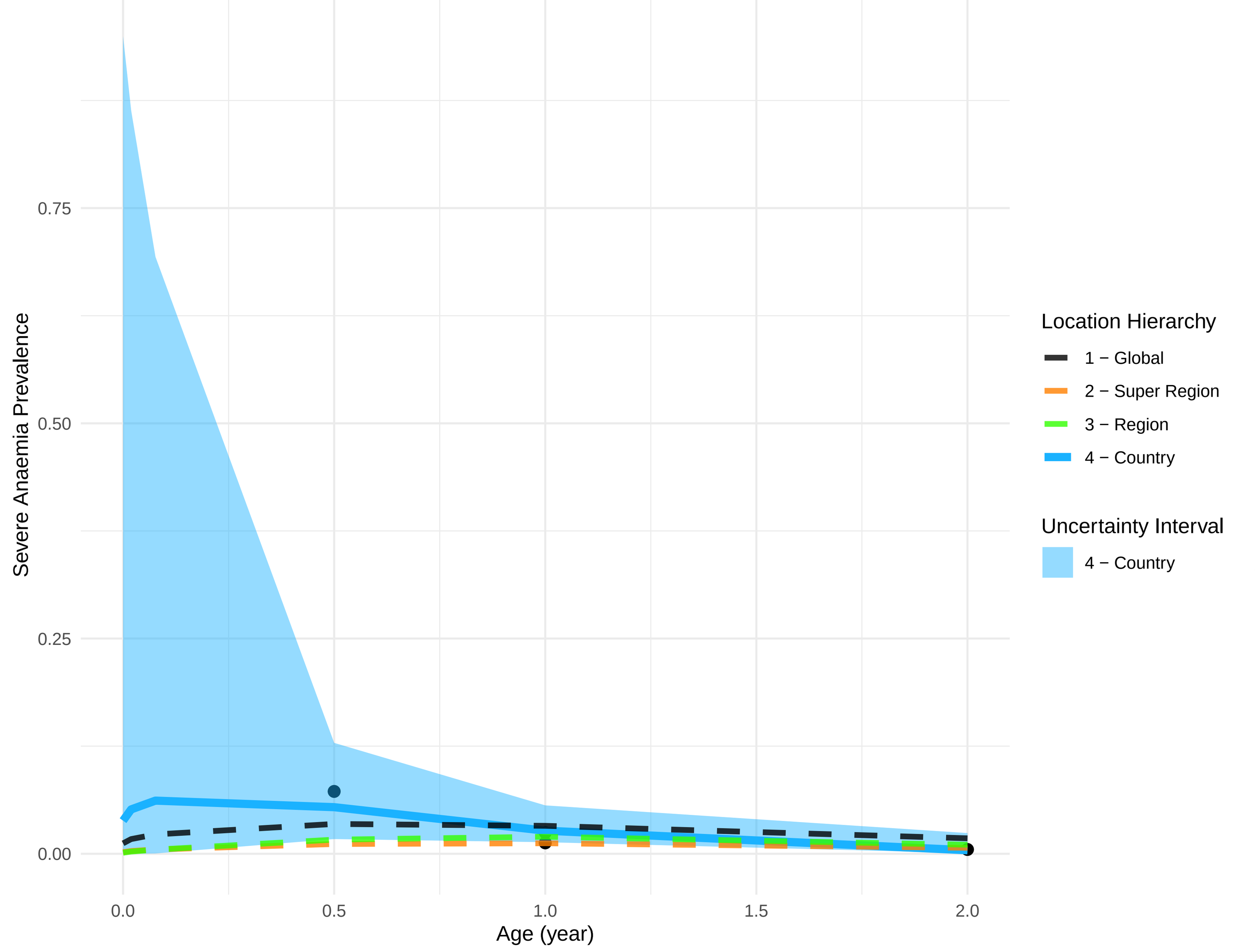

MR−BRT Age/Sex Fits − Males <2 Years Old − Severe Anaemia Prevalence − Azerbaijan
WHO−2001 Adjustment Method

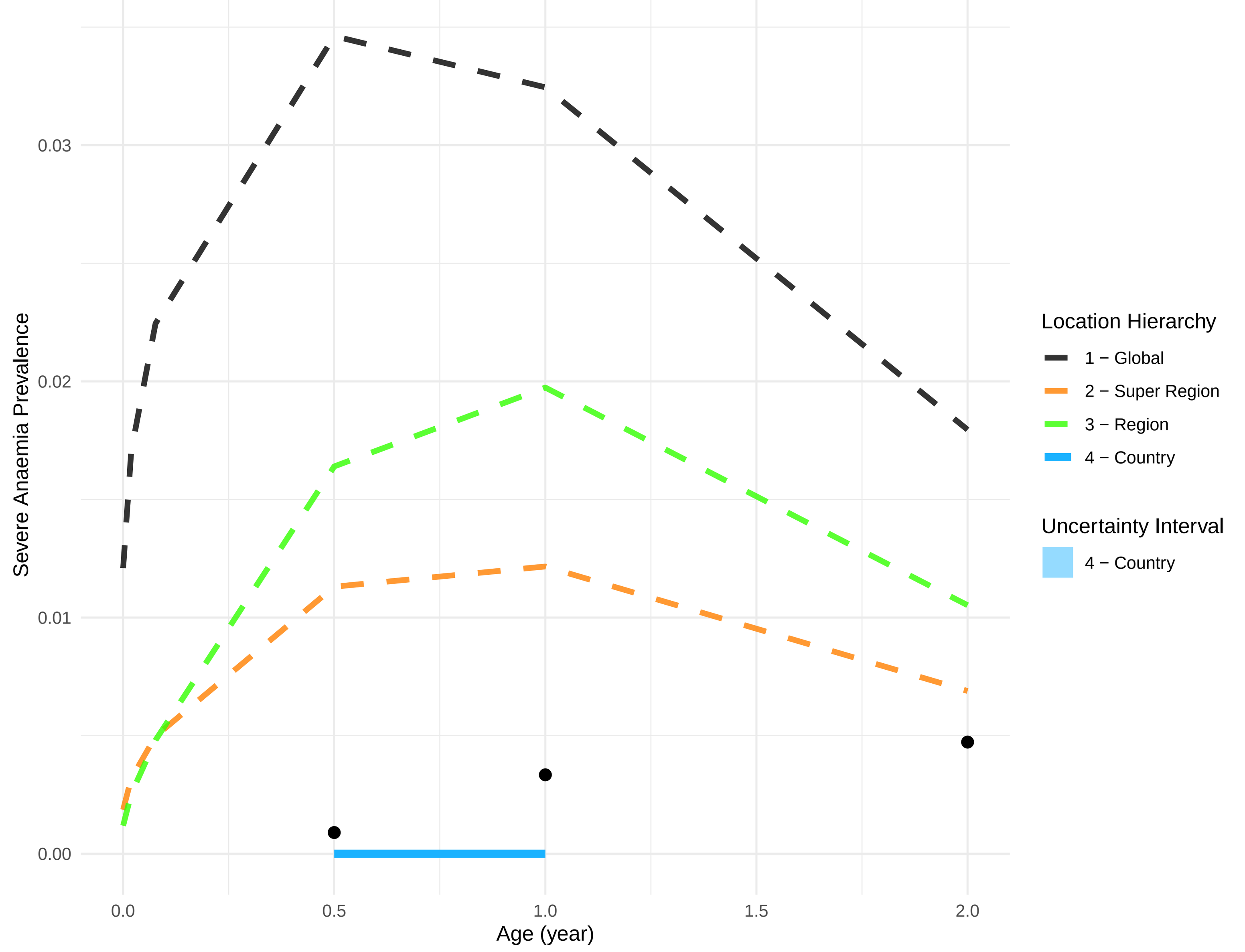

MR−BRT Age/Sex Fits − Males <2 Years Old − Severe Anaemia Prevalence − Kyrgyzstan
WHO−2001 Adjustment Method

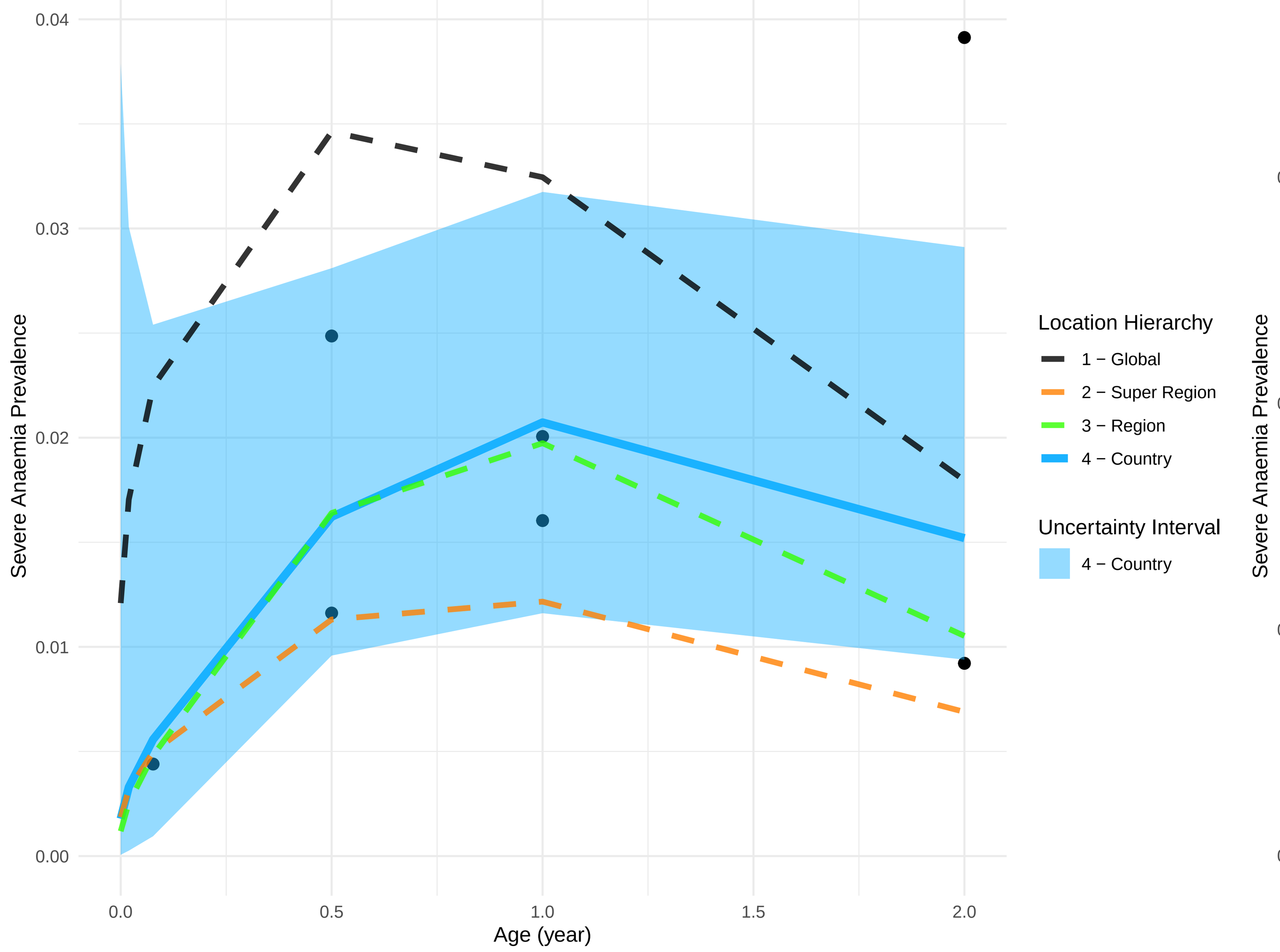

MR−BRT Age/Sex Fits − Males <2 Years Old − Severe Anaemia Prevalence − Uzbekistan
WHO−2001 Adjustment Method

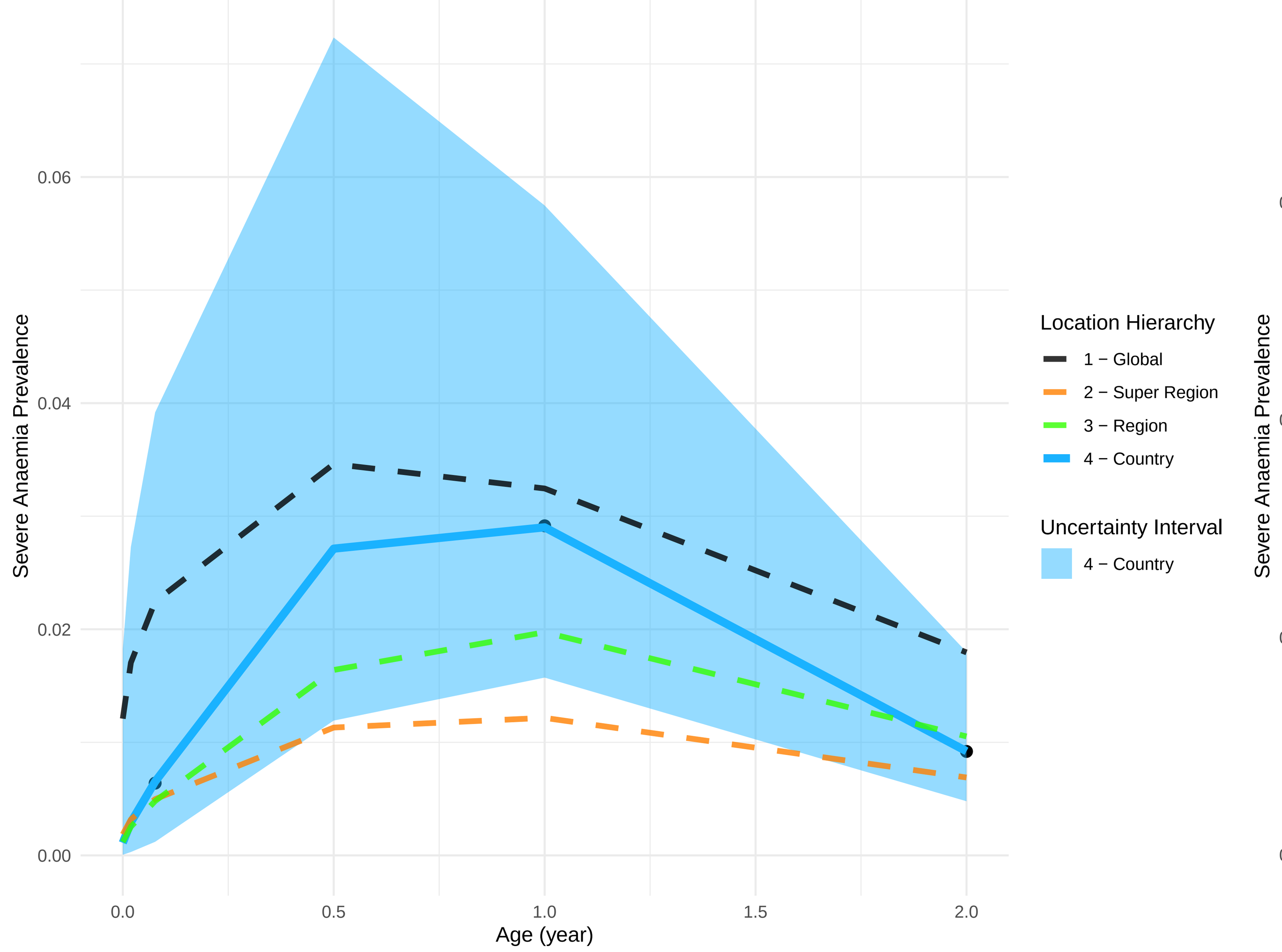

MR−BRT Age/Sex Fits − Males <2 Years Old − Severe Anaemia Prevalence − Central Europe
WHO−2001 Adjustment Method

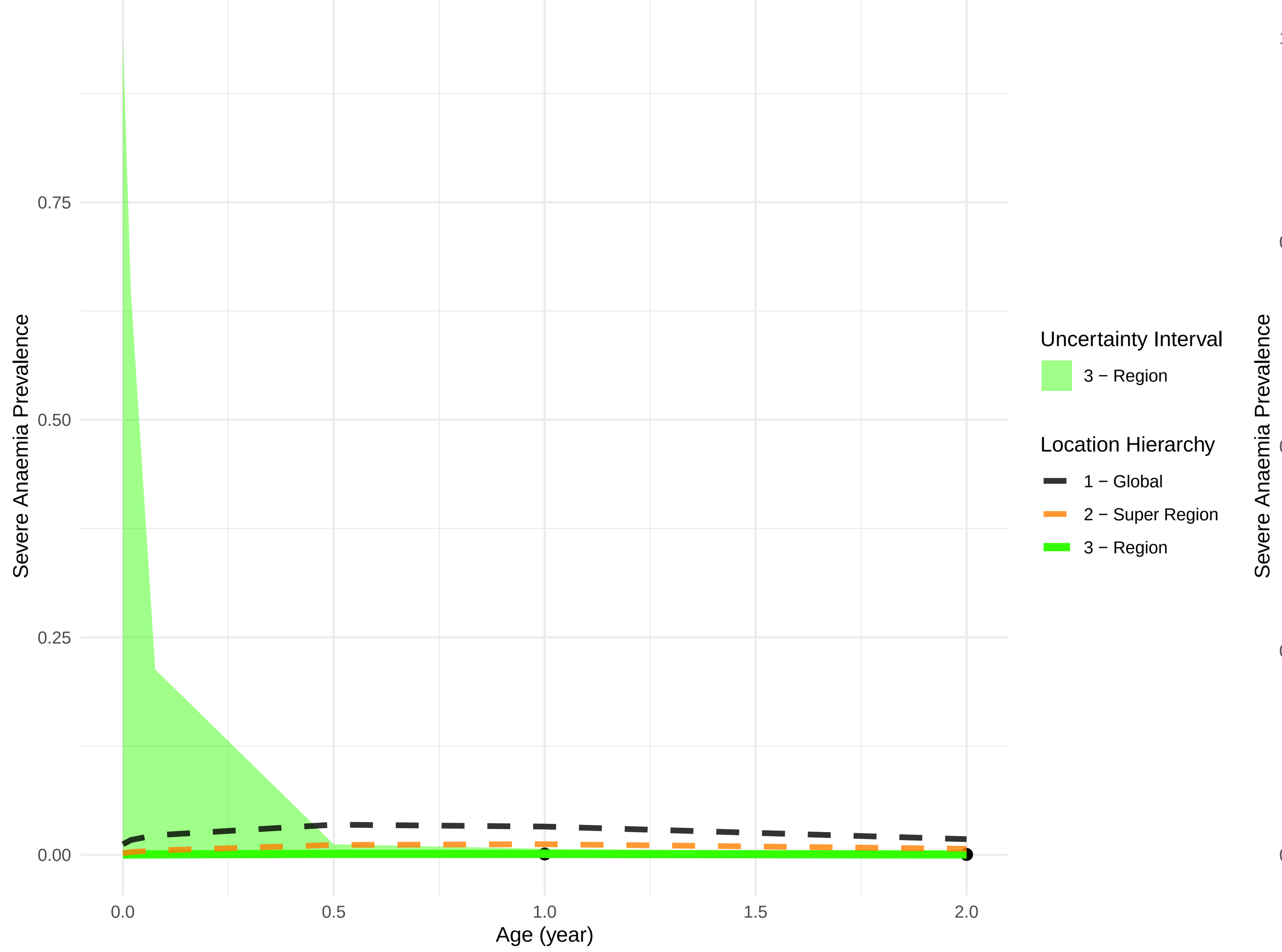

MR−BRT Age/Sex Fits − Males <2 Years Old − Severe Anaemia Prevalence − Albania
WHO−2001 Adjustment Method

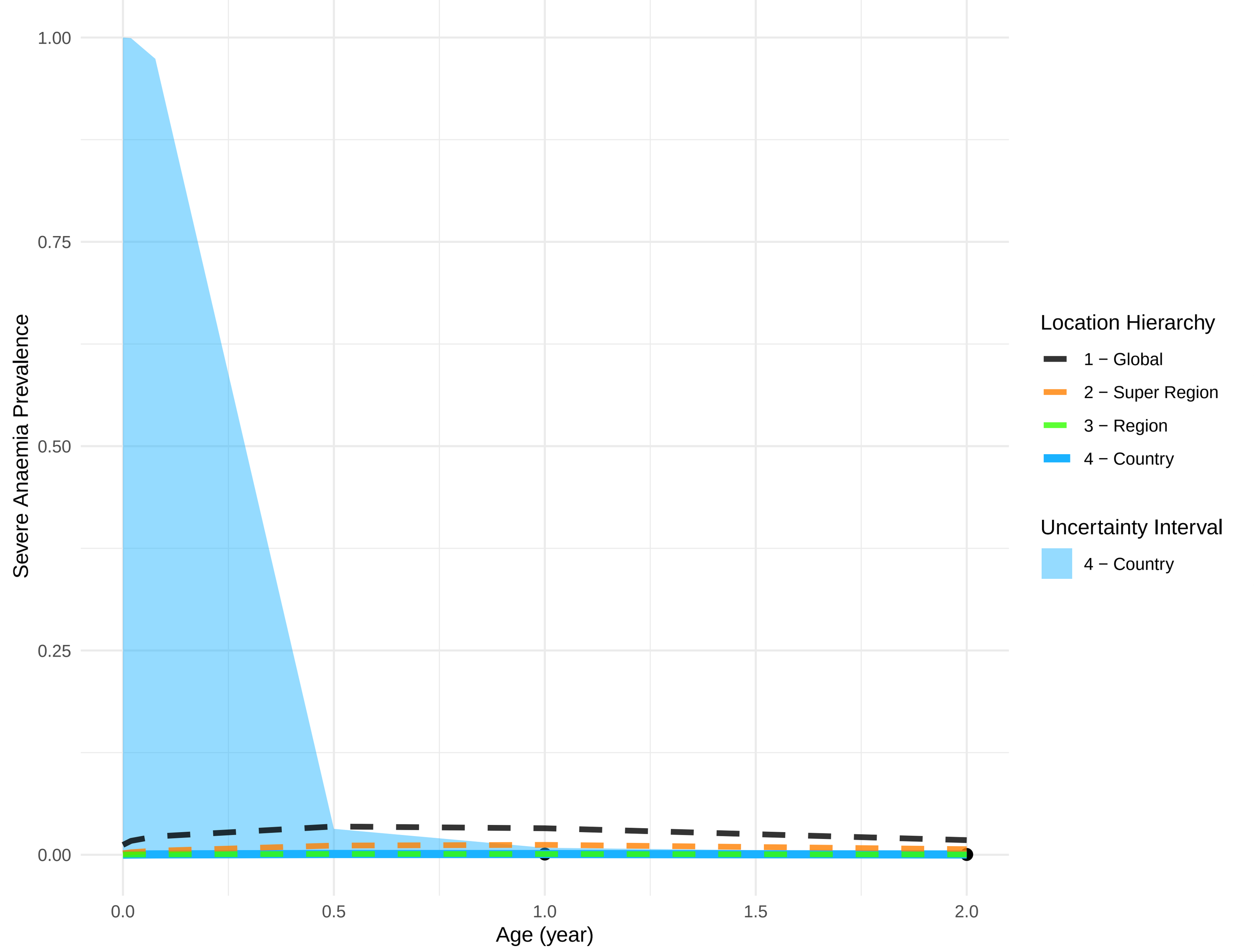

MR−BRT Age/Sex Fits − Males <2 Years Old − Severe Anaemia Prevalence − Latin America and Caribbean
WHO−2001 Adjustment Method

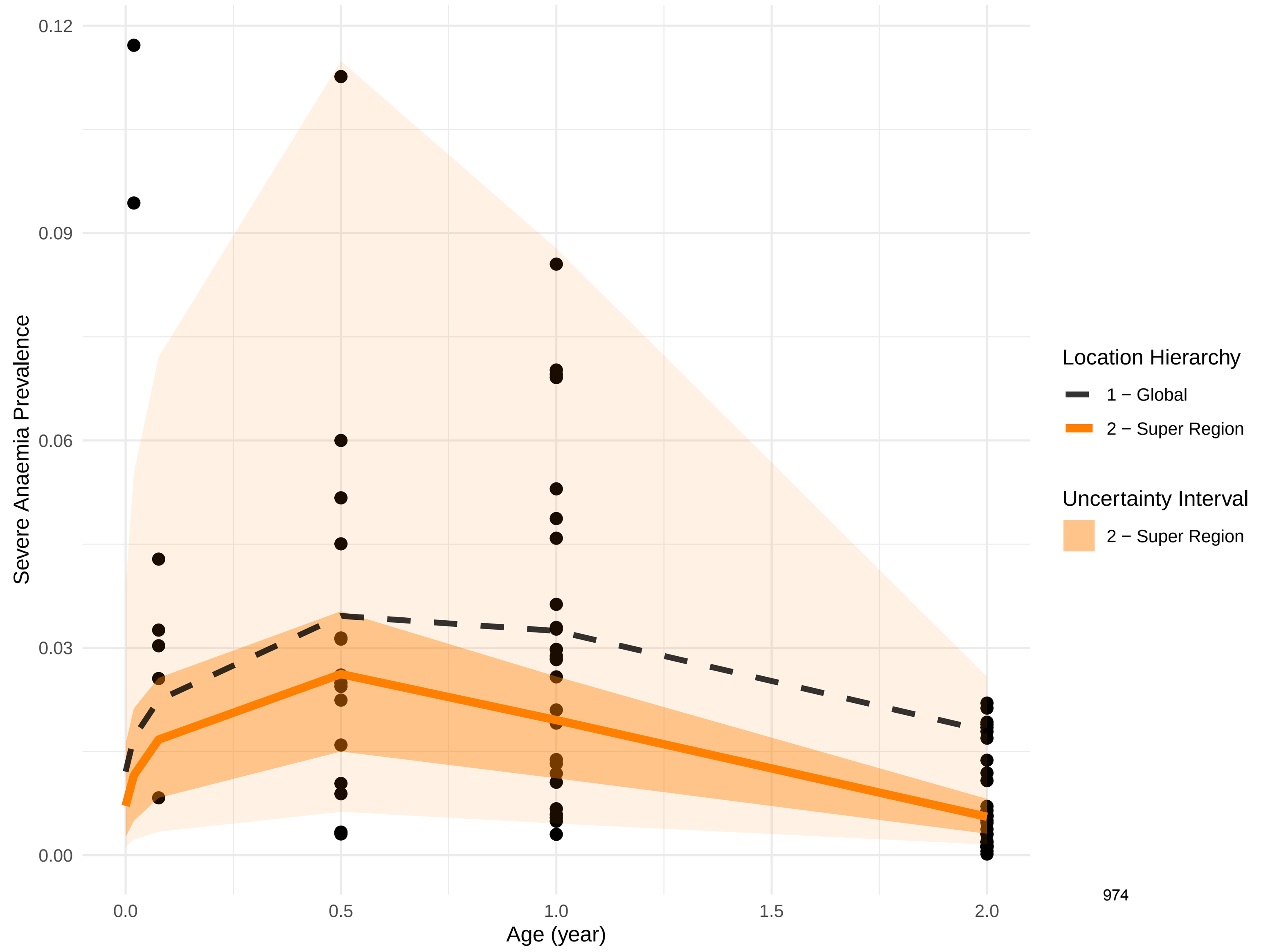

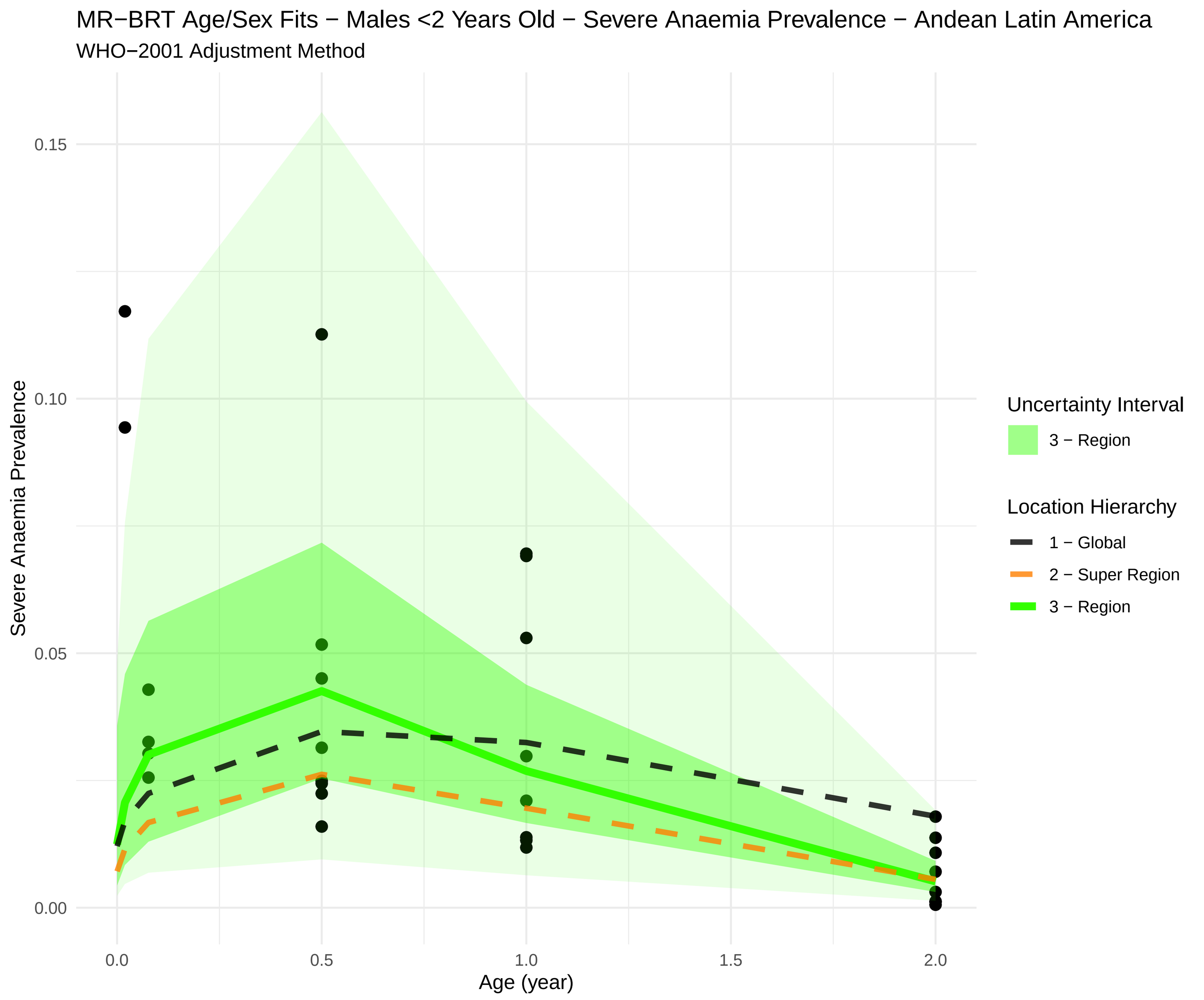
MR−BRT Age/Sex Fits − Males <2 Years Old − Severe Anaemia Prevalence − Andean Latin America
WHO−2001 Adjustment Method
Severe Anaemia Prevalence
Age (year)
Uncertainty Interval
3 − Region
Location Hierarchy
1 − Global
2 − Super Region
3 − Region

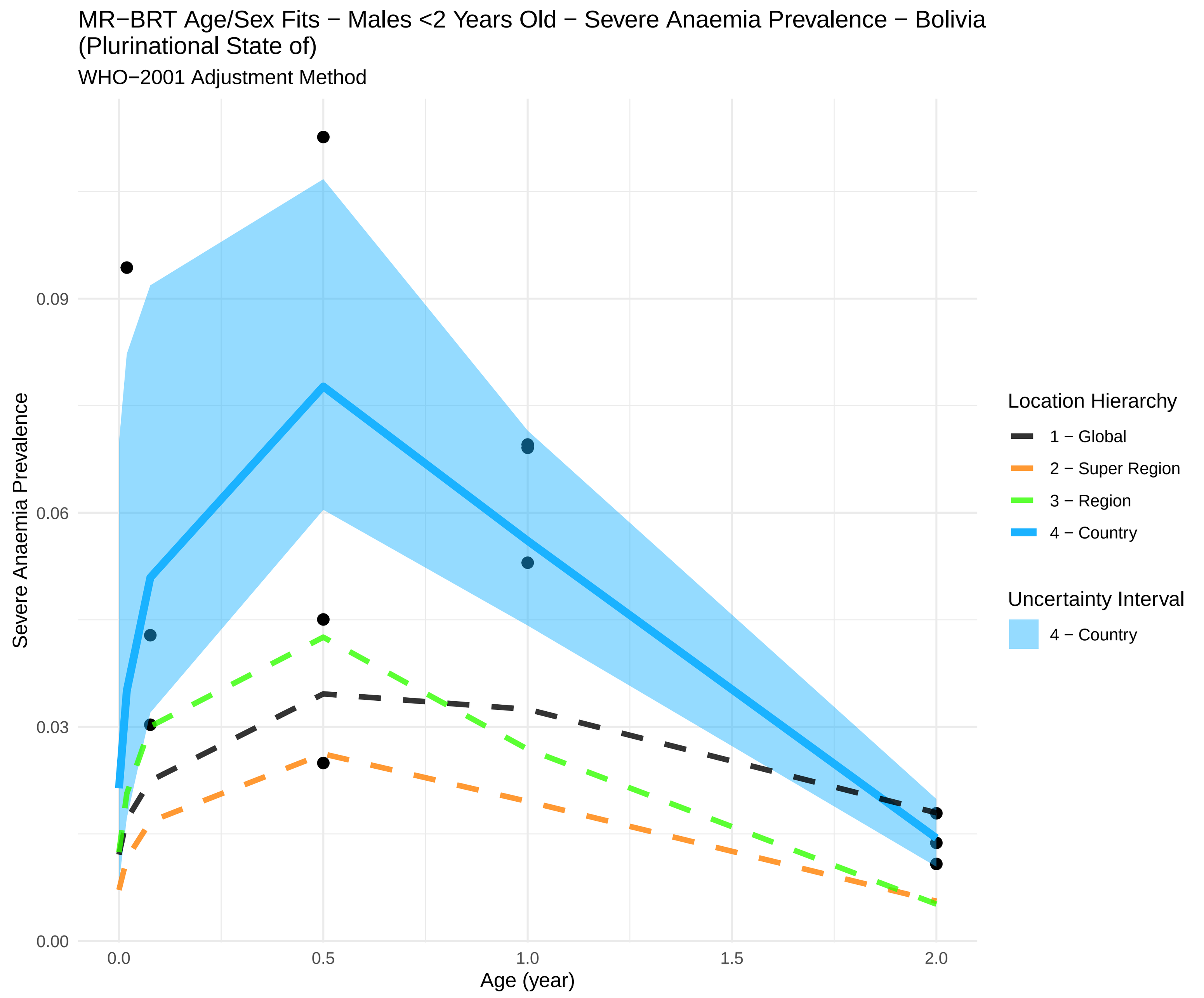
MR−BRT Age/Sex Fits − Males <2 Years Old − Severe Anaemia Prevalence − Bolivia (Plurinational State of)
WHO−2001 Adjustment Method
Severe Anaemia Prevalence
Age (year)
Location Hierarchy
1 − Global
2 − Super Region
3 − Region
4 − Country
Uncertainty Interval
4 − Country

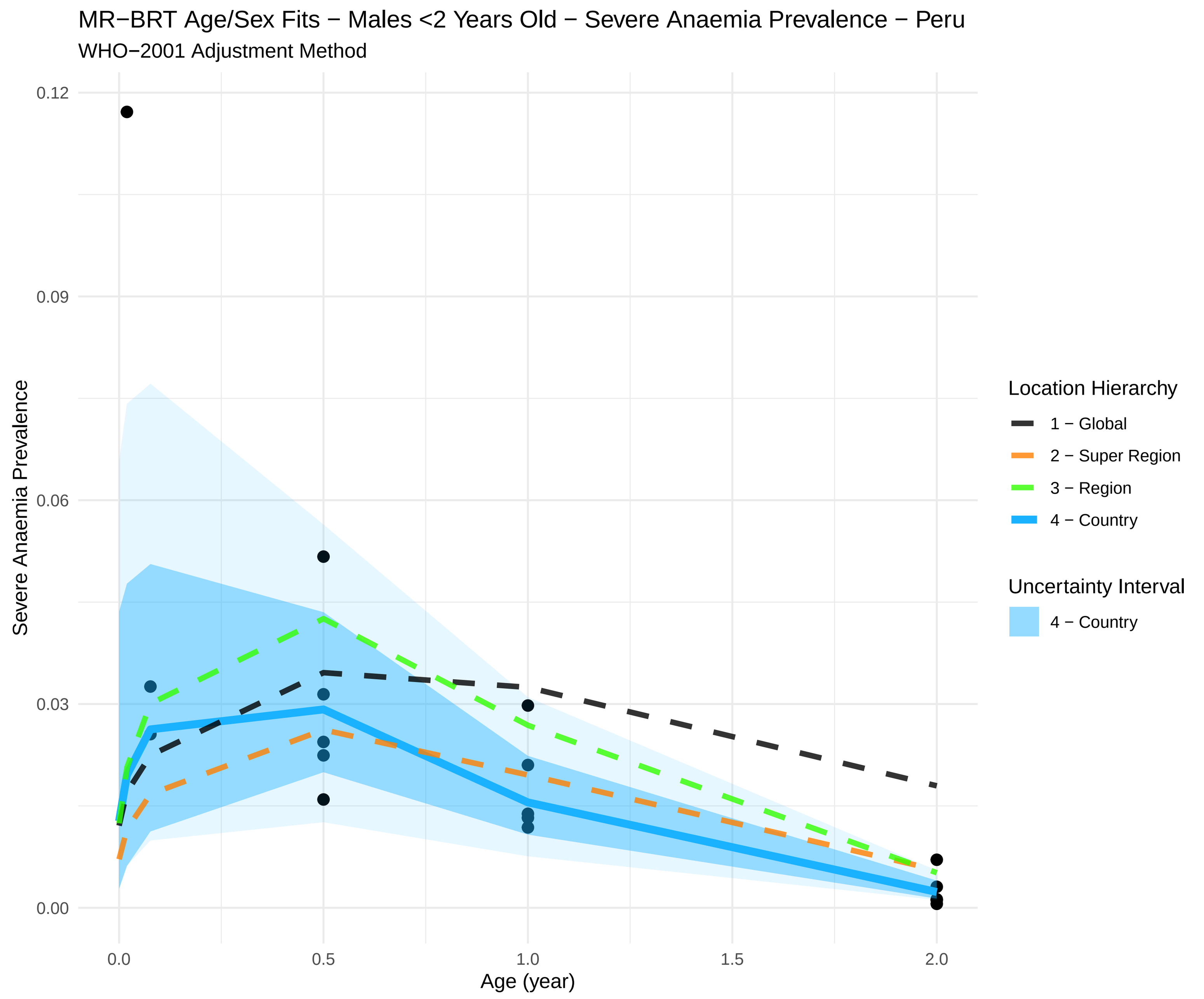
MR−BRT Age/Sex Fits − Males <2 Years Old − Severe Anaemia Prevalence − Peru
WHO−2001 Adjustment Method
Severe Anaemia Prevalence
Age (year)
Location Hierarchy
1 − Global
2 − Super Region
3 − Region
4 − Country
Uncertainty Interval
4 − Country

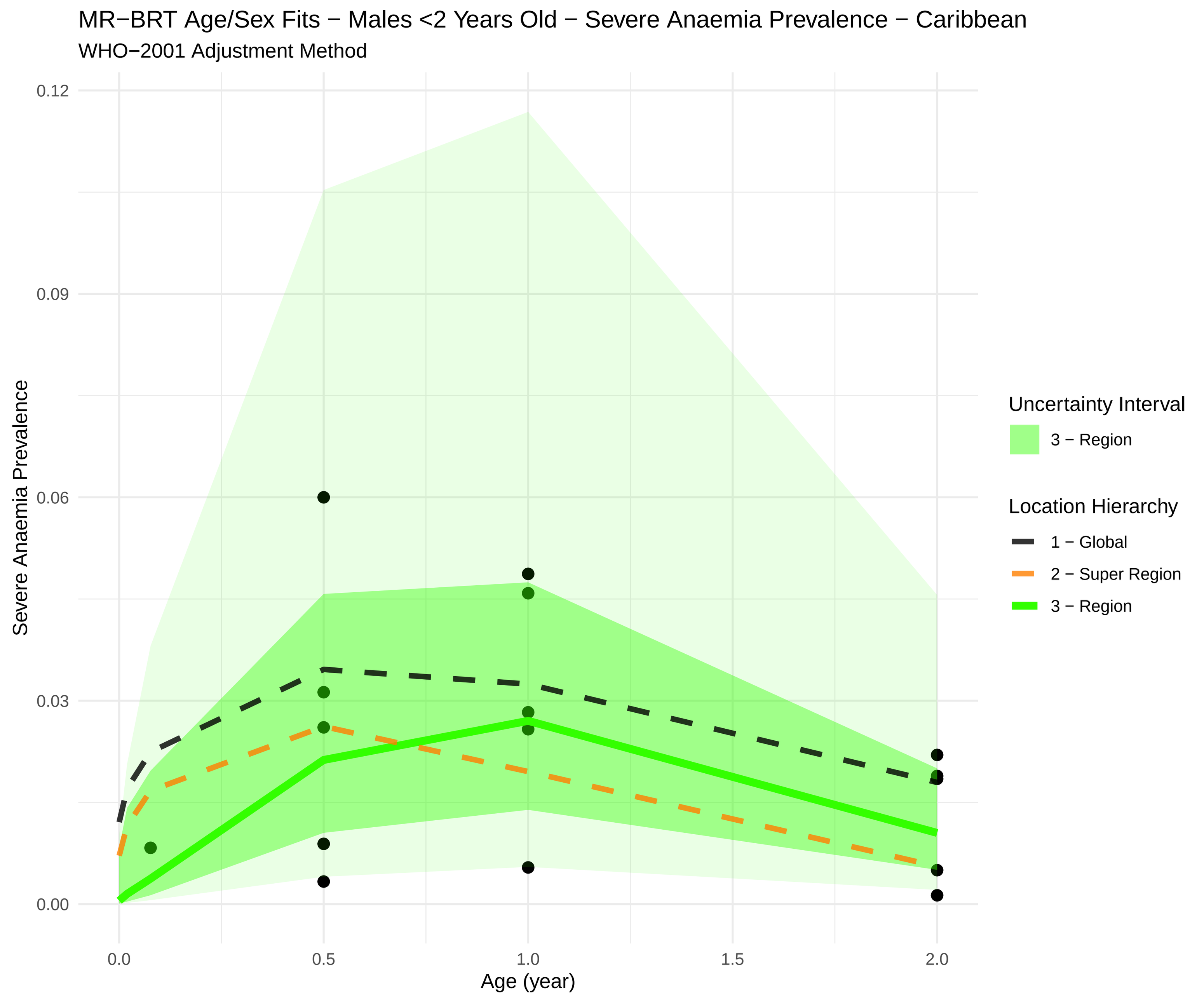
MR−BRT Age/Sex Fits − Males <2 Years Old − Severe Anaemia Prevalence − Caribbean
WHO−2001 Adjustment Method
Severe Anaemia Prevalence
Age (year)
Uncertainty Interval
3 − Region
Location Hierarchy
1 − Global
2 − Super Region
3 − Region

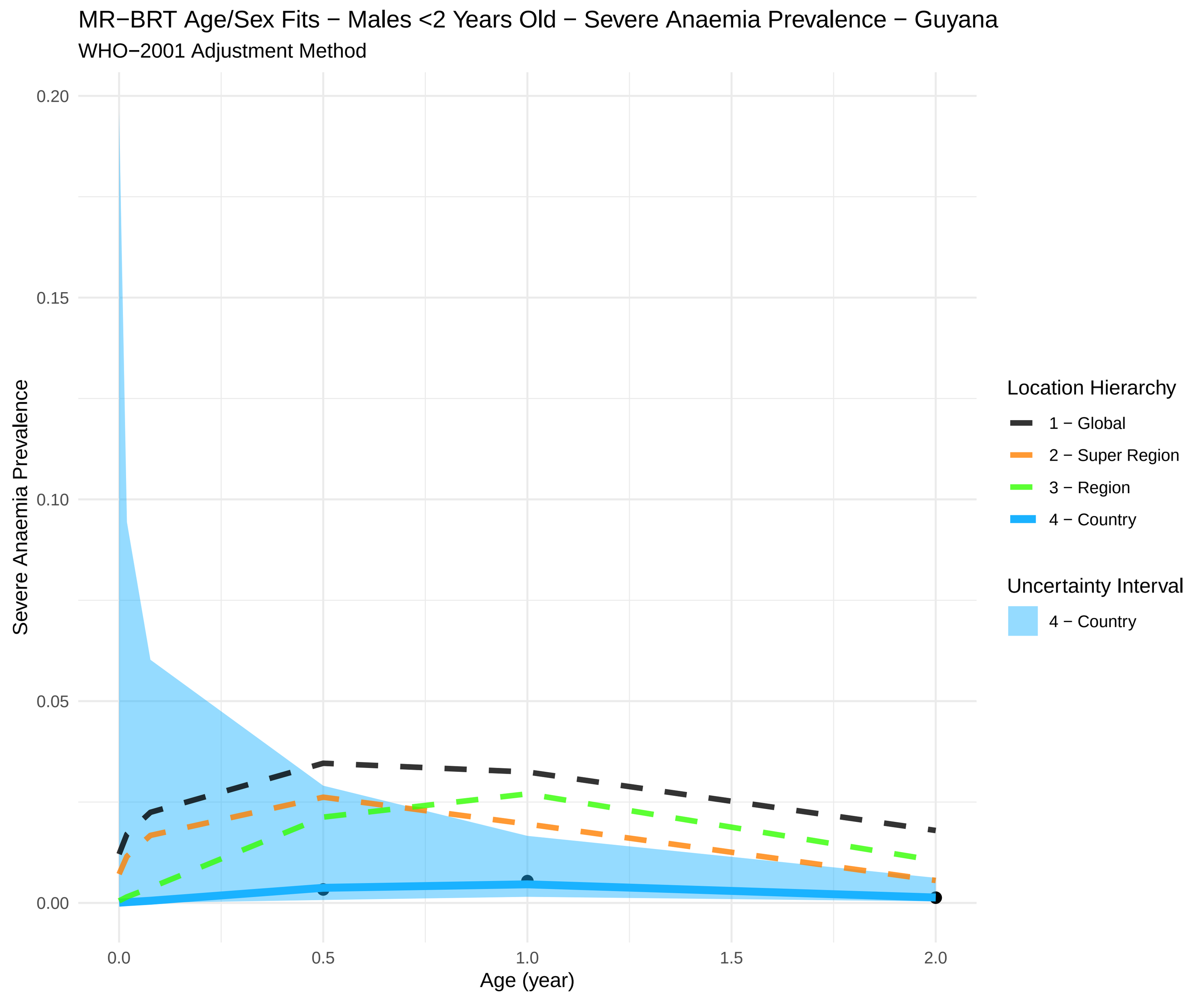
MR−BRT Age/Sex Fits − Males <2 Years Old − Severe Anaemia Prevalence − Guyana
WHO−2001 Adjustment Method
Severe Anaemia Prevalence
Age (year)
Location Hierarchy
1 − Global
2 − Super Region
3 − Region
4 − Country
Uncertainty Interval
4 − Country

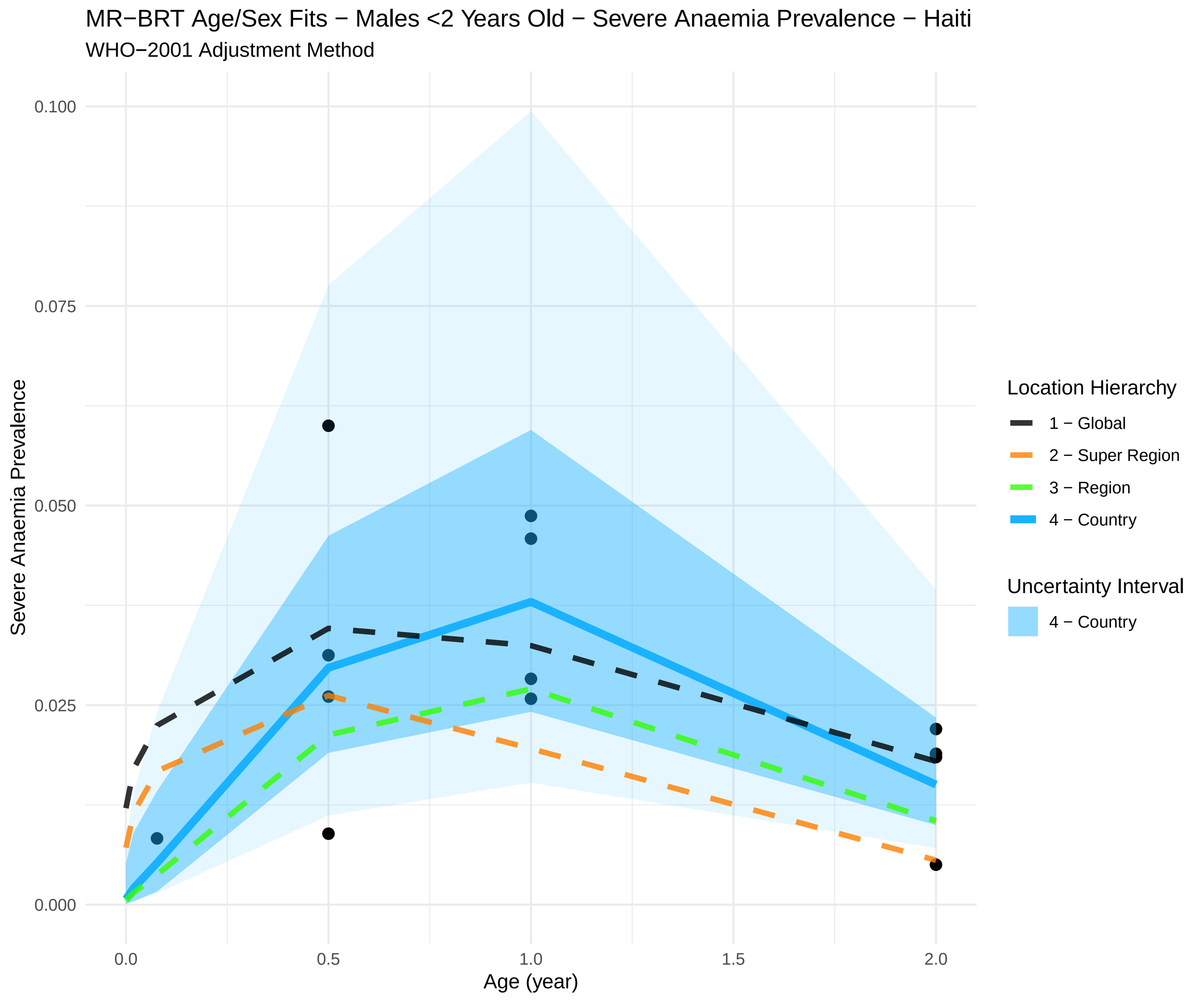
MR−BRT Age/Sex Fits − Males <2 Years Old − Severe Anaemia Prevalence − Haiti
WHO−2001 Adjustment Method
Severe Anaemia Prevalence
Age (year)
Location Hierarchy
1 − Global
2 − Super Region
3 − Region
4 − Country
Uncertainty Interval
4 − Country

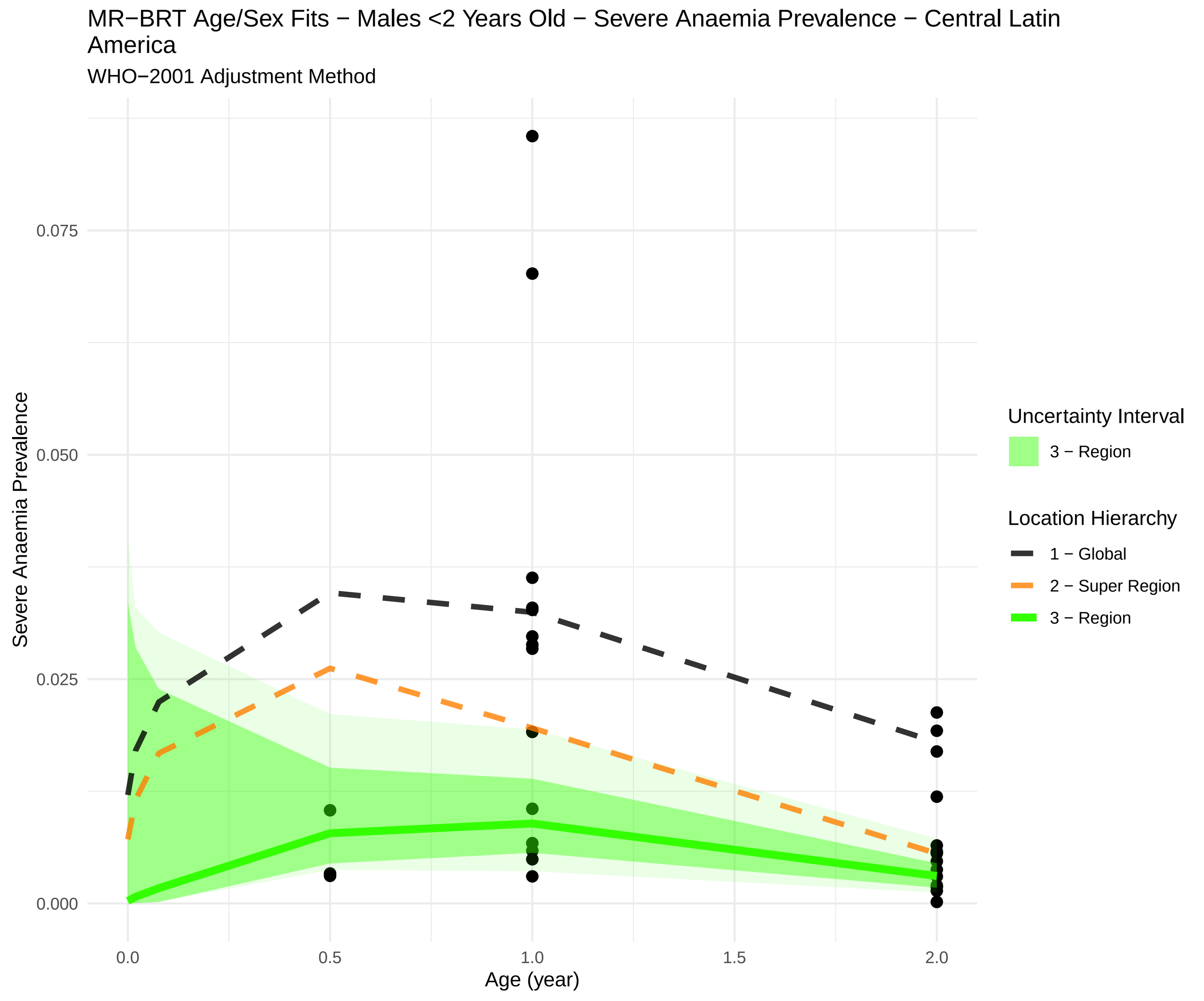
MR−BRT Age/Sex Fits − Males <2 Years Old − Severe Anaemia Prevalence − Central Latin America
WHO−2001 Adjustment Method
Severe Anaemia Prevalence
Age (year)
Uncertainty Interval
3 − Region
Location Hierarchy
1 − Global
2 − Super Region
3 − Region

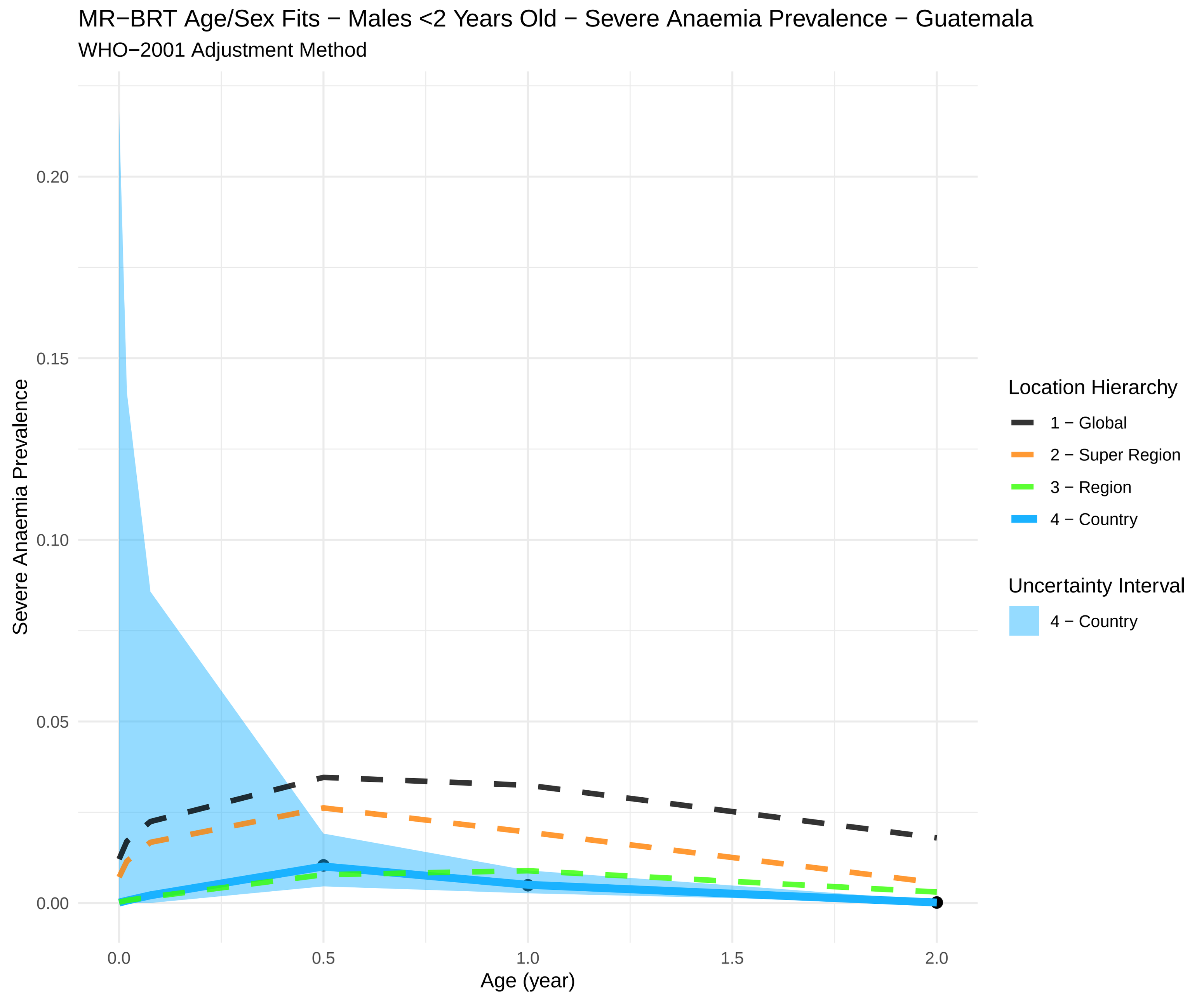
MR−BRT Age/Sex Fits − Males <2 Years Old − Severe Anaemia Prevalence − Guatemala
WHO−2001 Adjustment Method
Severe Anaemia Prevalence
Age (year)
Location Hierarchy
1 − Global
2 − Super Region
3 − Region
4 − Country
Uncertainty Interval
4 − Country

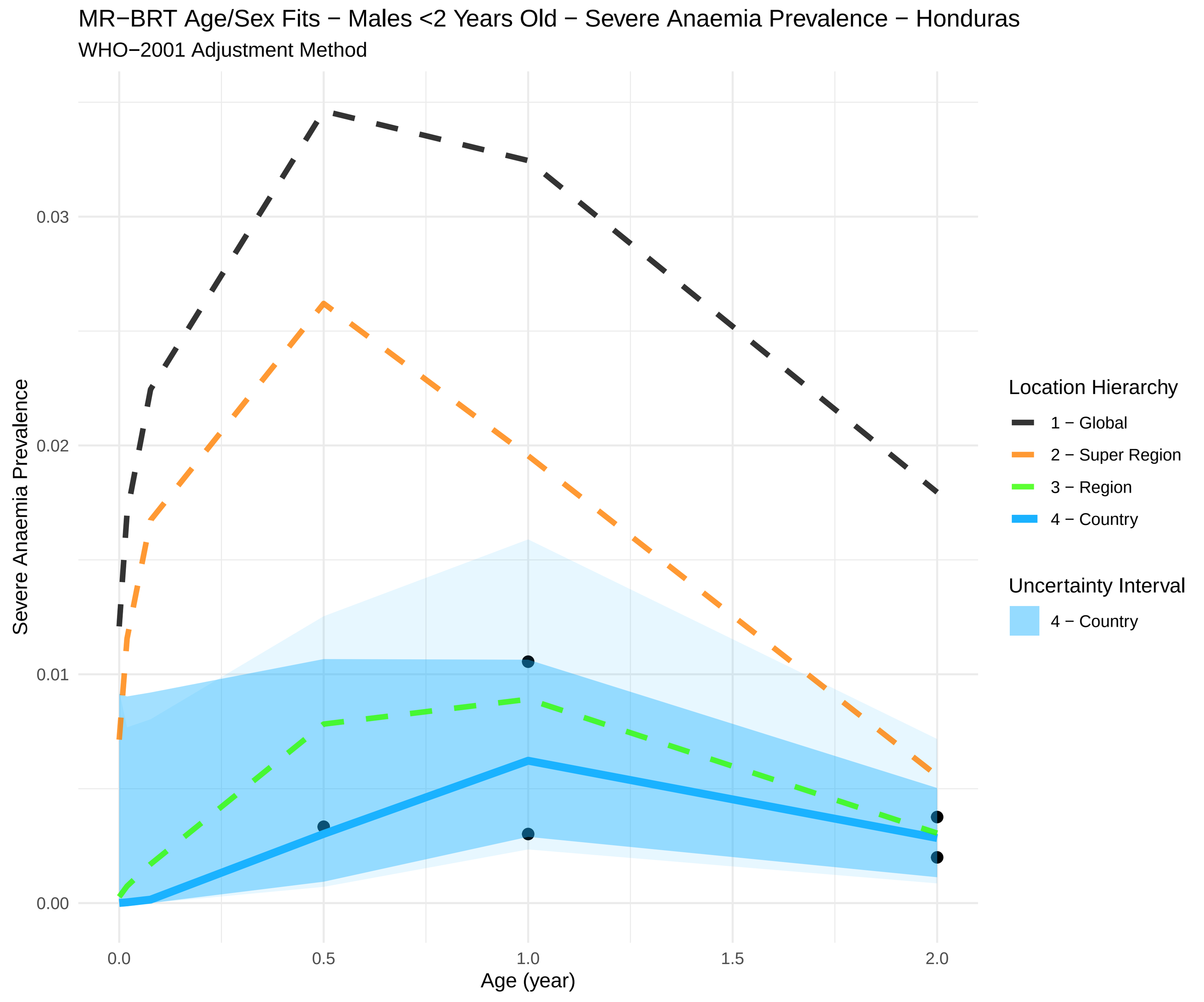
MR−BRT Age/Sex Fits − Males <2 Years Old − Severe Anaemia Prevalence − Honduras
WHO−2001 Adjustment Method
Severe Anaemia Prevalence
Age (year)
Location Hierarchy
1 − Global
2 − Super Region
3 − Region
4 − Country
Uncertainty Interval
4 − Country

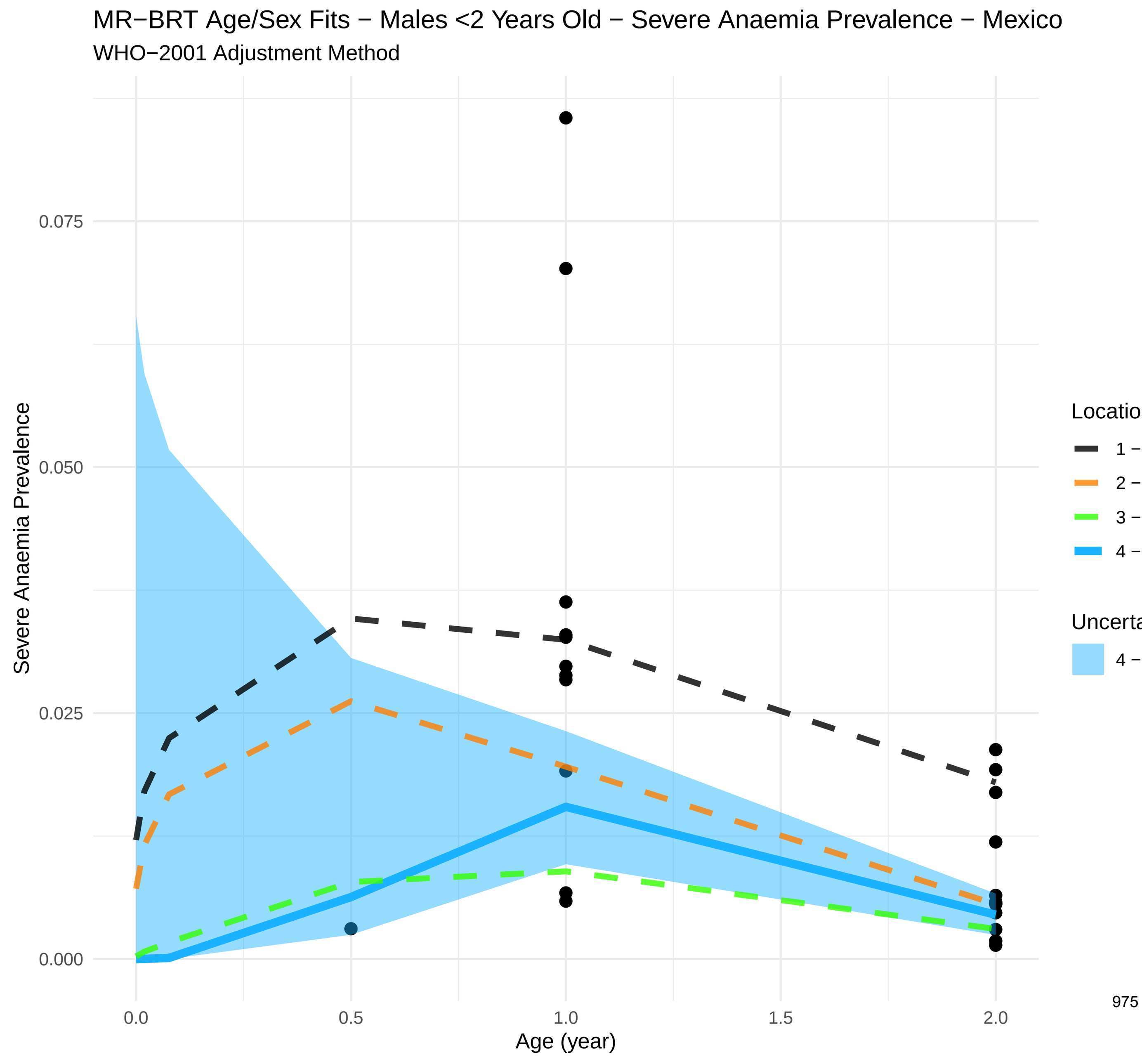
MR−BRT Age/Sex Fits − Males <2 Years Old − Severe Anaemia Prevalence − Mexico
WHO−2001 Adjustment Method
Severe Anaemia Prevalence
Age (year)
Location Hierarchy
1 − Global
2 − Super Region
3 − Region
4 − Country
Uncertainty Interval
4 − Country

MR−BRT Age/Sex Fits − Males <2 Years Old − Severe Anaemia Prevalence − Durango
WHO−2001 Adjustment Method

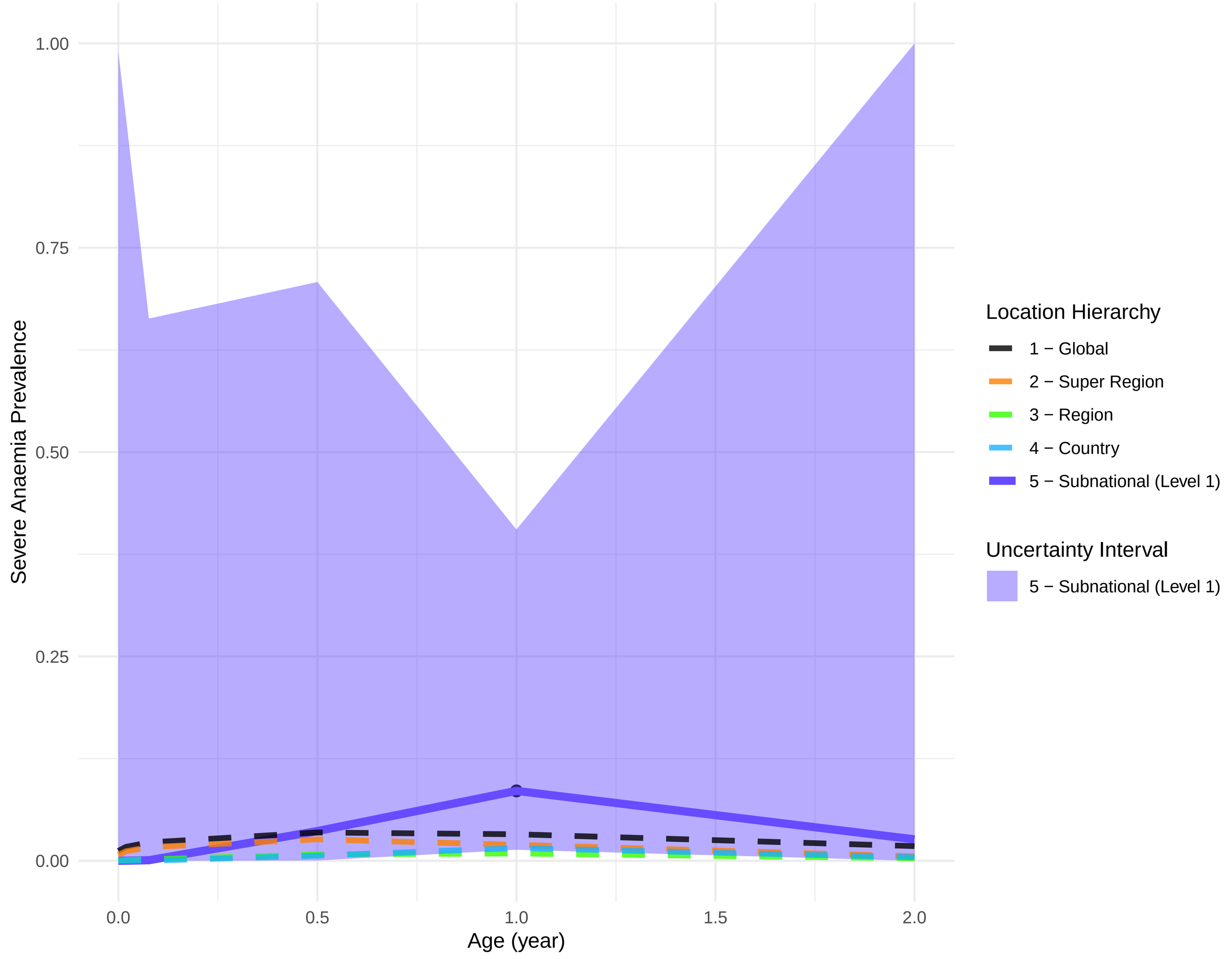

MR−BRT Age/Sex Fits − Males <2 Years Old − Severe Anaemia Prevalence − Guanajuato
WHO−2001 Adjustment Method

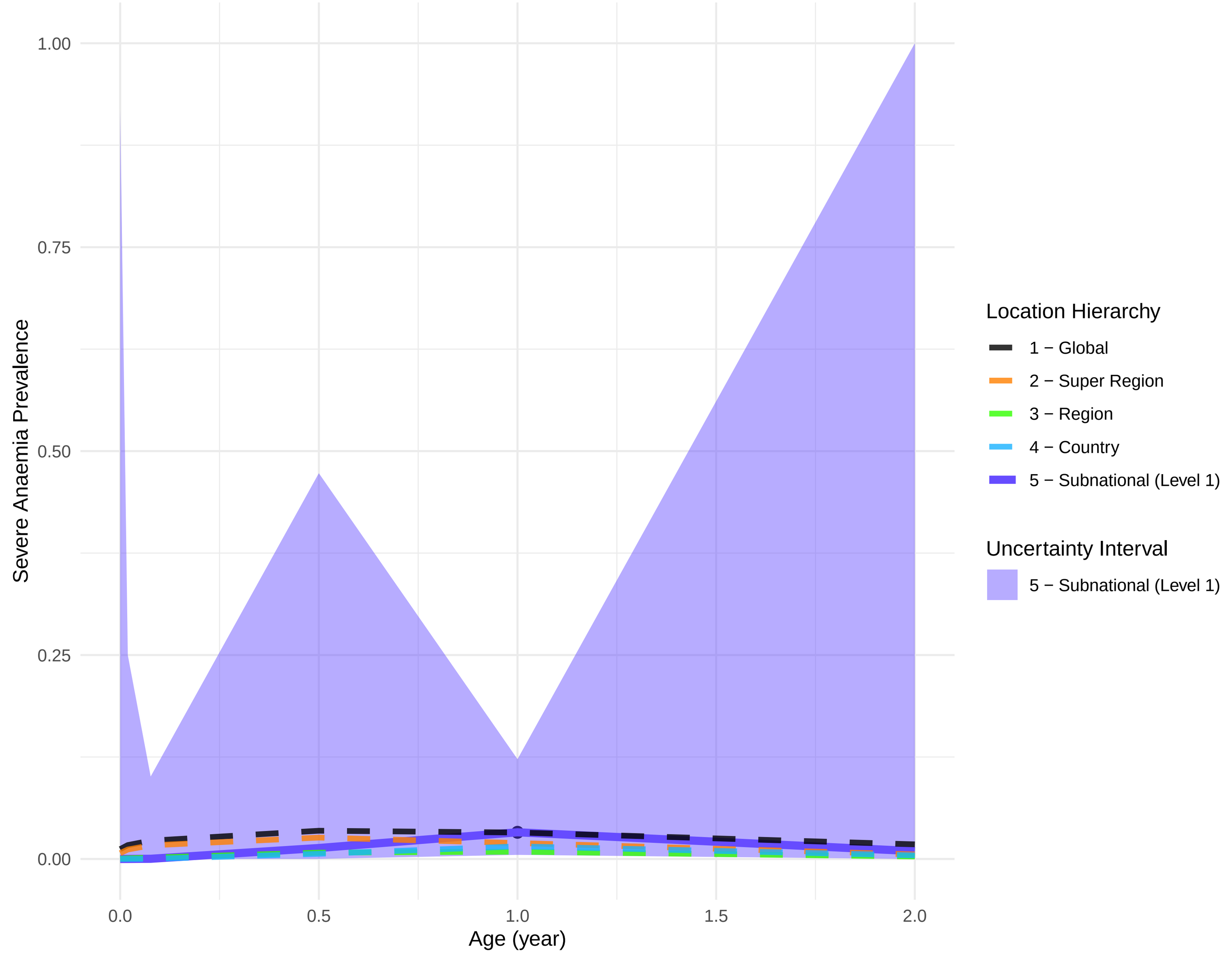

MR−BRT Age/Sex Fits − Males <2 Years Old − Severe Anaemia Prevalence − Guerrero
WHO−2001 Adjustment Method

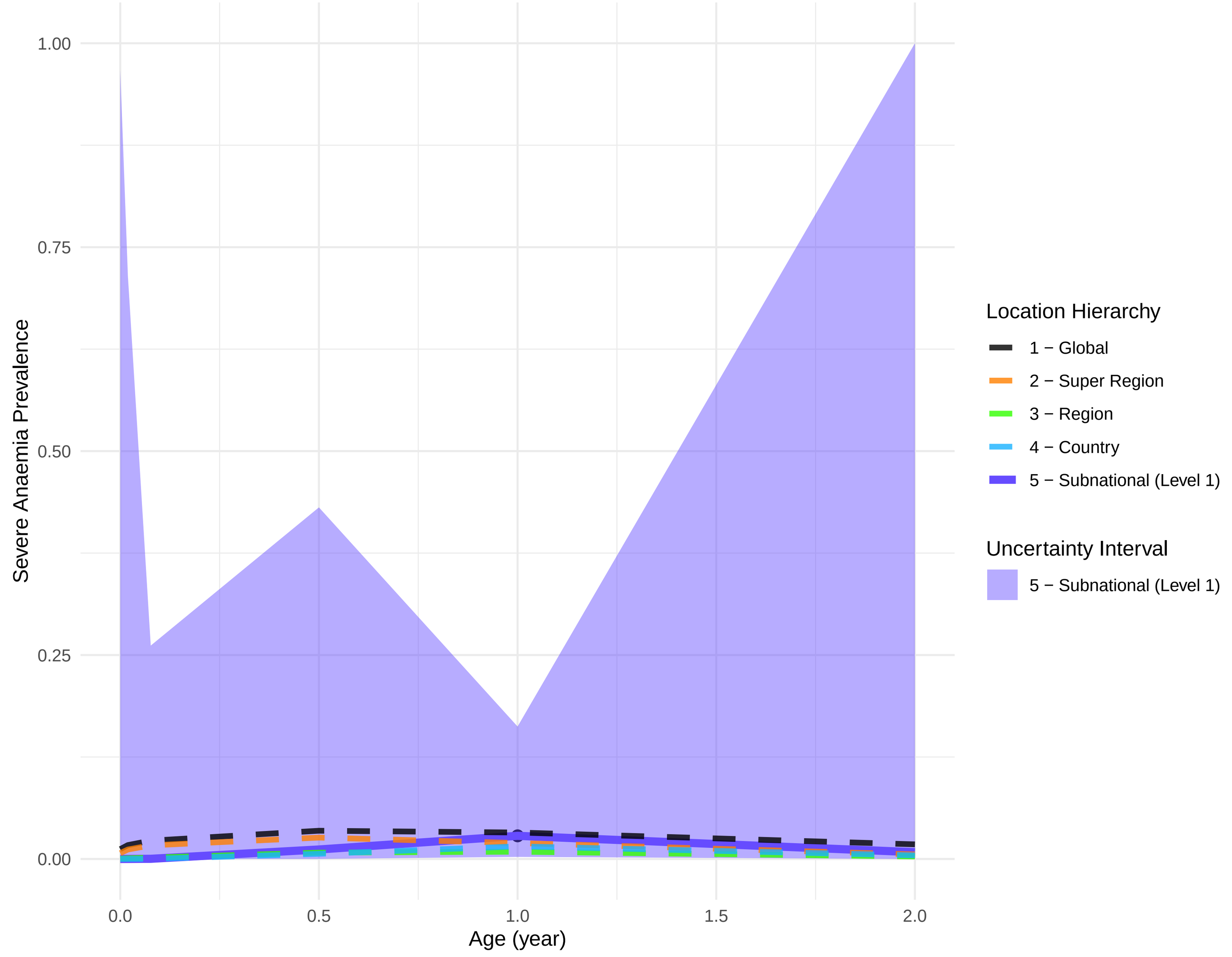

MR−BRT Age/Sex Fits − Males <2 Years Old − Severe Anaemia Prevalence − Hidalgo
WHO−2001 Adjustment Method

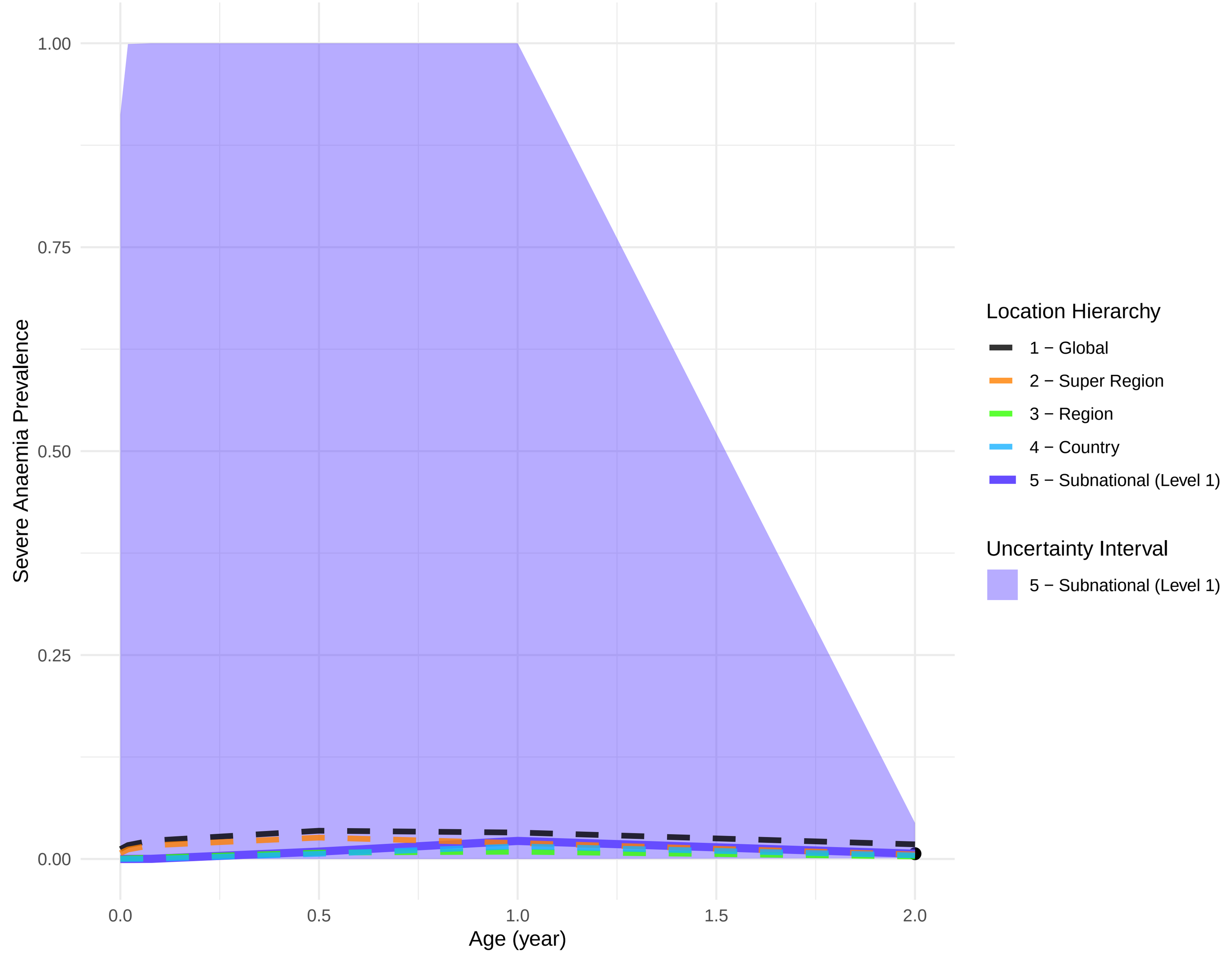

MR−BRT Age/Sex Fits − Males <2 Years Old − Severe Anaemia Prevalence − México
WHO−2001 Adjustment Method

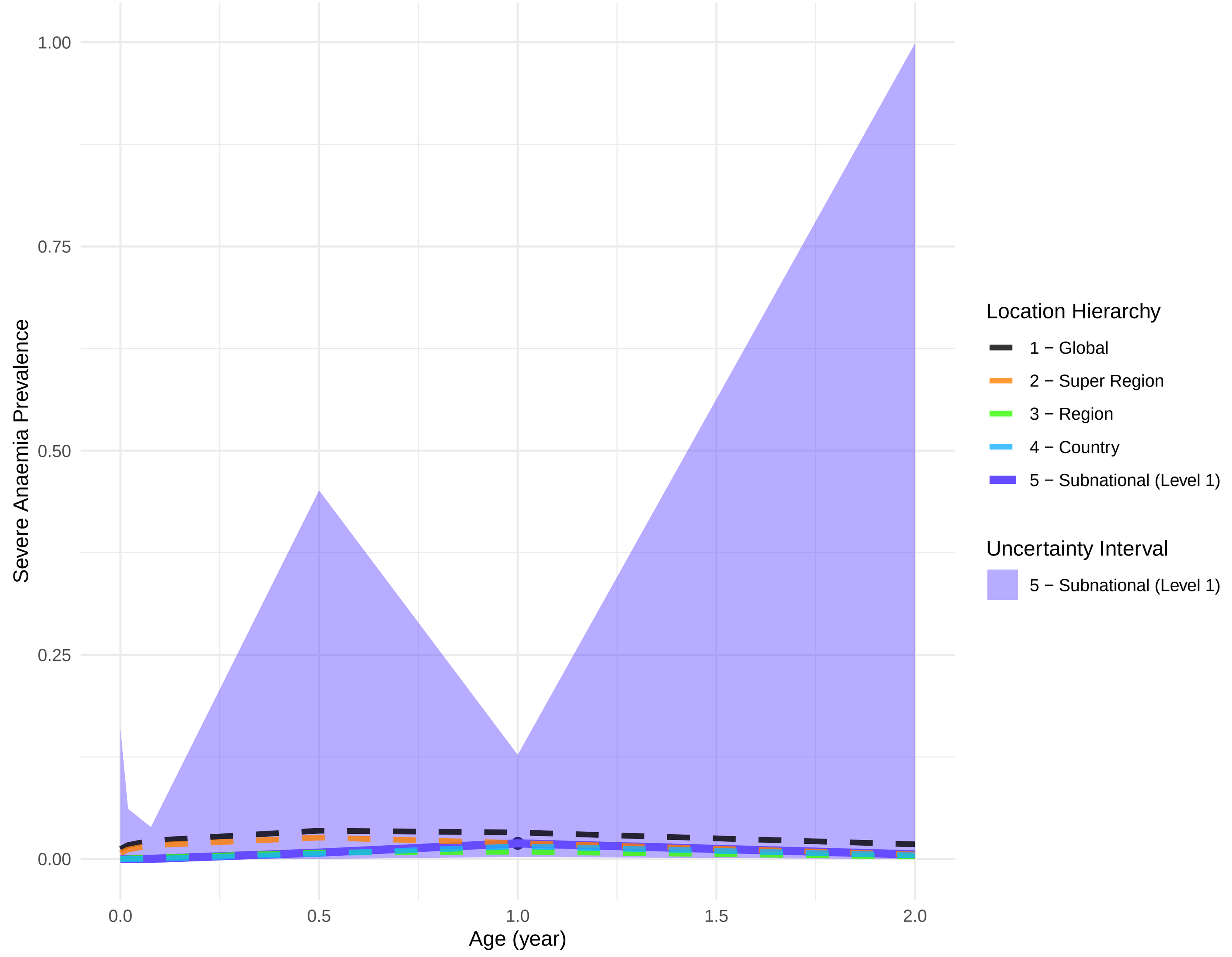

MR−BRT Age/Sex Fits − Males <2 Years Old − Severe Anaemia Prevalence − Mexico City
WHO−2001 Adjustment Method

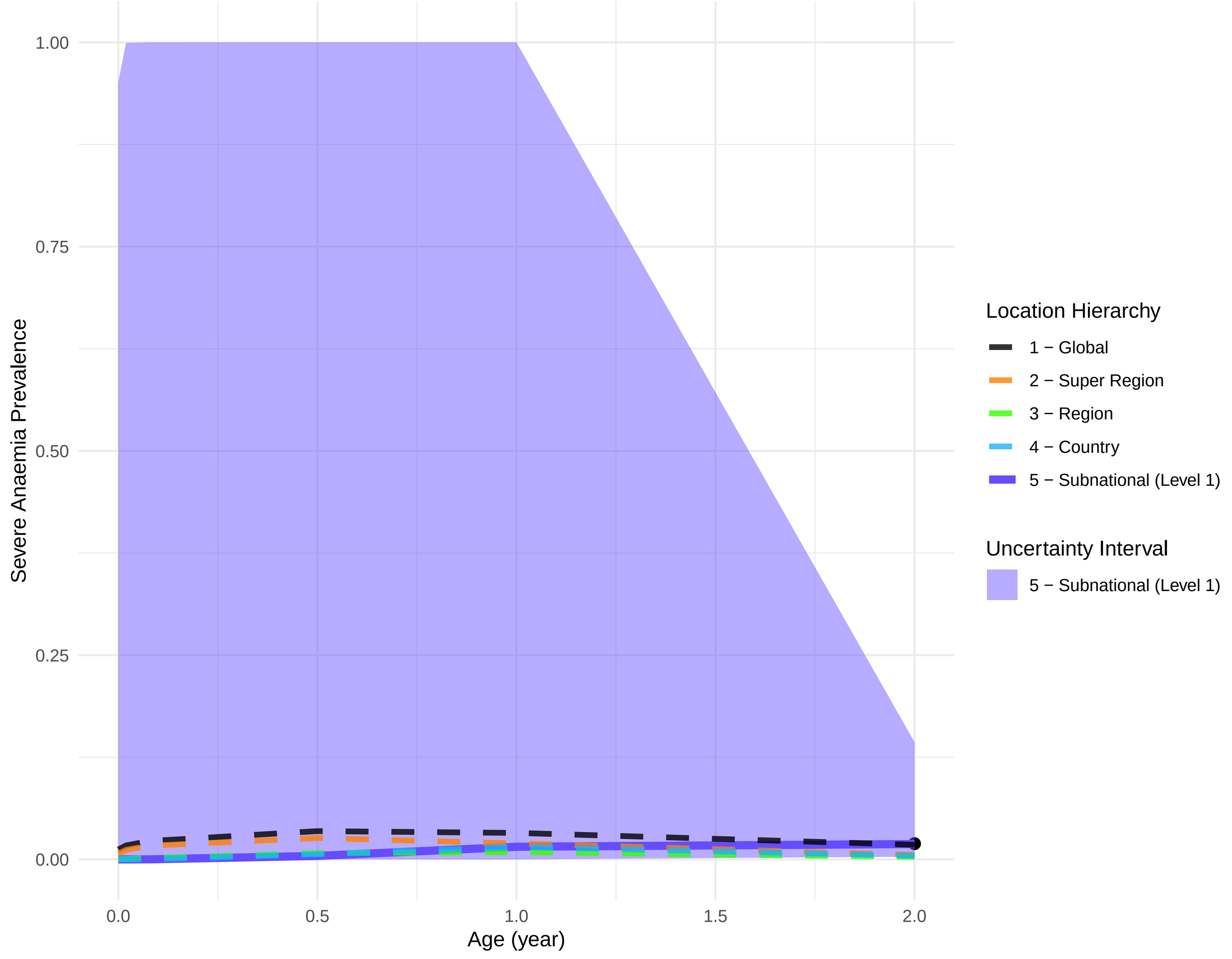

MR−BRT Age/Sex Fits − Males <2 Years Old − Severe Anaemia Prevalence − Michoacán de Ocampo
WHO−2001 Adjustment Method

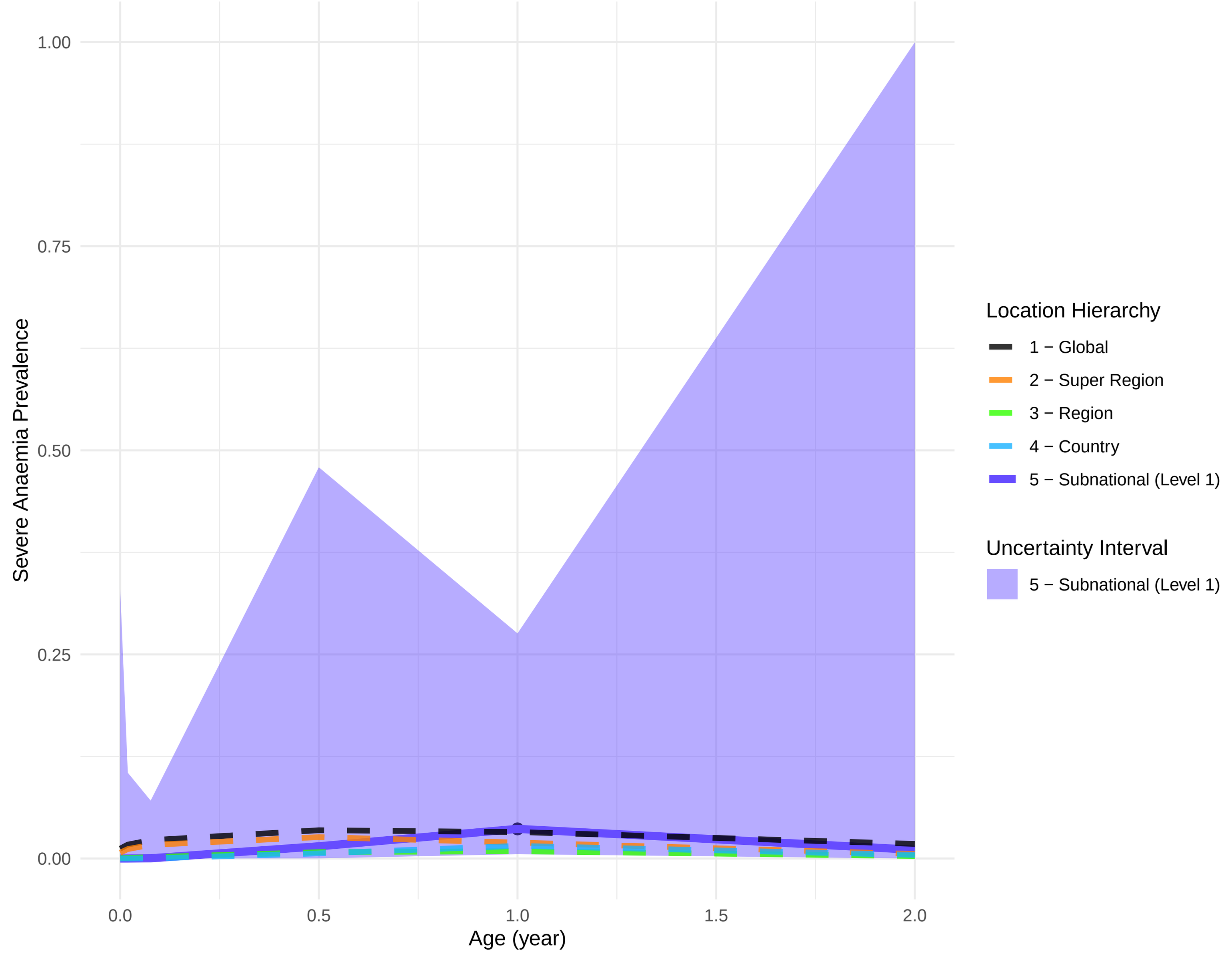

MR−BRT Age/Sex Fits − Males <2 Years Old − Severe Anaemia Prevalence − Nayarit
WHO−2001 Adjustment Method

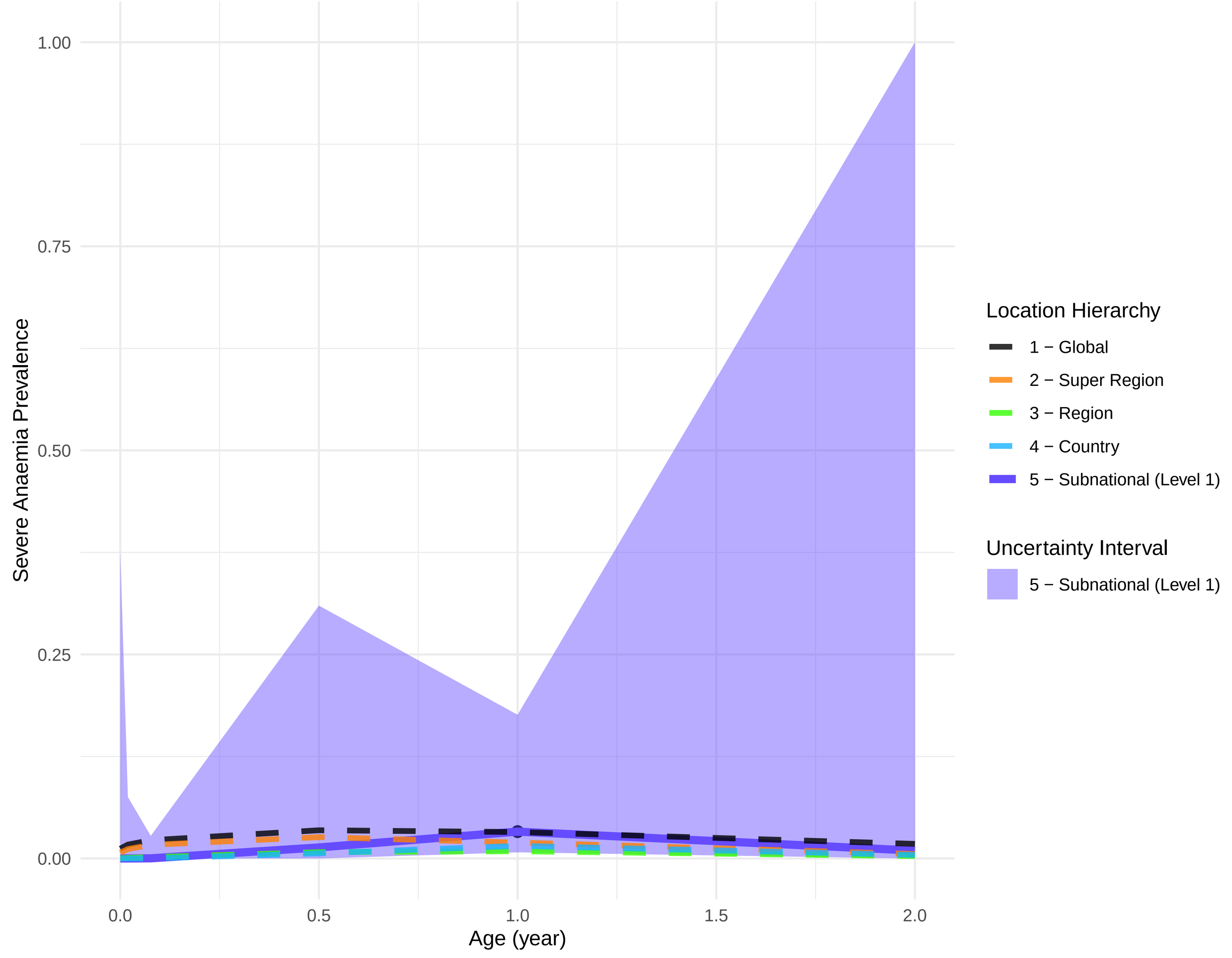

MR−BRT Age/Sex Fits − Males <2 Years Old − Severe Anaemia Prevalence − Oaxaca
WHO−2001 Adjustment Method

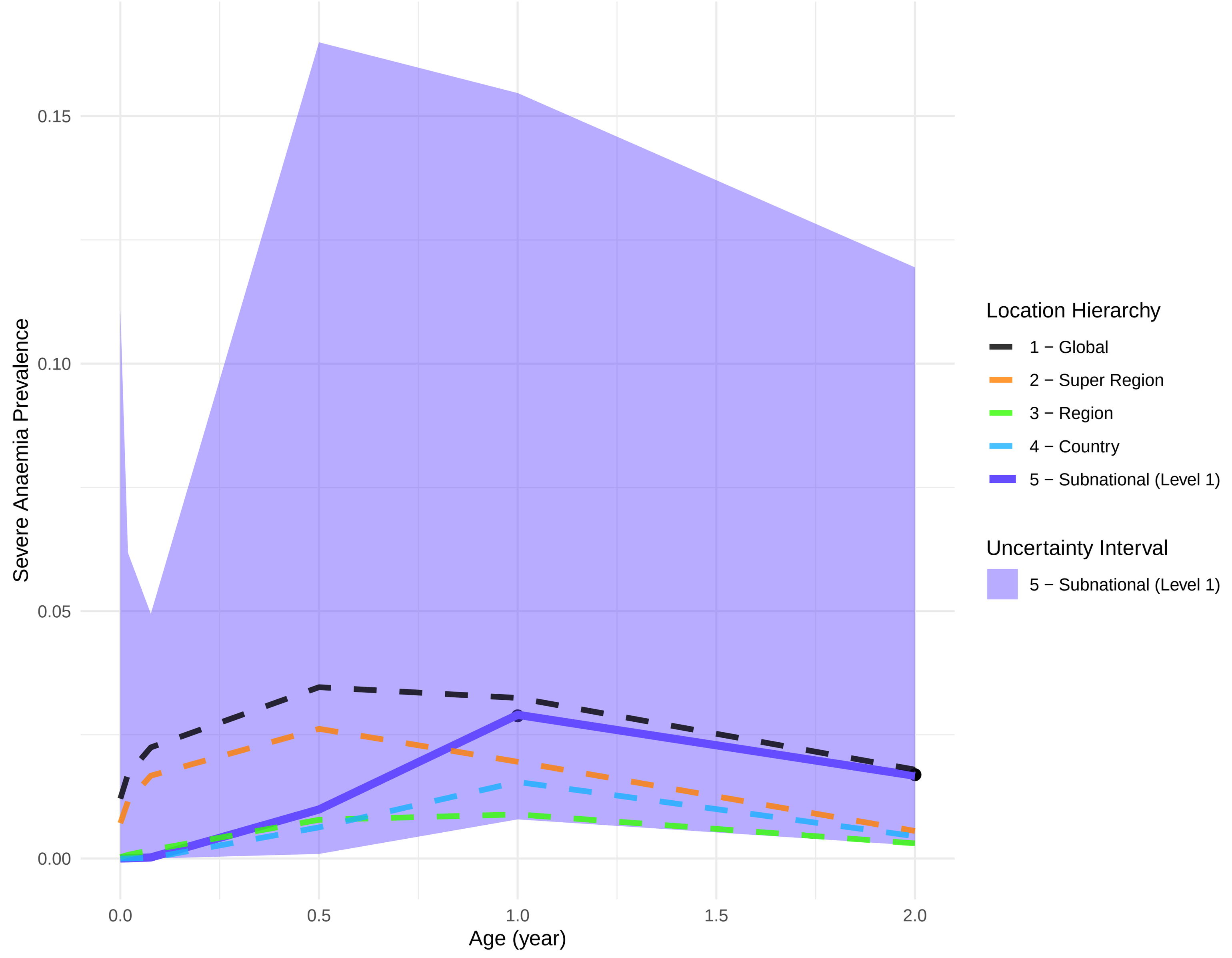

MR−BRT Age/Sex Fits − Males <2 Years Old − Severe Anaemia Prevalence − Puebla
WHO−2001 Adjustment Method

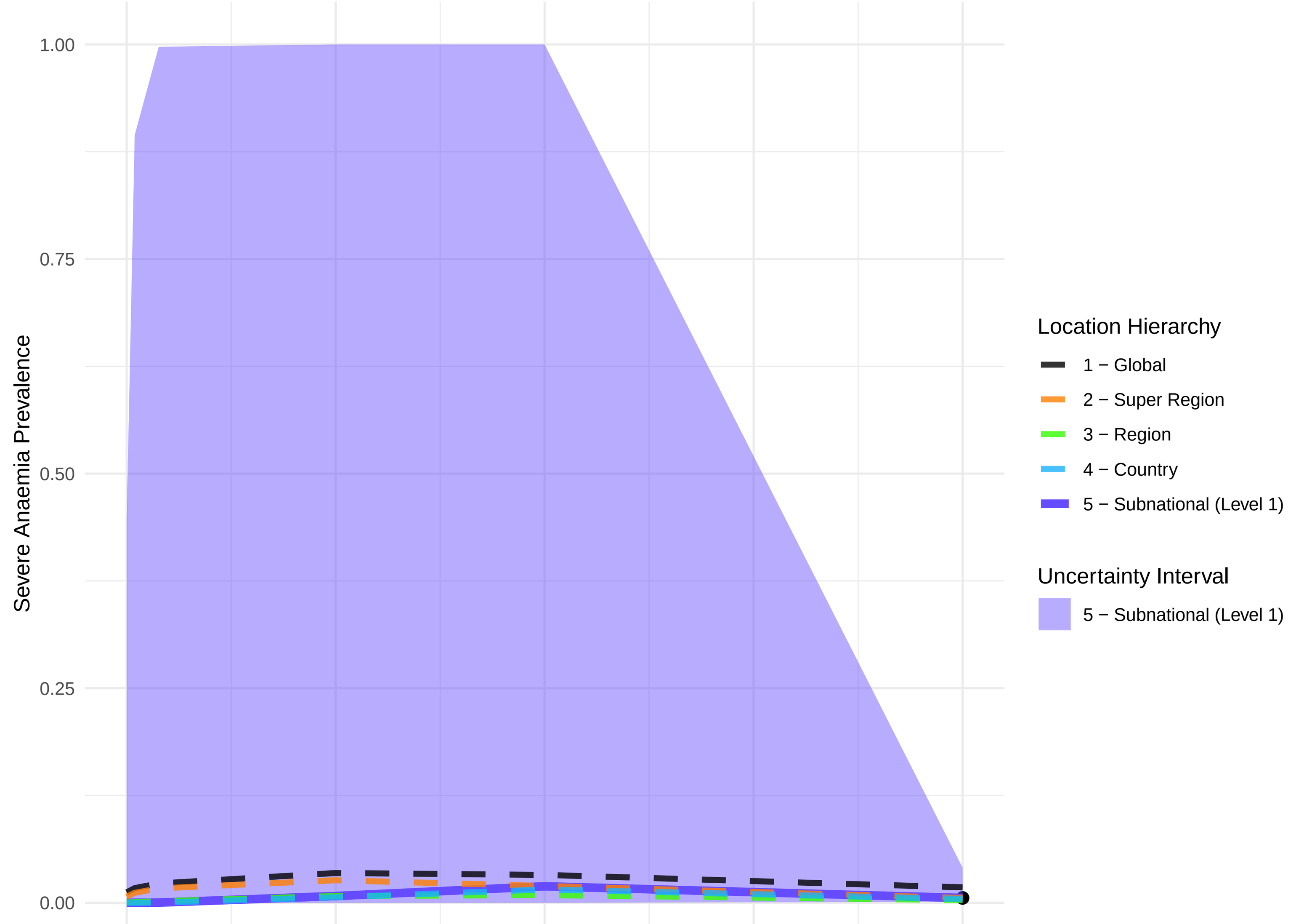

MR−BRT Age/Sex Fits − Males <2 Years Old − Severe Anaemia Prevalence − Quintana Roo
WHO−2001 Adjustment Method

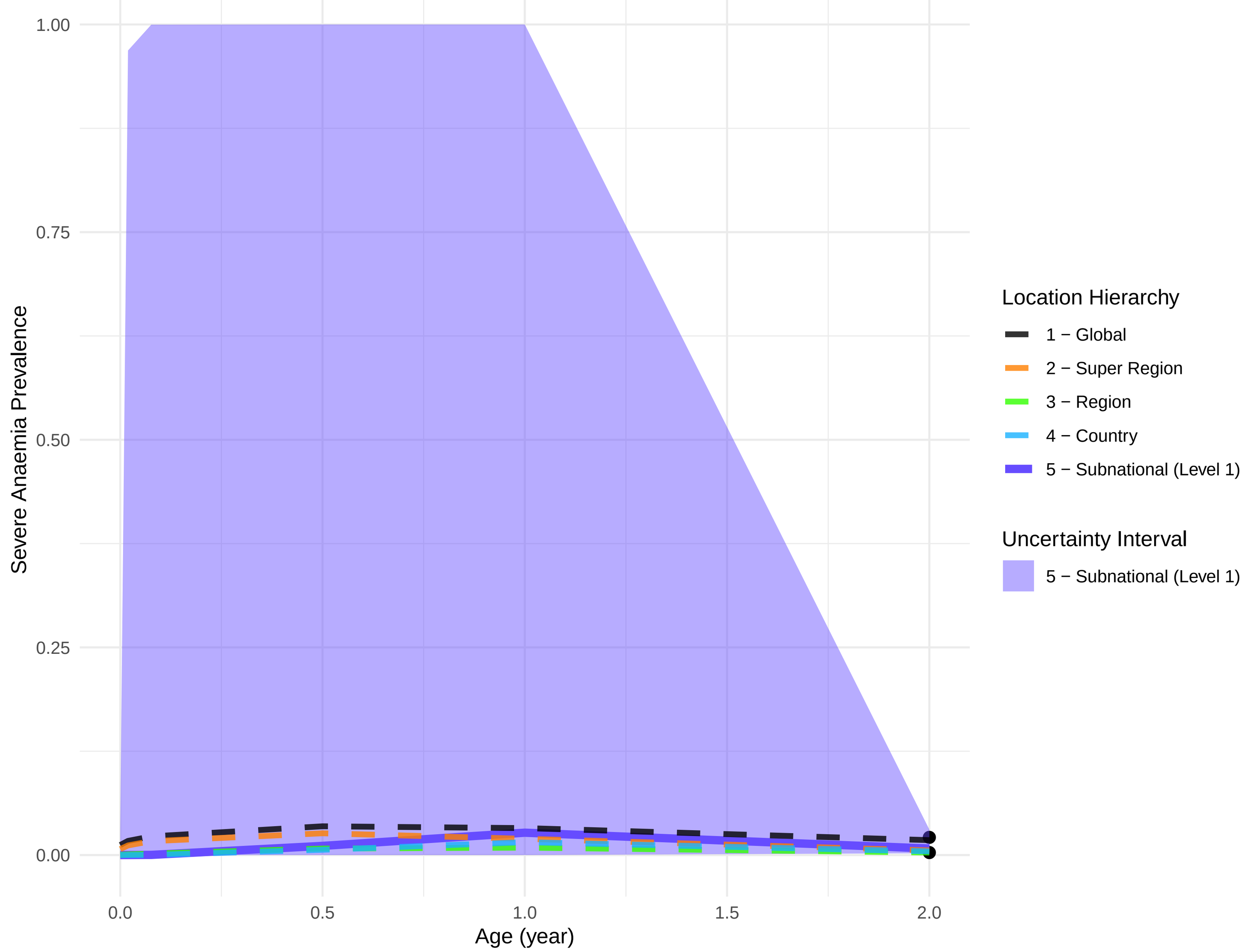

MR−BRT Age/Sex Fits − Males <2 Years Old − Severe Anaemia Prevalence − San Luis Potosí
WHO−2001 Adjustment Method

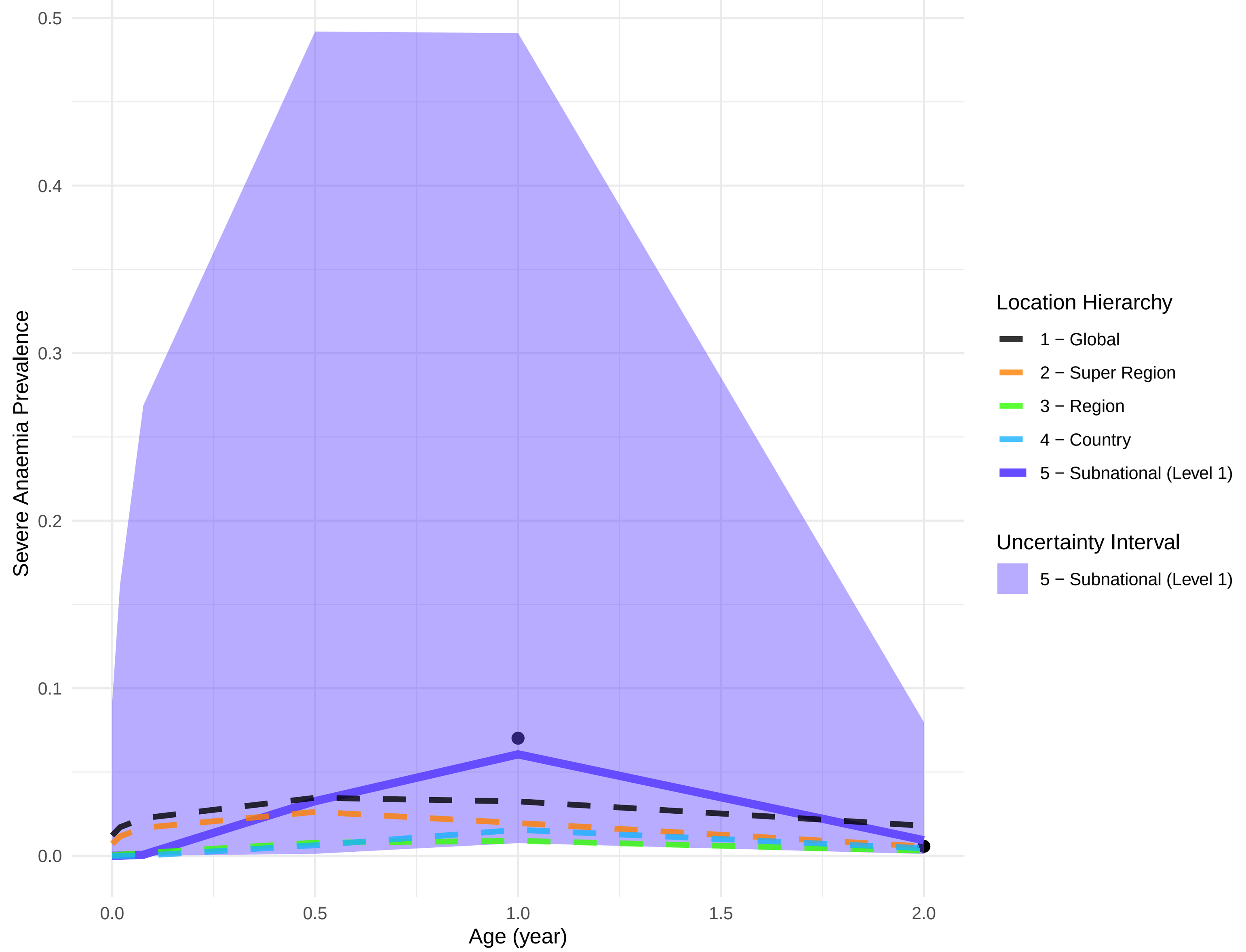

MR−BRT Age/Sex Fits − Males <2 Years Old − Severe Anaemia Prevalence − Sinaloa
WHO−2001 Adjustment Method

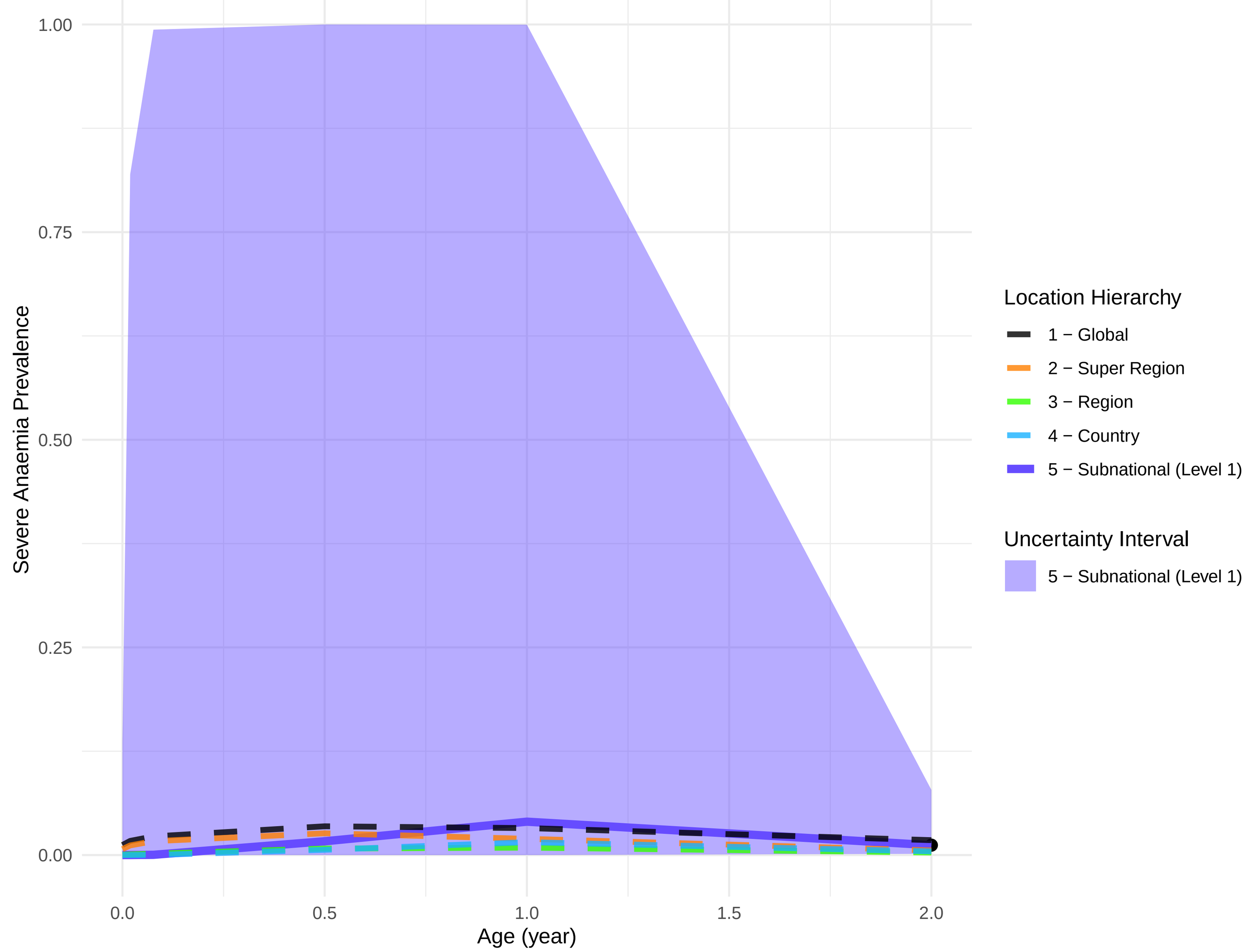

MR−BRT Age/Sex Fits − Males <2 Years Old − Severe Anaemia Prevalence − Tlaxcala
WHO−2001 Adjustment Method

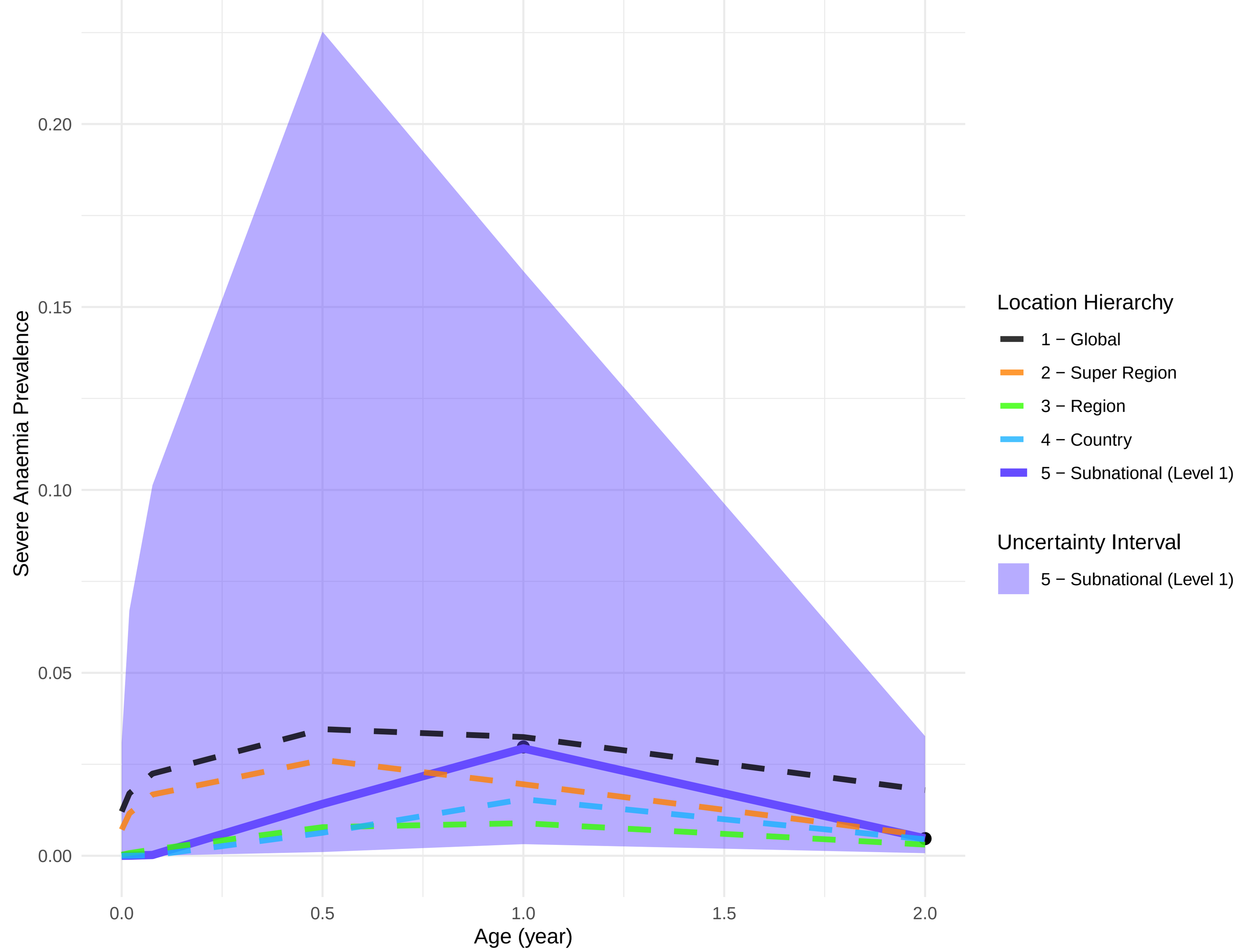

MR−BRT Age/Sex Fits − Males <2 Years Old − Severe Anaemia Prevalence − Veracruz de Ignacio de la Llave
WHO−2001 Adjustment Method

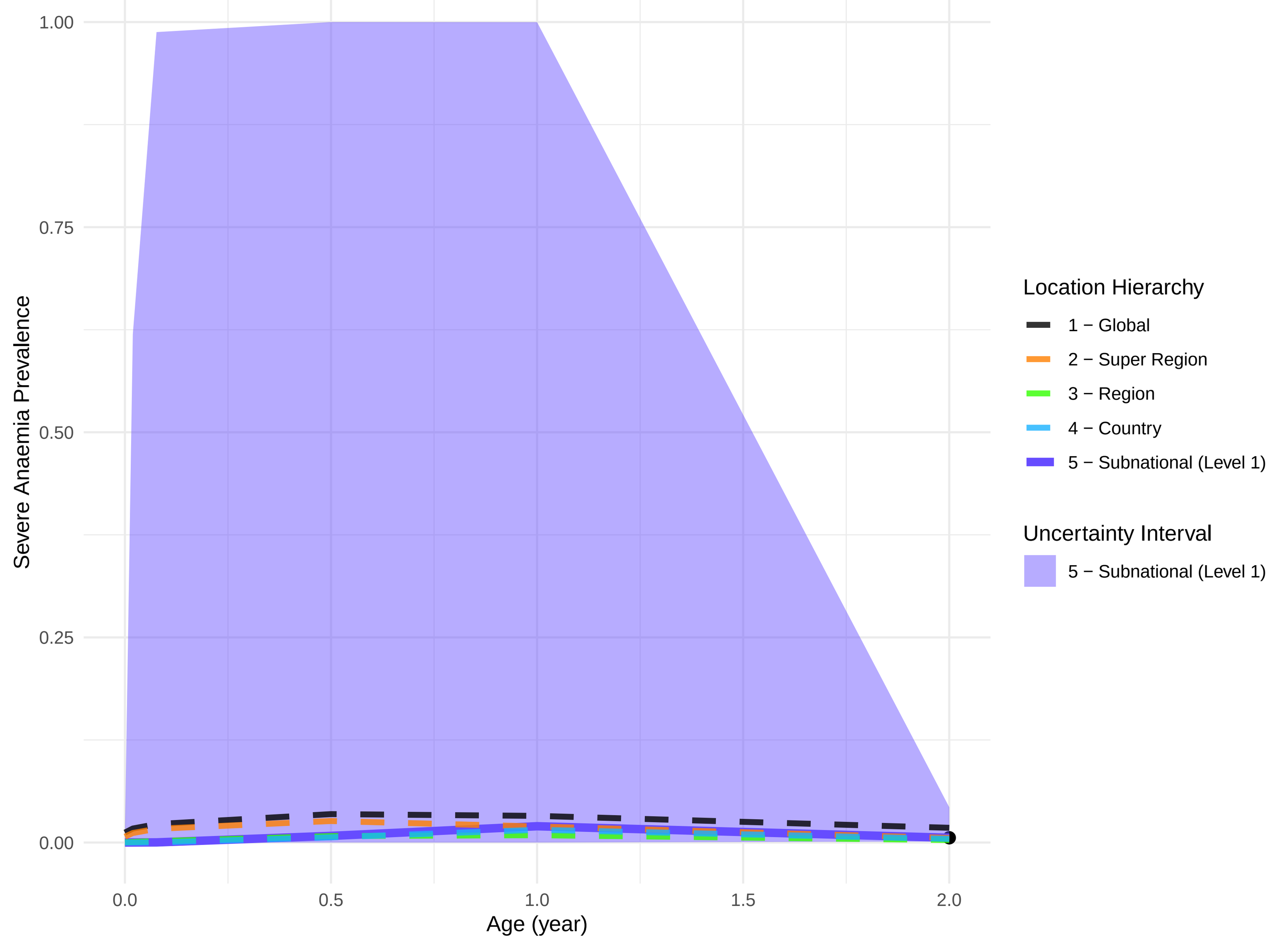

MR−BRT Age/Sex Fits − Males <2 Years Old − Severe Anaemia Prevalence − North Africa and Middle East
WHO−2001 Adjustment Method

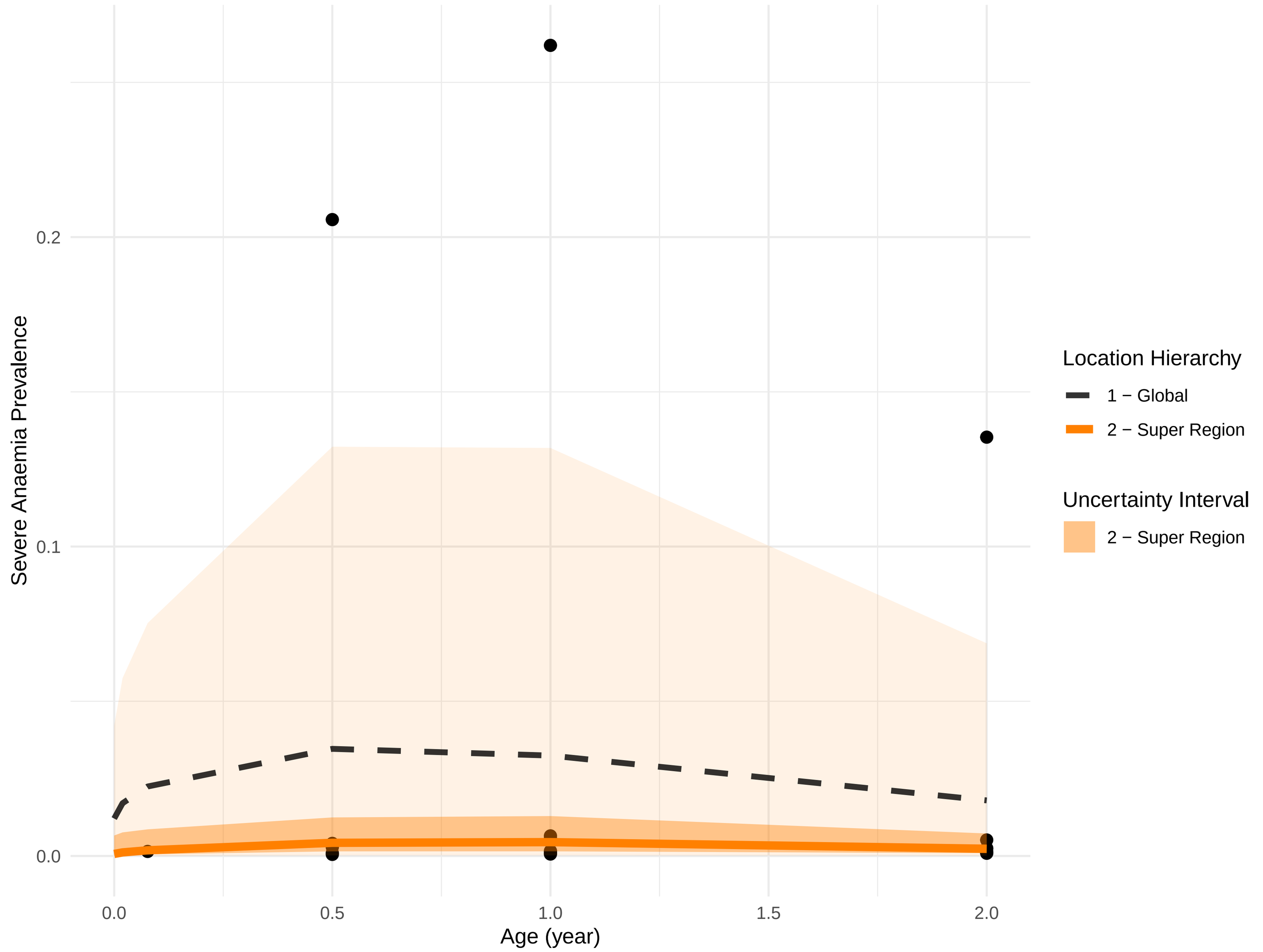

MR−BRT Age/Sex Fits − Males <2 Years Old − Severe Anaemia Prevalence − North Africa and Middle East
WHO−2001 Adjustment Method

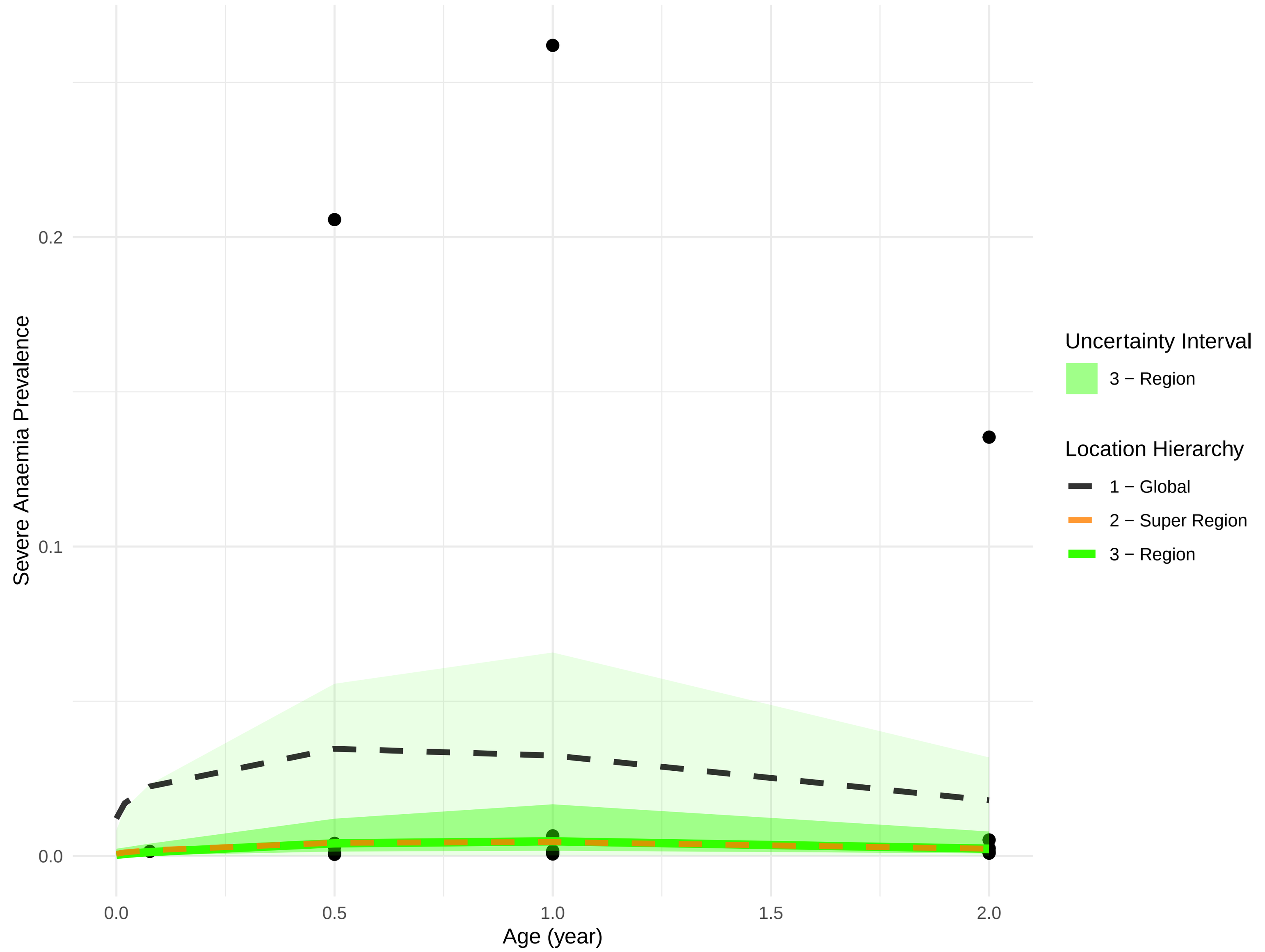

MR−BRT Age/Sex Fits − Males <2 Years Old − Severe Anaemia Prevalence − Egypt
WHO−2001 Adjustment Method

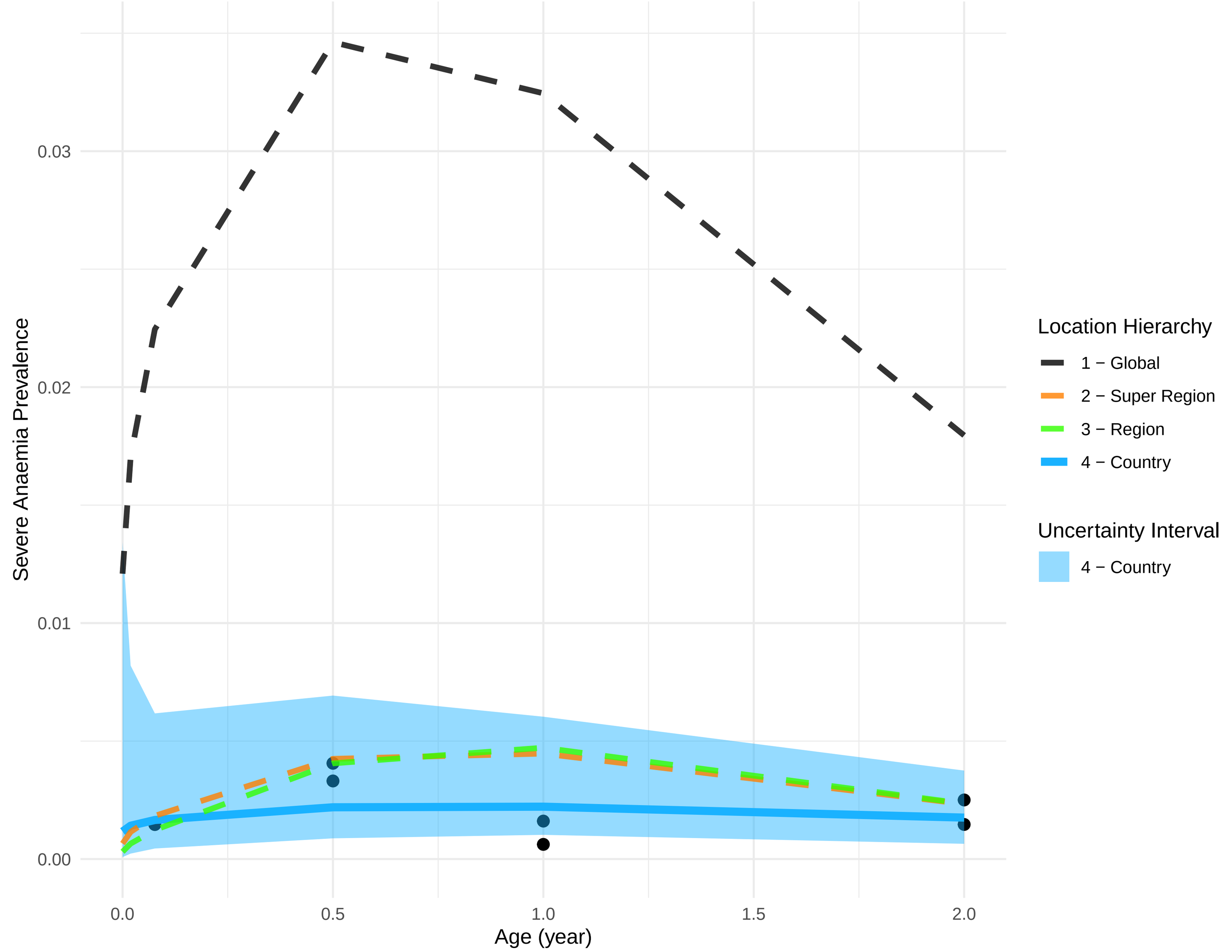

MR−BRT Age/Sex Fits − Males <2 Years Old − Severe Anaemia Prevalence − Jordan
WHO−2001 Adjustment Method

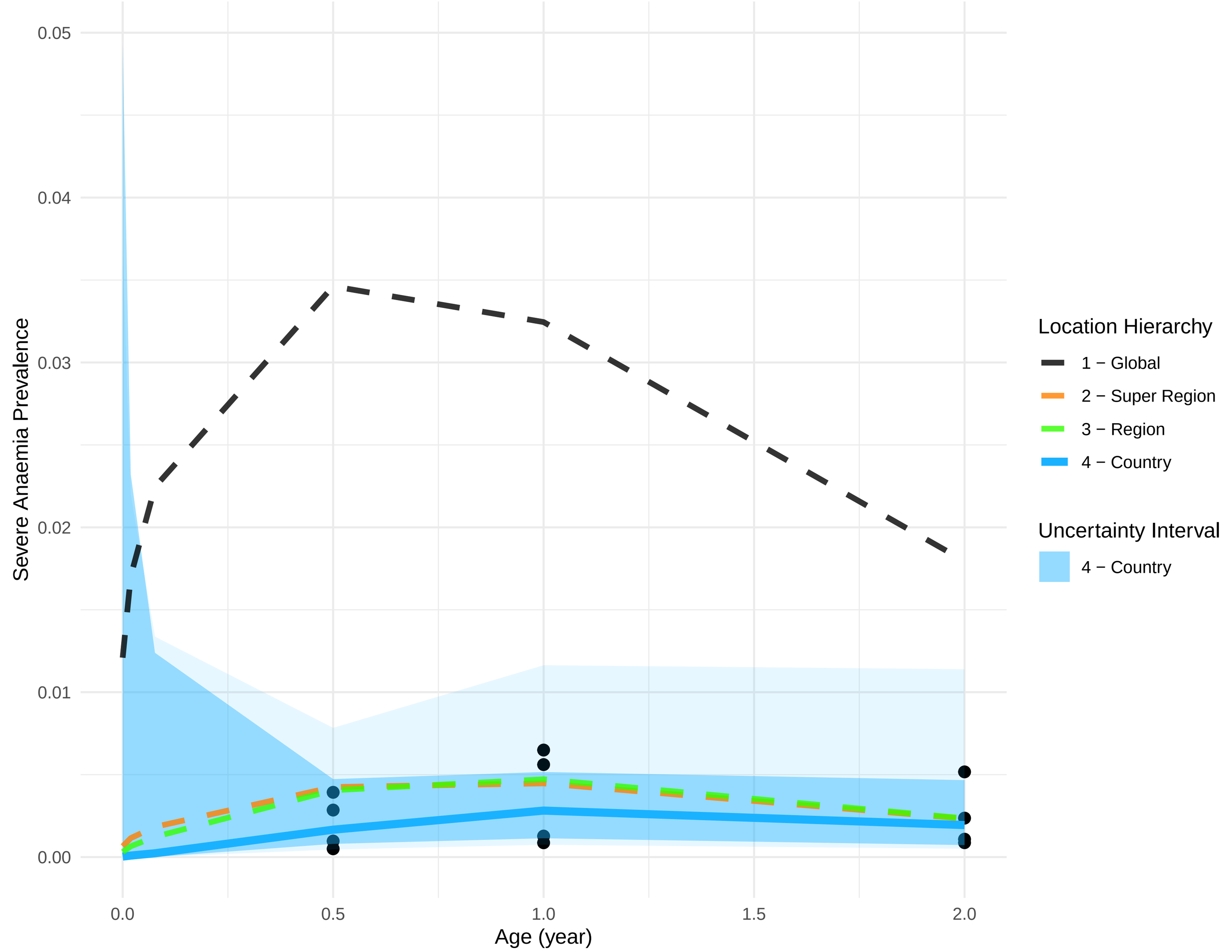

MR−BRT Age/Sex Fits − Males <2 Years Old − Severe Anaemia Prevalence − Yemen
WHO−2001 Adjustment Method

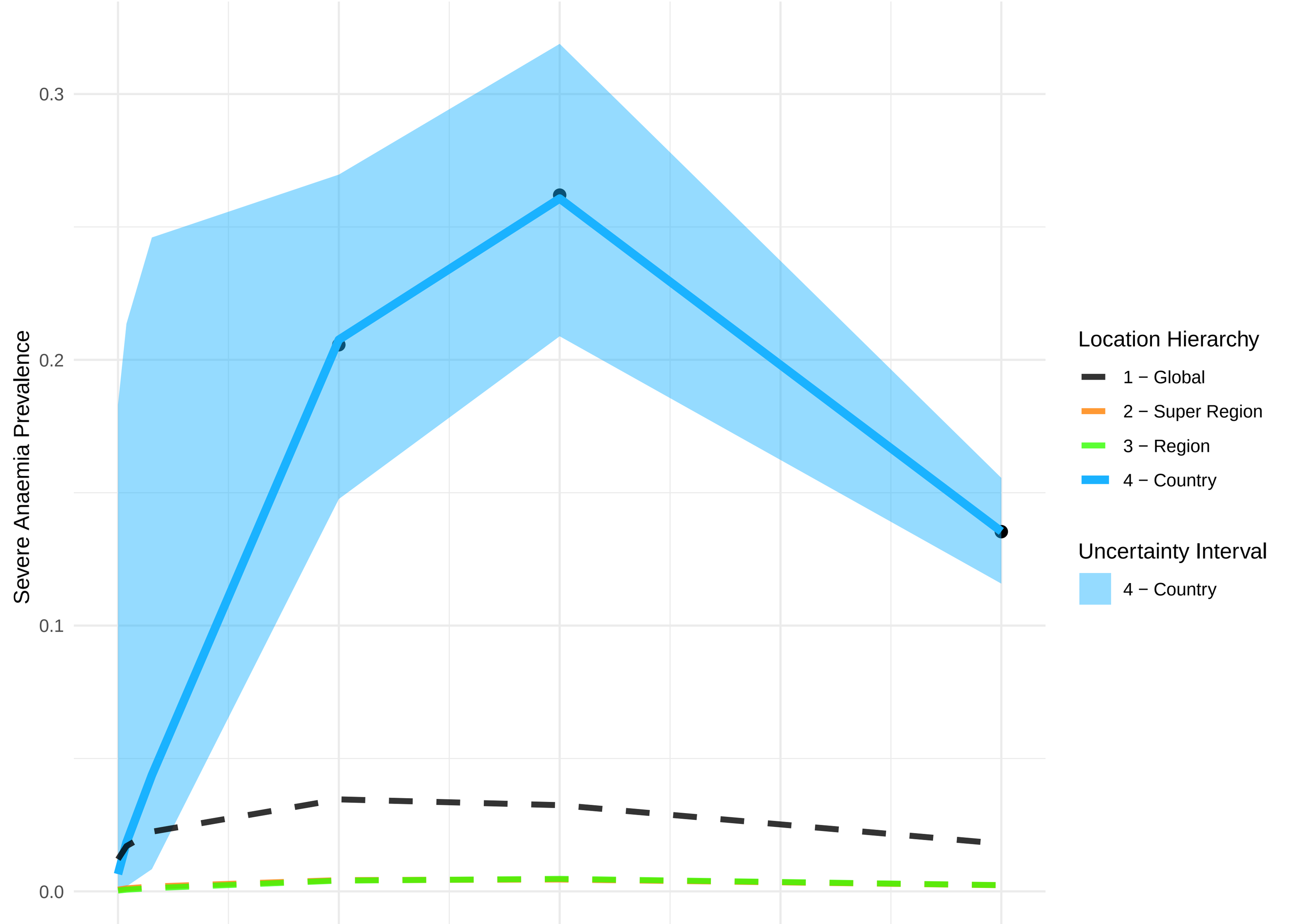

MR−BRT Age/Sex Fits − Males <2 Years Old − Severe Anaemia Prevalence − South Asia
WHO−2001 Adjustment Method

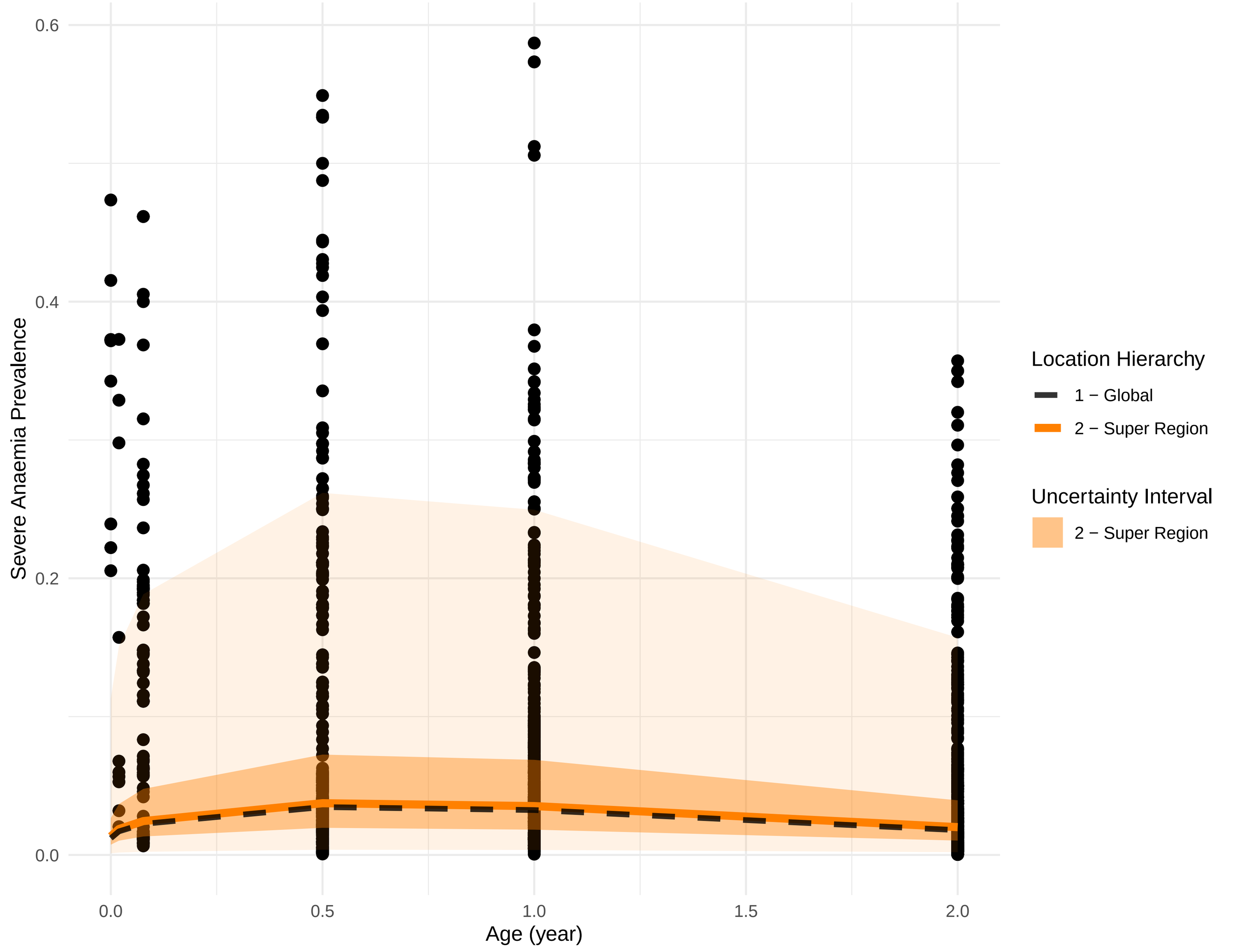

MR−BRT Age/Sex Fits − Males <2 Years Old − Severe Anaemia Prevalence − South Asia
WHO−2001 Adjustment Method

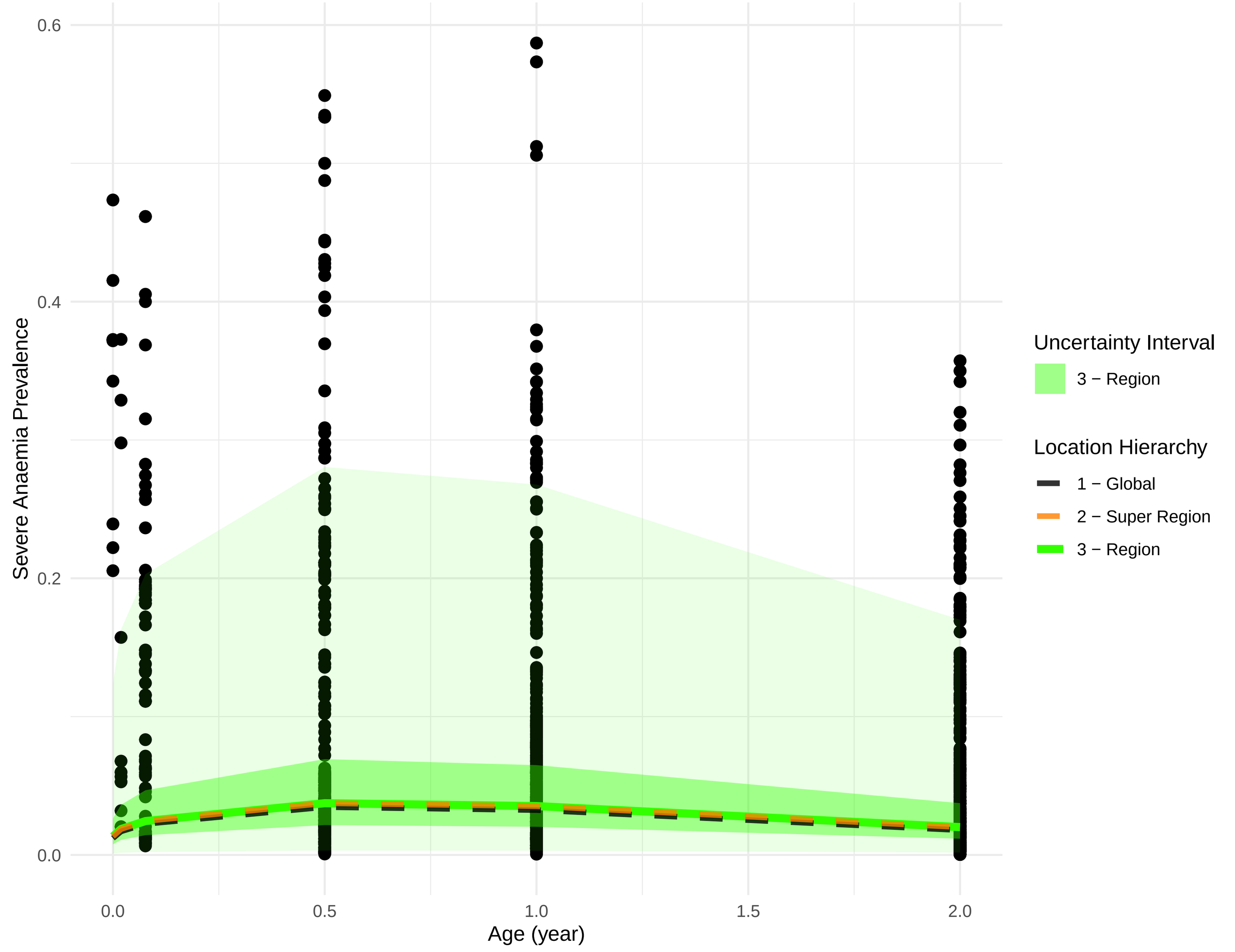

MR−BRT Age/Sex Fits − Males <2 Years Old − Severe Anaemia Prevalence − Bangladesh
WHO−2001 Adjustment Method

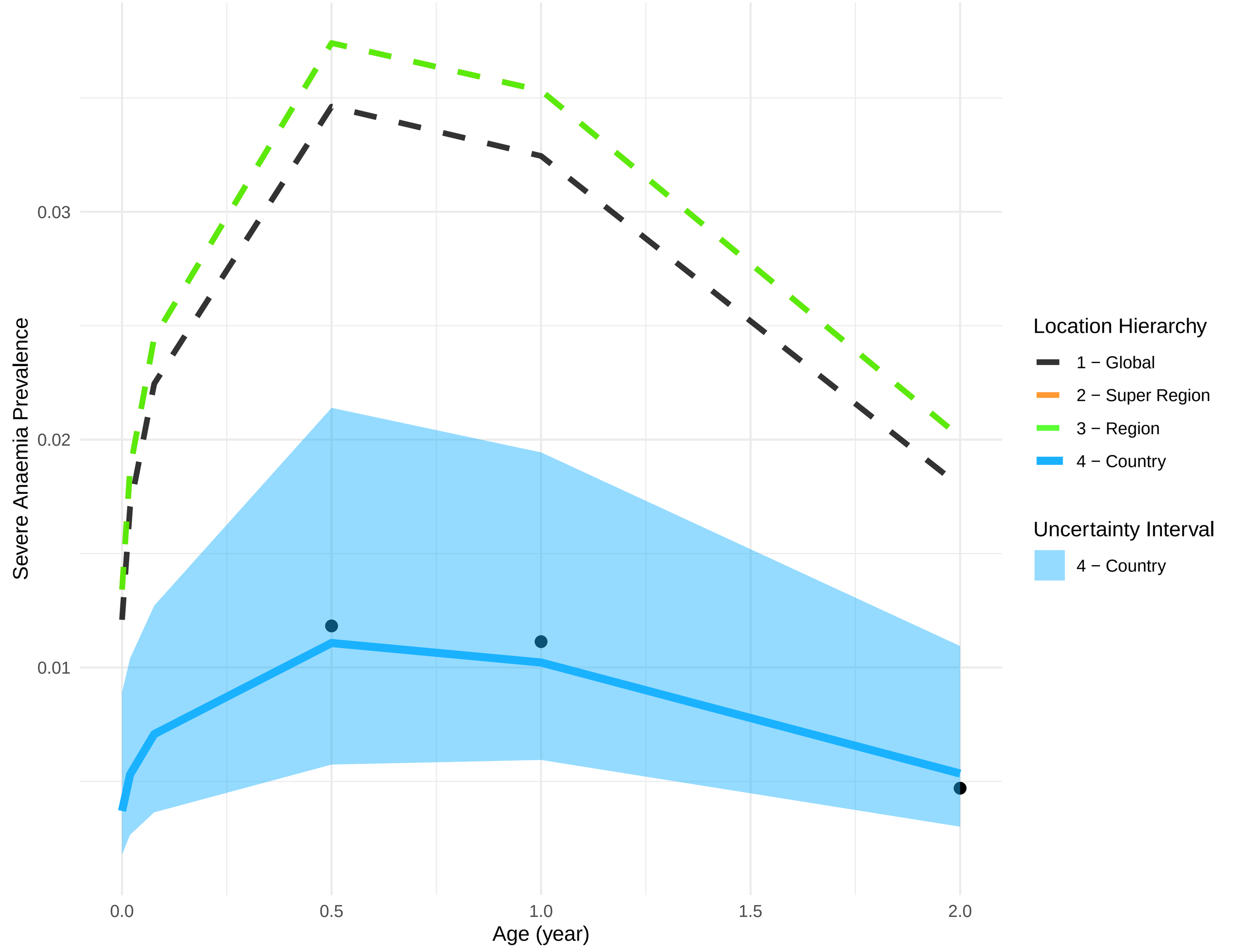

MR−BRT Age/Sex Fits − Males <2 Years Old − Severe Anaemia Prevalence − India
WHO−2001 Adjustment Method

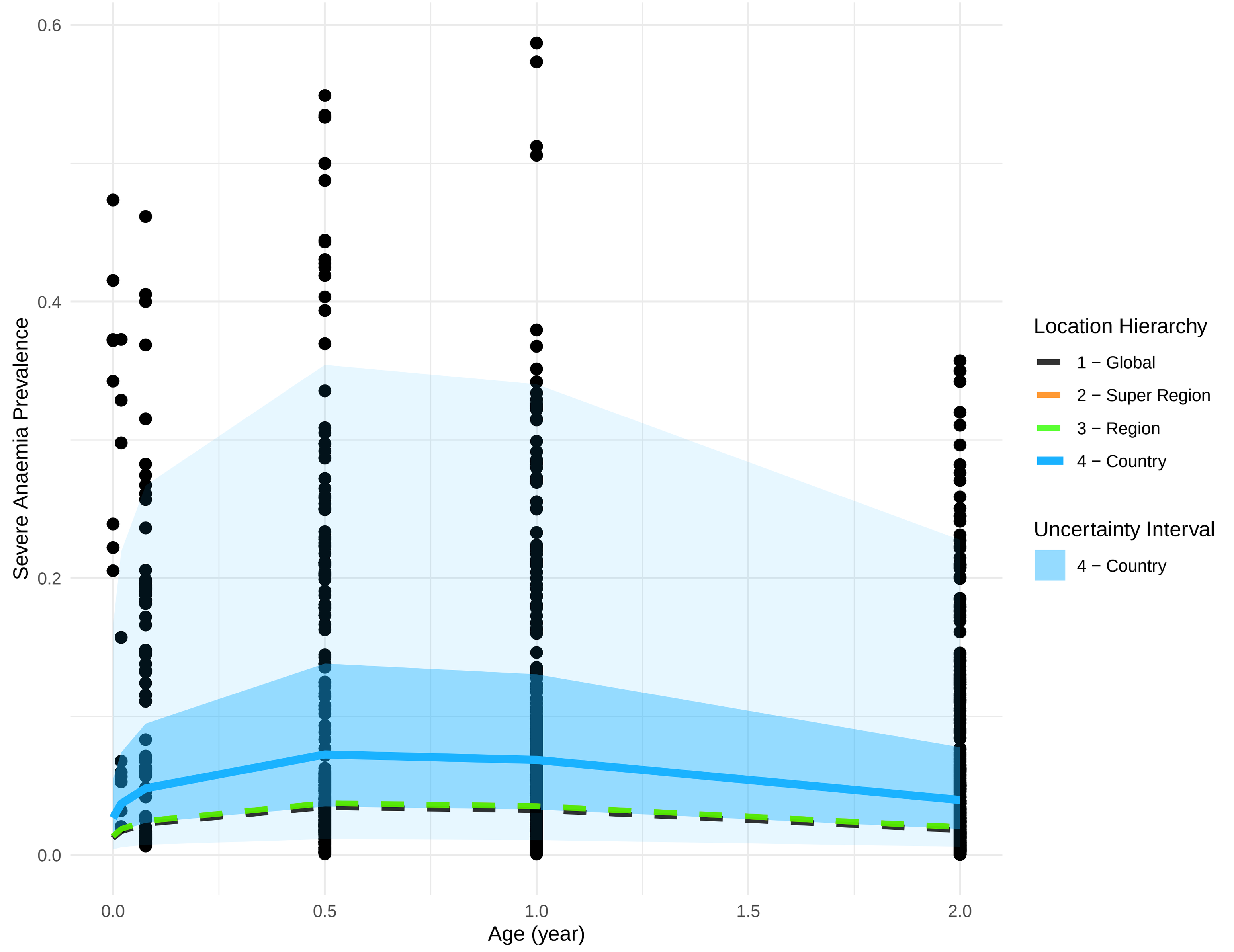

MR−BRT Age/Sex Fits − Males <2 Years Old − Severe Anaemia Prevalence − Andhra Pradesh
WHO−2001 Adjustment Method

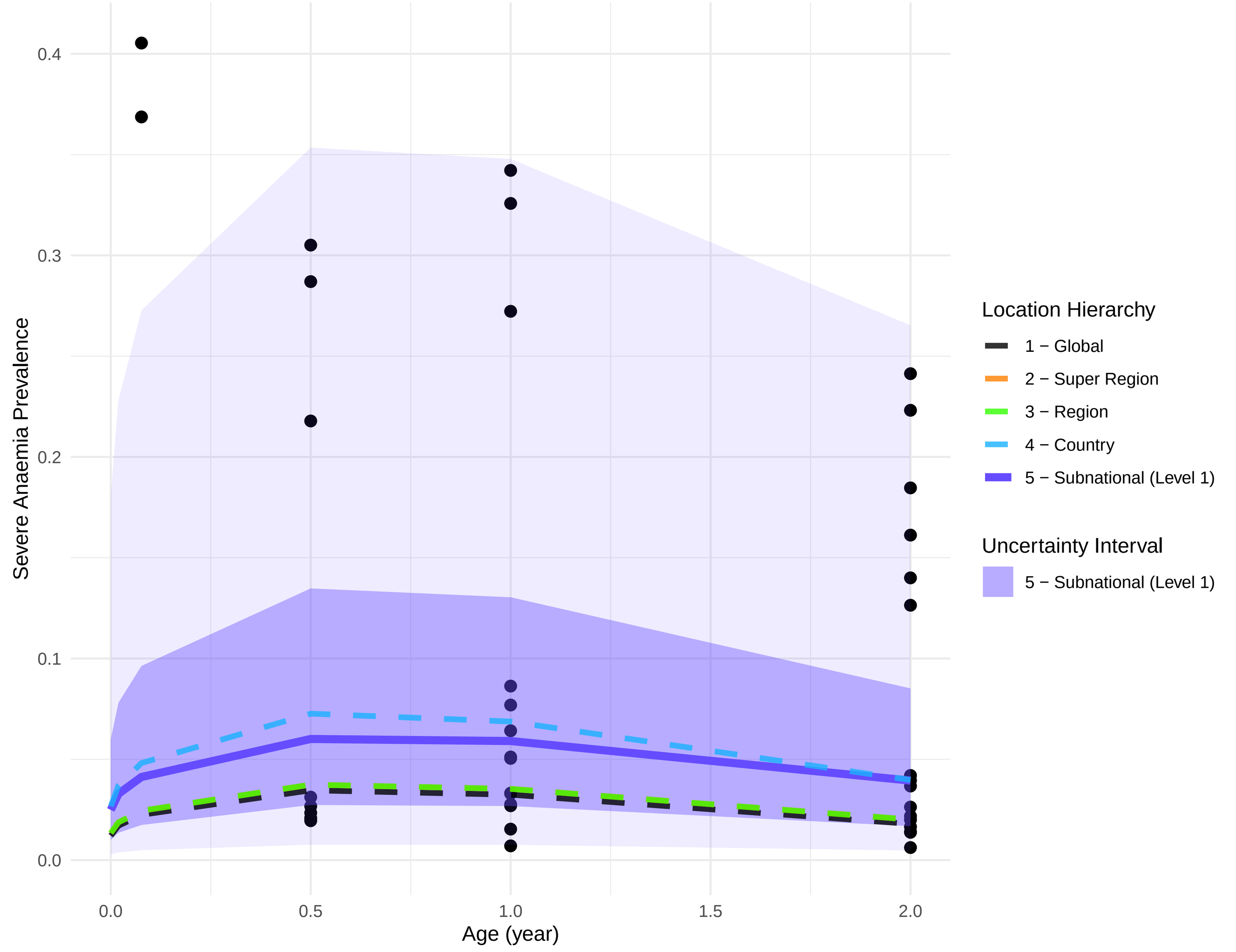

MR−BRT Age/Sex Fits − Males <2 Years Old − Severe Anaemia Prevalence − Andhra Pradesh, Rural
WHO−2001 Adjustment Method

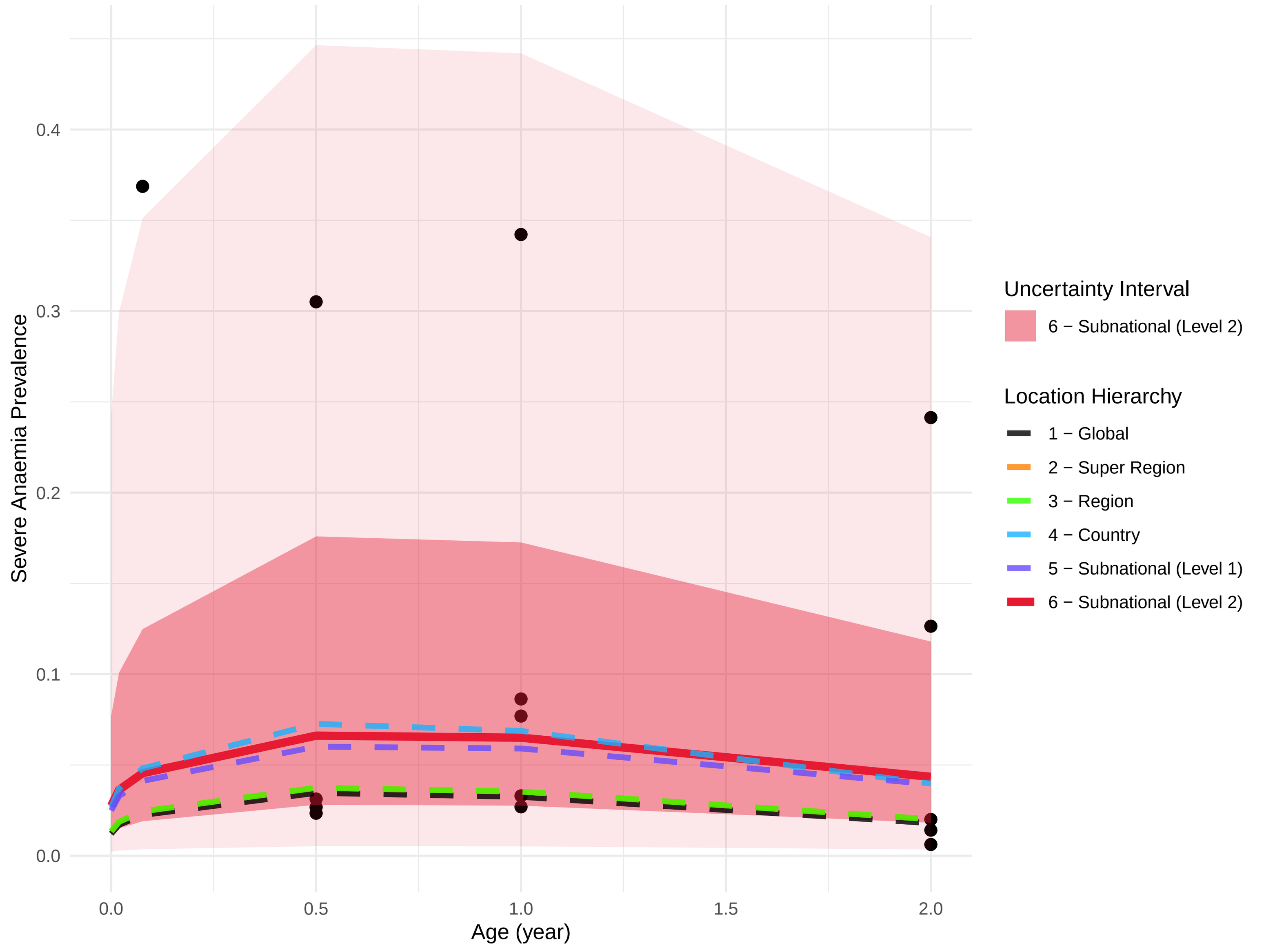

MR−BRT Age/Sex Fits − Males <2 Years Old − Severe Anaemia Prevalence − Andhra Pradesh, Urban
WHO−2001 Adjustment Method

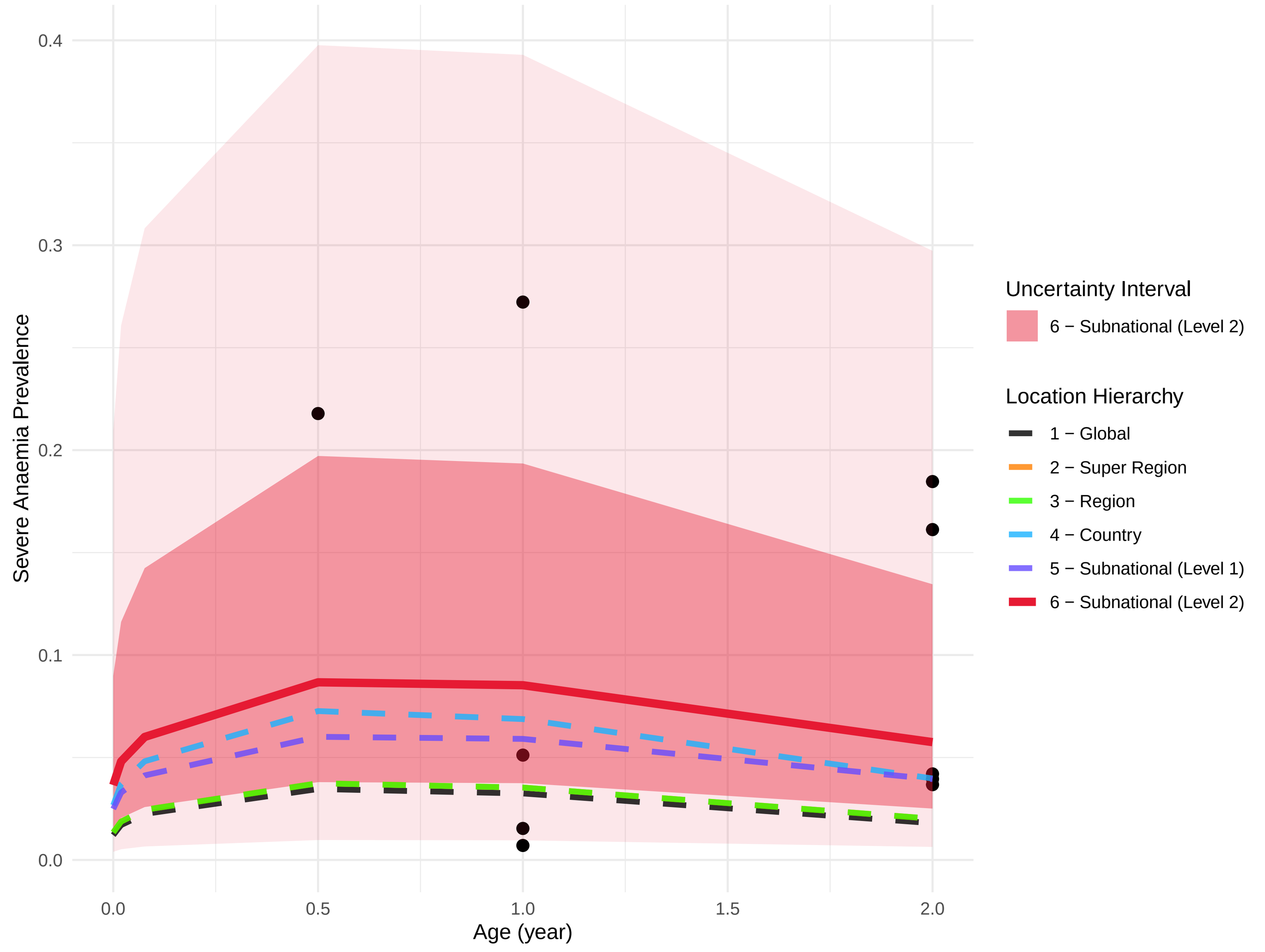

MR−BRT Age/Sex Fits − Males <2 Years Old − Severe Anaemia Prevalence − Arunachal Pradesh
WHO−2001 Adjustment Method

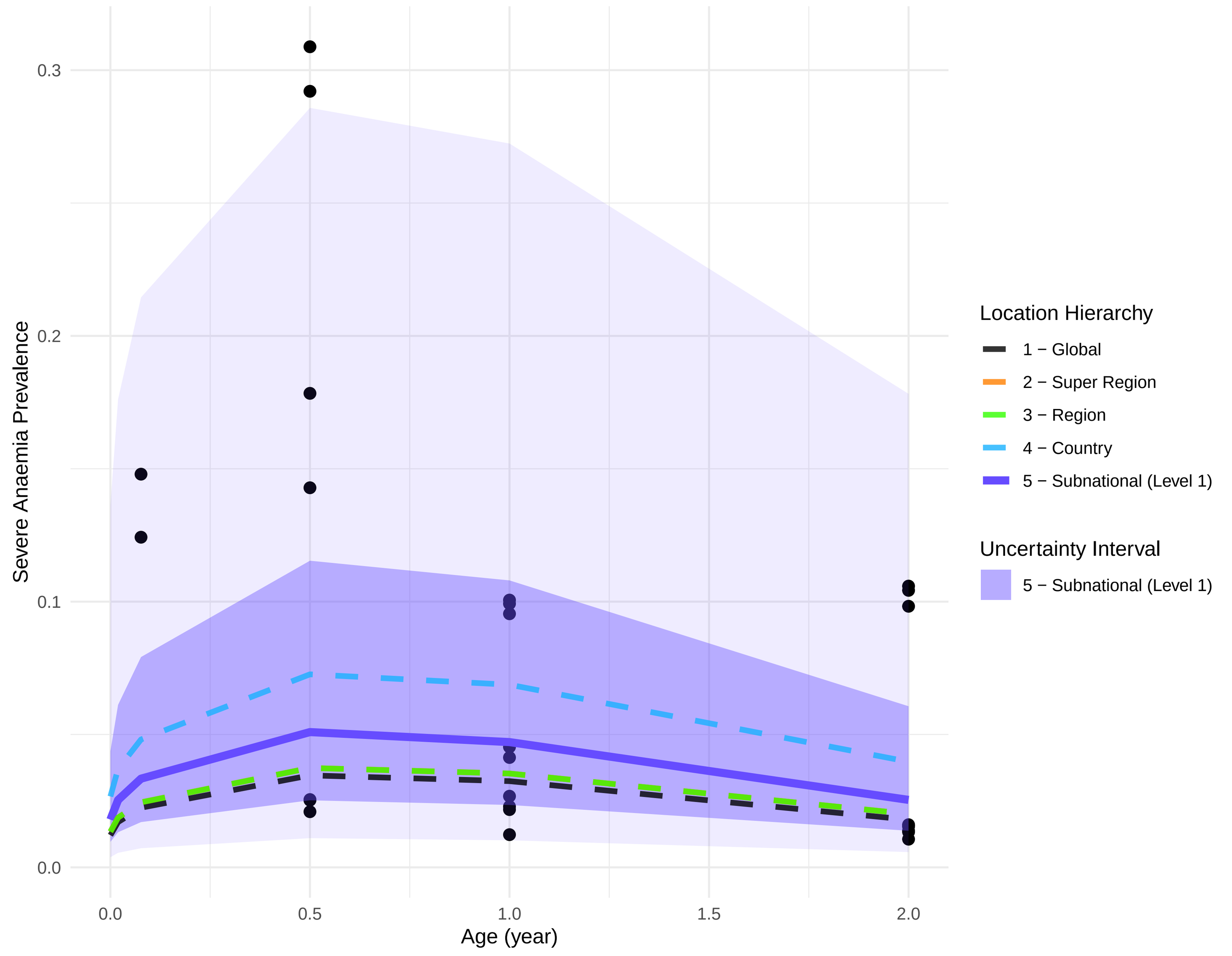

MR−BRT Age/Sex Fits − Males <2 Years Old − Severe Anaemia Prevalence − Arunachal Pradesh, Rural
WHO−2001 Adjustment Method

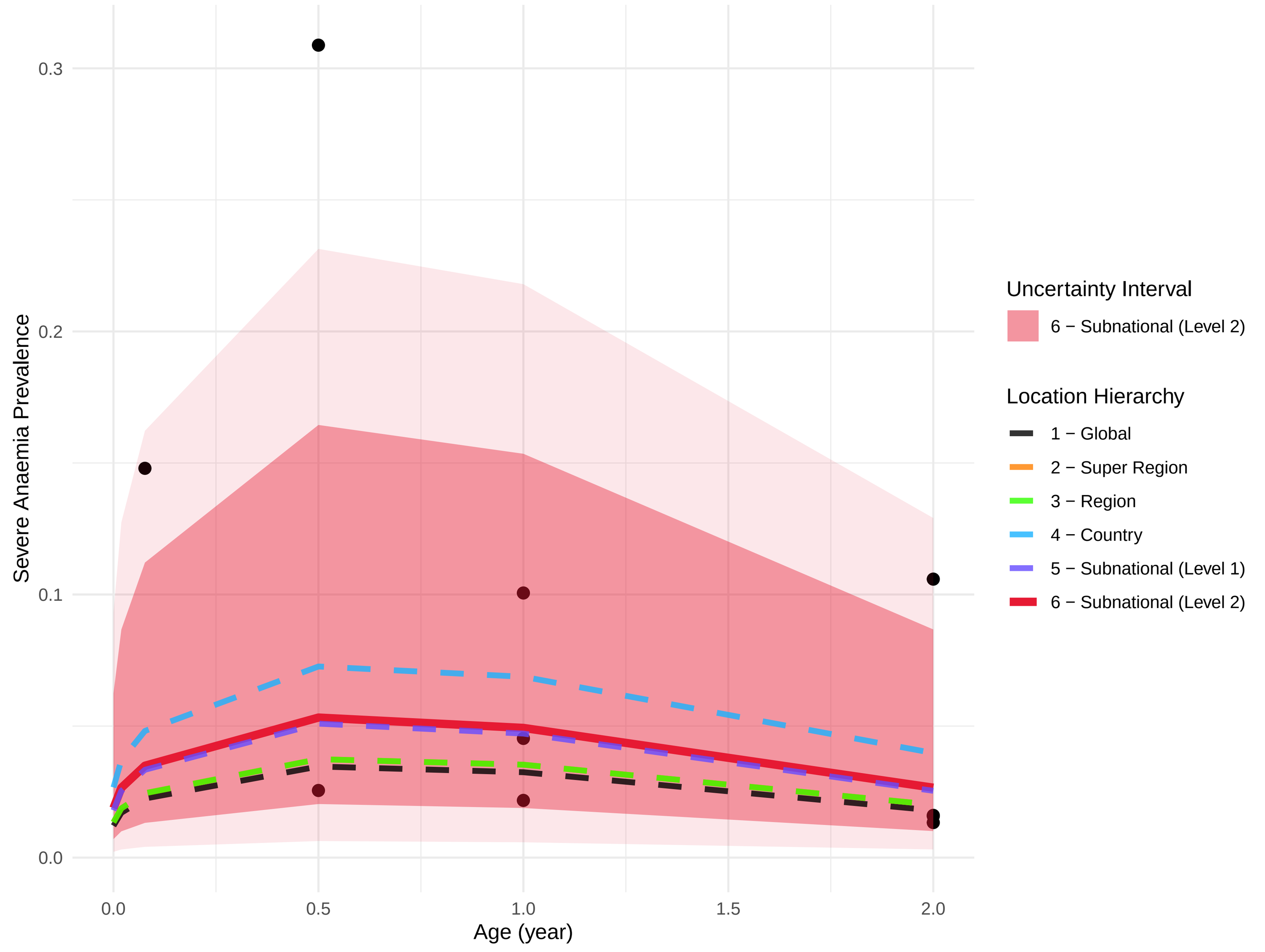

MR−BRT Age/Sex Fits − Males <2 Years Old − Severe Anaemia Prevalence − Arunachal Pradesh, Urban
WHO−2001 Adjustment Method

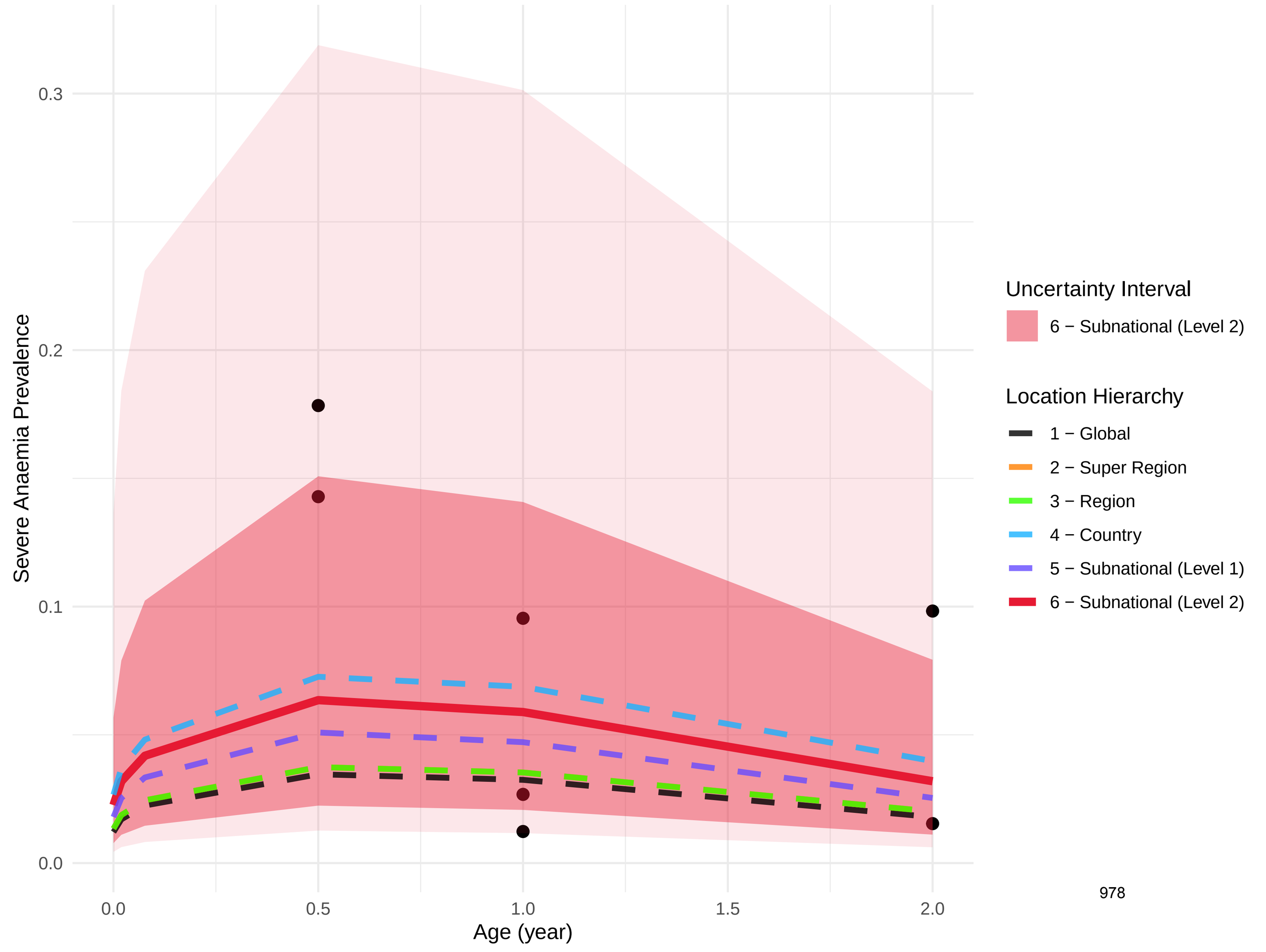

MR−BRT Age/Sex Fits − Males <2 Years Old − Severe Anaemia Prevalence − Assam
WHO−2001 Adjustment Method

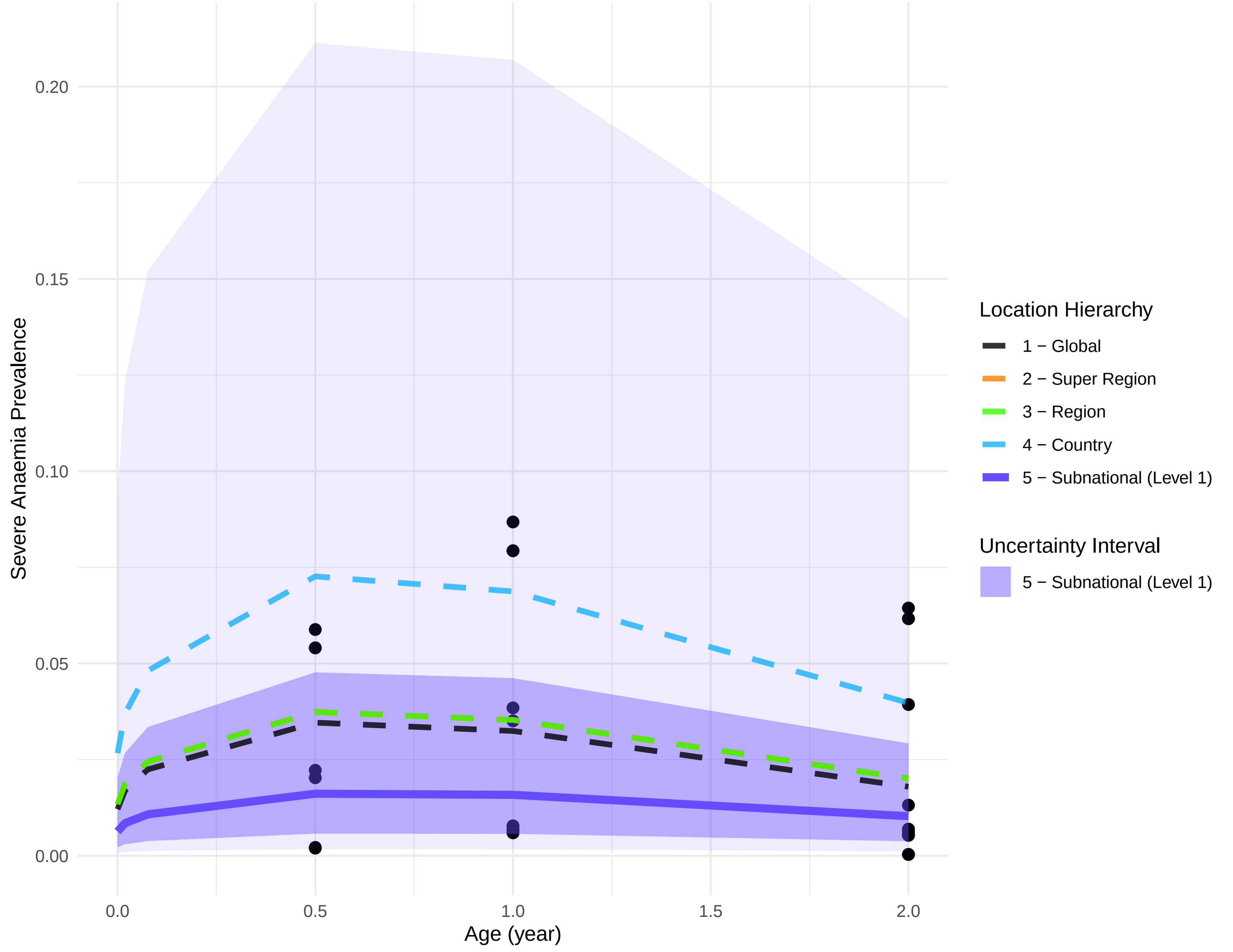

MR−BRT Age/Sex Fits − Males <2 Years Old − Severe Anaemia Prevalence − Assam, Rural
WHO−2001 Adjustment Method

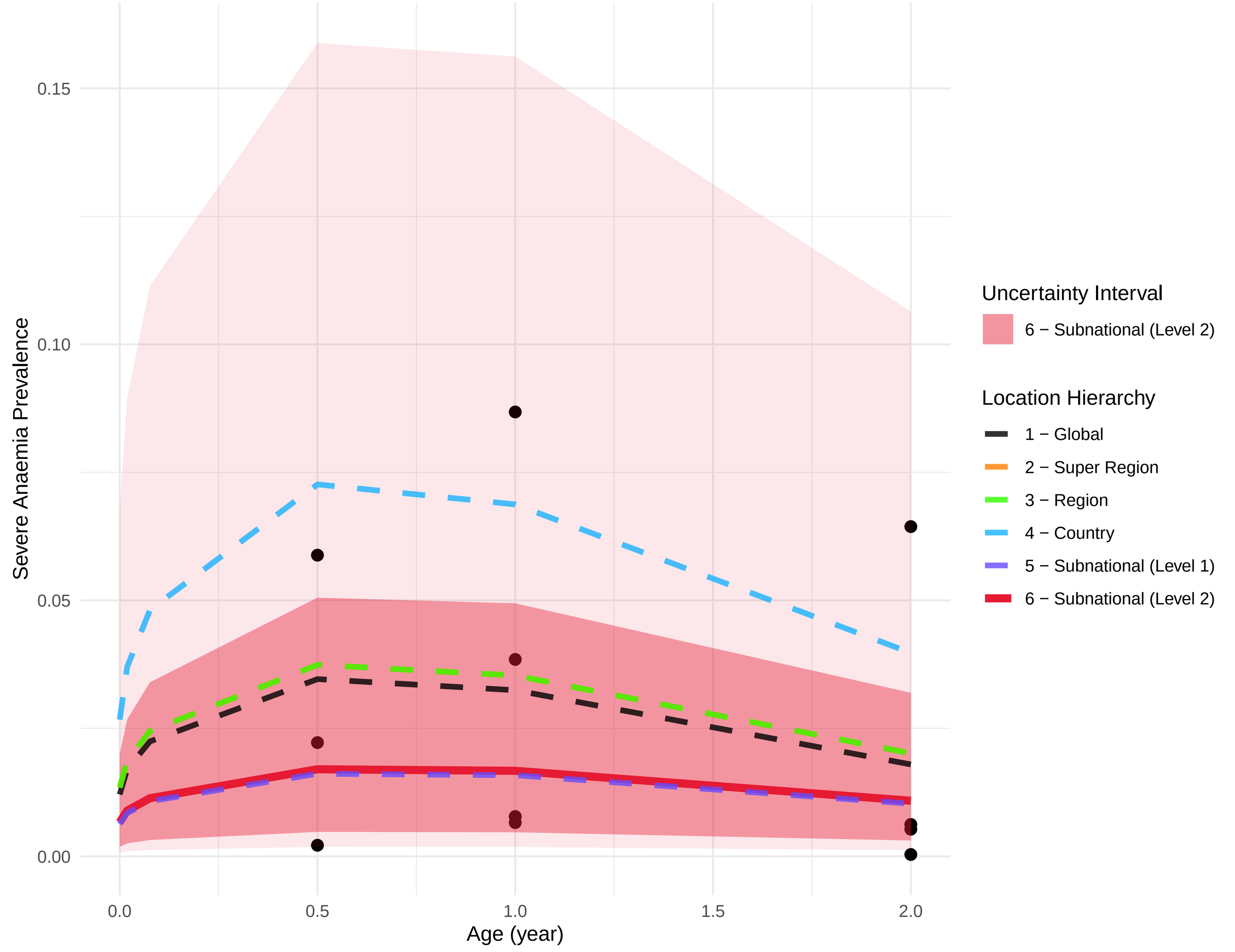

MR−BRT Age/Sex Fits − Males <2 Years Old − Severe Anaemia Prevalence − Assam, Urban
WHO−2001 Adjustment Method

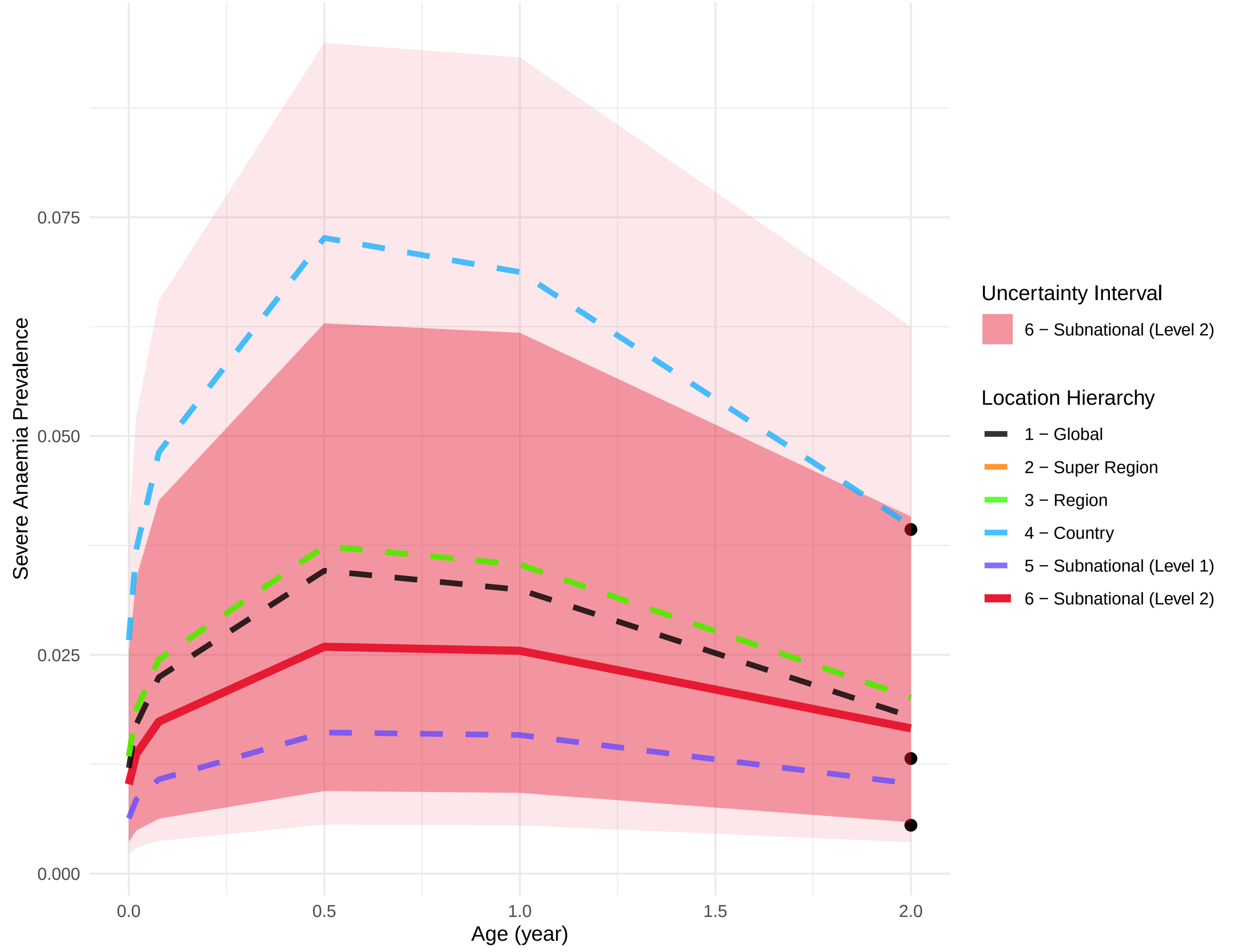

MR−BRT Age/Sex Fits − Males <2 Years Old − Severe Anaemia Prevalence − Bihar
WHO−2001 Adjustment Method

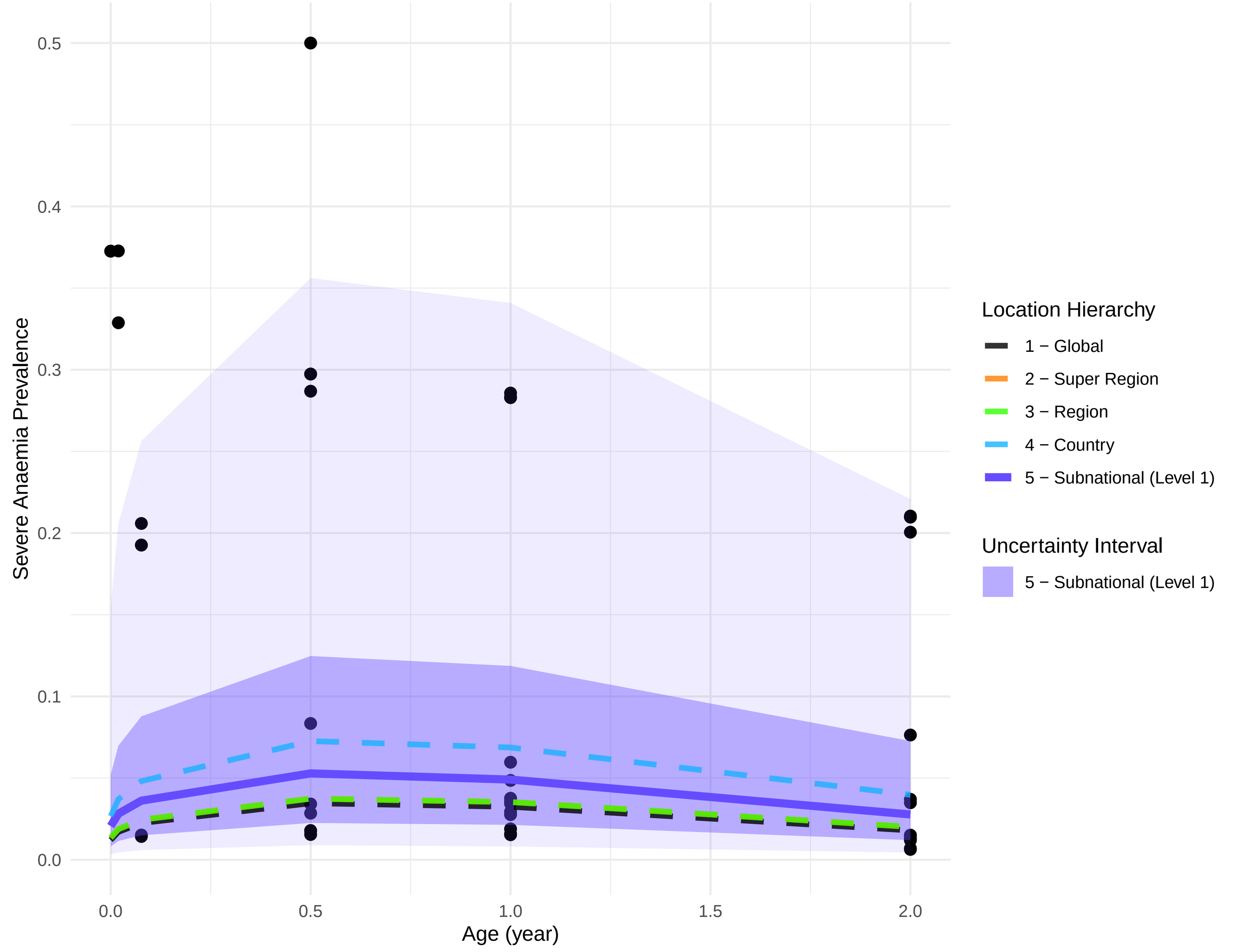

MR−BRT Age/Sex Fits − Males <2 Years Old − Severe Anaemia Prevalence − Bihar, Rural

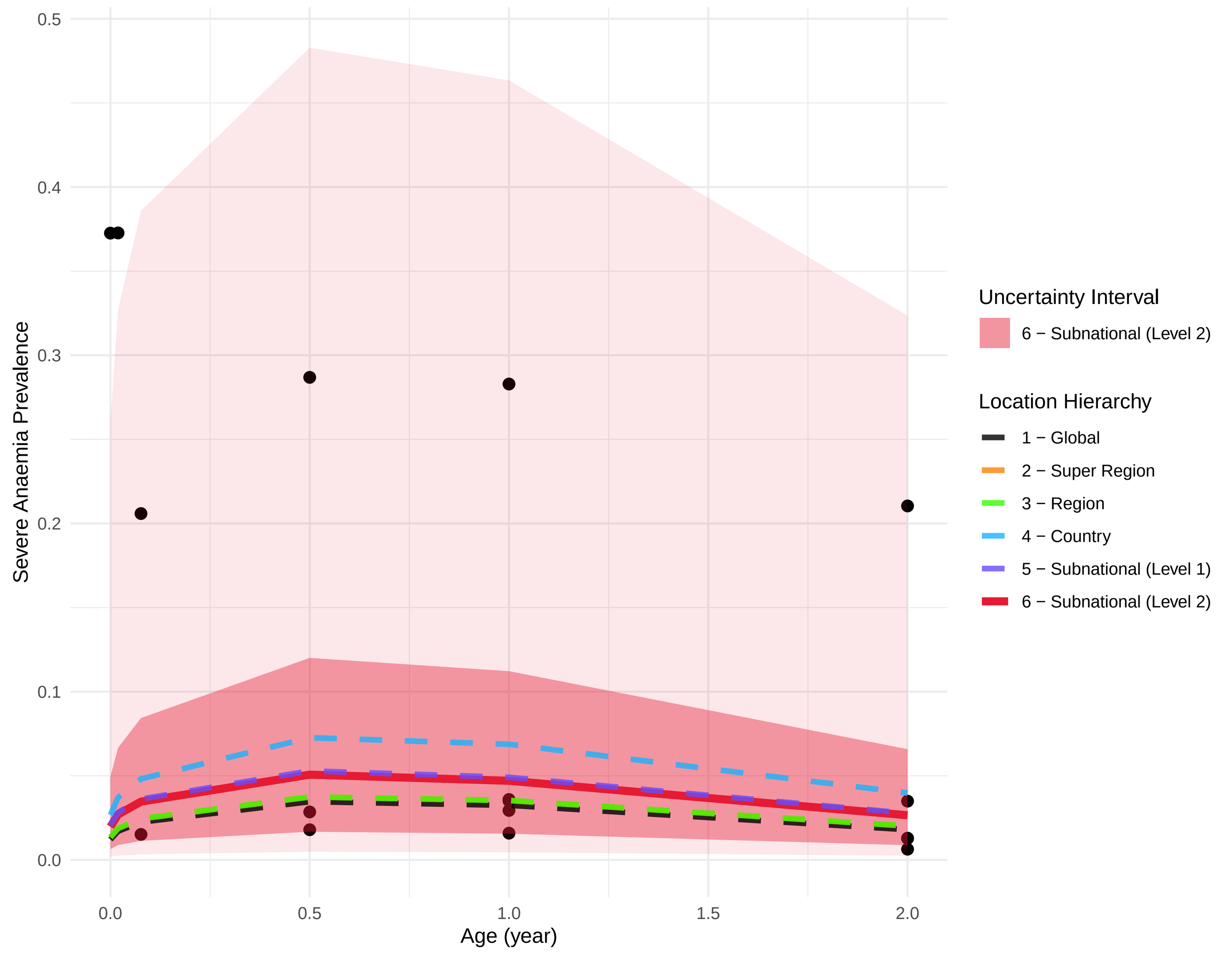

MR−BRT Age/Sex Fits − Males <2 Years Old − Severe Anaemia Prevalence − Bihar, Urban
WHO−2001 Adjustment Method

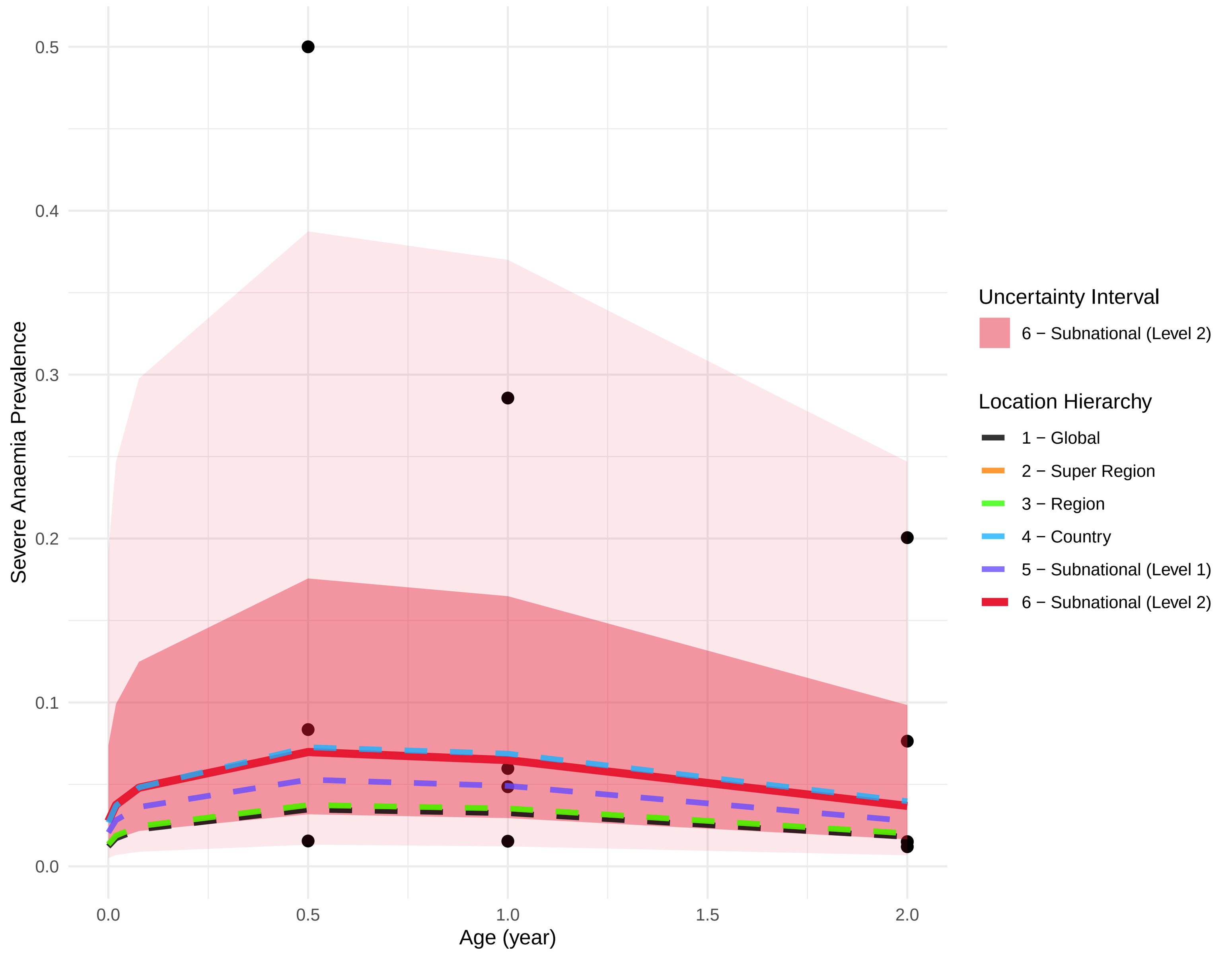

MR−BRT Age/Sex Fits − Males <2 Years Old − Severe Anaemia Prevalence − Chhattisgarh
WHO−2001 Adjustment Method

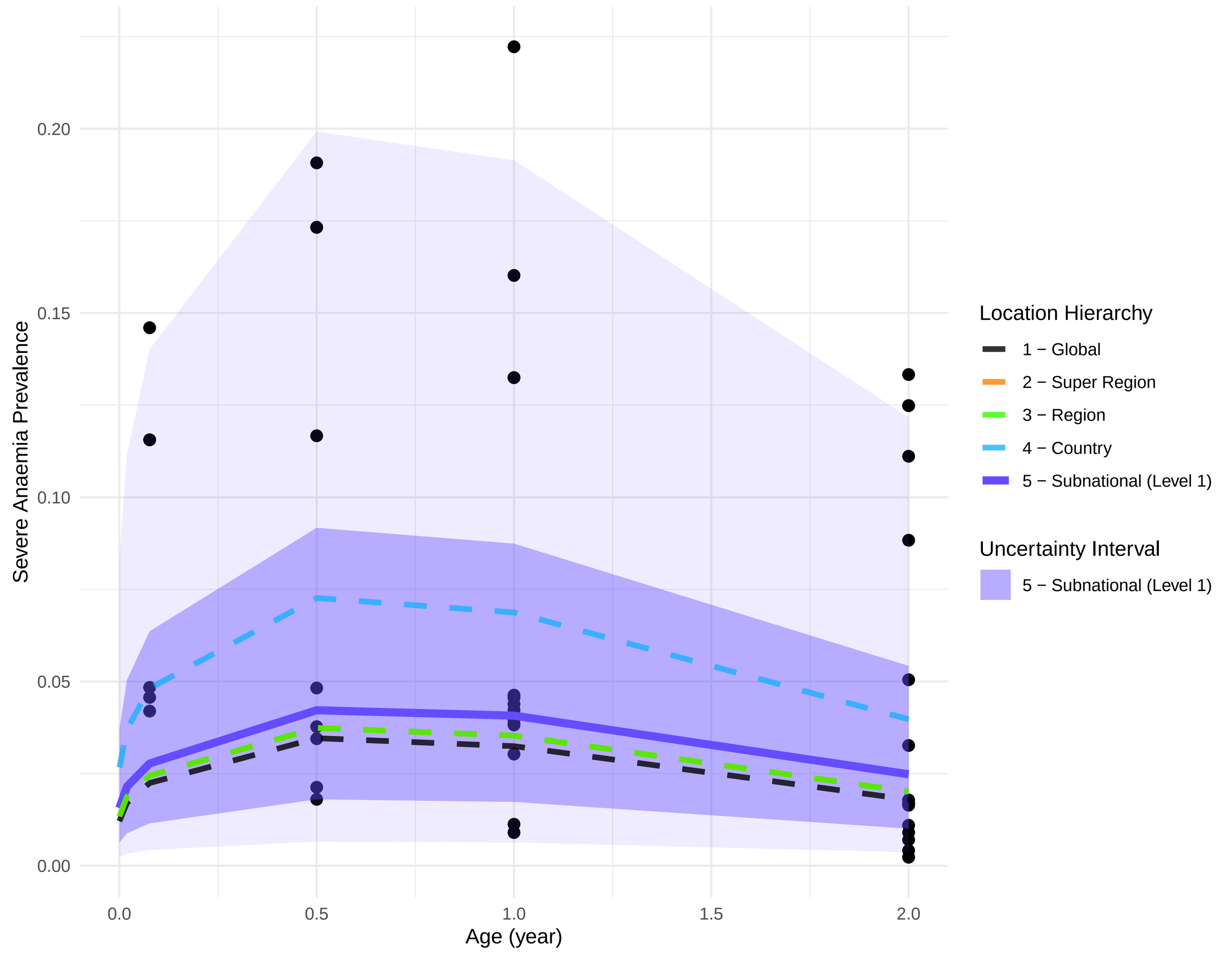

MR−BRT Age/Sex Fits − Males <2 Years Old − Severe Anaemia Prevalence − Chhattisgarh, Rural
WHO−2001 Adjustment Method

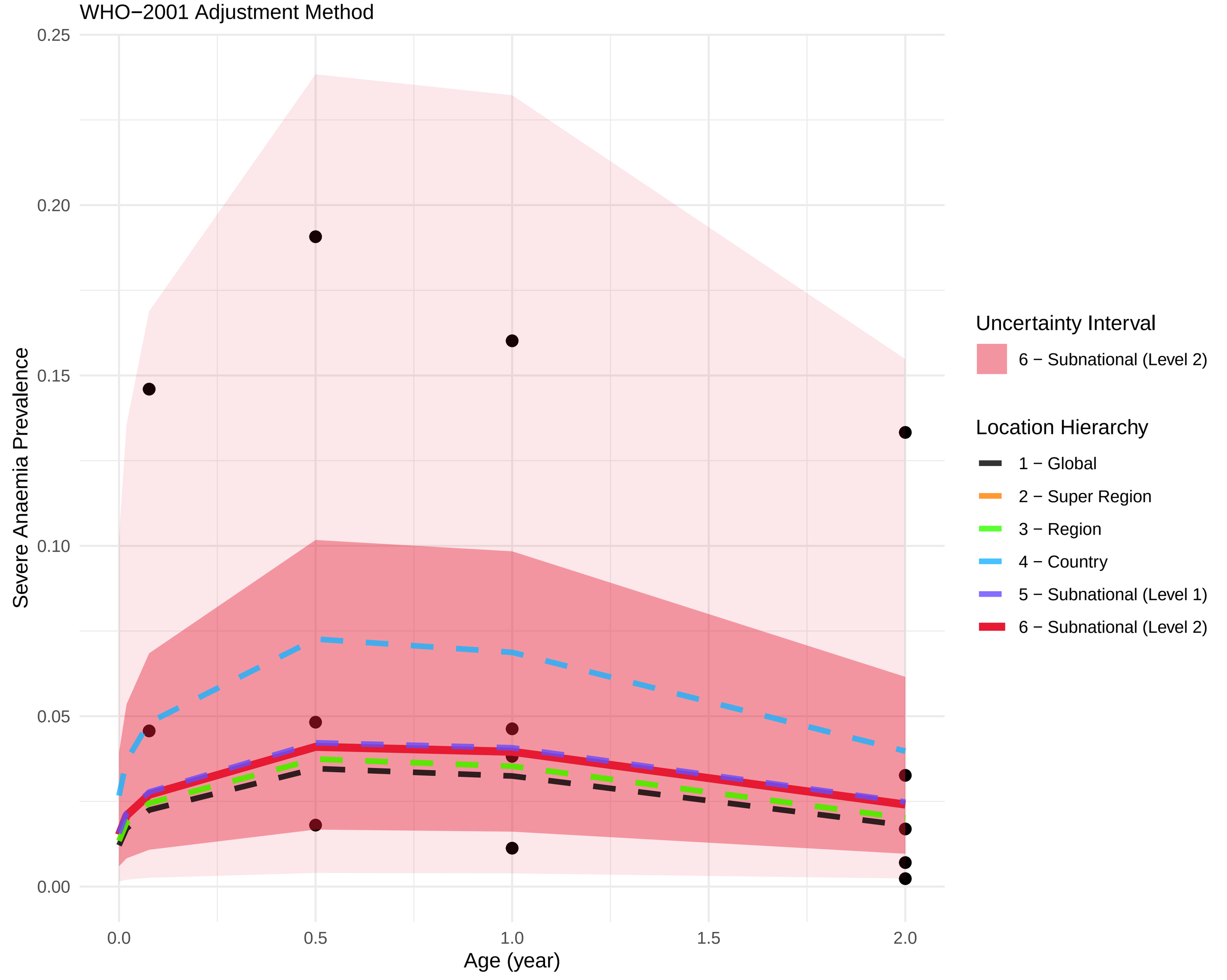

MR−BRT Age/Sex Fits − Males <2 Years Old − Severe Anaemia Prevalence − Chhattisgarh, Urban
WHO−2001 Adjustment Method

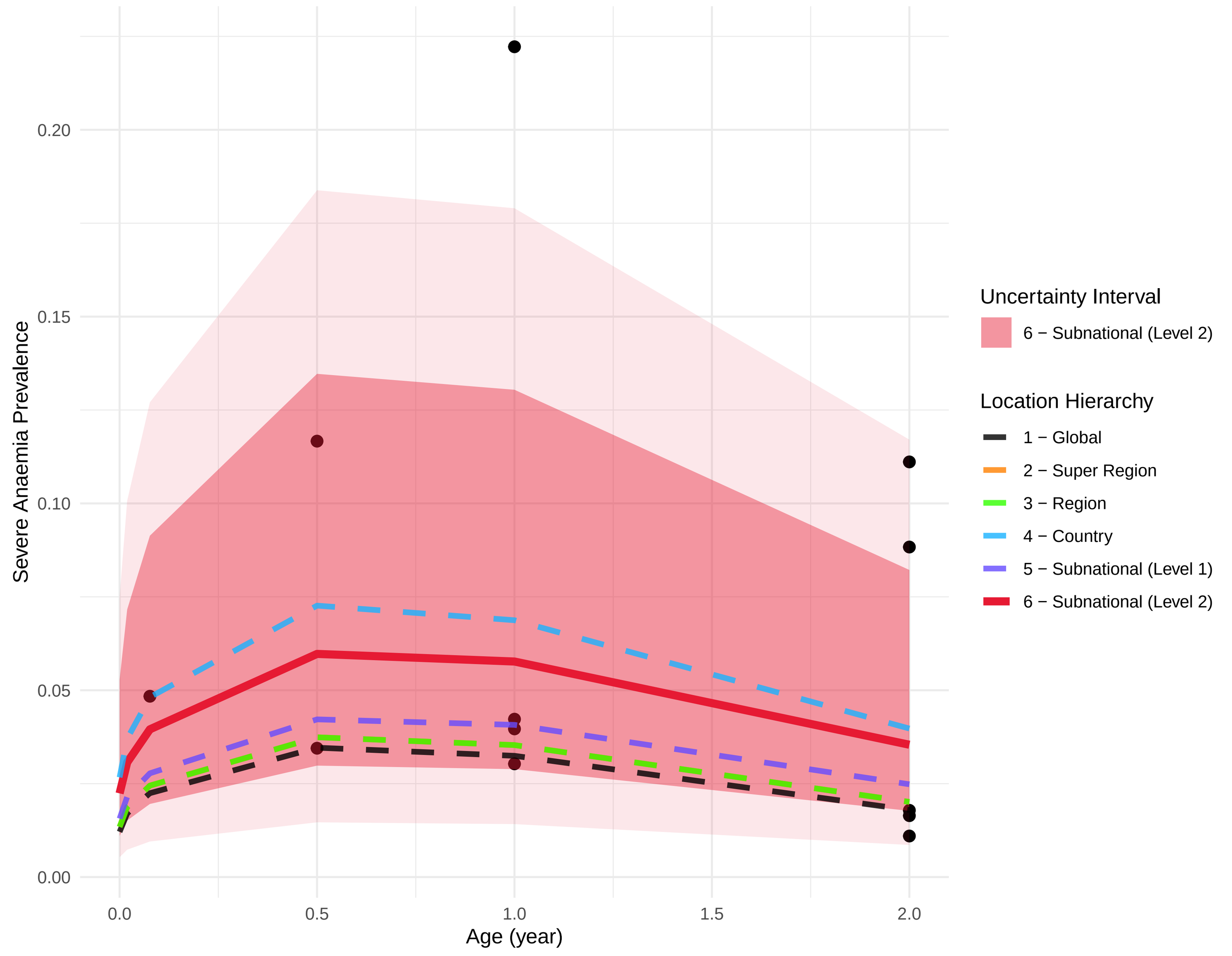

MR−BRT Age/Sex Fits − Males <2 Years Old − Severe Anaemia Prevalence − Delhi
WHO−2001 Adjustment Method

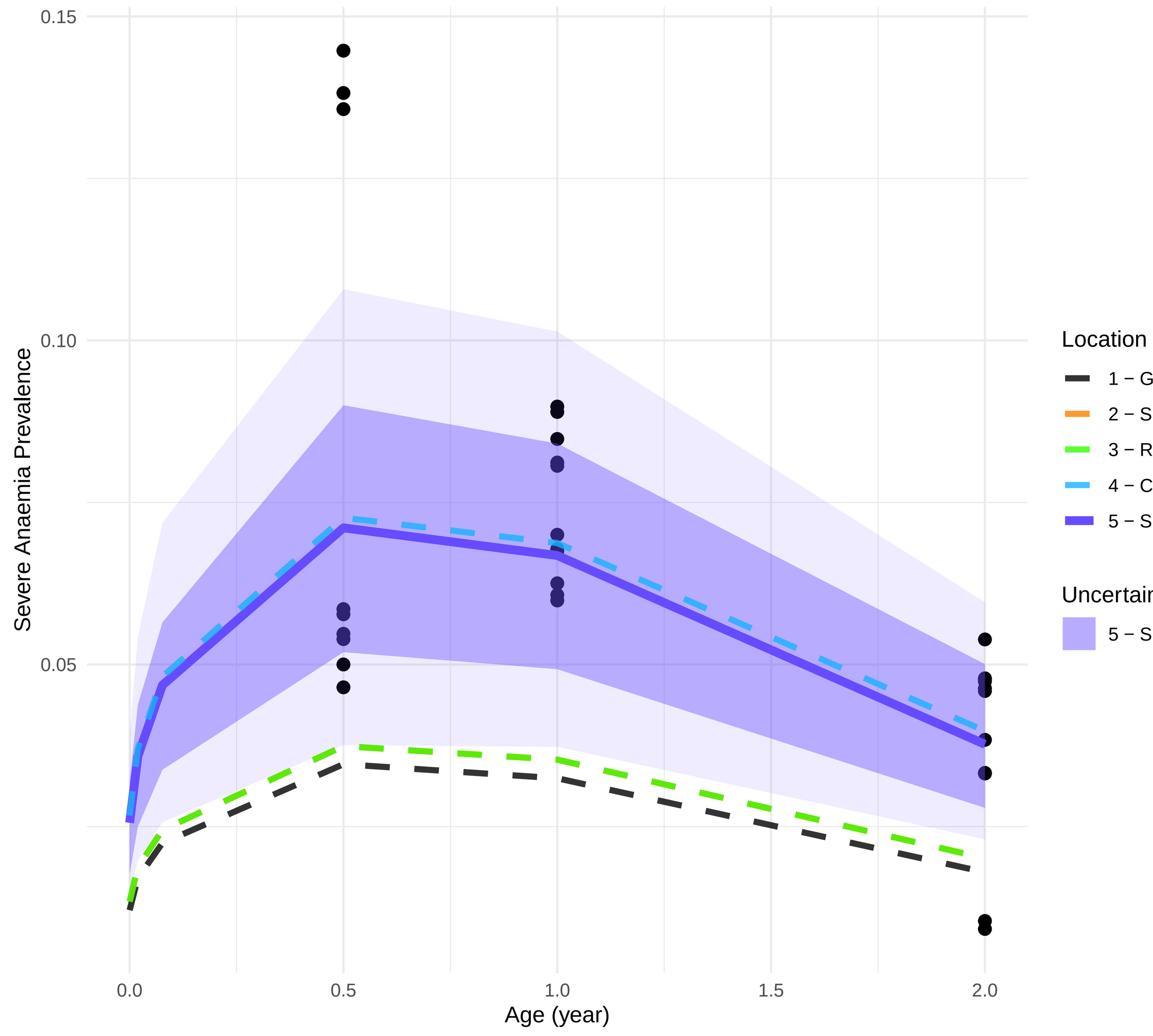

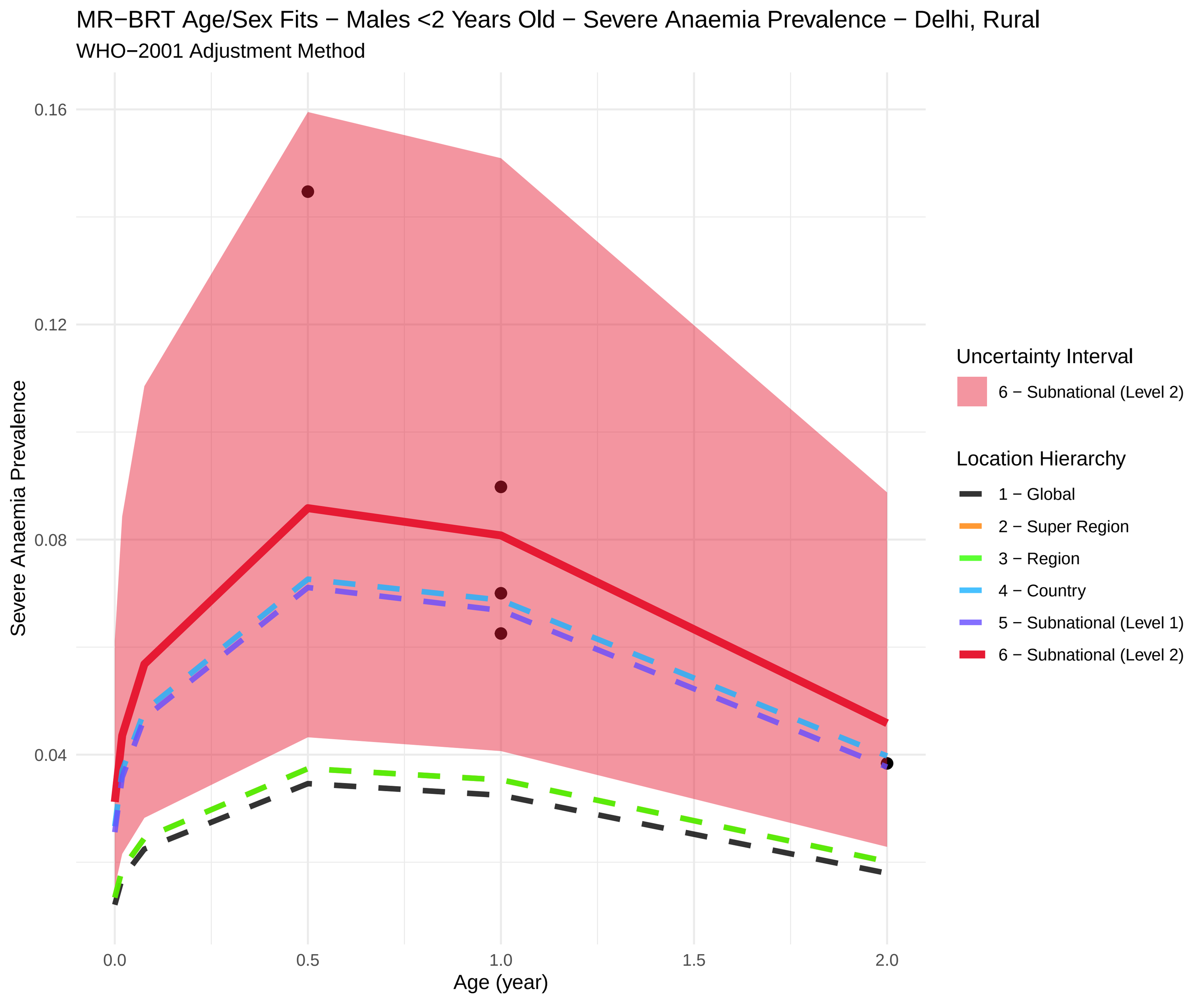
MR−BRT Age/Sex Fits − Males <2 Years Old − Severe Anaemia Prevalence − Delhi, Rural
WHO−2001 Adjustment Method
Severe Anaemia Prevalence
Age (year)
Uncertainty Interval
6 − Subnational (Level 2)
Location Hierarchy
1 − Global
2 − Super Region
3 − Region
4 − Country
5 − Subnational (Level 1)
6 − Subnational (Level 2)

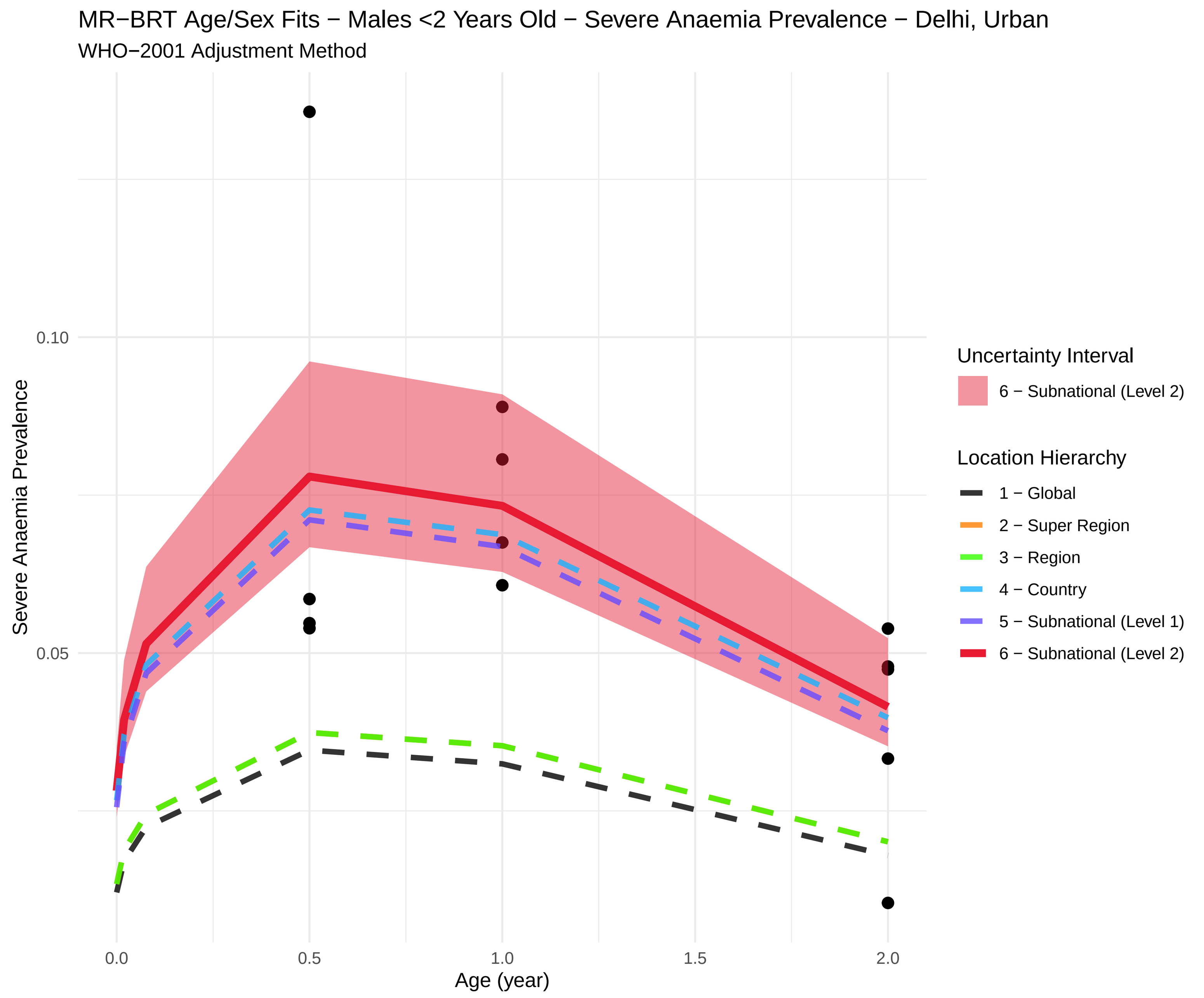
MR−BRT Age/Sex Fits − Males <2 Years Old − Severe Anaemia Prevalence − Delhi, Urban
WHO−2001 Adjustment Method
Severe Anaemia Prevalence
Age (year)
Uncertainty Interval
6 − Subnational (Level 2)
Location Hierarchy
1 − Global
2 − Super Region
3 − Region
4 − Country
5 − Subnational (Level 1)
6 − Subnational (Level 2)

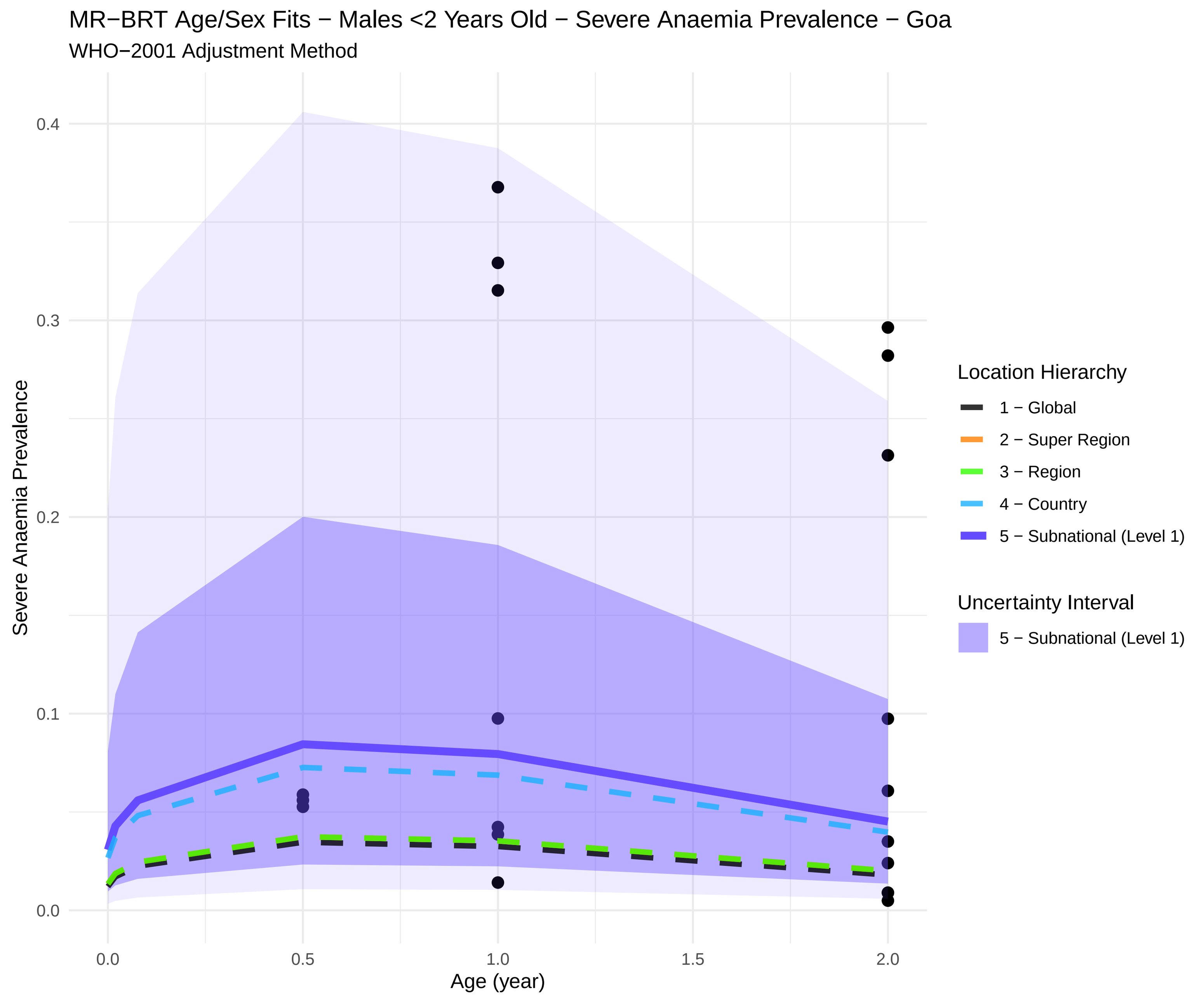
MR−BRT Age/Sex Fits − Males <2 Years Old − Severe Anaemia Prevalence − Goa
WHO−2001 Adjustment Method
Severe Anaemia Prevalence
Age (year)
Location Hierarchy
1 − Global
2 − Super Region
3 − Region
4 − Country
5 − Subnational (Level 1)
Uncertainty Interval
5 − Subnational (Level 1)

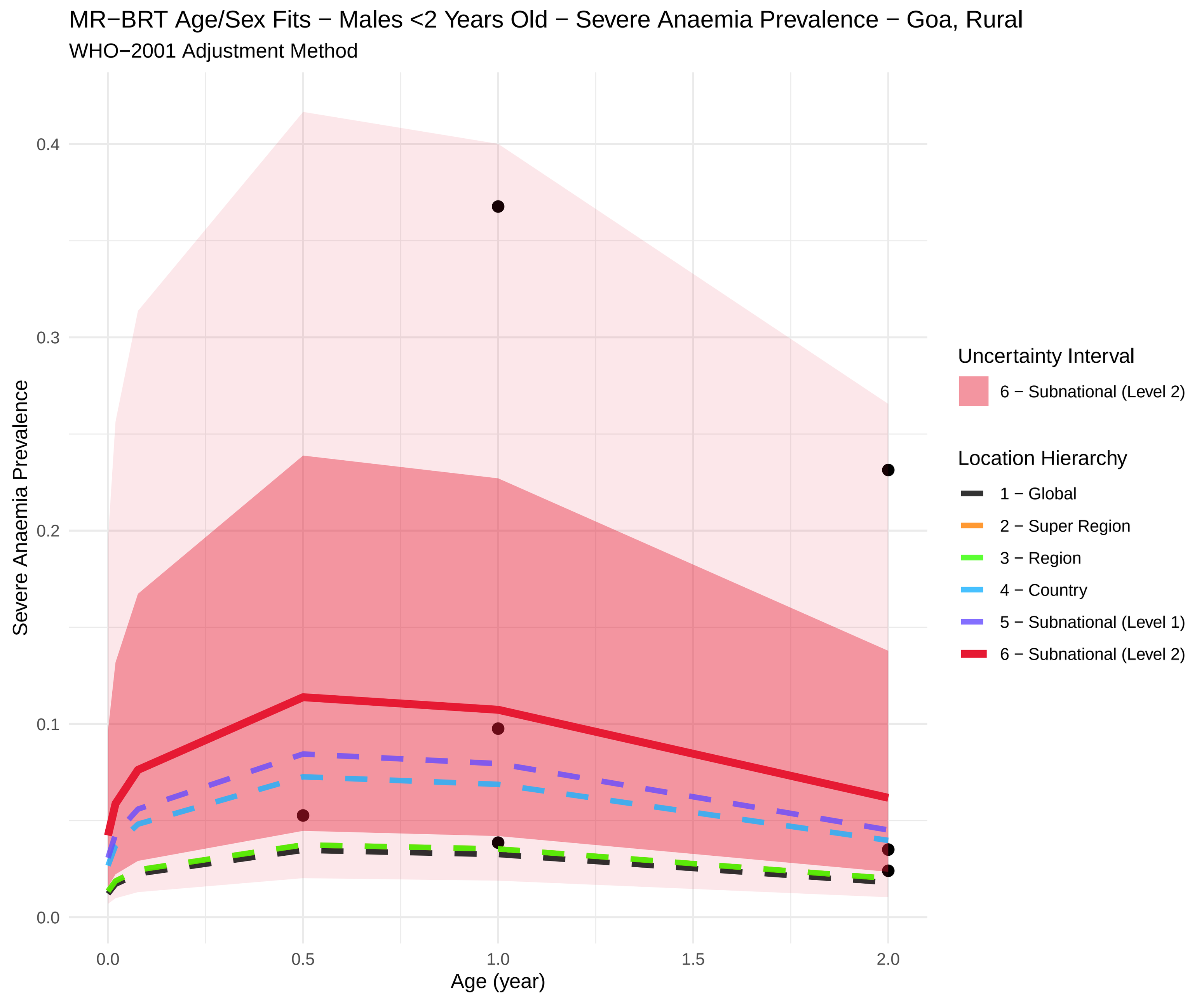
MR−BRT Age/Sex Fits − Males <2 Years Old − Severe Anaemia Prevalence − Goa, Rural
WHO−2001 Adjustment Method
Severe Anaemia Prevalence
Age (year)
Uncertainty Interval
6 − Subnational (Level 2)
Location Hierarchy
1 − Global
2 − Super Region
3 − Region
4 − Country
5 − Subnational (Level 1)
6 − Subnational (Level 2)

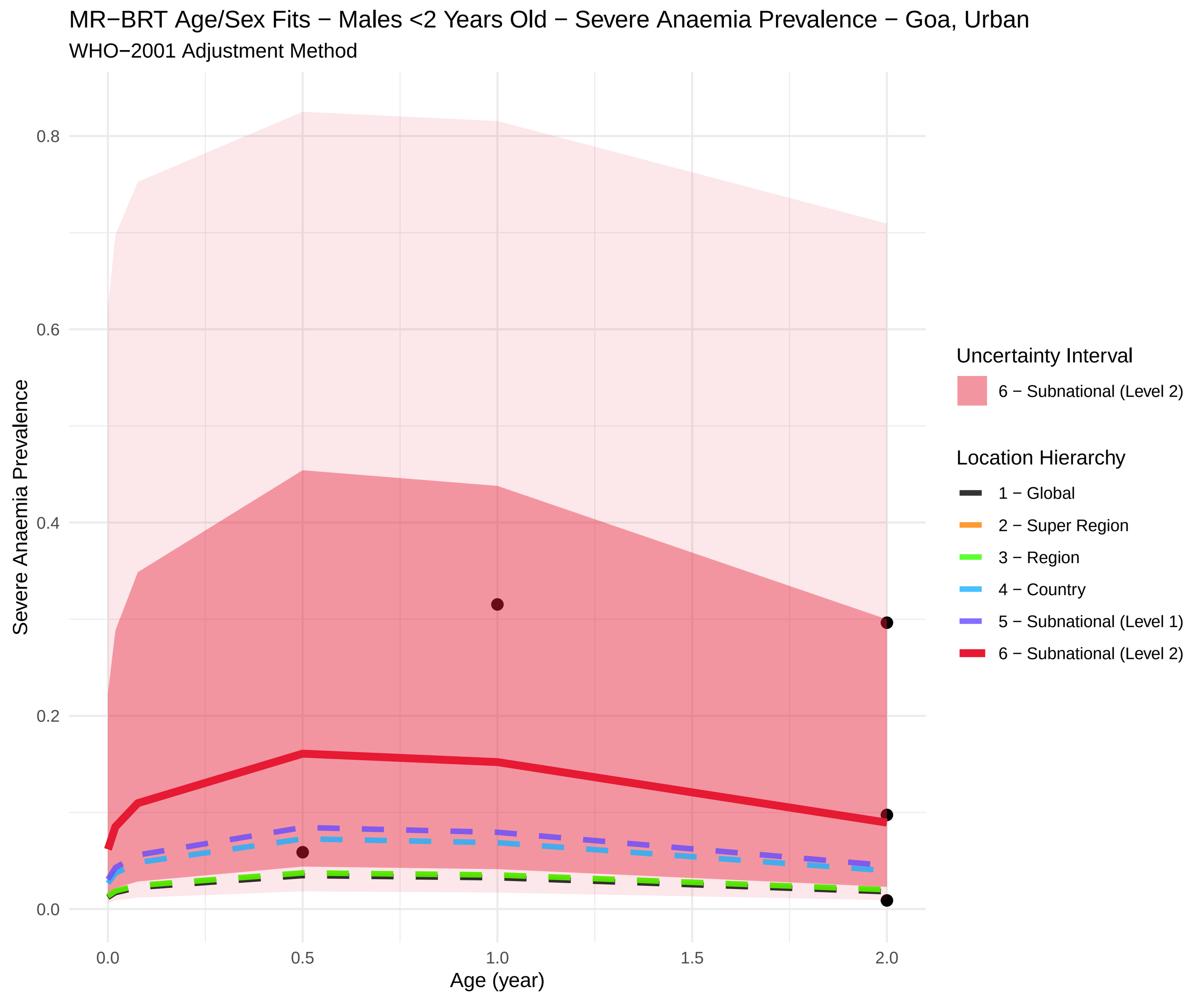
MR−BRT Age/Sex Fits − Males <2 Years Old − Severe Anaemia Prevalence − Goa, Urban
WHO−2001 Adjustment Method
Severe Anaemia Prevalence
Age (year)
Uncertainty Interval
6 − Subnational (Level 2)
Location Hierarchy
1 − Global
2 − Super Region
3 − Region
4 − Country
5 − Subnational (Level 1)
6 − Subnational (Level 2)

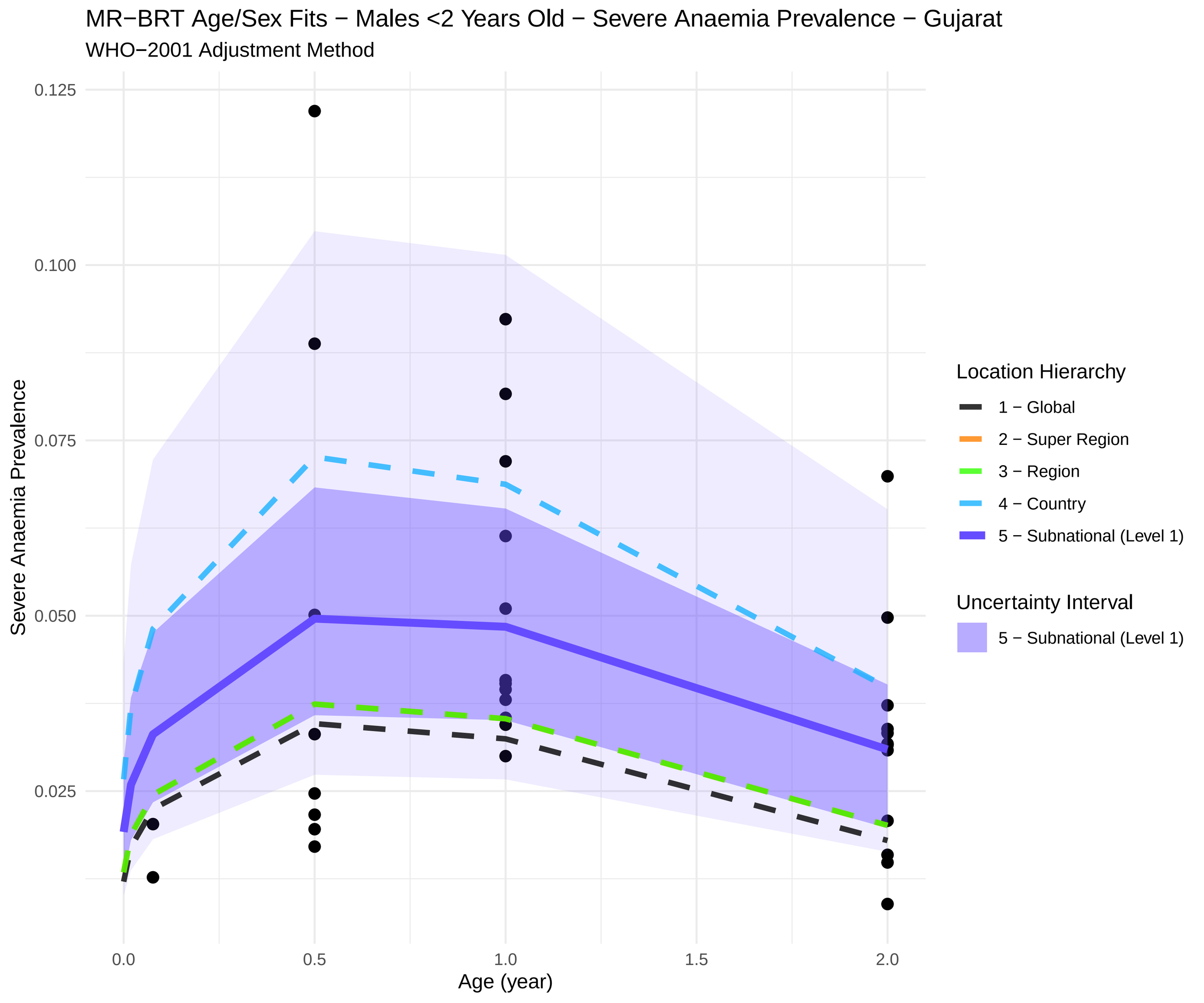
MR−BRT Age/Sex Fits − Males <2 Years Old − Severe Anaemia Prevalence − Gujarat
WHO−2001 Adjustment Method
Severe Anaemia Prevalence
Age (year)
Location Hierarchy
1 − Global
2 − Super Region
3 − Region
4 − Country
5 − Subnational (Level 1)
Uncertainty Interval
5 − Subnational (Level 1)

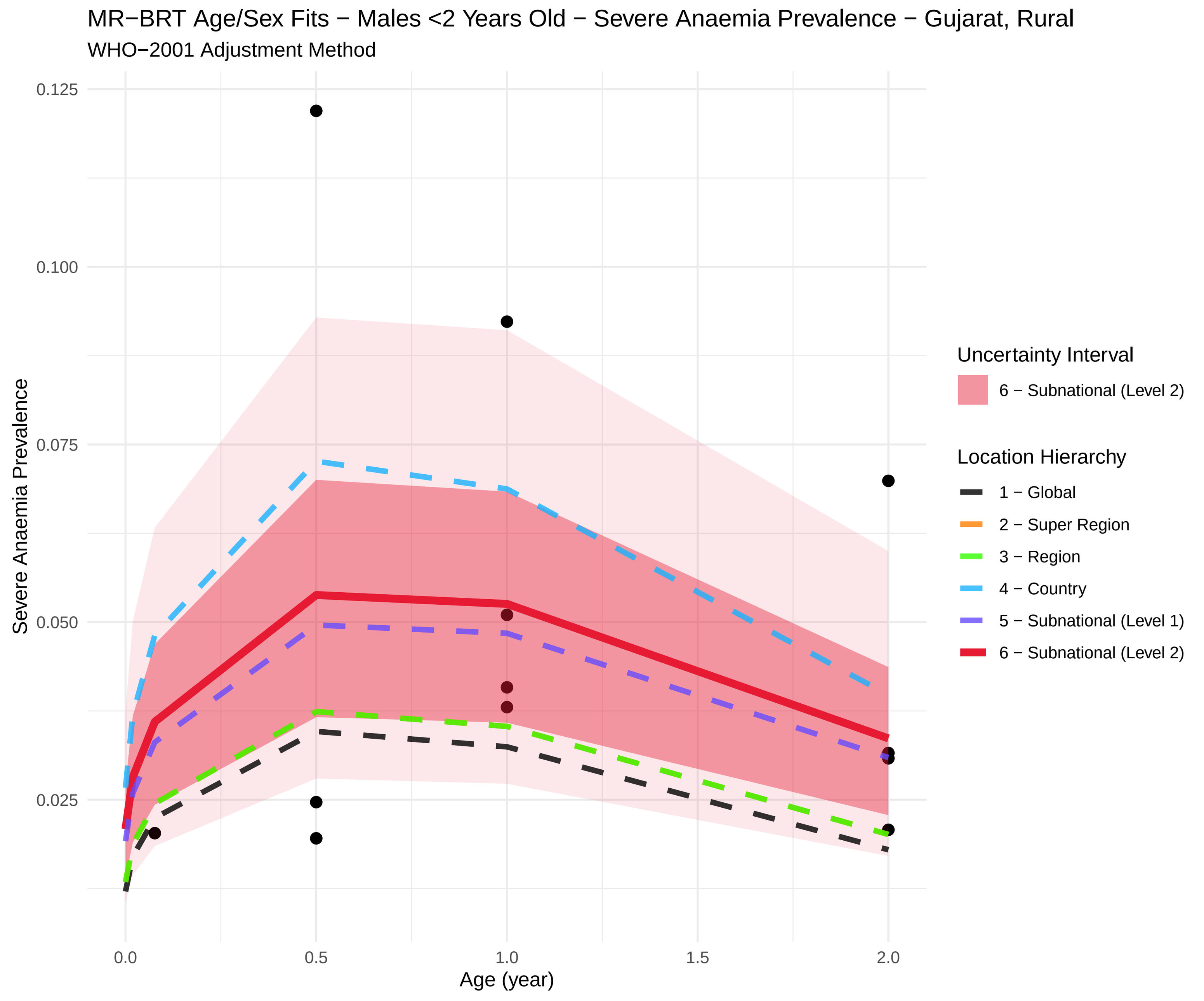
MR−BRT Age/Sex Fits − Males <2 Years Old − Severe Anaemia Prevalence − Gujarat, Rural
WHO−2001 Adjustment Method
Severe Anaemia Prevalence
Age (year)
Uncertainty Interval
6 − Subnational (Level 2)
Location Hierarchy
1 − Global
2 − Super Region
3 − Region
4 − Country
5 − Subnational (Level 1)
6 − Subnational (Level 2)

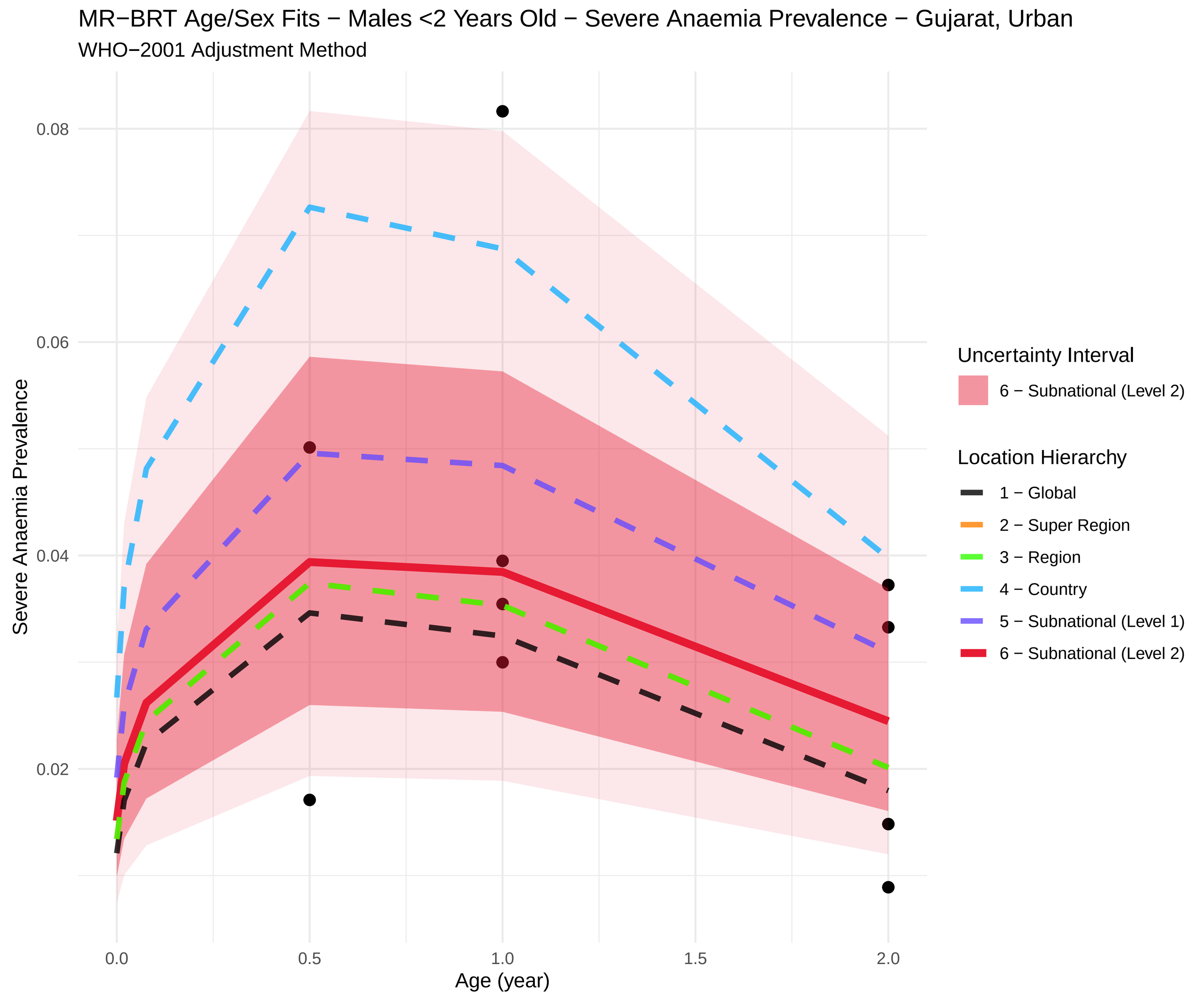
MR−BRT Age/Sex Fits − Males <2 Years Old − Severe Anaemia Prevalence − Gujarat, Urban
WHO−2001 Adjustment Method
Severe Anaemia Prevalence
Age (year)
Uncertainty Interval
6 − Subnational (Level 2)
Location Hierarchy
1 − Global
2 − Super Region
3 − Region
4 − Country
5 − Subnational (Level 1)
6 − Subnational (Level 2)

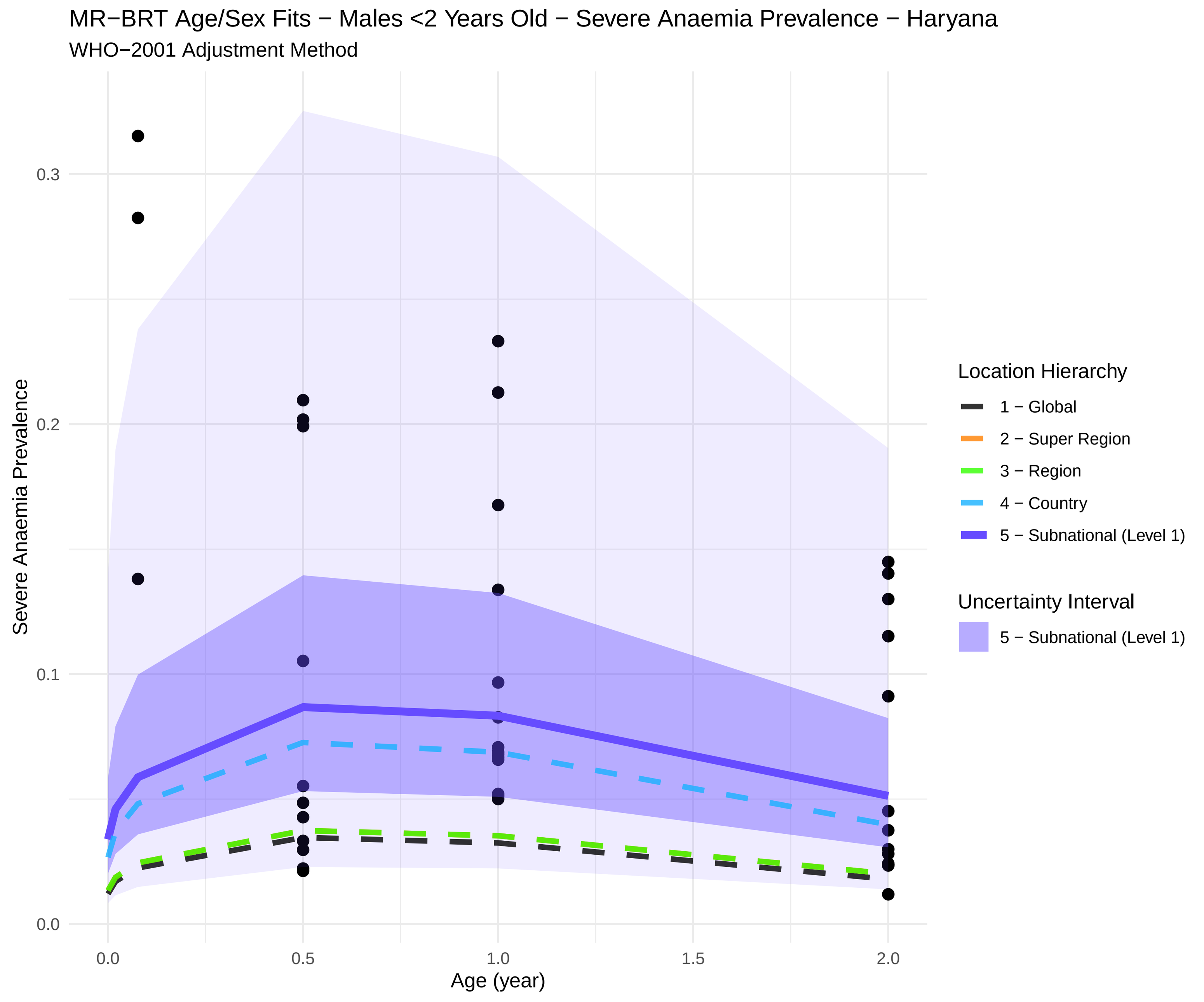
MR−BRT Age/Sex Fits − Males <2 Years Old − Severe Anaemia Prevalence − Haryana
WHO−2001 Adjustment Method
Severe Anaemia Prevalence
Age (year)
Location Hierarchy
1 − Global
2 − Super Region
3 − Region
4 − Country
5 − Subnational (Level 1)
Uncertainty Interval
5 − Subnational (Level 1)

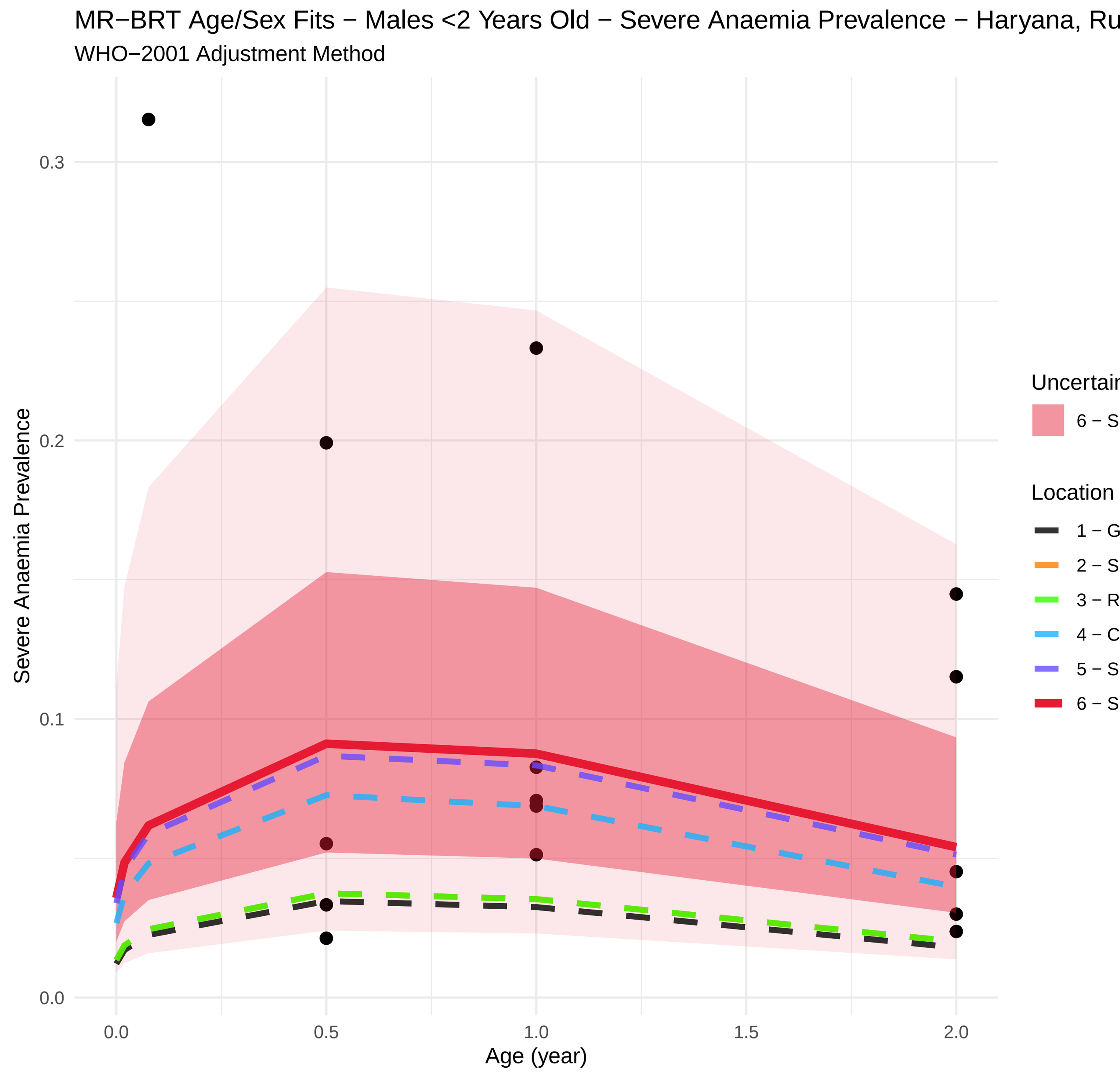
MR−BRT Age/Sex Fits − Males <2 Years Old − Severe Anaemia Prevalence − Haryana, Rural
WHO−2001 Adjustment Method
Severe Anaemia Prevalence
Age (year)
Uncertainty Interval
6 − Subnational (Level 2)
Location Hierarchy
1 − Global
2 − Super Region
3 − Region
4 − Country
5 − Subnational (Level 1)
6 − Subnational (Level 2)

MR−BRT Age/Sex Fits − Males <2 Years Old − Severe Anaemia Prevalence − Haryana, Urban
WHO−2001 Adjustment Method

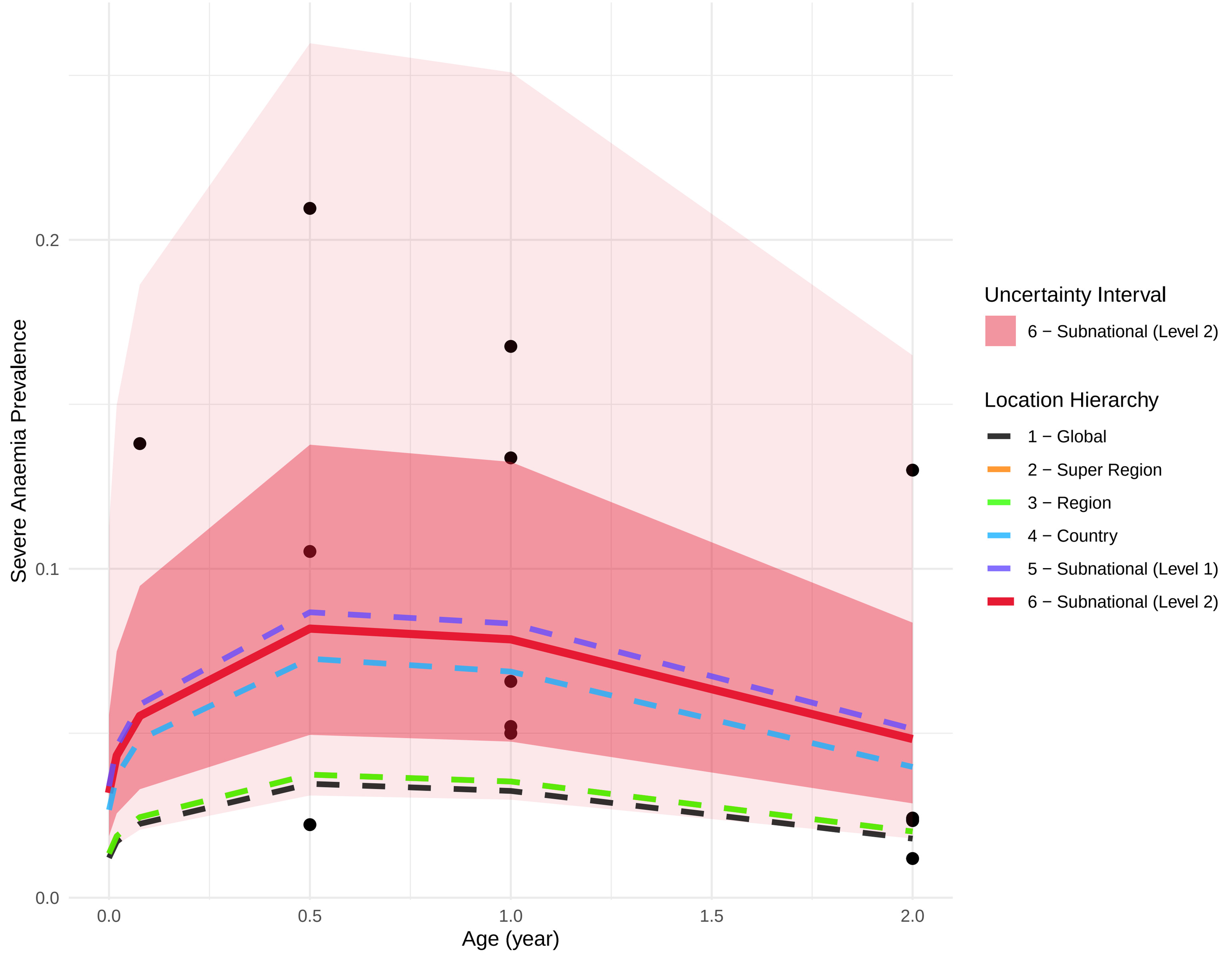

MR−BRT Age/Sex Fits − Males <2 Years Old − Severe Anaemia Prevalence − Himachal Pradesh
WHO−2001 Adjustment Method

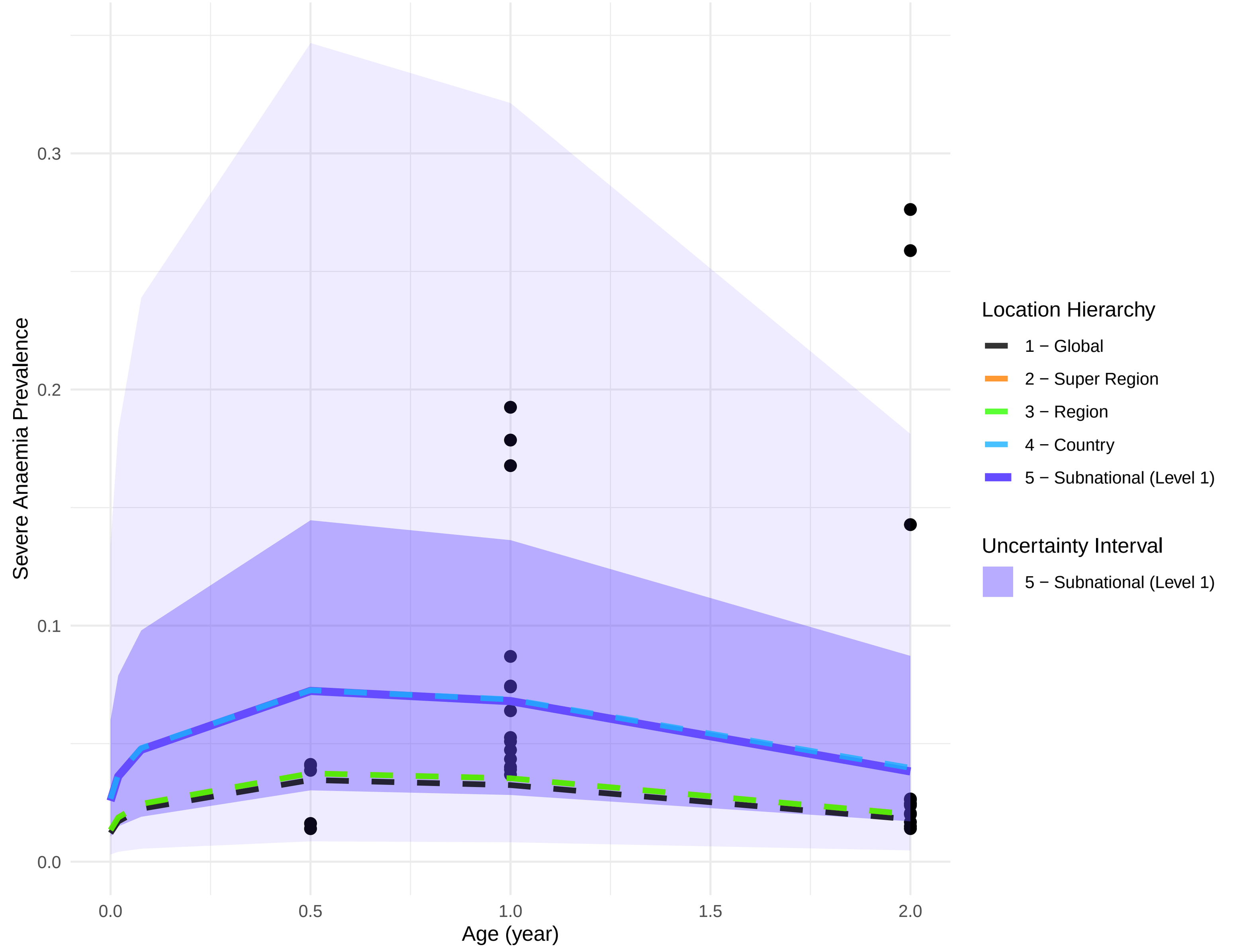

MR−BRT Age/Sex Fits − Males <2 Years Old − Severe Anaemia Prevalence − Himachal Pradesh, Rural
WHO−2001 Adjustment Method

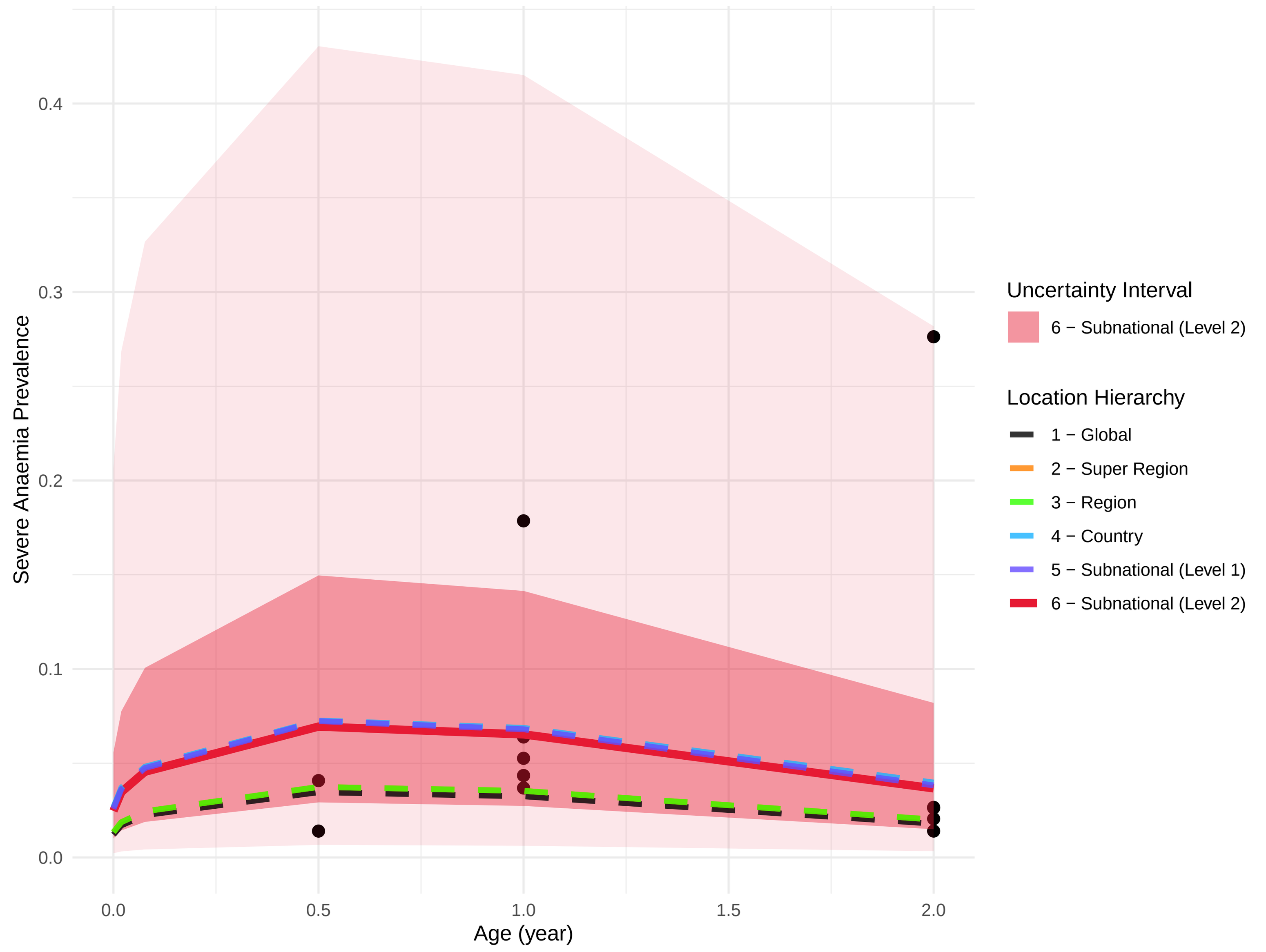

MR−BRT Age/Sex Fits − Males <2 Years Old − Severe Anaemia Prevalence − Himachal Pradesh, Urban
WHO−2001 Adjustment Method

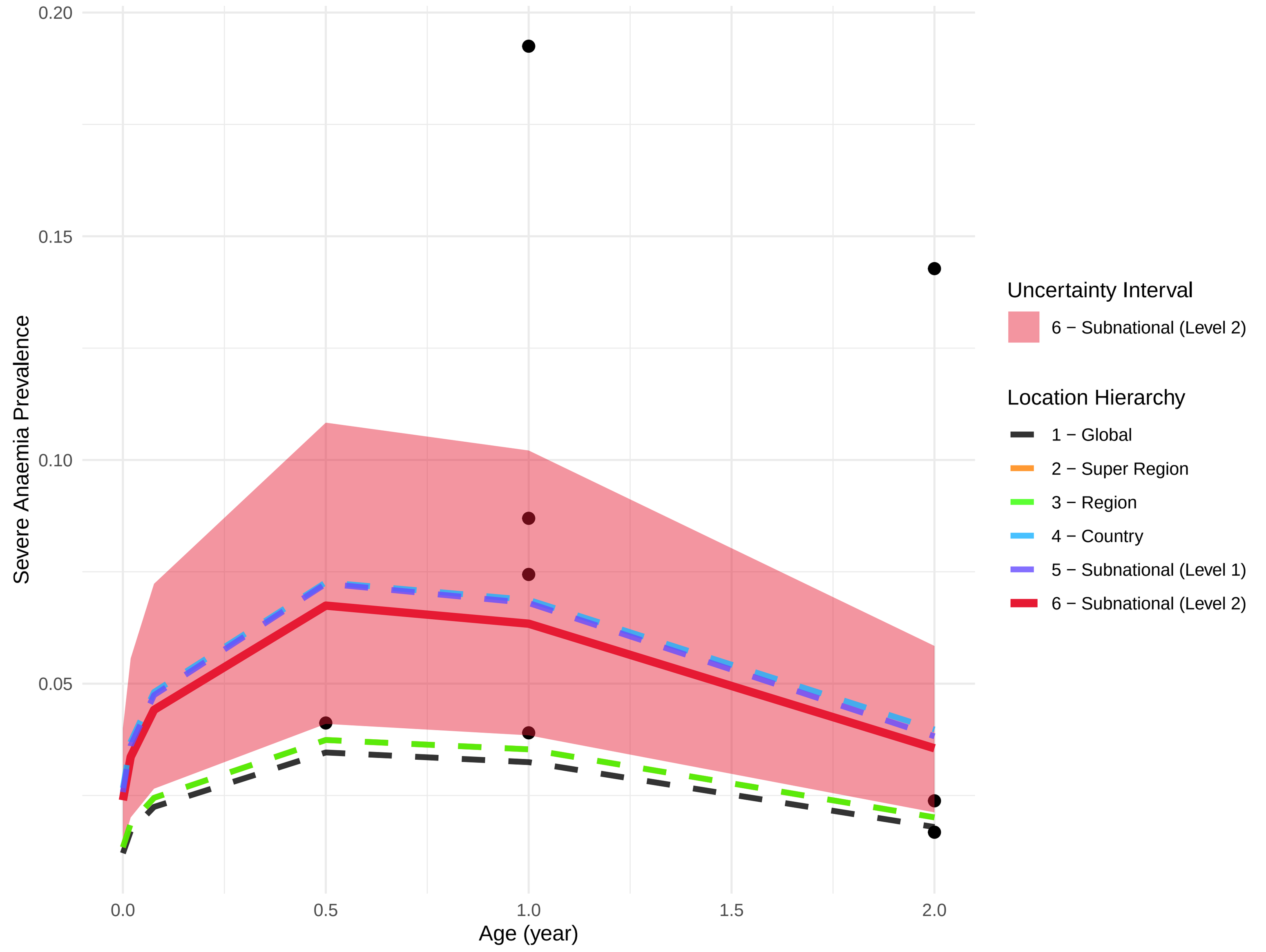

MR−BRT Age/Sex Fits − Males <2 Years Old − Severe Anaemia Prevalence − Jammu & Kashmir and Ladakh
WHO−2001 Adjustment Method

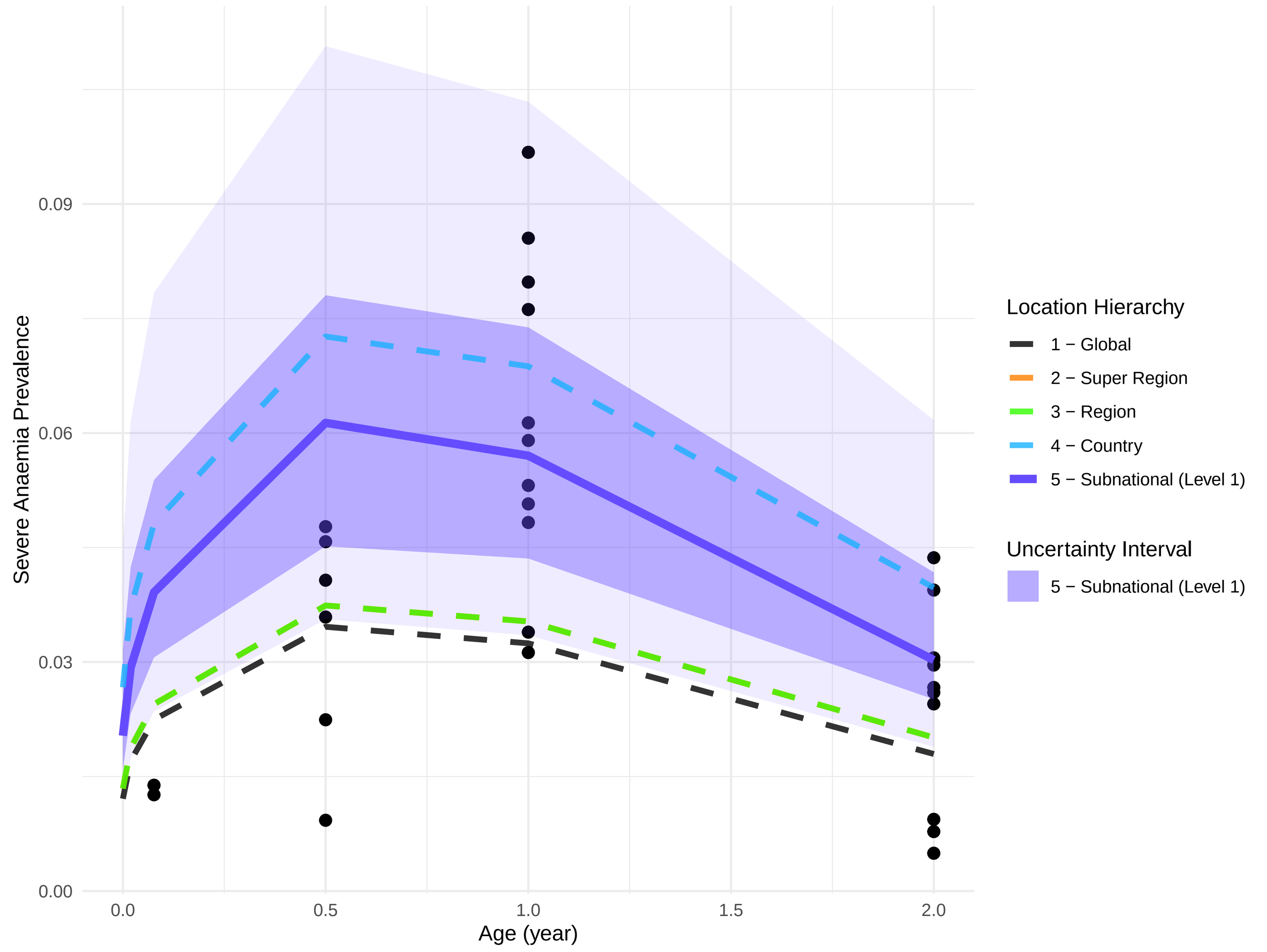

MR−BRT Age/Sex Fits − Males <2 Years Old − Severe Anaemia Prevalence − Jammu & Kashmir and Ladakh, Rural
WHO−2001 Adjustment Method

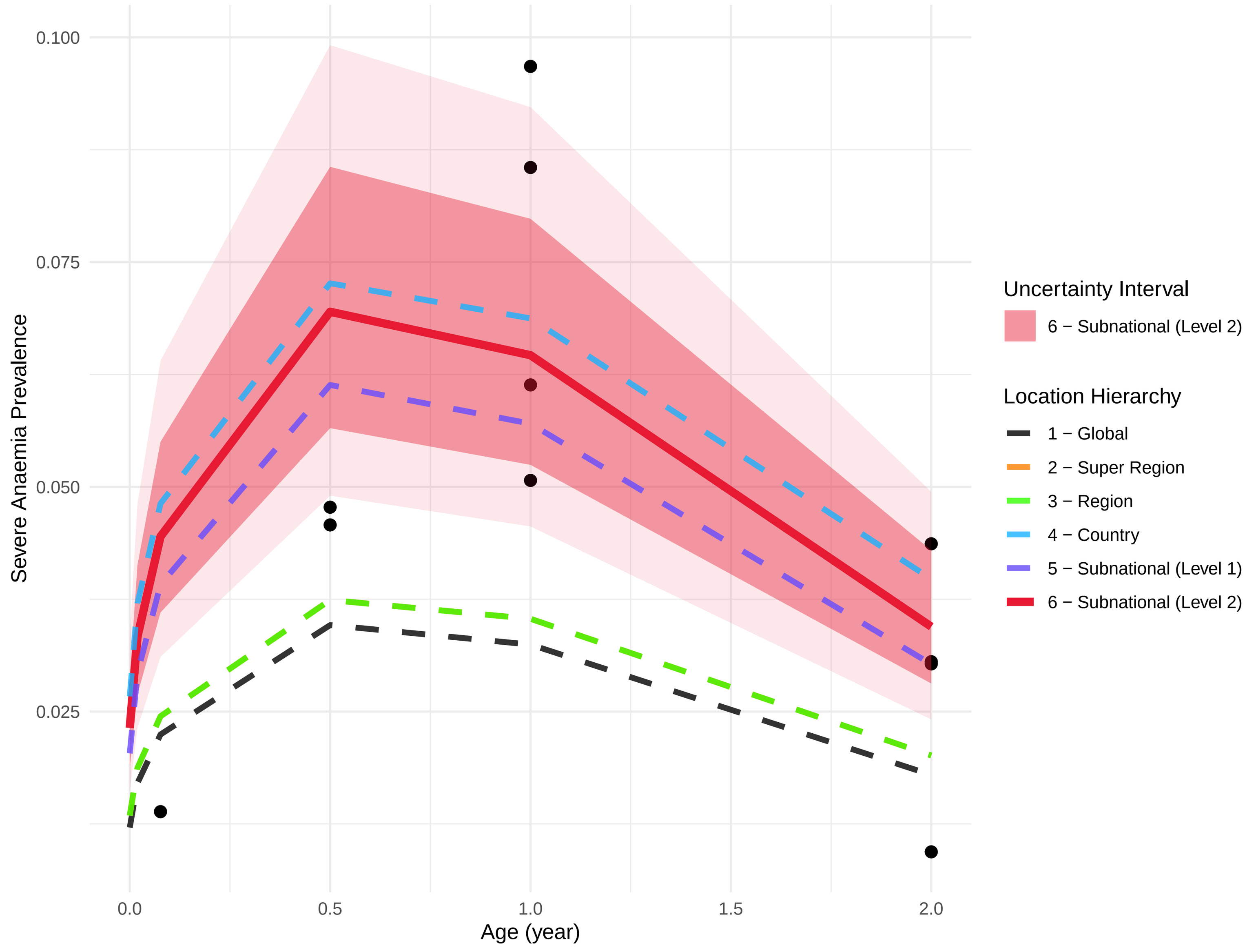

MR−BRT Age/Sex Fits − Males <2 Years Old − Severe Anaemia Prevalence − Jammu & Kashmir and Ladakh, Urban
WHO−2001 Adjustment Method

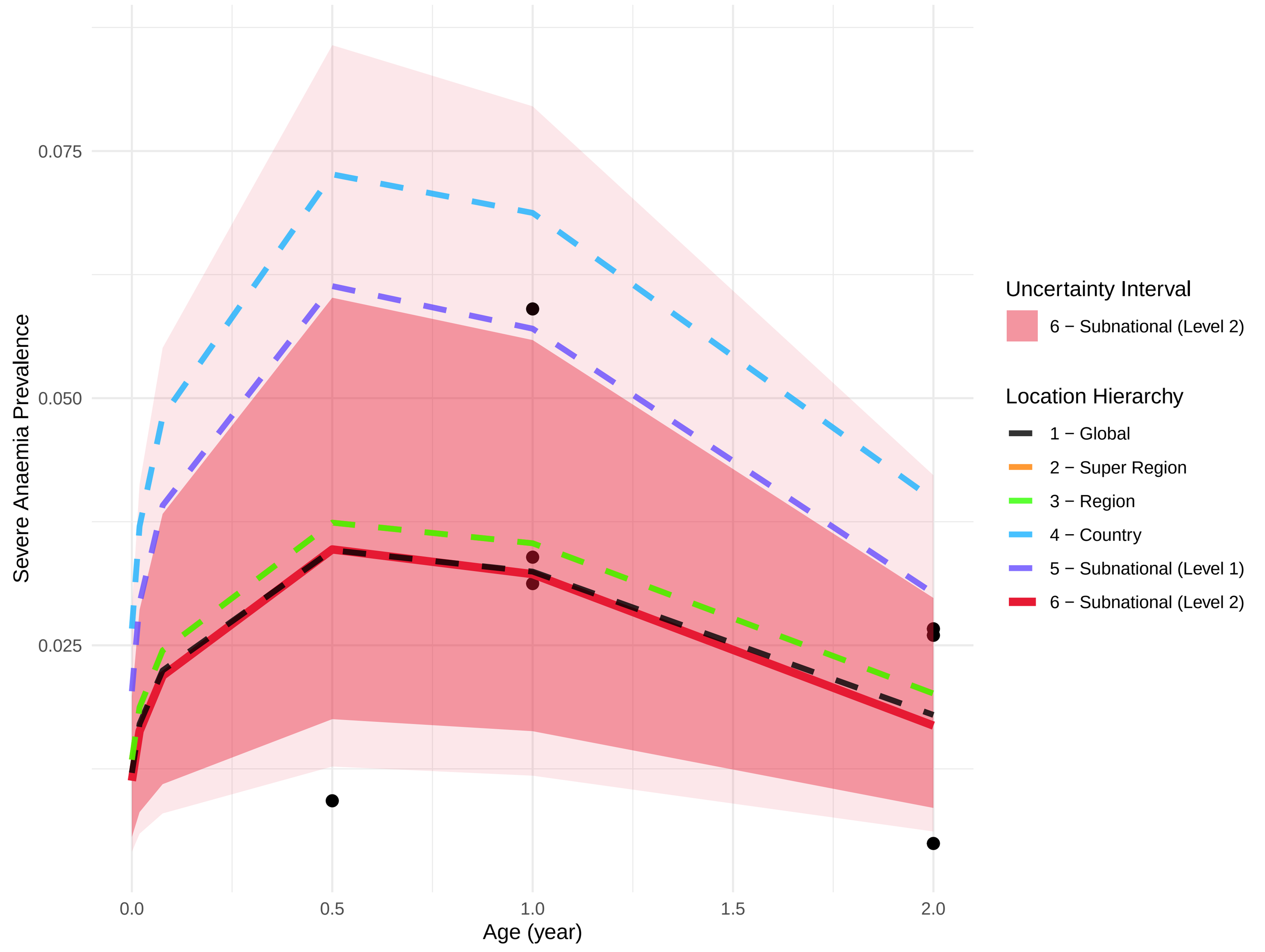

MR−BRT Age/Sex Fits − Males <2 Years Old − Severe Anaemia Prevalence − Jharkhand
WHO−2001 Adjustment Method

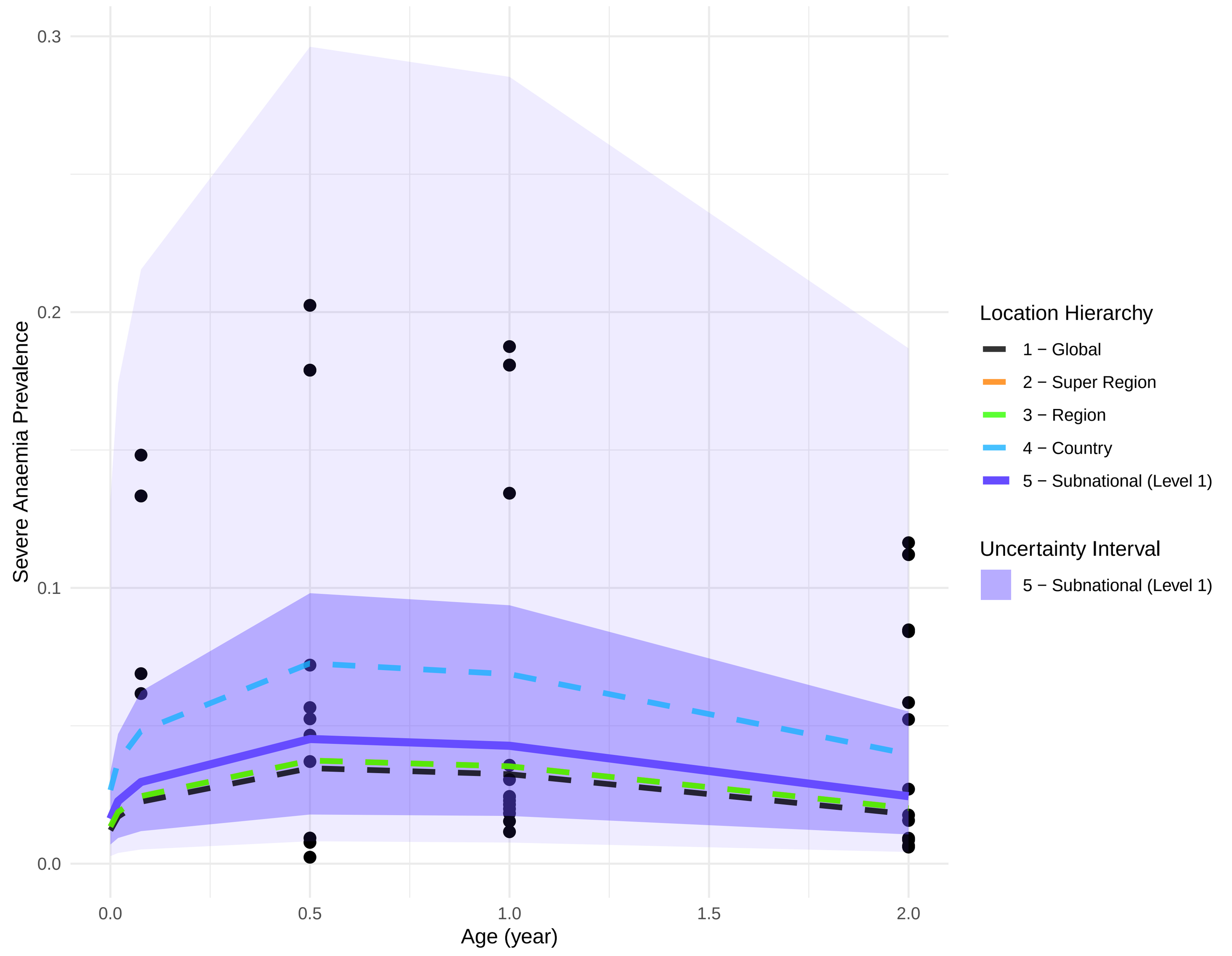

MR−BRT Age/Sex Fits − Males <2 Years Old − Severe Anaemia Prevalence − Jharkhand, Rural
WHO−2001 Adjustment Method

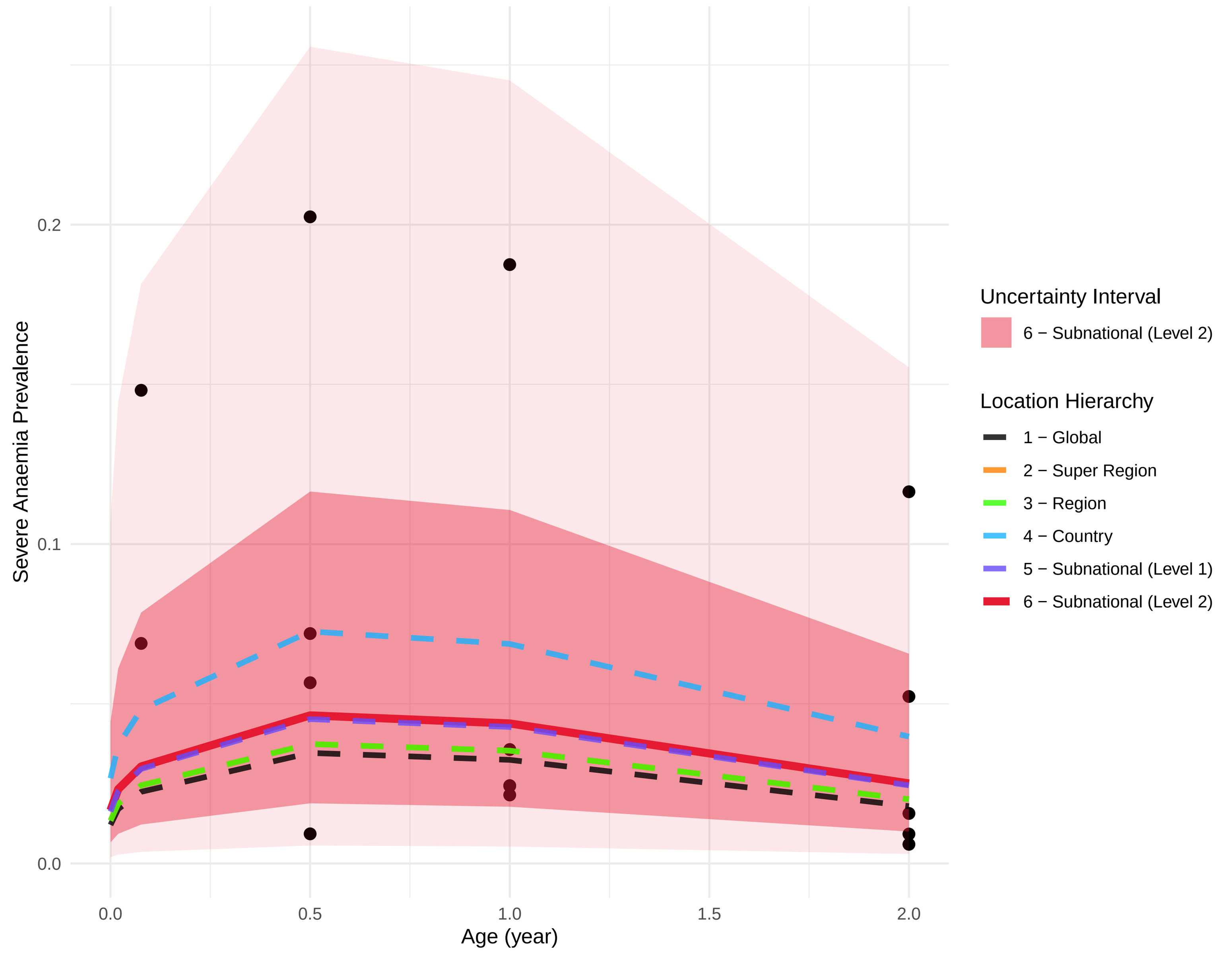

MR−BRT Age/Sex Fits − Males <2 Years Old − Severe Anaemia Prevalence − Jharkhand, Urban
WHO−2001 Adjustment Method

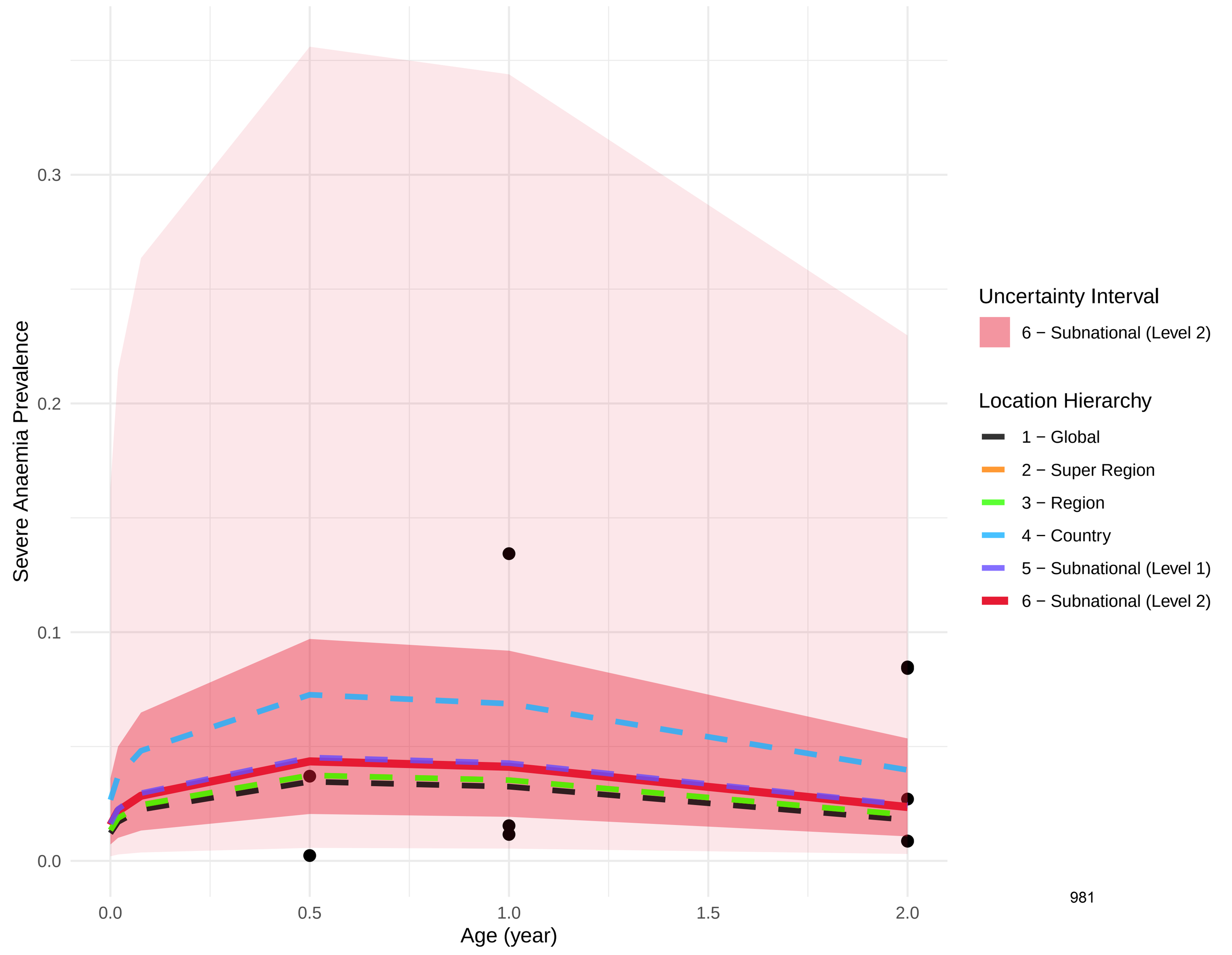

MR−BRT Age/Sex Fits − Males <2 Years Old − Severe Anaemia Prevalence − Karnataka
WHO−2001 Adjustment Method

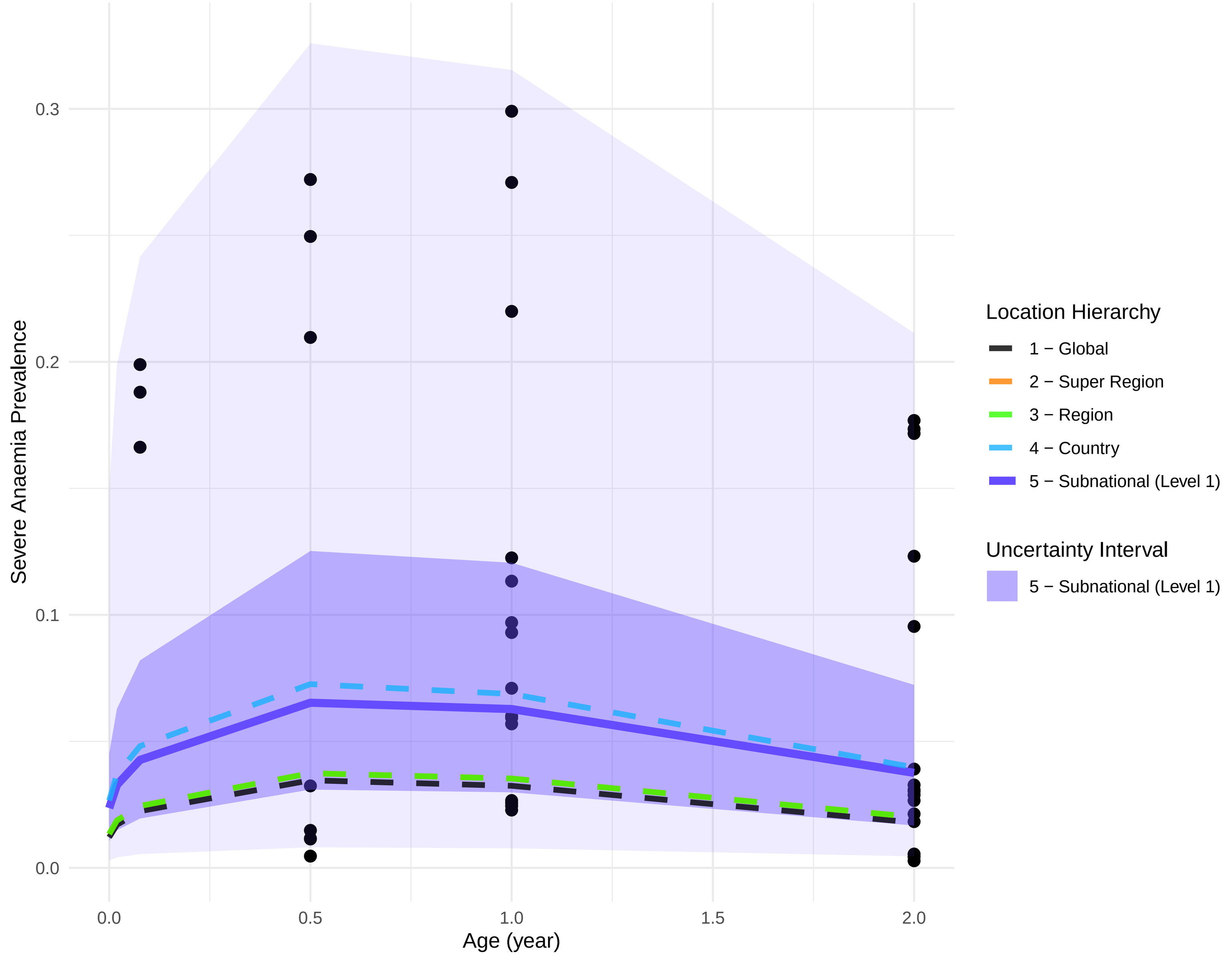

MR−BRT Age/Sex Fits − Males <2 Years Old − Severe Anaemia Prevalence − Karnataka, Rural
WHO−2001 Adjustment Method

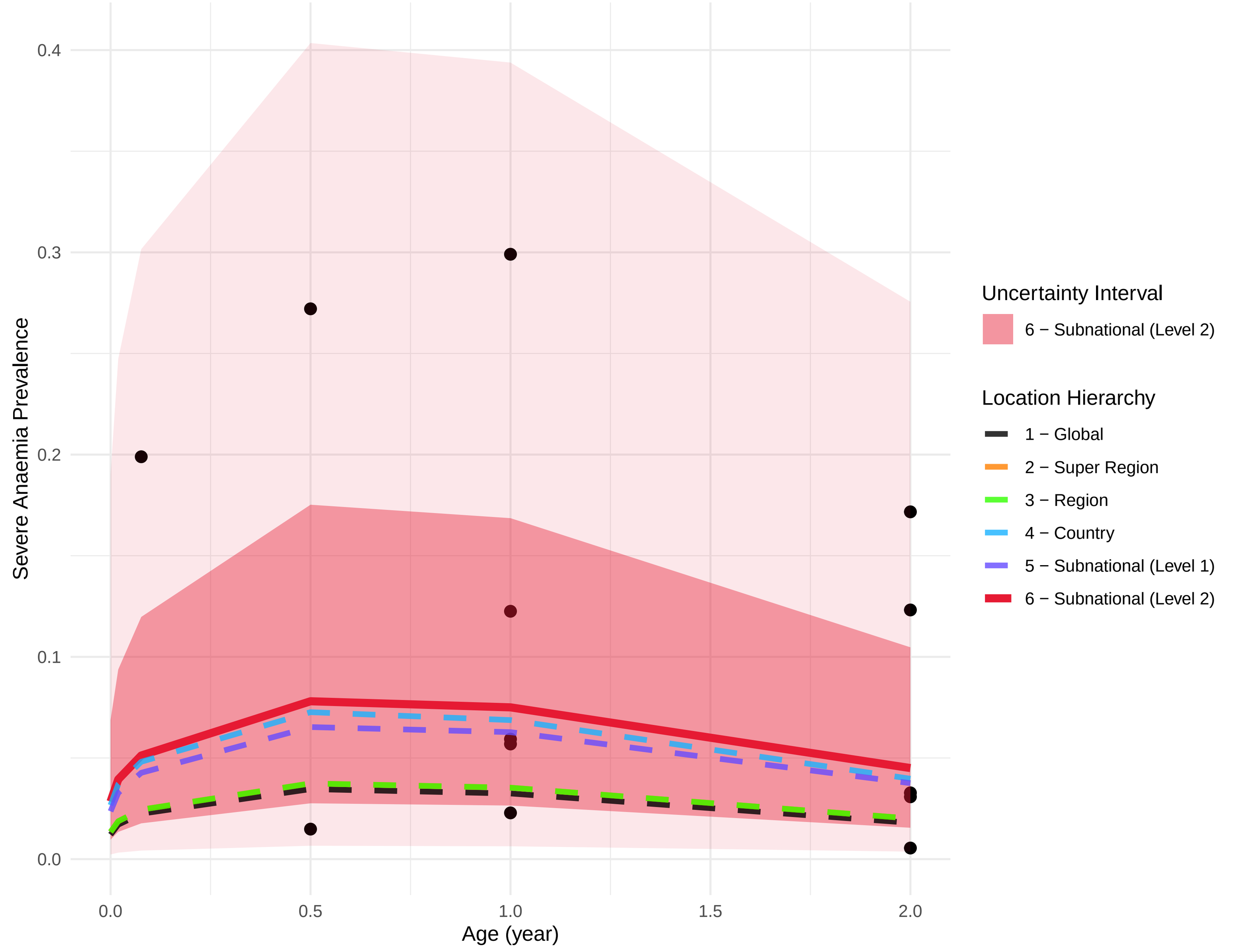

MR−BRT Age/Sex Fits − Males <2 Years Old − Severe Anaemia Prevalence − Karnataka, Urban
WHO−2001 Adjustment Method

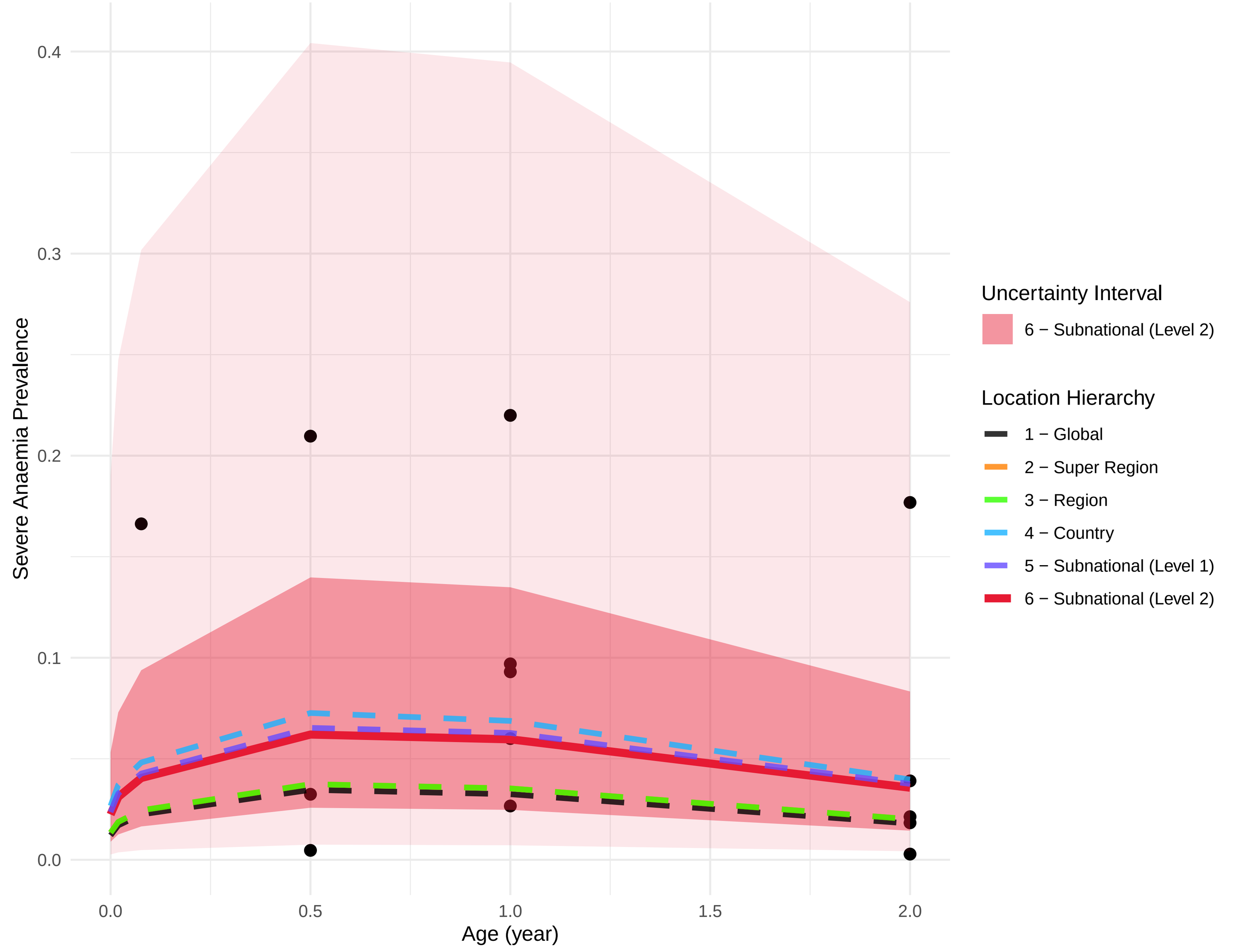

MR−BRT Age/Sex Fits − Males <2 Years Old − Severe Anaemia Prevalence − Kerala
WHO−2001 Adjustment Method

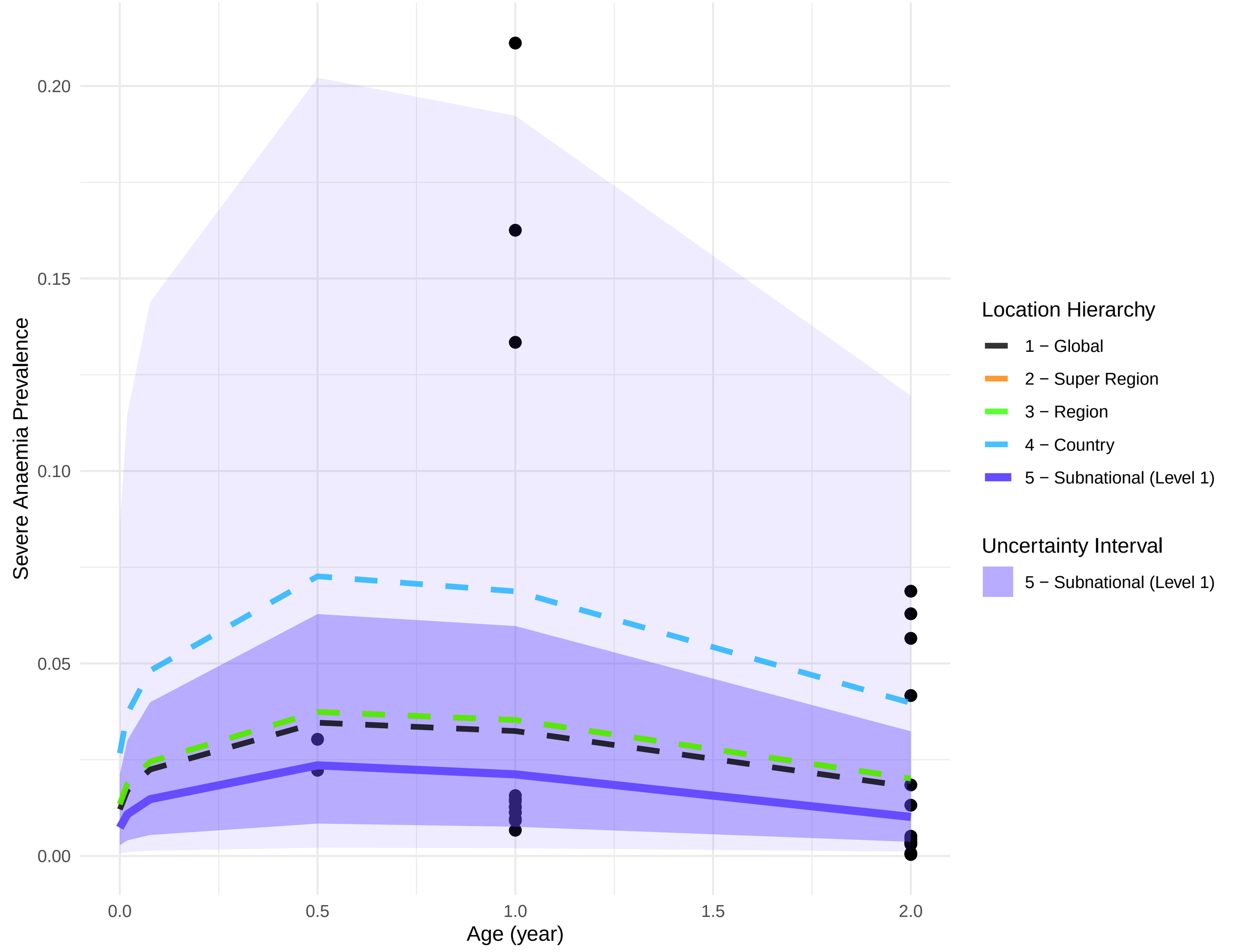

MR−BRT Age/Sex Fits − Males <2 Years Old − Severe Anaemia Prevalence − Kerala, Rural
WHO−2001 Adjustment Method

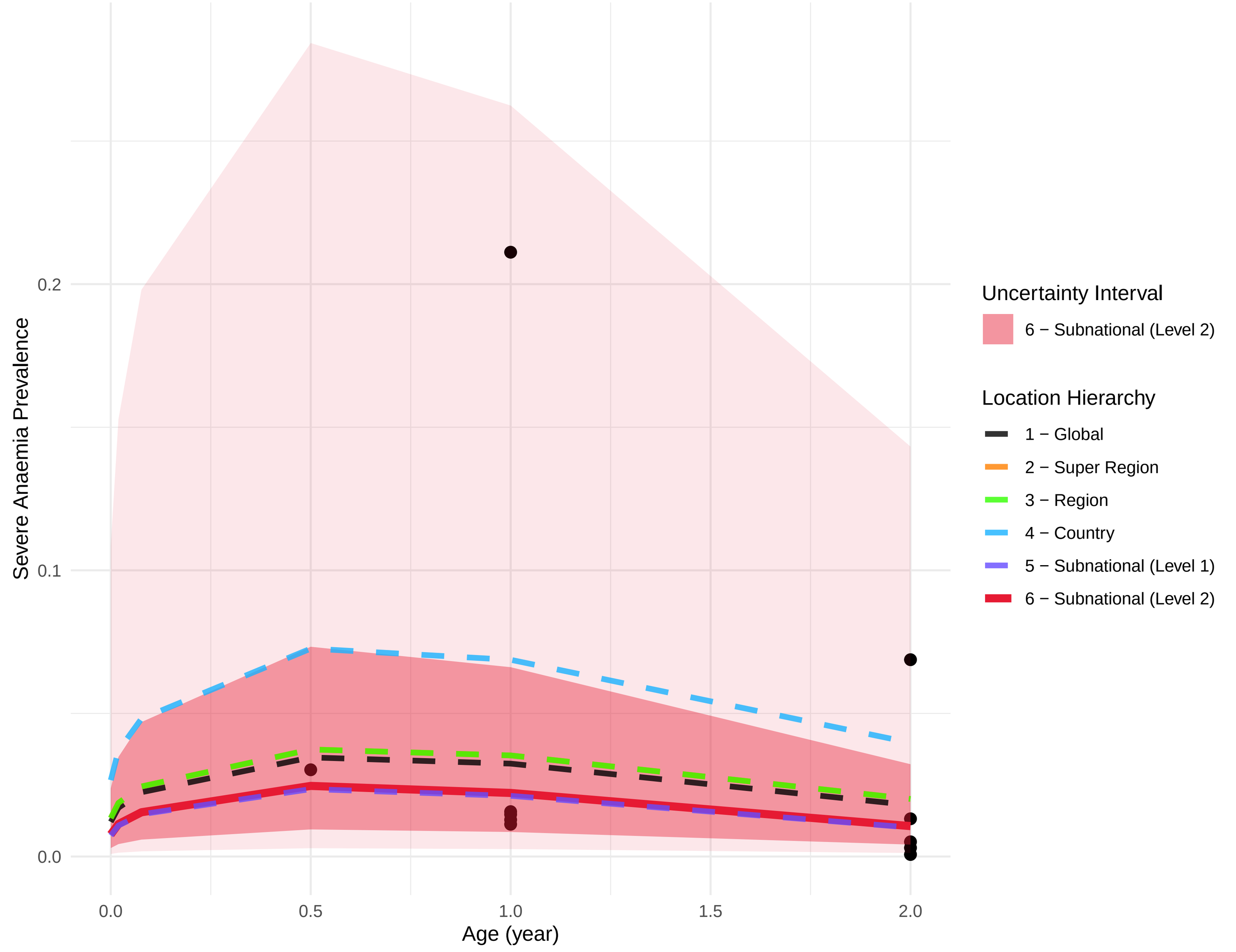

MR−BRT Age/Sex Fits − Males <2 Years Old − Severe Anaemia Prevalence − Kerala, Urban
WHO−2001 Adjustment Method

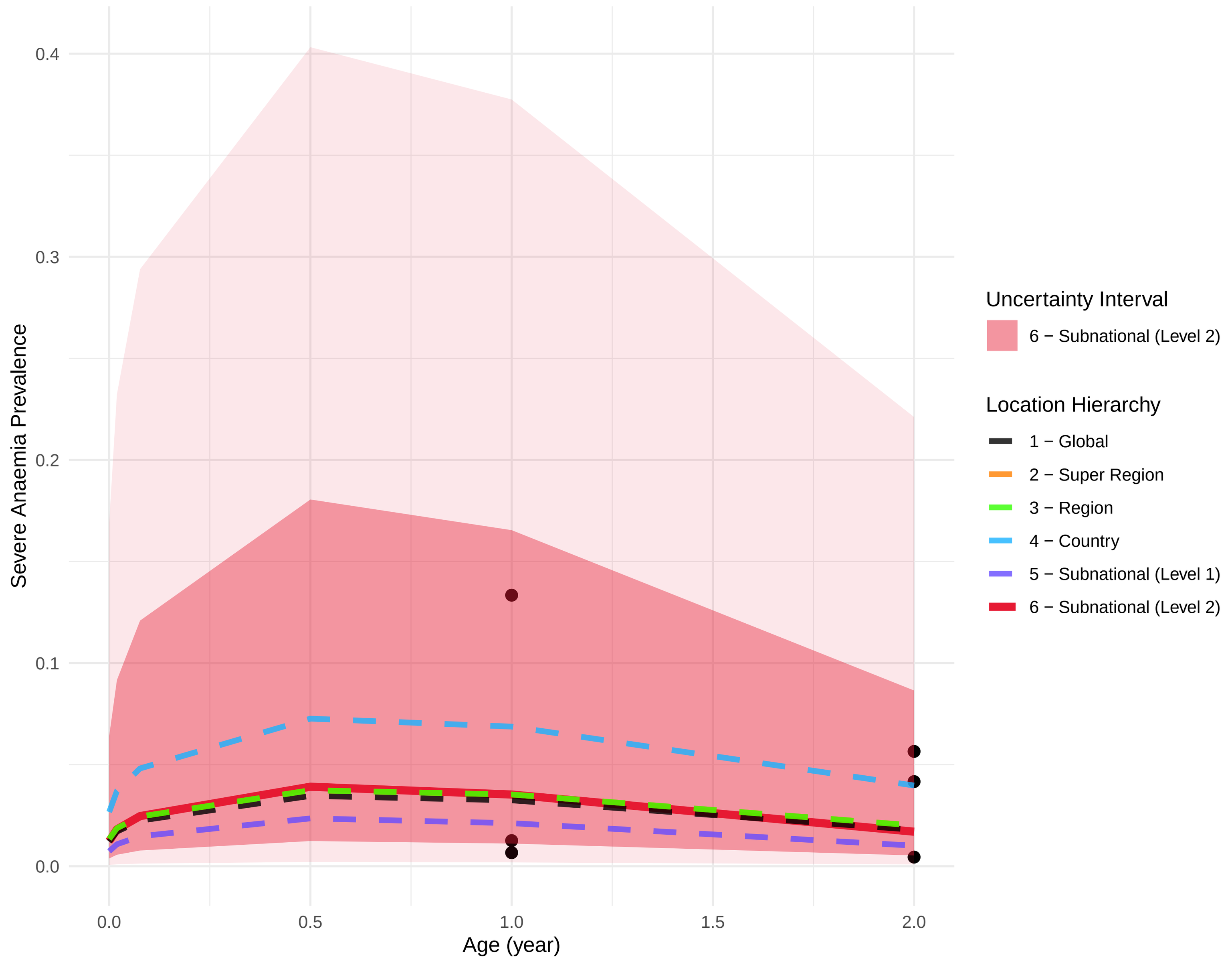

MR−BRT Age/Sex Fits − Males <2 Years Old − Severe Anaemia Prevalence − Madhya Pradesh
WHO−2001 Adjustment Method

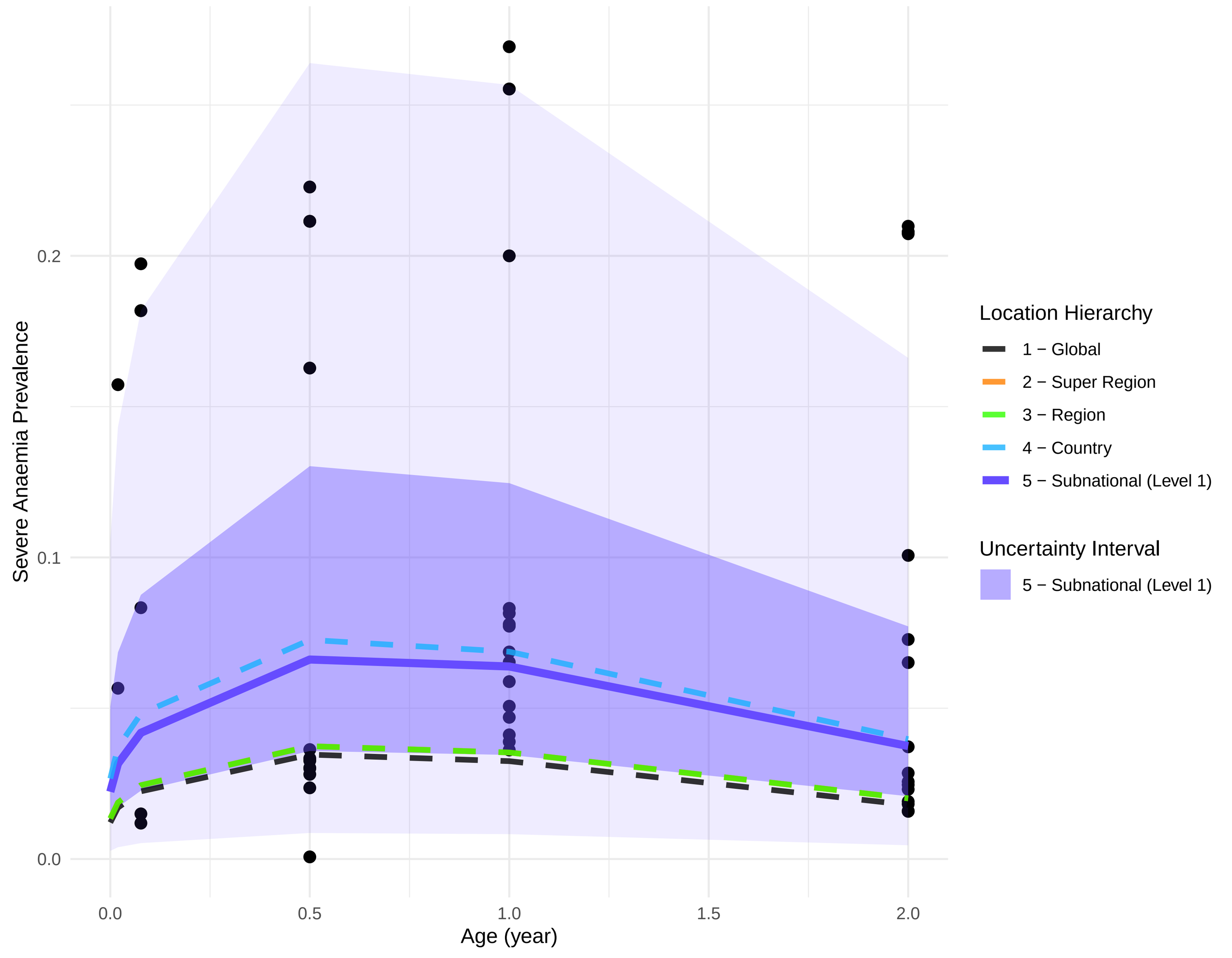

MR−BRT Age/Sex Fits − Males <2 Years Old − Severe Anaemia Prevalence − Madhya Pradesh, Rural
WHO−2001 Adjustment Method

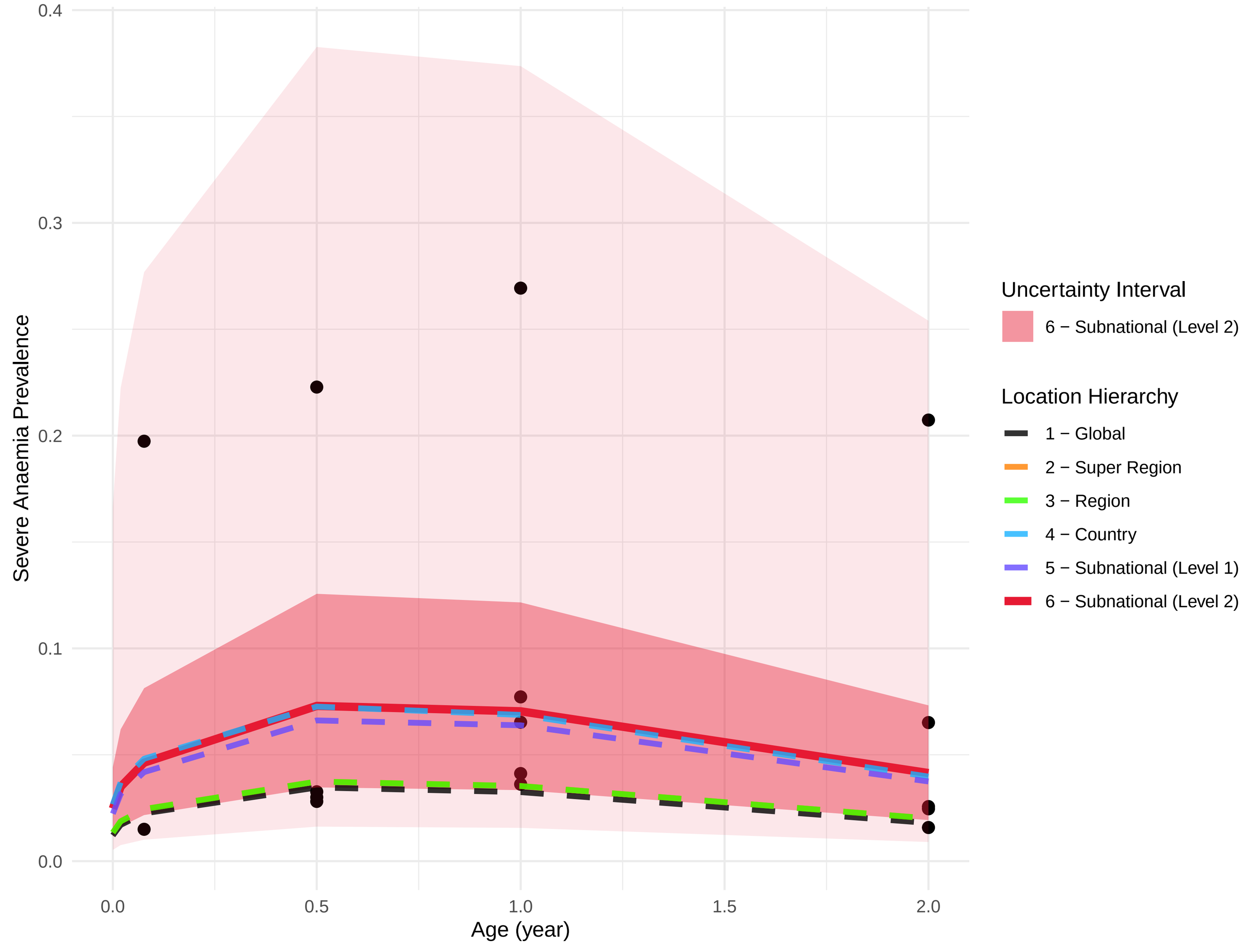

MR−BRT Age/Sex Fits − Males <2 Years Old − Severe Anaemia Prevalence − Madhya Pradesh, Urban
WHO−2001 Adjustment Method

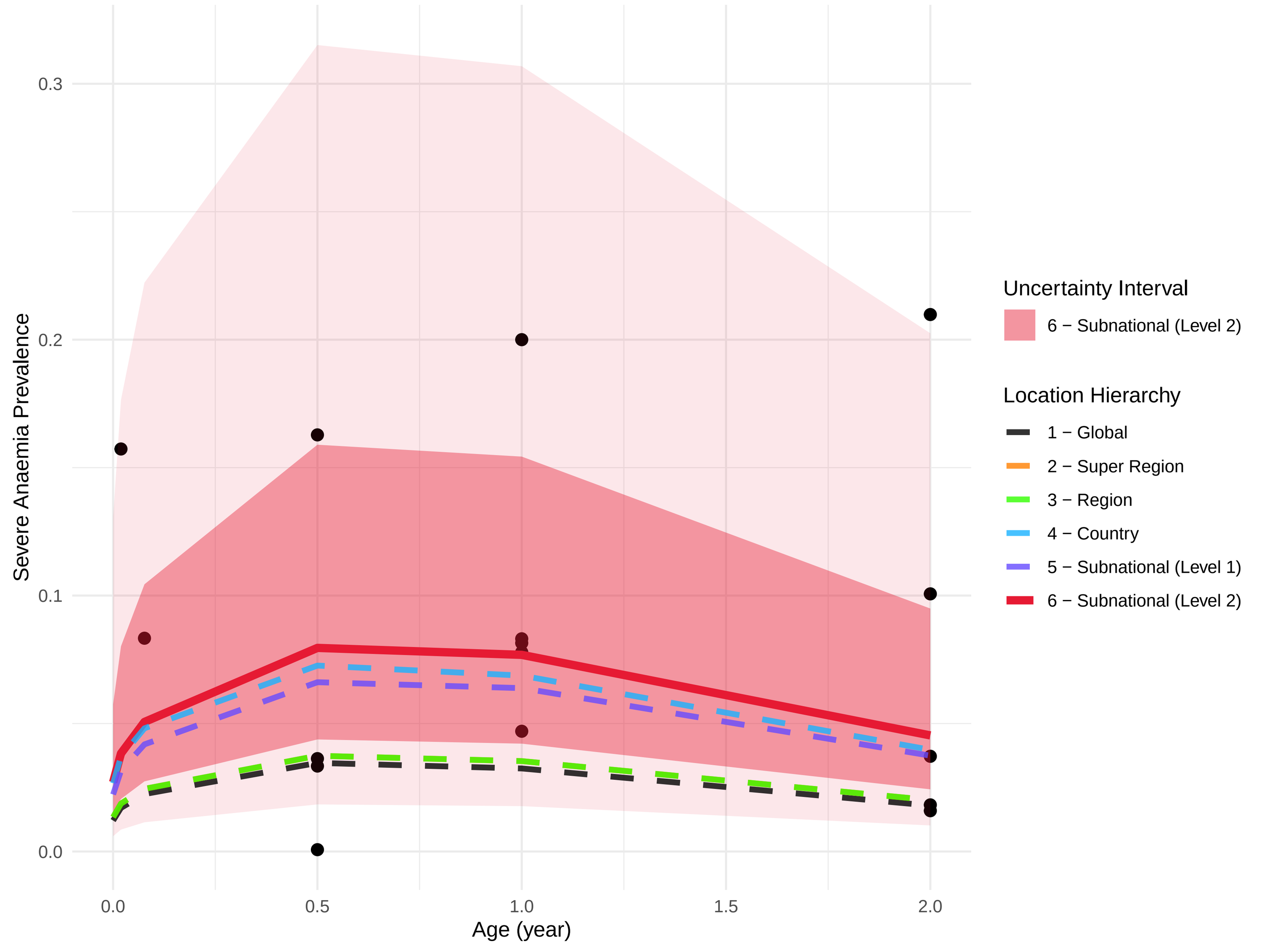

MR−BRT Age/Sex Fits − Males <2 Years Old − Severe Anaemia Prevalence − Maharashtra
WHO−2001 Adjustment Method

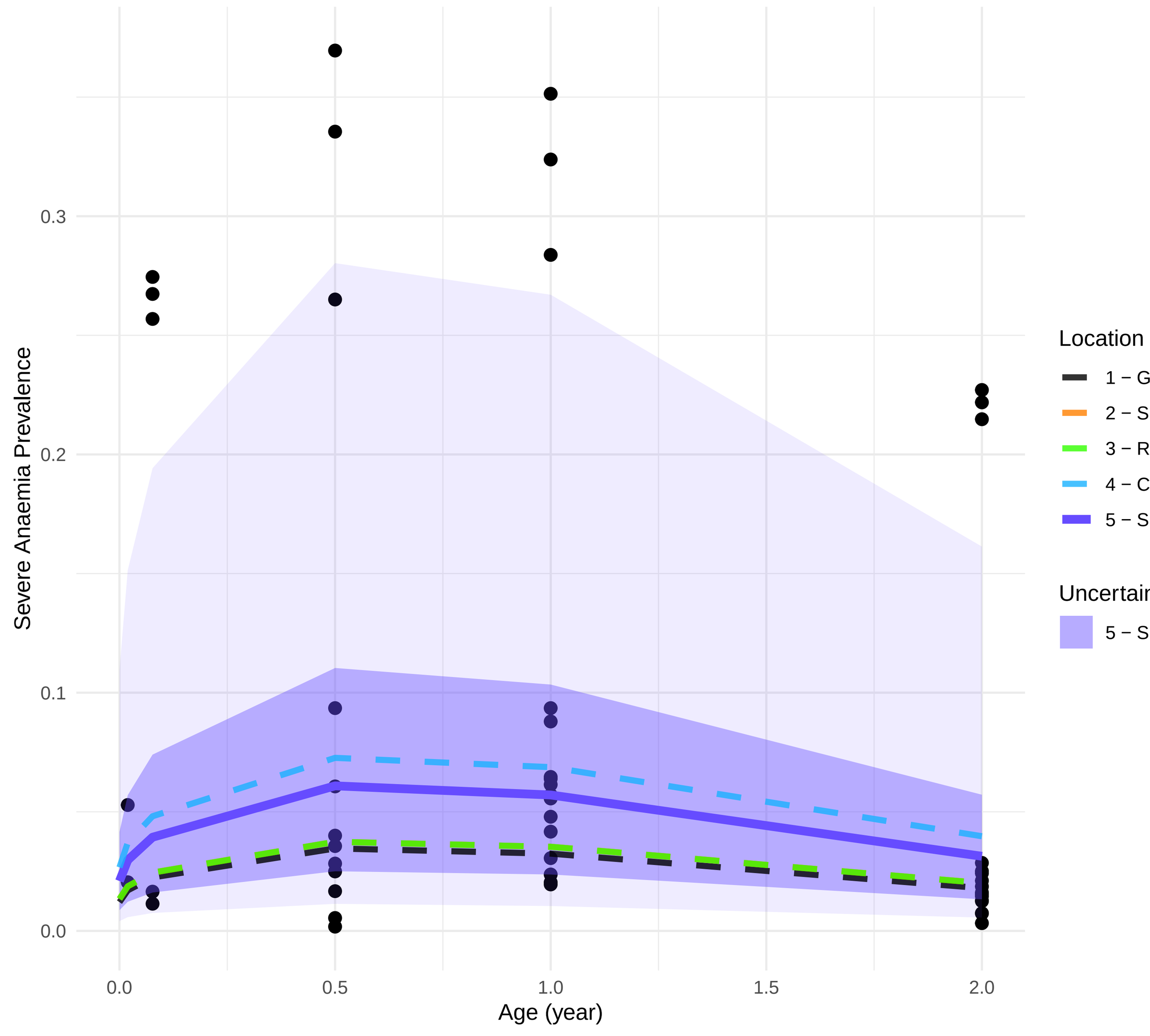

MR−BRT Age/Sex Fits − Males <2 Years Old − Severe Anaemia Prevalence − Maharashtra, Rural
WHO−2001 Adjustment Method

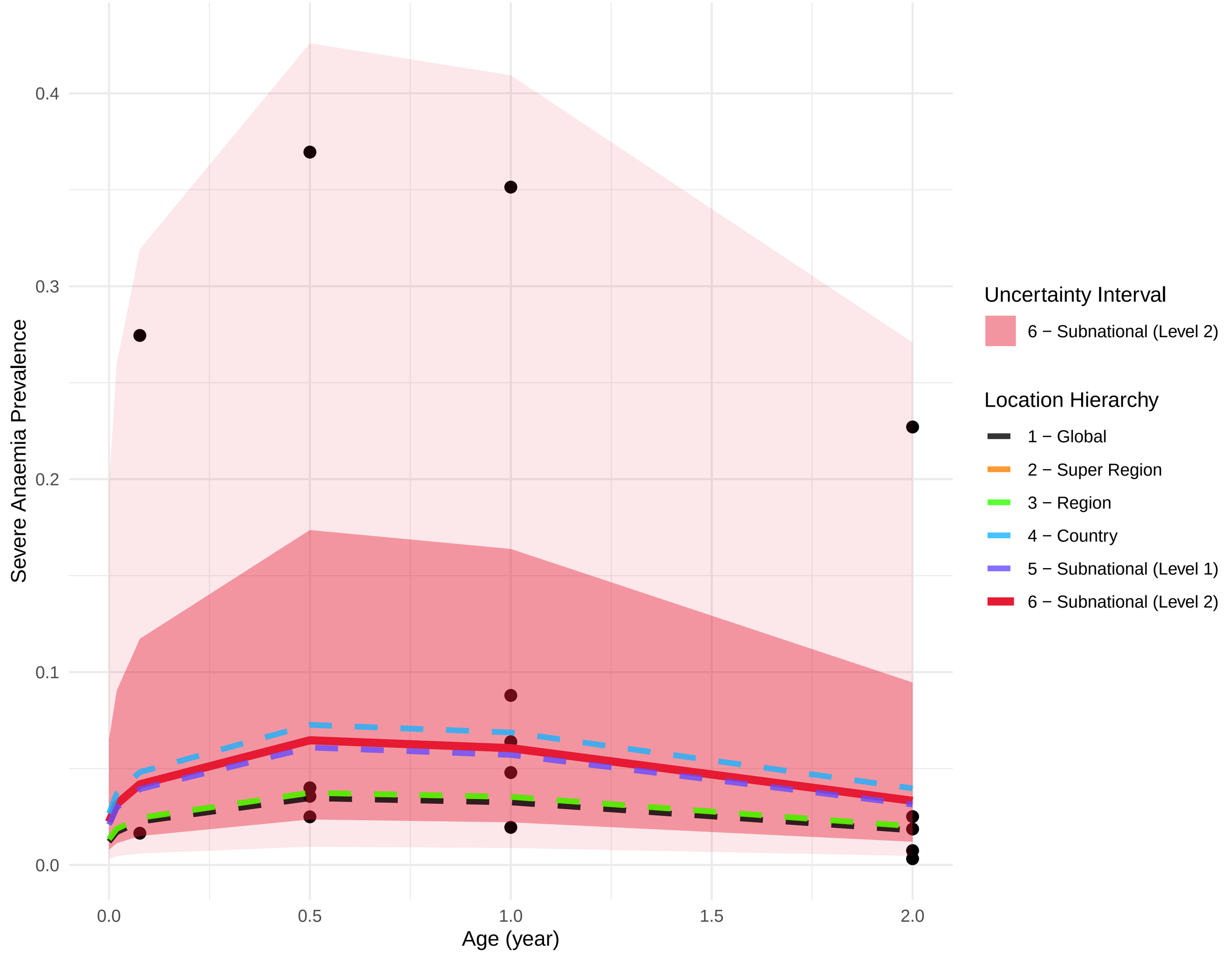

MR−BRT Age/Sex Fits − Males <2 Years Old − Severe Anaemia Prevalence − Maharashtra, Urban
WHO−2001 Adjustment Method

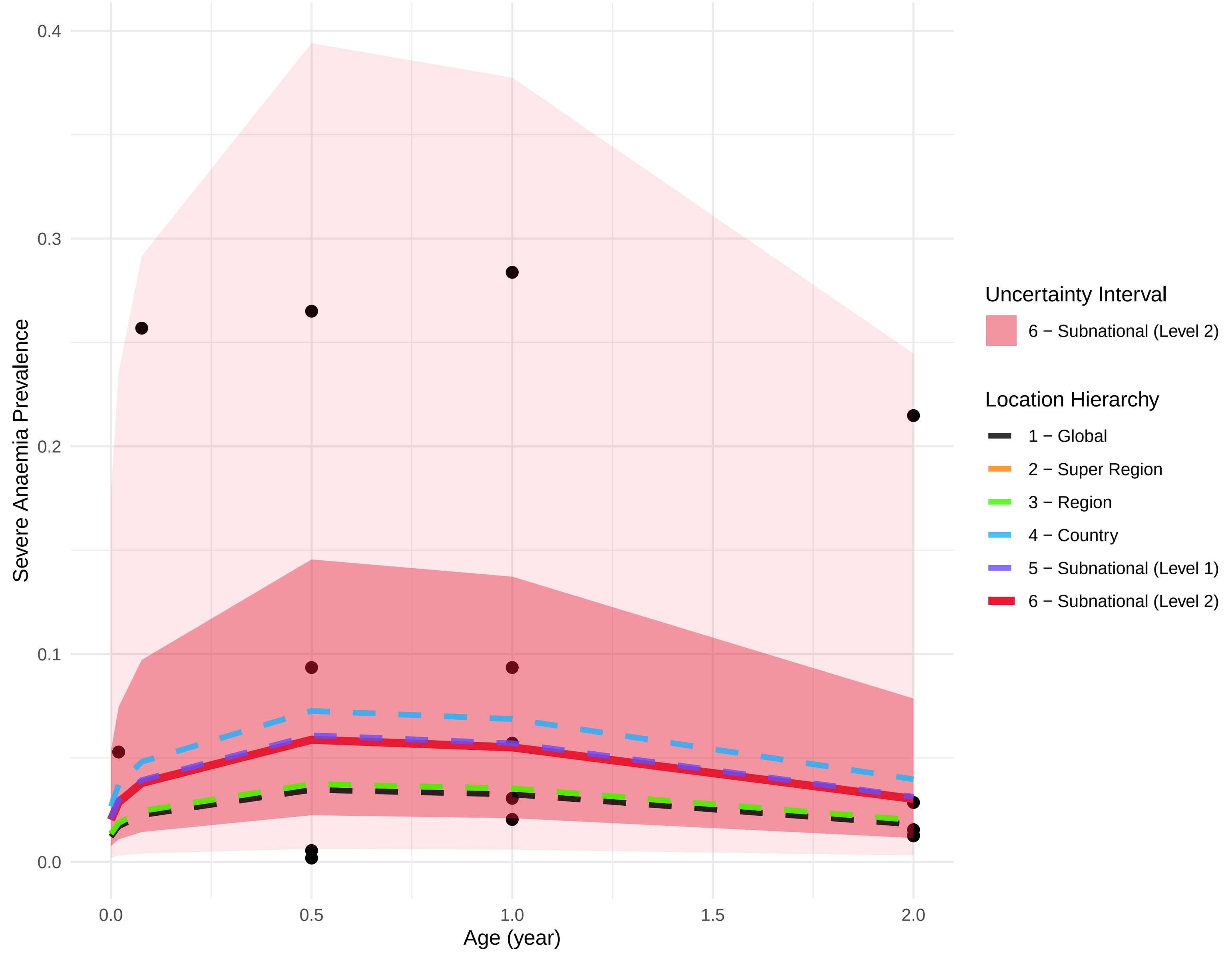

MR−BRT Age/Sex Fits − Males <2 Years Old − Severe Anaemia Prevalence − Manipur
WHO−2001 Adjustment Method

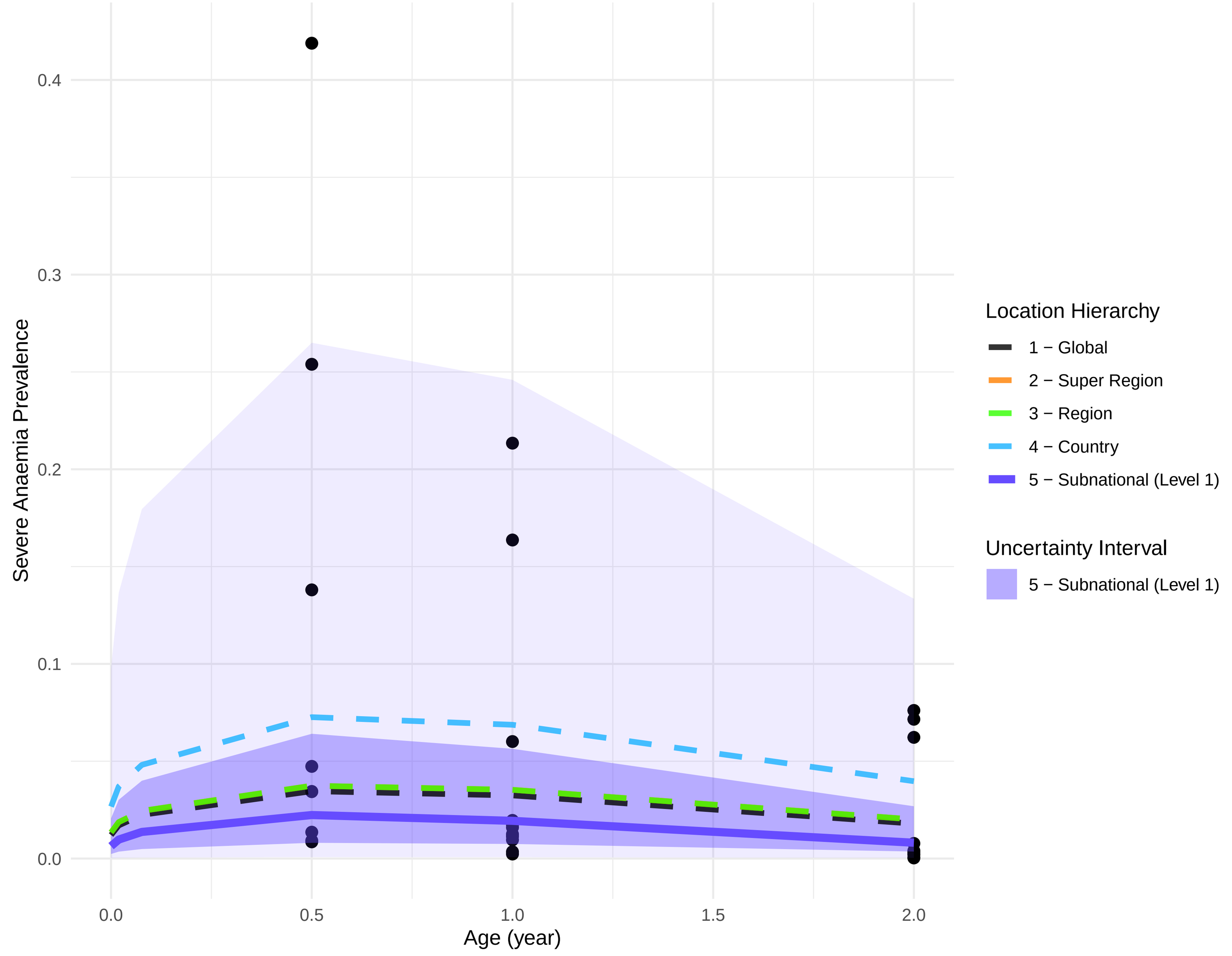

MR−BRT Age/Sex Fits − Males <2 Years Old − Severe Anaemia Prevalence − Manipur, Rural
WHO−2001 Adjustment Method

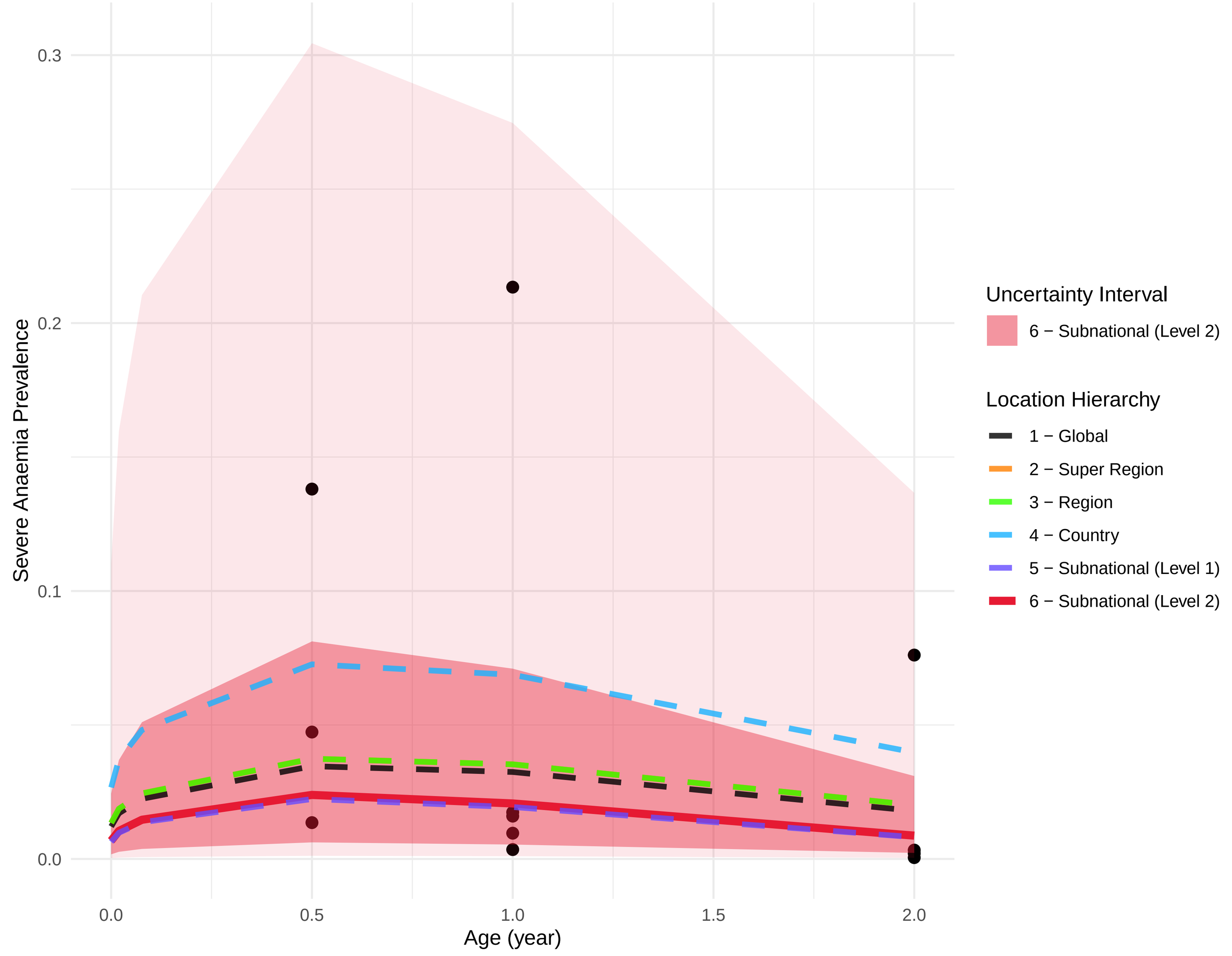

MR−BRT Age/Sex Fits − Males <2 Years Old − Severe Anaemia Prevalence − Manipur, Urban
WHO−2001 Adjustment Method

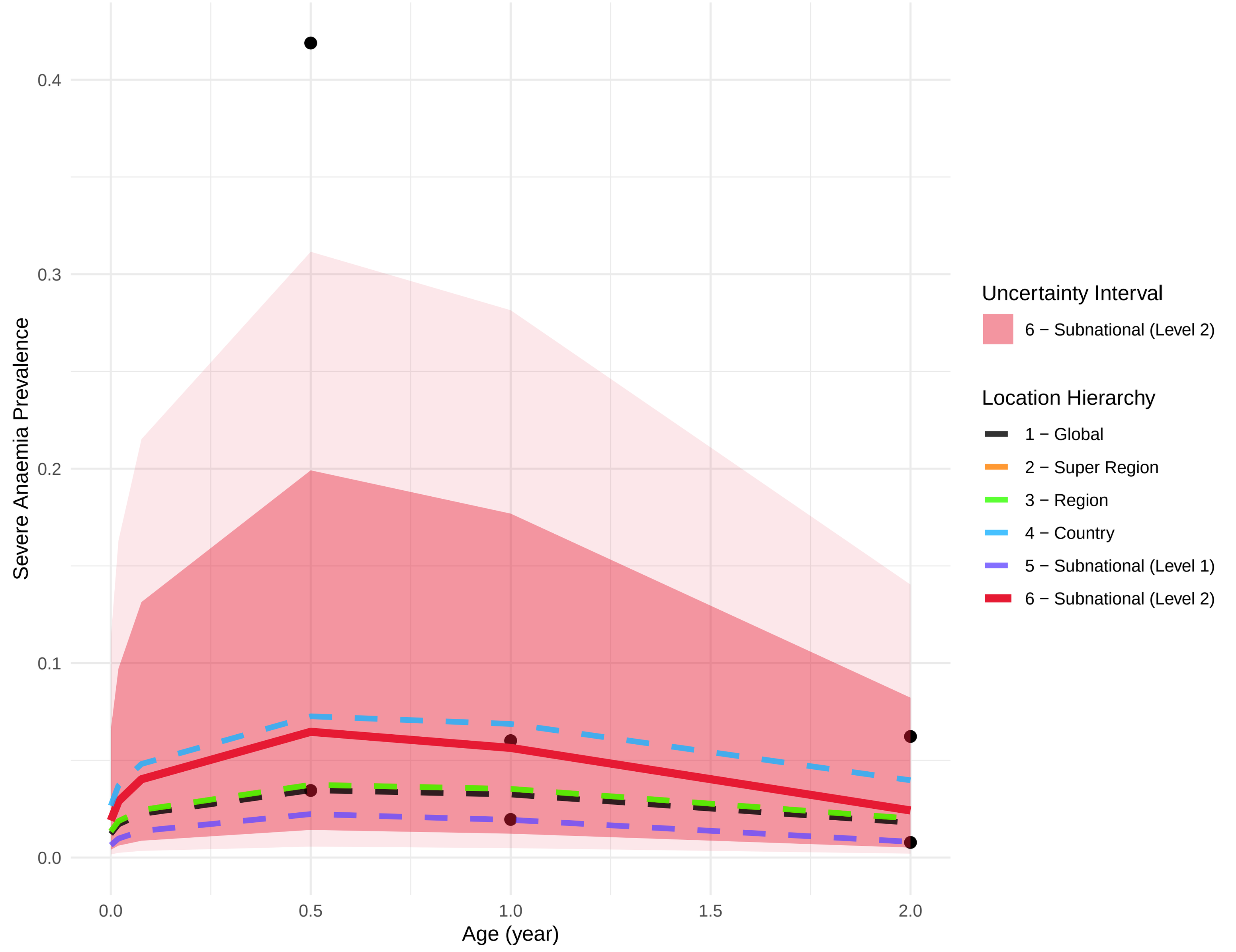

MR−BRT Age/Sex Fits − Males <2 Years Old − Severe Anaemia Prevalence − Meghalaya
WHO−2001 Adjustment Method

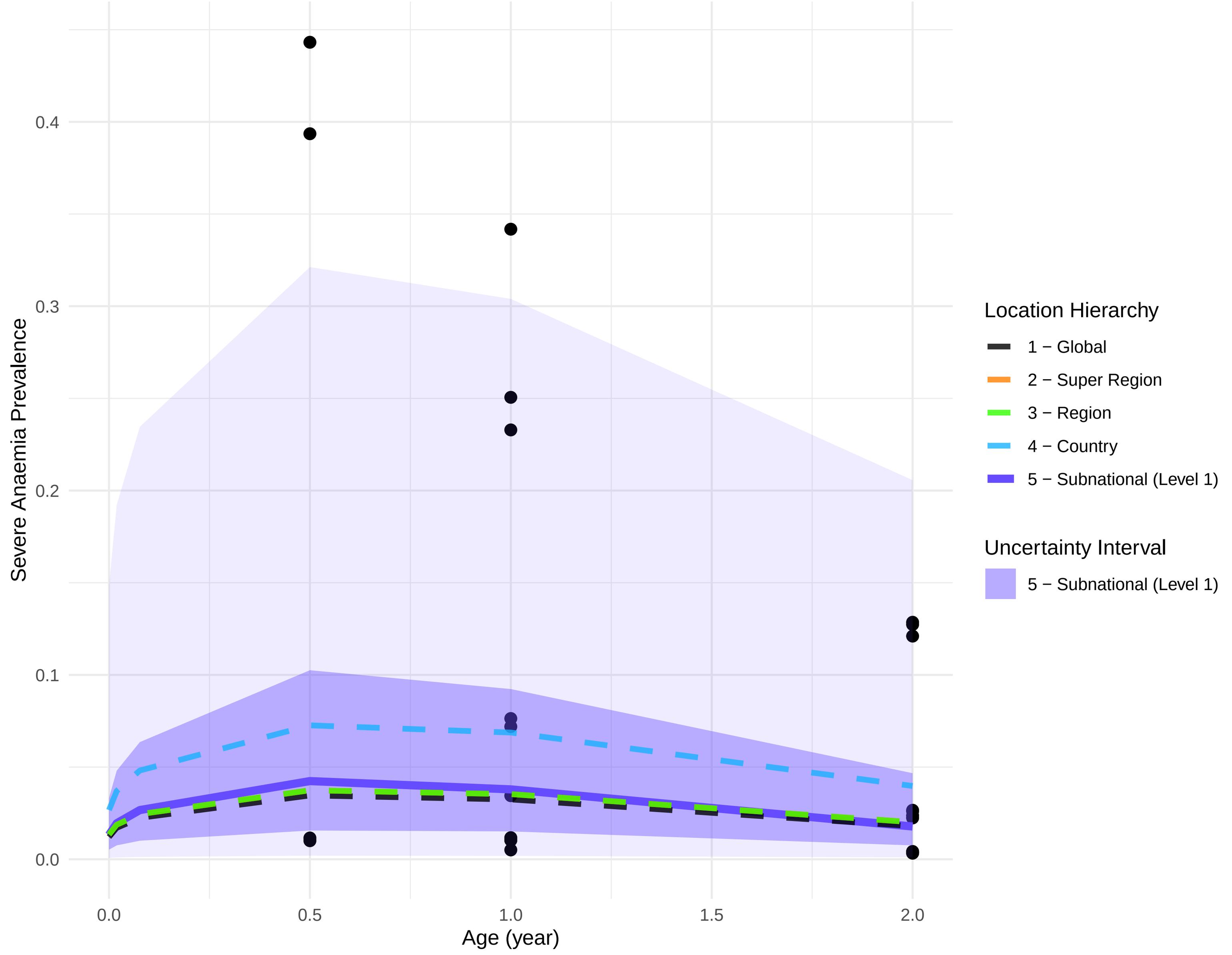

MR−BRT Age/Sex Fits − Males <2 Years Old − Severe Anaemia Prevalence − Meghalaya, Rural
WHO−2001 Adjustment Method

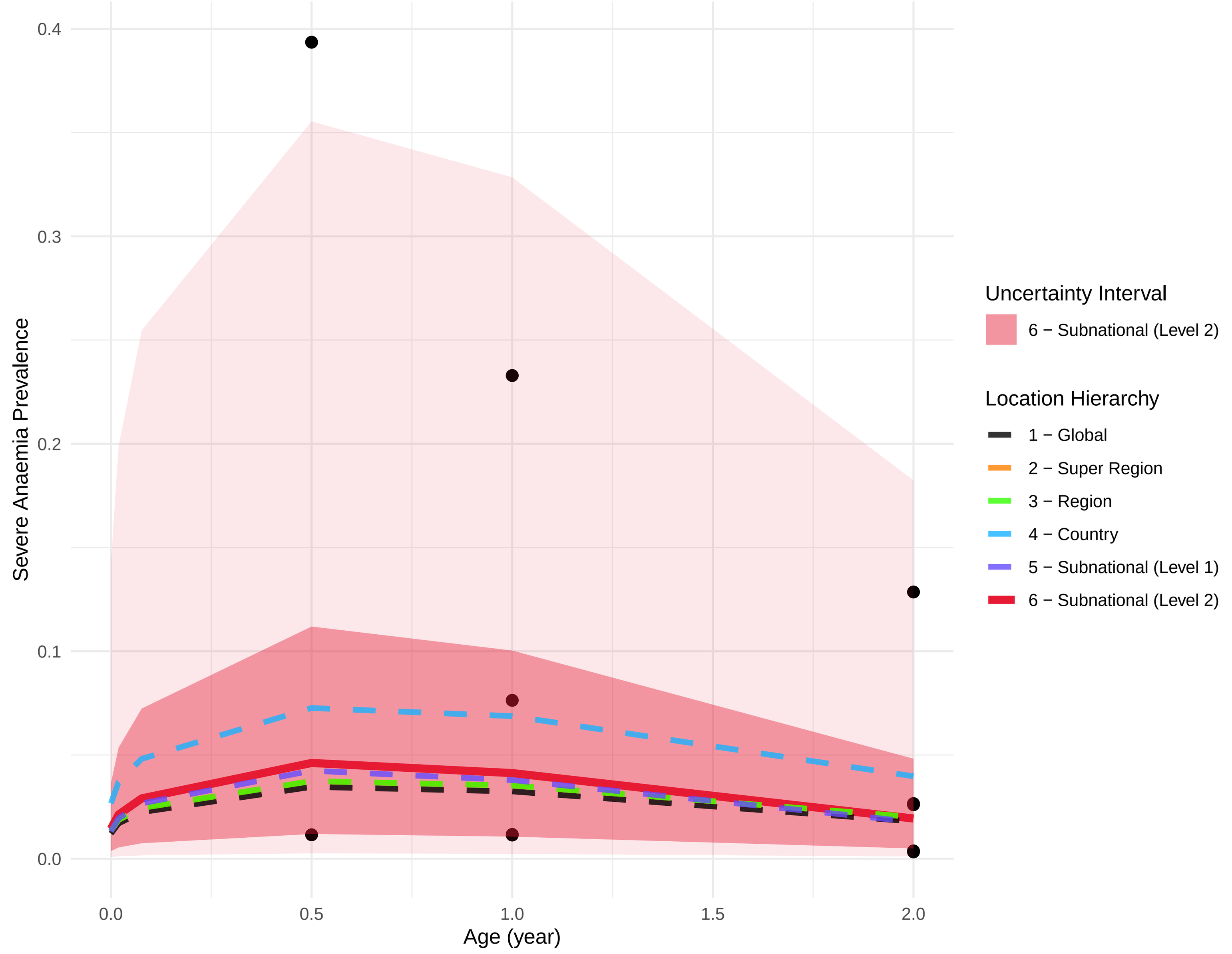

MR−BRT Age/Sex Fits − Males <2 Years Old − Severe Anaemia Prevalence − Meghalaya, Urban
WHO−2001 Adjustment Method

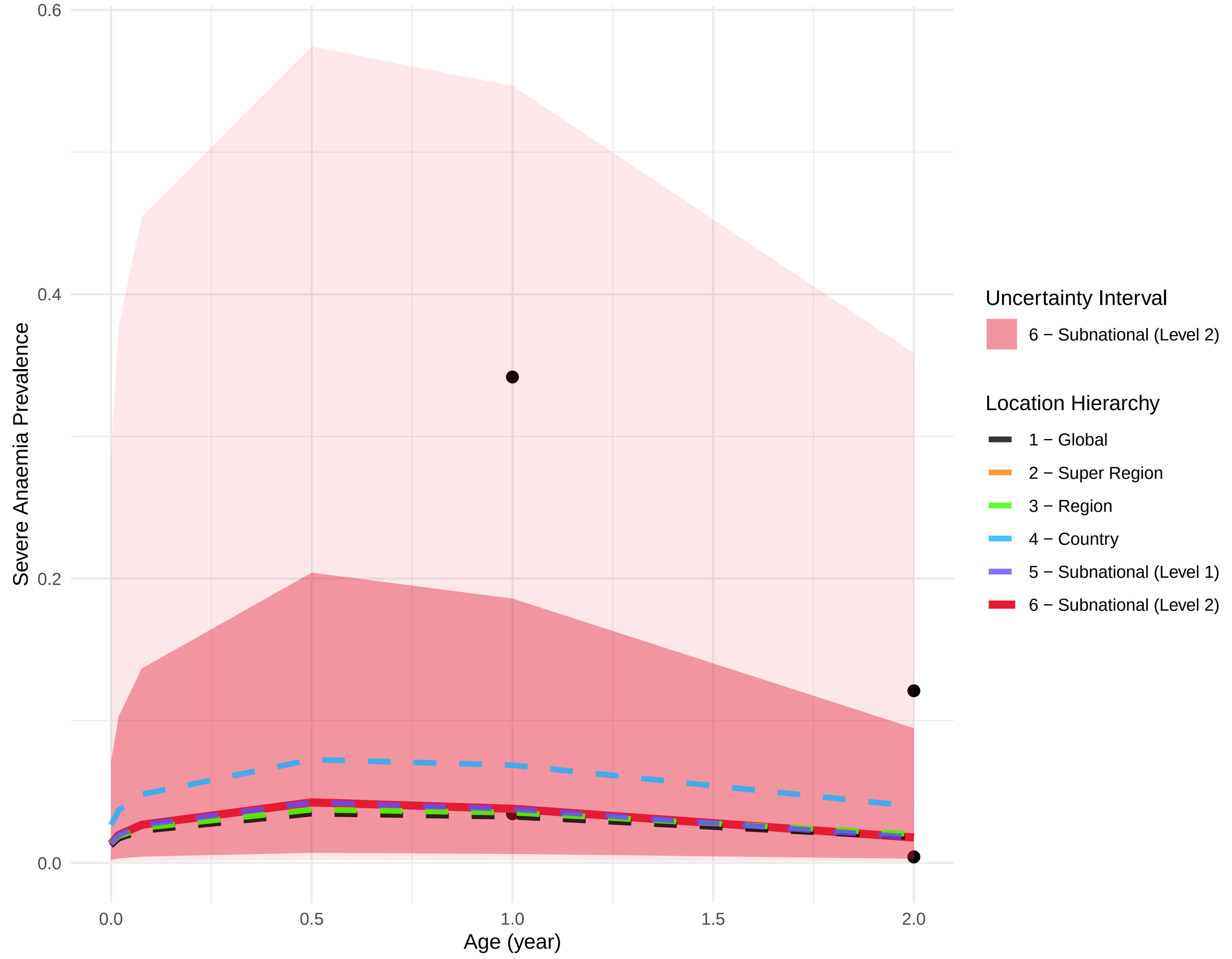

MR−BRT Age/Sex Fits − Males <2 Years Old − Severe Anaemia Prevalence − Mizoram
WHO−2001 Adjustment Method

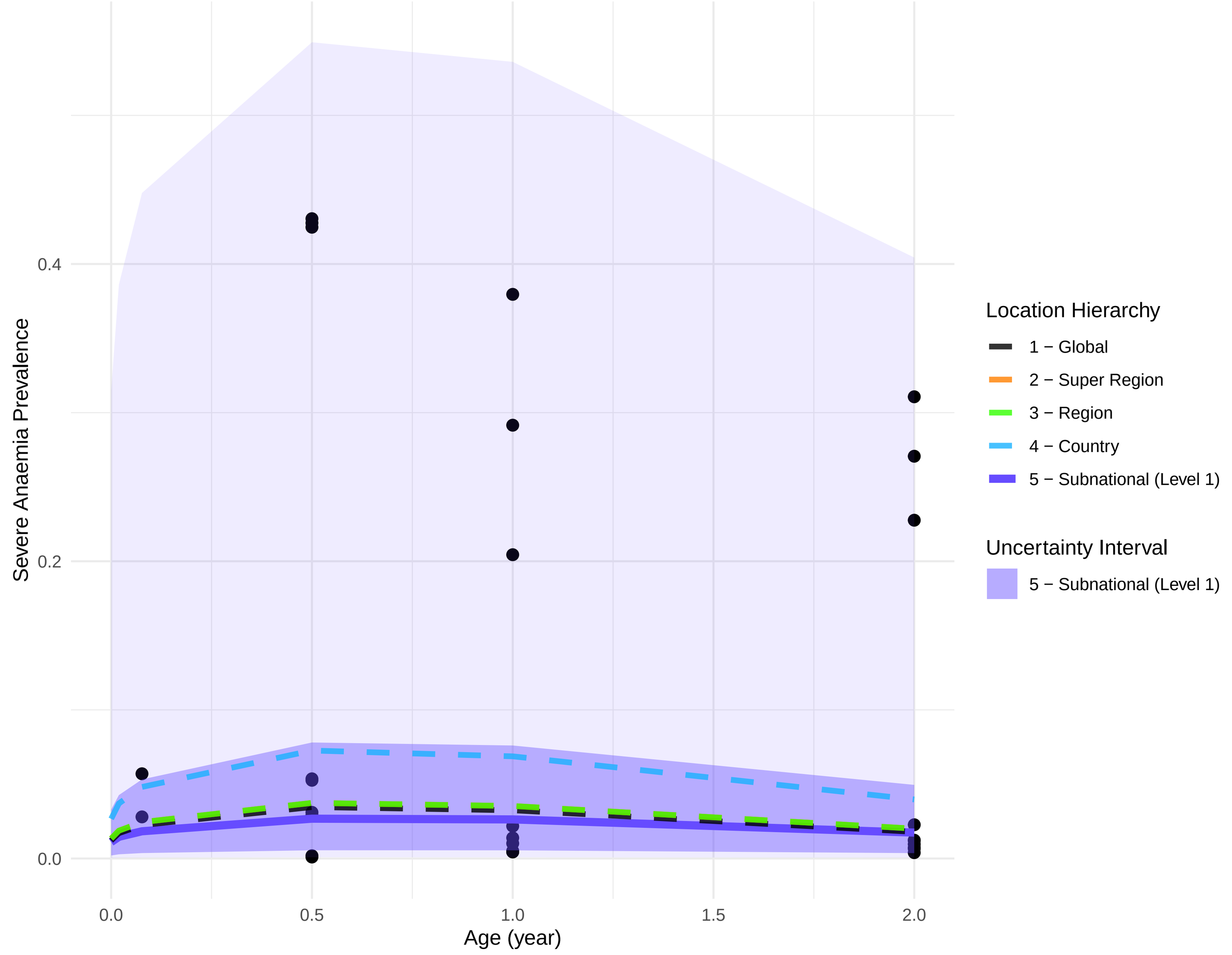

MR−BRT Age/Sex Fits − Males <2 Years Old − Severe Anaemia Prevalence − Mizoram, Rural
WHO−2001 Adjustment Method

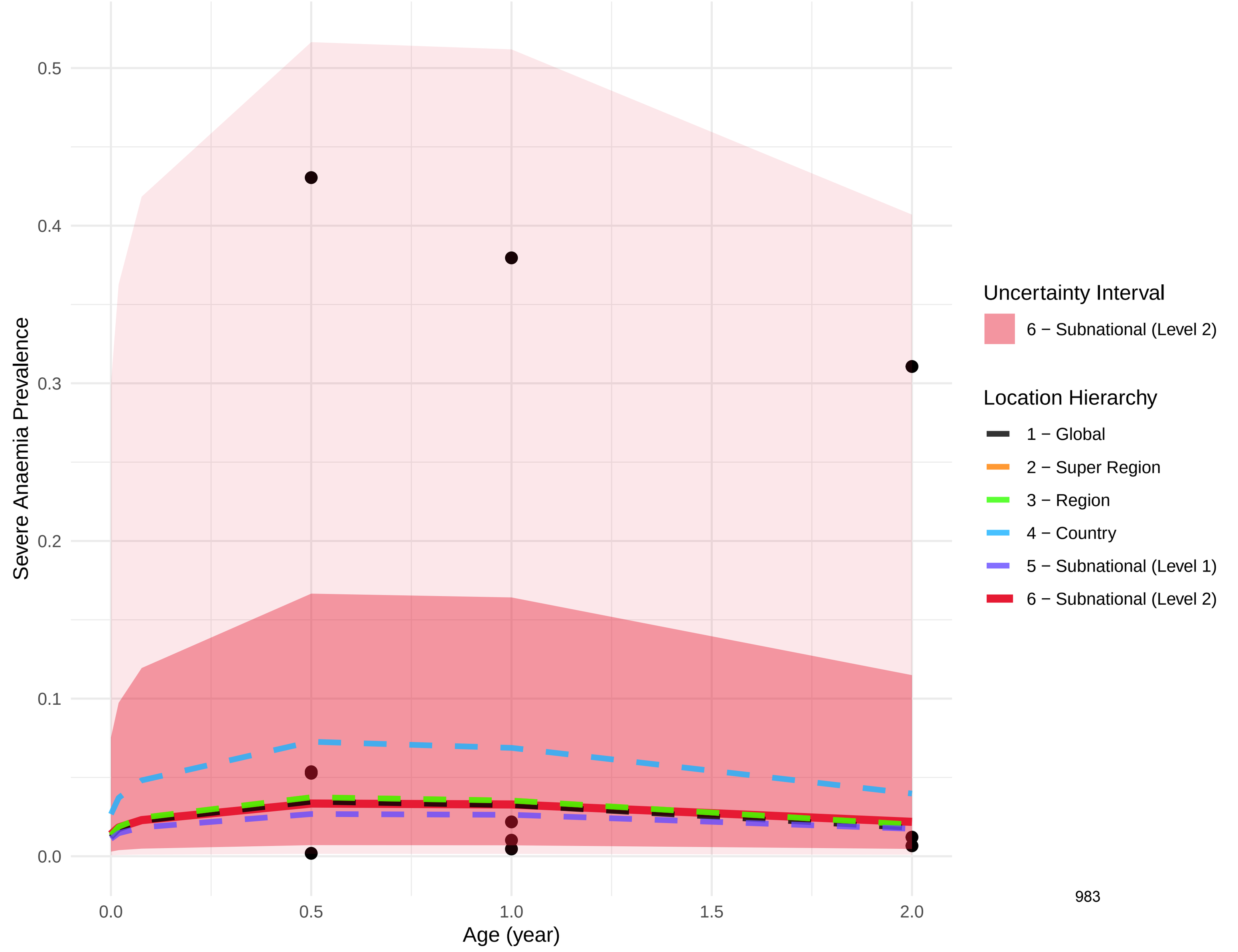

MR−BRT Age/Sex Fits − Males <2 Years Old − Severe Anaemia Prevalence − Mizoram, Urban
WHO−2001 Adjustment Method

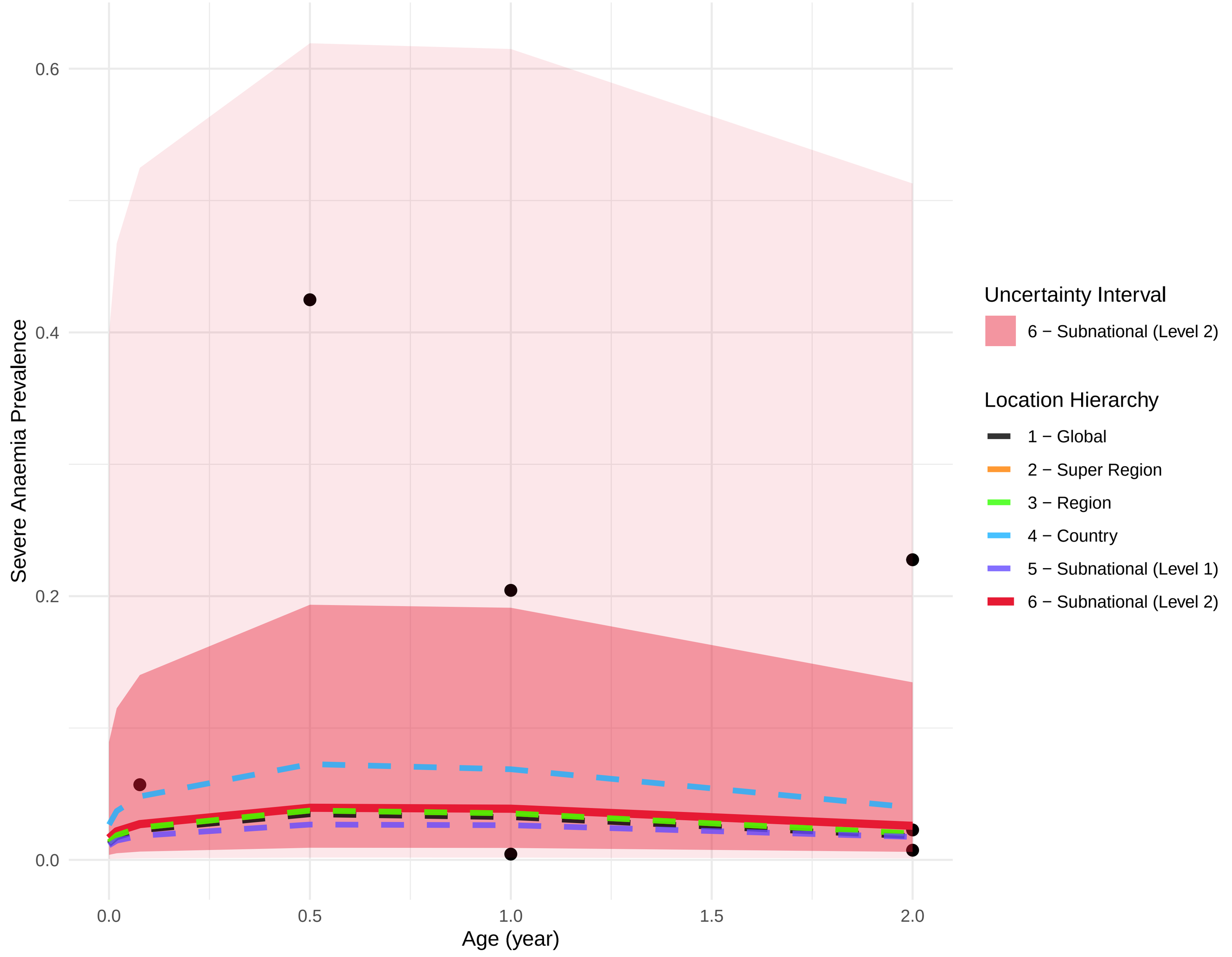

MR−BRT Age/Sex Fits − Males <2 Years Old − Severe Anaemia Prevalence − Nagaland
WHO−2001 Adjustment Method

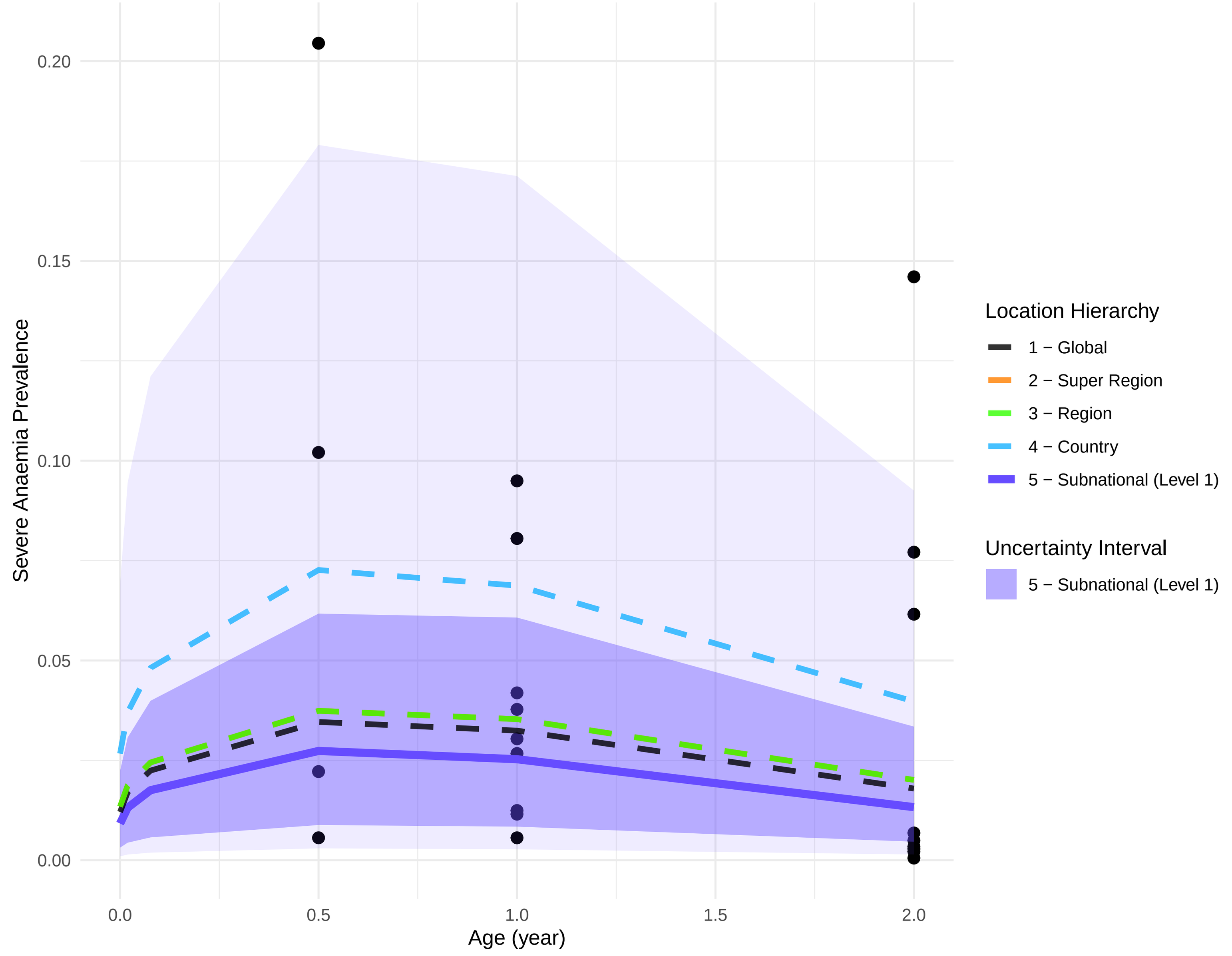

MR−BRT Age/Sex Fits − Males <2 Years Old − Severe Anaemia Prevalence − Nagaland, Rural
WHO−2001 Adjustment Method

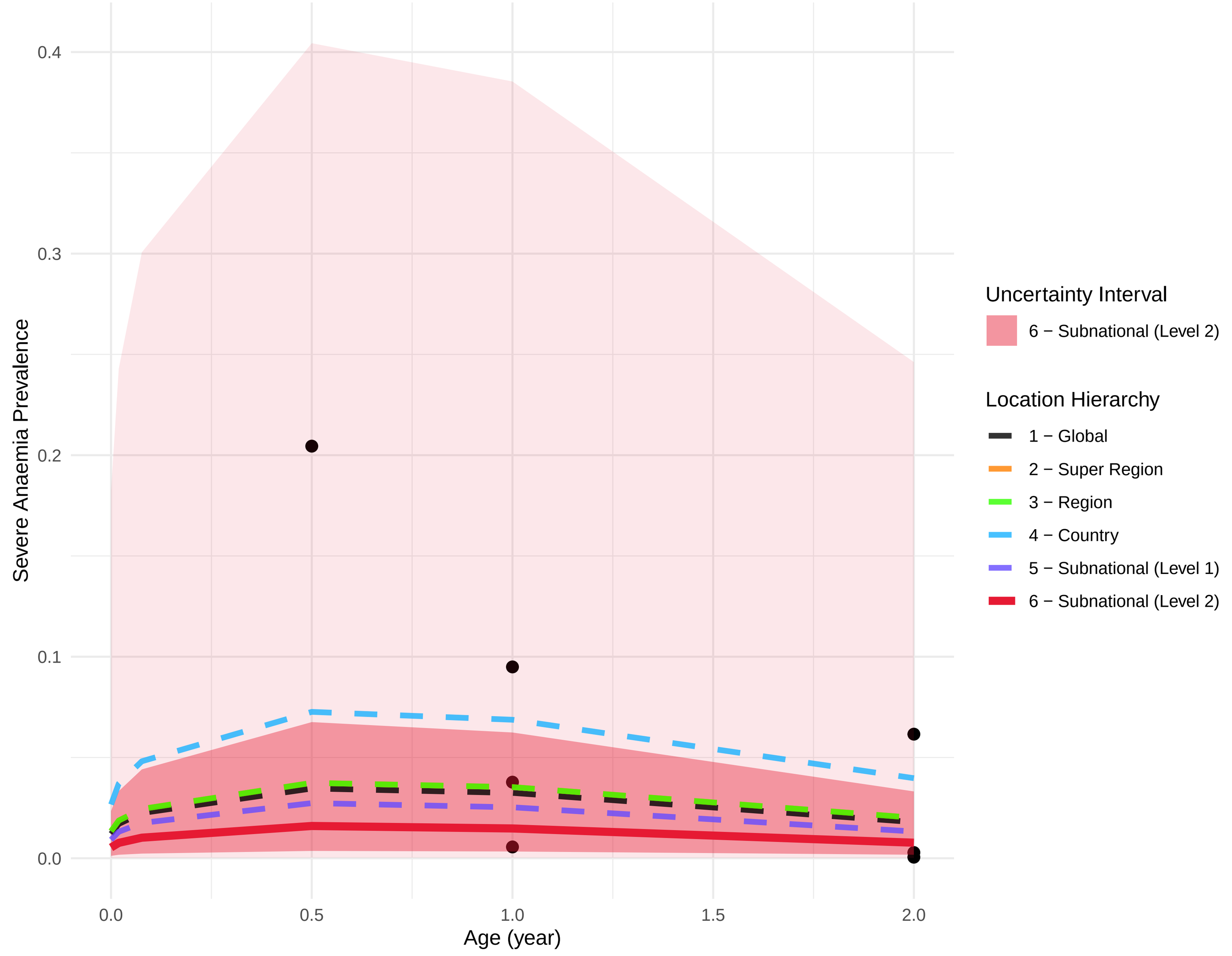

MR−BRT Age/Sex Fits − Males <2 Years Old − Severe Anaemia Prevalence − Nagaland, Urban
WHO−2001 Adjustment Method

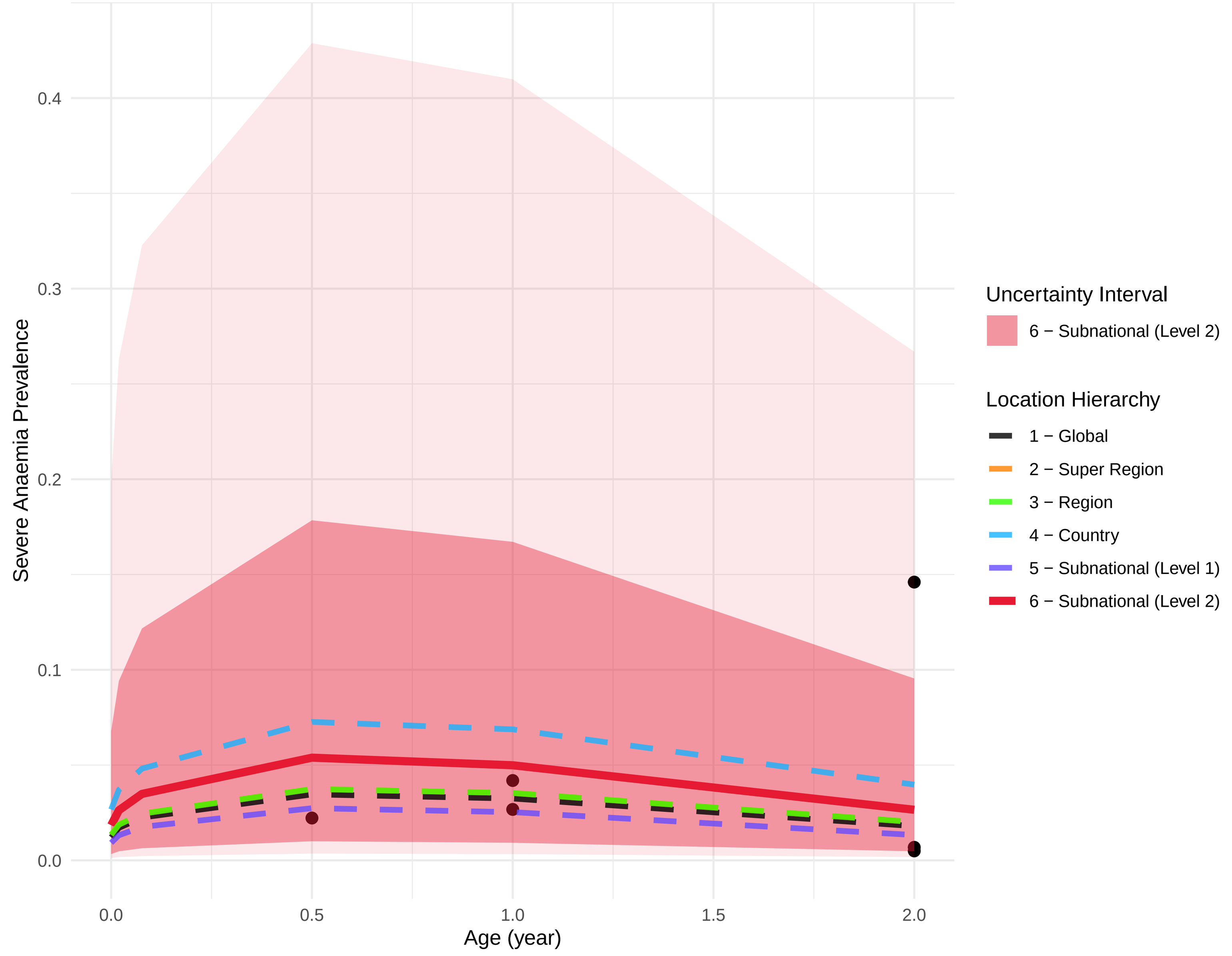

MR−BRT Age/Sex Fits − Males <2 Years Old − Severe Anaemia Prevalence − Odisha
WHO−2001 Adjustment Method

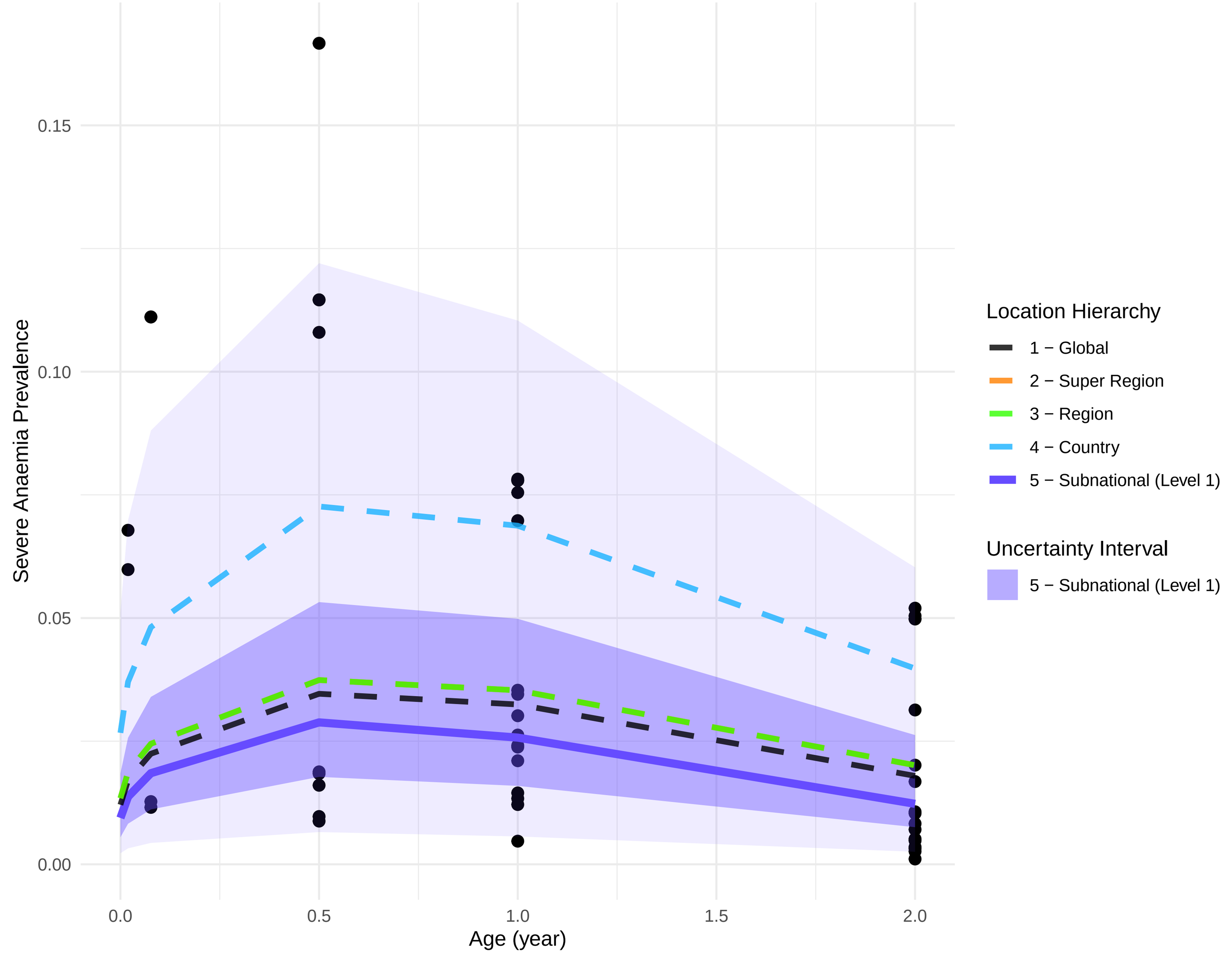

MR−BRT Age/Sex Fits − Males <2 Years Old − Severe Anaemia Prevalence − Odisha, Rural
WHO−2001 Adjustment Method

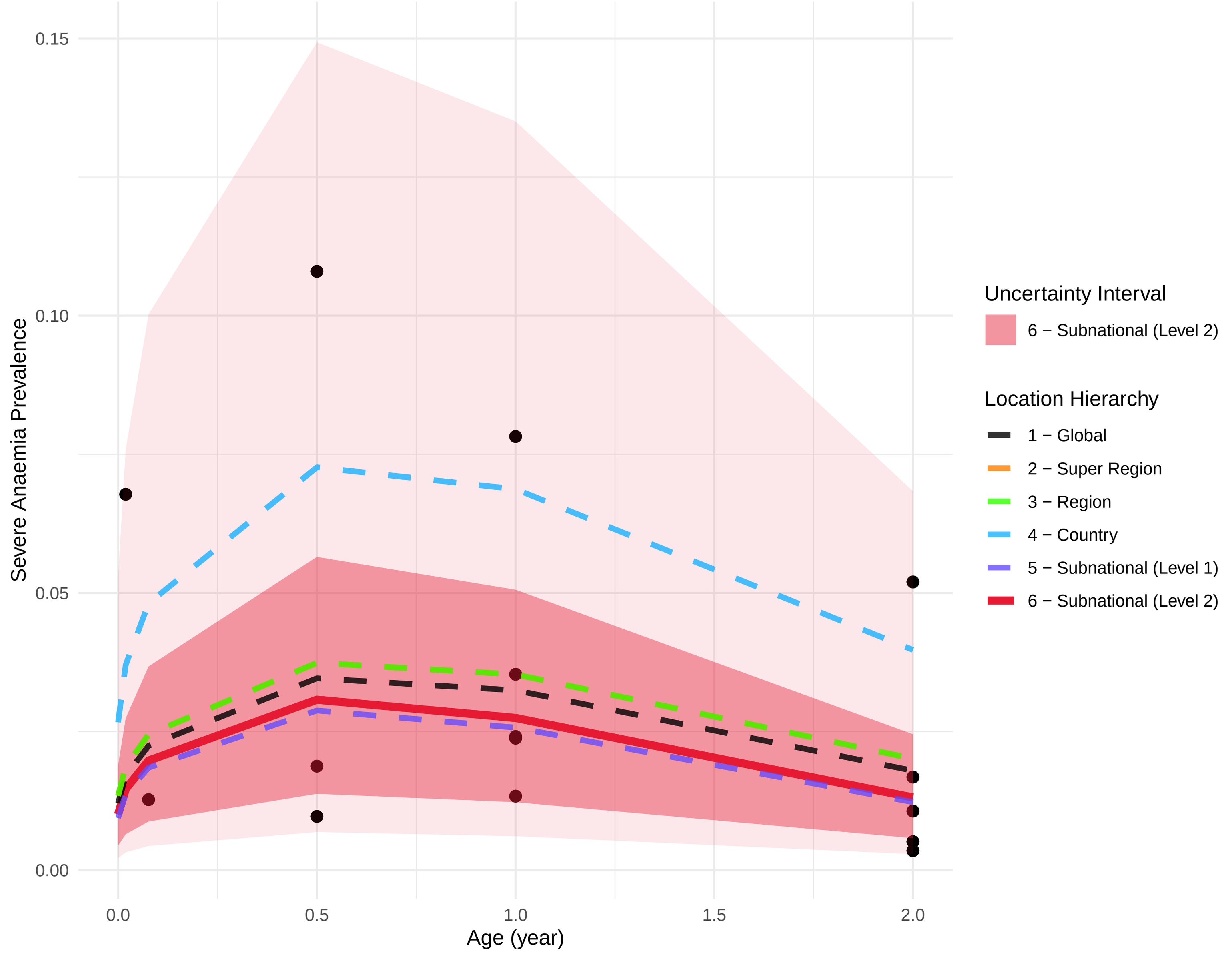

MR−BRT Age/Sex Fits − Males <2 Years Old − Severe Anaemia Prevalence − Odisha, Urban
WHO−2001 Adjustment Method

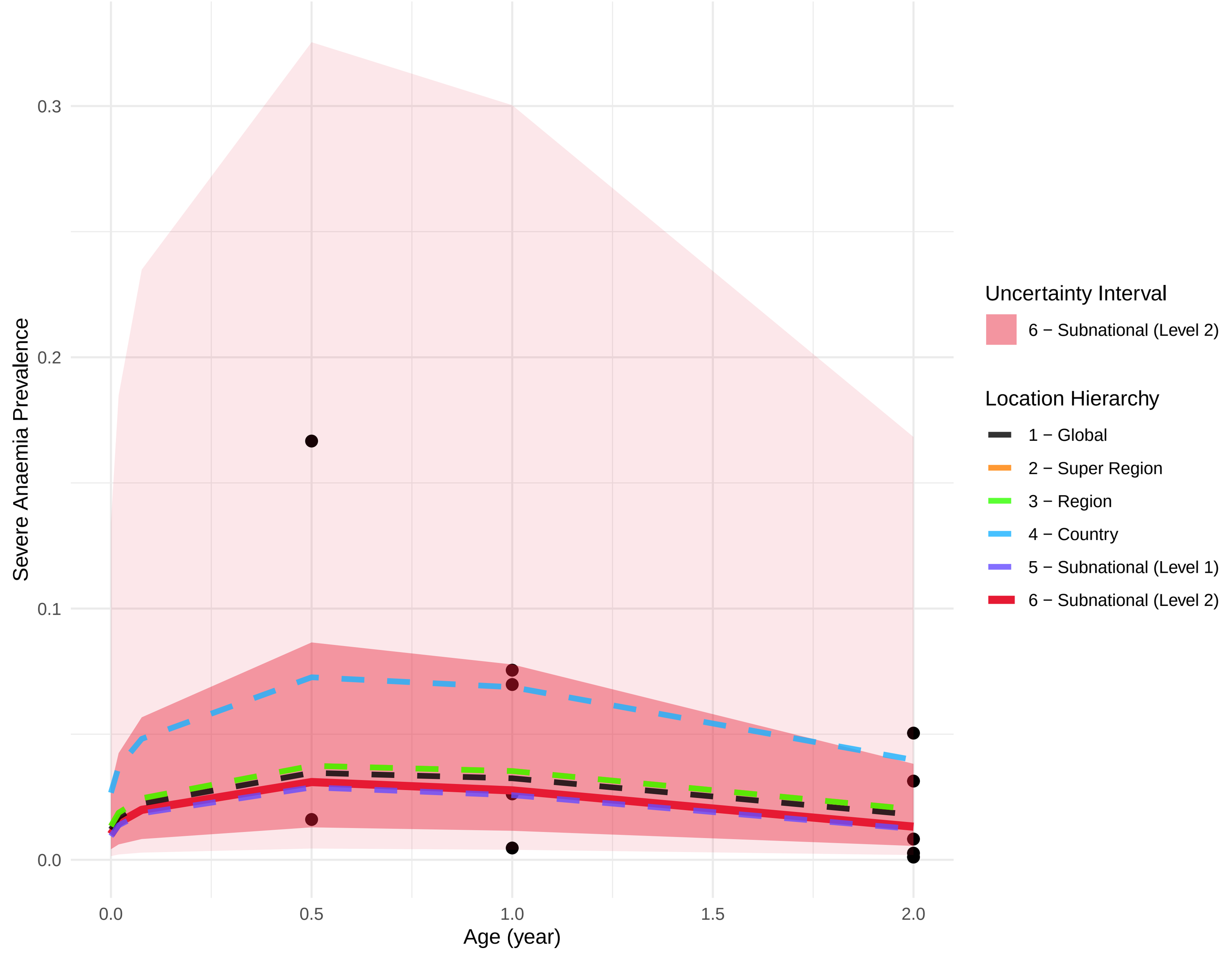

MR−BRT Age/Sex Fits − Males <2 Years Old − Severe Anaemia Prevalence − Other Union Territories
WHO−2001 Adjustment Method

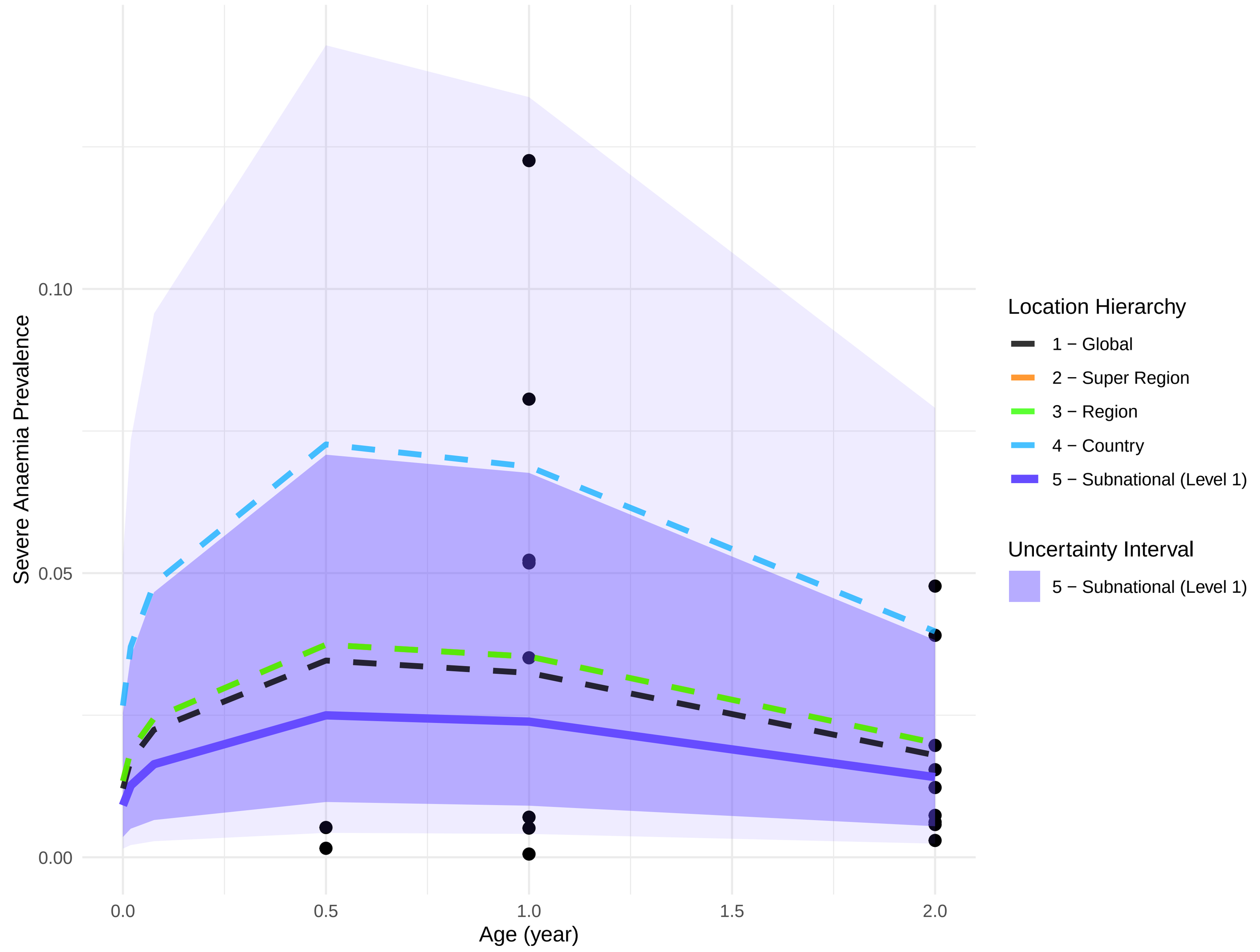

MR−BRT Age/Sex Fits − Males <2 Years Old − Severe Anaemia Prevalence − Other Union Territories, Rural
WHO−2001 Adjustment Method

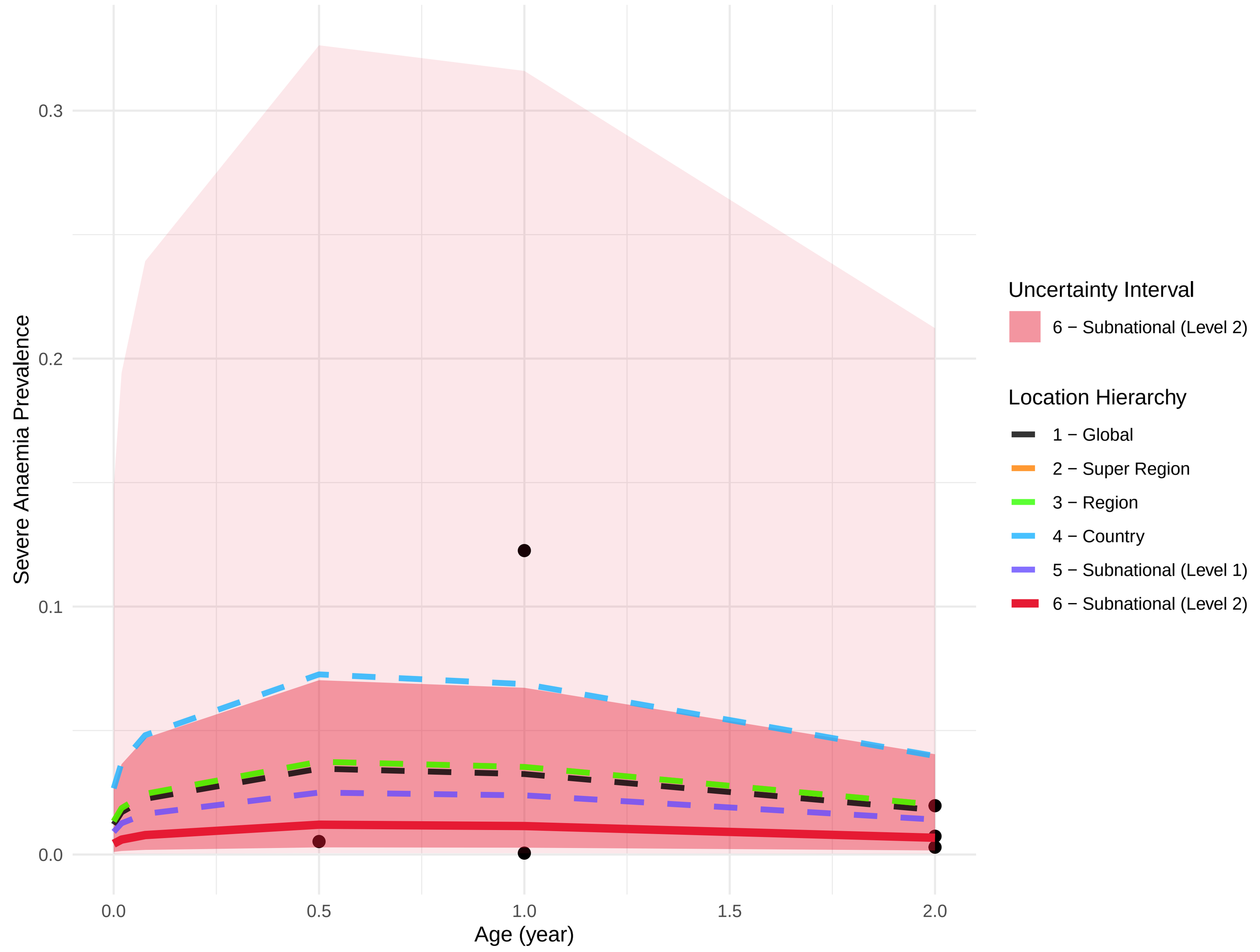

MR−BRT Age/Sex Fits − Males <2 Years Old − Severe Anaemia Prevalence − Other Union Territories, Urban
WHO−2001 Adjustment Method

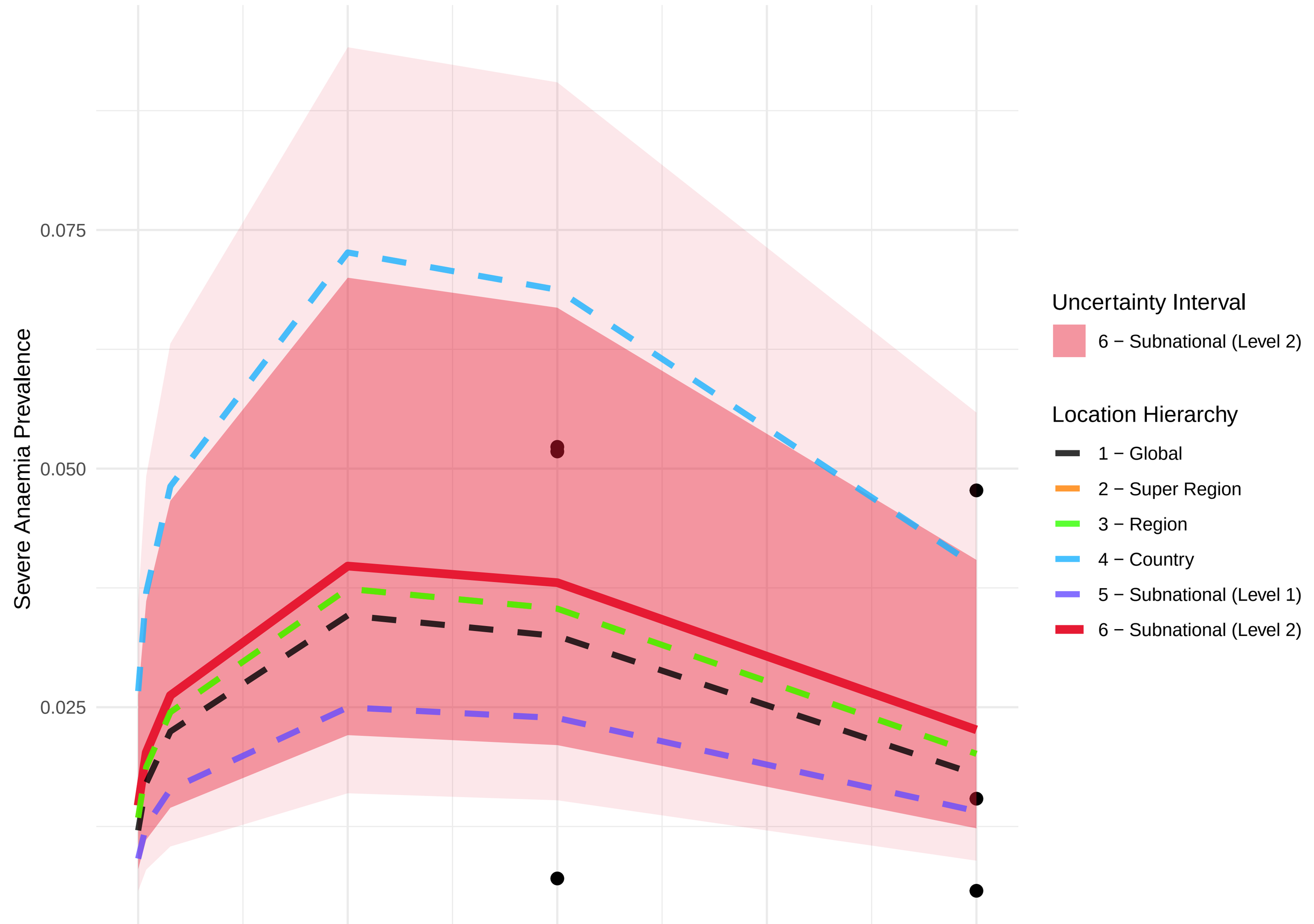

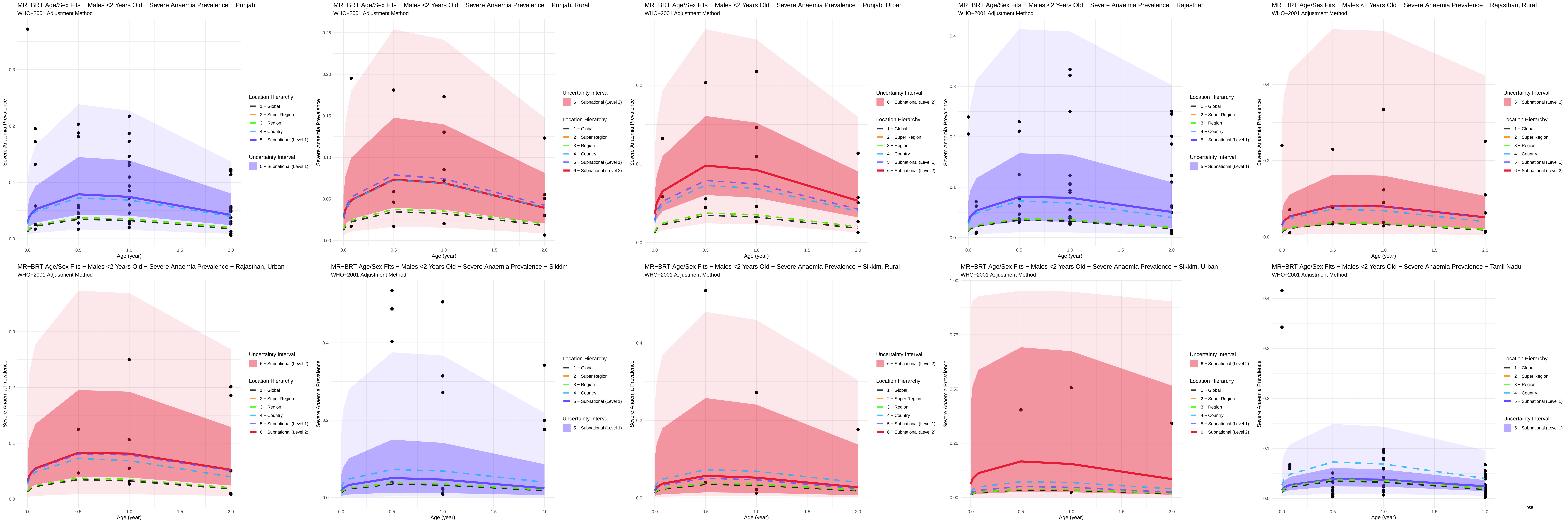
MR−BRT Age/Sex Fits − Males <2 Years Old − Severe Anaemia Prevalence − Punjab
WHO−2001 Adjustment Method
Severe Anaemia Prevalence
Age (year)
Location Hierarchy
1 − Global
2 − Super Region
3 − Region
4 − Country
5 − Subnational (Level 1)
Uncertainty Interval
5 − Subnational (Level 1)
MR−BRT Age/Sex Fits − Males <2 Years Old − Severe Anaemia Prevalence − Punjab, Rural
WHO−2001 Adjustment Method
Uncertainty Interval
6 − Subnational (Level 2)
Location Hierarchy
1 − Global
2 − Super Region
3 − Region
4 − Country
5 − Subnational (Level 1)
6 − Subnational (Level 2)
MR−BRT Age/Sex Fits − Males <2 Years Old − Severe Anaemia Prevalence − Punjab, Urban
WHO−2001 Adjustment Method
MR−BRT Age/Sex Fits − Males <2 Years Old − Severe Anaemia Prevalence − Rajasthan
WHO−2001 Adjustment Method
MR−BRT Age/Sex Fits − Males <2 Years Old − Severe Anaemia Prevalence − Rajasthan, Rural
WHO−2001 Adjustment Method
MR−BRT Age/Sex Fits − Males <2 Years Old − Severe Anaemia Prevalence − Rajasthan, Urban
WHO−2001 Adjustment Method
MR−BRT Age/Sex Fits − Males <2 Years Old − Severe Anaemia Prevalence − Sikkim
WHO−2001 Adjustment Method
MR−BRT Age/Sex Fits − Males <2 Years Old − Severe Anaemia Prevalence − Sikkim, Rural
WHO−2001 Adjustment Method
MR−BRT Age/Sex Fits − Males <2 Years Old − Severe Anaemia Prevalence − Sikkim, Urban
WHO−2001 Adjustment Method
MR−BRT Age/Sex Fits − Males <2 Years Old − Severe Anaemia Prevalence − Tamil Nadu
WHO−2001 Adjustment Method

MR−BRT Age/Sex Fits − Males <2 Years Old − Severe Anaemia Prevalence − Tamil Nadu, Rural
WHO−2001 Adjustment Method

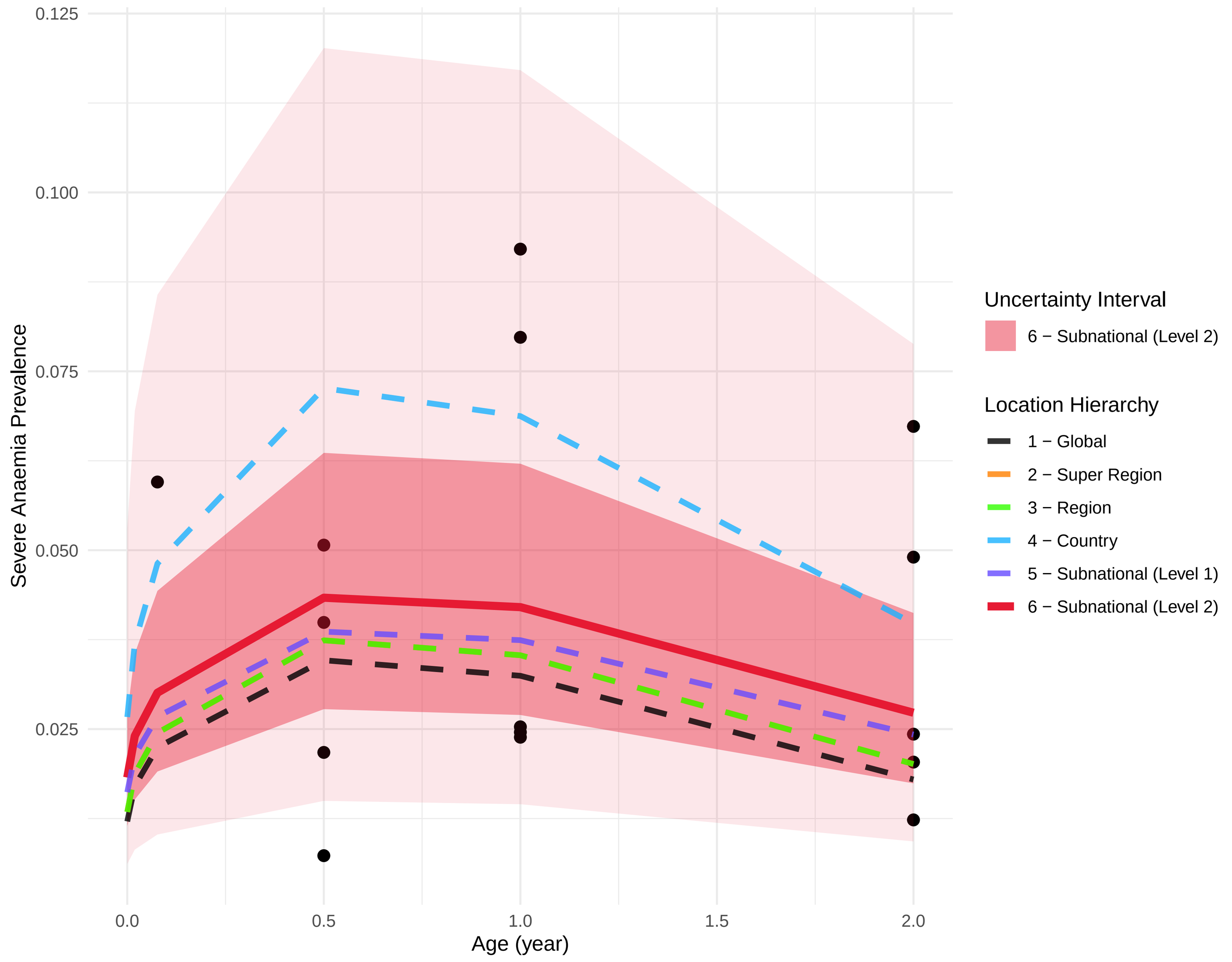

MR−BRT Age/Sex Fits − Males <2 Years Old − Severe Anaemia Prevalence − Tamil Nadu, Urban
WHO−2001 Adjustment Method

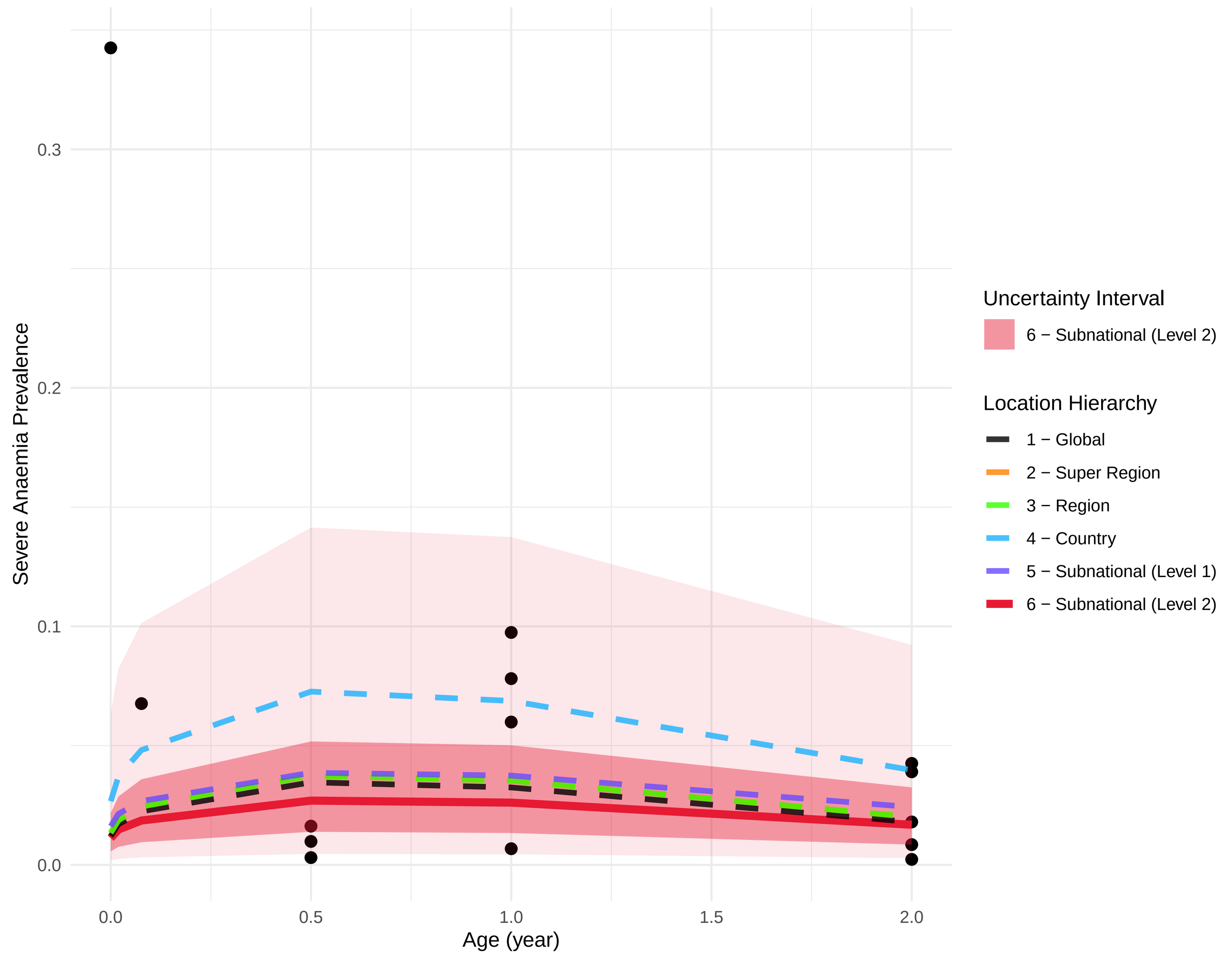

MR−BRT Age/Sex Fits − Males <2 Years Old − Severe Anaemia Prevalence − Telangana
WHO−2001 Adjustment Method

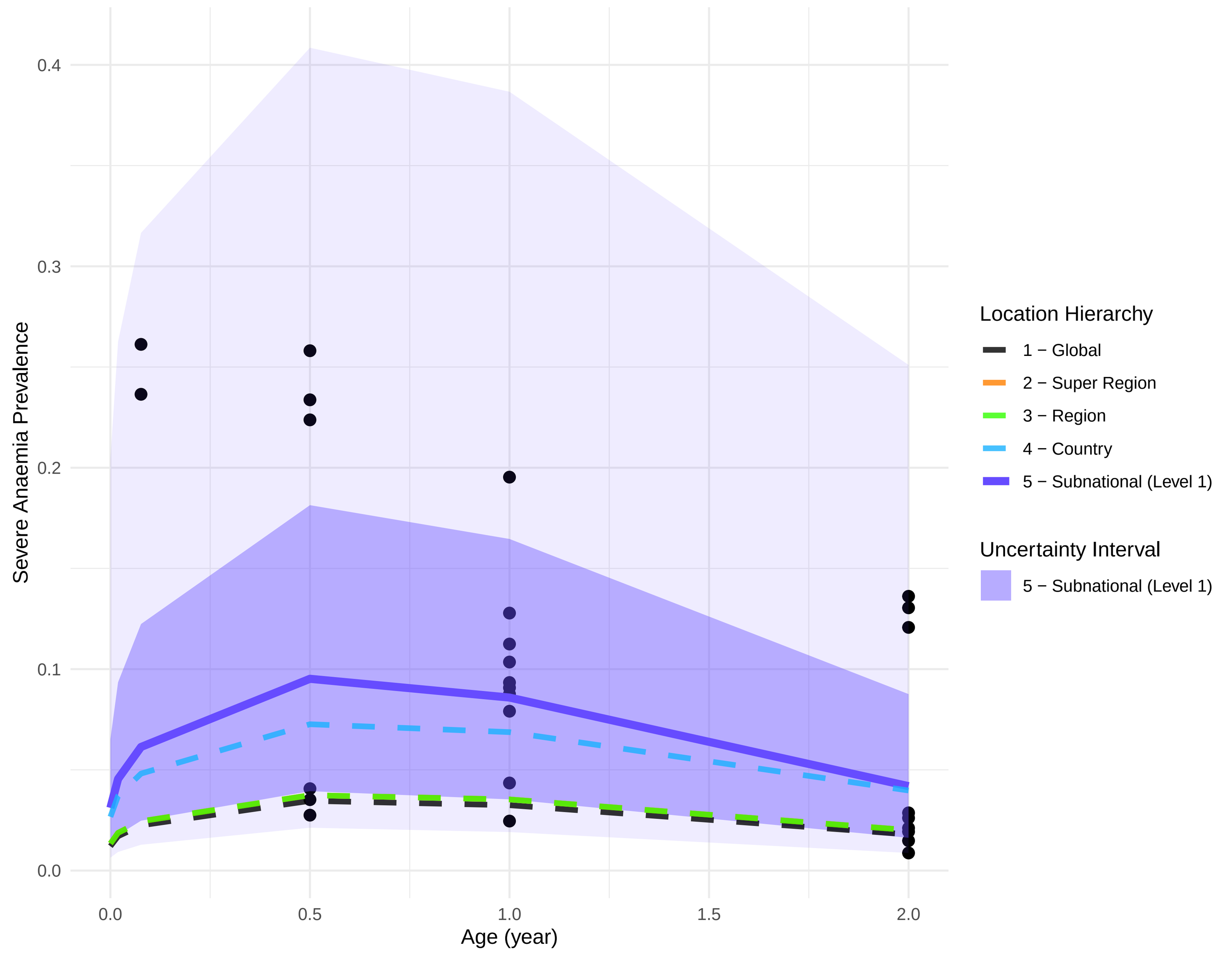

MR−BRT Age/Sex Fits − Males <2 Years Old − Severe Anaemia Prevalence − Telangana, Rural
WHO−2001 Adjustment Method

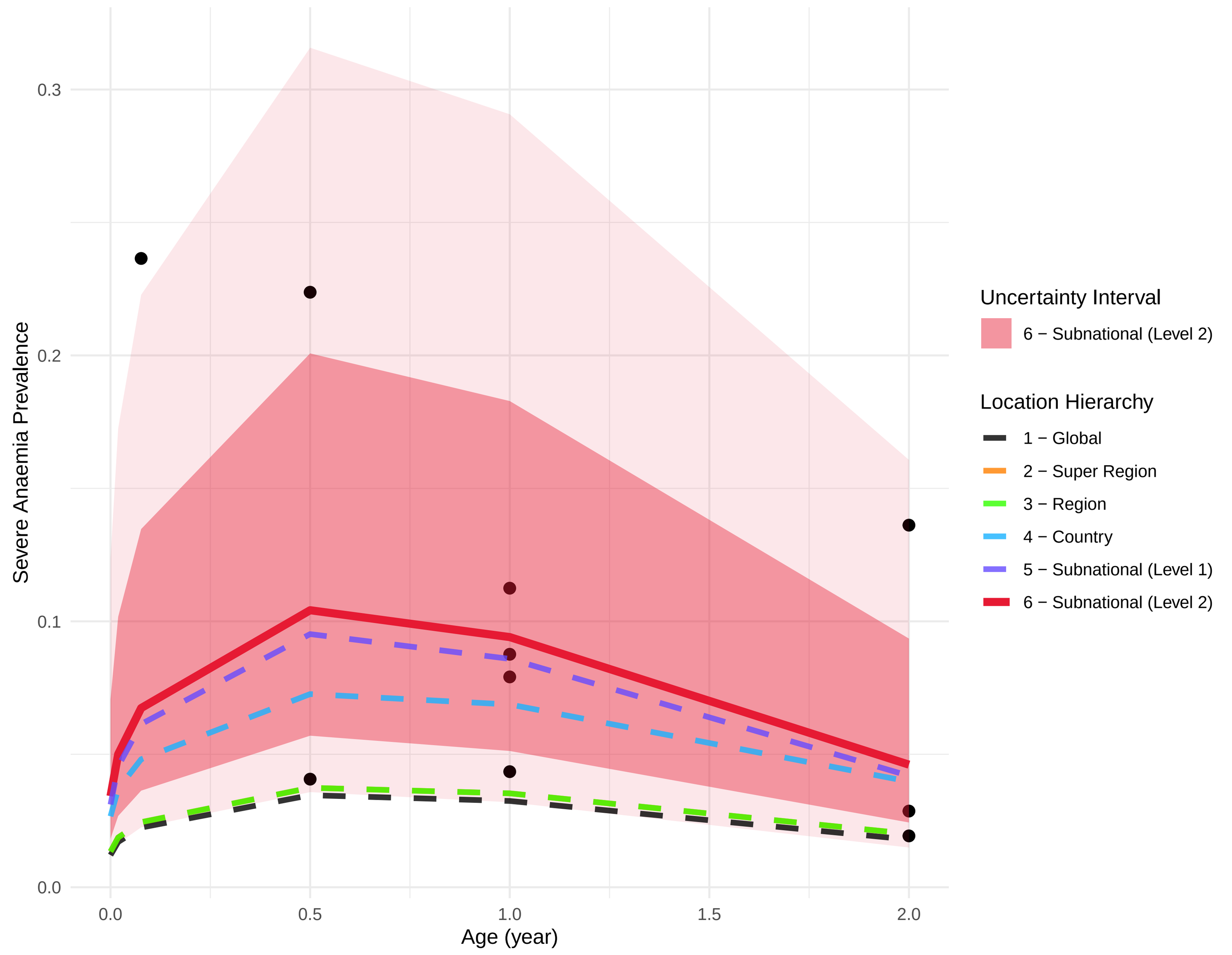

MR−BRT Age/Sex Fits − Males <2 Years Old − Severe Anaemia Prevalence − Telangana, Urban
WHO−2001 Adjustment Method

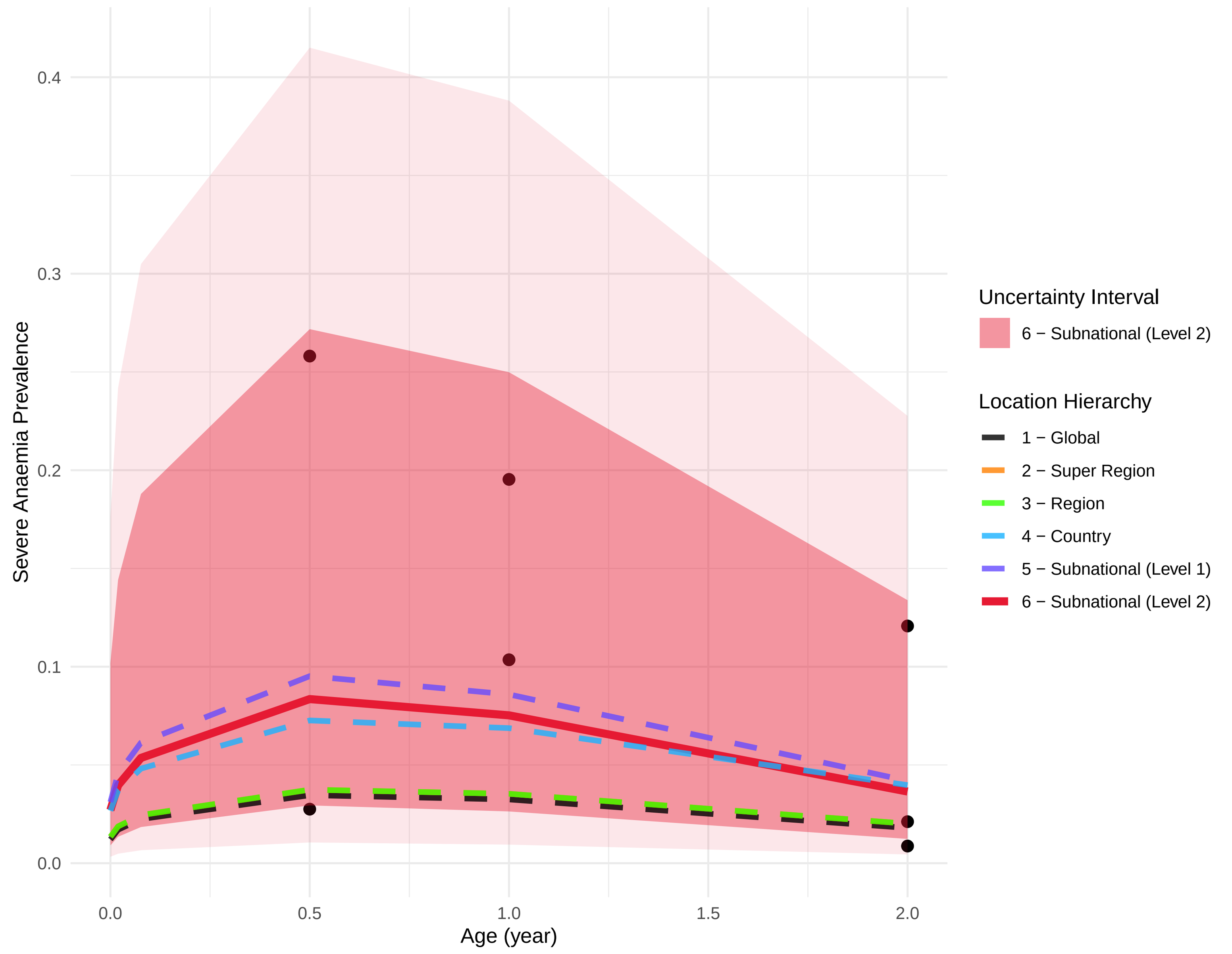

MR−BRT Age/Sex Fits − Males <2 Years Old − Severe Anaemia Prevalence − Tripura
WHO−2001 Adjustment Method

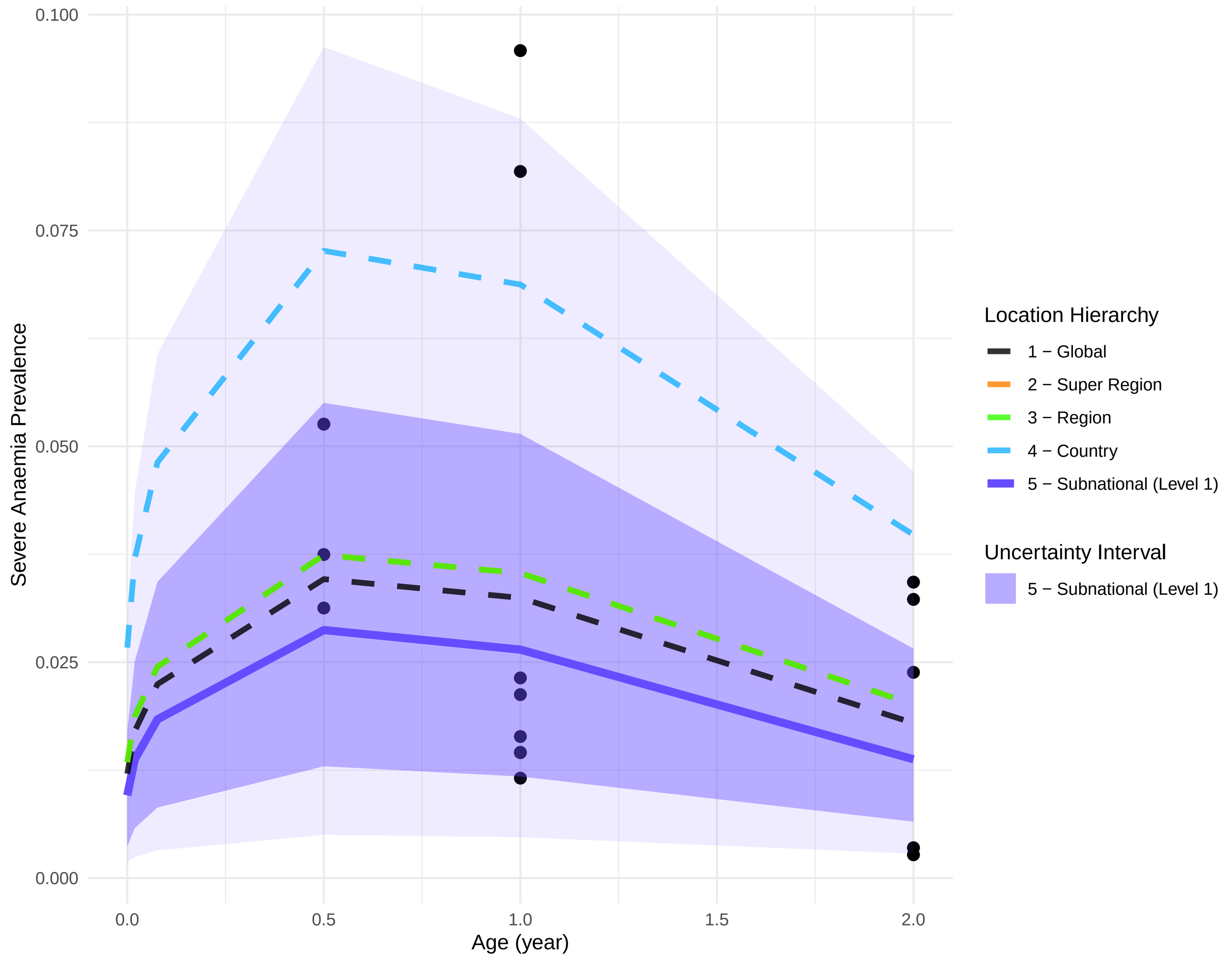

MR−BRT Age/Sex Fits − Males <2 Years Old − Severe Anaemia Prevalence − Tripura, Rural
WHO−2001 Adjustment Method

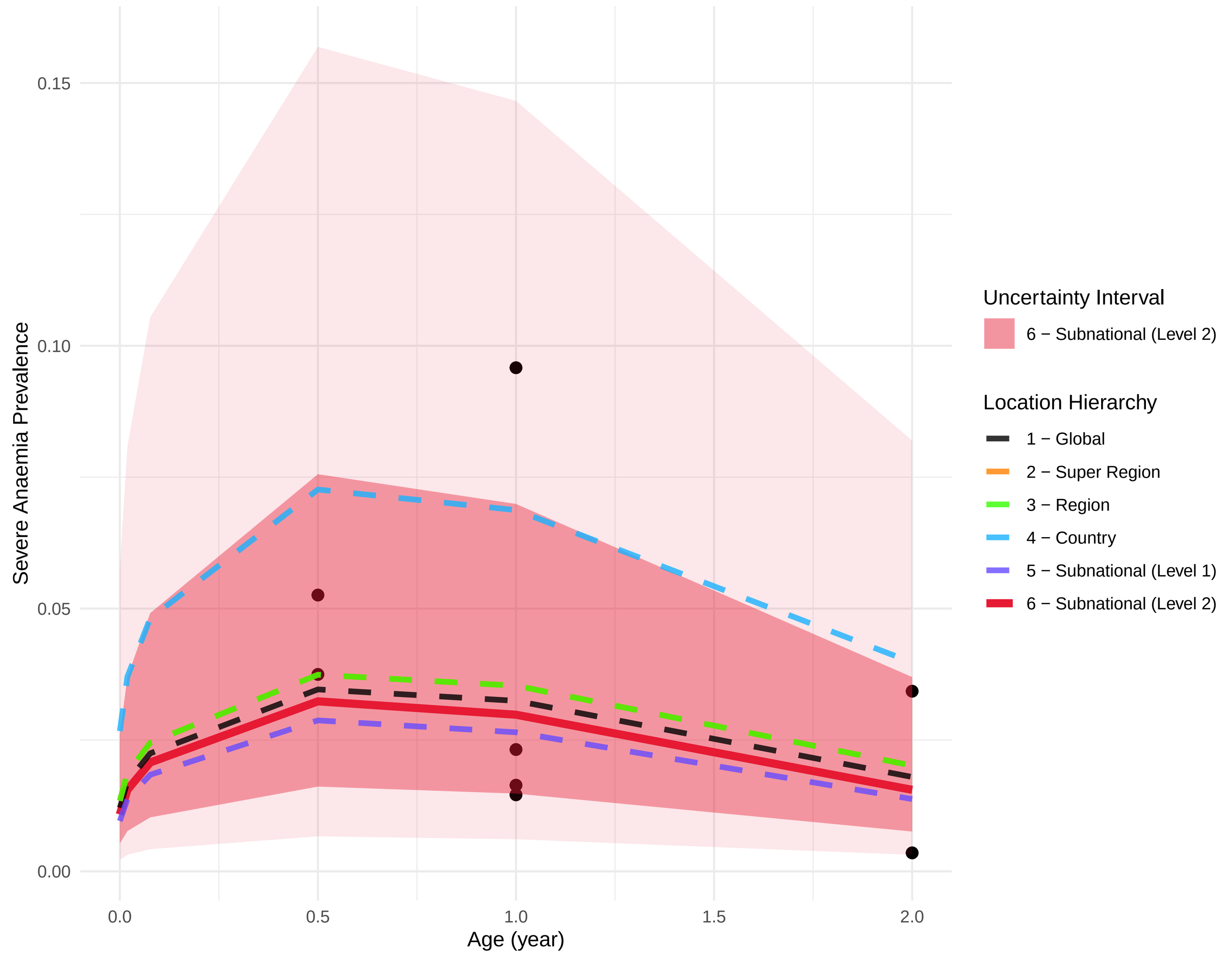

MR−BRT Age/Sex Fits − Males <2 Years Old − Severe Anaemia Prevalence − Tripura, Urban
WHO−2001 Adjustment Method

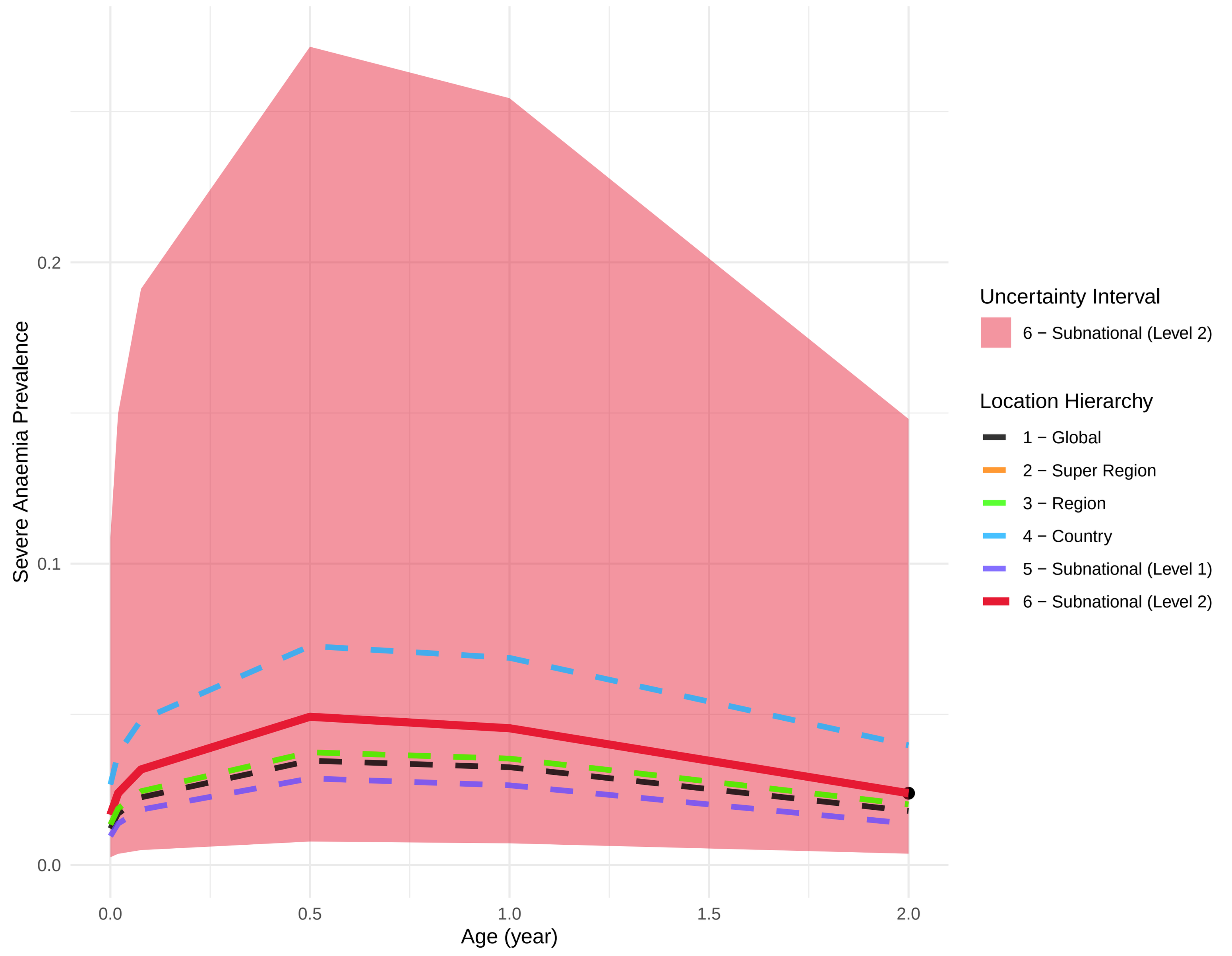

MR−BRT Age/Sex Fits − Males <2 Years Old − Severe Anaemia Prevalence − Uttar Pradesh
WHO−2001 Adjustment Method

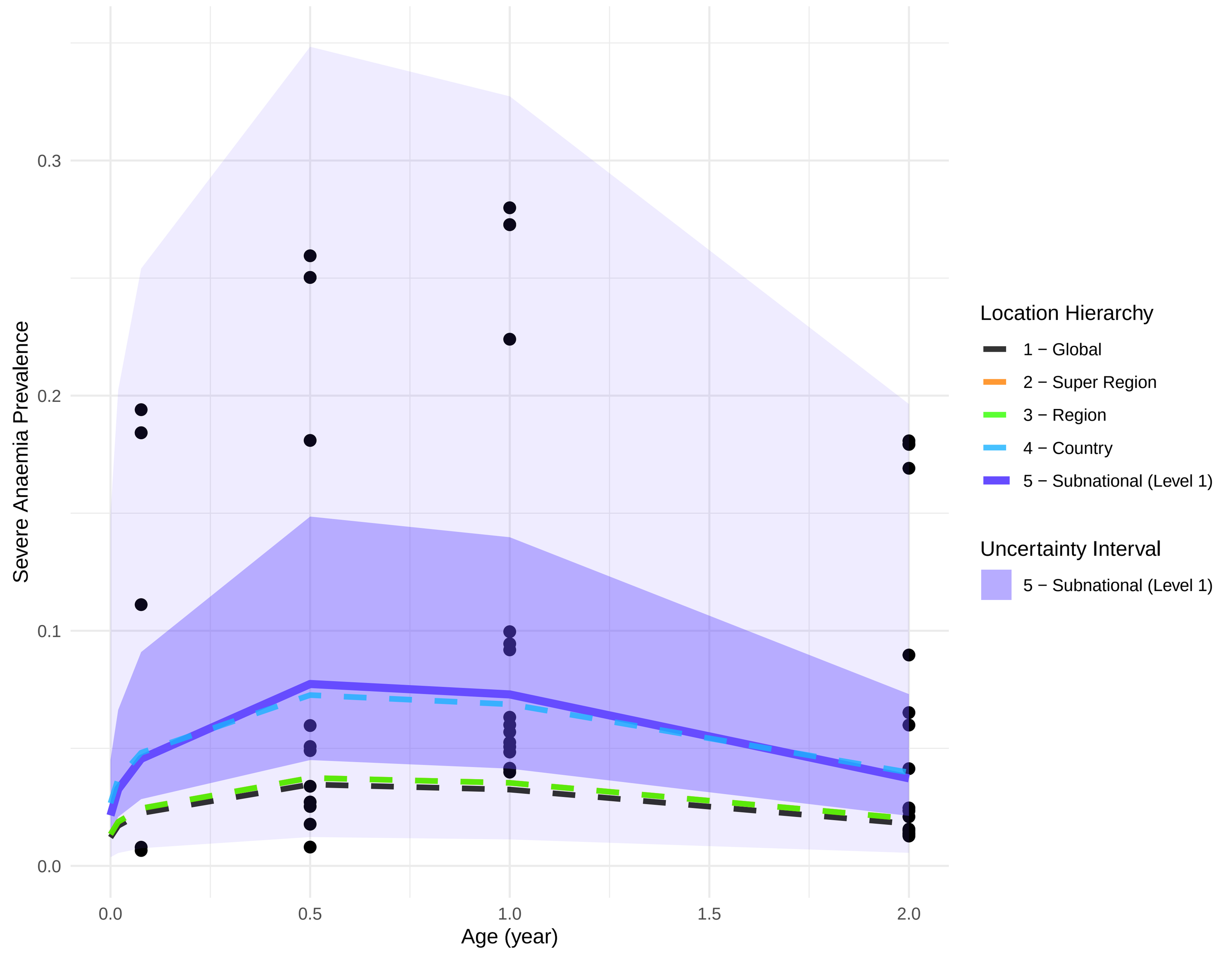

MR−BRT Age/Sex Fits − Males <2 Years Old − Severe Anaemia Prevalence − Uttar Pradesh, Rural
WHO−2001 Adjustment Method

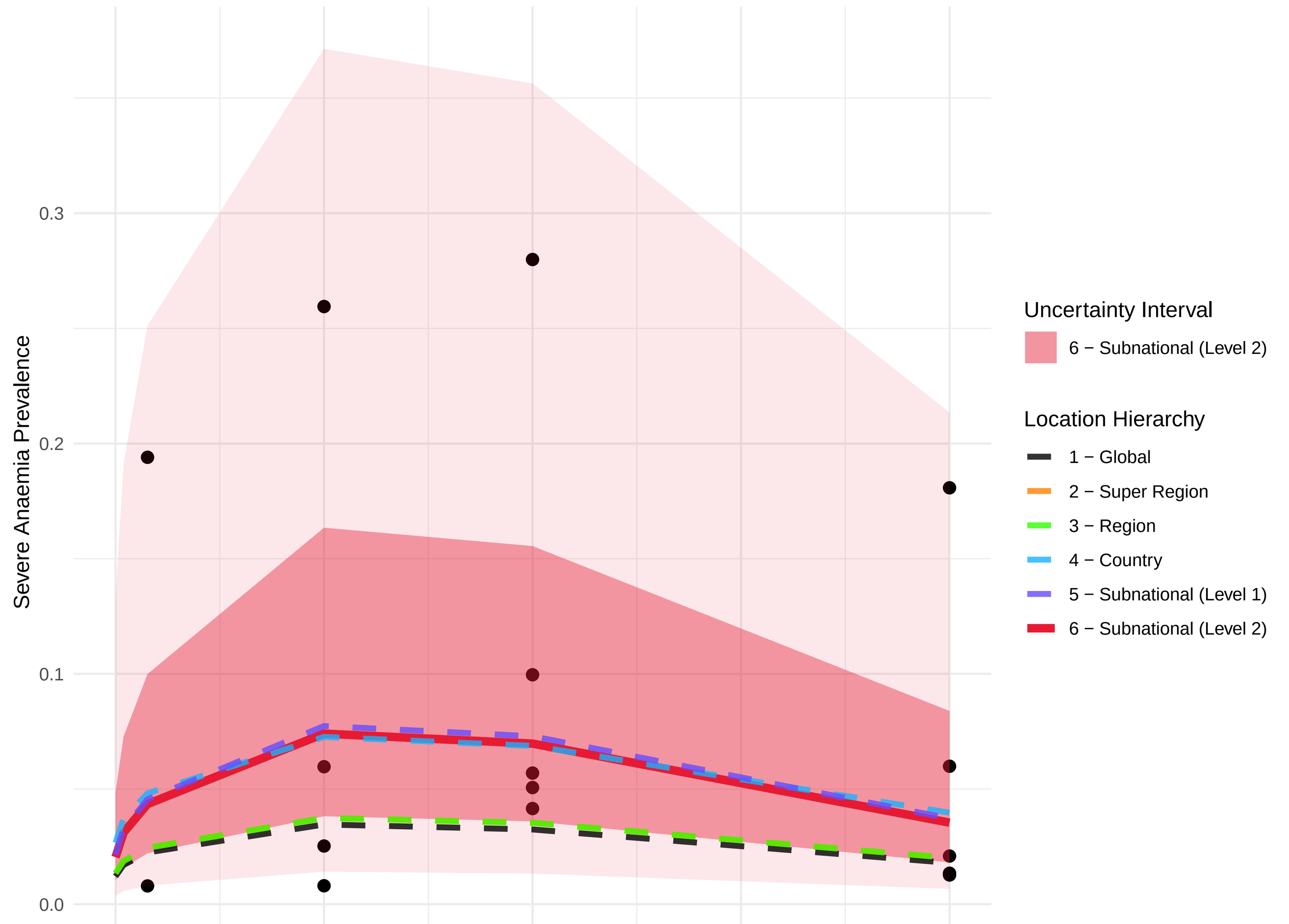

MR−BRT Age/Sex Fits − Males <2 Years Old − Severe Anaemia Prevalence − Uttar Pradesh, Urban
WHO−2001 Adjustment Method

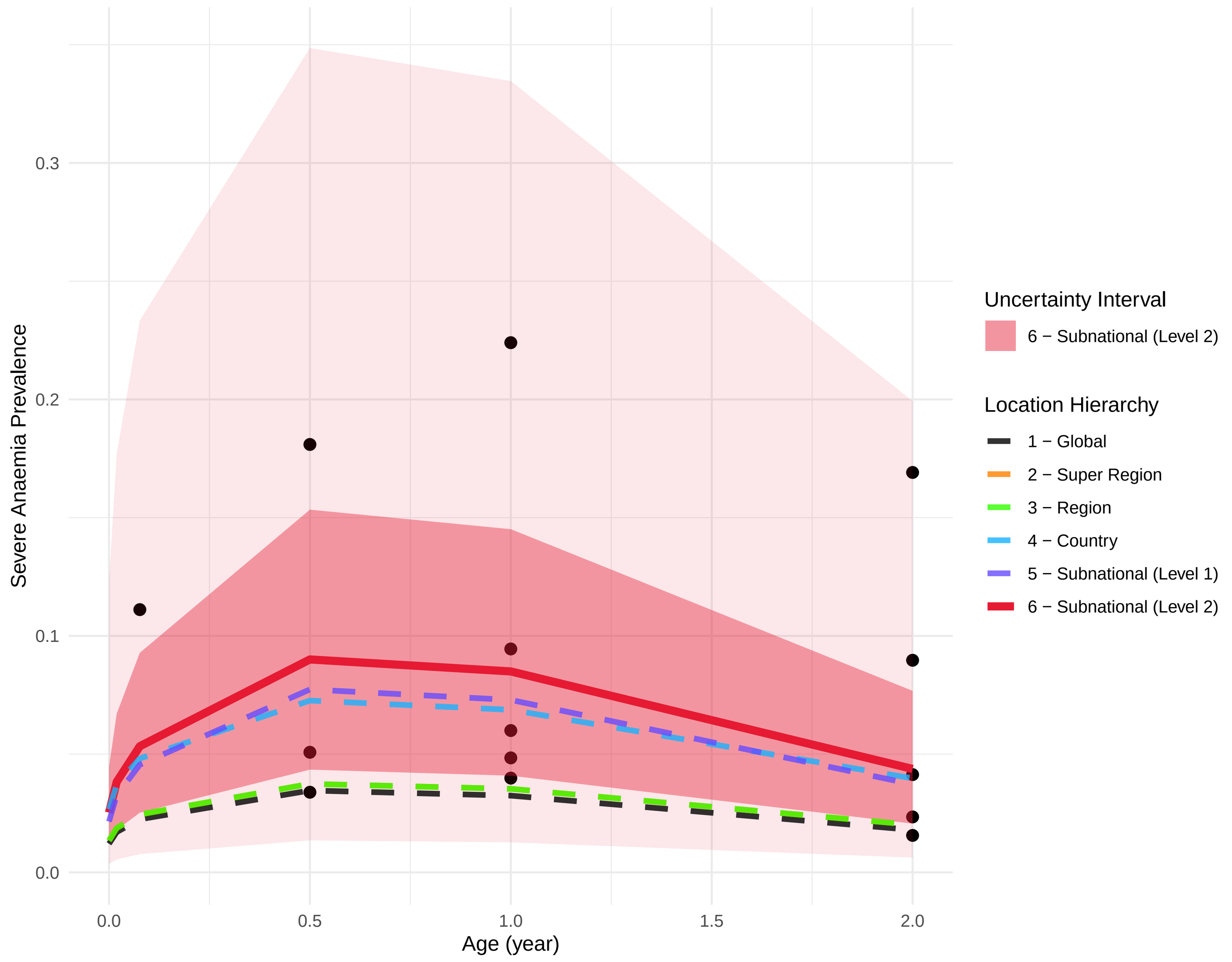

MR−BRT Age/Sex Fits − Males <2 Years Old − Severe Anaemia Prevalence − Uttarakhand
WHO−2001 Adjustment Method

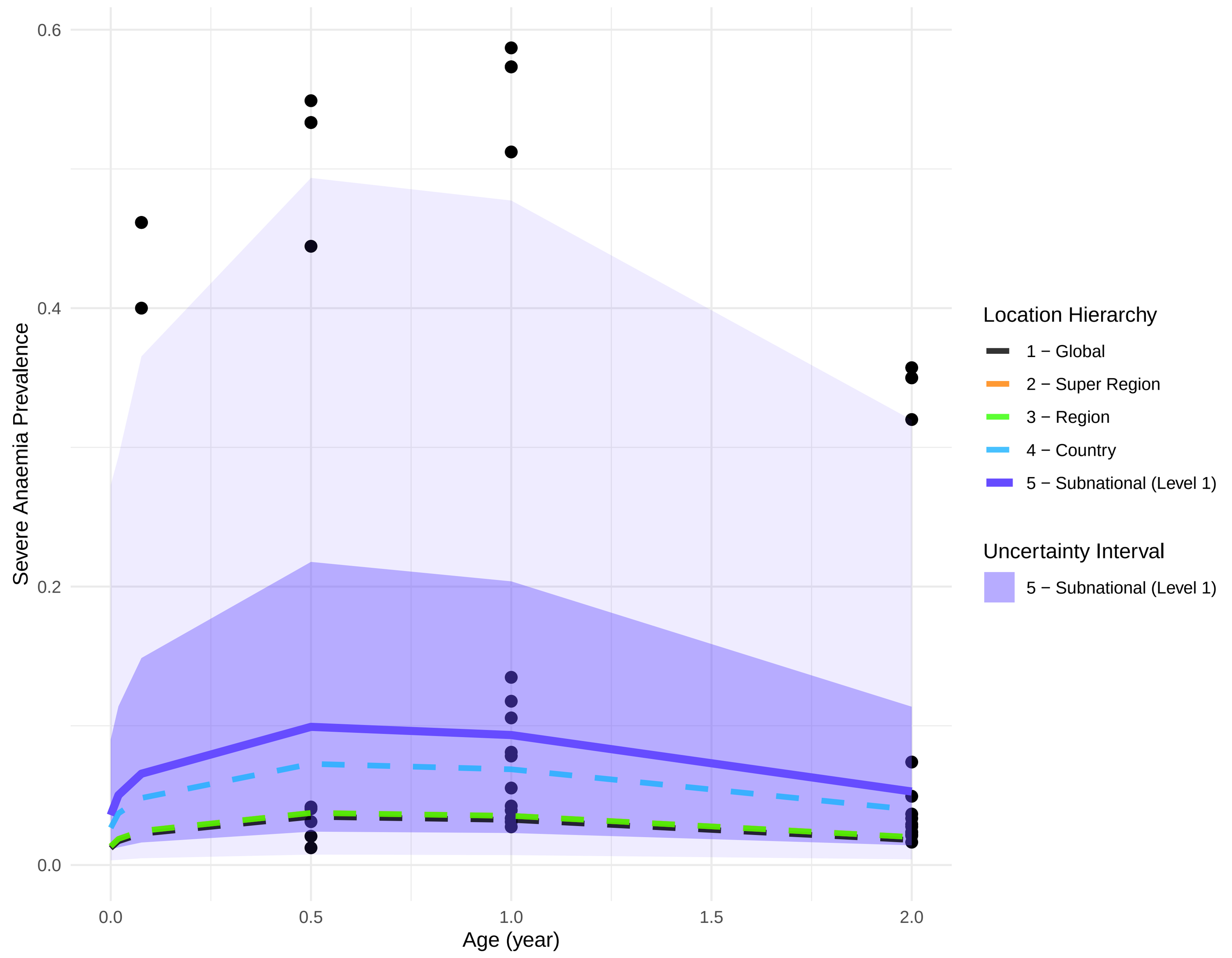

MR−BRT Age/Sex Fits − Males <2 Years Old − Severe Anaemia Prevalence − Uttarakhand, Rural
WHO−2001 Adjustment Method

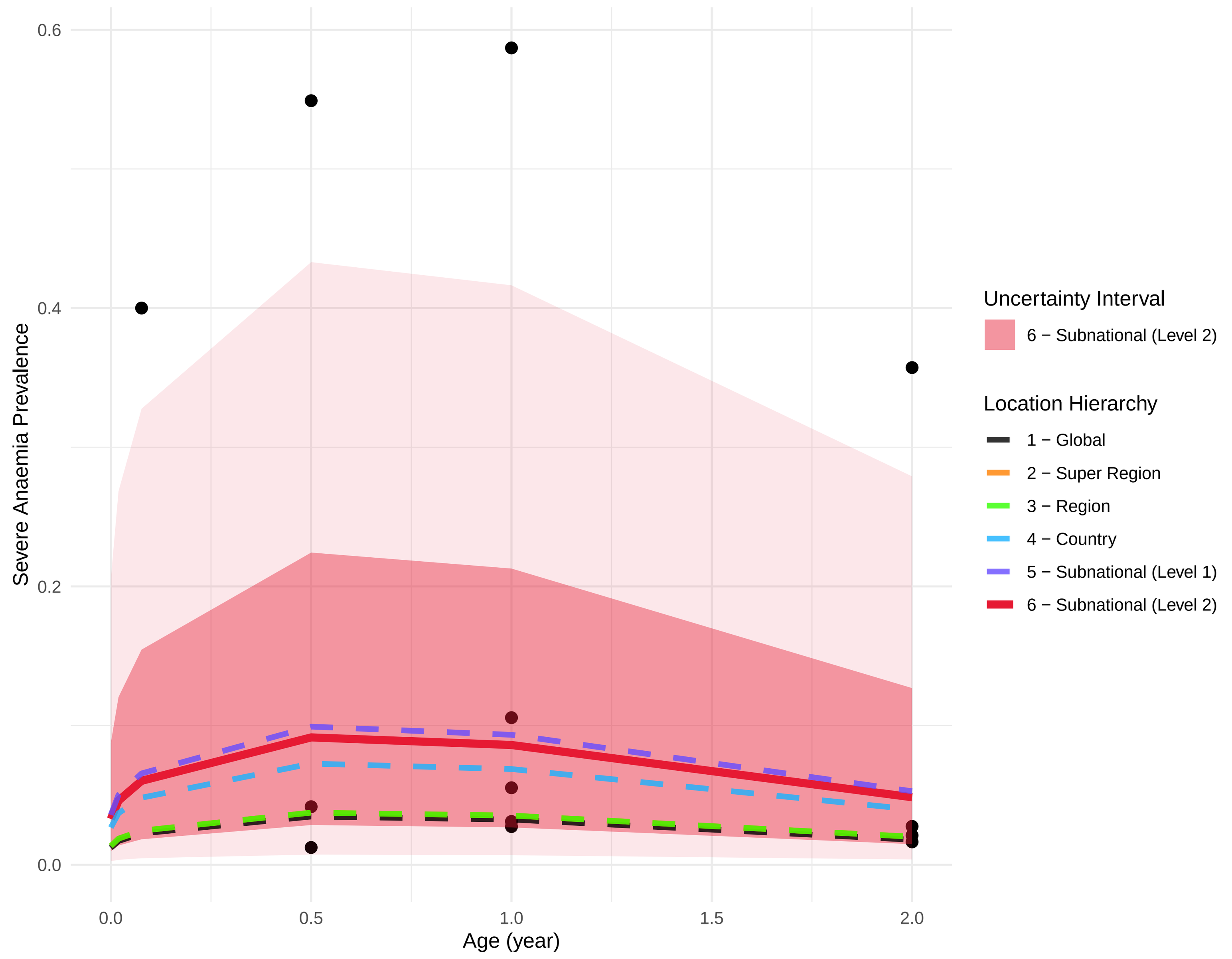

MR−BRT Age/Sex Fits − Males <2 Years Old − Severe Anaemia Prevalence − Uttarakhand, Urban
WHO−2001 Adjustment Method

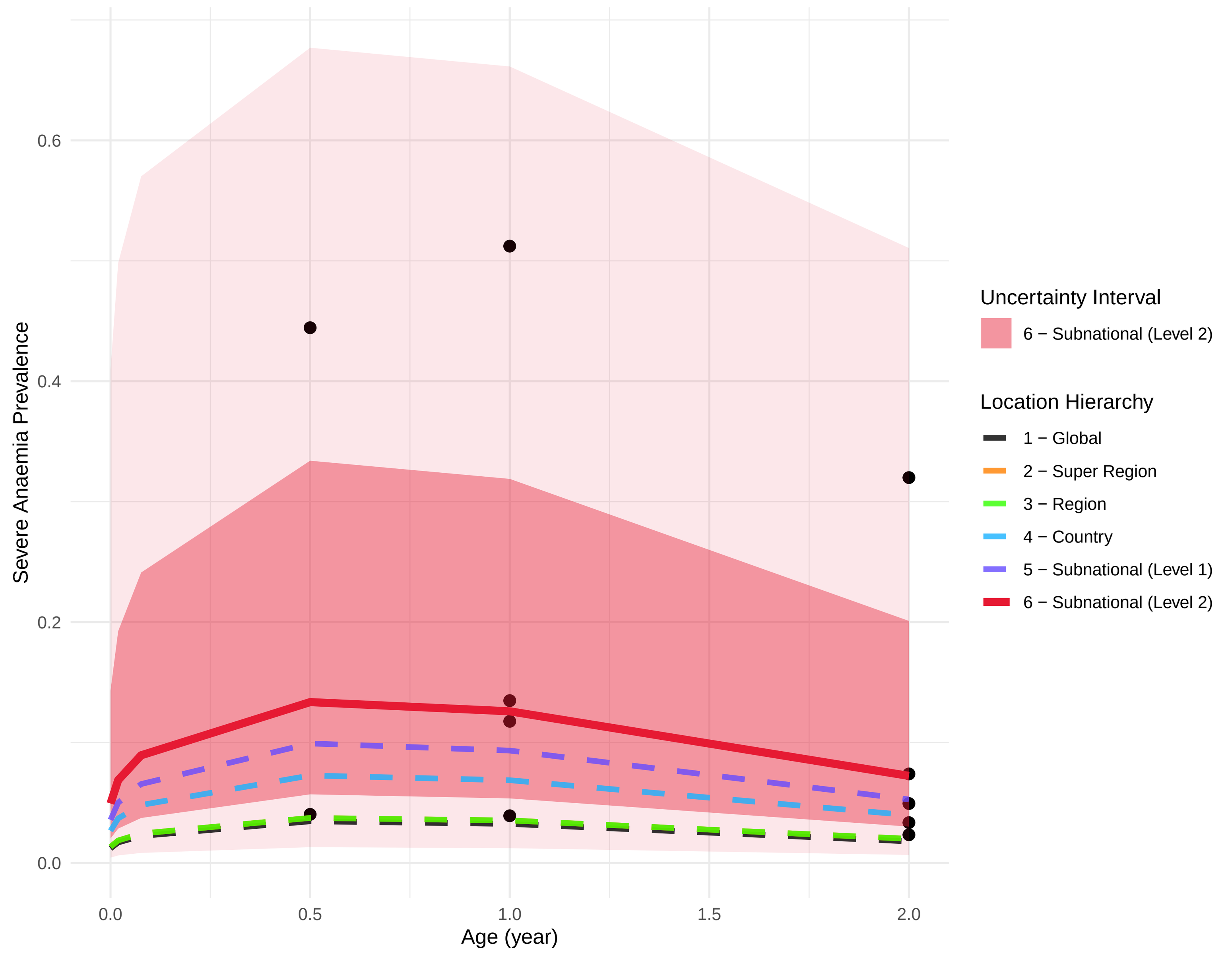

MR−BRT Age/Sex Fits − Males <2 Years Old − Severe Anaemia Prevalence − West Bengal
WHO−2001 Adjustment Method

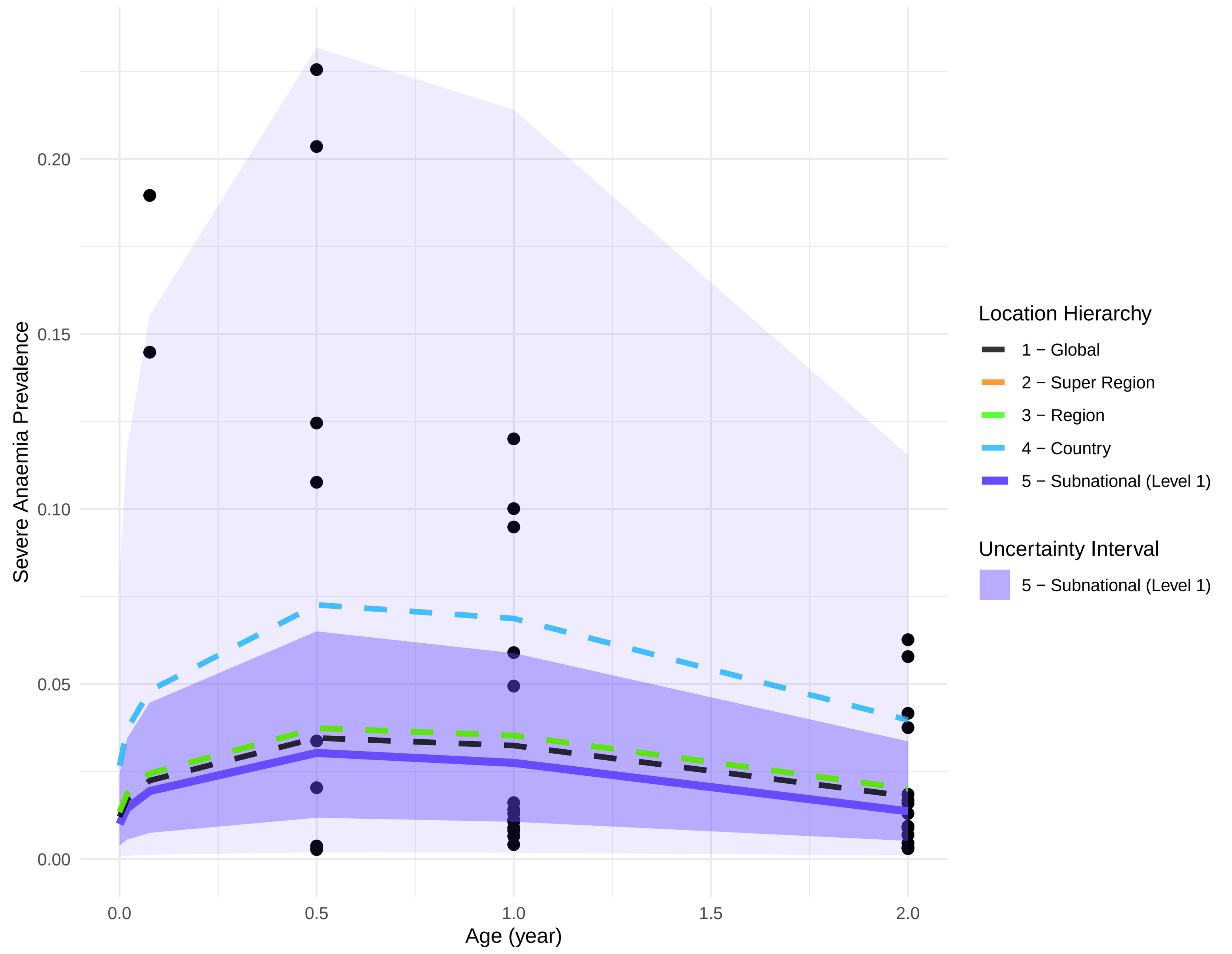

MR−BRT Age/Sex Fits − Males <2 Years Old − Severe Anaemia Prevalence − West Bengal, Rural
WHO−2001 Adjustment Method

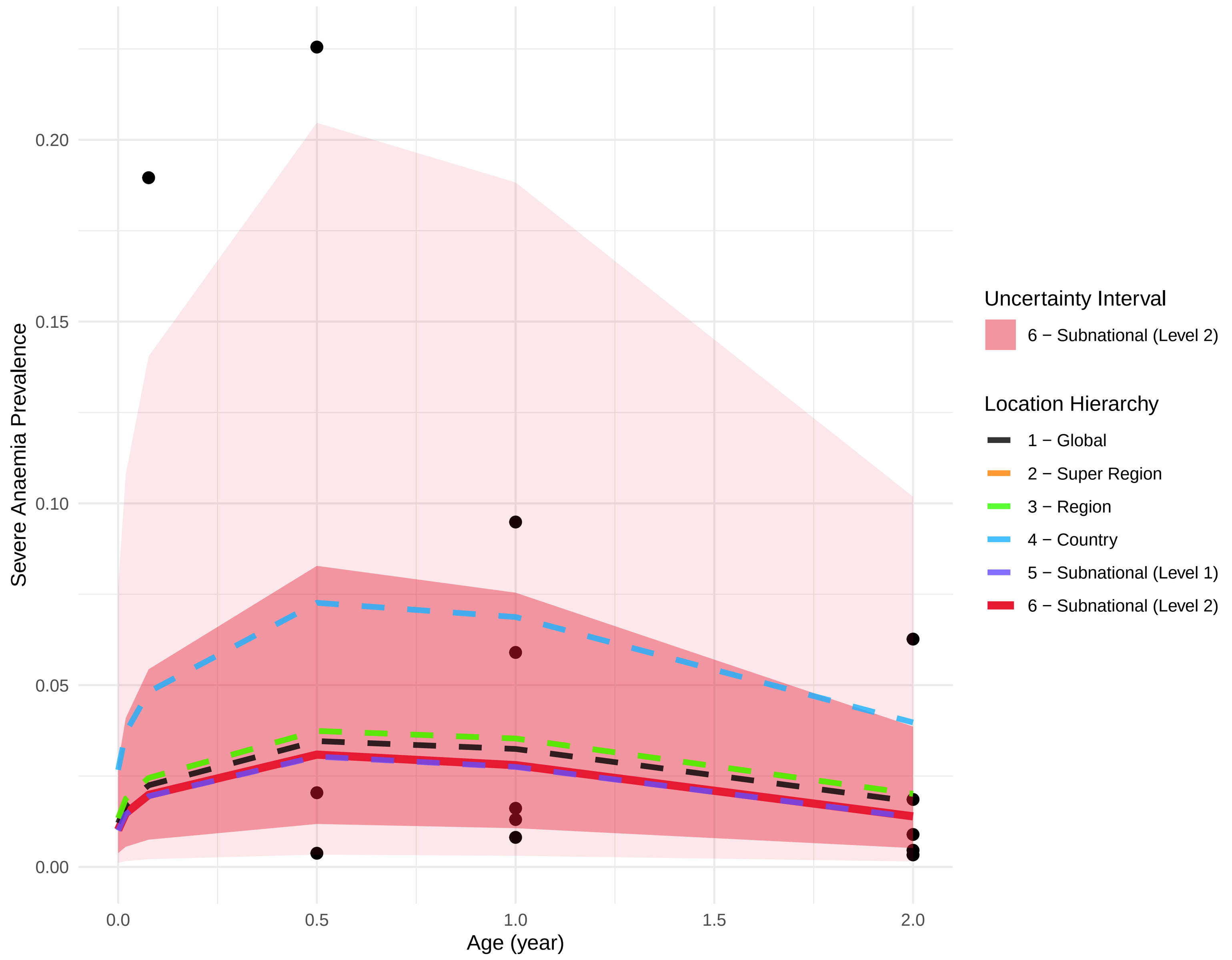

MR−BRT Age/Sex Fits − Males <2 Years Old − Severe Anaemia Prevalence − West Bengal, Urban
WHO−2001 Adjustment Method

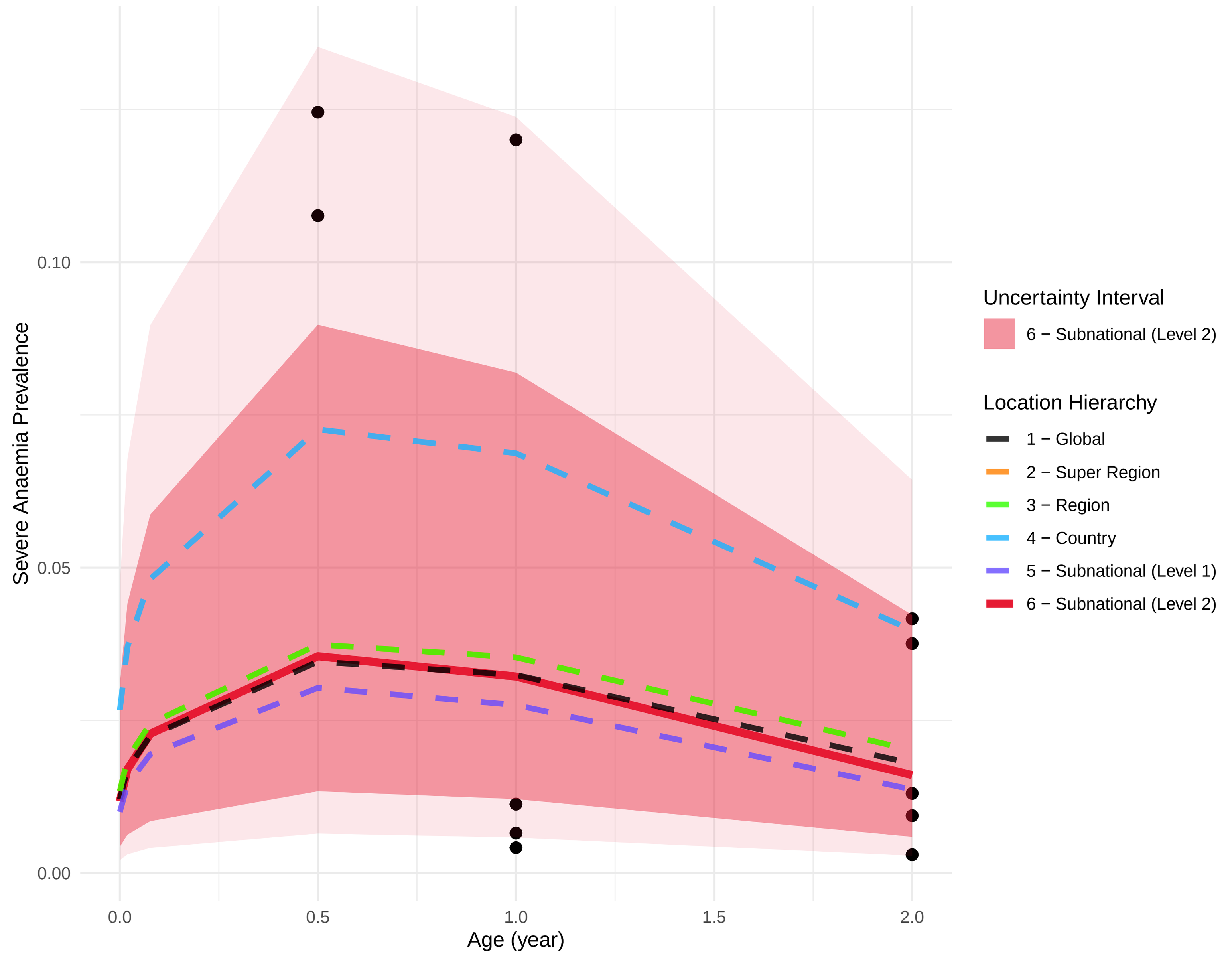

MR−BRT Age/Sex Fits − Males <2 Years Old − Severe Anaemia Prevalence − Nepal
WHO−2001 Adjustment Method

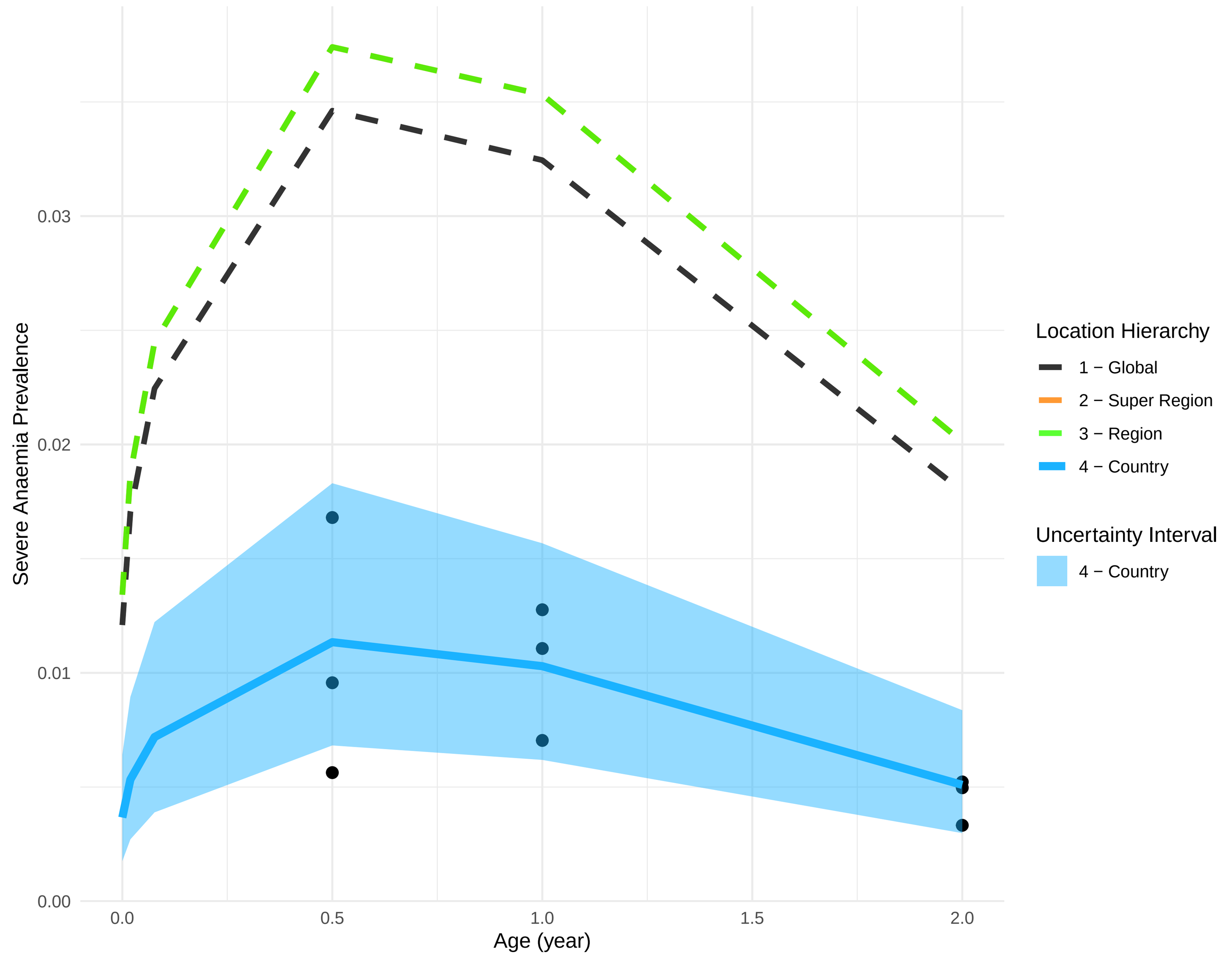

MR−BRT Age/Sex Fits − Males <2 Years Old − Severe Anaemia Prevalence − Southeast Asia, East Asia, and Oceania
WHO−2001 Adjustment Method

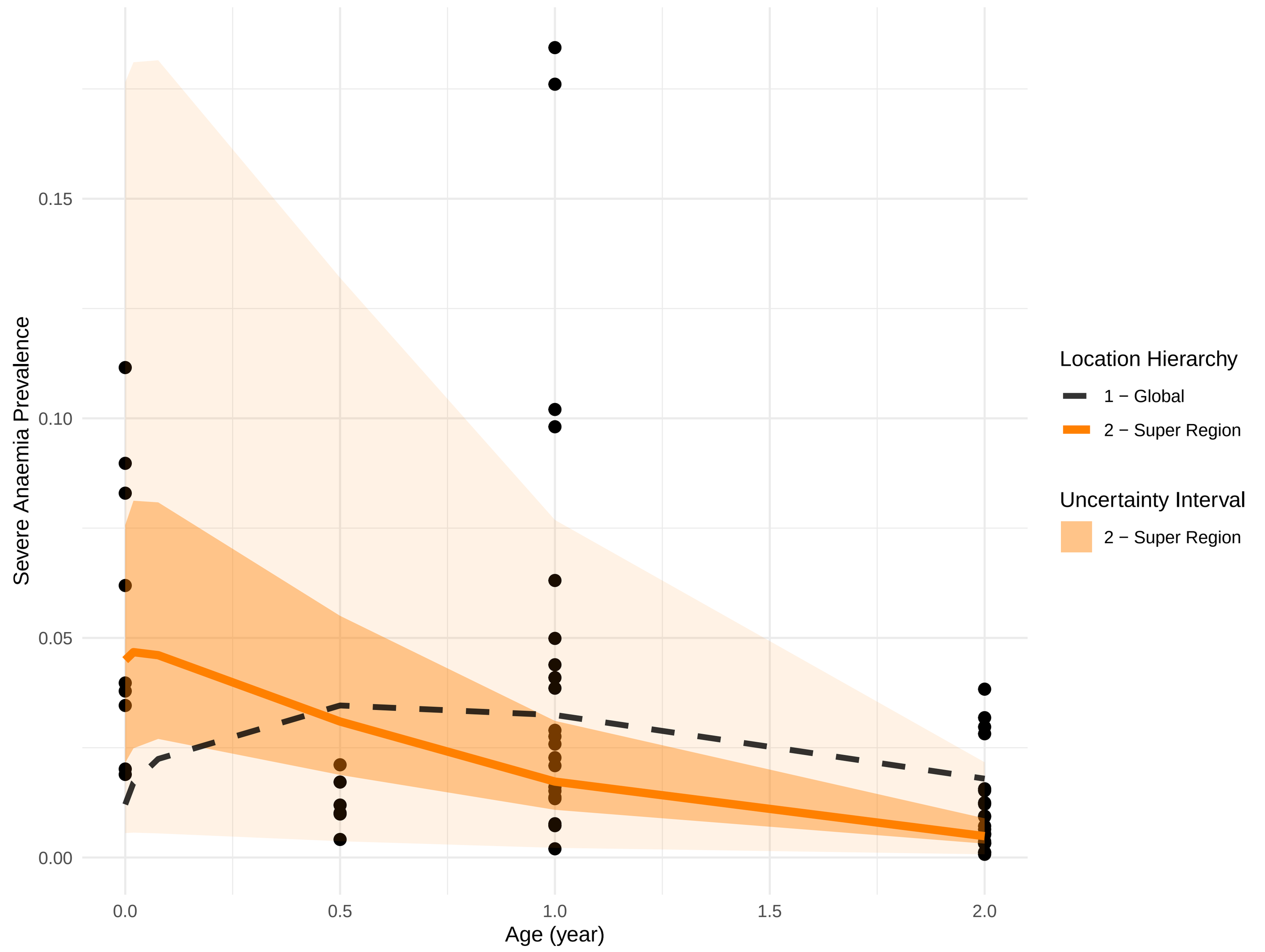

MR−BRT Age/Sex Fits − Males <2 Years Old − Severe Anaemia Prevalence − Southeast Asia
WHO−2001 Adjustment Method

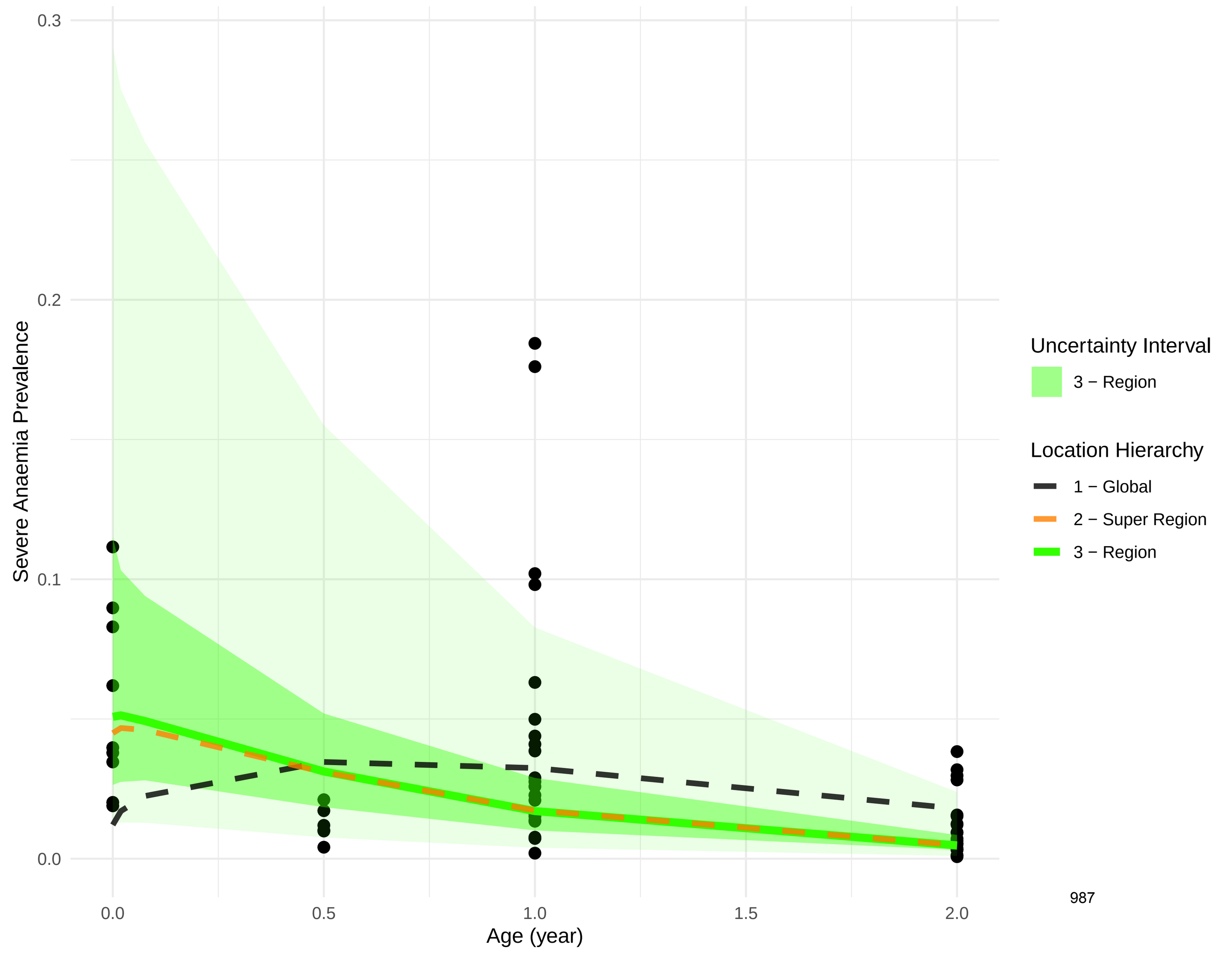

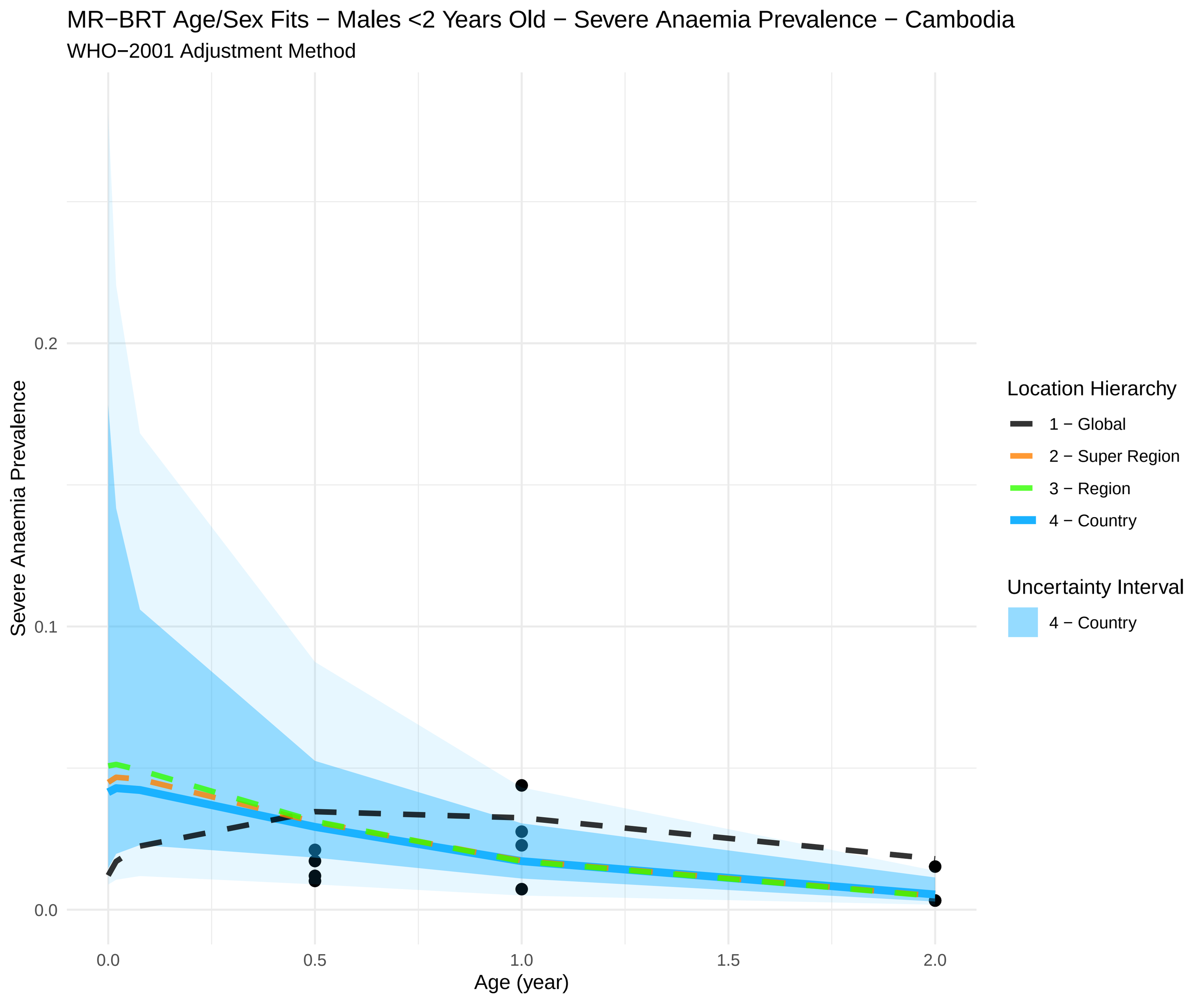
MR−BRT Age/Sex Fits − Males <2 Years Old − Severe Anaemia Prevalence − Cambodia
WHO−2001 Adjustment Method
Severe Anaemia Prevalence
Age (year)
Location Hierarchy
1 − Global
2 − Super Region
3 − Region
4 − Country
Uncertainty Interval
4 − Country

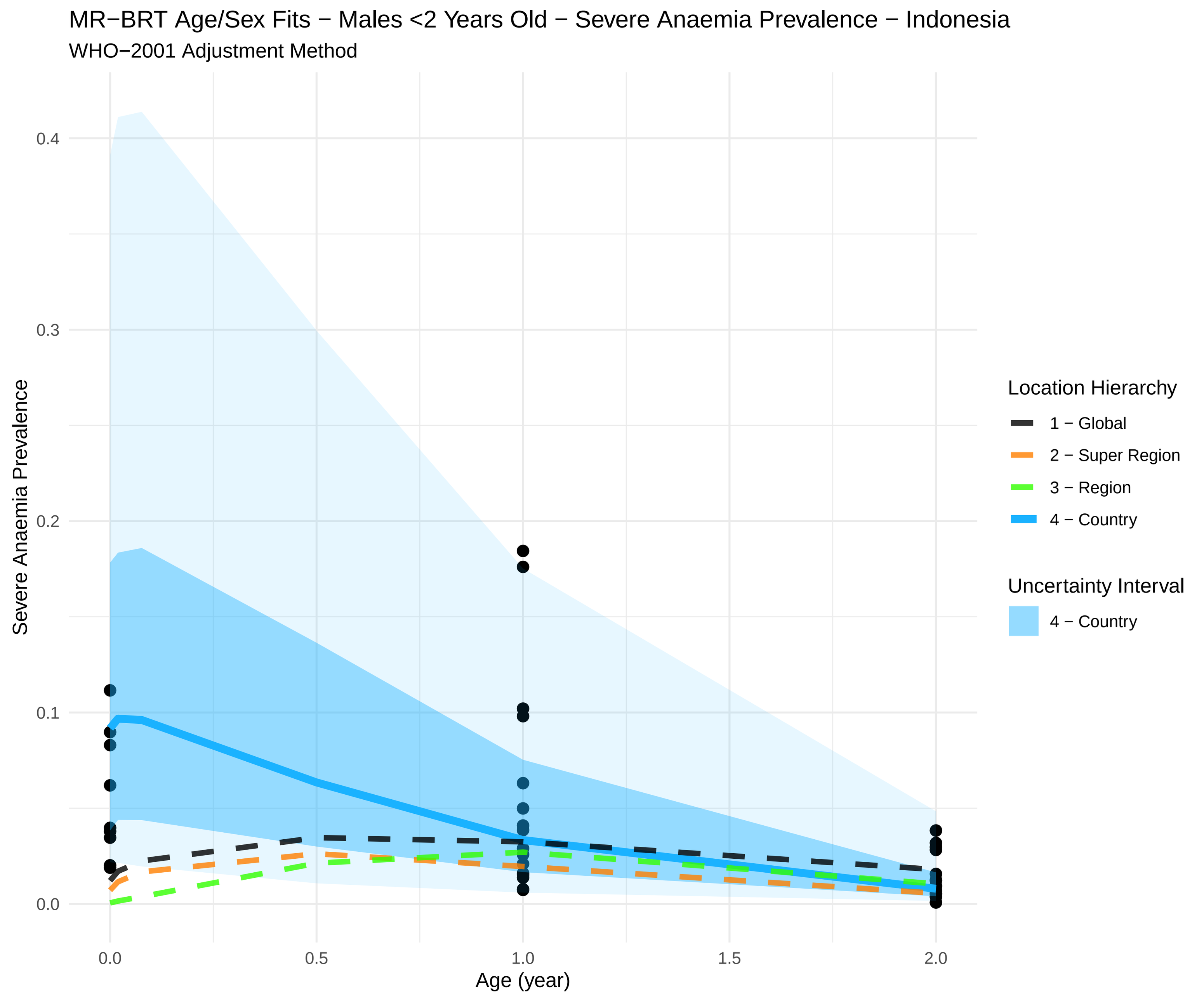
MR−BRT Age/Sex Fits − Males <2 Years Old − Severe Anaemia Prevalence − Indonesia
WHO−2001 Adjustment Method
Severe Anaemia Prevalence
Age (year)
Location Hierarchy
1 − Global
2 − Super Region
3 − Region
4 − Country
Uncertainty Interval
4 − Country

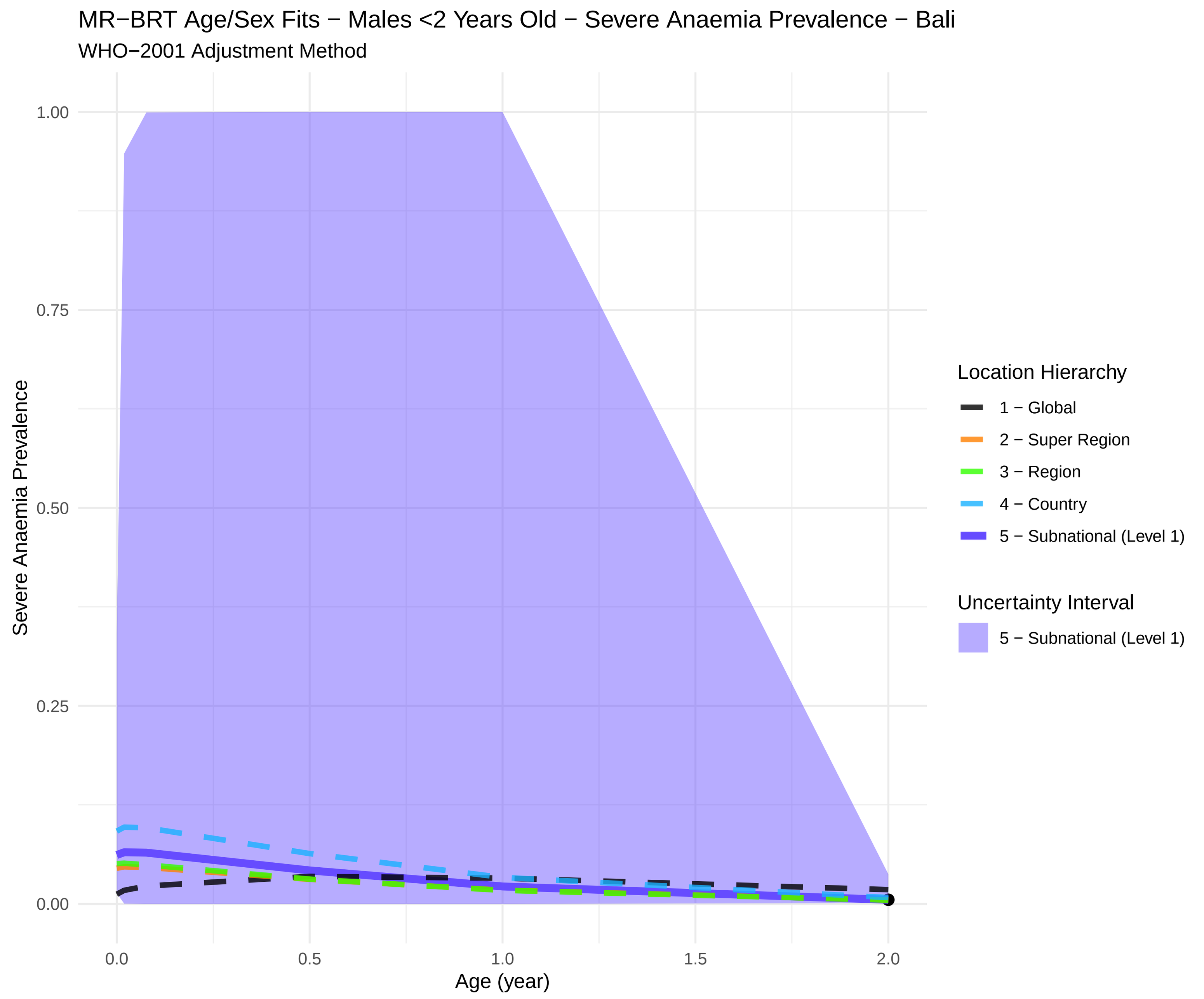
MR−BRT Age/Sex Fits − Males <2 Years Old − Severe Anaemia Prevalence − Bali
WHO−2001 Adjustment Method
Severe Anaemia Prevalence
Age (year)
Location Hierarchy
1 − Global
2 − Super Region
3 − Region
4 − Country
5 − Subnational (Level 1)
Uncertainty Interval
5 − Subnational (Level 1)

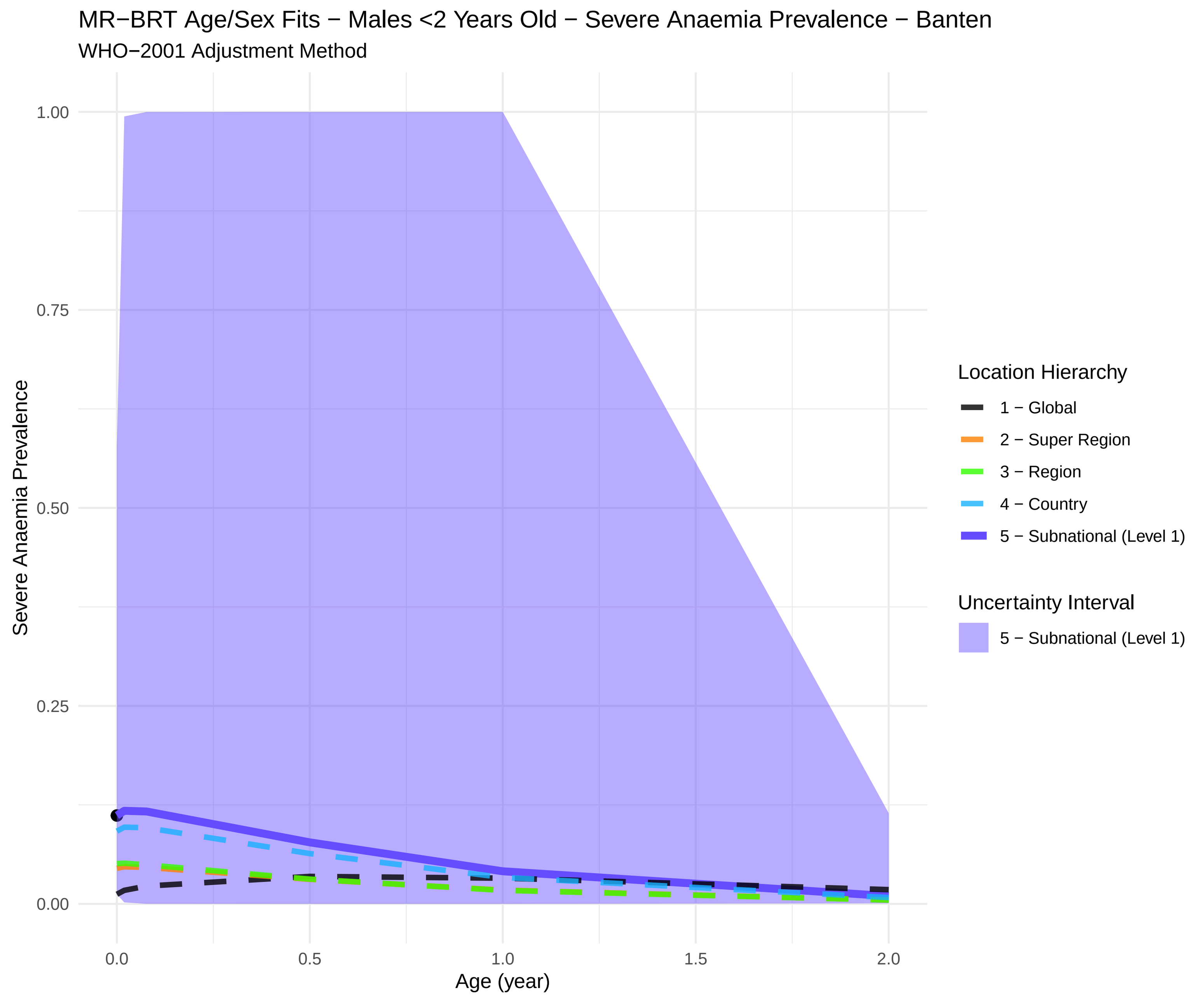
MR−BRT Age/Sex Fits − Males <2 Years Old − Severe Anaemia Prevalence − Banten
WHO−2001 Adjustment Method
Severe Anaemia Prevalence
Age (year)
Location Hierarchy
1 − Global
2 − Super Region
3 − Region
4 − Country
5 − Subnational (Level 1)
Uncertainty Interval
5 − Subnational (Level 1)

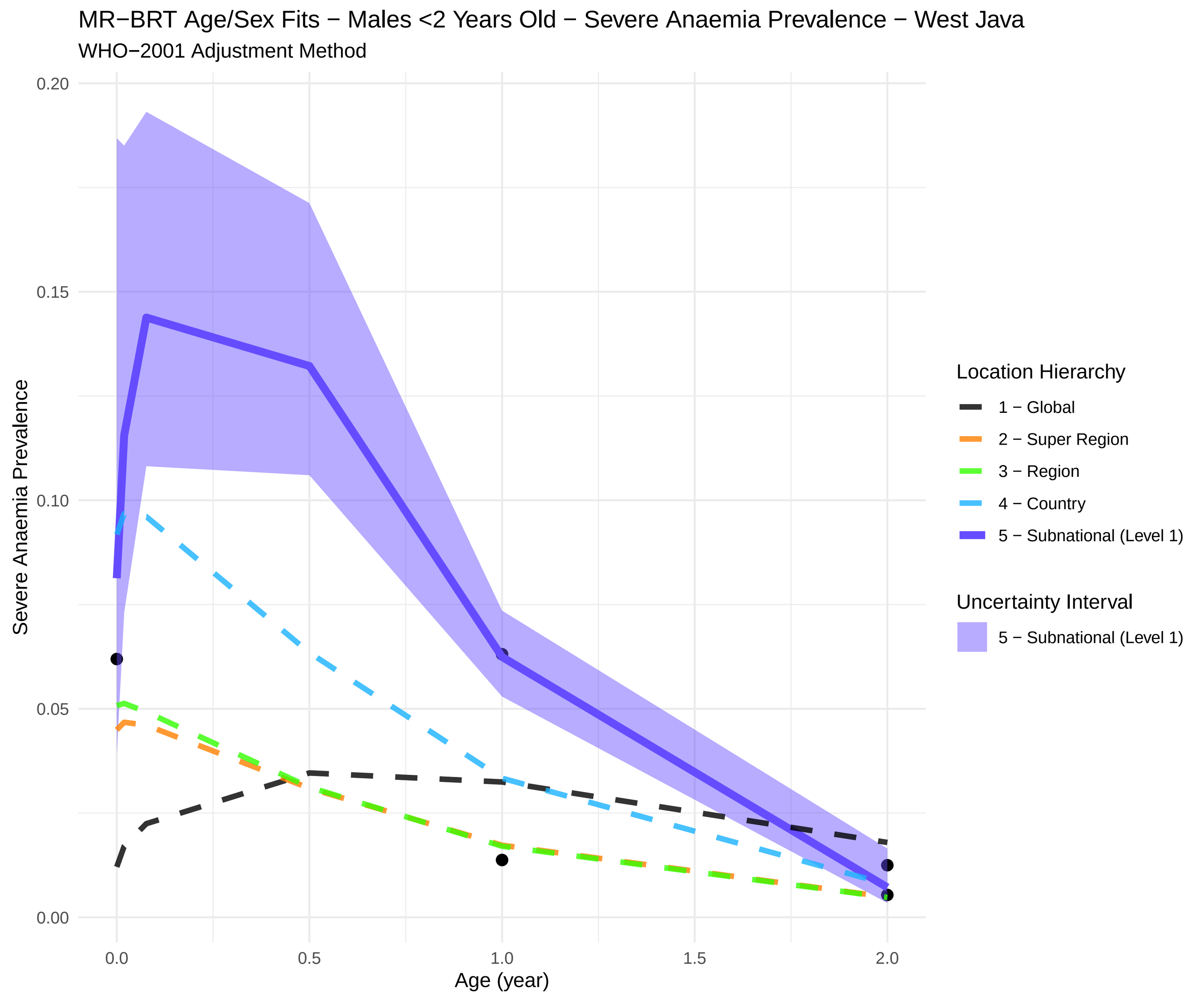
MR−BRT Age/Sex Fits − Males <2 Years Old − Severe Anaemia Prevalence − West Java
WHO−2001 Adjustment Method
Severe Anaemia Prevalence
Age (year)
Location Hierarchy
1 − Global
2 − Super Region
3 − Region
4 − Country
5 − Subnational (Level 1)
Uncertainty Interval
5 − Subnational (Level 1)

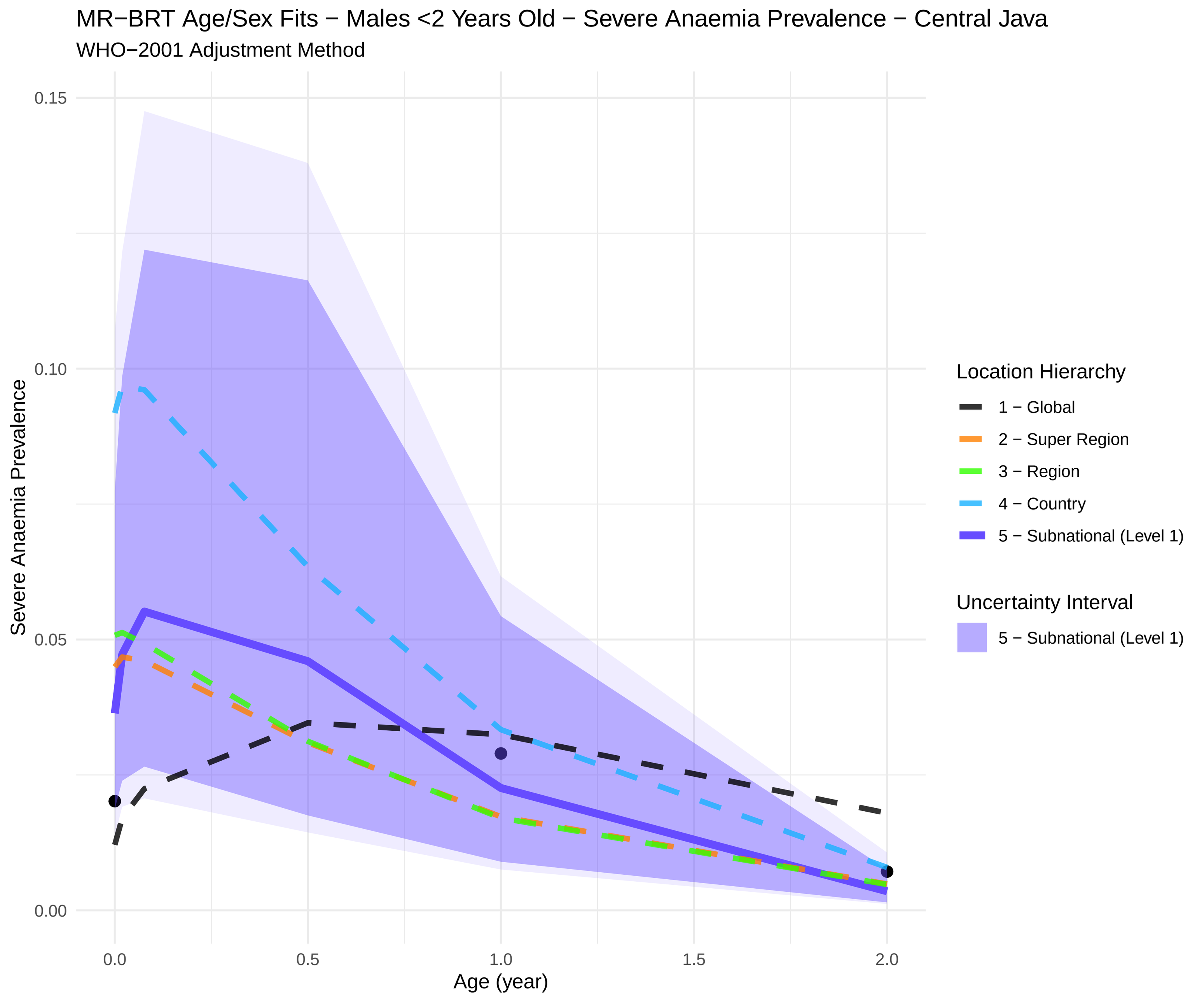
MR−BRT Age/Sex Fits − Males <2 Years Old − Severe Anaemia Prevalence − Central Java
WHO−2001 Adjustment Method
Severe Anaemia Prevalence
Age (year)
Location Hierarchy
1 − Global
2 − Super Region
3 − Region
4 − Country
5 − Subnational (Level 1)
Uncertainty Interval
5 − Subnational (Level 1)

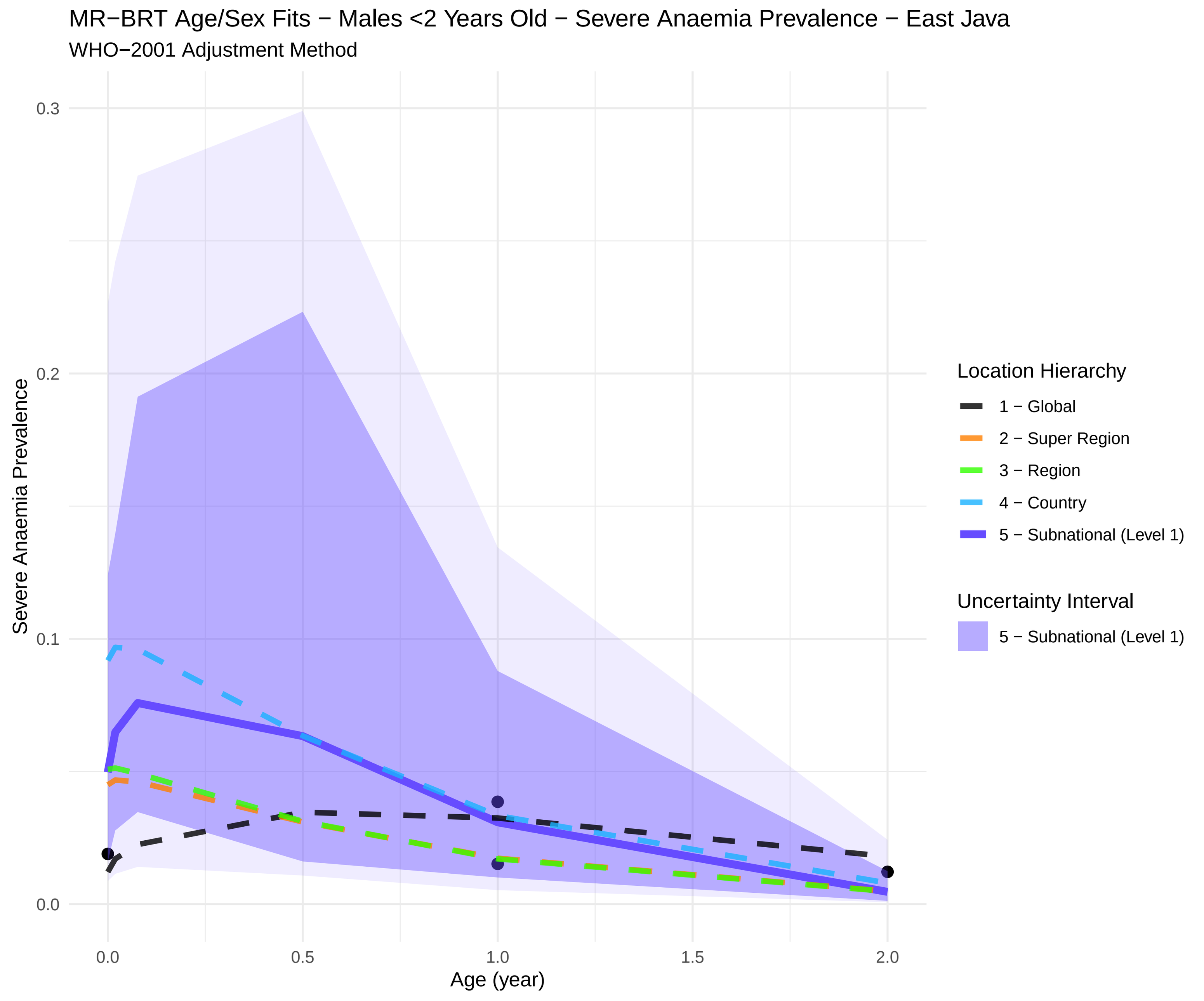
MR−BRT Age/Sex Fits − Males <2 Years Old − Severe Anaemia Prevalence − East Java
WHO−2001 Adjustment Method
Severe Anaemia Prevalence
Age (year)
Location Hierarchy
1 − Global
2 − Super Region
3 − Region
4 − Country
5 − Subnational (Level 1)
Uncertainty Interval
5 − Subnational (Level 1)

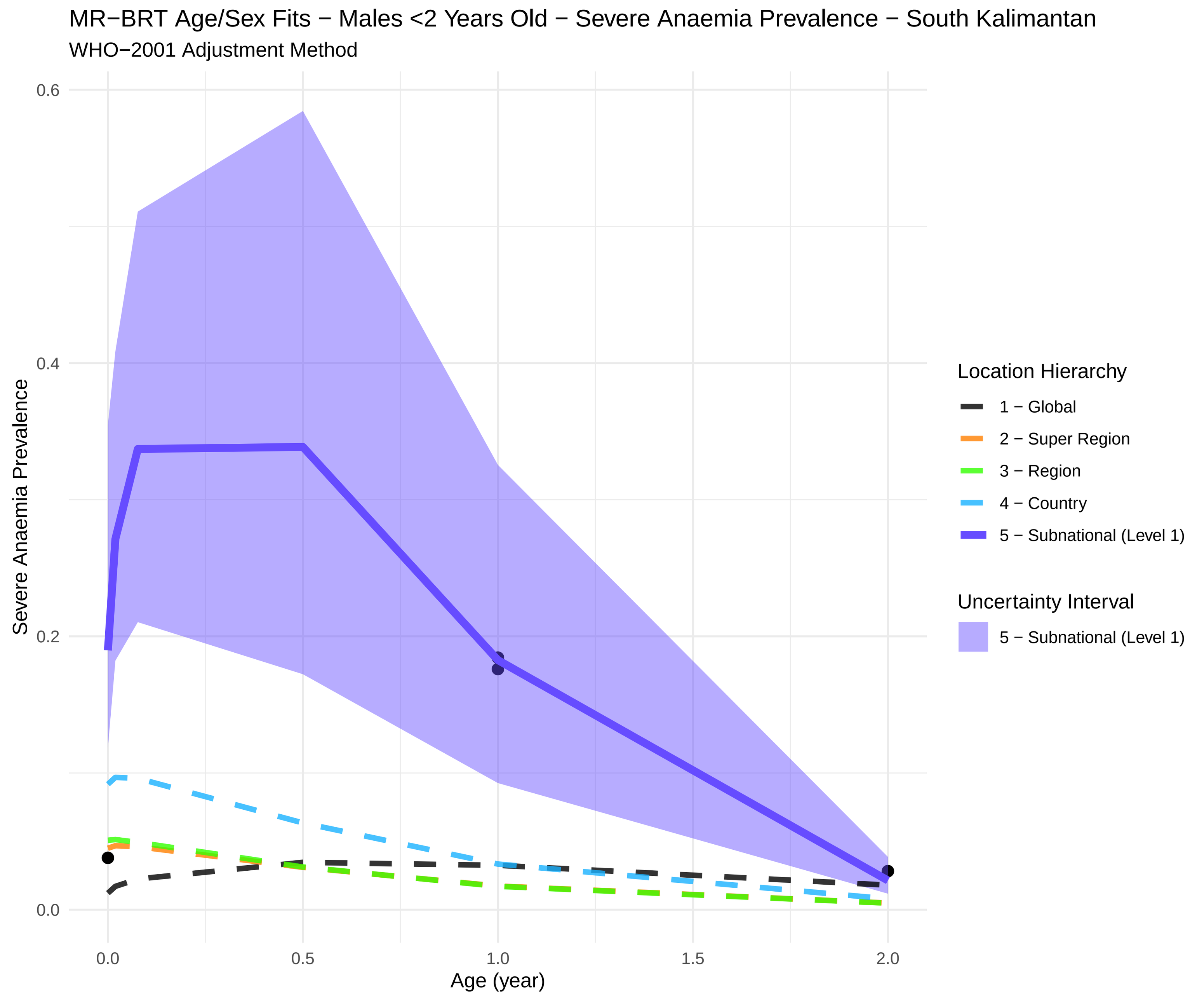
MR−BRT Age/Sex Fits − Males <2 Years Old − Severe Anaemia Prevalence − South Kalimantan
WHO−2001 Adjustment Method
Severe Anaemia Prevalence
Age (year)
Location Hierarchy
1 − Global
2 − Super Region
3 − Region
4 − Country
5 − Subnational (Level 1)
Uncertainty Interval
5 − Subnational (Level 1)

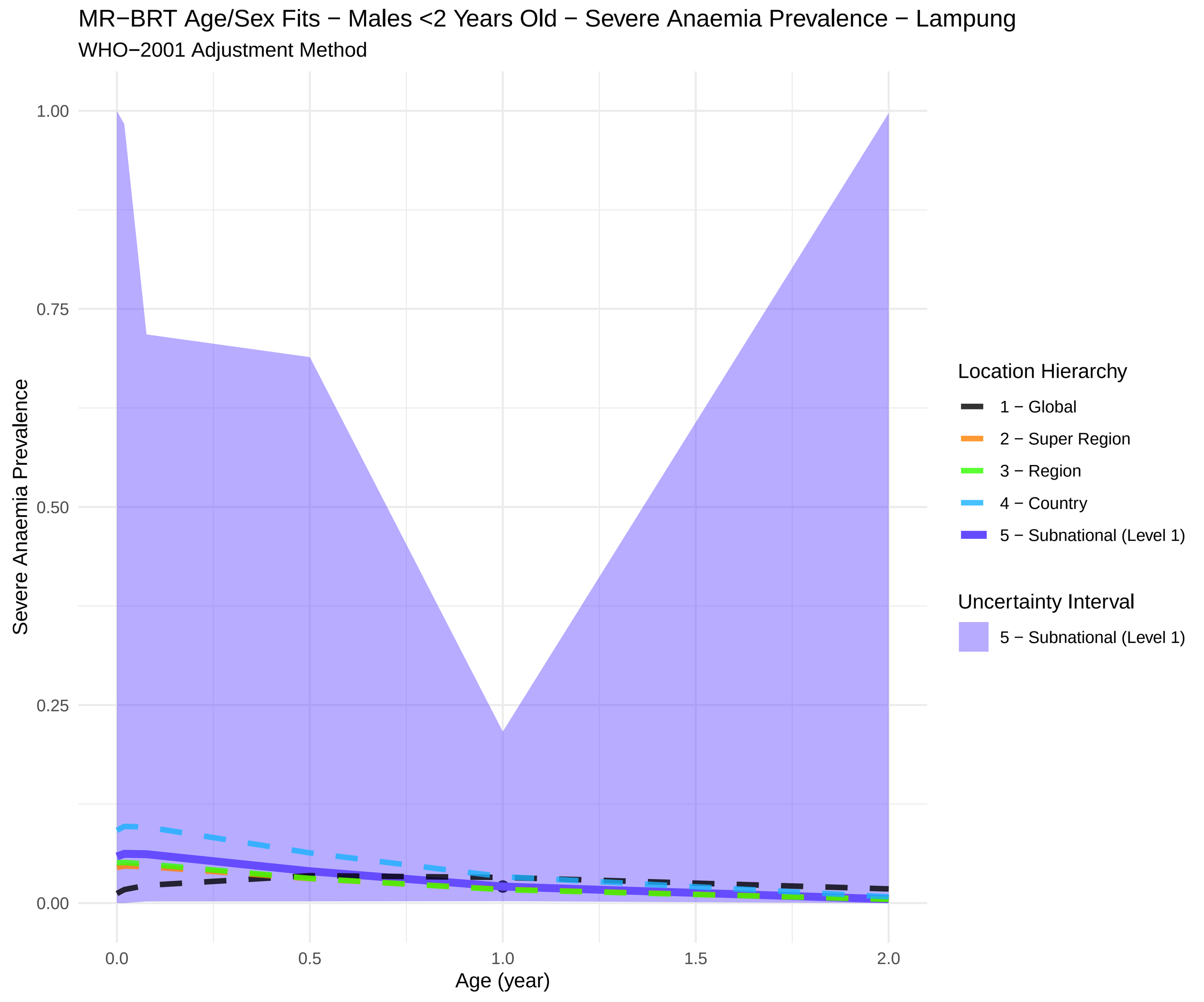
MR−BRT Age/Sex Fits − Males <2 Years Old − Severe Anaemia Prevalence − Lampung
WHO−2001 Adjustment Method
Severe Anaemia Prevalence
Age (year)
Location Hierarchy
1 − Global
2 − Super Region
3 − Region
4 − Country
5 − Subnational (Level 1)
Uncertainty Interval
5 − Subnational (Level 1)

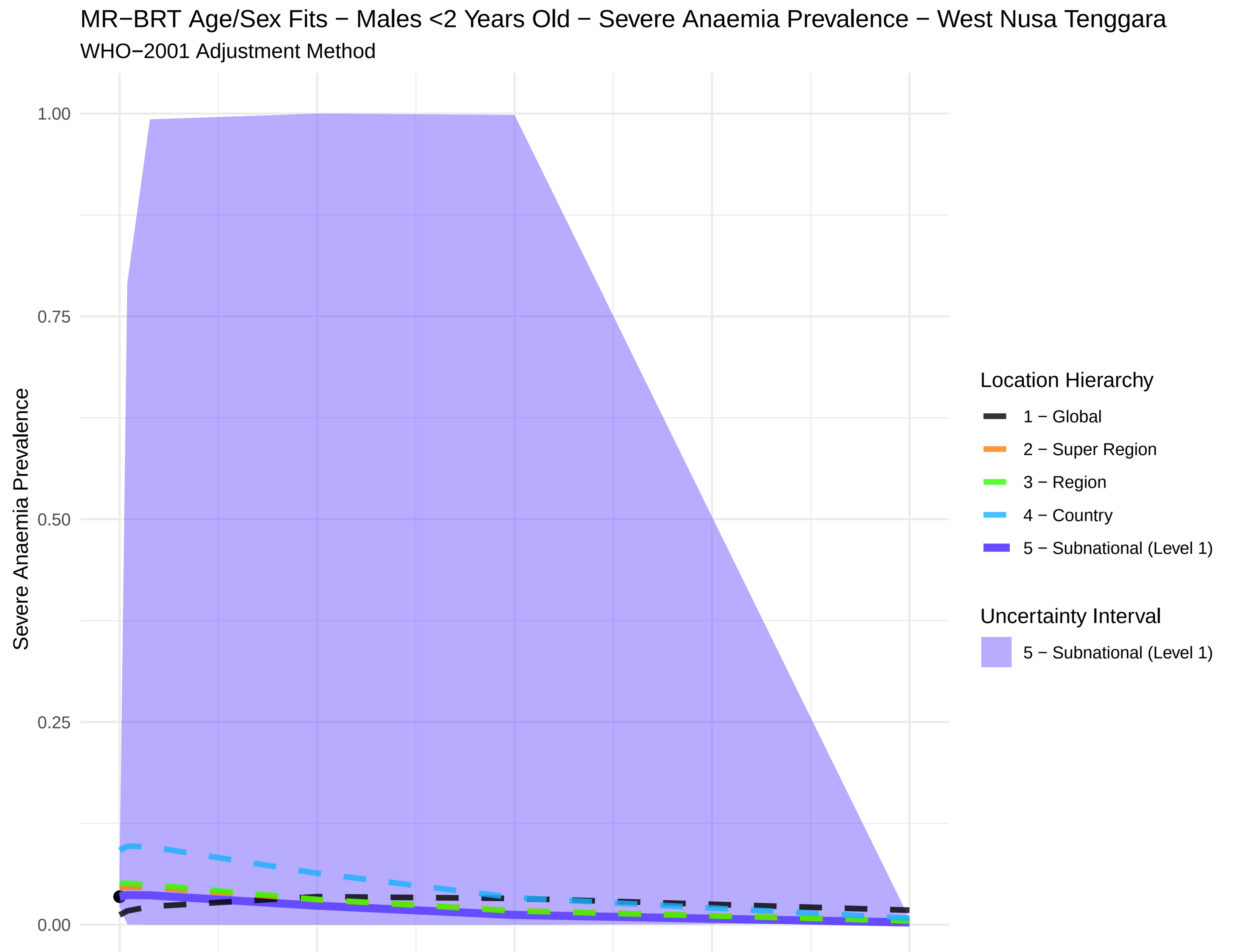
MR−BRT Age/Sex Fits − Males <2 Years Old − Severe Anaemia Prevalence − West Nusa Tenggara
WHO−2001 Adjustment Method
Severe Anaemia Prevalence
Age (year)
Location Hierarchy
1 − Global
2 − Super Region
3 − Region
4 − Country
5 − Subnational (Level 1)
Uncertainty Interval
5 − Subnational (Level 1)

MR−BRT Age/Sex Fits − Males <2 Years Old − Severe Anaemia Prevalence − South Sulawesi
WHO−2001 Adjustment Method

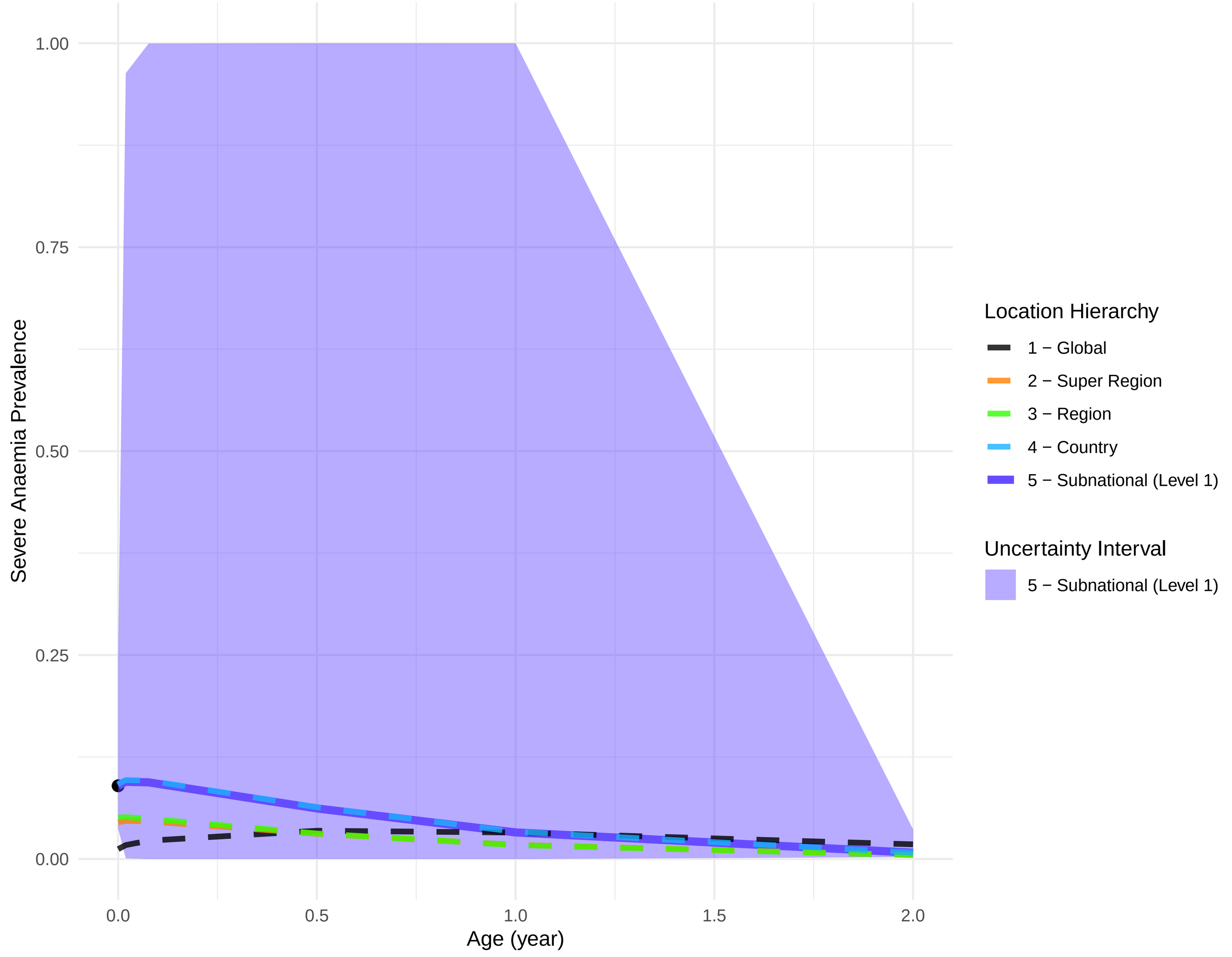

MR−BRT Age/Sex Fits − Males <2 Years Old − Severe Anaemia Prevalence − West Sumatra
WHO−2001 Adjustment Method

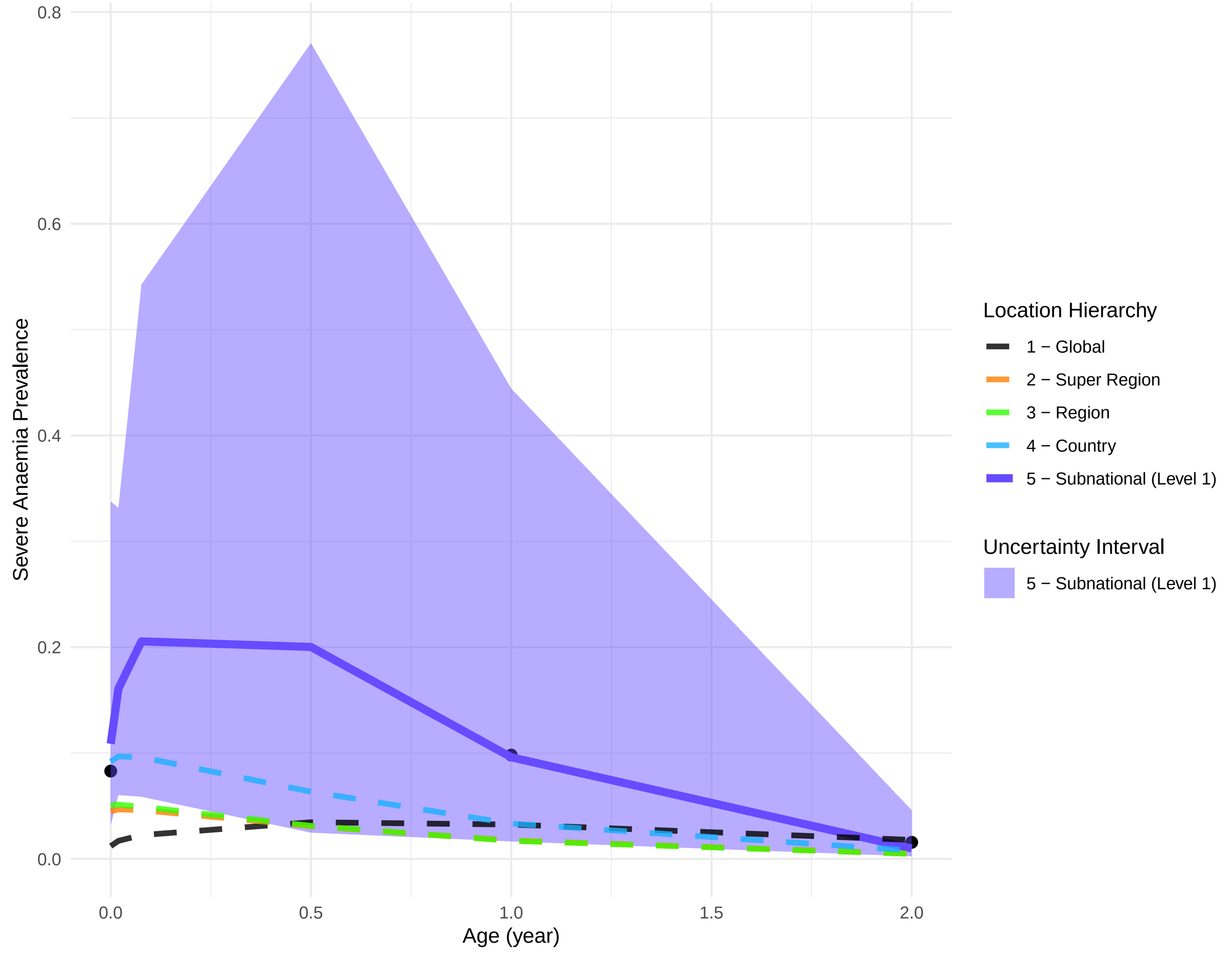

MR−BRT Age/Sex Fits − Males <2 Years Old − Severe Anaemia Prevalence − South Sumatra
WHO−2001 Adjustment Method

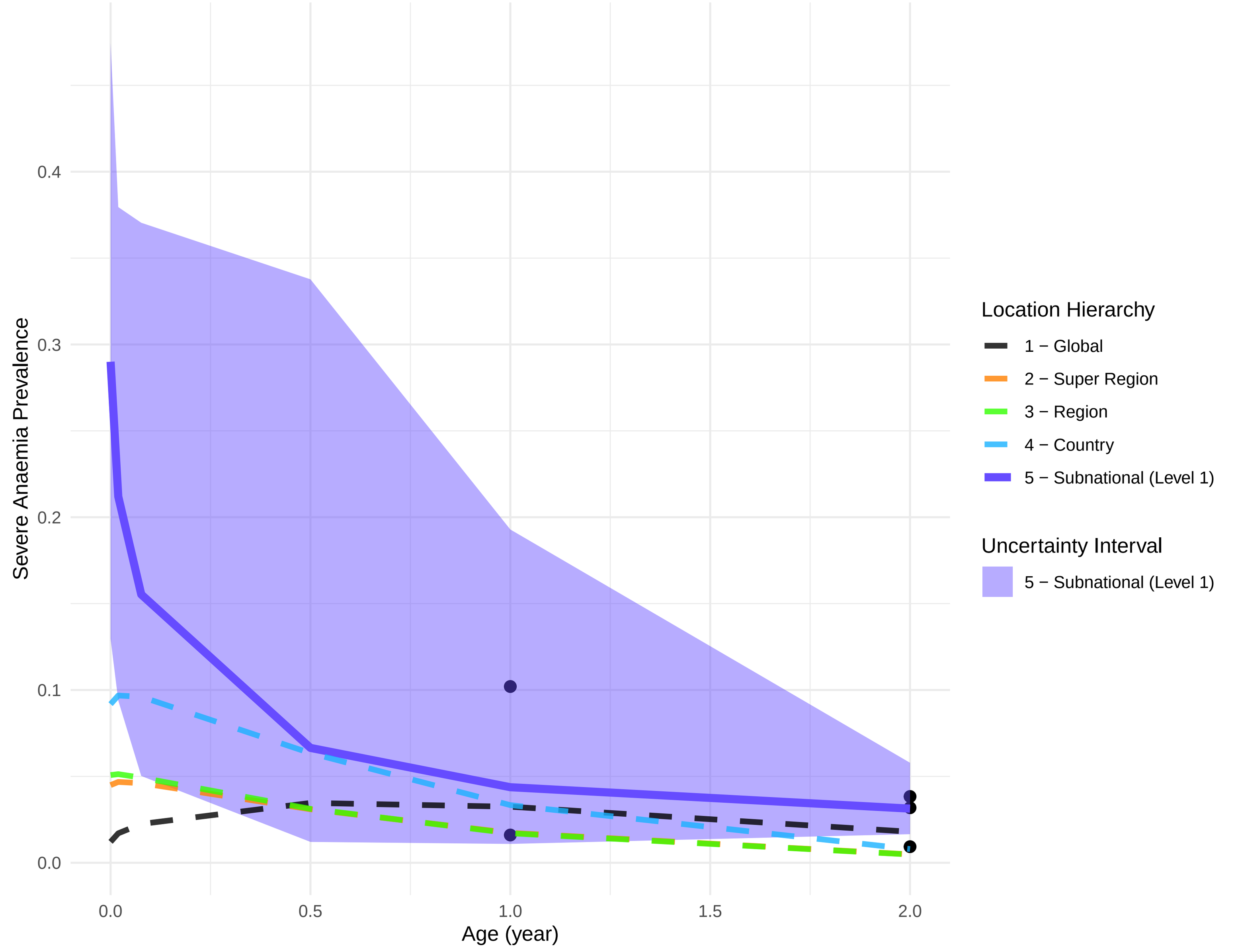

MR−BRT Age/Sex Fits − Males <2 Years Old − Severe Anaemia Prevalence − North Sumatra
WHO−2001 Adjustment Method

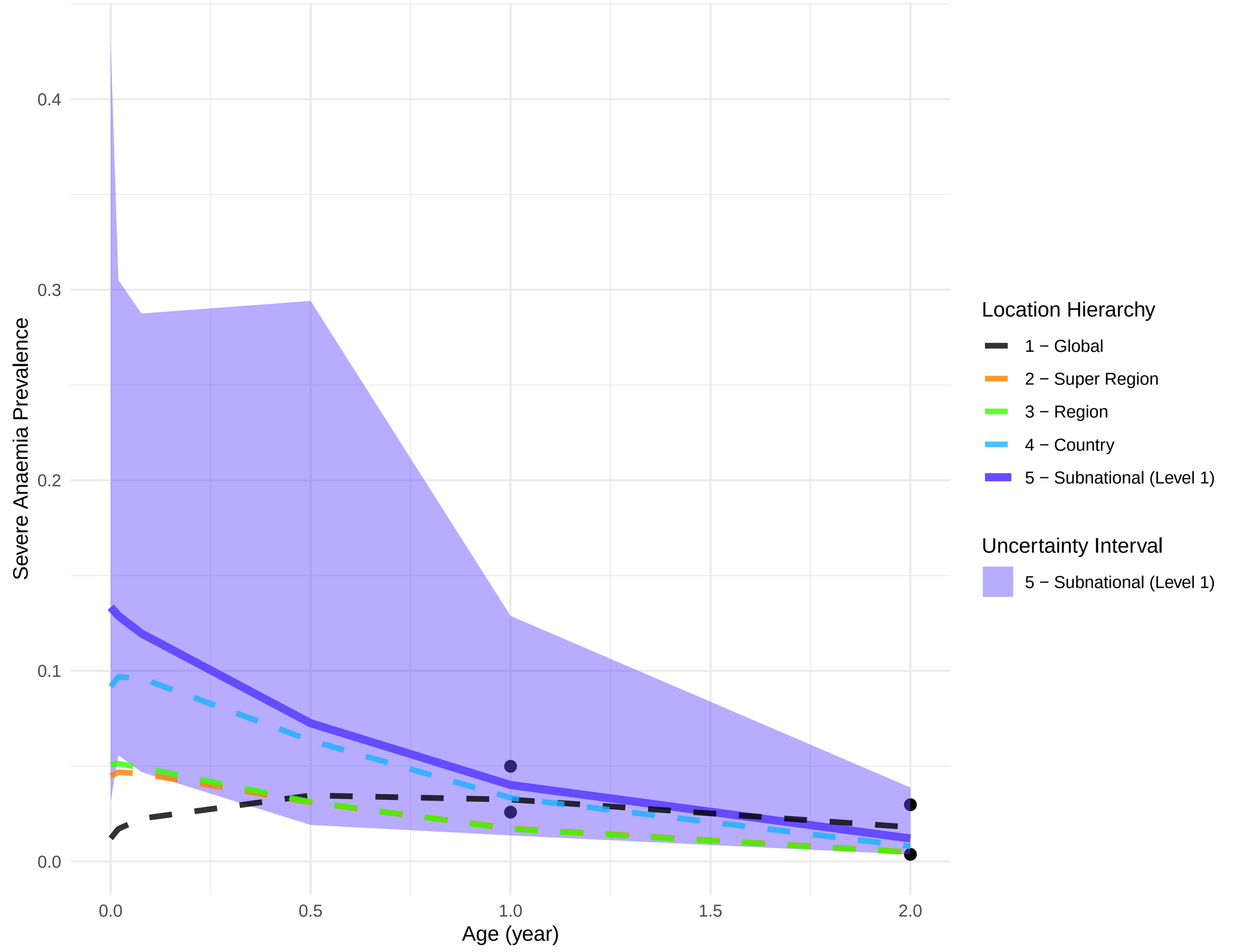

MR−BRT Age/Sex Fits − Males <2 Years Old − Severe Anaemia Prevalence − Myanmar
WHO−2001 Adjustment Method

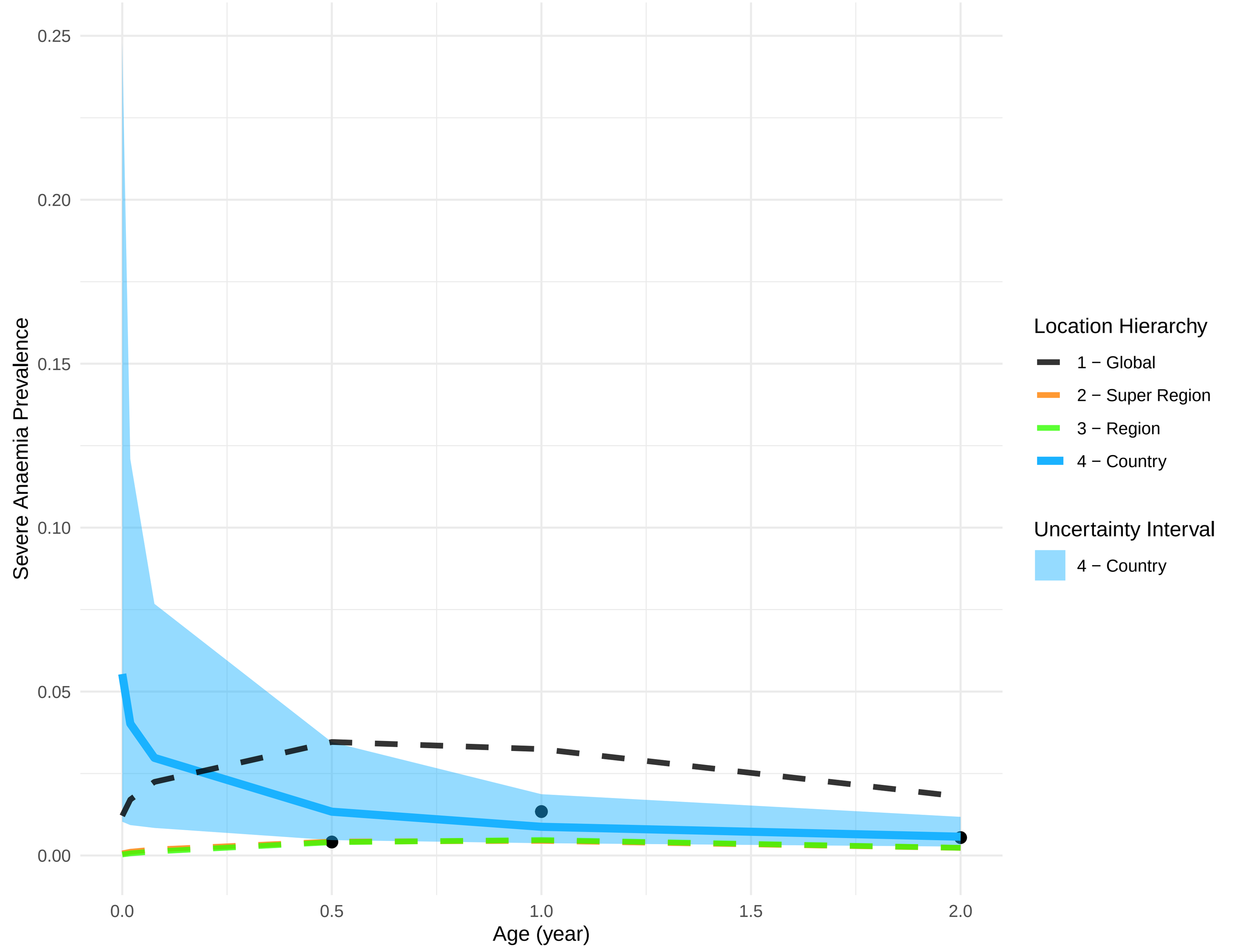

MR−BRT Age/Sex Fits − Males <2 Years Old − Severe Anaemia Prevalence − Timor−Leste
WHO−2001 Adjustment Method

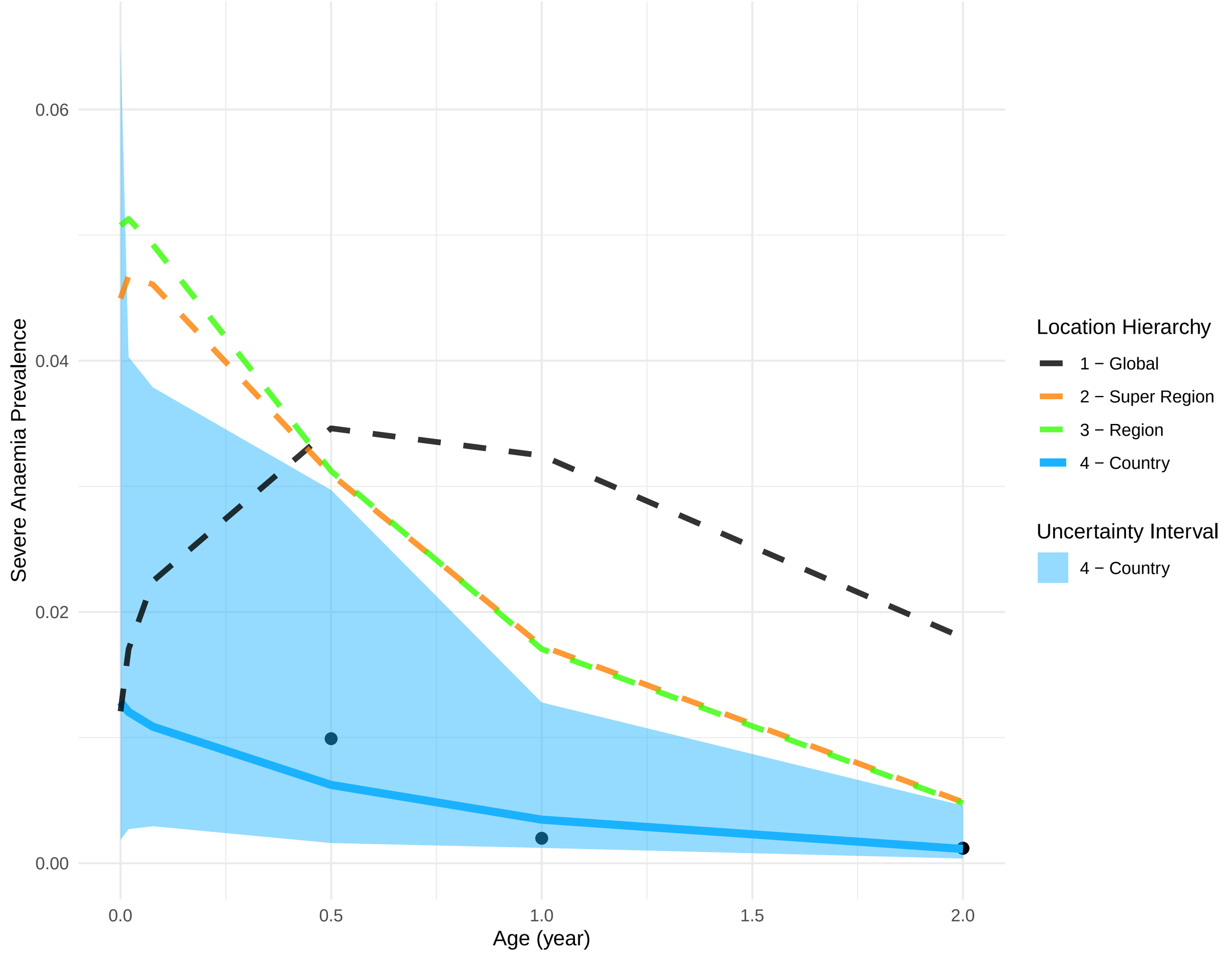

MR−BRT Age/Sex Fits − Males <2 Years Old − Severe Anaemia Prevalence − Sub−Saharan Africa
WHO−2001 Adjustment Method

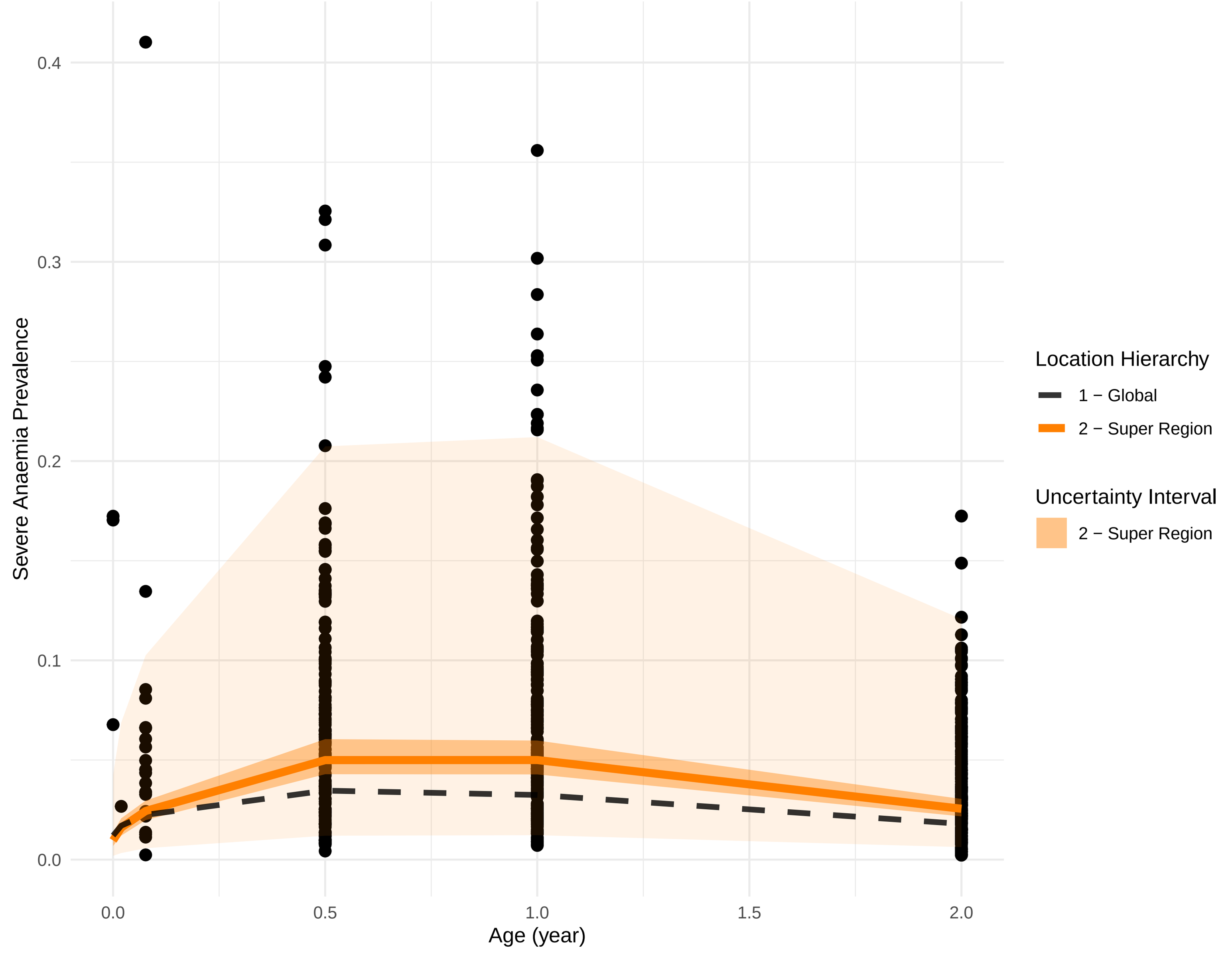

MR−BRT Age/Sex Fits − Males <2 Years Old − Severe Anaemia Prevalence − Central Sub−Saharan Africa
WHO−2001 Adjustment Method

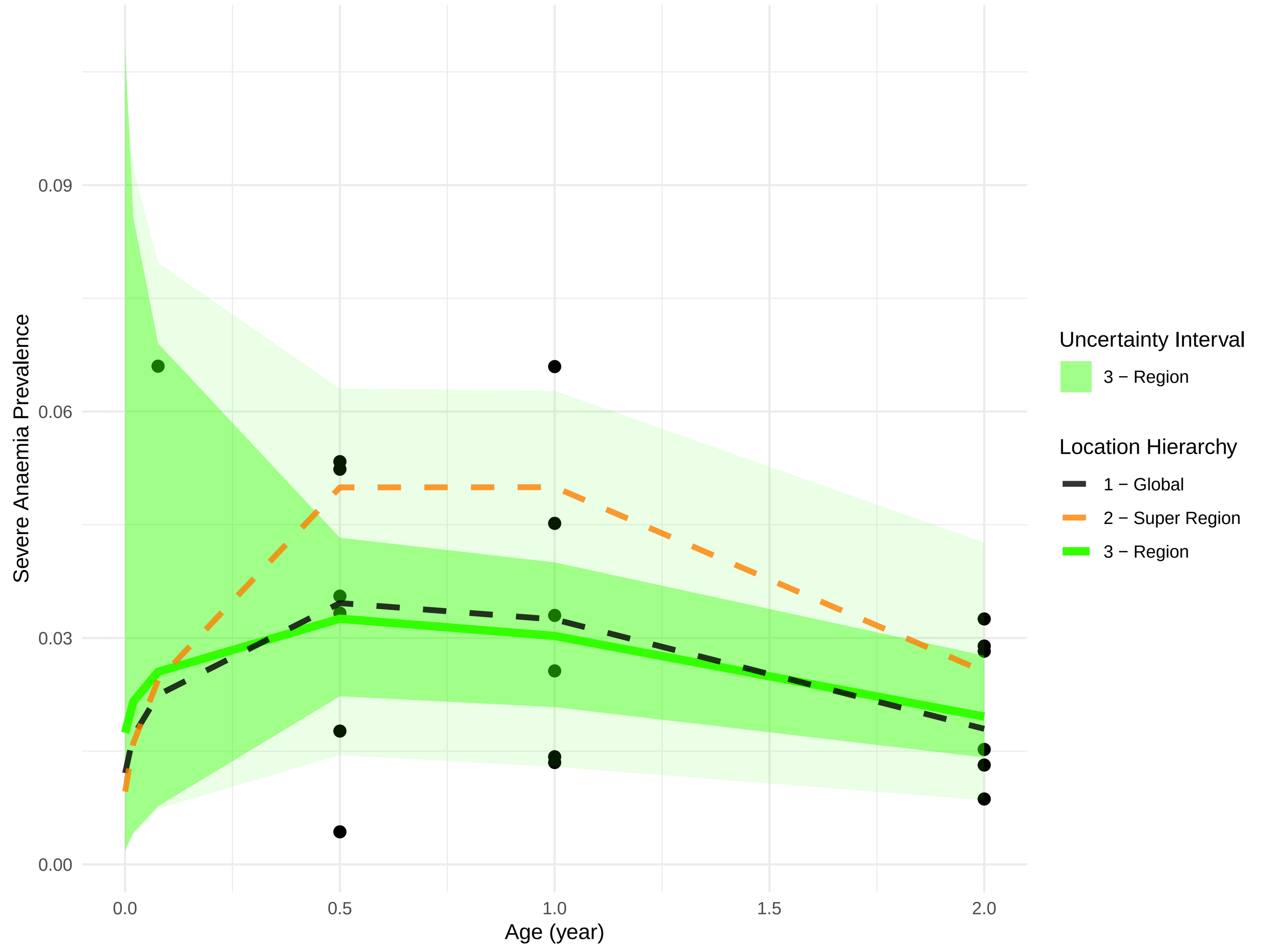

MR−BRT Age/Sex Fits − Males <2 Years Old − Severe Anaemia Prevalence − Angola
WHO−2001 Adjustment Method

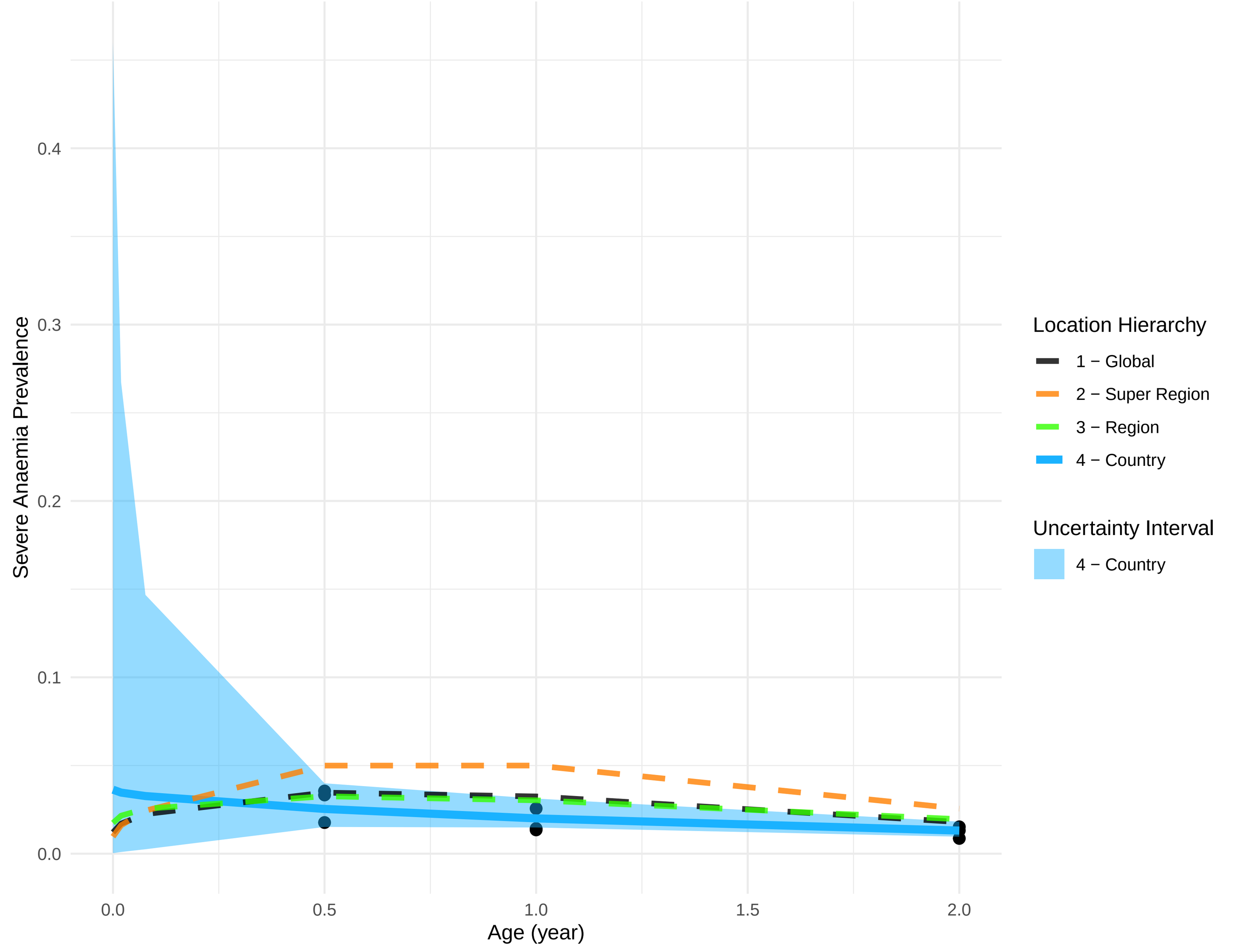

MR−BRT Age/Sex Fits − Males <2 Years Old − Severe Anaemia Prevalence − Democratic Republic of the Congo
WHO−2001 Adjustment Method

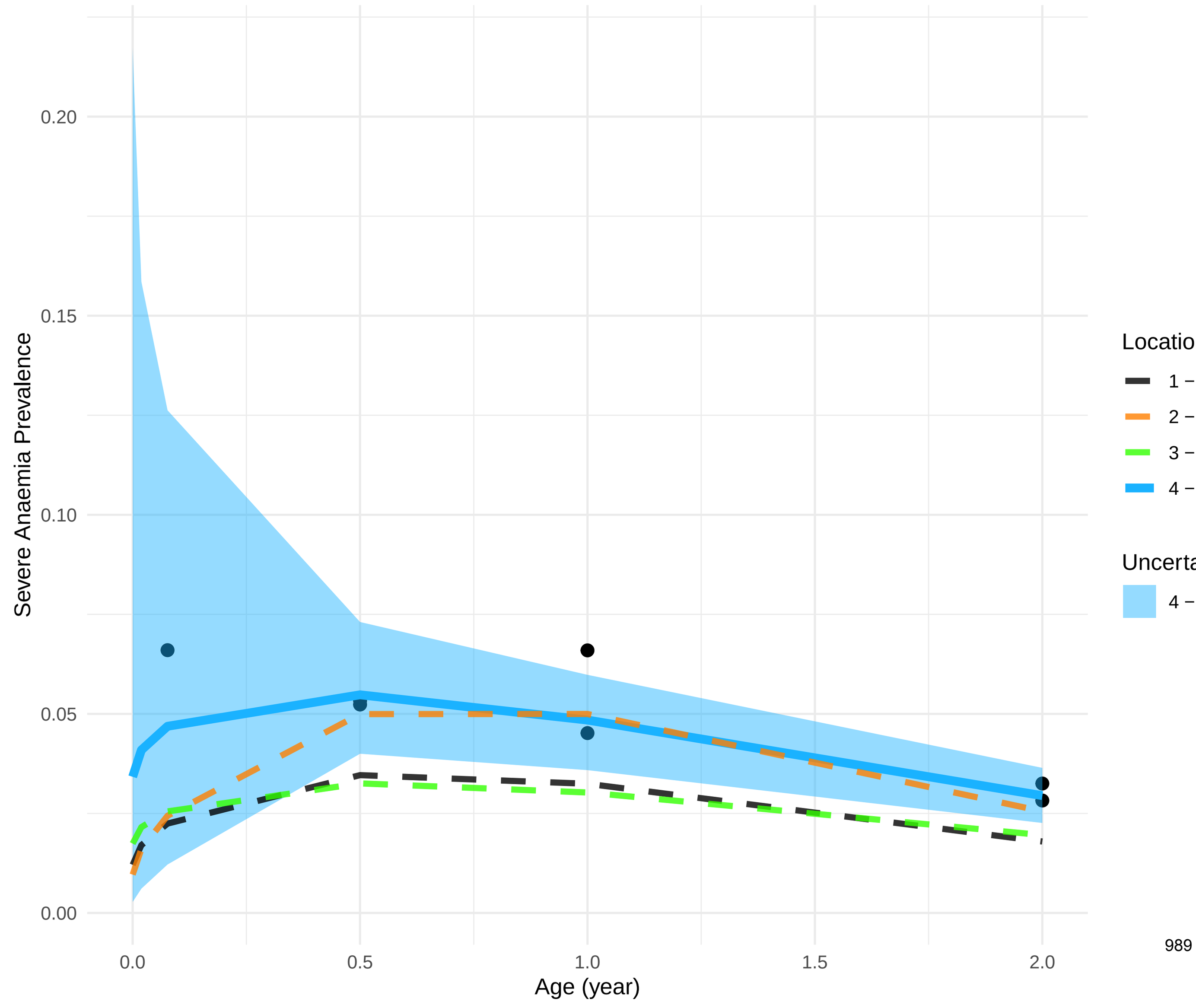

MR−BRT Age/Sex Fits − Males <2 Years Old − Severe Anaemia Prevalence − Gabon
WHO−2001 Adjustment Method

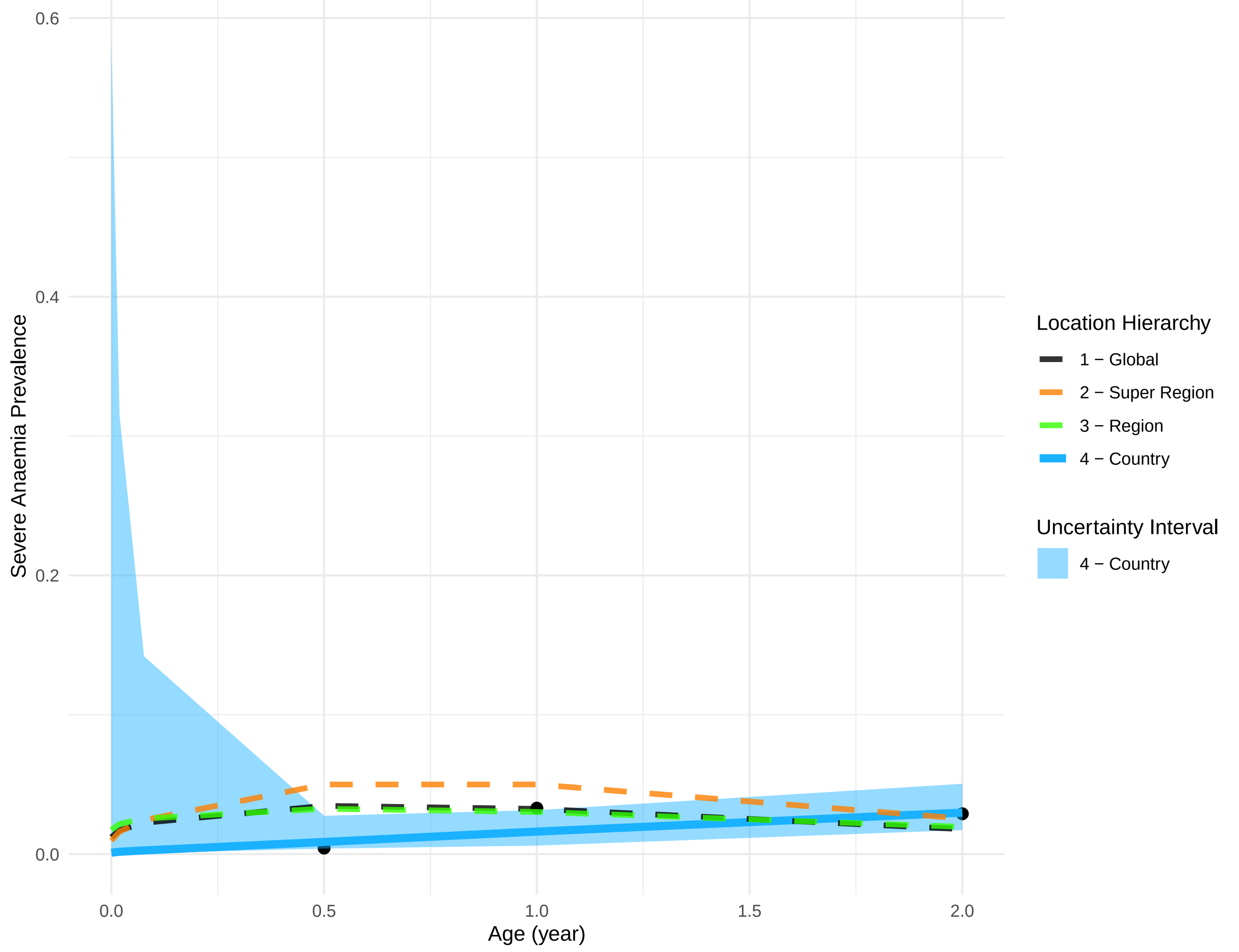

MR−BRT Age/Sex Fits − Males <2 Years Old − Severe Anaemia Prevalence − Eastern Sub−Saharan Africa
WHO−2001 Adjustment Method

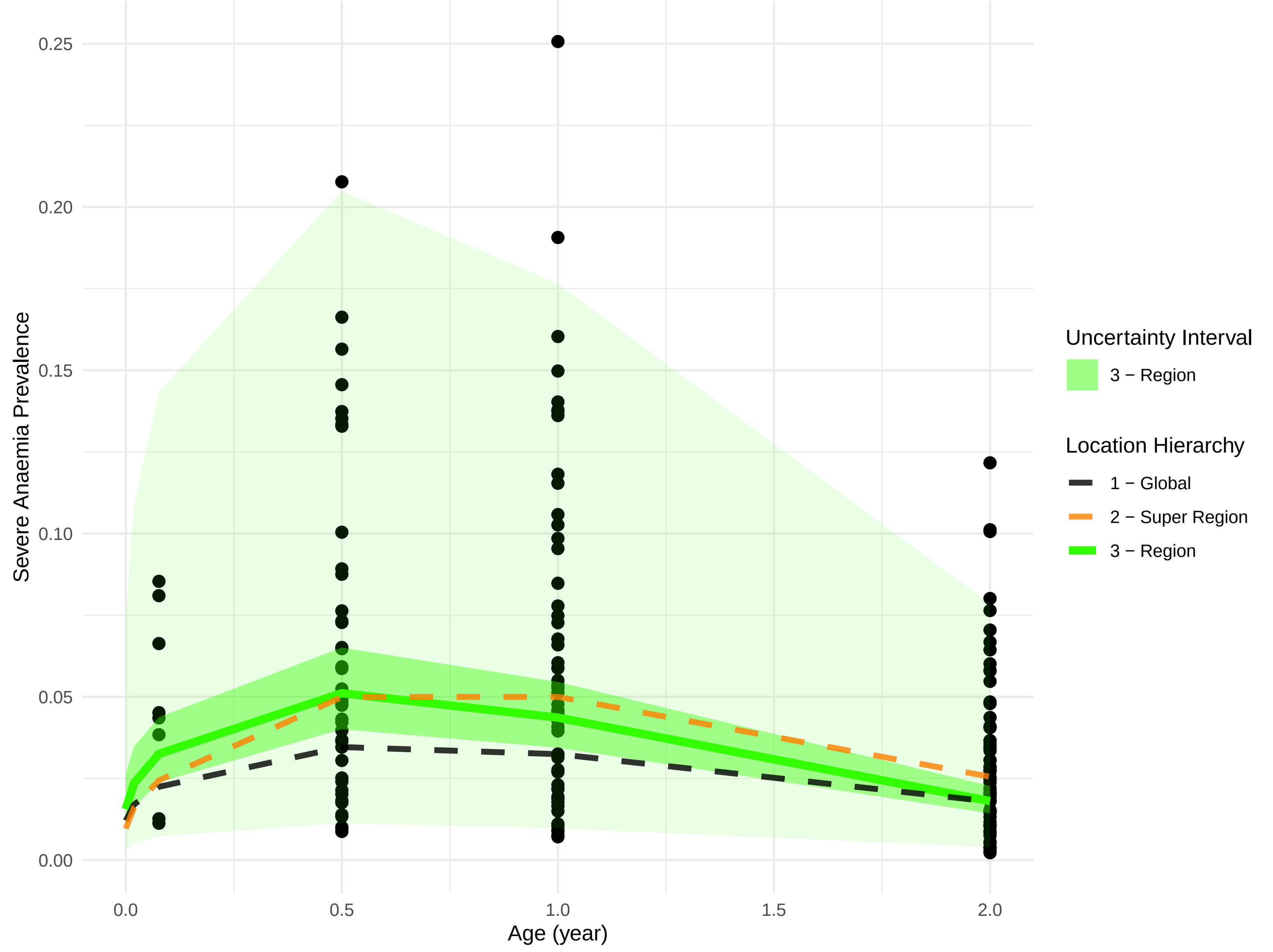

MR−BRT Age/Sex Fits − Males <2 Years Old − Severe Anaemia Prevalence − Burundi
WHO−2001 Adjustment Method

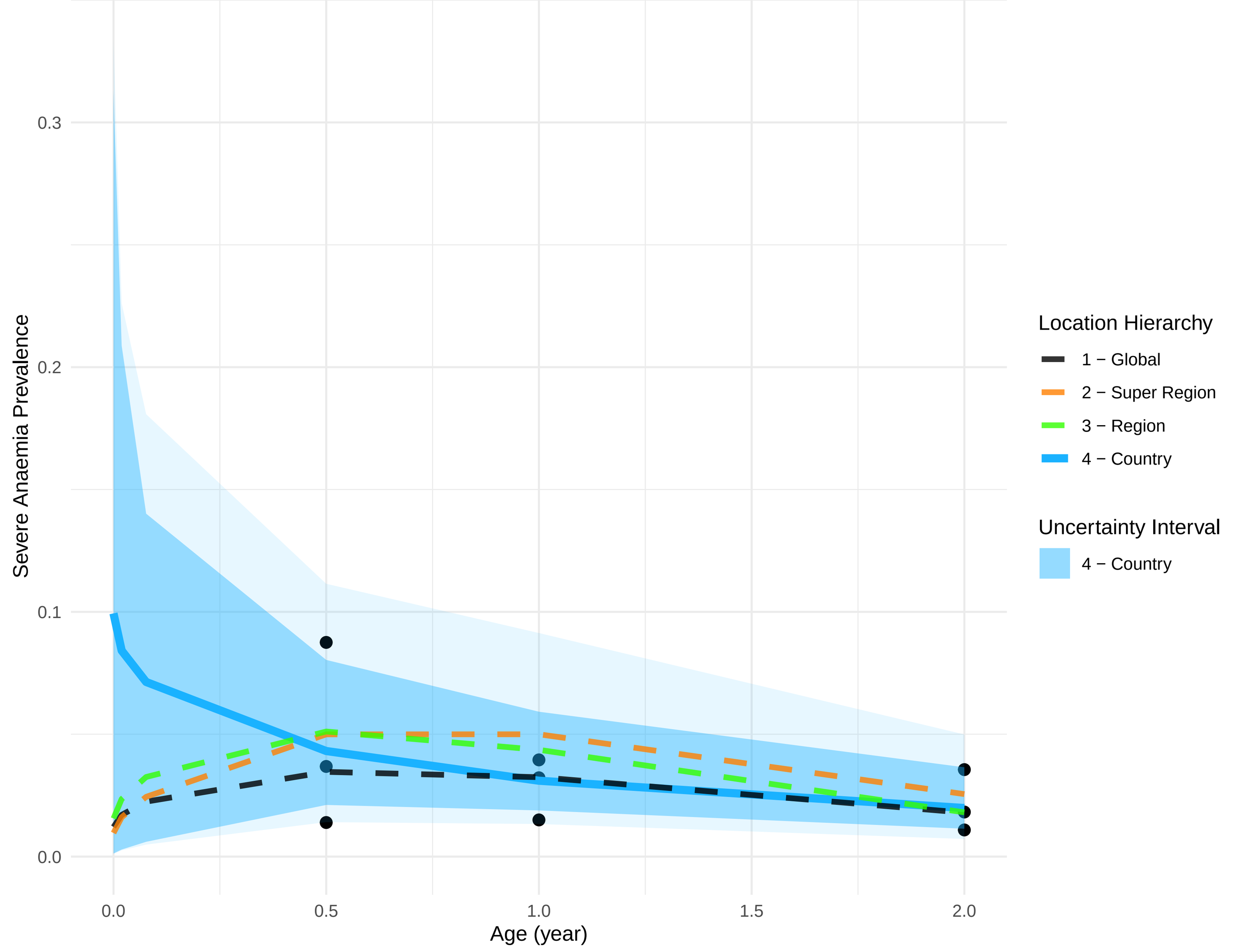

MR−BRT Age/Sex Fits − Males <2 Years Old − Severe Anaemia Prevalence − Ethiopia
WHO−2001 Adjustment Method

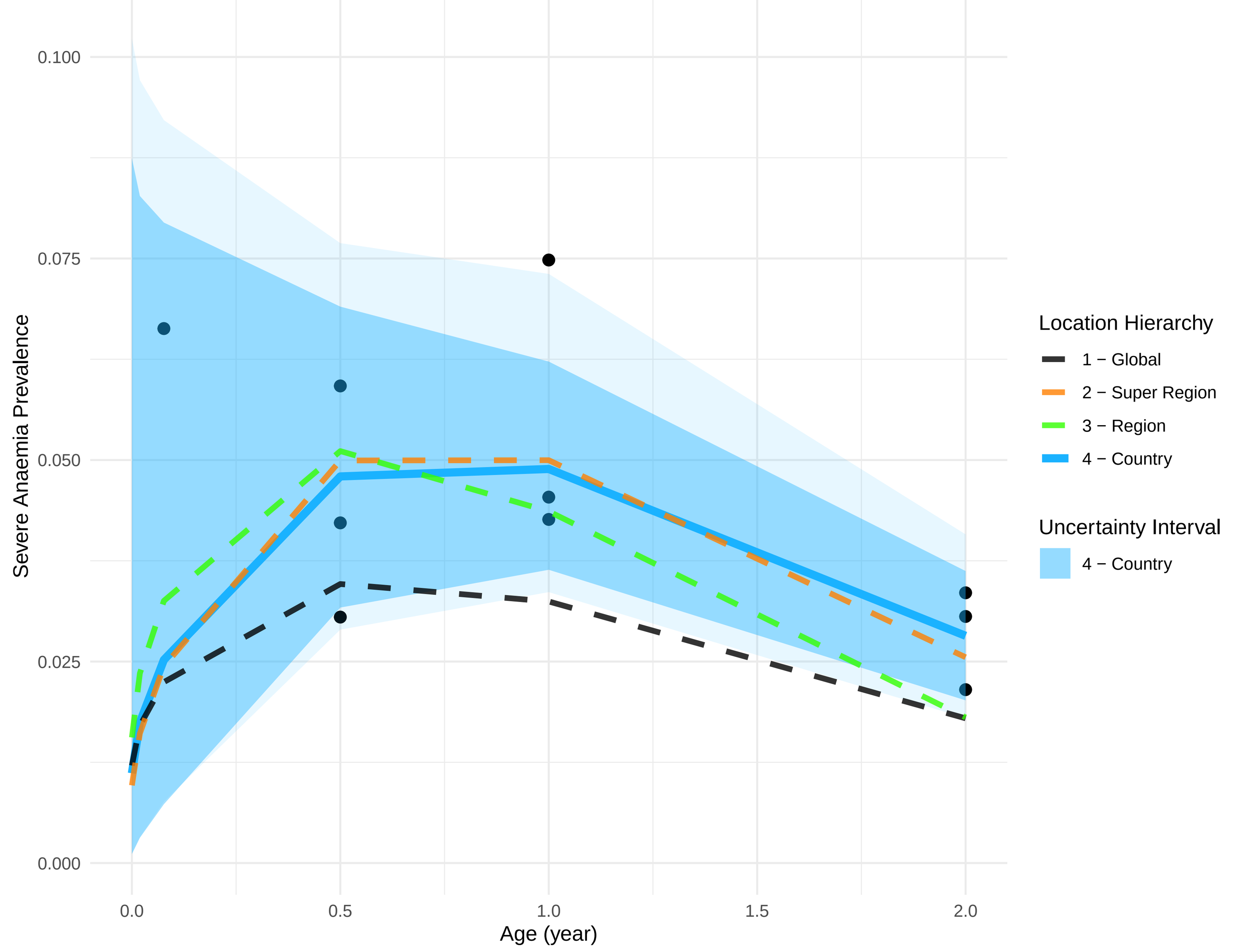

MR−BRT Age/Sex Fits − Males <2 Years Old − Severe Anaemia Prevalence − Kenya
WHO−2001 Adjustment Method

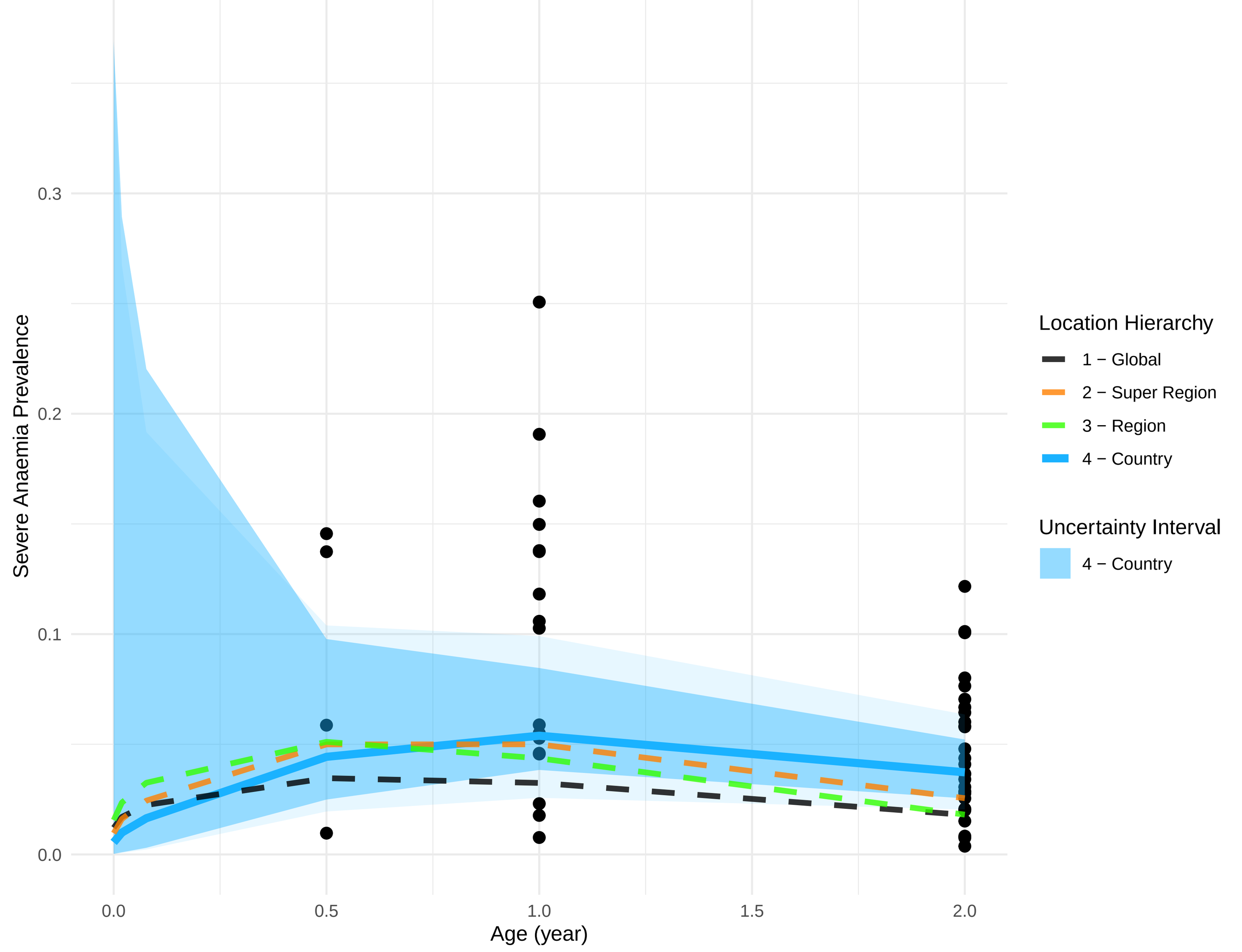

MR−BRT Age/Sex Fits − Males <2 Years Old − Severe Anaemia Prevalence − Baringo
WHO−2001 Adjustment Method

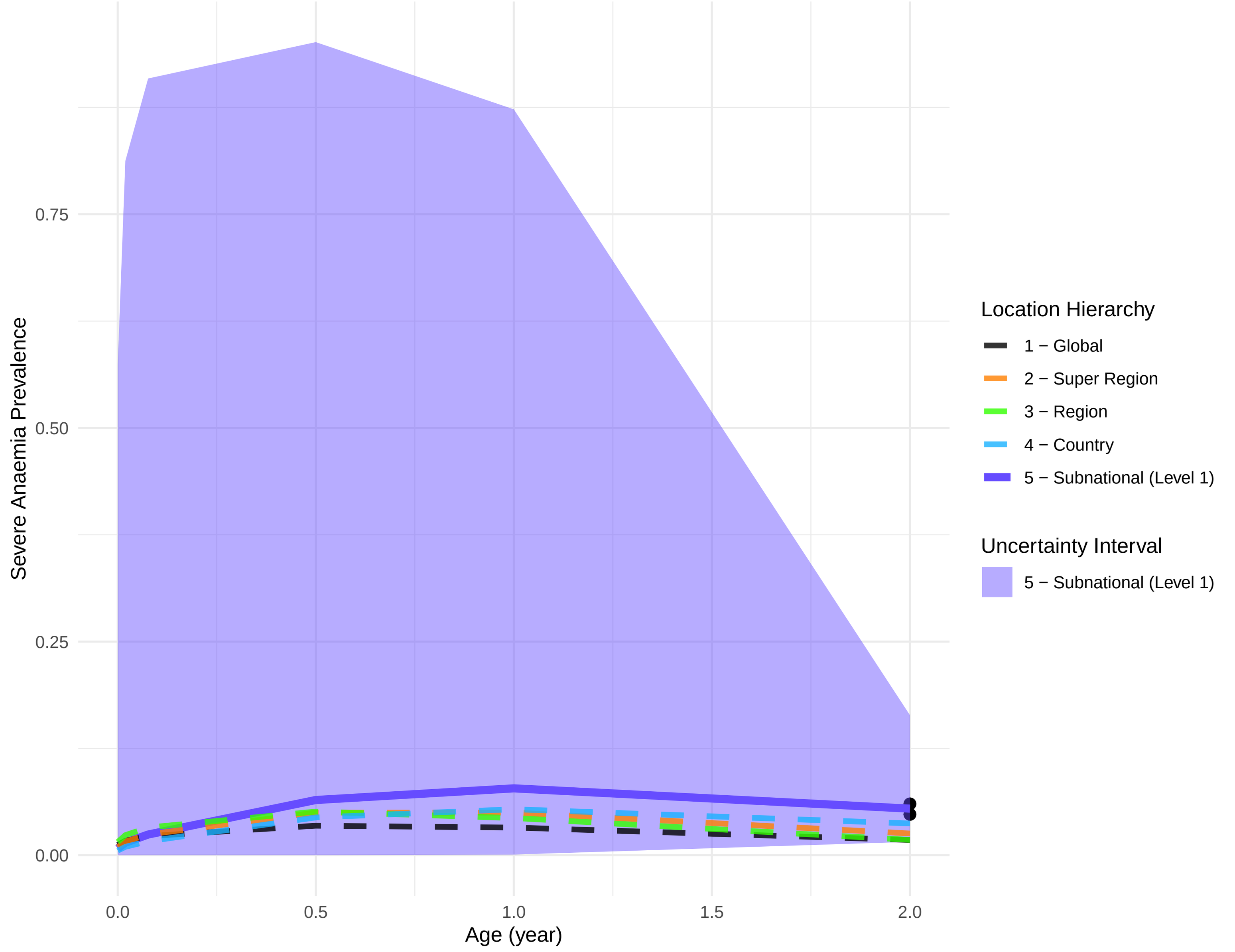

MR−BRT Age/Sex Fits − Males <2 Years Old − Severe Anaemia Prevalence − Bungoma
WHO−2001 Adjustment Method

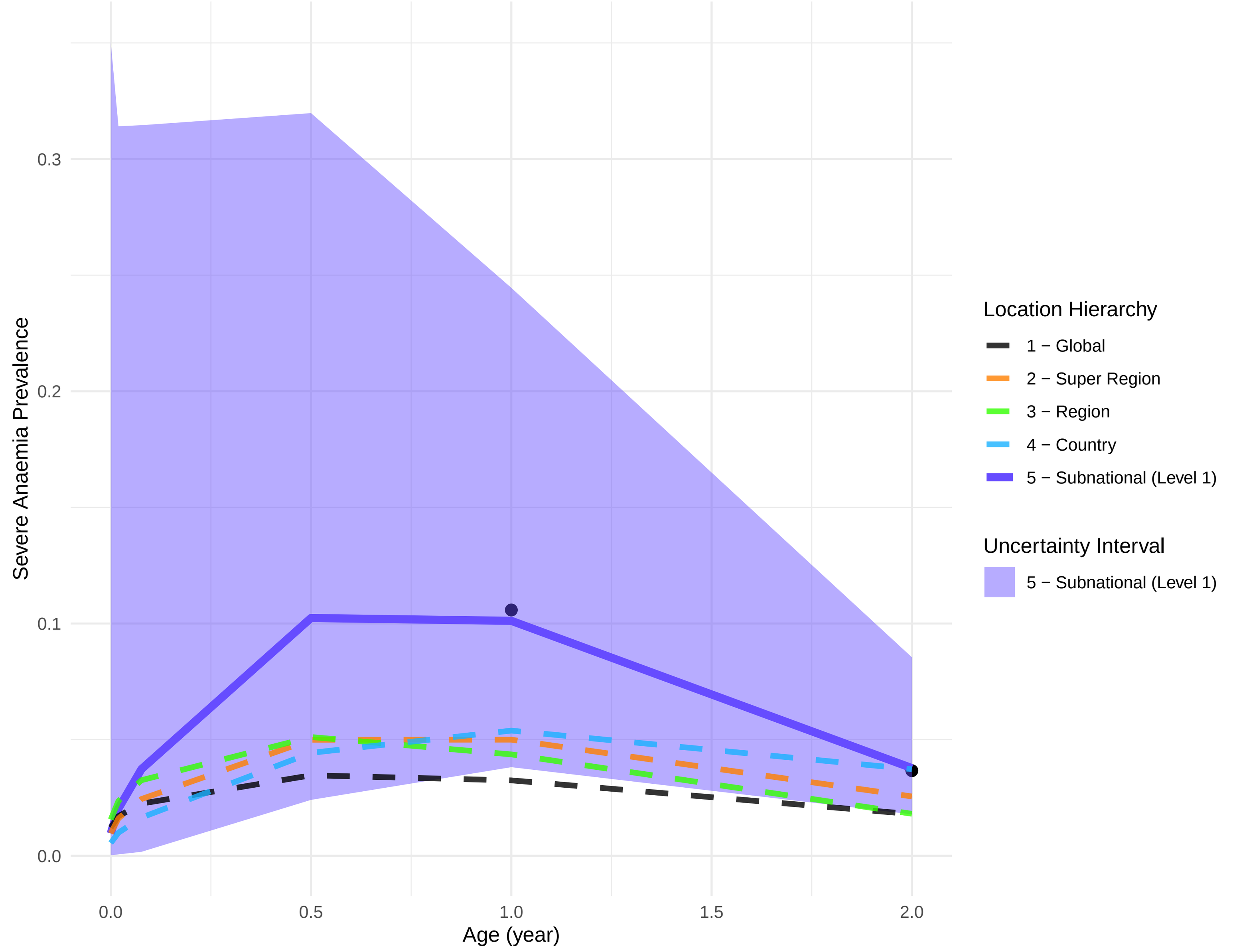

MR−BRT Age/Sex Fits − Males <2 Years Old − Severe Anaemia Prevalence − Elgeyo−Marakwet
WHO−2001 Adjustment Method

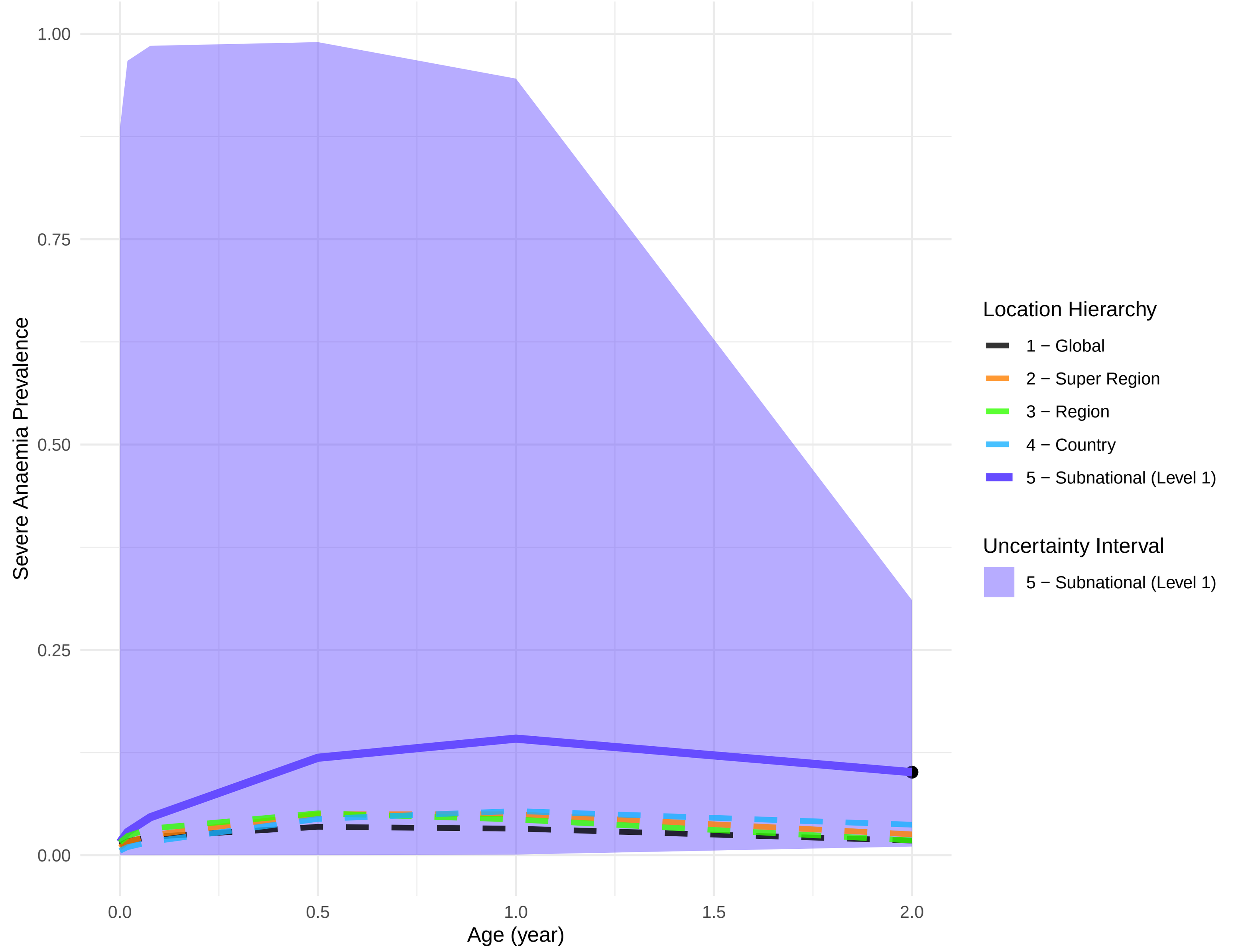

MR−BRT Age/Sex Fits − Males <2 Years Old − Severe Anaemia Prevalence − Garissa
WHO−2001 Adjustment Method

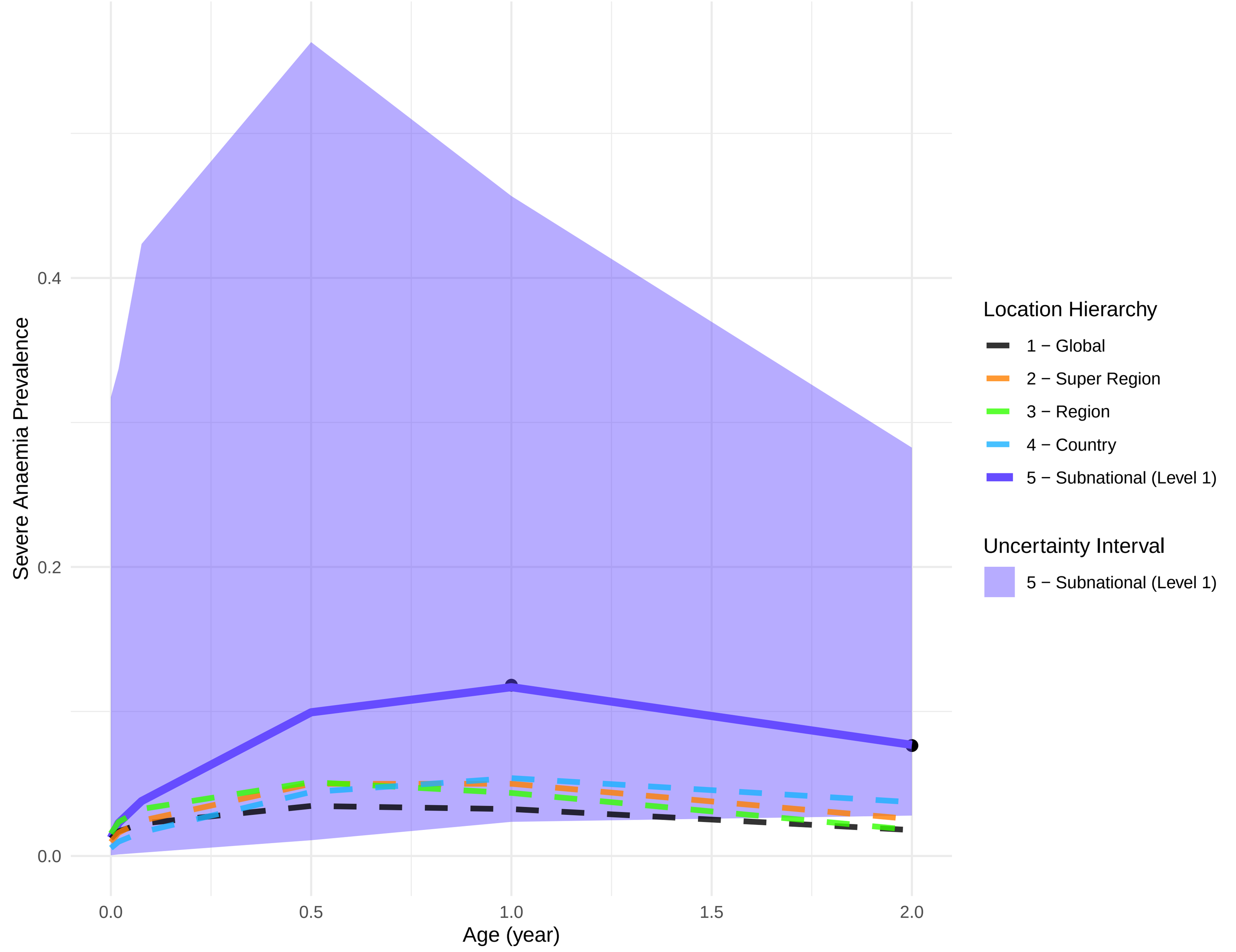

MR−BRT Age/Sex Fits − Males <2 Years Old − Severe Anaemia Prevalence − Homa Bay
WHO−2001 Adjustment Method

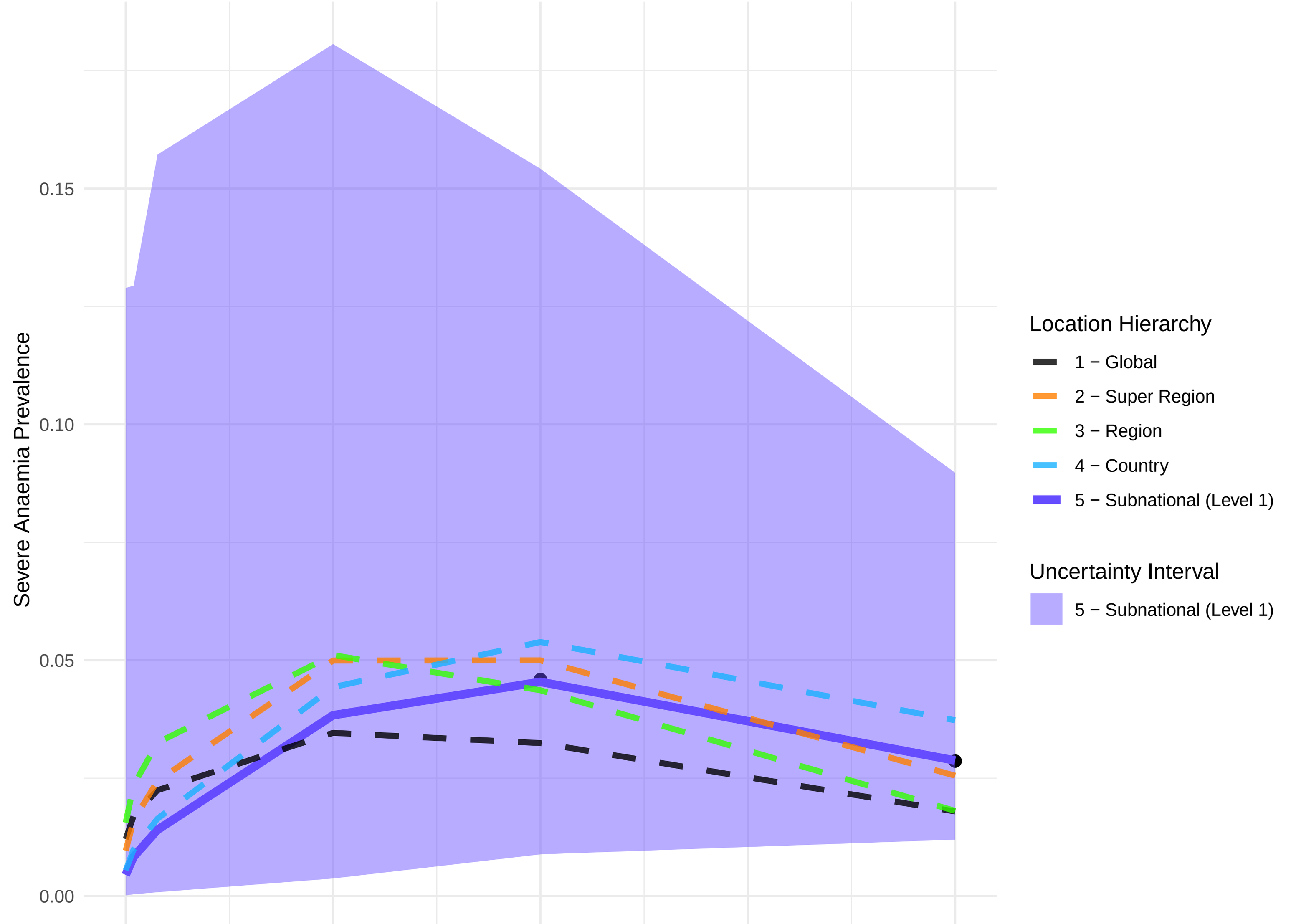

MR−BRT Age/Sex Fits − Males <2 Years Old − Severe Anaemia Prevalence − Isiolo
WHO−2001 Adjustment Method

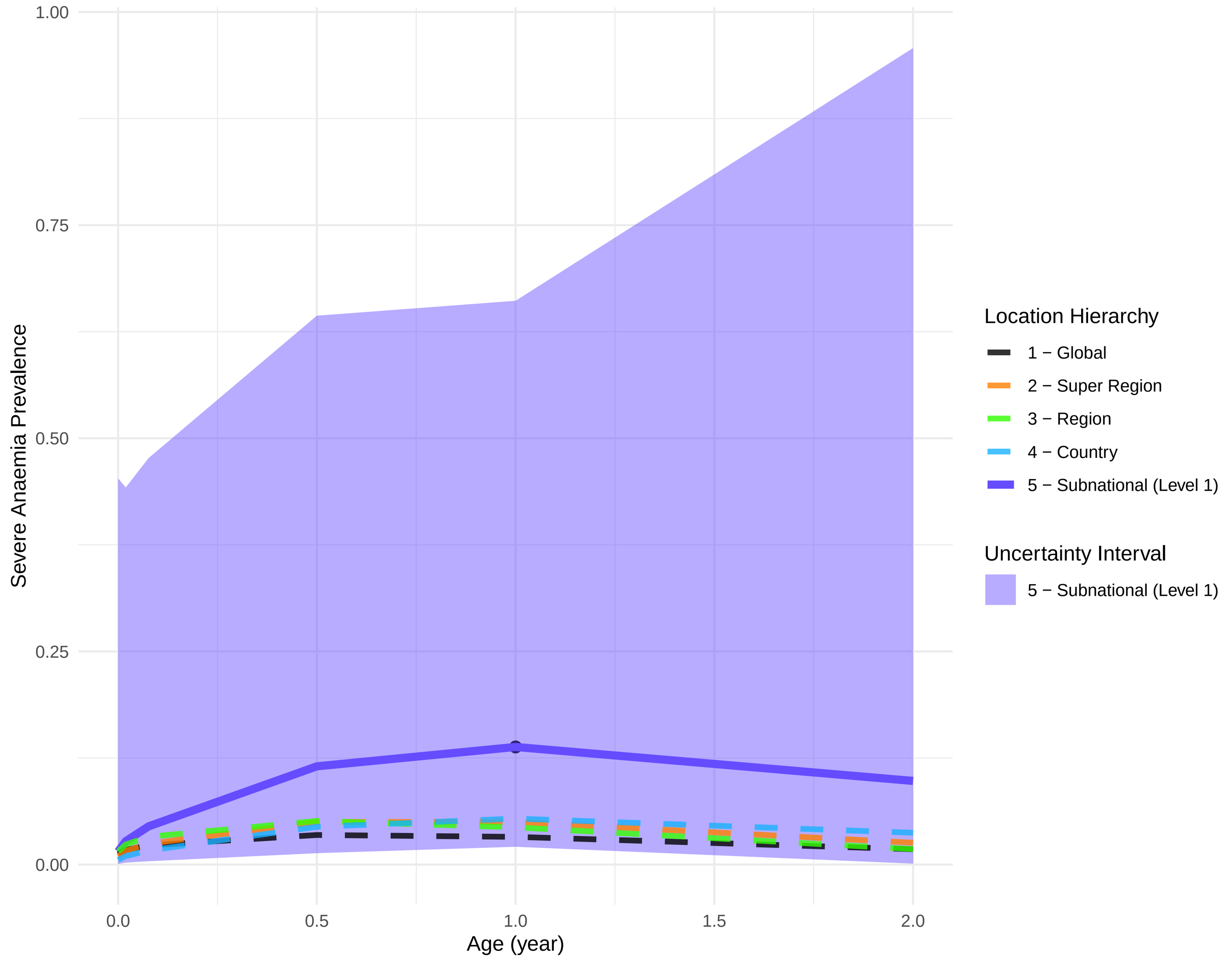

MR−BRT Age/Sex Fits − Males <2 Years Old − Severe Anaemia Prevalence − Kajiado
WHO−2001 Adjustment Method

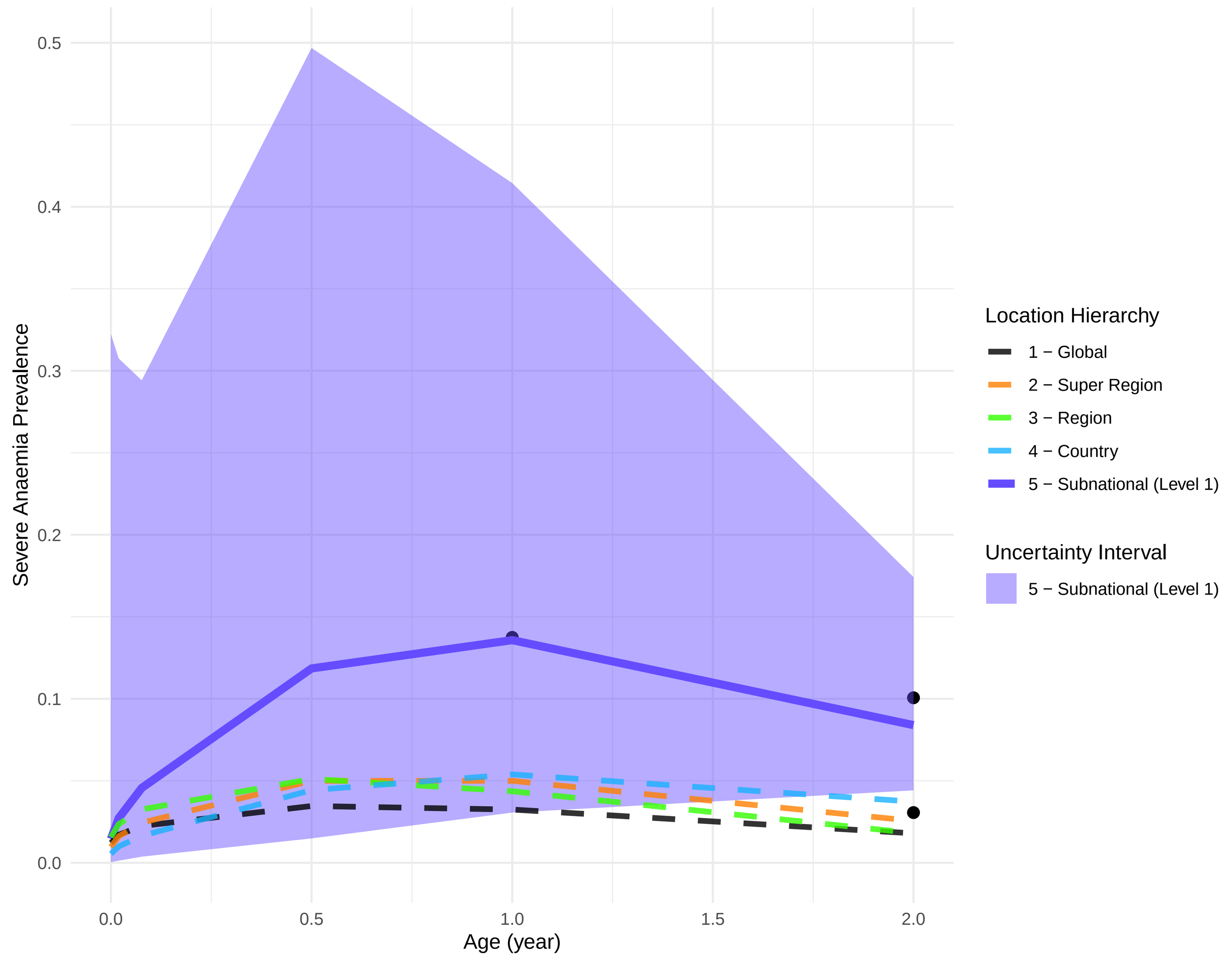

MR−BRT Age/Sex Fits − Males <2 Years Old − Severe Anaemia Prevalence − Kakamega
WHO−2001 Adjustment Method

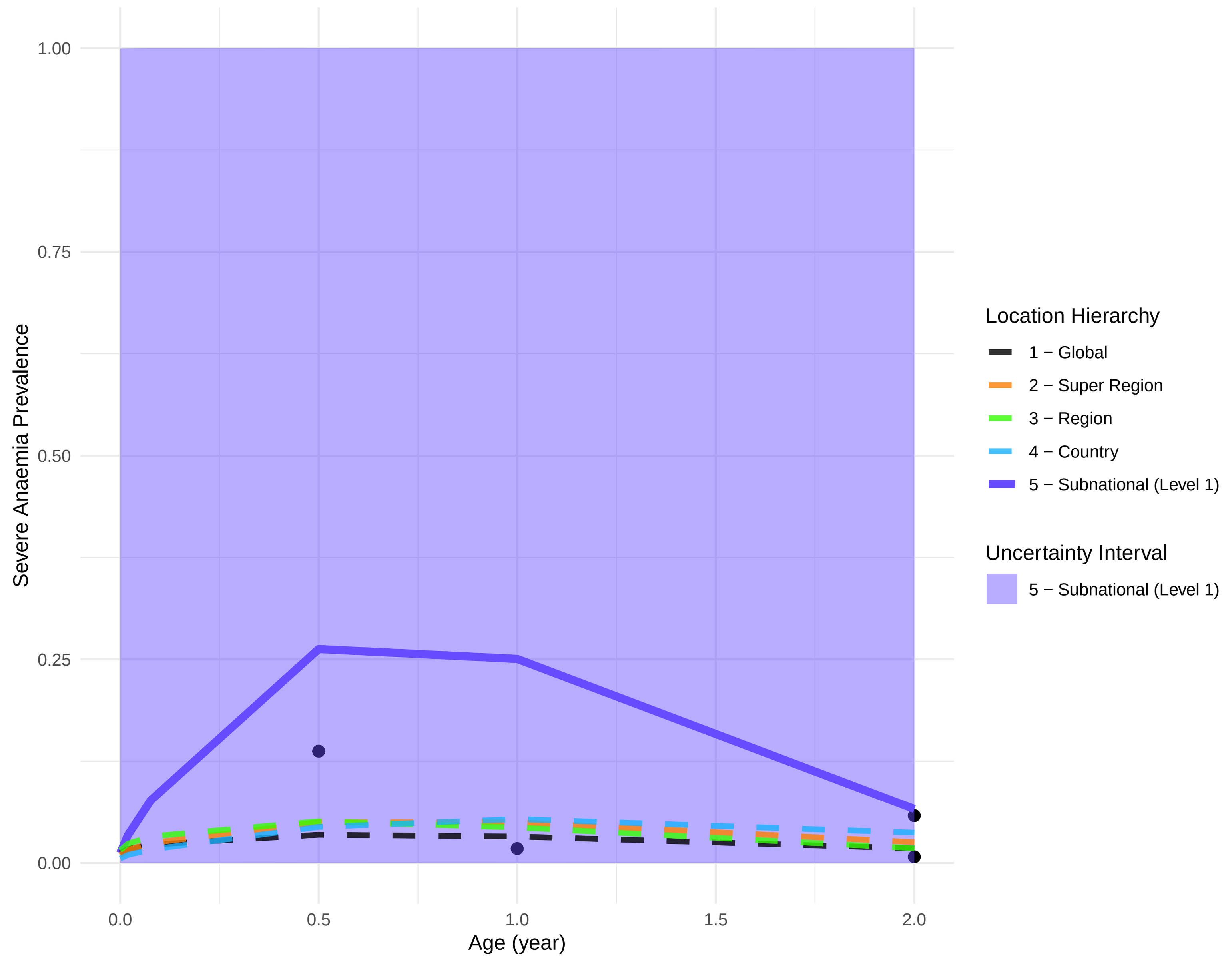

MR−BRT Age/Sex Fits − Males <2 Years Old − Severe Anaemia Prevalence − Kericho
WHO−2001 Adjustment Method

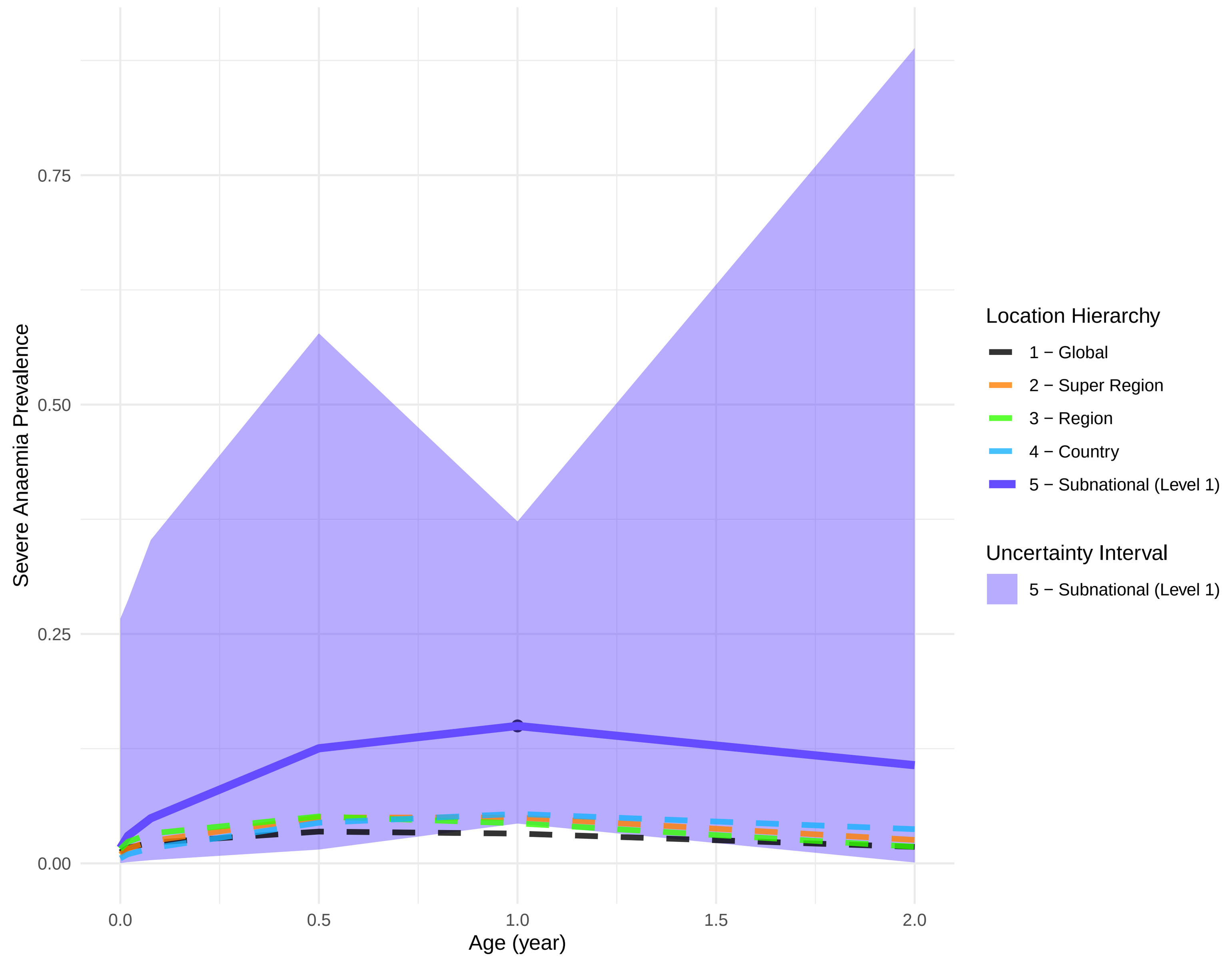

MR−BRT Age/Sex Fits − Males <2 Years Old − Severe Anaemia Prevalence − Kilifi
WHO−2001 Adjustment Method

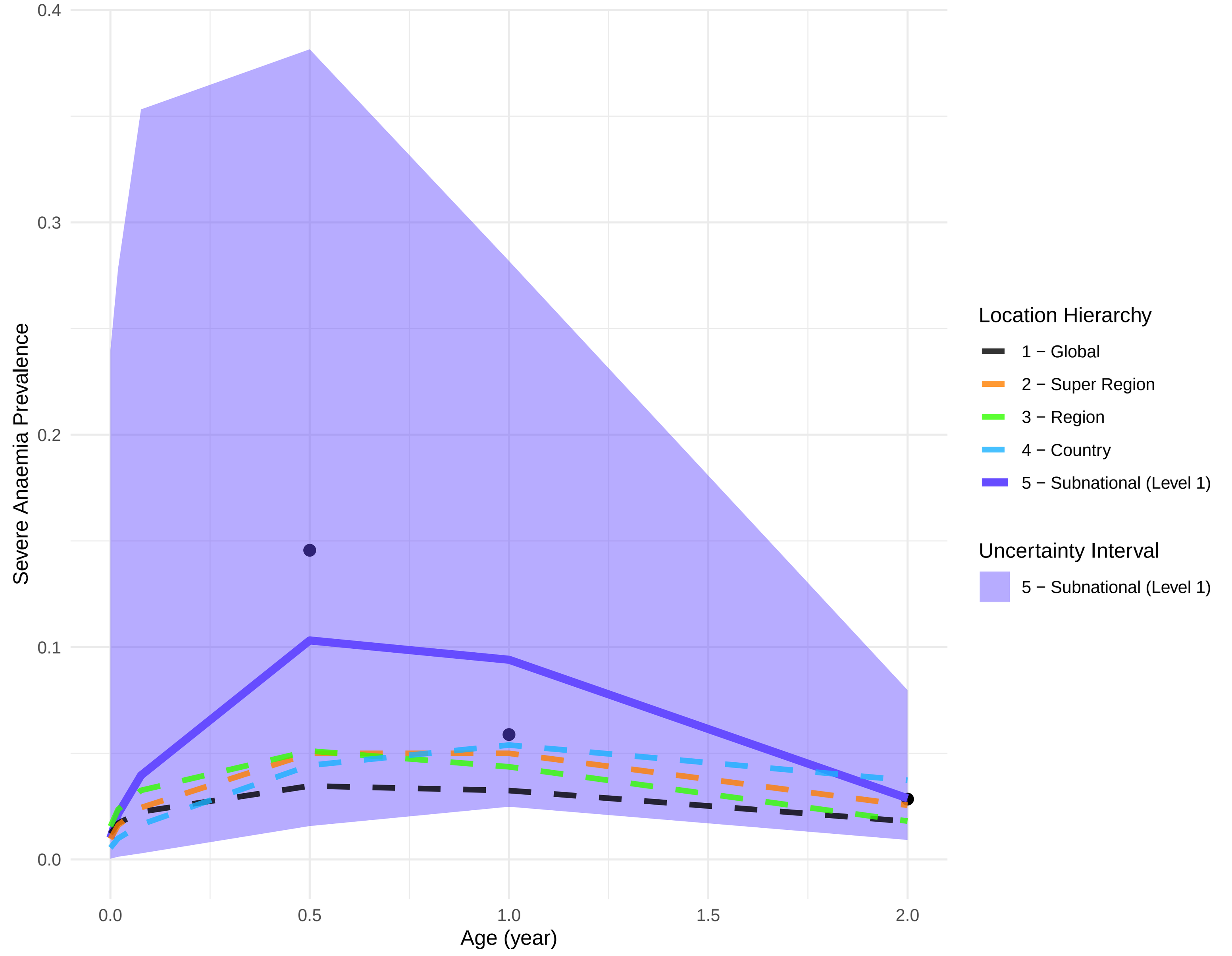

MR−BRT Age/Sex Fits − Males <2 Years Old − Severe Anaemia Prevalence − Kitui
WHO−2001 Adjustment Method

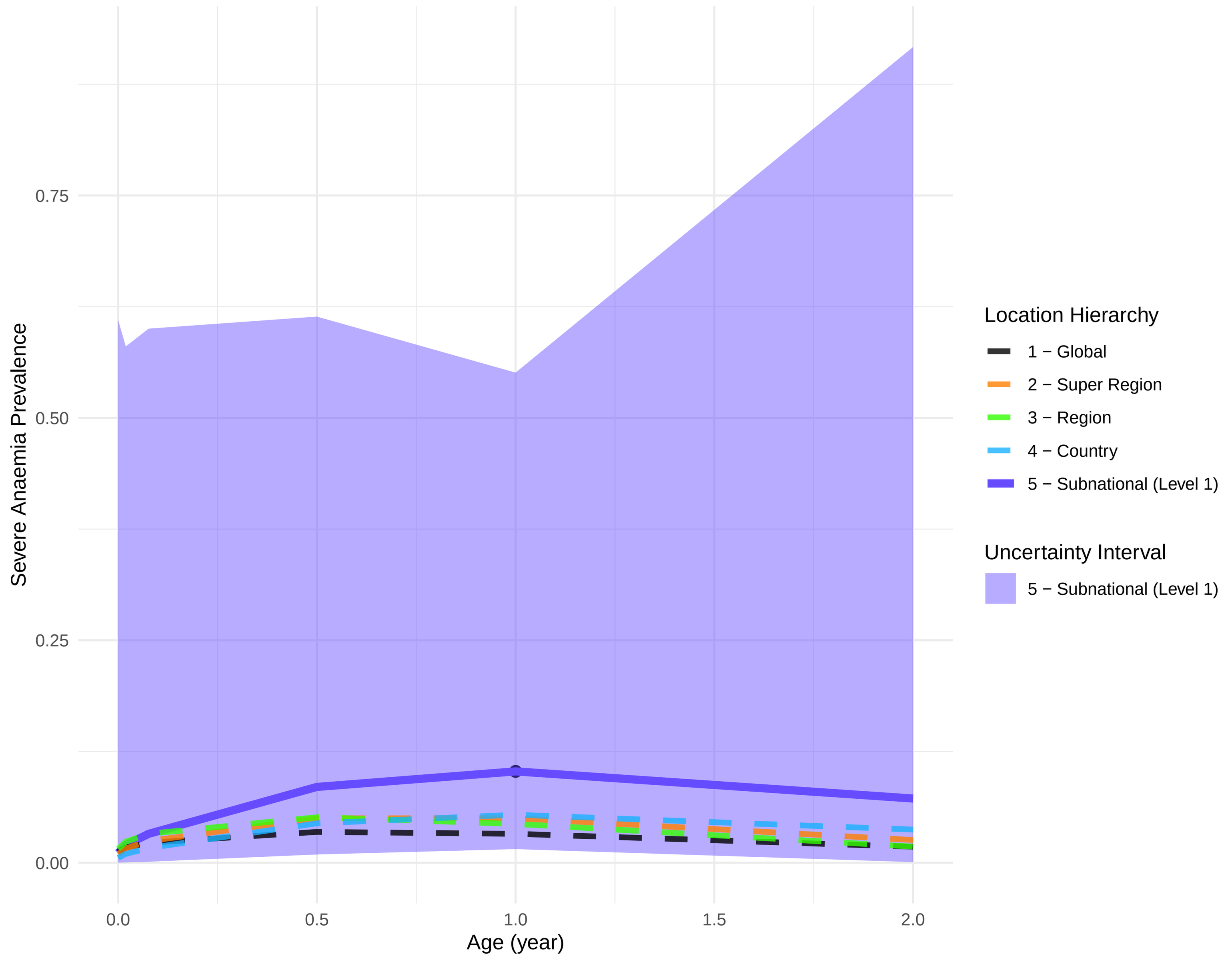

MR−BRT Age/Sex Fits − Males <2 Years Old − Severe Anaemia Prevalence − Kwale
WHO−2001 Adjustment Method

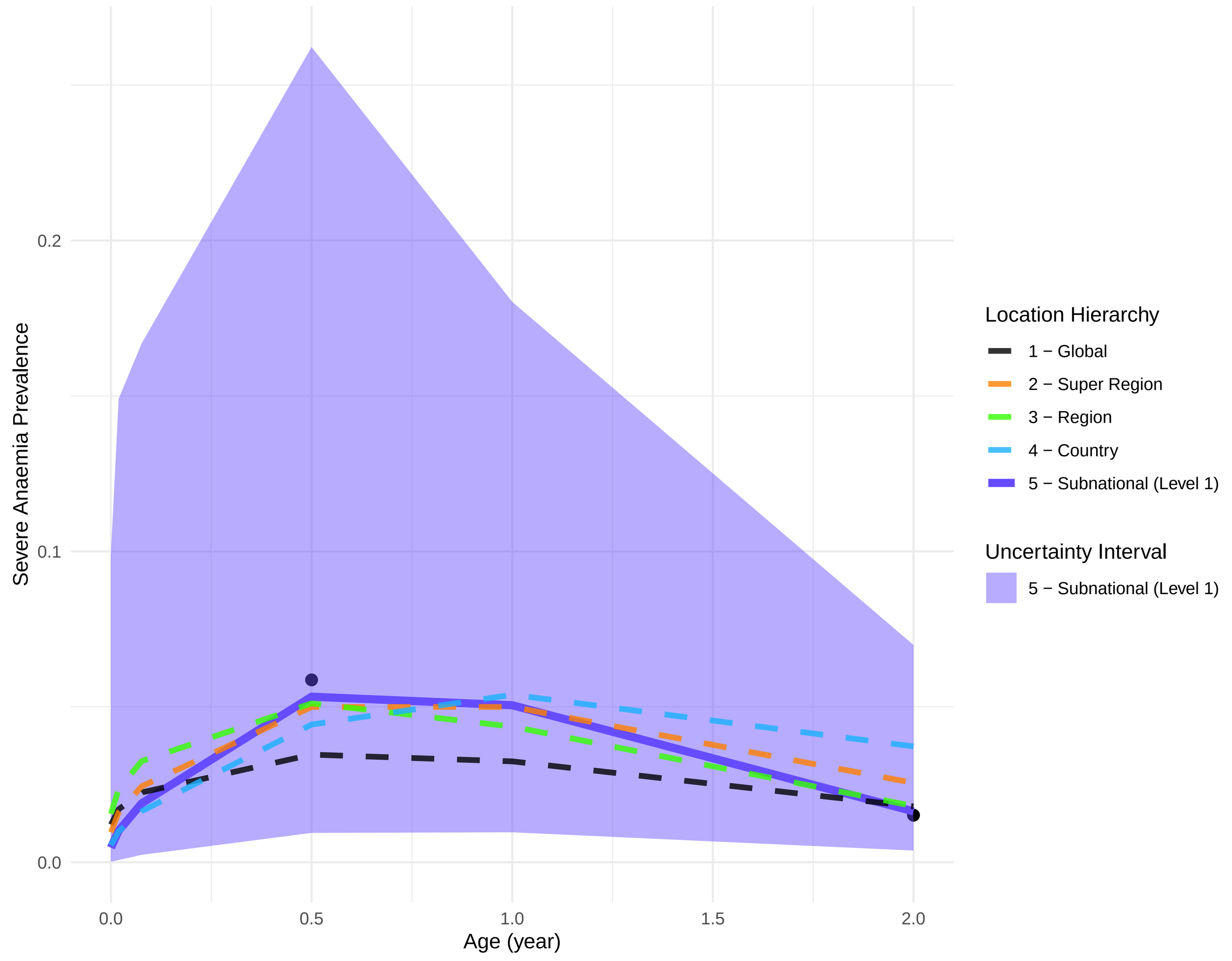

MR−BRT Age/Sex Fits − Males <2 Years Old − Severe Anaemia Prevalence − Machakos
WHO−2001 Adjustment Method

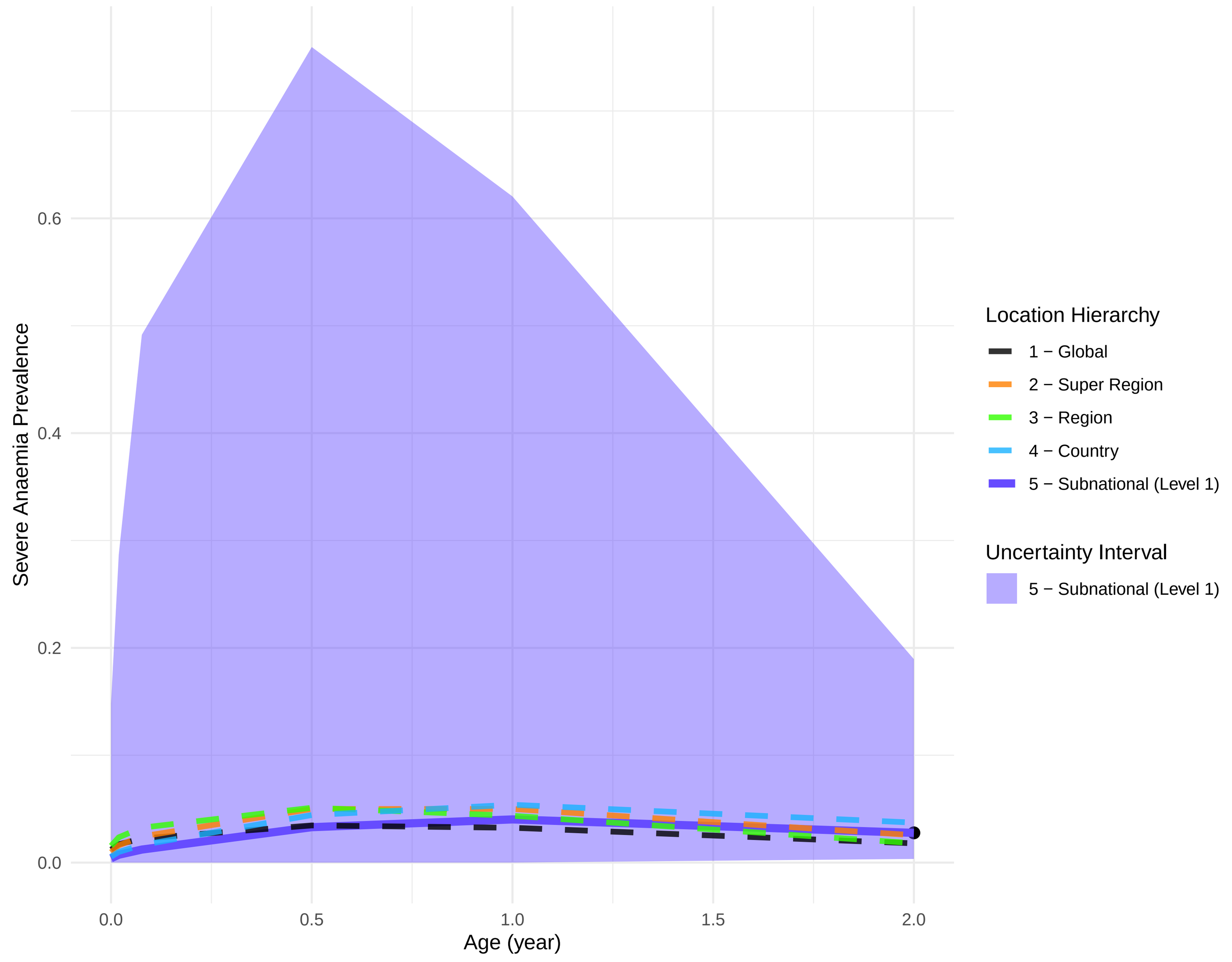

MR−BRT Age/Sex Fits − Males <2 Years Old − Severe Anaemia Prevalence − Mandera
WHO−2001 Adjustment Method

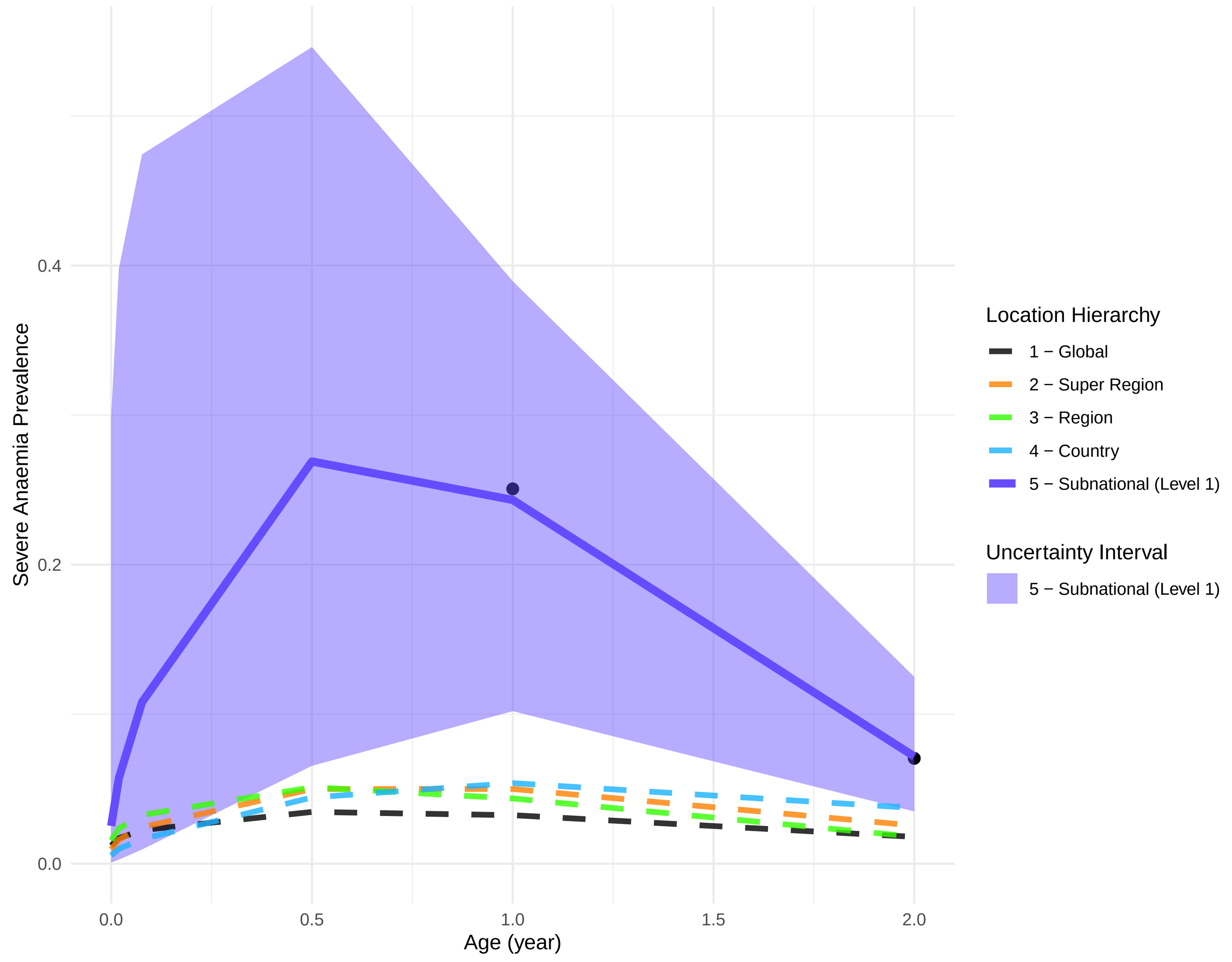

MR−BRT Age/Sex Fits − Males <2 Years Old − Severe Anaemia Prevalence − Meru
WHO−2001 Adjustment Method

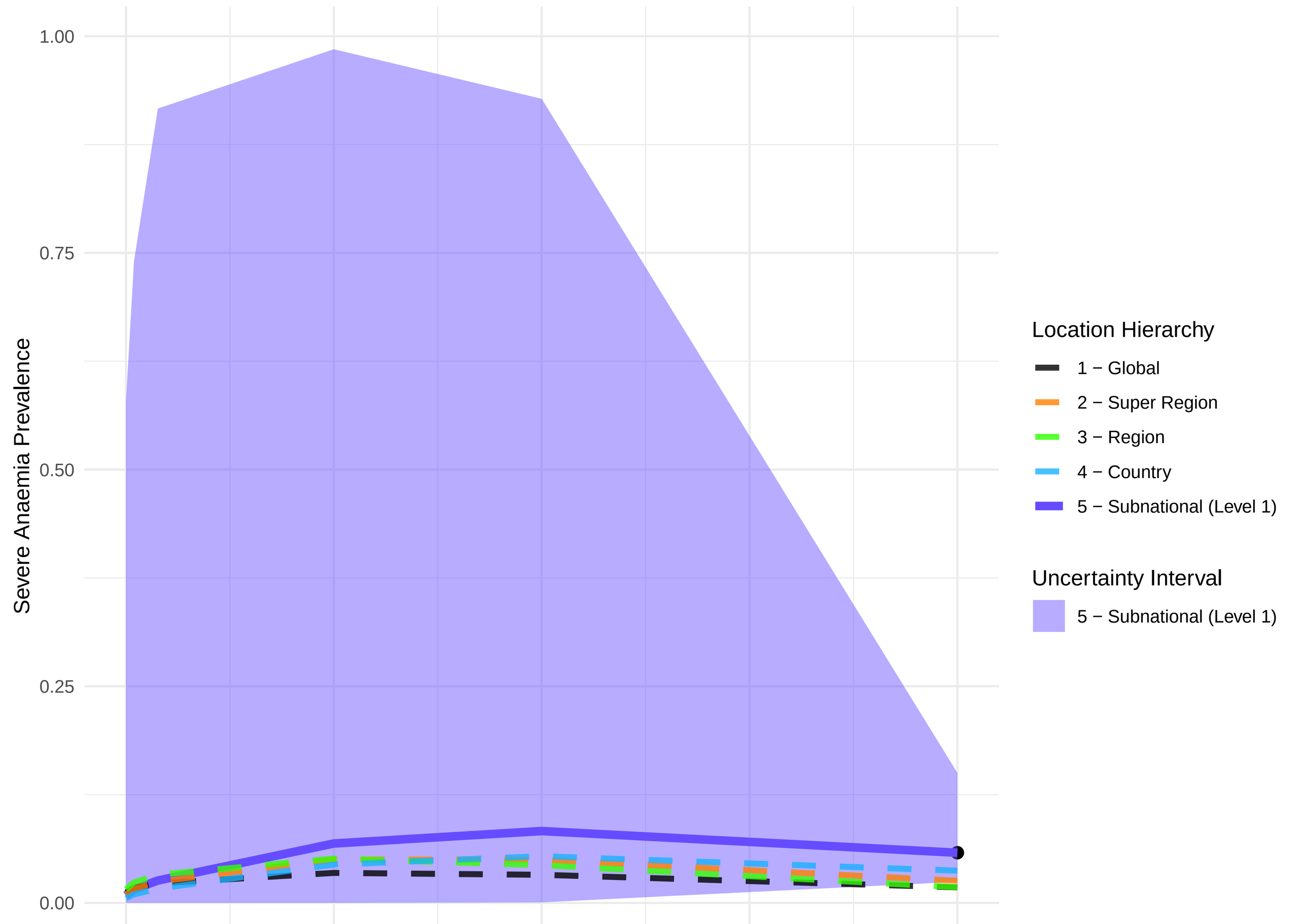

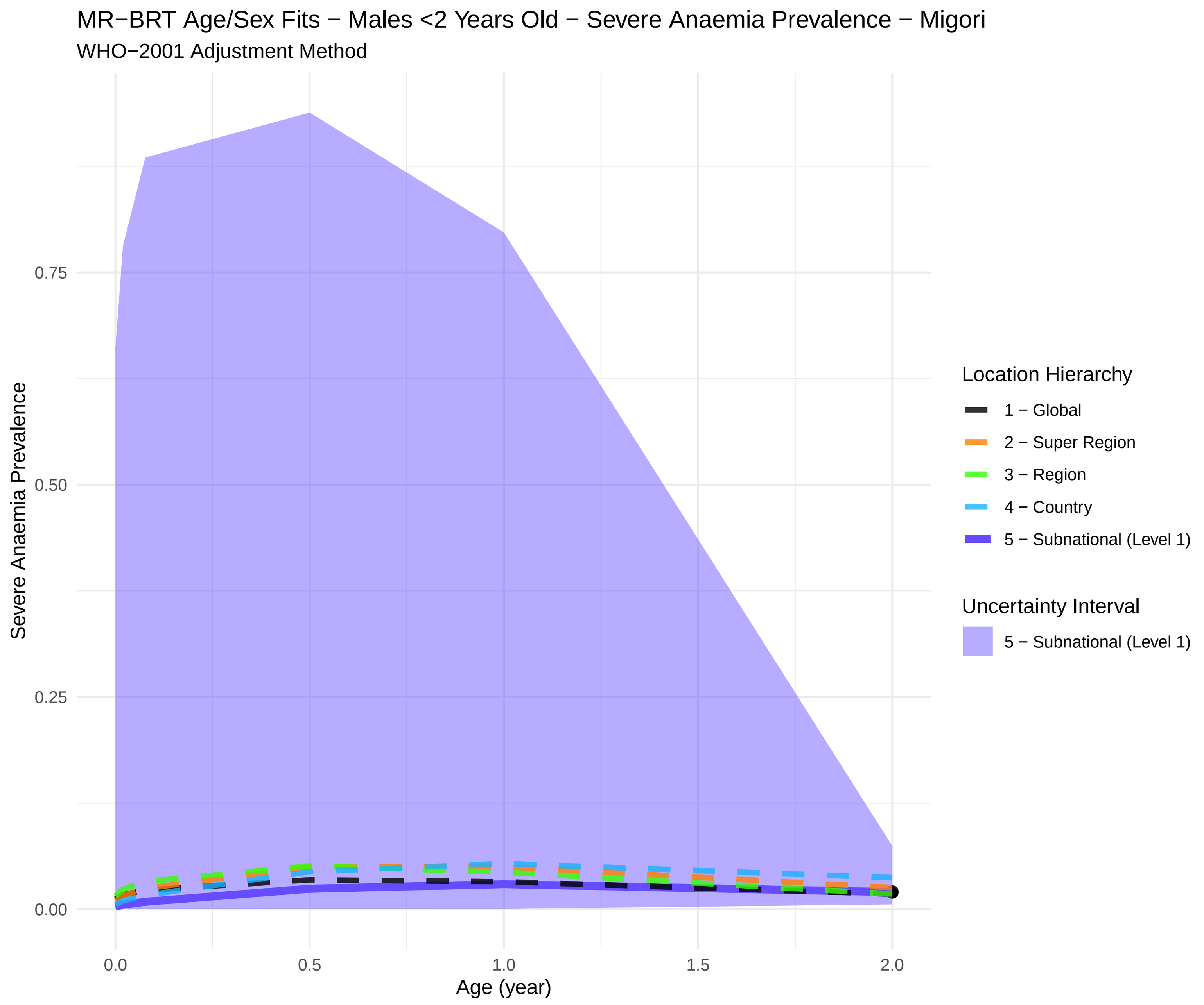
MR−BRT Age/Sex Fits − Males <2 Years Old − Severe Anaemia Prevalence − Migori
WHO−2001 Adjustment Method
Severe Anaemia Prevalence
Age (year)
Location Hierarchy
1 − Global
2 − Super Region
3 − Region
4 − Country
5 − Subnational (Level 1)
Uncertainty Interval
5 − Subnational (Level 1)

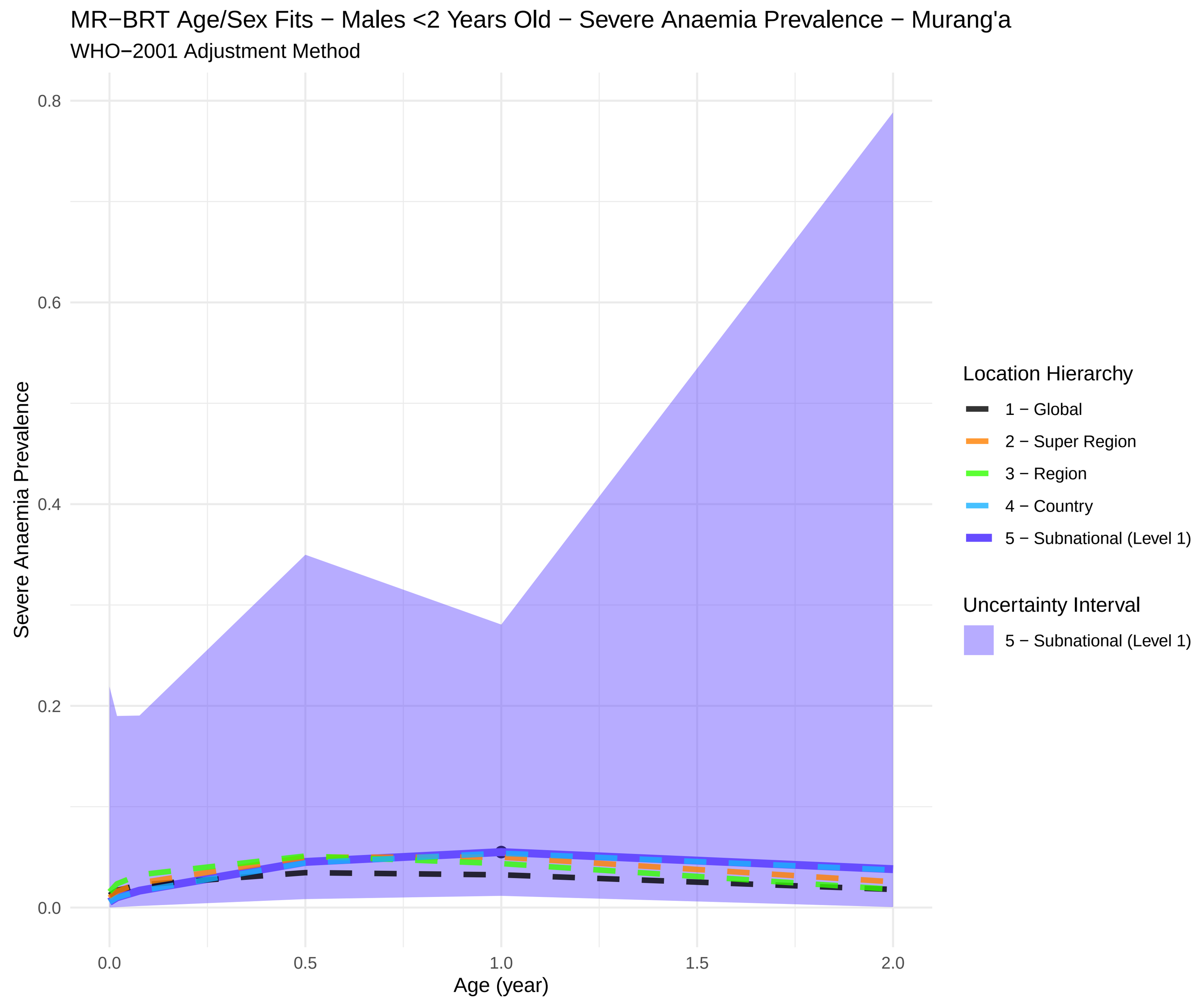
MR−BRT Age/Sex Fits − Males <2 Years Old − Severe Anaemia Prevalence − Murang'a
WHO−2001 Adjustment Method
Severe Anaemia Prevalence
Age (year)
Location Hierarchy
1 − Global
2 − Super Region
3 − Region
4 − Country
5 − Subnational (Level 1)
Uncertainty Interval
5 − Subnational (Level 1)

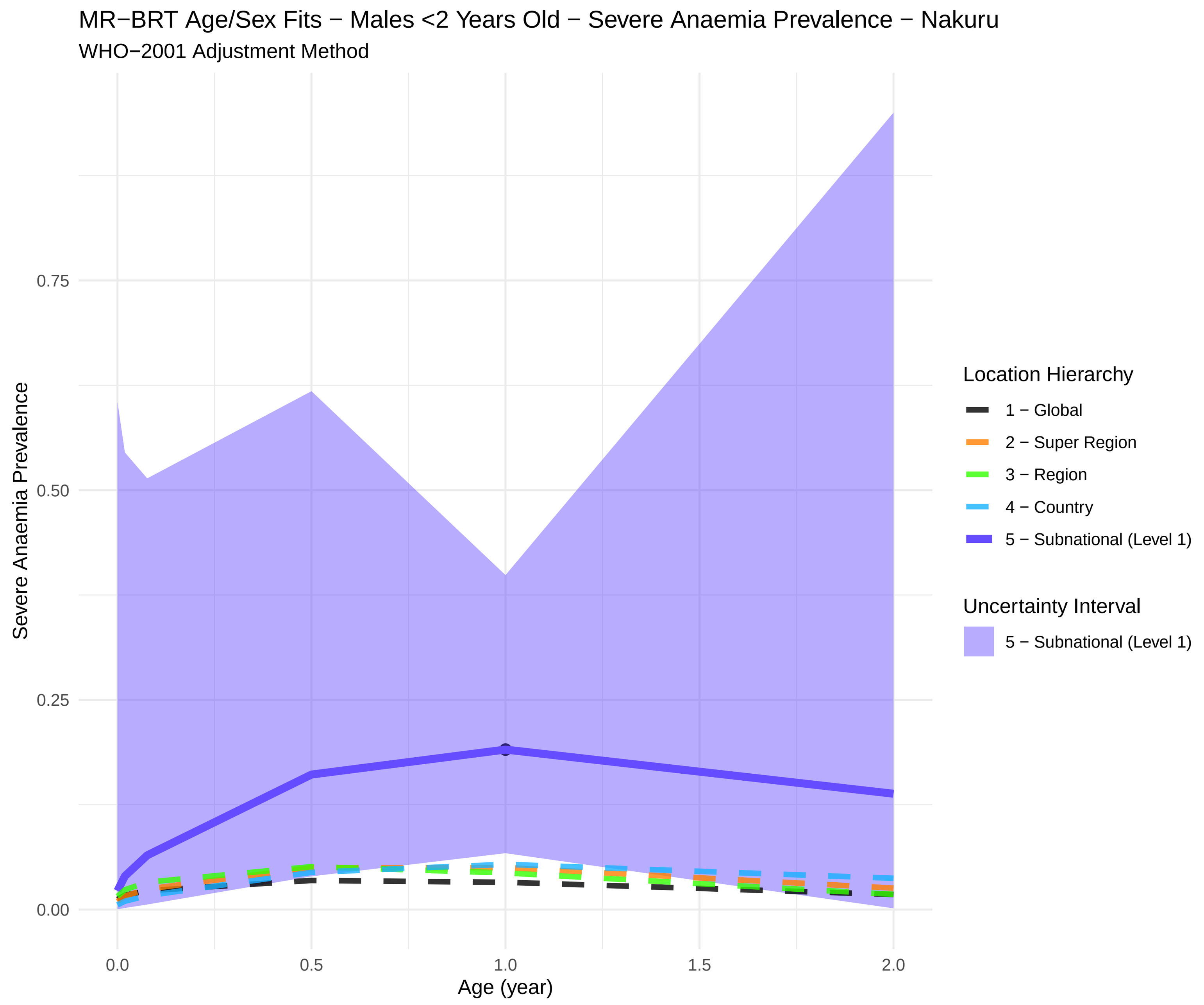
MR−BRT Age/Sex Fits − Males <2 Years Old − Severe Anaemia Prevalence − Nakuru
WHO−2001 Adjustment Method
Severe Anaemia Prevalence
Age (year)
Location Hierarchy
1 − Global
2 − Super Region
3 − Region
4 − Country
5 − Subnational (Level 1)
Uncertainty Interval
5 − Subnational (Level 1)

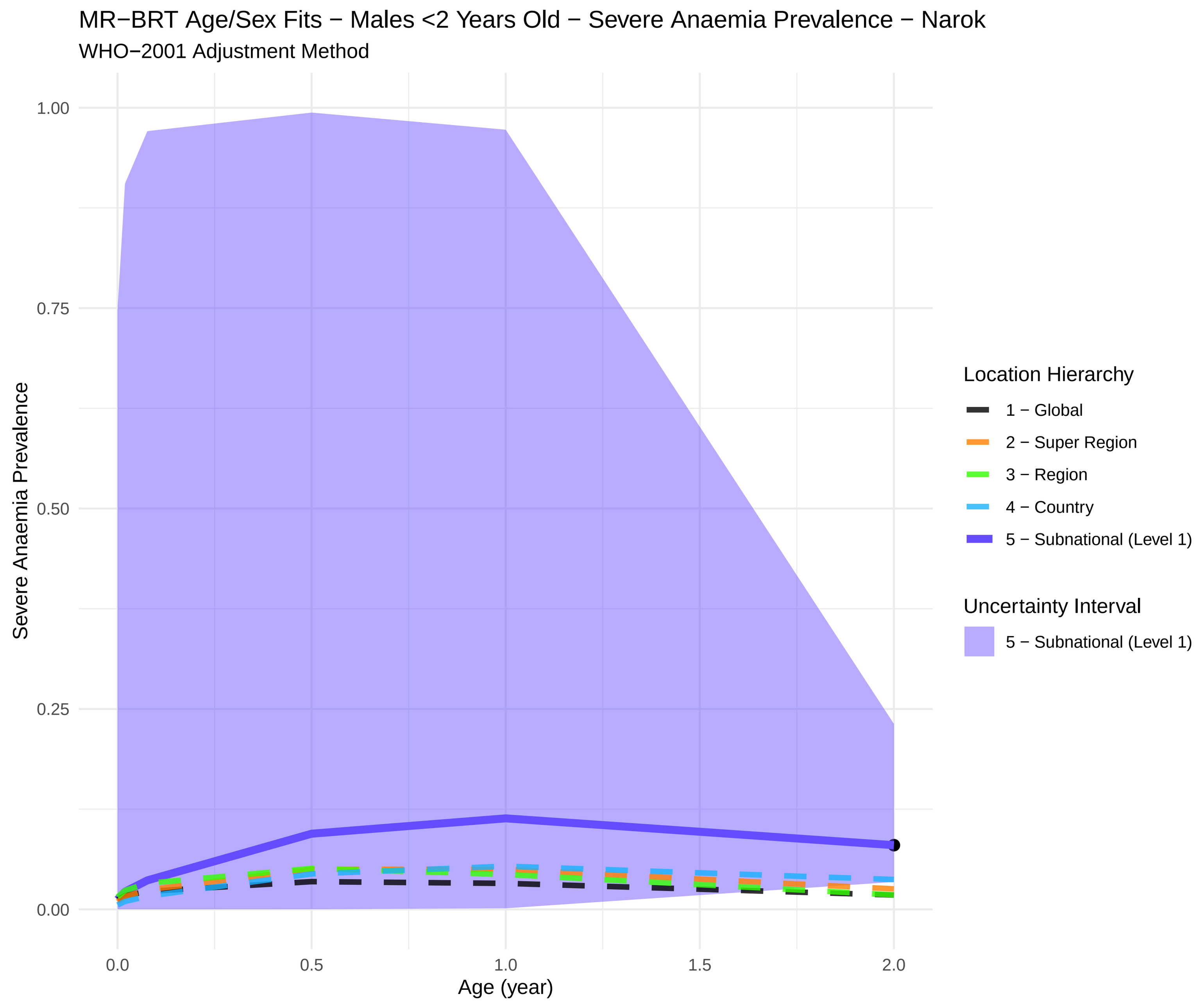
MR−BRT Age/Sex Fits − Males <2 Years Old − Severe Anaemia Prevalence − Narok
WHO−2001 Adjustment Method
Severe Anaemia Prevalence
Age (year)
Location Hierarchy
1 − Global
2 − Super Region
3 − Region
4 − Country
5 − Subnational (Level 1)
Uncertainty Interval
5 − Subnational (Level 1)

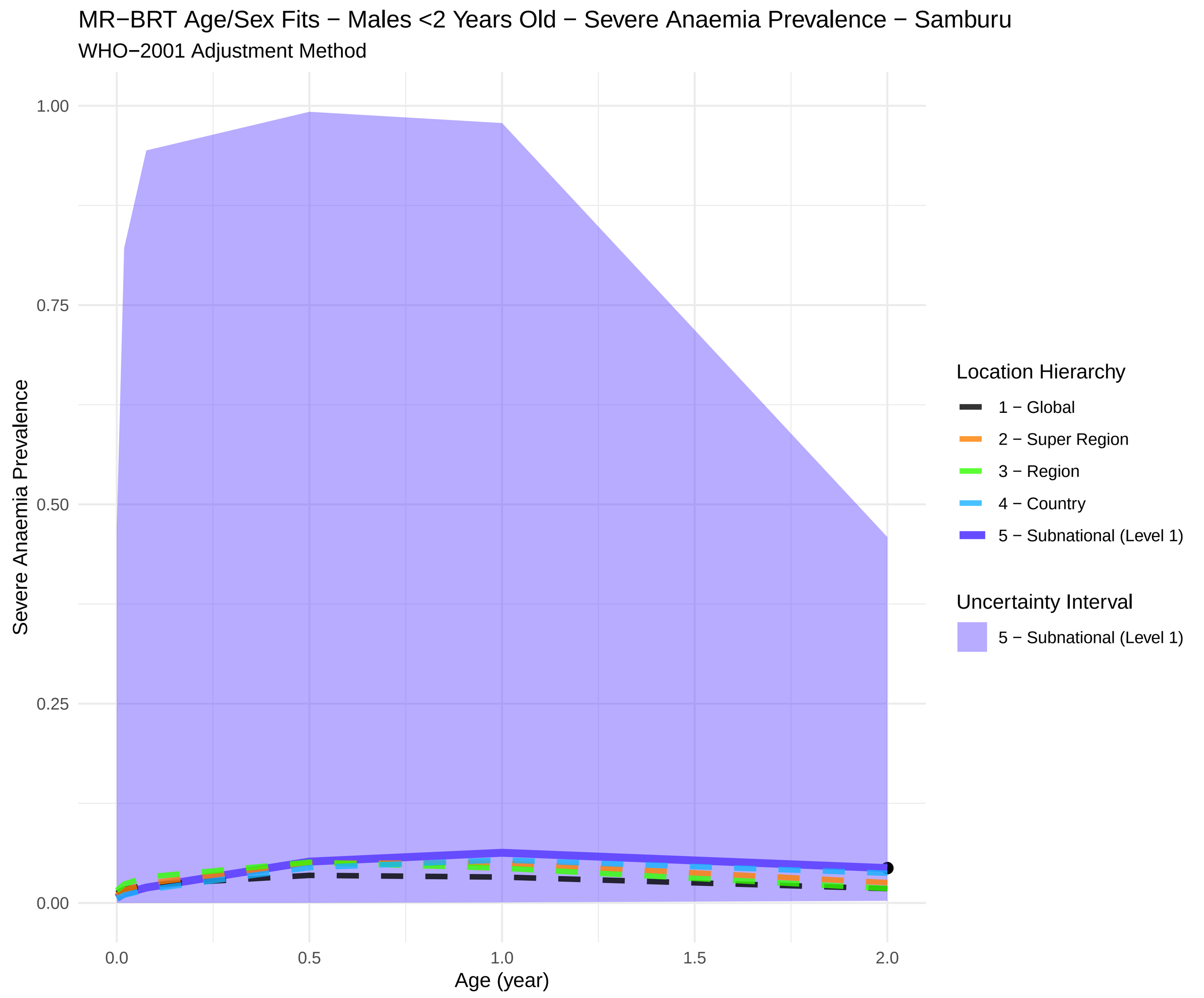
MR−BRT Age/Sex Fits − Males <2 Years Old − Severe Anaemia Prevalence − Samburu
WHO−2001 Adjustment Method
Severe Anaemia Prevalence
Age (year)
Location Hierarchy
1 − Global
2 − Super Region
3 − Region
4 − Country
5 − Subnational (Level 1)
Uncertainty Interval
5 − Subnational (Level 1)

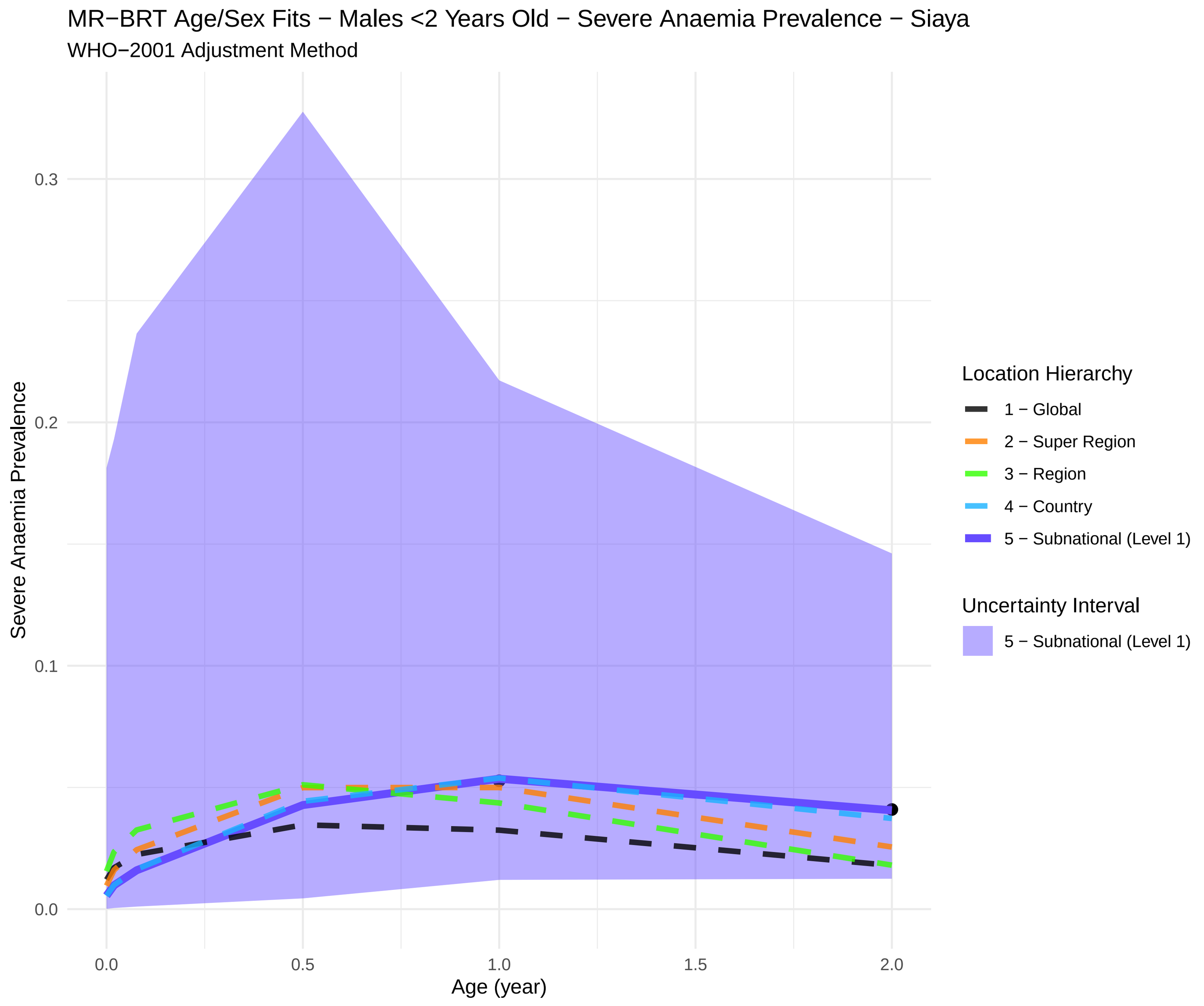
MR−BRT Age/Sex Fits − Males <2 Years Old − Severe Anaemia Prevalence − Siaya
WHO−2001 Adjustment Method
Severe Anaemia Prevalence
Age (year)
Location Hierarchy
1 − Global
2 − Super Region
3 − Region
4 − Country
5 − Subnational (Level 1)
Uncertainty Interval
5 − Subnational (Level 1)

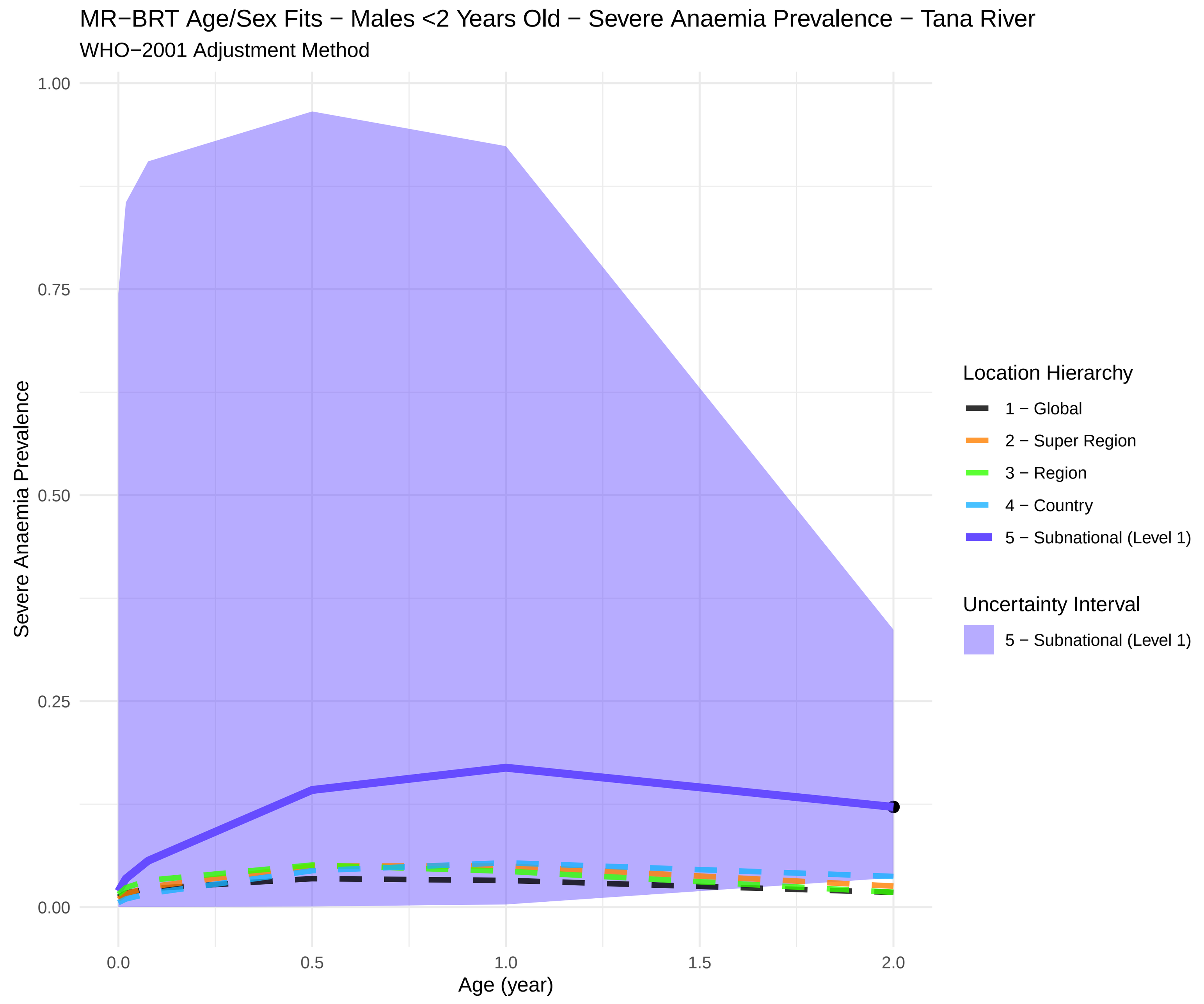
MR−BRT Age/Sex Fits − Males <2 Years Old − Severe Anaemia Prevalence − Tana River
WHO−2001 Adjustment Method
Severe Anaemia Prevalence
Age (year)
Location Hierarchy
1 − Global
2 − Super Region
3 − Region
4 − Country
5 − Subnational (Level 1)
Uncertainty Interval
5 − Subnational (Level 1)

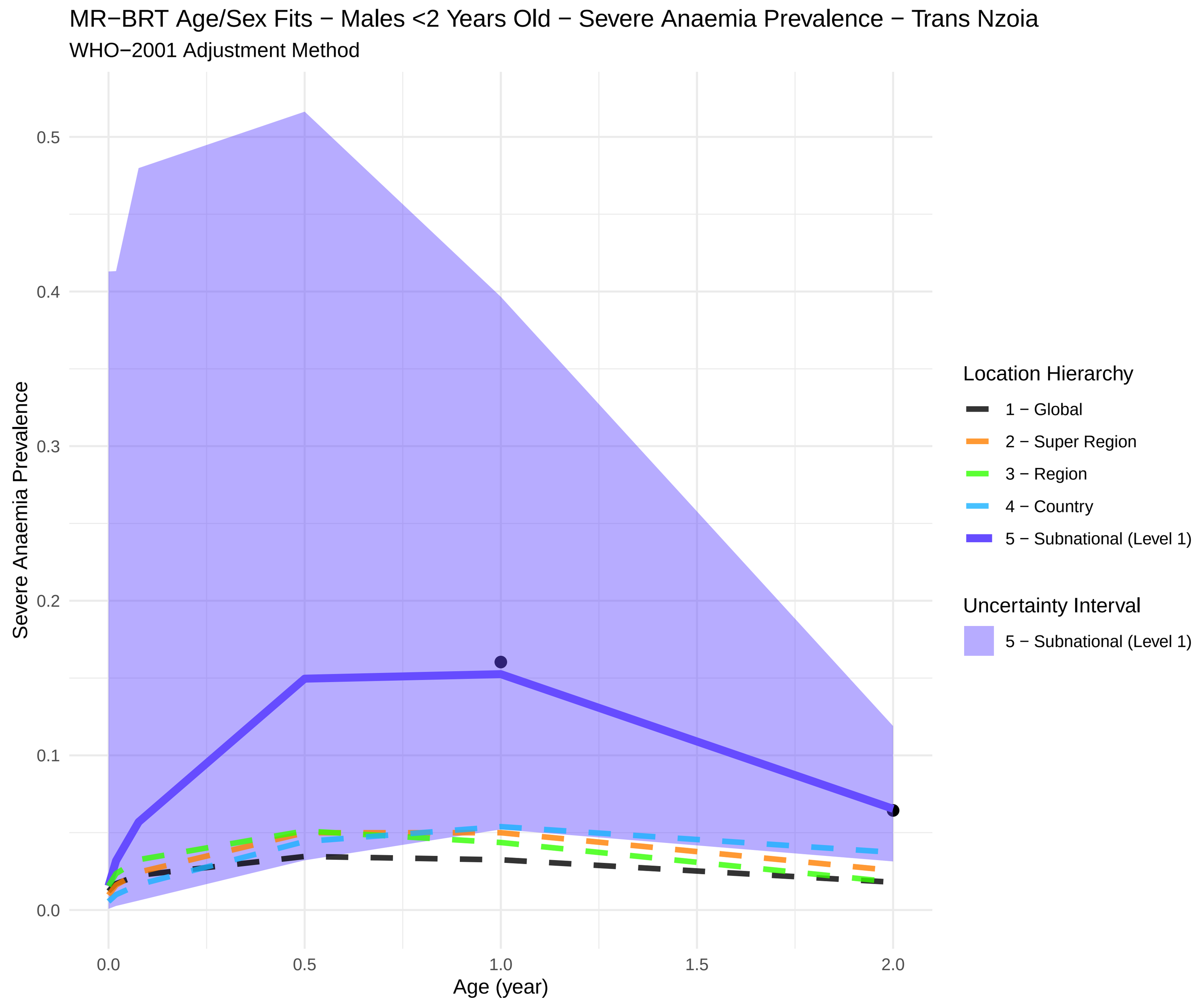
MR−BRT Age/Sex Fits − Males <2 Years Old − Severe Anaemia Prevalence − Trans Nzoia
WHO−2001 Adjustment Method
Severe Anaemia Prevalence
Age (year)
Location Hierarchy
1 − Global
2 − Super Region
3 − Region
4 − Country
5 − Subnational (Level 1)
Uncertainty Interval
5 − Subnational (Level 1)

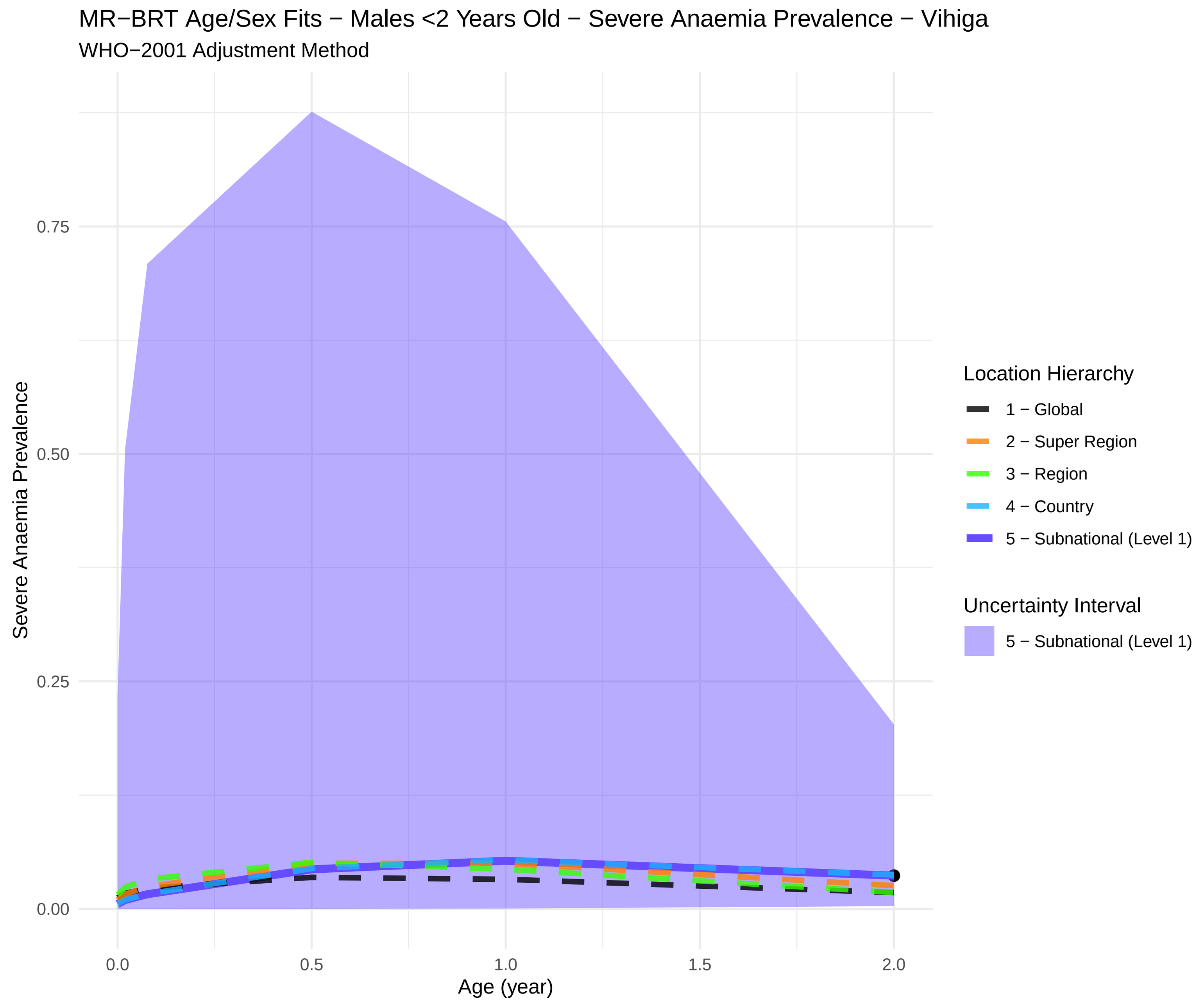
MR−BRT Age/Sex Fits − Males <2 Years Old − Severe Anaemia Prevalence − Vihiga
WHO−2001 Adjustment Method
Severe Anaemia Prevalence
Age (year)
Location Hierarchy
1 − Global
2 − Super Region
3 − Region
4 − Country
5 − Subnational (Level 1)
Uncertainty Interval
5 − Subnational (Level 1)

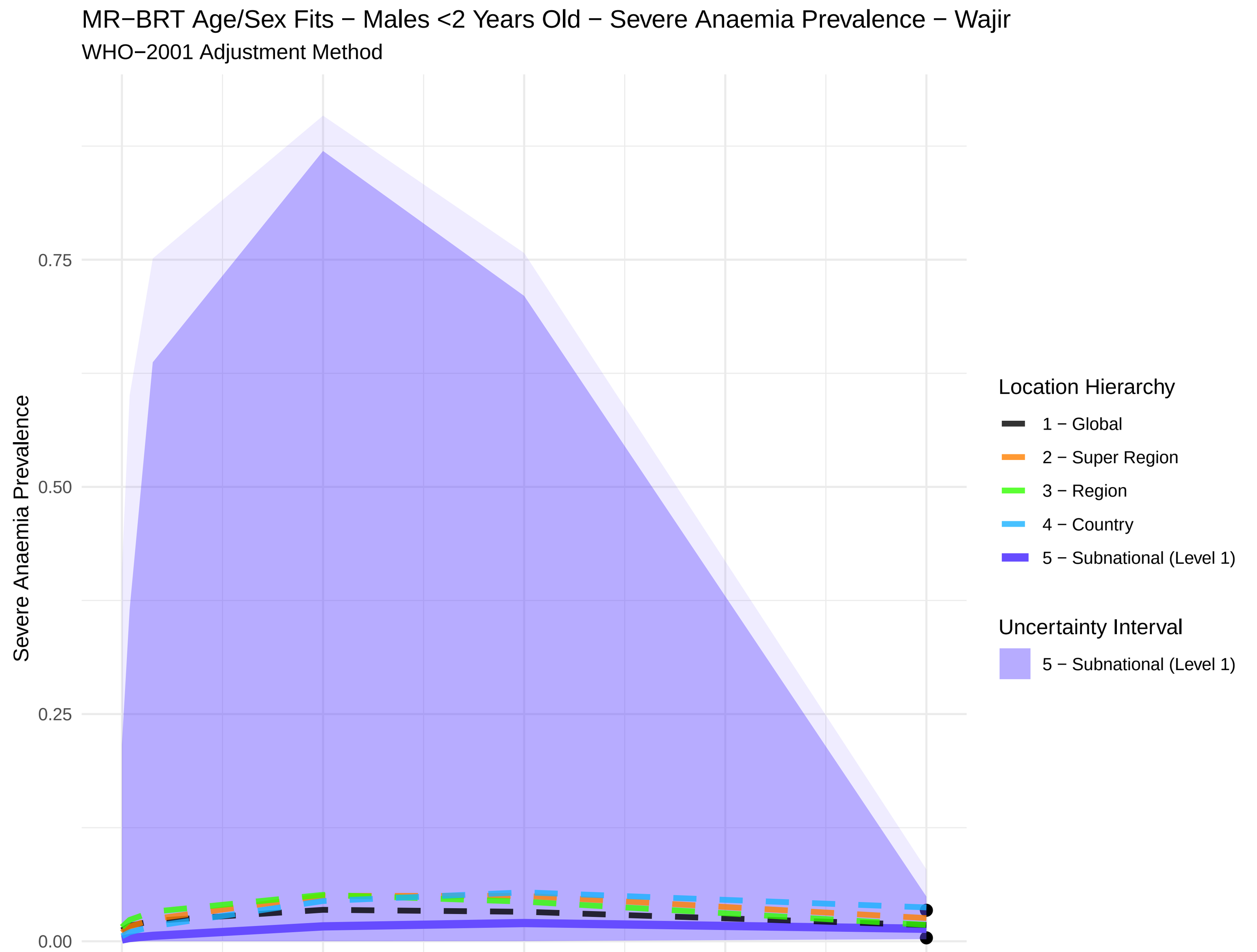
MR−BRT Age/Sex Fits − Males <2 Years Old − Severe Anaemia Prevalence − Wajir
WHO−2001 Adjustment Method
Severe Anaemia Prevalence
Age (year)
Location Hierarchy
1 − Global
2 − Super Region
3 − Region
4 − Country
5 − Subnational (Level 1)
Uncertainty Interval
5 − Subnational (Level 1)

MR−BRT Age/Sex Fits − Males <2 Years Old − Severe Anaemia Prevalence − West Pokot
WHO−2001 Adjustment Method

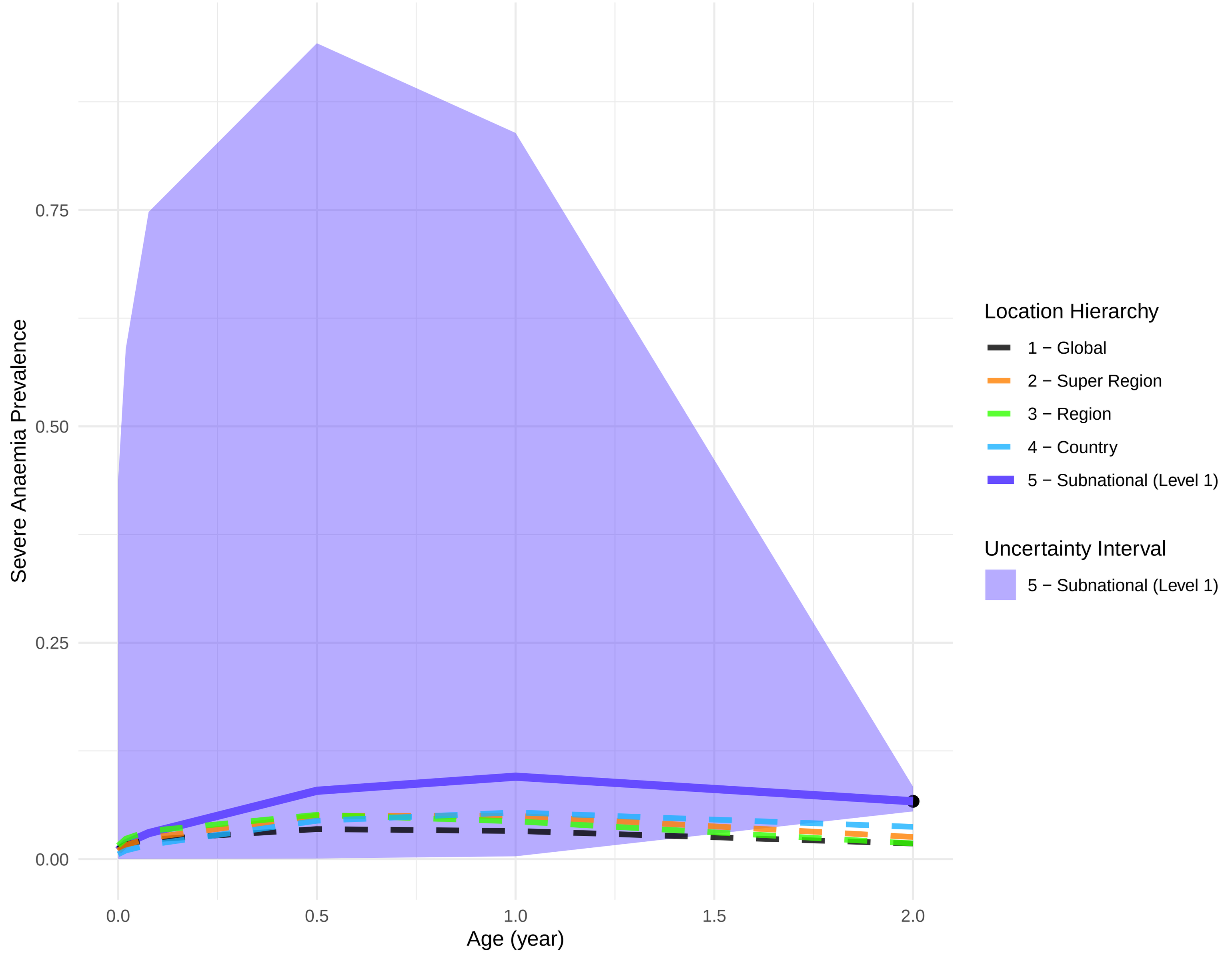

MR−BRT Age/Sex Fits − Males <2 Years Old − Severe Anaemia Prevalence − Madagascar
WHO−2001 Adjustment Method

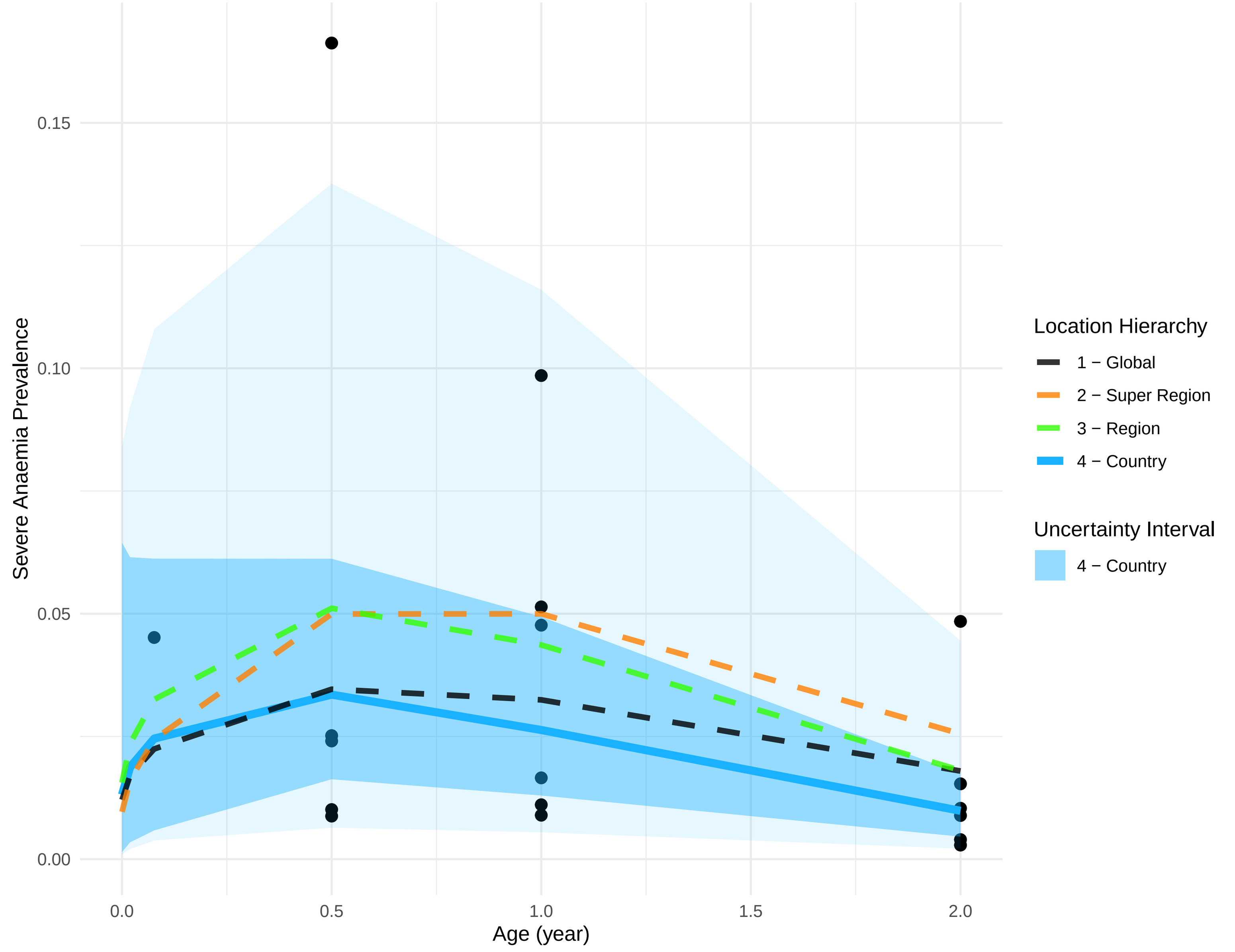

MR−BRT Age/Sex Fits − Males <2 Years Old − Severe Anaemia Prevalence − Malawi
WHO−2001 Adjustment Method

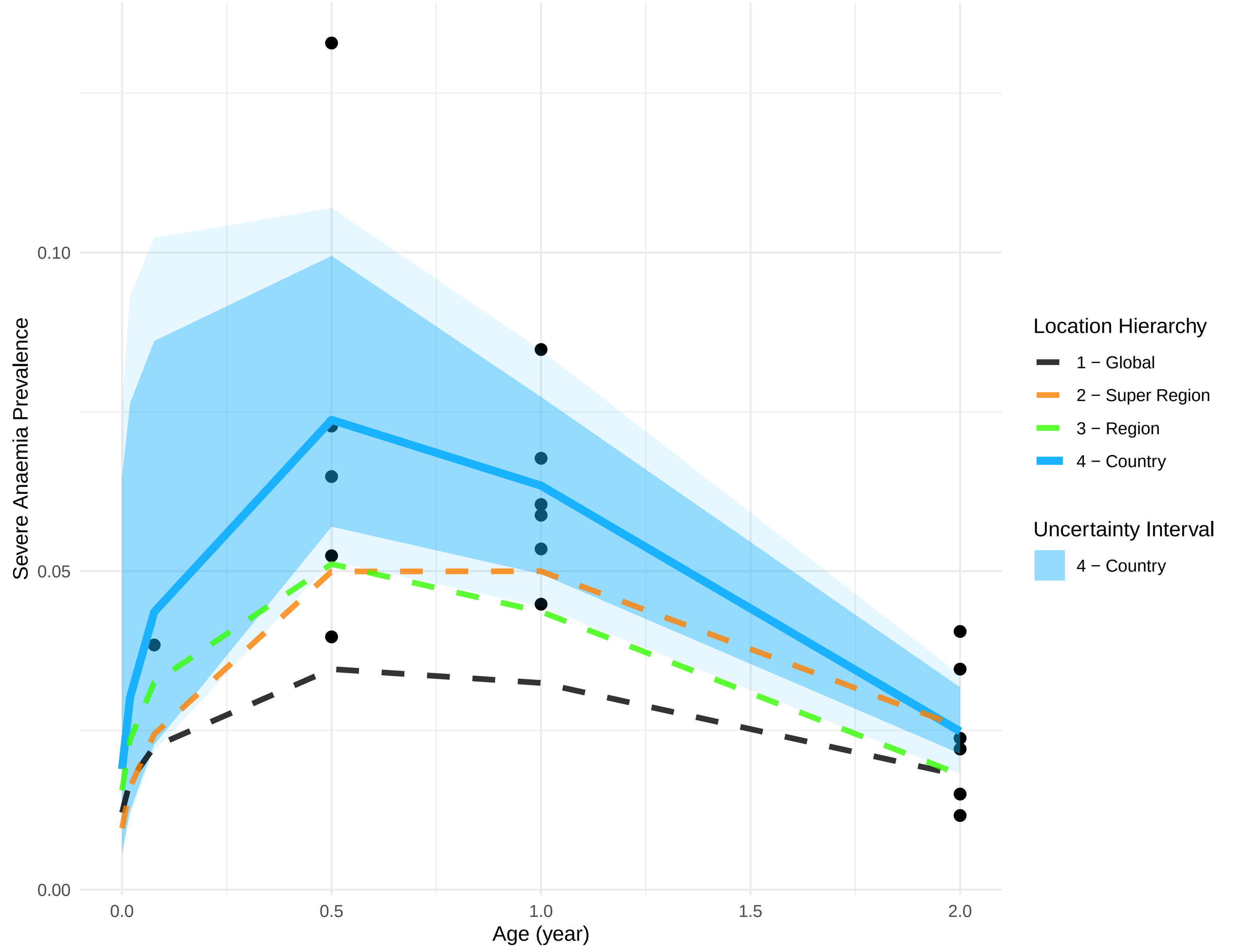

MR−BRT Age/Sex Fits − Males <2 Years Old − Severe Anaemia Prevalence − Mozambique
WHO−2001 Adjustment Method

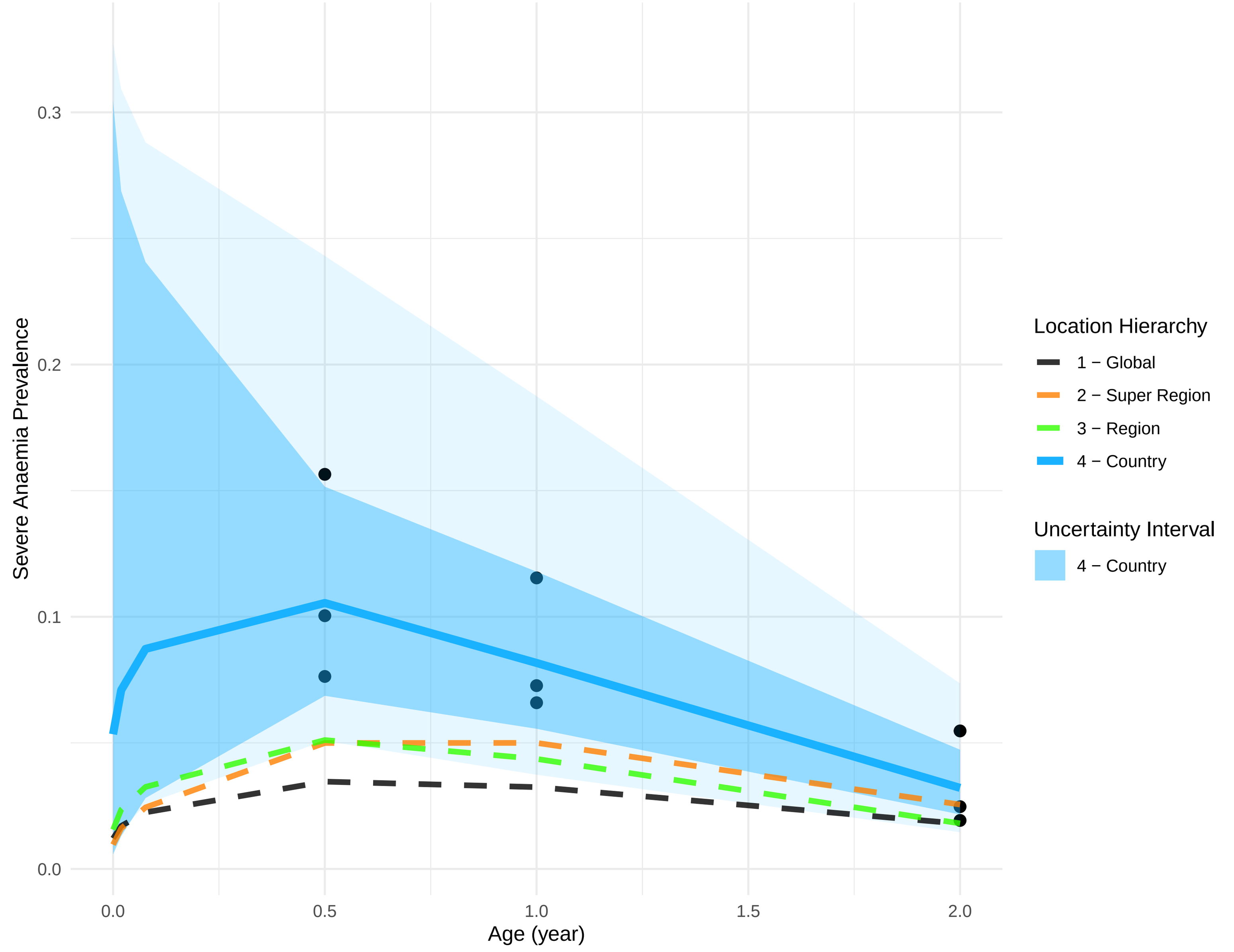

MR−BRT Age/Sex Fits − Males <2 Years Old − Severe Anaemia Prevalence − Rwanda
WHO−2001 Adjustment Method

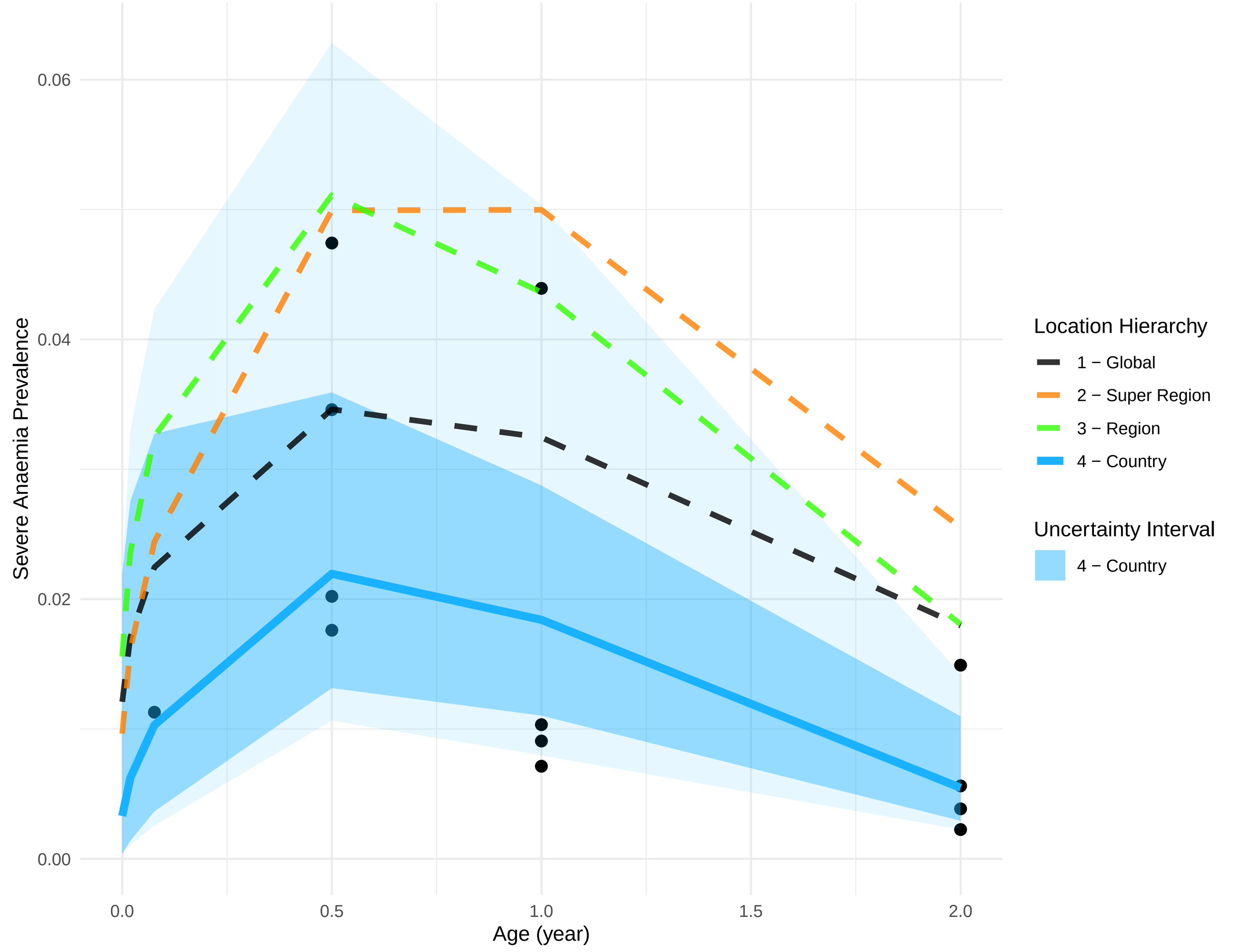

MR−BRT Age/Sex Fits − Males <2 Years Old − Severe Anaemia Prevalence − Uganda
WHO−2001 Adjustment Method

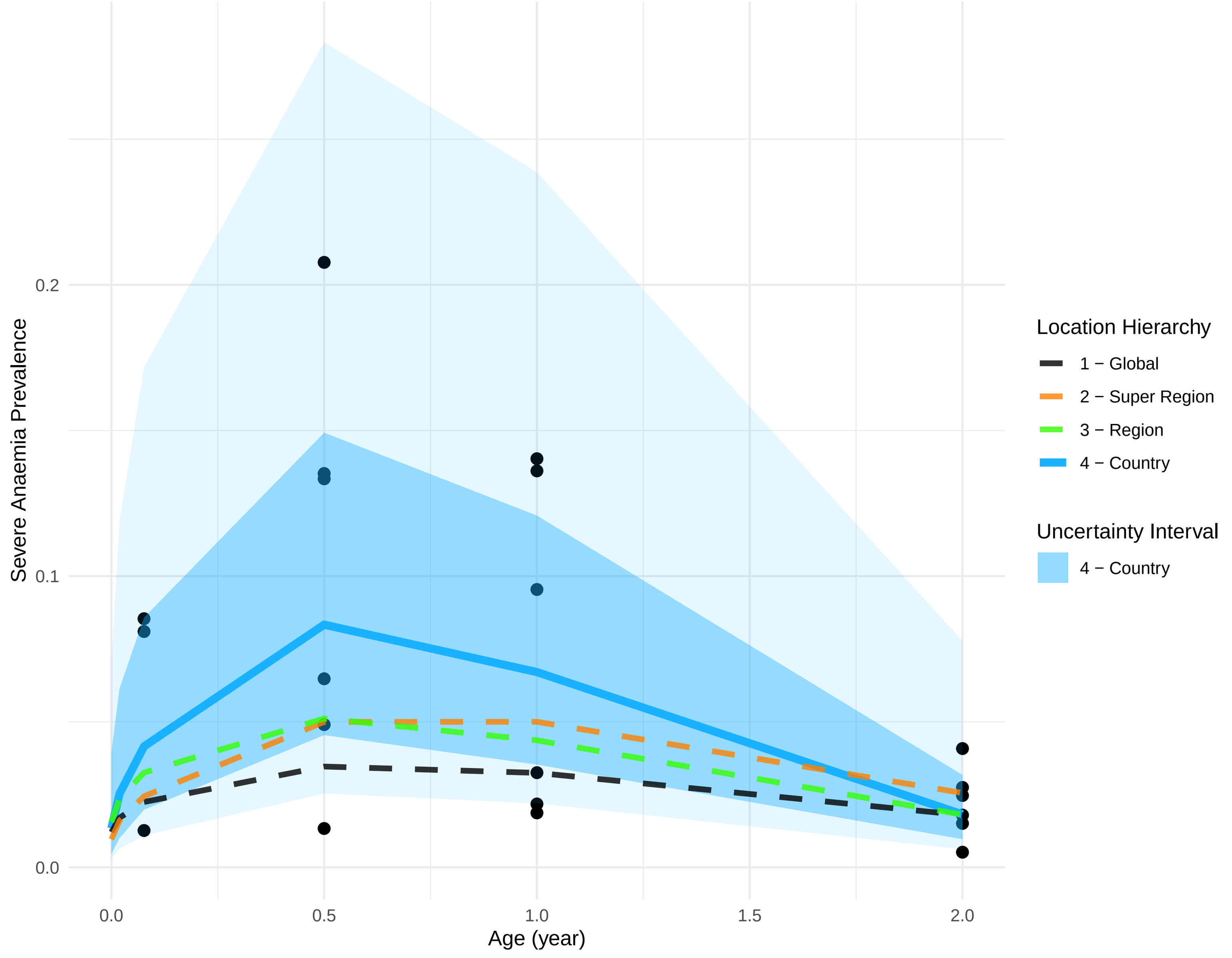

MR−BRT Age/Sex Fits − Males <2 Years Old − Severe Anaemia Prevalence − United Republic of Tanzania
WHO−2001 Adjustment Method

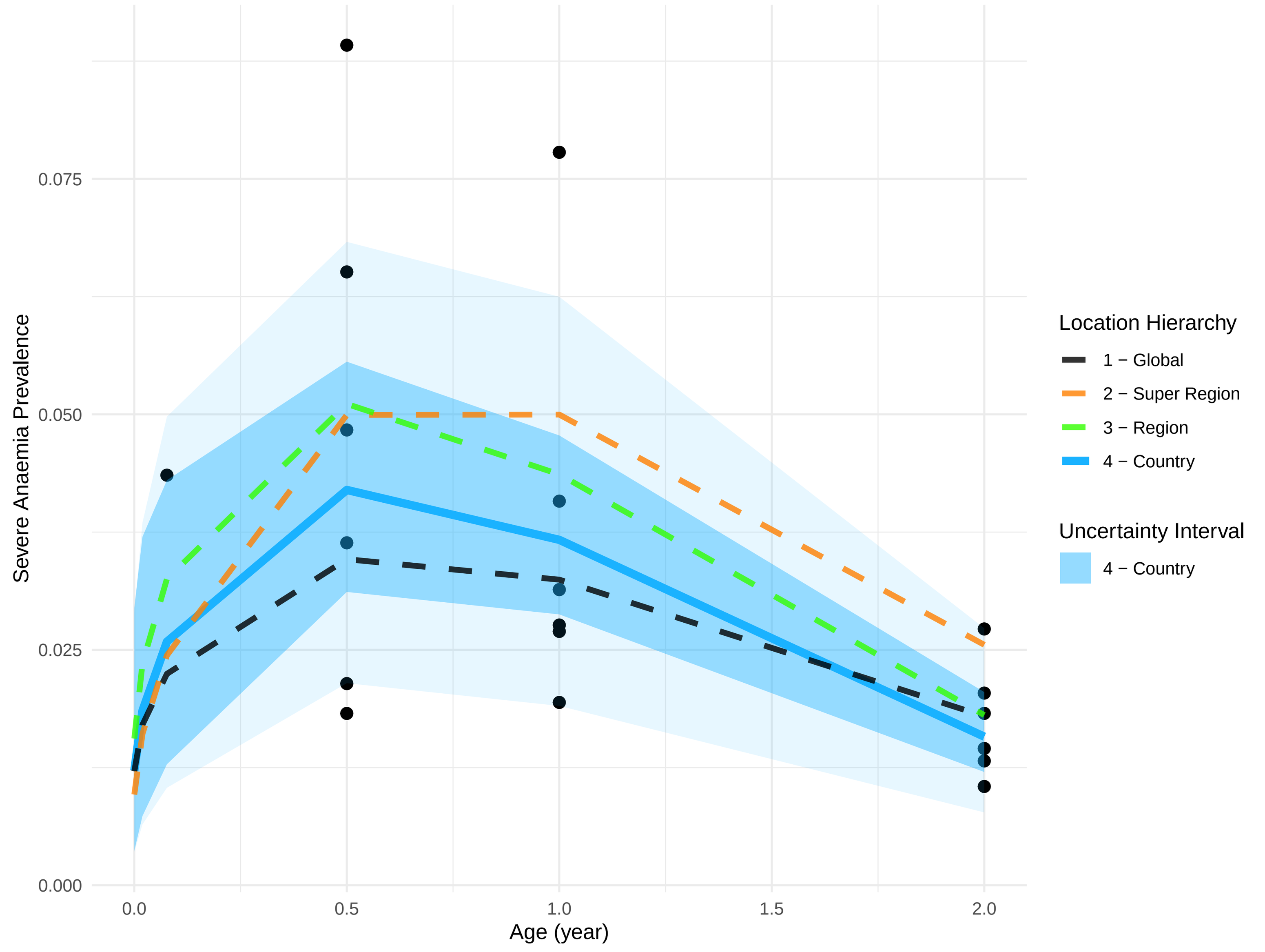

MR−BRT Age/Sex Fits − Males <2 Years Old − Severe Anaemia Prevalence − Zambia
WHO−2001 Adjustment Method

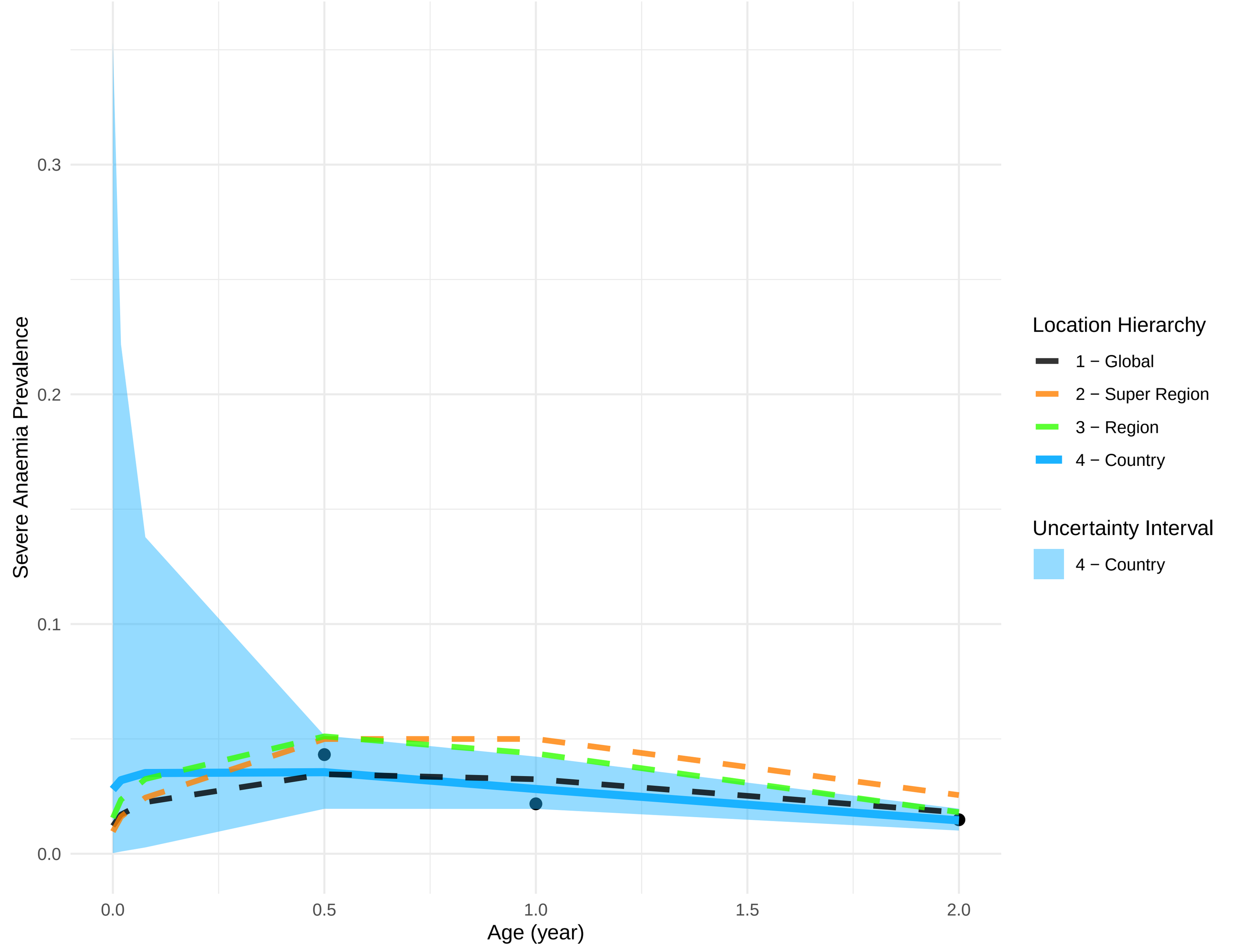

MR−BRT Age/Sex Fits − Males <2 Years Old − Severe Anaemia Prevalence − Southern Sub−Saharan Africa
WHO−2001 Adjustment Method

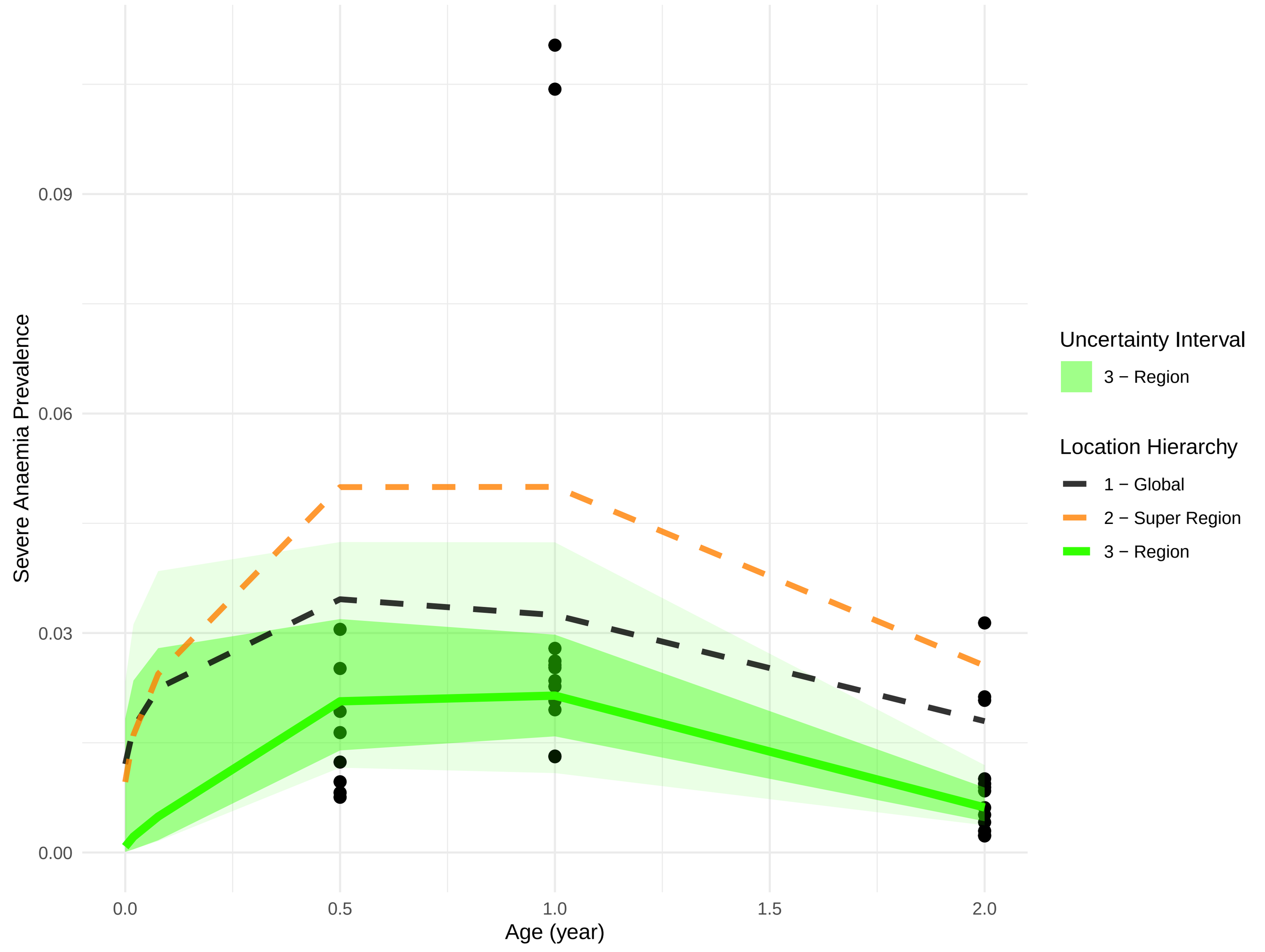

MR−BRT Age/Sex Fits − Males <2 Years Old − Severe Anaemia Prevalence − Eswatini
WHO−2001 Adjustment Method

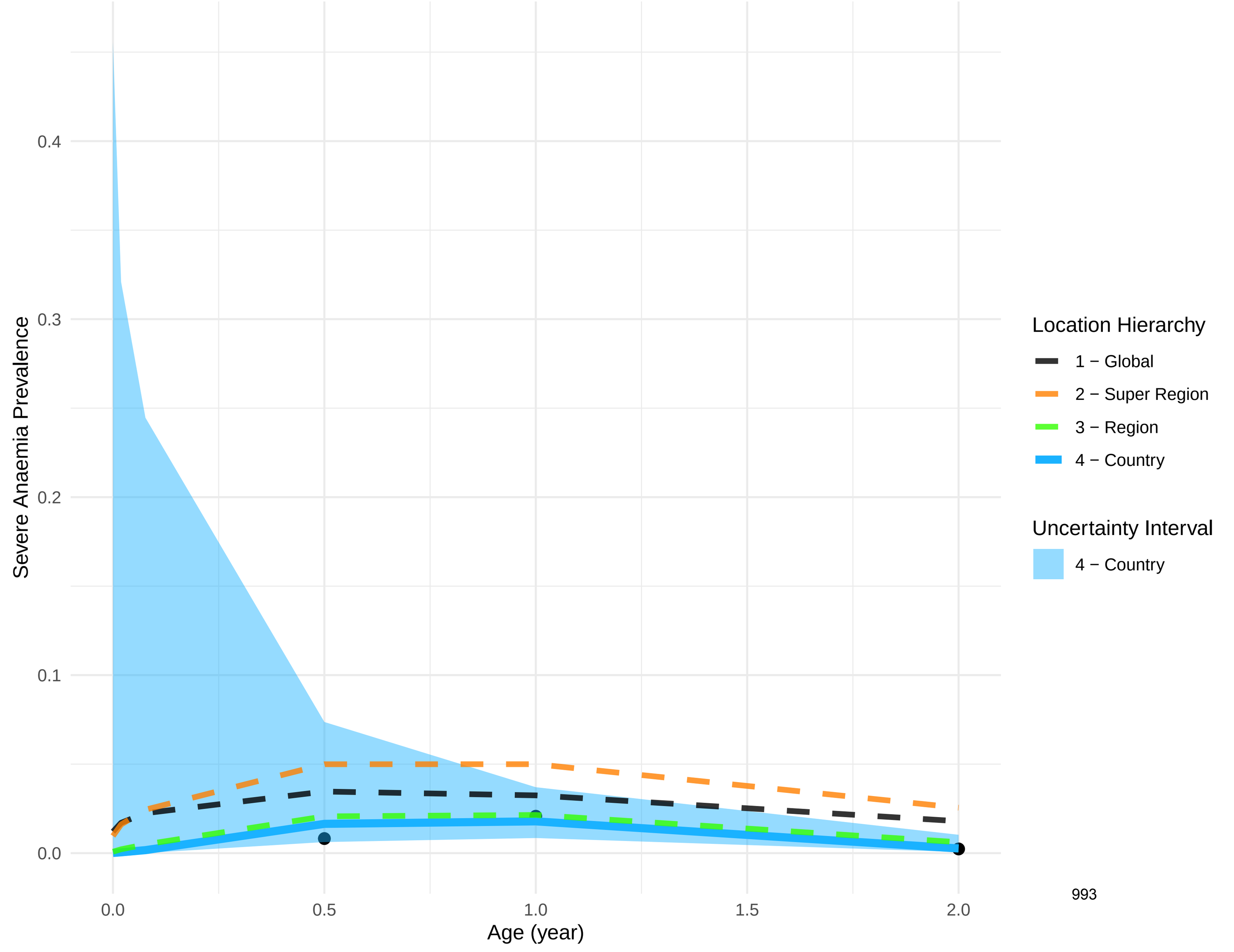

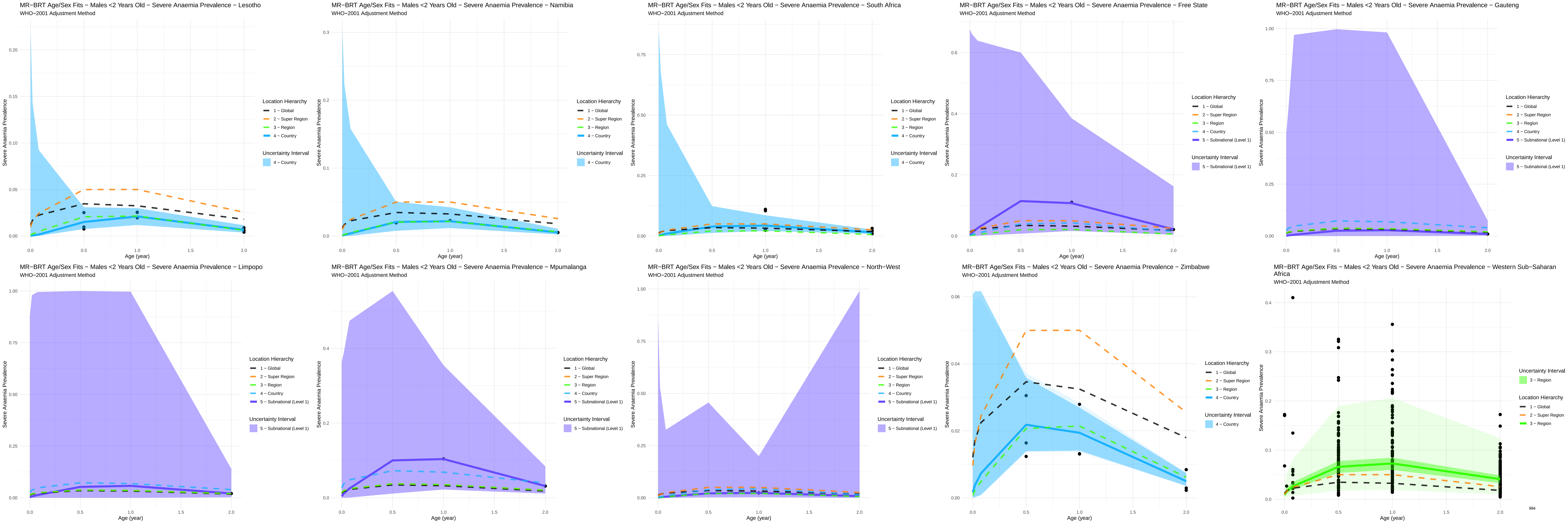
MR−BRT Age/Sex Fits − Males <2 Years Old − Severe Anaemia Prevalence − Lesotho
WHO−2001 Adjustment Method
MR−BRT Age/Sex Fits − Males <2 Years Old − Severe Anaemia Prevalence − Namibia
WHO−2001 Adjustment Method
MR−BRT Age/Sex Fits − Males <2 Years Old − Severe Anaemia Prevalence − South Africa
WHO−2001 Adjustment Method
MR−BRT Age/Sex Fits − Males <2 Years Old − Severe Anaemia Prevalence − Free State
WHO−2001 Adjustment Method
MR−BRT Age/Sex Fits − Males <2 Years Old − Severe Anaemia Prevalence − Gauteng
WHO−2001 Adjustment Method
MR−BRT Age/Sex Fits − Males <2 Years Old − Severe Anaemia Prevalence − Limpopo
WHO−2001 Adjustment Method
MR−BRT Age/Sex Fits − Males <2 Years Old − Severe Anaemia Prevalence − Mpumalanga
WHO−2001 Adjustment Method
MR−BRT Age/Sex Fits − Males <2 Years Old − Severe Anaemia Prevalence − North−West
WHO−2001 Adjustment Method
MR−BRT Age/Sex Fits − Males <2 Years Old − Severe Anaemia Prevalence − Zimbabwe
WHO−2001 Adjustment Method
MR−BRT Age/Sex Fits − Males <2 Years Old − Severe Anaemia Prevalence − Western Sub−Saharan Africa
WHO−2001 Adjustment Method
Severe Anaemia Prevalence
Age (year)
Location Hierarchy
1 − Global
2 − Super Region
3 − Region
4 − Country
5 − Subnational (Level 1)
Uncertainty Interval
4 − Country
5 − Subnational (Level 1)
3 − Region

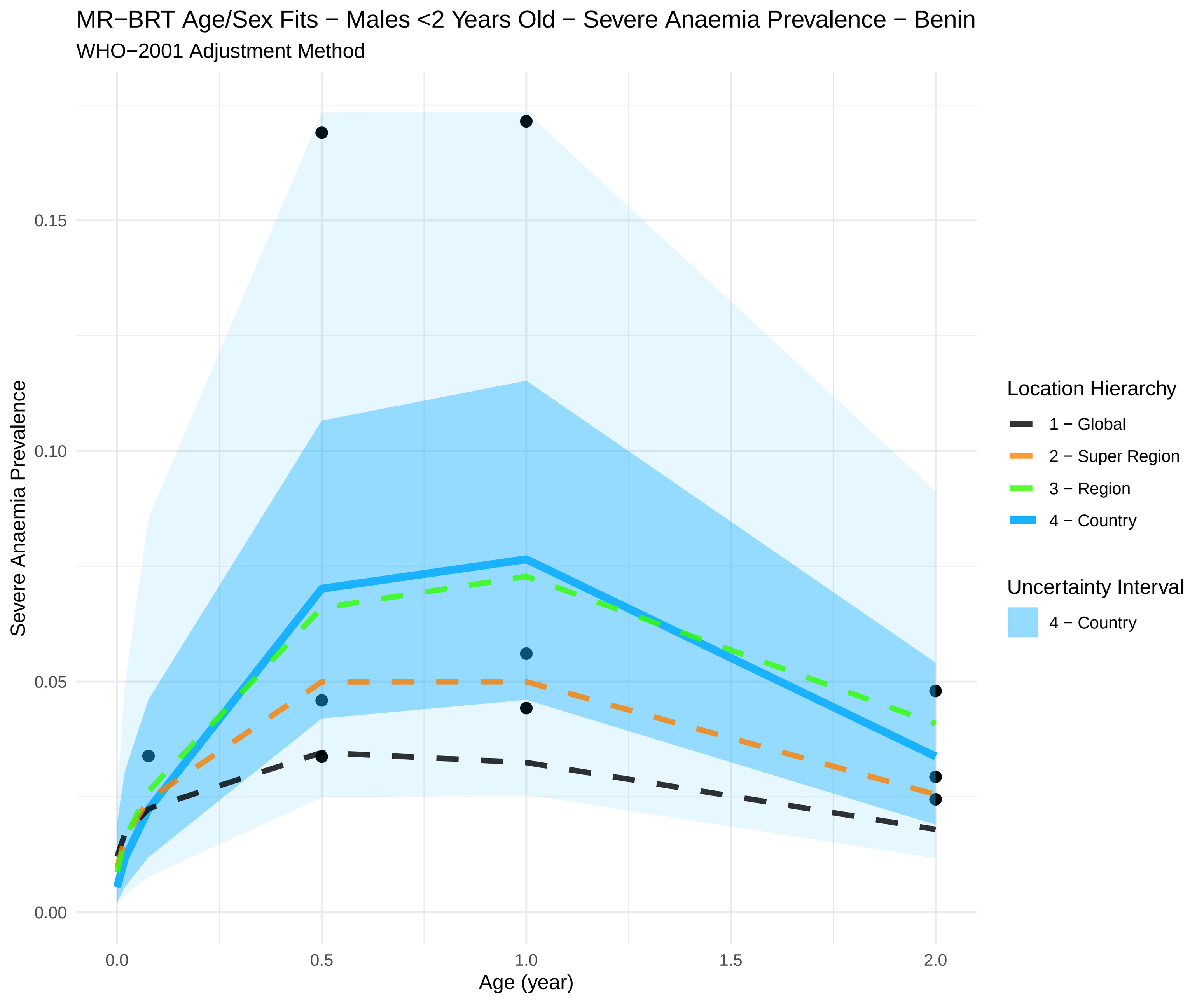
MR−BRT Age/Sex Fits − Males <2 Years Old − Severe Anaemia Prevalence − Benin
WHO−2001 Adjustment Method
Severe Anaemia Prevalence
Age (year)
Location Hierarchy
1 − Global
2 − Super Region
3 − Region
4 − Country
Uncertainty Interval
4 − Country

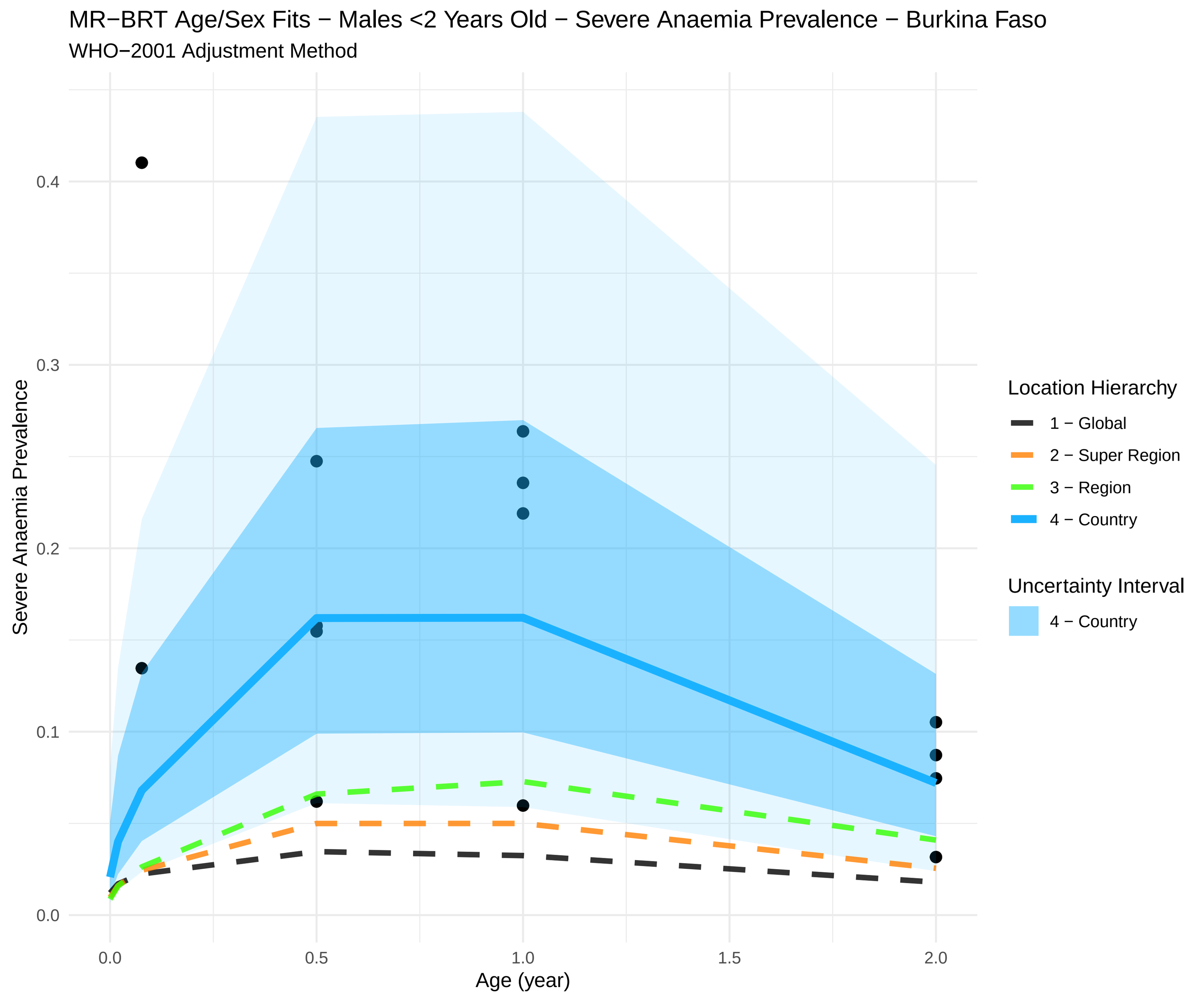
MR−BRT Age/Sex Fits − Males <2 Years Old − Severe Anaemia Prevalence − Burkina Faso
WHO−2001 Adjustment Method
Severe Anaemia Prevalence
Age (year)
Location Hierarchy
1 − Global
2 − Super Region
3 − Region
4 − Country
Uncertainty Interval
4 − Country

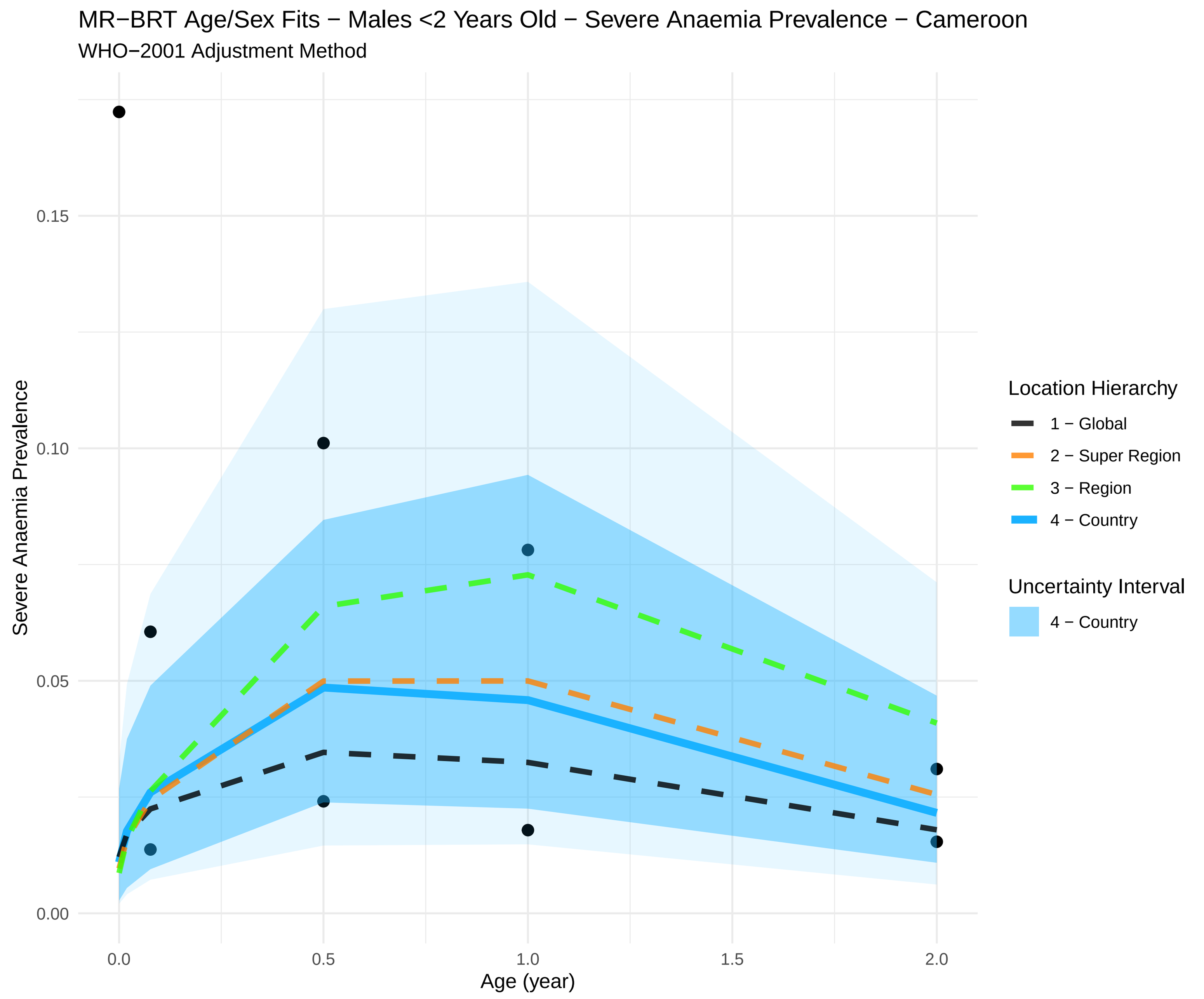
MR−BRT Age/Sex Fits − Males <2 Years Old − Severe Anaemia Prevalence − Cameroon
WHO−2001 Adjustment Method
Severe Anaemia Prevalence
Age (year)
Location Hierarchy
1 − Global
2 − Super Region
3 − Region
4 − Country
Uncertainty Interval
4 − Country

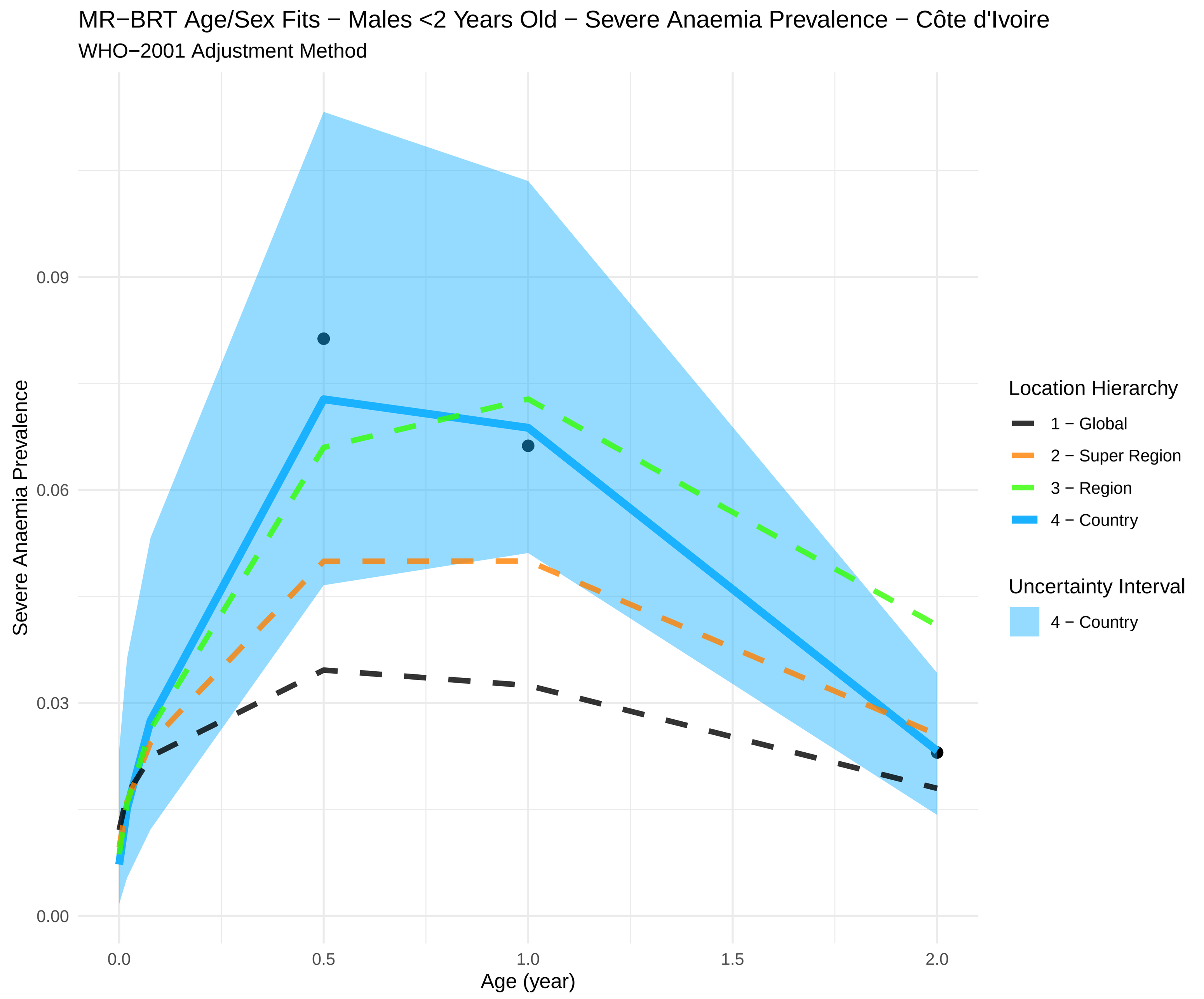
MR−BRT Age/Sex Fits − Males <2 Years Old − Severe Anaemia Prevalence − Côte d'Ivoire
WHO−2001 Adjustment Method
Severe Anaemia Prevalence
Age (year)
Location Hierarchy
1 − Global
2 − Super Region
3 − Region
4 − Country
Uncertainty Interval
4 − Country

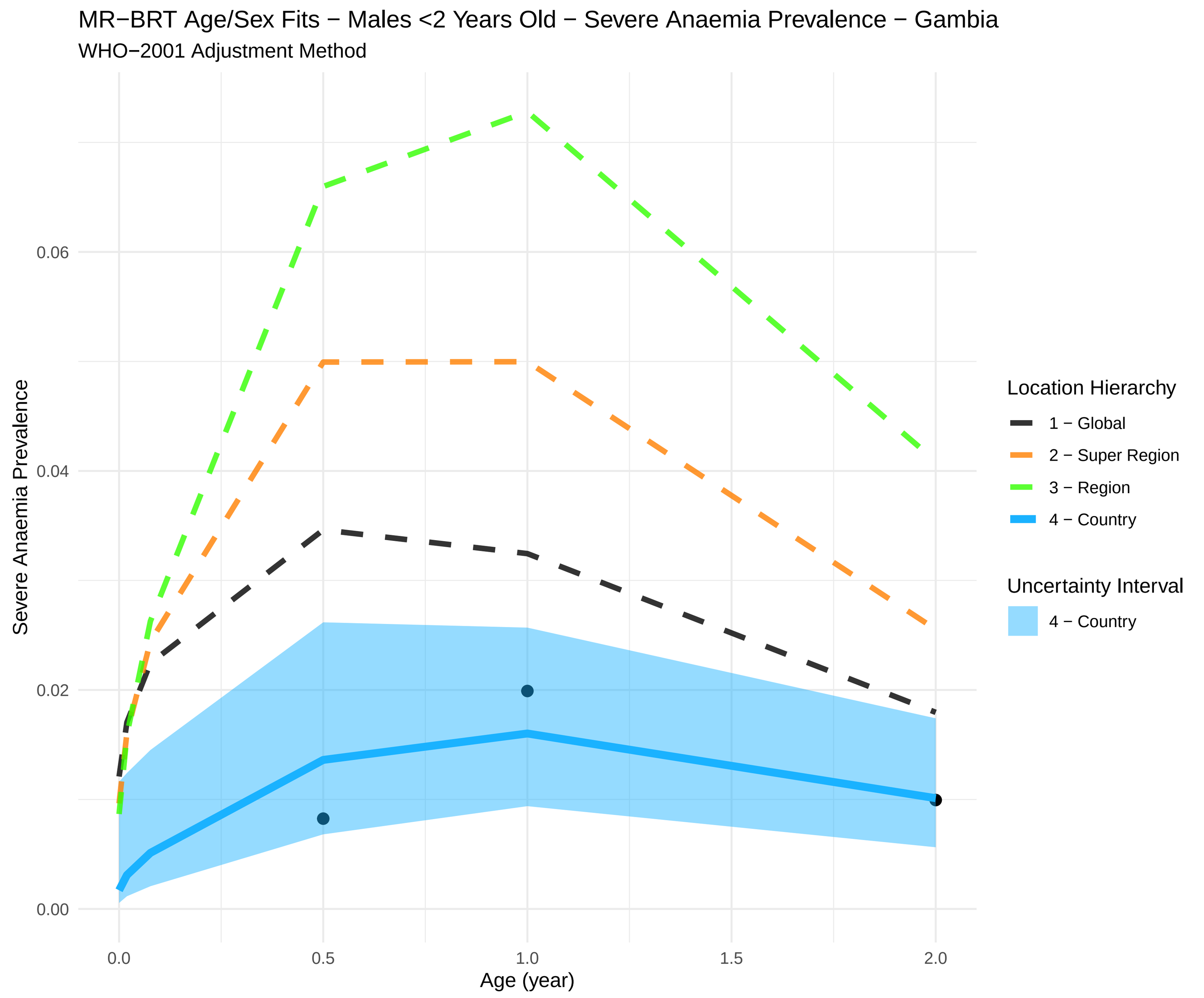
MR−BRT Age/Sex Fits − Males <2 Years Old − Severe Anaemia Prevalence − Gambia
WHO−2001 Adjustment Method
Severe Anaemia Prevalence
Age (year)
Location Hierarchy
1 − Global
2 − Super Region
3 − Region
4 − Country
Uncertainty Interval
4 − Country

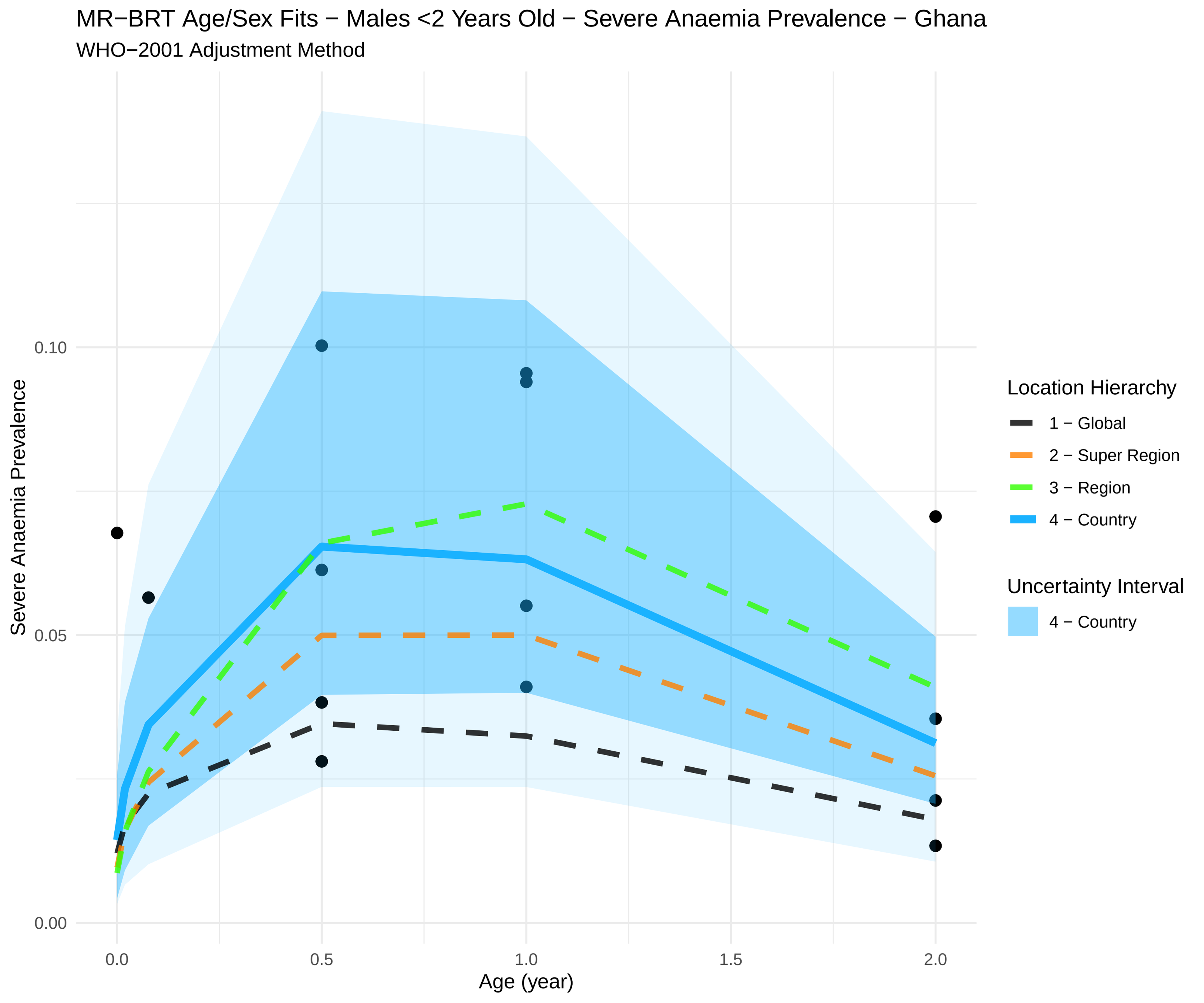
MR−BRT Age/Sex Fits − Males <2 Years Old − Severe Anaemia Prevalence − Ghana
WHO−2001 Adjustment Method
Severe Anaemia Prevalence
Age (year)
Location Hierarchy
1 − Global
2 − Super Region
3 − Region
4 − Country
Uncertainty Interval
4 − Country

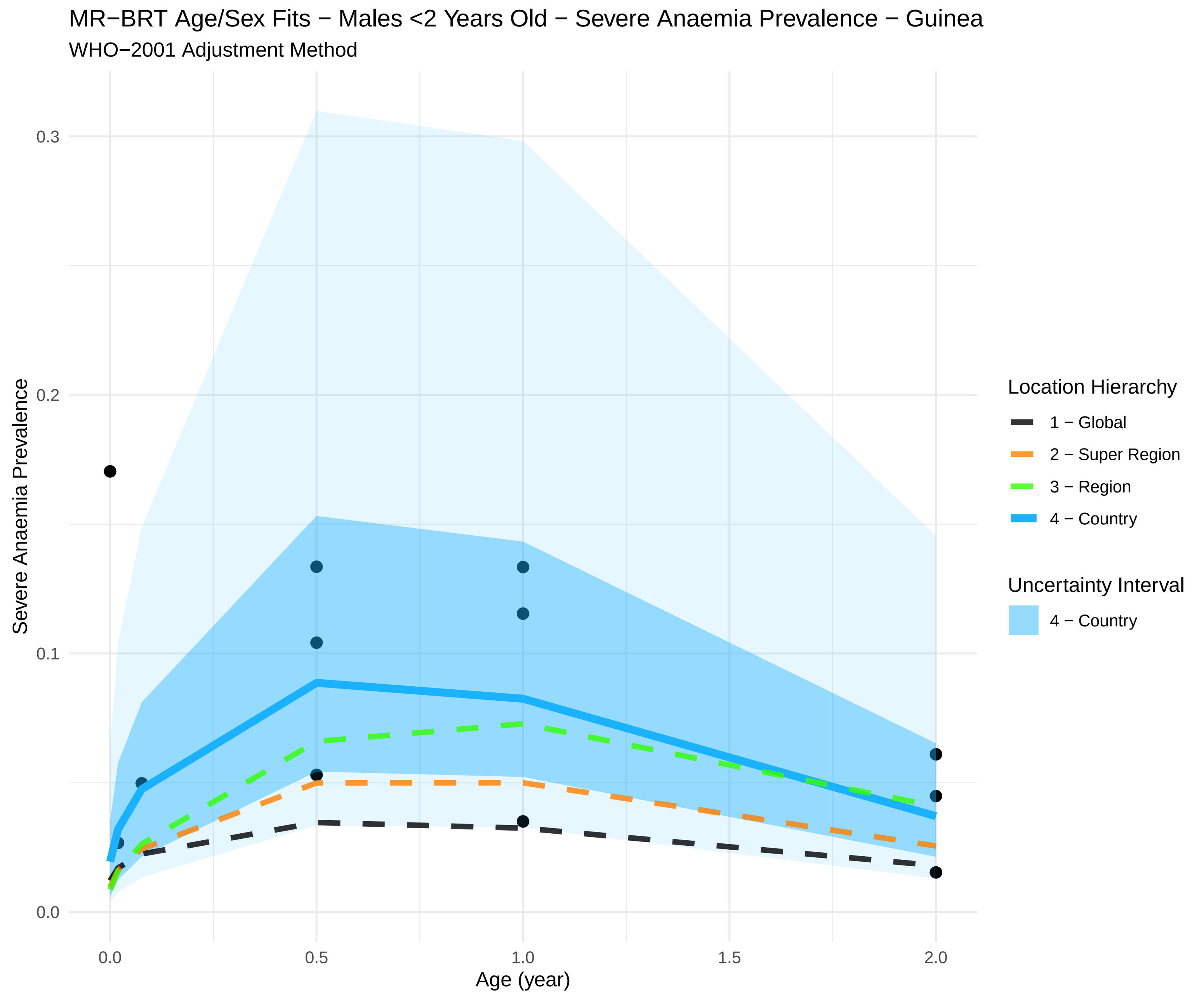
MR−BRT Age/Sex Fits − Males <2 Years Old − Severe Anaemia Prevalence − Guinea
WHO−2001 Adjustment Method
Severe Anaemia Prevalence
Age (year)
Location Hierarchy
1 − Global
2 − Super Region
3 − Region
4 − Country
Uncertainty Interval
4 − Country

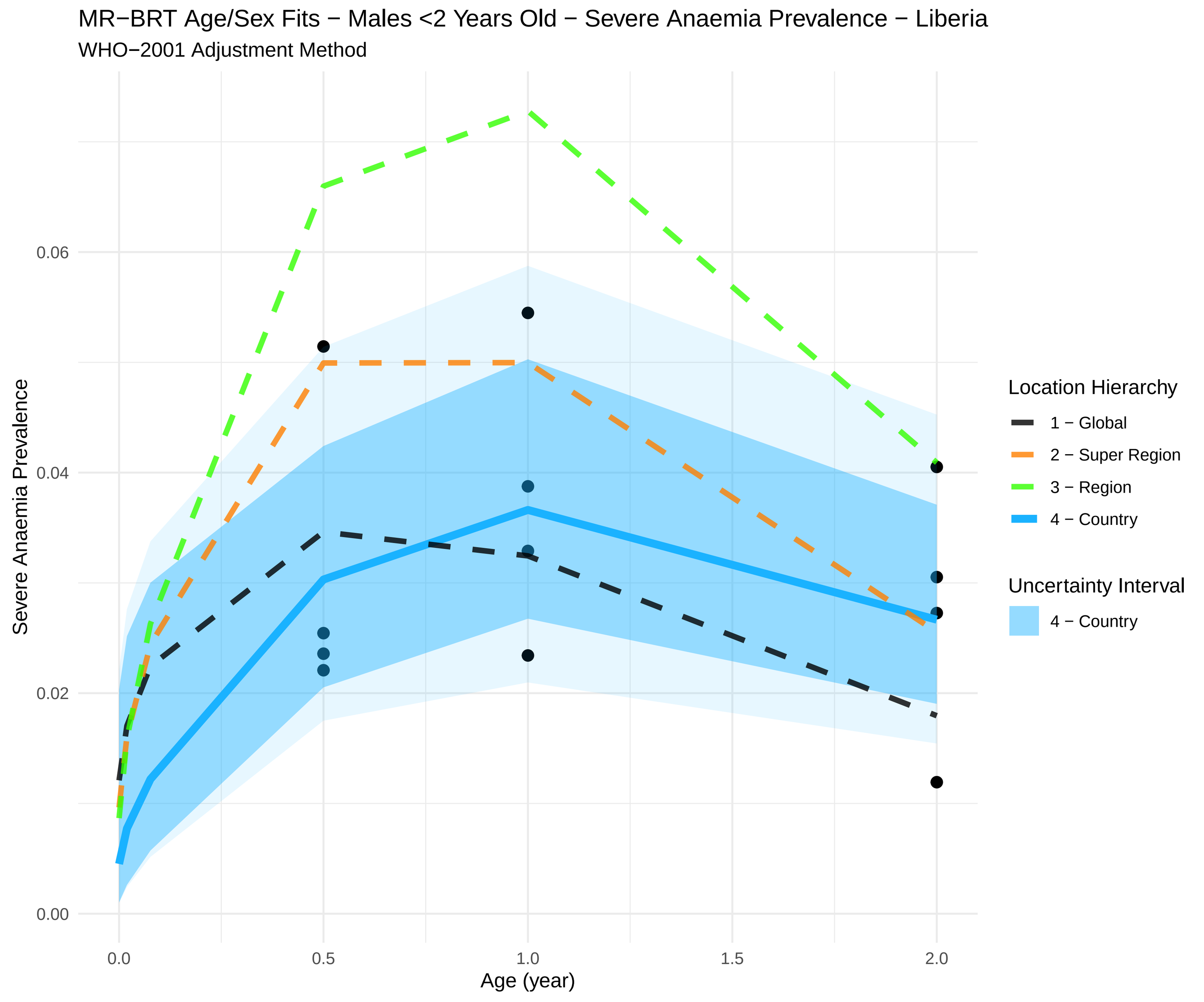
MR−BRT Age/Sex Fits − Males <2 Years Old − Severe Anaemia Prevalence − Liberia
WHO−2001 Adjustment Method
Severe Anaemia Prevalence
Age (year)
Location Hierarchy
1 − Global
2 − Super Region
3 − Region
4 − Country
Uncertainty Interval
4 − Country

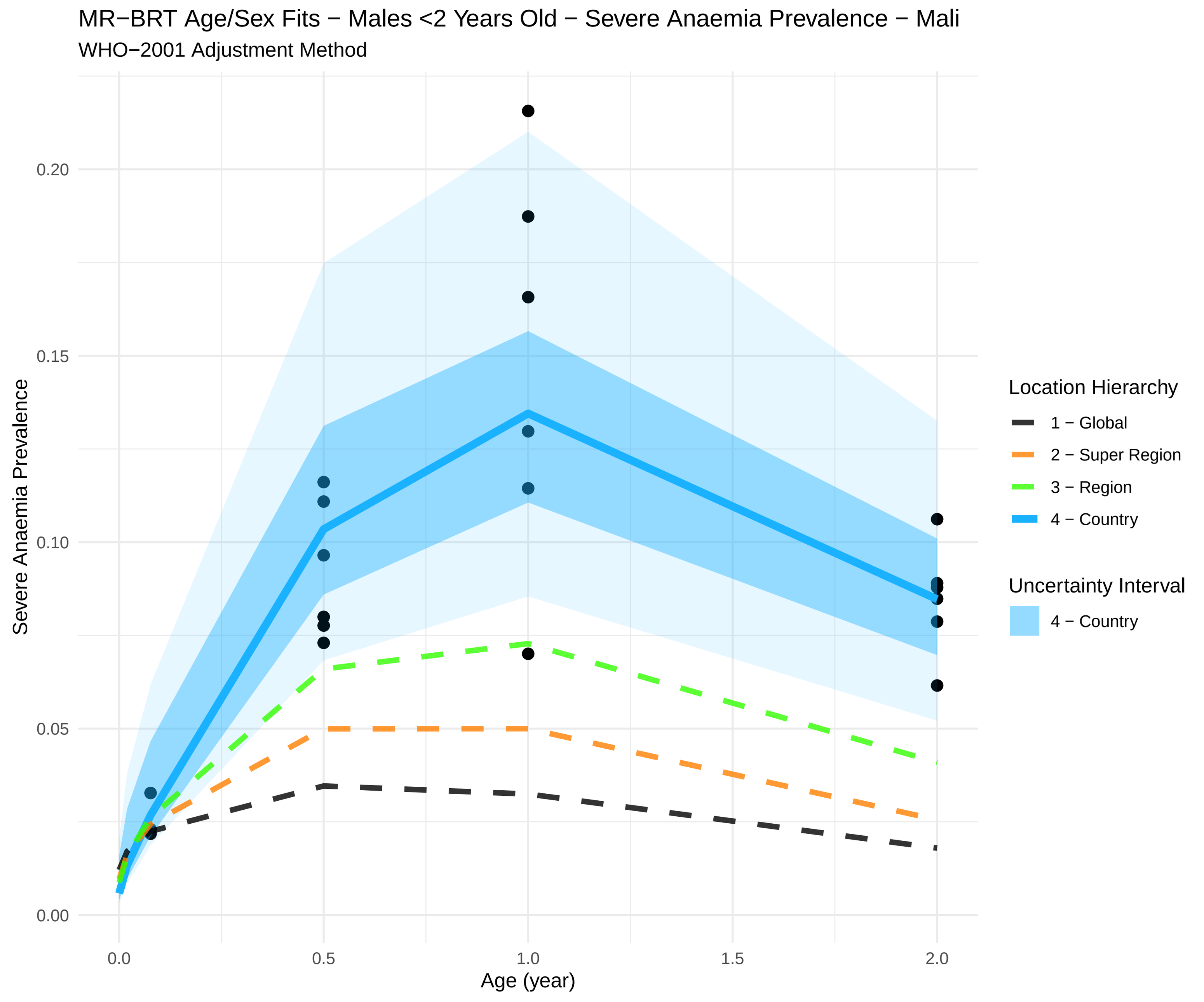
MR−BRT Age/Sex Fits − Males <2 Years Old − Severe Anaemia Prevalence − Mali
WHO−2001 Adjustment Method
Severe Anaemia Prevalence
Age (year)
Location Hierarchy
1 − Global
2 − Super Region
3 − Region
4 − Country
Uncertainty Interval
4 − Country

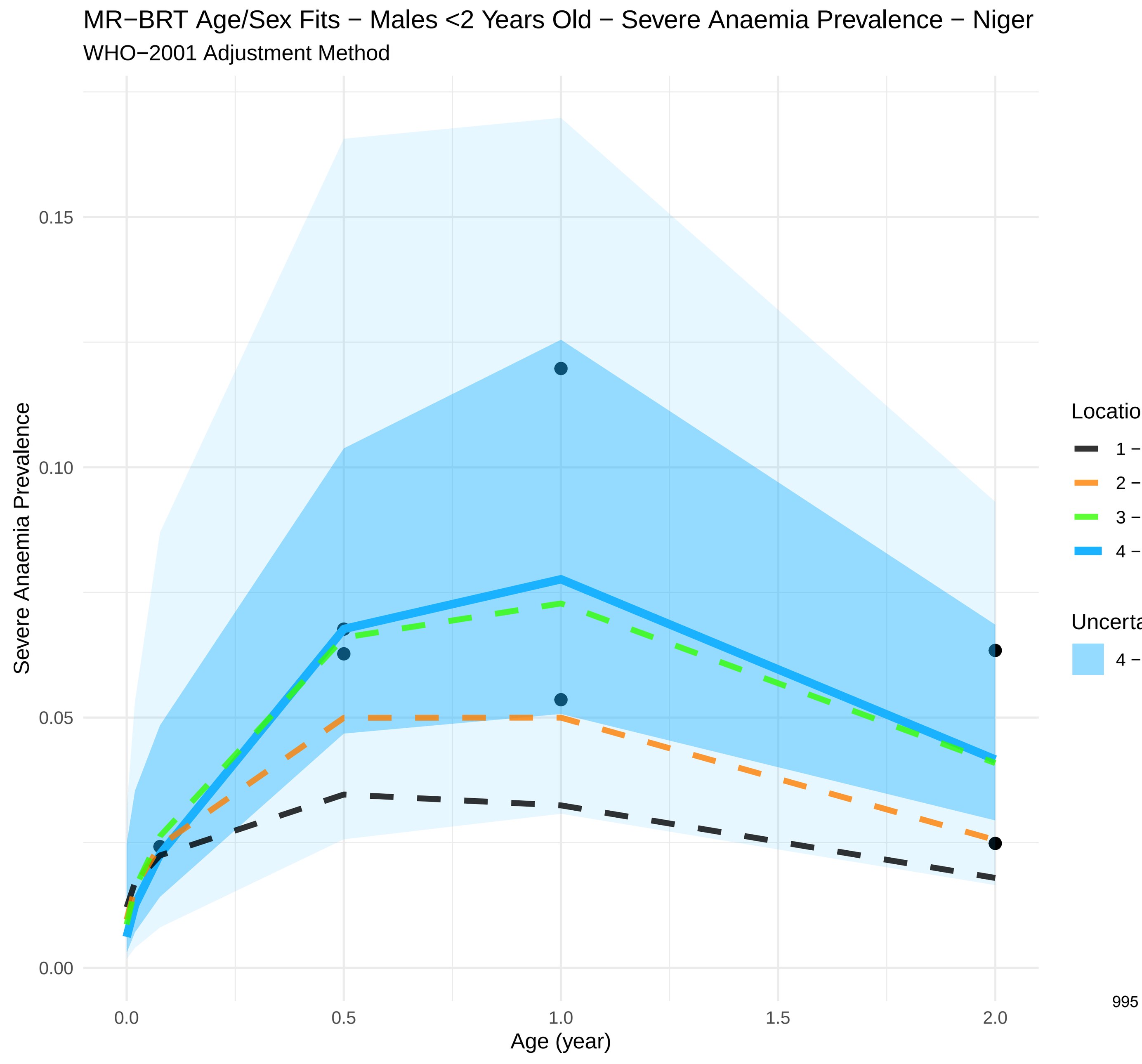
MR−BRT Age/Sex Fits − Males <2 Years Old − Severe Anaemia Prevalence − Niger
WHO−2001 Adjustment Method
Severe Anaemia Prevalence
Age (year)
Location Hierarchy
1 − Global
2 − Super Region
3 − Region
4 − Country
Uncertainty Interval
4 − Country

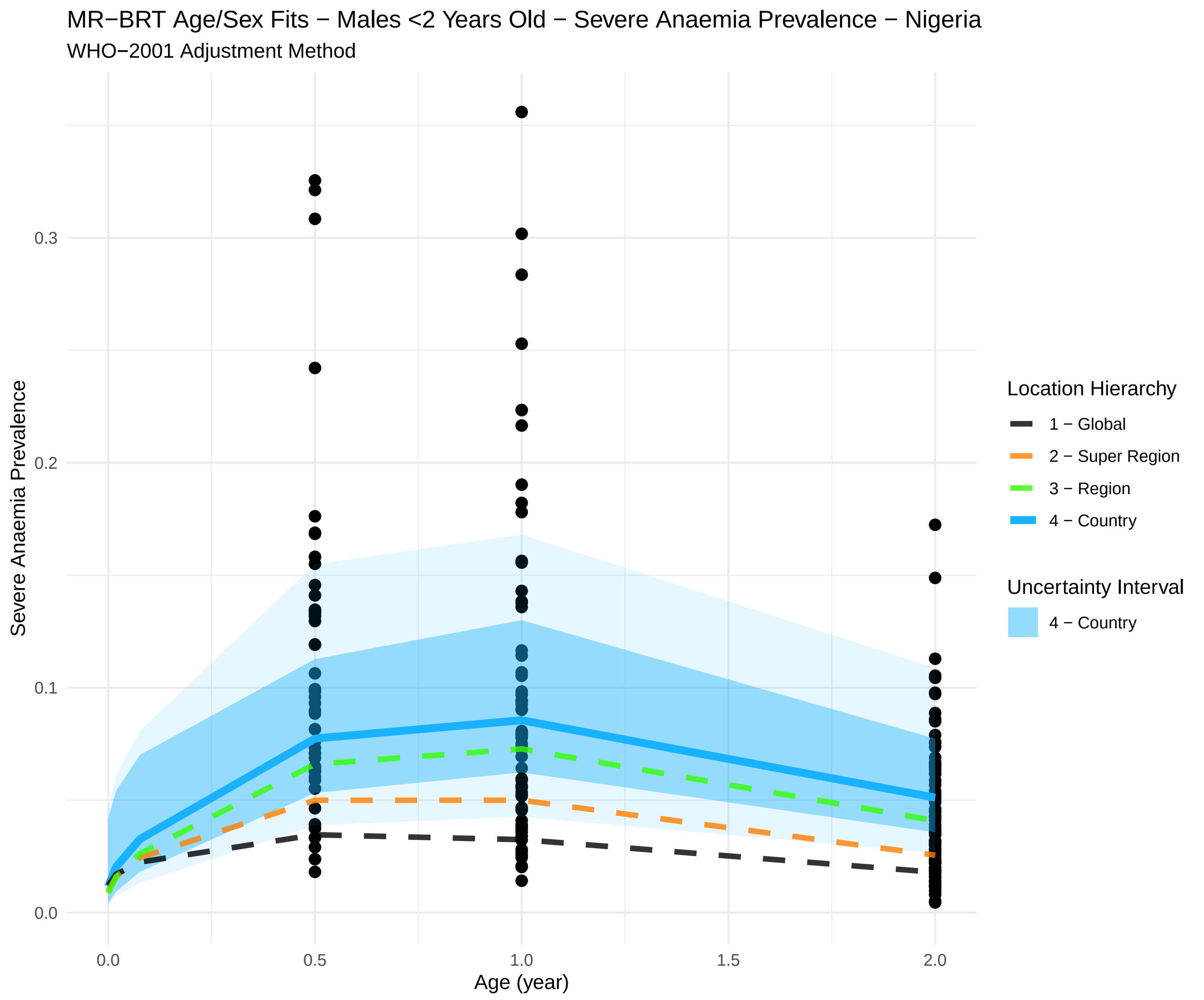
MR−BRT Age/Sex Fits − Males <2 Years Old − Severe Anaemia Prevalence − Nigeria
WHO−2001 Adjustment Method
Severe Anaemia Prevalence
Age (year)
Location Hierarchy
1 − Global
2 − Super Region
3 − Region
4 − Country
Uncertainty Interval
4 − Country

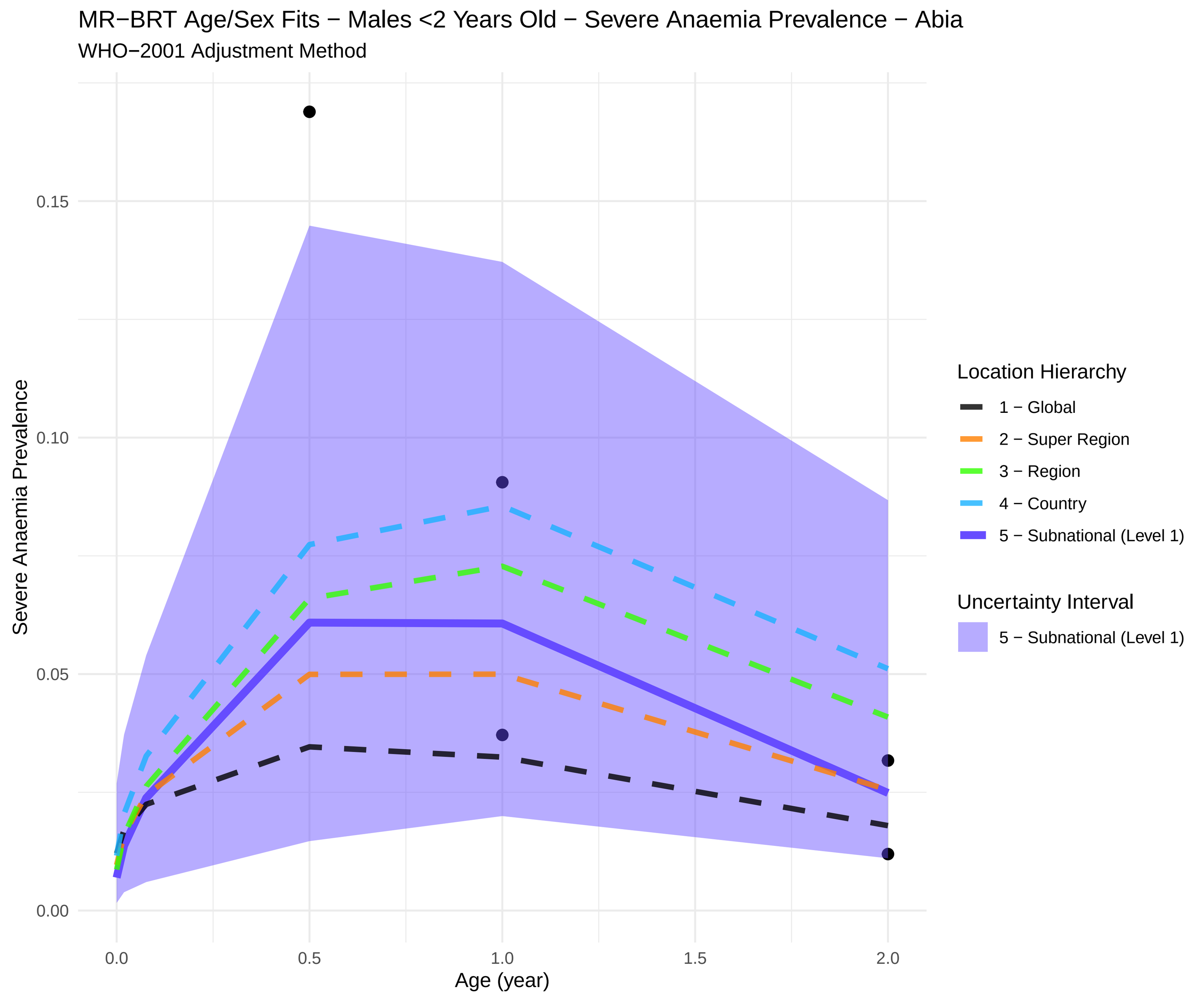
MR−BRT Age/Sex Fits − Males <2 Years Old − Severe Anaemia Prevalence − Abia
WHO−2001 Adjustment Method
Severe Anaemia Prevalence
Age (year)
Location Hierarchy
1 − Global
2 − Super Region
3 − Region
4 − Country
5 − Subnational (Level 1)
Uncertainty Interval
5 − Subnational (Level 1)

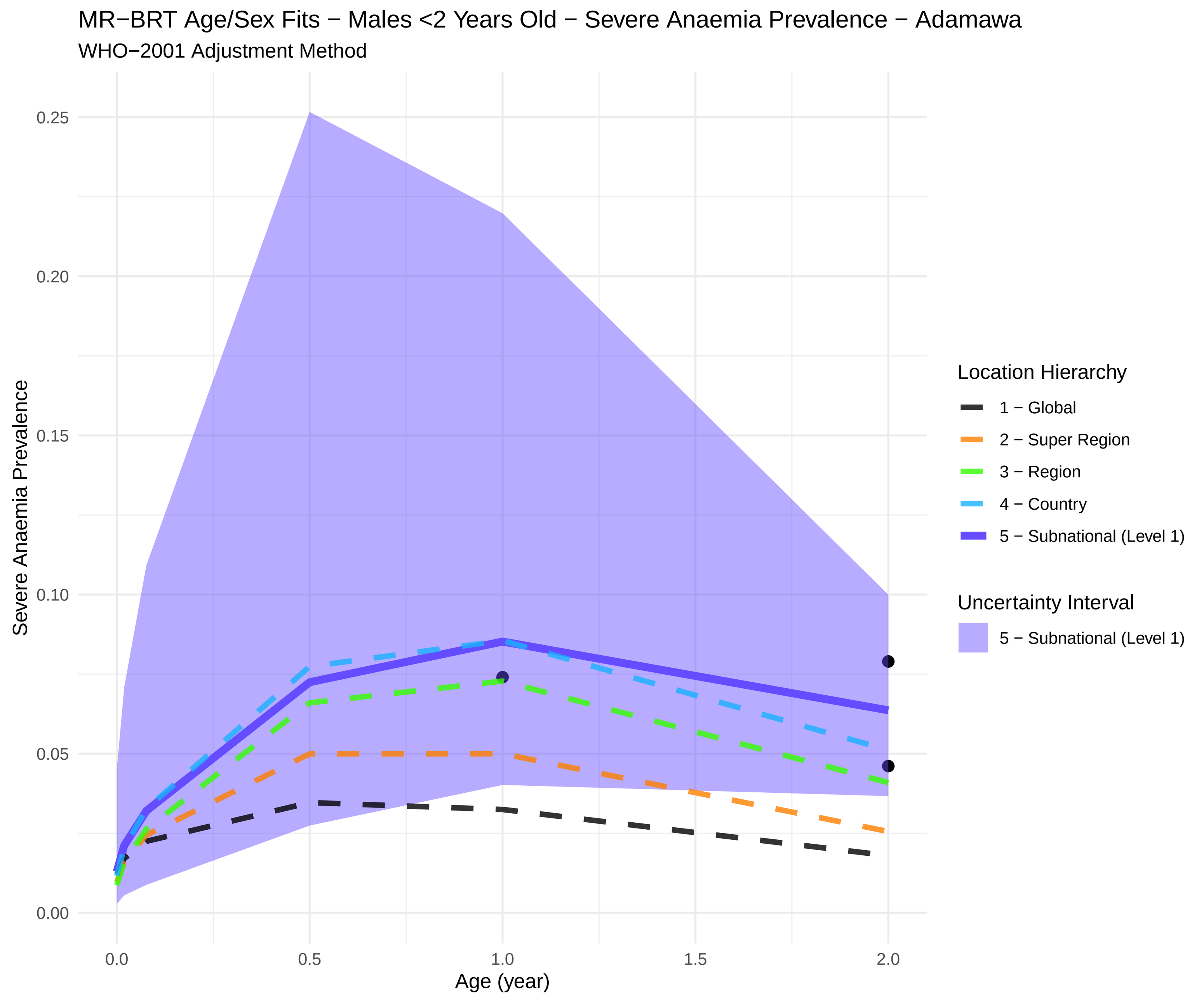
MR−BRT Age/Sex Fits − Males <2 Years Old − Severe Anaemia Prevalence − Adamawa
WHO−2001 Adjustment Method
Severe Anaemia Prevalence
Age (year)
Location Hierarchy
1 − Global
2 − Super Region
3 − Region
4 − Country
5 − Subnational (Level 1)
Uncertainty Interval
5 − Subnational (Level 1)

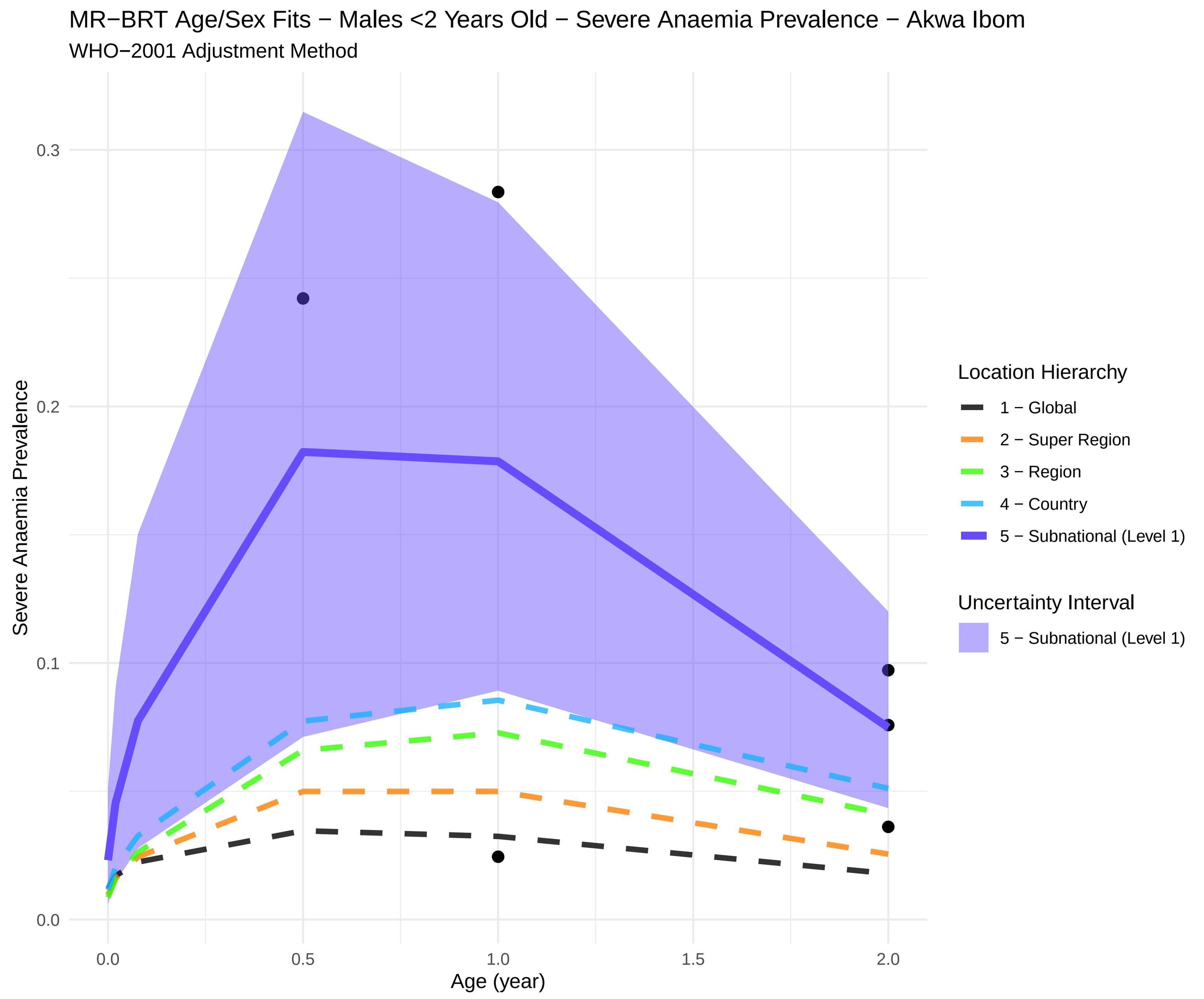
MR−BRT Age/Sex Fits − Males <2 Years Old − Severe Anaemia Prevalence − Akwa Ibom
WHO−2001 Adjustment Method
Severe Anaemia Prevalence
Age (year)
Location Hierarchy
1 − Global
2 − Super Region
3 − Region
4 − Country
5 − Subnational (Level 1)
Uncertainty Interval
5 − Subnational (Level 1)

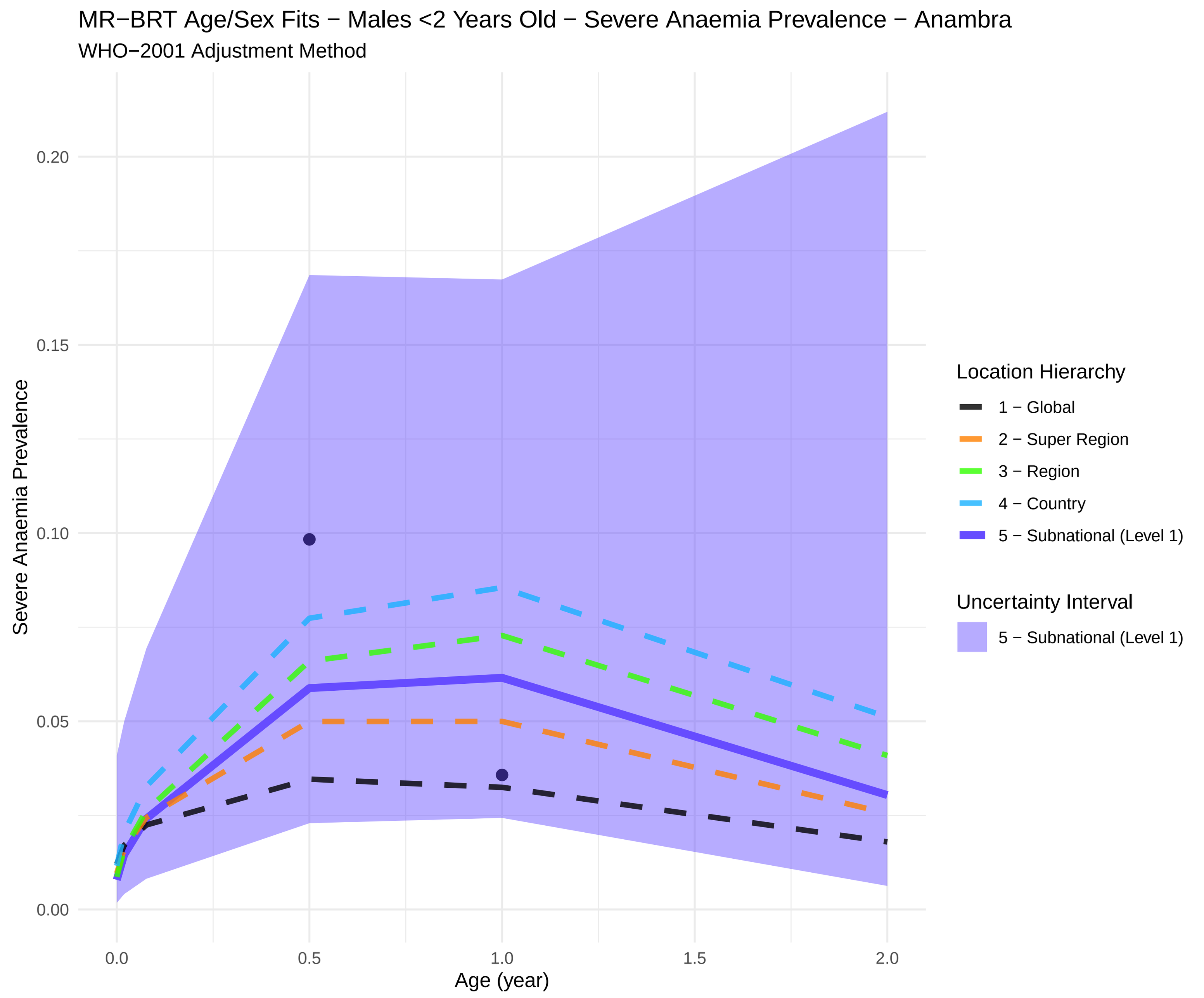
MR−BRT Age/Sex Fits − Males <2 Years Old − Severe Anaemia Prevalence − Anambra
WHO−2001 Adjustment Method
Severe Anaemia Prevalence
Age (year)
Location Hierarchy
1 − Global
2 − Super Region
3 − Region
4 − Country
5 − Subnational (Level 1)
Uncertainty Interval
5 − Subnational (Level 1)

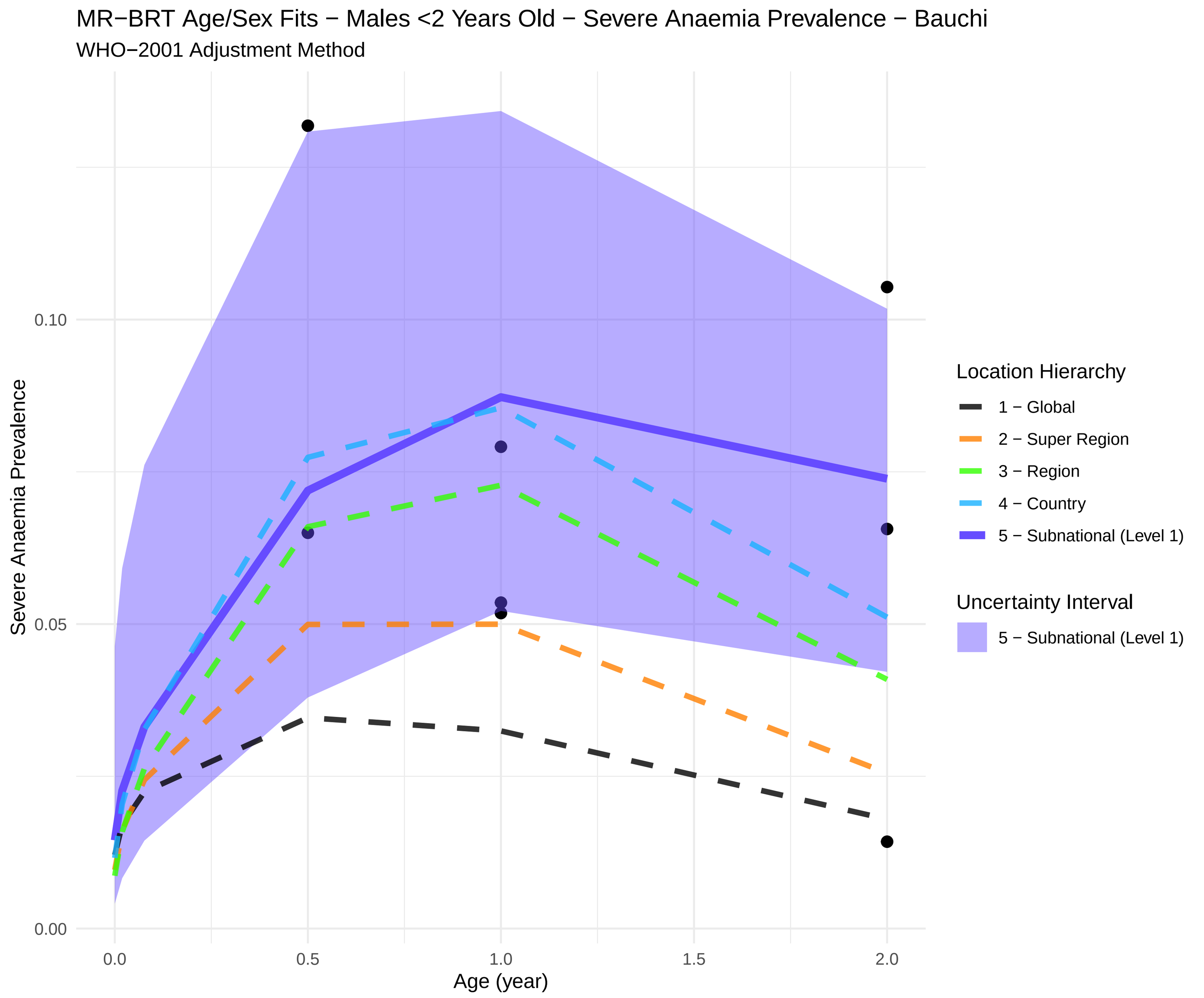
MR−BRT Age/Sex Fits − Males <2 Years Old − Severe Anaemia Prevalence − Bauchi
WHO−2001 Adjustment Method
Severe Anaemia Prevalence
Age (year)
Location Hierarchy
1 − Global
2 − Super Region
3 − Region
4 − Country
5 − Subnational (Level 1)
Uncertainty Interval
5 − Subnational (Level 1)

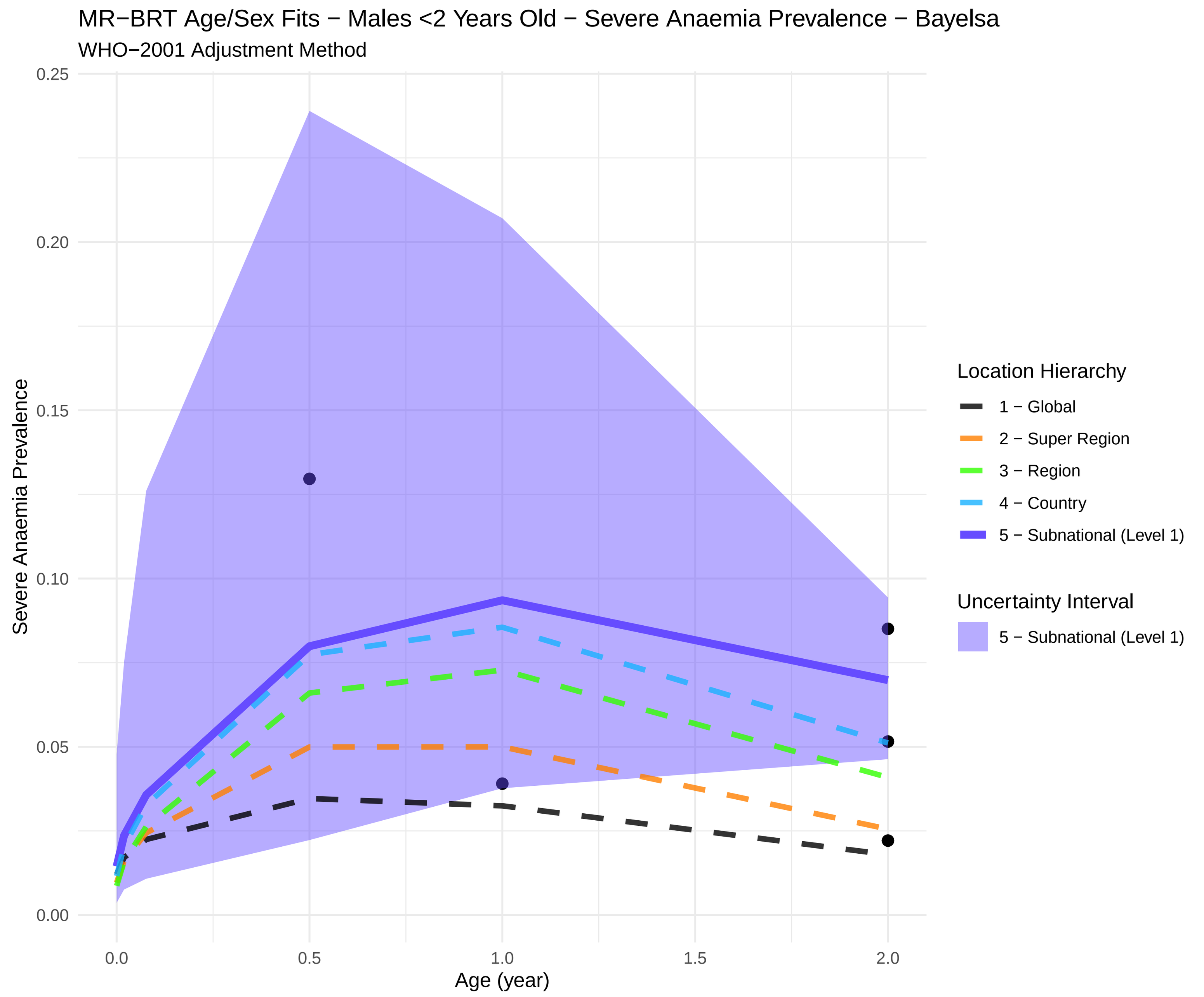
MR−BRT Age/Sex Fits − Males <2 Years Old − Severe Anaemia Prevalence − Bayelsa
WHO−2001 Adjustment Method
Severe Anaemia Prevalence
Age (year)
Location Hierarchy
1 − Global
2 − Super Region
3 − Region
4 − Country
5 − Subnational (Level 1)
Uncertainty Interval
5 − Subnational (Level 1)

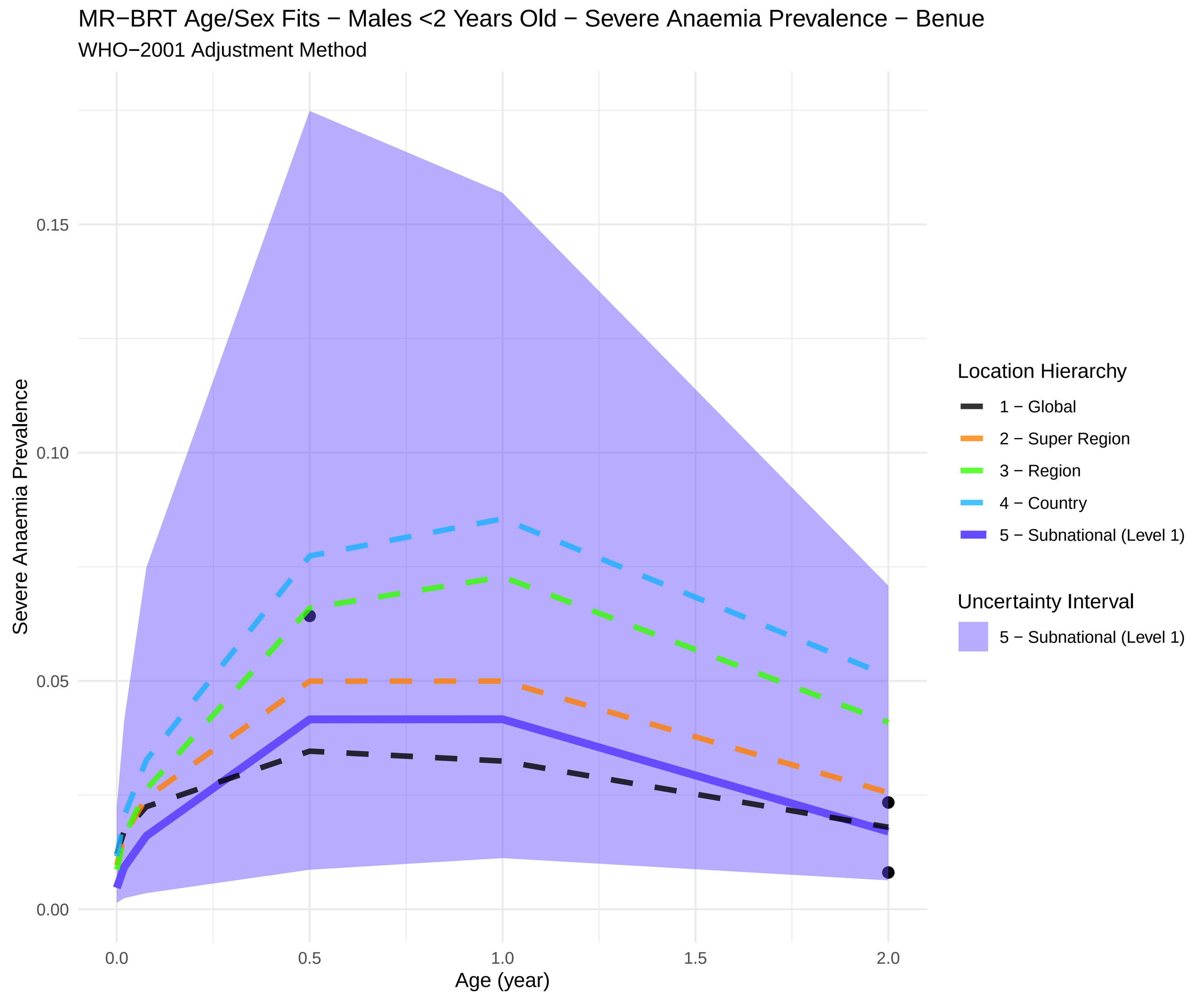
MR−BRT Age/Sex Fits − Males <2 Years Old − Severe Anaemia Prevalence − Benue
WHO−2001 Adjustment Method
Severe Anaemia Prevalence
Age (year)
Location Hierarchy
1 − Global
2 − Super Region
3 − Region
4 − Country
5 − Subnational (Level 1)
Uncertainty Interval
5 − Subnational (Level 1)

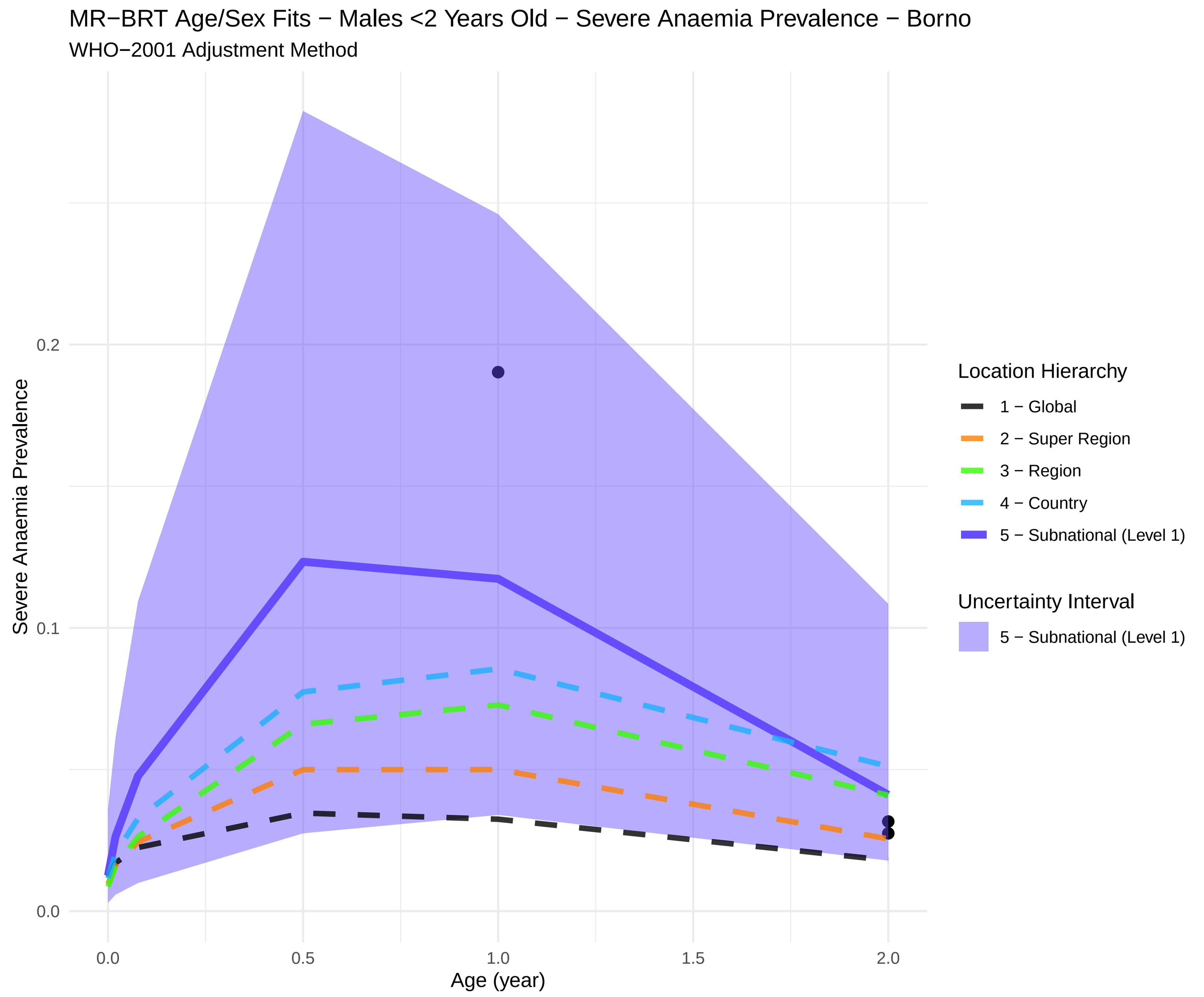
MR−BRT Age/Sex Fits − Males <2 Years Old − Severe Anaemia Prevalence − Borno
WHO−2001 Adjustment Method
Severe Anaemia Prevalence
Age (year)
Location Hierarchy
1 − Global
2 − Super Region
3 − Region
4 − Country
5 − Subnational (Level 1)
Uncertainty Interval
5 − Subnational (Level 1)

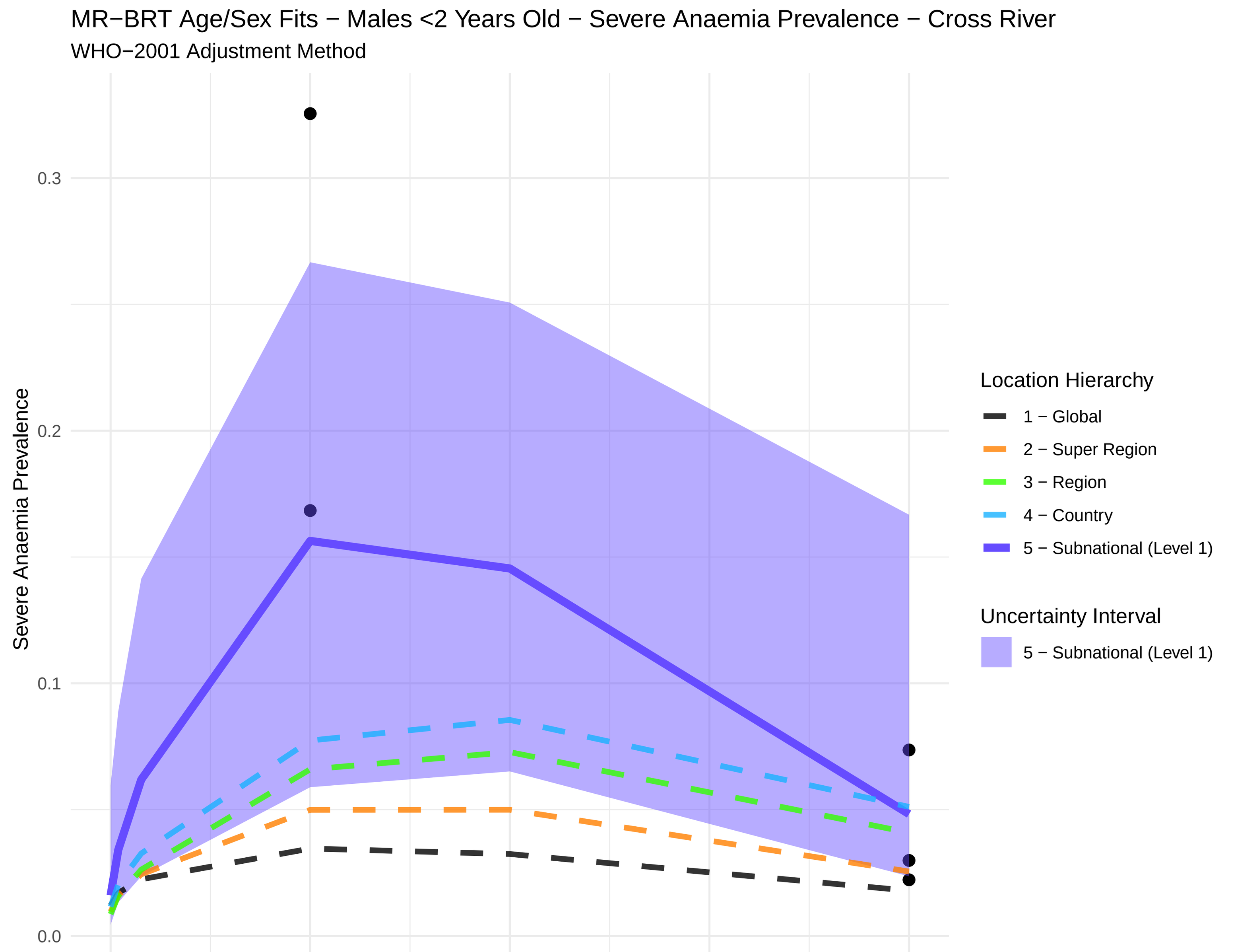
MR−BRT Age/Sex Fits − Males <2 Years Old − Severe Anaemia Prevalence − Cross River
WHO−2001 Adjustment Method
Severe Anaemia Prevalence
Age (year)
Location Hierarchy
1 − Global
2 − Super Region
3 − Region
4 − Country
5 − Subnational (Level 1)
Uncertainty Interval
5 − Subnational (Level 1)

MR−BRT Age/Sex Fits − Males <2 Years Old − Severe Anaemia Prevalence − Delta
WHO−2001 Adjustment Method

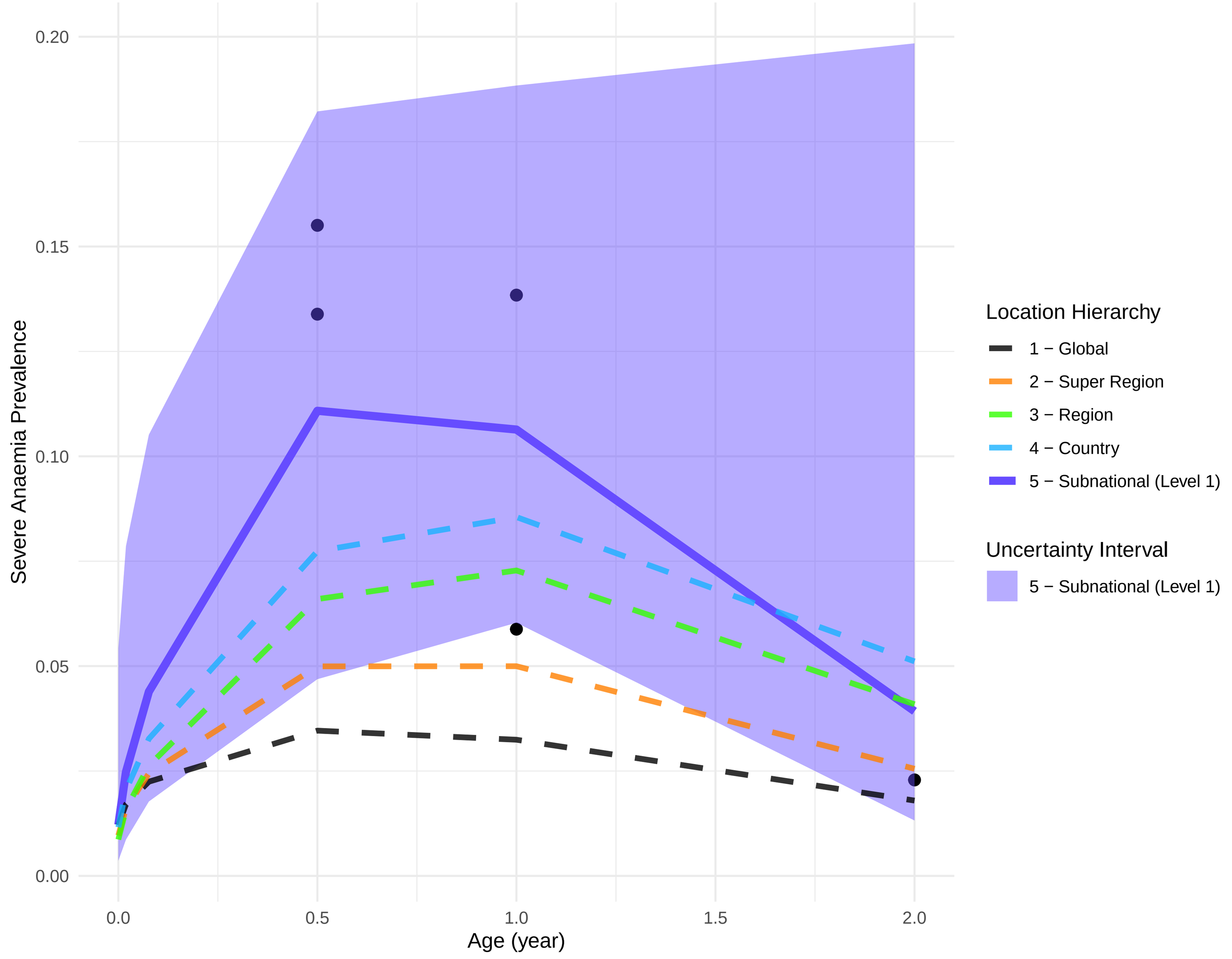

MR−BRT Age/Sex Fits − Males <2 Years Old − Severe Anaemia Prevalence − Ebonyi
WHO−2001 Adjustment Method

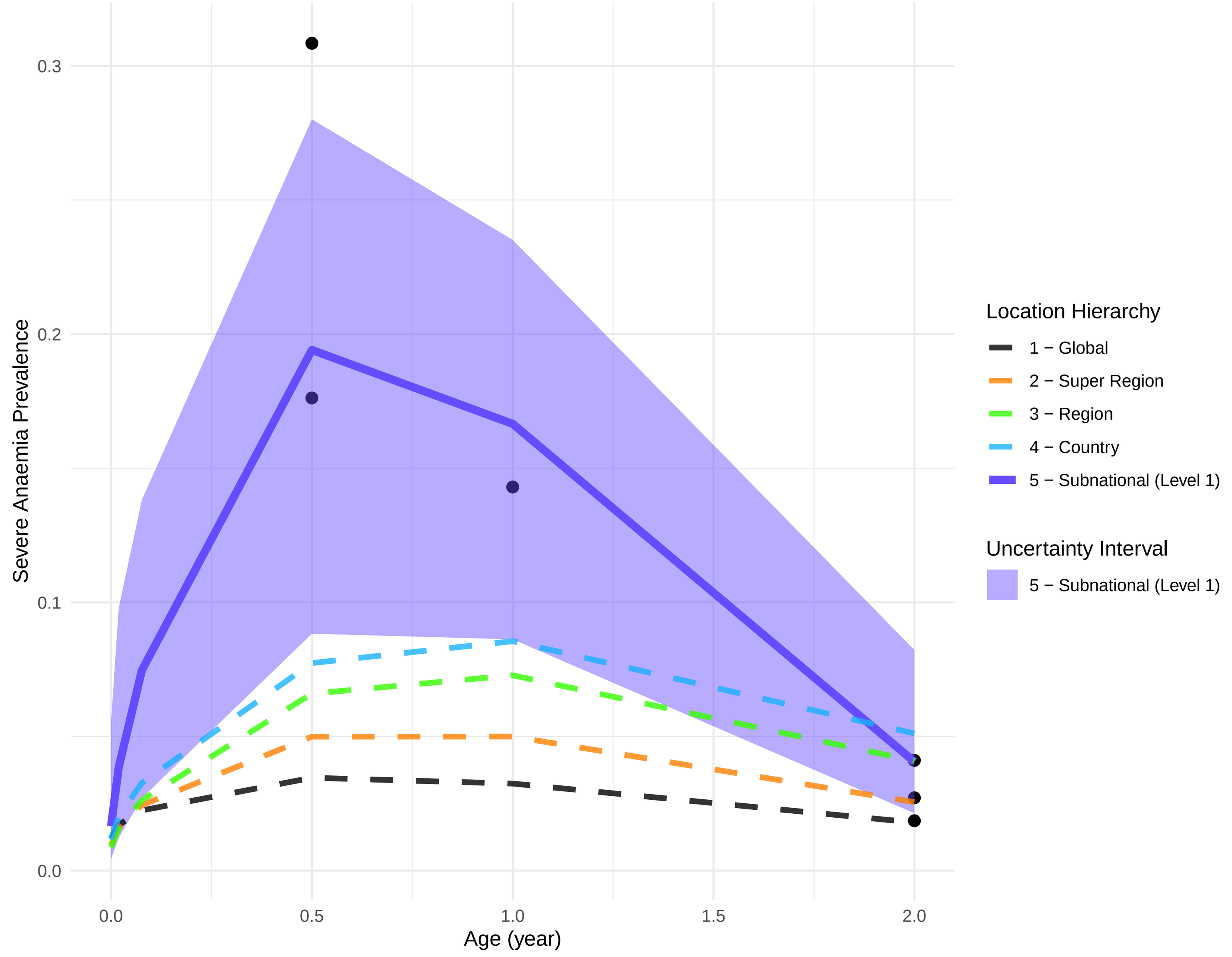

MR−BRT Age/Sex Fits − Males <2 Years Old − Severe Anaemia Prevalence − Edo
WHO−2001 Adjustment Method

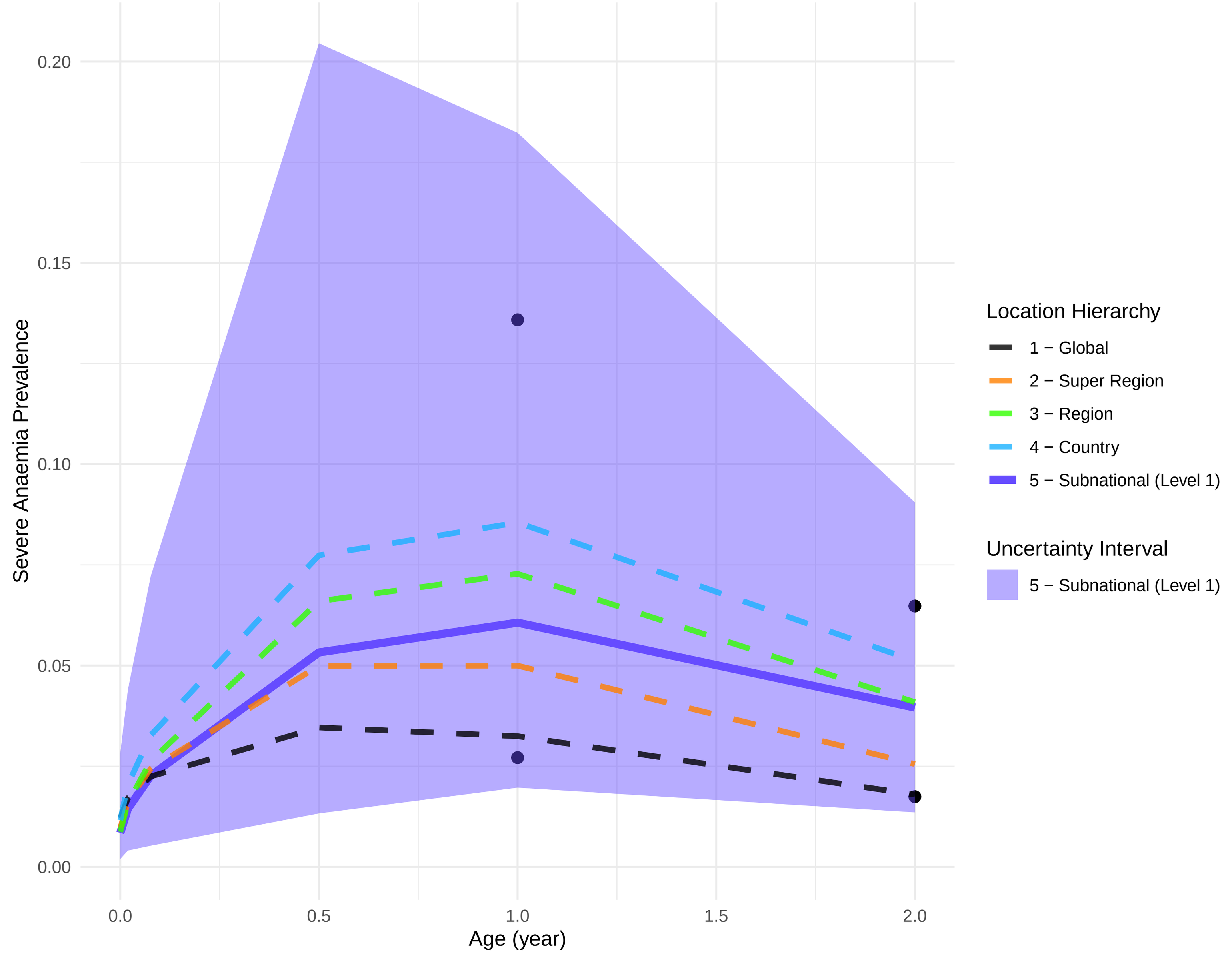

MR−BRT Age/Sex Fits − Males <2 Years Old − Severe Anaemia Prevalence − Ekiti
WHO−2001 Adjustment Method

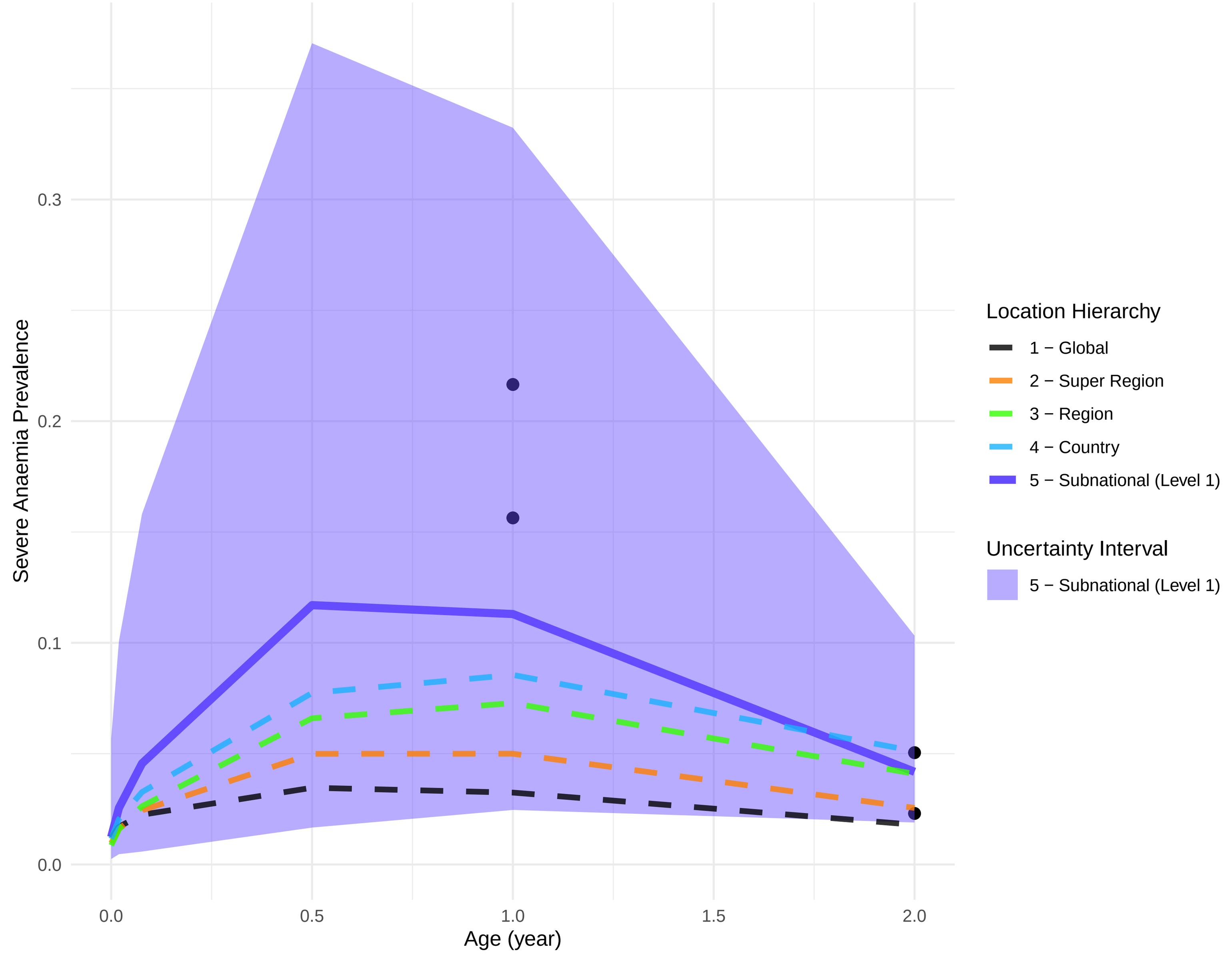

MR−BRT Age/Sex Fits − Males <2 Years Old − Severe Anaemia Prevalence − Enugu
WHO−2001 Adjustment Method

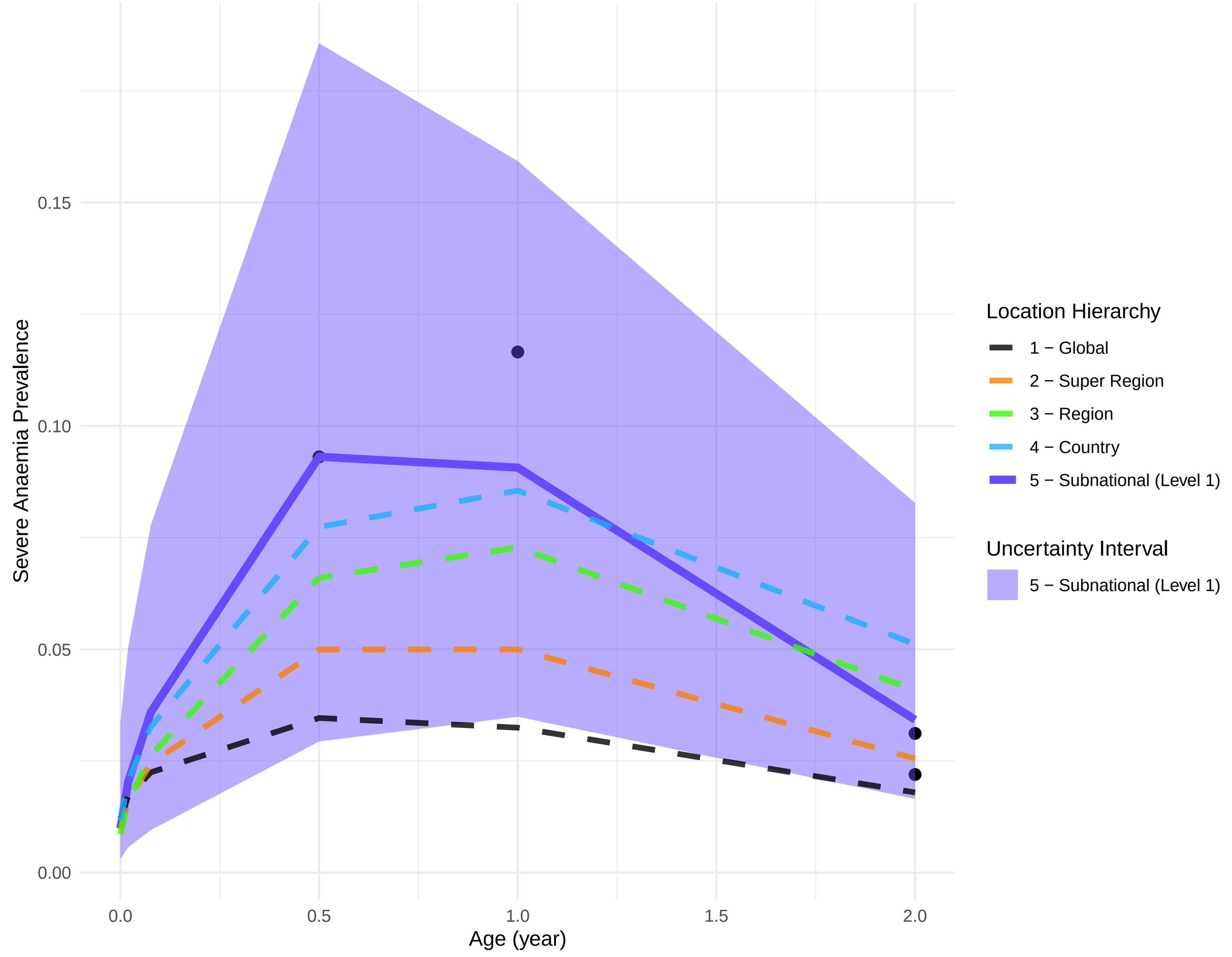

MR−BRT Age/Sex Fits − Males <2 Years Old − Severe Anaemia Prevalence − FCT (Abuja)
WHO−2001 Adjustment Method

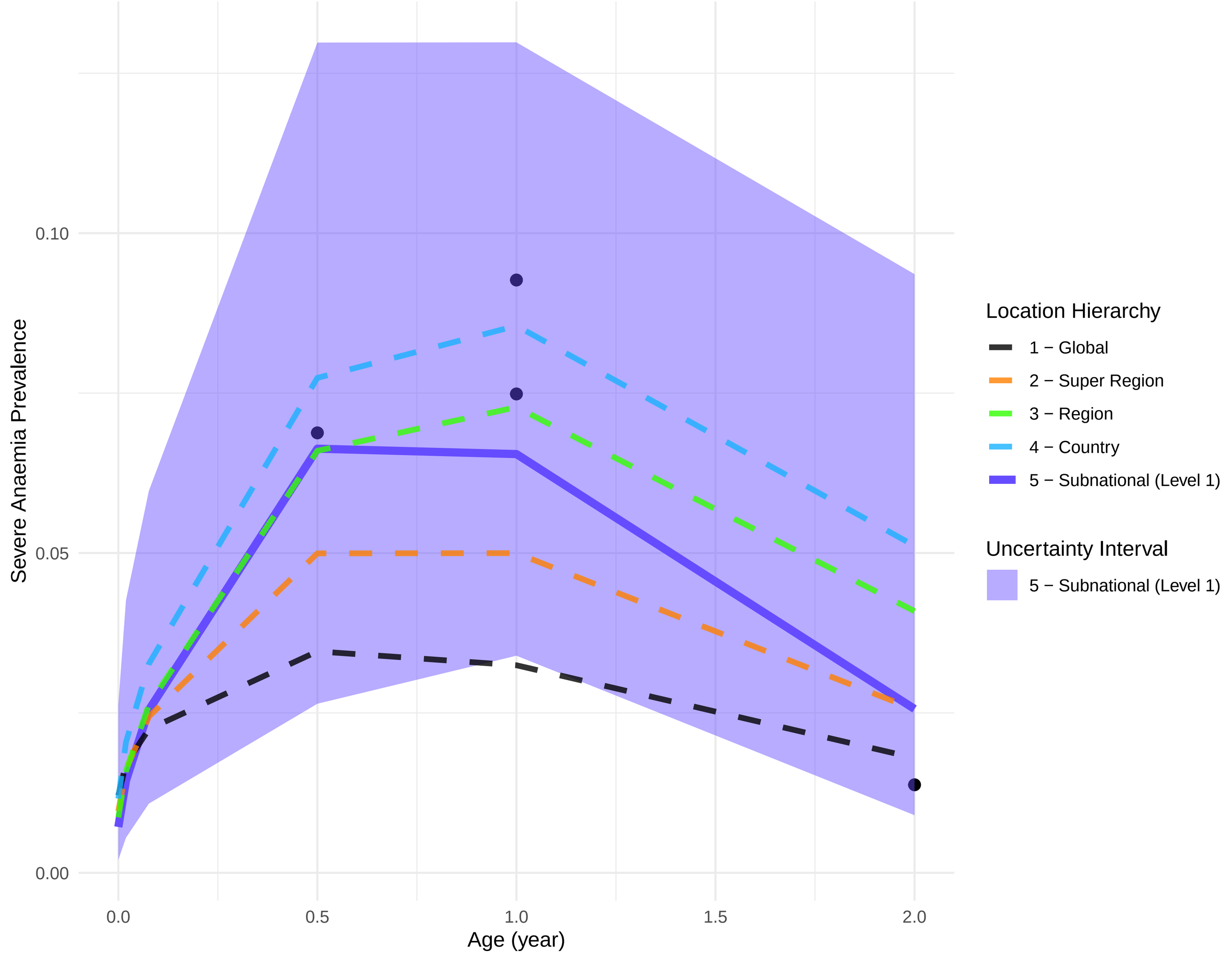

MR−BRT Age/Sex Fits − Males <2 Years Old − Severe Anaemia Prevalence − Gombe
WHO−2001 Adjustment Method

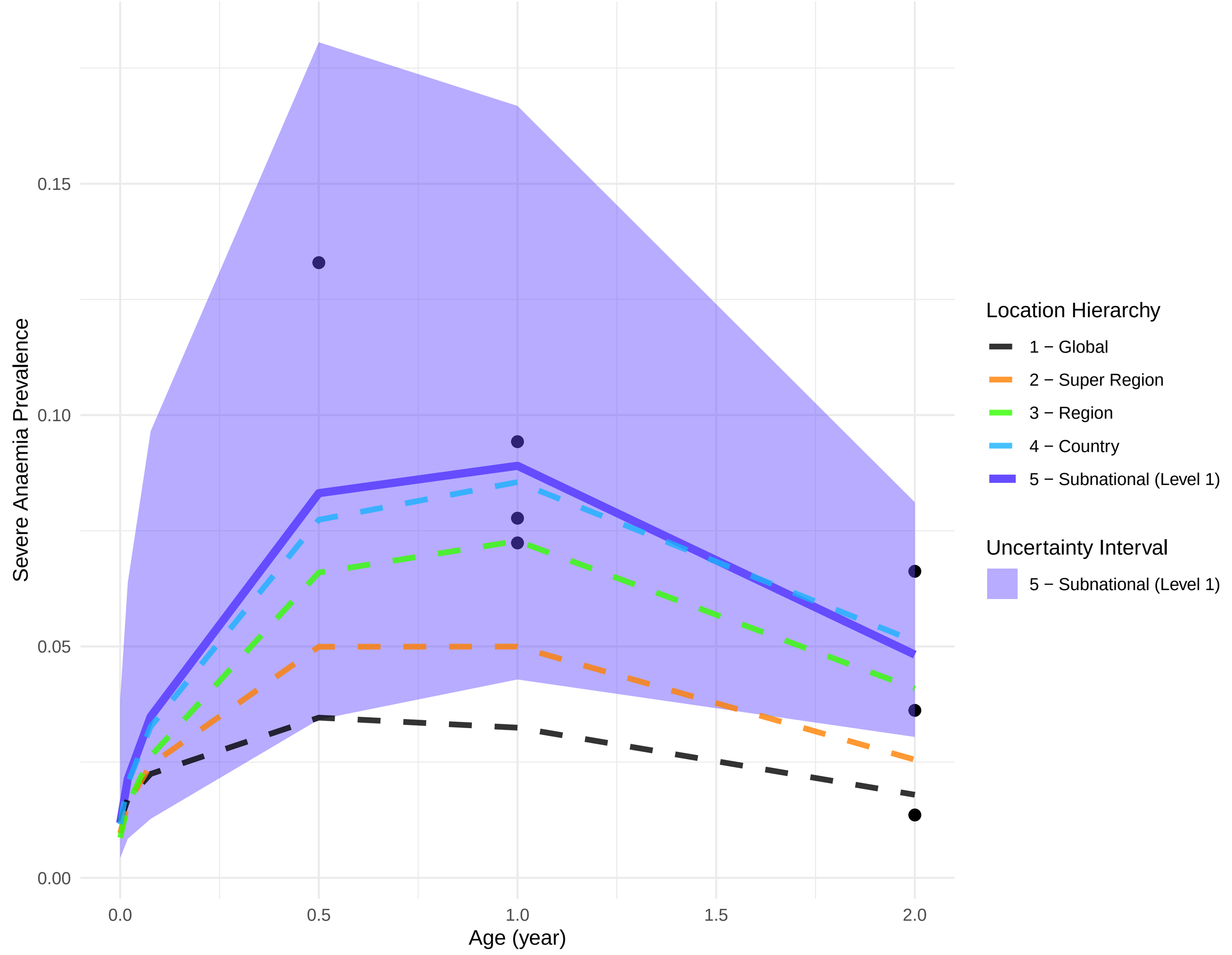

MR−BRT Age/Sex Fits − Males <2 Years Old − Severe Anaemia Prevalence − Imo
WHO−2001 Adjustment Method

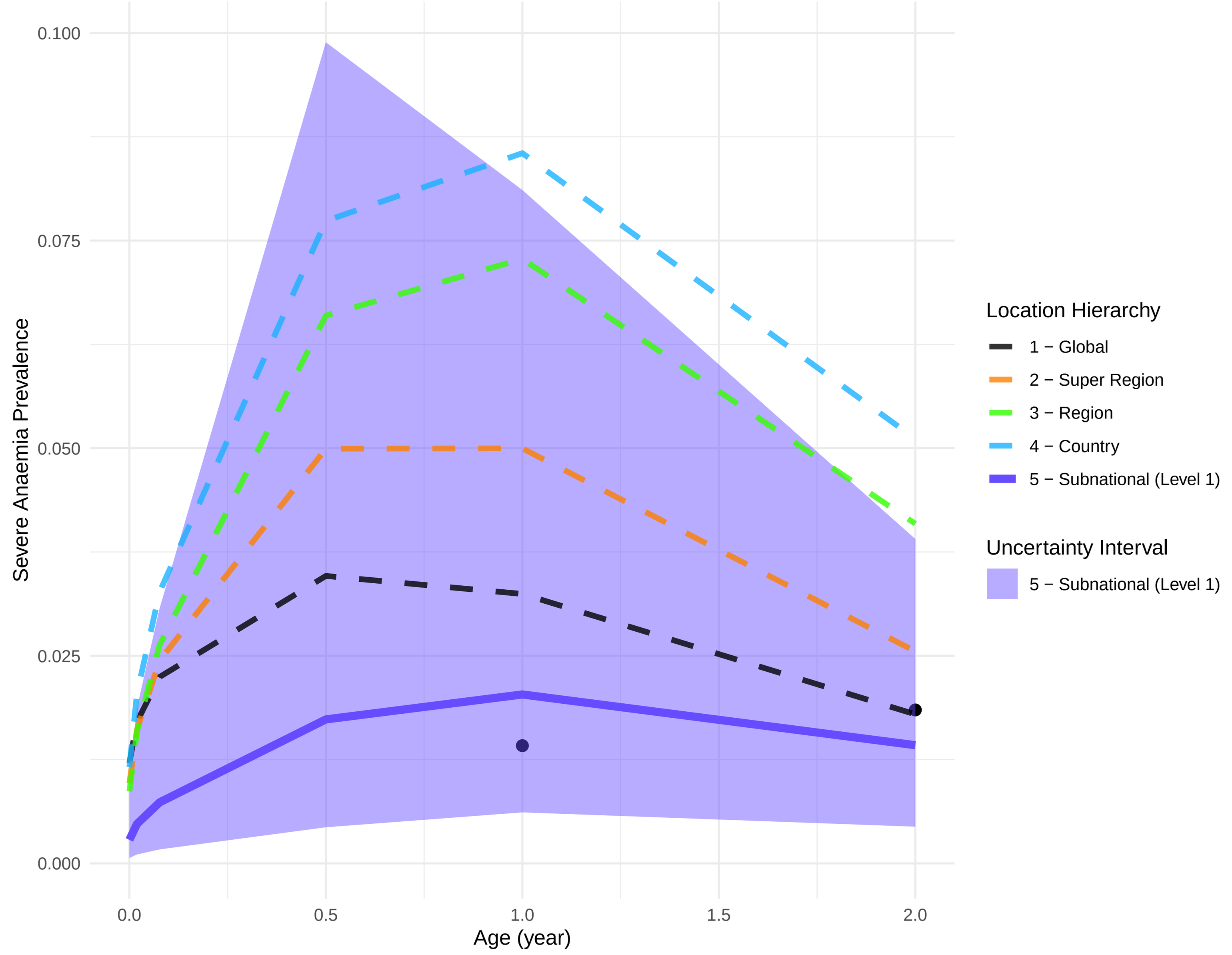

MR−BRT Age/Sex Fits − Males <2 Years Old − Severe Anaemia Prevalence − Jigawa
WHO−2001 Adjustment Method

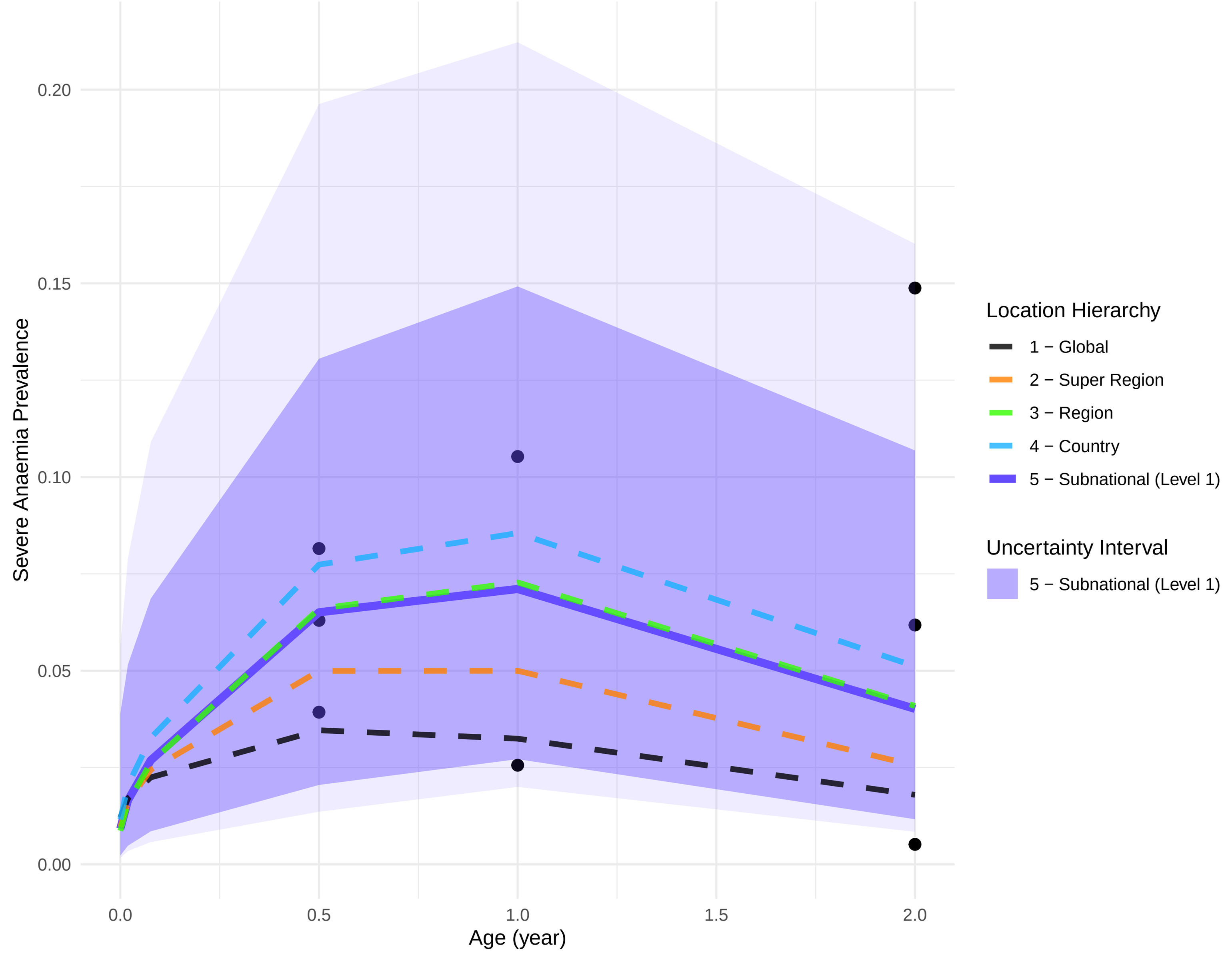

MR−BRT Age/Sex Fits − Males <2 Years Old − Severe Anaemia Prevalence − Kaduna
WHO−2001 Adjustment Method

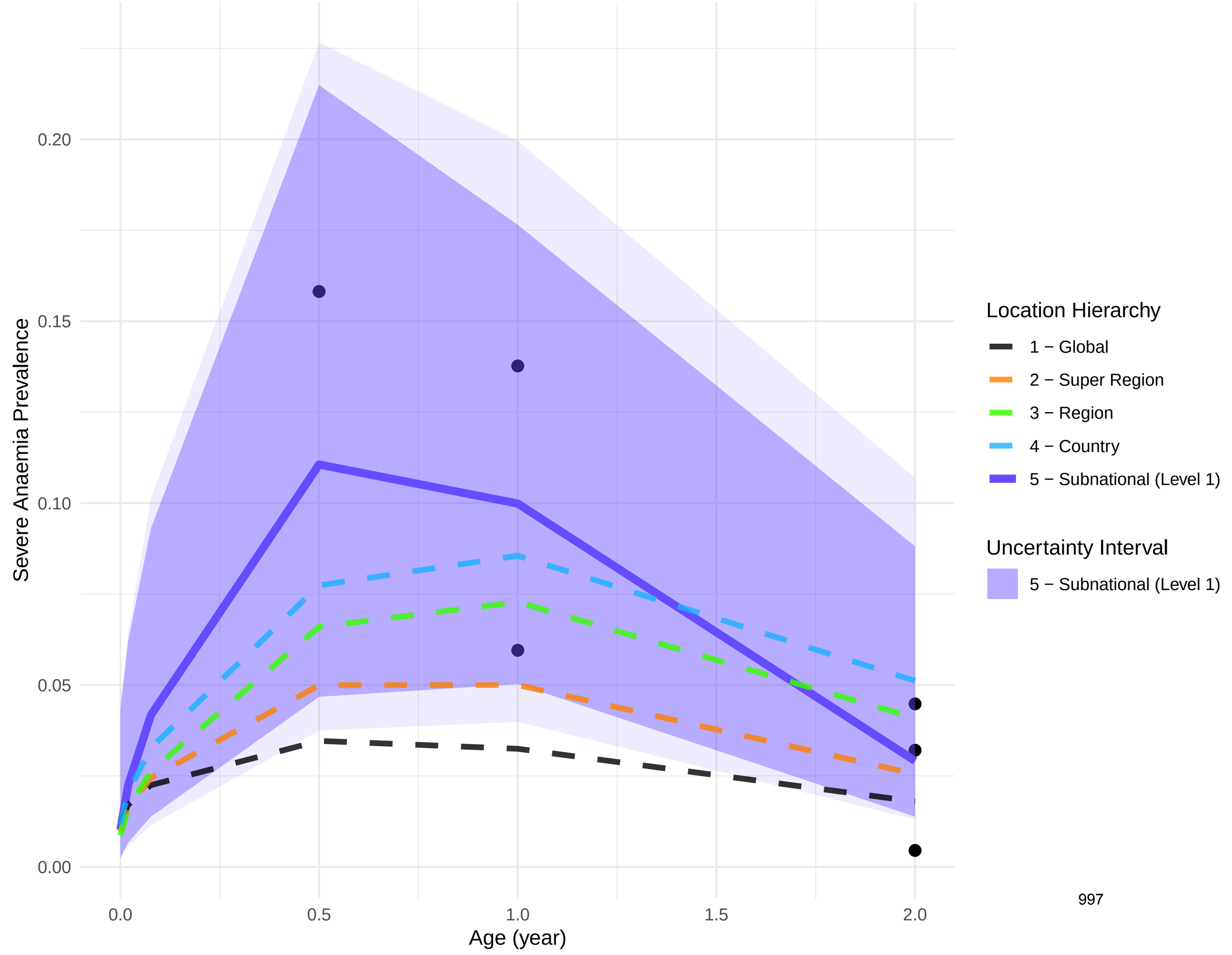

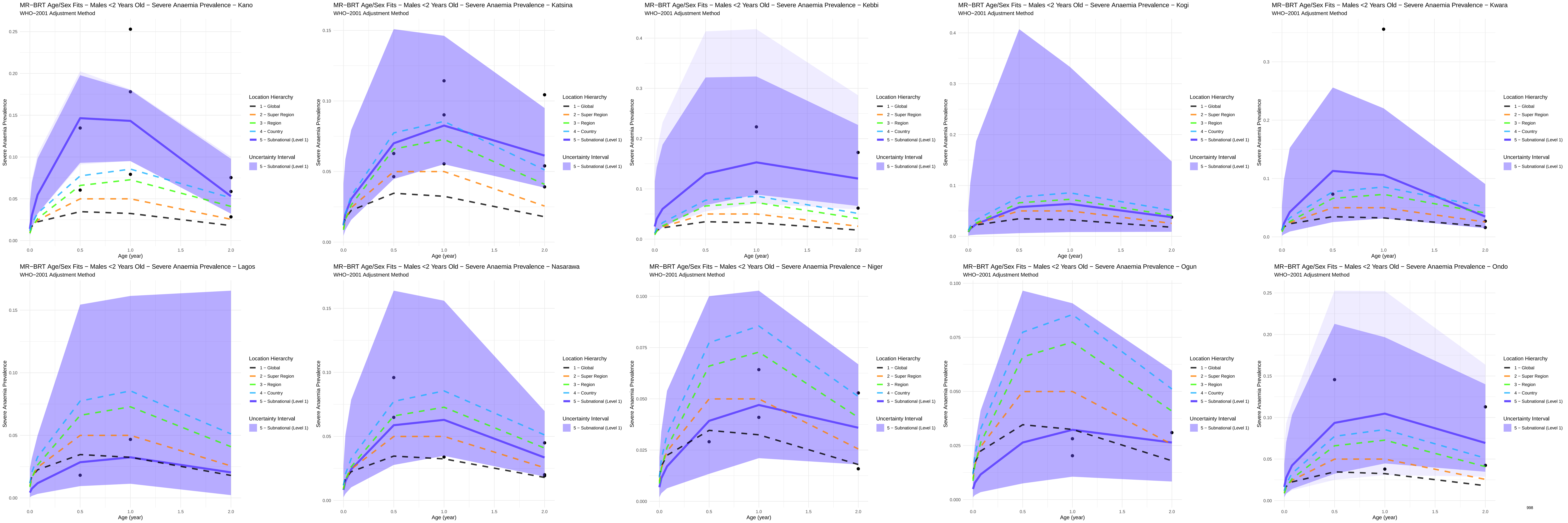
MR−BRT Age/Sex Fits − Males <2 Years Old − Severe Anaemia Prevalence − Kano
WHO−2001 Adjustment Method
MR−BRT Age/Sex Fits − Males <2 Years Old − Severe Anaemia Prevalence − Katsina
WHO−2001 Adjustment Method
MR−BRT Age/Sex Fits − Males <2 Years Old − Severe Anaemia Prevalence − Kebbi
WHO−2001 Adjustment Method
MR−BRT Age/Sex Fits − Males <2 Years Old − Severe Anaemia Prevalence − Kogi
WHO−2001 Adjustment Method
MR−BRT Age/Sex Fits − Males <2 Years Old − Severe Anaemia Prevalence − Kwara
WHO−2001 Adjustment Method
MR−BRT Age/Sex Fits − Males <2 Years Old − Severe Anaemia Prevalence − Lagos
WHO−2001 Adjustment Method
MR−BRT Age/Sex Fits − Males <2 Years Old − Severe Anaemia Prevalence − Nasarawa
WHO−2001 Adjustment Method
MR−BRT Age/Sex Fits − Males <2 Years Old − Severe Anaemia Prevalence − Niger
WHO−2001 Adjustment Method
MR−BRT Age/Sex Fits − Males <2 Years Old − Severe Anaemia Prevalence − Ogun
WHO−2001 Adjustment Method
MR−BRT Age/Sex Fits − Males <2 Years Old − Severe Anaemia Prevalence − Ondo
WHO−2001 Adjustment Method
Severe Anaemia Prevalence
Age (year)
Location Hierarchy
1 − Global
2 − Super Region
3 − Region
4 − Country
5 − Subnational (Level 1)
Uncertainty Interval
5 − Subnational (Level 1)

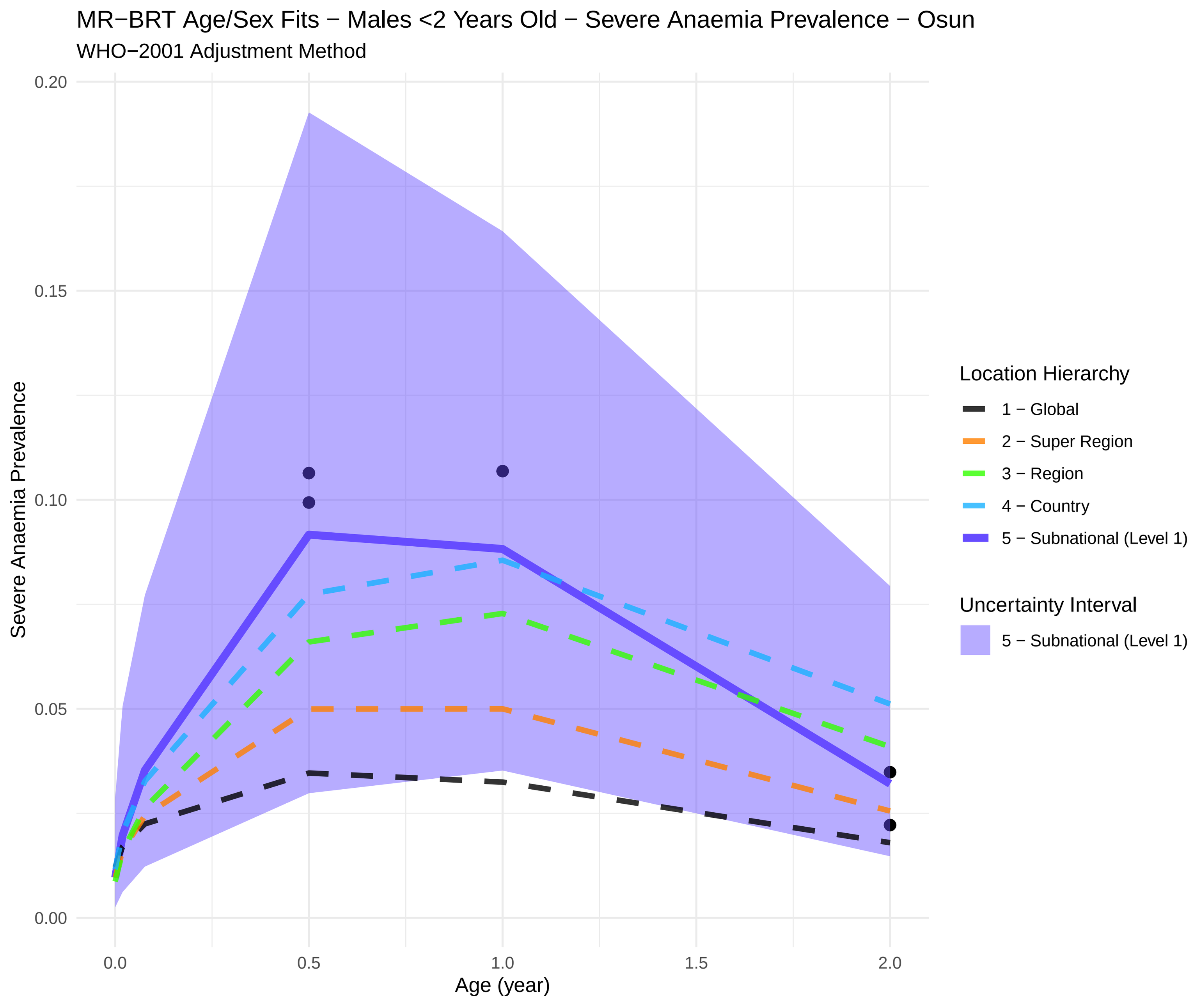
MR−BRT Age/Sex Fits − Males <2 Years Old − Severe Anaemia Prevalence − Osun
WHO−2001 Adjustment Method
Severe Anaemia Prevalence
Age (year)
Location Hierarchy
1 − Global
2 − Super Region
3 − Region
4 − Country
5 − Subnational (Level 1)
Uncertainty Interval
5 − Subnational (Level 1)

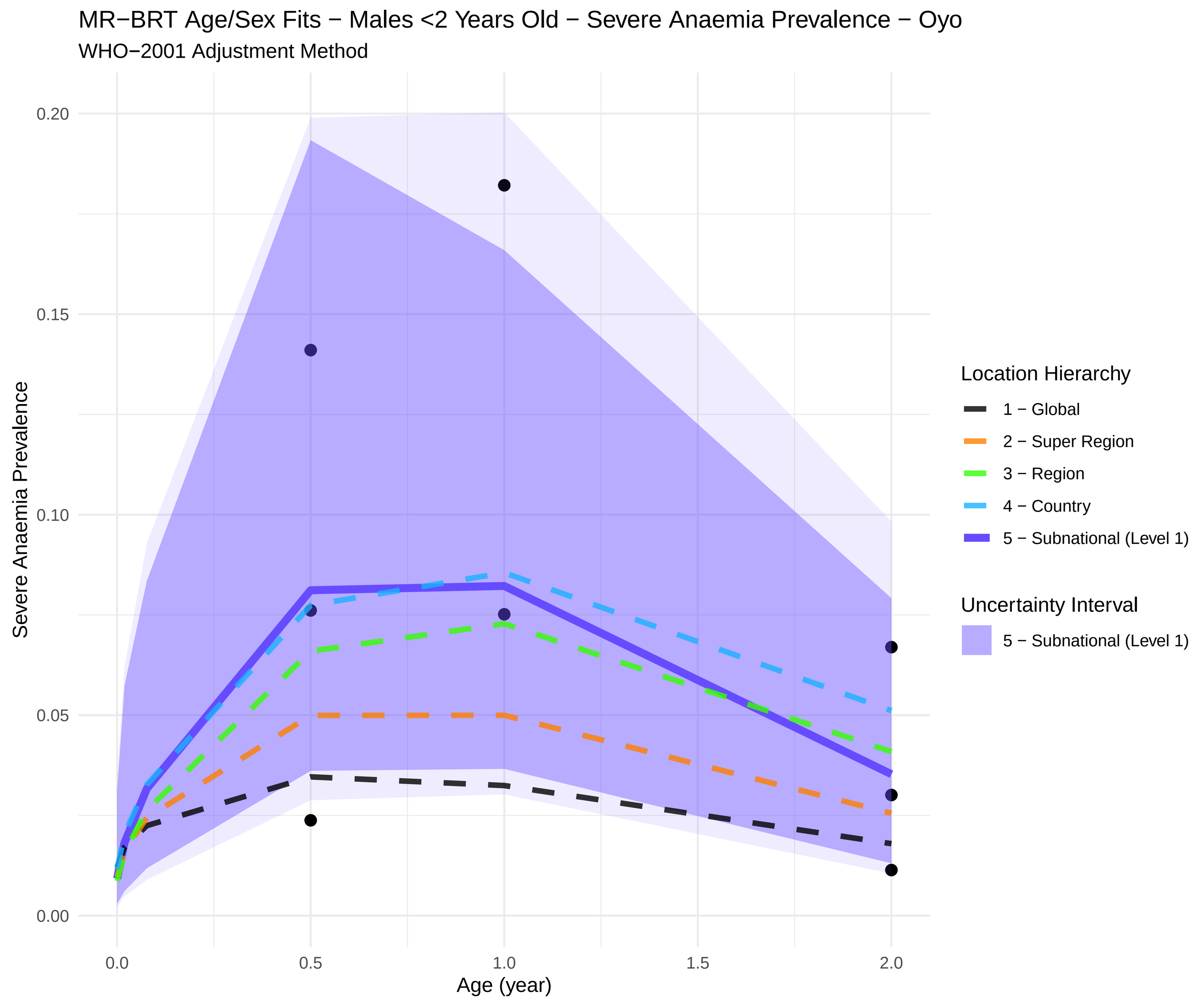
MR−BRT Age/Sex Fits − Males <2 Years Old − Severe Anaemia Prevalence − Oyo
WHO−2001 Adjustment Method
Severe Anaemia Prevalence
Age (year)
Location Hierarchy
1 − Global
2 − Super Region
3 − Region
4 − Country
5 − Subnational (Level 1)
Uncertainty Interval
5 − Subnational (Level 1)

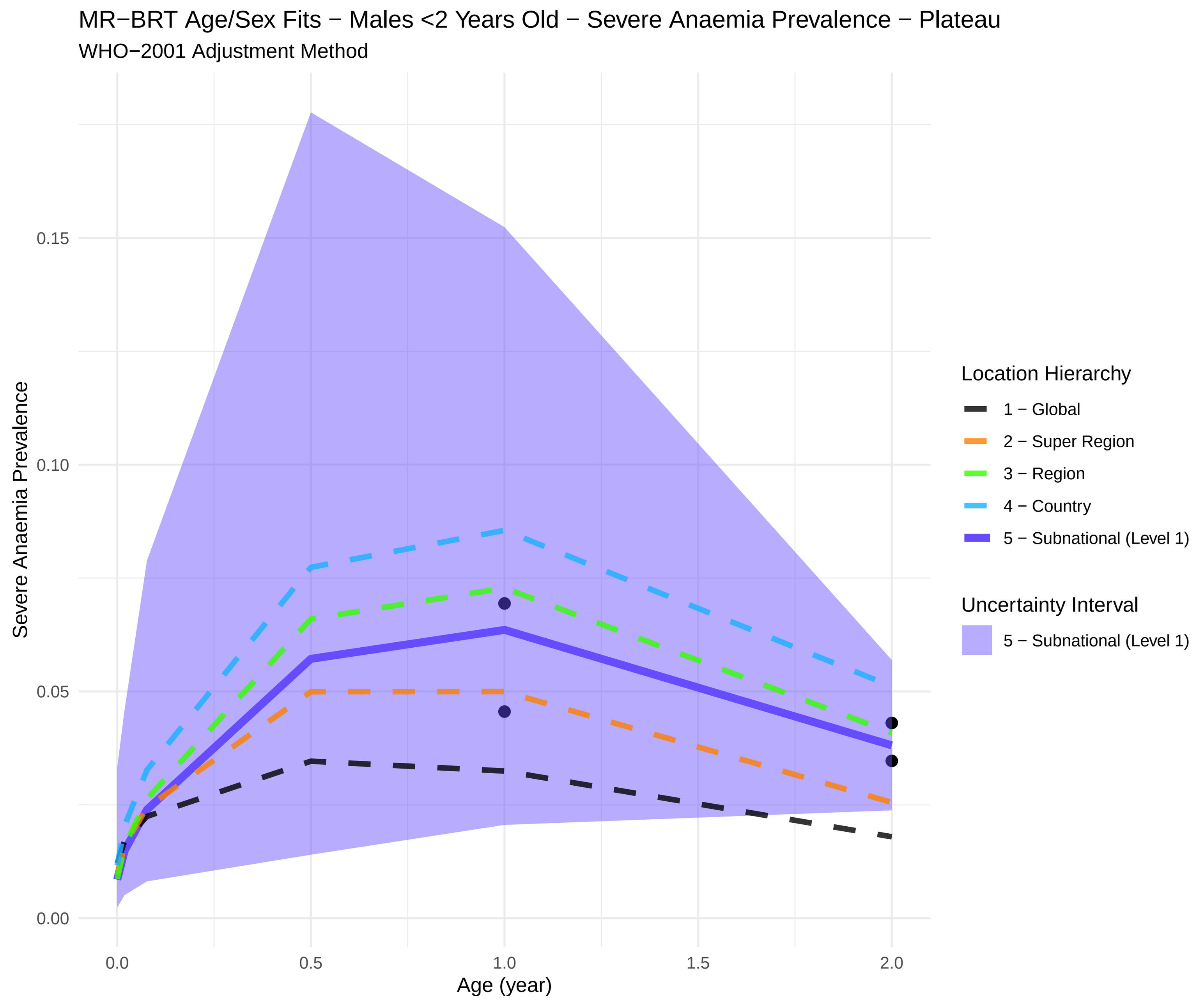
MR−BRT Age/Sex Fits − Males <2 Years Old − Severe Anaemia Prevalence − Plateau
WHO−2001 Adjustment Method
Severe Anaemia Prevalence
Age (year)
Location Hierarchy
1 − Global
2 − Super Region
3 − Region
4 − Country
5 − Subnational (Level 1)
Uncertainty Interval
5 − Subnational (Level 1)

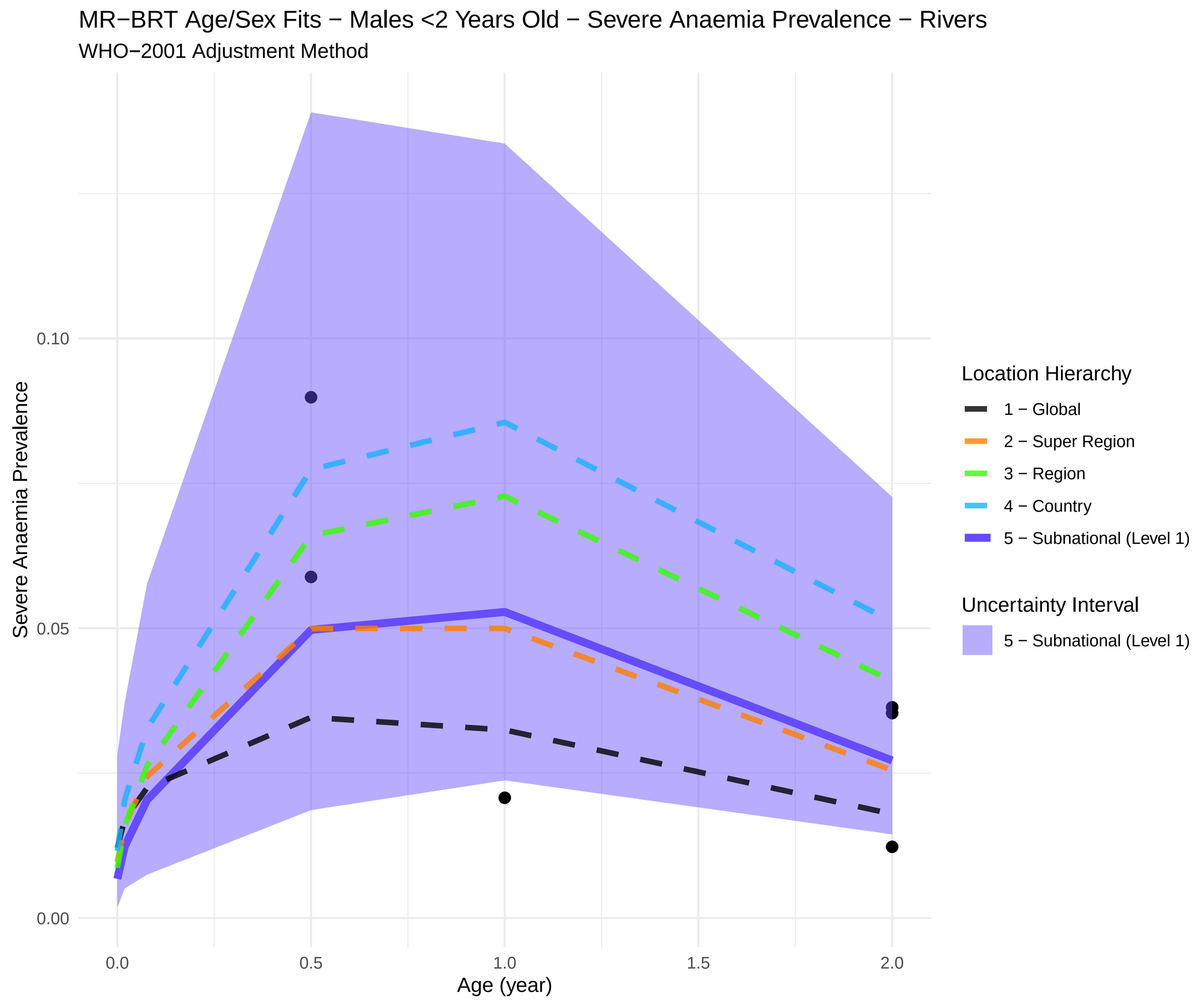
MR−BRT Age/Sex Fits − Males <2 Years Old − Severe Anaemia Prevalence − Rivers
WHO−2001 Adjustment Method
Severe Anaemia Prevalence
Age (year)
Location Hierarchy
1 − Global
2 − Super Region
3 − Region
4 − Country
5 − Subnational (Level 1)
Uncertainty Interval
5 − Subnational (Level 1)

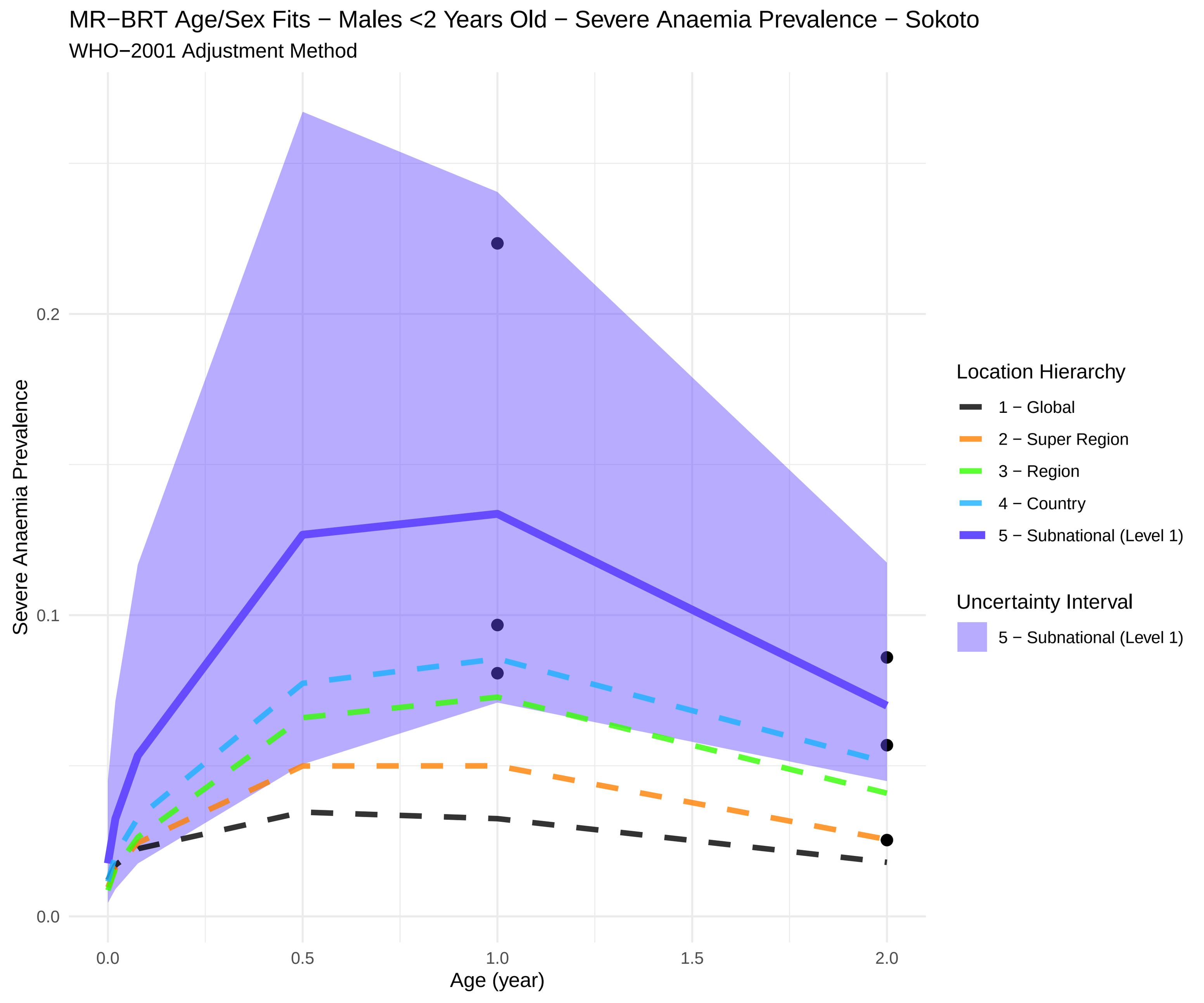
MR−BRT Age/Sex Fits − Males <2 Years Old − Severe Anaemia Prevalence − Sokoto
WHO−2001 Adjustment Method
Severe Anaemia Prevalence
Age (year)
Location Hierarchy
1 − Global
2 − Super Region
3 − Region
4 − Country
5 − Subnational (Level 1)
Uncertainty Interval
5 − Subnational (Level 1)

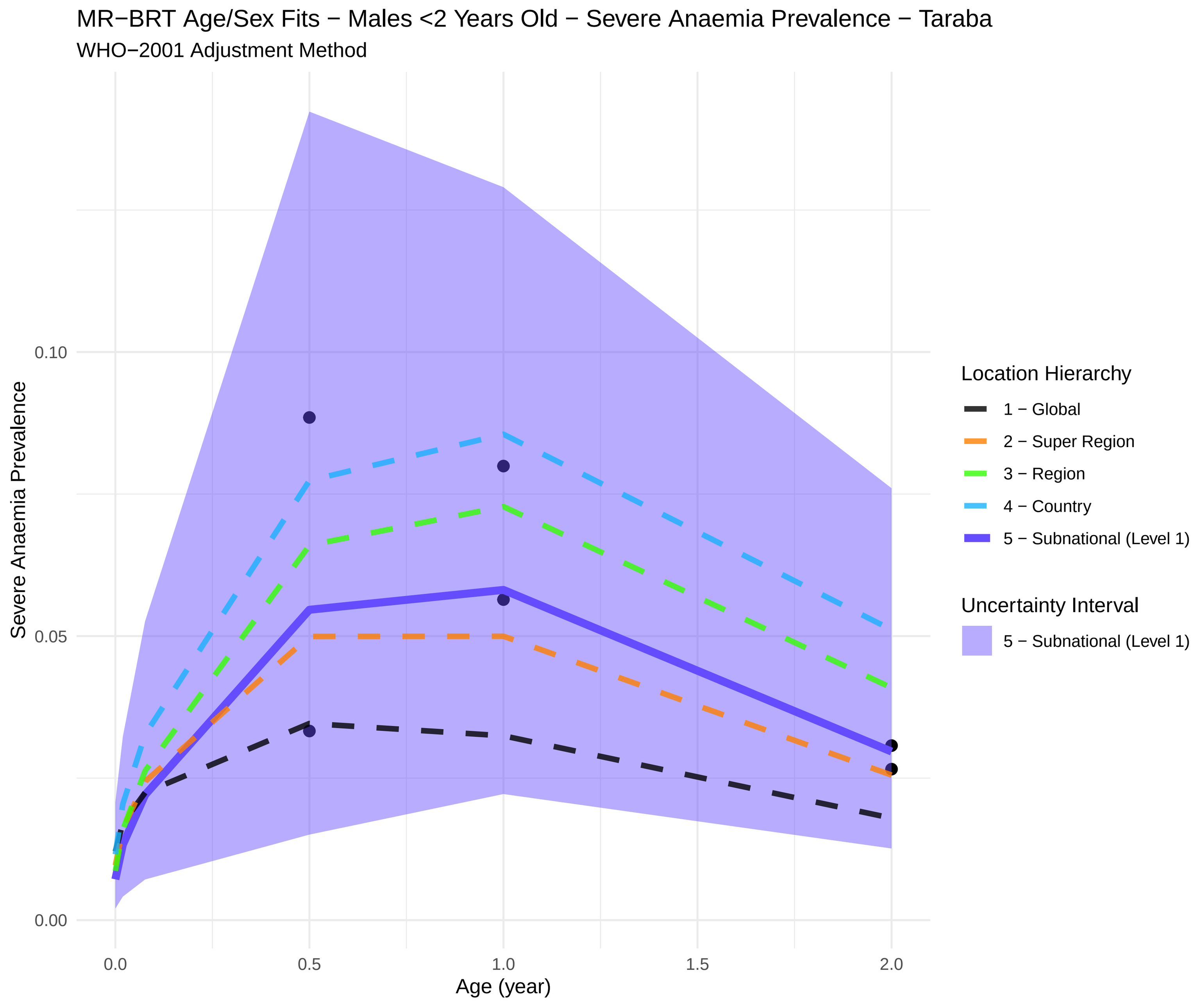
MR−BRT Age/Sex Fits − Males <2 Years Old − Severe Anaemia Prevalence − Taraba
WHO−2001 Adjustment Method
Severe Anaemia Prevalence
Age (year)
Location Hierarchy
1 − Global
2 − Super Region
3 − Region
4 − Country
5 − Subnational (Level 1)
Uncertainty Interval
5 − Subnational (Level 1)

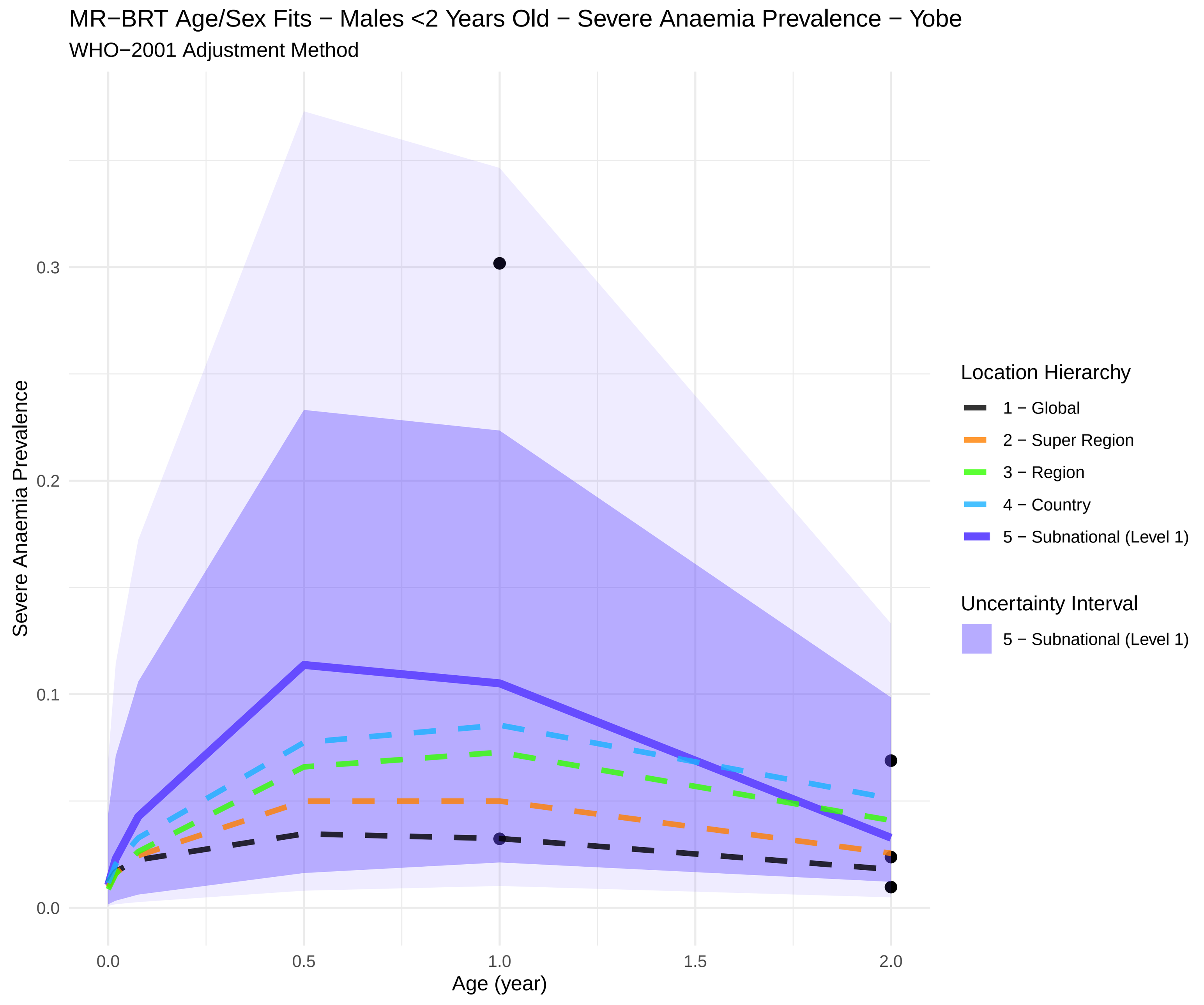
MR−BRT Age/Sex Fits − Males <2 Years Old − Severe Anaemia Prevalence − Yobe
WHO−2001 Adjustment Method
Severe Anaemia Prevalence
Age (year)
Location Hierarchy
1 − Global
2 − Super Region
3 − Region
4 − Country
5 − Subnational (Level 1)
Uncertainty Interval
5 − Subnational (Level 1)

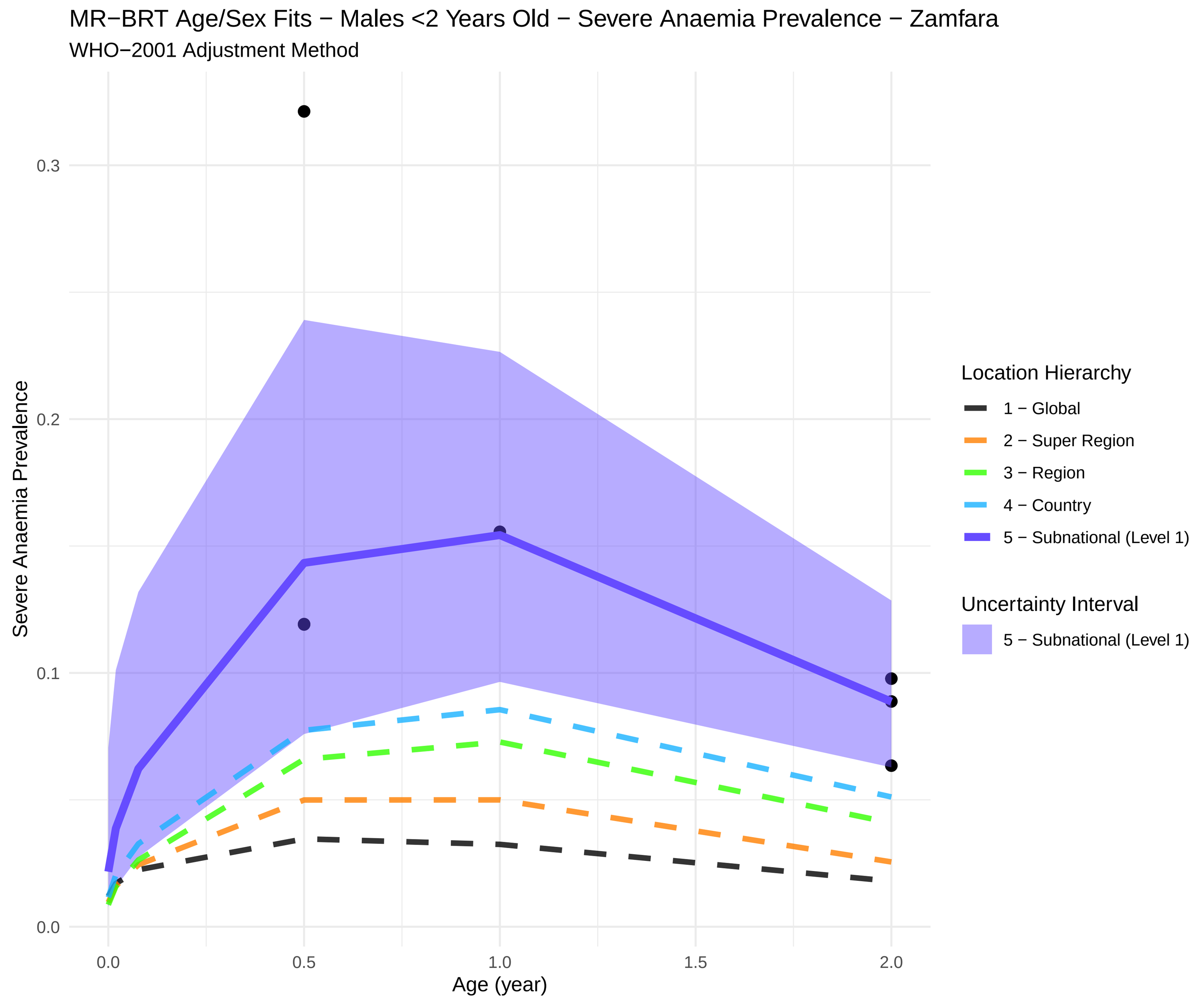
MR−BRT Age/Sex Fits − Males <2 Years Old − Severe Anaemia Prevalence − Zamfara
WHO−2001 Adjustment Method
Severe Anaemia Prevalence
Age (year)
Location Hierarchy
1 − Global
2 − Super Region
3 − Region
4 − Country
5 − Subnational (Level 1)
Uncertainty Interval
5 − Subnational (Level 1)

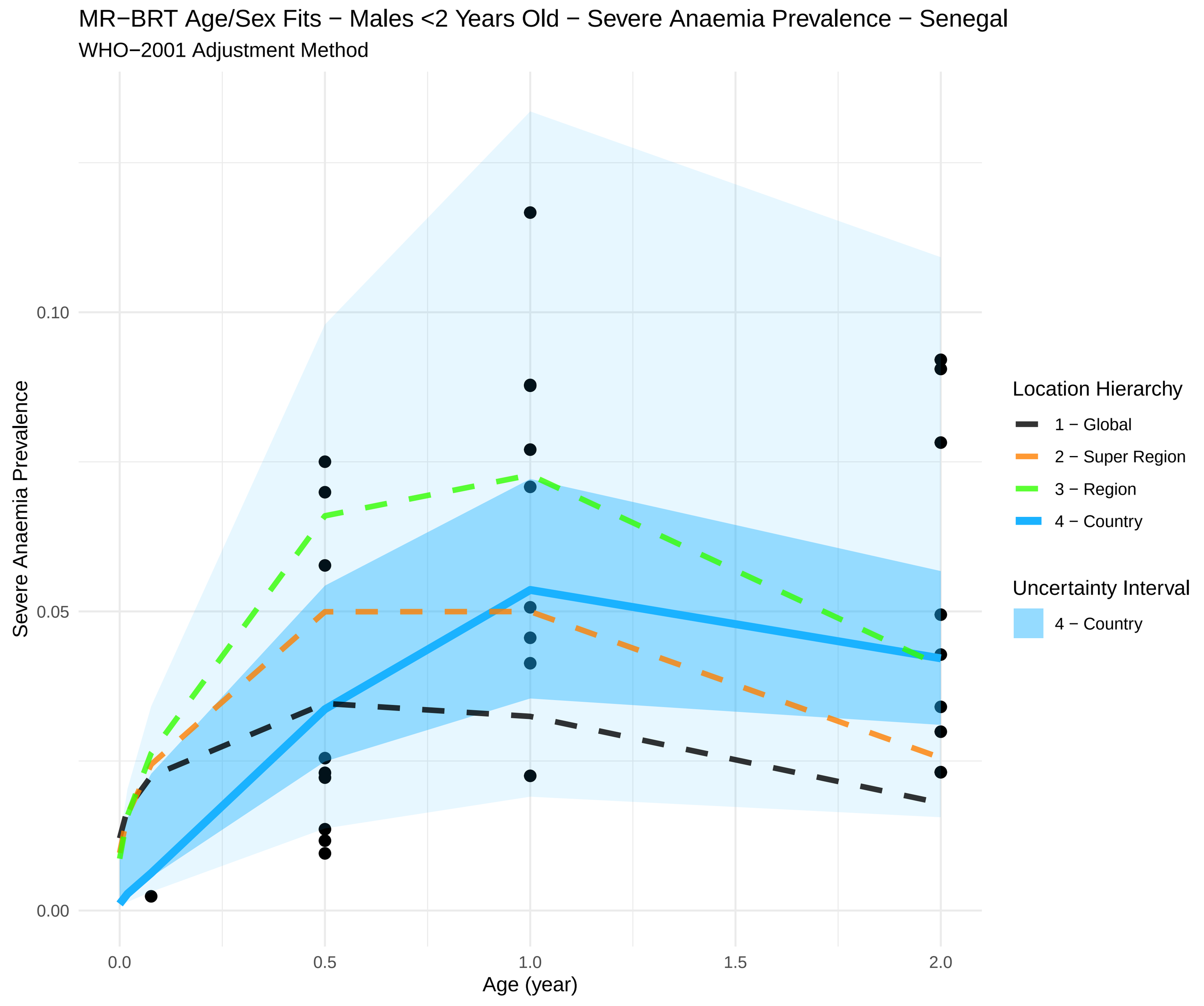
MR−BRT Age/Sex Fits − Males <2 Years Old − Severe Anaemia Prevalence − Senegal
WHO−2001 Adjustment Method
Severe Anaemia Prevalence
Age (year)
Location Hierarchy
1 − Global
2 − Super Region
3 − Region
4 − Country
Uncertainty Interval
4 − Country

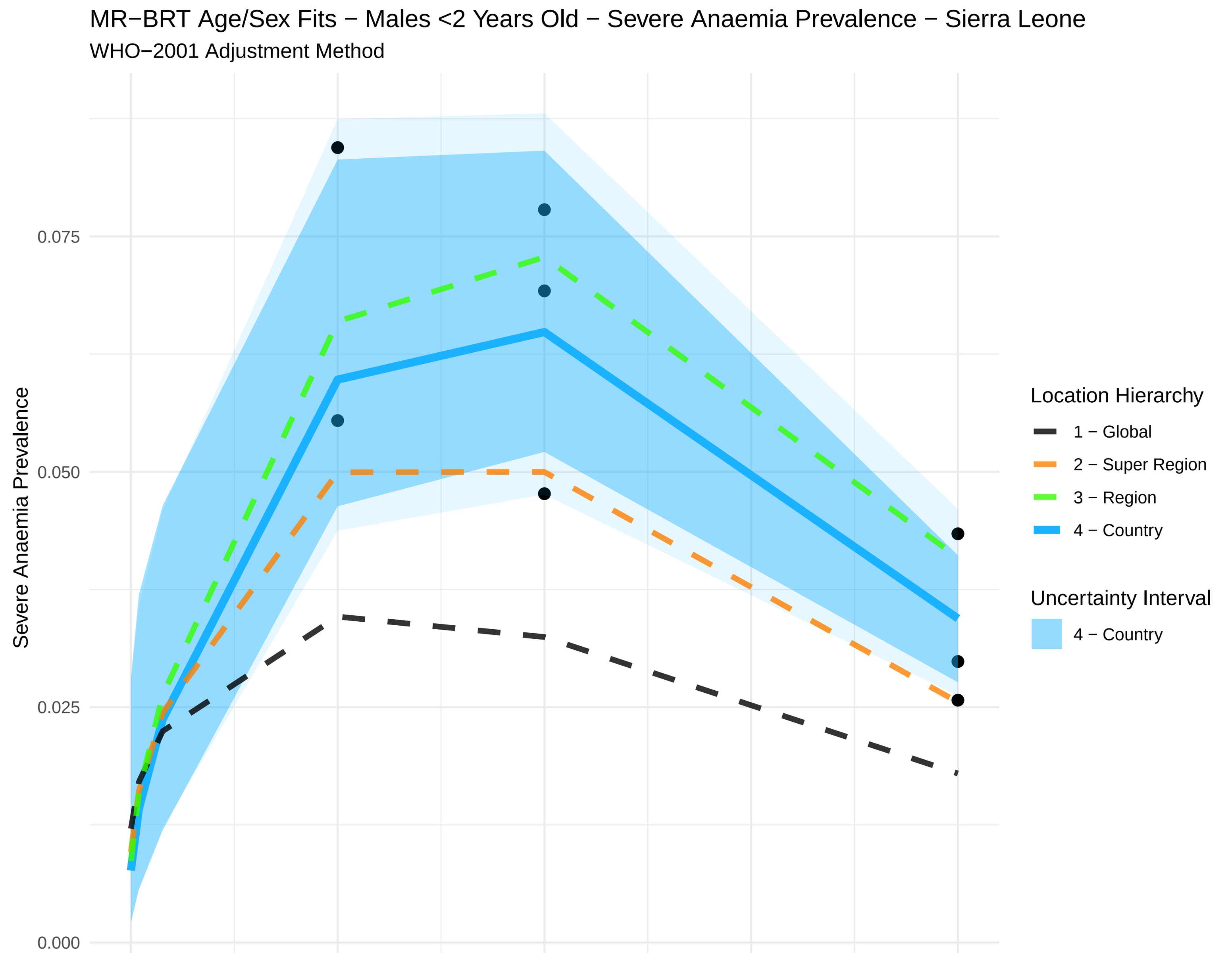
MR−BRT Age/Sex Fits − Males <2 Years Old − Severe Anaemia Prevalence − Sierra Leone
WHO−2001 Adjustment Method
Severe Anaemia Prevalence
Age (year)
Location Hierarchy
1 − Global
2 − Super Region
3 − Region
4 − Country
Uncertainty Interval
4 − Country

MR−BRT Age/Sex Fits − Males <2 Years Old − Severe Anaemia Prevalence − Togo

WHO−2001 Adjustment Method

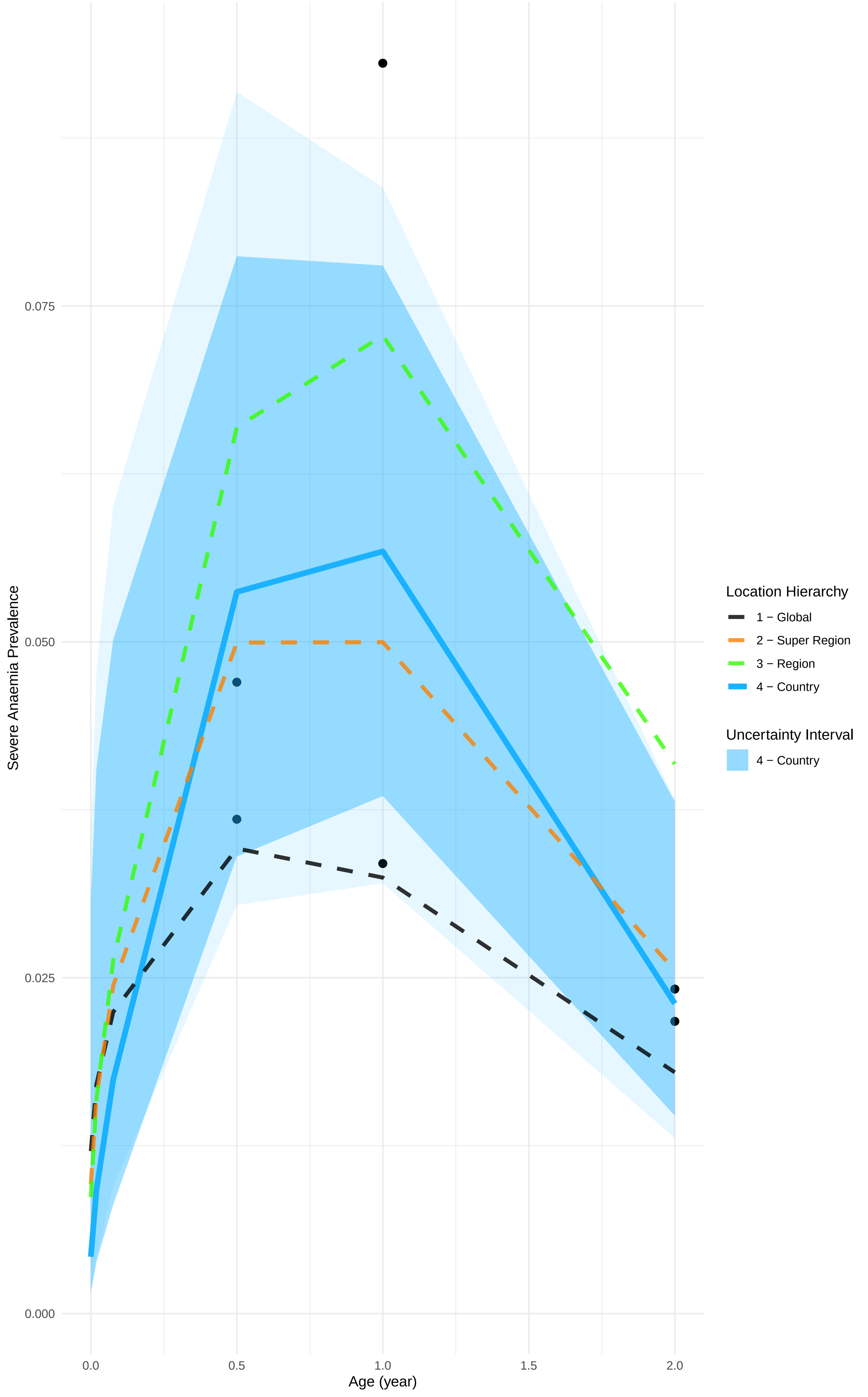

MR−BRT Age/Sex Fits − Non-pregnant Females <2 Years Old − Severe Anaemia Prevalence − Global
WHO−2001 Adjustment Method

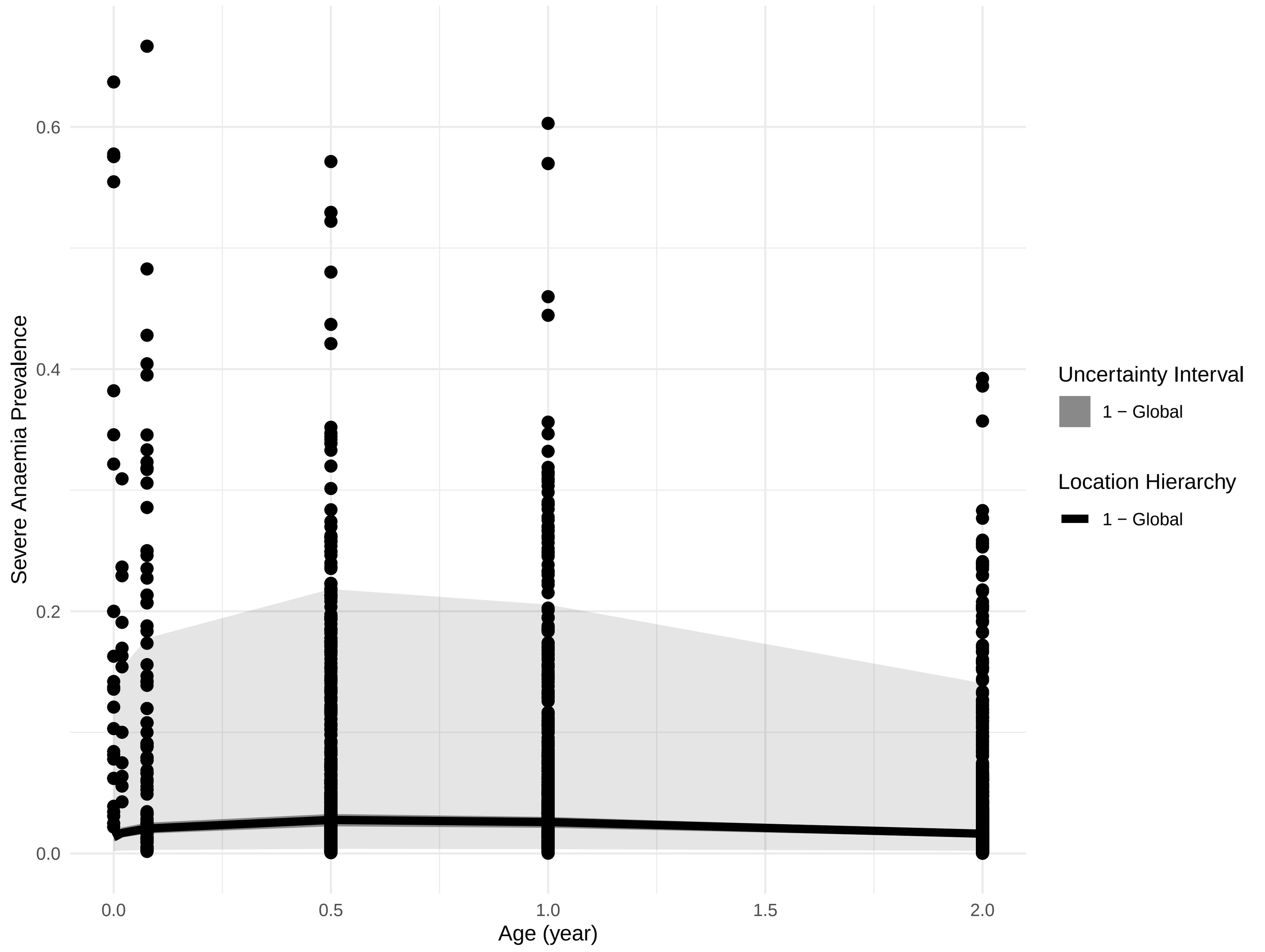

MR−BRT Age/Sex Fits − Non-pregnant Females <2 Years Old − Severe Anaemia Prevalence − Central Europe, Eastern Europe, and Central Asia
WHO−2001 Adjustment Method

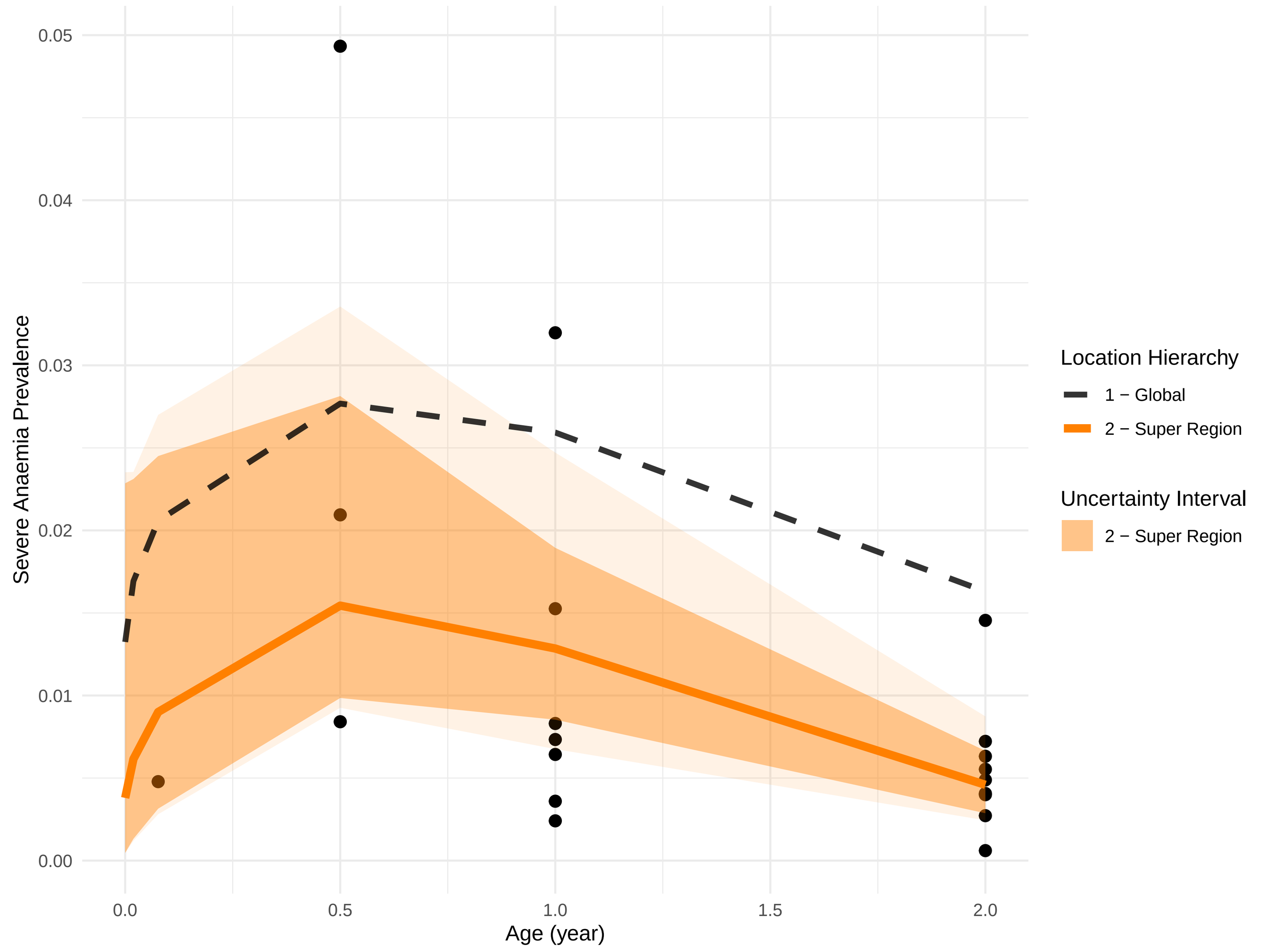

MR−BRT Age/Sex Fits − Non-pregnant Females <2 Years Old − Severe Anaemia Prevalence − Central Asia
WHO−2001 Adjustment Method

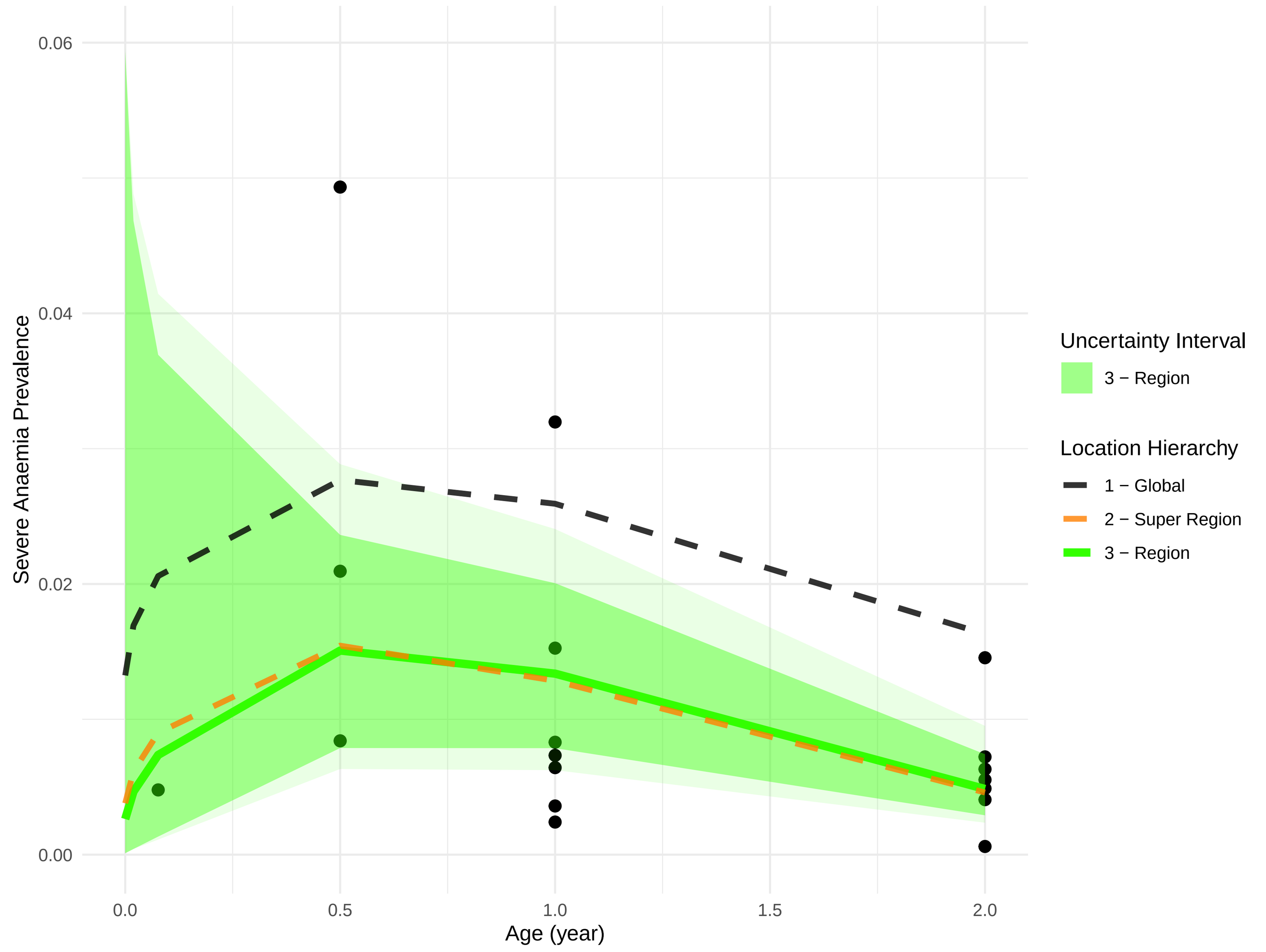

MR−BRT Age/Sex Fits − Non-pregnant Females <2 Years Old − Severe Anaemia Prevalence − Armenia
WHO−2001 Adjustment Method

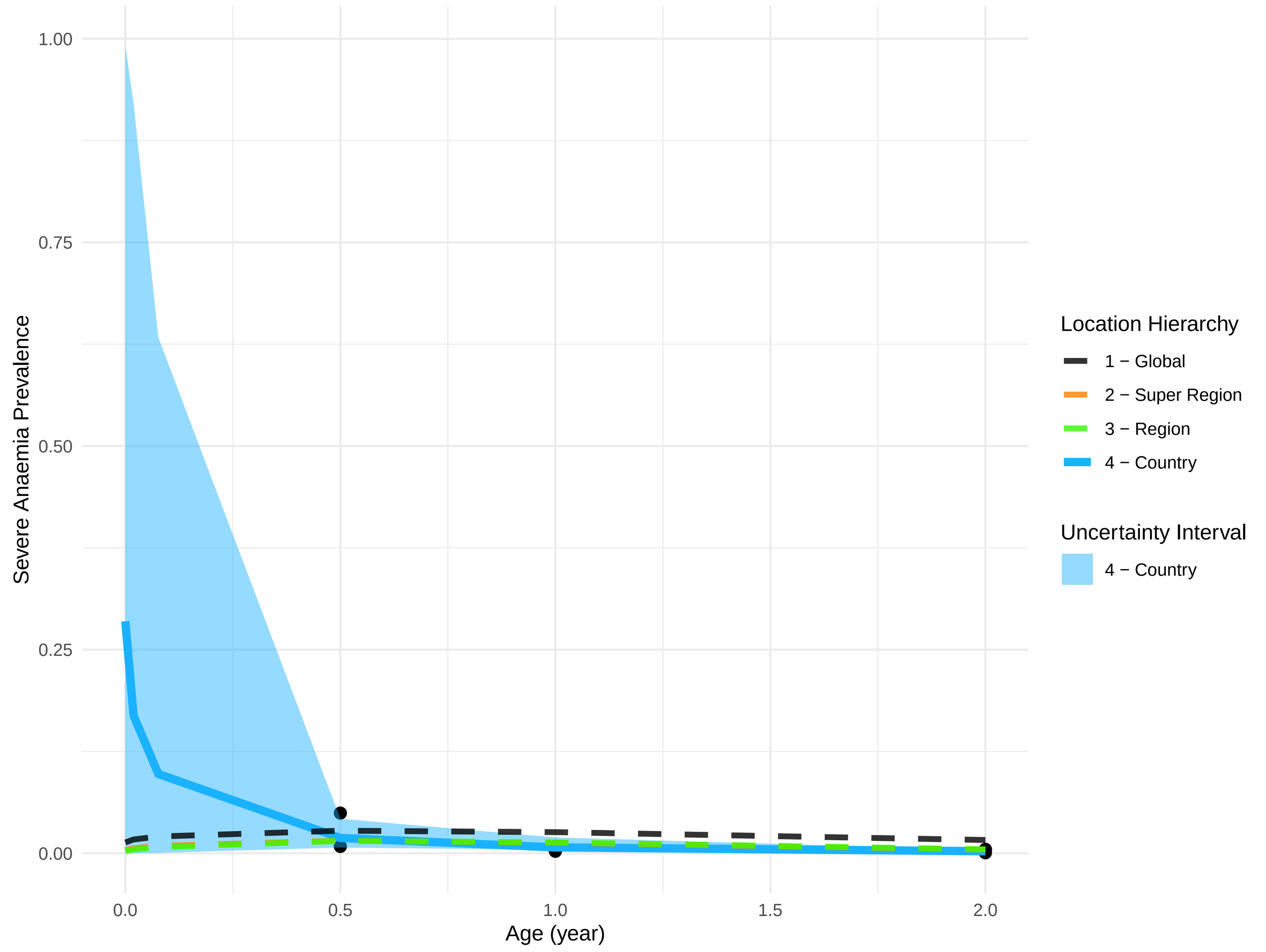

MR−BRT Age/Sex Fits − Non-pregnant Females <2 Years Old − Severe Anaemia Prevalence − Azerbaijan
WHO−2001 Adjustment Method

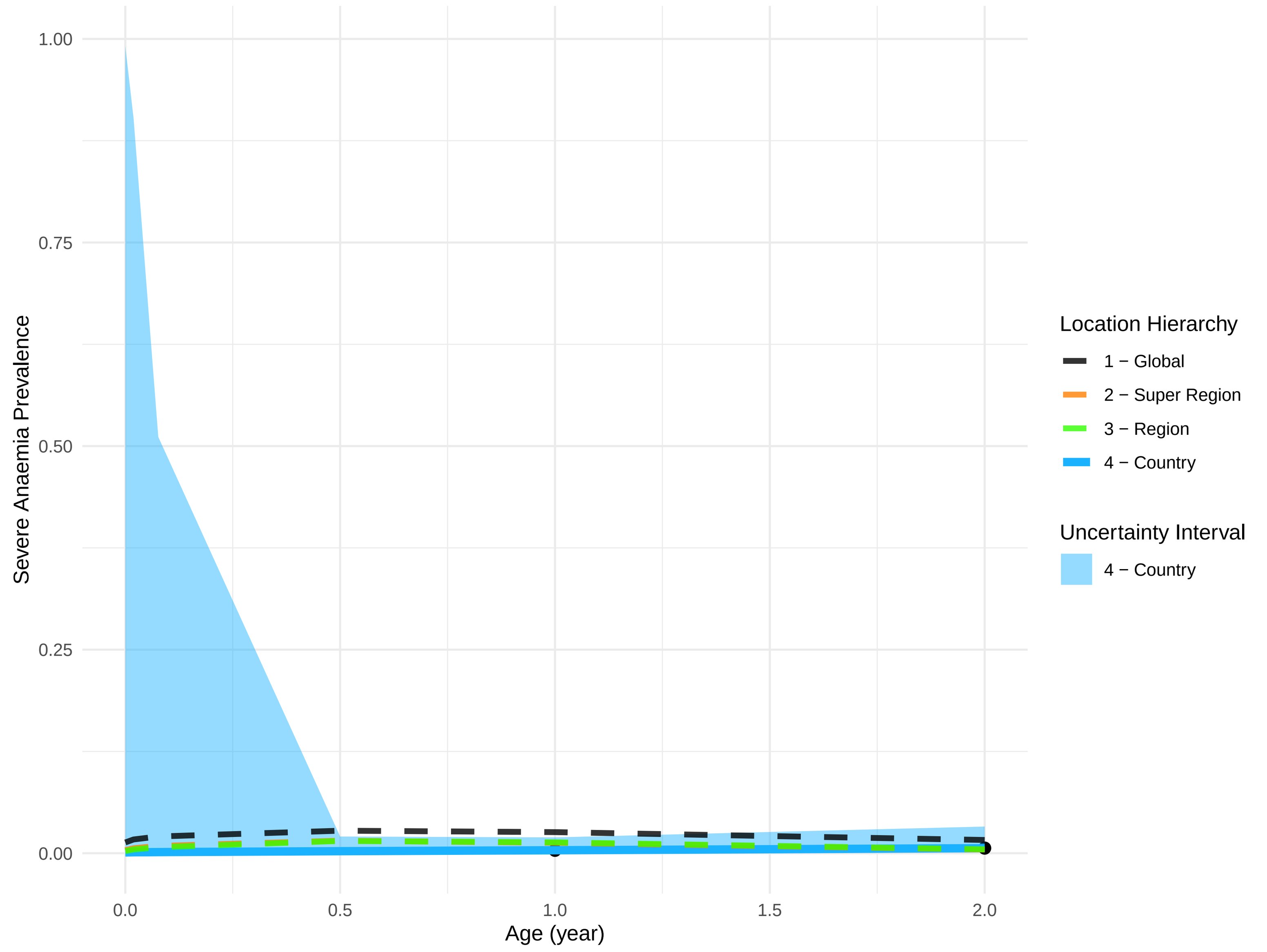

MR−BRT Age/Sex Fits − Non-pregnant Females <2 Years Old − Severe Anaemia Prevalence − Kyrgyzstan
WHO−2001 Adjustment Method

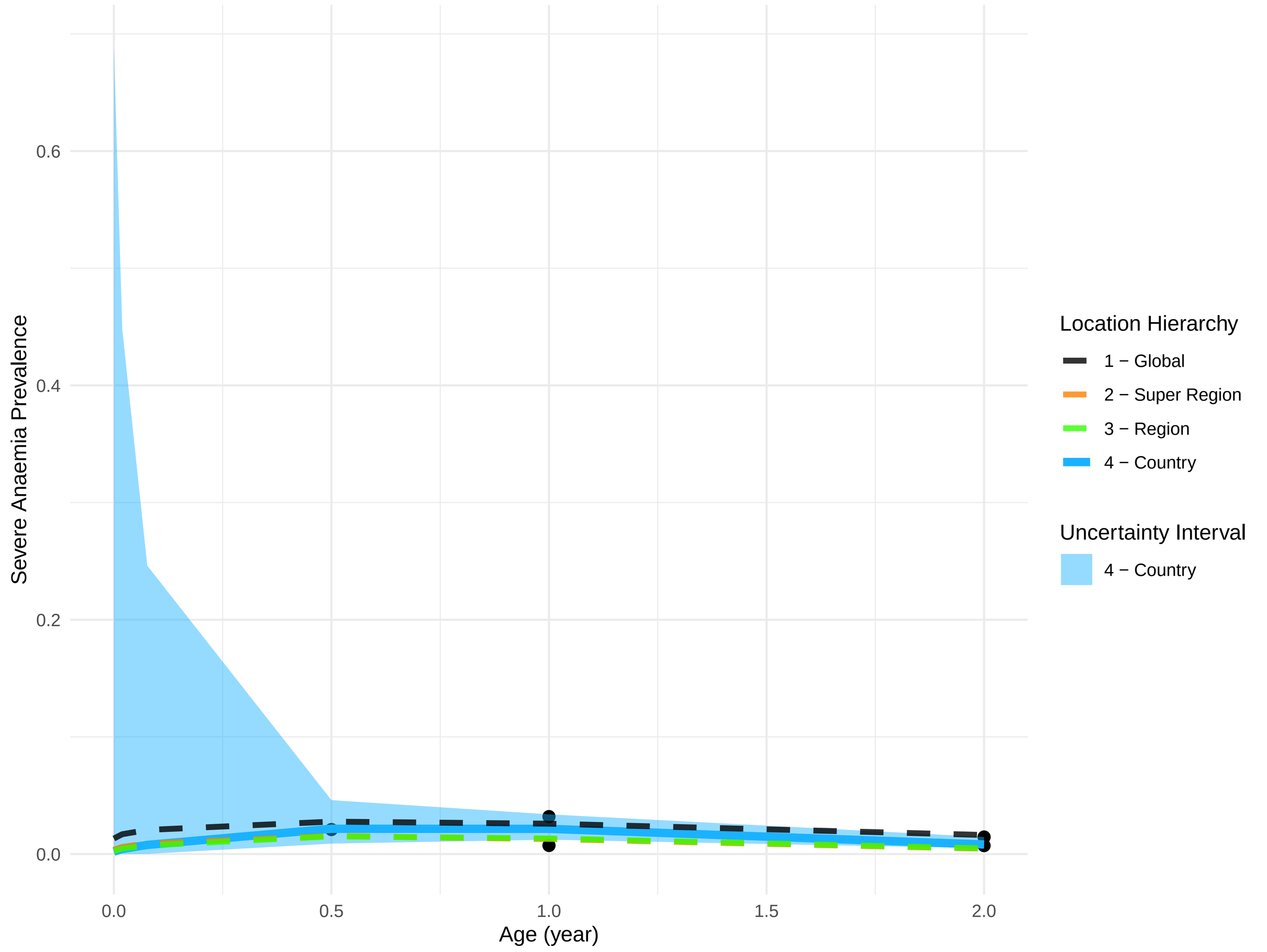

MR−BRT Age/Sex Fits − Non-pregnant Females <2 Years Old − Severe Anaemia Prevalence − Uzbekistan
WHO−2001 Adjustment Method

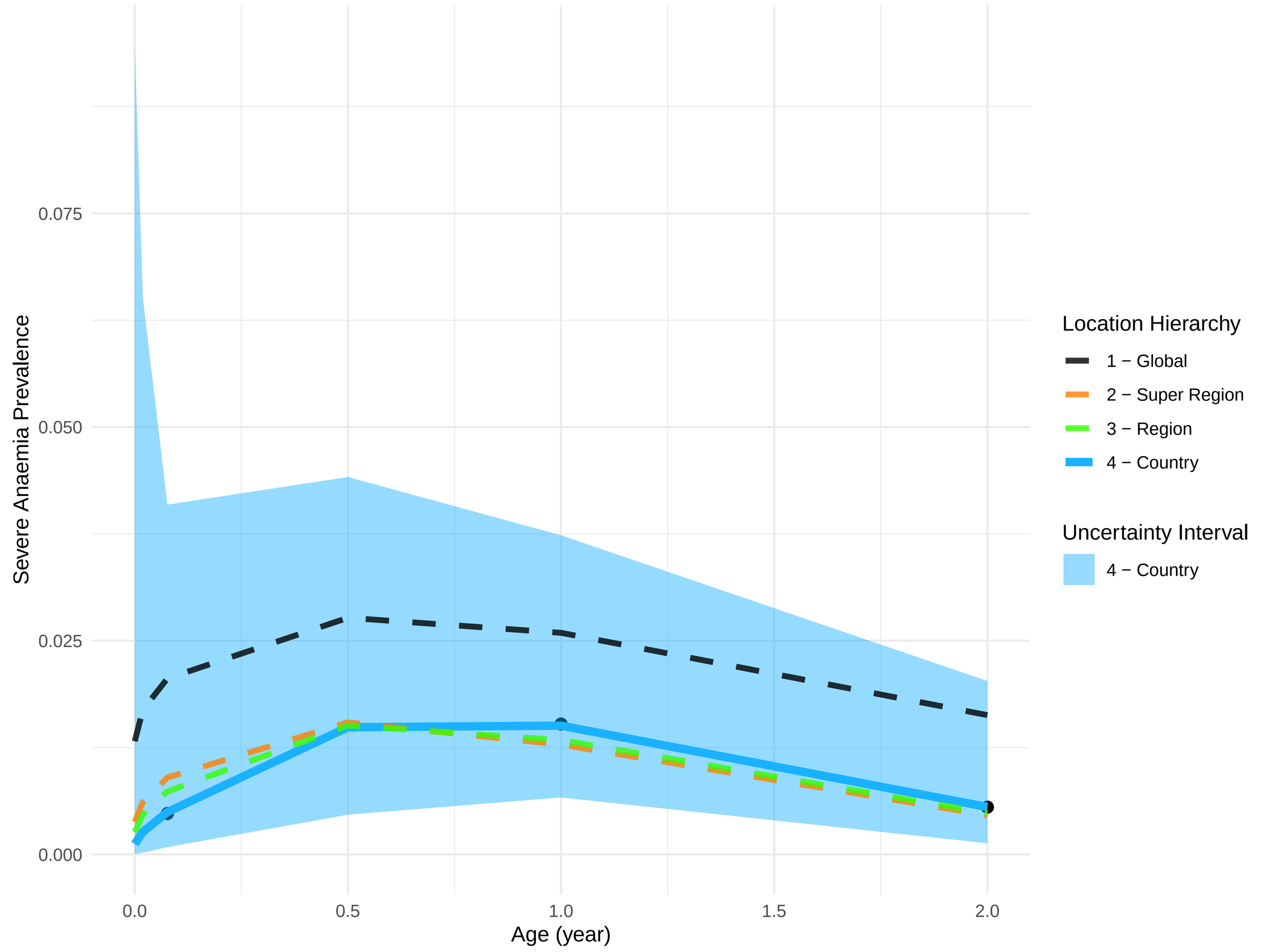

MR−BRT Age/Sex Fits − Non-pregnant Females <2 Years Old − Severe Anaemia Prevalence − Central Europe
WHO−2001 Adjustment Method

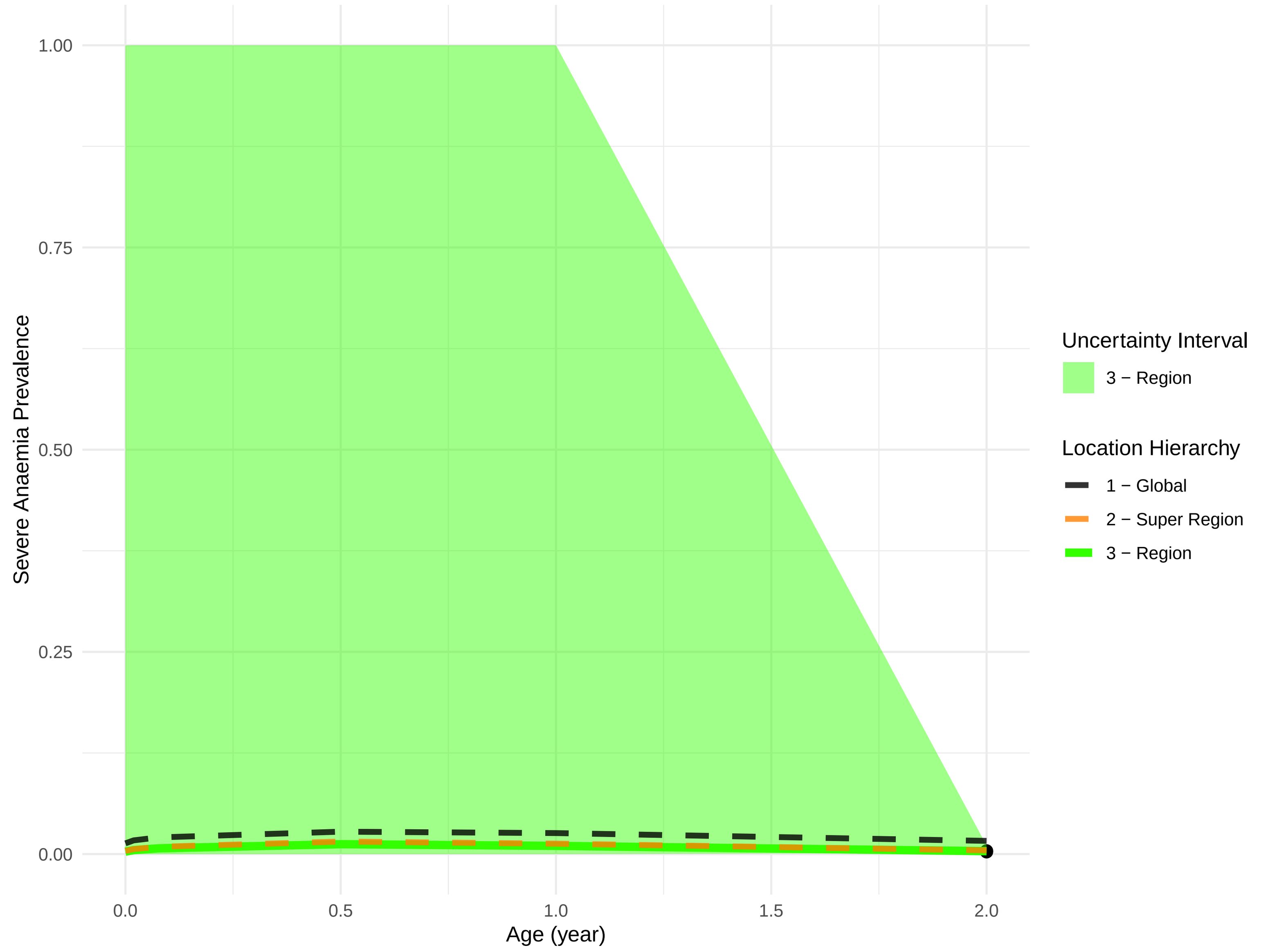

MR−BRT Age/Sex Fits − Non-pregnant Females <2 Years Old − Severe Anaemia Prevalence − Albania
WHO−2001 Adjustment Method

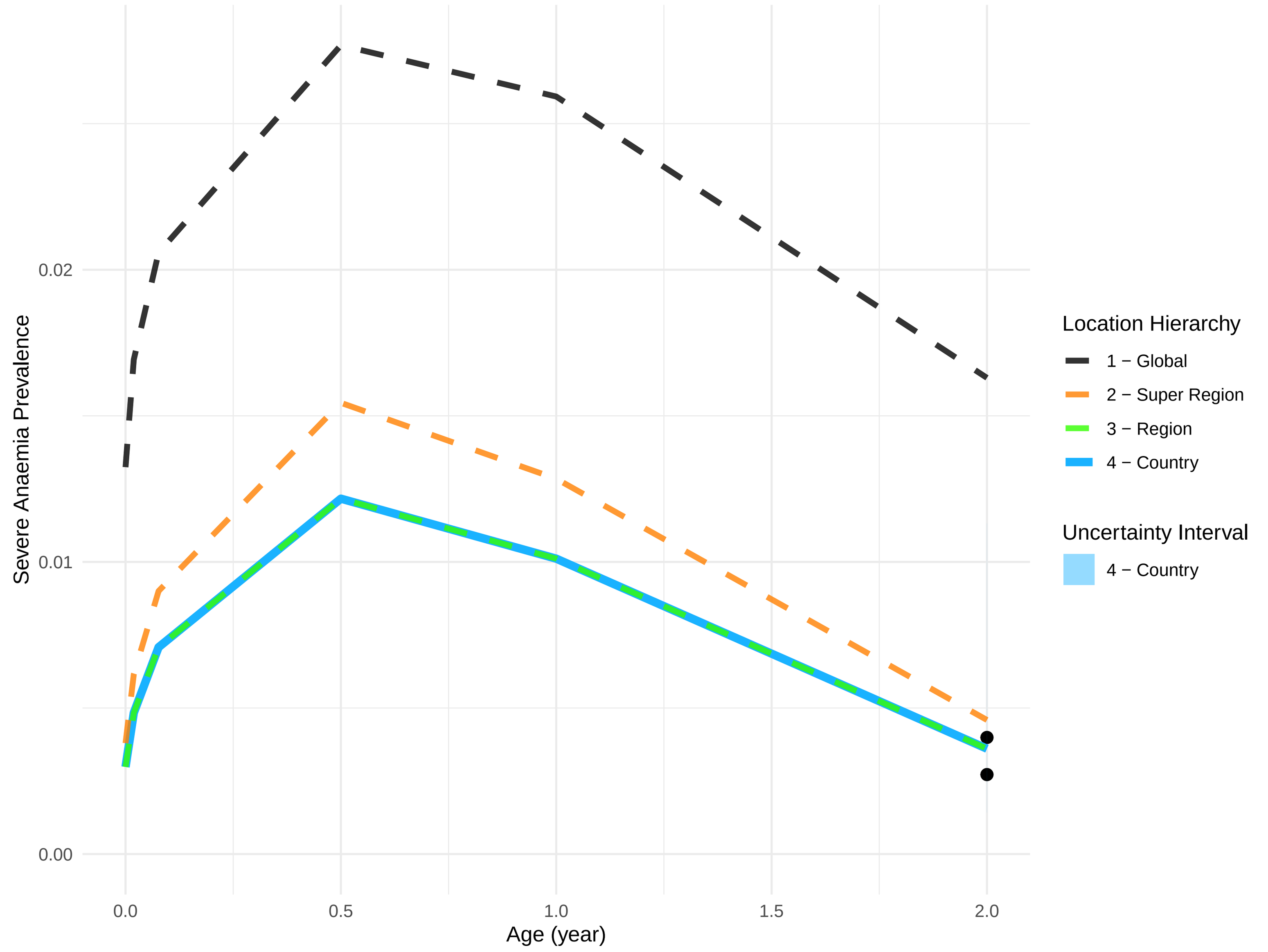

MR−BRT Age/Sex Fits − Non-pregnant Females <2 Years Old − Severe Anaemia Prevalence − High−income
WHO−2001 Adjustment Method

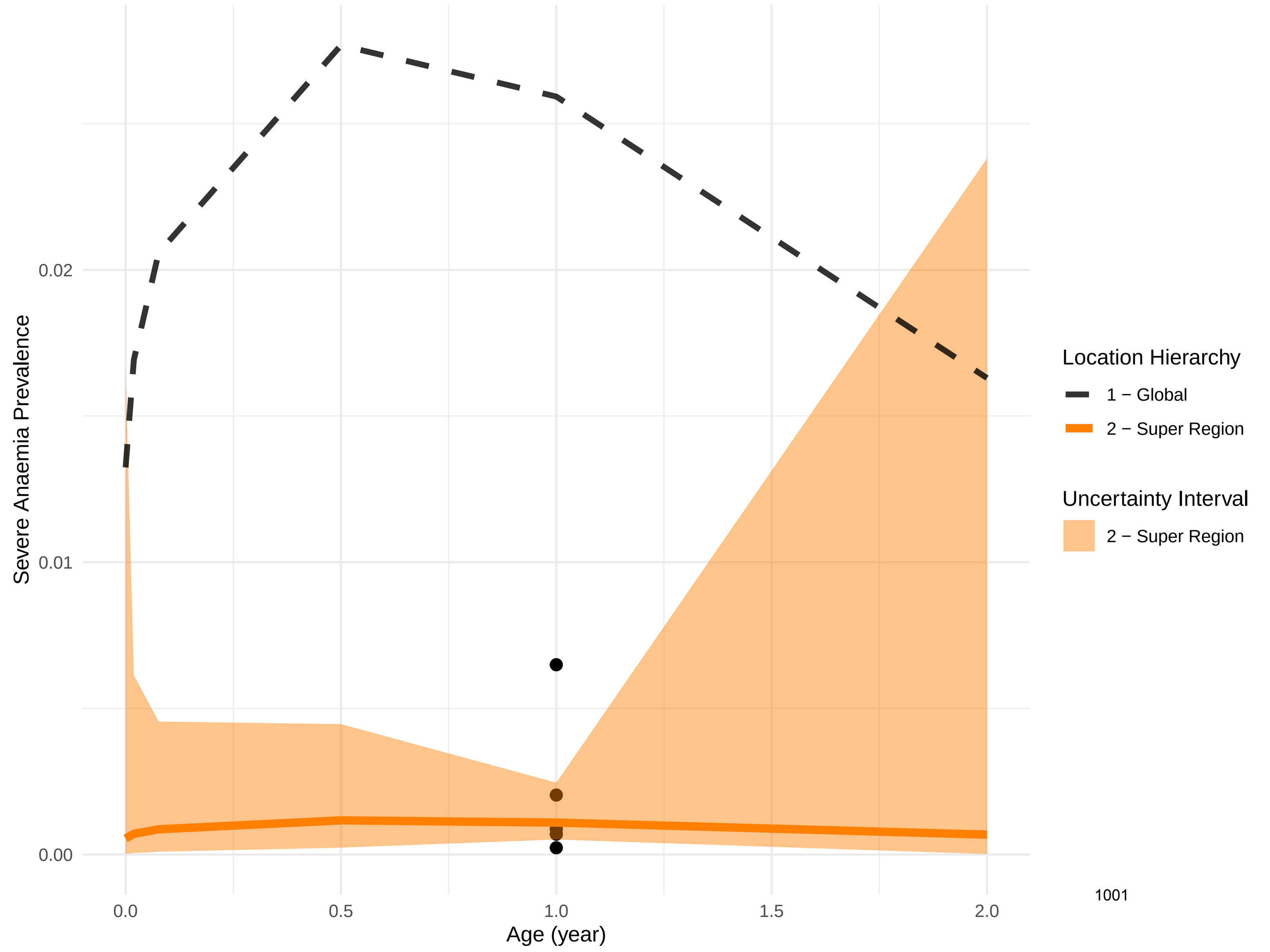

MR−BRT Age/Sex Fits − Non−pregnant Females <2 Years Old − Severe Anaemia Prevalence − Western Europe
WHO−2001 Adjustment Method

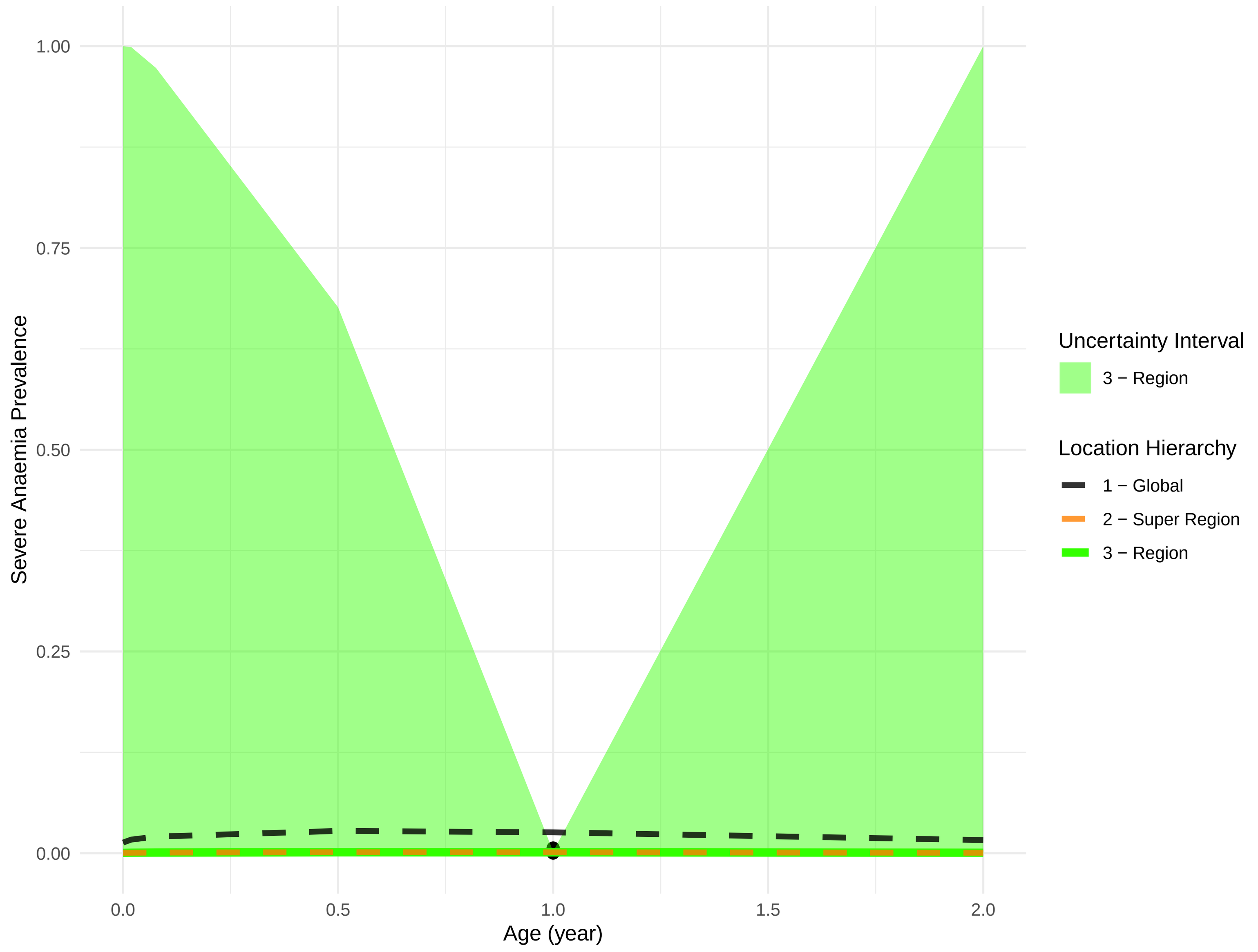

MR−BRT Age/Sex Fits − Non−pregnant Females <2 Years Old − Severe Anaemia Prevalence − United Kingdom
WHO−2001 Adjustment Method

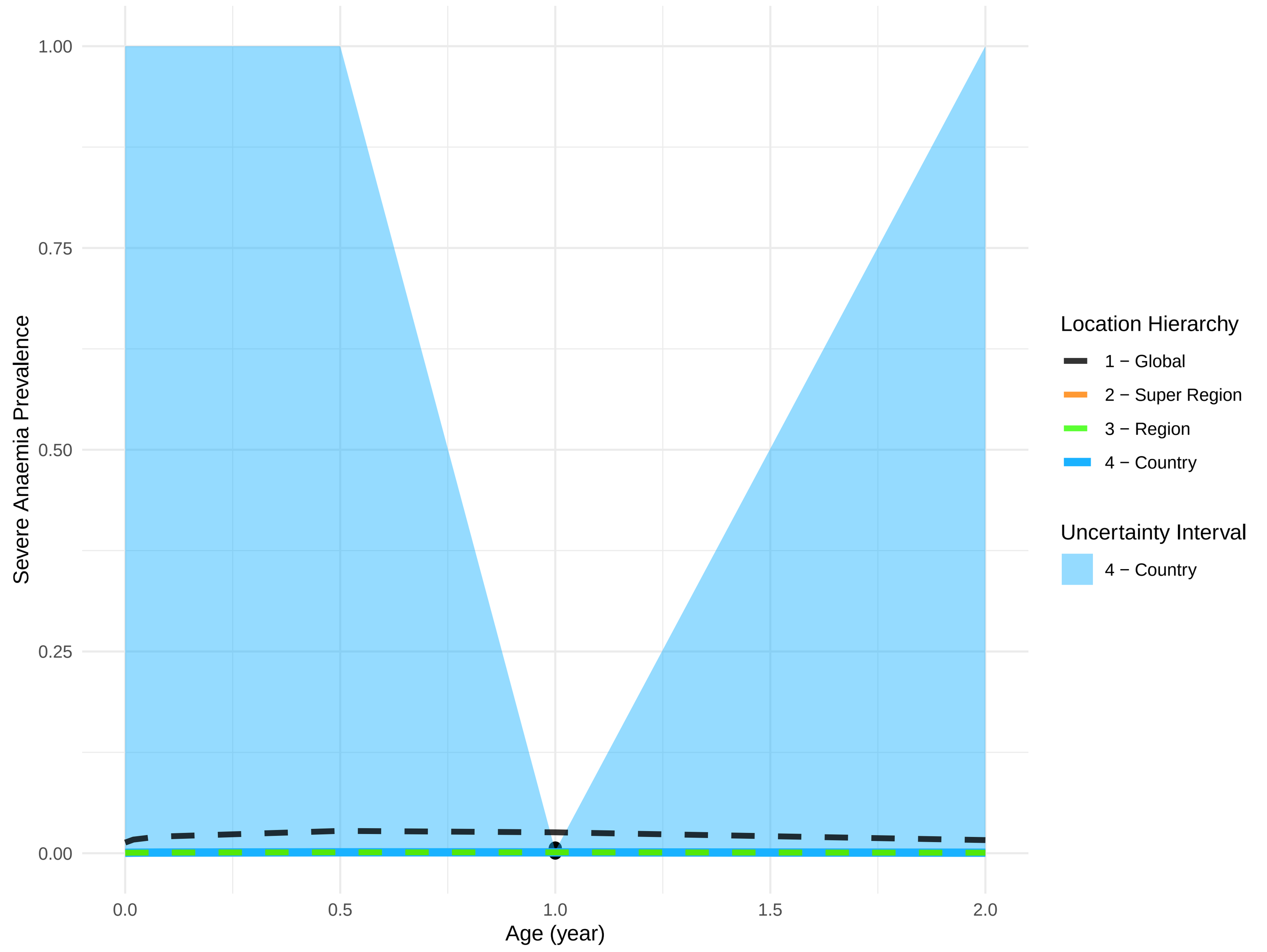

MR−BRT Age/Sex Fits − Non−pregnant Females <2 Years Old − Severe Anaemia Prevalence − England
WHO−2001 Adjustment Method

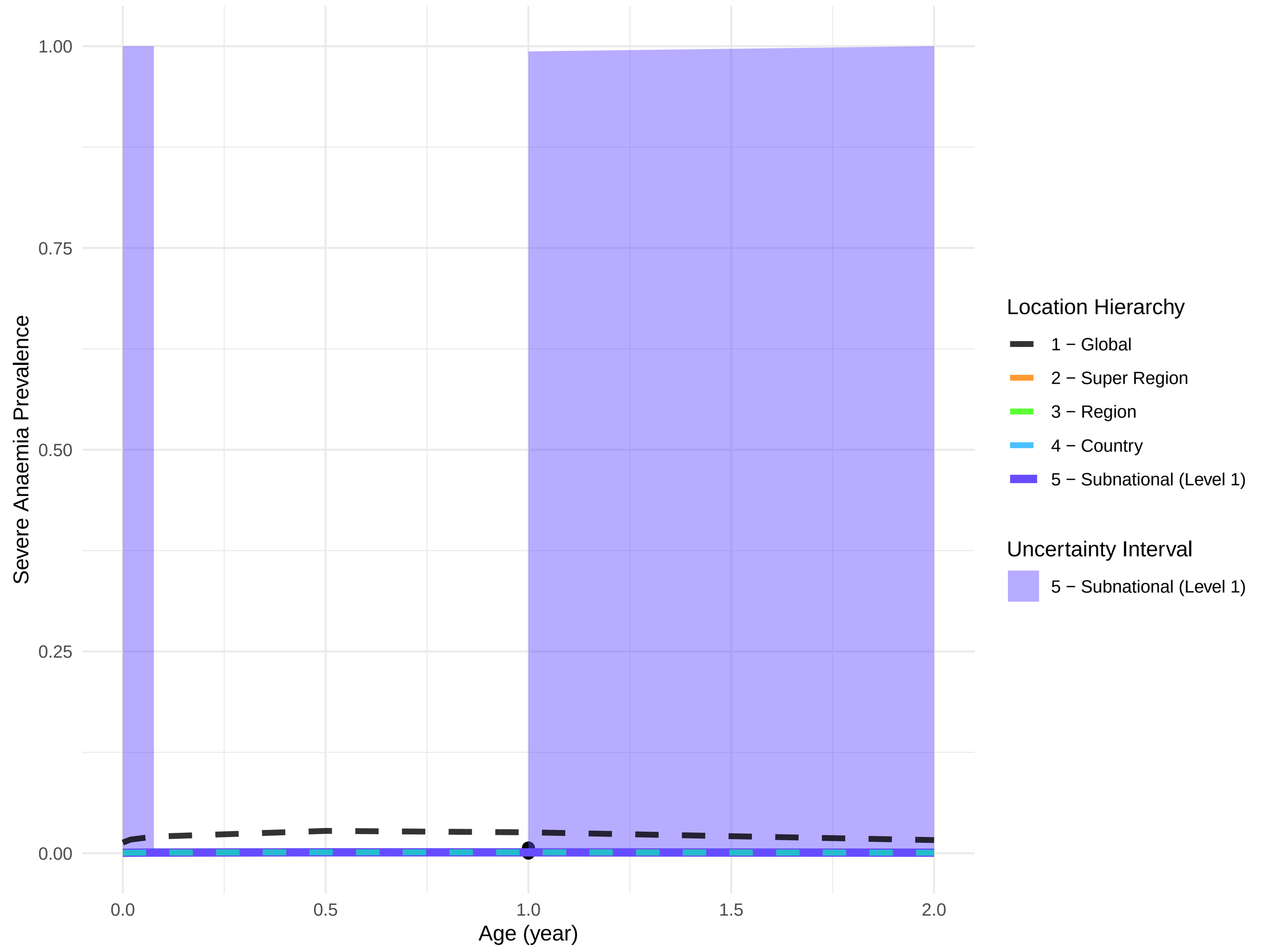

MR−BRT Age/Sex Fits − Non−pregnant Females <2 Years Old − Severe Anaemia Prevalence − East Midlands
WHO−2001 Adjustment Method

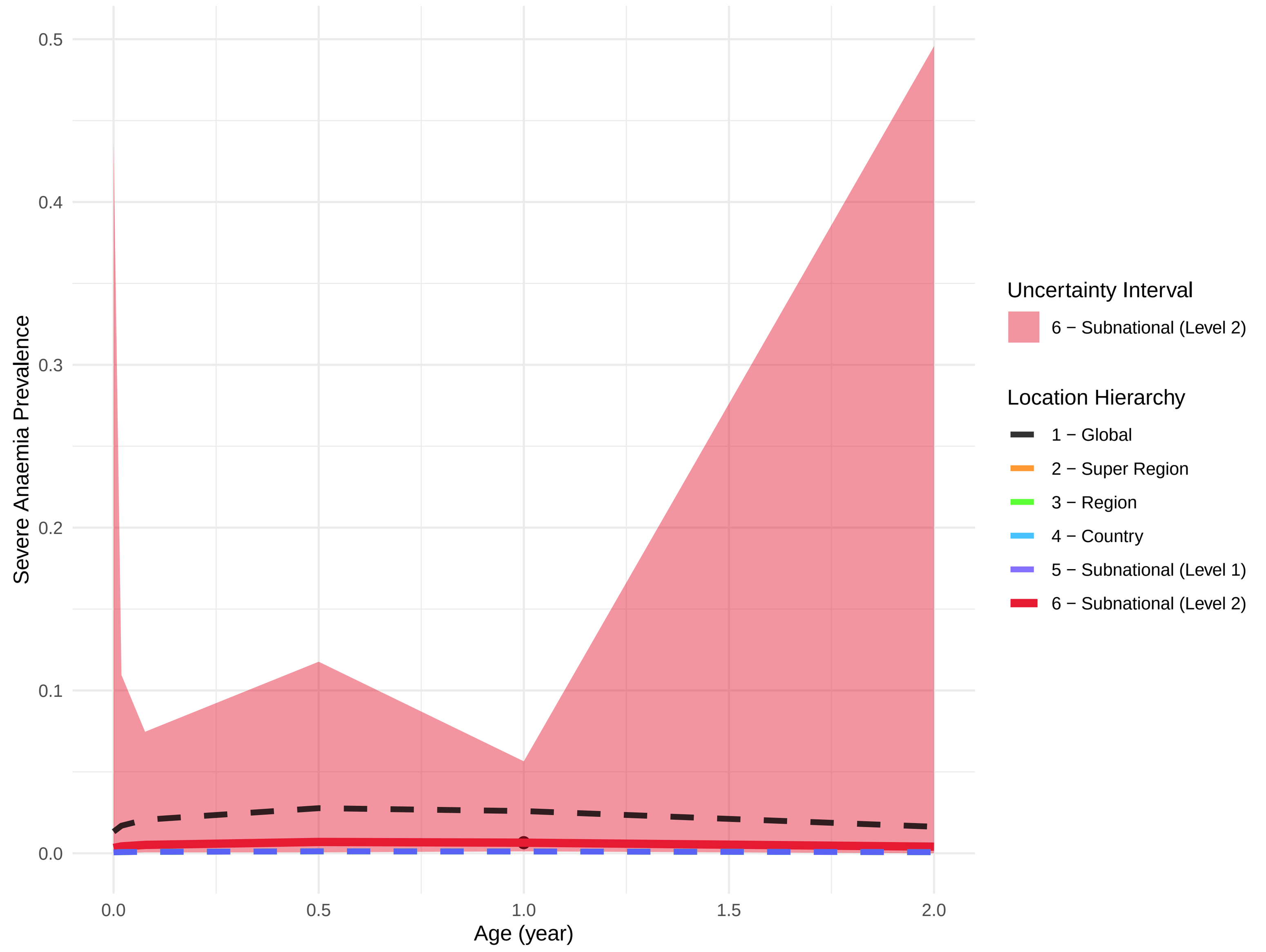

MR−BRT Age/Sex Fits − Non−pregnant Females <2 Years Old − Severe Anaemia Prevalence − East of England
WHO−2001 Adjustment Method

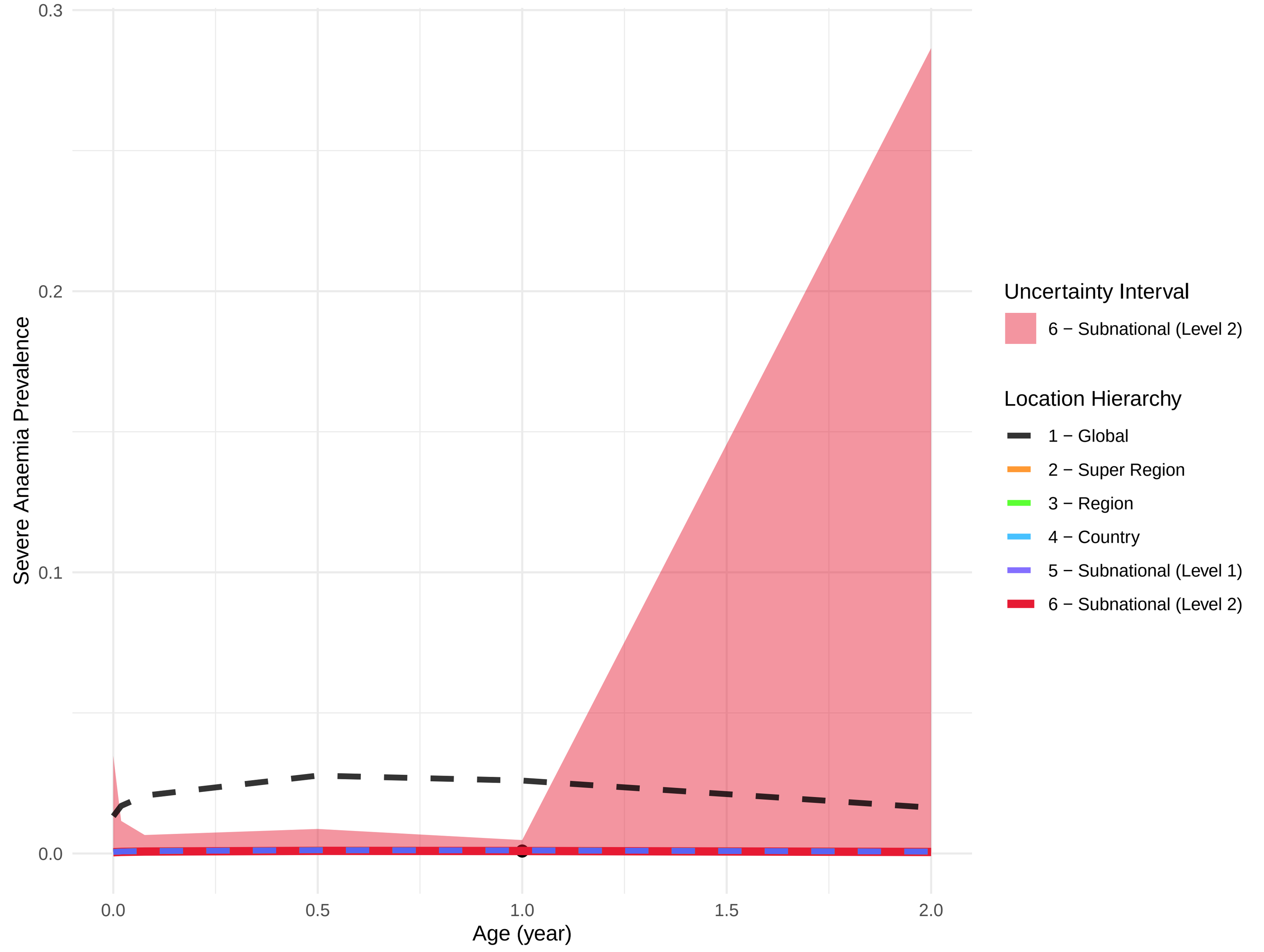

MR−BRT Age/Sex Fits − Non−pregnant Females <2 Years Old − Severe Anaemia Prevalence − West Midlands
WHO−2001 Adjustment Method

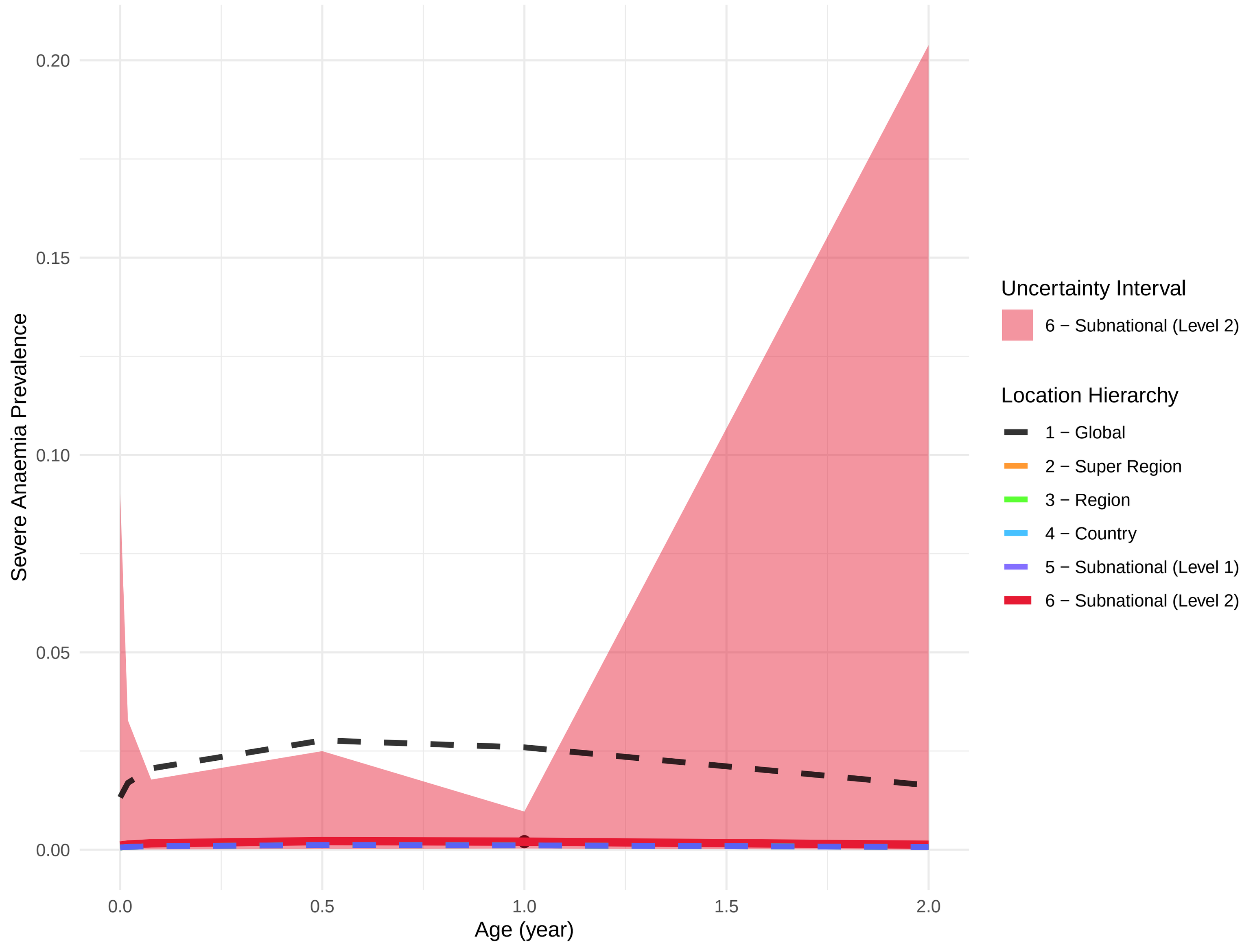

MR−BRT Age/Sex Fits − Non−pregnant Females <2 Years Old − Severe Anaemia Prevalence − Latin America and Caribbean
WHO−2001 Adjustment Method

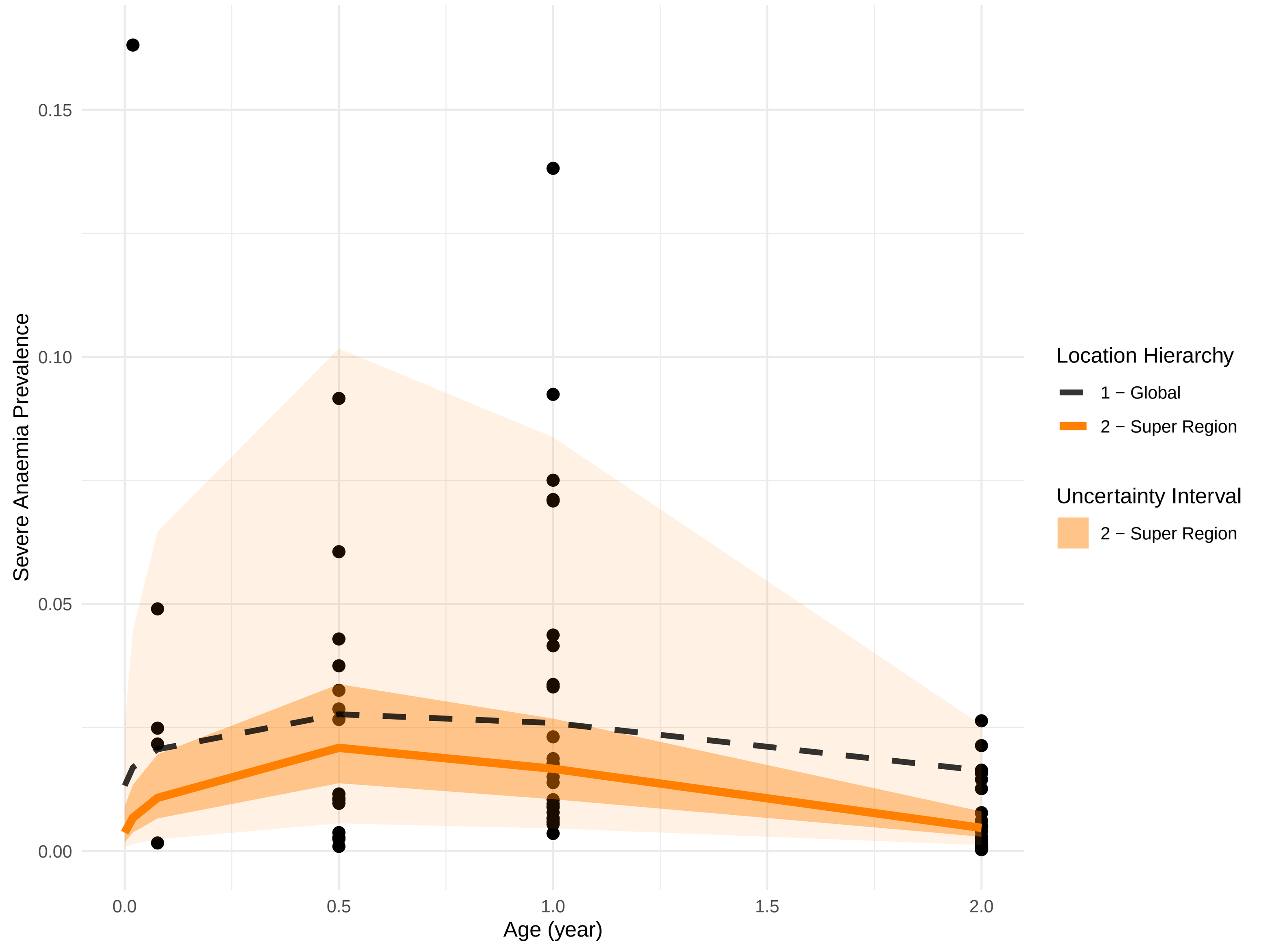

MR−BRT Age/Sex Fits − Non−pregnant Females <2 Years Old − Severe Anaemia Prevalence − Andean Latin America
WHO−2001 Adjustment Method

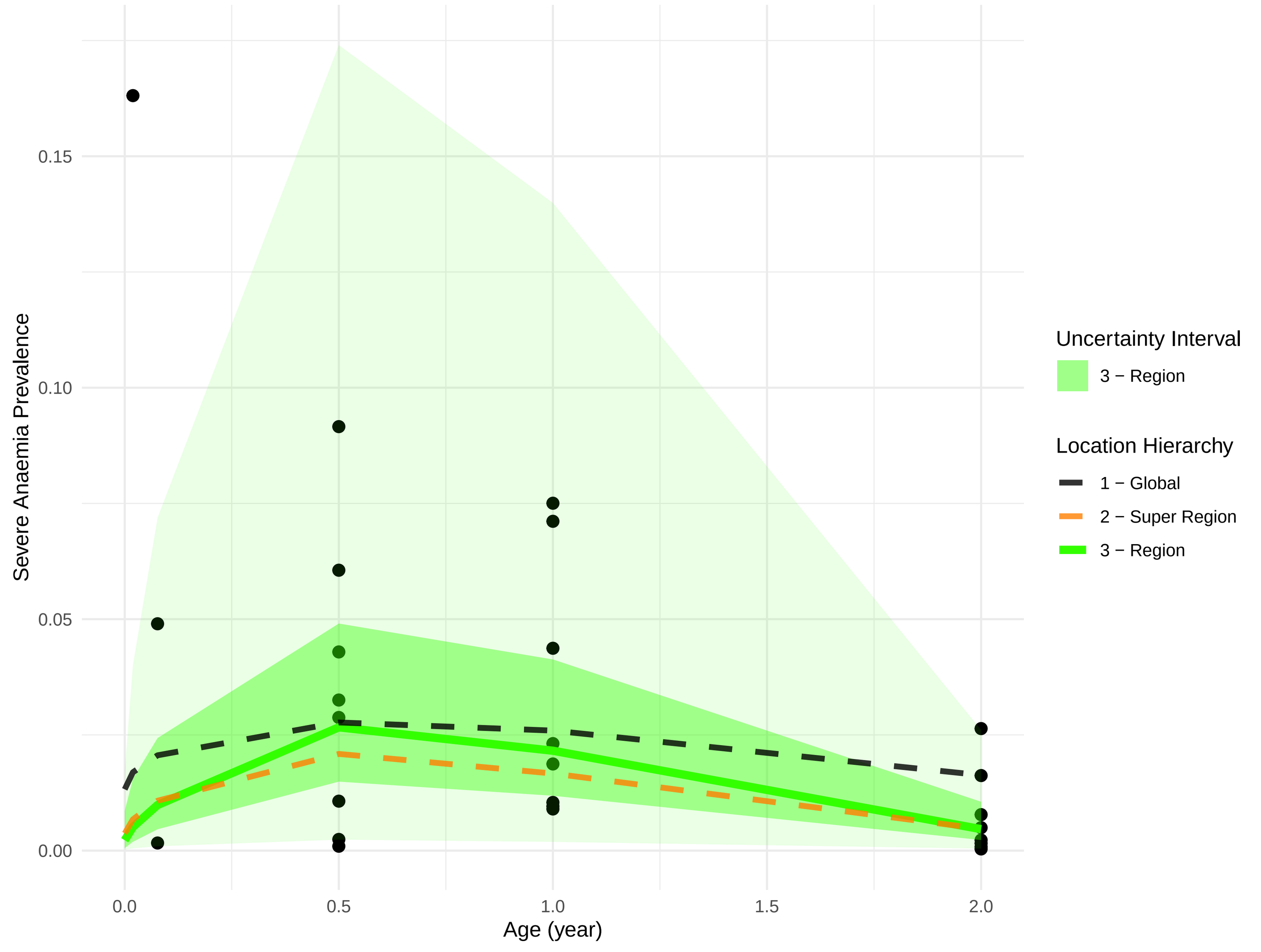

MR−BRT Age/Sex Fits − Non−pregnant Females <2 Years Old − Severe Anaemia Prevalence − Bolivia (Plurinational State of)
WHO−2001 Adjustment Method

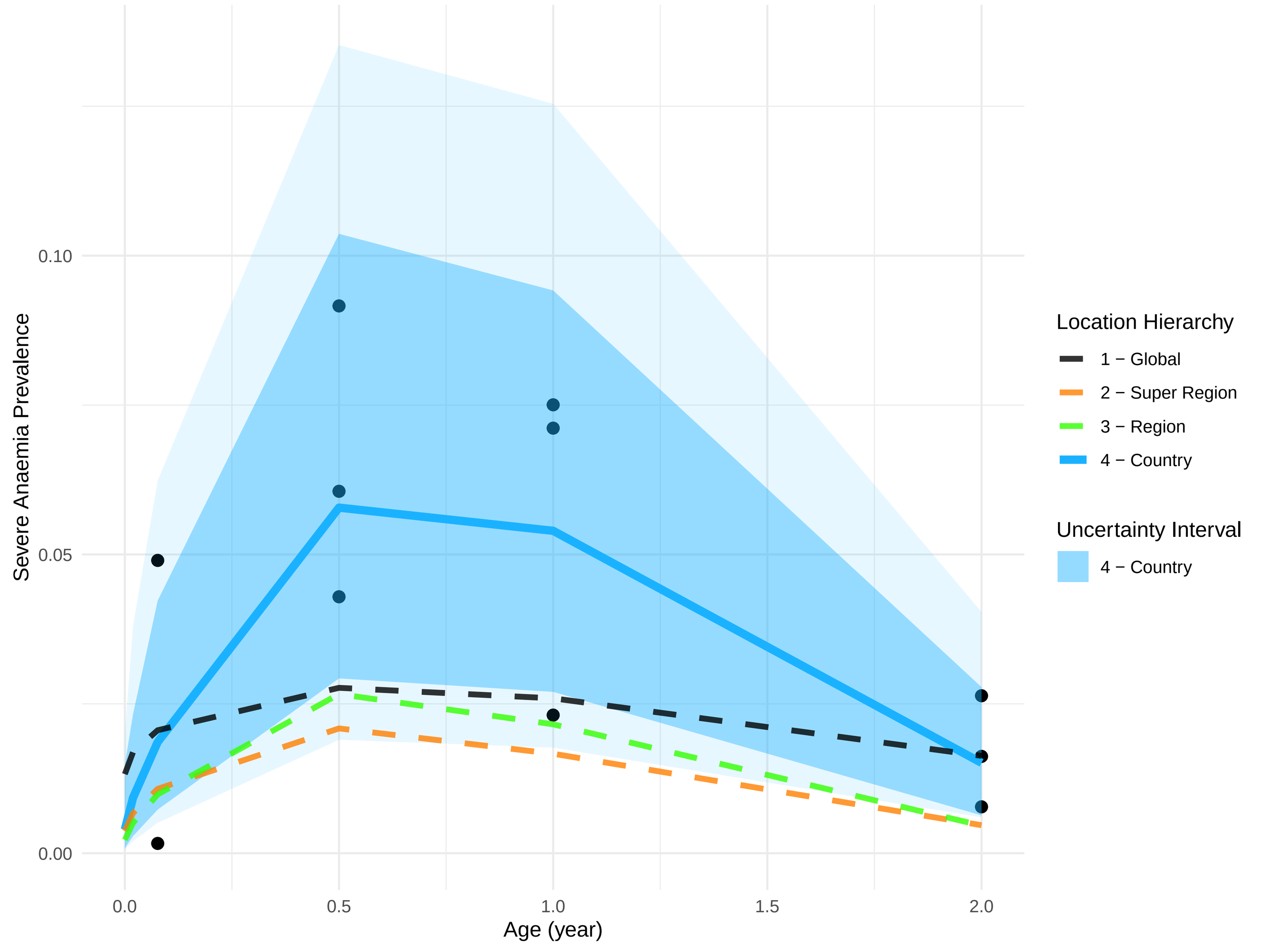

MR−BRT Age/Sex Fits − Non−pregnant Females <2 Years Old − Severe Anaemia Prevalence − Peru
WHO−2001 Adjustment Method

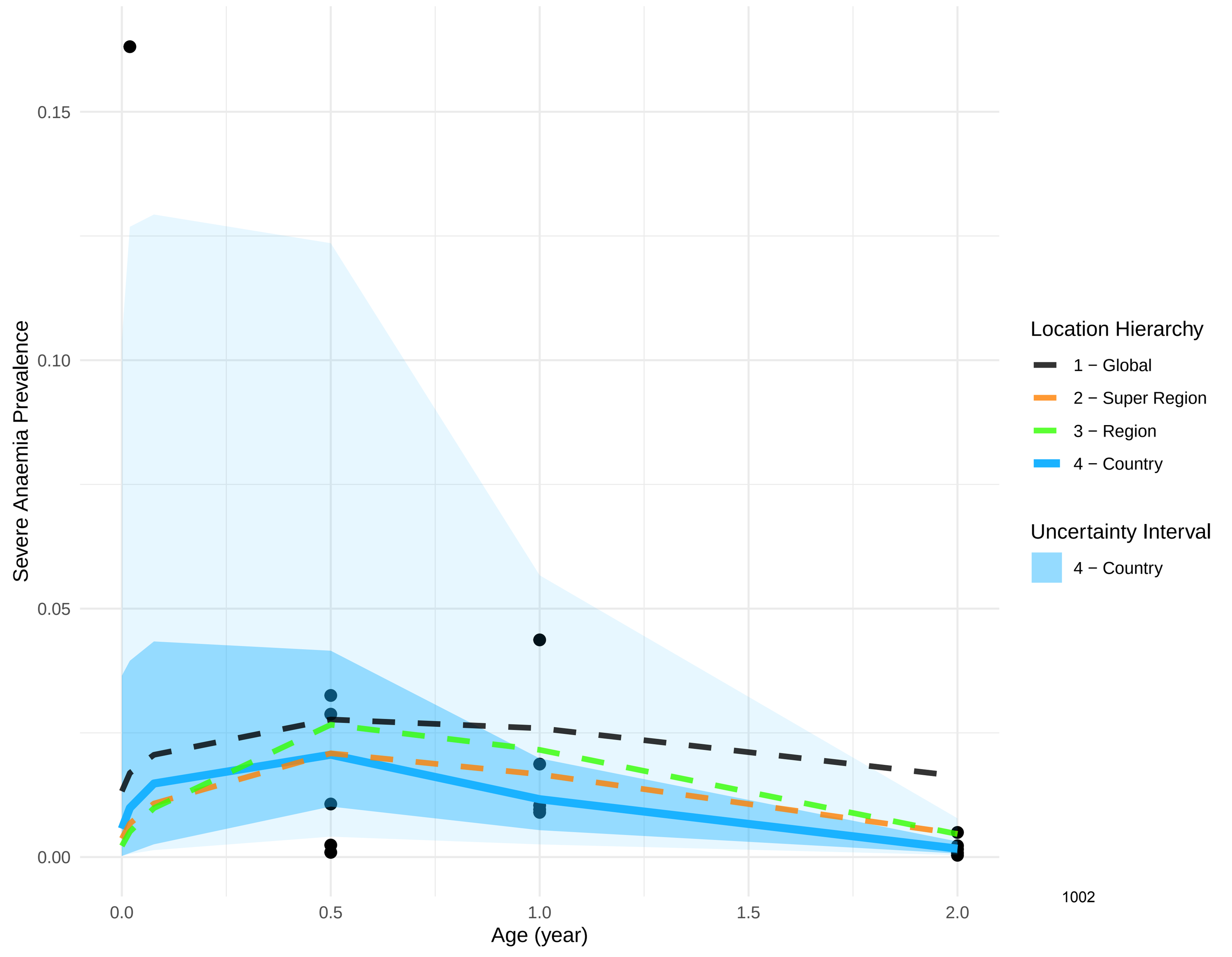

MR−BRT Age/Sex Fits − Non−pregnant Females <2 Years Old − Severe Anaemia Prevalence − Caribbean
WHO−2001 Adjustment Method

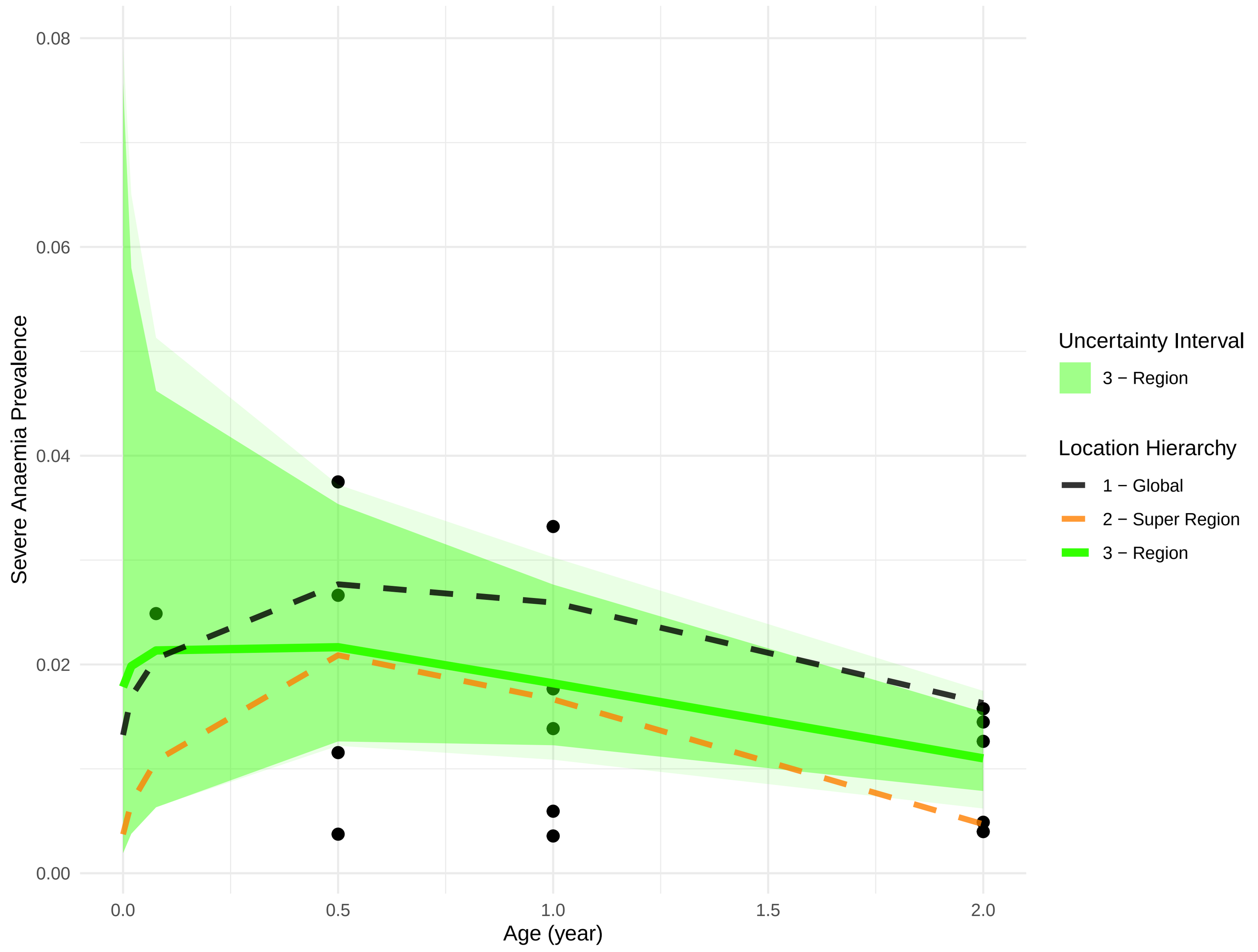

MR−BRT Age/Sex Fits − Non−pregnant Females <2 Years Old − Severe Anaemia Prevalence − Guyana
WHO−2001 Adjustment Method

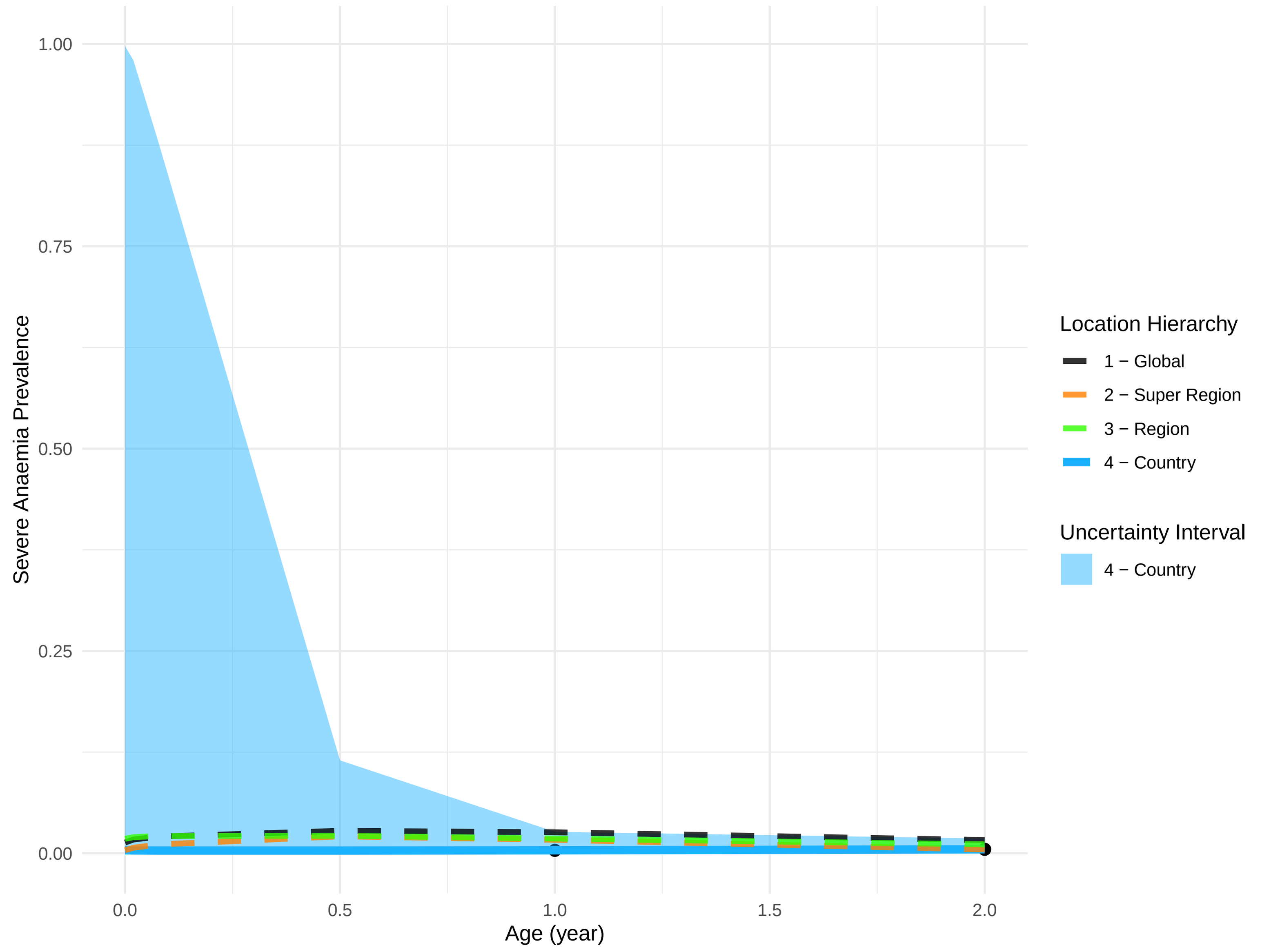

MR−BRT Age/Sex Fits − Non−pregnant Females <2 Years Old − Severe Anaemia Prevalence − Haiti
WHO−2001 Adjustment Method

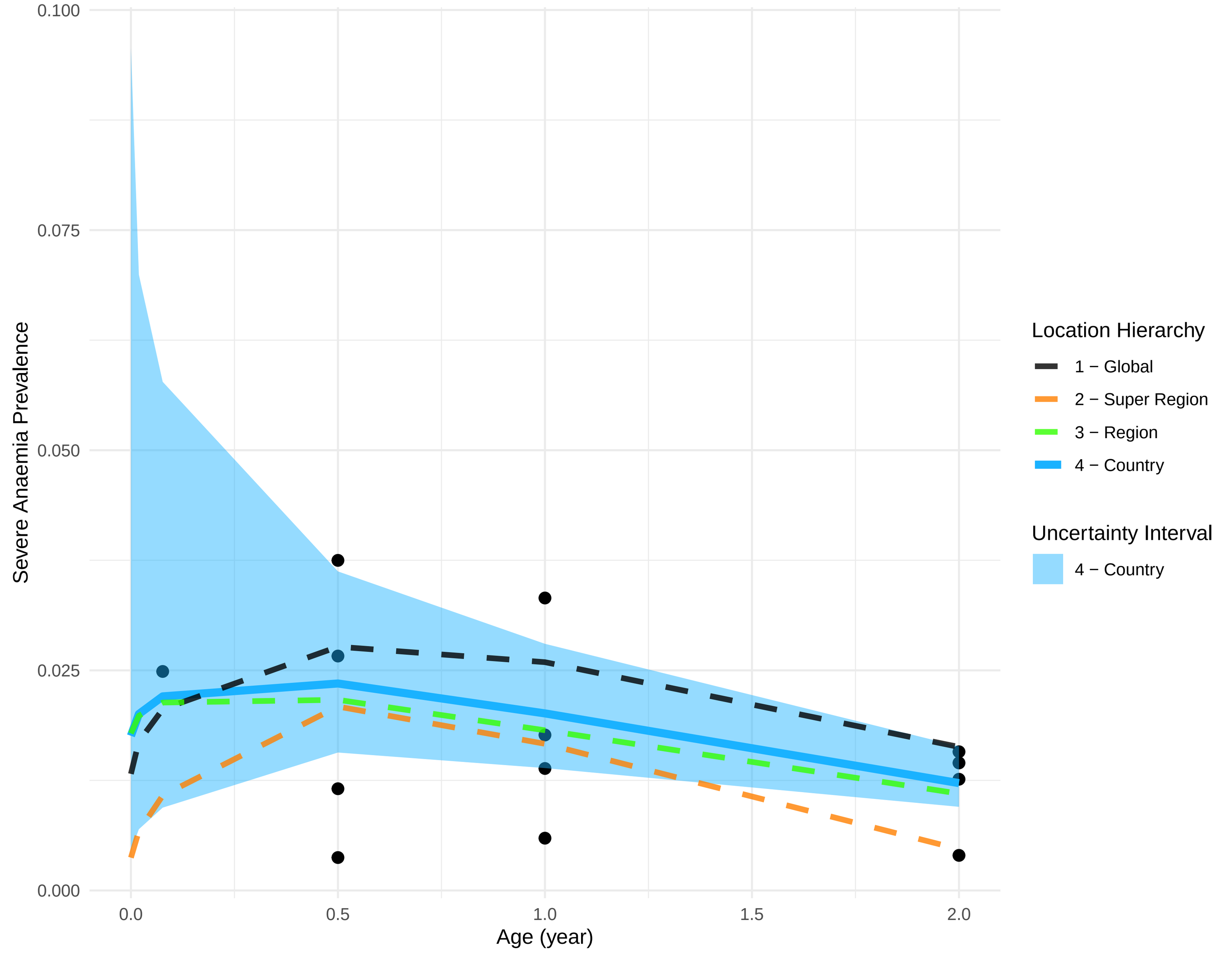

MR−BRT Age/Sex Fits − Non−pregnant Females <2 Years Old − Severe Anaemia Prevalence − Central Latin America
WHO−2001 Adjustment Method

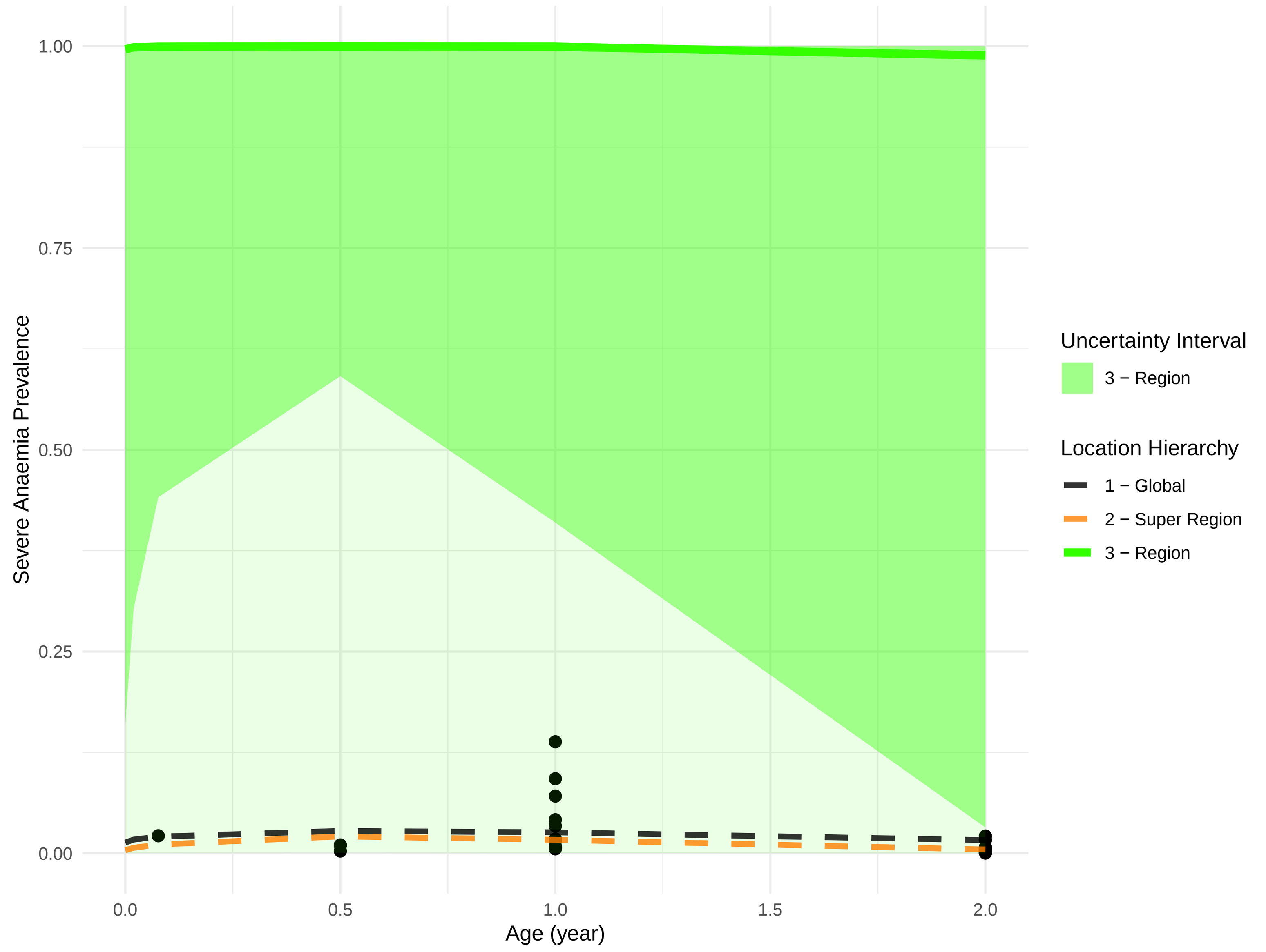

MR−BRT Age/Sex Fits − Non−pregnant Females <2 Years Old − Severe Anaemia Prevalence − Guatemala
WHO−2001 Adjustment Method

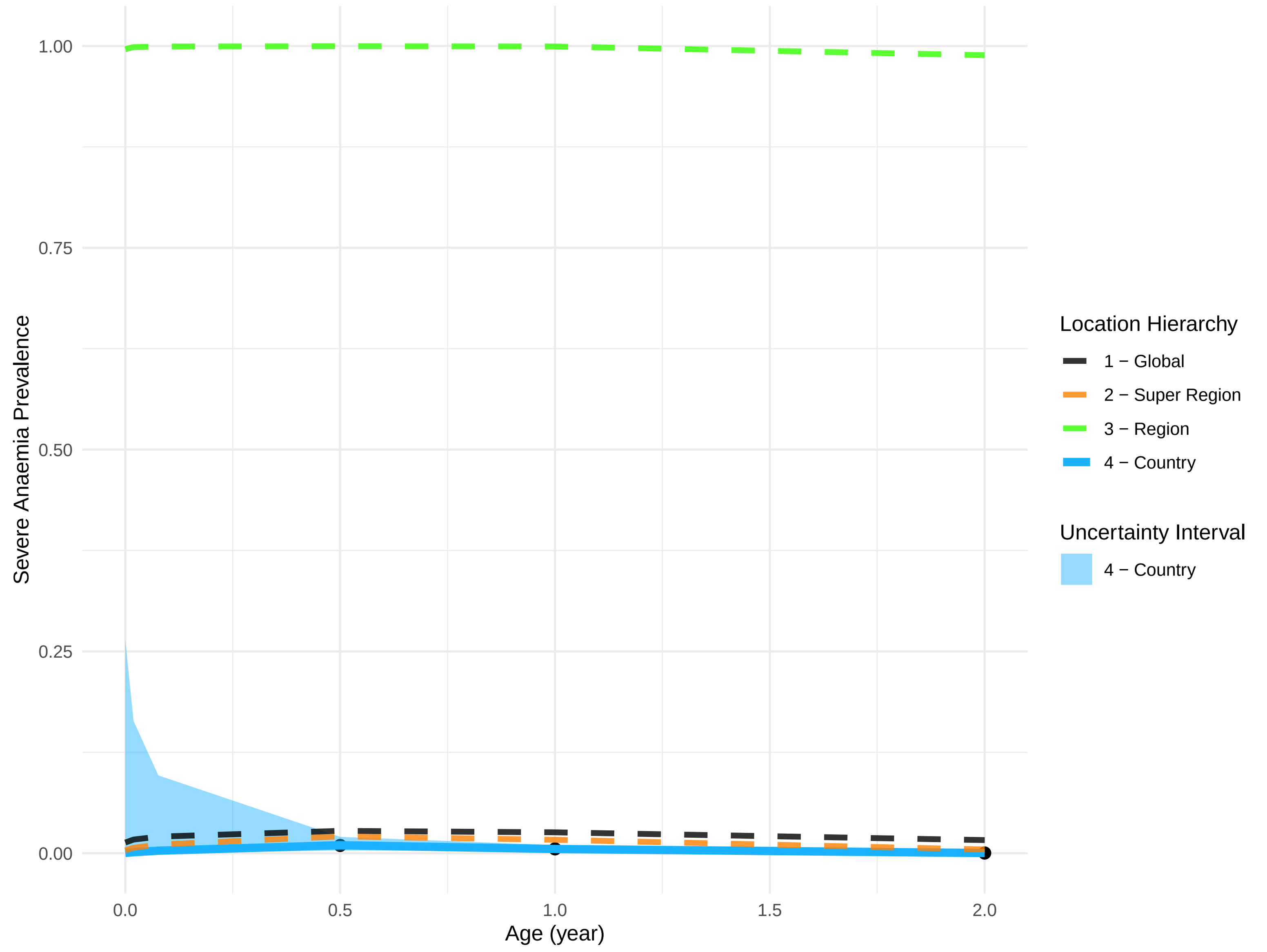

MR−BRT Age/Sex Fits − Non−pregnant Females <2 Years Old − Severe Anaemia Prevalence − Honduras
WHO−2001 Adjustment Method

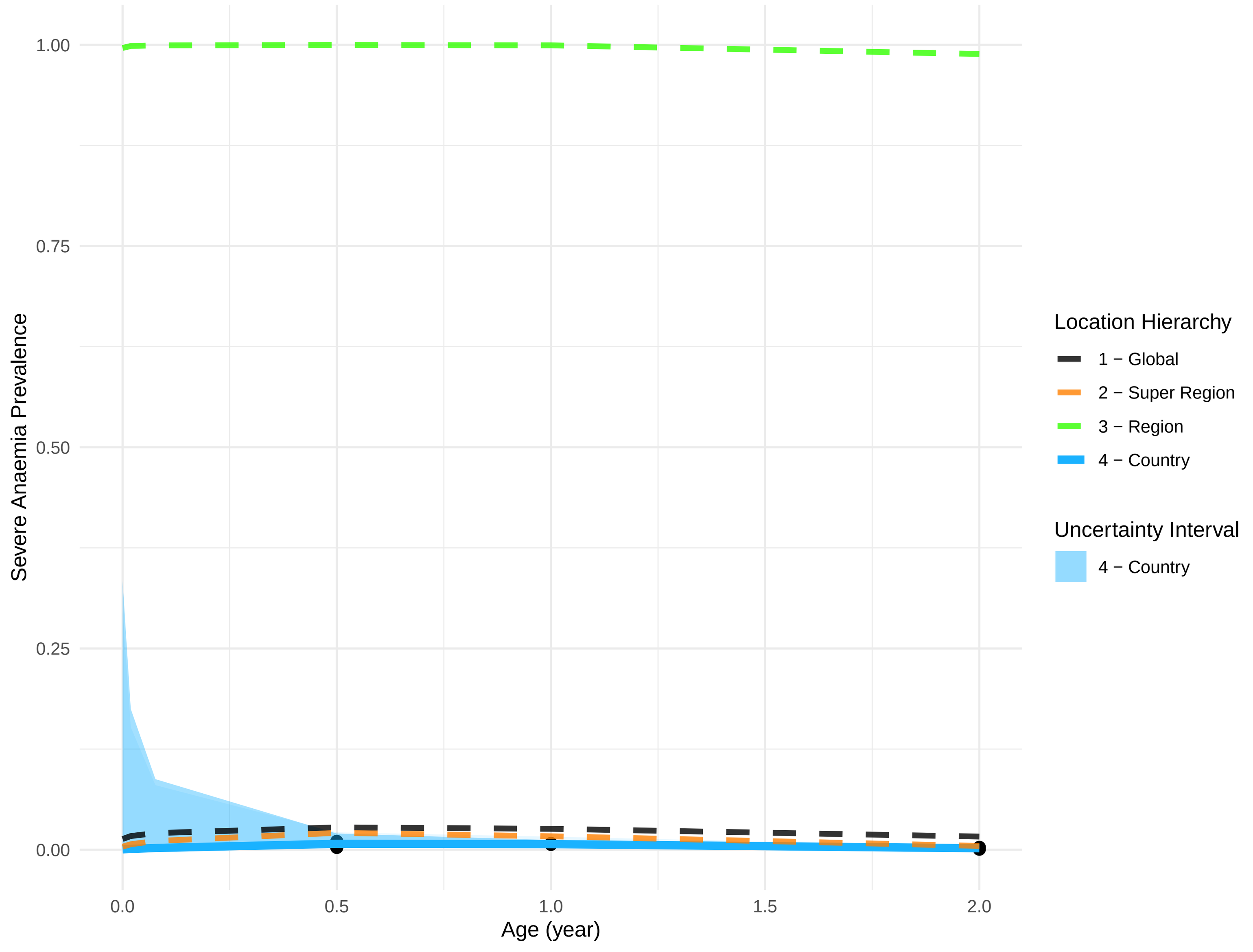

MR−BRT Age/Sex Fits − Non−pregnant Females <2 Years Old − Severe Anaemia Prevalence − Mexico
WHO−2001 Adjustment Method

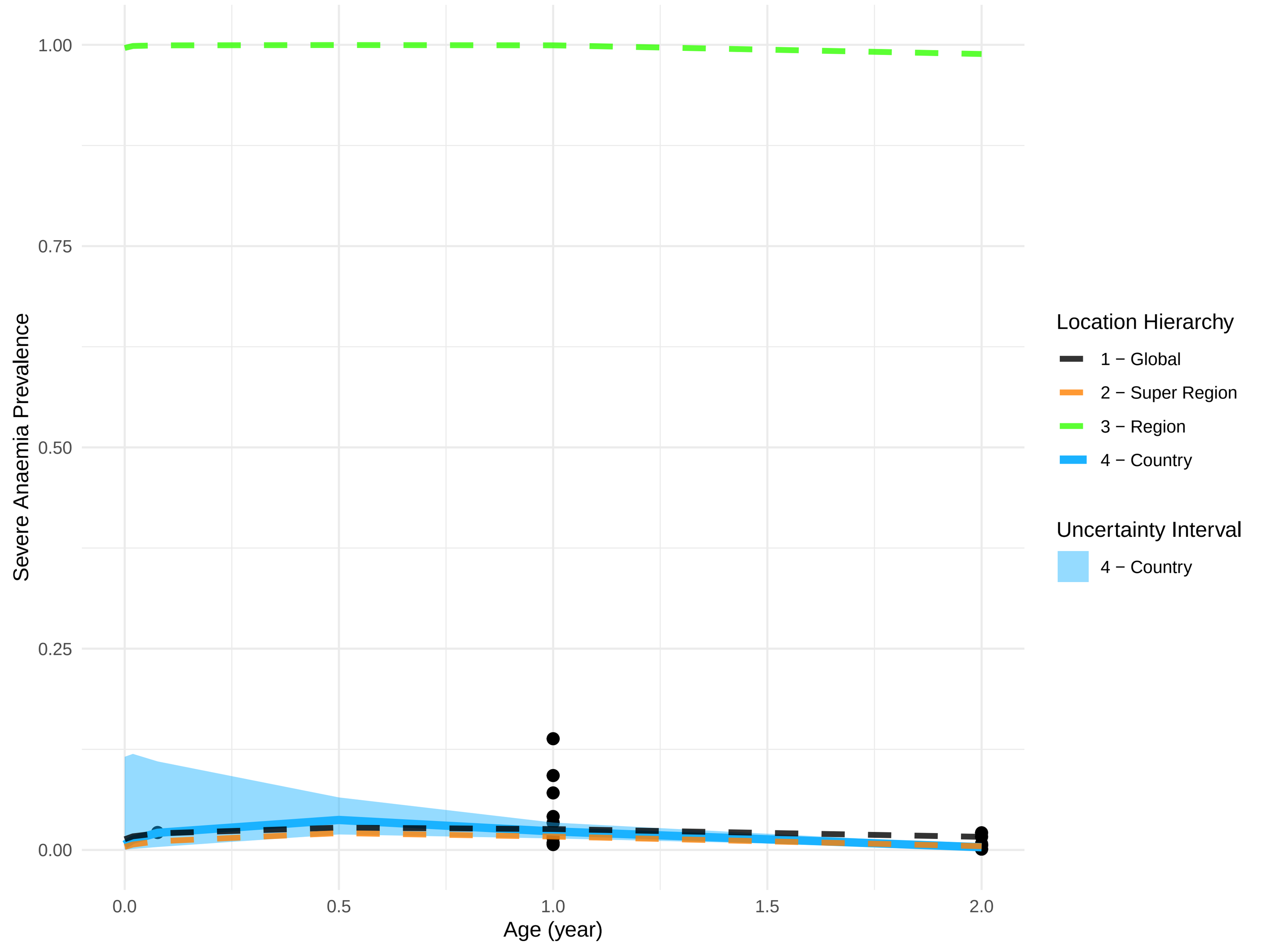

MR−BRT Age/Sex Fits − Non−pregnant Females <2 Years Old − Severe Anaemia Prevalence − Baja California
WHO−2001 Adjustment Method

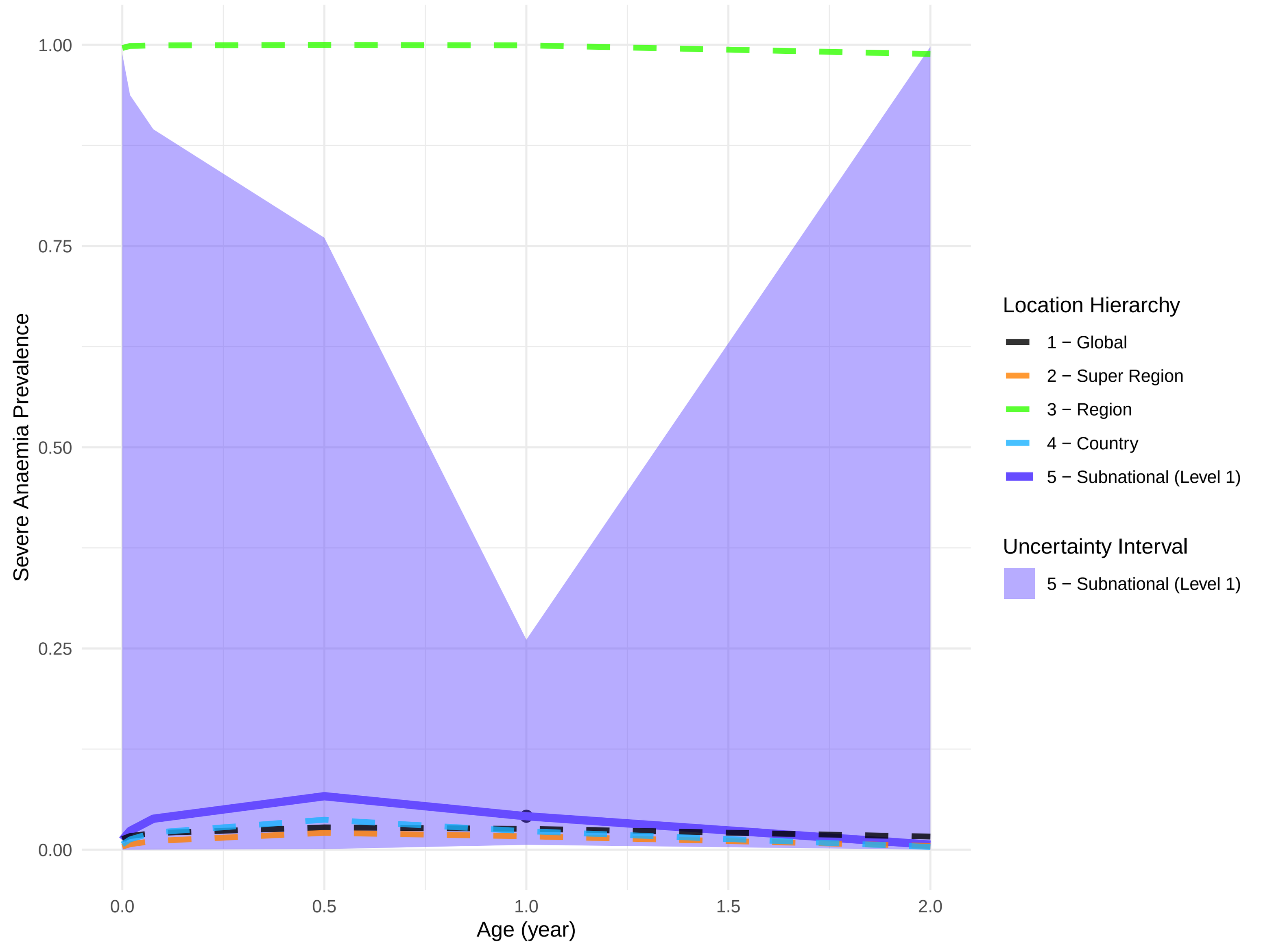

MR−BRT Age/Sex Fits − Non−pregnant Females <2 Years Old − Severe Anaemia Prevalence − Baja California Sur
WHO−2001 Adjustment Method

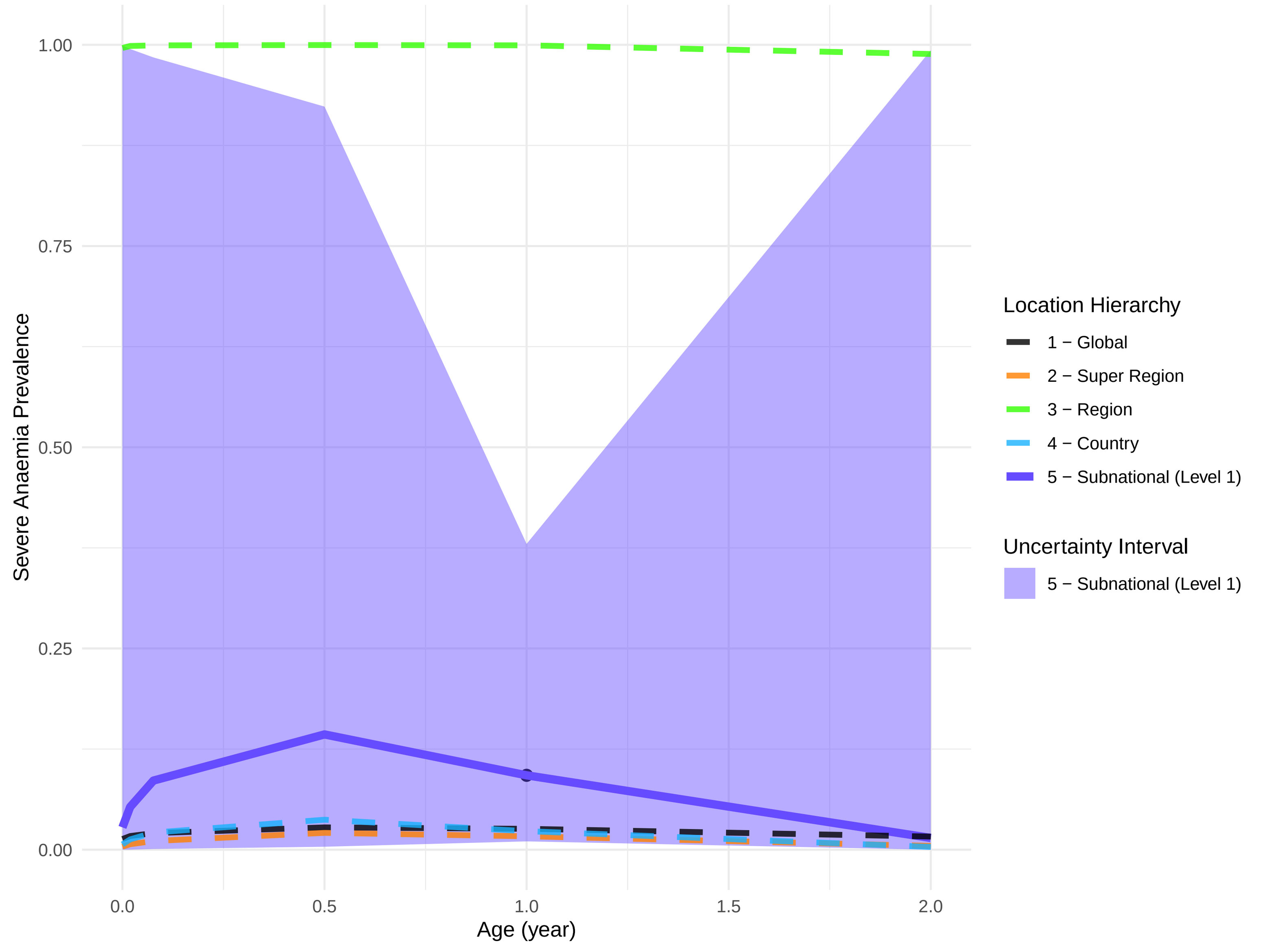

MR−BRT Age/Sex Fits − Non−pregnant Females <2 Years Old − Severe Anaemia Prevalence − Guanajuato
WHO−2001 Adjustment Method

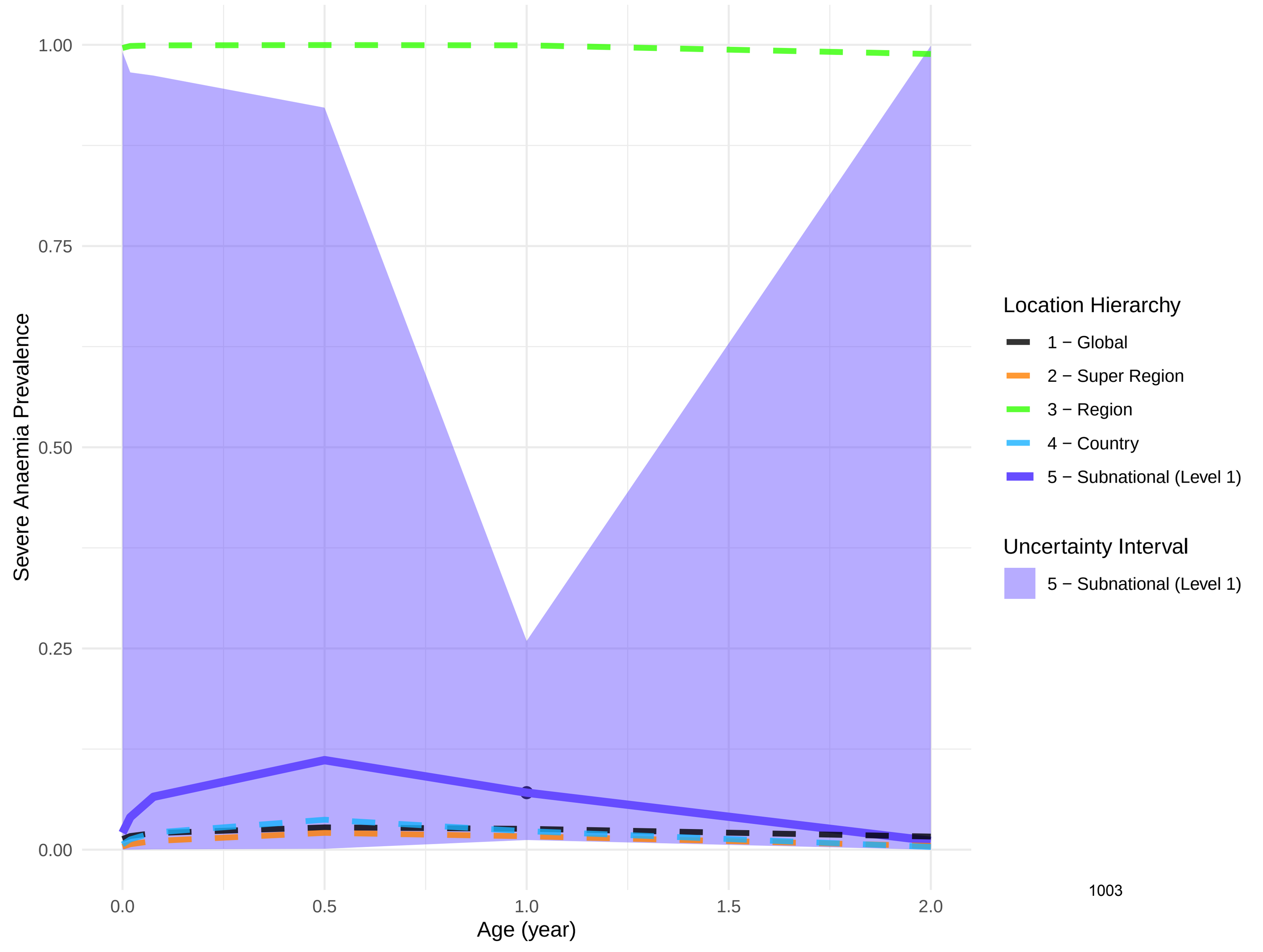

MR−BRT Age/Sex Fits − Non−pregnant Females <2 Years Old − Severe Anaemia Prevalence − Guerrero
WHO−2001 Adjustment Method

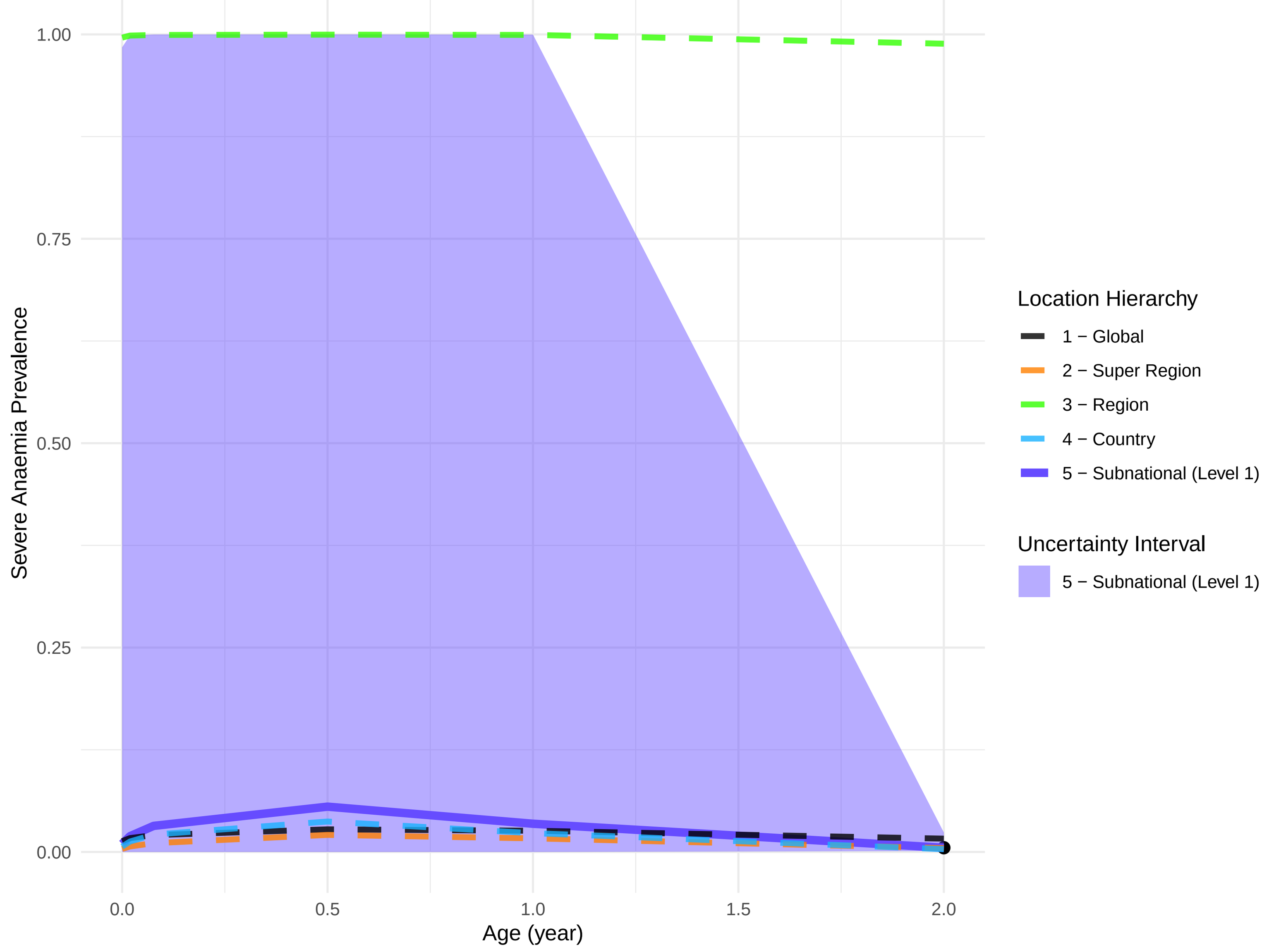

MR−BRT Age/Sex Fits − Non−pregnant Females <2 Years Old − Severe Anaemia Prevalence − Jalisco
WHO−2001 Adjustment Method

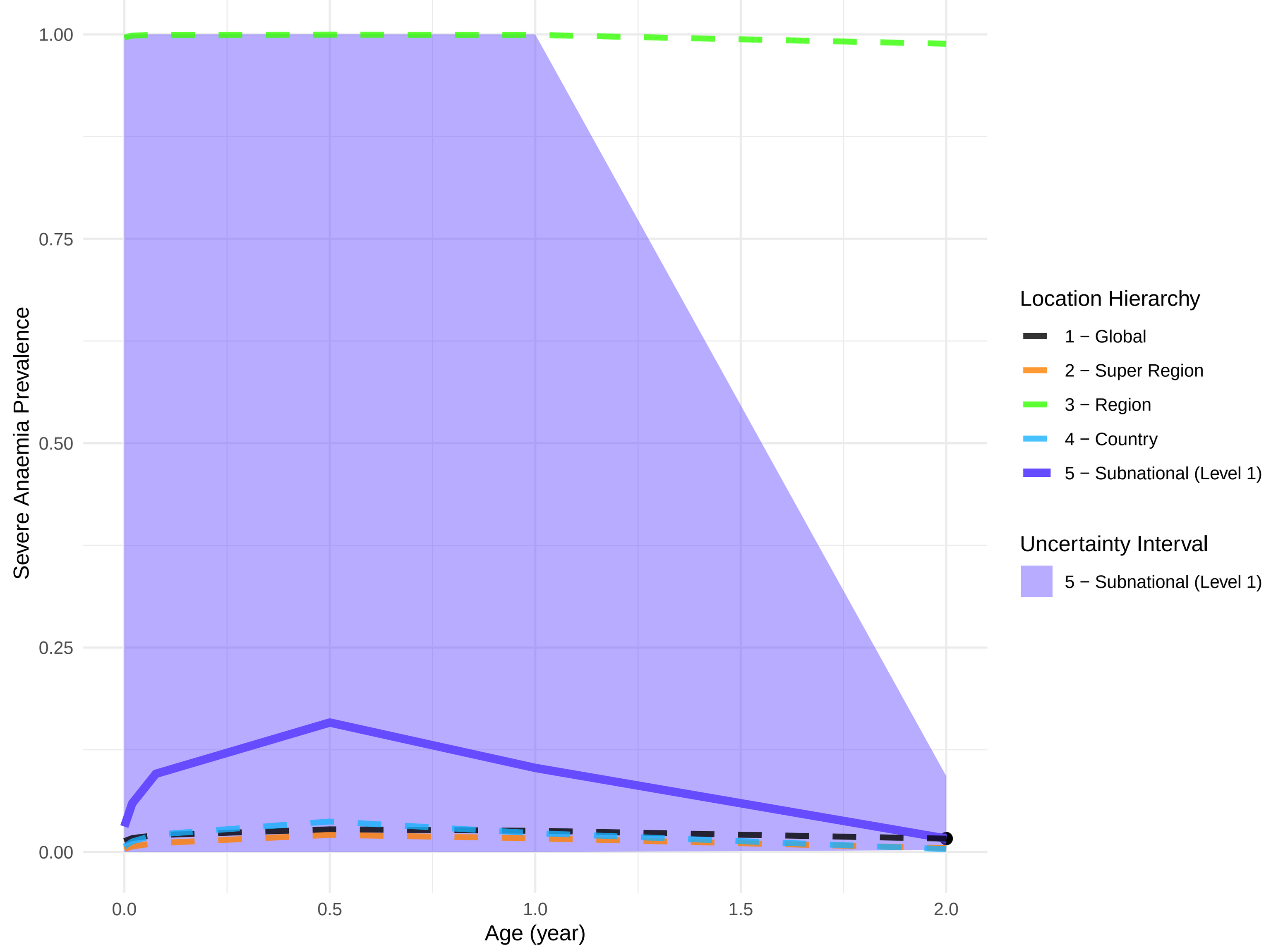

MR−BRT Age/Sex Fits − Non−pregnant Females <2 Years Old − Severe Anaemia Prevalence − México
WHO−2001 Adjustment Method

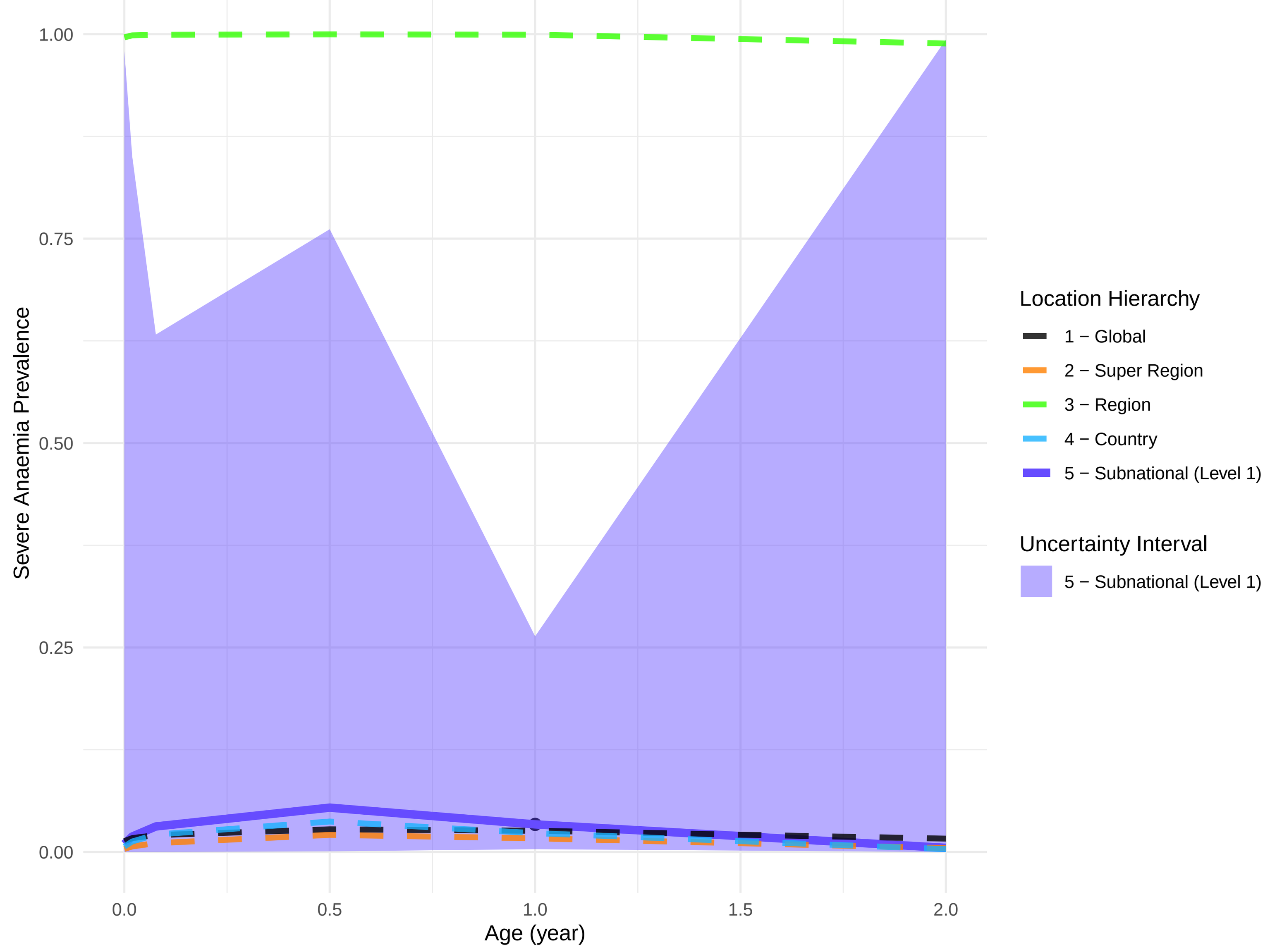

MR−BRT Age/Sex Fits − Non−pregnant Females <2 Years Old − Severe Anaemia Prevalence − Michoacán de Ocampo
WHO−2001 Adjustment Method

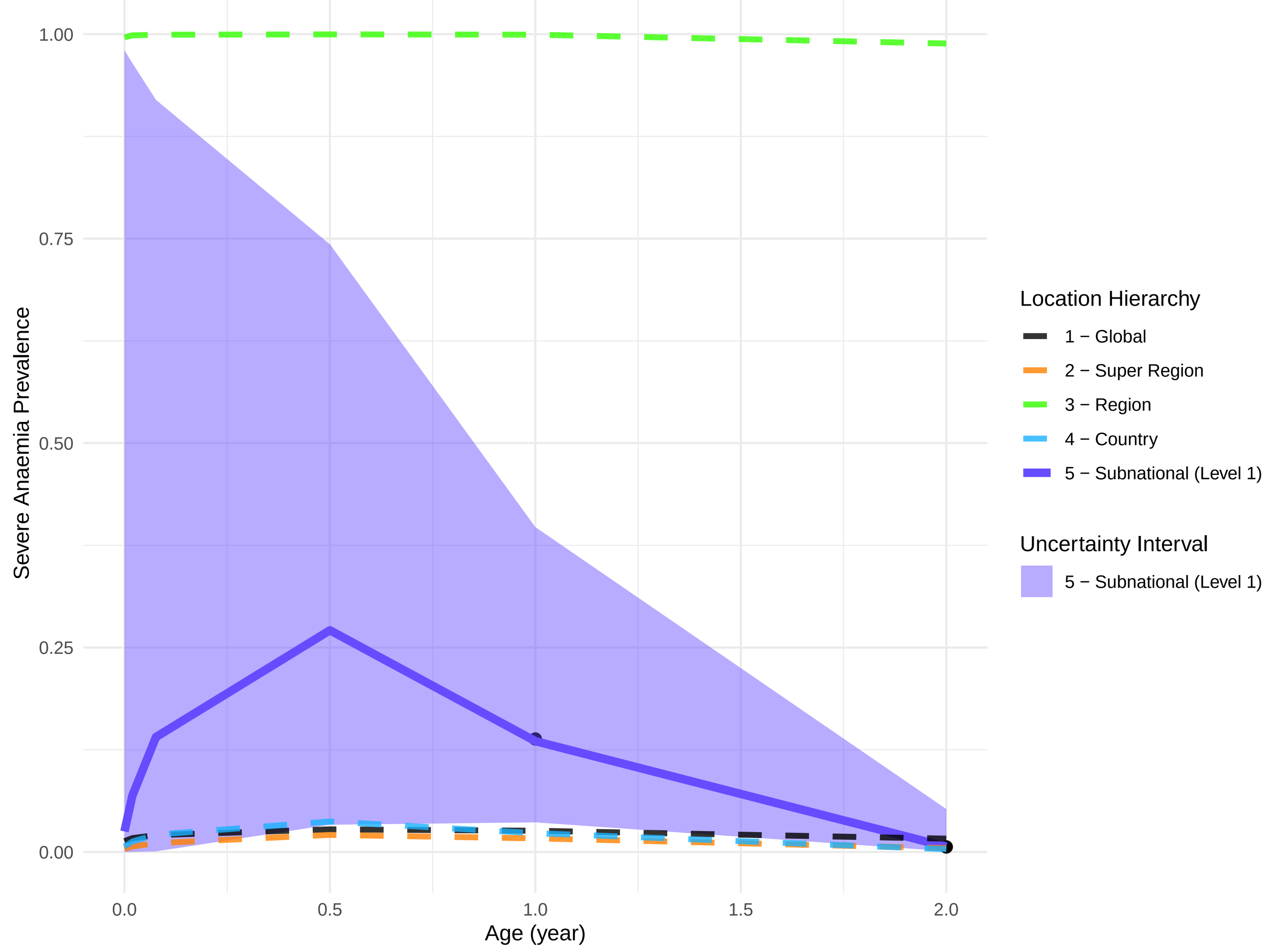

MR−BRT Age/Sex Fits − Non−pregnant Females <2 Years Old − Severe Anaemia Prevalence − San Luis Potosí
WHO−2001 Adjustment Method

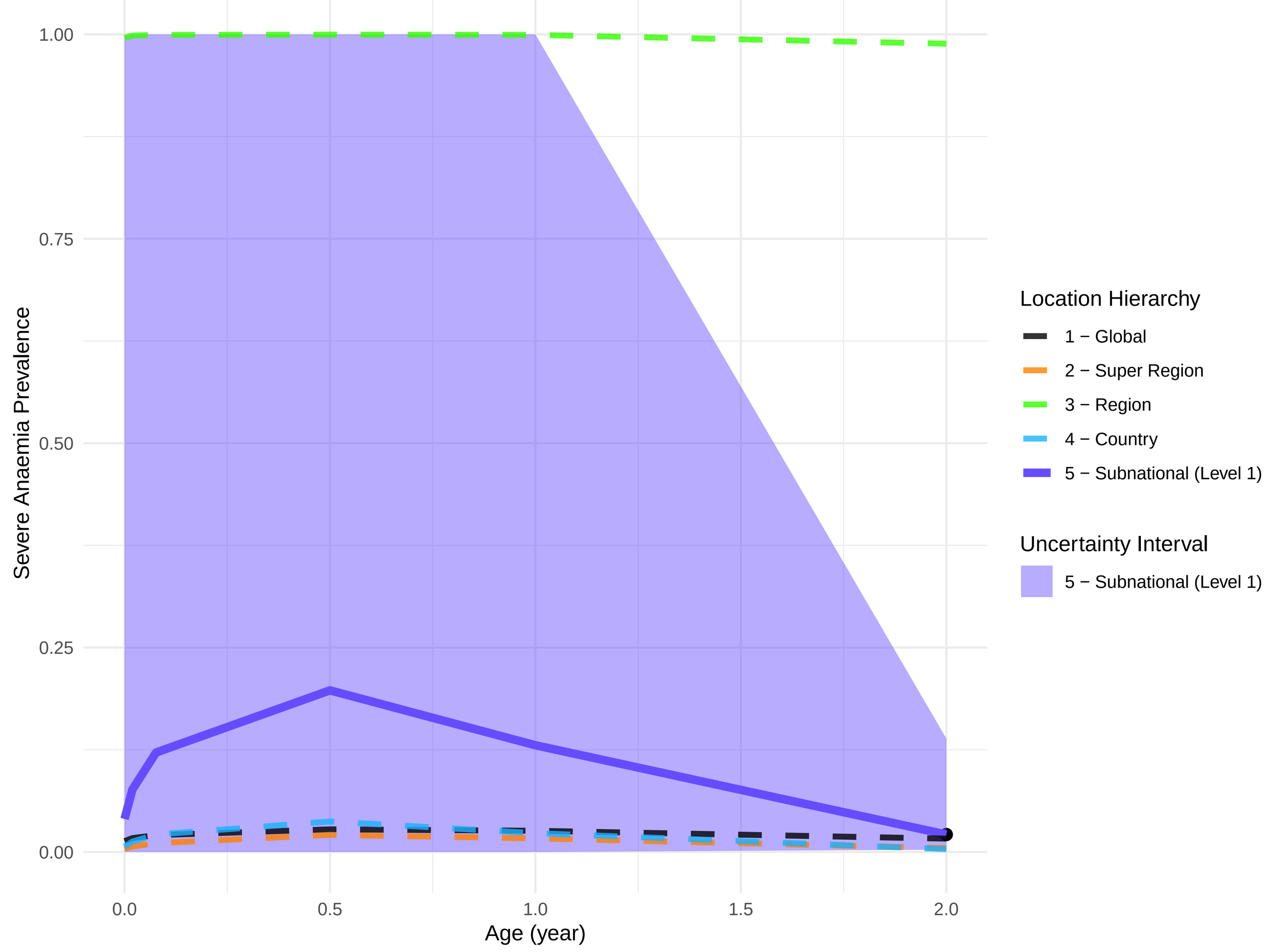

MR−BRT Age/Sex Fits − Non−pregnant Females <2 Years Old − Severe Anaemia Prevalence − Sinaloa
WHO−2001 Adjustment Method

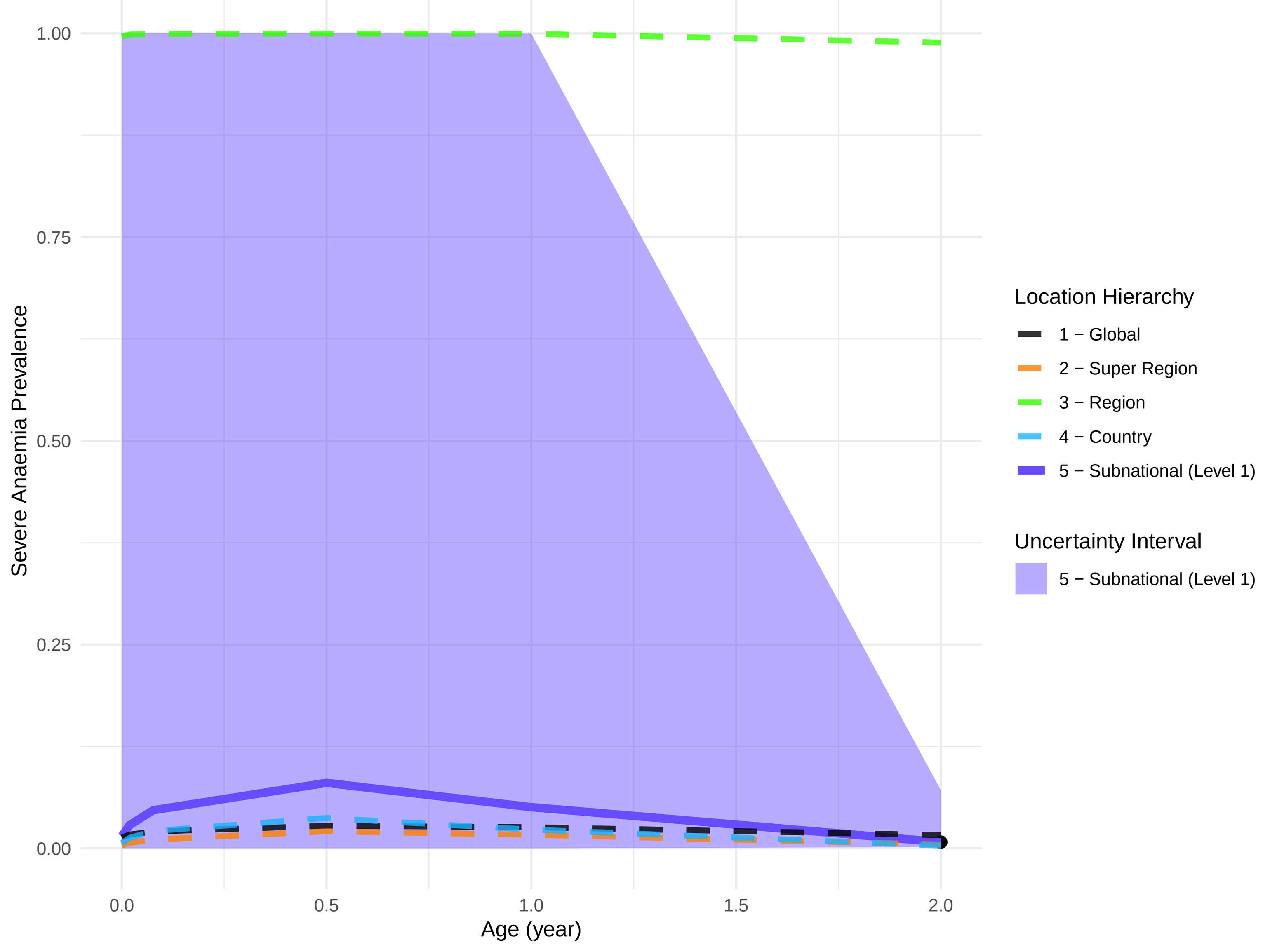

MR−BRT Age/Sex Fits − Non−pregnant Females <2 Years Old − Severe Anaemia Prevalence − Sonora
WHO−2001 Adjustment Method

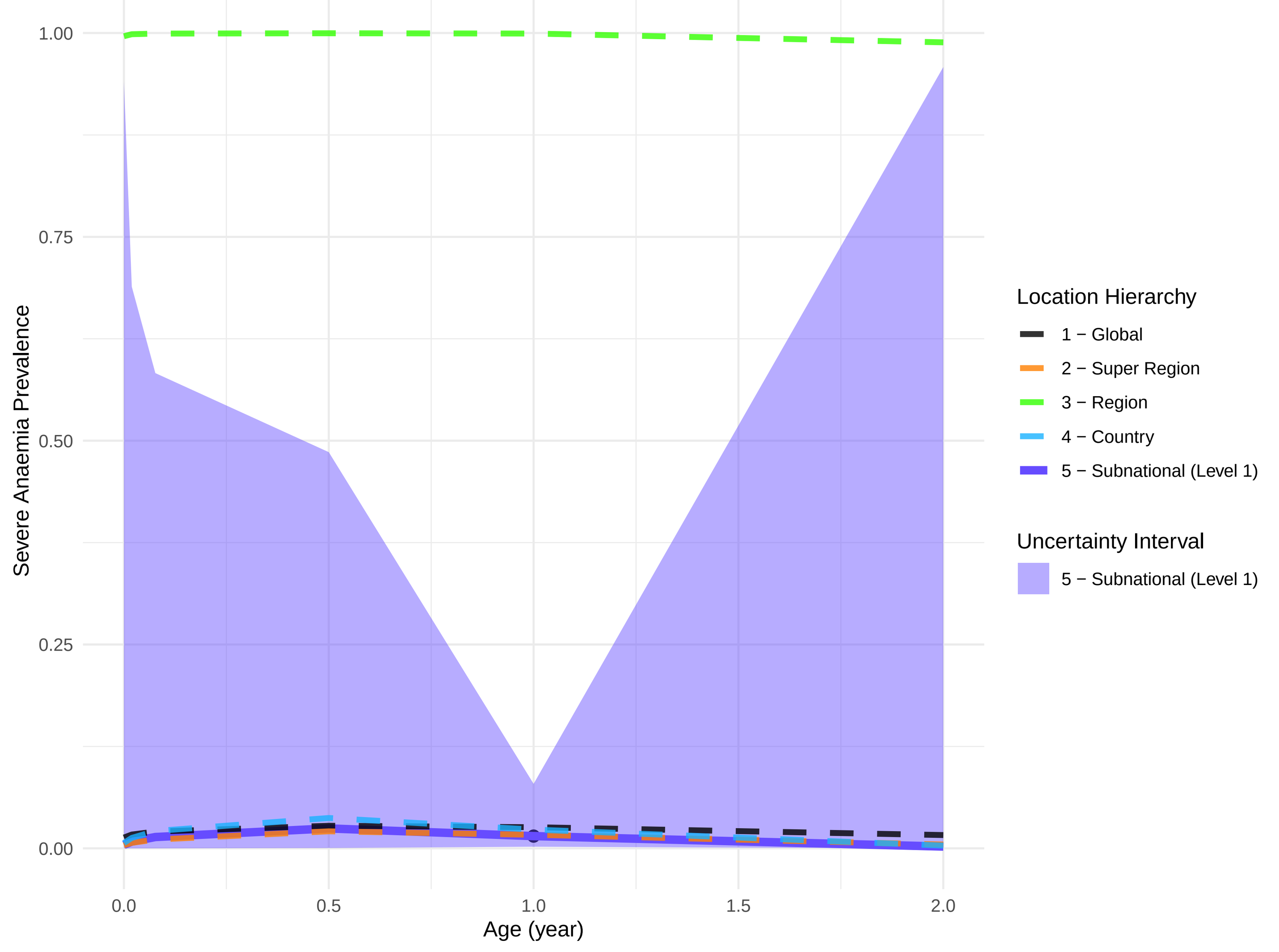

MR−BRT Age/Sex Fits − Non−pregnant Females <2 Years Old − Severe Anaemia Prevalence − Veracruz de Ignacio de la Llave
WHO−2001 Adjustment Method

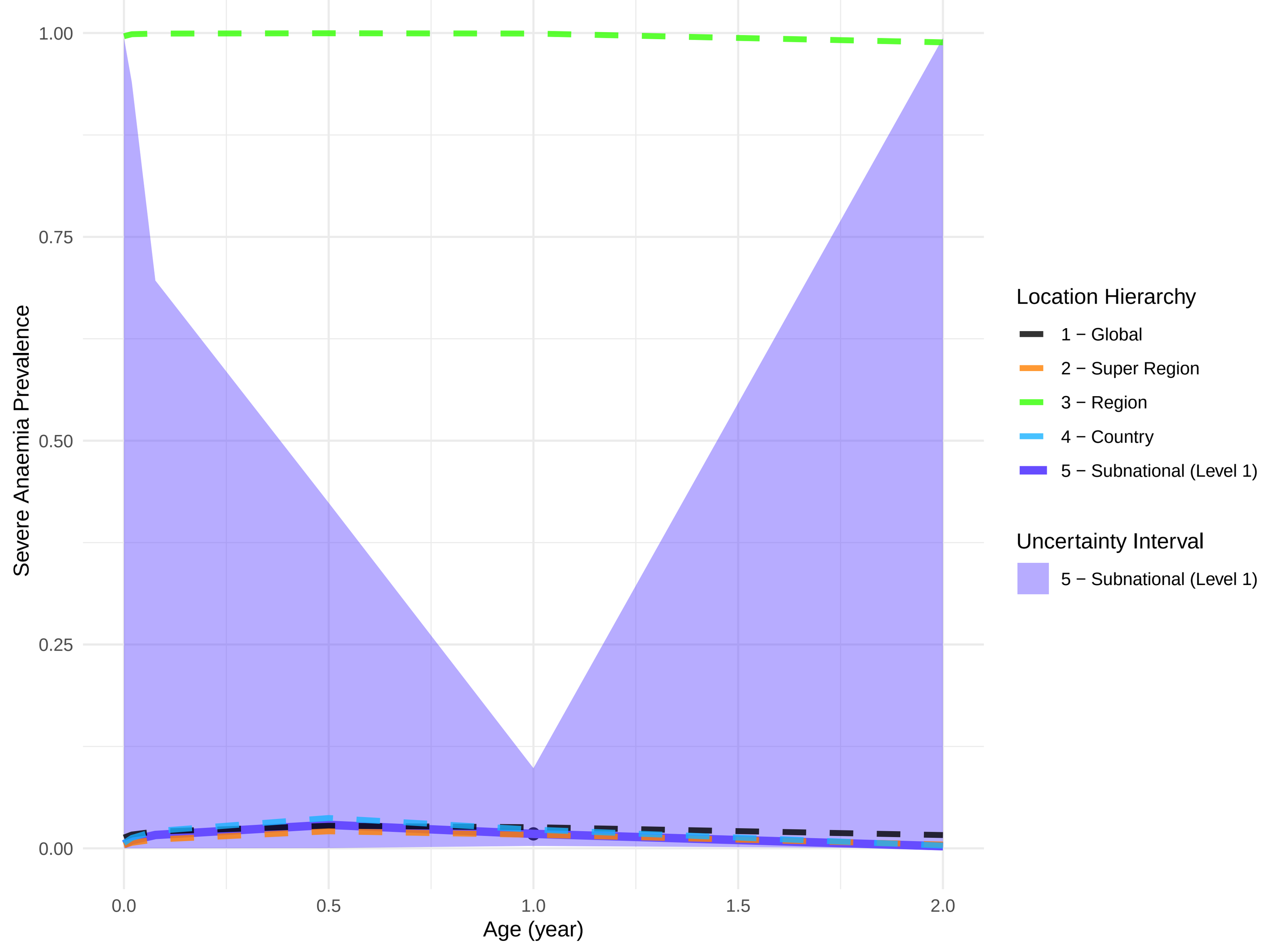

MR−BRT Age/Sex Fits − Non−pregnant Females <2 Years Old − Severe Anaemia Prevalence − North Africa and Middle East
WHO−2001 Adjustment Method

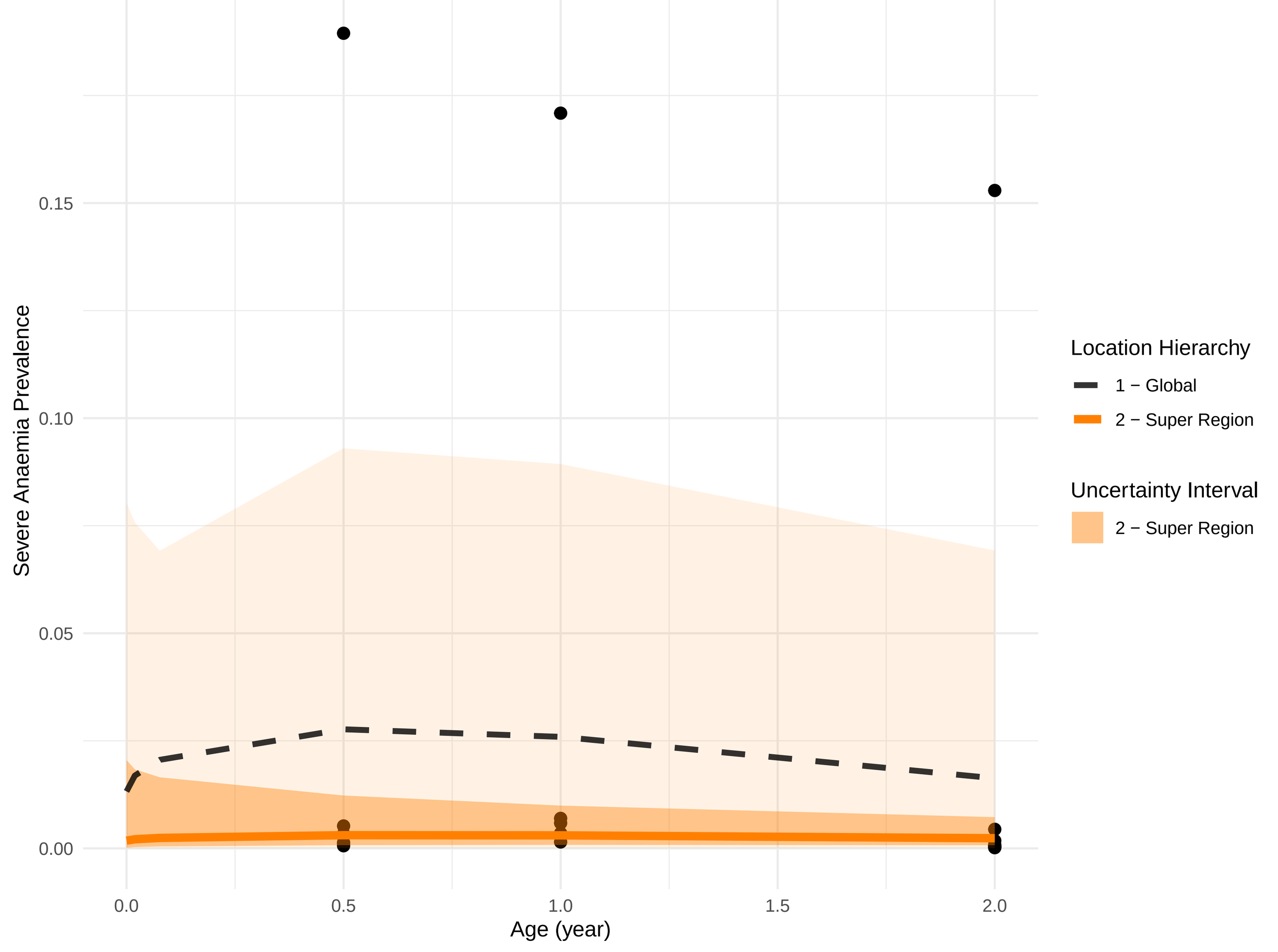

MR−BRT Age/Sex Fits − Non−pregnant Females <2 Years Old − Severe Anaemia Prevalence − North Africa and Middle East
WHO−2001 Adjustment Method

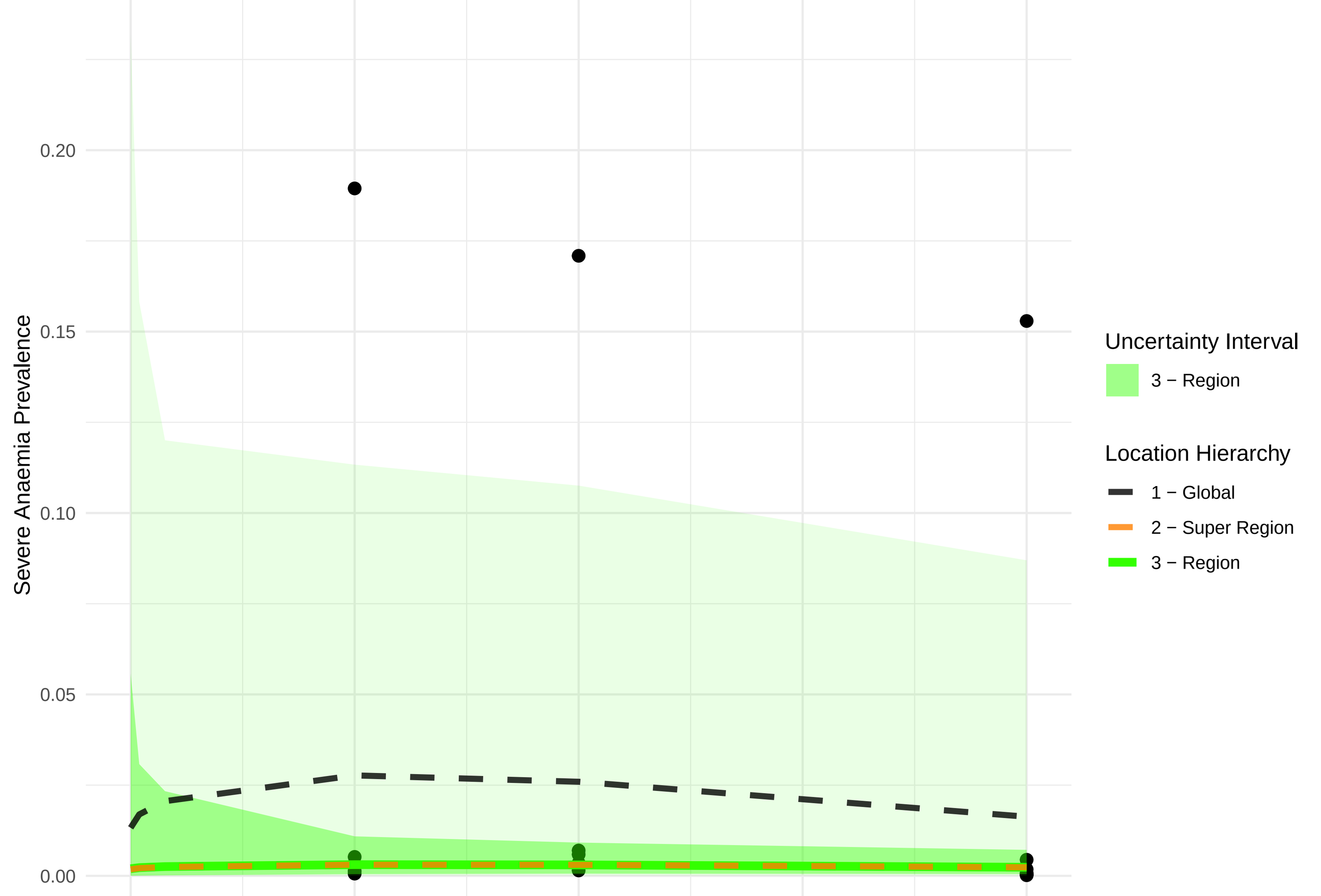

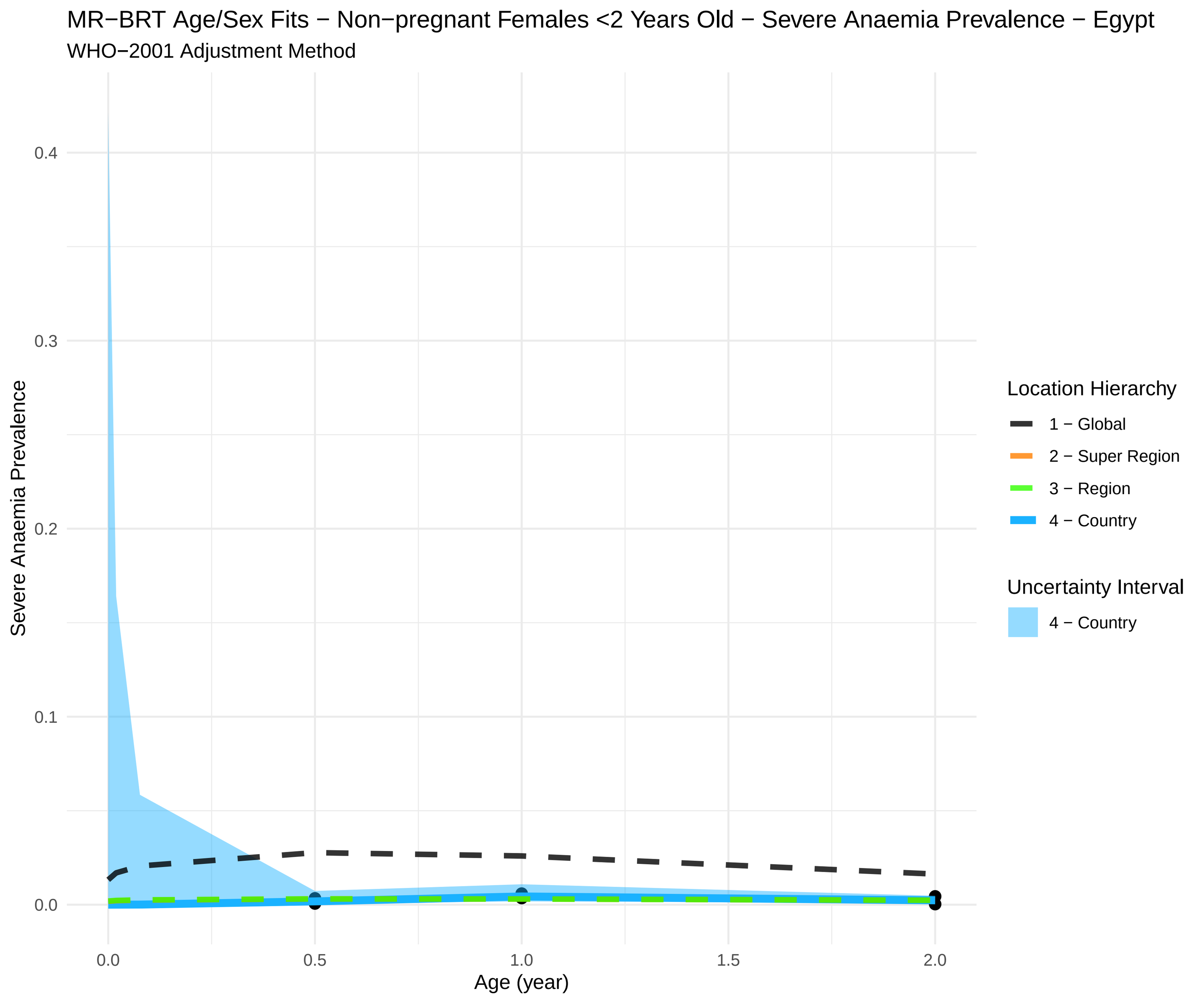
MR−BRT Age/Sex Fits − Non−pregnant Females <2 Years Old − Severe Anaemia Prevalence − Egypt
WHO−2001 Adjustment Method
Severe Anaemia Prevalence
Age (year)
Location Hierarchy
1 − Global
2 − Super Region
3 − Region
4 − Country
Uncertainty Interval
4 − Country

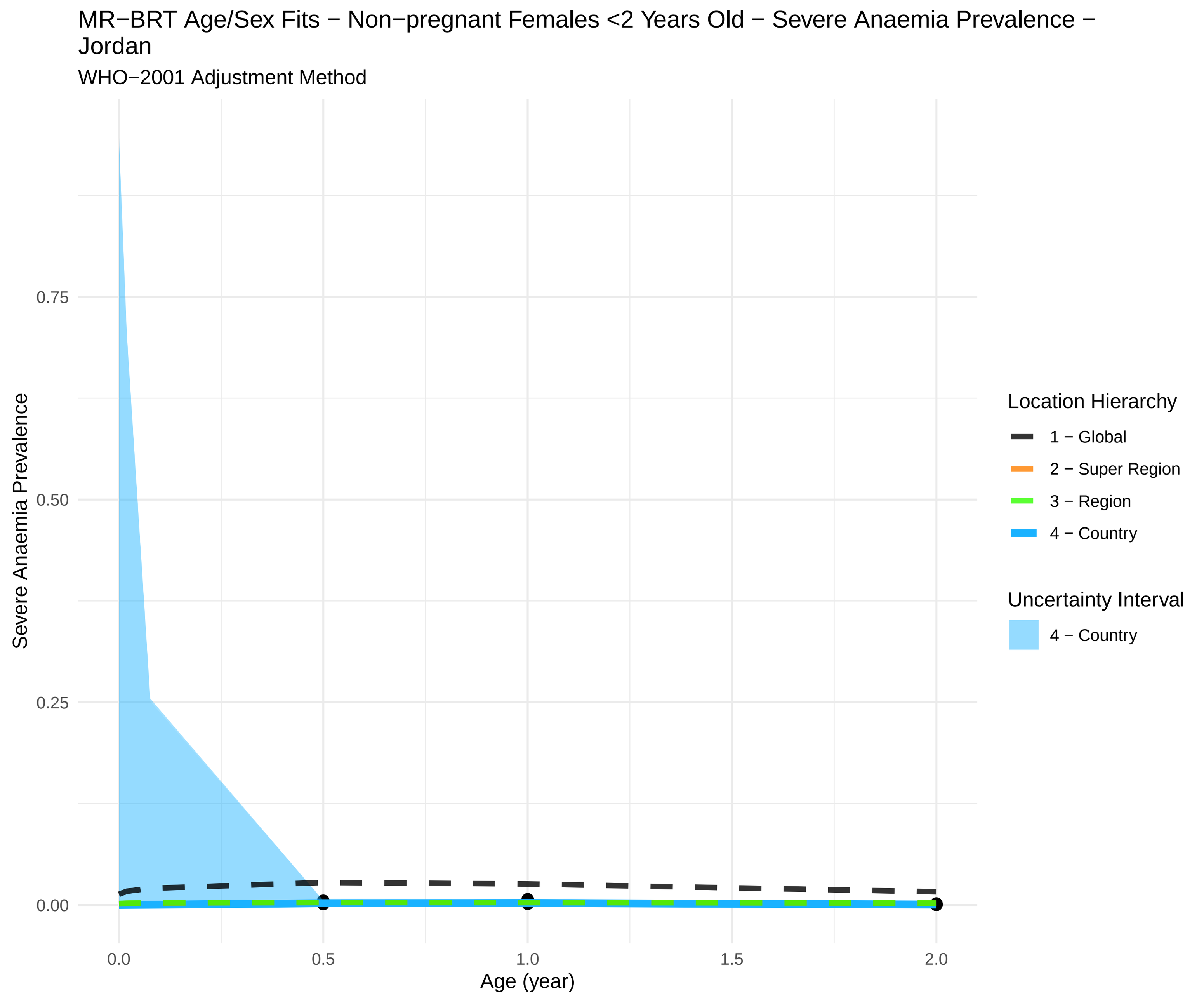
MR−BRT Age/Sex Fits − Non−pregnant Females <2 Years Old − Severe Anaemia Prevalence − Jordan
WHO−2001 Adjustment Method
Severe Anaemia Prevalence
Age (year)
Location Hierarchy
1 − Global
2 − Super Region
3 − Region
4 − Country
Uncertainty Interval
4 − Country

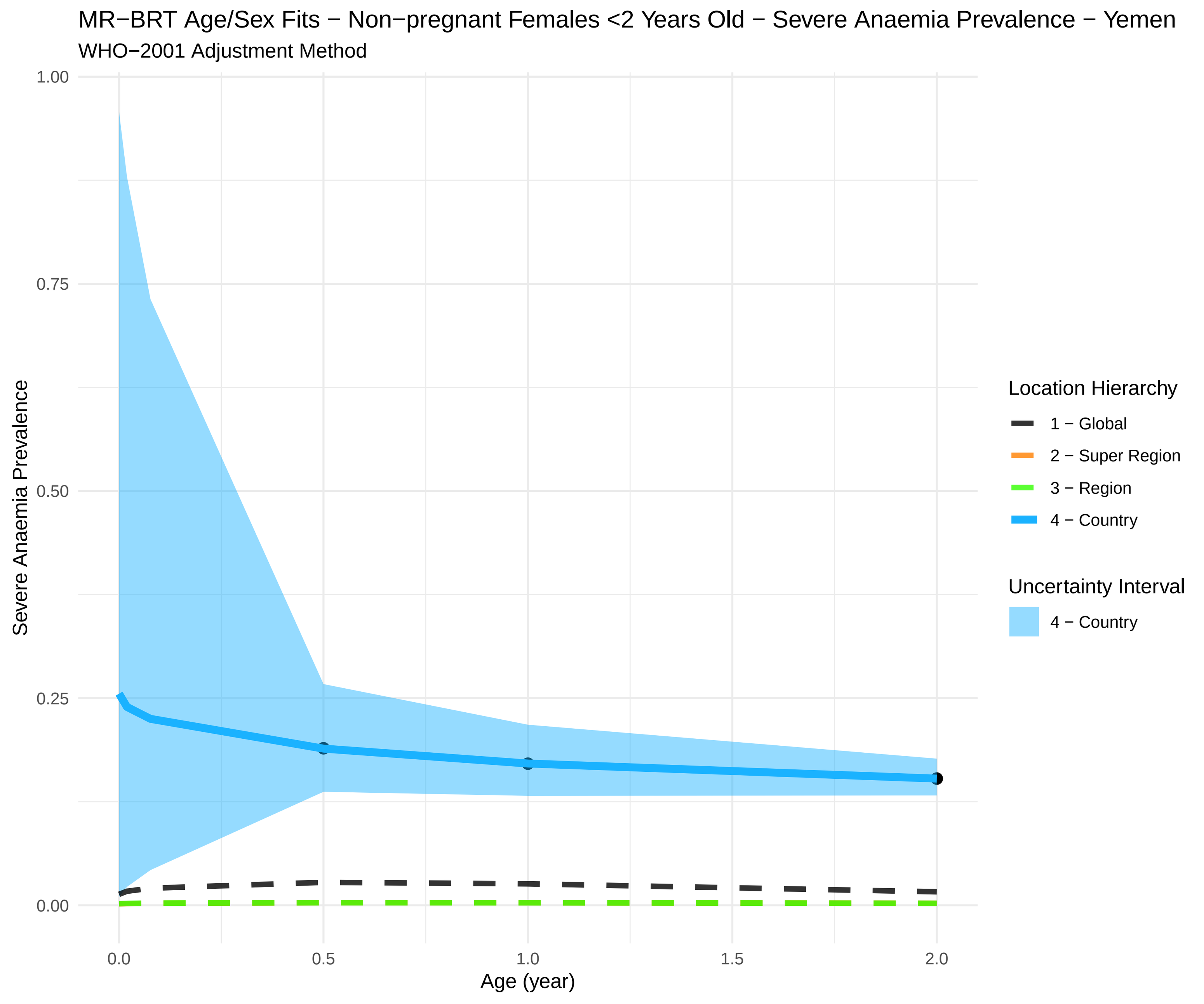
MR−BRT Age/Sex Fits − Non−pregnant Females <2 Years Old − Severe Anaemia Prevalence − Yemen
WHO−2001 Adjustment Method
Severe Anaemia Prevalence
Age (year)
Location Hierarchy
1 − Global
2 − Super Region
3 − Region
4 − Country
Uncertainty Interval
4 − Country

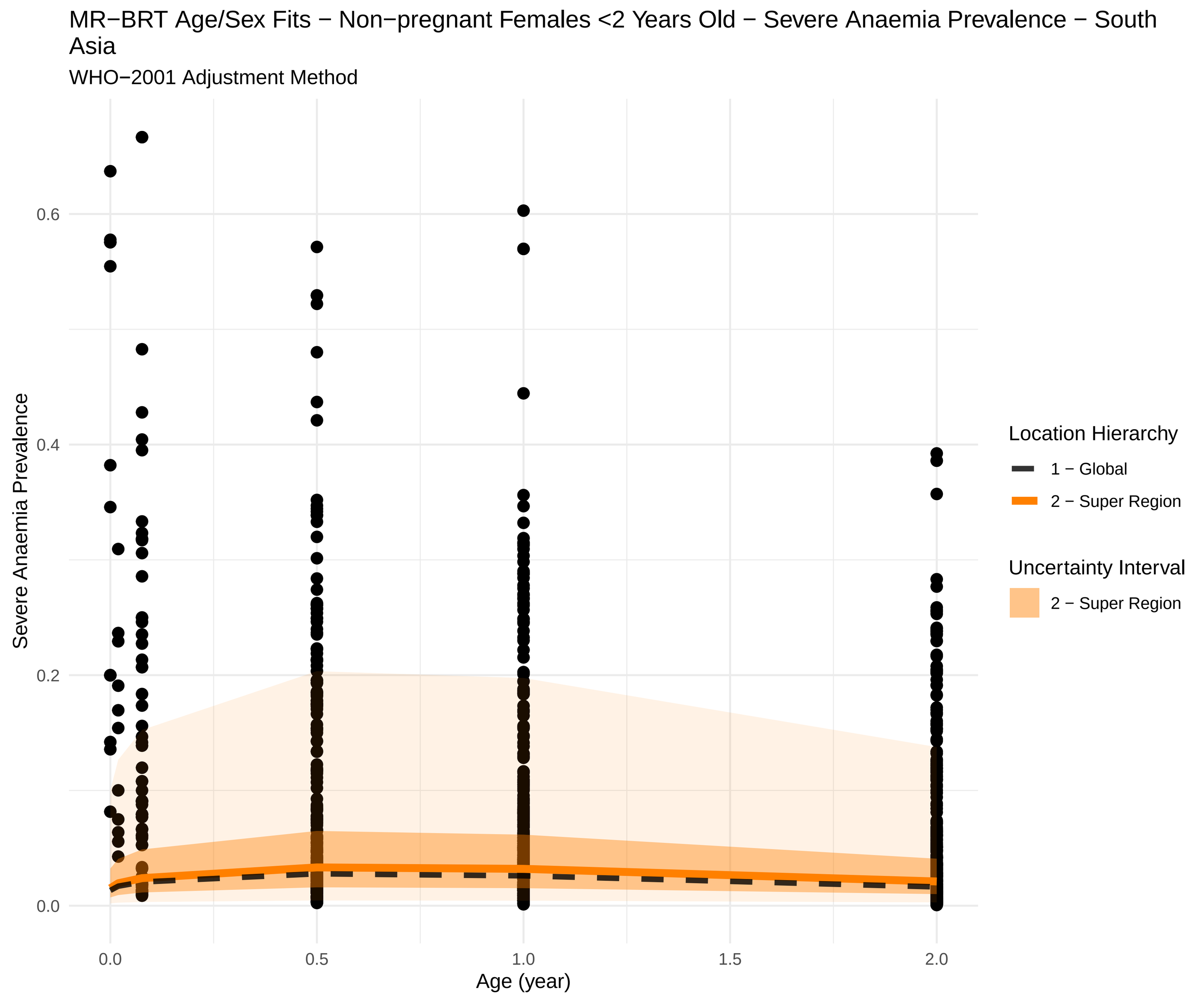
MR−BRT Age/Sex Fits − Non−pregnant Females <2 Years Old − Severe Anaemia Prevalence − South Asia
WHO−2001 Adjustment Method
Severe Anaemia Prevalence
Age (year)
Location Hierarchy
1 − Global
2 − Super Region
Uncertainty Interval
2 − Super Region

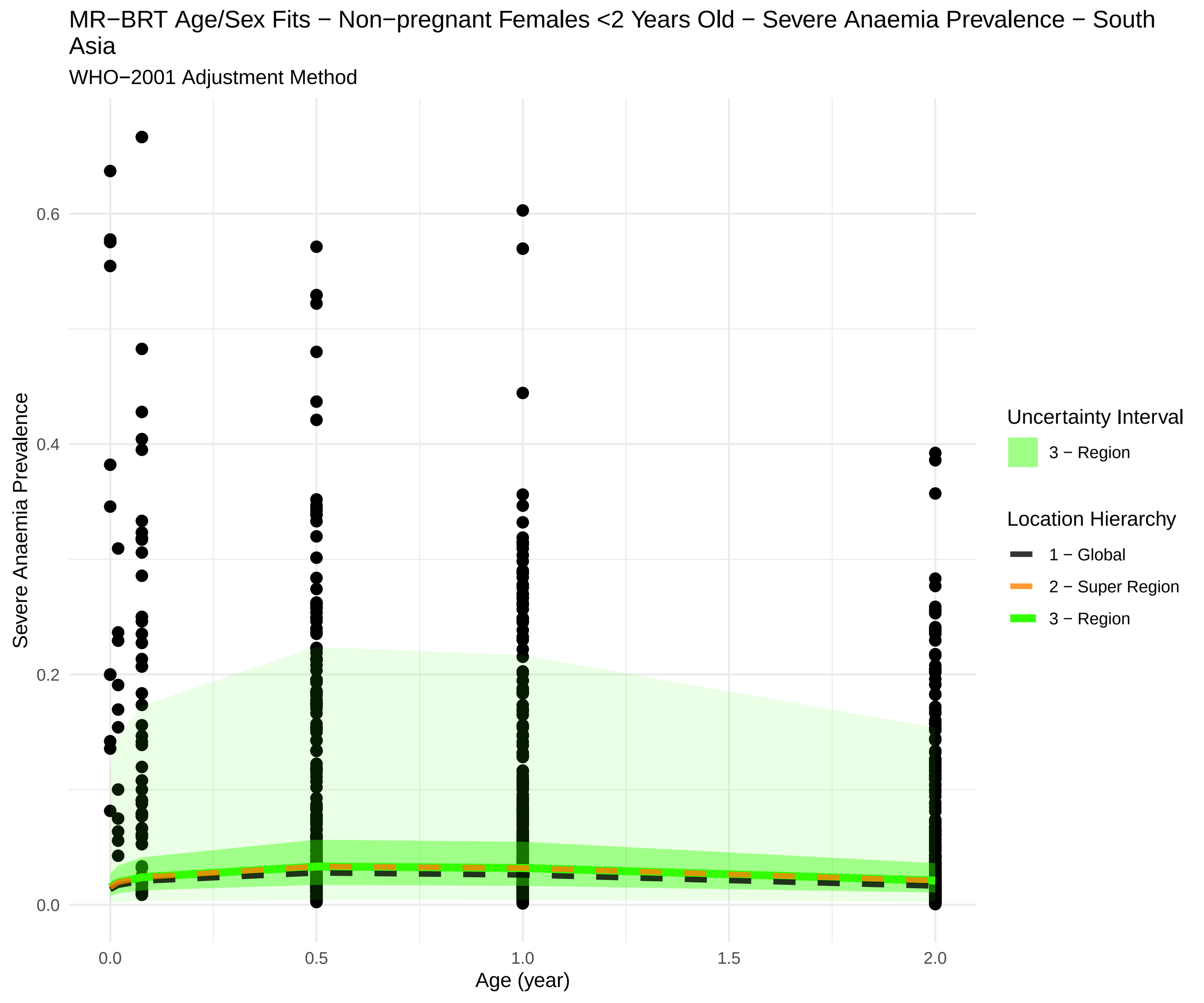
MR−BRT Age/Sex Fits − Non−pregnant Females <2 Years Old − Severe Anaemia Prevalence − South Asia
WHO−2001 Adjustment Method
Severe Anaemia Prevalence
Age (year)
Uncertainty Interval
3 − Region
Location Hierarchy
1 − Global
2 − Super Region
3 − Region

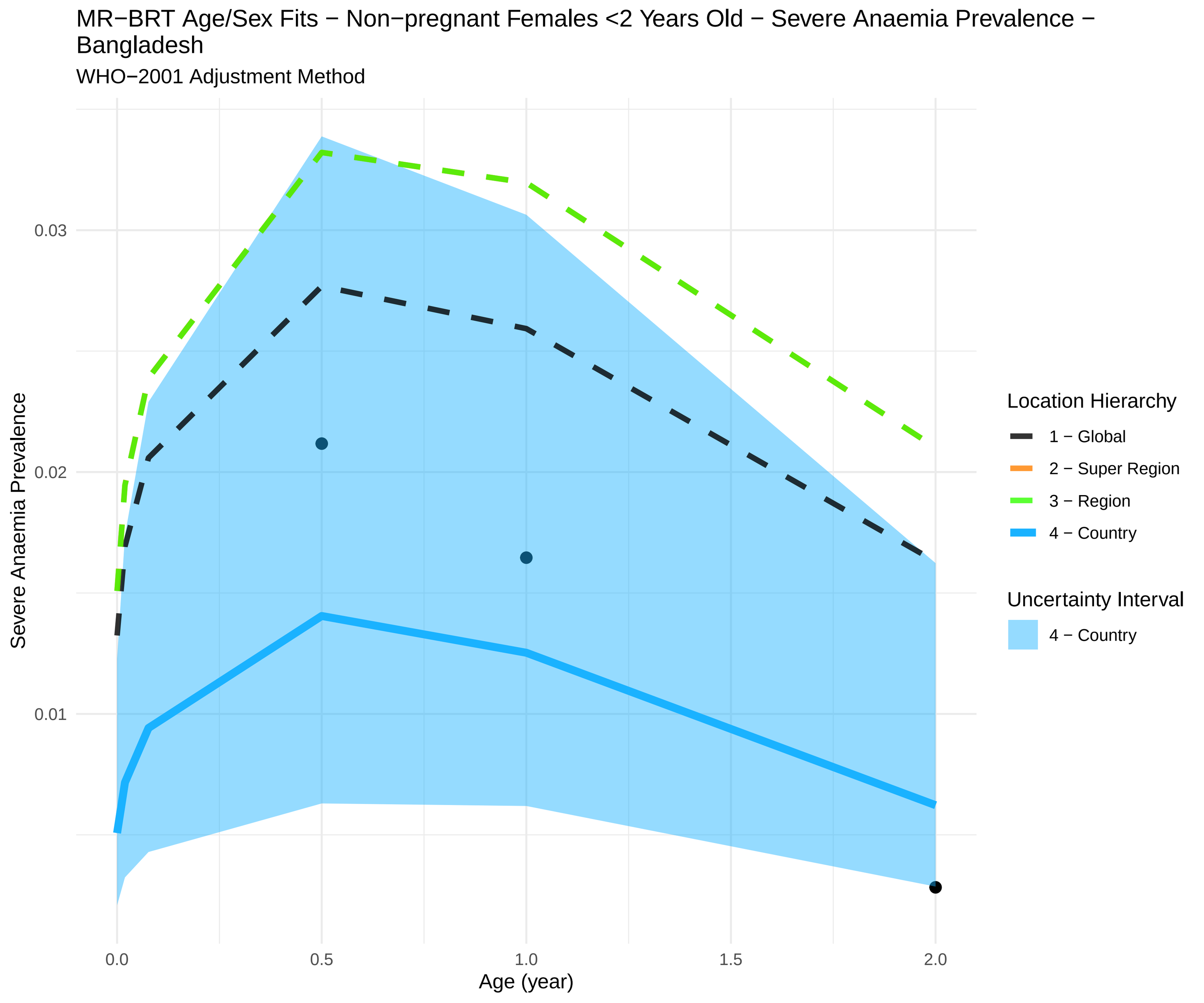
MR−BRT Age/Sex Fits − Non−pregnant Females <2 Years Old − Severe Anaemia Prevalence − Bangladesh
WHO−2001 Adjustment Method
Severe Anaemia Prevalence
Age (year)
Location Hierarchy
1 − Global
2 − Super Region
3 − Region
4 − Country
Uncertainty Interval
4 − Country

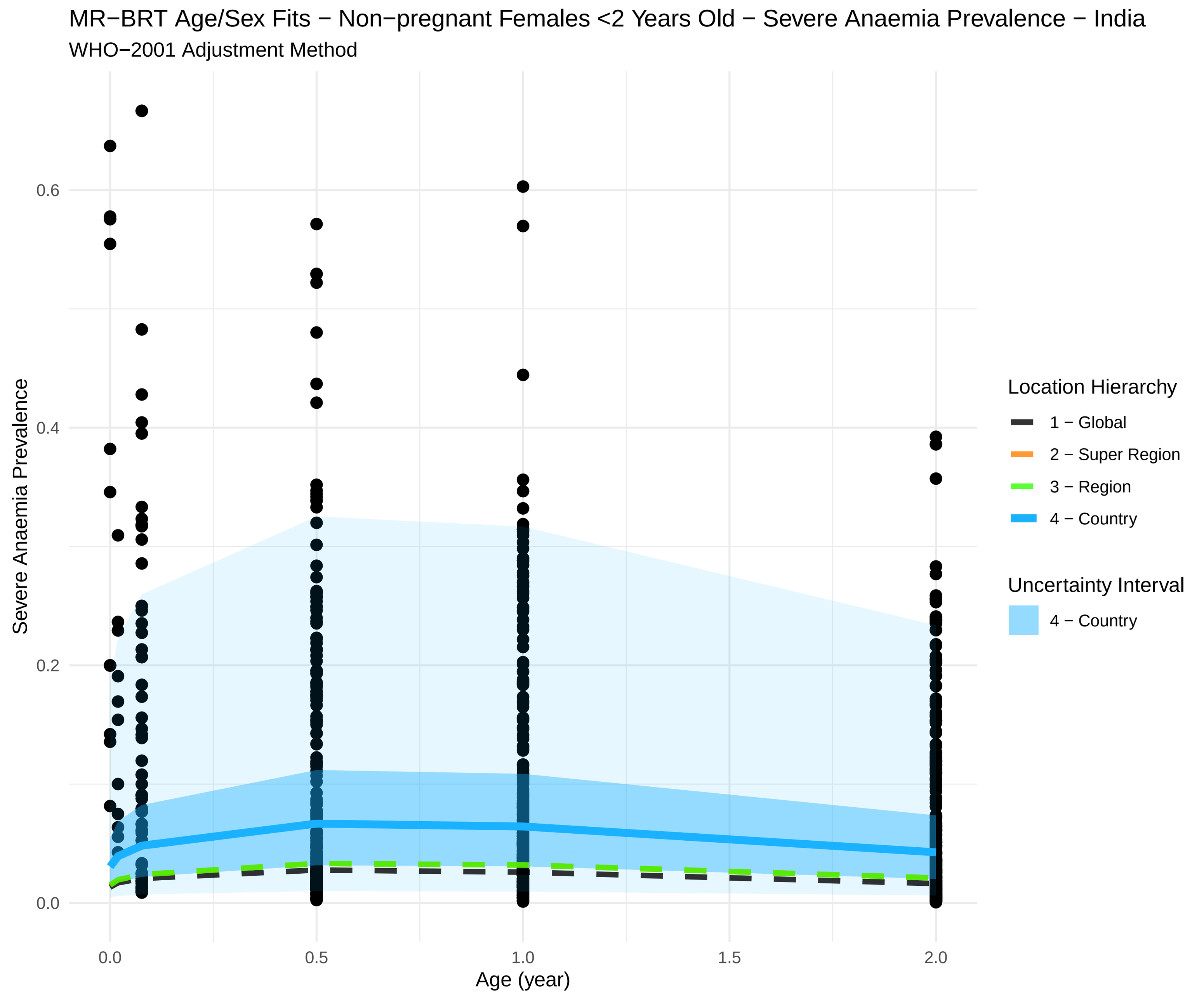
MR−BRT Age/Sex Fits − Non−pregnant Females <2 Years Old − Severe Anaemia Prevalence − India
WHO−2001 Adjustment Method
Severe Anaemia Prevalence
Age (year)
Location Hierarchy
1 − Global
2 − Super Region
3 − Region
4 − Country
Uncertainty Interval
4 − Country

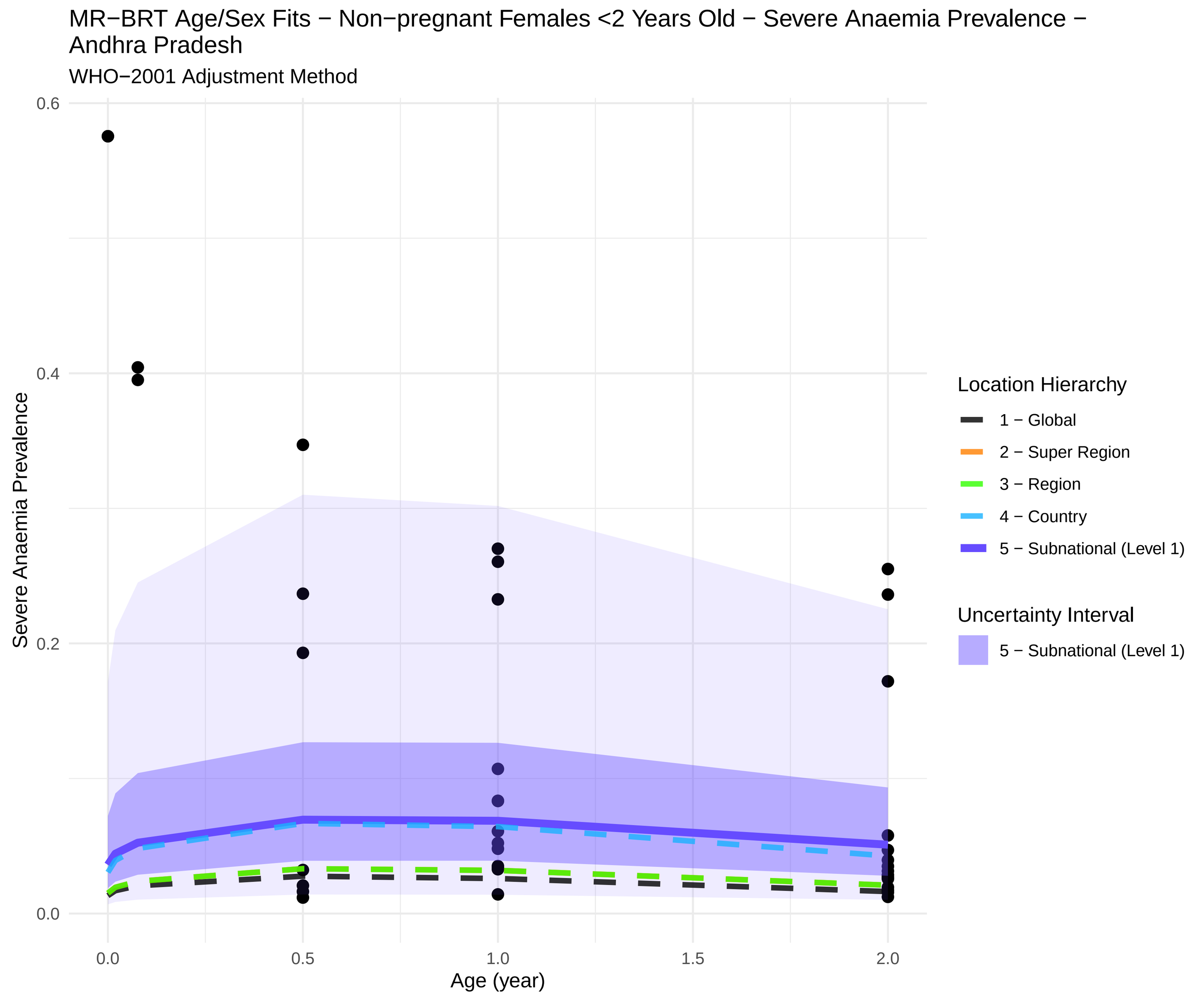
MR−BRT Age/Sex Fits − Non−pregnant Females <2 Years Old − Severe Anaemia Prevalence − Andhra Pradesh
WHO−2001 Adjustment Method
Severe Anaemia Prevalence
Age (year)
Location Hierarchy
1 − Global
2 − Super Region
3 − Region
4 − Country
5 − Subnational (Level 1)
Uncertainty Interval
5 − Subnational (Level 1)

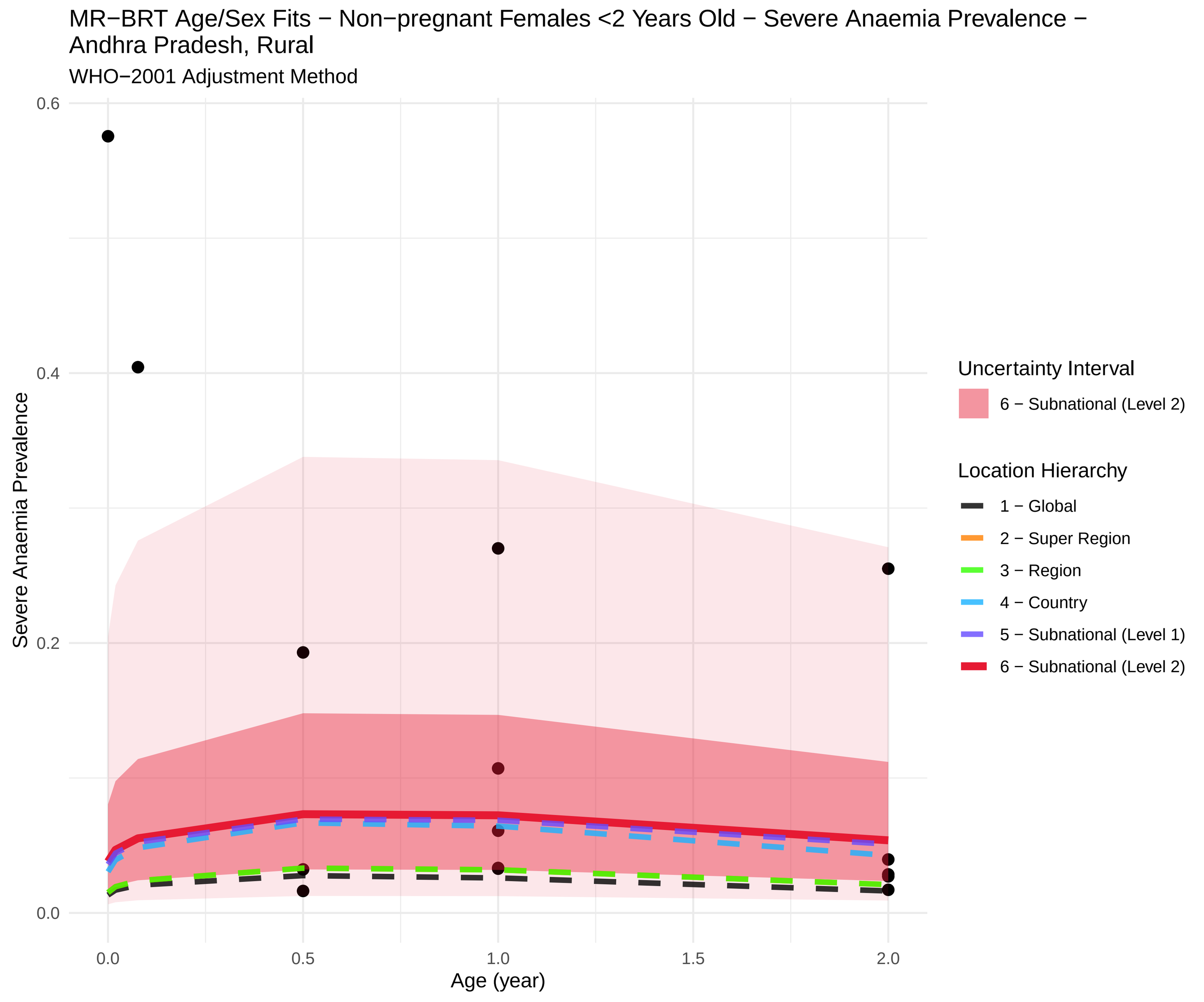
MR−BRT Age/Sex Fits − Non−pregnant Females <2 Years Old − Severe Anaemia Prevalence − Andhra Pradesh, Rural
WHO−2001 Adjustment Method
Severe Anaemia Prevalence
Age (year)
Uncertainty Interval
6 − Subnational (Level 2)
Location Hierarchy
1 − Global
2 − Super Region
3 − Region
4 − Country
5 − Subnational (Level 1)
6 − Subnational (Level 2)

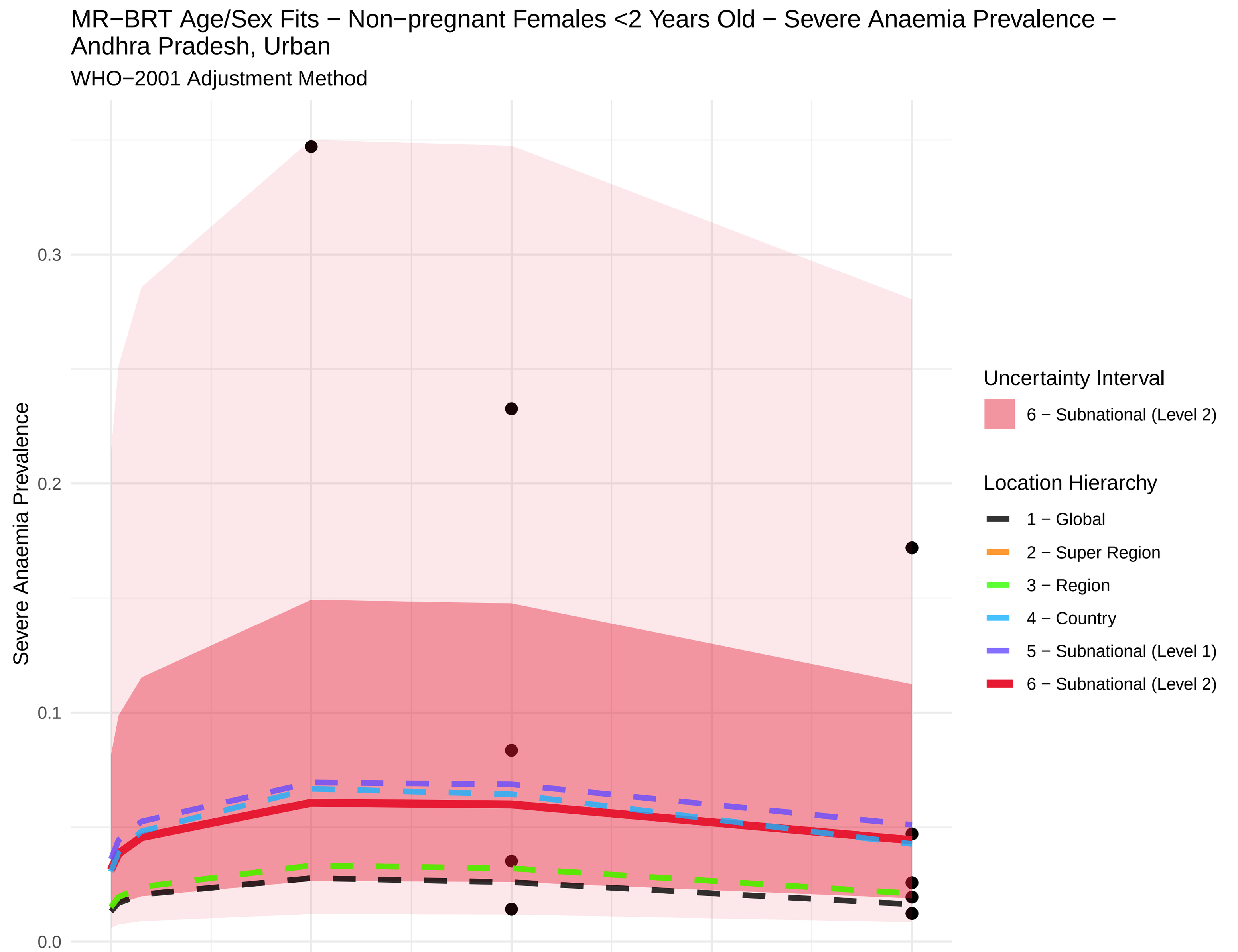
MR−BRT Age/Sex Fits − Non−pregnant Females <2 Years Old − Severe Anaemia Prevalence − Andhra Pradesh, Urban
WHO−2001 Adjustment Method
Severe Anaemia Prevalence
Age (year)
Uncertainty Interval
6 − Subnational (Level 2)
Location Hierarchy
1 − Global
2 − Super Region
3 − Region
4 − Country
5 − Subnational (Level 1)
6 − Subnational (Level 2)

MR−BRT Age/Sex Fits − Non−pregnant Females <2 Years Old − Severe Anaemia Prevalence − Arunachal Pradesh
WHO−2001 Adjustment Method

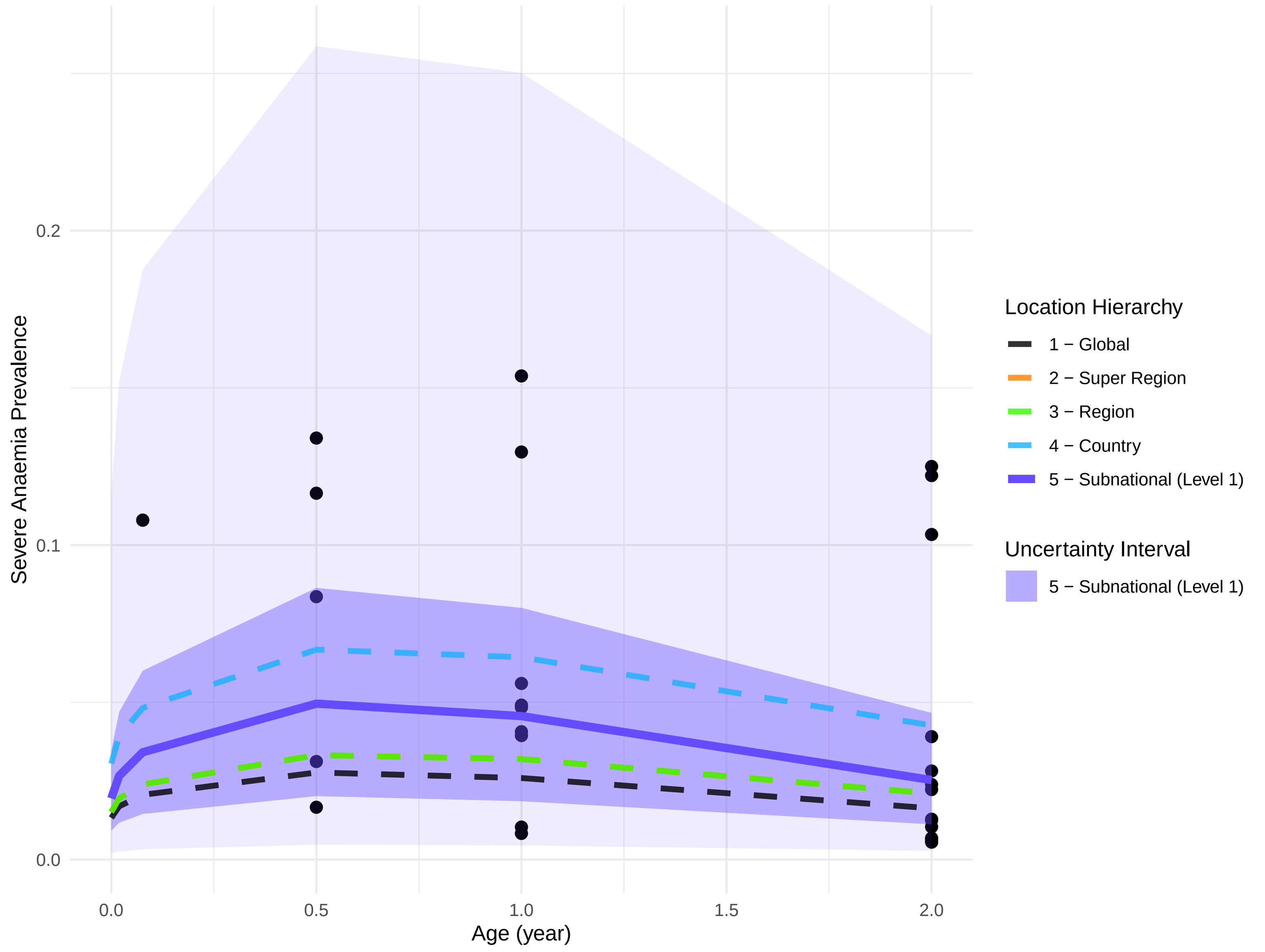

MR−BRT Age/Sex Fits − Non−pregnant Females <2 Years Old − Severe Anaemia Prevalence − Arunachal Pradesh, Rural
WHO−2001 Adjustment Method

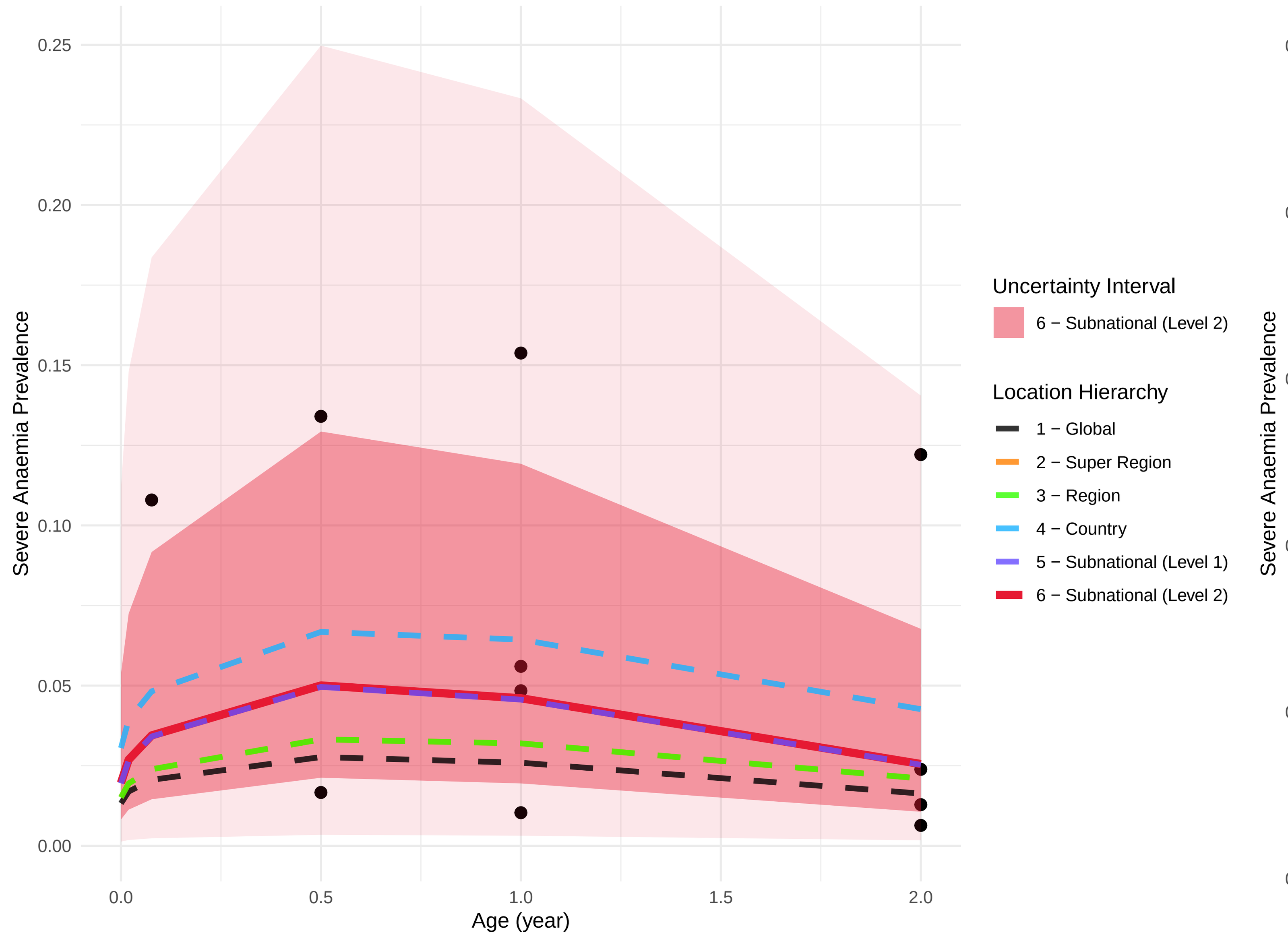

MR−BRT Age/Sex Fits − Non−pregnant Females <2 Years Old − Severe Anaemia Prevalence − Arunachal Pradesh, Urban
WHO−2001 Adjustment Method

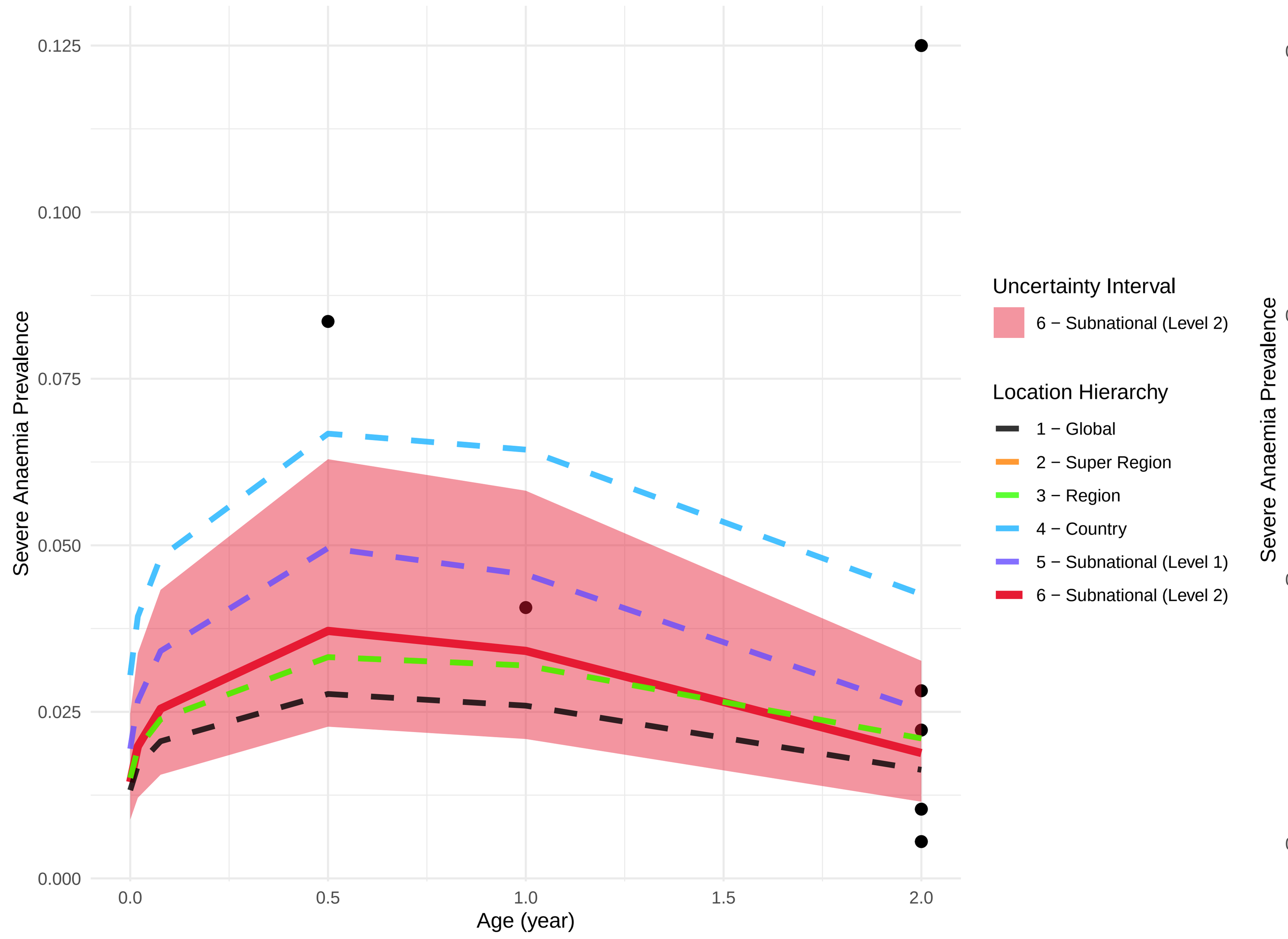

MR−BRT Age/Sex Fits − Non−pregnant Females <2 Years Old − Severe Anaemia Prevalence − Assam
WHO−2001 Adjustment Method

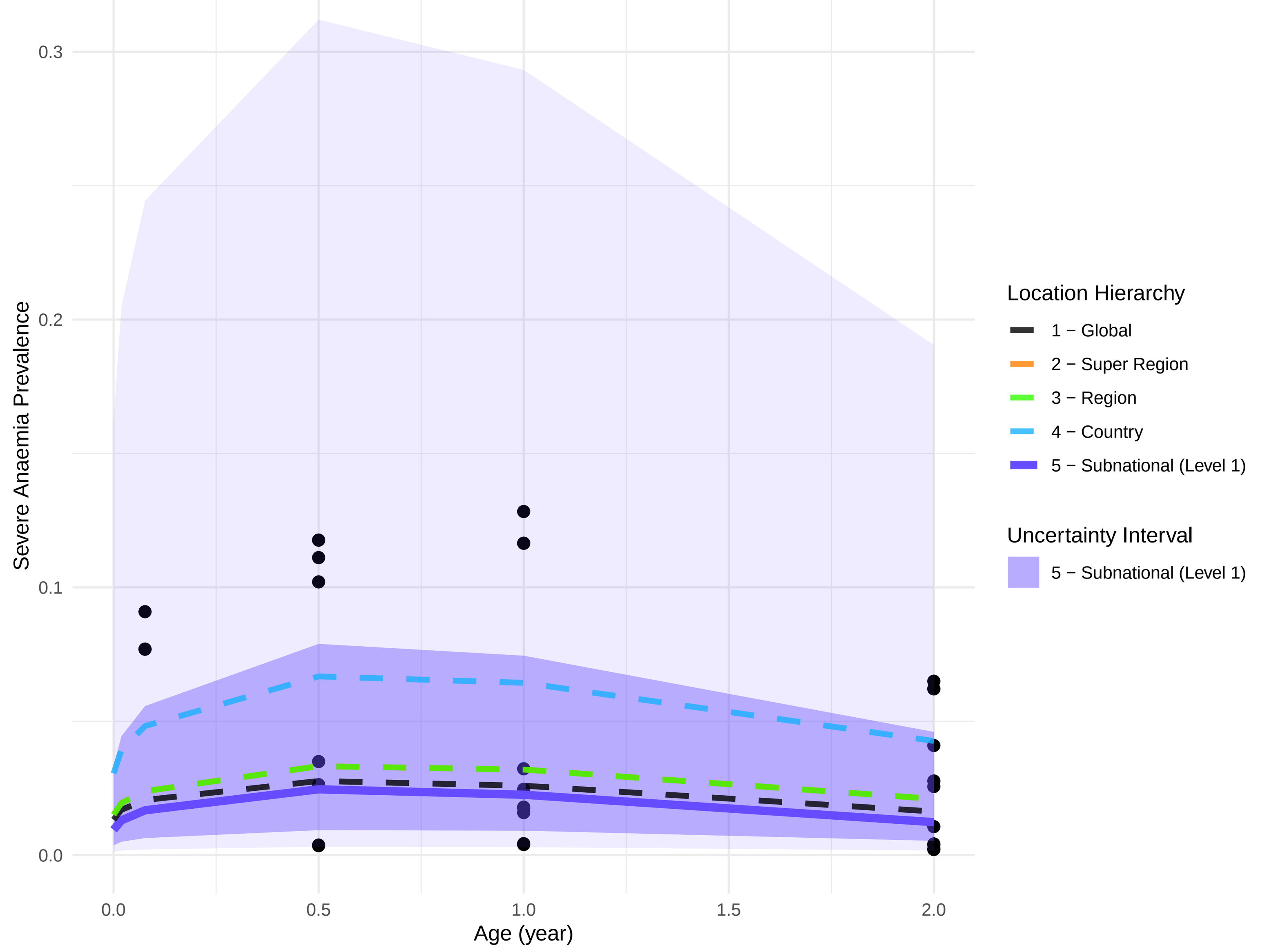

MR−BRT Age/Sex Fits − Non−pregnant Females <2 Years Old − Severe Anaemia Prevalence − Assam, Rural
WHO−2001 Adjustment Method

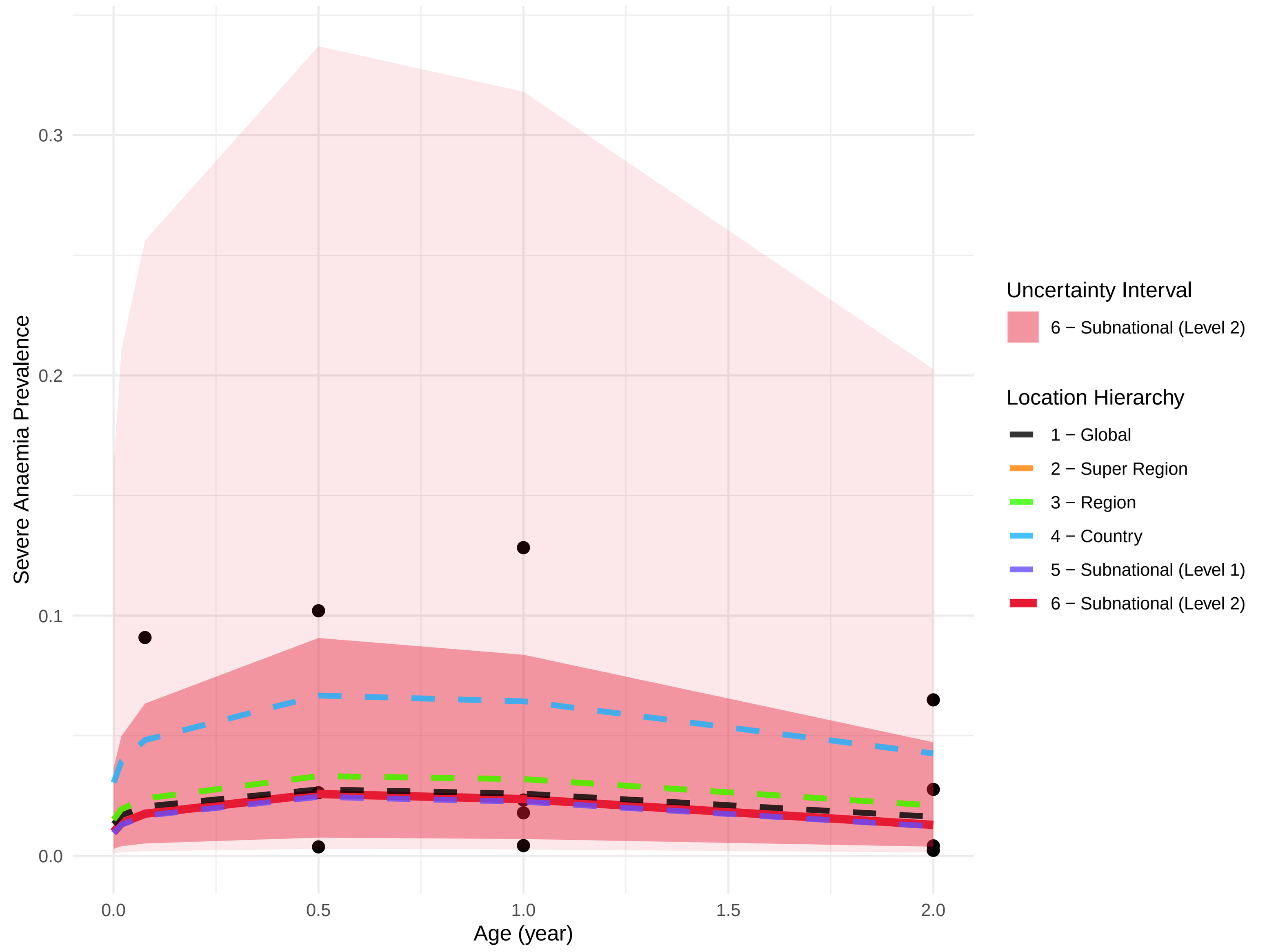

MR−BRT Age/Sex Fits − Non−pregnant Females <2 Years Old − Severe Anaemia Prevalence − Assam, Urban
WHO−2001 Adjustment Method

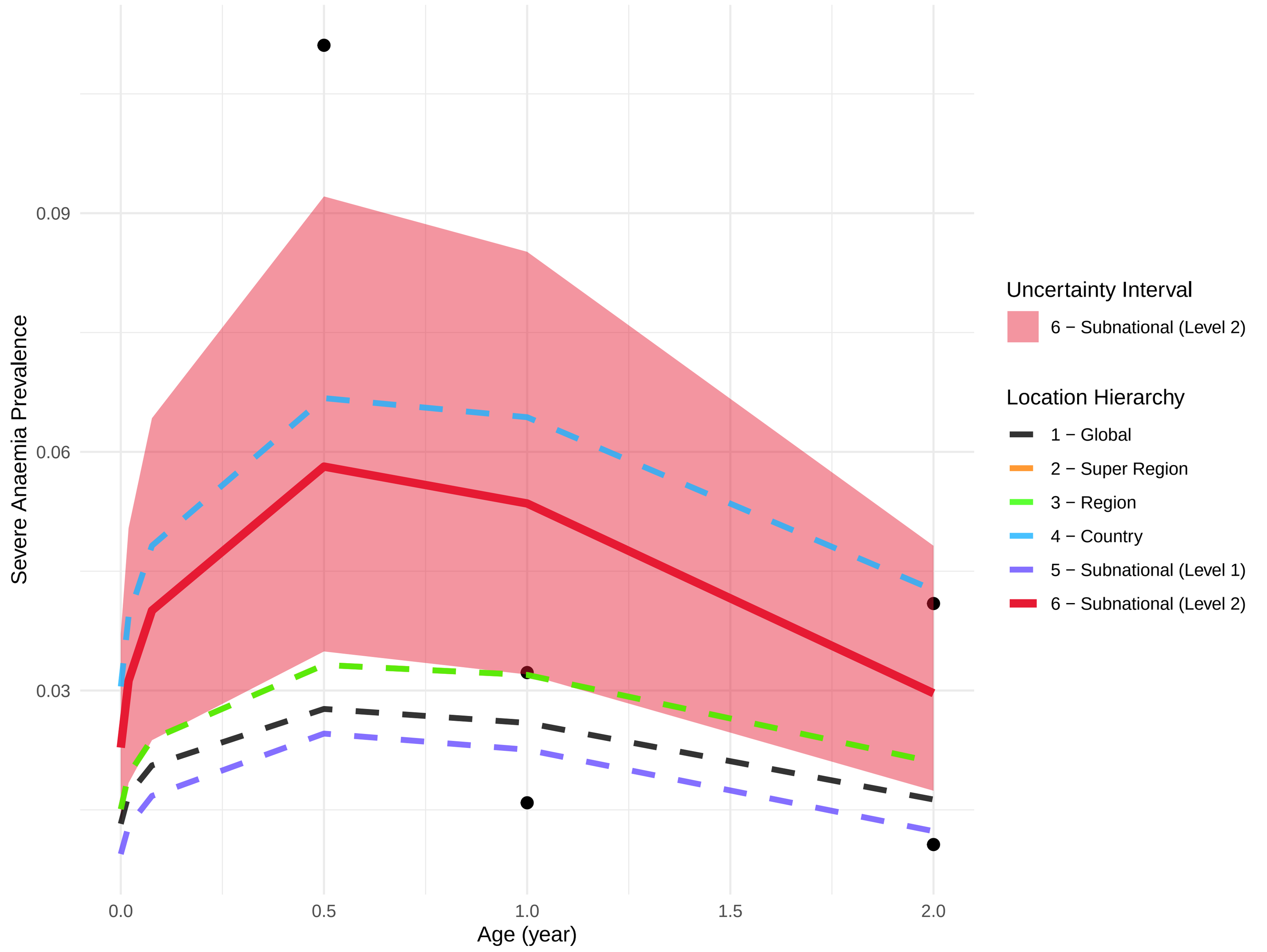

MR−BRT Age/Sex Fits − Non−pregnant Females <2 Years Old − Severe Anaemia Prevalence − Bihar
WHO−2001 Adjustment Method

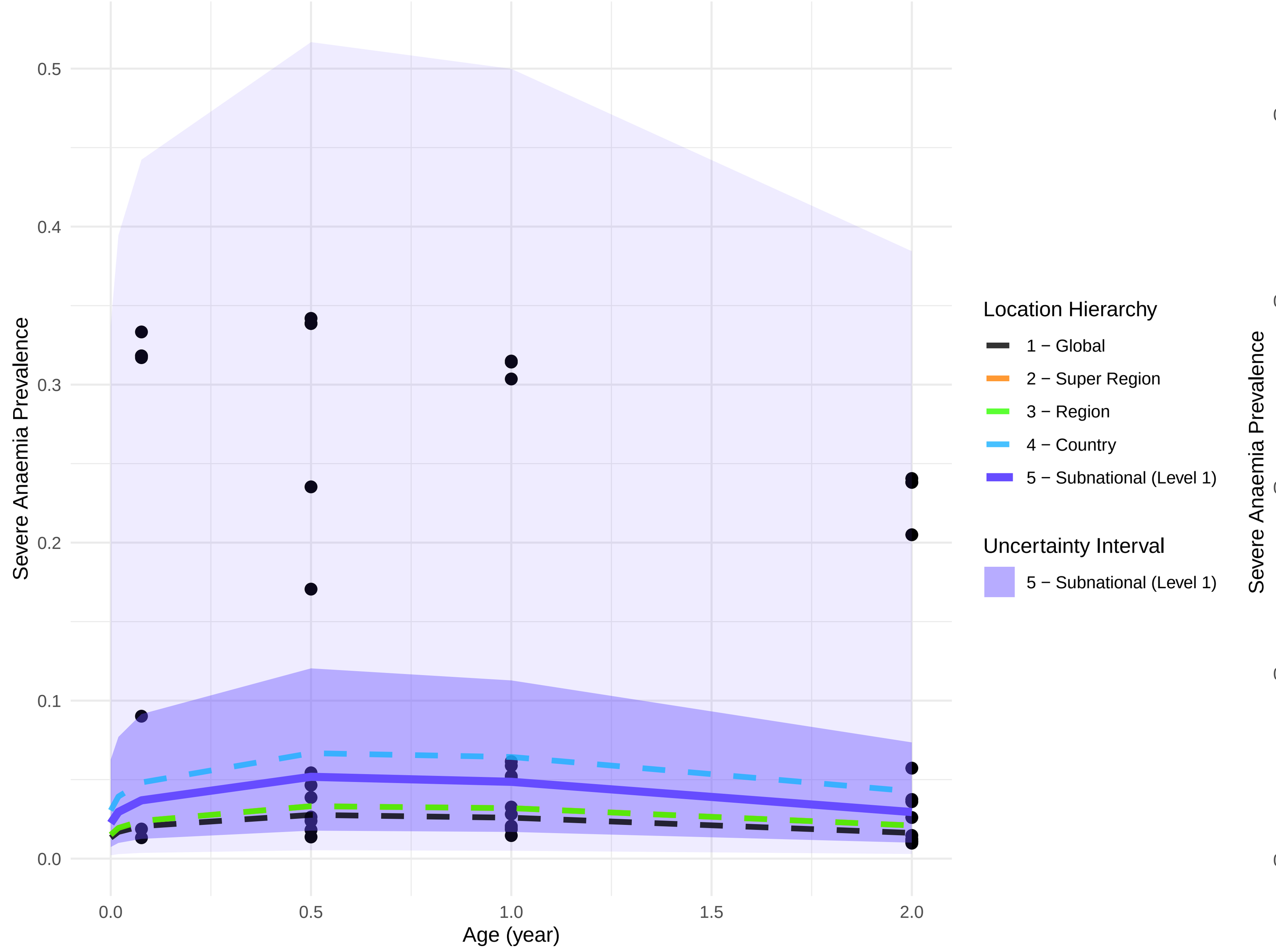

MR−BRT Age/Sex Fits − Non−pregnant Females <2 Years Old − Severe Anaemia Prevalence − Bihar, Rural
WHO−2001 Adjustment Method

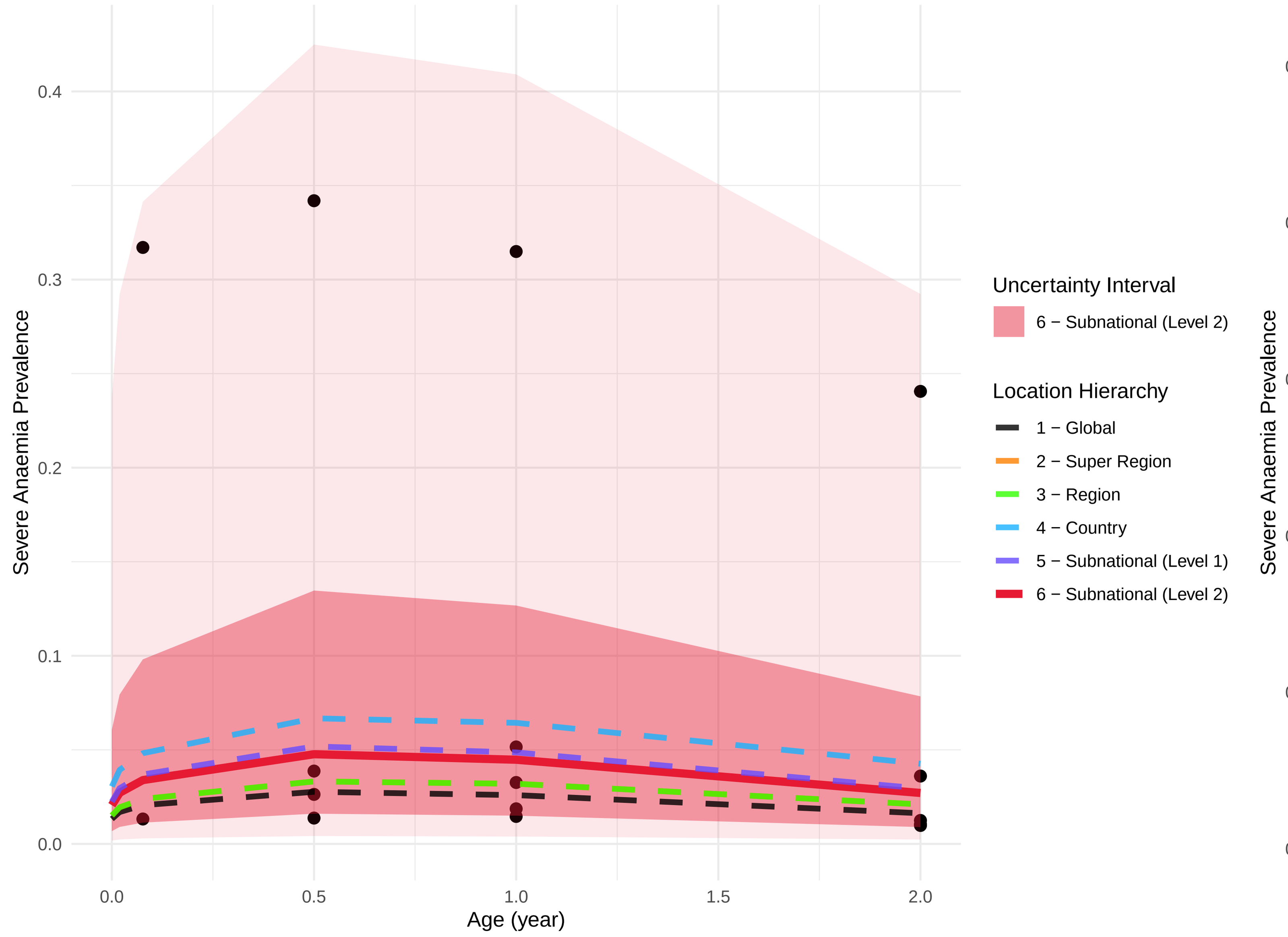

MR−BRT Age/Sex Fits − Non−pregnant Females <2 Years Old − Severe Anaemia Prevalence − Bihar, Urban
WHO−2001 Adjustment Method

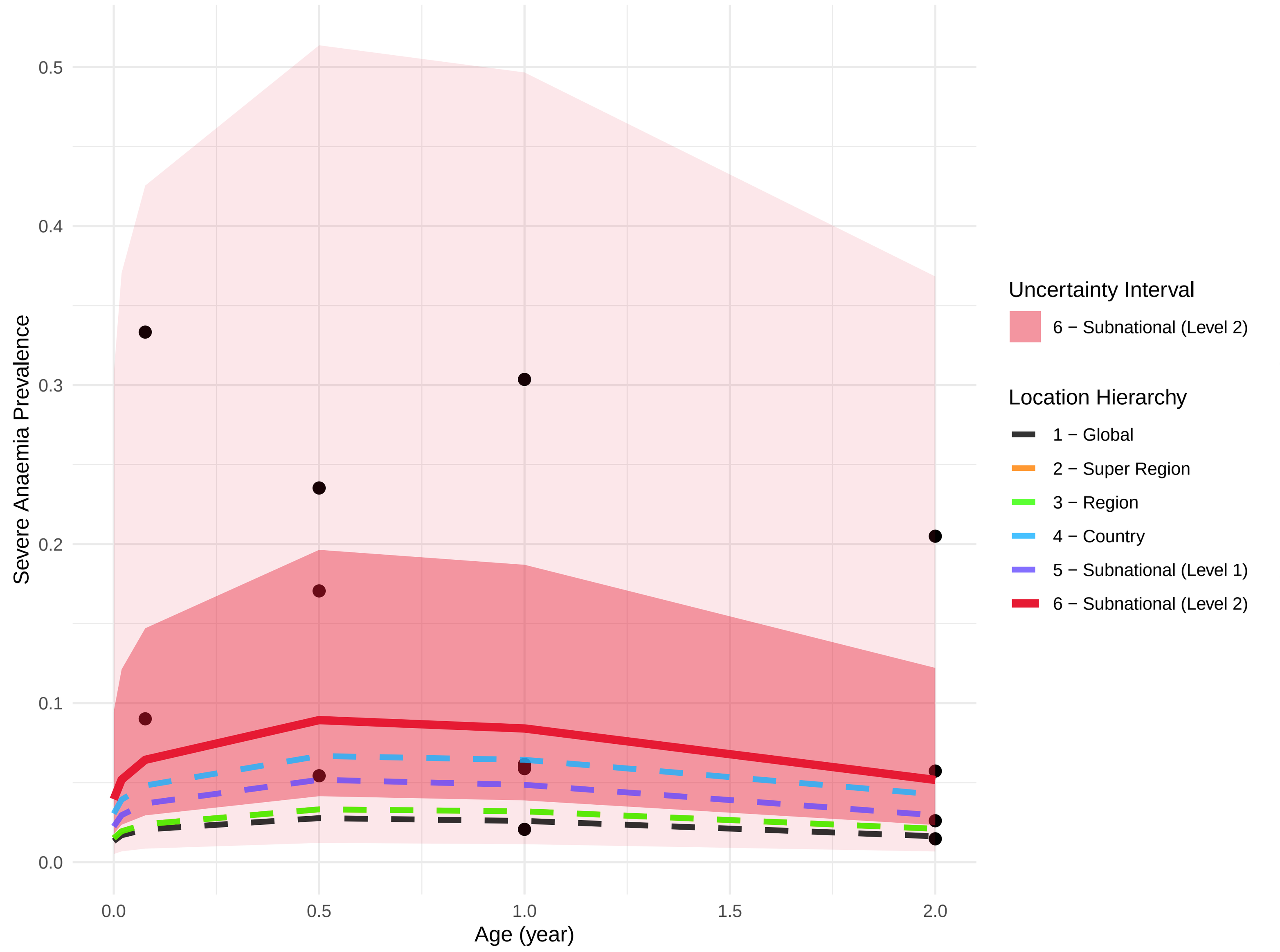

MR−BRT Age/Sex Fits − Non−pregnant Females <2 Years Old − Severe Anaemia Prevalence − Chhattisgarh
WHO−2001 Adjustment Method

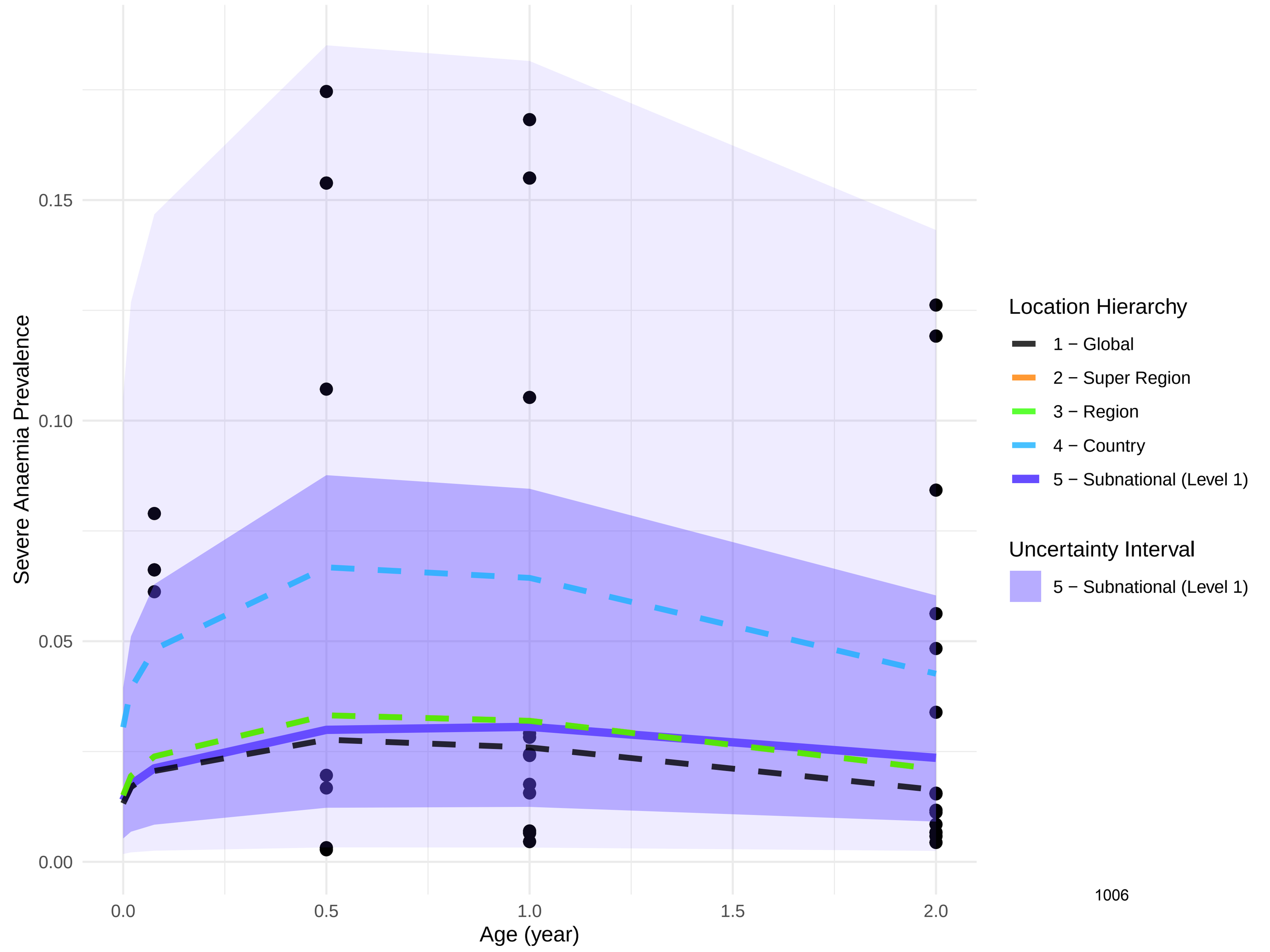

MR−BRT Age/Sex Fits − Non-pregnant Females <2 Years Old − Severe Anaemia Prevalence − Chhattisgarh, Rural
WHO−2001 Adjustment Method

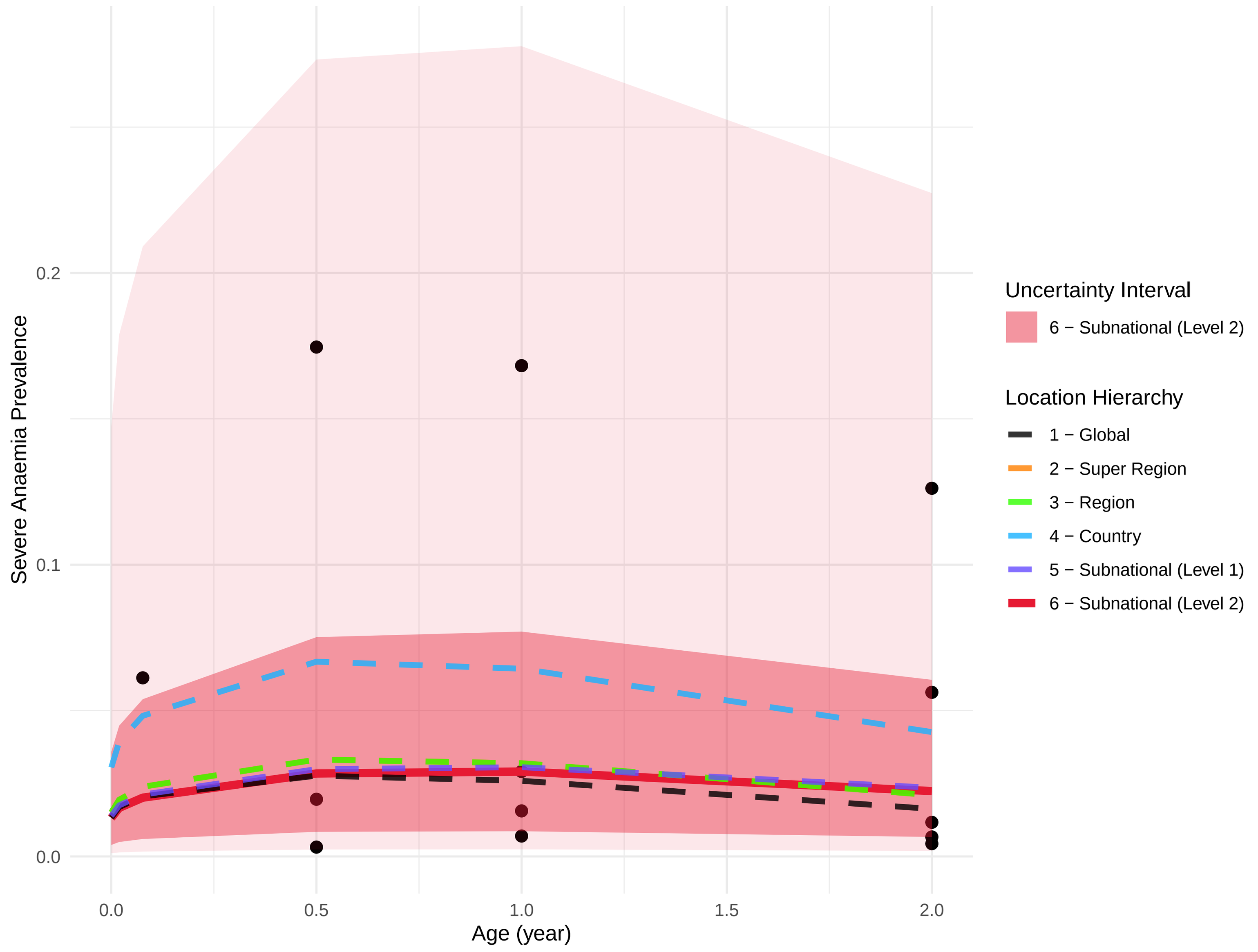

MR−BRT Age/Sex Fits − Non-pregnant Females <2 Years Old − Severe Anaemia Prevalence − Chhattisgarh, Urban
WHO−2001 Adjustment Method

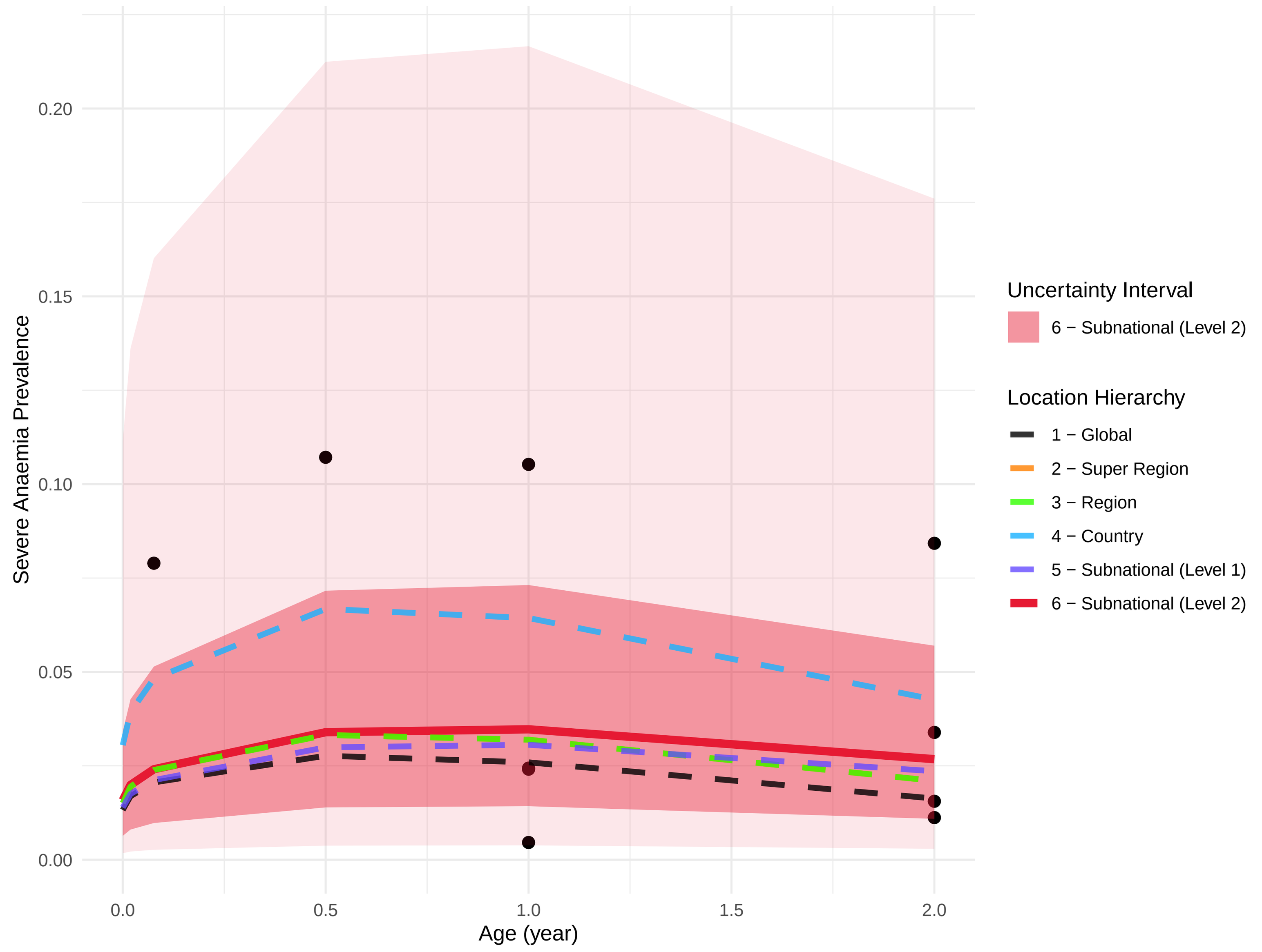

MR−BRT Age/Sex Fits − Non-pregnant Females <2 Years Old − Severe Anaemia Prevalence − Delhi
WHO−2001 Adjustment Method

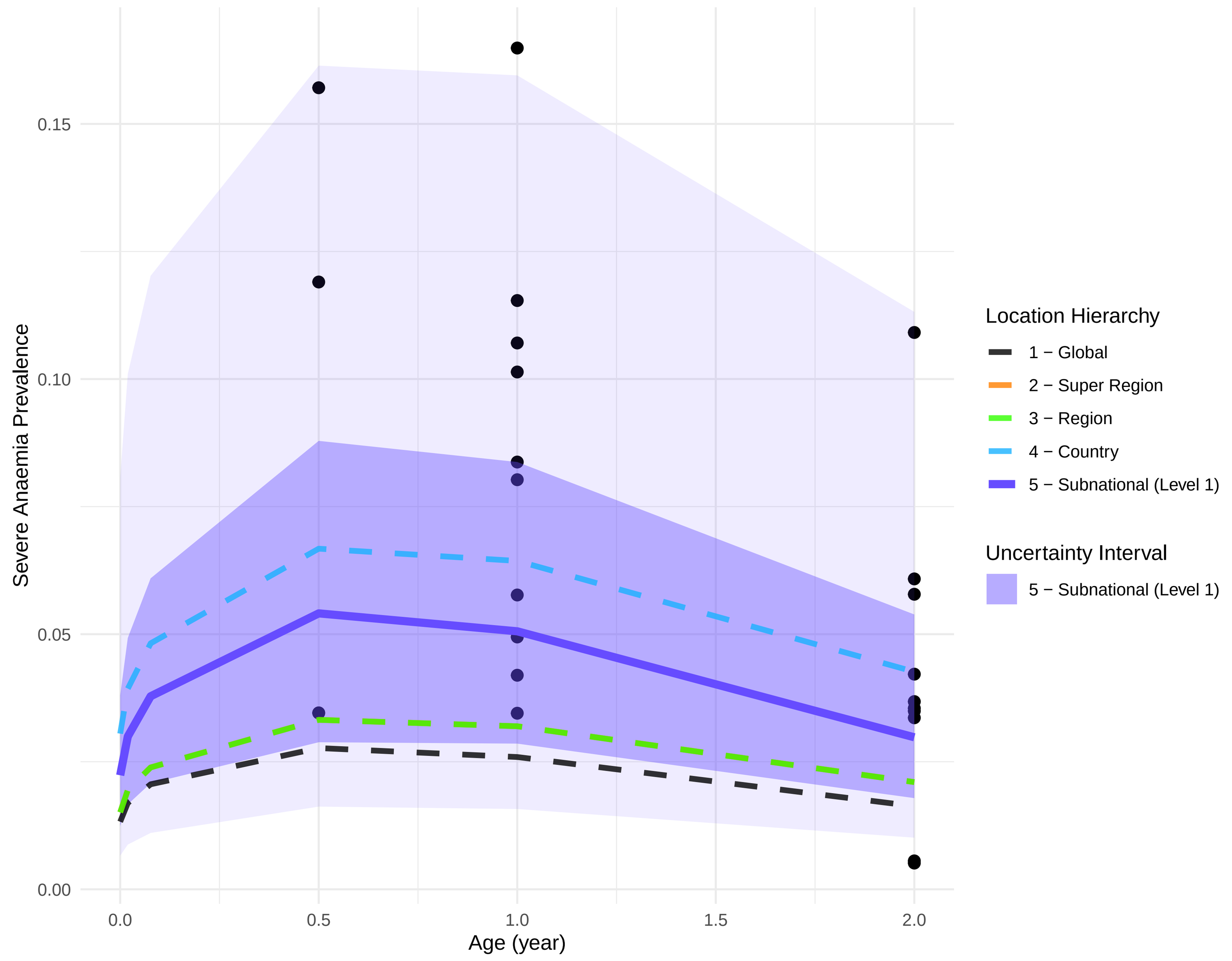

MR−BRT Age/Sex Fits − Non-pregnant Females <2 Years Old − Severe Anaemia Prevalence − Delhi, Rural
WHO−2001 Adjustment Method

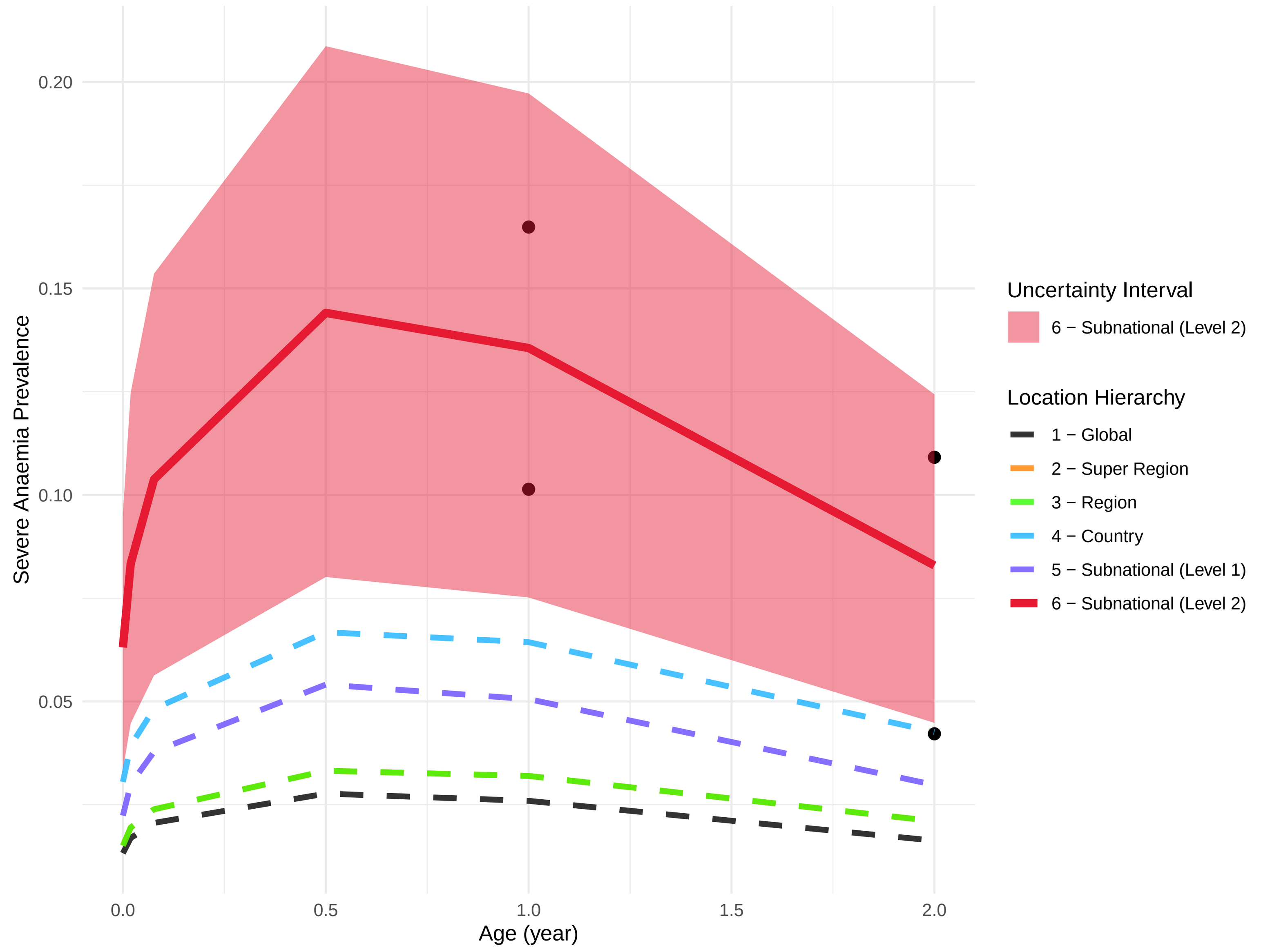

MR−BRT Age/Sex Fits − Non-pregnant Females <2 Years Old − Severe Anaemia Prevalence − Delhi, Urban
WHO−2001 Adjustment Method

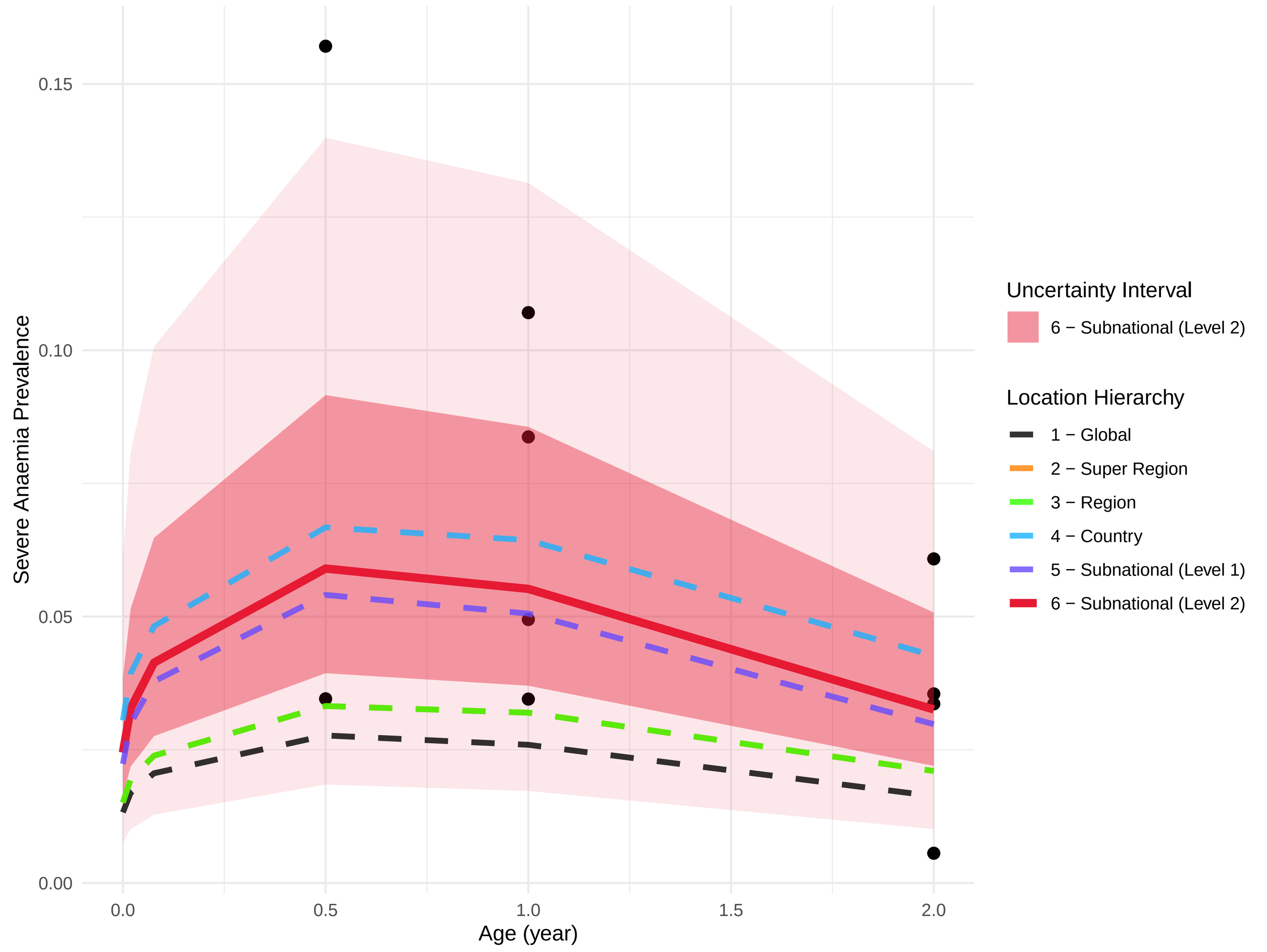

MR−BRT Age/Sex Fits − Non-pregnant Females <2 Years Old − Severe Anaemia Prevalence − Goa
WHO−2001 Adjustment Method

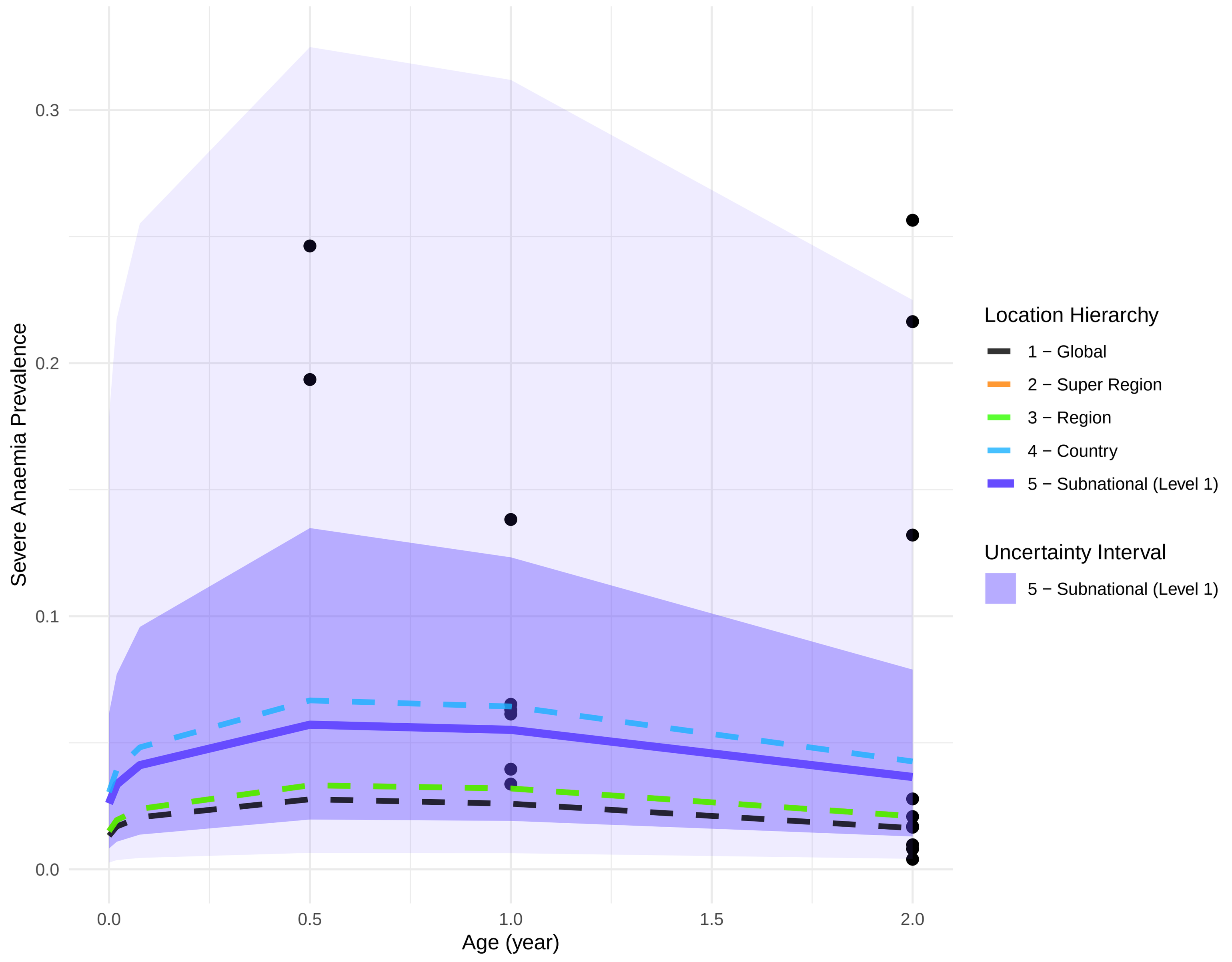

MR−BRT Age/Sex Fits − Non-pregnant Females <2 Years Old − Severe Anaemia Prevalence − Goa, Rural
WHO−2001 Adjustment Method

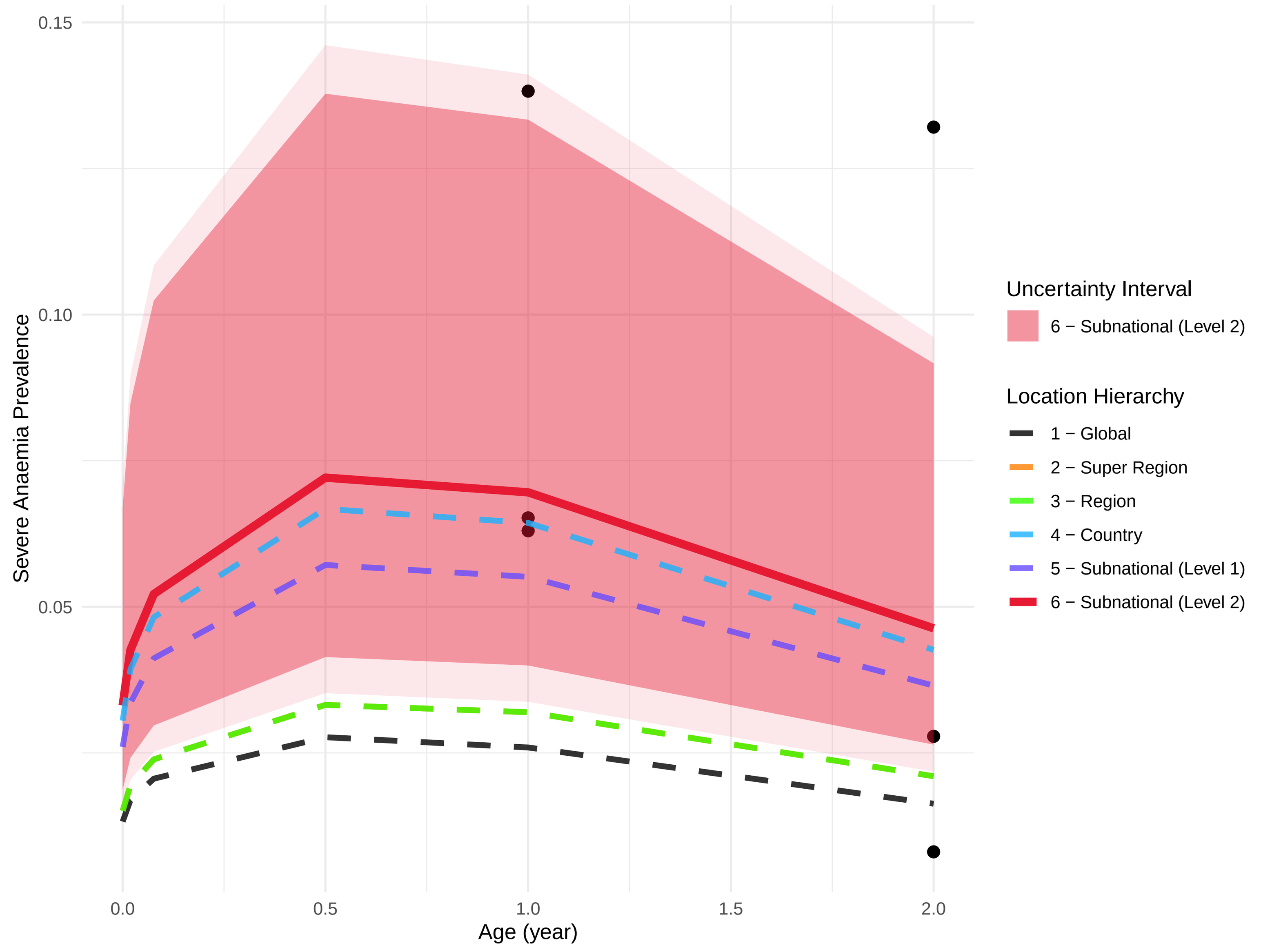

MR−BRT Age/Sex Fits − Non-pregnant Females <2 Years Old − Severe Anaemia Prevalence − Goa, Urban
WHO−2001 Adjustment Method

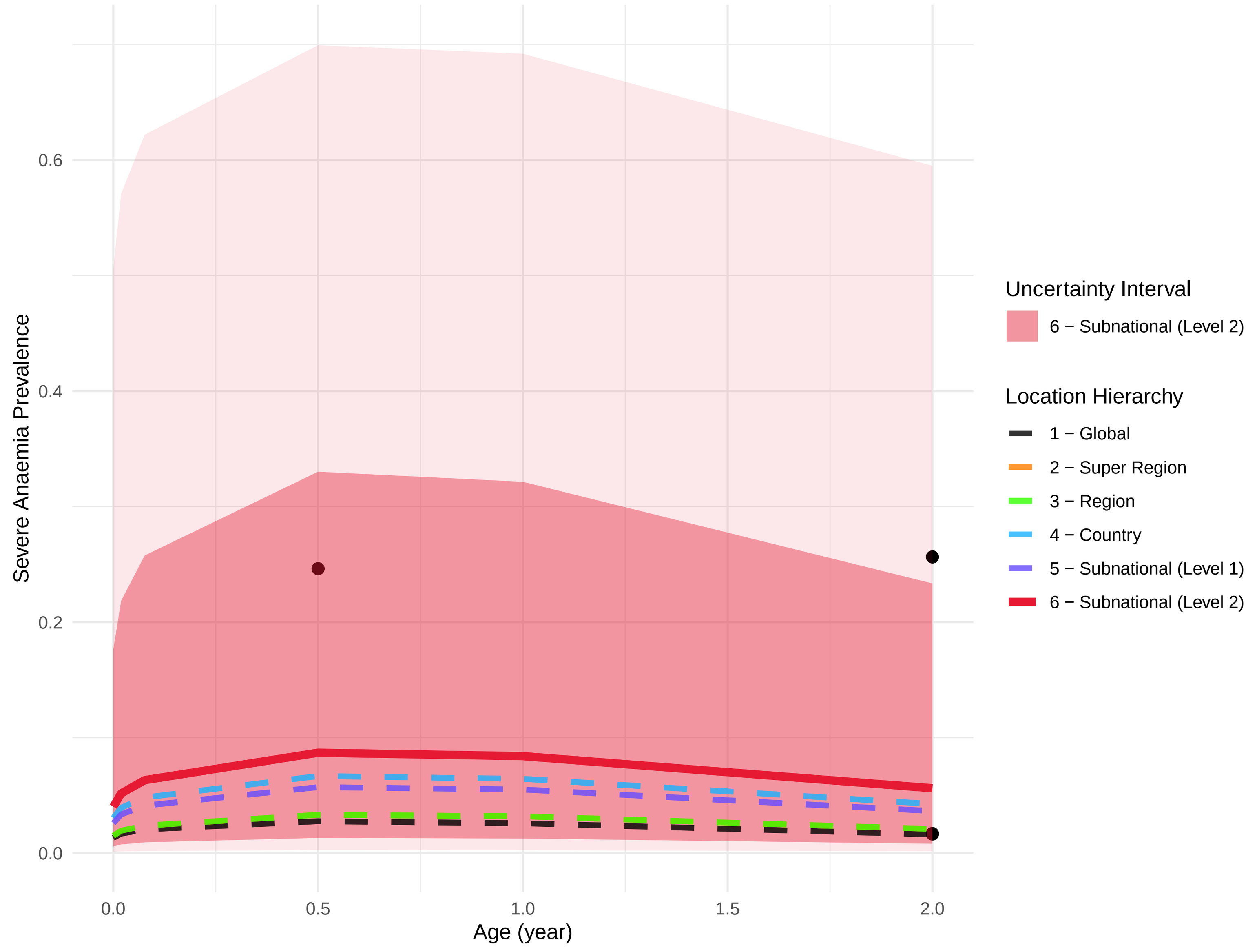

MR−BRT Age/Sex Fits − Non-pregnant Females <2 Years Old − Severe Anaemia Prevalence − Gujarat
WHO−2001 Adjustment Method

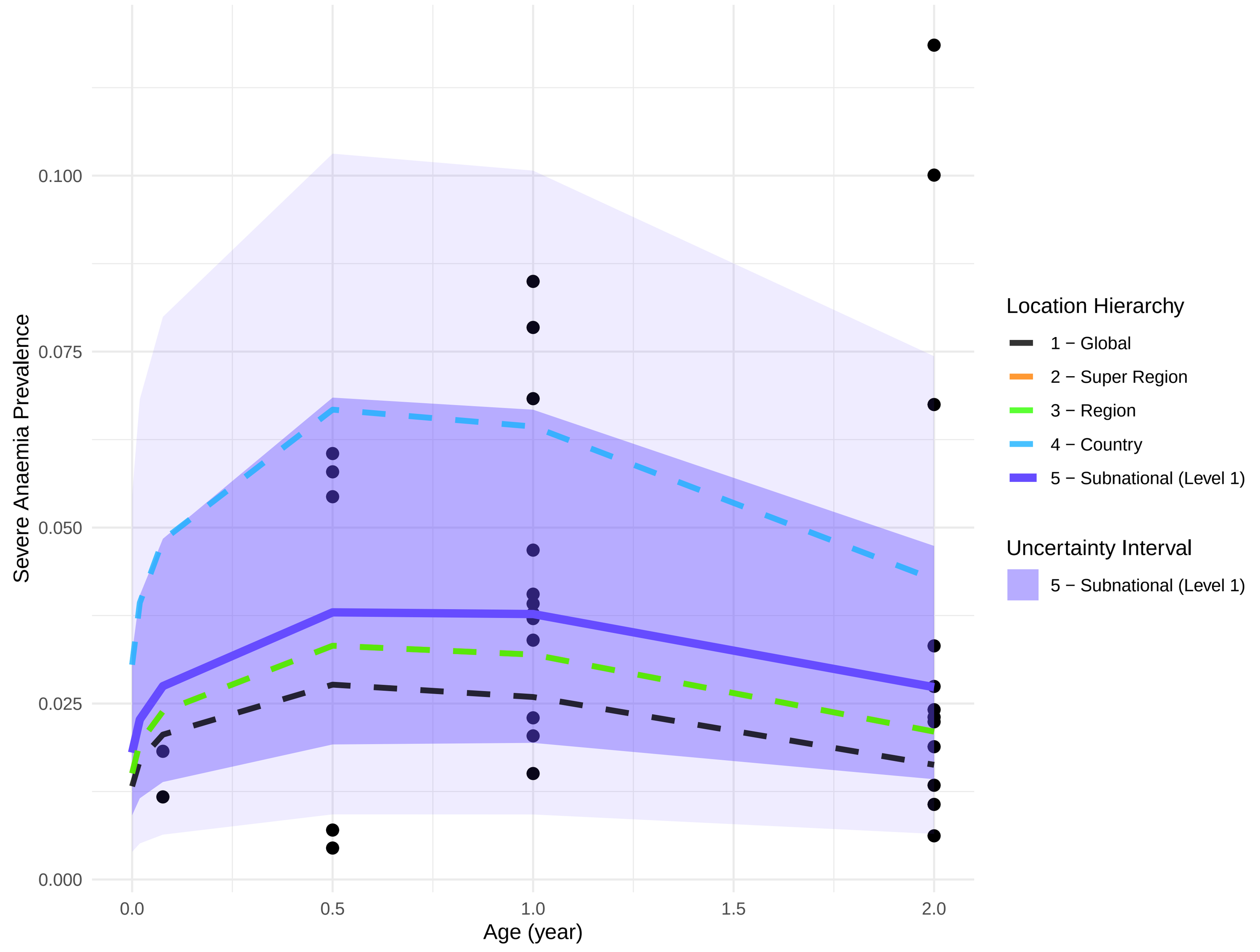

MR−BRT Age/Sex Fits − Non-pregnant Females <2 Years Old − Severe Anaemia Prevalence − Gujarat, Rural
WHO−2001 Adjustment Method

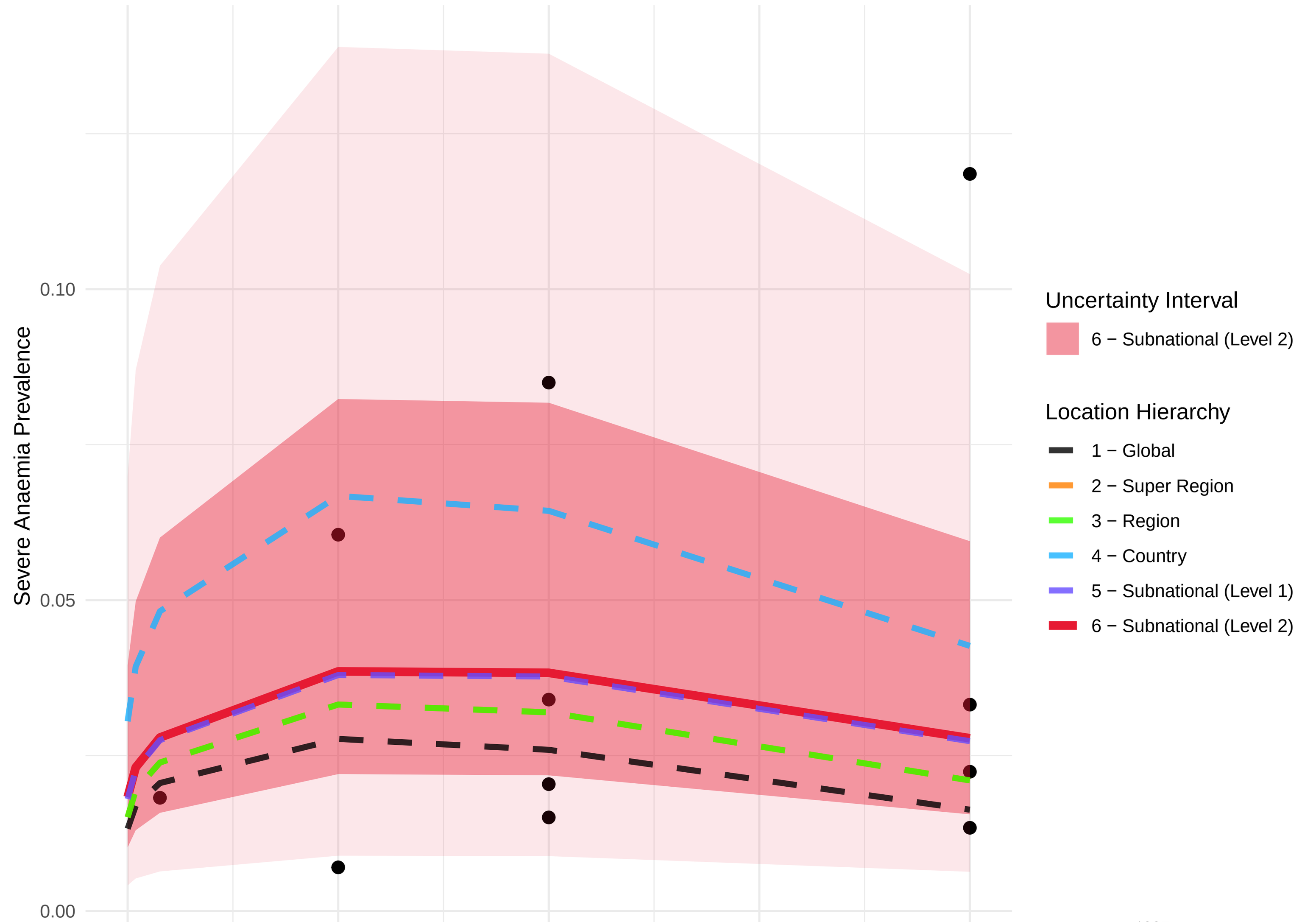

MR−BRT Age/Sex Fits − Non−pregnant Females <2 Years Old − Severe Anaemia Prevalence − Gujarat, Urban
WHO−2001 Adjustment Method

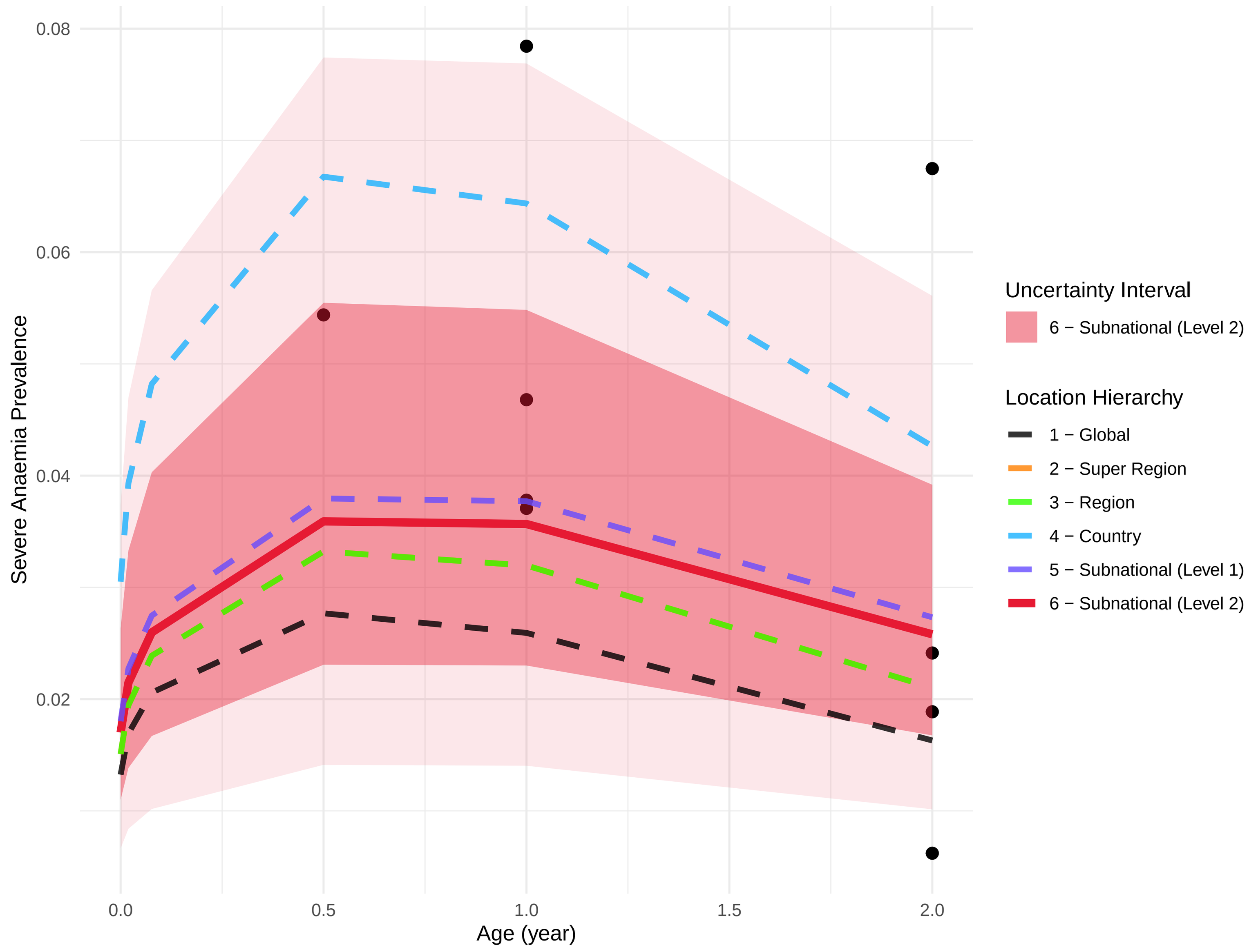

MR−BRT Age/Sex Fits − Non−pregnant Females <2 Years Old − Severe Anaemia Prevalence − Haryana
WHO−2001 Adjustment Method

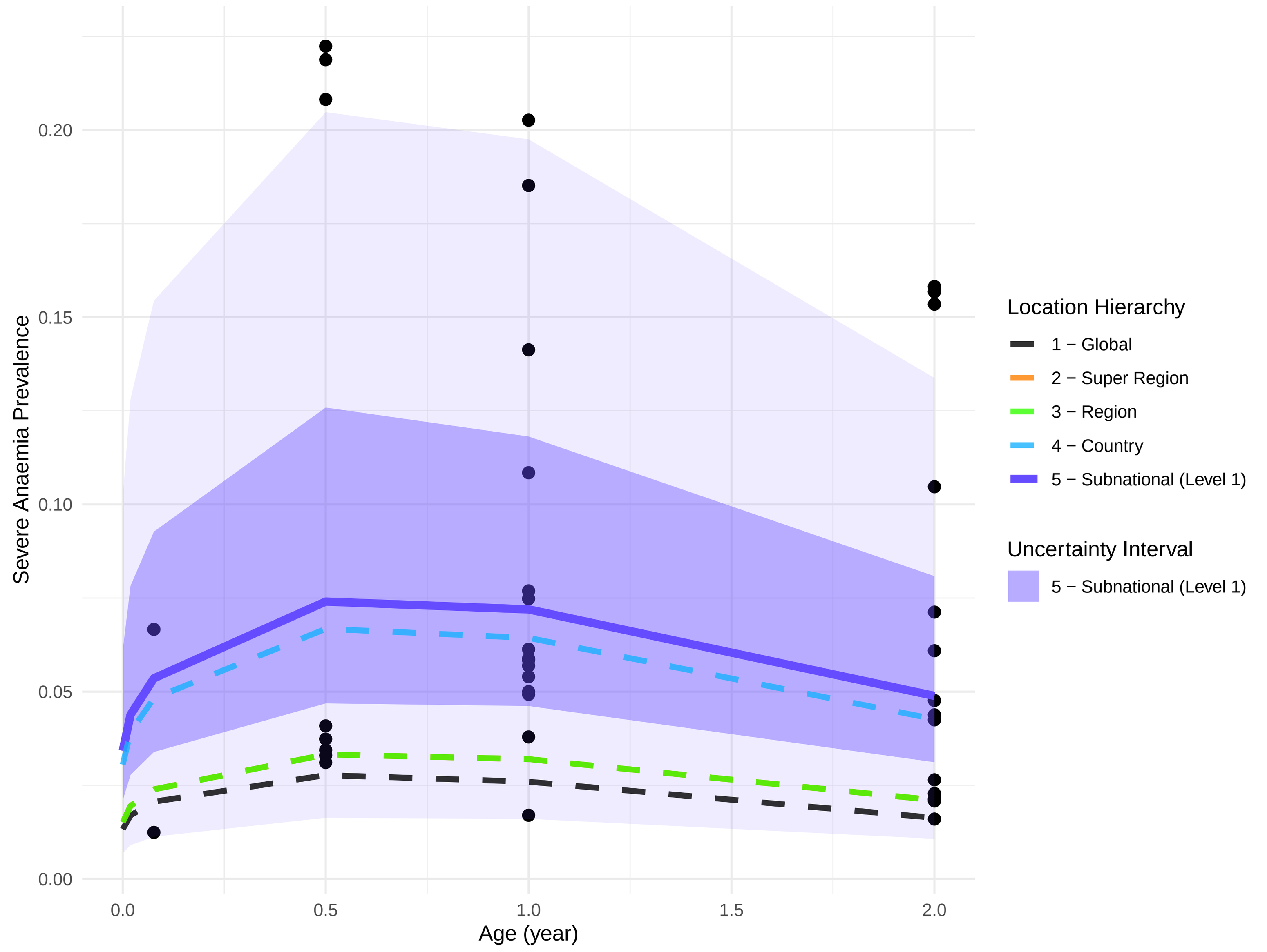

MR−BRT Age/Sex Fits − Non−pregnant Females <2 Years Old − Severe Anaemia Prevalence − Haryana, Rural
WHO−2001 Adjustment Method

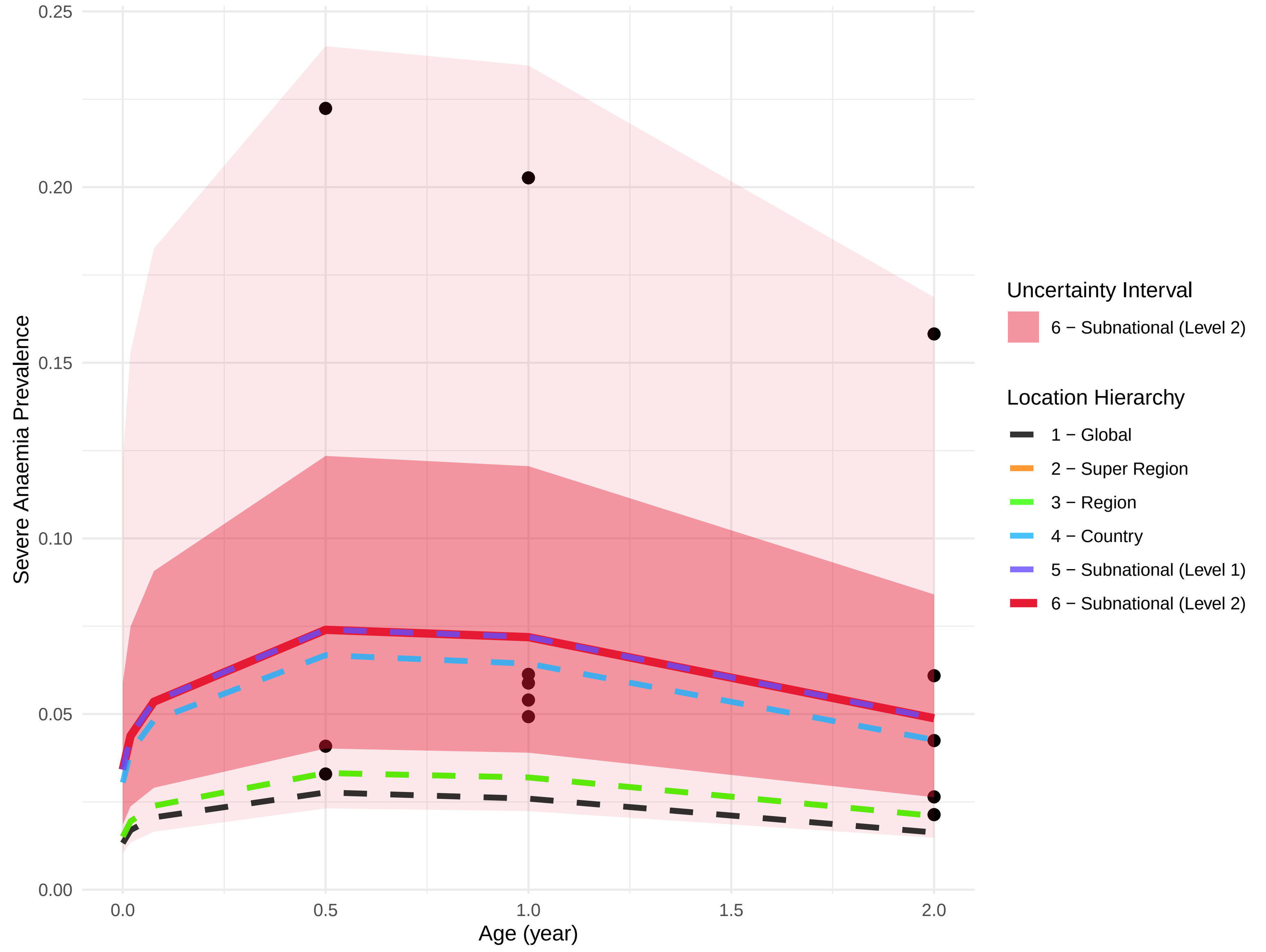

MR−BRT Age/Sex Fits − Non−pregnant Females <2 Years Old − Severe Anaemia Prevalence − Haryana, Urban
WHO−2001 Adjustment Method

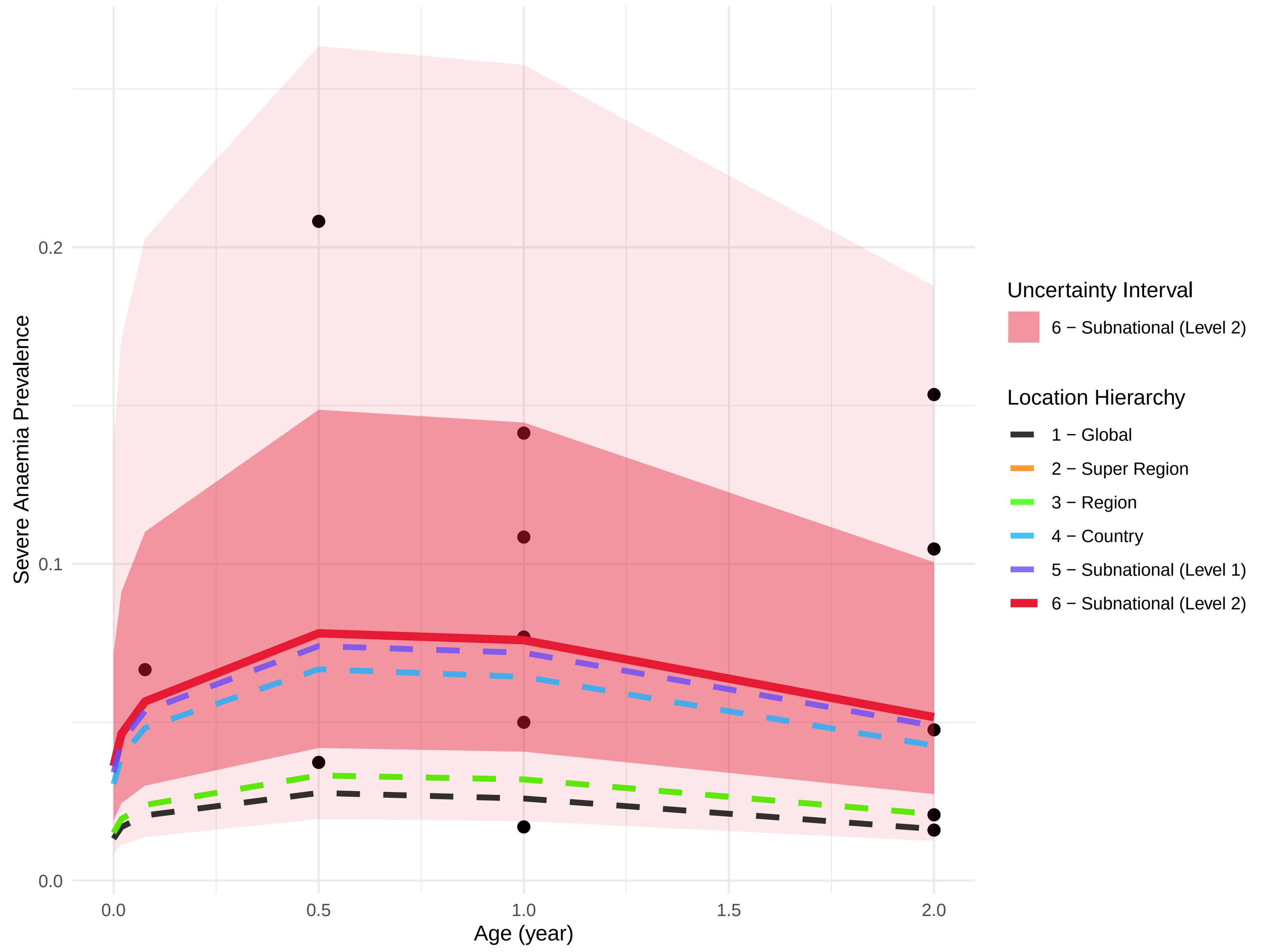

MR−BRT Age/Sex Fits − Non−pregnant Females <2 Years Old − Severe Anaemia Prevalence − Himachal Pradesh
WHO−2001 Adjustment Method

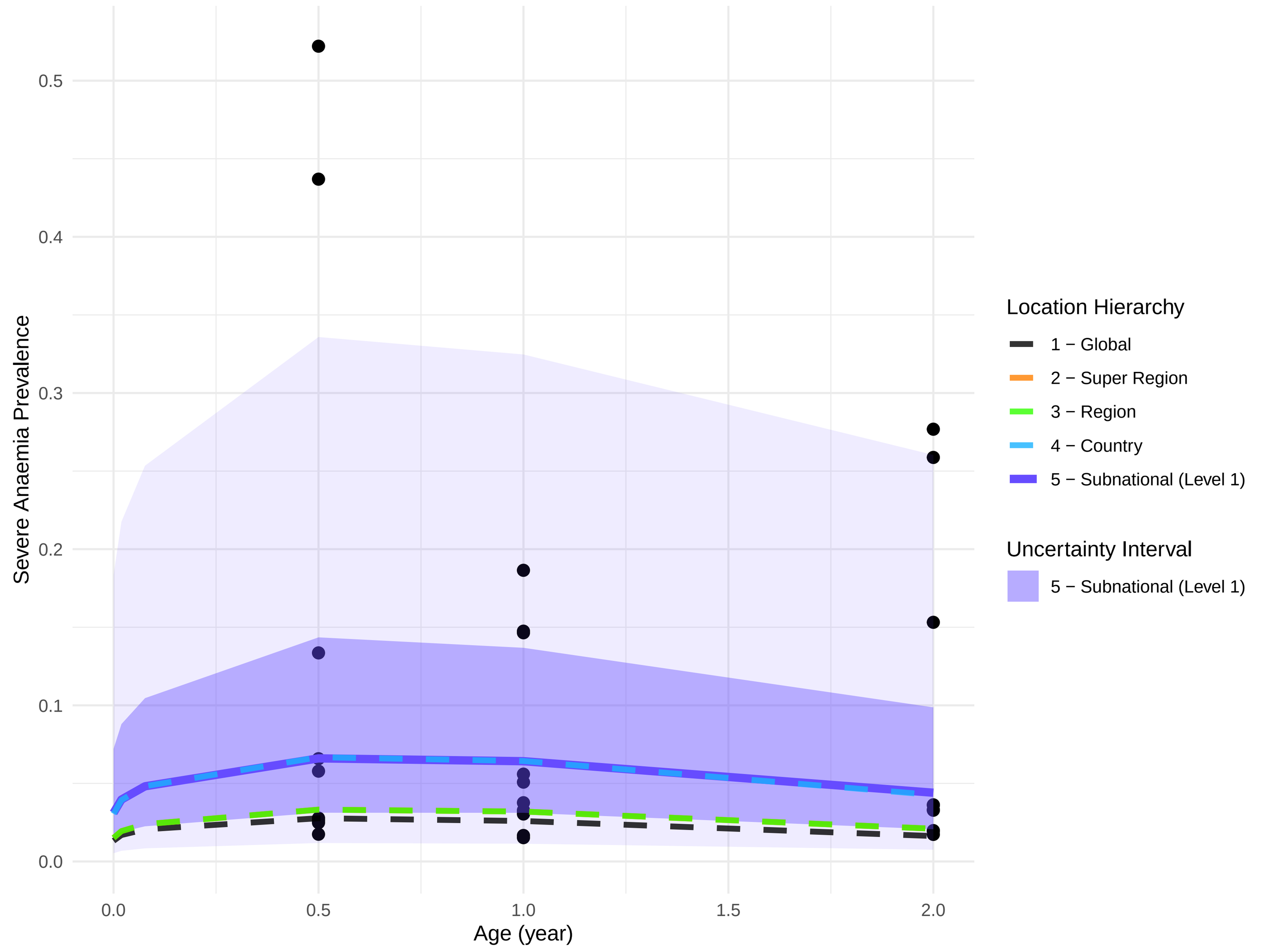

MR−BRT Age/Sex Fits − Non−pregnant Females <2 Years Old − Severe Anaemia Prevalence − Himachal Pradesh, Rural
WHO−2001 Adjustment Method

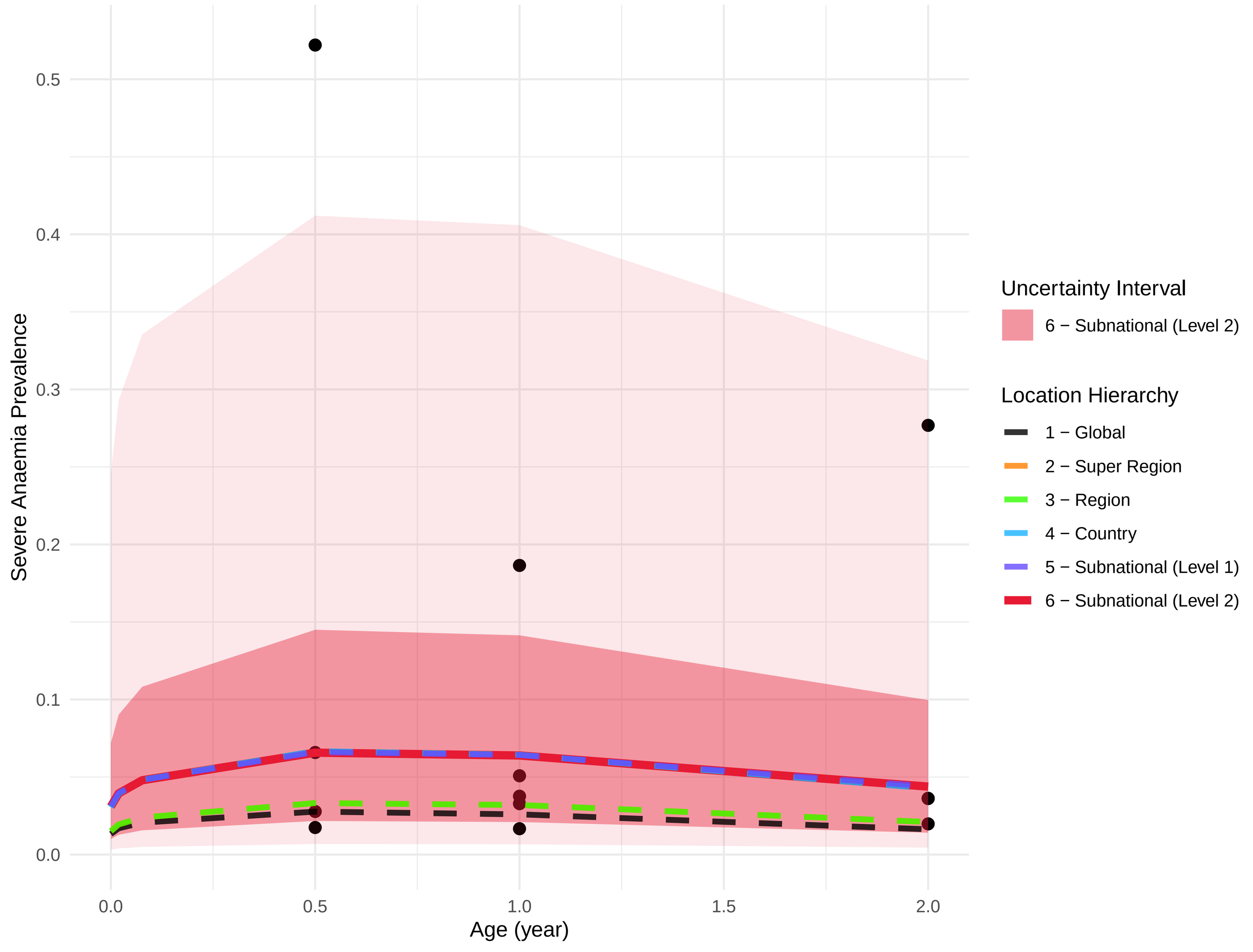

MR−BRT Age/Sex Fits − Non−pregnant Females <2 Years Old − Severe Anaemia Prevalence − Himachal Pradesh, Urban
WHO−2001 Adjustment Method

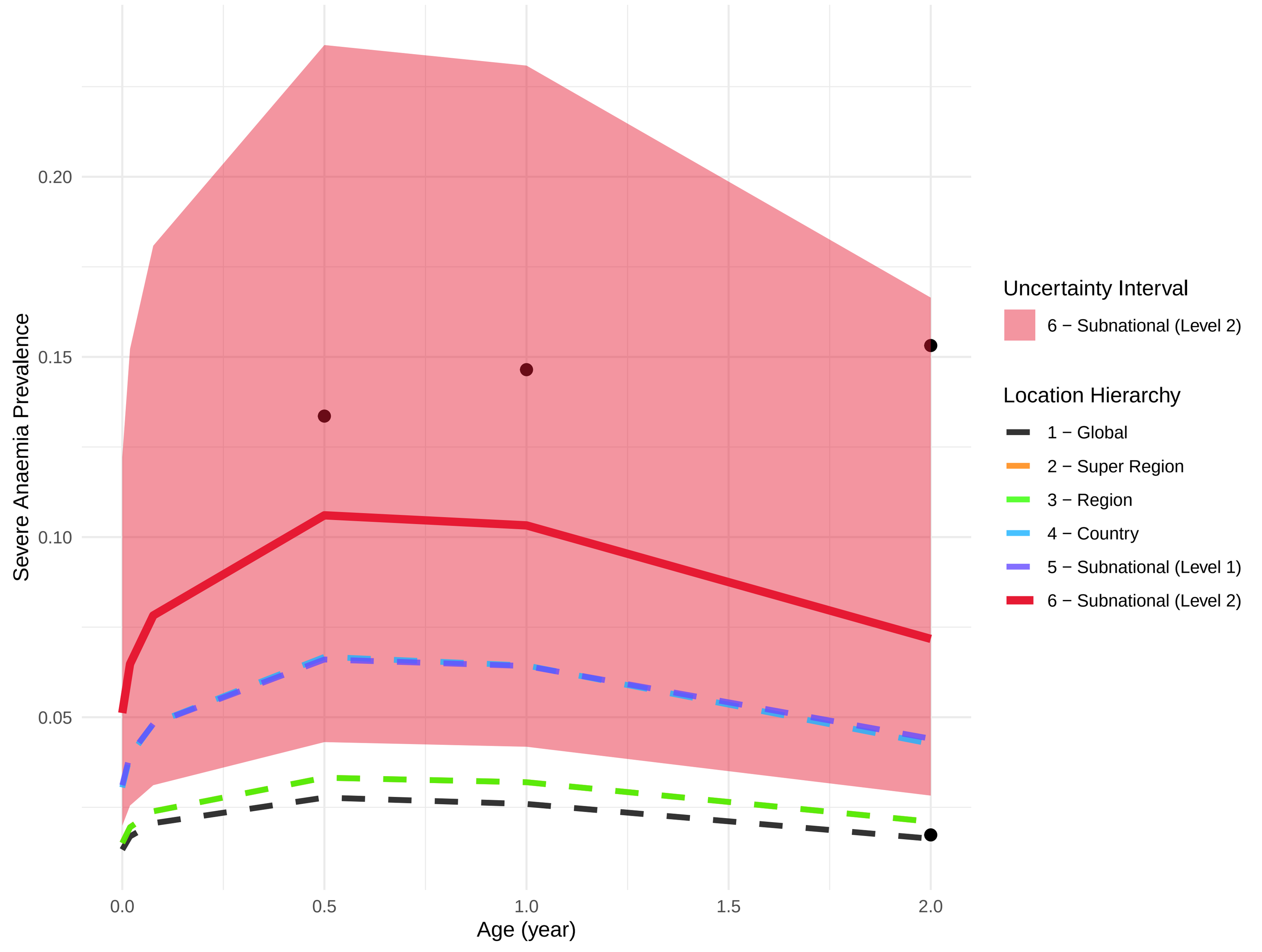

MR−BRT Age/Sex Fits − Non−pregnant Females <2 Years Old − Severe Anaemia Prevalence − Jammu & Kashmir and Ladakh
WHO−2001 Adjustment Method

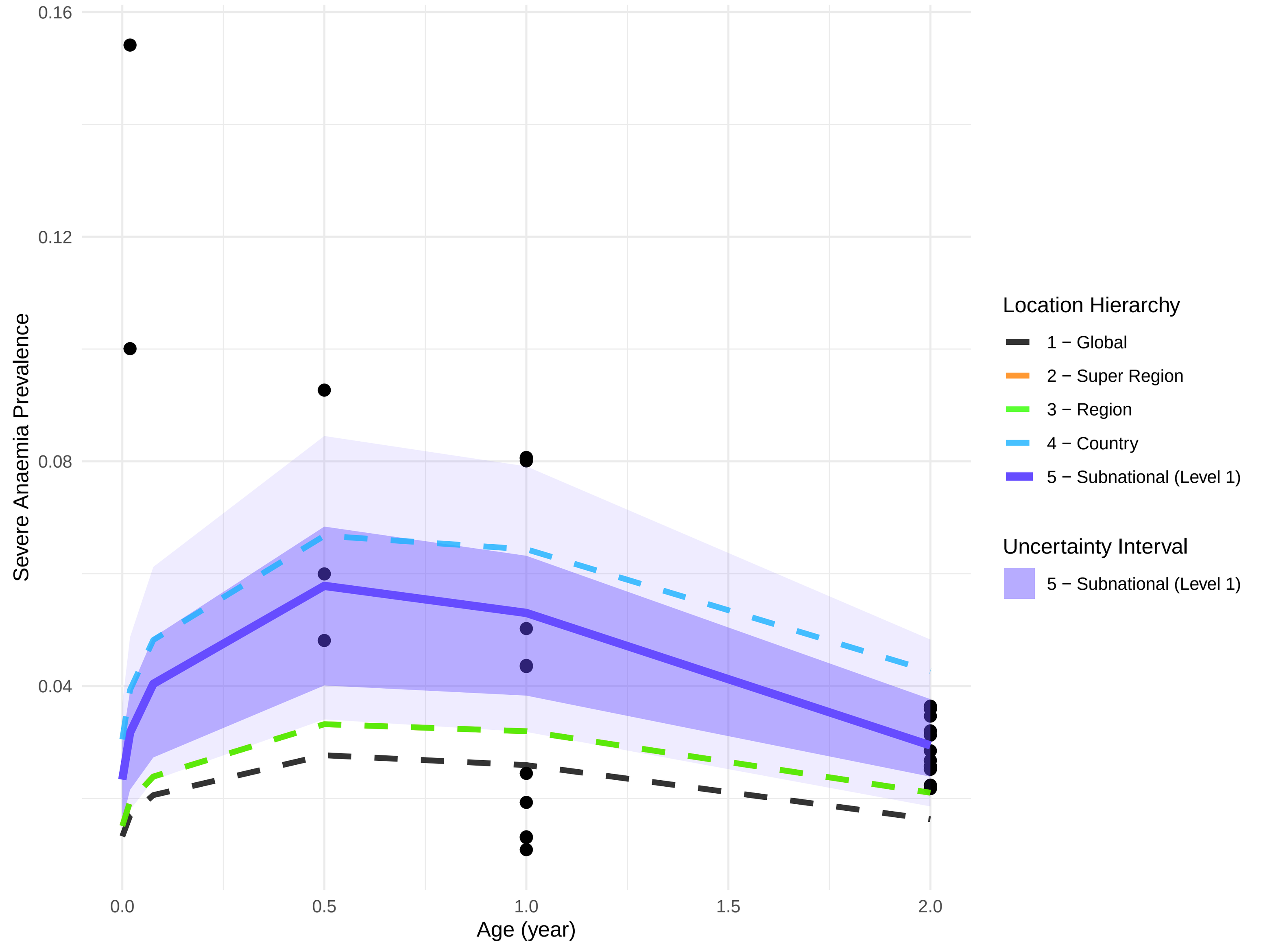

MR−BRT Age/Sex Fits − Non−pregnant Females <2 Years Old − Severe Anaemia Prevalence − Jammu & Kashmir and Ladakh, Rural
WHO−2001 Adjustment Method

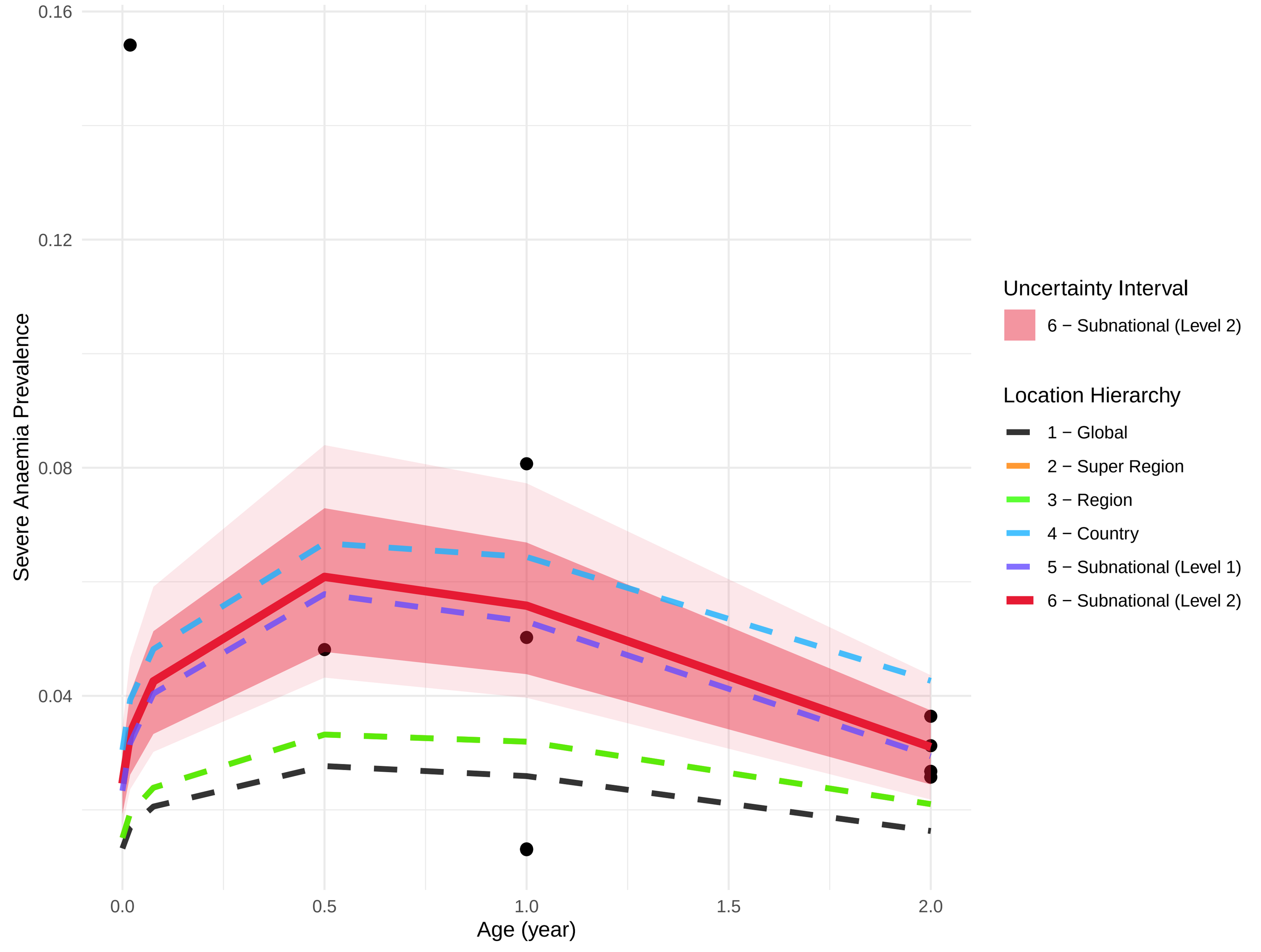

MR−BRT Age/Sex Fits − Non−pregnant Females <2 Years Old − Severe Anaemia Prevalence − Jammu & Kashmir and Ladakh, Urban
WHO−2001 Adjustment Method

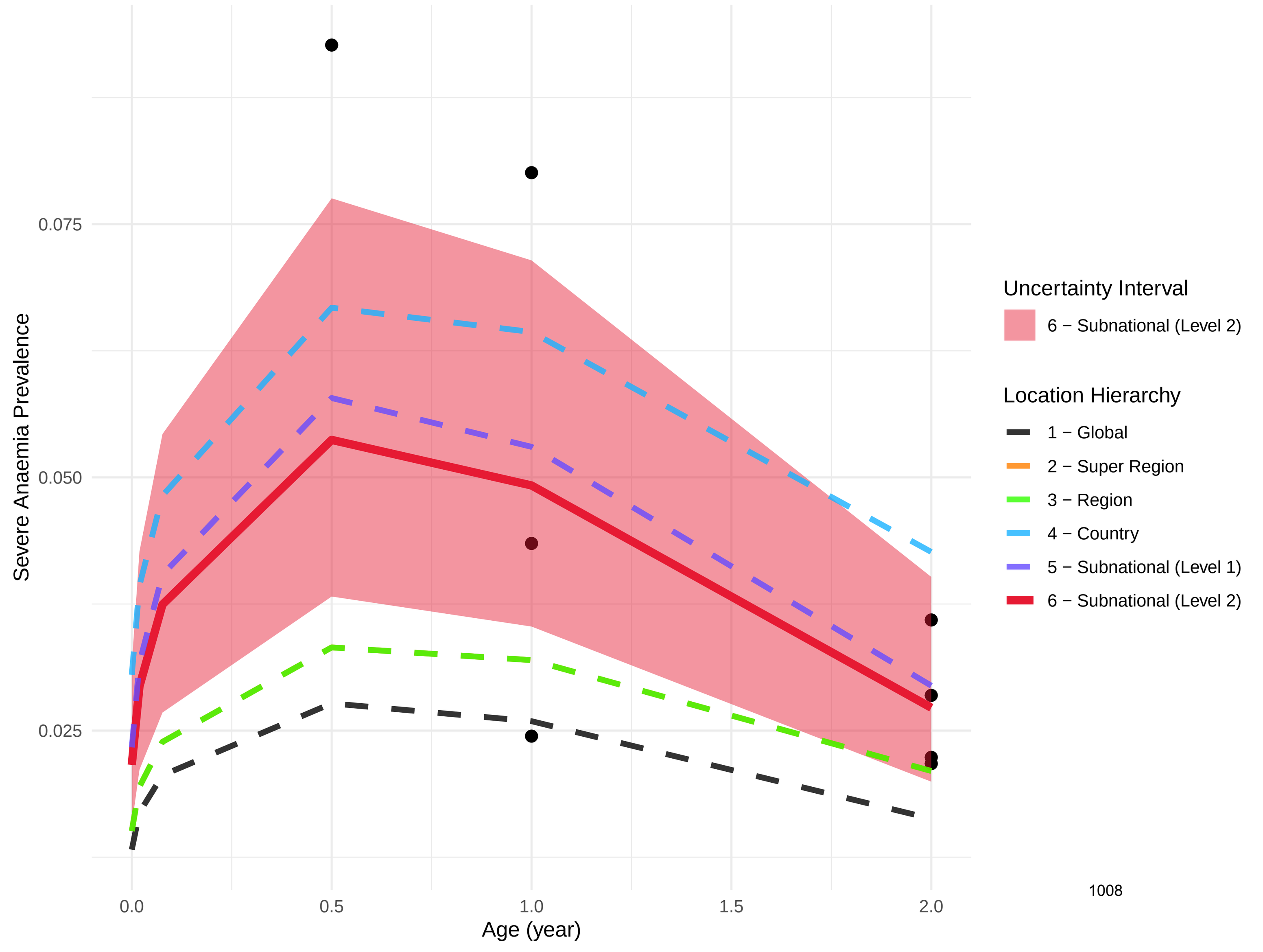

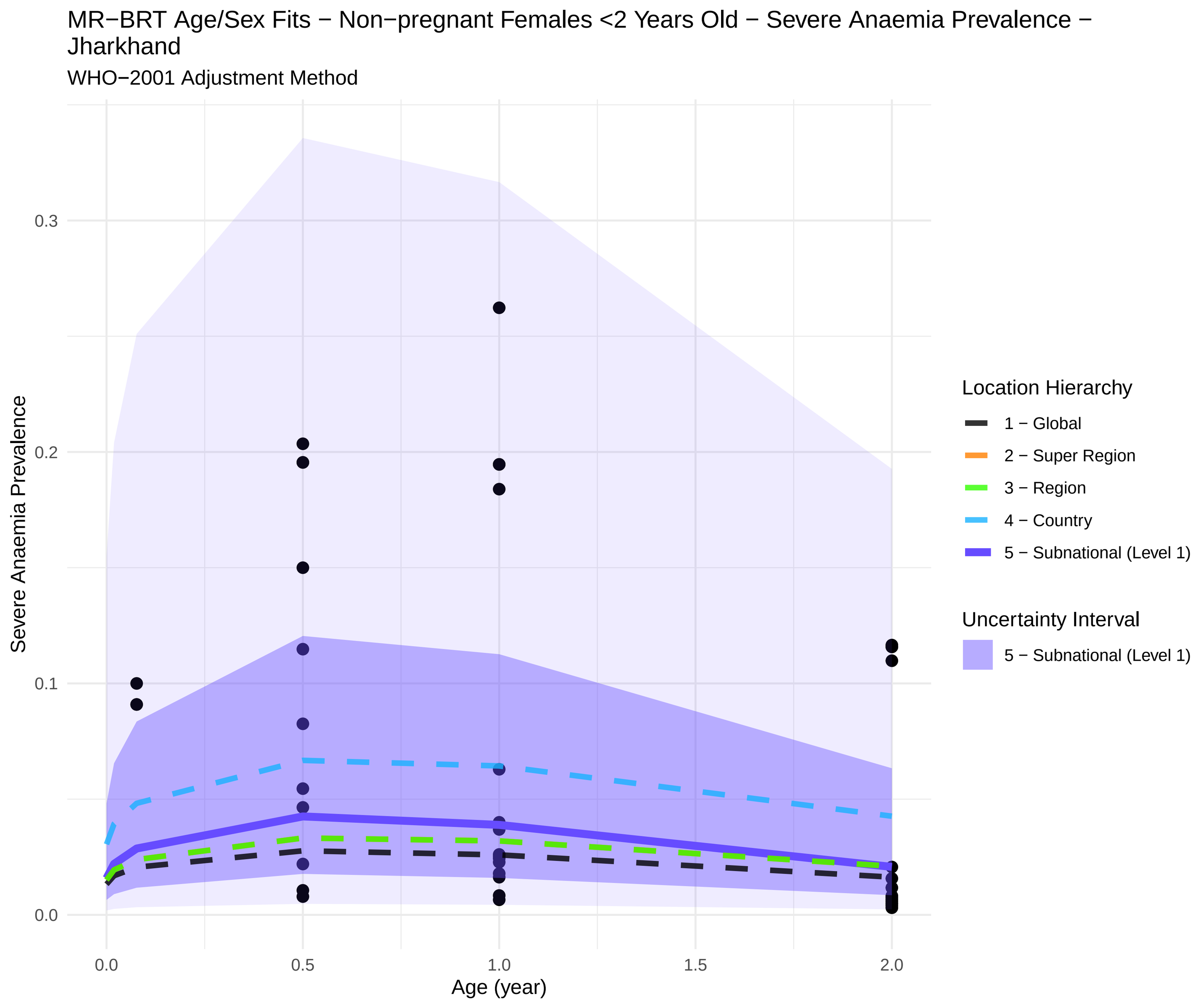
MR−BRT Age/Sex Fits − Non−pregnant Females <2 Years Old − Severe Anaemia Prevalence − Jharkhand
WHO−2001 Adjustment Method
Severe Anaemia Prevalence
Age (year)
Location Hierarchy
1 − Global
2 − Super Region
3 − Region
4 − Country
5 − Subnational (Level 1)
Uncertainty Interval
5 − Subnational (Level 1)

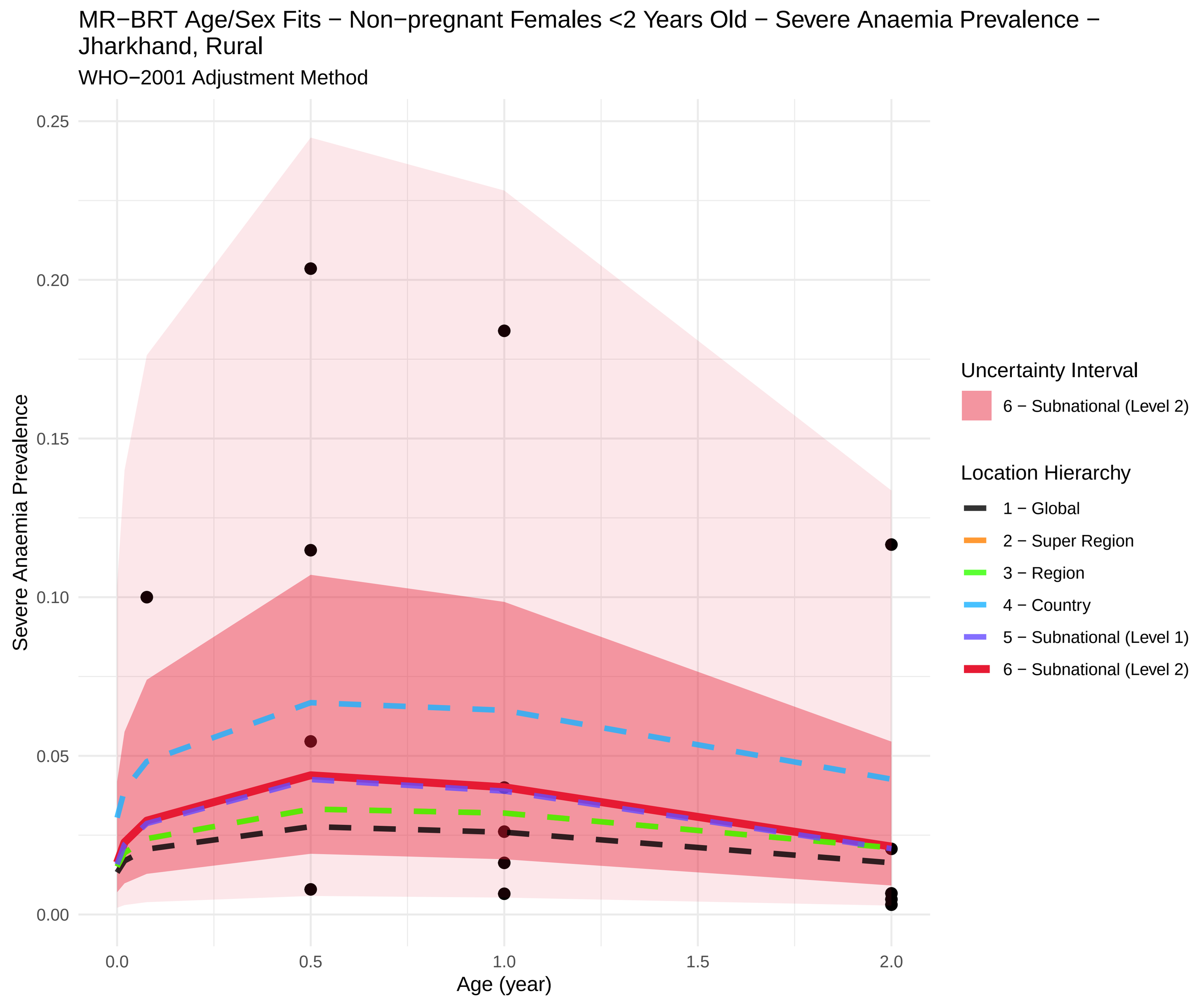
MR−BRT Age/Sex Fits − Non−pregnant Females <2 Years Old − Severe Anaemia Prevalence − Jharkhand, Rural
WHO−2001 Adjustment Method
Severe Anaemia Prevalence
Age (year)
Uncertainty Interval
6 − Subnational (Level 2)
Location Hierarchy
1 − Global
2 − Super Region
3 − Region
4 − Country
5 − Subnational (Level 1)
6 − Subnational (Level 2)

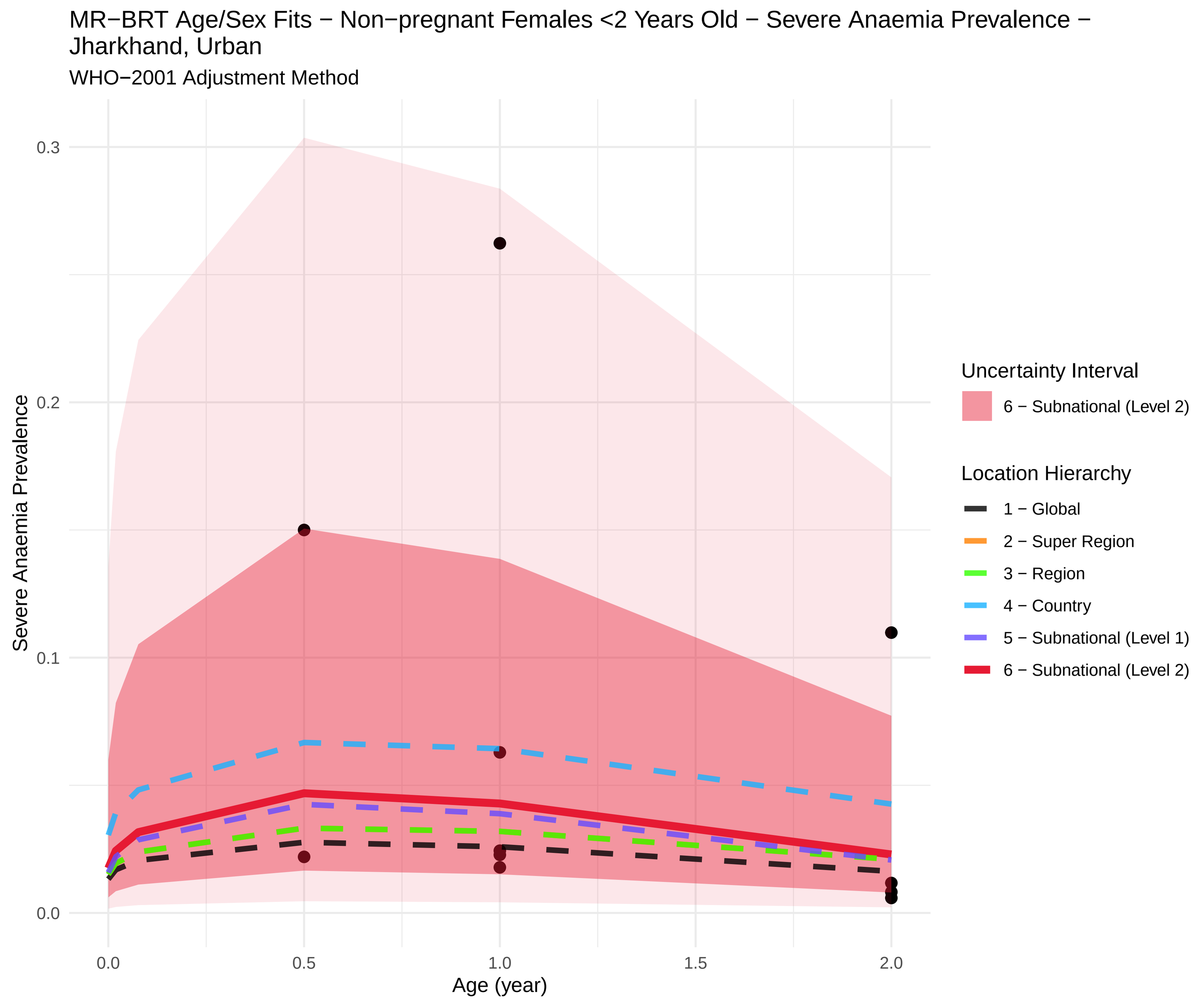
MR−BRT Age/Sex Fits − Non−pregnant Females <2 Years Old − Severe Anaemia Prevalence − Jharkhand, Urban
WHO−2001 Adjustment Method
Severe Anaemia Prevalence
Age (year)
Uncertainty Interval
6 − Subnational (Level 2)
Location Hierarchy
1 − Global
2 − Super Region
3 − Region
4 − Country
5 − Subnational (Level 1)
6 − Subnational (Level 2)

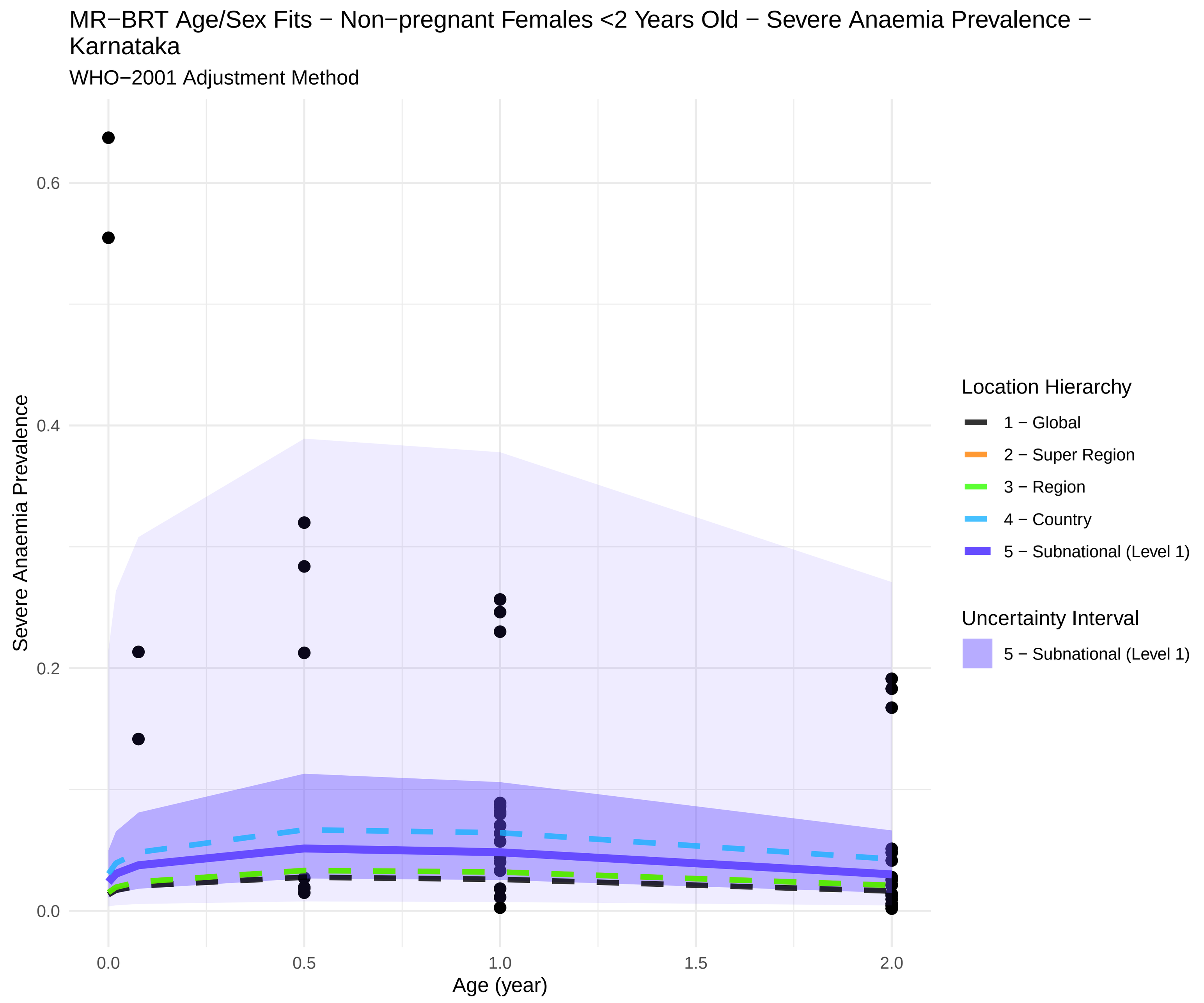
MR−BRT Age/Sex Fits − Non−pregnant Females <2 Years Old − Severe Anaemia Prevalence − Karnataka
WHO−2001 Adjustment Method
Severe Anaemia Prevalence
Age (year)
Location Hierarchy
1 − Global
2 − Super Region
3 − Region
4 − Country
5 − Subnational (Level 1)
Uncertainty Interval
5 − Subnational (Level 1)

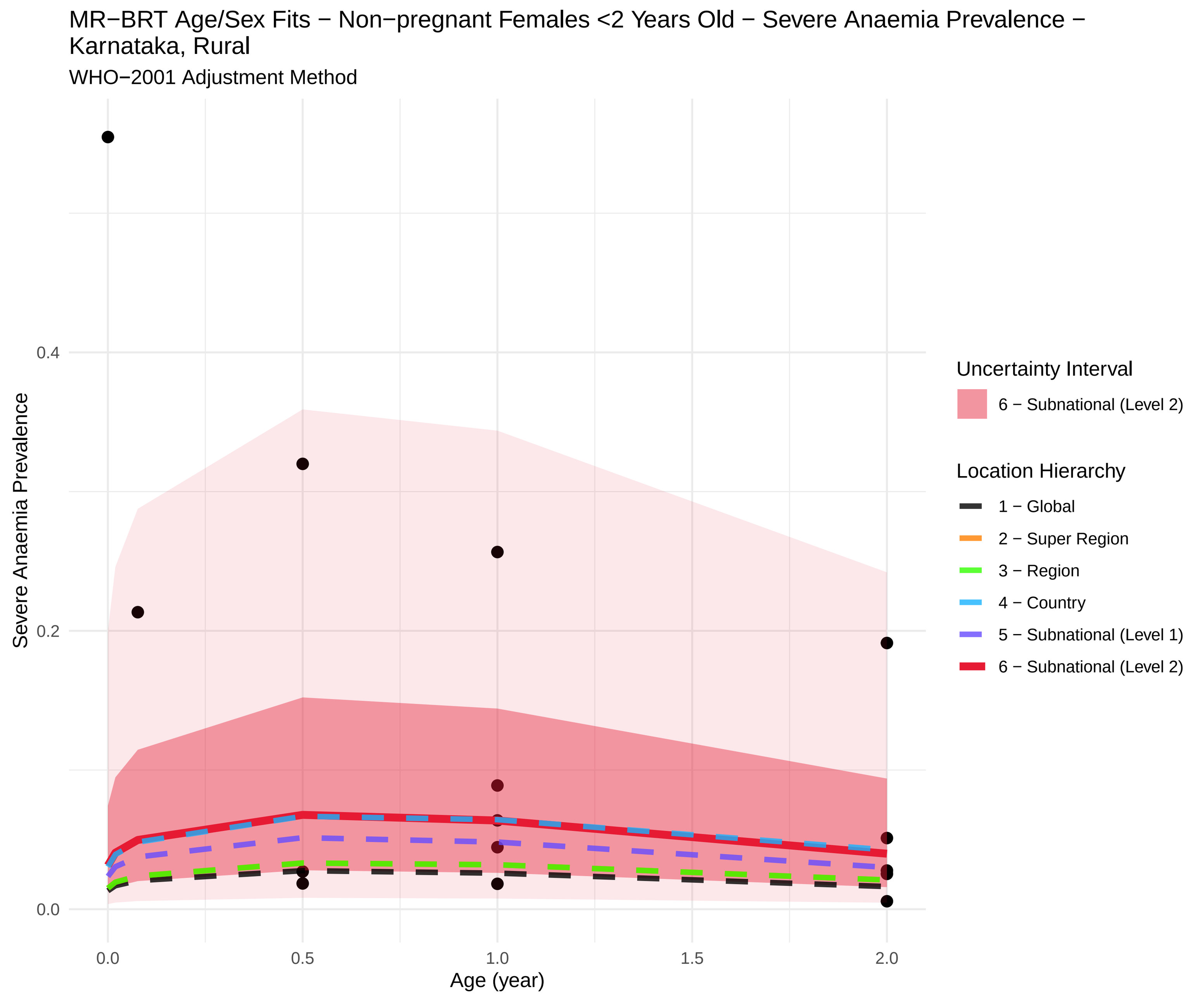
MR−BRT Age/Sex Fits − Non−pregnant Females <2 Years Old − Severe Anaemia Prevalence − Karnataka, Rural
WHO−2001 Adjustment Method
Severe Anaemia Prevalence
Age (year)
Uncertainty Interval
6 − Subnational (Level 2)
Location Hierarchy
1 − Global
2 − Super Region
3 − Region
4 − Country
5 − Subnational (Level 1)
6 − Subnational (Level 2)

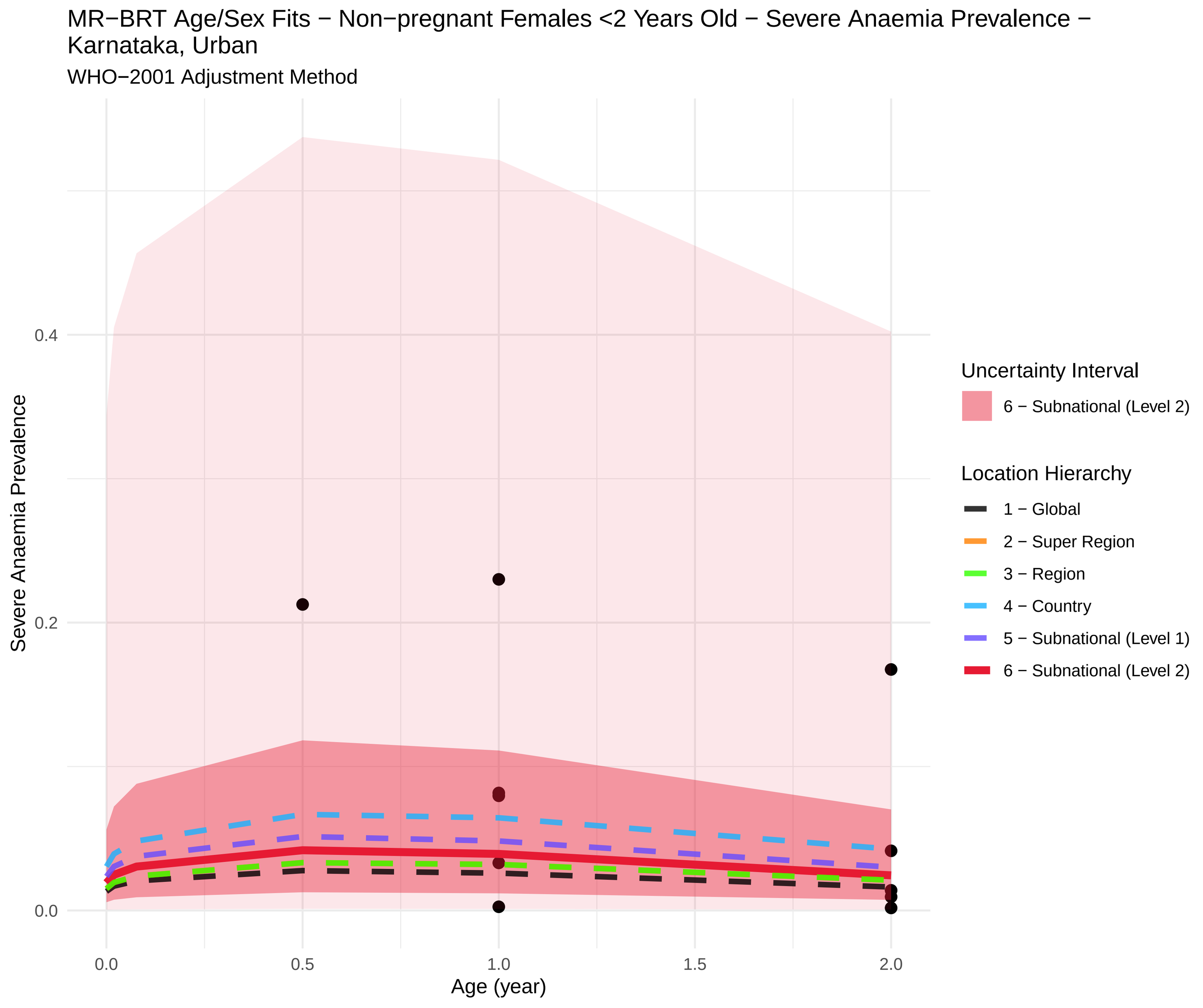
MR−BRT Age/Sex Fits − Non−pregnant Females <2 Years Old − Severe Anaemia Prevalence − Karnataka, Urban
WHO−2001 Adjustment Method
Severe Anaemia Prevalence
Age (year)
Uncertainty Interval
6 − Subnational (Level 2)
Location Hierarchy
1 − Global
2 − Super Region
3 − Region
4 − Country
5 − Subnational (Level 1)
6 − Subnational (Level 2)

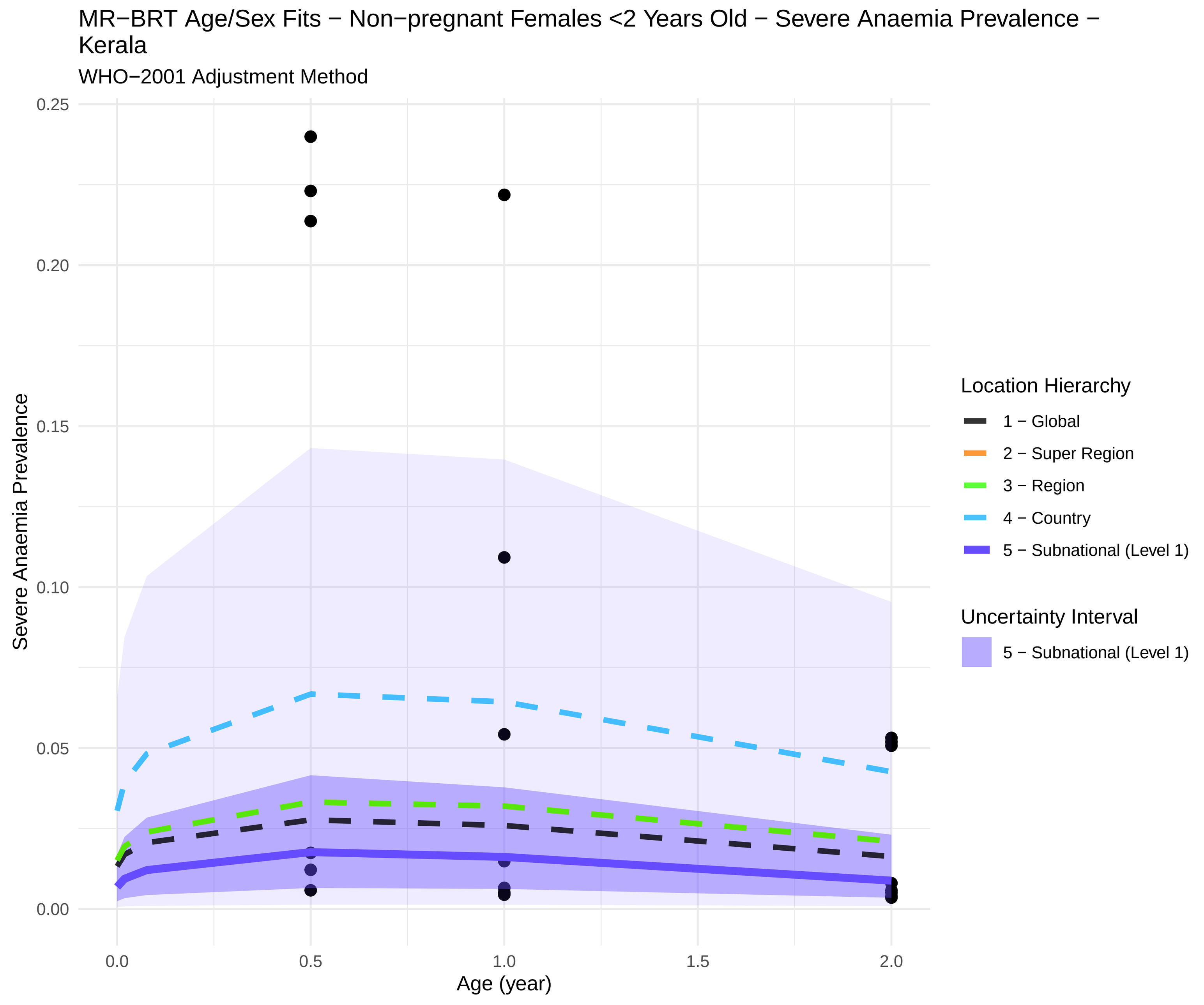
MR−BRT Age/Sex Fits − Non−pregnant Females <2 Years Old − Severe Anaemia Prevalence − Kerala
WHO−2001 Adjustment Method
Severe Anaemia Prevalence
Age (year)
Location Hierarchy
1 − Global
2 − Super Region
3 − Region
4 − Country
5 − Subnational (Level 1)
Uncertainty Interval
5 − Subnational (Level 1)

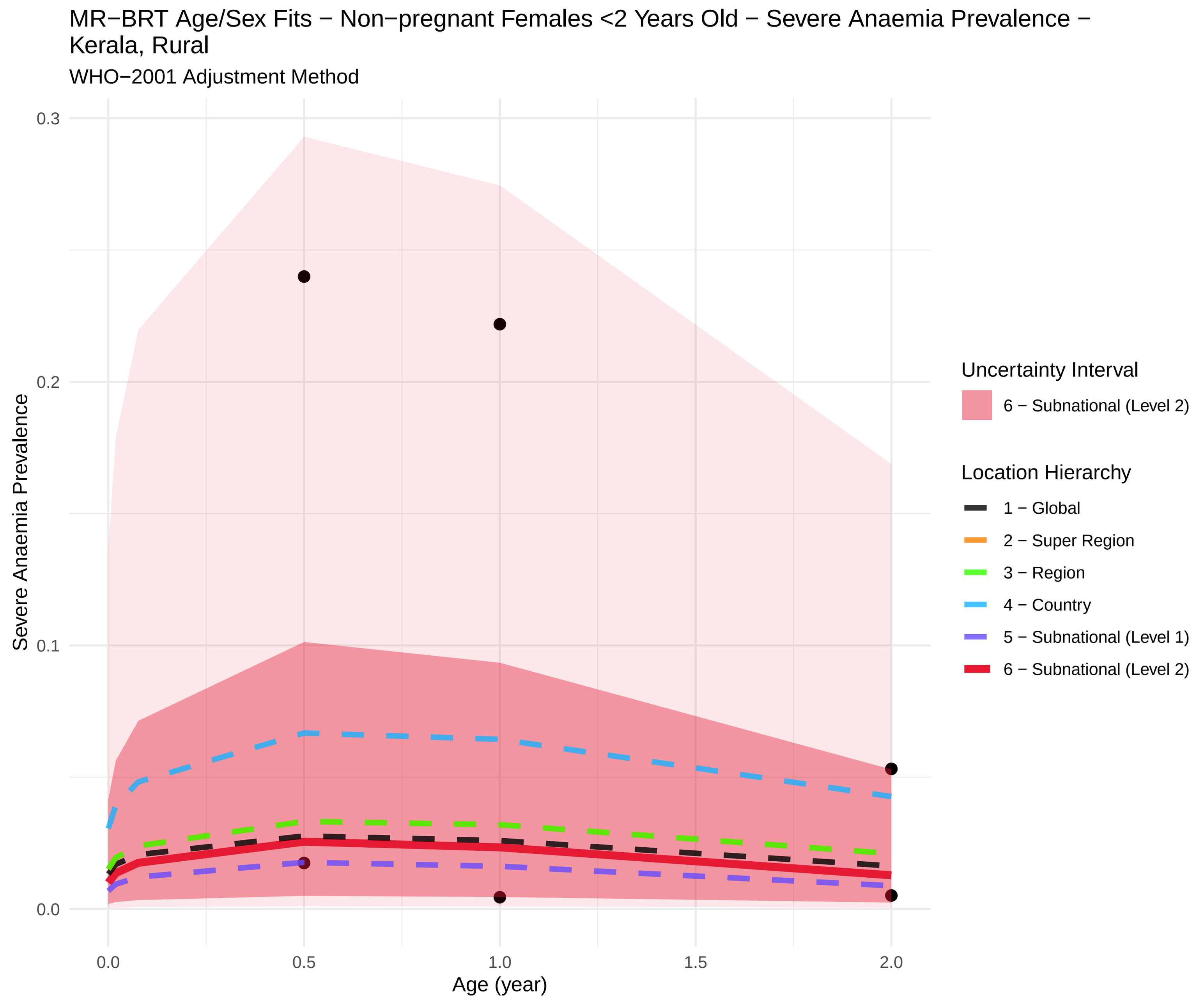
MR−BRT Age/Sex Fits − Non−pregnant Females <2 Years Old − Severe Anaemia Prevalence − Kerala, Rural
WHO−2001 Adjustment Method
Severe Anaemia Prevalence
Age (year)
Uncertainty Interval
6 − Subnational (Level 2)
Location Hierarchy
1 − Global
2 − Super Region
3 − Region
4 − Country
5 − Subnational (Level 1)
6 − Subnational (Level 2)

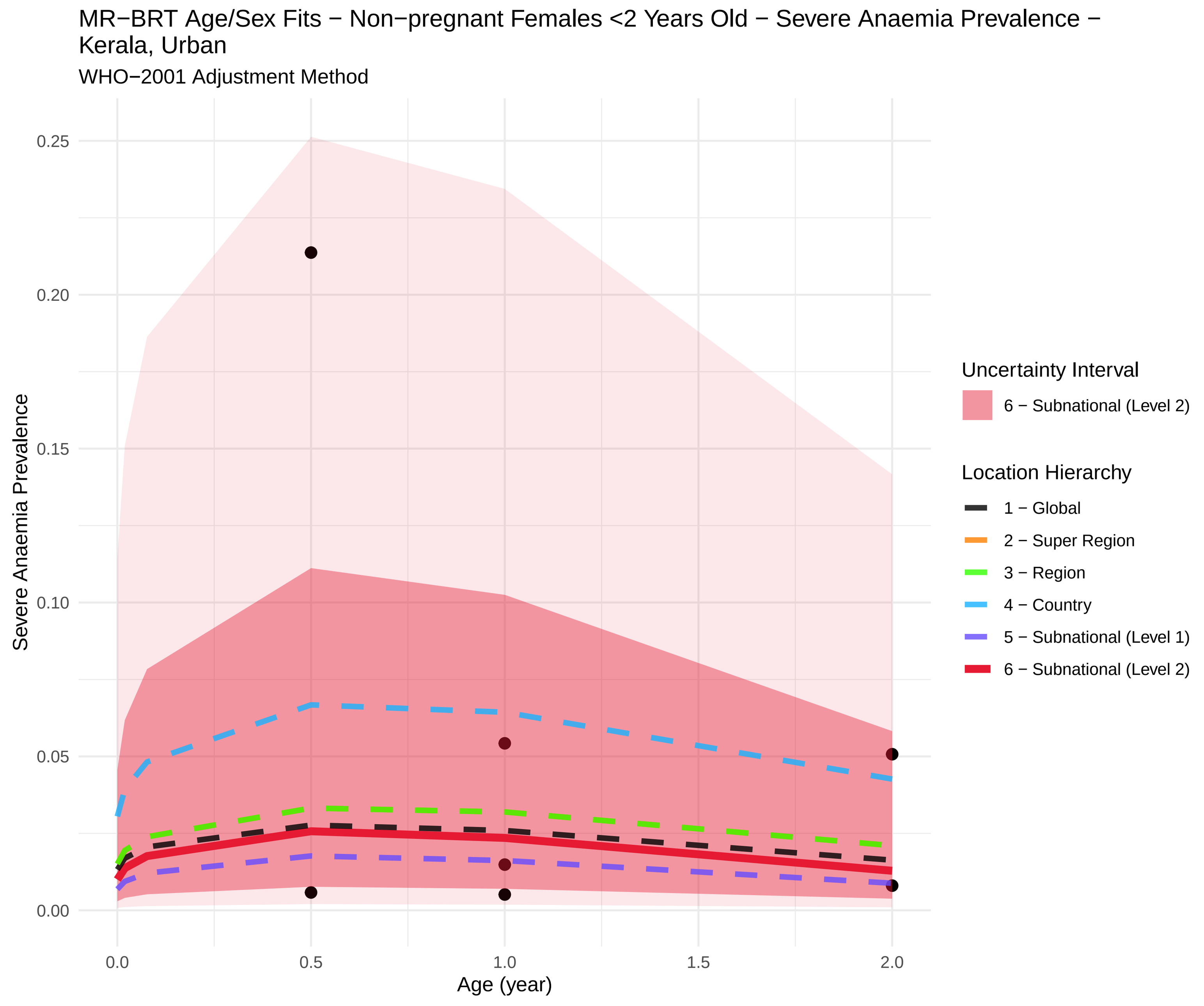
MR−BRT Age/Sex Fits − Non−pregnant Females <2 Years Old − Severe Anaemia Prevalence − Kerala, Urban
WHO−2001 Adjustment Method
Severe Anaemia Prevalence
Age (year)
Uncertainty Interval
6 − Subnational (Level 2)
Location Hierarchy
1 − Global
2 − Super Region
3 − Region
4 − Country
5 − Subnational (Level 1)
6 − Subnational (Level 2)

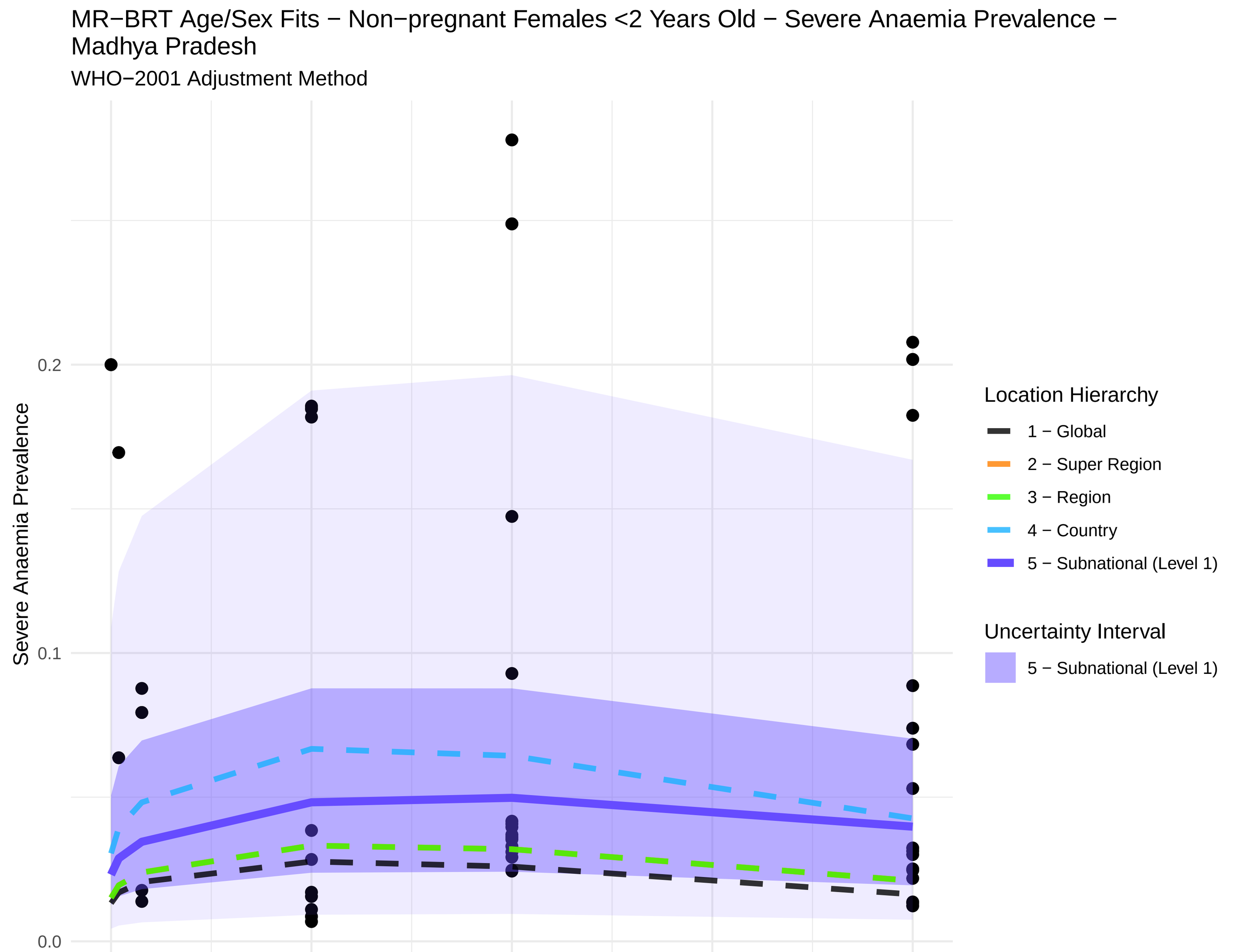
MR−BRT Age/Sex Fits − Non−pregnant Females <2 Years Old − Severe Anaemia Prevalence − Madhya Pradesh
WHO−2001 Adjustment Method
Severe Anaemia Prevalence
Age (year)
Location Hierarchy
1 − Global
2 − Super Region
3 − Region
4 − Country
5 − Subnational (Level 1)
Uncertainty Interval
5 − Subnational (Level 1)

MR−BRT Age/Sex Fits − Non−pregnant Females <2 Years Old − Severe Anaemia Prevalence − Madhya Pradesh, Rural
WHO−2001 Adjustment Method

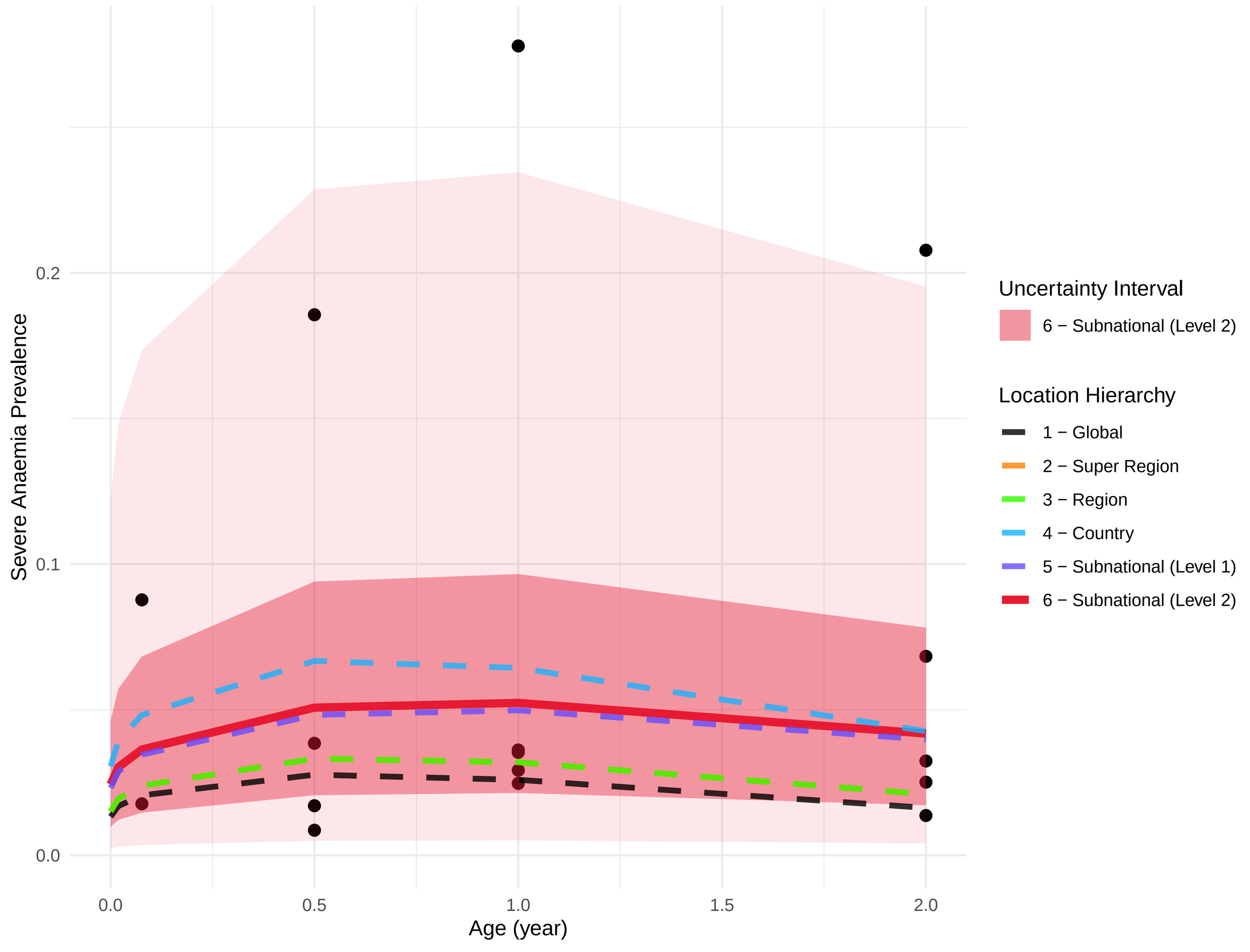

MR−BRT Age/Sex Fits − Non−pregnant Females <2 Years Old − Severe Anaemia Prevalence − Madhya Pradesh, Urban
WHO−2001 Adjustment Method

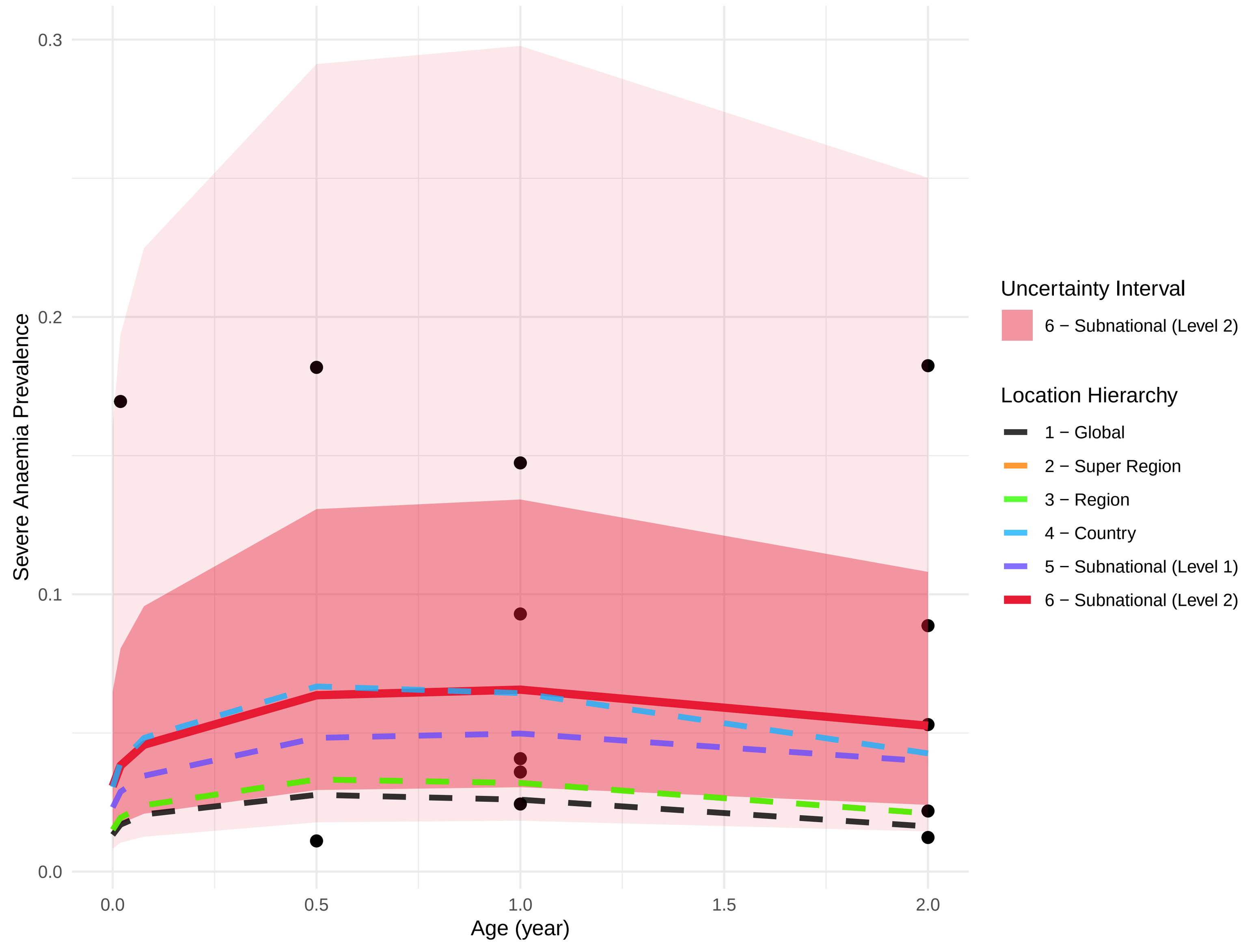

MR−BRT Age/Sex Fits − Non−pregnant Females <2 Years Old − Severe Anaemia Prevalence − Maharashtra
WHO−2001 Adjustment Method

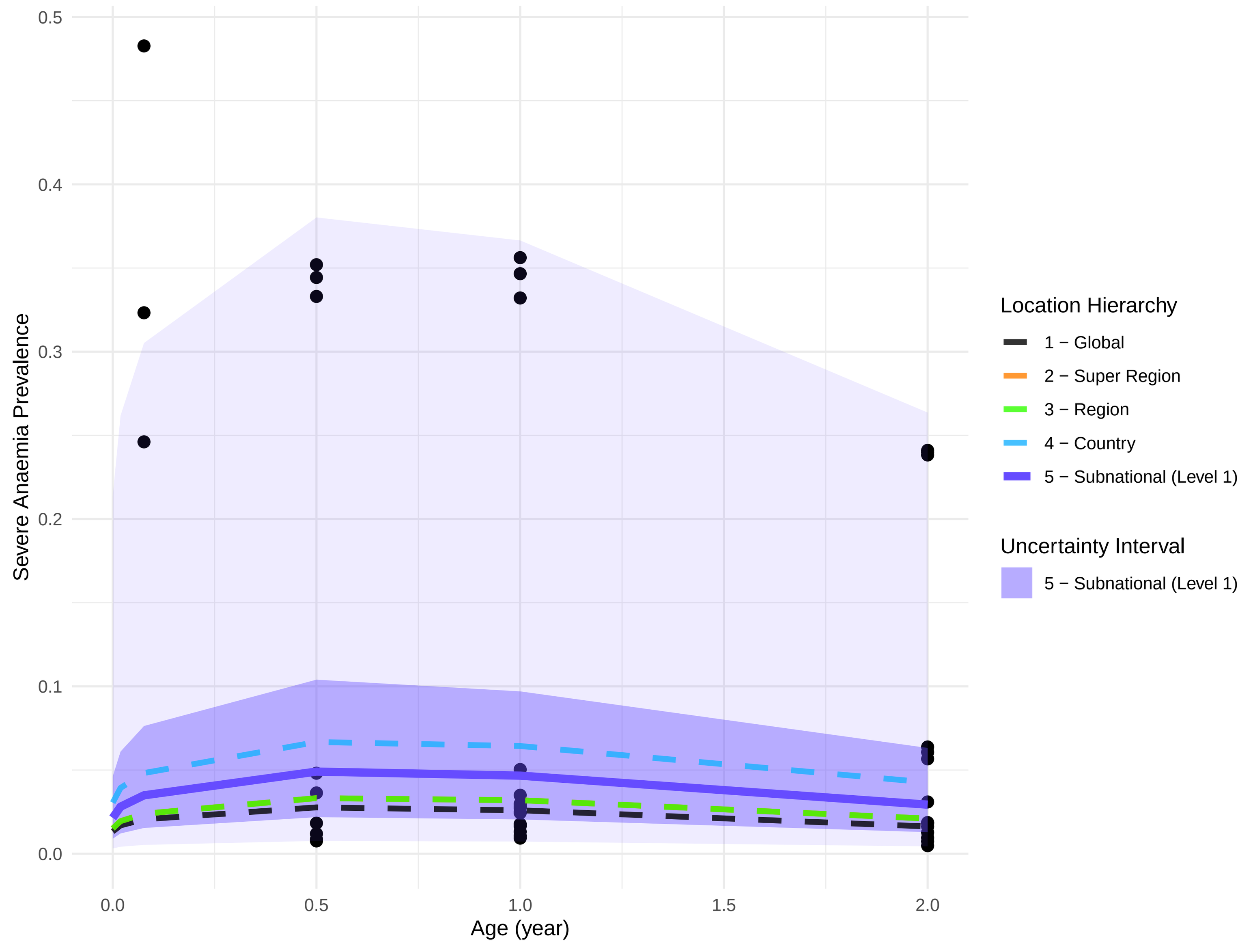

MR−BRT Age/Sex Fits − Non−pregnant Females <2 Years Old − Severe Anaemia Prevalence − Maharashtra, Rural
WHO−2001 Adjustment Method

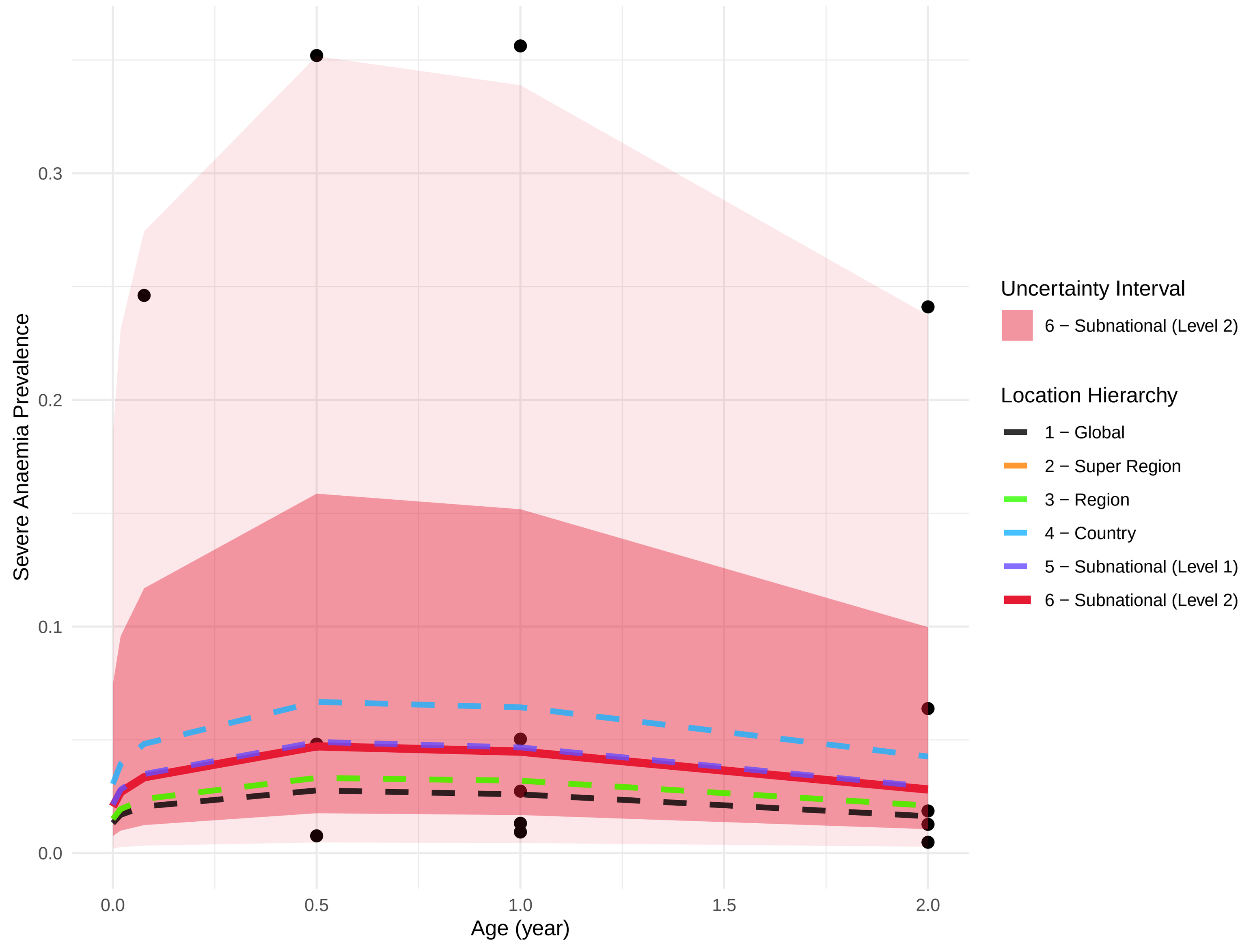

MR−BRT Age/Sex Fits − Non−pregnant Females <2 Years Old − Severe Anaemia Prevalence − Maharashtra, Urban
WHO−2001 Adjustment Method

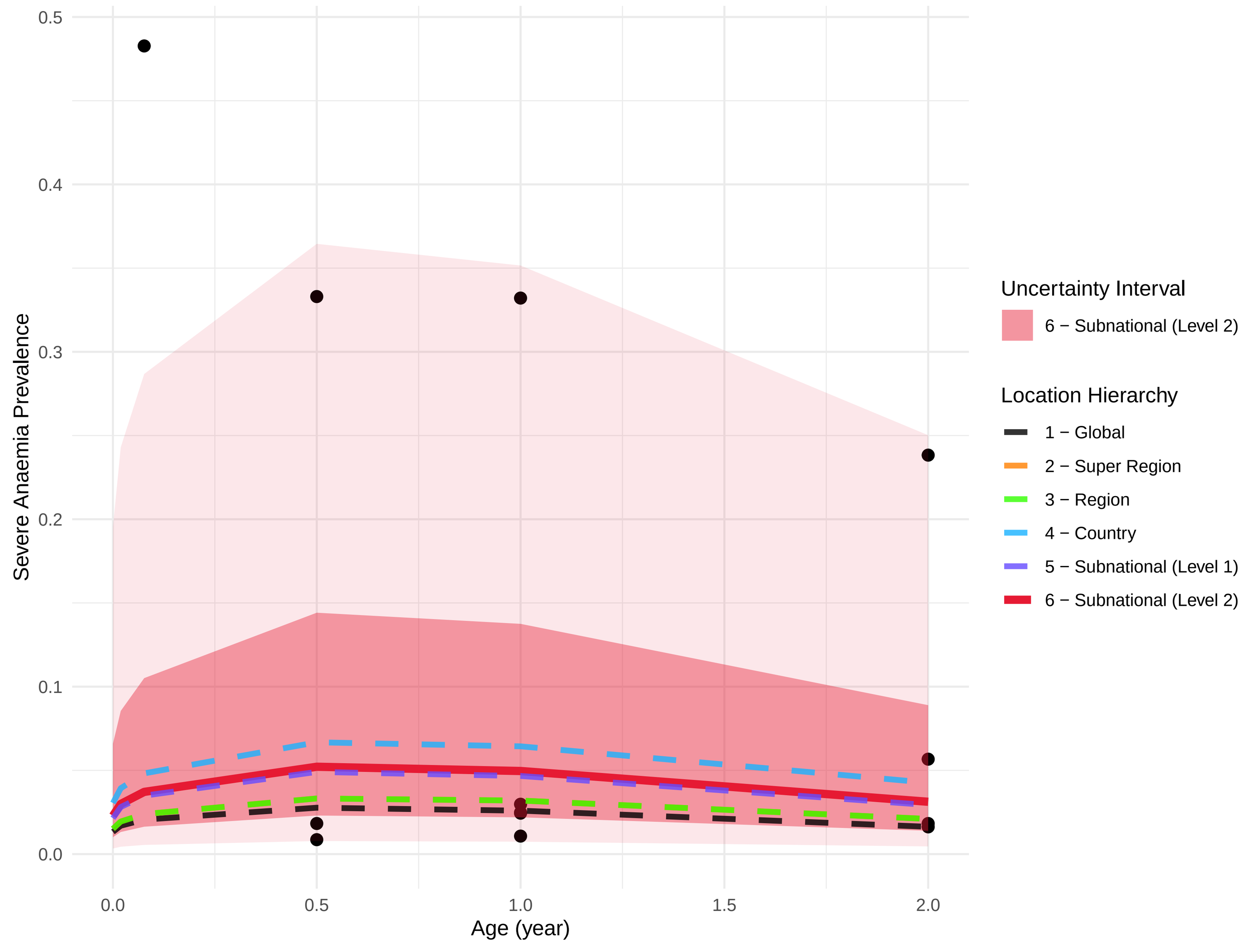

MR−BRT Age/Sex Fits − Non−pregnant Females <2 Years Old − Severe Anaemia Prevalence − Manipur
WHO−2001 Adjustment Method

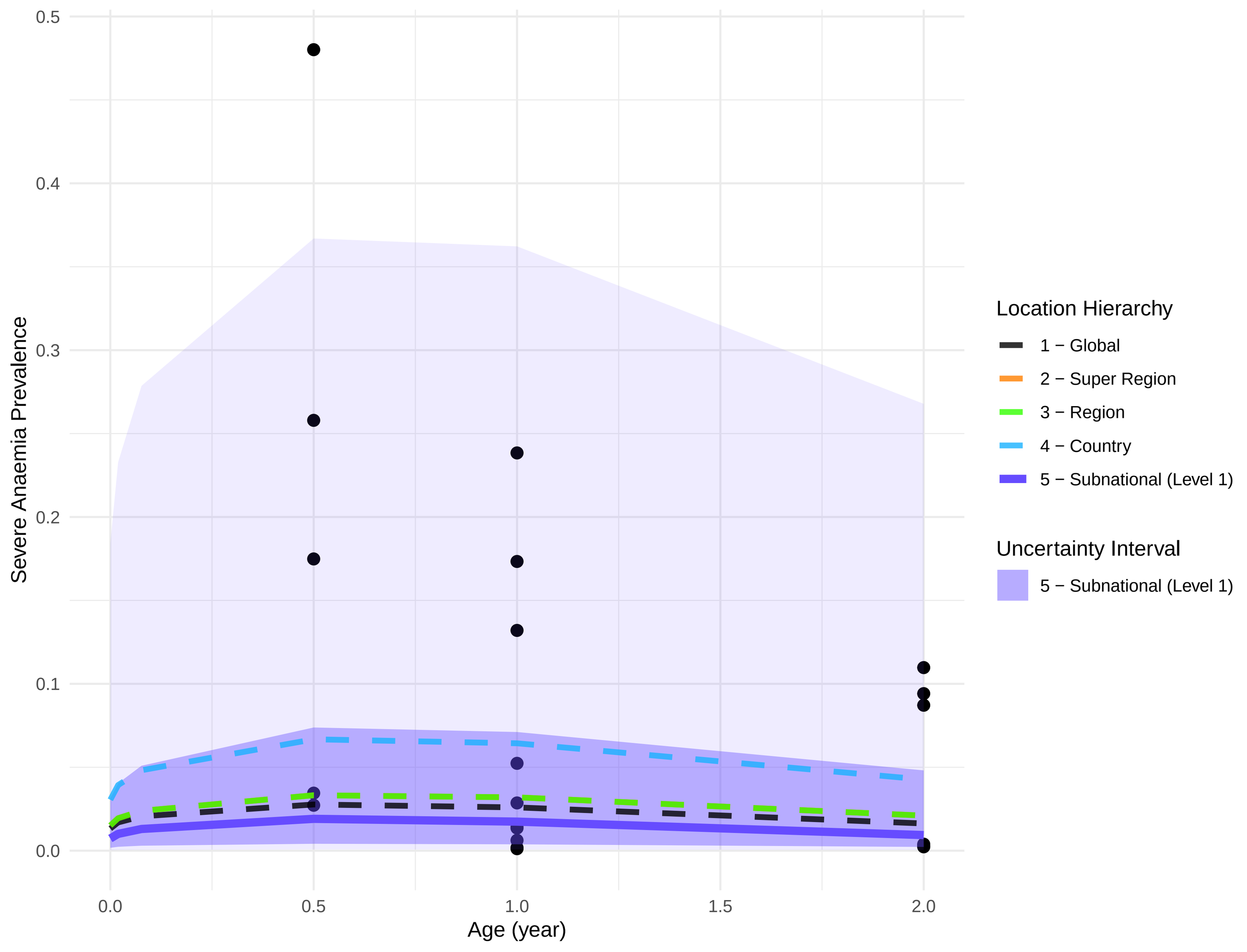

MR−BRT Age/Sex Fits − Non−pregnant Females <2 Years Old − Severe Anaemia Prevalence − Manipur, Rural
WHO−2001 Adjustment Method

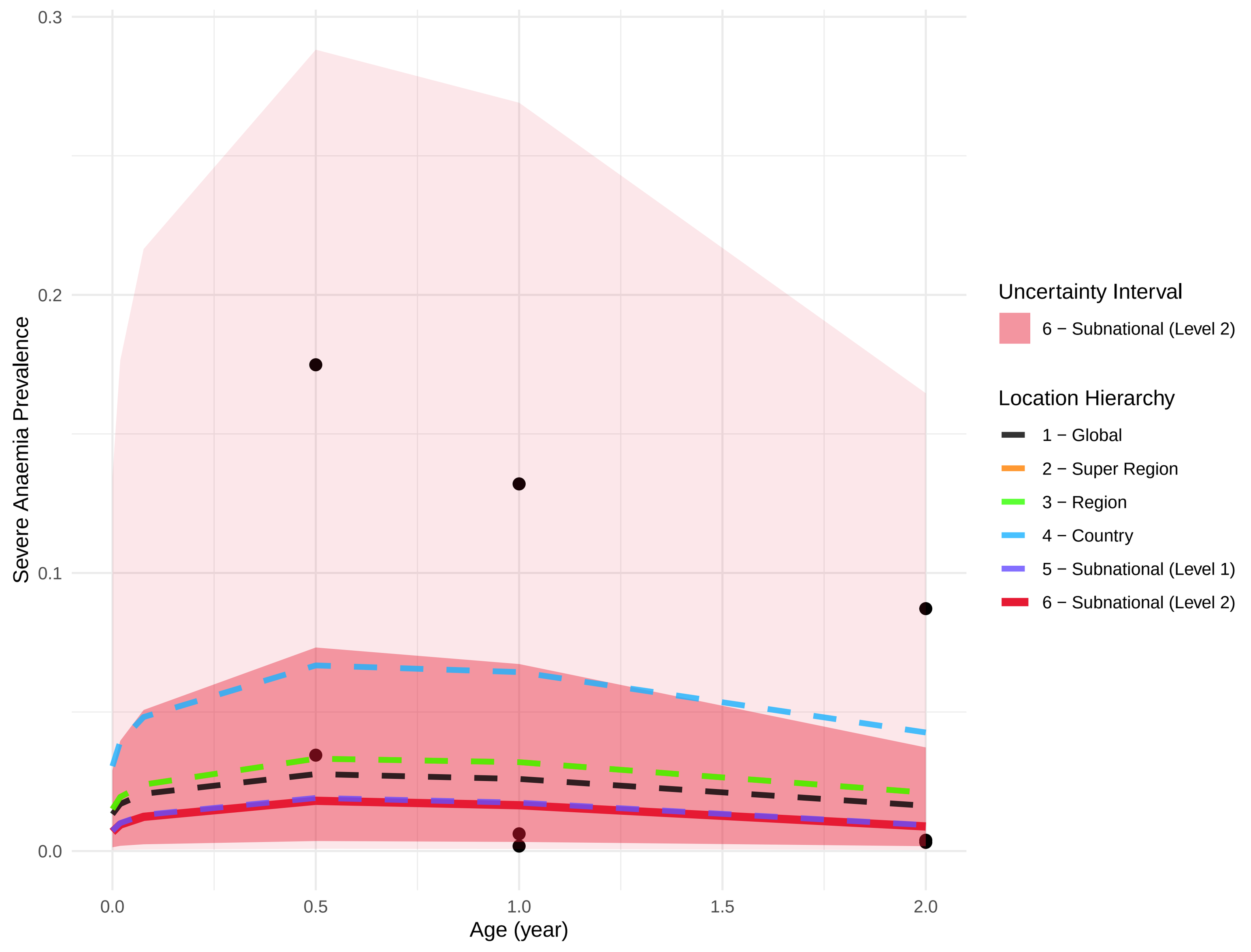

MR−BRT Age/Sex Fits − Non−pregnant Females <2 Years Old − Severe Anaemia Prevalence − Manipur, Urban
WHO−2001 Adjustment Method

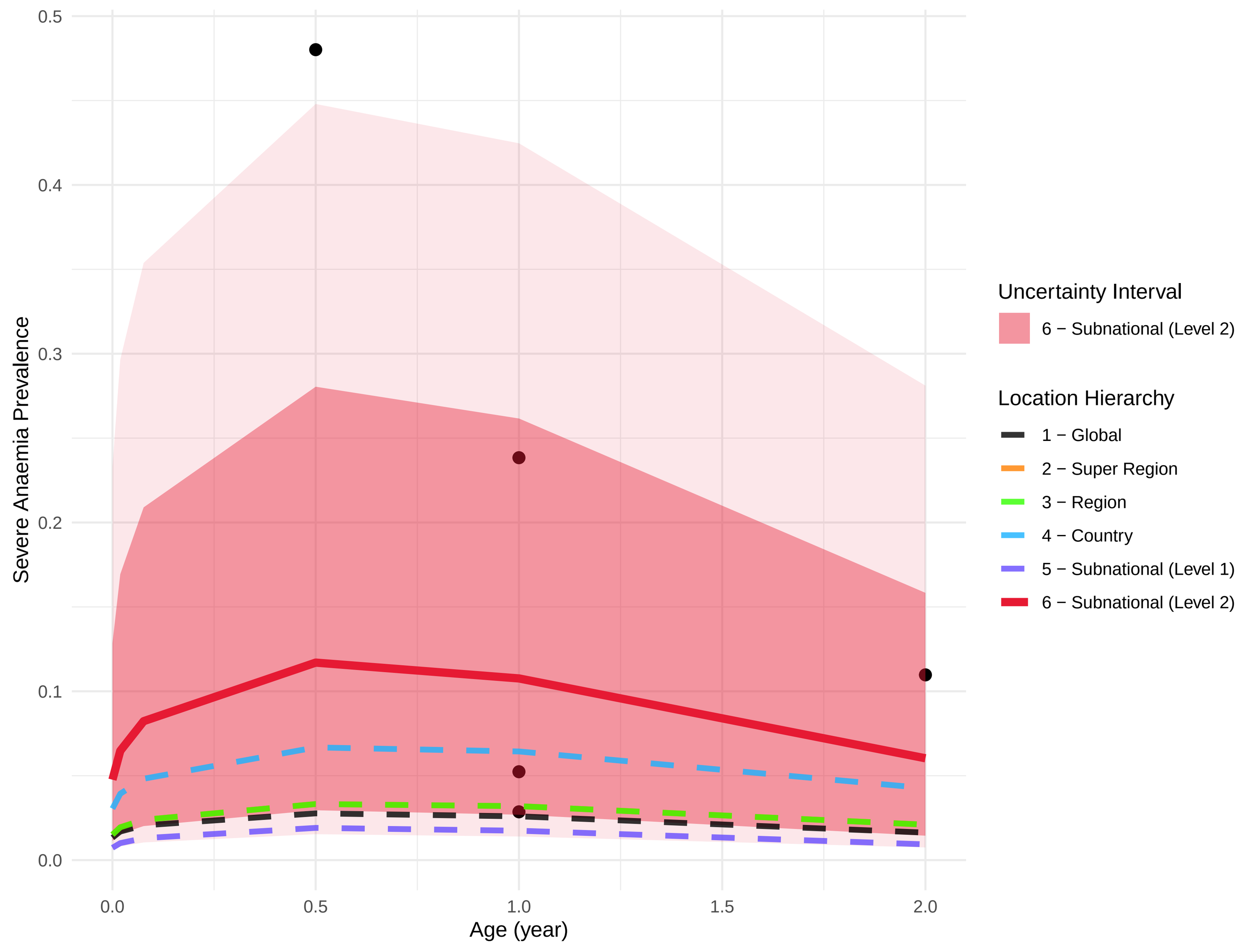

MR−BRT Age/Sex Fits − Non−pregnant Females <2 Years Old − Severe Anaemia Prevalence − Meghalaya
WHO−2001 Adjustment Method

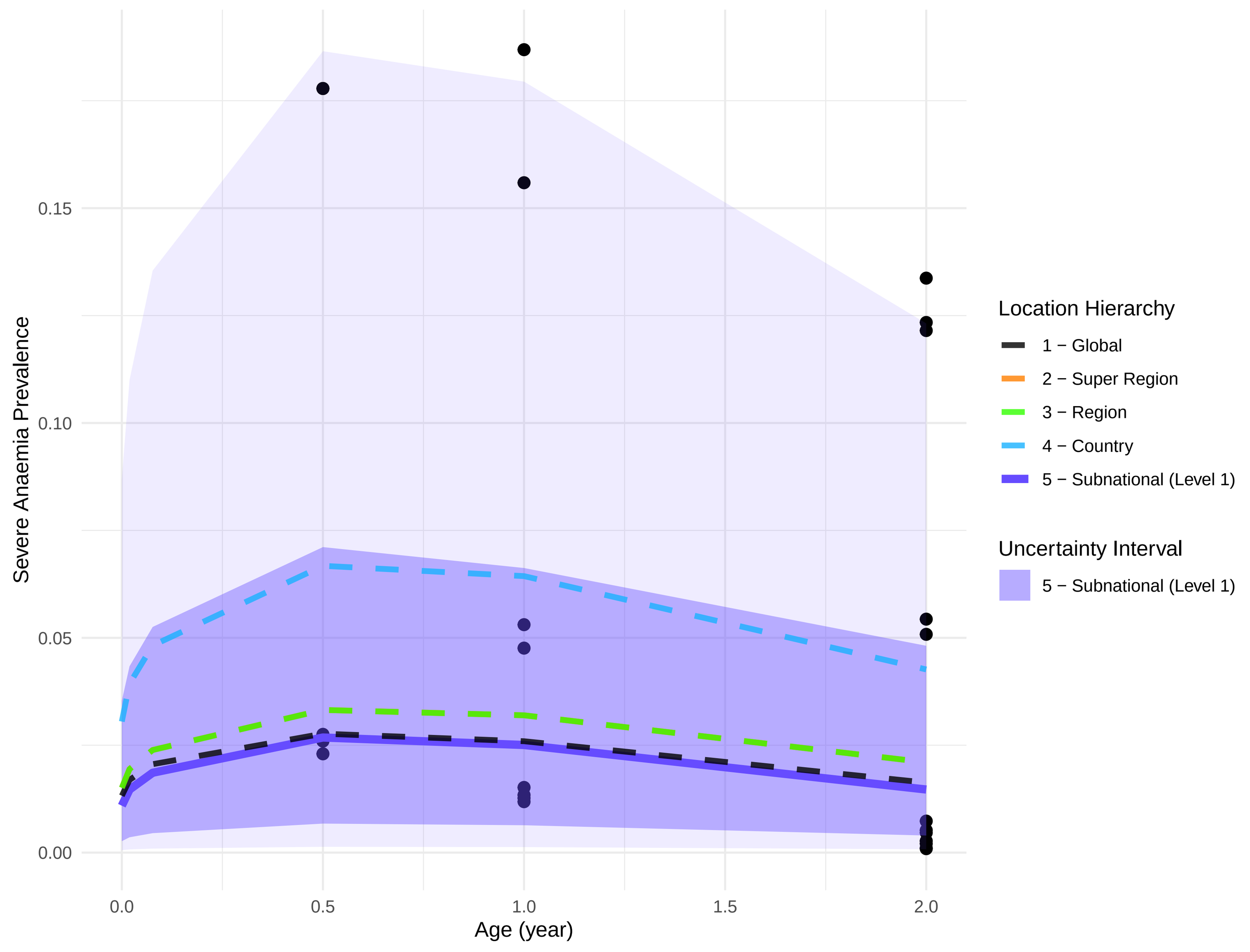

MR−BRT Age/Sex Fits − Non−pregnant Females <2 Years Old − Severe Anaemia Prevalence − Meghalaya, Rural
WHO−2001 Adjustment Method

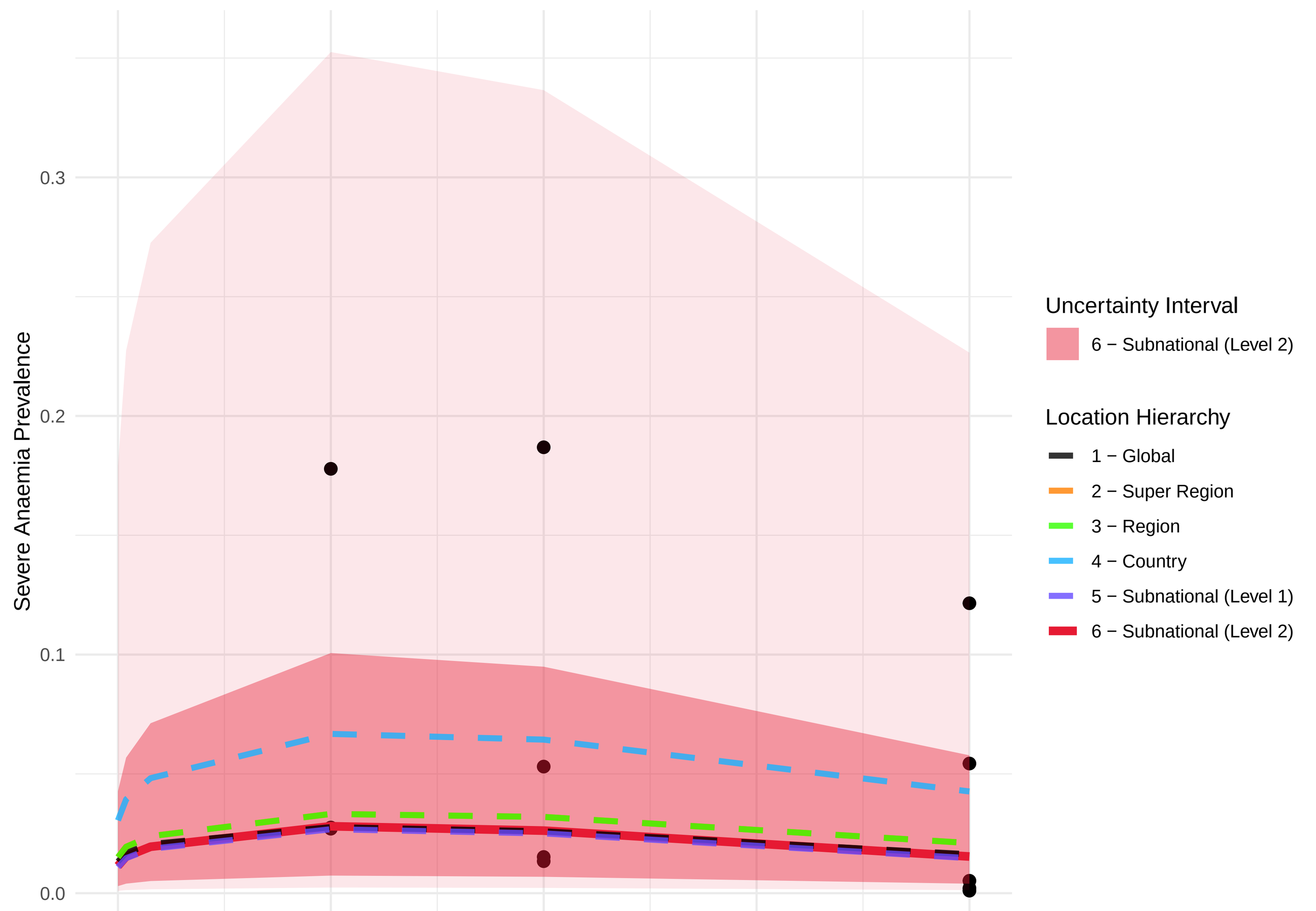

MR−BRT Age/Sex Fits − Non−pregnant Females <2 Years Old − Severe Anaemia Prevalence − Meghalaya, Urban
WHO−2001 Adjustment Method

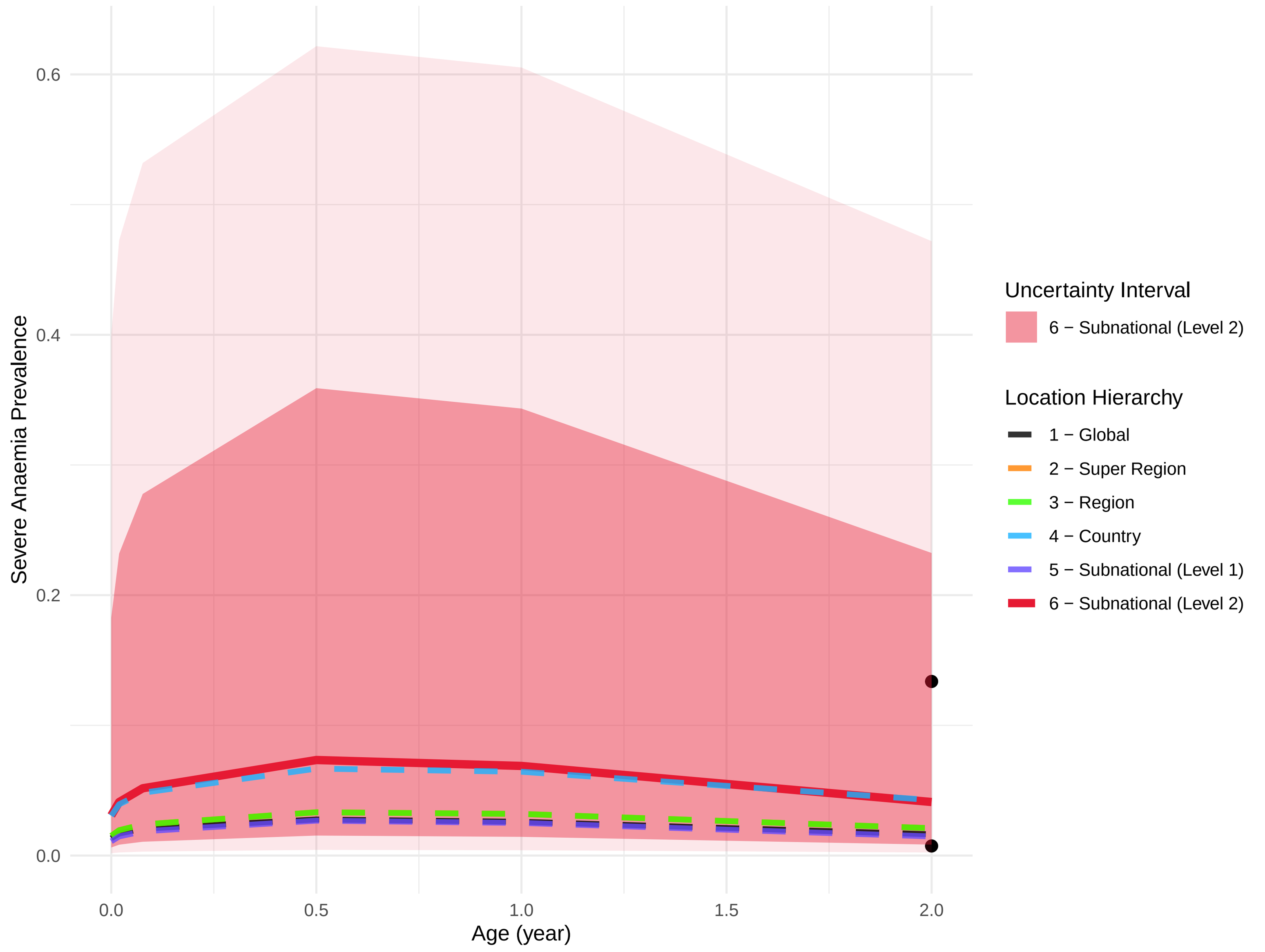

MR−BRT Age/Sex Fits − Non−pregnant Females <2 Years Old − Severe Anaemia Prevalence − Mizoram
WHO−2001 Adjustment Method

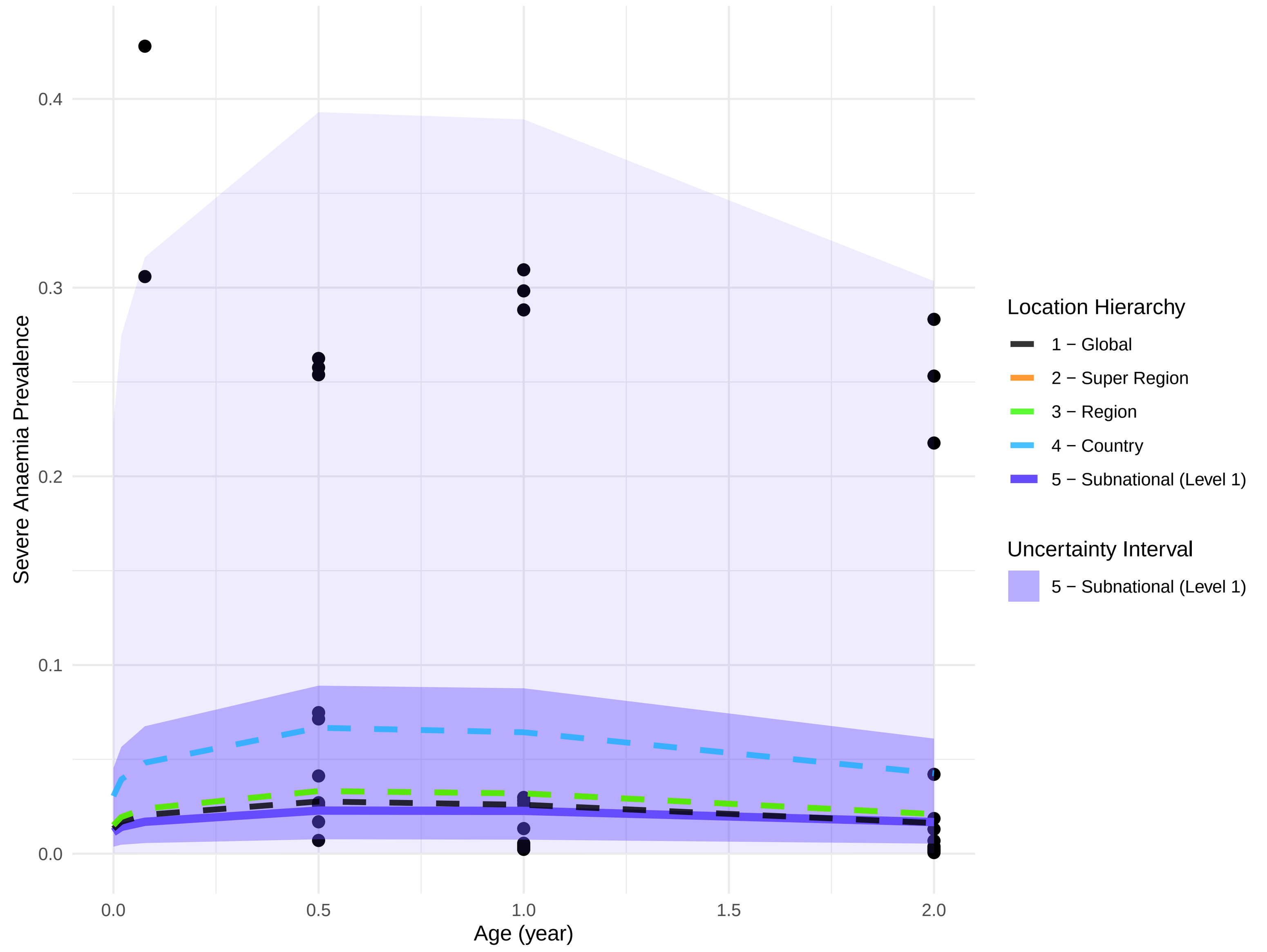

MR−BRT Age/Sex Fits − Non−pregnant Females <2 Years Old − Severe Anaemia Prevalence − Mizoram, Rural
WHO−2001 Adjustment Method

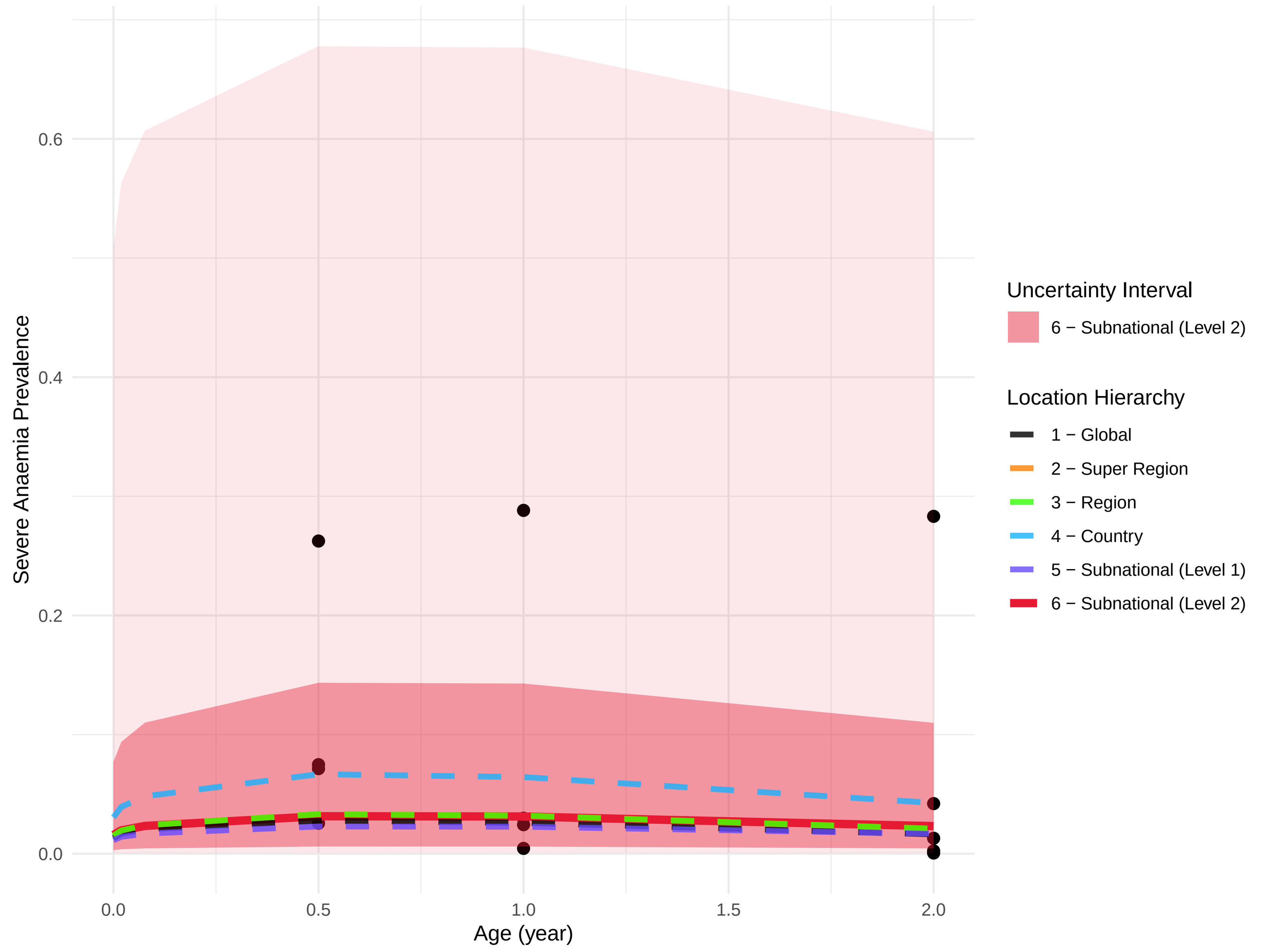

MR−BRT Age/Sex Fits − Non−pregnant Females <2 Years Old − Severe Anaemia Prevalence − Mizoram, Urban
WHO−2001 Adjustment Method

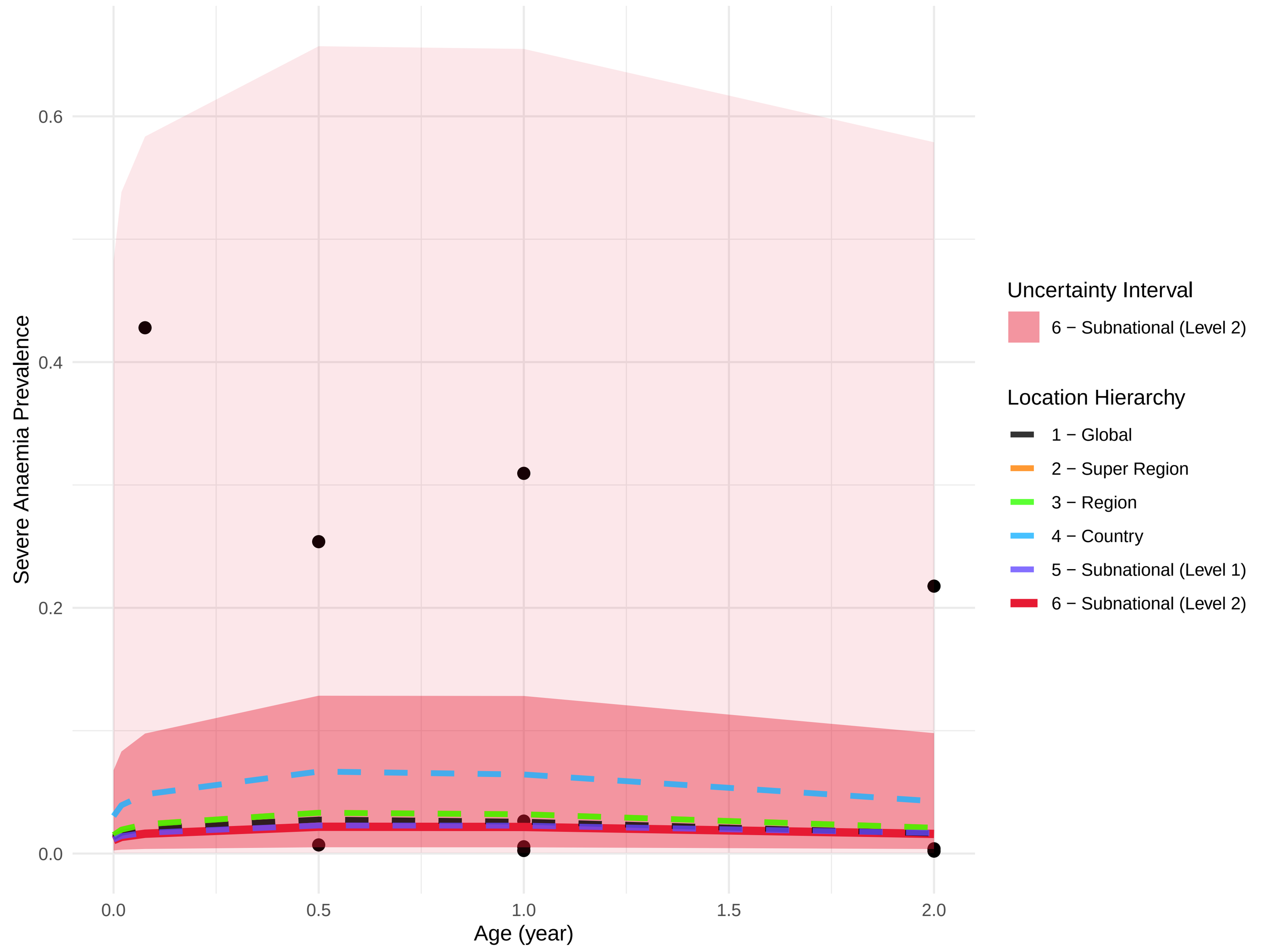

MR−BRT Age/Sex Fits − Non−pregnant Females <2 Years Old − Severe Anaemia Prevalence − Nagaland
WHO−2001 Adjustment Method

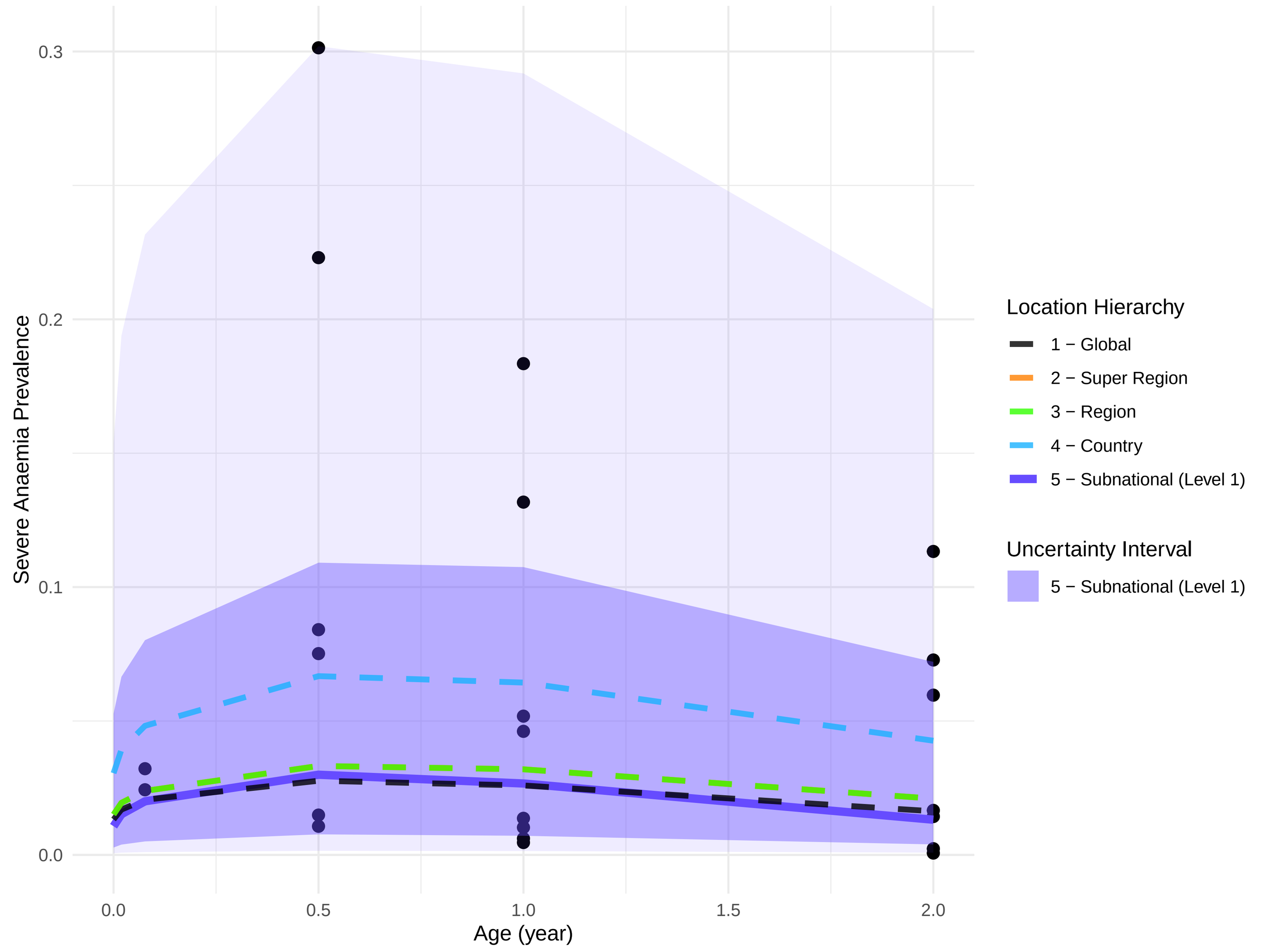

MR−BRT Age/Sex Fits − Non−pregnant Females <2 Years Old − Severe Anaemia Prevalence − Nagaland, Rural
WHO−2001 Adjustment Method

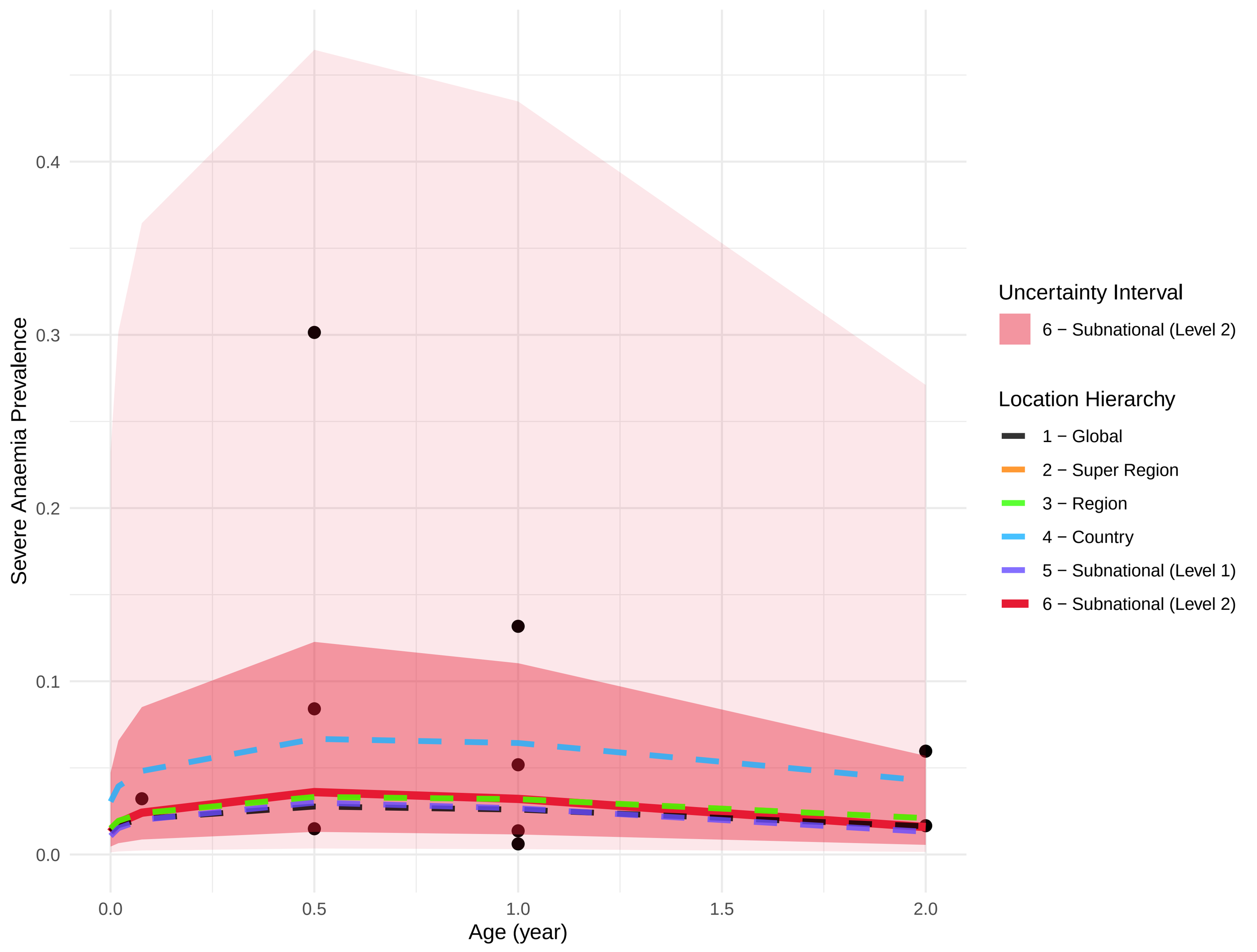

MR−BRT Age/Sex Fits − Non−pregnant Females <2 Years Old − Severe Anaemia Prevalence − Nagaland, Urban
WHO−2001 Adjustment Method

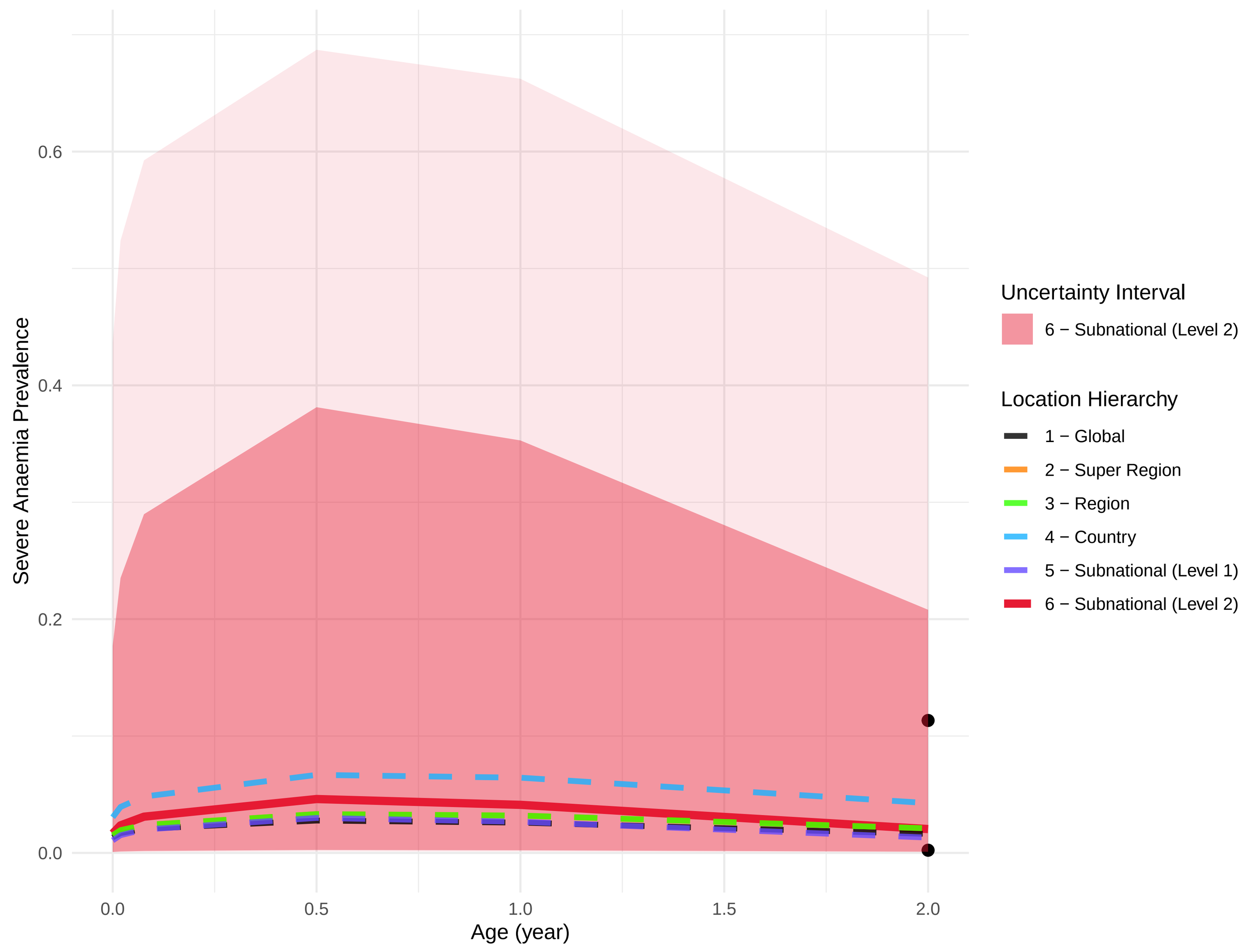

MR−BRT Age/Sex Fits − Non−pregnant Females <2 Years Old − Severe Anaemia Prevalence − Odisha
WHO−2001 Adjustment Method

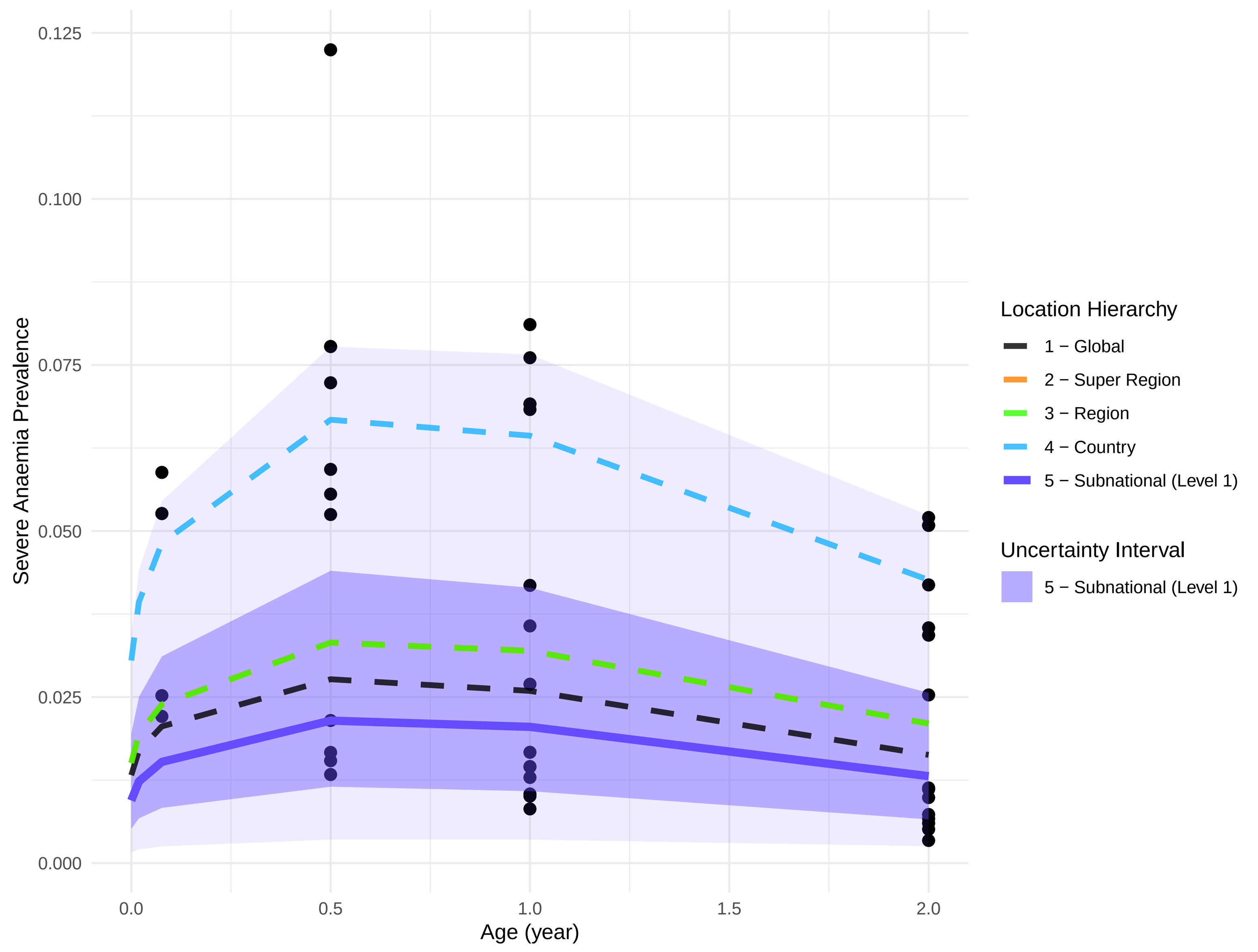

MR−BRT Age/Sex Fits − Non−pregnant Females <2 Years Old − Severe Anaemia Prevalence − Odisha, Rural
WHO−2001 Adjustment Method

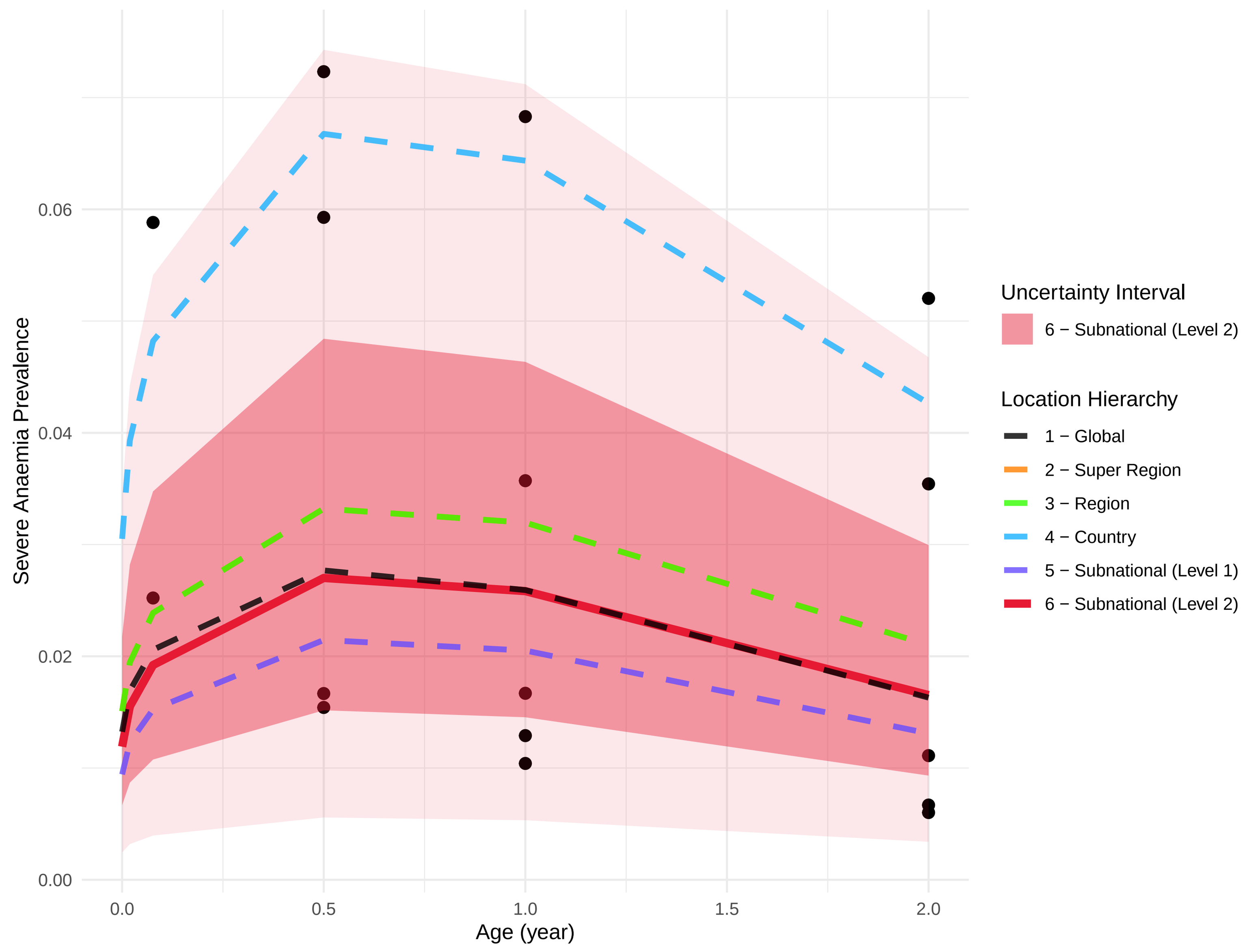

MR−BRT Age/Sex Fits − Non−pregnant Females <2 Years Old − Severe Anaemia Prevalence − Odisha, Urban
WHO−2001 Adjustment Method

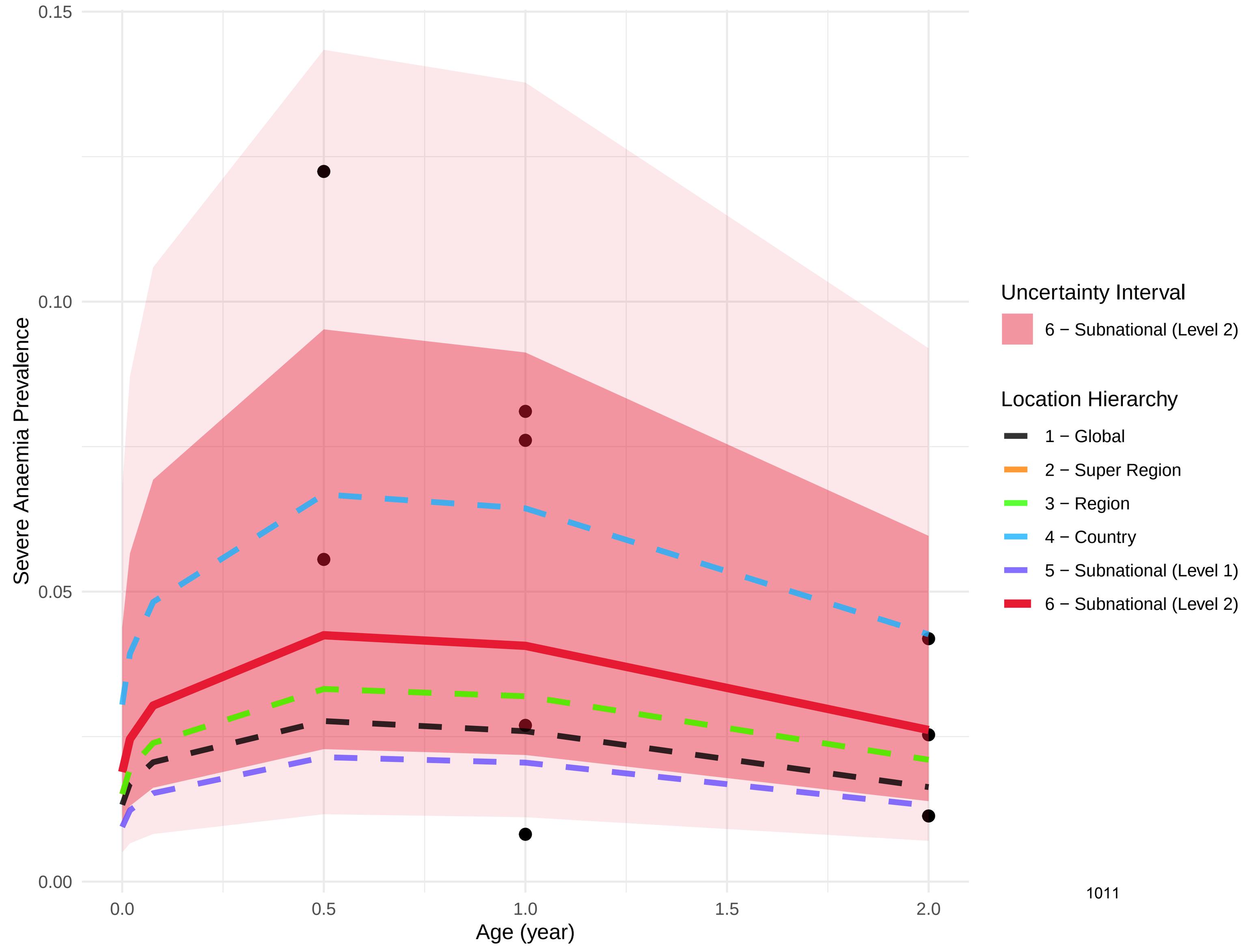

MR−BRT Age/Sex Fits − Non−pregnant Females <2 Years Old − Severe Anaemia Prevalence − Other Union Territories
WHO−2001 Adjustment Method

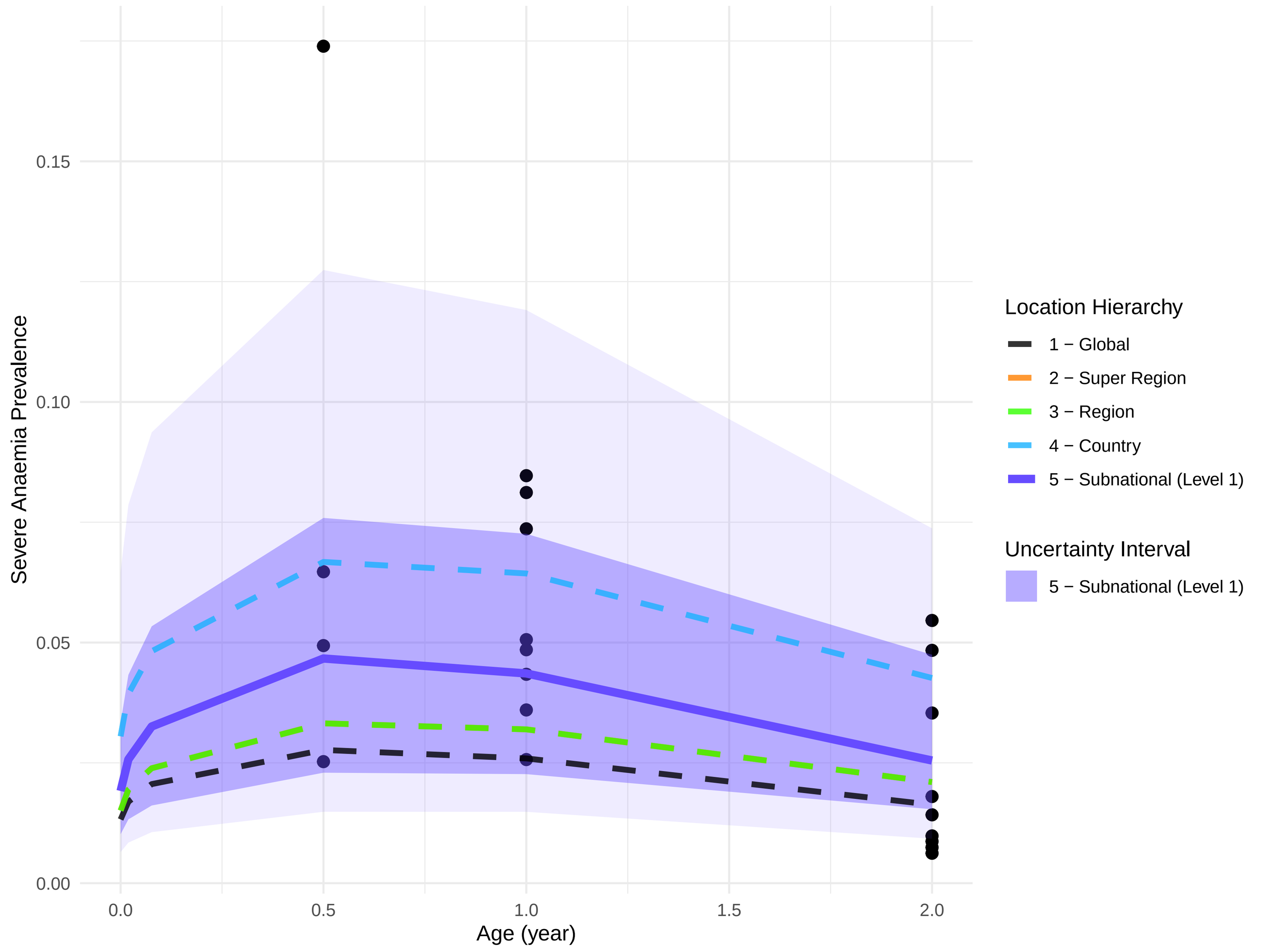

MR−BRT Age/Sex Fits − Non−pregnant Females <2 Years Old − Severe Anaemia Prevalence − Other Union Territories, Rural
WHO−2001 Adjustment Method

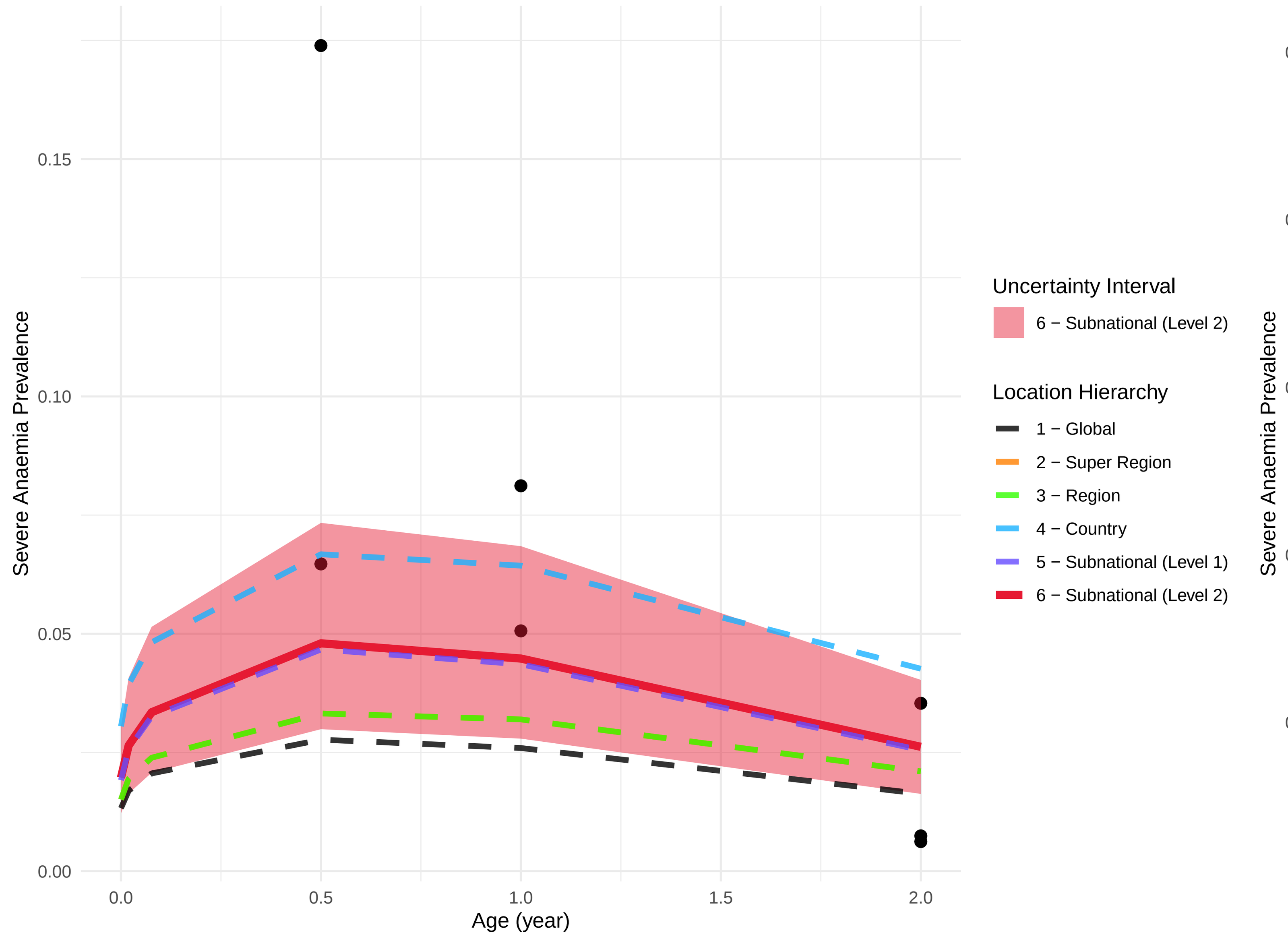

MR−BRT Age/Sex Fits − Non−pregnant Females <2 Years Old − Severe Anaemia Prevalence − Other Union Territories, Urban
WHO−2001 Adjustment Method

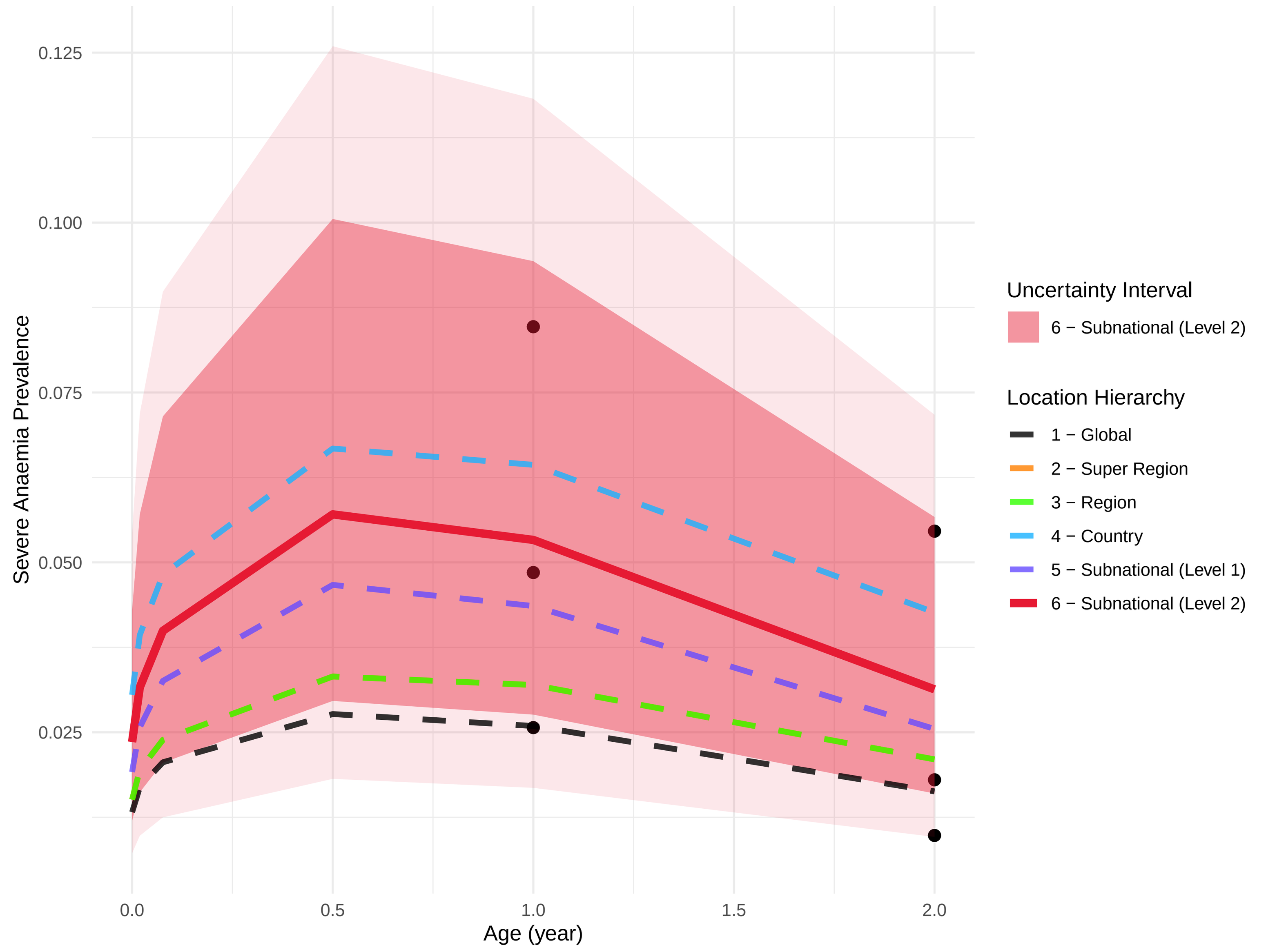

MR−BRT Age/Sex Fits − Non−pregnant Females <2 Years Old − Severe Anaemia Prevalence − Punjab
WHO−2001 Adjustment Method

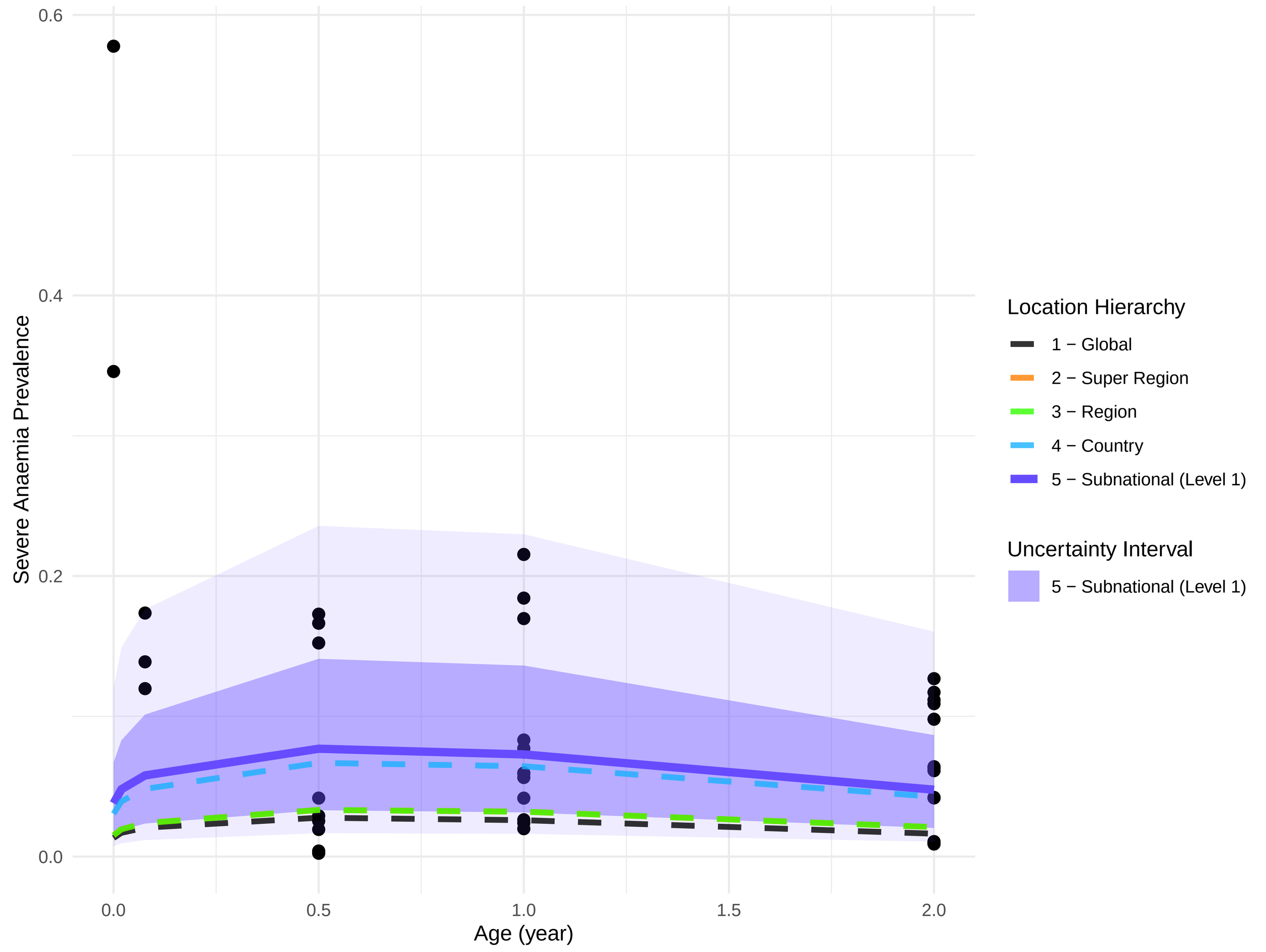

MR−BRT Age/Sex Fits − Non−pregnant Females <2 Years Old − Severe Anaemia Prevalence − Punjab, Rural
WHO−2001 Adjustment Method

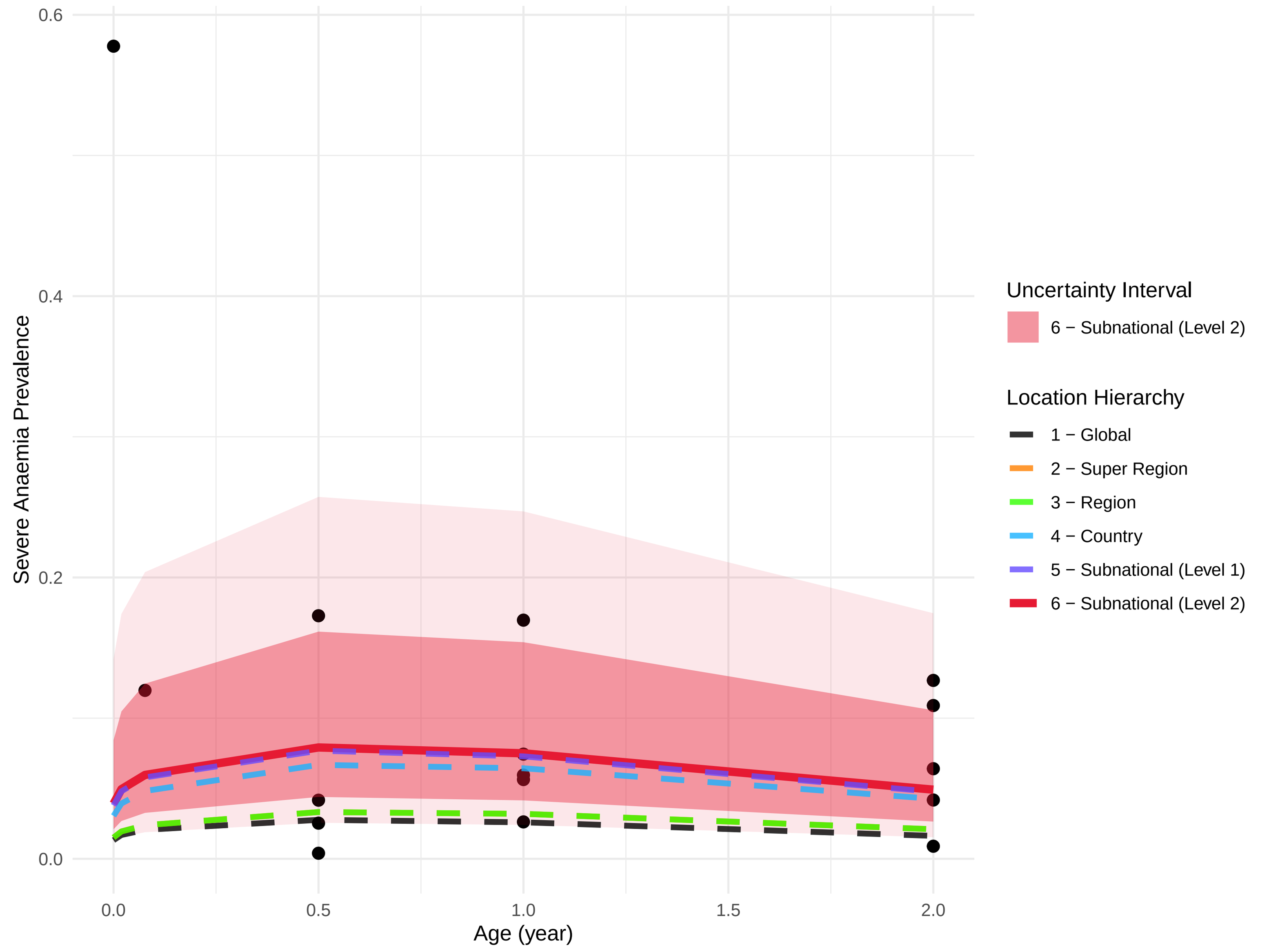

MR−BRT Age/Sex Fits − Non−pregnant Females <2 Years Old − Severe Anaemia Prevalence − Punjab, Urban
WHO−2001 Adjustment Method

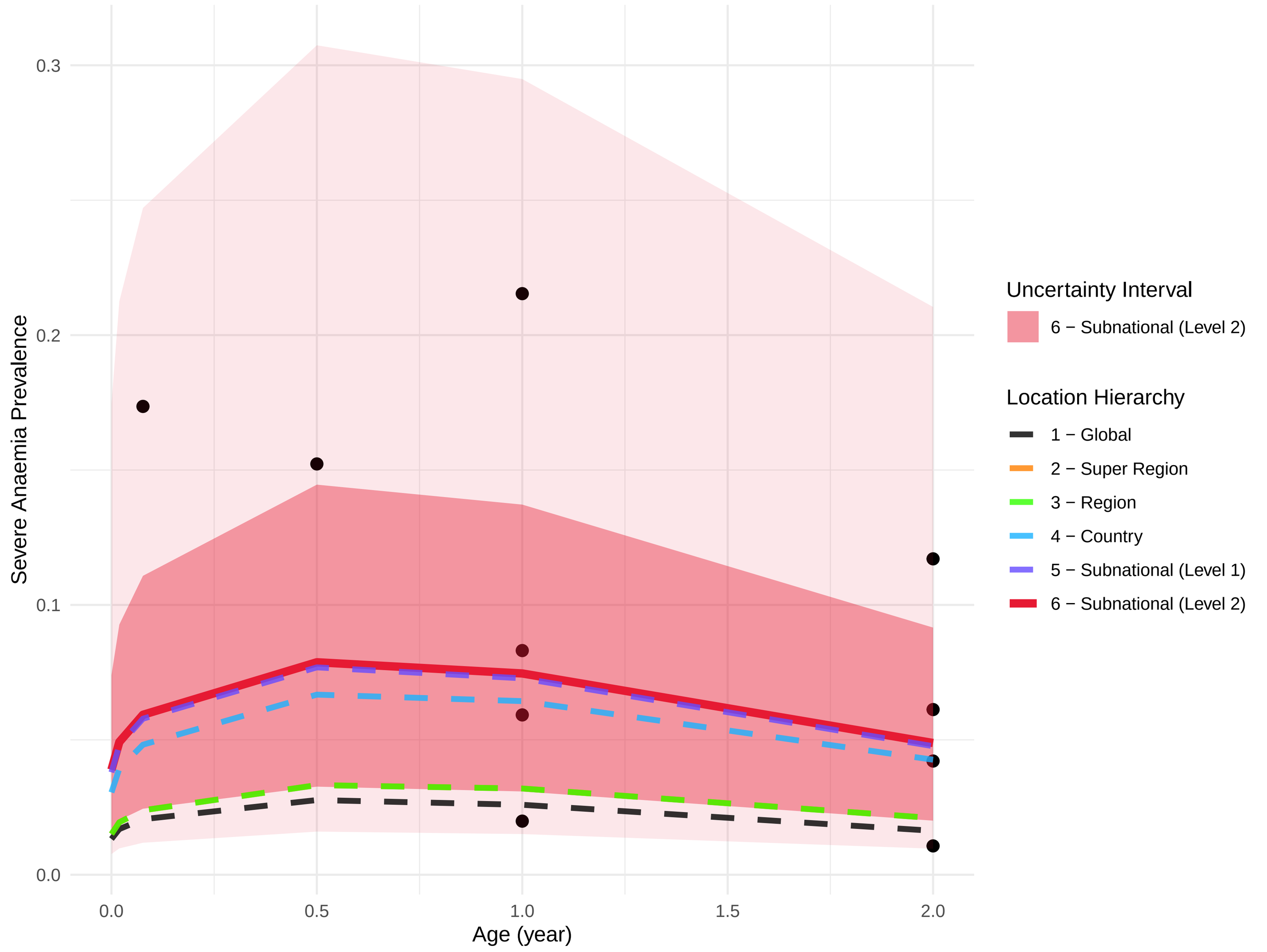

MR−BRT Age/Sex Fits − Non−pregnant Females <2 Years Old − Severe Anaemia Prevalence − Rajasthan
WHO−2001 Adjustment Method

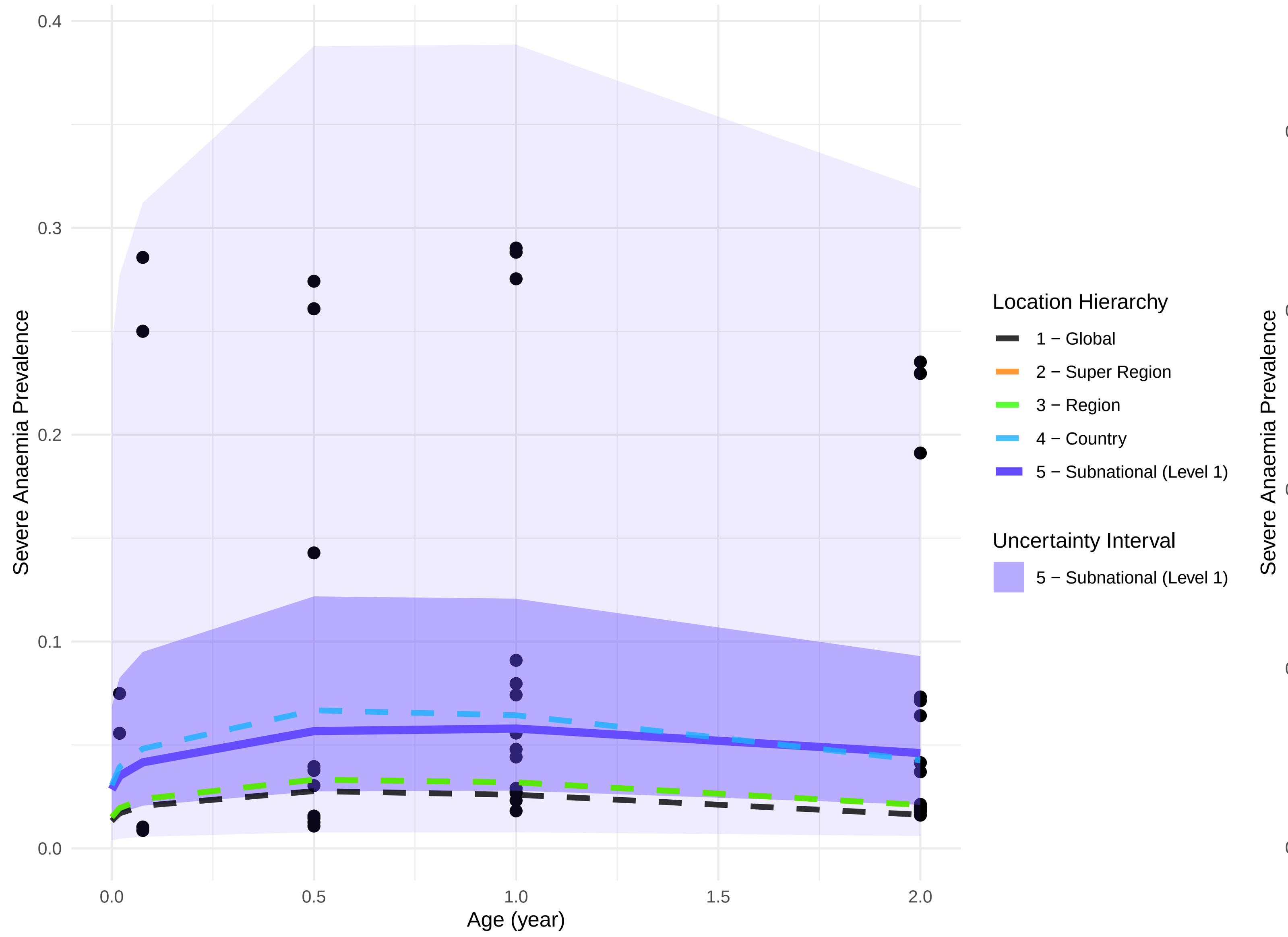

MR−BRT Age/Sex Fits − Non−pregnant Females <2 Years Old − Severe Anaemia Prevalence − Rajasthan, Rural
WHO−2001 Adjustment Method

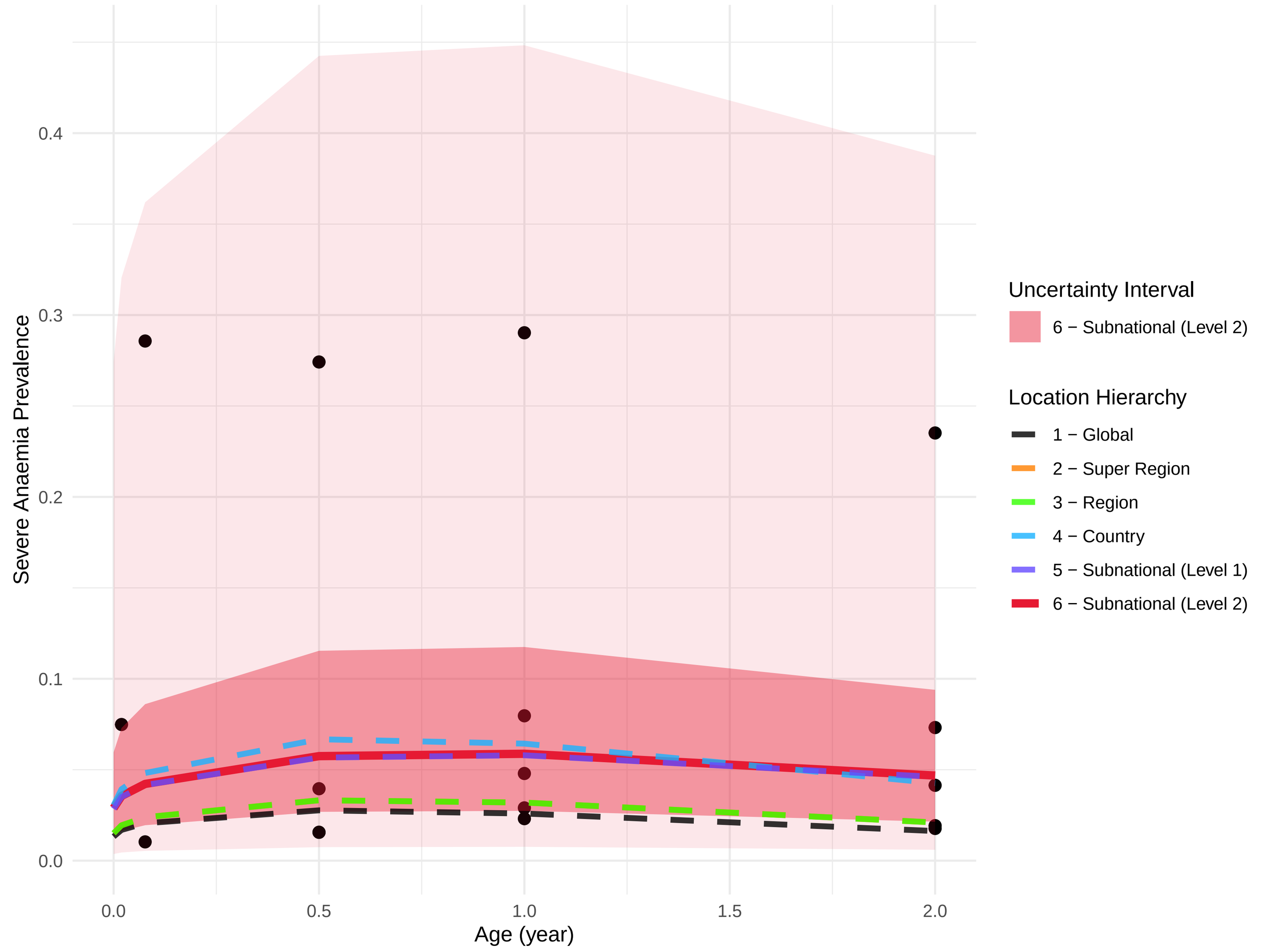

MR−BRT Age/Sex Fits − Non−pregnant Females <2 Years Old − Severe Anaemia Prevalence − Rajasthan, Urban
WHO−2001 Adjustment Method

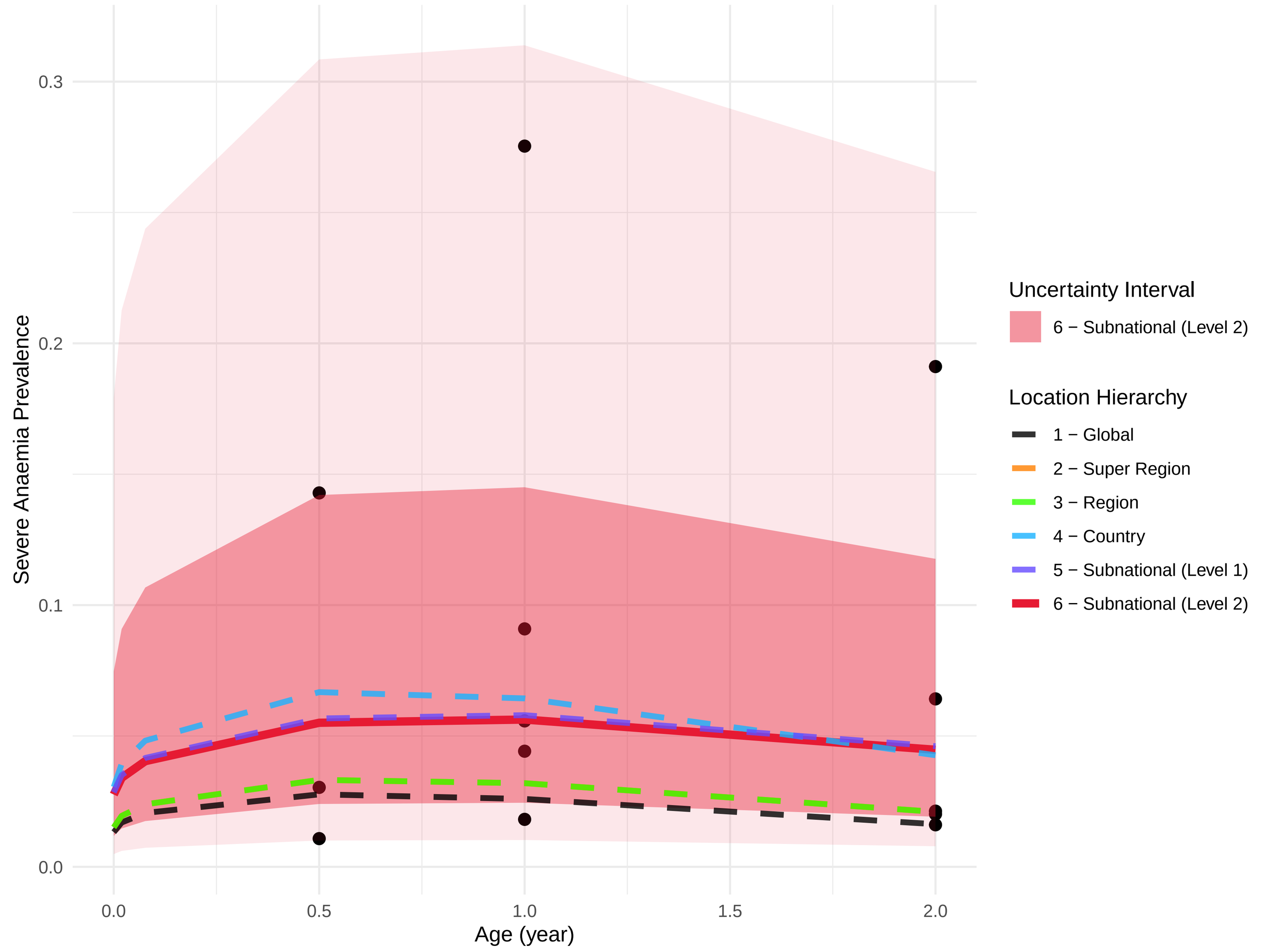

MR−BRT Age/Sex Fits − Non−pregnant Females <2 Years Old − Severe Anaemia Prevalence − Sikkim
WHO−2001 Adjustment Method

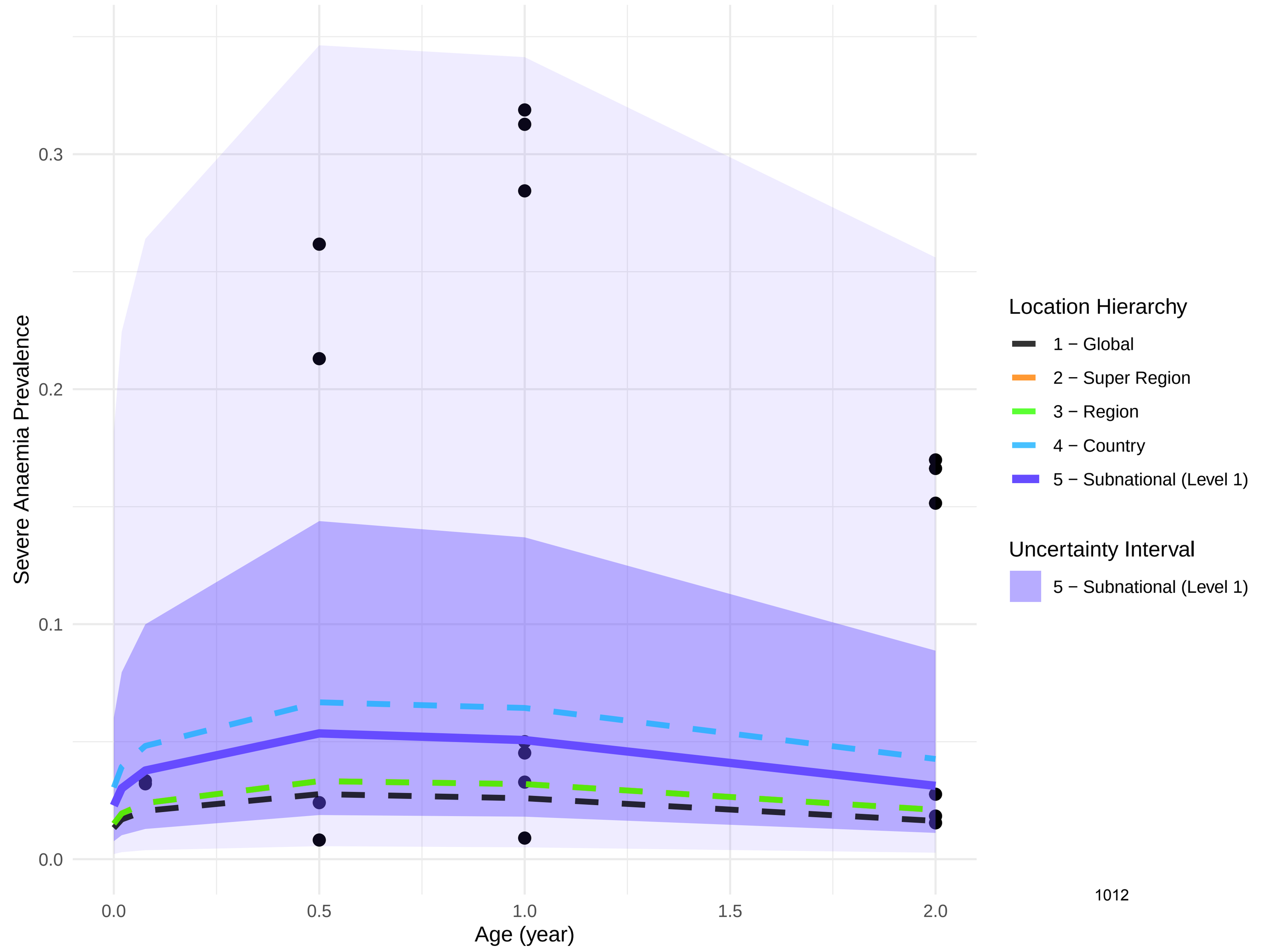

MR−BRT Age/Sex Fits − Non−pregnant Females <2 Years Old − Severe Anaemia Prevalence − Sikkim, Rural
WHO−2001 Adjustment Method

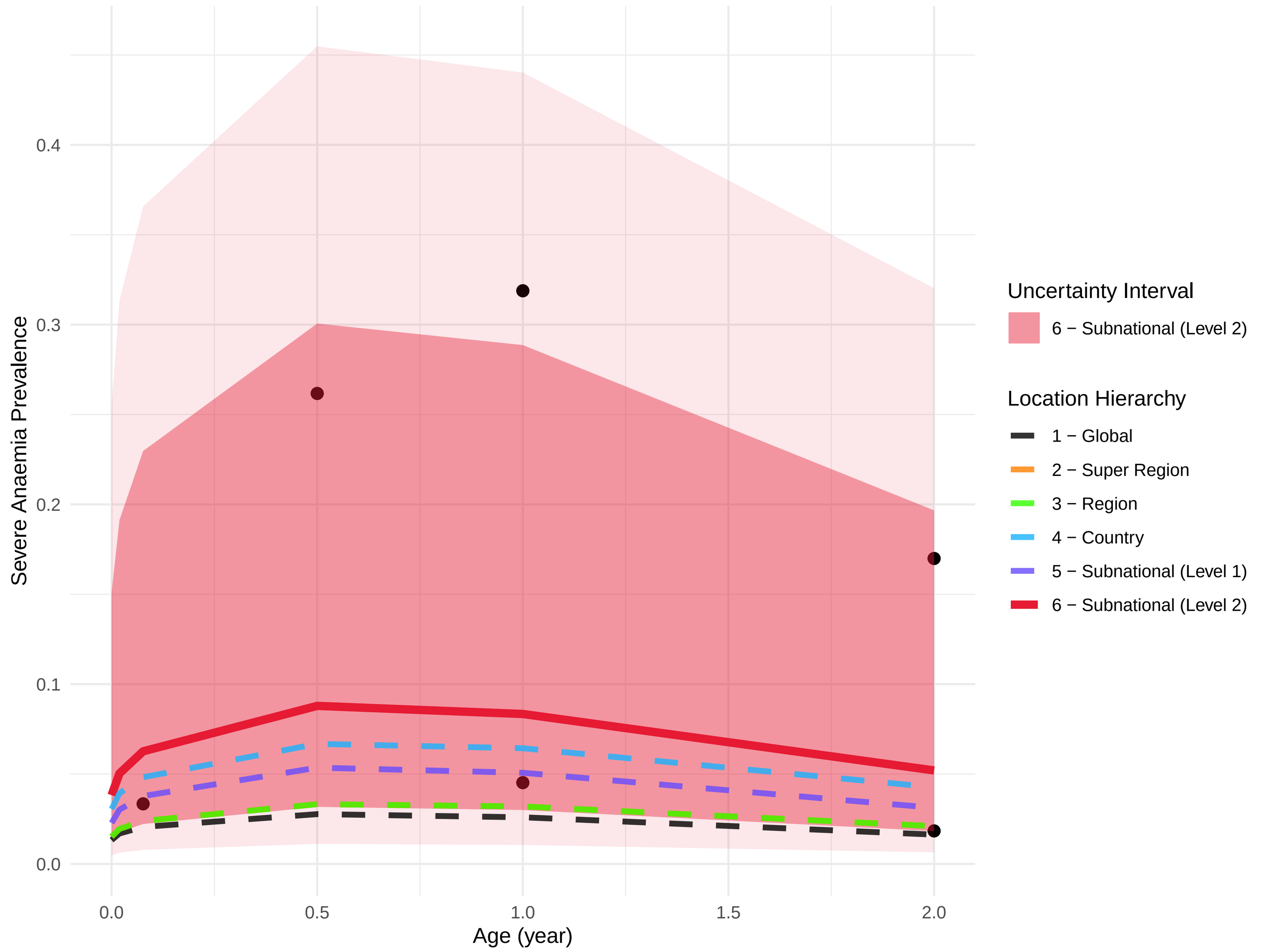

MR−BRT Age/Sex Fits − Non−pregnant Females <2 Years Old − Severe Anaemia Prevalence − Sikkim, Urban
WHO−2001 Adjustment Method

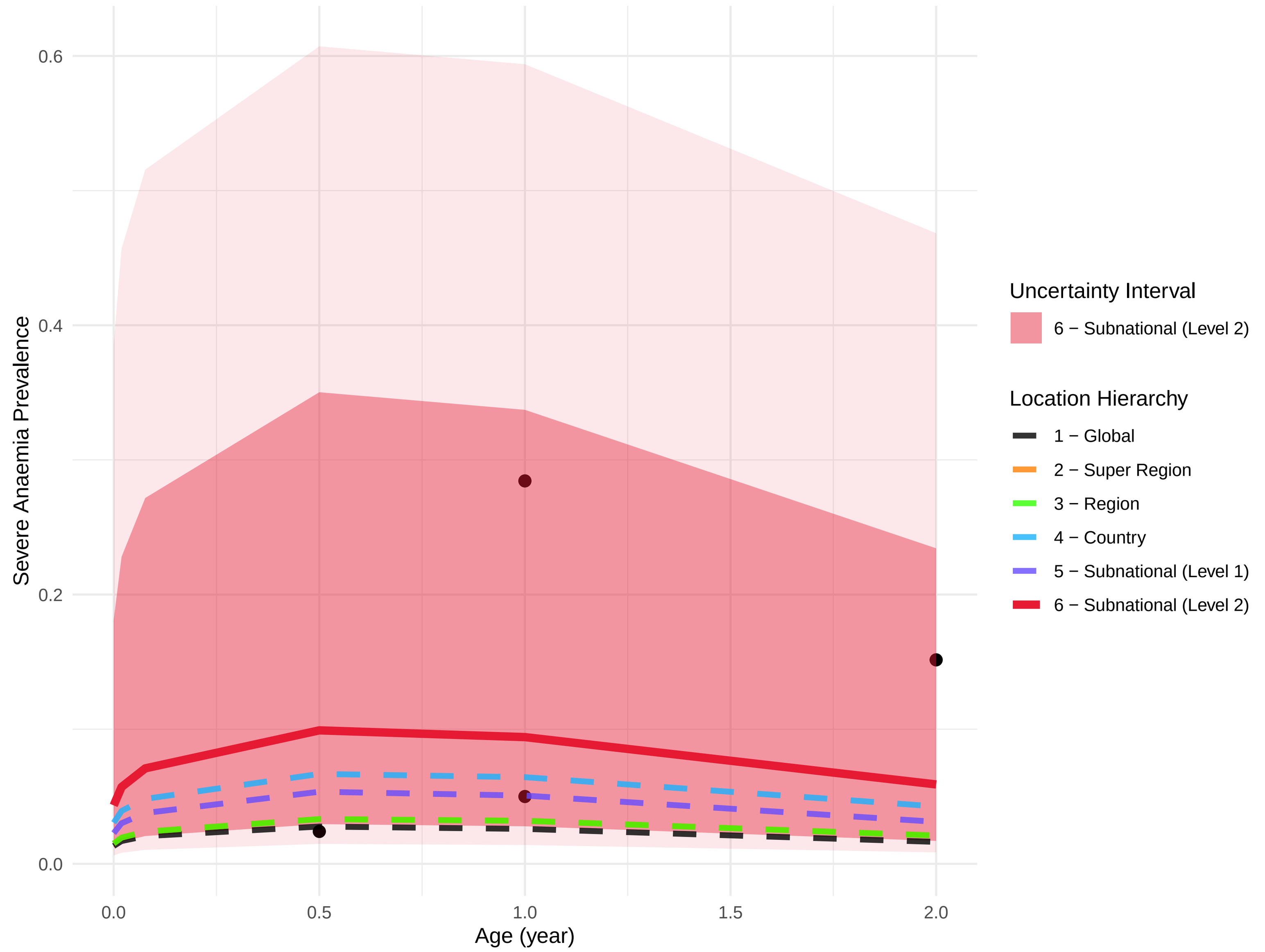

MR−BRT Age/Sex Fits − Non−pregnant Females <2 Years Old − Severe Anaemia Prevalence − Tamil Nadu
WHO−2001 Adjustment Method

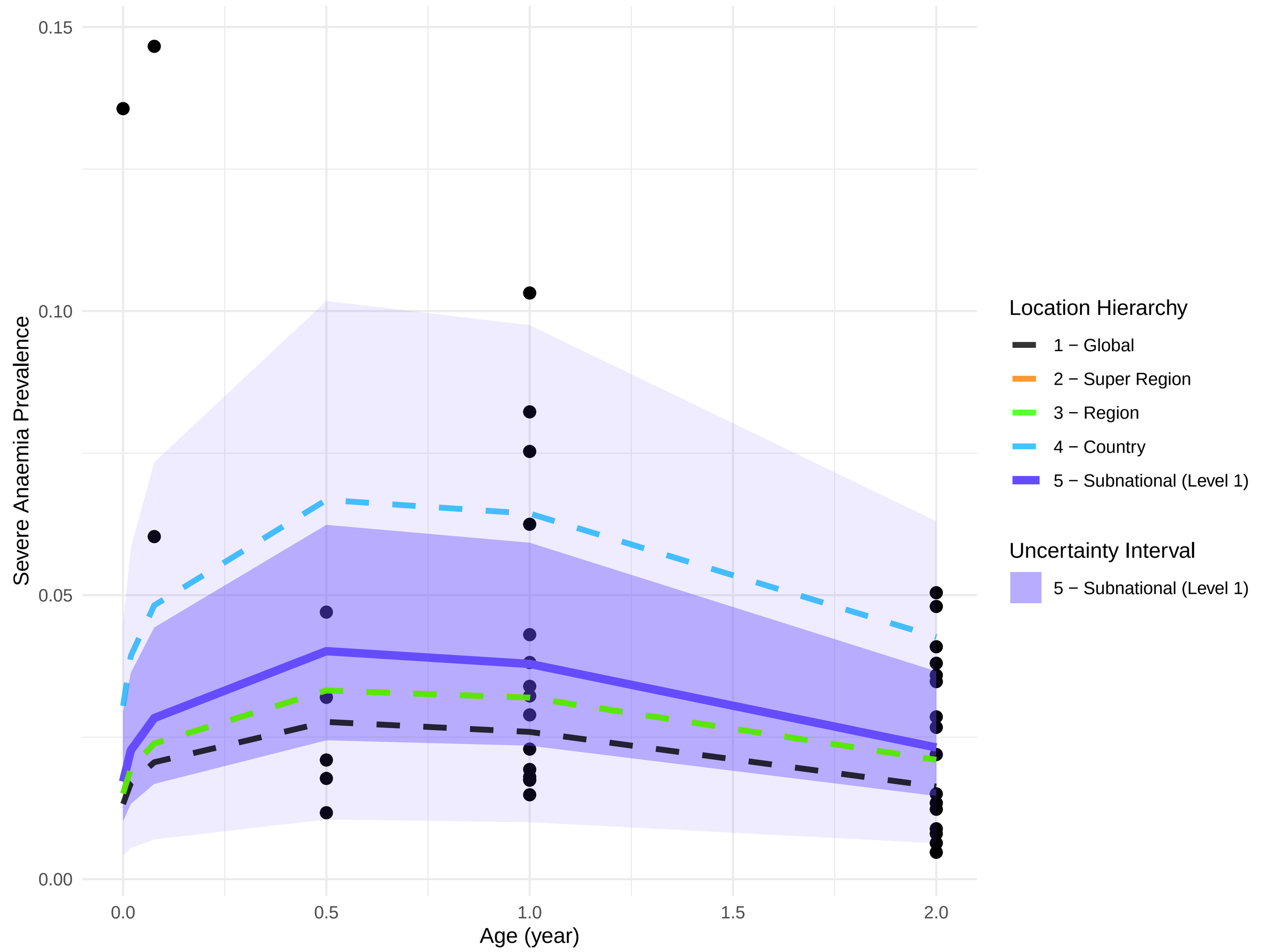

MR−BRT Age/Sex Fits − Non−pregnant Females <2 Years Old − Severe Anaemia Prevalence − Tamil Nadu, Rural
WHO−2001 Adjustment Method

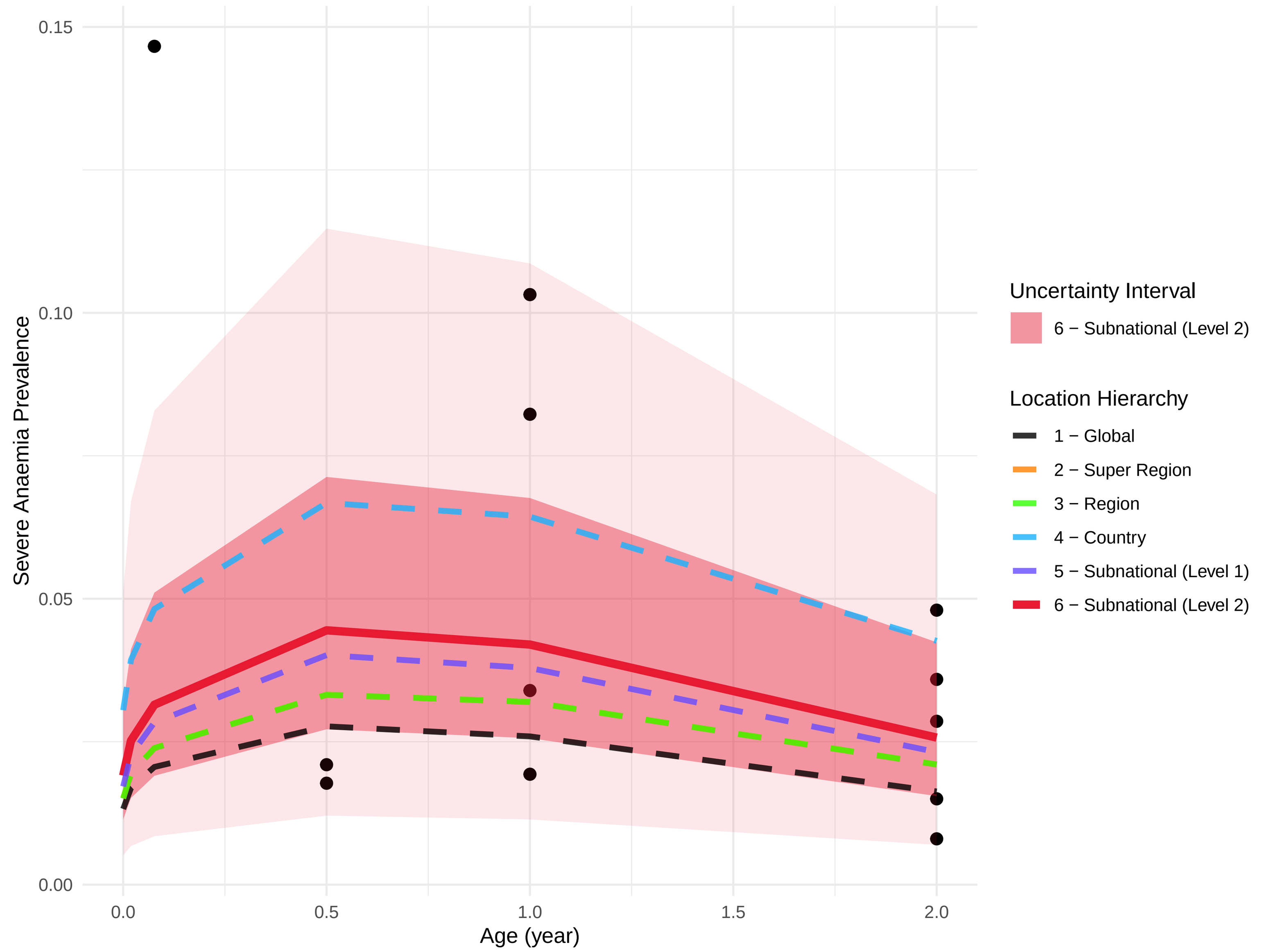

MR−BRT Age/Sex Fits − Non−pregnant Females <2 Years Old − Severe Anaemia Prevalence − Tamil Nadu, Urban
WHO−2001 Adjustment Method

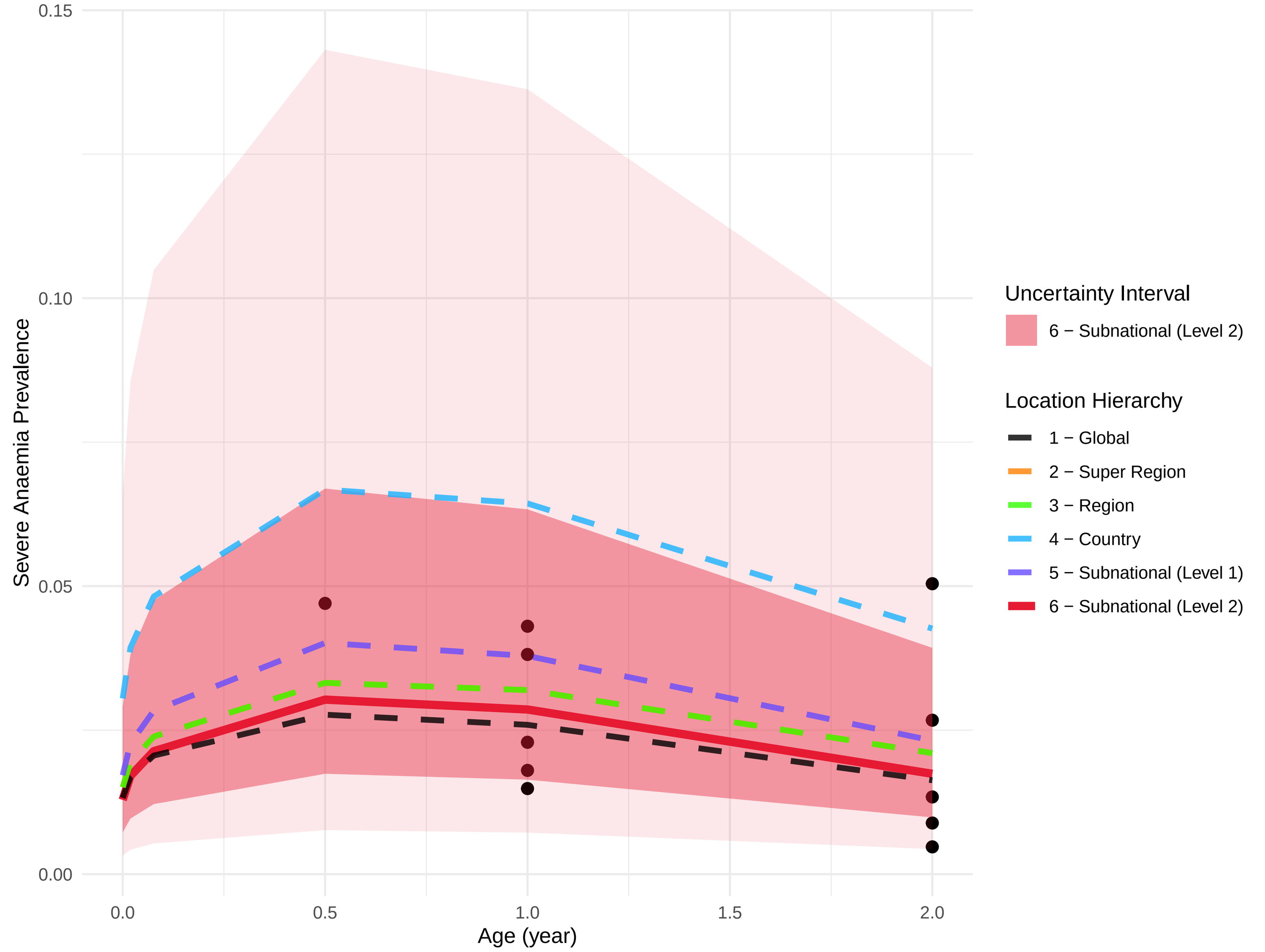

MR−BRT Age/Sex Fits − Non−pregnant Females <2 Years Old − Severe Anaemia Prevalence − Telangana
WHO−2001 Adjustment Method

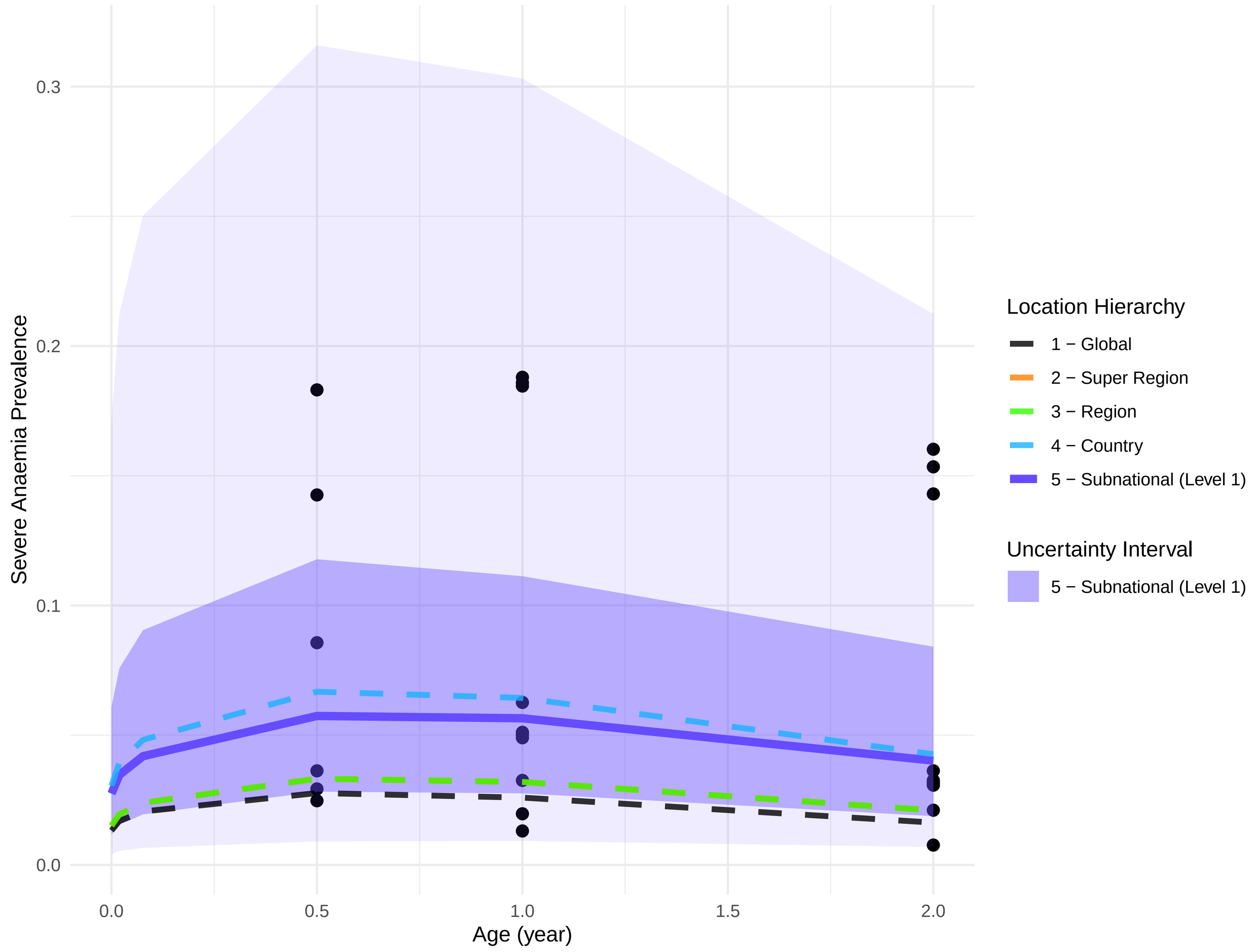

MR−BRT Age/Sex Fits − Non−pregnant Females <2 Years Old − Severe Anaemia Prevalence − Telangana, Rural
WHO−2001 Adjustment Method

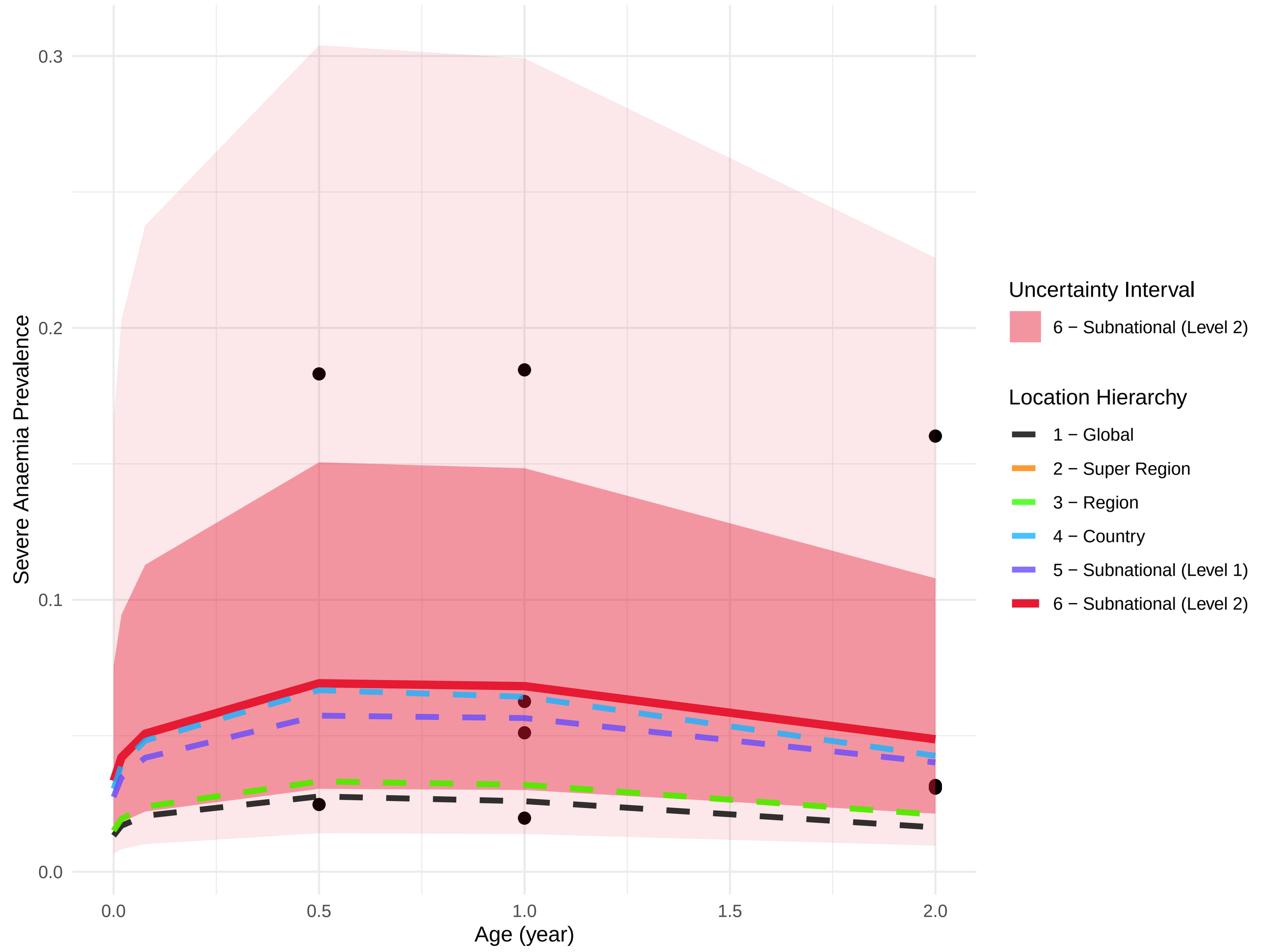

MR−BRT Age/Sex Fits − Non−pregnant Females <2 Years Old − Severe Anaemia Prevalence − Telangana, Urban
WHO−2001 Adjustment Method

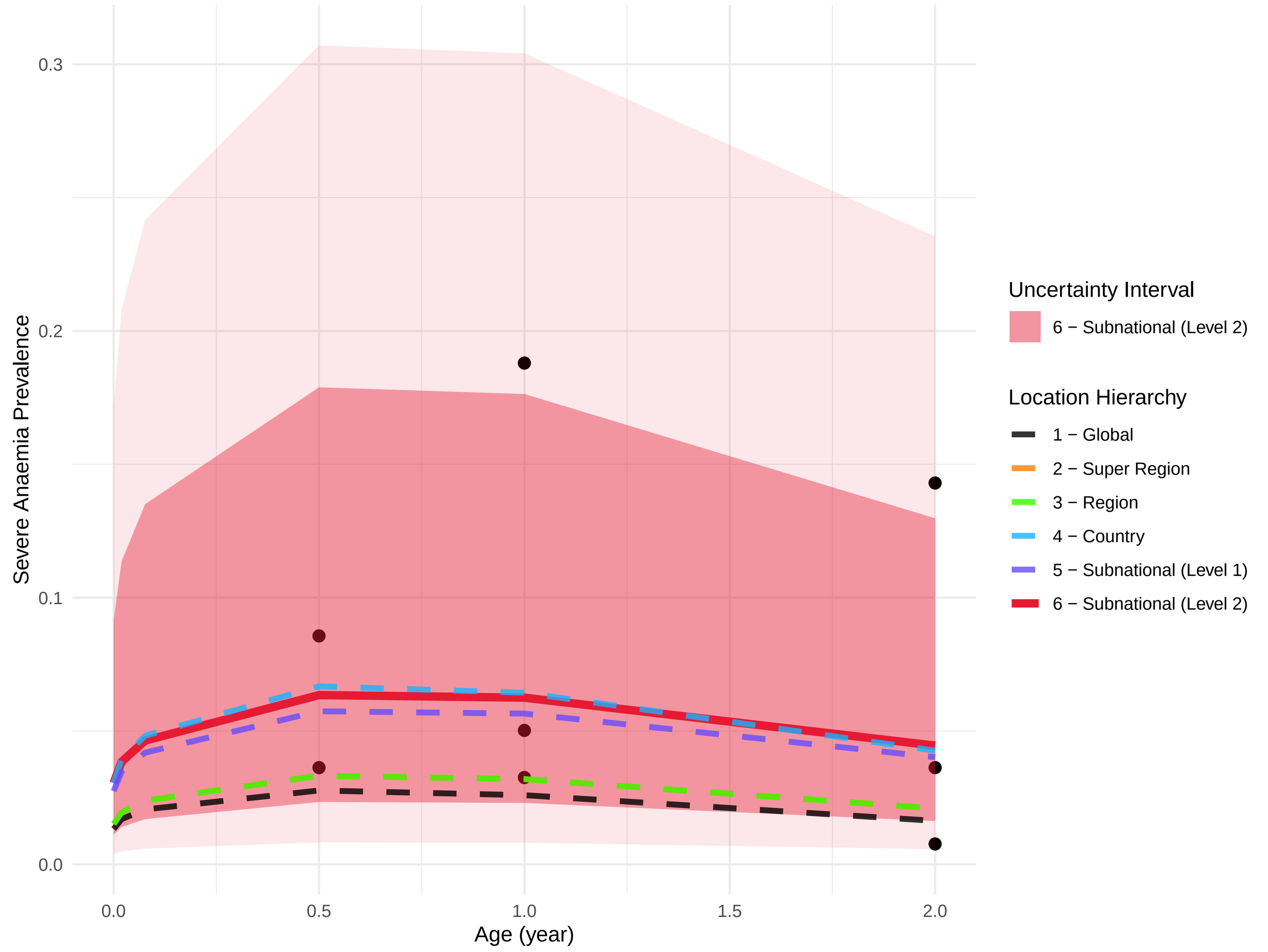

MR−BRT Age/Sex Fits − Non−pregnant Females <2 Years Old − Severe Anaemia Prevalence − Tripura
WHO−2001 Adjustment Method

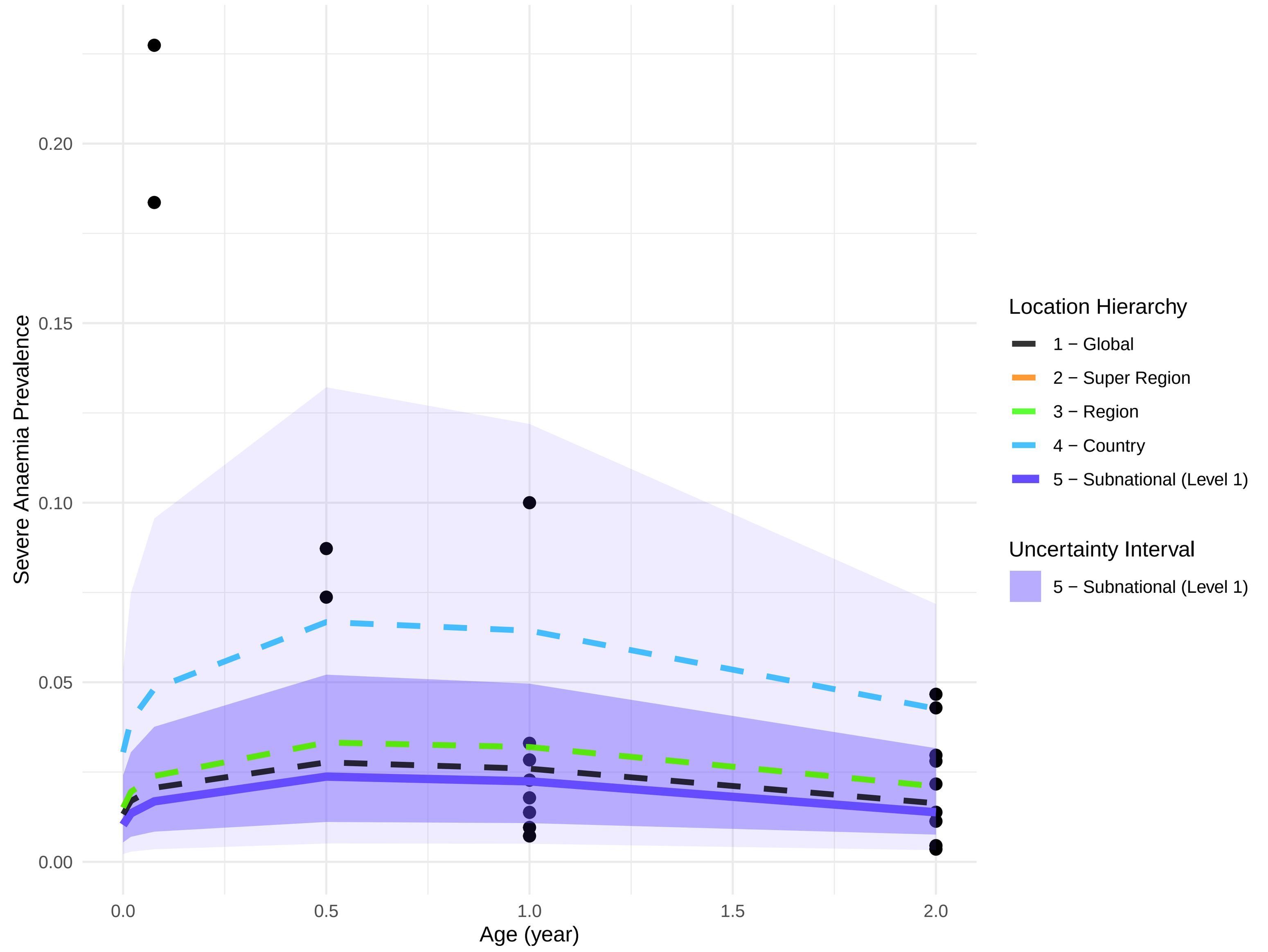

MR−BRT Age/Sex Fits − Non−pregnant Females <2 Years Old − Severe Anaemia Prevalence − Tripura, Rural
WHO−2001 Adjustment Method

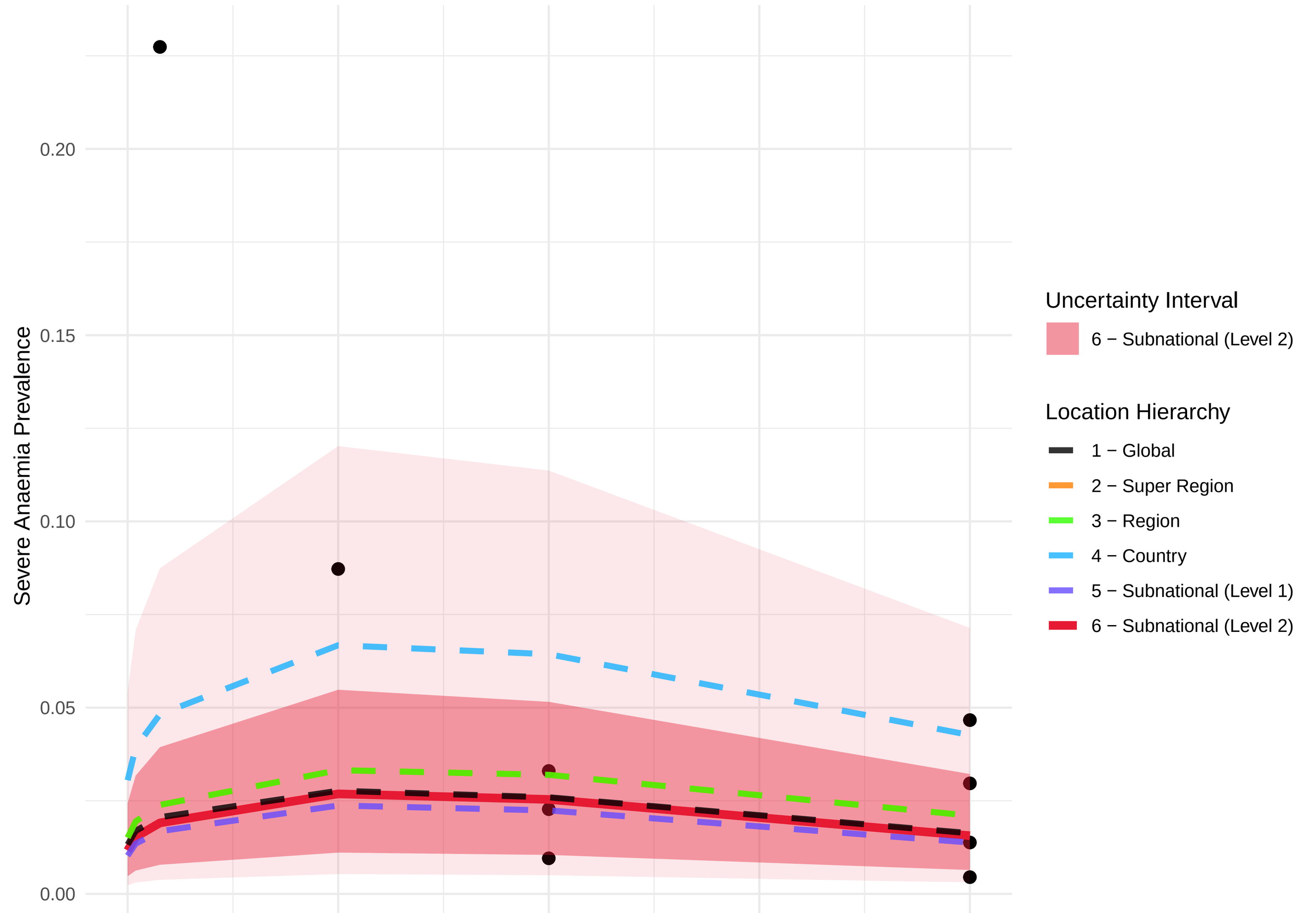

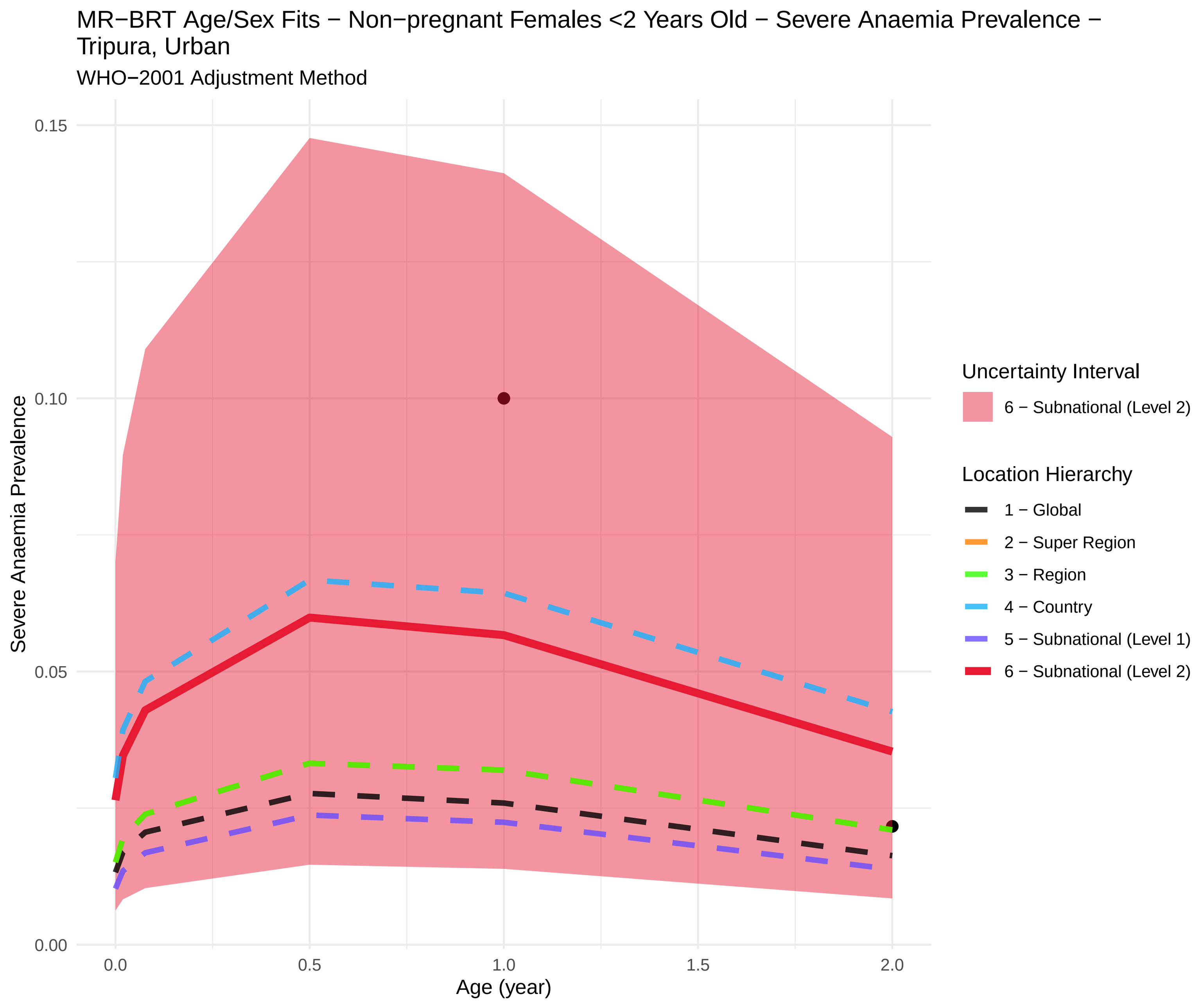
MR−BRT Age/Sex Fits − Non−pregnant Females <2 Years Old − Severe Anaemia Prevalence − Tripura, Urban
WHO−2001 Adjustment Method
Uncertainty Interval
6 − Subnational (Level 2)
Location Hierarchy
1 − Global
2 − Super Region
3 − Region
4 − Country
5 − Subnational (Level 1)
6 − Subnational (Level 2)
Severe Anaemia Prevalence
Age (year)

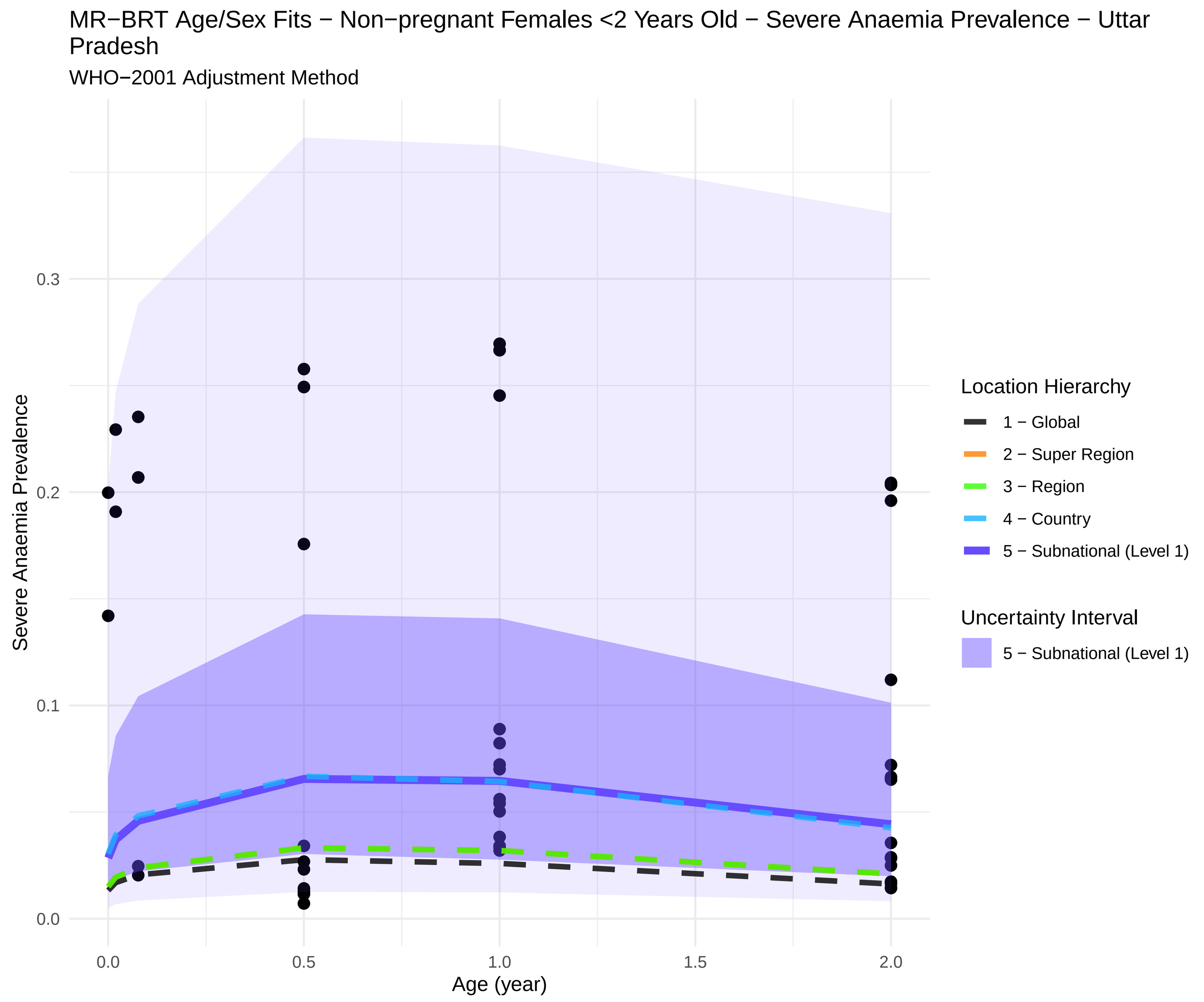
MR−BRT Age/Sex Fits − Non−pregnant Females <2 Years Old − Severe Anaemia Prevalence − Uttar Pradesh
WHO−2001 Adjustment Method
Location Hierarchy
1 − Global
2 − Super Region
3 − Region
4 − Country
5 − Subnational (Level 1)
Uncertainty Interval
5 − Subnational (Level 1)
Severe Anaemia Prevalence
Age (year)

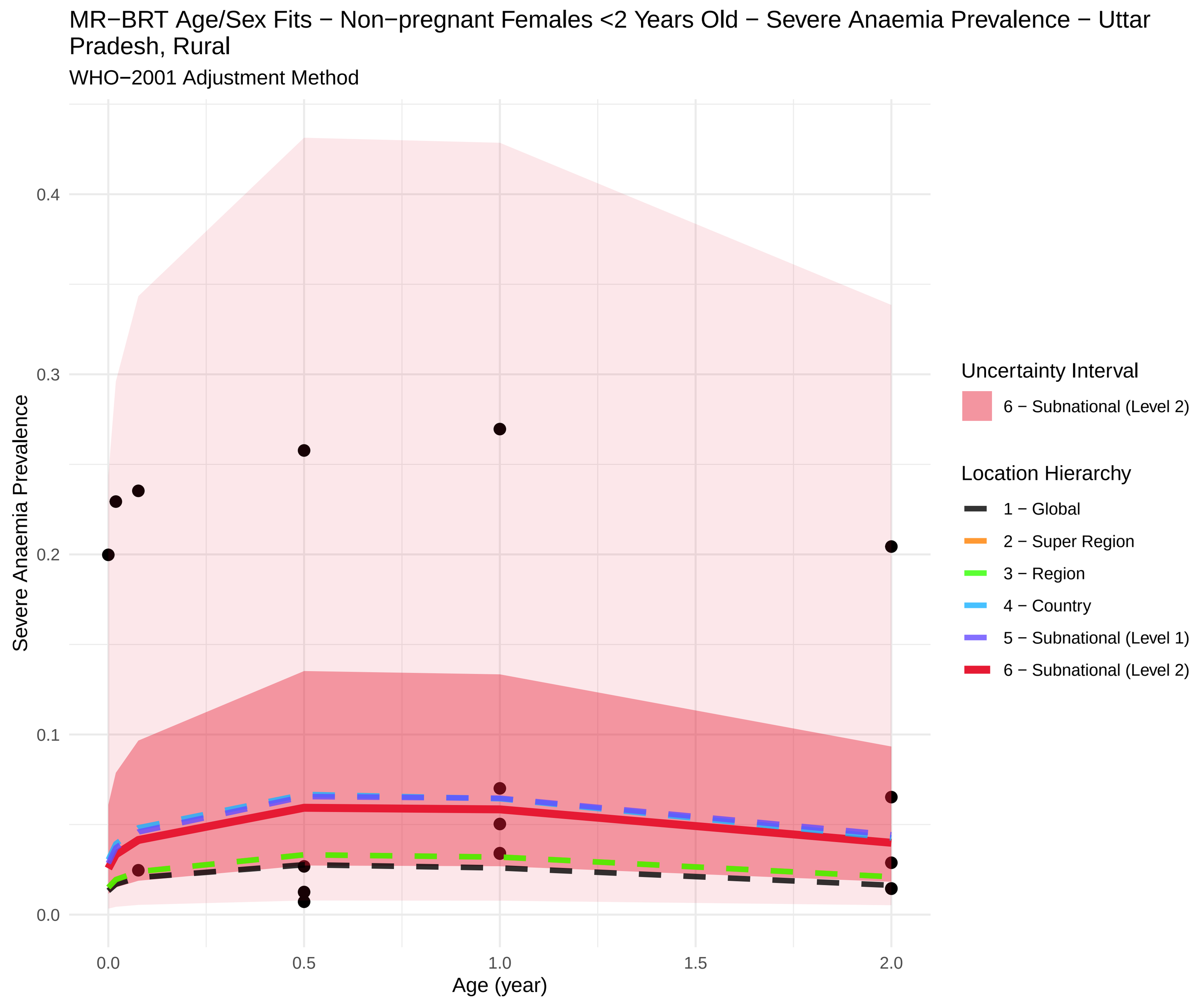
MR−BRT Age/Sex Fits − Non−pregnant Females <2 Years Old − Severe Anaemia Prevalence − Uttar Pradesh, Rural
WHO−2001 Adjustment Method
Uncertainty Interval
6 − Subnational (Level 2)
Location Hierarchy
1 − Global
2 − Super Region
3 − Region
4 − Country
5 − Subnational (Level 1)
6 − Subnational (Level 2)
Severe Anaemia Prevalence
Age (year)

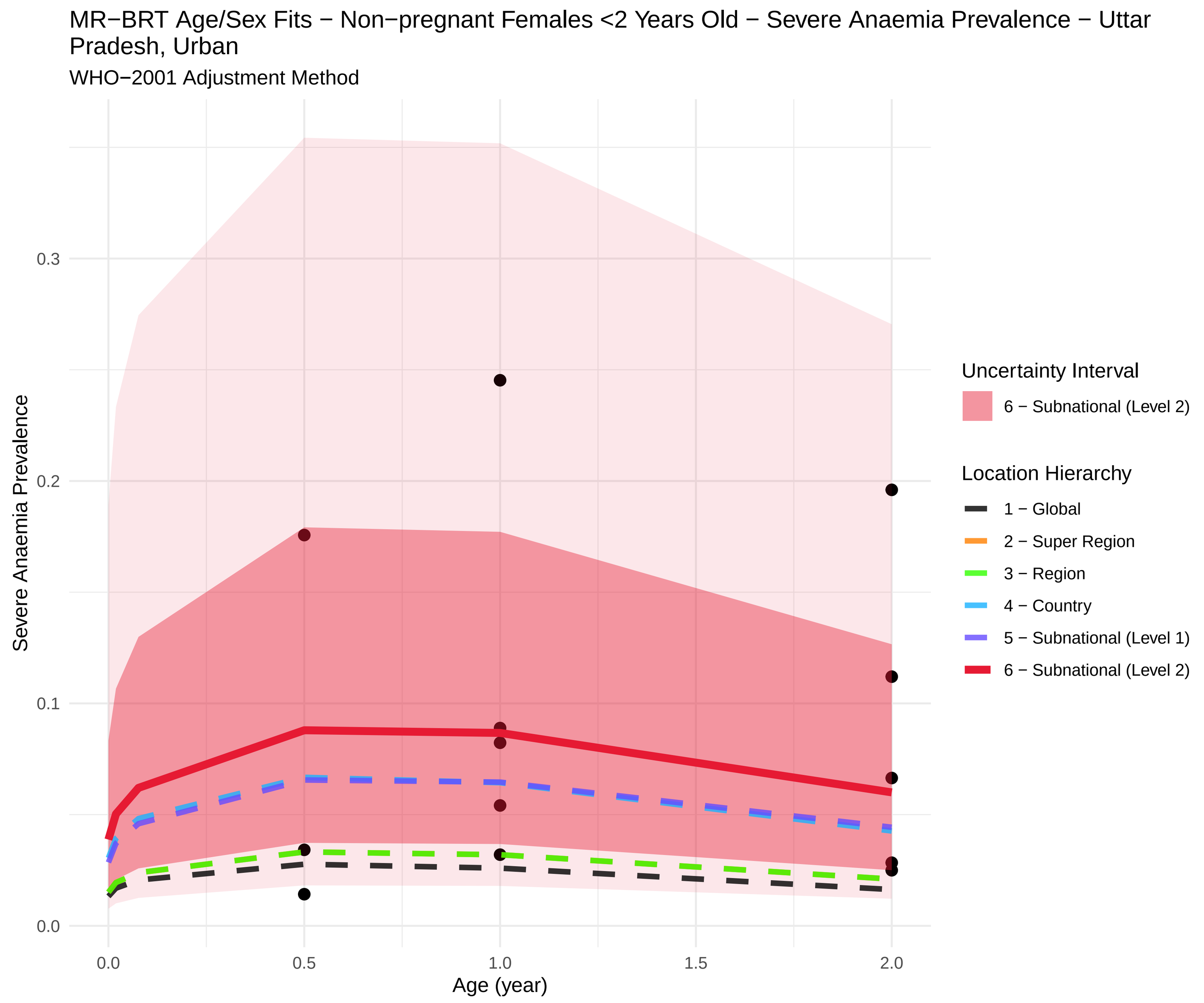
MR−BRT Age/Sex Fits − Non−pregnant Females <2 Years Old − Severe Anaemia Prevalence − Uttar Pradesh, Urban
WHO−2001 Adjustment Method
Uncertainty Interval
6 − Subnational (Level 2)
Location Hierarchy
1 − Global
2 − Super Region
3 − Region
4 − Country
5 − Subnational (Level 1)
6 − Subnational (Level 2)
Severe Anaemia Prevalence
Age (year)

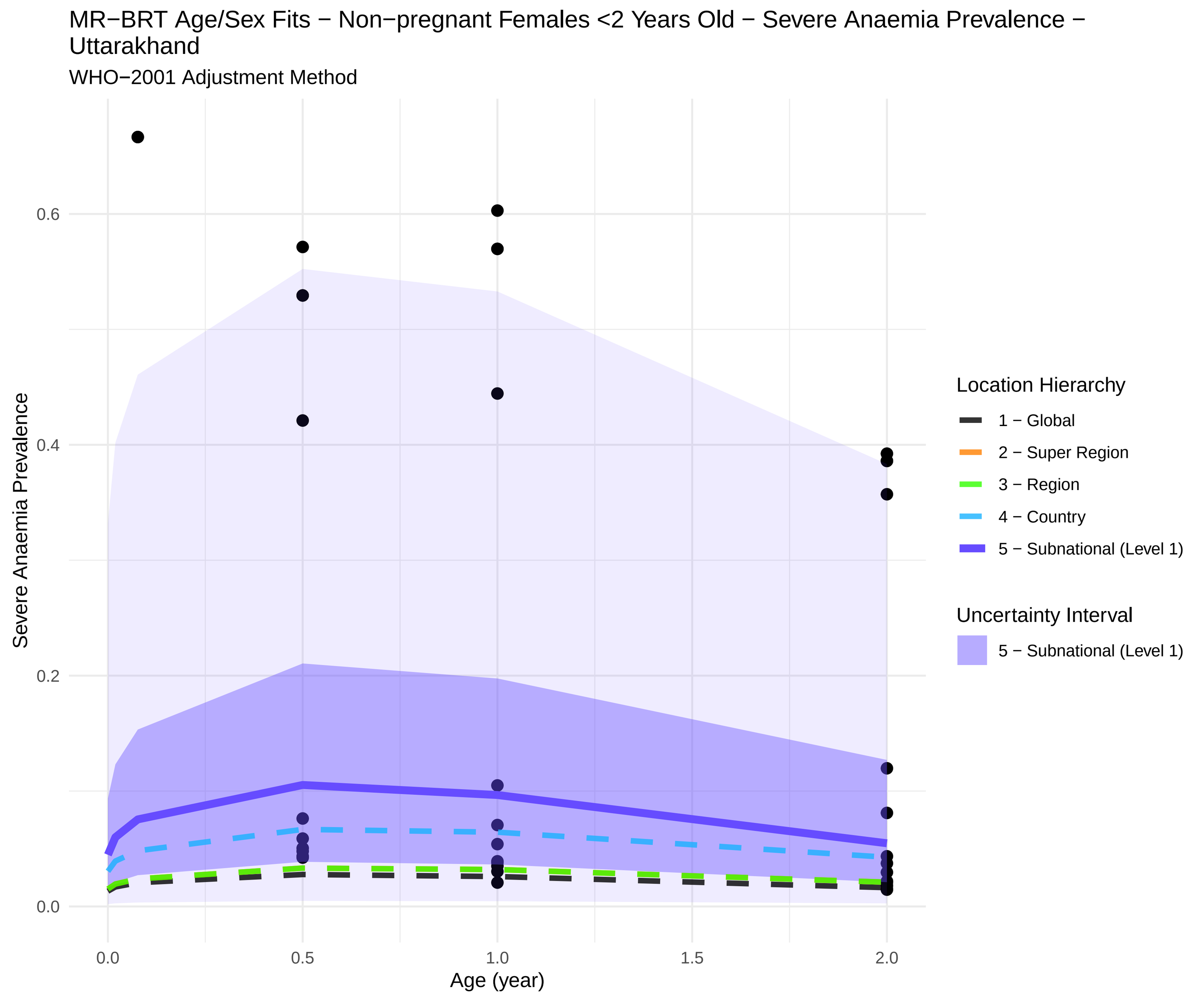
MR−BRT Age/Sex Fits − Non−pregnant Females <2 Years Old − Severe Anaemia Prevalence − Uttarakhand
WHO−2001 Adjustment Method
Location Hierarchy
1 − Global
2 − Super Region
3 − Region
4 − Country
5 − Subnational (Level 1)
Uncertainty Interval
5 − Subnational (Level 1)
Severe Anaemia Prevalence
Age (year)

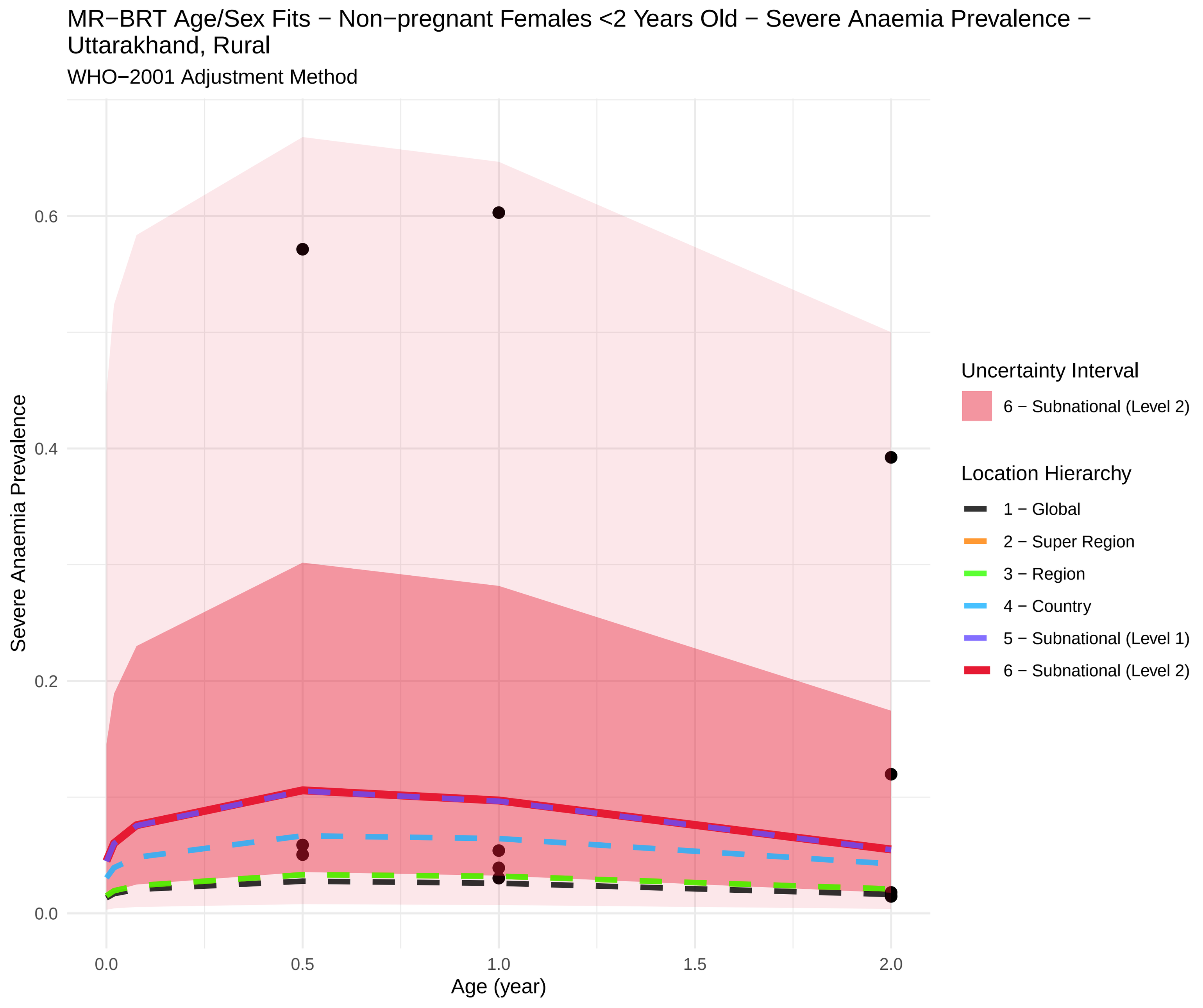
MR−BRT Age/Sex Fits − Non−pregnant Females <2 Years Old − Severe Anaemia Prevalence − Uttarakhand, Rural
WHO−2001 Adjustment Method
Uncertainty Interval
6 − Subnational (Level 2)
Location Hierarchy
1 − Global
2 − Super Region
3 − Region
4 − Country
5 − Subnational (Level 1)
6 − Subnational (Level 2)
Severe Anaemia Prevalence
Age (year)

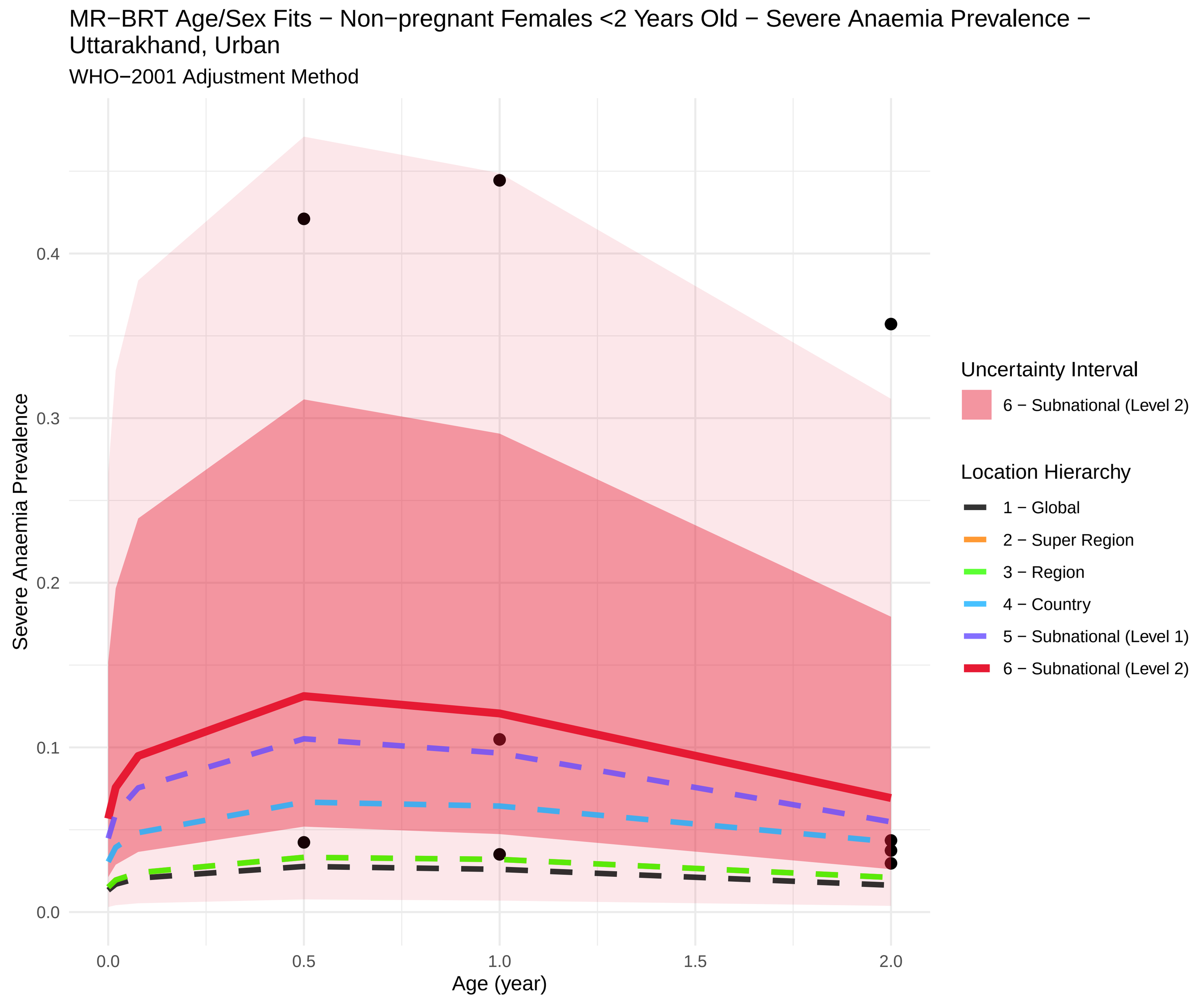
MR−BRT Age/Sex Fits − Non−pregnant Females <2 Years Old − Severe Anaemia Prevalence − Uttarakhand, Urban
WHO−2001 Adjustment Method
Uncertainty Interval
6 − Subnational (Level 2)
Location Hierarchy
1 − Global
2 − Super Region
3 − Region
4 − Country
5 − Subnational (Level 1)
6 − Subnational (Level 2)
Severe Anaemia Prevalence
Age (year)

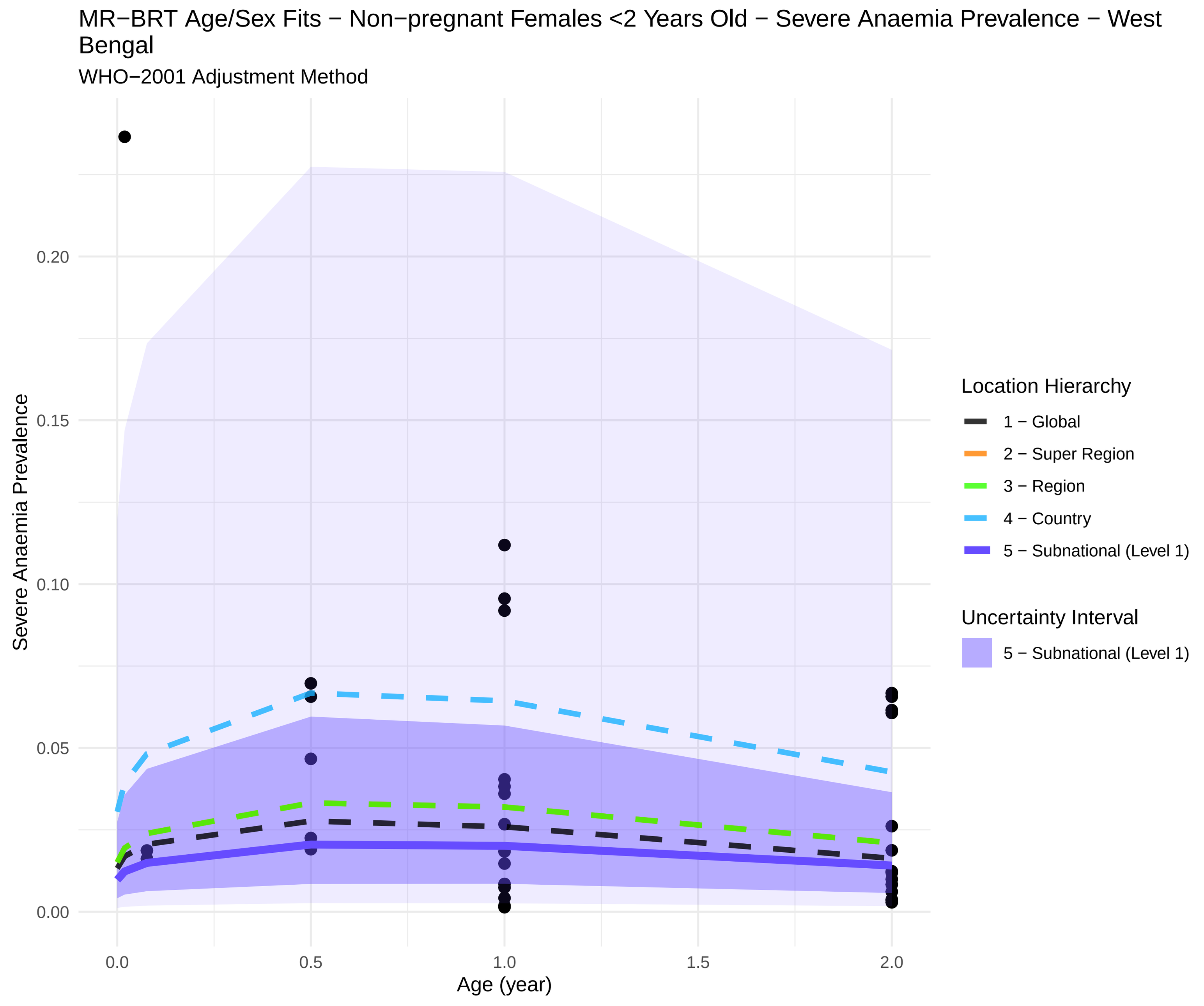
MR−BRT Age/Sex Fits − Non−pregnant Females <2 Years Old − Severe Anaemia Prevalence − West Bengal
WHO−2001 Adjustment Method
Location Hierarchy
1 − Global
2 − Super Region
3 − Region
4 − Country
5 − Subnational (Level 1)
Uncertainty Interval
5 − Subnational (Level 1)
Severe Anaemia Prevalence
Age (year)

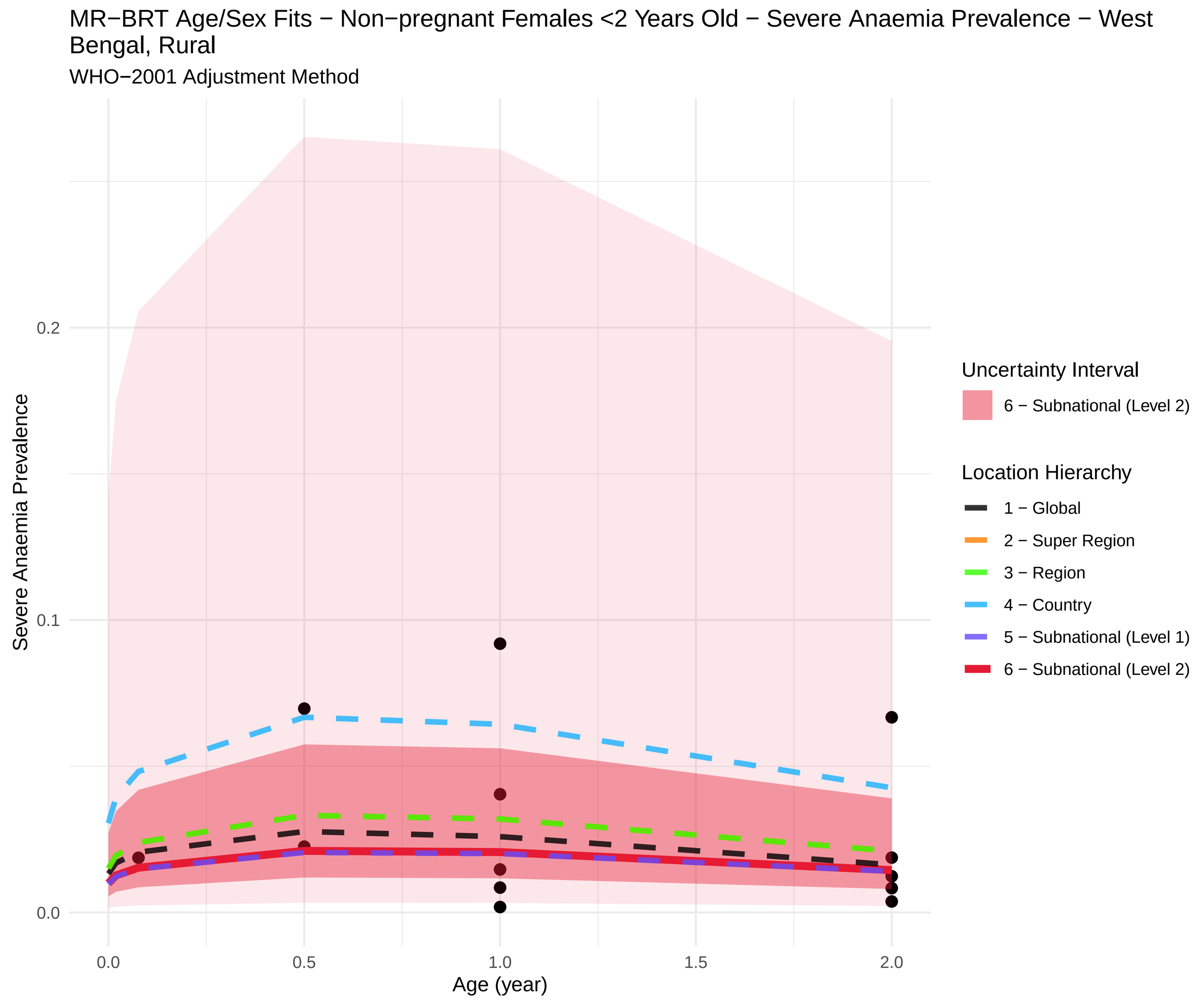
MR−BRT Age/Sex Fits − Non−pregnant Females <2 Years Old − Severe Anaemia Prevalence − West Bengal, Rural
WHO−2001 Adjustment Method
Uncertainty Interval
6 − Subnational (Level 2)
Location Hierarchy
1 − Global
2 − Super Region
3 − Region
4 − Country
5 − Subnational (Level 1)
6 − Subnational (Level 2)
Severe Anaemia Prevalence
Age (year)

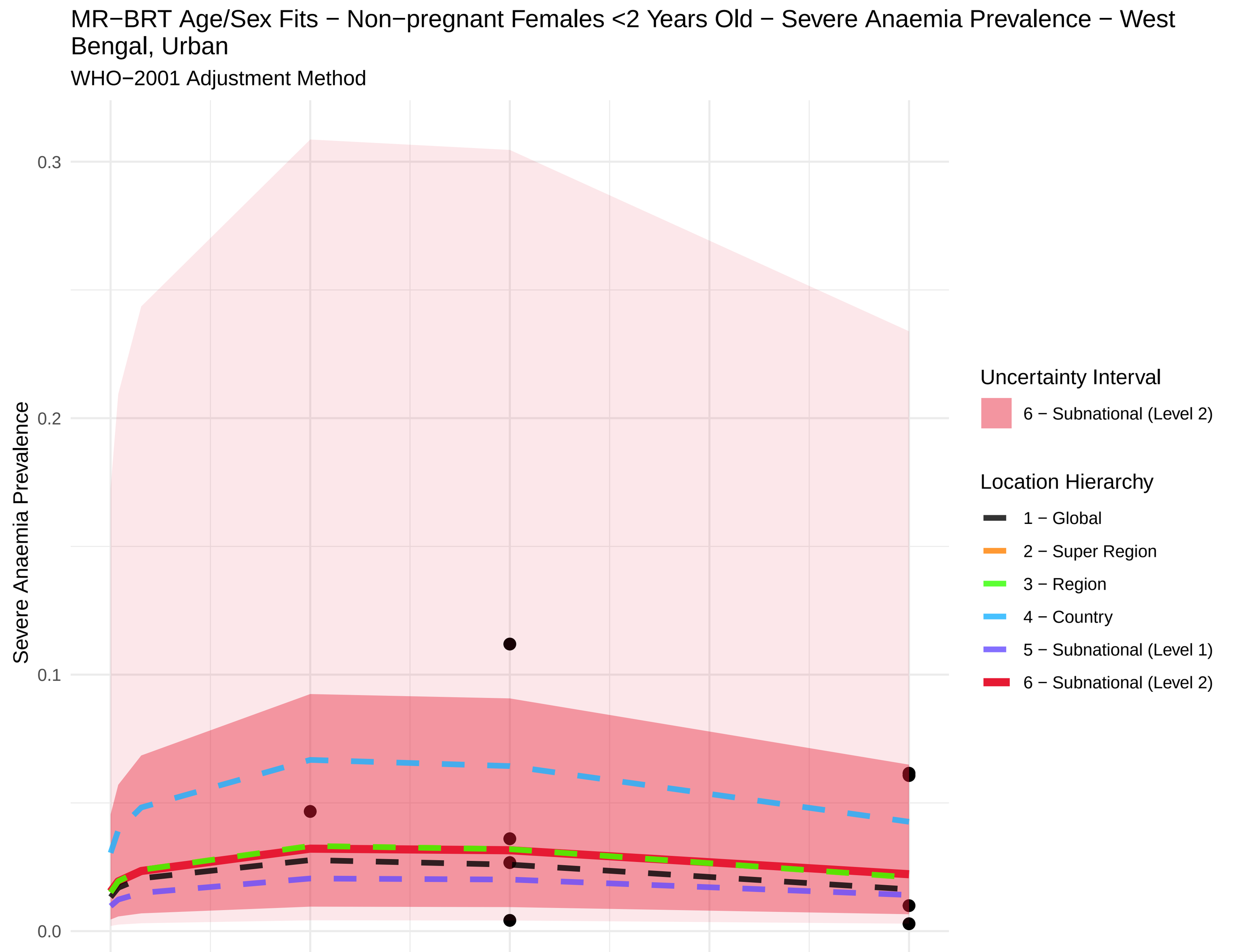
MR−BRT Age/Sex Fits − Non−pregnant Females <2 Years Old − Severe Anaemia Prevalence − West Bengal, Urban
WHO−2001 Adjustment Method
Uncertainty Interval
6 − Subnational (Level 2)
Location Hierarchy
1 − Global
2 − Super Region
3 − Region
4 − Country
5 − Subnational (Level 1)
6 − Subnational (Level 2)
Severe Anaemia Prevalence
Age (year)

MR−BRT Age/Sex Fits − Non−pregnant Females <2 Years Old − Severe Anaemia Prevalence − Nepal
WHO−2001 Adjustment Method

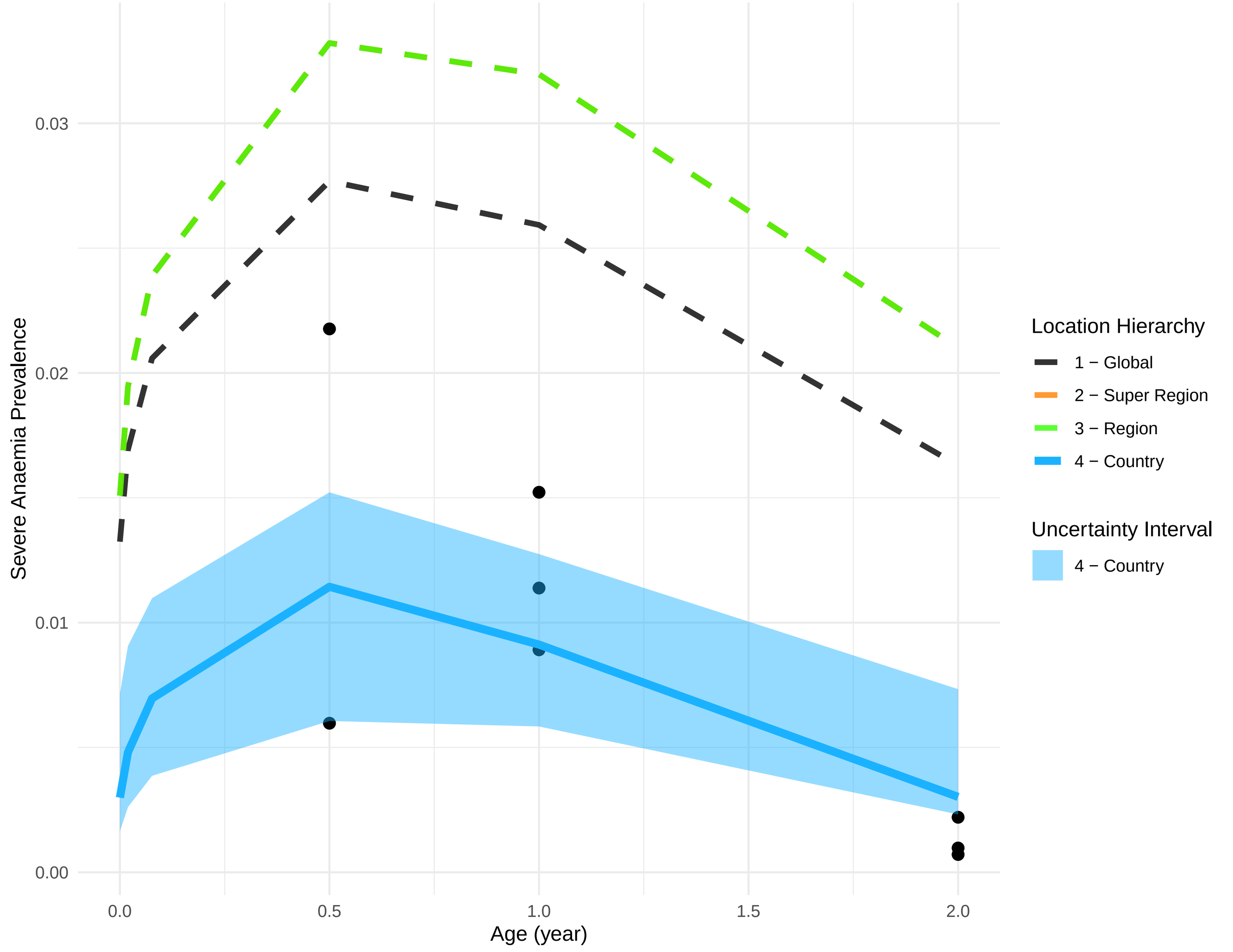

MR−BRT Age/Sex Fits − Non−pregnant Females <2 Years Old − Severe Anaemia Prevalence − Pakistan
WHO−2001 Adjustment Method

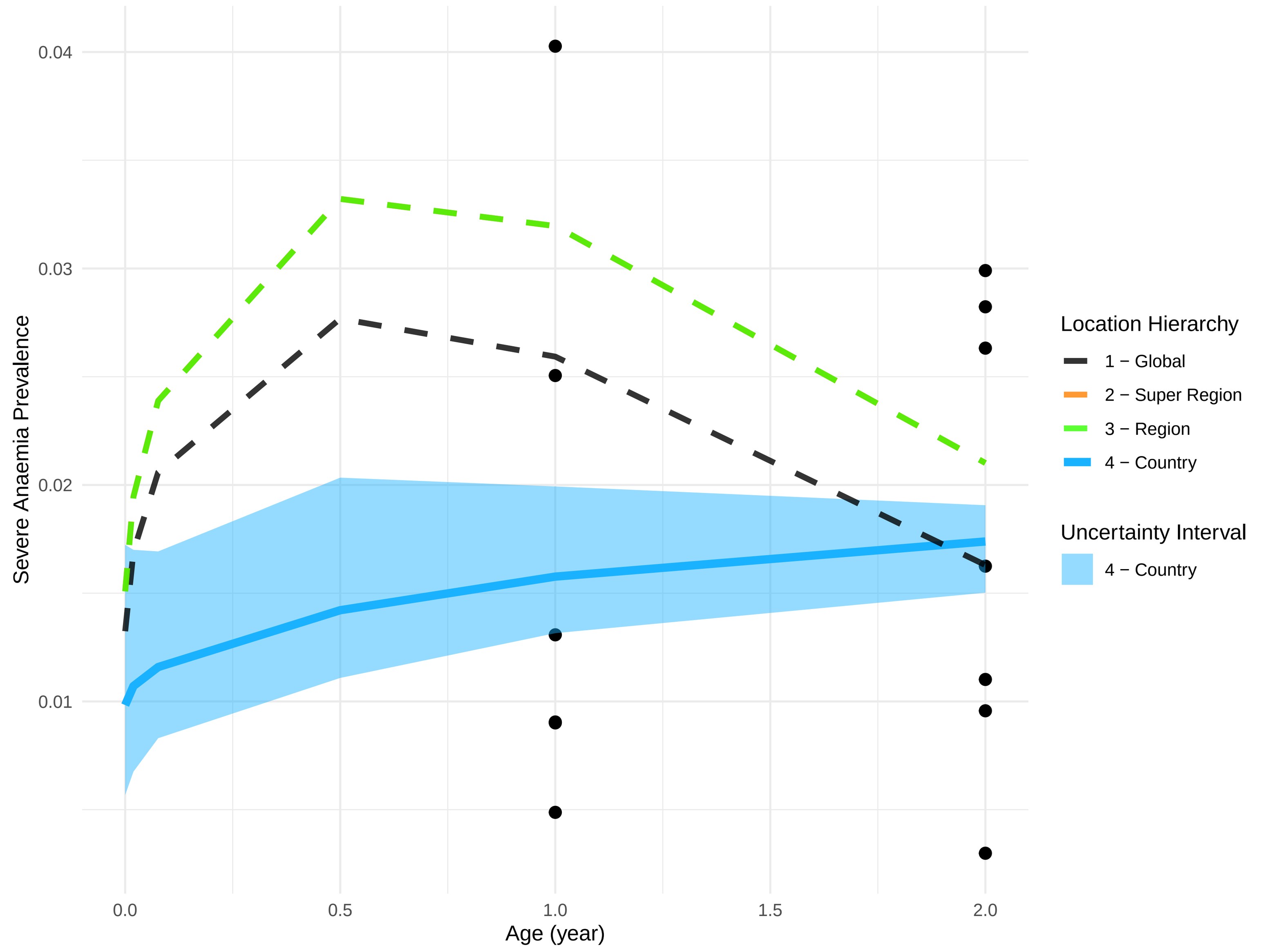

MR−BRT Age/Sex Fits − Non−pregnant Females <2 Years Old − Severe Anaemia Prevalence − Azad Jammu & Kashmir
WHO−2001 Adjustment Method

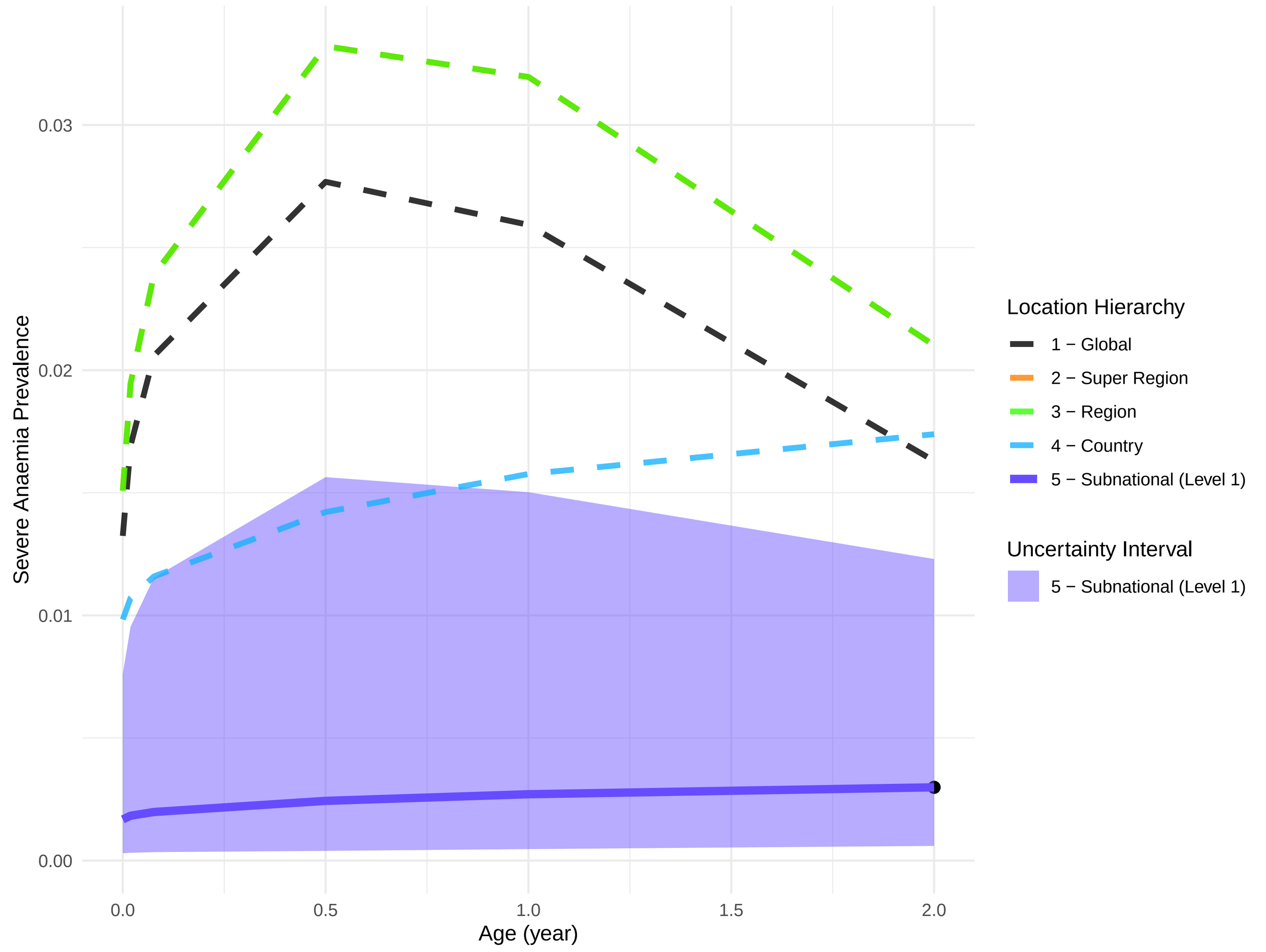

MR−BRT Age/Sex Fits − Non−pregnant Females <2 Years Old − Severe Anaemia Prevalence − Balochistan
WHO−2001 Adjustment Method

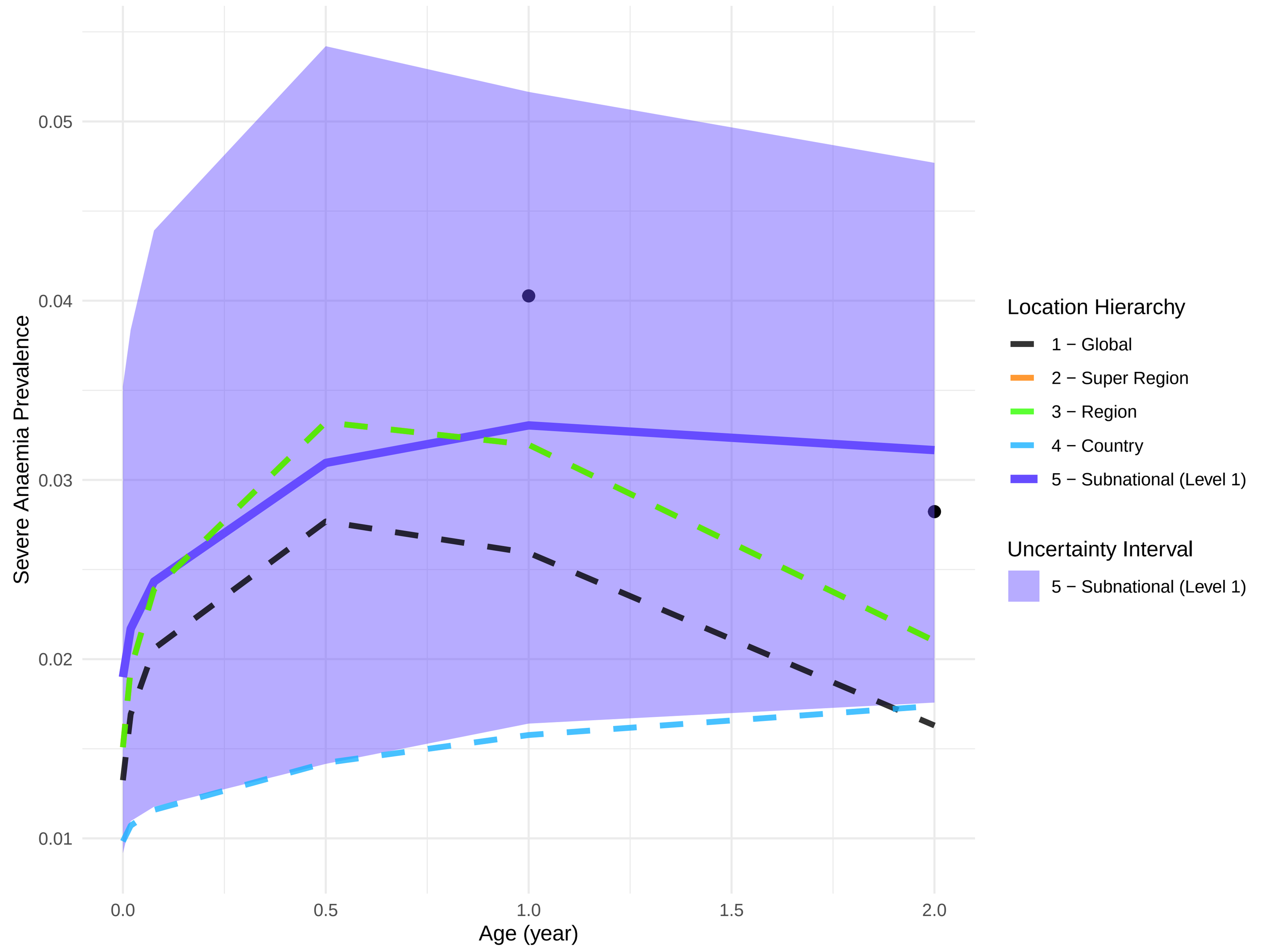

MR−BRT Age/Sex Fits − Non−pregnant Females <2 Years Old − Severe Anaemia Prevalence − Gilgit−Baltistan
WHO−2001 Adjustment Method

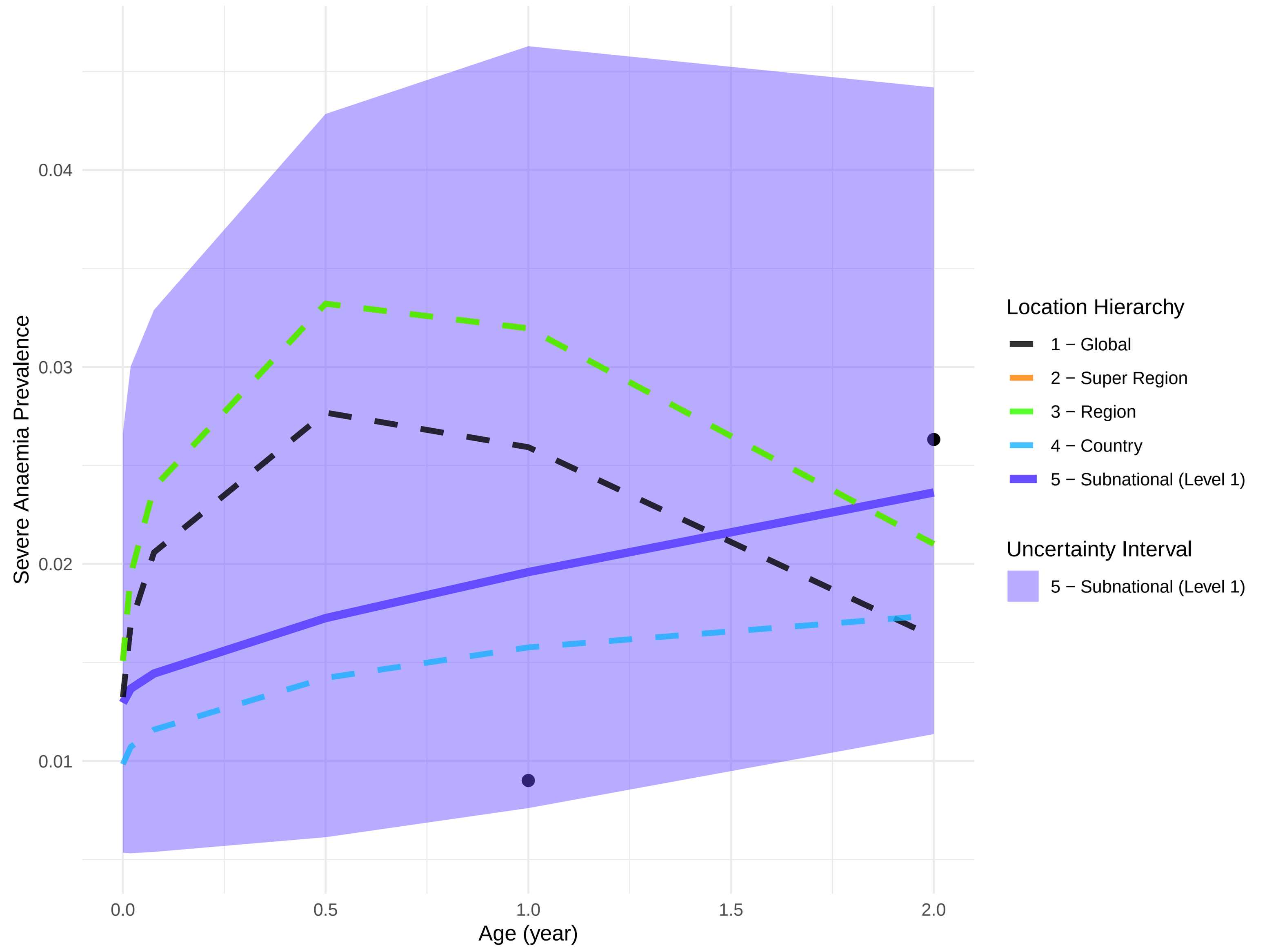

MR−BRT Age/Sex Fits − Non−pregnant Females <2 Years Old − Severe Anaemia Prevalence − Khyber Pakhtunkhwa
WHO−2001 Adjustment Method

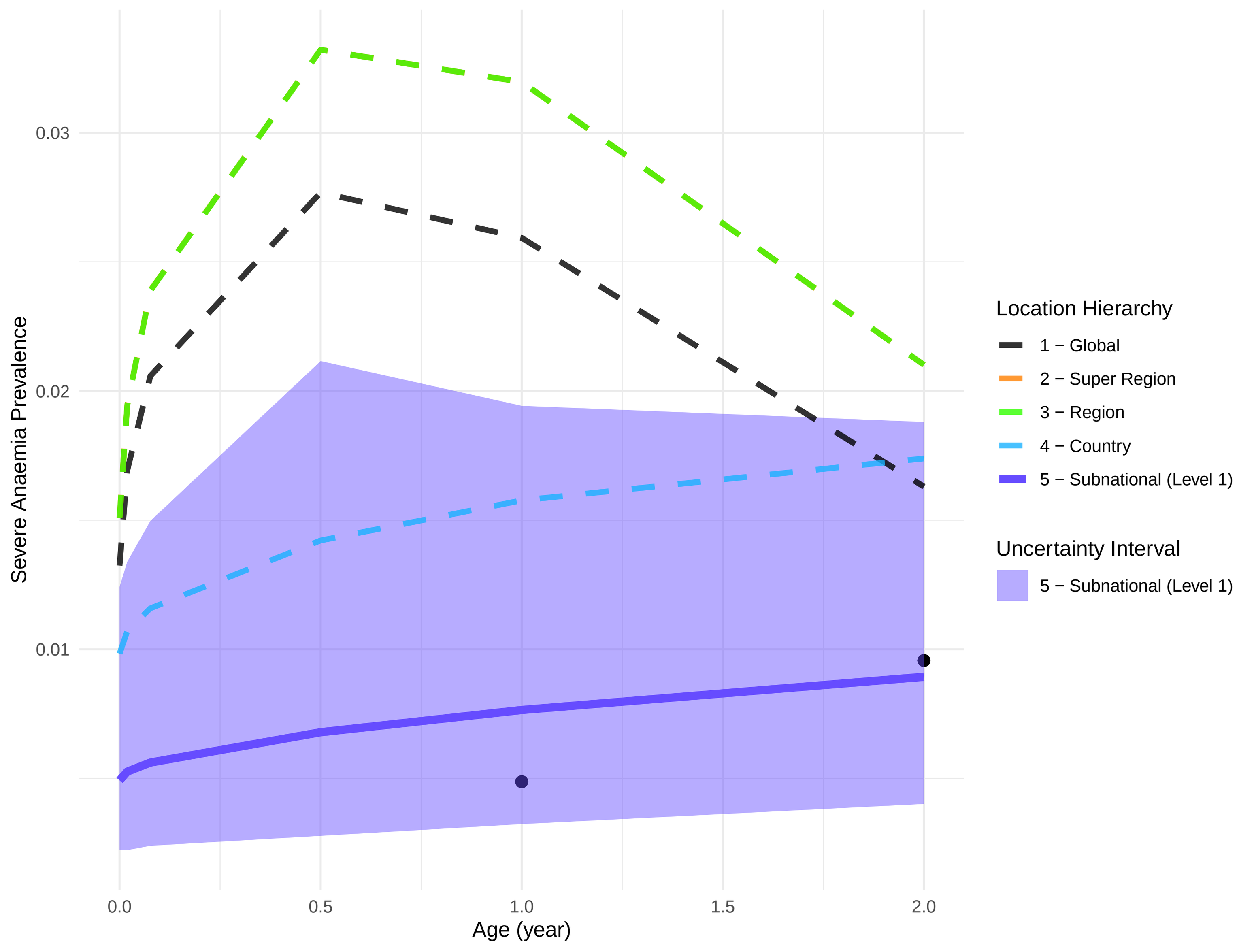

MR−BRT Age/Sex Fits − Non−pregnant Females <2 Years Old − Severe Anaemia Prevalence − Punjab
WHO−2001 Adjustment Method

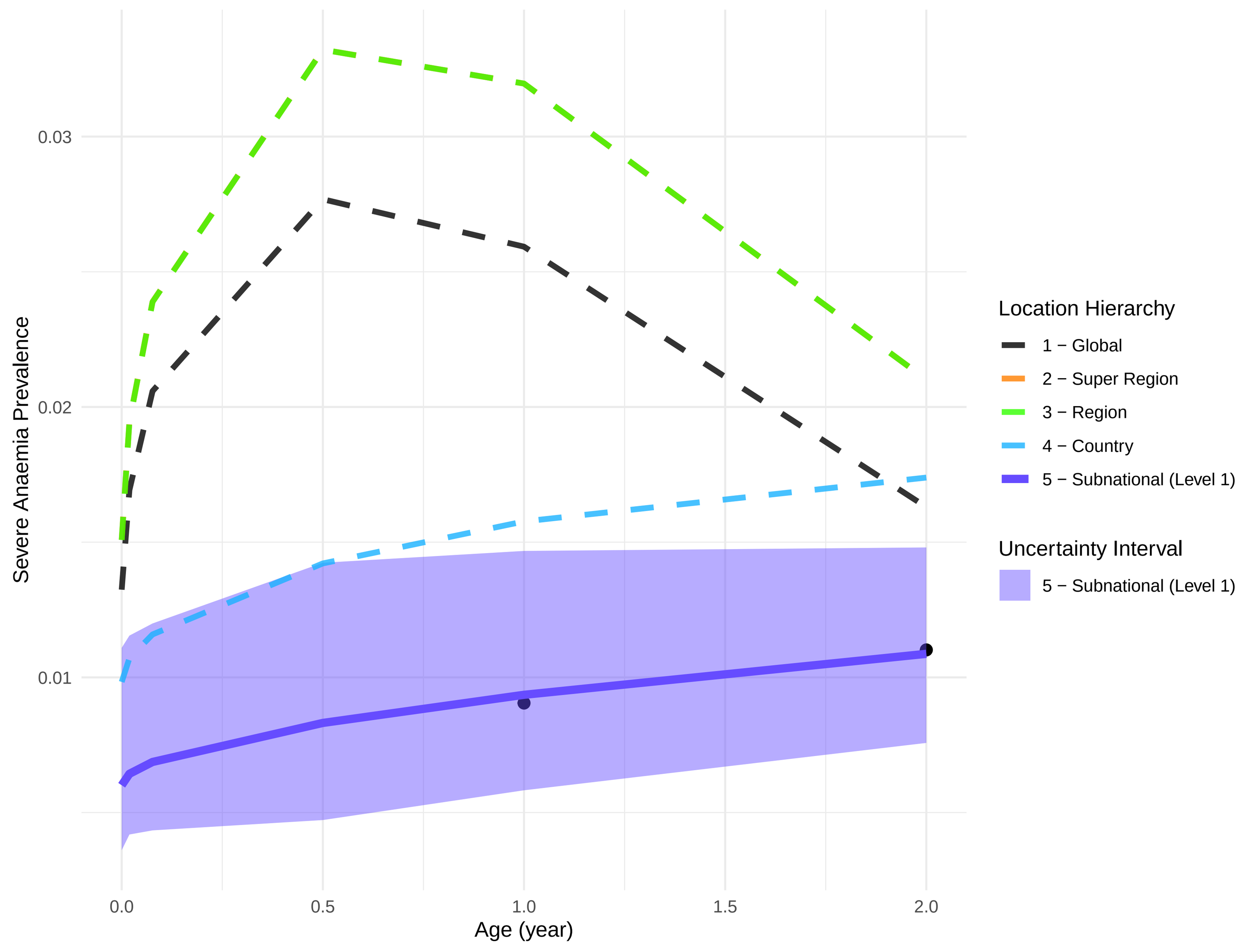

MR−BRT Age/Sex Fits − Non−pregnant Females <2 Years Old − Severe Anaemia Prevalence − Sindh
WHO−2001 Adjustment Method

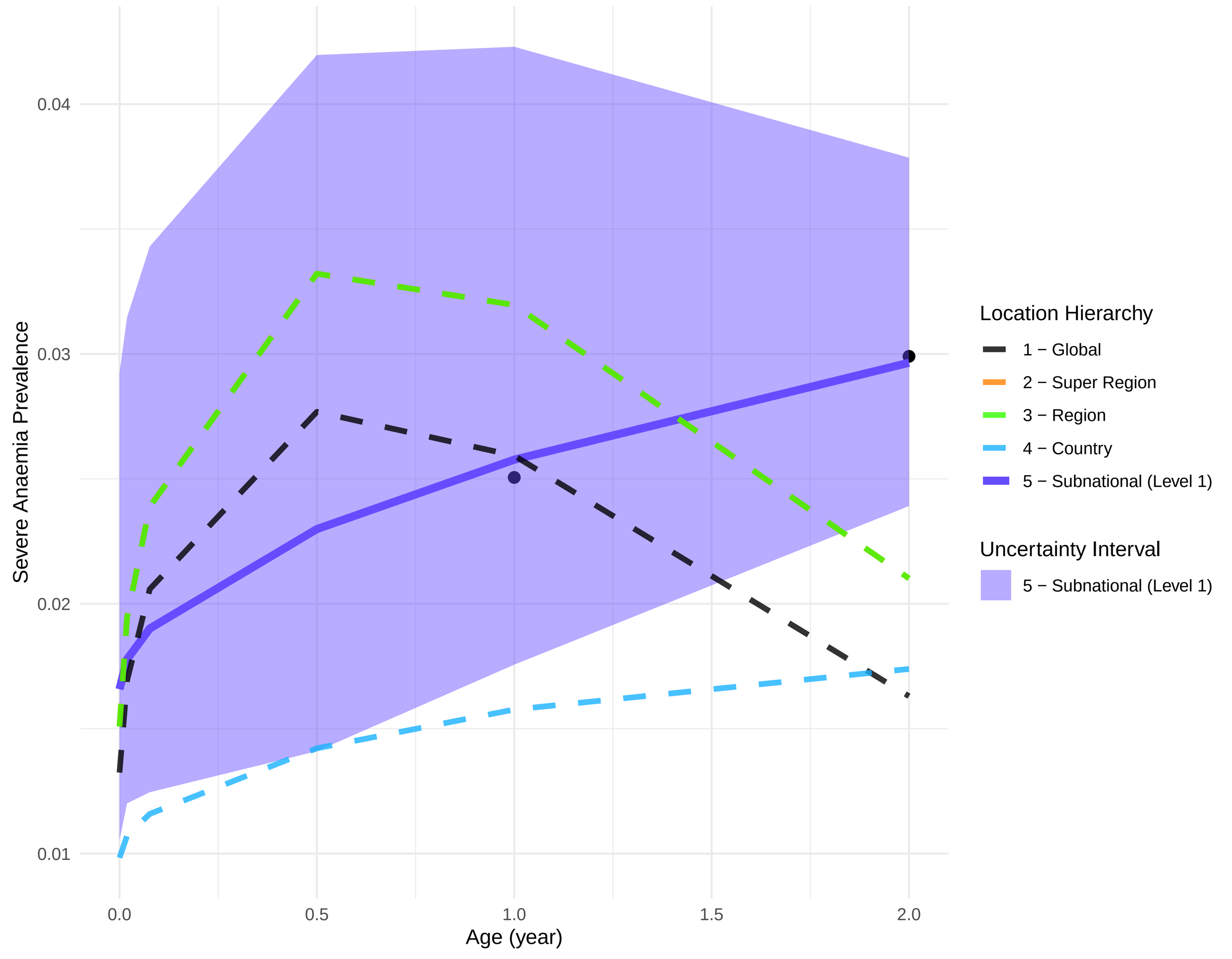

MR−BRT Age/Sex Fits − Non−pregnant Females <2 Years Old − Severe Anaemia Prevalence − Southeast Asia, East Asia, and Oceania
WHO−2001 Adjustment Method

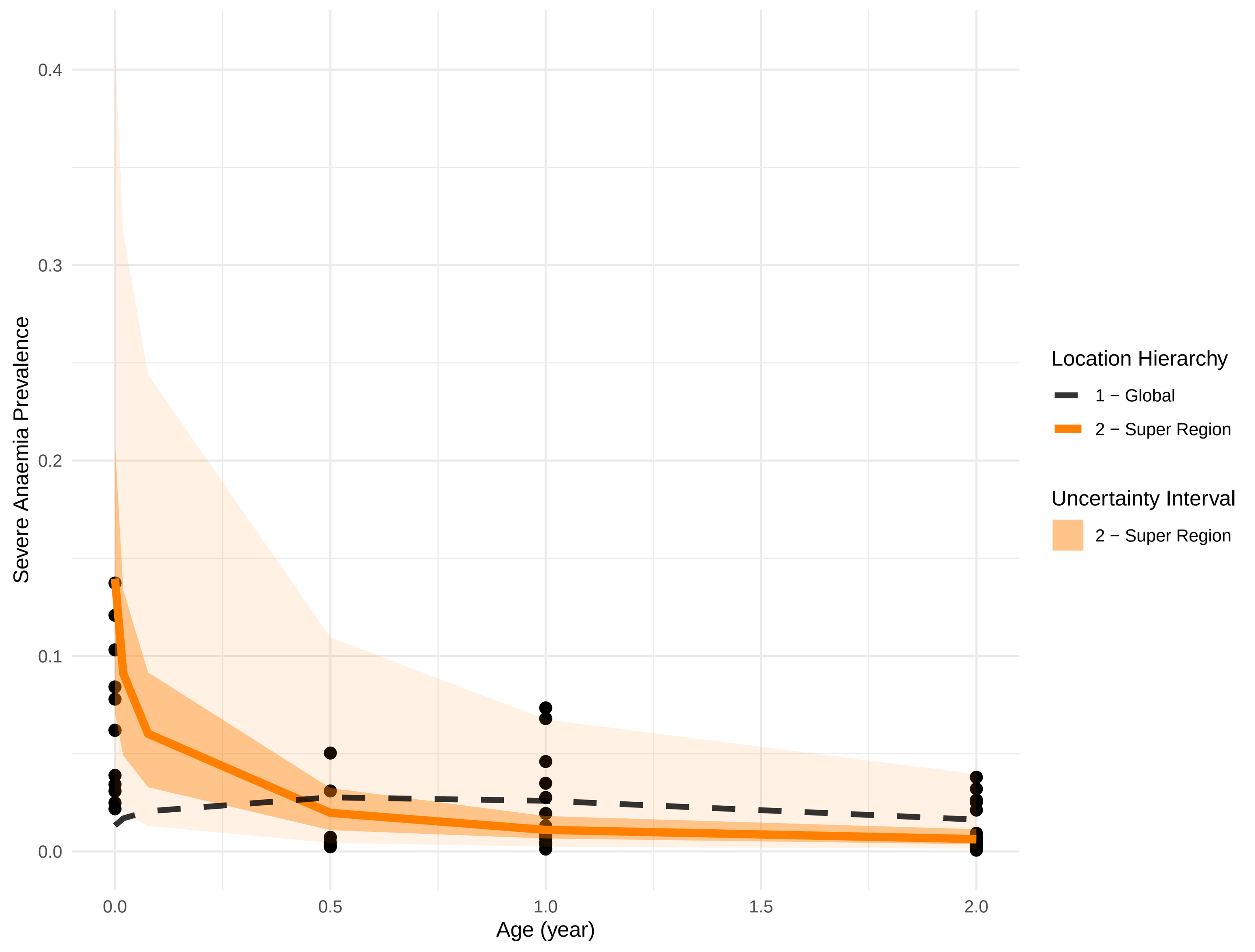

MR−BRT Age/Sex Fits − Non−pregnant Females <2 Years Old − Severe Anaemia Prevalence − Southeast Asia
WHO−2001 Adjustment Method

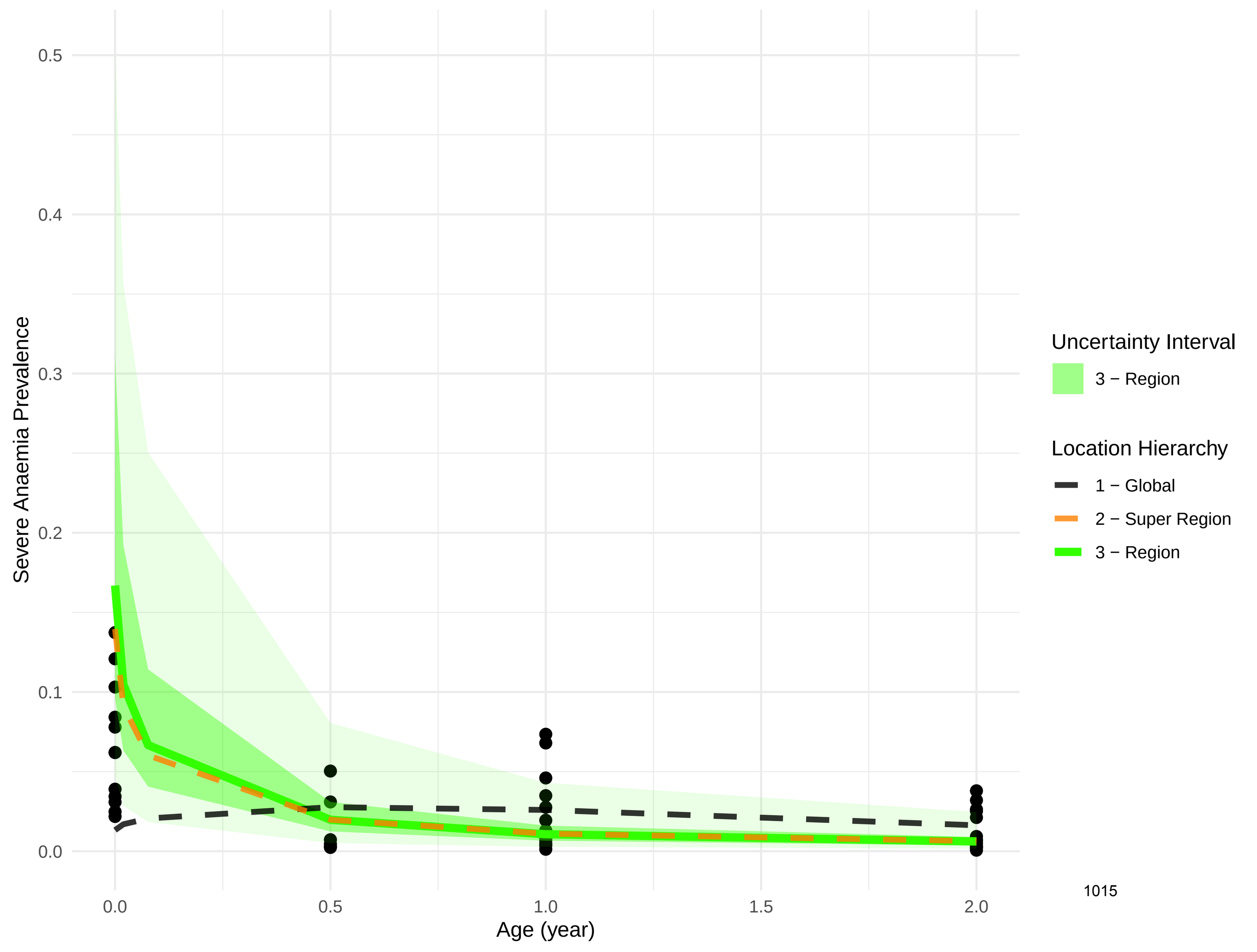

MR−BRT Age/Sex Fits − Non−pregnant Females <2 Years Old − Severe Anaemia Prevalence − Cambodia
WHO−2001 Adjustment Method

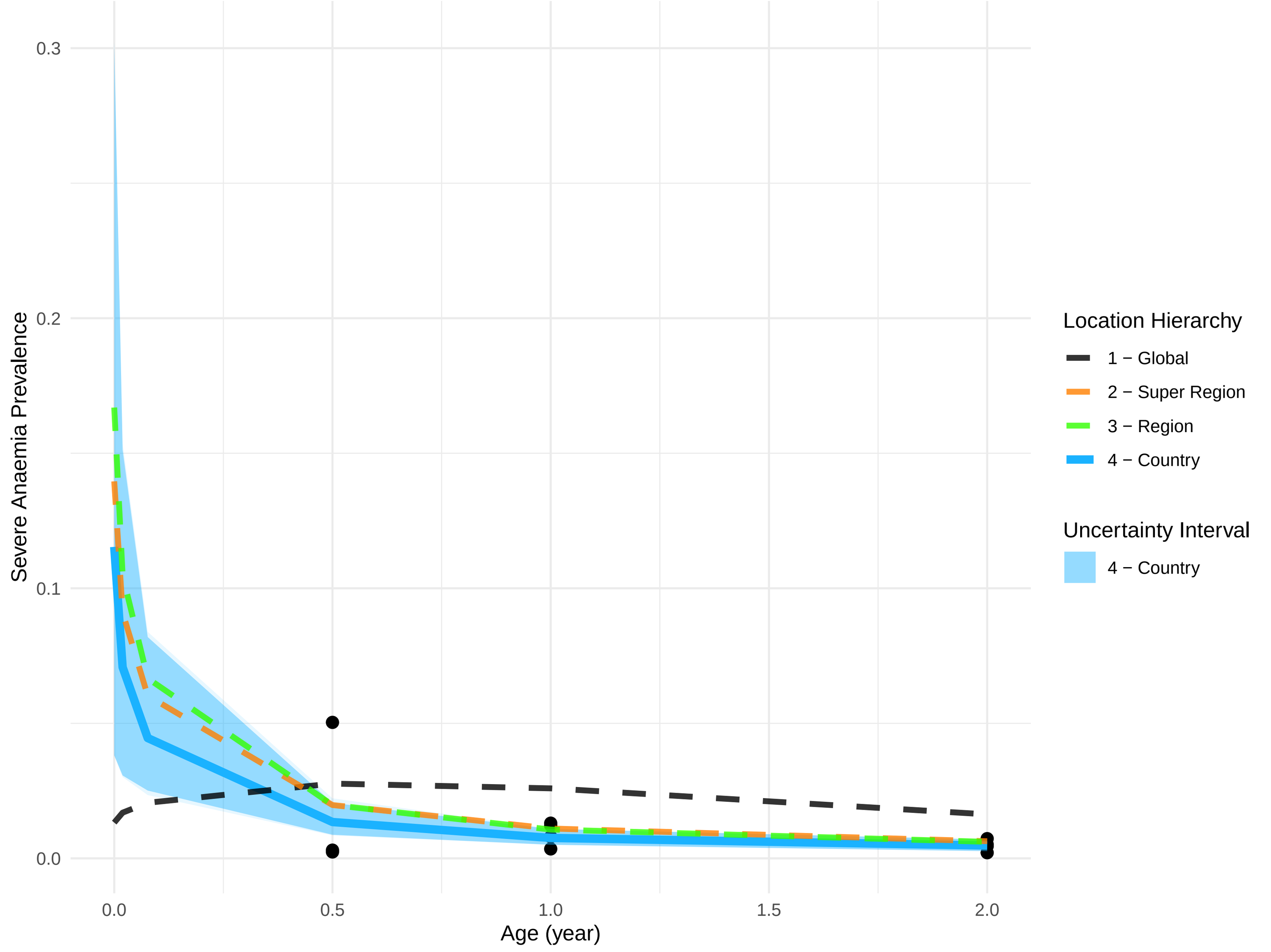

MR−BRT Age/Sex Fits − Non−pregnant Females <2 Years Old − Severe Anaemia Prevalence − Indonesia
WHO−2001 Adjustment Method

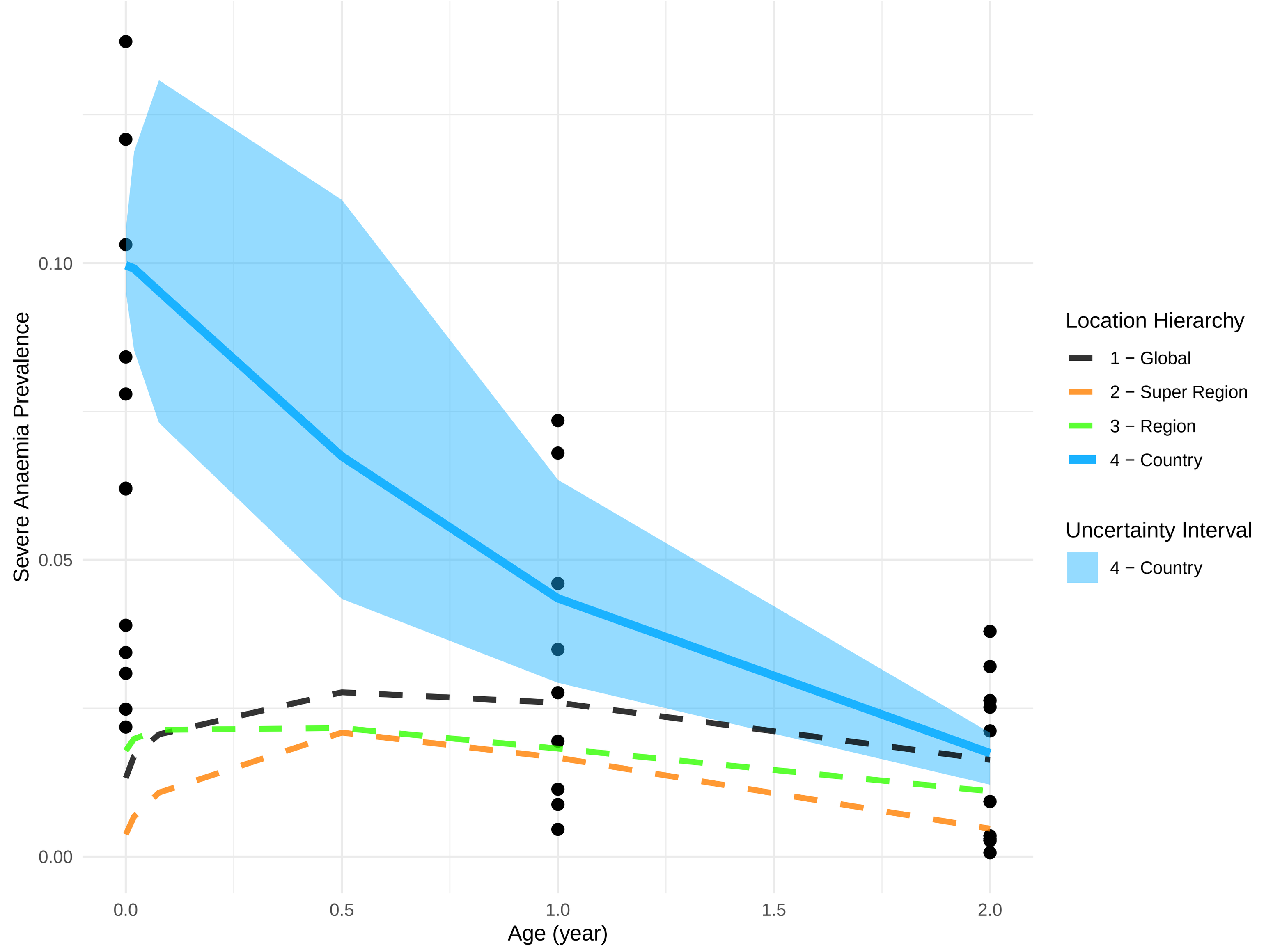

MR−BRT Age/Sex Fits − Non−pregnant Females <2 Years Old − Severe Anaemia Prevalence − Banten
WHO−2001 Adjustment Method

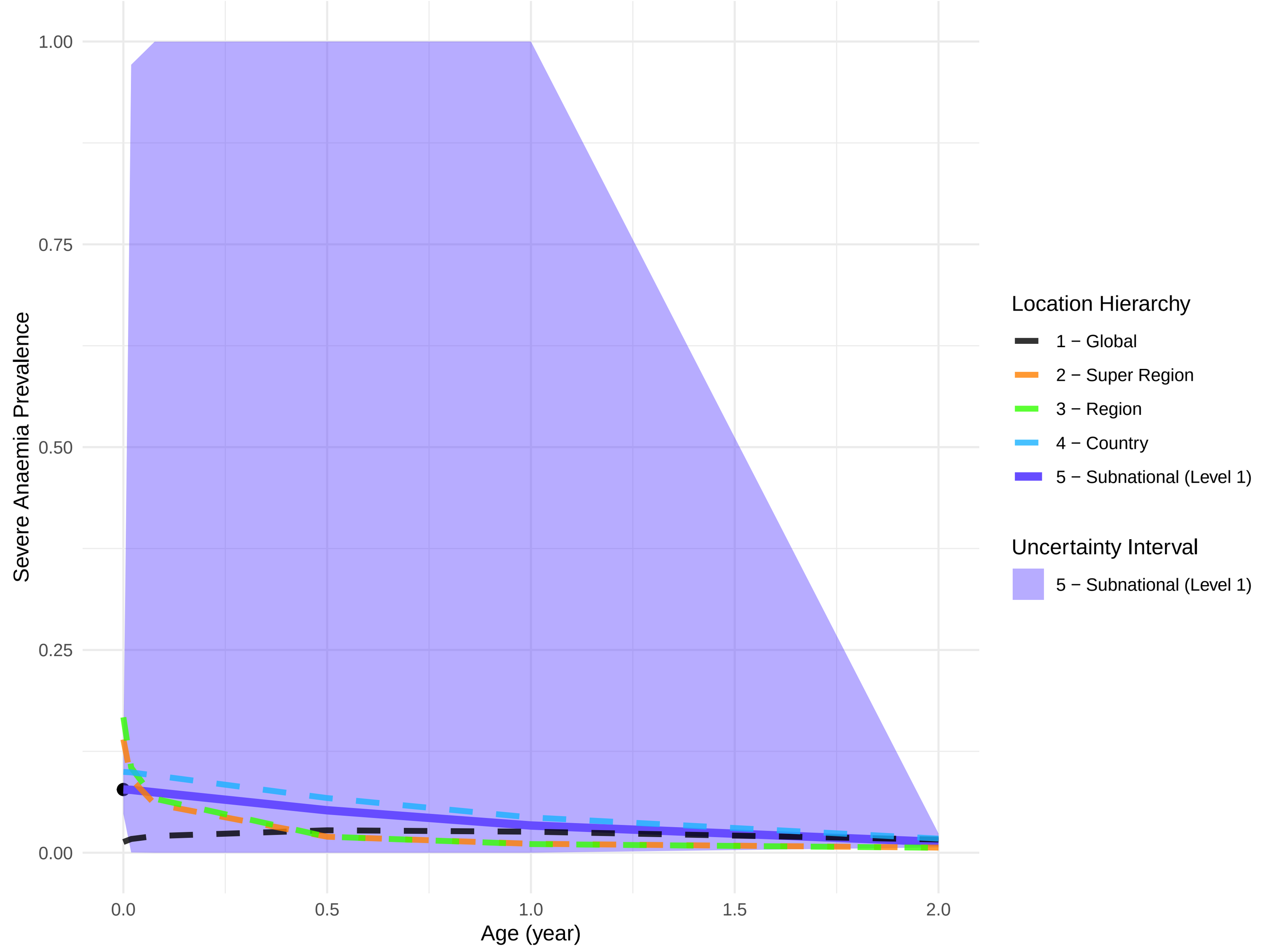

MR−BRT Age/Sex Fits − Non−pregnant Females <2 Years Old − Severe Anaemia Prevalence − West Java
WHO−2001 Adjustment Method

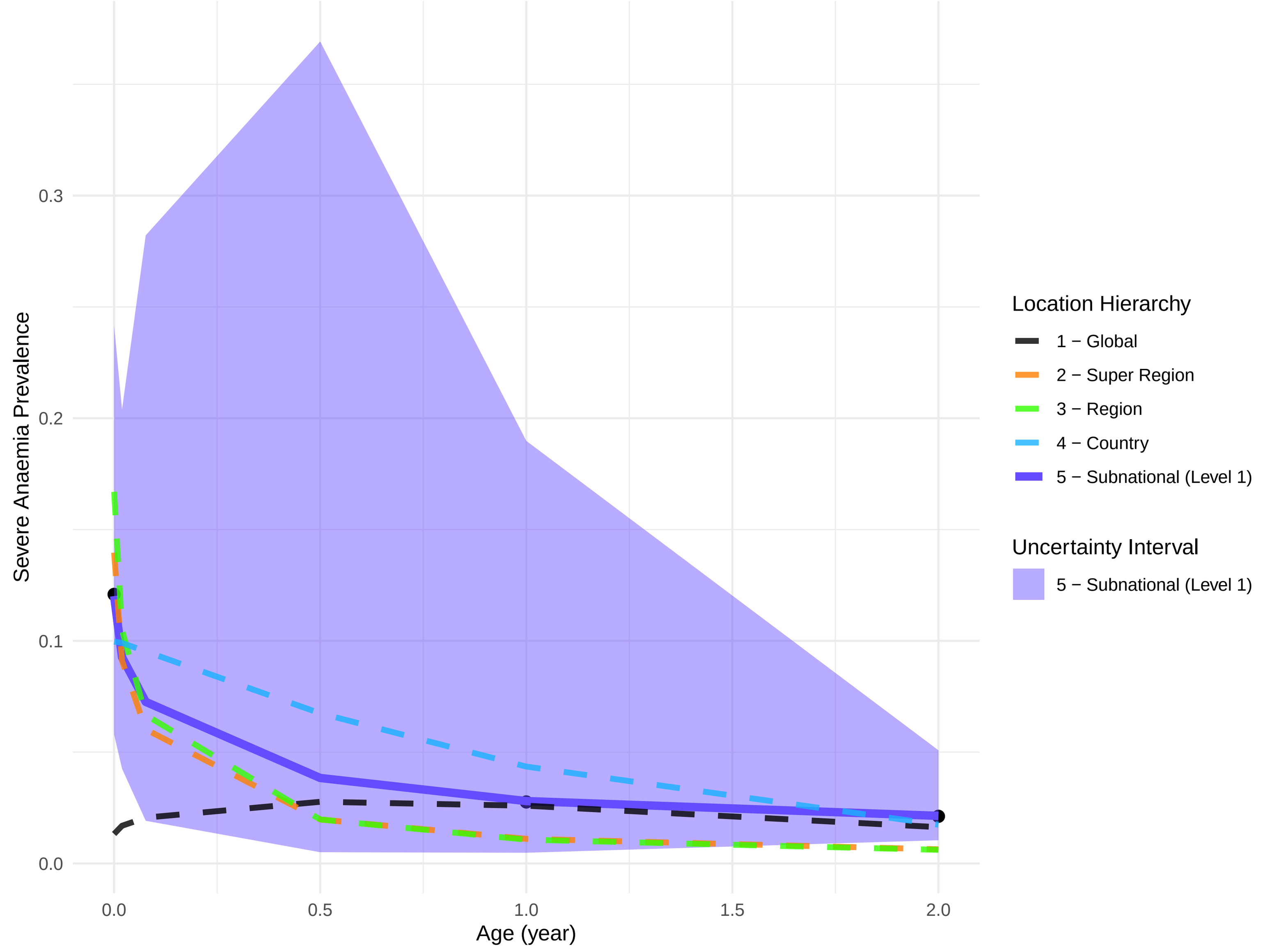

MR−BRT Age/Sex Fits − Non−pregnant Females <2 Years Old − Severe Anaemia Prevalence − Central Java
WHO−2001 Adjustment Method

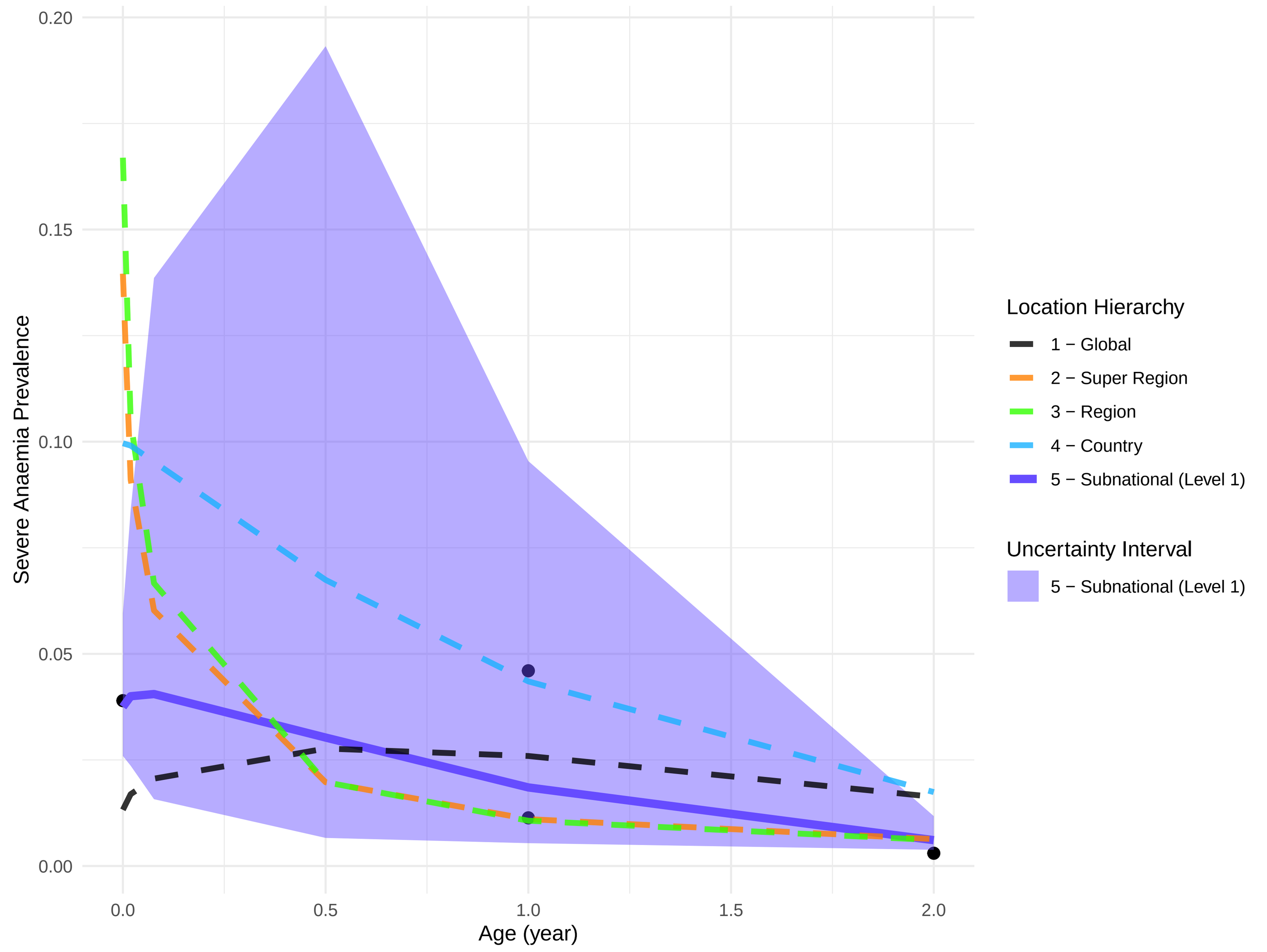

MR−BRT Age/Sex Fits − Non−pregnant Females <2 Years Old − Severe Anaemia Prevalence − East Java
WHO−2001 Adjustment Method

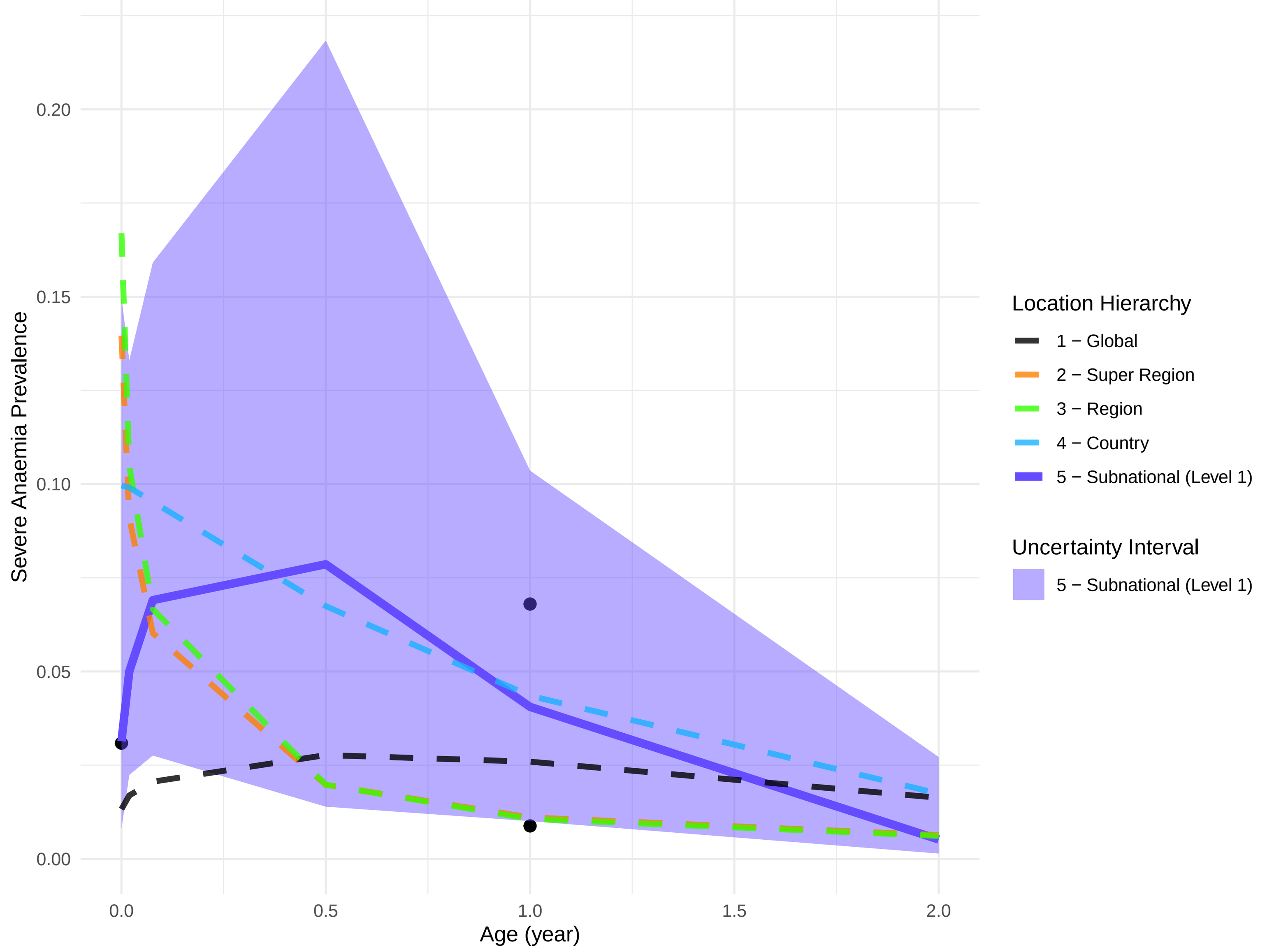

MR−BRT Age/Sex Fits − Non−pregnant Females <2 Years Old − Severe Anaemia Prevalence − South Kalimantan
WHO−2001 Adjustment Method

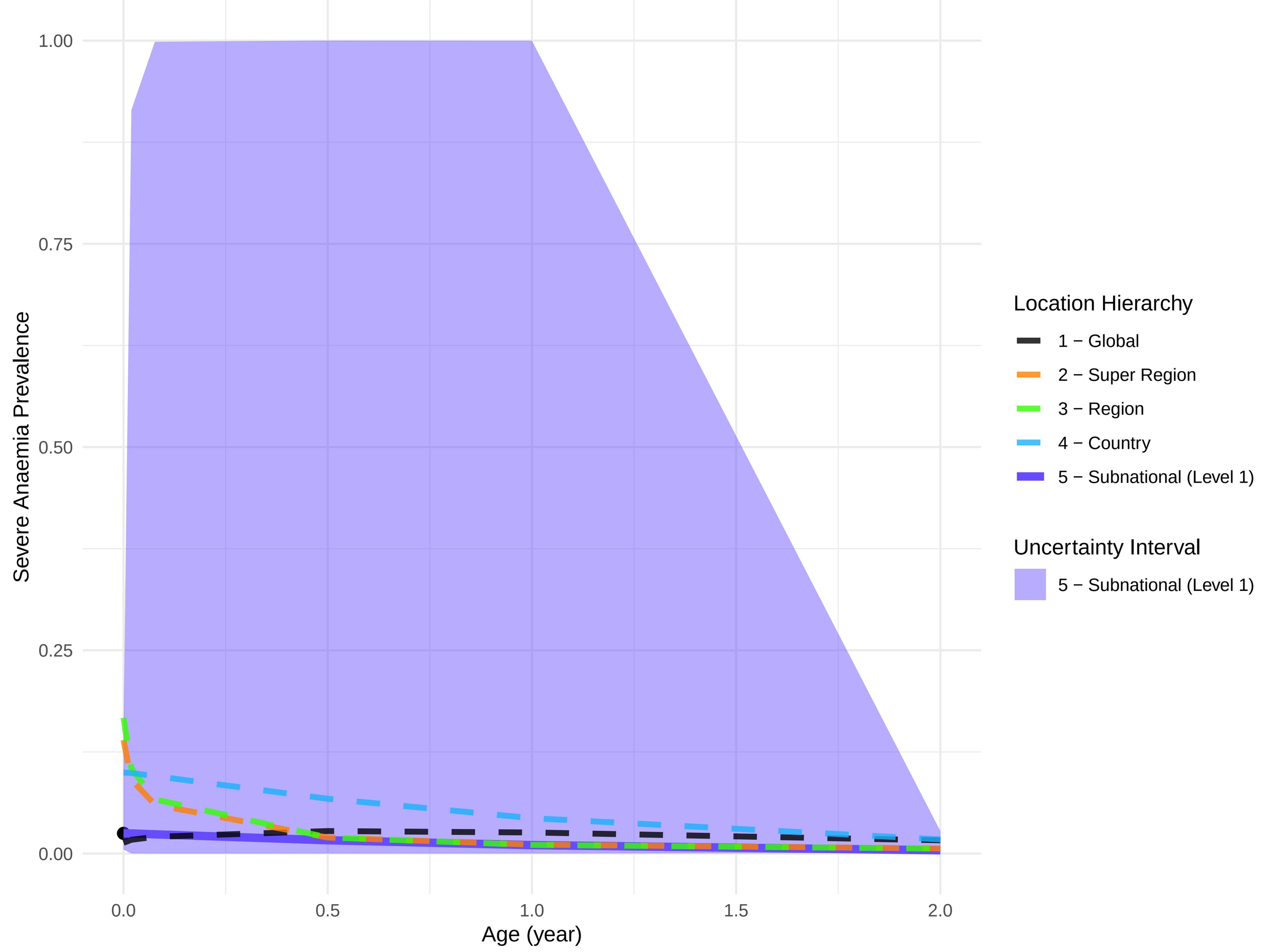

MR−BRT Age/Sex Fits − Non−pregnant Females <2 Years Old − Severe Anaemia Prevalence − Lampung
WHO−2001 Adjustment Method

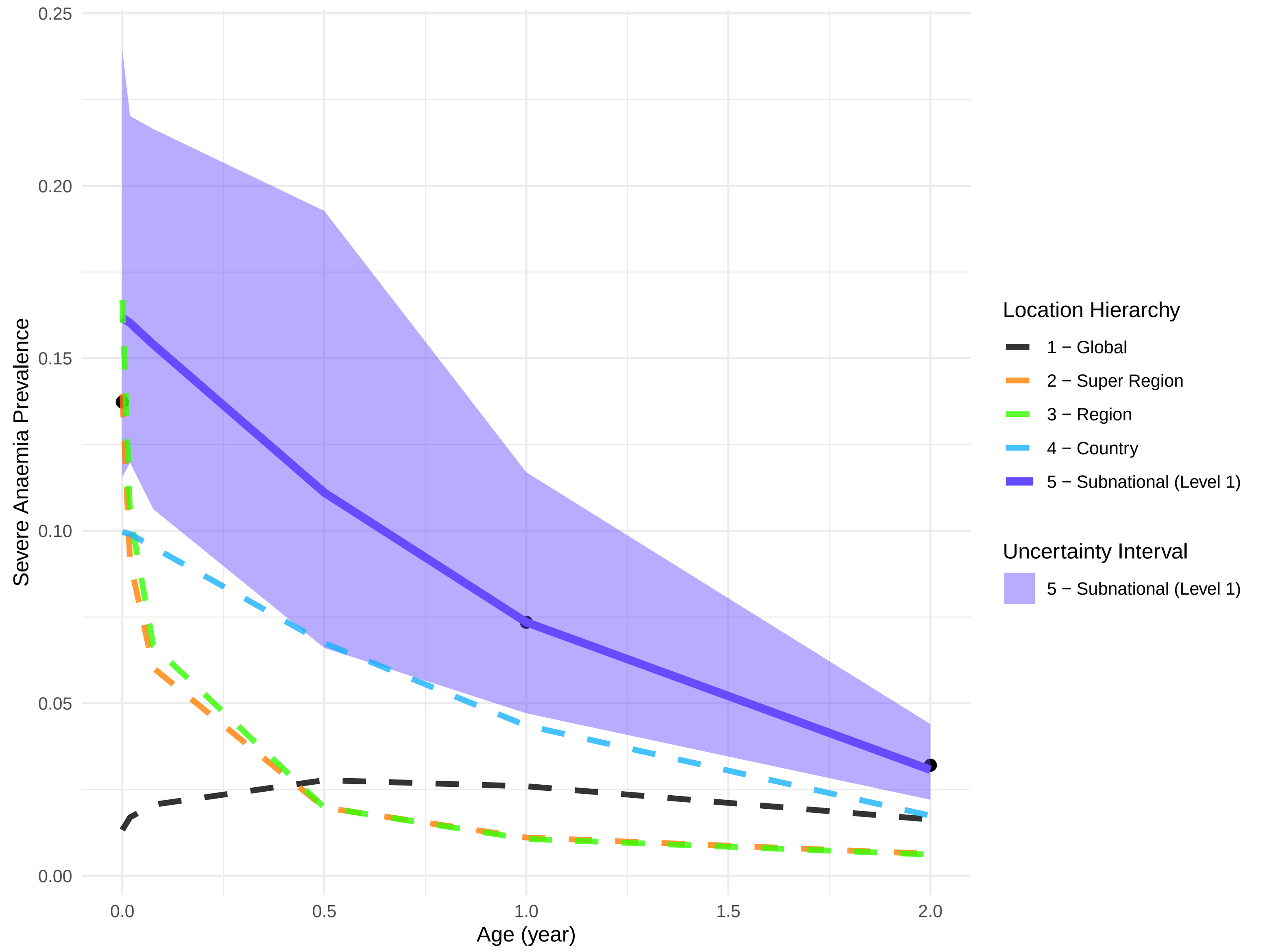

MR−BRT Age/Sex Fits − Non−pregnant Females <2 Years Old − Severe Anaemia Prevalence − West Nusa Tenggara
WHO−2001 Adjustment Method

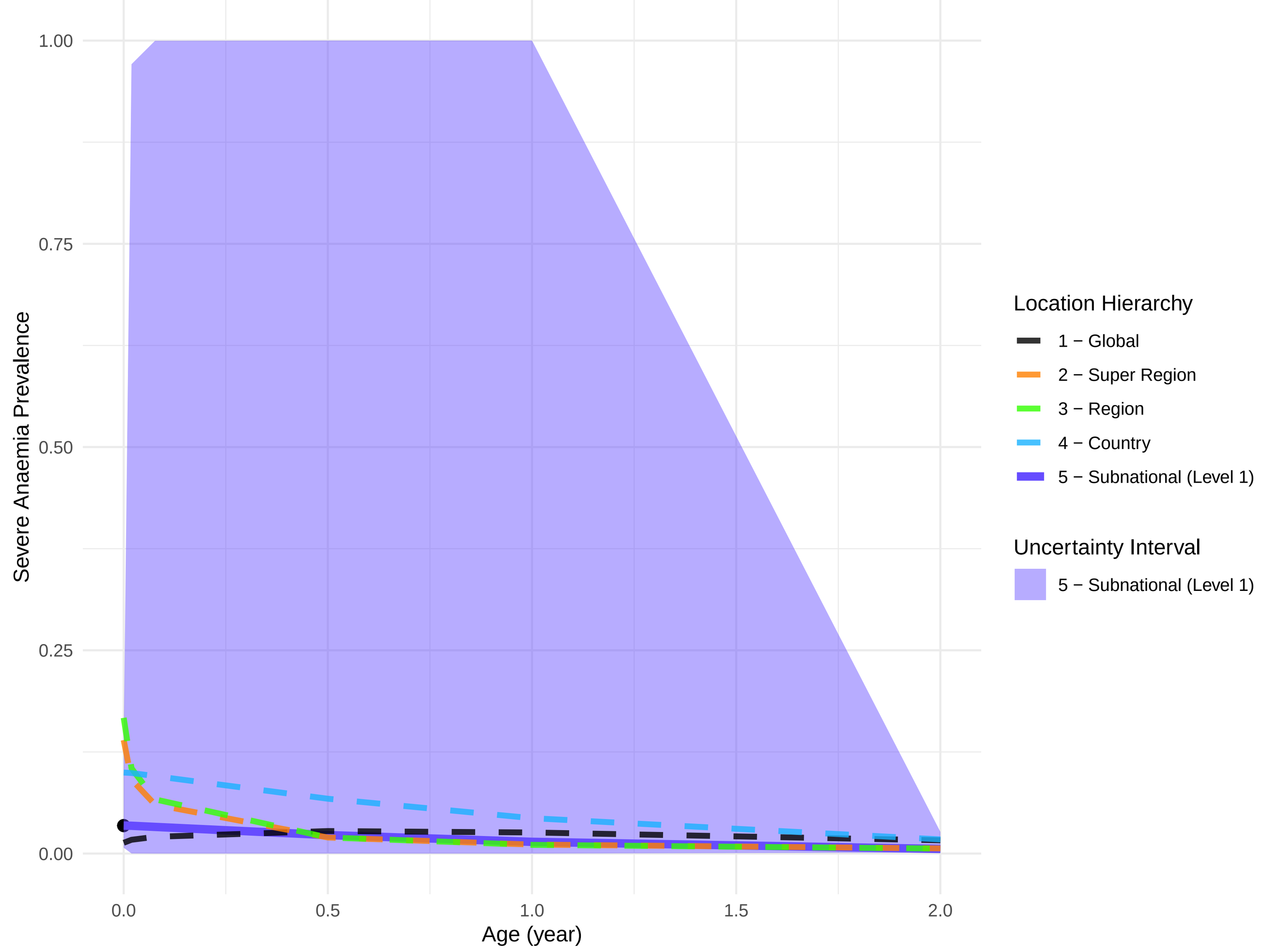

MR−BRT Age/Sex Fits − Non−pregnant Females <2 Years Old − Severe Anaemia Prevalence − South Sulawesi
WHO−2001 Adjustment Method

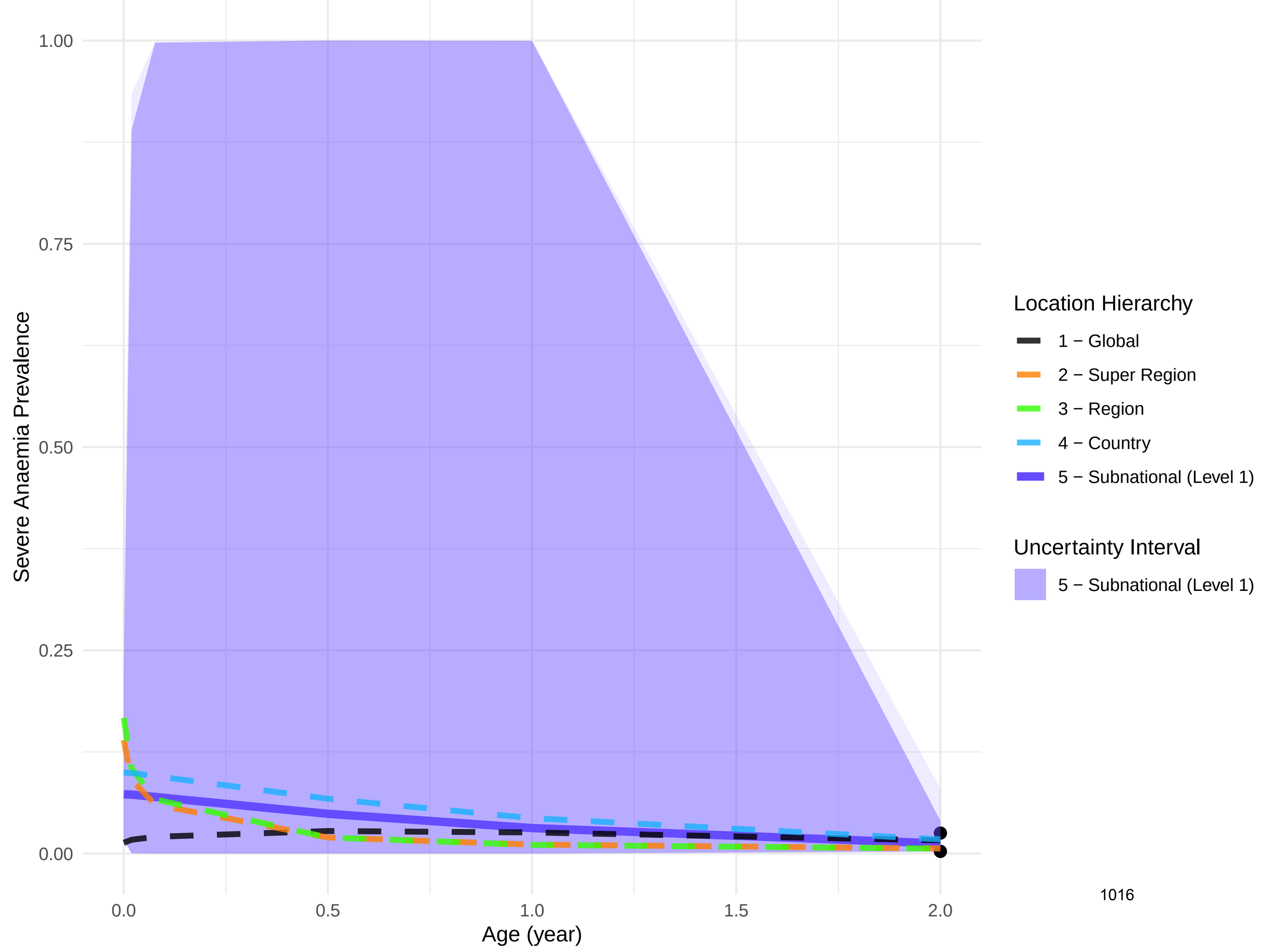

MR−BRT Age/Sex Fits − Non−pregnant Females <2 Years Old − Severe Anaemia Prevalence − West Sumatra
WHO−2001 Adjustment Method

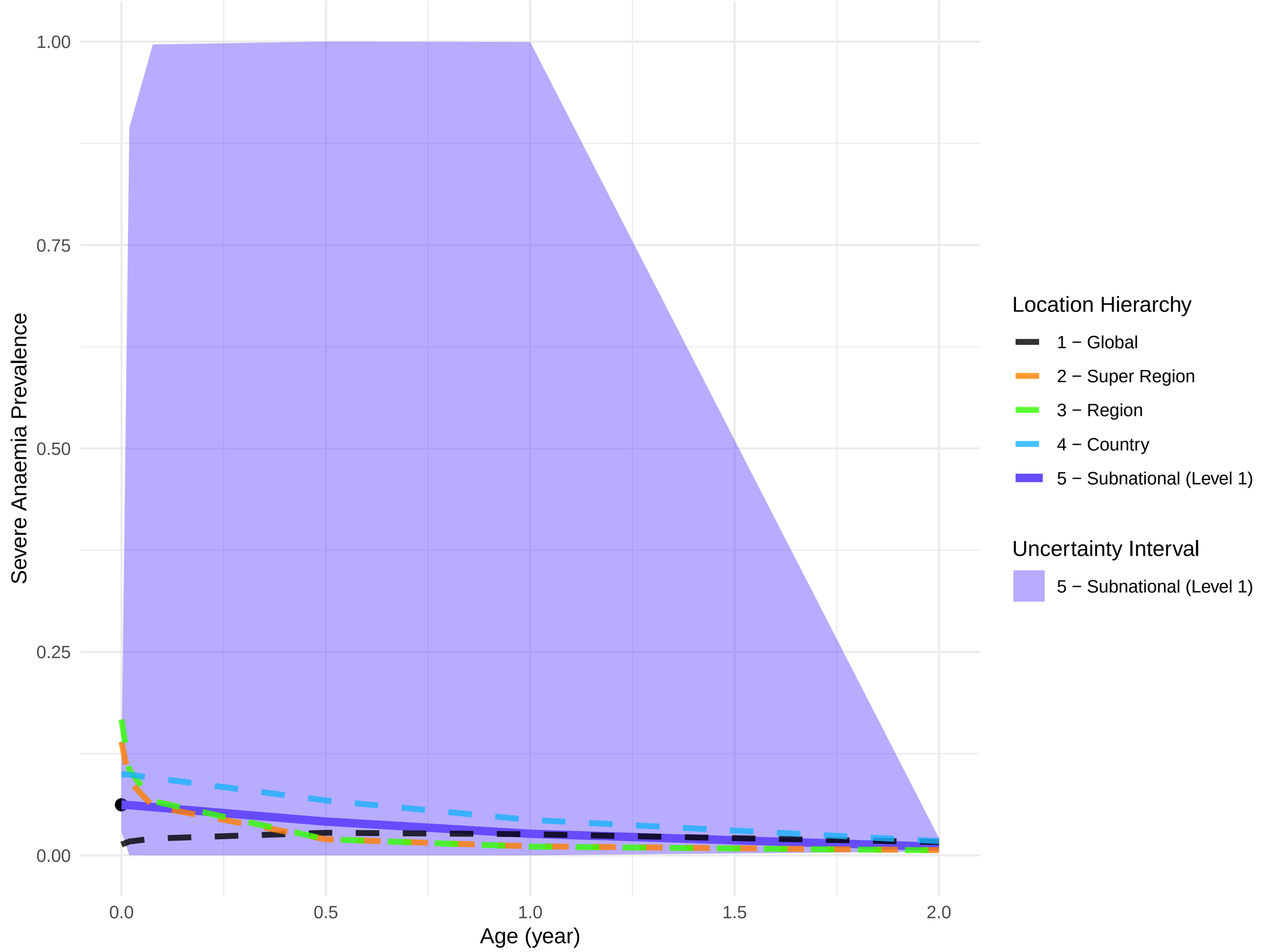

MR−BRT Age/Sex Fits − Non−pregnant Females <2 Years Old − Severe Anaemia Prevalence − South Sumatra
WHO−2001 Adjustment Method

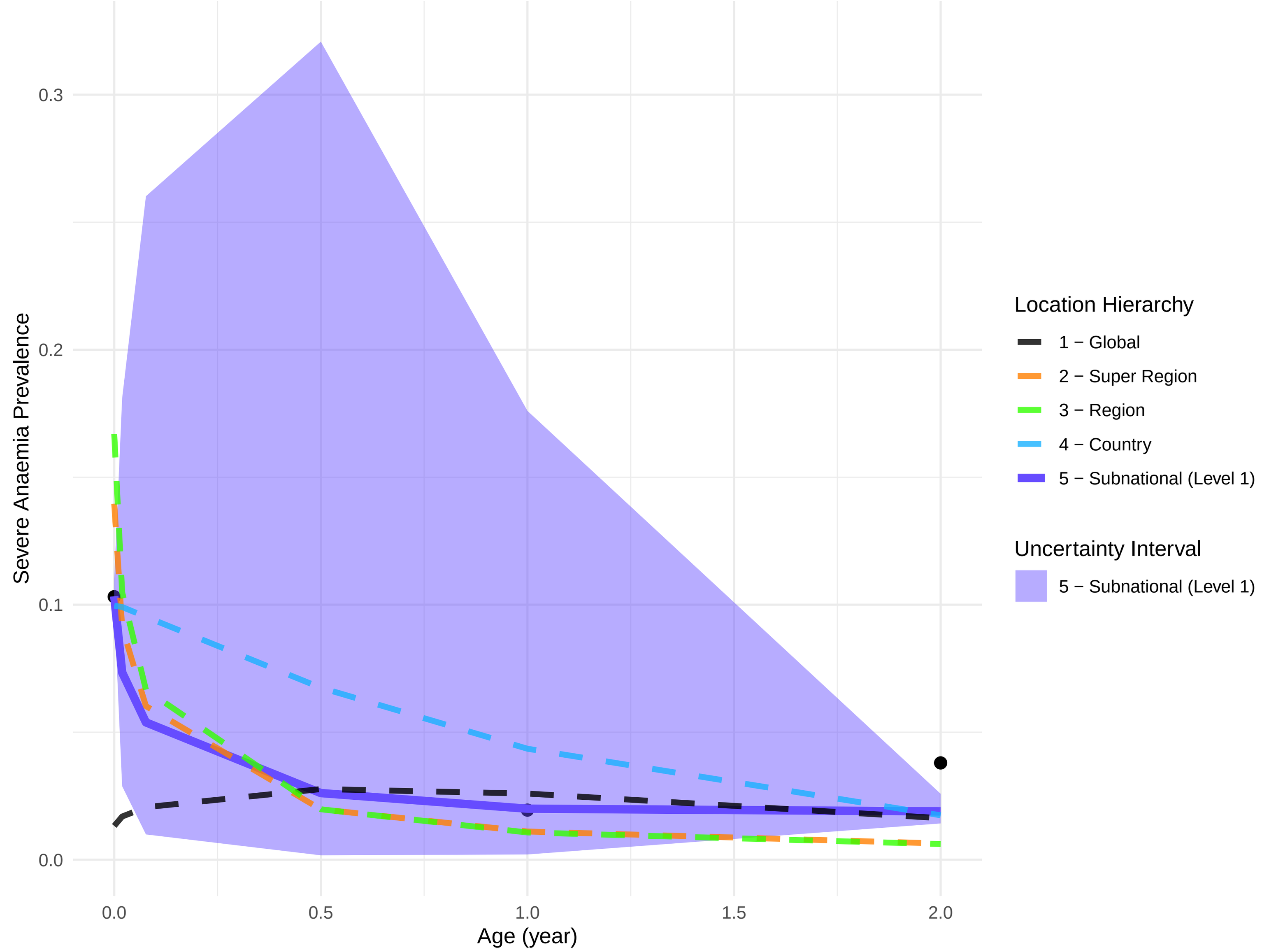

MR−BRT Age/Sex Fits − Non−pregnant Females <2 Years Old − Severe Anaemia Prevalence − North Sumatra
WHO−2001 Adjustment Method

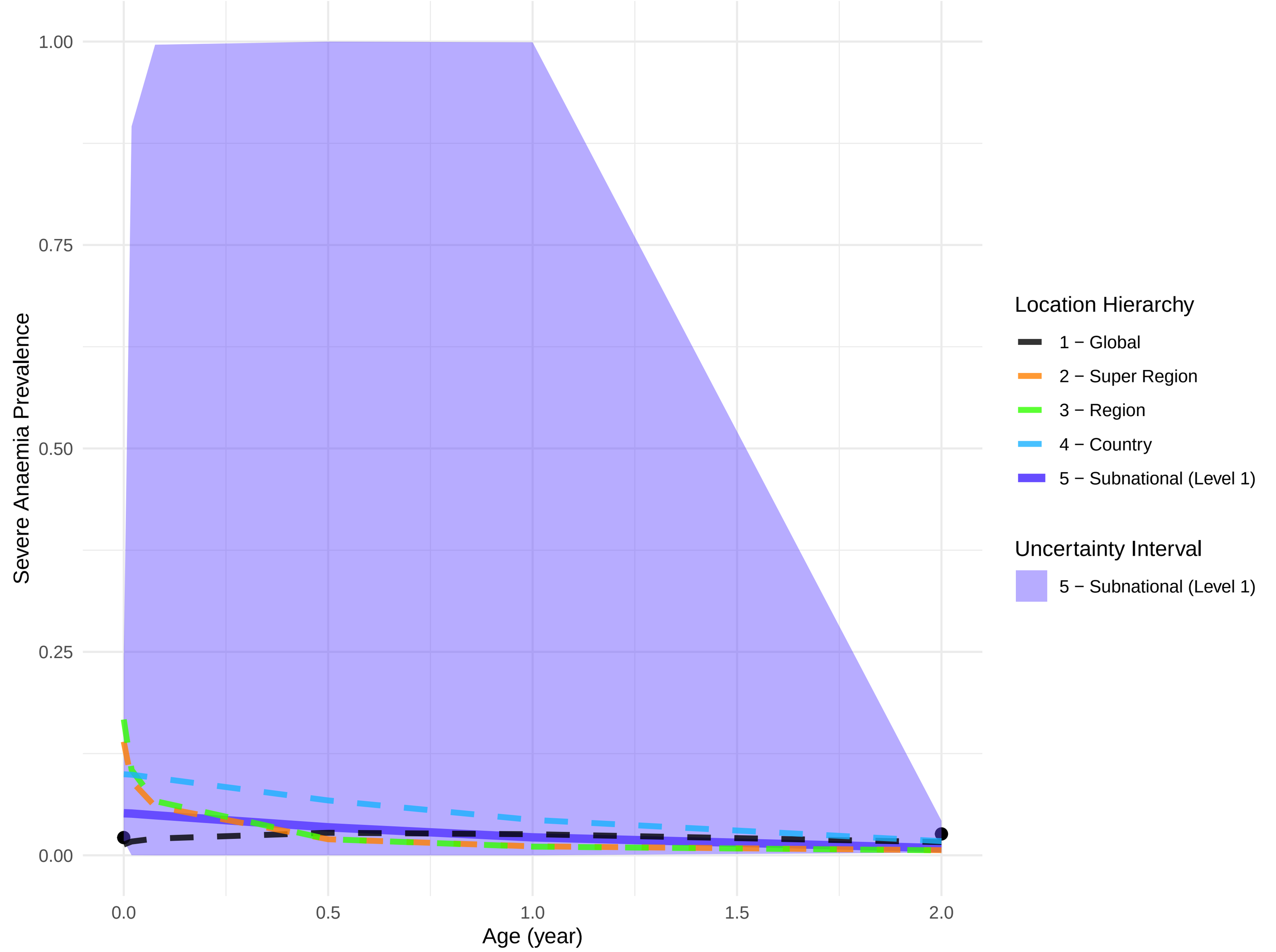

MR−BRT Age/Sex Fits − Non−pregnant Females <2 Years Old − Severe Anaemia Prevalence − Yogyakarta
WHO−2001 Adjustment Method

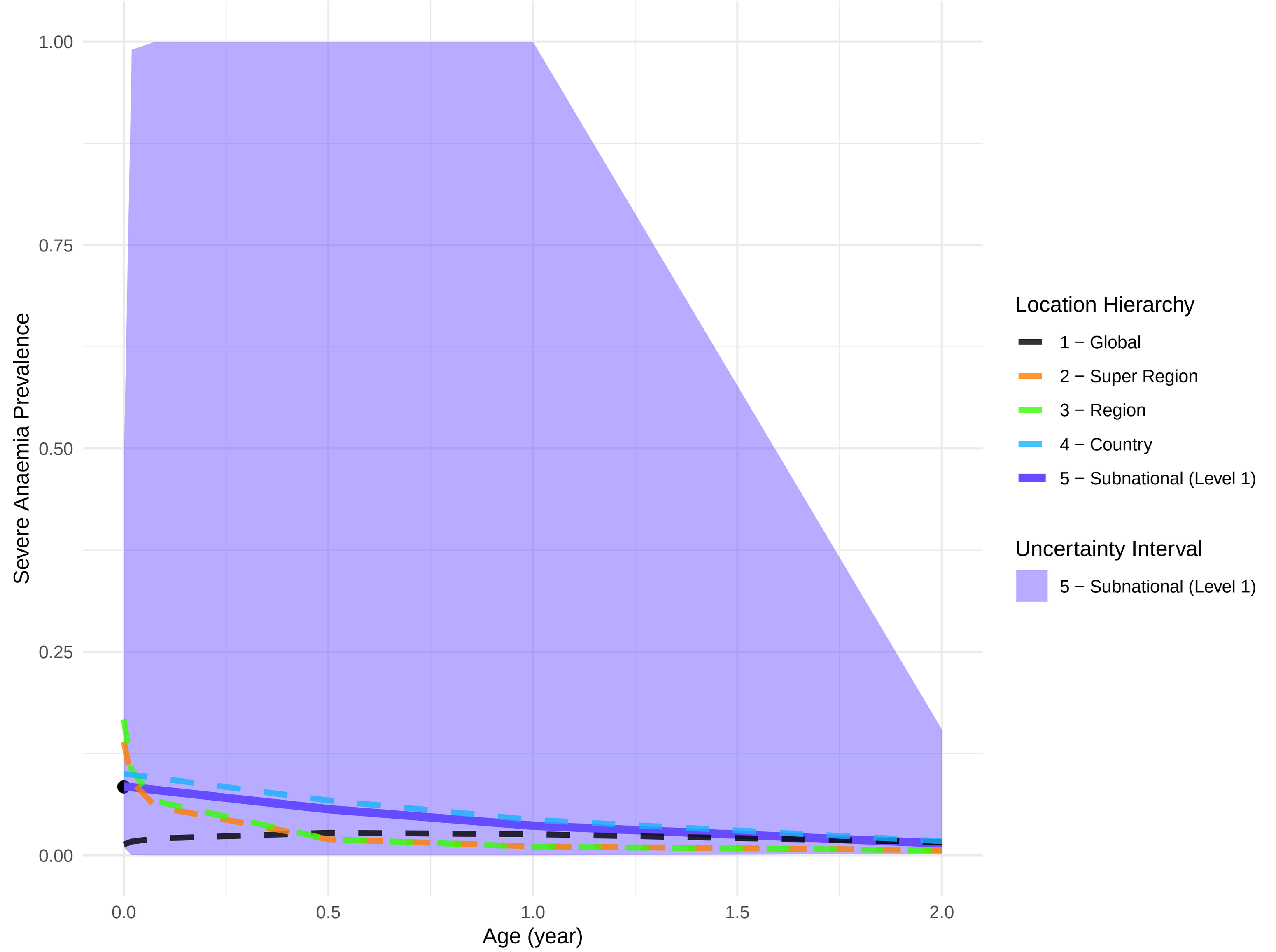

MR−BRT Age/Sex Fits − Non−pregnant Females <2 Years Old − Severe Anaemia Prevalence − Myanmar
WHO−2001 Adjustment Method

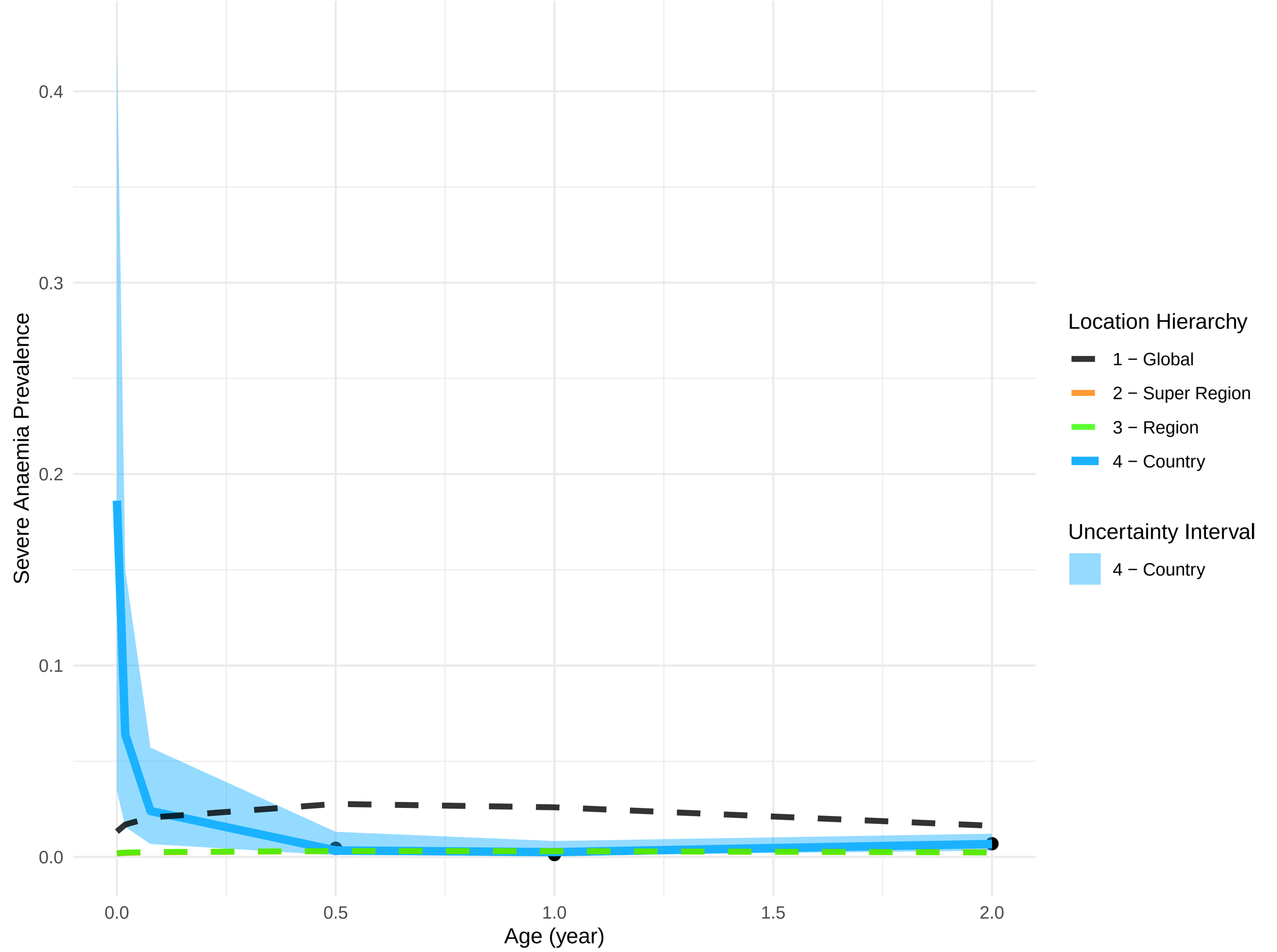

MR−BRT Age/Sex Fits − Non−pregnant Females <2 Years Old − Severe Anaemia Prevalence − Timor−Leste
WHO−2001 Adjustment Method

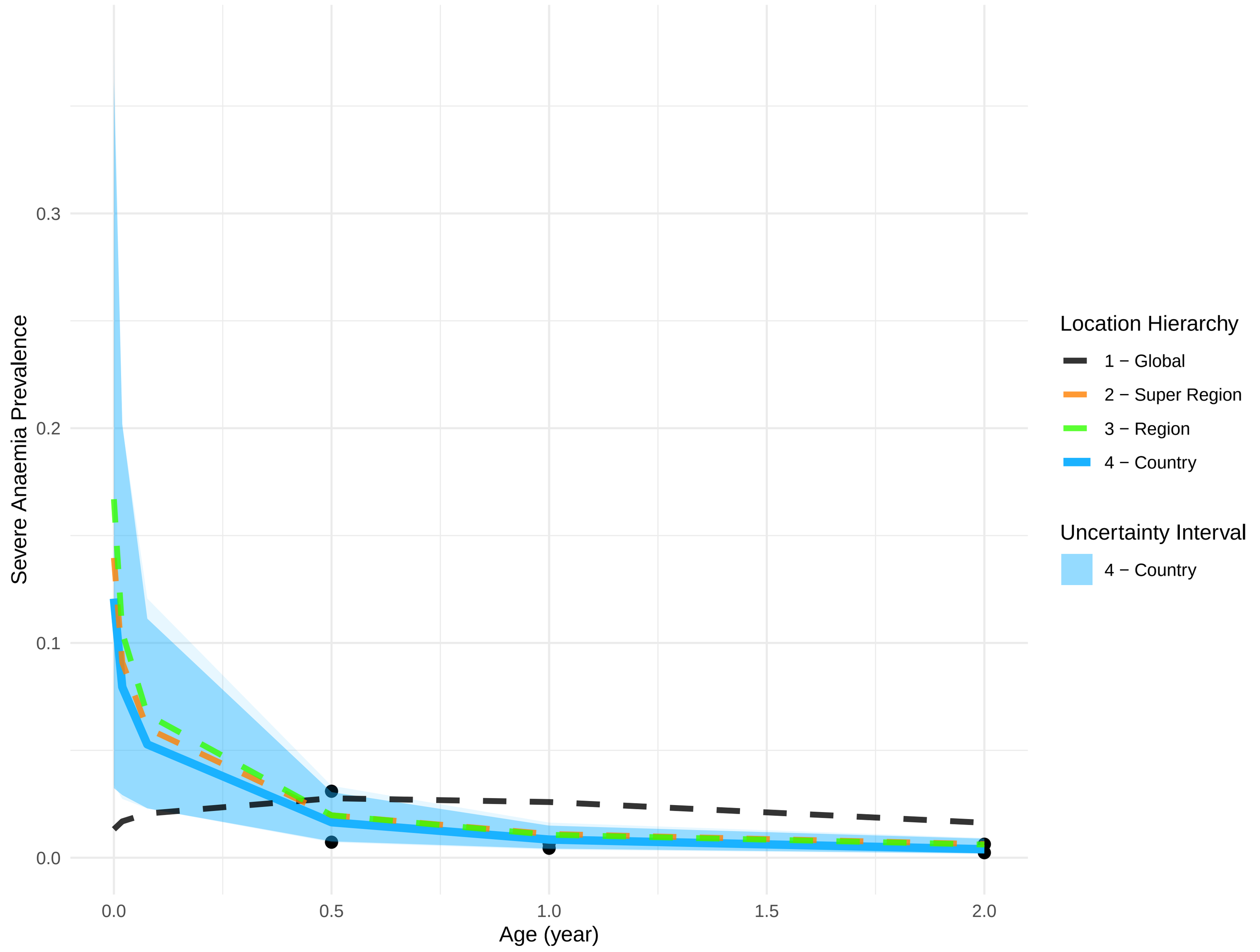

MR−BRT Age/Sex Fits − Non−pregnant Females <2 Years Old − Severe Anaemia Prevalence − Sub−Saharan Africa
WHO−2001 Adjustment Method

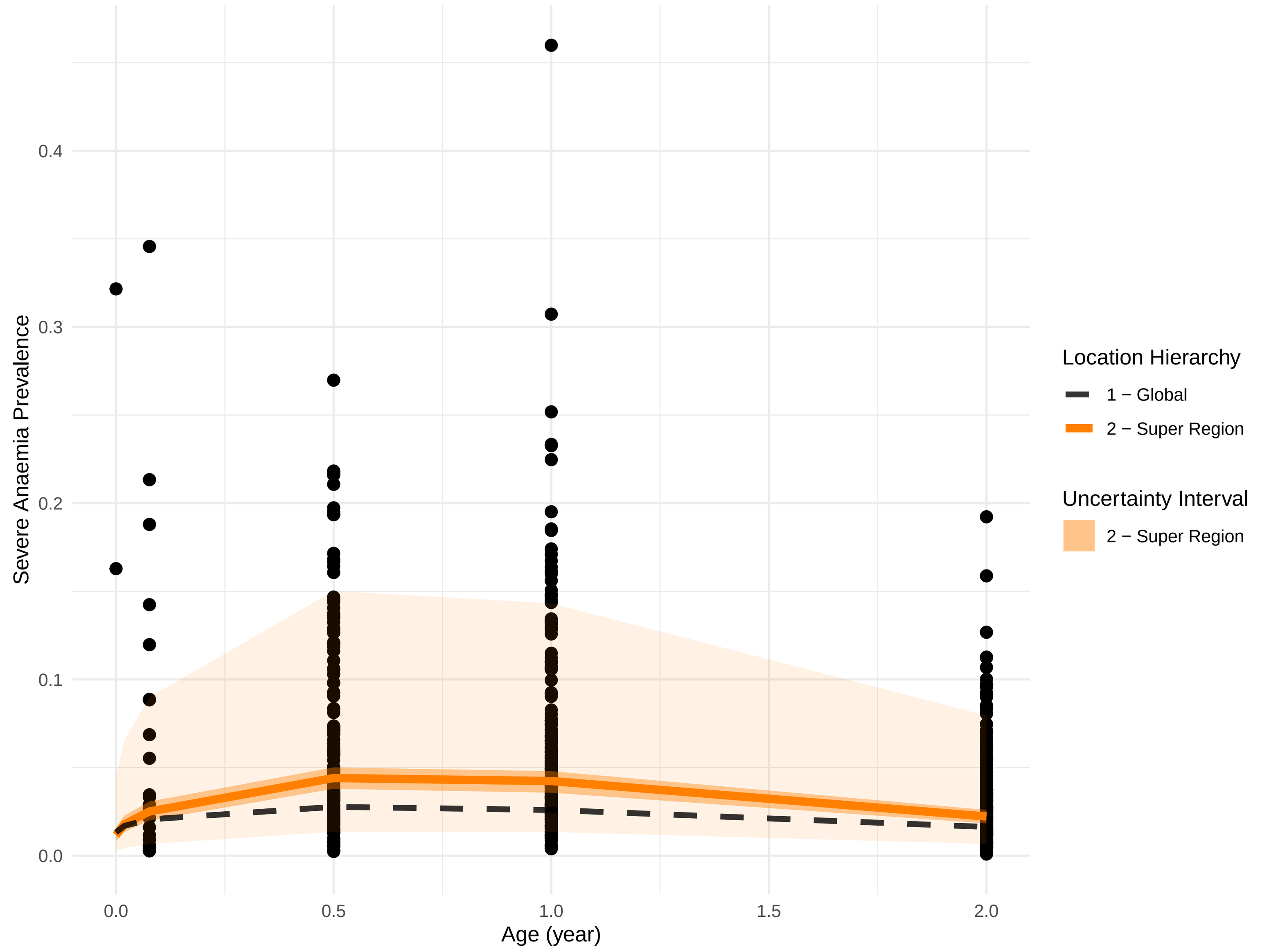

MR−BRT Age/Sex Fits − Non−pregnant Females <2 Years Old − Severe Anaemia Prevalence − Central Sub−Saharan Africa
WHO−2001 Adjustment Method

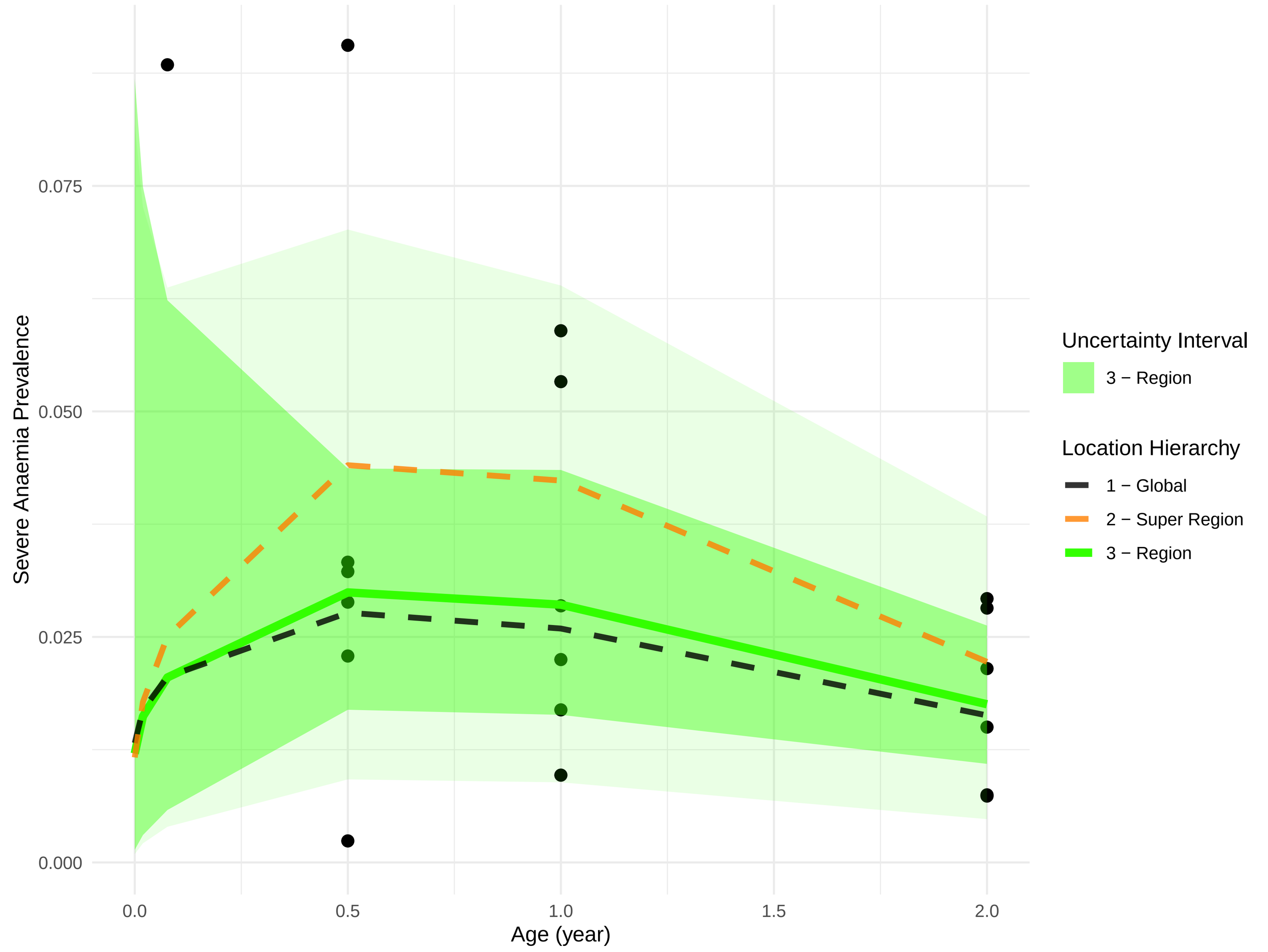

MR−BRT Age/Sex Fits − Non−pregnant Females <2 Years Old − Severe Anaemia Prevalence − Angola
WHO−2001 Adjustment Method

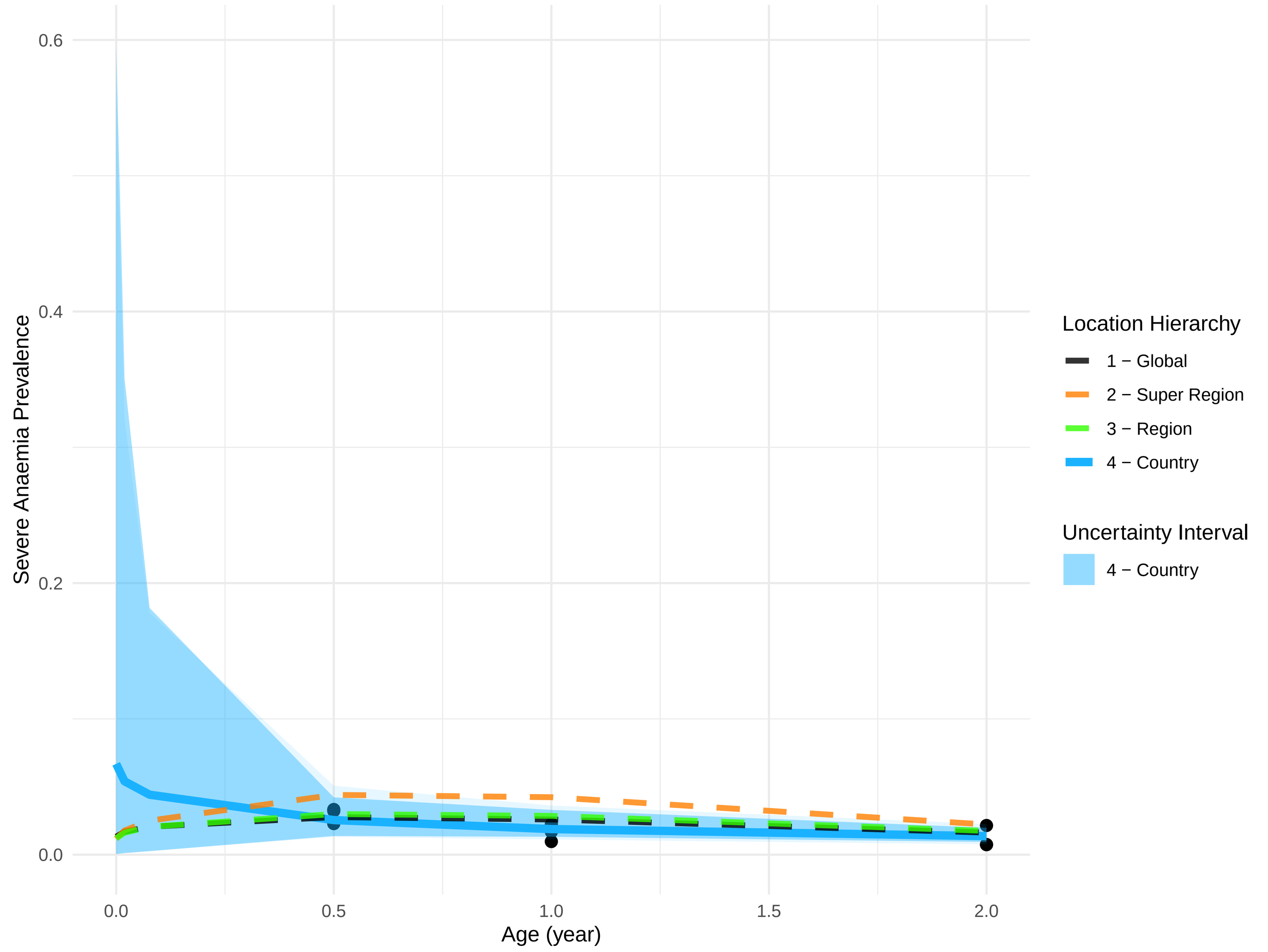

MR−BRT Age/Sex Fits − Non−pregnant Females <2 Years Old − Severe Anaemia Prevalence − Democratic Republic of the Congo
WHO−2001 Adjustment Method

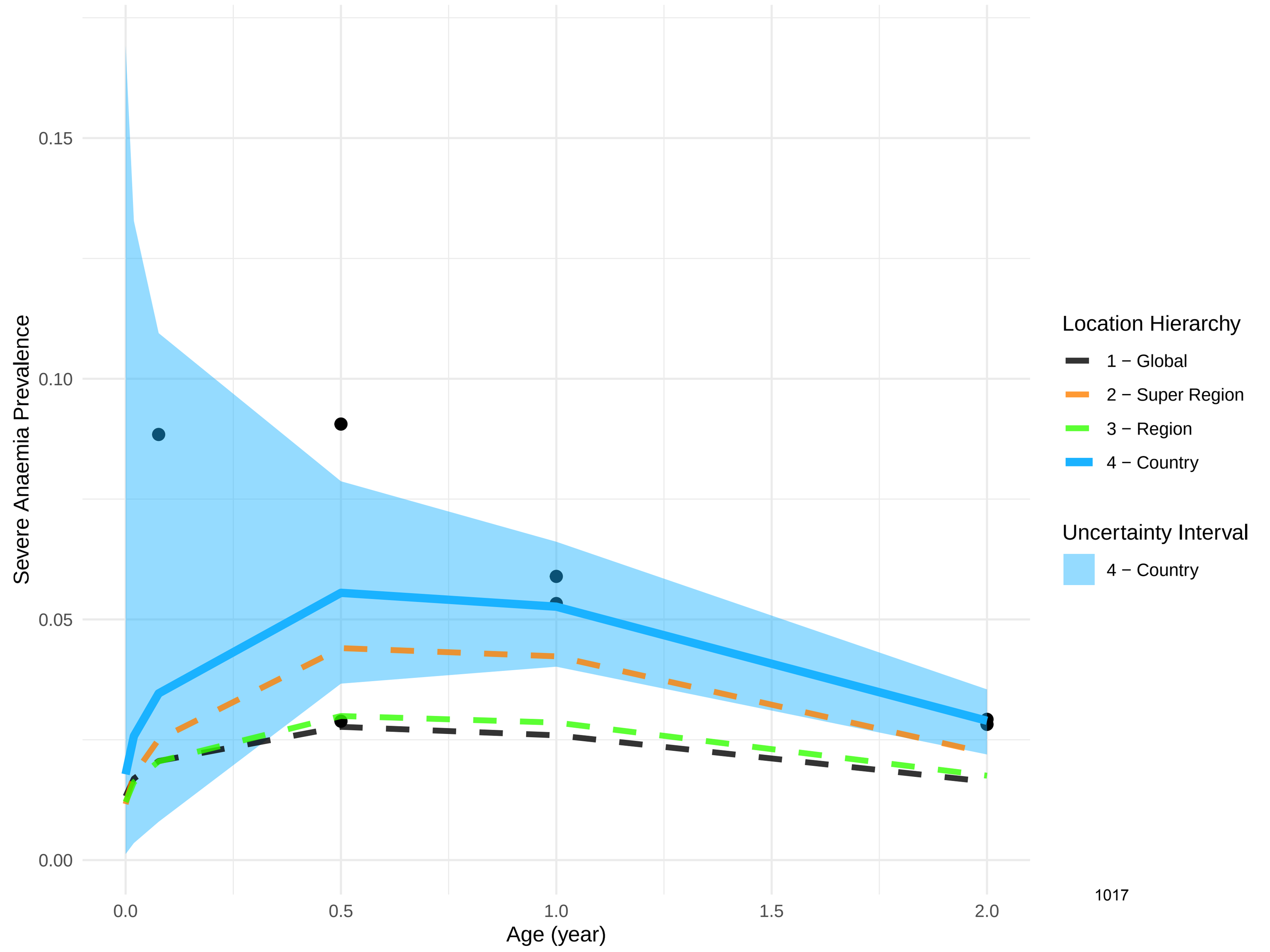

MR−BRT Age/Sex Fits − Non−pregnant Females <2 Years Old − Severe Anaemia Prevalence − Gabon
WHO−2001 Adjustment Method

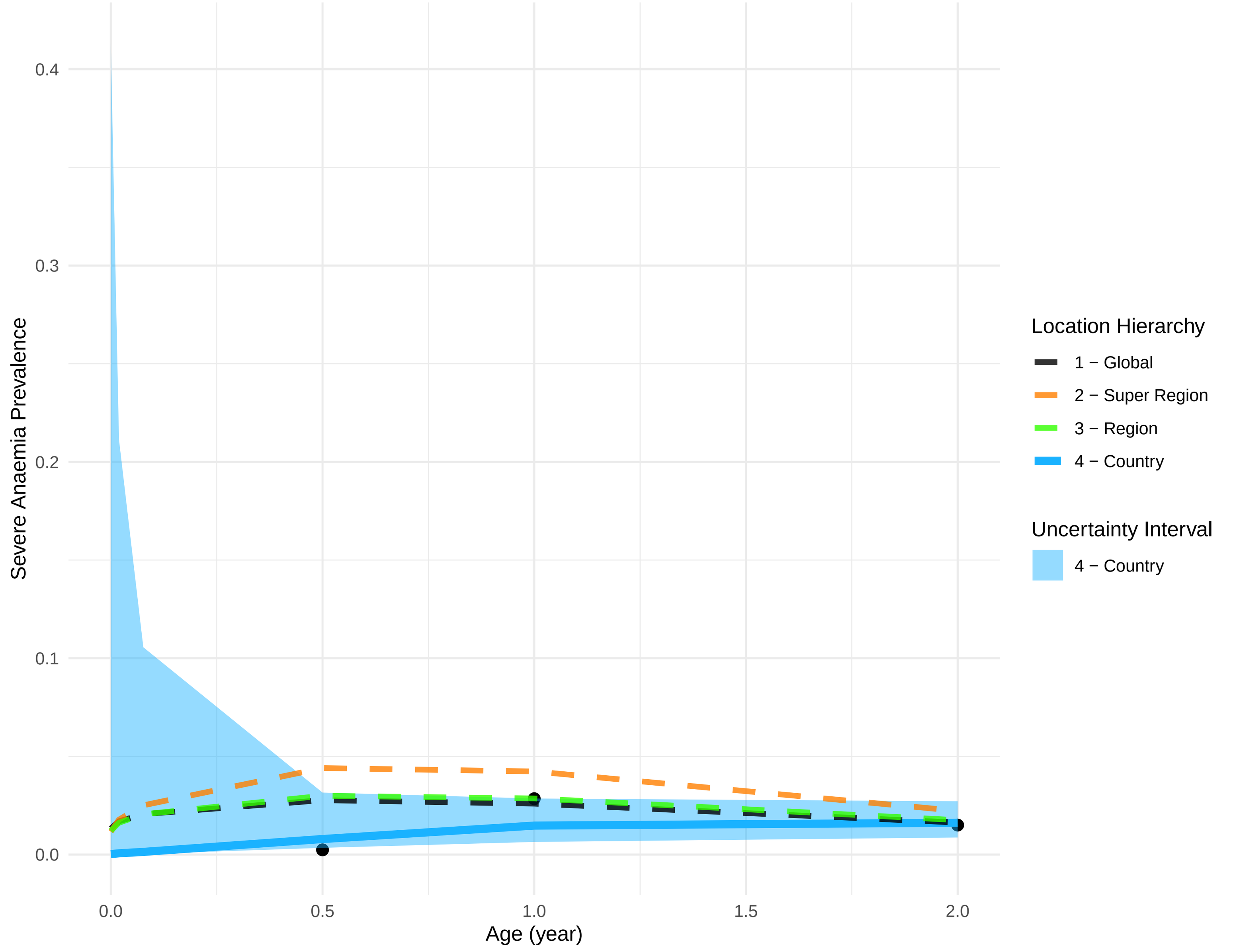

MR−BRT Age/Sex Fits − Non−pregnant Females <2 Years Old − Severe Anaemia Prevalence − Eastern Sub−Saharan Africa
WHO−2001 Adjustment Method

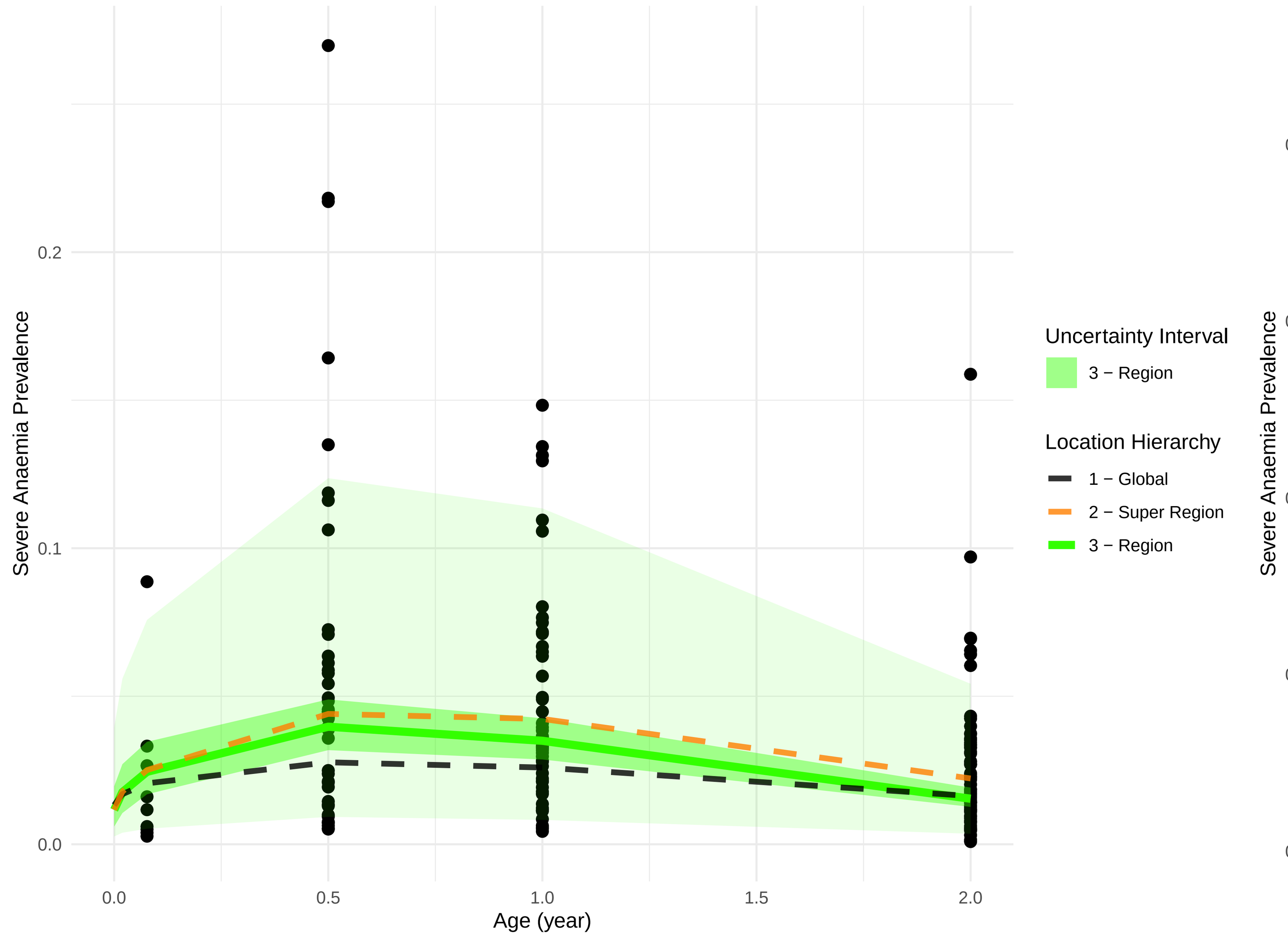

MR−BRT Age/Sex Fits − Non−pregnant Females <2 Years Old − Severe Anaemia Prevalence − Burundi
WHO−2001 Adjustment Method

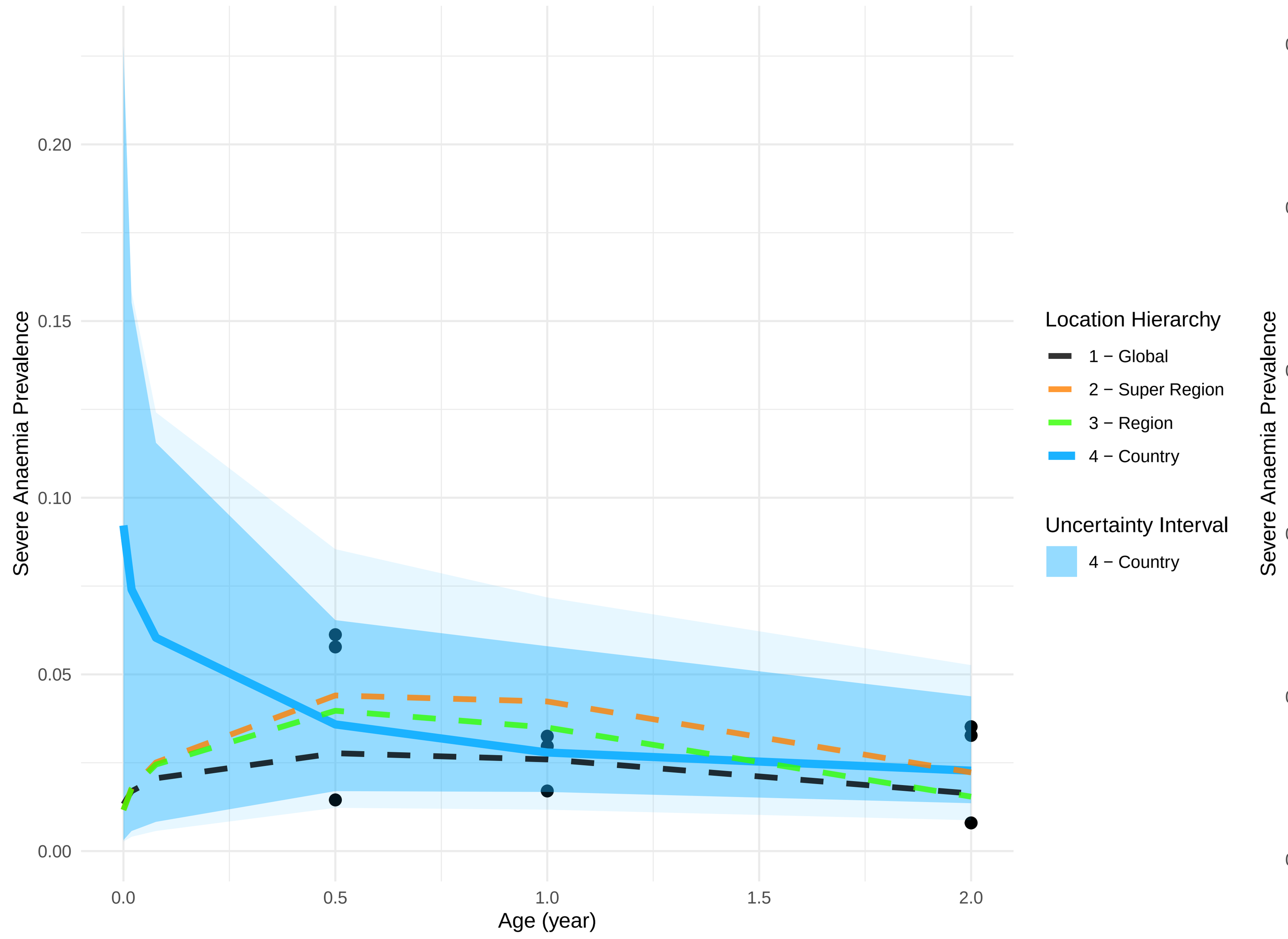

MR−BRT Age/Sex Fits − Non−pregnant Females <2 Years Old − Severe Anaemia Prevalence − Ethiopia
WHO−2001 Adjustment Method

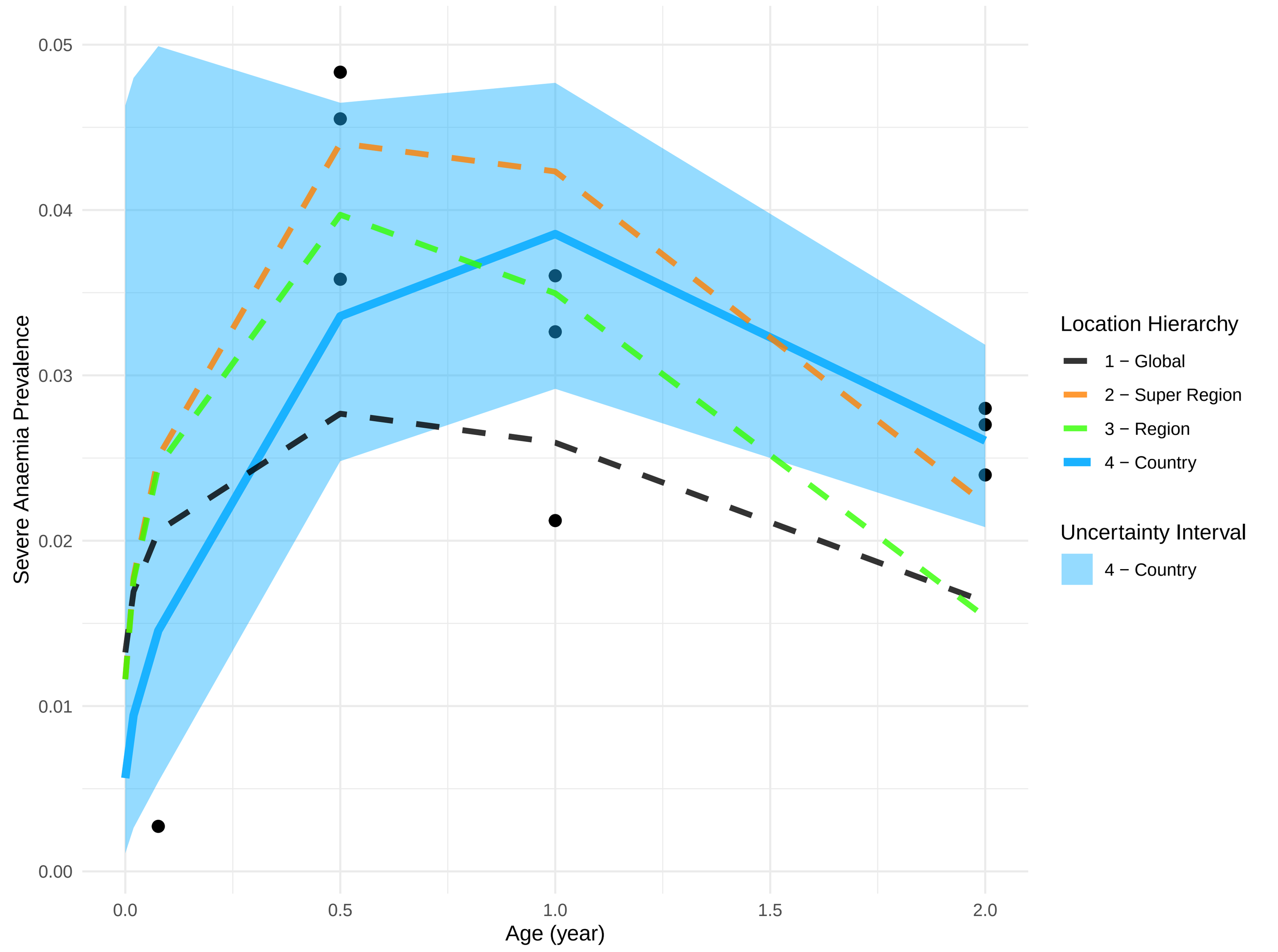

MR−BRT Age/Sex Fits − Non−pregnant Females <2 Years Old − Severe Anaemia Prevalence − Kenya
WHO−2001 Adjustment Method

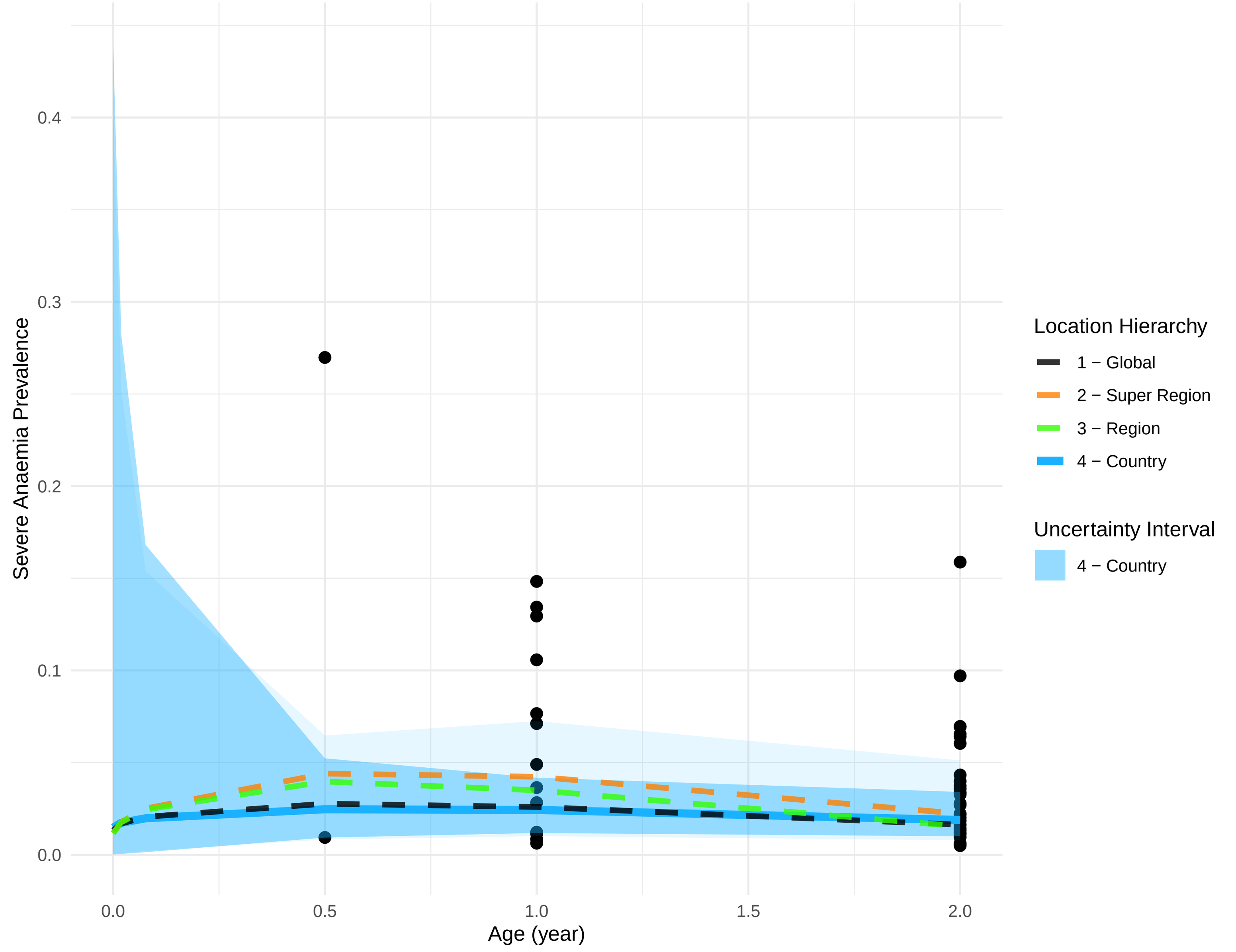

MR−BRT Age/Sex Fits − Non−pregnant Females <2 Years Old − Severe Anaemia Prevalence − Bungoma
WHO−2001 Adjustment Method

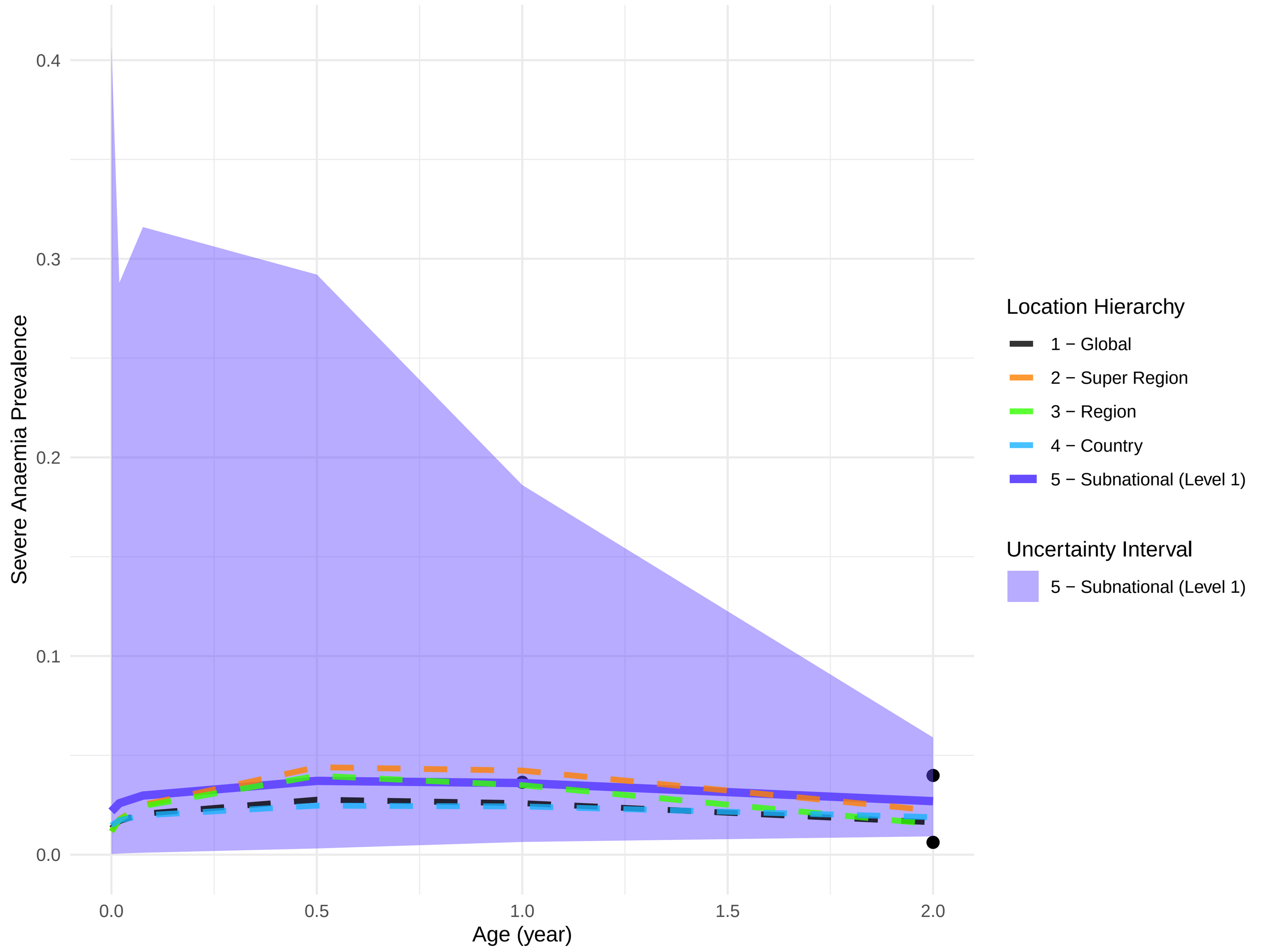

MR−BRT Age/Sex Fits − Non−pregnant Females <2 Years Old − Severe Anaemia Prevalence − Busia
WHO−2001 Adjustment Method

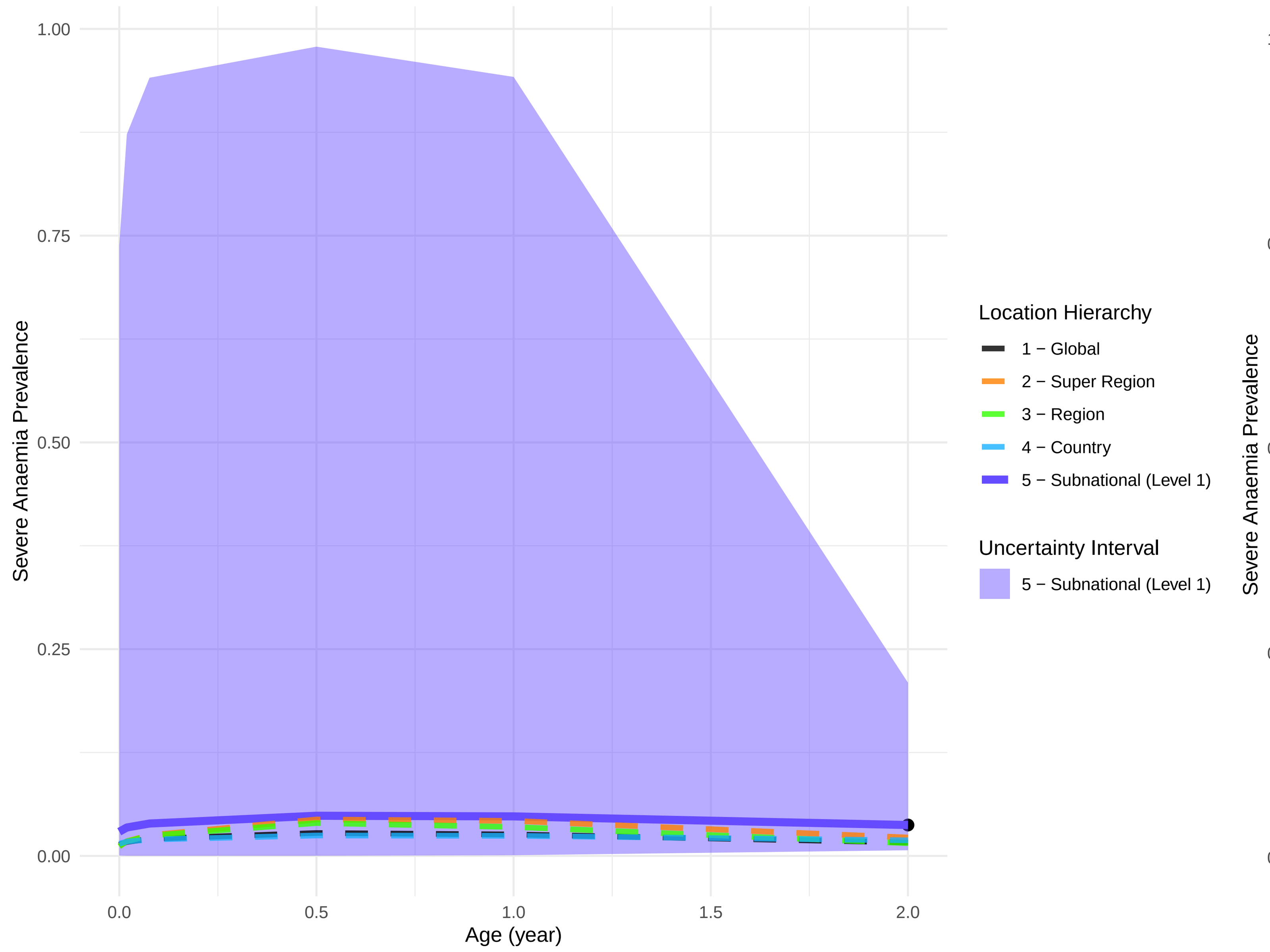

MR−BRT Age/Sex Fits − Non−pregnant Females <2 Years Old − Severe Anaemia Prevalence − Elgeyo−Marakwet
WHO−2001 Adjustment Method

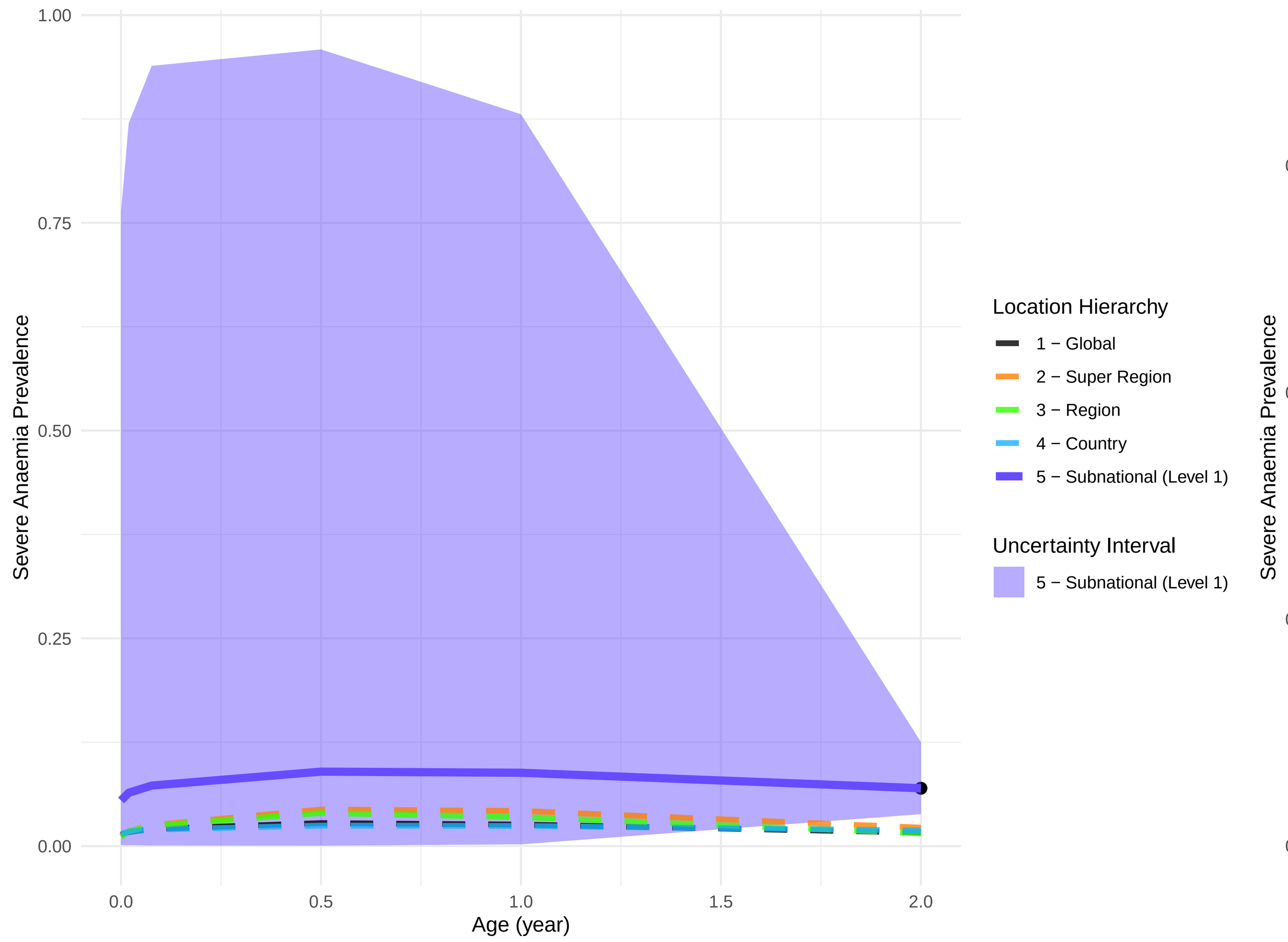

MR−BRT Age/Sex Fits − Non−pregnant Females <2 Years Old − Severe Anaemia Prevalence − Garissa
WHO−2001 Adjustment Method

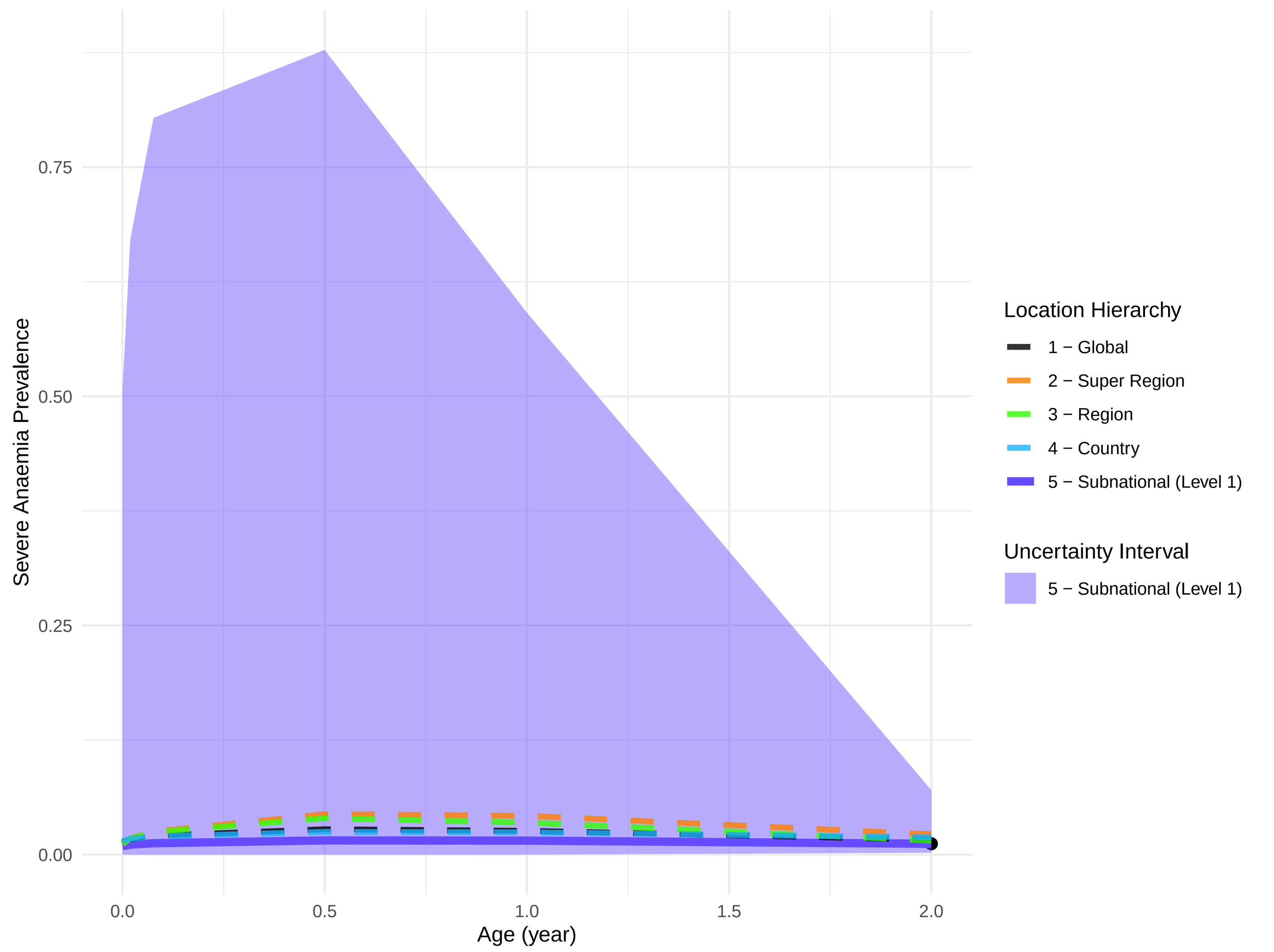

MR−BRT Age/Sex Fits − Non−pregnant Females <2 Years Old − Severe Anaemia Prevalence − Homa Bay
WHO−2001 Adjustment Method

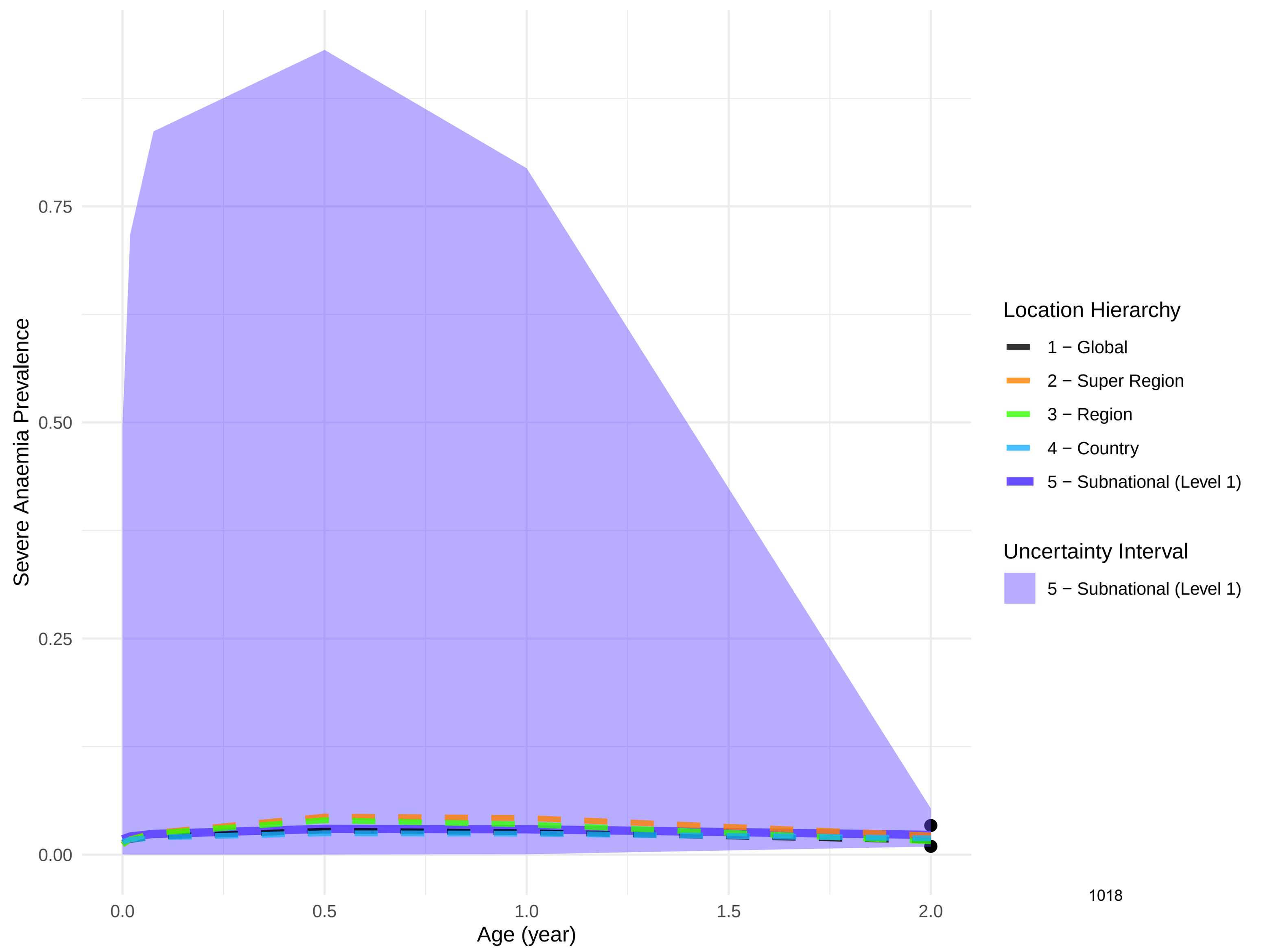

MR−BRT Age/Sex Fits − Non−pregnant Females <2 Years Old − Severe Anaemia Prevalence − Kajiado
WHO−2001 Adjustment Method

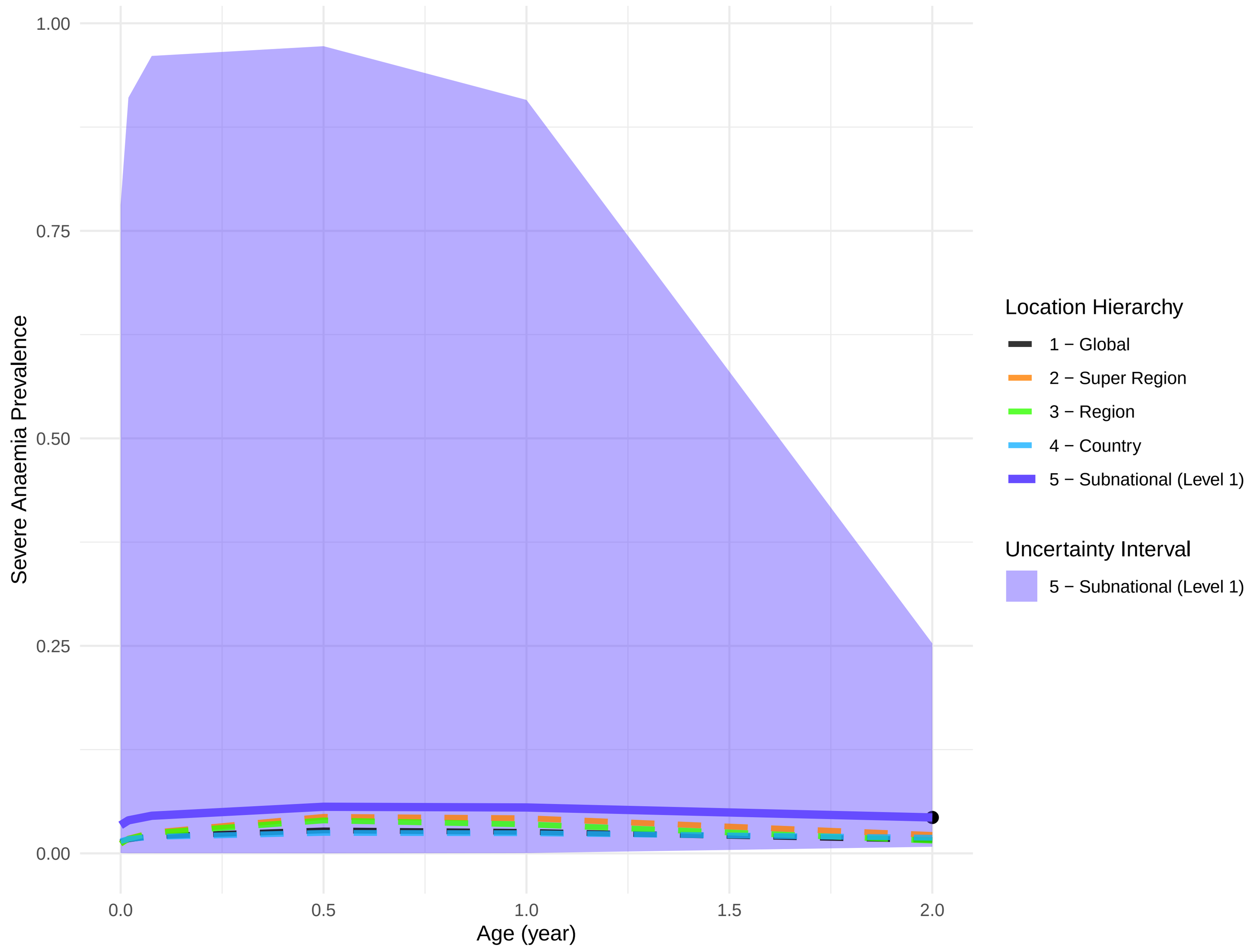

MR−BRT Age/Sex Fits − Non−pregnant Females <2 Years Old − Severe Anaemia Prevalence − Kakamega
WHO−2001 Adjustment Method

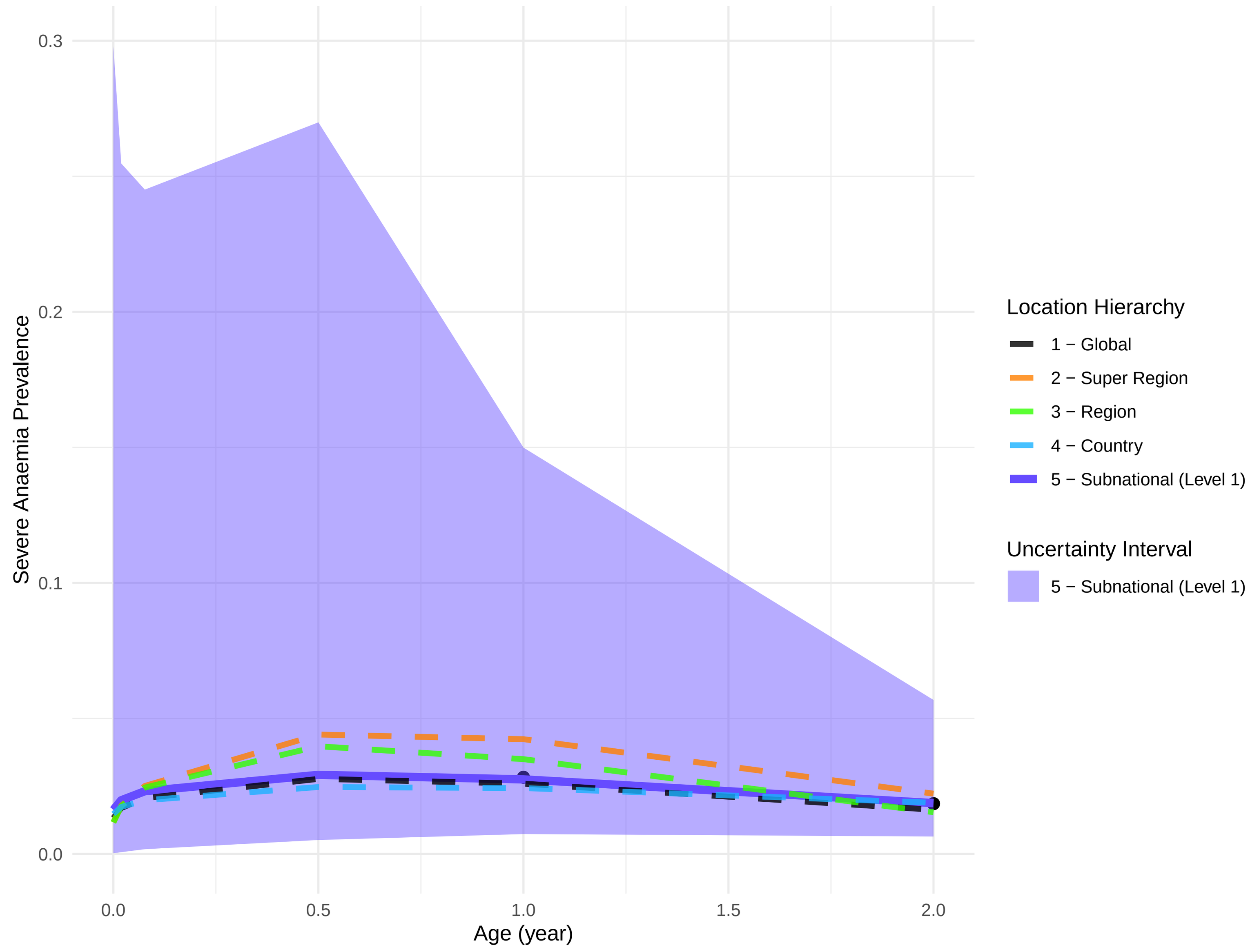

MR−BRT Age/Sex Fits − Non−pregnant Females <2 Years Old − Severe Anaemia Prevalence − Kiambu
WHO−2001 Adjustment Method

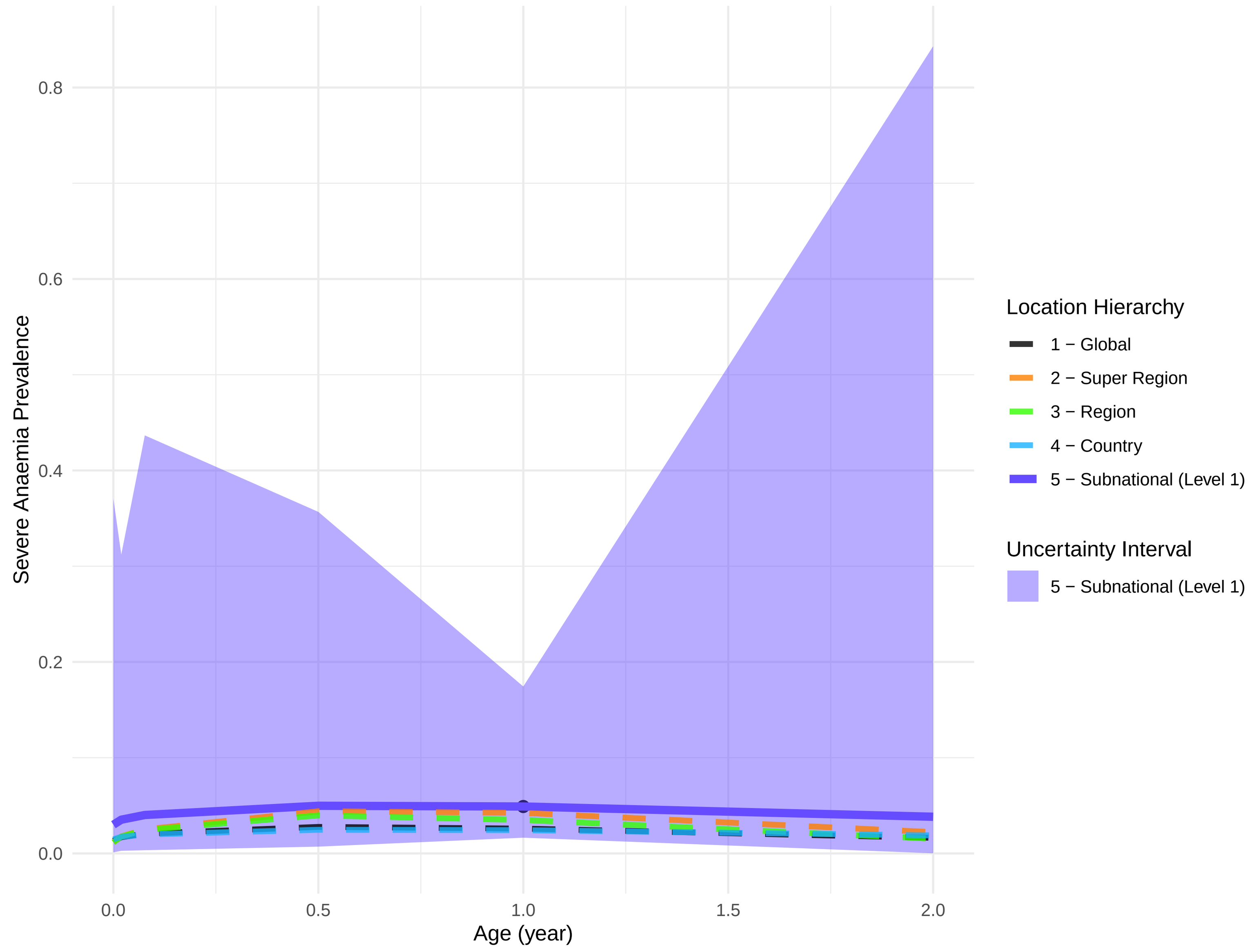

MR−BRT Age/Sex Fits − Non−pregnant Females <2 Years Old − Severe Anaemia Prevalence − Kilifi
WHO−2001 Adjustment Method

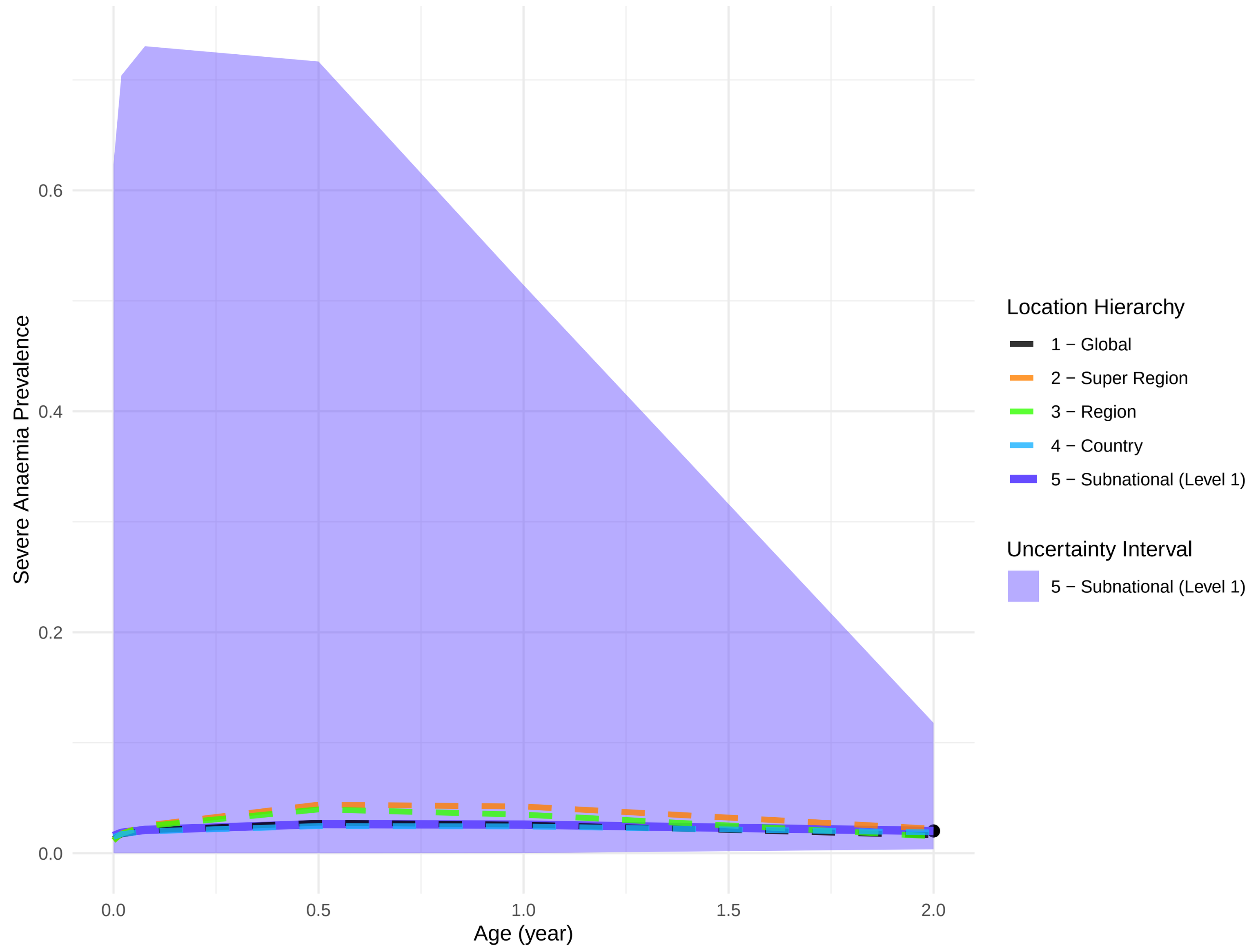

MR−BRT Age/Sex Fits − Non−pregnant Females <2 Years Old − Severe Anaemia Prevalence − Kisumu
WHO−2001 Adjustment Method

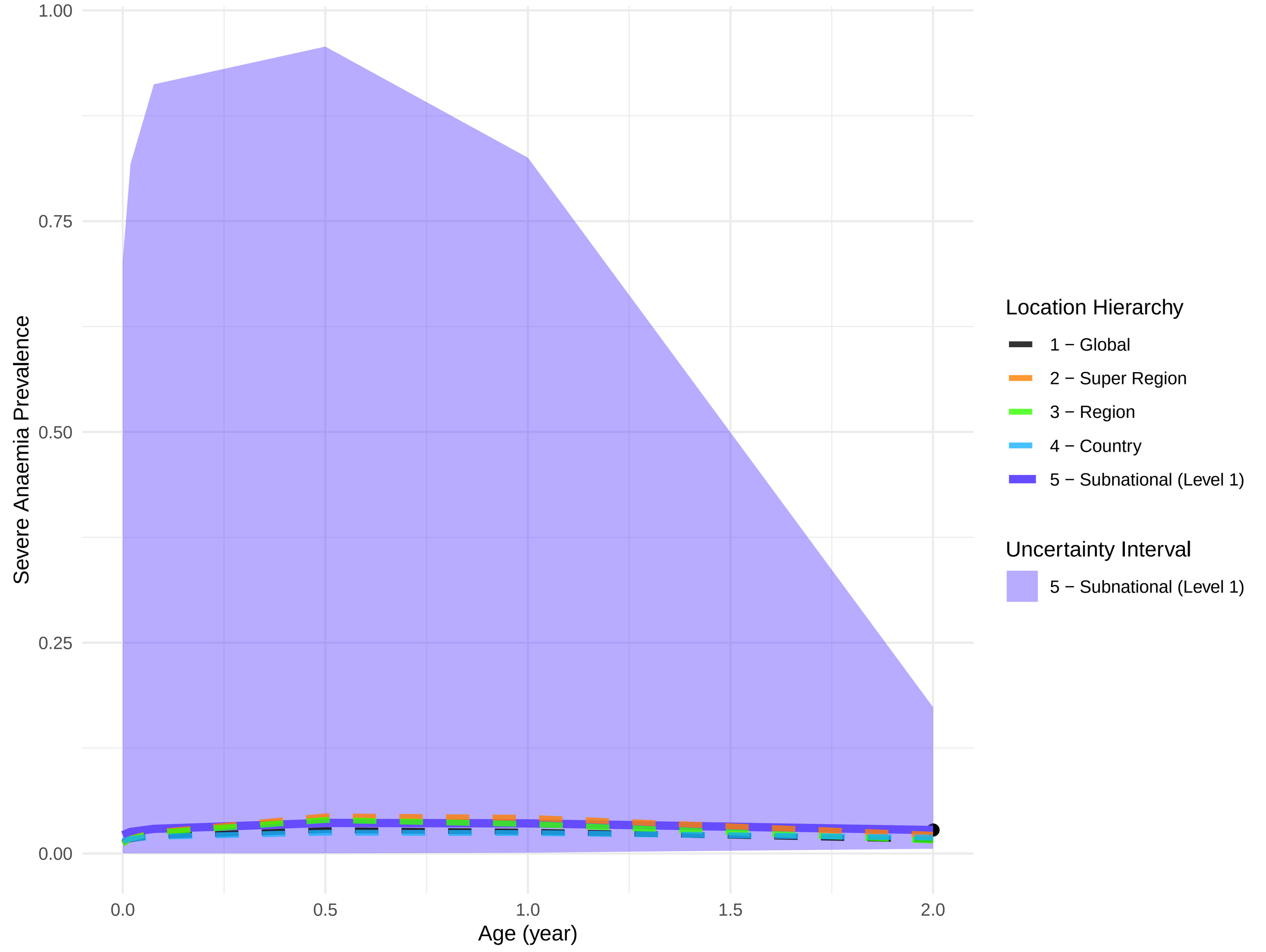

MR−BRT Age/Sex Fits − Non−pregnant Females <2 Years Old − Severe Anaemia Prevalence − Kwale
WHO−2001 Adjustment Method

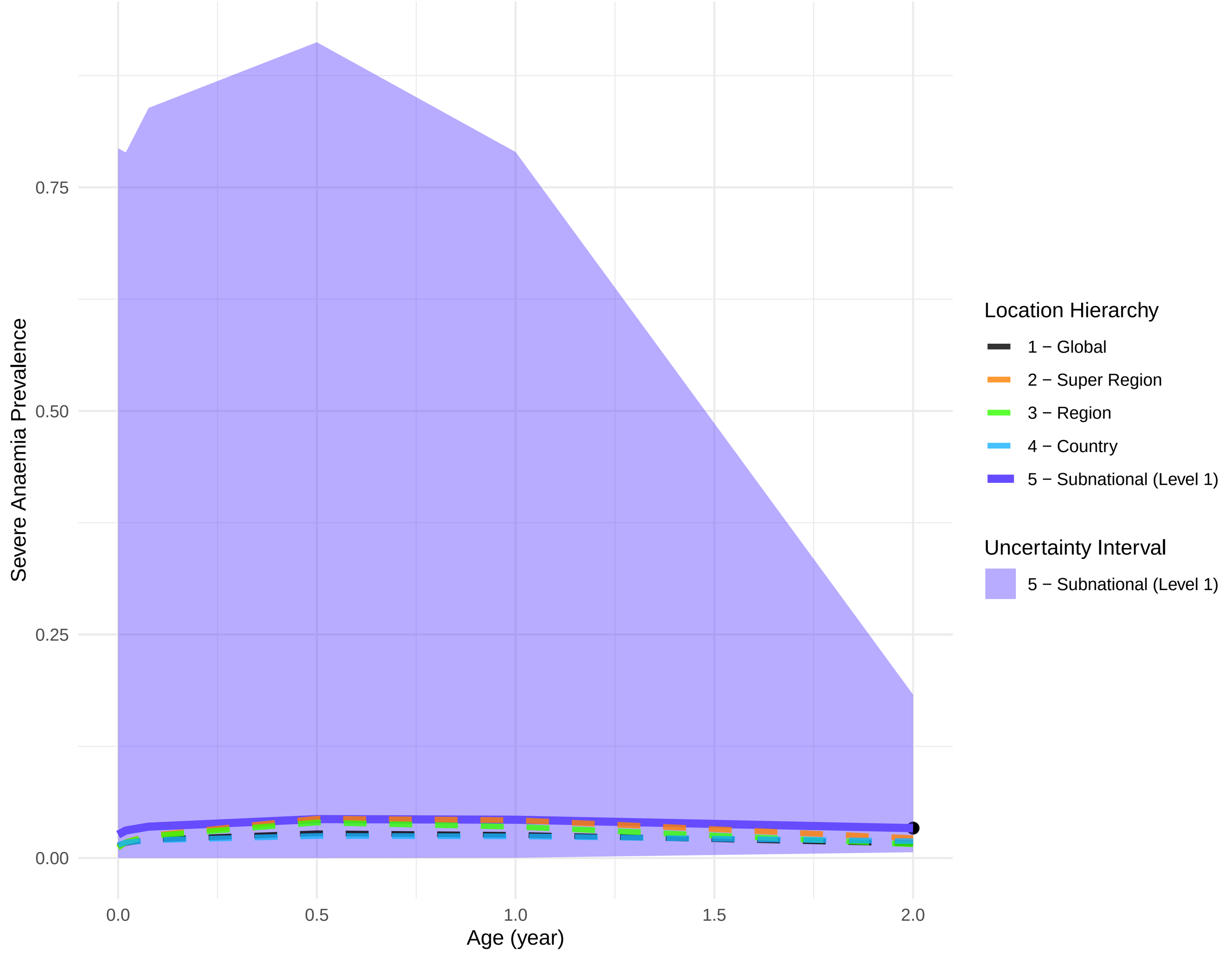

MR−BRT Age/Sex Fits − Non−pregnant Females <2 Years Old − Severe Anaemia Prevalence − Lamu
WHO−2001 Adjustment Method

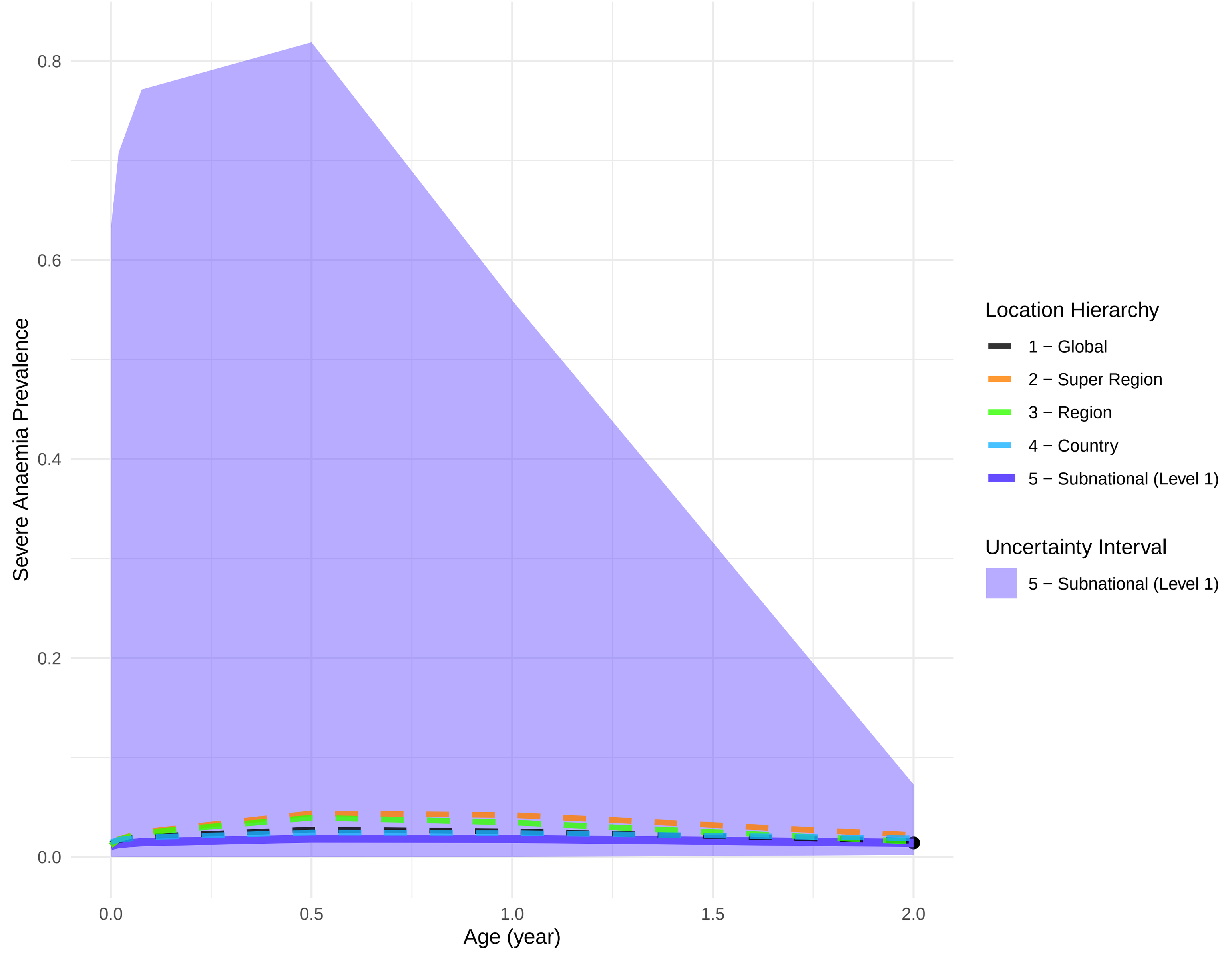

MR−BRT Age/Sex Fits − Non−pregnant Females <2 Years Old − Severe Anaemia Prevalence − Machakos
WHO−2001 Adjustment Method

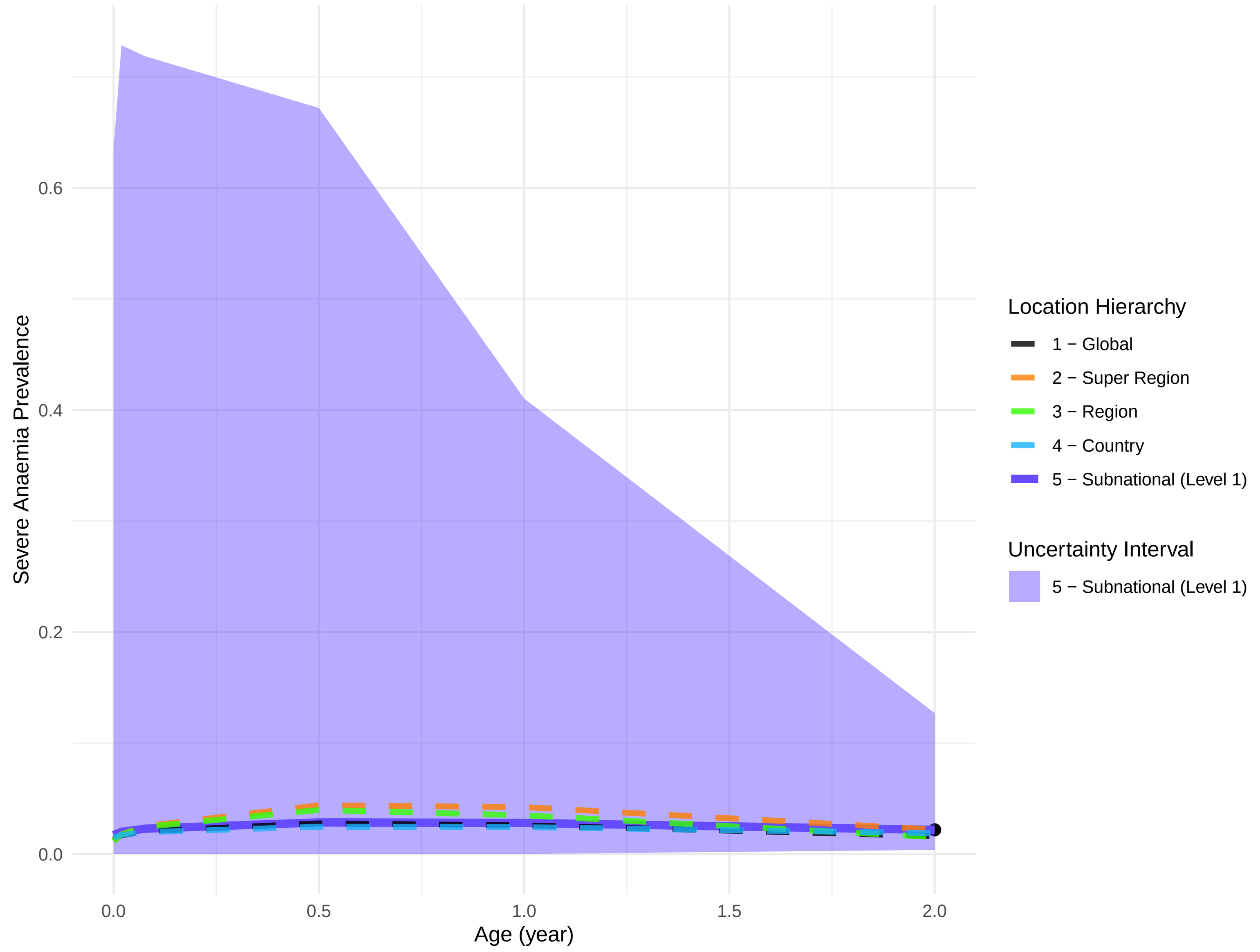

MR−BRT Age/Sex Fits − Non−pregnant Females <2 Years Old − Severe Anaemia Prevalence − Mandera
WHO−2001 Adjustment Method

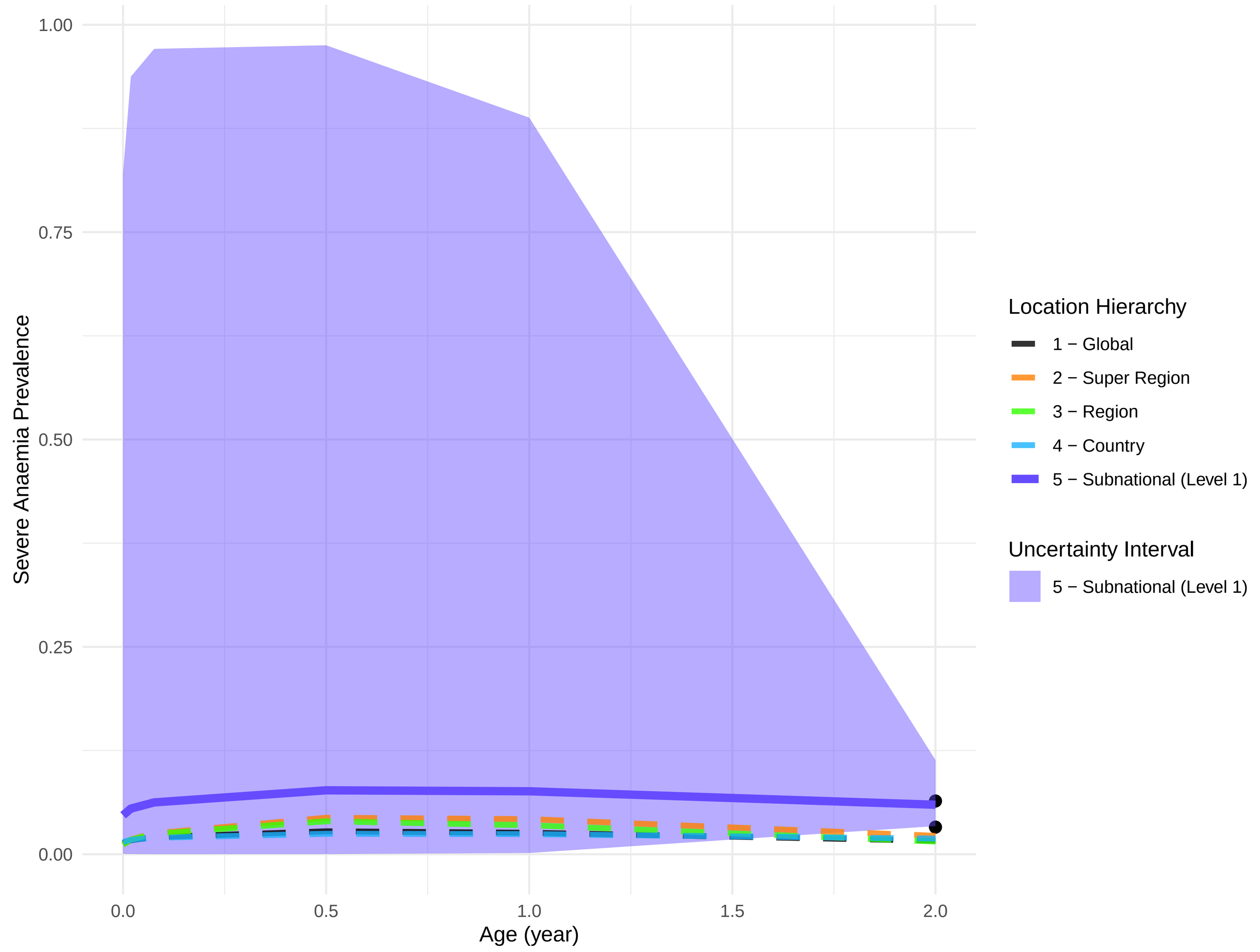

MR−BRT Age/Sex Fits − Non−pregnant Females <2 Years Old − Severe Anaemia Prevalence − Marsabit
WHO−2001 Adjustment Method

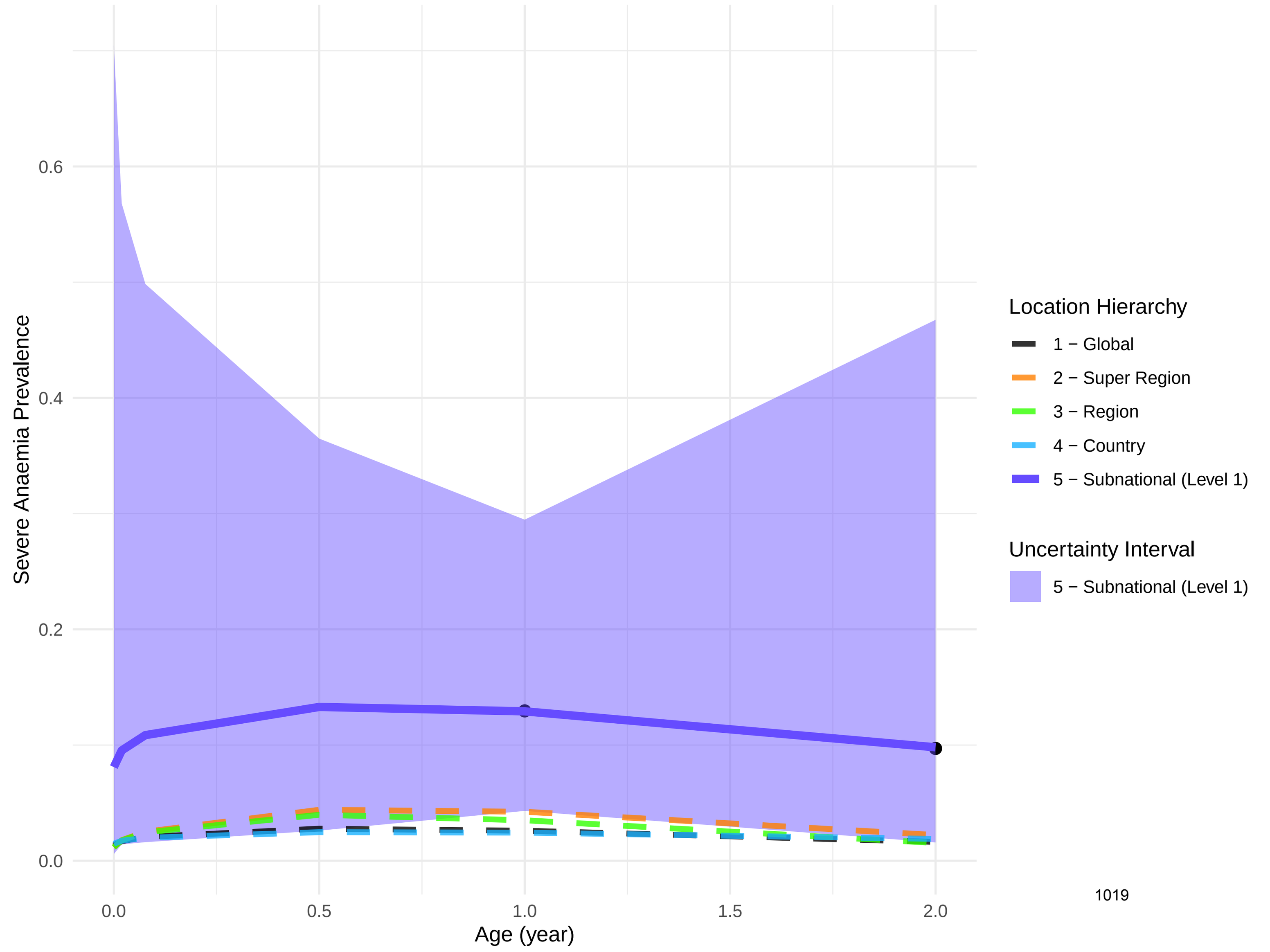

MR−BRT Age/Sex Fits − Non−pregnant Females <2 Years Old − Severe Anaemia Prevalence − Migori
WHO−2001 Adjustment Method

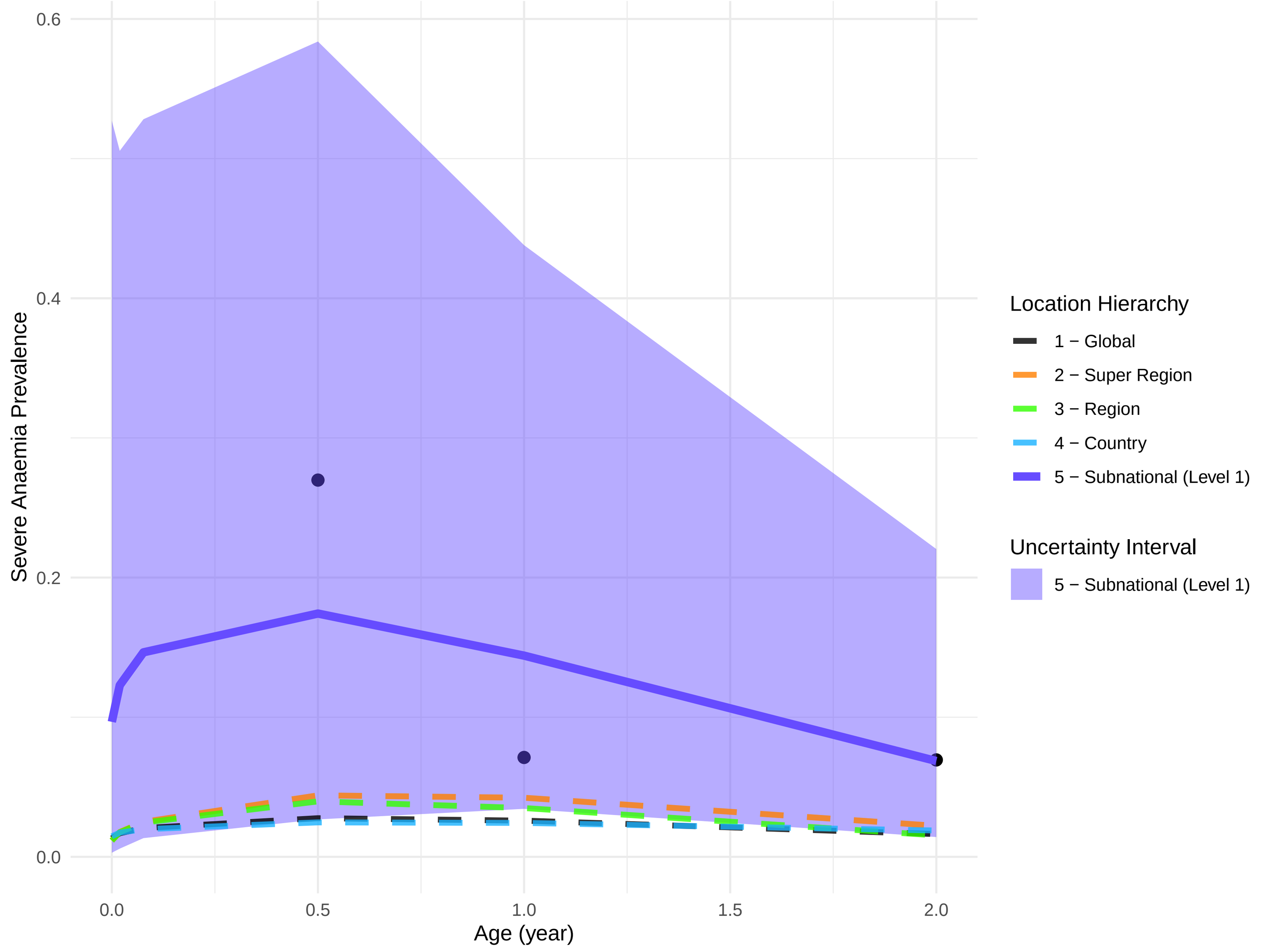

MR−BRT Age/Sex Fits − Non−pregnant Females <2 Years Old − Severe Anaemia Prevalence − Mombasa
WHO−2001 Adjustment Method

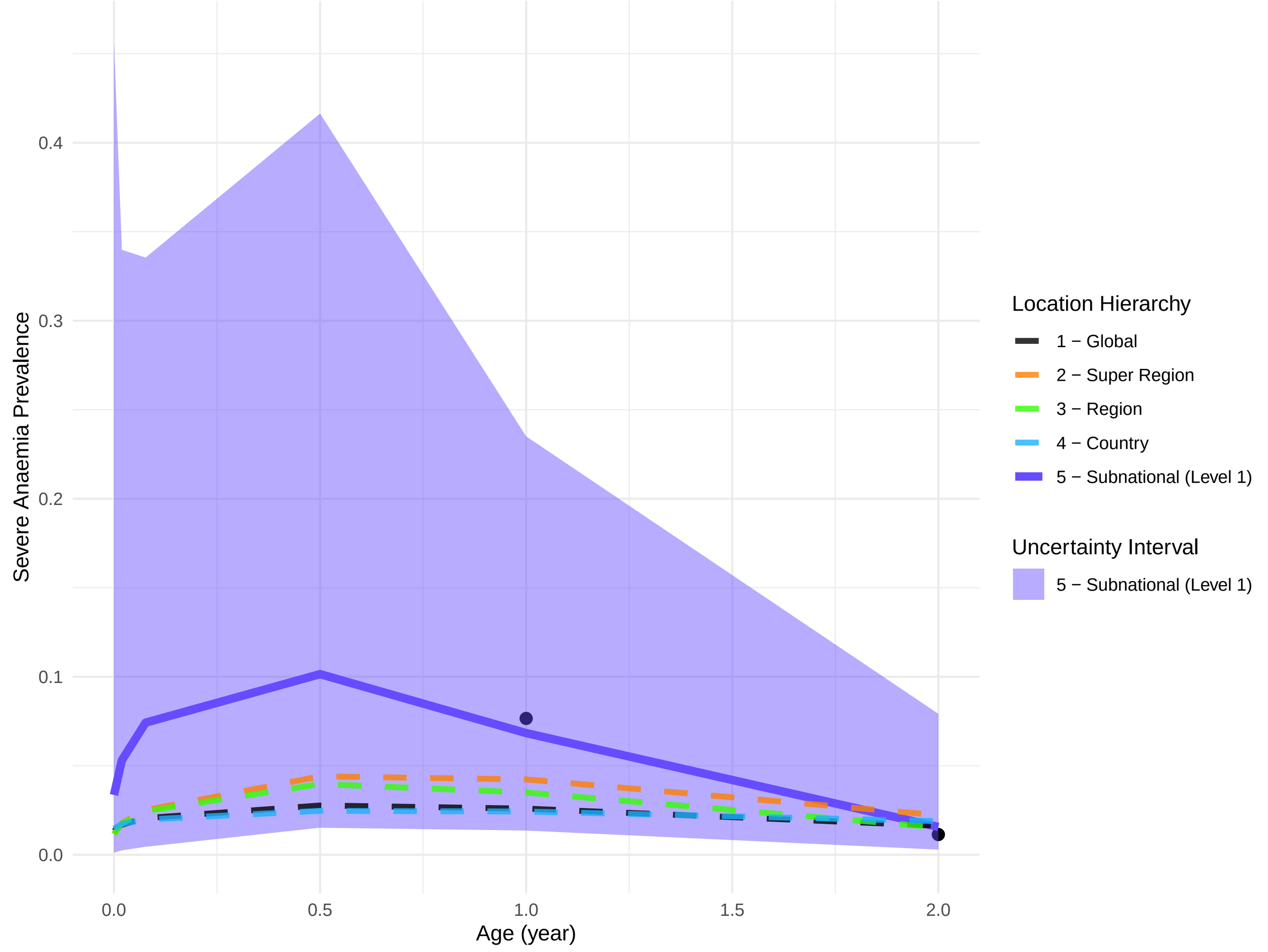

MR−BRT Age/Sex Fits − Non−pregnant Females <2 Years Old − Severe Anaemia Prevalence − Narok
WHO−2001 Adjustment Method

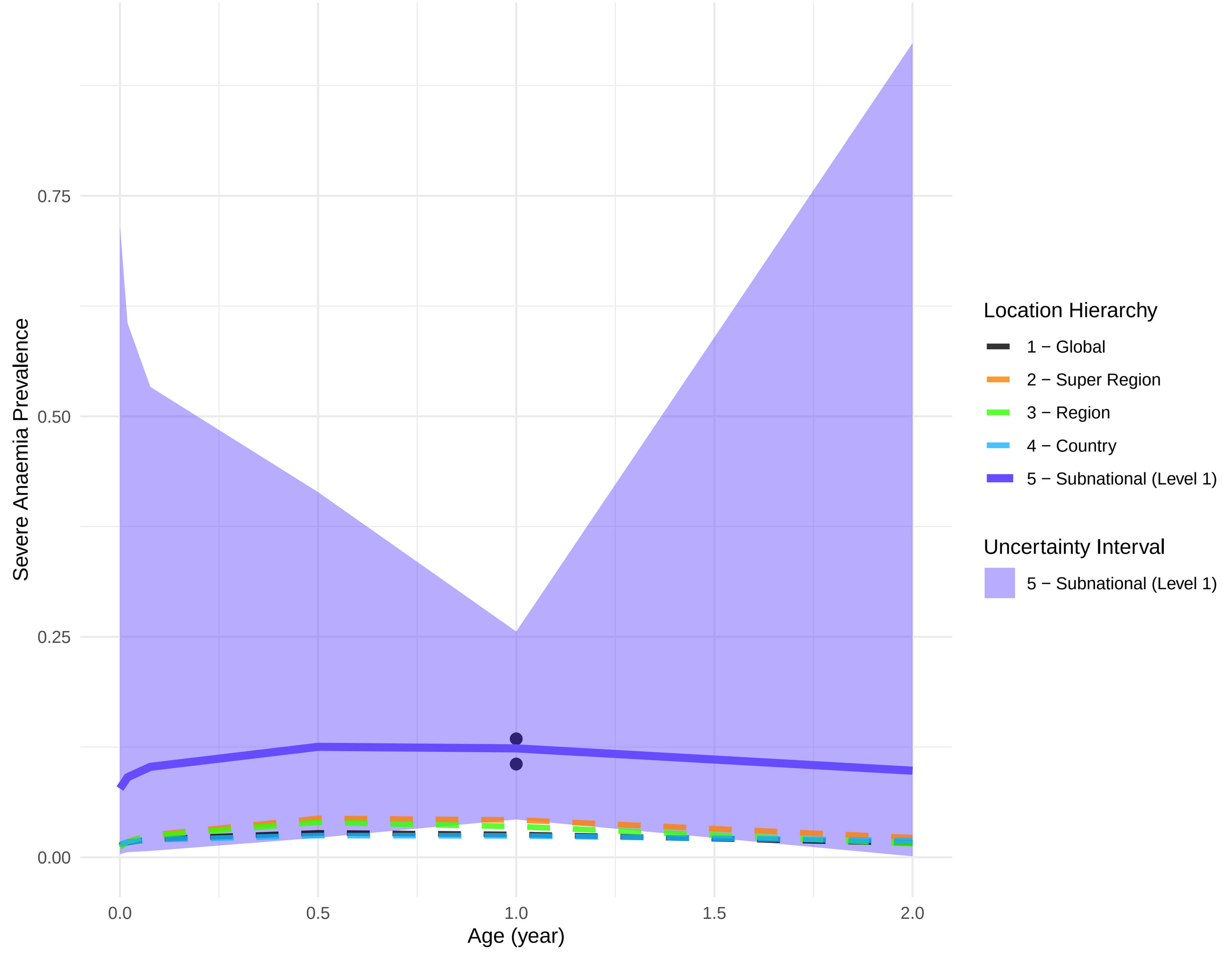

MR−BRT Age/Sex Fits − Non−pregnant Females <2 Years Old − Severe Anaemia Prevalence − Siaya
WHO−2001 Adjustment Method

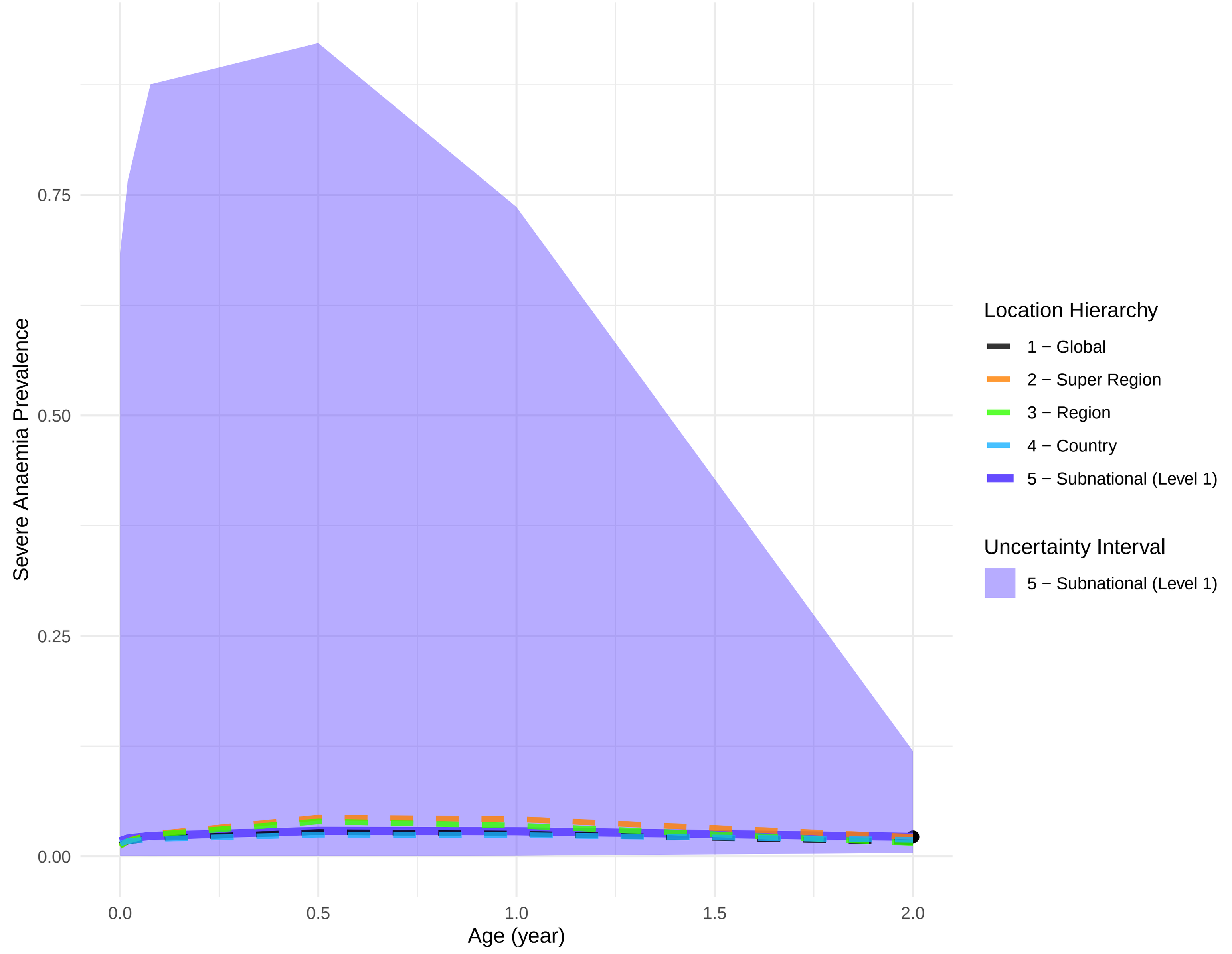

MR−BRT Age/Sex Fits − Non−pregnant Females <2 Years Old − Severe Anaemia Prevalence − Tana River
WHO−2001 Adjustment Method

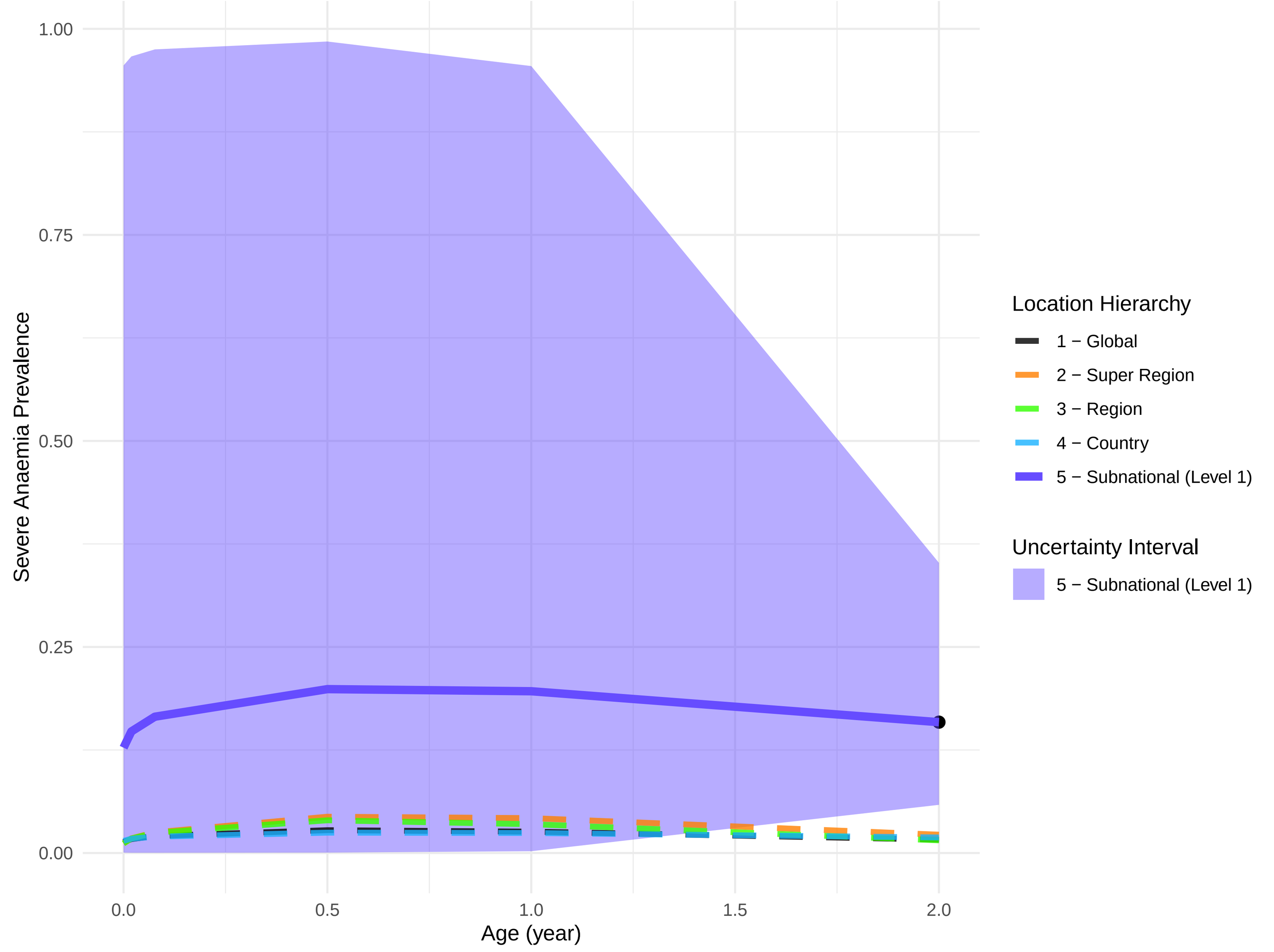

MR−BRT Age/Sex Fits − Non−pregnant Females <2 Years Old − Severe Anaemia Prevalence − Vihiga
WHO−2001 Adjustment Method

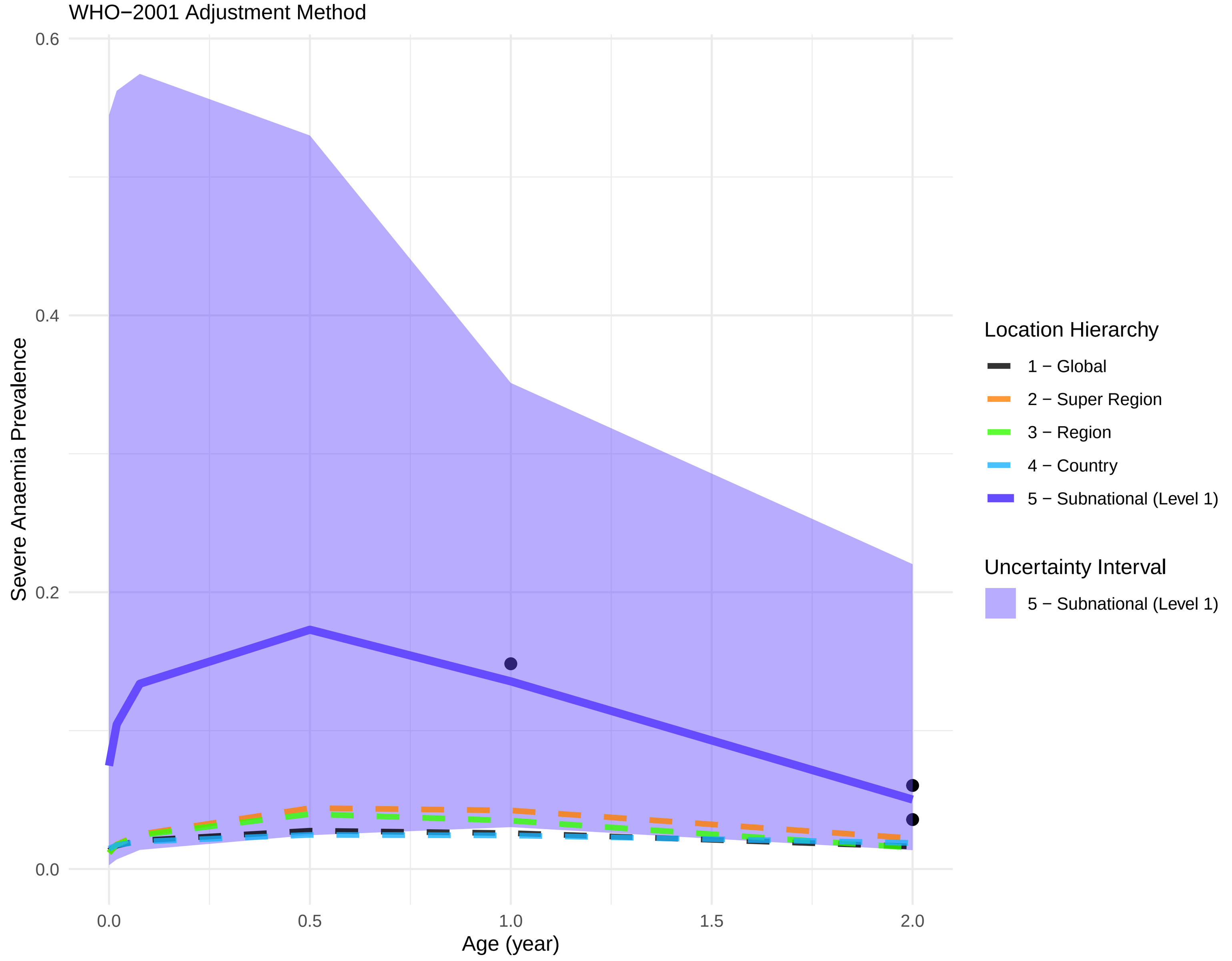

MR−BRT Age/Sex Fits − Non−pregnant Females <2 Years Old − Severe Anaemia Prevalence − Wajir
WHO−2001 Adjustment Method

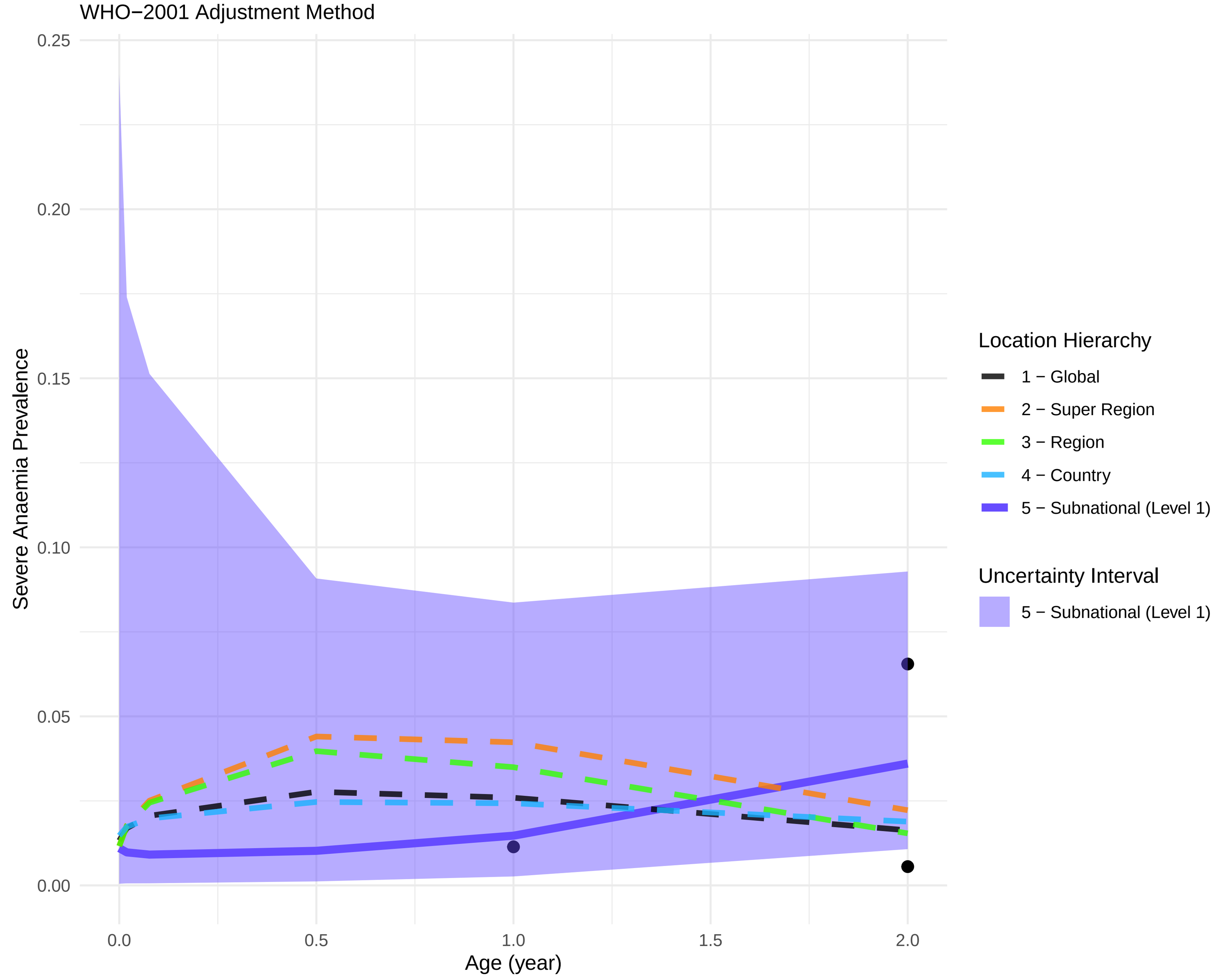

MR−BRT Age/Sex Fits − Non−pregnant Females <2 Years Old − Severe Anaemia Prevalence − West Pokot
WHO−2001 Adjustment Method

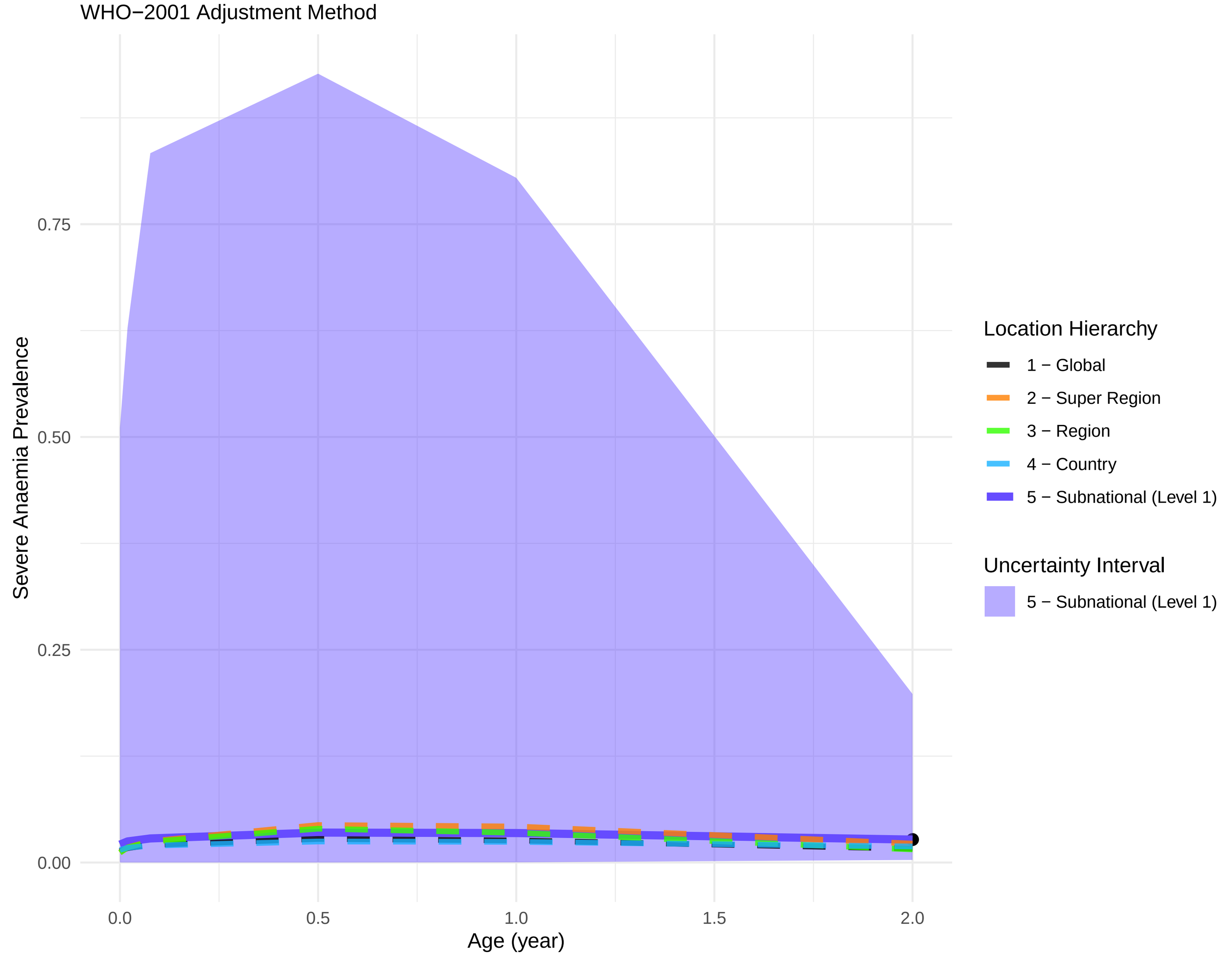

MR−BRT Age/Sex Fits − Non−pregnant Females <2 Years Old − Severe Anaemia Prevalence − Madagascar
WHO−2001 Adjustment Method

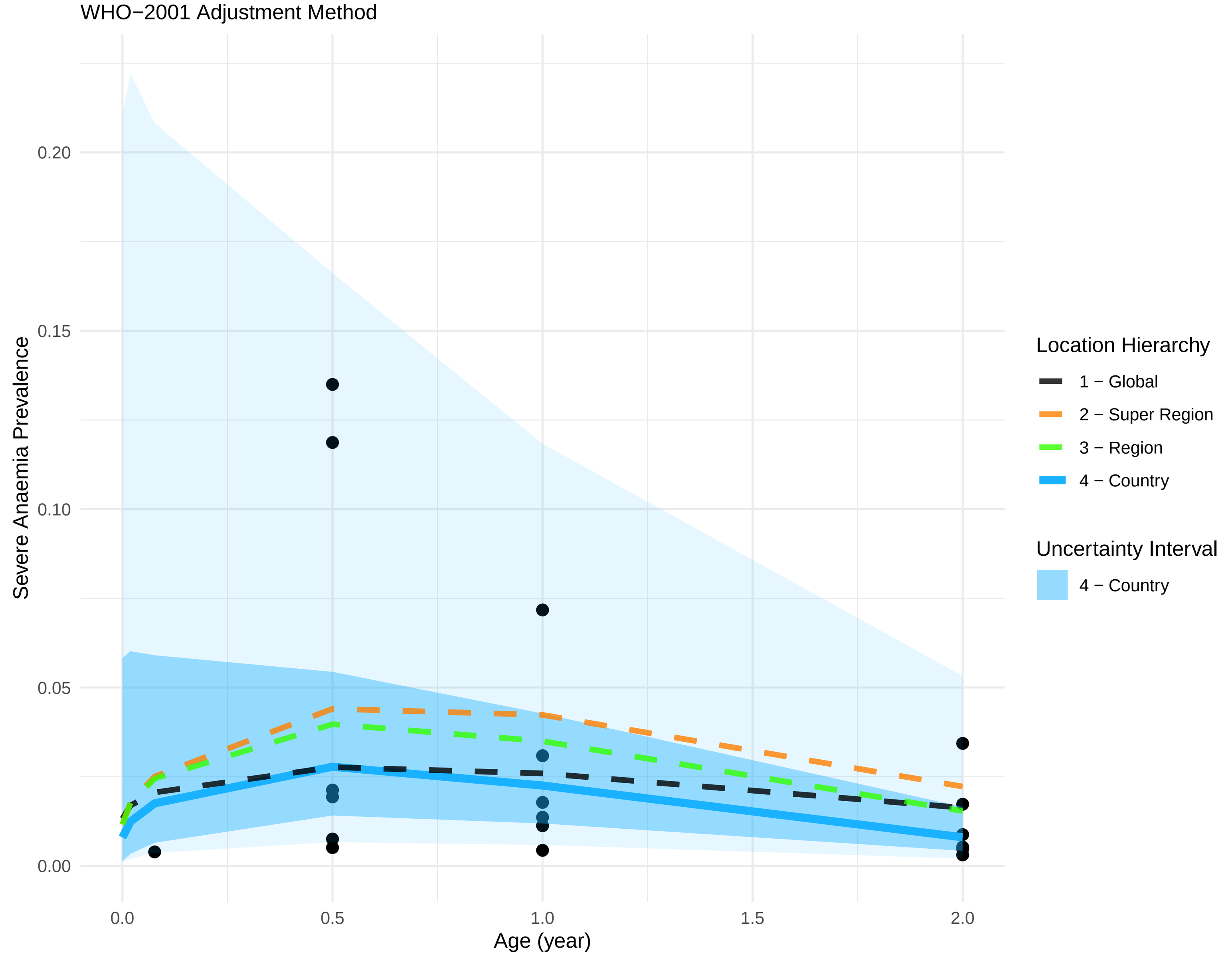

MR−BRT Age/Sex Fits − Non−pregnant Females <2 Years Old − Severe Anaemia Prevalence − Malawi
WHO−2001 Adjustment Method

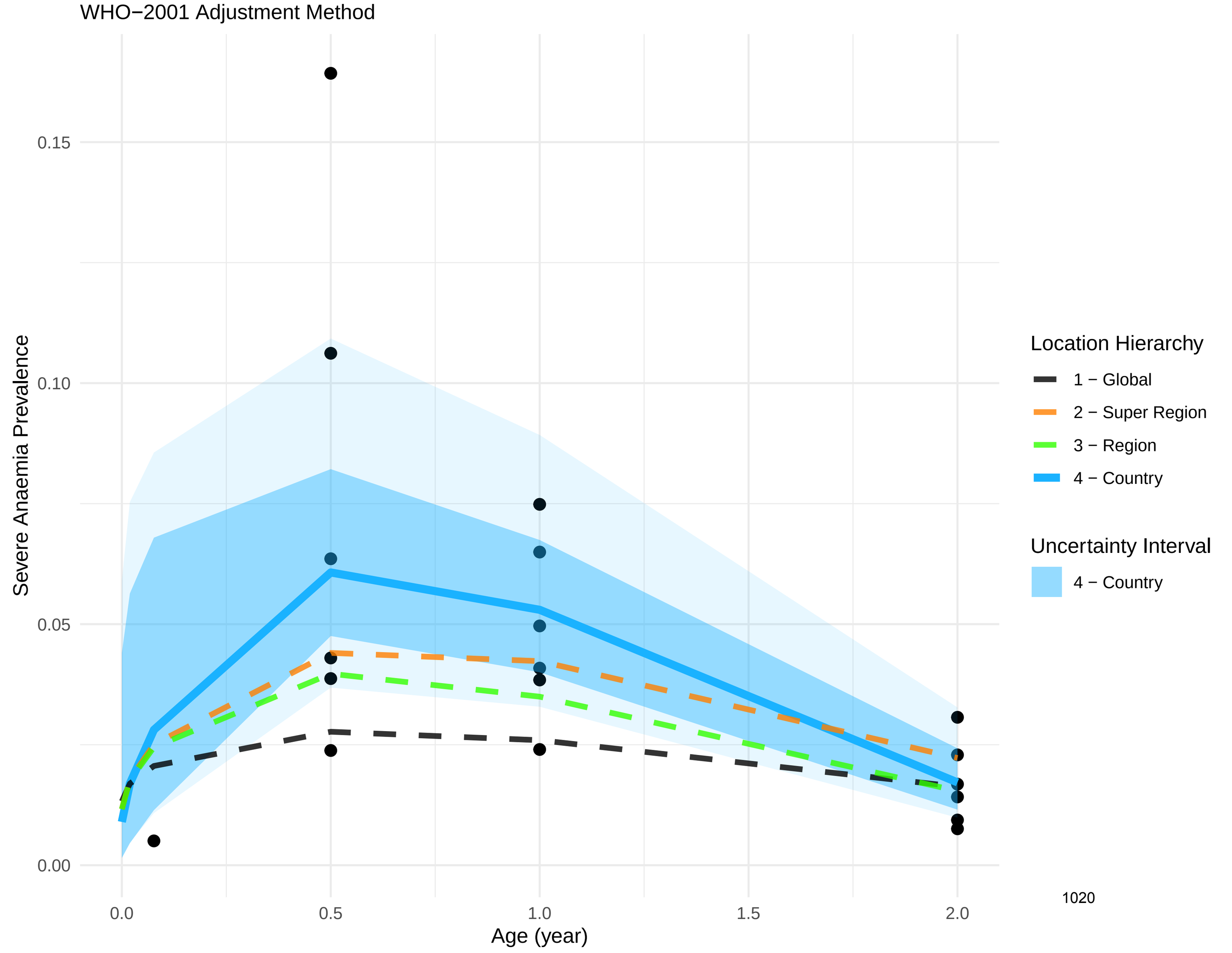

MR−BRT Age/Sex Fits − Non−pregnant Females <2 Years Old − Severe Anaemia Prevalence − Mozambique
WHO−2001 Adjustment Method

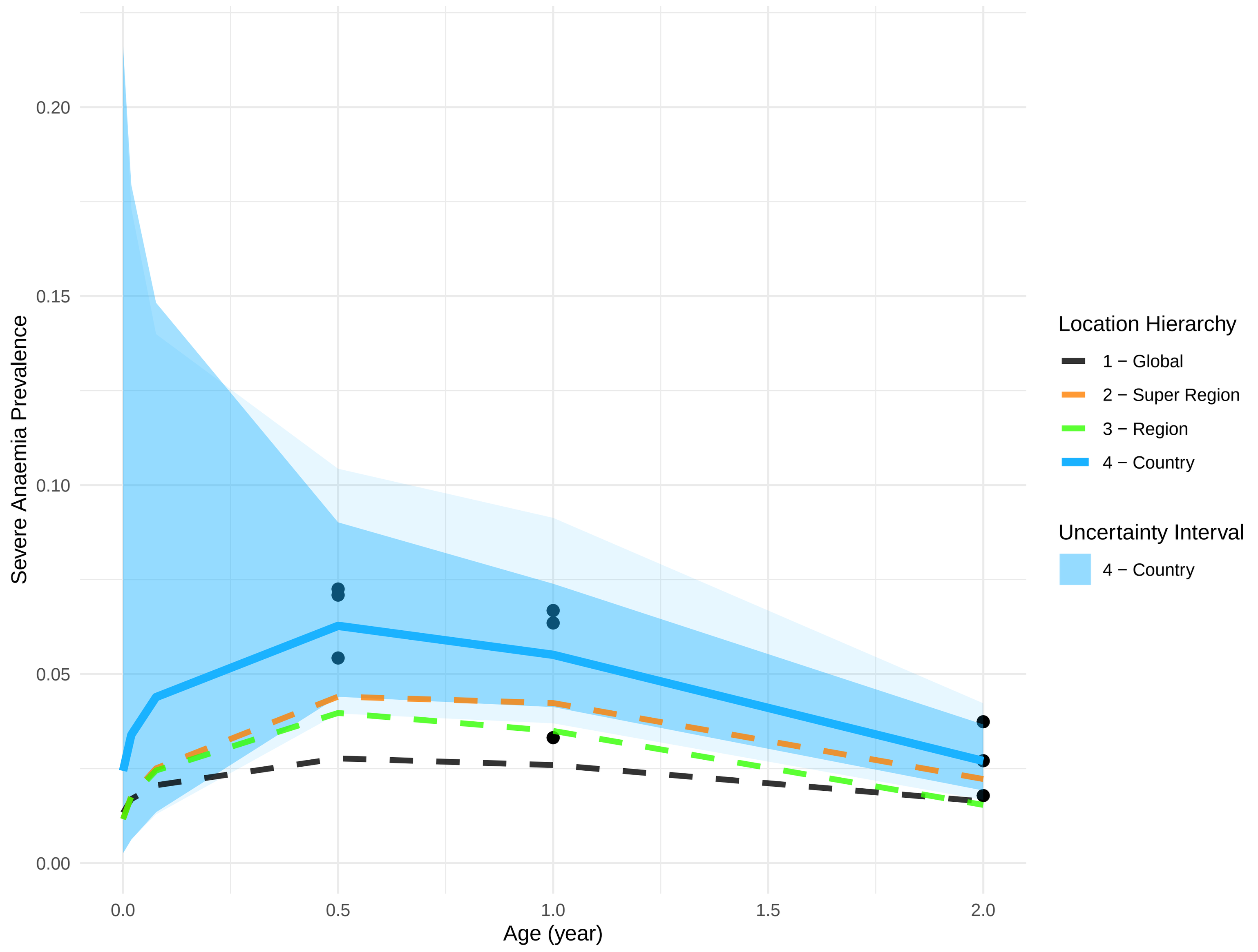

MR−BRT Age/Sex Fits − Non−pregnant Females <2 Years Old − Severe Anaemia Prevalence − Rwanda
WHO−2001 Adjustment Method

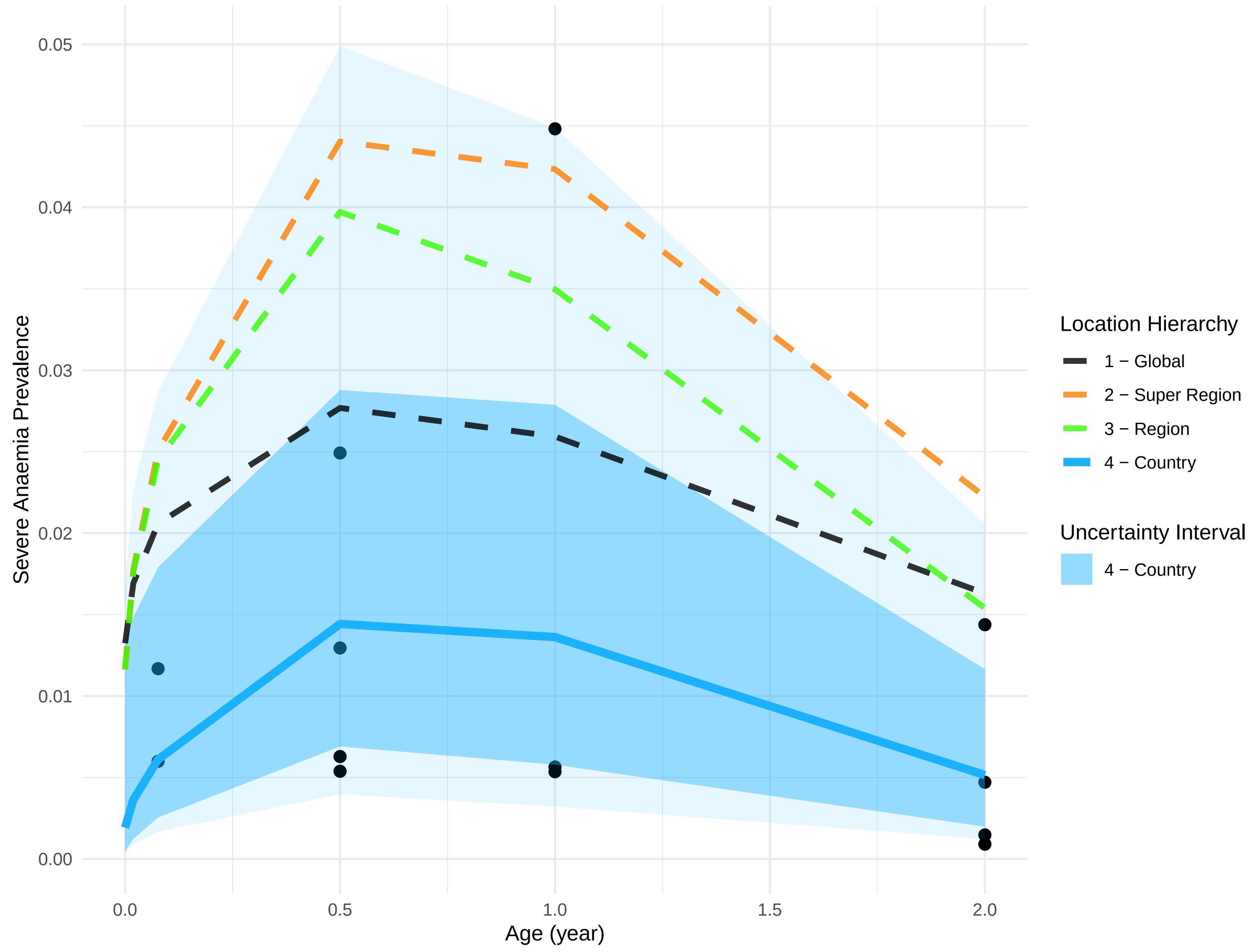

MR−BRT Age/Sex Fits − Non−pregnant Females <2 Years Old − Severe Anaemia Prevalence − Uganda
WHO−2001 Adjustment Method

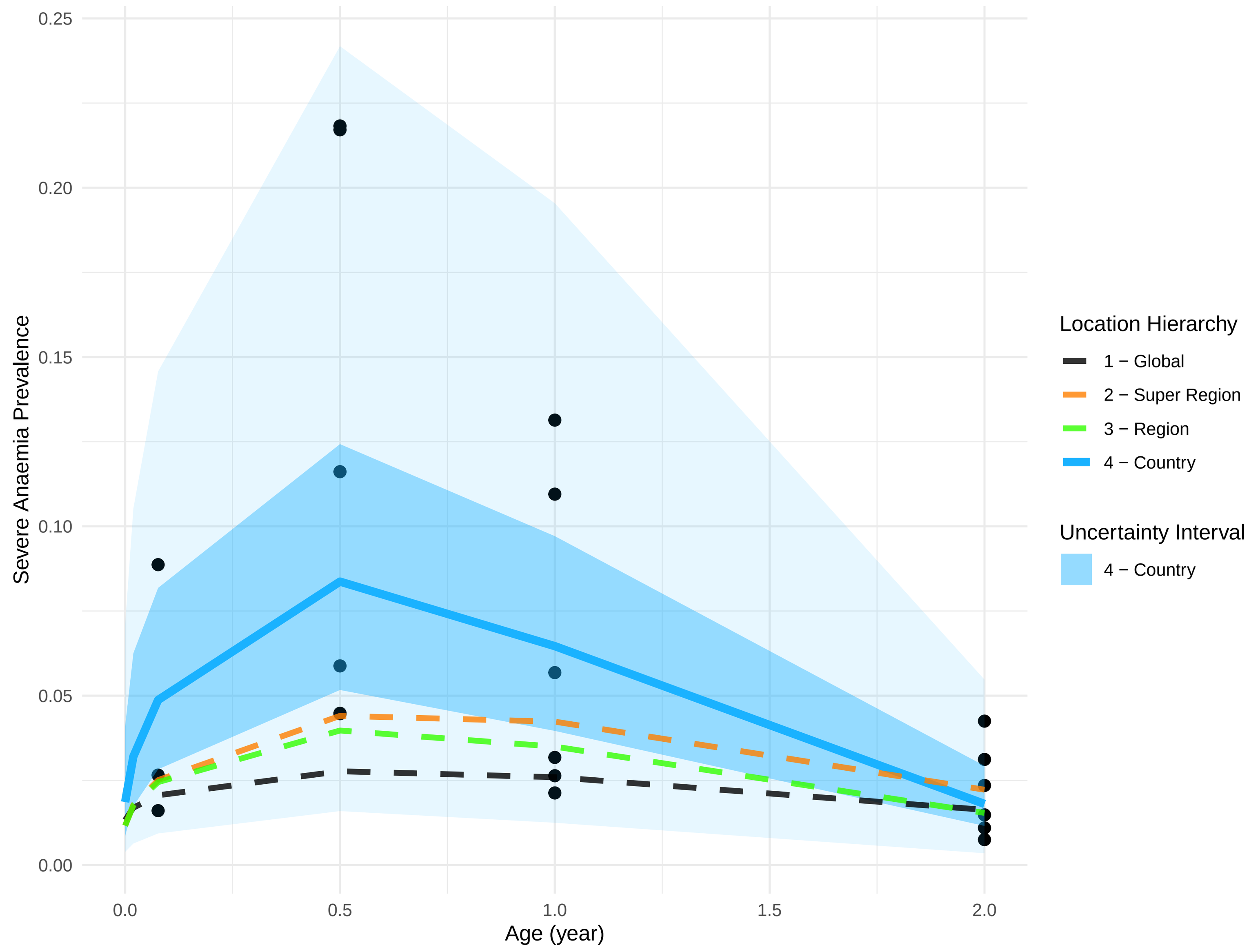

MR−BRT Age/Sex Fits − Non−pregnant Females <2 Years Old − Severe Anaemia Prevalence − United Republic of Tanzania
WHO−2001 Adjustment Method

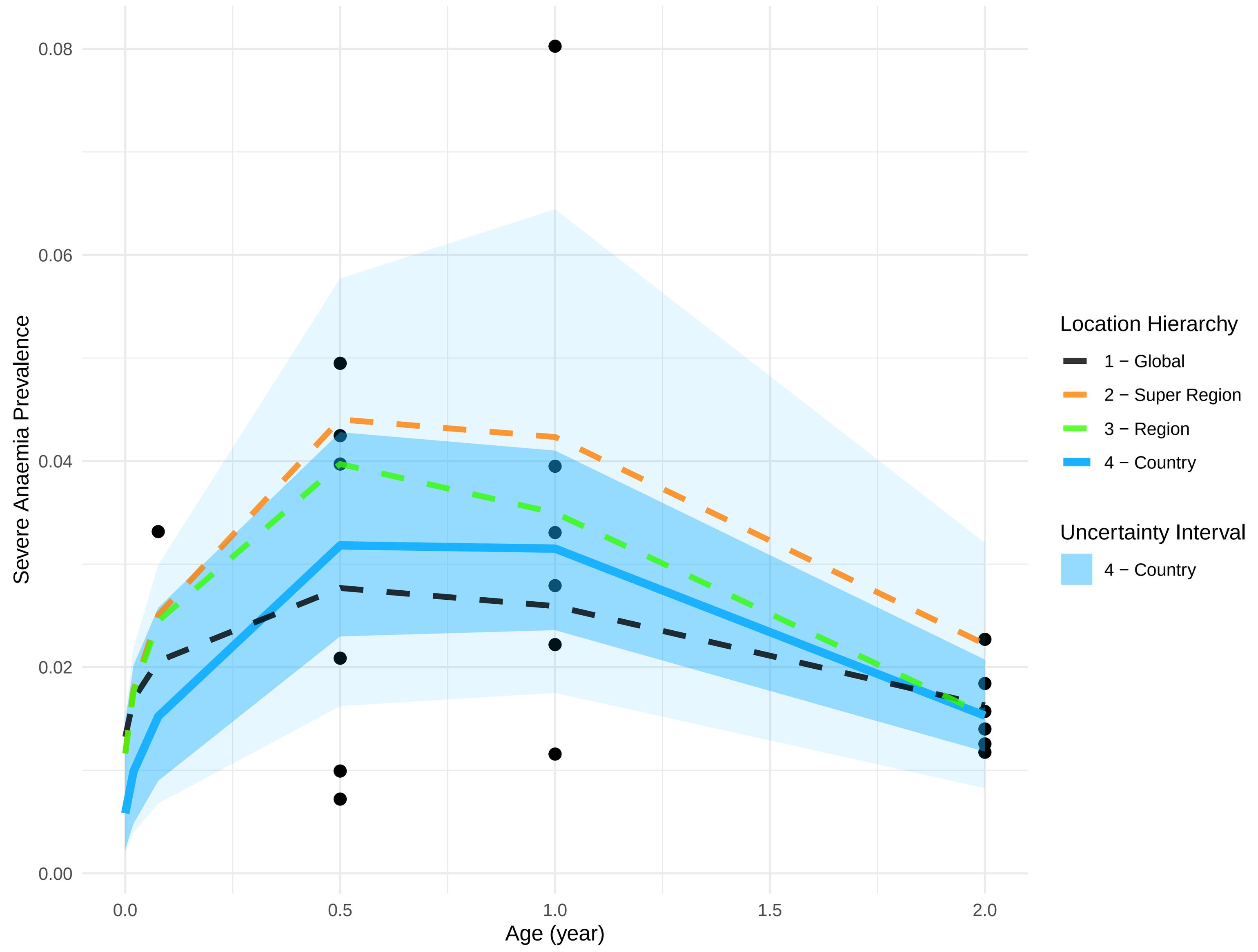

MR−BRT Age/Sex Fits − Non−pregnant Females <2 Years Old − Severe Anaemia Prevalence − Zambia
WHO−2001 Adjustment Method

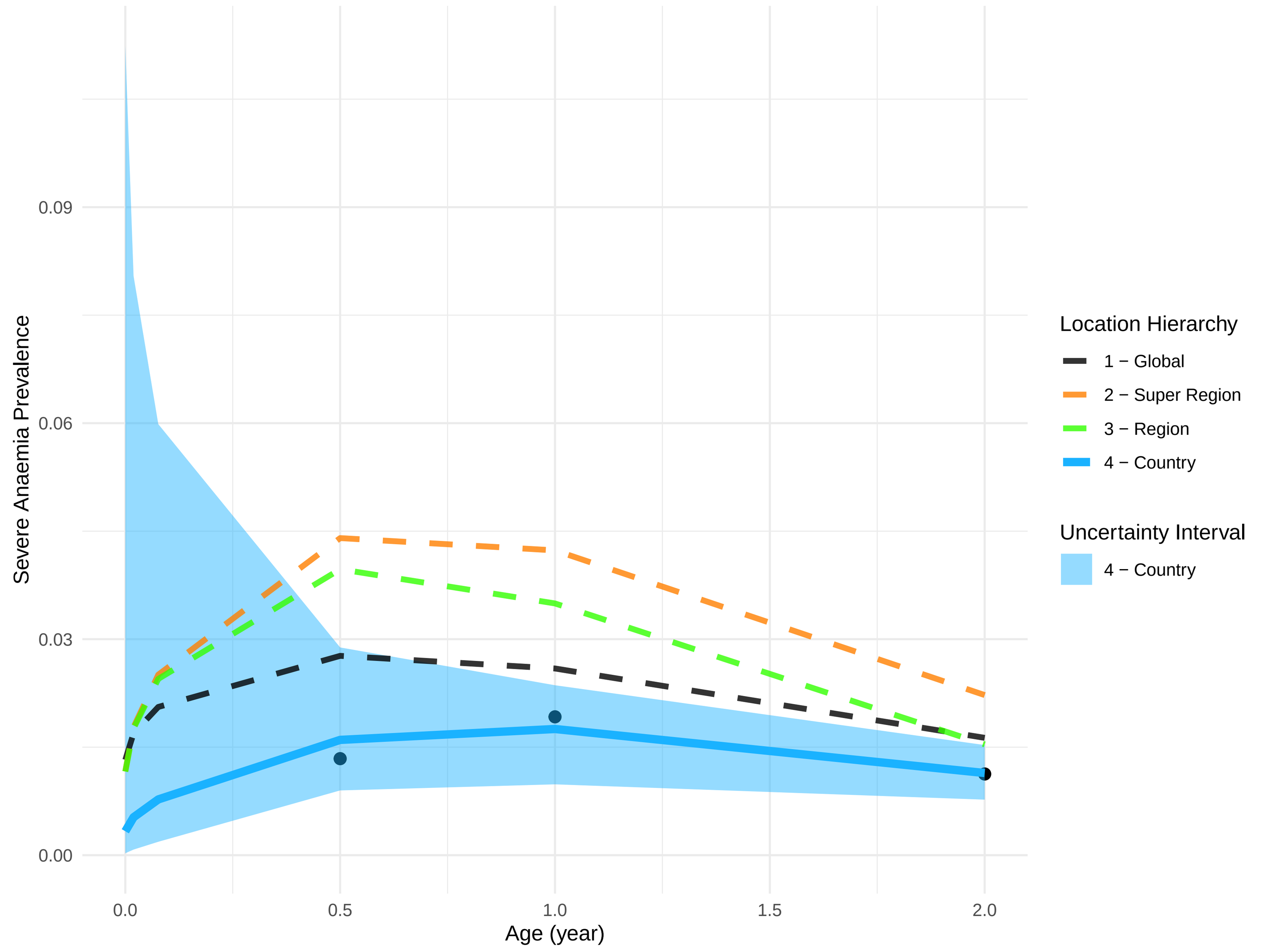

MR−BRT Age/Sex Fits − Non−pregnant Females <2 Years Old − Severe Anaemia Prevalence − Southern Sub−Saharan Africa
WHO−2001 Adjustment Method

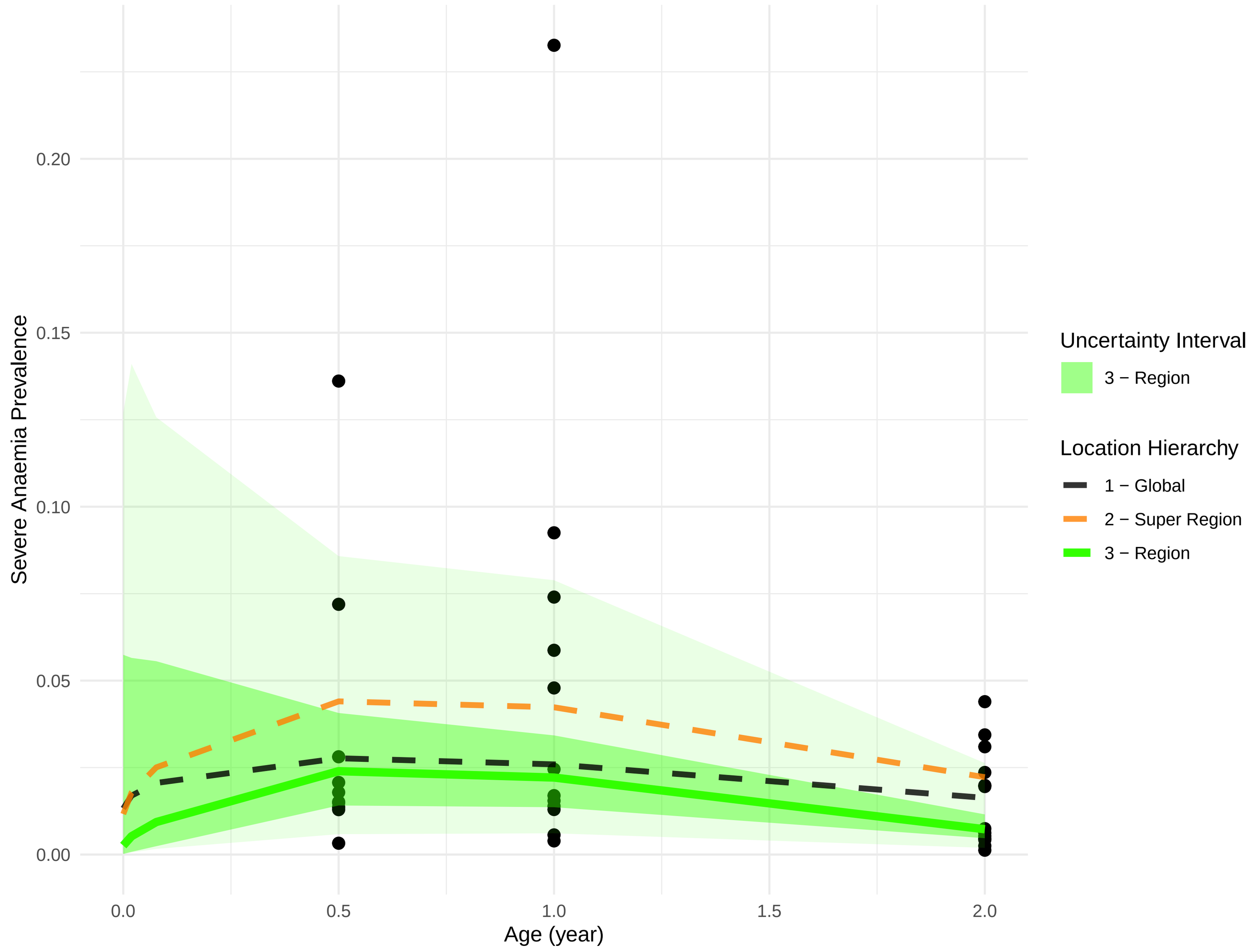

MR−BRT Age/Sex Fits − Non−pregnant Females <2 Years Old − Severe Anaemia Prevalence − Eswatini
WHO−2001 Adjustment Method

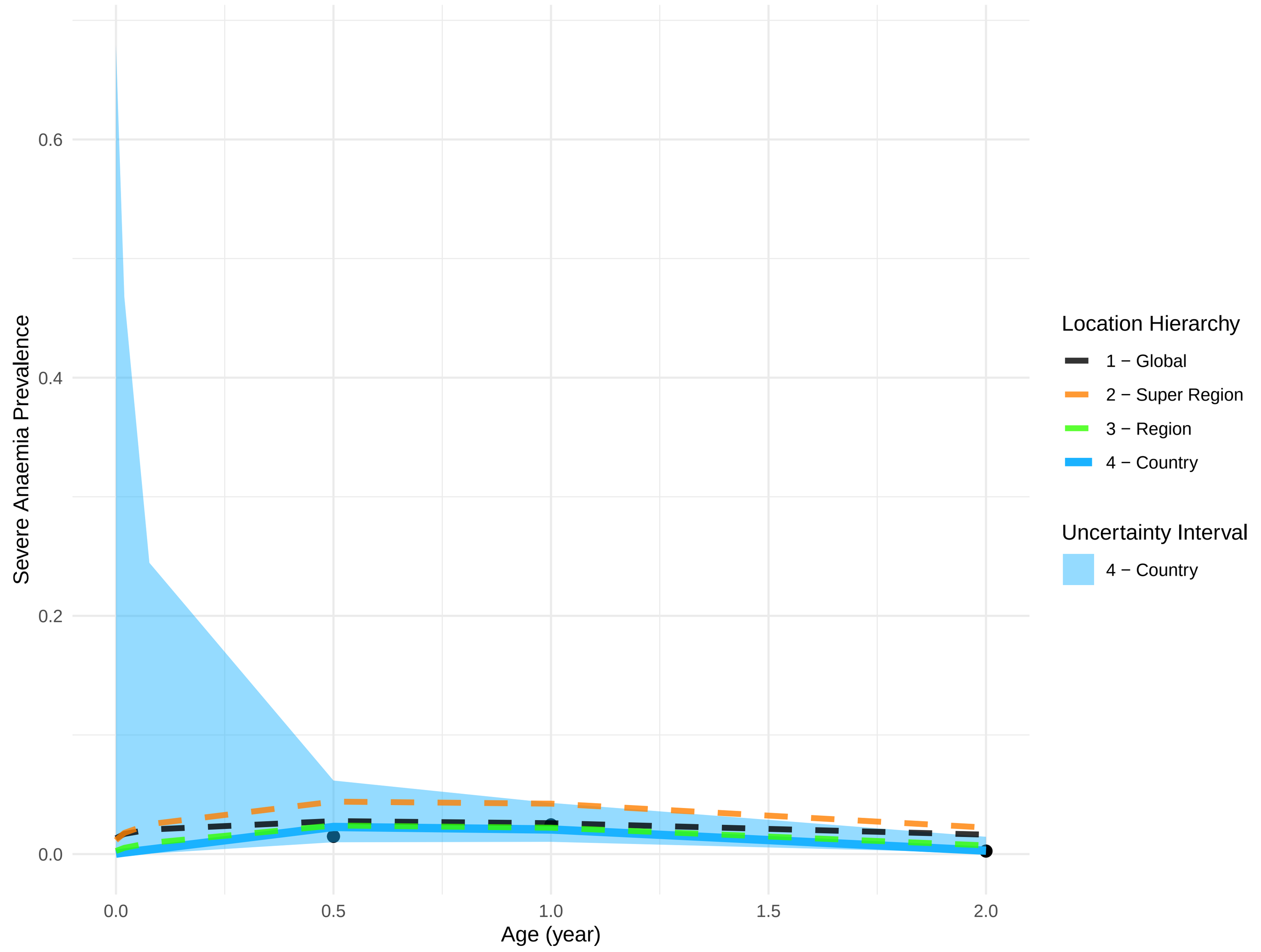

MR−BRT Age/Sex Fits − Non−pregnant Females <2 Years Old − Severe Anaemia Prevalence − Lesotho
WHO−2001 Adjustment Method

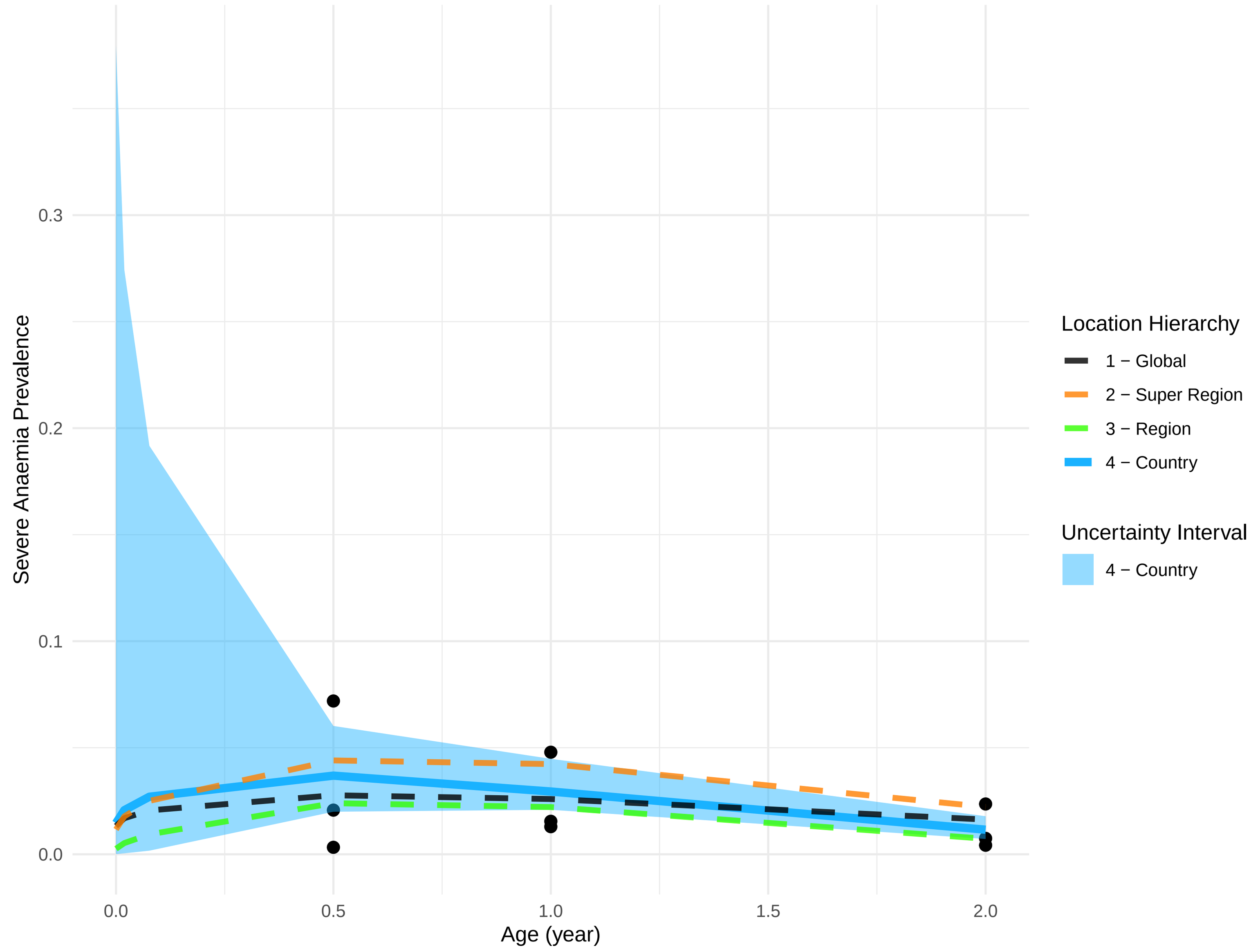

MR−BRT Age/Sex Fits − Non−pregnant Females <2 Years Old − Severe Anaemia Prevalence − Namibia
WHO−2001 Adjustment Method

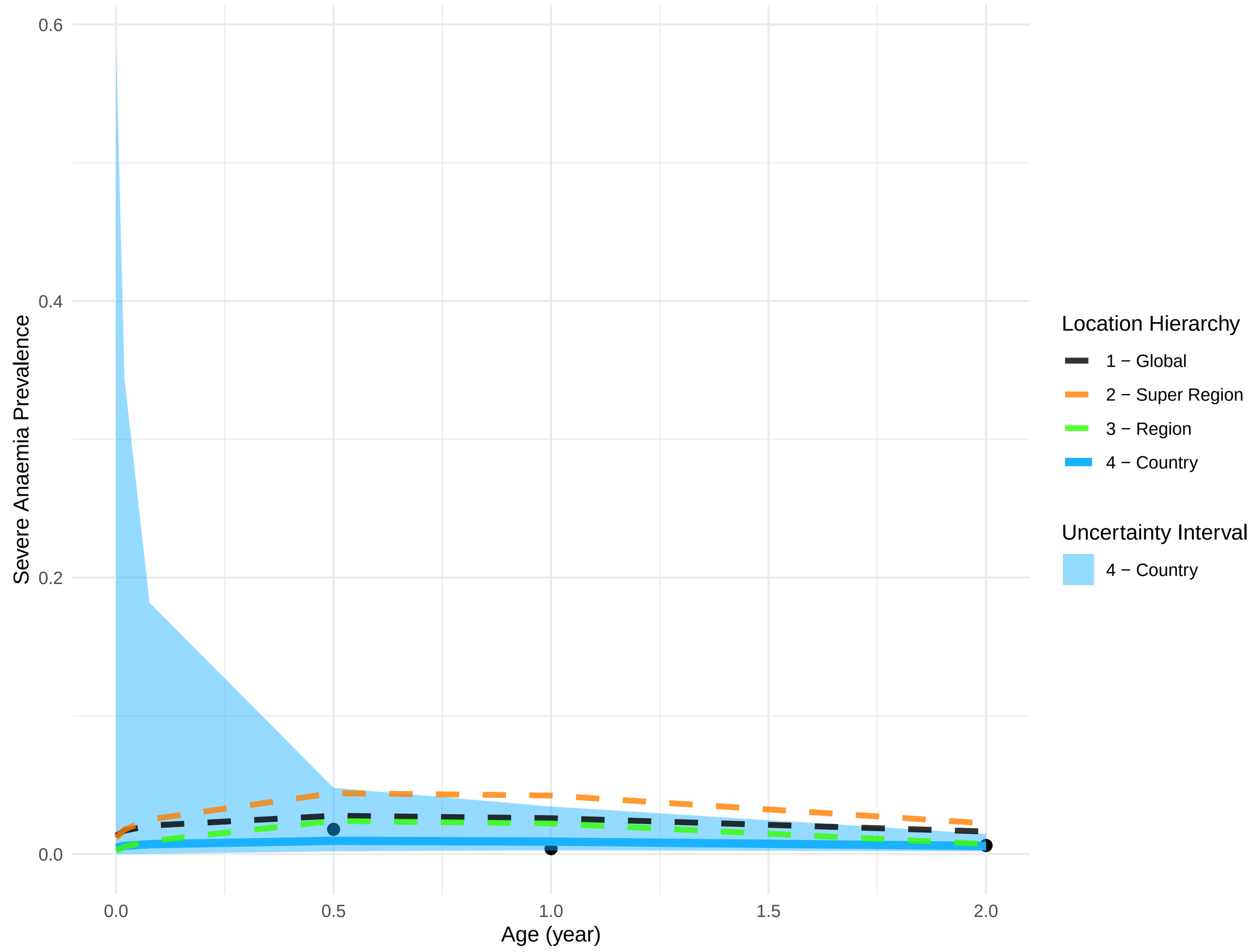

MR−BRT Age/Sex Fits − Non−pregnant Females <2 Years Old − Severe Anaemia Prevalence − South Africa
WHO−2001 Adjustment Method

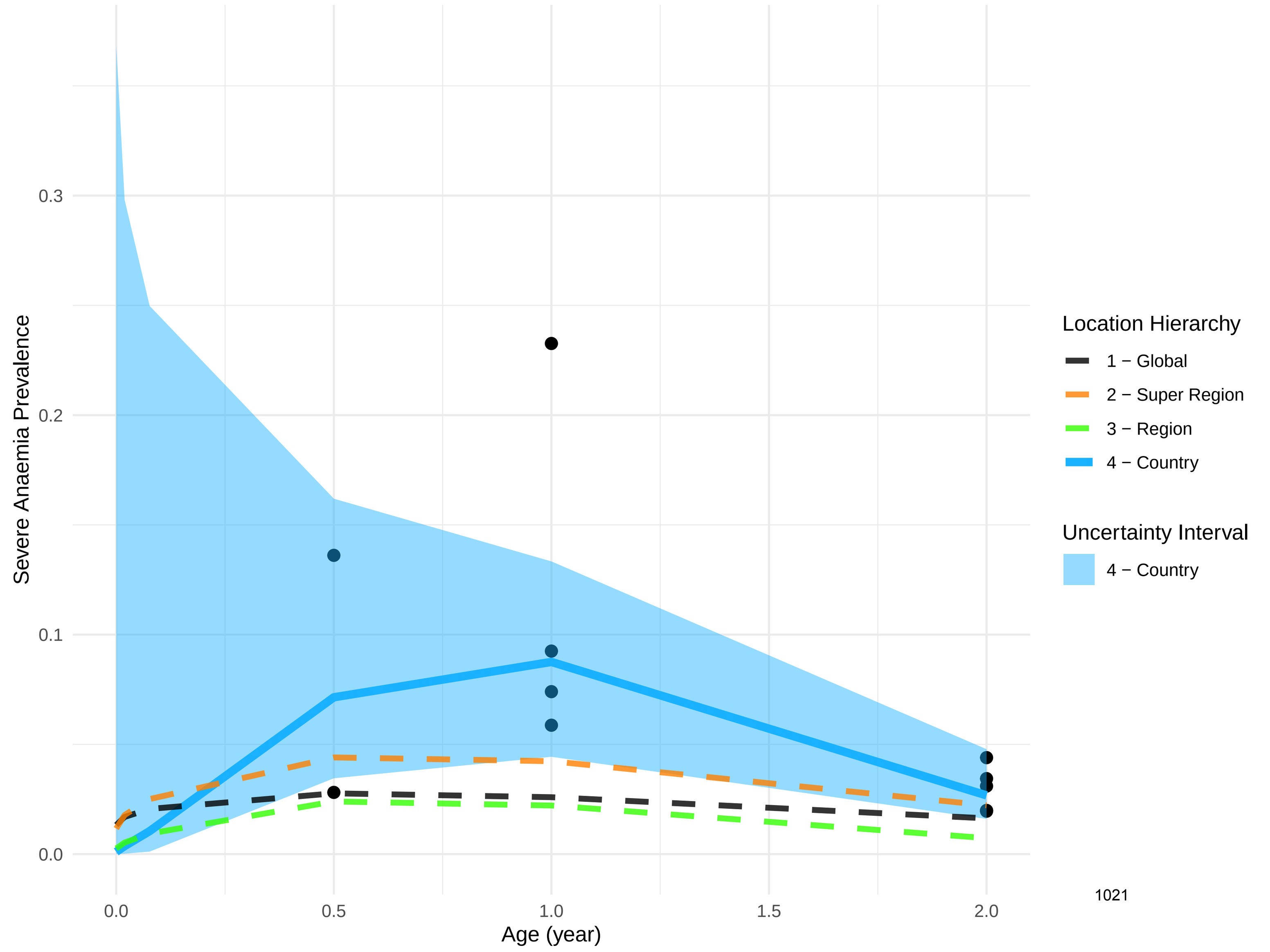

MR−BRT Age/Sex Fits − Non−pregnant Females <2 Years Old − Severe Anaemia Prevalence − Free State
WHO−2001 Adjustment Method

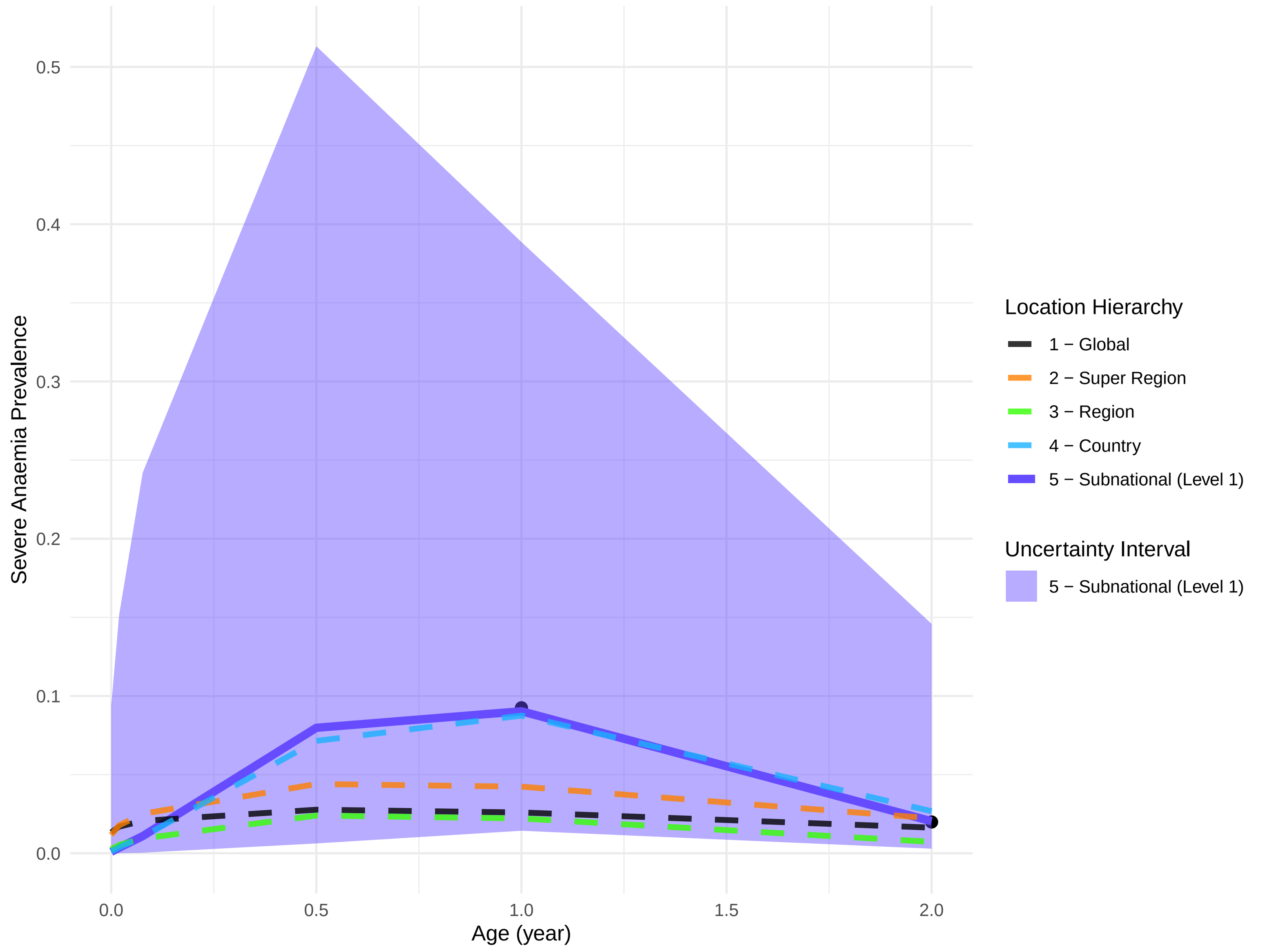

MR−BRT Age/Sex Fits − Non−pregnant Females <2 Years Old − Severe Anaemia Prevalence − Gauteng
WHO−2001 Adjustment Method

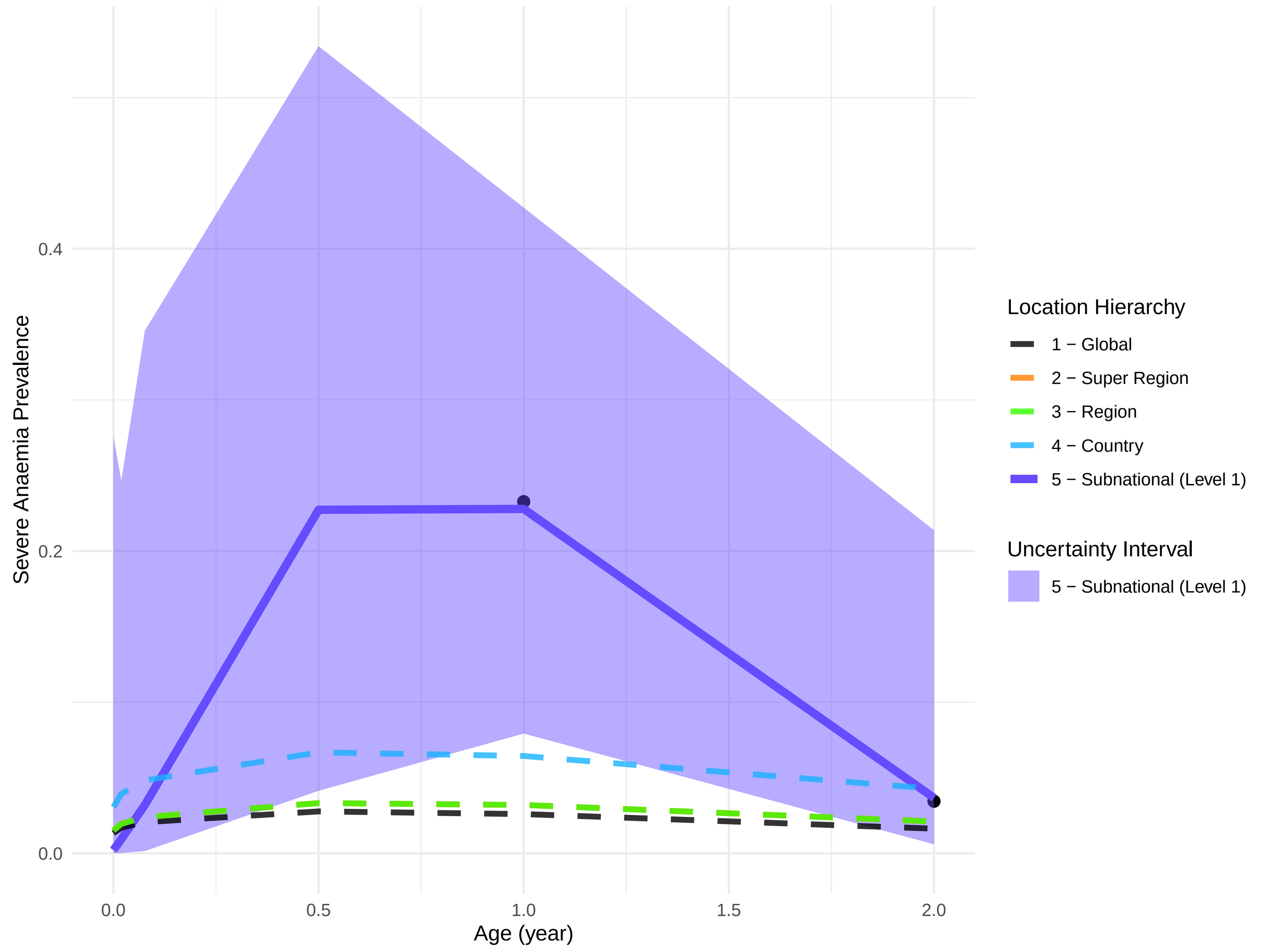

MR−BRT Age/Sex Fits − Non−pregnant Females <2 Years Old − Severe Anaemia Prevalence − KwaZulu−Natal
WHO−2001 Adjustment Method

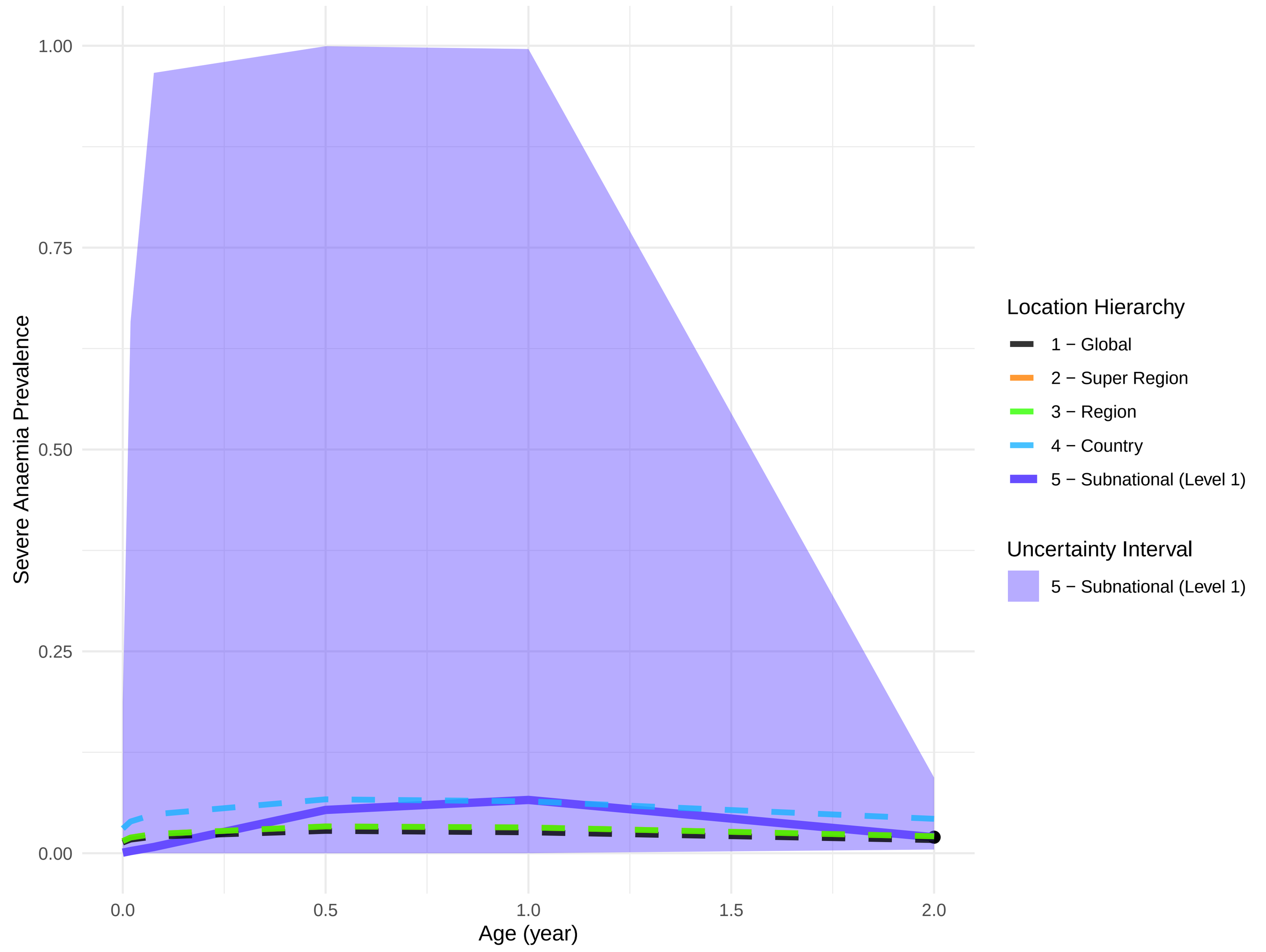

MR−BRT Age/Sex Fits − Non−pregnant Females <2 Years Old − Severe Anaemia Prevalence − Mpumalanga
WHO−2001 Adjustment Method

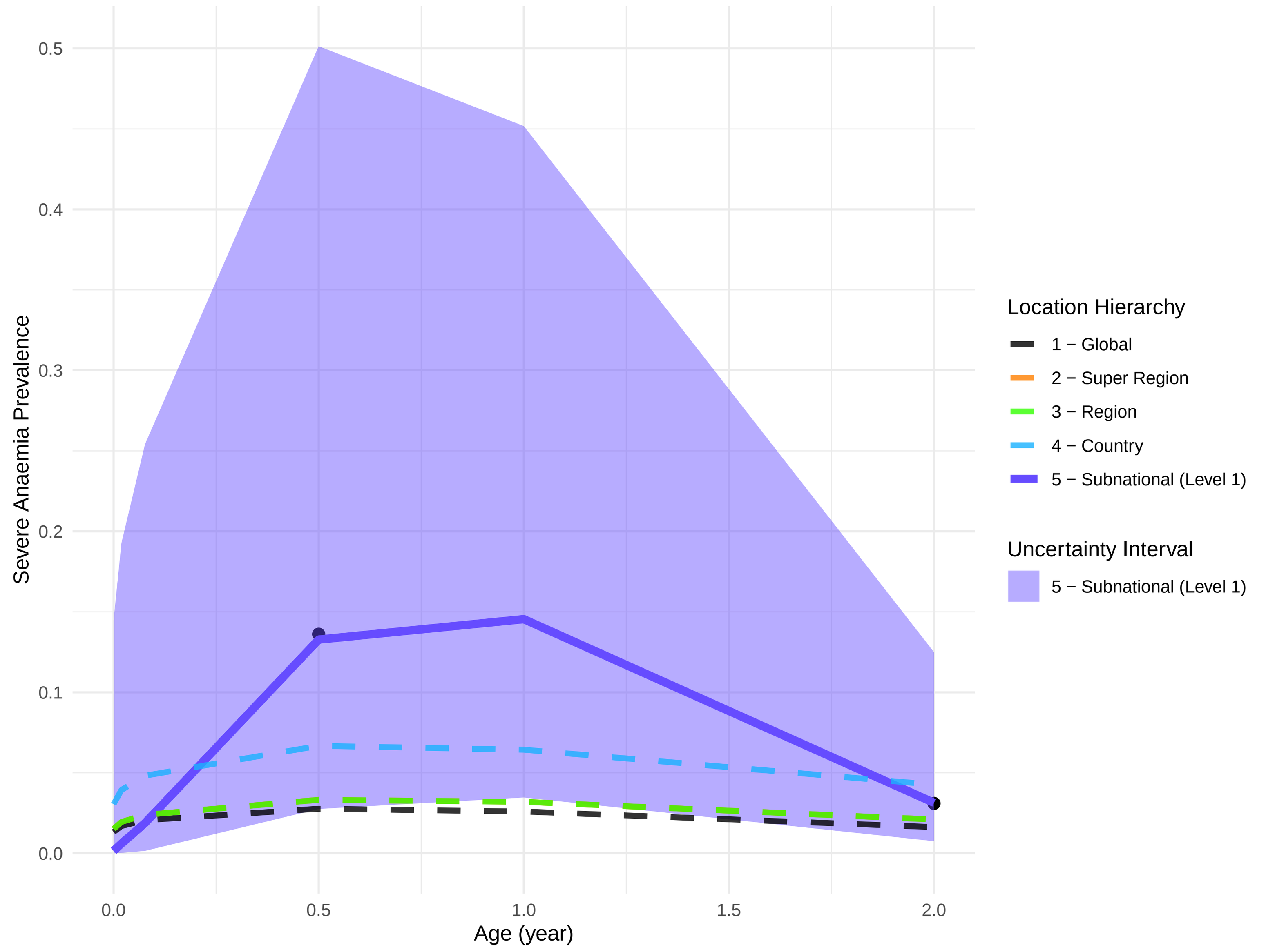

MR−BRT Age/Sex Fits − Non−pregnant Females <2 Years Old − Severe Anaemia Prevalence − North−West
WHO−2001 Adjustment Method

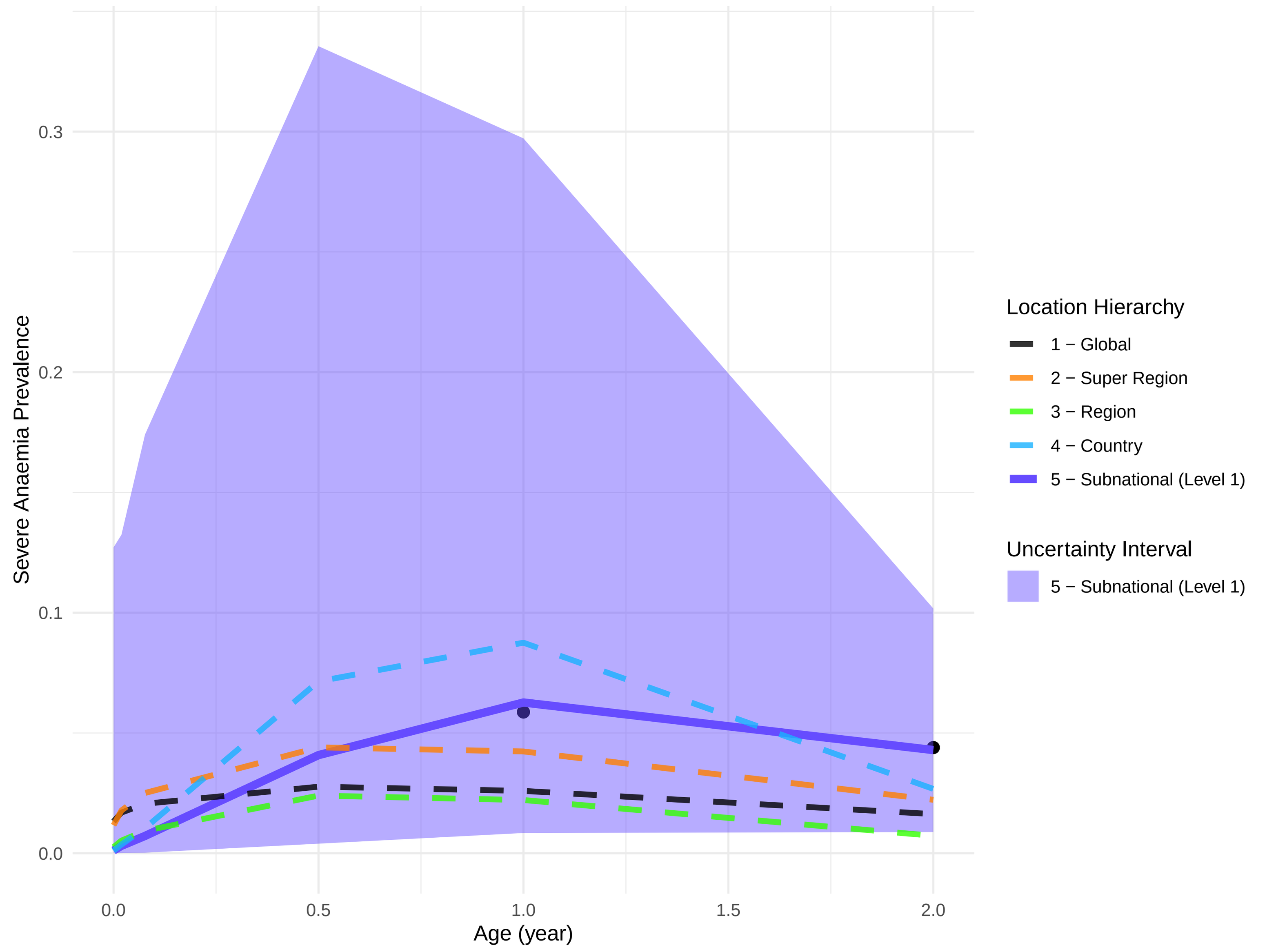

MR−BRT Age/Sex Fits − Non−pregnant Females <2 Years Old − Severe Anaemia Prevalence − Zimbabwe
WHO−2001 Adjustment Method

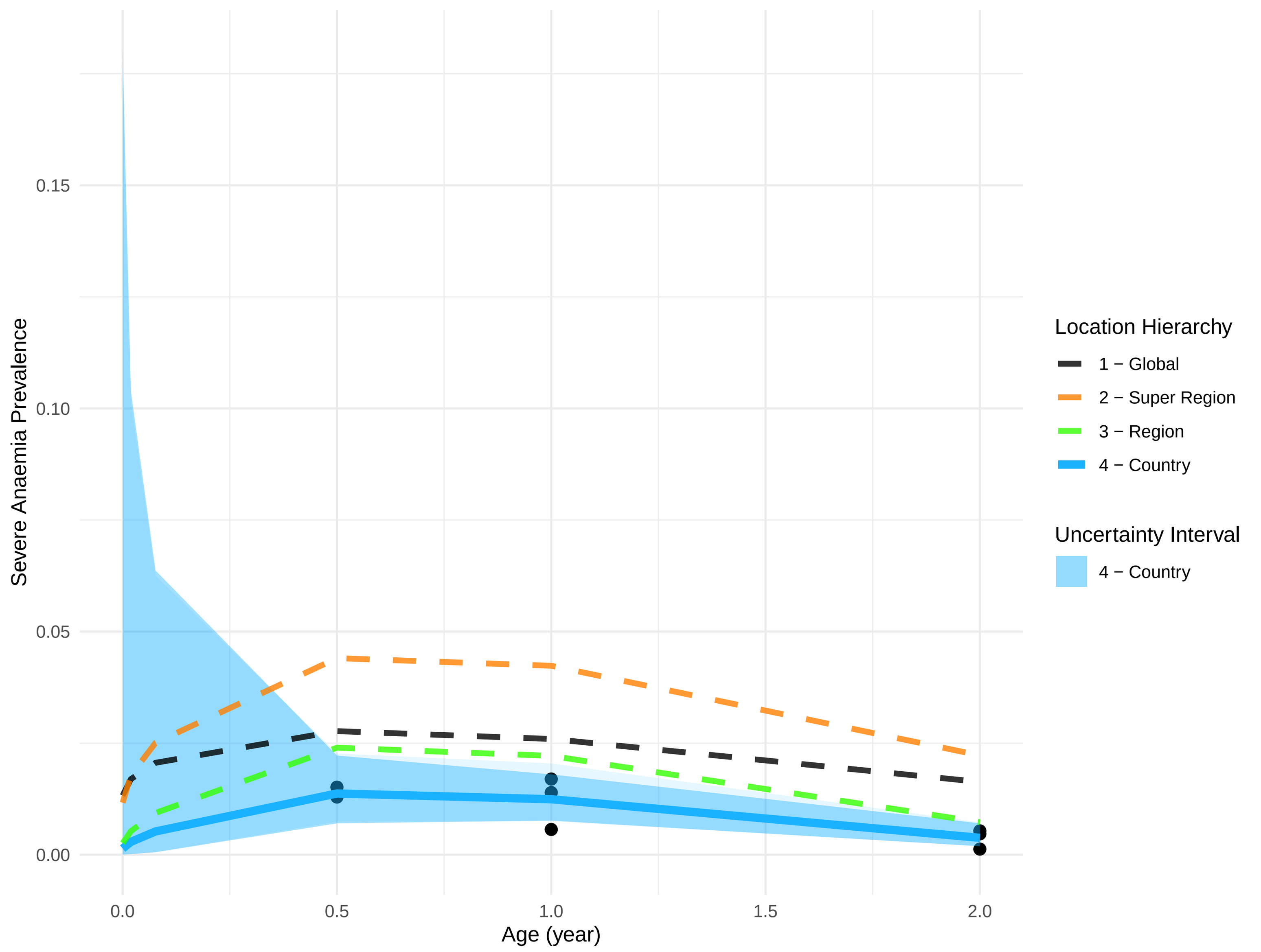

MR−BRT Age/Sex Fits − Non−pregnant Females <2 Years Old − Severe Anaemia Prevalence − Western Sub−Saharan Africa
WHO−2001 Adjustment Method

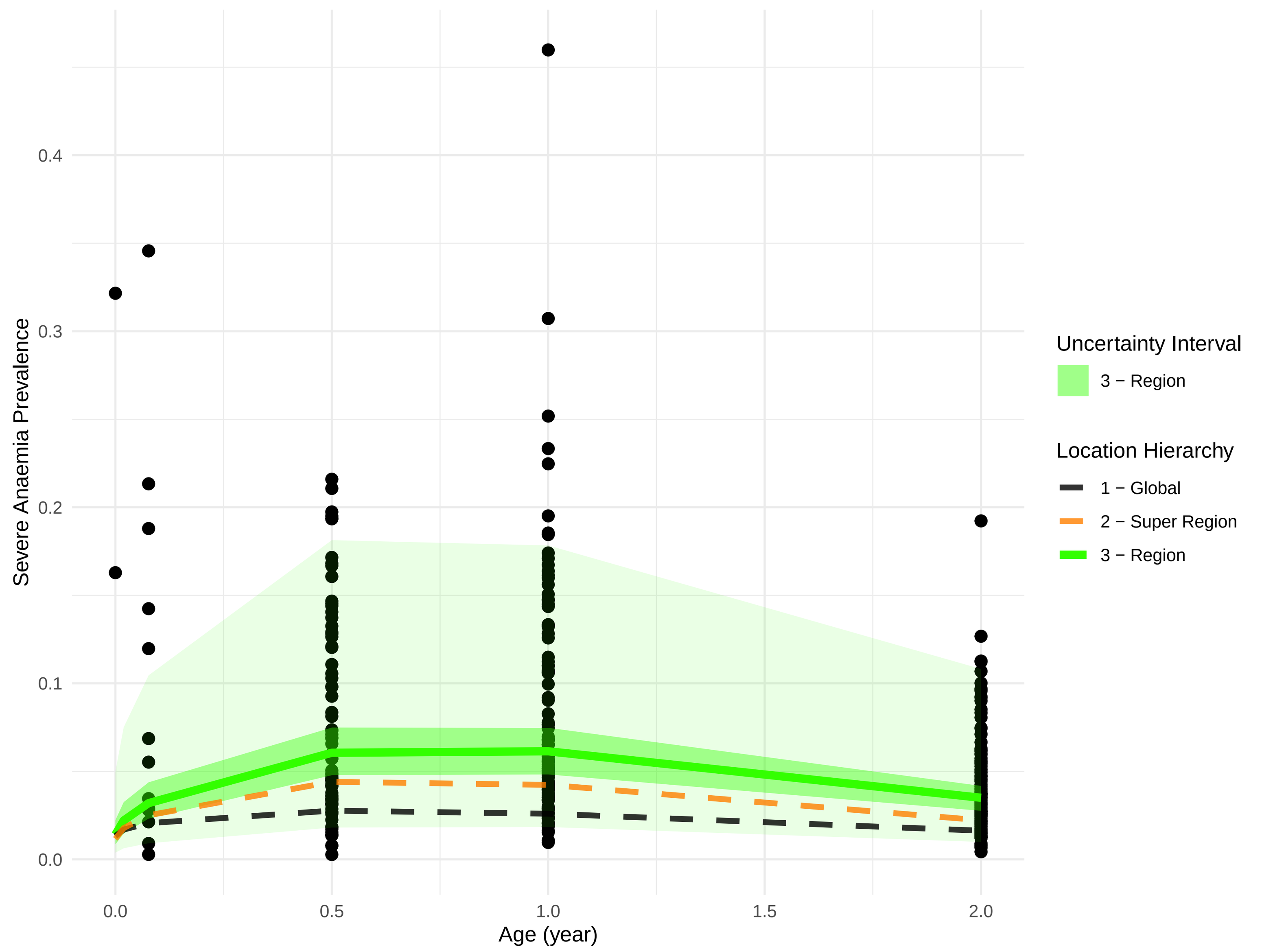

MR−BRT Age/Sex Fits − Non−pregnant Females <2 Years Old − Severe Anaemia Prevalence − Benin
WHO−2001 Adjustment Method

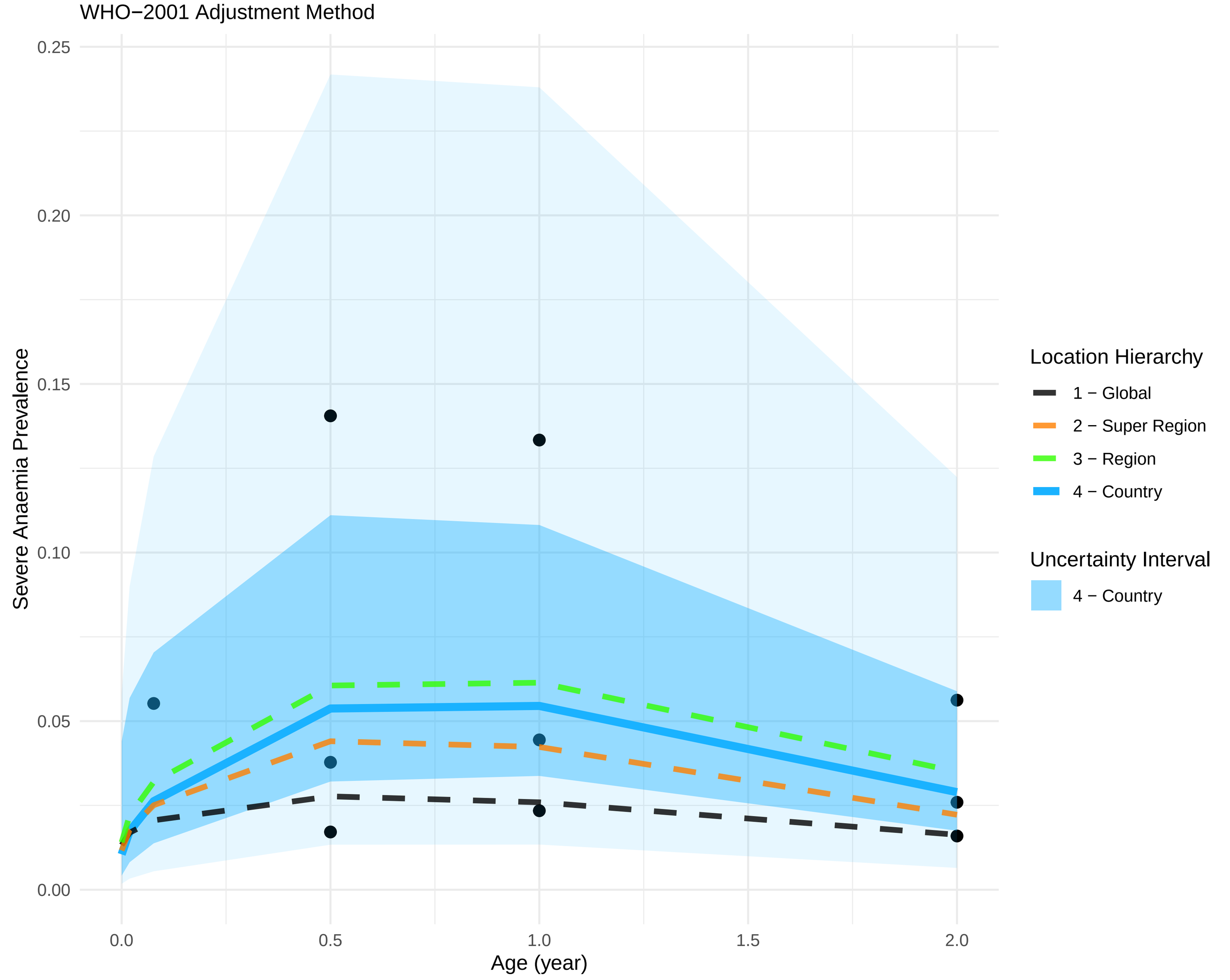

MR−BRT Age/Sex Fits − Non−pregnant Females <2 Years Old − Severe Anaemia Prevalence − Burkina Faso
WHO−2001 Adjustment Method

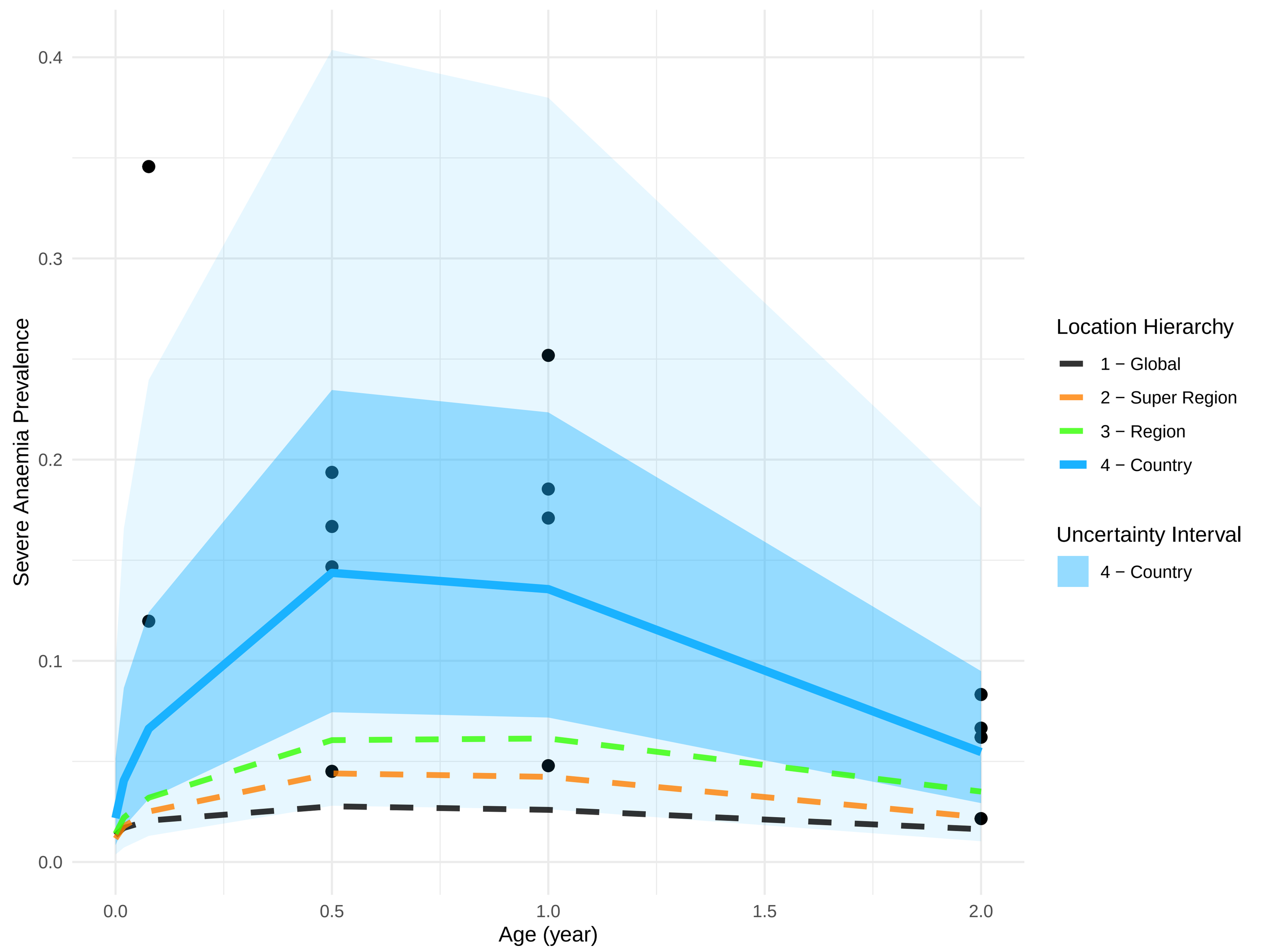

MR−BRT Age/Sex Fits − Non−pregnant Females <2 Years Old − Severe Anaemia Prevalence − Cameroon
WHO−2001 Adjustment Method

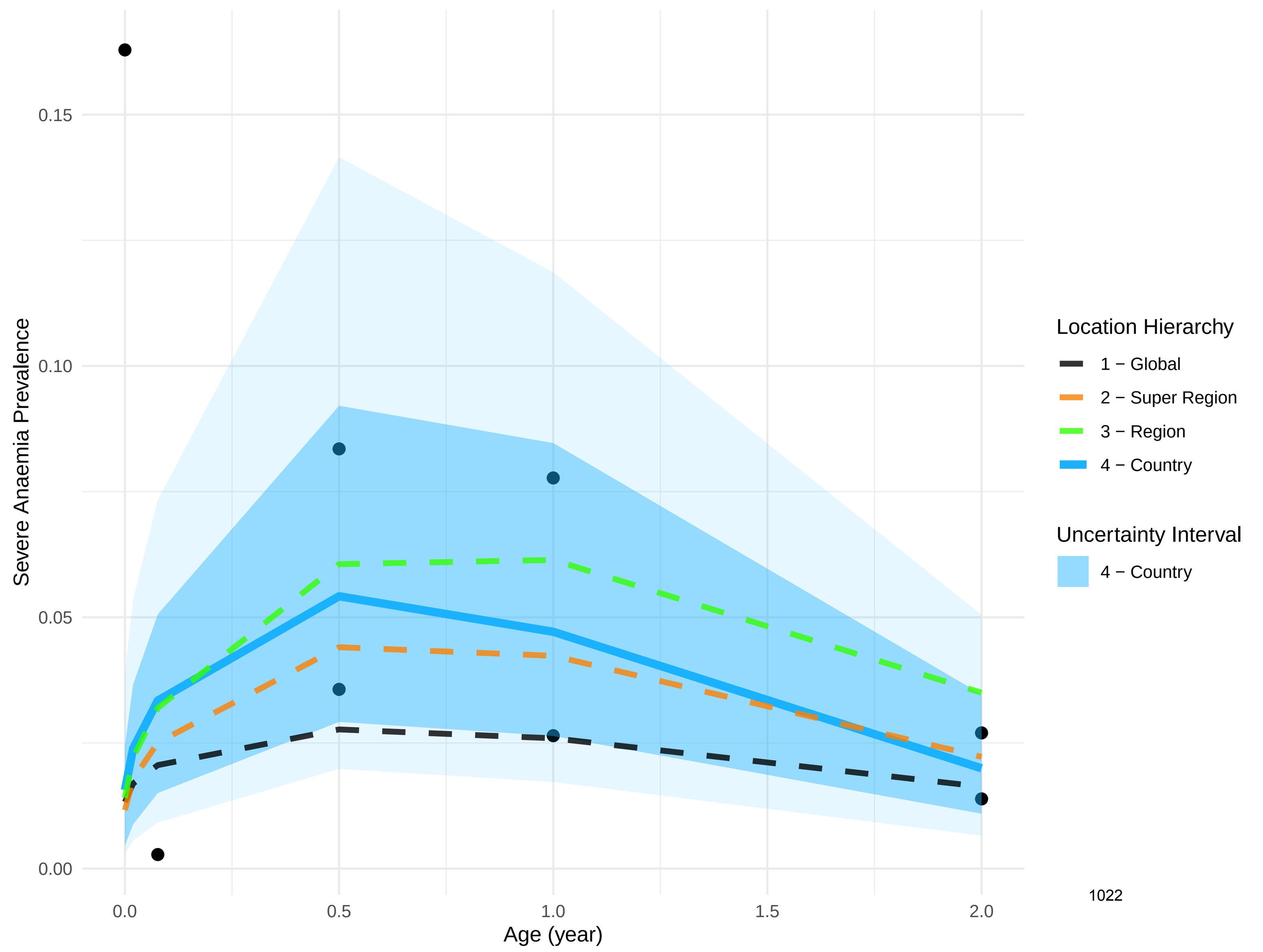

MR−BRT Age/Sex Fits − Non−pregnant Females <2 Years Old − Severe Anaemia Prevalence − Côte d'Ivoire
WHO−2001 Adjustment Method

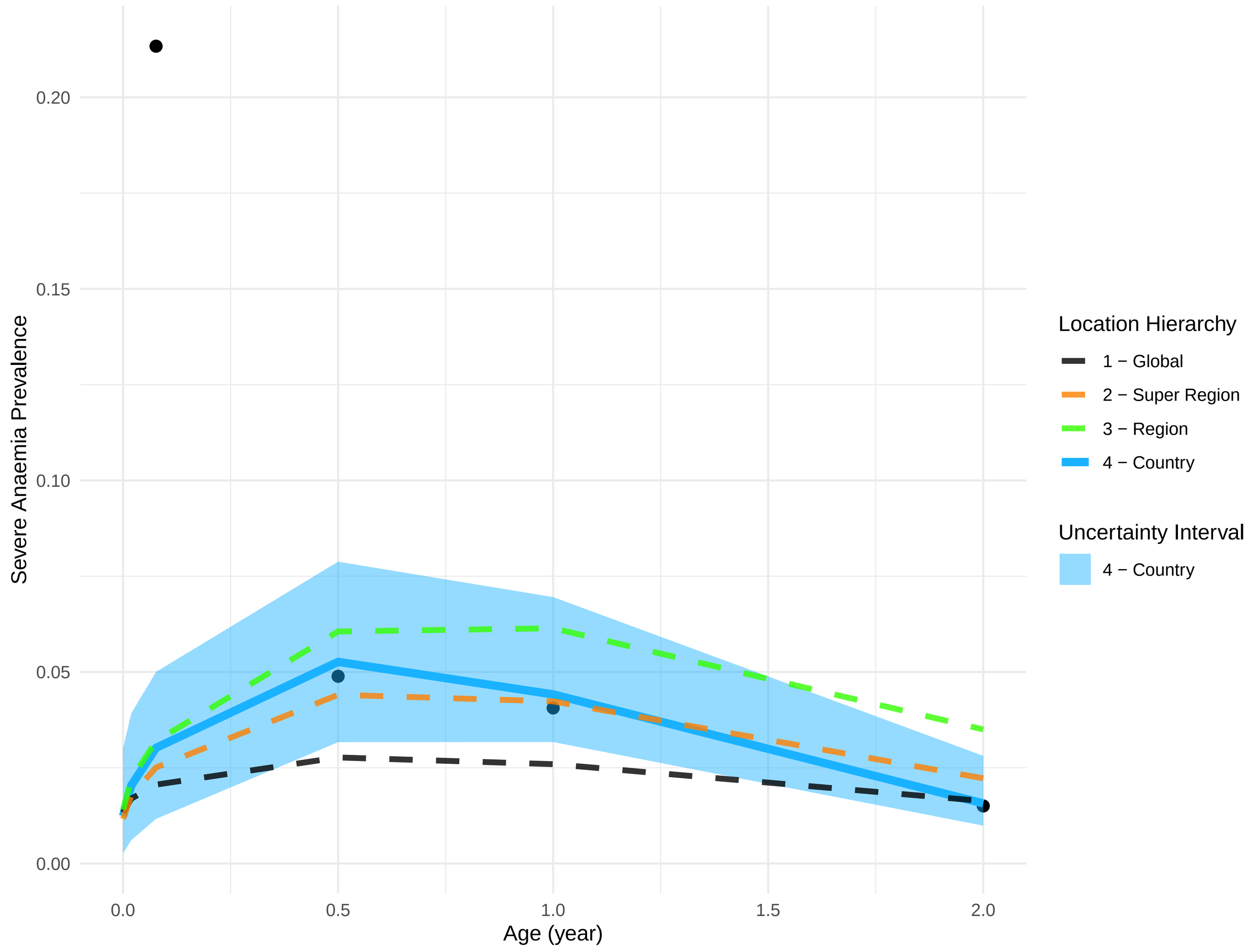

MR−BRT Age/Sex Fits − Non−pregnant Females <2 Years Old − Severe Anaemia Prevalence − Gambia
WHO−2001 Adjustment Method

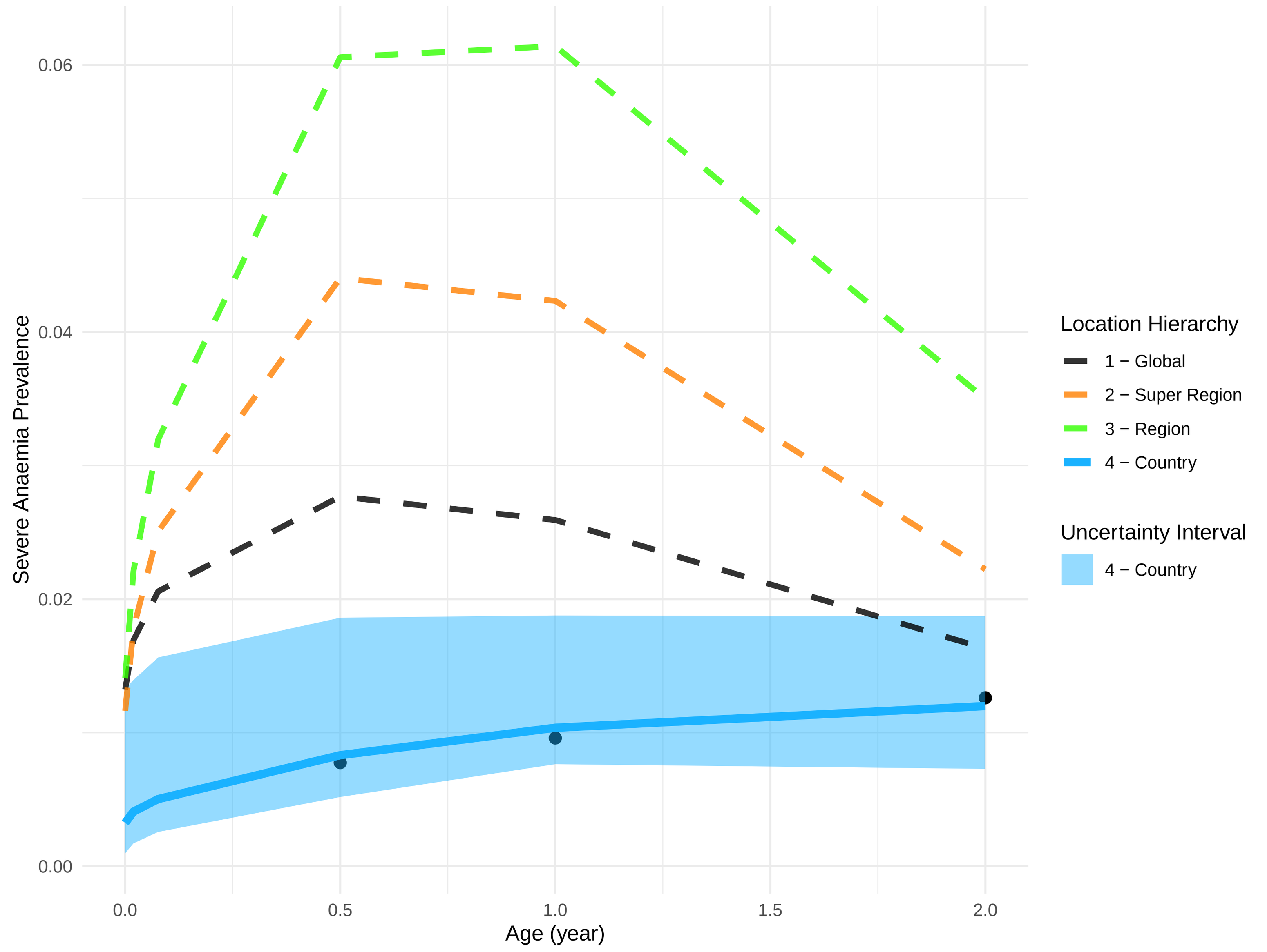

MR−BRT Age/Sex Fits − Non−pregnant Females <2 Years Old − Severe Anaemia Prevalence − Ghana
WHO−2001 Adjustment Method

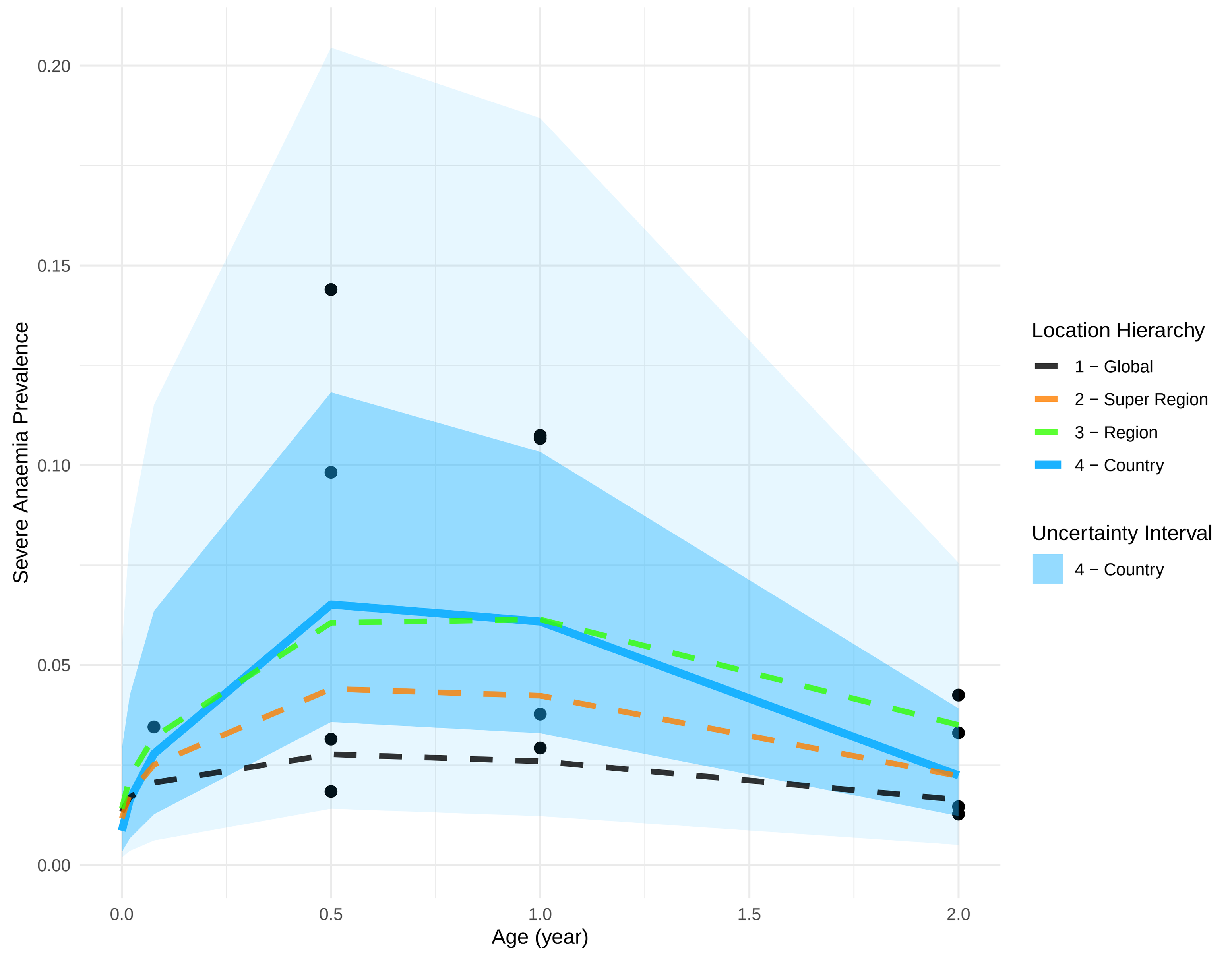

MR−BRT Age/Sex Fits − Non−pregnant Females <2 Years Old − Severe Anaemia Prevalence − Guinea
WHO−2001 Adjustment Method

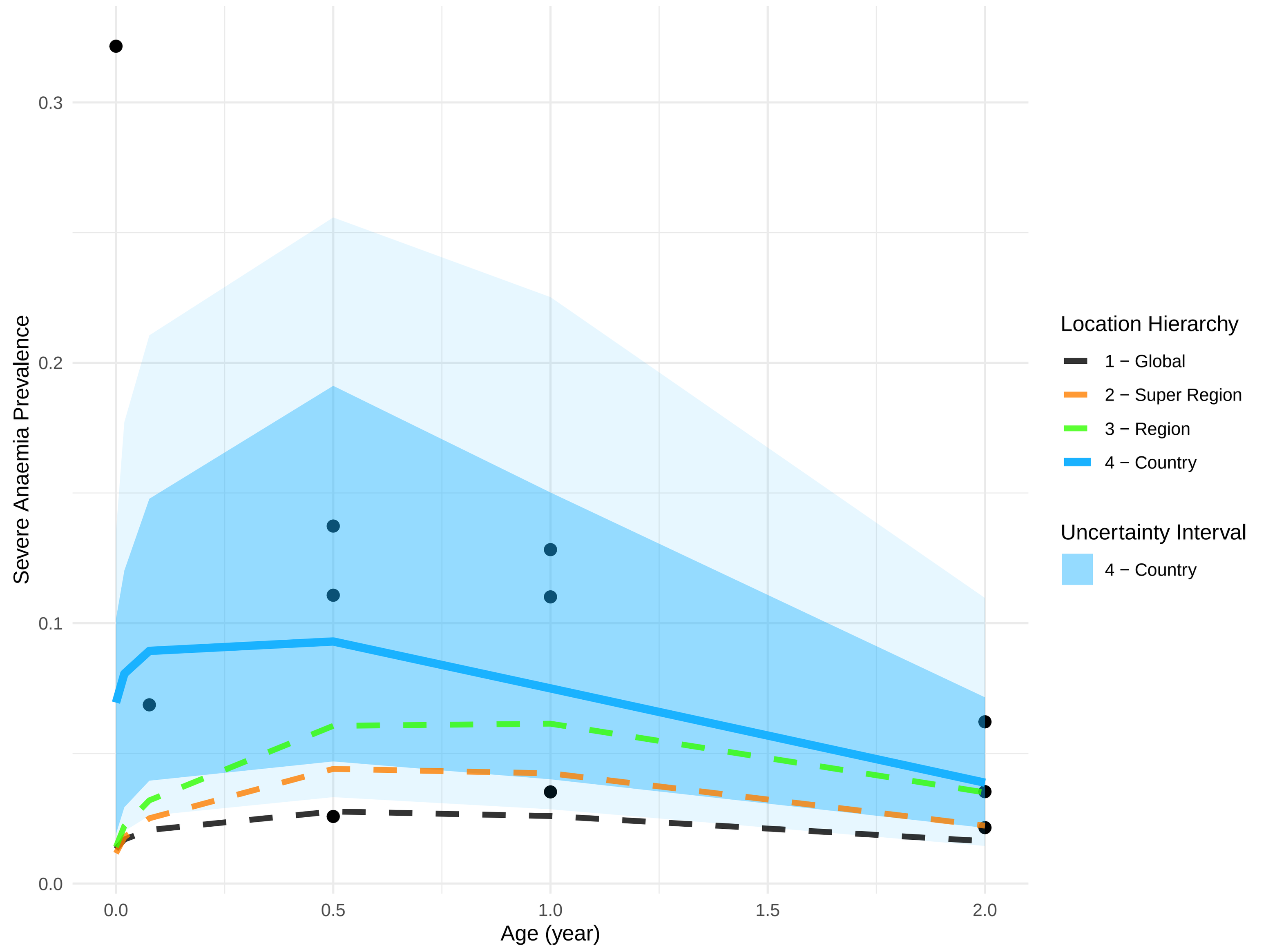

MR−BRT Age/Sex Fits − Non−pregnant Females <2 Years Old − Severe Anaemia Prevalence − Liberia
WHO−2001 Adjustment Method

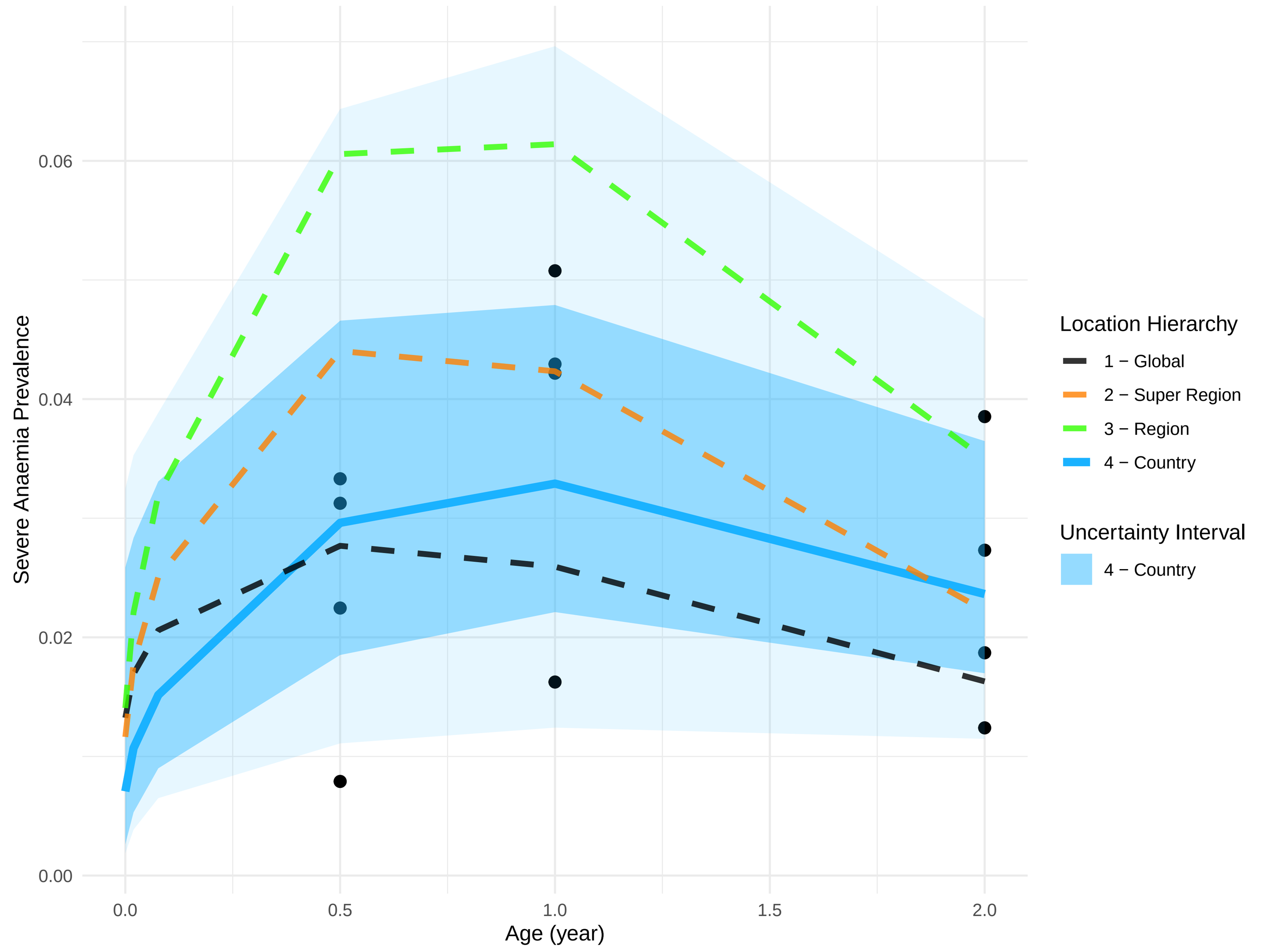

MR−BRT Age/Sex Fits − Non−pregnant Females <2 Years Old − Severe Anaemia Prevalence − Mali
WHO−2001 Adjustment Method

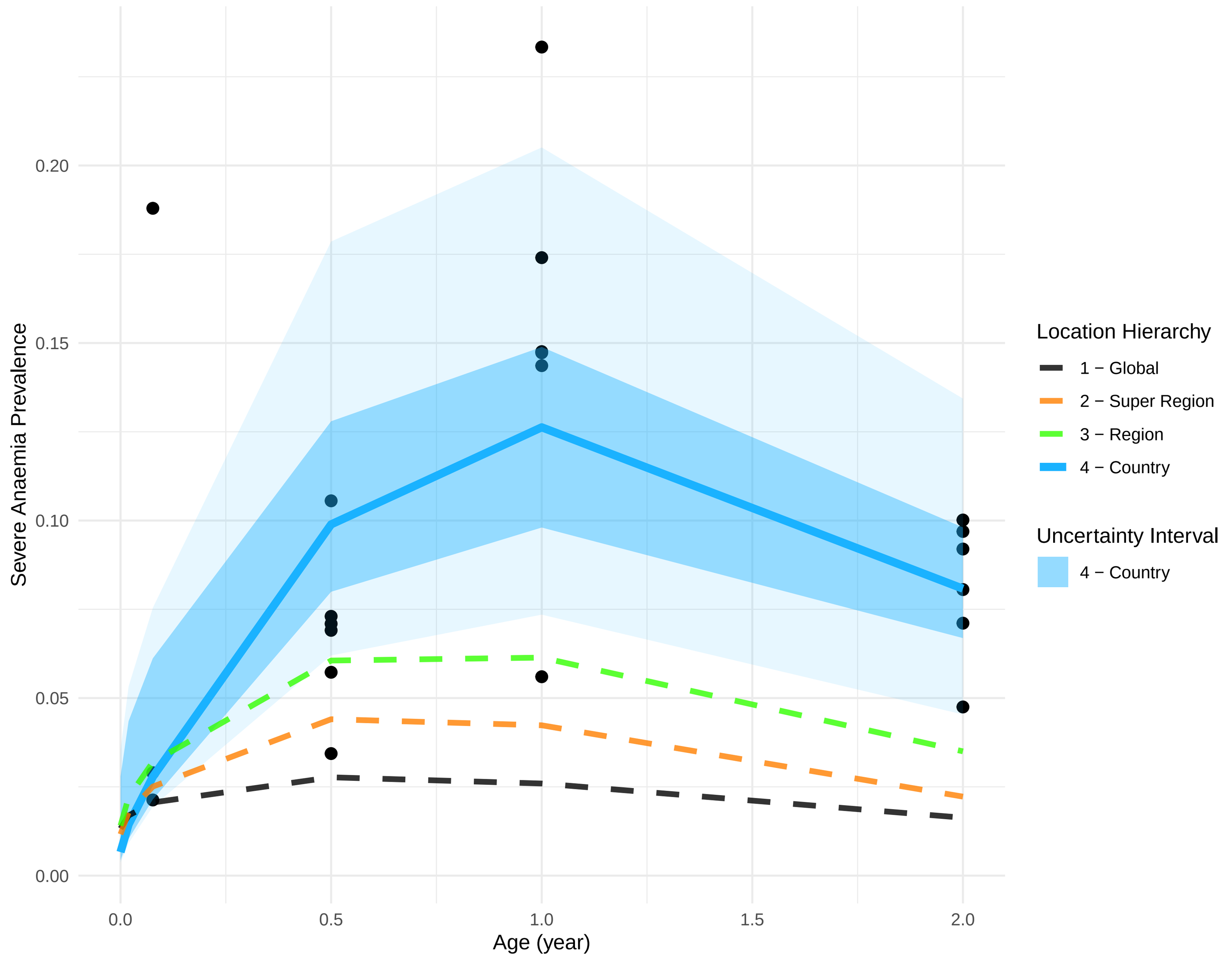

MR−BRT Age/Sex Fits − Non−pregnant Females <2 Years Old − Severe Anaemia Prevalence − Niger
WHO−2001 Adjustment Method

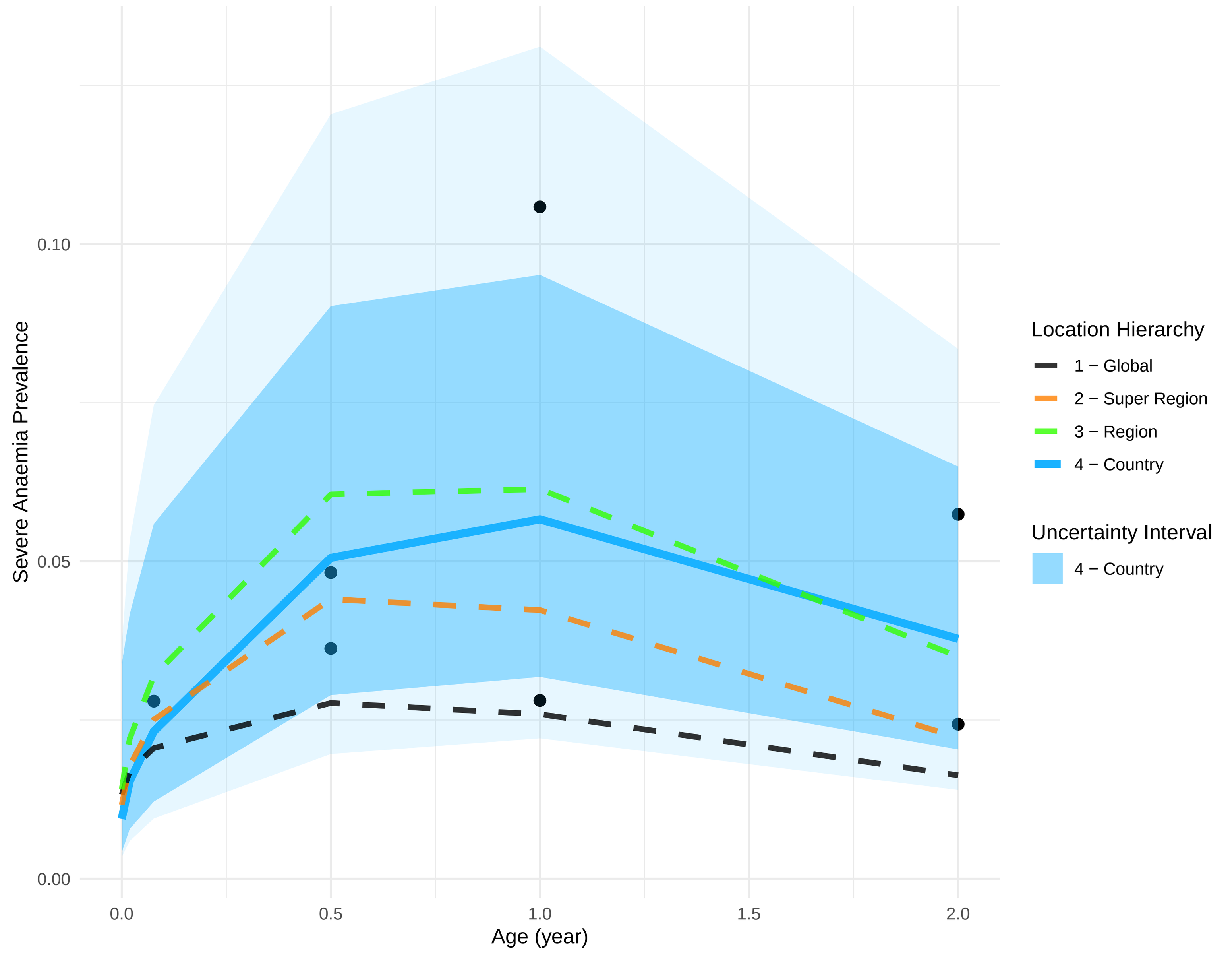

MR−BRT Age/Sex Fits − Non−pregnant Females <2 Years Old − Severe Anaemia Prevalence − Nigeria
WHO−2001 Adjustment Method

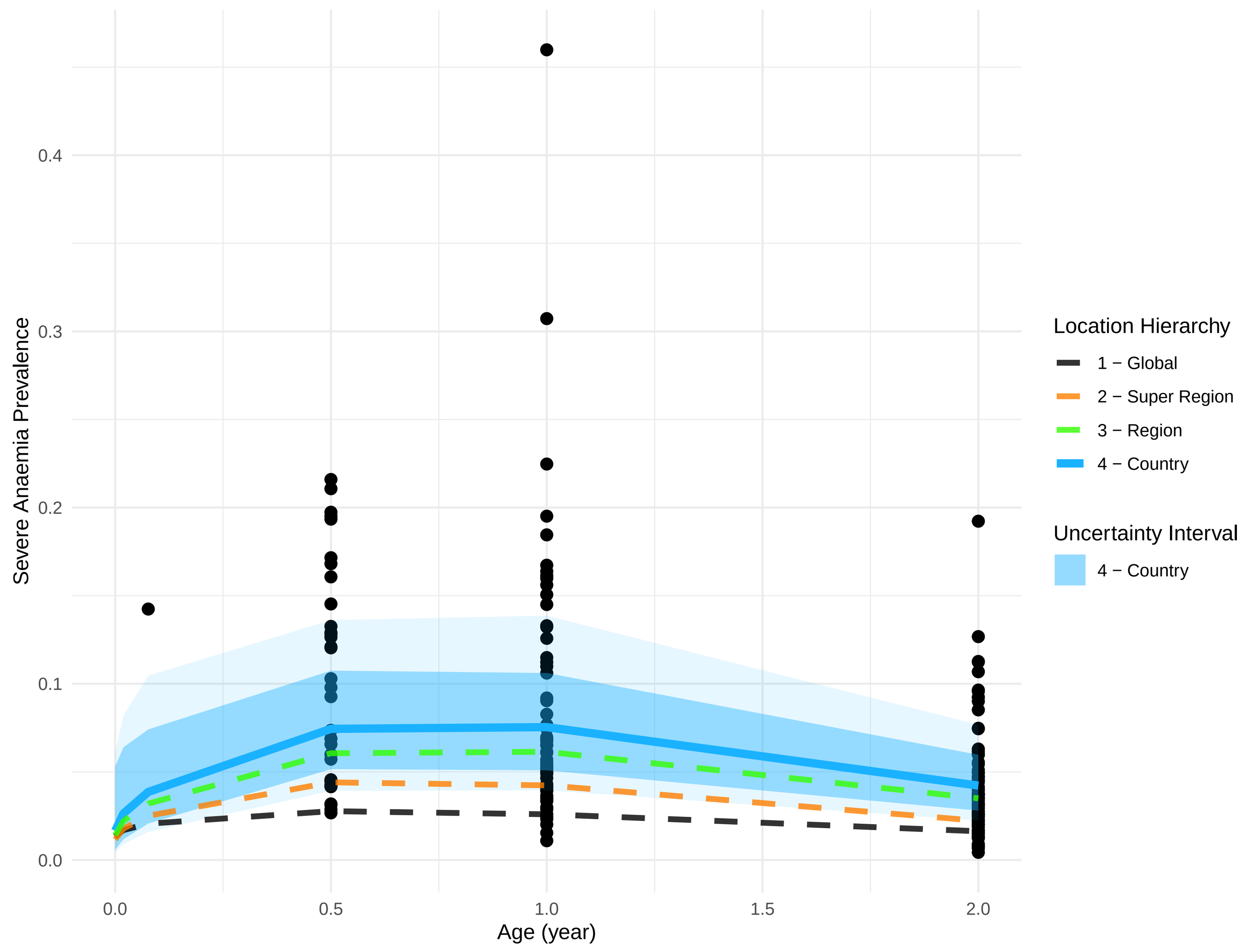

MR−BRT Age/Sex Fits − Non−pregnant Females <2 Years Old − Severe Anaemia Prevalence − Abia
WHO−2001 Adjustment Method

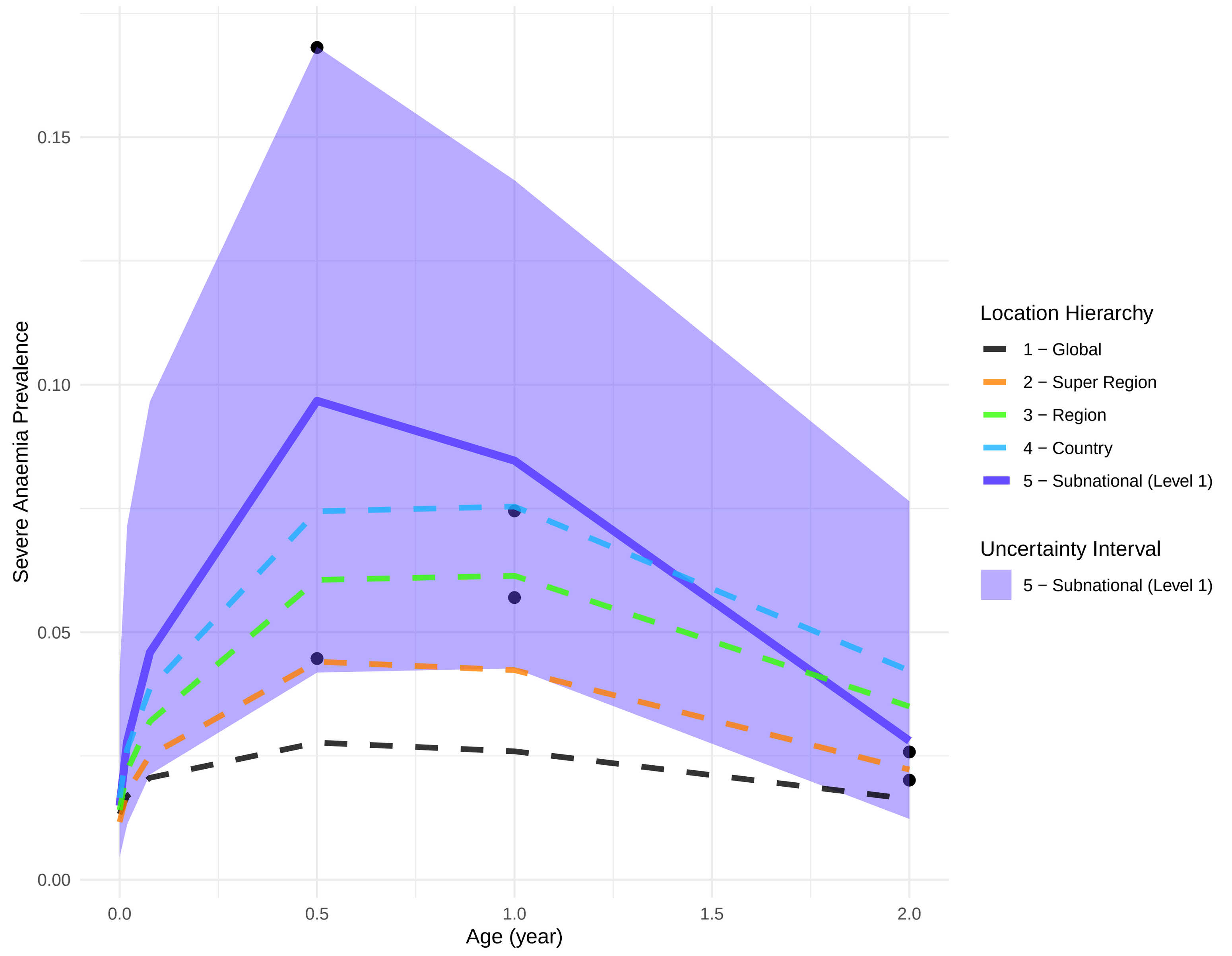

MR−BRT Age/Sex Fits − Non−pregnant Females <2 Years Old − Severe Anaemia Prevalence − Adamawa
WHO−2001 Adjustment Method

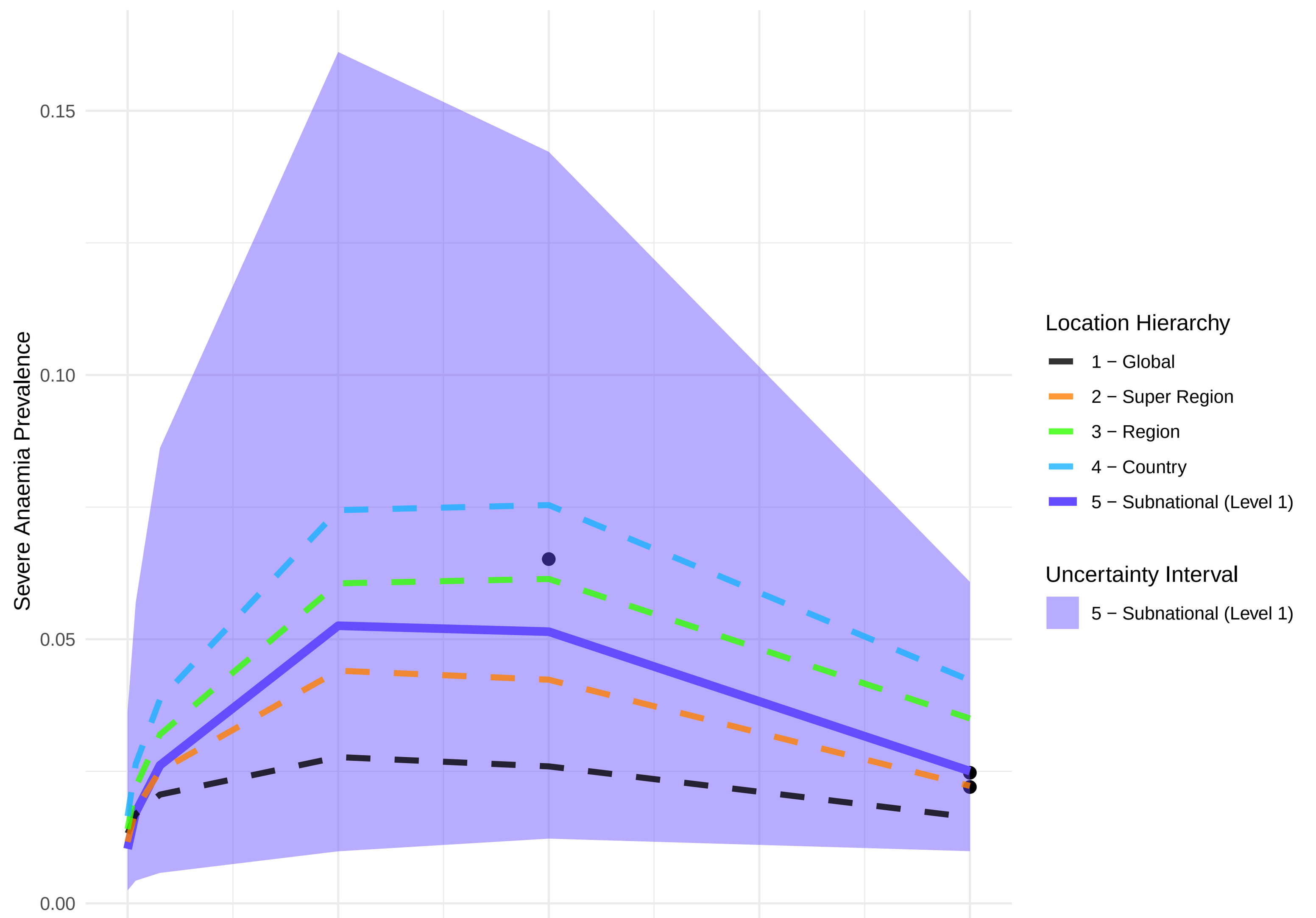

MR−BRT Age/Sex Fits − Non−pregnant Females <2 Years Old − Severe Anaemia Prevalence − Akwa Ibom
WHO−2001 Adjustment Method

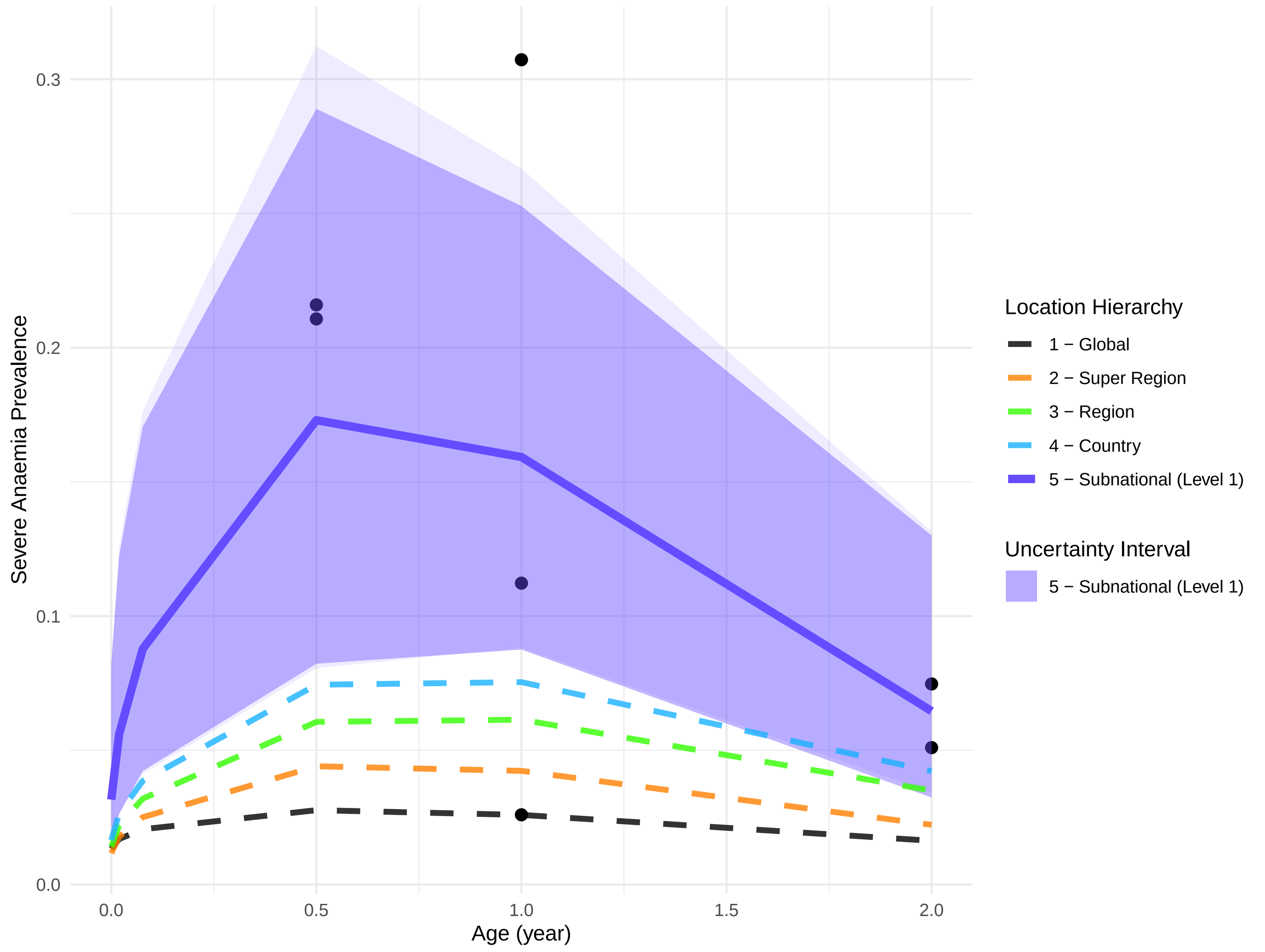

MR−BRT Age/Sex Fits − Non−pregnant Females <2 Years Old − Severe Anaemia Prevalence − Anambra
WHO−2001 Adjustment Method

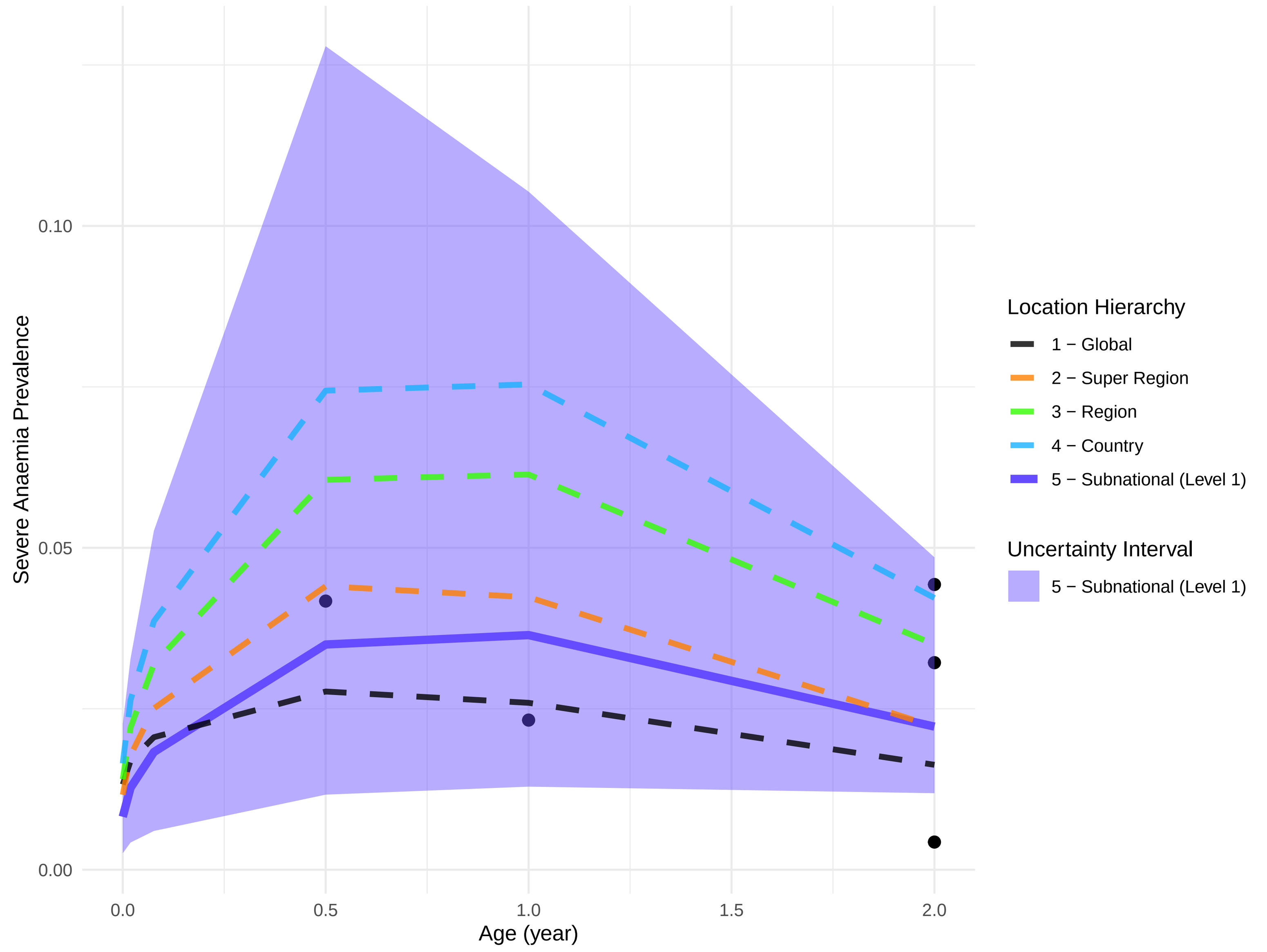

MR−BRT Age/Sex Fits − Non−pregnant Females <2 Years Old − Severe Anaemia Prevalence − Bauchi
WHO−2001 Adjustment Method

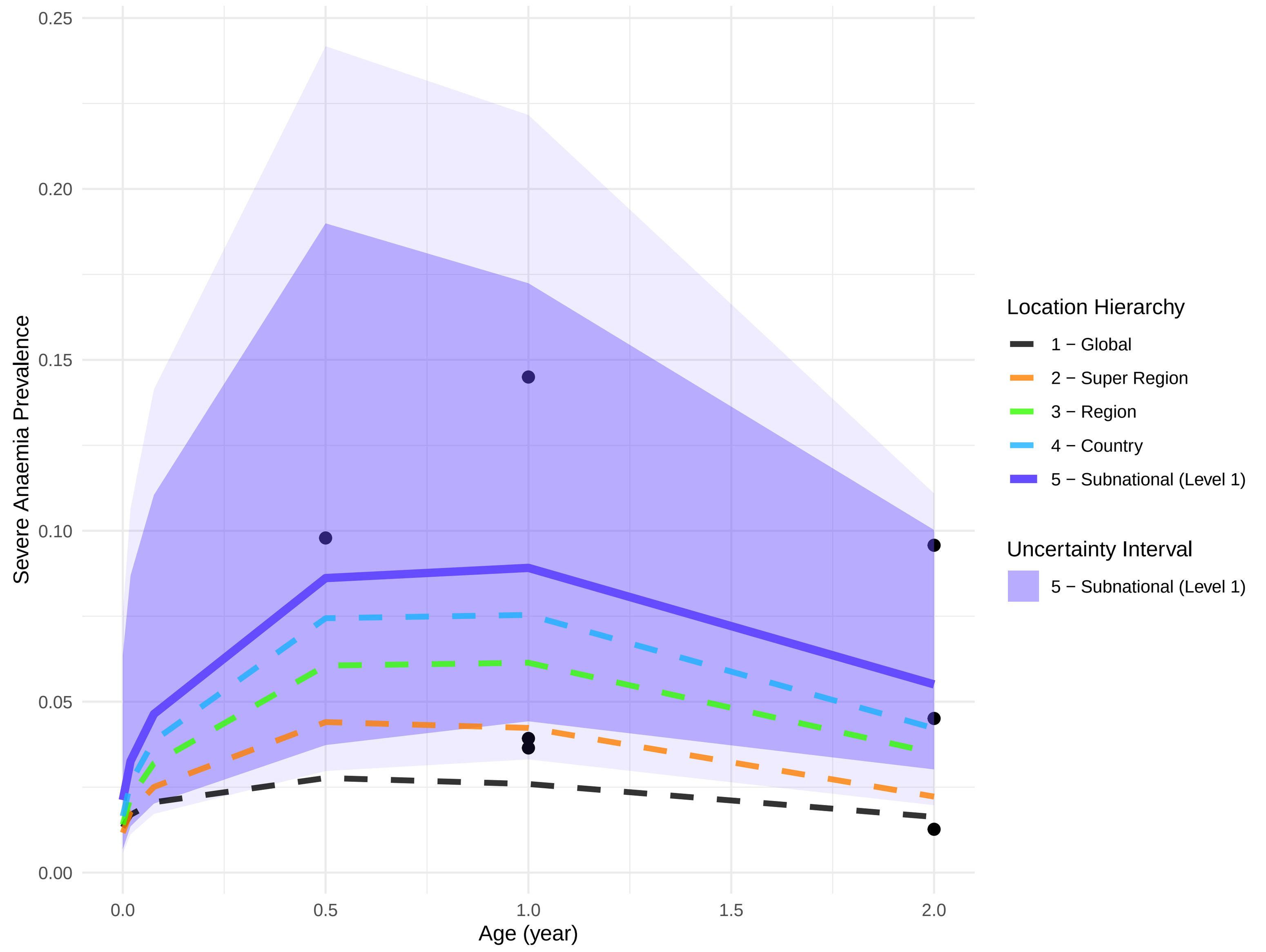

MR−BRT Age/Sex Fits − Non−pregnant Females <2 Years Old − Severe Anaemia Prevalence − Bayelsa
WHO−2001 Adjustment Method

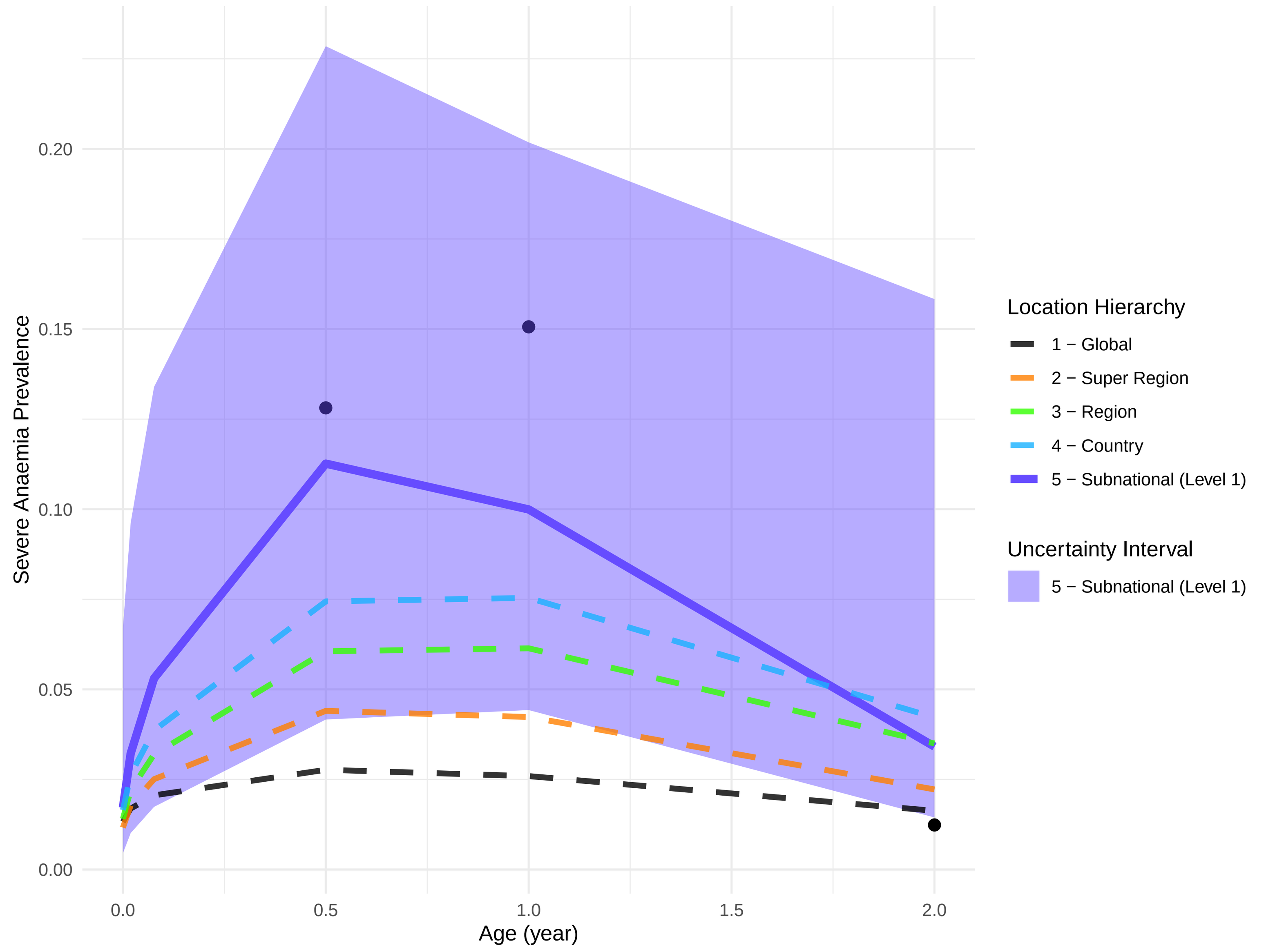

MR−BRT Age/Sex Fits − Non−pregnant Females <2 Years Old − Severe Anaemia Prevalence − Benue
WHO−2001 Adjustment Method

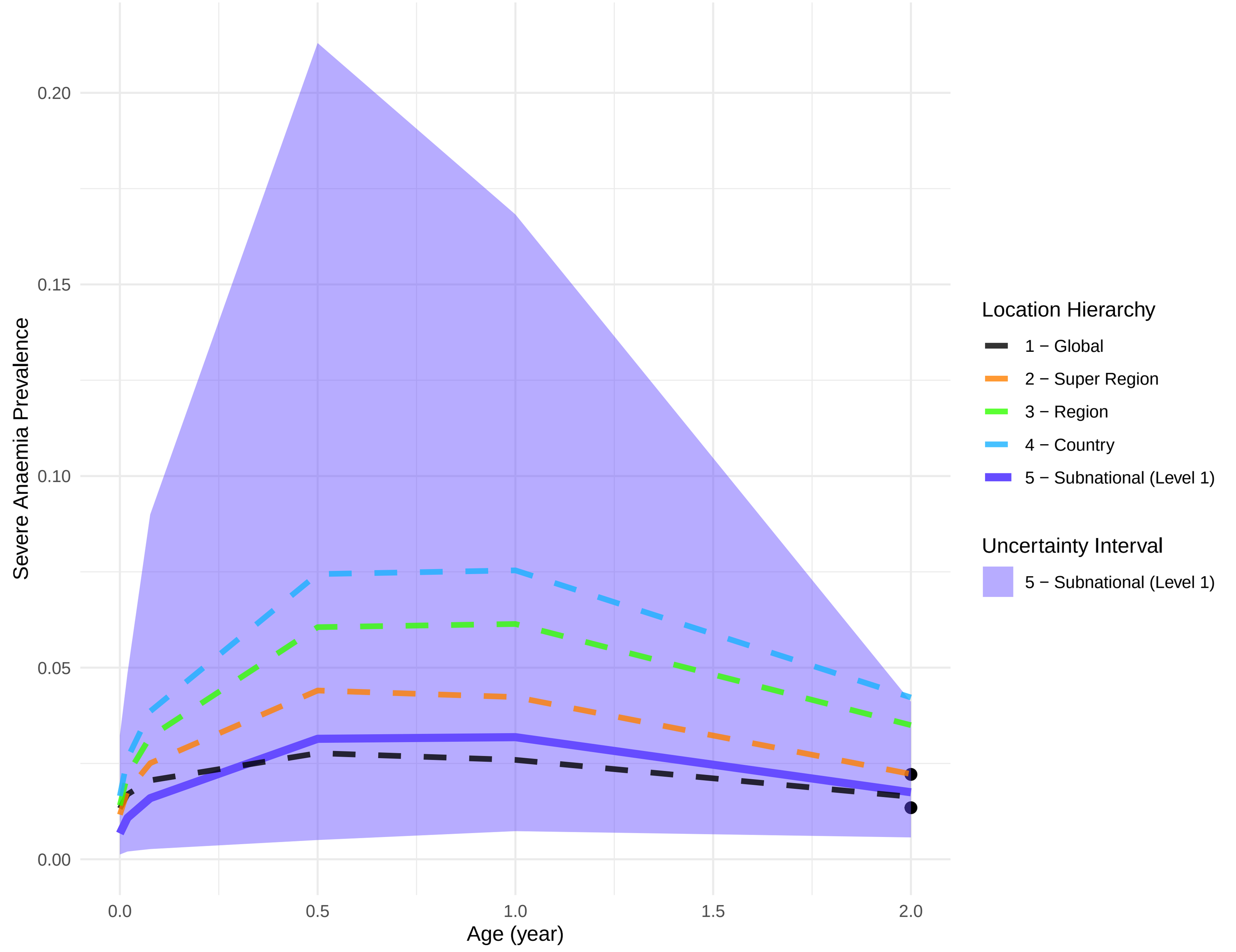

MR−BRT Age/Sex Fits − Non−pregnant Females <2 Years Old − Severe Anaemia Prevalence − Borno
WHO−2001 Adjustment Method

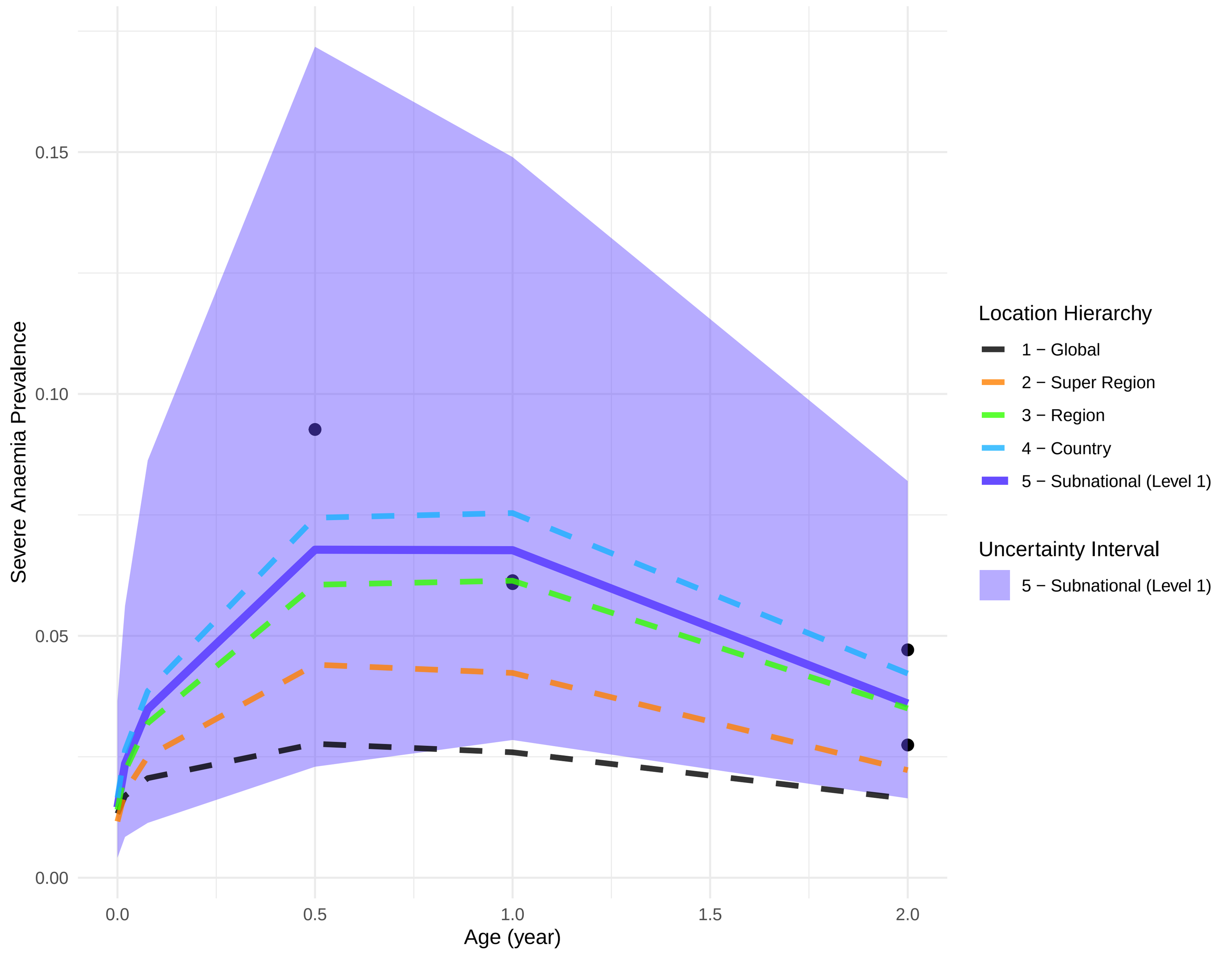

MR−BRT Age/Sex Fits − Non−pregnant Females <2 Years Old − Severe Anaemia Prevalence − Cross River
WHO−2001 Adjustment Method

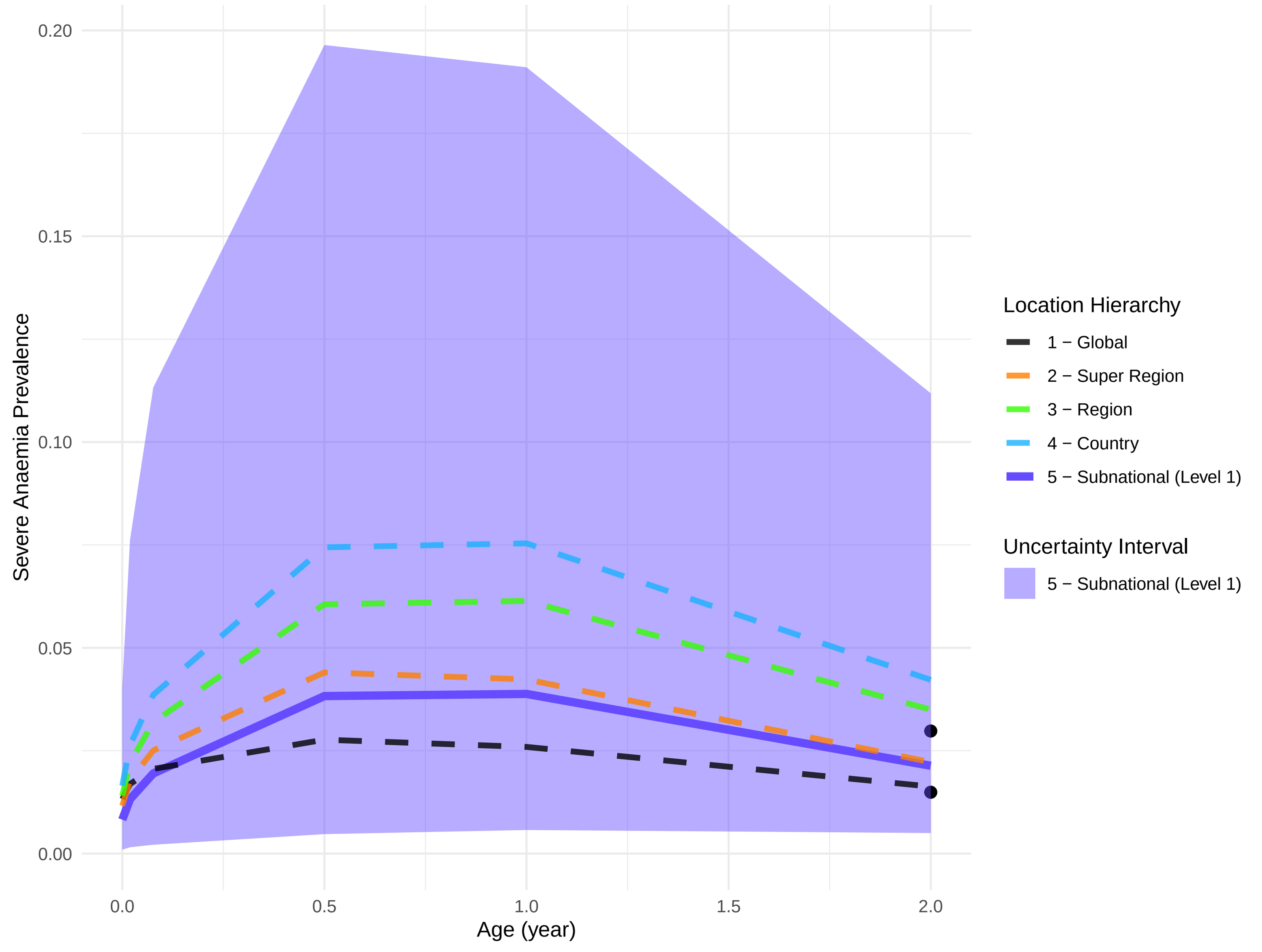

MR−BRT Age/Sex Fits − Non−pregnant Females <2 Years Old − Severe Anaemia Prevalence − Delta
WHO−2001 Adjustment Method

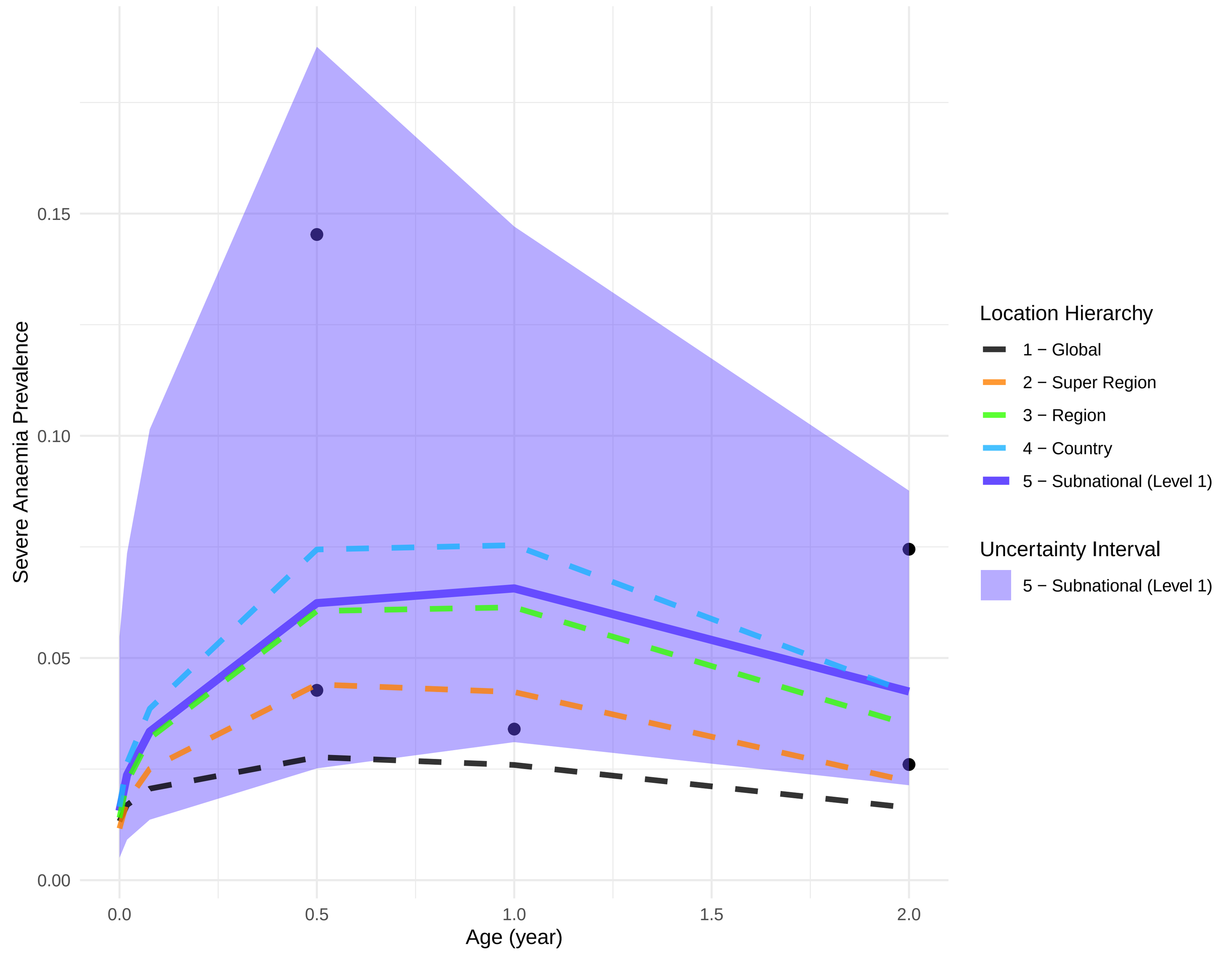

MR−BRT Age/Sex Fits − Non−pregnant Females <2 Years Old − Severe Anaemia Prevalence − Ebonyi
WHO−2001 Adjustment Method

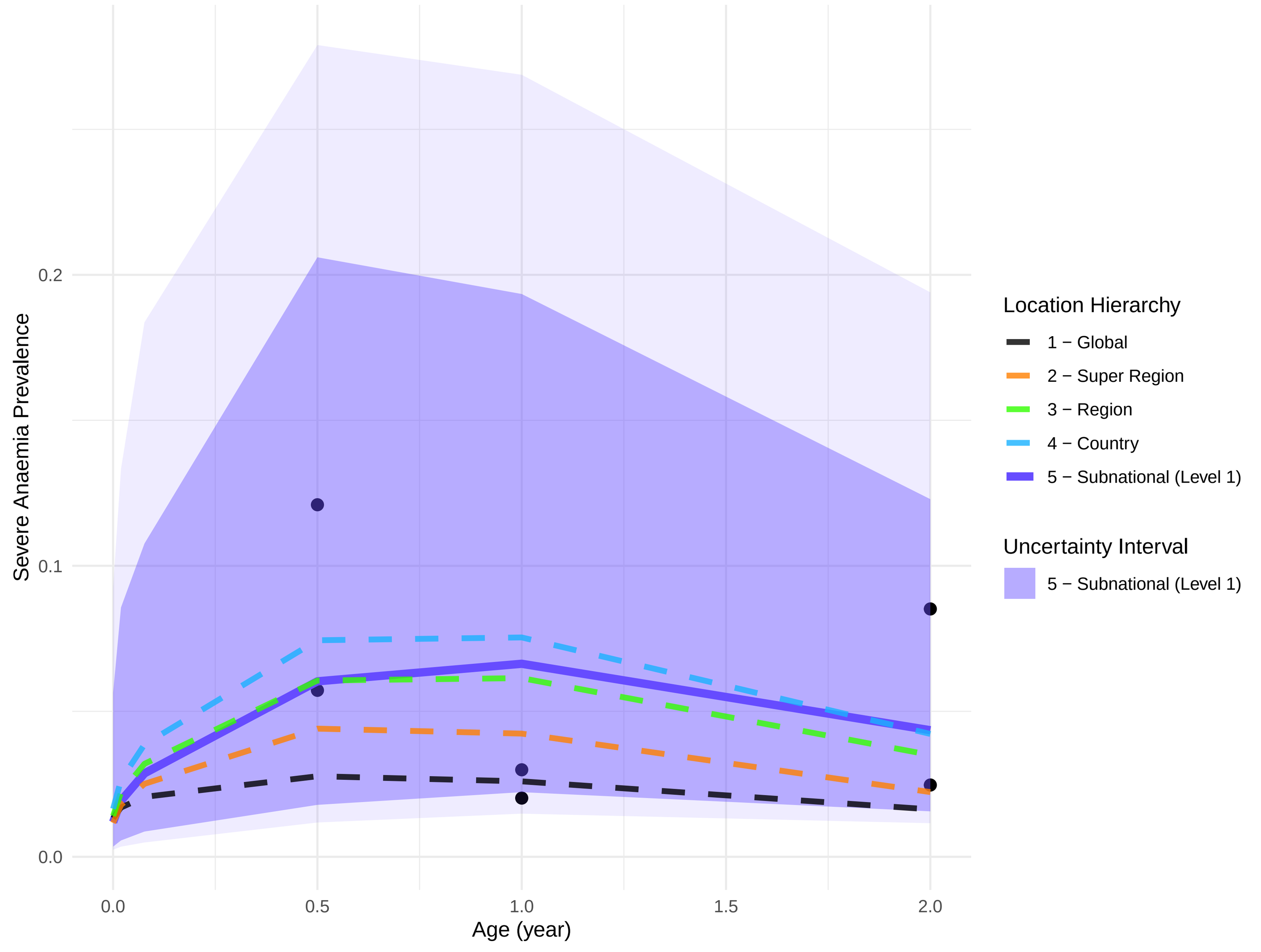

MR−BRT Age/Sex Fits − Non−pregnant Females <2 Years Old − Severe Anaemia Prevalence − Edo
WHO−2001 Adjustment Method

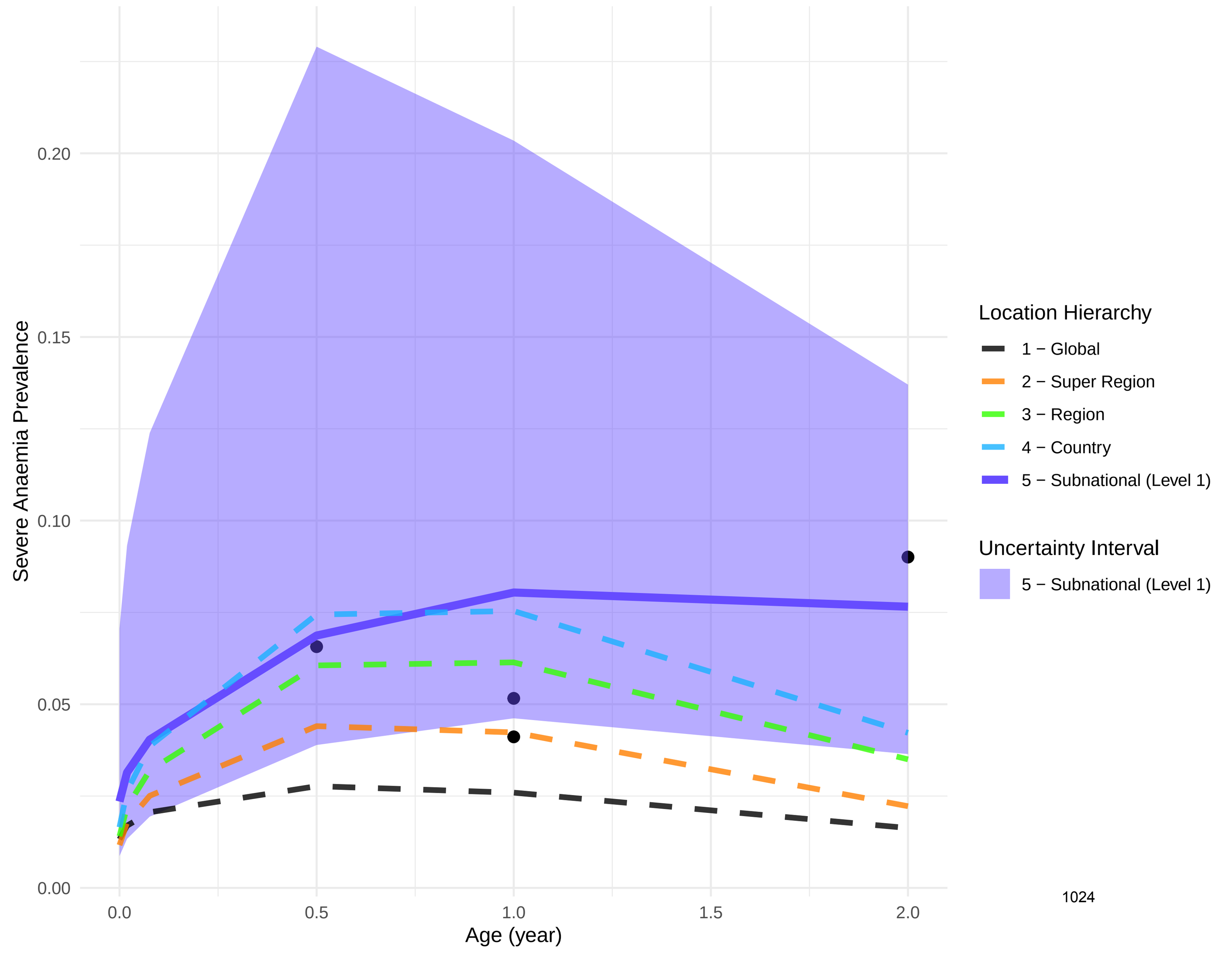

MR−BRT Age/Sex Fits − Non−pregnant Females <2 Years Old − Severe Anaemia Prevalence − Ekiti
WHO−2001 Adjustment Method

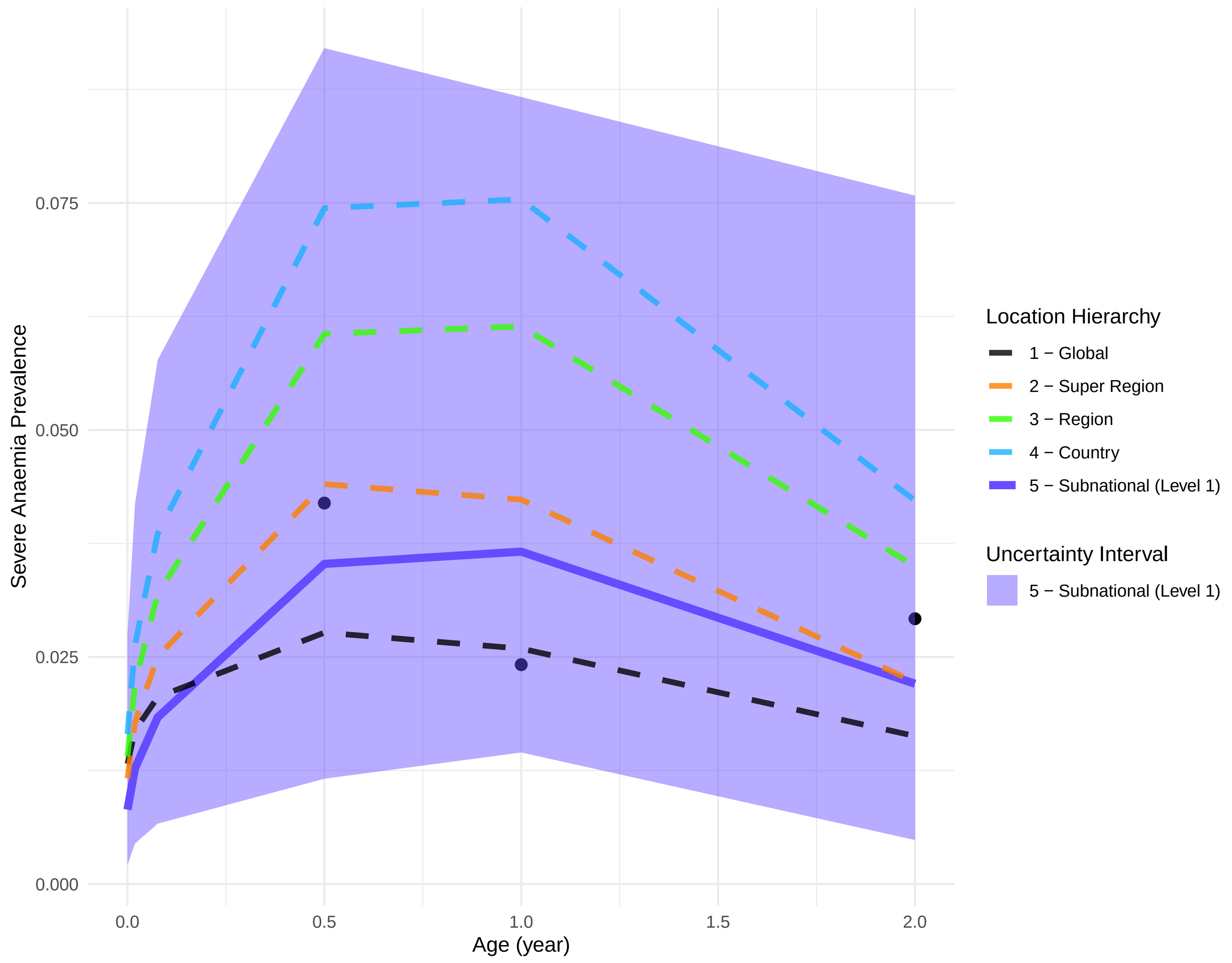

MR−BRT Age/Sex Fits − Non−pregnant Females <2 Years Old − Severe Anaemia Prevalence − Enugu
WHO−2001 Adjustment Method

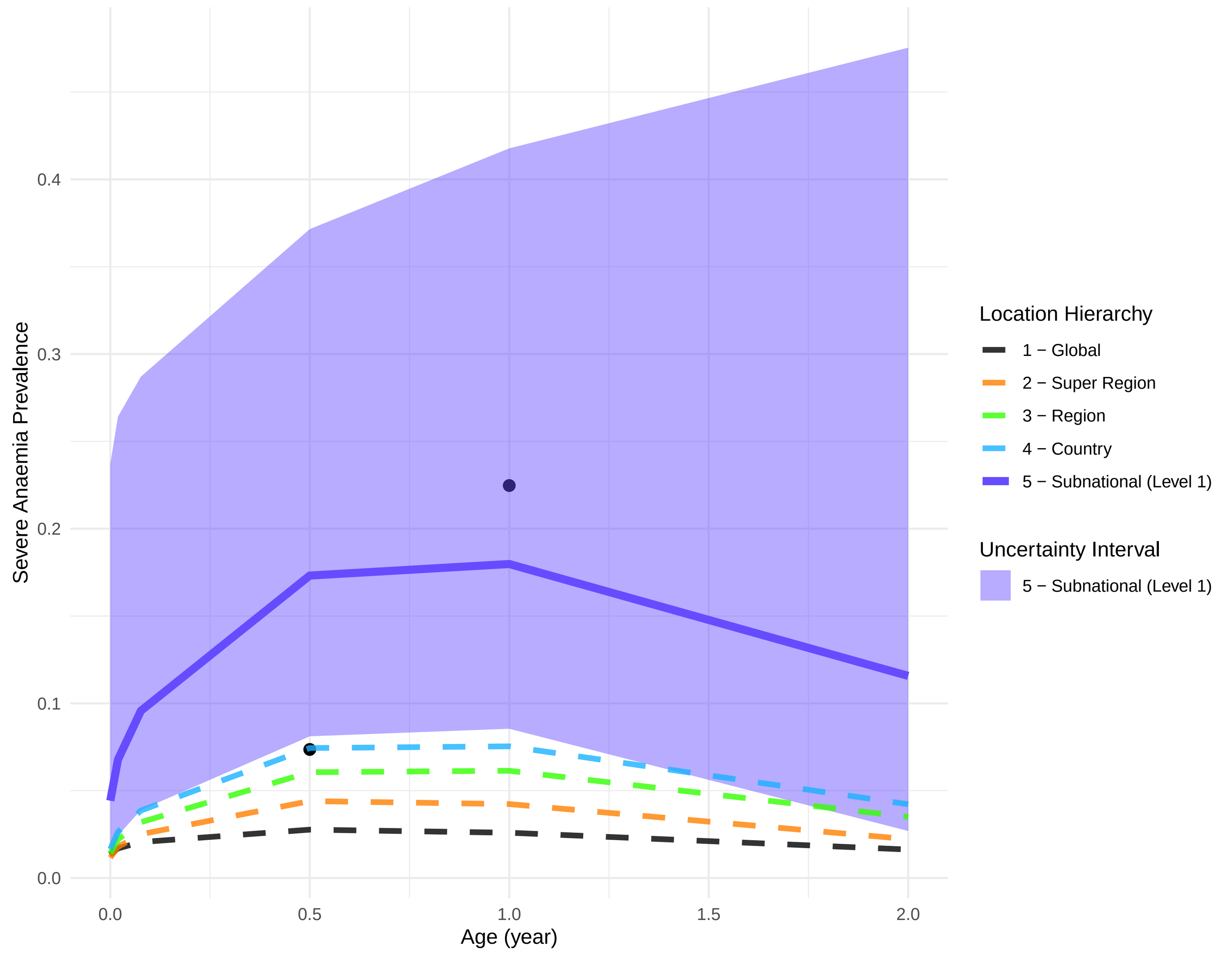

MR−BRT Age/Sex Fits − Non−pregnant Females <2 Years Old − Severe Anaemia Prevalence − FCT (Abuja)
WHO−2001 Adjustment Method

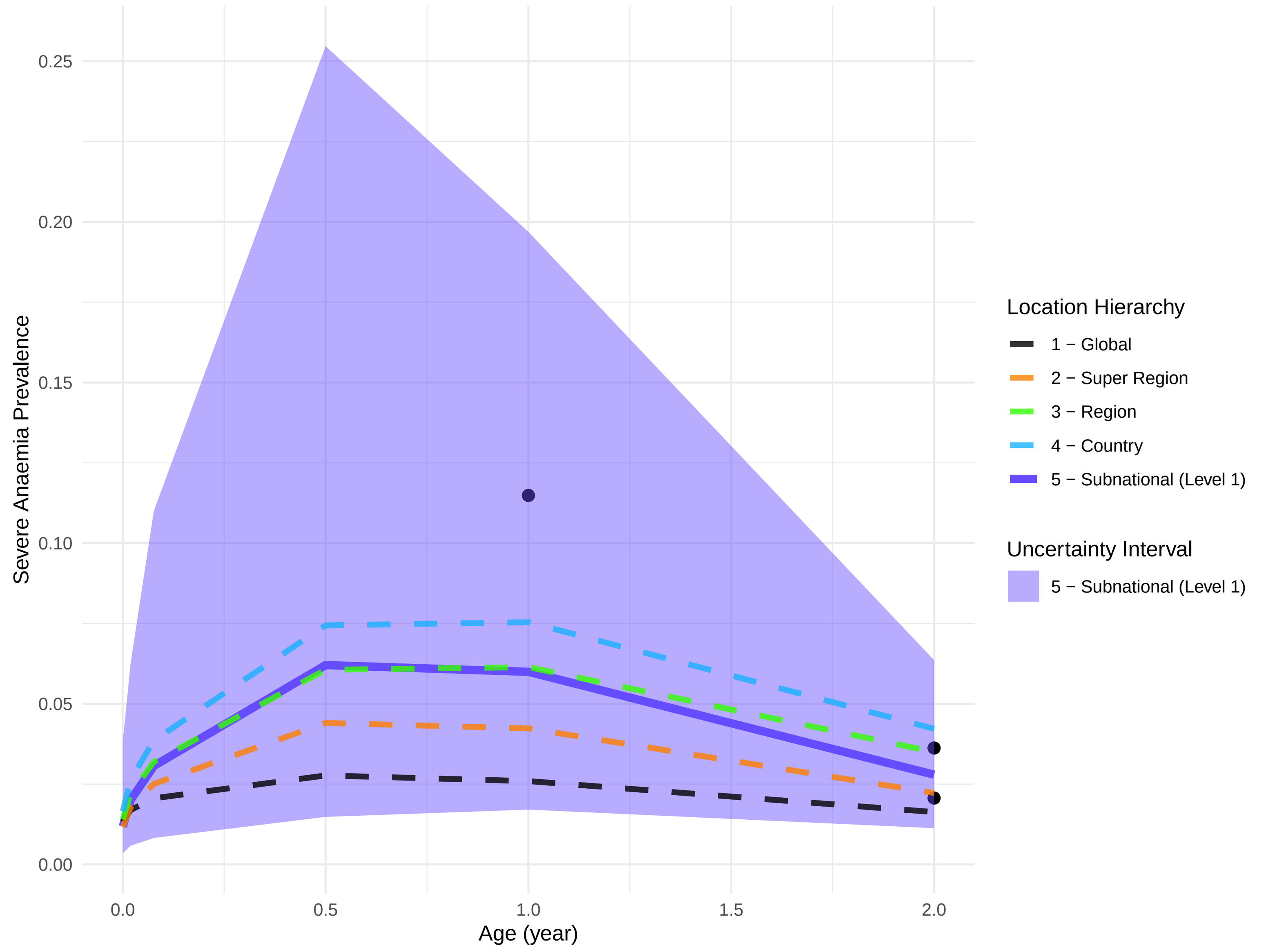

MR−BRT Age/Sex Fits − Non−pregnant Females <2 Years Old − Severe Anaemia Prevalence − Gombe
WHO−2001 Adjustment Method

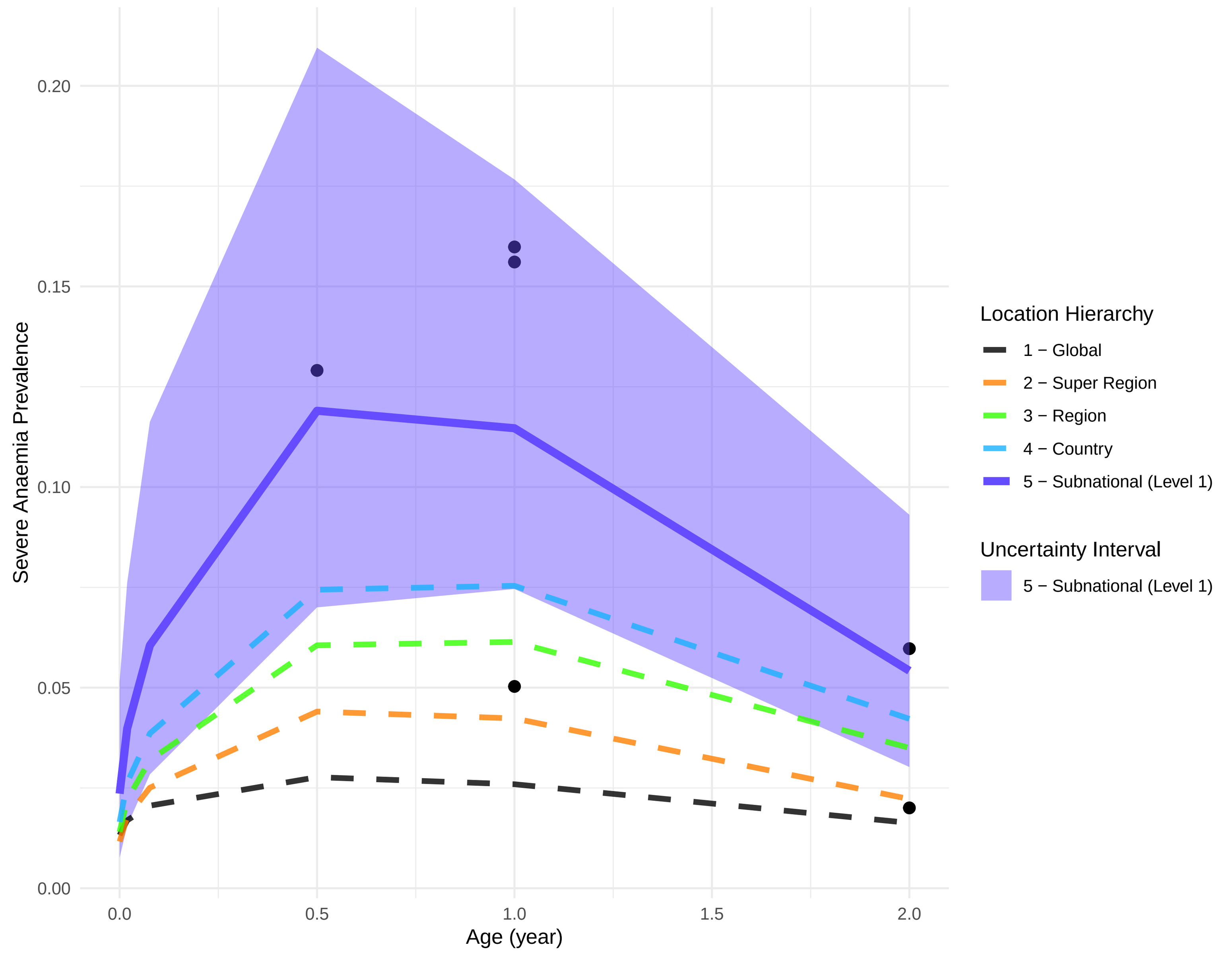

MR−BRT Age/Sex Fits − Non−pregnant Females <2 Years Old − Severe Anaemia Prevalence − Imo
WHO−2001 Adjustment Method

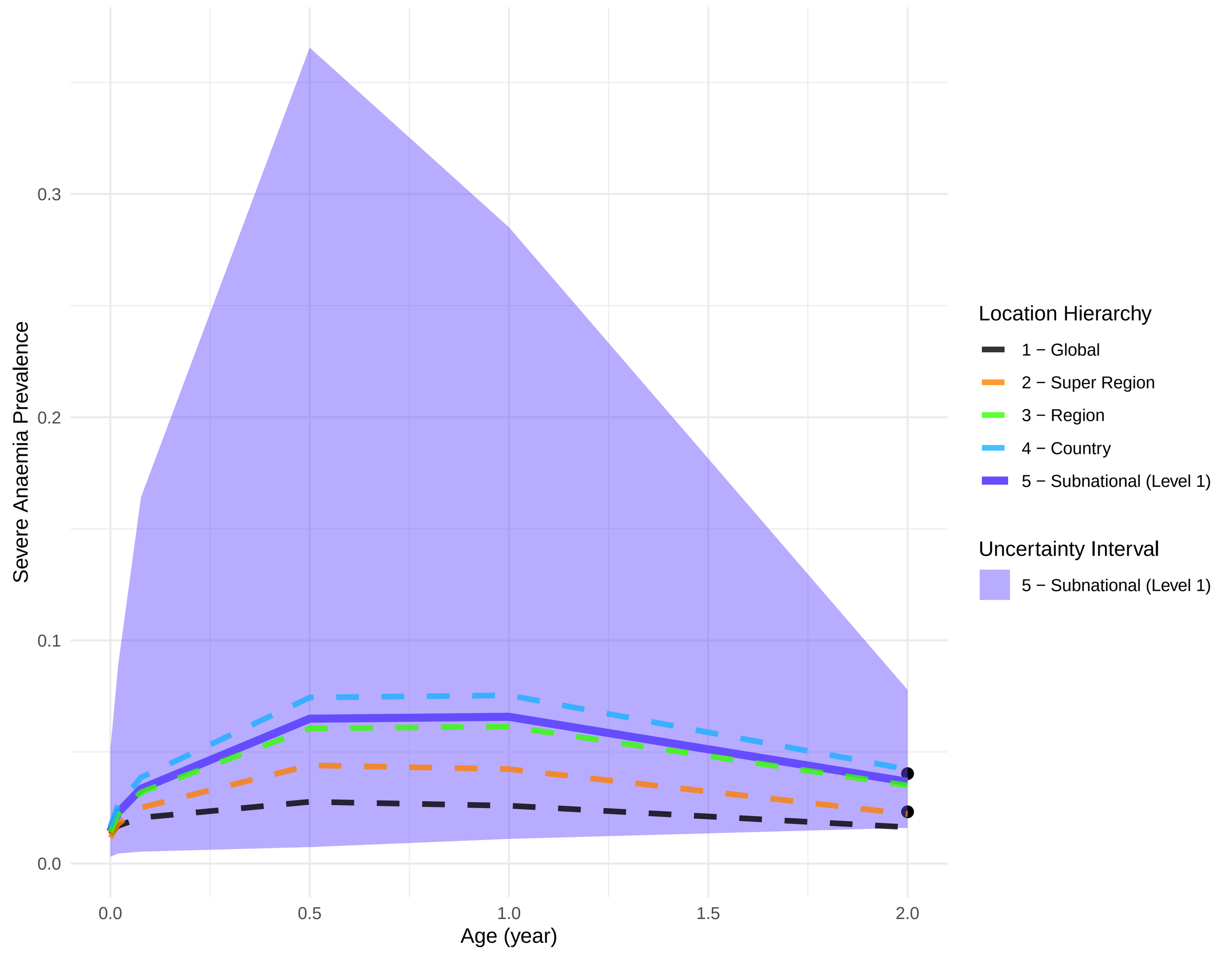

MR−BRT Age/Sex Fits − Non−pregnant Females <2 Years Old − Severe Anaemia Prevalence − Jigawa
WHO−2001 Adjustment Method

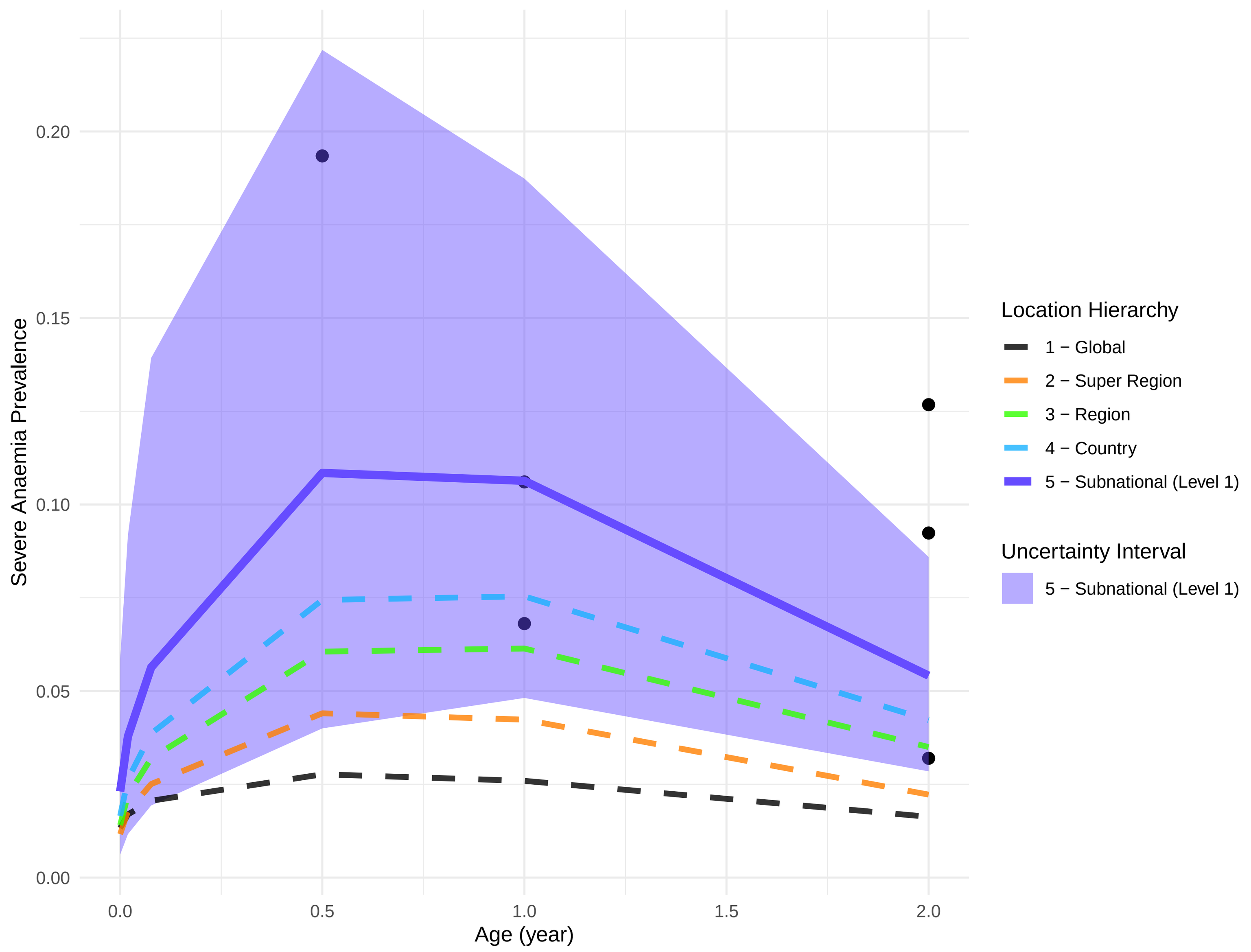

MR−BRT Age/Sex Fits − Non−pregnant Females <2 Years Old − Severe Anaemia Prevalence − Kaduna
WHO−2001 Adjustment Method

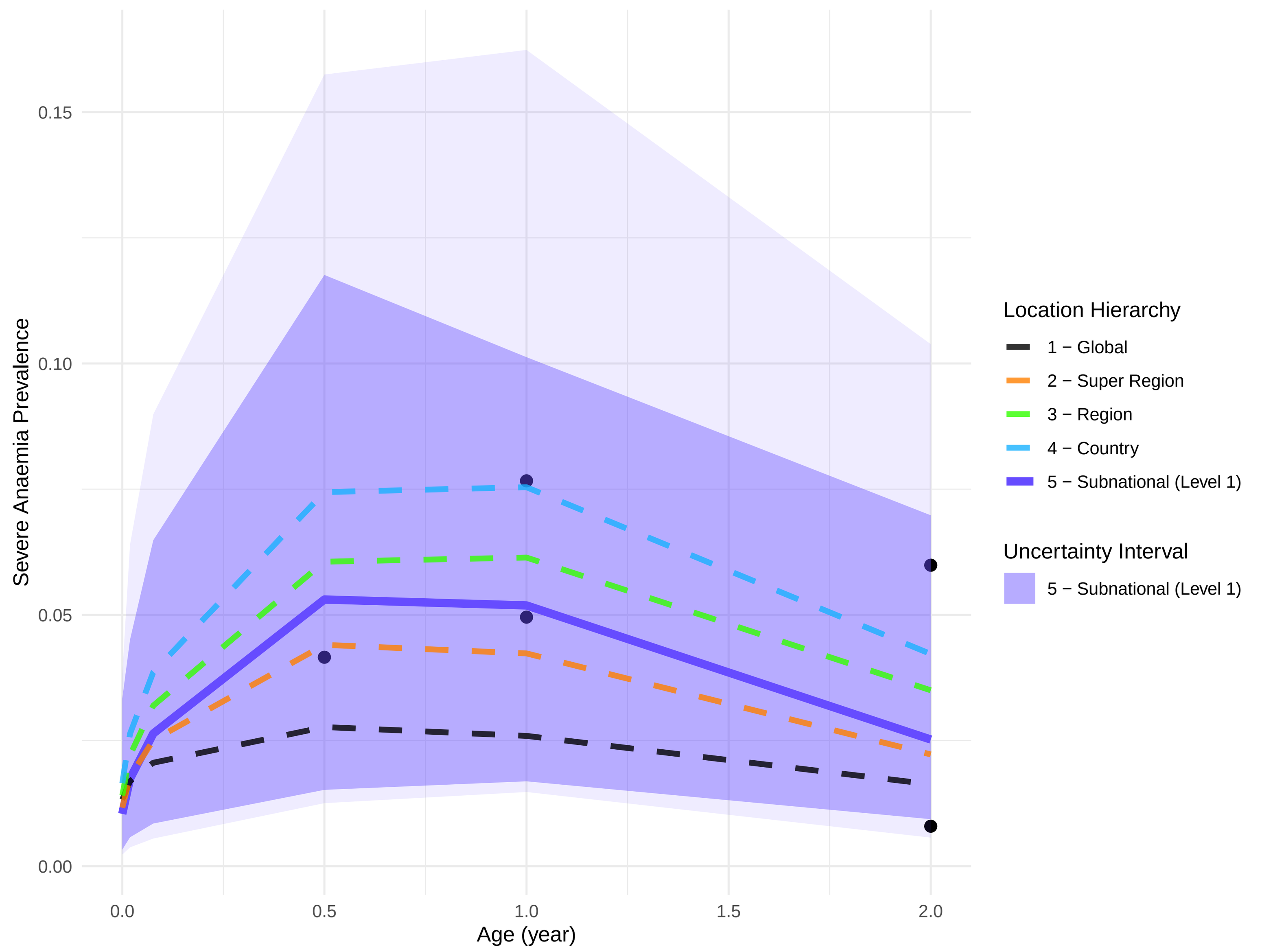

MR−BRT Age/Sex Fits − Non−pregnant Females <2 Years Old − Severe Anaemia Prevalence − Kano
WHO−2001 Adjustment Method

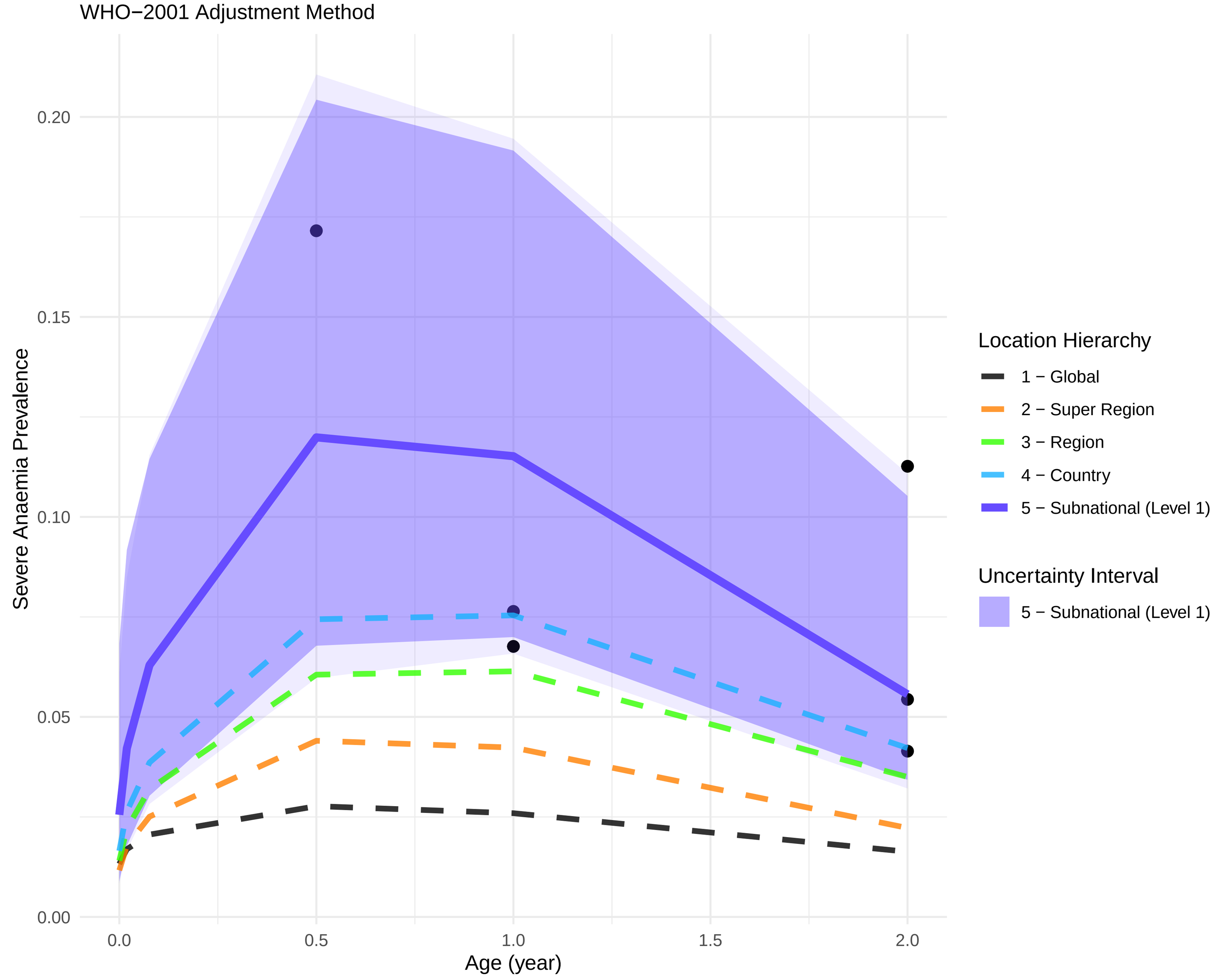

MR−BRT Age/Sex Fits − Non−pregnant Females <2 Years Old − Severe Anaemia Prevalence − Katsina
WHO−2001 Adjustment Method

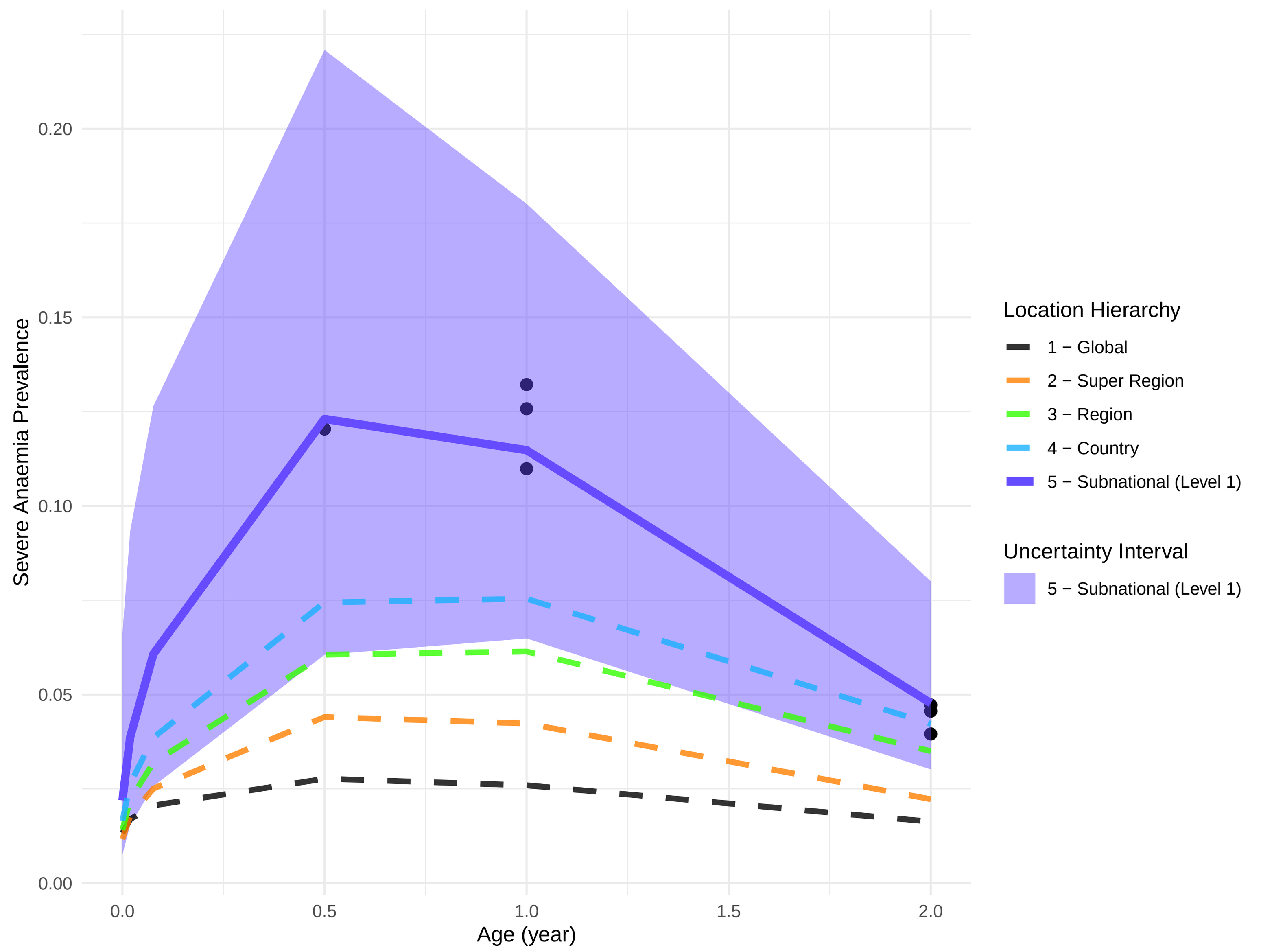

MR−BRT Age/Sex Fits − Non−pregnant Females <2 Years Old − Severe Anaemia Prevalence − Kebbi
WHO−2001 Adjustment Method

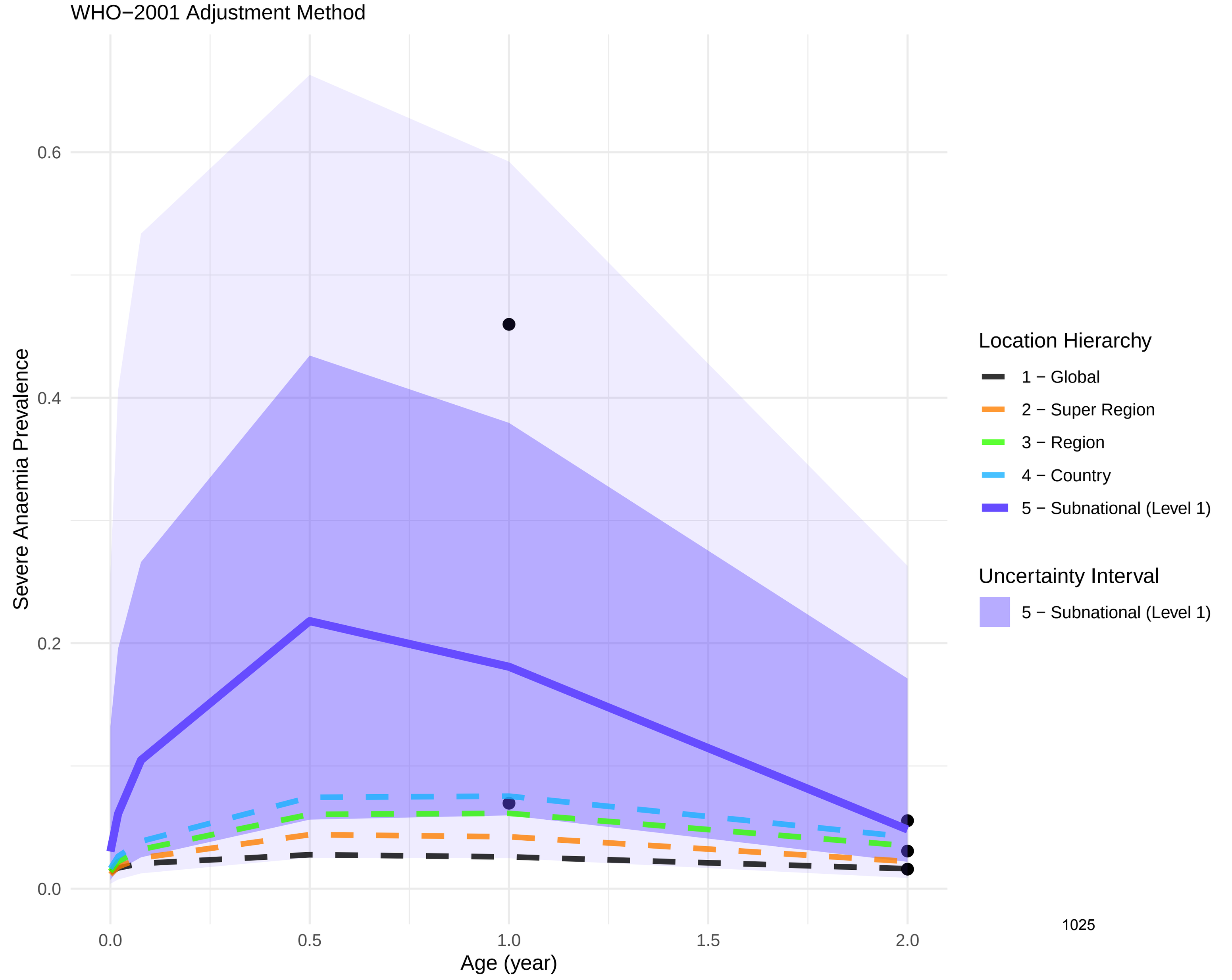

MR−BRT Age/Sex Fits − Non−pregnant Females <2 Years Old − Severe Anaemia Prevalence − Kogi
WHO−2001 Adjustment Method

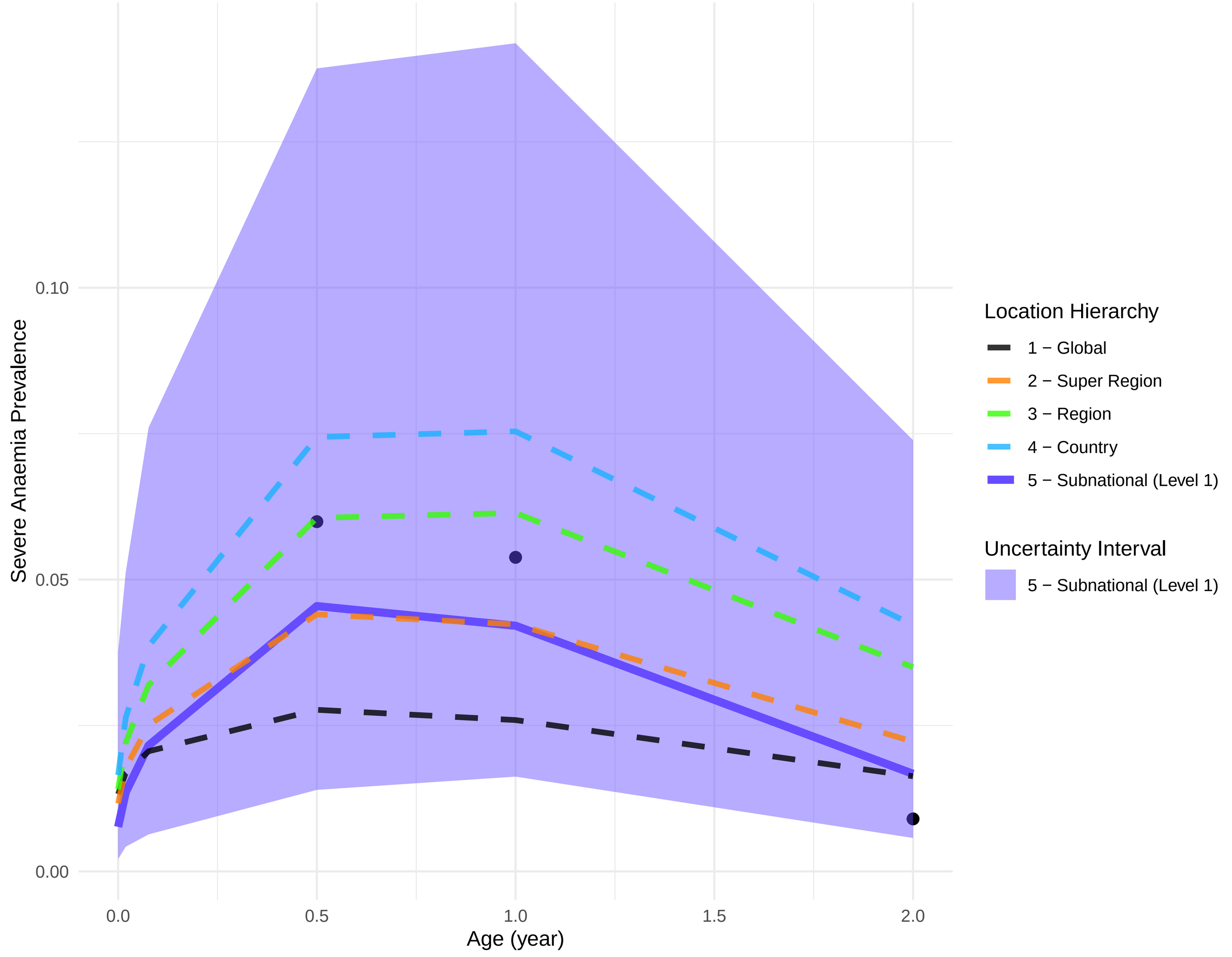

MR−BRT Age/Sex Fits − Non−pregnant Females <2 Years Old − Severe Anaemia Prevalence − Kwara
WHO−2001 Adjustment Method

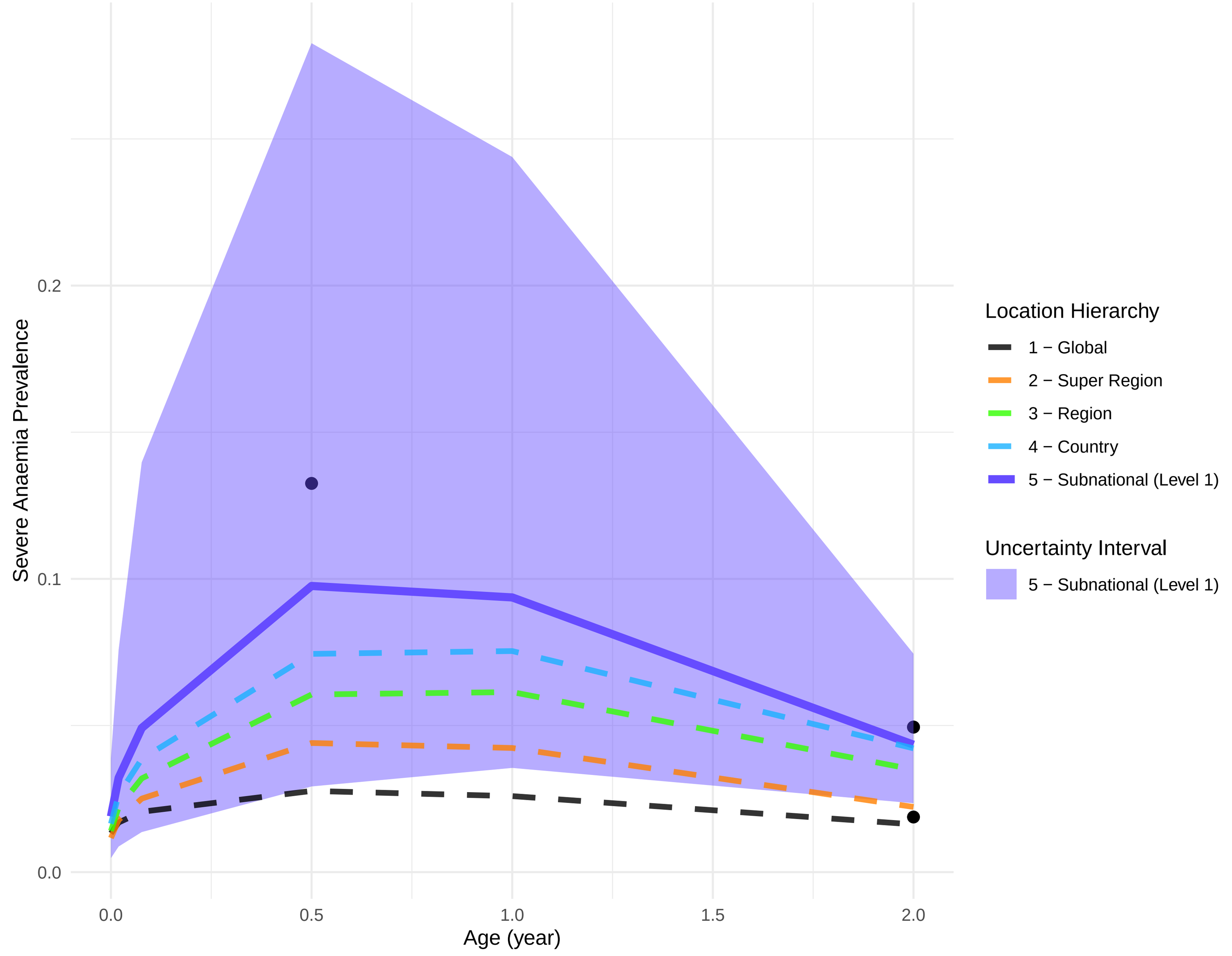

MR−BRT Age/Sex Fits − Non−pregnant Females <2 Years Old − Severe Anaemia Prevalence − Lagos
WHO−2001 Adjustment Method

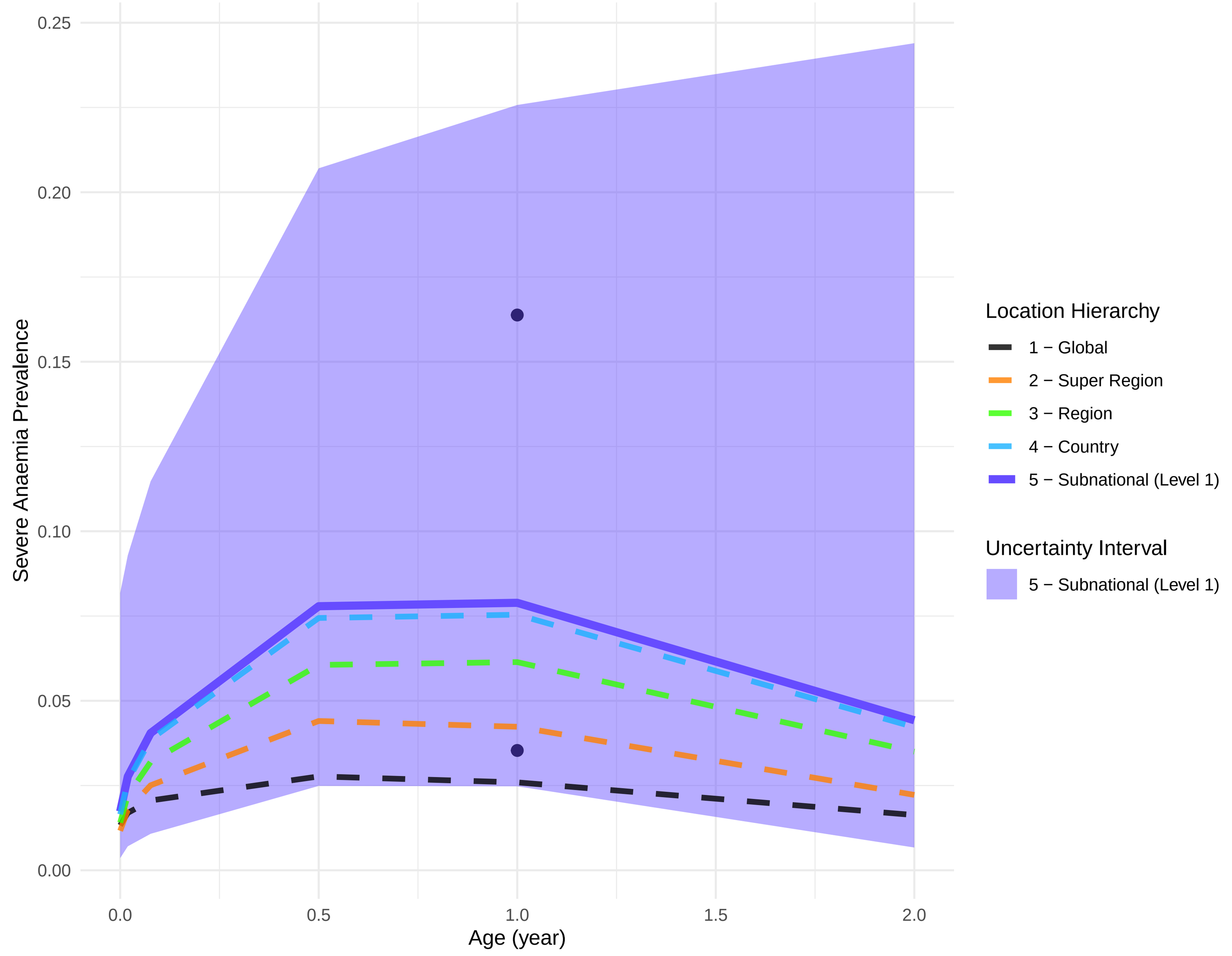

MR−BRT Age/Sex Fits − Non−pregnant Females <2 Years Old − Severe Anaemia Prevalence − Nasarawa
WHO−2001 Adjustment Method

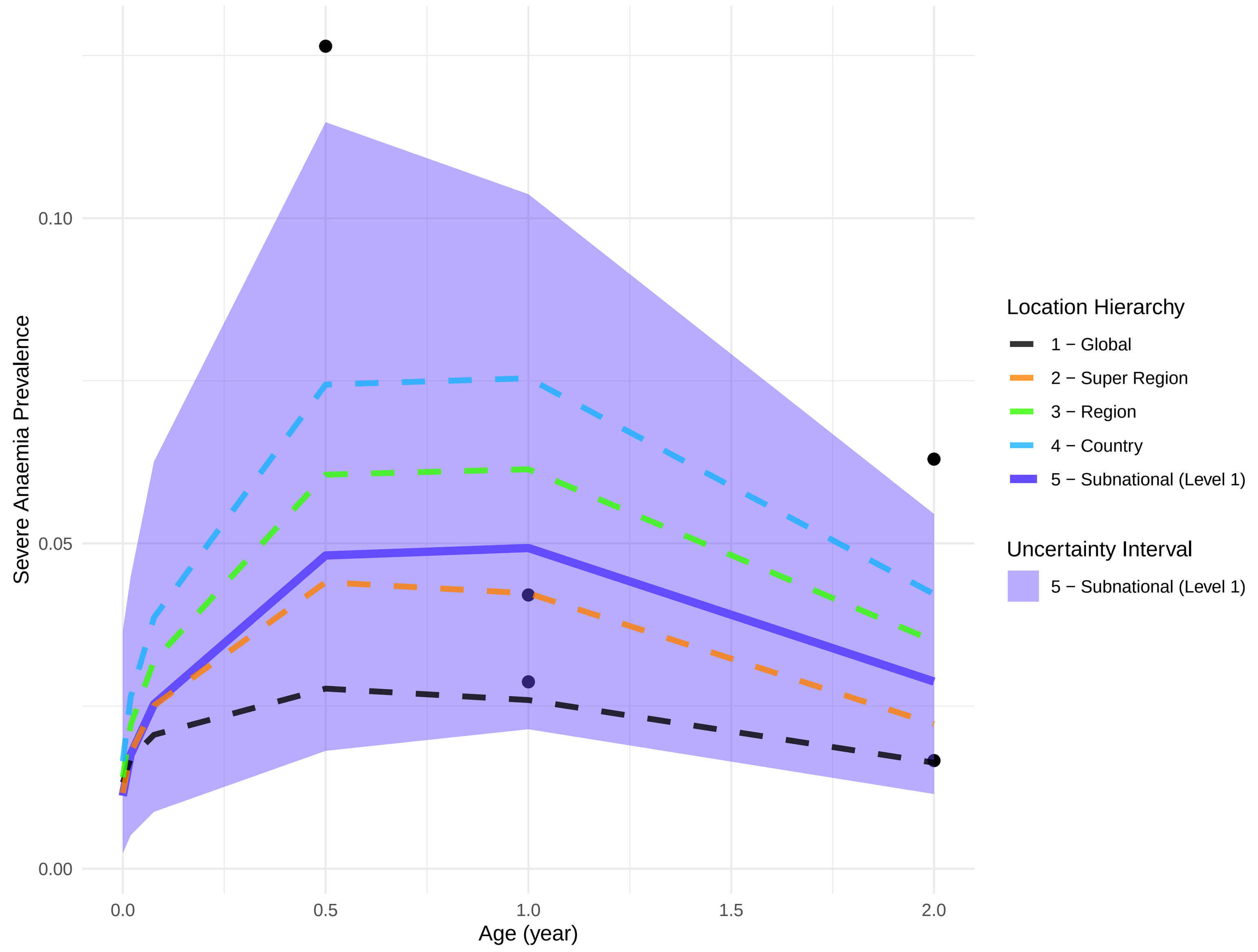

MR−BRT Age/Sex Fits − Non−pregnant Females <2 Years Old − Severe Anaemia Prevalence − Niger
WHO−2001 Adjustment Method

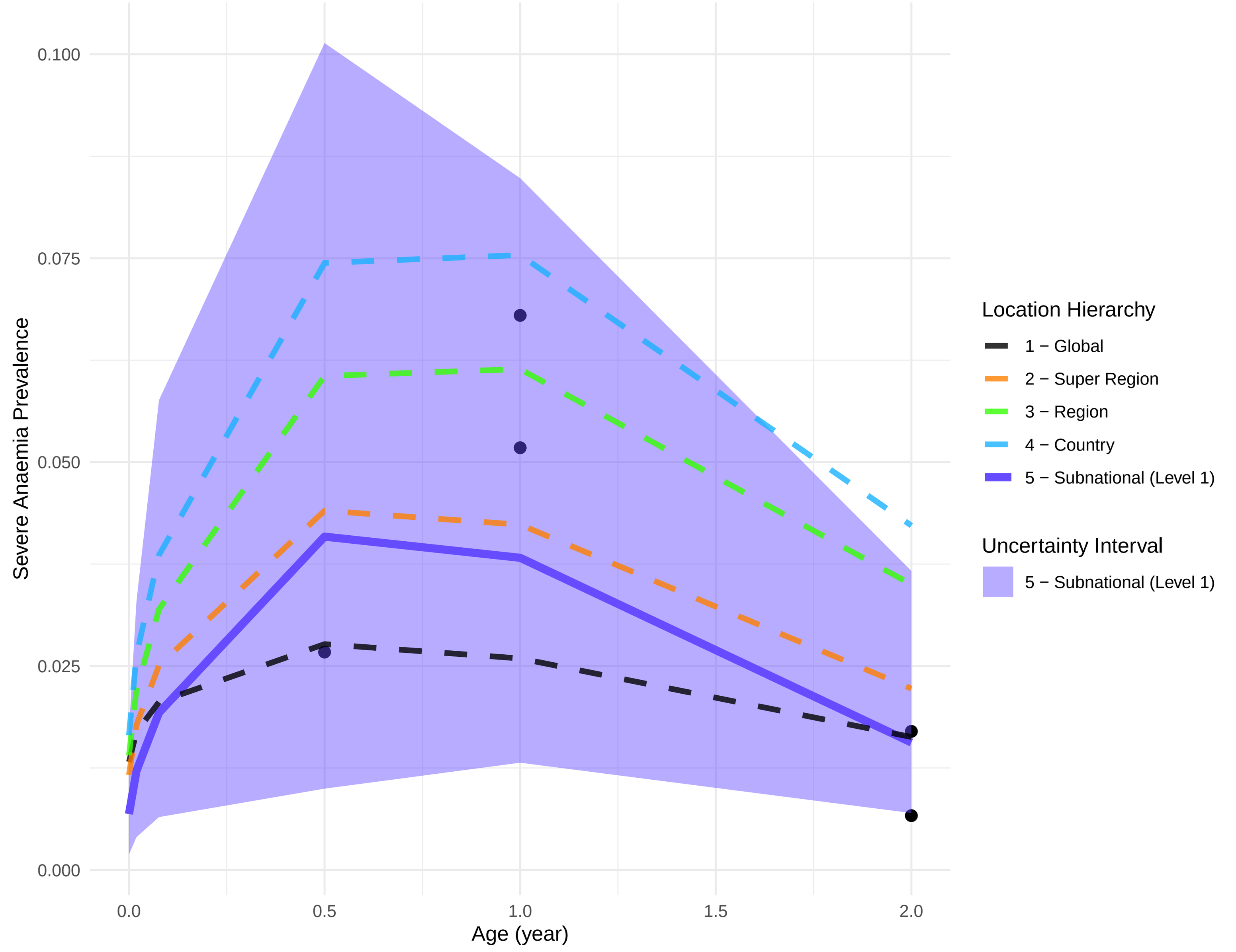

MR−BRT Age/Sex Fits − Non−pregnant Females <2 Years Old − Severe Anaemia Prevalence − Ogun
WHO−2001 Adjustment Method

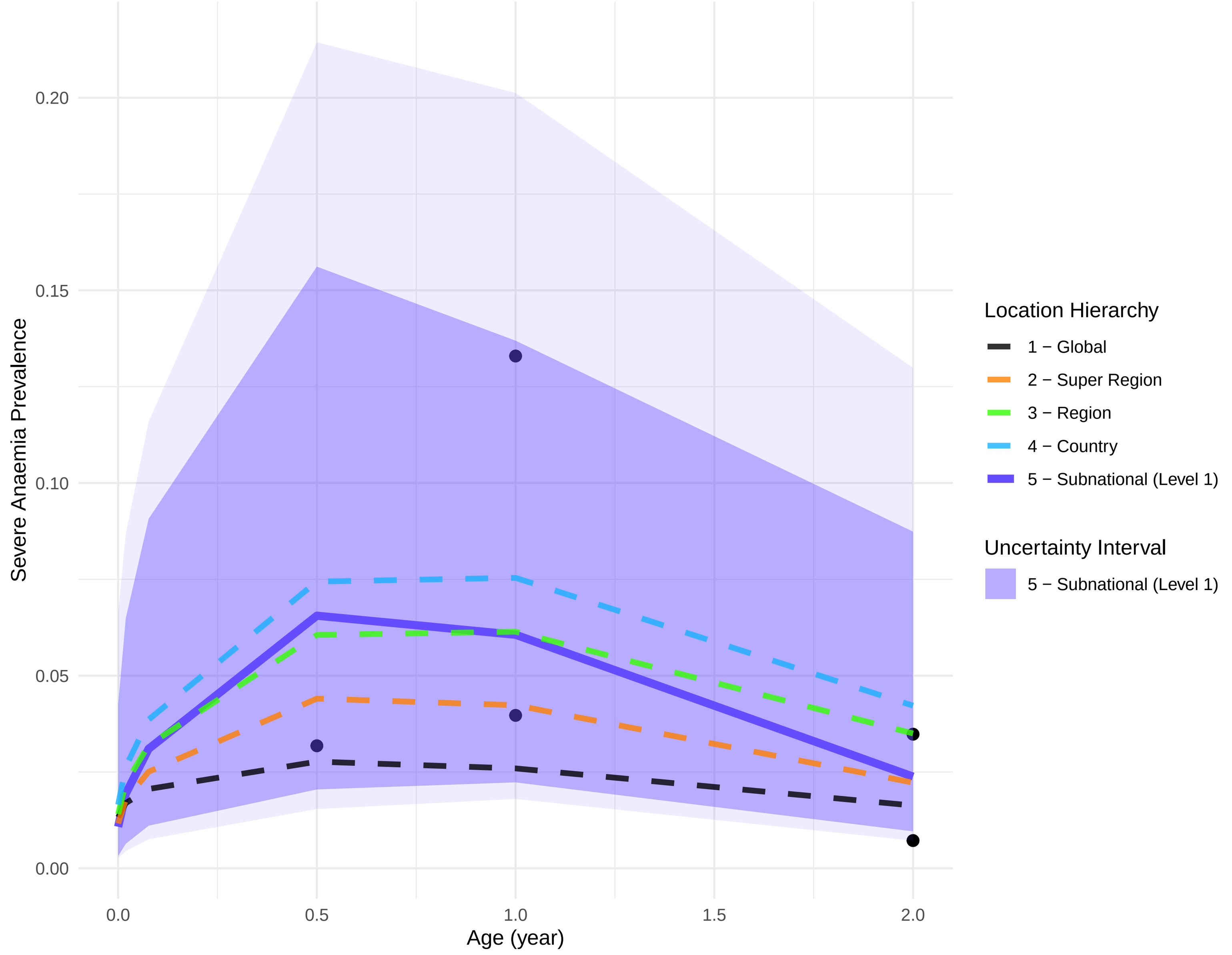

MR−BRT Age/Sex Fits − Non−pregnant Females <2 Years Old − Severe Anaemia Prevalence − Ondo
WHO−2001 Adjustment Method

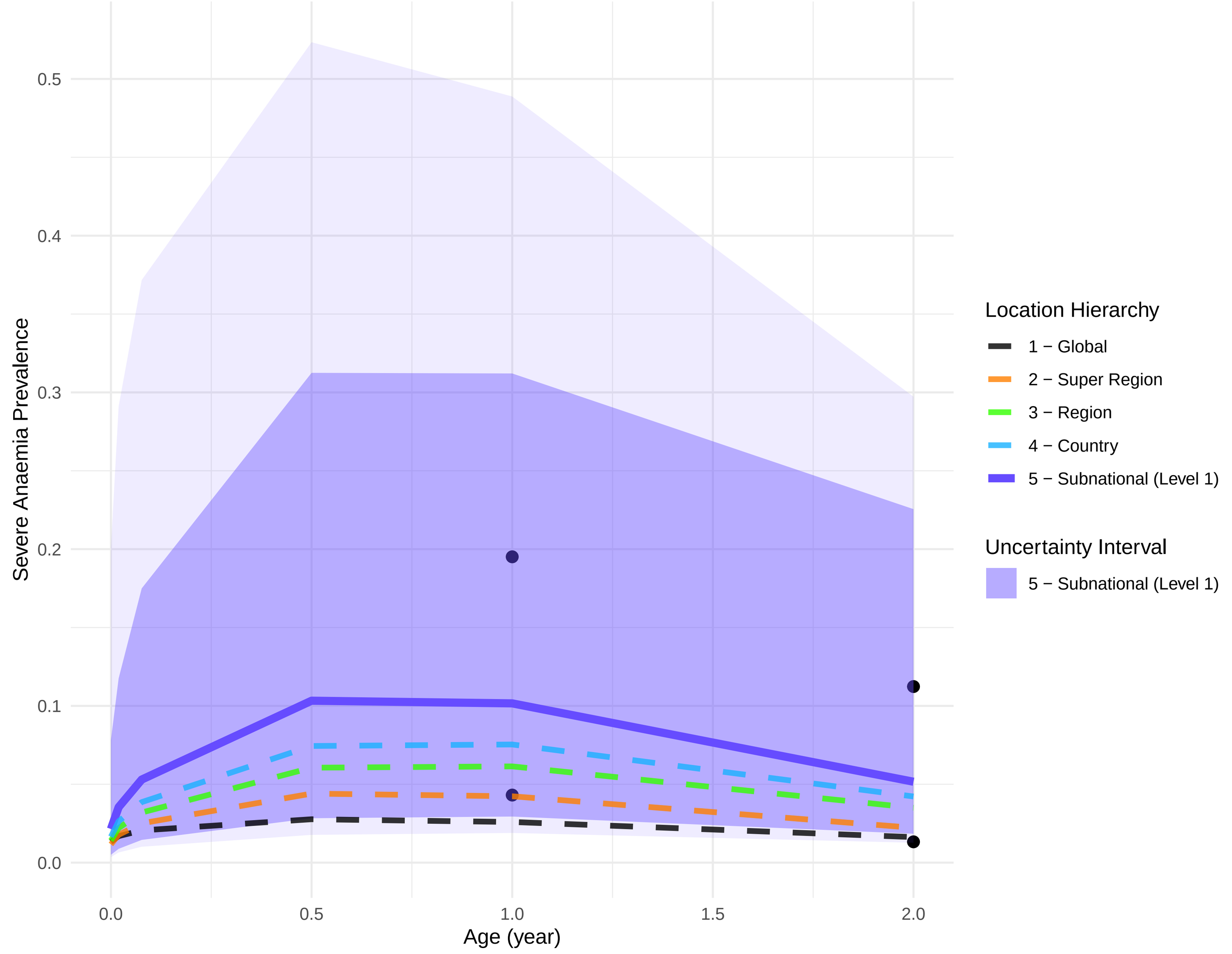

MR−BRT Age/Sex Fits − Non−pregnant Females <2 Years Old − Severe Anaemia Prevalence − Osun
WHO−2001 Adjustment Method

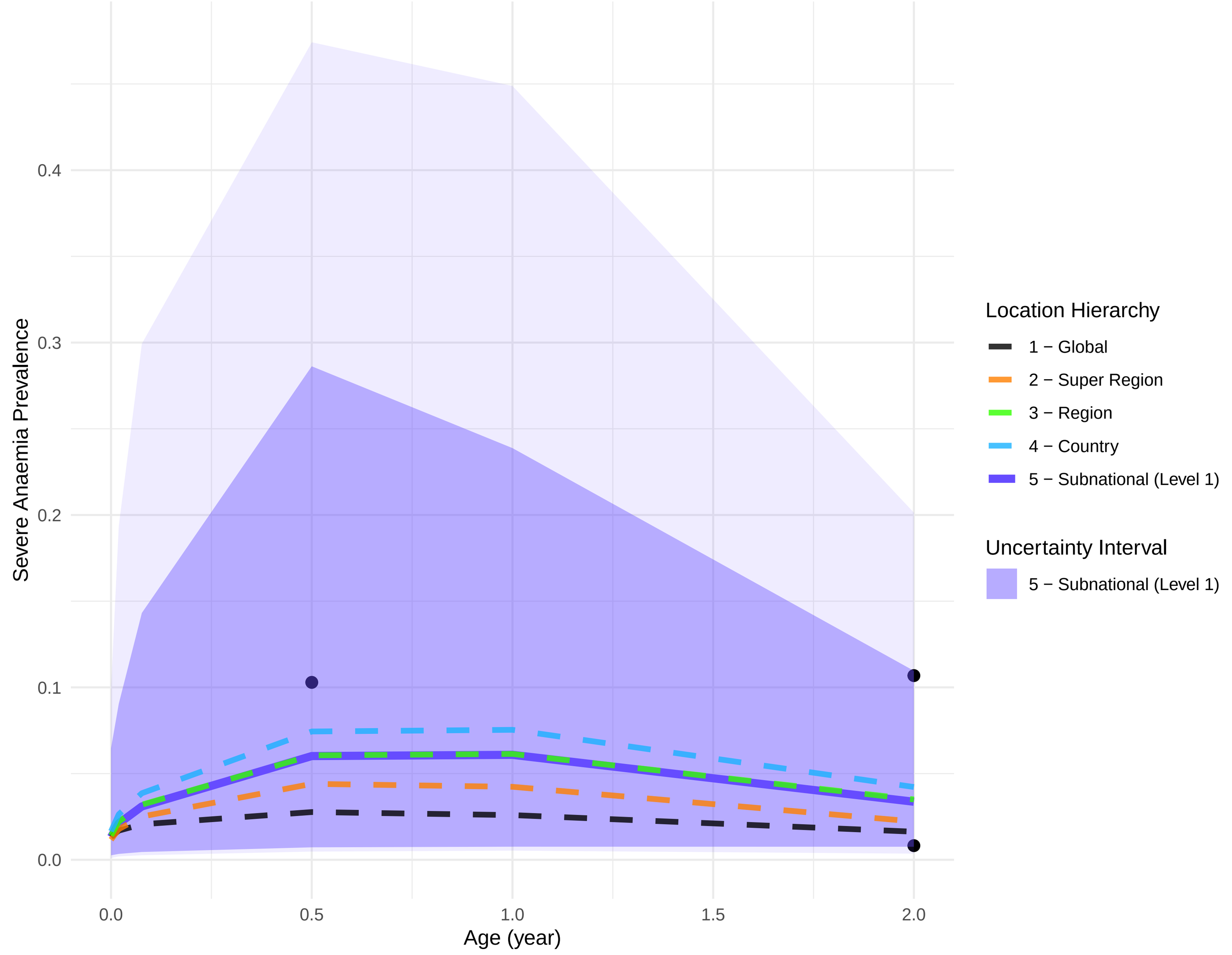

MR−BRT Age/Sex Fits − Non−pregnant Females <2 Years Old − Severe Anaemia Prevalence − Oyo
WHO−2001 Adjustment Method

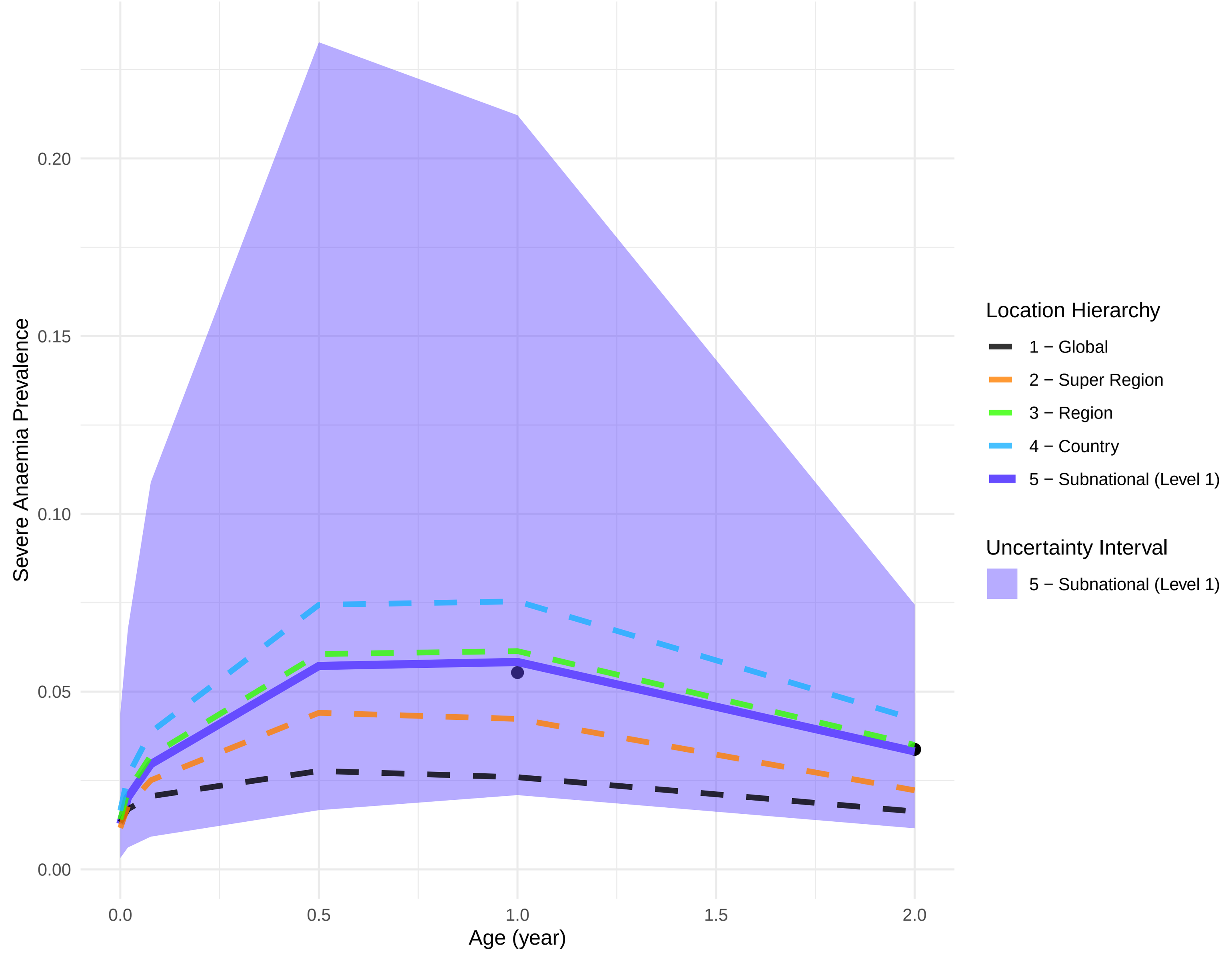

MR−BRT Age/Sex Fits − Non−pregnant Females <2 Years Old − Severe Anaemia Prevalence − Plateau
WHO−2001 Adjustment Method

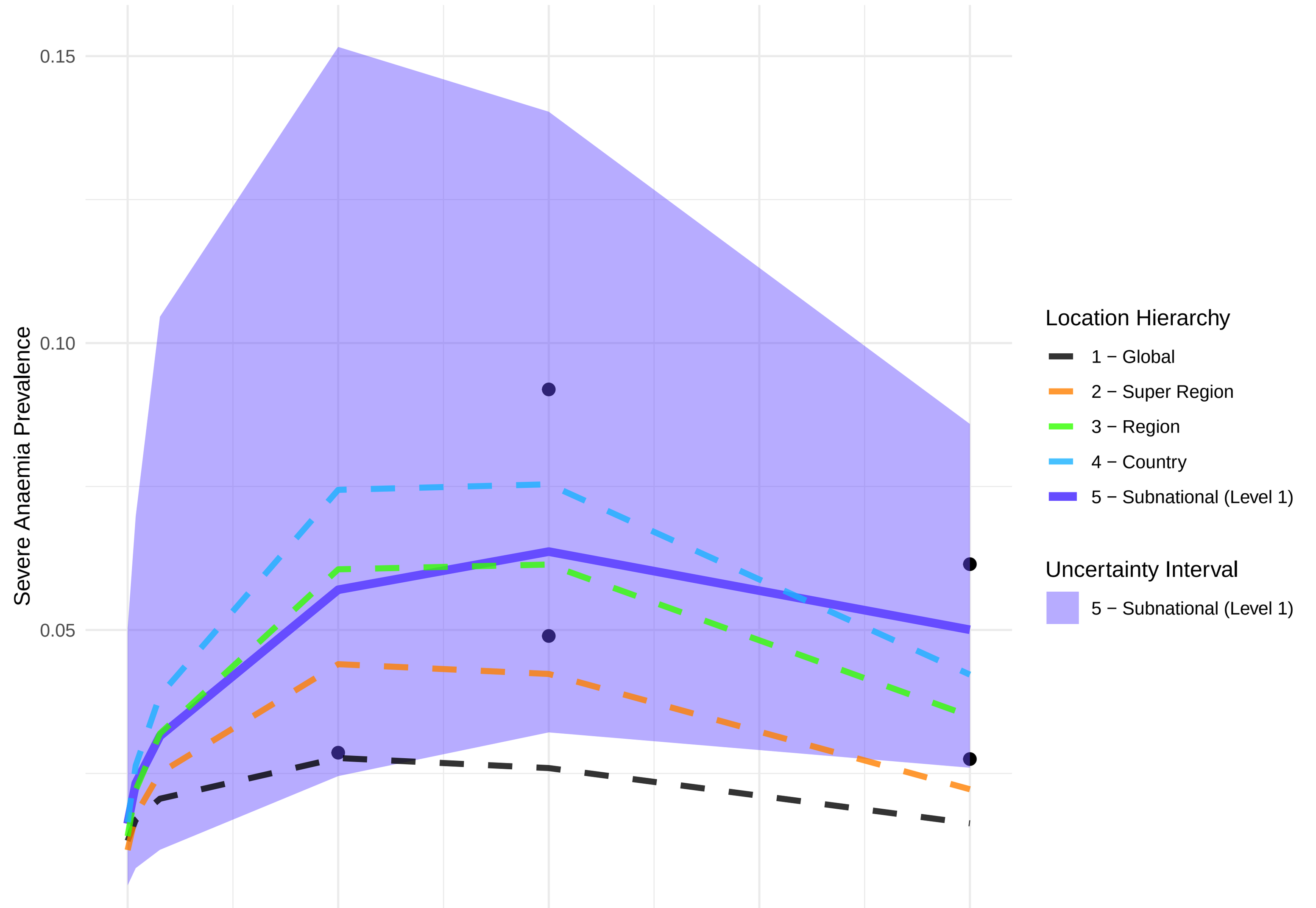

MR−BRT Age/Sex Fits − Non−pregnant Females <2 Years Old − Severe Anaemia Prevalence − Rivers
WHO−2001 Adjustment Method

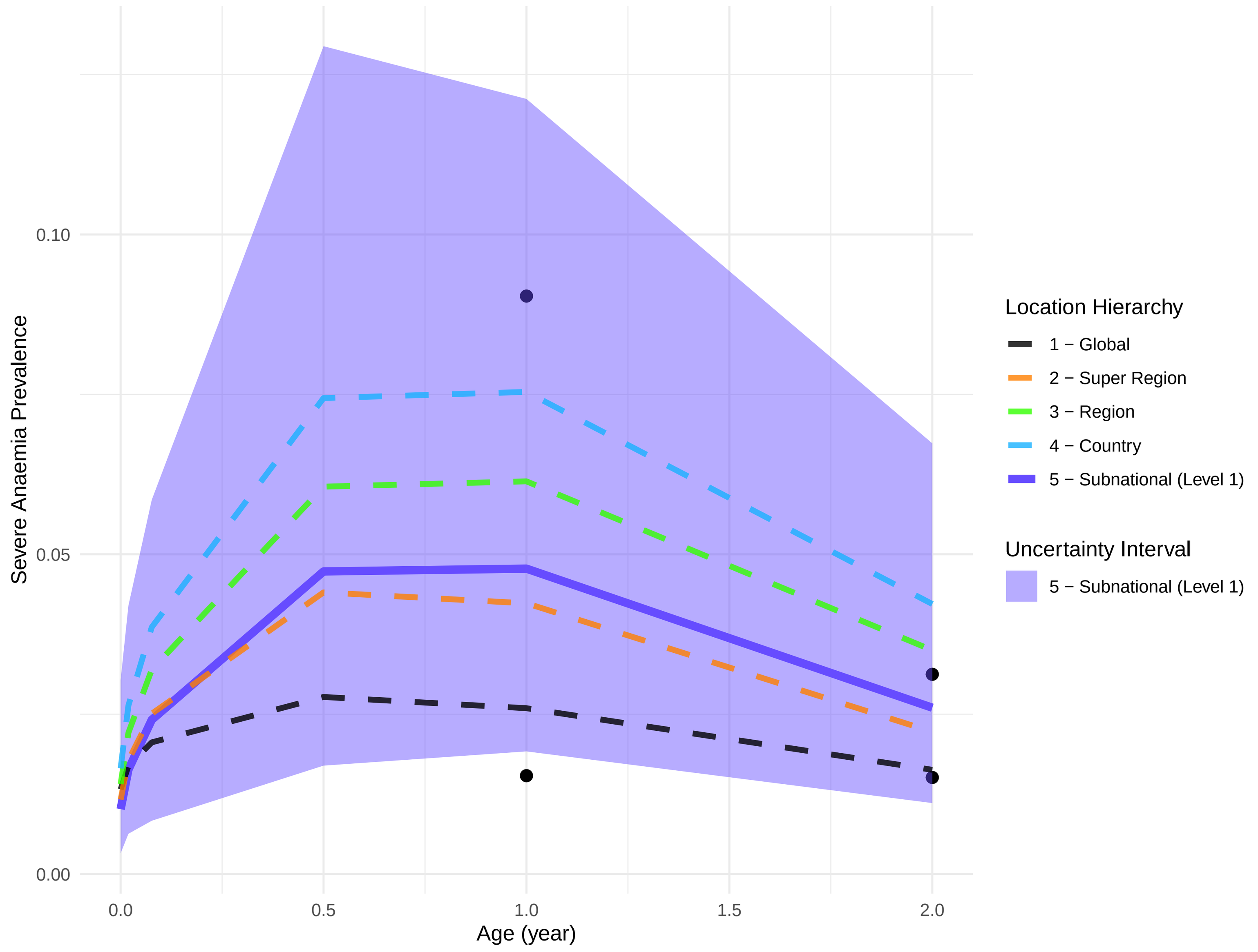

MR−BRT Age/Sex Fits − Non−pregnant Females <2 Years Old − Severe Anaemia Prevalence − Sokoto
WHO−2001 Adjustment Method

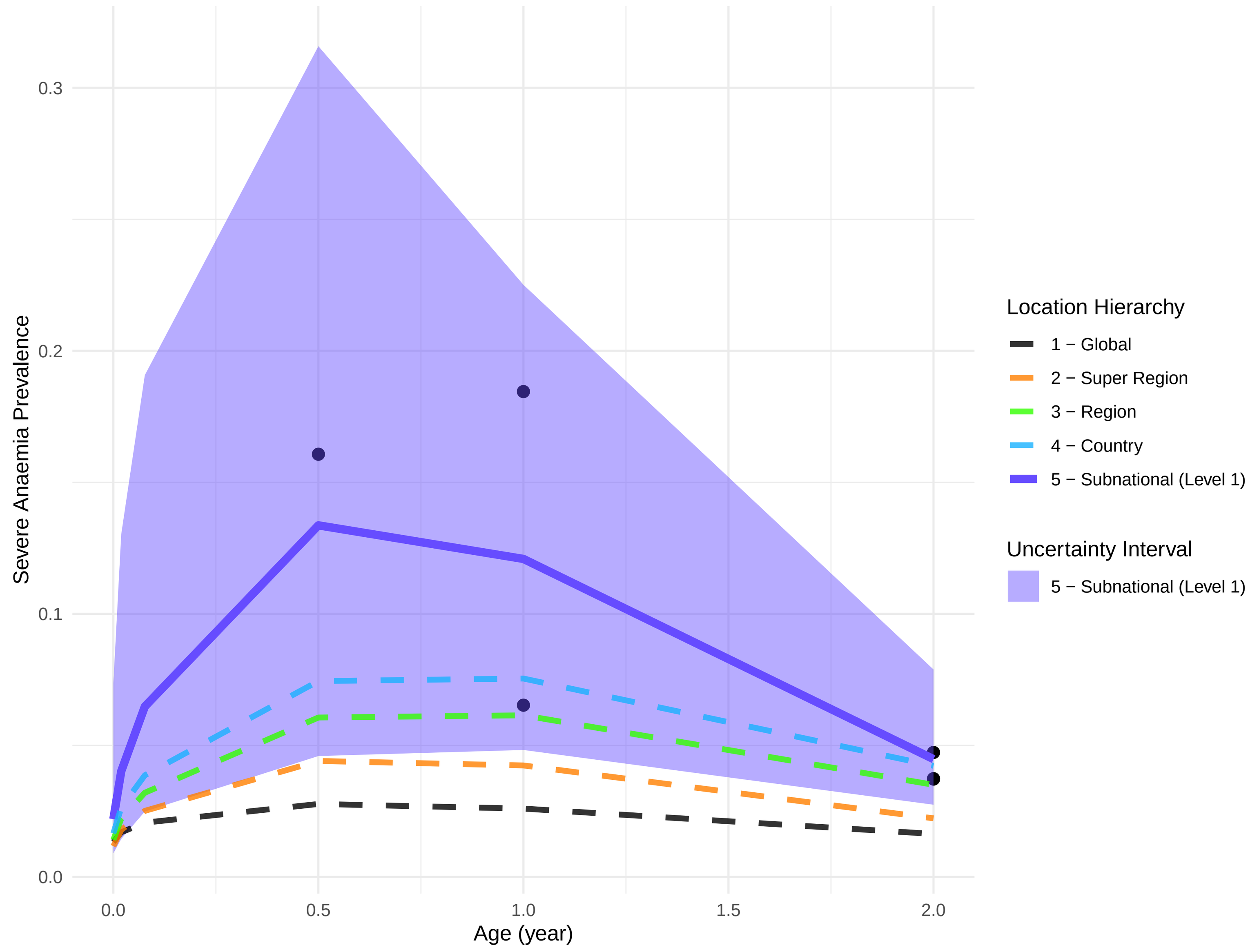

MR−BRT Age/Sex Fits − Non−pregnant Females <2 Years Old − Severe Anaemia Prevalence − Taraba
WHO−2001 Adjustment Method

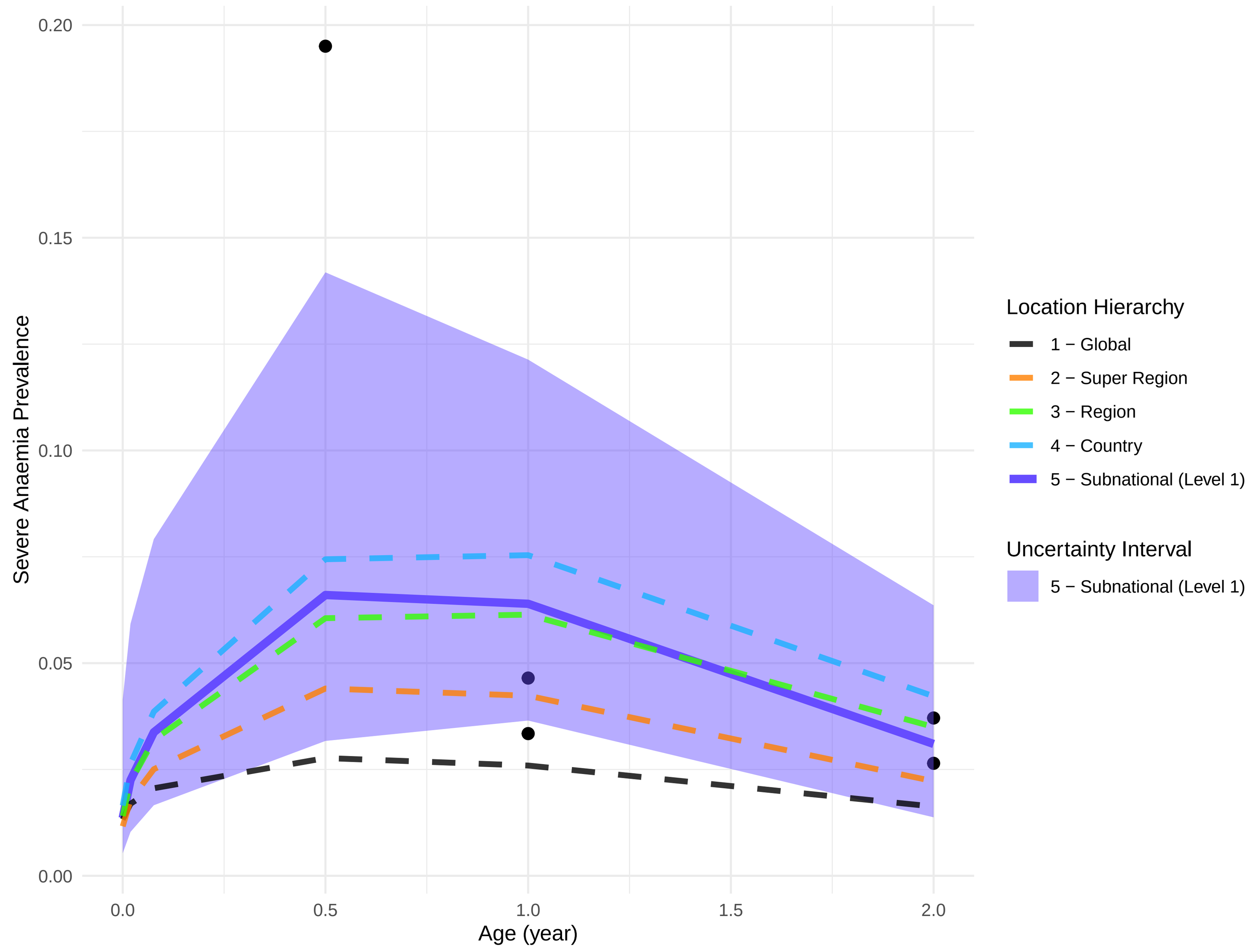

MR−BRT Age/Sex Fits − Non−pregnant Females <2 Years Old − Severe Anaemia Prevalence − Yobe
WHO−2001 Adjustment Method

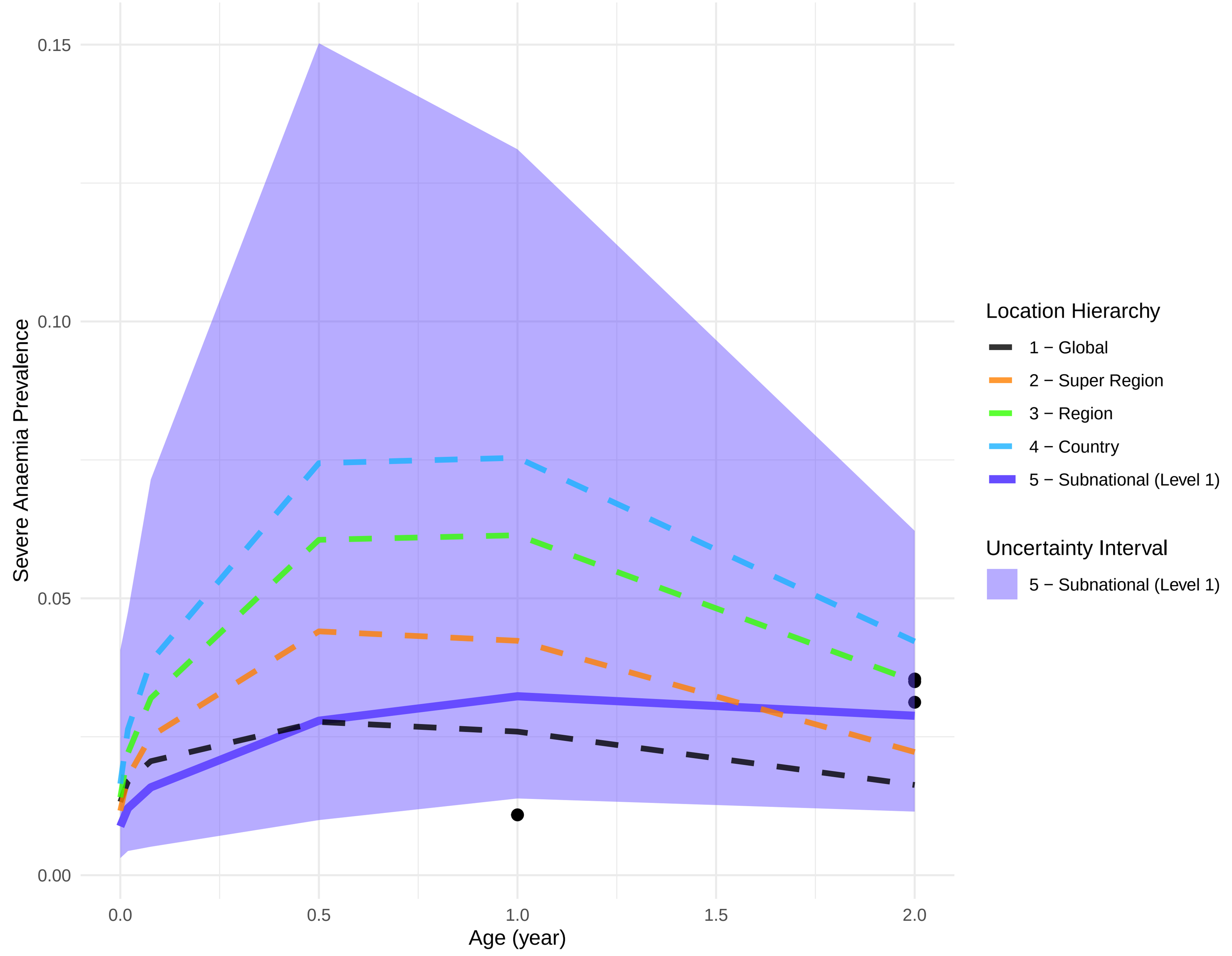

MR−BRT Age/Sex Fits − Non−pregnant Females <2 Years Old − Severe Anaemia Prevalence − Zamfara
WHO−2001 Adjustment Method

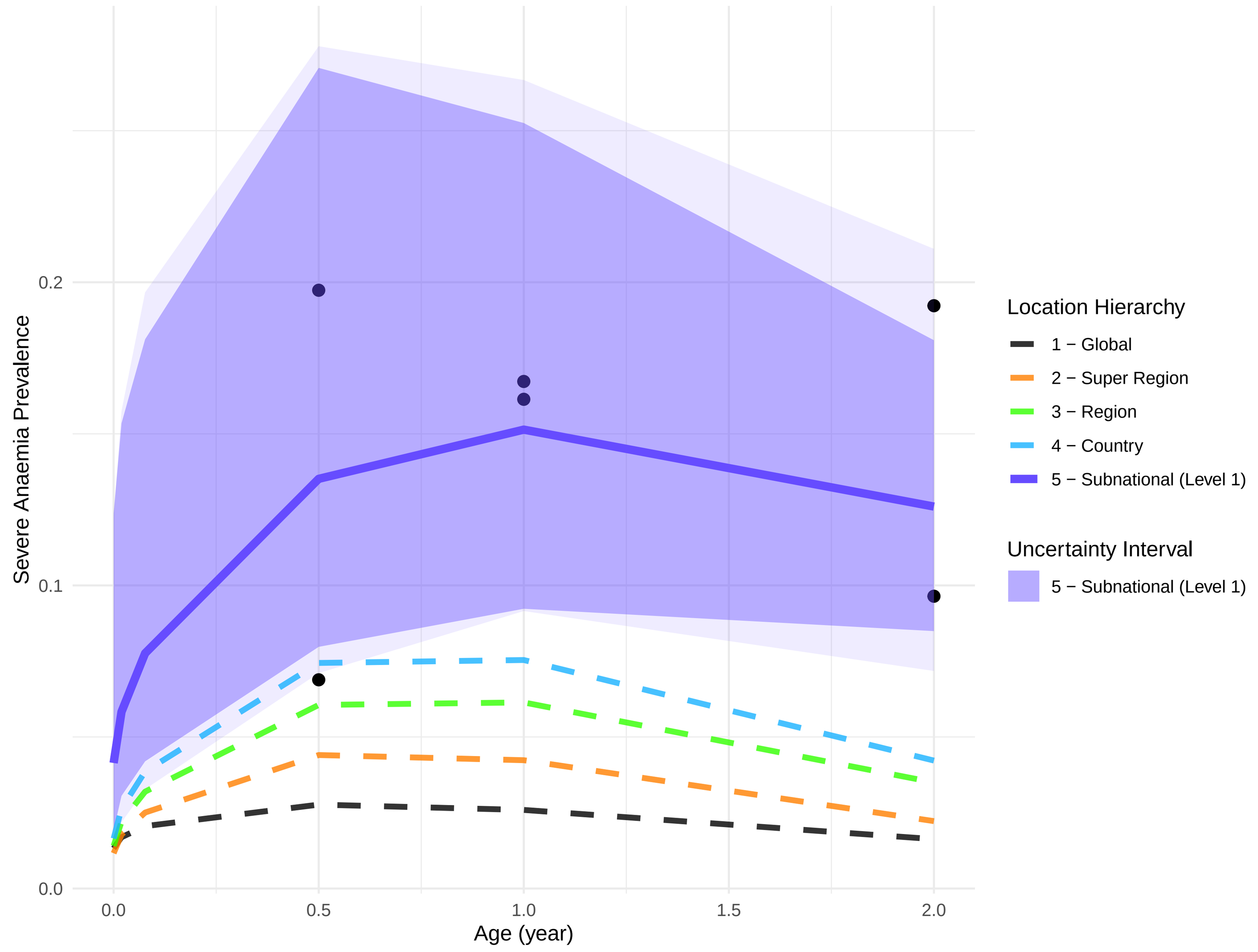

MR−BRT Age/Sex Fits − Non−pregnant Females <2 Years Old − Severe Anaemia Prevalence − Senegal
WHO−2001 Adjustment Method

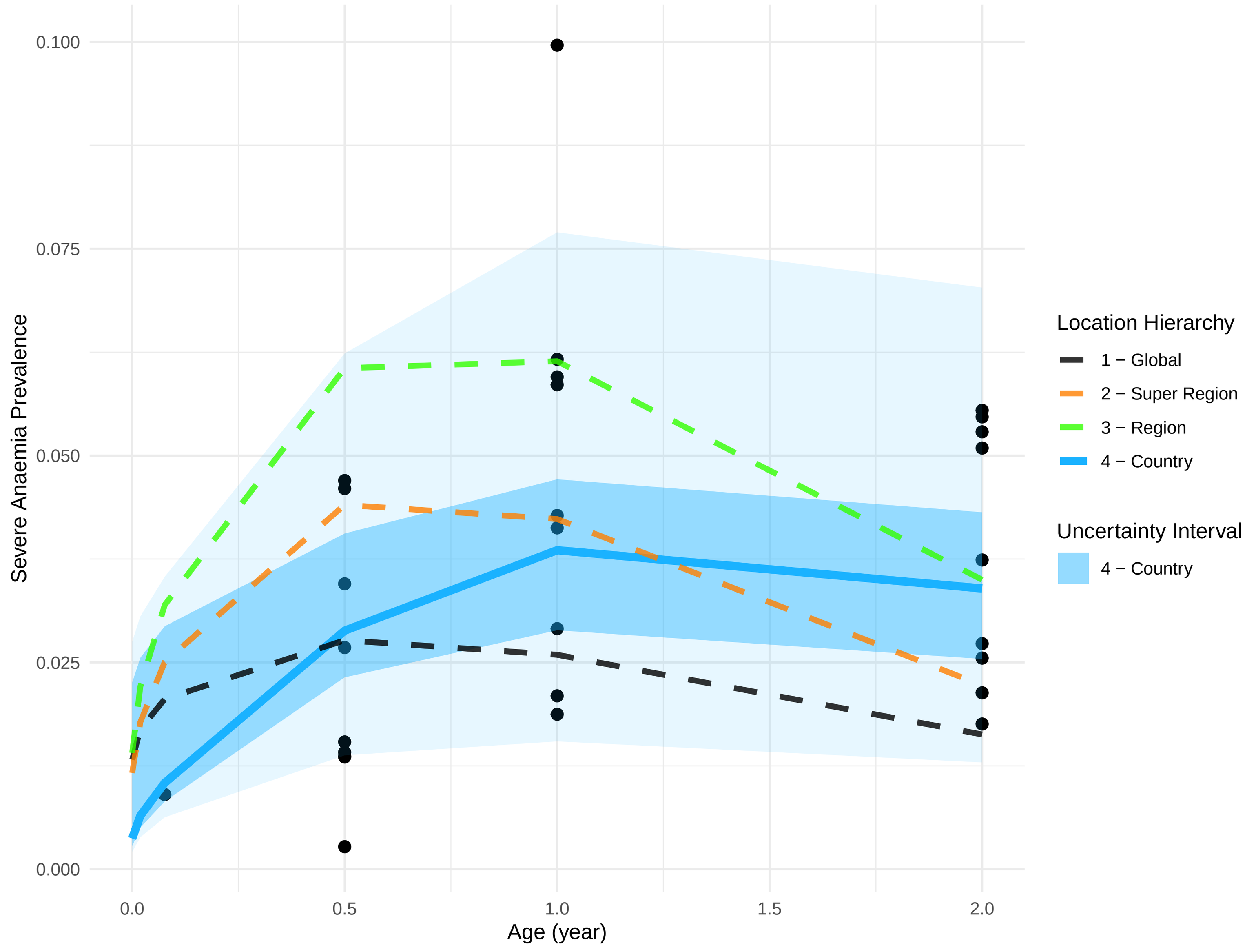

MR−BRT Age/Sex Fits − Non−pregnant Females <2 Years Old − Severe Anaemia Prevalence − Sierra Leone
WHO−2001 Adjustment Method

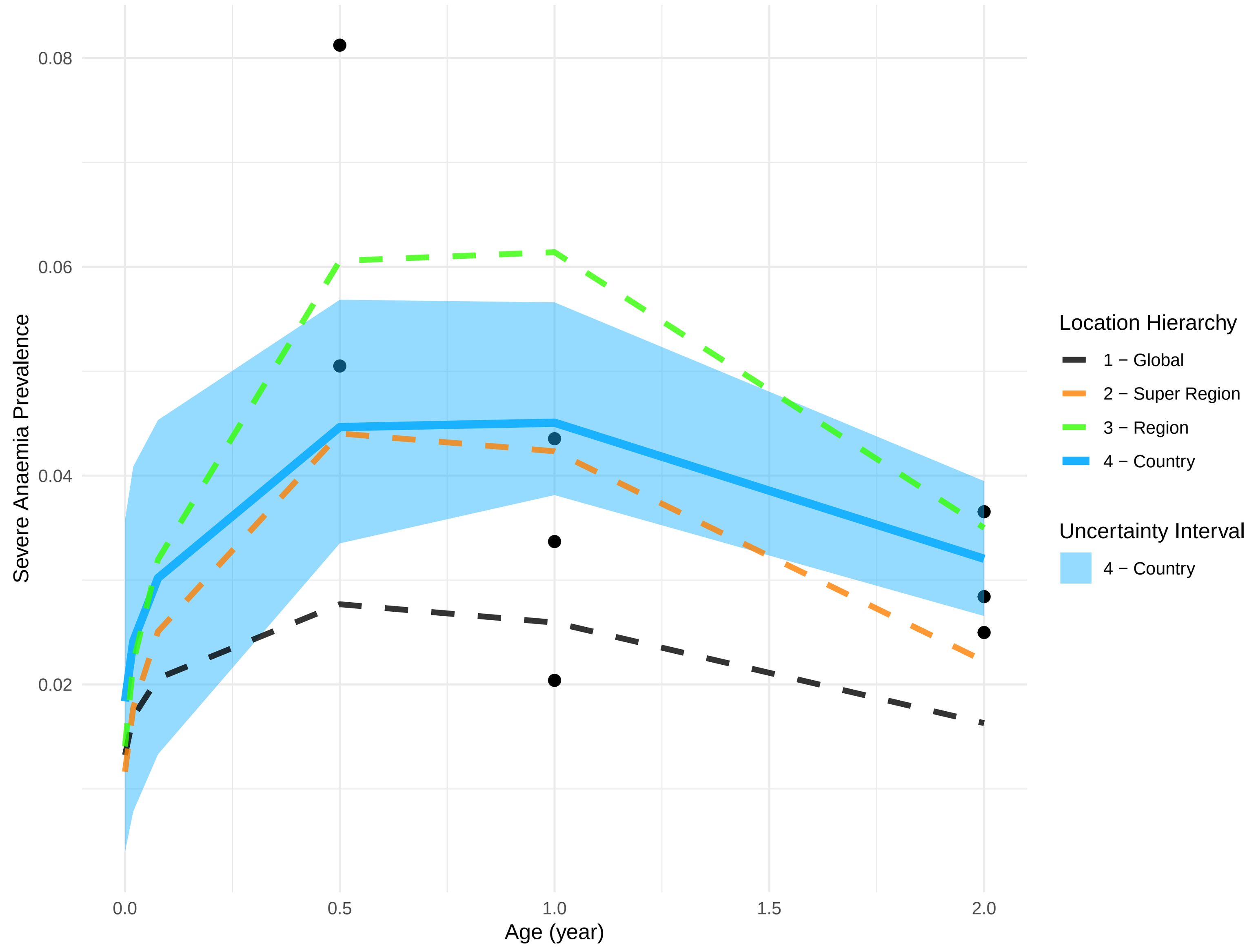

MR−BRT Age/Sex Fits − Non−pregnant Females <2 Years Old − Severe Anaemia Prevalence − Togo
WHO−2001 Adjustment Method

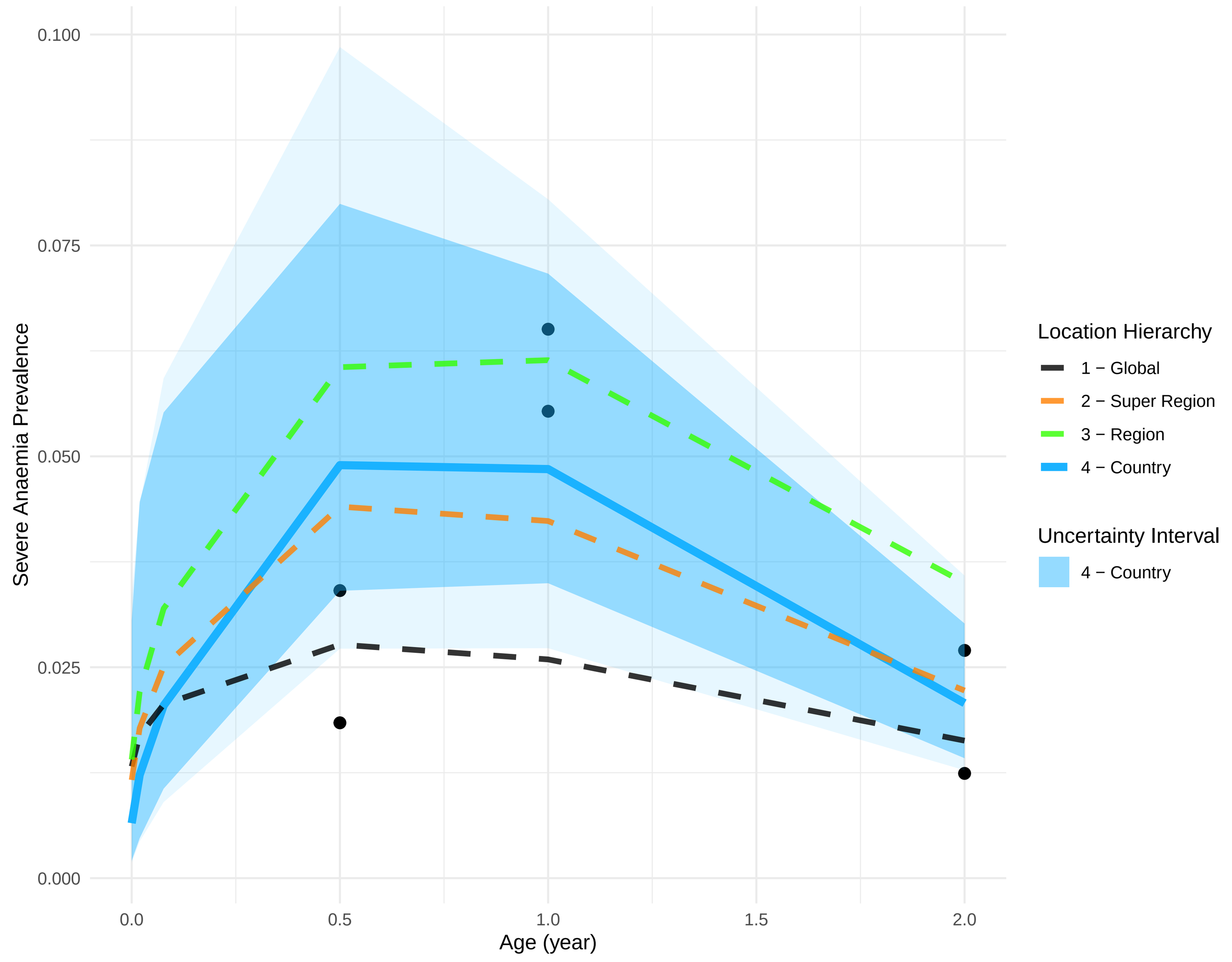

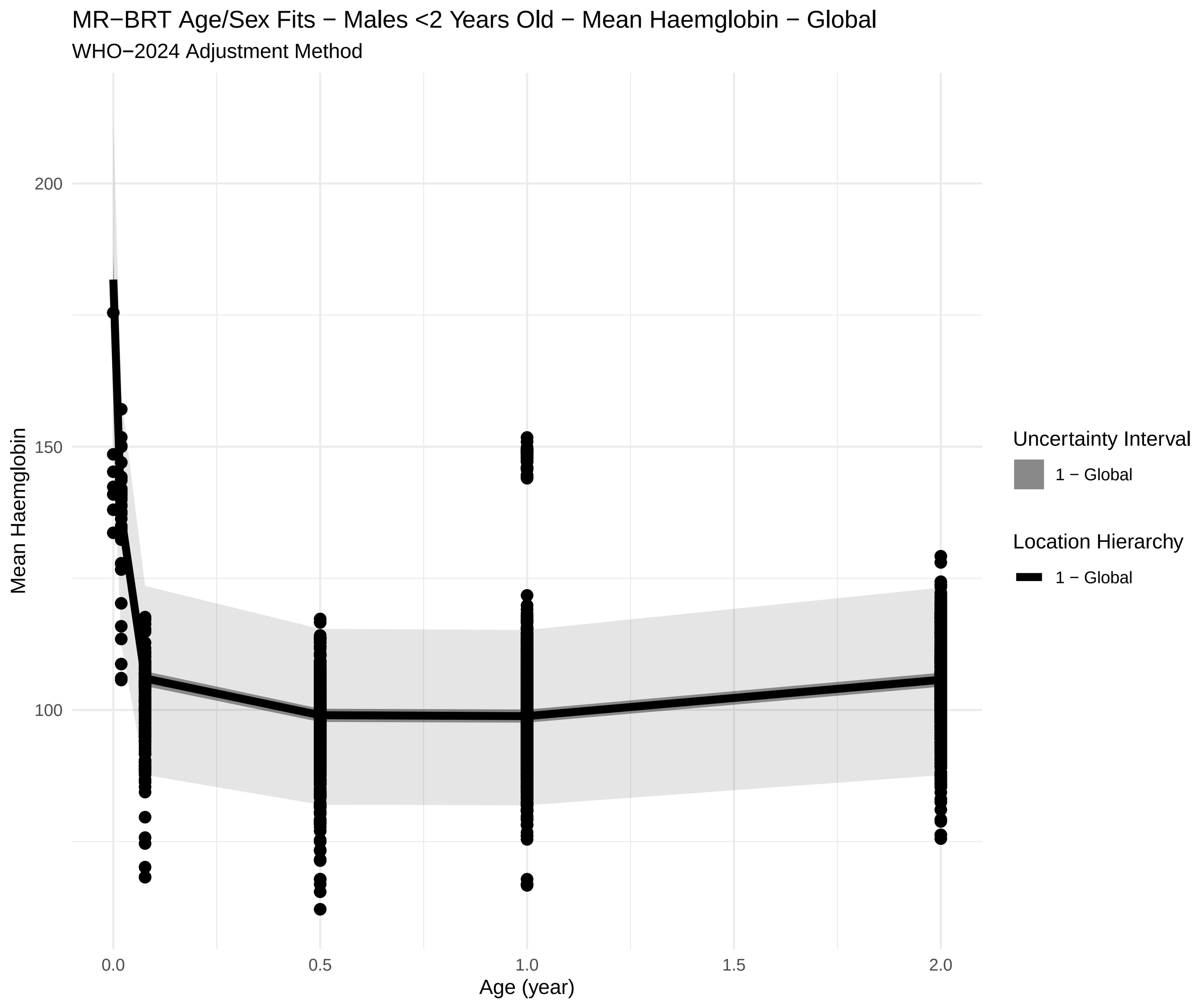
MR−BRT Age/Sex Fits − Males <2 Years Old − Mean Haemoglobin − Global
WHO−2024 Adjustment Method
Mean Haemoglobin
Age (year)
Uncertainty Interval
1 − Global
Location Hierarchy
1 − Global

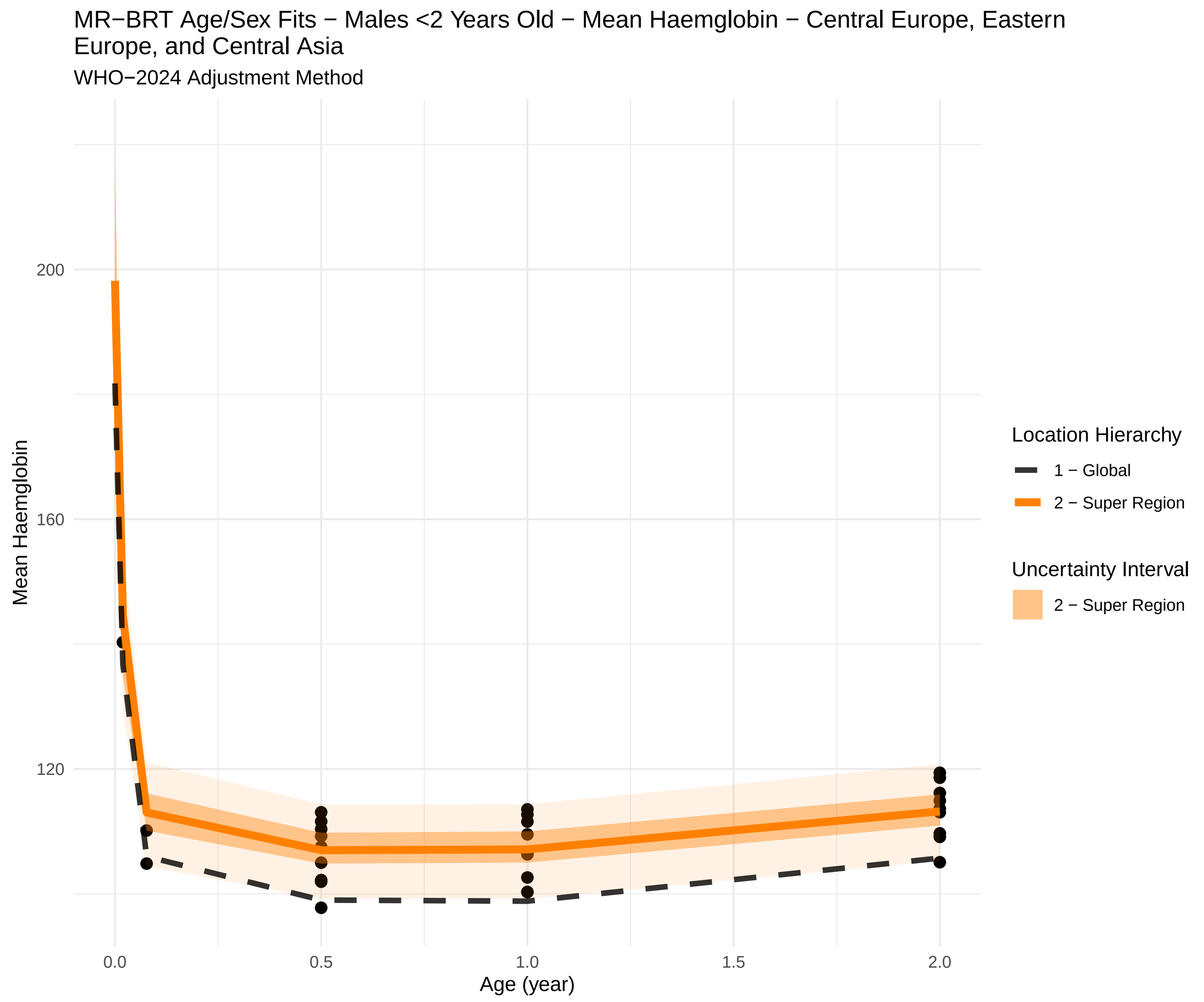
MR−BRT Age/Sex Fits − Males <2 Years Old − Mean Haemoglobin − Central Europe, Eastern Europe, and Central Asia
WHO−2024 Adjustment Method
Mean Haemoglobin
Age (year)
Location Hierarchy
1 − Global
2 − Super Region
Uncertainty Interval
2 − Super Region

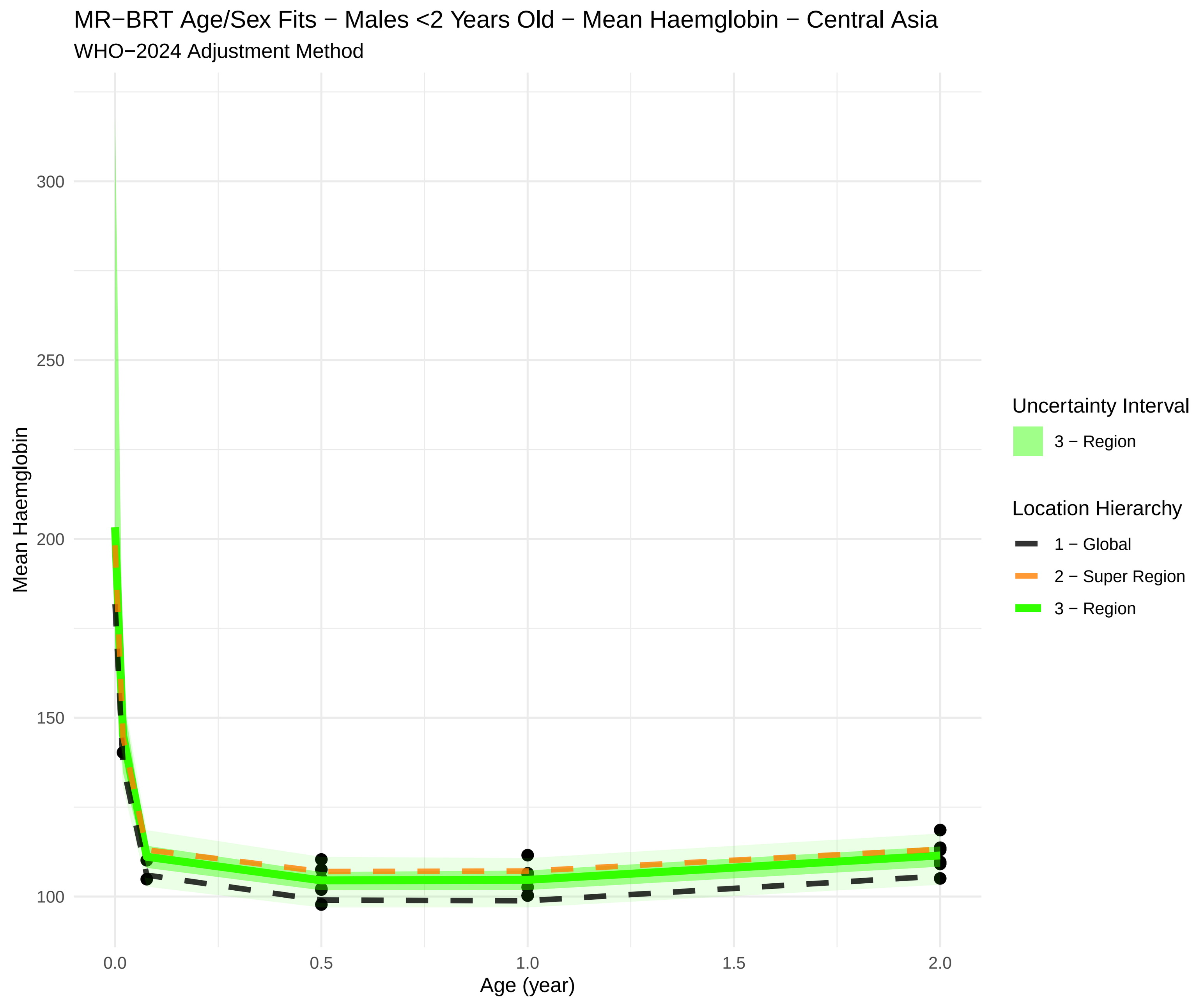
MR−BRT Age/Sex Fits − Males <2 Years Old − Mean Haemoglobin − Central Asia
WHO−2024 Adjustment Method
Mean Haemoglobin
Age (year)
Uncertainty Interval
3 − Region
Location Hierarchy
1 − Global
2 − Super Region
3 − Region

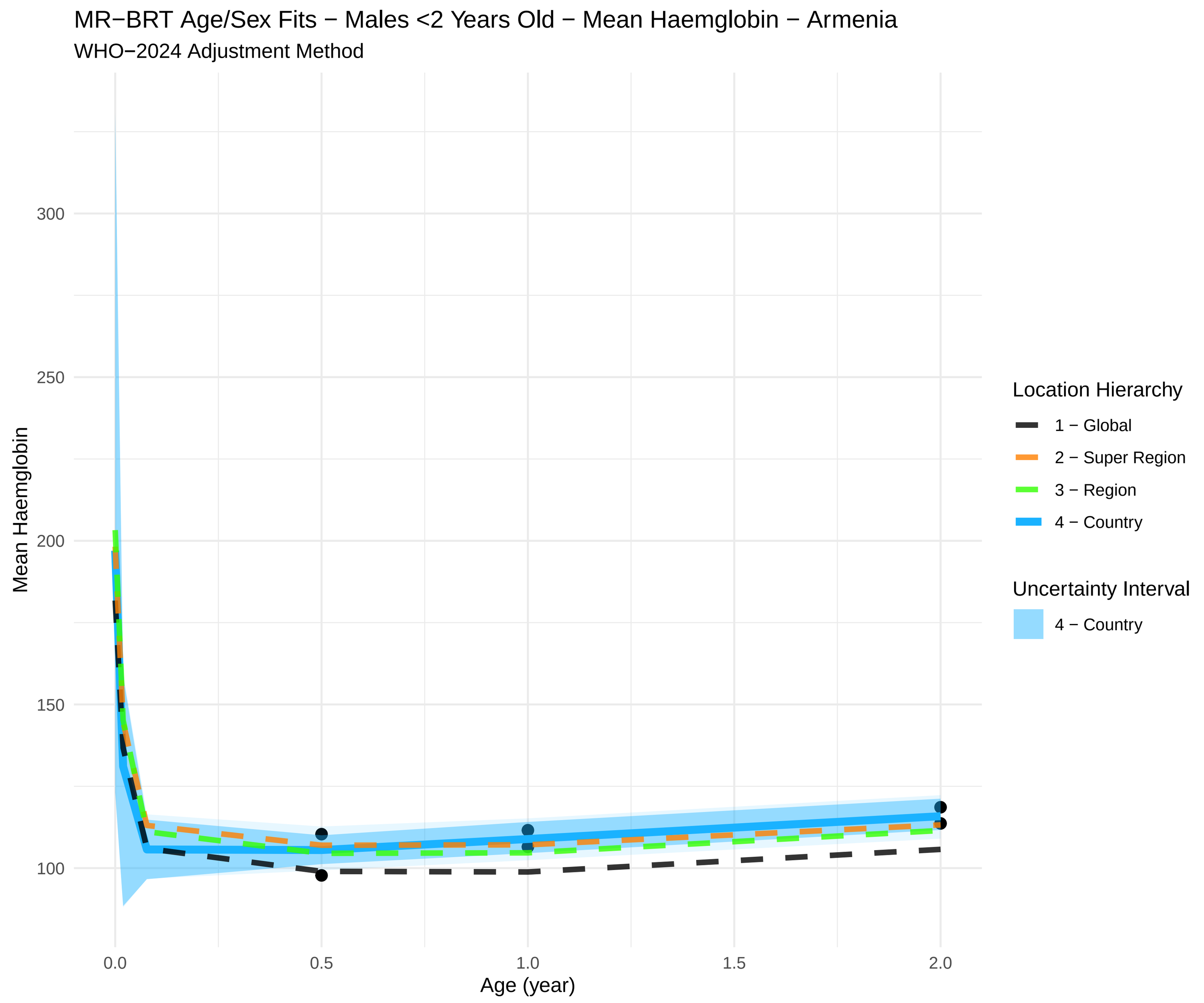
MR−BRT Age/Sex Fits − Males <2 Years Old − Mean Haemoglobin − Armenia
WHO−2024 Adjustment Method
Mean Haemoglobin
Age (year)
Location Hierarchy
1 − Global
2 − Super Region
3 − Region
4 − Country
Uncertainty Interval
4 − Country

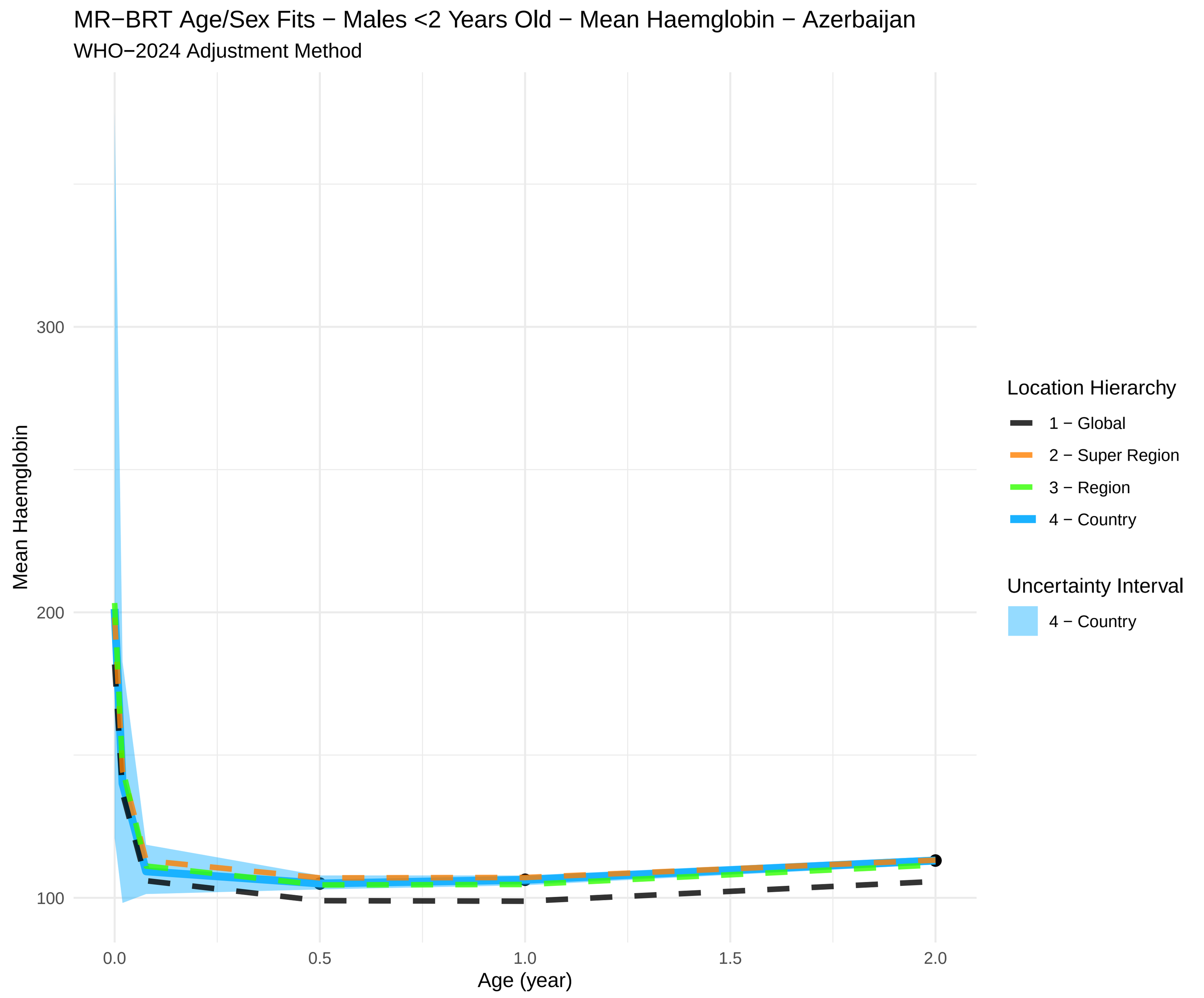
MR−BRT Age/Sex Fits − Males <2 Years Old − Mean Haemoglobin − Azerbaijan
WHO−2024 Adjustment Method
Mean Haemoglobin
Age (year)
Location Hierarchy
1 − Global
2 − Super Region
3 − Region
4 − Country
Uncertainty Interval
4 − Country

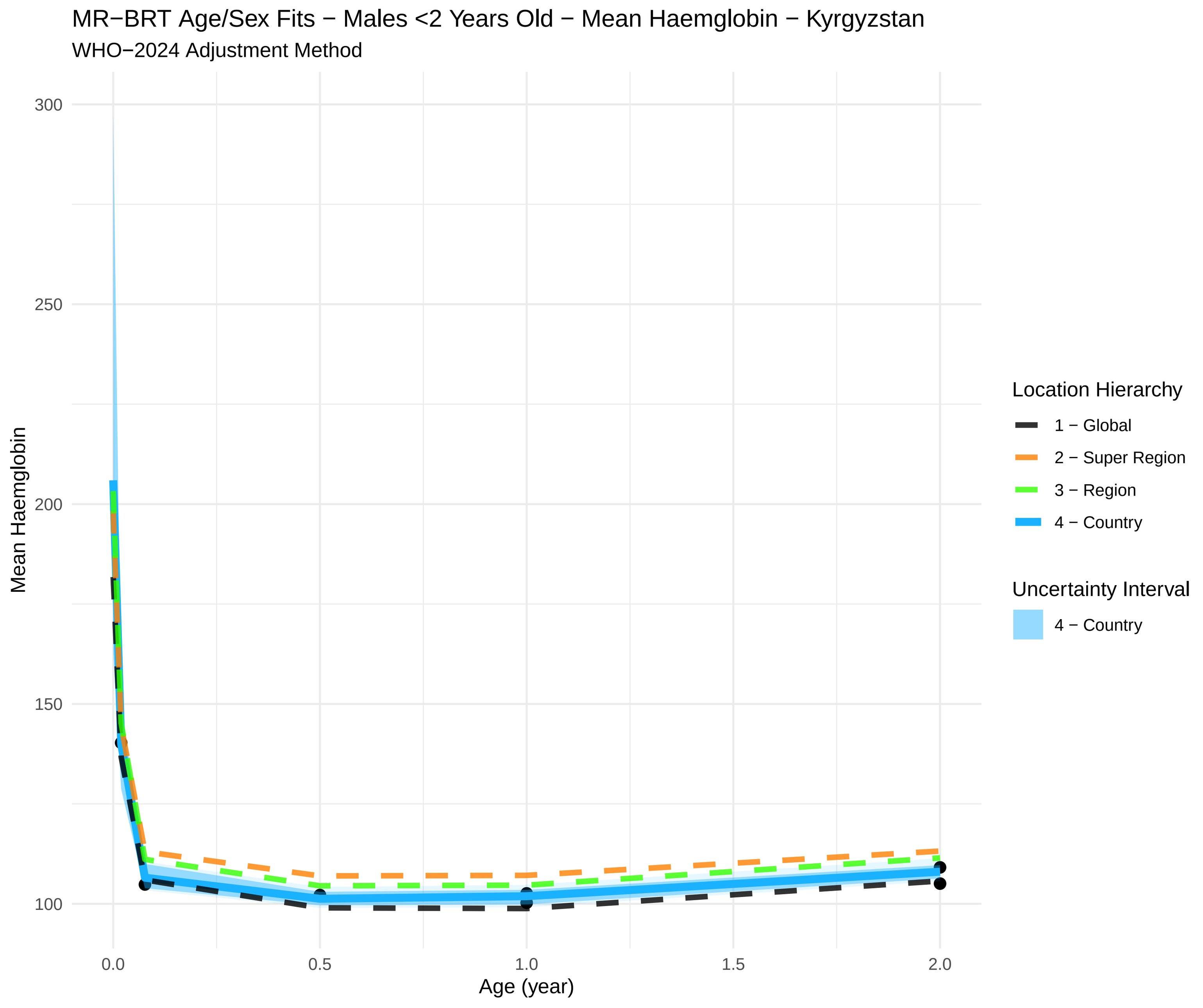
MR−BRT Age/Sex Fits − Males <2 Years Old − Mean Haemoglobin − Kyrgyzstan
WHO−2024 Adjustment Method
Mean Haemoglobin
Age (year)
Location Hierarchy
1 − Global
2 − Super Region
3 − Region
4 − Country
Uncertainty Interval
4 − Country

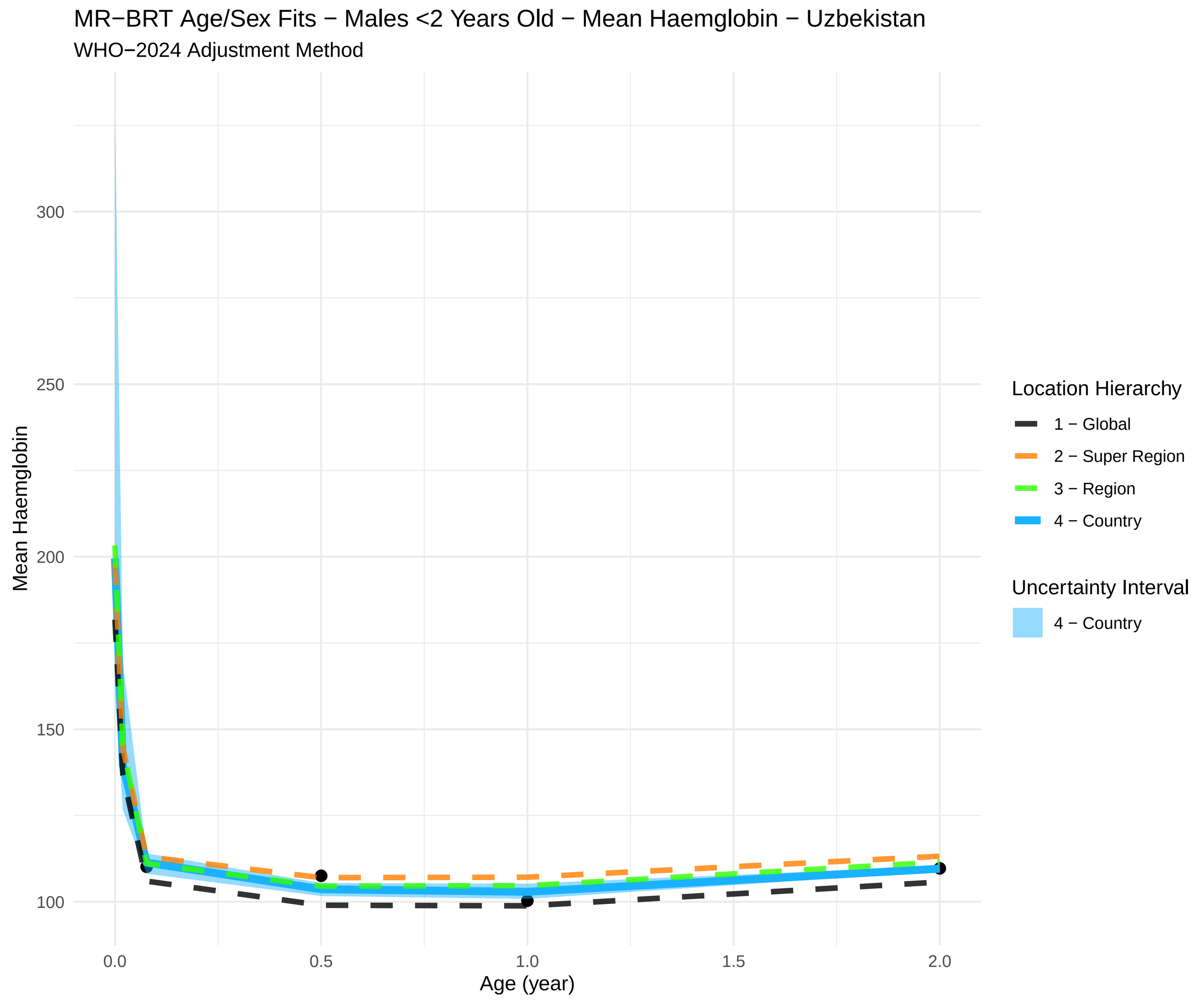
MR−BRT Age/Sex Fits − Males <2 Years Old − Mean Haemoglobin − Uzbekistan
WHO−2024 Adjustment Method
Mean Haemoglobin
Age (year)
Location Hierarchy
1 − Global
2 − Super Region
3 − Region
4 − Country
Uncertainty Interval
4 − Country

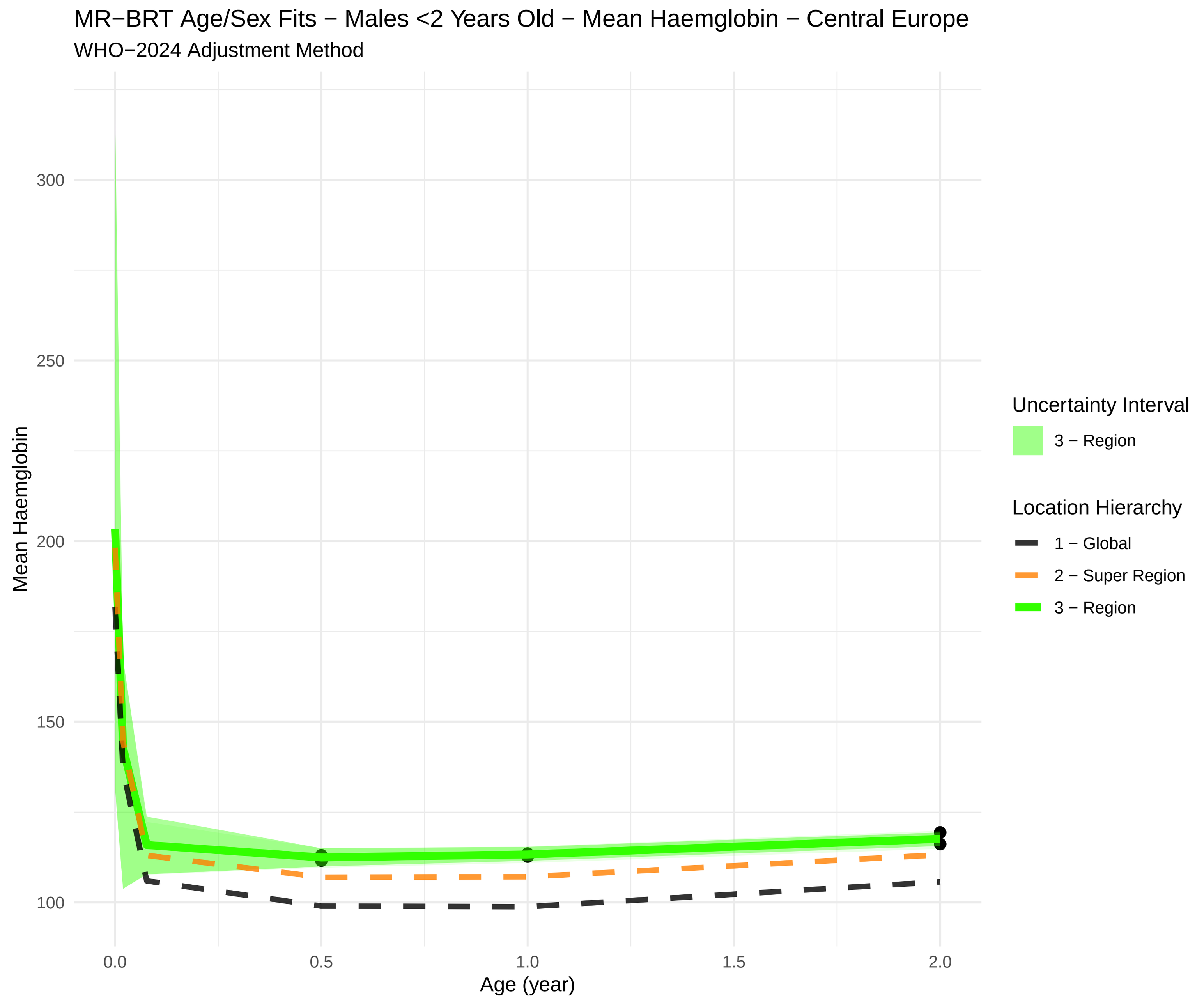
MR−BRT Age/Sex Fits − Males <2 Years Old − Mean Haemoglobin − Central Europe
WHO−2024 Adjustment Method
Mean Haemoglobin
Age (year)
Uncertainty Interval
3 − Region
Location Hierarchy
1 − Global
2 − Super Region
3 − Region

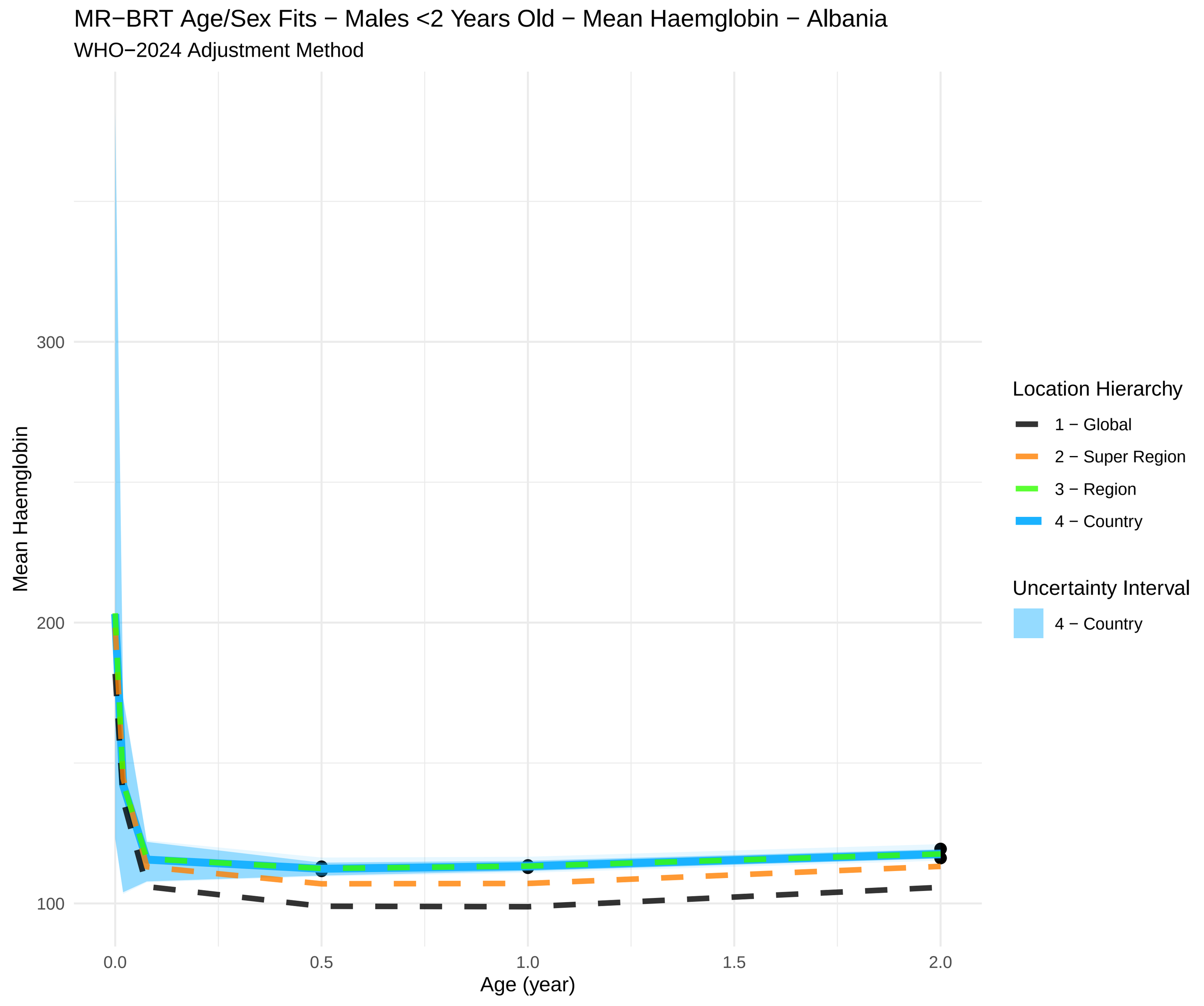
MR−BRT Age/Sex Fits − Males <2 Years Old − Mean Haemoglobin − Albania
WHO−2024 Adjustment Method
Mean Haemoglobin
Age (year)
Location Hierarchy
1 − Global
2 − Super Region
3 − Region
4 − Country
Uncertainty Interval
4 − Country

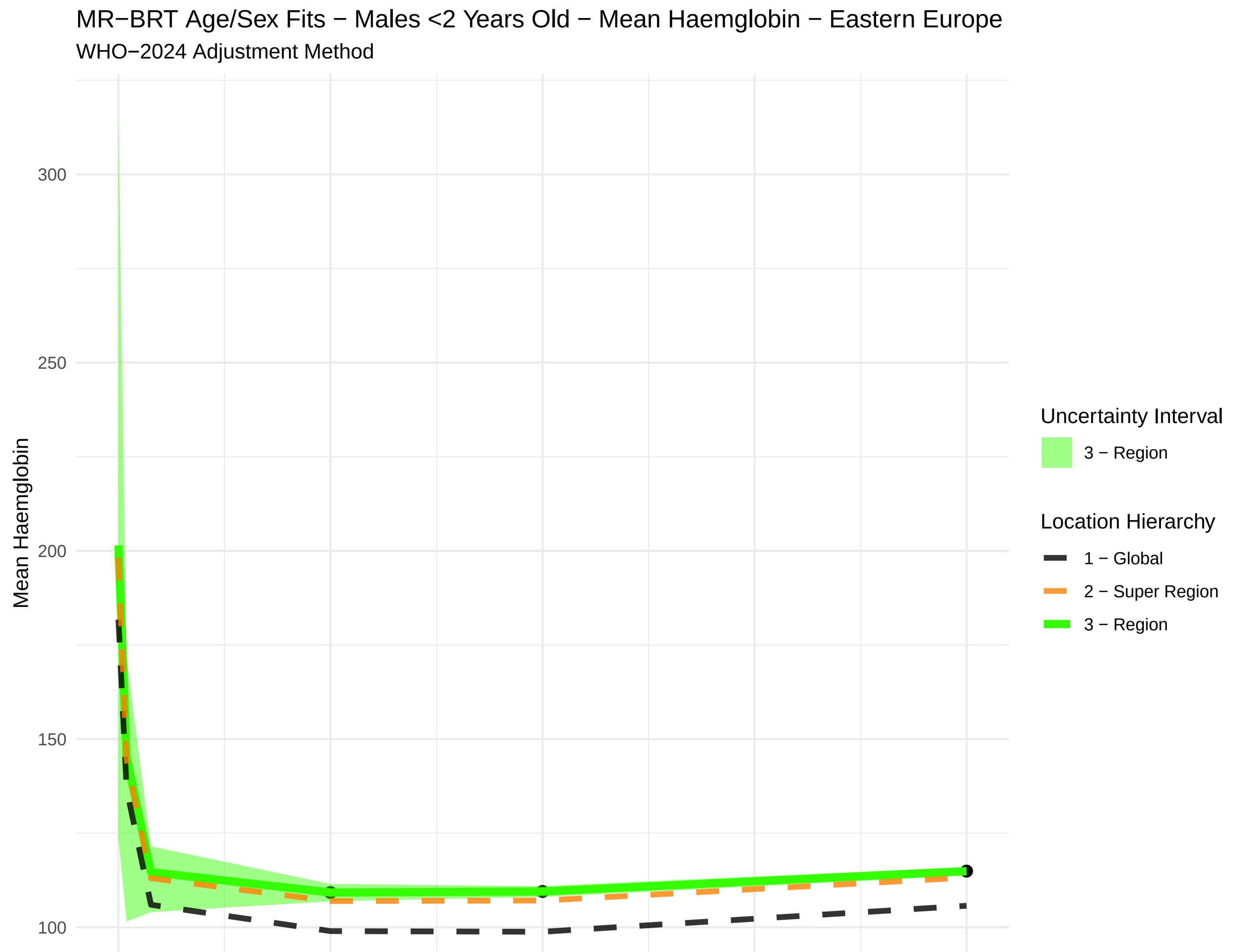
MR−BRT Age/Sex Fits − Males <2 Years Old − Mean Haemoglobin − Eastern Europe
WHO−2024 Adjustment Method
Mean Haemoglobin
Age (year)
Uncertainty Interval
3 − Region
Location Hierarchy
1 − Global
2 − Super Region
3 − Region

MR−BRT Age/Sex Fits − Males <2 Years Old − Mean Haemoglobin − Republic of Moldova
WHO−2024 Adjustment Method

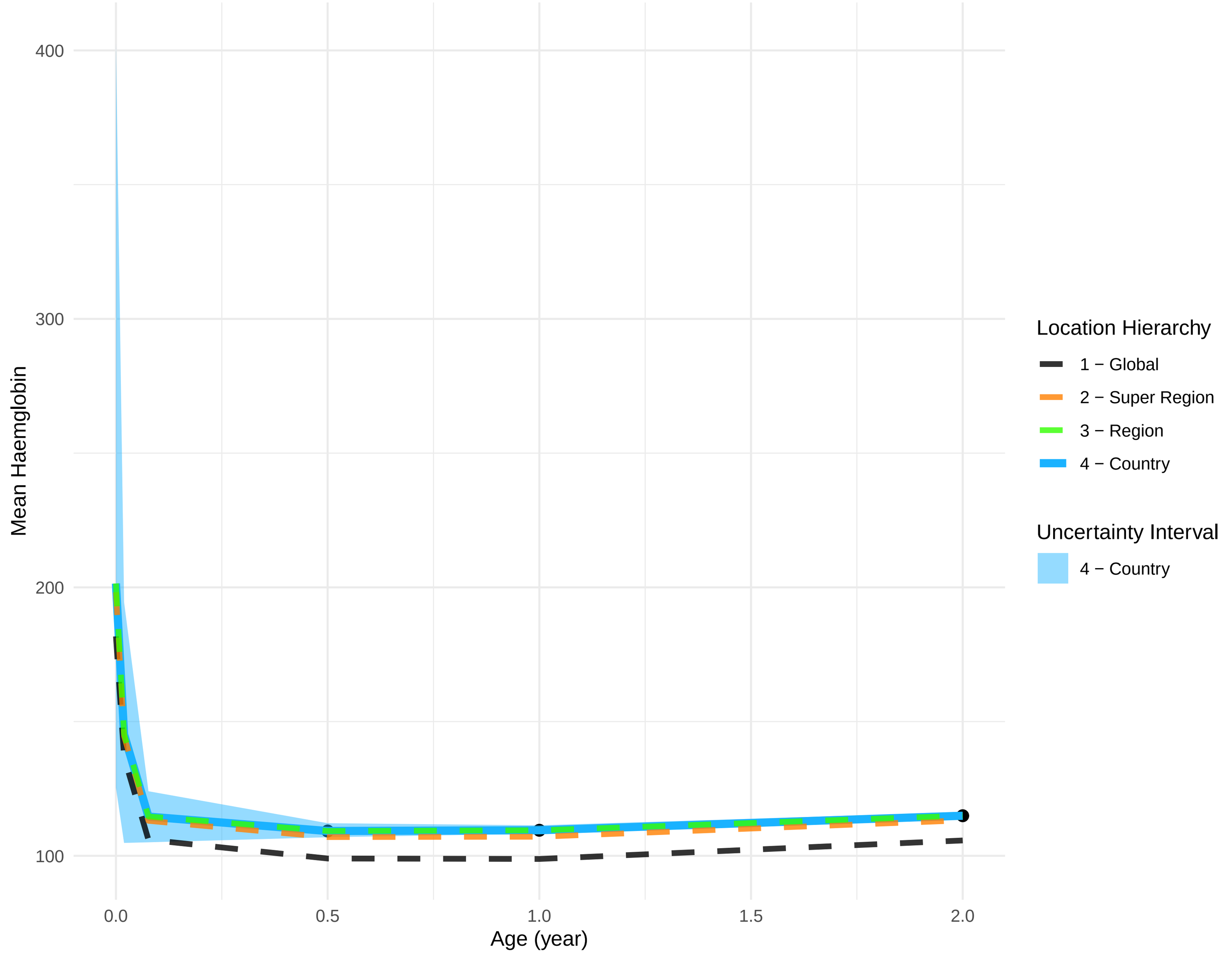

MR−BRT Age/Sex Fits − Males <2 Years Old − Mean Haemoglobin − High−income
WHO−2024 Adjustment Method

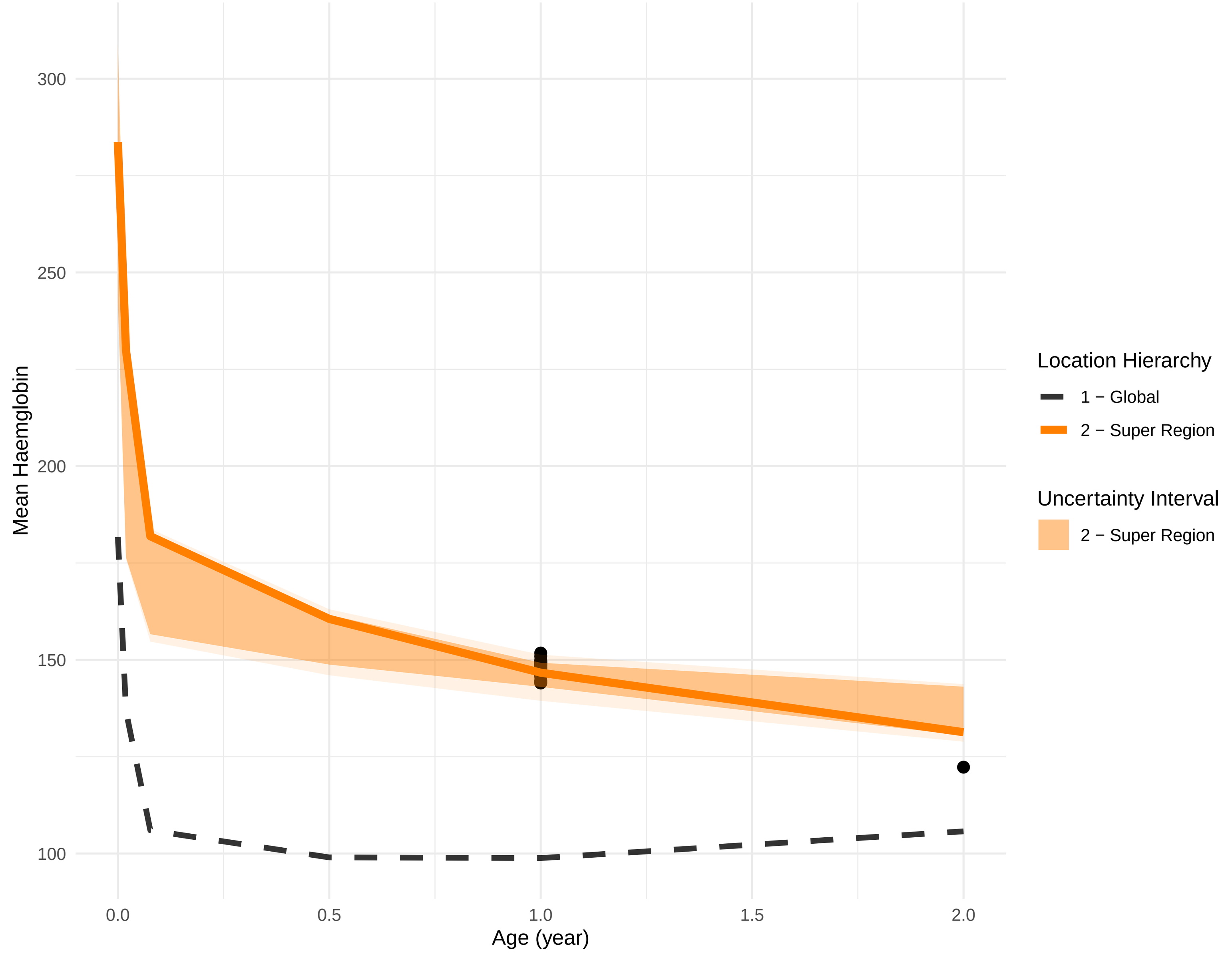

MR−BRT Age/Sex Fits − Males <2 Years Old − Mean Haemoglobin − Western Europe
WHO−2024 Adjustment Method

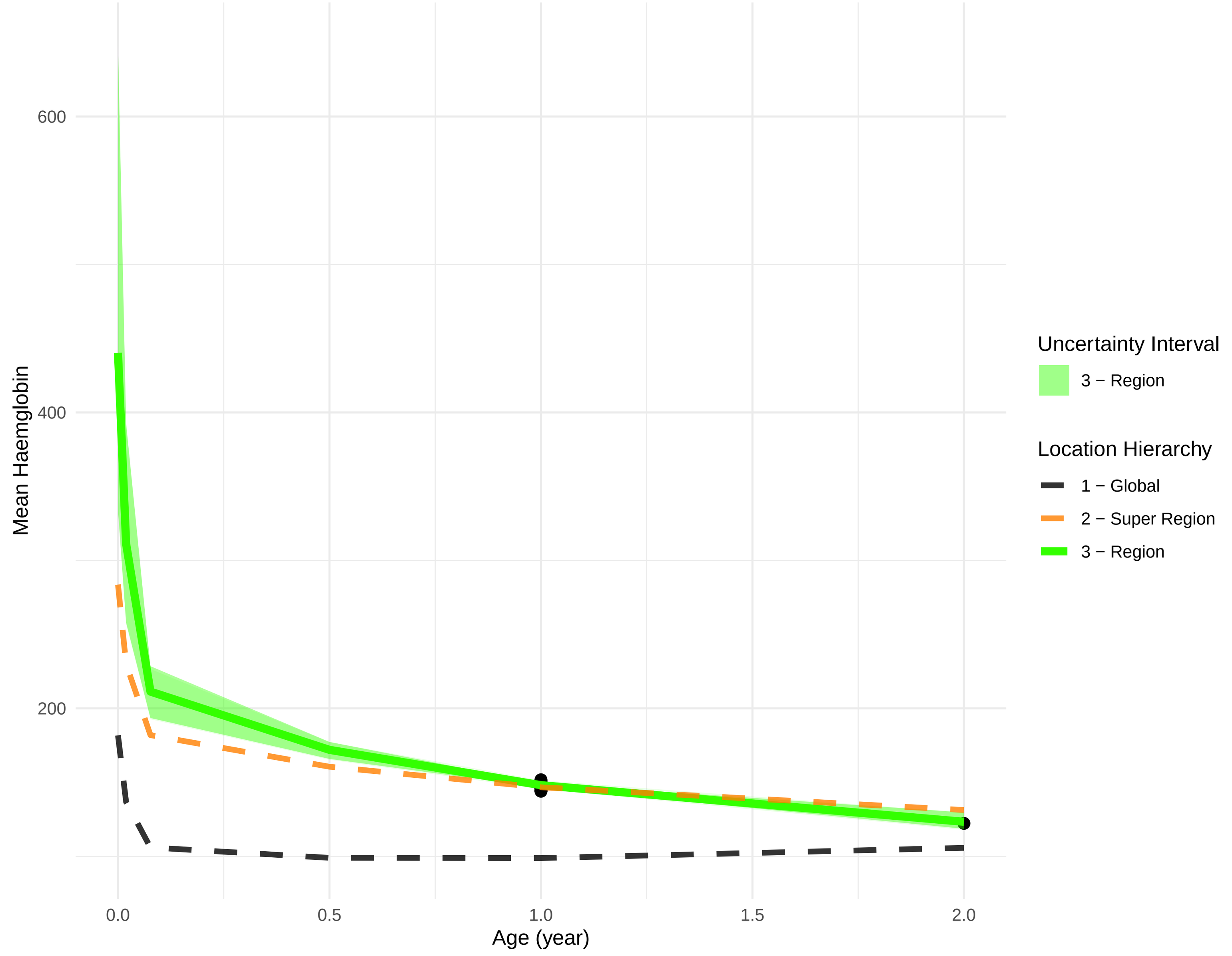

MR−BRT Age/Sex Fits − Males <2 Years Old − Mean Haemoglobin − United Kingdom
WHO−2024 Adjustment Method

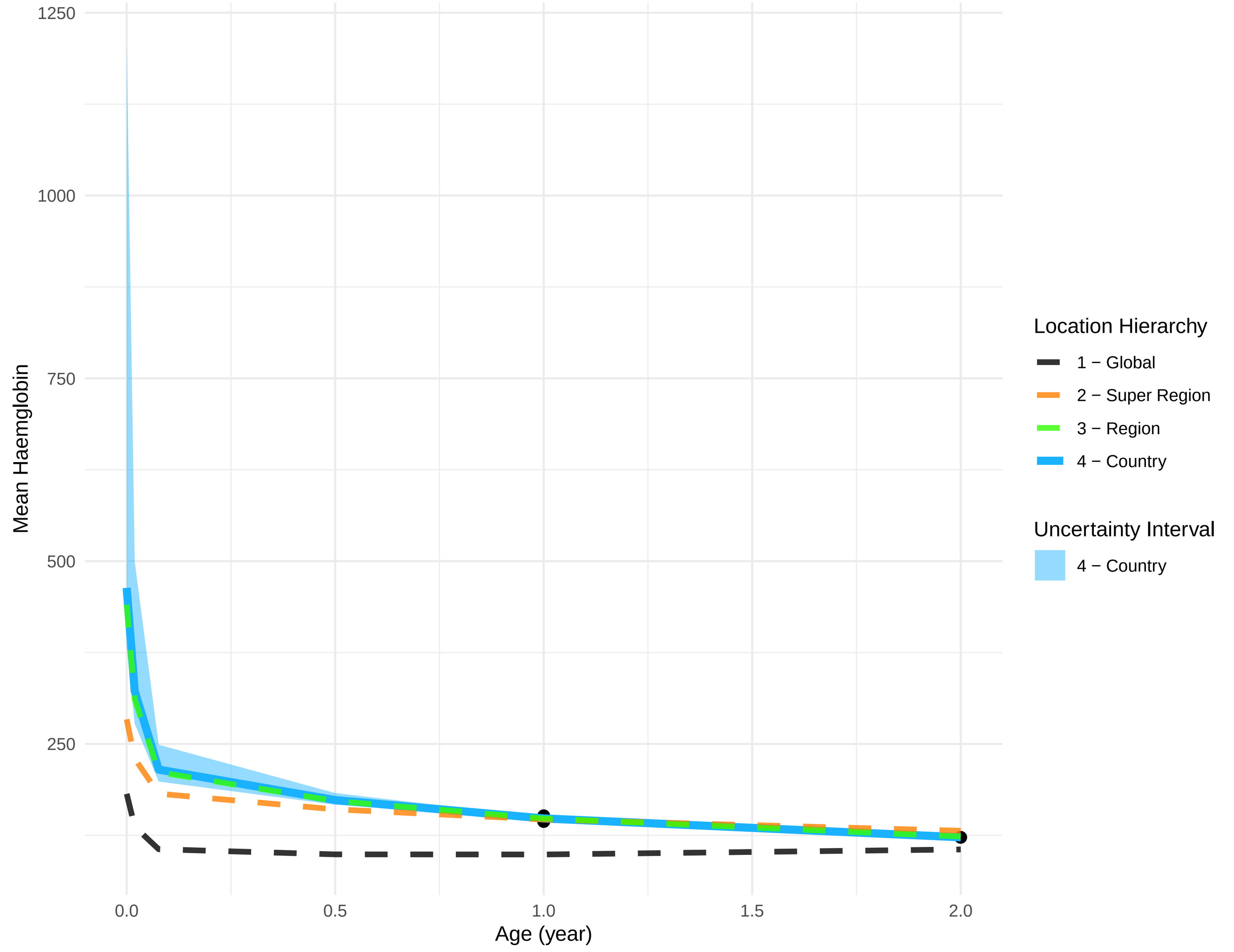

MR−BRT Age/Sex Fits − Males <2 Years Old − Mean Haemoglobin − England
WHO−2024 Adjustment Method

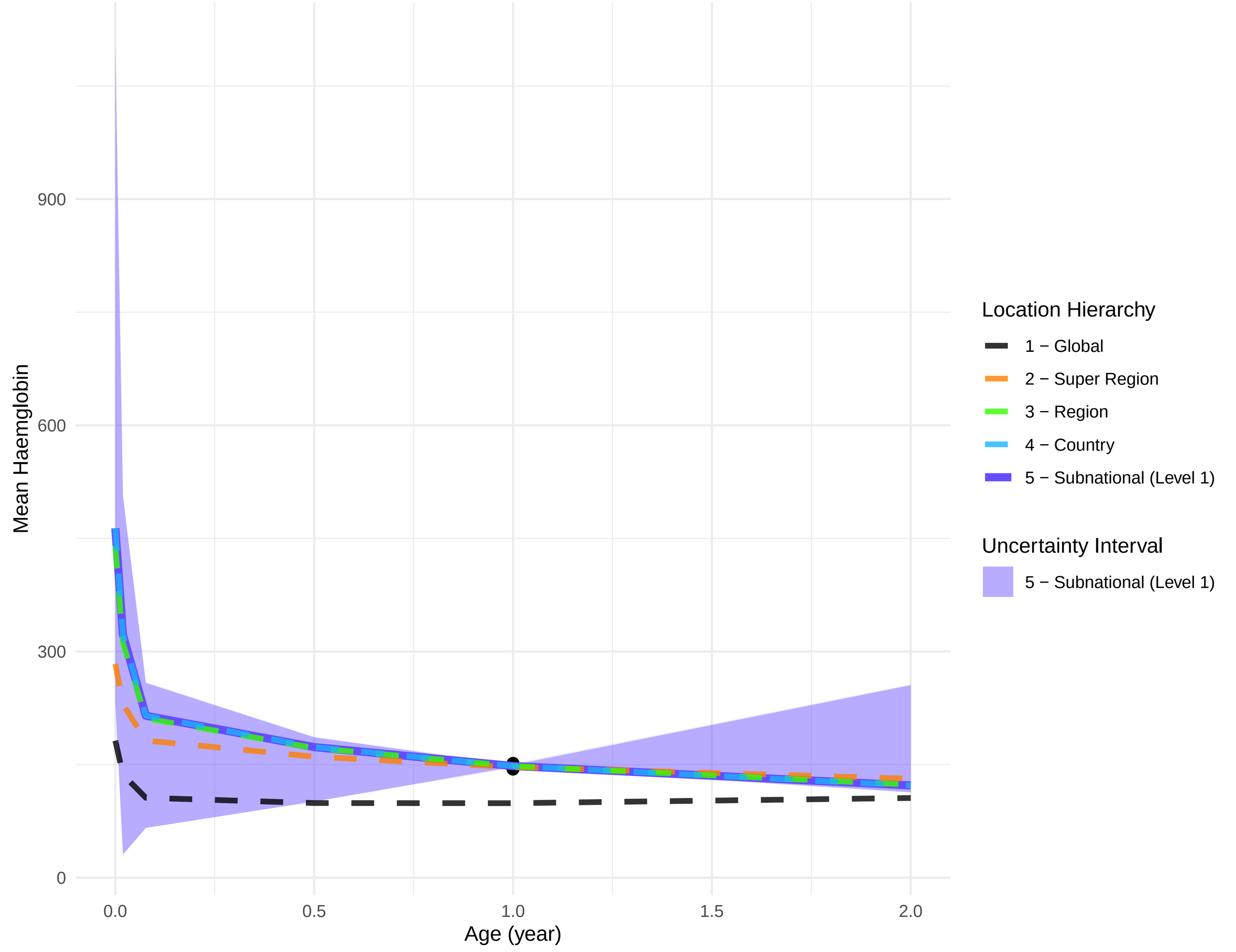

MR−BRT Age/Sex Fits − Males <2 Years Old − Mean Haemoglobin − East Midlands
WHO−2024 Adjustment Method

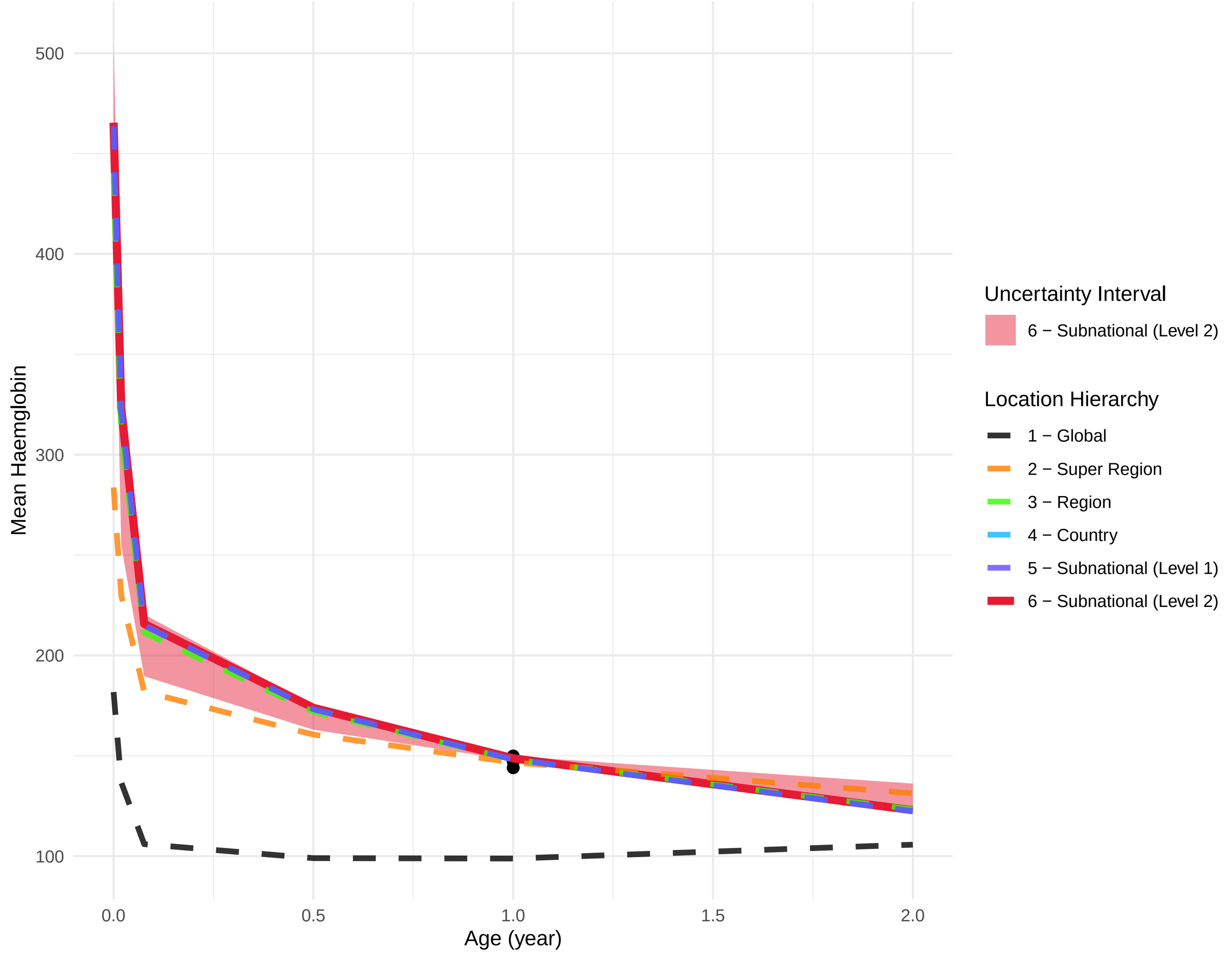

MR−BRT Age/Sex Fits − Males <2 Years Old − Mean Haemoglobin − East of England
WHO−2024 Adjustment Method

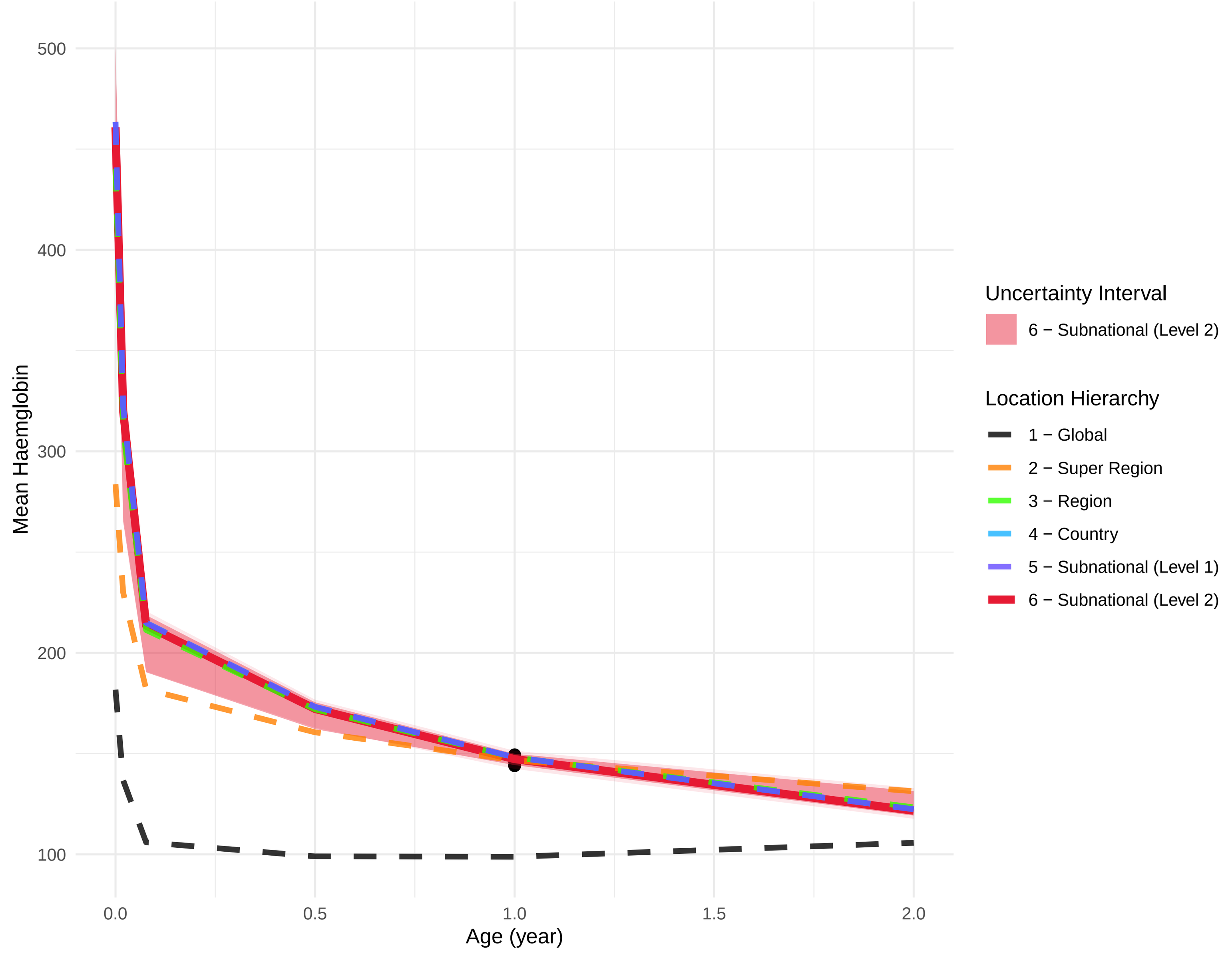

MR−BRT Age/Sex Fits − Males <2 Years Old − Mean Haemoglobin − Greater London
WHO−2024 Adjustment Method

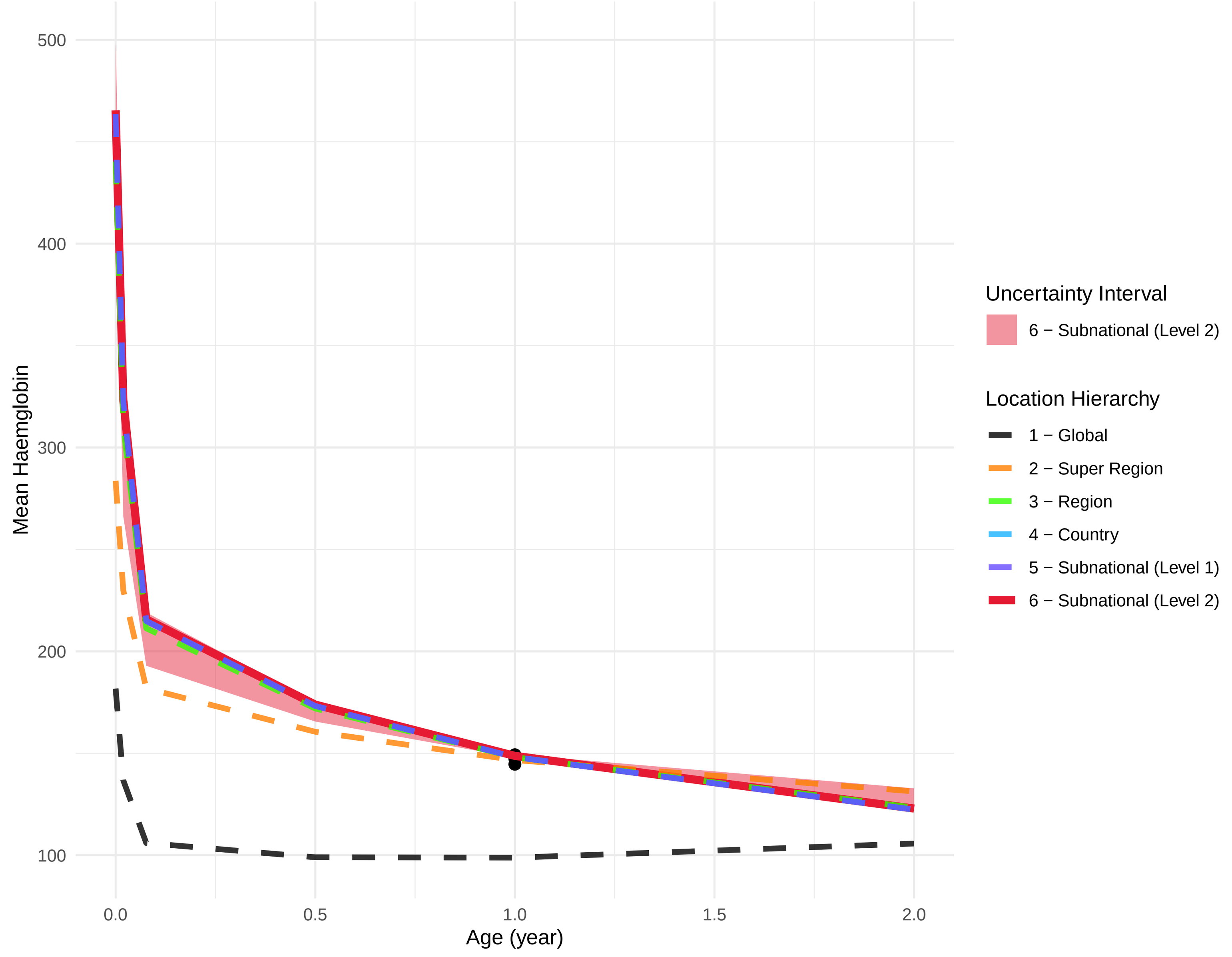

MR−BRT Age/Sex Fits − Males <2 Years Old − Mean Haemoglobin − North East England
WHO−2024 Adjustment Method

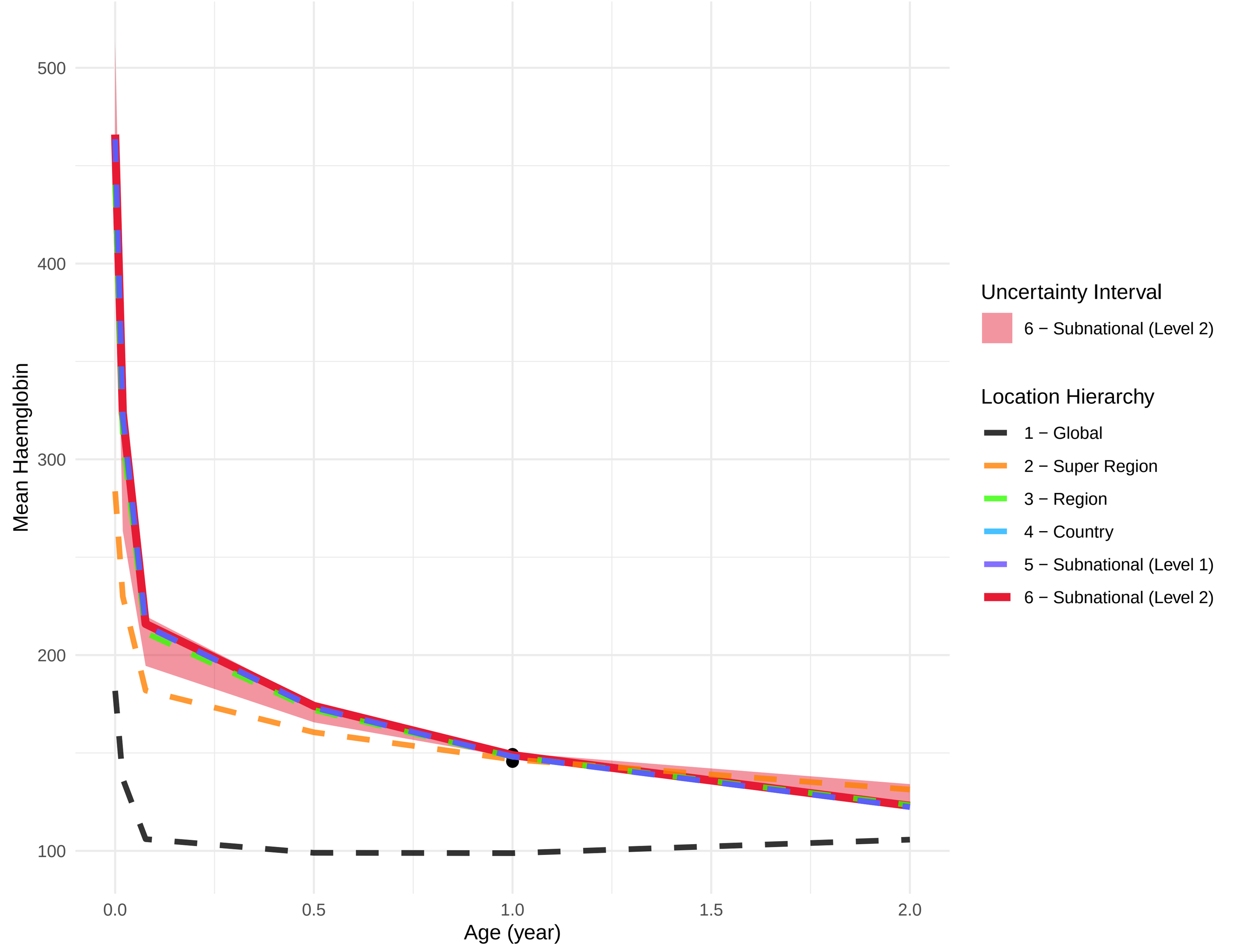

MR−BRT Age/Sex Fits − Males <2 Years Old − Mean Haemoglobin − North West England
WHO−2024 Adjustment Method

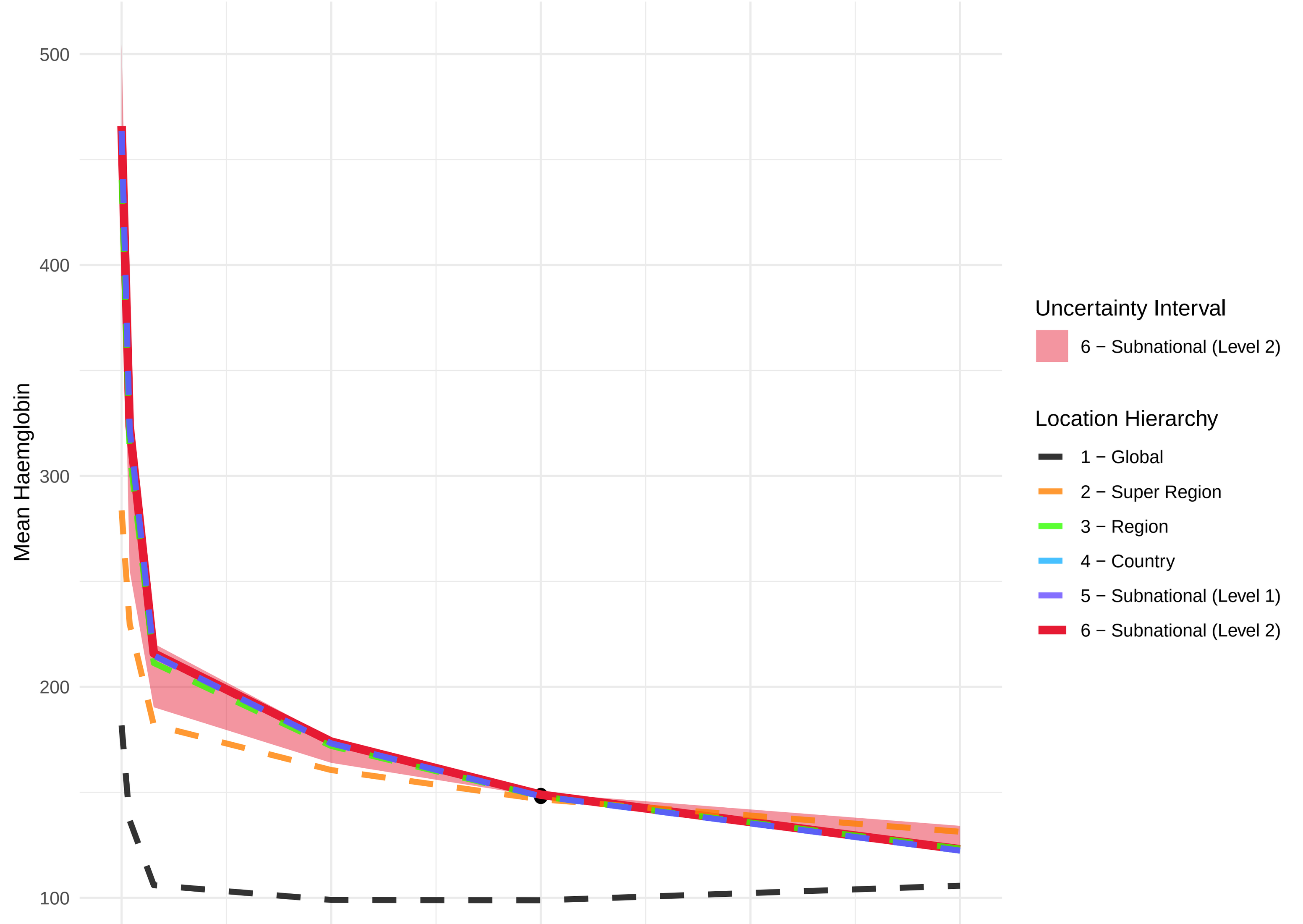

MR−BRT Age/Sex Fits − Males <2 Years Old − Mean Haemglobin − South East England
WHO−2024 Adjustment Method

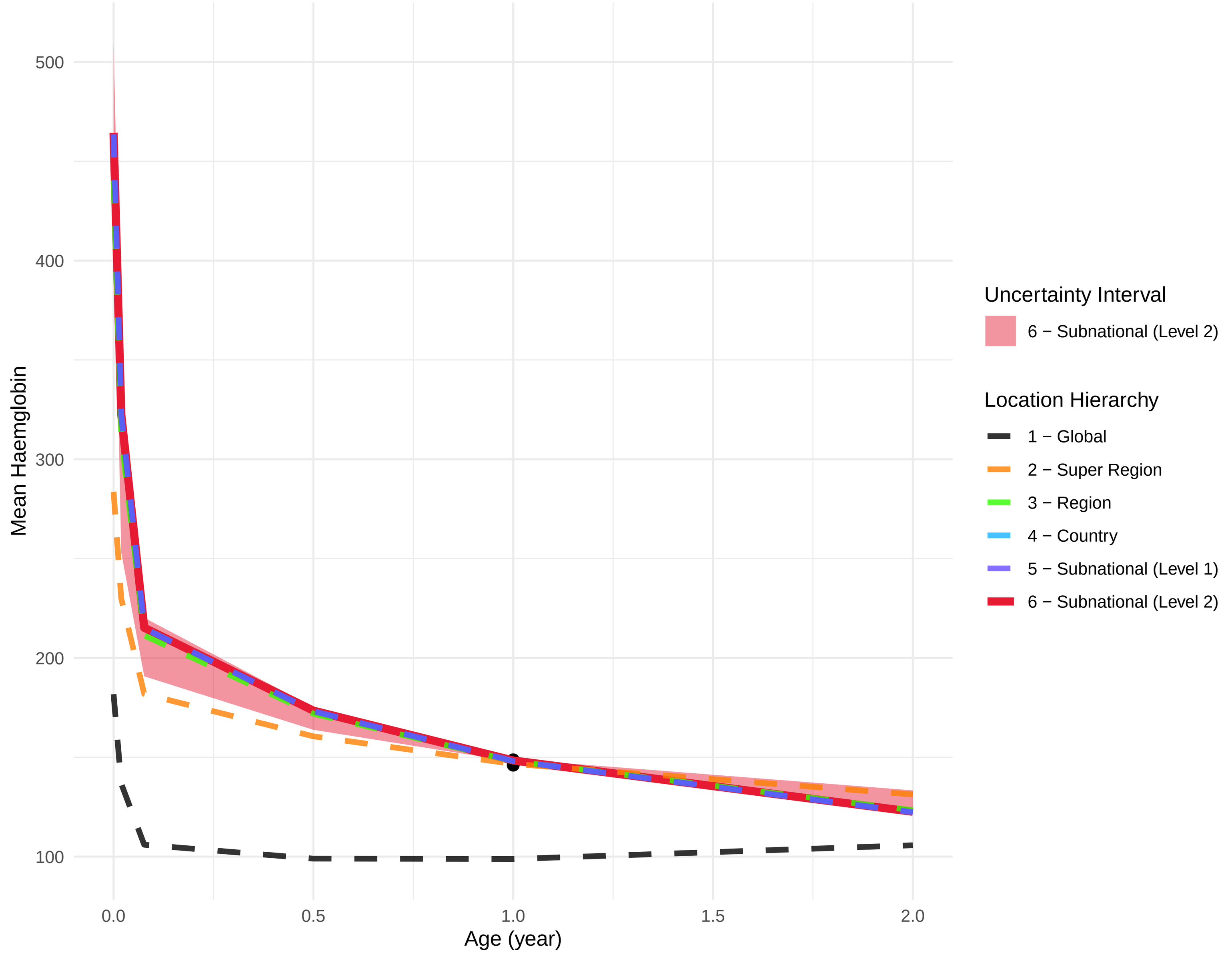

MR−BRT Age/Sex Fits − Males <2 Years Old − Mean Haemglobin − South West England
WHO−2024 Adjustment Method

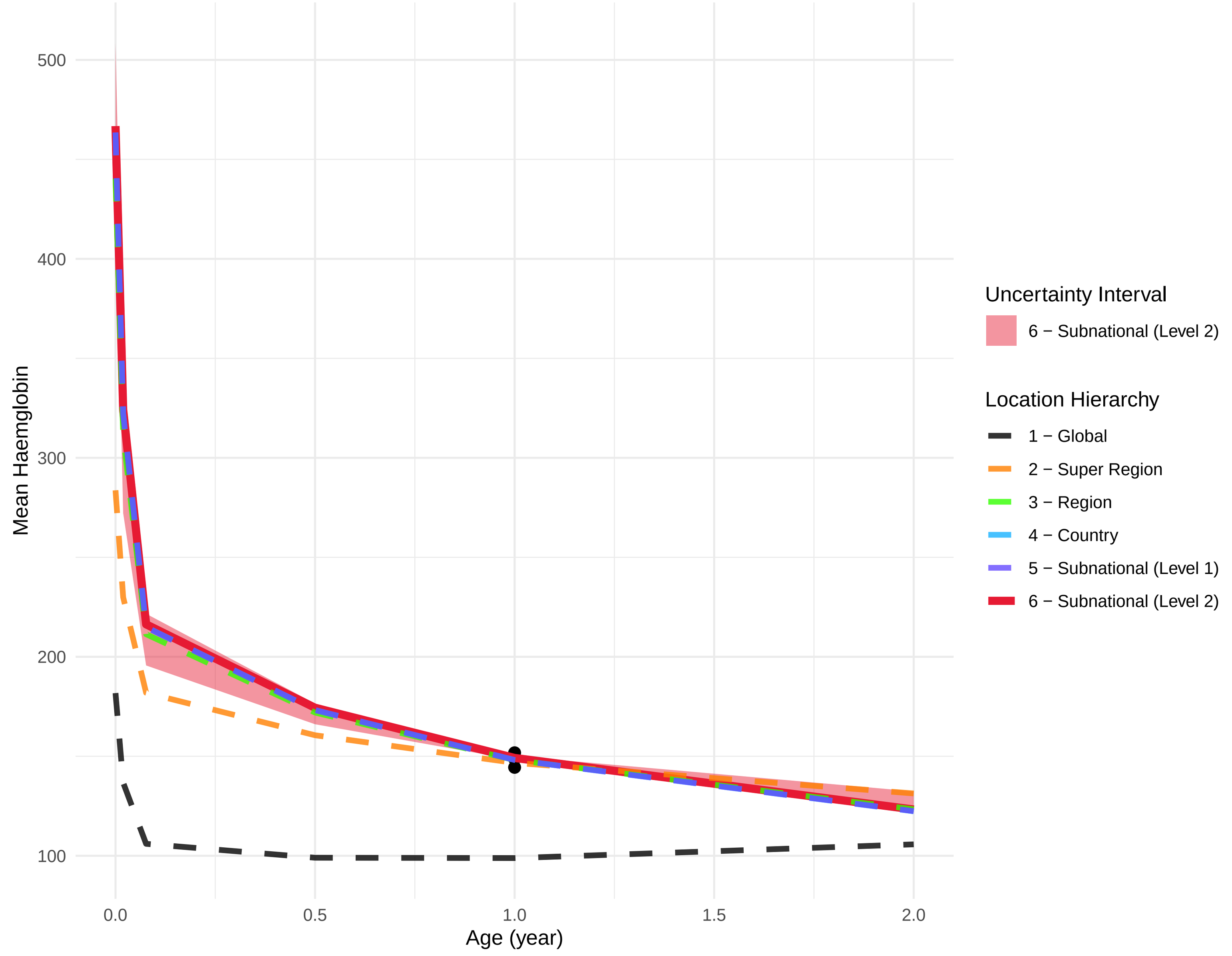

MR−BRT Age/Sex Fits − Males <2 Years Old − Mean Haemglobin − West Midlands
WHO−2024 Adjustment Method

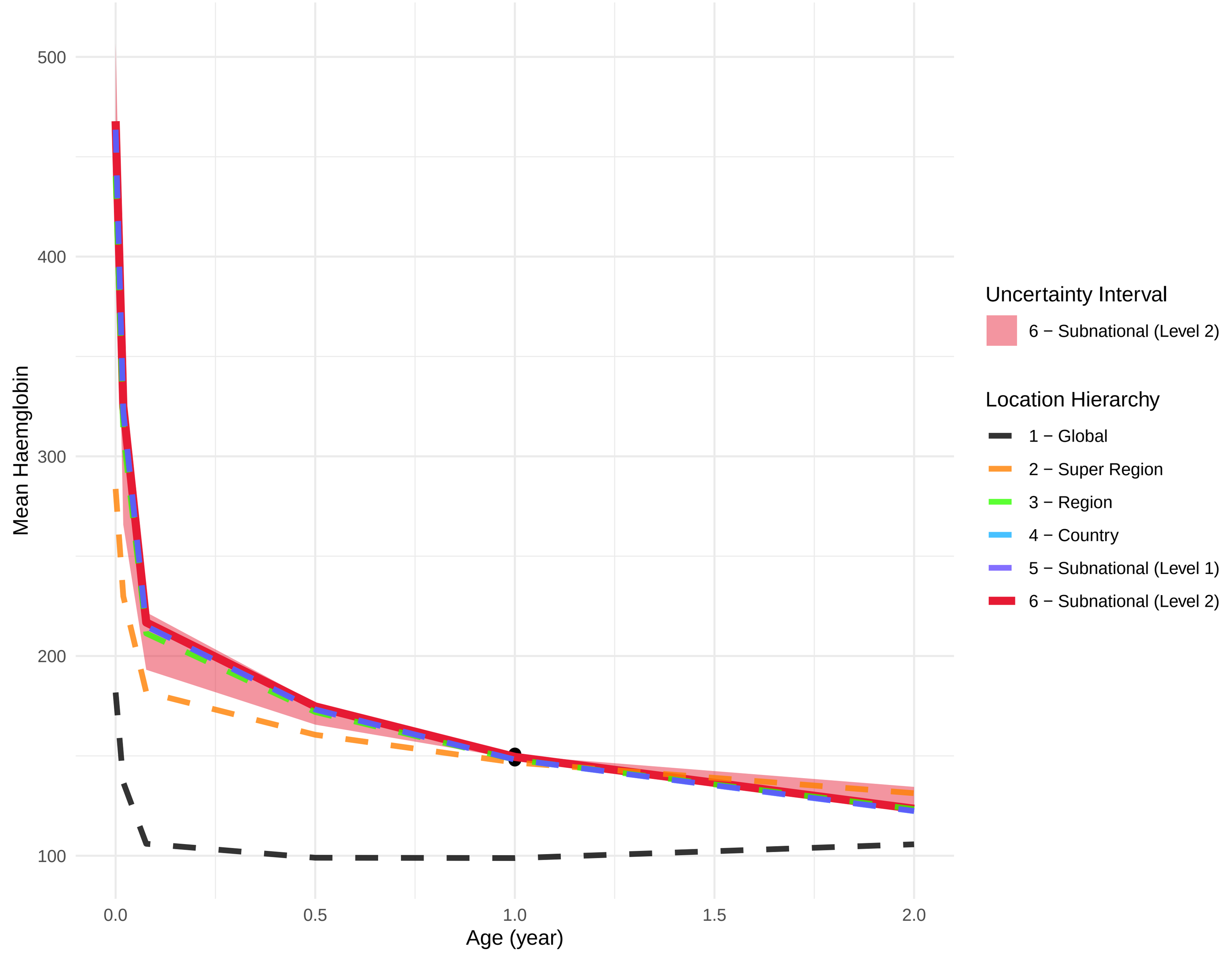

MR−BRT Age/Sex Fits − Males <2 Years Old − Mean Haemglobin − Yorkshire and the Humber
WHO−2024 Adjustment Method

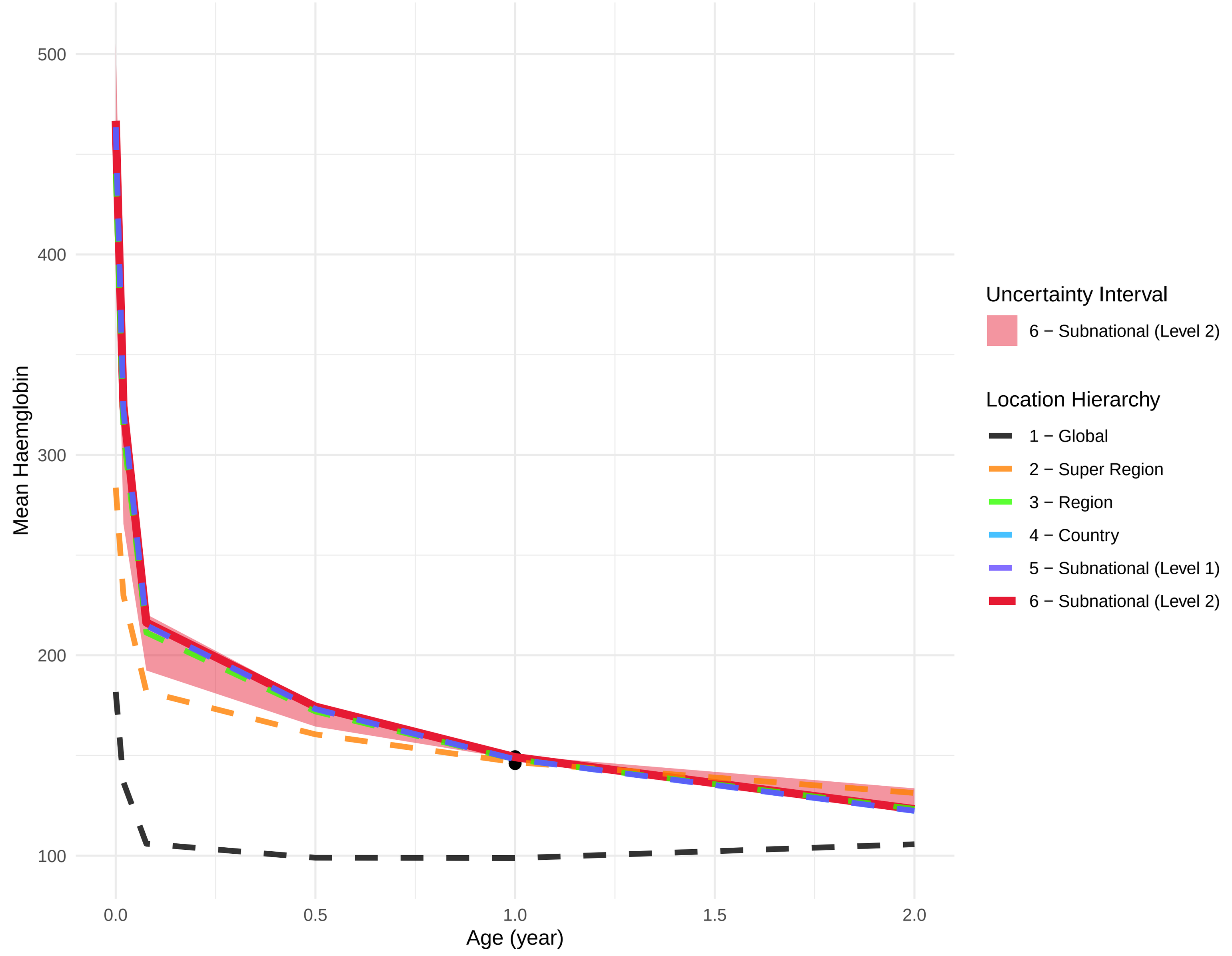

MR−BRT Age/Sex Fits − Males <2 Years Old − Mean Haemglobin − Latin America and Caribbean
WHO−2024 Adjustment Method

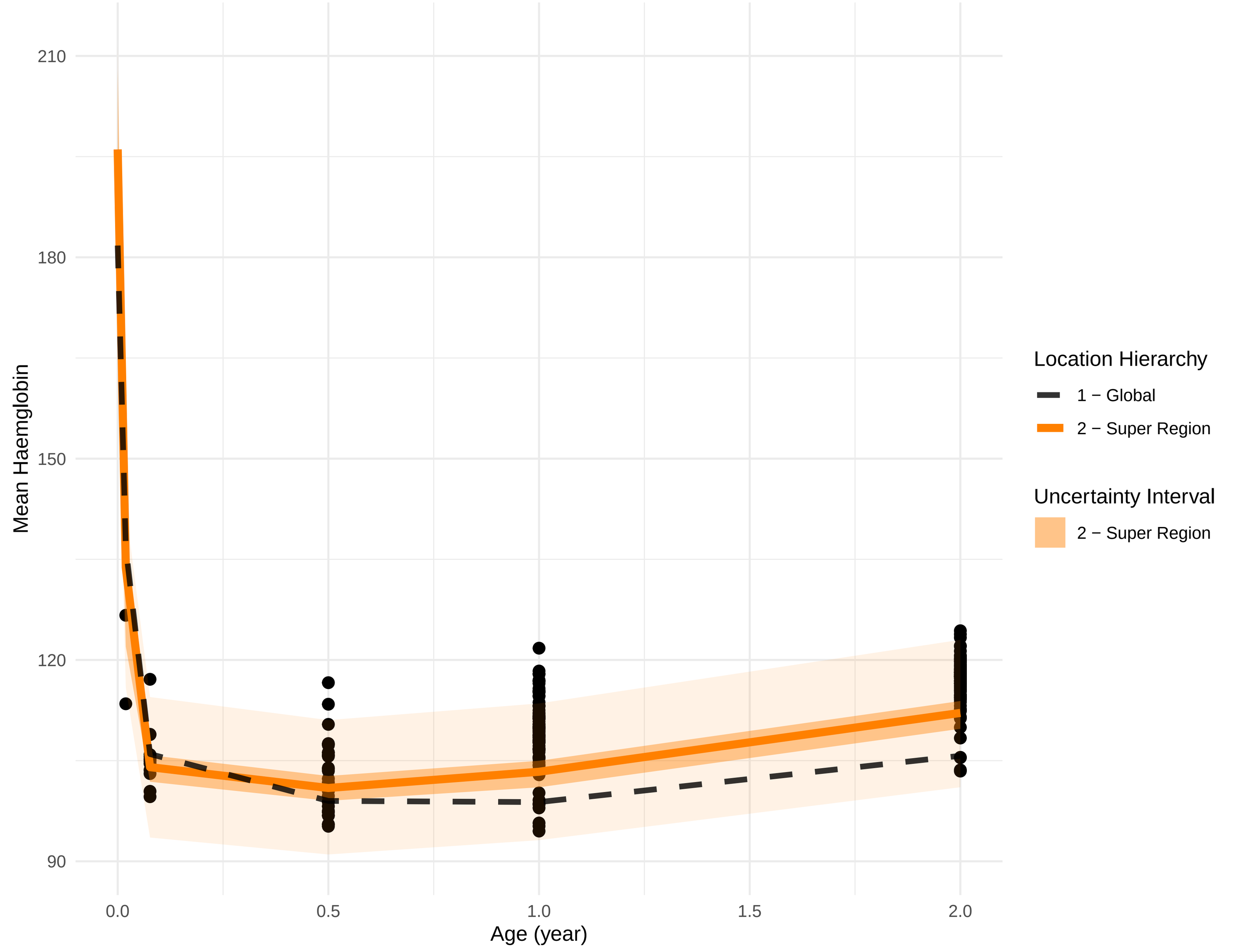

MR−BRT Age/Sex Fits − Males <2 Years Old − Mean Haemglobin − Andean Latin America
WHO−2024 Adjustment Method

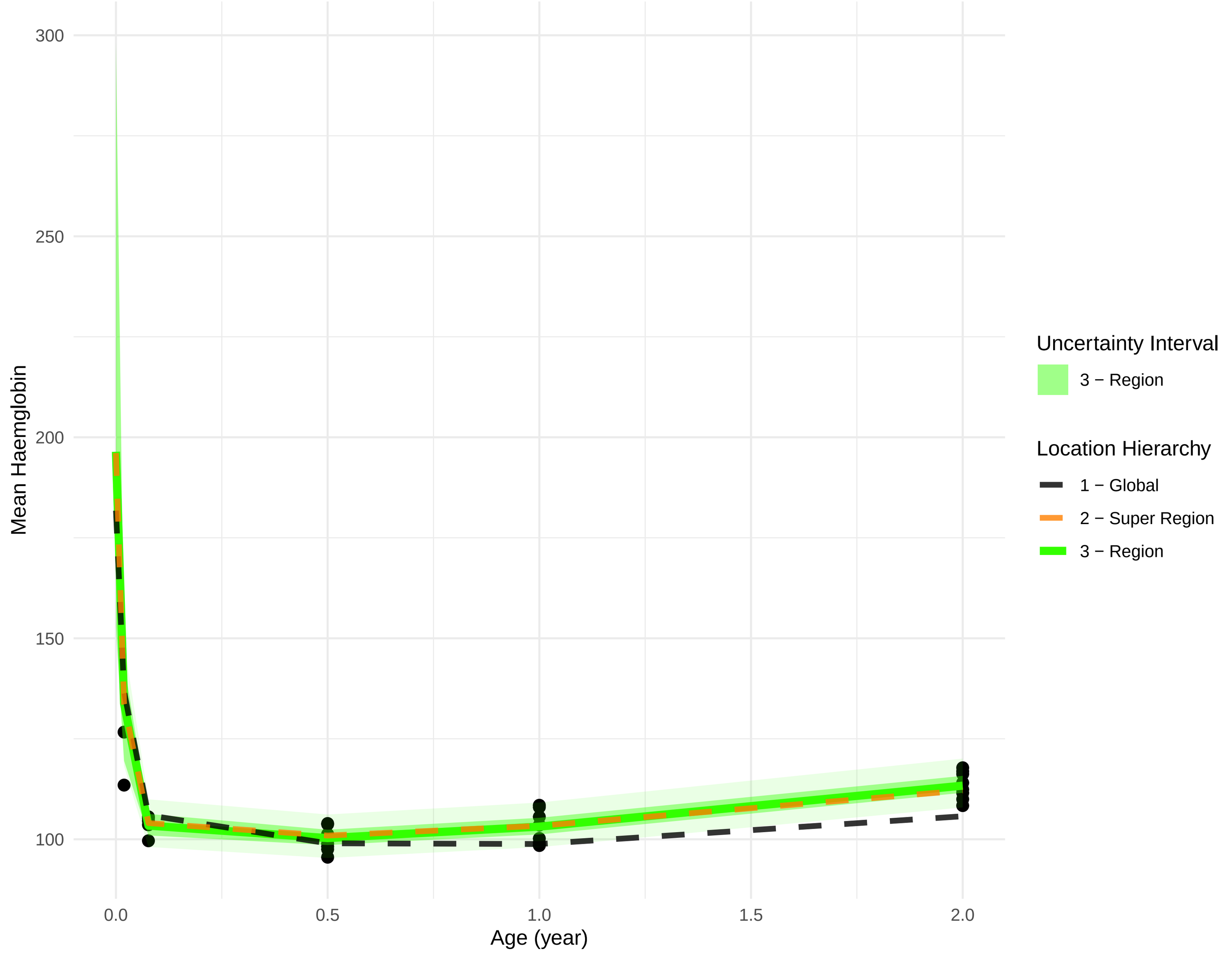

MR−BRT Age/Sex Fits − Males <2 Years Old − Mean Haemglobin − Bolivia (Plurinational State of)
WHO−2024 Adjustment Method

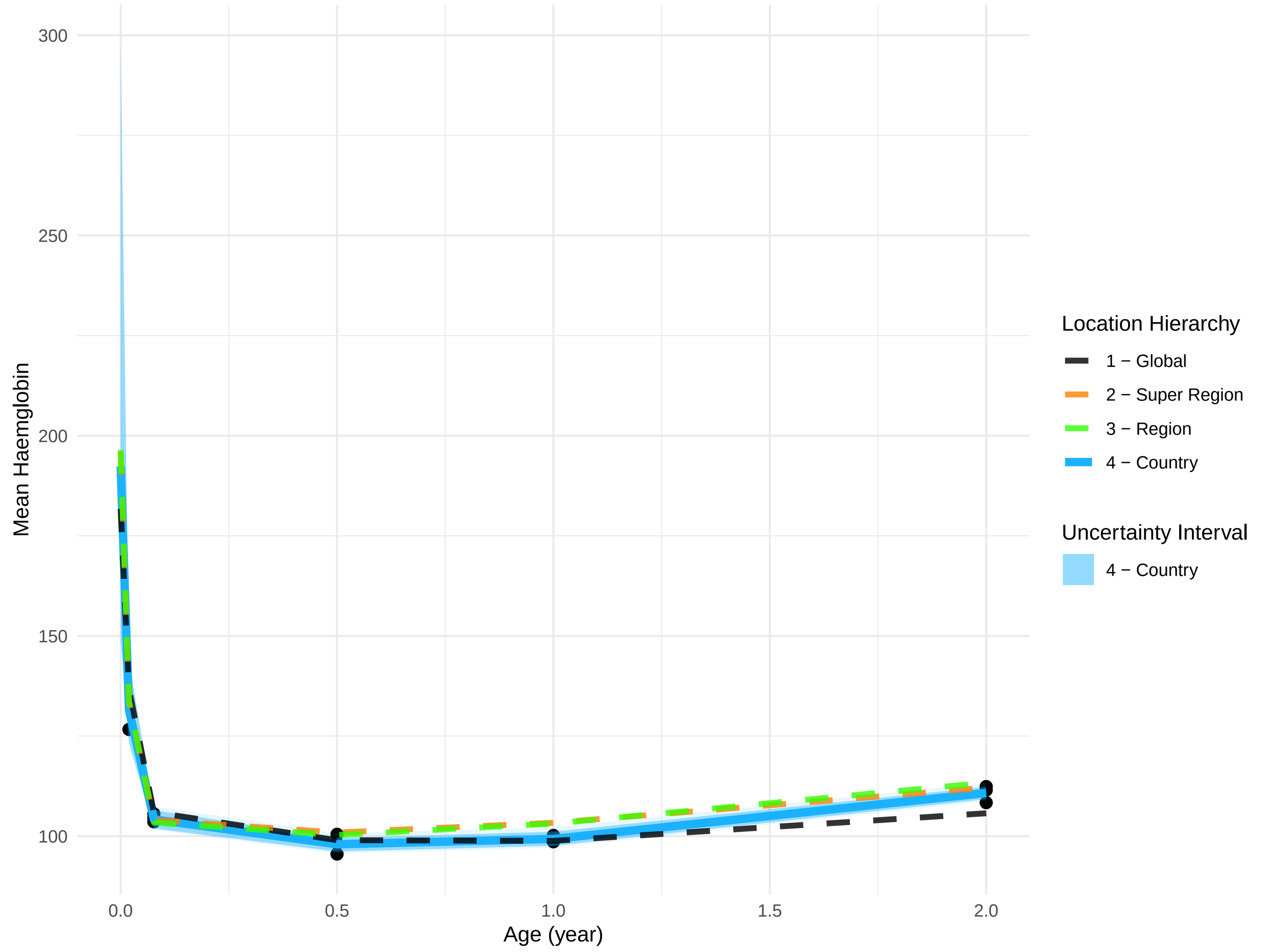

MR−BRT Age/Sex Fits − Males <2 Years Old − Mean Haemglobin − Peru
WHO−2024 Adjustment Method

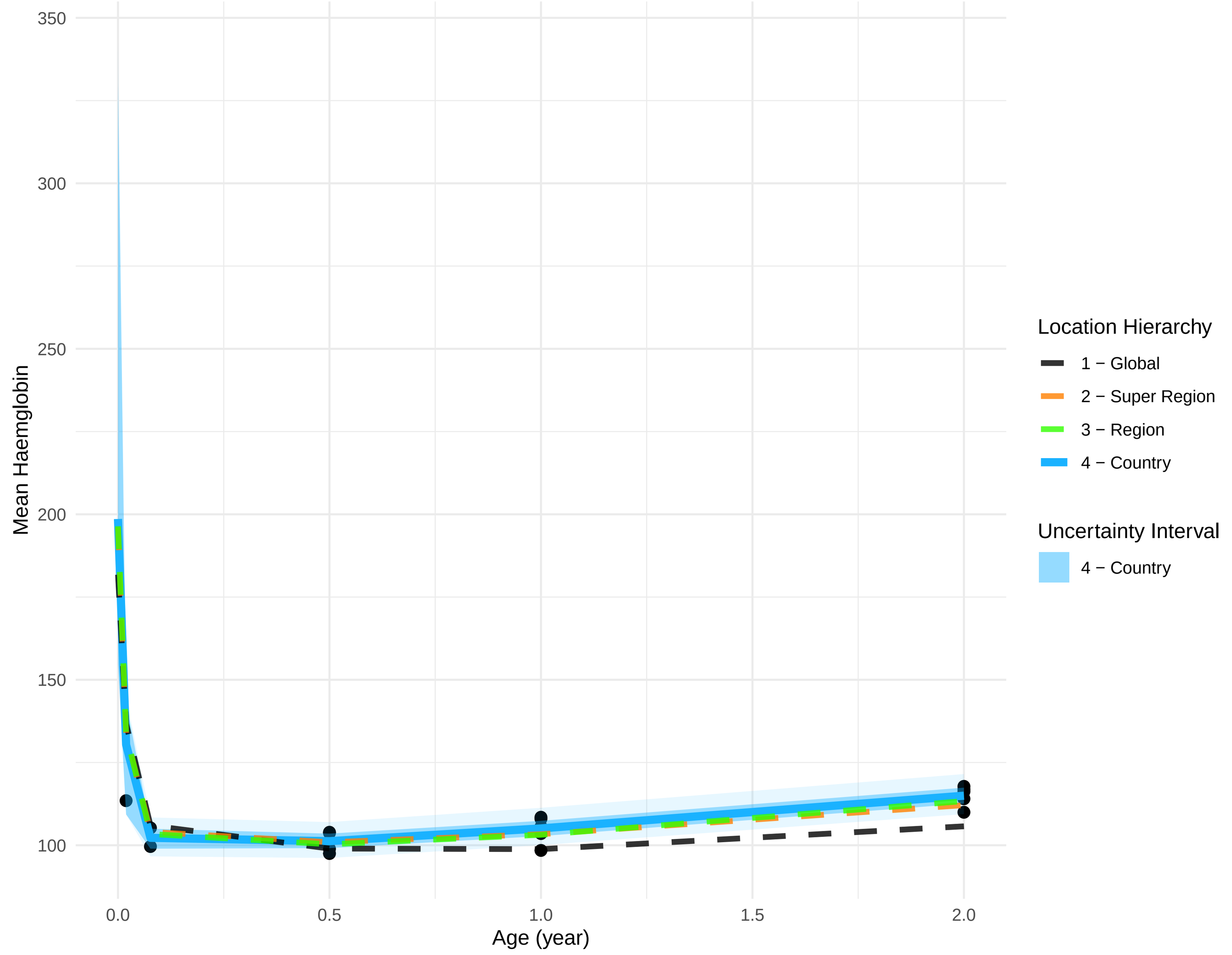

MR−BRT Age/Sex Fits − Males <2 Years Old − Mean Haemglobin − Caribbean
WHO−2024 Adjustment Method

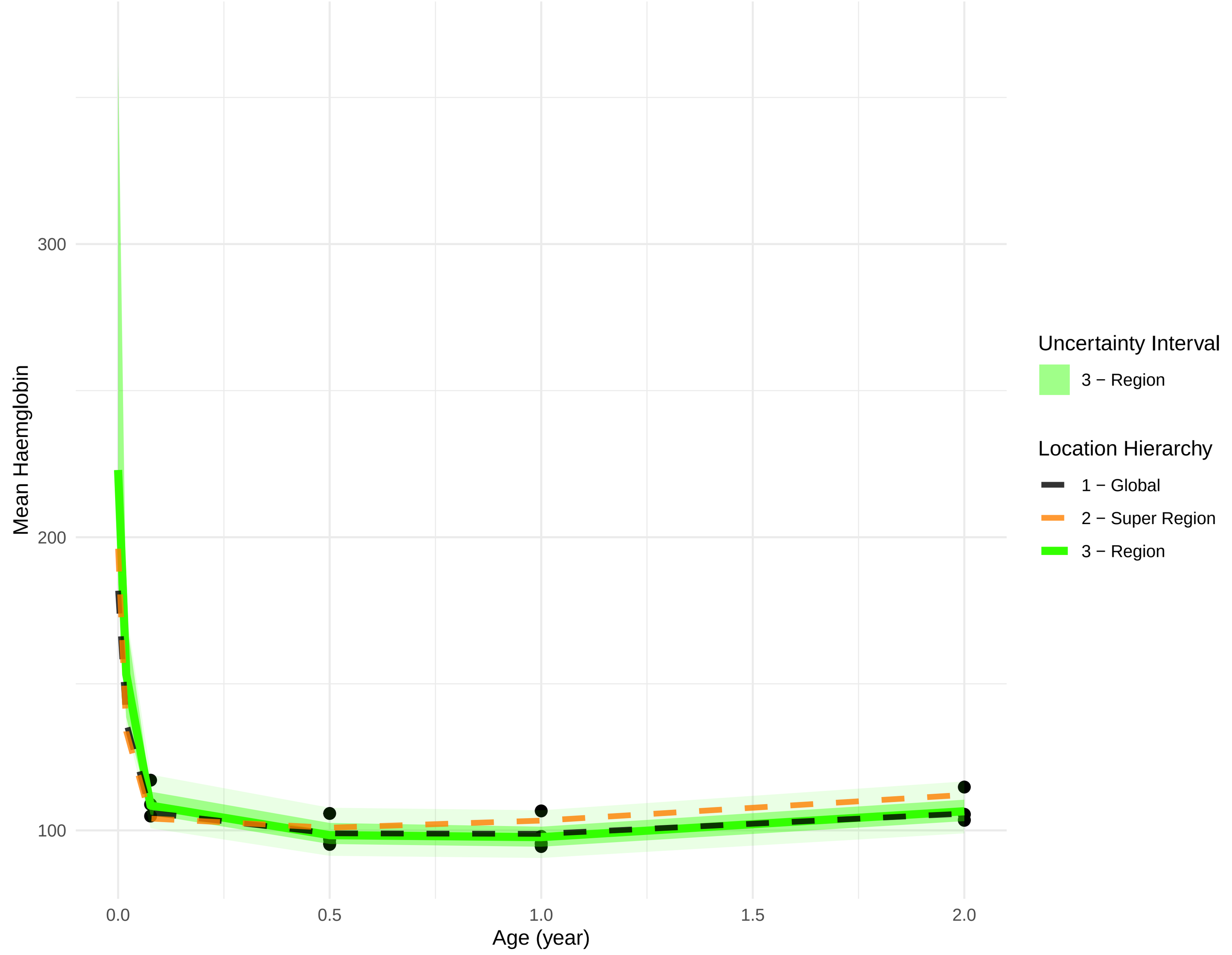

MR−BRT Age/Sex Fits − Males <2 Years Old − Mean Haemglobin − Guyana
WHO−2024 Adjustment Method

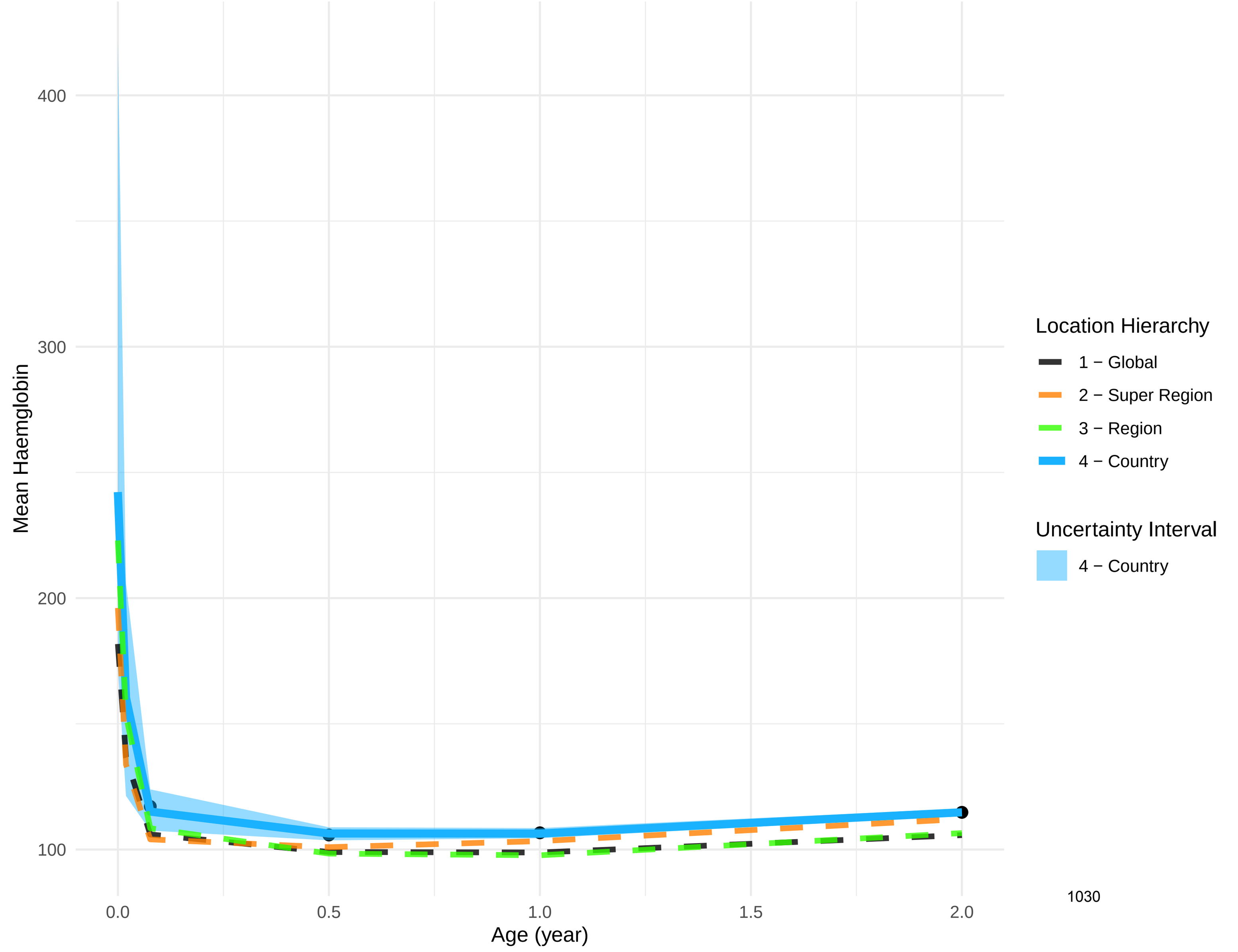

MR−BRT Age/Sex Fits − Males <2 Years Old − Mean Haemglobin − Haiti
WHO−2024 Adjustment Method

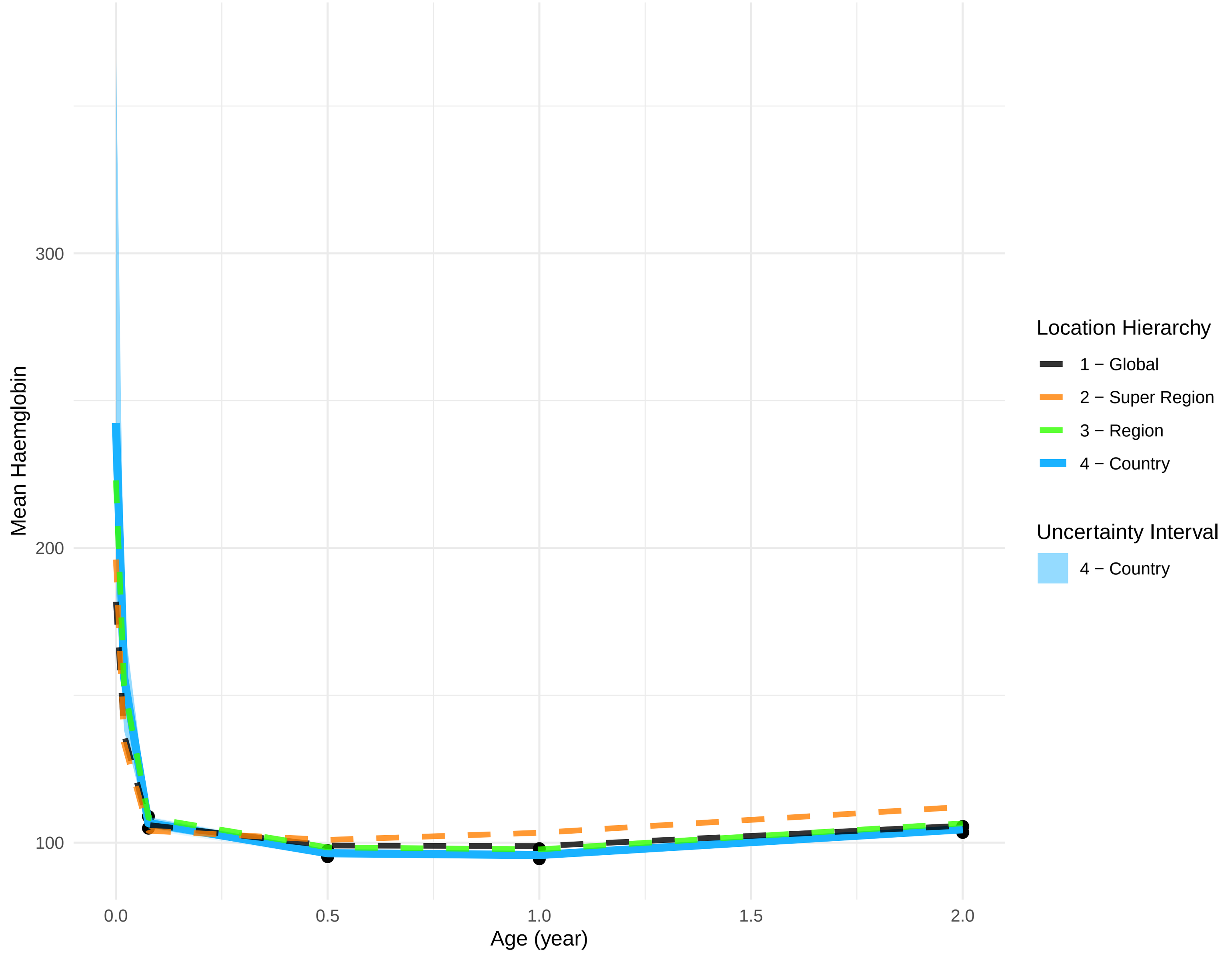

MR−BRT Age/Sex Fits − Males <2 Years Old − Mean Haemglobin − Central Latin America
WHO−2024 Adjustment Method

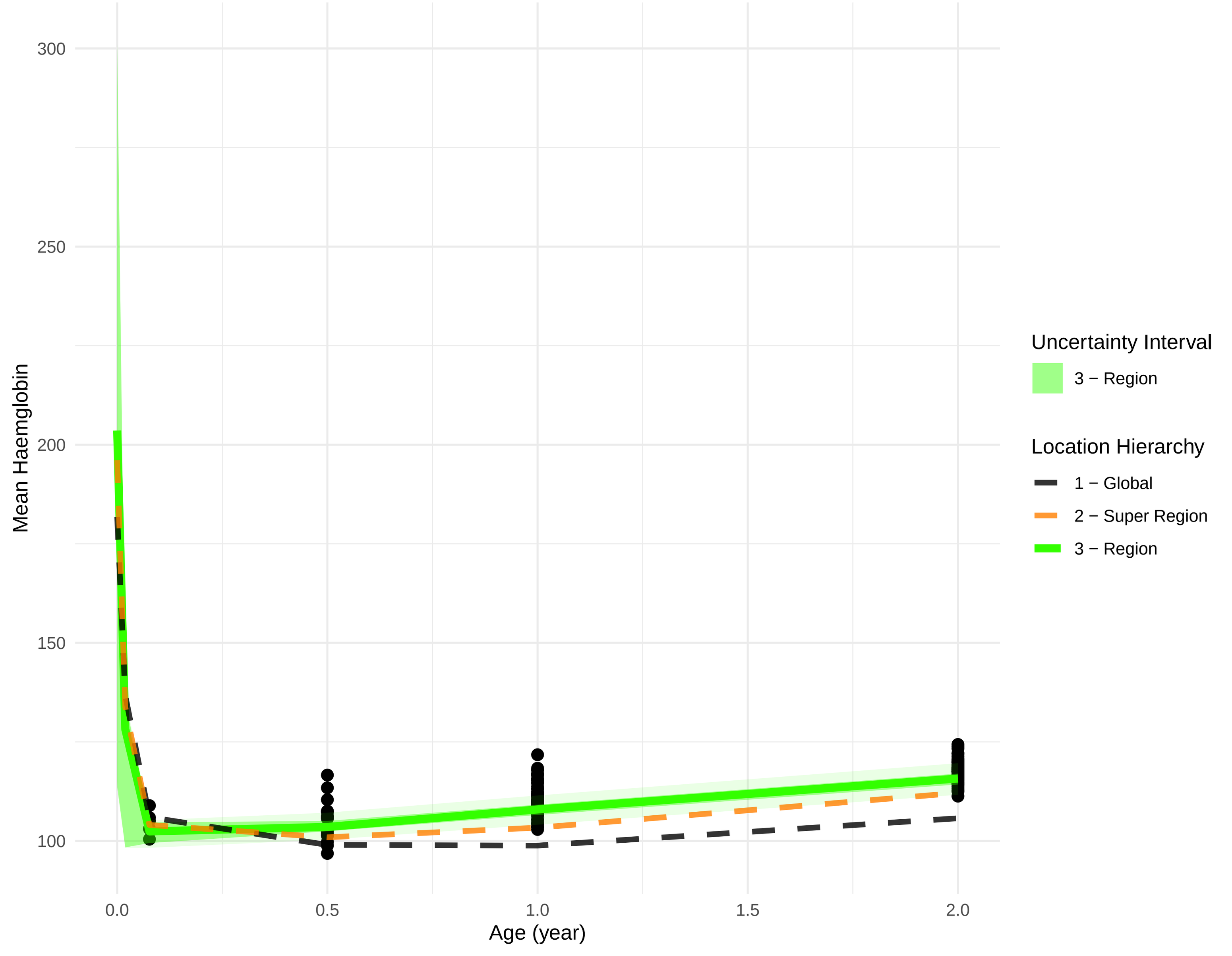

MR−BRT Age/Sex Fits − Males <2 Years Old − Mean Haemglobin − Guatemala
WHO−2024 Adjustment Method

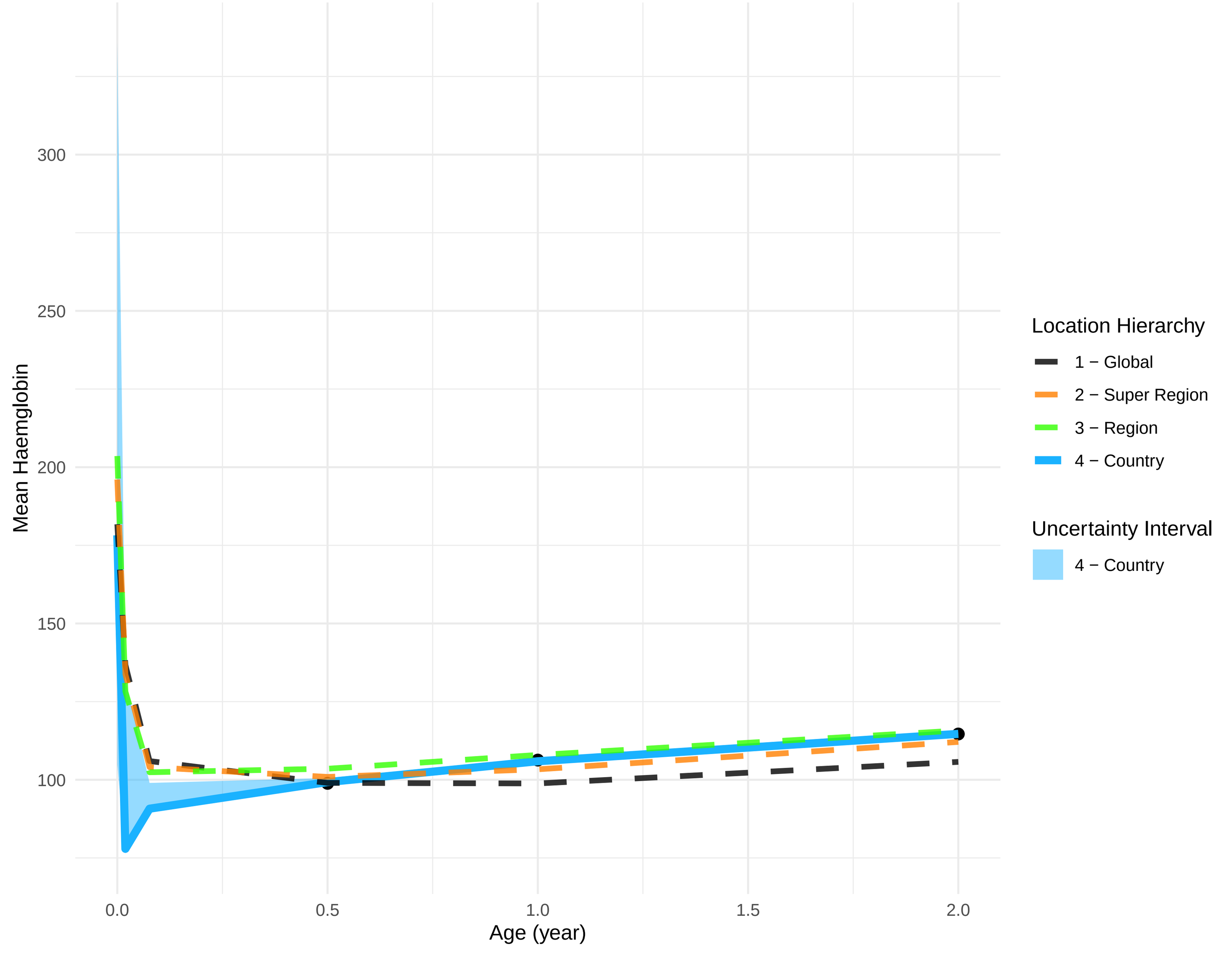

MR−BRT Age/Sex Fits − Males <2 Years Old − Mean Haemglobin − Honduras
WHO−2024 Adjustment Method

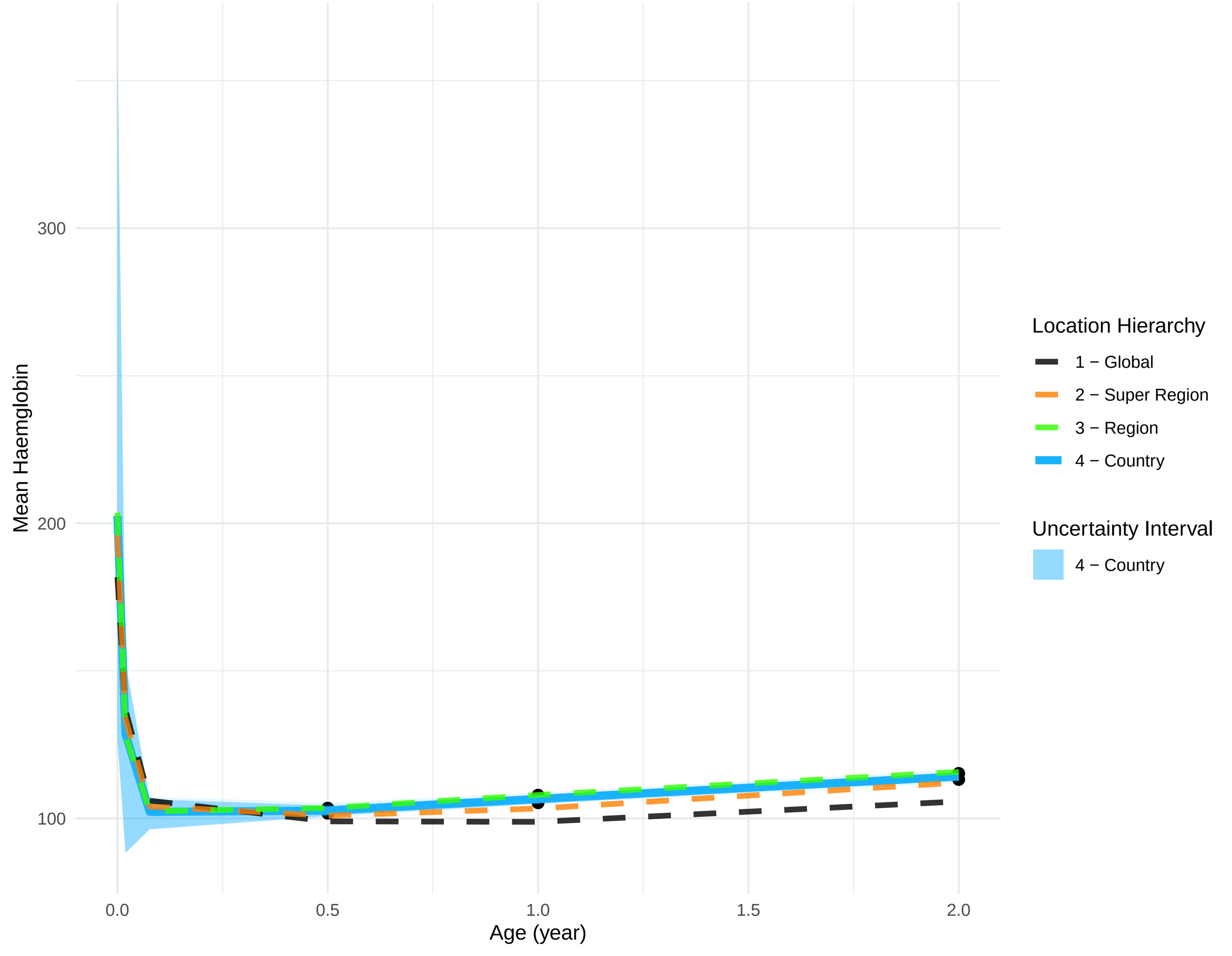

MR−BRT Age/Sex Fits − Males <2 Years Old − Mean Haemglobin − Mexico
WHO−2024 Adjustment Method

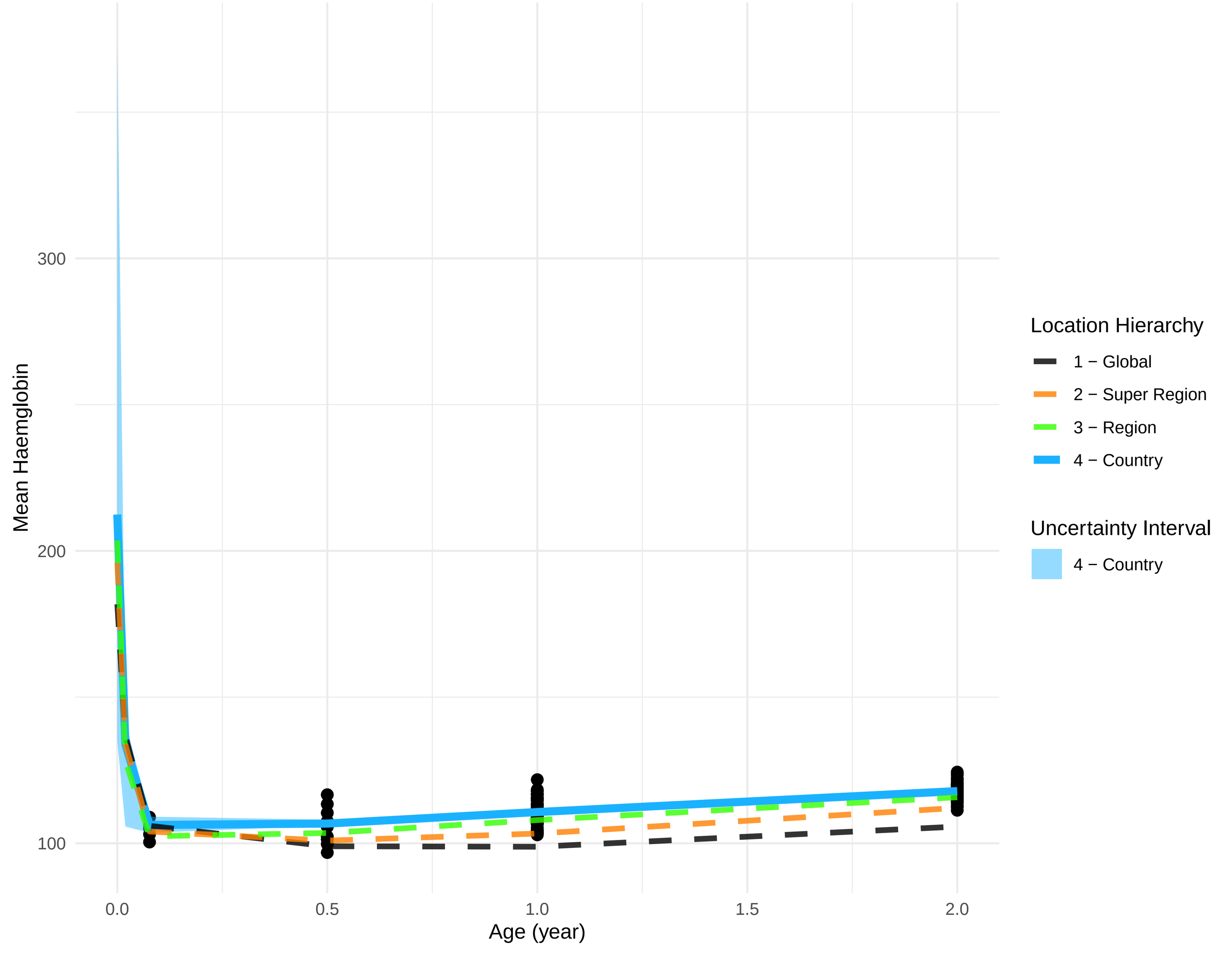

MR−BRT Age/Sex Fits − Males <2 Years Old − Mean Haemglobin − Aguascalientes
WHO−2024 Adjustment Method

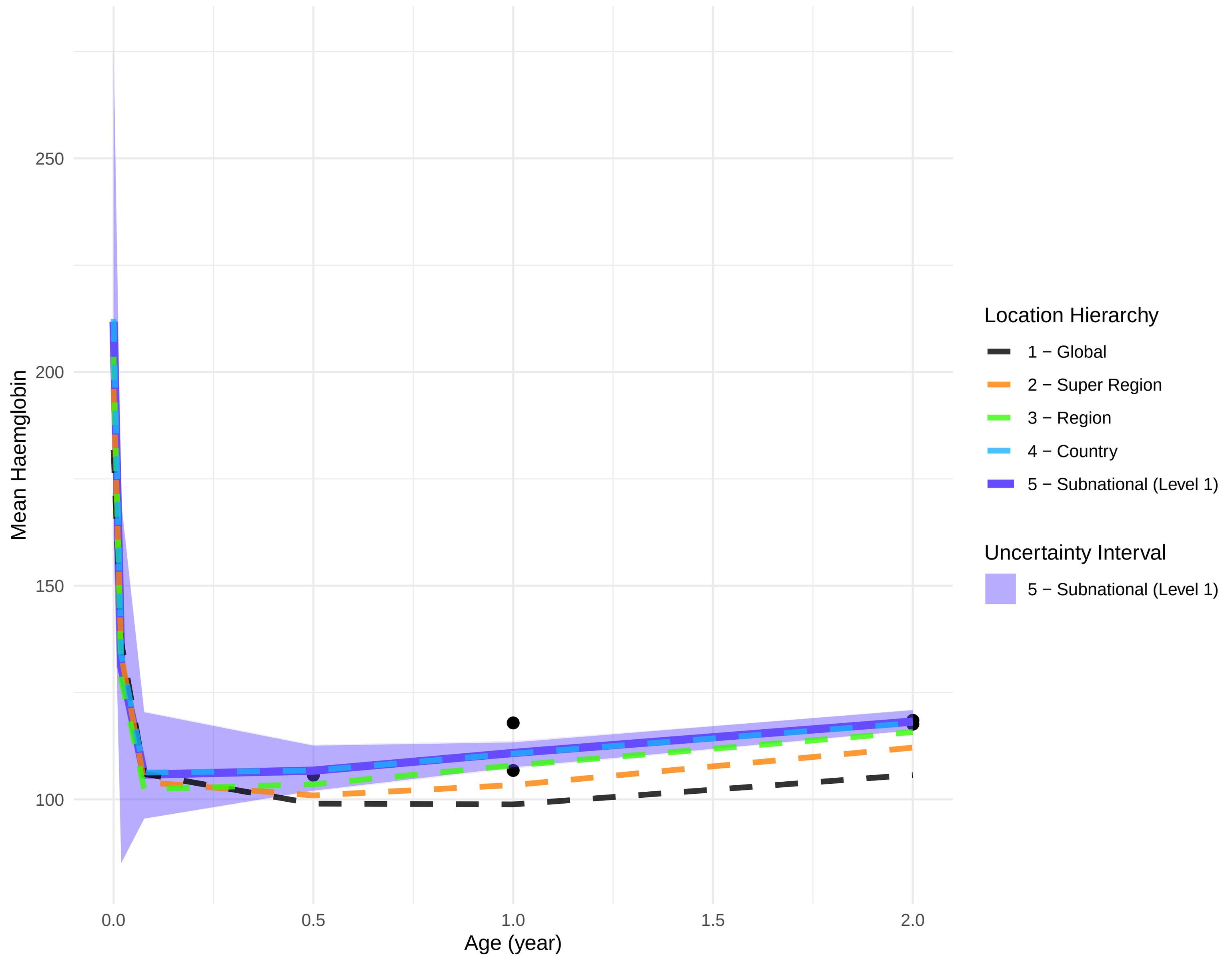

MR−BRT Age/Sex Fits − Males <2 Years Old − Mean Haemglobin − Baja California
WHO−2024 Adjustment Method

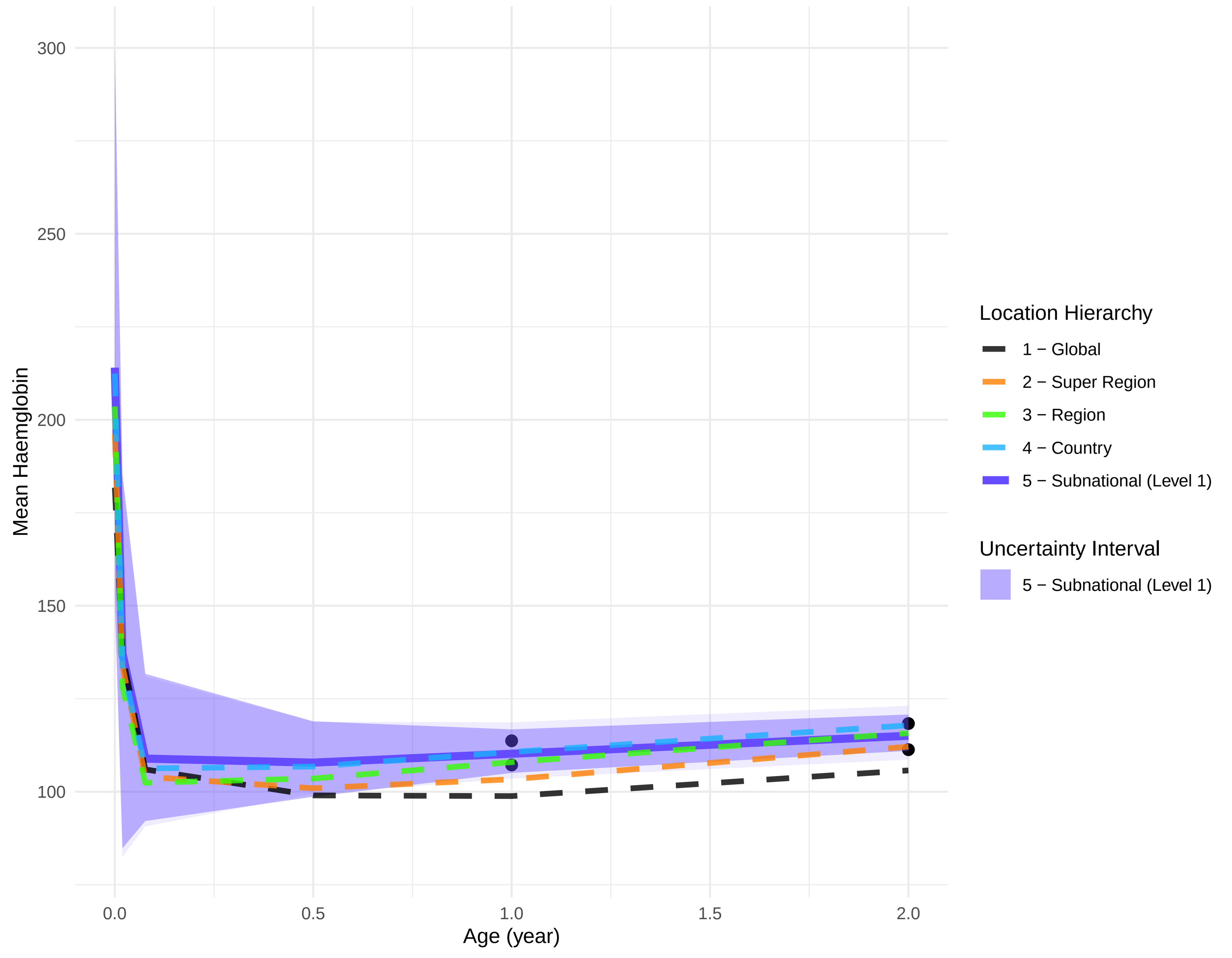

MR−BRT Age/Sex Fits − Males <2 Years Old − Mean Haemglobin − Baja California Sur
WHO−2024 Adjustment Method

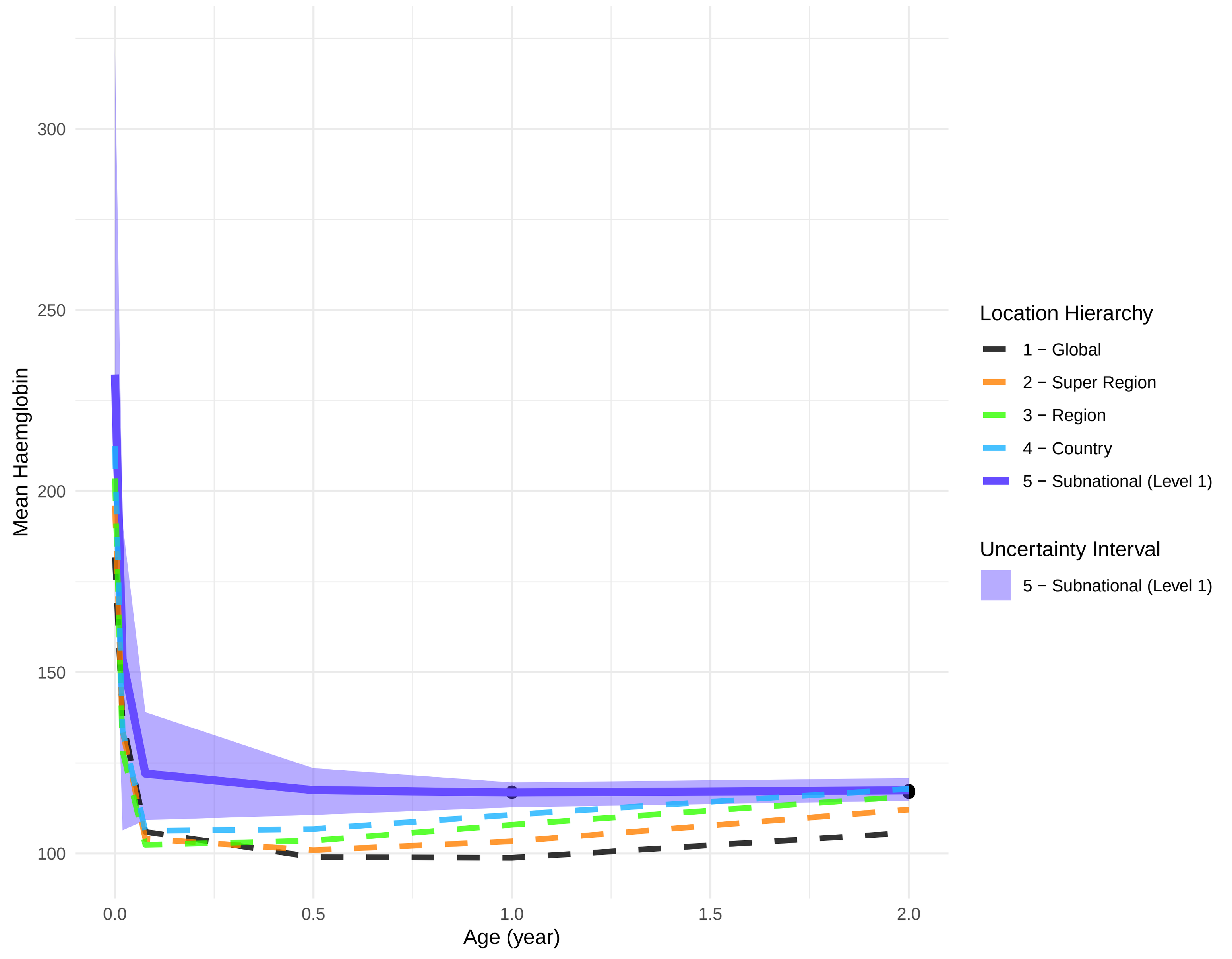

MR−BRT Age/Sex Fits − Males <2 Years Old − Mean Haemglobin − Campeche
WHO−2024 Adjustment Method

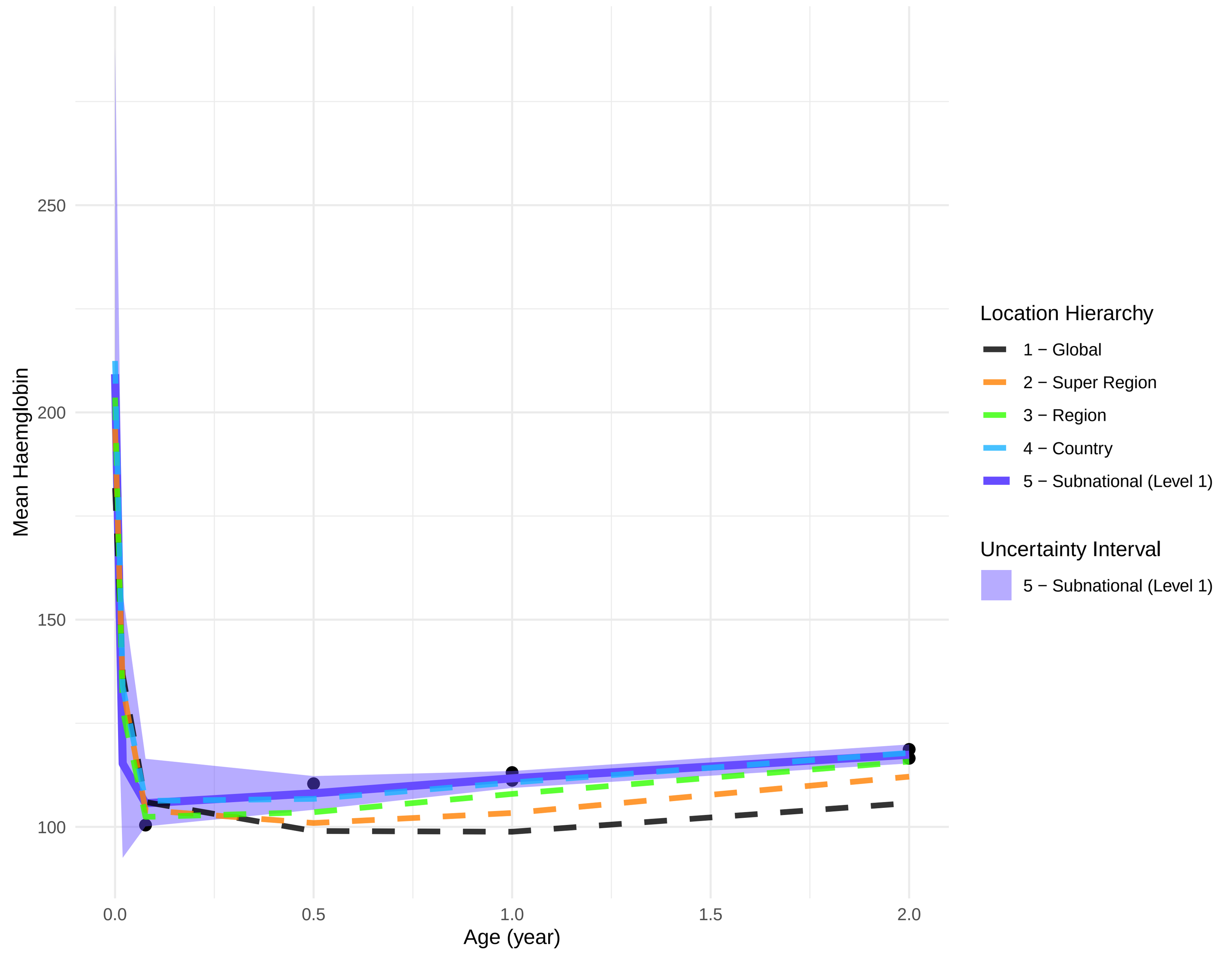

MR−BRT Age/Sex Fits − Males <2 Years Old − Mean Haemglobin − Chiapas
WHO−2024 Adjustment Method

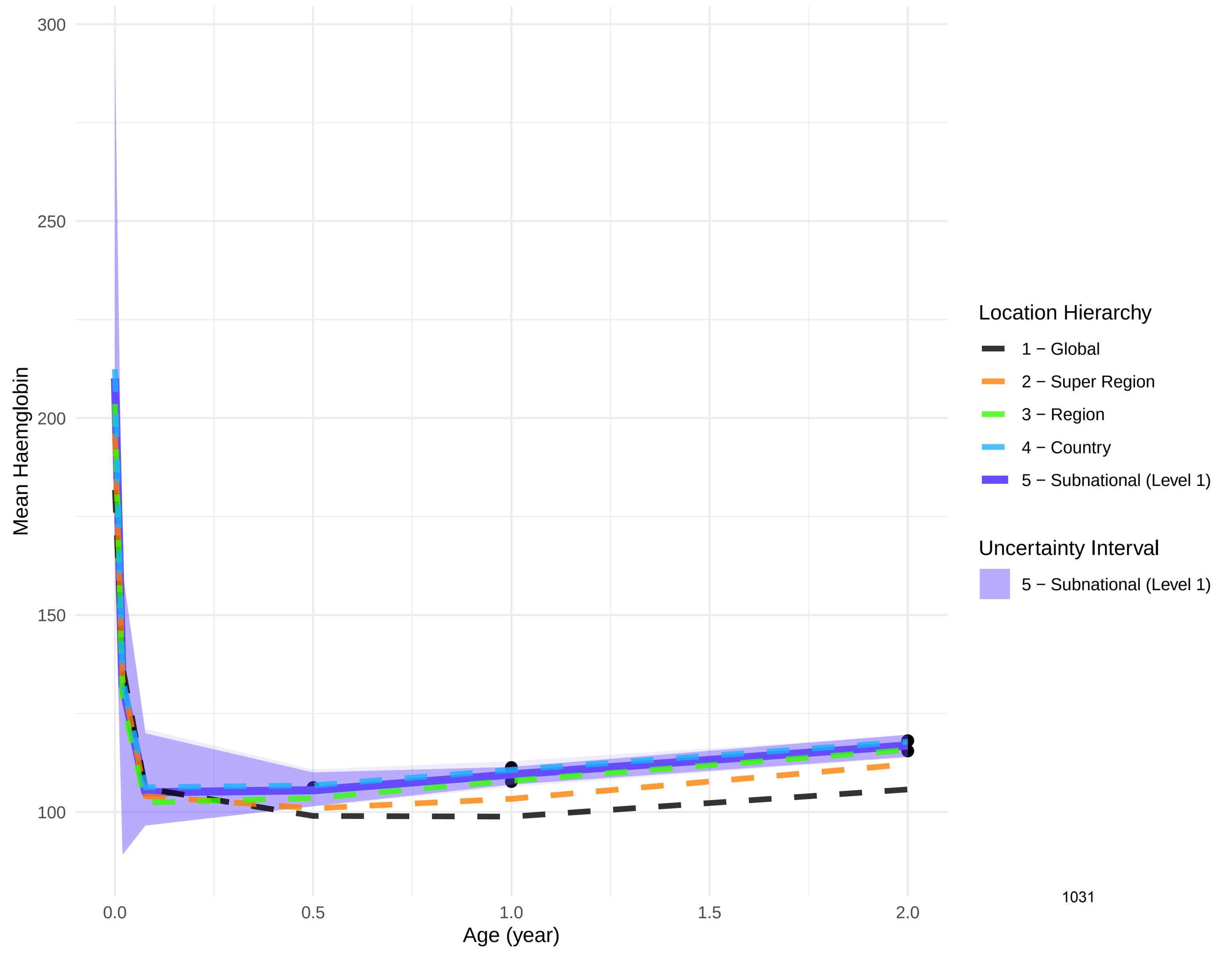

MR−BRT Age/Sex Fits − Males <2 Years Old − Mean Haemglobin − Chihuahua
WHO−2024 Adjustment Method

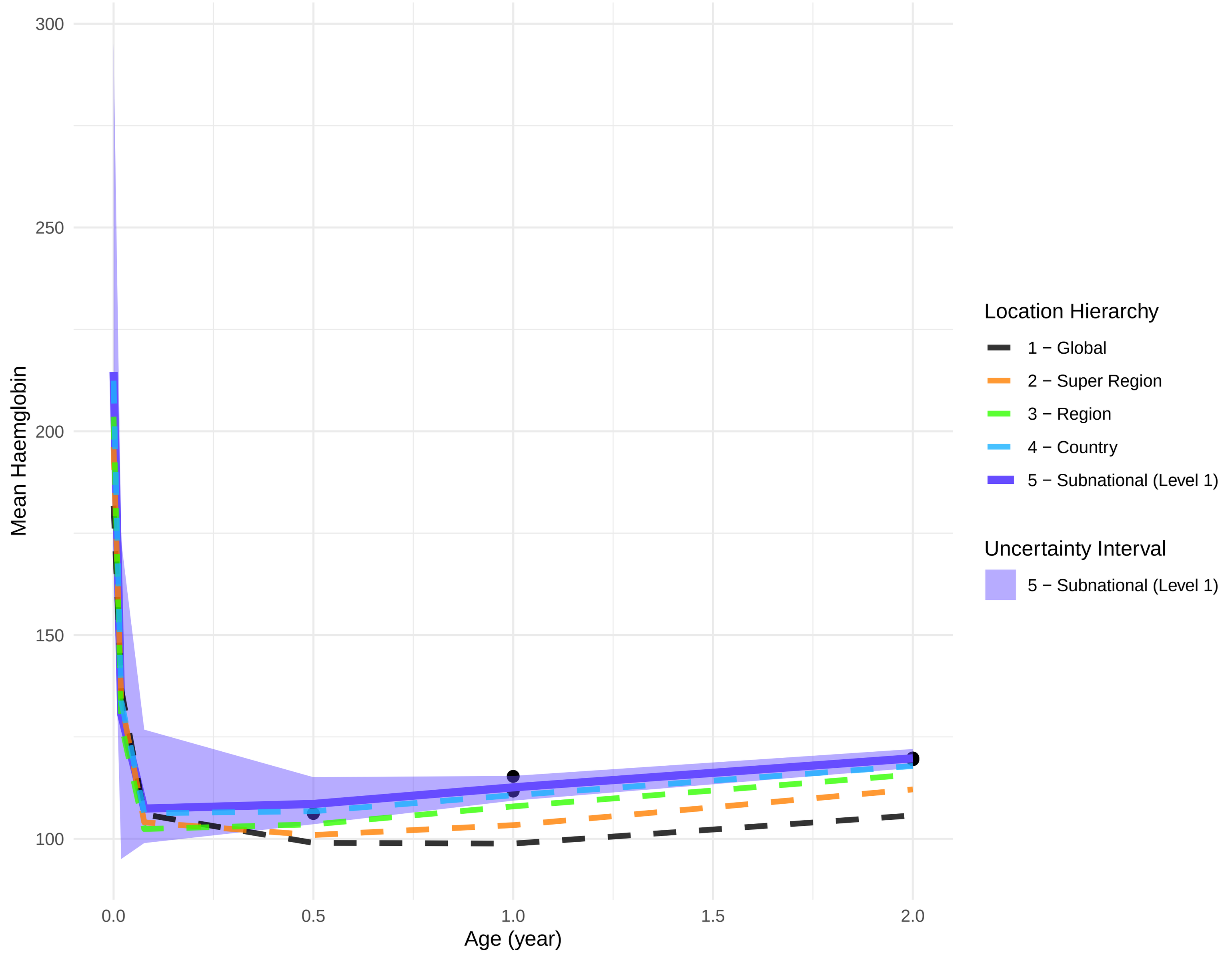

MR−BRT Age/Sex Fits − Males <2 Years Old − Mean Haemglobin − Coahuila
WHO−2024 Adjustment Method

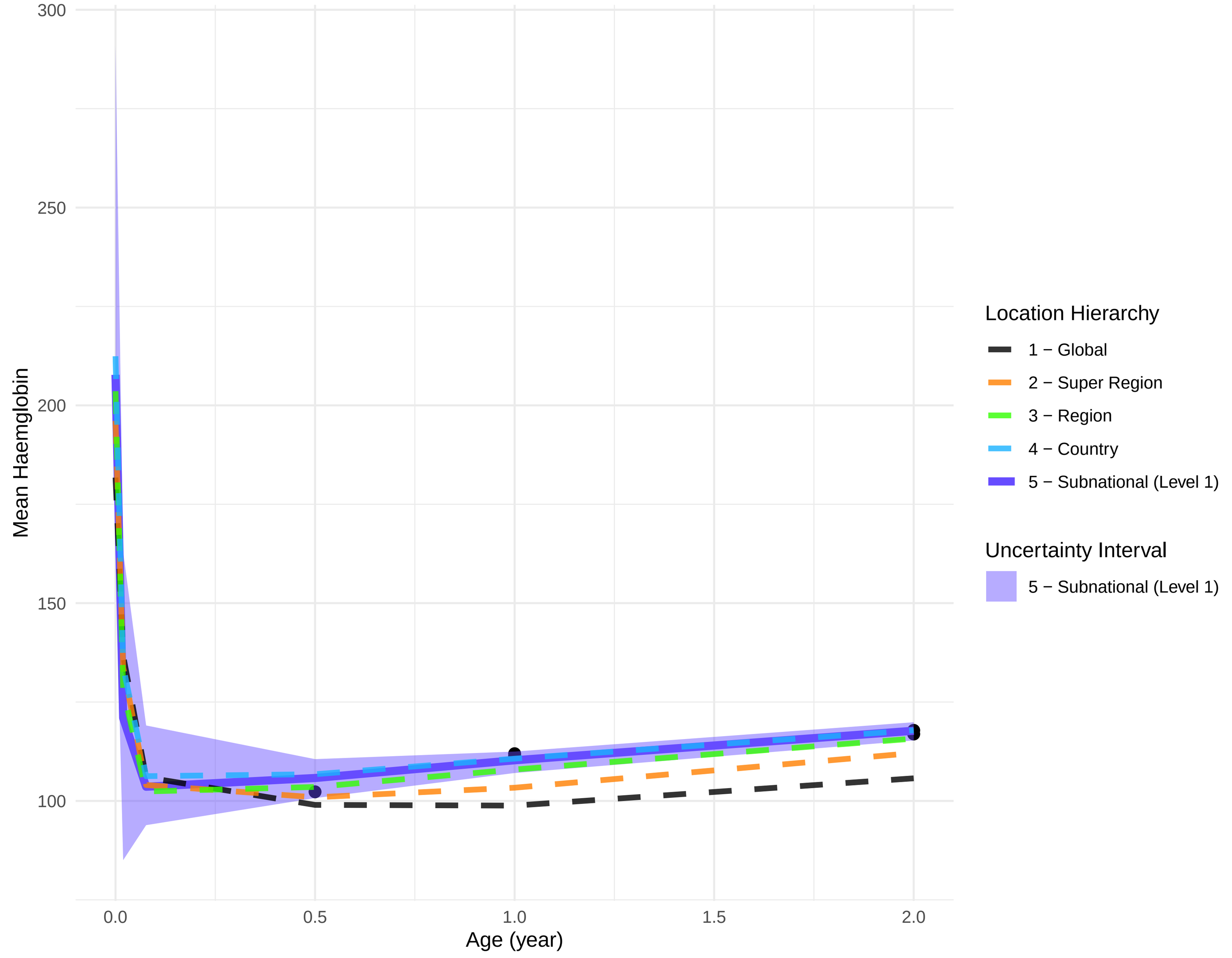

MR−BRT Age/Sex Fits − Males <2 Years Old − Mean Haemglobin − Colima
WHO−2024 Adjustment Method

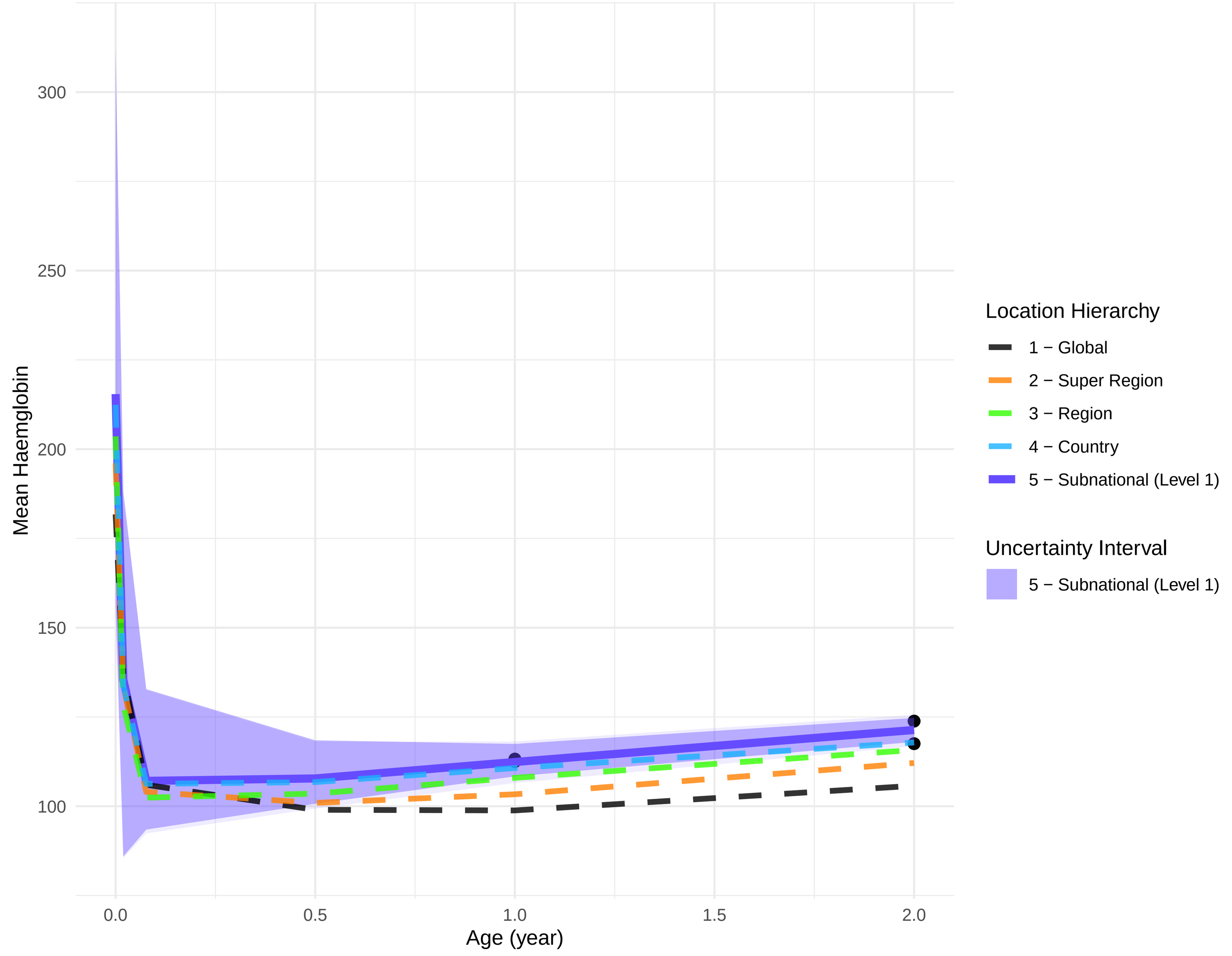

MR−BRT Age/Sex Fits − Males <2 Years Old − Mean Haemglobin − Durango
WHO−2024 Adjustment Method

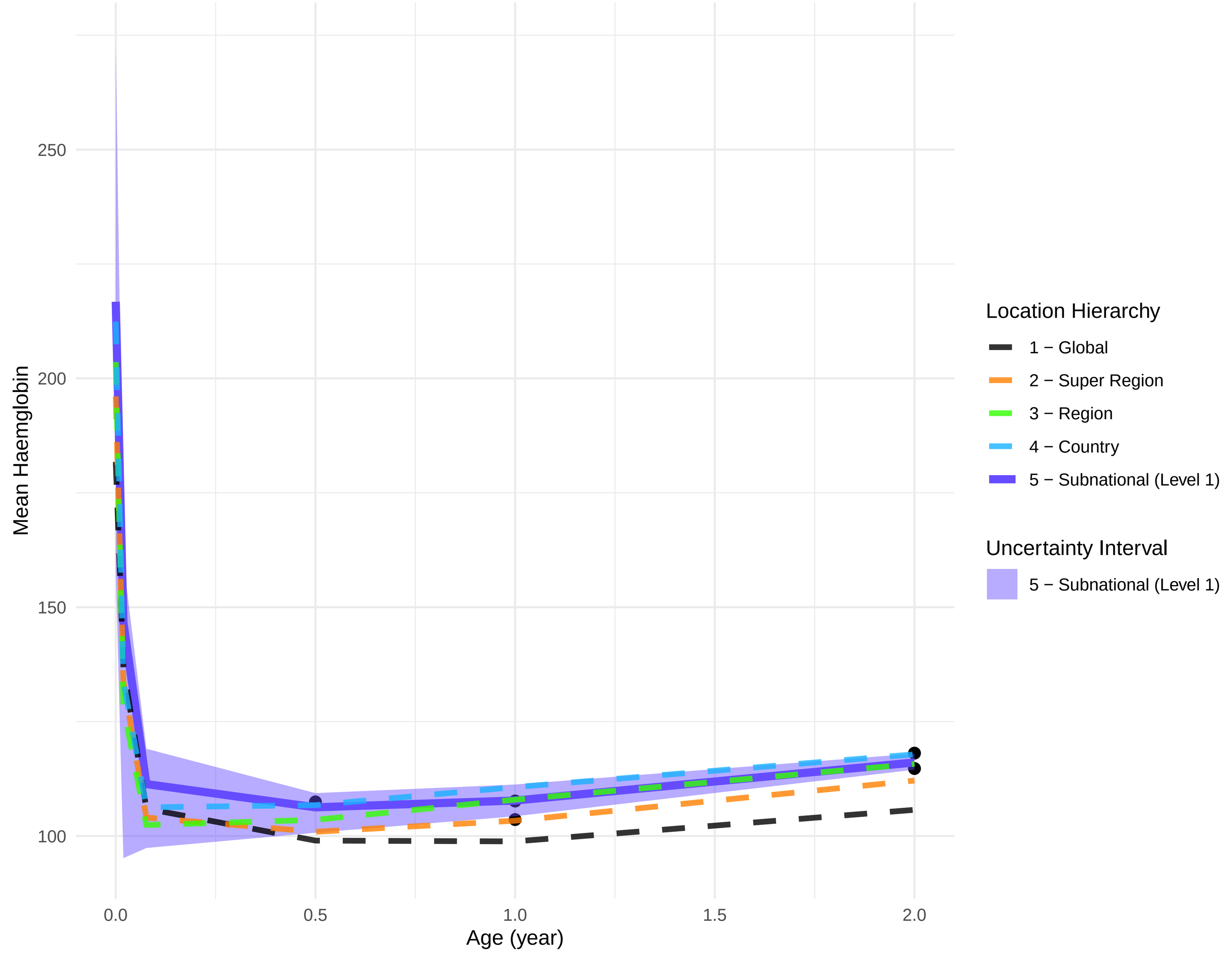

MR−BRT Age/Sex Fits − Males <2 Years Old − Mean Haemglobin − Guanajuato
WHO−2024 Adjustment Method

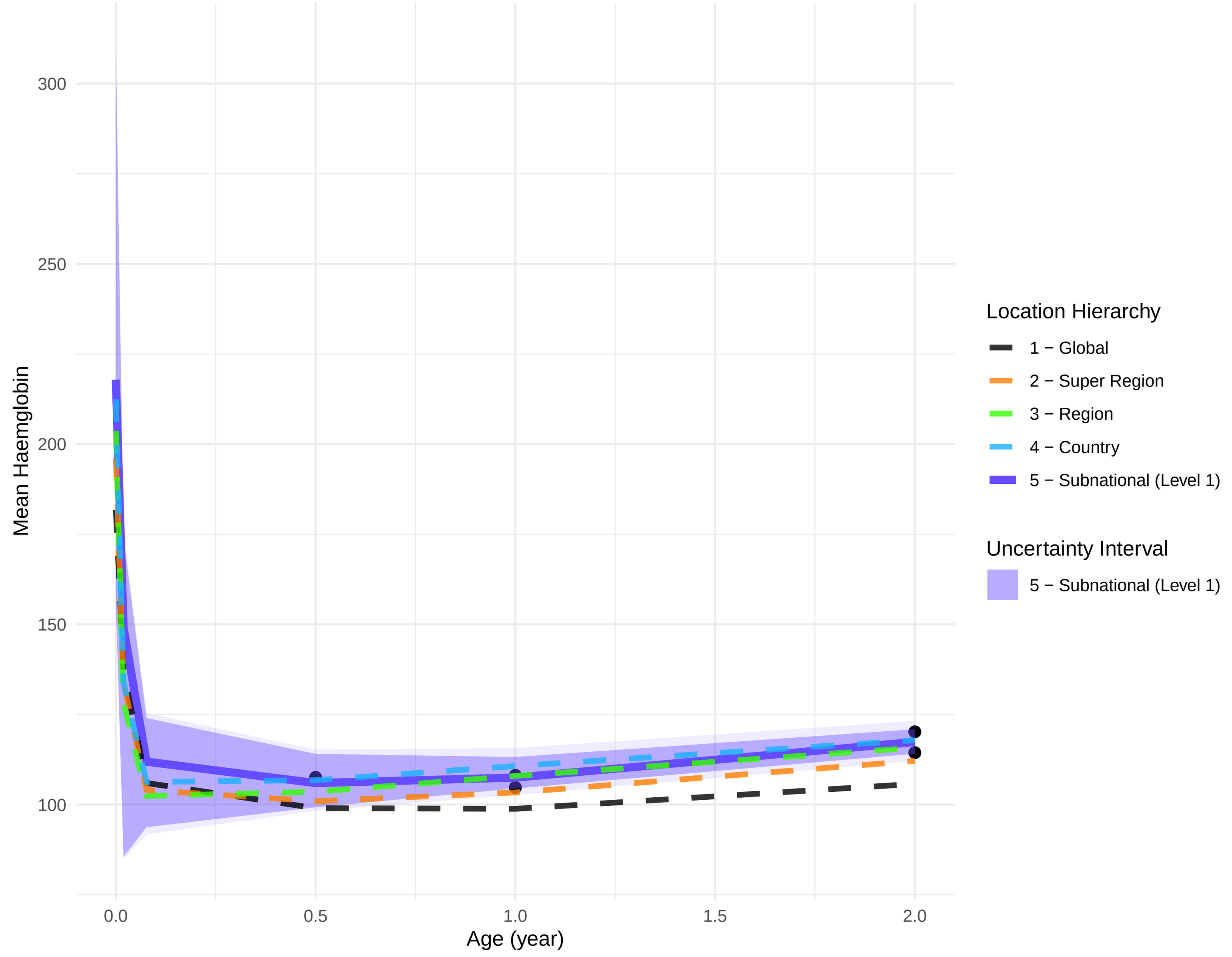

MR−BRT Age/Sex Fits − Males <2 Years Old − Mean Haemglobin − Guerrero
WHO−2024 Adjustment Method

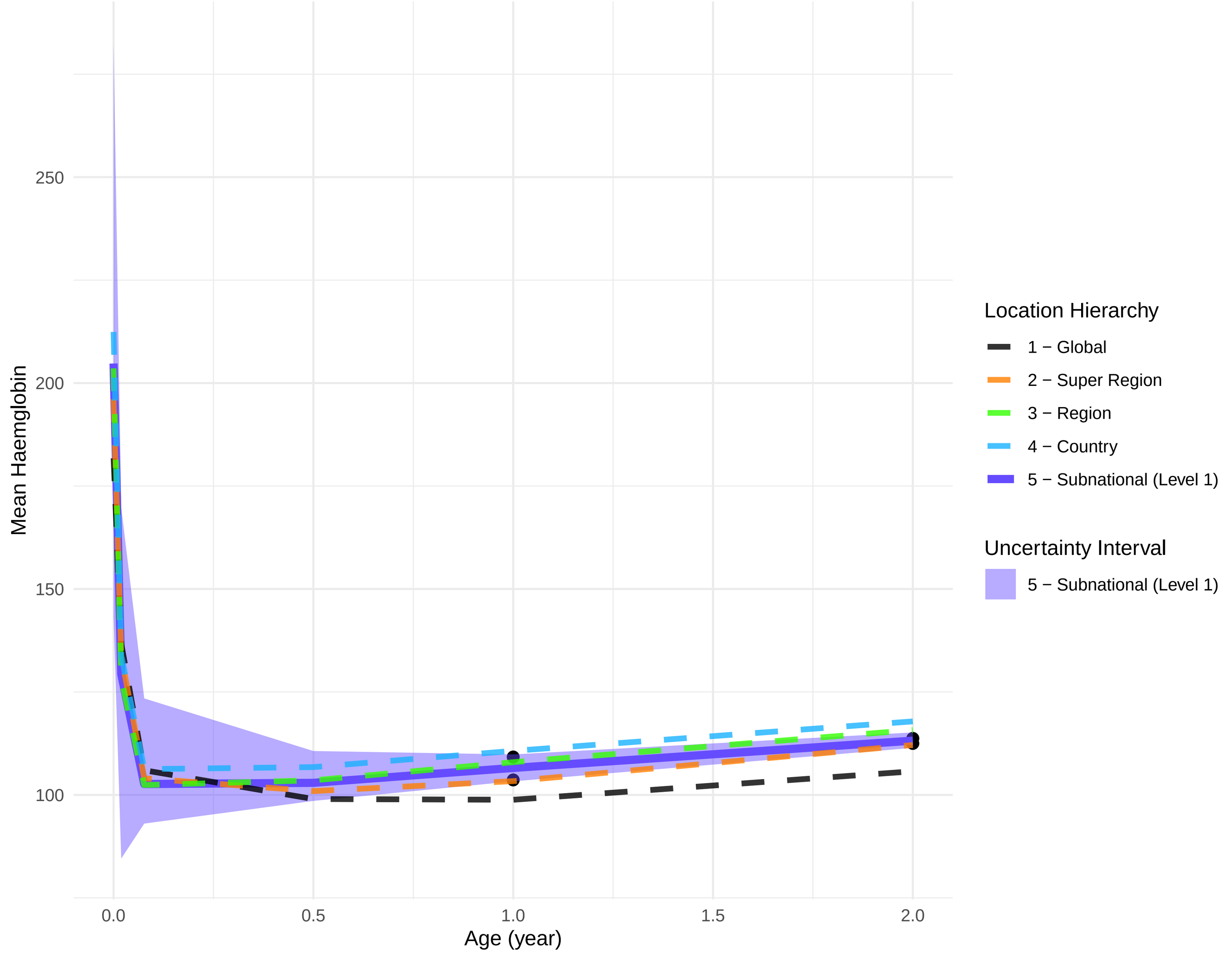

MR−BRT Age/Sex Fits − Males <2 Years Old − Mean Haemglobin − Hidalgo
WHO−2024 Adjustment Method

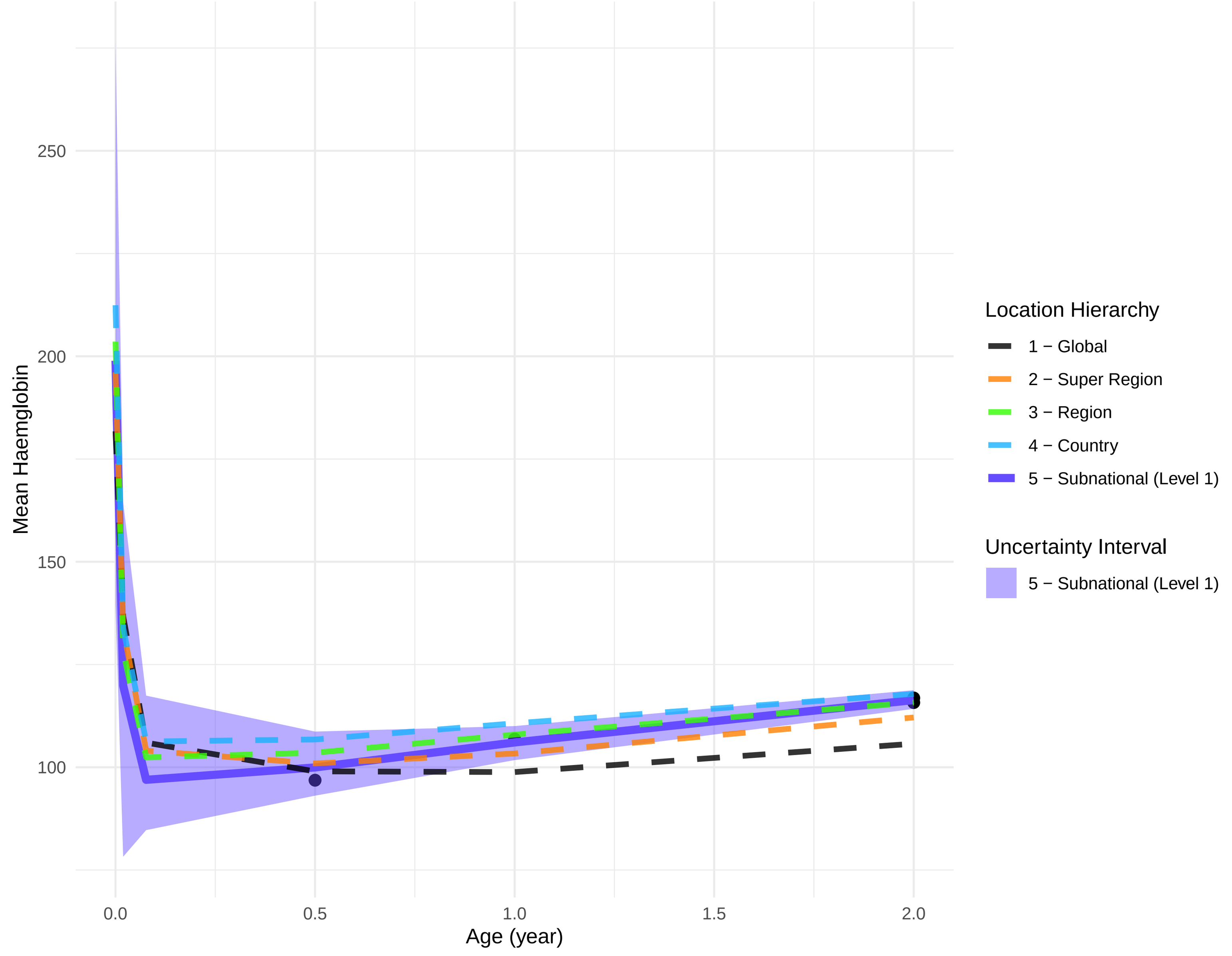

MR−BRT Age/Sex Fits − Males <2 Years Old − Mean Haemglobin − Jalisco
WHO−2024 Adjustment Method

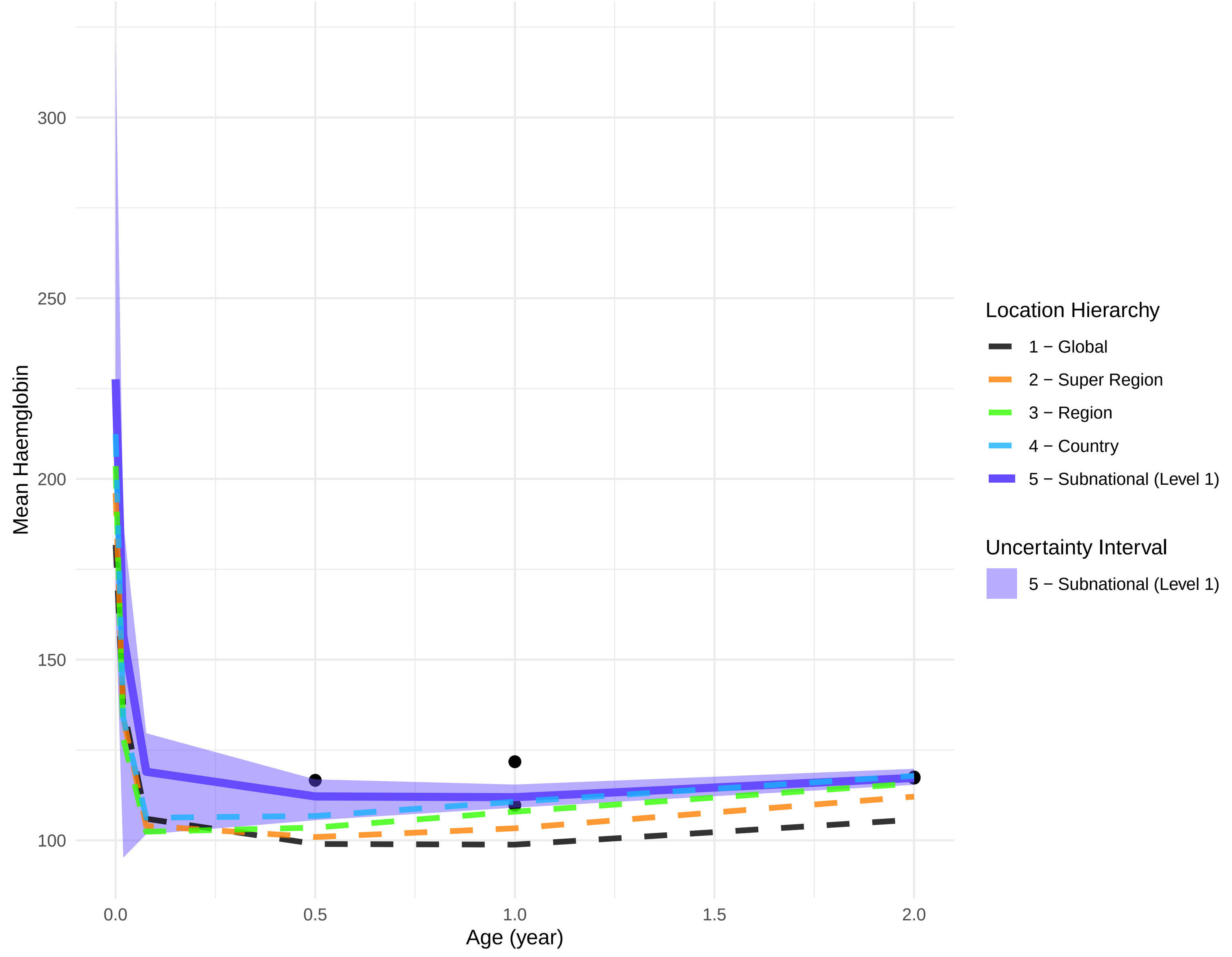

MR−BRT Age/Sex Fits − Males <2 Years Old − Mean Haemglobin − México
WHO−2024 Adjustment Method

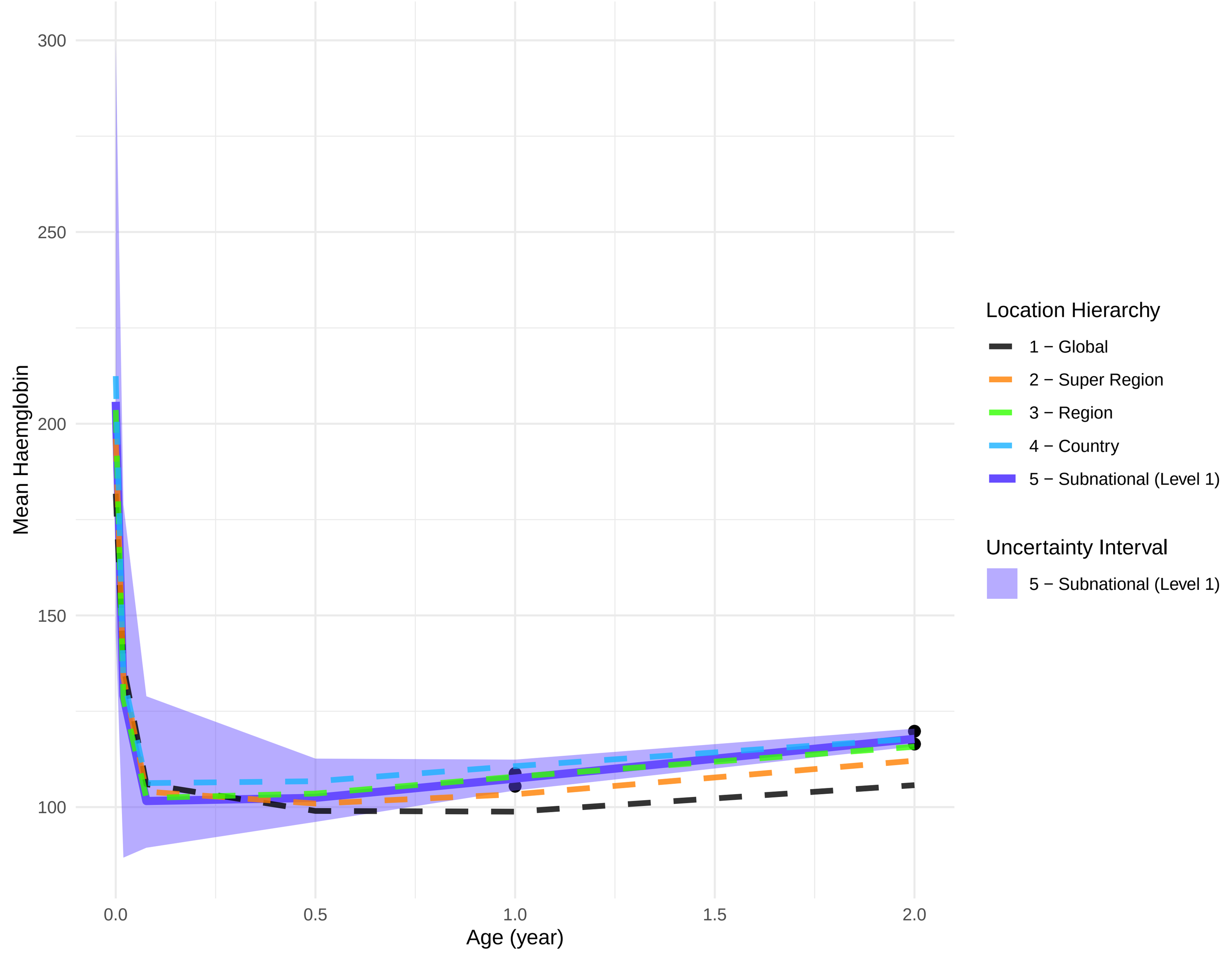

MR−BRT Age/Sex Fits − Males <2 Years Old − Mean Haemglobin − Mexico City
WHO−2024 Adjustment Method

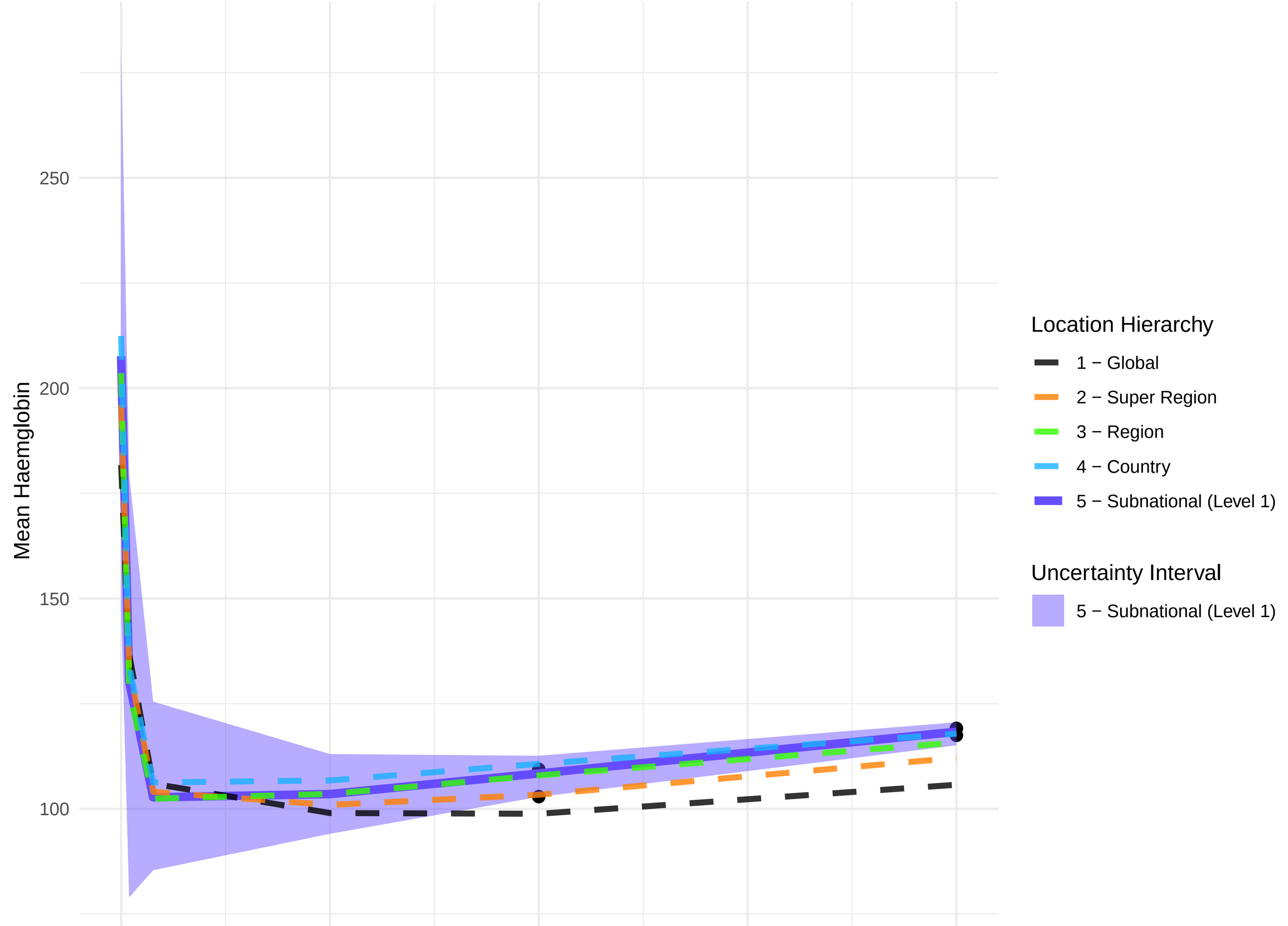

MR−BRT Age/Sex Fits − Males <2 Years Old − Mean Haemglobin − Michoacán de Ocampo
WHO−2024 Adjustment Method

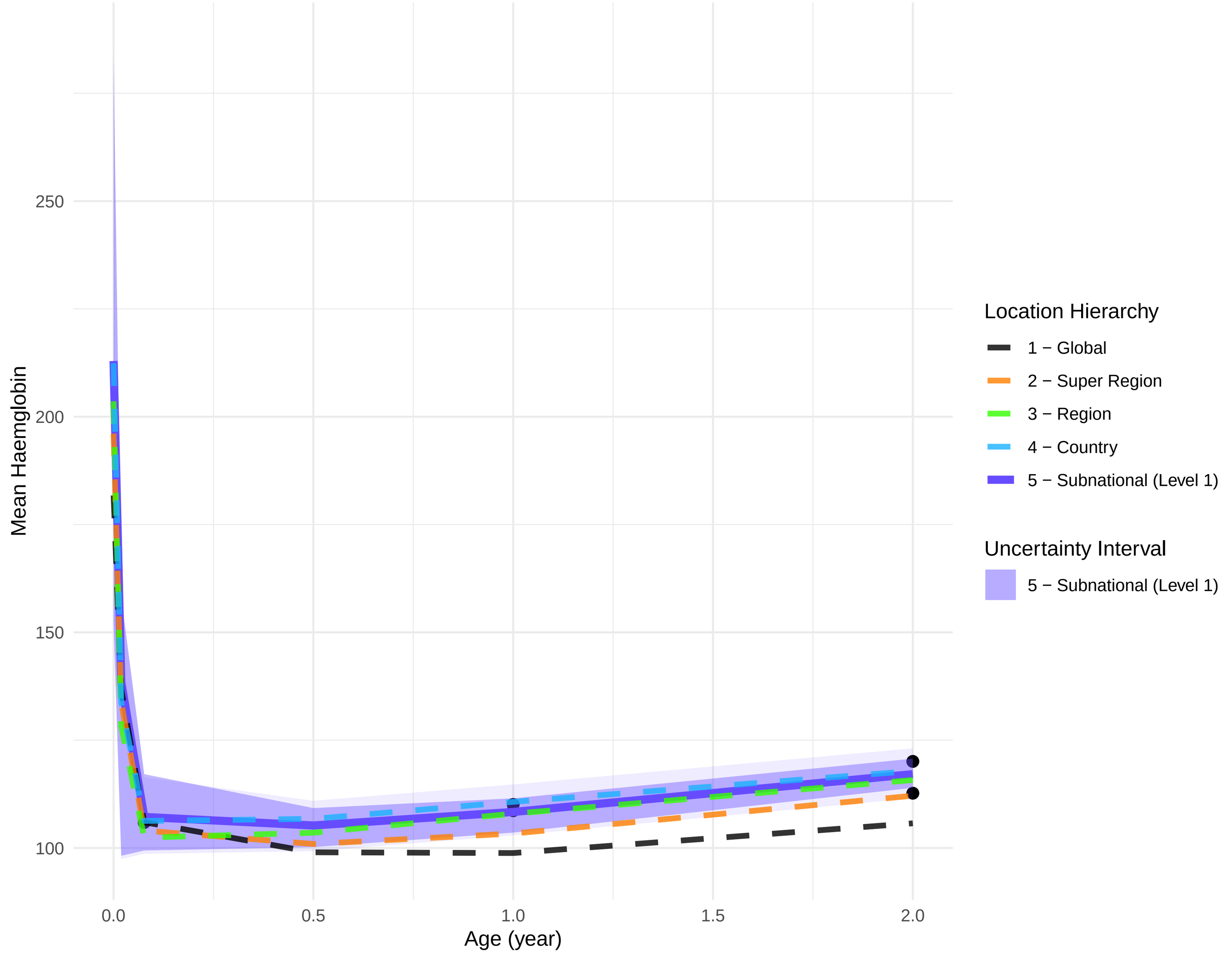

MR−BRT Age/Sex Fits − Males <2 Years Old − Mean Haemglobin − Morelos
WHO−2024 Adjustment Method

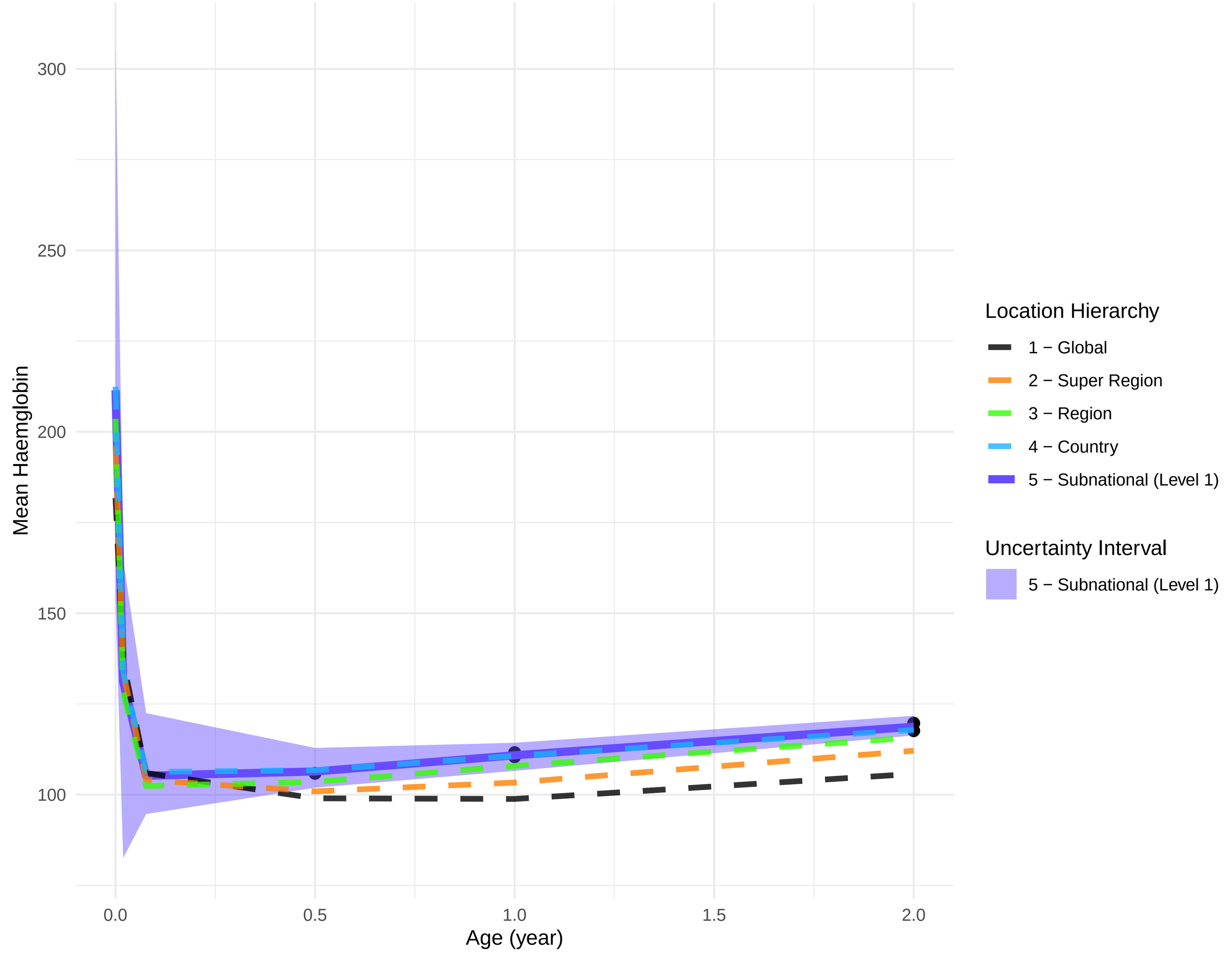

MR−BRT Age/Sex Fits − Males <2 Years Old − Mean Haemglobin − Nayarit
WHO−2024 Adjustment Method

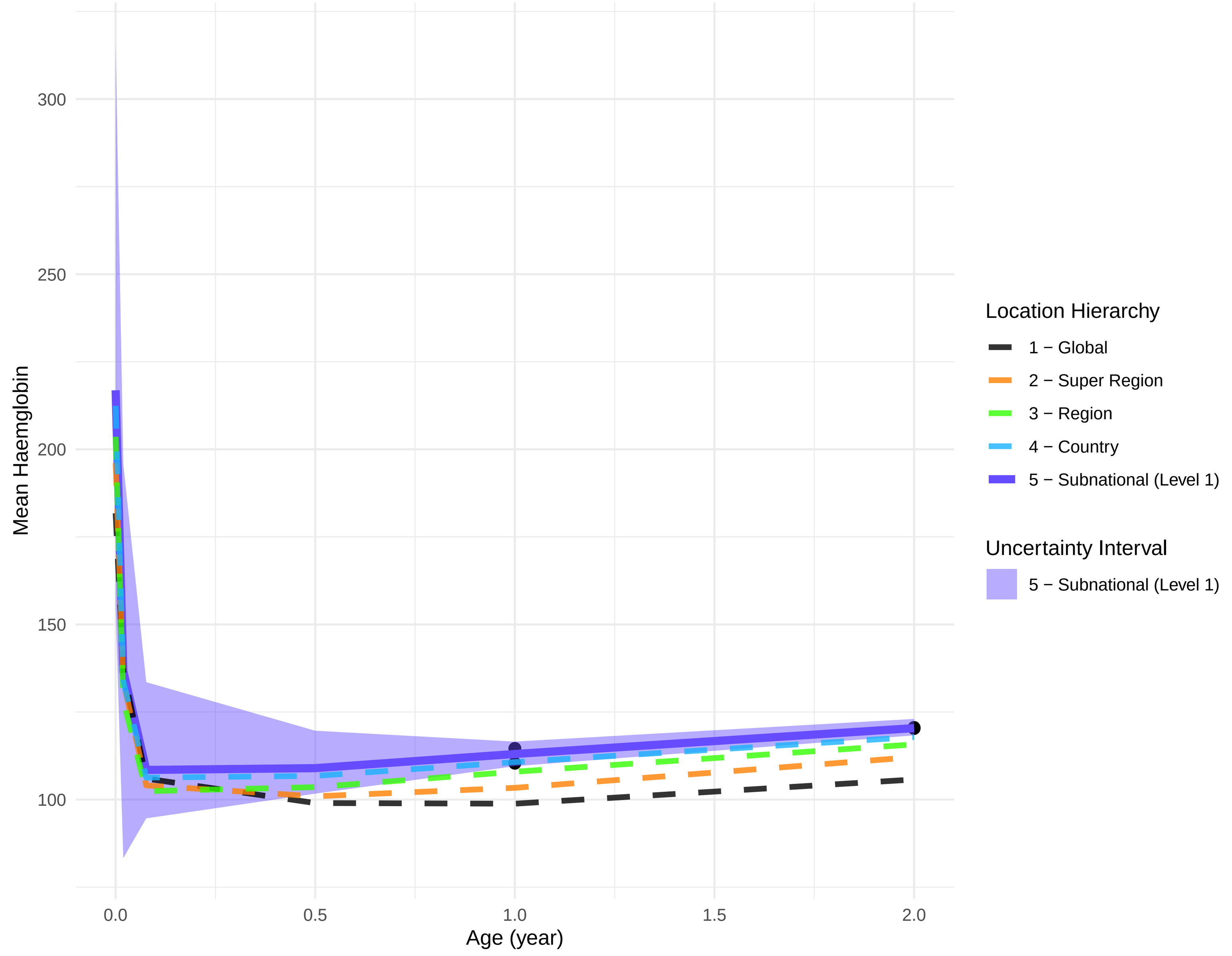

MR−BRT Age/Sex Fits − Males <2 Years Old − Mean Haemglobin − Nuevo León
WHO−2024 Adjustment Method

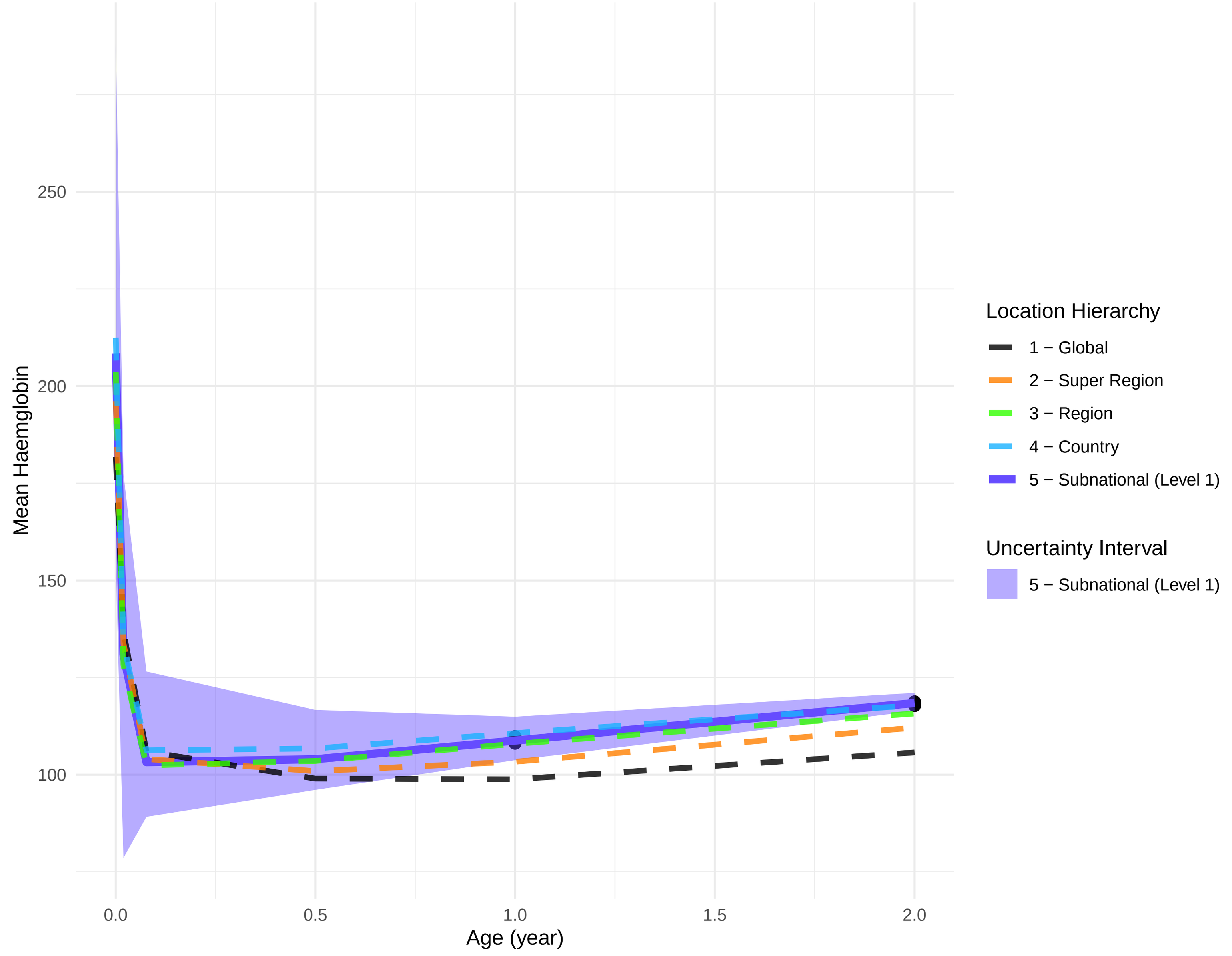

MR−BRT Age/Sex Fits − Males <2 Years Old − Mean Haemglobin − Oaxaca
WHO−2024 Adjustment Method

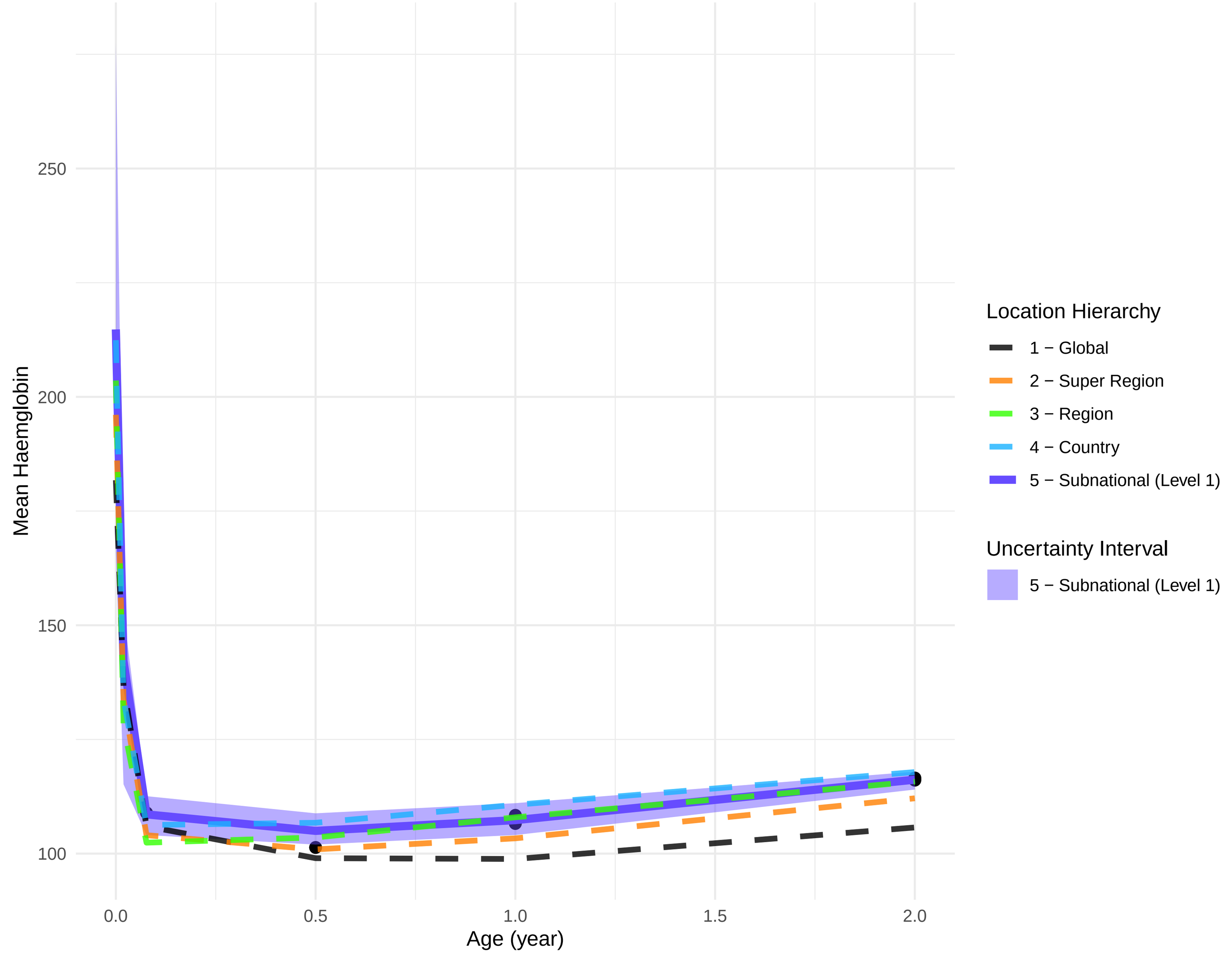

MR−BRT Age/Sex Fits − Males <2 Years Old − Mean Haemglobin − Puebla
WHO−2024 Adjustment Method

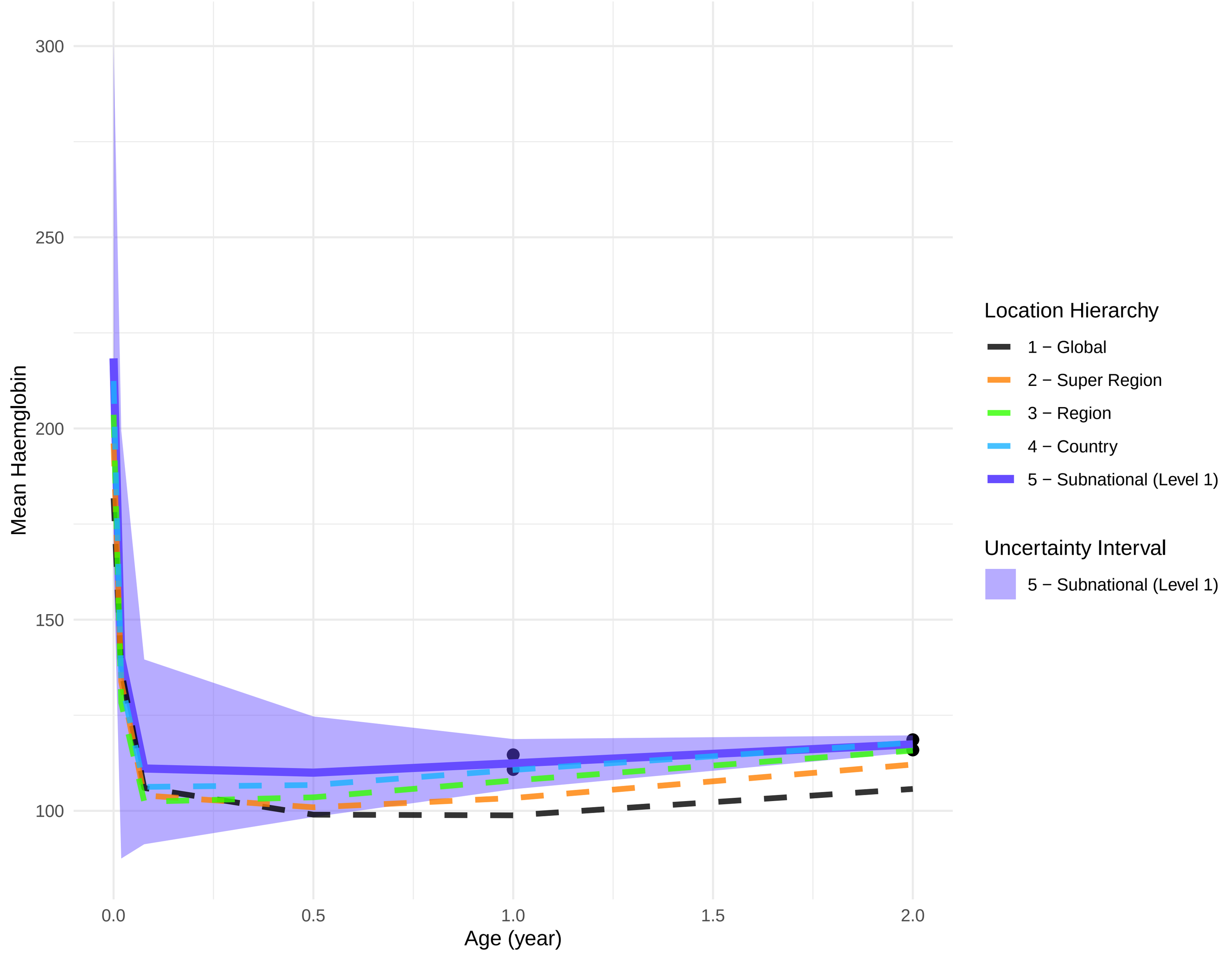

MR−BRT Age/Sex Fits − Males <2 Years Old − Mean Haemglobin − Querétaro
WHO−2024 Adjustment Method

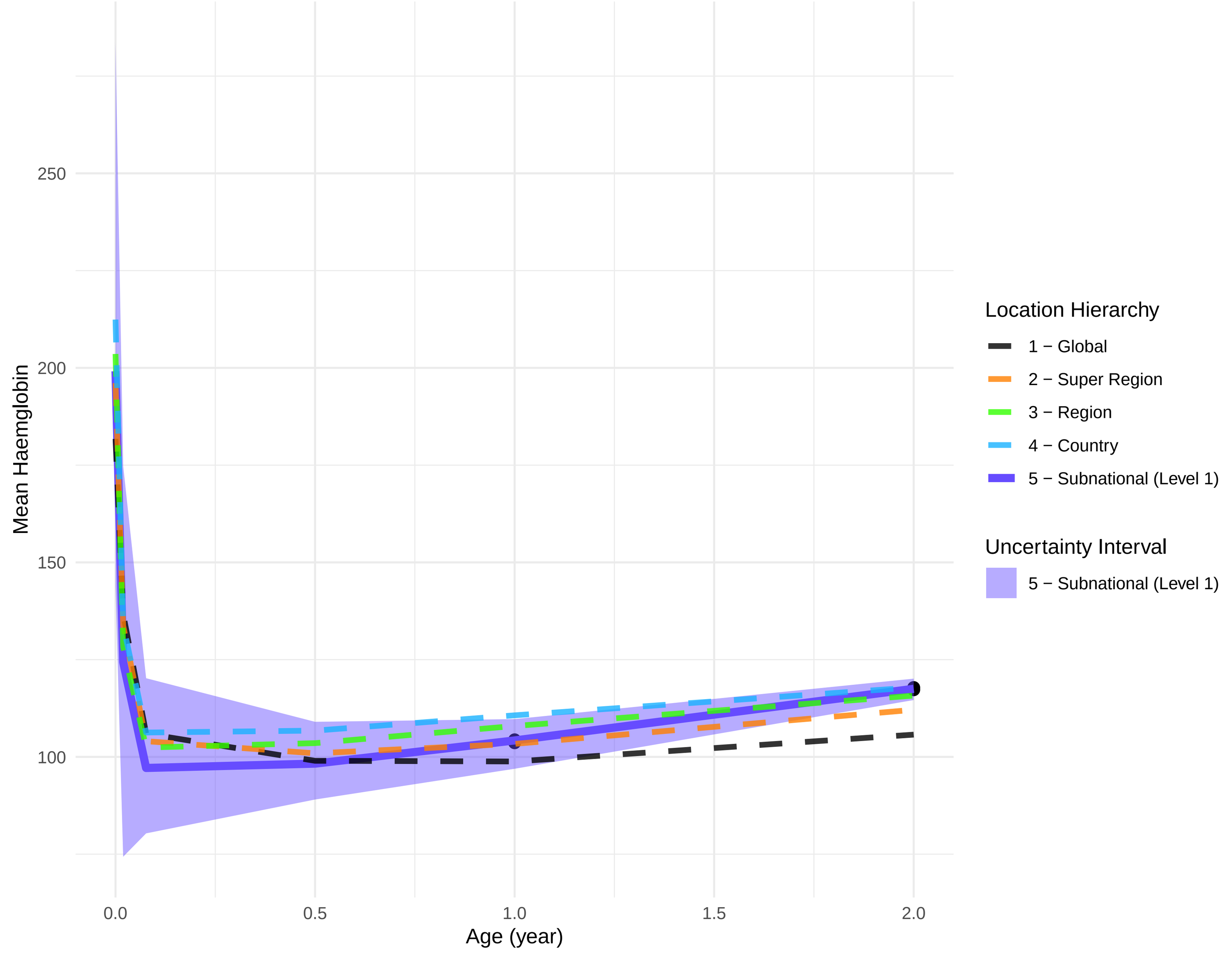

MR−BRT Age/Sex Fits − Males <2 Years Old − Mean Haemglobin − Quintana Roo
WHO−2024 Adjustment Method

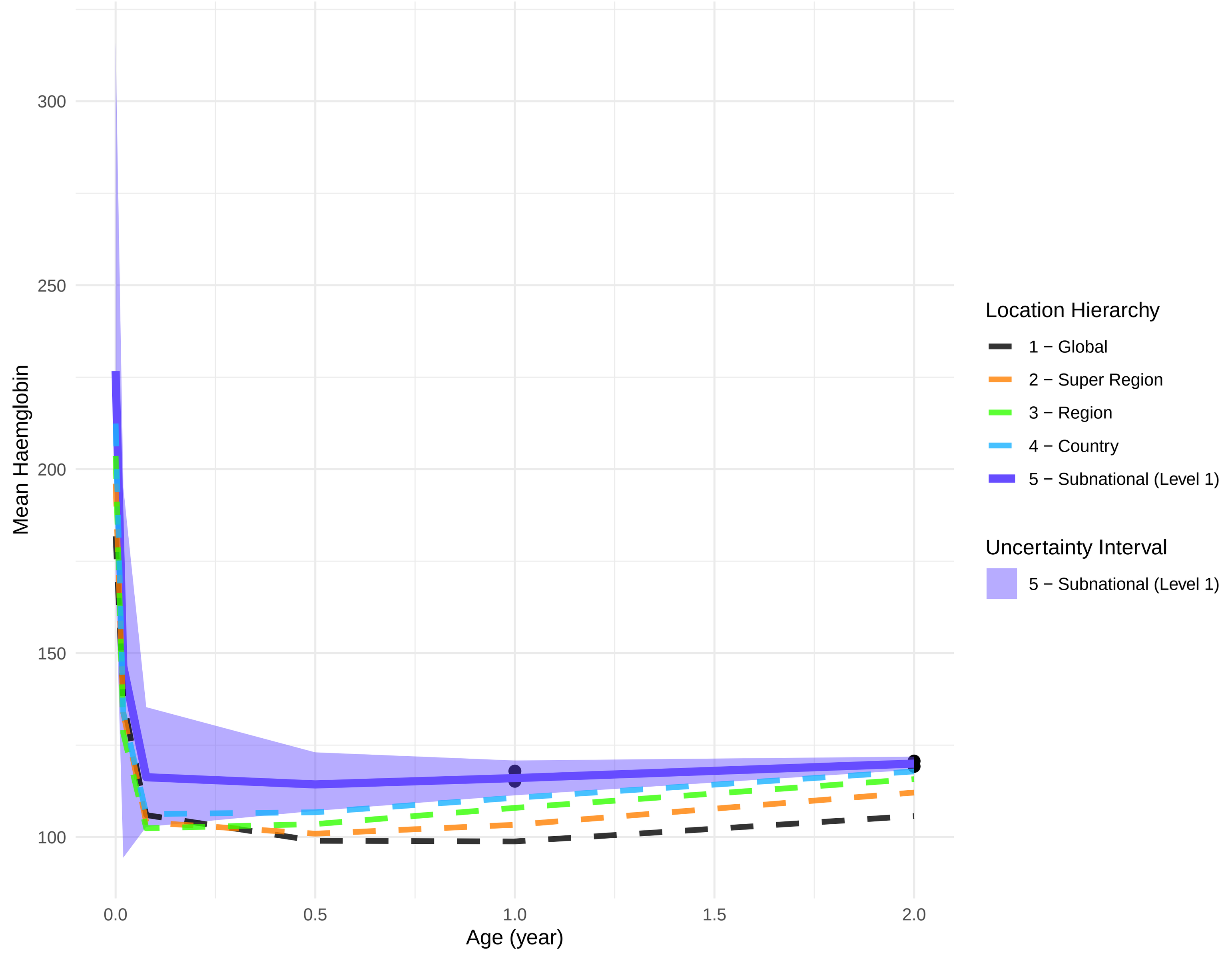

MR−BRT Age/Sex Fits − Males <2 Years Old − Mean Haemglobin − San Luis Potosí
WHO−2024 Adjustment Method

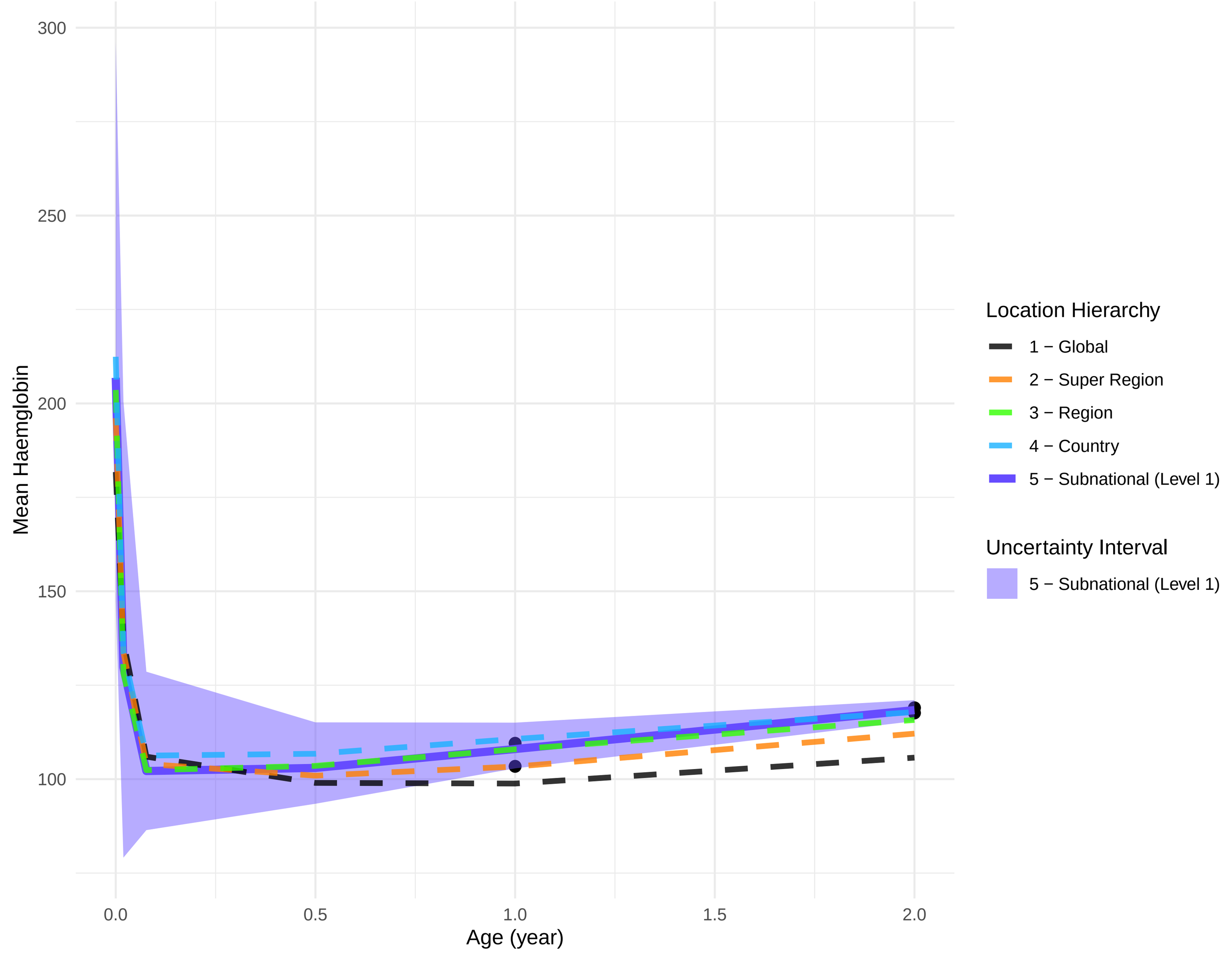

MR−BRT Age/Sex Fits − Males <2 Years Old − Mean Haemglobin − Sinaloa
WHO−2024 Adjustment Method

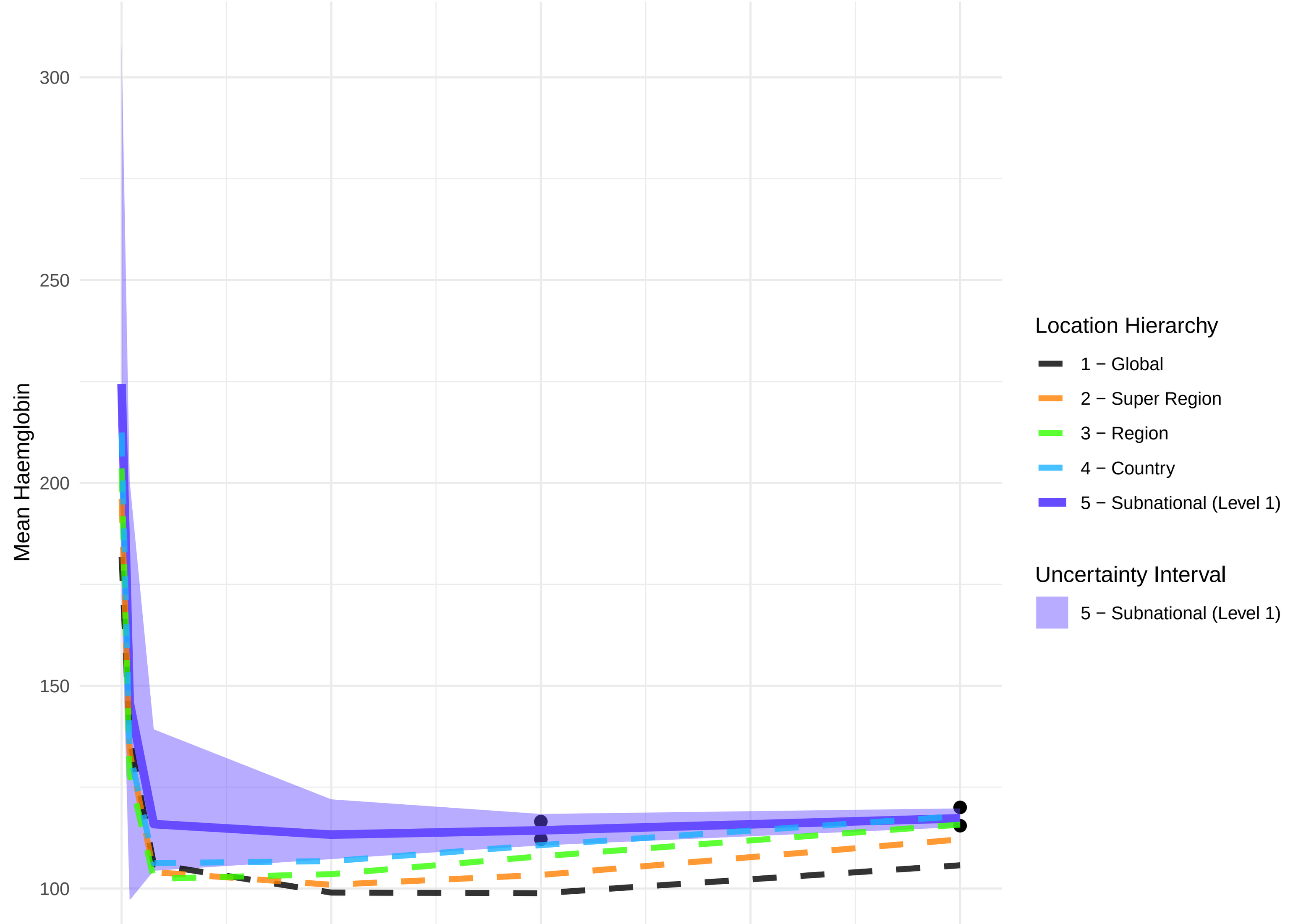

MR−BRT Age/Sex Fits − Males <2 Years Old − Mean Haemglobin − Sonora
WHO−2024 Adjustment Method

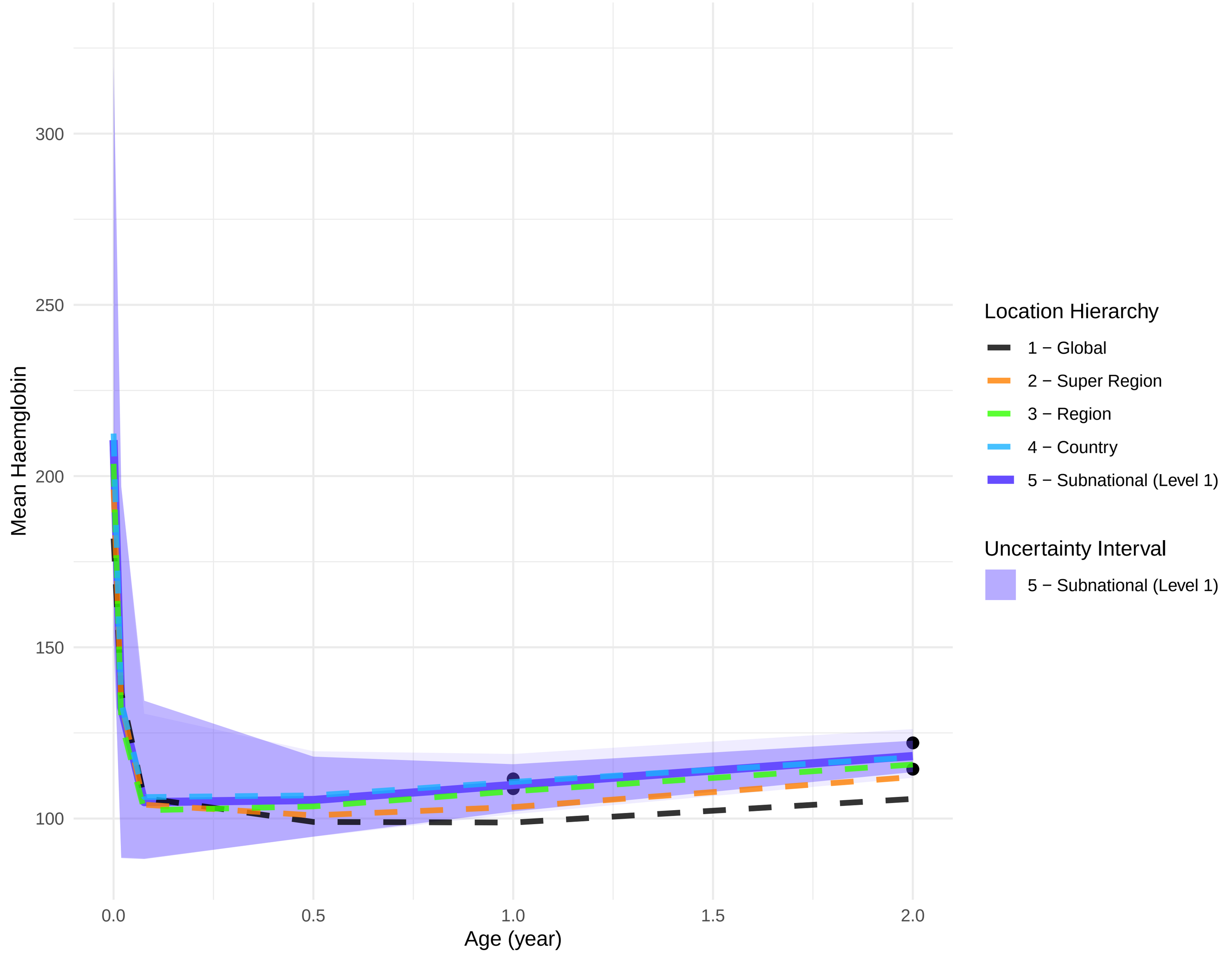

MR−BRT Age/Sex Fits − Males <2 Years Old − Mean Haemglobin − Tabasco
WHO−2024 Adjustment Method

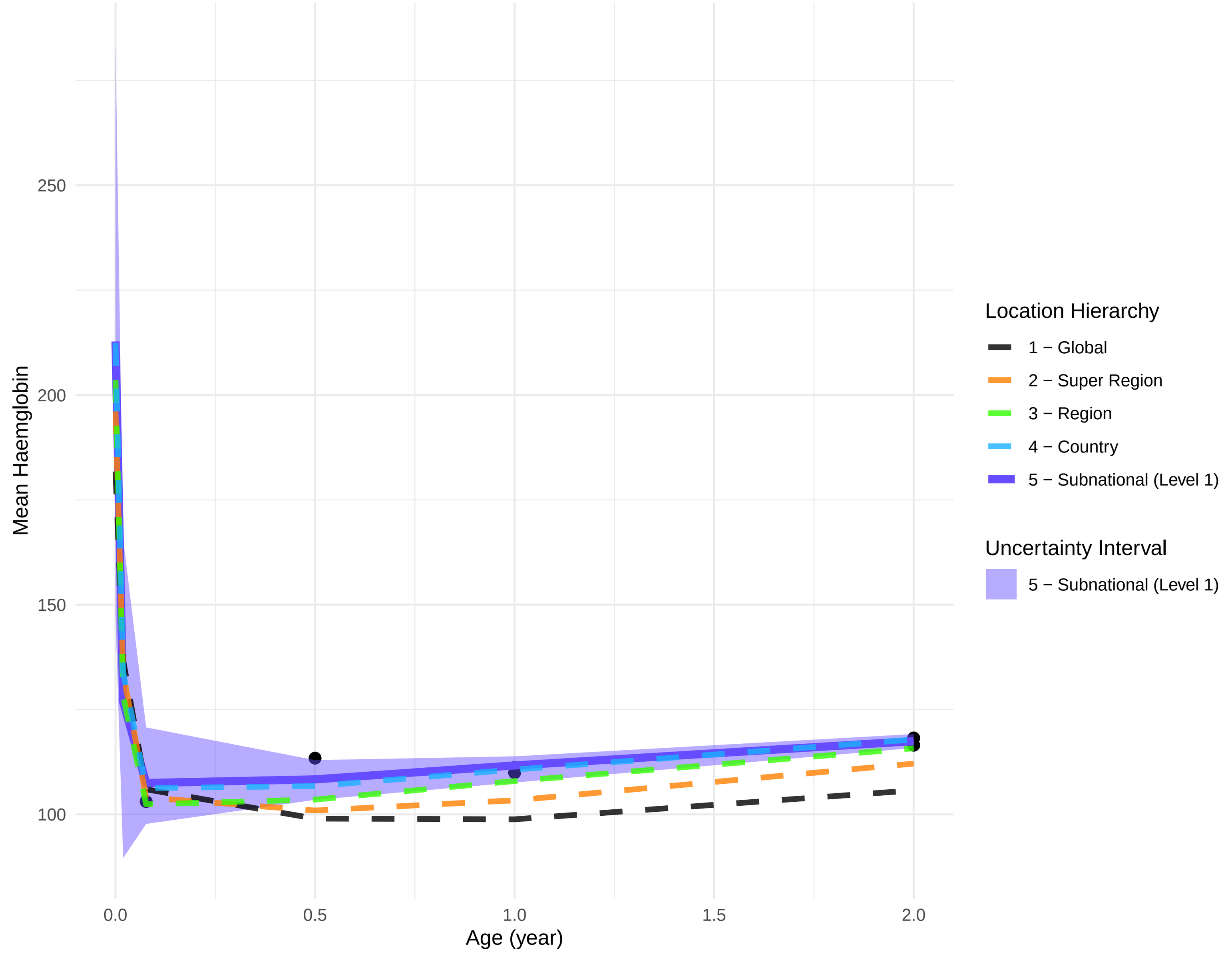

MR−BRT Age/Sex Fits − Males <2 Years Old − Mean Haemglobin − Tamaulipas
WHO−2024 Adjustment Method

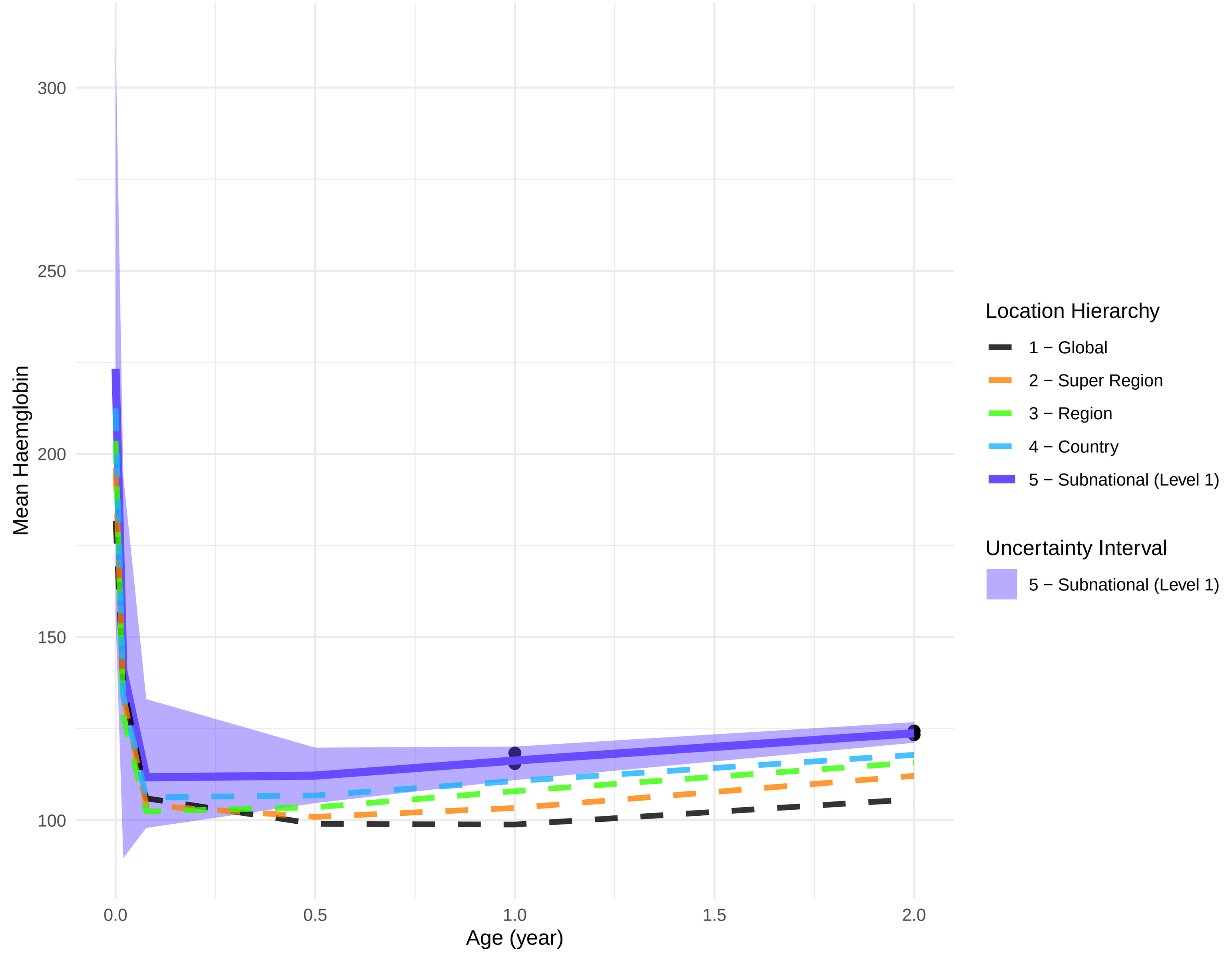

MR−BRT Age/Sex Fits − Males <2 Years Old − Mean Haemglobin − Tlaxcala
WHO−2024 Adjustment Method

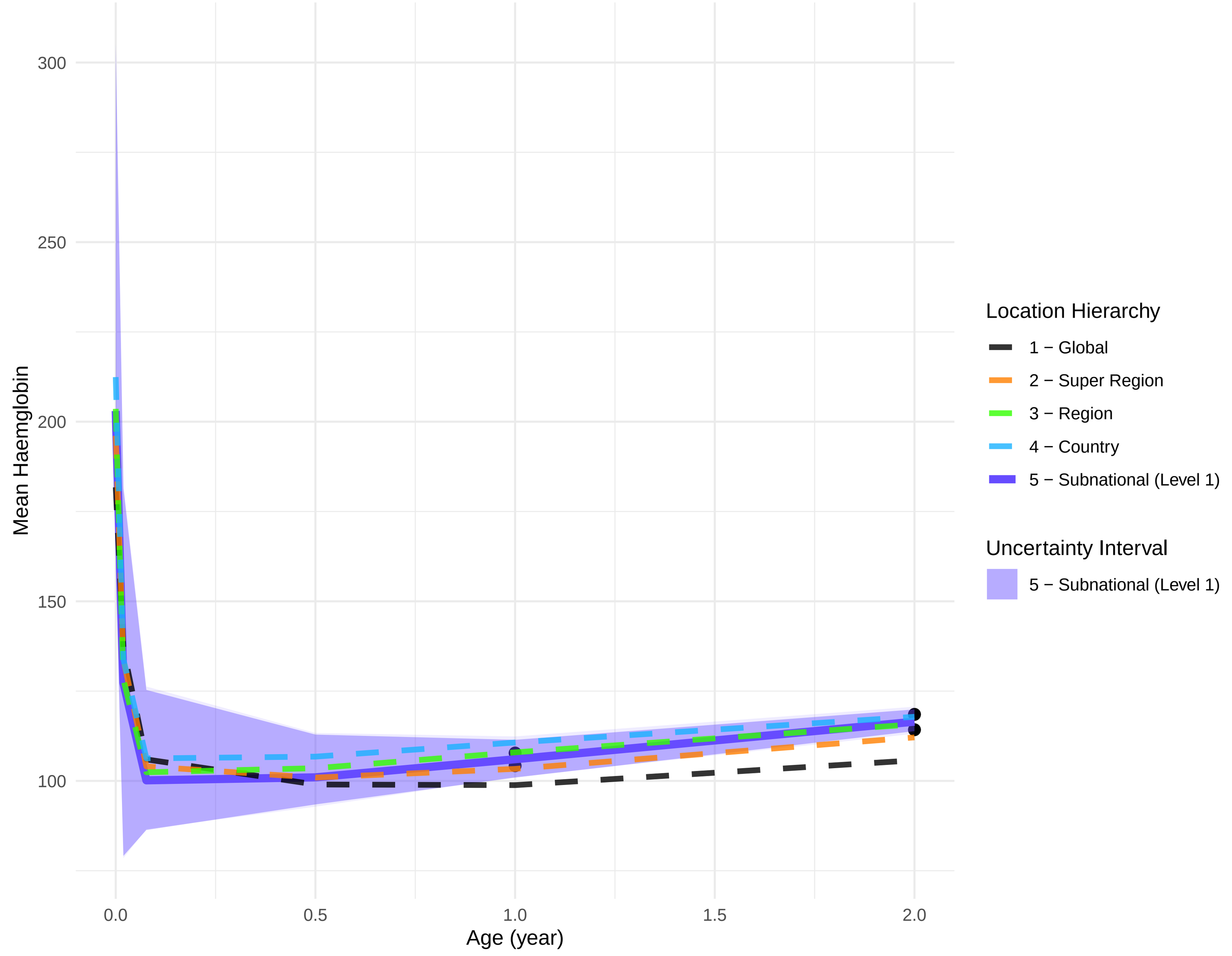

MR−BRT Age/Sex Fits − Males <2 Years Old − Mean Haemglobin − Veracruz de Ignacio de la Llave
WHO−2024 Adjustment Method

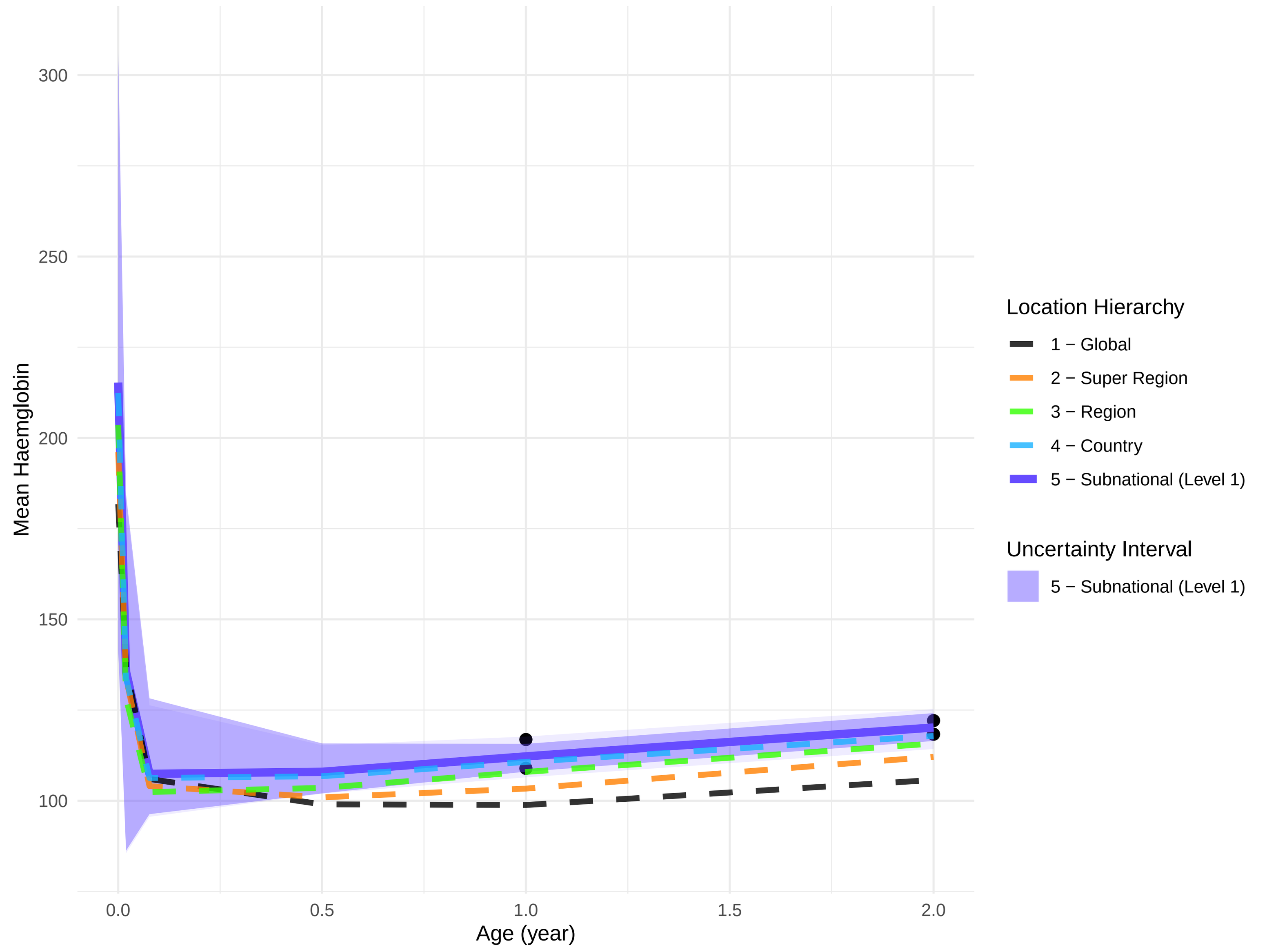

MR−BRT Age/Sex Fits − Males <2 Years Old − Mean Haemglobin − Yucatán
WHO−2024 Adjustment Method

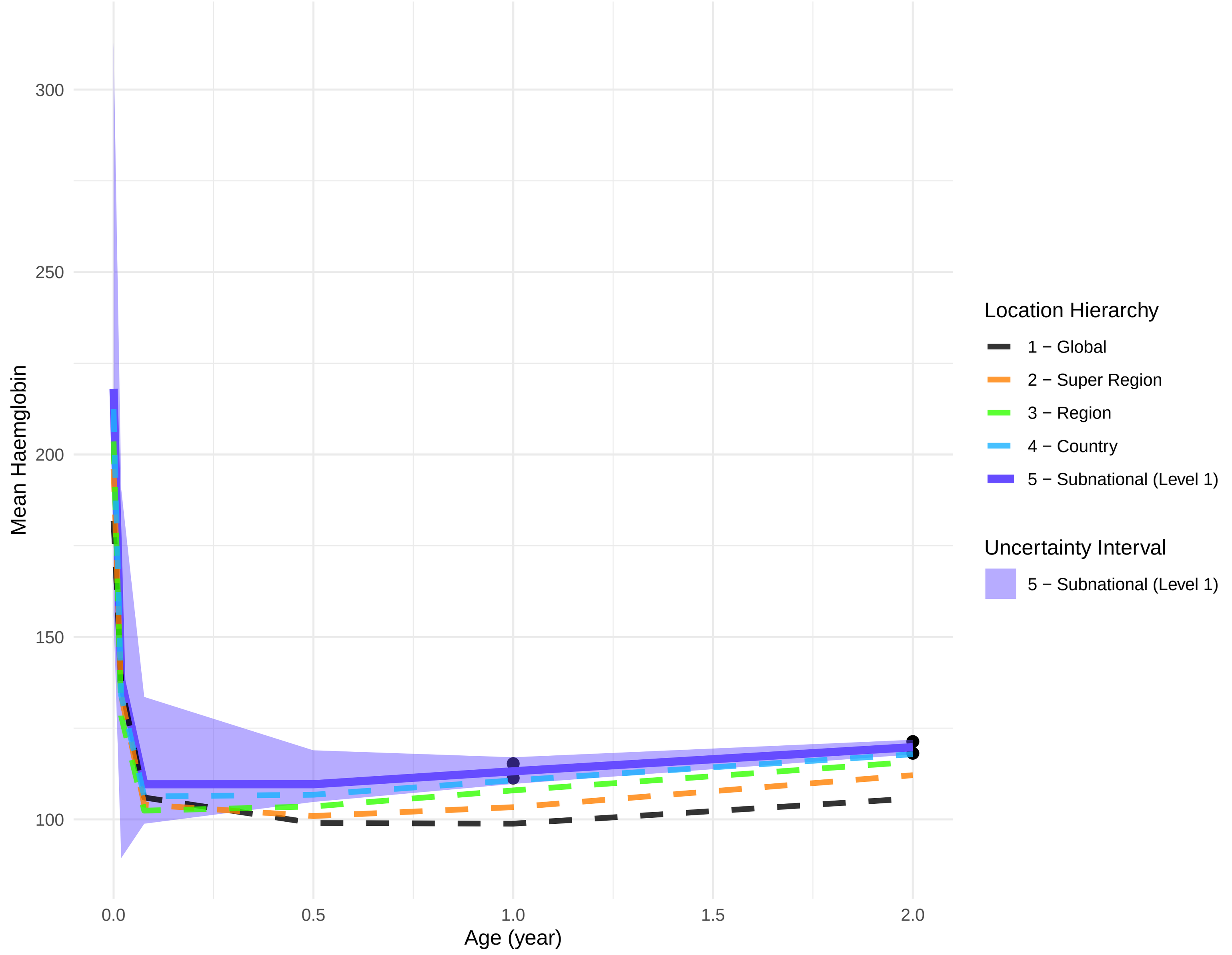

MR−BRT Age/Sex Fits − Males <2 Years Old − Mean Haemglobin − Zacatecas
WHO−2024 Adjustment Method

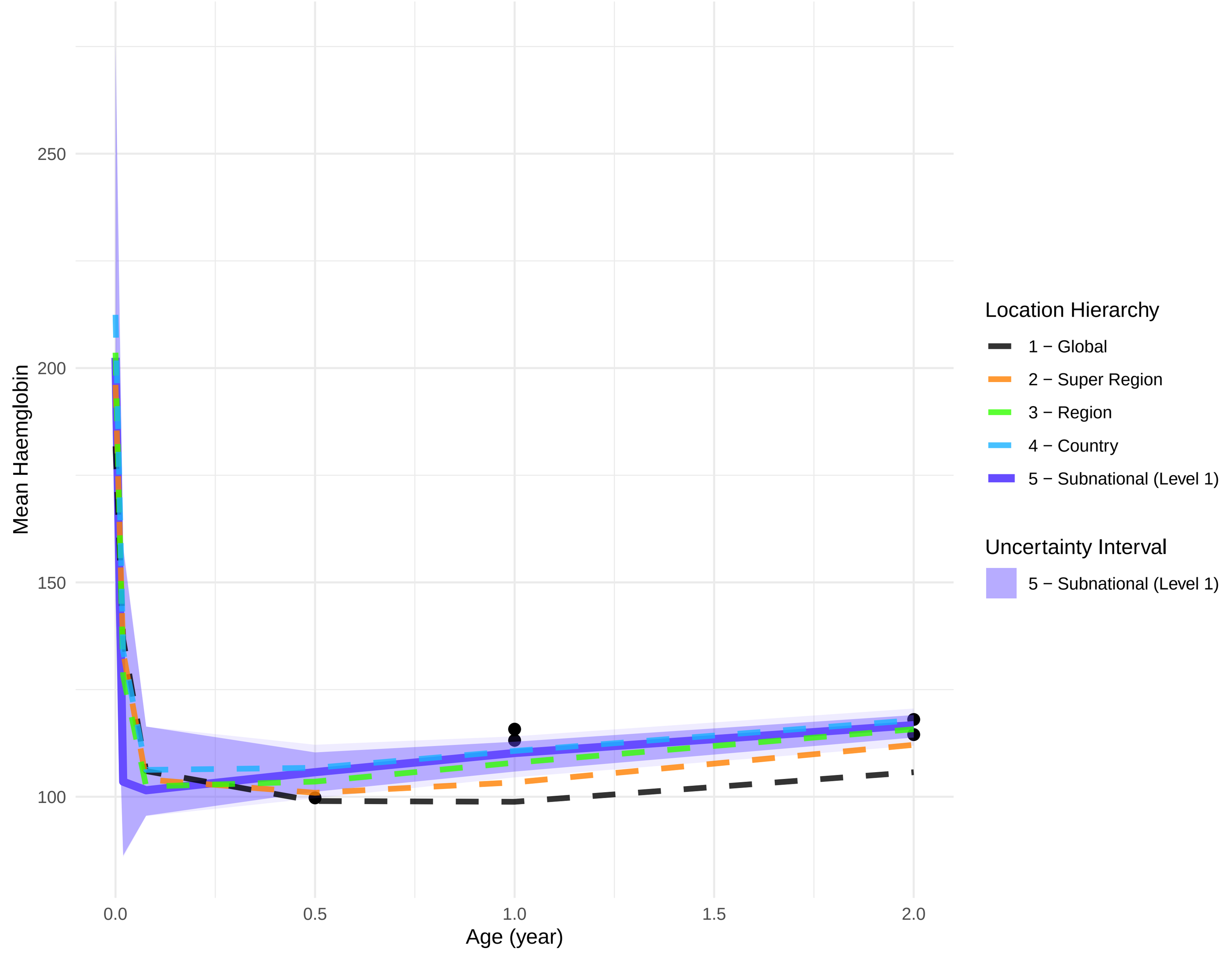

MR−BRT Age/Sex Fits − Males <2 Years Old − Mean Haemglobin − North Africa and Middle East
WHO−2024 Adjustment Method

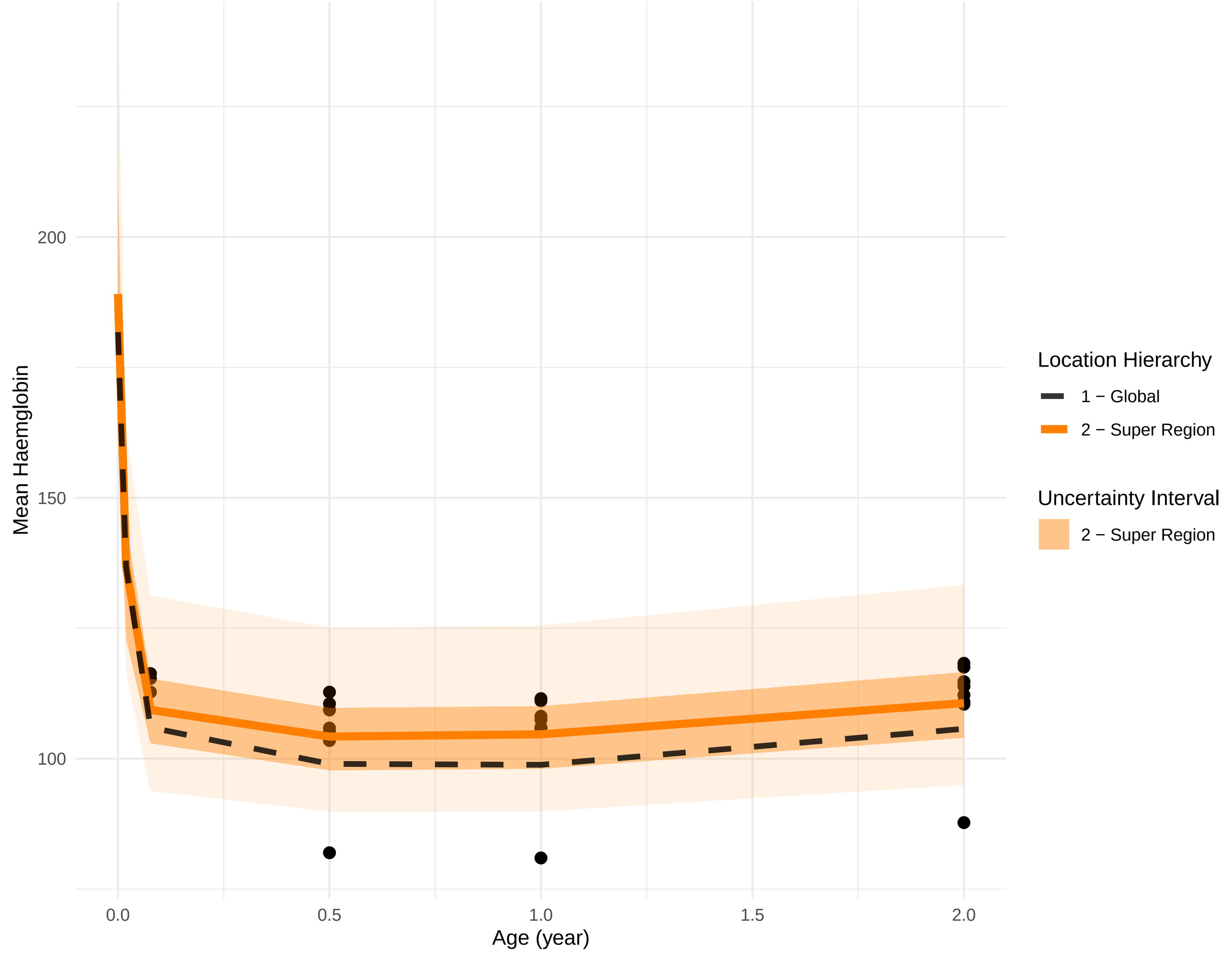

MR−BRT Age/Sex Fits − Males <2 Years Old − Mean Haemglobin − North Africa and Middle East
WHO−2024 Adjustment Method

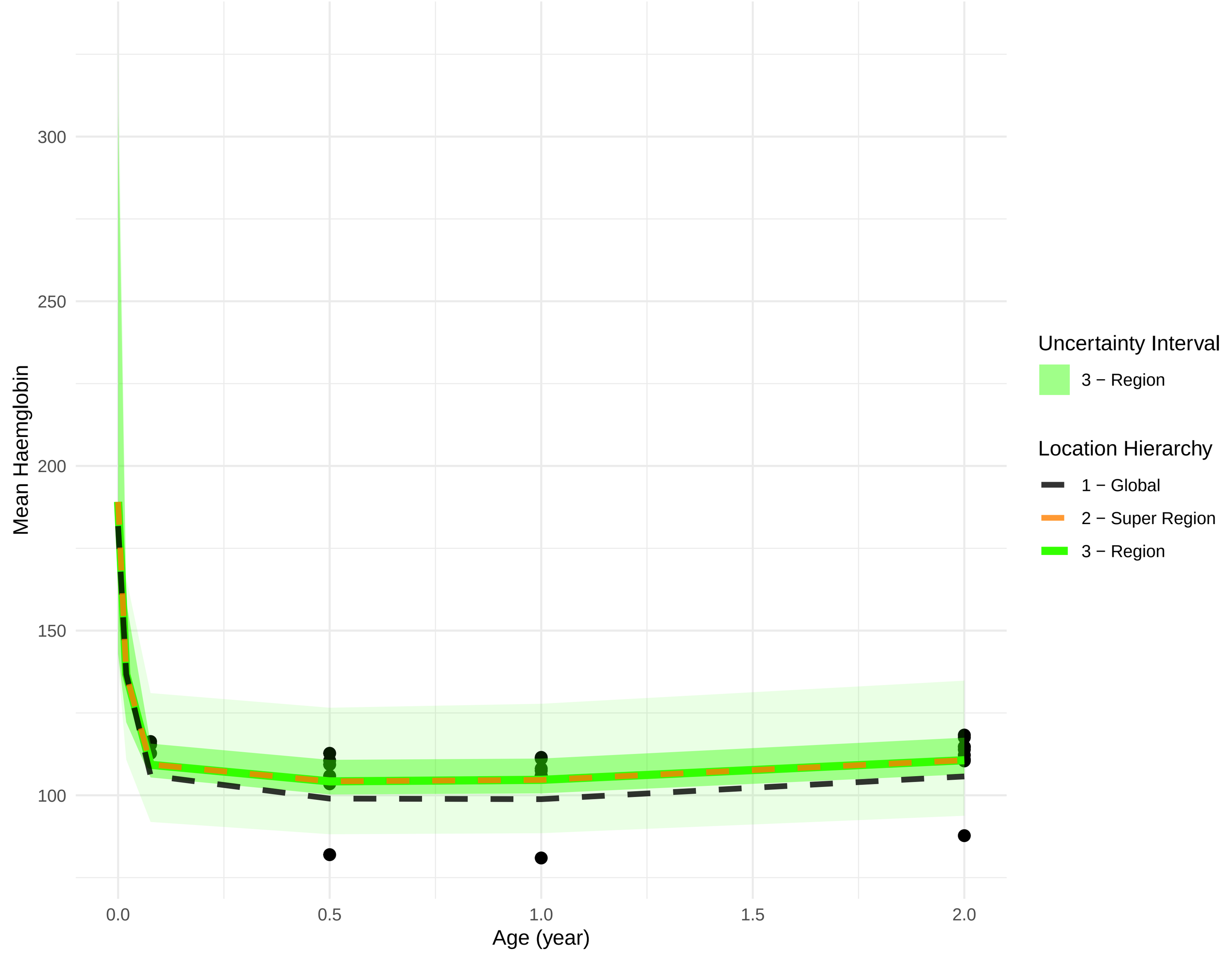

MR−BRT Age/Sex Fits − Males <2 Years Old − Mean Haemglobin − Egypt
WHO−2024 Adjustment Method

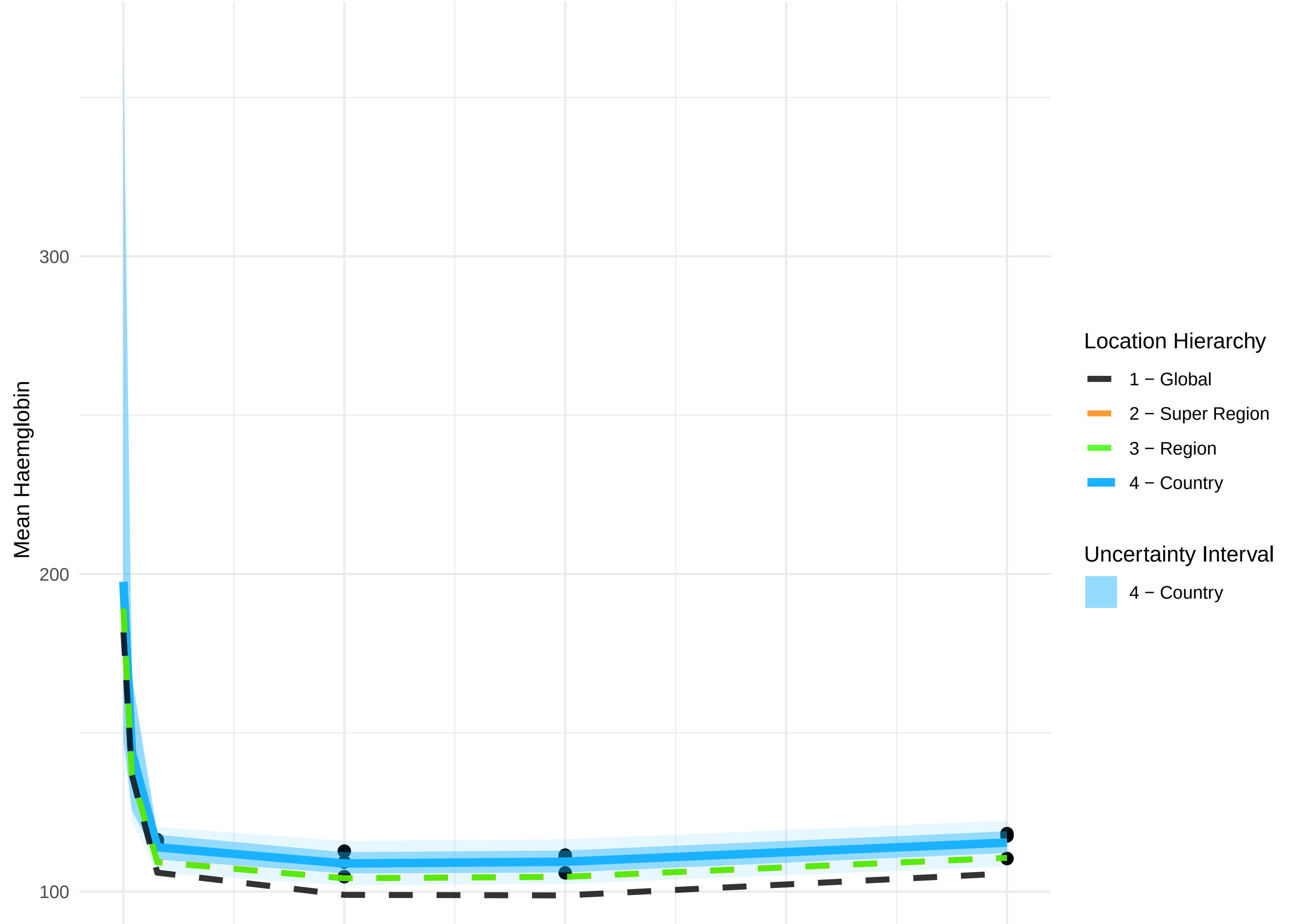

MR−BRT Age/Sex Fits − Males <2 Years Old − Mean Haemglobin − Jordan
WHO−2024 Adjustment Method

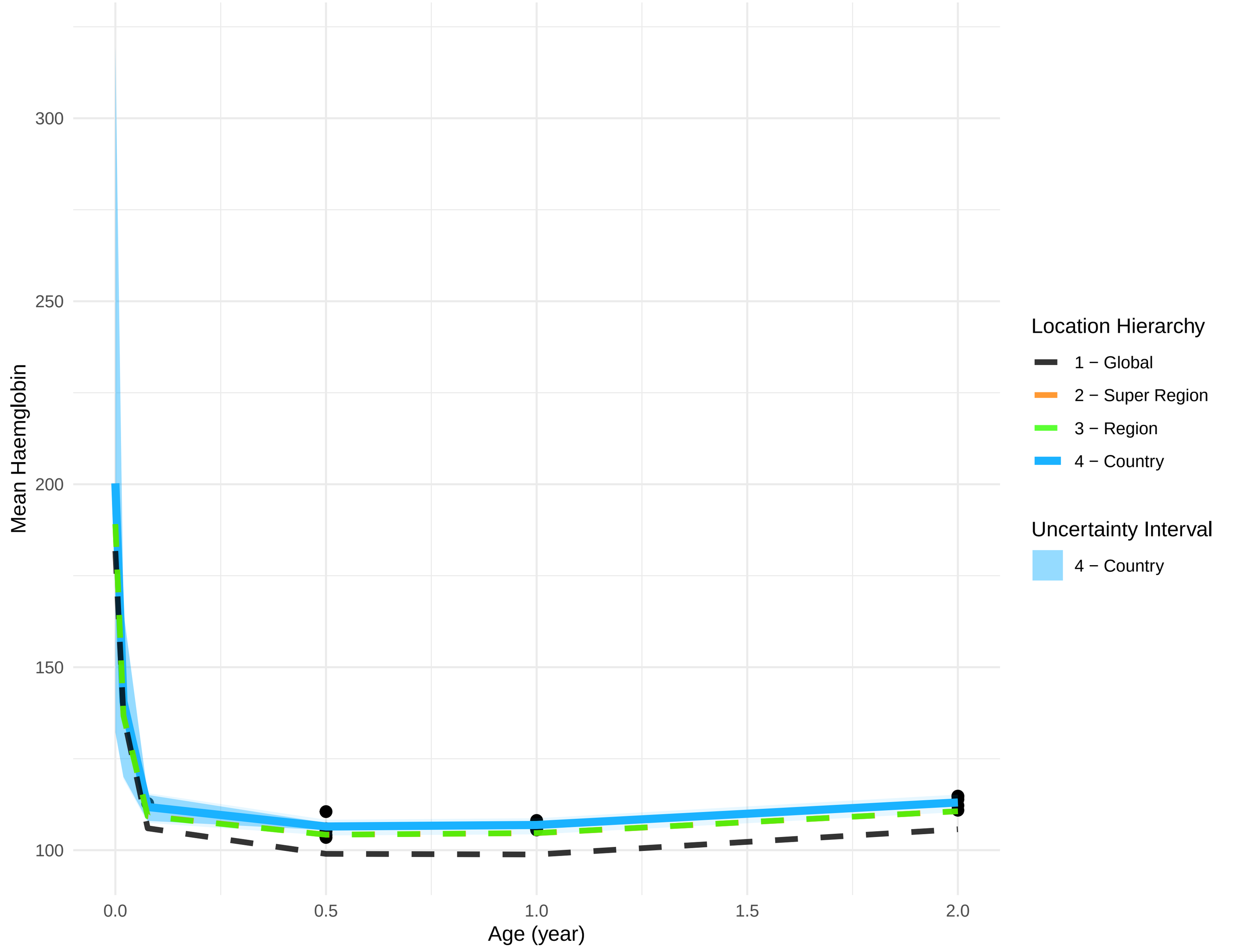

MR−BRT Age/Sex Fits − Males <2 Years Old − Mean Haemglobin − Yemen
WHO−2024 Adjustment Method

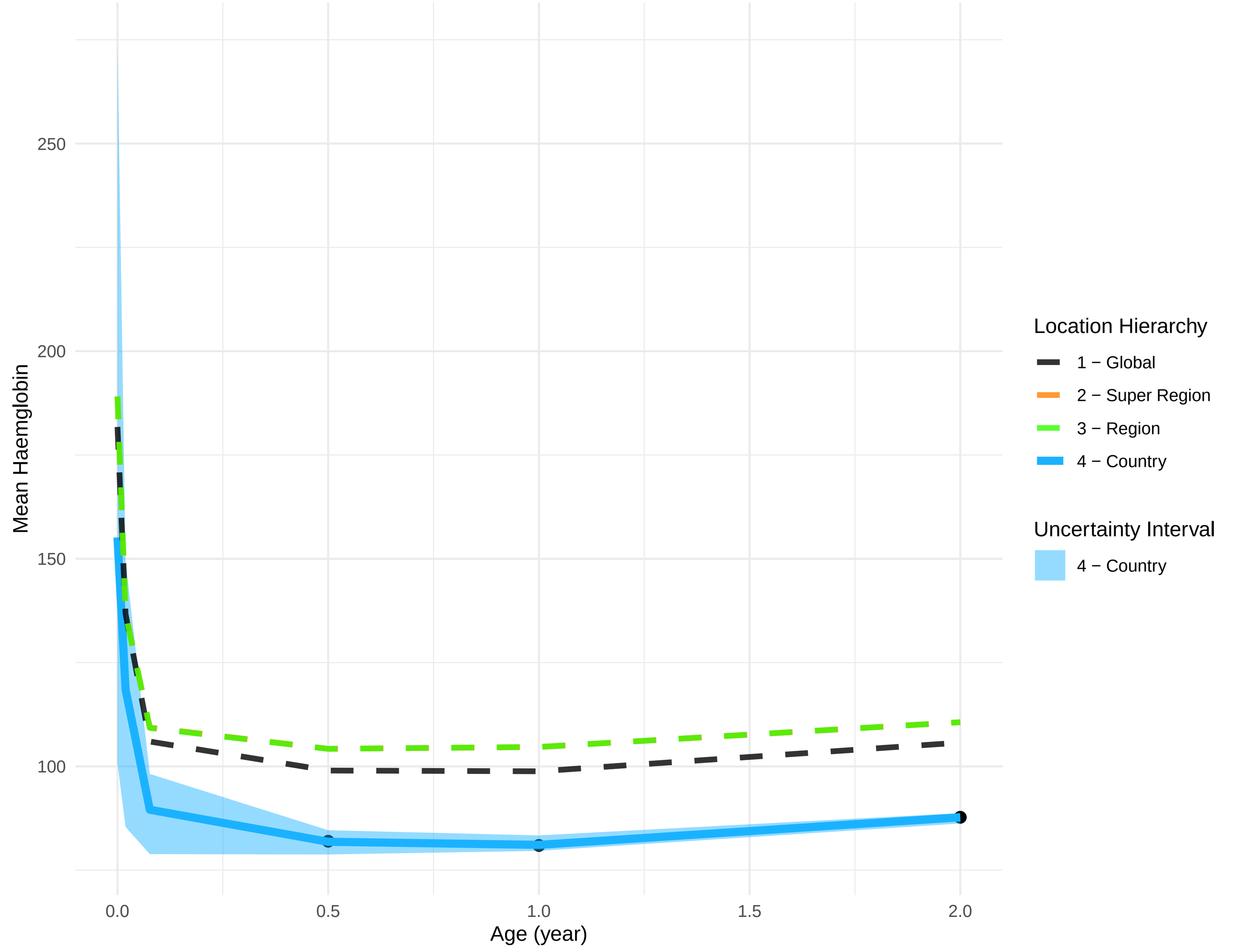

MR−BRT Age/Sex Fits − Males <2 Years Old − Mean Haemglobin − South Asia
WHO−2024 Adjustment Method

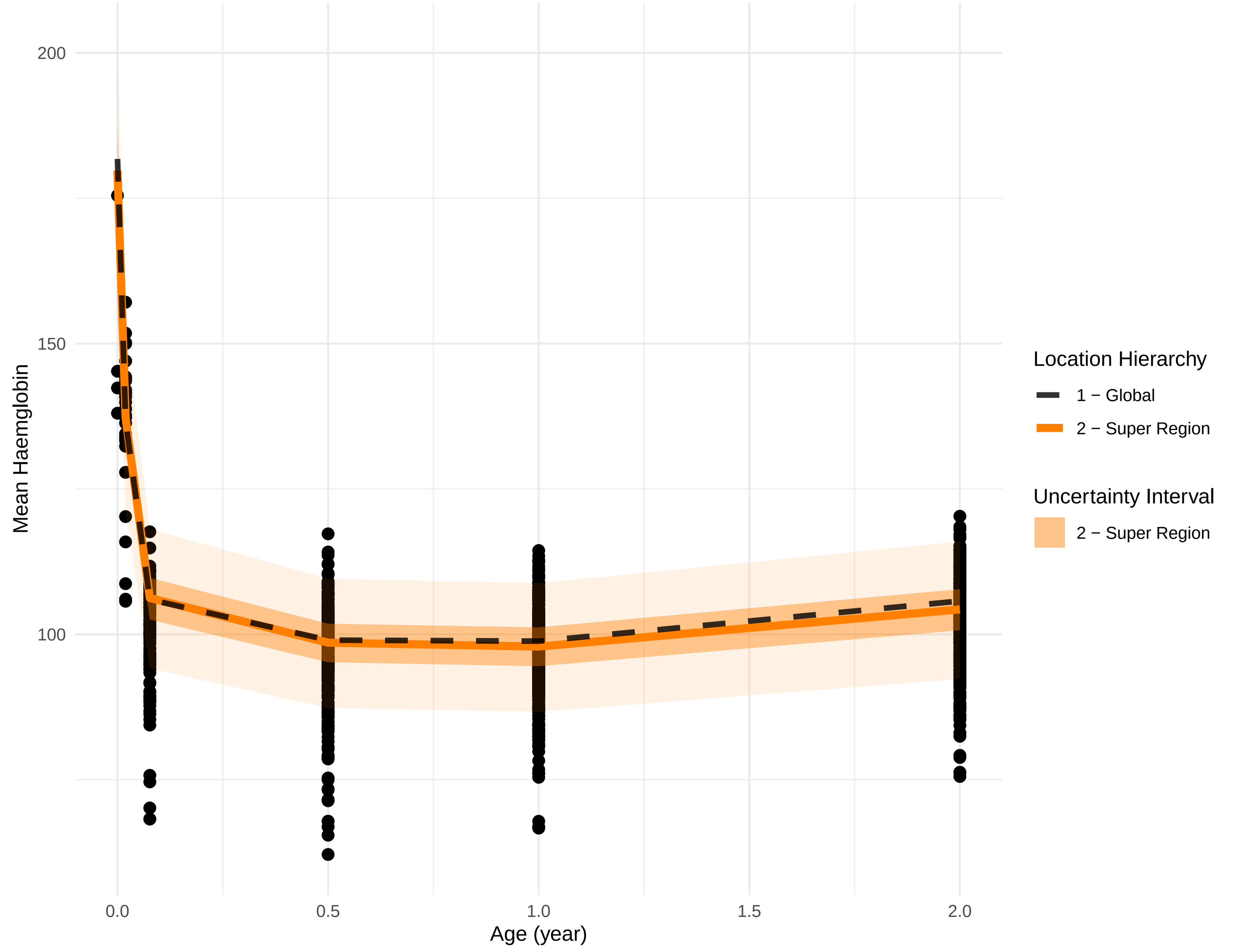

MR−BRT Age/Sex Fits − Males <2 Years Old − Mean Haemglobin − South Asia
WHO−2024 Adjustment Method

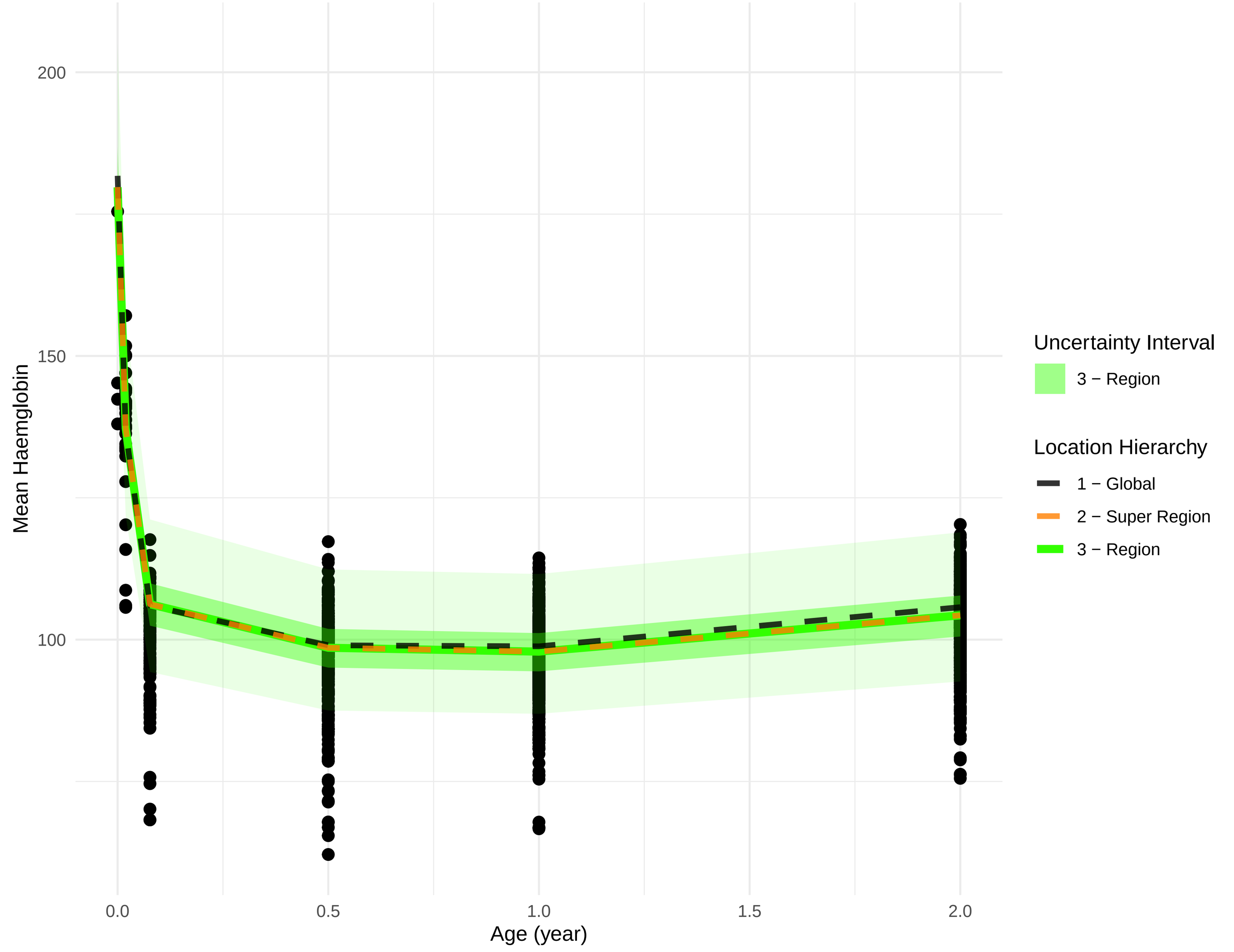

MR−BRT Age/Sex Fits − Males <2 Years Old − Mean Haemglobin − Bangladesh
WHO−2024 Adjustment Method

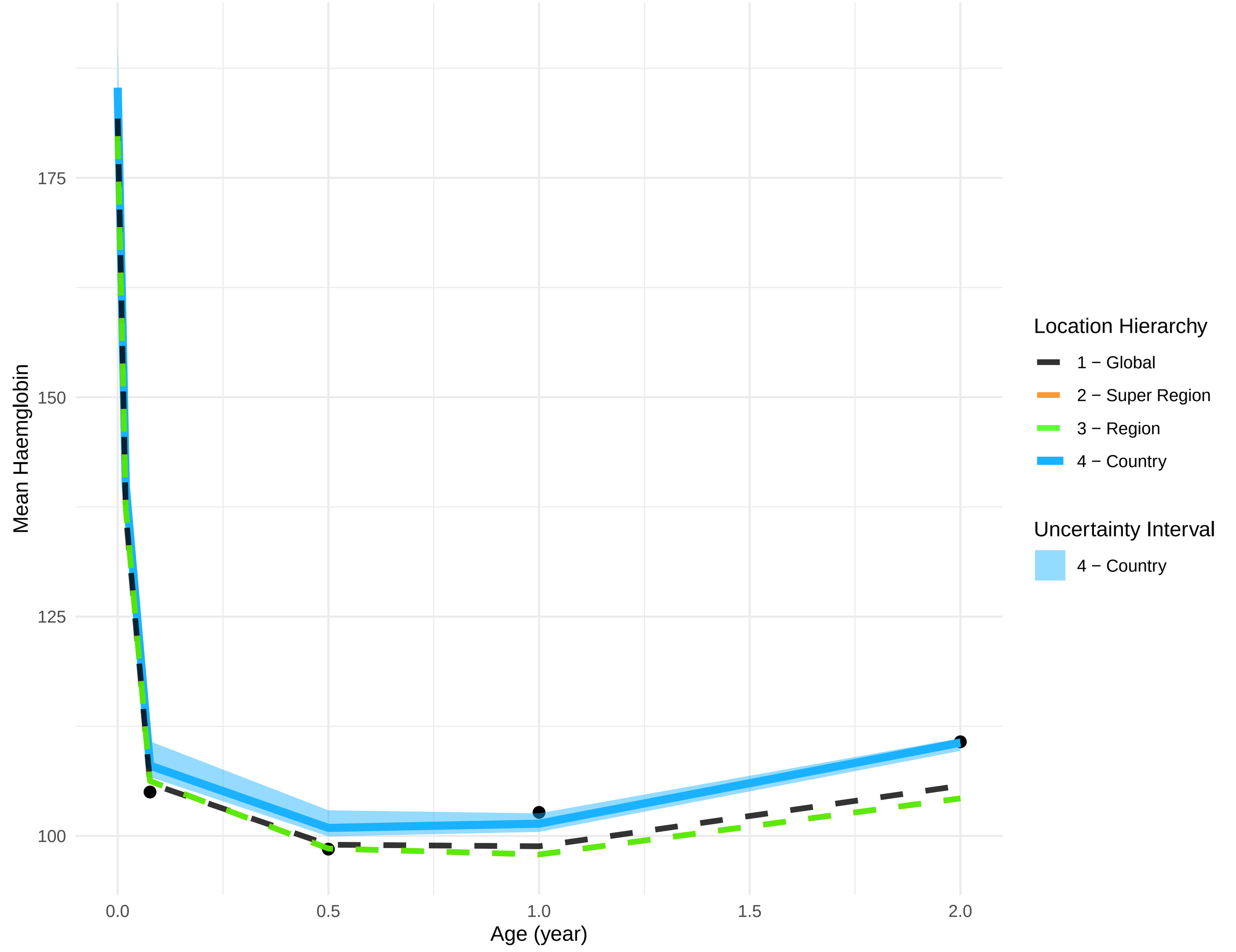

MR−BRT Age/Sex Fits − Males <2 Years Old − Mean Haemglobin − India
WHO−2024 Adjustment Method

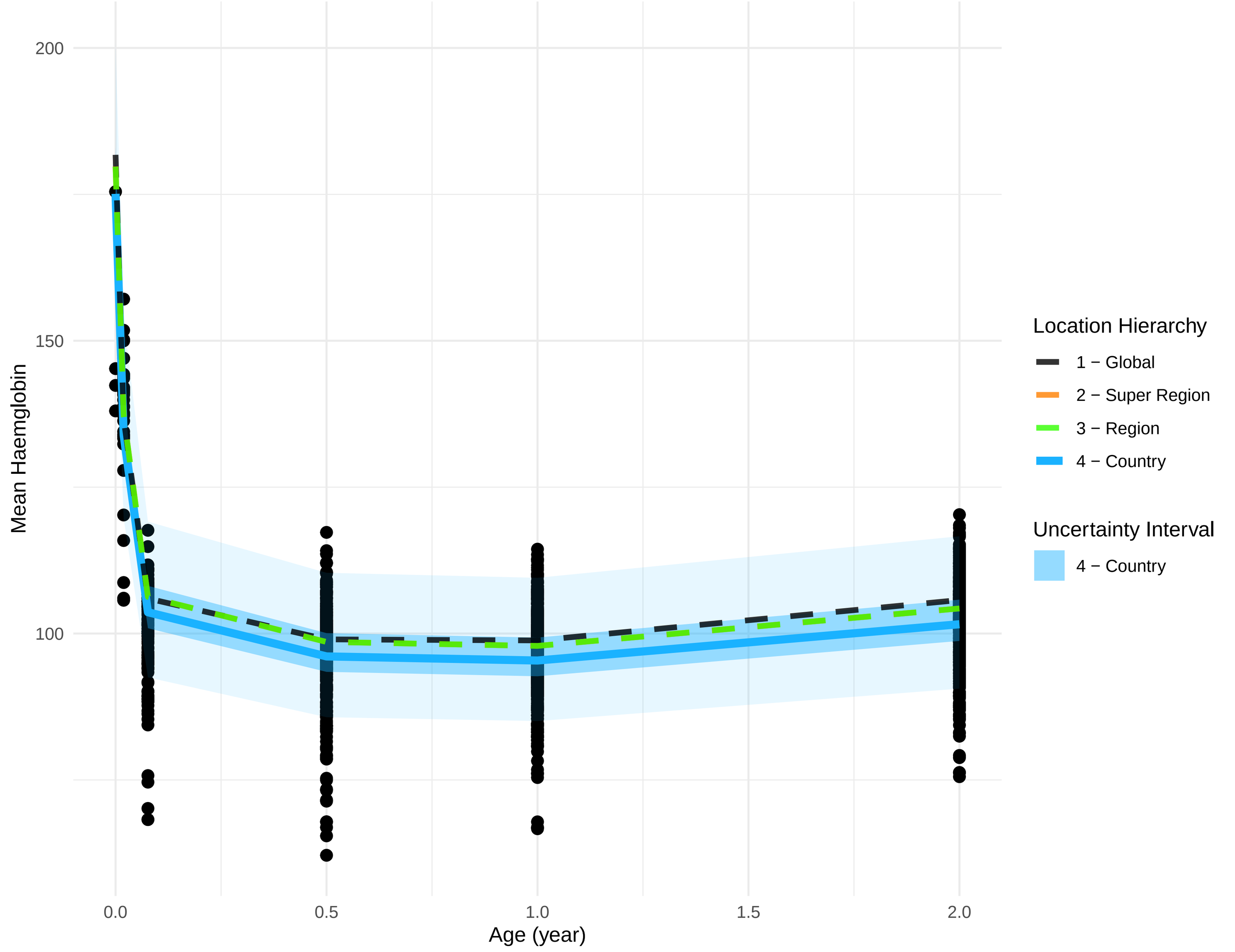

MR−BRT Age/Sex Fits − Males <2 Years Old − Mean Haemglobin − Andhra Pradesh
WHO−2024 Adjustment Method

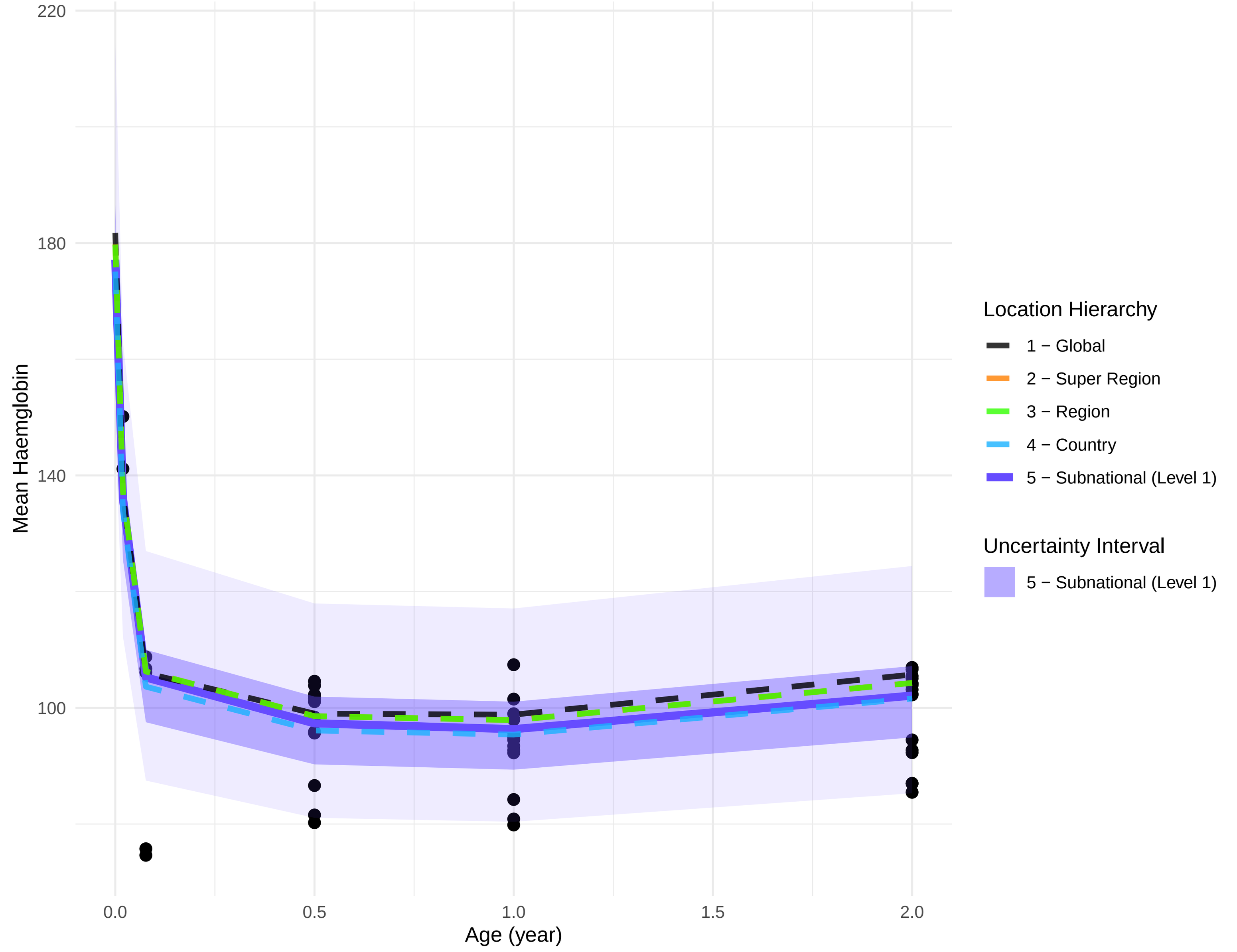

MR−BRT Age/Sex Fits − Males <2 Years Old − Mean Haemglobin − Andhra Pradesh, Rural
WHO−2024 Adjustment Method

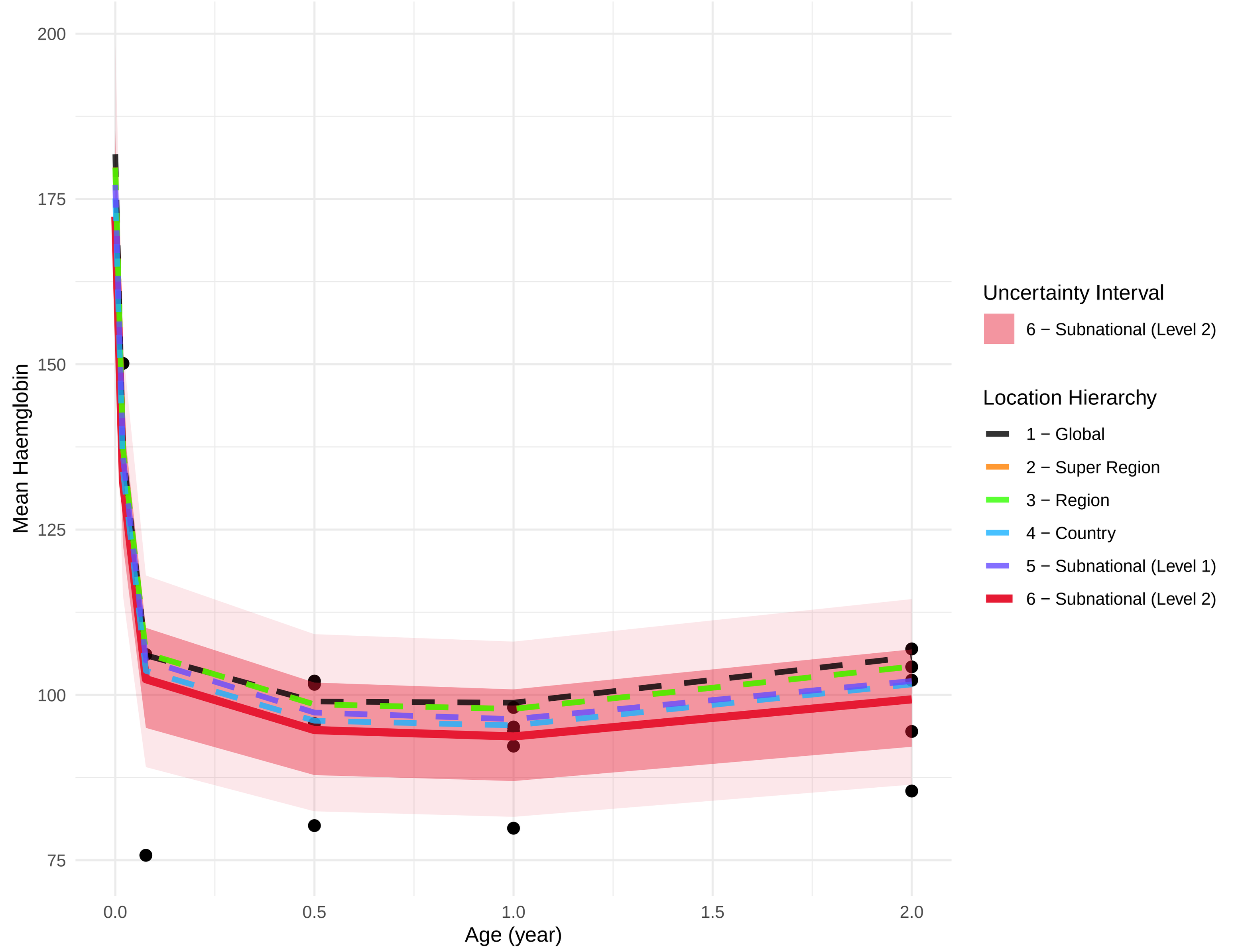

MR−BRT Age/Sex Fits − Males <2 Years Old − Mean Haemglobin − Andhra Pradesh, Urban
WHO−2024 Adjustment Method

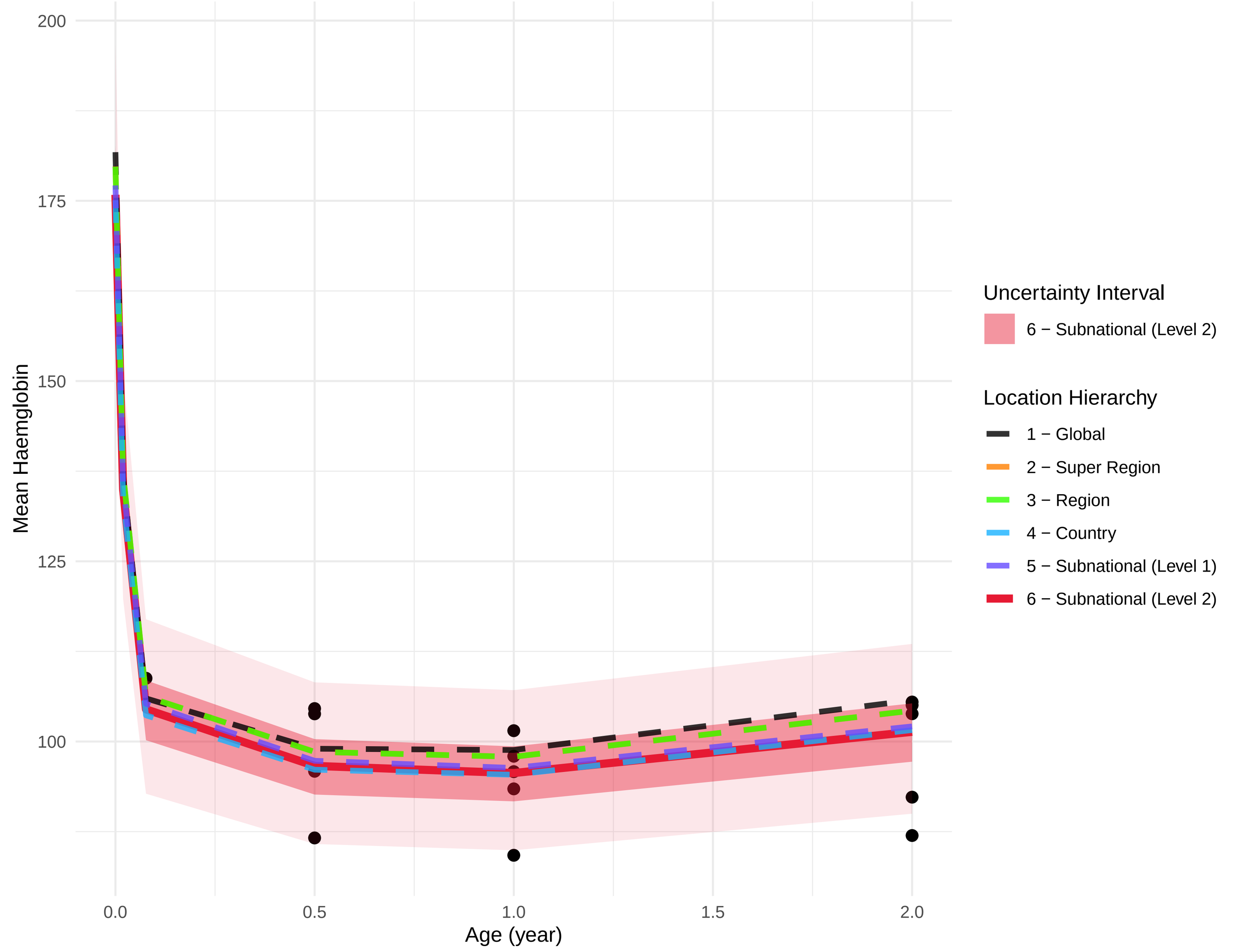

MR−BRT Age/Sex Fits − Males <2 Years Old − Mean Haemglobin − Arunachal Pradesh
WHO−2024 Adjustment Method

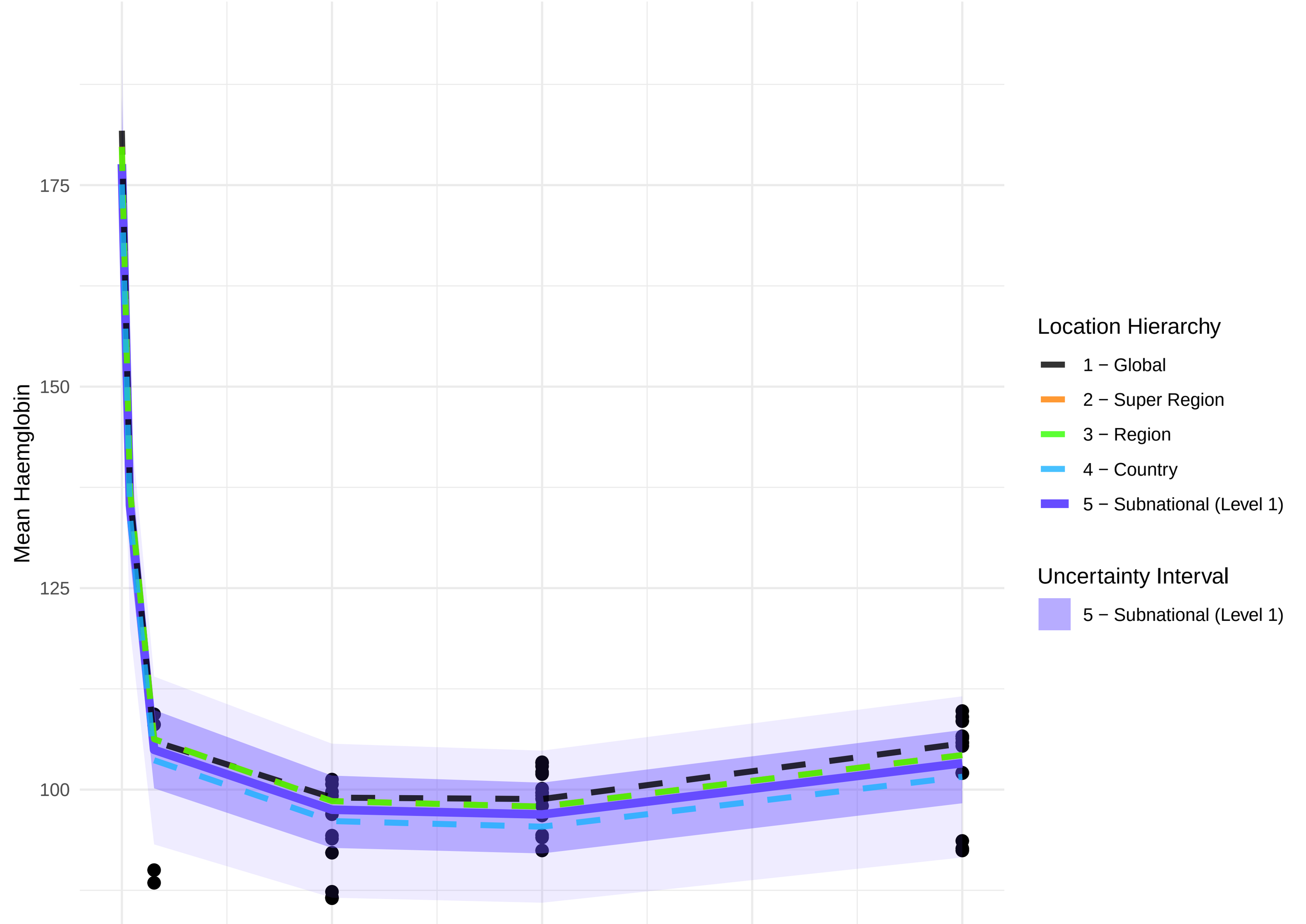

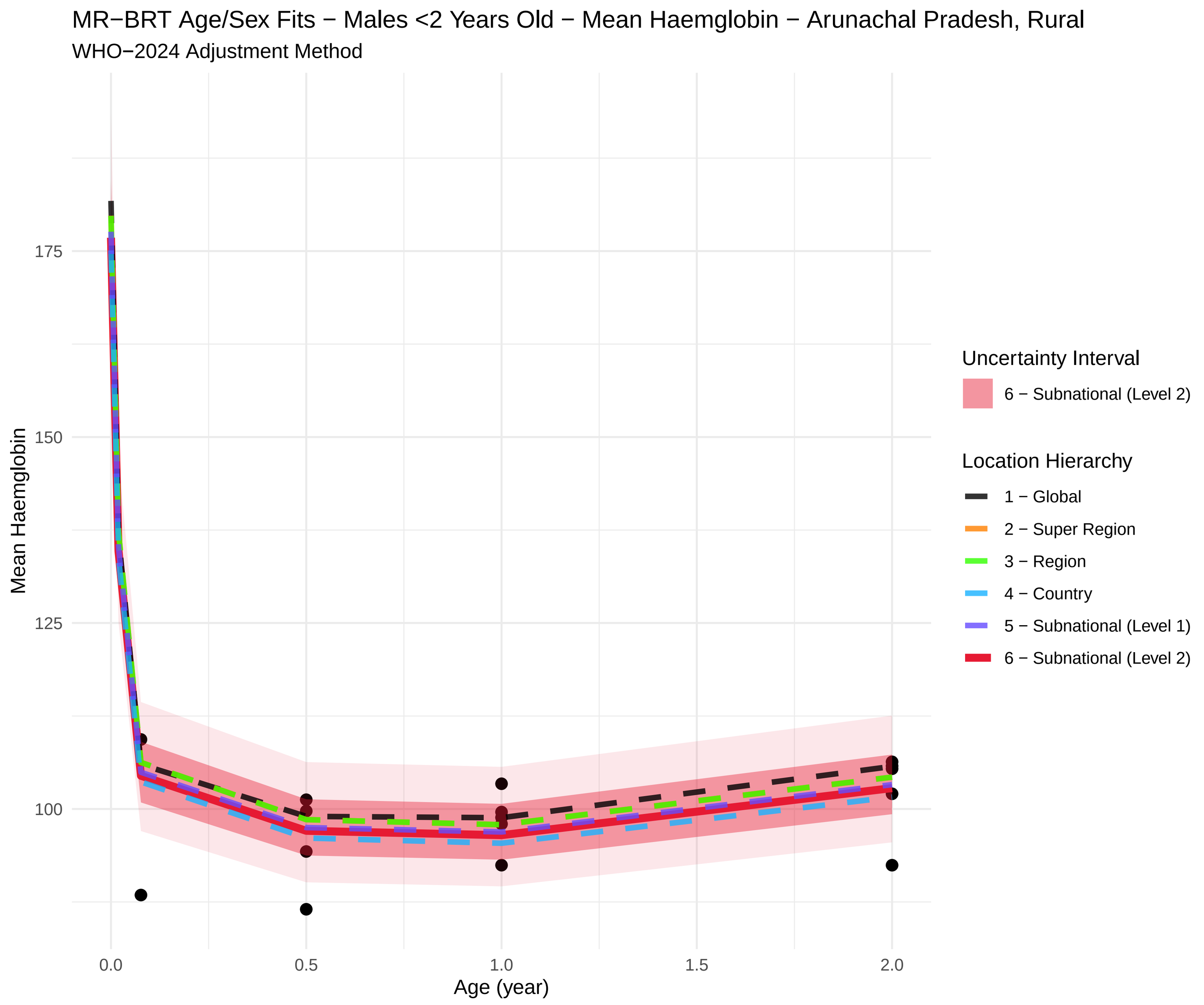
MR−BRT Age/Sex Fits − Males <2 Years Old − Mean Haemglobin − Arunachal Pradesh, Rural
WHO−2024 Adjustment Method
Mean Haemglobin
Age (year)
Uncertainty Interval
6 − Subnational (Level 2)
Location Hierarchy
1 − Global
2 − Super Region
3 − Region
4 − Country
5 − Subnational (Level 1)
6 − Subnational (Level 2)

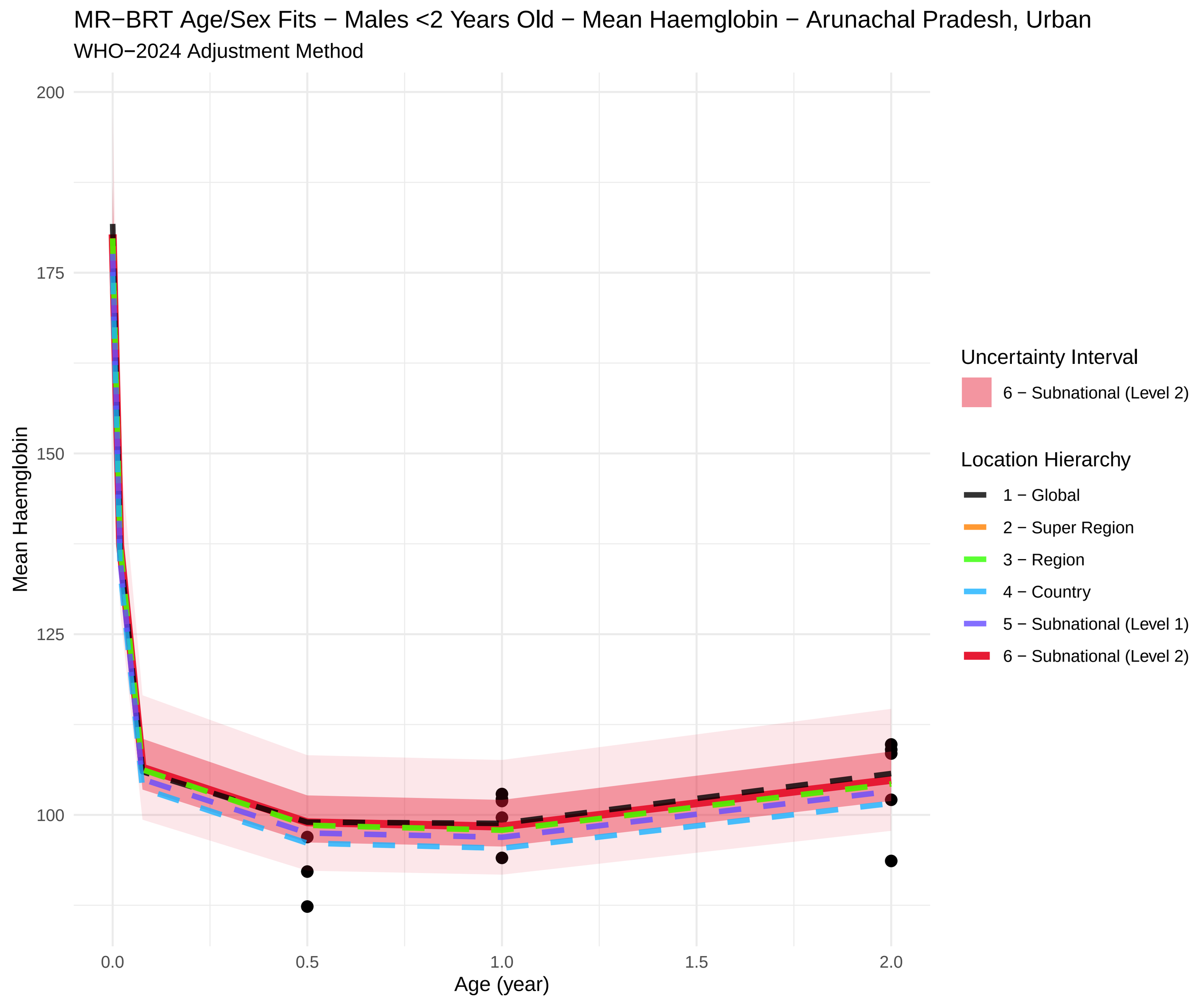
MR−BRT Age/Sex Fits − Males <2 Years Old − Mean Haemglobin − Arunachal Pradesh, Urban
WHO−2024 Adjustment Method
Mean Haemglobin
Age (year)
Uncertainty Interval
6 − Subnational (Level 2)
Location Hierarchy
1 − Global
2 − Super Region
3 − Region
4 − Country
5 − Subnational (Level 1)
6 − Subnational (Level 2)

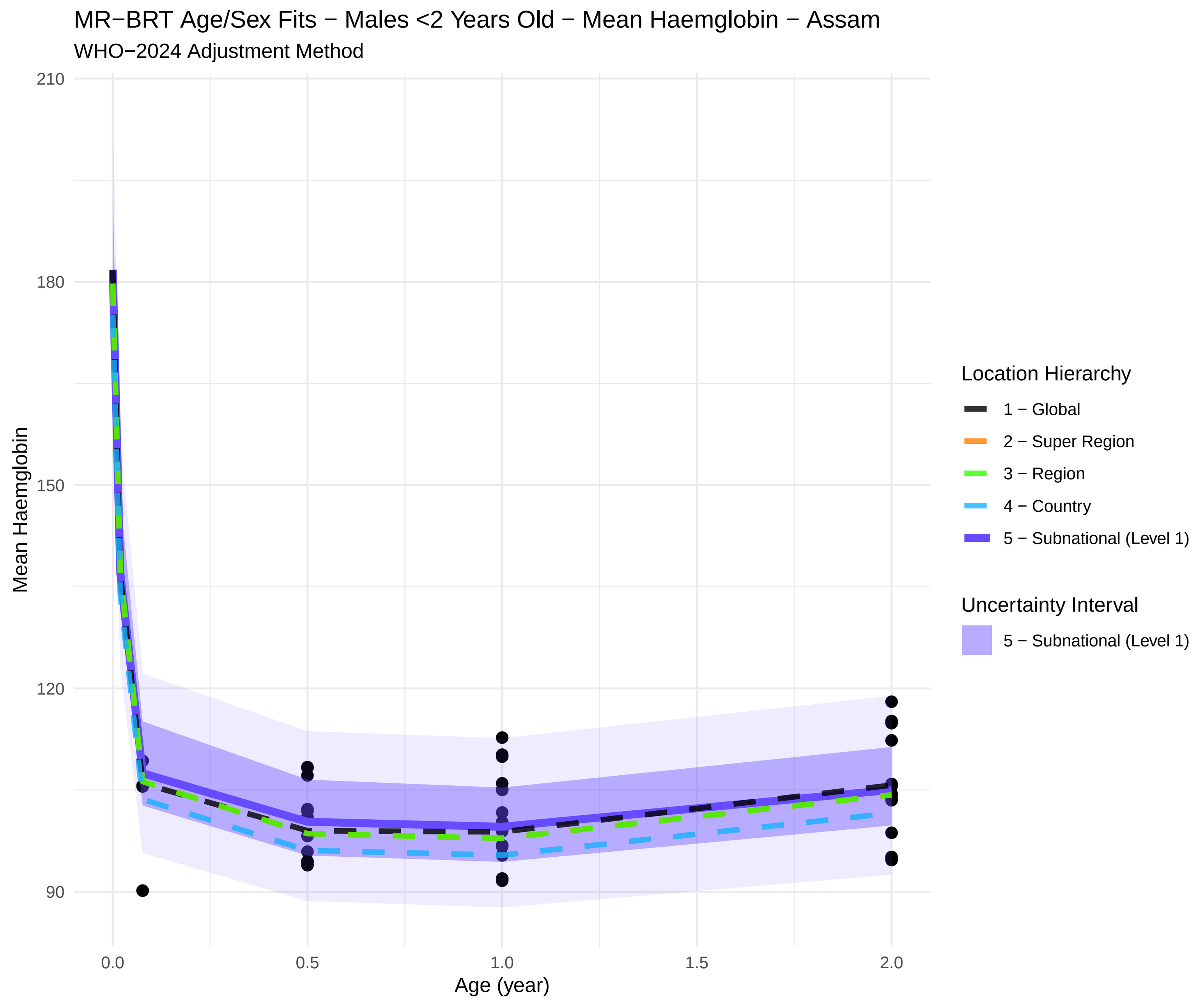
MR−BRT Age/Sex Fits − Males <2 Years Old − Mean Haemglobin − Assam
WHO−2024 Adjustment Method
Mean Haemglobin
Age (year)
Location Hierarchy
1 − Global
2 − Super Region
3 − Region
4 − Country
5 − Subnational (Level 1)
Uncertainty Interval
5 − Subnational (Level 1)

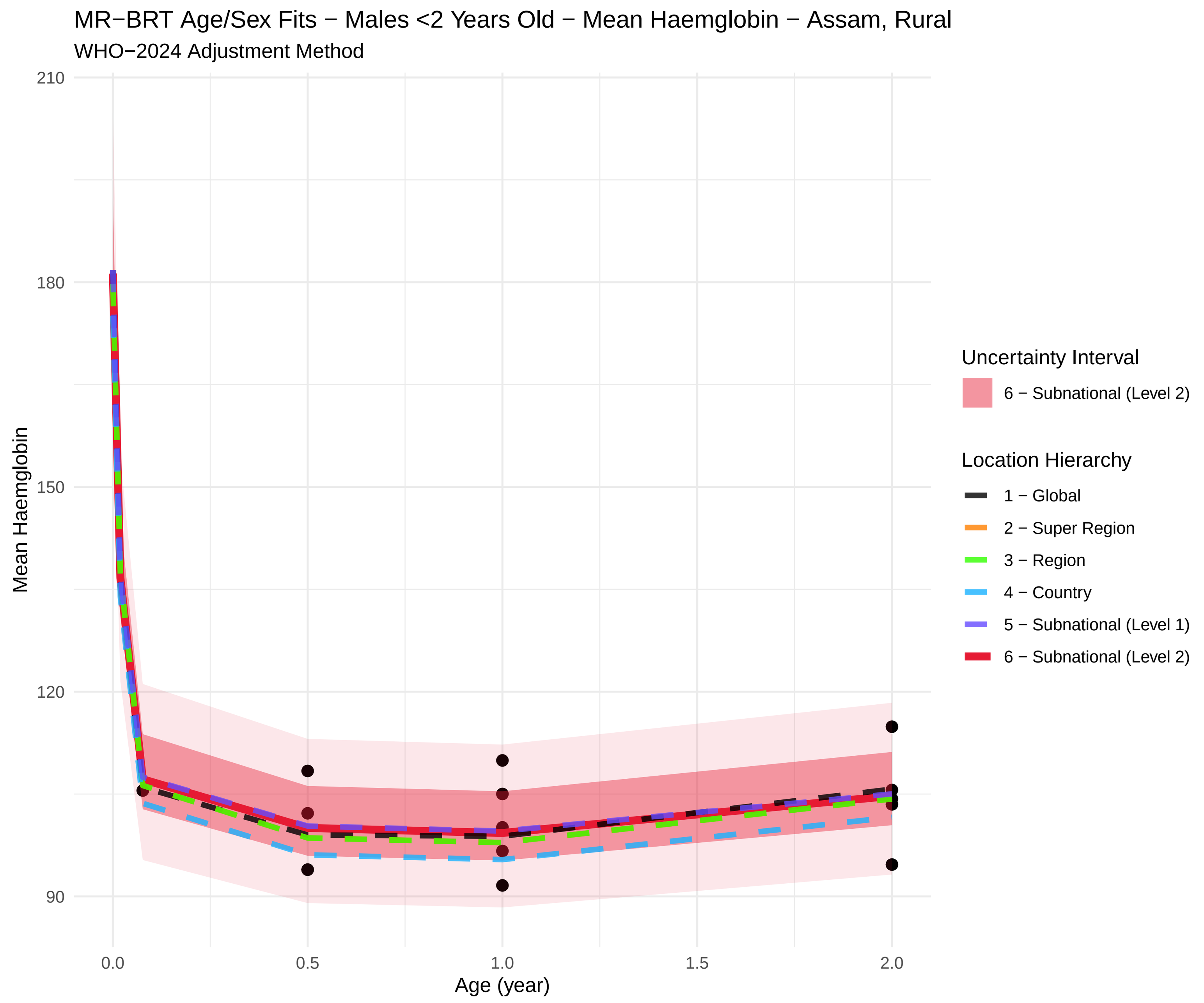
MR−BRT Age/Sex Fits − Males <2 Years Old − Mean Haemglobin − Assam, Rural
WHO−2024 Adjustment Method
Mean Haemglobin
Age (year)
Uncertainty Interval
6 − Subnational (Level 2)
Location Hierarchy
1 − Global
2 − Super Region
3 − Region
4 − Country
5 − Subnational (Level 1)
6 − Subnational (Level 2)

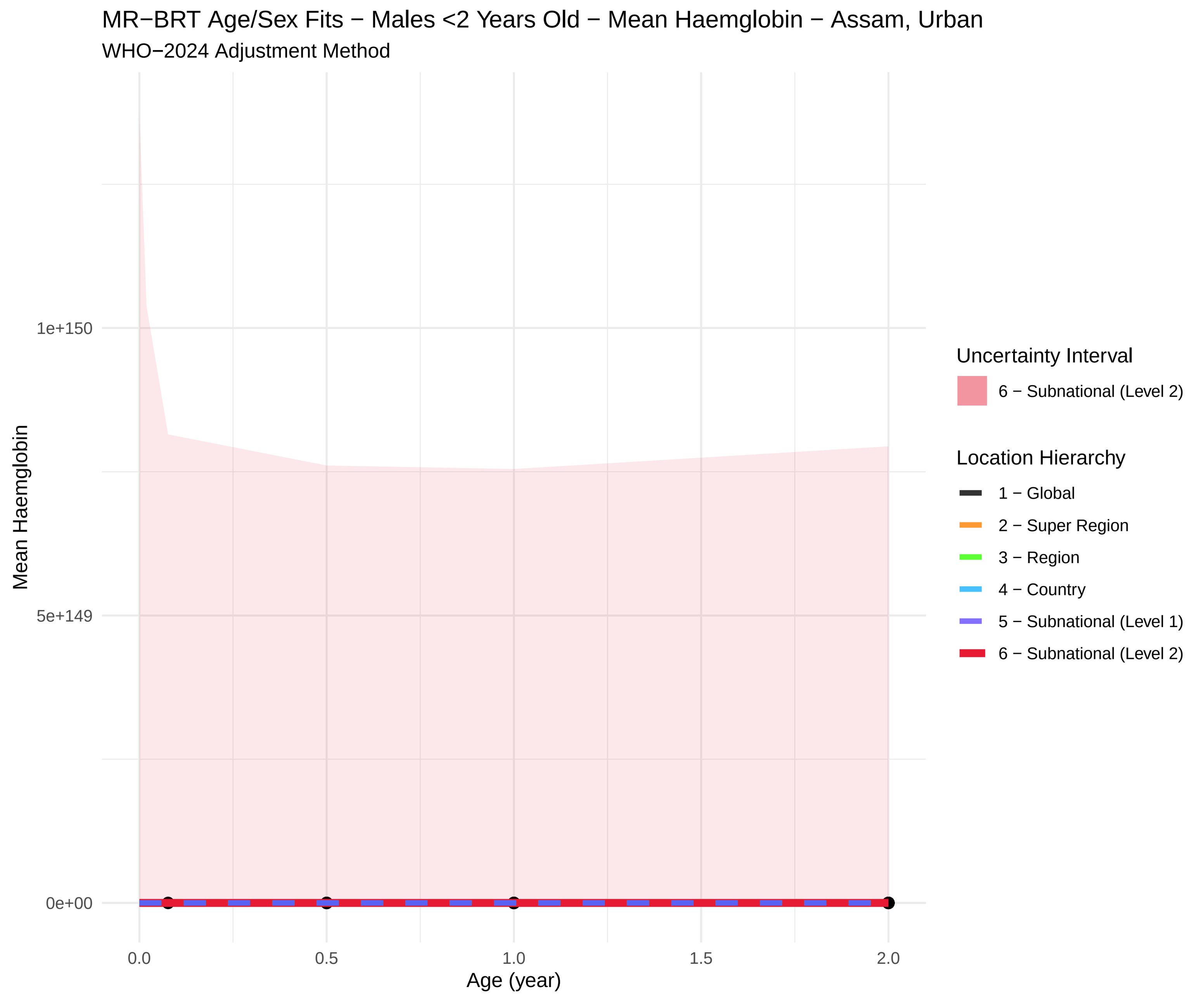
MR−BRT Age/Sex Fits − Males <2 Years Old − Mean Haemglobin − Assam, Urban
WHO−2024 Adjustment Method
Mean Haemglobin
Age (year)
Uncertainty Interval
6 − Subnational (Level 2)
Location Hierarchy
1 − Global
2 − Super Region
3 − Region
4 − Country
5 − Subnational (Level 1)
6 − Subnational (Level 2)

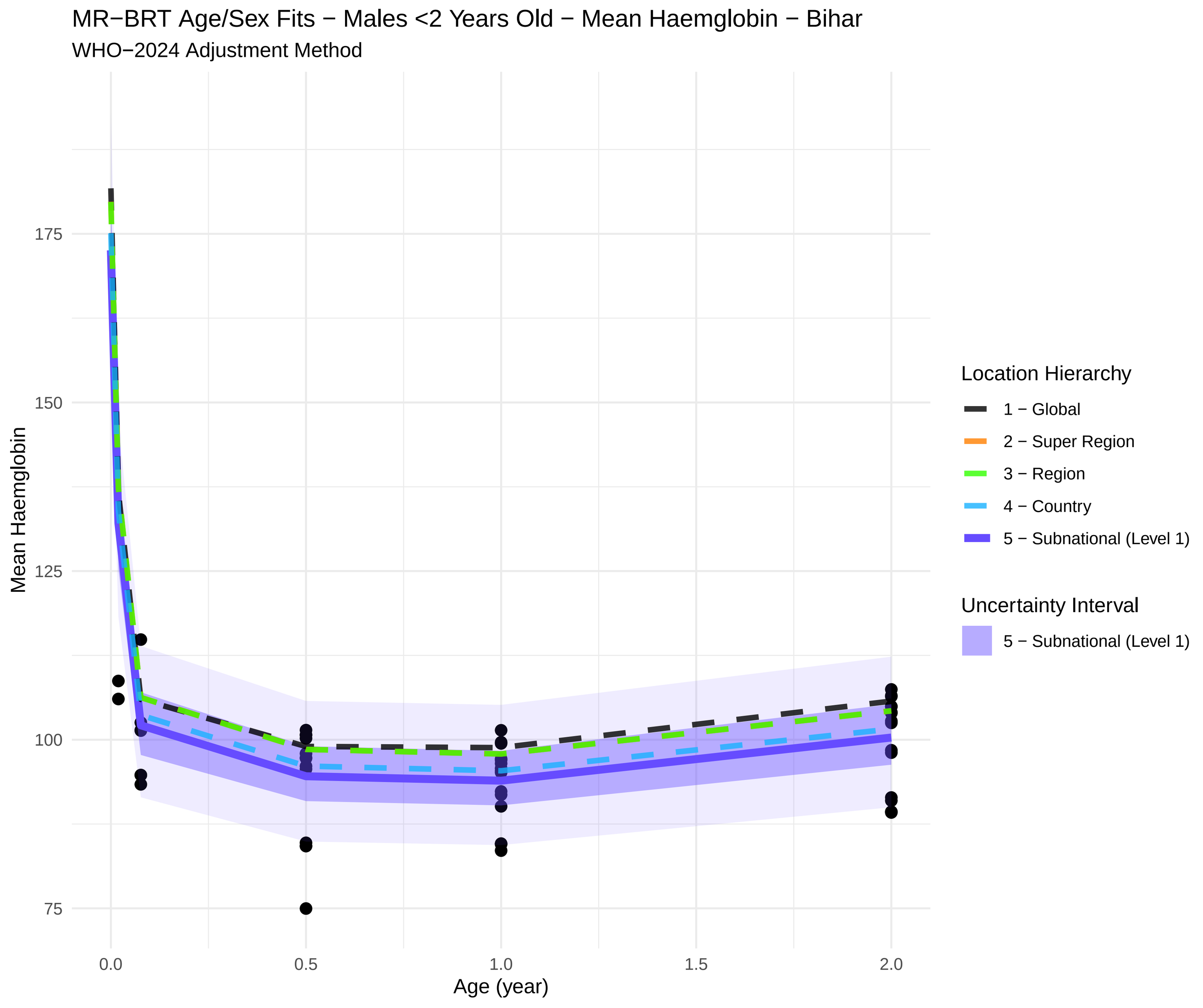
MR−BRT Age/Sex Fits − Males <2 Years Old − Mean Haemglobin − Bihar
WHO−2024 Adjustment Method
Mean Haemglobin
Age (year)
Location Hierarchy
1 − Global
2 − Super Region
3 − Region
4 − Country
5 − Subnational (Level 1)
Uncertainty Interval
5 − Subnational (Level 1)

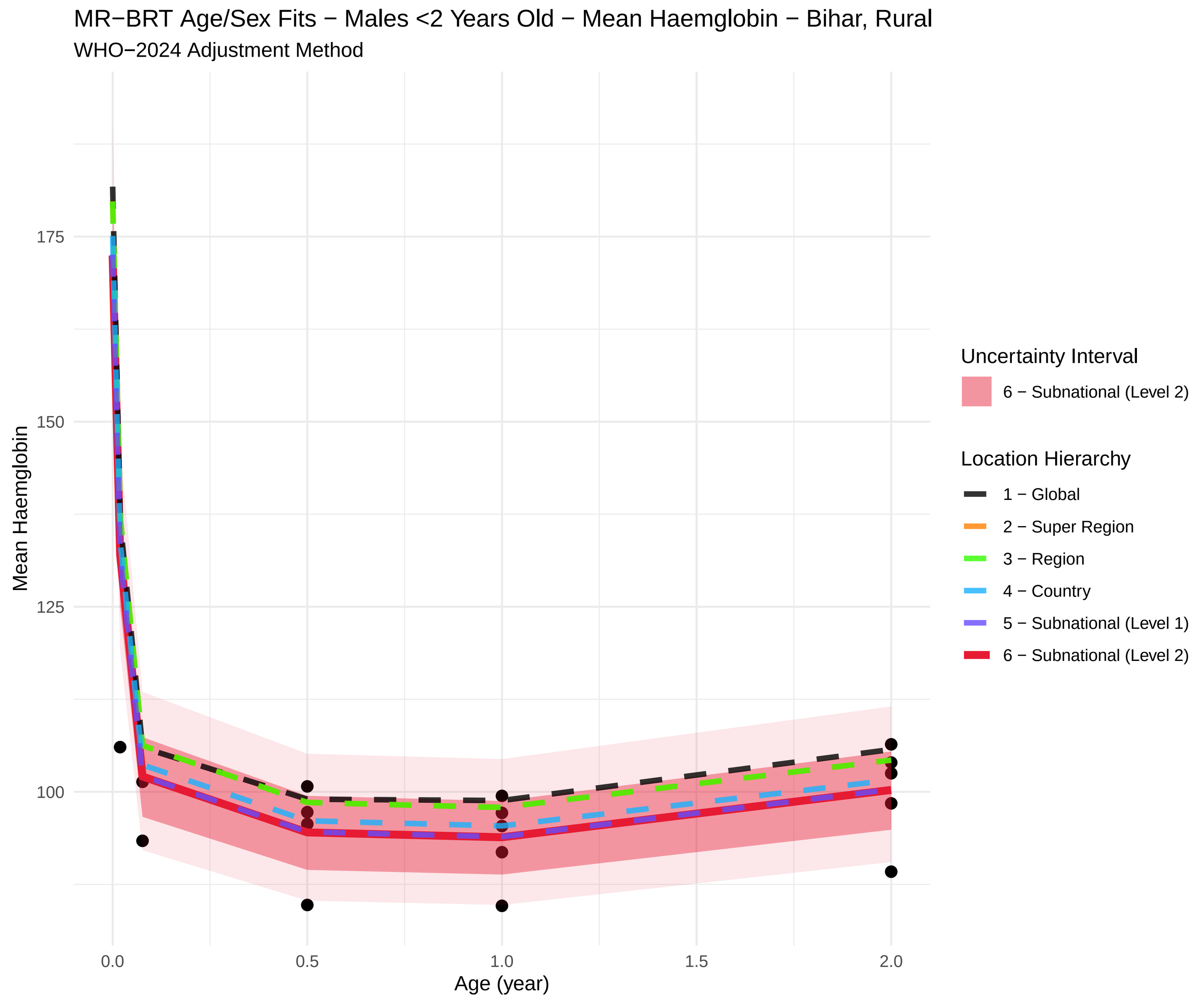
MR−BRT Age/Sex Fits − Males <2 Years Old − Mean Haemglobin − Bihar, Rural
WHO−2024 Adjustment Method
Mean Haemglobin
Age (year)
Uncertainty Interval
6 − Subnational (Level 2)
Location Hierarchy
1 − Global
2 − Super Region
3 − Region
4 − Country
5 − Subnational (Level 1)
6 − Subnational (Level 2)

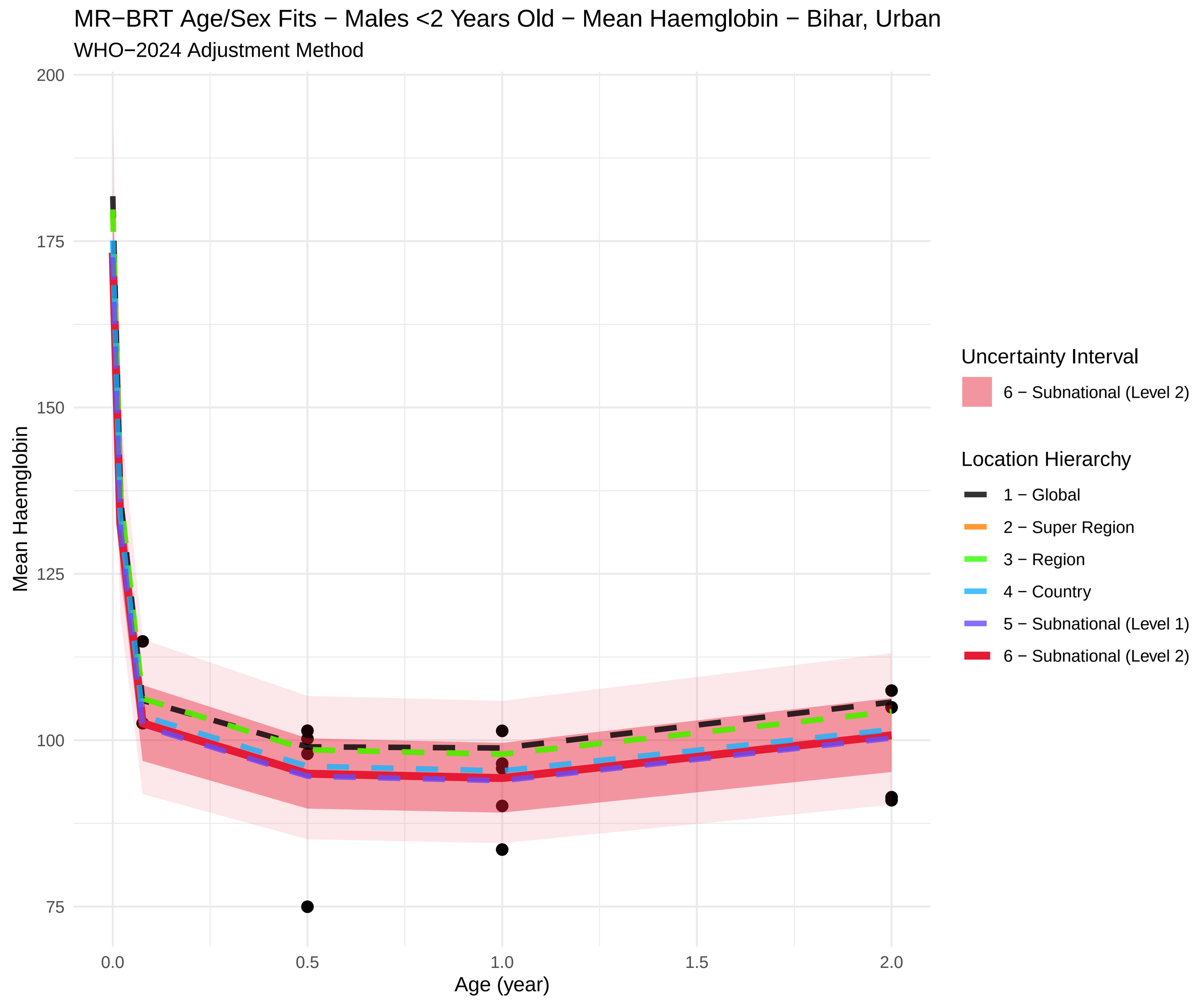
MR−BRT Age/Sex Fits − Males <2 Years Old − Mean Haemglobin − Bihar, Urban
WHO−2024 Adjustment Method
Mean Haemglobin
Age (year)
Uncertainty Interval
6 − Subnational (Level 2)
Location Hierarchy
1 − Global
2 − Super Region
3 − Region
4 − Country
5 − Subnational (Level 1)
6 − Subnational (Level 2)

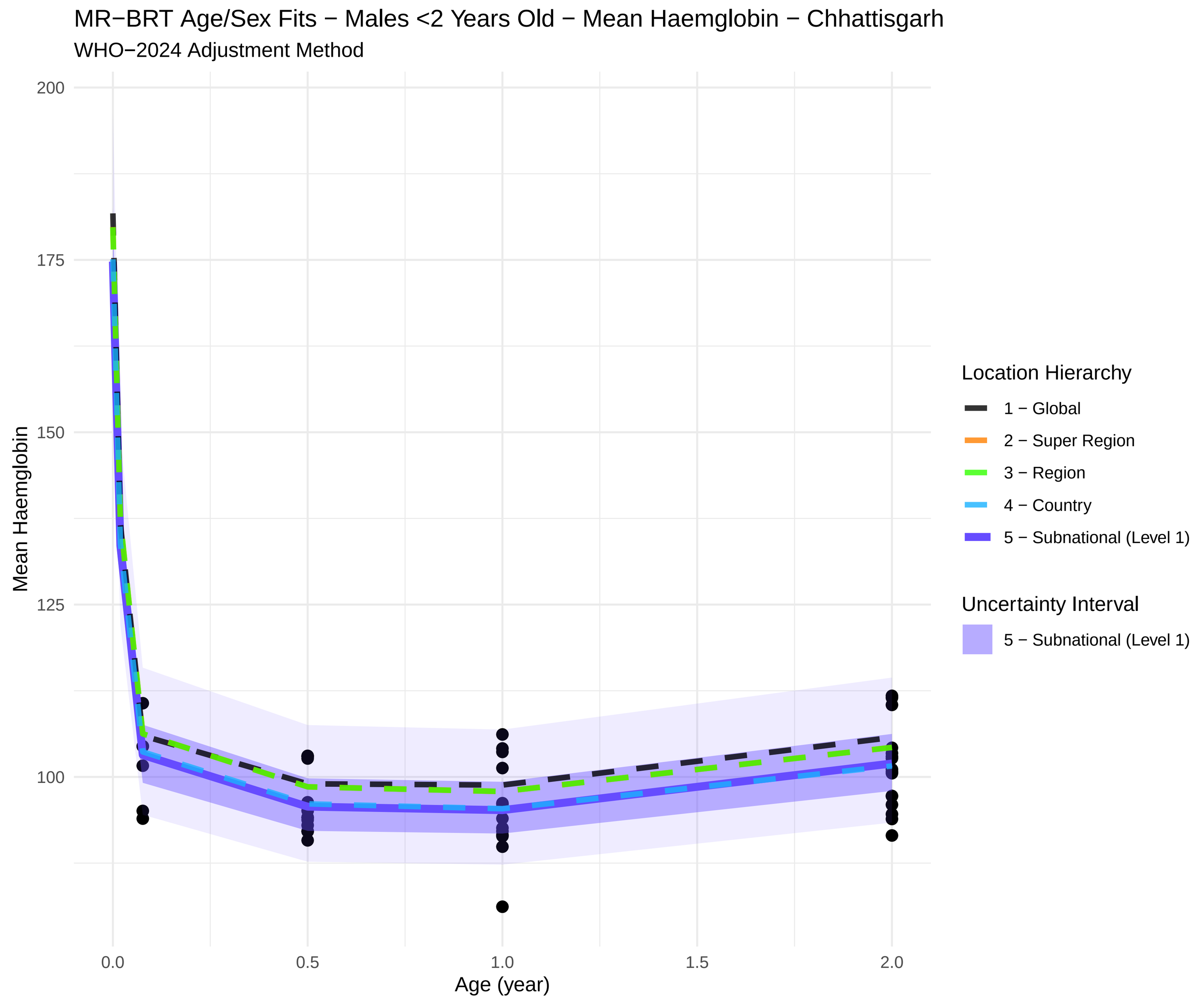
MR−BRT Age/Sex Fits − Males <2 Years Old − Mean Haemglobin − Chhattisgarh
WHO−2024 Adjustment Method
Mean Haemglobin
Age (year)
Location Hierarchy
1 − Global
2 − Super Region
3 − Region
4 − Country
5 − Subnational (Level 1)
Uncertainty Interval
5 − Subnational (Level 1)

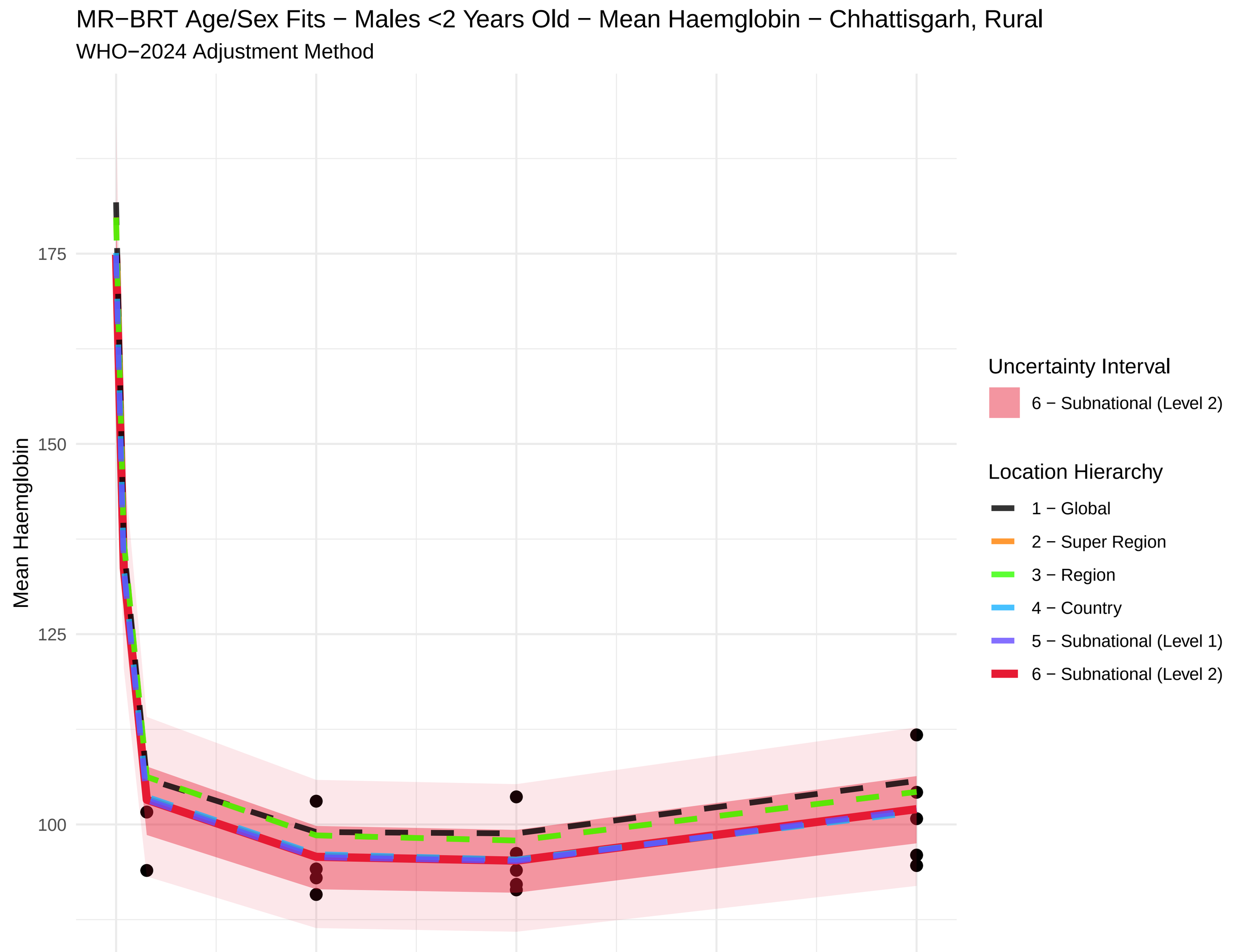
MR−BRT Age/Sex Fits − Males <2 Years Old − Mean Haemglobin − Chhattisgarh, Rural
WHO−2024 Adjustment Method
Mean Haemglobin
Age (year)
Uncertainty Interval
6 − Subnational (Level 2)
Location Hierarchy
1 − Global
2 − Super Region
3 − Region
4 − Country
5 − Subnational (Level 1)
6 − Subnational (Level 2)

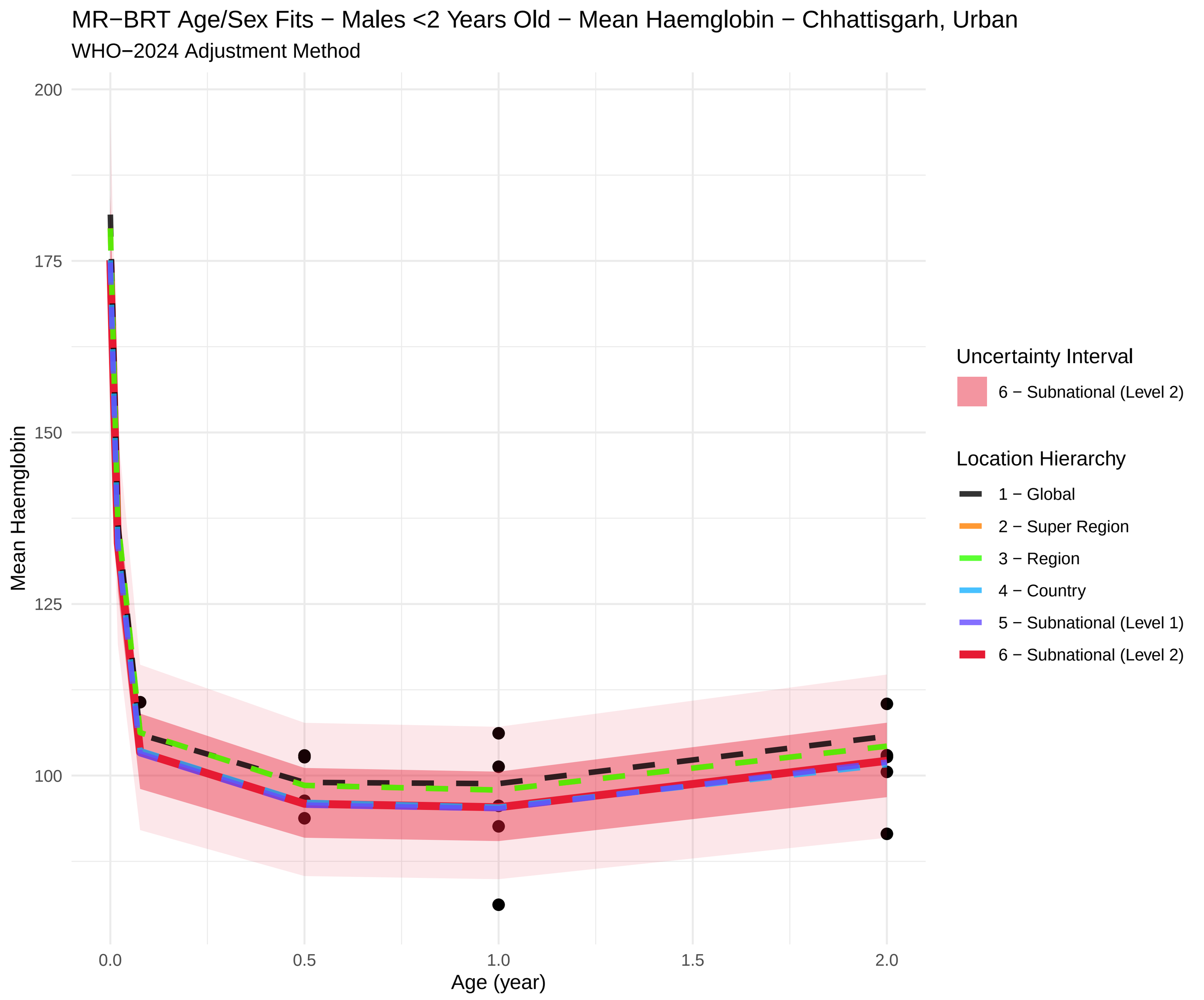
MR−BRT Age/Sex Fits − Males <2 Years Old − Mean Haemglobin − Chhattisgarh, Urban
WHO−2024 Adjustment Method
Mean Haemglobin
Age (year)
Uncertainty Interval
6 − Subnational (Level 2)
Location Hierarchy
1 − Global
2 − Super Region
3 − Region
4 − Country
5 − Subnational (Level 1)
6 − Subnational (Level 2)

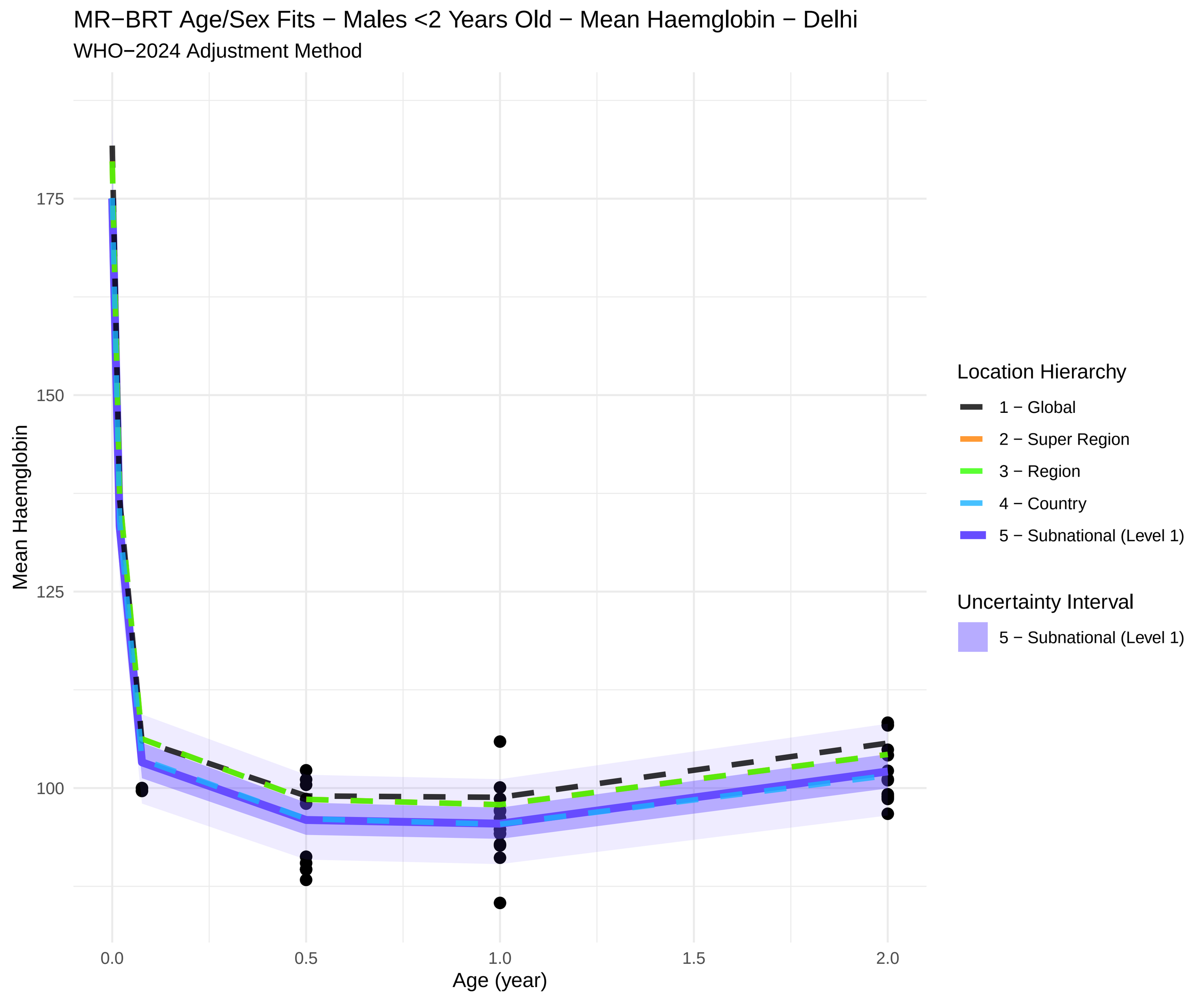
MR−BRT Age/Sex Fits − Males <2 Years Old − Mean Haemglobin − Delhi
WHO−2024 Adjustment Method
Mean Haemglobin
Age (year)
Location Hierarchy
1 − Global
2 − Super Region
3 − Region
4 − Country
5 − Subnational (Level 1)
Uncertainty Interval
5 − Subnational (Level 1)

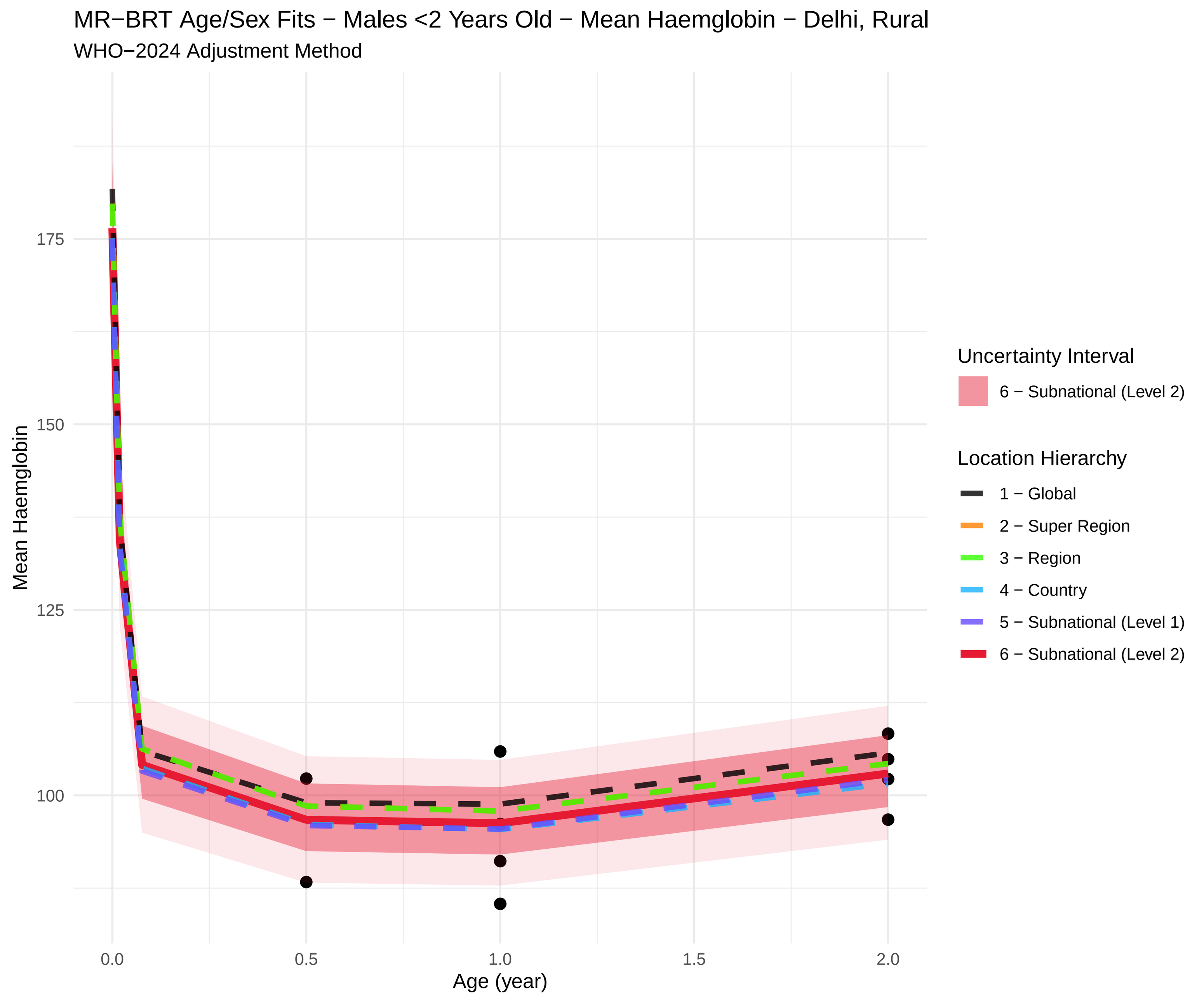
MR−BRT Age/Sex Fits − Males <2 Years Old − Mean Haemglobin − Delhi, Rural
WHO−2024 Adjustment Method
Mean Haemglobin
Age (year)
Uncertainty Interval
6 − Subnational (Level 2)
Location Hierarchy
1 − Global
2 − Super Region
3 − Region
4 − Country
5 − Subnational (Level 1)
6 − Subnational (Level 2)

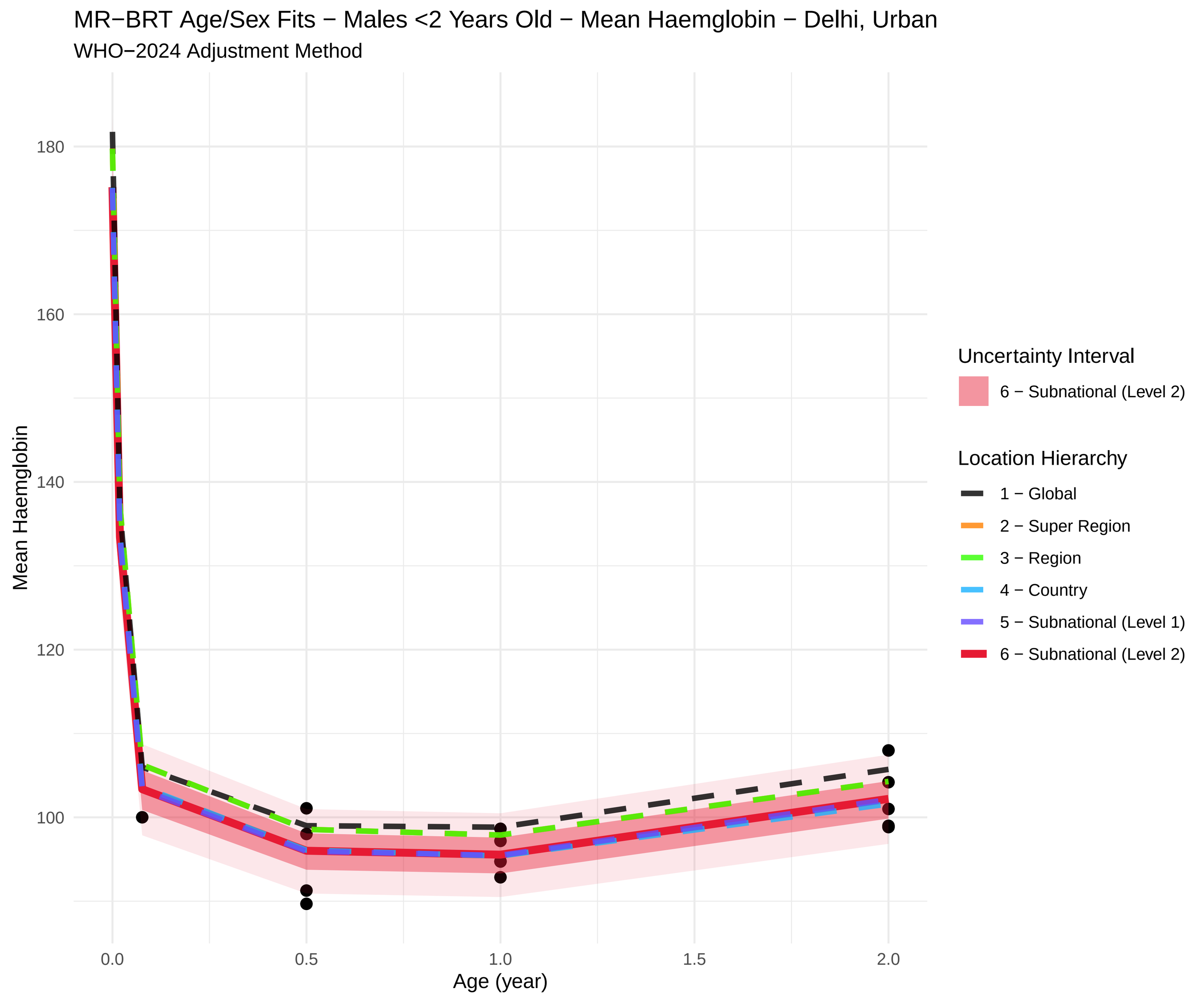
MR−BRT Age/Sex Fits − Males <2 Years Old − Mean Haemglobin − Delhi, Urban
WHO−2024 Adjustment Method
Mean Haemglobin
Age (year)
Uncertainty Interval
6 − Subnational (Level 2)
Location Hierarchy
1 − Global
2 − Super Region
3 − Region
4 − Country
5 − Subnational (Level 1)
6 − Subnational (Level 2)

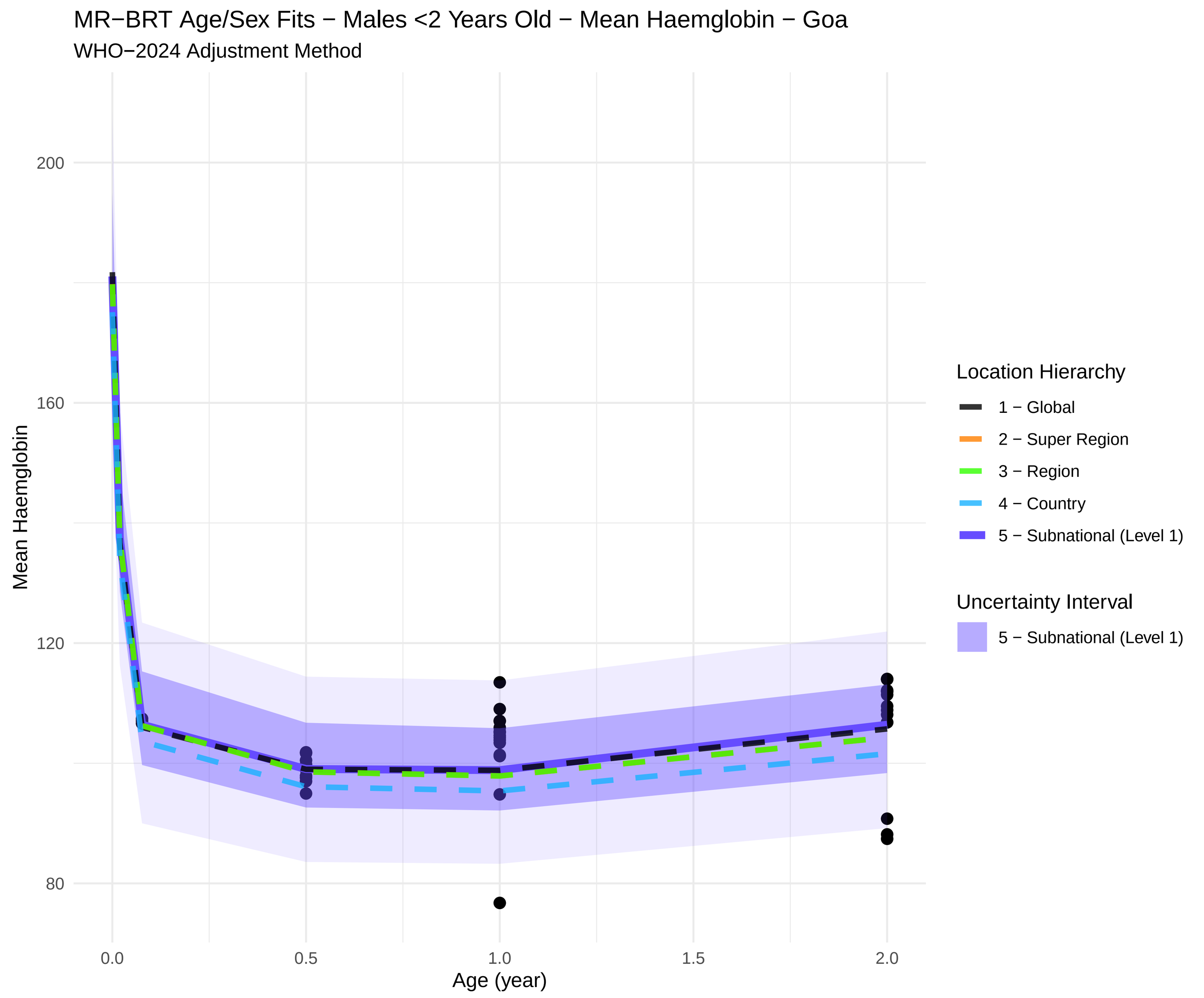
MR−BRT Age/Sex Fits − Males <2 Years Old − Mean Haemglobin − Goa
WHO−2024 Adjustment Method
Mean Haemglobin
Age (year)
Location Hierarchy
1 − Global
2 − Super Region
3 − Region
4 − Country
5 − Subnational (Level 1)
Uncertainty Interval
5 − Subnational (Level 1)

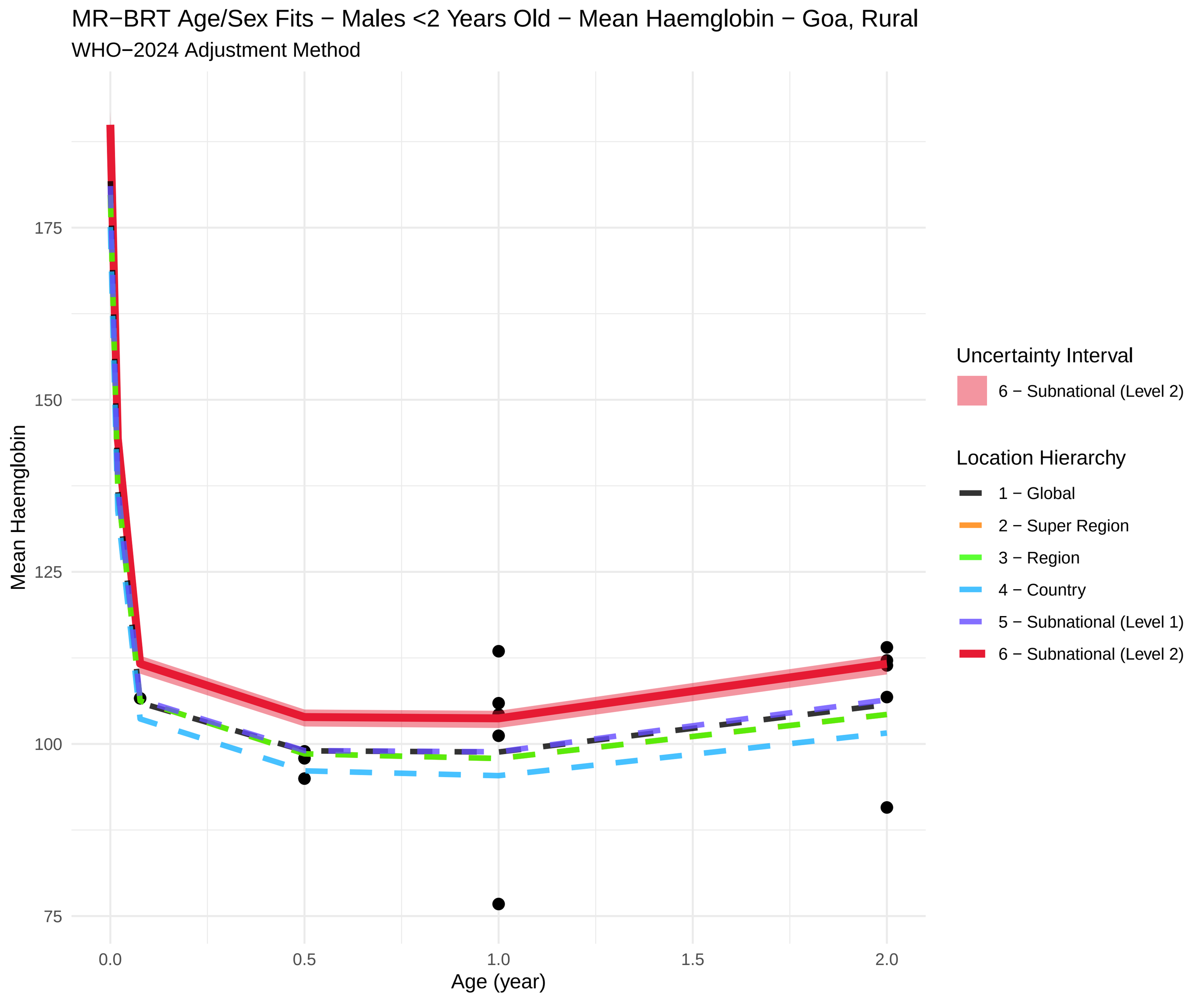
MR−BRT Age/Sex Fits − Males <2 Years Old − Mean Haemglobin − Goa, Rural
WHO−2024 Adjustment Method
Mean Haemglobin
Age (year)
Uncertainty Interval
6 − Subnational (Level 2)
Location Hierarchy
1 − Global
2 − Super Region
3 − Region
4 − Country
5 − Subnational (Level 1)
6 − Subnational (Level 2)

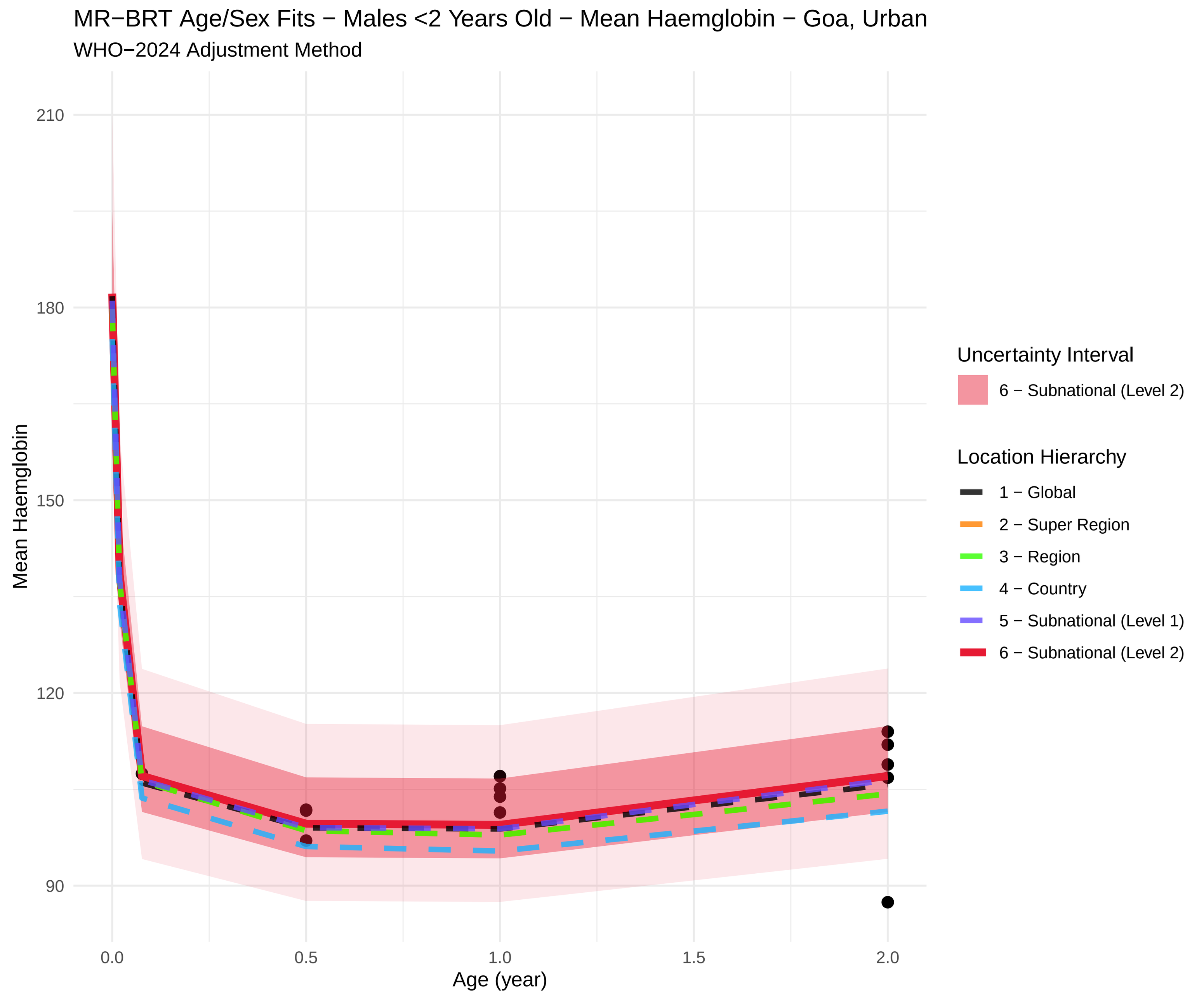
MR−BRT Age/Sex Fits − Males <2 Years Old − Mean Haemglobin − Goa, Urban
WHO−2024 Adjustment Method
Mean Haemglobin
Age (year)
Uncertainty Interval
6 − Subnational (Level 2)
Location Hierarchy
1 − Global
2 − Super Region
3 − Region
4 − Country
5 − Subnational (Level 1)
6 − Subnational (Level 2)

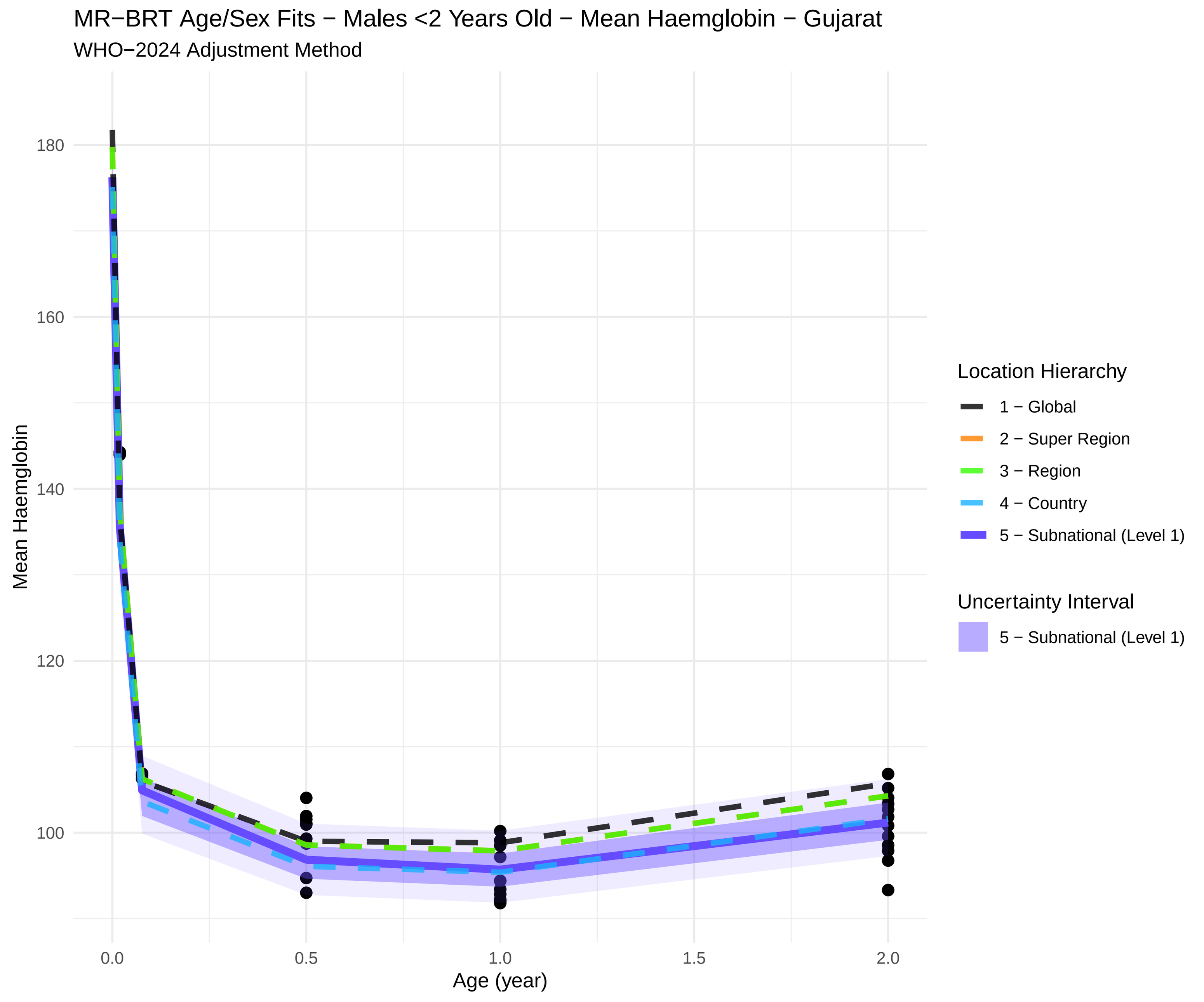
MR−BRT Age/Sex Fits − Males <2 Years Old − Mean Haemglobin − Gujarat
WHO−2024 Adjustment Method
Mean Haemglobin
Age (year)
Location Hierarchy
1 − Global
2 − Super Region
3 − Region
4 − Country
5 − Subnational (Level 1)
Uncertainty Interval
5 − Subnational (Level 1)

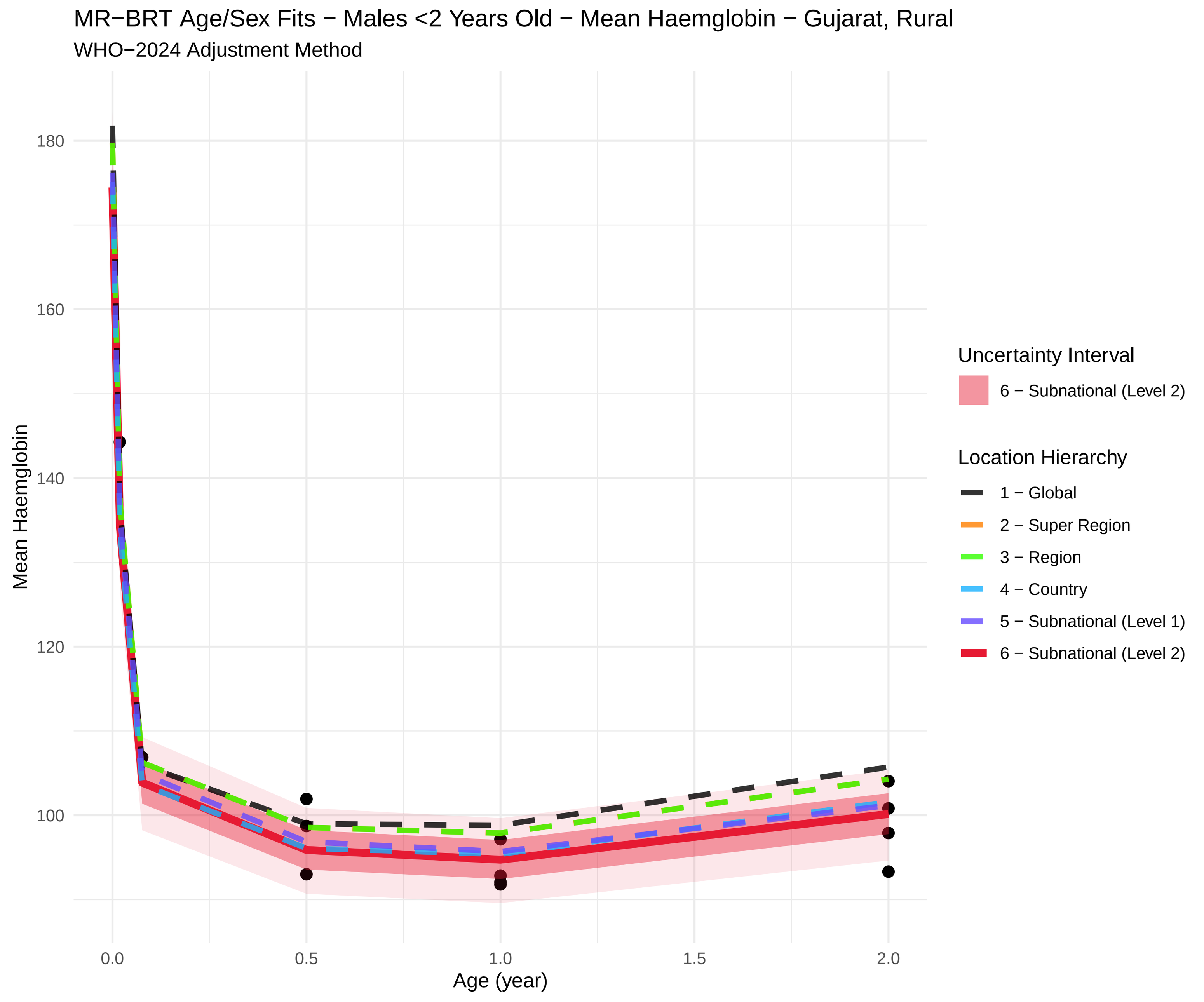
MR−BRT Age/Sex Fits − Males <2 Years Old − Mean Haemglobin − Gujarat, Rural
WHO−2024 Adjustment Method
Mean Haemglobin
Age (year)
Uncertainty Interval
6 − Subnational (Level 2)
Location Hierarchy
1 − Global
2 − Super Region
3 − Region
4 − Country
5 − Subnational (Level 1)
6 − Subnational (Level 2)

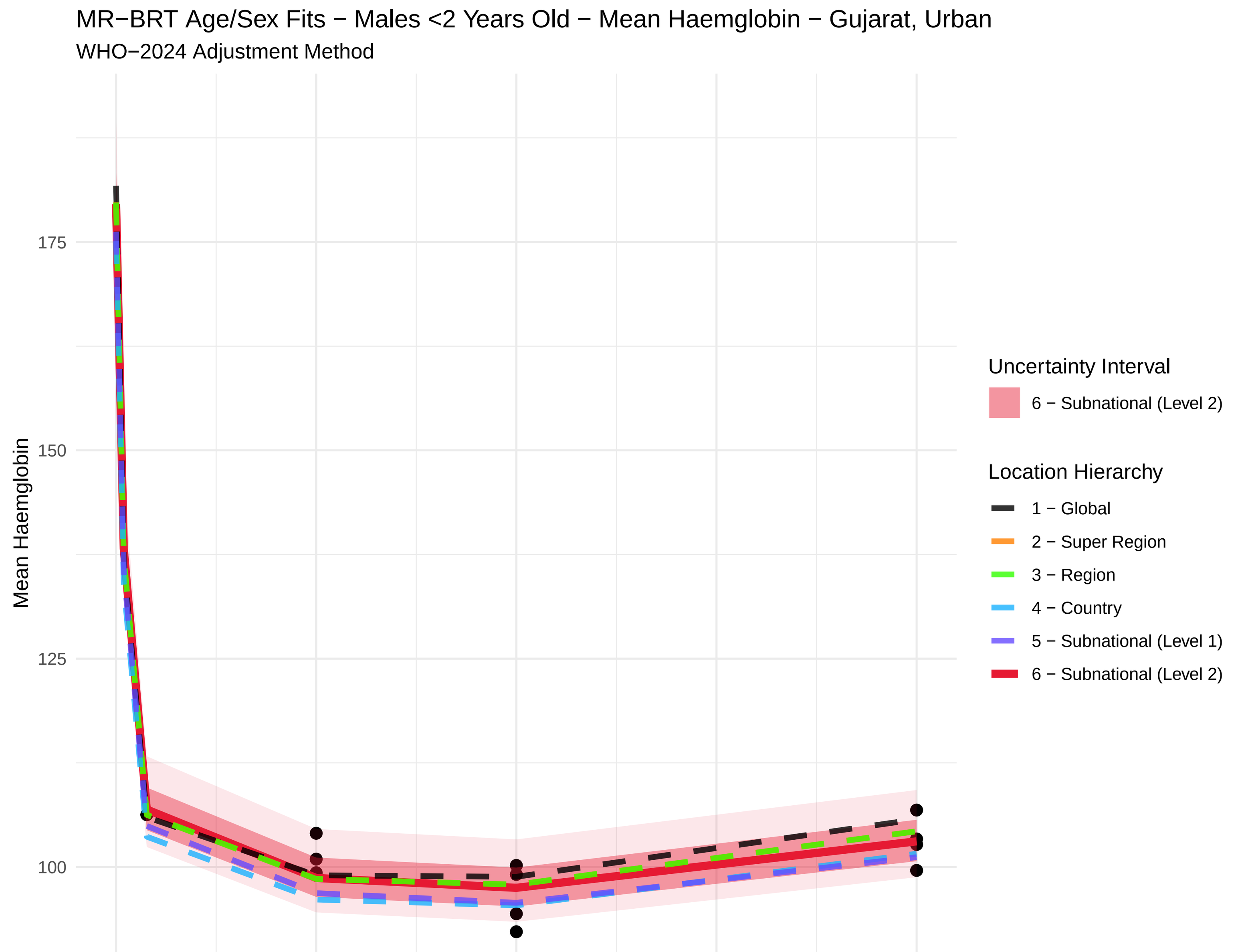
MR−BRT Age/Sex Fits − Males <2 Years Old − Mean Haemglobin − Gujarat, Urban
WHO−2024 Adjustment Method
Mean Haemglobin
Age (year)
Uncertainty Interval
6 − Subnational (Level 2)
Location Hierarchy
1 − Global
2 − Super Region
3 − Region
4 − Country
5 − Subnational (Level 1)
6 − Subnational (Level 2)

MR−BRT Age/Sex Fits − Males <2 Years Old − Mean Haemglobin − Haryana
WHO−2024 Adjustment Method

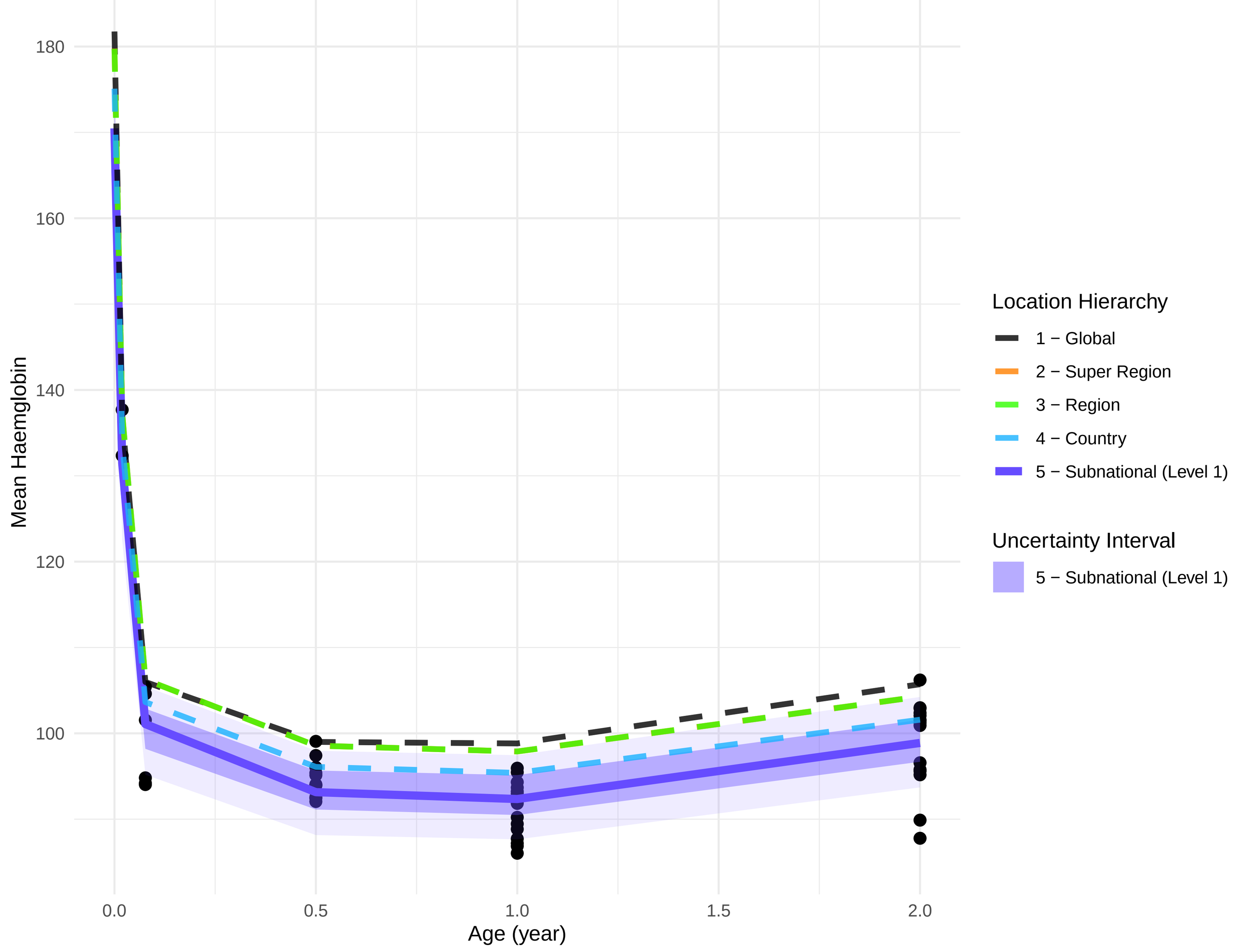

MR−BRT Age/Sex Fits − Males <2 Years Old − Mean Haemglobin − Haryana, Rural
WHO−2024 Adjustment Method

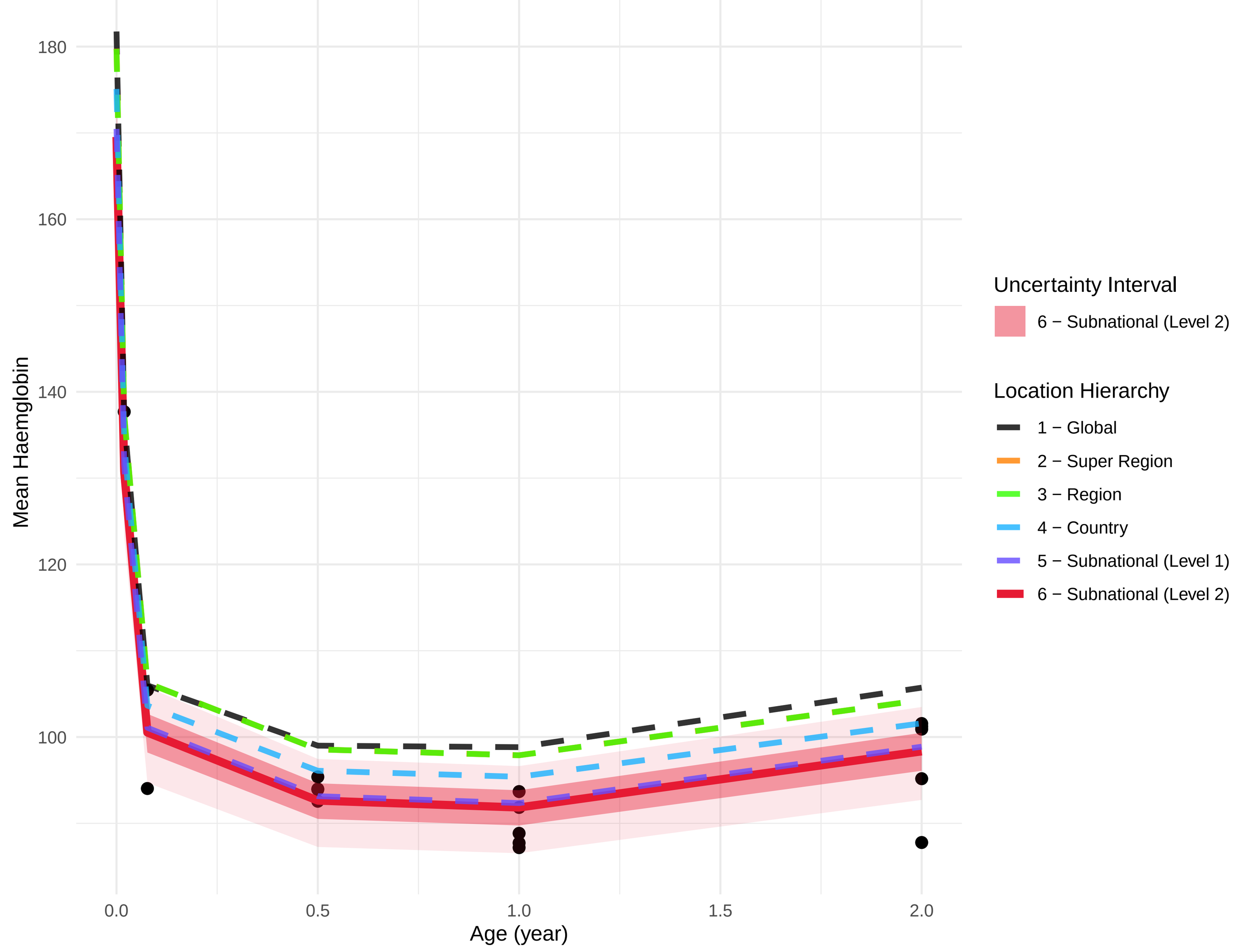

MR−BRT Age/Sex Fits − Males <2 Years Old − Mean Haemglobin − Haryana, Urban
WHO−2024 Adjustment Method

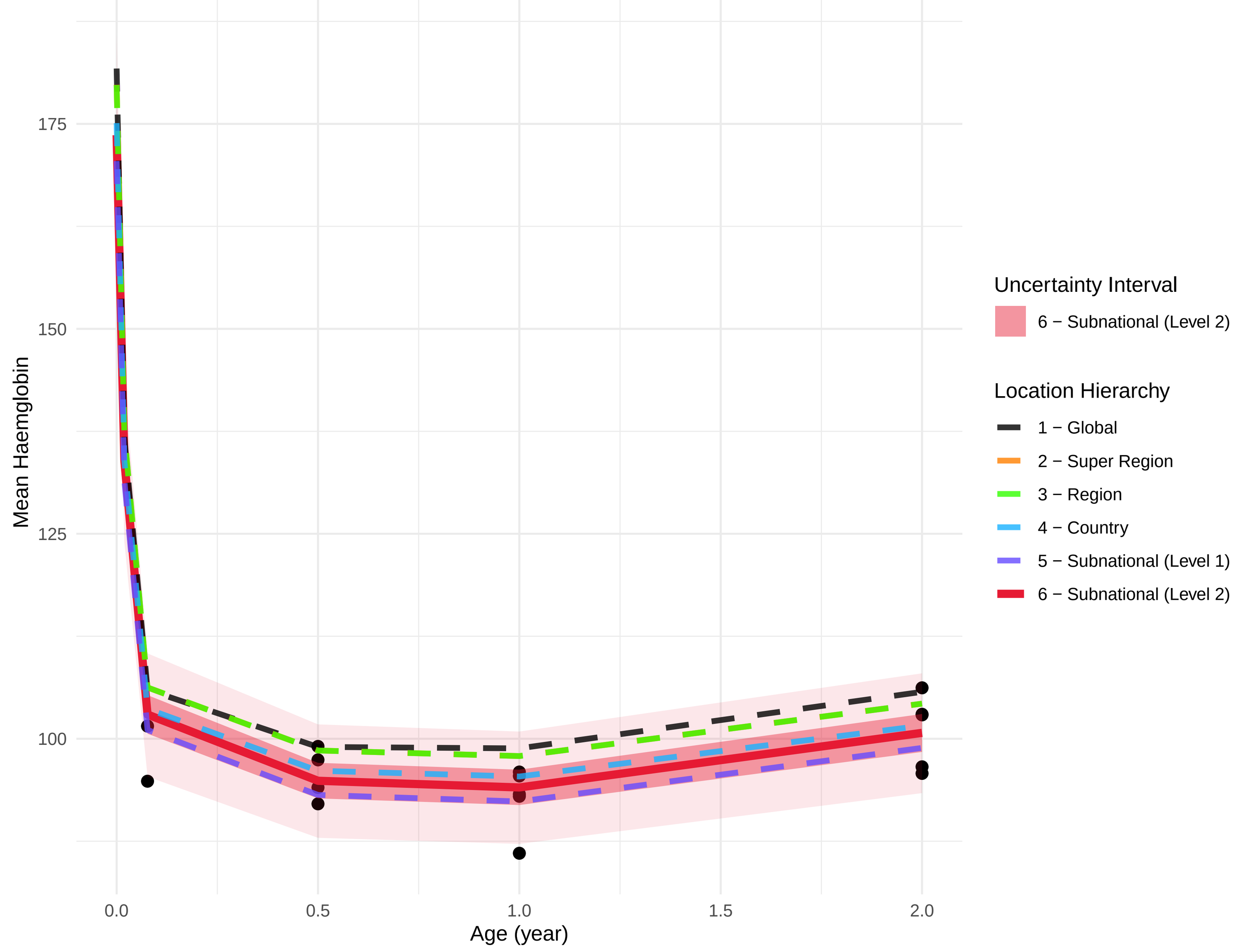

MR−BRT Age/Sex Fits − Males <2 Years Old − Mean Haemglobin − Himachal Pradesh
WHO−2024 Adjustment Method

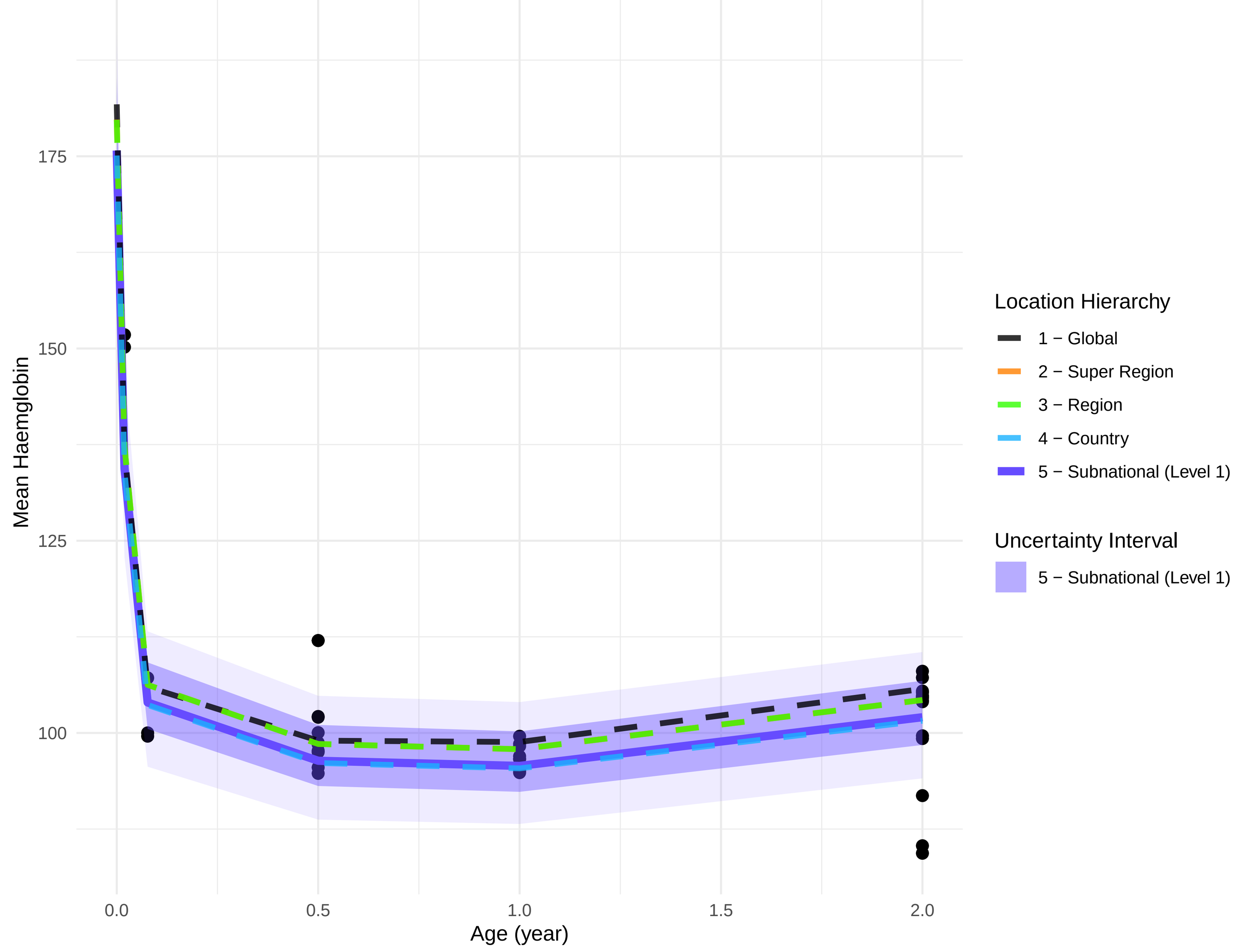

MR−BRT Age/Sex Fits − Males <2 Years Old − Mean Haemglobin − Himachal Pradesh, Rural
WHO−2024 Adjustment Method

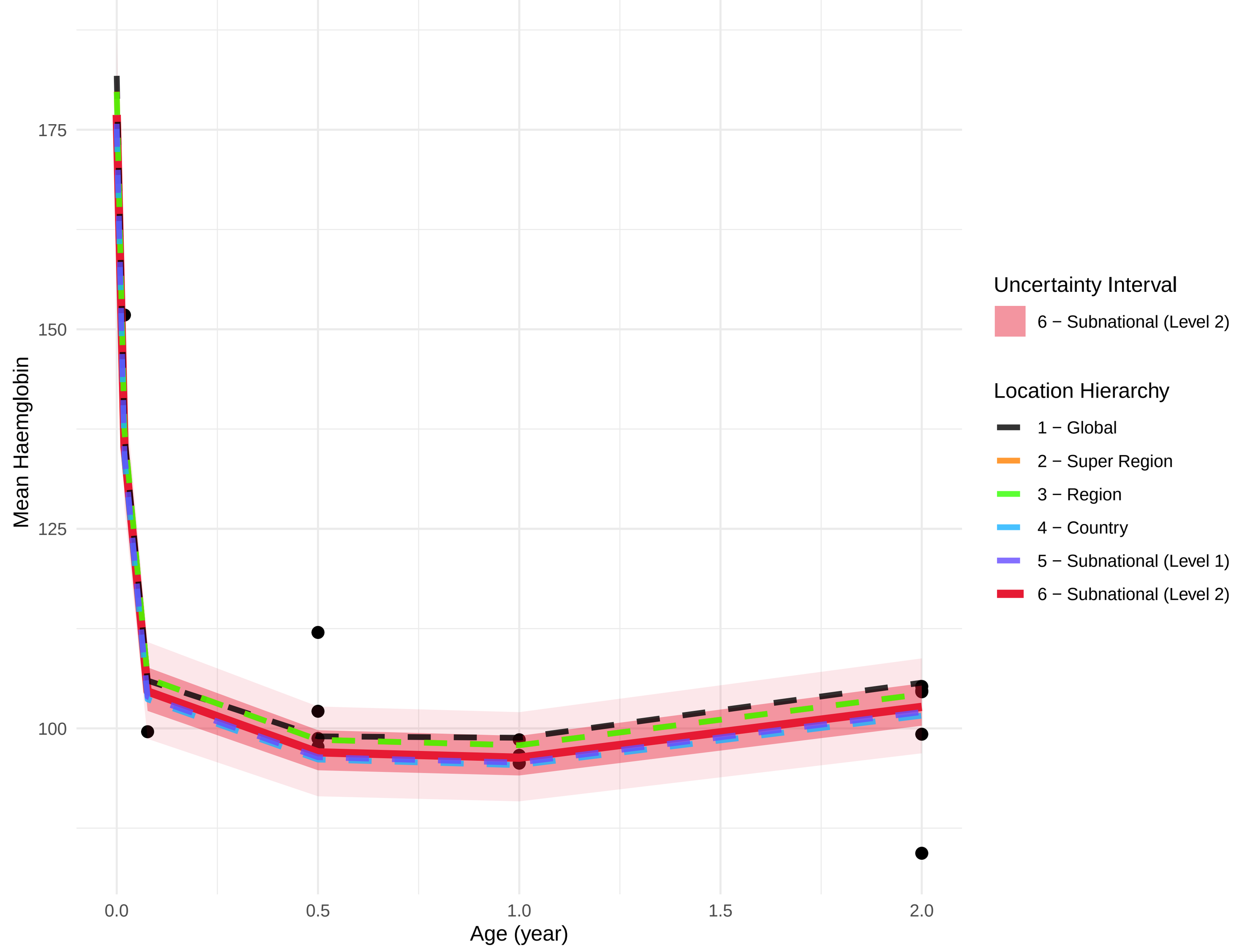

MR−BRT Age/Sex Fits − Males <2 Years Old − Mean Haemglobin − Himachal Pradesh, Urban
WHO−2024 Adjustment Method

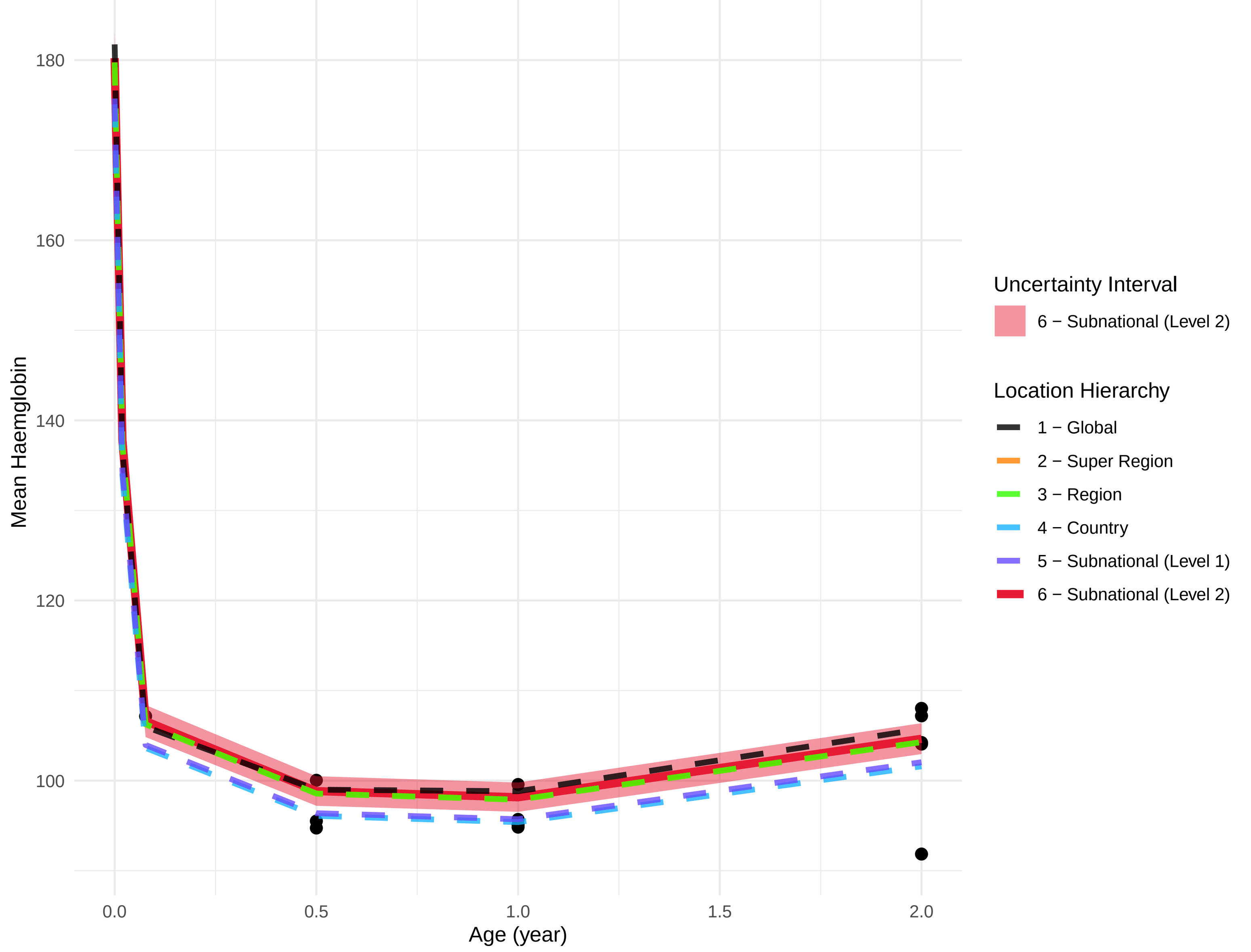

MR−BRT Age/Sex Fits − Males <2 Years Old − Mean Haemglobin − Jammu & Kashmir and Ladakh
WHO−2024 Adjustment Method

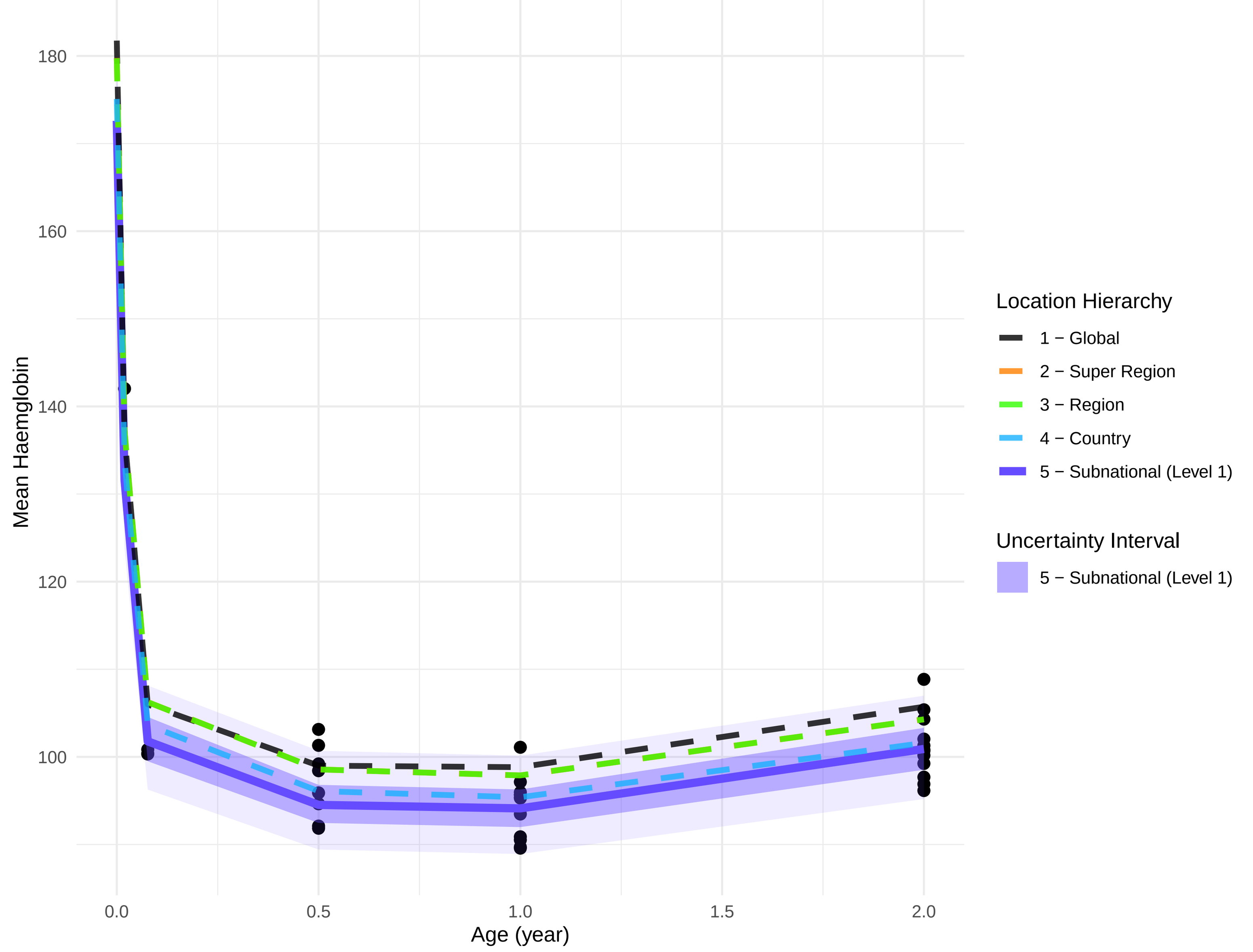

MR−BRT Age/Sex Fits − Males <2 Years Old − Mean Haemglobin − Jammu & Kashmir and Ladakh, Rural
WHO−2024 Adjustment Method

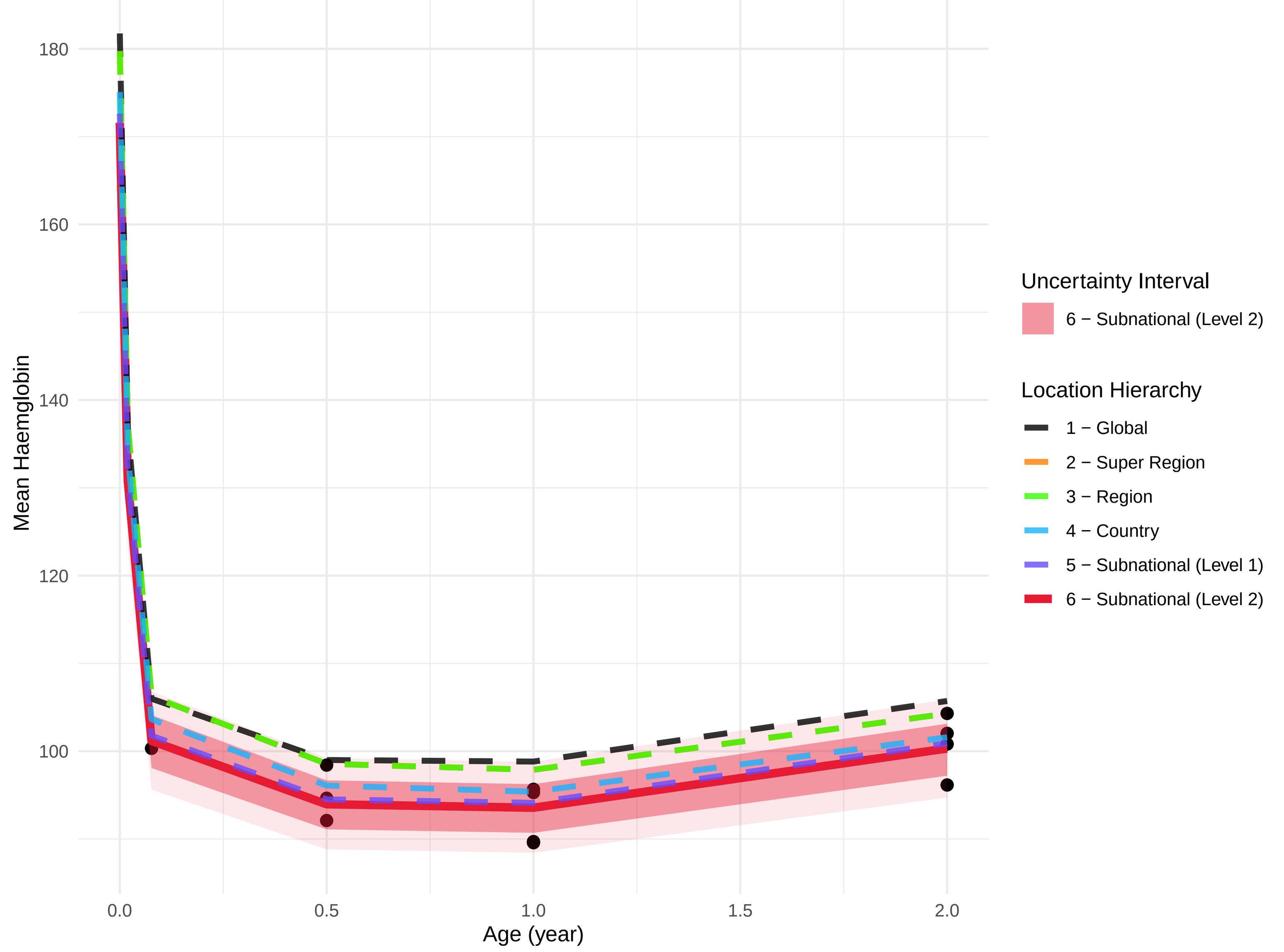

MR−BRT Age/Sex Fits − Males <2 Years Old − Mean Haemglobin − Jammu & Kashmir and Ladakh, Urban
WHO−2024 Adjustment Method

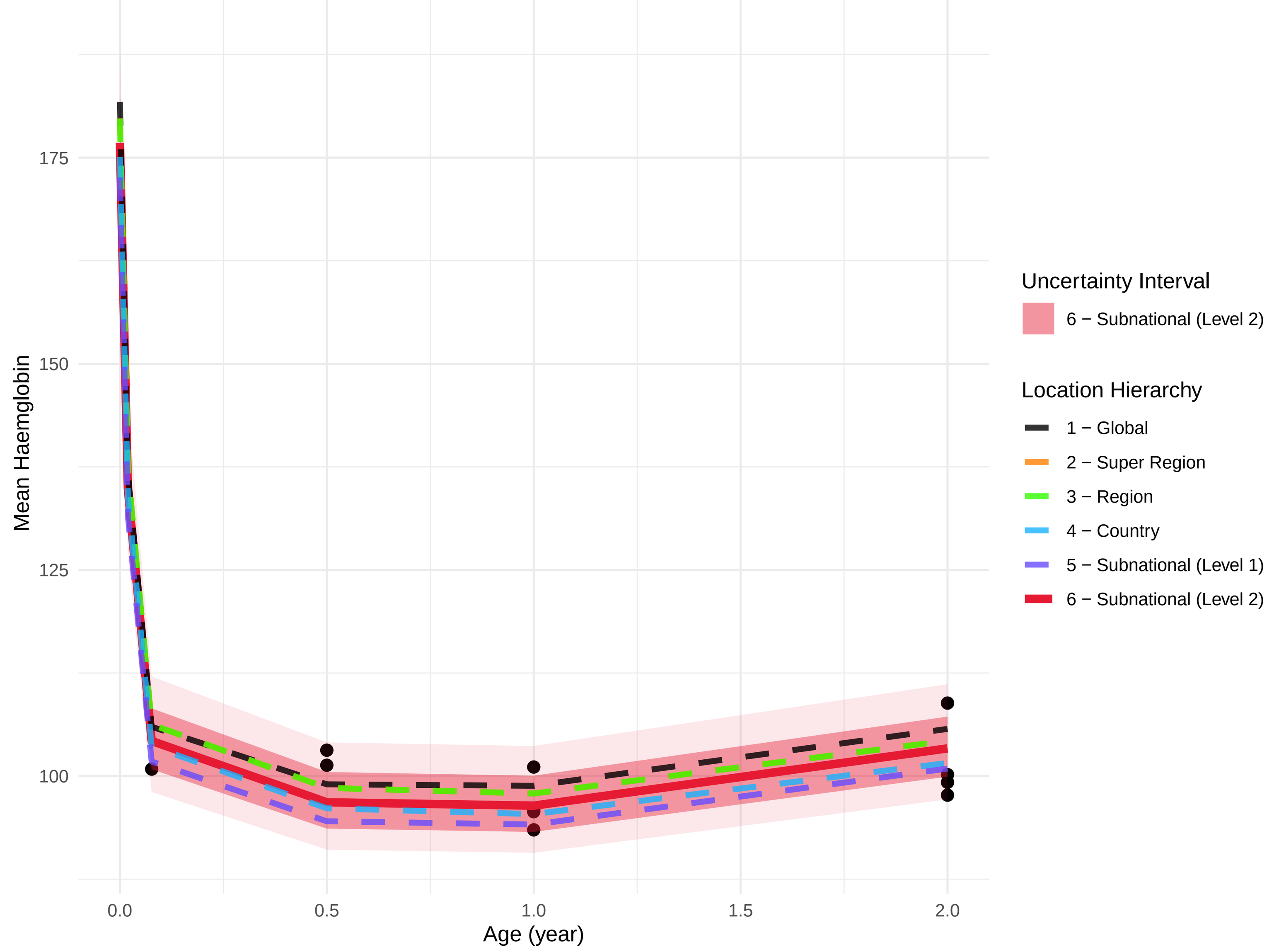

MR−BRT Age/Sex Fits − Males <2 Years Old − Mean Haemglobin − Jharkhand
WHO−2024 Adjustment Method

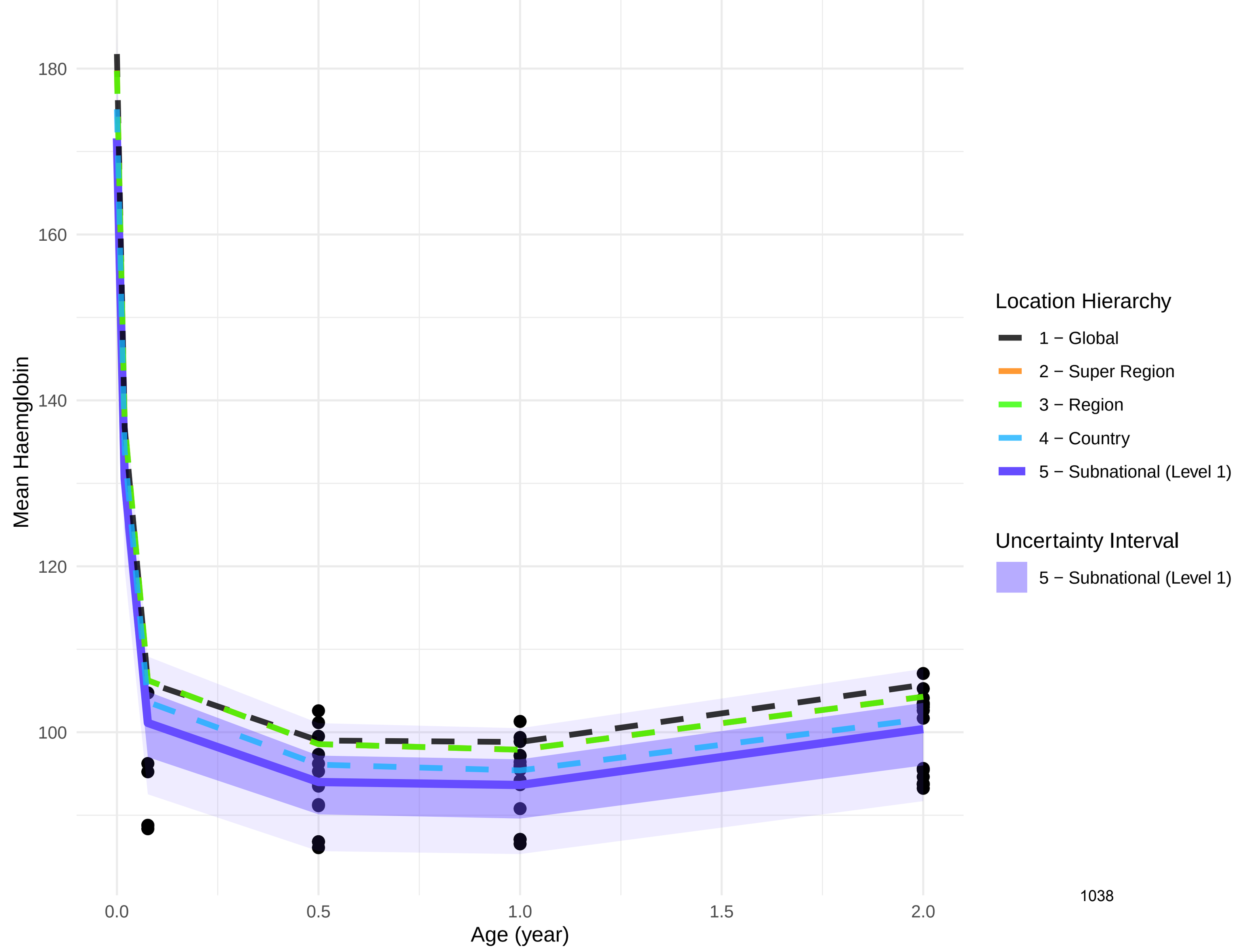

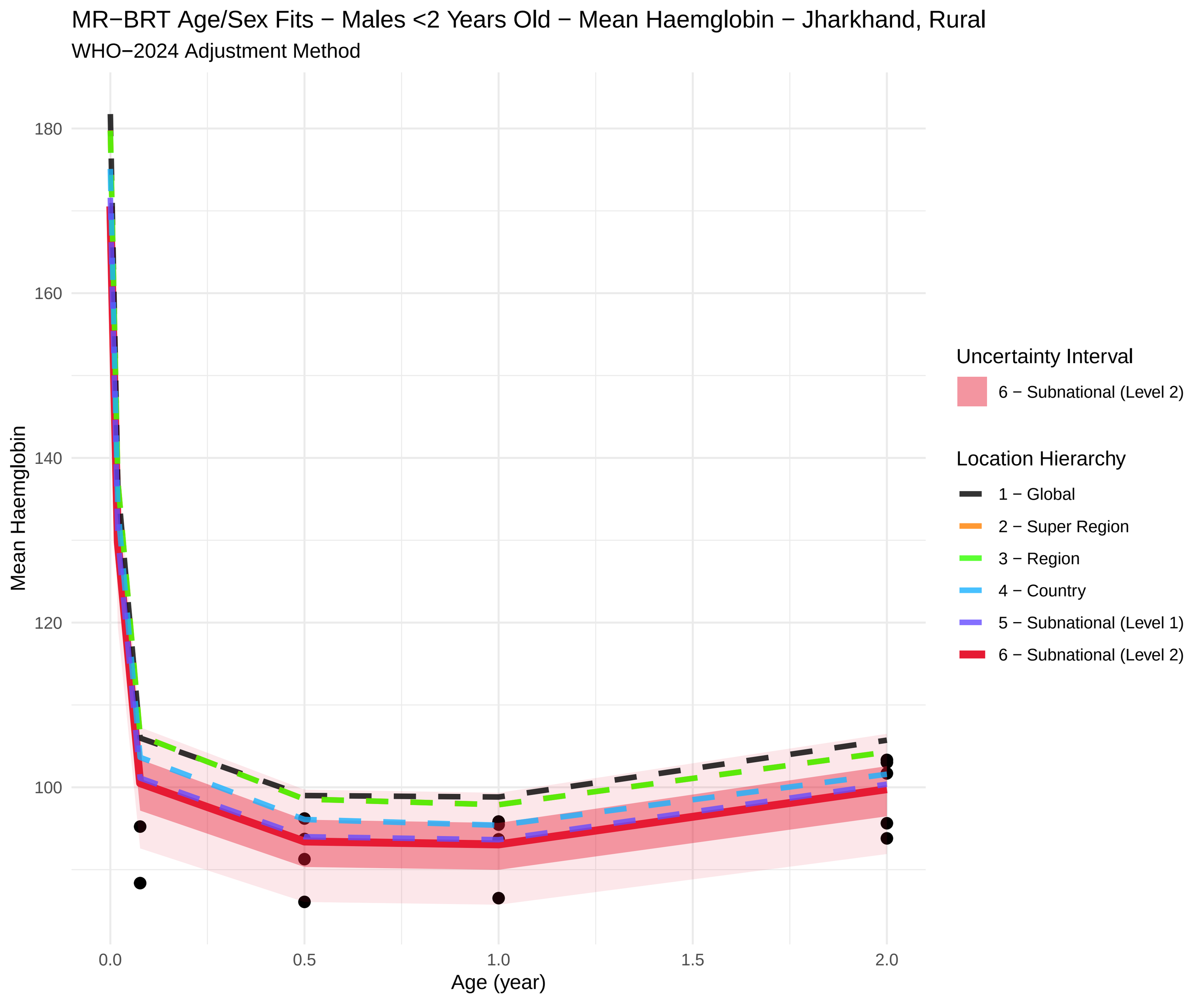
MR−BRT Age/Sex Fits − Males <2 Years Old − Mean Haemglobin − Jharkhand, Rural
WHO−2024 Adjustment Method
Mean Haemglobin
Age (year)
Uncertainty Interval
6 − Subnational (Level 2)
Location Hierarchy
1 − Global
2 − Super Region
3 − Region
4 − Country
5 − Subnational (Level 1)
6 − Subnational (Level 2)

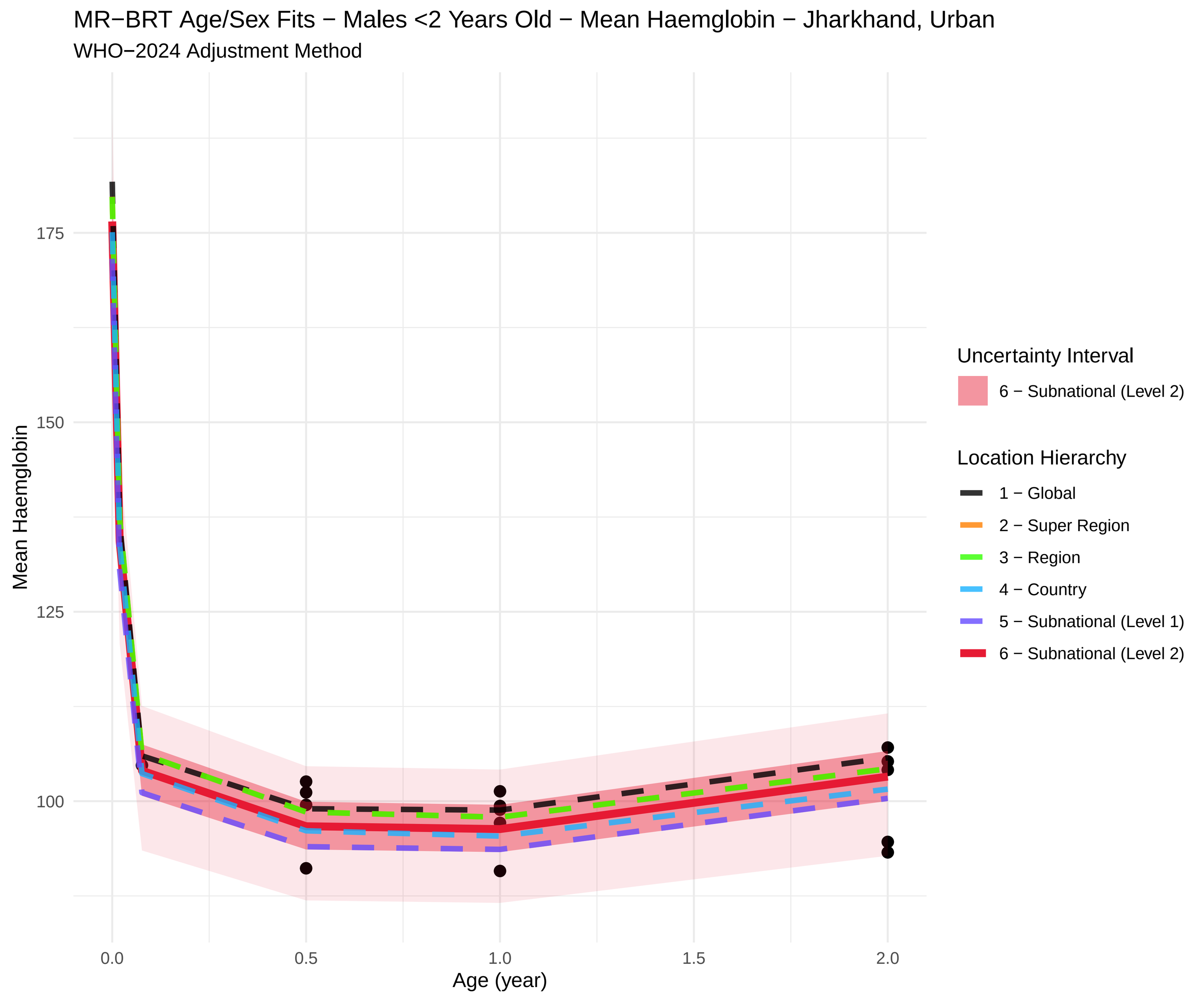
MR−BRT Age/Sex Fits − Males <2 Years Old − Mean Haemglobin − Jharkhand, Urban
WHO−2024 Adjustment Method
Mean Haemglobin
Age (year)
Uncertainty Interval
6 − Subnational (Level 2)
Location Hierarchy
1 − Global
2 − Super Region
3 − Region
4 − Country
5 − Subnational (Level 1)
6 − Subnational (Level 2)

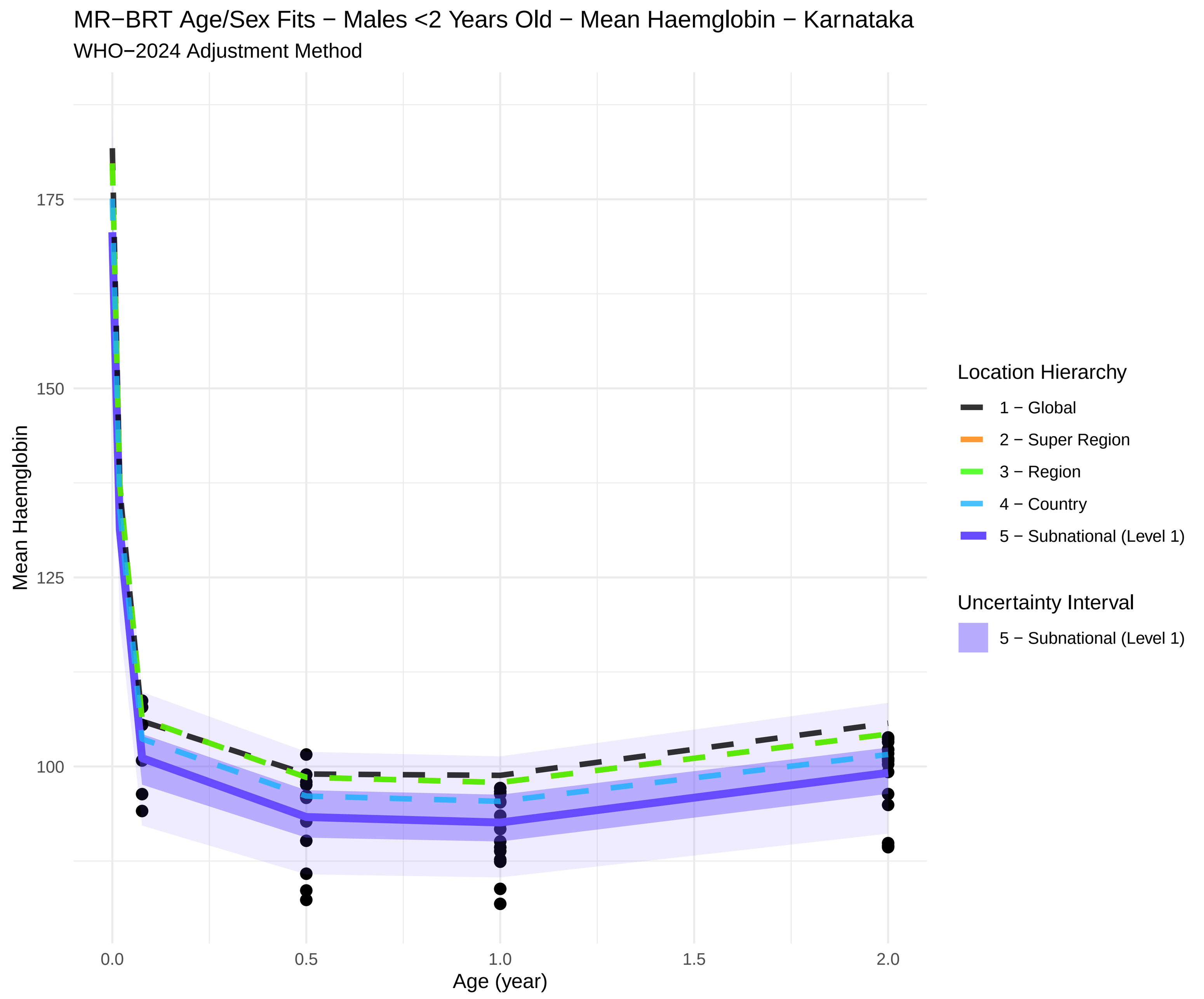
MR−BRT Age/Sex Fits − Males <2 Years Old − Mean Haemglobin − Karnataka
WHO−2024 Adjustment Method
Mean Haemglobin
Age (year)
Location Hierarchy
1 − Global
2 − Super Region
3 − Region
4 − Country
5 − Subnational (Level 1)
Uncertainty Interval
5 − Subnational (Level 1)

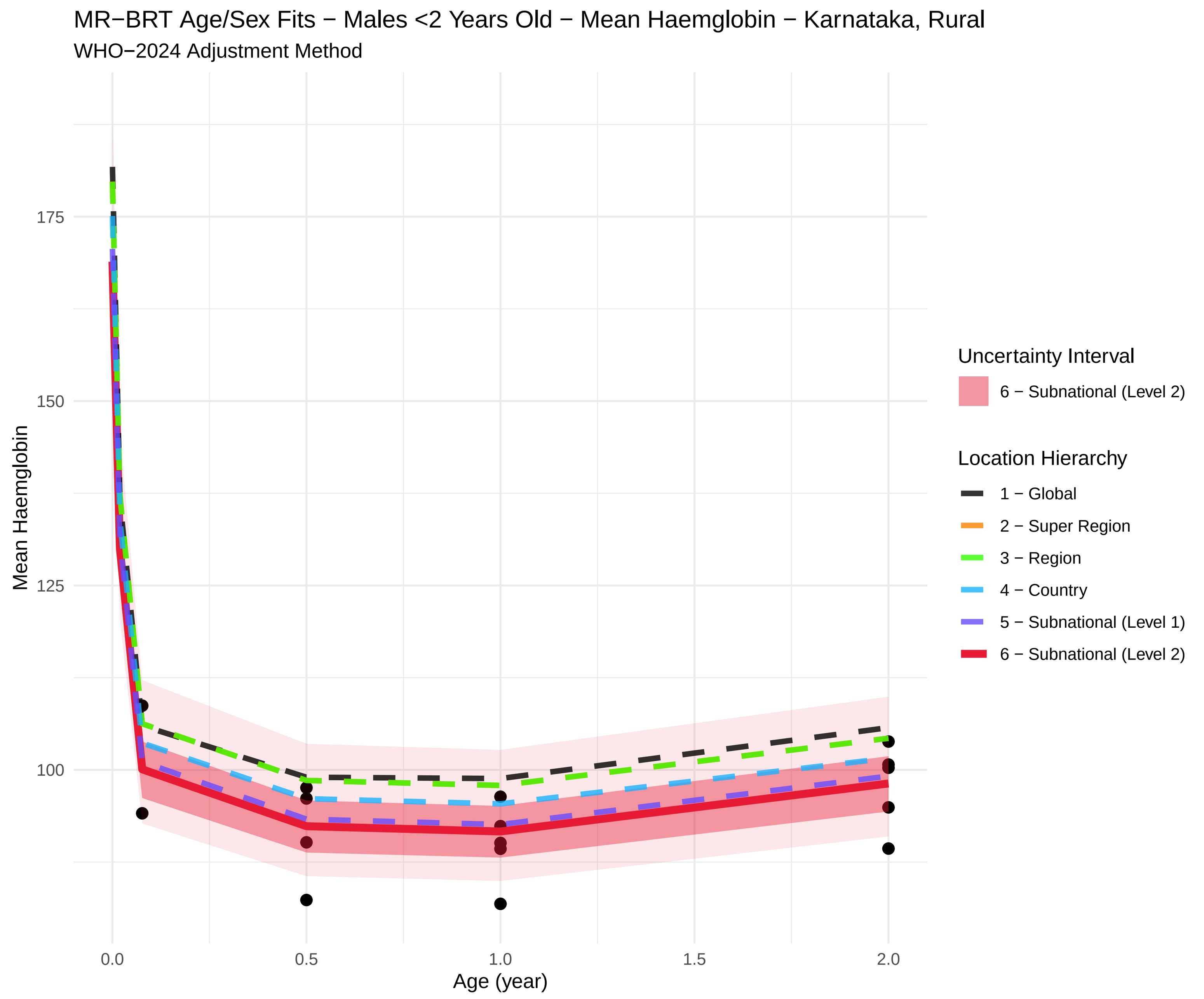
MR−BRT Age/Sex Fits − Males <2 Years Old − Mean Haemglobin − Karnataka, Rural
WHO−2024 Adjustment Method
Mean Haemglobin
Age (year)
Uncertainty Interval
6 − Subnational (Level 2)
Location Hierarchy
1 − Global
2 − Super Region
3 − Region
4 − Country
5 − Subnational (Level 1)
6 − Subnational (Level 2)

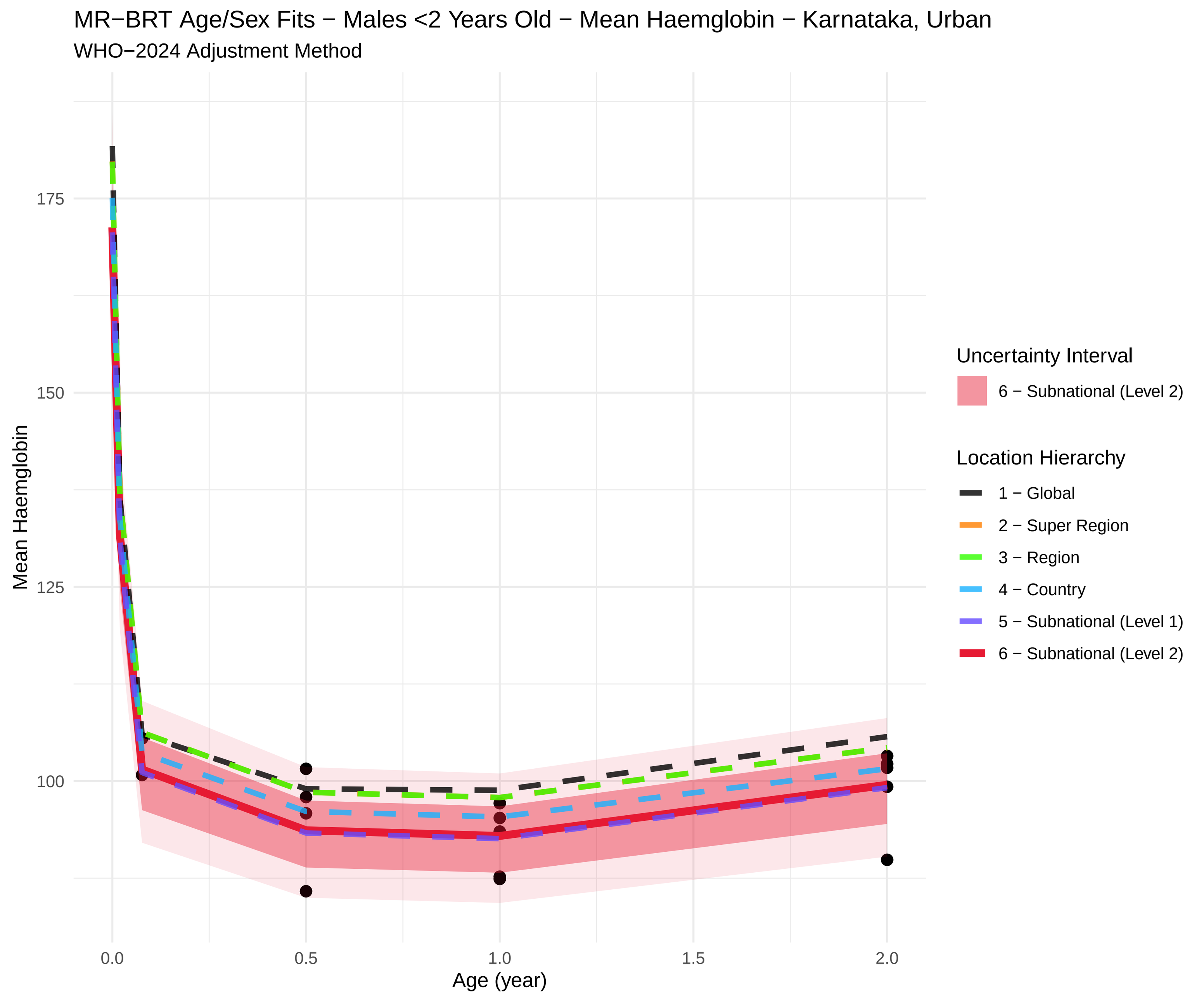
MR−BRT Age/Sex Fits − Males <2 Years Old − Mean Haemglobin − Karnataka, Urban
WHO−2024 Adjustment Method
Mean Haemglobin
Age (year)
Uncertainty Interval
6 − Subnational (Level 2)
Location Hierarchy
1 − Global
2 − Super Region
3 − Region
4 − Country
5 − Subnational (Level 1)
6 − Subnational (Level 2)

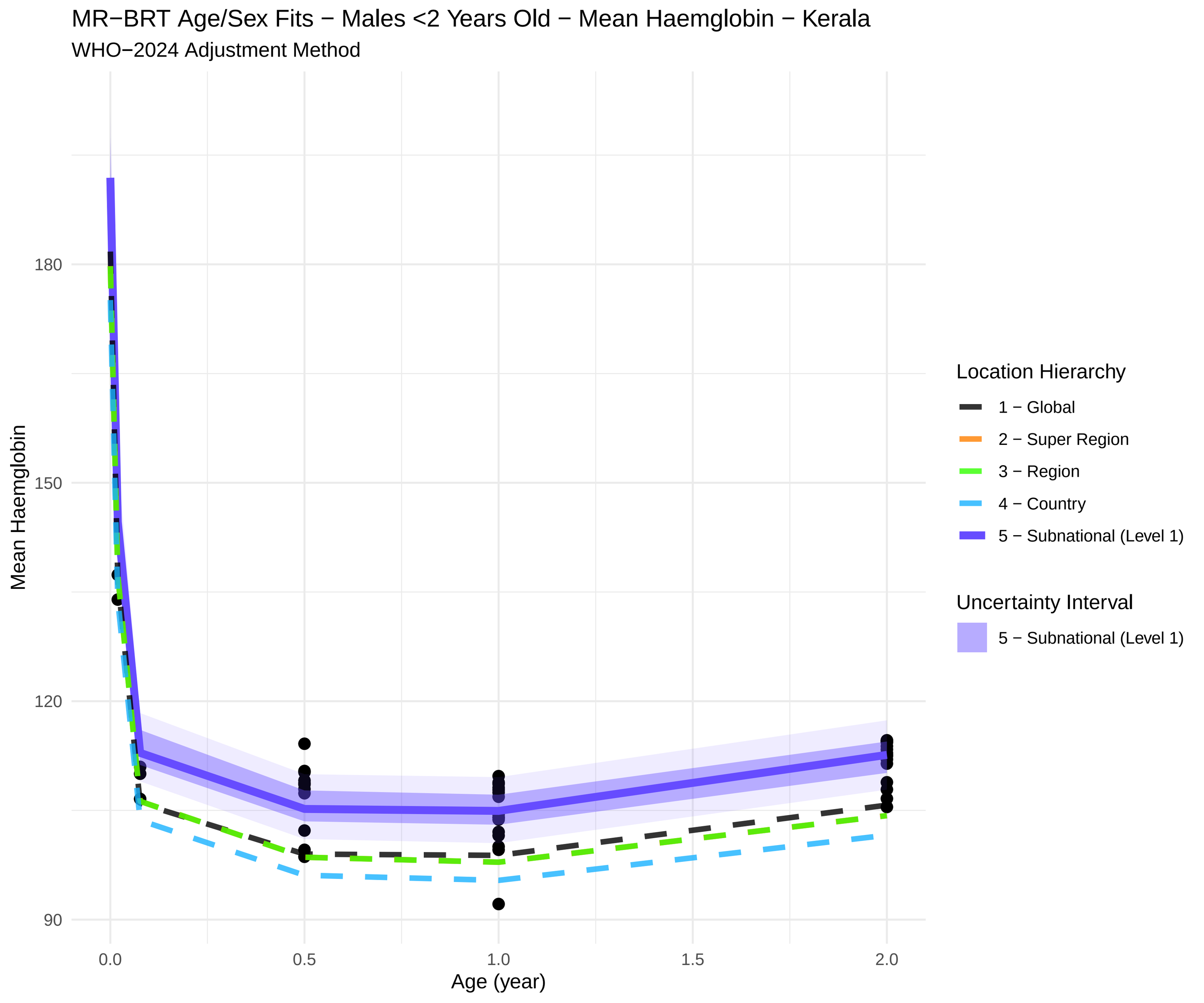
MR−BRT Age/Sex Fits − Males <2 Years Old − Mean Haemglobin − Kerala
WHO−2024 Adjustment Method
Mean Haemglobin
Age (year)
Location Hierarchy
1 − Global
2 − Super Region
3 − Region
4 − Country
5 − Subnational (Level 1)
Uncertainty Interval
5 − Subnational (Level 1)

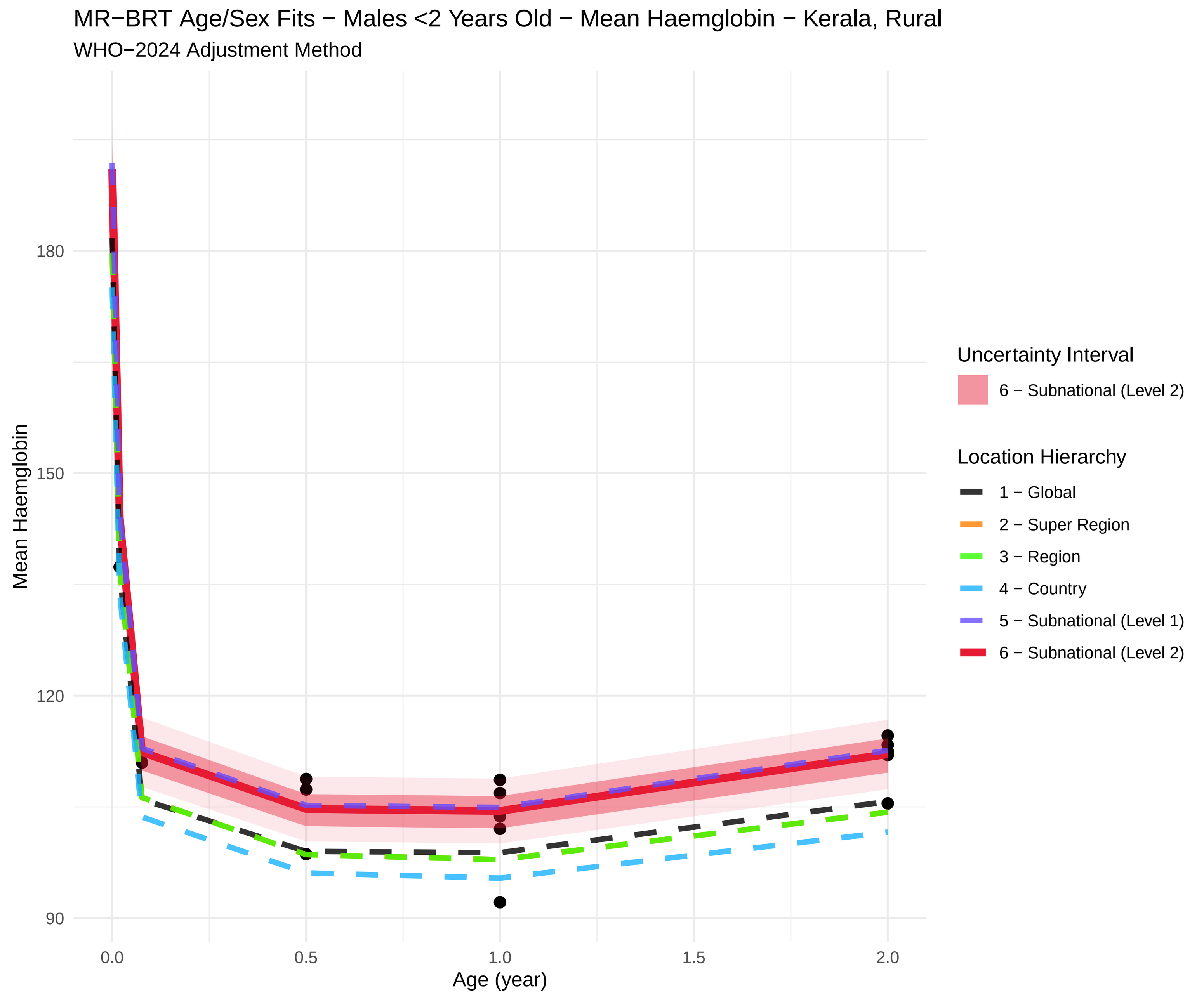
MR−BRT Age/Sex Fits − Males <2 Years Old − Mean Haemglobin − Kerala, Rural
WHO−2024 Adjustment Method
Mean Haemglobin
Age (year)
Uncertainty Interval
6 − Subnational (Level 2)
Location Hierarchy
1 − Global
2 − Super Region
3 − Region
4 − Country
5 − Subnational (Level 1)
6 − Subnational (Level 2)

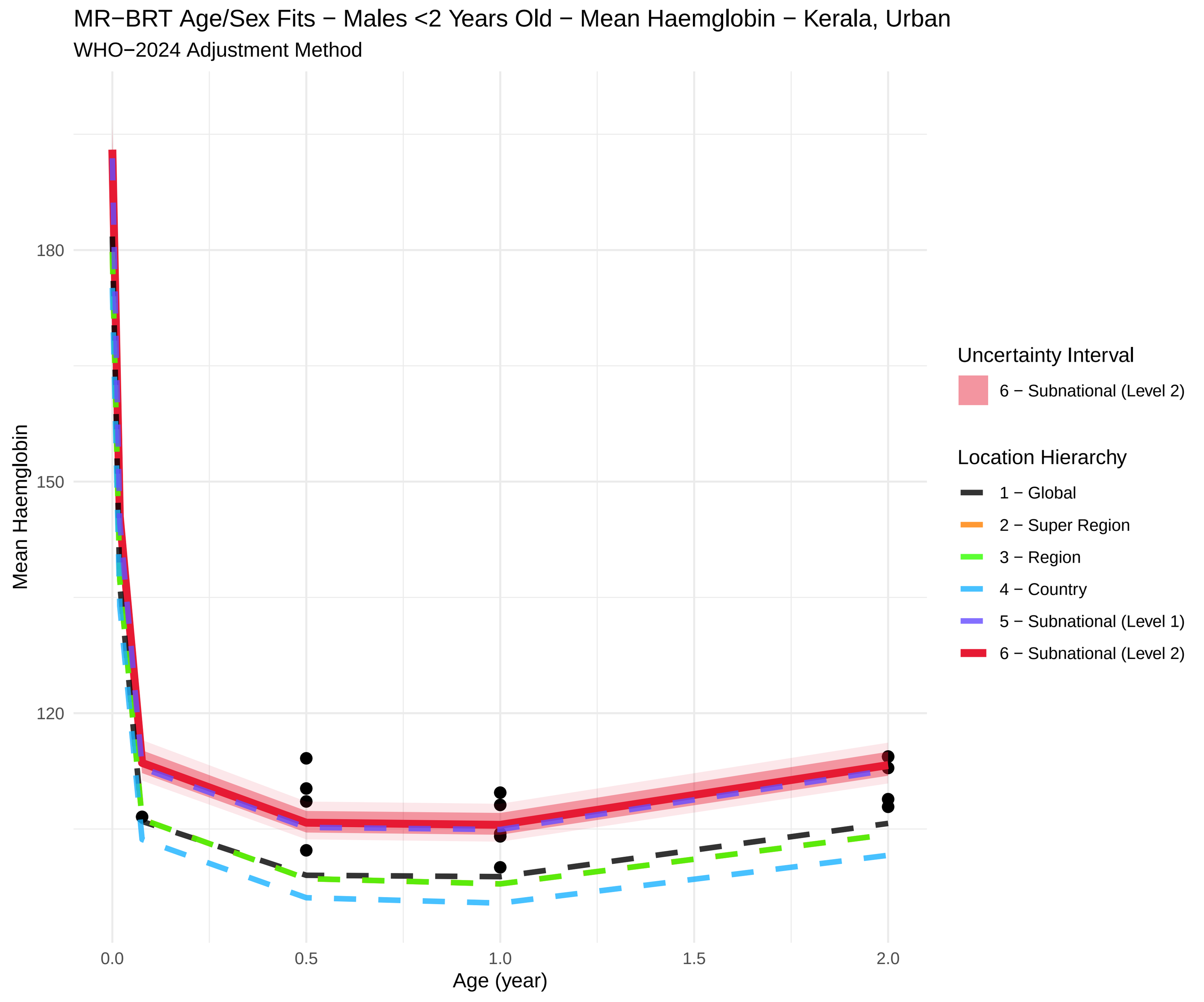
MR−BRT Age/Sex Fits − Males <2 Years Old − Mean Haemglobin − Kerala, Urban
WHO−2024 Adjustment Method
Mean Haemglobin
Age (year)
Uncertainty Interval
6 − Subnational (Level 2)
Location Hierarchy
1 − Global
2 − Super Region
3 − Region
4 − Country
5 − Subnational (Level 1)
6 − Subnational (Level 2)

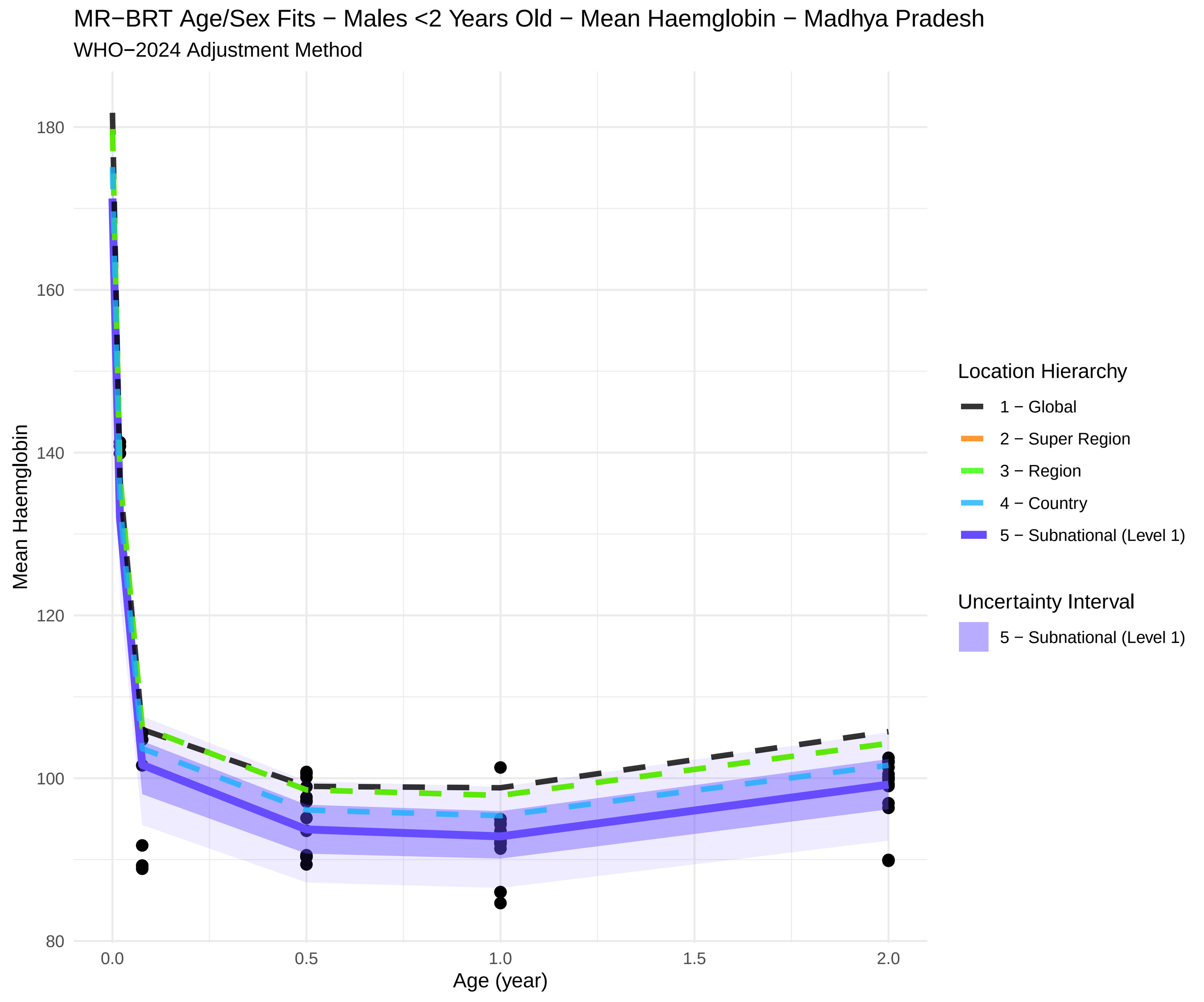
MR−BRT Age/Sex Fits − Males <2 Years Old − Mean Haemglobin − Madhya Pradesh
WHO−2024 Adjustment Method
Mean Haemglobin
Age (year)
Location Hierarchy
1 − Global
2 − Super Region
3 − Region
4 − Country
5 − Subnational (Level 1)
Uncertainty Interval
5 − Subnational (Level 1)

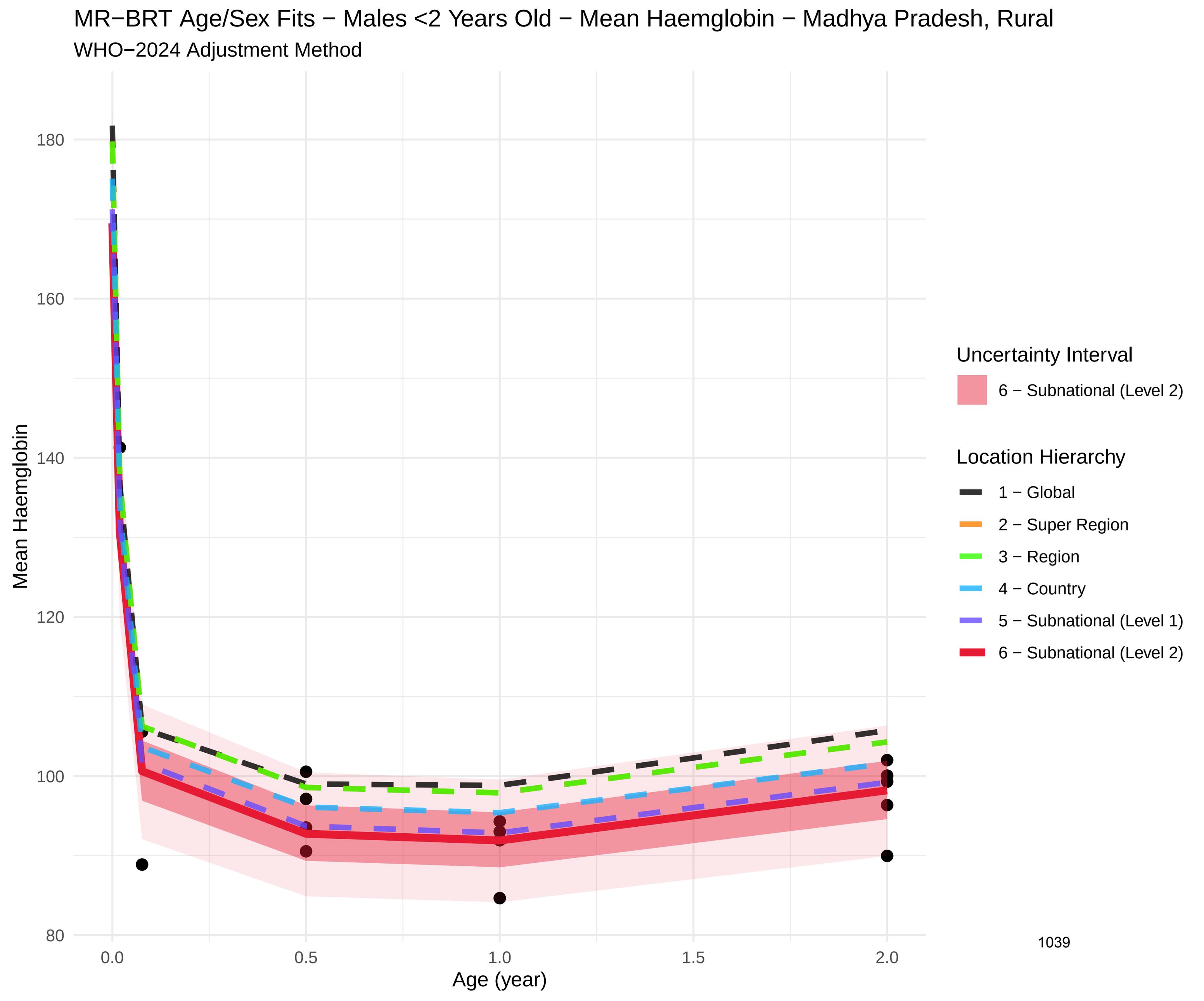
MR−BRT Age/Sex Fits − Males <2 Years Old − Mean Haemglobin − Madhya Pradesh, Rural
WHO−2024 Adjustment Method
Mean Haemglobin
Age (year)
Uncertainty Interval
6 − Subnational (Level 2)
Location Hierarchy
1 − Global
2 − Super Region
3 − Region
4 − Country
5 − Subnational (Level 1)
6 − Subnational (Level 2)

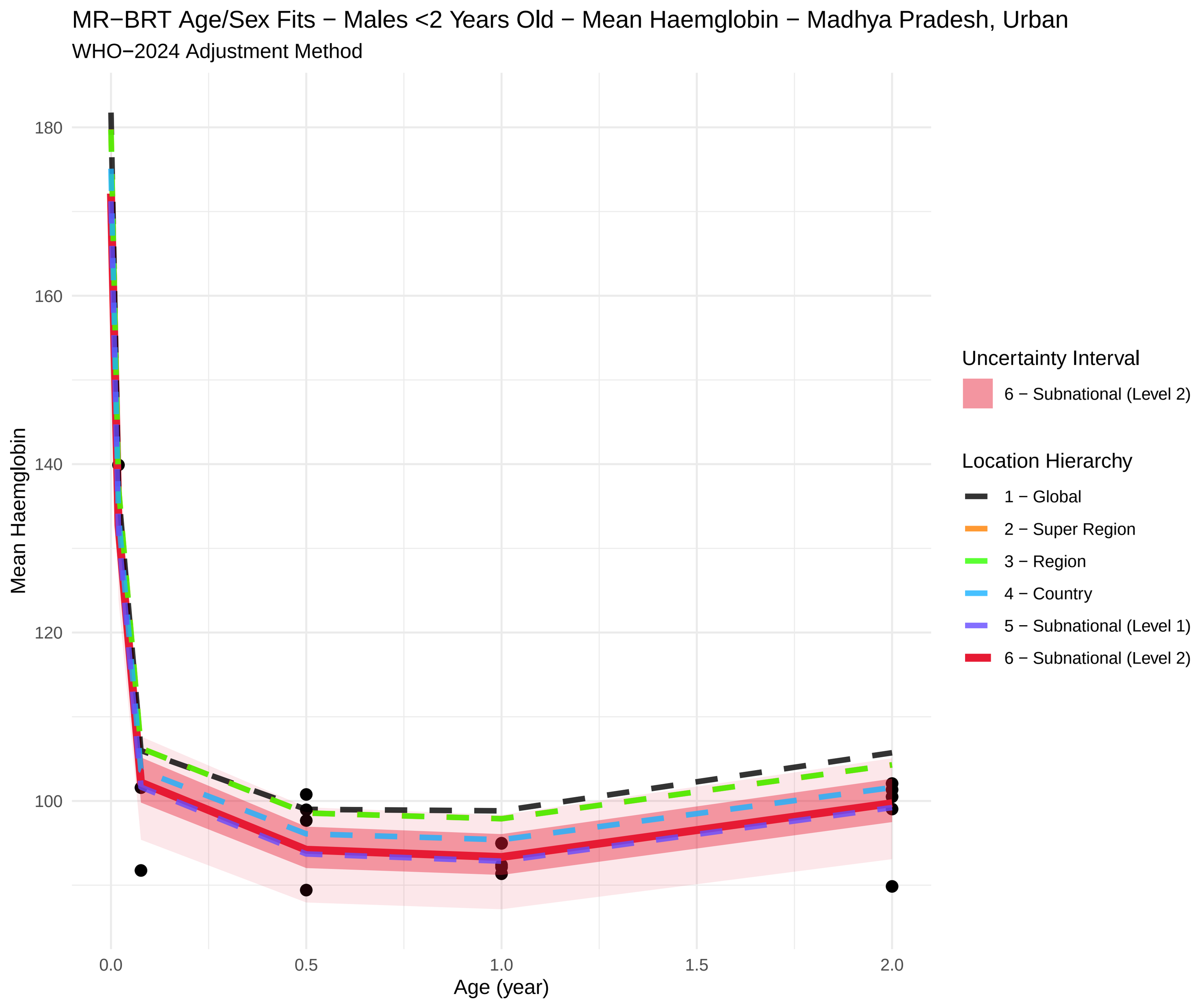
MR−BRT Age/Sex Fits − Males <2 Years Old − Mean Haemglobin − Madhya Pradesh, Urban
WHO−2024 Adjustment Method
Mean Haemglobin
Age (year)
Uncertainty Interval
6 − Subnational (Level 2)
Location Hierarchy
1 − Global
2 − Super Region
3 − Region
4 − Country
5 − Subnational (Level 1)
6 − Subnational (Level 2)

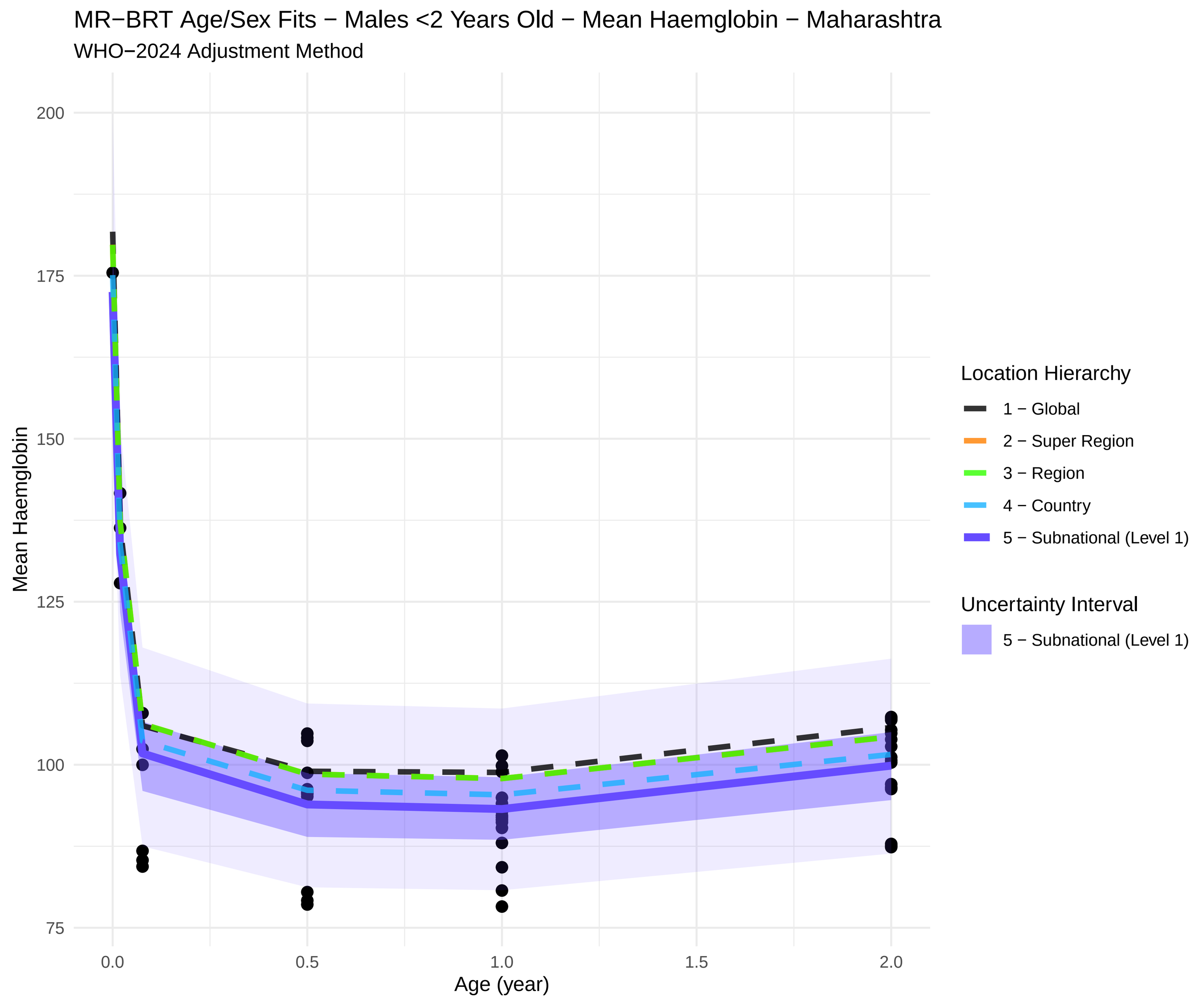
MR−BRT Age/Sex Fits − Males <2 Years Old − Mean Haemglobin − Maharashtra
WHO−2024 Adjustment Method
Mean Haemglobin
Age (year)
Location Hierarchy
1 − Global
2 − Super Region
3 − Region
4 − Country
5 − Subnational (Level 1)
Uncertainty Interval
5 − Subnational (Level 1)

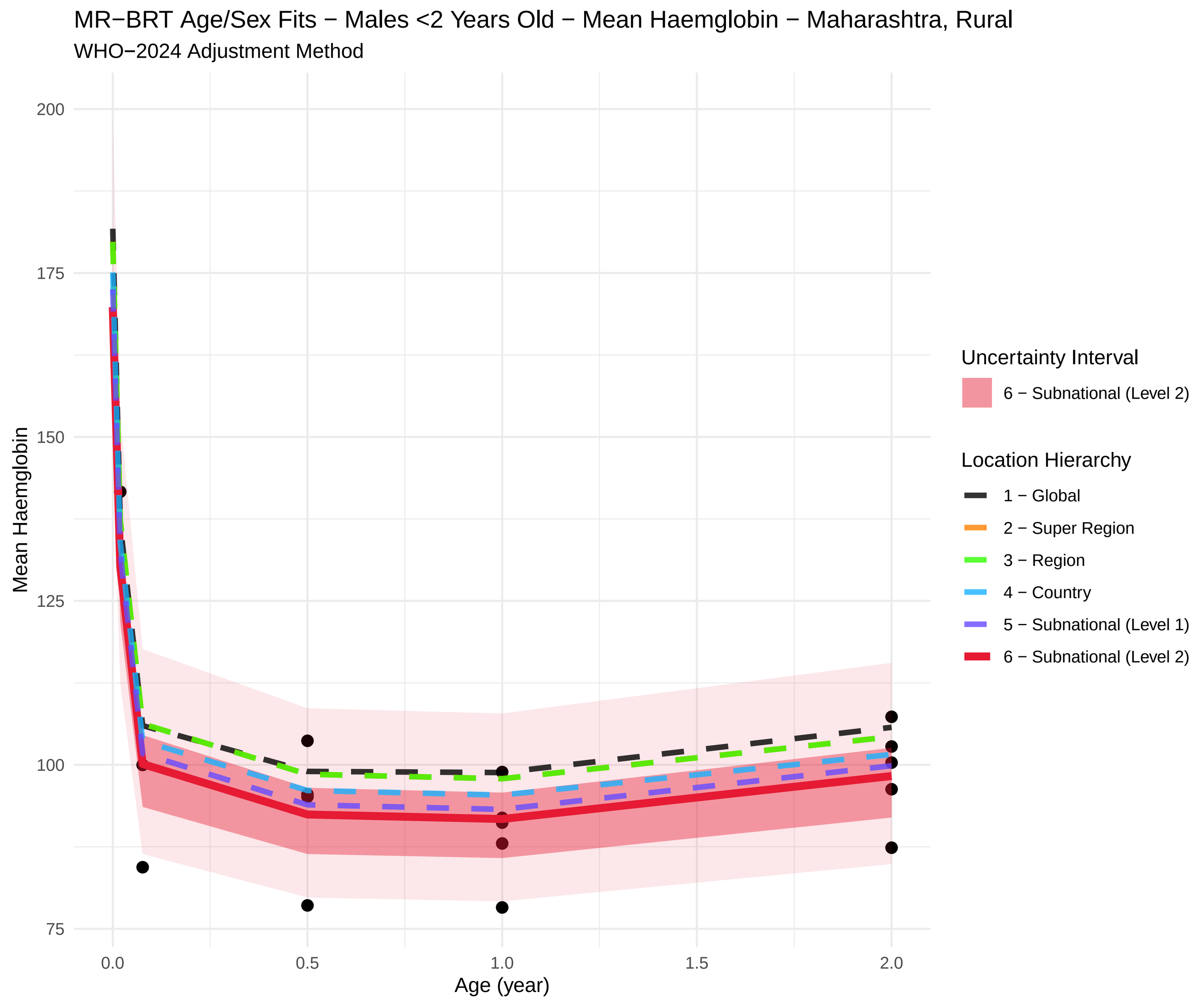
MR−BRT Age/Sex Fits − Males <2 Years Old − Mean Haemglobin − Maharashtra, Rural
WHO−2024 Adjustment Method
Mean Haemglobin
Age (year)
Uncertainty Interval
6 − Subnational (Level 2)
Location Hierarchy
1 − Global
2 − Super Region
3 − Region
4 − Country
5 − Subnational (Level 1)
6 − Subnational (Level 2)

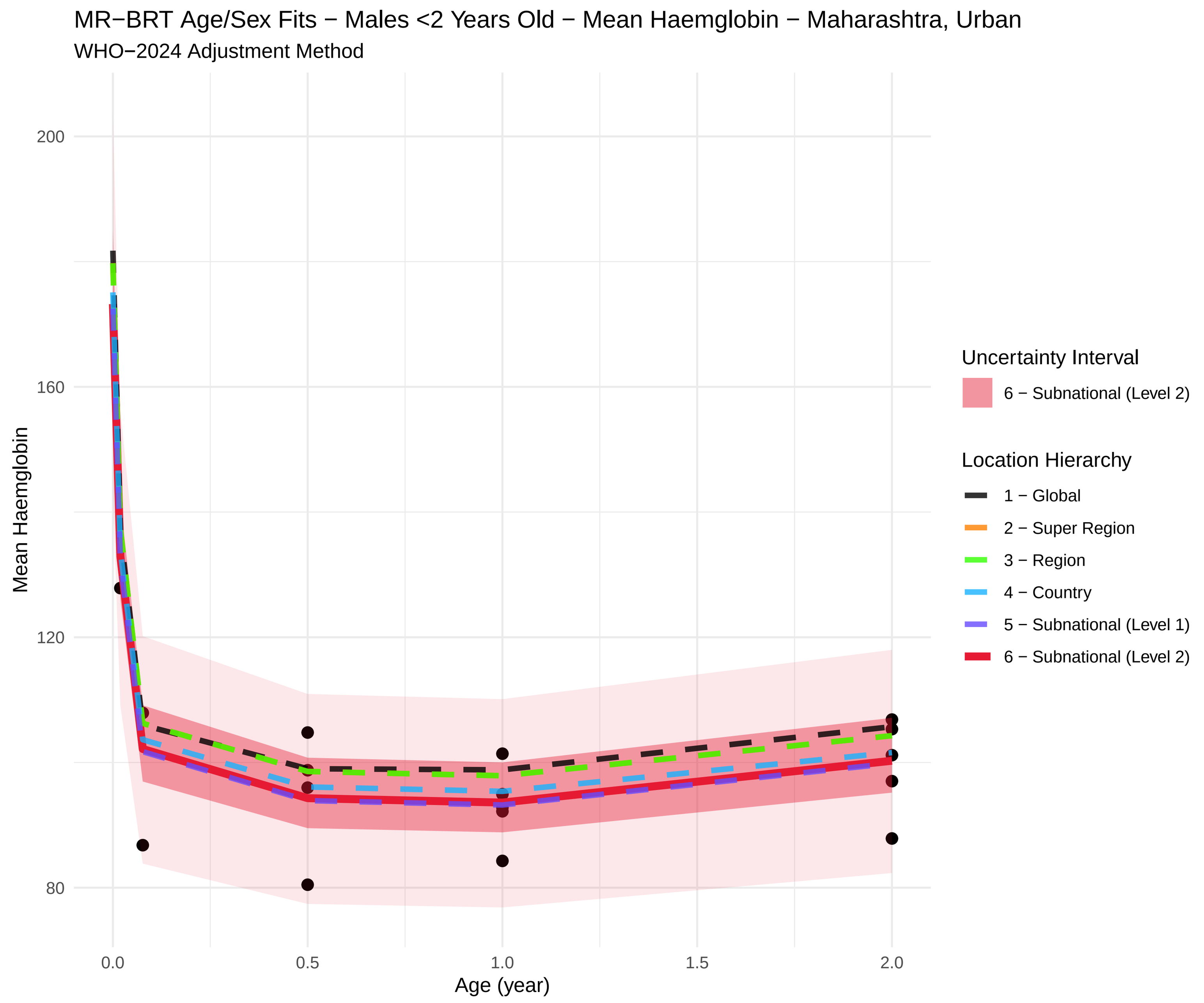
MR−BRT Age/Sex Fits − Males <2 Years Old − Mean Haemglobin − Maharashtra, Urban
WHO−2024 Adjustment Method
Mean Haemglobin
Age (year)
Uncertainty Interval
6 − Subnational (Level 2)
Location Hierarchy
1 − Global
2 − Super Region
3 − Region
4 − Country
5 − Subnational (Level 1)
6 − Subnational (Level 2)

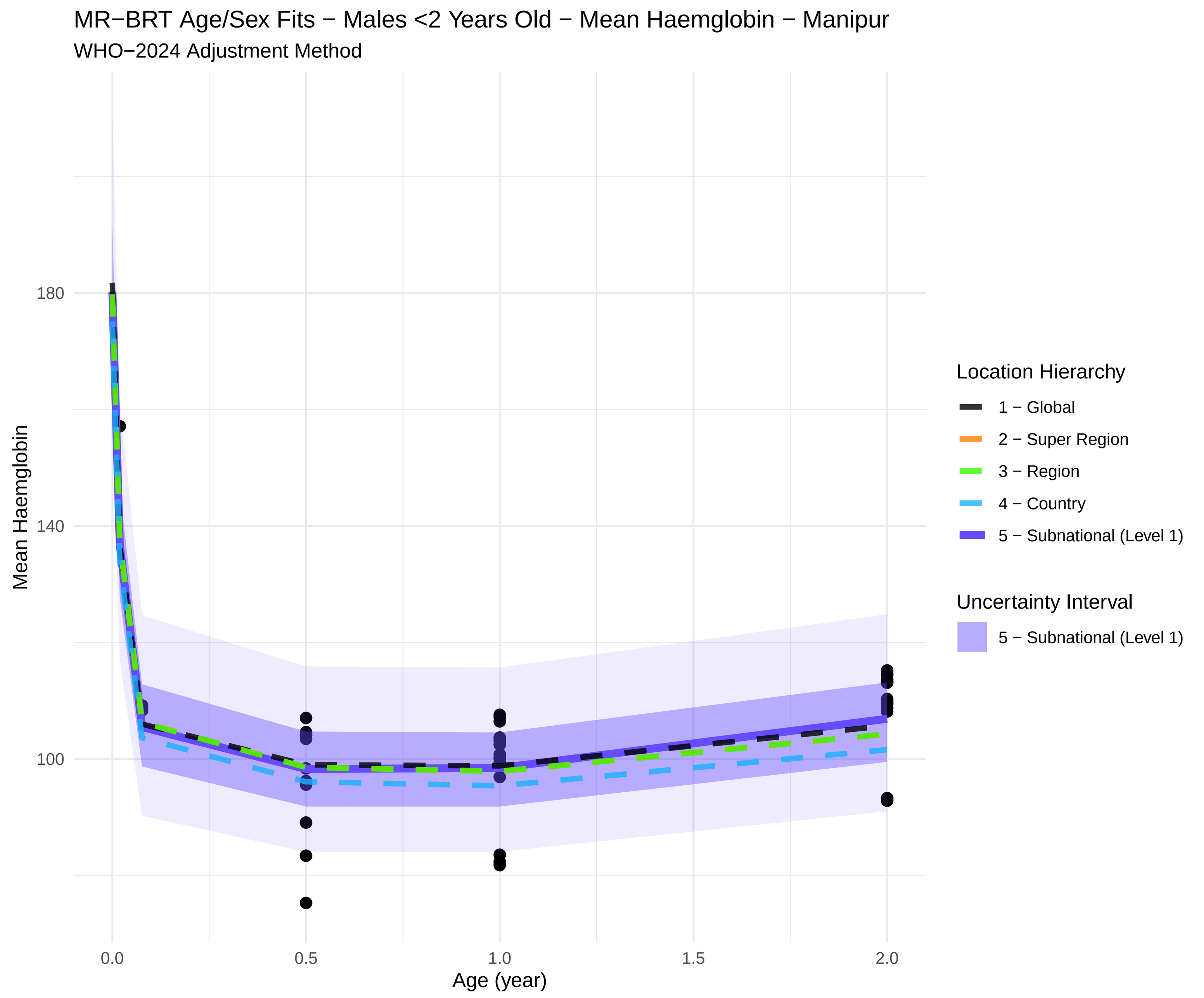
MR−BRT Age/Sex Fits − Males <2 Years Old − Mean Haemglobin − Manipur
WHO−2024 Adjustment Method
Mean Haemglobin
Age (year)
Location Hierarchy
1 − Global
2 − Super Region
3 − Region
4 − Country
5 − Subnational (Level 1)
Uncertainty Interval
5 − Subnational (Level 1)

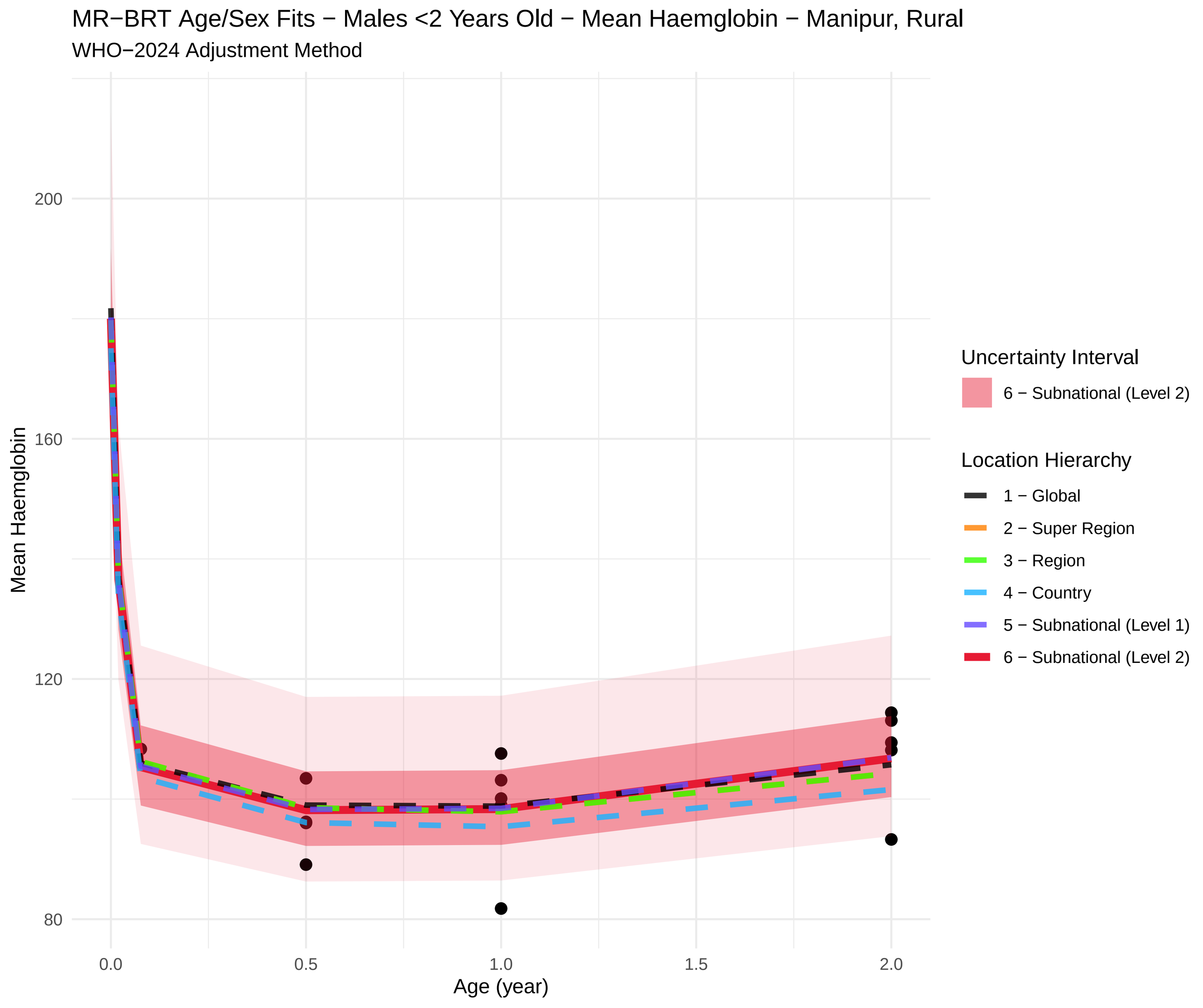
MR−BRT Age/Sex Fits − Males <2 Years Old − Mean Haemglobin − Manipur, Rural
WHO−2024 Adjustment Method
Mean Haemglobin
Age (year)
Uncertainty Interval
6 − Subnational (Level 2)
Location Hierarchy
1 − Global
2 − Super Region
3 − Region
4 − Country
5 − Subnational (Level 1)
6 − Subnational (Level 2)

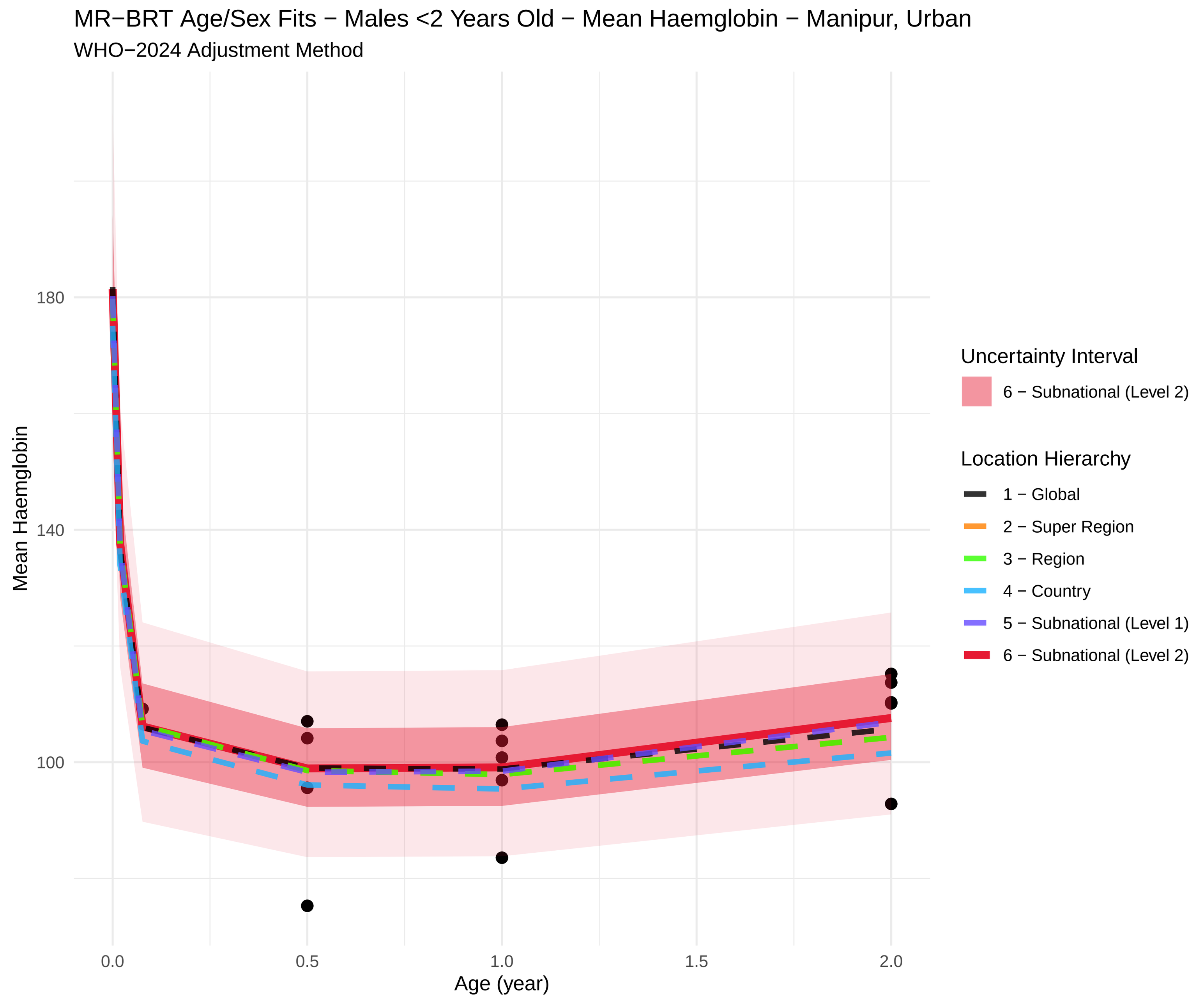
MR−BRT Age/Sex Fits − Males <2 Years Old − Mean Haemglobin − Manipur, Urban
WHO−2024 Adjustment Method
Mean Haemglobin
Age (year)
Uncertainty Interval
6 − Subnational (Level 2)
Location Hierarchy
1 − Global
2 − Super Region
3 − Region
4 − Country
5 − Subnational (Level 1)
6 − Subnational (Level 2)

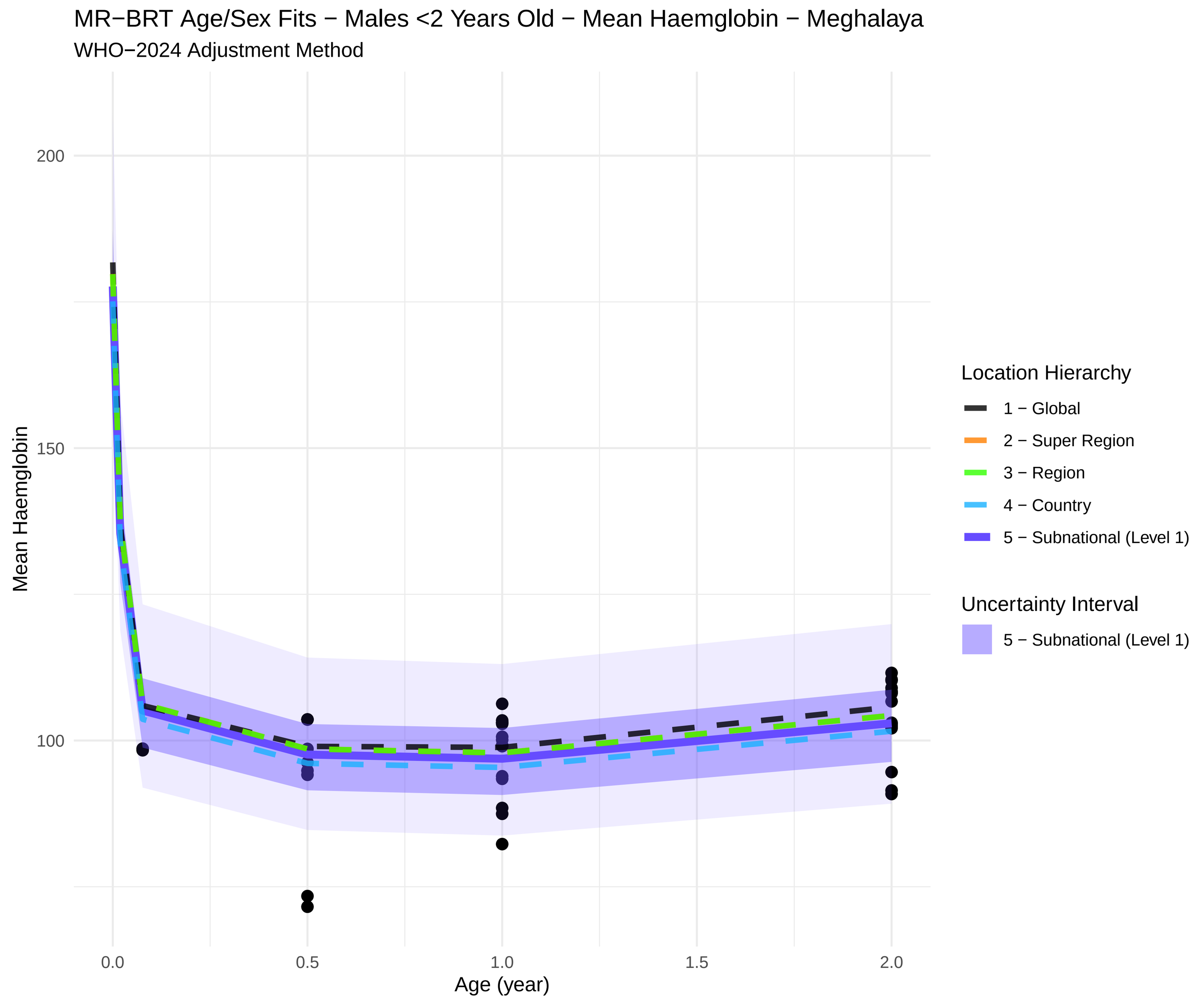
MR−BRT Age/Sex Fits − Males <2 Years Old − Mean Haemglobin − Meghalaya
WHO−2024 Adjustment Method
Mean Haemglobin
Age (year)
Location Hierarchy
1 − Global
2 − Super Region
3 − Region
4 − Country
5 − Subnational (Level 1)
Uncertainty Interval
5 − Subnational (Level 1)

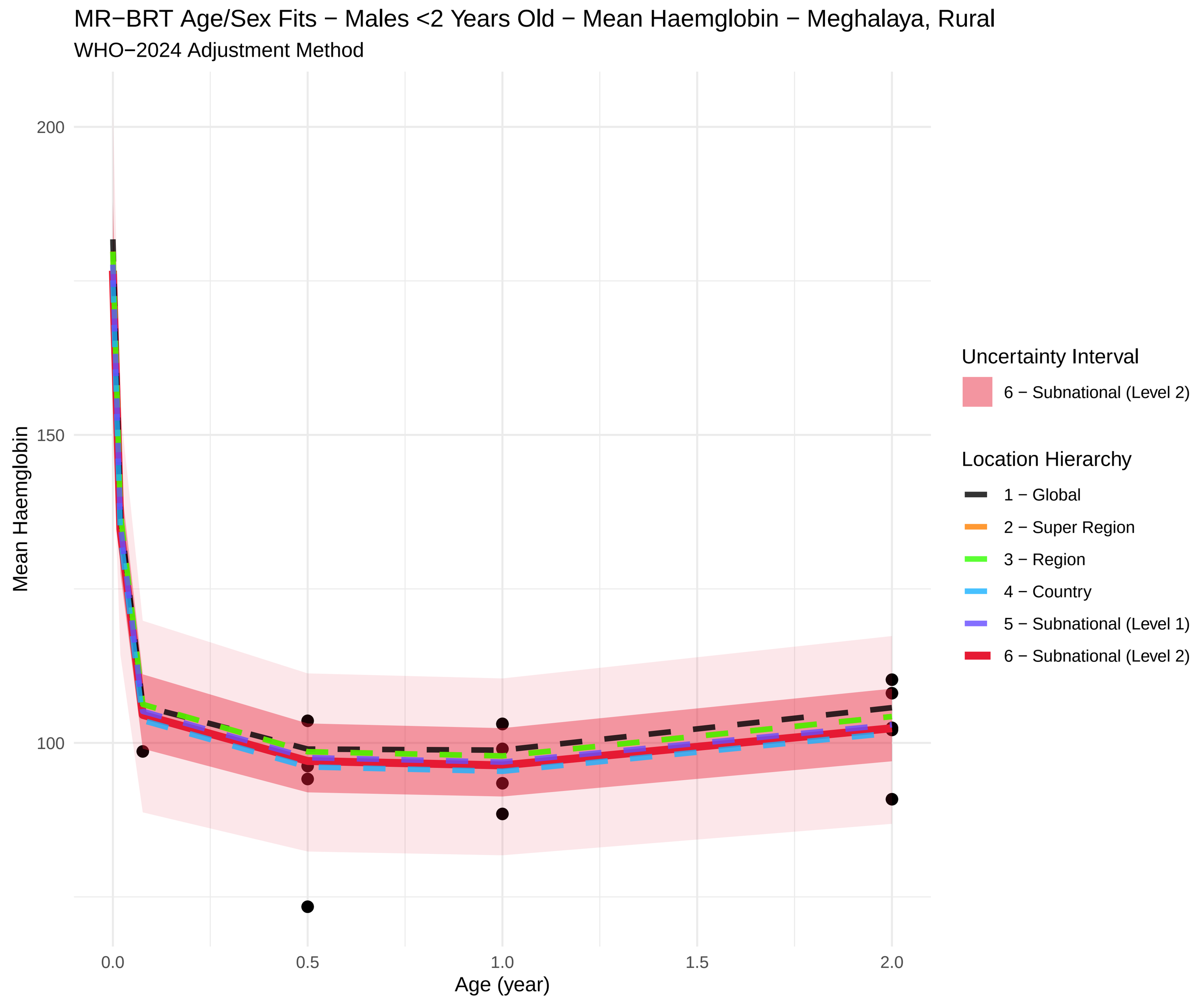
MR−BRT Age/Sex Fits − Males <2 Years Old − Mean Haemglobin − Meghalaya, Rural
WHO−2024 Adjustment Method
Mean Haemglobin
Age (year)
Uncertainty Interval
6 − Subnational (Level 2)
Location Hierarchy
1 − Global
2 − Super Region
3 − Region
4 − Country
5 − Subnational (Level 1)
6 − Subnational (Level 2)

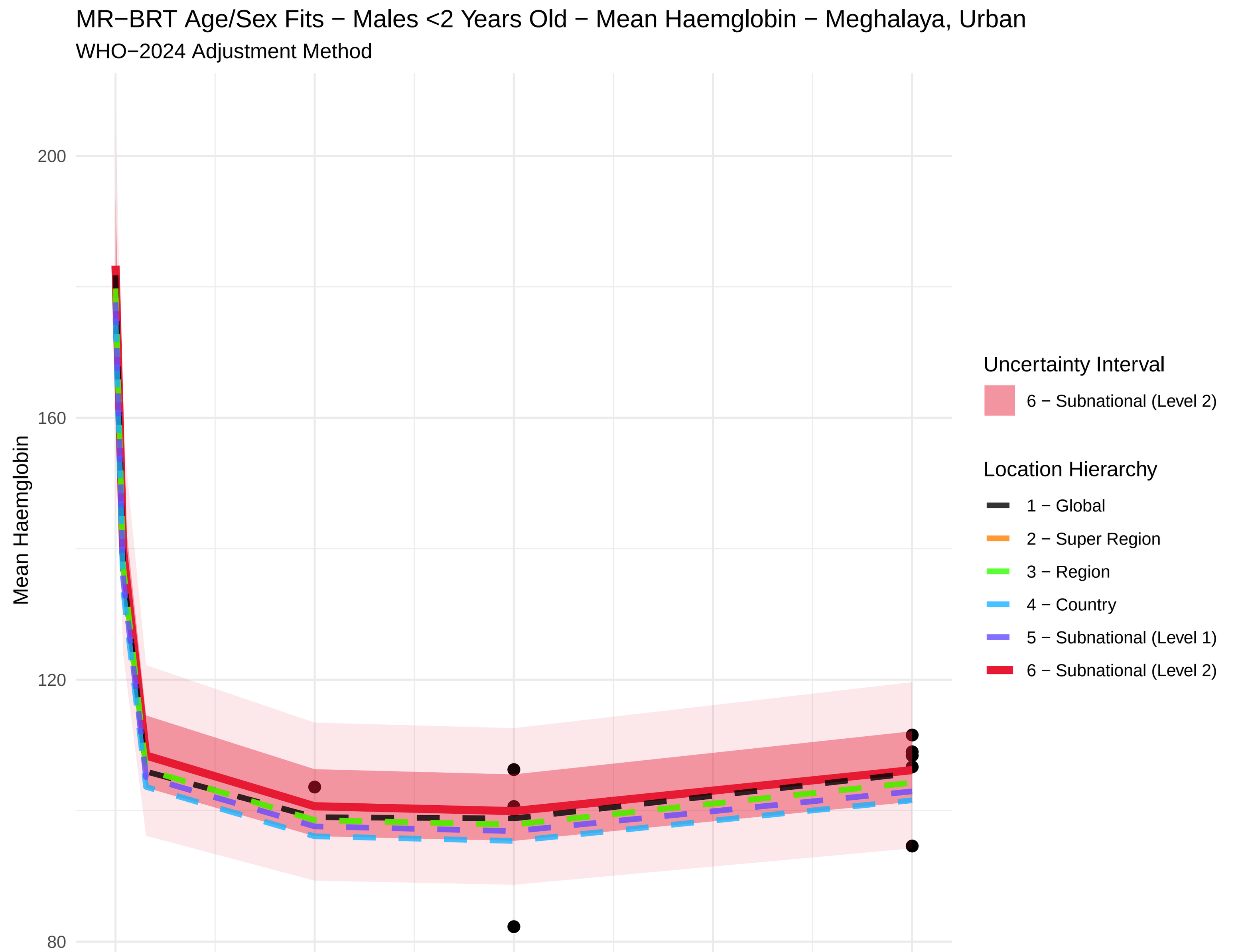
MR−BRT Age/Sex Fits − Males <2 Years Old − Mean Haemglobin − Meghalaya, Urban
WHO−2024 Adjustment Method
Mean Haemglobin
Age (year)
Uncertainty Interval
6 − Subnational (Level 2)
Location Hierarchy
1 − Global
2 − Super Region
3 − Region
4 − Country
5 − Subnational (Level 1)
6 − Subnational (Level 2)

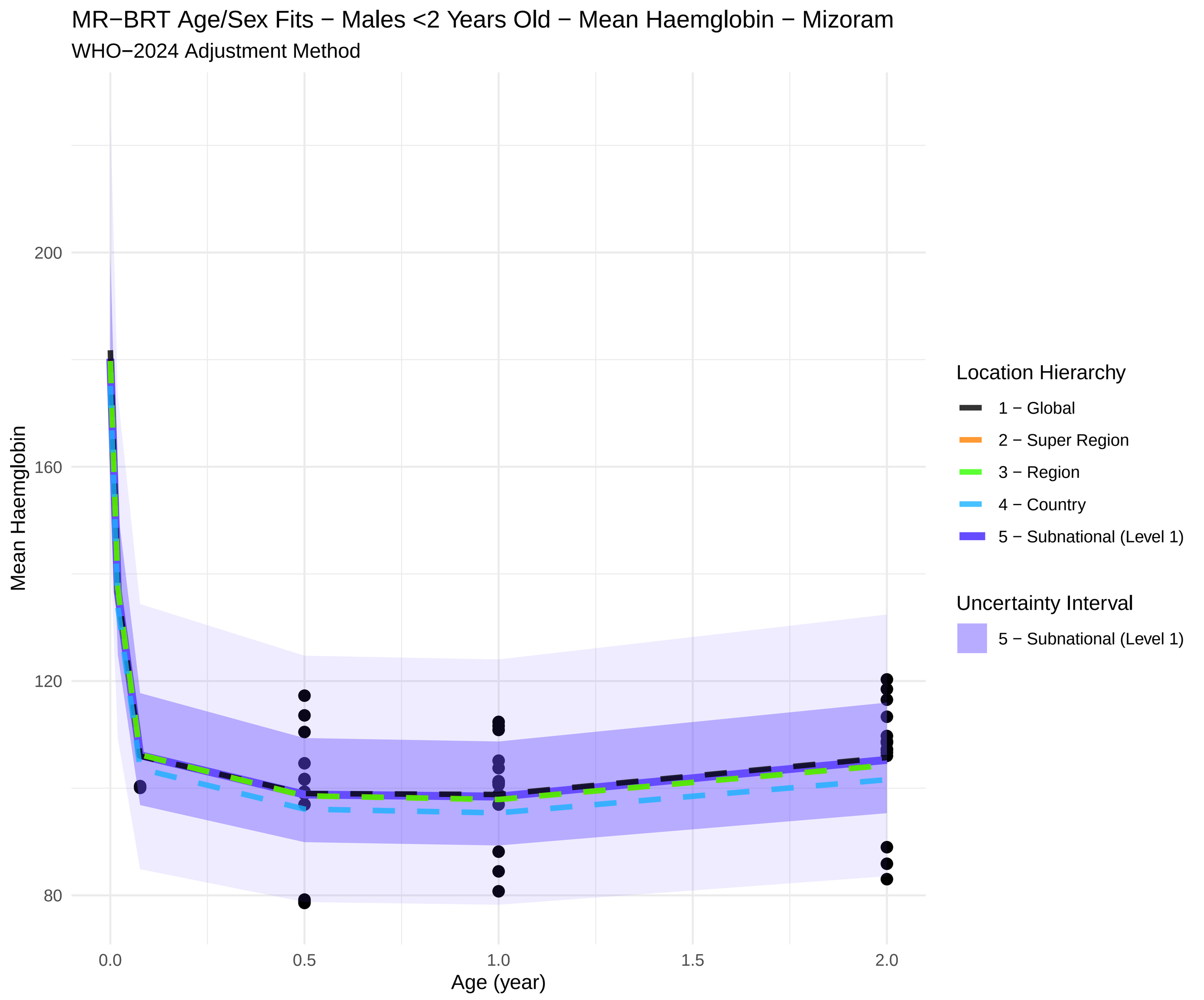
MR−BRT Age/Sex Fits − Males <2 Years Old − Mean Haemglobin − Mizoram
WHO−2024 Adjustment Method
Mean Haemglobin
Age (year)
Location Hierarchy
1 − Global
2 − Super Region
3 − Region
4 − Country
5 − Subnational (Level 1)
Uncertainty Interval
5 − Subnational (Level 1)

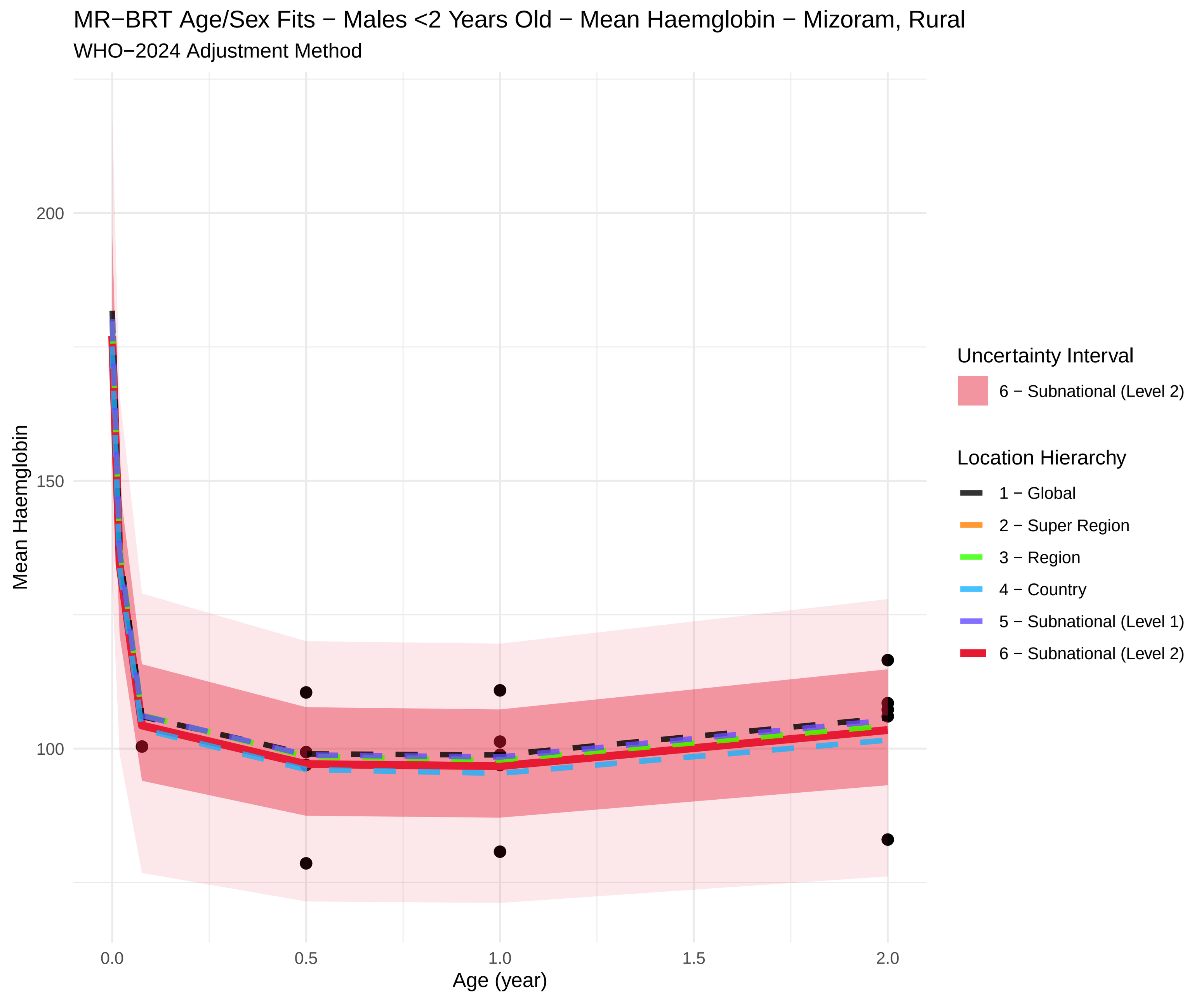
MR−BRT Age/Sex Fits − Males <2 Years Old − Mean Haemglobin − Mizoram, Rural
WHO−2024 Adjustment Method
Mean Haemglobin
Age (year)
Uncertainty Interval
6 − Subnational (Level 2)
Location Hierarchy
1 − Global
2 − Super Region
3 − Region
4 − Country
5 − Subnational (Level 1)
6 − Subnational (Level 2)

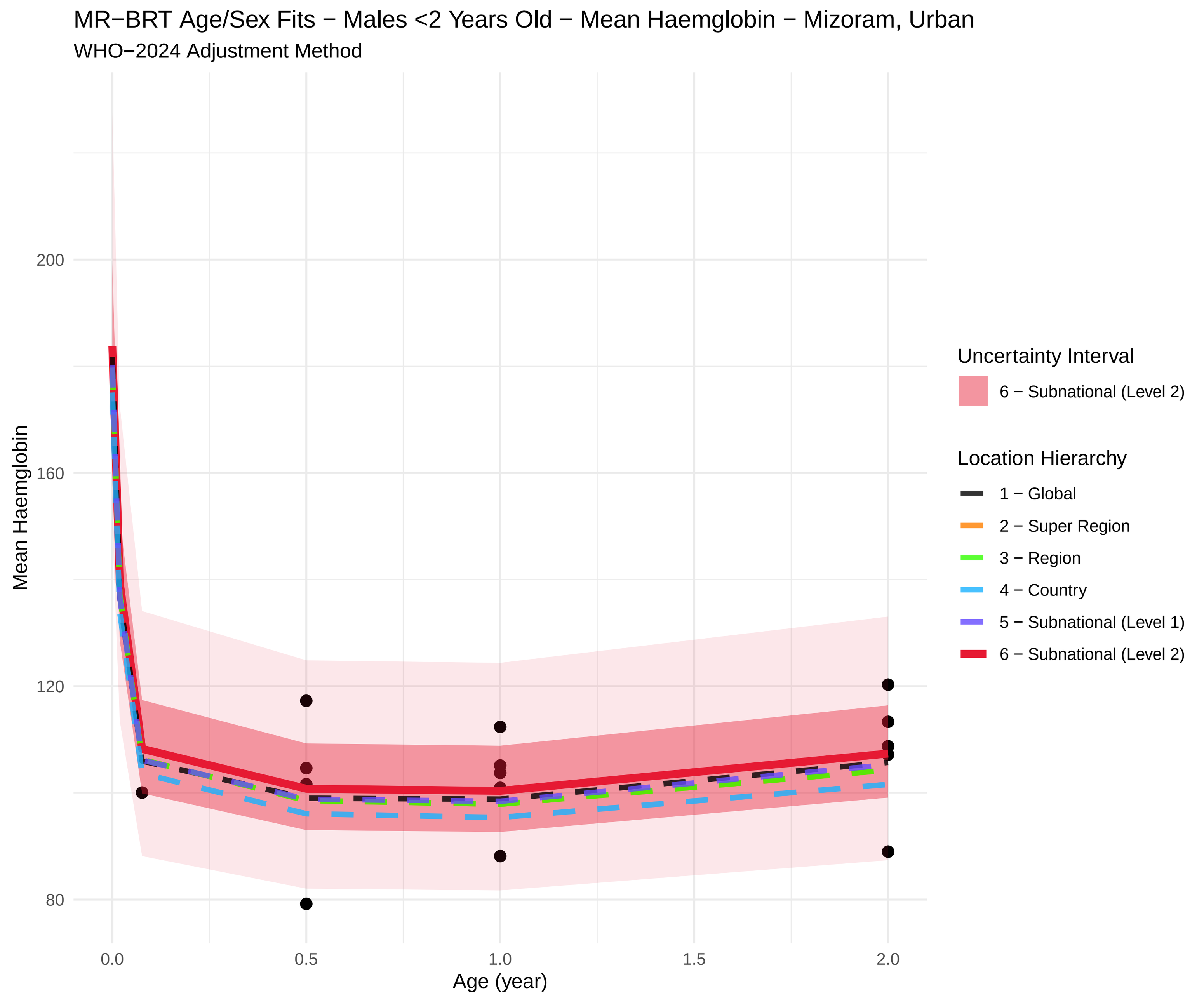
MR−BRT Age/Sex Fits − Males <2 Years Old − Mean Haemglobin − Mizoram, Urban
WHO−2024 Adjustment Method
Mean Haemglobin
Age (year)
Uncertainty Interval
6 − Subnational (Level 2)
Location Hierarchy
1 − Global
2 − Super Region
3 − Region
4 − Country
5 − Subnational (Level 1)
6 − Subnational (Level 2)

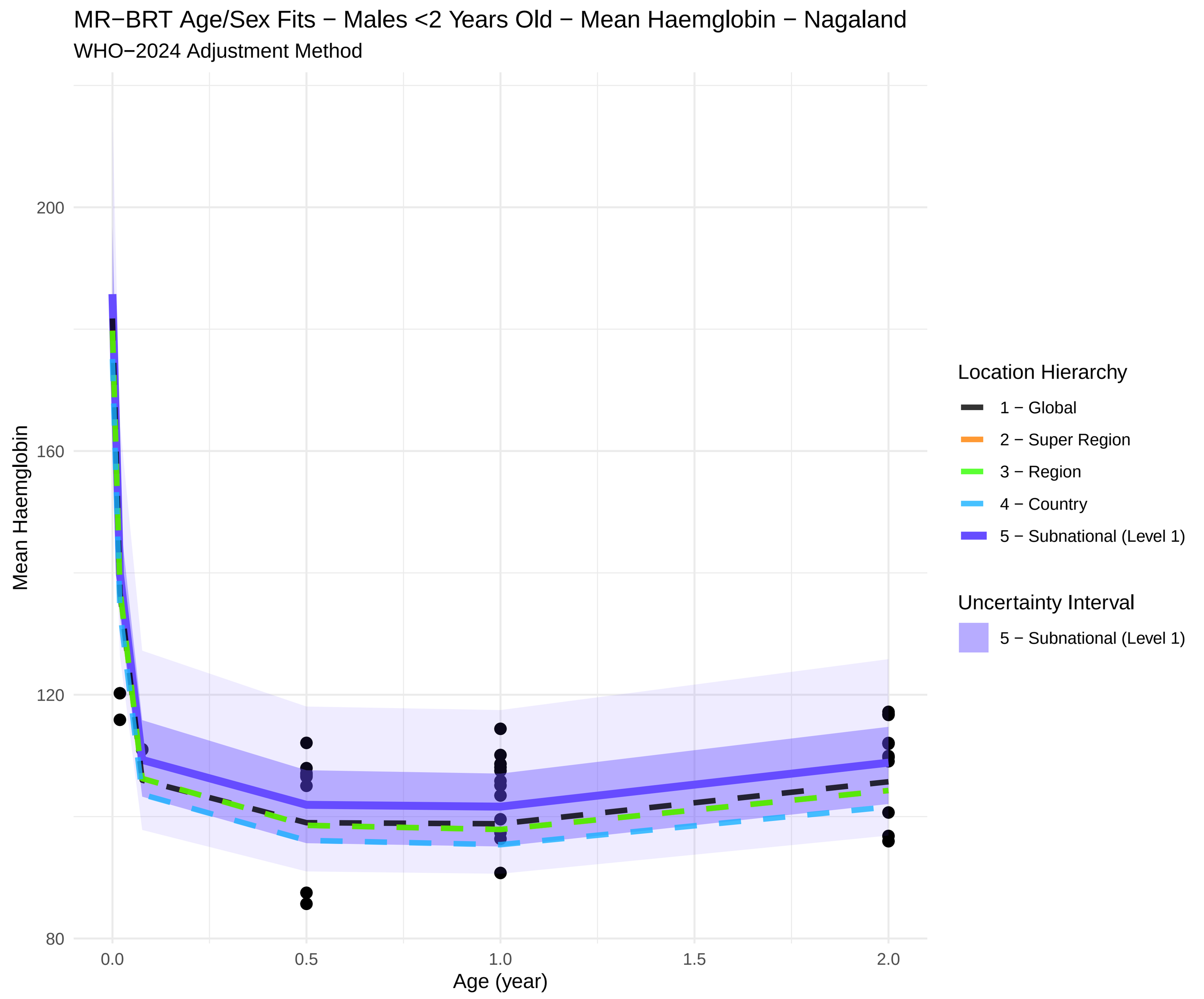
MR−BRT Age/Sex Fits − Males <2 Years Old − Mean Haemglobin − Nagaland
WHO−2024 Adjustment Method
Mean Haemglobin
Age (year)
Location Hierarchy
1 − Global
2 − Super Region
3 − Region
4 − Country
5 − Subnational (Level 1)
Uncertainty Interval
5 − Subnational (Level 1)

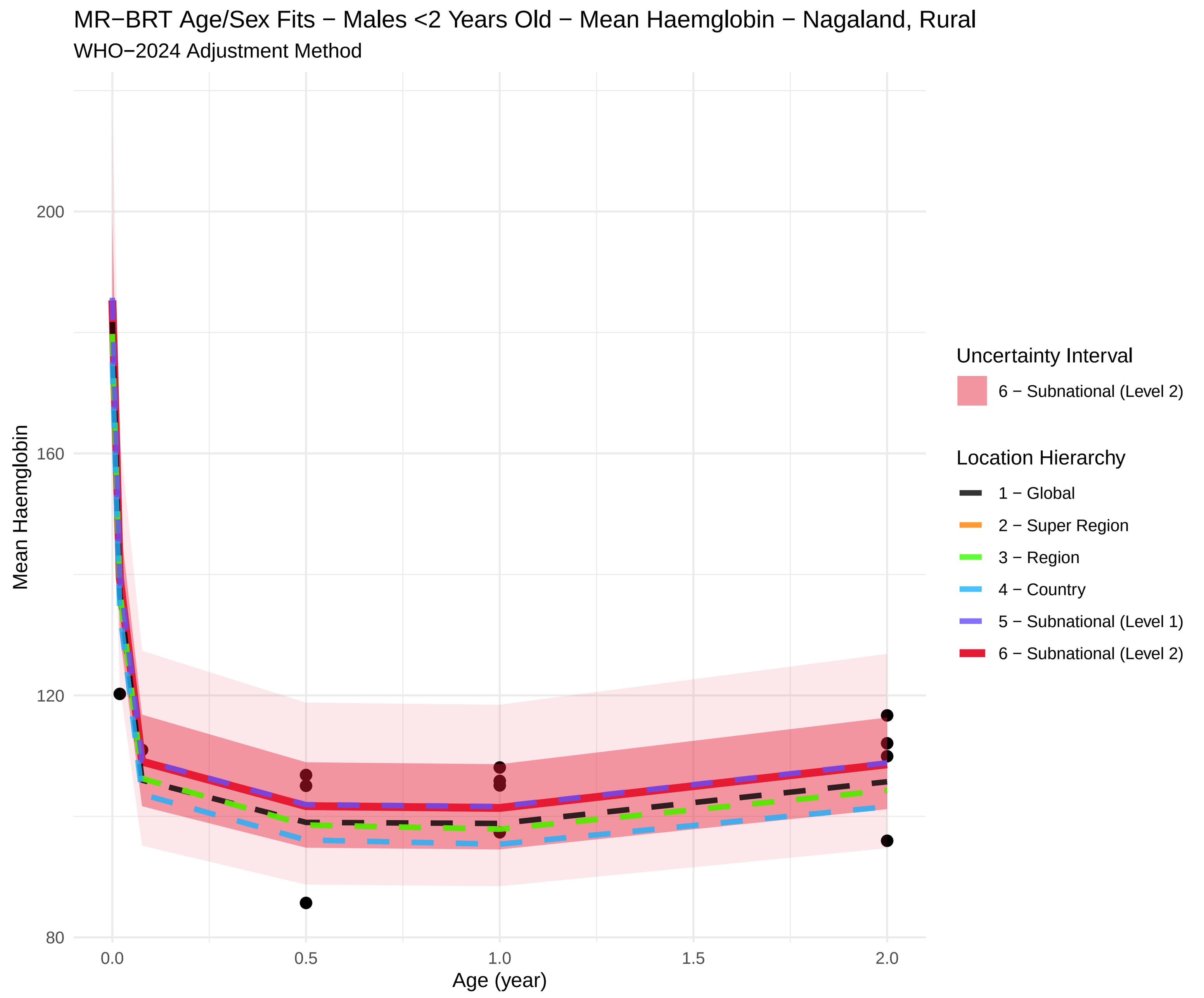
MR−BRT Age/Sex Fits − Males <2 Years Old − Mean Haemglobin − Nagaland, Rural
WHO−2024 Adjustment Method
Mean Haemglobin
Age (year)
Uncertainty Interval
6 − Subnational (Level 2)
Location Hierarchy
1 − Global
2 − Super Region
3 − Region
4 − Country
5 − Subnational (Level 1)
6 − Subnational (Level 2)

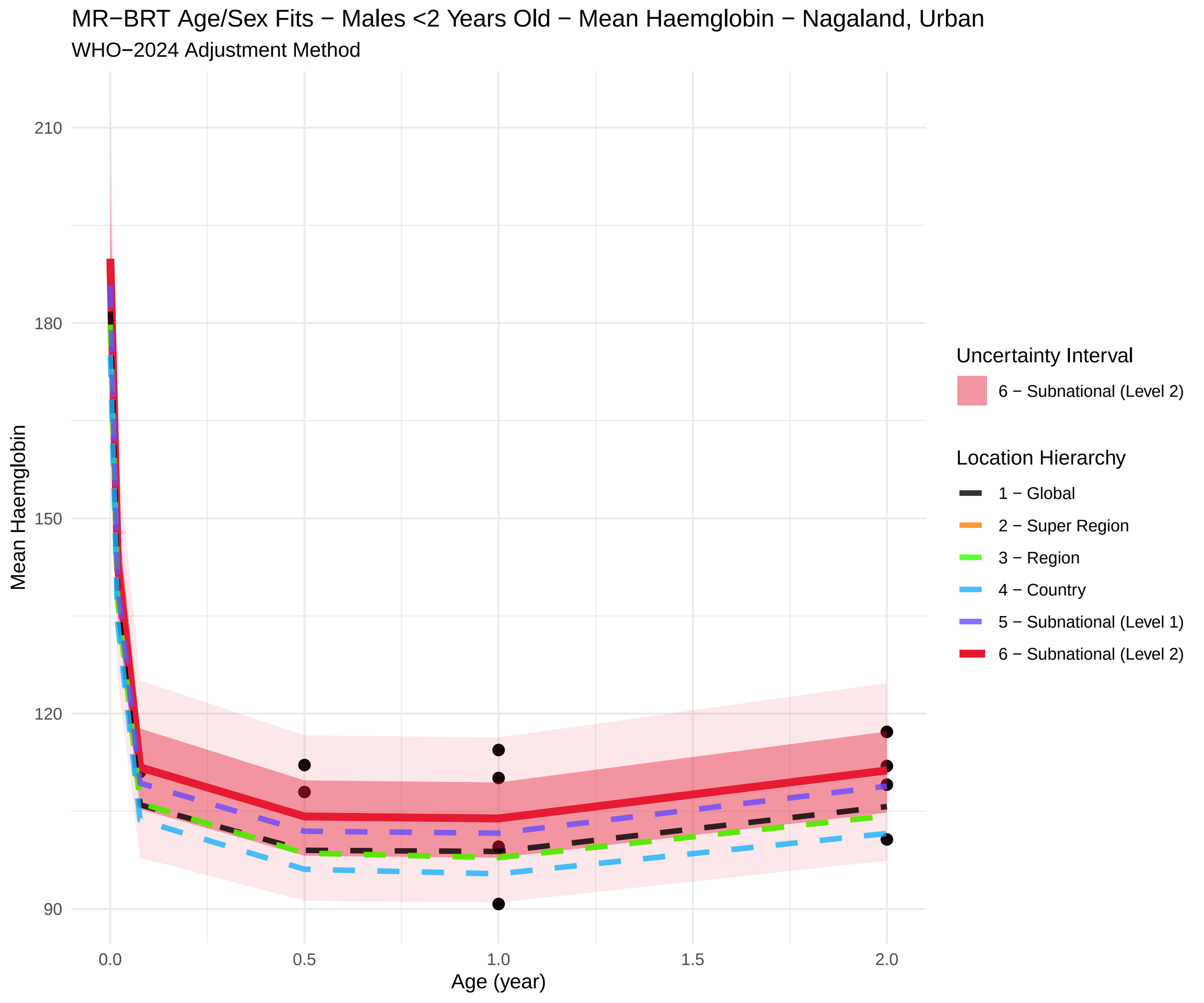
MR−BRT Age/Sex Fits − Males <2 Years Old − Mean Haemglobin − Nagaland, Urban
WHO−2024 Adjustment Method
Mean Haemglobin
Age (year)
Uncertainty Interval
6 − Subnational (Level 2)
Location Hierarchy
1 − Global
2 − Super Region
3 − Region
4 − Country
5 − Subnational (Level 1)
6 − Subnational (Level 2)

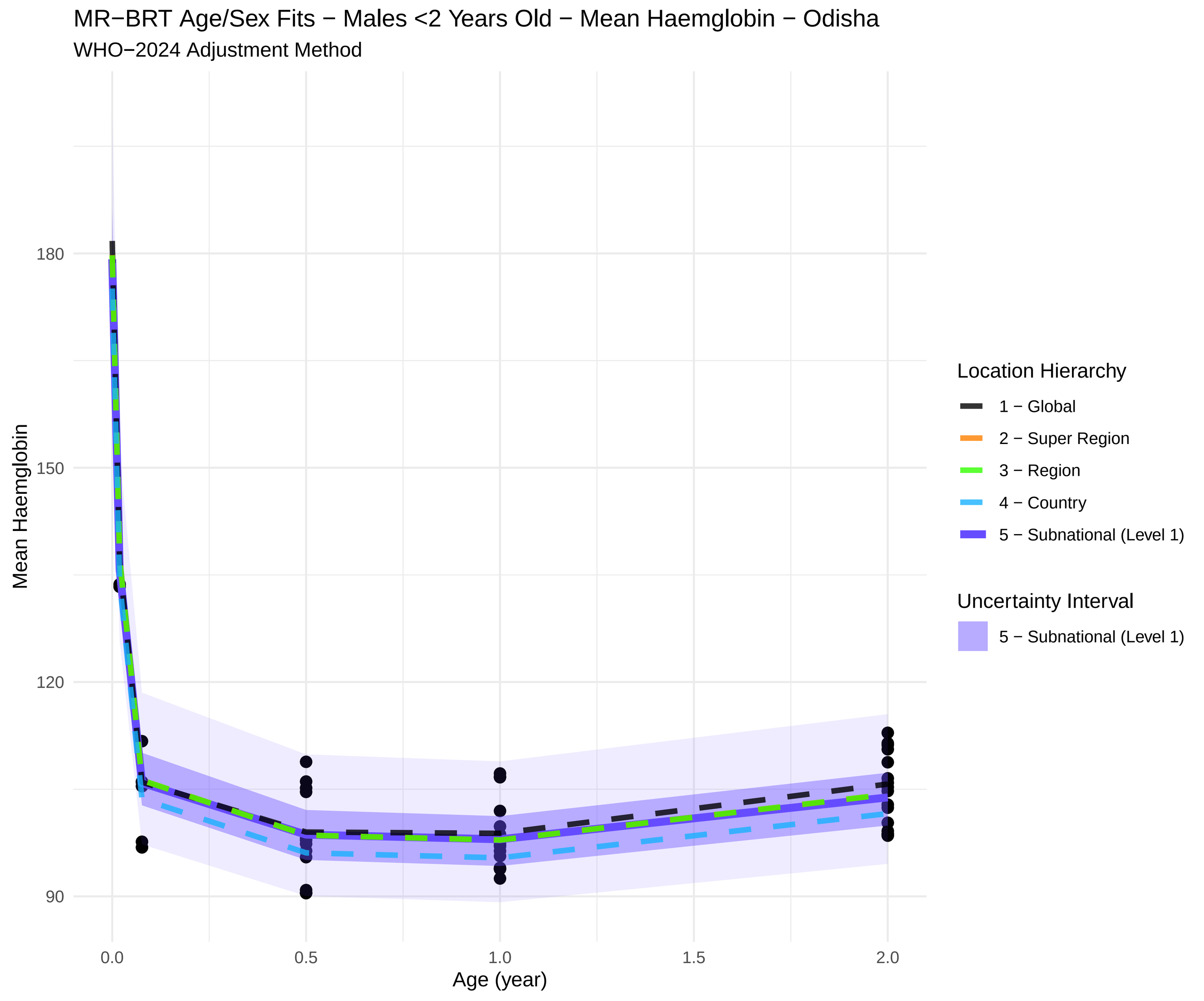
MR−BRT Age/Sex Fits − Males <2 Years Old − Mean Haemglobin − Odisha
WHO−2024 Adjustment Method
Mean Haemglobin
Age (year)
Location Hierarchy
1 − Global
2 − Super Region
3 − Region
4 − Country
5 − Subnational (Level 1)
Uncertainty Interval
5 − Subnational (Level 1)

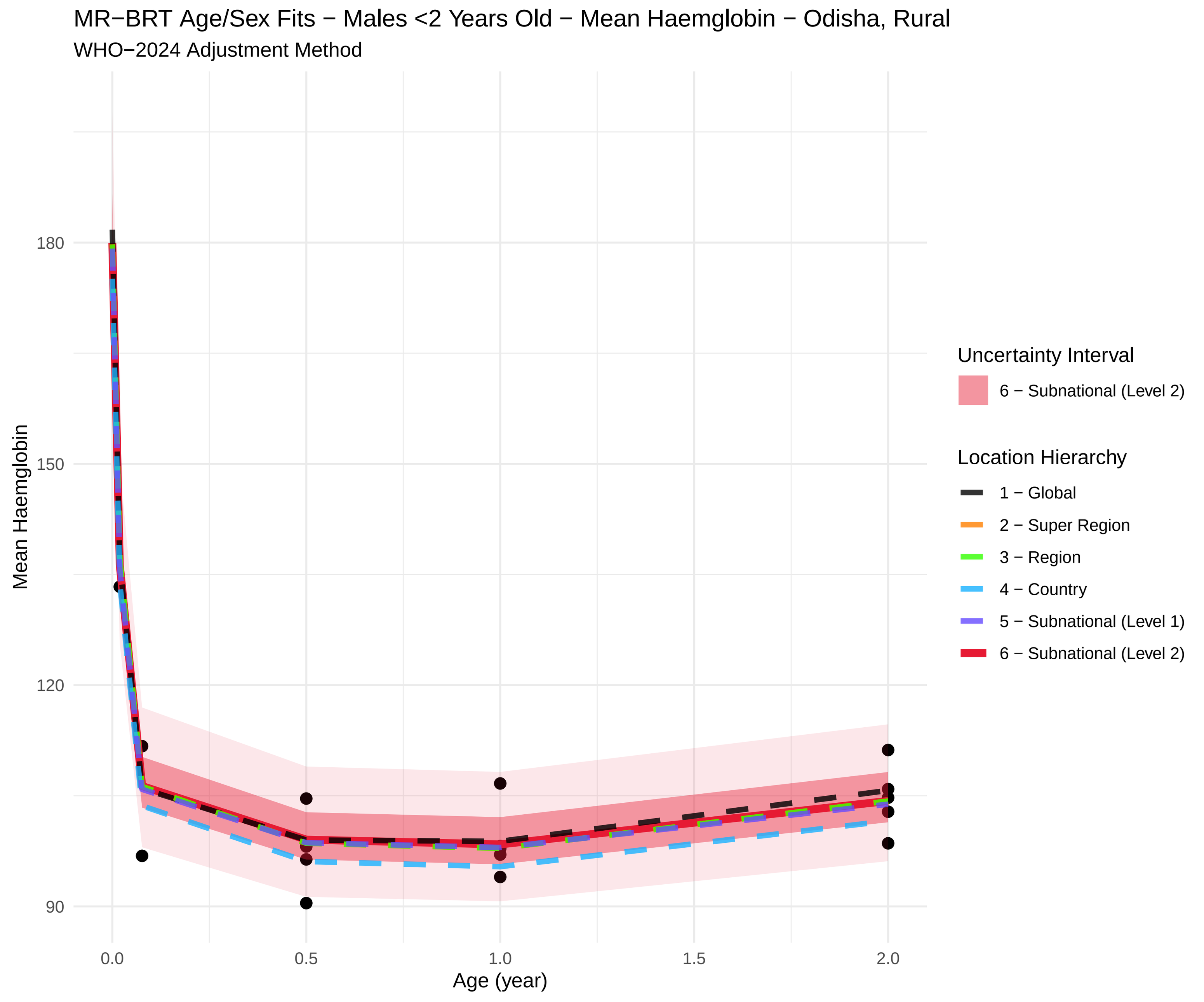
MR−BRT Age/Sex Fits − Males <2 Years Old − Mean Haemglobin − Odisha, Rural
WHO−2024 Adjustment Method
Mean Haemglobin
Age (year)
Uncertainty Interval
6 − Subnational (Level 2)
Location Hierarchy
1 − Global
2 − Super Region
3 − Region
4 − Country
5 − Subnational (Level 1)
6 − Subnational (Level 2)

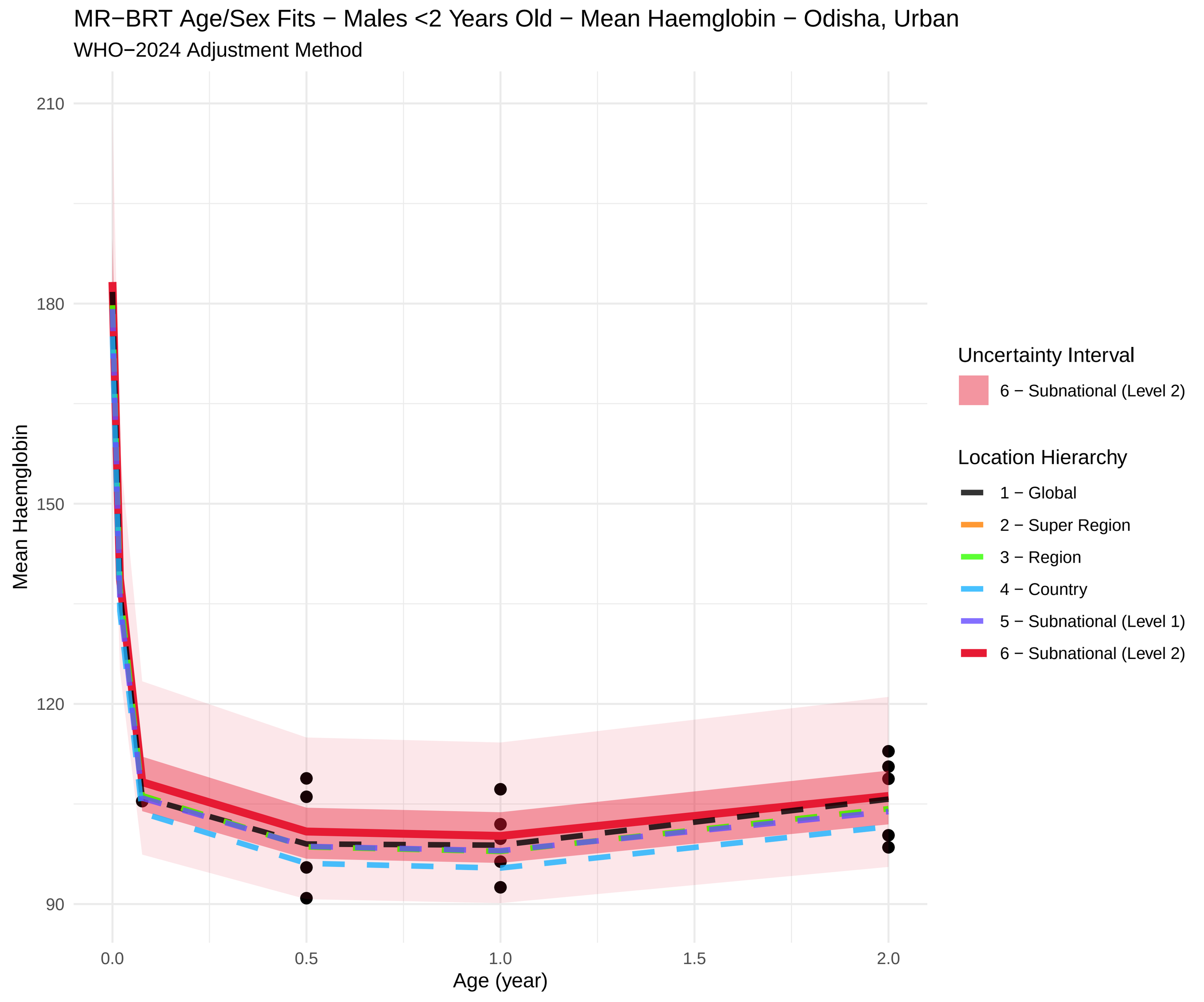
MR−BRT Age/Sex Fits − Males <2 Years Old − Mean Haemglobin − Odisha, Urban
WHO−2024 Adjustment Method
Mean Haemglobin
Age (year)
Uncertainty Interval
6 − Subnational (Level 2)
Location Hierarchy
1 − Global
2 − Super Region
3 − Region
4 − Country
5 − Subnational (Level 1)
6 − Subnational (Level 2)

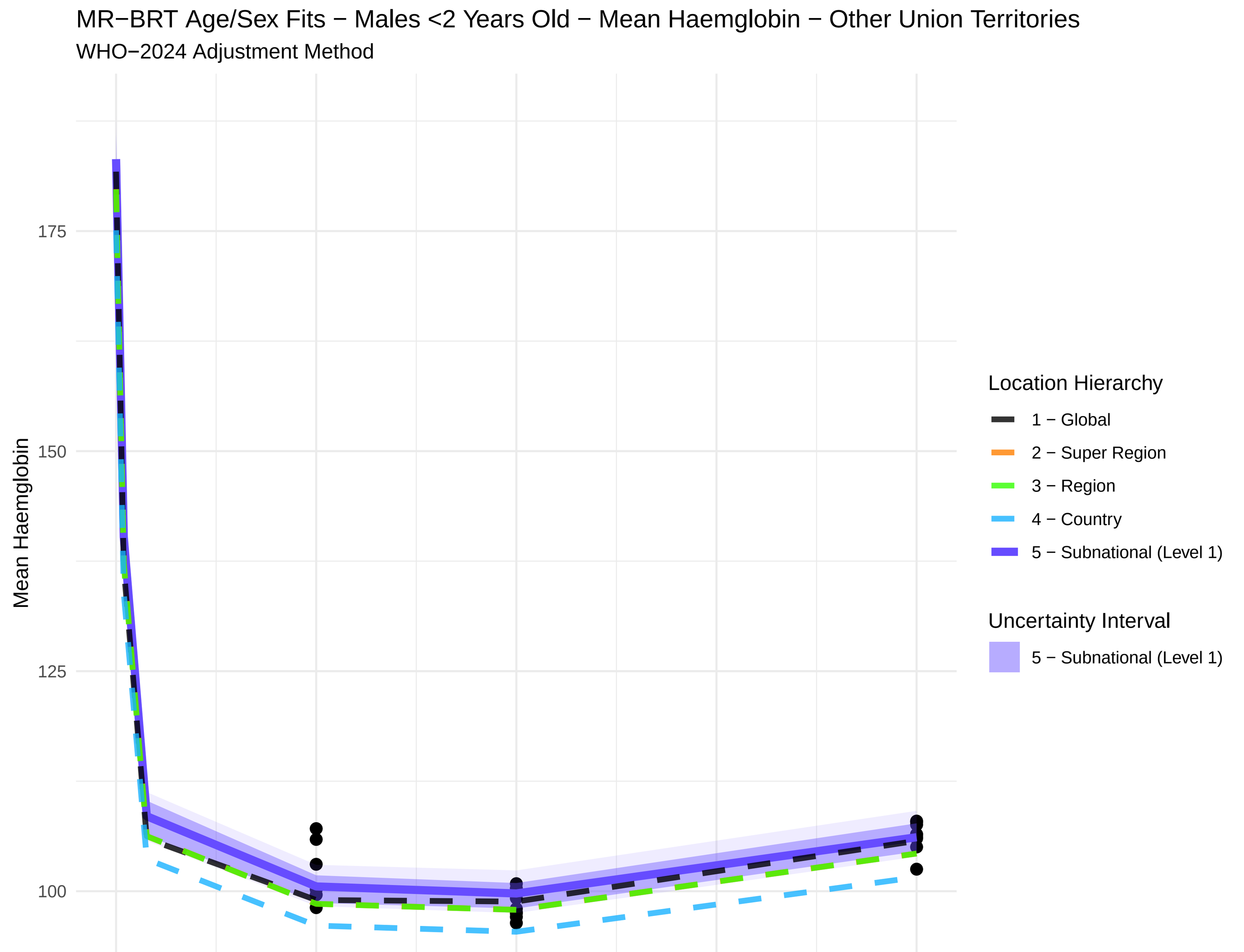
MR−BRT Age/Sex Fits − Males <2 Years Old − Mean Haemglobin − Other Union Territories
WHO−2024 Adjustment Method
Mean Haemglobin
Age (year)
Location Hierarchy
1 − Global
2 − Super Region
3 − Region
4 − Country
5 − Subnational (Level 1)
Uncertainty Interval
5 − Subnational (Level 1)

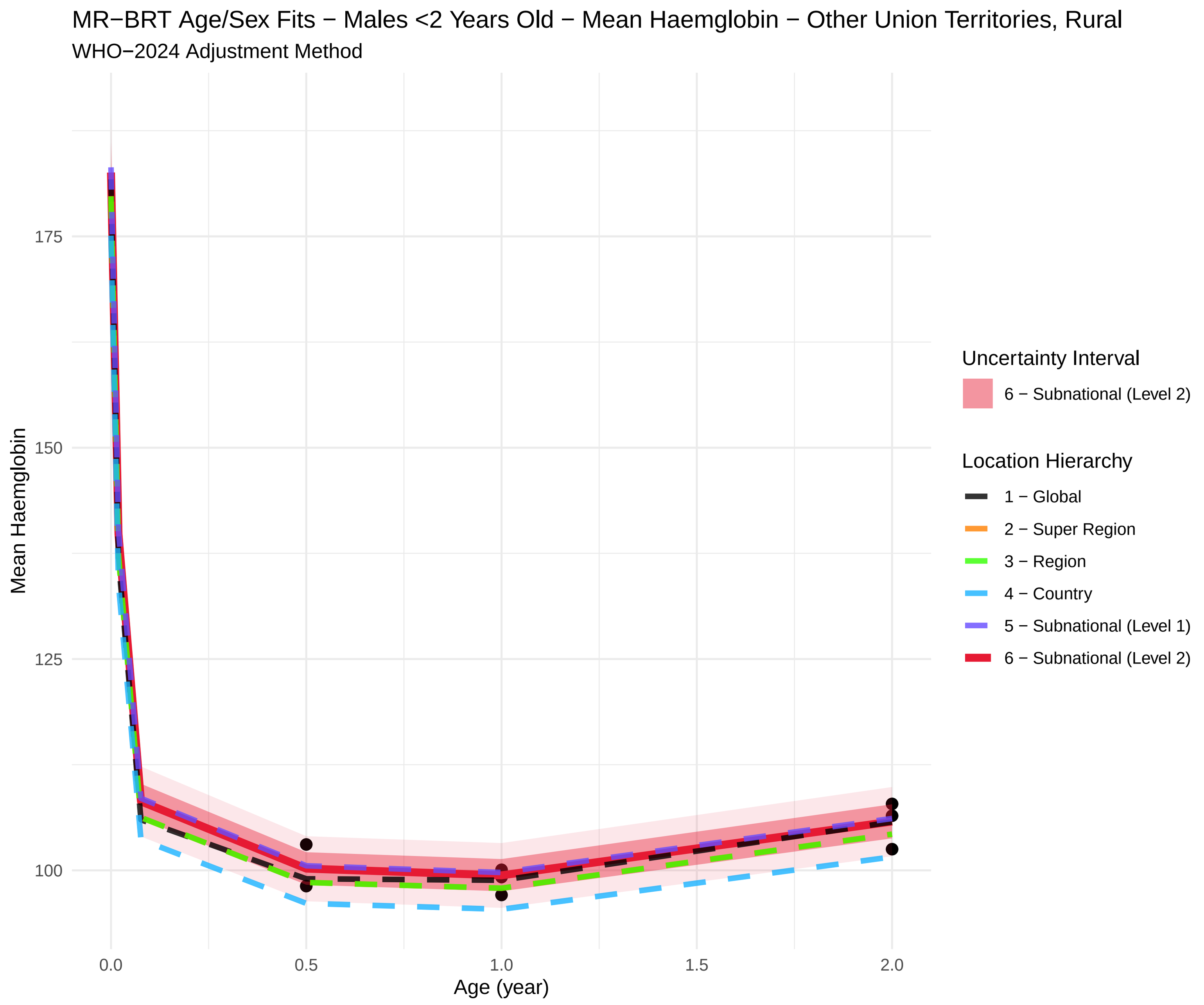
MR−BRT Age/Sex Fits − Males <2 Years Old − Mean Haemglobin − Other Union Territories, Rural
WHO−2024 Adjustment Method
Mean Haemglobin
Age (year)
Uncertainty Interval
6 − Subnational (Level 2)
Location Hierarchy
1 − Global
2 − Super Region
3 − Region
4 − Country
5 − Subnational (Level 1)
6 − Subnational (Level 2)

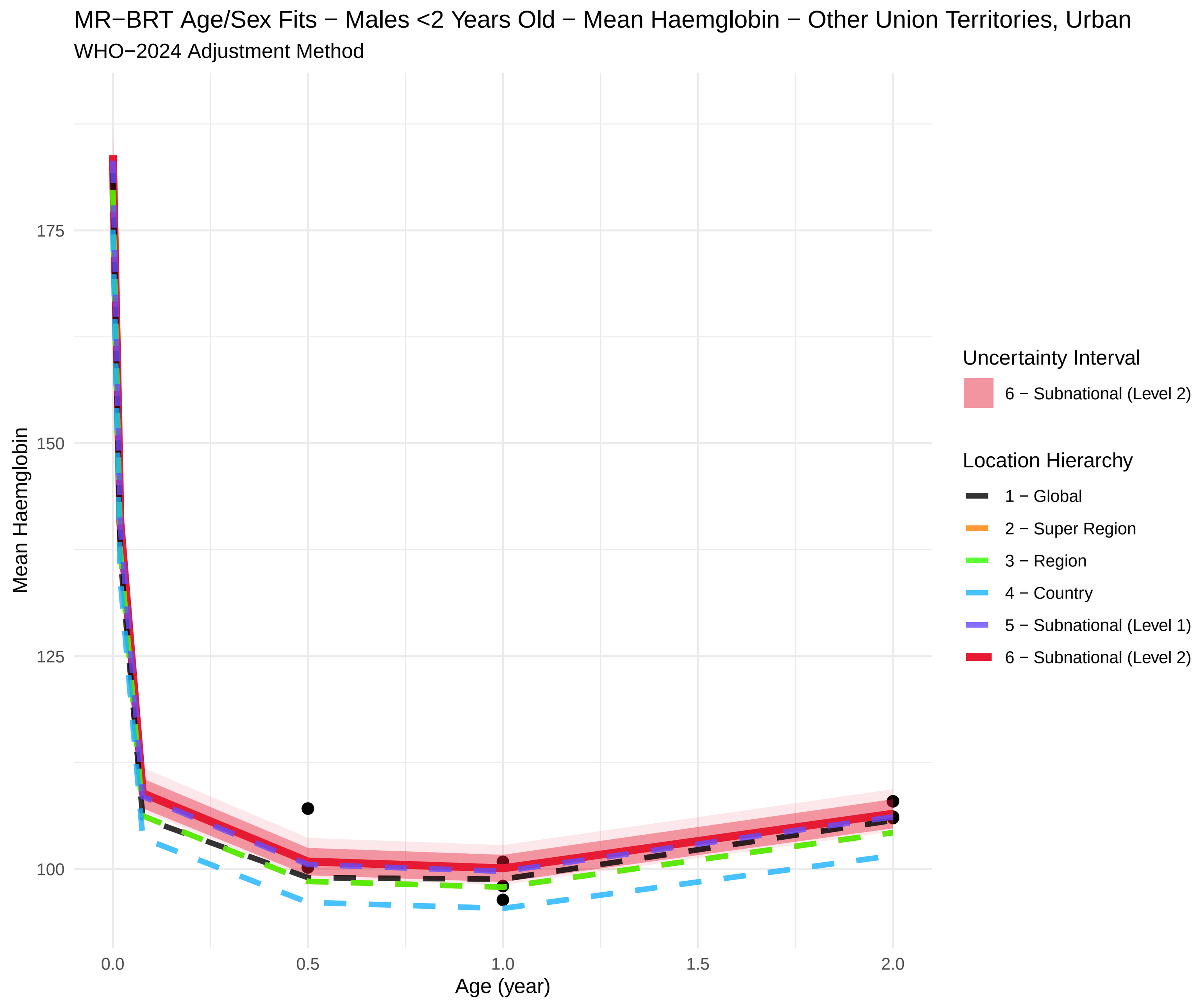
MR−BRT Age/Sex Fits − Males <2 Years Old − Mean Haemglobin − Other Union Territories, Urban
WHO−2024 Adjustment Method
Mean Haemglobin
Age (year)
Uncertainty Interval
6 − Subnational (Level 2)
Location Hierarchy
1 − Global
2 − Super Region
3 − Region
4 − Country
5 − Subnational (Level 1)
6 − Subnational (Level 2)

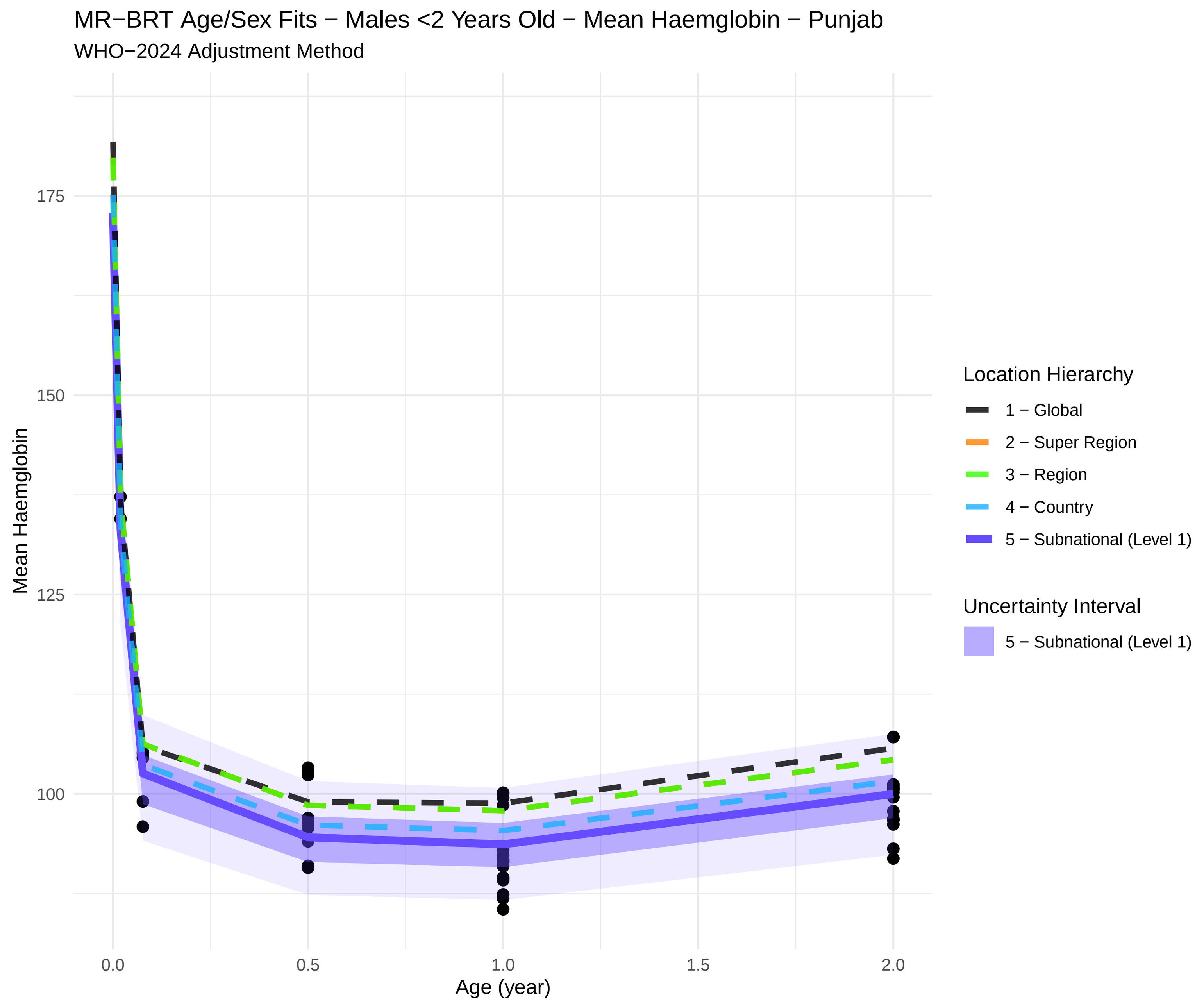
MR−BRT Age/Sex Fits − Males <2 Years Old − Mean Haemglobin − Punjab
WHO−2024 Adjustment Method
Mean Haemglobin
Age (year)
Location Hierarchy
1 − Global
2 − Super Region
3 − Region
4 − Country
5 − Subnational (Level 1)
Uncertainty Interval
5 − Subnational (Level 1)

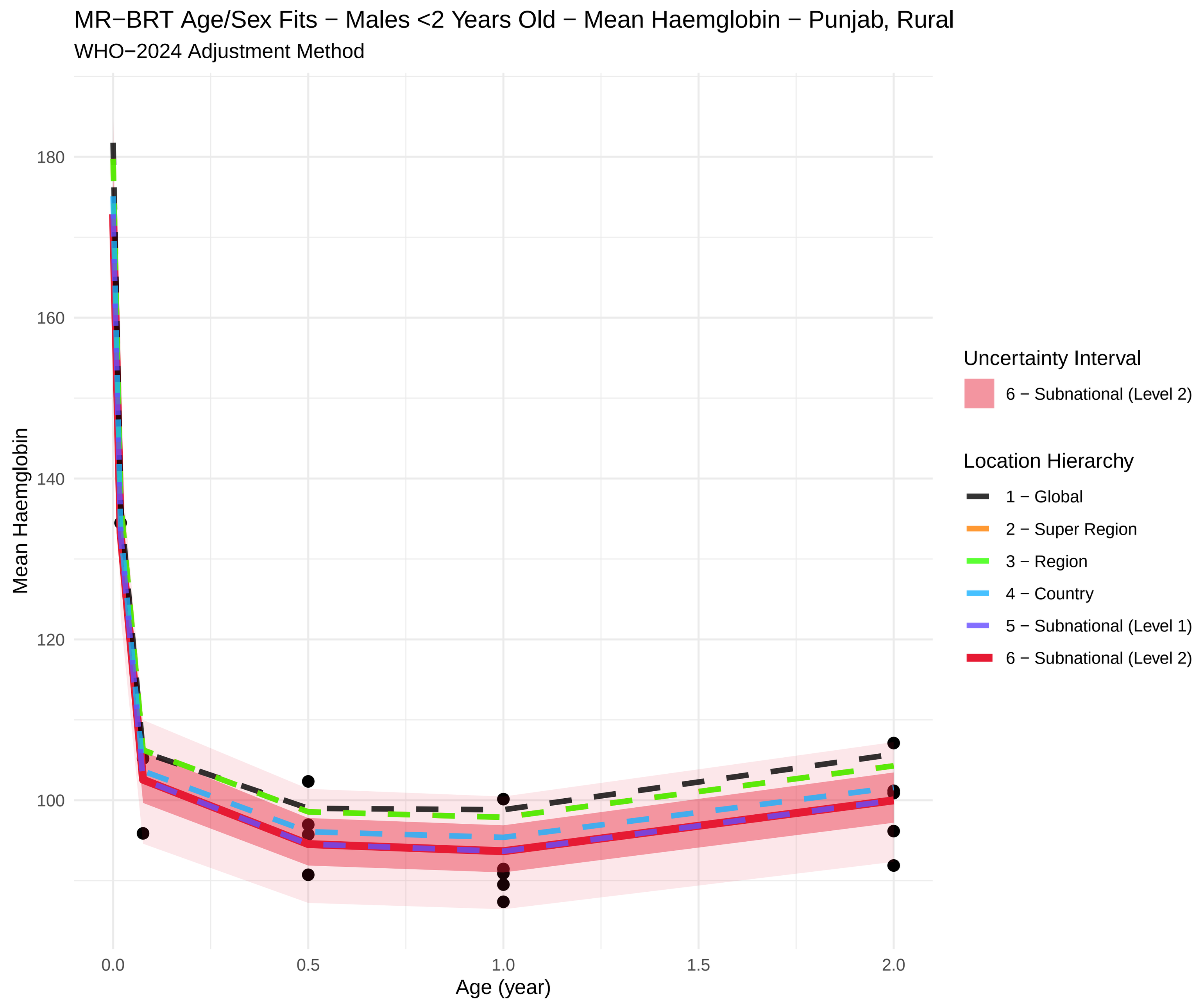
MR−BRT Age/Sex Fits − Males <2 Years Old − Mean Haemglobin − Punjab, Rural
WHO−2024 Adjustment Method
Mean Haemglobin
Age (year)
Uncertainty Interval
6 − Subnational (Level 2)
Location Hierarchy
1 − Global
2 − Super Region
3 − Region
4 − Country
5 − Subnational (Level 1)
6 − Subnational (Level 2)

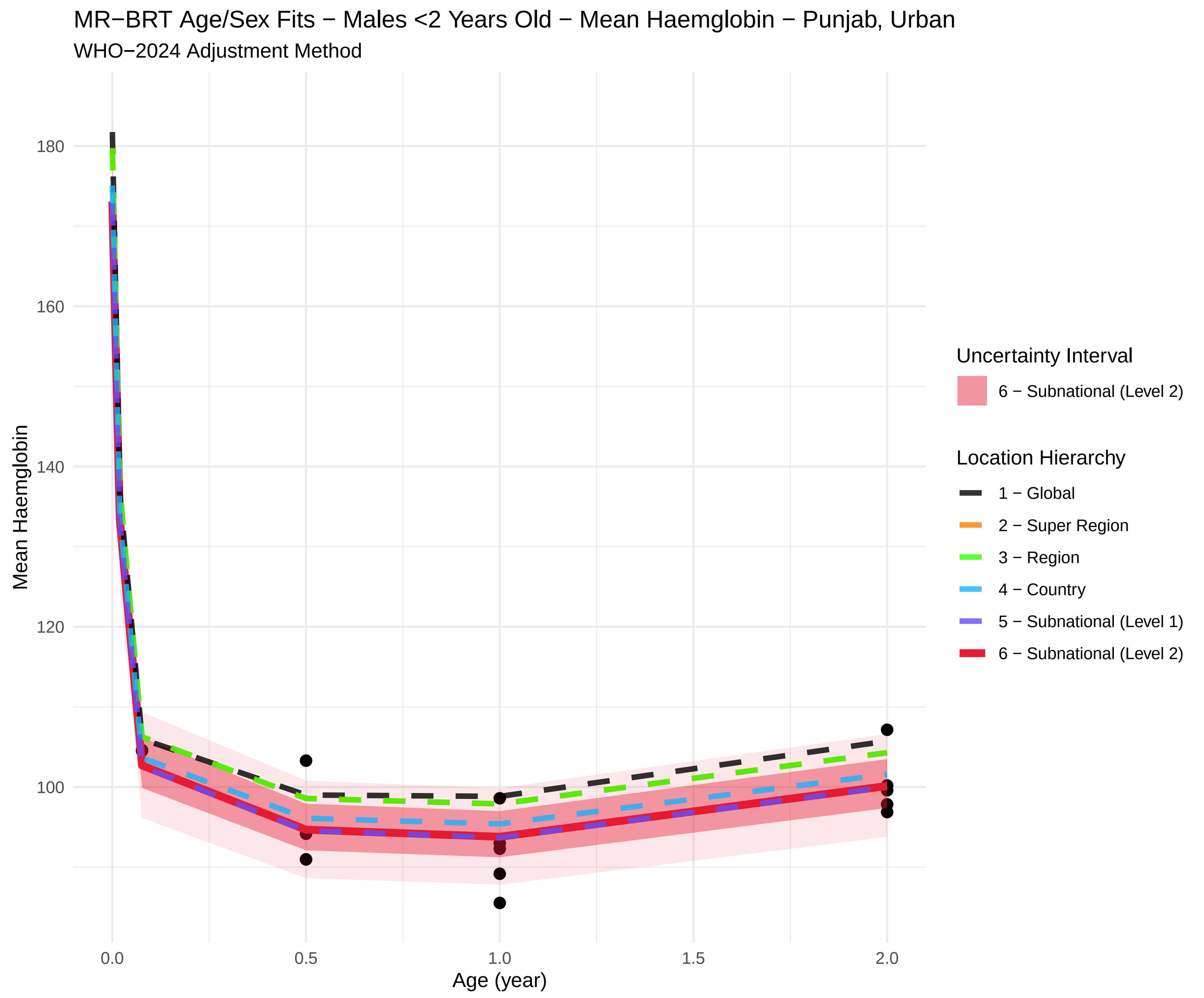
MR−BRT Age/Sex Fits − Males <2 Years Old − Mean Haemglobin − Punjab, Urban
WHO−2024 Adjustment Method
Mean Haemglobin
Age (year)
Uncertainty Interval
6 − Subnational (Level 2)
Location Hierarchy
1 − Global
2 − Super Region
3 − Region
4 − Country
5 − Subnational (Level 1)
6 − Subnational (Level 2)

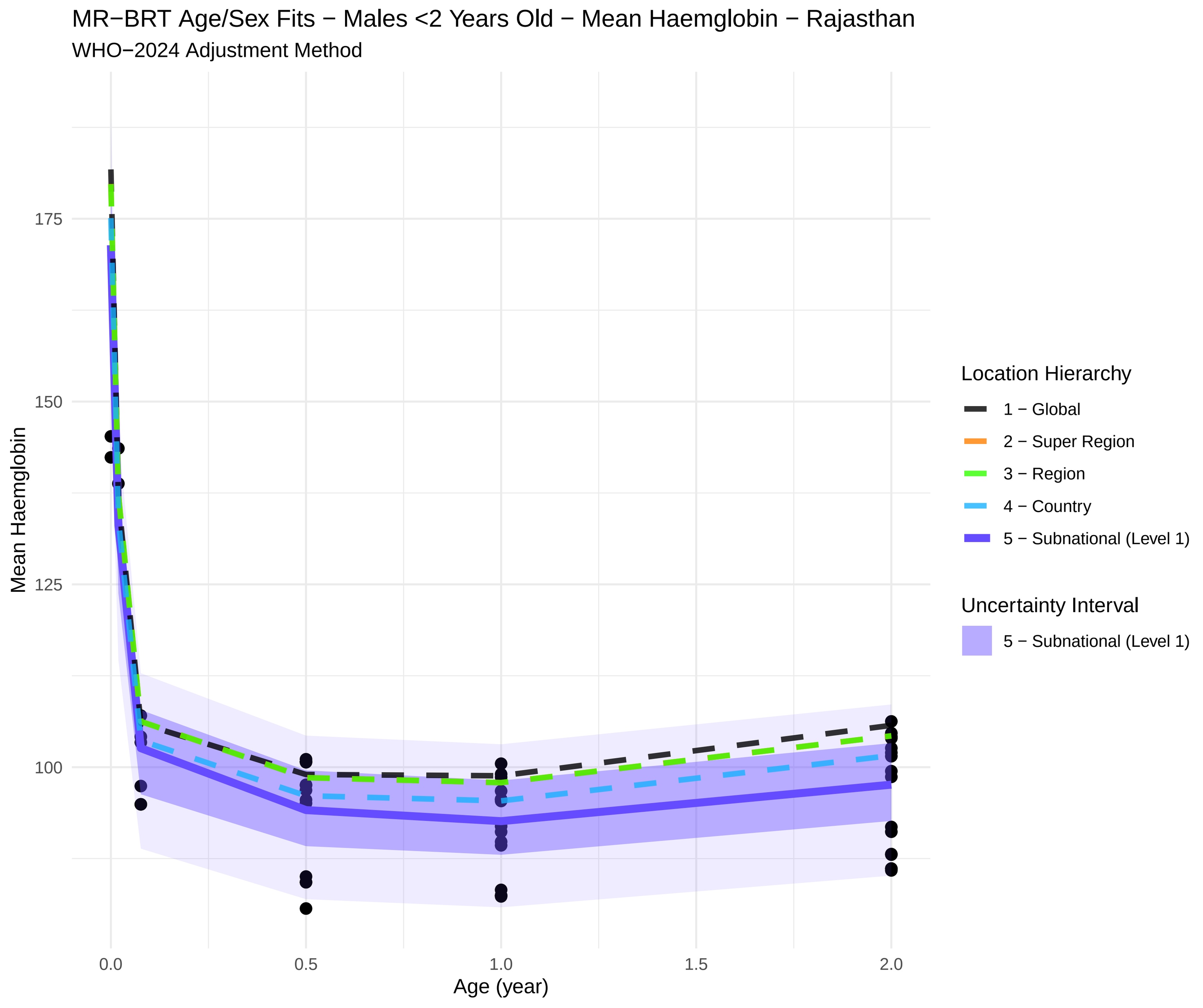
MR−BRT Age/Sex Fits − Males <2 Years Old − Mean Haemglobin − Rajasthan
WHO−2024 Adjustment Method
Mean Haemglobin
Age (year)
Location Hierarchy
1 − Global
2 − Super Region
3 − Region
4 − Country
5 − Subnational (Level 1)
Uncertainty Interval
5 − Subnational (Level 1)

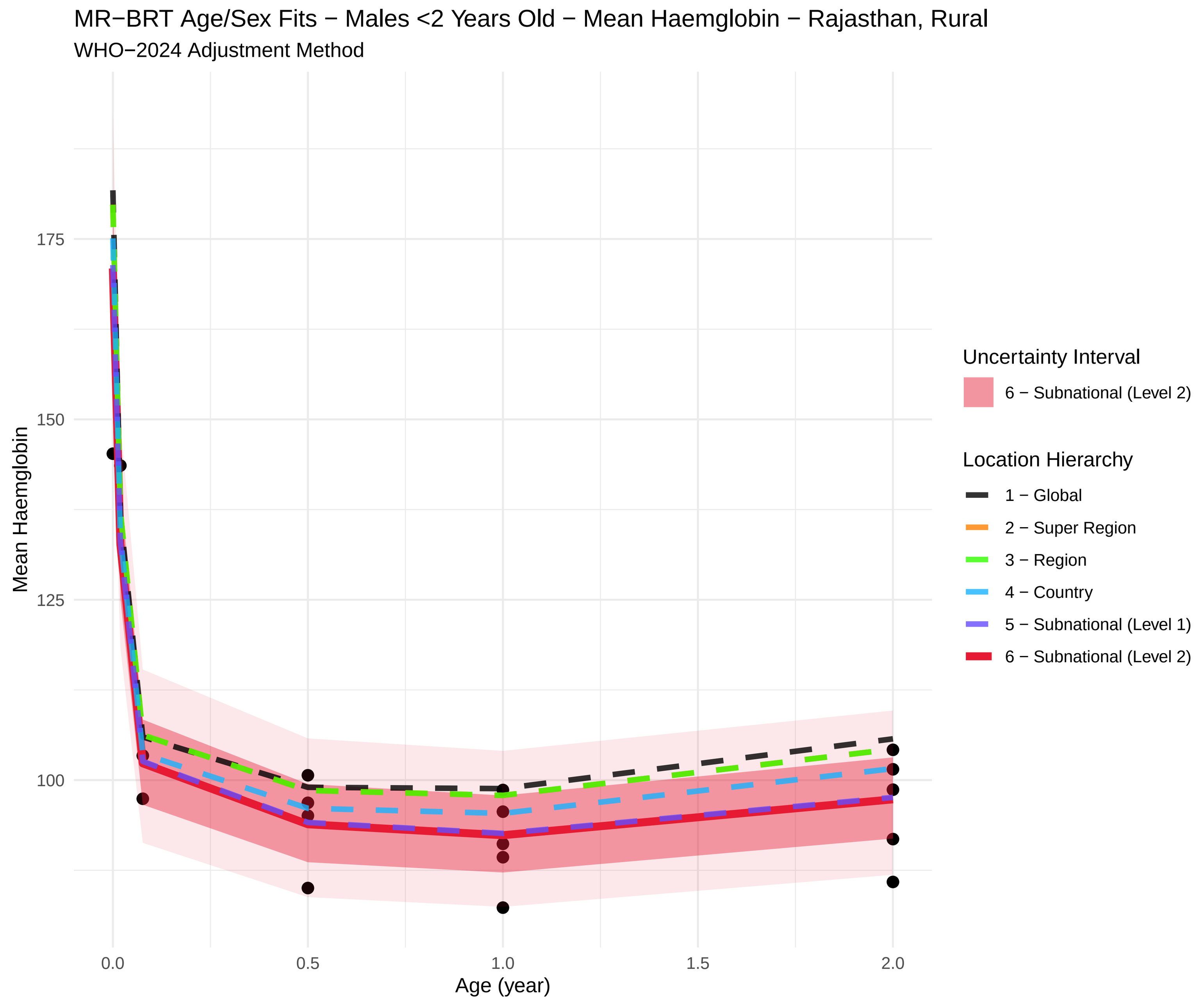
MR−BRT Age/Sex Fits − Males <2 Years Old − Mean Haemglobin − Rajasthan, Rural
WHO−2024 Adjustment Method
Mean Haemglobin
Age (year)
Uncertainty Interval
6 − Subnational (Level 2)
Location Hierarchy
1 − Global
2 − Super Region
3 − Region
4 − Country
5 − Subnational (Level 1)
6 − Subnational (Level 2)

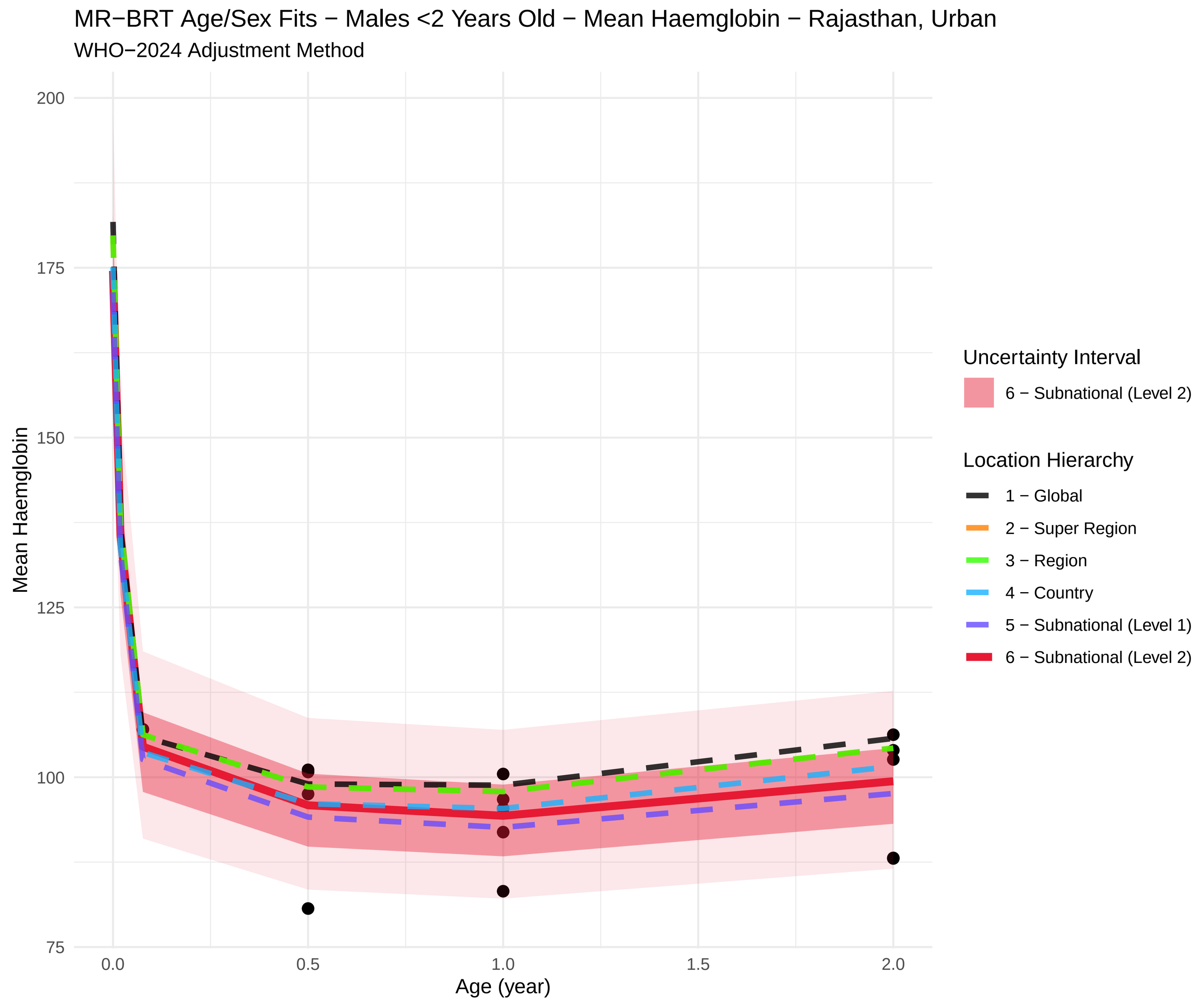
MR−BRT Age/Sex Fits − Males <2 Years Old − Mean Haemglobin − Rajasthan, Urban
WHO−2024 Adjustment Method
Mean Haemglobin
Age (year)
Uncertainty Interval
6 − Subnational (Level 2)
Location Hierarchy
1 − Global
2 − Super Region
3 − Region
4 − Country
5 − Subnational (Level 1)
6 − Subnational (Level 2)

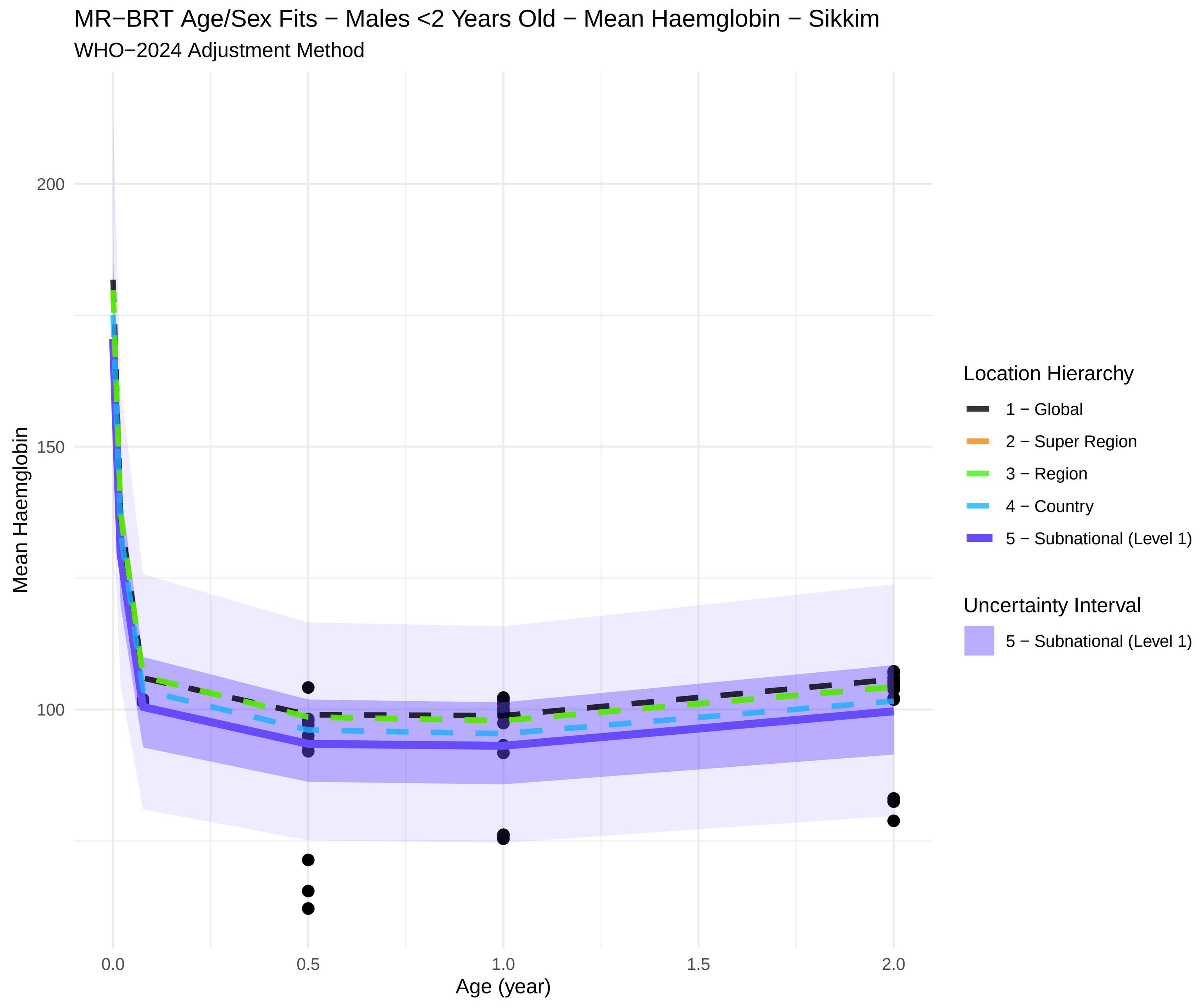
MR−BRT Age/Sex Fits − Males <2 Years Old − Mean Haemglobin − Sikkim
WHO−2024 Adjustment Method
Mean Haemglobin
Age (year)
Location Hierarchy
1 − Global
2 − Super Region
3 − Region
4 − Country
5 − Subnational (Level 1)
Uncertainty Interval
5 − Subnational (Level 1)

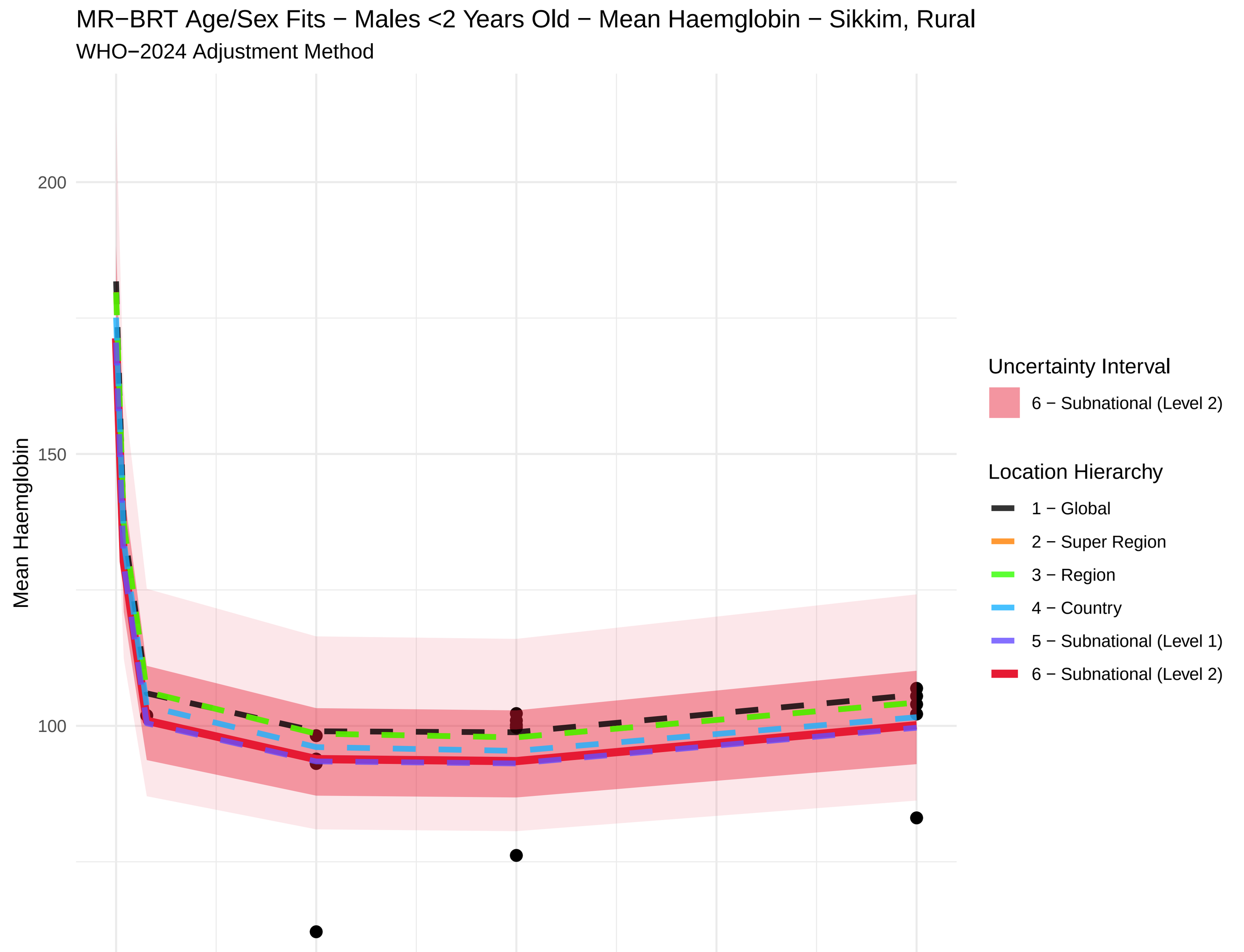
MR−BRT Age/Sex Fits − Males <2 Years Old − Mean Haemglobin − Sikkim, Rural
WHO−2024 Adjustment Method
Mean Haemglobin
Age (year)
Uncertainty Interval
6 − Subnational (Level 2)
Location Hierarchy
1 − Global
2 − Super Region
3 − Region
4 − Country
5 − Subnational (Level 1)
6 − Subnational (Level 2)

MR−BRT Age/Sex Fits − Males <2 Years Old − Mean Haemglobin − Sikkim, Urban
WHO−2024 Adjustment Method

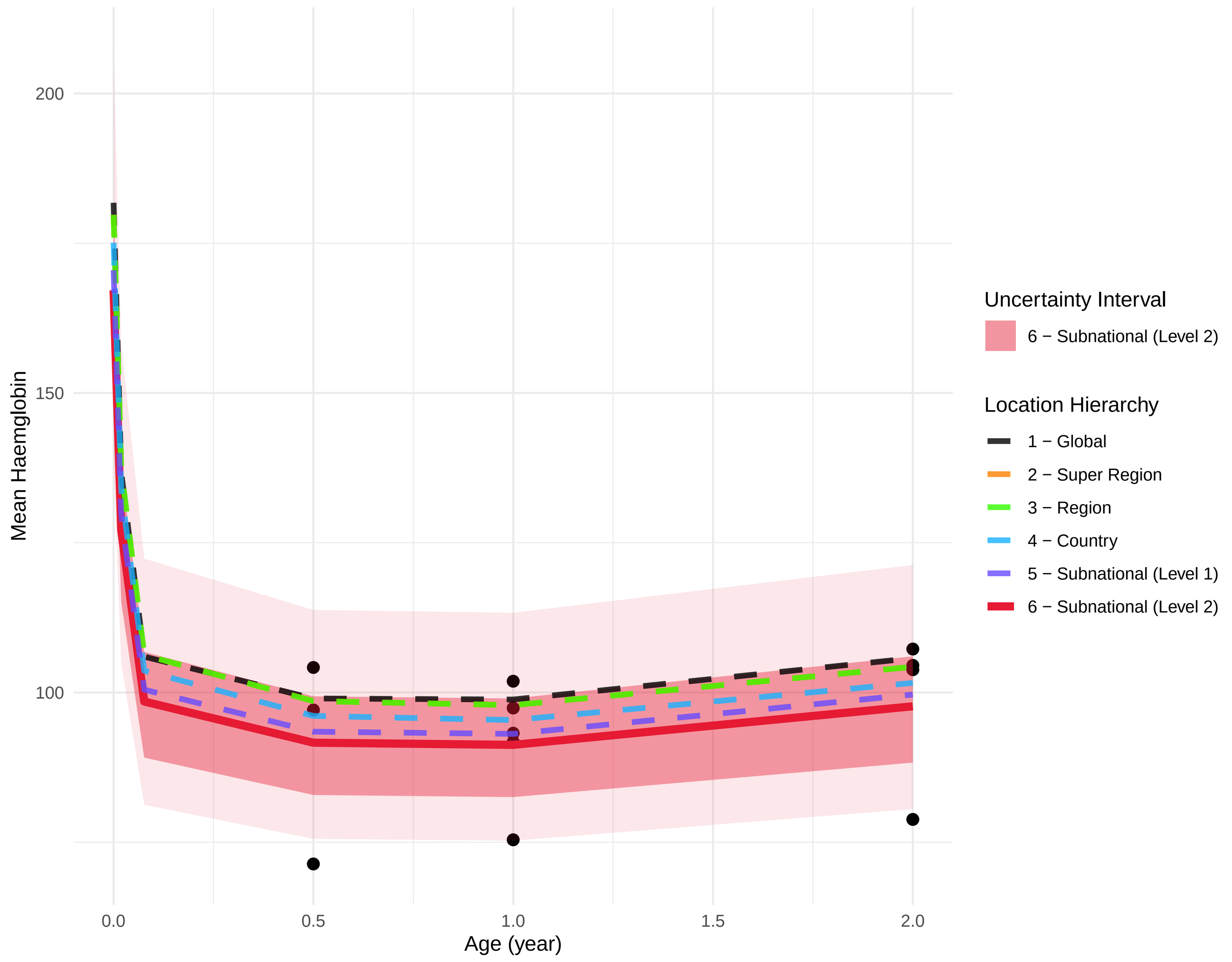

MR−BRT Age/Sex Fits − Males <2 Years Old − Mean Haemglobin − Tamil Nadu
WHO−2024 Adjustment Method

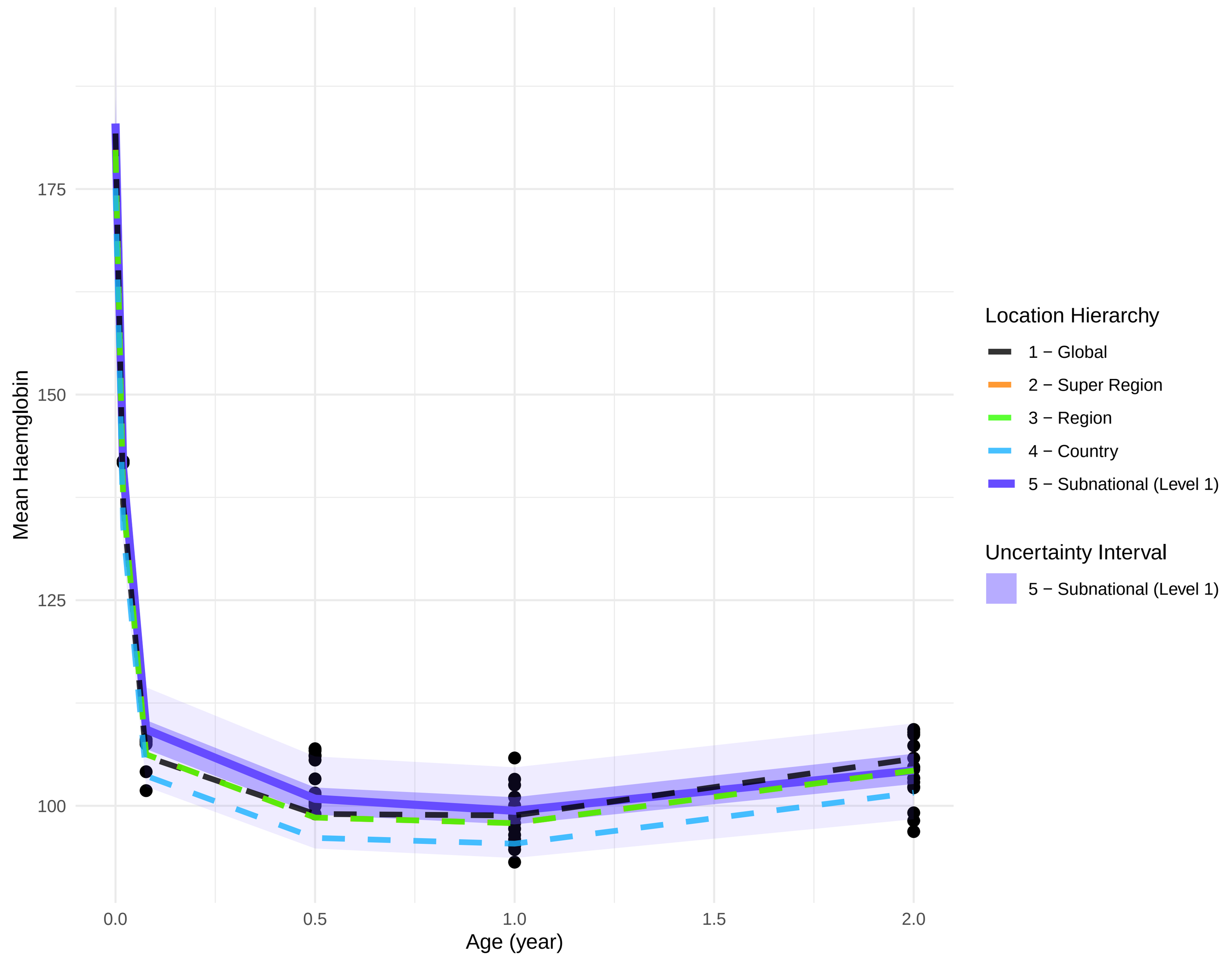

MR−BRT Age/Sex Fits − Males <2 Years Old − Mean Haemglobin − Tamil Nadu, Rural
WHO−2024 Adjustment Method

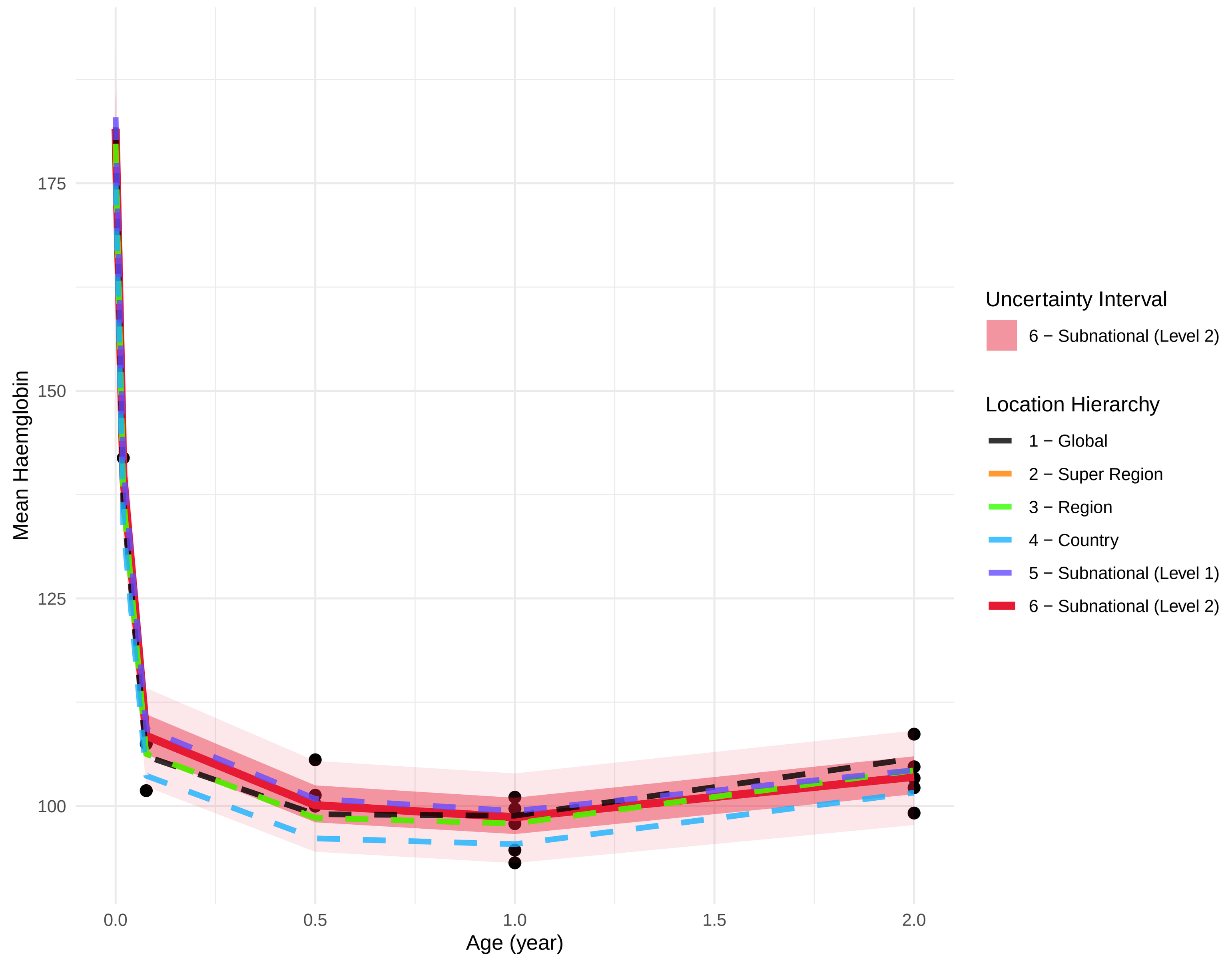

MR−BRT Age/Sex Fits − Males <2 Years Old − Mean Haemglobin − Tamil Nadu, Urban
WHO−2024 Adjustment Method

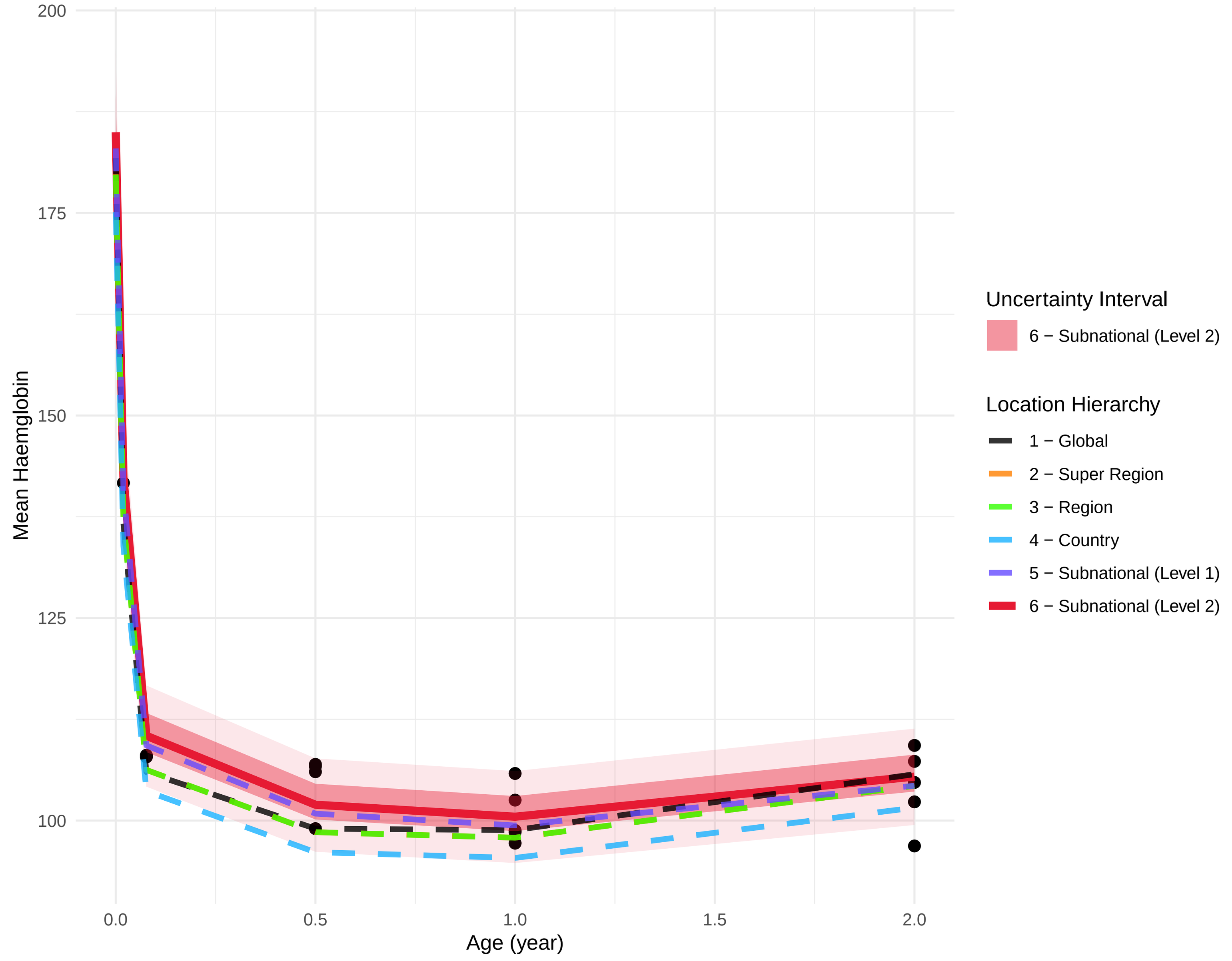

MR−BRT Age/Sex Fits − Males <2 Years Old − Mean Haemglobin − Telangana
WHO−2024 Adjustment Method

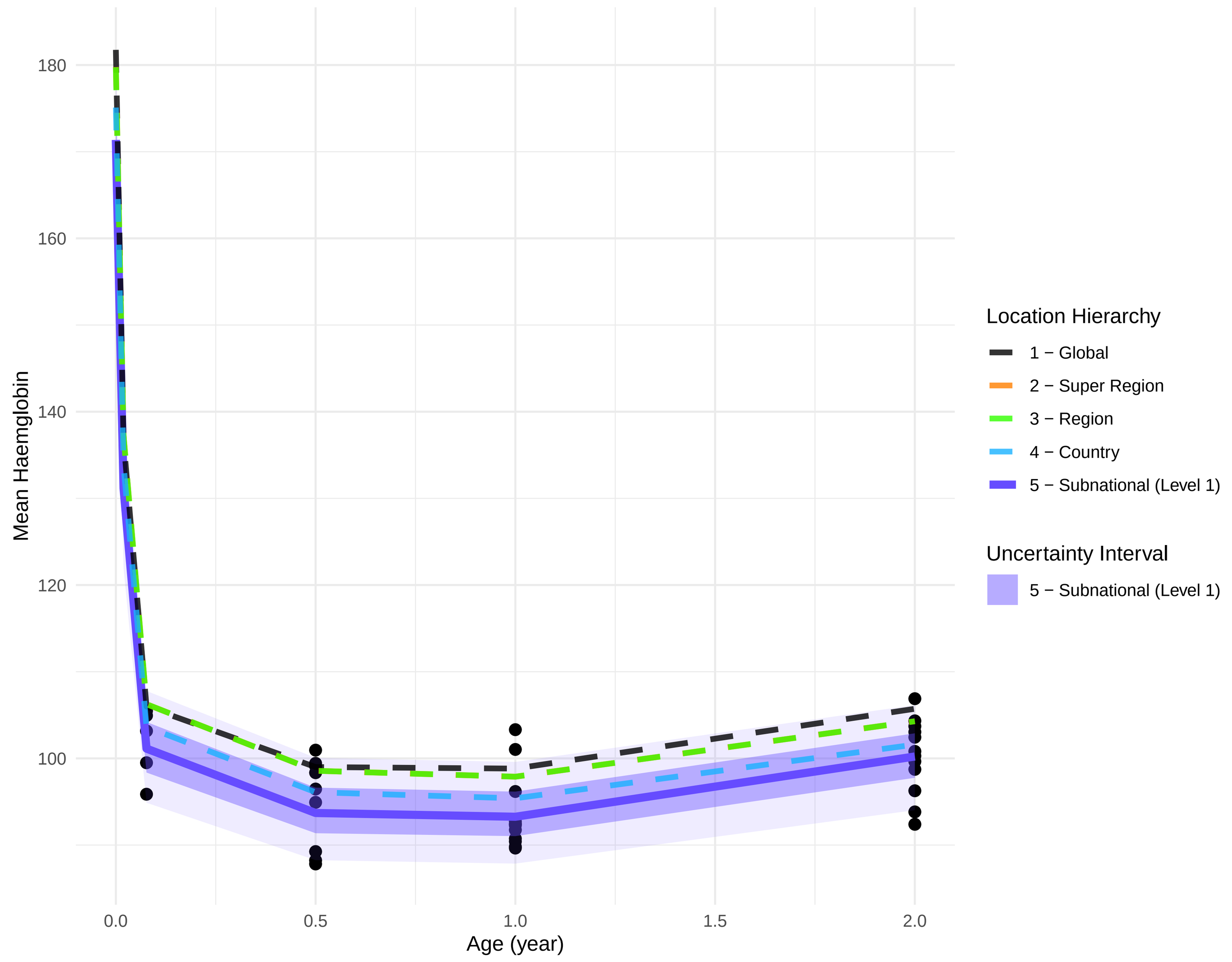

MR−BRT Age/Sex Fits − Males <2 Years Old − Mean Haemglobin − Telangana, Rural
WHO−2024 Adjustment Method

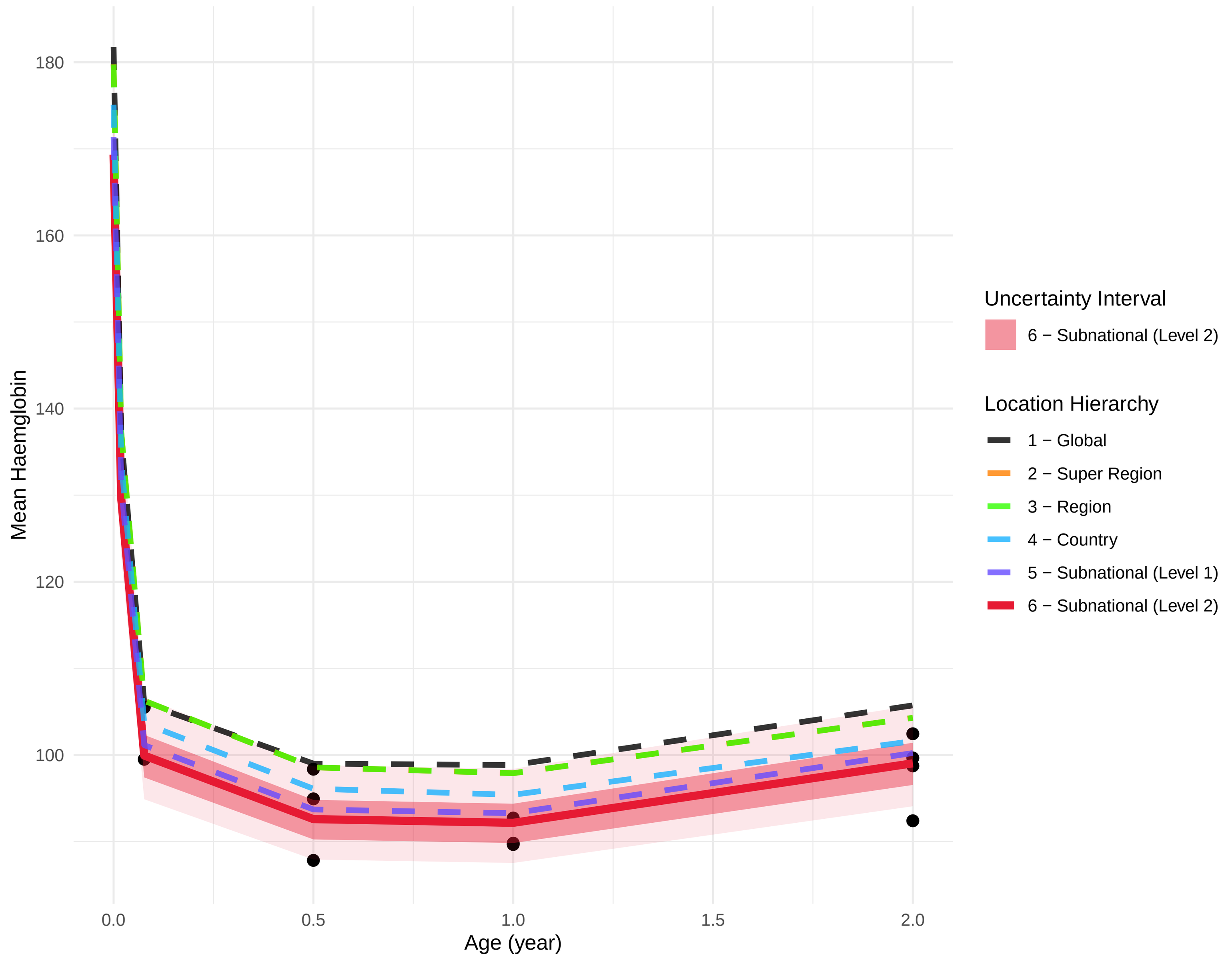

MR−BRT Age/Sex Fits − Males <2 Years Old − Mean Haemglobin − Telangana, Urban
WHO−2024 Adjustment Method

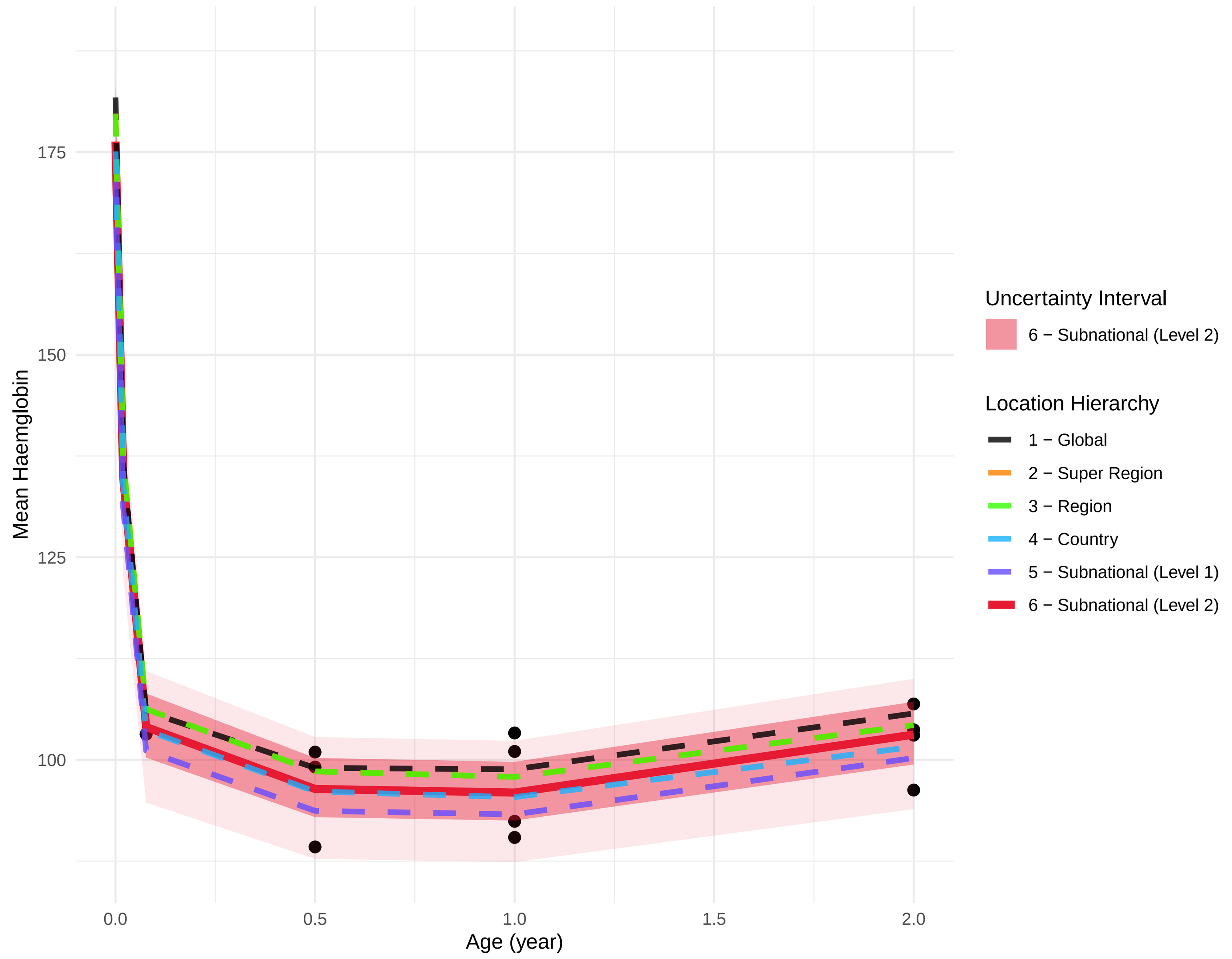

MR−BRT Age/Sex Fits − Males <2 Years Old − Mean Haemglobin − Tripura
WHO−2024 Adjustment Method

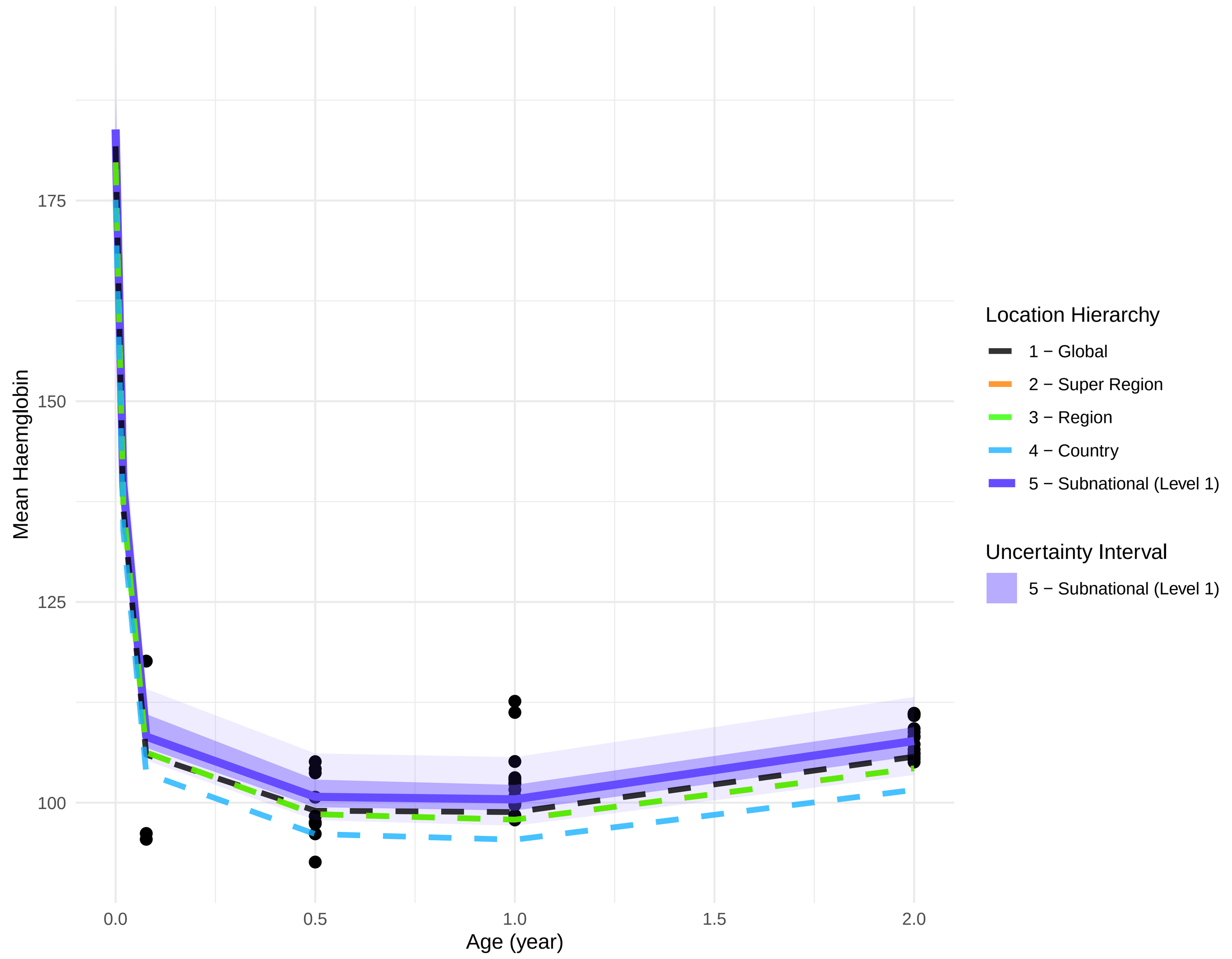

MR−BRT Age/Sex Fits − Males <2 Years Old − Mean Haemglobin − Tripura, Rural
WHO−2024 Adjustment Method

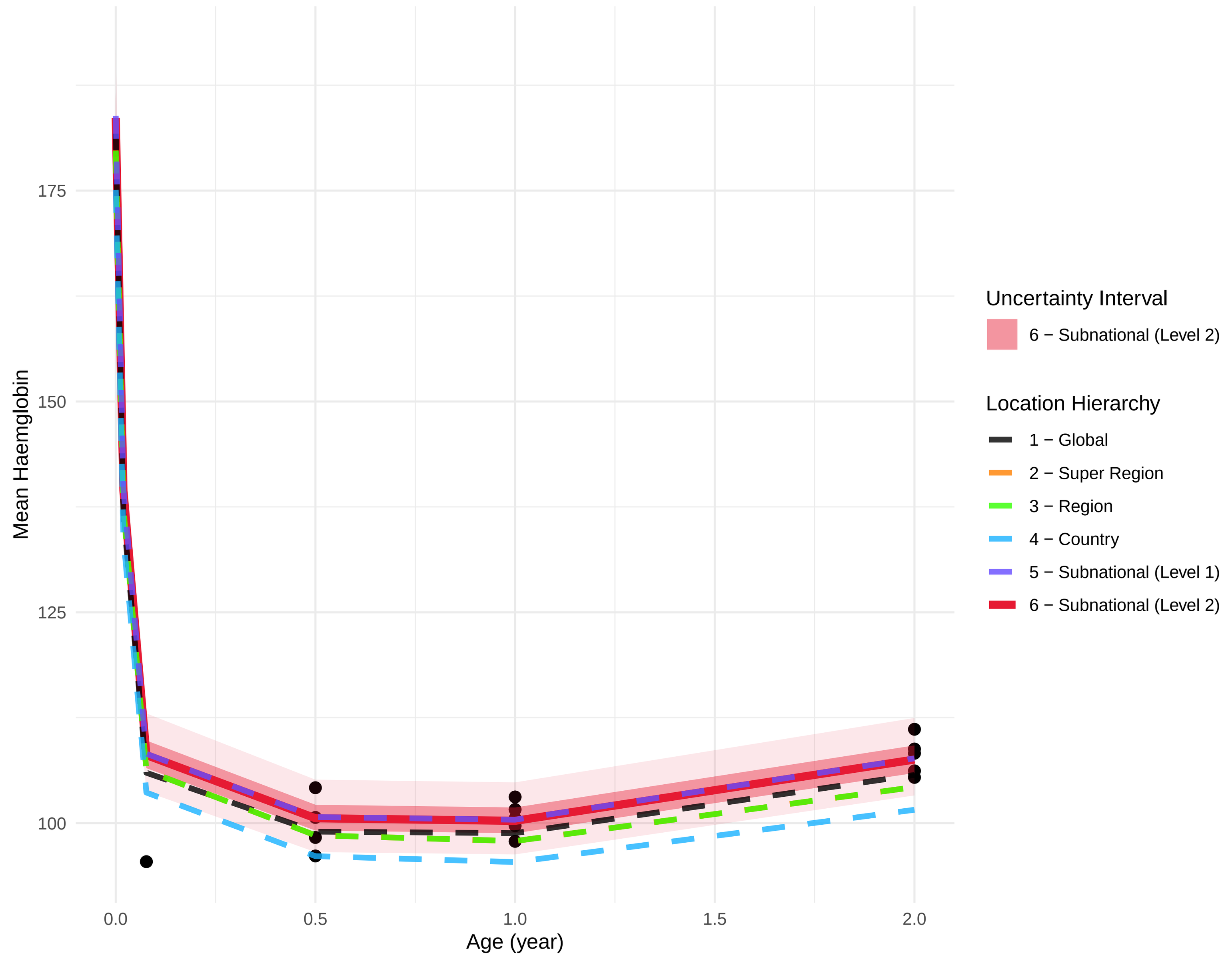

MR−BRT Age/Sex Fits − Males <2 Years Old − Mean Haemglobin − Tripura, Urban
WHO−2024 Adjustment Method

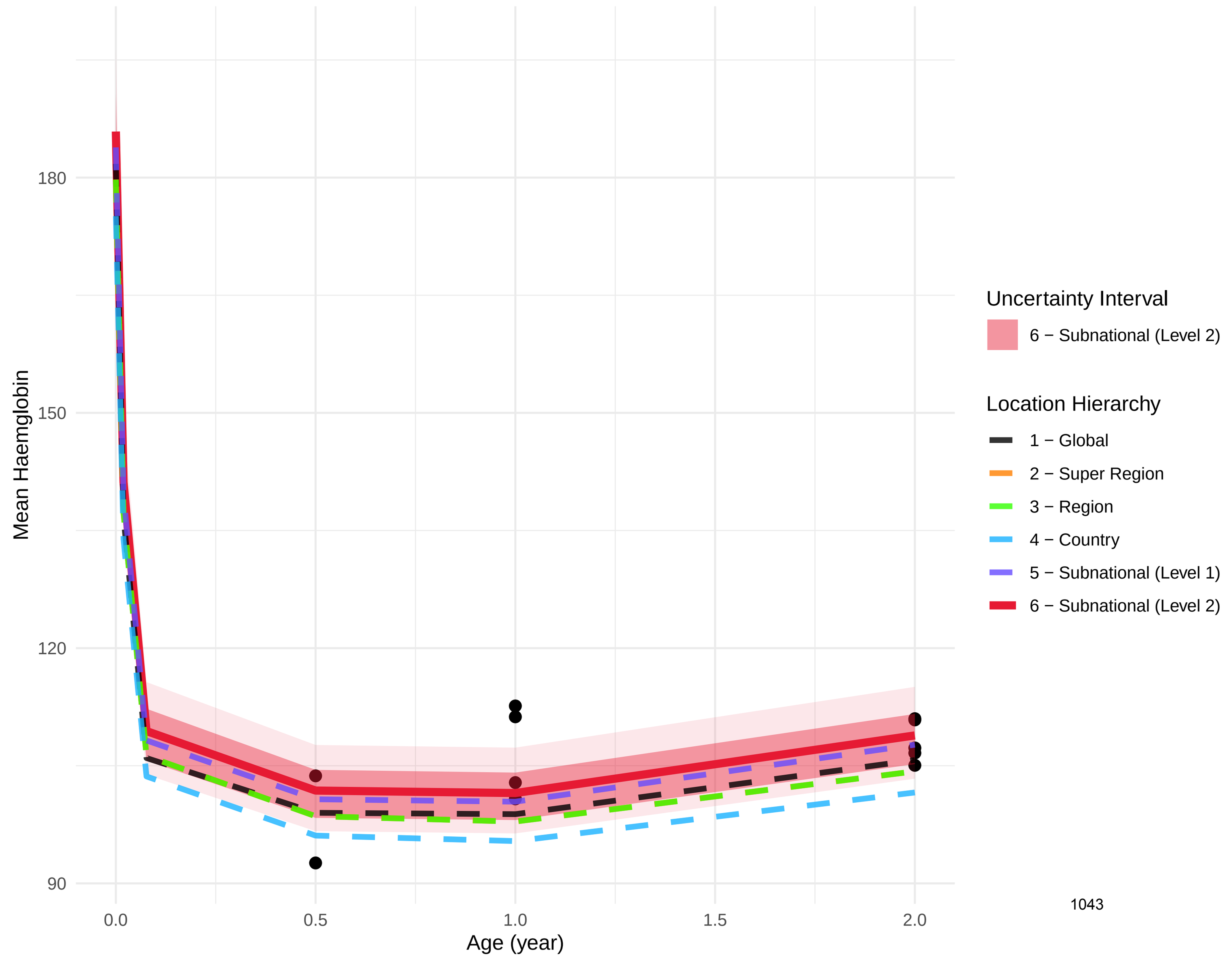

MR−BRT Age/Sex Fits − Males <2 Years Old − Mean Haemglobin − Uttar Pradesh

WHO−2024 Adjustment Method

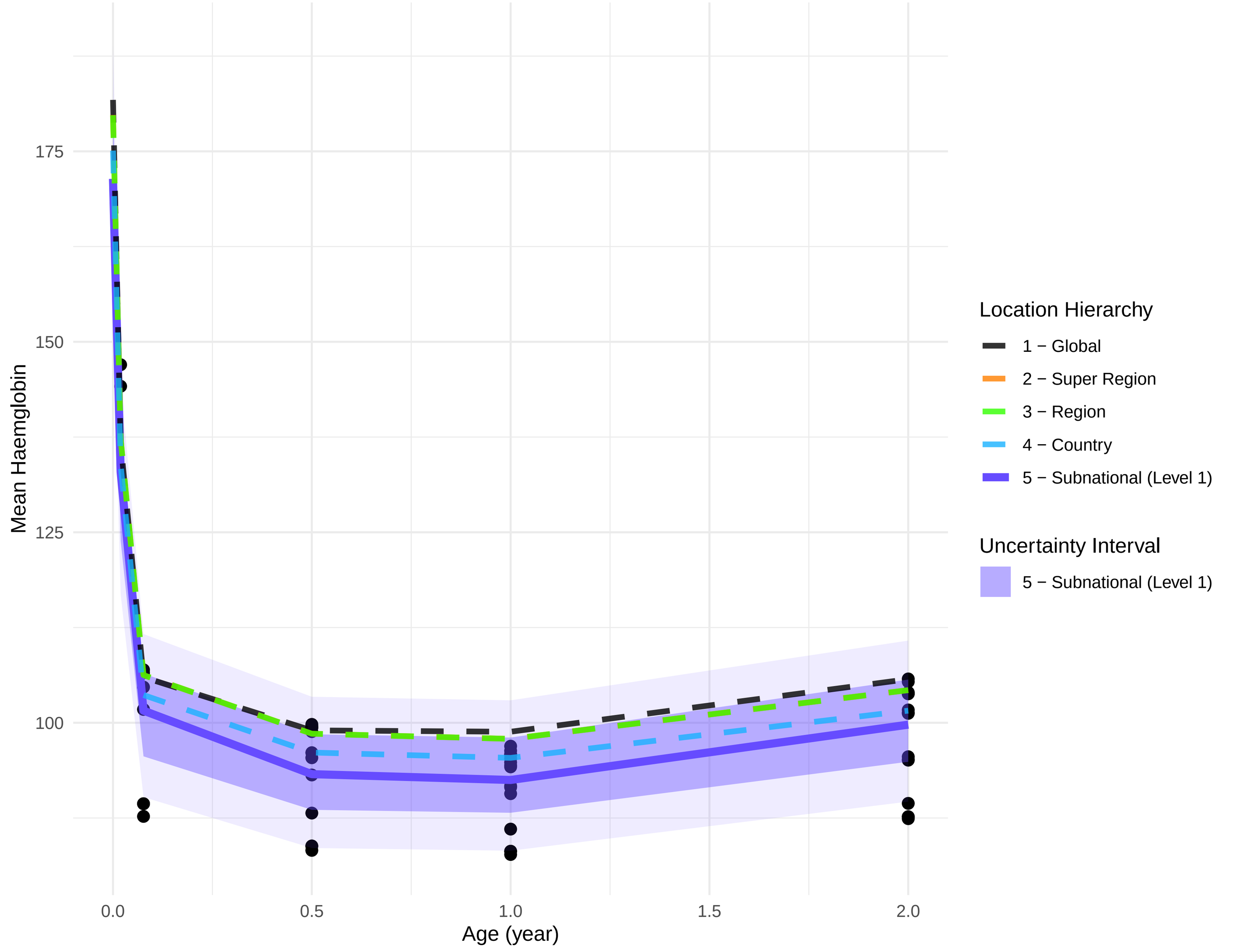

MR−BRT Age/Sex Fits − Males <2 Years Old − Mean Haemglobin − Uttar Pradesh, Rural

WHO−2024 Adjustment Method

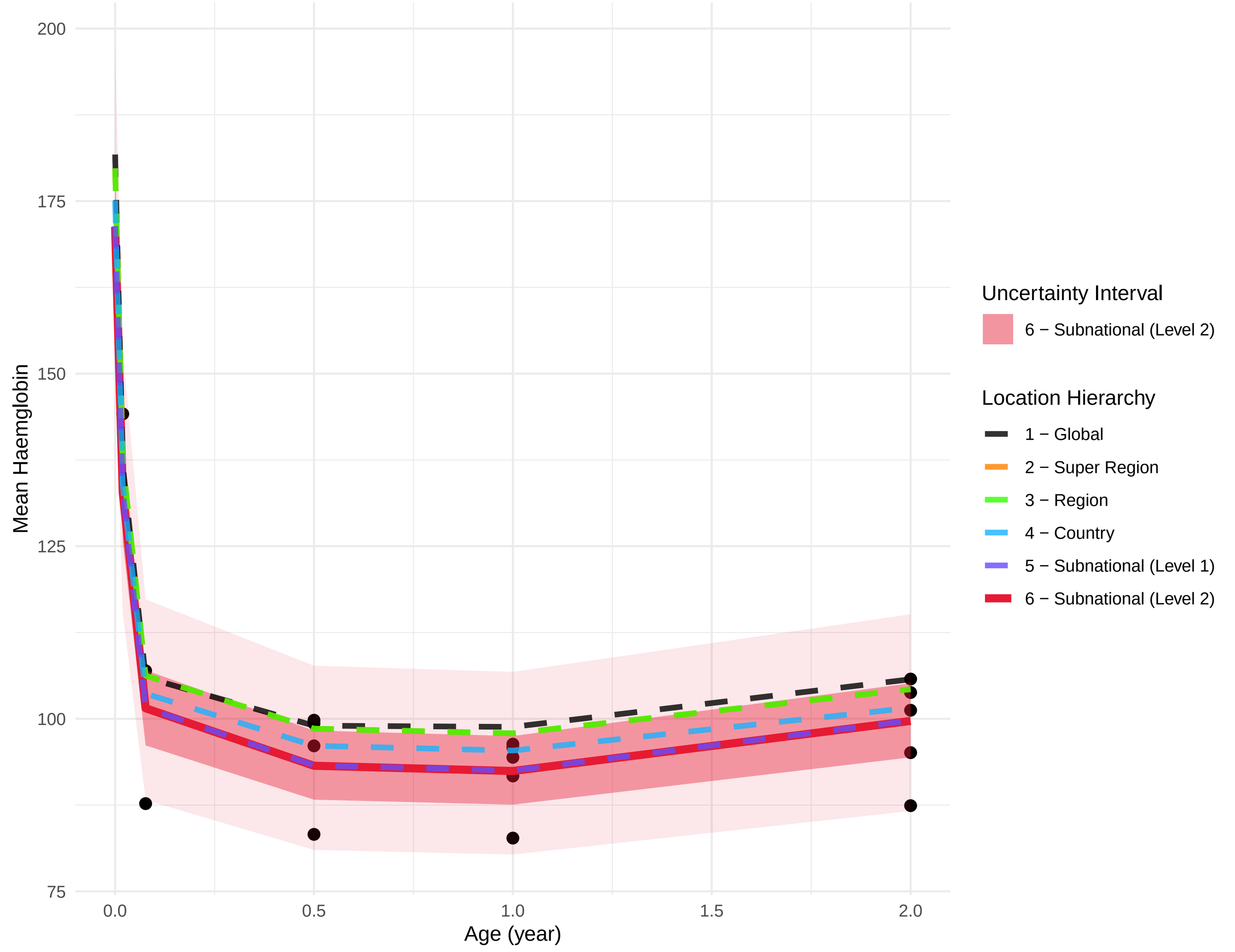

MR−BRT Age/Sex Fits − Males <2 Years Old − Mean Haemglobin − Uttar Pradesh, Urban

WHO−2024 Adjustment Method

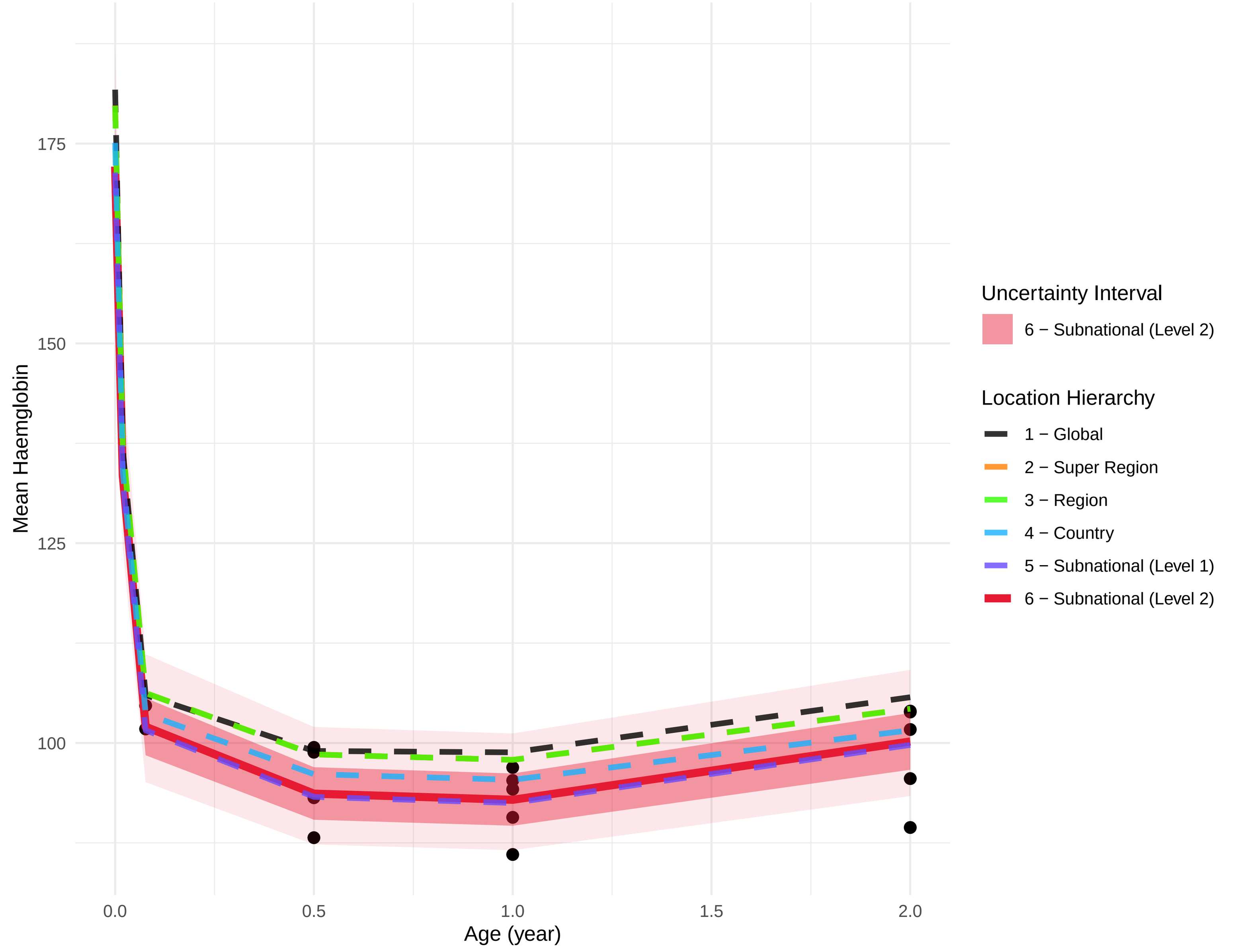

MR−BRT Age/Sex Fits − Males <2 Years Old − Mean Haemglobin − Uttarakhand

WHO−2024 Adjustment Method

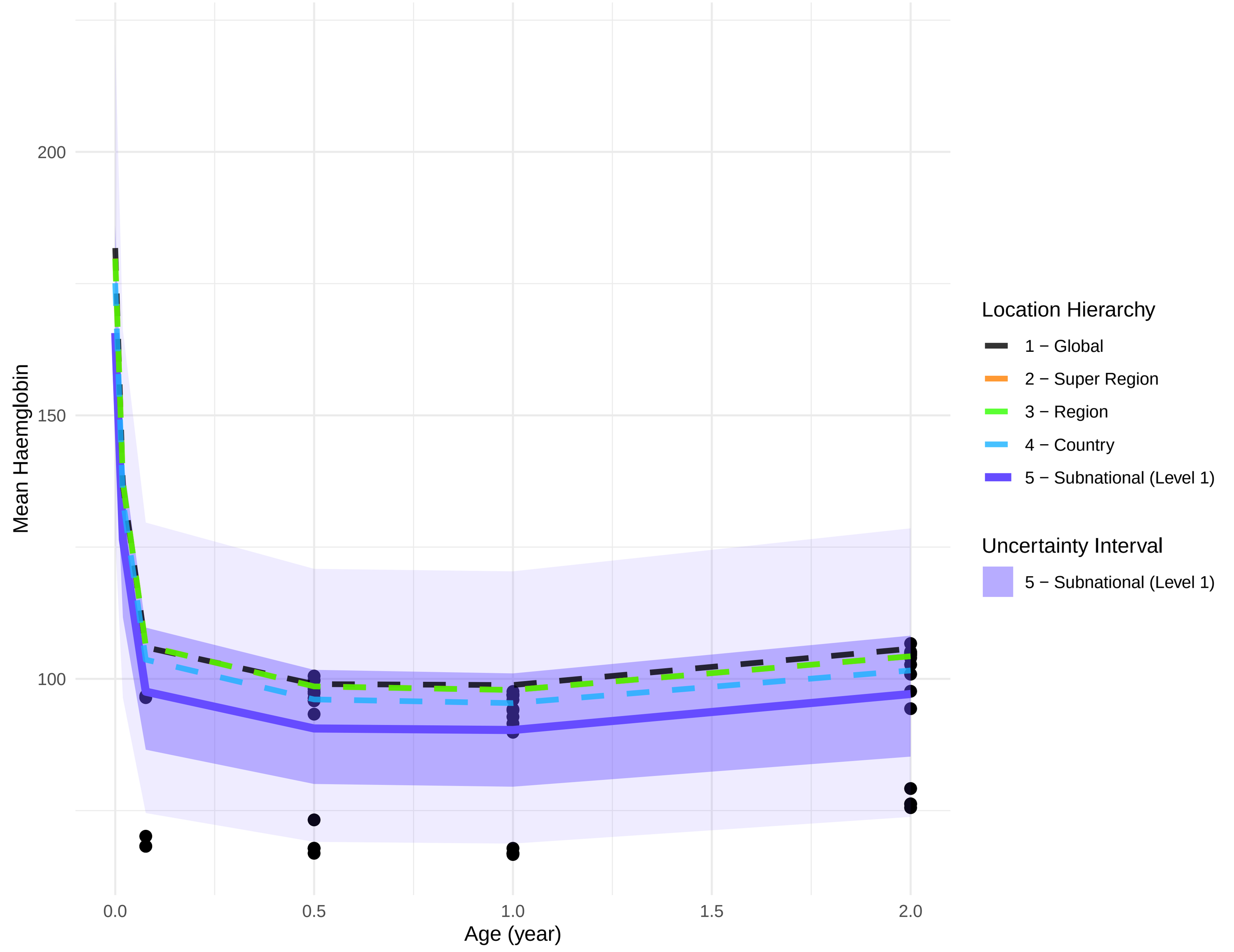

MR−BRT Age/Sex Fits − Males <2 Years Old − Mean Haemglobin − Uttarakhand, Rural

WHO−2024 Adjustment Method

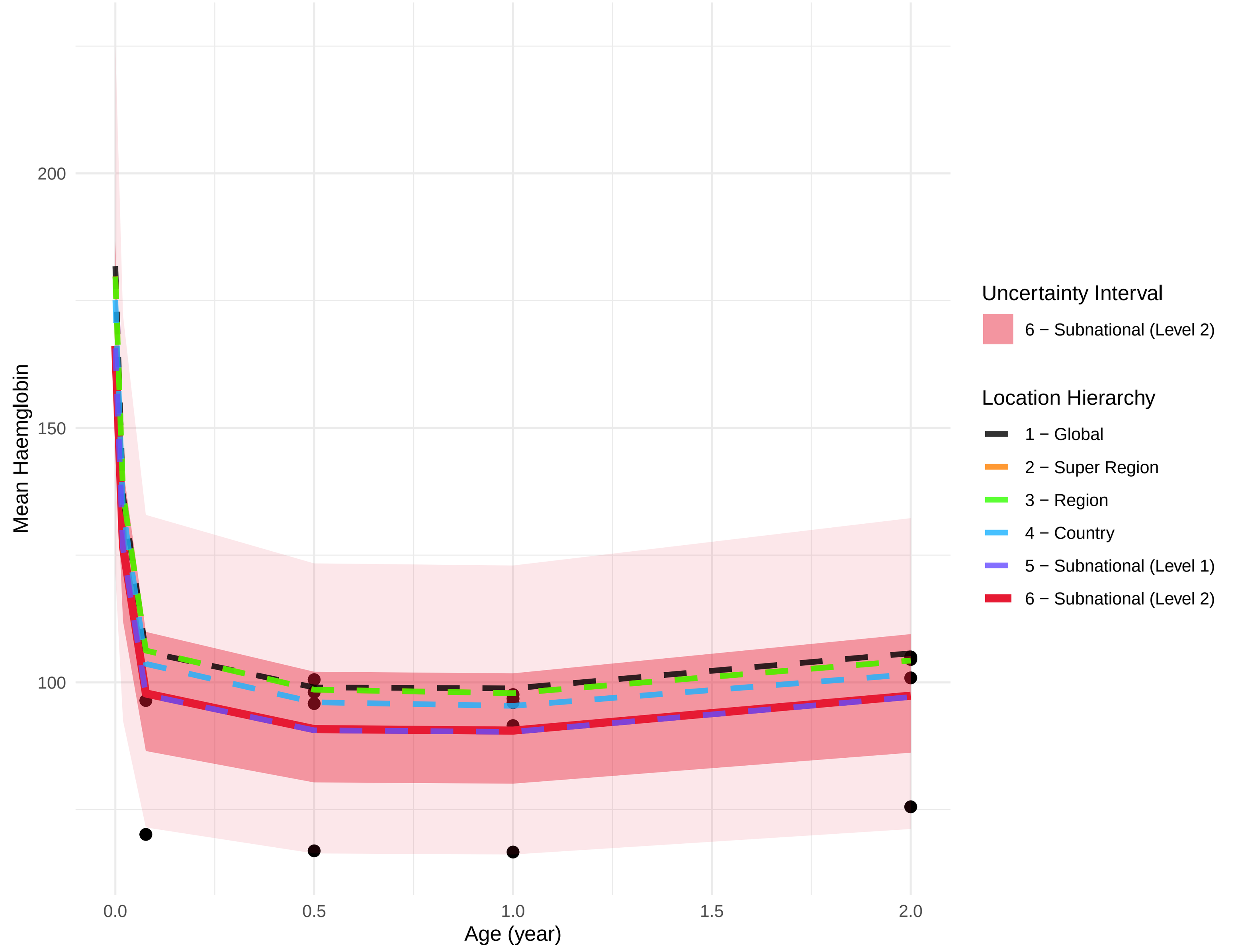

MR−BRT Age/Sex Fits − Males <2 Years Old − Mean Haemglobin − Uttarakhand, Urban

WHO−2024 Adjustment Method

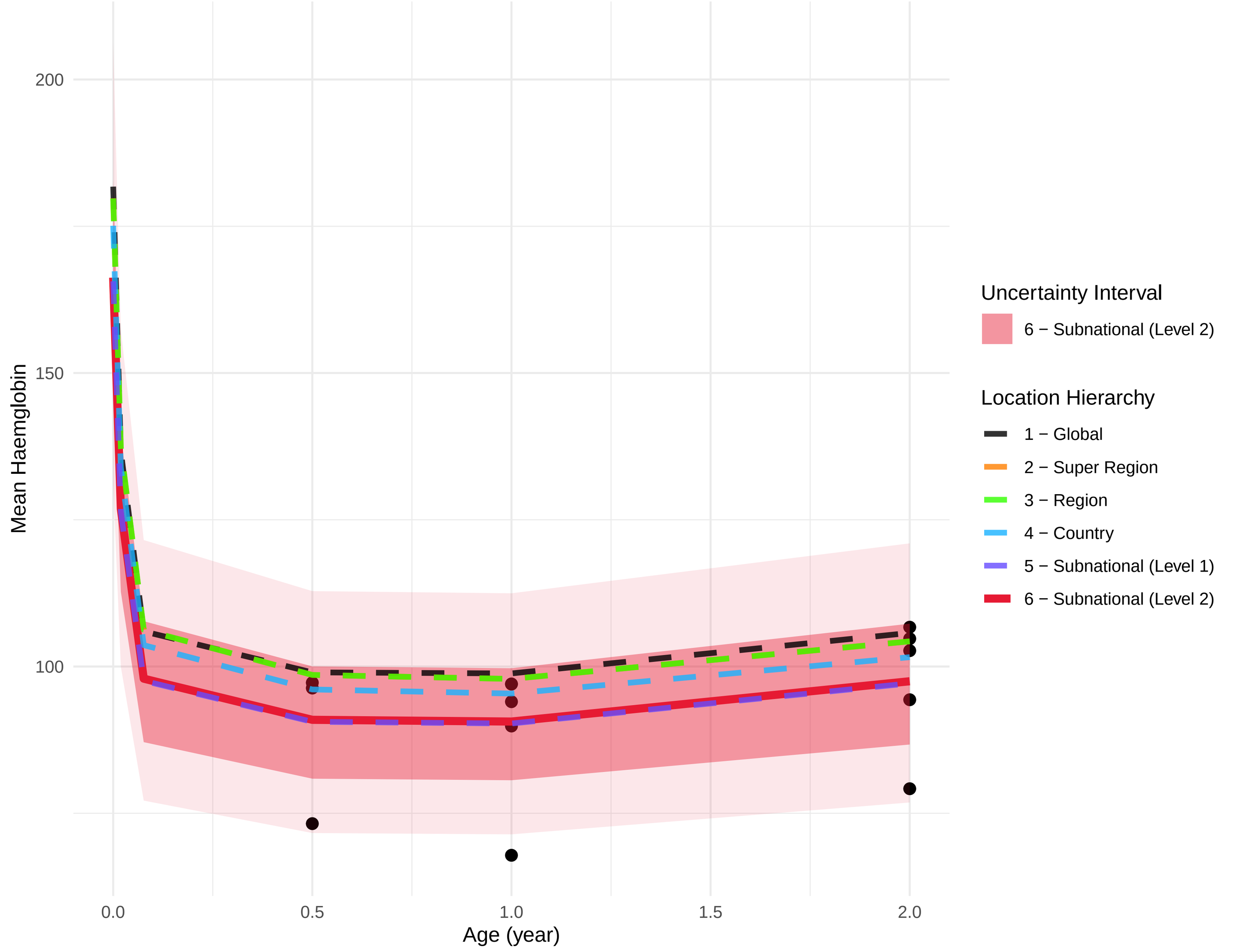

MR−BRT Age/Sex Fits − Males <2 Years Old − Mean Haemglobin − West Bengal

WHO−2024 Adjustment Method

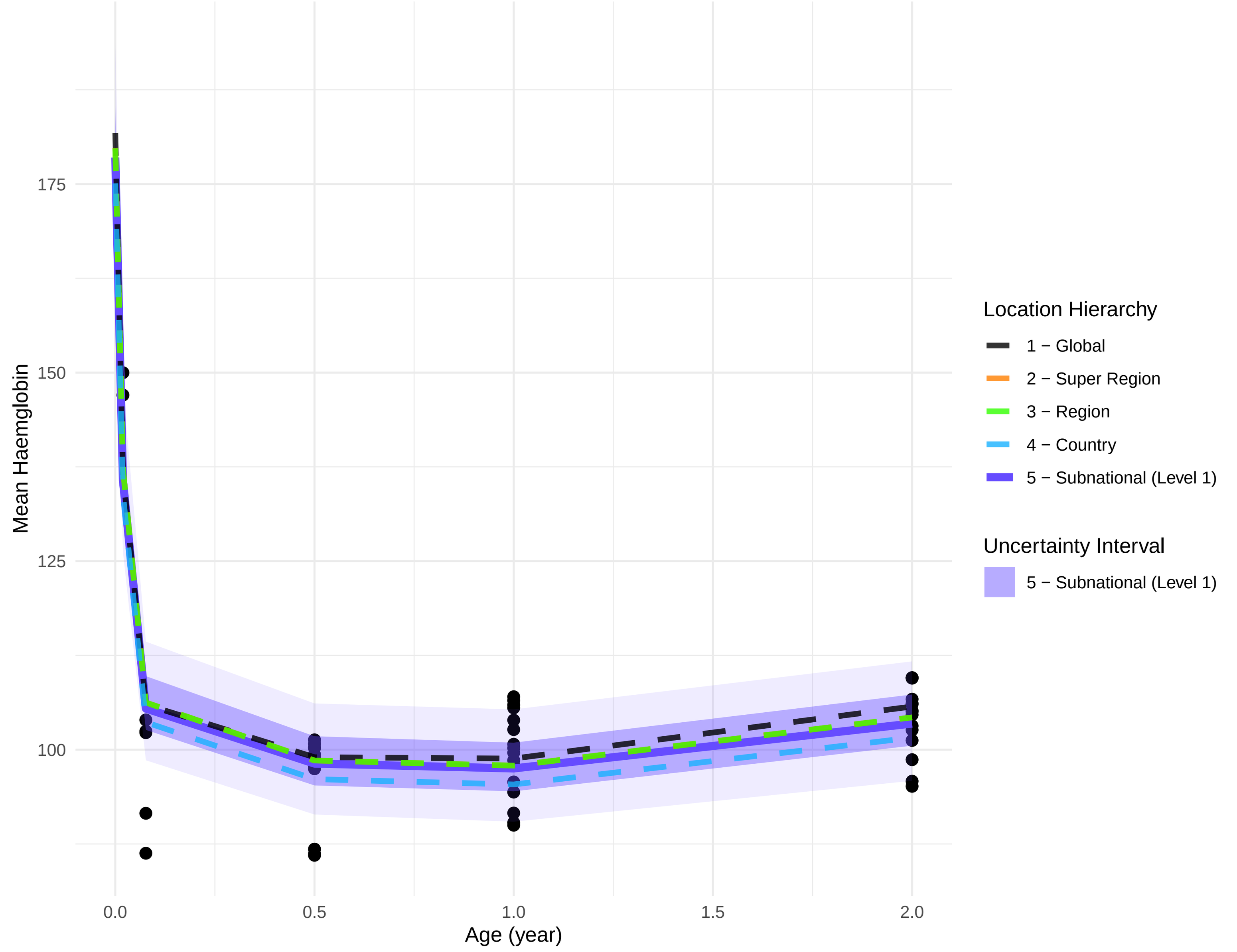

MR−BRT Age/Sex Fits − Males <2 Years Old − Mean Haemglobin − West Bengal, Rural

WHO−2024 Adjustment Method

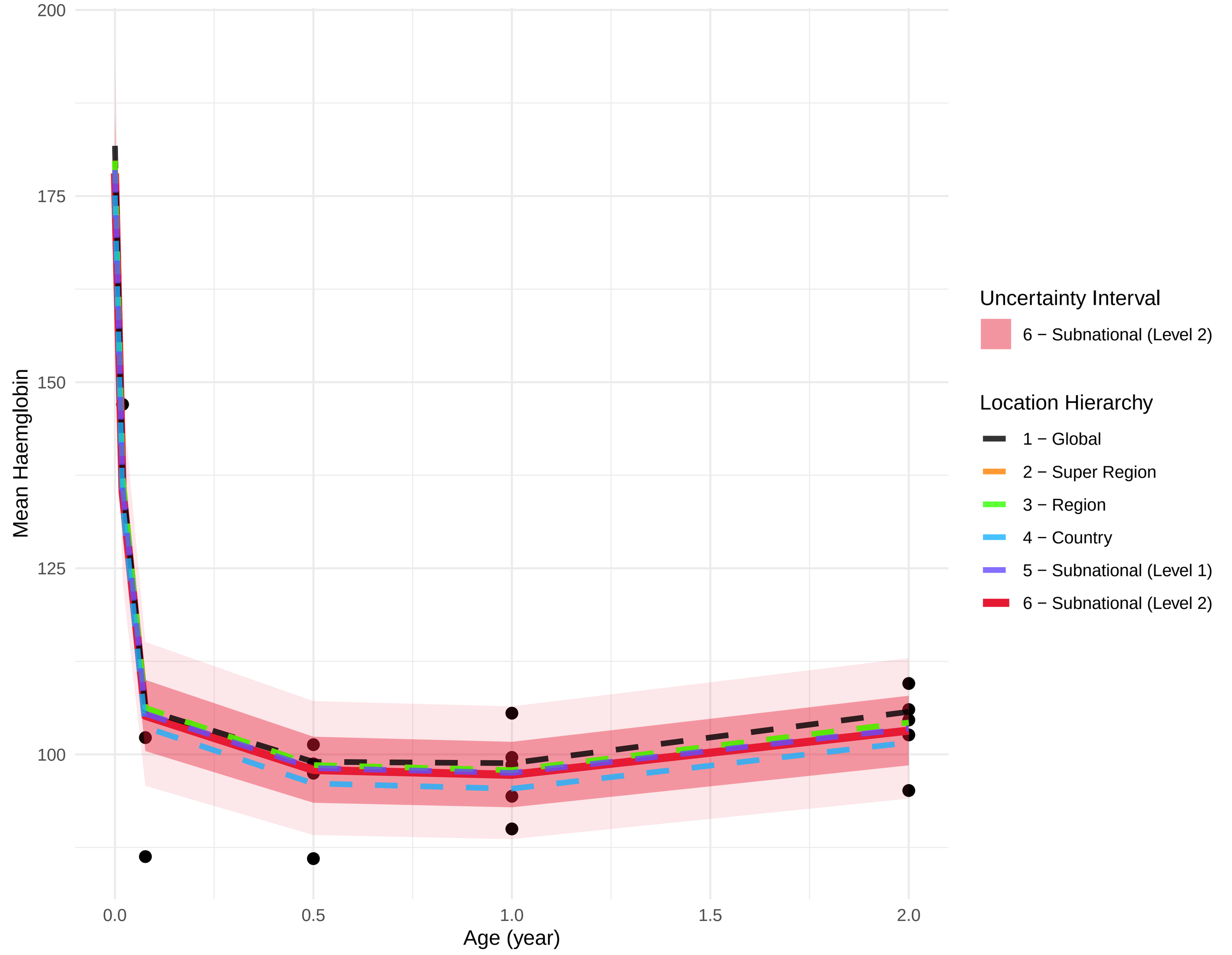

MR−BRT Age/Sex Fits − Males <2 Years Old − Mean Haemglobin − West Bengal, Urban

WHO−2024 Adjustment Method

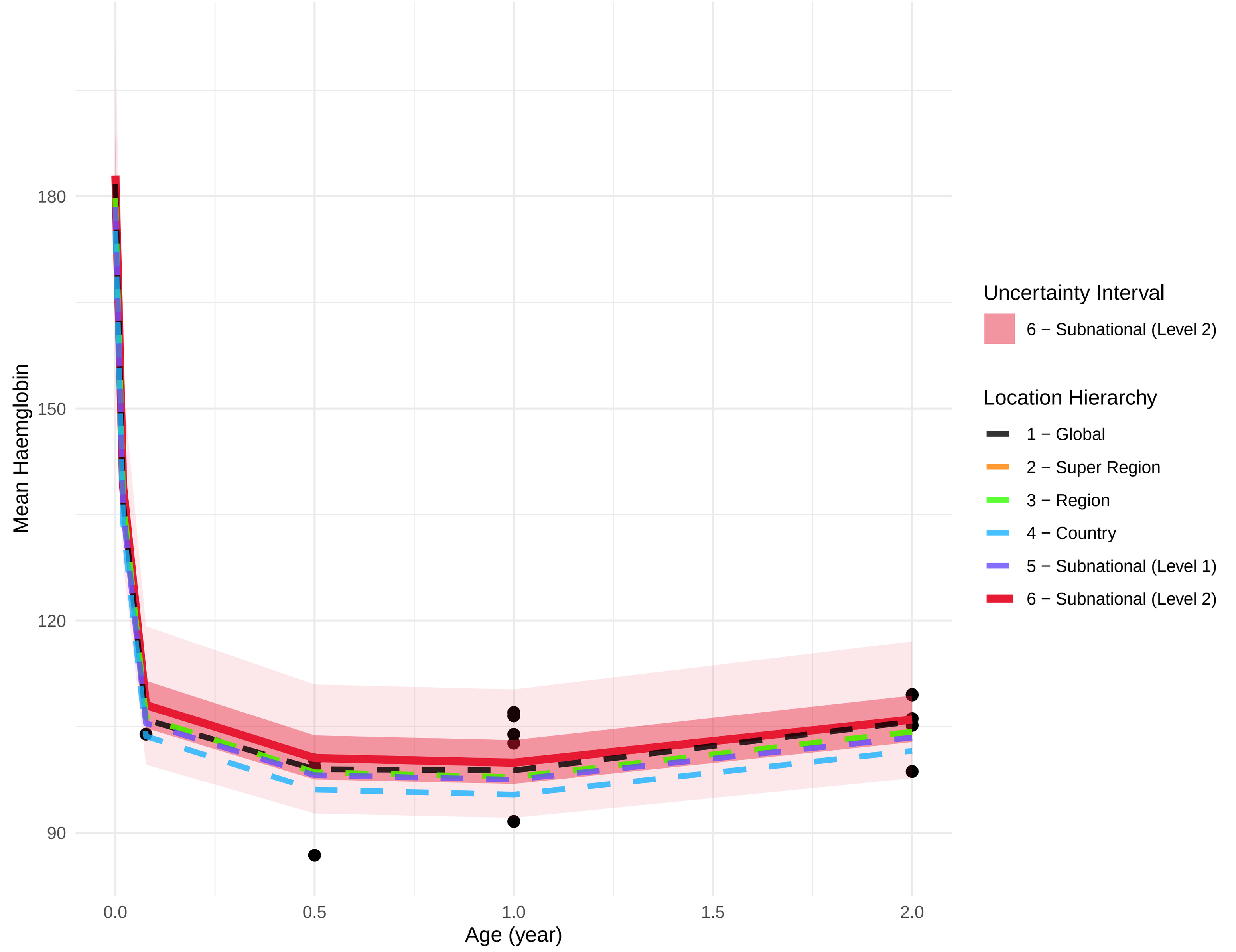

MR−BRT Age/Sex Fits − Males <2 Years Old − Mean Haemglobin − Nepal

WHO−2024 Adjustment Method

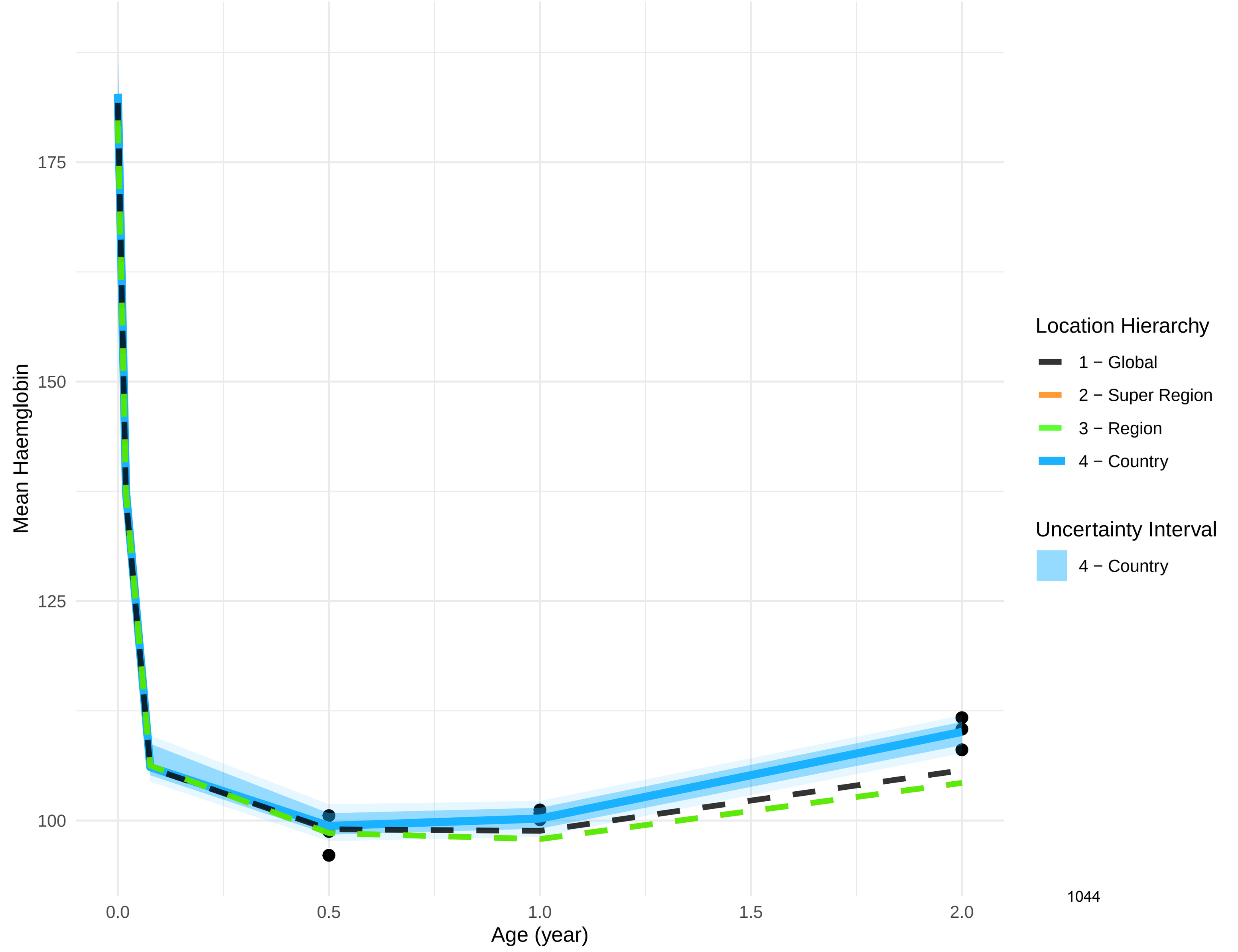

MR−BRT Age/Sex Fits − Males <2 Years Old − Mean Haemglobin − Southeast Asia, East Asia, and Oceania
WHO−2024 Adjustment Method

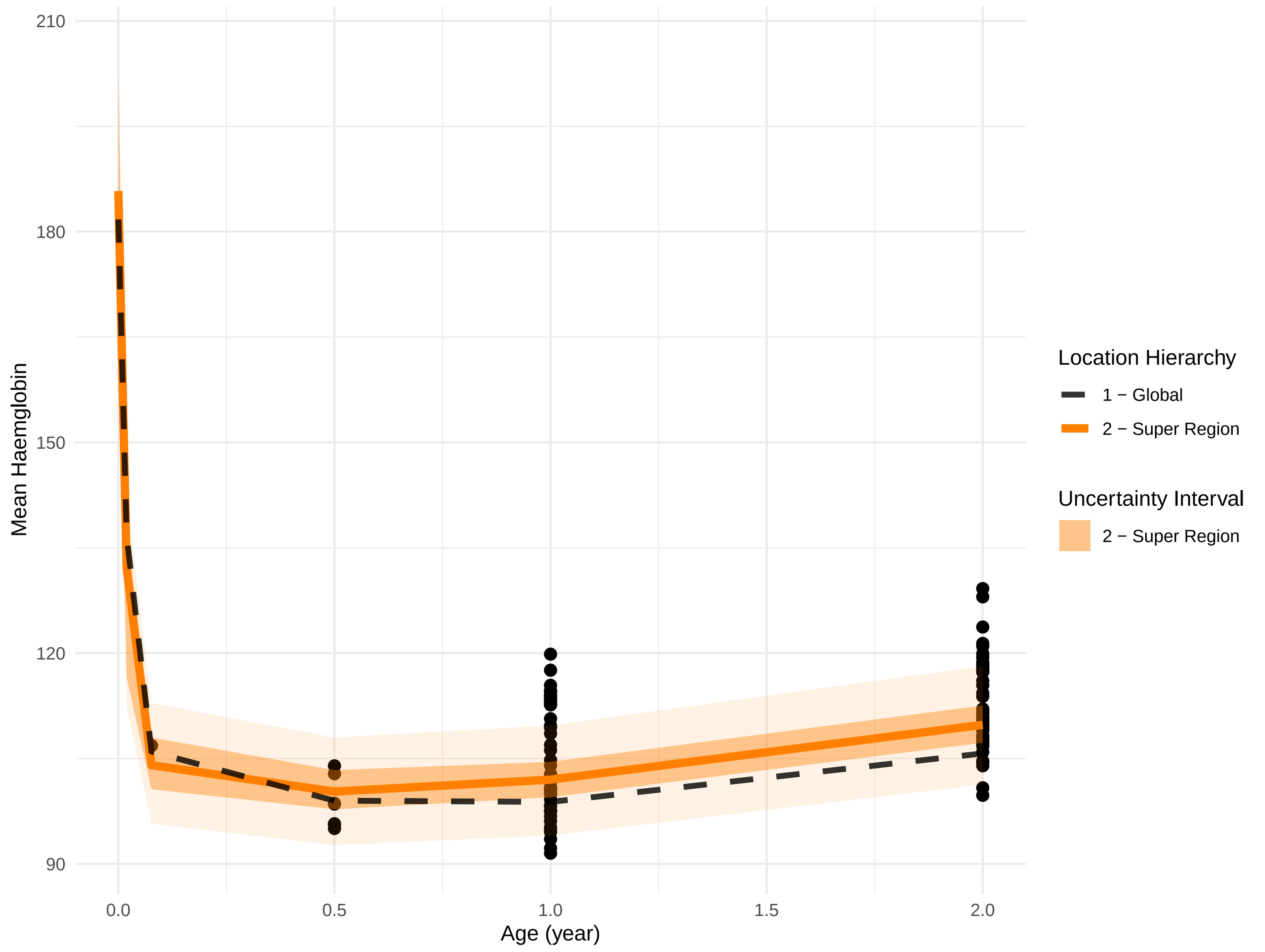

MR−BRT Age/Sex Fits − Males <2 Years Old − Mean Haemglobin − Southeast Asia
WHO−2024 Adjustment Method

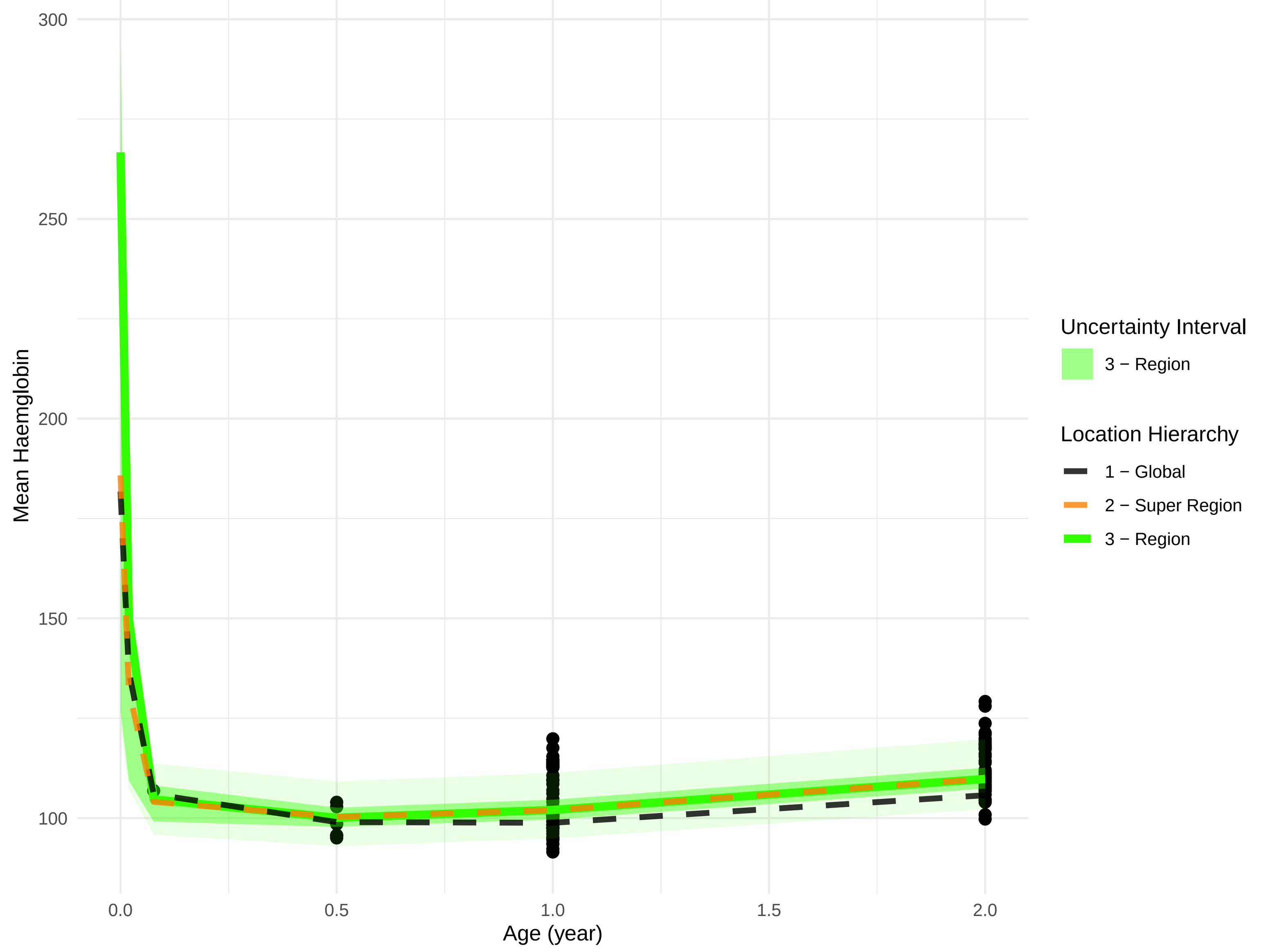

MR−BRT Age/Sex Fits − Males <2 Years Old − Mean Haemglobin − Cambodia
WHO−2024 Adjustment Method

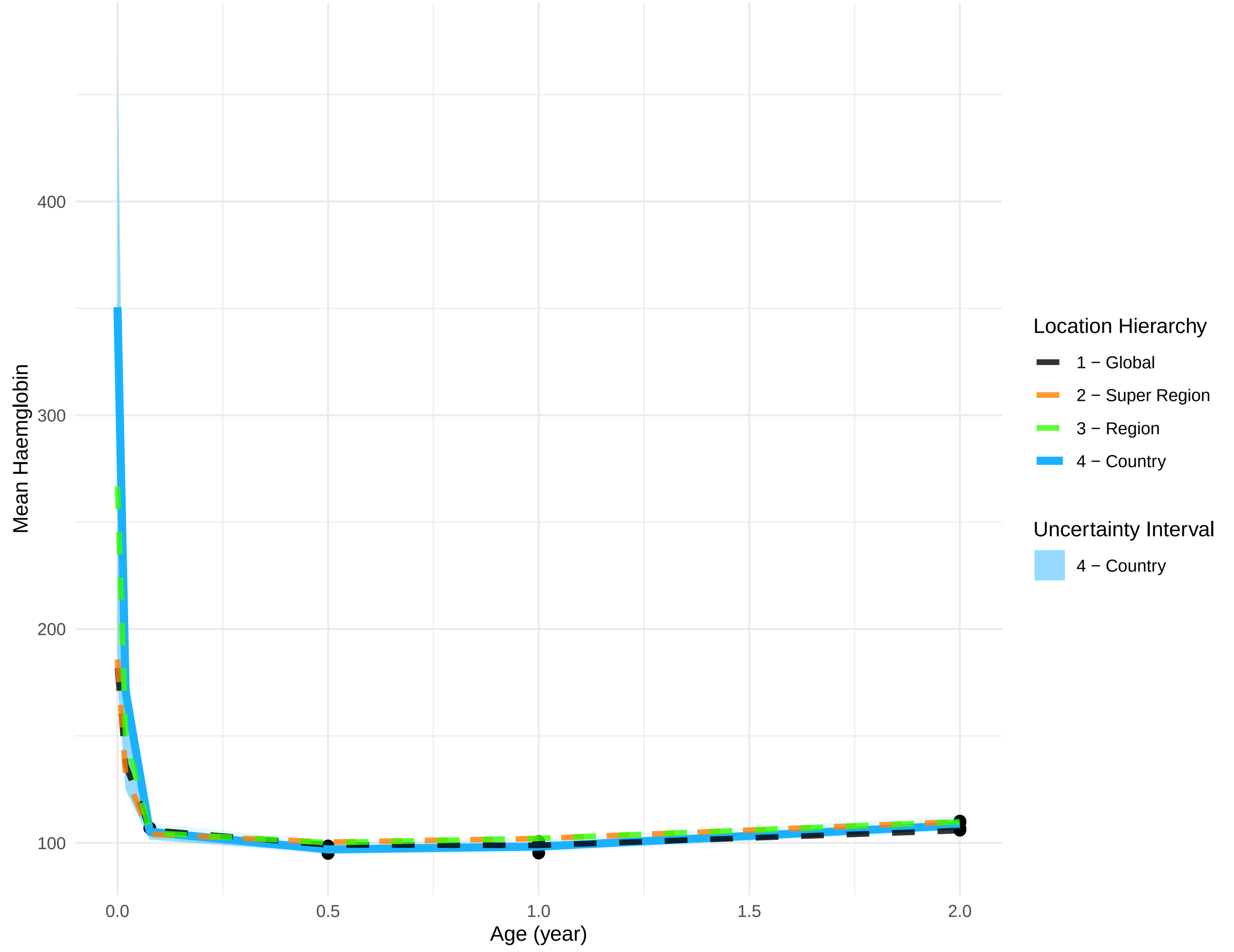

MR−BRT Age/Sex Fits − Males <2 Years Old − Mean Haemglobin − Indonesia
WHO−2024 Adjustment Method

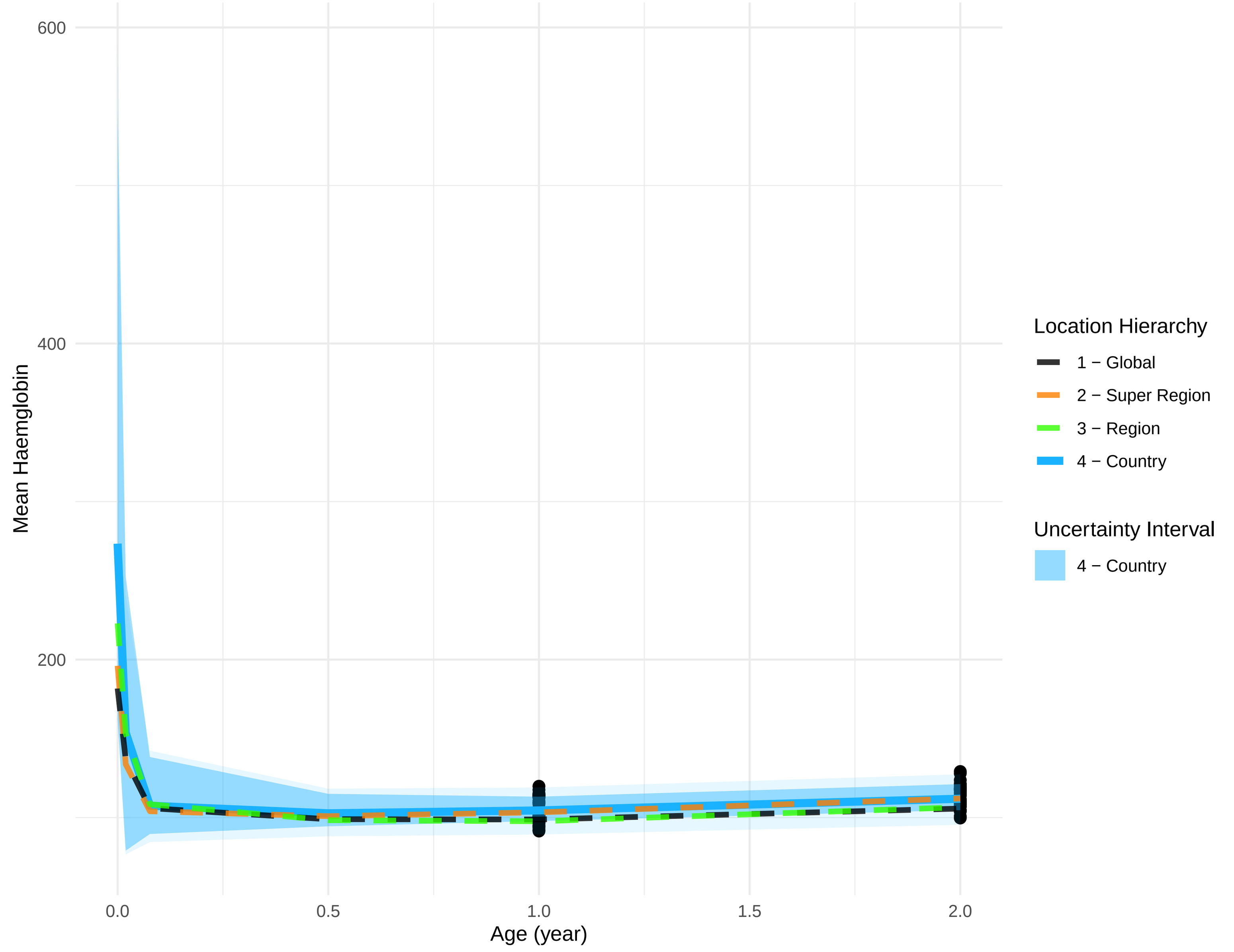

MR−BRT Age/Sex Fits − Males <2 Years Old − Mean Haemglobin − Bali
WHO−2024 Adjustment Method

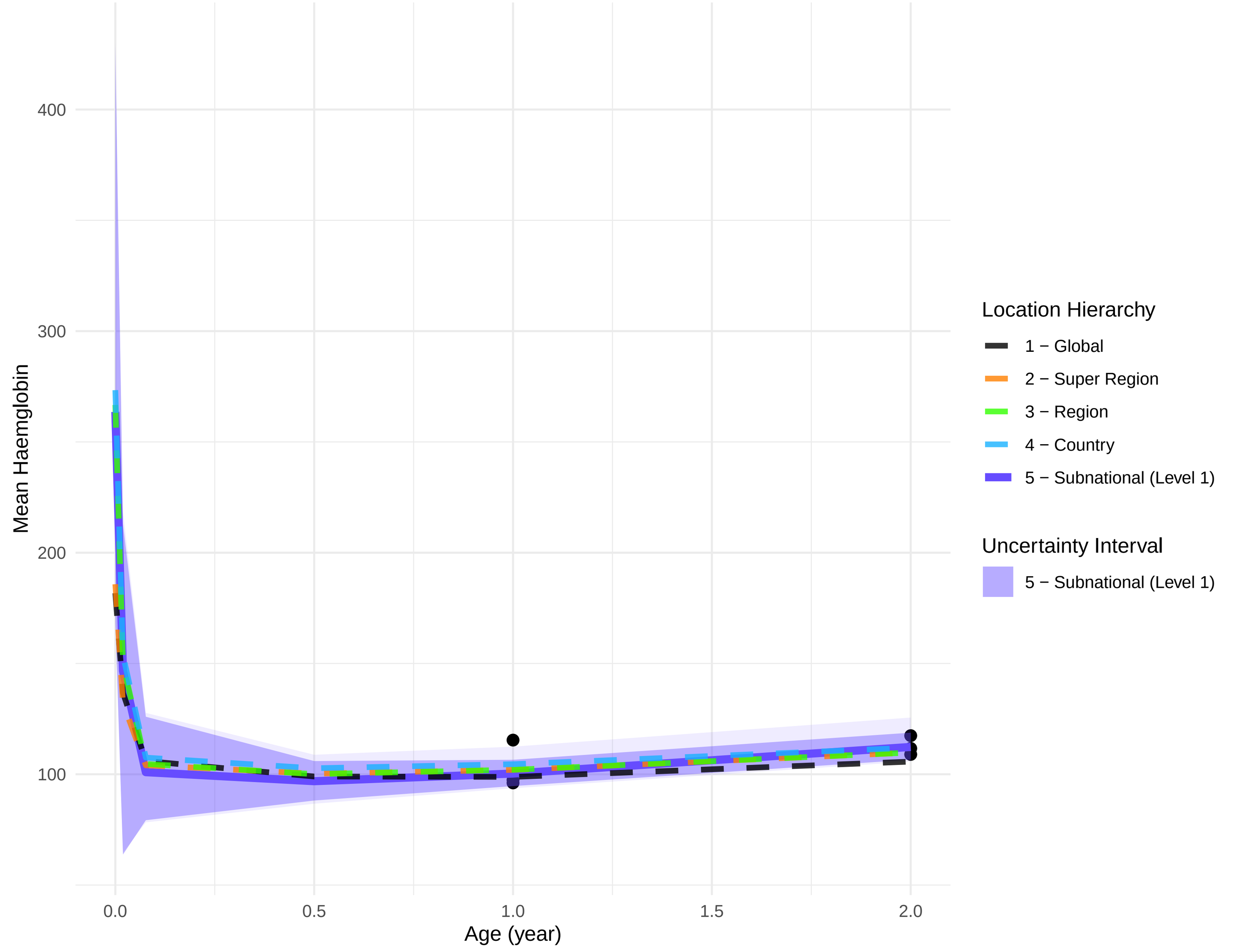

MR−BRT Age/Sex Fits − Males <2 Years Old − Mean Haemglobin − Bangka−Belitung Islands
WHO−2024 Adjustment Method

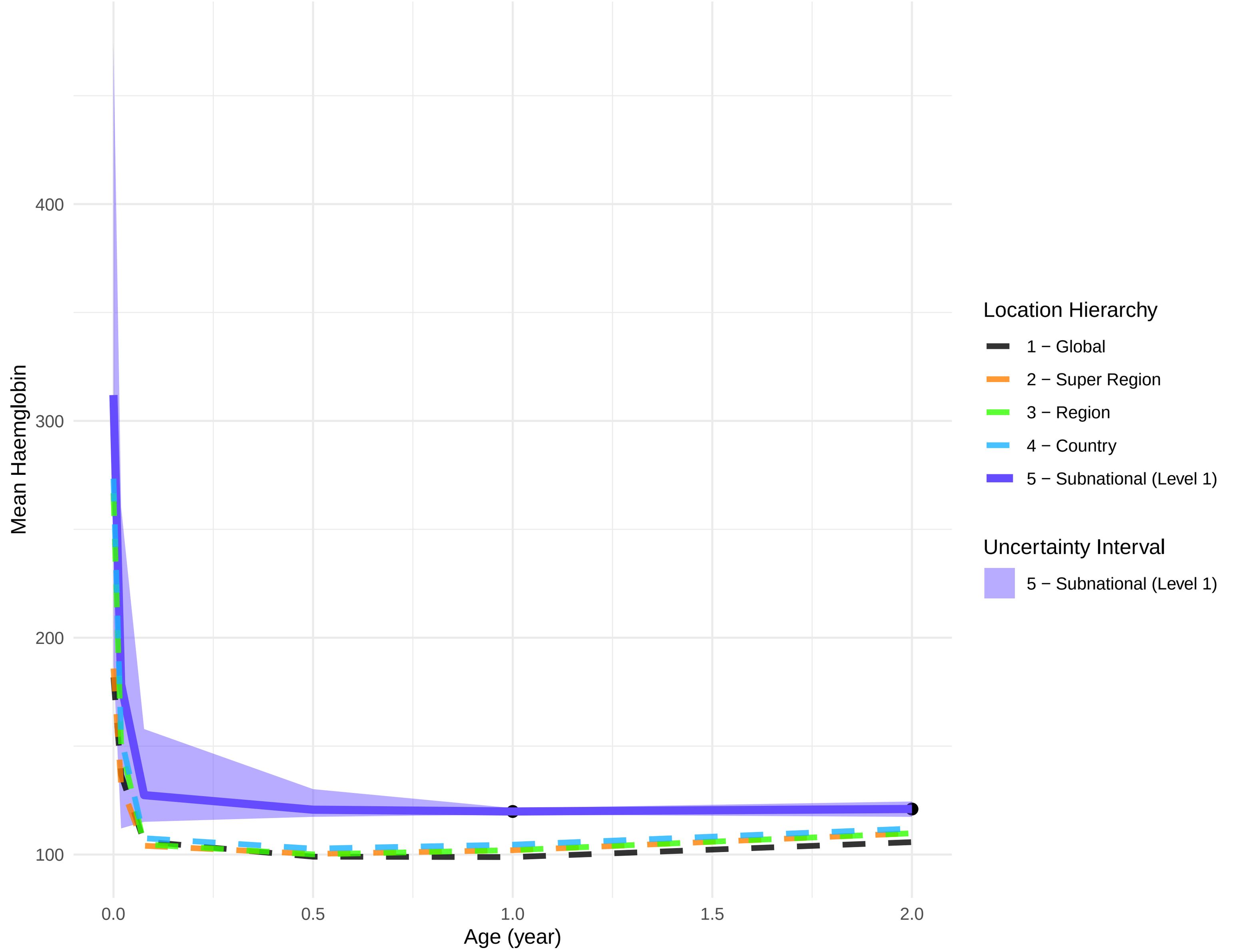

MR−BRT Age/Sex Fits − Males <2 Years Old − Mean Haemglobin − Banten
WHO−2024 Adjustment Method

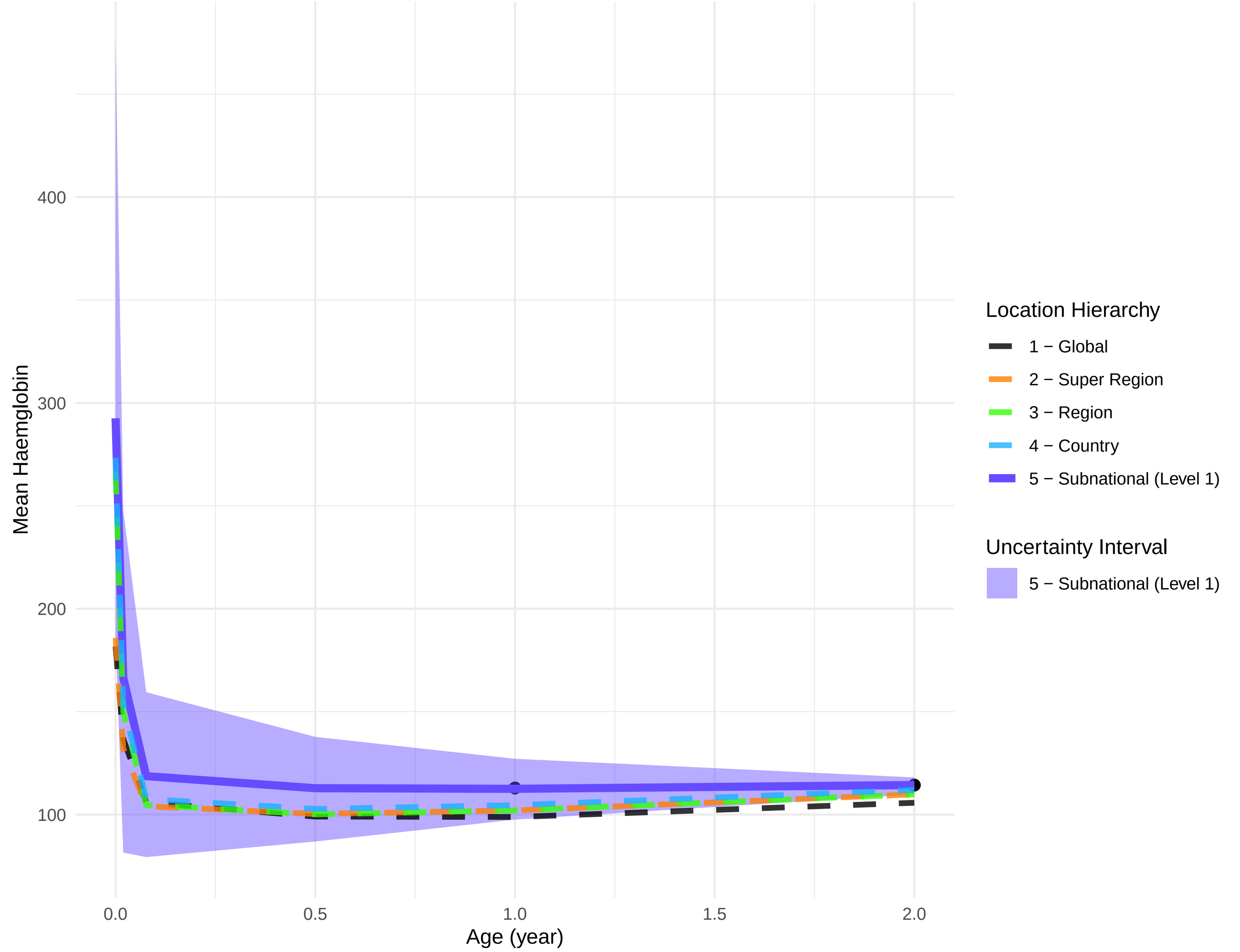

MR−BRT Age/Sex Fits − Males <2 Years Old − Mean Haemglobin − Jakarta
WHO−2024 Adjustment Method

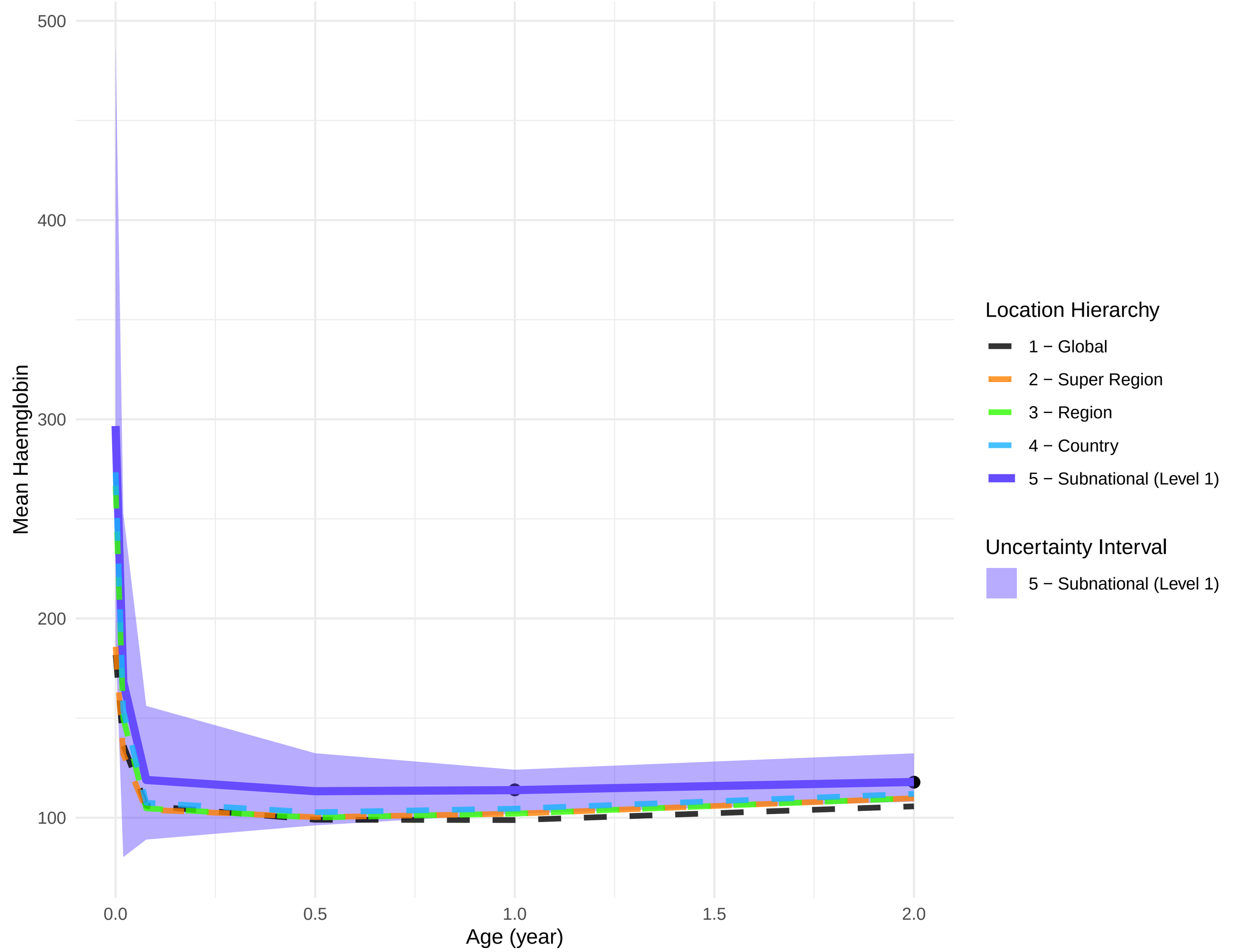

MR−BRT Age/Sex Fits − Males <2 Years Old − Mean Haemglobin − West Java
WHO−2024 Adjustment Method

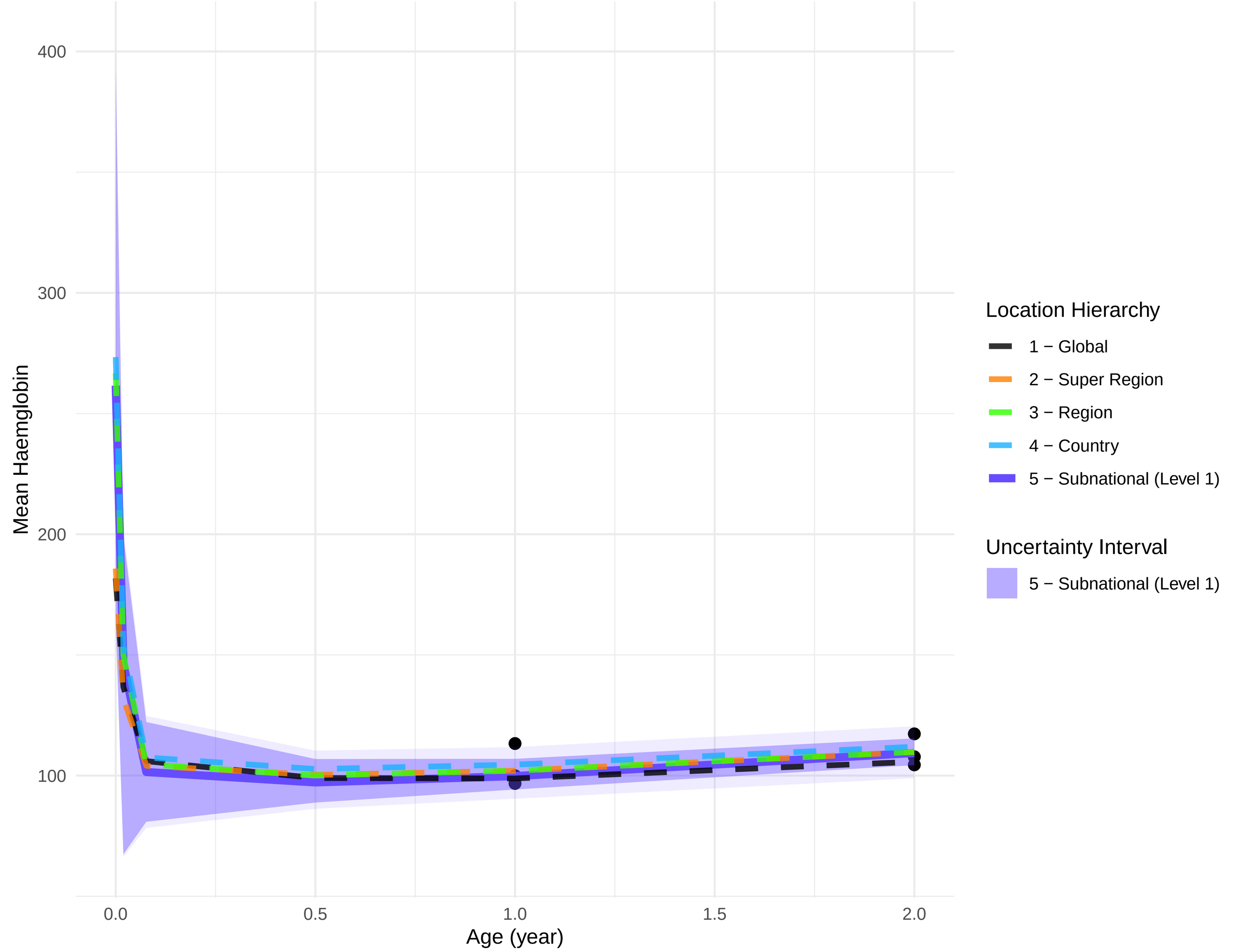

MR−BRT Age/Sex Fits − Males <2 Years Old − Mean Haemglobin − Central Java
WHO−2024 Adjustment Method

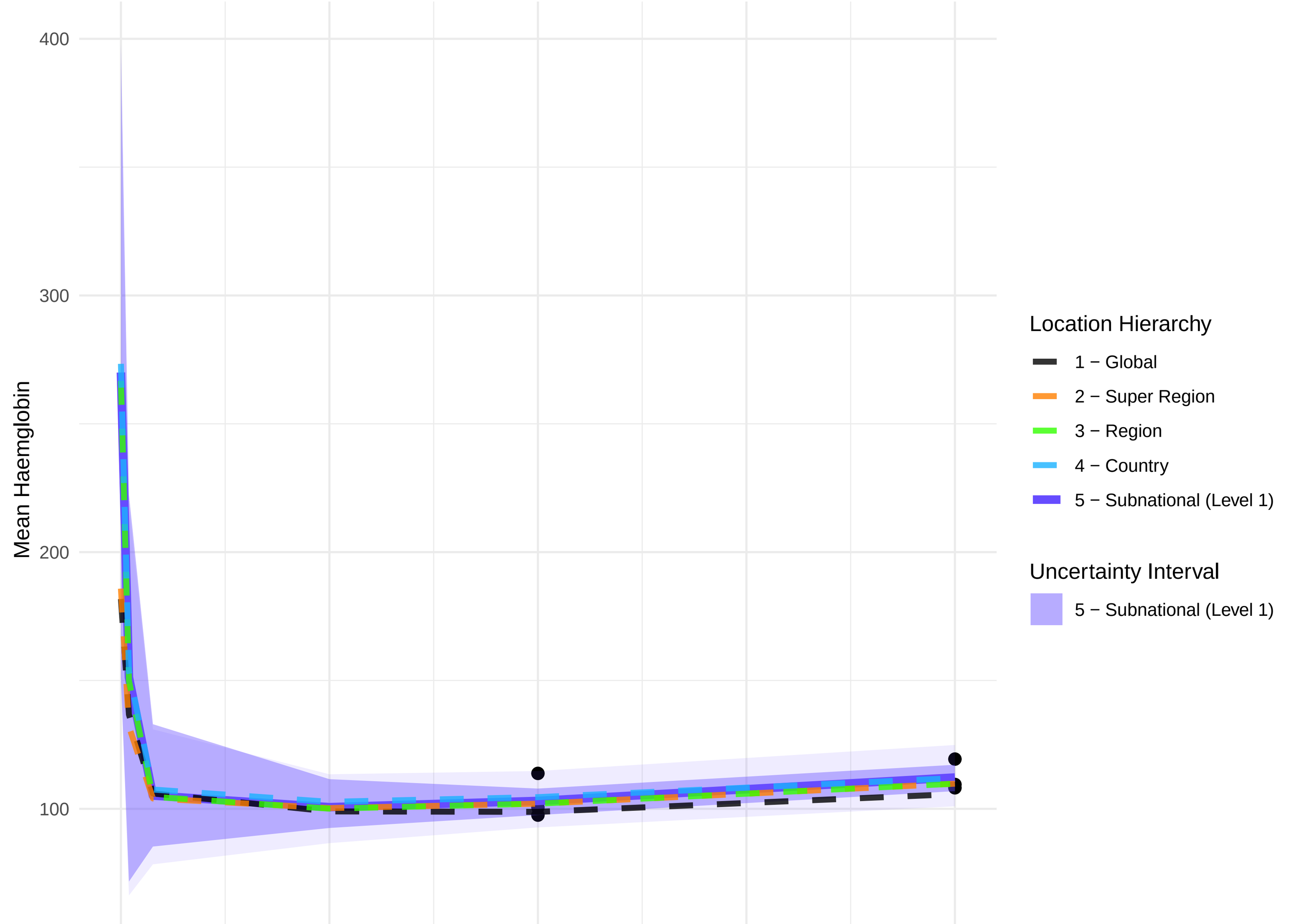

MR−BRT Age/Sex Fits − Males <2 Years Old − Mean Haemglobin − East Java
WHO−2024 Adjustment Method

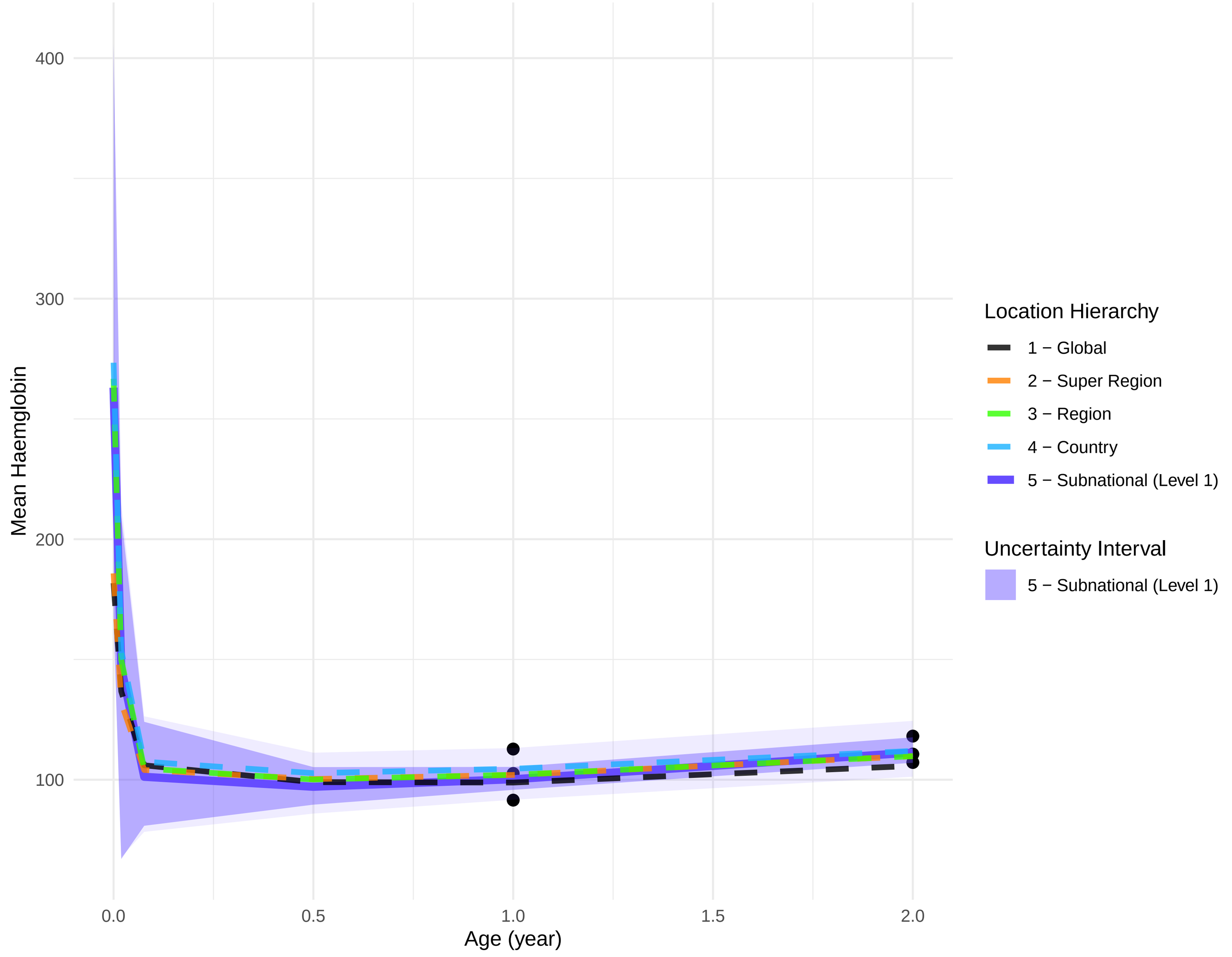

MR−BRT Age/Sex Fits − Males <2 Years Old − Mean Haemglobin − South Kalimantan
WHO−2024 Adjustment Method

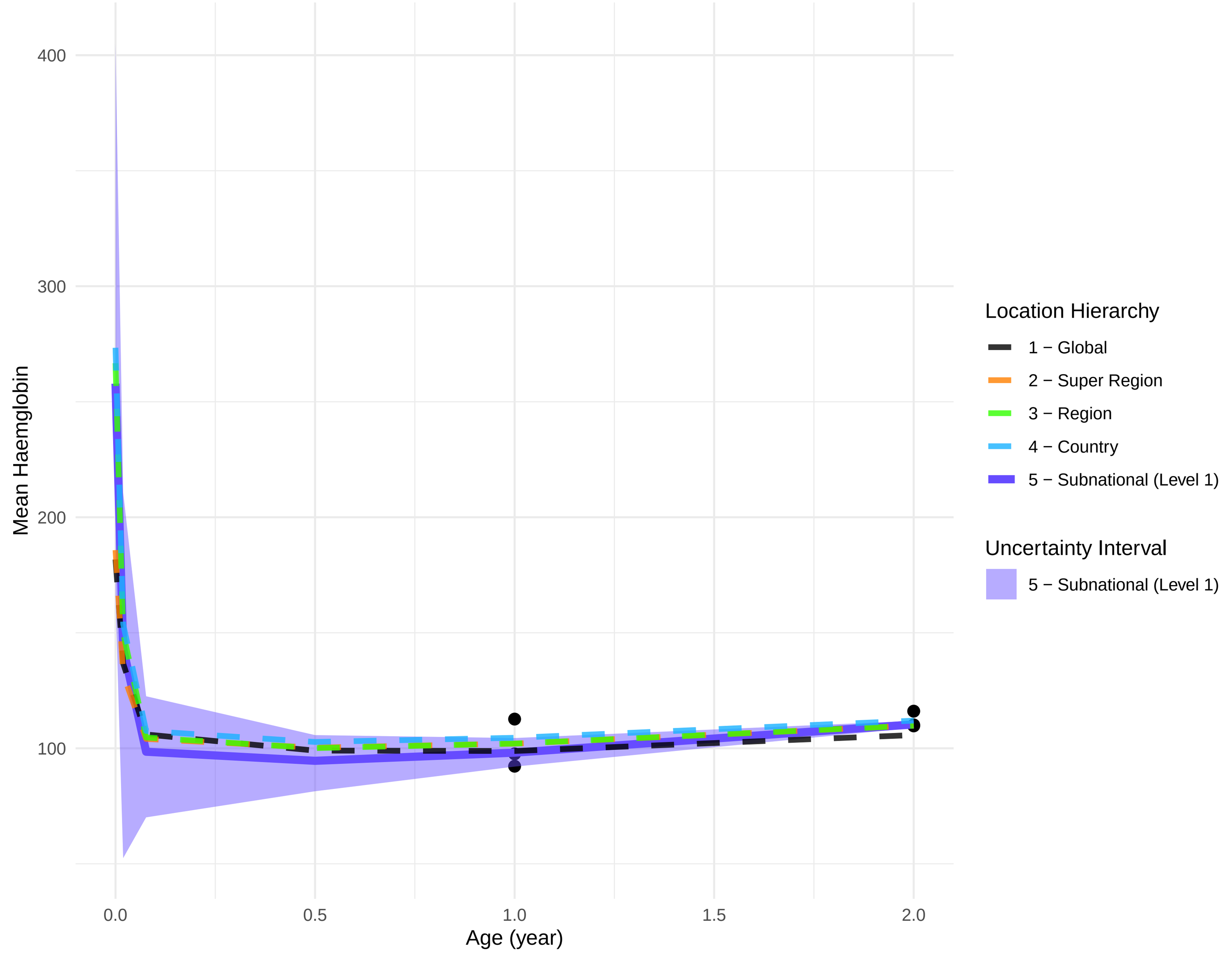

MR−BRT Age/Sex Fits − Males <2 Years Old − Mean Haemglobin − Riau Islands
WHO−2024 Adjustment Method

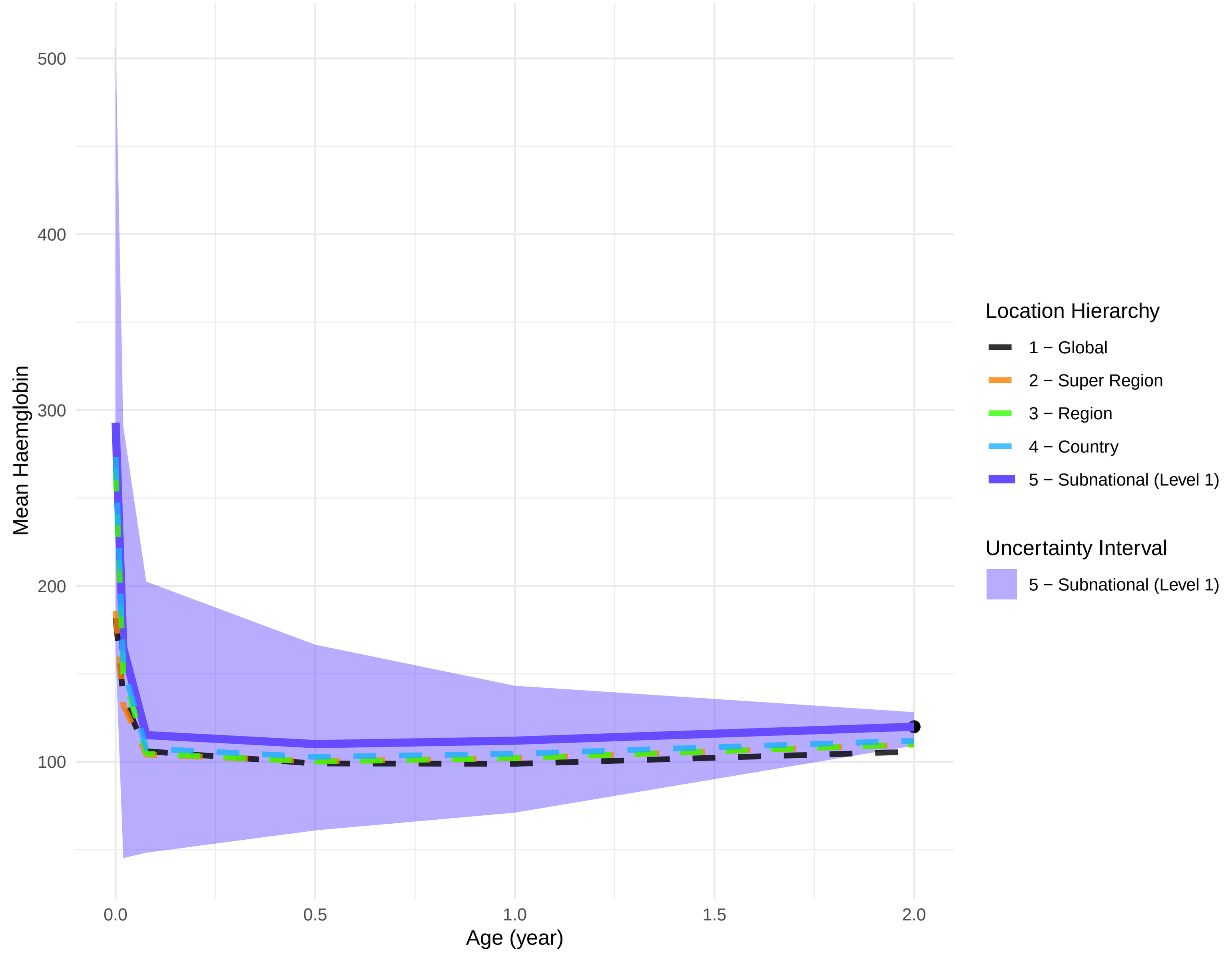

MR−BRT Age/Sex Fits − Males <2 Years Old − Mean Haemglobin − Lampung
WHO−2024 Adjustment Method

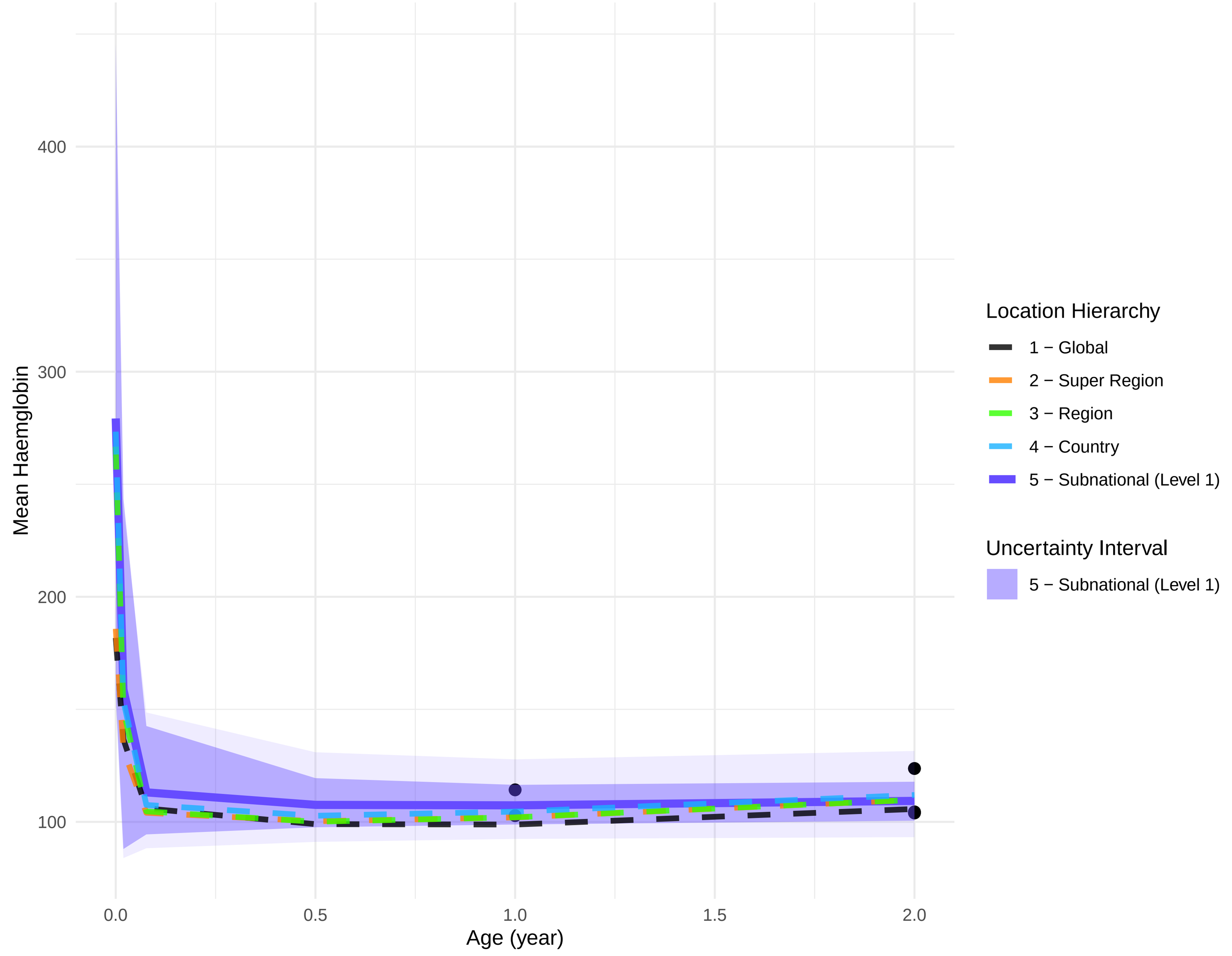

MR−BRT Age/Sex Fits − Males <2 Years Old − Mean Haemglobin − West Nusa Tenggara
WHO−2024 Adjustment Method

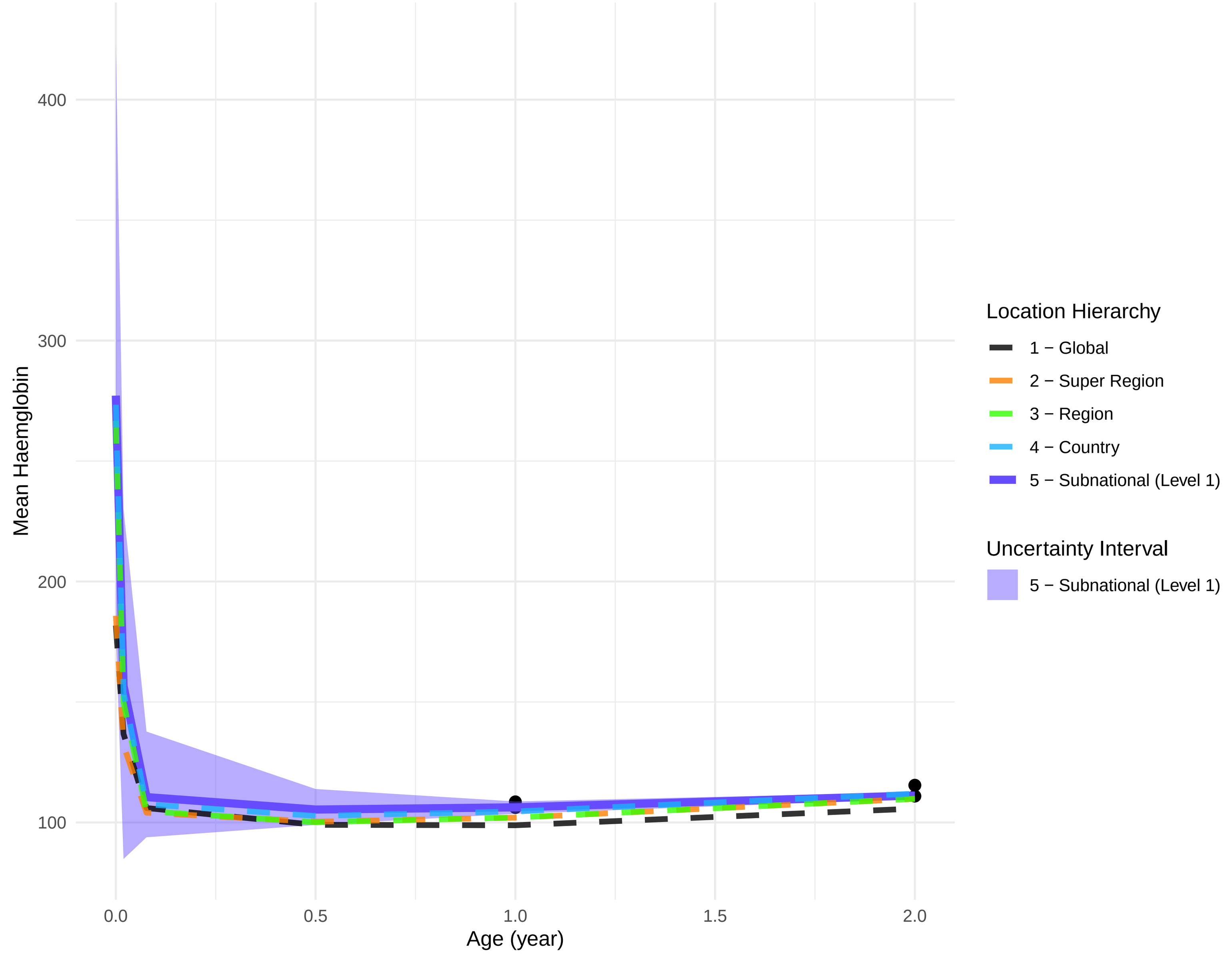

MR−BRT Age/Sex Fits − Males <2 Years Old − Mean Haemglobin − Riau
WHO−2024 Adjustment Method

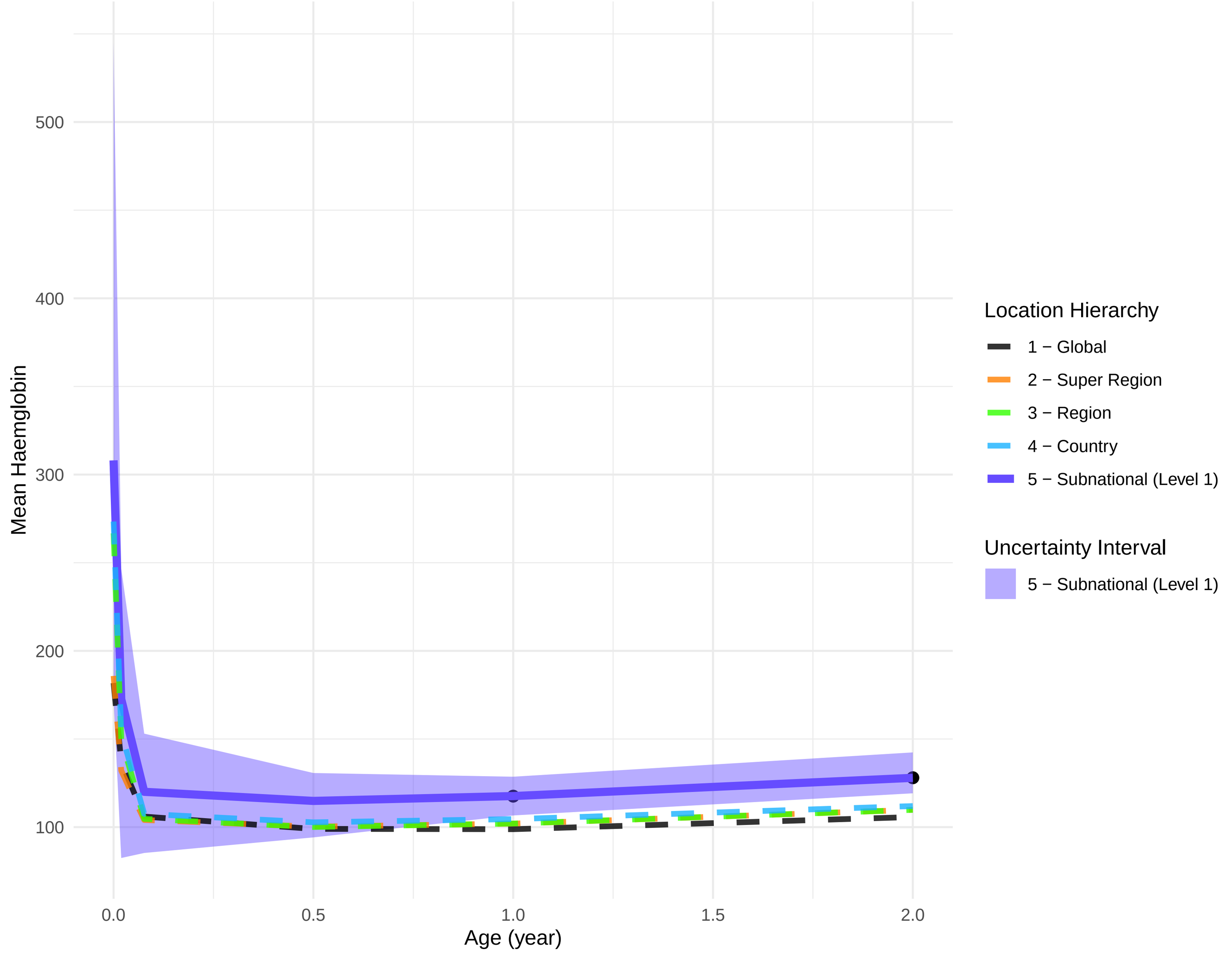

MR−BRT Age/Sex Fits − Males <2 Years Old − Mean Haemglobin − West Sulawesi
WHO−2024 Adjustment Method

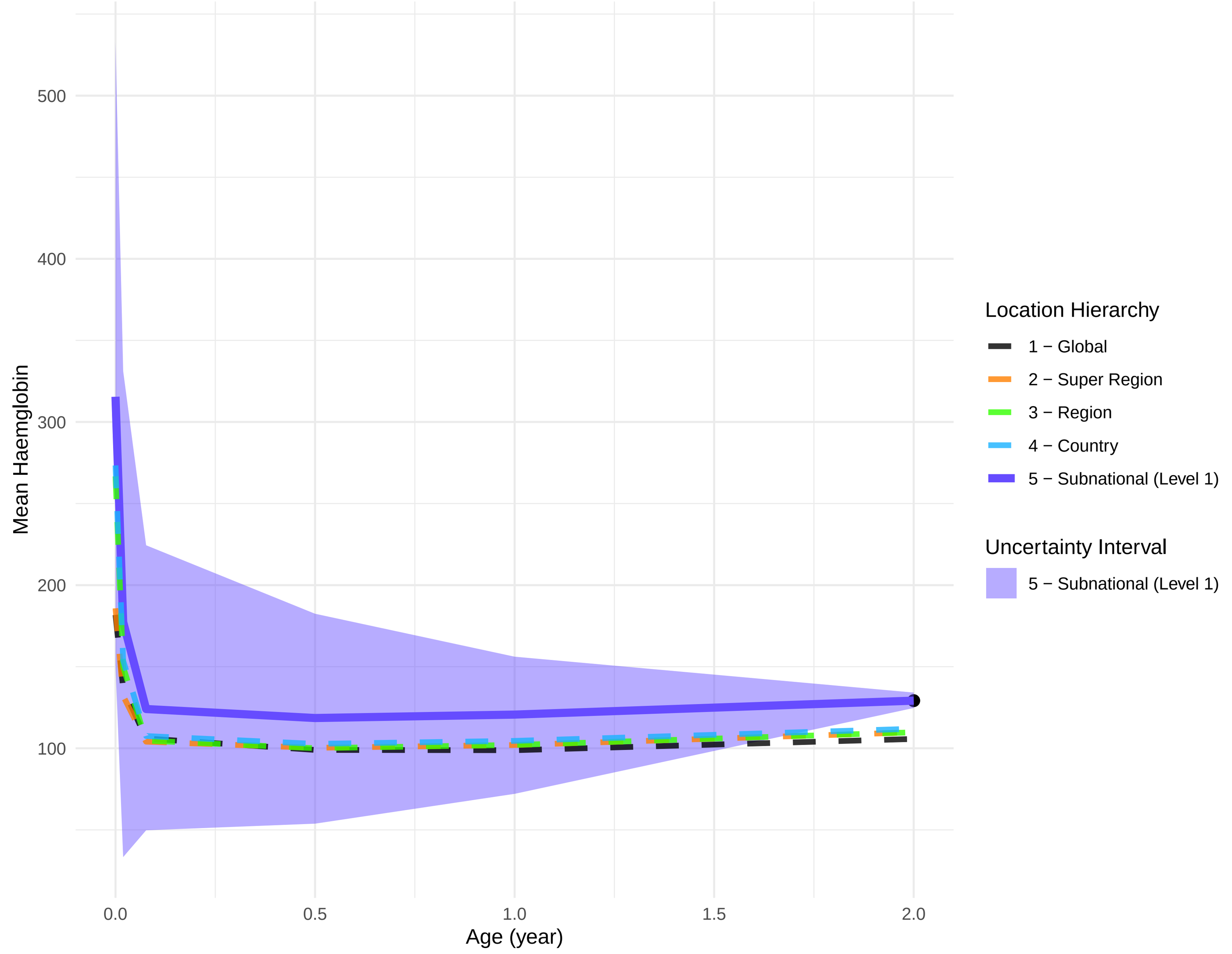

MR−BRT Age/Sex Fits − Males <2 Years Old − Mean Haemglobin − South Sulawesi
WHO−2024 Adjustment Method

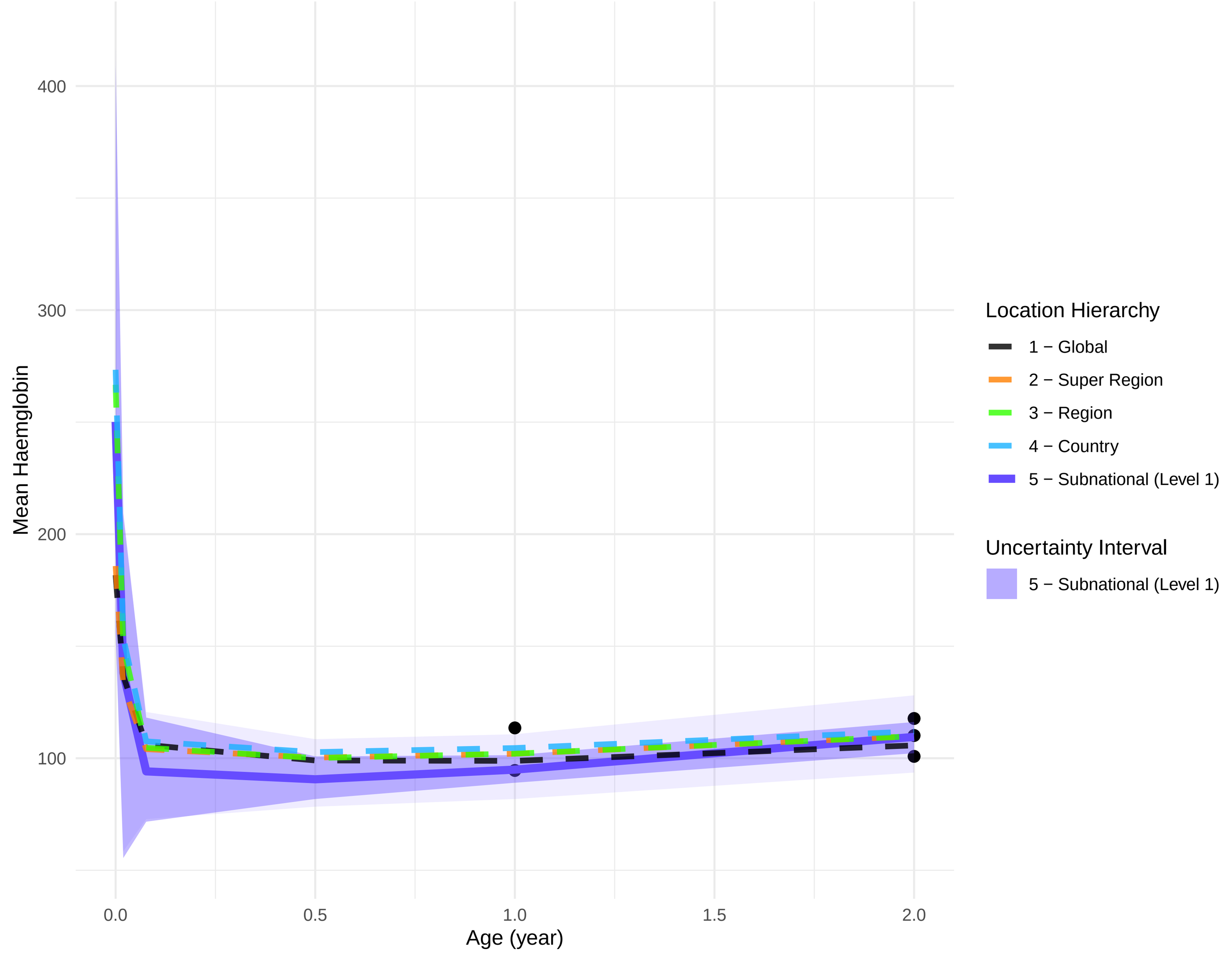

MR−BRT Age/Sex Fits − Males <2 Years Old − Mean Haemglobin − West Sumatra
WHO−2024 Adjustment Method

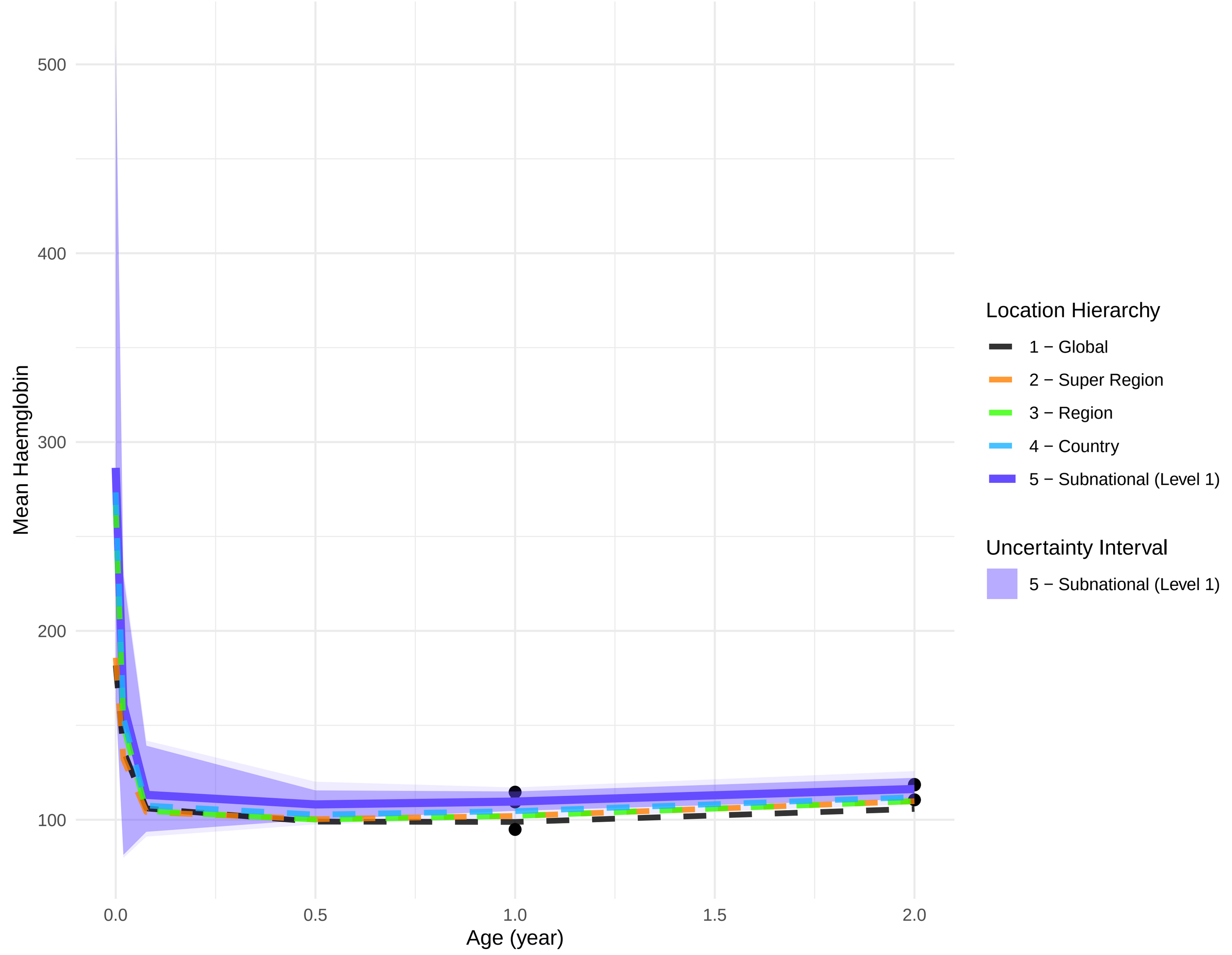

MR−BRT Age/Sex Fits − Males <2 Years Old − Mean Haemglobin − South Sumatra
WHO−2024 Adjustment Method

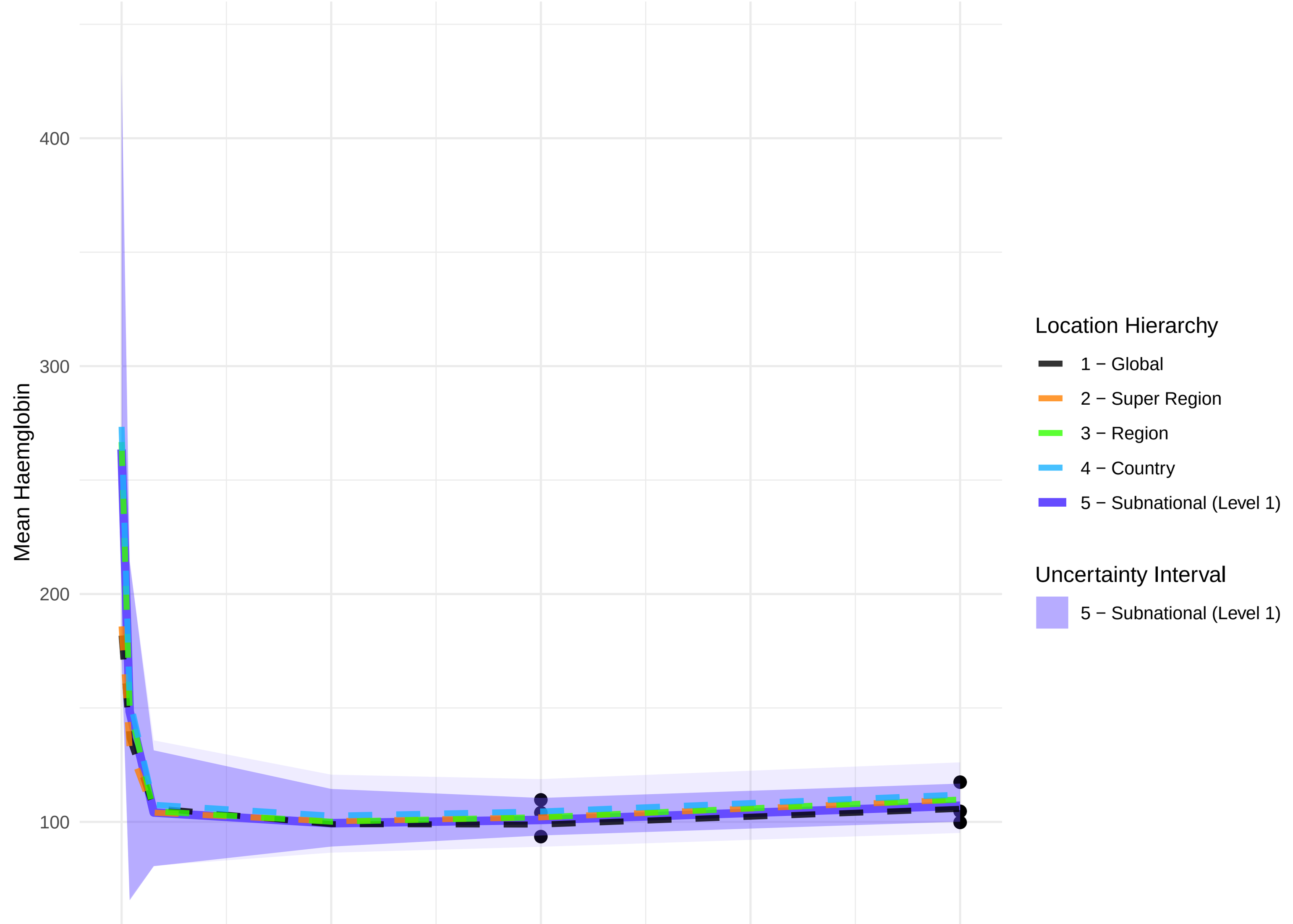

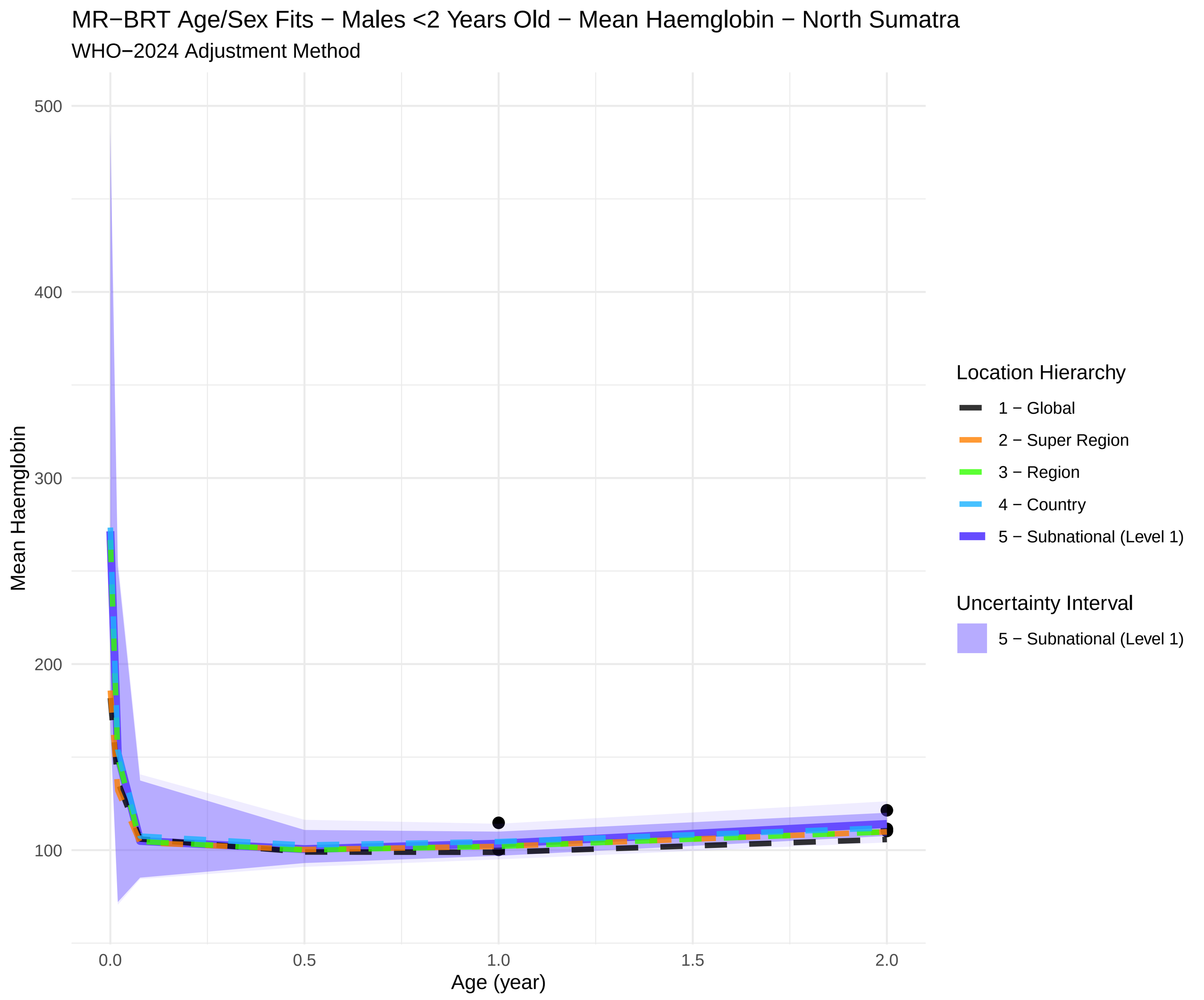

MR−BRT Age/Sex Fits − Males <2 Years Old − Mean Haemglobin − North Sumatra
WHO−2024 Adjustment Method
Mean Haemglobin
Age (year)
Location Hierarchy
1 − Global
2 − Super Region
3 − Region
4 − Country
5 − Subnational (Level 1)
Uncertainty Interval
5 − Subnational (Level 1)

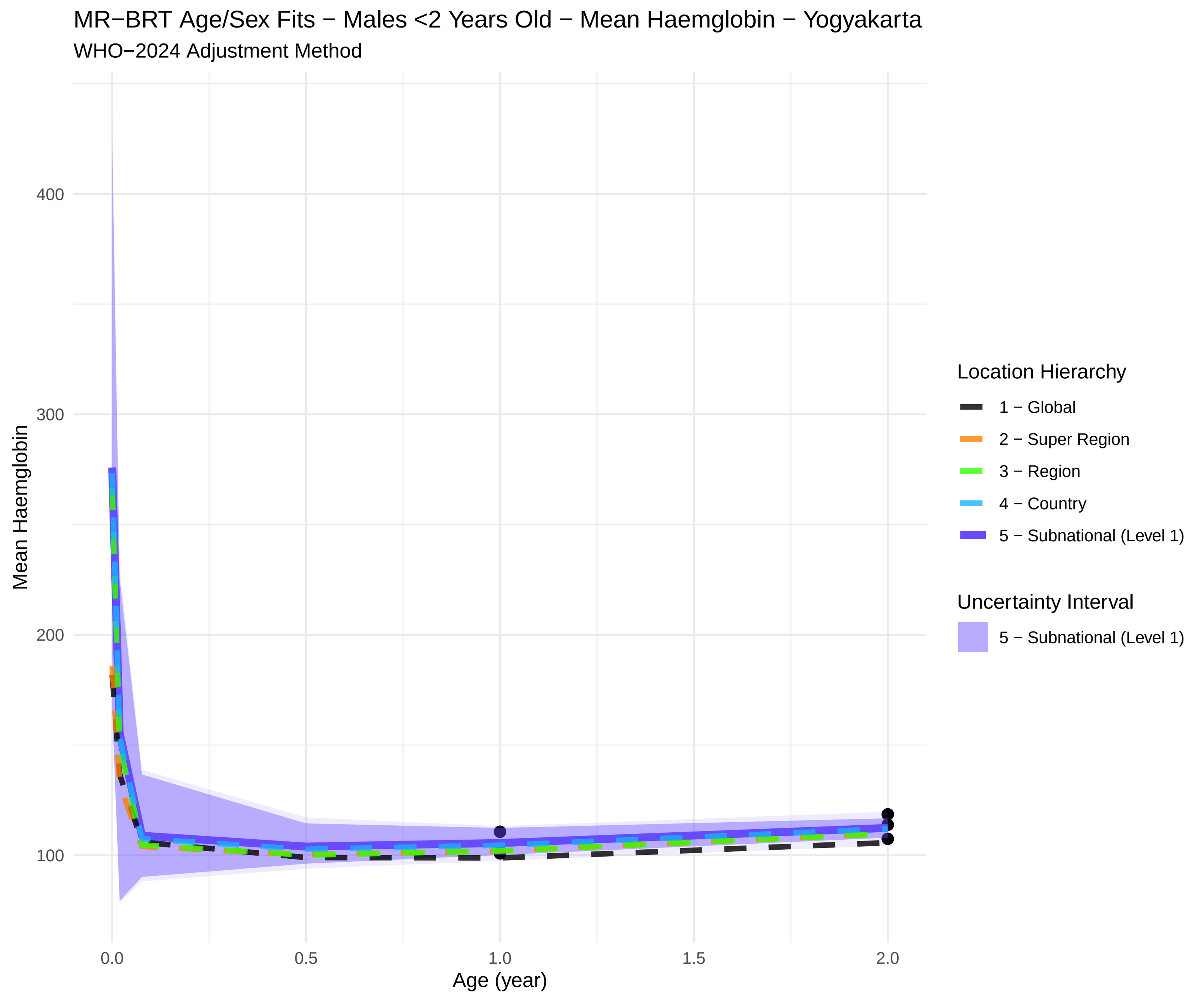

MR−BRT Age/Sex Fits − Males <2 Years Old − Mean Haemglobin − Yogyakarta
WHO−2024 Adjustment Method
Mean Haemglobin
Age (year)
Location Hierarchy
1 − Global
2 − Super Region
3 − Region
4 − Country
5 − Subnational (Level 1)
Uncertainty Interval
5 − Subnational (Level 1)

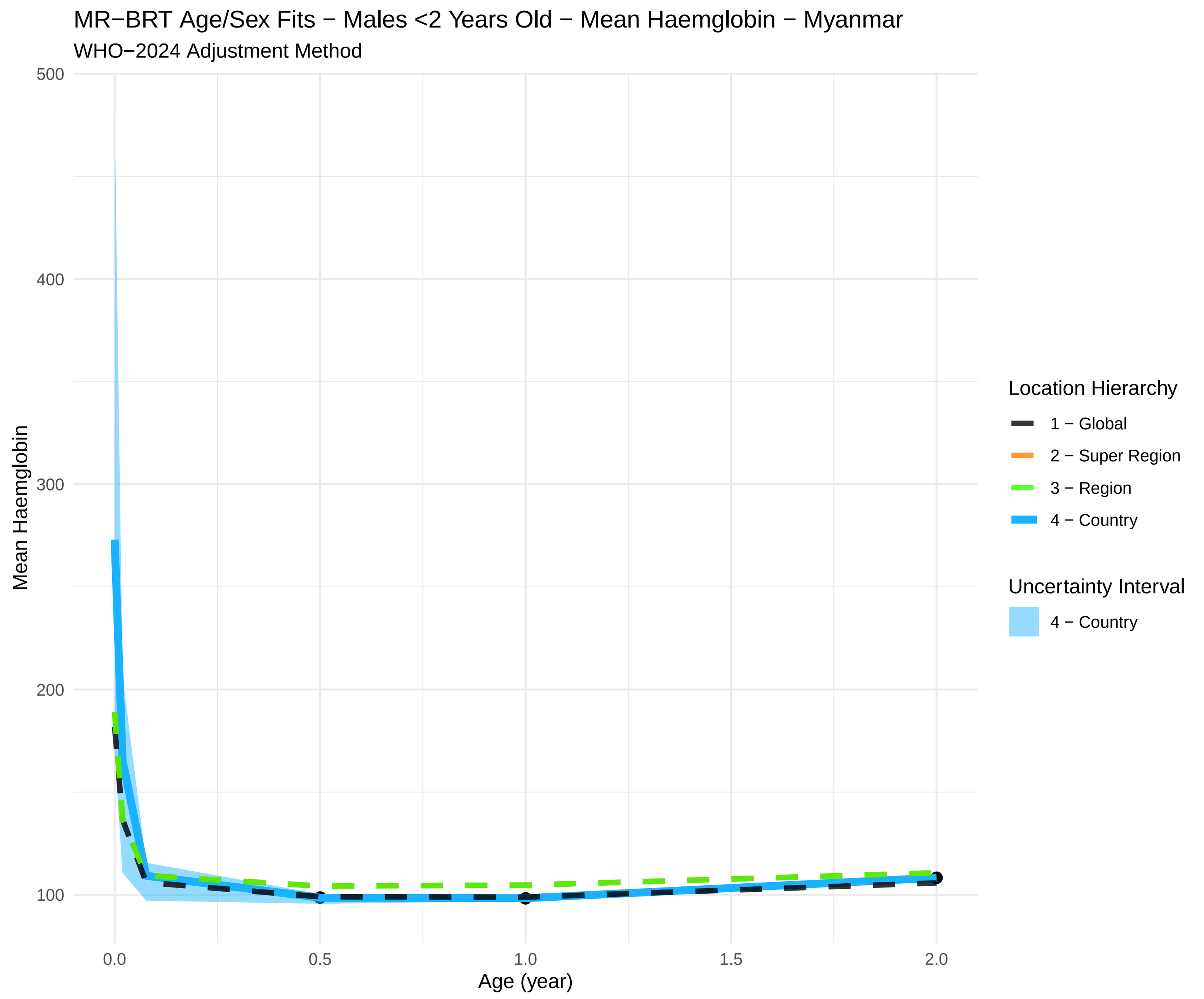

MR−BRT Age/Sex Fits − Males <2 Years Old − Mean Haemglobin − Myanmar
WHO−2024 Adjustment Method
Mean Haemglobin
Age (year)
Location Hierarchy
1 − Global
2 − Super Region
3 − Region
4 − Country
Uncertainty Interval
4 − Country

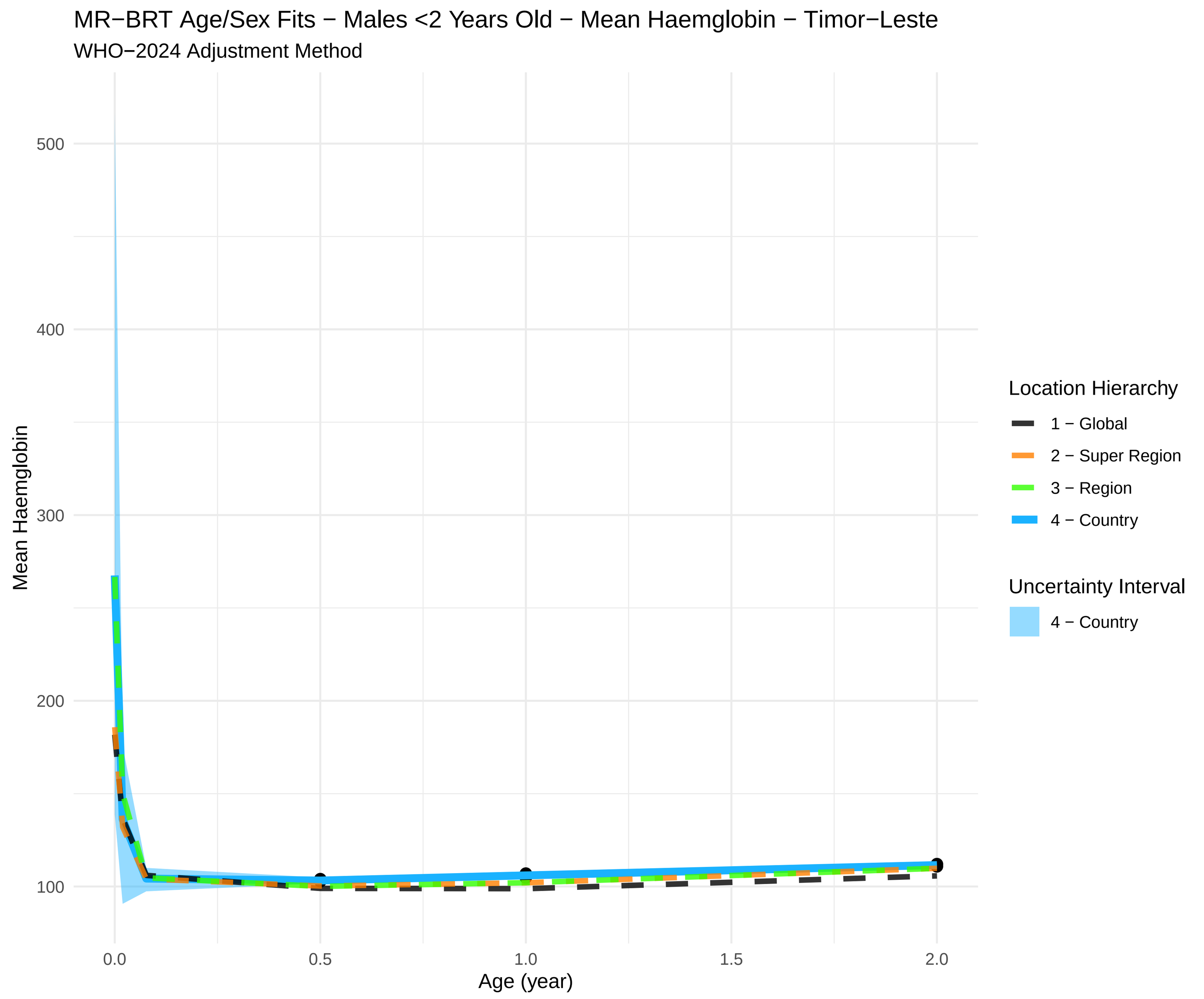

MR−BRT Age/Sex Fits − Males <2 Years Old − Mean Haemglobin − Timor−Leste
WHO−2024 Adjustment Method
Mean Haemglobin
Age (year)
Location Hierarchy
1 − Global
2 − Super Region
3 − Region
4 − Country
Uncertainty Interval
4 − Country

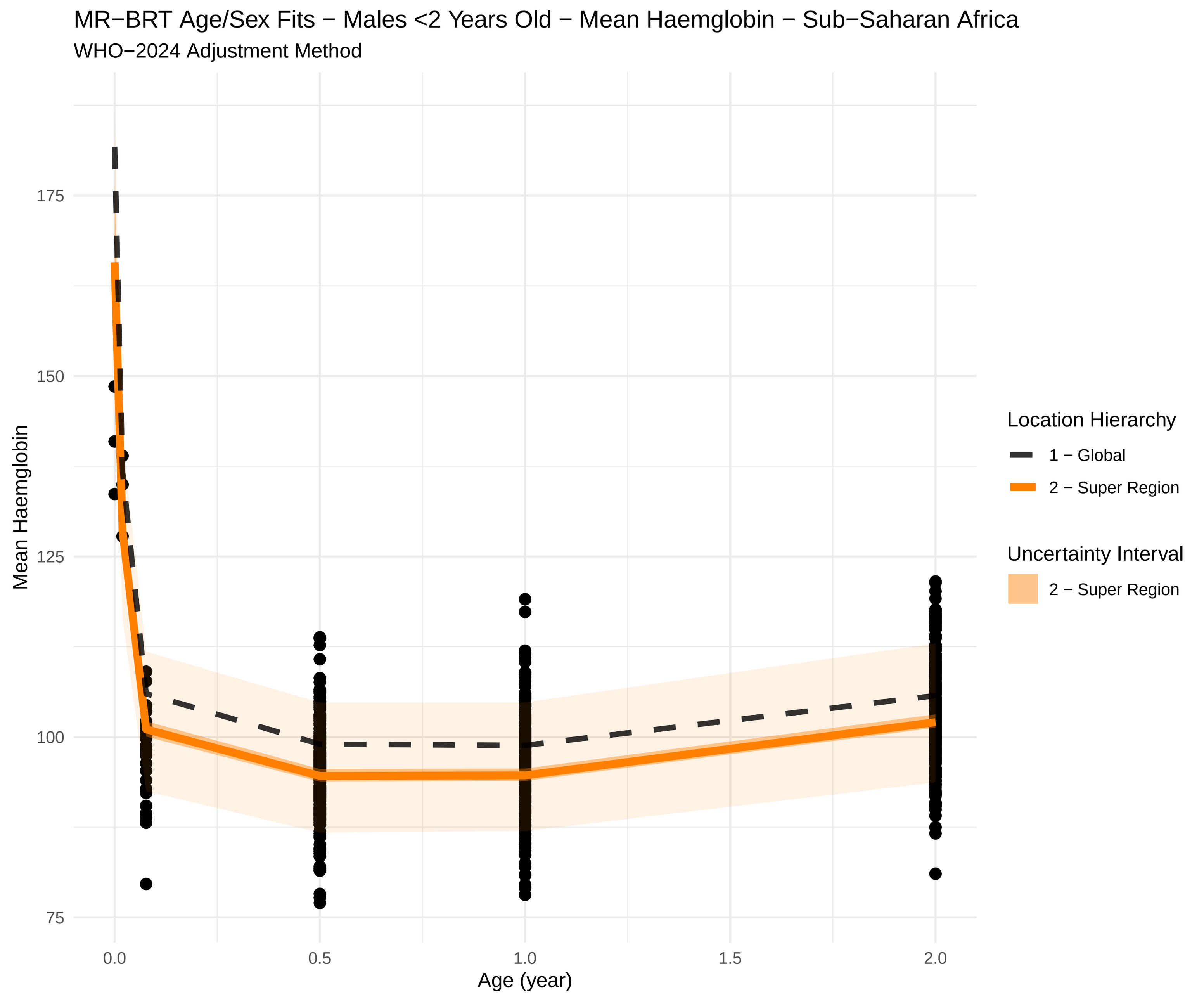

MR−BRT Age/Sex Fits − Males <2 Years Old − Mean Haemglobin − Sub−Saharan Africa
WHO−2024 Adjustment Method
Mean Haemglobin
Age (year)
Location Hierarchy
1 − Global
2 − Super Region
Uncertainty Interval
2 − Super Region

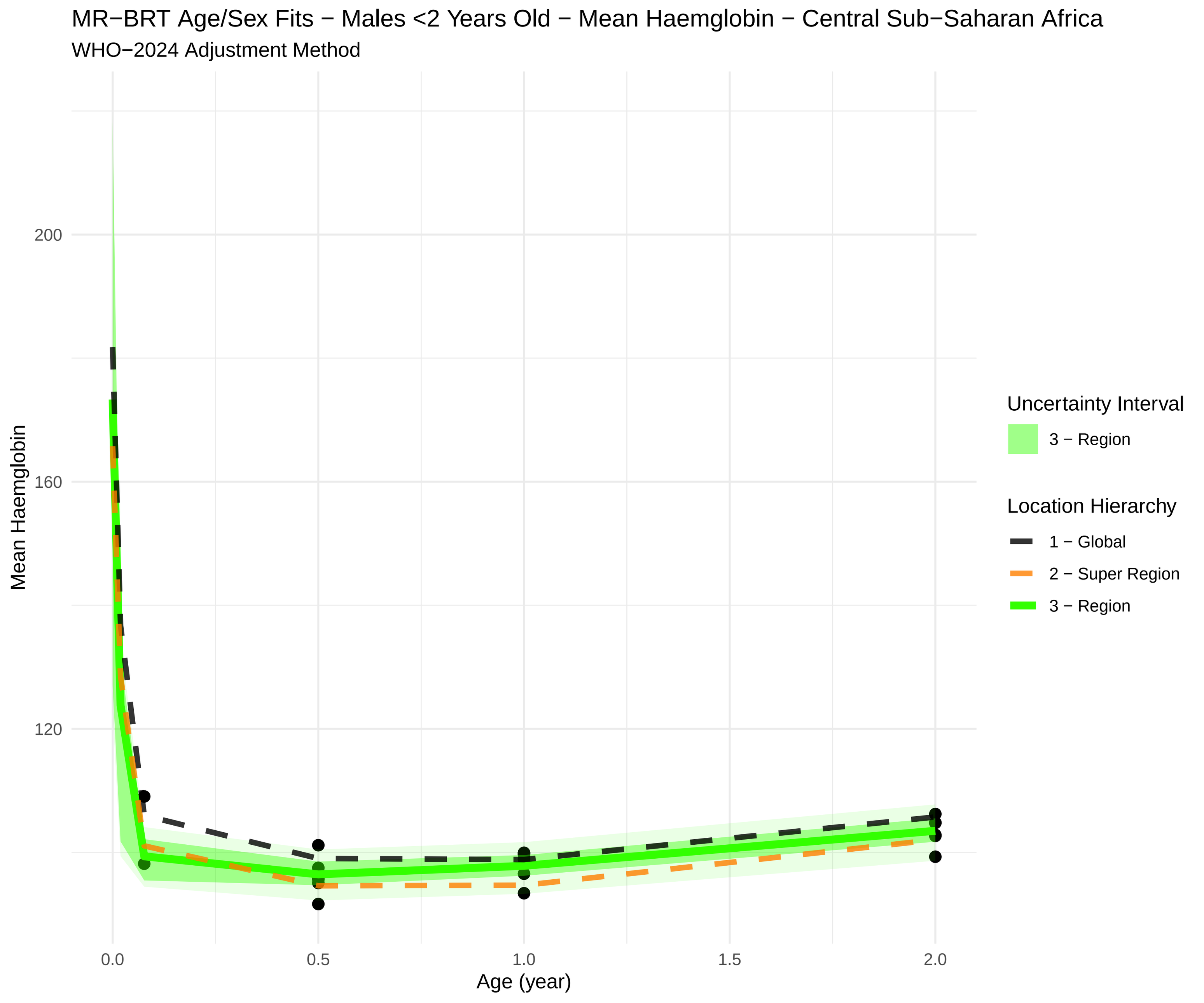

MR−BRT Age/Sex Fits − Males <2 Years Old − Mean Haemglobin − Central Sub−Saharan Africa
WHO−2024 Adjustment Method
Mean Haemglobin
Age (year)
Uncertainty Interval
3 − Region
Location Hierarchy
1 − Global
2 − Super Region
3 − Region

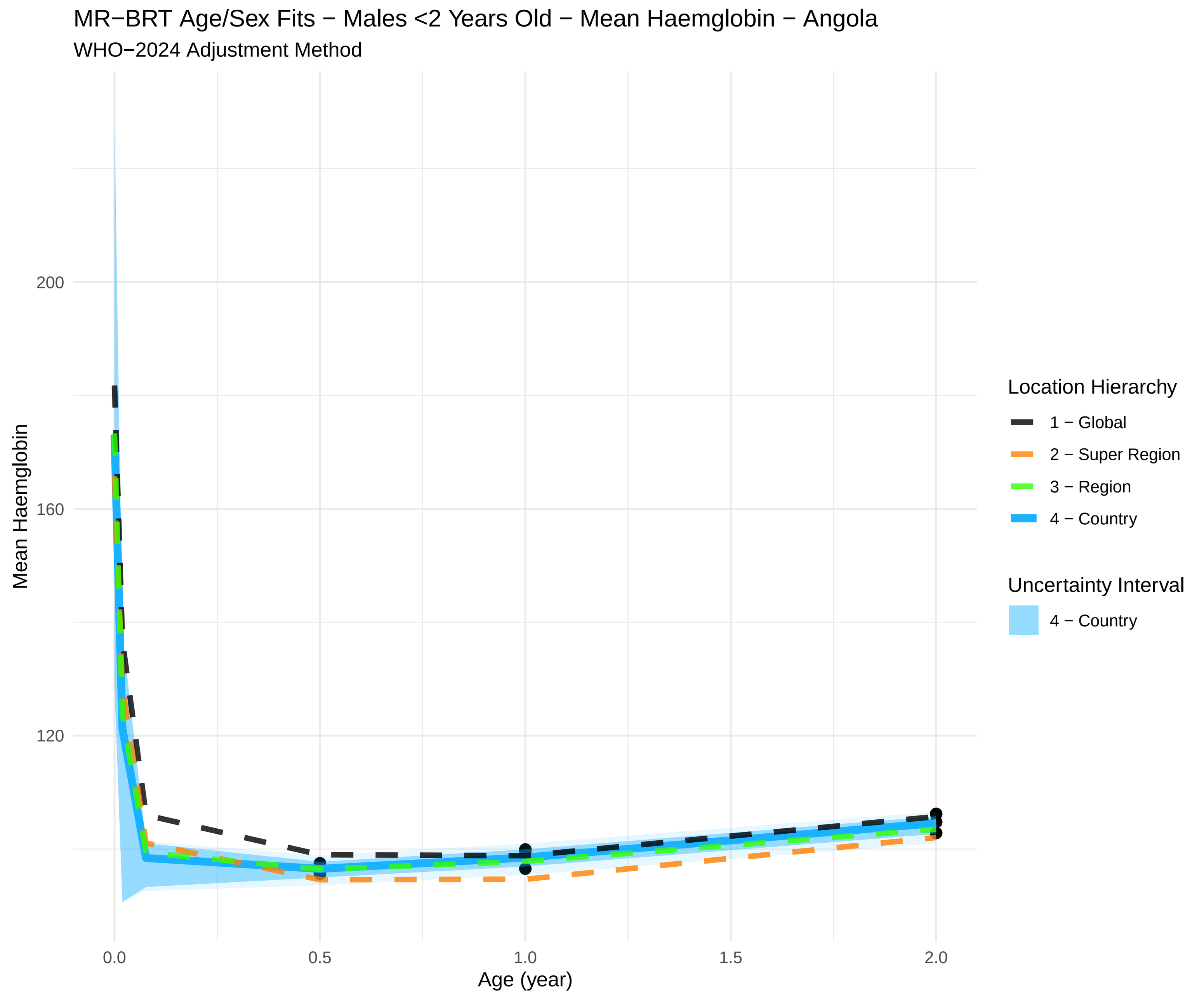

MR−BRT Age/Sex Fits − Males <2 Years Old − Mean Haemglobin − Angola
WHO−2024 Adjustment Method
Mean Haemglobin
Age (year)
Location Hierarchy
1 − Global
2 − Super Region
3 − Region
4 − Country
Uncertainty Interval
4 − Country

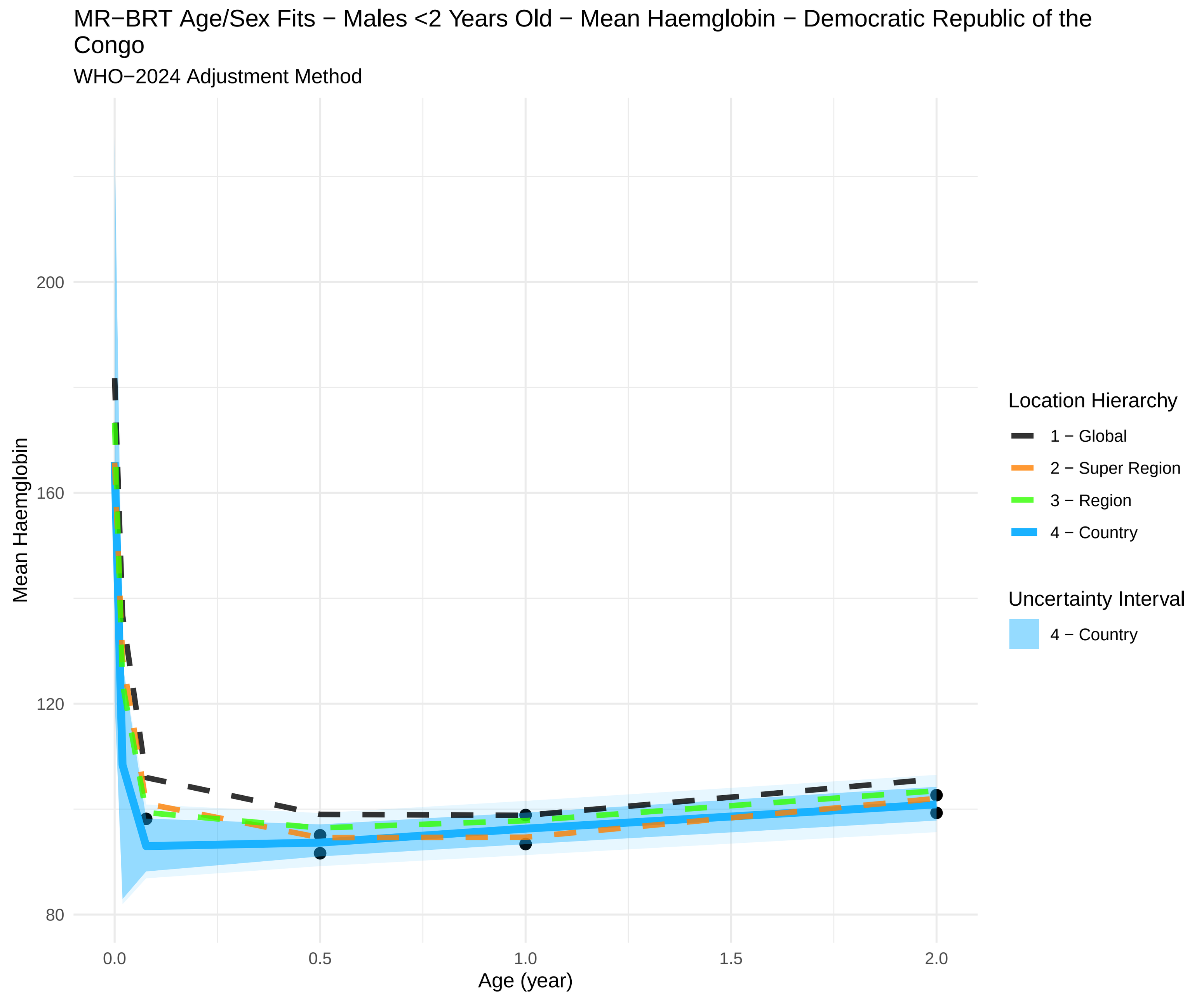

MR−BRT Age/Sex Fits − Males <2 Years Old − Mean Haemglobin − Democratic Republic of the Congo
WHO−2024 Adjustment Method
Mean Haemglobin
Age (year)
Location Hierarchy
1 − Global
2 − Super Region
3 − Region
4 − Country
Uncertainty Interval
4 − Country

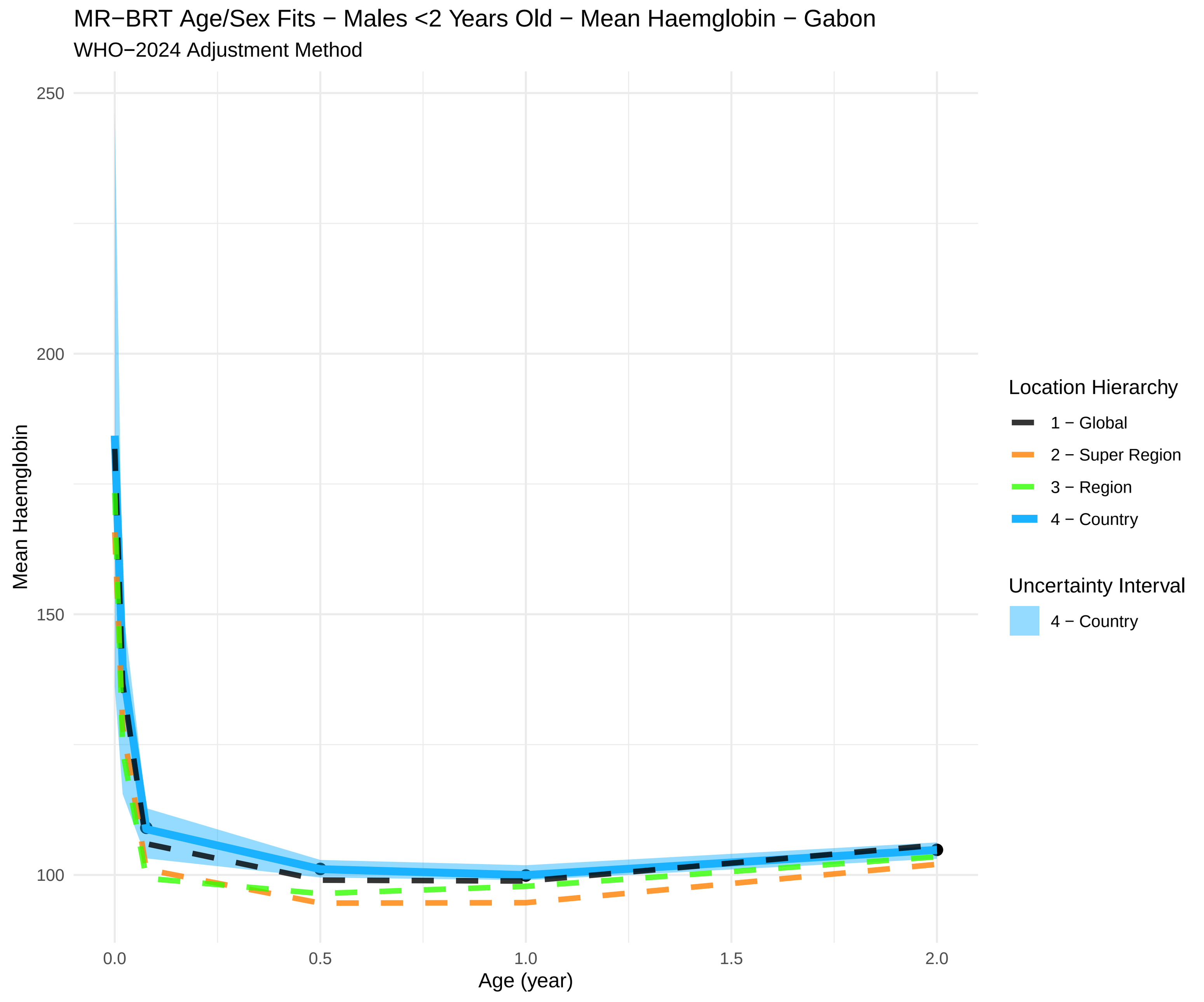

MR−BRT Age/Sex Fits − Males <2 Years Old − Mean Haemglobin − Gabon
WHO−2024 Adjustment Method
Mean Haemglobin
Age (year)
Location Hierarchy
1 − Global
2 − Super Region
3 − Region
4 − Country
Uncertainty Interval
4 − Country

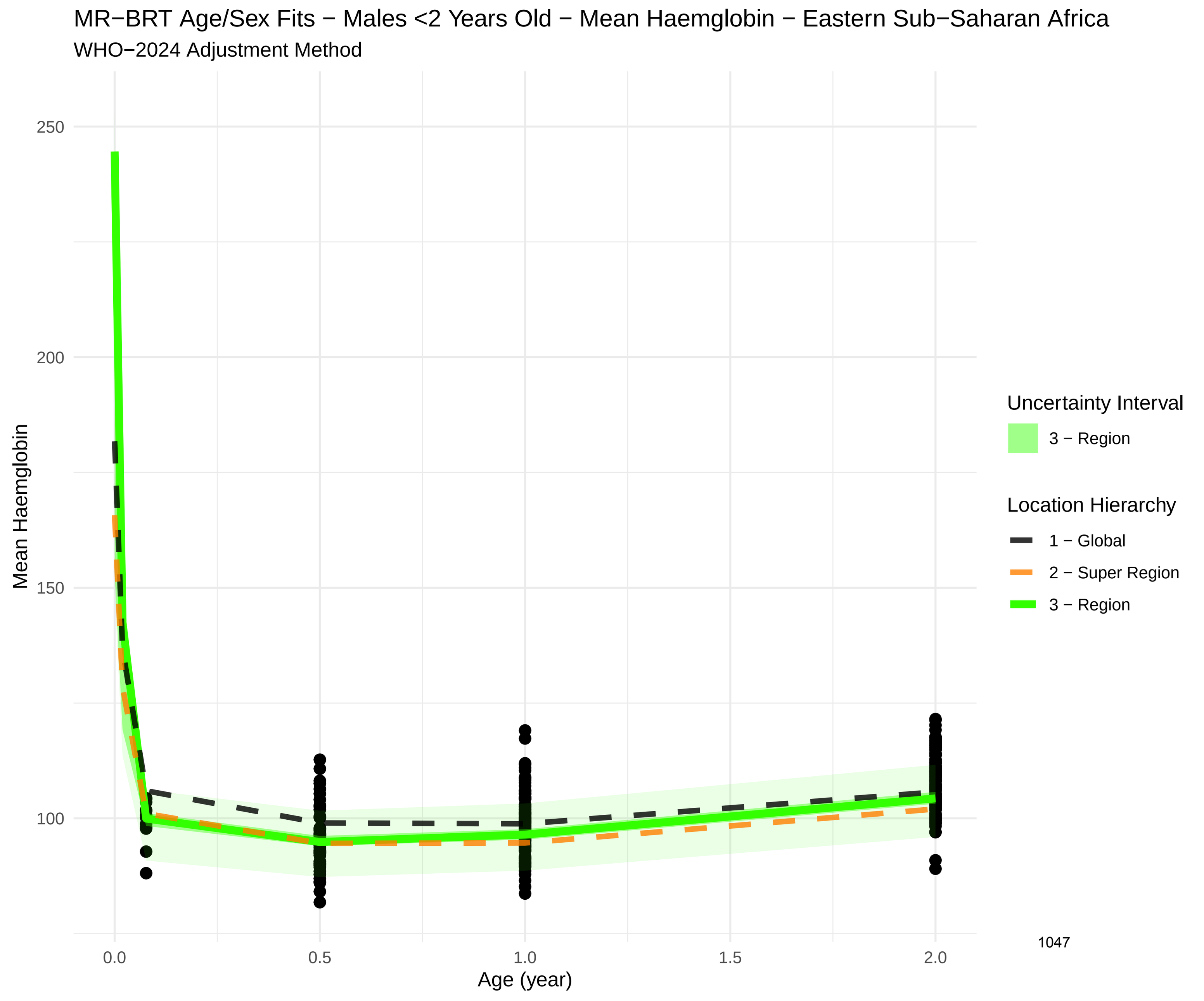

MR−BRT Age/Sex Fits − Males <2 Years Old − Mean Haemglobin − Eastern Sub−Saharan Africa
WHO−2024 Adjustment Method
Mean Haemglobin
Age (year)
Uncertainty Interval
3 − Region
Location Hierarchy
1 − Global
2 − Super Region
3 − Region

MR−BRT Age/Sex Fits − Males <2 Years Old − Mean Haemglobin − Burundi
WHO−2024 Adjustment Method

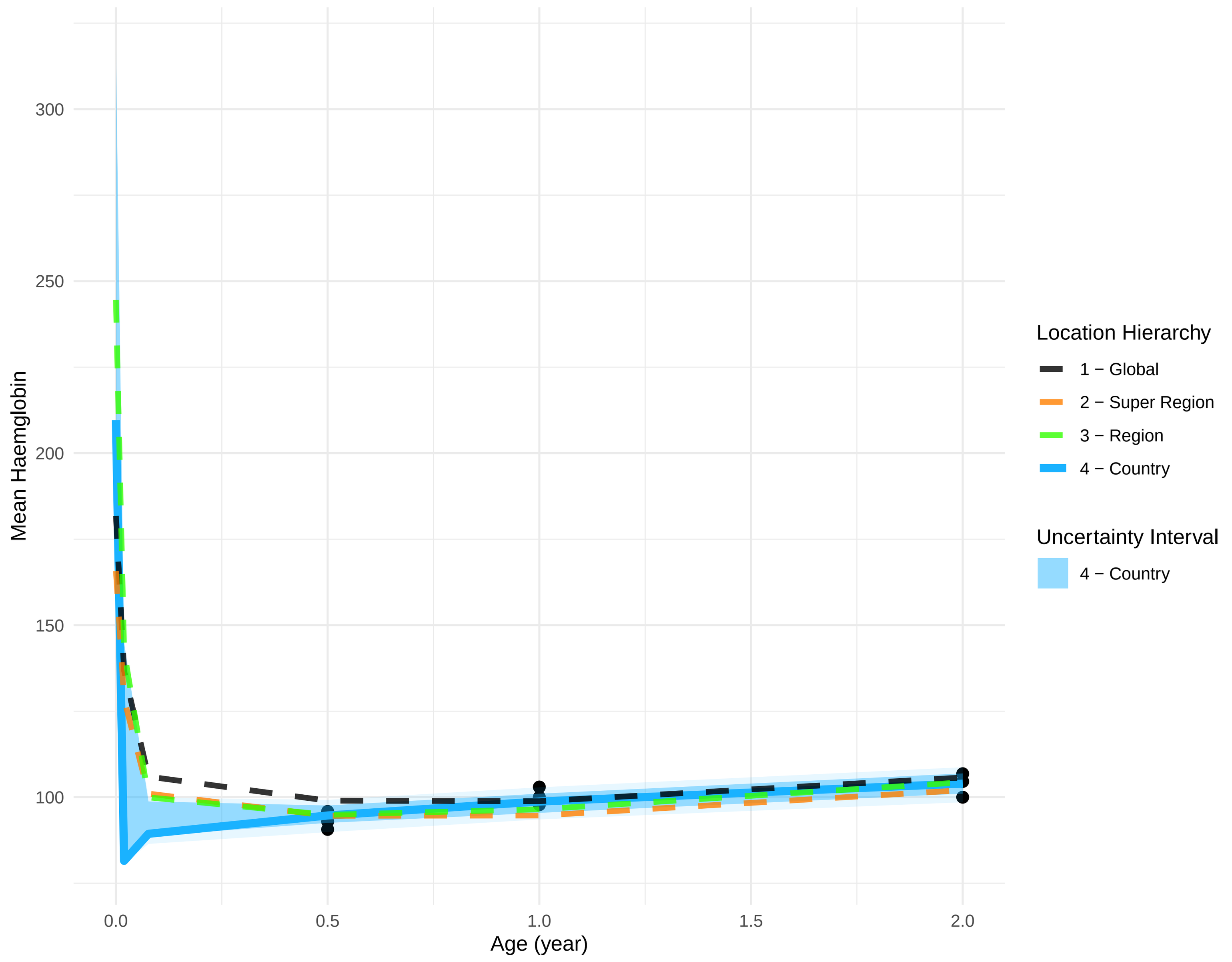

MR−BRT Age/Sex Fits − Males <2 Years Old − Mean Haemglobin − Ethiopia
WHO−2024 Adjustment Method

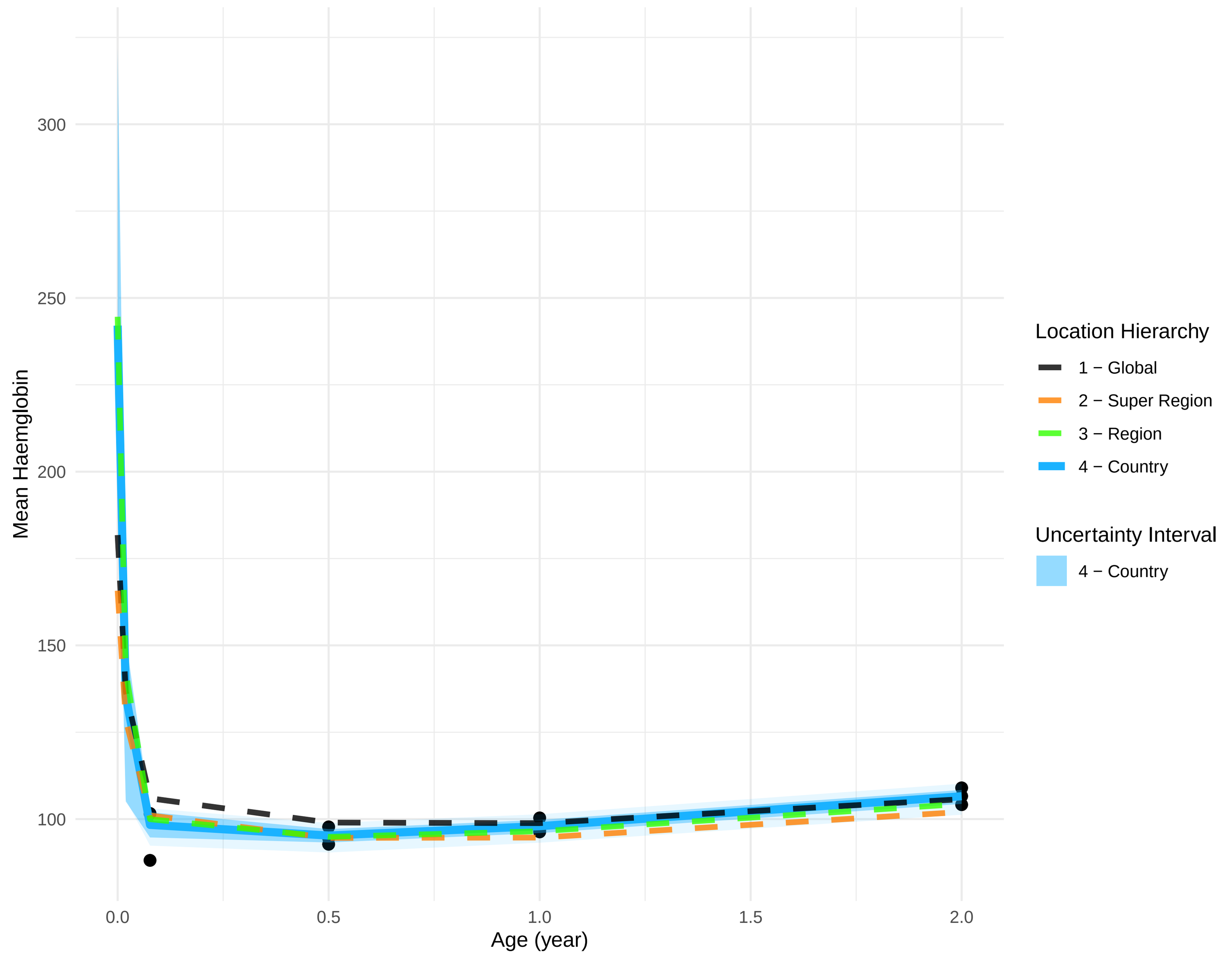

MR−BRT Age/Sex Fits − Males <2 Years Old − Mean Haemglobin − Kenya
WHO−2024 Adjustment Method

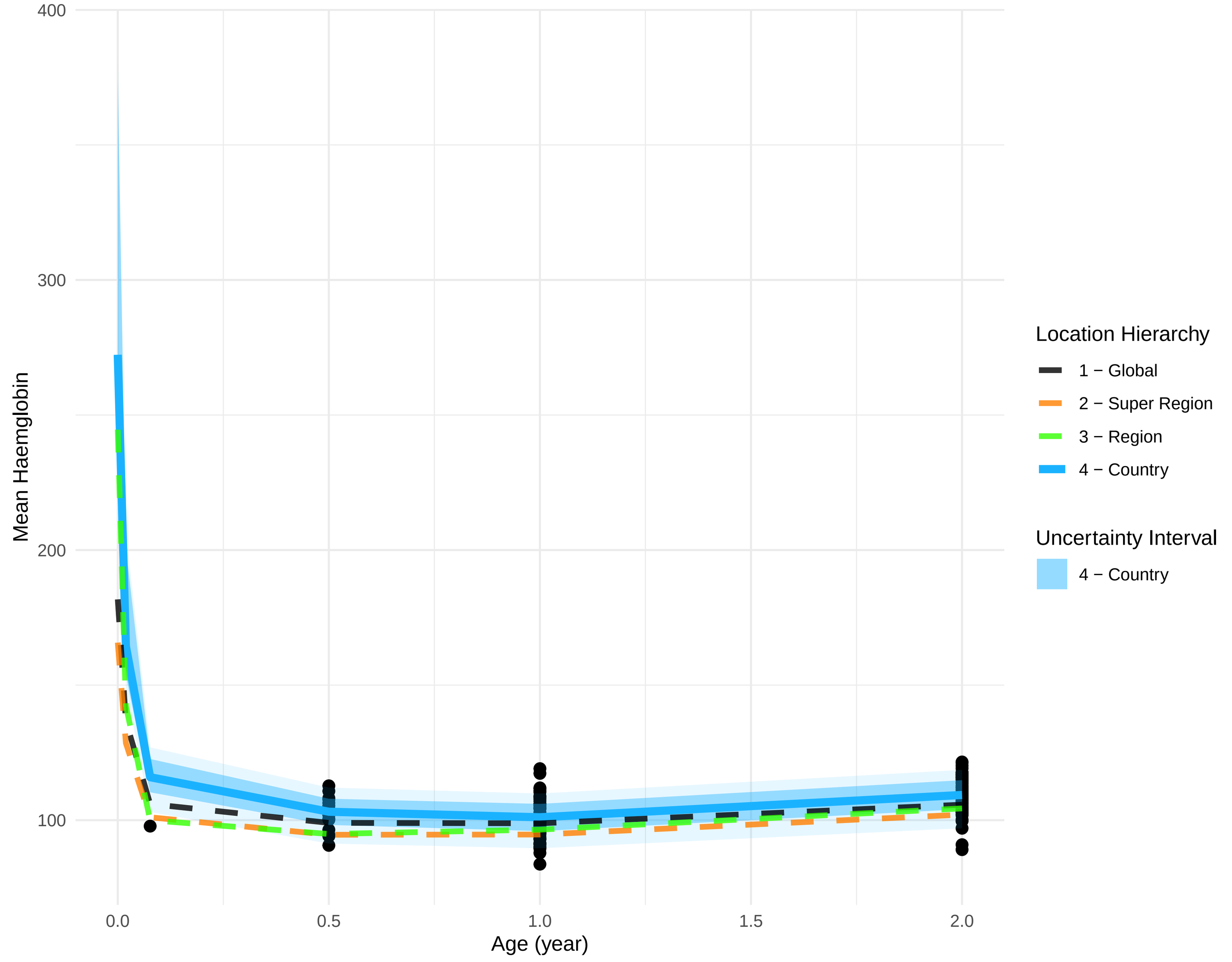

MR−BRT Age/Sex Fits − Males <2 Years Old − Mean Haemglobin − Baringo
WHO−2024 Adjustment Method

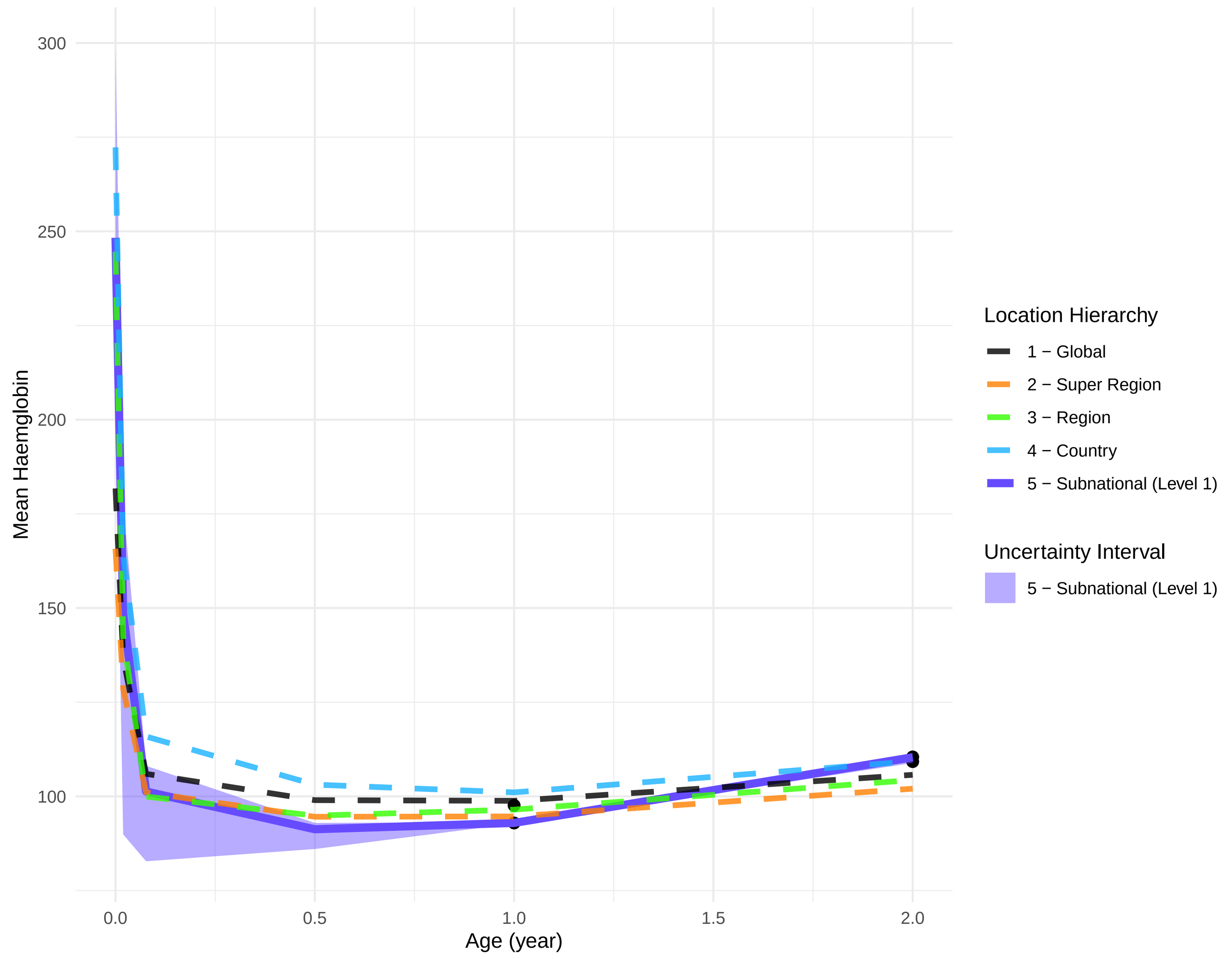

MR−BRT Age/Sex Fits − Males <2 Years Old − Mean Haemglobin − Bomet
WHO−2024 Adjustment Method

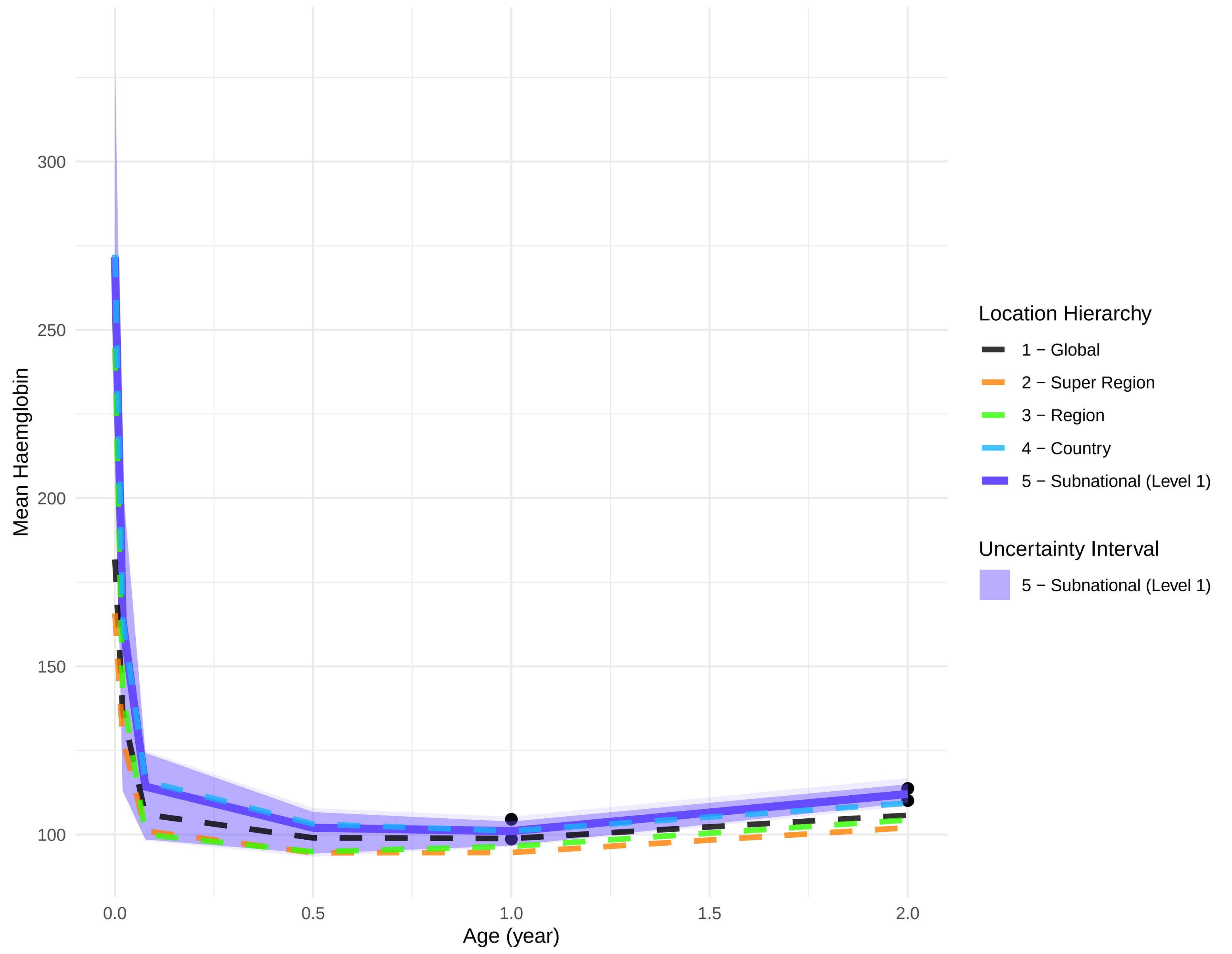

MR−BRT Age/Sex Fits − Males <2 Years Old − Mean Haemglobin − Bungoma
WHO−2024 Adjustment Method

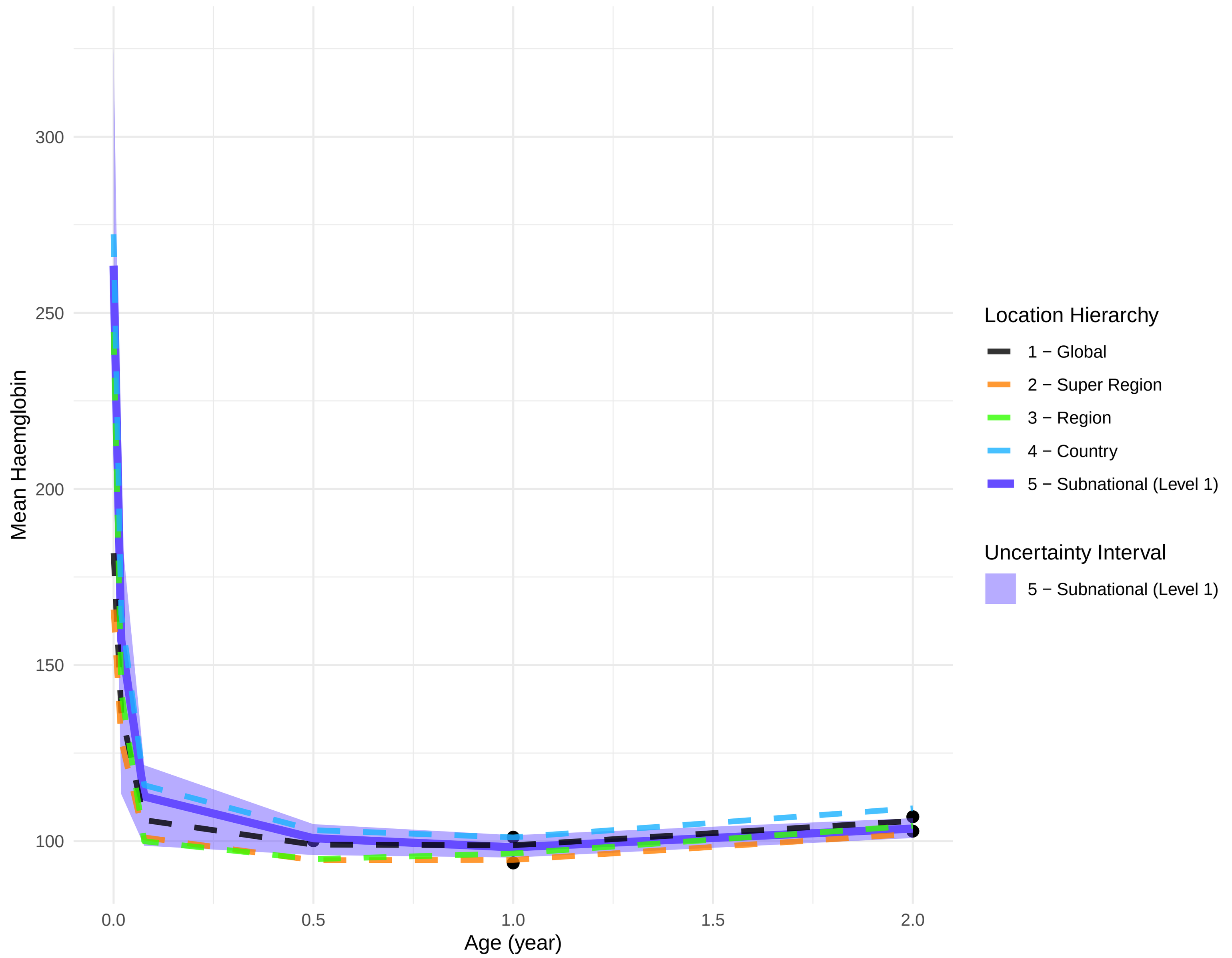

MR−BRT Age/Sex Fits − Males <2 Years Old − Mean Haemglobin − Busia
WHO−2024 Adjustment Method

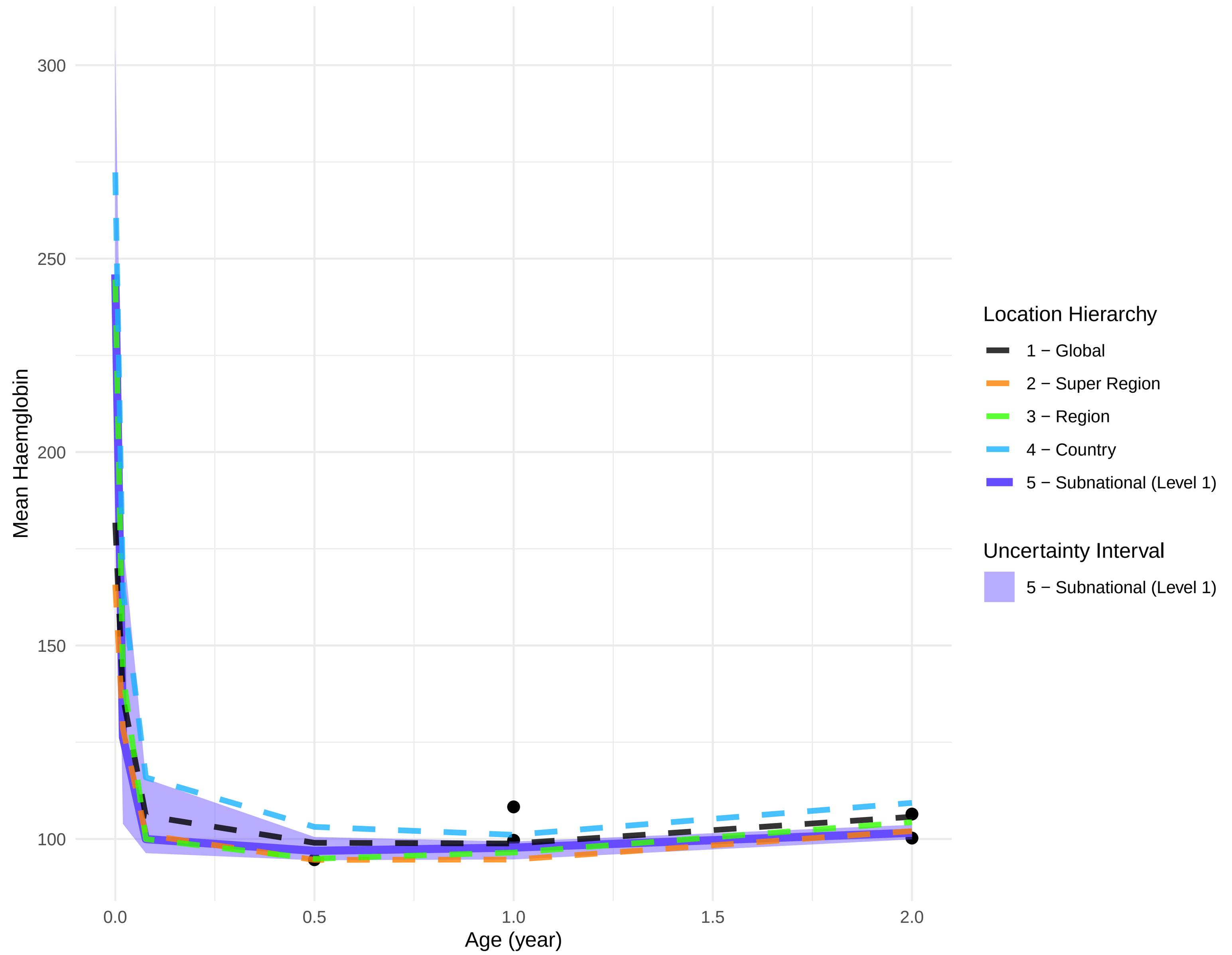

MR−BRT Age/Sex Fits − Males <2 Years Old − Mean Haemglobin − Elgeyo−Marakwet
WHO−2024 Adjustment Method

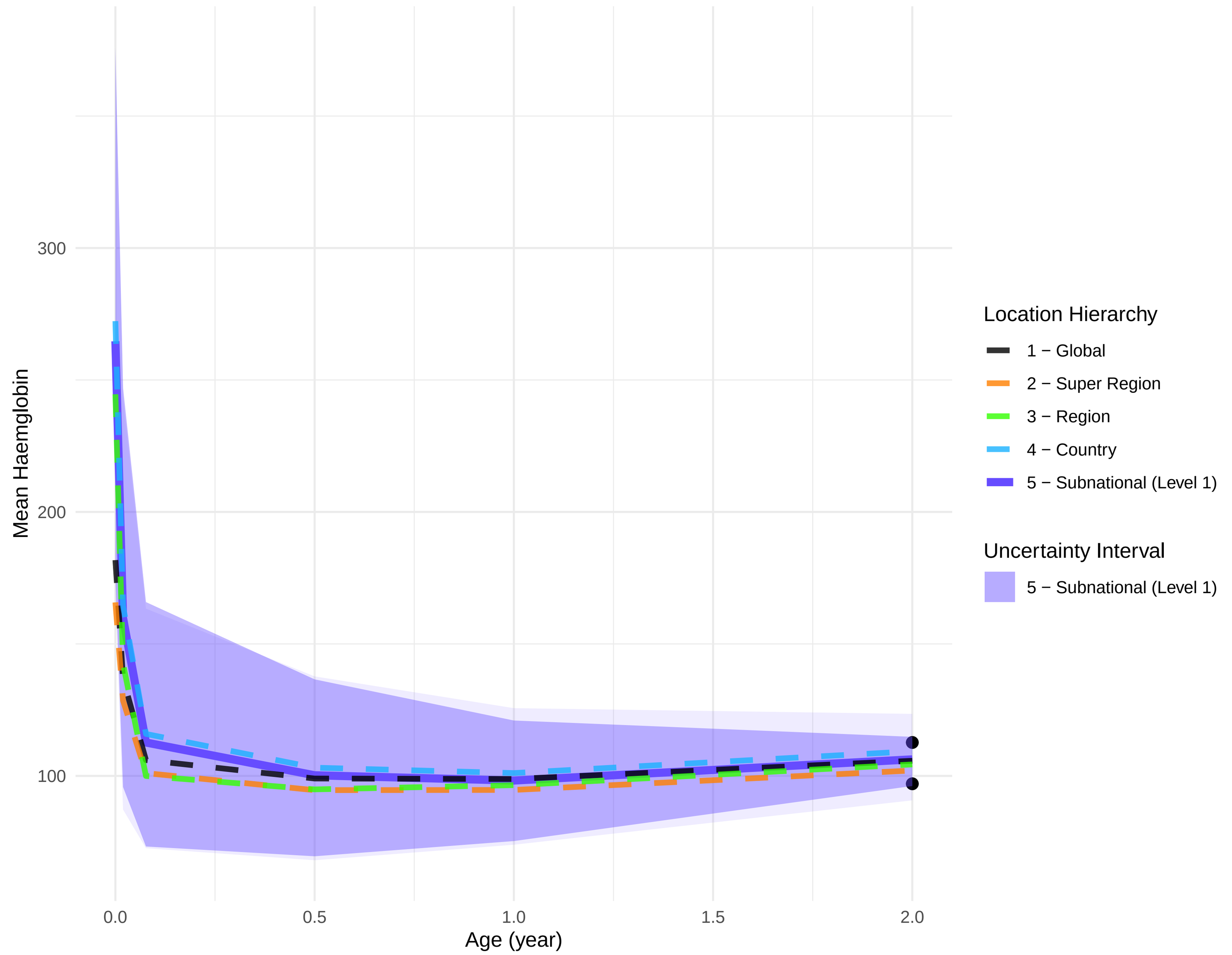

MR−BRT Age/Sex Fits − Males <2 Years Old − Mean Haemglobin − Embu
WHO−2024 Adjustment Method

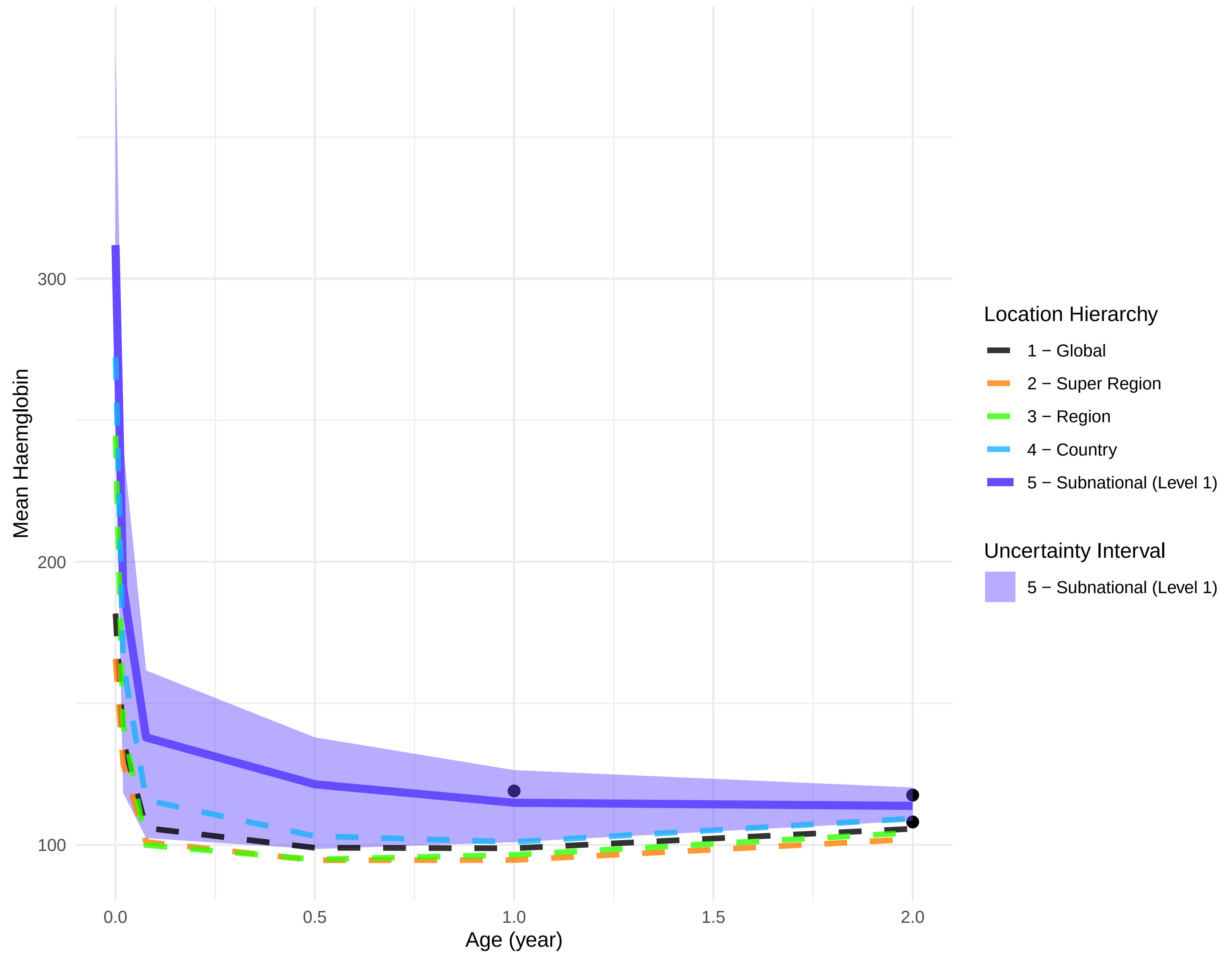

MR−BRT Age/Sex Fits − Males <2 Years Old − Mean Haemglobin − Garissa
WHO−2024 Adjustment Method

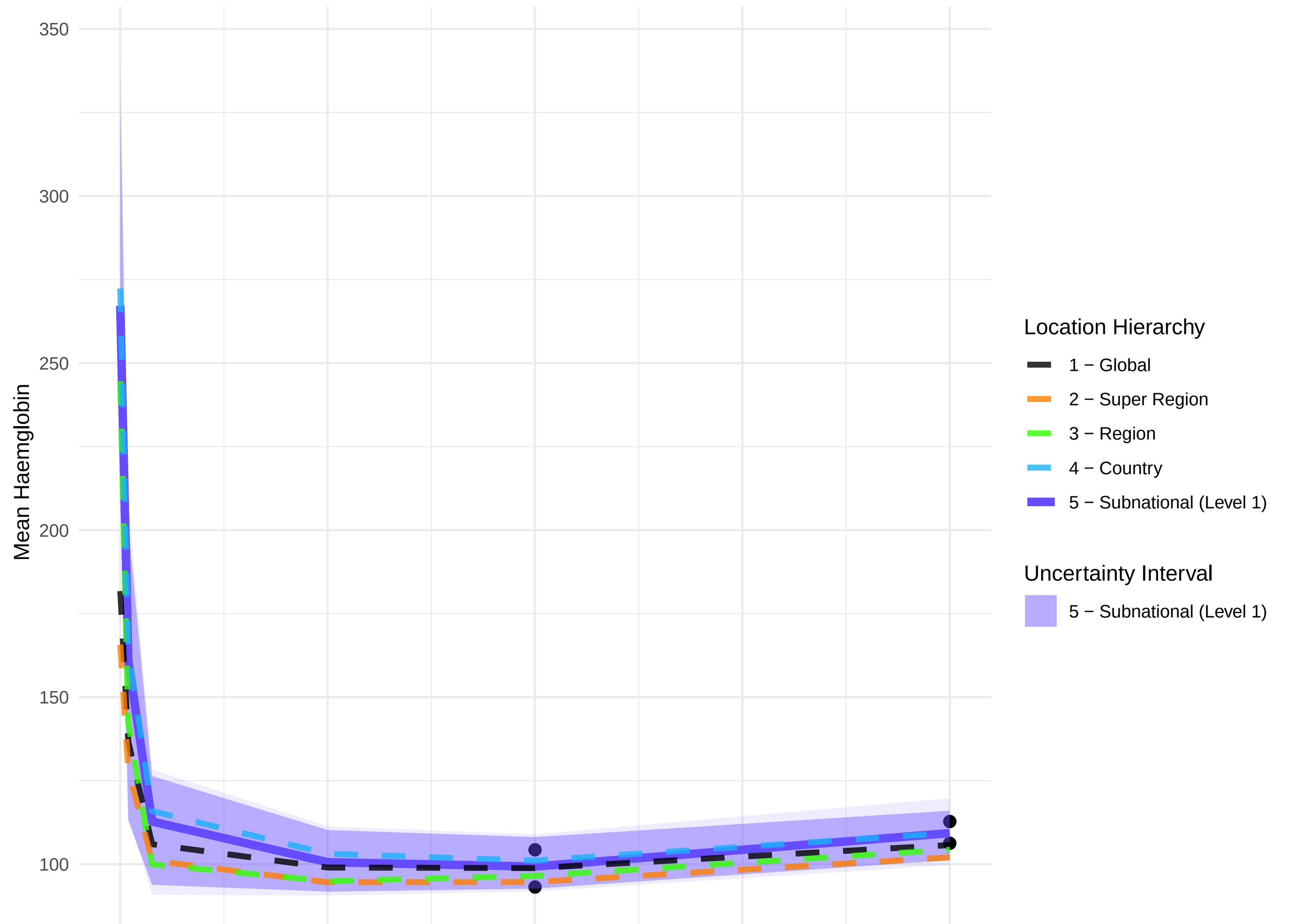

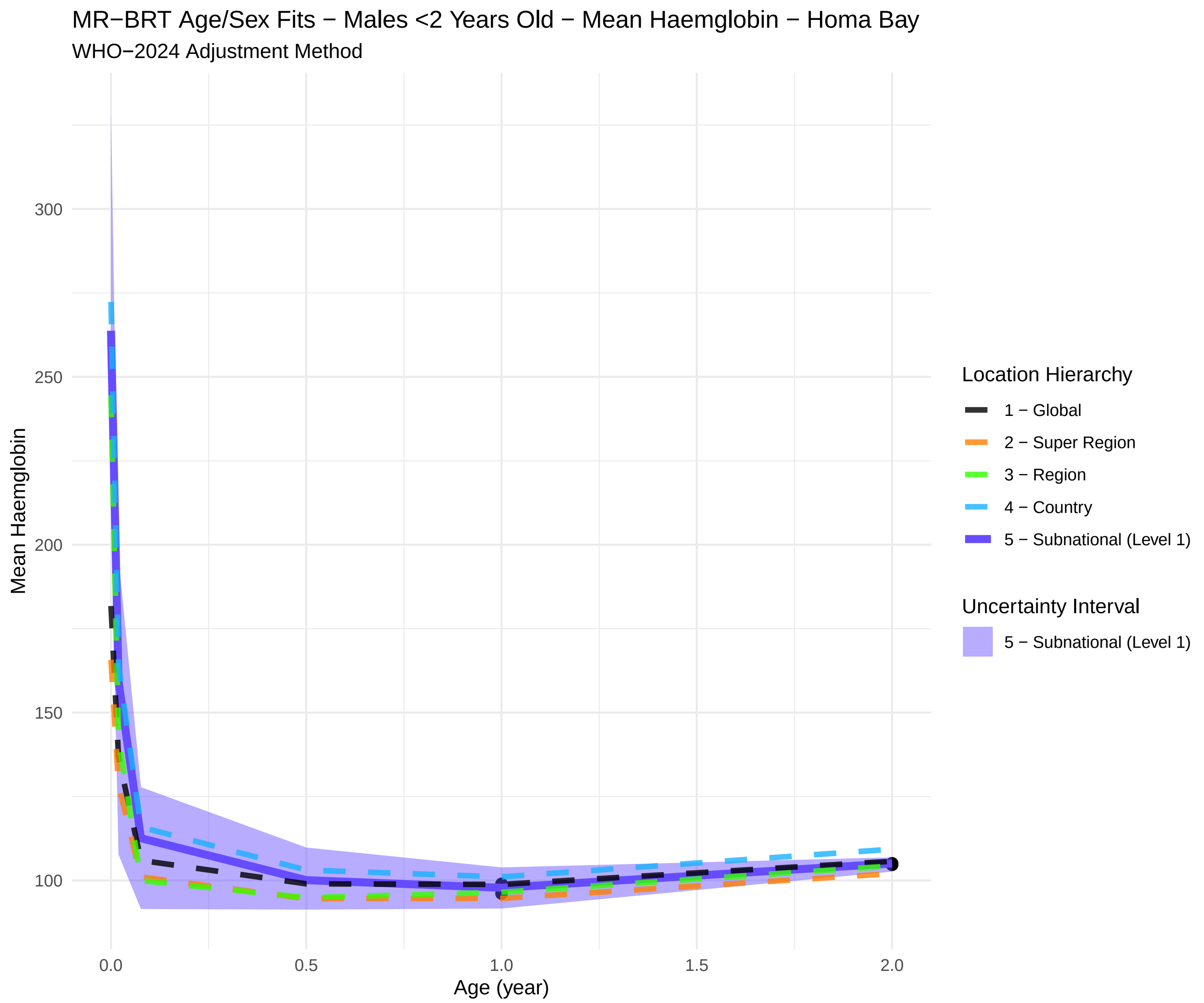
MR−BRT Age/Sex Fits − Males <2 Years Old − Mean Haemglobin − Homa Bay
WHO−2024 Adjustment Method
Mean Haemglobin
Age (year)
Location Hierarchy
1 − Global
2 − Super Region
3 − Region
4 − Country
5 − Subnational (Level 1)
Uncertainty Interval
5 − Subnational (Level 1)

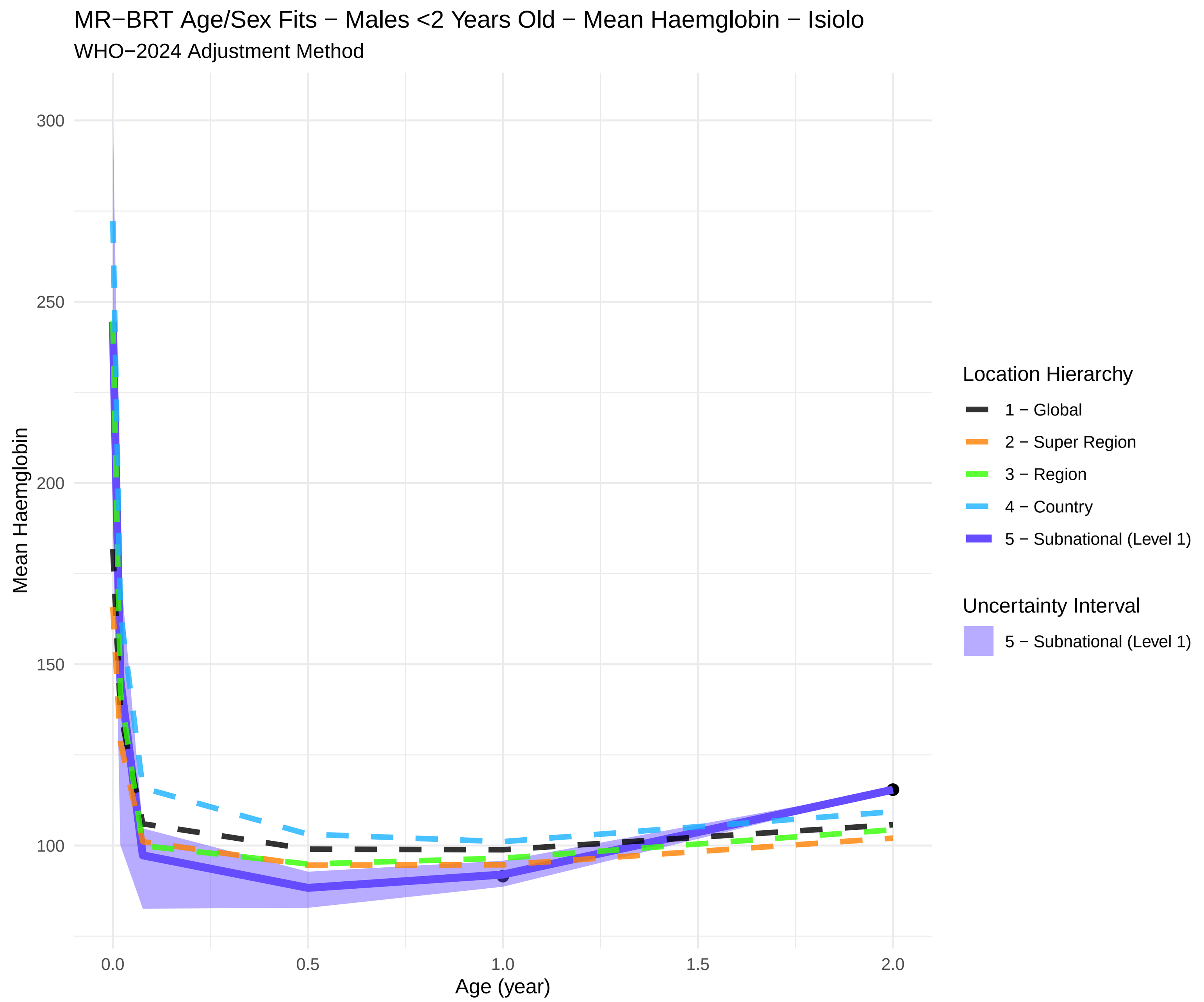
MR−BRT Age/Sex Fits − Males <2 Years Old − Mean Haemglobin − Isiolo
WHO−2024 Adjustment Method
Mean Haemglobin
Age (year)
Location Hierarchy
1 − Global
2 − Super Region
3 − Region
4 − Country
5 − Subnational (Level 1)
Uncertainty Interval
5 − Subnational (Level 1)

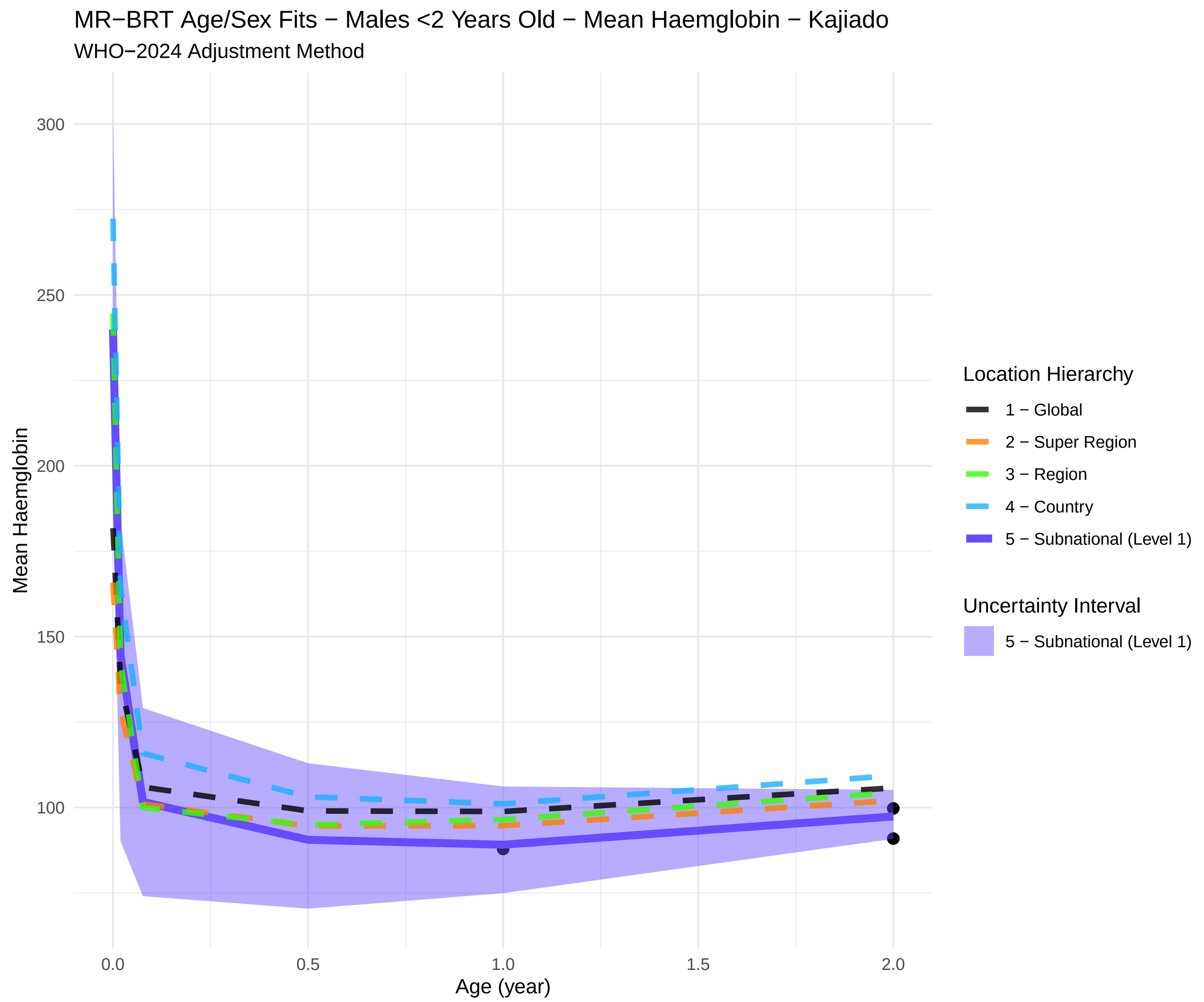
MR−BRT Age/Sex Fits − Males <2 Years Old − Mean Haemglobin − Kajiado
WHO−2024 Adjustment Method
Mean Haemglobin
Age (year)
Location Hierarchy
1 − Global
2 − Super Region
3 − Region
4 − Country
5 − Subnational (Level 1)
Uncertainty Interval
5 − Subnational (Level 1)

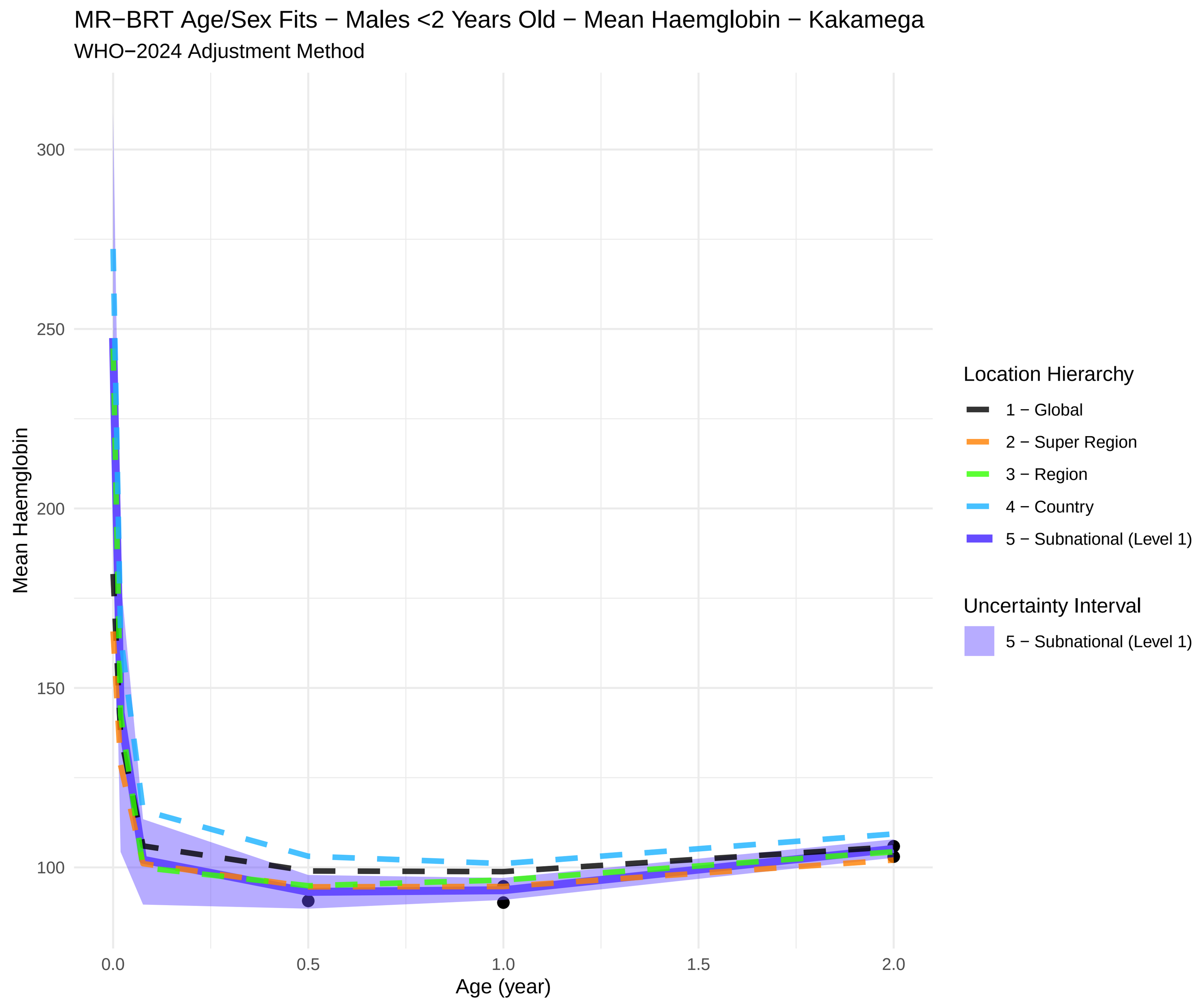
MR−BRT Age/Sex Fits − Males <2 Years Old − Mean Haemglobin − Kakamega
WHO−2024 Adjustment Method
Mean Haemglobin
Age (year)
Location Hierarchy
1 − Global
2 − Super Region
3 − Region
4 − Country
5 − Subnational (Level 1)
Uncertainty Interval
5 − Subnational (Level 1)

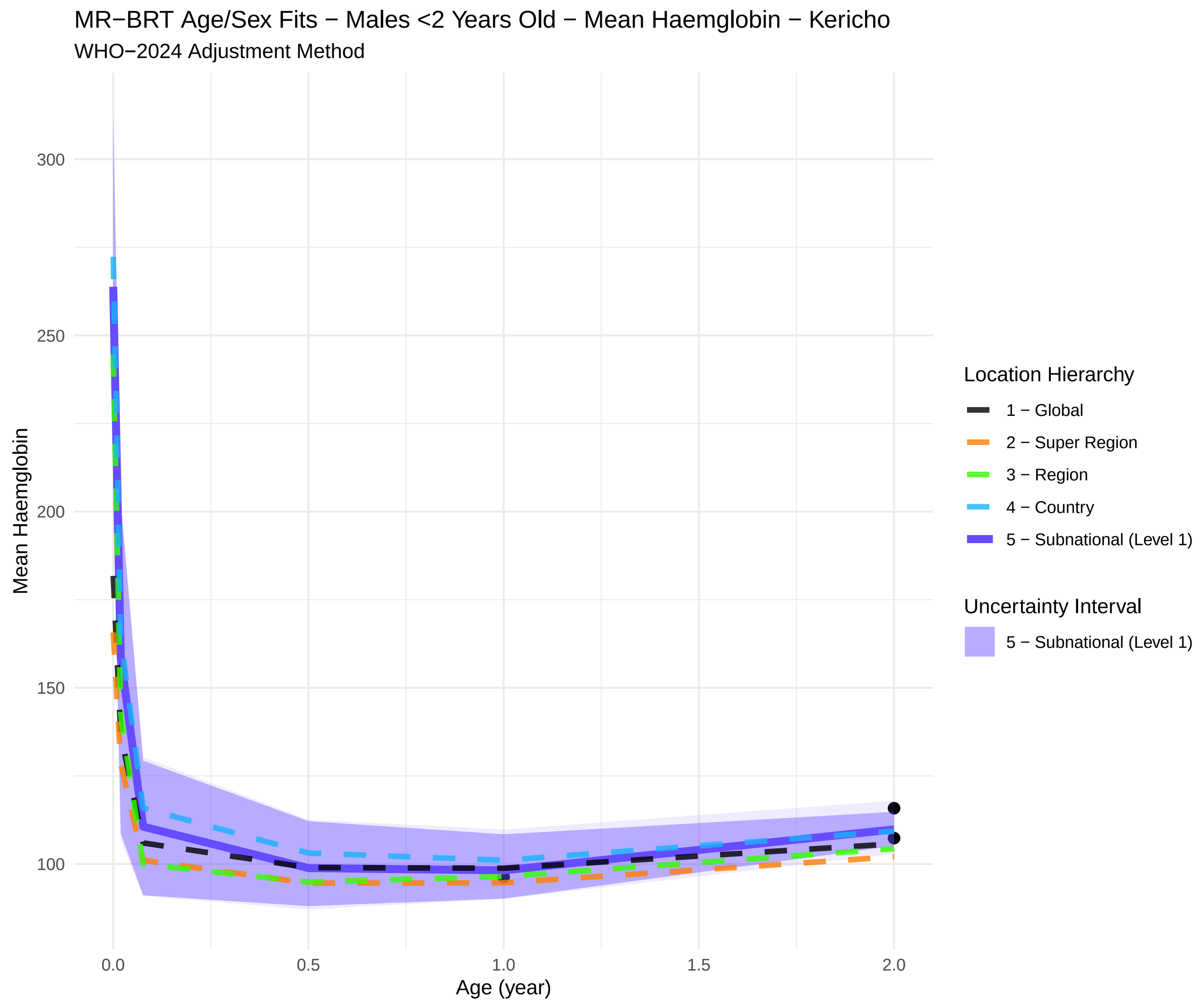
MR−BRT Age/Sex Fits − Males <2 Years Old − Mean Haemglobin − Kericho
WHO−2024 Adjustment Method
Mean Haemglobin
Age (year)
Location Hierarchy
1 − Global
2 − Super Region
3 − Region
4 − Country
5 − Subnational (Level 1)
Uncertainty Interval
5 − Subnational (Level 1)

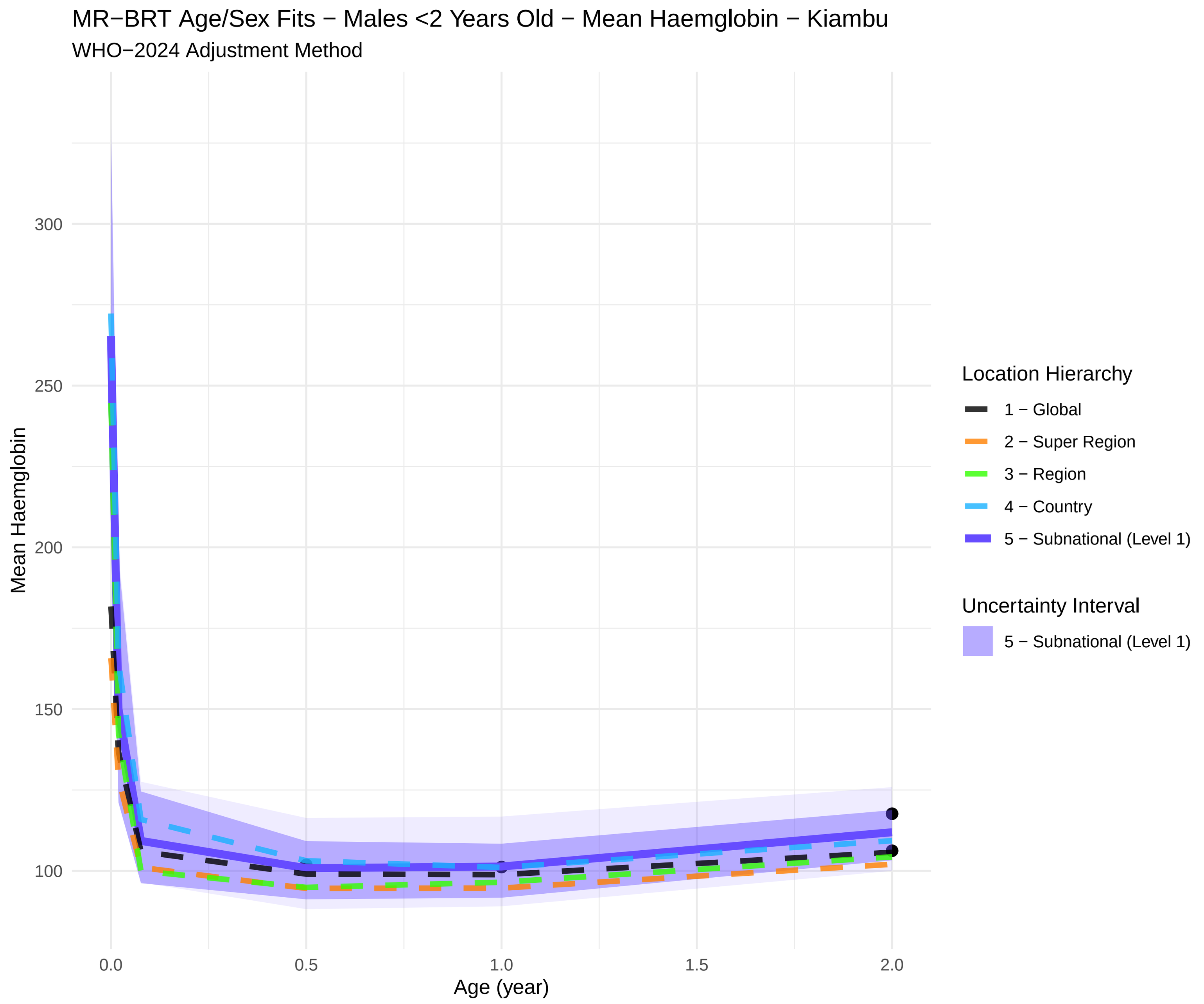
MR−BRT Age/Sex Fits − Males <2 Years Old − Mean Haemglobin − Kiambu
WHO−2024 Adjustment Method
Mean Haemglobin
Age (year)
Location Hierarchy
1 − Global
2 − Super Region
3 − Region
4 − Country
5 − Subnational (Level 1)
Uncertainty Interval
5 − Subnational (Level 1)

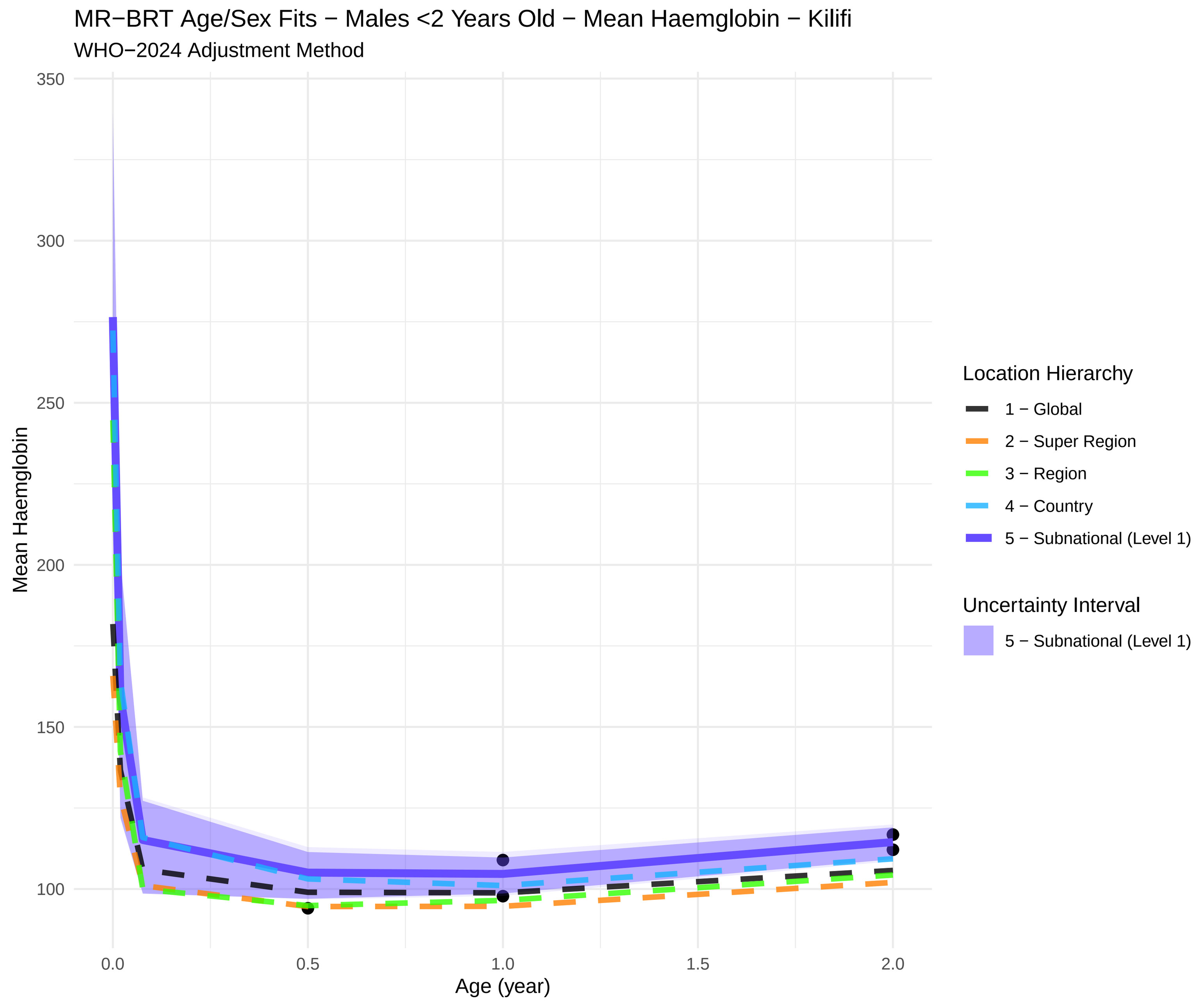
MR−BRT Age/Sex Fits − Males <2 Years Old − Mean Haemglobin − Kilifi
WHO−2024 Adjustment Method
Mean Haemglobin
Age (year)
Location Hierarchy
1 − Global
2 − Super Region
3 − Region
4 − Country
5 − Subnational (Level 1)
Uncertainty Interval
5 − Subnational (Level 1)

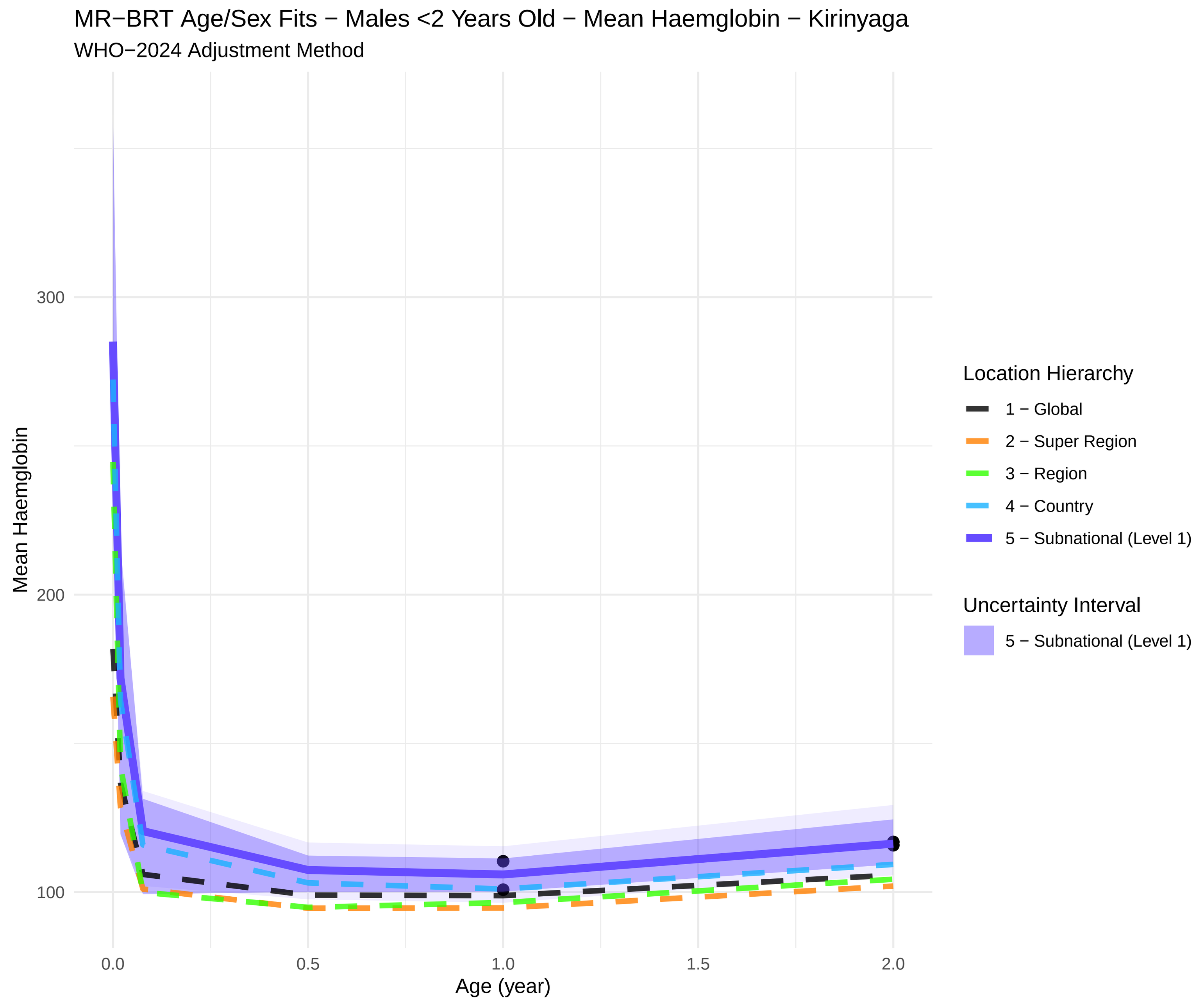
MR−BRT Age/Sex Fits − Males <2 Years Old − Mean Haemglobin − Kirinyaga
WHO−2024 Adjustment Method
Mean Haemglobin
Age (year)
Location Hierarchy
1 − Global
2 − Super Region
3 − Region
4 − Country
5 − Subnational (Level 1)
Uncertainty Interval
5 − Subnational (Level 1)

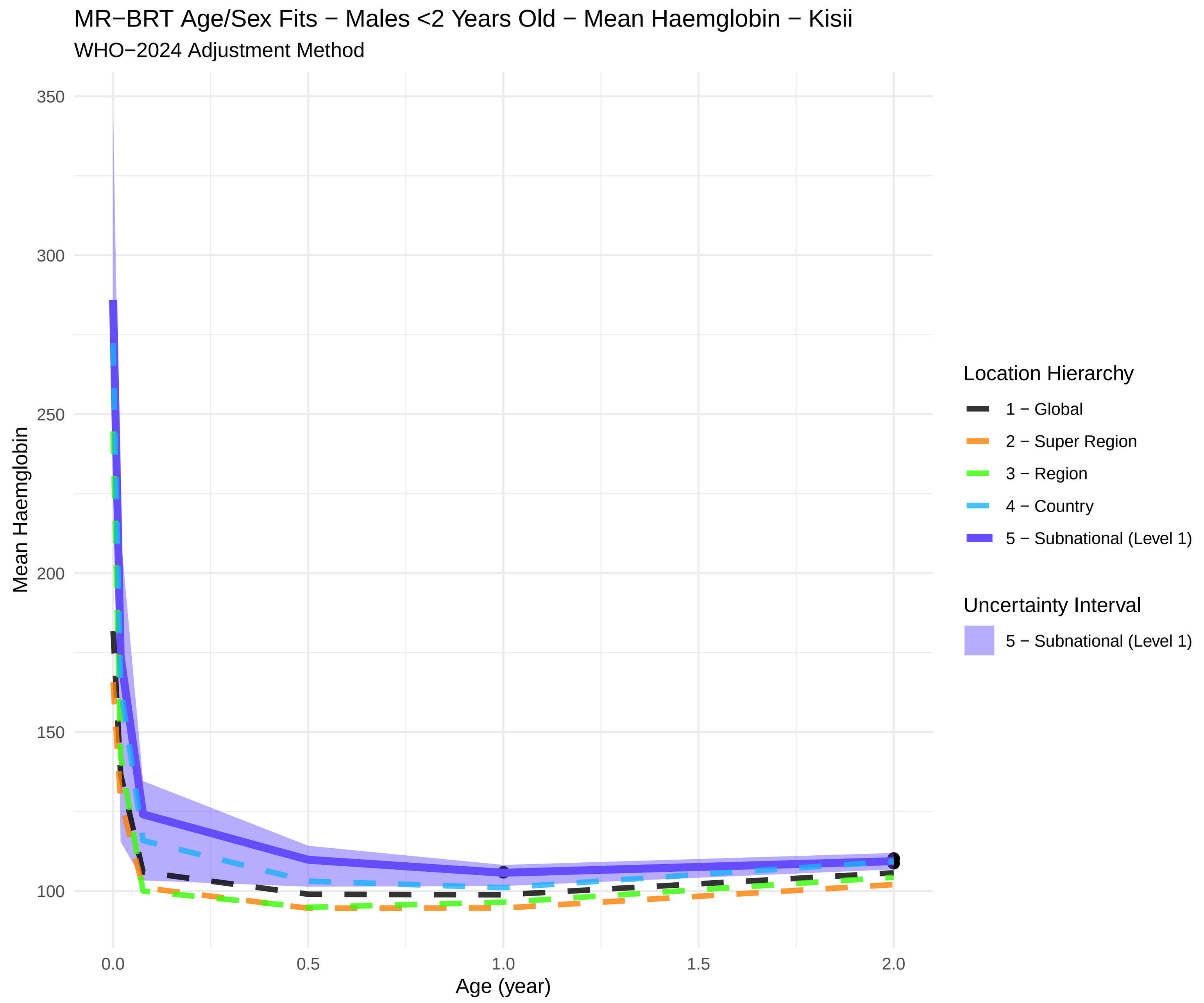
MR−BRT Age/Sex Fits − Males <2 Years Old − Mean Haemglobin − Kisii
WHO−2024 Adjustment Method
Mean Haemglobin
Age (year)
Location Hierarchy
1 − Global
2 − Super Region
3 − Region
4 − Country
5 − Subnational (Level 1)
Uncertainty Interval
5 − Subnational (Level 1)

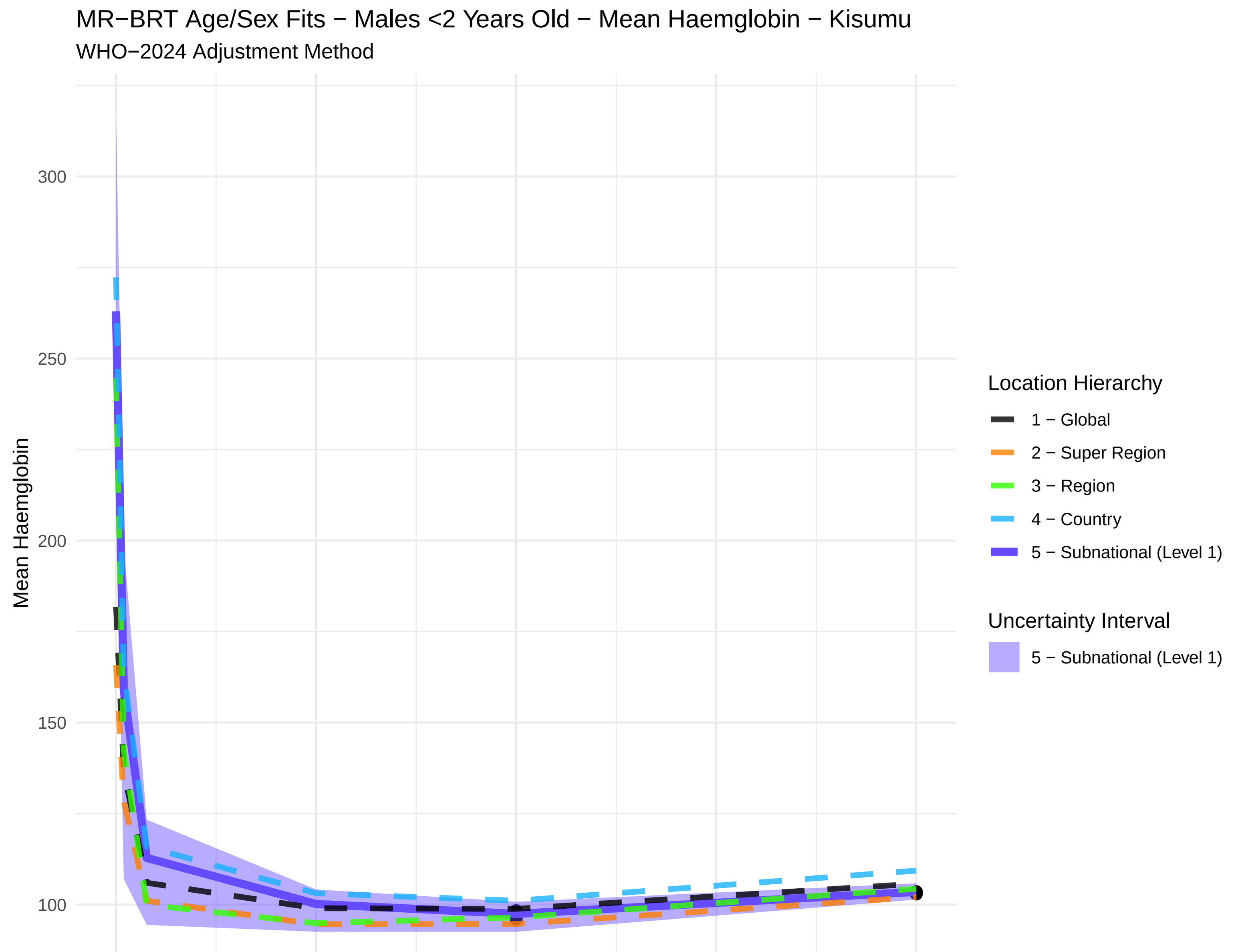
MR−BRT Age/Sex Fits − Males <2 Years Old − Mean Haemglobin − Kisumu
WHO−2024 Adjustment Method
Mean Haemglobin
Age (year)
Location Hierarchy
1 − Global
2 − Super Region
3 − Region
4 − Country
5 − Subnational (Level 1)
Uncertainty Interval
5 − Subnational (Level 1)

MR−BRT Age/Sex Fits − Males <2 Years Old − Mean Haemglobin − Kitui
WHO−2024 Adjustment Method

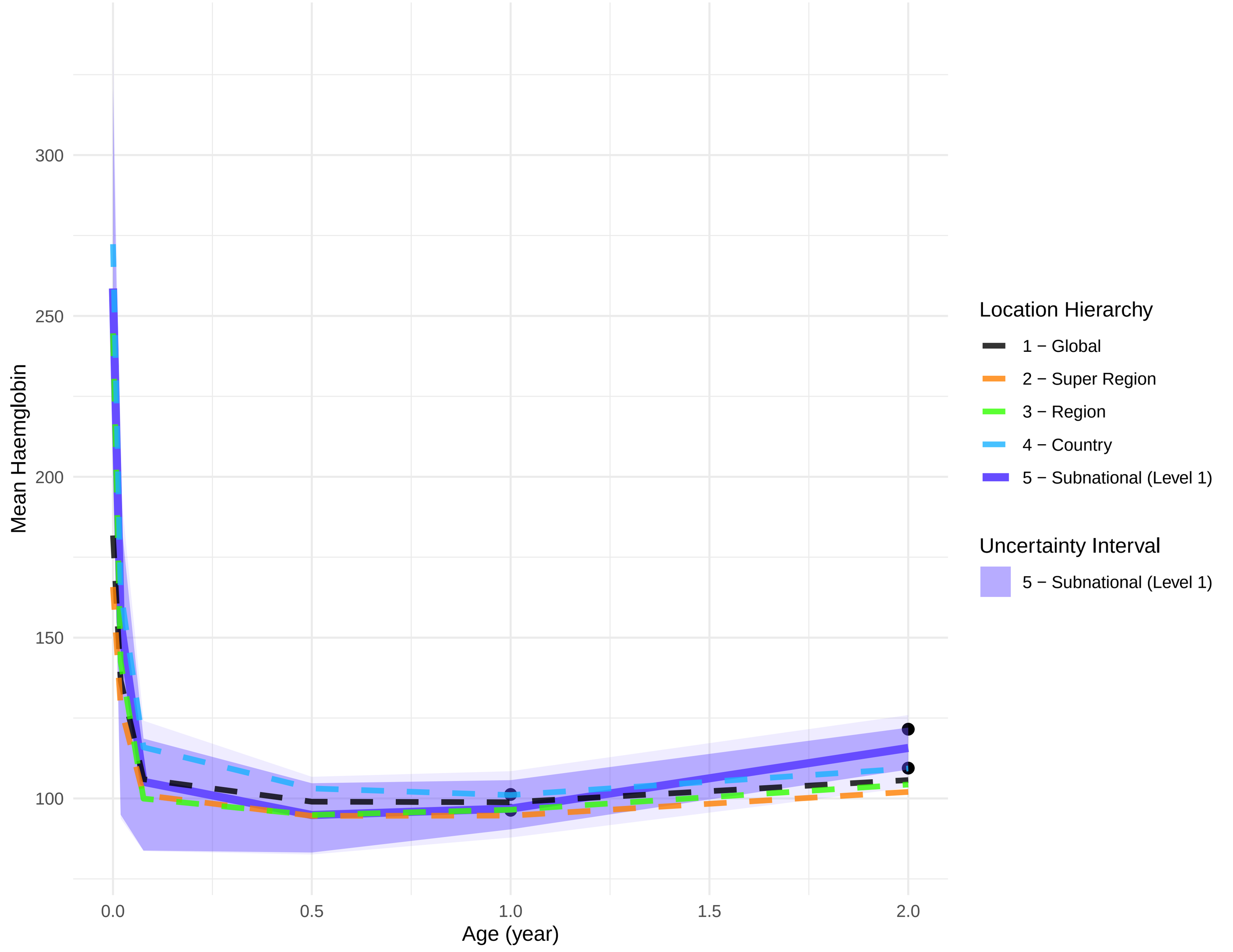

MR−BRT Age/Sex Fits − Males <2 Years Old − Mean Haemglobin − Kwale
WHO−2024 Adjustment Method

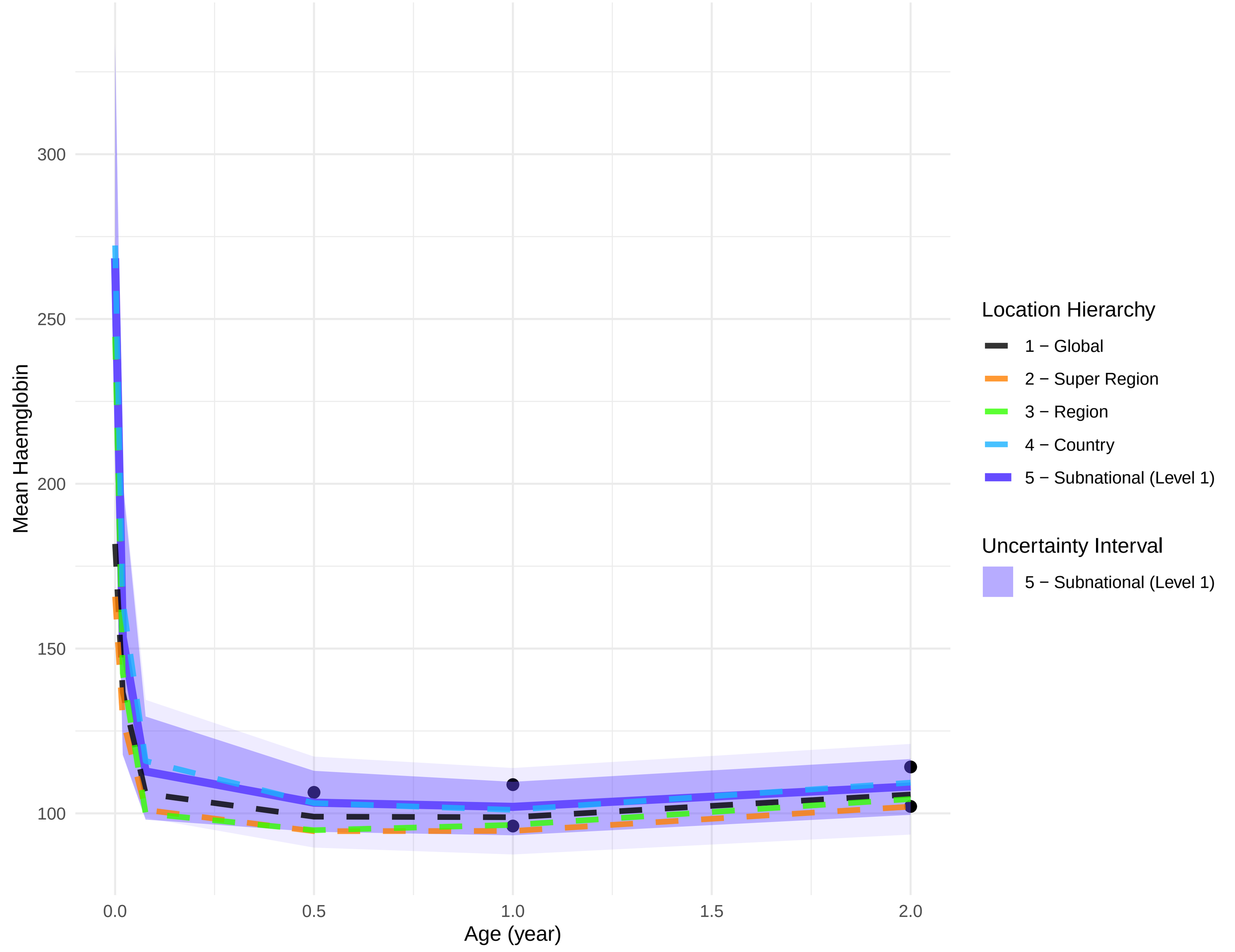

MR−BRT Age/Sex Fits − Males <2 Years Old − Mean Haemglobin − Laikipia
WHO−2024 Adjustment Method

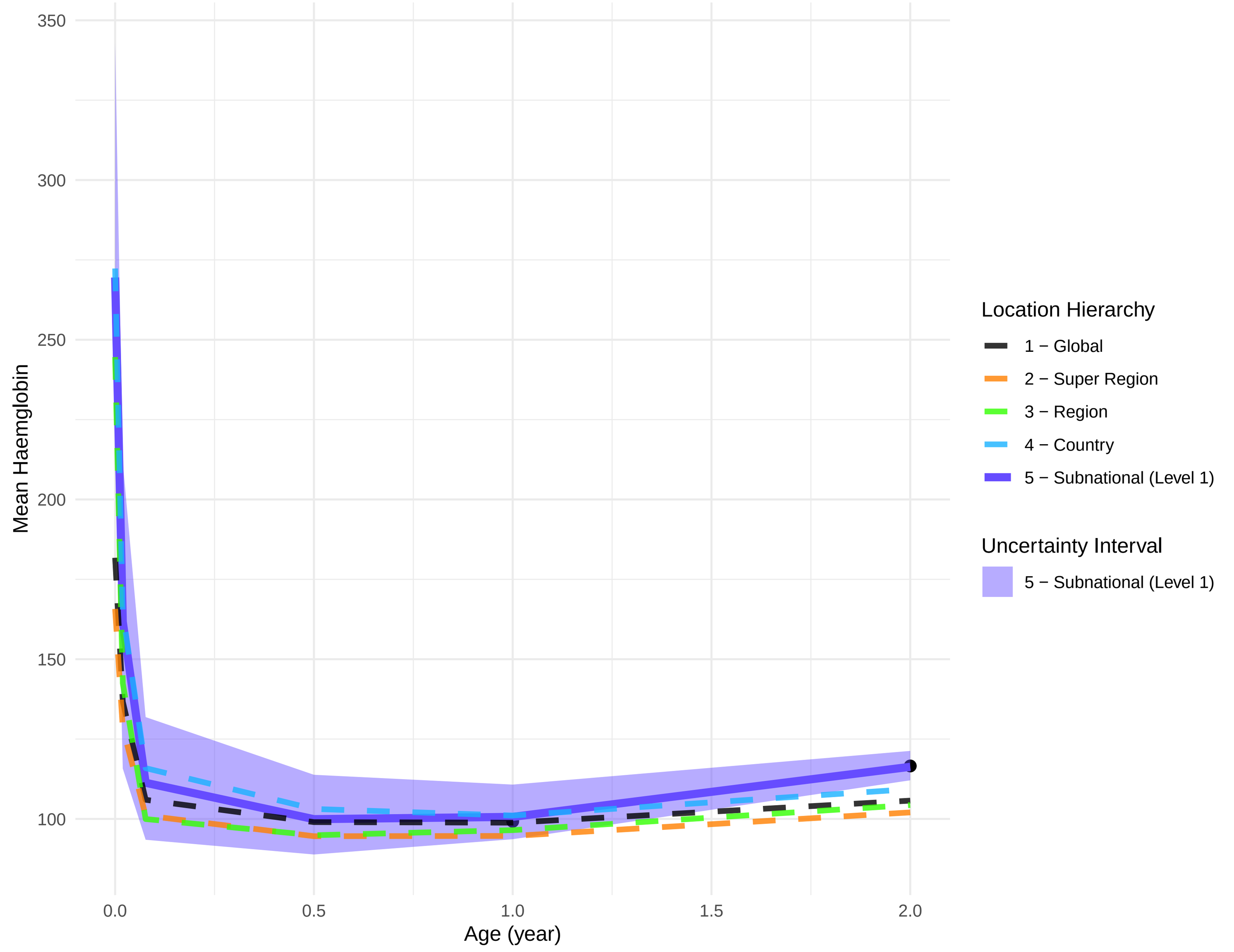

MR−BRT Age/Sex Fits − Males <2 Years Old − Mean Haemglobin − Lamu
WHO−2024 Adjustment Method

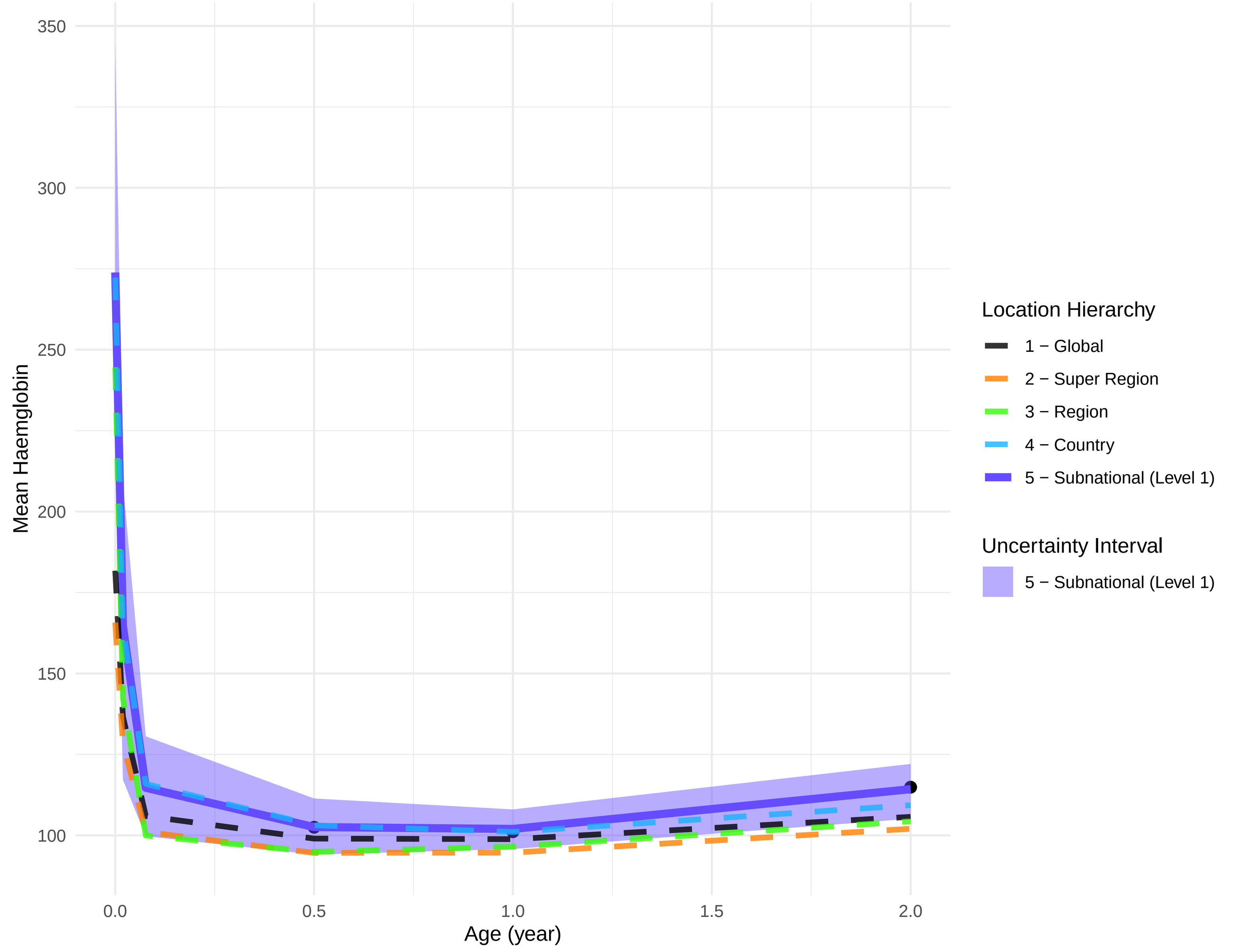

MR−BRT Age/Sex Fits − Males <2 Years Old − Mean Haemglobin − Machakos
WHO−2024 Adjustment Method

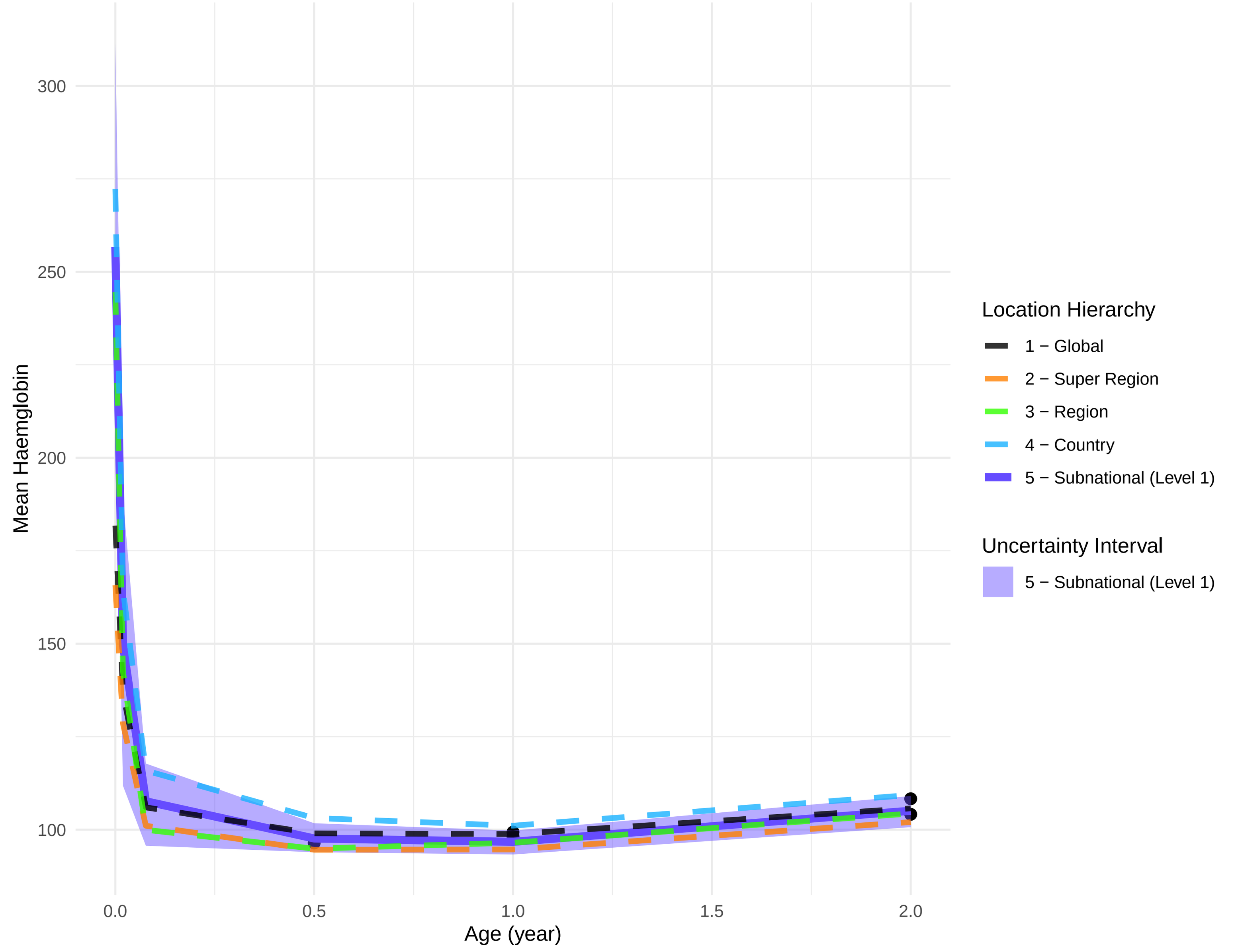

MR−BRT Age/Sex Fits − Males <2 Years Old − Mean Haemglobin − Makueni
WHO−2024 Adjustment Method

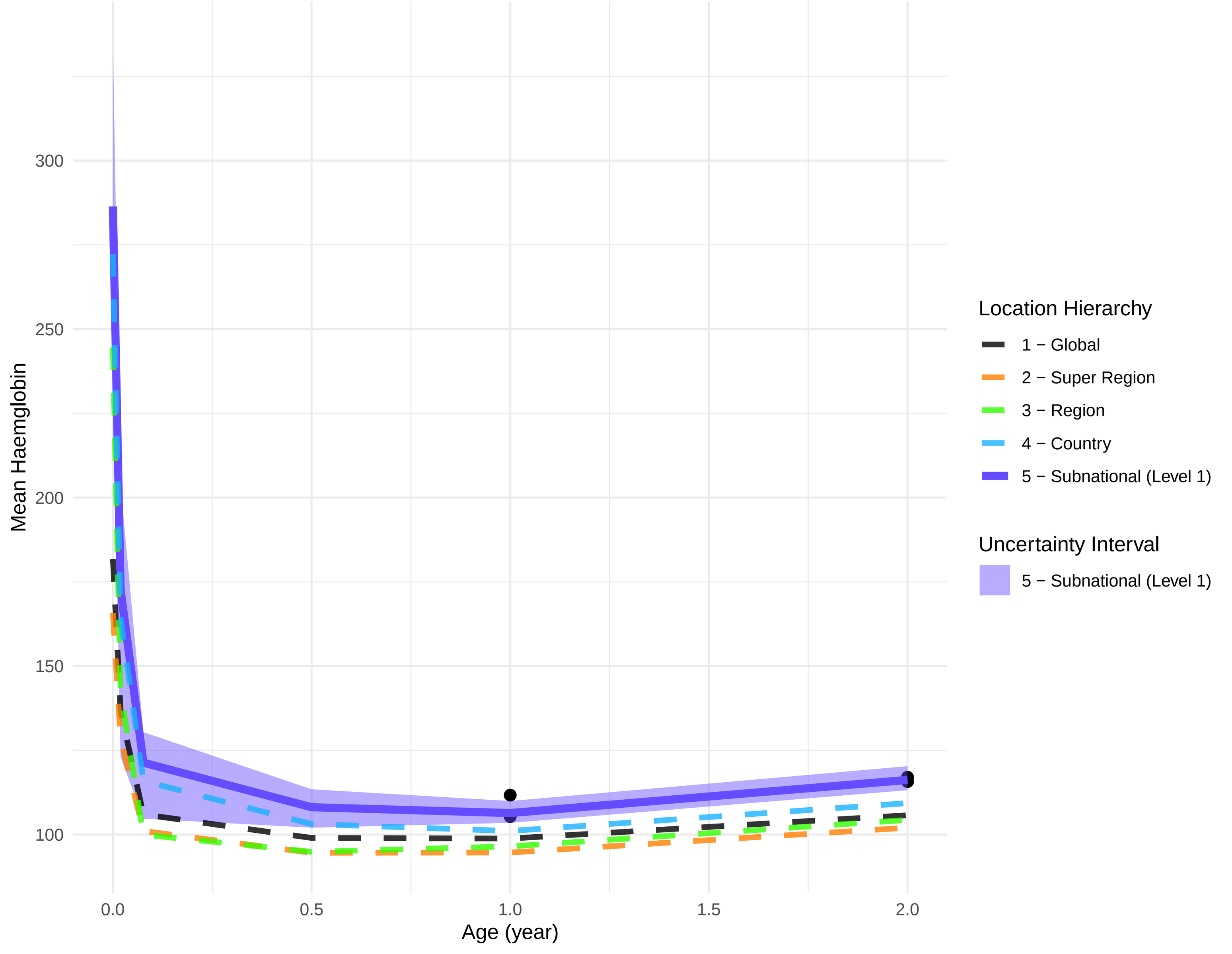

MR−BRT Age/Sex Fits − Males <2 Years Old − Mean Haemglobin − Mandera
WHO−2024 Adjustment Method

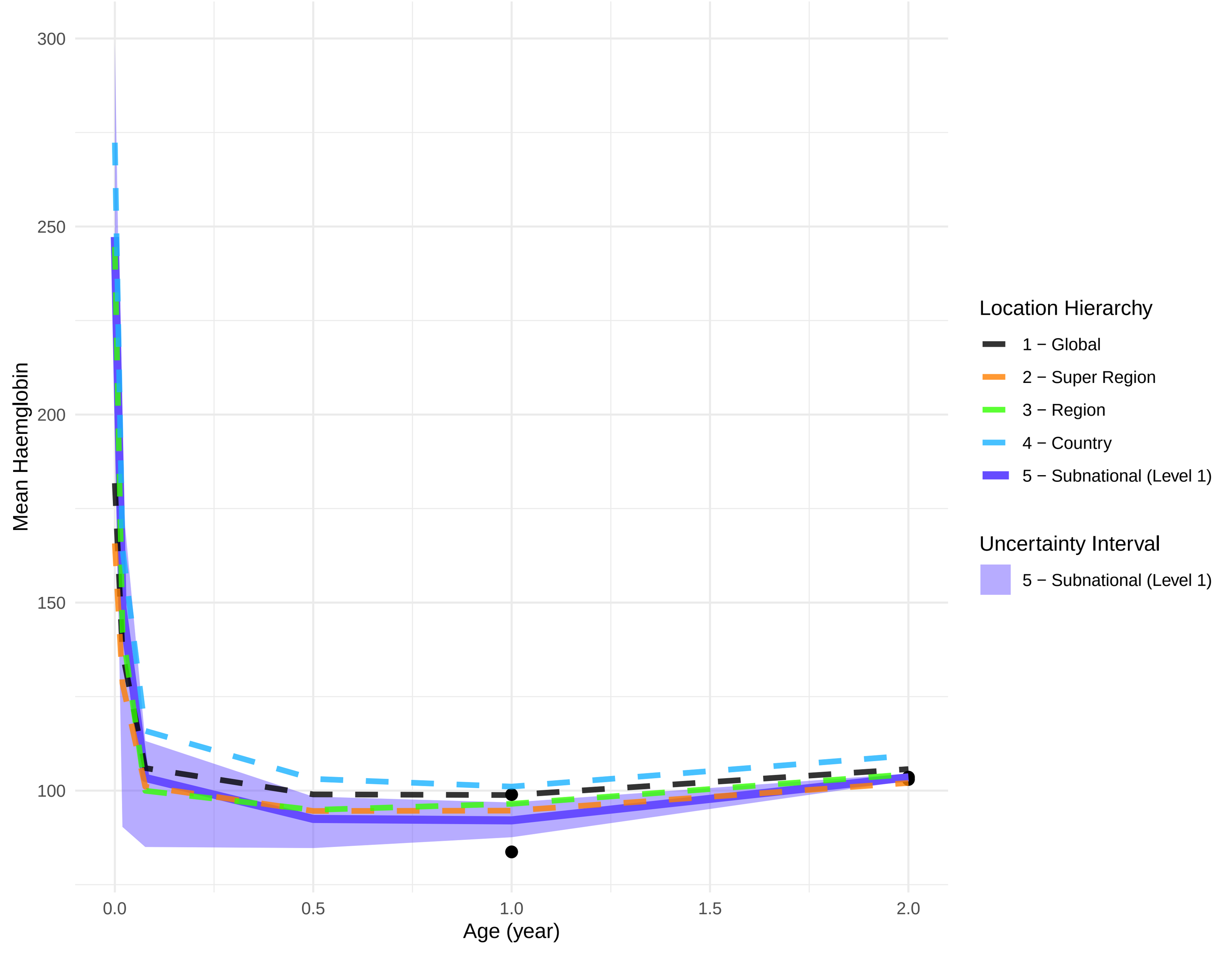

MR−BRT Age/Sex Fits − Males <2 Years Old − Mean Haemglobin − Marsabit
WHO−2024 Adjustment Method

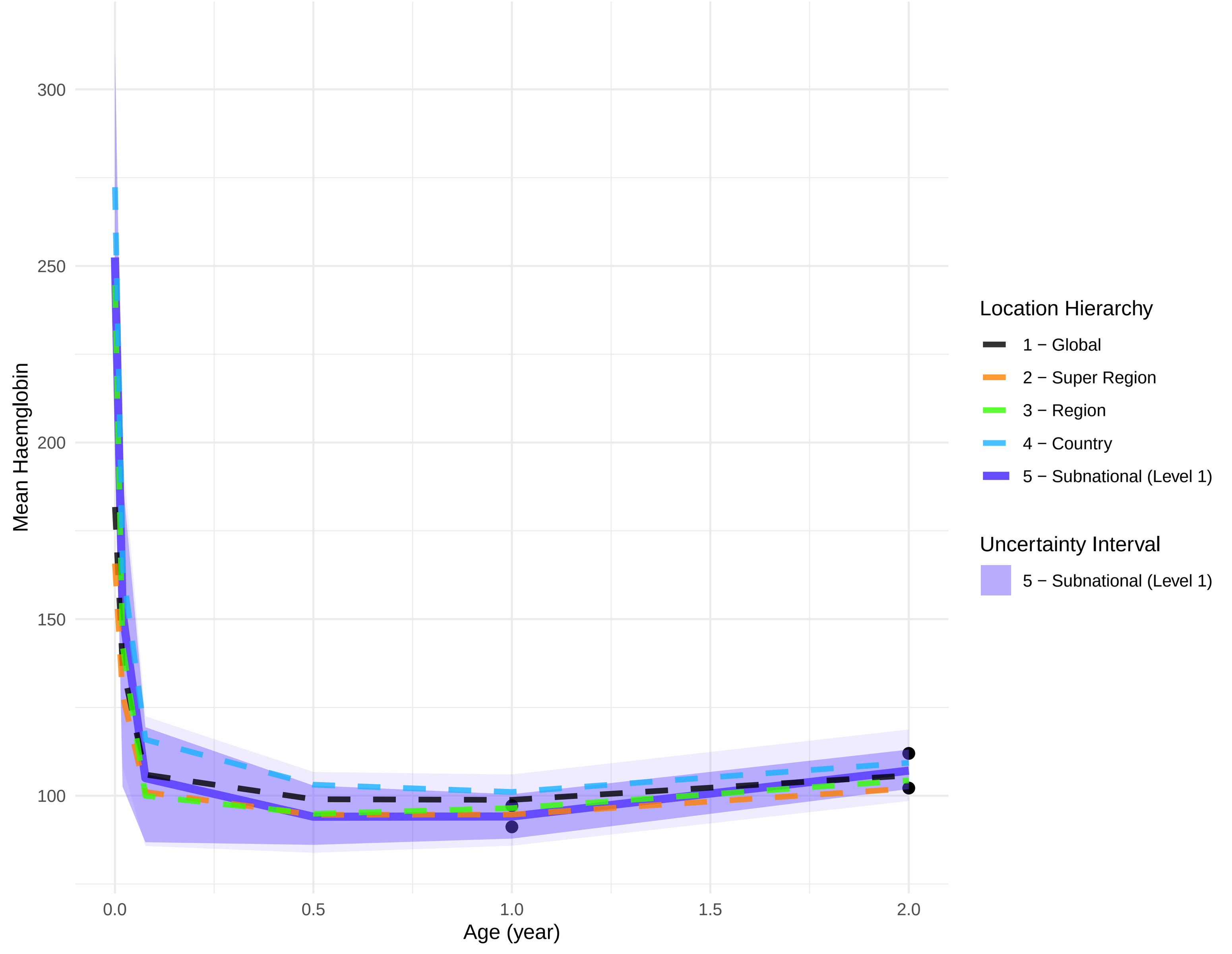

MR−BRT Age/Sex Fits − Males <2 Years Old − Mean Haemglobin − Meru
WHO−2024 Adjustment Method

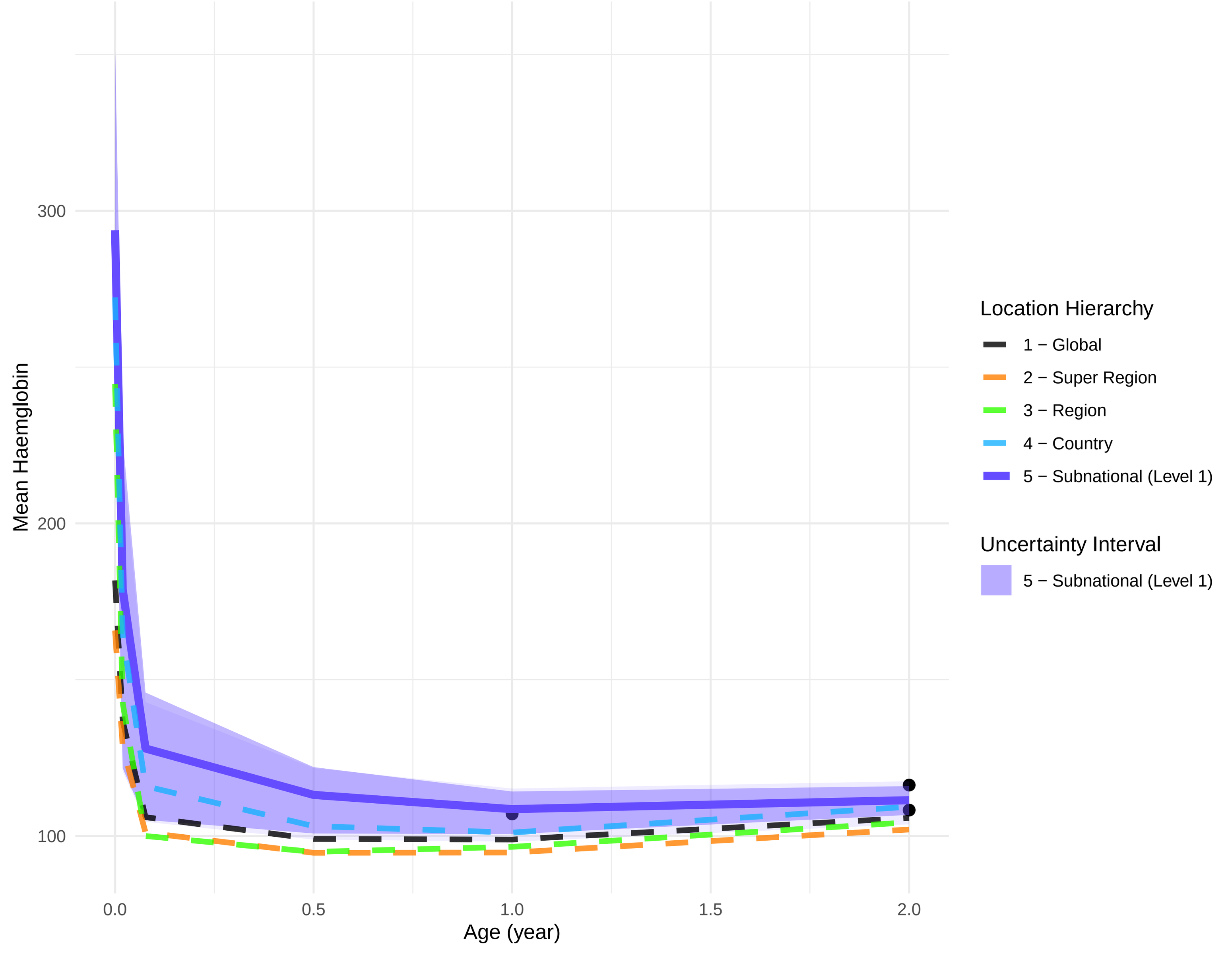

MR−BRT Age/Sex Fits − Males <2 Years Old − Mean Haemglobin − Migori
WHO−2024 Adjustment Method

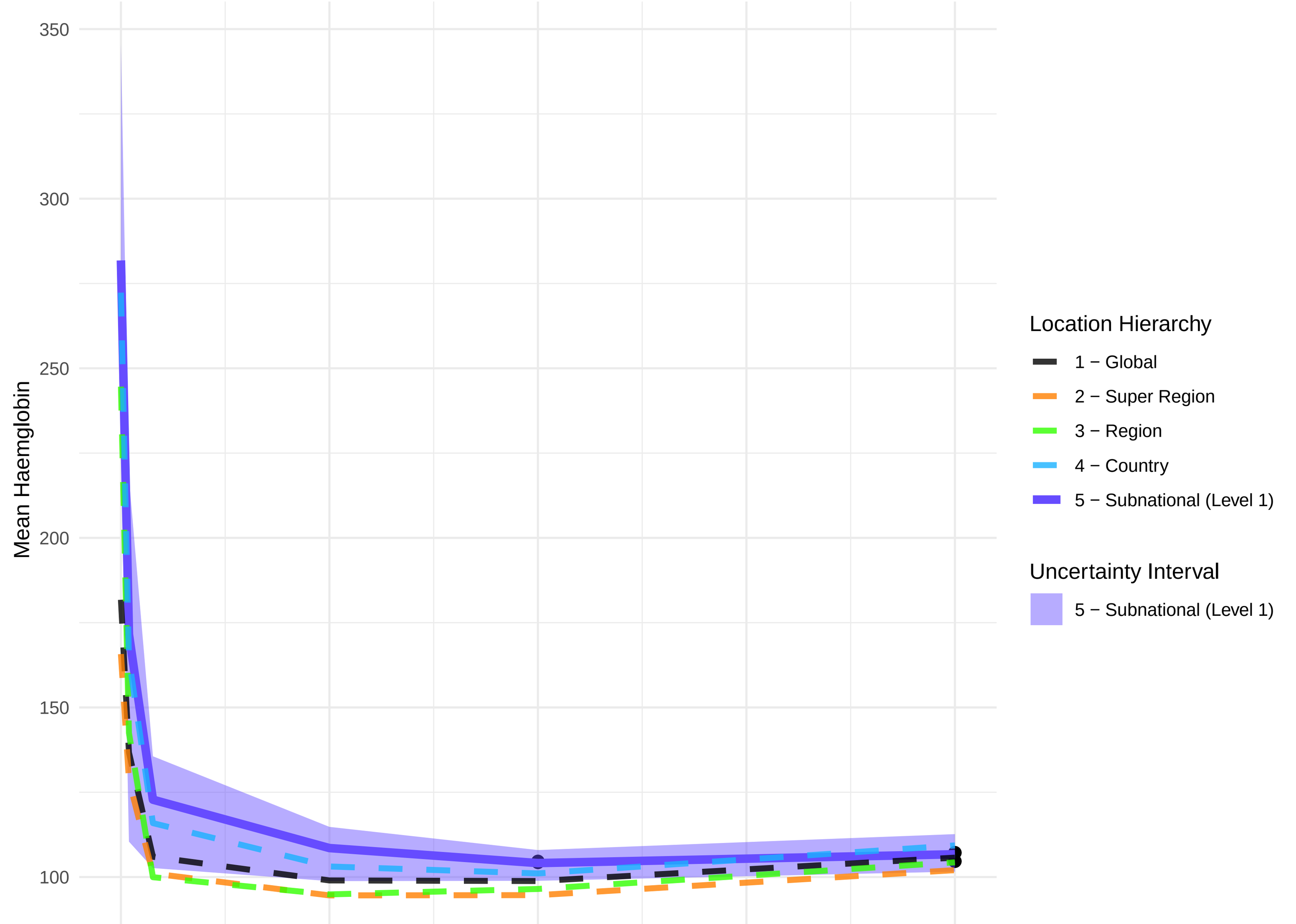

MR−BRT Age/Sex Fits − Males <2 Years Old − Mean Haemglobin − Mombasa
WHO−2024 Adjustment Method

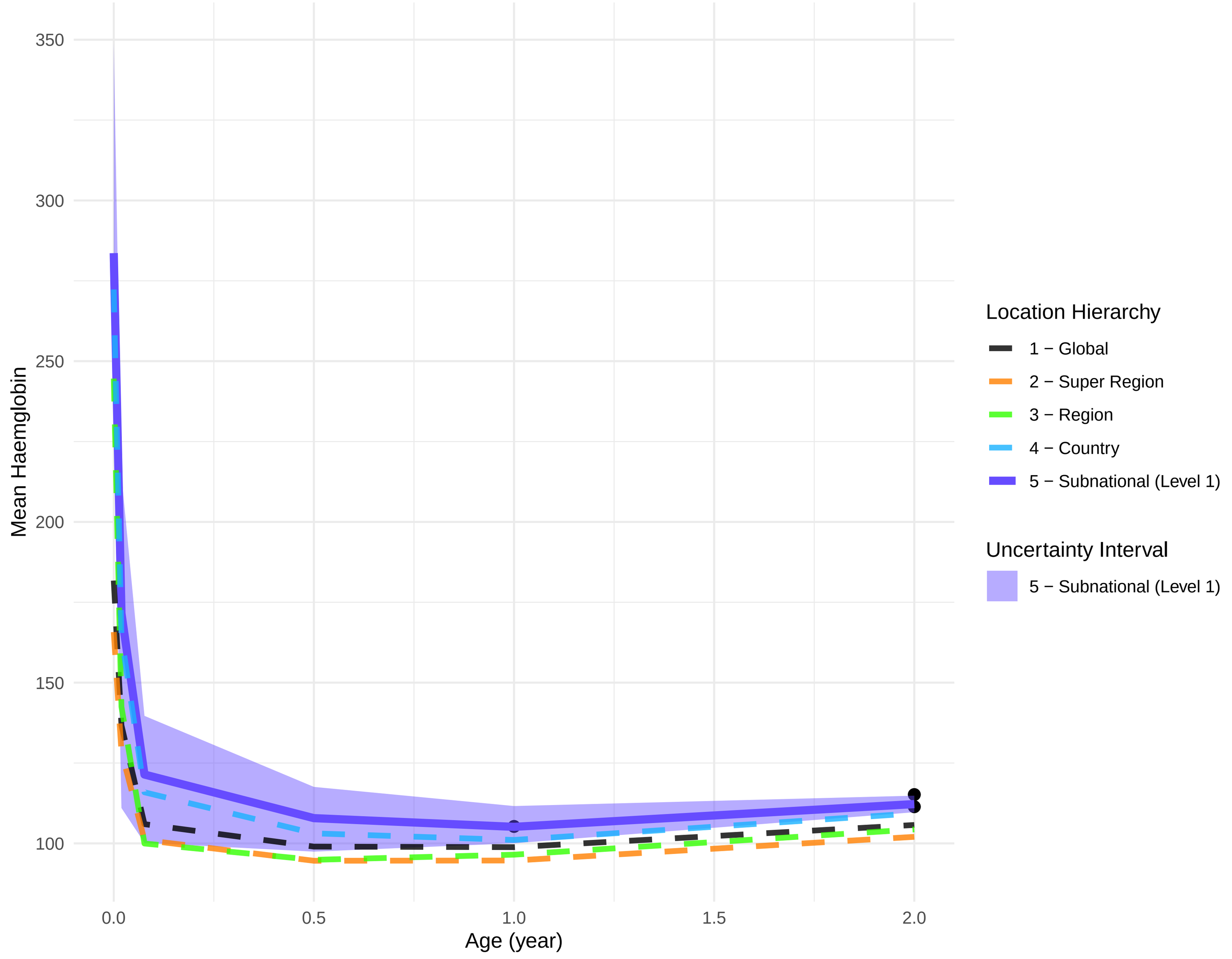

MR−BRT Age/Sex Fits − Males <2 Years Old − Mean Haemglobin − Murang'a
WHO−2024 Adjustment Method

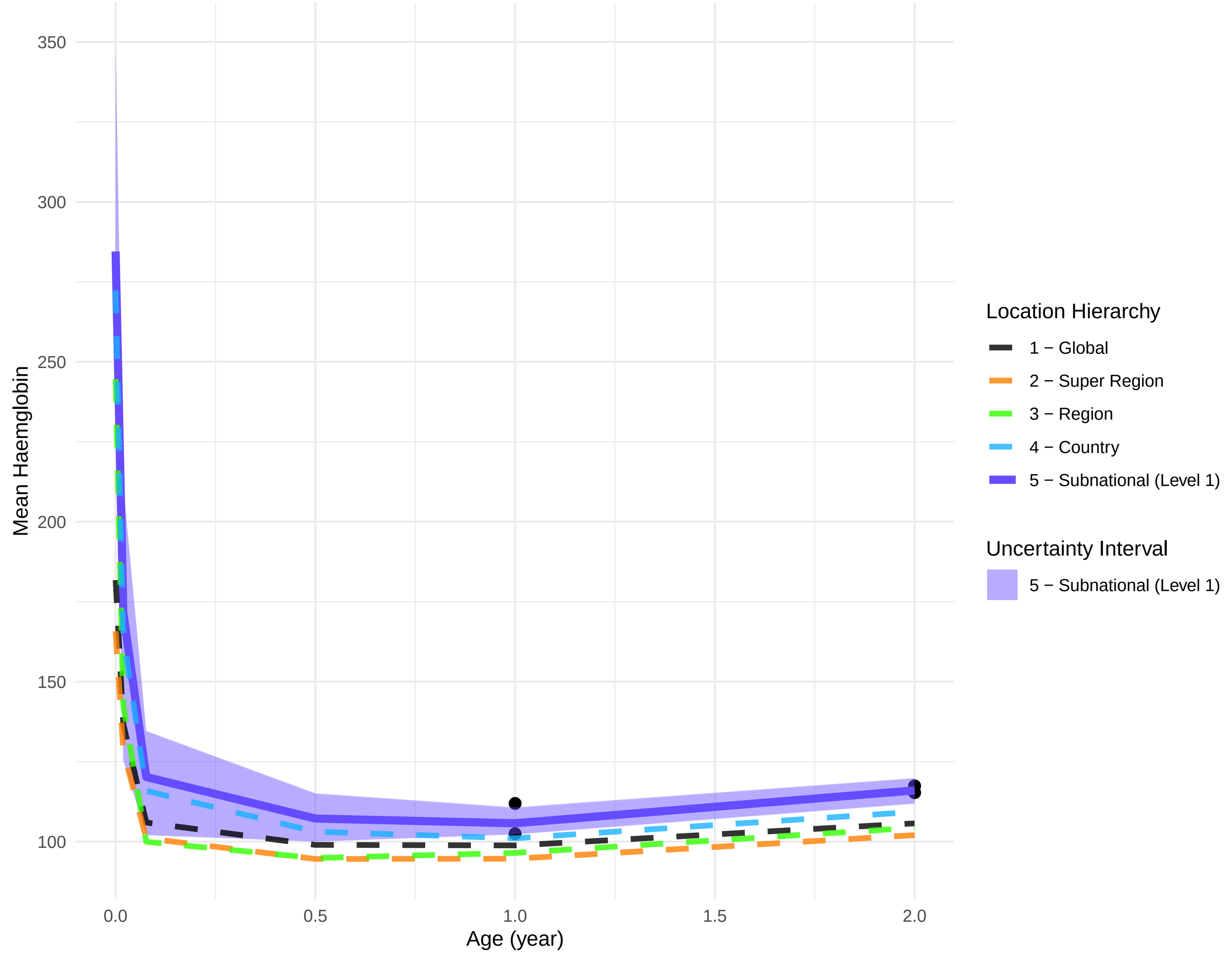

MR−BRT Age/Sex Fits − Males <2 Years Old − Mean Haemglobin − Nairobi
WHO−2024 Adjustment Method

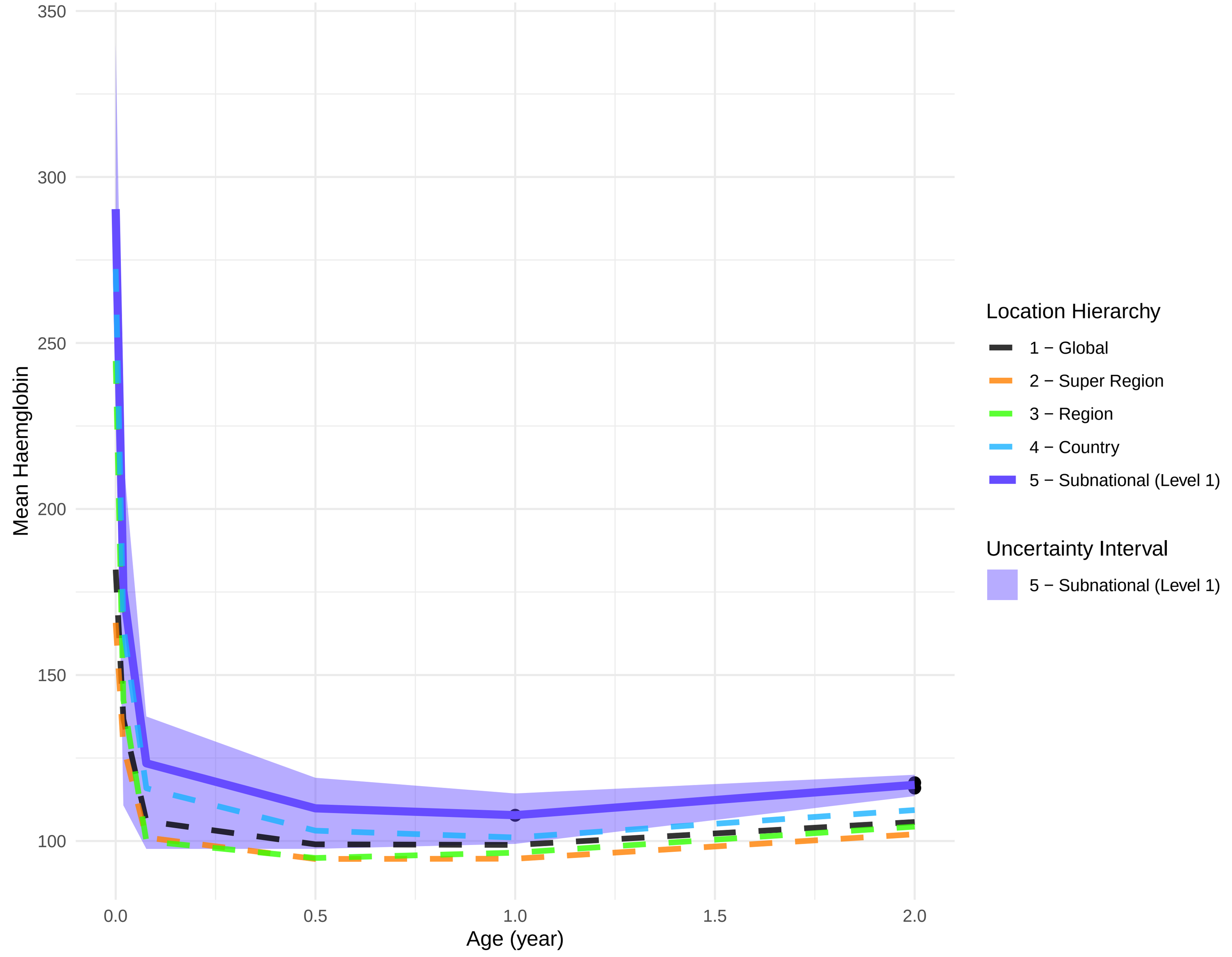

MR−BRT Age/Sex Fits − Males <2 Years Old − Mean Haemglobin − Nakuru
WHO−2024 Adjustment Method

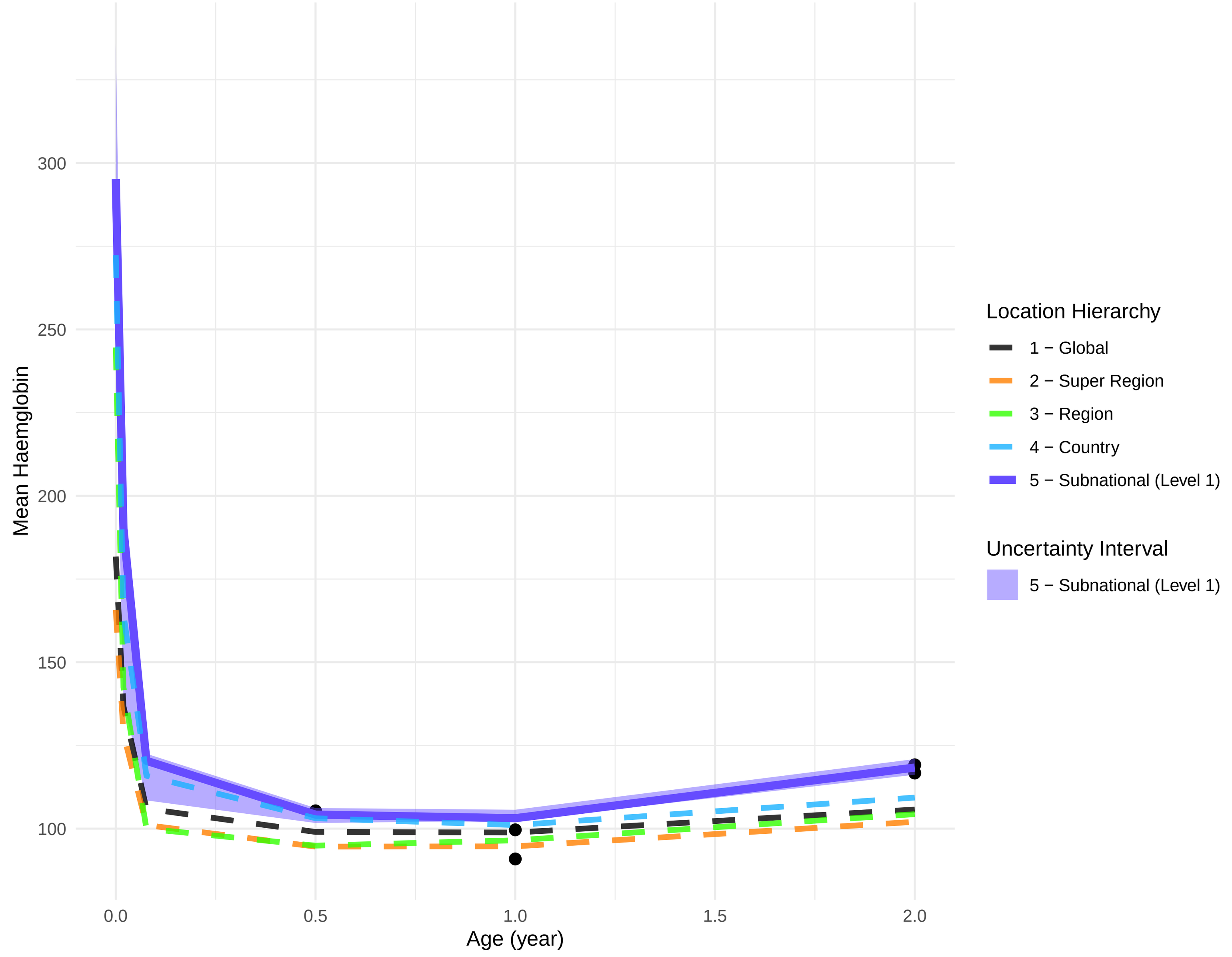

MR−BRT Age/Sex Fits − Males <2 Years Old − Mean Haemglobin − Nandi
WHO−2024 Adjustment Method

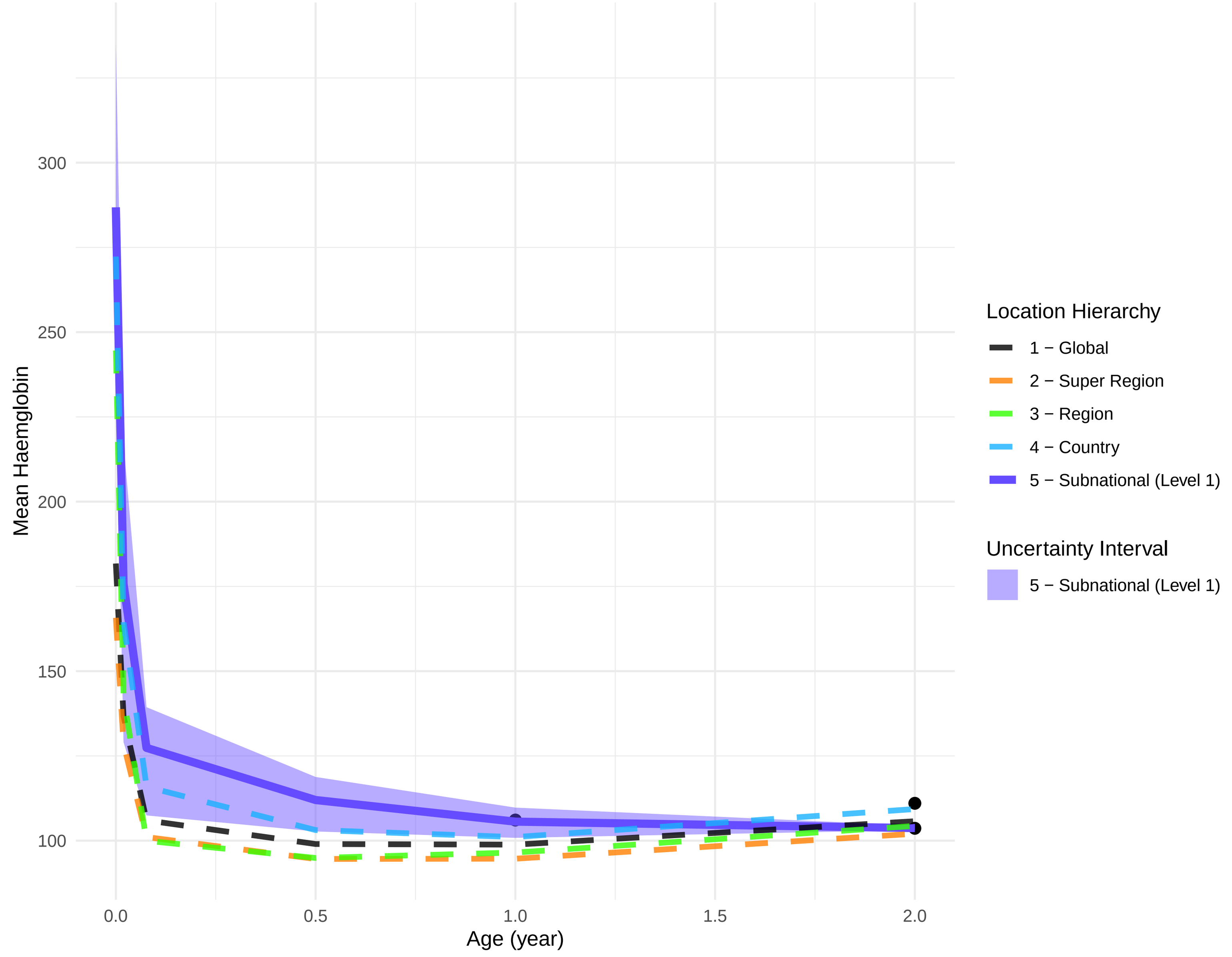

MR−BRT Age/Sex Fits − Males <2 Years Old − Mean Haemglobin − Narok
WHO−2024 Adjustment Method

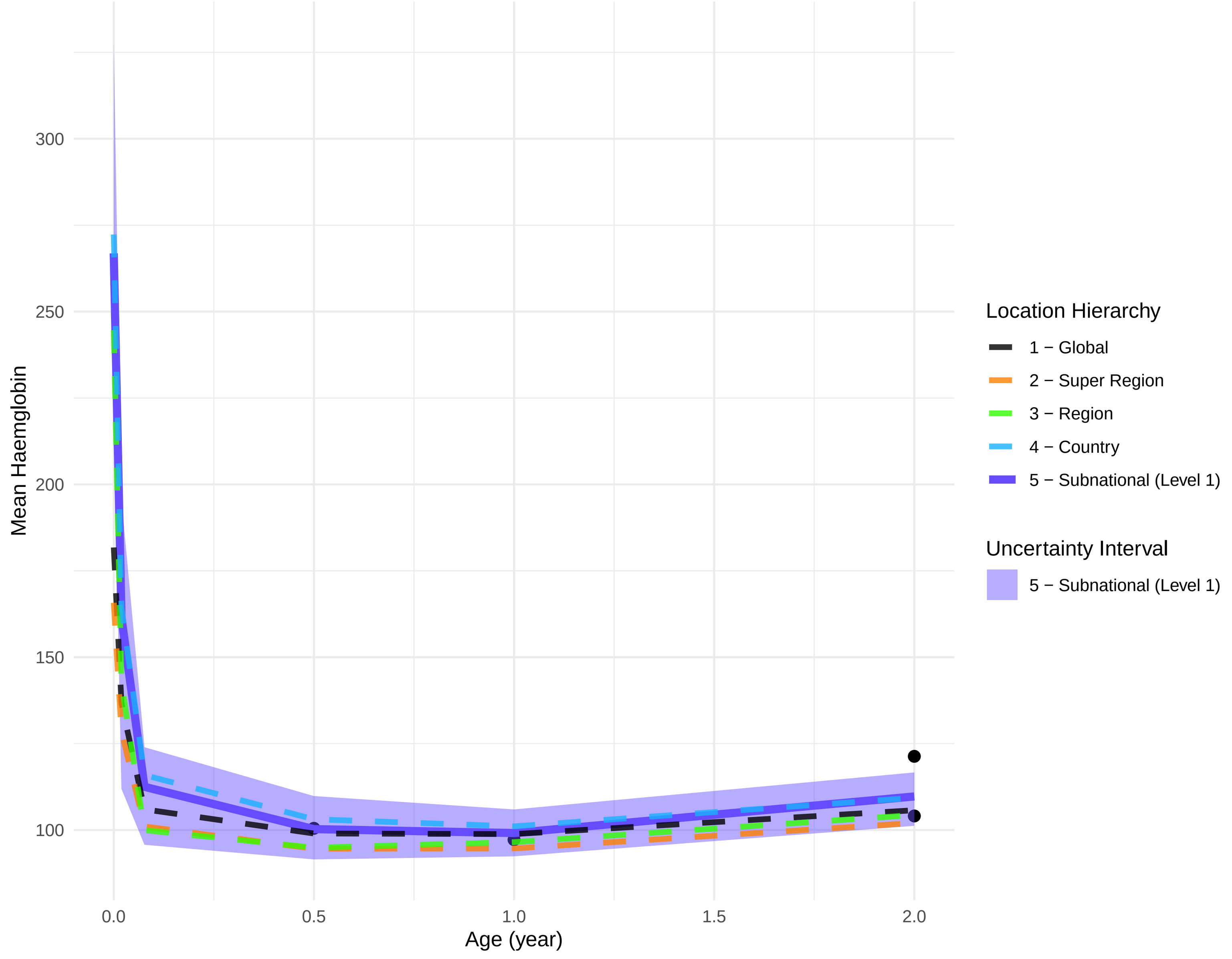

MR−BRT Age/Sex Fits − Males <2 Years Old − Mean Haemglobin − Nyamira
WHO−2024 Adjustment Method

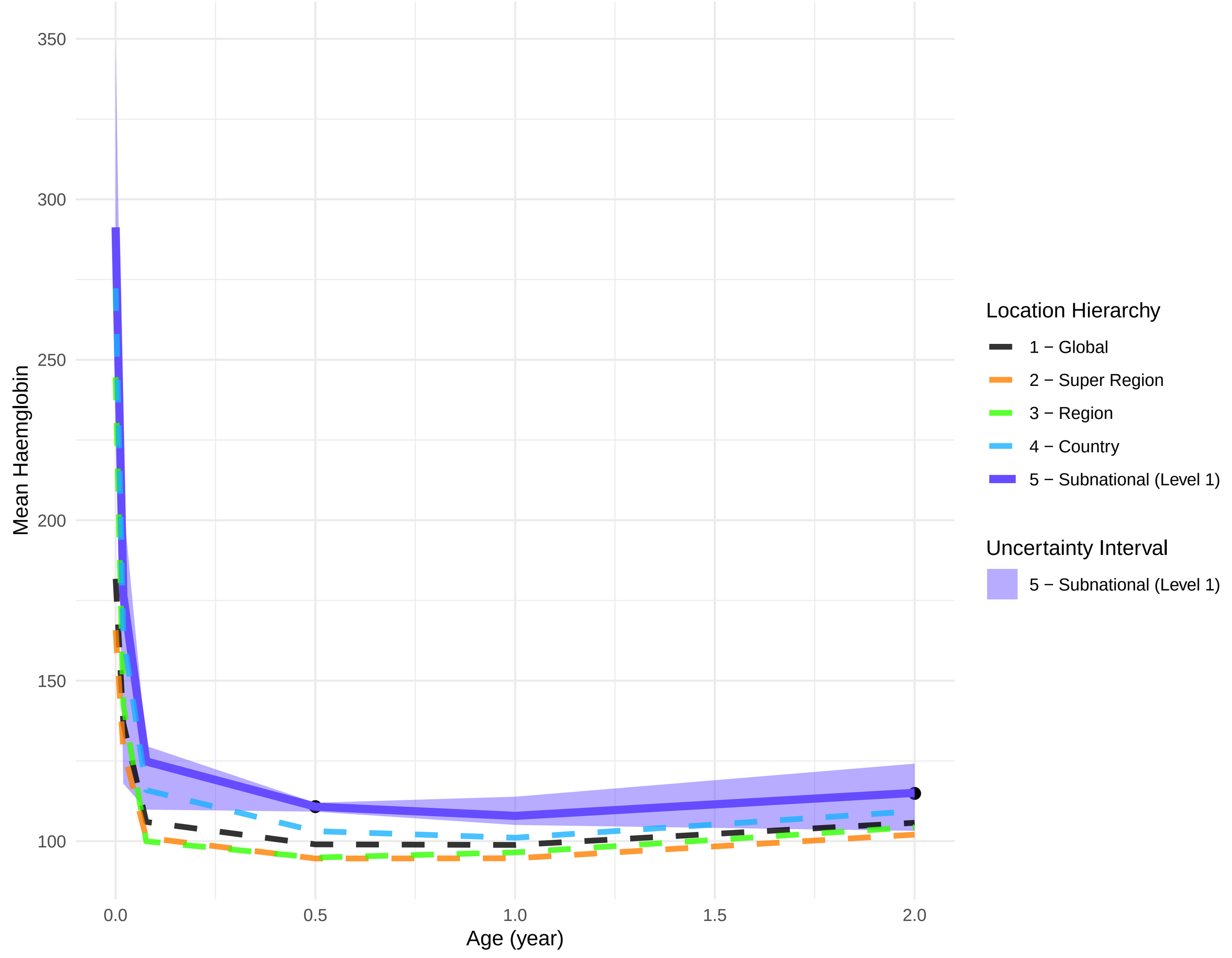

MR−BRT Age/Sex Fits − Males <2 Years Old − Mean Haemglobin − Nyandarua
WHO−2024 Adjustment Method

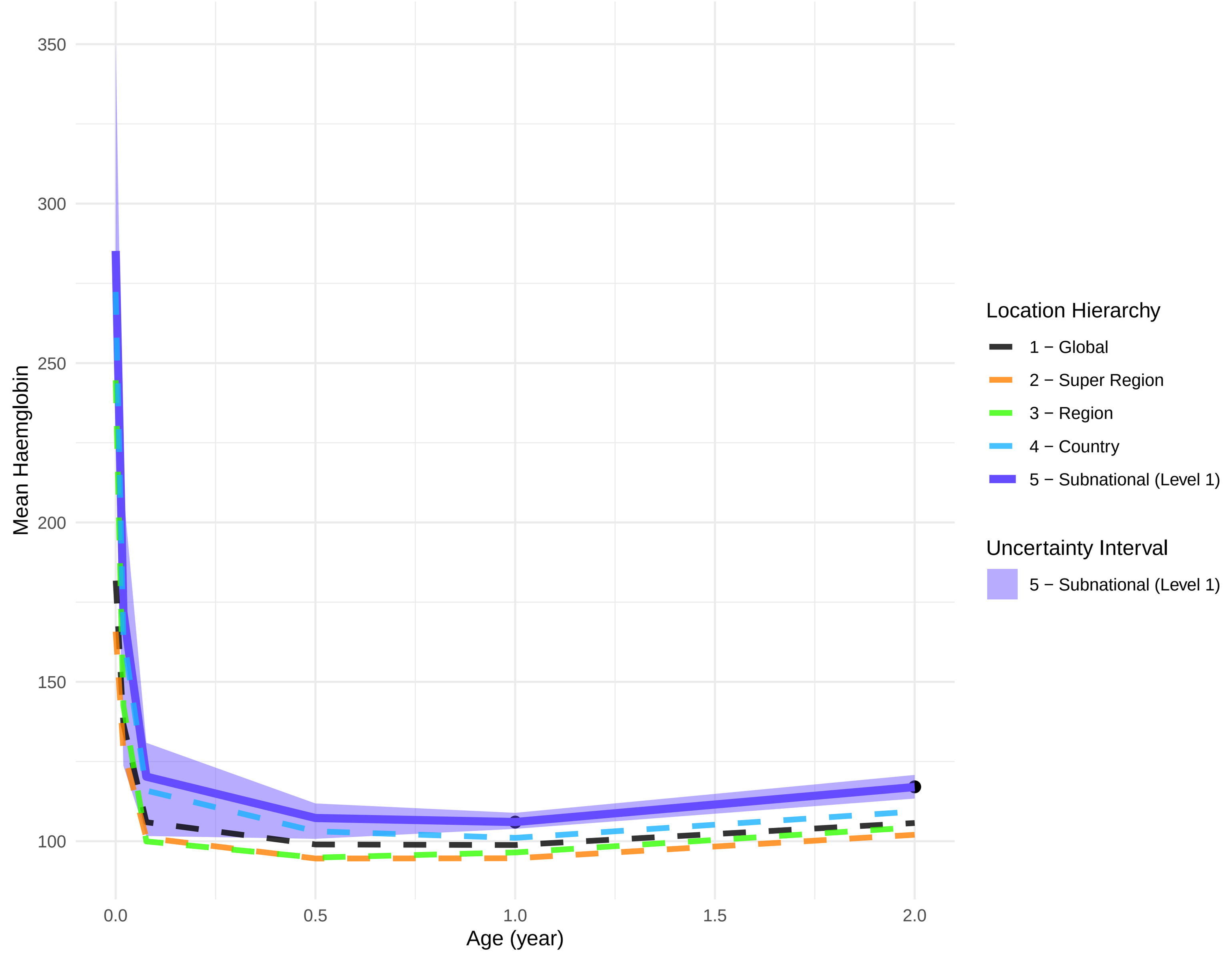

MR−BRT Age/Sex Fits − Males <2 Years Old − Mean Haemglobin − Nyeri
WHO−2024 Adjustment Method

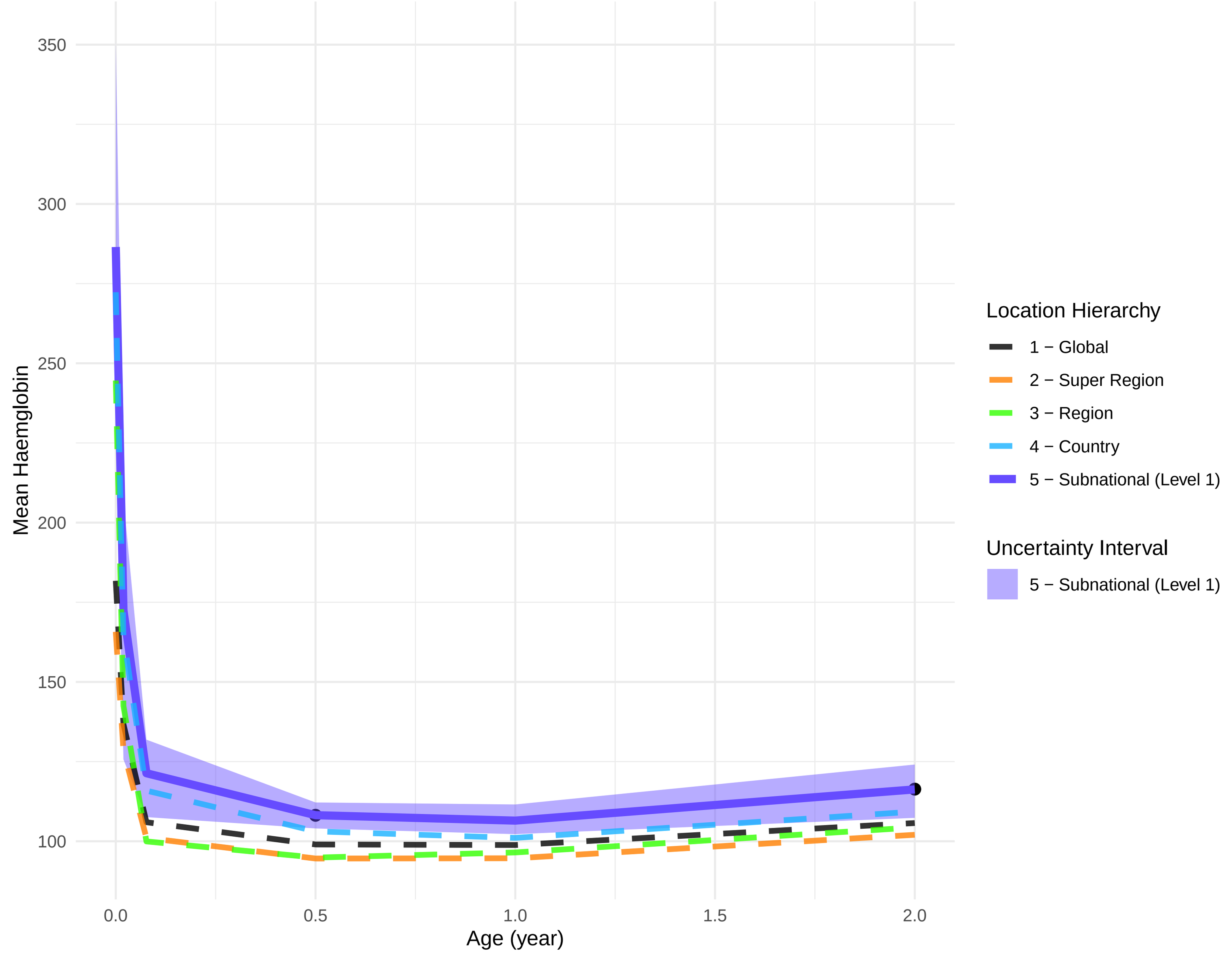

MR−BRT Age/Sex Fits − Males <2 Years Old − Mean Haemglobin − Samburu
WHO−2024 Adjustment Method

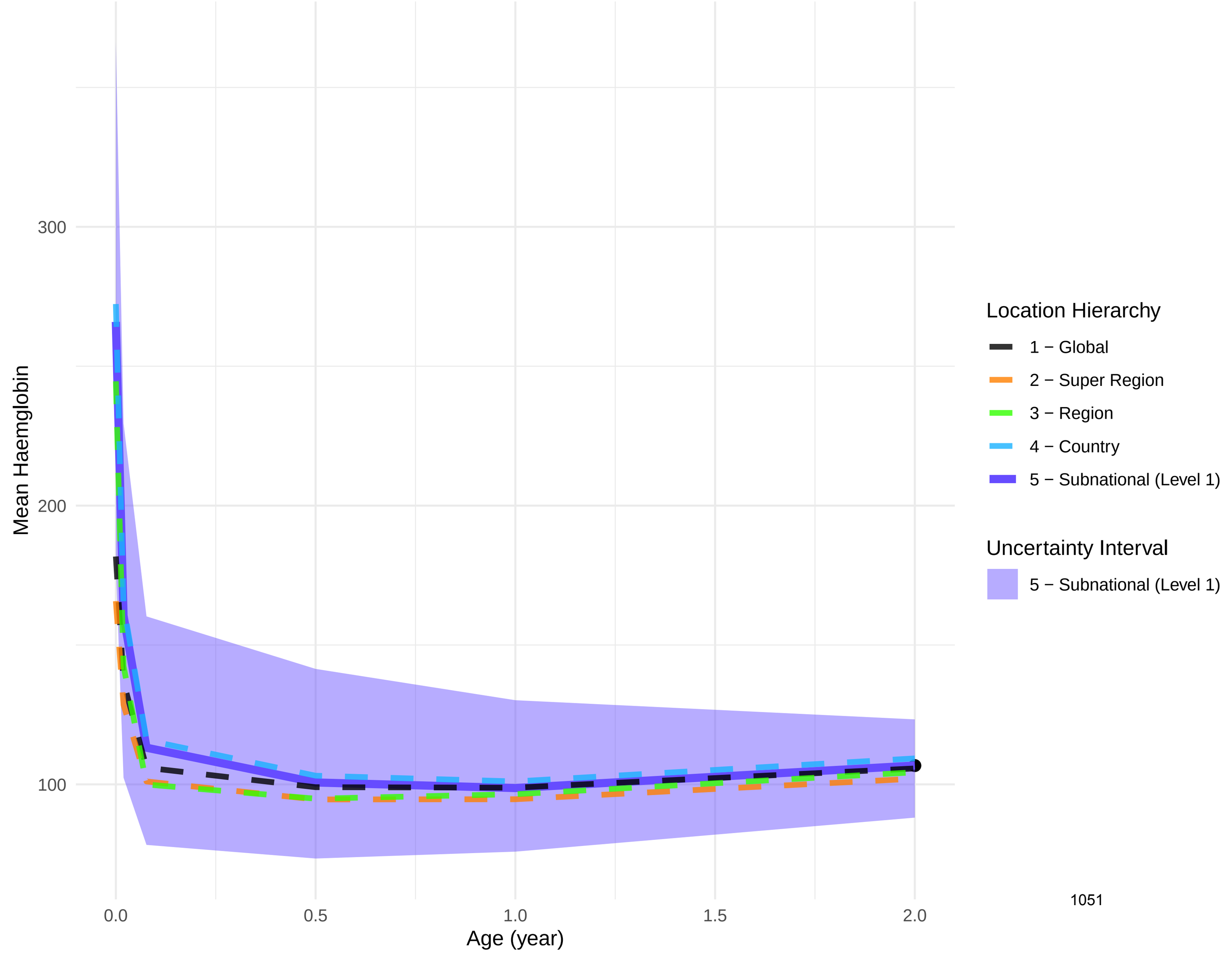

MR−BRT Age/Sex Fits − Males <2 Years Old − Mean Haemglobin − Siaya
WHO−2024 Adjustment Method

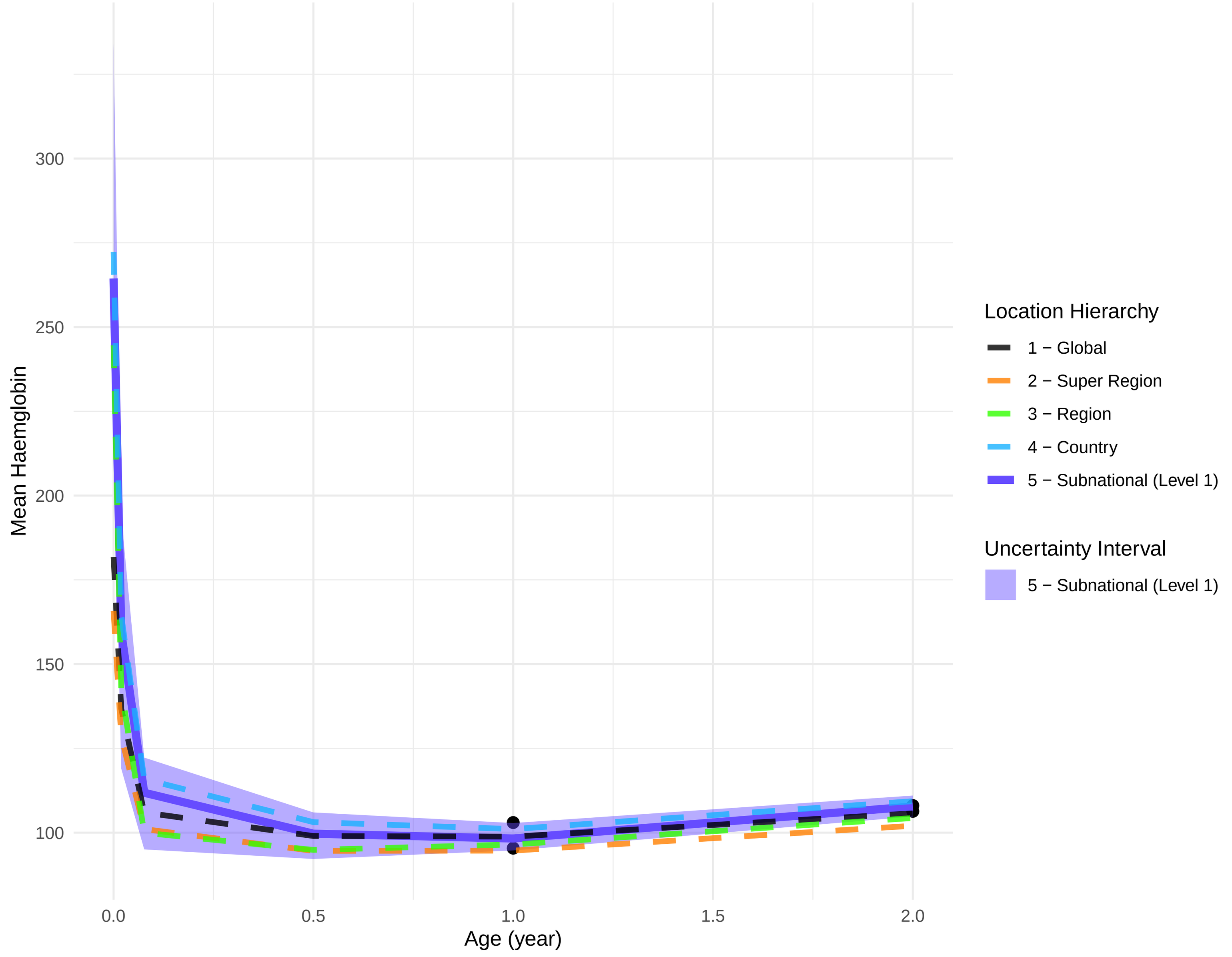

MR−BRT Age/Sex Fits − Males <2 Years Old − Mean Haemglobin − Taita Taveta
WHO−2024 Adjustment Method

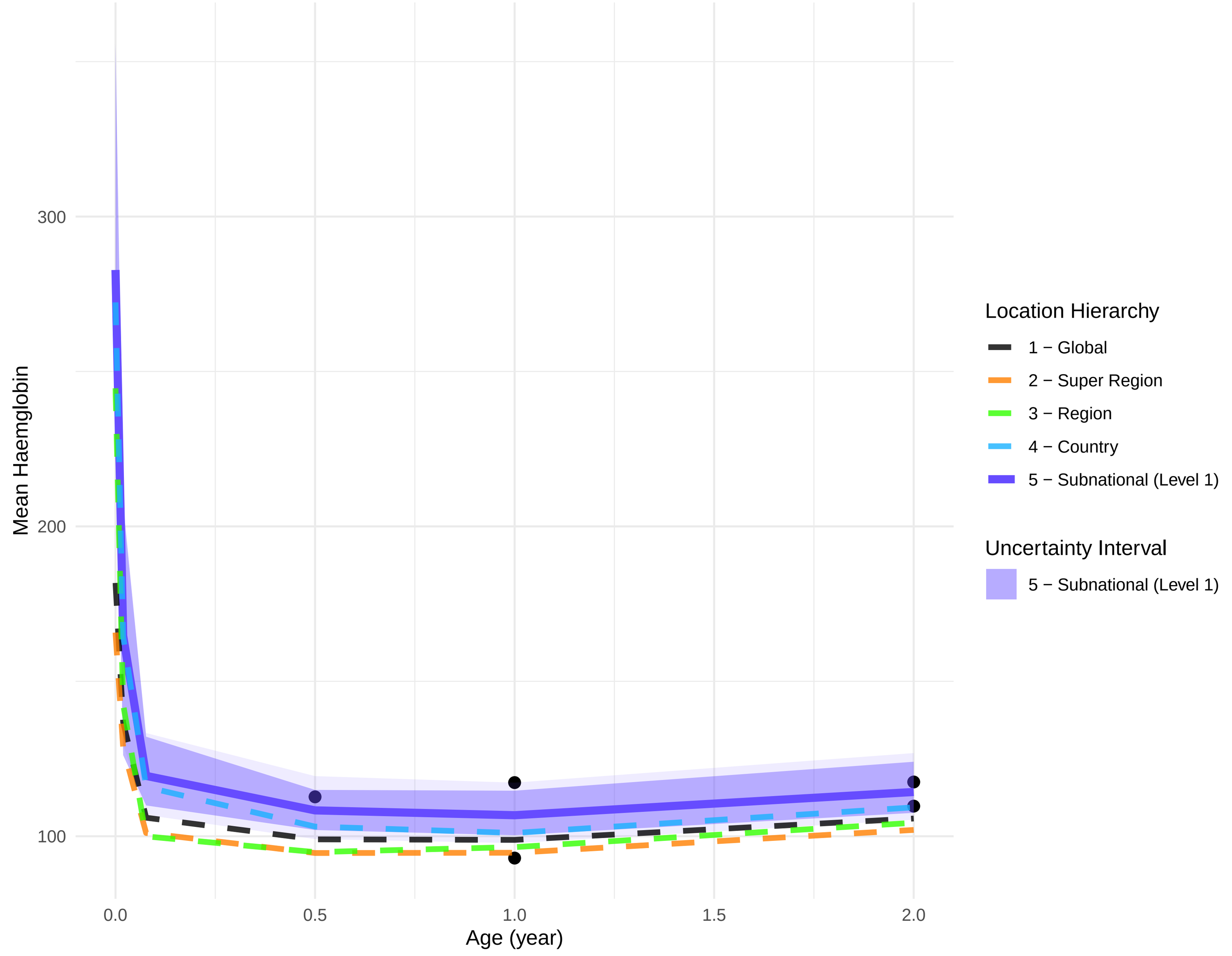

MR−BRT Age/Sex Fits − Males <2 Years Old − Mean Haemglobin − Tana River
WHO−2024 Adjustment Method

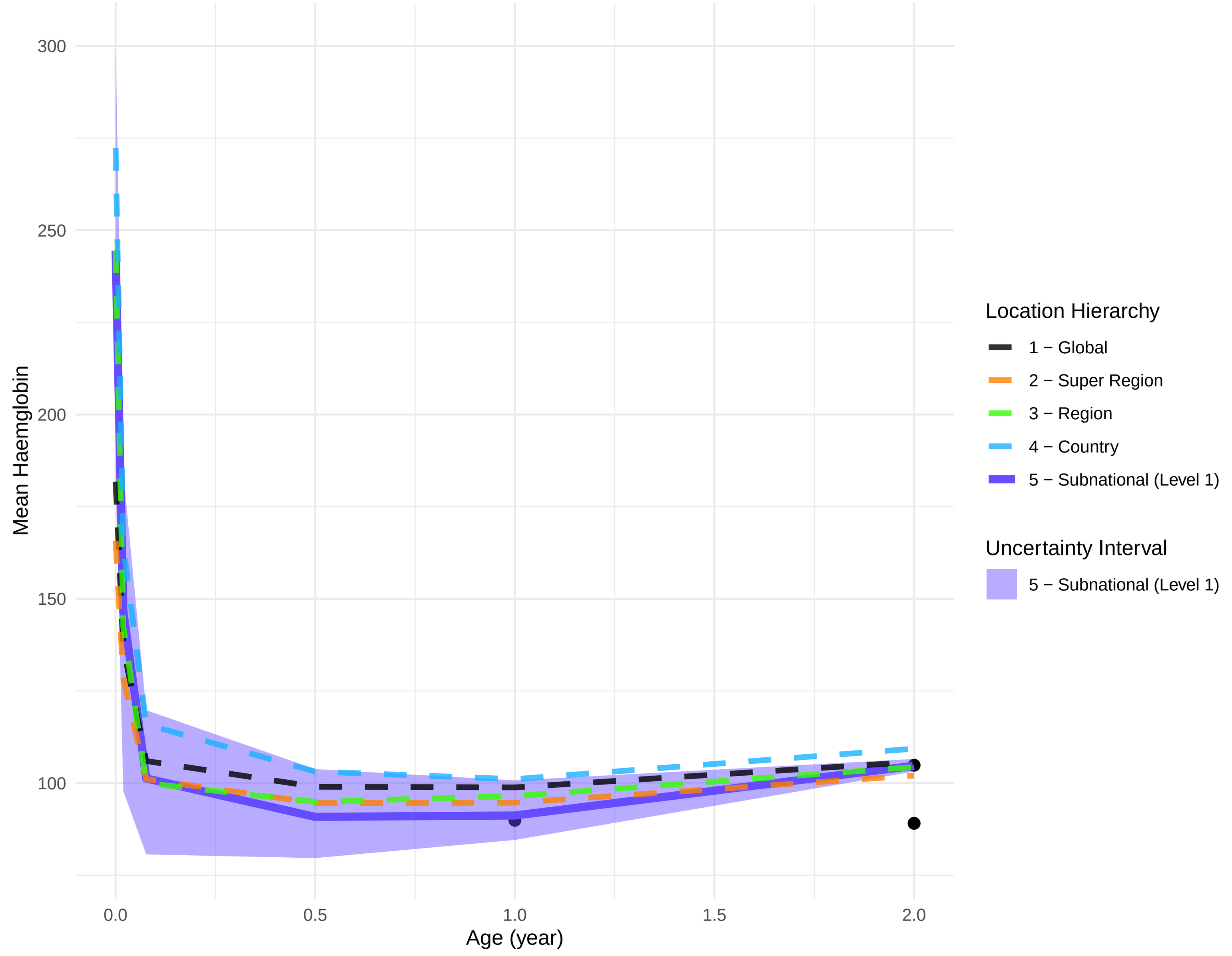

MR−BRT Age/Sex Fits − Males <2 Years Old − Mean Haemglobin − Tharaka Nithi
WHO−2024 Adjustment Method

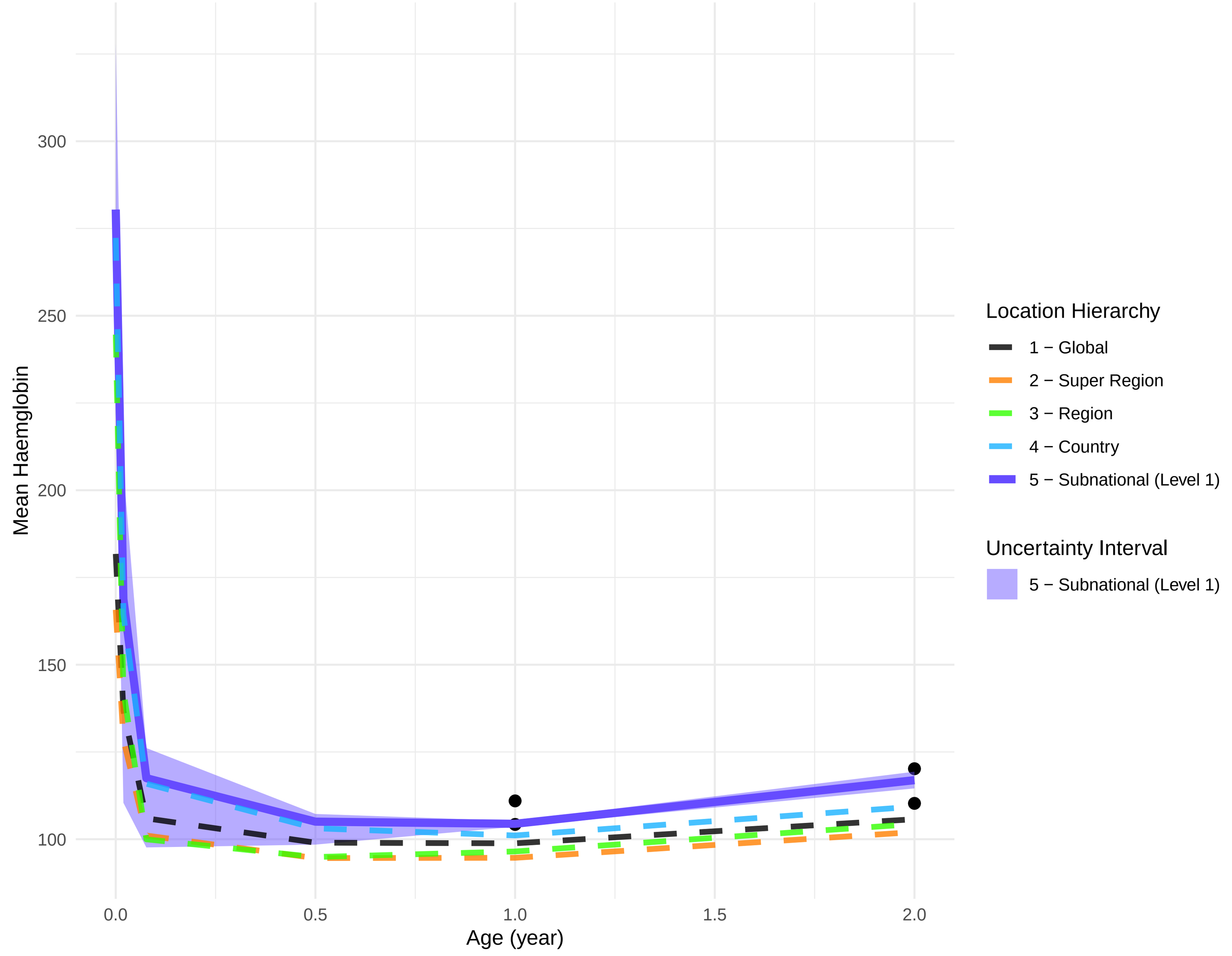

MR−BRT Age/Sex Fits − Males <2 Years Old − Mean Haemglobin − Trans Nzoia
WHO−2024 Adjustment Method

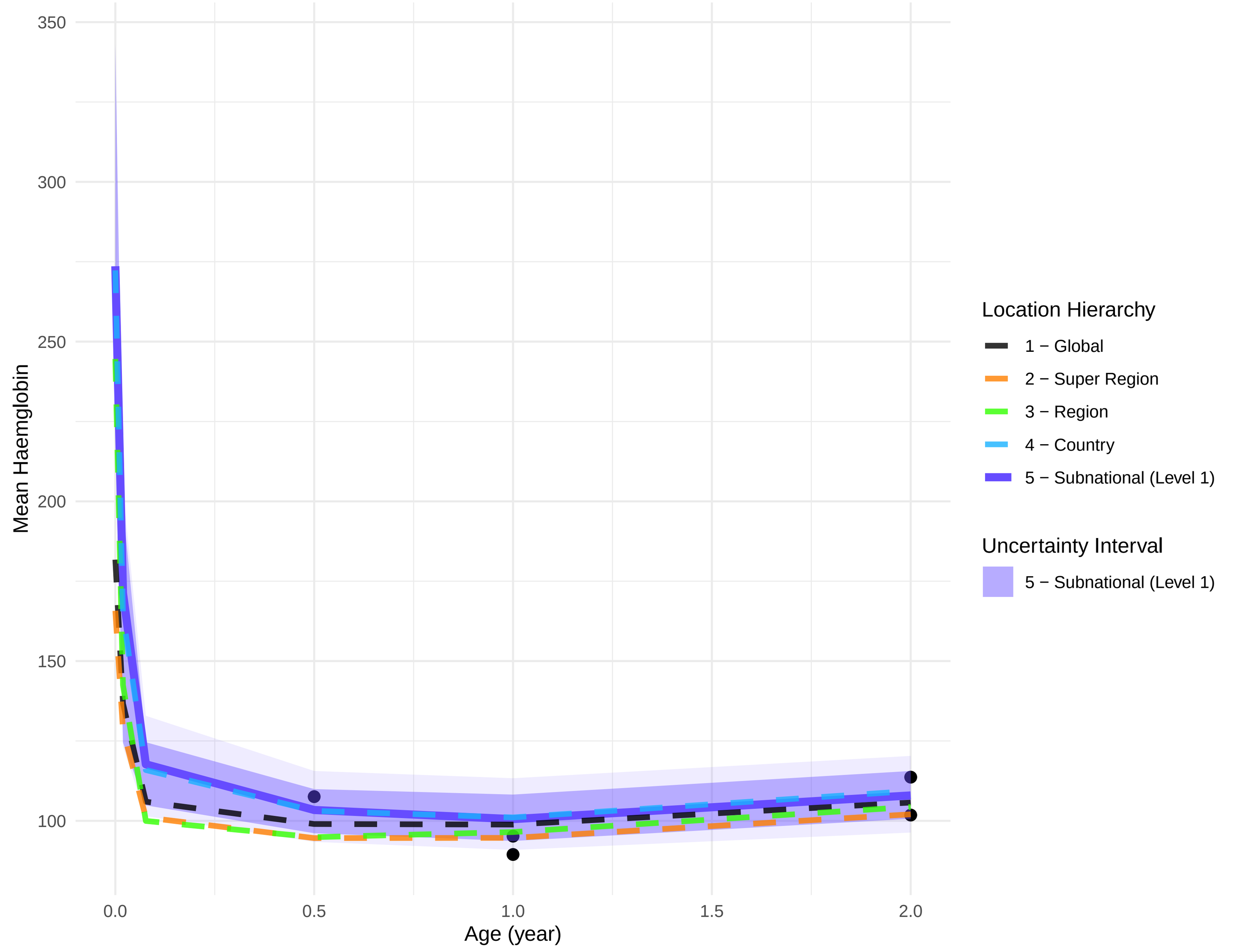

MR−BRT Age/Sex Fits − Males <2 Years Old − Mean Haemglobin − Turkana
WHO−2024 Adjustment Method

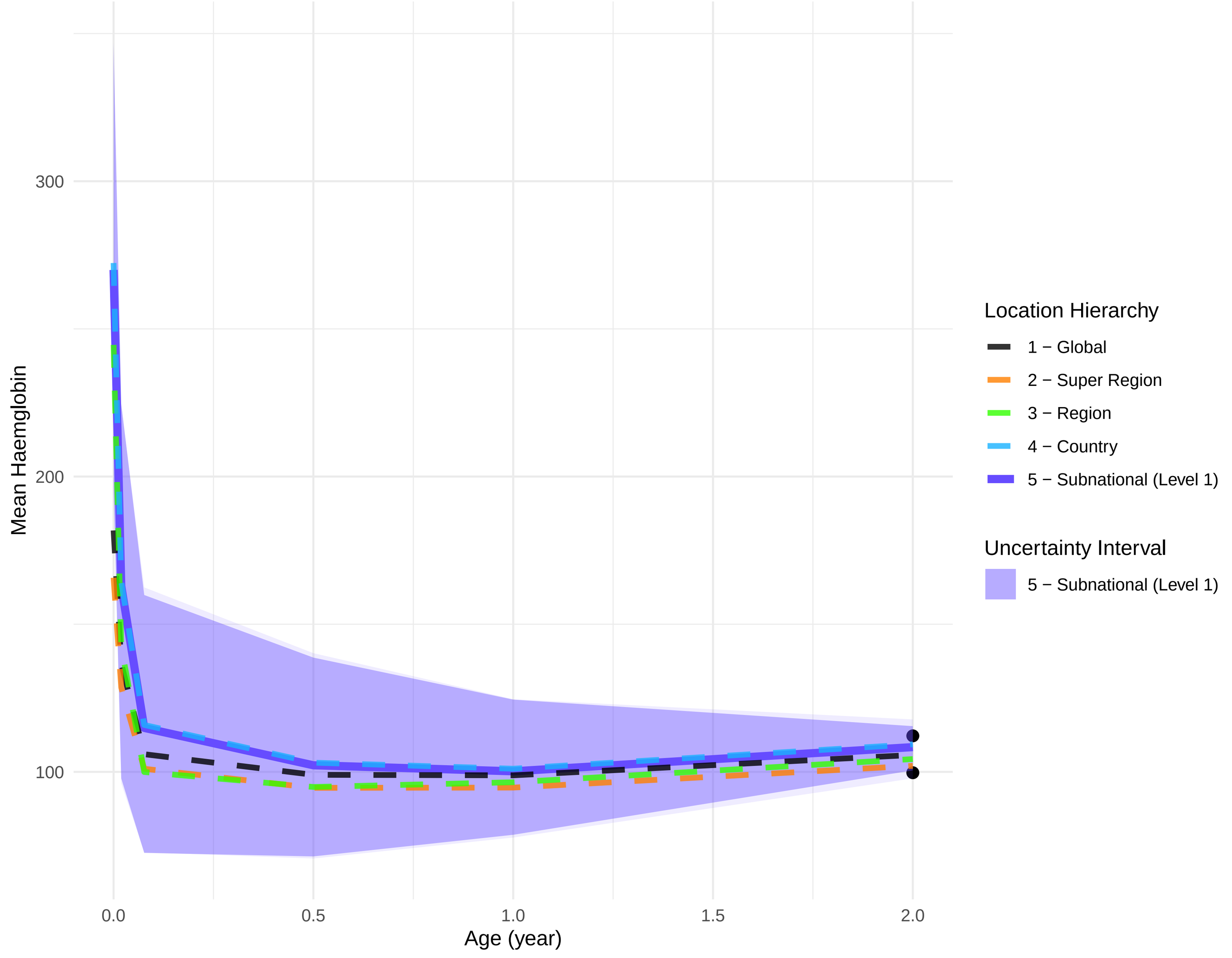

MR−BRT Age/Sex Fits − Males <2 Years Old − Mean Haemglobin − Uasin Gishu
WHO−2024 Adjustment Method

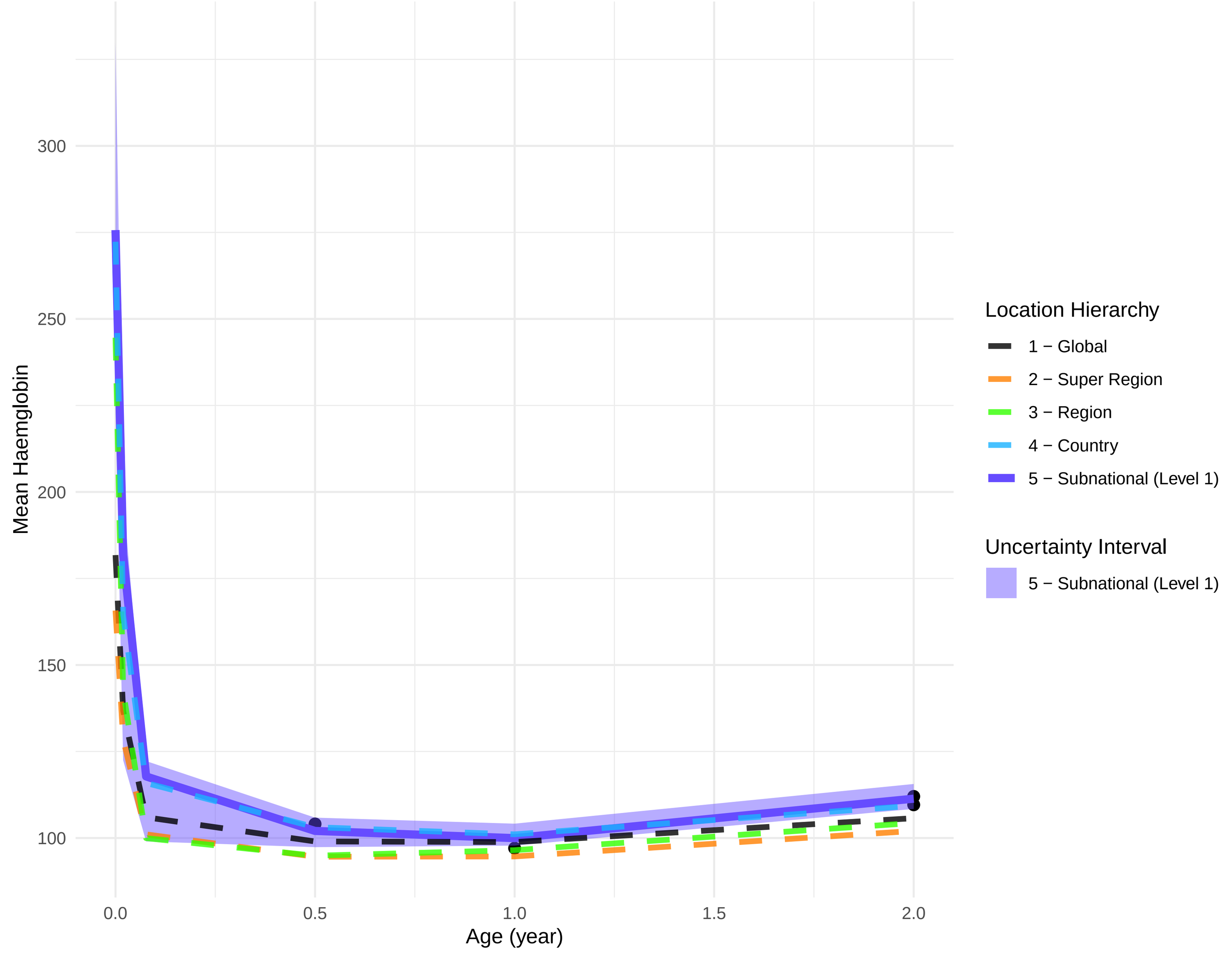

MR−BRT Age/Sex Fits − Males <2 Years Old − Mean Haemglobin − Vihiga
WHO−2024 Adjustment Method

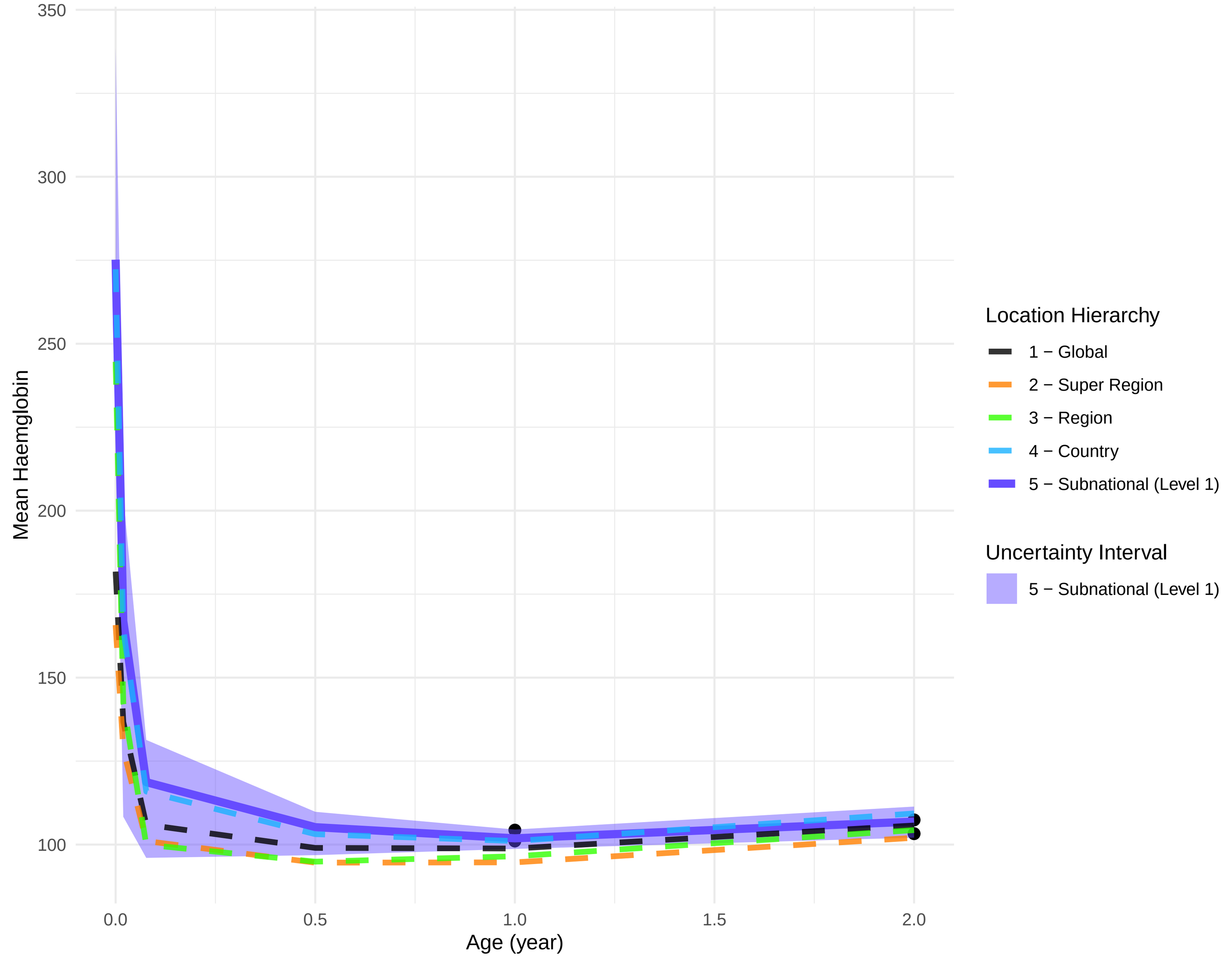

MR−BRT Age/Sex Fits − Males <2 Years Old − Mean Haemglobin − Wajir
WHO−2024 Adjustment Method

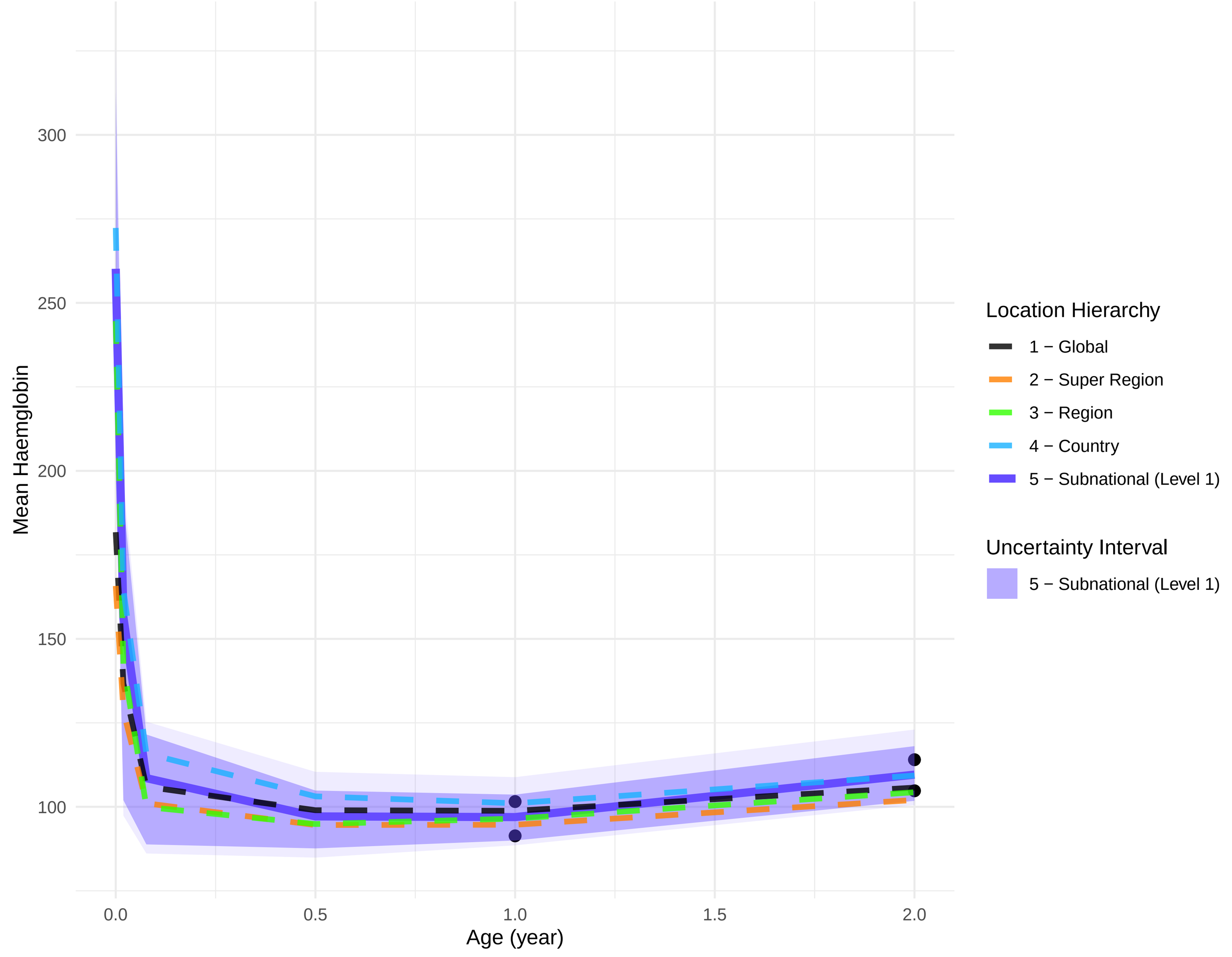

MR−BRT Age/Sex Fits − Males <2 Years Old − Mean Haemglobin − West Pokot
WHO−2024 Adjustment Method

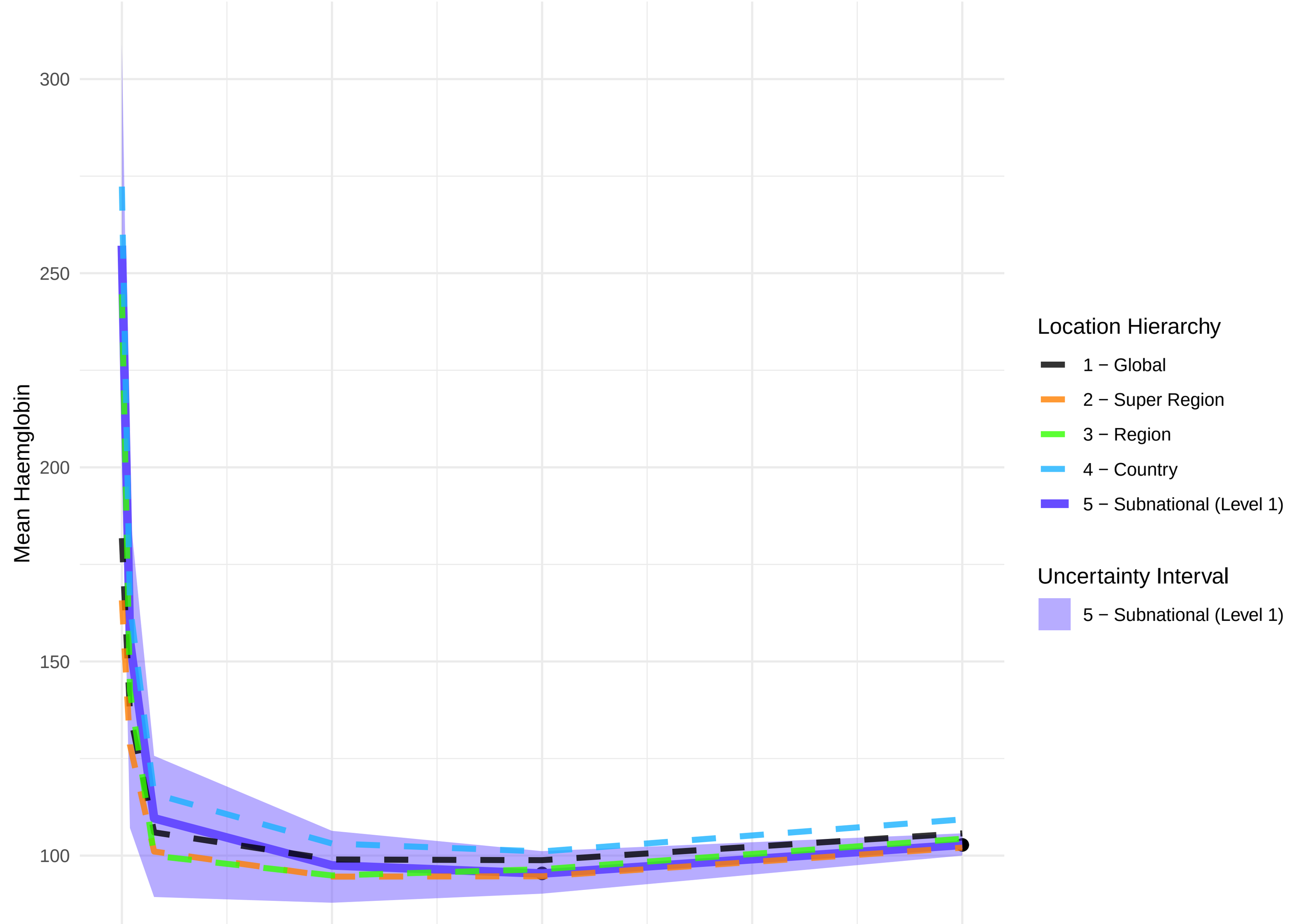

MR−BRT Age/Sex Fits − Males <2 Years Old − Mean Haemglobin − Madagascar
WHO−2024 Adjustment Method

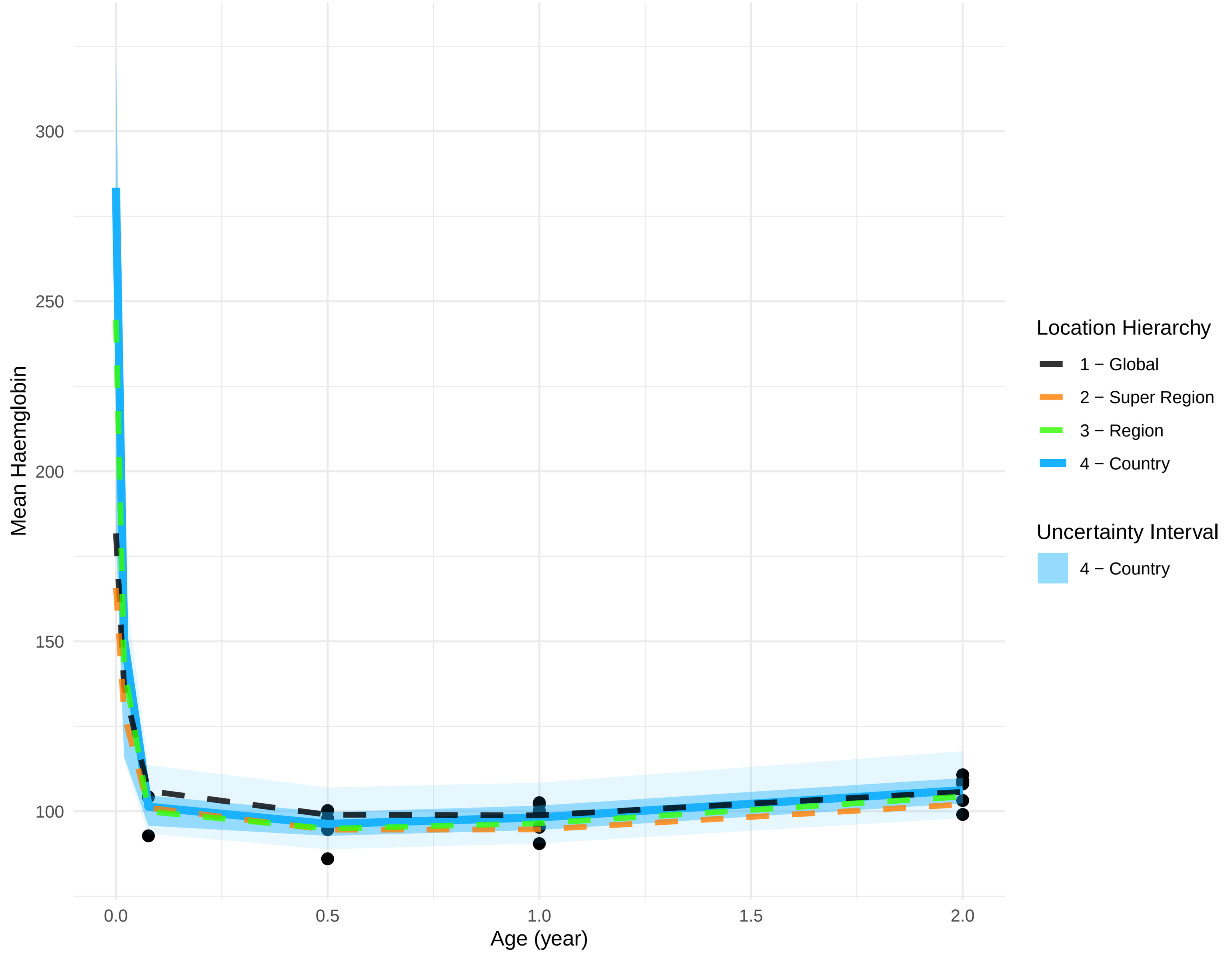

MR−BRT Age/Sex Fits − Males <2 Years Old − Mean Haemglobin − Malawi
WHO−2024 Adjustment Method

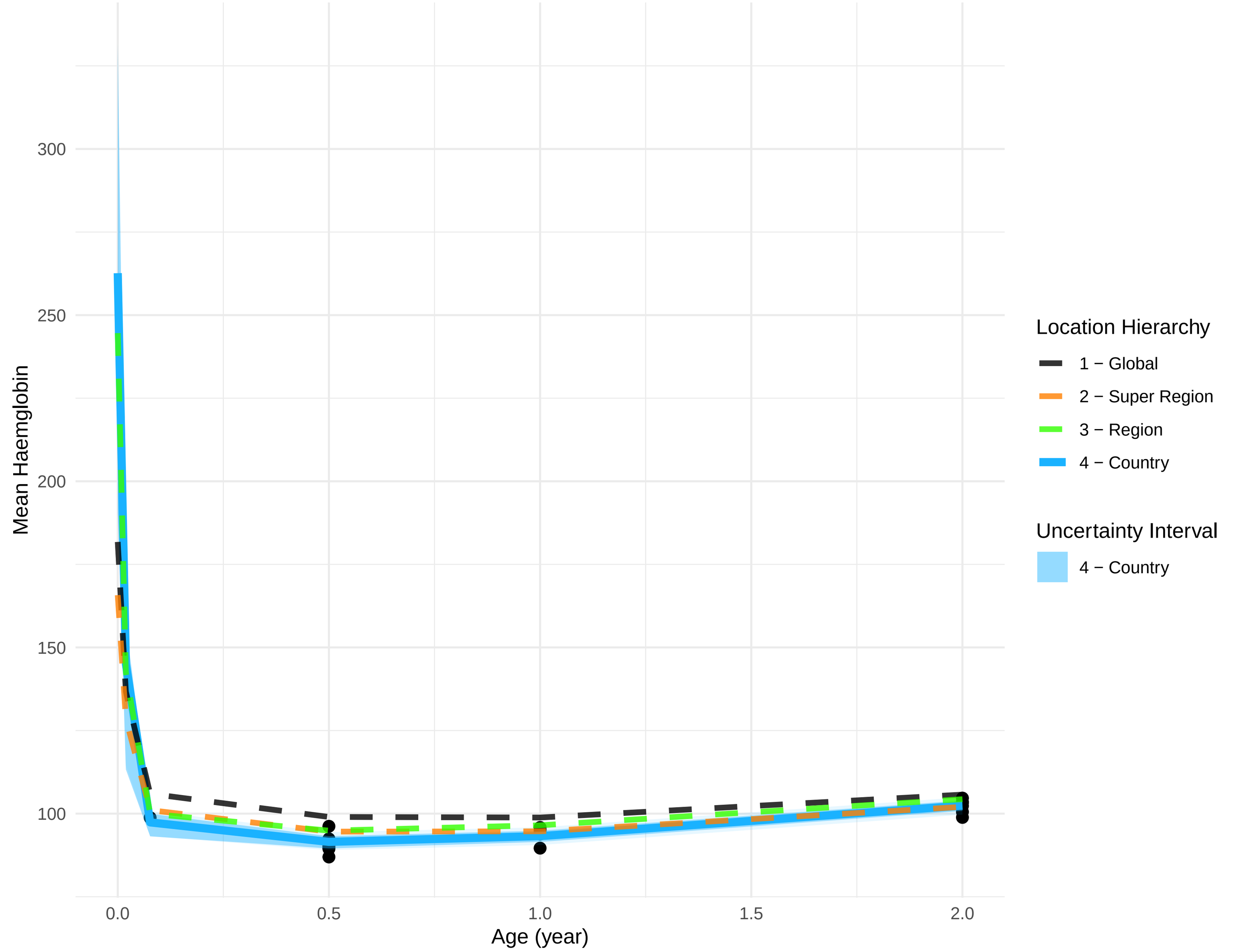

MR−BRT Age/Sex Fits − Males <2 Years Old − Mean Haemglobin − Mozambique
WHO−2024 Adjustment Method

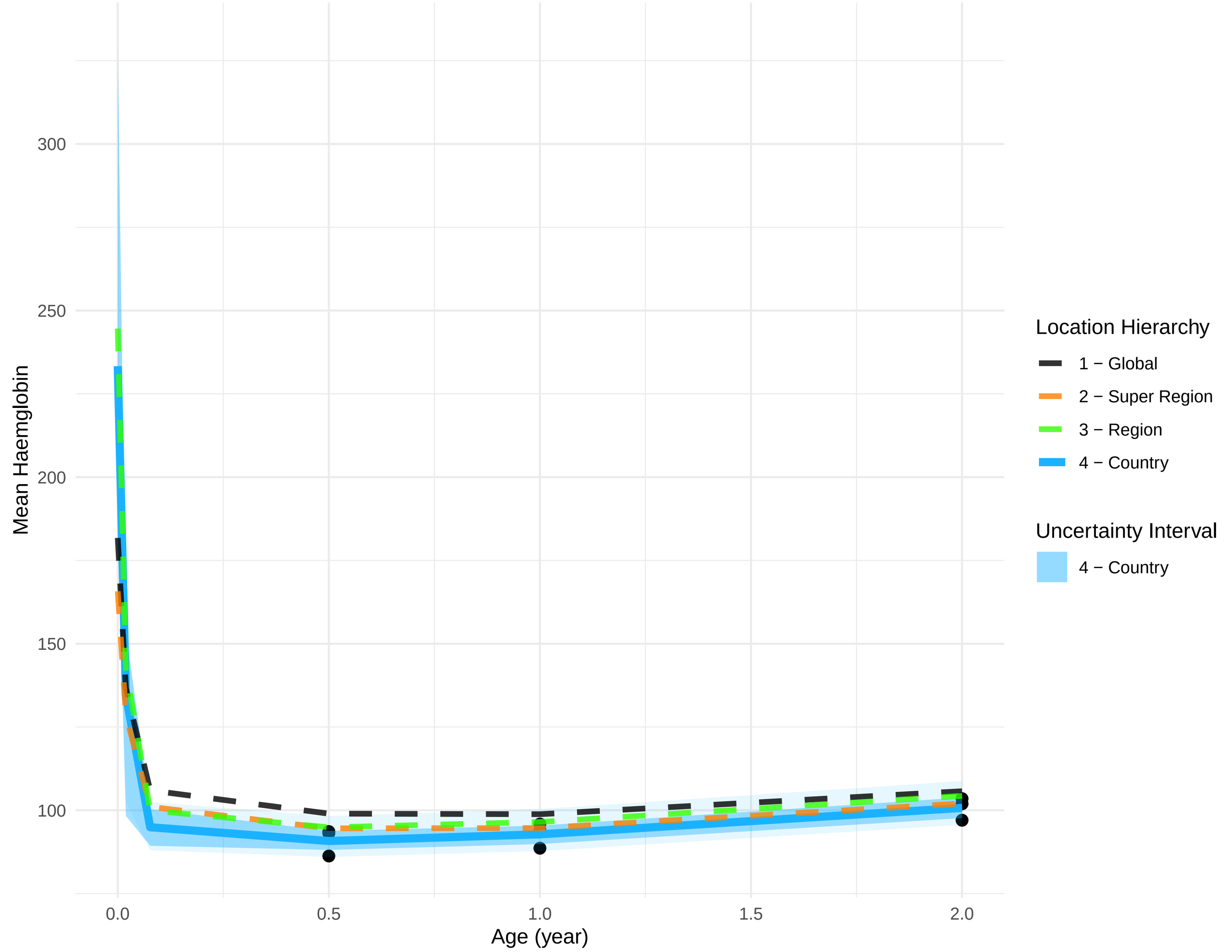

MR−BRT Age/Sex Fits − Males <2 Years Old − Mean Haemglobin − Rwanda
WHO−2024 Adjustment Method

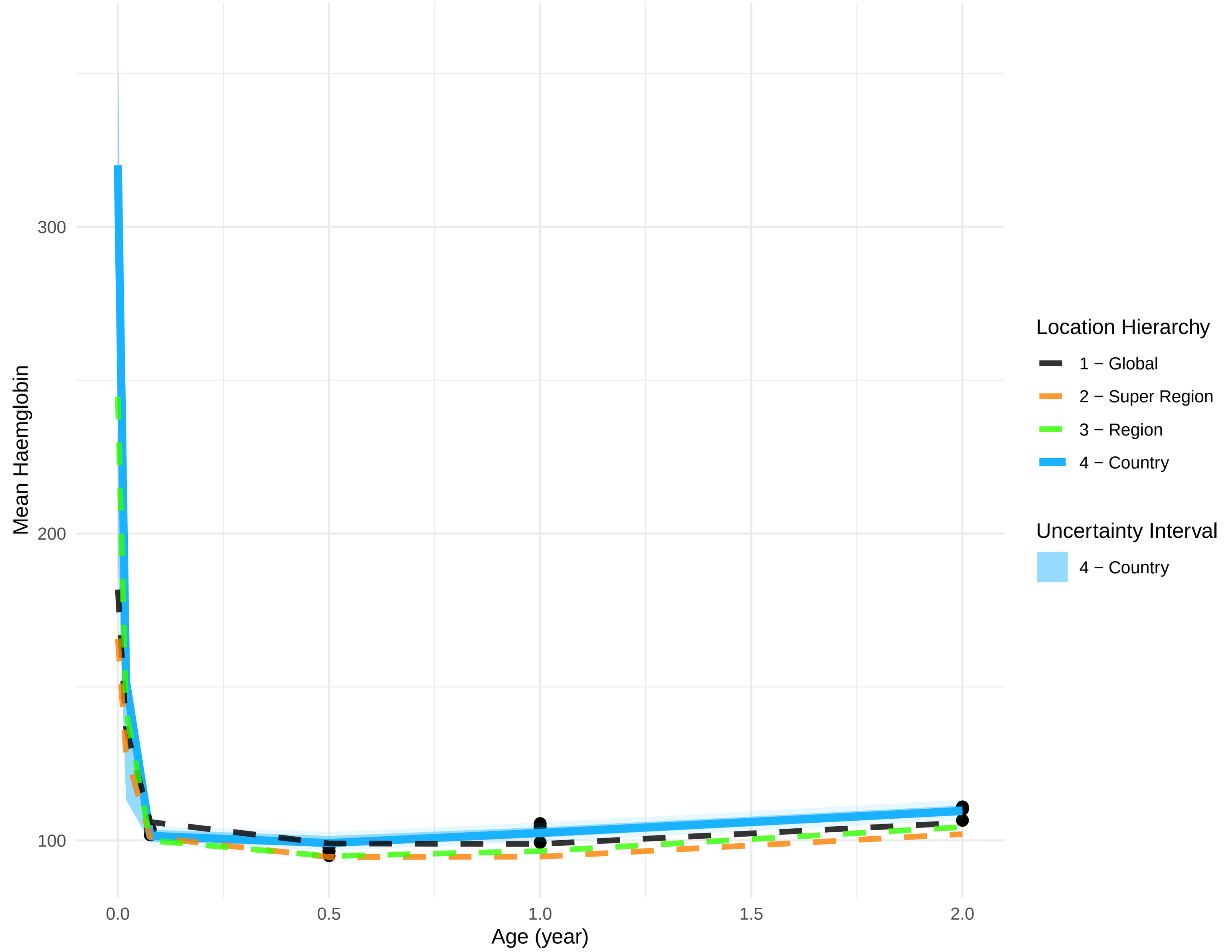

MR−BRT Age/Sex Fits − Males <2 Years Old − Mean Haemglobin − Uganda
WHO−2024 Adjustment Method

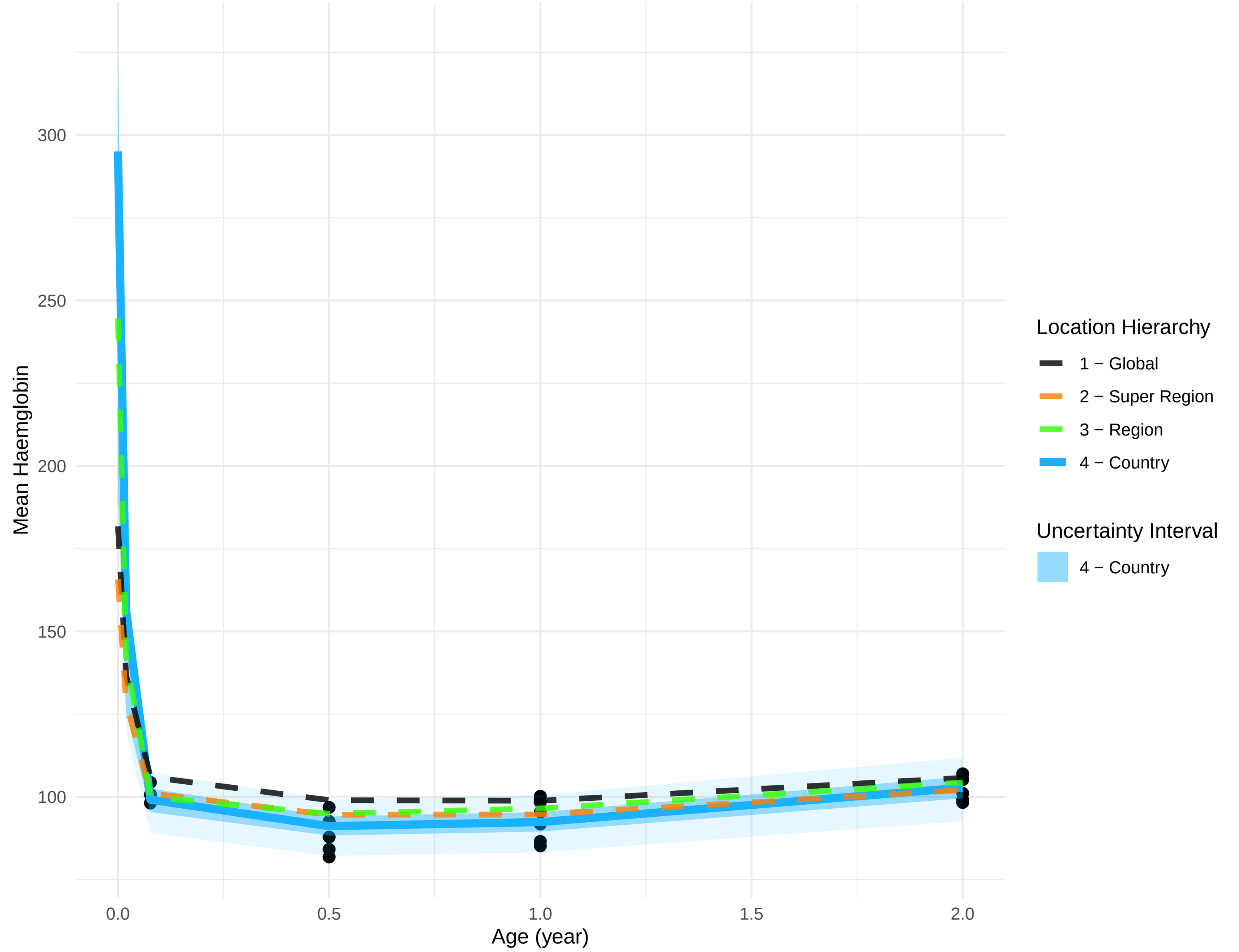

MR−BRT Age/Sex Fits − Males <2 Years Old − Mean Haemglobin − United Republic of Tanzania
WHO−2024 Adjustment Method

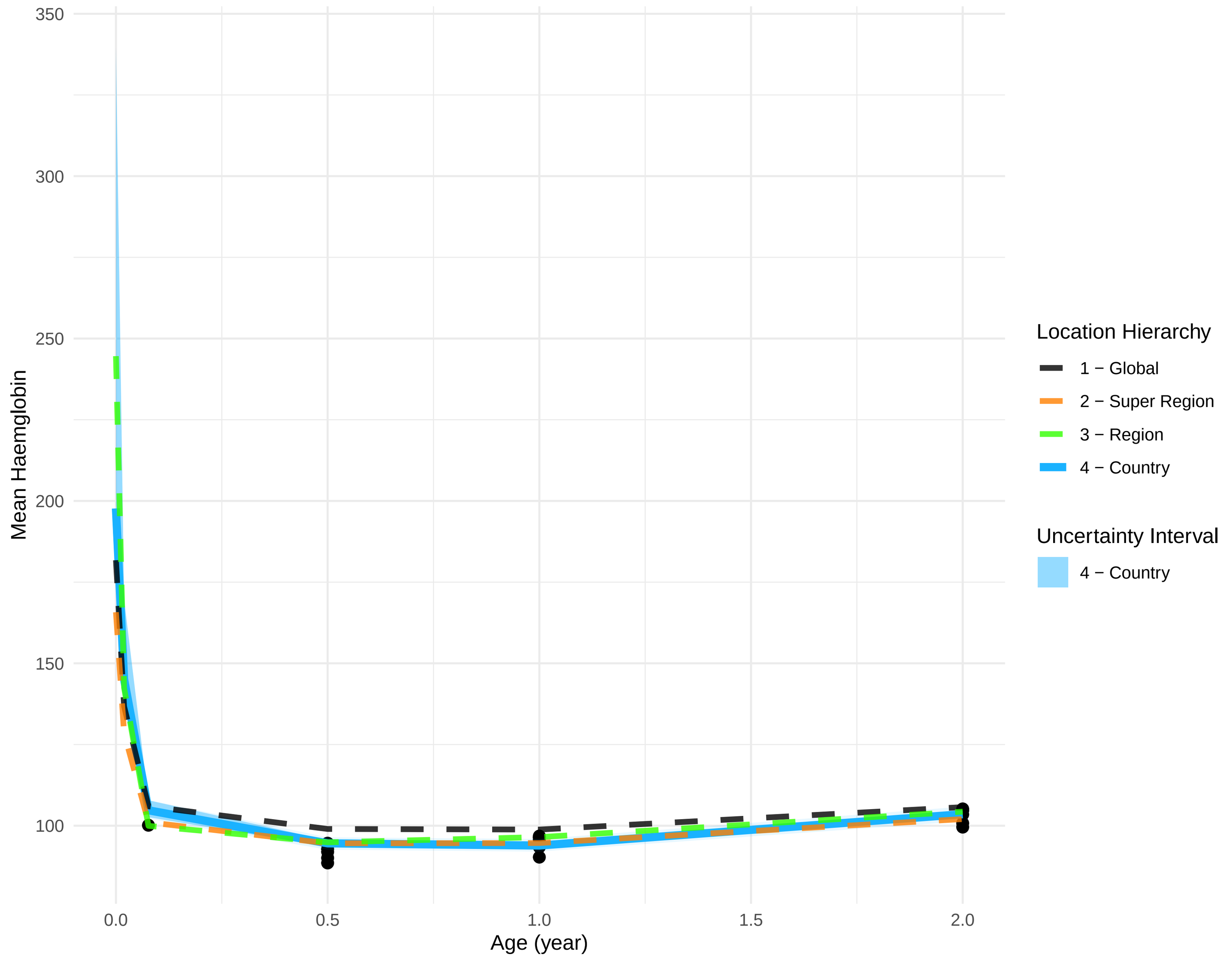

MR−BRT Age/Sex Fits − Males <2 Years Old − Mean Haemglobin − Zambia
WHO−2024 Adjustment Method

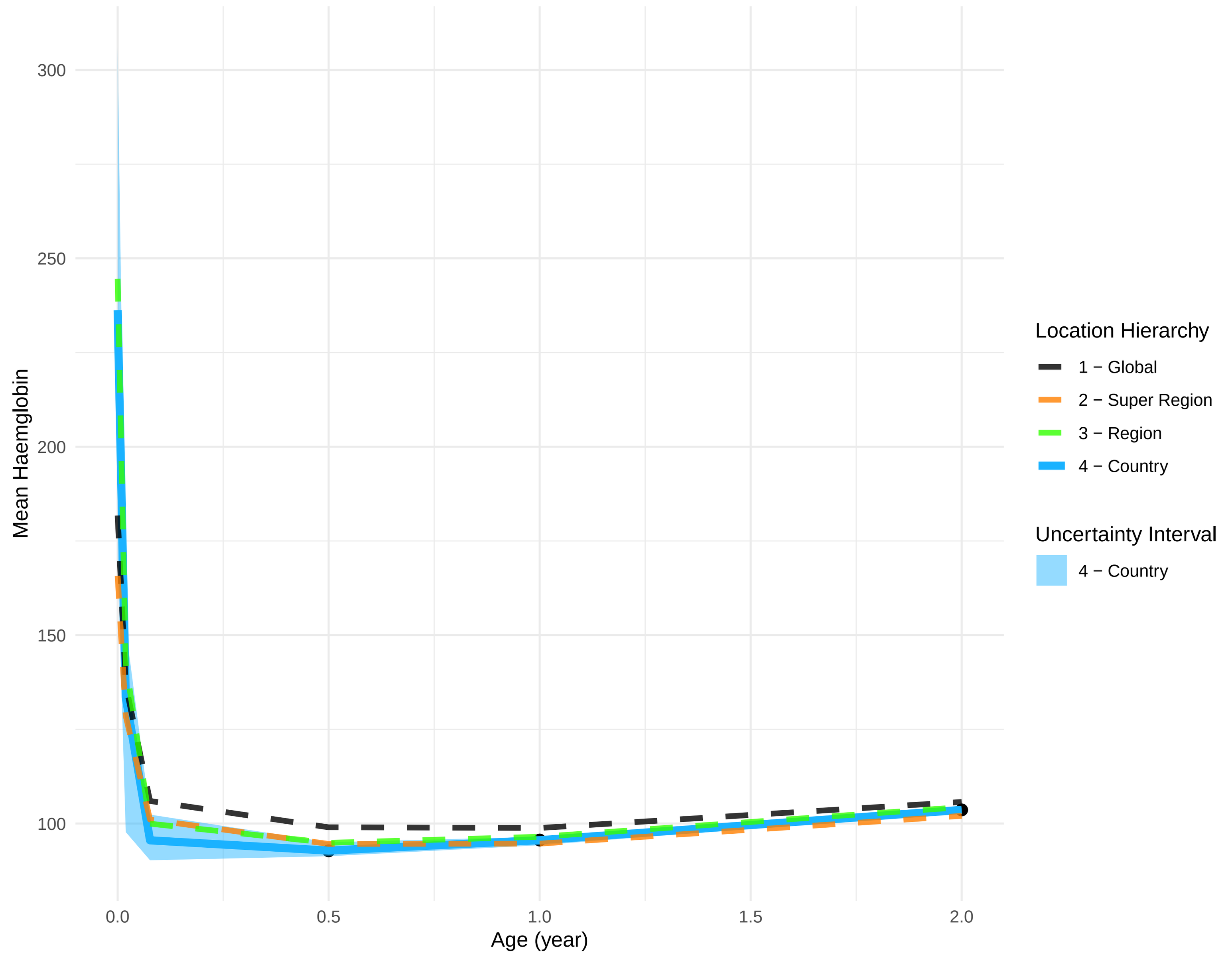

MR−BRT Age/Sex Fits − Males <2 Years Old − Mean Haemglobin − Southern Sub−Saharan Africa
WHO−2024 Adjustment Method

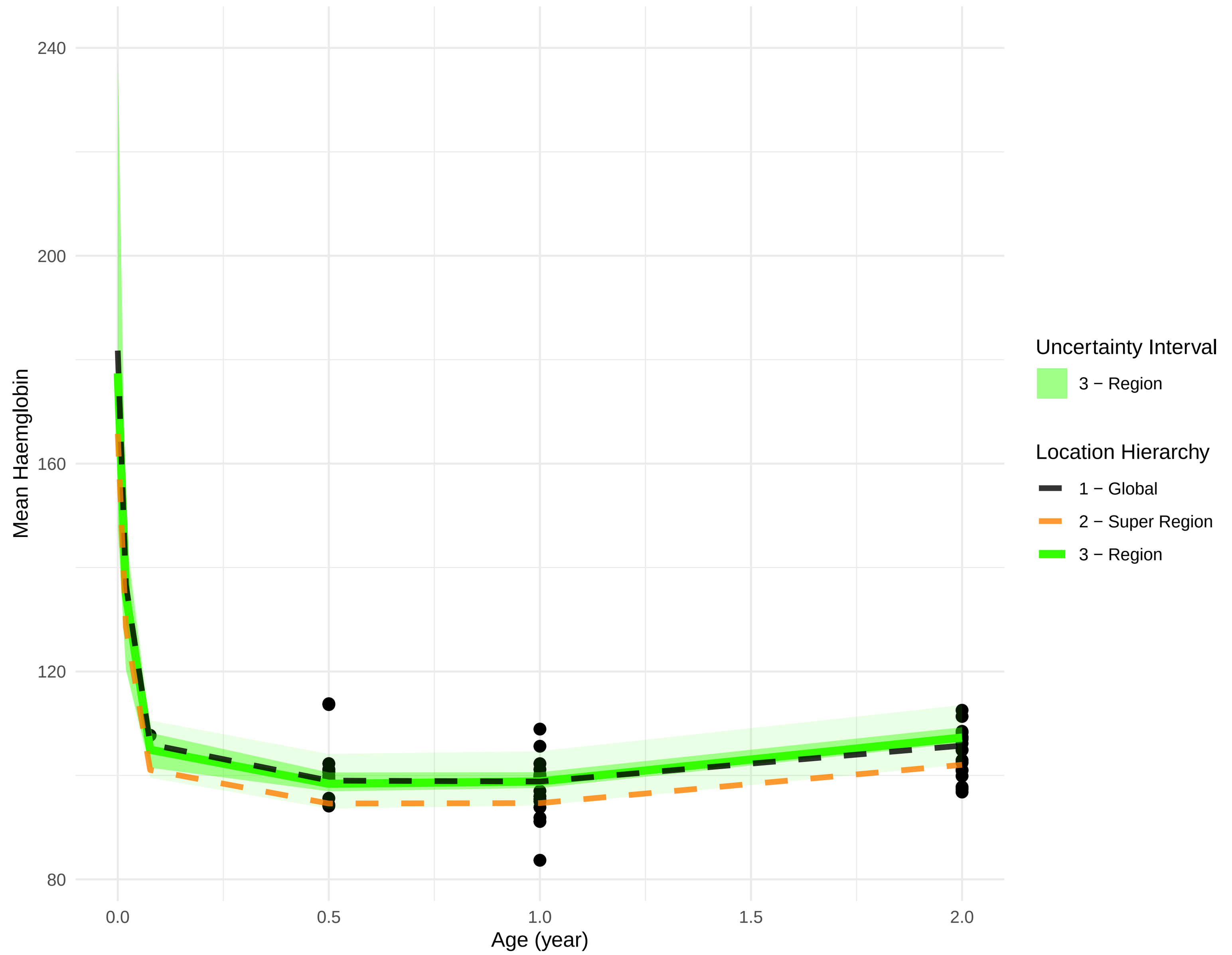

MR−BRT Age/Sex Fits − Males <2 Years Old − Mean Haemglobin − Eswatini
WHO−2024 Adjustment Method

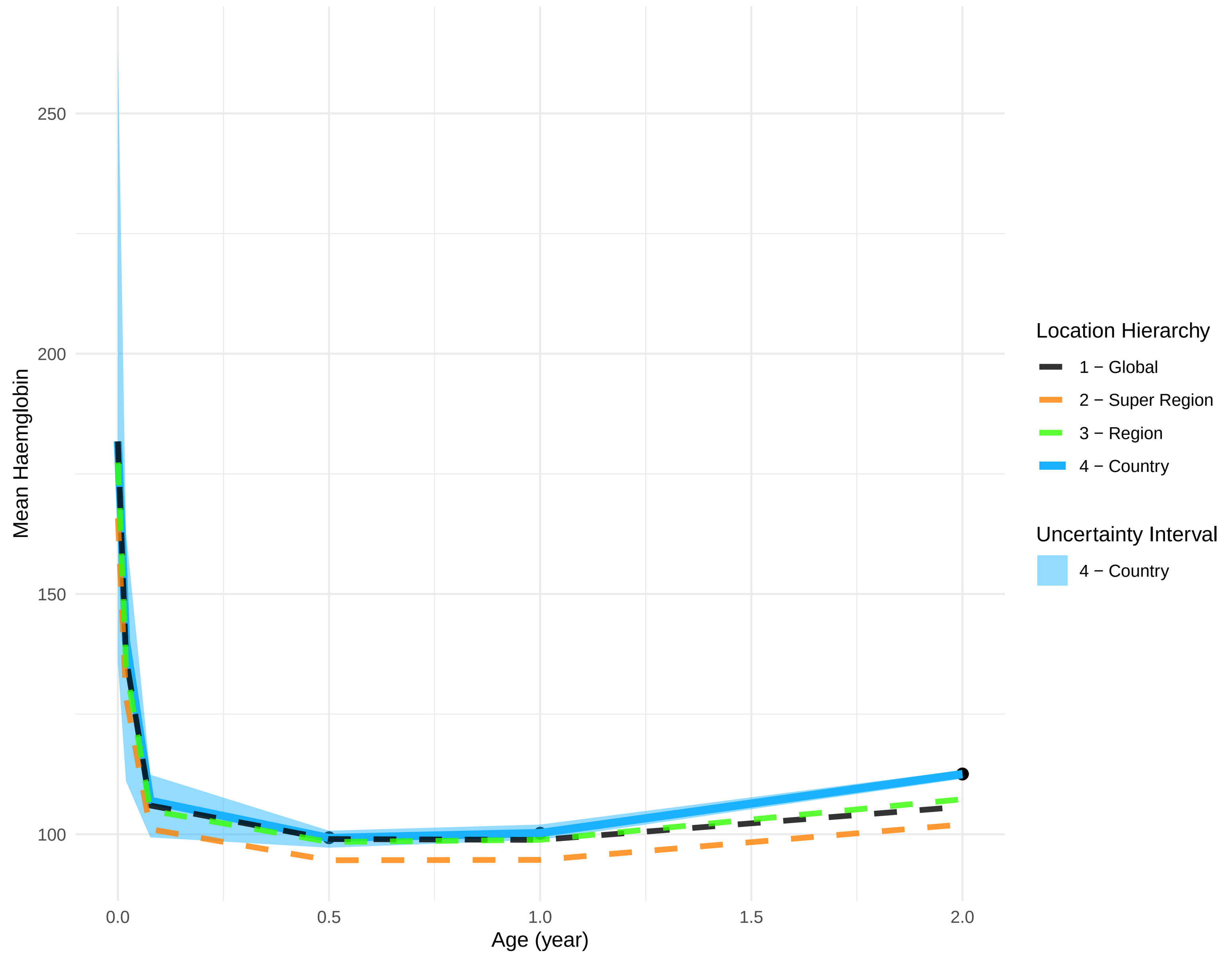

MR−BRT Age/Sex Fits − Males <2 Years Old − Mean Haemglobin − Lesotho
WHO−2024 Adjustment Method

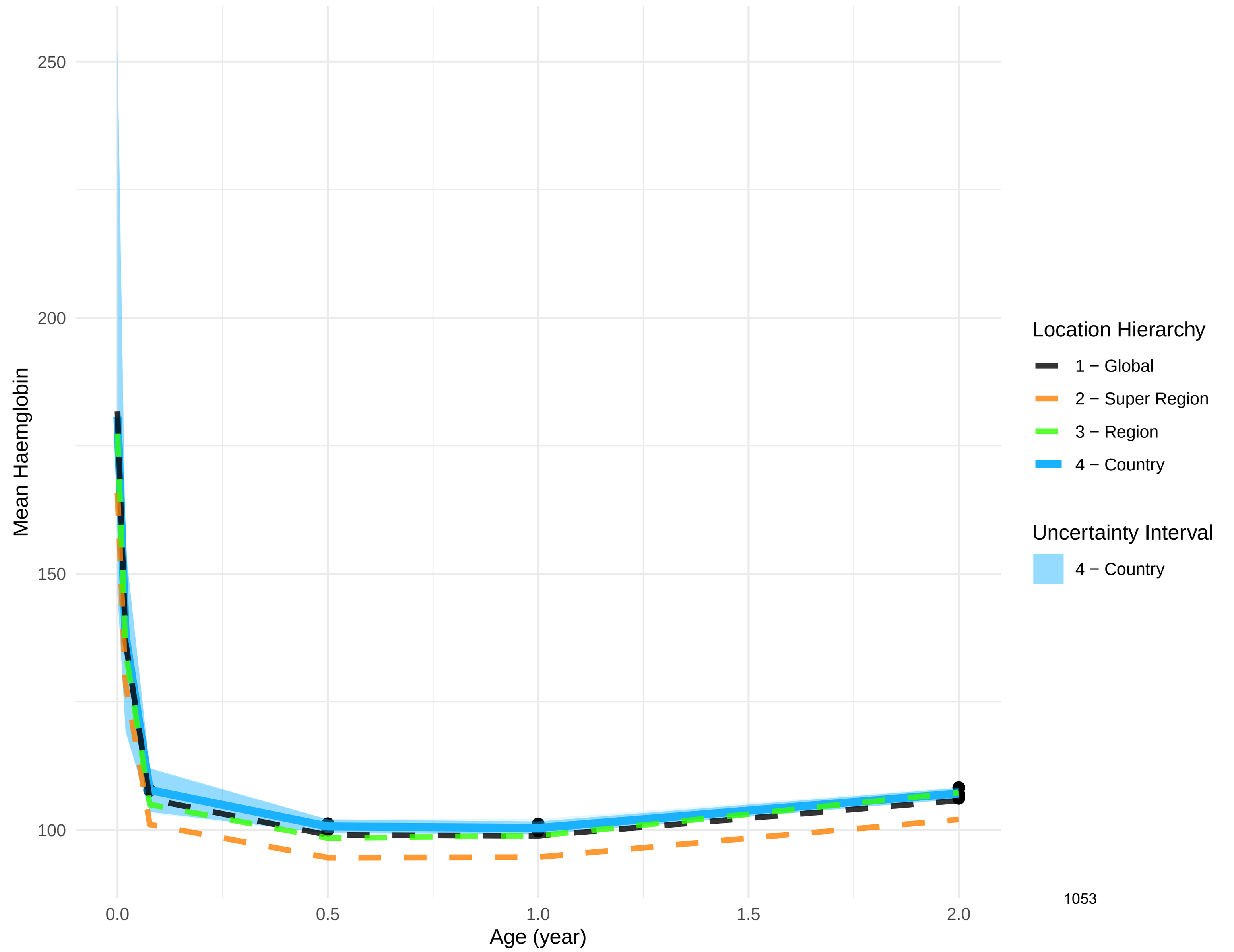

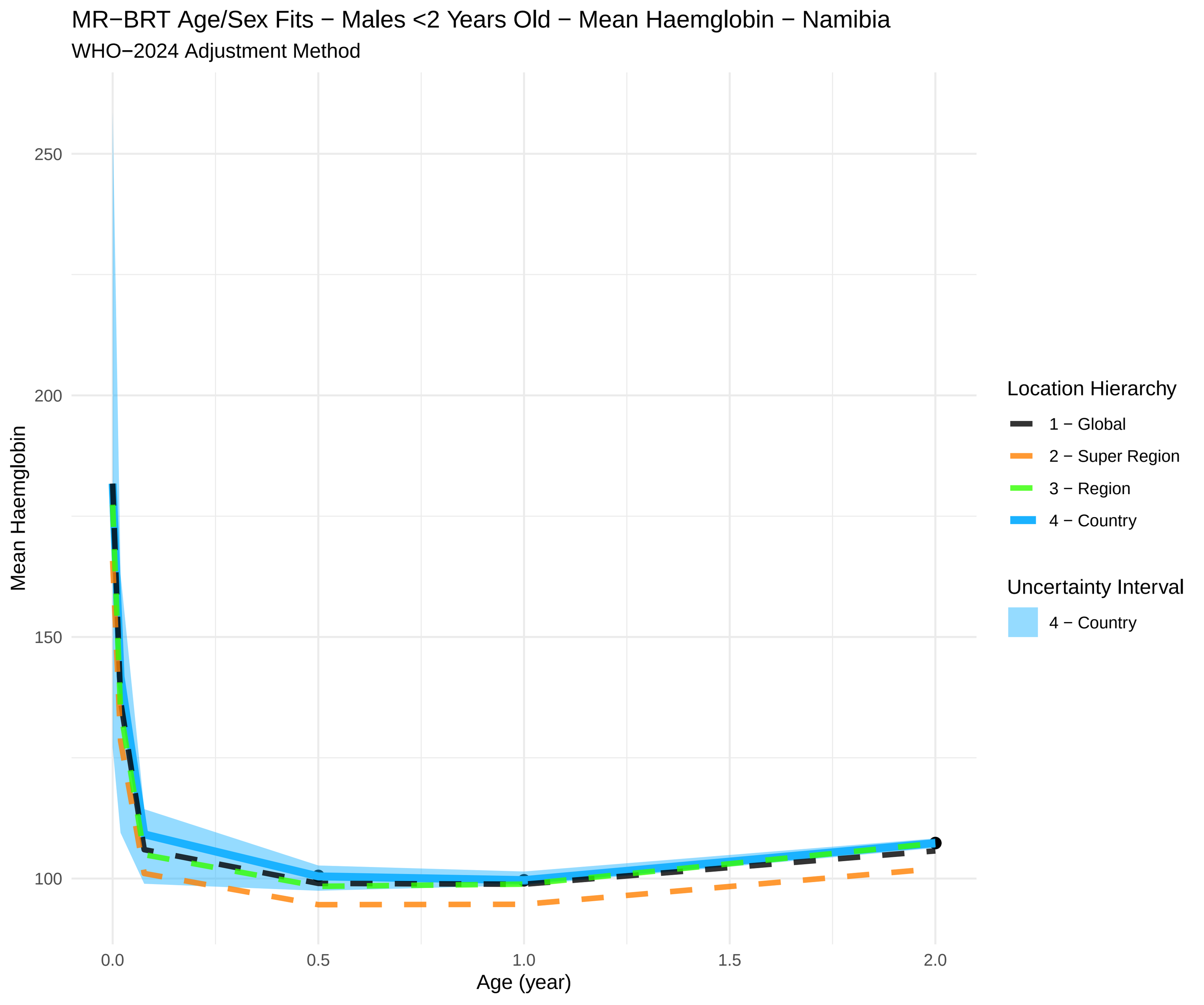
MR−BRT Age/Sex Fits − Males <2 Years Old − Mean Haemglobin − Namibia
WHO−2024 Adjustment Method
Mean Haemglobin
Age (year)
Location Hierarchy
1 − Global
2 − Super Region
3 − Region
4 − Country
Uncertainty Interval
4 − Country
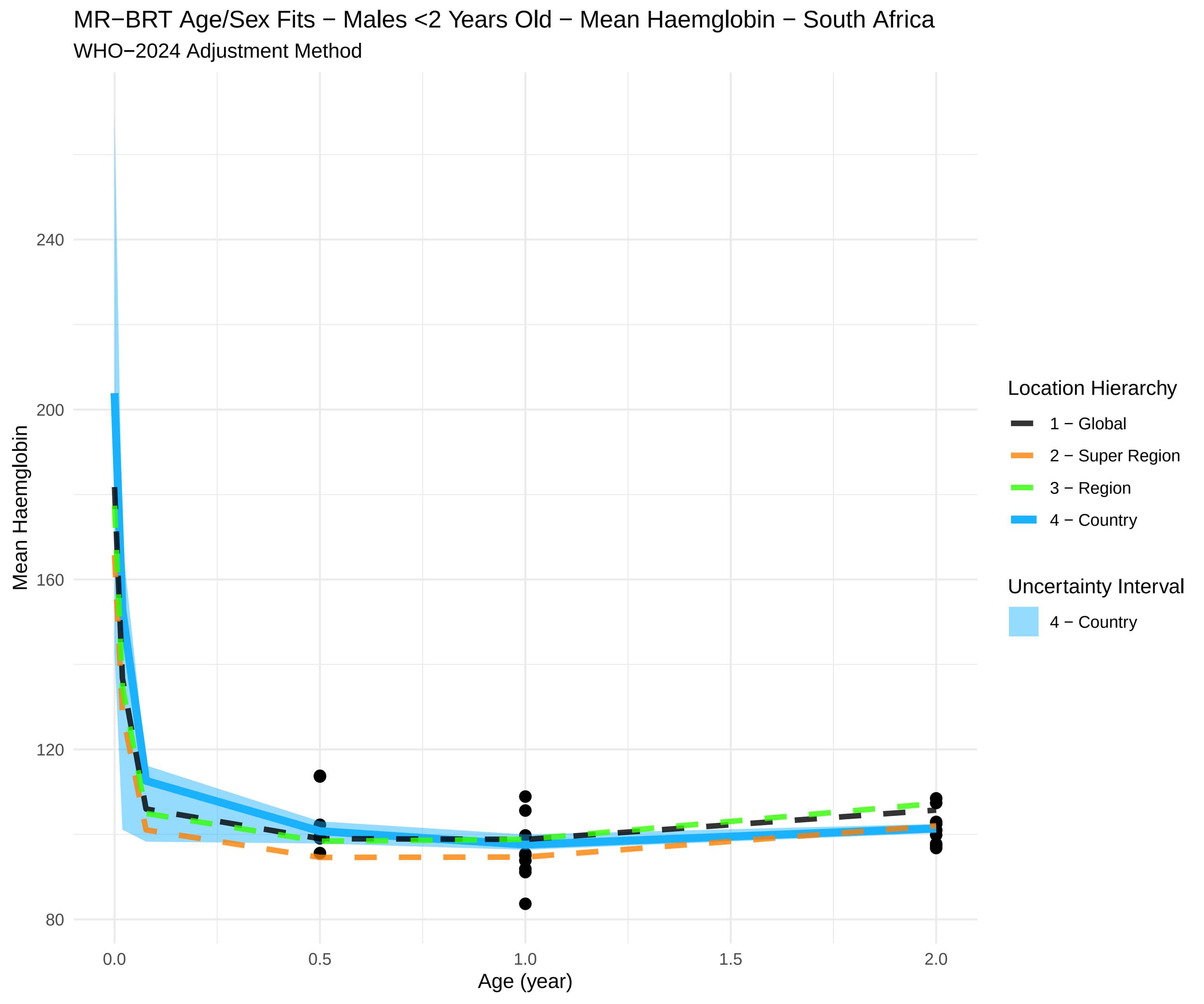
MR−BRT Age/Sex Fits − Males <2 Years Old − Mean Haemglobin − South Africa
WHO−2024 Adjustment Method
Mean Haemglobin
Age (year)
Location Hierarchy
1 − Global
2 − Super Region
3 − Region
4 − Country
Uncertainty Interval
4 − Country
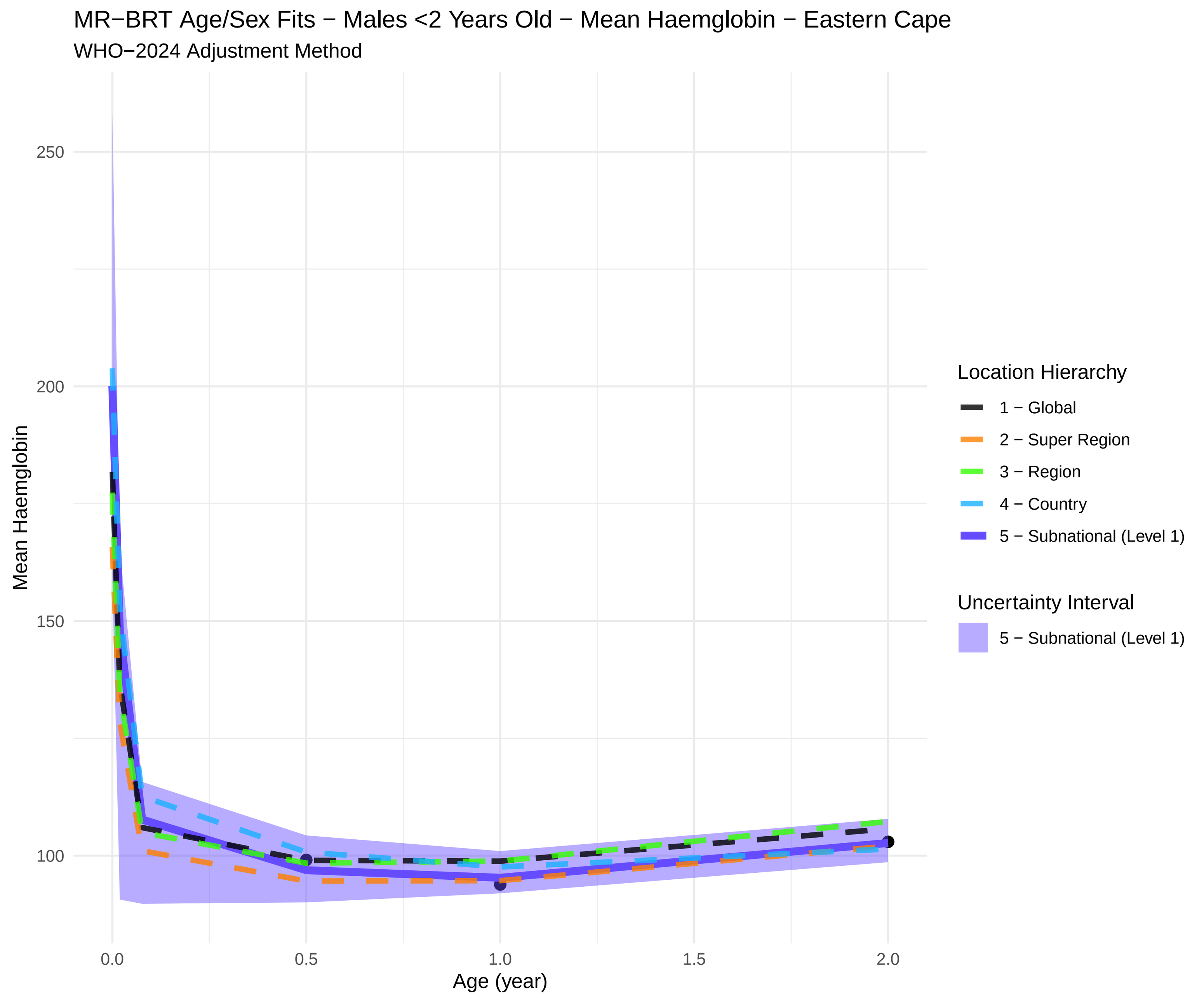
MR−BRT Age/Sex Fits − Males <2 Years Old − Mean Haemglobin − Eastern Cape
WHO−2024 Adjustment Method
Mean Haemglobin
Age (year)
Location Hierarchy
1 − Global
2 − Super Region
3 − Region
4 − Country
5 − Subnational (Level 1)
Uncertainty Interval
5 − Subnational (Level 1)
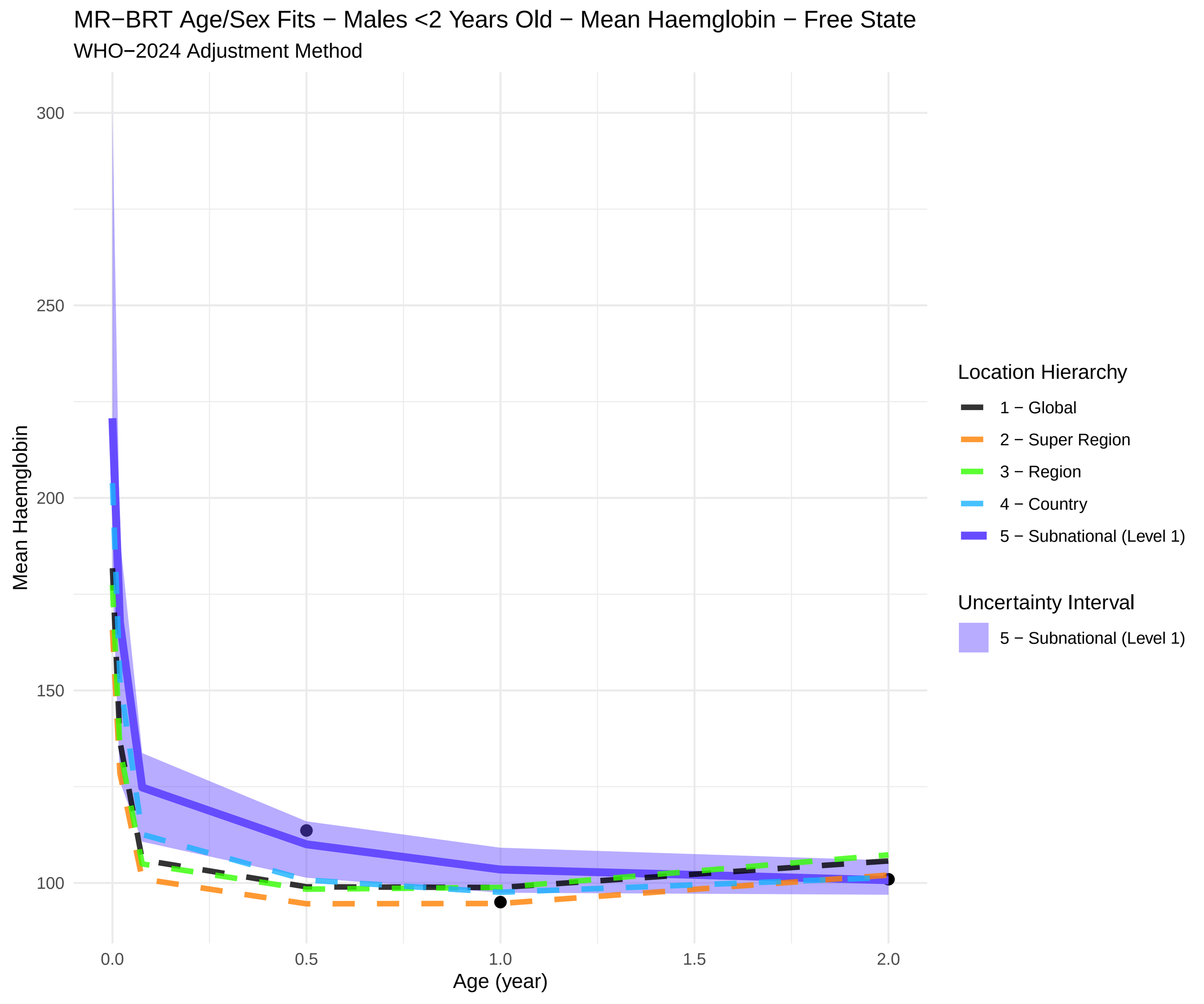
MR−BRT Age/Sex Fits − Males <2 Years Old − Mean Haemglobin − Free State
WHO−2024 Adjustment Method
Mean Haemglobin
Age (year)
Location Hierarchy
1 − Global
2 − Super Region
3 − Region
4 − Country
5 − Subnational (Level 1)
Uncertainty Interval
5 − Subnational (Level 1)
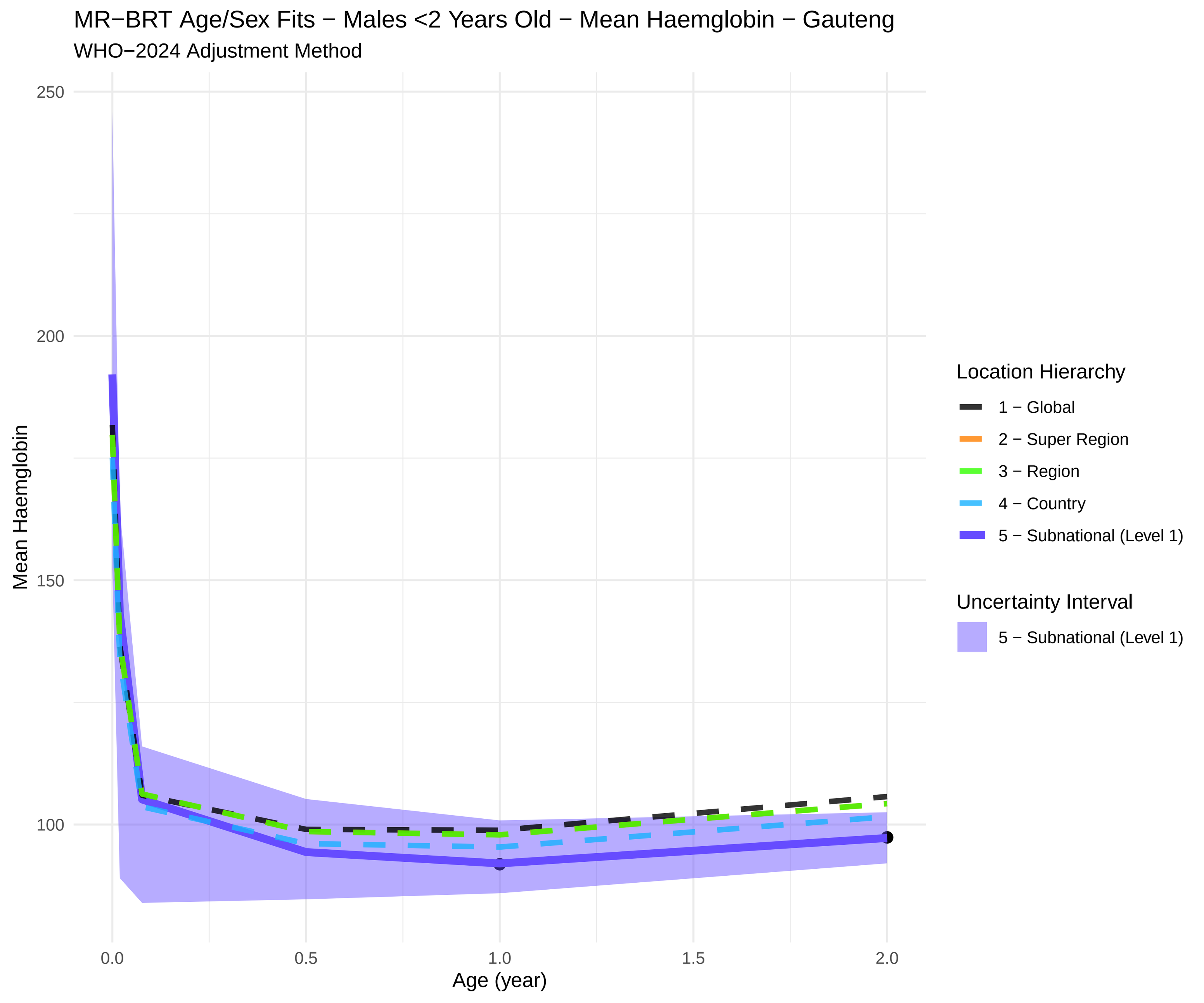
MR−BRT Age/Sex Fits − Males <2 Years Old − Mean Haemglobin − Gauteng
WHO−2024 Adjustment Method
Mean Haemglobin
Age (year)
Location Hierarchy
1 − Global
2 − Super Region
3 − Region
4 − Country
5 − Subnational (Level 1)
Uncertainty Interval
5 − Subnational (Level 1)
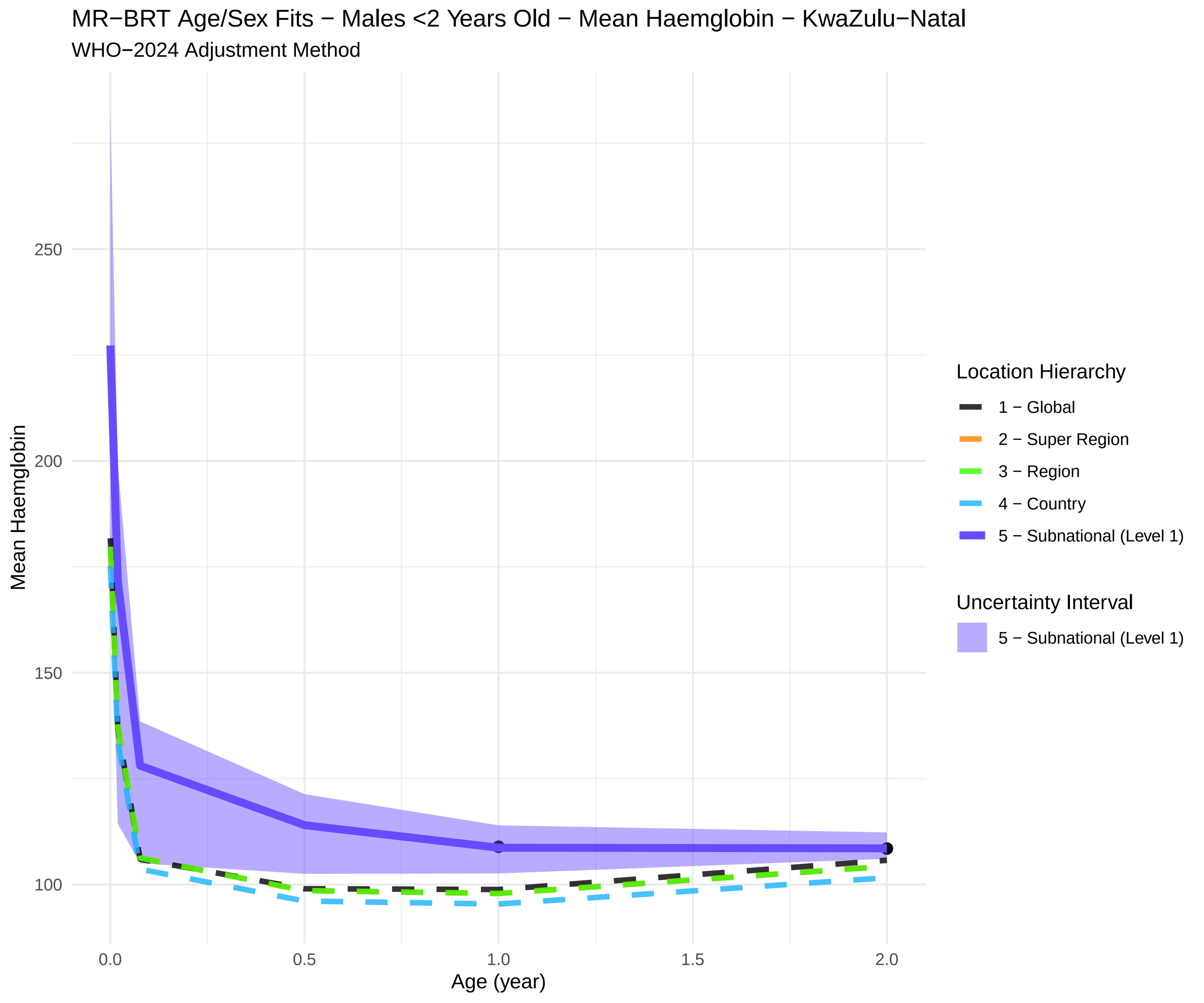
MR−BRT Age/Sex Fits − Males <2 Years Old − Mean Haemglobin − KwaZulu−Natal
WHO−2024 Adjustment Method
Mean Haemglobin
Age (year)
Location Hierarchy
1 − Global
2 − Super Region
3 − Region
4 − Country
5 − Subnational (Level 1)
Uncertainty Interval
5 − Subnational (Level 1)
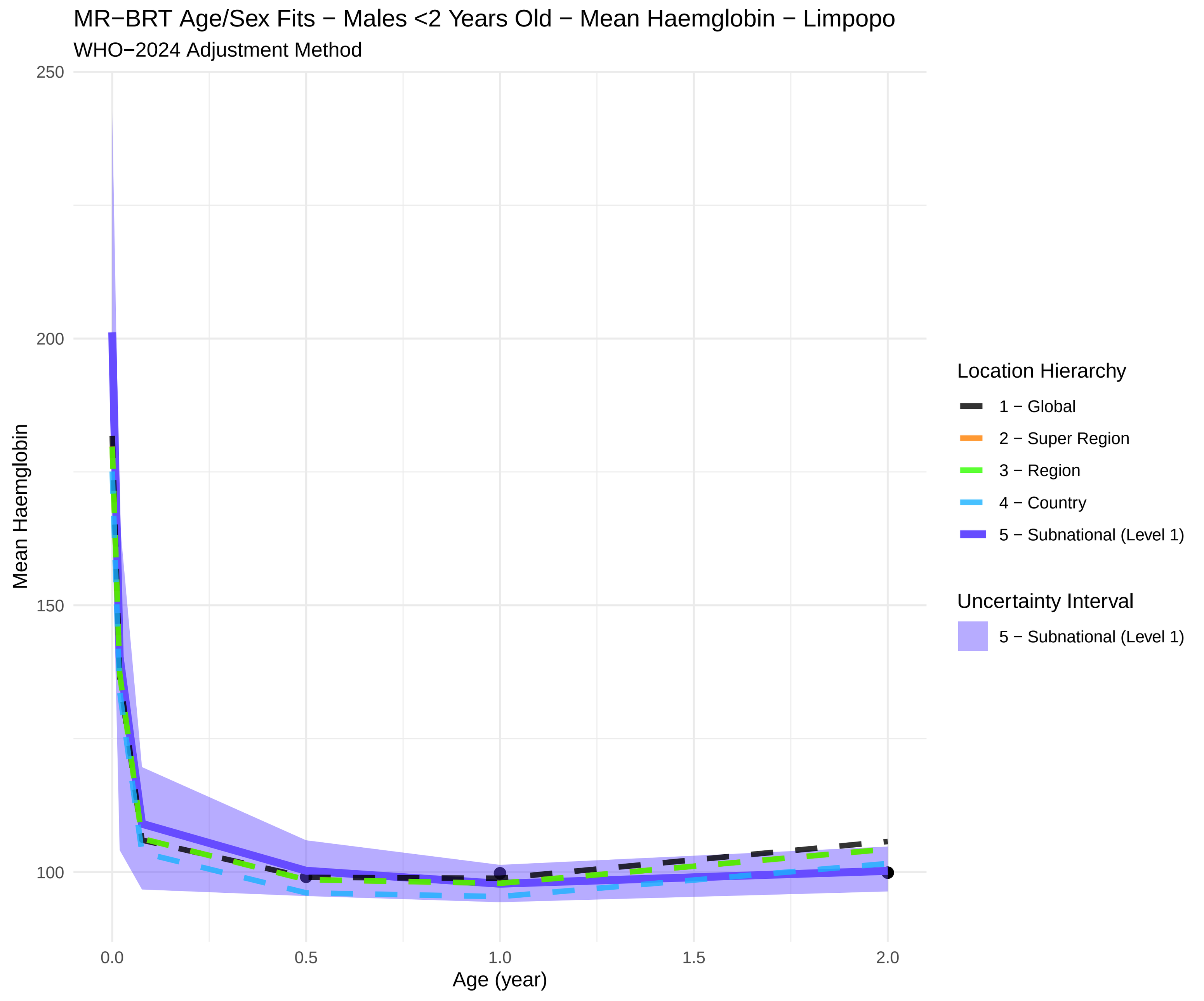
MR−BRT Age/Sex Fits − Males <2 Years Old − Mean Haemglobin − Limpopo
WHO−2024 Adjustment Method
Mean Haemglobin
Age (year)
Location Hierarchy
1 − Global
2 − Super Region
3 − Region
4 − Country
5 − Subnational (Level 1)
Uncertainty Interval
5 − Subnational (Level 1)
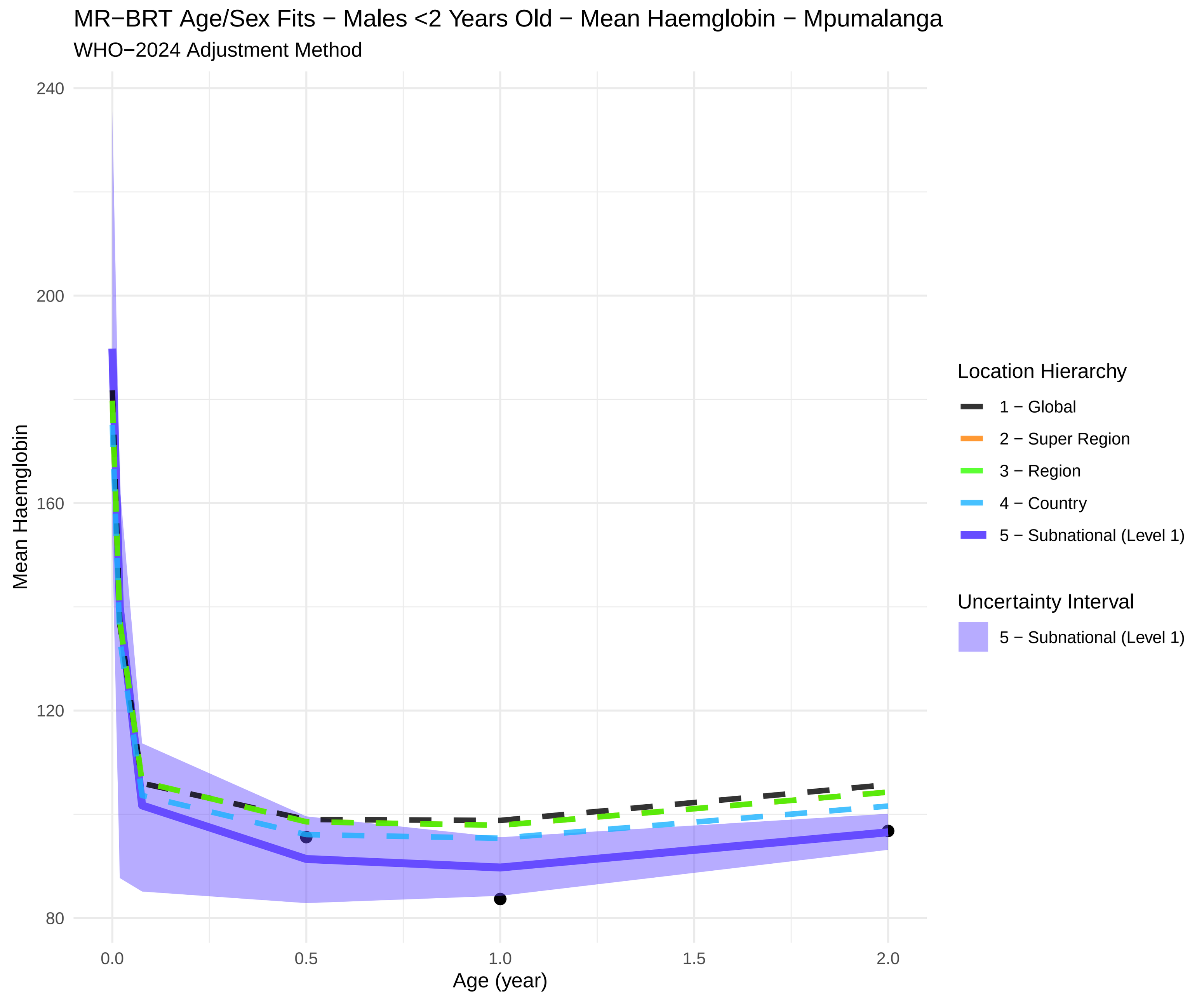
MR−BRT Age/Sex Fits − Males <2 Years Old − Mean Haemglobin − Mpumalanga
WHO−2024 Adjustment Method
Mean Haemglobin
Age (year)
Location Hierarchy
1 − Global
2 − Super Region
3 − Region
4 − Country
5 − Subnational (Level 1)
Uncertainty Interval
5 − Subnational (Level 1)
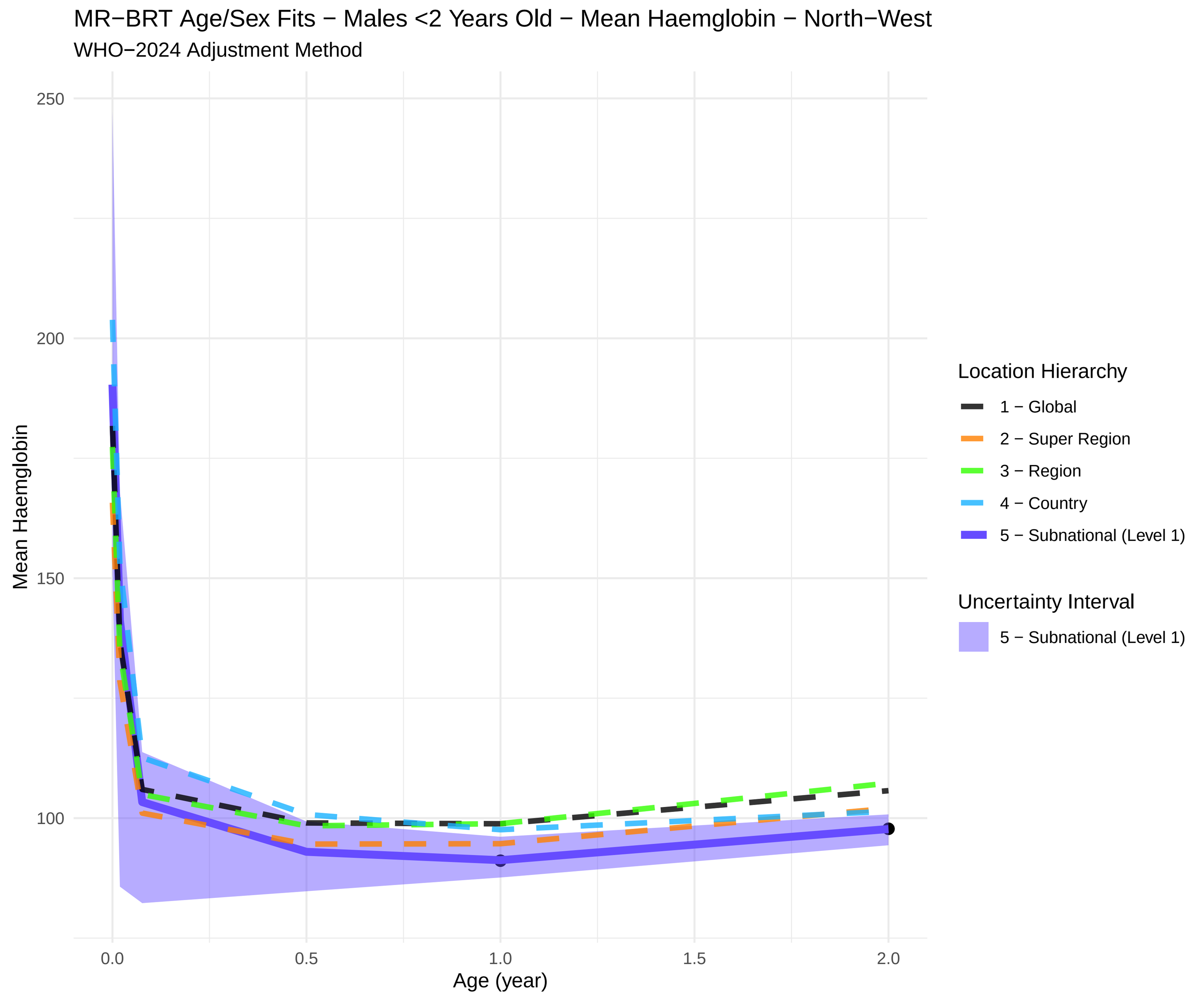
MR−BRT Age/Sex Fits − Males <2 Years Old − Mean Haemglobin − North−West
WHO−2024 Adjustment Method
Mean Haemglobin
Age (year)
Location Hierarchy
1 − Global
2 − Super Region
3 − Region
4 − Country
5 − Subnational (Level 1)
Uncertainty Interval
5 − Subnational (Level 1)
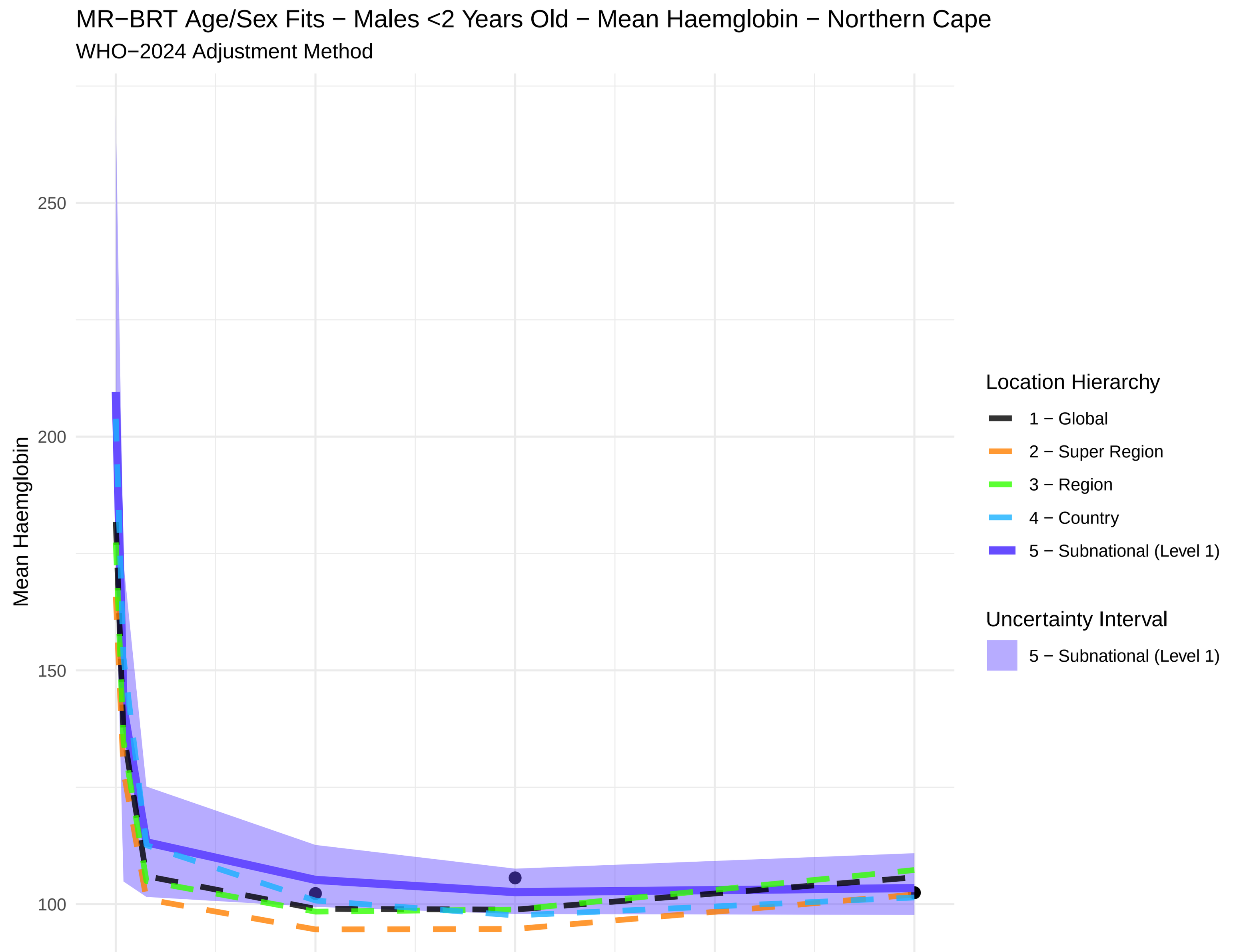
MR−BRT Age/Sex Fits − Males <2 Years Old − Mean Haemglobin − Northern Cape
WHO−2024 Adjustment Method
Mean Haemglobin
Age (year)
Location Hierarchy
1 − Global
2 − Super Region
3 − Region
4 − Country
5 − Subnational (Level 1)
Uncertainty Interval
5 − Subnational (Level 1)

MR−BRT Age/Sex Fits − Males <2 Years Old − Mean Haemglobin − Western Cape
WHO−2024 Adjustment Method

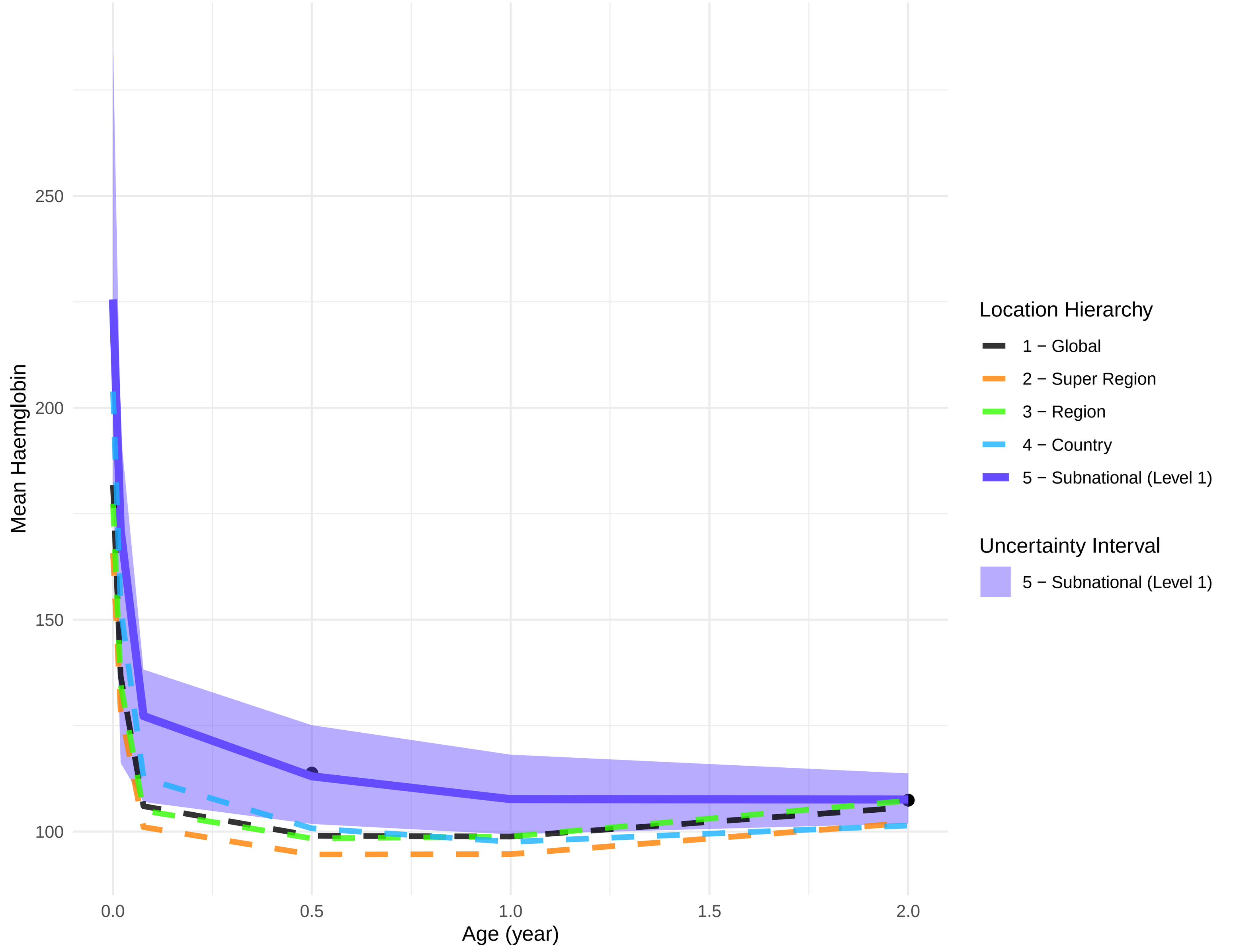

MR−BRT Age/Sex Fits − Males <2 Years Old − Mean Haemglobin − Zimbabwe
WHO−2024 Adjustment Method

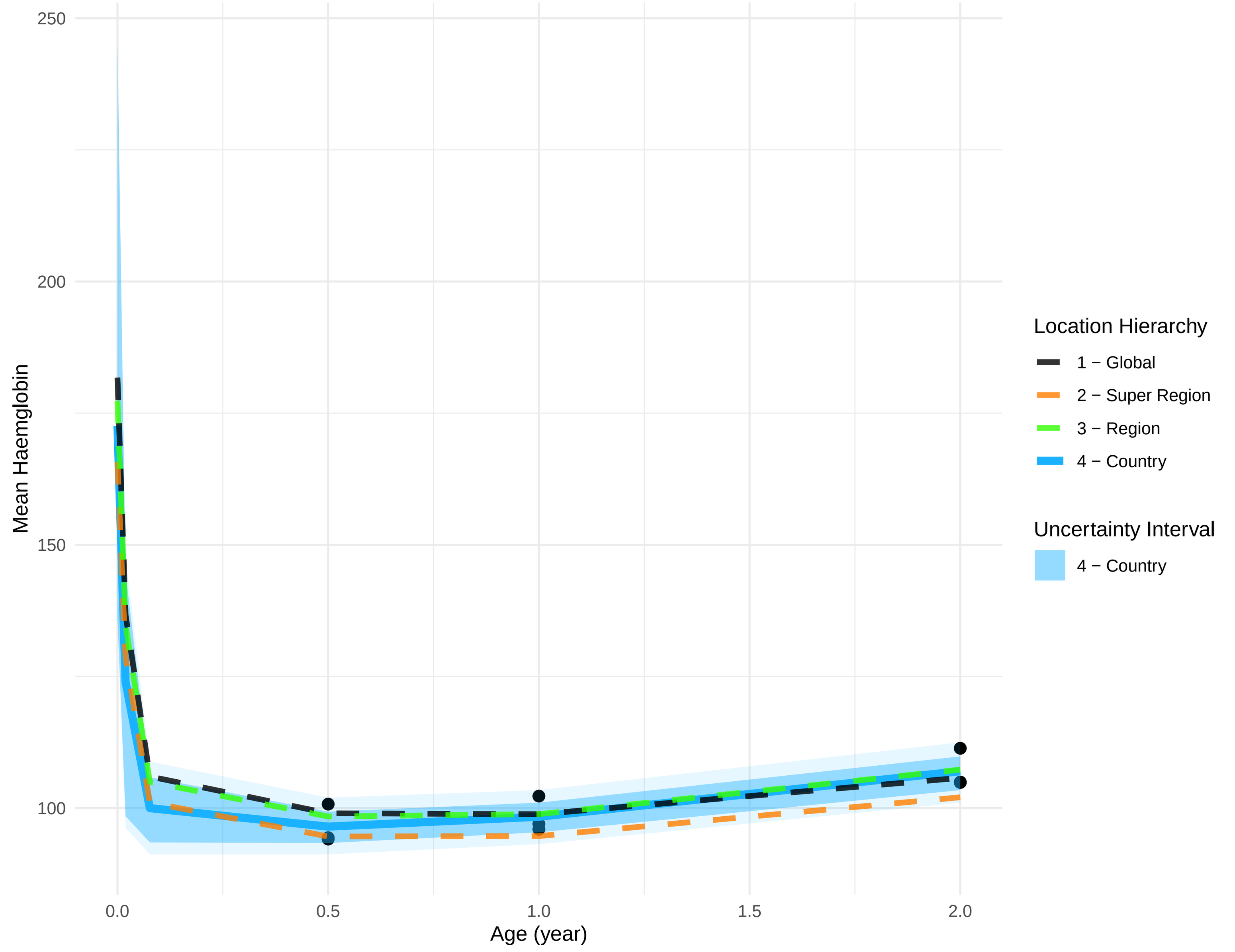

MR−BRT Age/Sex Fits − Males <2 Years Old − Mean Haemglobin − Western Sub−Saharan Africa
WHO−2024 Adjustment Method

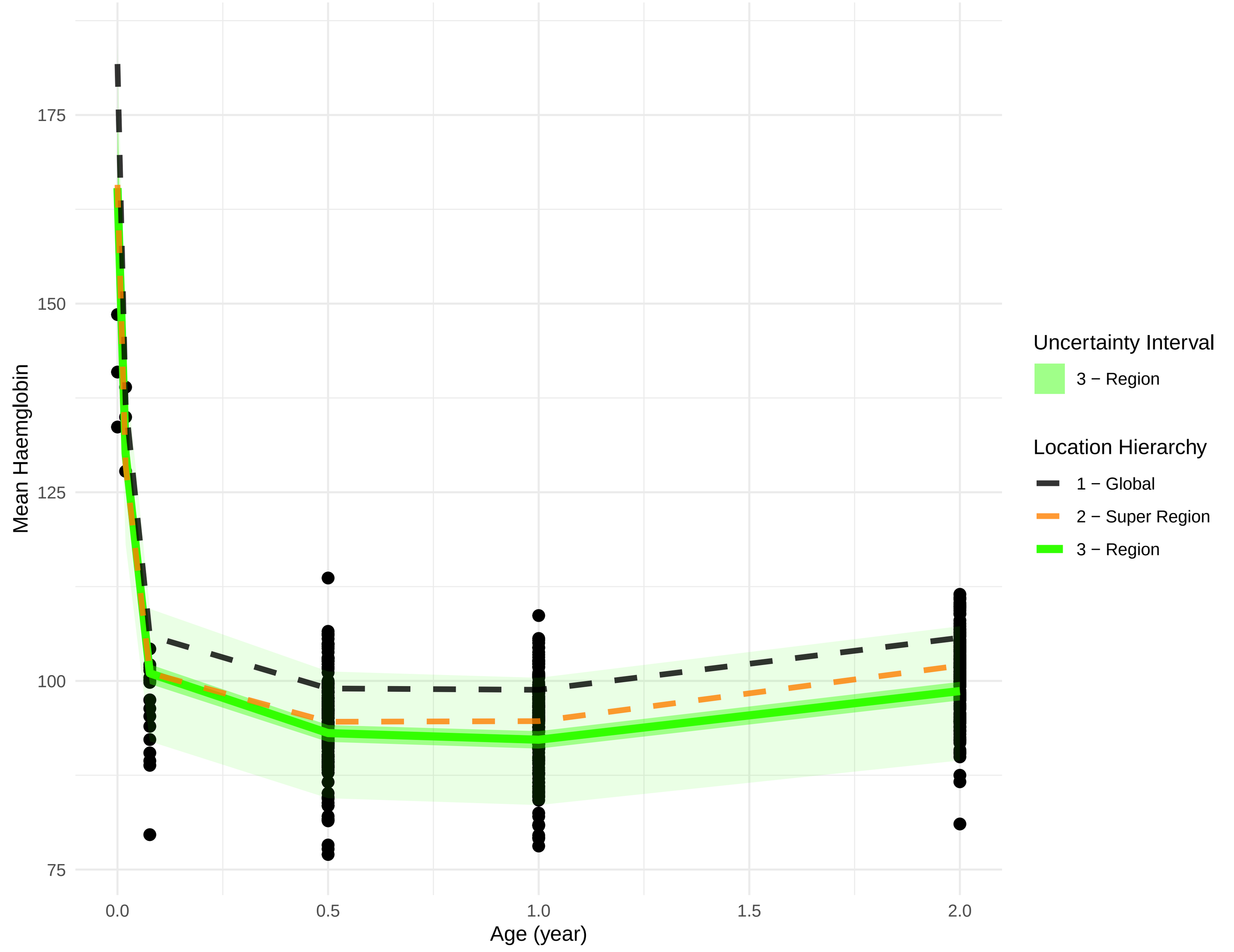

MR−BRT Age/Sex Fits − Males <2 Years Old − Mean Haemglobin − Benin
WHO−2024 Adjustment Method

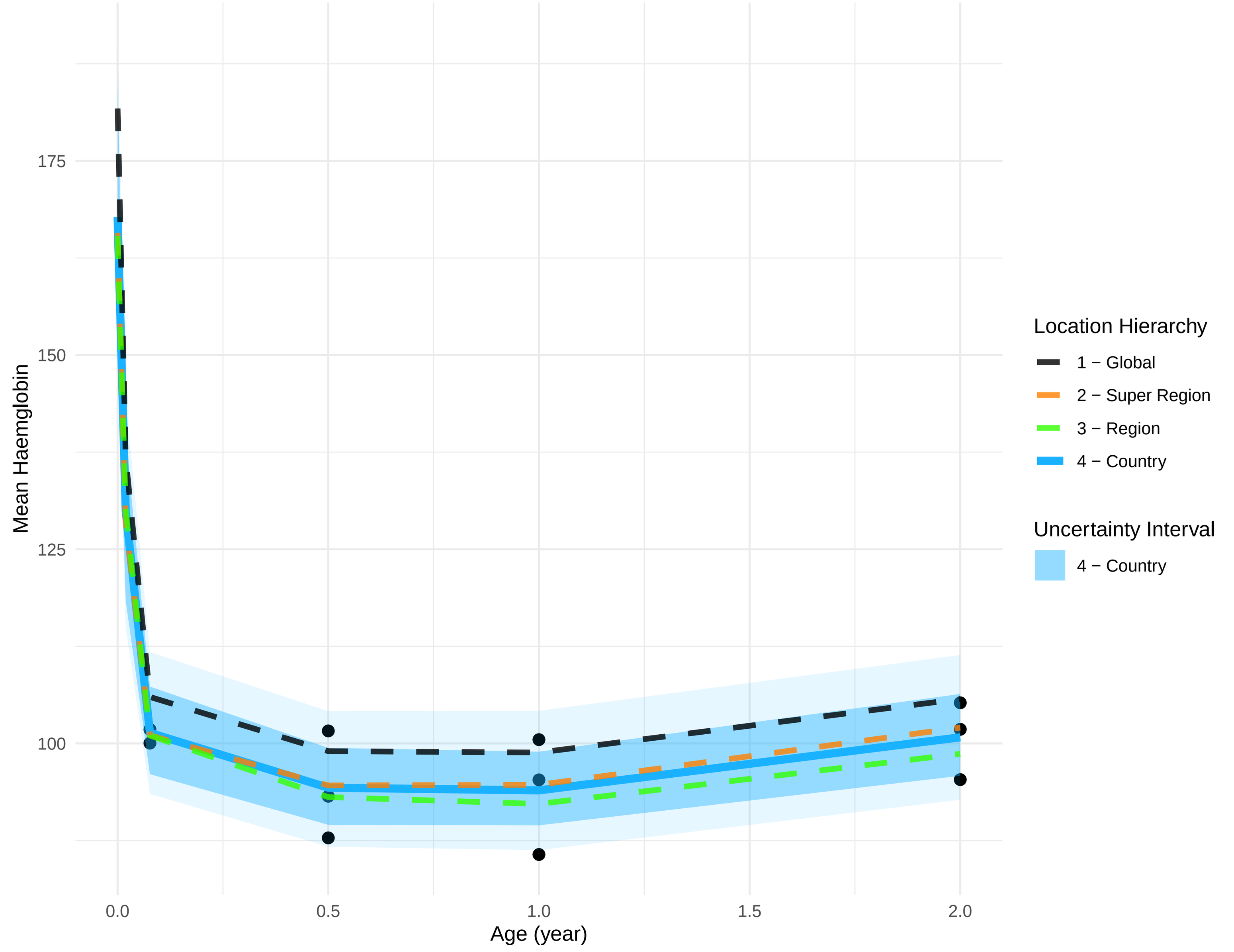

MR−BRT Age/Sex Fits − Males <2 Years Old − Mean Haemglobin − Burkina Faso
WHO−2024 Adjustment Method

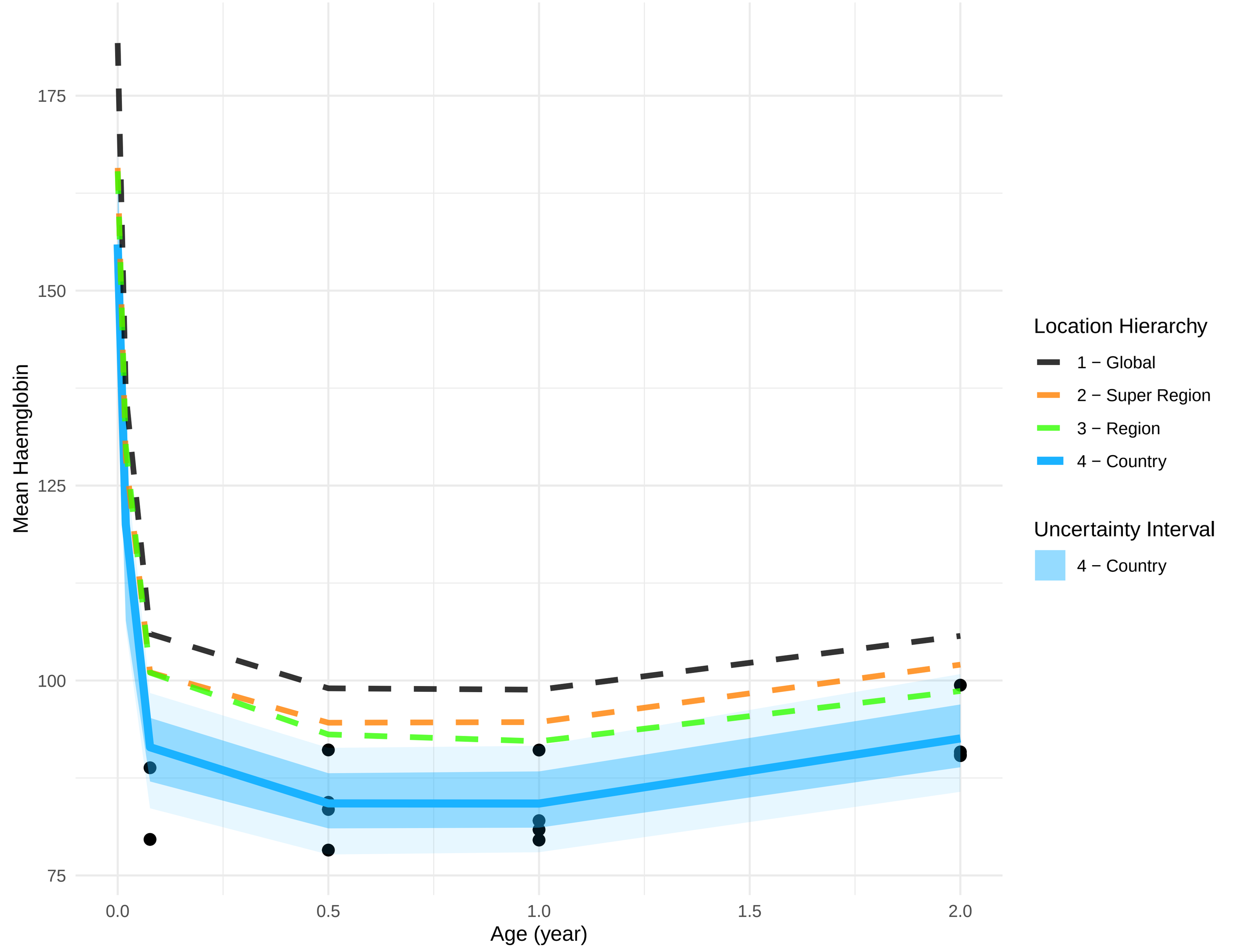

MR−BRT Age/Sex Fits − Males <2 Years Old − Mean Haemglobin − Cameroon
WHO−2024 Adjustment Method

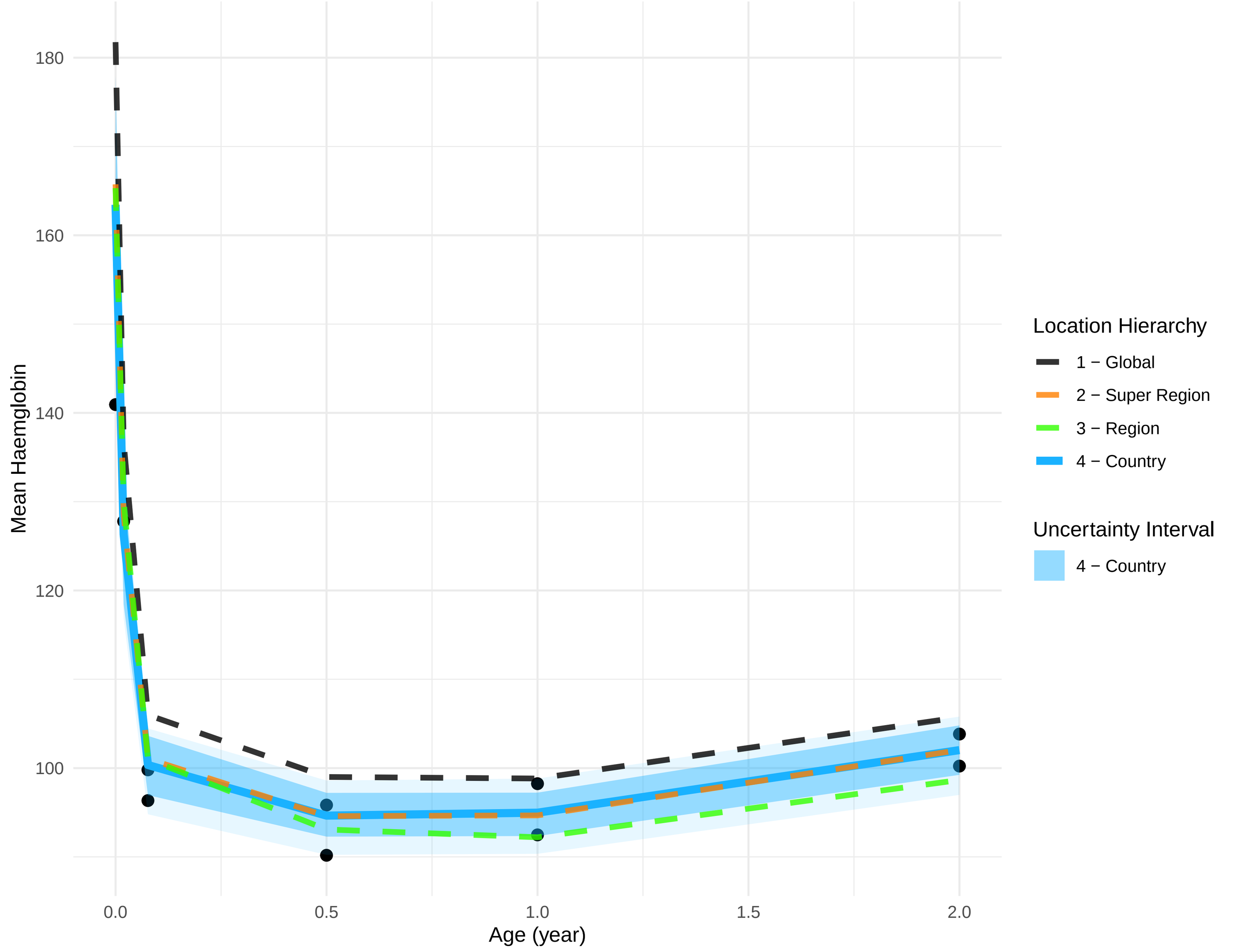

MR−BRT Age/Sex Fits − Males <2 Years Old − Mean Haemglobin − Côte d'Ivoire
WHO−2024 Adjustment Method

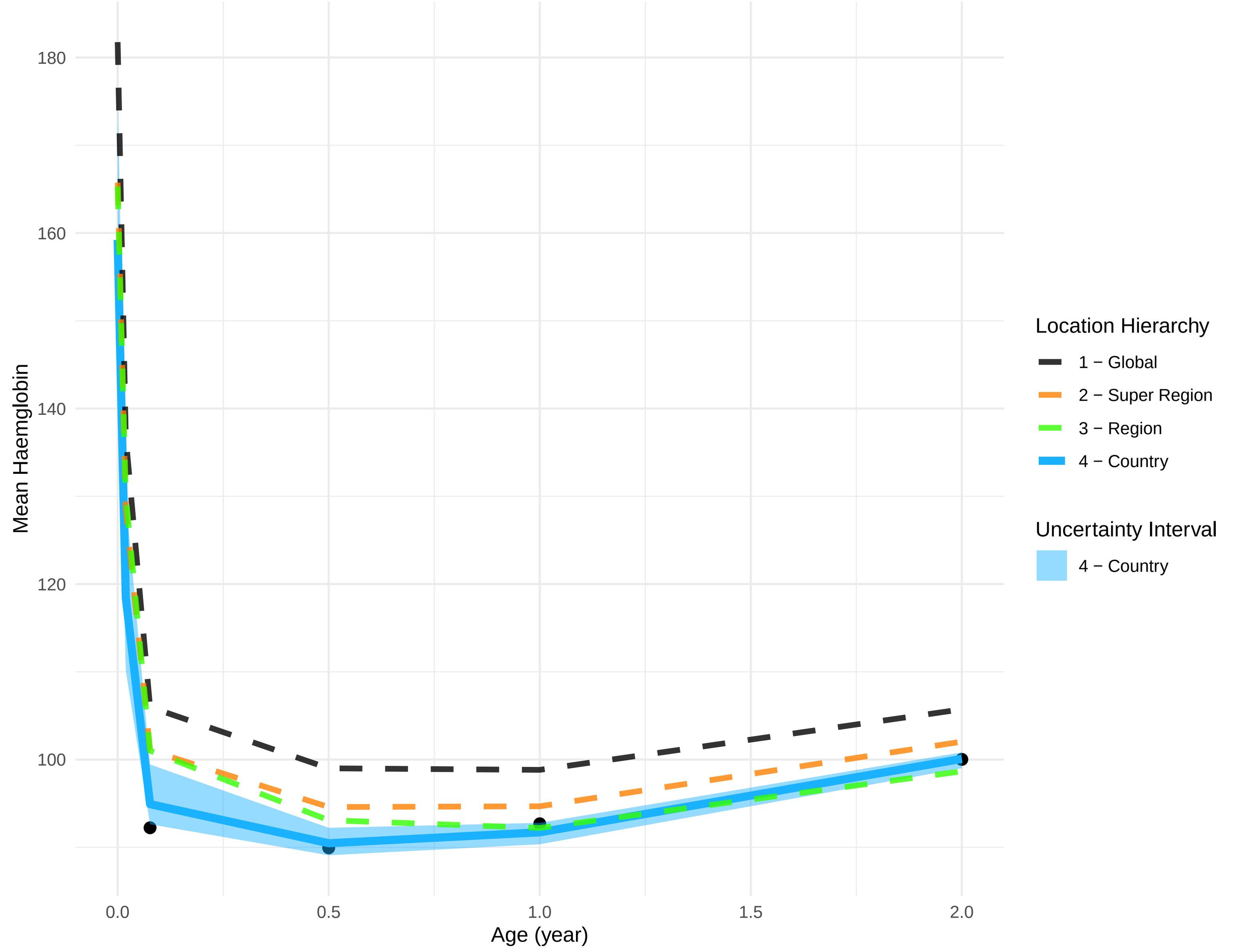

MR−BRT Age/Sex Fits − Males <2 Years Old − Mean Haemglobin − Gambia
WHO−2024 Adjustment Method

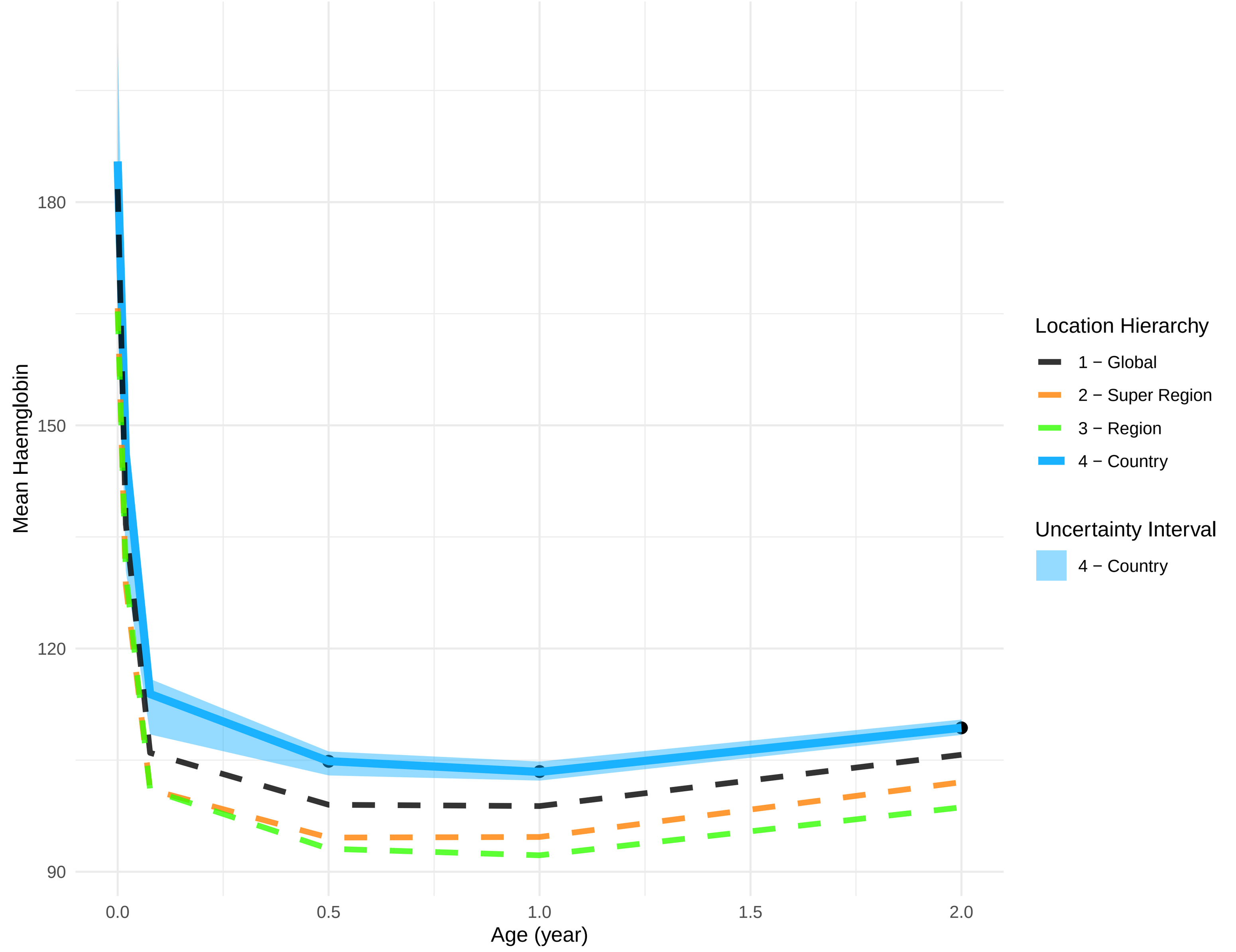

MR−BRT Age/Sex Fits − Males <2 Years Old − Mean Haemglobin − Ghana
WHO−2024 Adjustment Method

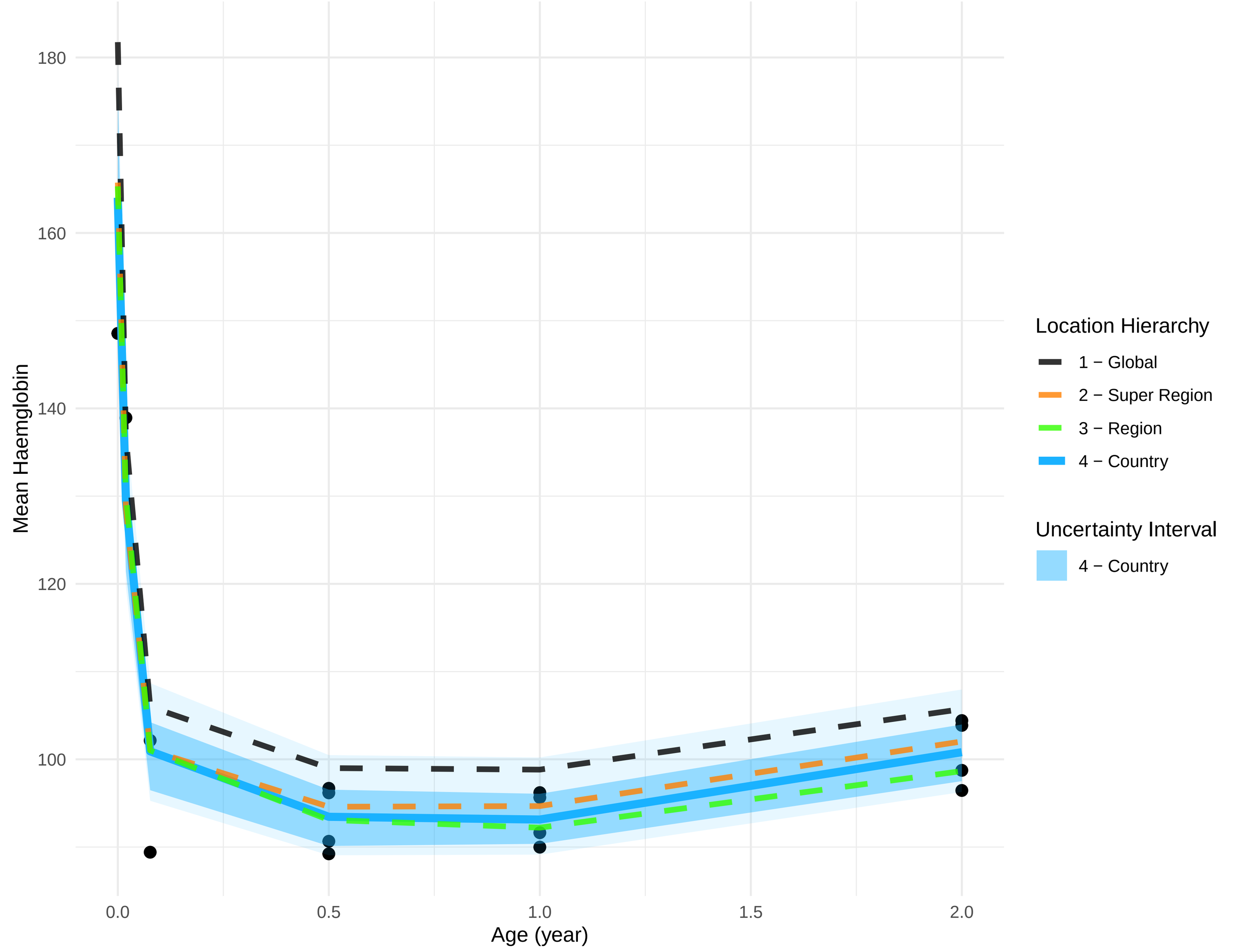

MR−BRT Age/Sex Fits − Males <2 Years Old − Mean Haemglobin − Guinea
WHO−2024 Adjustment Method

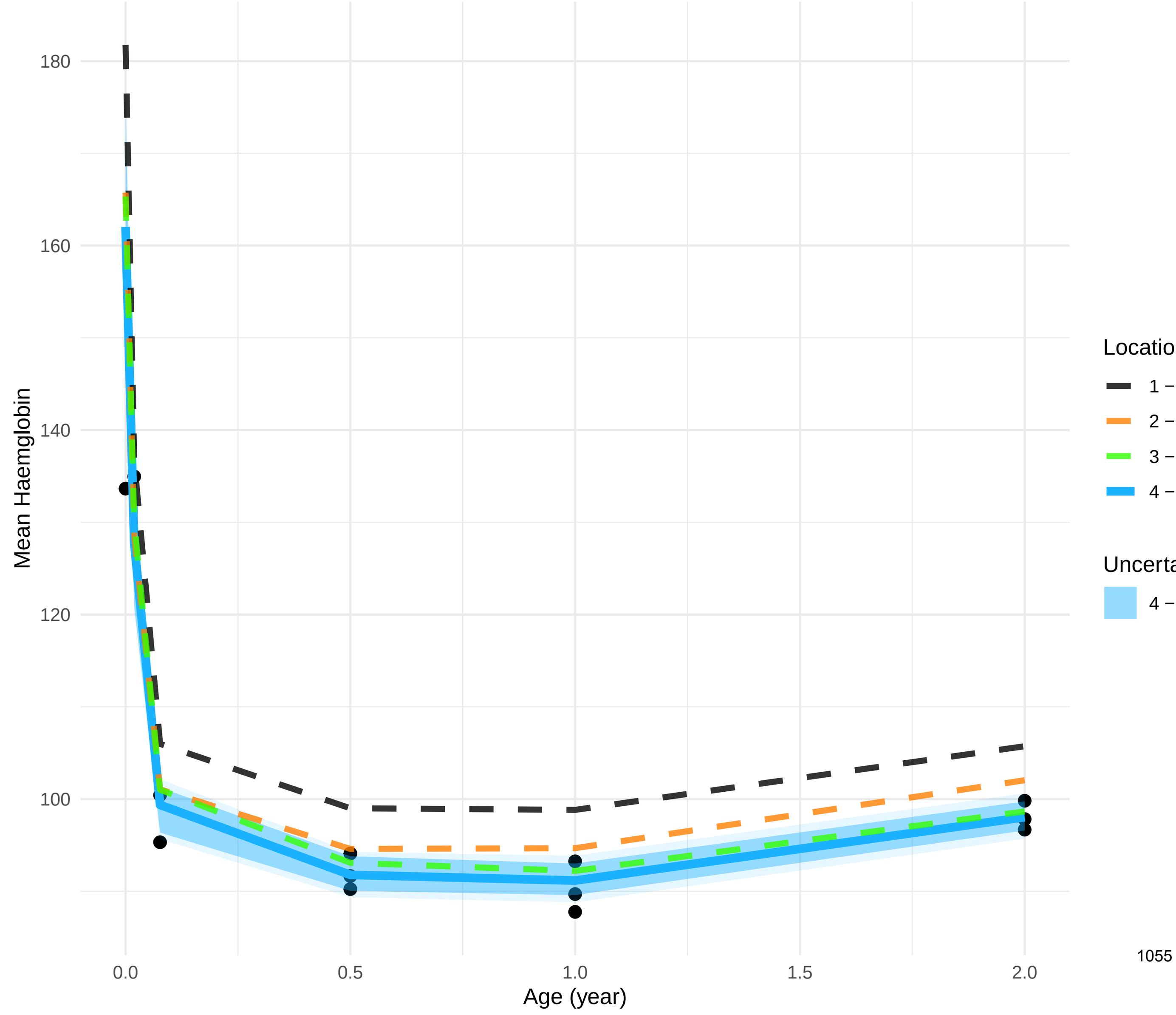

MR−BRT Age/Sex Fits − Males <2 Years Old − Mean Haemoglobin − Liberia
WHO−2024 Adjustment Method

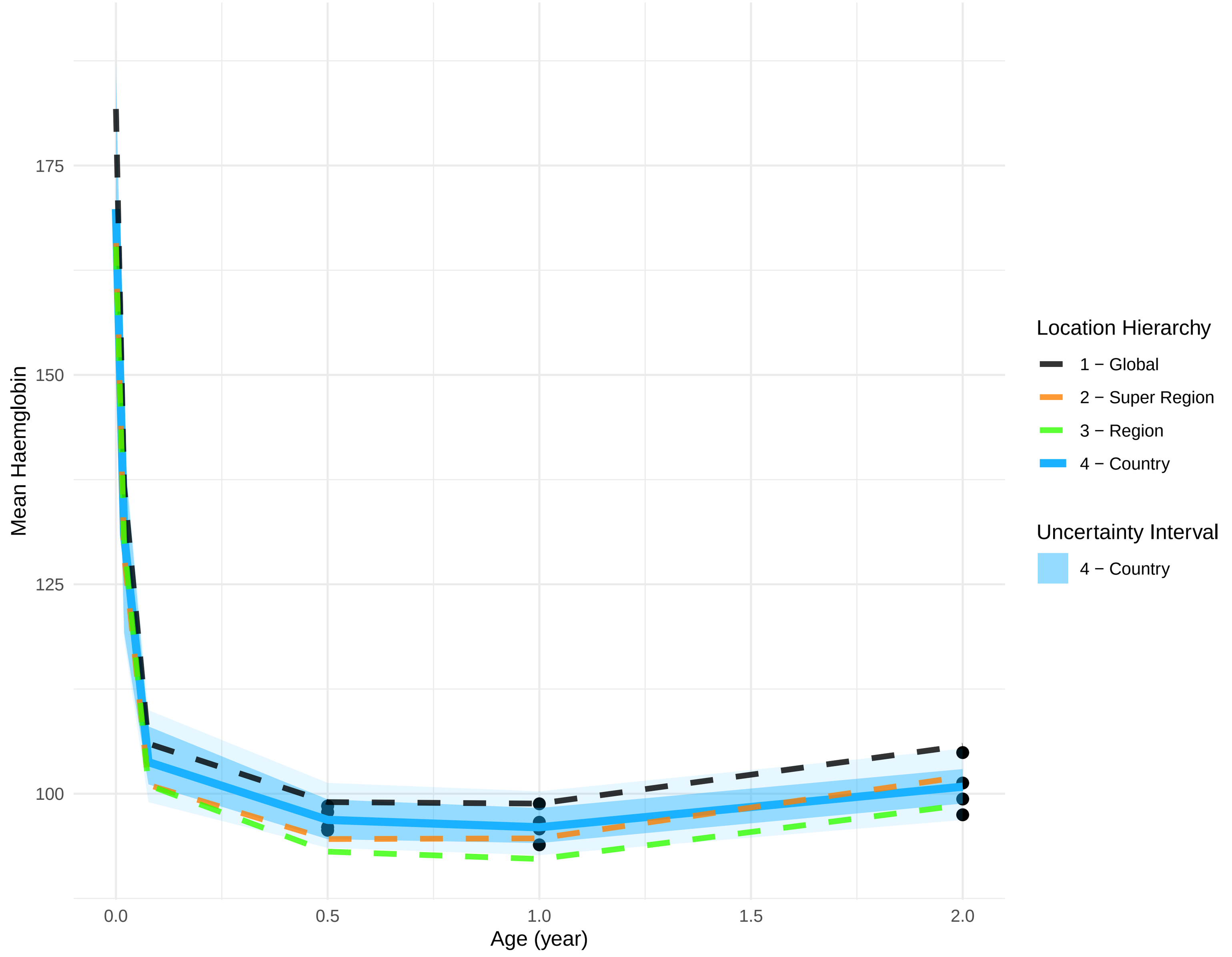

MR−BRT Age/Sex Fits − Males <2 Years Old − Mean Haemoglobin − Mali
WHO−2024 Adjustment Method

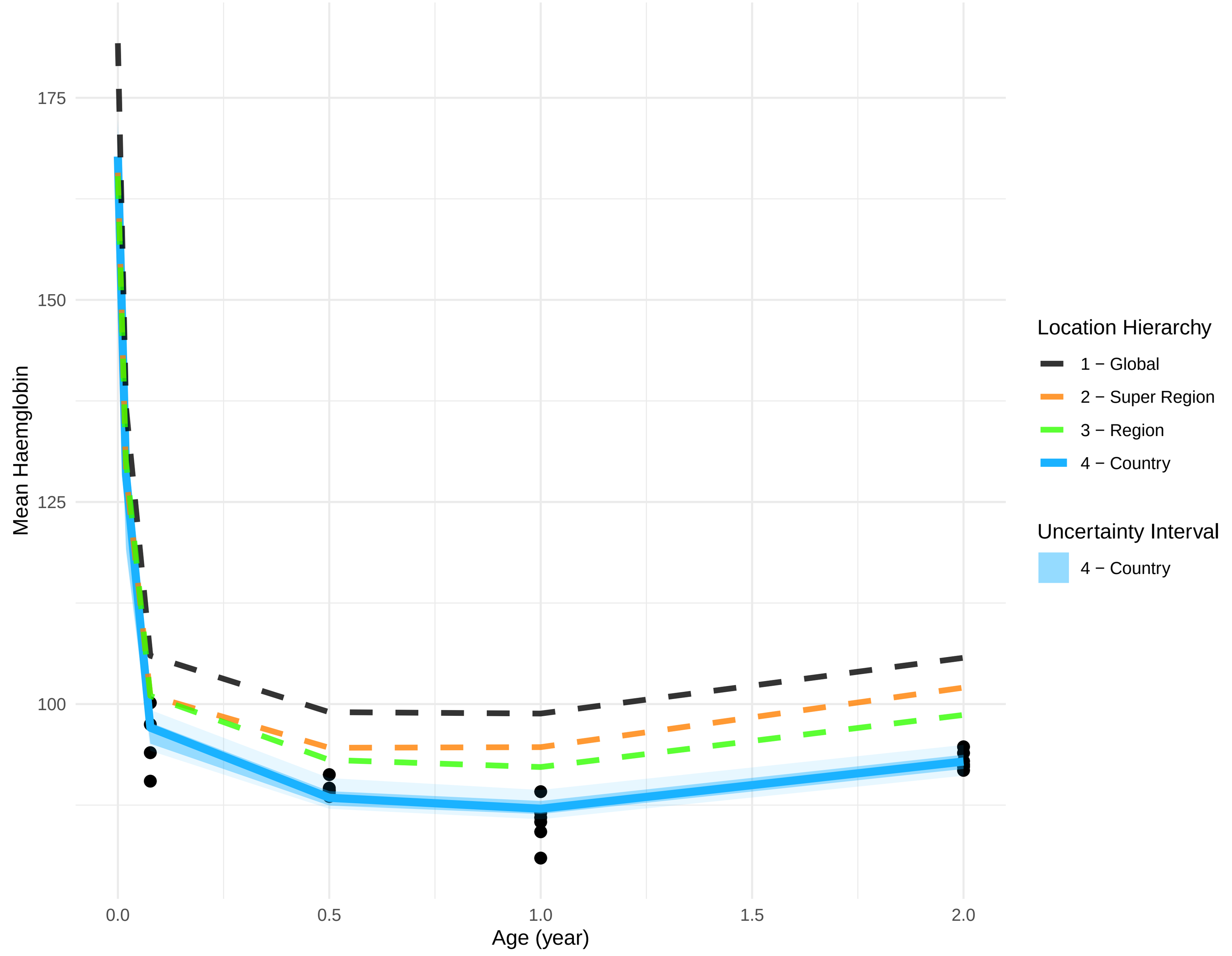

MR−BRT Age/Sex Fits − Males <2 Years Old − Mean Haemoglobin − Niger
WHO−2024 Adjustment Method

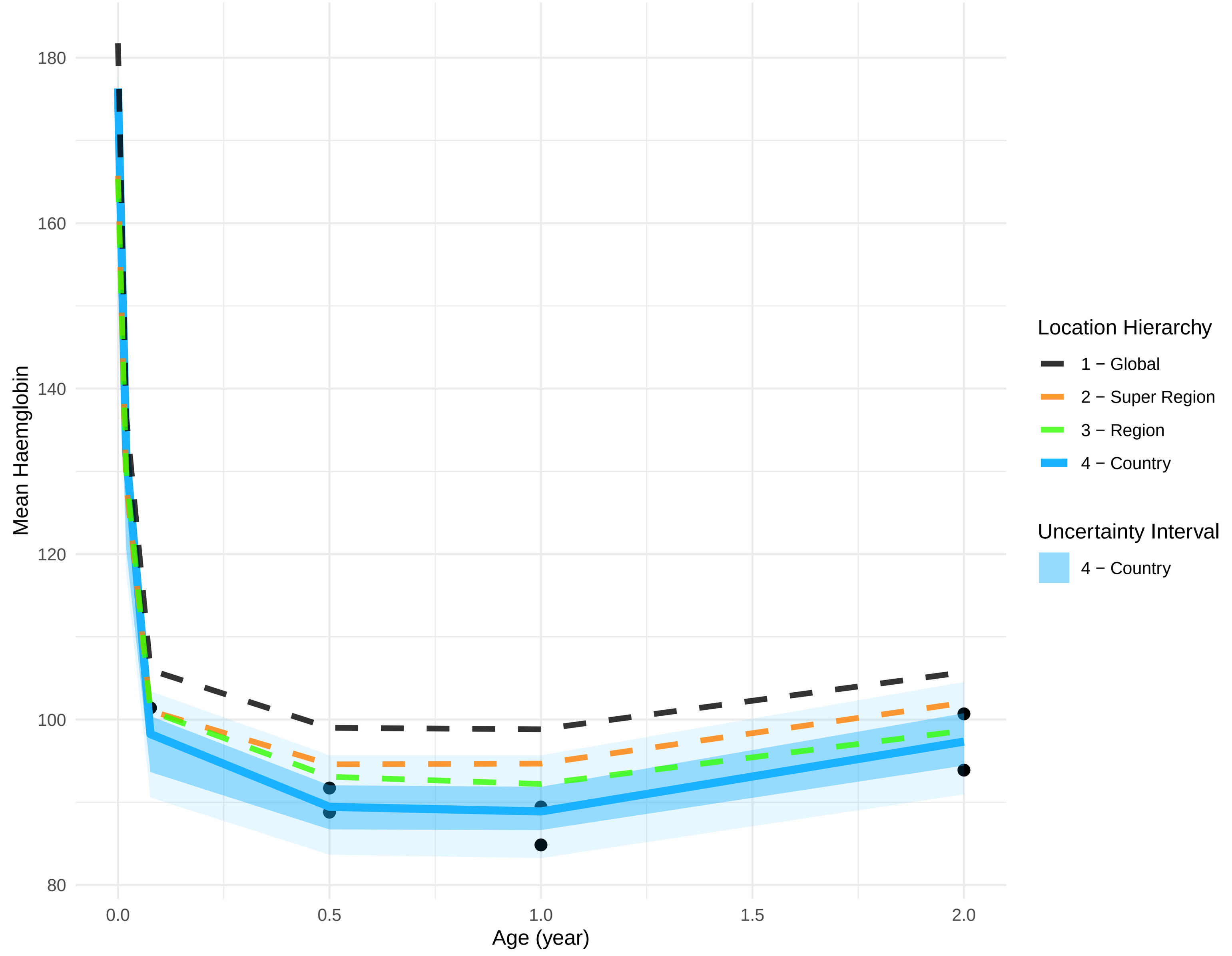

MR−BRT Age/Sex Fits − Males <2 Years Old − Mean Haemoglobin − Nigeria
WHO−2024 Adjustment Method

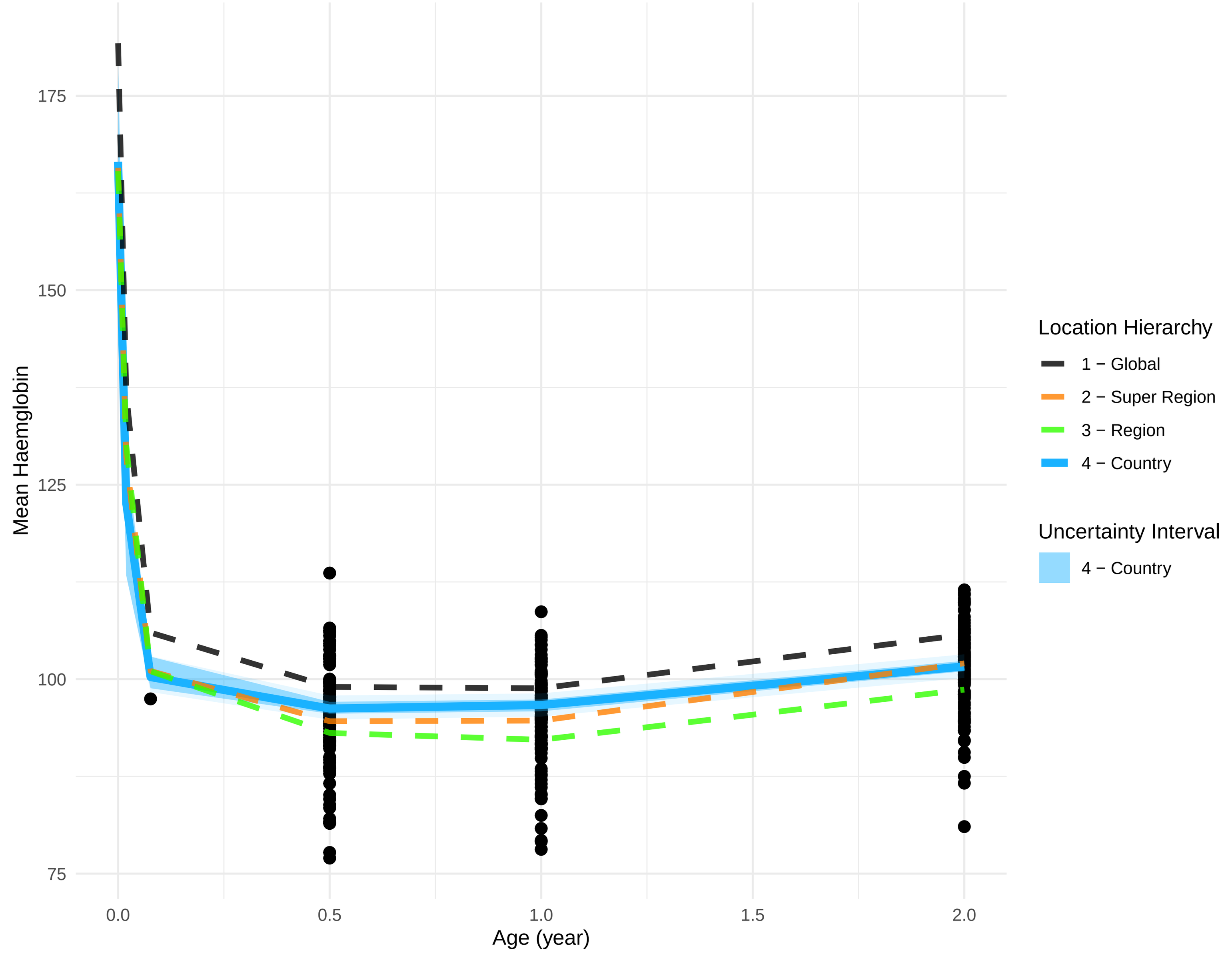

MR−BRT Age/Sex Fits − Males <2 Years Old − Mean Haemoglobin − Abia
WHO−2024 Adjustment Method

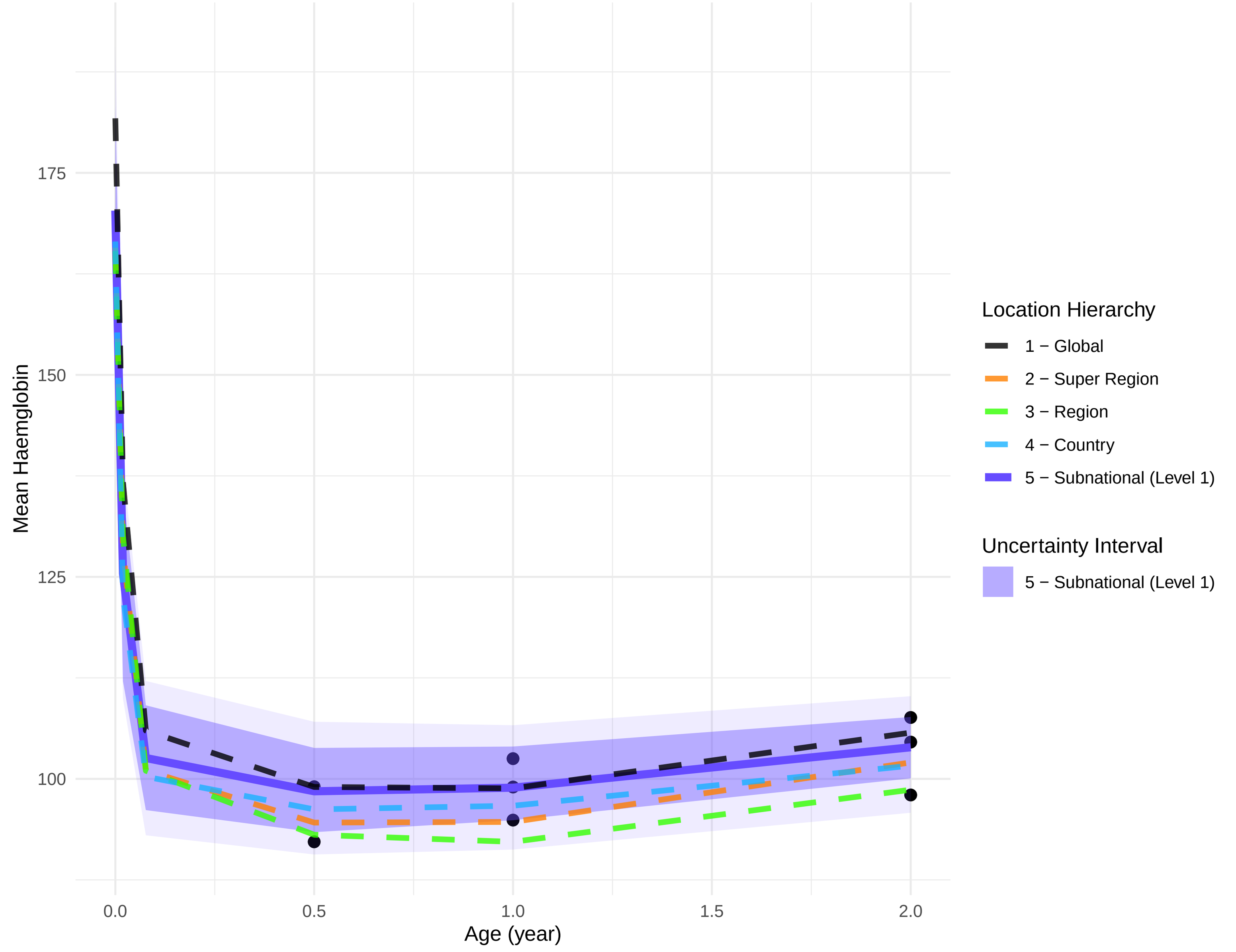

MR−BRT Age/Sex Fits − Males <2 Years Old − Mean Haemoglobin − Adamawa
WHO−2024 Adjustment Method

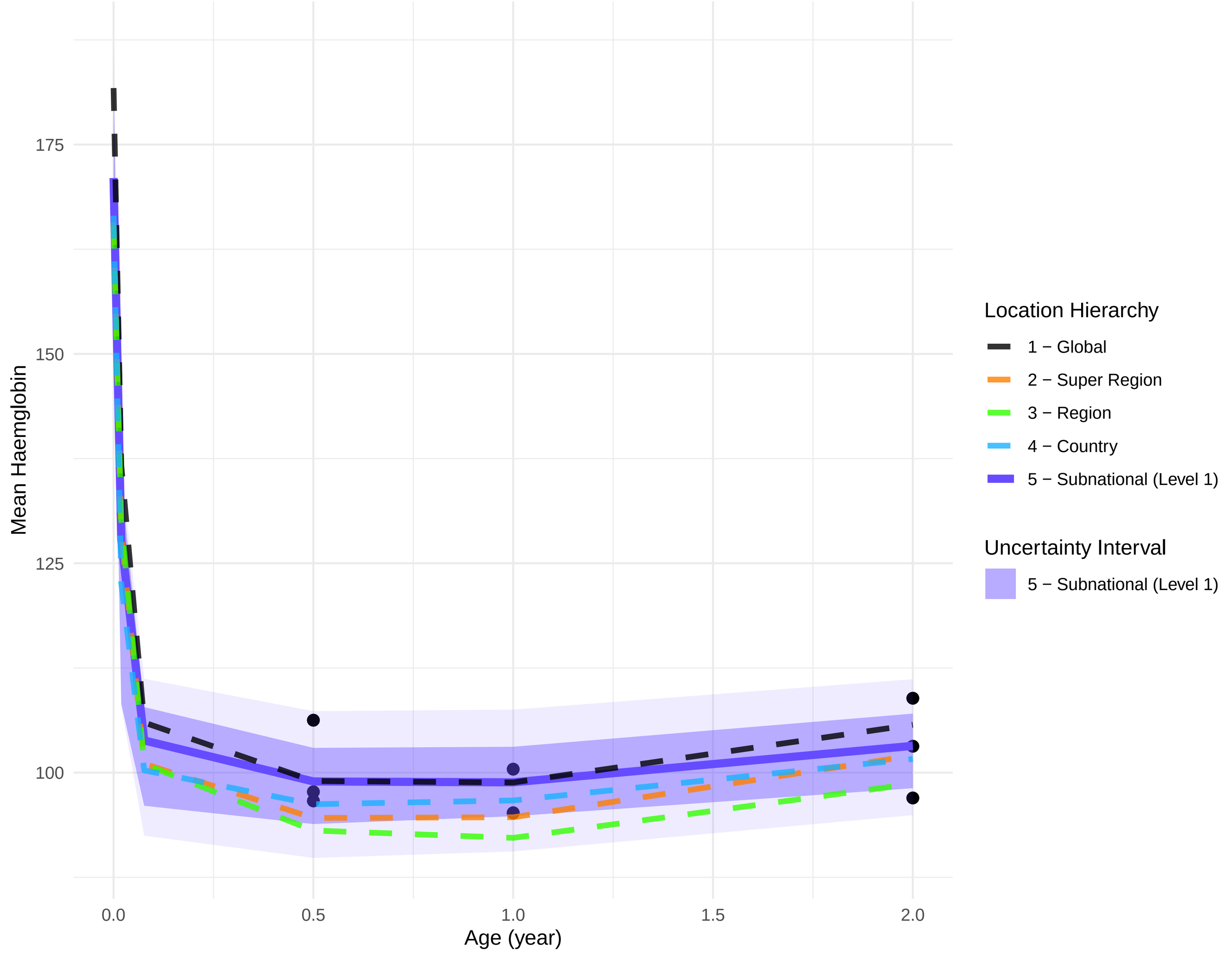

MR−BRT Age/Sex Fits − Males <2 Years Old − Mean Haemoglobin − Akwa Ibom
WHO−2024 Adjustment Method

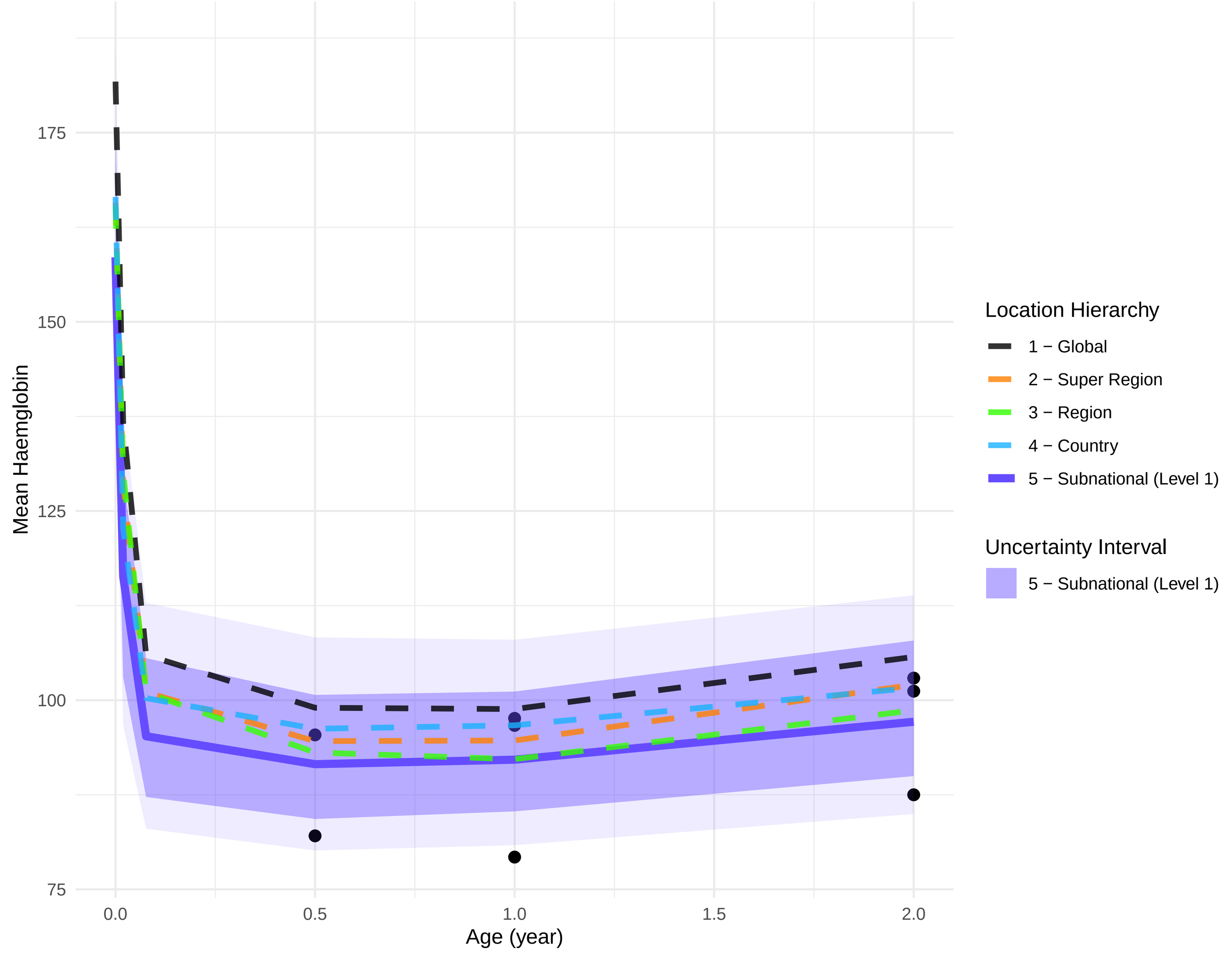

MR−BRT Age/Sex Fits − Males <2 Years Old − Mean Haemoglobin − Anambra
WHO−2024 Adjustment Method

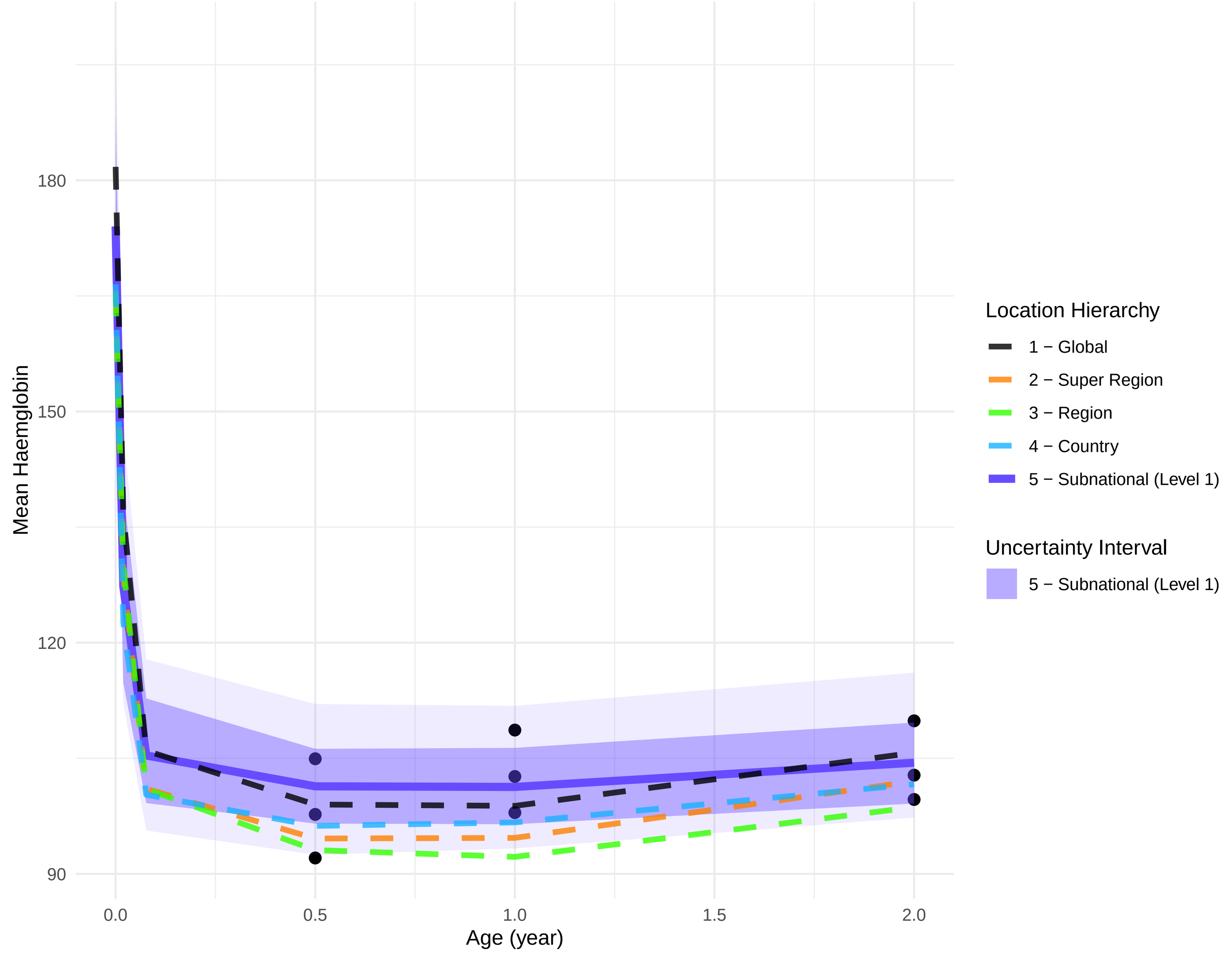

MR−BRT Age/Sex Fits − Males <2 Years Old − Mean Haemoglobin − Bauchi
WHO−2024 Adjustment Method

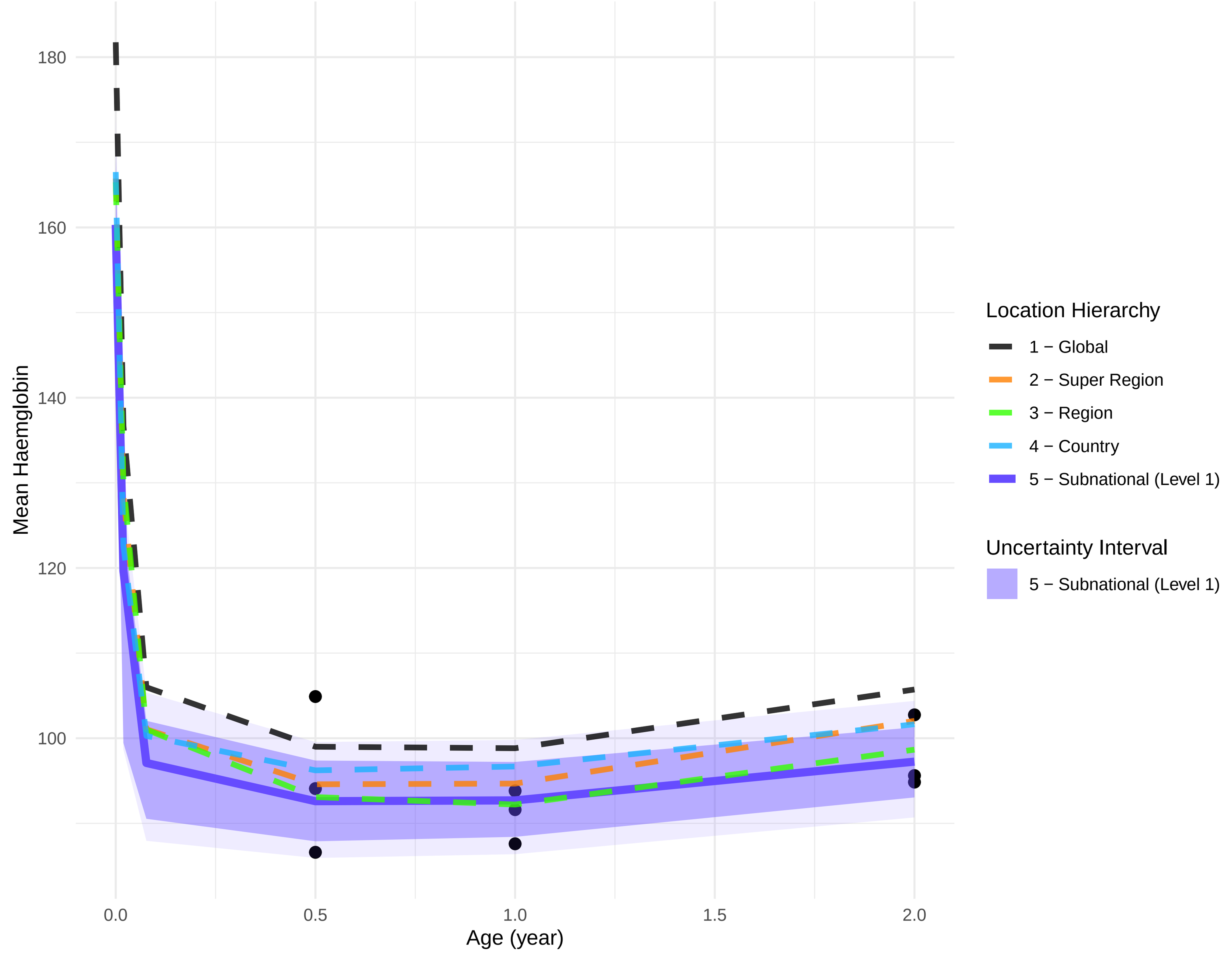

MR−BRT Age/Sex Fits − Males <2 Years Old − Mean Haemoglobin − Bayelsa
WHO−2024 Adjustment Method

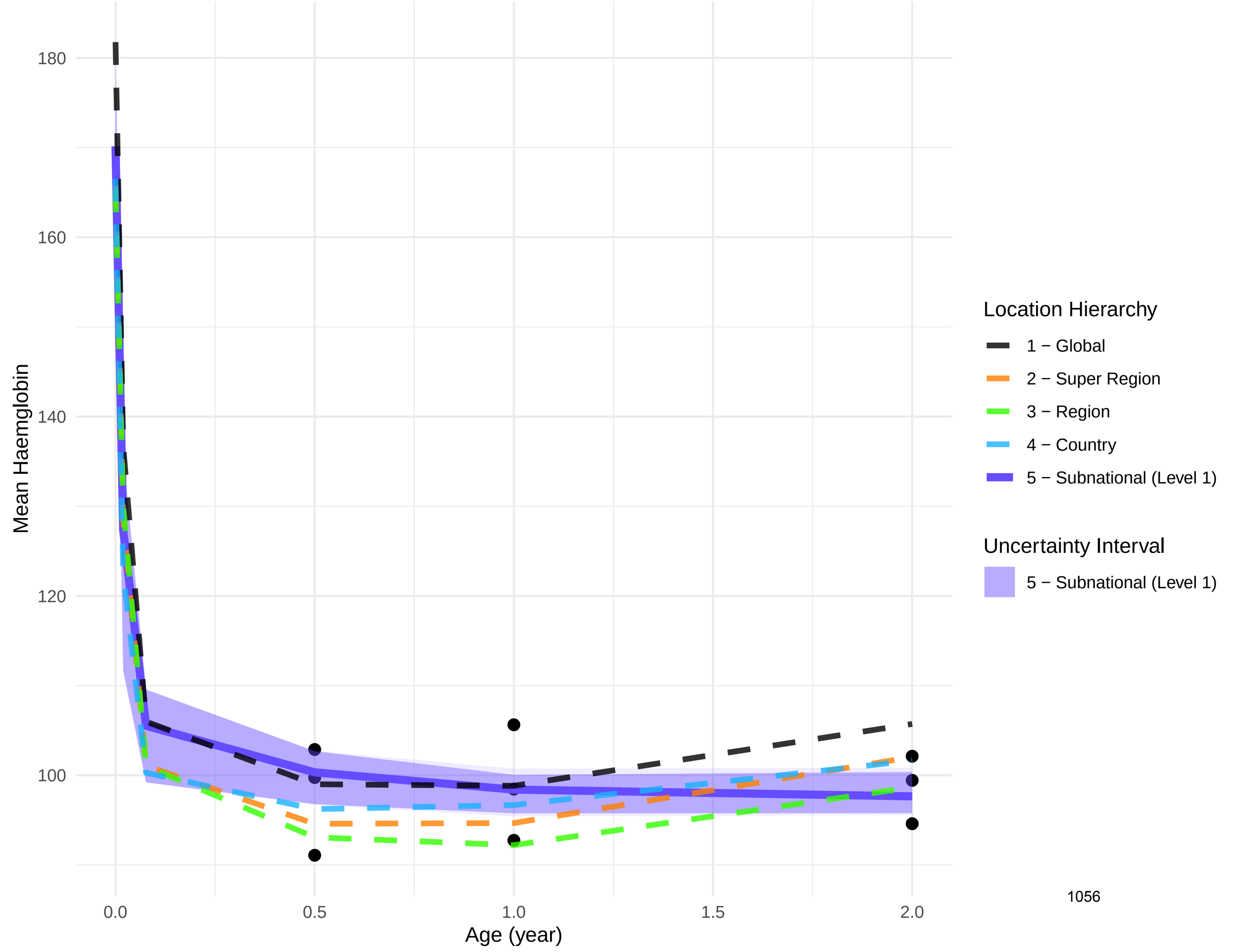

MR−BRT Age/Sex Fits − Males <2 Years Old − Mean Haemglobin − Benue
WHO−2024 Adjustment Method

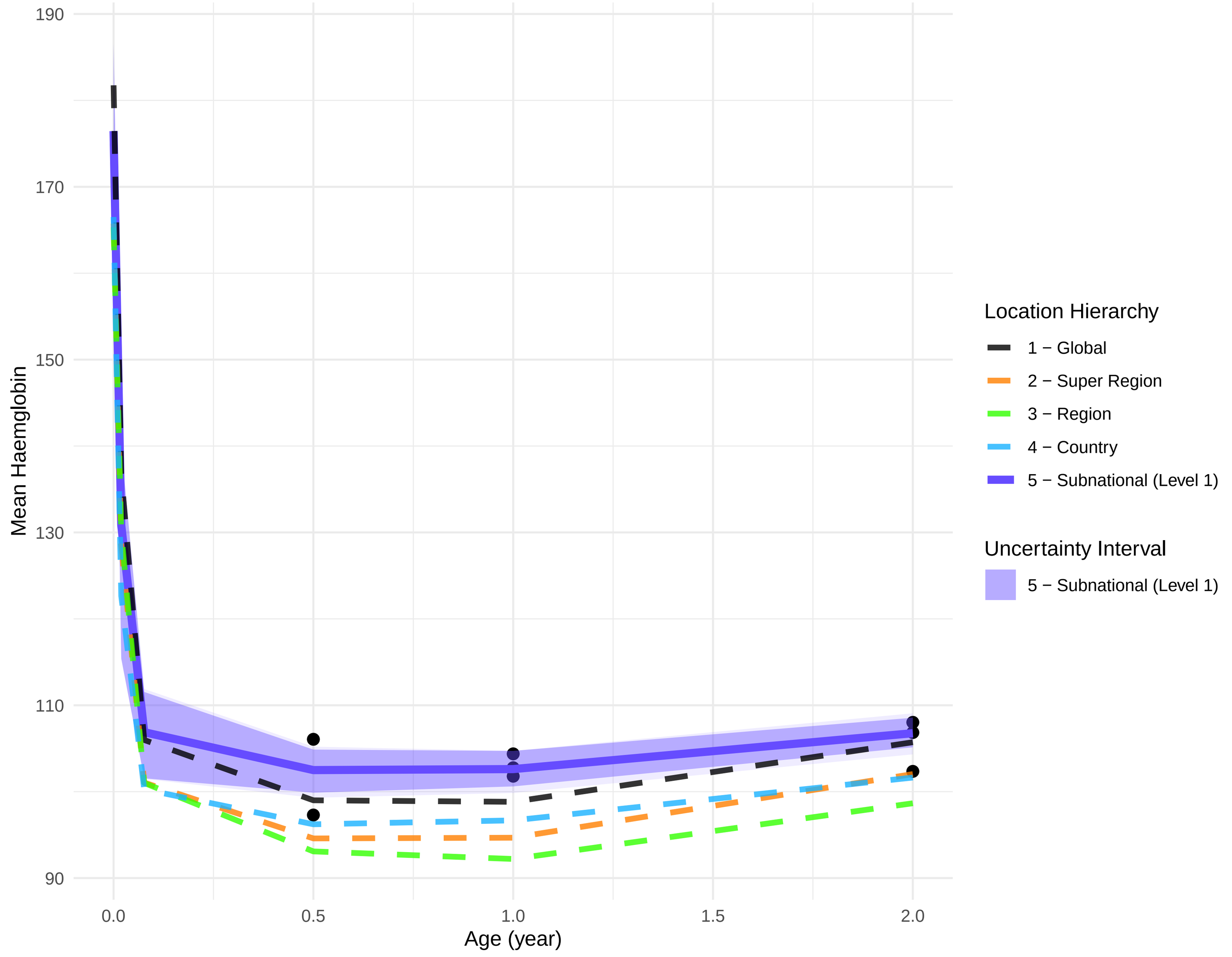

MR−BRT Age/Sex Fits − Males <2 Years Old − Mean Haemglobin − Borno
WHO−2024 Adjustment Method

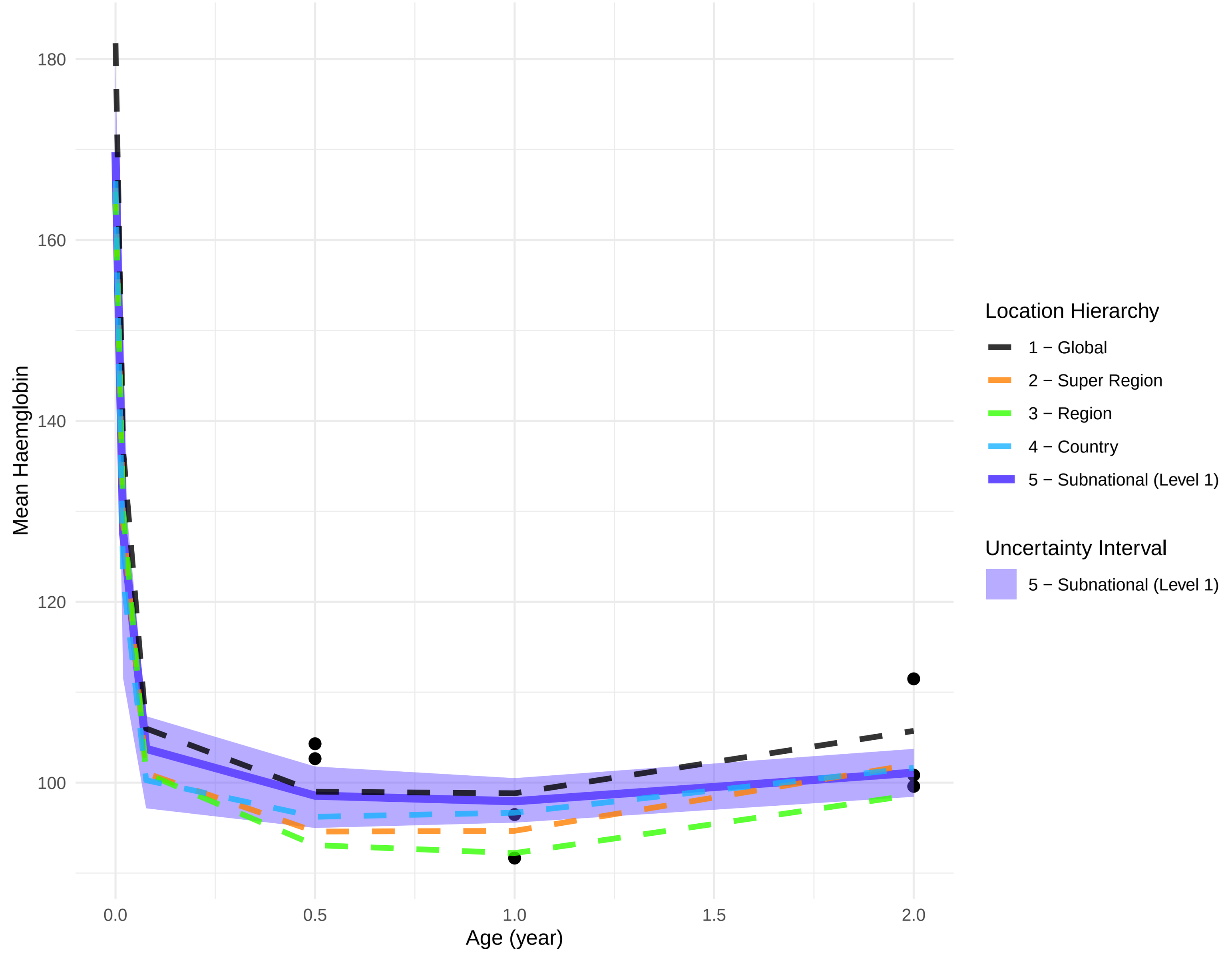

MR−BRT Age/Sex Fits − Males <2 Years Old − Mean Haemglobin − Cross River
WHO−2024 Adjustment Method

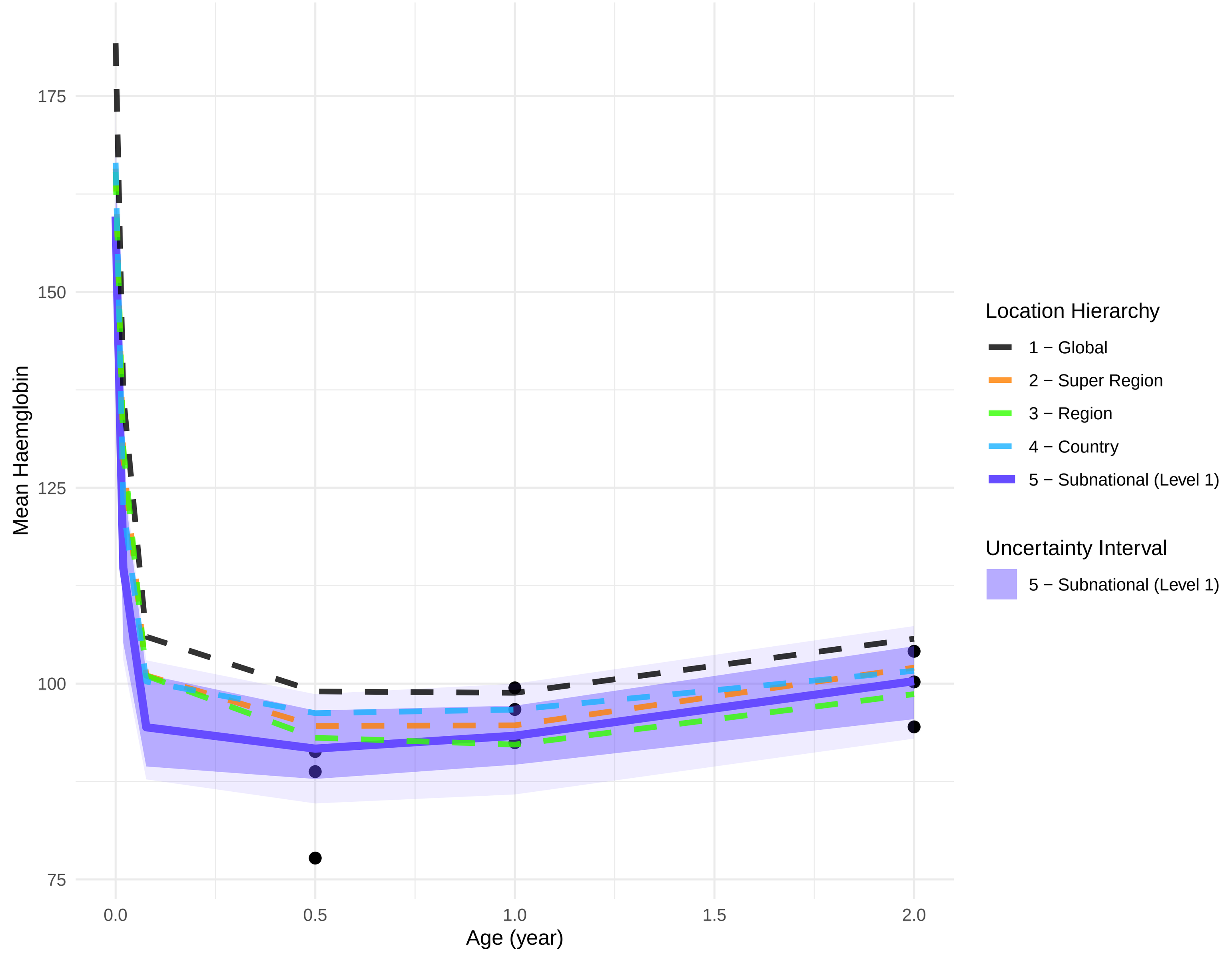

MR−BRT Age/Sex Fits − Males <2 Years Old − Mean Haemglobin − Delta
WHO−2024 Adjustment Method

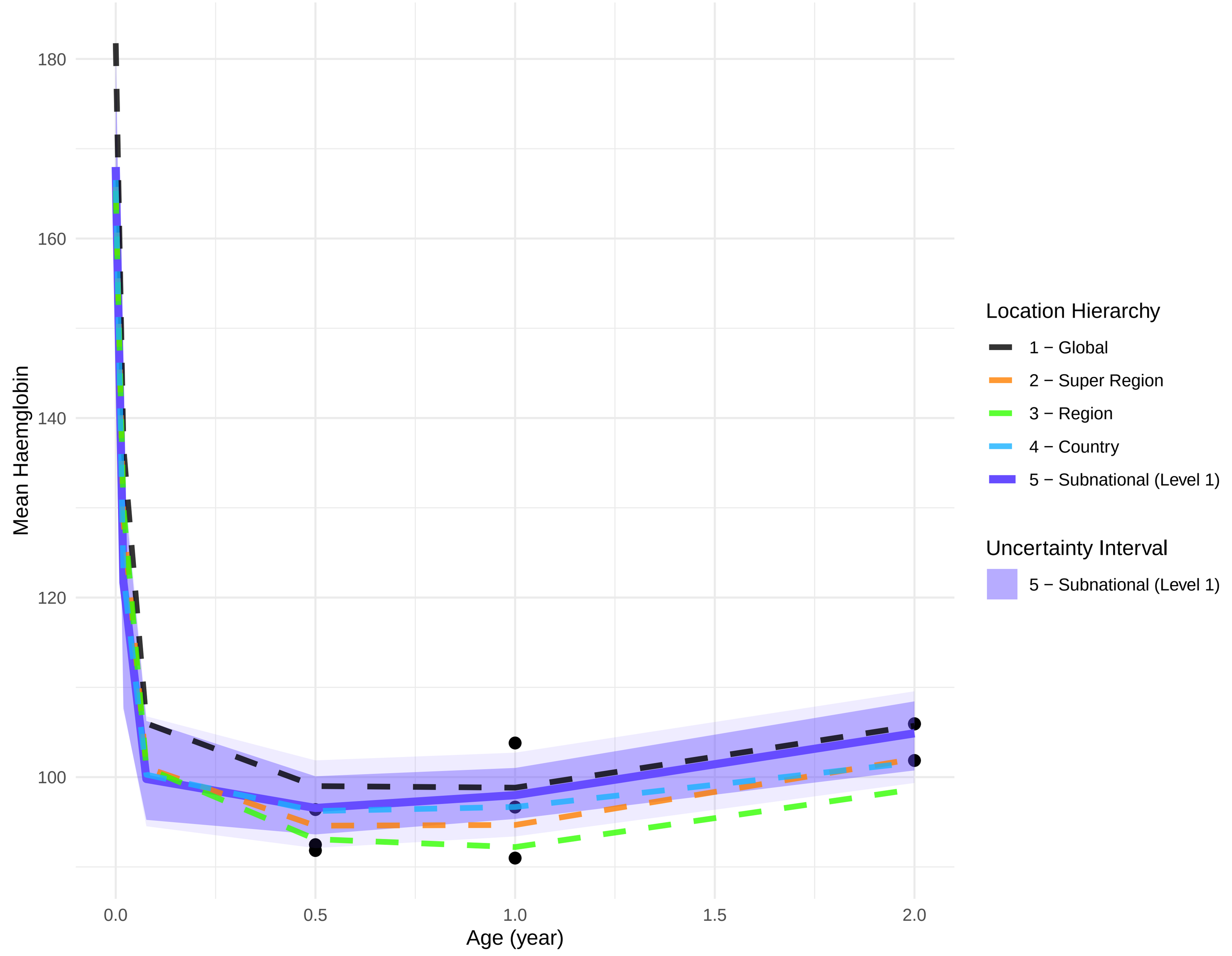

MR−BRT Age/Sex Fits − Males <2 Years Old − Mean Haemglobin − Ebonyi
WHO−2024 Adjustment Method

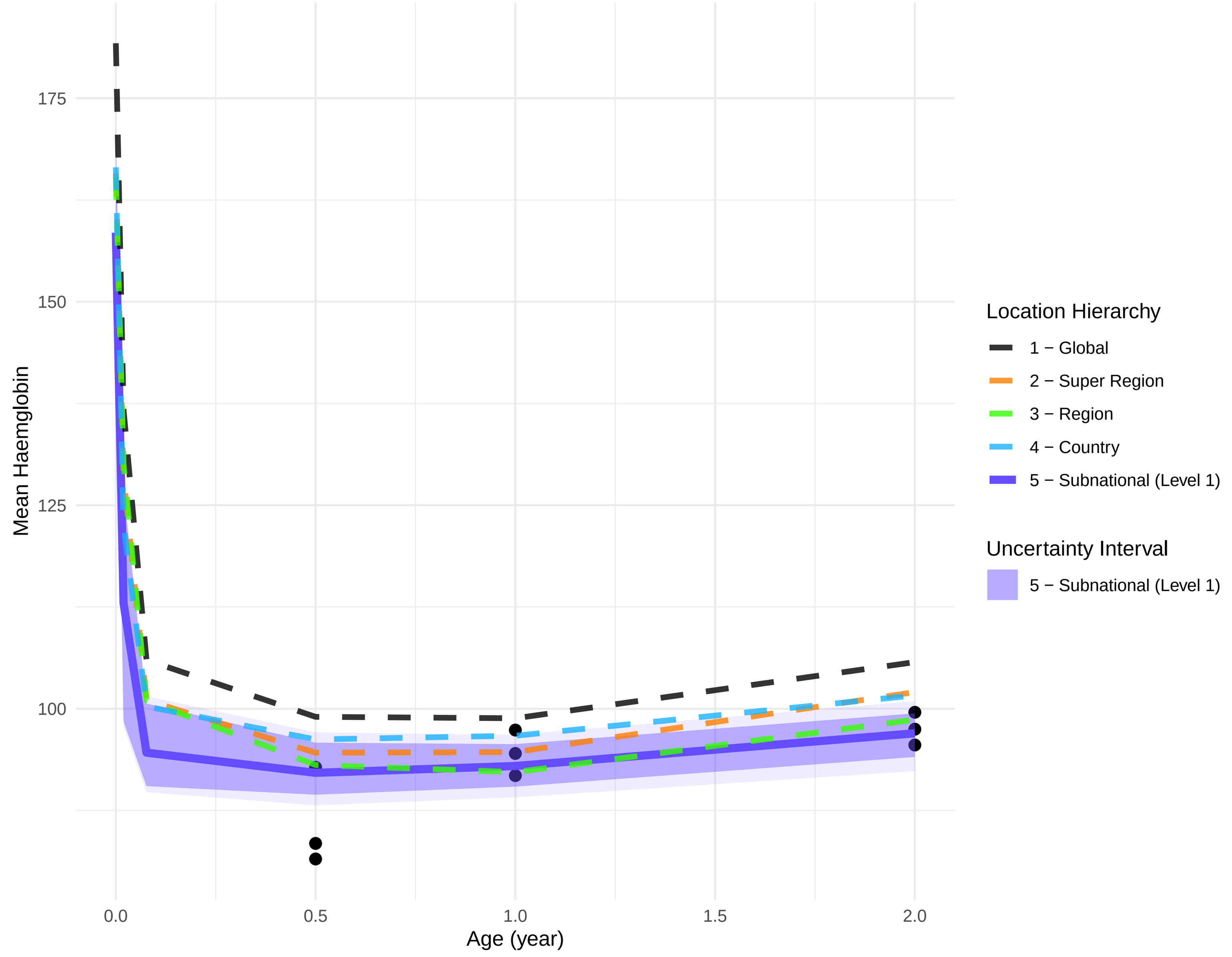

MR−BRT Age/Sex Fits − Males <2 Years Old − Mean Haemglobin − Edo
WHO−2024 Adjustment Method

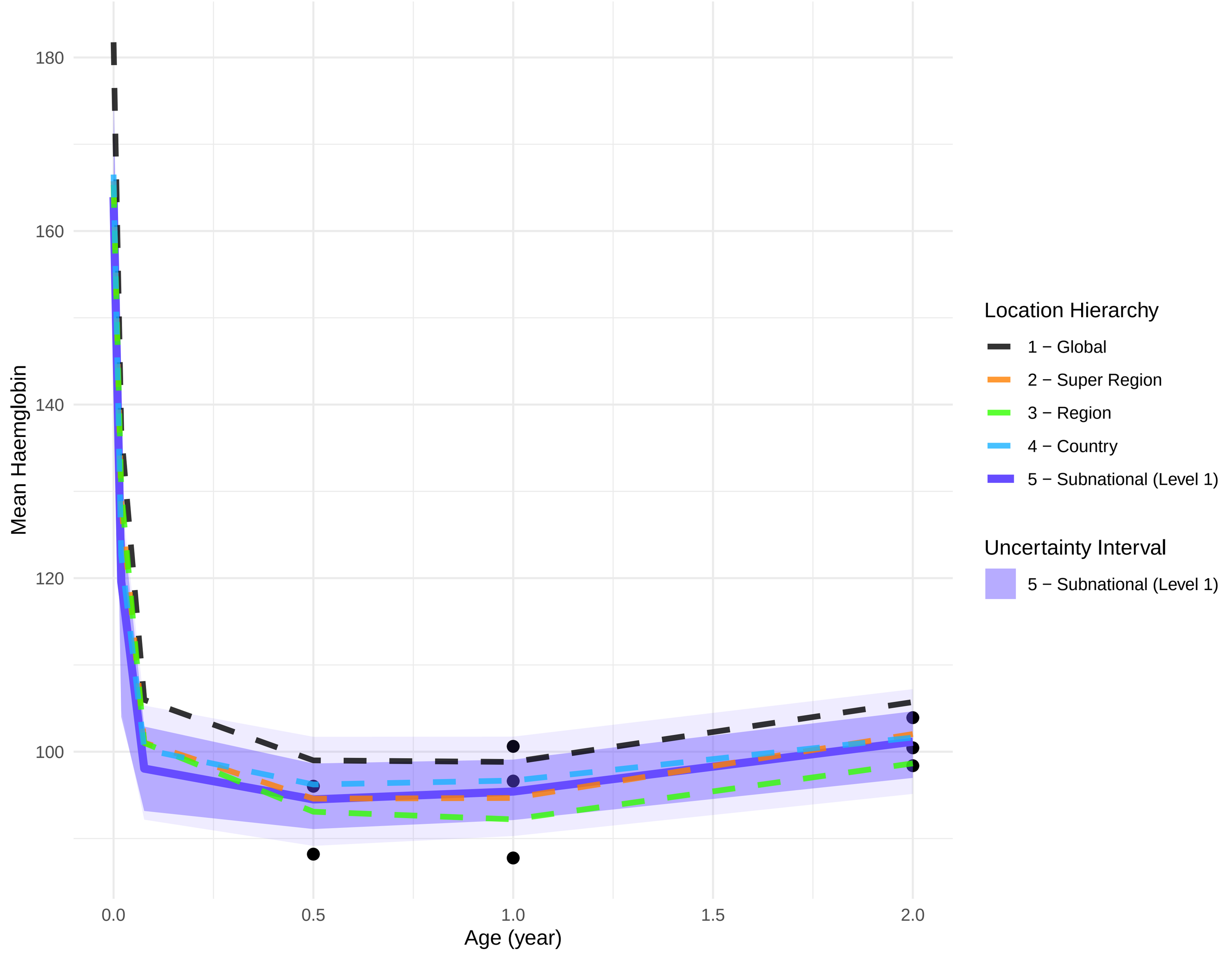

MR−BRT Age/Sex Fits − Males <2 Years Old − Mean Haemglobin − Ekiti
WHO−2024 Adjustment Method

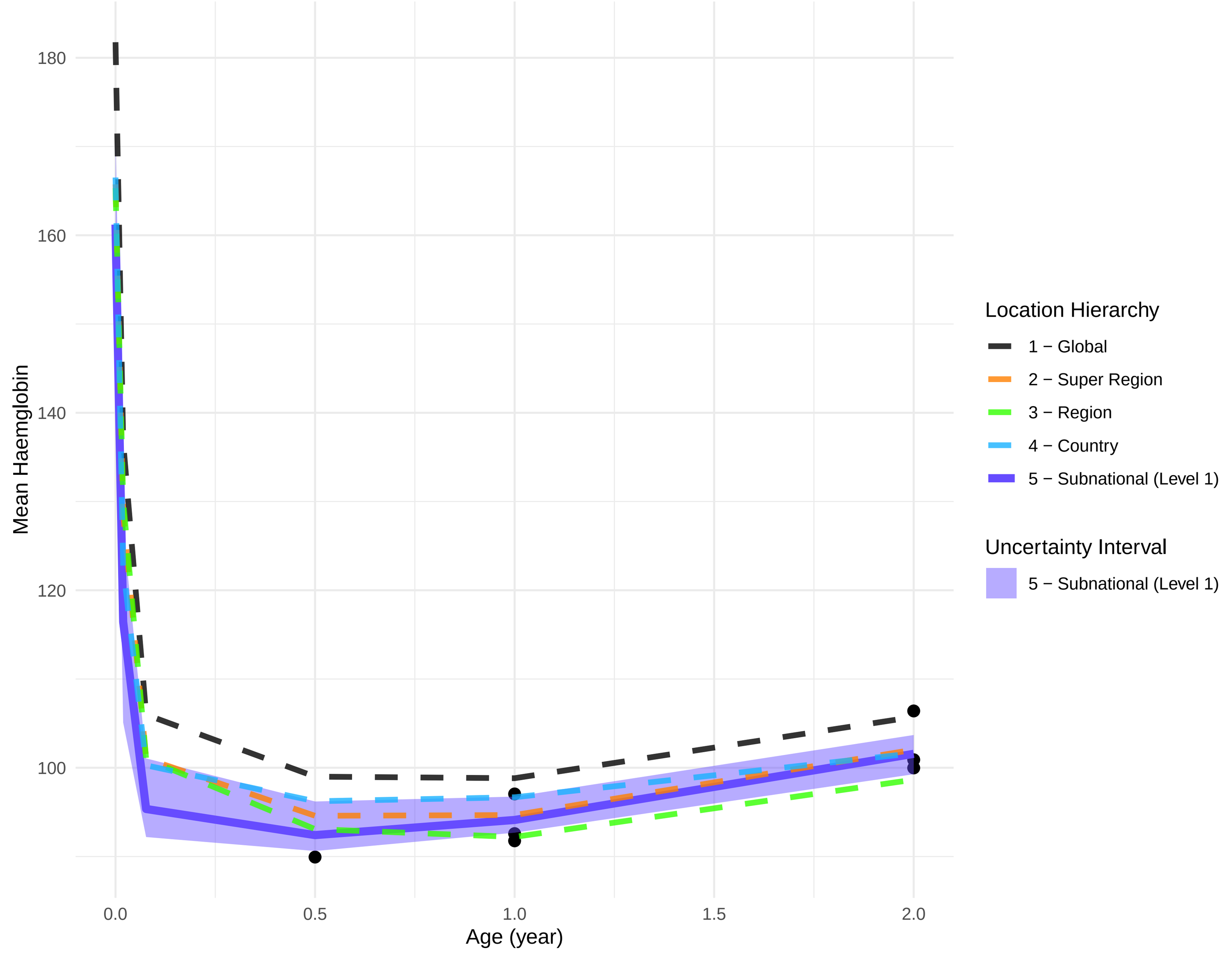

MR−BRT Age/Sex Fits − Males <2 Years Old − Mean Haemglobin − Enugu
WHO−2024 Adjustment Method

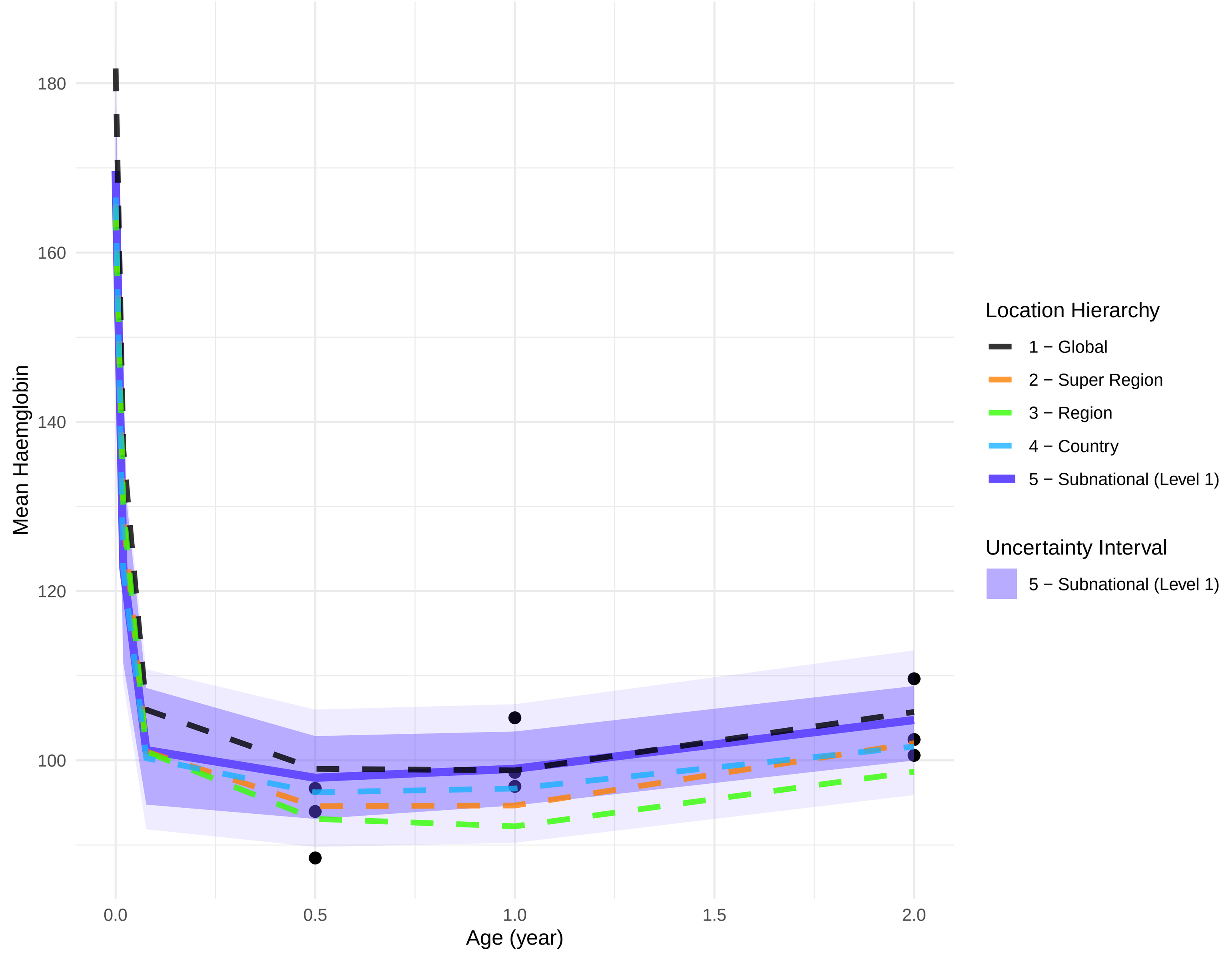

MR−BRT Age/Sex Fits − Males <2 Years Old − Mean Haemglobin − FCT (Abuja)
WHO−2024 Adjustment Method

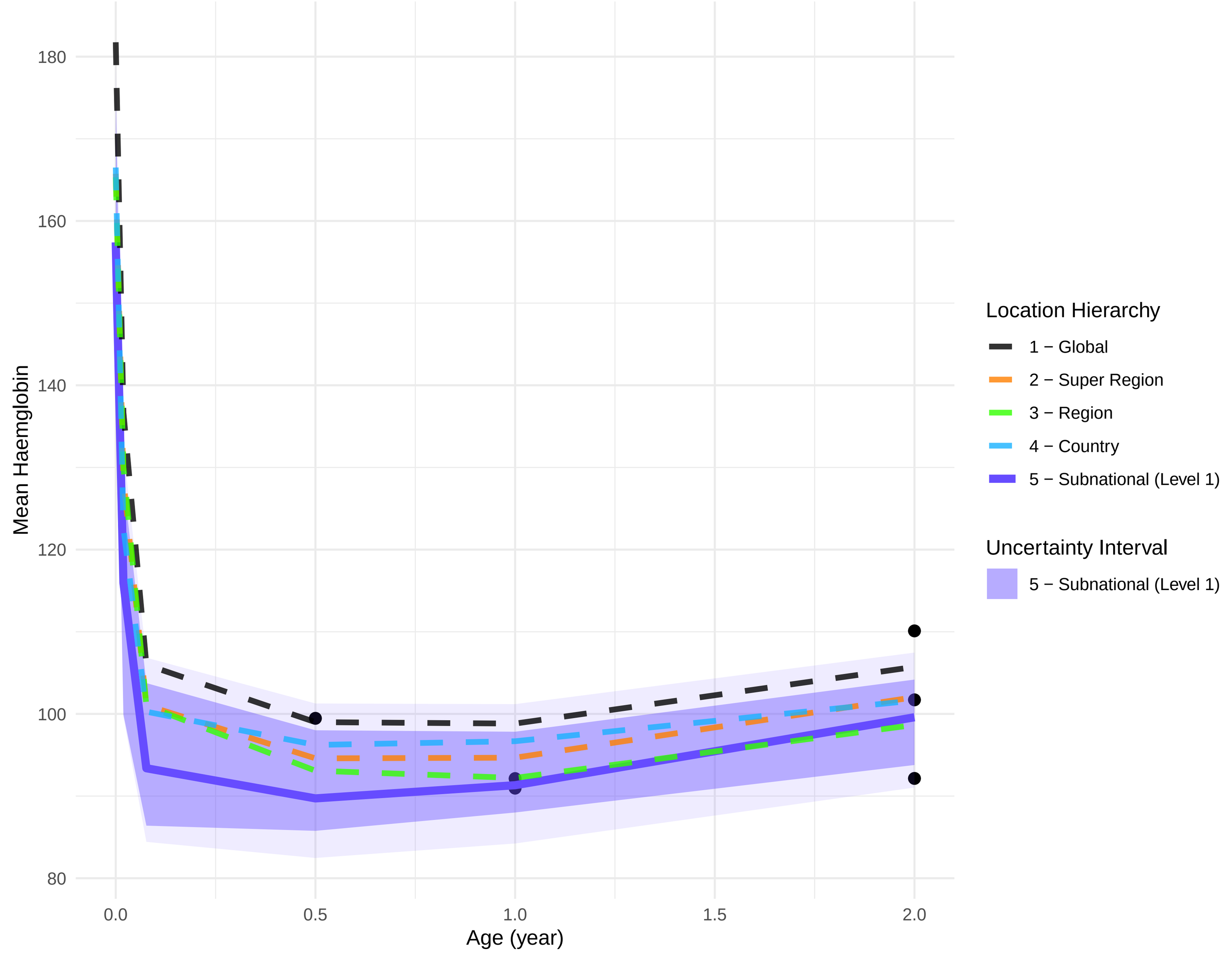

MR−BRT Age/Sex Fits − Males <2 Years Old − Mean Haemglobin − Gombe
WHO−2024 Adjustment Method

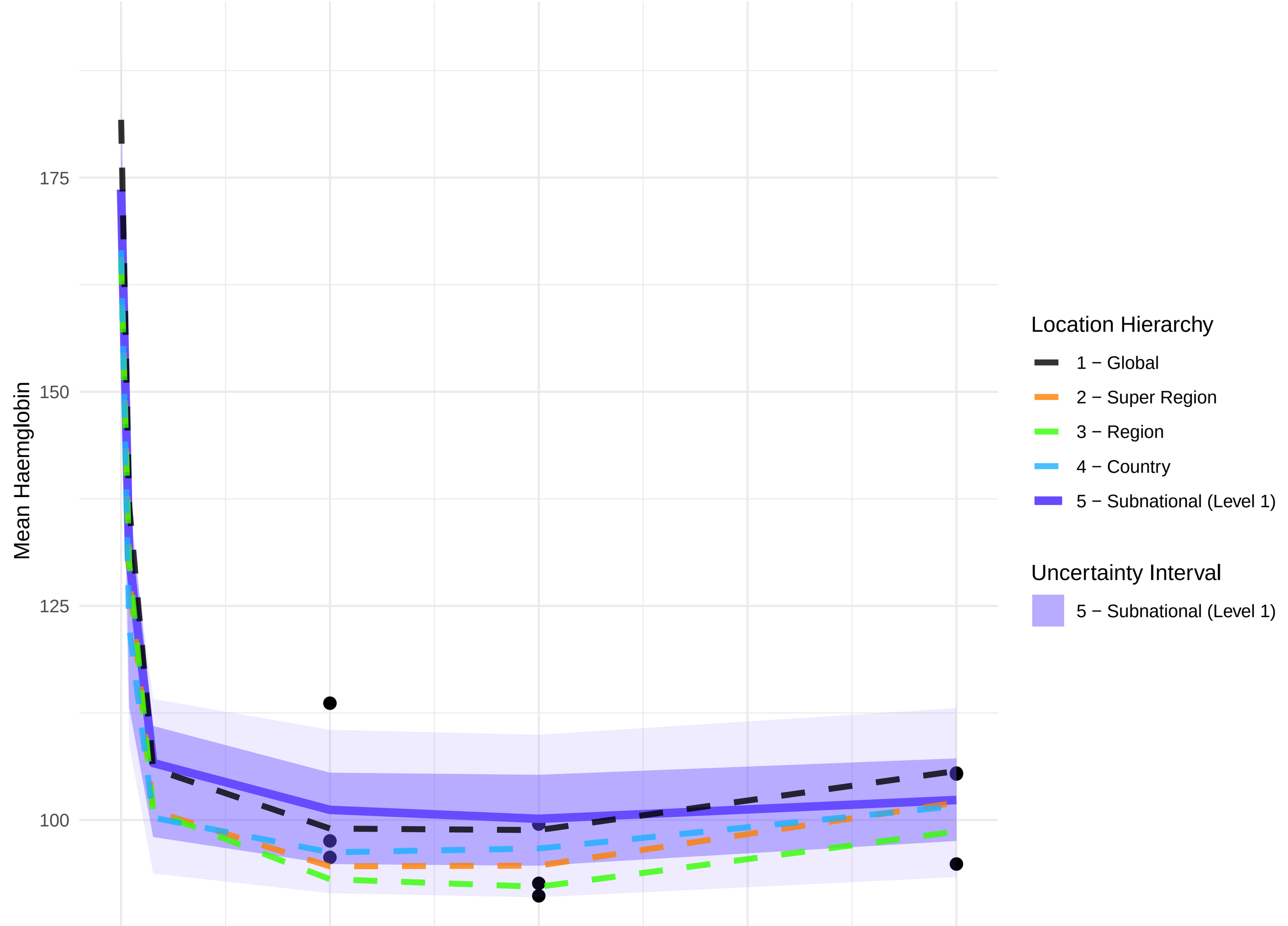

MR−BRT Age/Sex Fits − Males <2 Years Old − Mean Haemglobin − Imo
WHO−2024 Adjustment Method

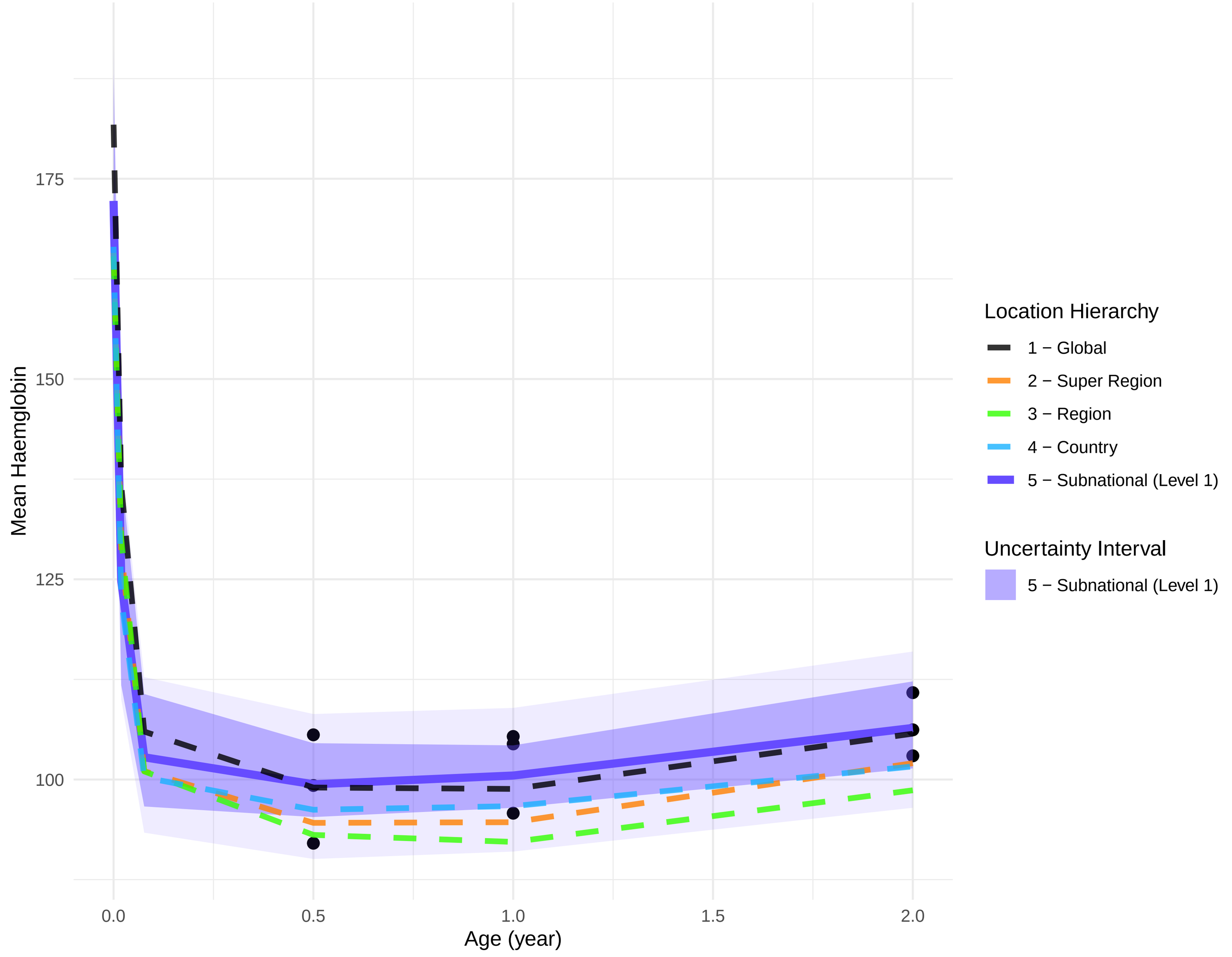

MR−BRT Age/Sex Fits − Males <2 Years Old − Mean Haemglobin − Jigawa
WHO−2024 Adjustment Method

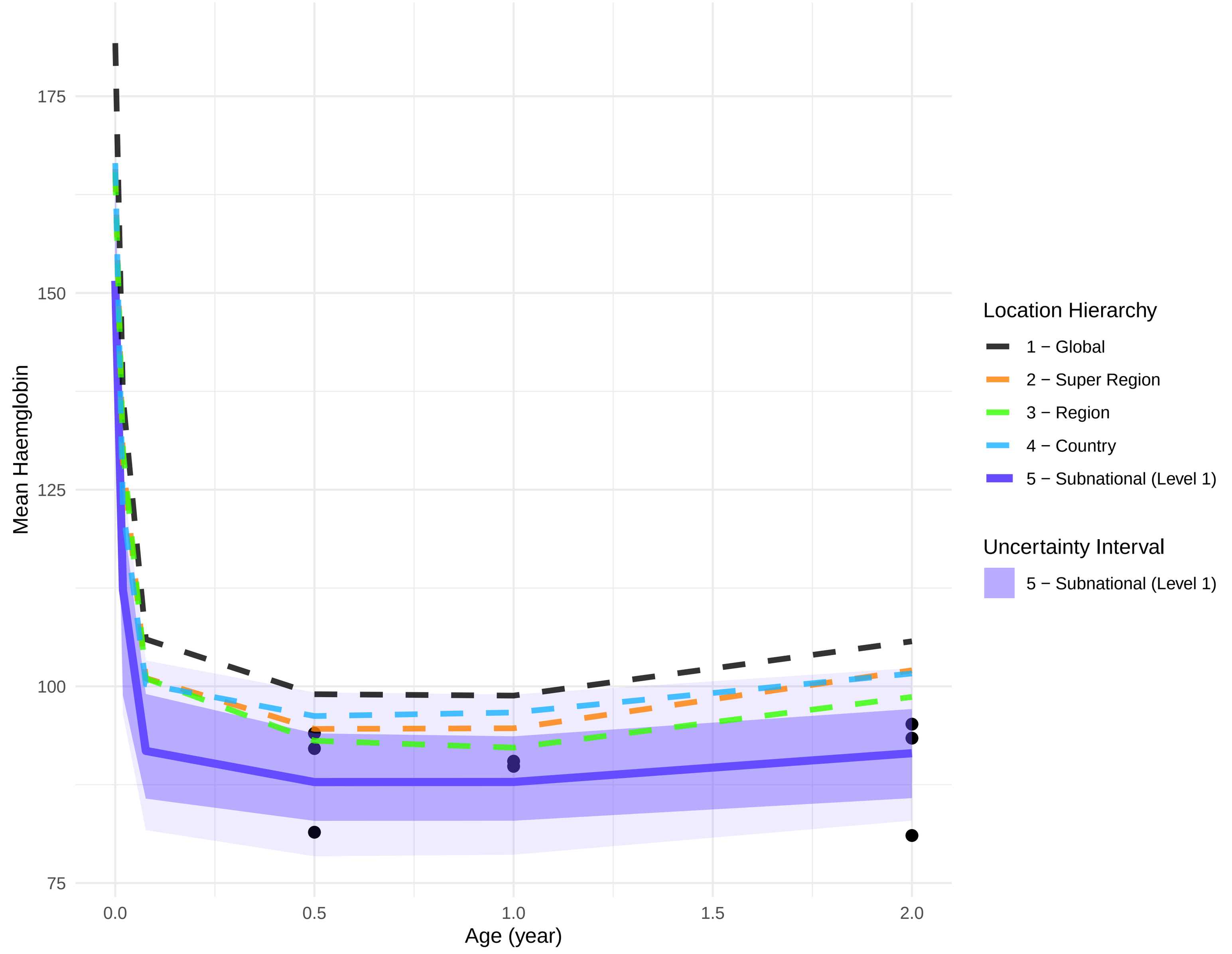

MR−BRT Age/Sex Fits − Males <2 Years Old − Mean Haemglobin − Kaduna
WHO−2024 Adjustment Method

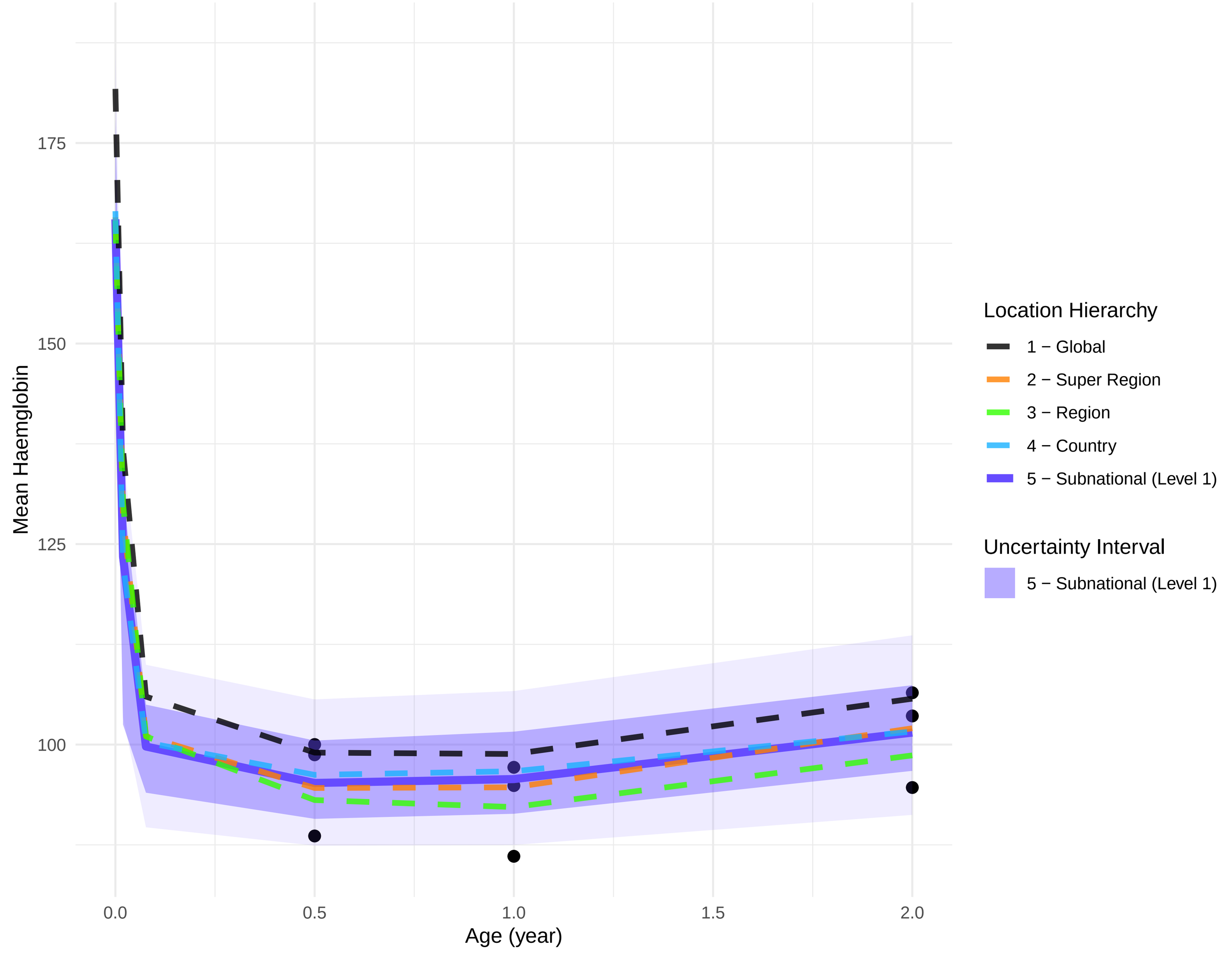

MR−BRT Age/Sex Fits − Males <2 Years Old − Mean Haemglobin − Kano
WHO−2024 Adjustment Method

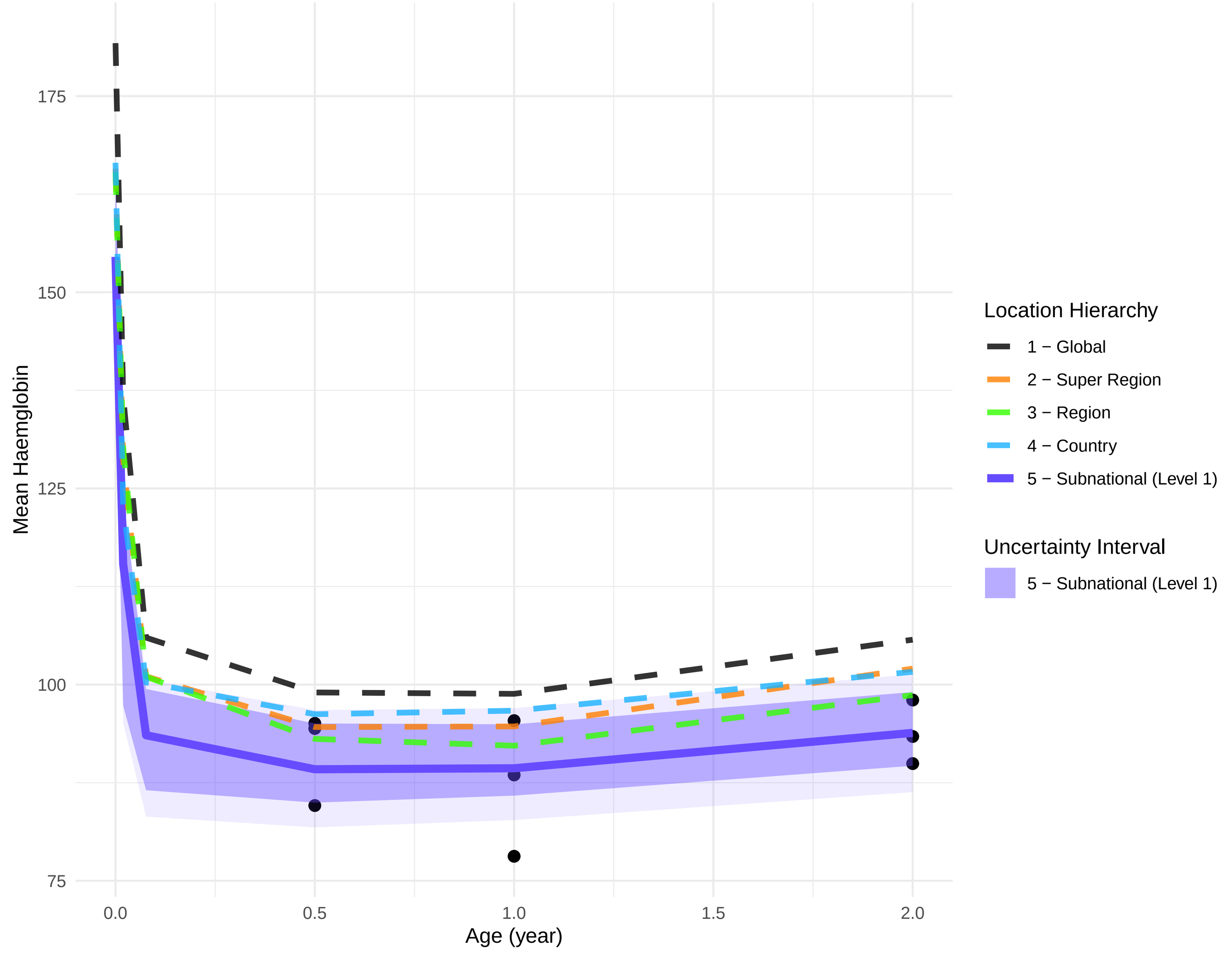

MR−BRT Age/Sex Fits − Males <2 Years Old − Mean Haemglobin − Katsina
WHO−2024 Adjustment Method

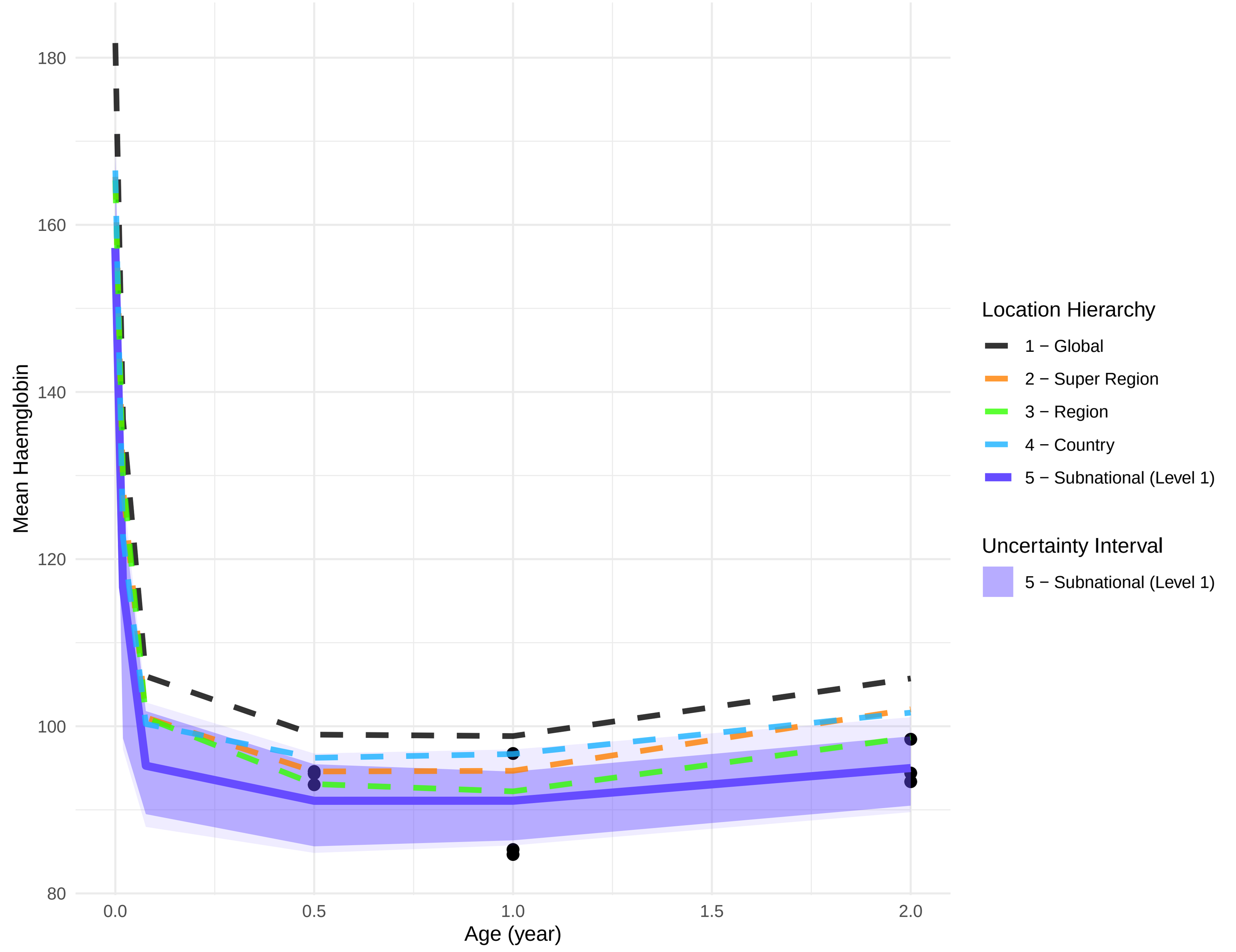

MR−BRT Age/Sex Fits − Males <2 Years Old − Mean Haemglobin − Kebbi
WHO−2024 Adjustment Method

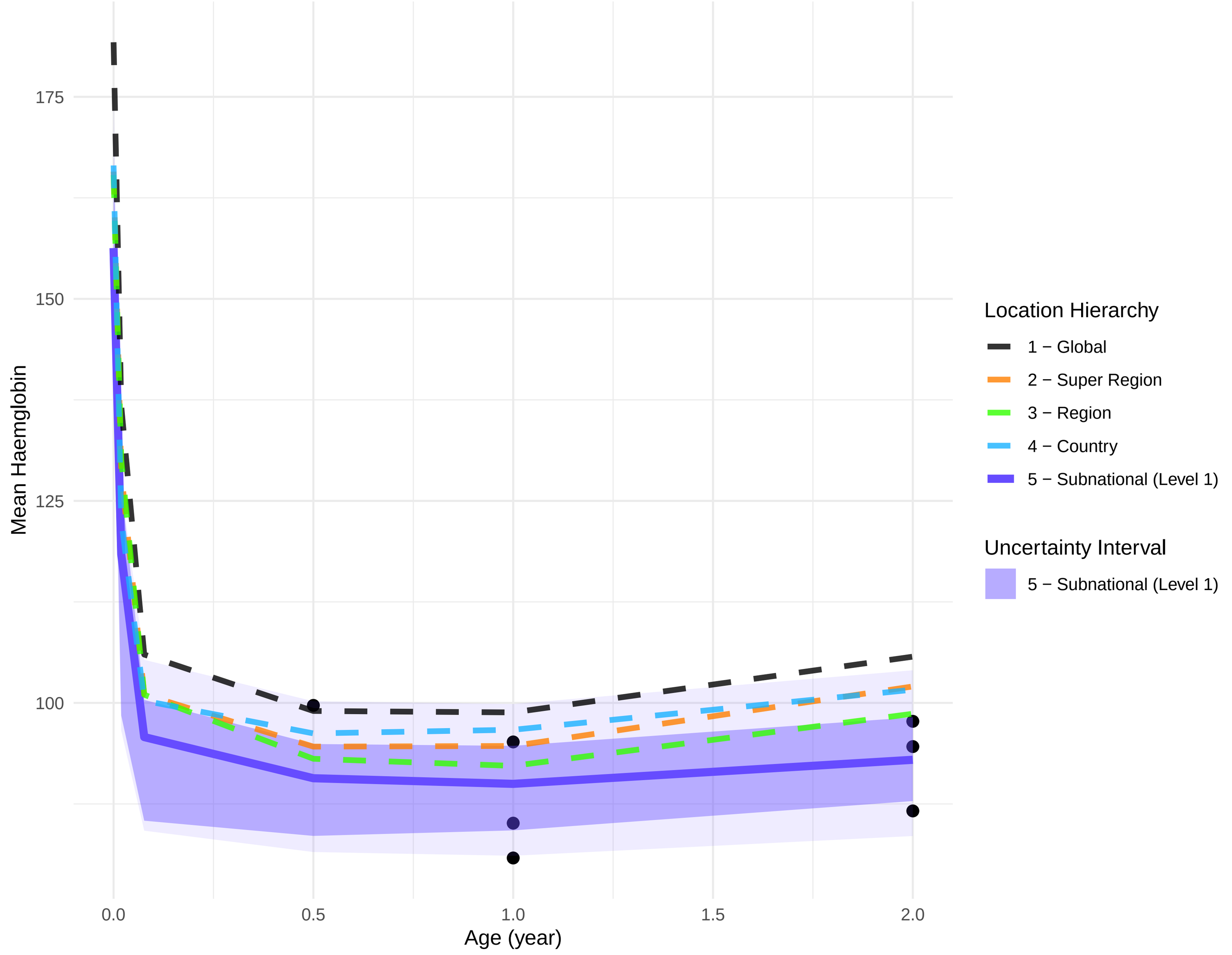

MR−BRT Age/Sex Fits − Males <2 Years Old − Mean Haemglobin − Kogi
WHO−2024 Adjustment Method

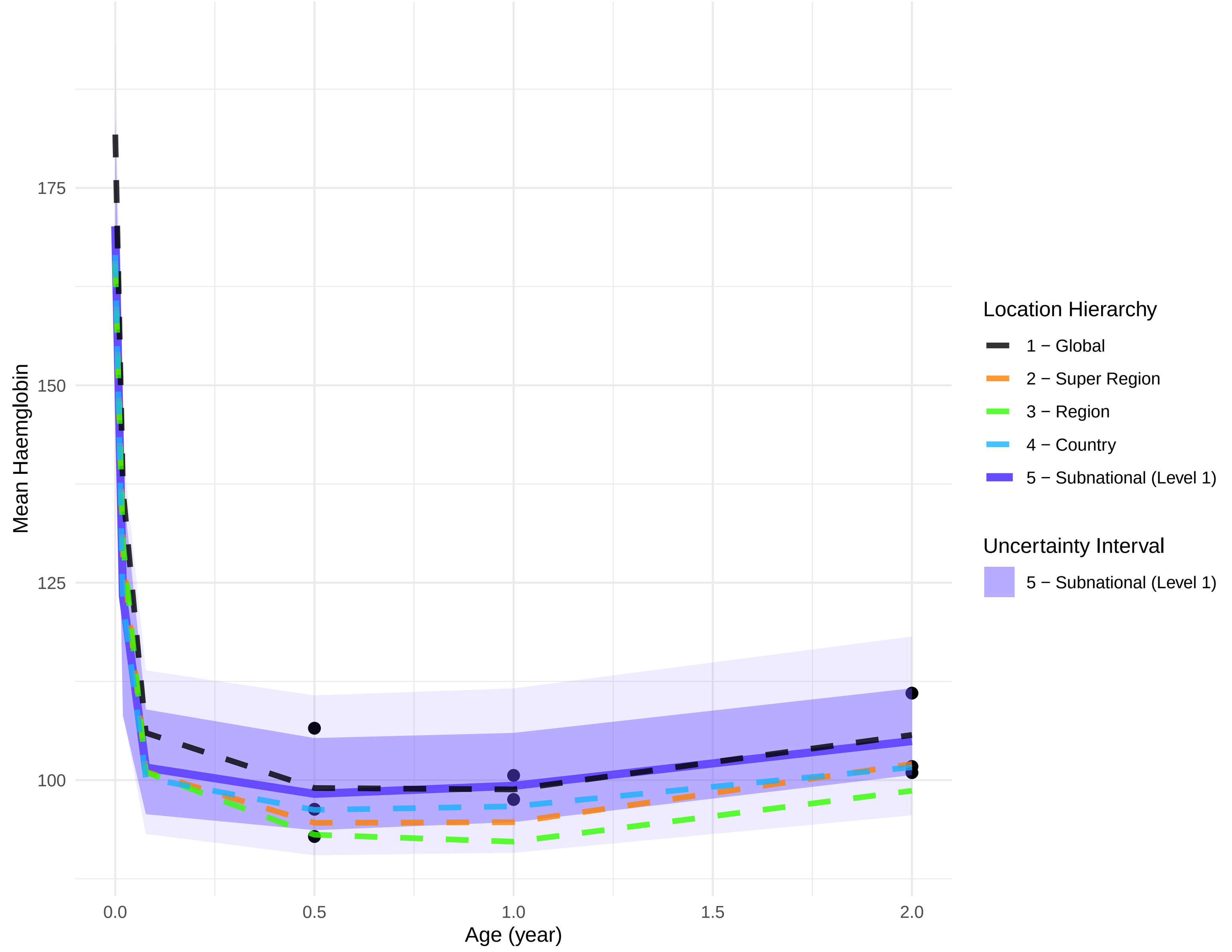

MR−BRT Age/Sex Fits − Males <2 Years Old − Mean Haemglobin − Kwara
WHO−2024 Adjustment Method

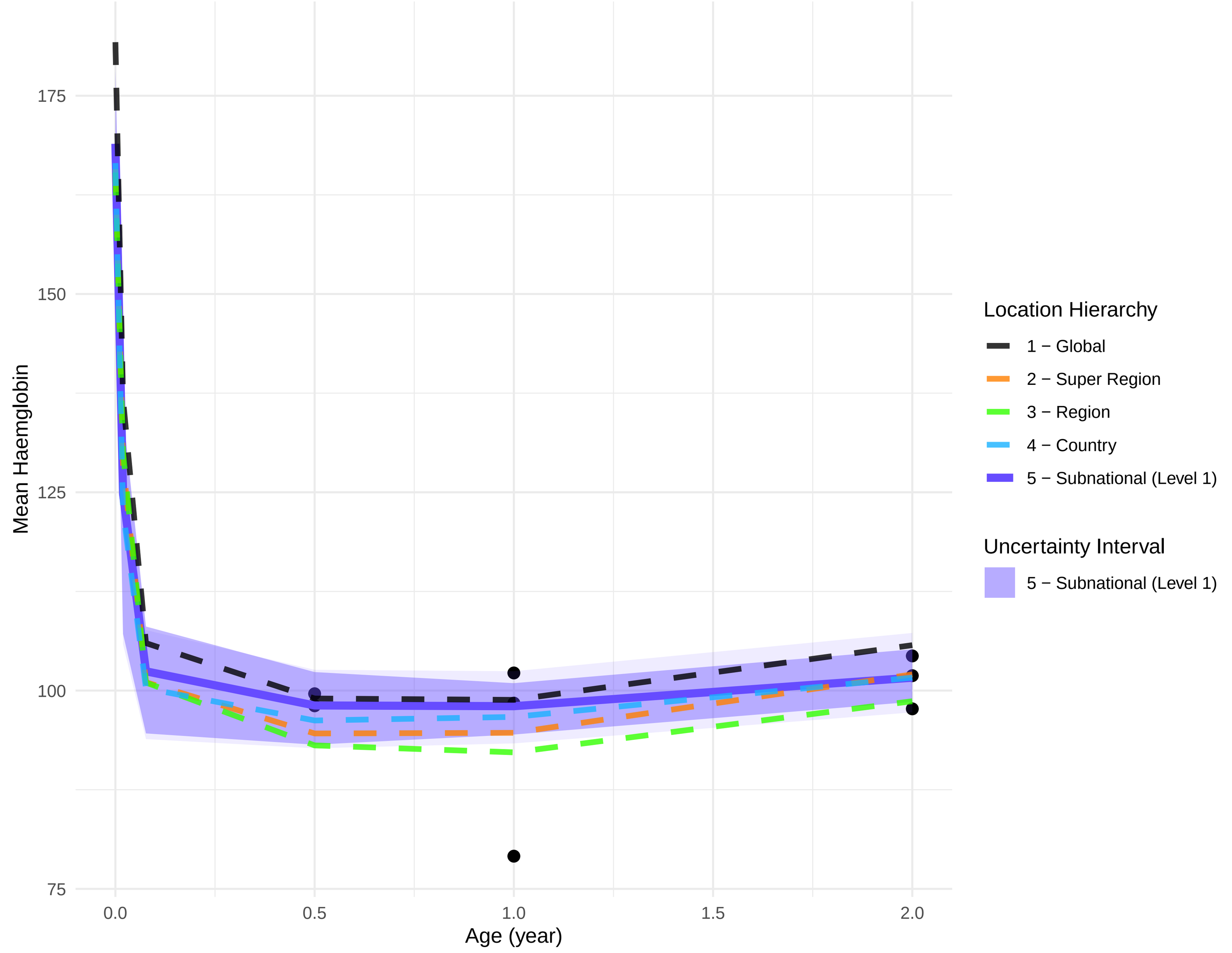

MR−BRT Age/Sex Fits − Males <2 Years Old − Mean Haemglobin − Lagos
WHO−2024 Adjustment Method

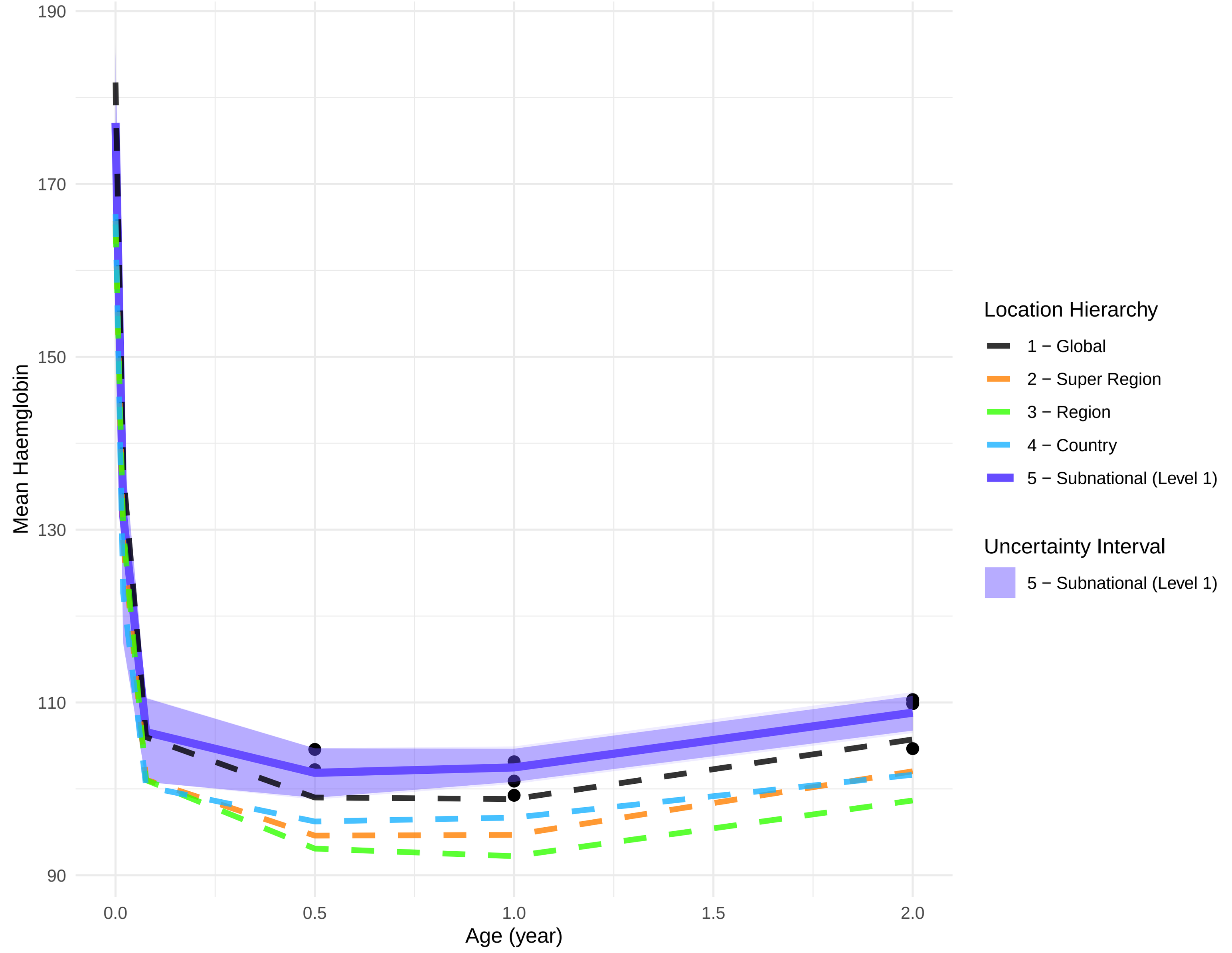

MR−BRT Age/Sex Fits − Males <2 Years Old − Mean Haemglobin − Nasarawa
WHO−2024 Adjustment Method

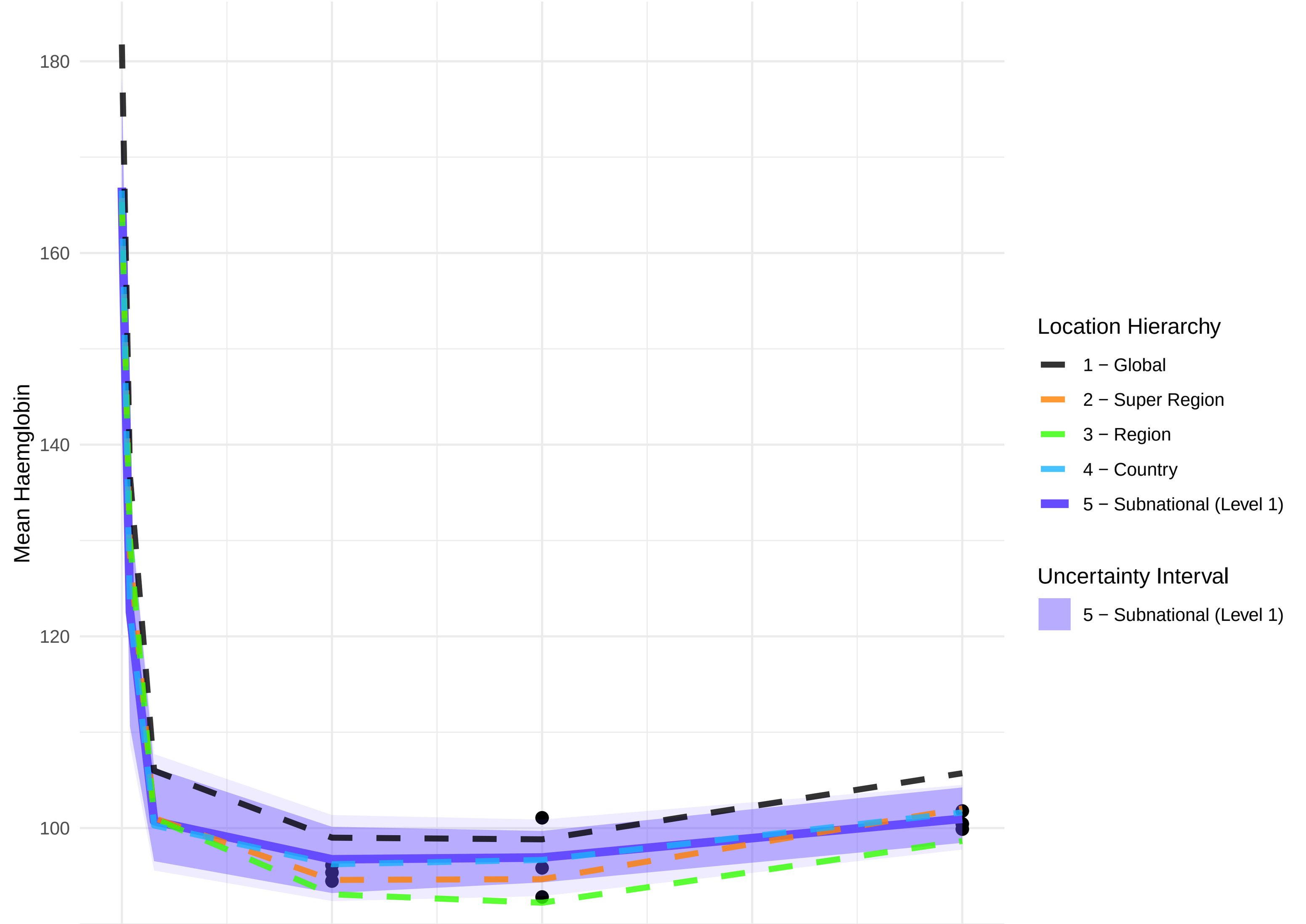

MR−BRT Age/Sex Fits − Males <2 Years Old − Mean Haemglobin − Niger
WHO−2024 Adjustment Method

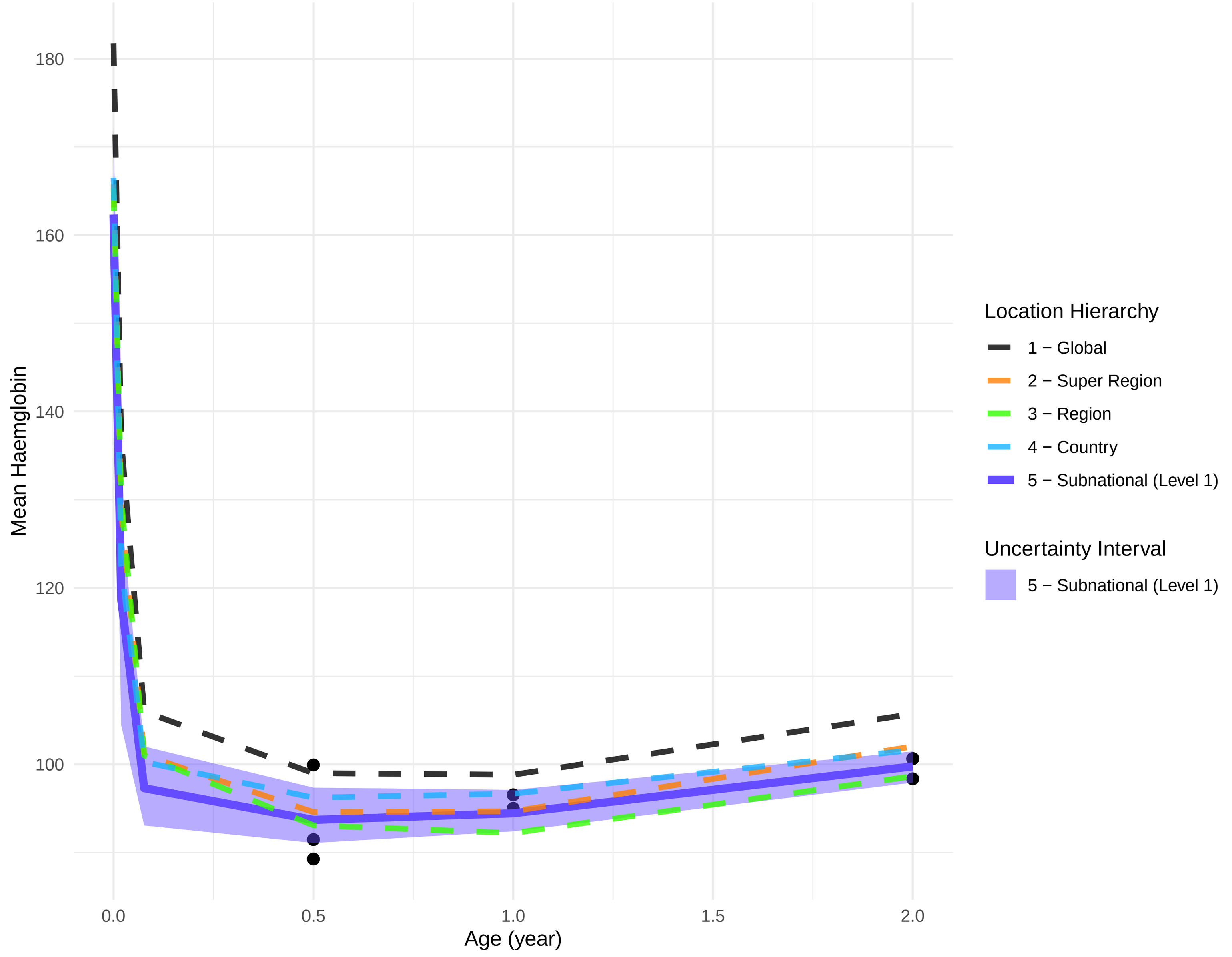

MR−BRT Age/Sex Fits − Males <2 Years Old − Mean Haemglobin − Ogun
WHO−2024 Adjustment Method

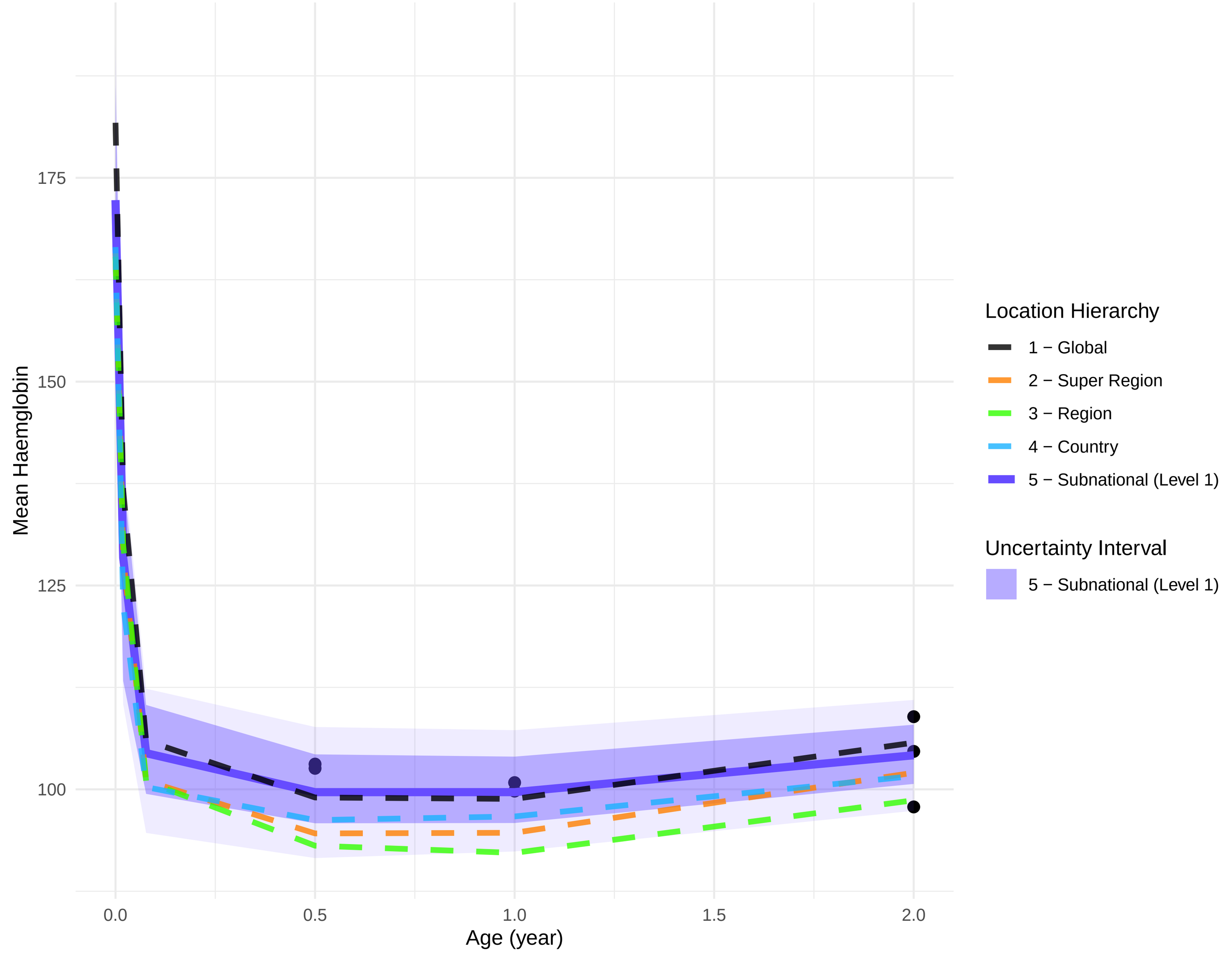

MR−BRT Age/Sex Fits − Males <2 Years Old − Mean Haemglobin − Ondo
WHO−2024 Adjustment Method

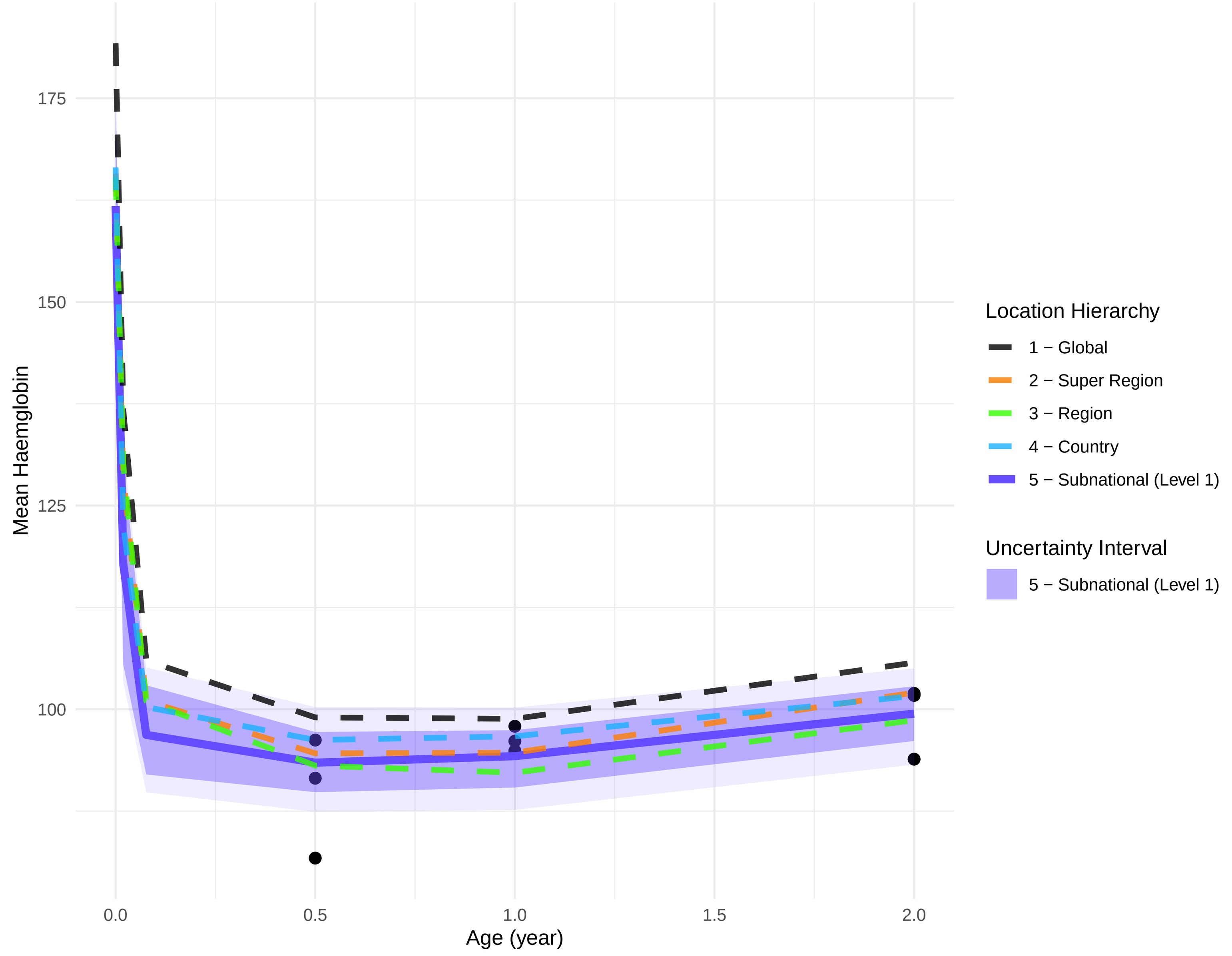

MR−BRT Age/Sex Fits − Males <2 Years Old − Mean Haemglobin − Osun
WHO−2024 Adjustment Method

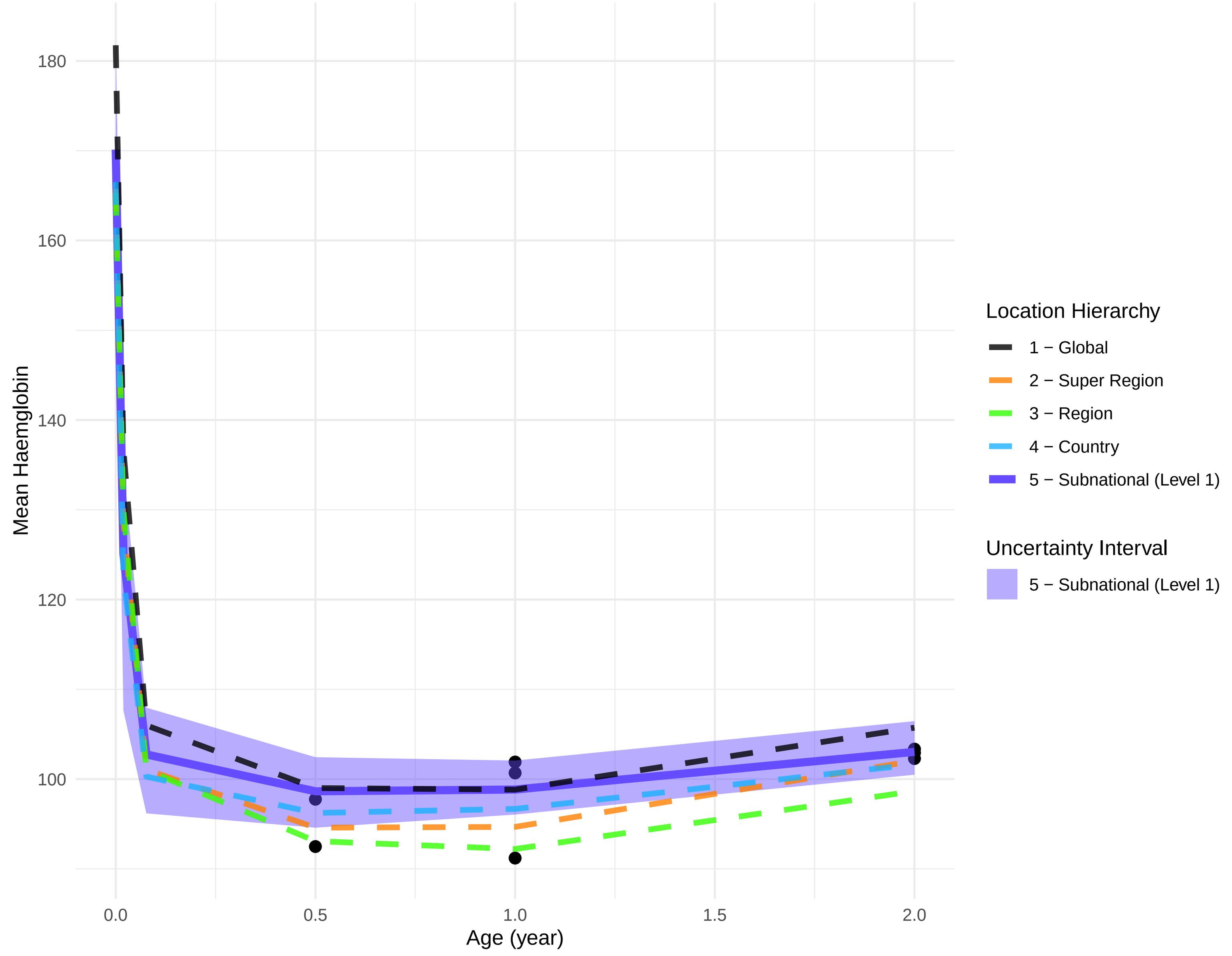

MR−BRT Age/Sex Fits − Males <2 Years Old − Mean Haemglobin − Oyo
WHO−2024 Adjustment Method

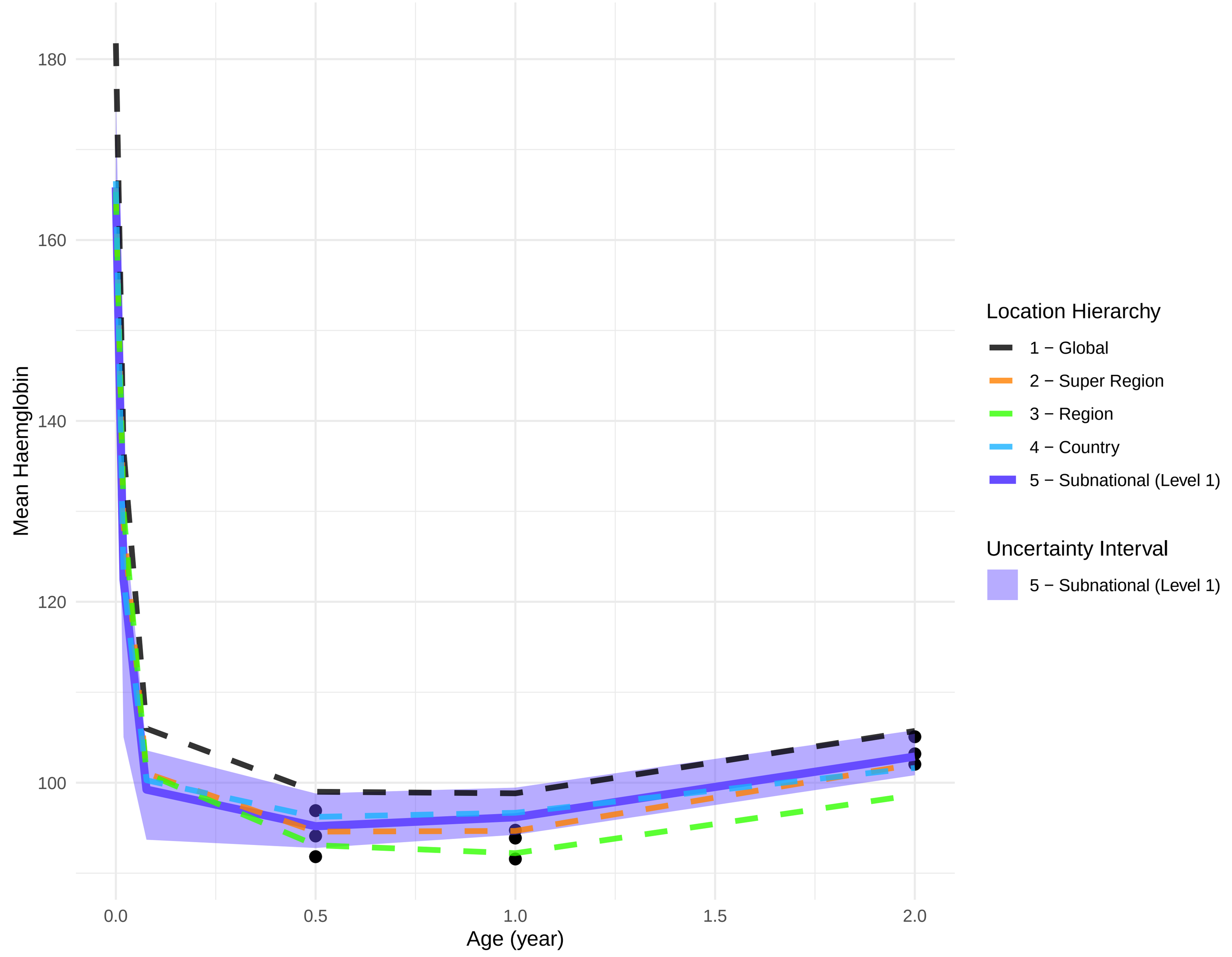

MR−BRT Age/Sex Fits − Males <2 Years Old − Mean Haemglobin − Plateau
WHO−2024 Adjustment Method

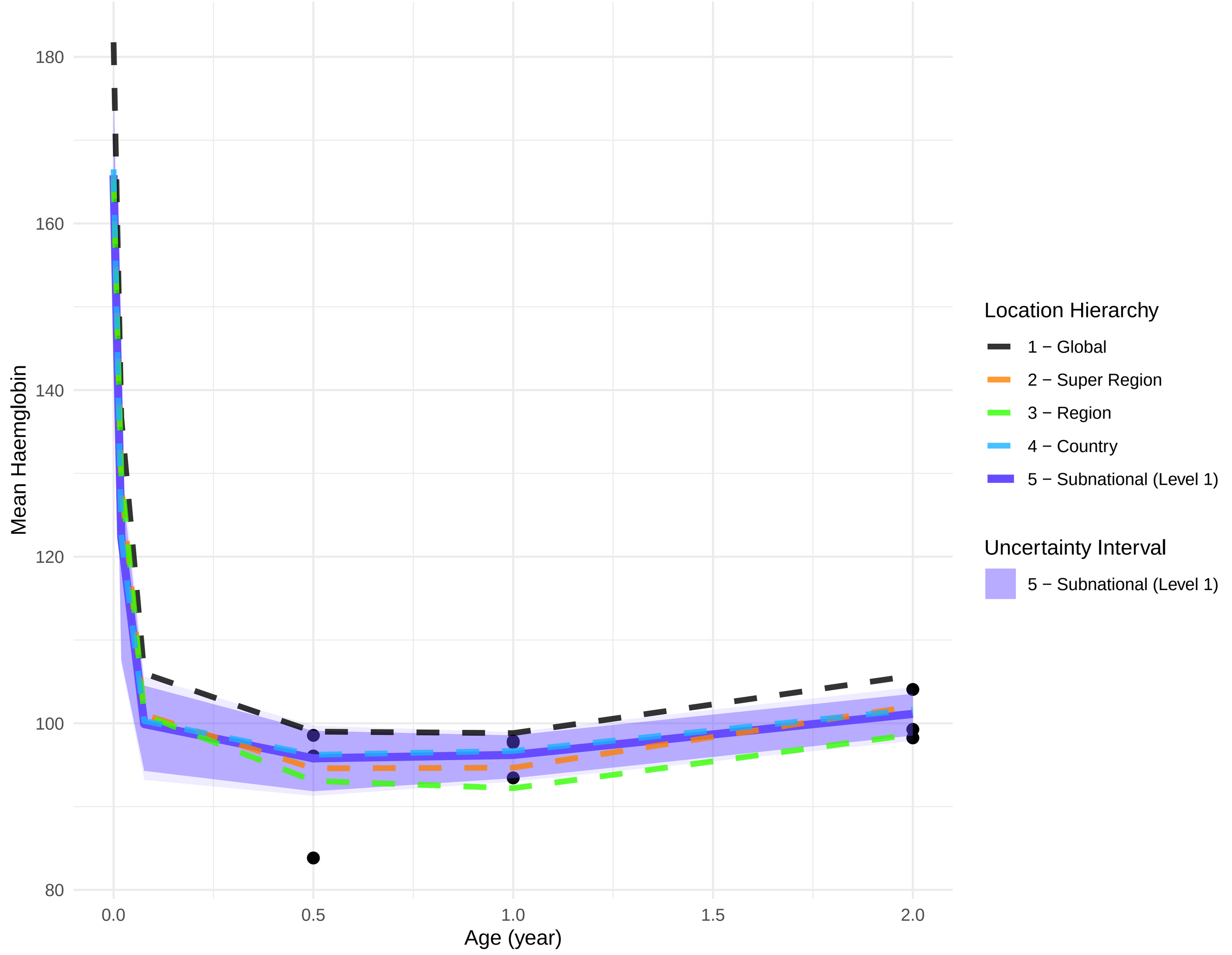

MR−BRT Age/Sex Fits − Males <2 Years Old − Mean Haemglobin − Rivers
WHO−2024 Adjustment Method

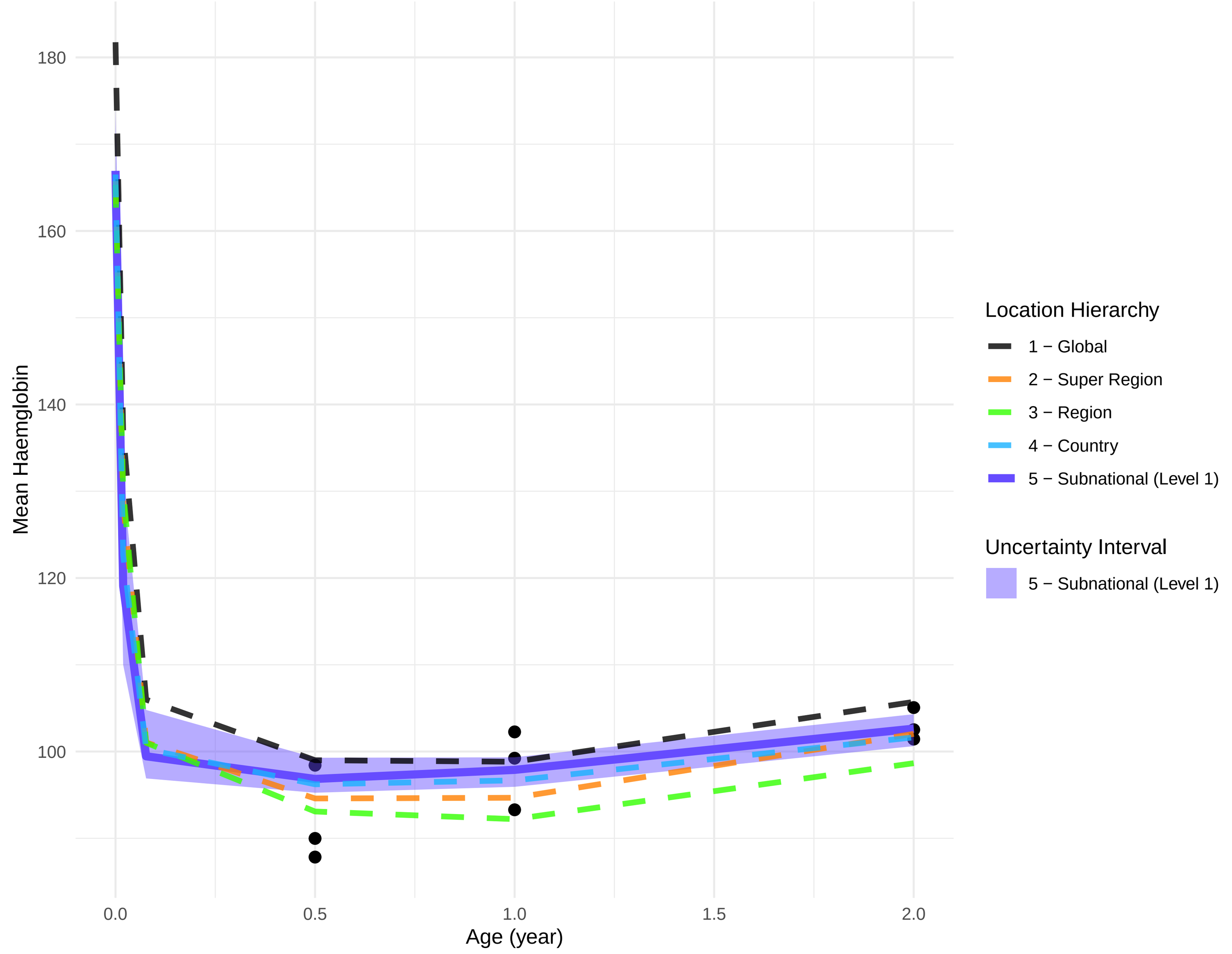

MR−BRT Age/Sex Fits − Males <2 Years Old − Mean Haemglobin − Sokoto
WHO−2024 Adjustment Method

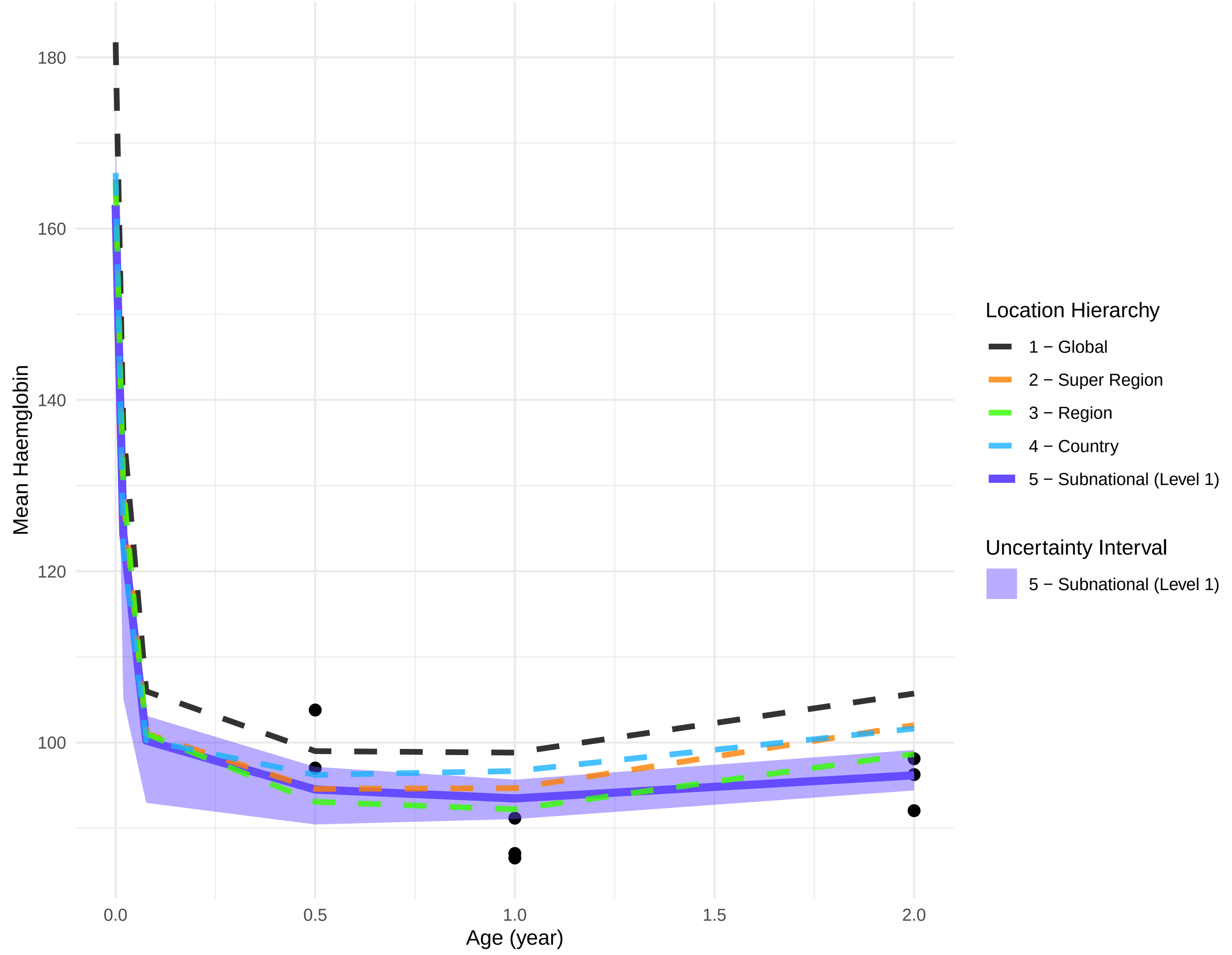

MR−BRT Age/Sex Fits − Males <2 Years Old − Mean Haemglobin − Taraba
WHO−2024 Adjustment Method

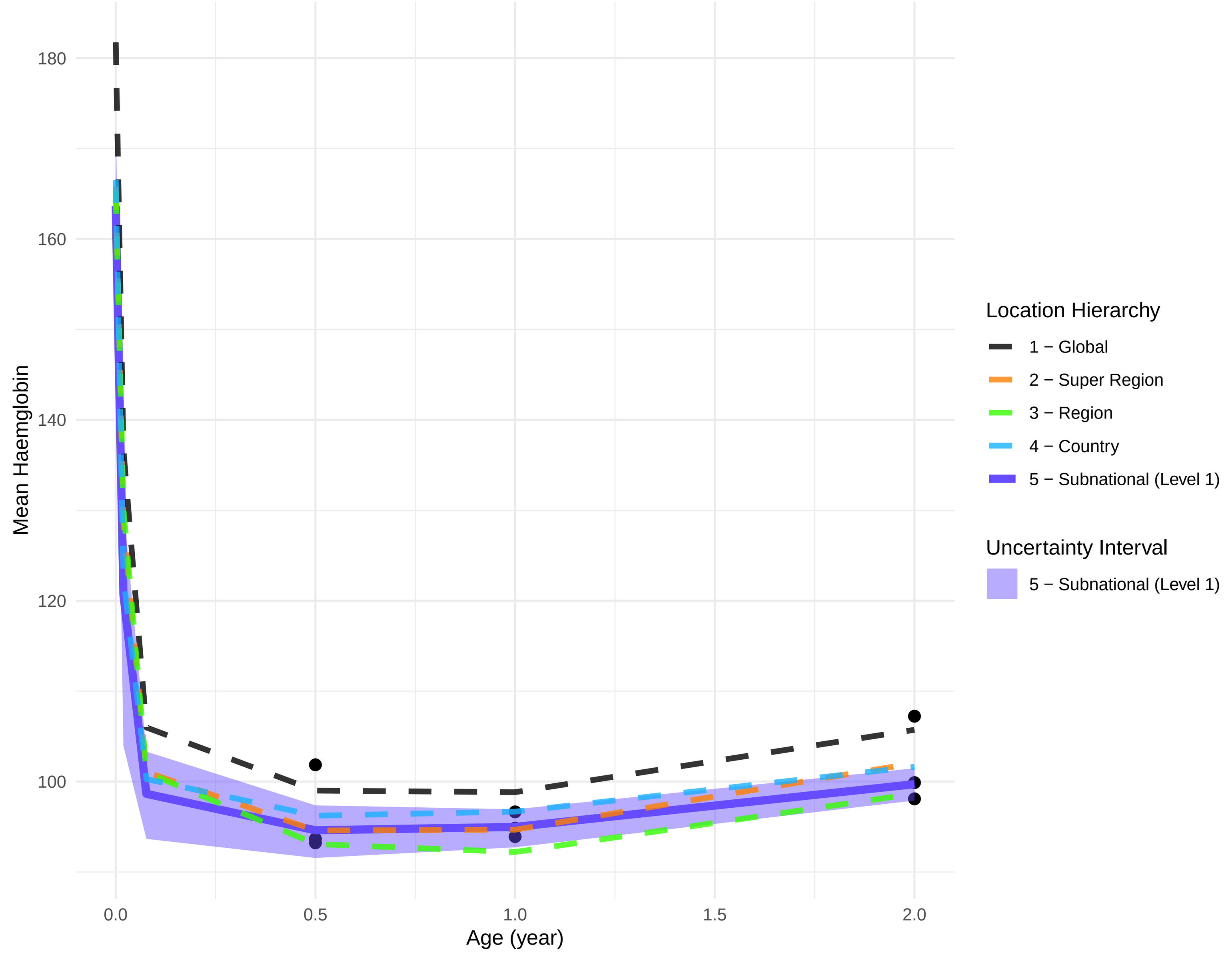

MR−BRT Age/Sex Fits − Males <2 Years Old − Mean Haemglobin − Yobe
WHO−2024 Adjustment Method

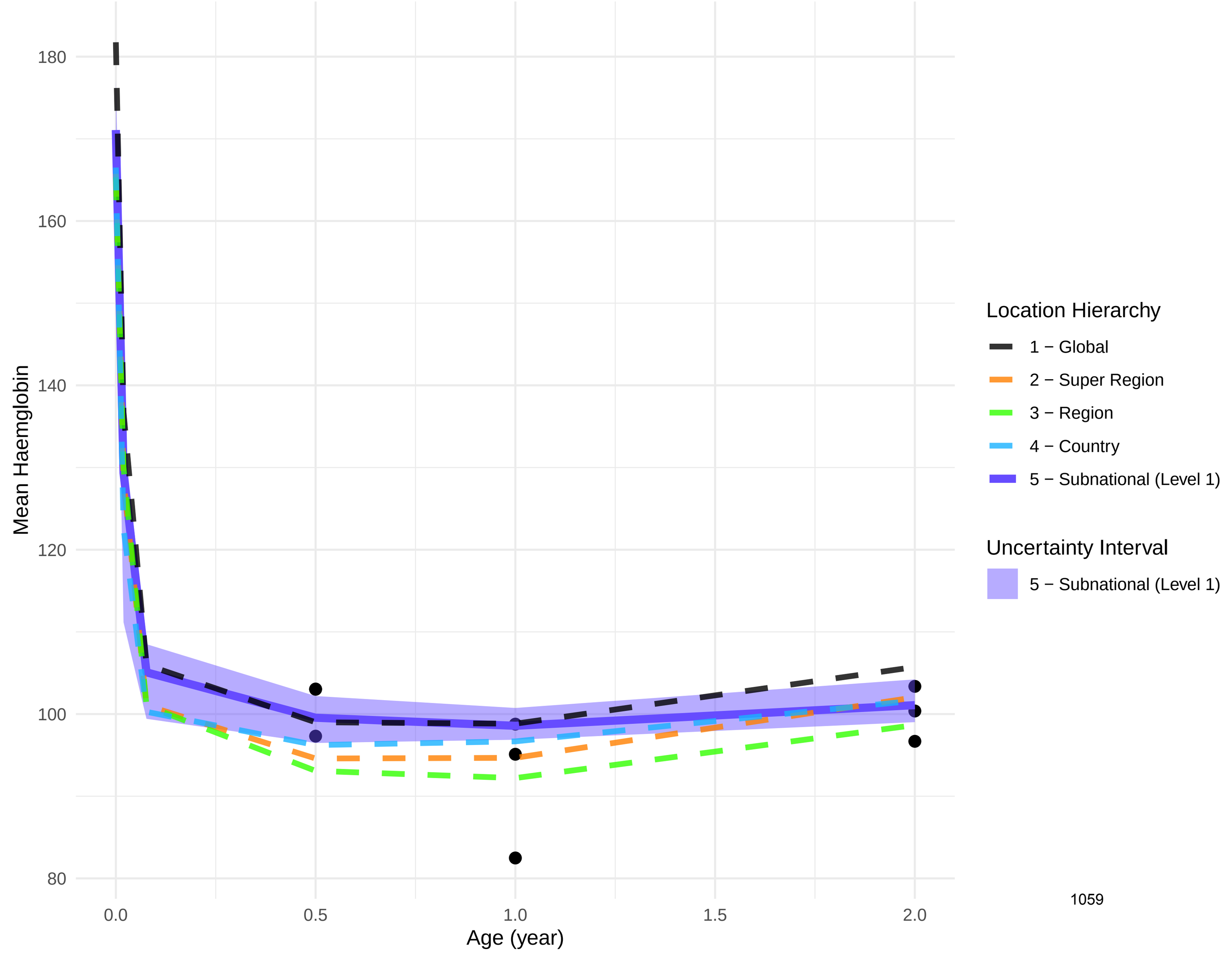

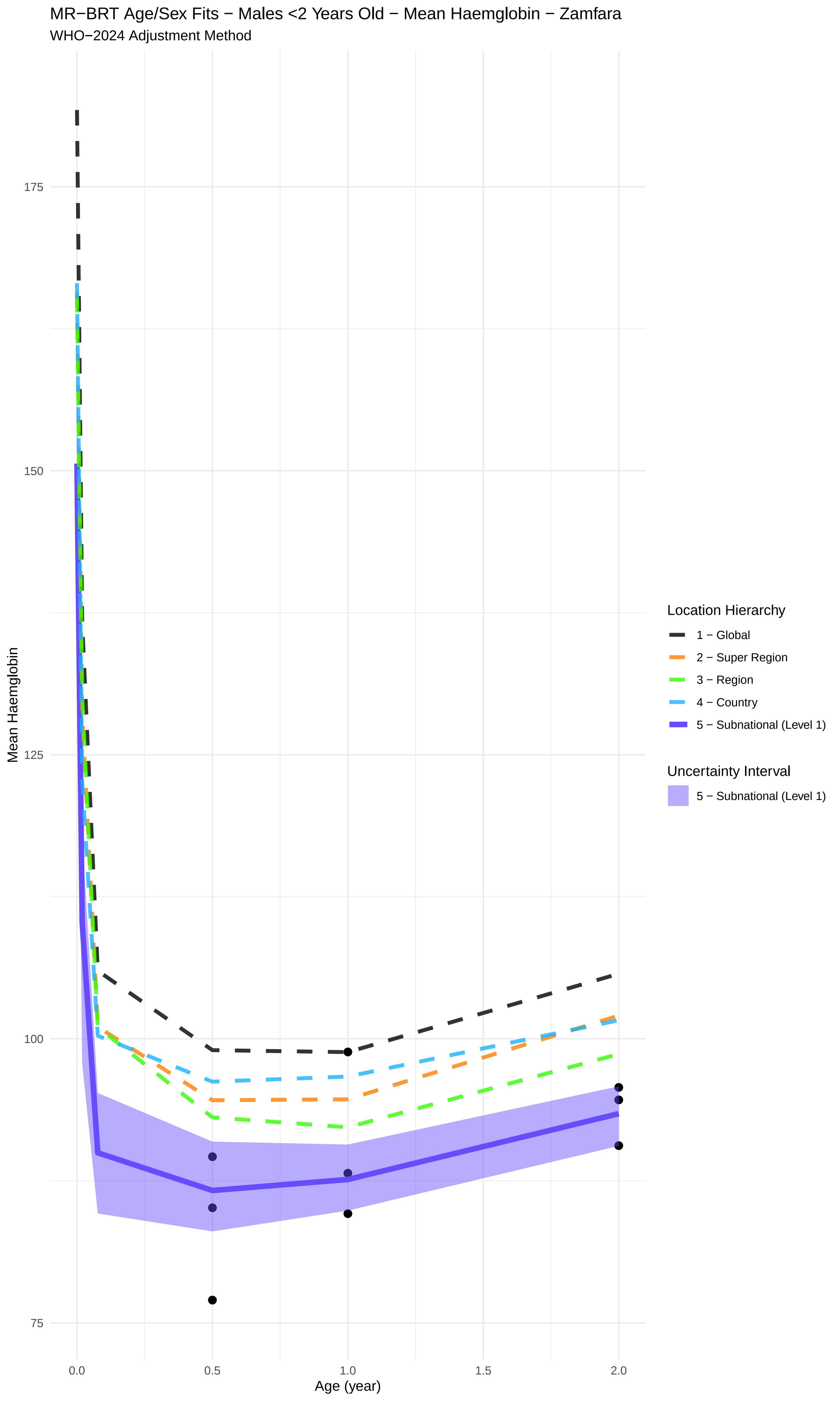
MR−BRT Age/Sex Fits − Males <2 Years Old − Mean Haemglobin − Zamfara
WHO−2024 Adjustment Method
Mean Haemglobin
Age (year)
Location Hierarchy
1 − Global
2 − Super Region
3 − Region
4 − Country
5 − Subnational (Level 1)
Uncertainty Interval
5 − Subnational (Level 1)

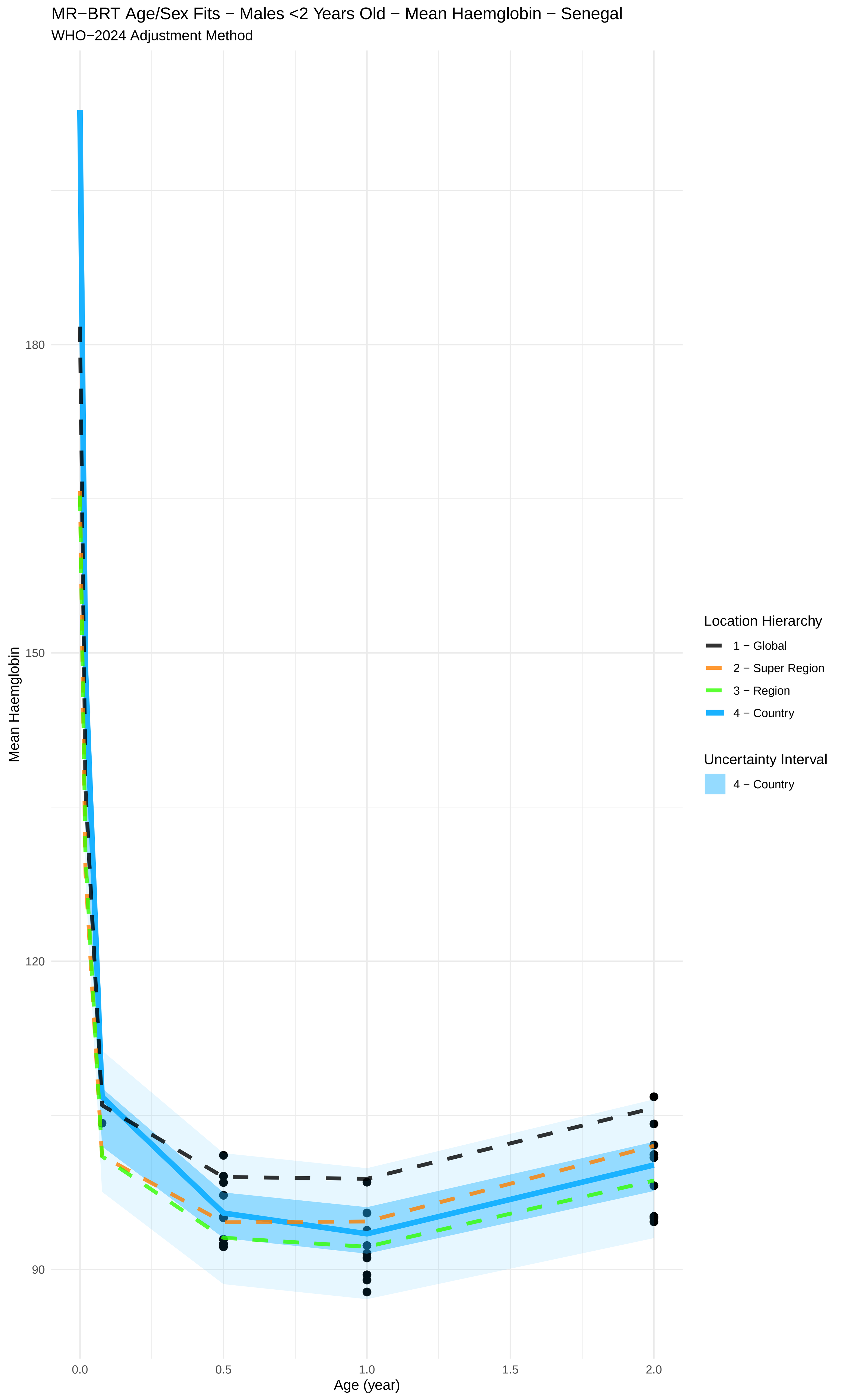
MR−BRT Age/Sex Fits − Males <2 Years Old − Mean Haemglobin − Senegal
WHO−2024 Adjustment Method
Mean Haemglobin
Age (year)
Location Hierarchy
1 − Global
2 − Super Region
3 − Region
4 − Country
Uncertainty Interval
4 − Country

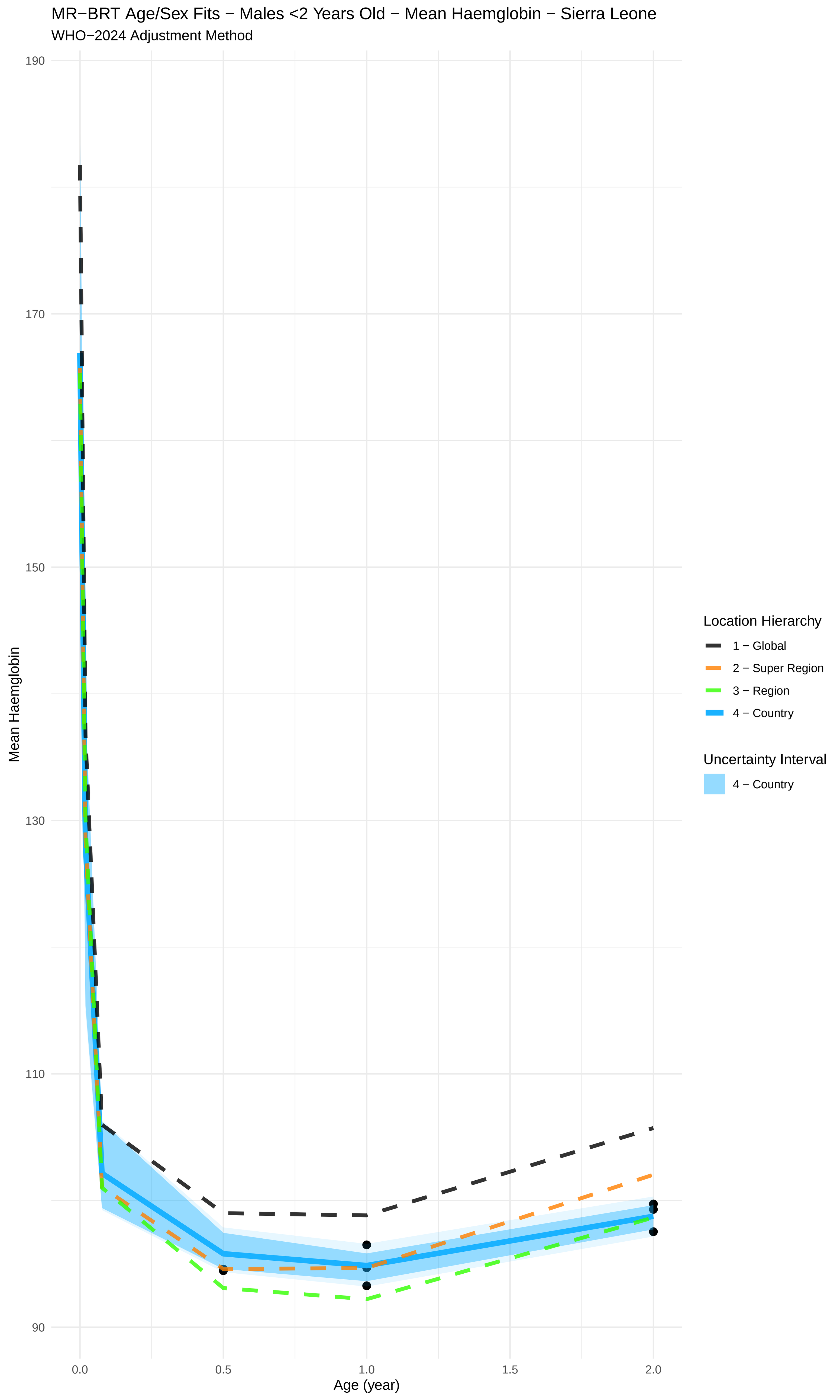
MR−BRT Age/Sex Fits − Males <2 Years Old − Mean Haemglobin − Sierra Leone
WHO−2024 Adjustment Method
Mean Haemglobin
Age (year)
Location Hierarchy
1 − Global
2 − Super Region
3 − Region
4 − Country
Uncertainty Interval
4 − Country

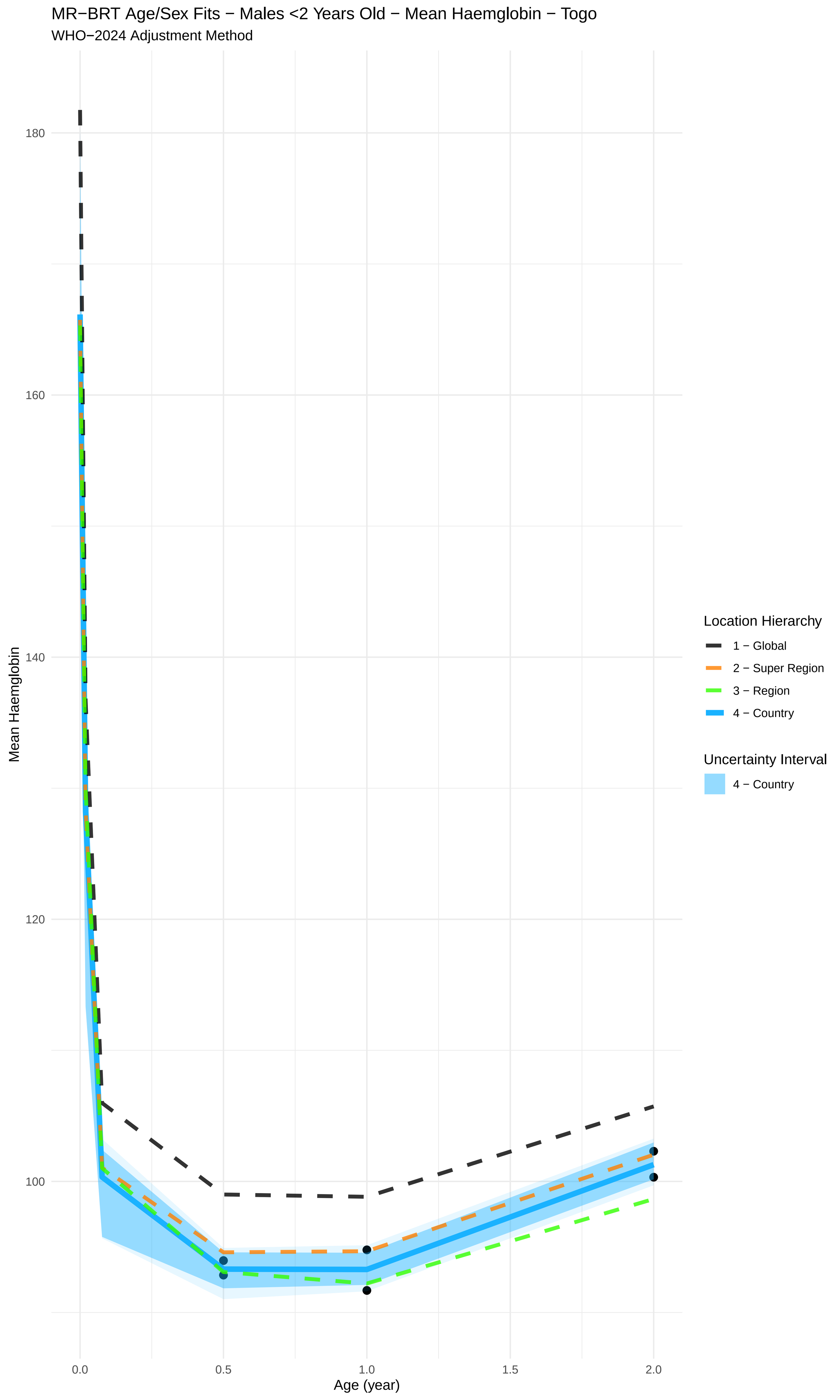
MR−BRT Age/Sex Fits − Males <2 Years Old − Mean Haemglobin − Togo
WHO−2024 Adjustment Method
Mean Haemglobin
Age (year)
Location Hierarchy
1 − Global
2 − Super Region
3 − Region
4 − Country
Uncertainty Interval
4 − Country

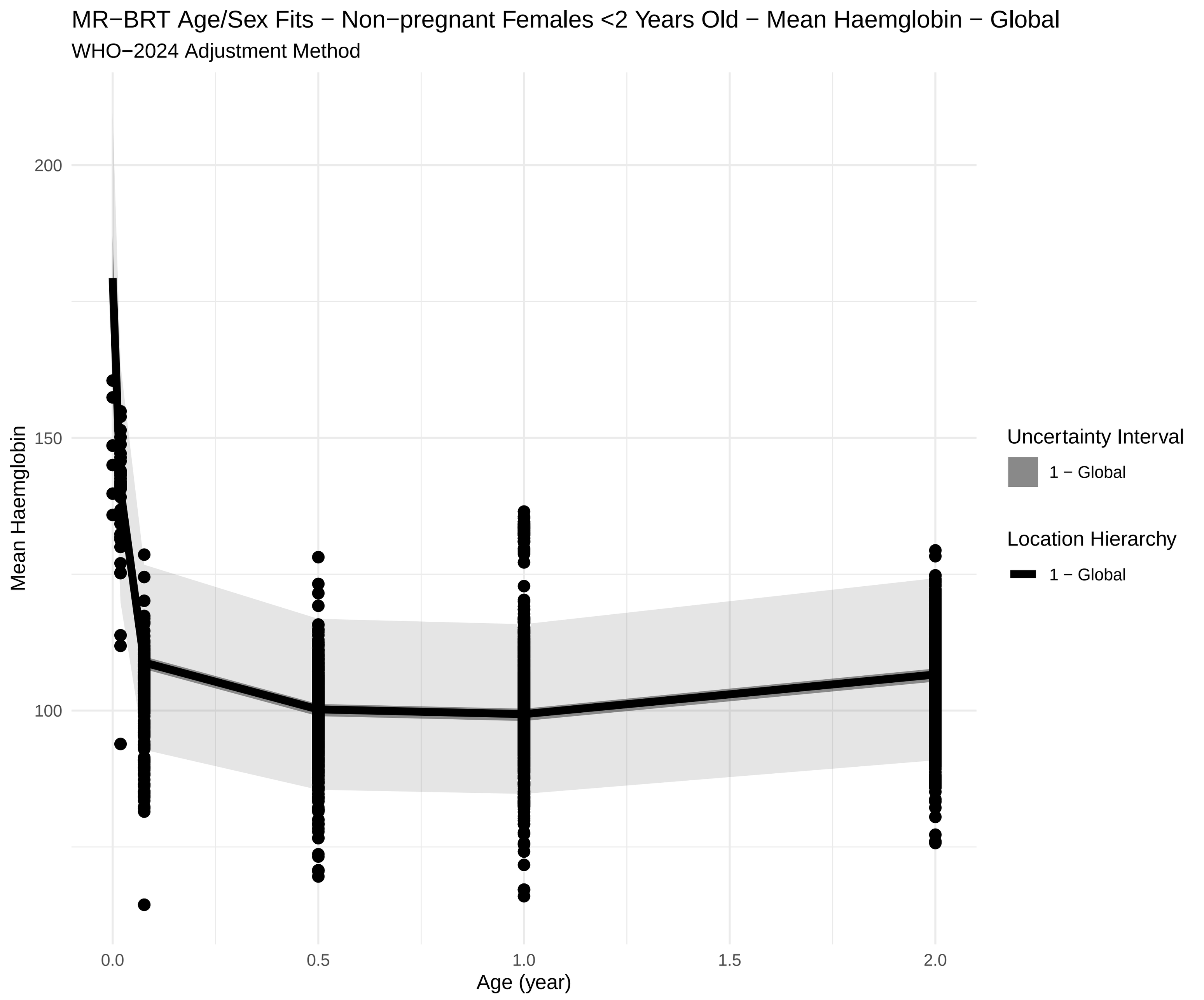
MR−BRT Age/Sex Fits − Non−pregnant Females <2 Years Old − Mean Haemglobin − Global
WHO−2024 Adjustment Method
Mean Haemglobin
Age (year)
Uncertainty Interval
1 − Global
Location Hierarchy
1 − Global

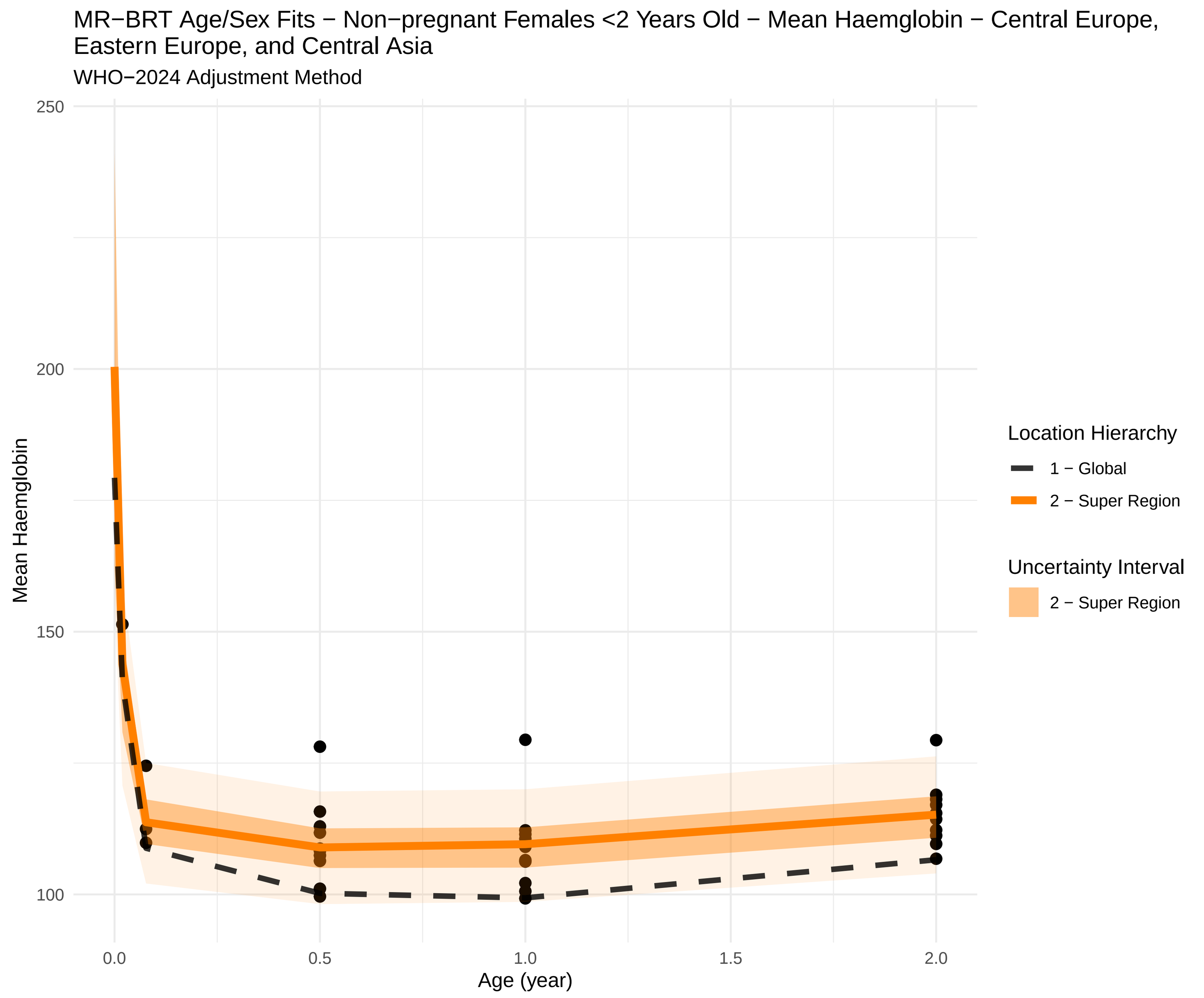
MR−BRT Age/Sex Fits − Non−pregnant Females <2 Years Old − Mean Haemglobin − Central Europe, Eastern Europe, and Central Asia
WHO−2024 Adjustment Method
Mean Haemglobin
Age (year)
Location Hierarchy
1 − Global
2 − Super Region
Uncertainty Interval
2 − Super Region

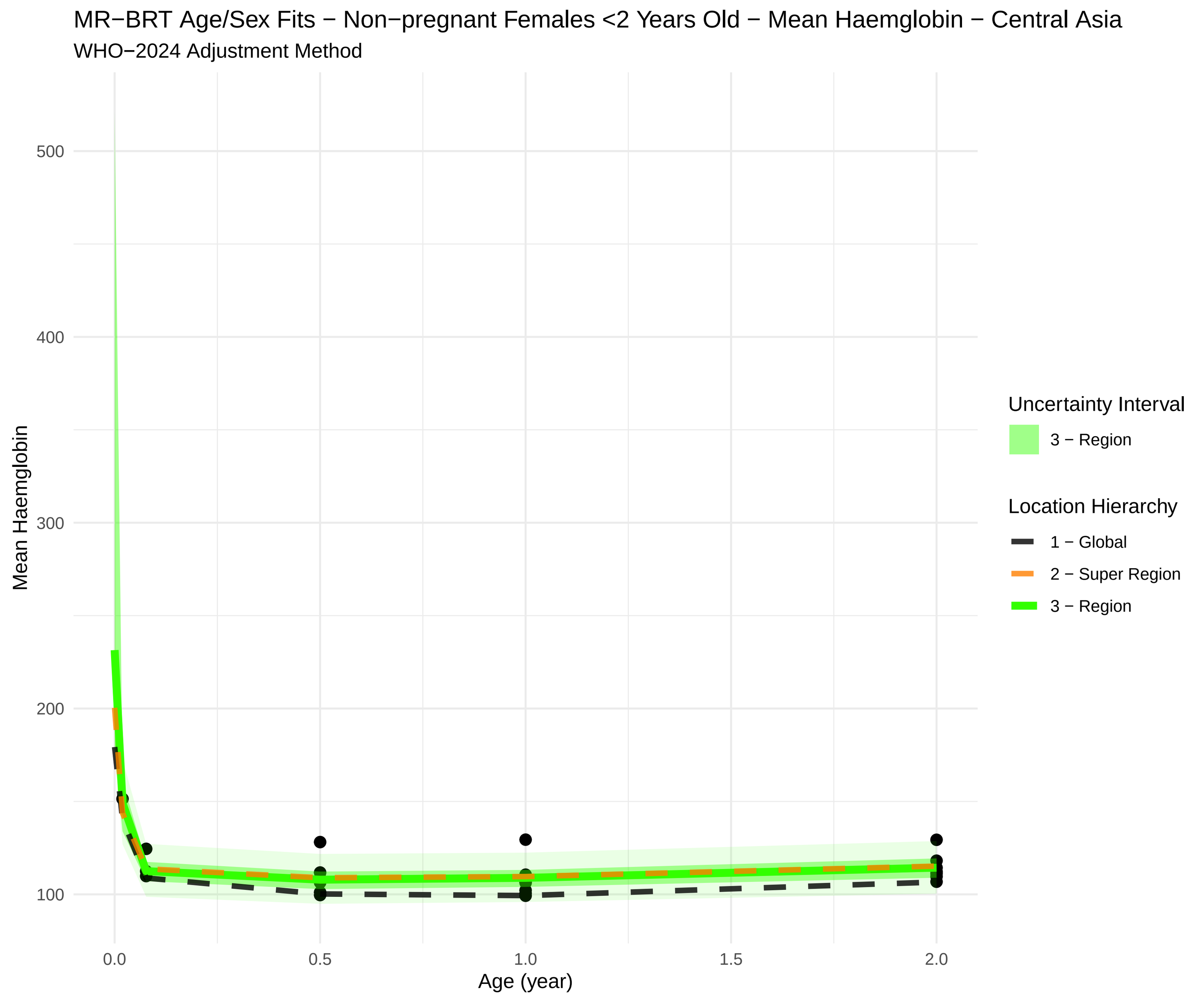
MR−BRT Age/Sex Fits − Non−pregnant Females <2 Years Old − Mean Haemglobin − Central Asia
WHO−2024 Adjustment Method
Mean Haemglobin
Age (year)
Uncertainty Interval
3 − Region
Location Hierarchy
1 − Global
2 − Super Region
3 − Region

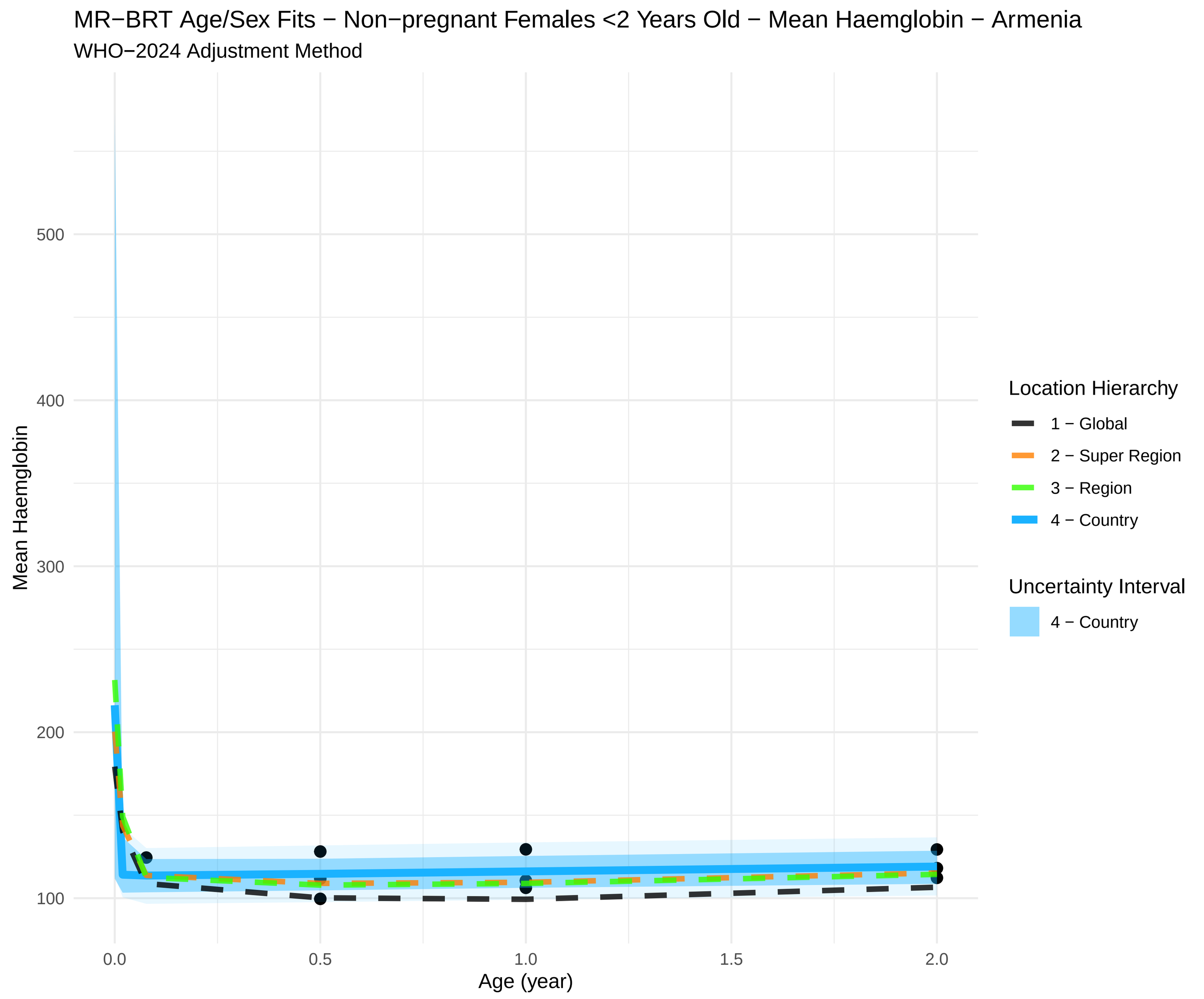
MR−BRT Age/Sex Fits − Non−pregnant Females <2 Years Old − Mean Haemglobin − Armenia
WHO−2024 Adjustment Method
Mean Haemglobin
Age (year)
Location Hierarchy
1 − Global
2 − Super Region
3 − Region
4 − Country
Uncertainty Interval
4 − Country

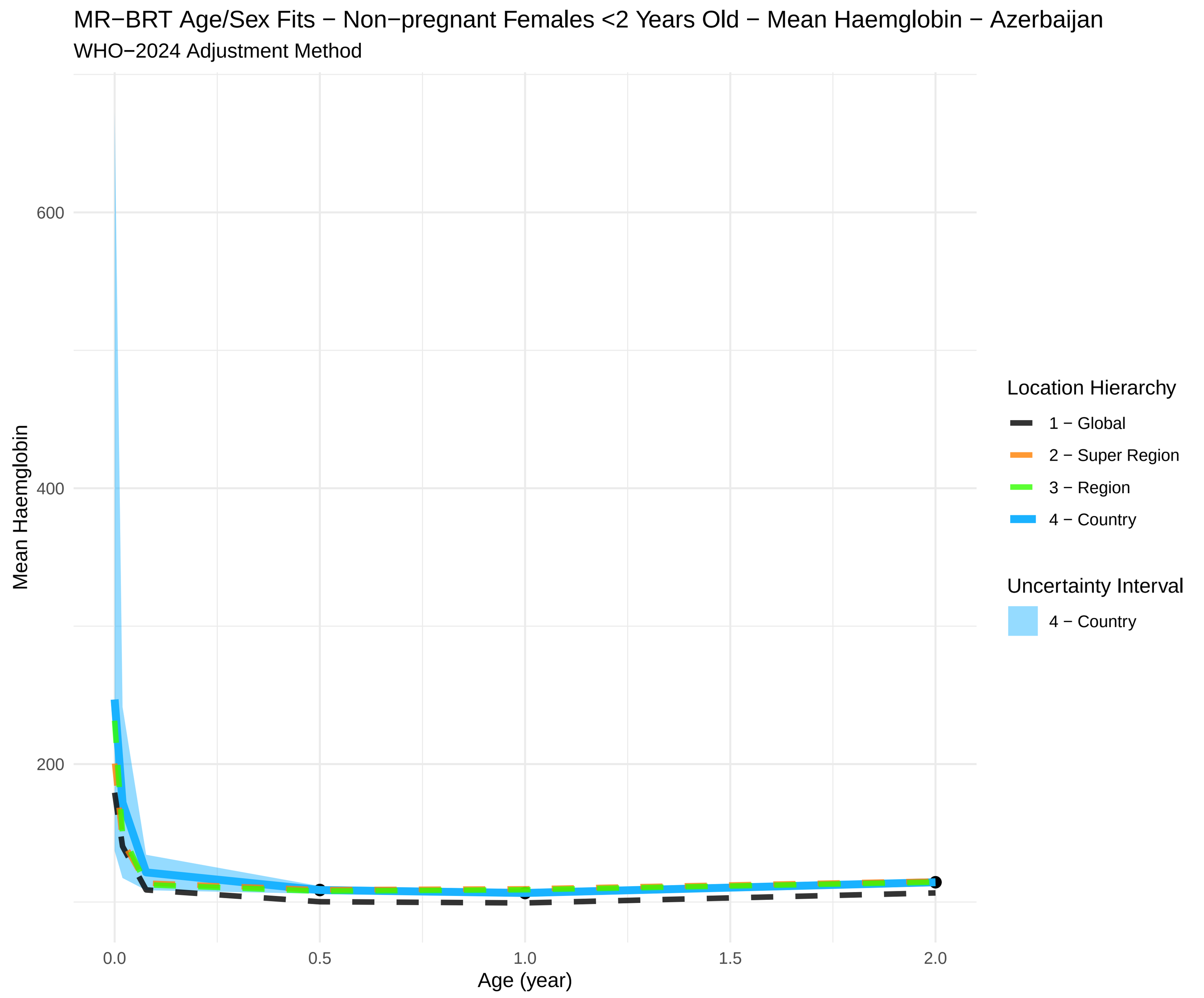
MR−BRT Age/Sex Fits − Non−pregnant Females <2 Years Old − Mean Haemglobin − Azerbaijan
WHO−2024 Adjustment Method
Mean Haemglobin
Age (year)
Location Hierarchy
1 − Global
2 − Super Region
3 − Region
4 − Country
Uncertainty Interval
4 − Country

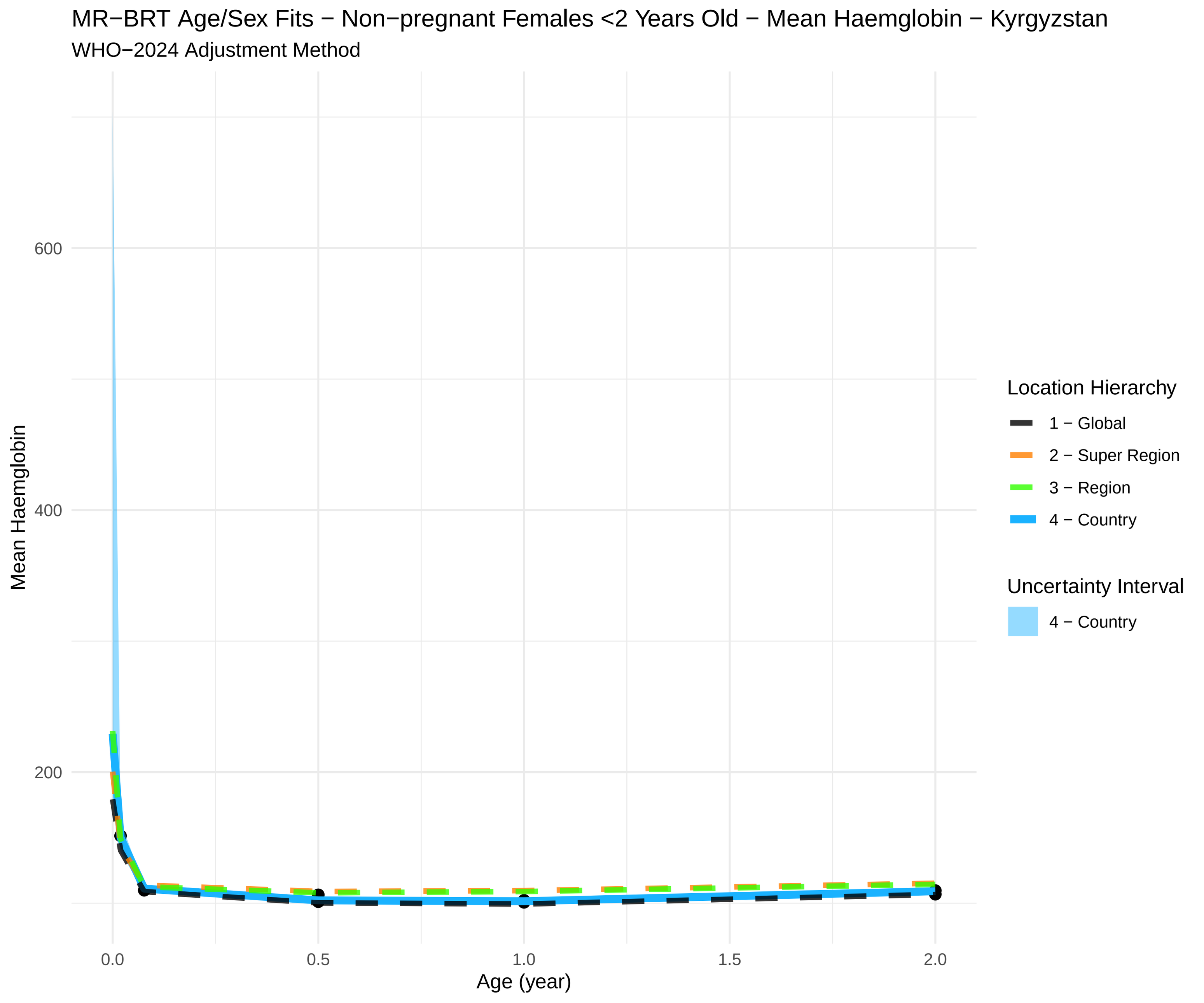
MR−BRT Age/Sex Fits − Non−pregnant Females <2 Years Old − Mean Haemglobin − Kyrgyzstan
WHO−2024 Adjustment Method
Mean Haemglobin
Age (year)
Location Hierarchy
1 − Global
2 − Super Region
3 − Region
4 − Country
Uncertainty Interval
4 − Country

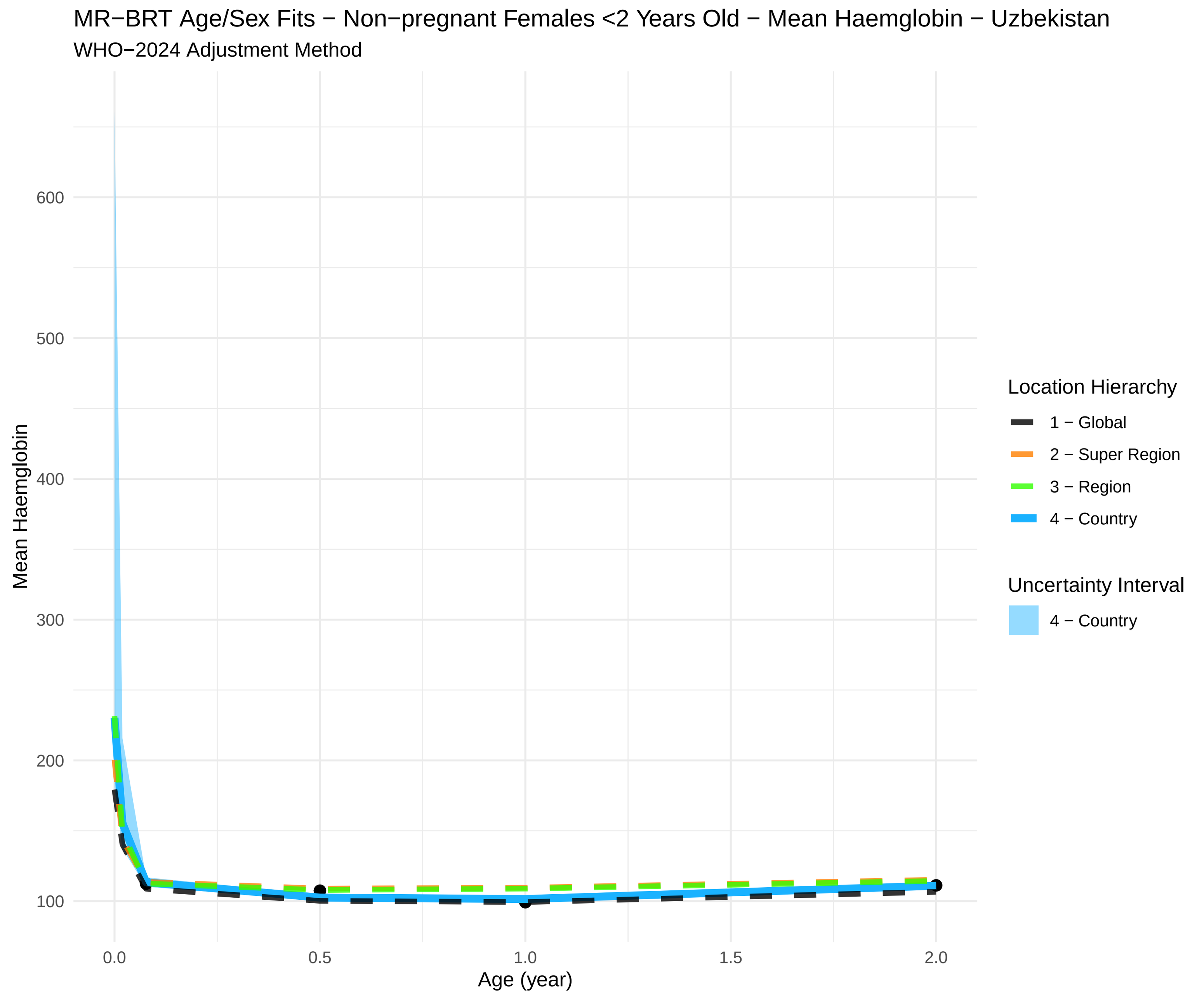
MR−BRT Age/Sex Fits − Non−pregnant Females <2 Years Old − Mean Haemglobin − Uzbekistan
WHO−2024 Adjustment Method
Mean Haemglobin
Age (year)
Location Hierarchy
1 − Global
2 − Super Region
3 − Region
4 − Country
Uncertainty Interval
4 − Country

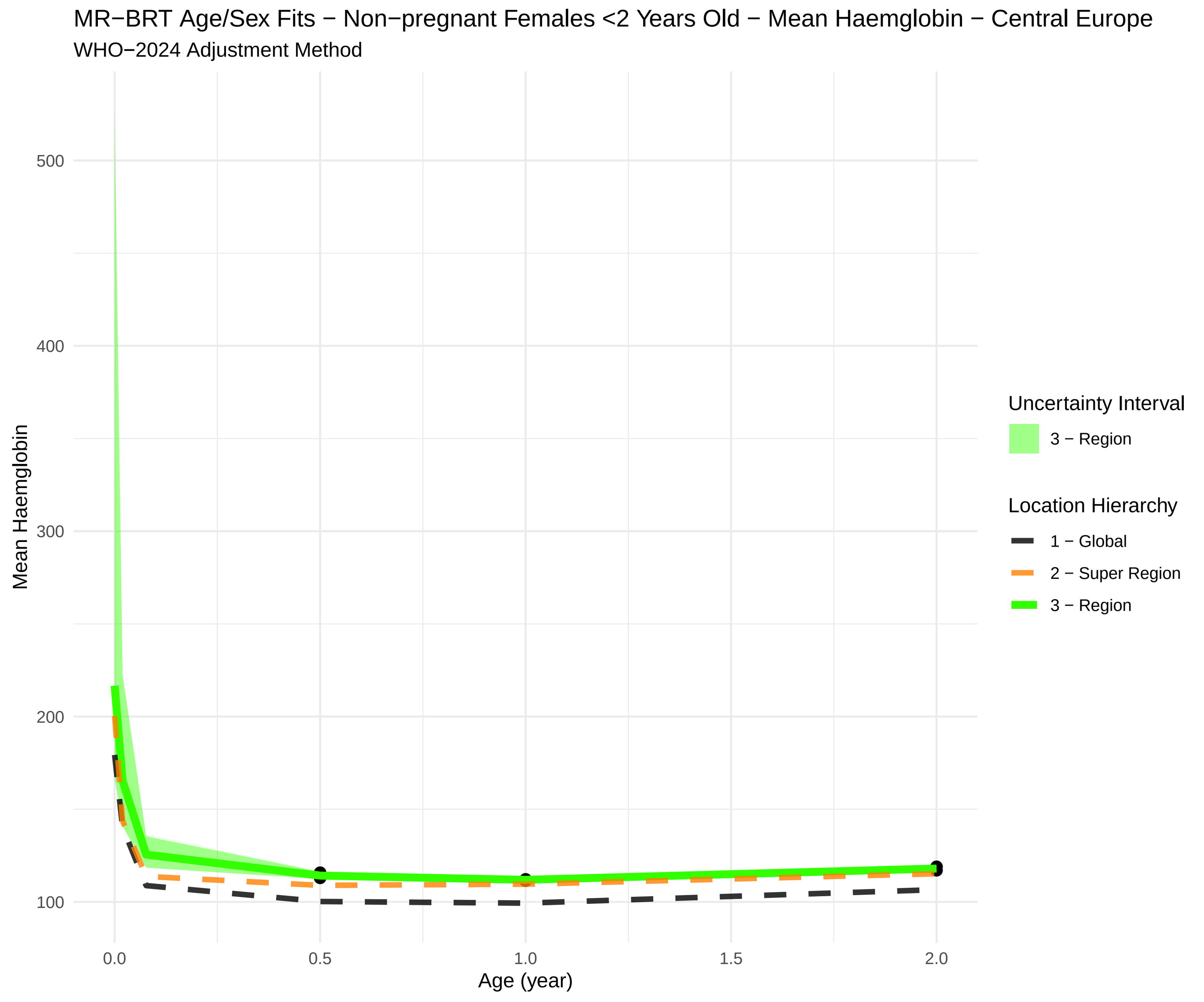
MR−BRT Age/Sex Fits − Non−pregnant Females <2 Years Old − Mean Haemglobin − Central Europe
WHO−2024 Adjustment Method
Mean Haemglobin
Age (year)
Uncertainty Interval
3 − Region
Location Hierarchy
1 − Global
2 − Super Region
3 − Region

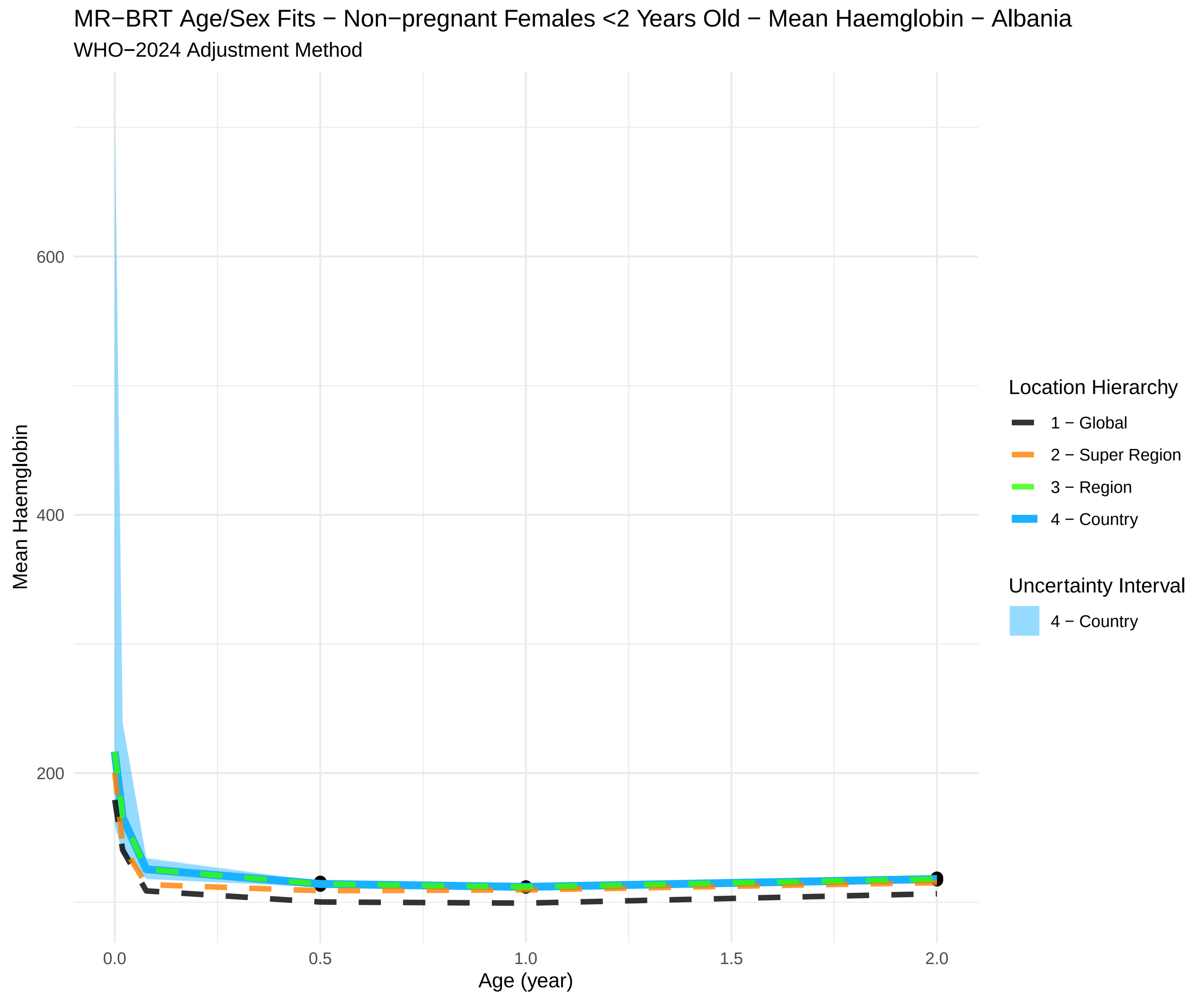
MR−BRT Age/Sex Fits − Non−pregnant Females <2 Years Old − Mean Haemglobin − Albania
WHO−2024 Adjustment Method
Mean Haemglobin
Age (year)
Location Hierarchy
1 − Global
2 − Super Region
3 − Region
4 − Country
Uncertainty Interval
4 − Country

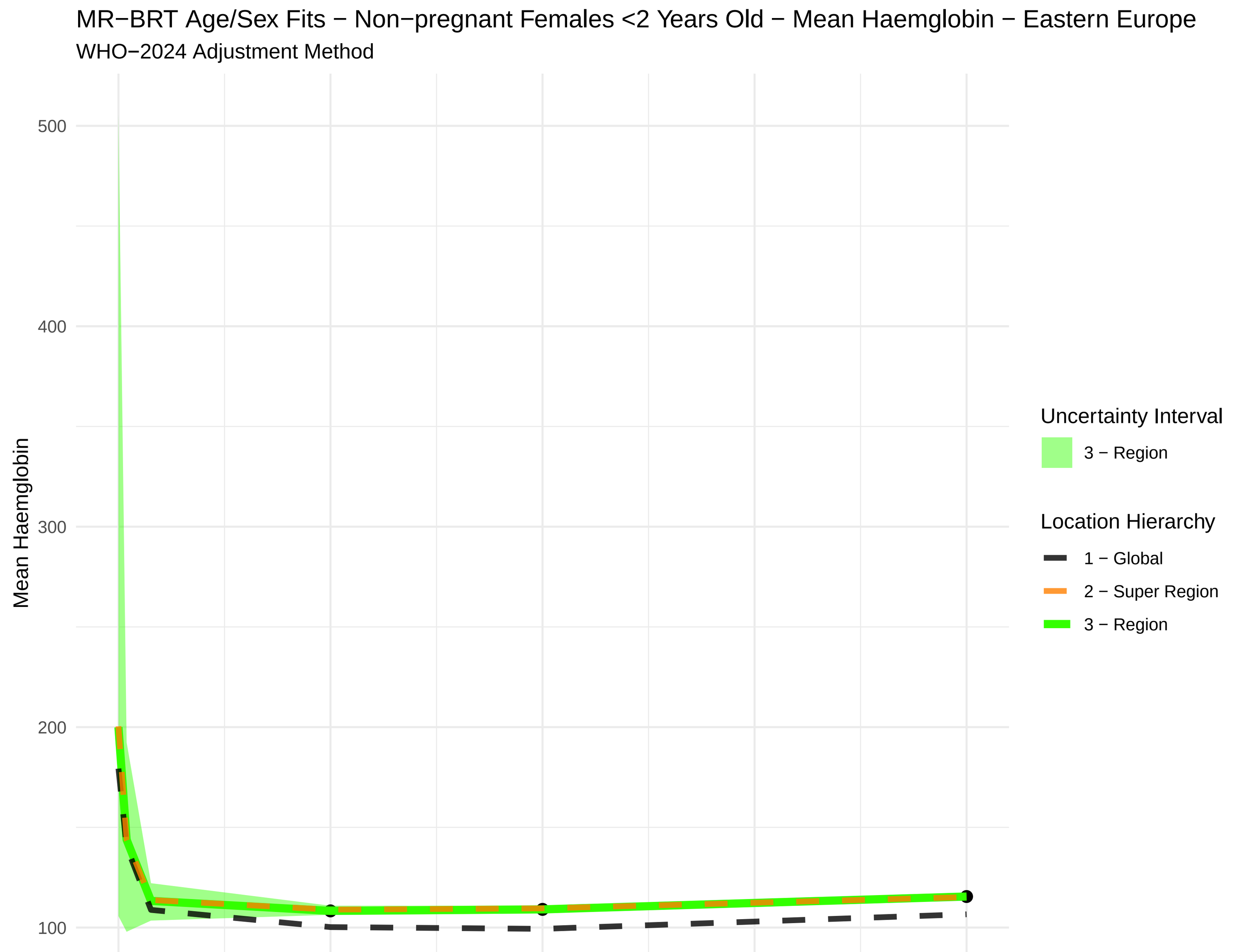
MR−BRT Age/Sex Fits − Non−pregnant Females <2 Years Old − Mean Haemglobin − Eastern Europe
WHO−2024 Adjustment Method
Mean Haemglobin
Age (year)
Uncertainty Interval
3 − Region
Location Hierarchy
1 − Global
2 − Super Region
3 − Region

MR−BRT Age/Sex Fits − Non−pregnant Females <2 Years Old − Mean Haemglobin − Republic of Moldova
WHO−2024 Adjustment Method

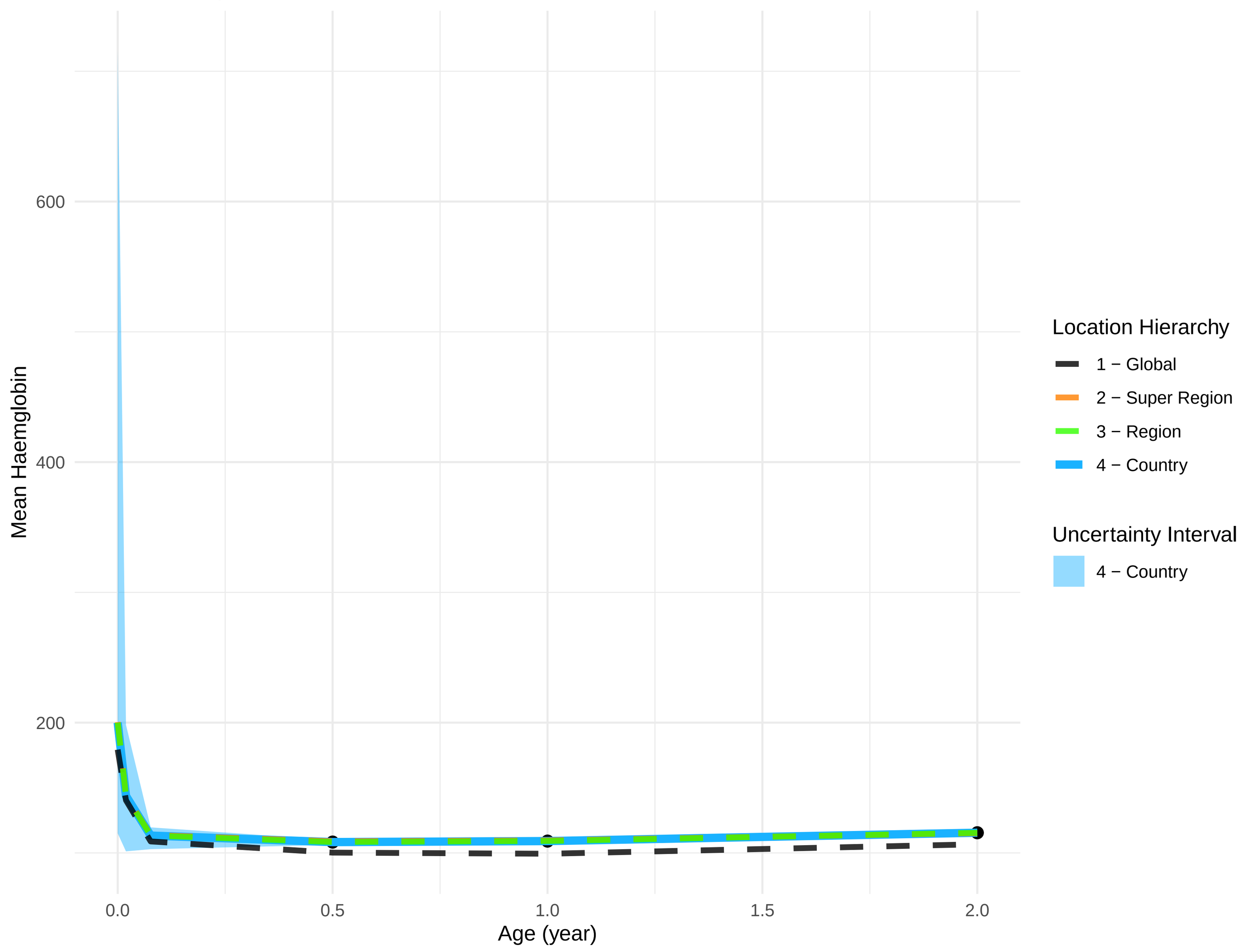

MR−BRT Age/Sex Fits − Non−pregnant Females <2 Years Old − Mean Haemglobin − High−income
WHO−2024 Adjustment Method

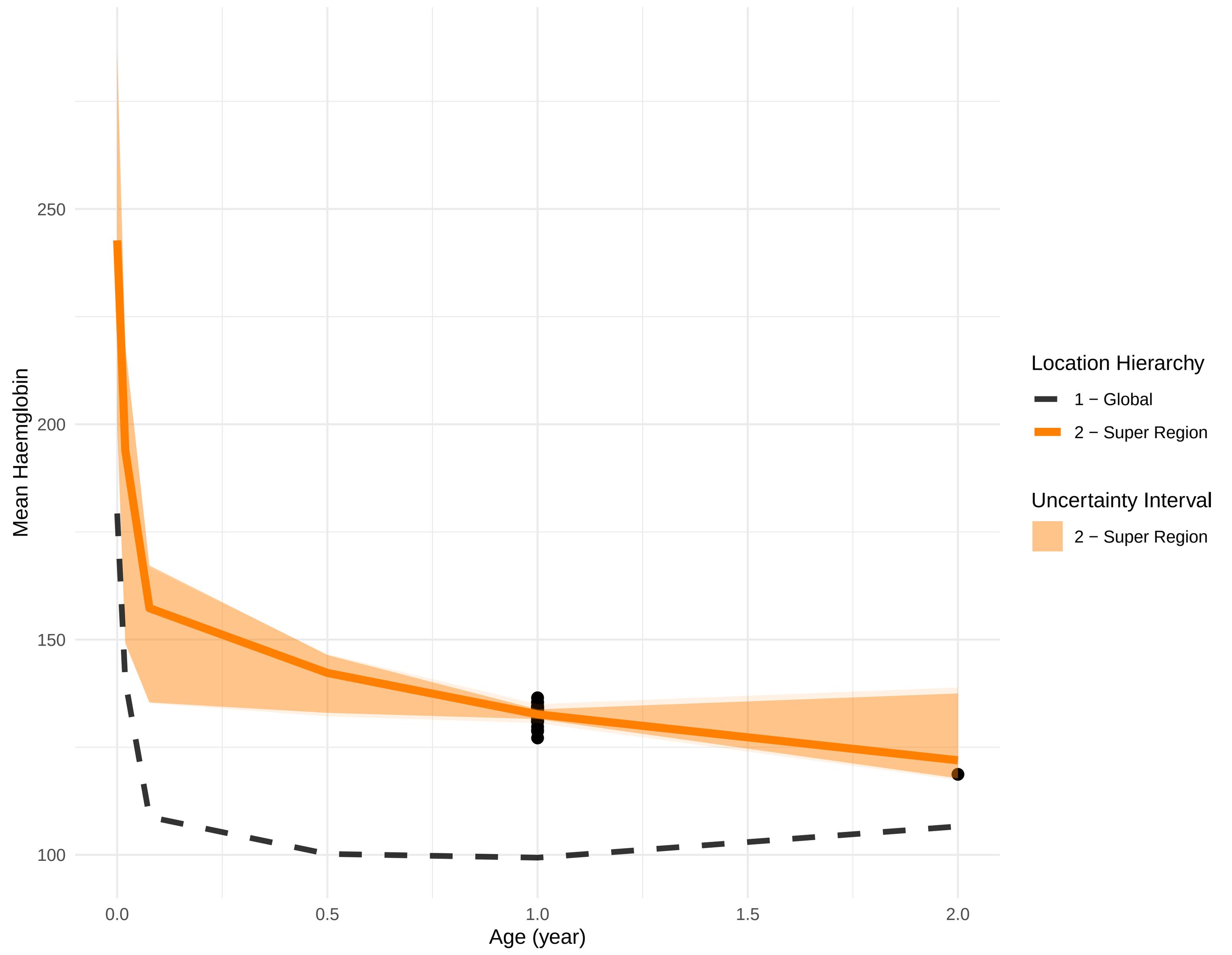

MR−BRT Age/Sex Fits − Non−pregnant Females <2 Years Old − Mean Haemglobin − Western Europe
WHO−2024 Adjustment Method

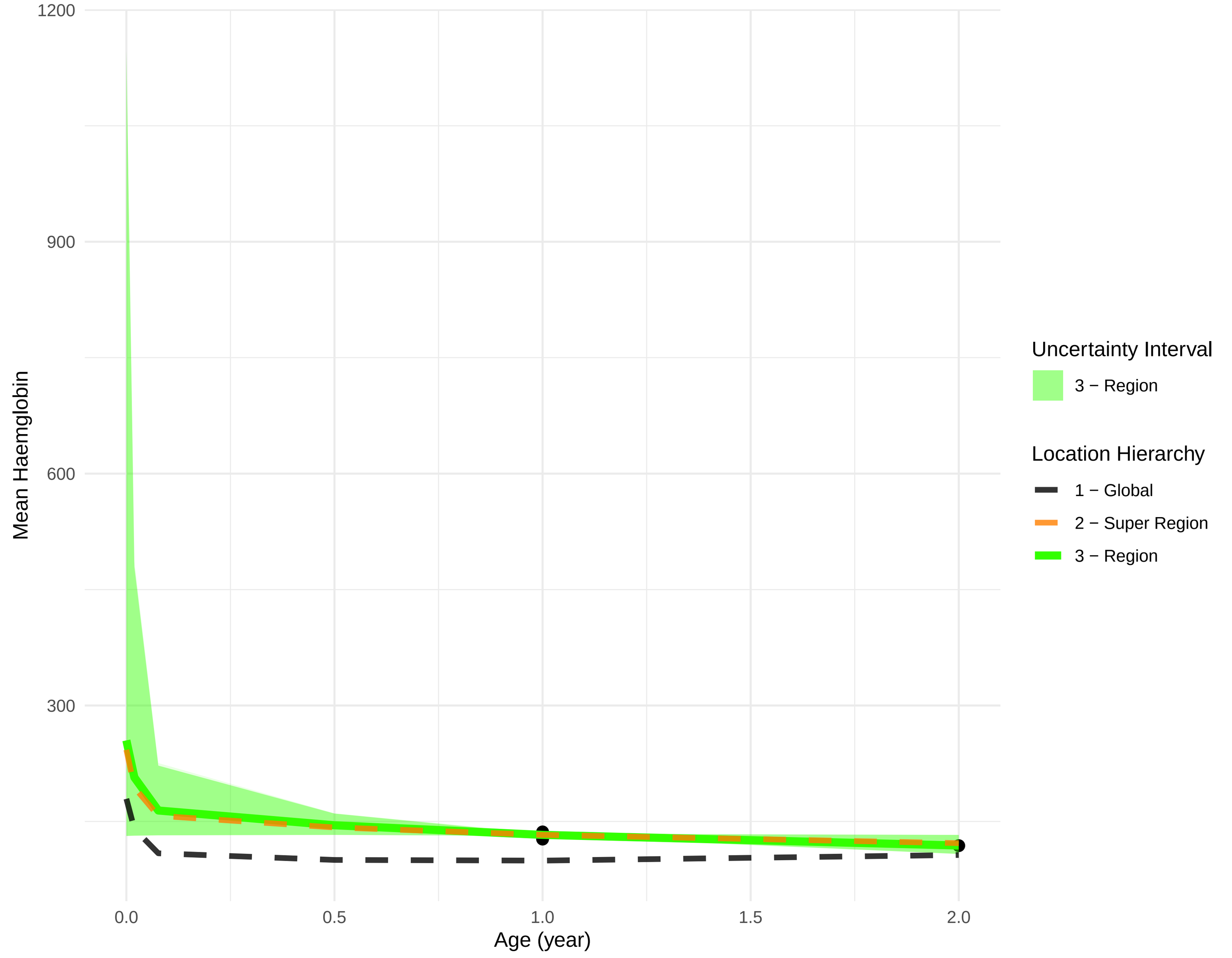

MR−BRT Age/Sex Fits − Non−pregnant Females <2 Years Old − Mean Haemglobin − United Kingdom
WHO−2024 Adjustment Method

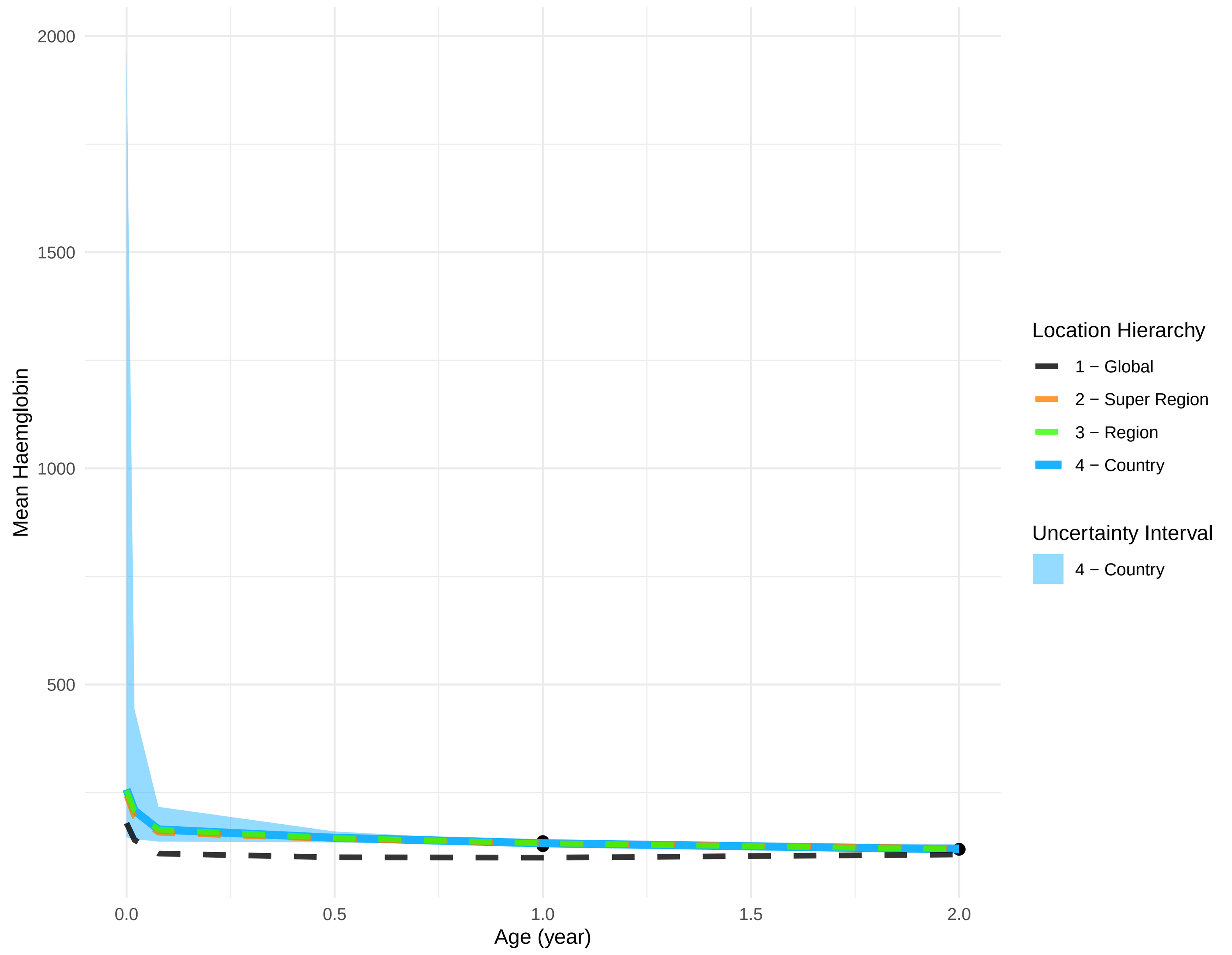

MR−BRT Age/Sex Fits − Non−pregnant Females <2 Years Old − Mean Haemglobin − England
WHO−2024 Adjustment Method

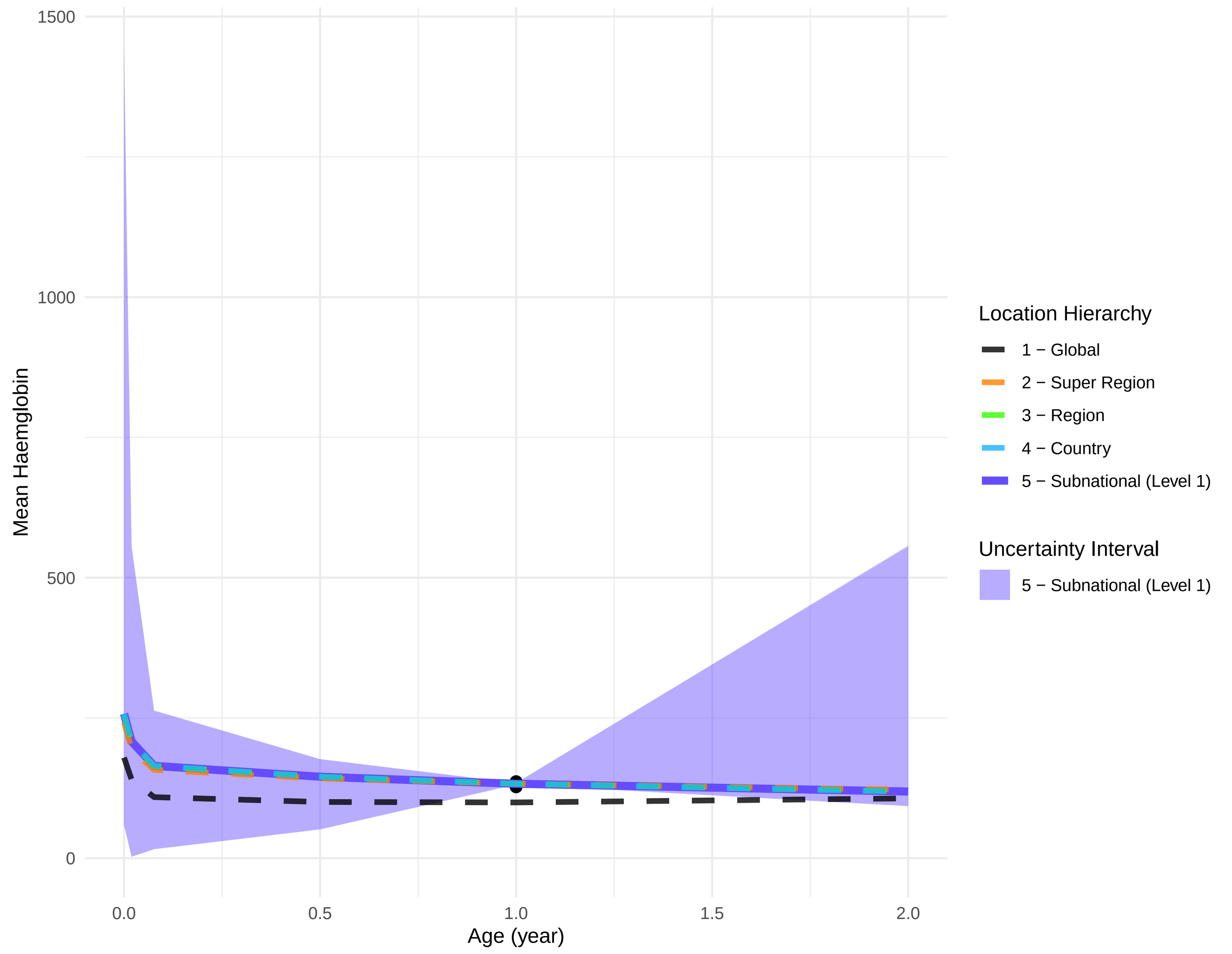

MR−BRT Age/Sex Fits − Non−pregnant Females <2 Years Old − Mean Haemglobin − East Midlands
WHO−2024 Adjustment Method

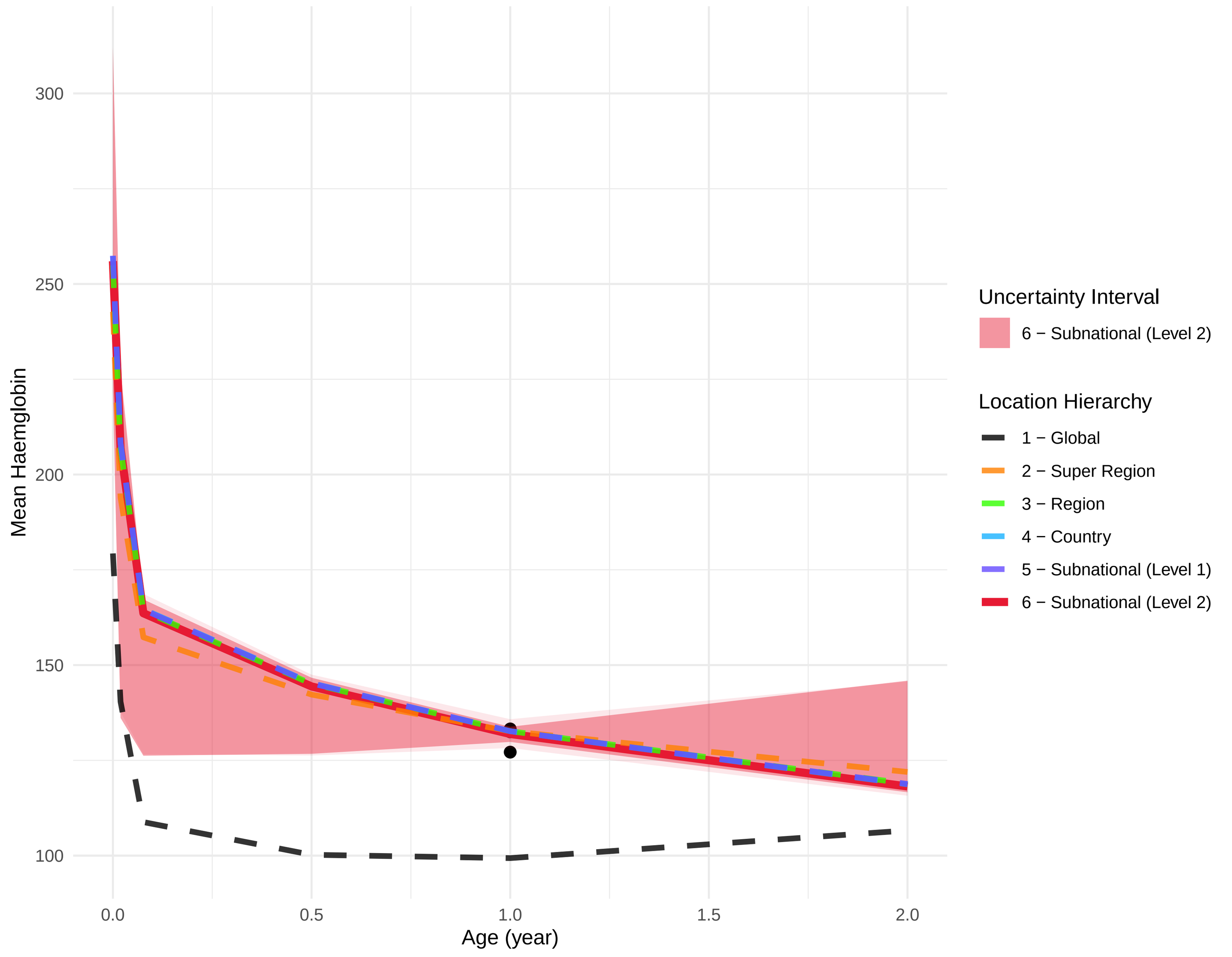

MR−BRT Age/Sex Fits − Non−pregnant Females <2 Years Old − Mean Haemglobin − East of England
WHO−2024 Adjustment Method

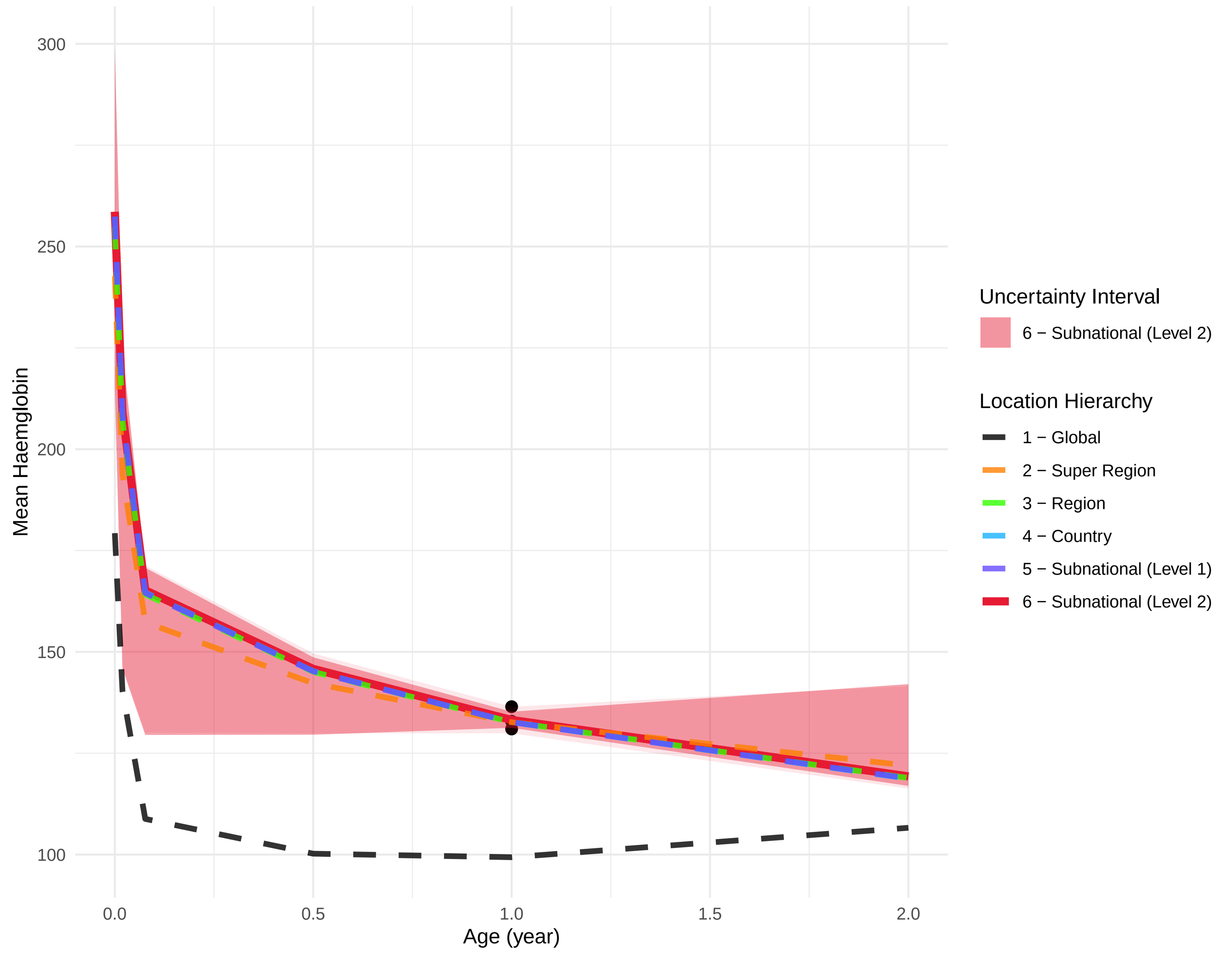

MR−BRT Age/Sex Fits − Non−pregnant Females <2 Years Old − Mean Haemglobin − Greater London
WHO−2024 Adjustment Method

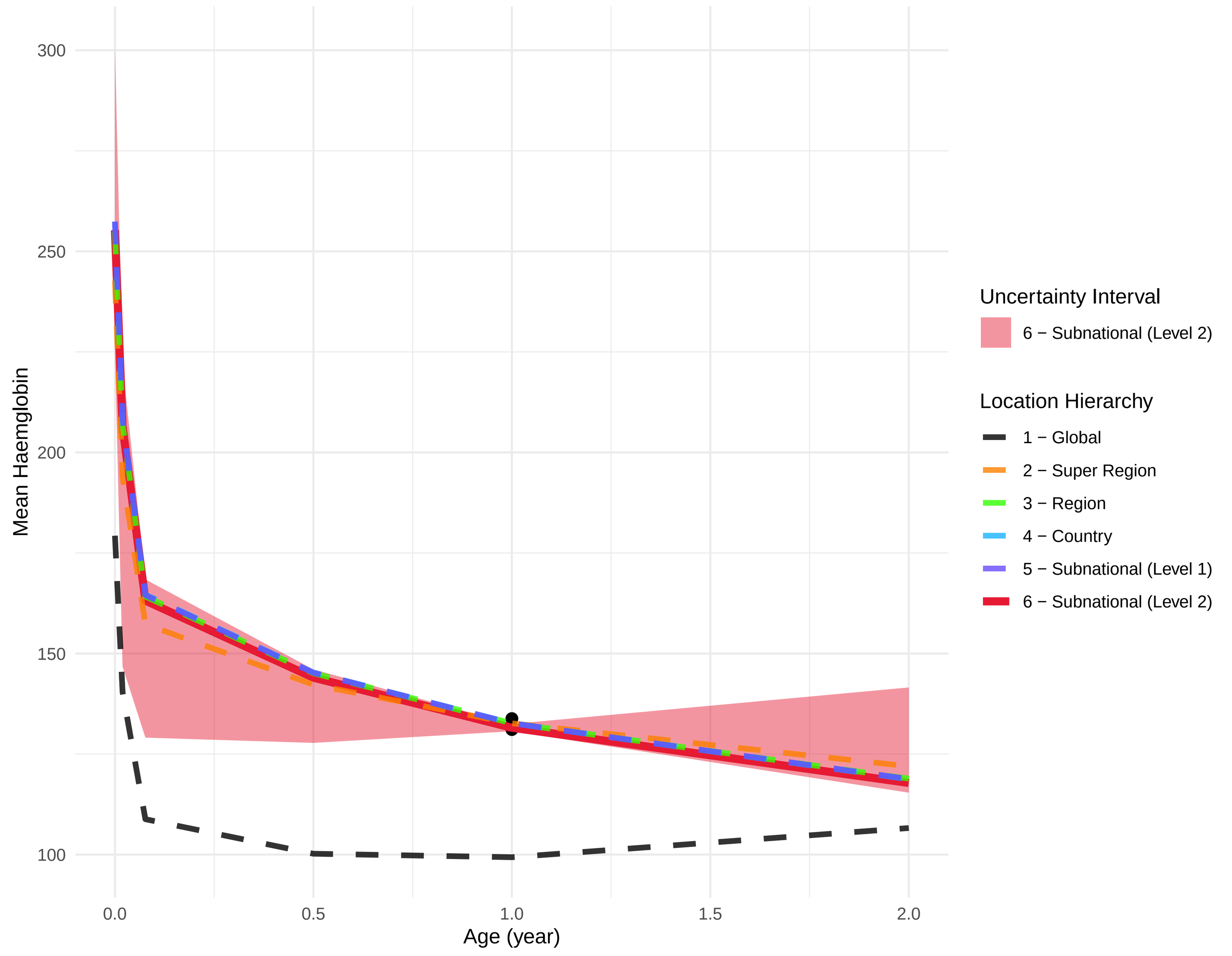

MR−BRT Age/Sex Fits − Non−pregnant Females <2 Years Old − Mean Haemglobin − North East England
WHO−2024 Adjustment Method

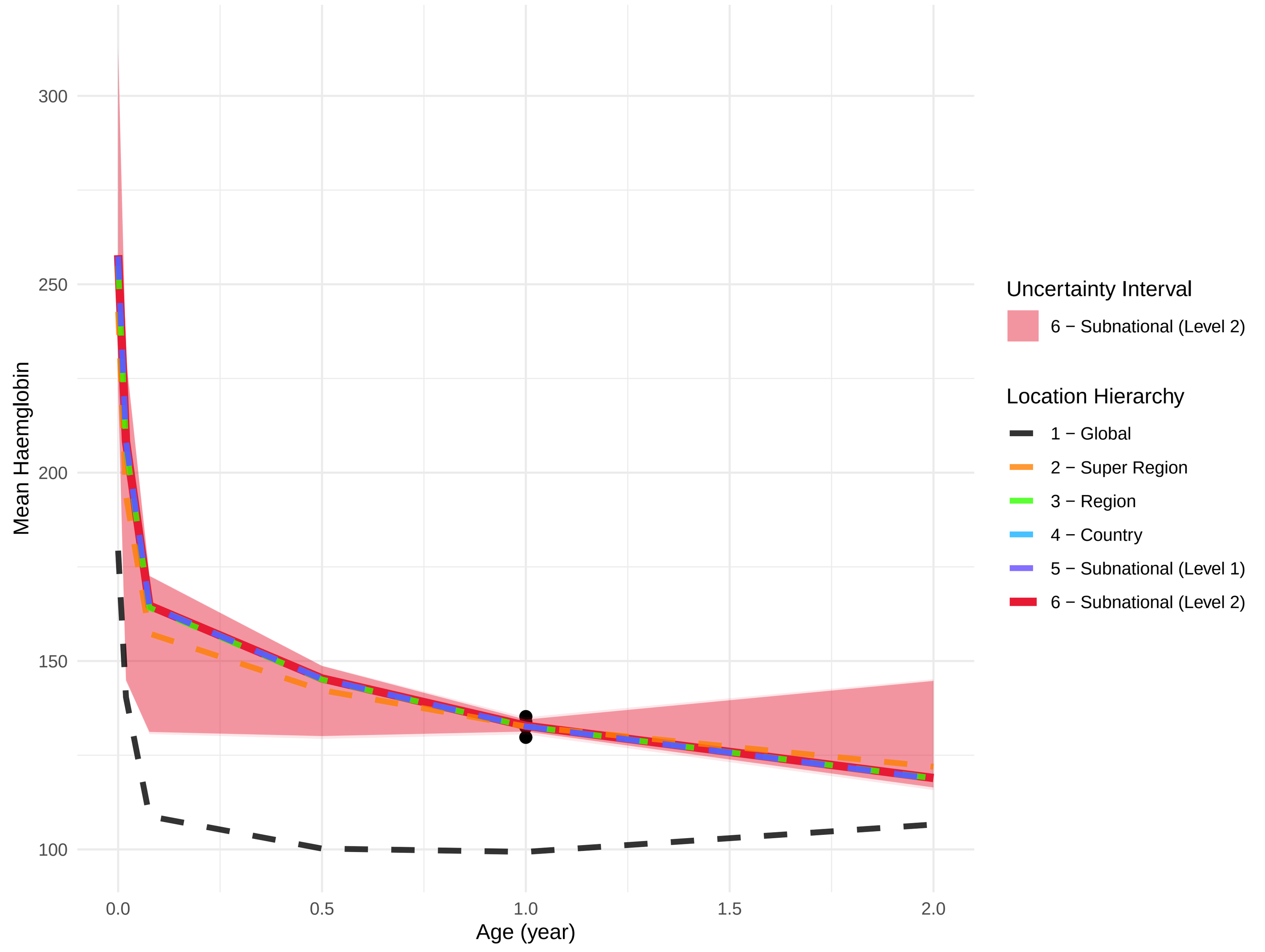

MR−BRT Age/Sex Fits − Non−pregnant Females <2 Years Old − Mean Haemglobin − North West England
WHO−2024 Adjustment Method

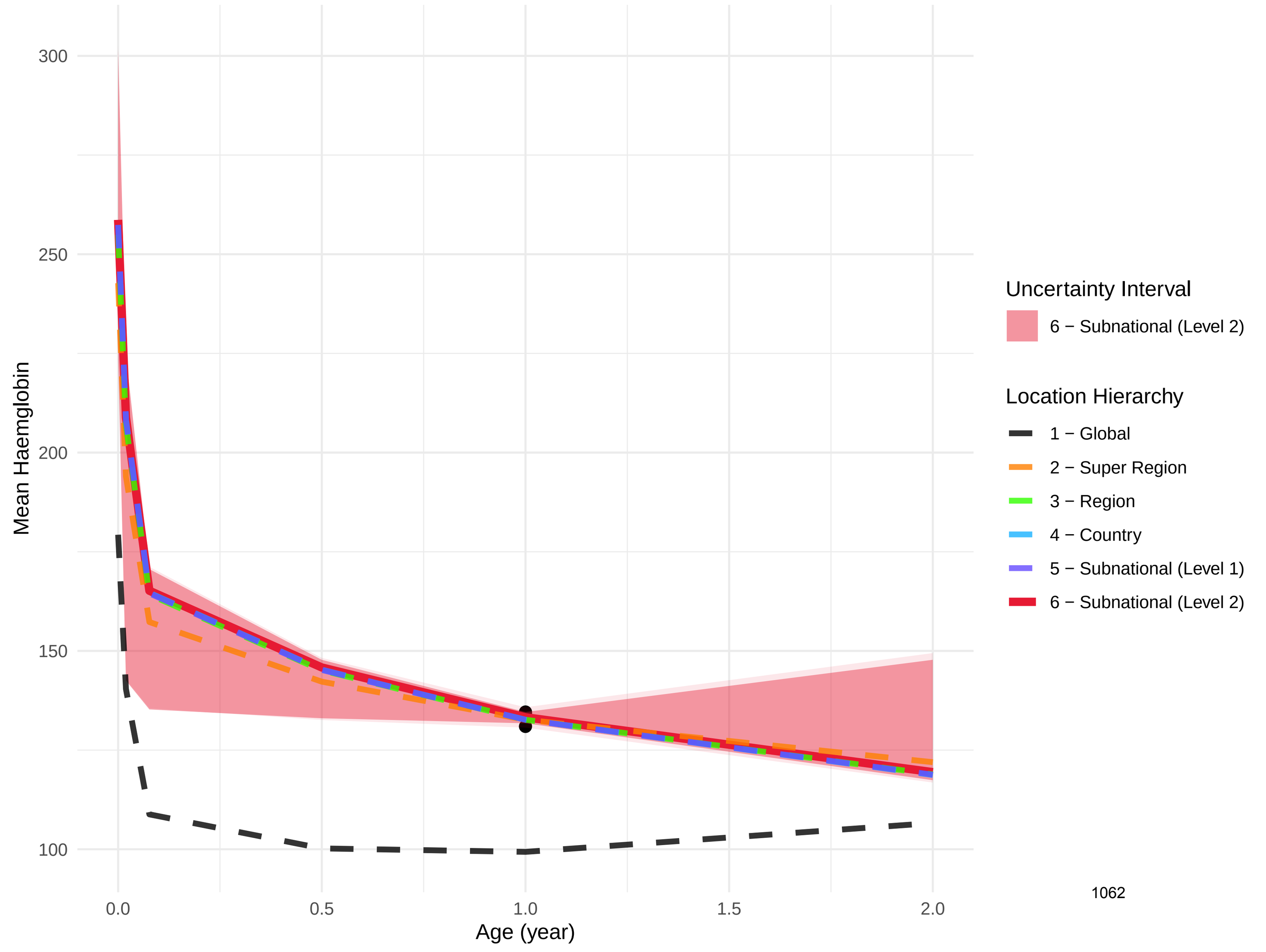

MR−BRT Age/Sex Fits − Non−pregnant Females <2 Years Old − Mean Haemglobin − South East England
WHO−2024 Adjustment Method

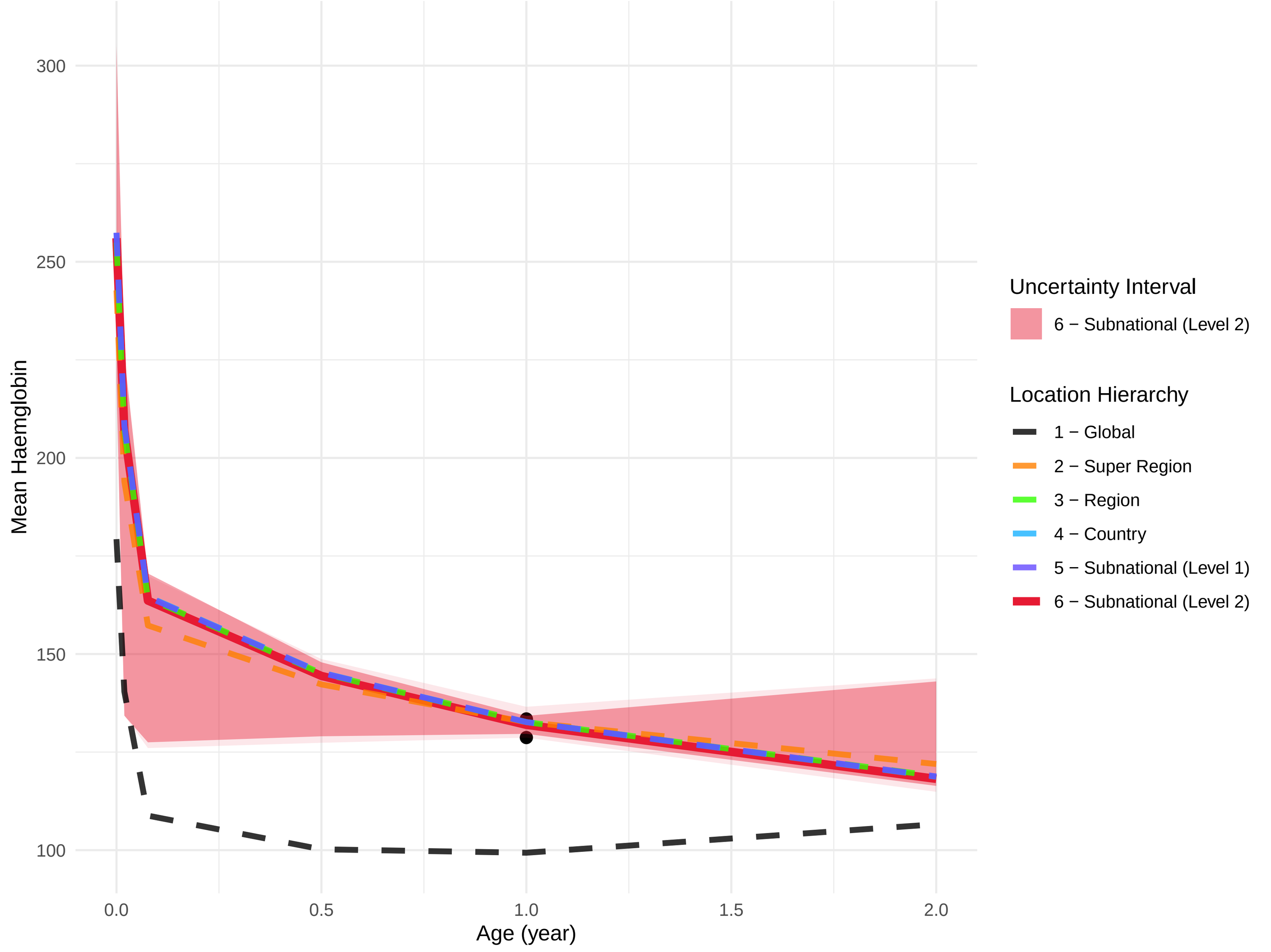

MR−BRT Age/Sex Fits − Non−pregnant Females <2 Years Old − Mean Haemglobin − South West England
WHO−2024 Adjustment Method

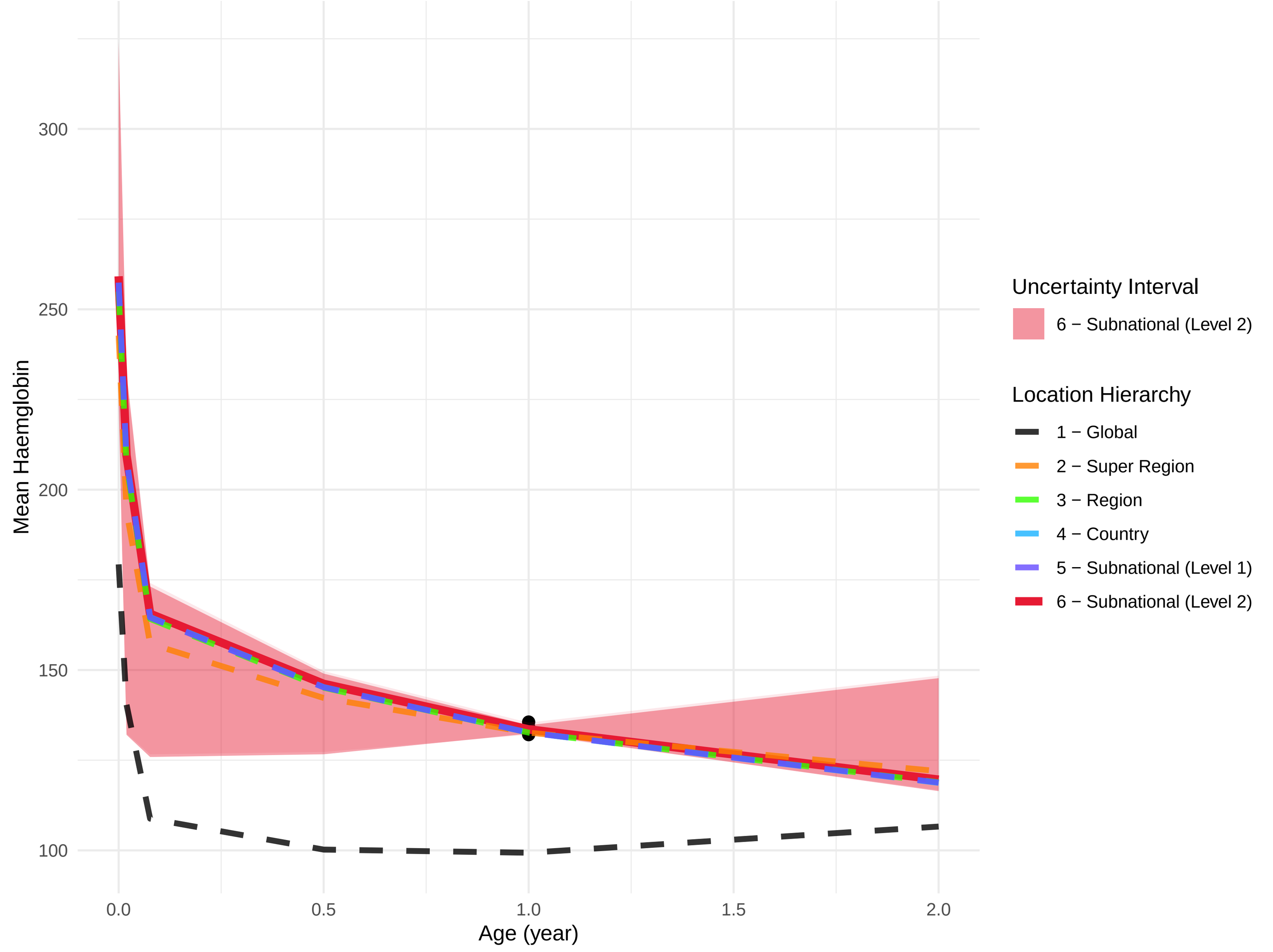

MR−BRT Age/Sex Fits − Non−pregnant Females <2 Years Old − Mean Haemglobin − West Midlands
WHO−2024 Adjustment Method

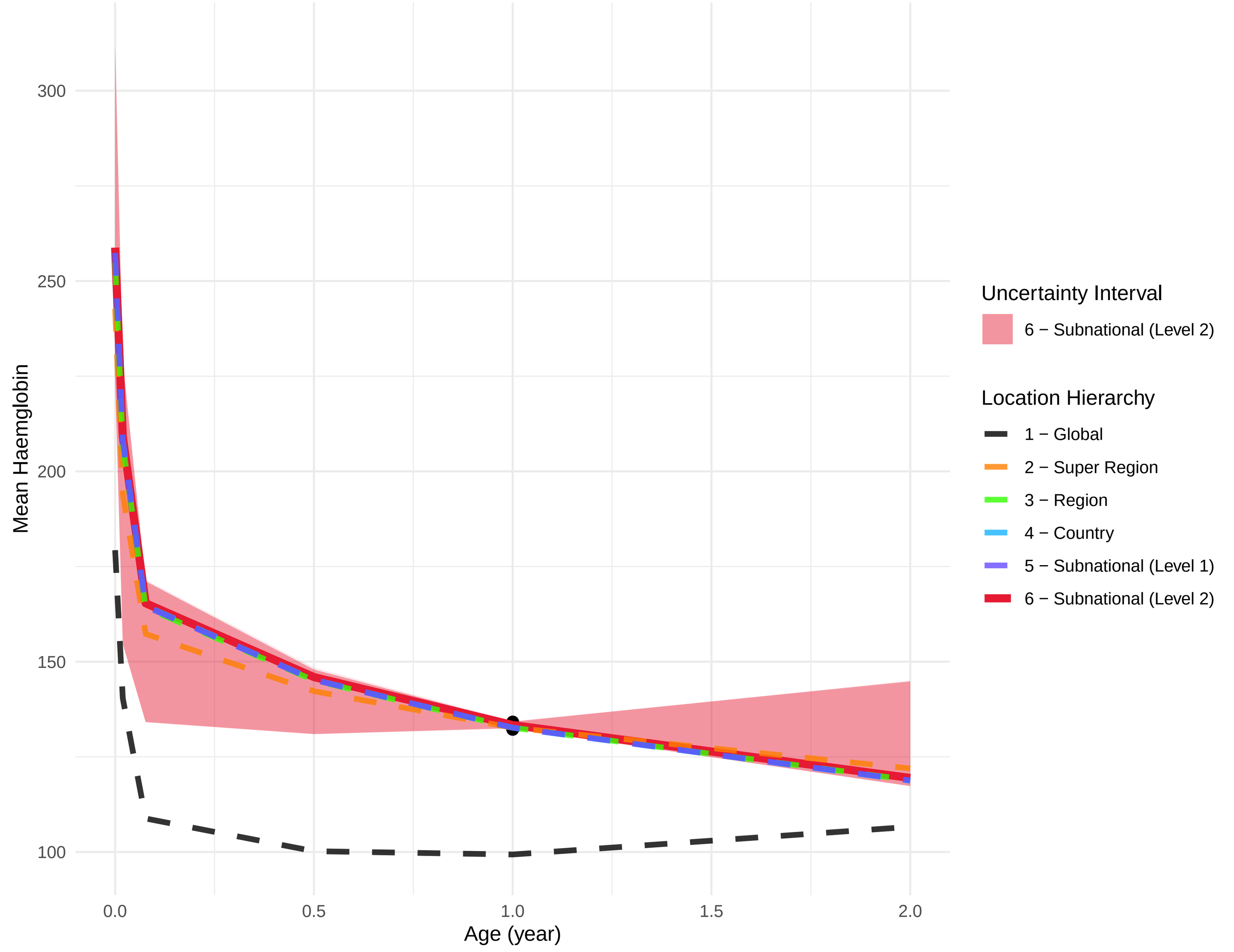

MR−BRT Age/Sex Fits − Non−pregnant Females <2 Years Old − Mean Haemglobin − Yorkshire and the Humber
WHO−2024 Adjustment Method

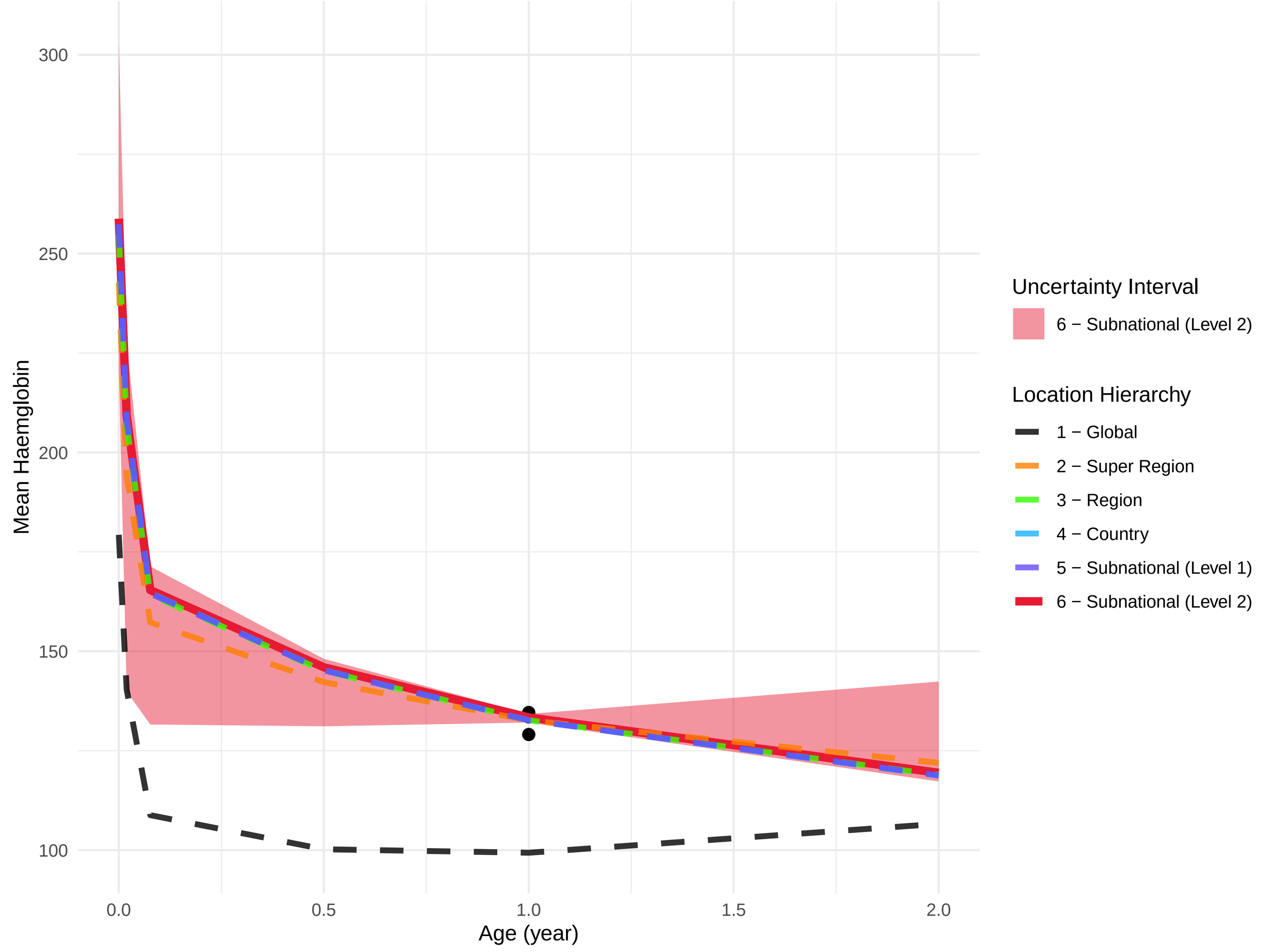

MR−BRT Age/Sex Fits − Non−pregnant Females <2 Years Old − Mean Haemglobin − Latin America and Caribbean
WHO−2024 Adjustment Method

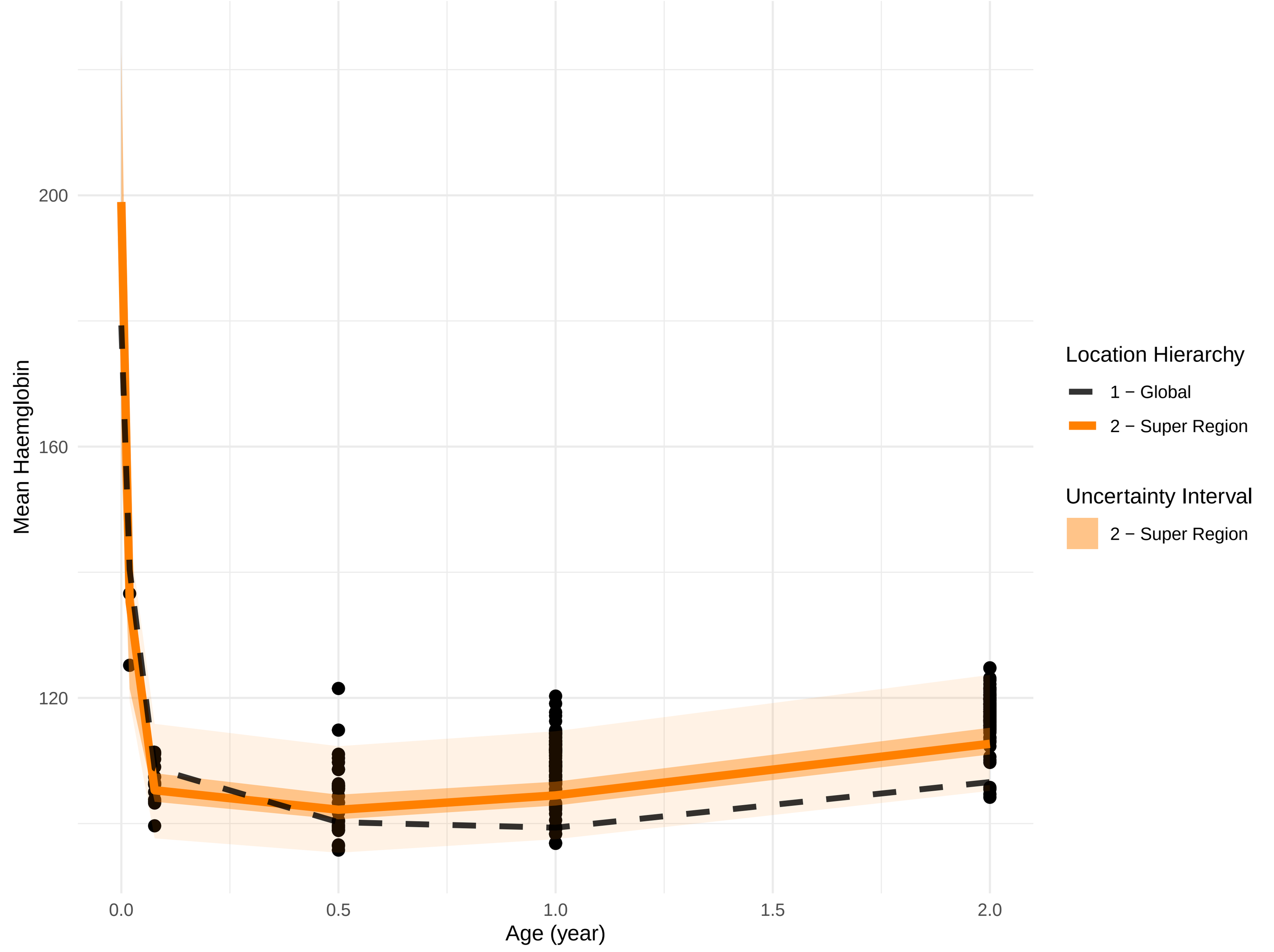

MR−BRT Age/Sex Fits − Non−pregnant Females <2 Years Old − Mean Haemglobin − Andean Latin America
WHO−2024 Adjustment Method

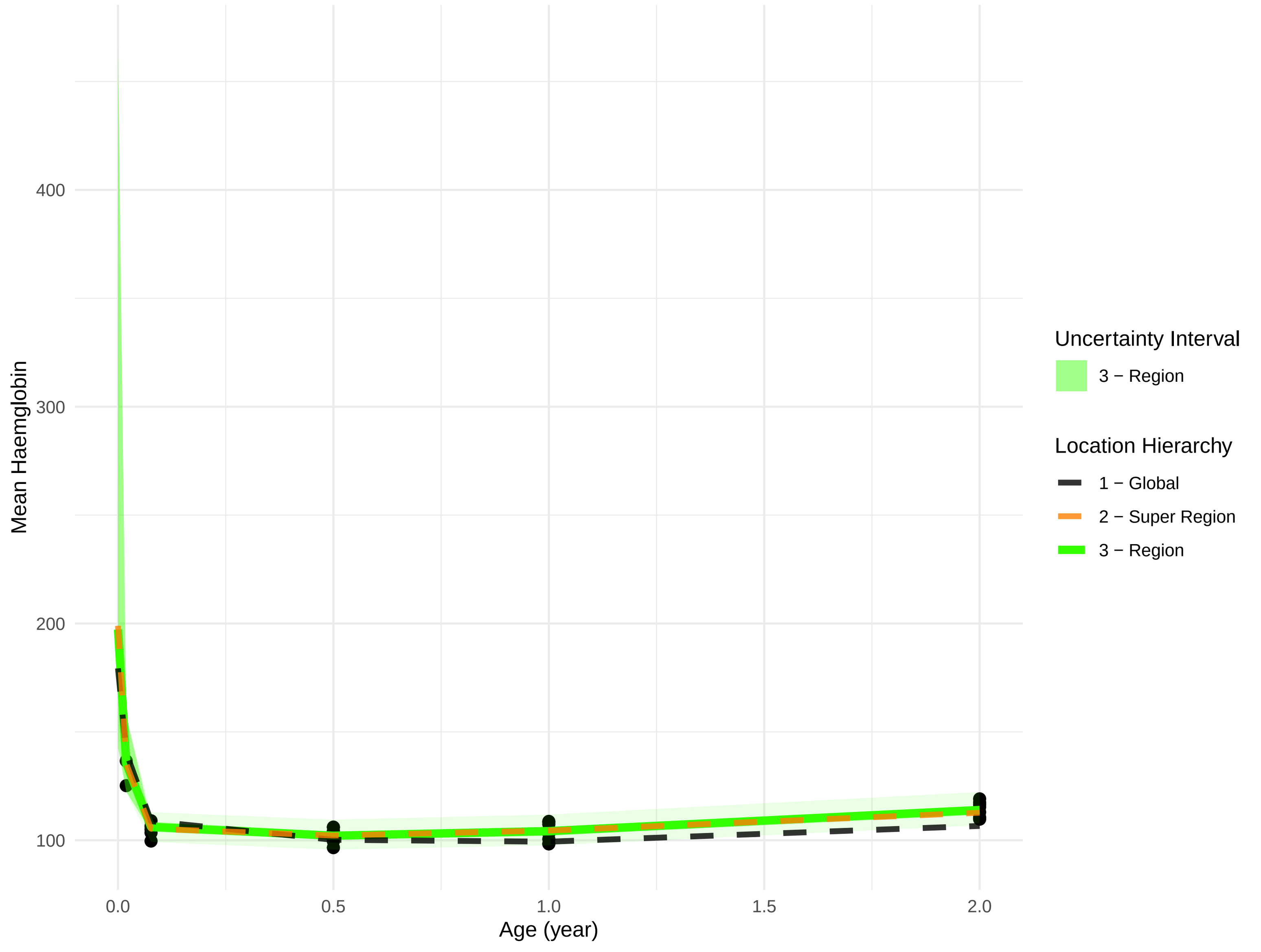

MR−BRT Age/Sex Fits − Non−pregnant Females <2 Years Old − Mean Haemglobin − Bolivia (Plurinational State of)
WHO−2024 Adjustment Method

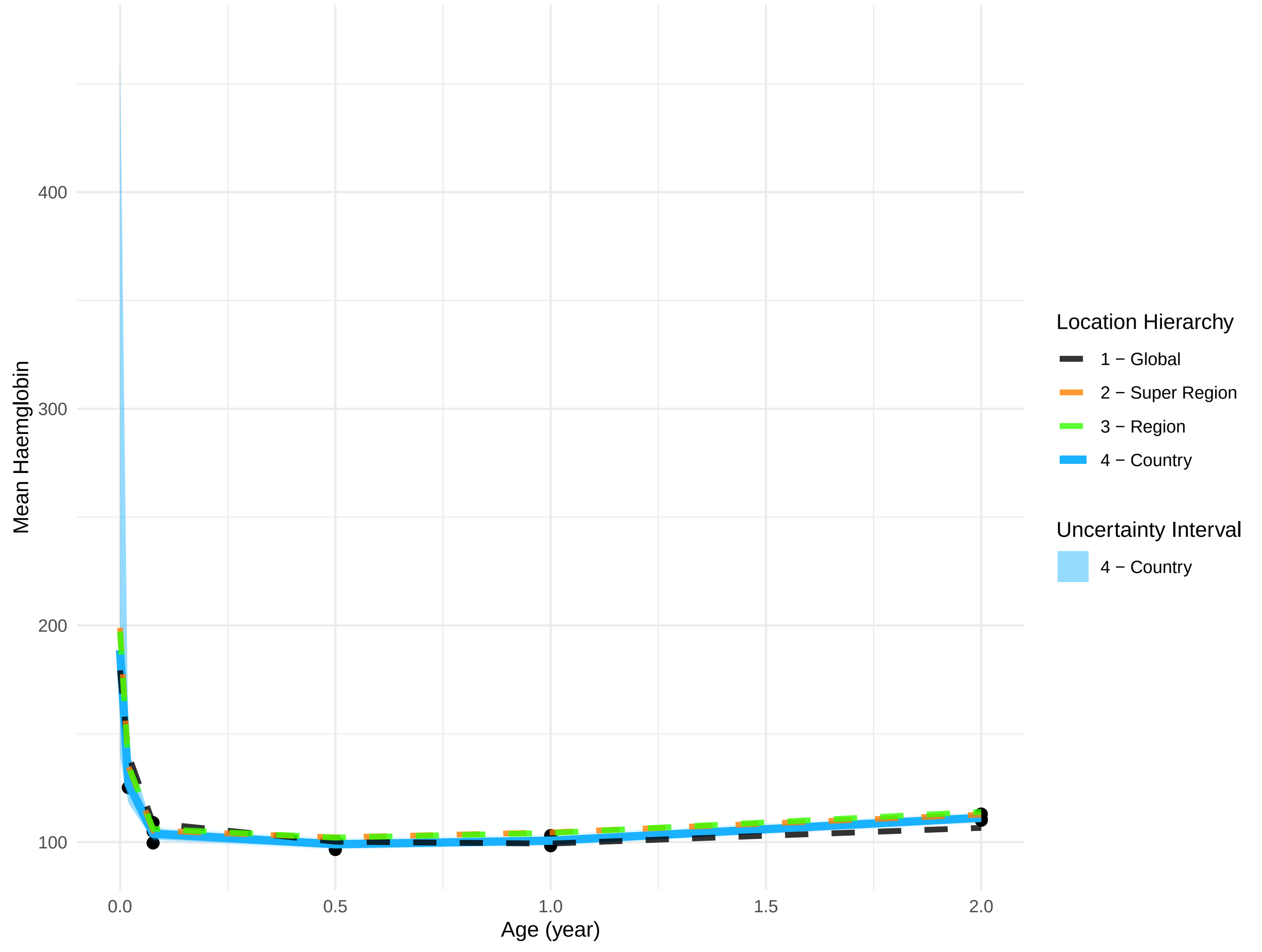

MR−BRT Age/Sex Fits − Non−pregnant Females <2 Years Old − Mean Haemglobin − Peru
WHO−2024 Adjustment Method

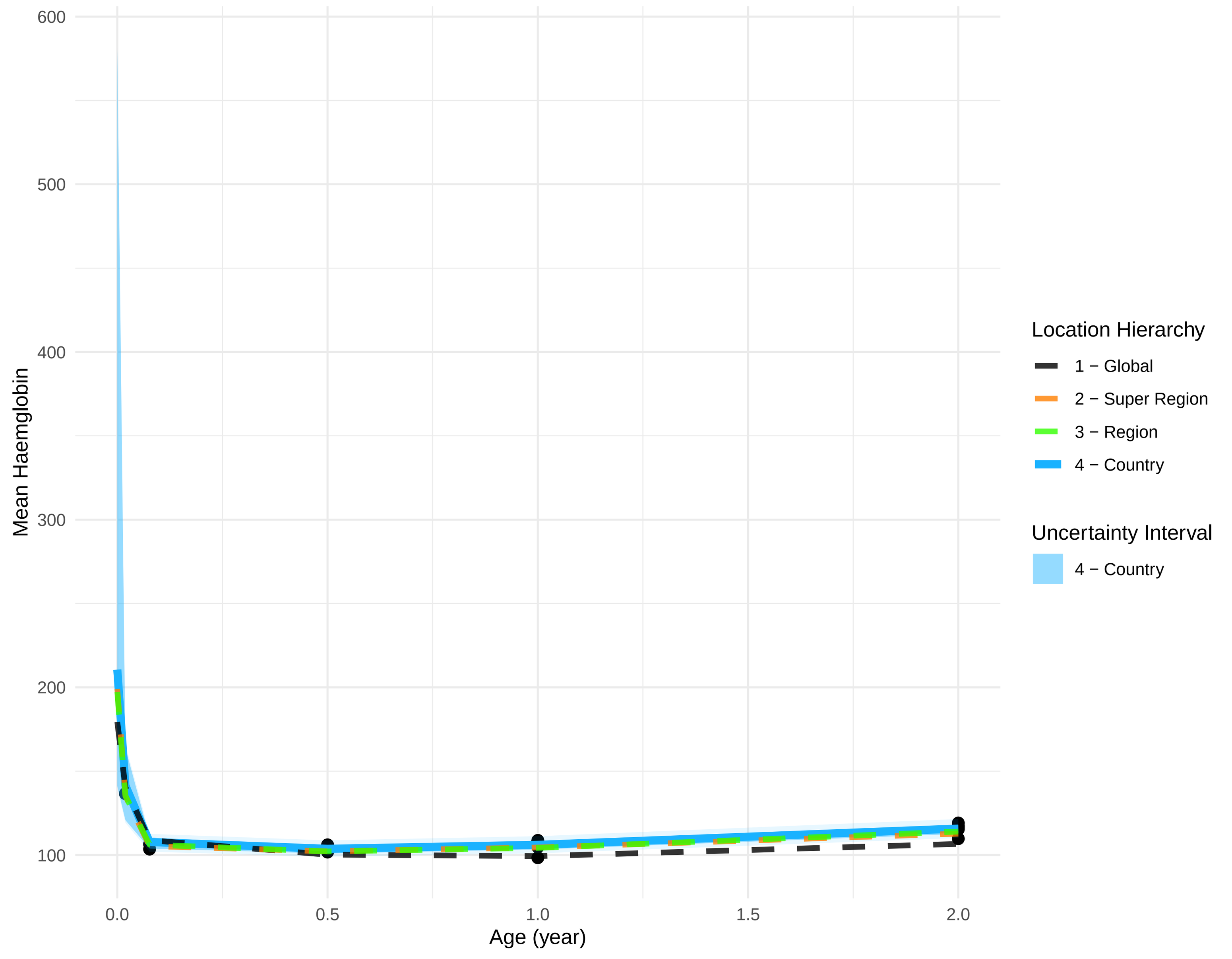

MR−BRT Age/Sex Fits − Non−pregnant Females <2 Years Old − Mean Haemglobin − Caribbean
WHO−2024 Adjustment Method

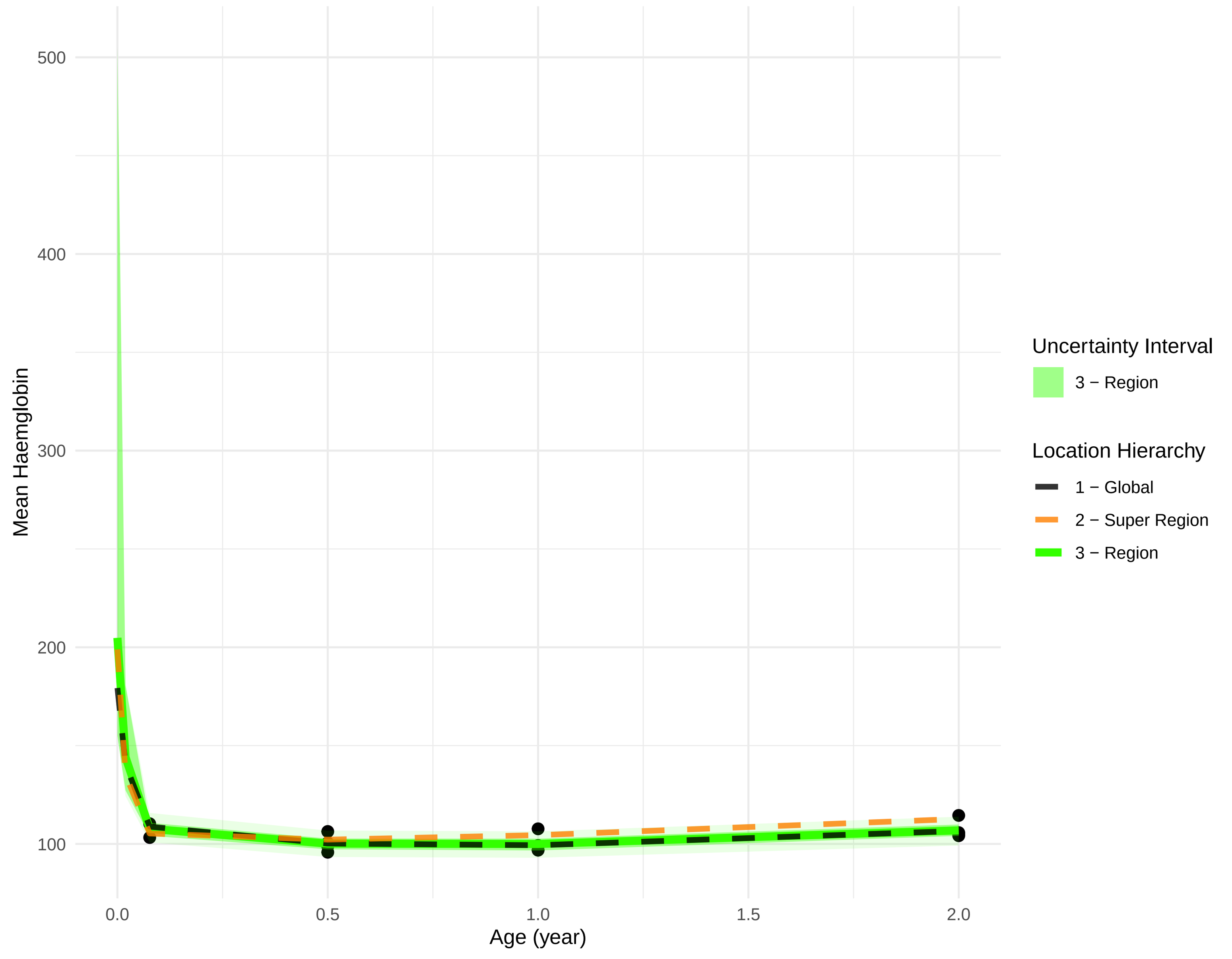

MR−BRT Age/Sex Fits − Non−pregnant Females <2 Years Old − Mean Haemglobin − Guyana
WHO−2024 Adjustment Method

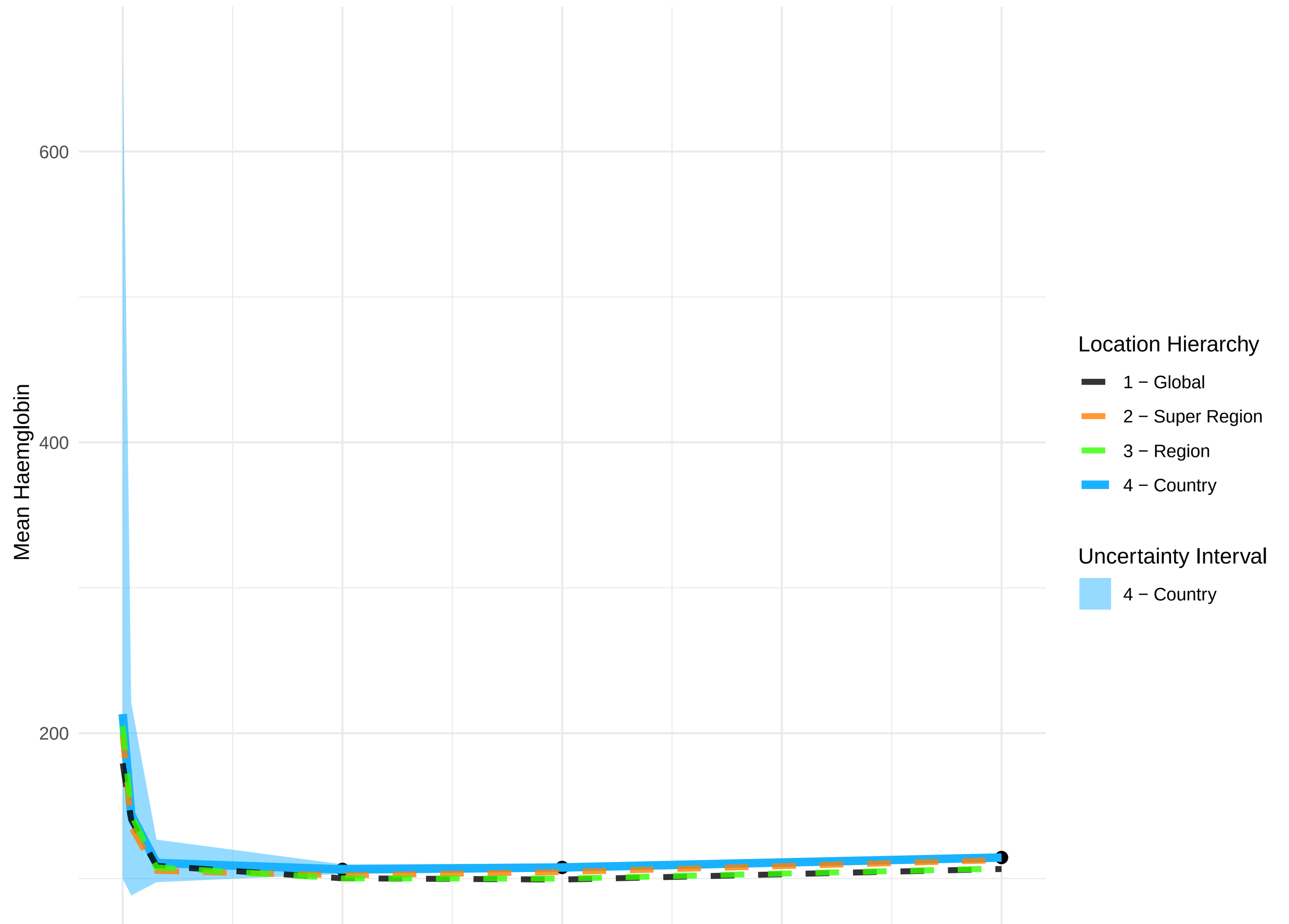

MR−BRT Age/Sex Fits − Non−pregnant Females <2 Years Old − Mean Haemglobin − Haiti
WHO−2024 Adjustment Method

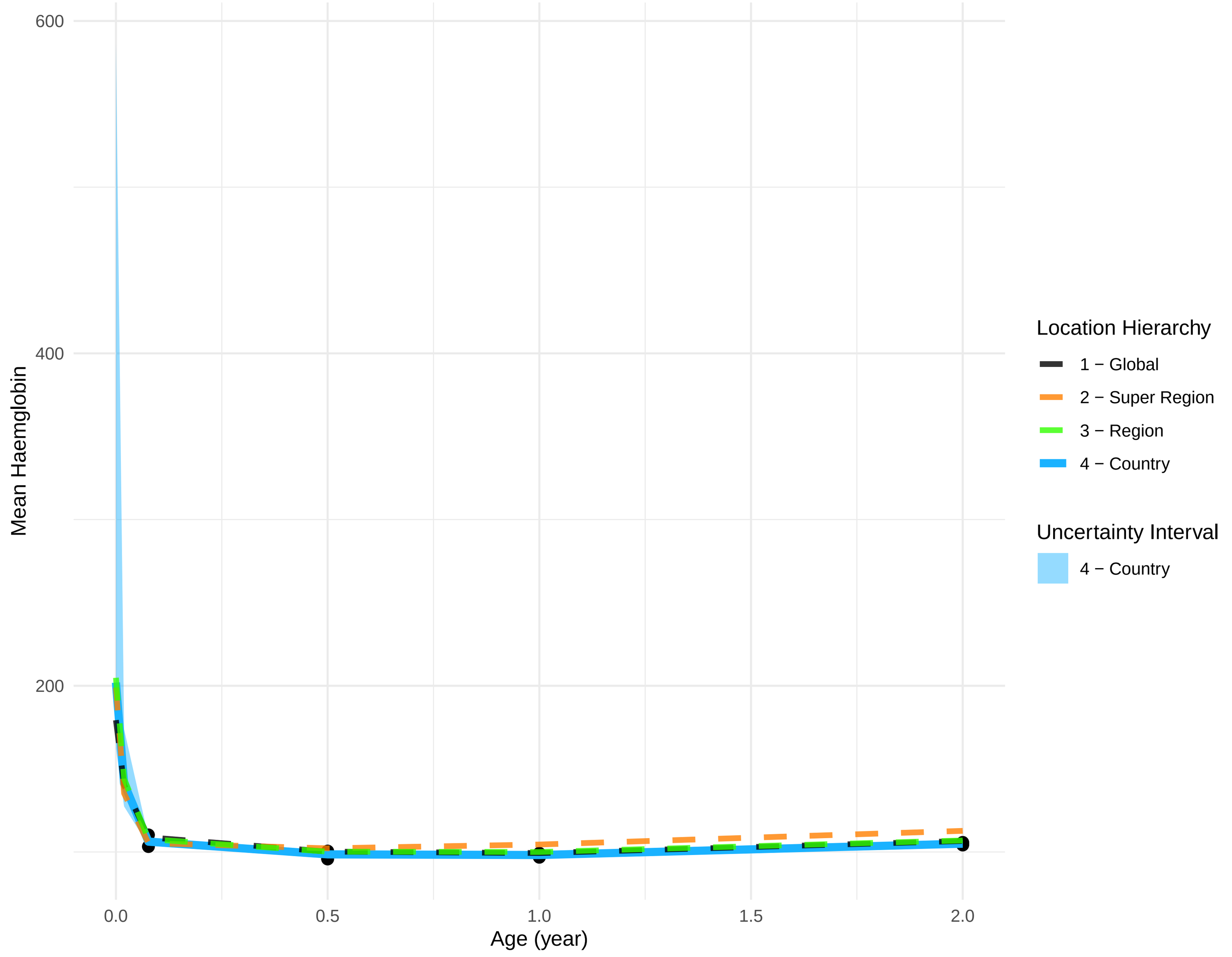

MR−BRT Age/Sex Fits − Non−pregnant Females <2 Years Old − Mean Haemglobin − Central Latin America
WHO−2024 Adjustment Method

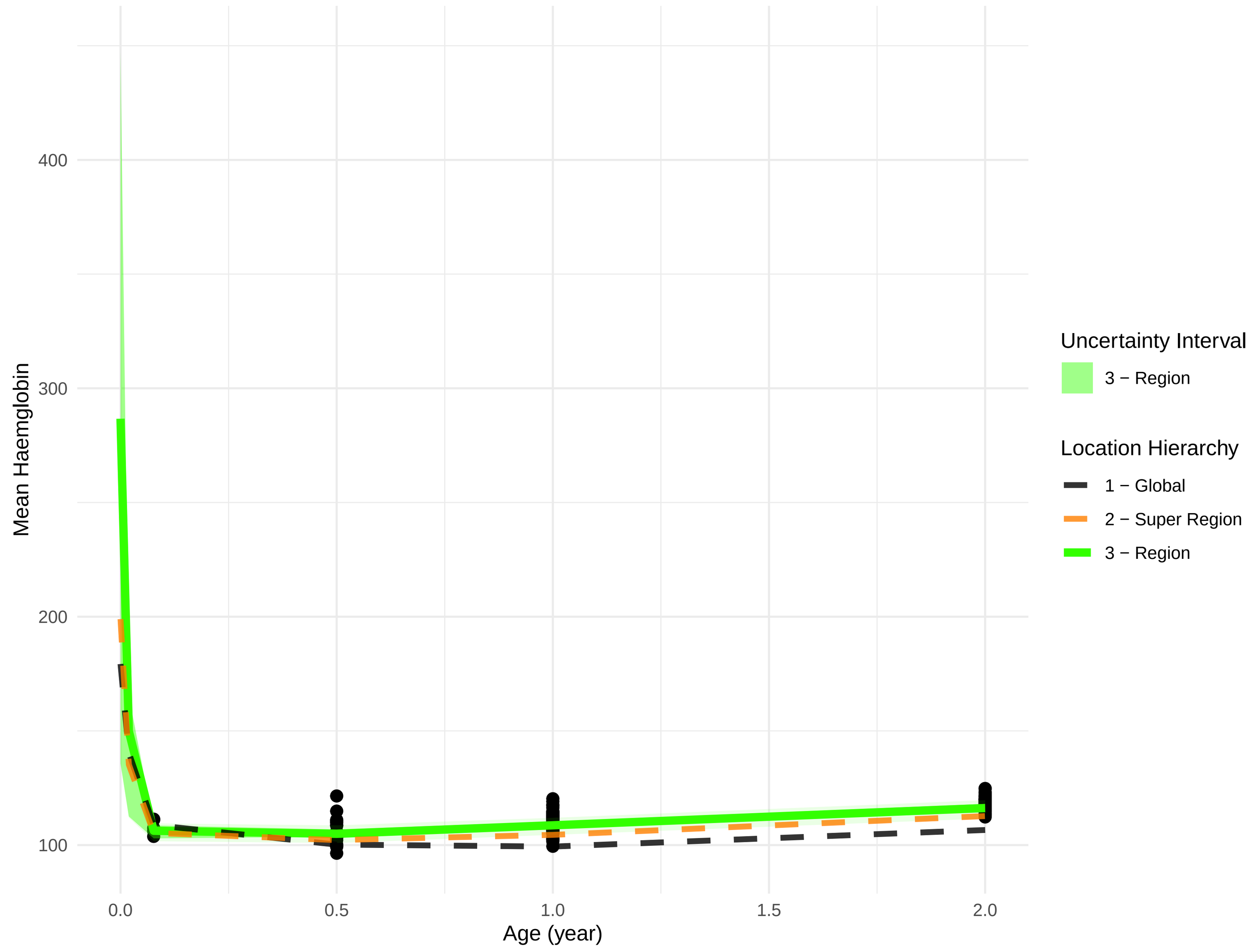

MR−BRT Age/Sex Fits − Non−pregnant Females <2 Years Old − Mean Haemglobin − Guatemala
WHO−2024 Adjustment Method

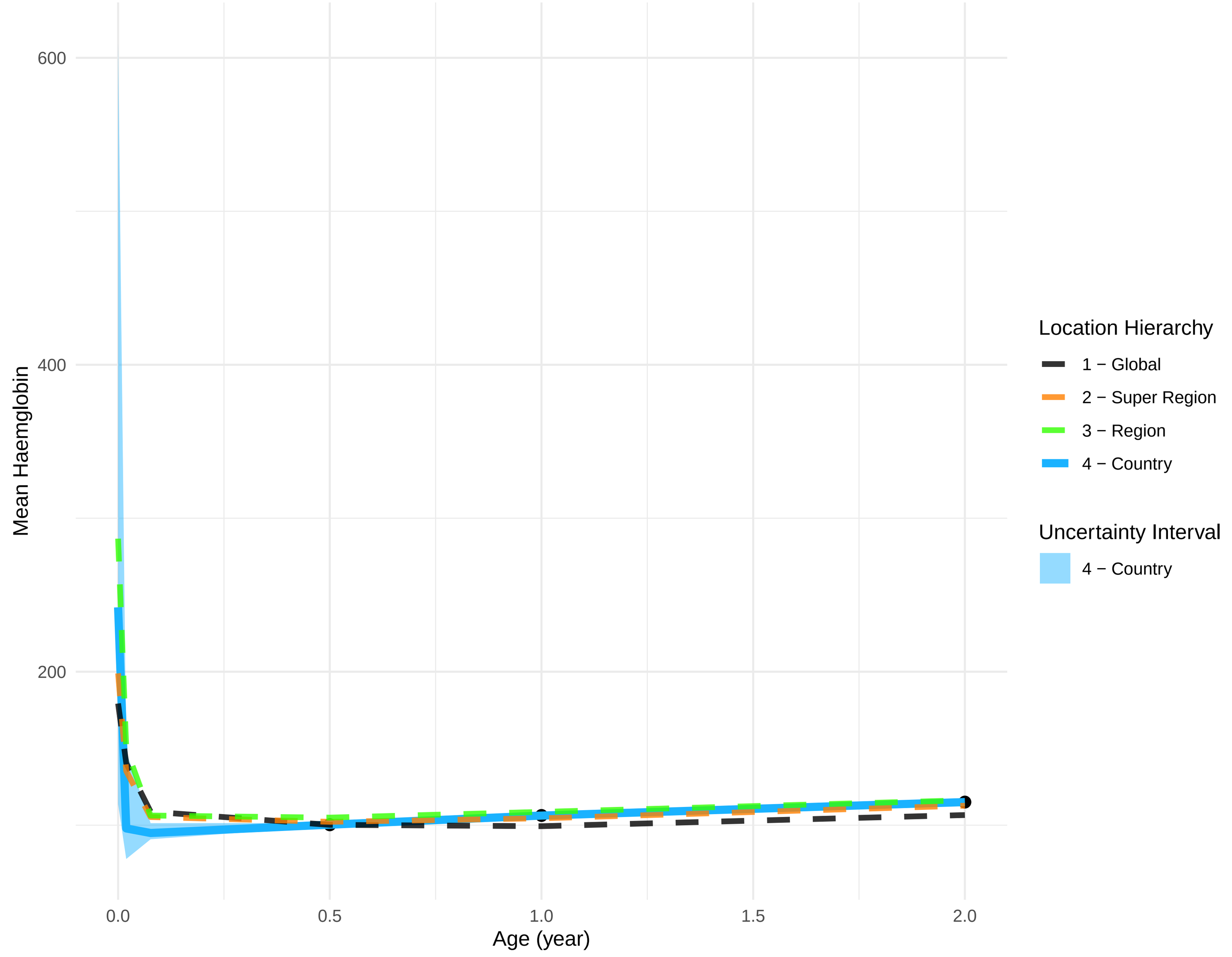

MR−BRT Age/Sex Fits − Non−pregnant Females <2 Years Old − Mean Haemglobin − Honduras
WHO−2024 Adjustment Method

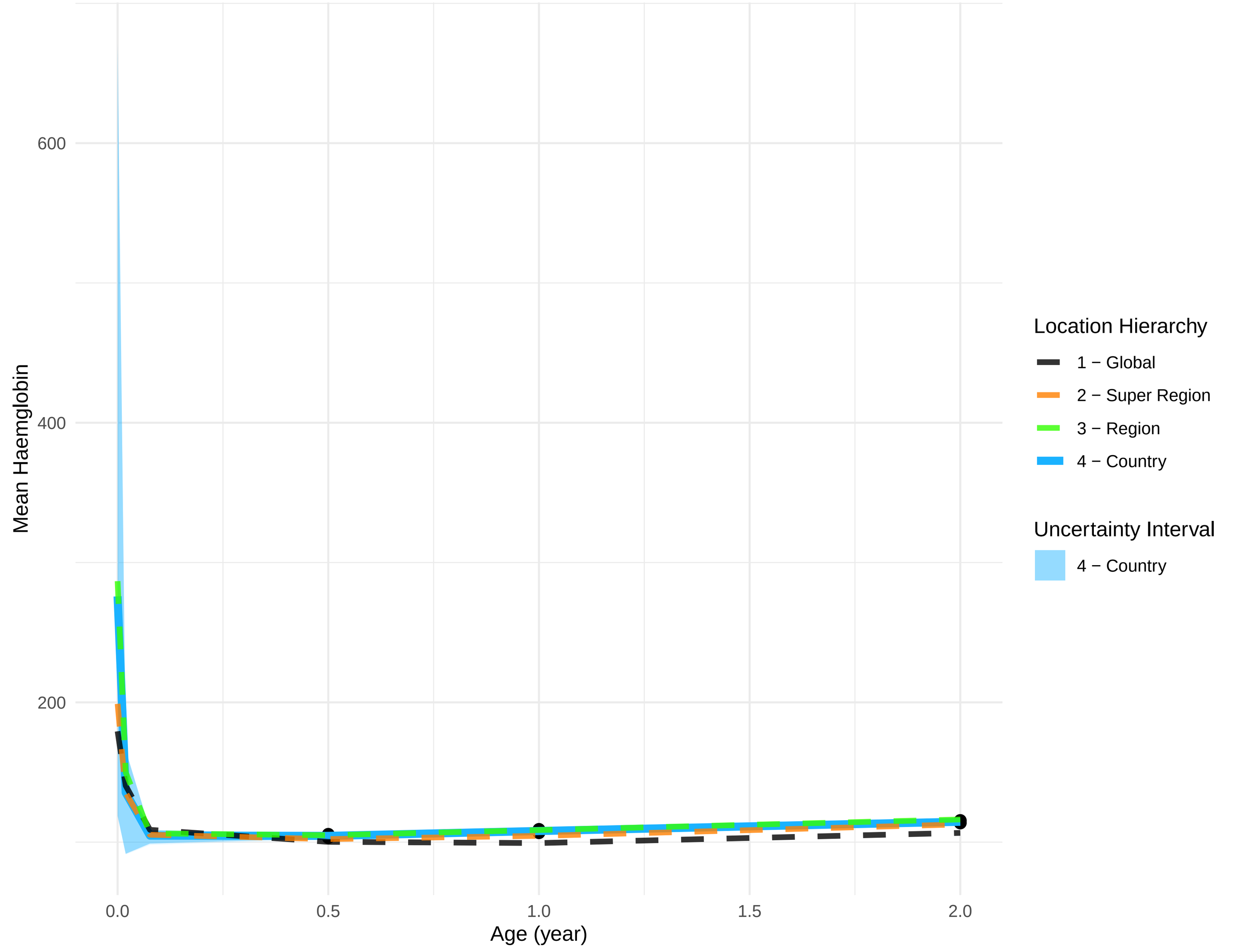

MR−BRT Age/Sex Fits − Non−pregnant Females <2 Years Old − Mean Haemglobin − Mexico
WHO−2024 Adjustment Method

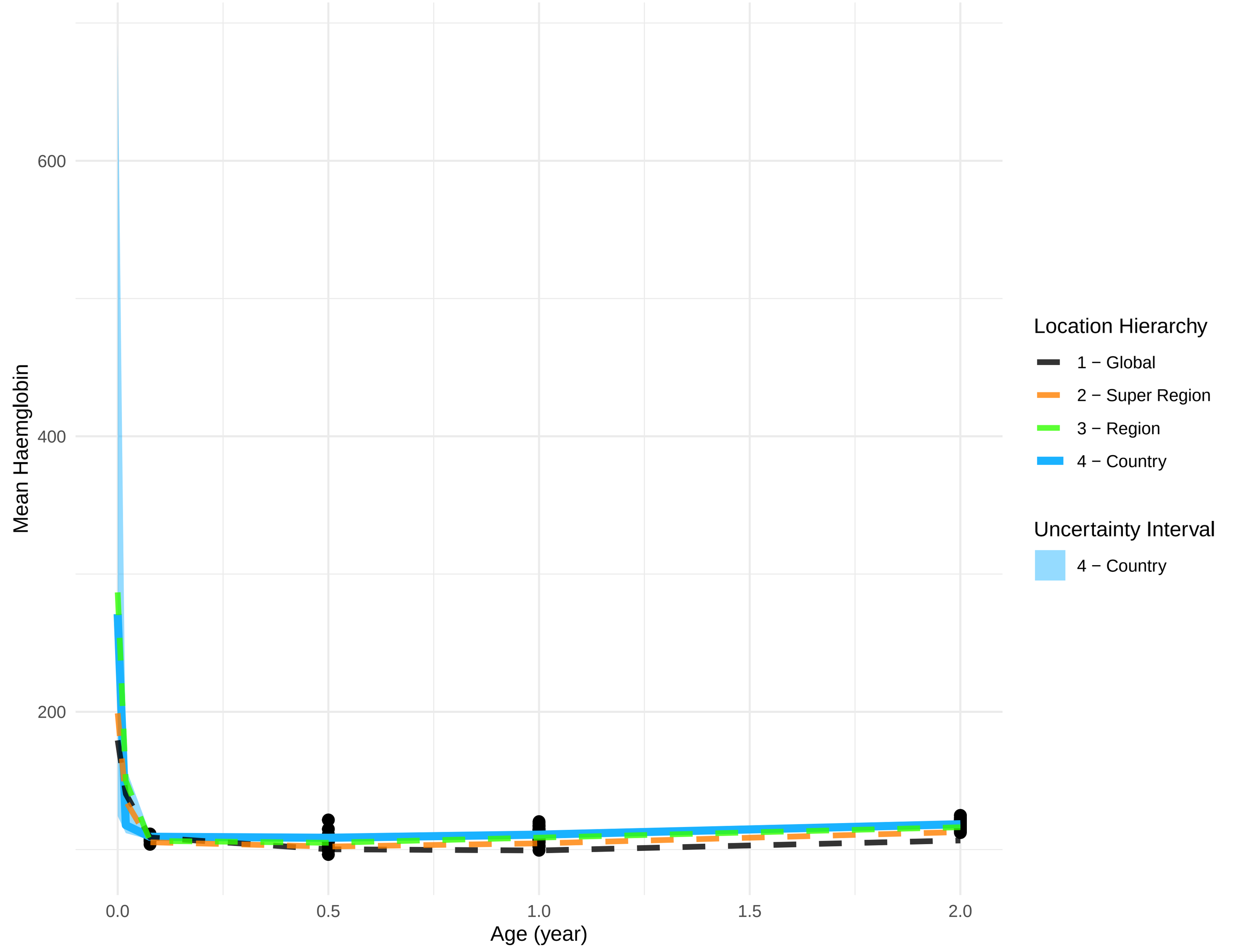

MR−BRT Age/Sex Fits − Non−pregnant Females <2 Years Old − Mean Haemglobin − Aguascalientes
WHO−2024 Adjustment Method

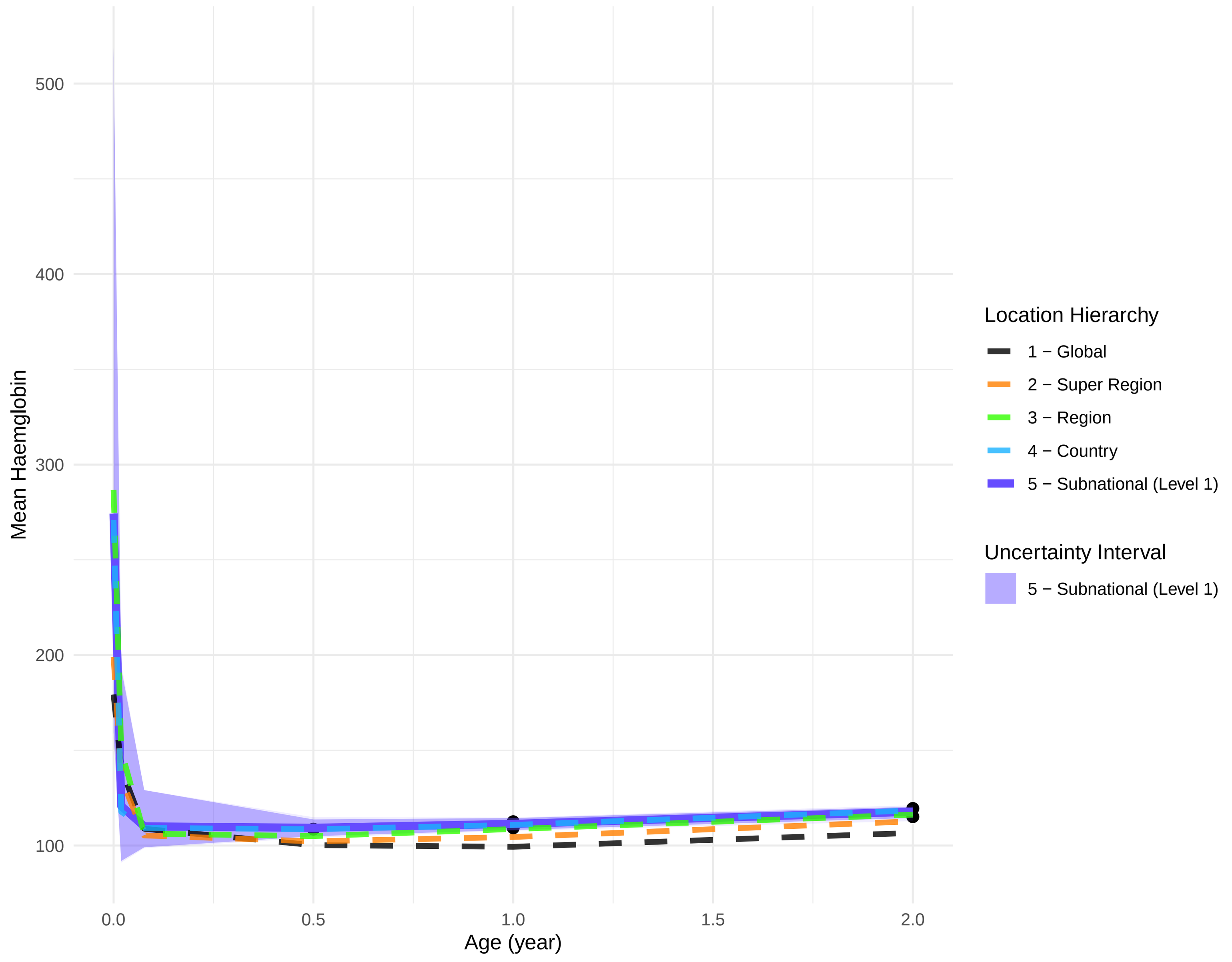

MR−BRT Age/Sex Fits − Non−pregnant Females <2 Years Old − Mean Haemglobin − Baja California
WHO−2024 Adjustment Method

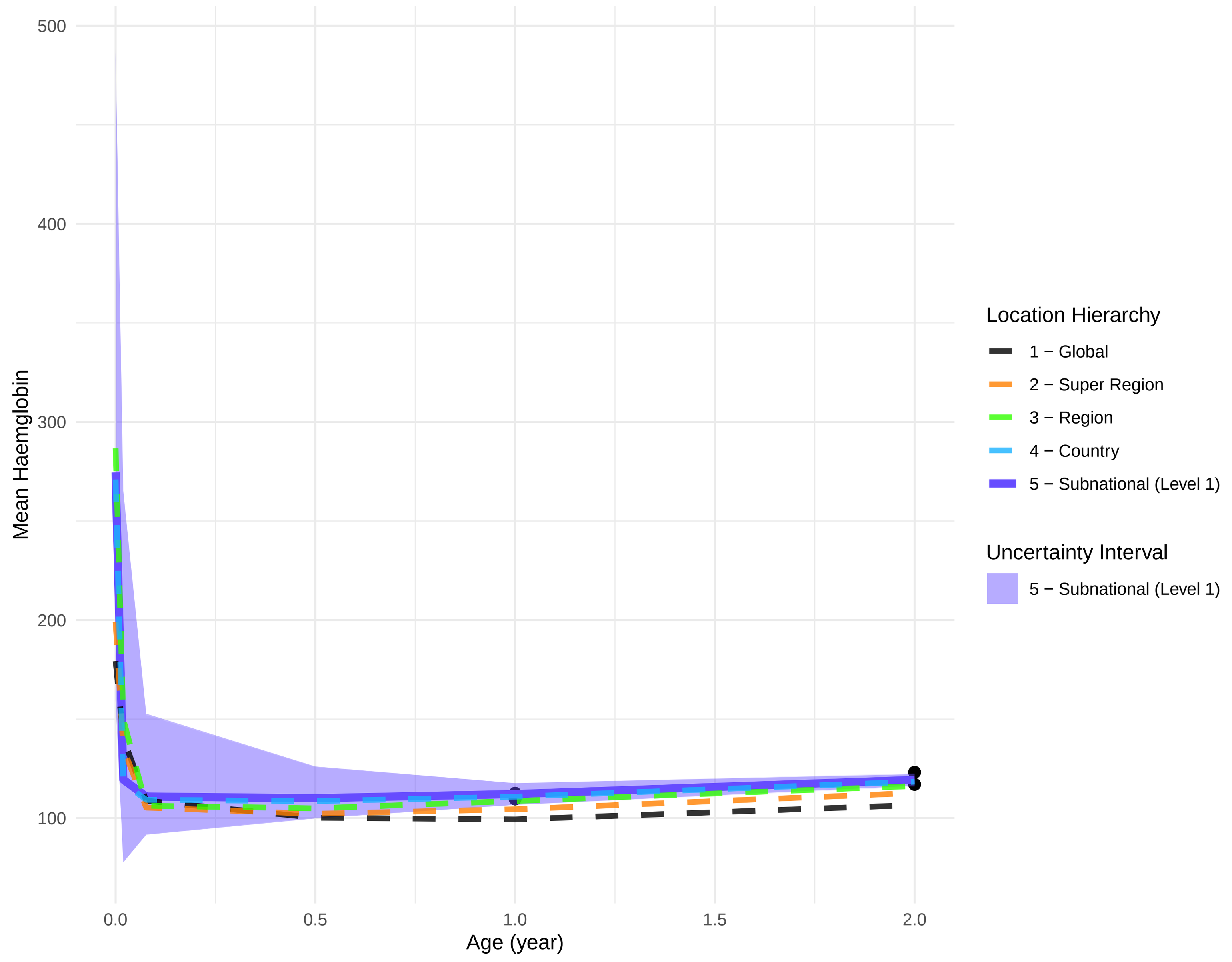

MR−BRT Age/Sex Fits − Non−pregnant Females <2 Years Old − Mean Haemglobin − Baja California Sur
WHO−2024 Adjustment Method

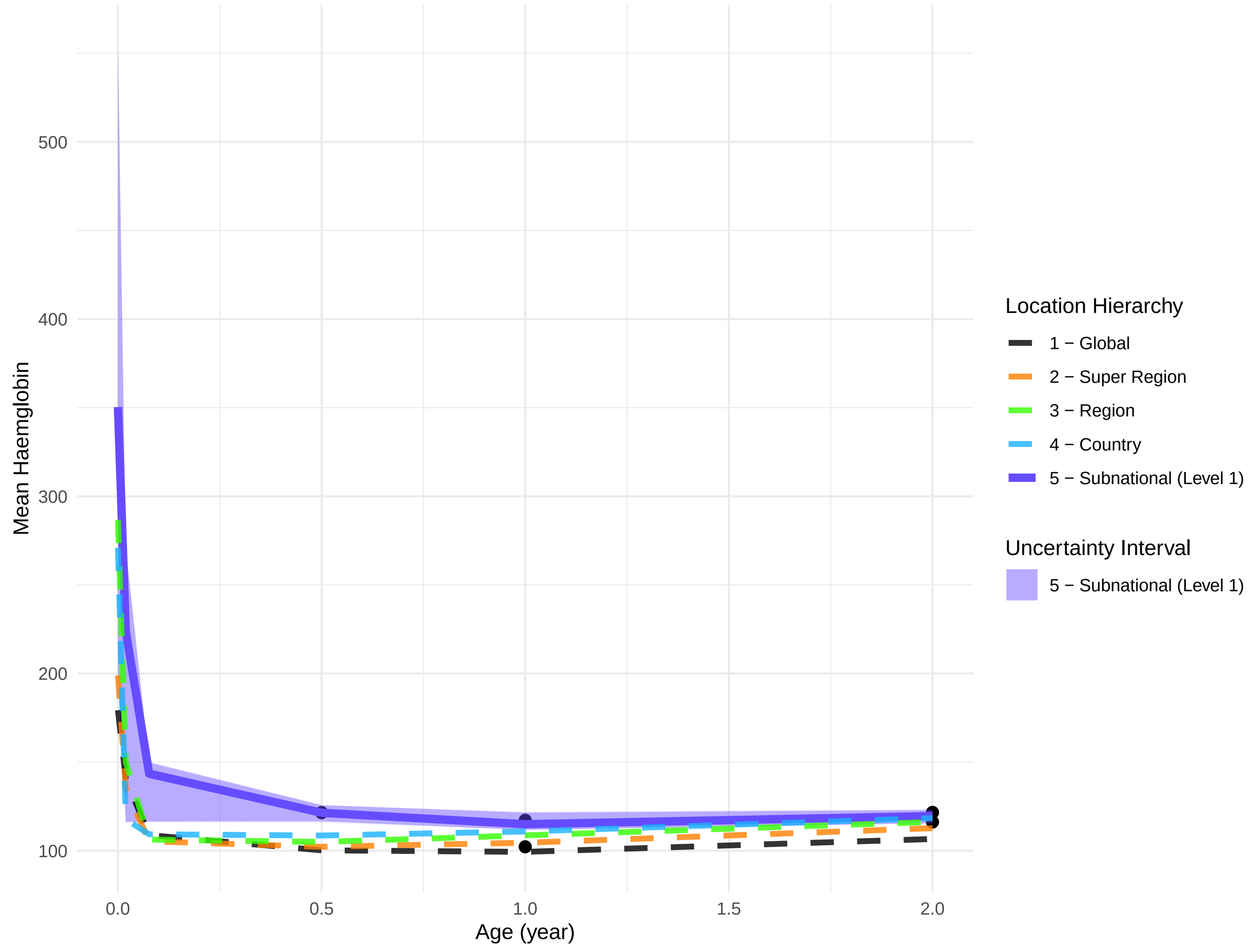

MR−BRT Age/Sex Fits − Non−pregnant Females <2 Years Old − Mean Haemglobin − Campeche
WHO−2024 Adjustment Method

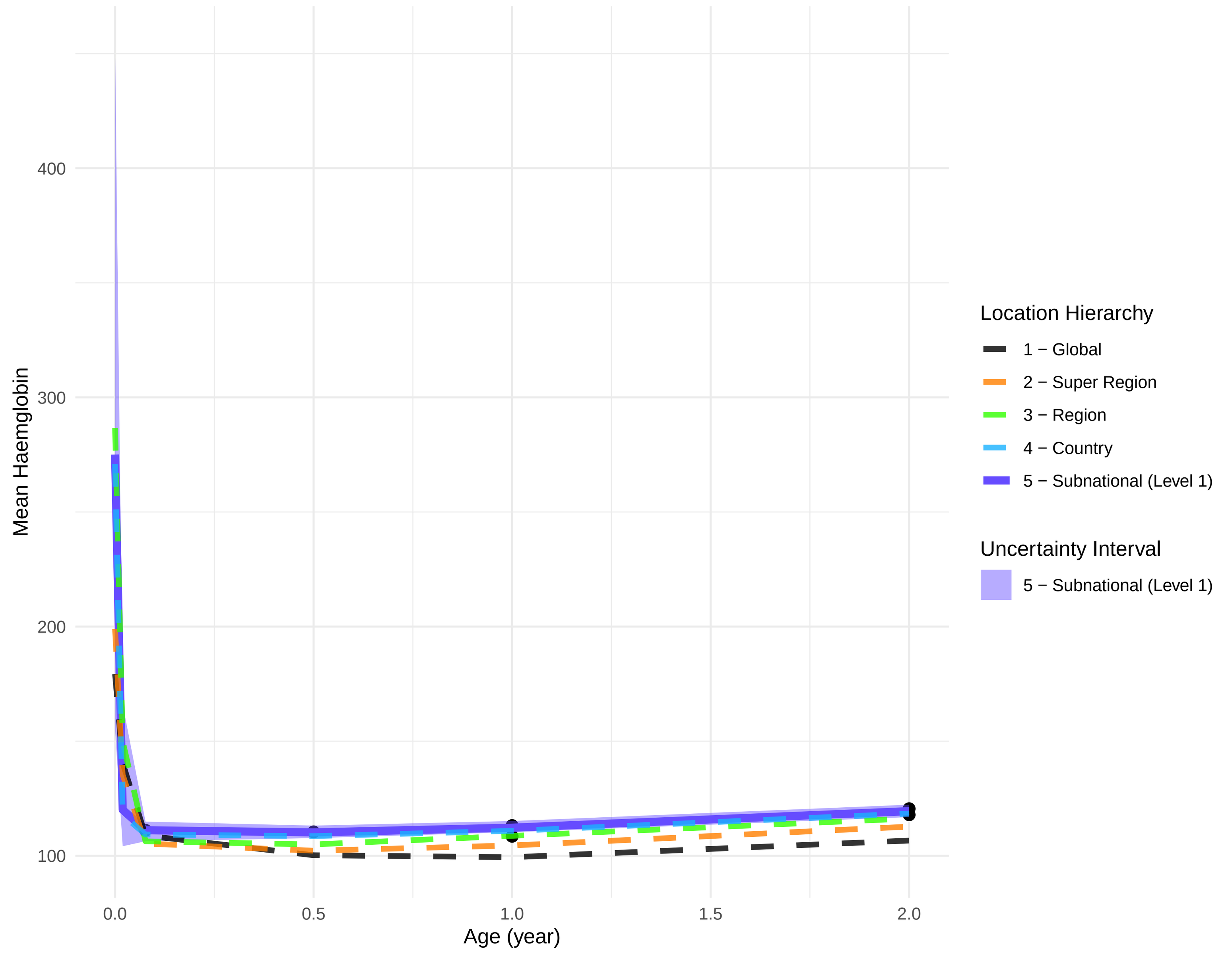

MR−BRT Age/Sex Fits − Non−pregnant Females <2 Years Old − Mean Haemglobin − Chiapas
WHO−2024 Adjustment Method

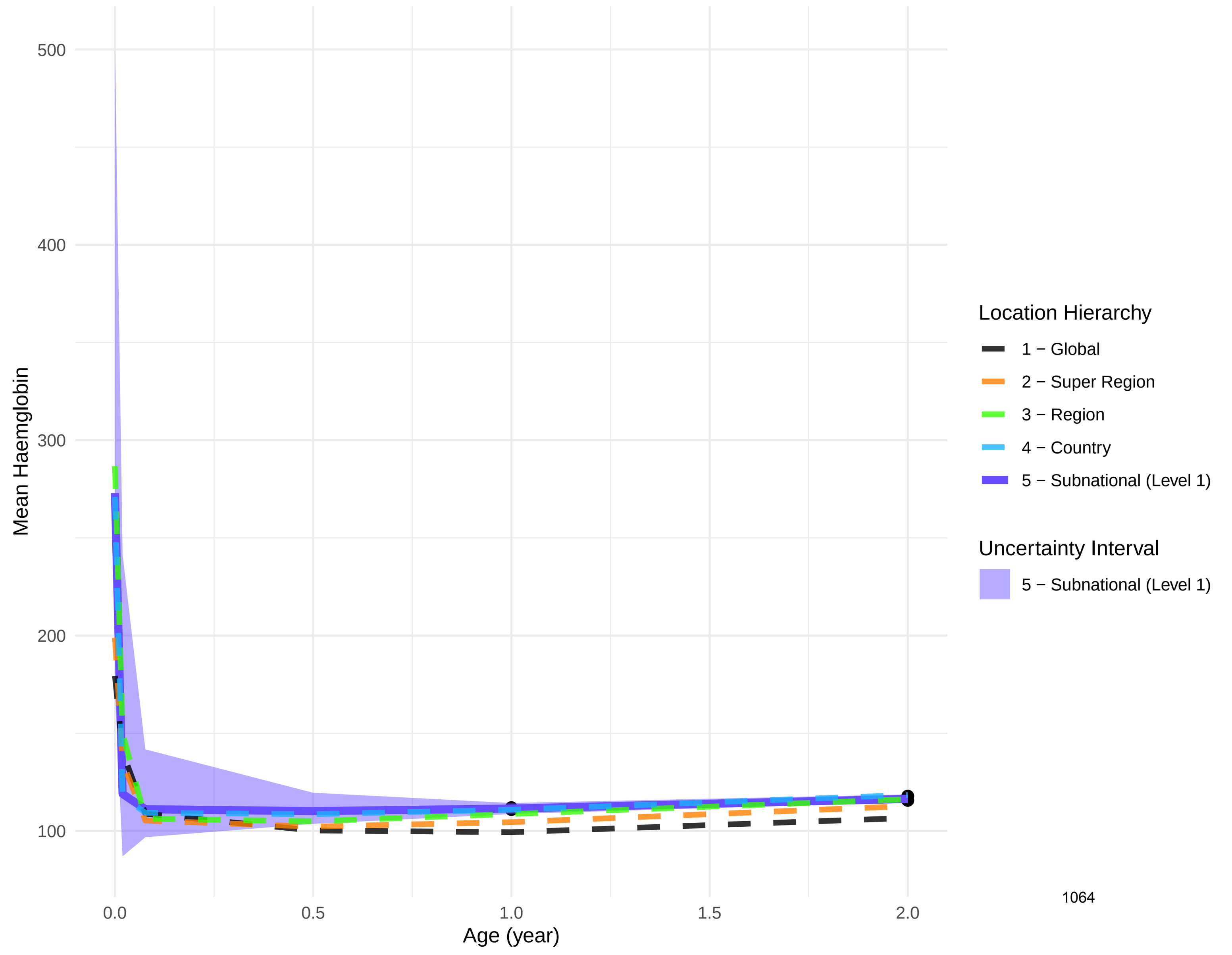

MR−BRT Age/Sex Fits − Non−pregnant Females <2 Years Old − Mean Haemglobin − Chihuahua
WHO−2024 Adjustment Method

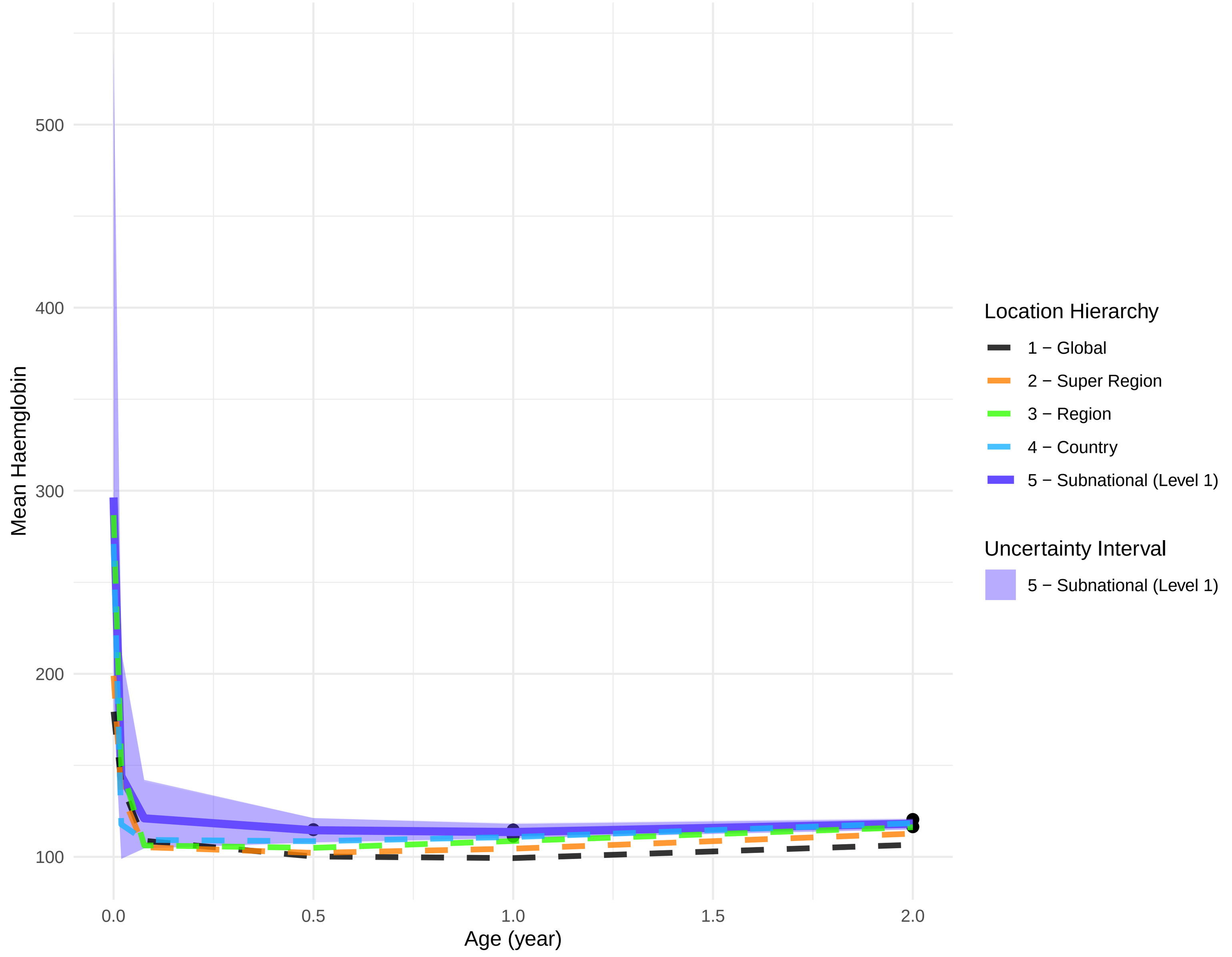

MR−BRT Age/Sex Fits − Non−pregnant Females <2 Years Old − Mean Haemglobin − Coahuila
WHO−2024 Adjustment Method

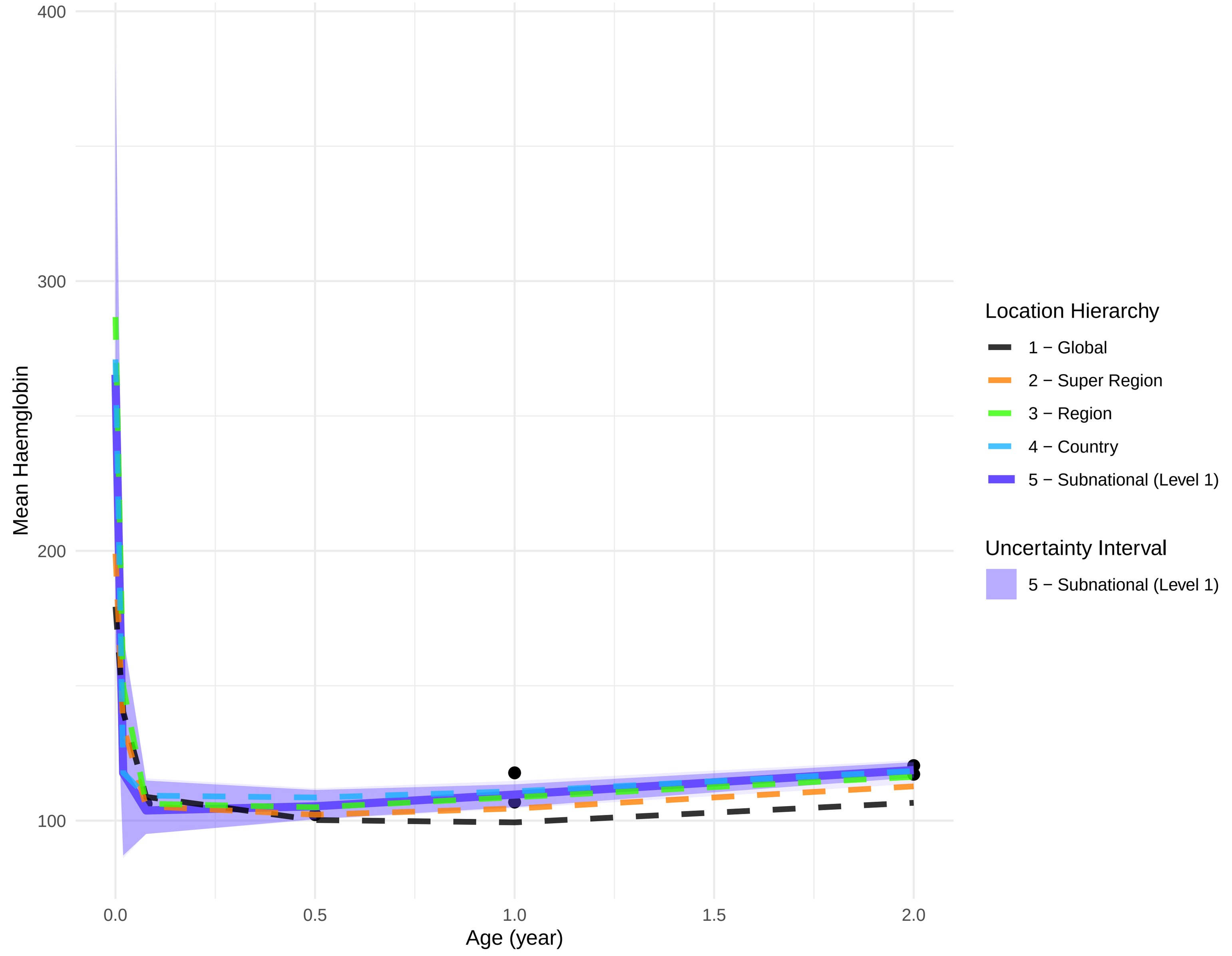

MR−BRT Age/Sex Fits − Non−pregnant Females <2 Years Old − Mean Haemglobin − Colima
WHO−2024 Adjustment Method

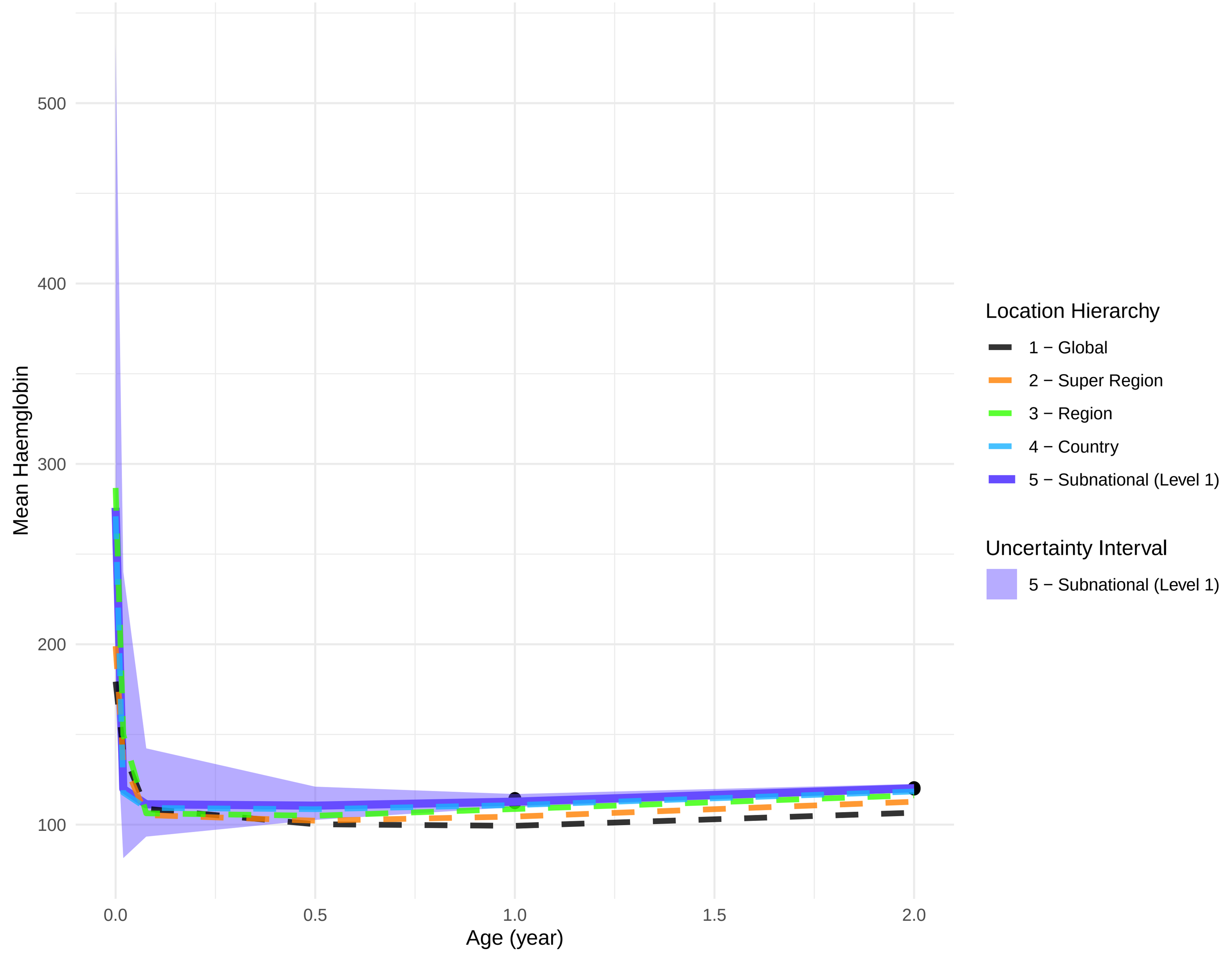

MR−BRT Age/Sex Fits − Non−pregnant Females <2 Years Old − Mean Haemglobin − Durango
WHO−2024 Adjustment Method

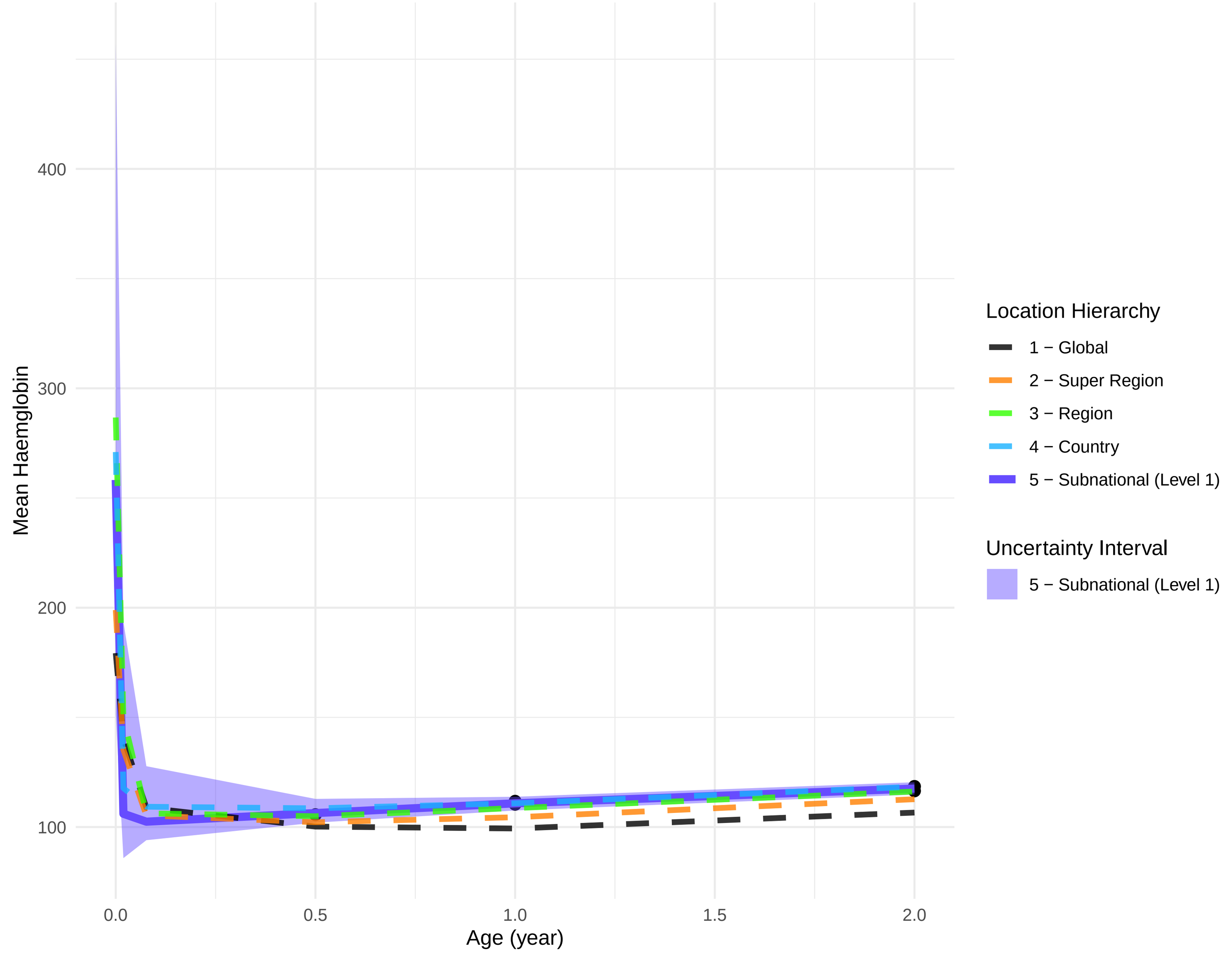

MR−BRT Age/Sex Fits − Non−pregnant Females <2 Years Old − Mean Haemglobin − Guanajuato
WHO−2024 Adjustment Method

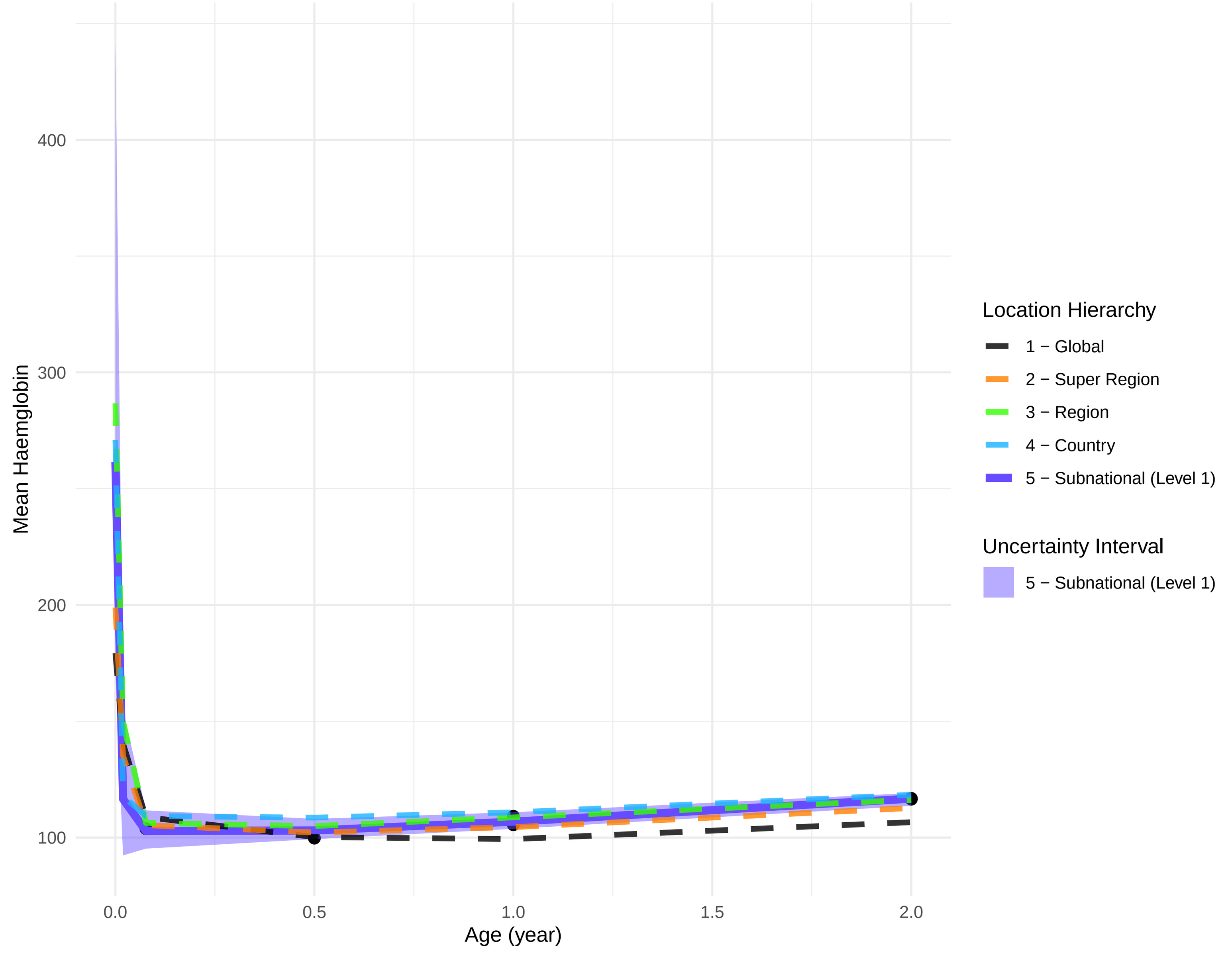

MR−BRT Age/Sex Fits − Non−pregnant Females <2 Years Old − Mean Haemglobin − Guerrero
WHO−2024 Adjustment Method

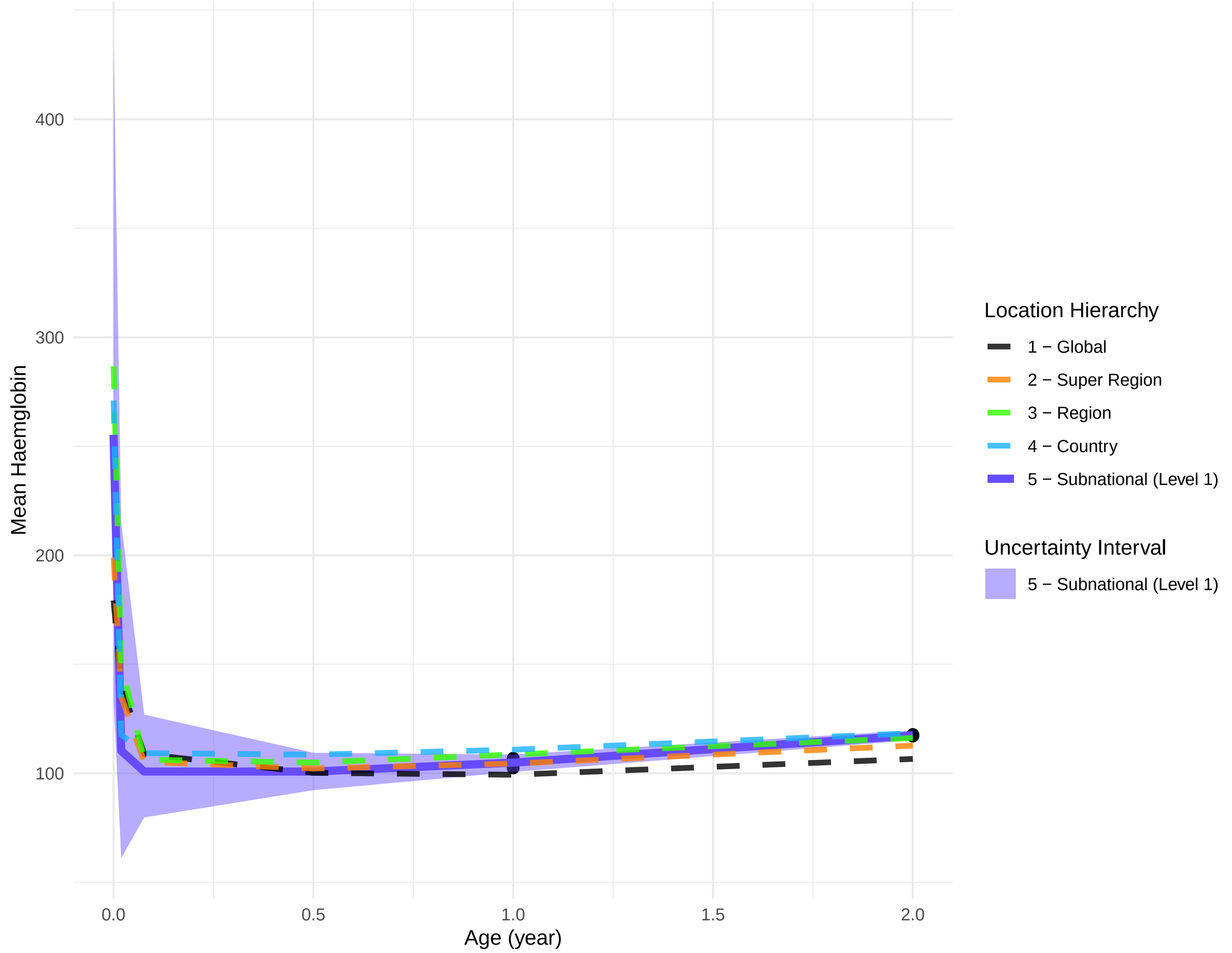

MR−BRT Age/Sex Fits − Non−pregnant Females <2 Years Old − Mean Haemglobin − Hidalgo
WHO−2024 Adjustment Method

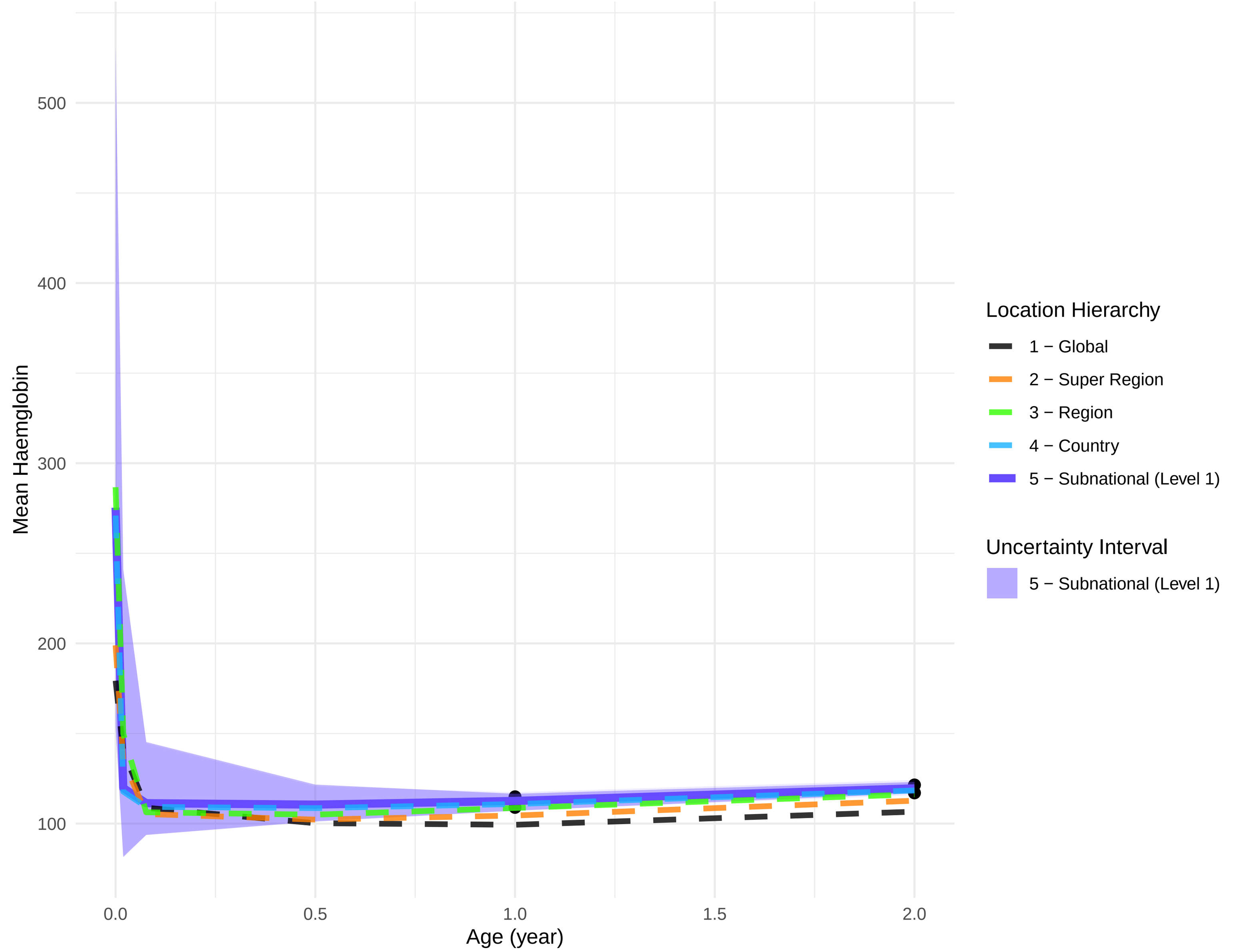

MR−BRT Age/Sex Fits − Non−pregnant Females <2 Years Old − Mean Haemglobin − Jalisco
WHO−2024 Adjustment Method

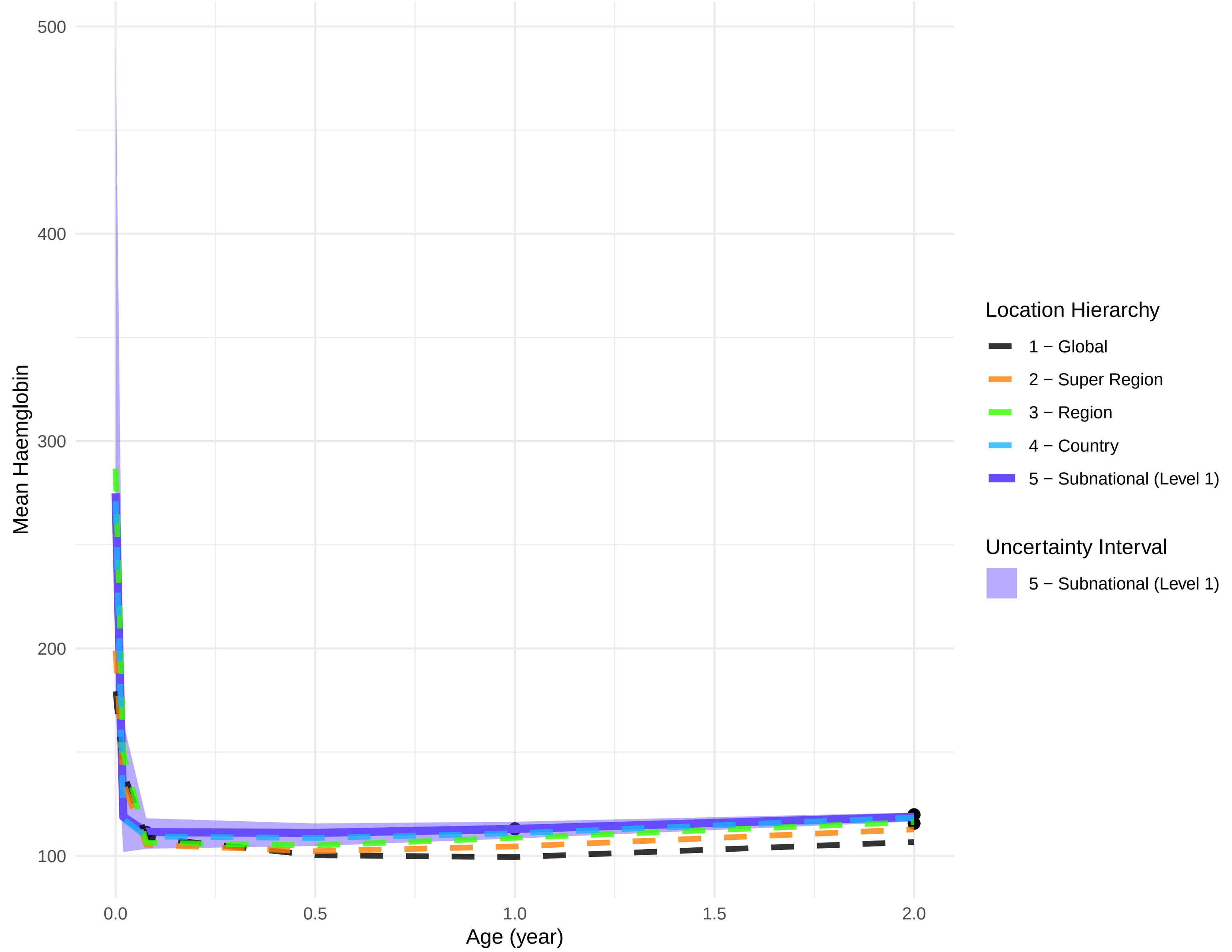

MR−BRT Age/Sex Fits − Non−pregnant Females <2 Years Old − Mean Haemglobin − México
WHO−2024 Adjustment Method

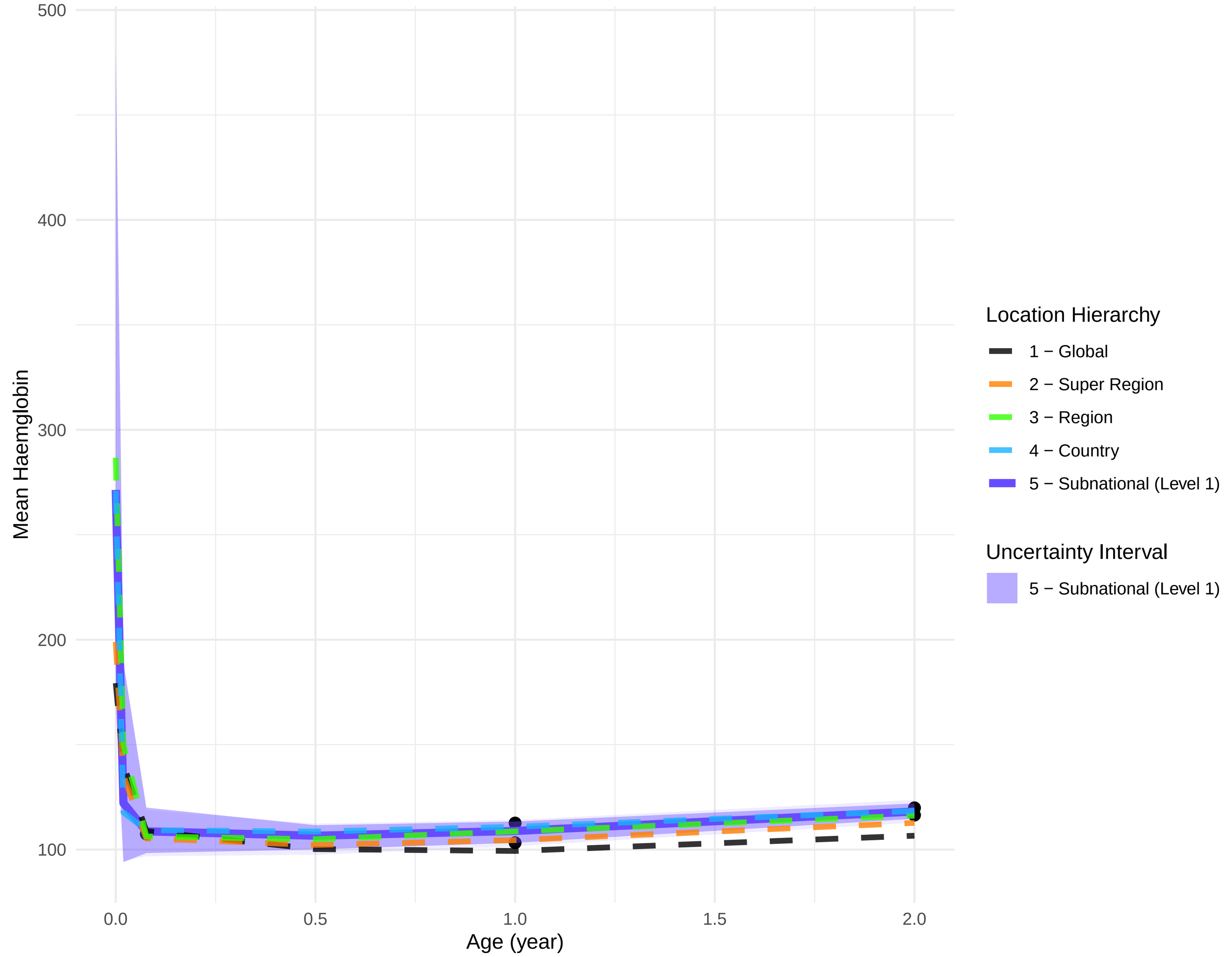

MR−BRT Age/Sex Fits − Non−pregnant Females <2 Years Old − Mean Haemglobin − Mexico City
WHO−2024 Adjustment Method

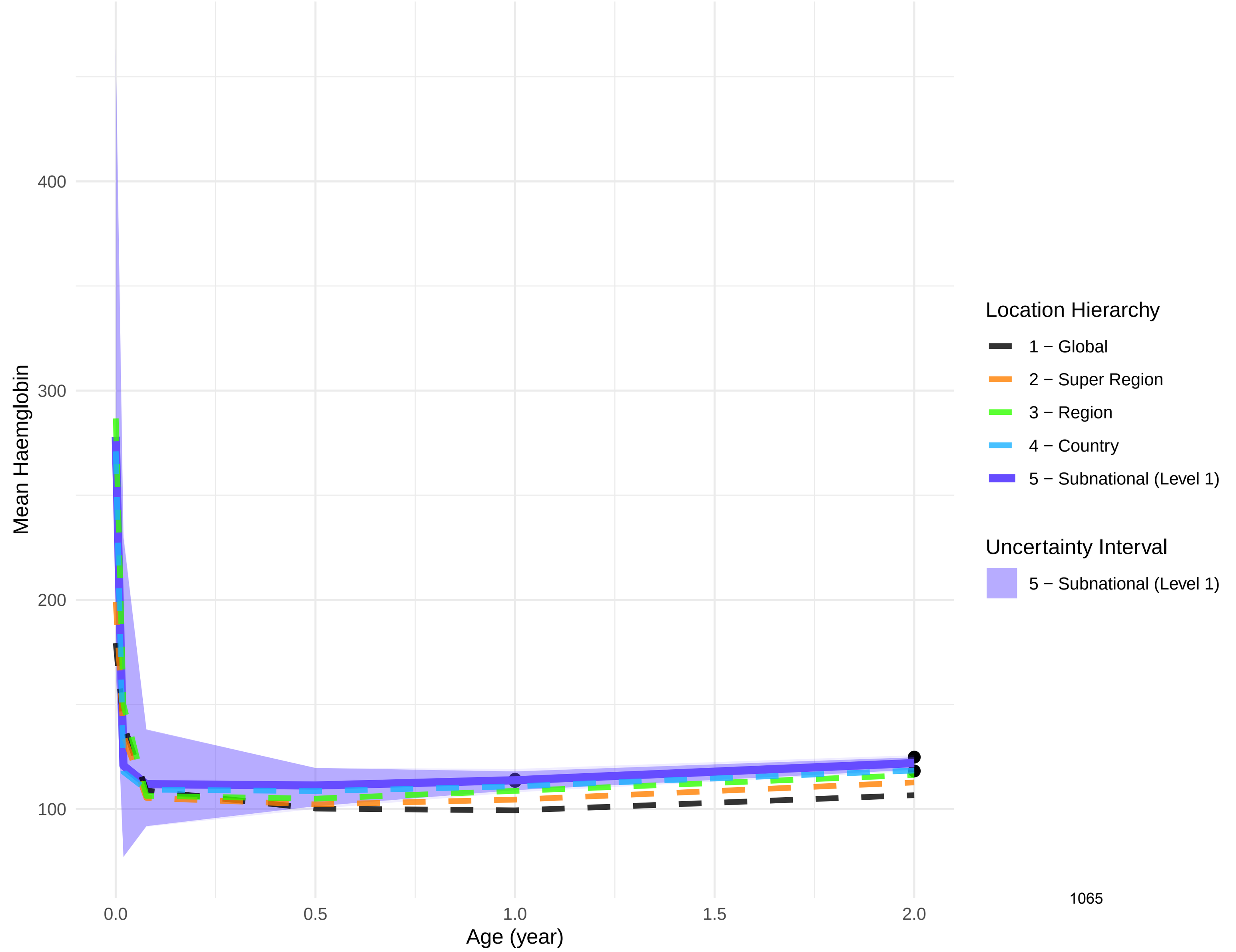

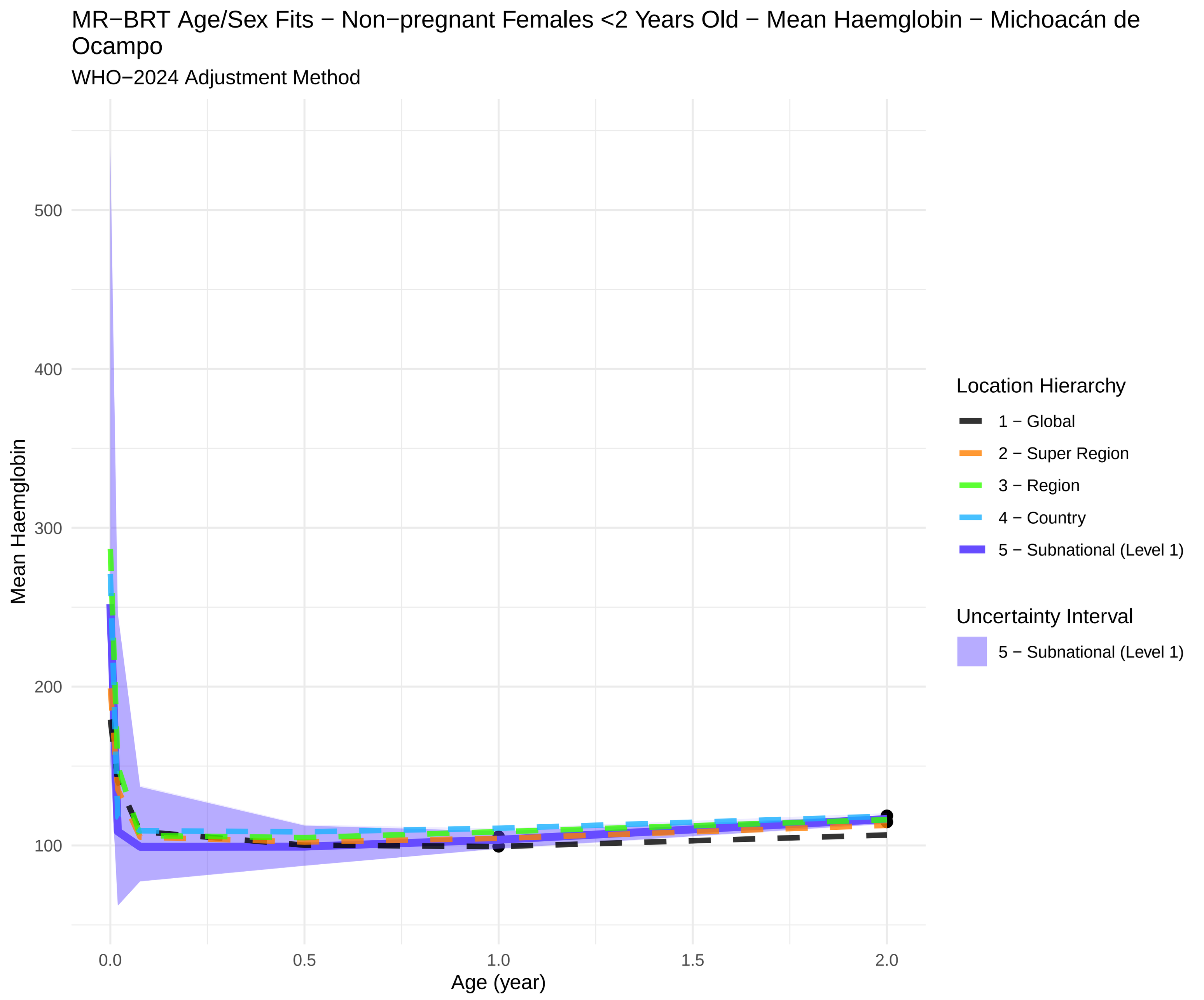
MR−BRT Age/Sex Fits − Non−pregnant Females <2 Years Old − Mean Haemglobin − Michoacán de Ocampo
WHO−2024 Adjustment Method
Mean Haemglobin
Age (year)
Location Hierarchy
1 − Global
2 − Super Region
3 − Region
4 − Country
5 − Subnational (Level 1)
Uncertainty Interval
5 − Subnational (Level 1)

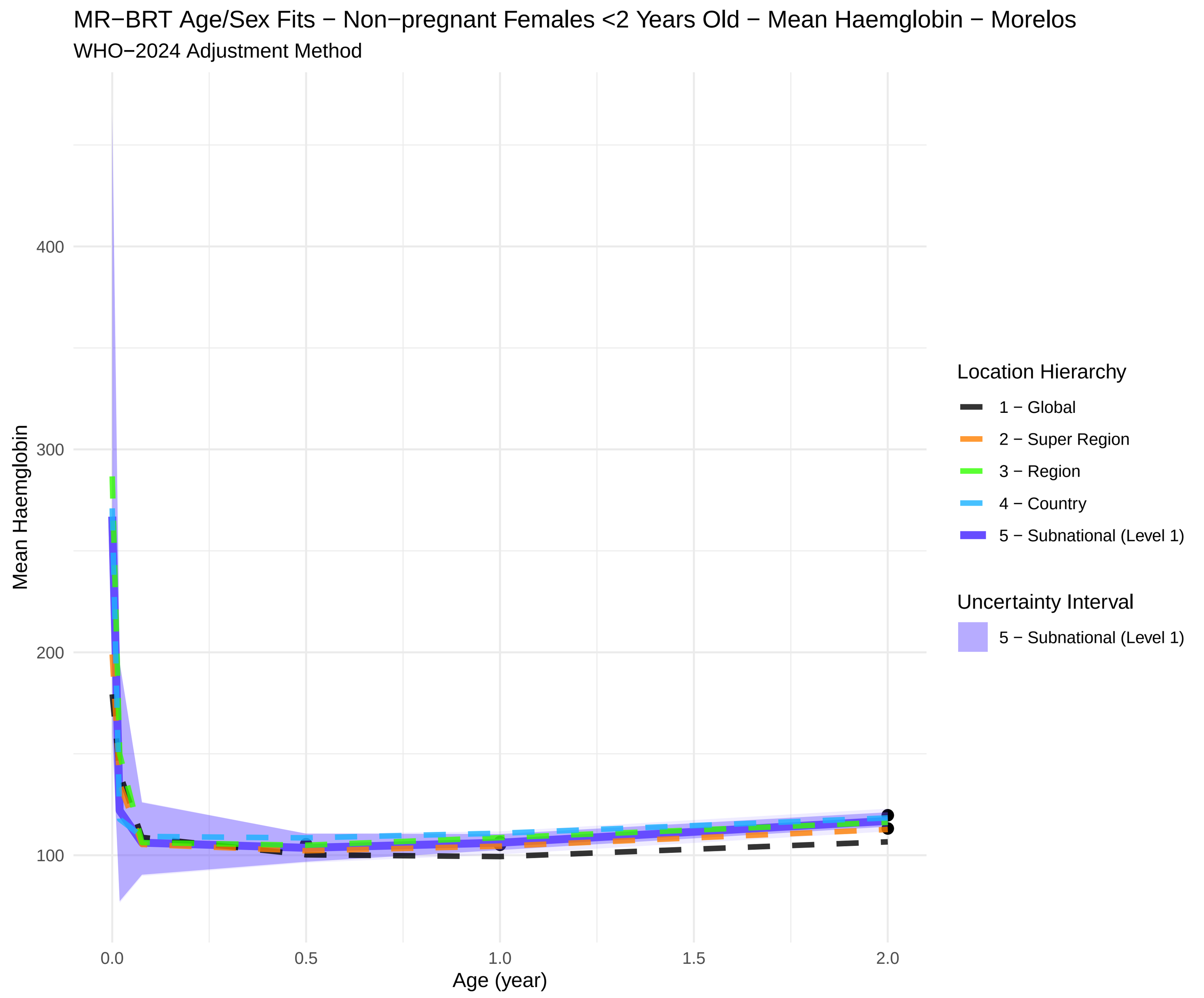
MR−BRT Age/Sex Fits − Non−pregnant Females <2 Years Old − Mean Haemglobin − Morelos
WHO−2024 Adjustment Method
Mean Haemglobin
Age (year)
Location Hierarchy
1 − Global
2 − Super Region
3 − Region
4 − Country
5 − Subnational (Level 1)
Uncertainty Interval
5 − Subnational (Level 1)

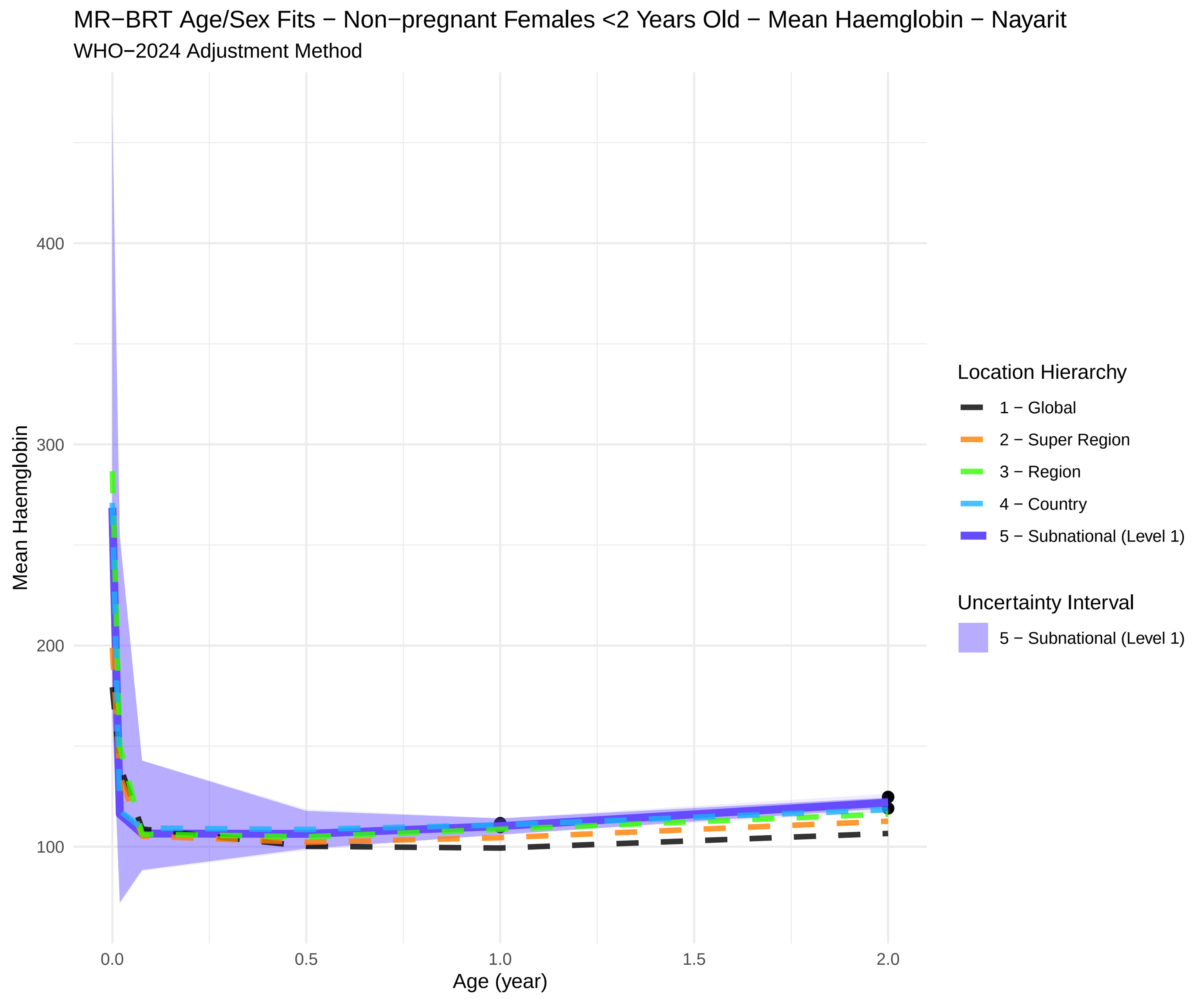
MR−BRT Age/Sex Fits − Non−pregnant Females <2 Years Old − Mean Haemglobin − Nayarit
WHO−2024 Adjustment Method
Mean Haemglobin
Age (year)
Location Hierarchy
1 − Global
2 − Super Region
3 − Region
4 − Country
5 − Subnational (Level 1)
Uncertainty Interval
5 − Subnational (Level 1)

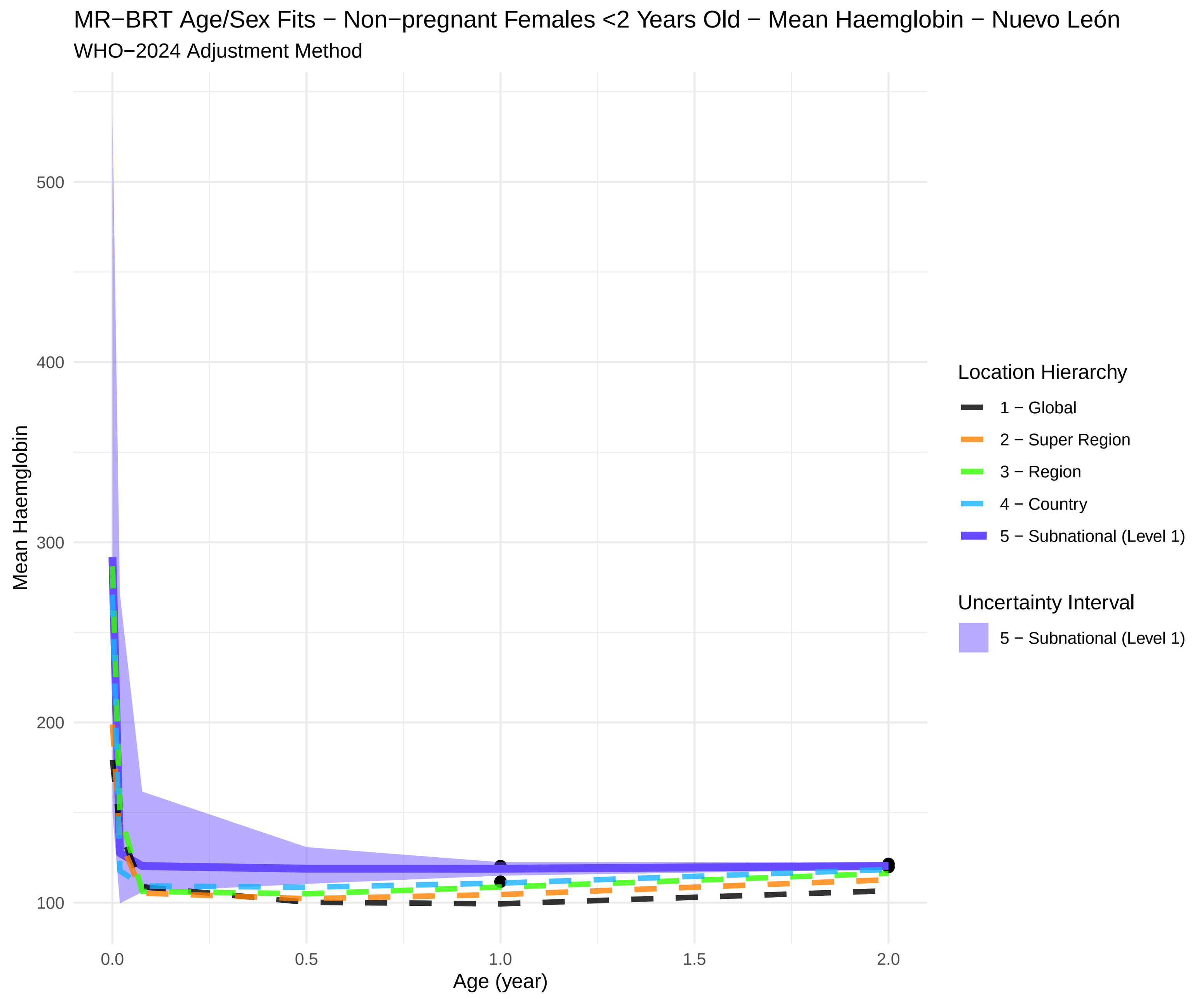
MR−BRT Age/Sex Fits − Non−pregnant Females <2 Years Old − Mean Haemglobin − Nuevo León
WHO−2024 Adjustment Method
Mean Haemglobin
Age (year)
Location Hierarchy
1 − Global
2 − Super Region
3 − Region
4 − Country
5 − Subnational (Level 1)
Uncertainty Interval
5 − Subnational (Level 1)

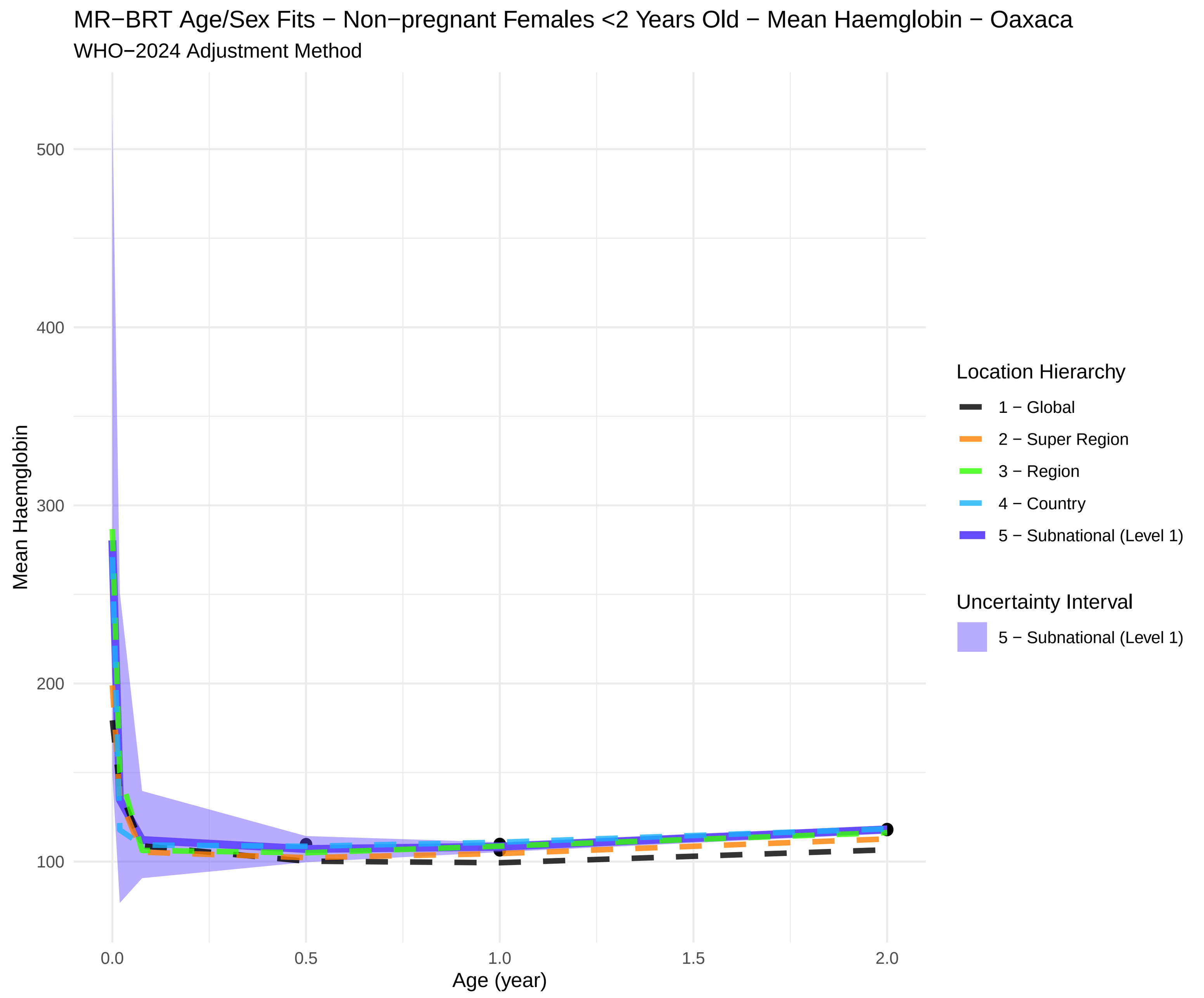
MR−BRT Age/Sex Fits − Non−pregnant Females <2 Years Old − Mean Haemglobin − Oaxaca
WHO−2024 Adjustment Method
Mean Haemglobin
Age (year)
Location Hierarchy
1 − Global
2 − Super Region
3 − Region
4 − Country
5 − Subnational (Level 1)
Uncertainty Interval
5 − Subnational (Level 1)

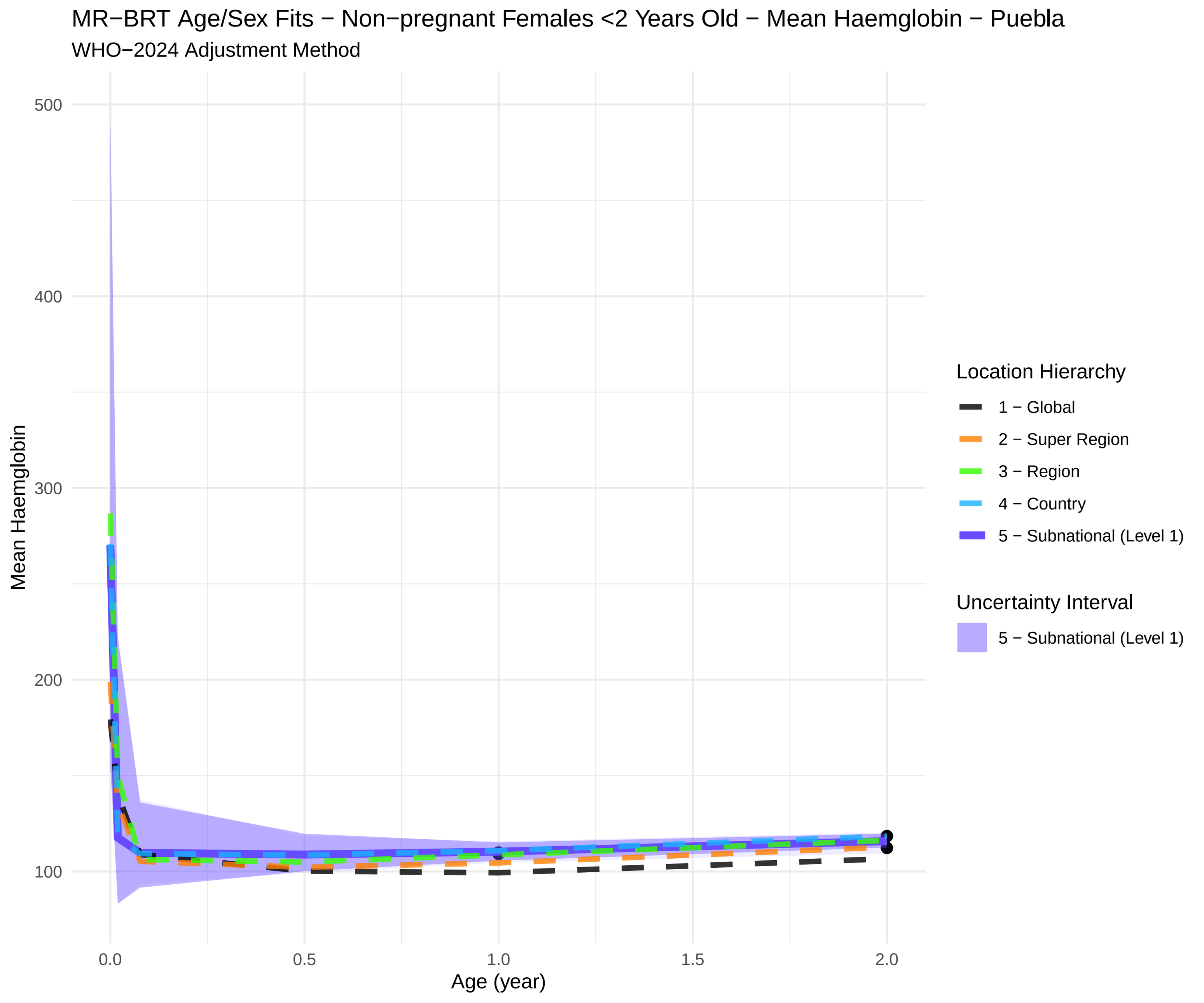
MR−BRT Age/Sex Fits − Non−pregnant Females <2 Years Old − Mean Haemglobin − Puebla
WHO−2024 Adjustment Method
Mean Haemglobin
Age (year)
Location Hierarchy
1 − Global
2 − Super Region
3 − Region
4 − Country
5 − Subnational (Level 1)
Uncertainty Interval
5 − Subnational (Level 1)

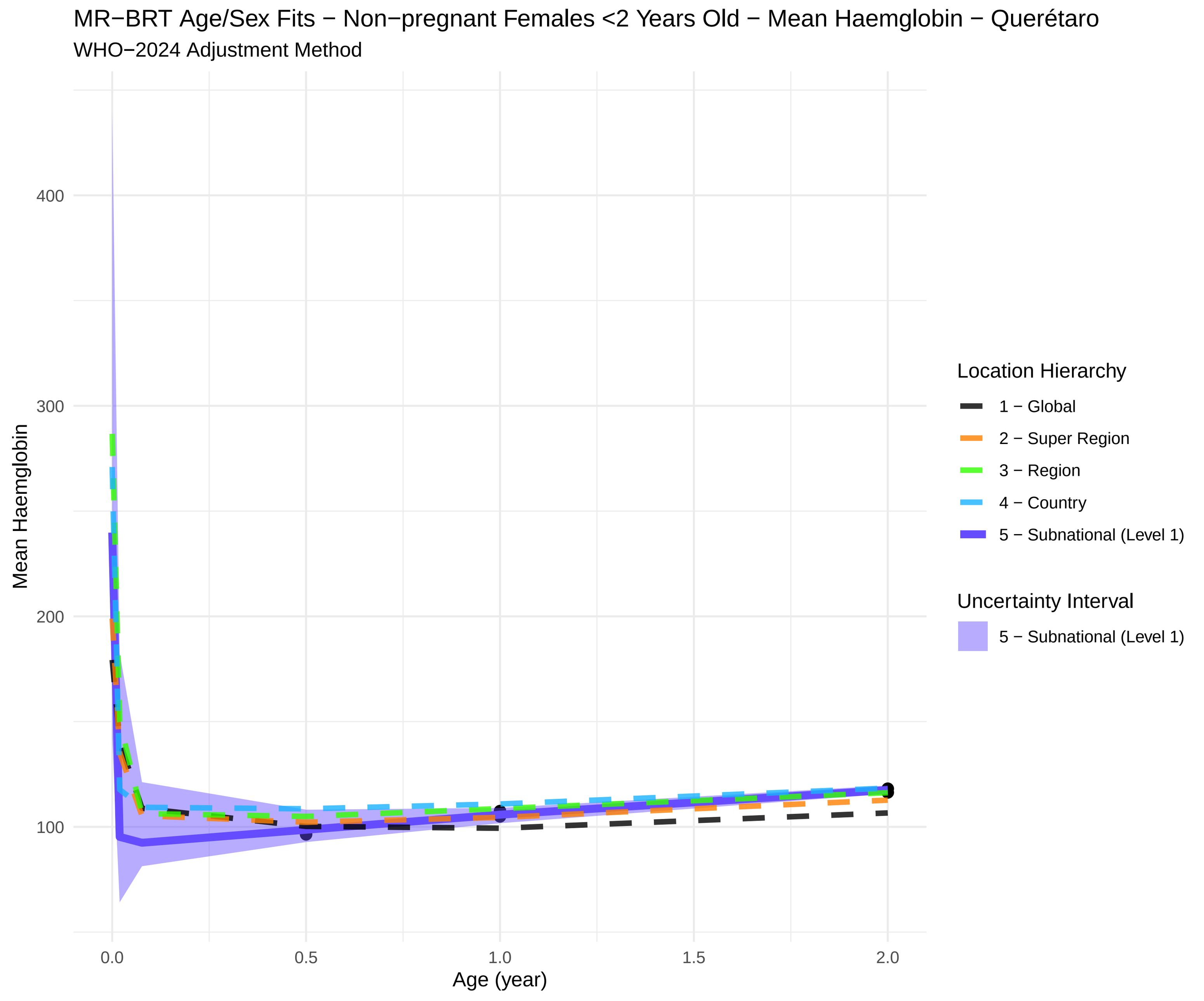
MR−BRT Age/Sex Fits − Non−pregnant Females <2 Years Old − Mean Haemglobin − Querétaro
WHO−2024 Adjustment Method
Mean Haemglobin
Age (year)
Location Hierarchy
1 − Global
2 − Super Region
3 − Region
4 − Country
5 − Subnational (Level 1)
Uncertainty Interval
5 − Subnational (Level 1)

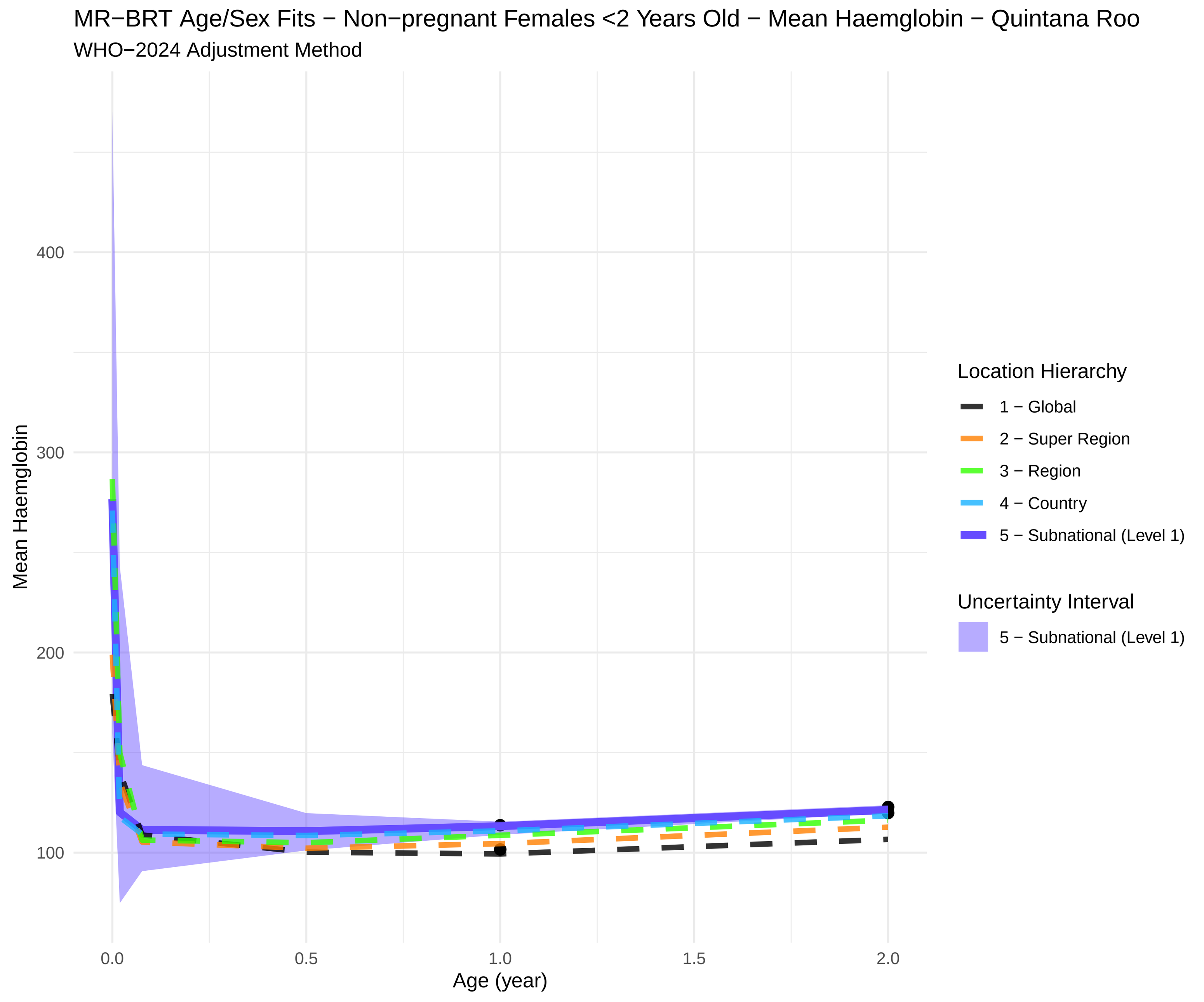
MR−BRT Age/Sex Fits − Non−pregnant Females <2 Years Old − Mean Haemglobin − Quintana Roo
WHO−2024 Adjustment Method
Mean Haemglobin
Age (year)
Location Hierarchy
1 − Global
2 − Super Region
3 − Region
4 − Country
5 − Subnational (Level 1)
Uncertainty Interval
5 − Subnational (Level 1)

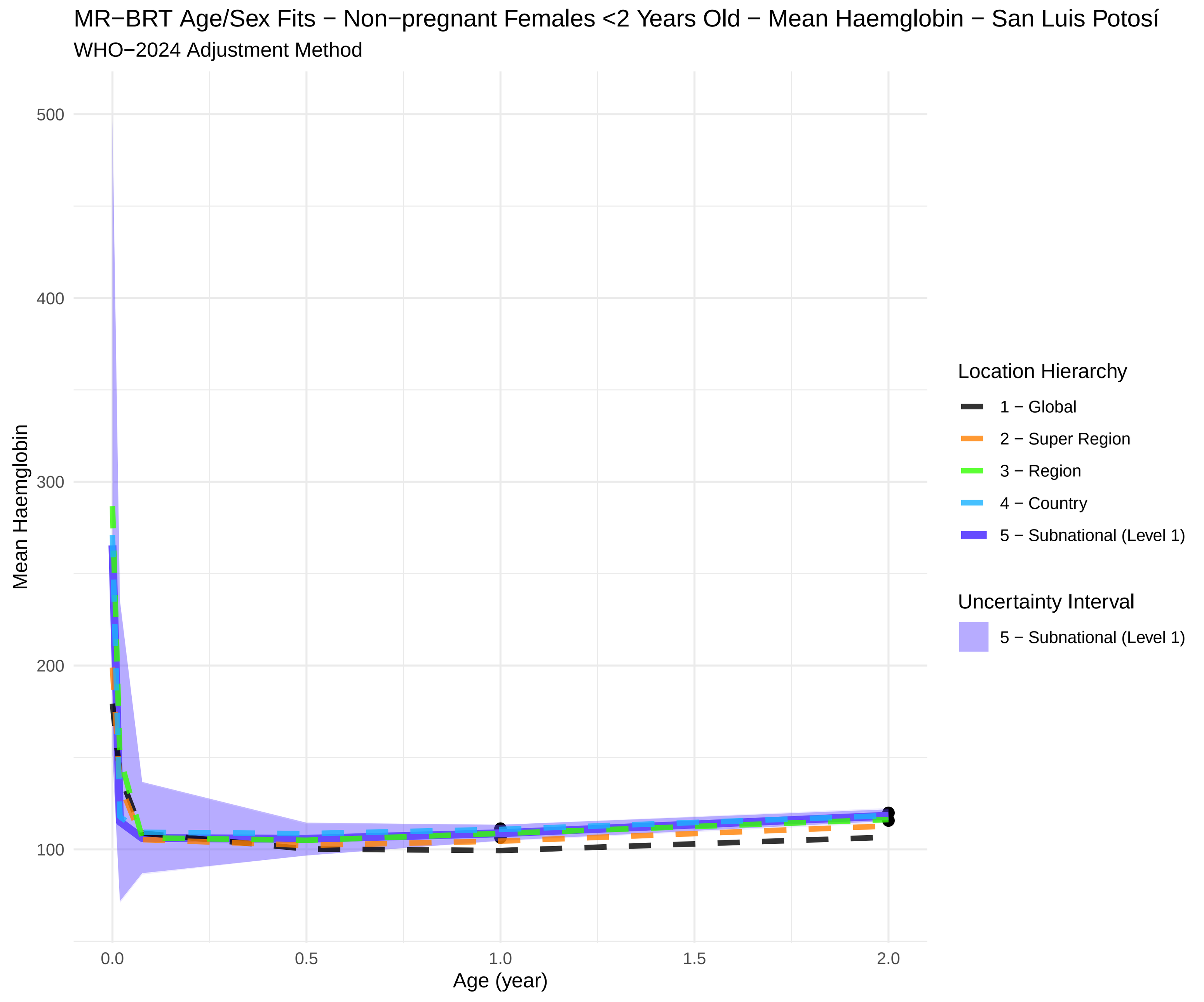
MR−BRT Age/Sex Fits − Non−pregnant Females <2 Years Old − Mean Haemglobin − San Luis Potosí
WHO−2024 Adjustment Method
Mean Haemglobin
Age (year)
Location Hierarchy
1 − Global
2 − Super Region
3 − Region
4 − Country
5 − Subnational (Level 1)
Uncertainty Interval
5 − Subnational (Level 1)

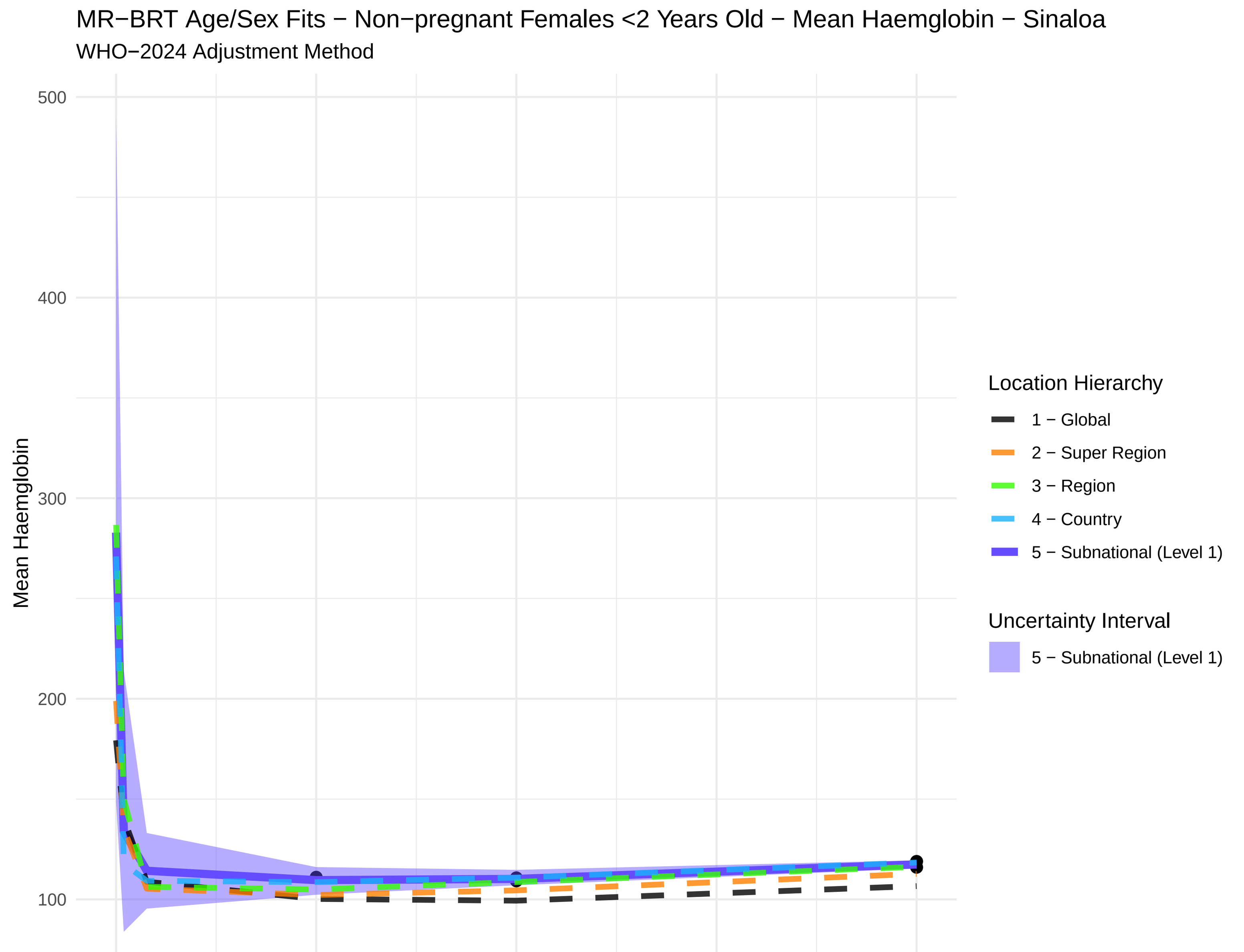
MR−BRT Age/Sex Fits − Non−pregnant Females <2 Years Old − Mean Haemglobin − Sinaloa
WHO−2024 Adjustment Method
Mean Haemglobin
Age (year)
Location Hierarchy
1 − Global
2 − Super Region
3 − Region
4 − Country
5 − Subnational (Level 1)
Uncertainty Interval
5 − Subnational (Level 1)

MR−BRT Age/Sex Fits − Non−pregnant Females <2 Years Old − Mean Haemglobin − Sonora
WHO−2024 Adjustment Method

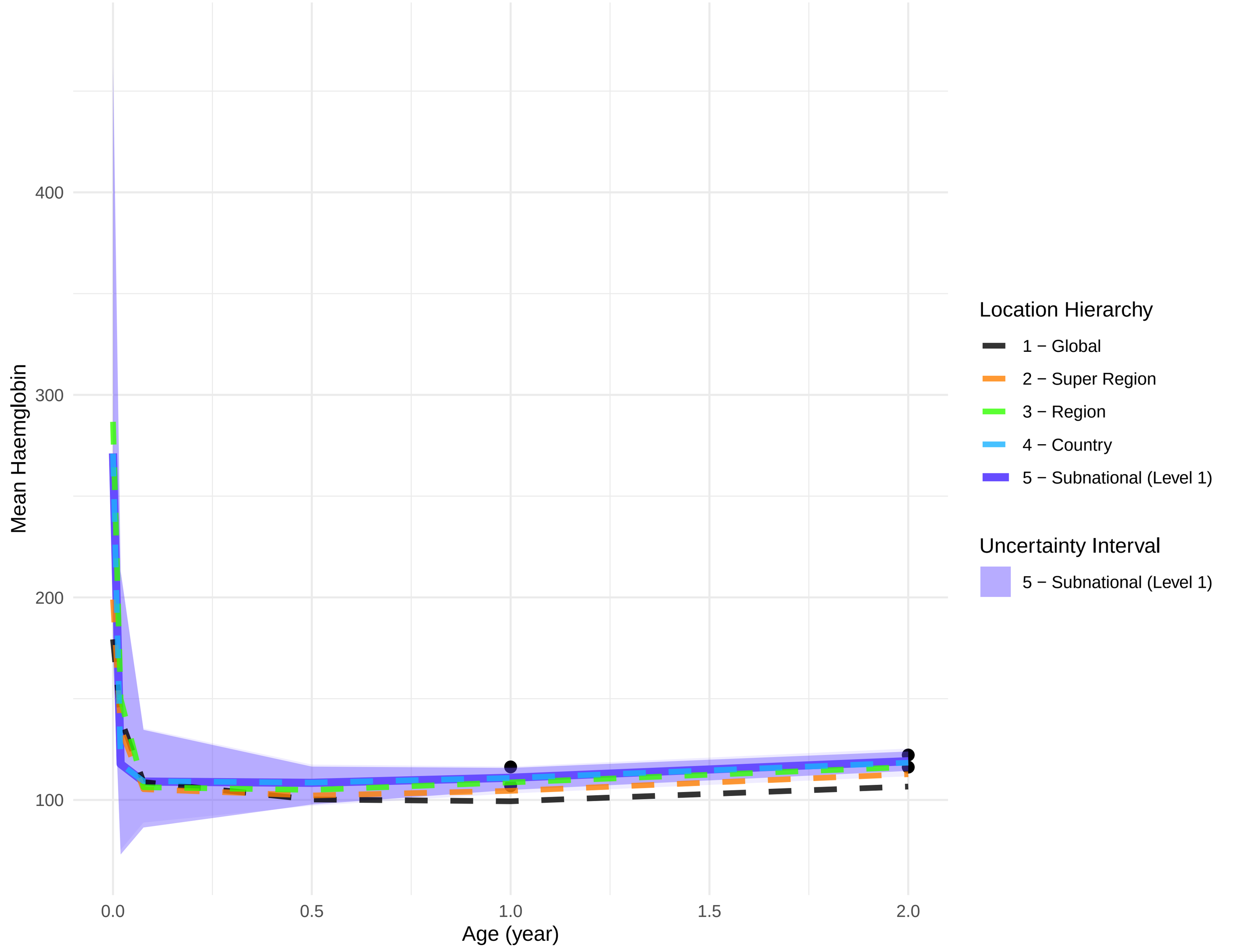

MR−BRT Age/Sex Fits − Non−pregnant Females <2 Years Old − Mean Haemglobin − Tabasco
WHO−2024 Adjustment Method

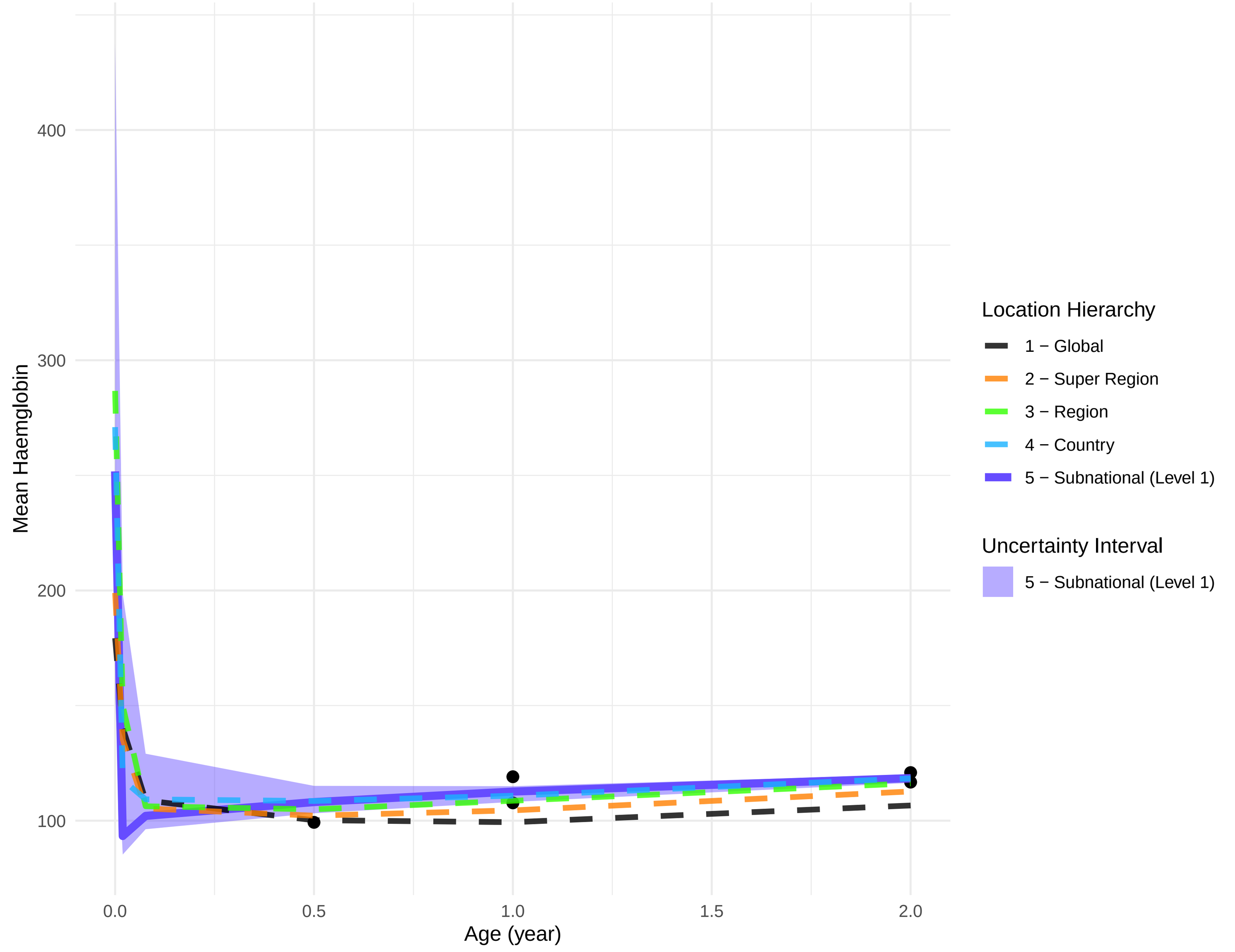

MR−BRT Age/Sex Fits − Non−pregnant Females <2 Years Old − Mean Haemglobin − Tamaulipas
WHO−2024 Adjustment Method

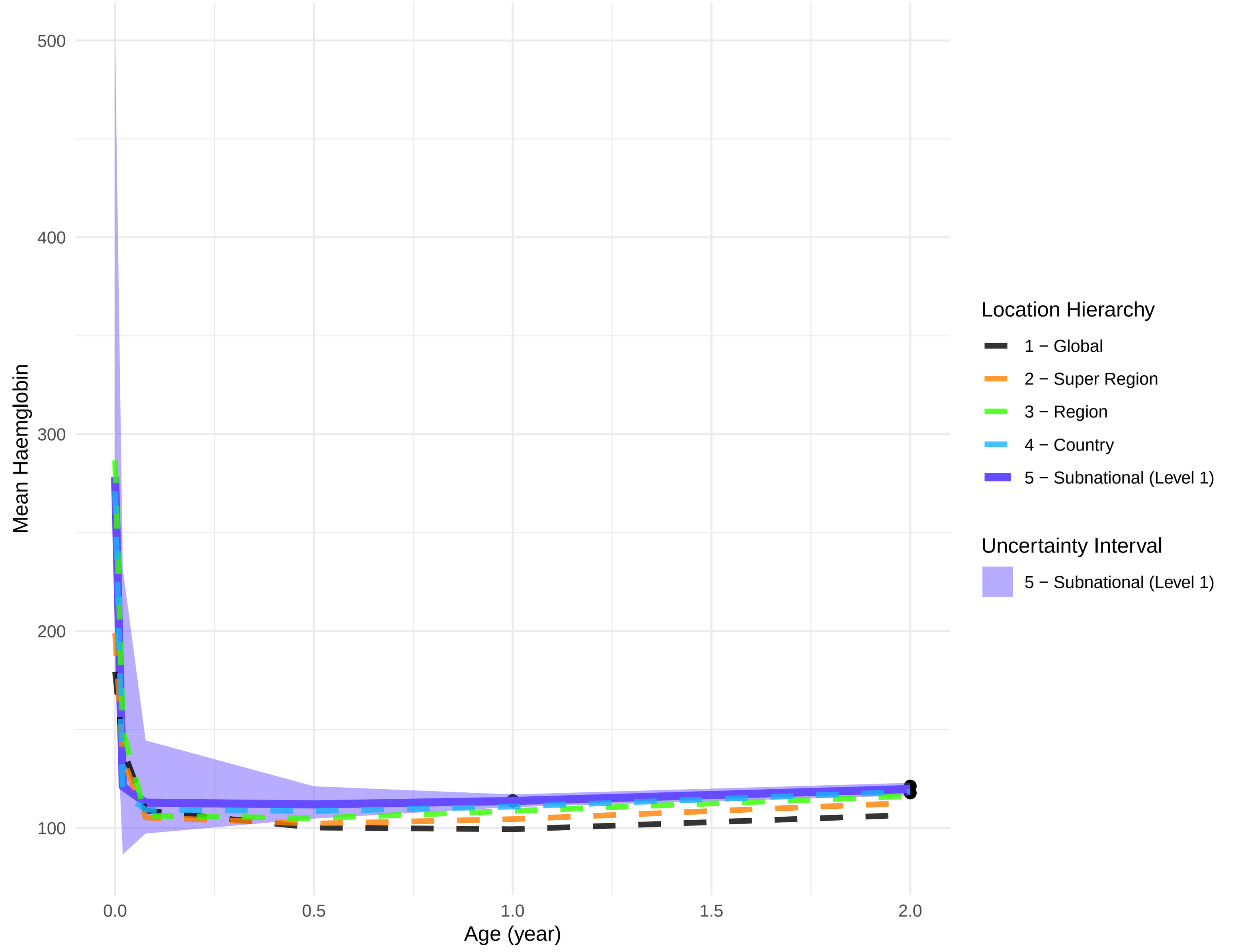

MR−BRT Age/Sex Fits − Non−pregnant Females <2 Years Old − Mean Haemglobin − Tlaxcala
WHO−2024 Adjustment Method

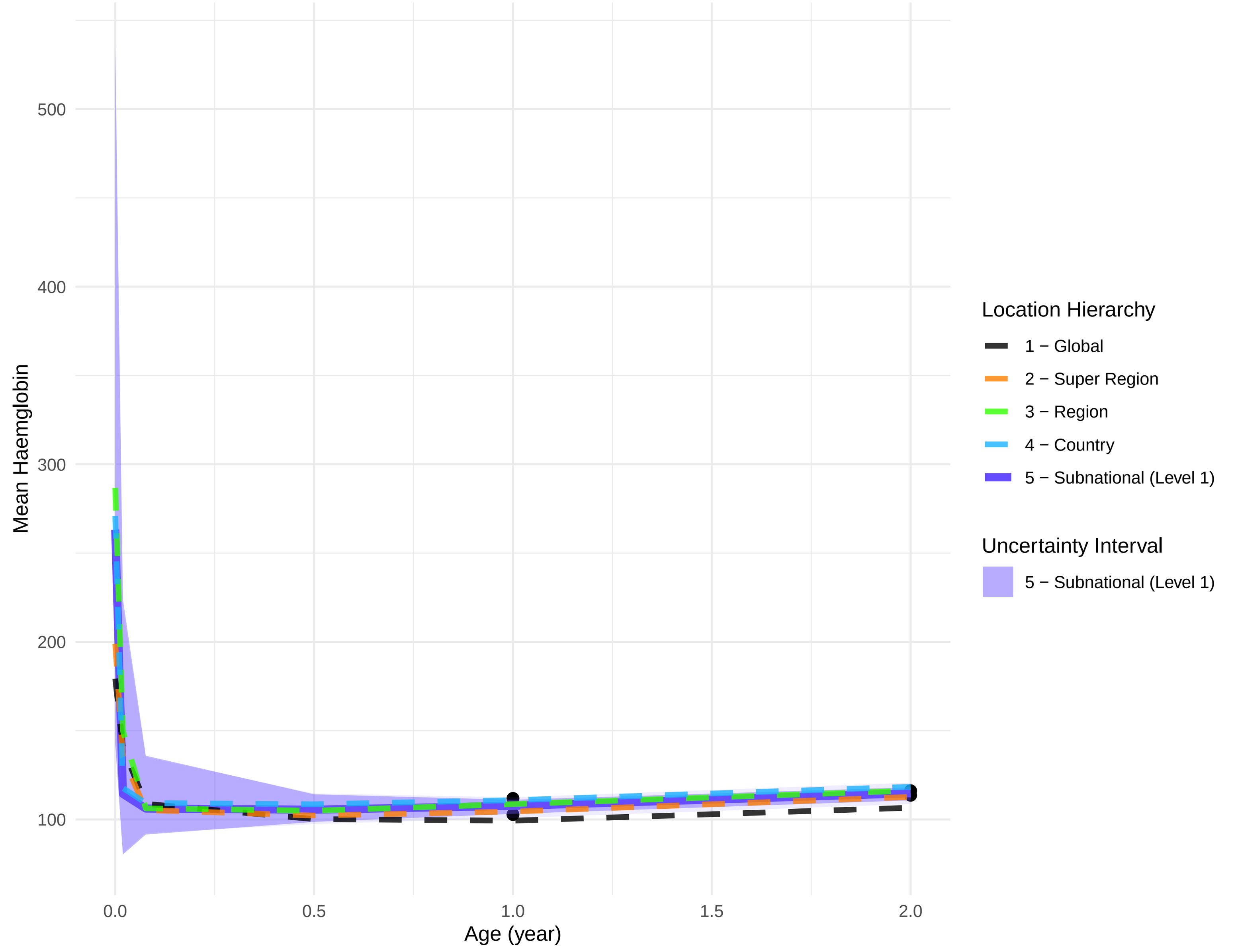

MR−BRT Age/Sex Fits − Non−pregnant Females <2 Years Old − Mean Haemglobin − Veracruz de Ignacio de la Llave
WHO−2024 Adjustment Method

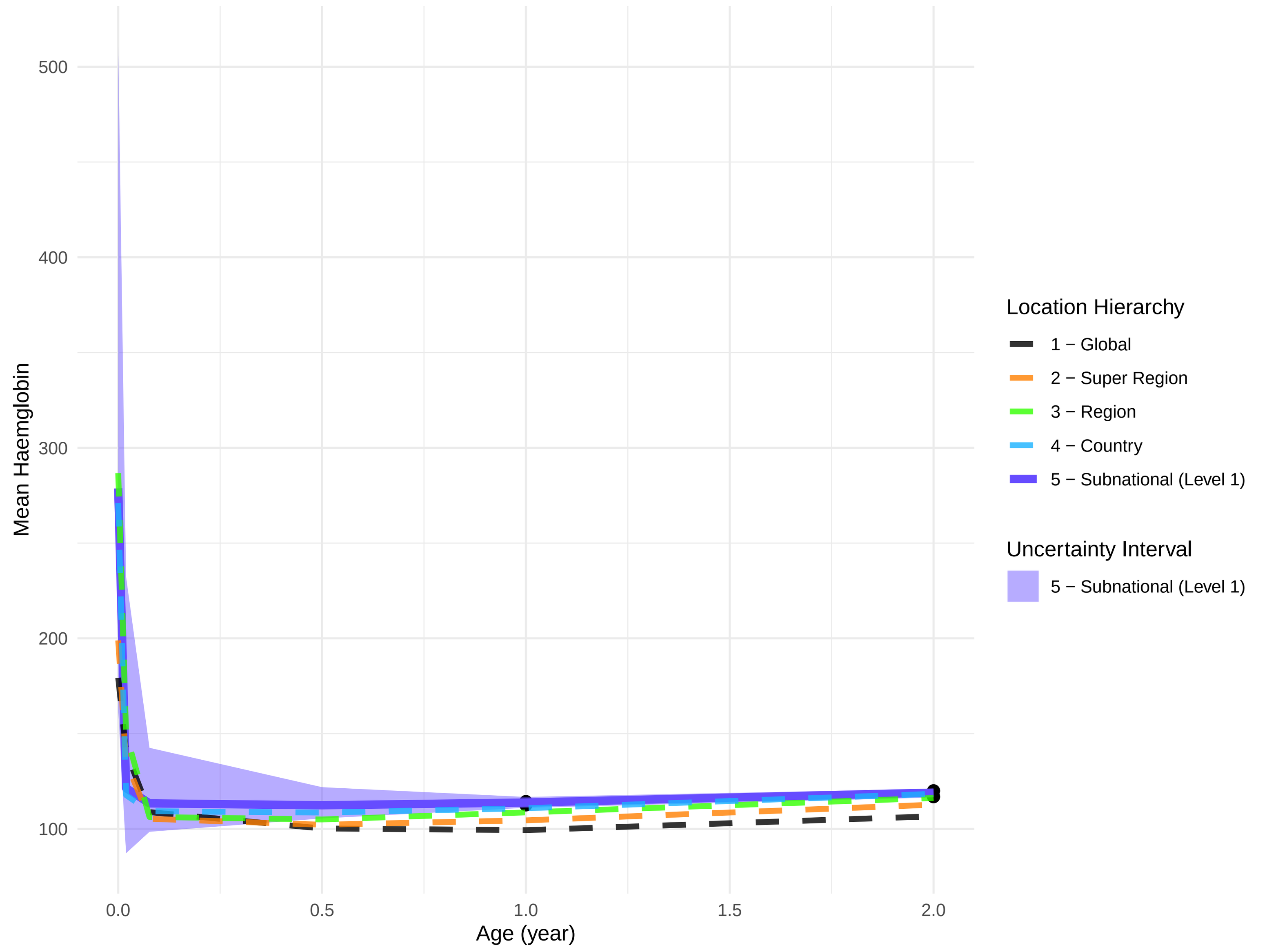

MR−BRT Age/Sex Fits − Non−pregnant Females <2 Years Old − Mean Haemglobin − Yucatán
WHO−2024 Adjustment Method

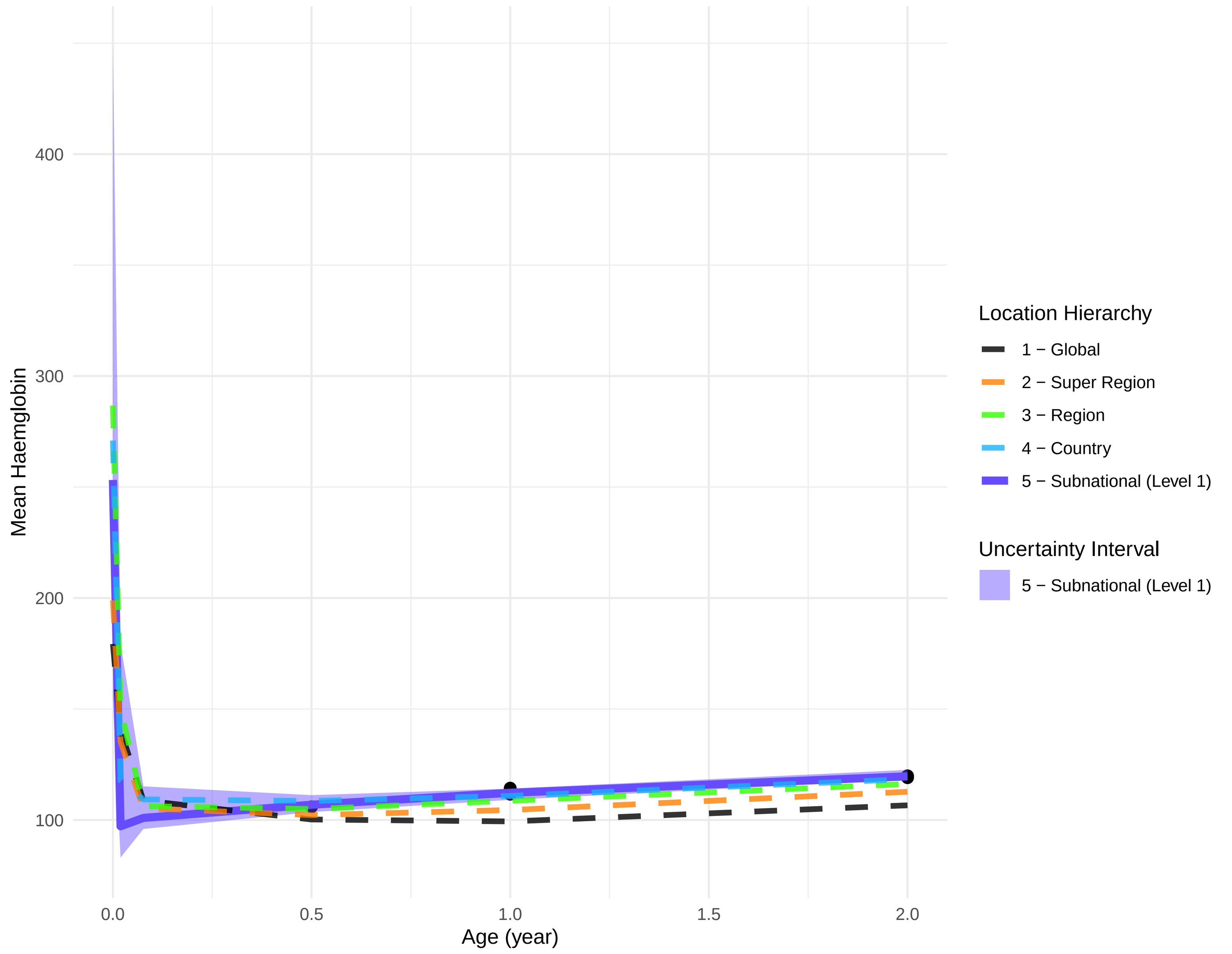

MR−BRT Age/Sex Fits − Non−pregnant Females <2 Years Old − Mean Haemglobin − Zacatecas
WHO−2024 Adjustment Method

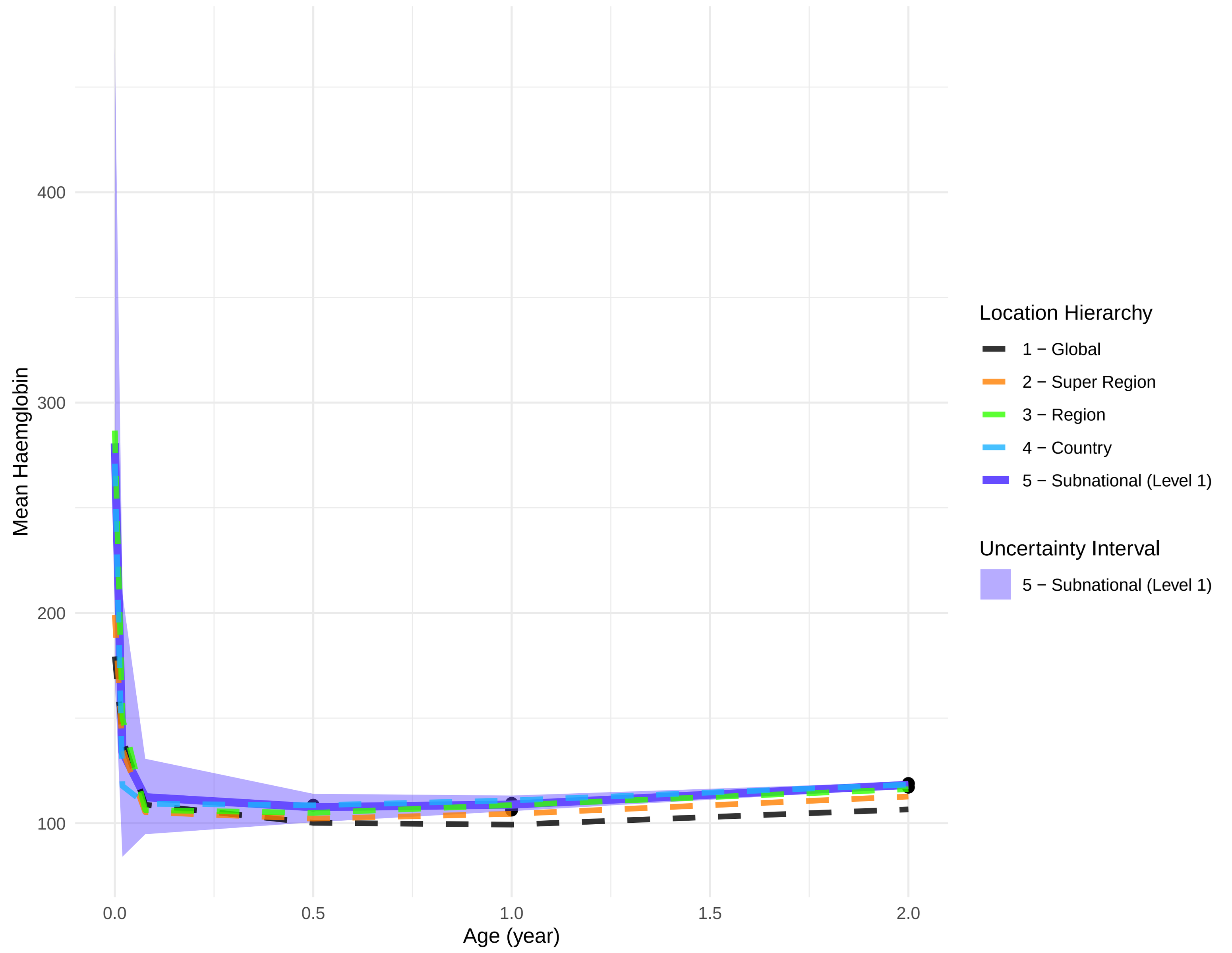

MR−BRT Age/Sex Fits − Non−pregnant Females <2 Years Old − Mean Haemglobin − North Africa and Middle East
WHO−2024 Adjustment Method

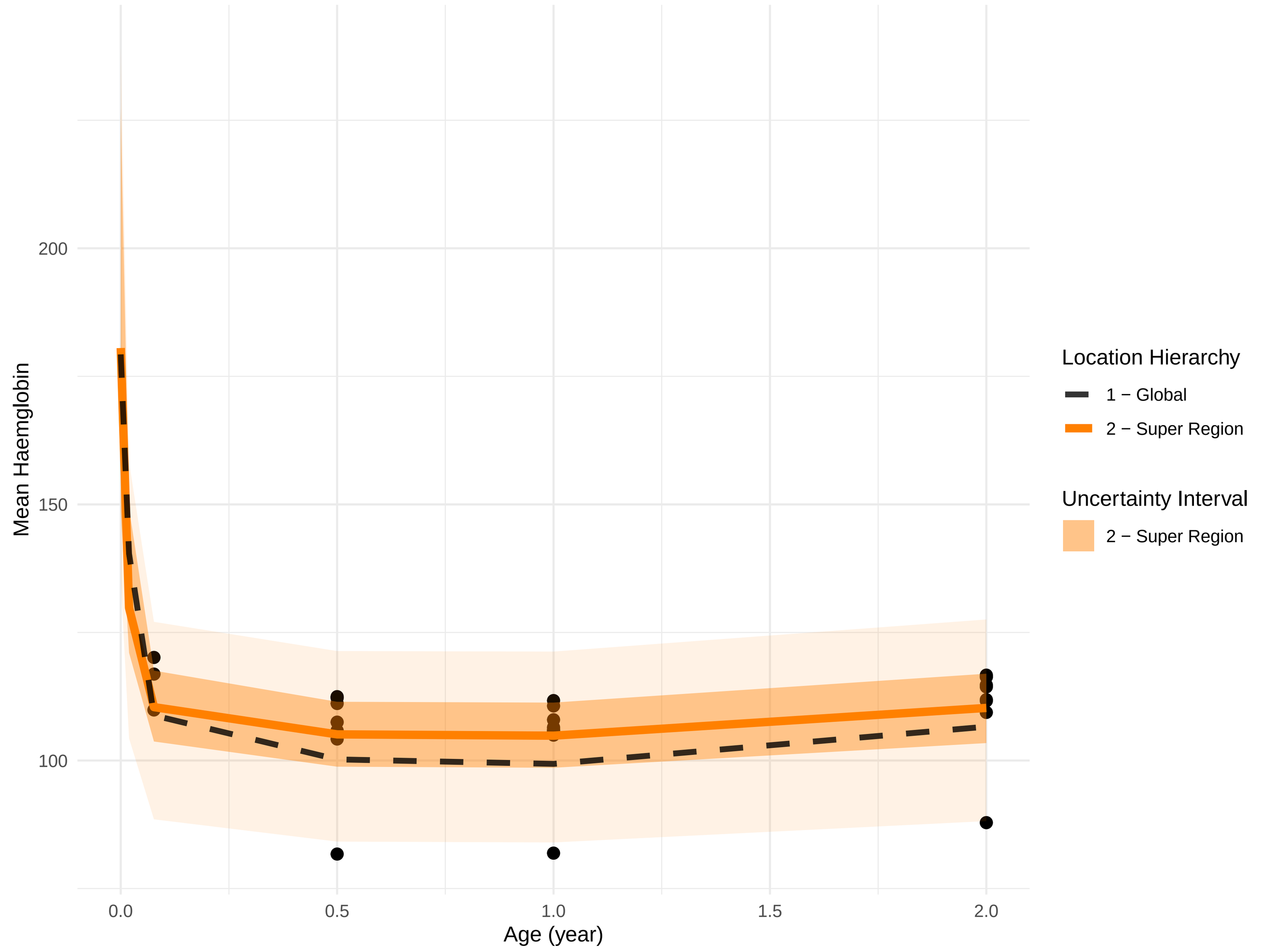

MR−BRT Age/Sex Fits − Non−pregnant Females <2 Years Old − Mean Haemglobin − North Africa and Middle East
WHO−2024 Adjustment Method

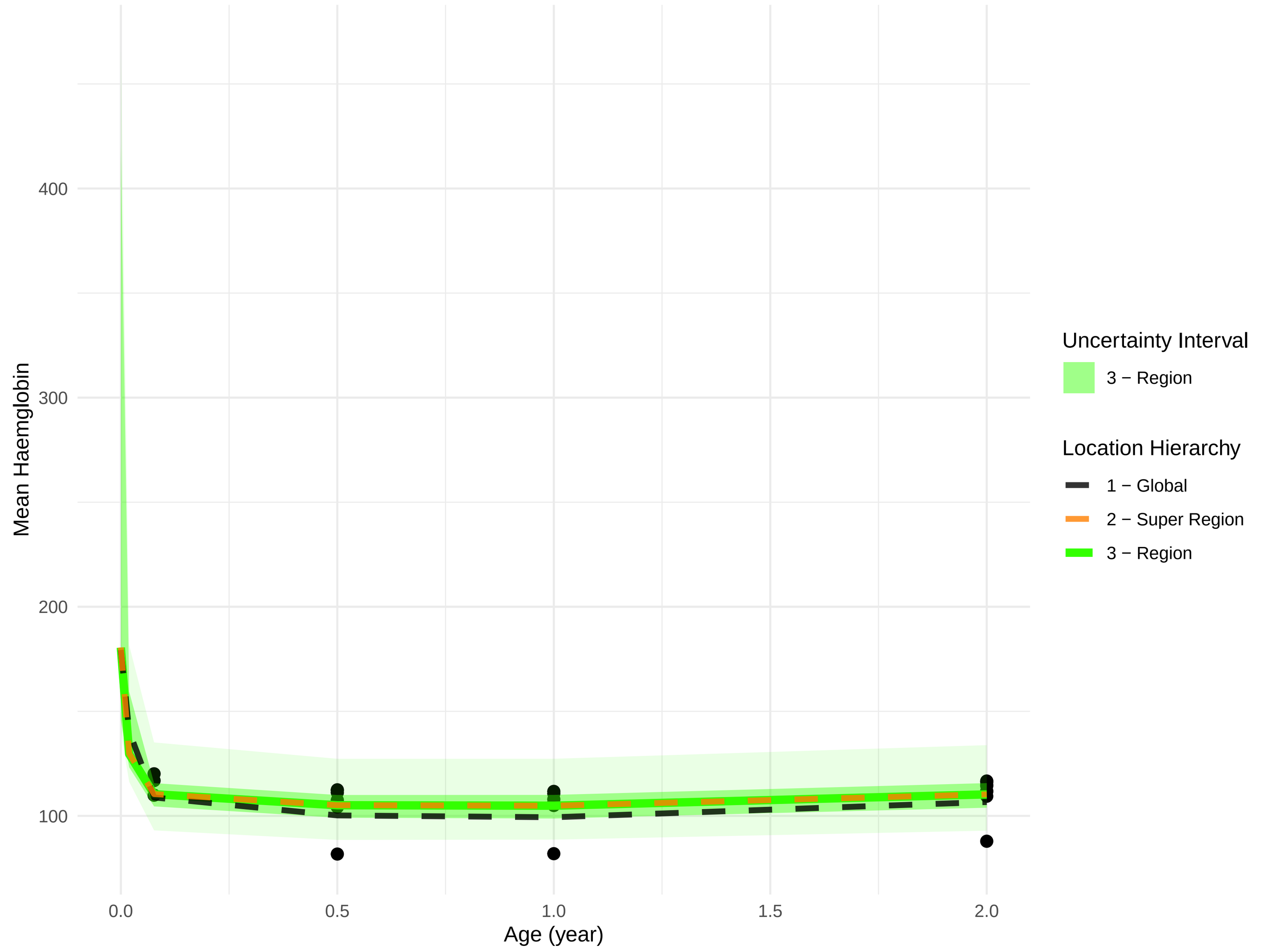

MR−BRT Age/Sex Fits − Non−pregnant Females <2 Years Old − Mean Haemglobin − Egypt
WHO−2024 Adjustment Method

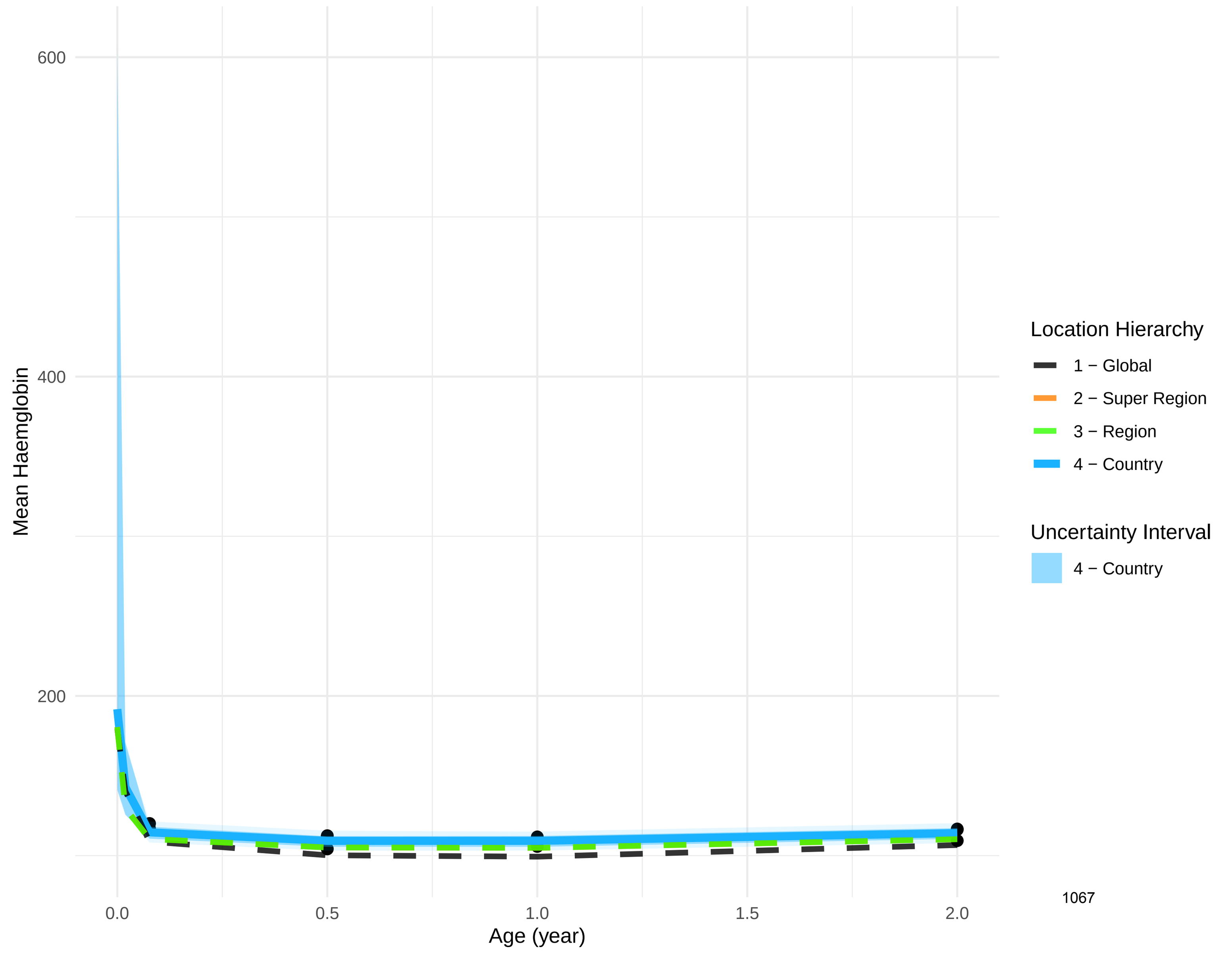

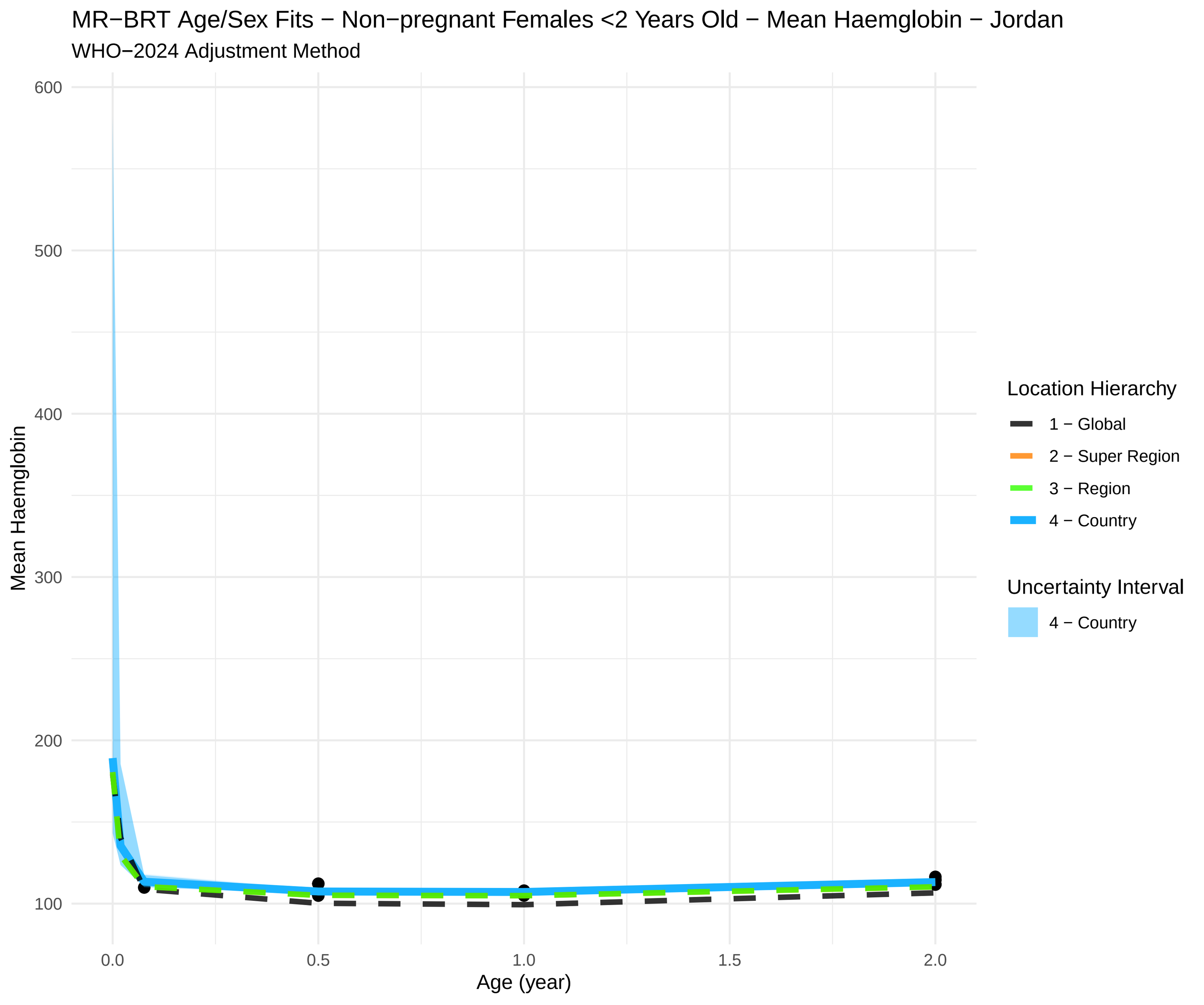
MR−BRT Age/Sex Fits − Non−pregnant Females <2 Years Old − Mean Haemglobin − Jordan
WHO−2024 Adjustment Method
Mean Haemglobin
Age (year)
Location Hierarchy
1 − Global
2 − Super Region
3 − Region
4 − Country
Uncertainty Interval
4 − Country

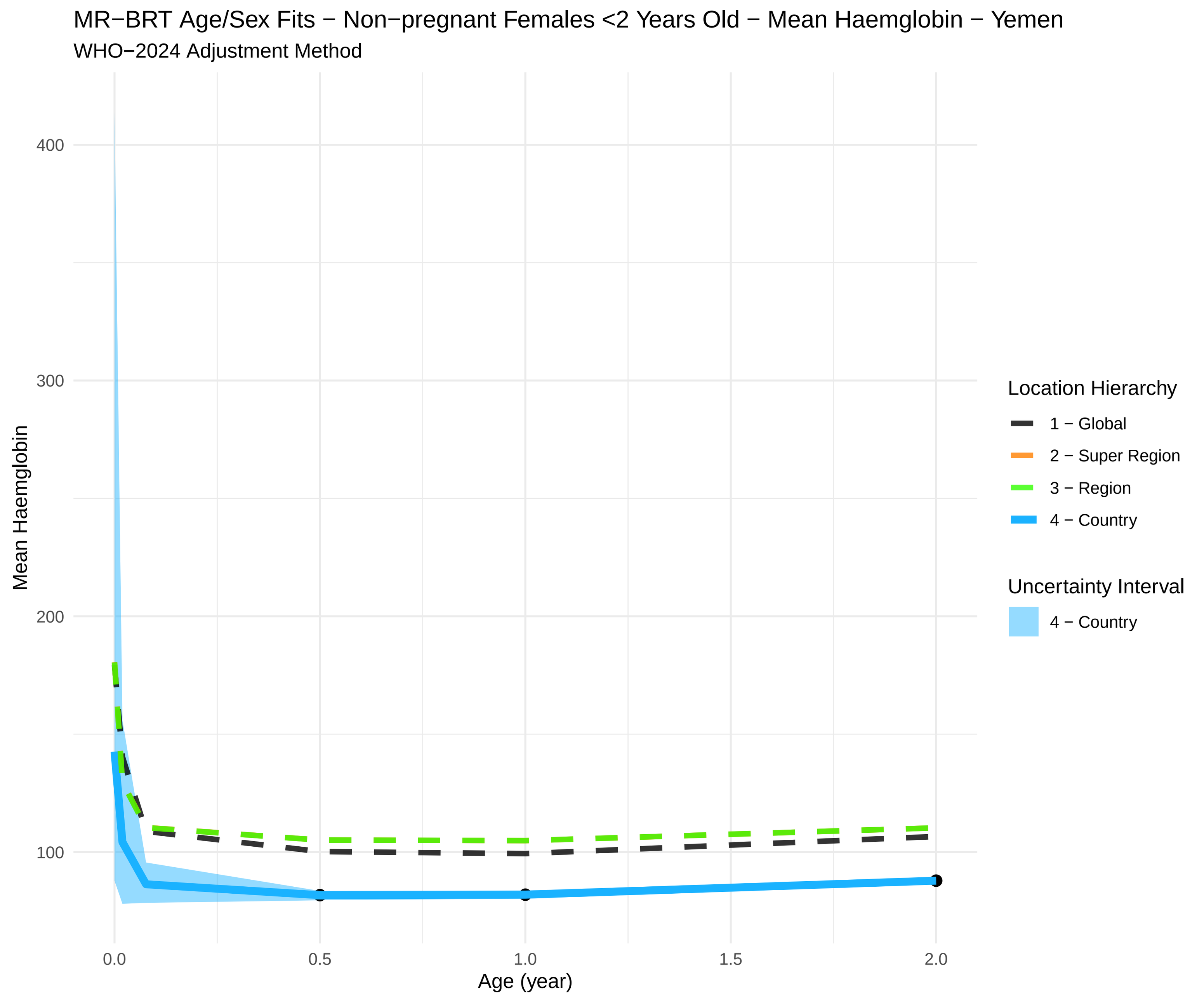
MR−BRT Age/Sex Fits − Non−pregnant Females <2 Years Old − Mean Haemglobin − Yemen
WHO−2024 Adjustment Method
Mean Haemglobin
Age (year)
Location Hierarchy
1 − Global
2 − Super Region
3 − Region
4 − Country
Uncertainty Interval
4 − Country

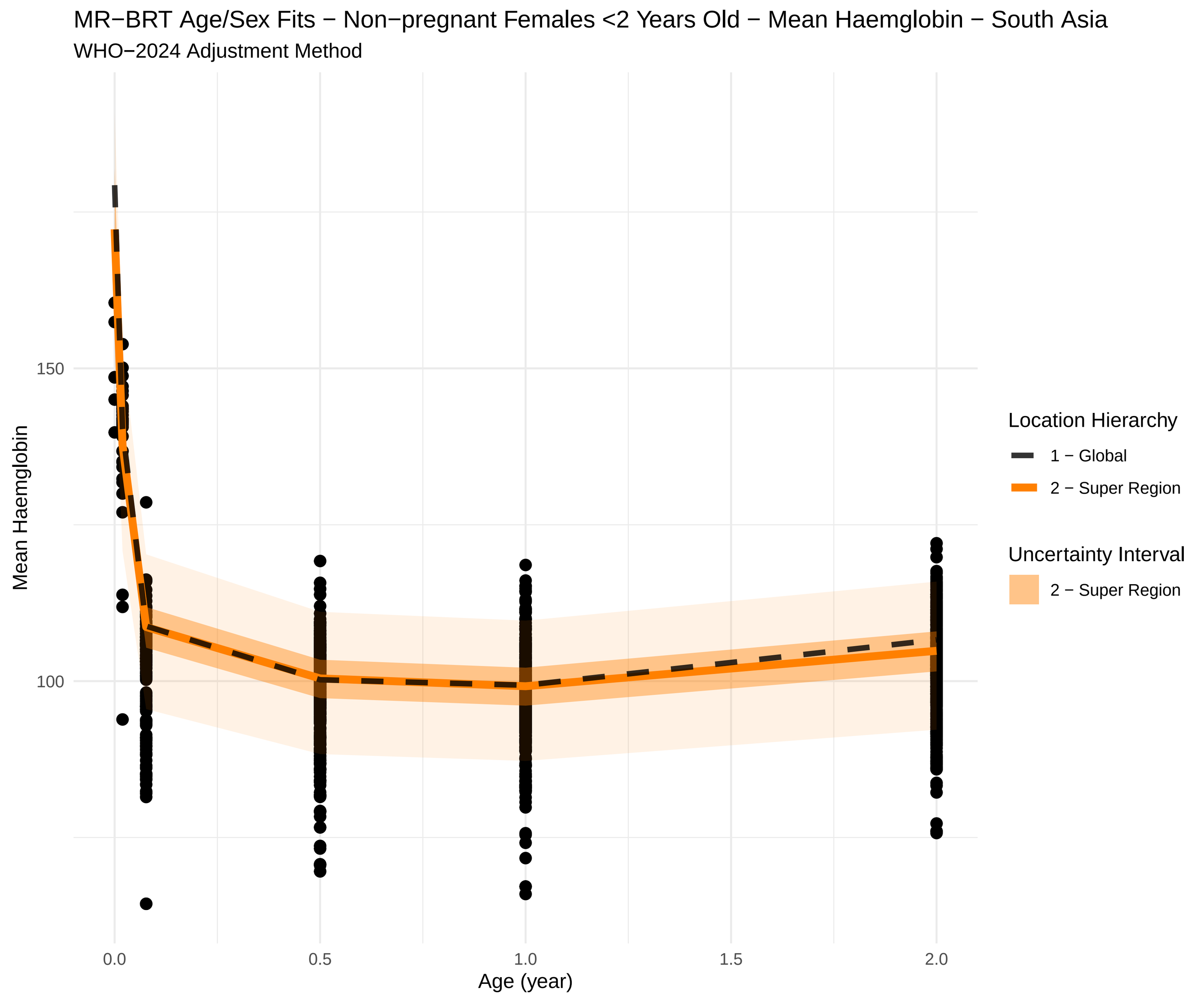
MR−BRT Age/Sex Fits − Non−pregnant Females <2 Years Old − Mean Haemglobin − South Asia
WHO−2024 Adjustment Method
Mean Haemglobin
Age (year)
Location Hierarchy
1 − Global
2 − Super Region
Uncertainty Interval
2 − Super Region

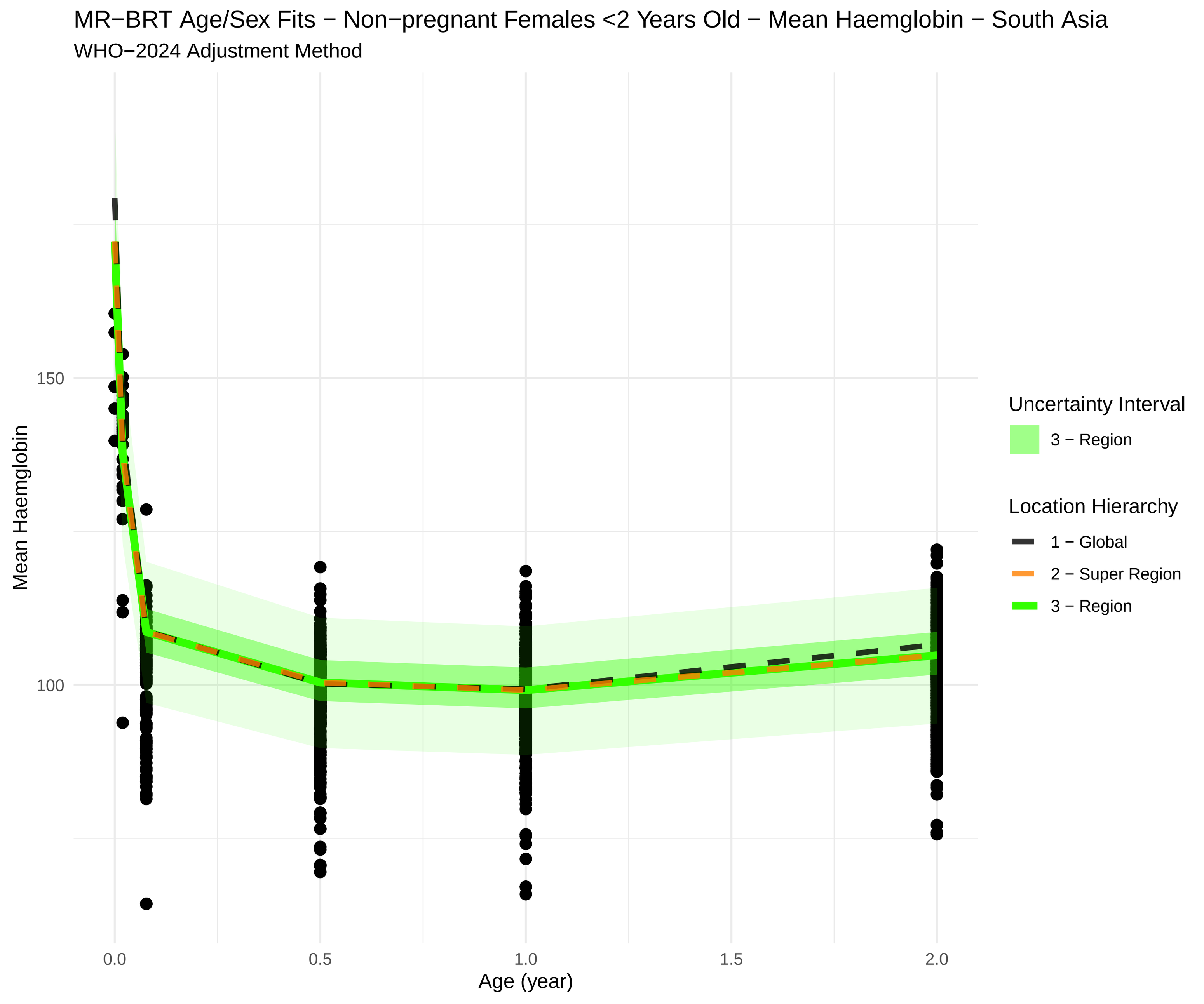
MR−BRT Age/Sex Fits − Non−pregnant Females <2 Years Old − Mean Haemglobin − South Asia
WHO−2024 Adjustment Method
Mean Haemglobin
Age (year)
Uncertainty Interval
3 − Region
Location Hierarchy
1 − Global
2 − Super Region
3 − Region

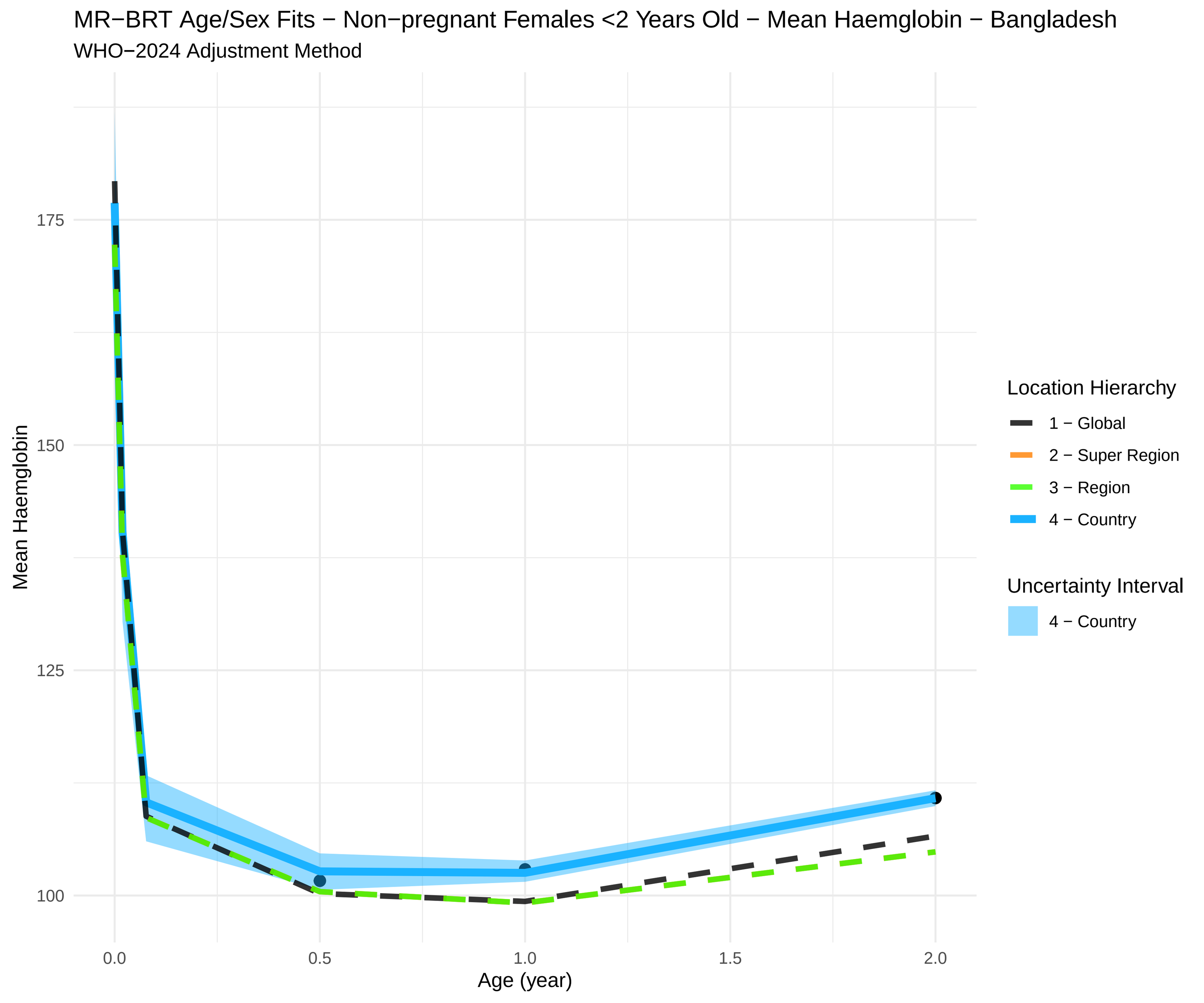
MR−BRT Age/Sex Fits − Non−pregnant Females <2 Years Old − Mean Haemglobin − Bangladesh
WHO−2024 Adjustment Method
Mean Haemglobin
Age (year)
Location Hierarchy
1 − Global
2 − Super Region
3 − Region
4 − Country
Uncertainty Interval
4 − Country

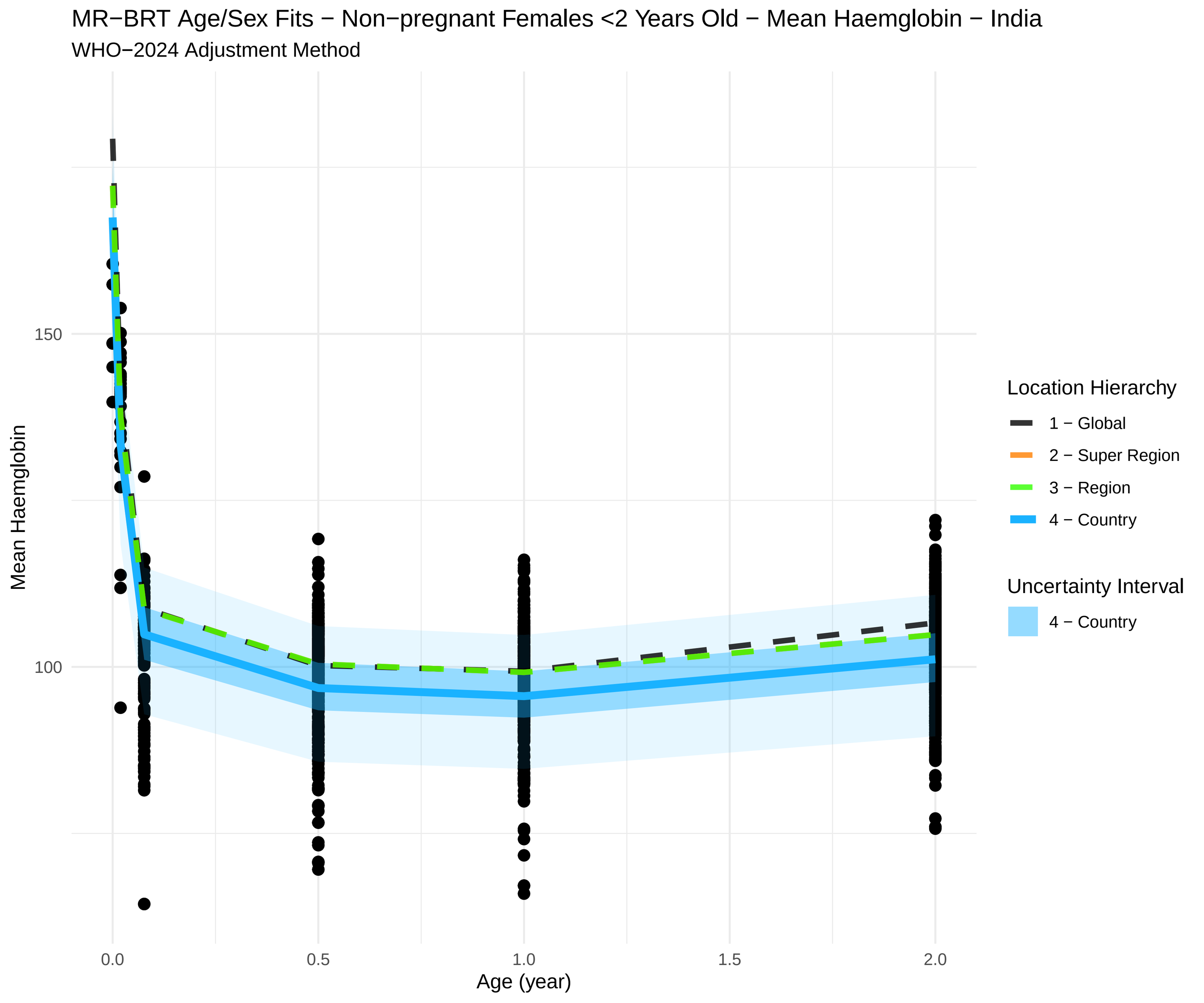
MR−BRT Age/Sex Fits − Non−pregnant Females <2 Years Old − Mean Haemglobin − India
WHO−2024 Adjustment Method
Mean Haemglobin
Age (year)
Location Hierarchy
1 − Global
2 − Super Region
3 − Region
4 − Country
Uncertainty Interval
4 − Country

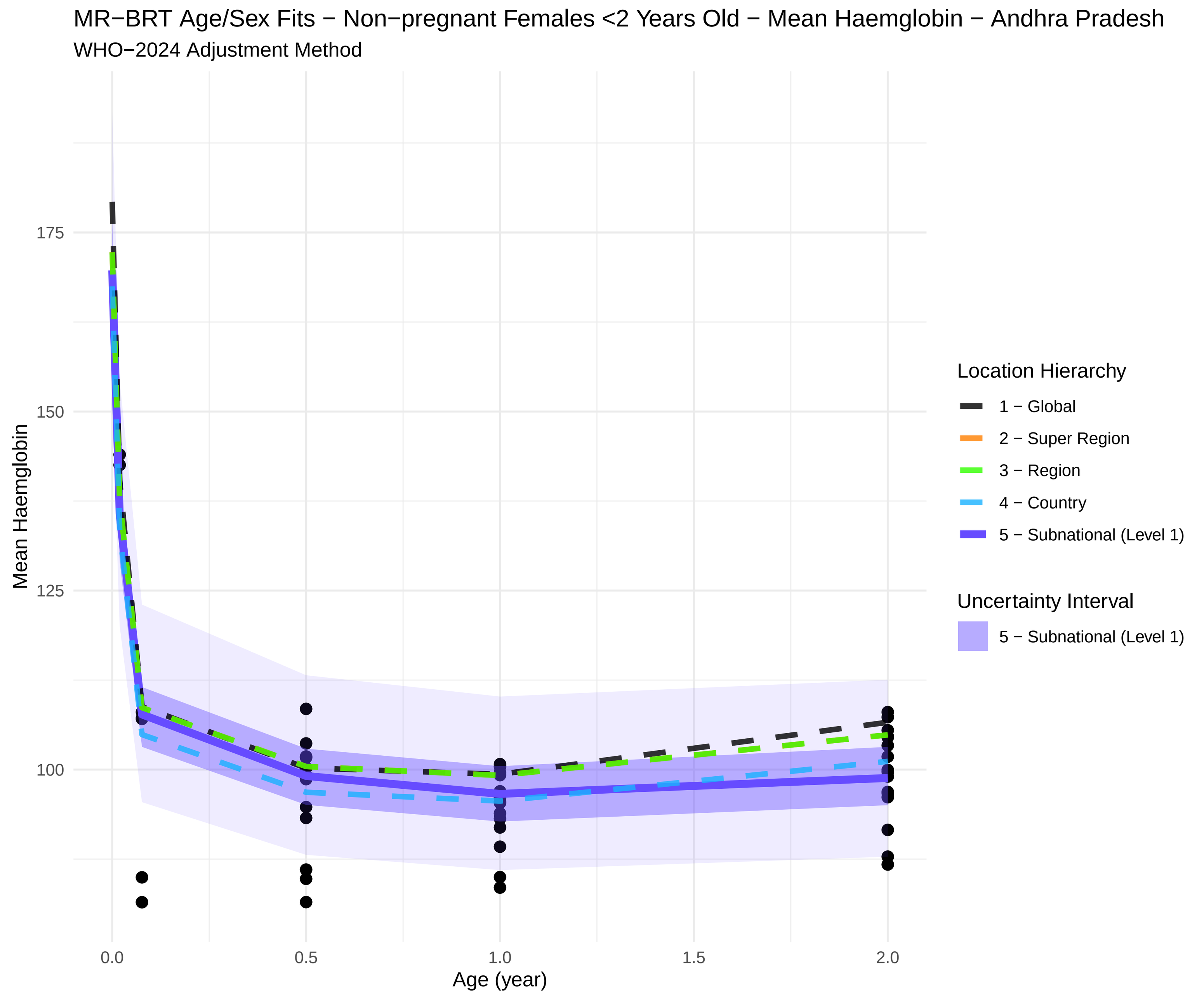
MR−BRT Age/Sex Fits − Non−pregnant Females <2 Years Old − Mean Haemglobin − Andhra Pradesh
WHO−2024 Adjustment Method
Mean Haemglobin
Age (year)
Location Hierarchy
1 − Global
2 − Super Region
3 − Region
4 − Country
5 − Subnational (Level 1)
Uncertainty Interval
5 − Subnational (Level 1)

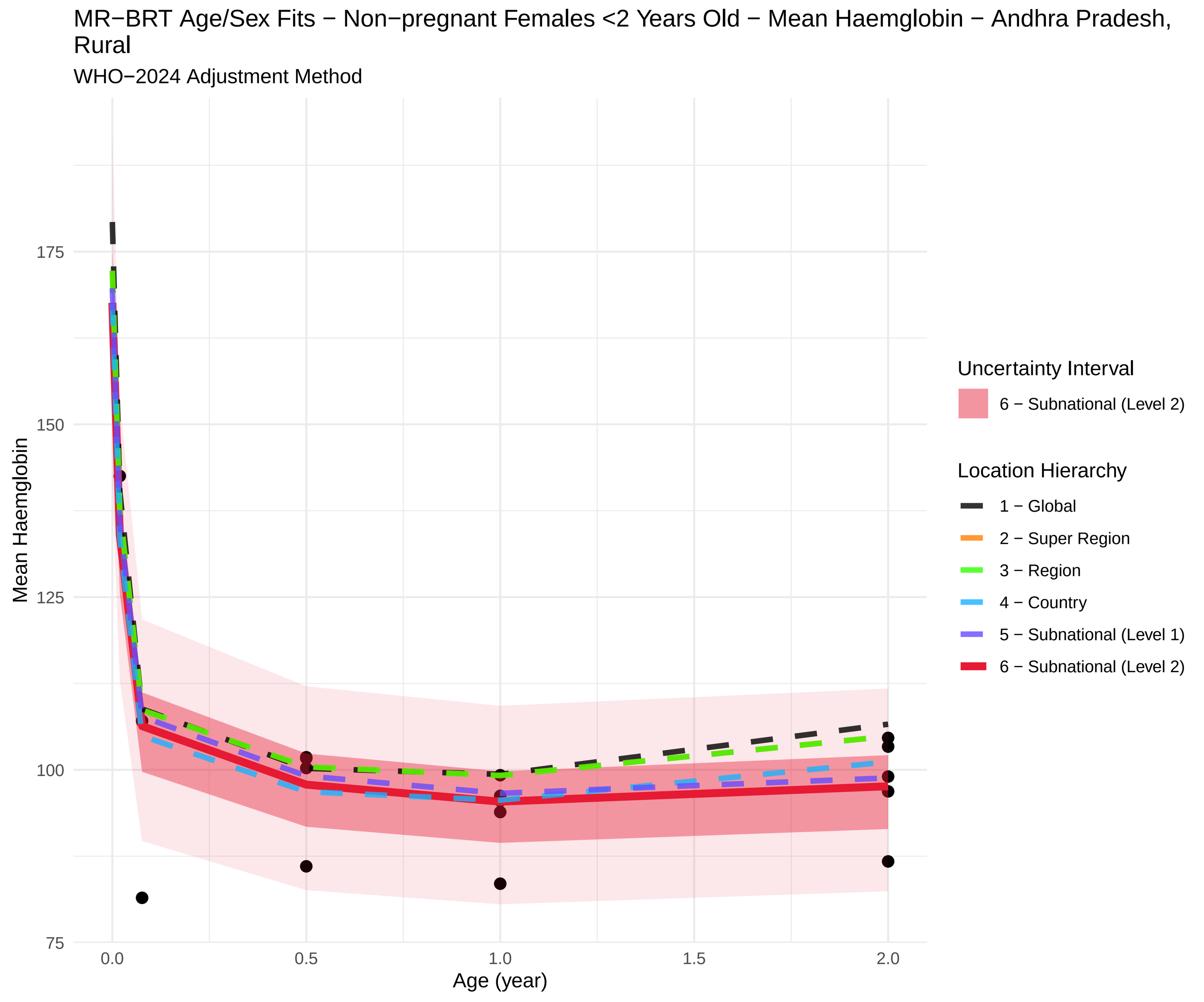
MR−BRT Age/Sex Fits − Non−pregnant Females <2 Years Old − Mean Haemglobin − Andhra Pradesh, Rural
WHO−2024 Adjustment Method
Mean Haemglobin
Age (year)
Uncertainty Interval
6 − Subnational (Level 2)
Location Hierarchy
1 − Global
2 − Super Region
3 − Region
4 − Country
5 − Subnational (Level 1)
6 − Subnational (Level 2)

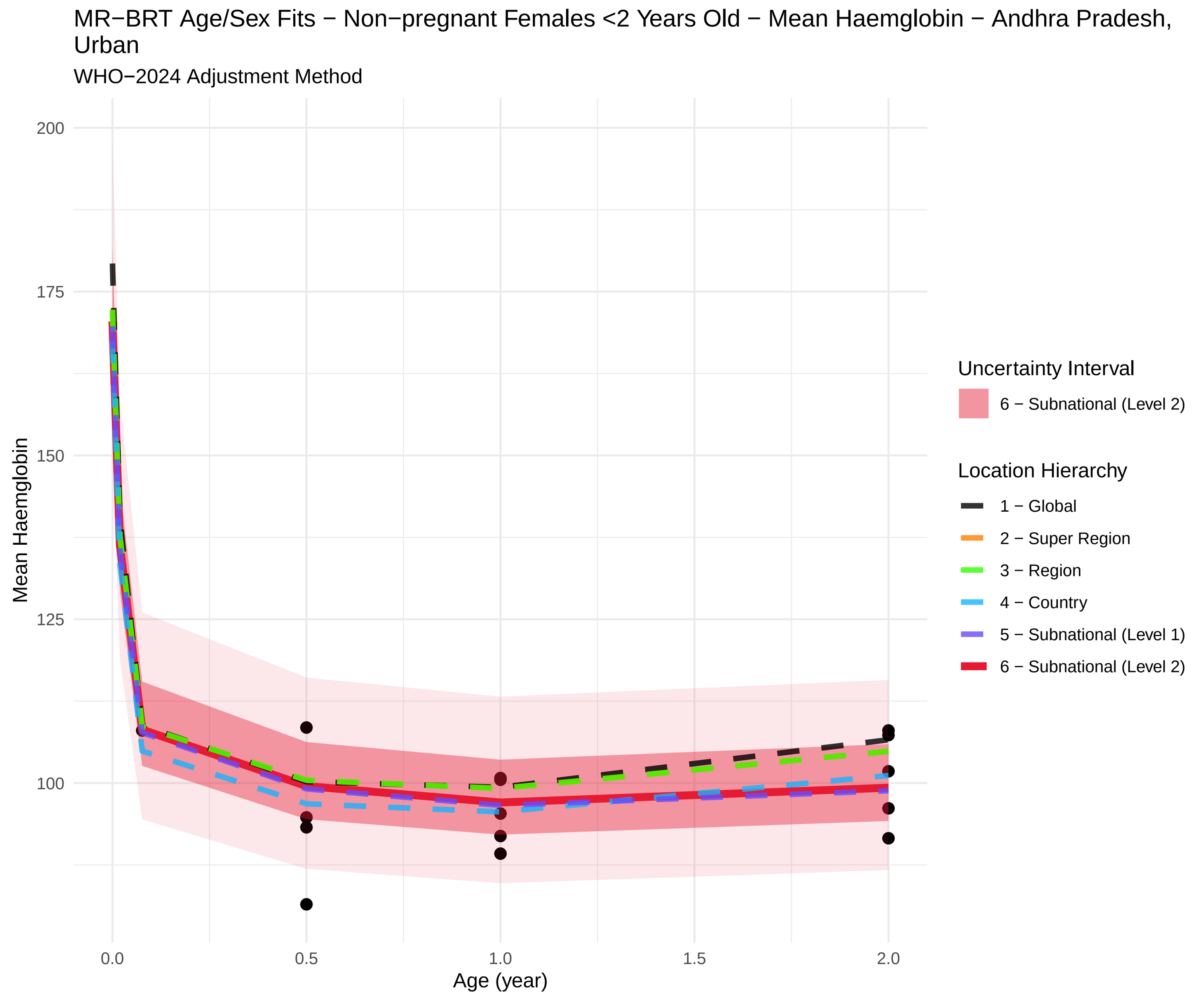
MR−BRT Age/Sex Fits − Non−pregnant Females <2 Years Old − Mean Haemglobin − Andhra Pradesh, Urban
WHO−2024 Adjustment Method
Mean Haemglobin
Age (year)
Uncertainty Interval
6 − Subnational (Level 2)
Location Hierarchy
1 − Global
2 − Super Region
3 − Region
4 − Country
5 − Subnational (Level 1)
6 − Subnational (Level 2)

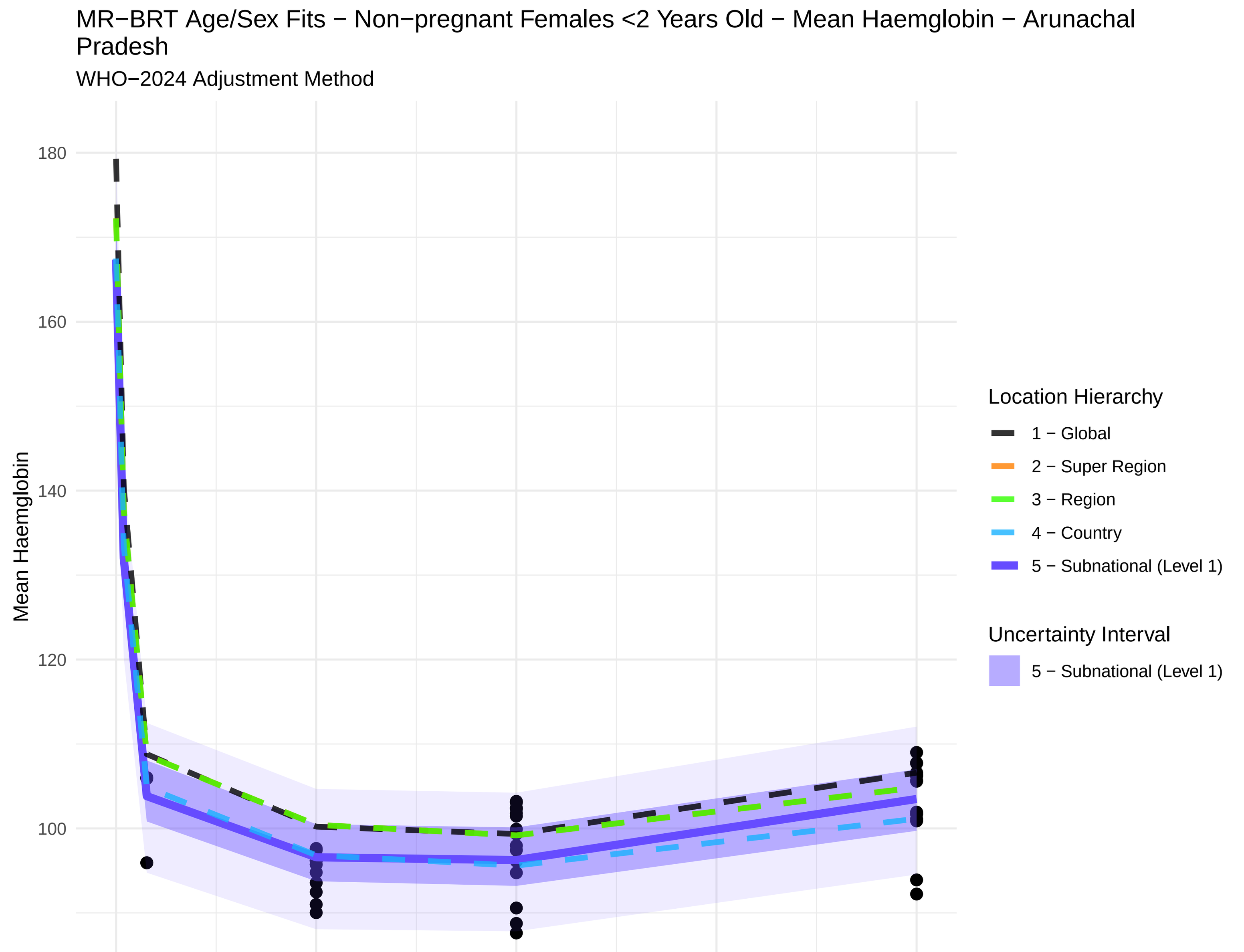
MR−BRT Age/Sex Fits − Non−pregnant Females <2 Years Old − Mean Haemglobin − Arunachal Pradesh
WHO−2024 Adjustment Method
Mean Haemglobin
Age (year)
Location Hierarchy
1 − Global
2 − Super Region
3 − Region
4 − Country
5 − Subnational (Level 1)
Uncertainty Interval
5 − Subnational (Level 1)

MR−BRT Age/Sex Fits − Non−pregnant Females <2 Years Old − Mean Haemglobin − Arunachal Pradesh, Rural

WHO−2024 Adjustment Method

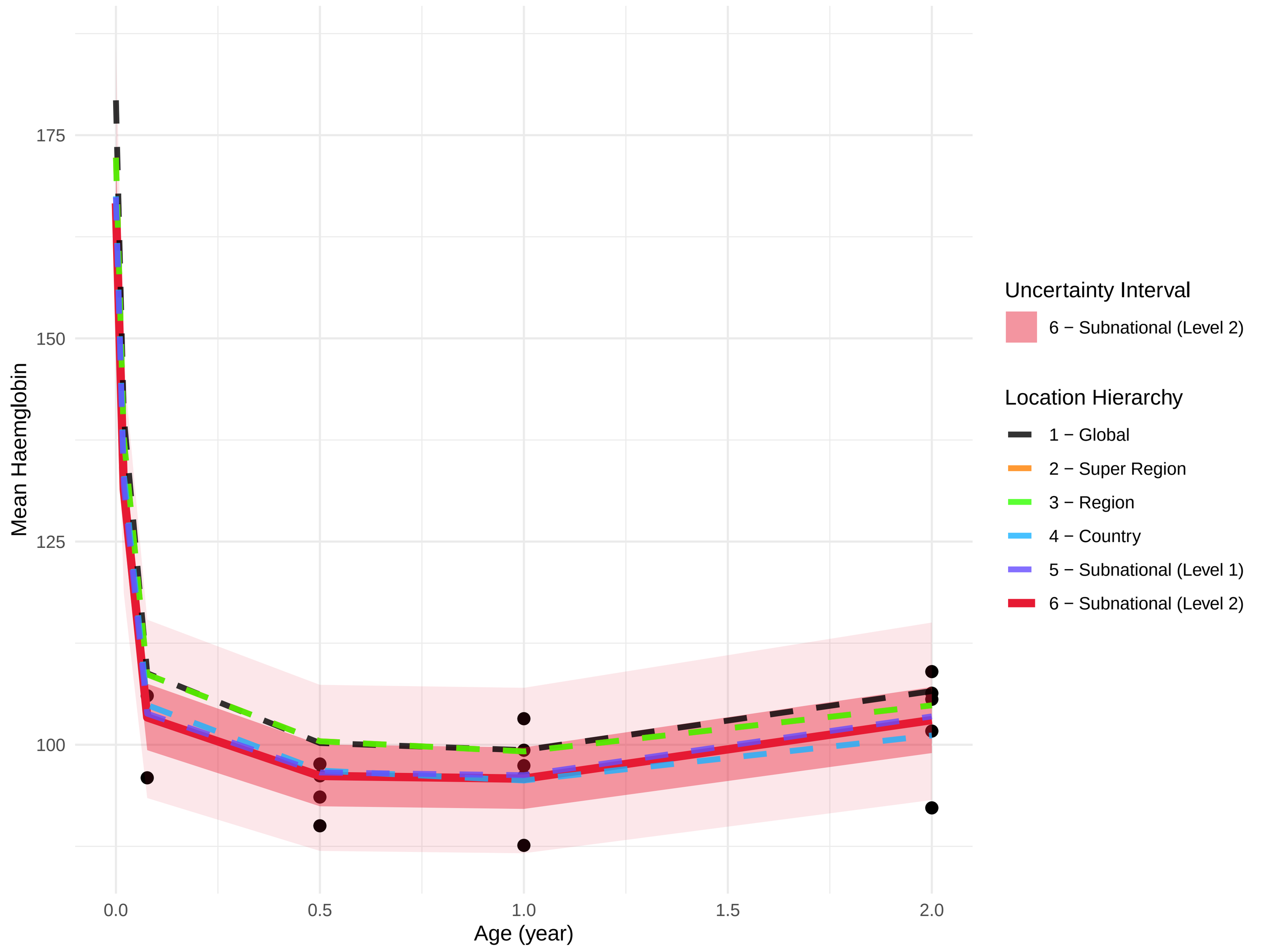

MR−BRT Age/Sex Fits − Non−pregnant Females <2 Years Old − Mean Haemglobin − Arunachal Pradesh, Urban

WHO−2024 Adjustment Method

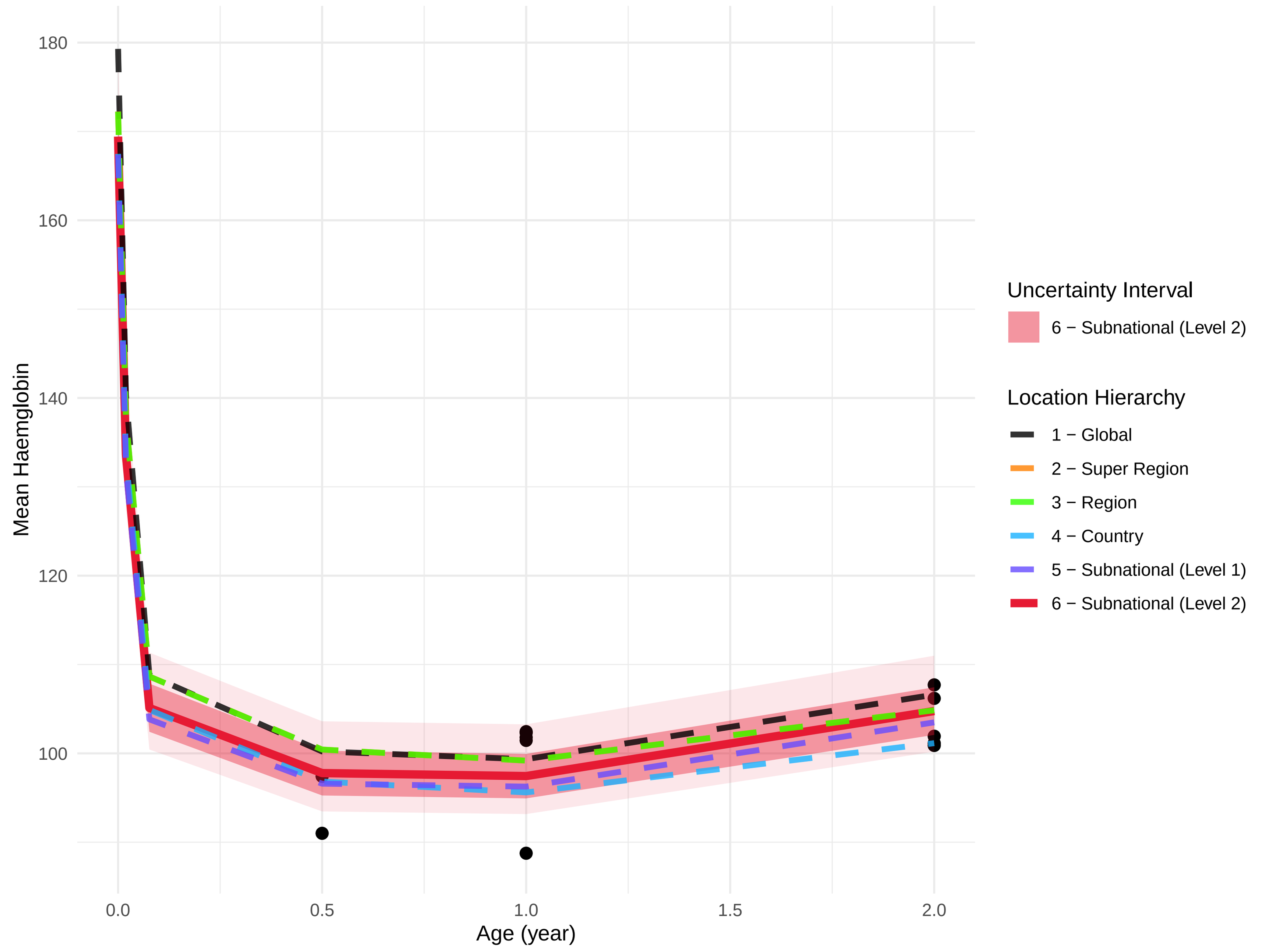

MR−BRT Age/Sex Fits − Non−pregnant Females <2 Years Old − Mean Haemglobin − Assam

WHO−2024 Adjustment Method

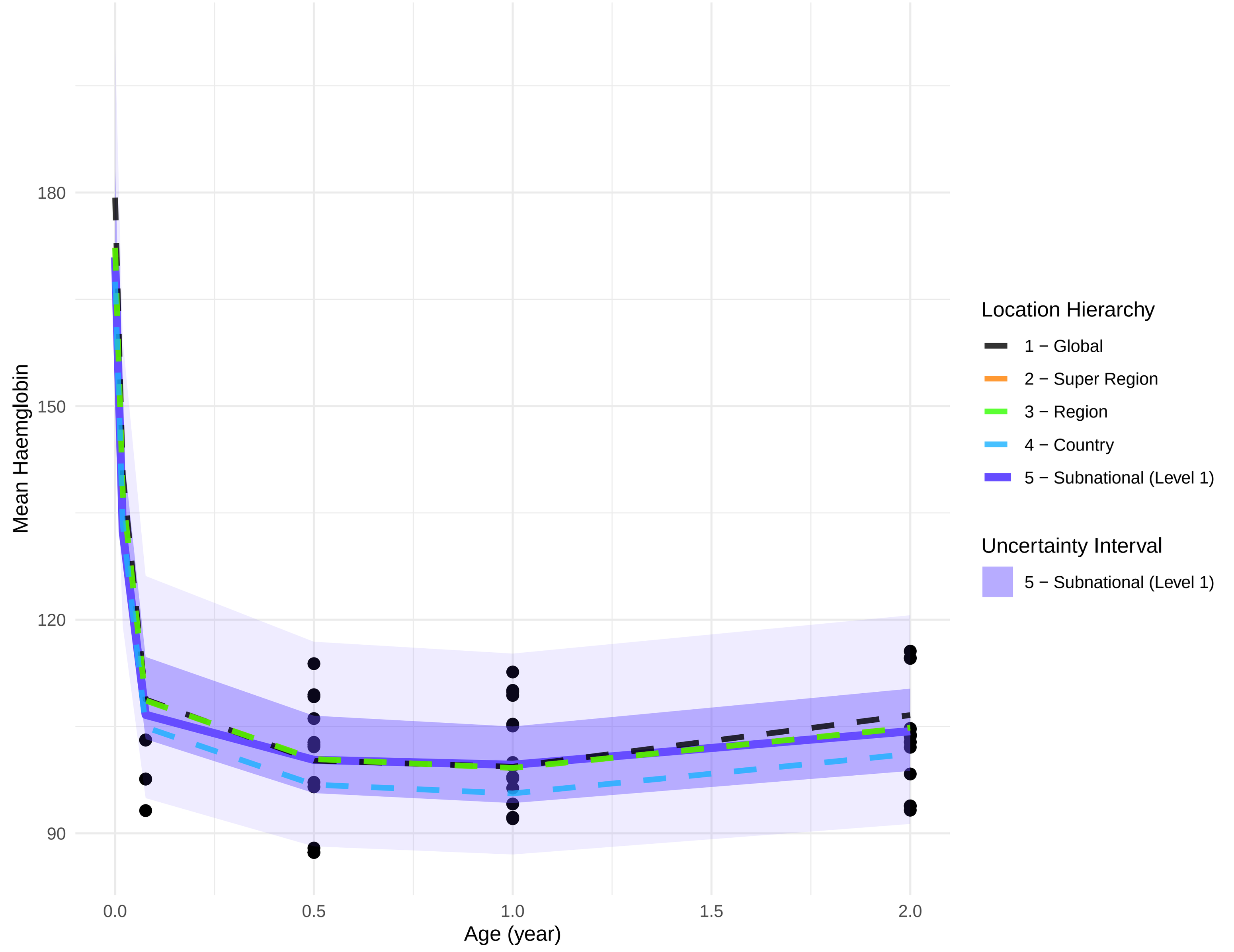

MR−BRT Age/Sex Fits − Non−pregnant Females <2 Years Old − Mean Haemglobin − Assam, Rural

WHO−2024 Adjustment Method

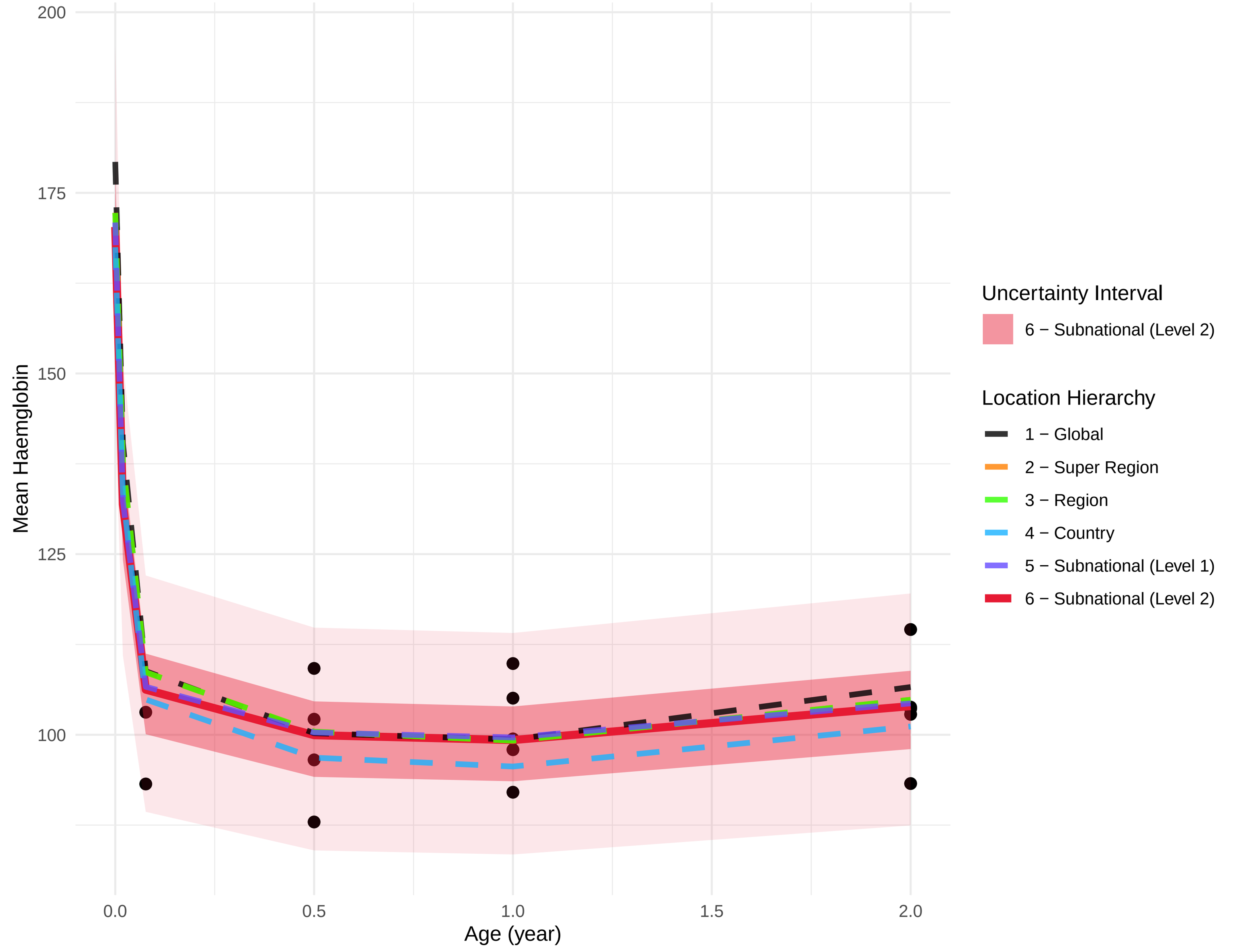

MR−BRT Age/Sex Fits − Non−pregnant Females <2 Years Old − Mean Haemglobin − Assam, Urban

WHO−2024 Adjustment Method

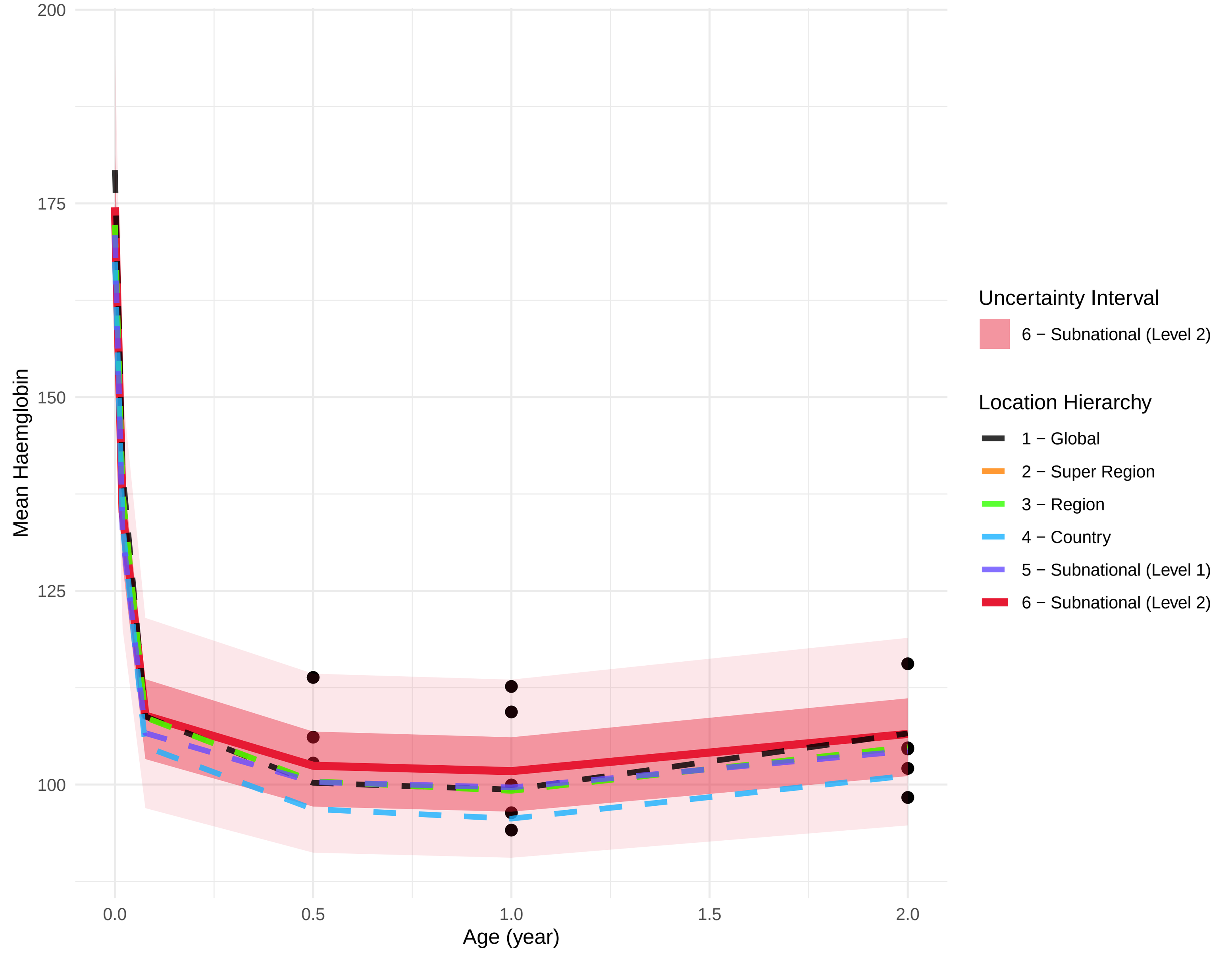

MR−BRT Age/Sex Fits − Non−pregnant Females <2 Years Old − Mean Haemglobin − Bihar

WHO−2024 Adjustment Method

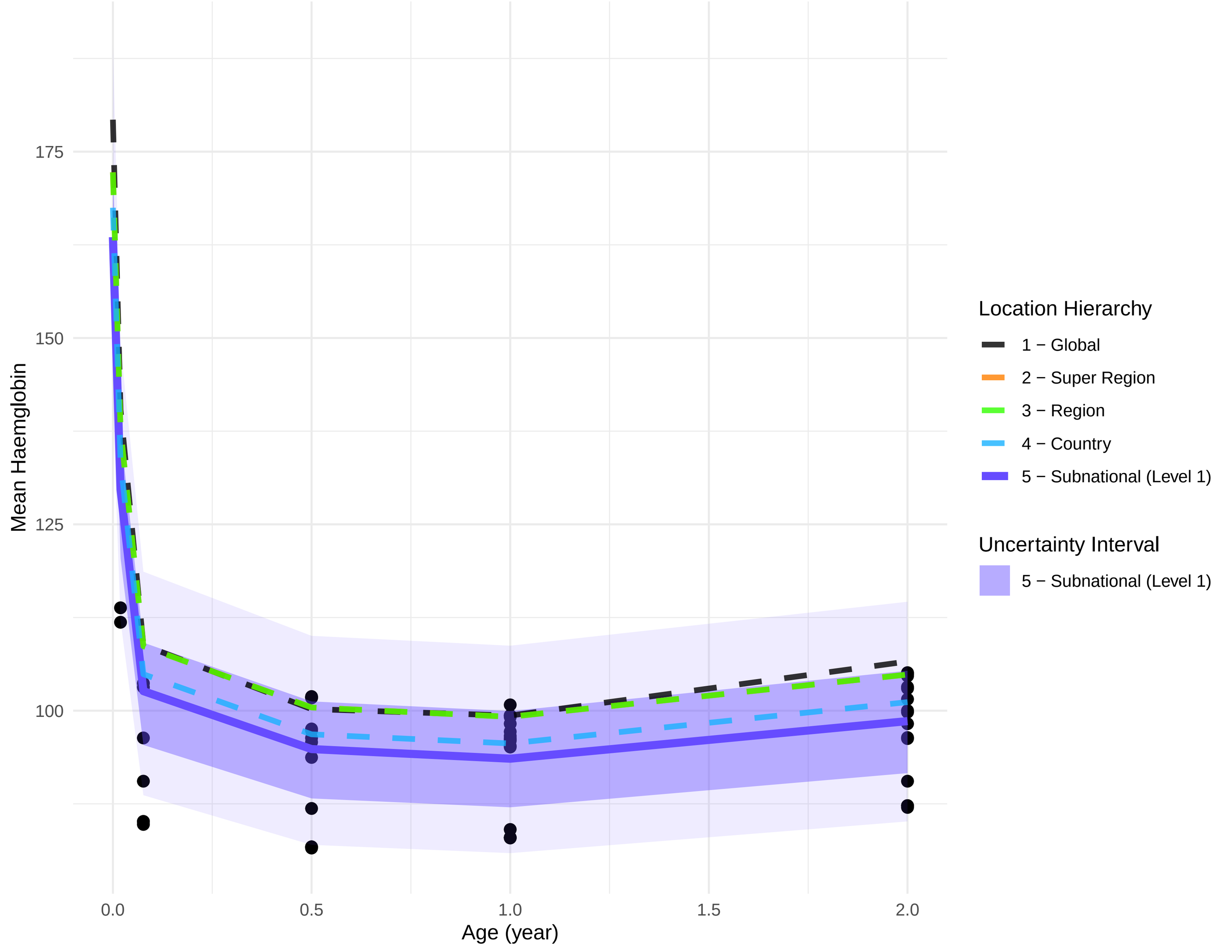

MR−BRT Age/Sex Fits − Non−pregnant Females <2 Years Old − Mean Haemglobin − Bihar, Rural

WHO−2024 Adjustment Method

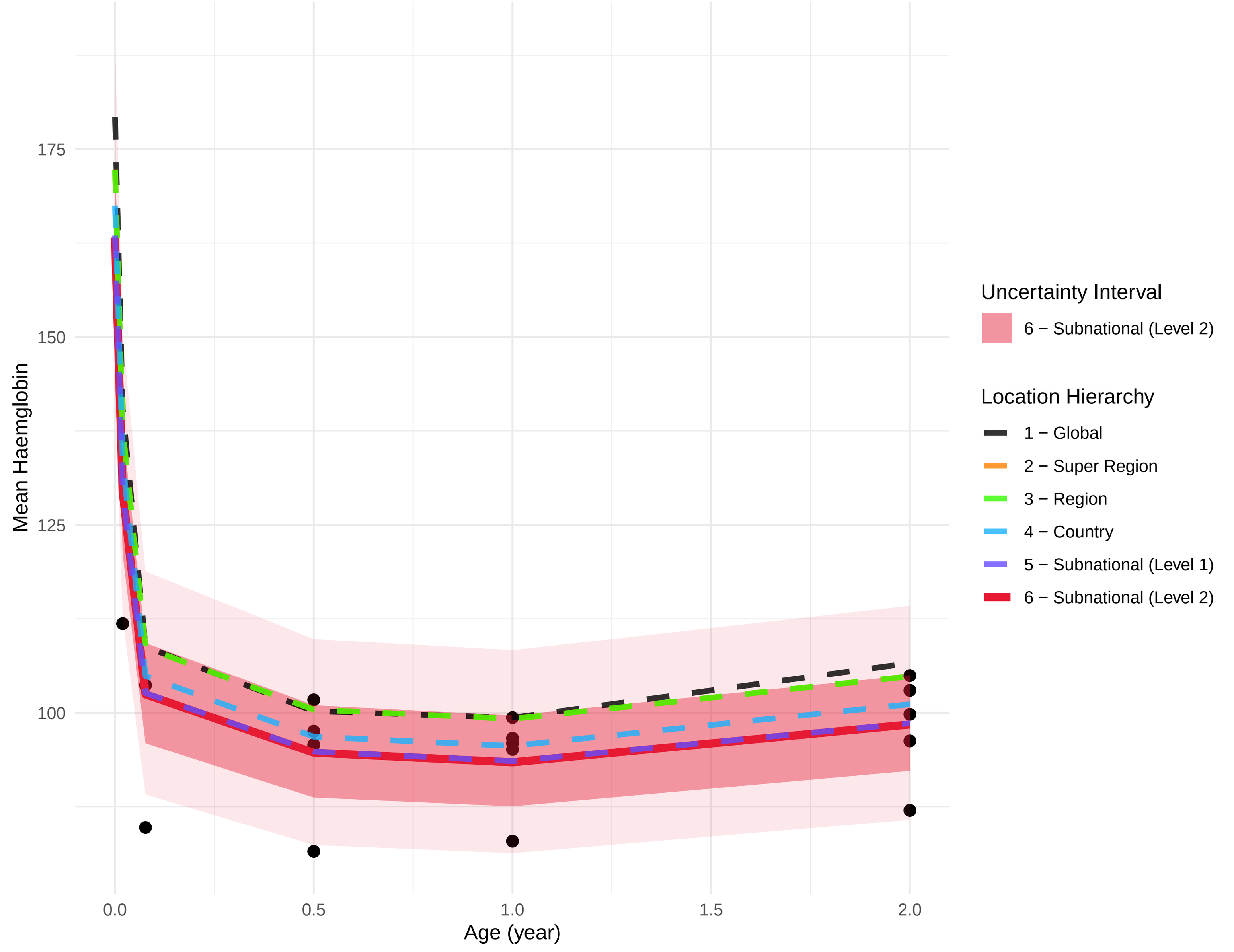

MR−BRT Age/Sex Fits − Non−pregnant Females <2 Years Old − Mean Haemglobin − Bihar, Urban

WHO−2024 Adjustment Method

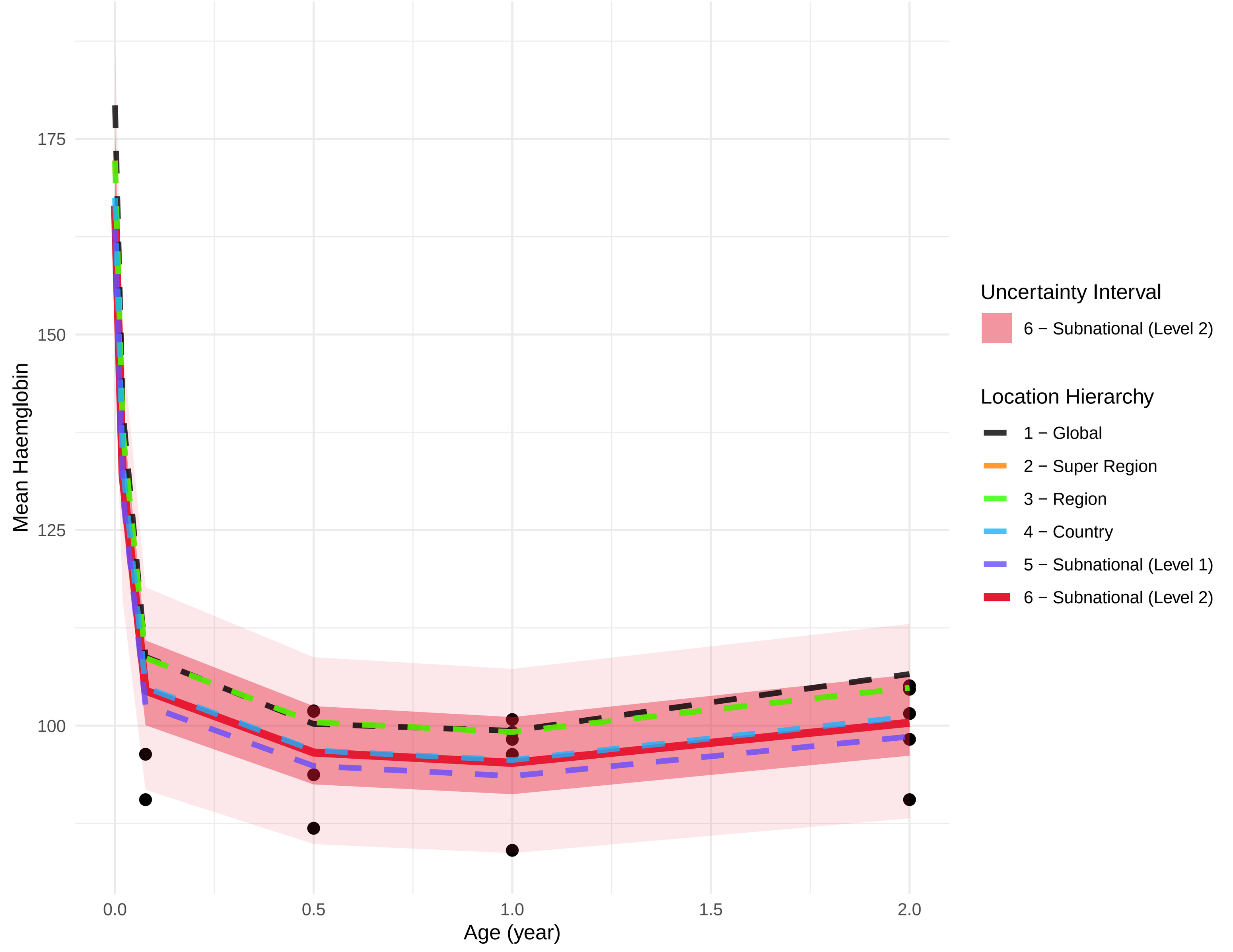

MR−BRT Age/Sex Fits − Non−pregnant Females <2 Years Old − Mean Haemglobin − Chhattisgarh

WHO−2024 Adjustment Method

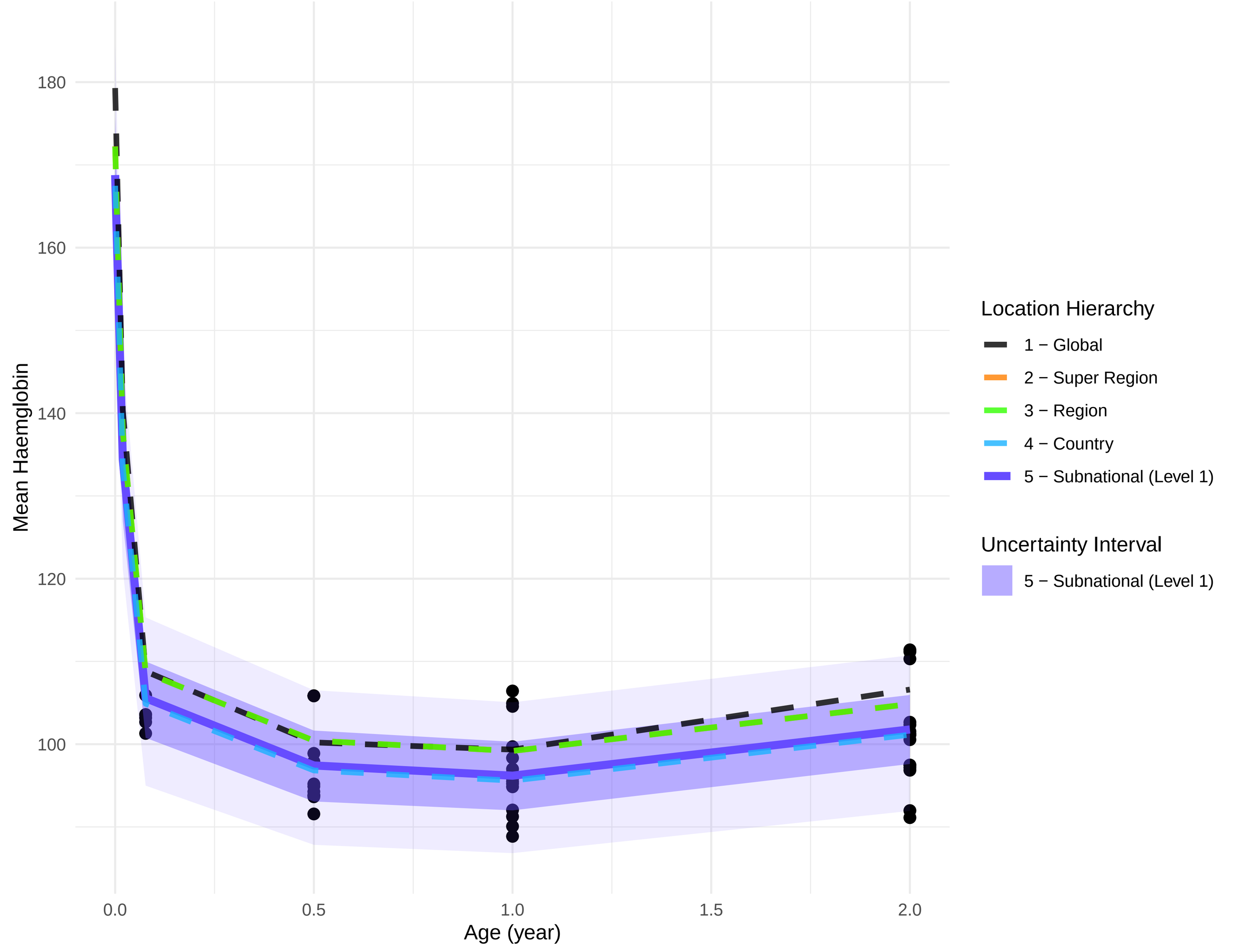

MR−BRT Age/Sex Fits − Non−pregnant Females <2 Years Old − Mean Haemglobin − Chhattisgarh, Rural

WHO−2024 Adjustment Method

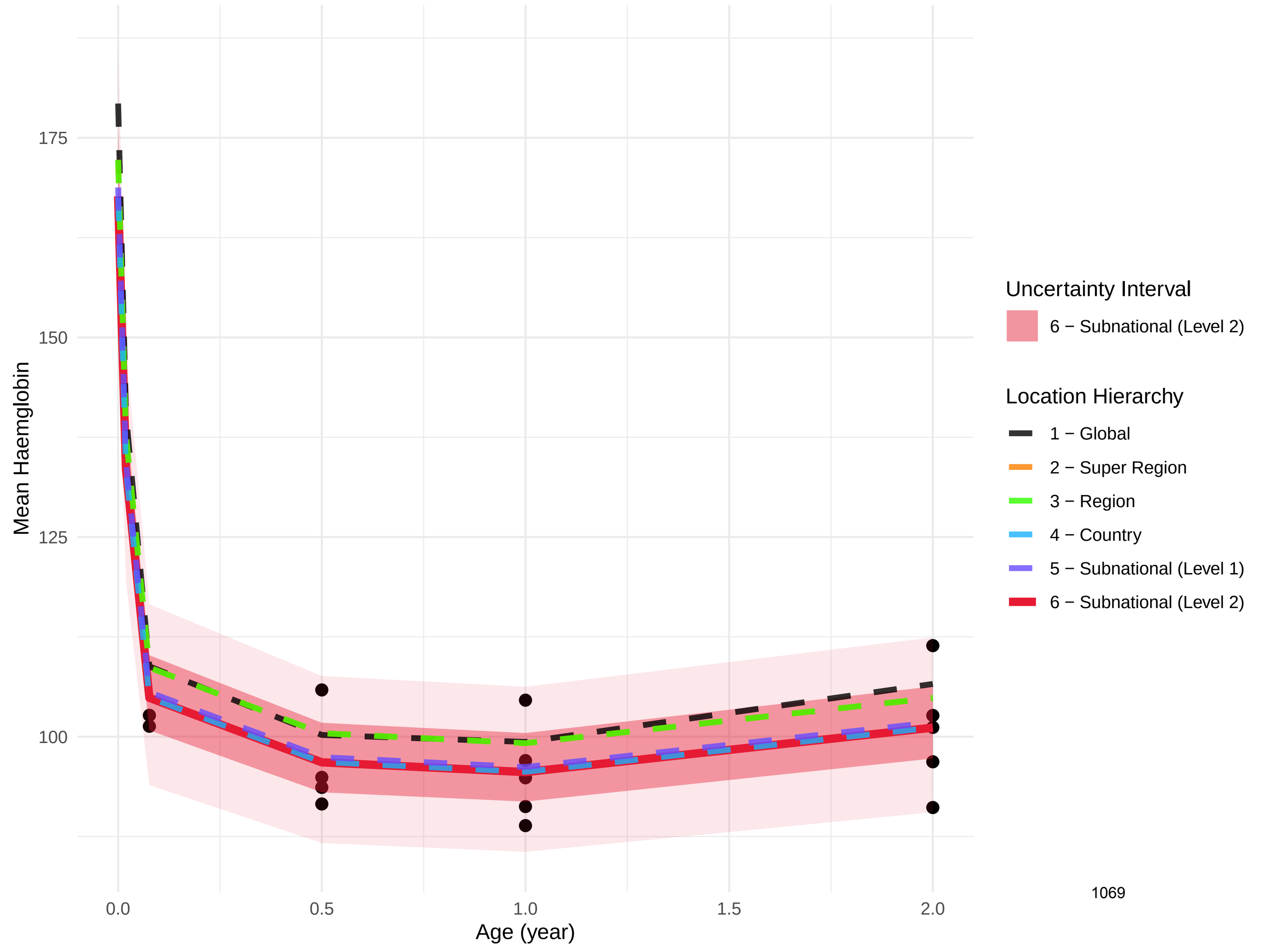

MR−BRT Age/Sex Fits − Non−pregnant Females <2 Years Old − Mean Haemglobin − Chhattisgarh, Urban
WHO−2024 Adjustment Method

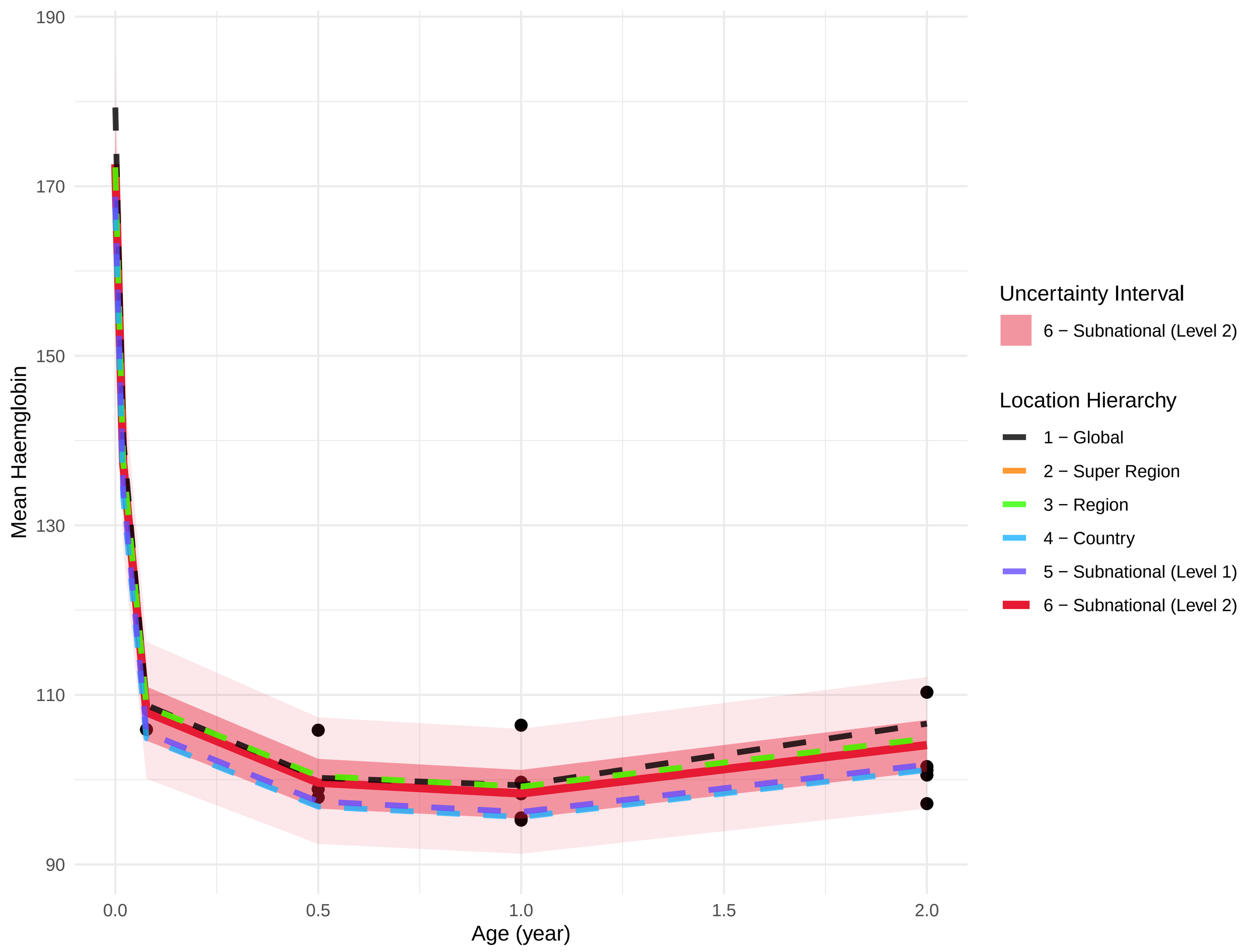

MR−BRT Age/Sex Fits − Non−pregnant Females <2 Years Old − Mean Haemglobin − Delhi
WHO−2024 Adjustment Method

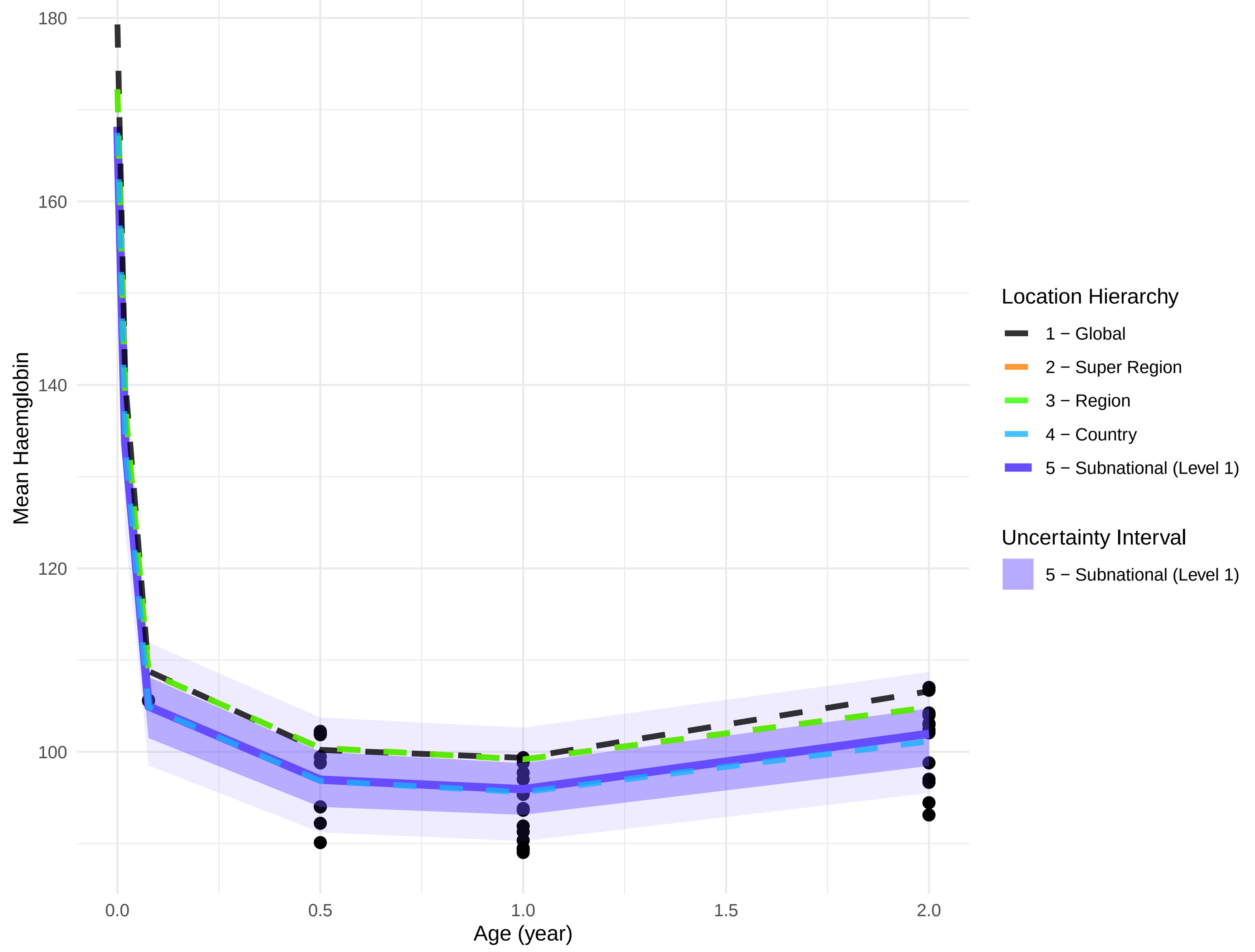

MR−BRT Age/Sex Fits − Non−pregnant Females <2 Years Old − Mean Haemglobin − Delhi, Rural
WHO−2024 Adjustment Method

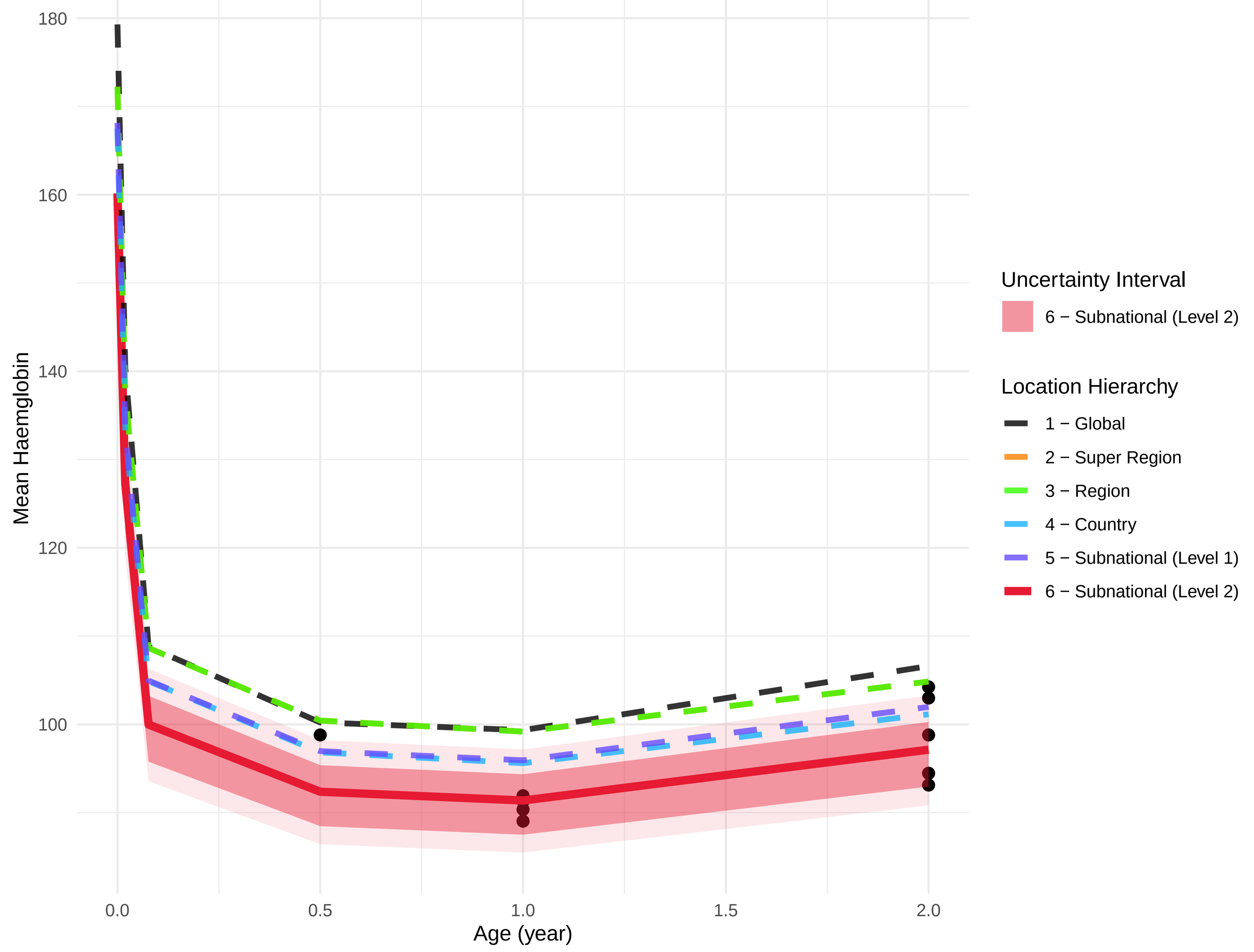

MR−BRT Age/Sex Fits − Non−pregnant Females <2 Years Old − Mean Haemglobin − Delhi, Urban
WHO−2024 Adjustment Method

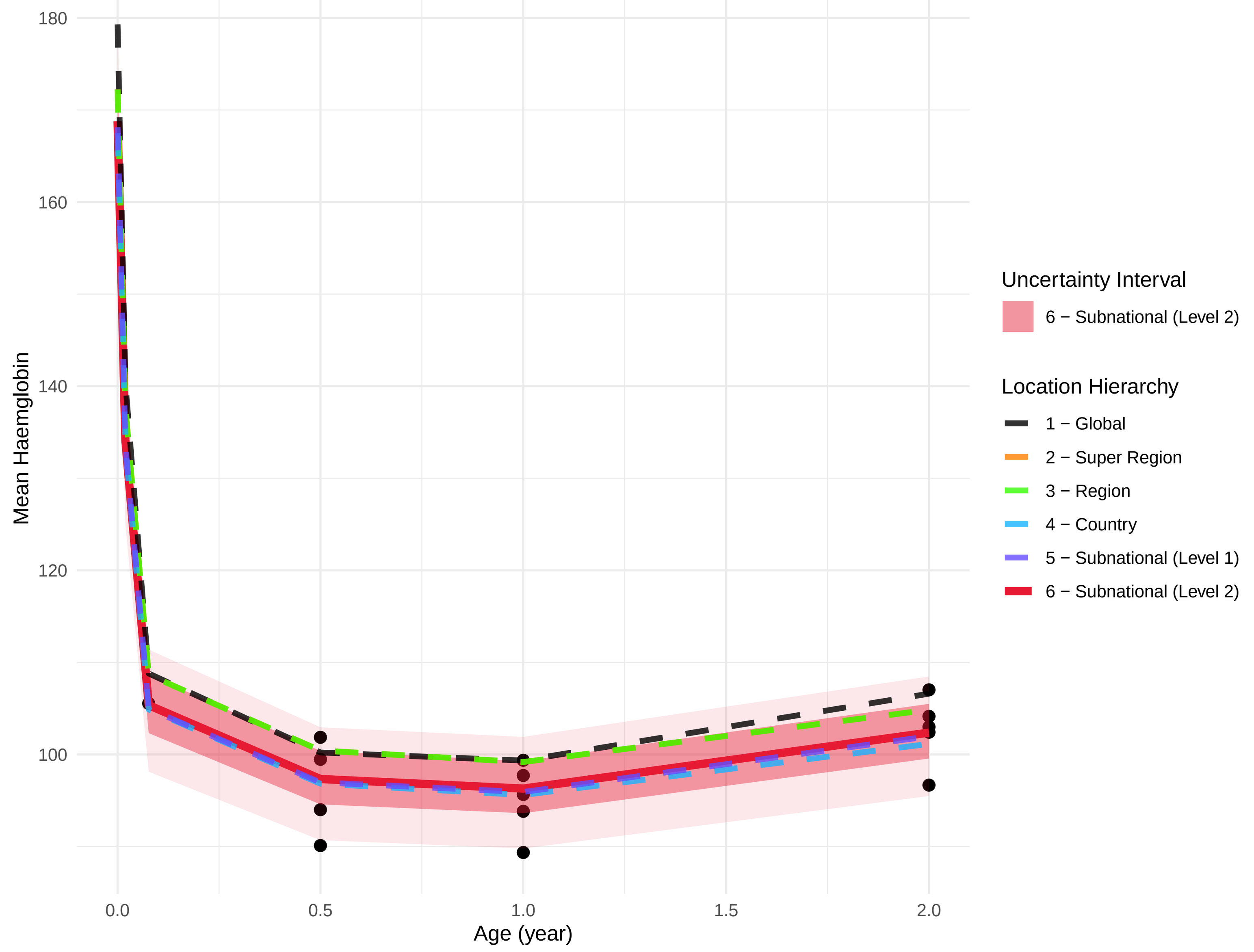

MR−BRT Age/Sex Fits − Non−pregnant Females <2 Years Old − Mean Haemglobin − Goa
WHO−2024 Adjustment Method

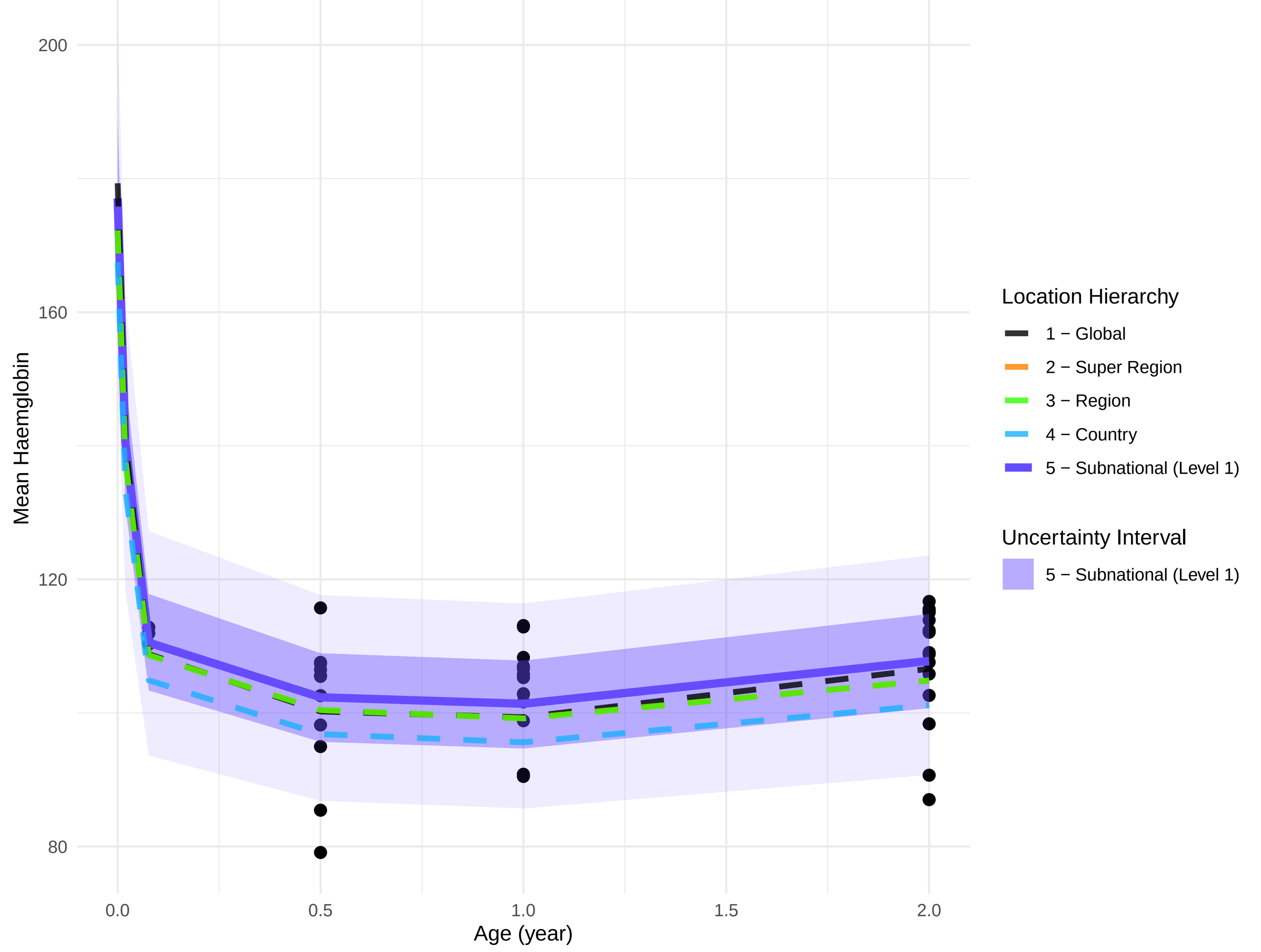

MR−BRT Age/Sex Fits − Non−pregnant Females <2 Years Old − Mean Haemglobin − Goa, Rural
WHO−2024 Adjustment Method

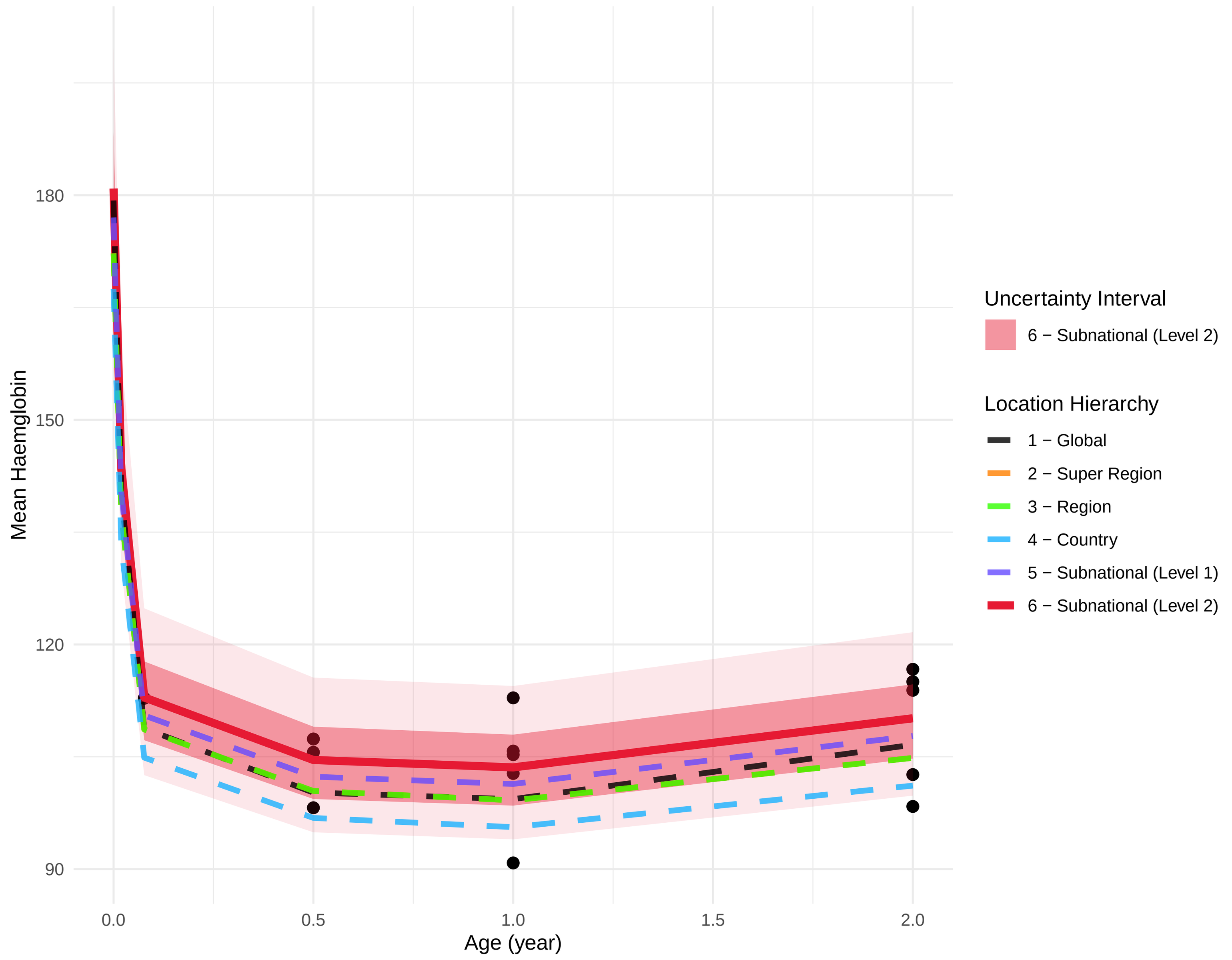

MR−BRT Age/Sex Fits − Non−pregnant Females <2 Years Old − Mean Haemglobin − Goa, Urban
WHO−2024 Adjustment Method

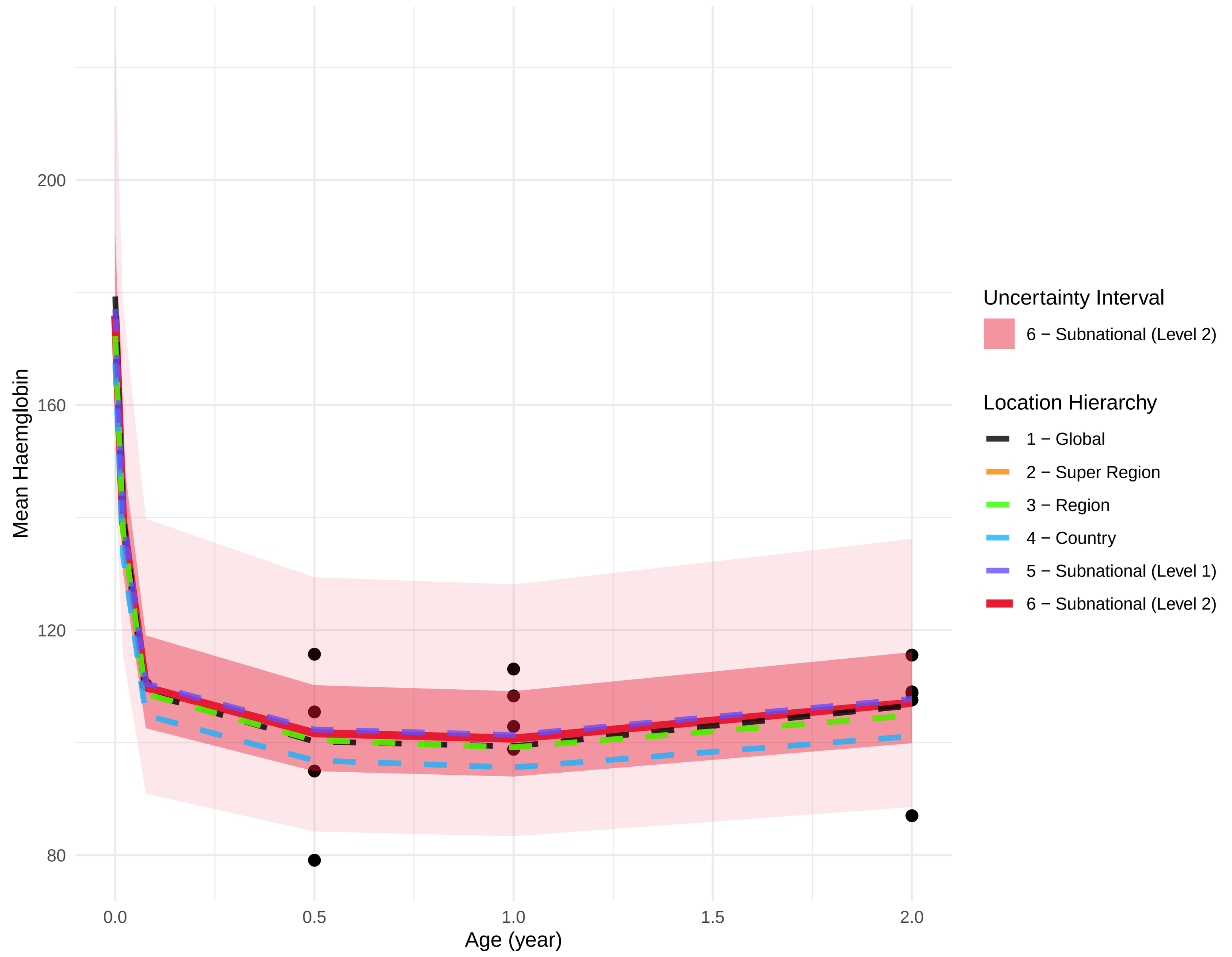

MR−BRT Age/Sex Fits − Non−pregnant Females <2 Years Old − Mean Haemglobin − Gujarat
WHO−2024 Adjustment Method

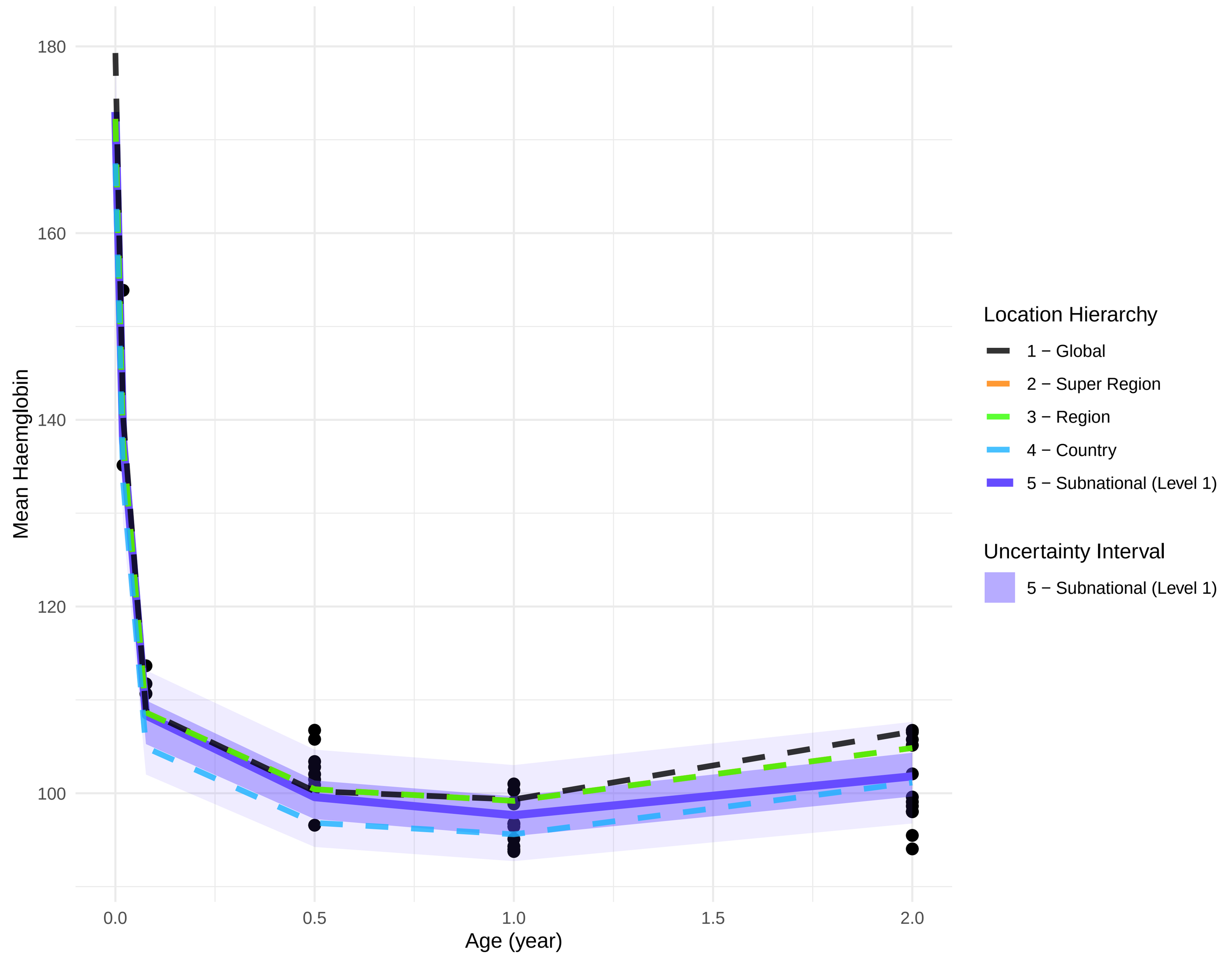

MR−BRT Age/Sex Fits − Non−pregnant Females <2 Years Old − Mean Haemglobin − Gujarat, Rural
WHO−2024 Adjustment Method

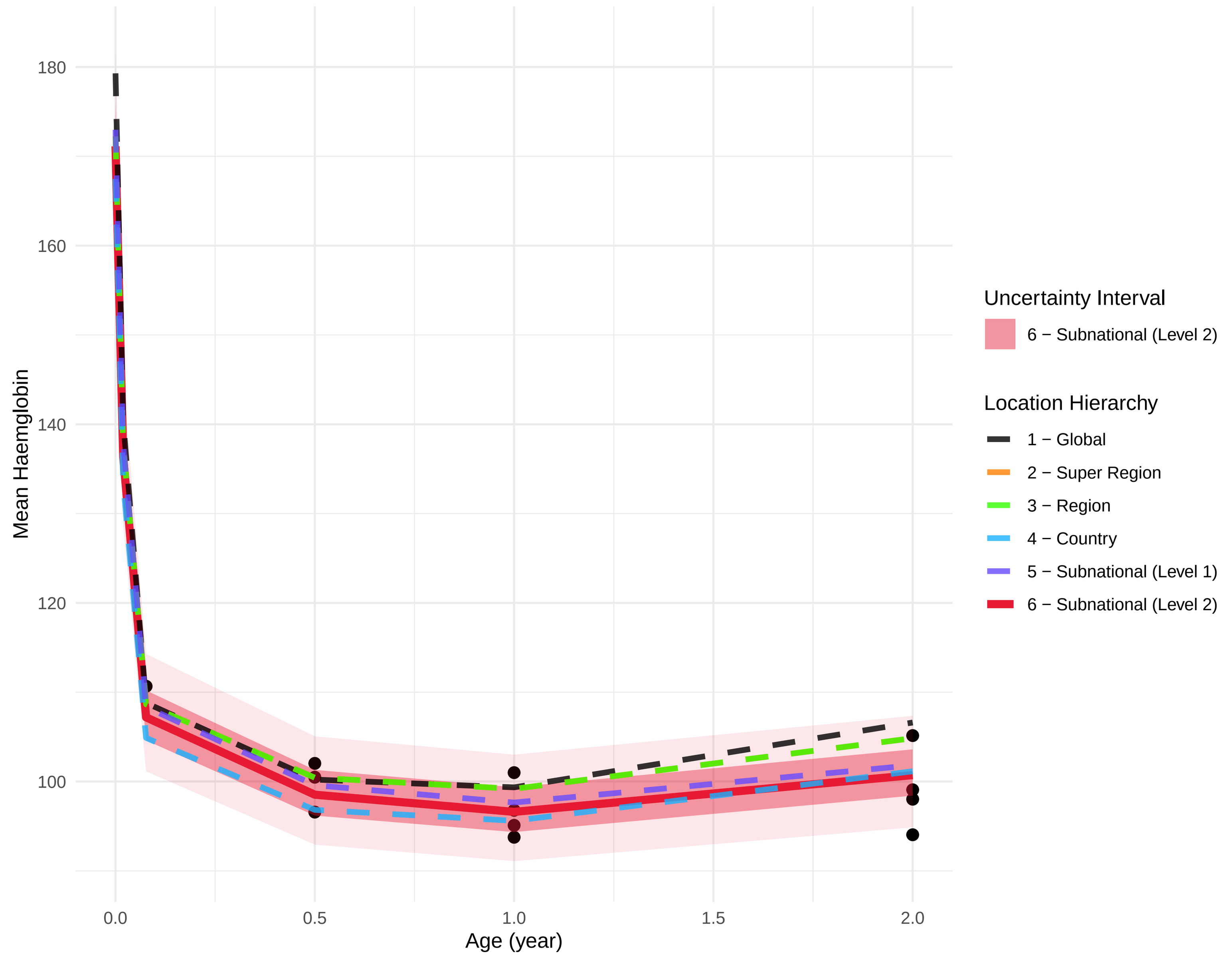

MR−BRT Age/Sex Fits − Non−pregnant Females <2 Years Old − Mean Haemglobin − Gujarat, Urban
WHO−2024 Adjustment Method

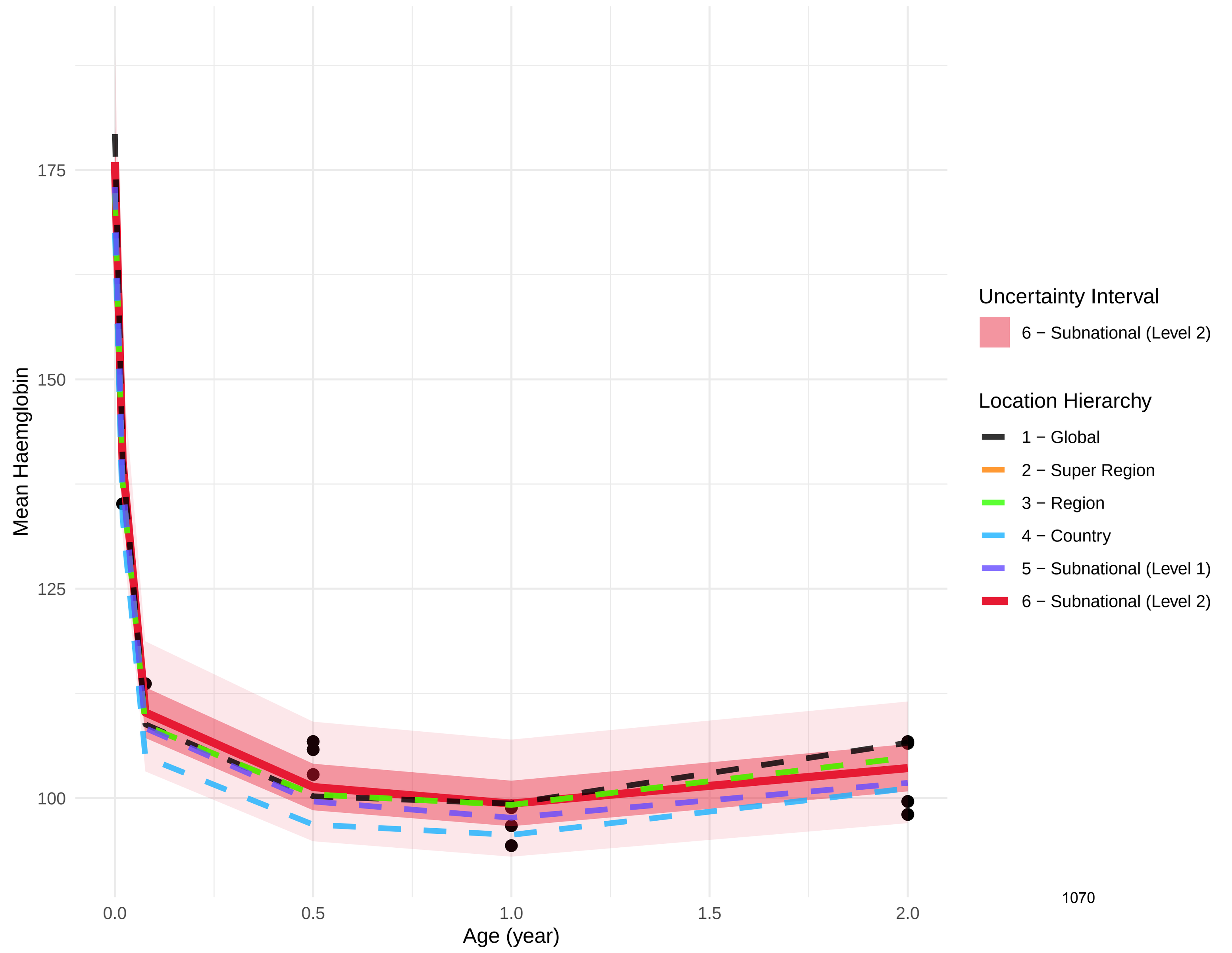

MR−BRT Age/Sex Fits − Non−pregnant Females <2 Years Old − Mean Haemglobin − Haryana
WHO−2024 Adjustment Method

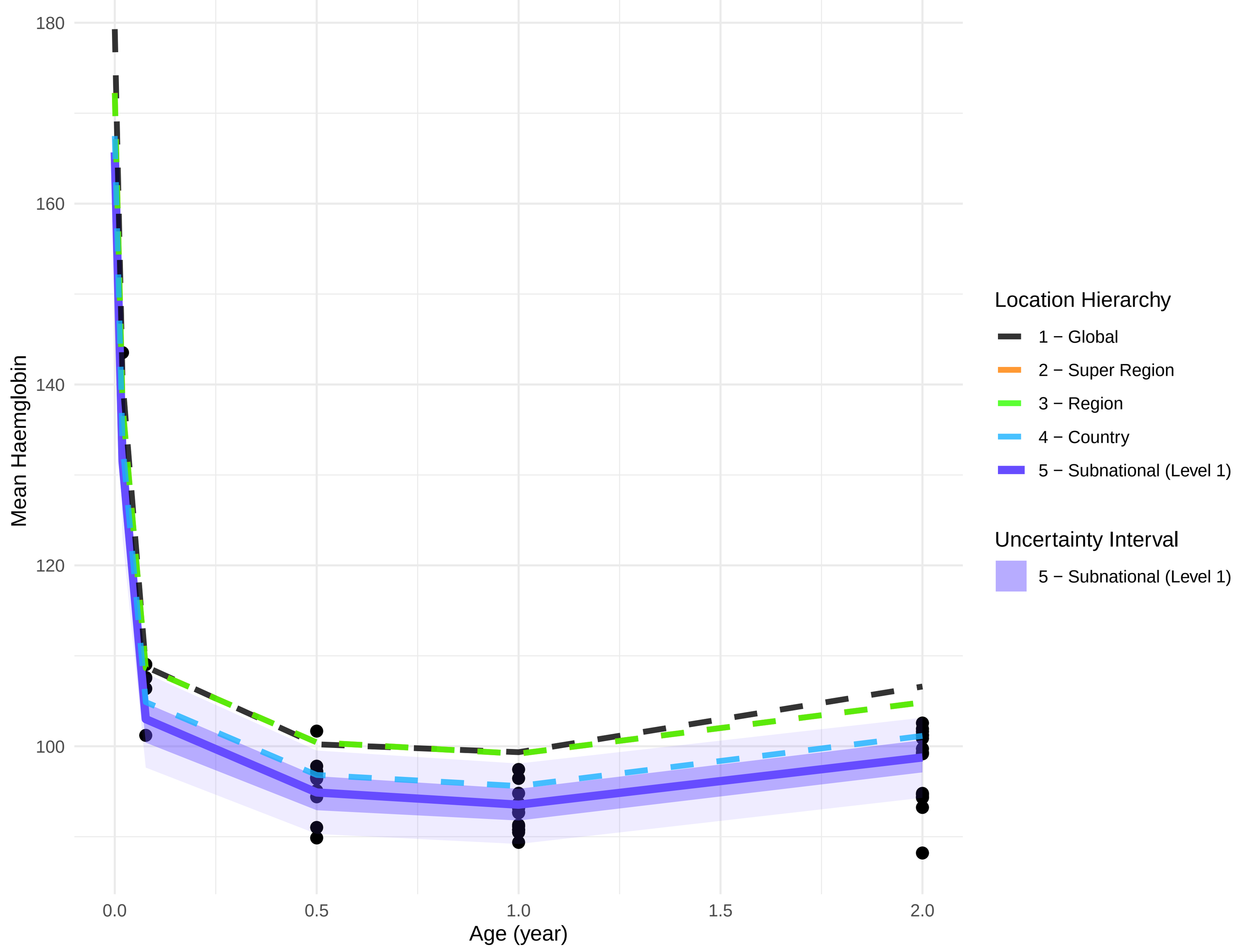

MR−BRT Age/Sex Fits − Non−pregnant Females <2 Years Old − Mean Haemglobin − Haryana, Rural
WHO−2024 Adjustment Method

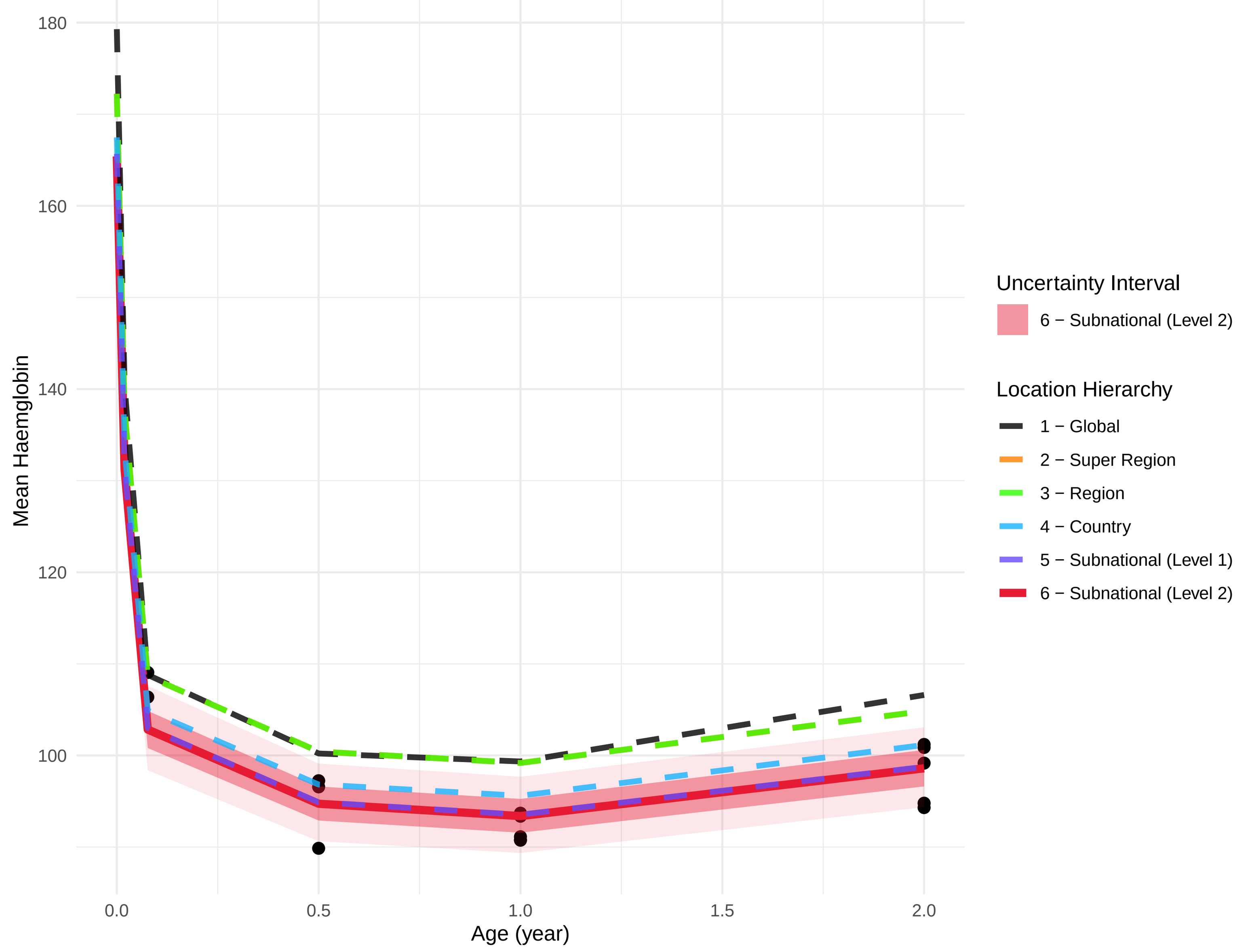

MR−BRT Age/Sex Fits − Non−pregnant Females <2 Years Old − Mean Haemglobin − Haryana, Urban
WHO−2024 Adjustment Method

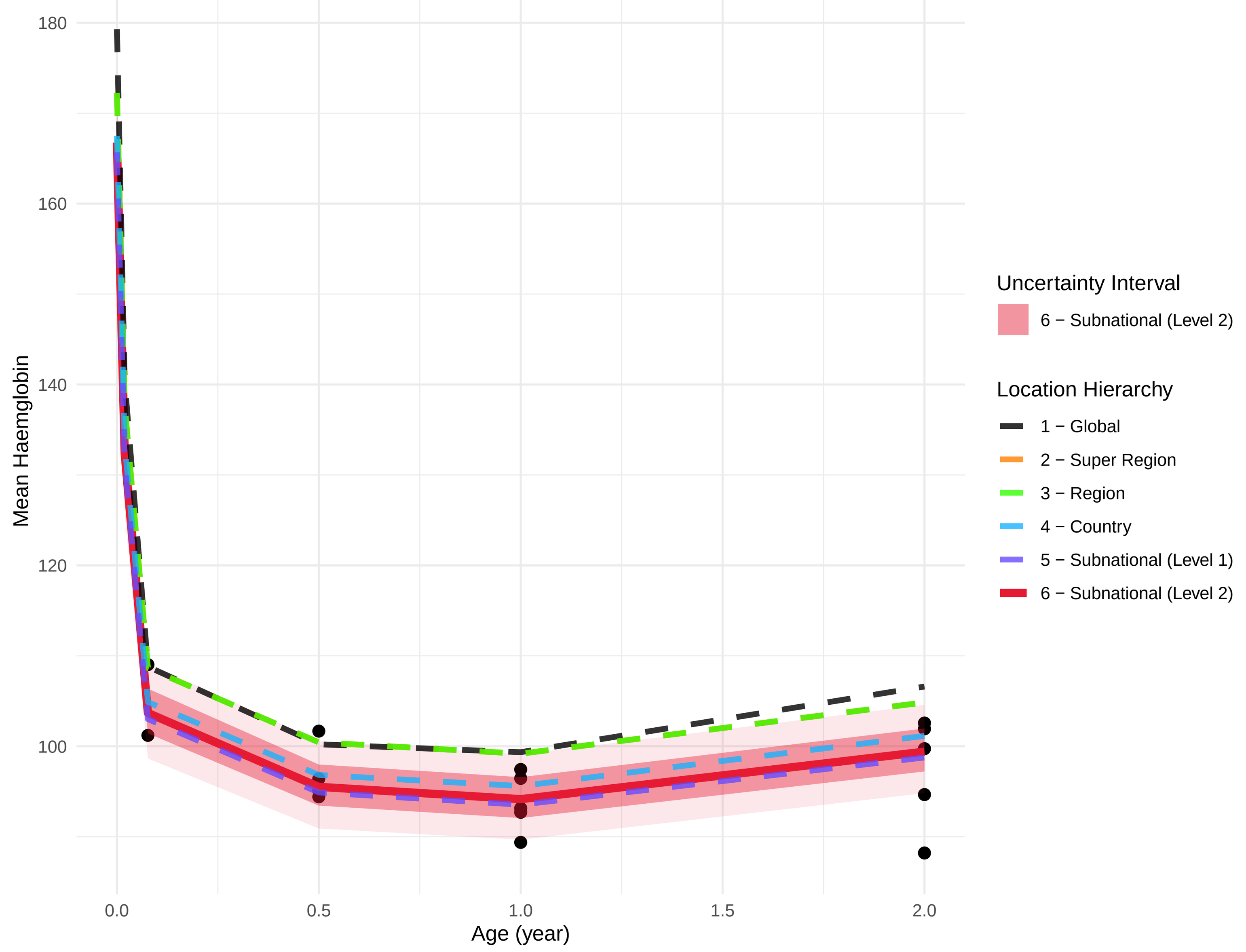

MR−BRT Age/Sex Fits − Non−pregnant Females <2 Years Old − Mean Haemglobin − Himachal Pradesh
WHO−2024 Adjustment Method

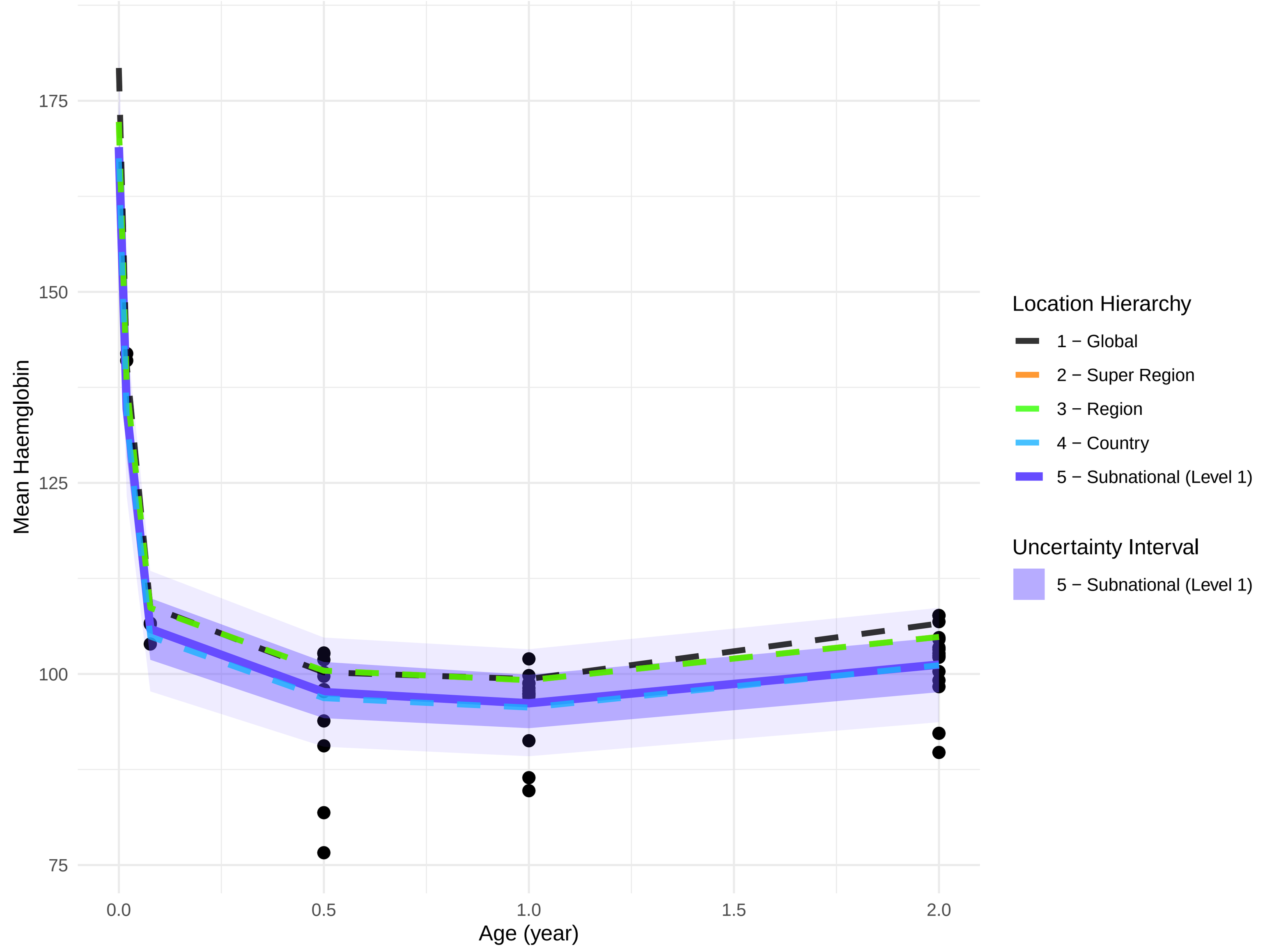

MR−BRT Age/Sex Fits − Non−pregnant Females <2 Years Old − Mean Haemglobin − Himachal Pradesh, Rural
WHO−2024 Adjustment Method

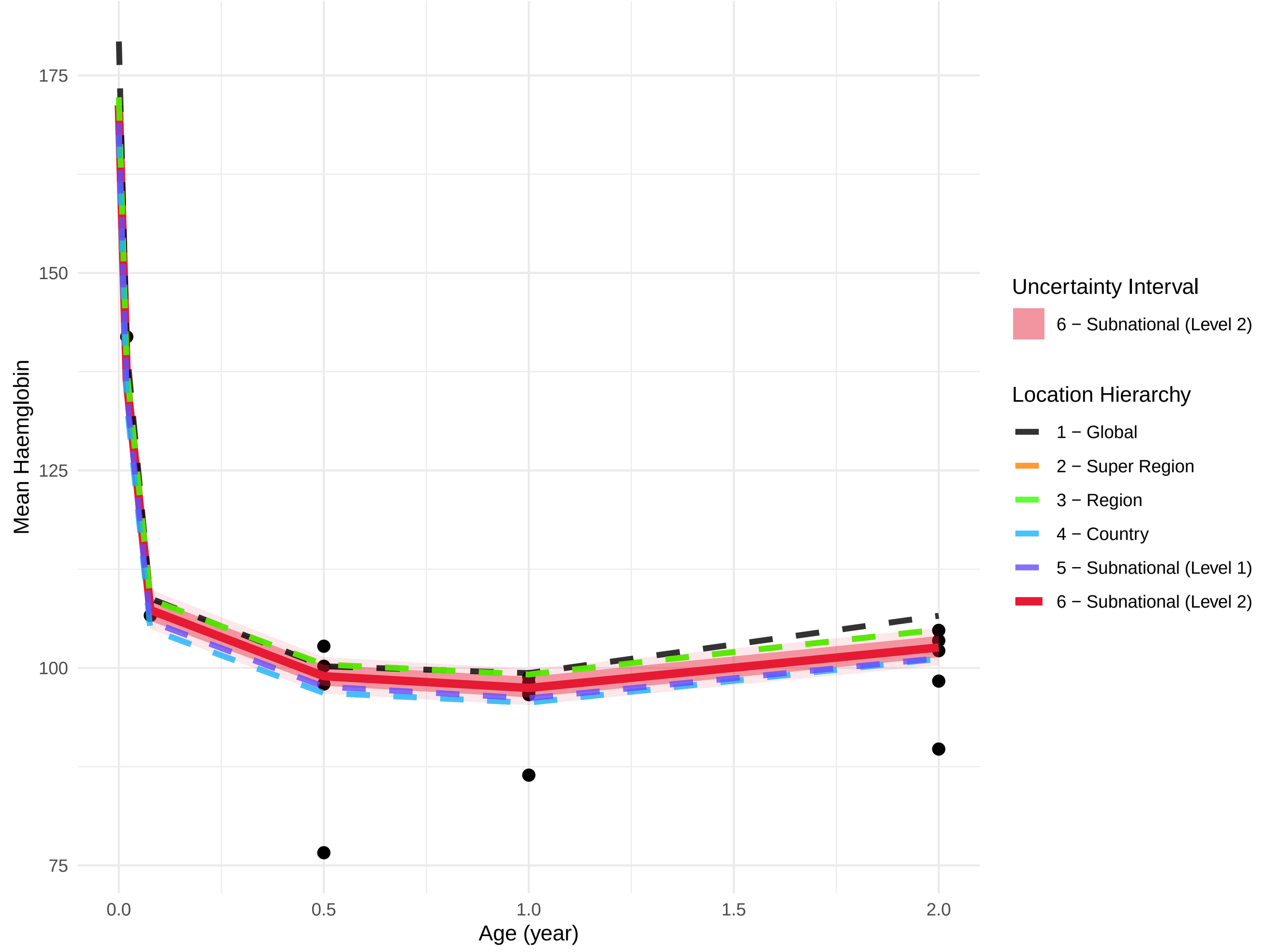

MR−BRT Age/Sex Fits − Non−pregnant Females <2 Years Old − Mean Haemglobin − Himachal Pradesh, Urban
WHO−2024 Adjustment Method

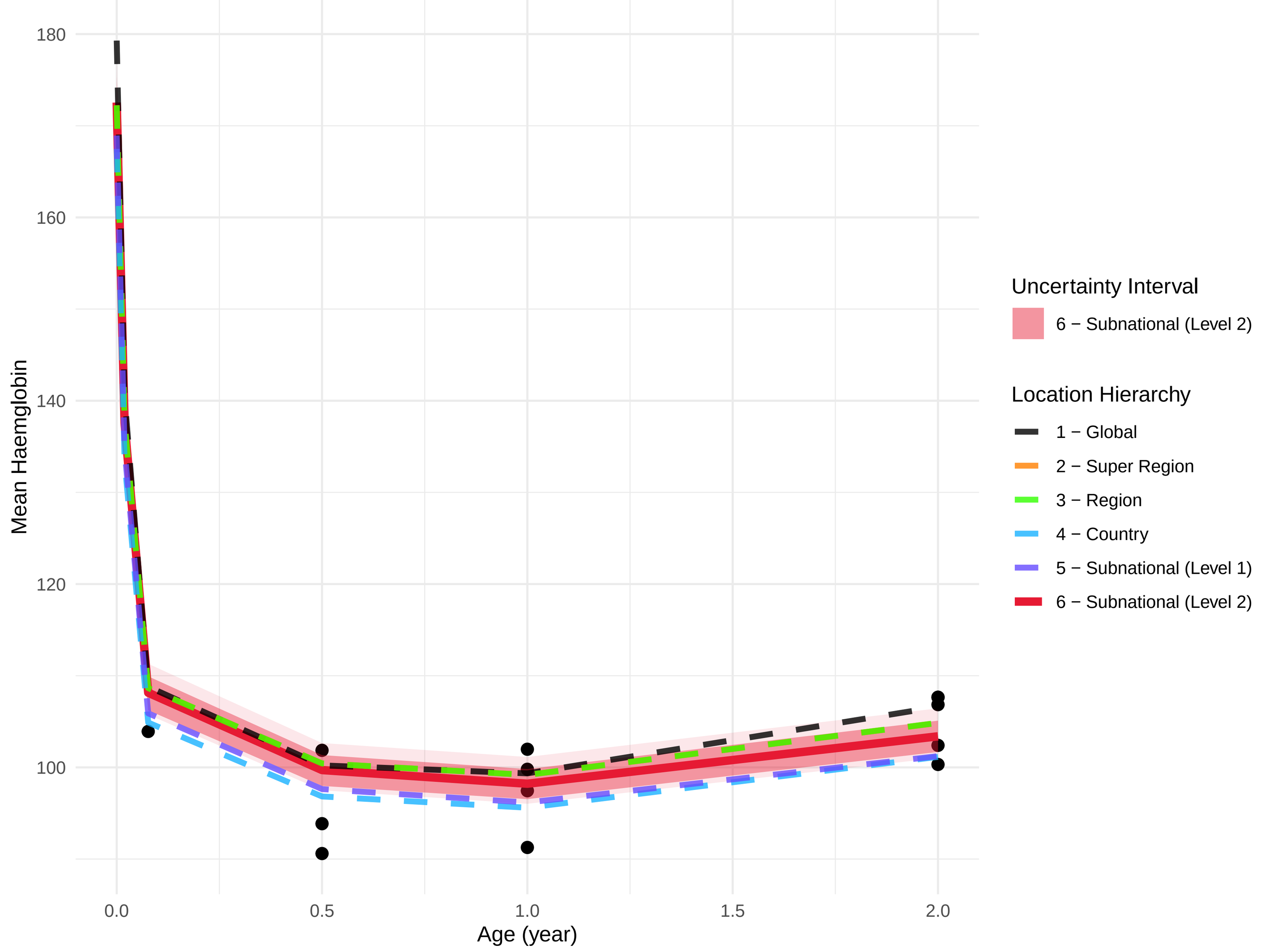

MR−BRT Age/Sex Fits − Non−pregnant Females <2 Years Old − Mean Haemglobin − Jammu & Kashmir and Ladakh
WHO−2024 Adjustment Method

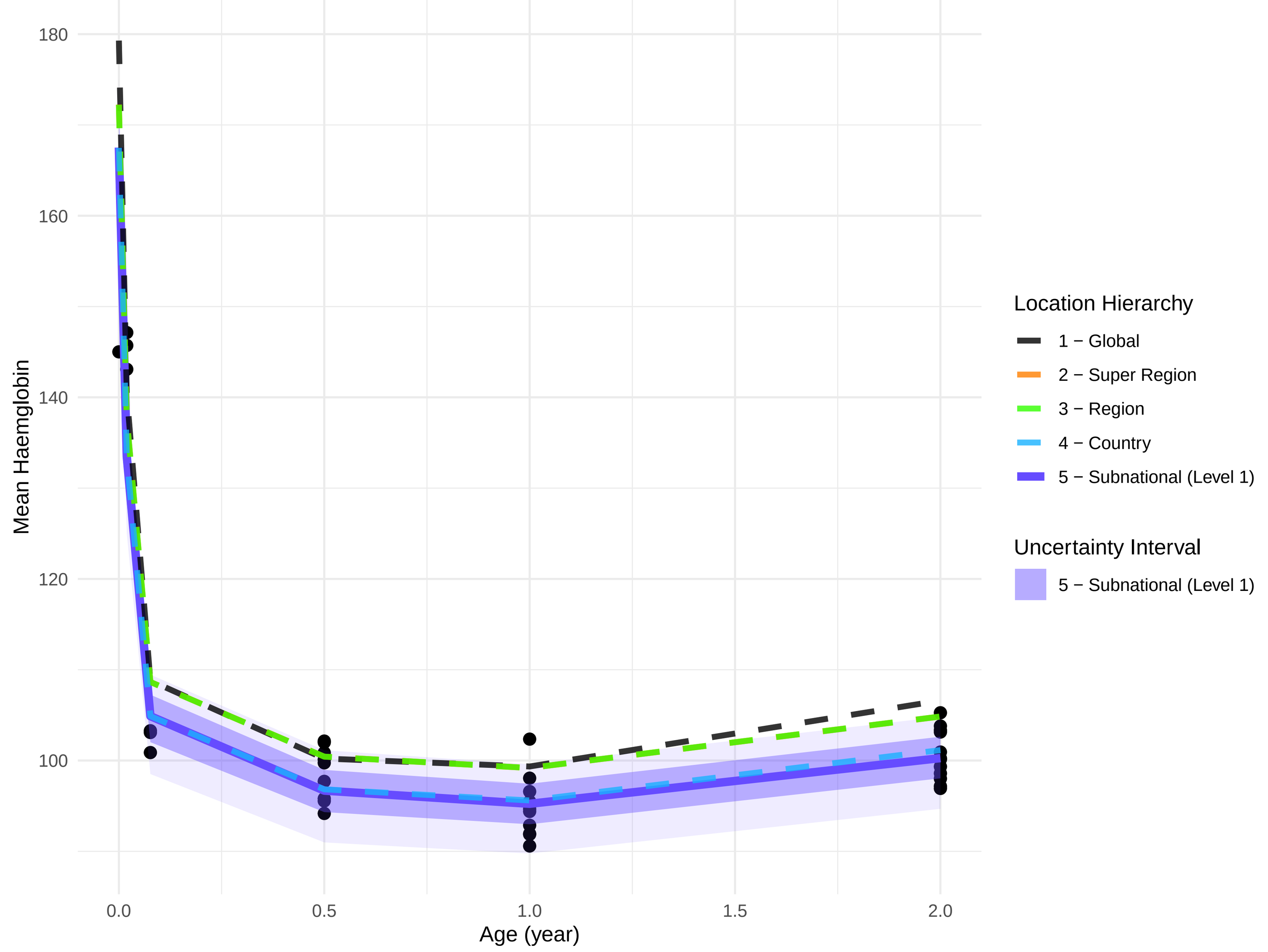

MR−BRT Age/Sex Fits − Non−pregnant Females <2 Years Old − Mean Haemglobin − Jammu & Kashmir and Ladakh, Rural
WHO−2024 Adjustment Method

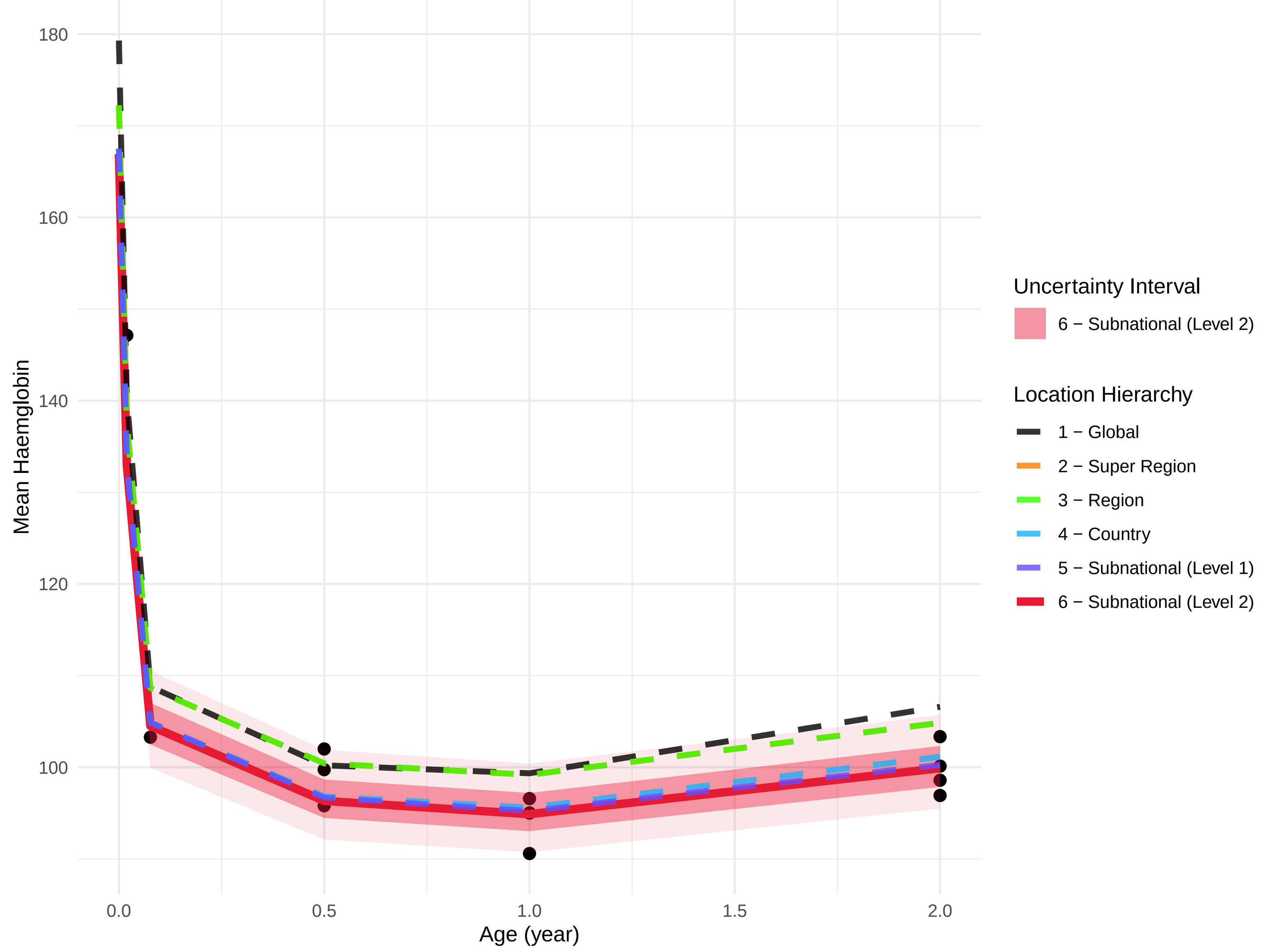

MR−BRT Age/Sex Fits − Non−pregnant Females <2 Years Old − Mean Haemglobin − Jammu & Kashmir and Ladakh, Urban
WHO−2024 Adjustment Method

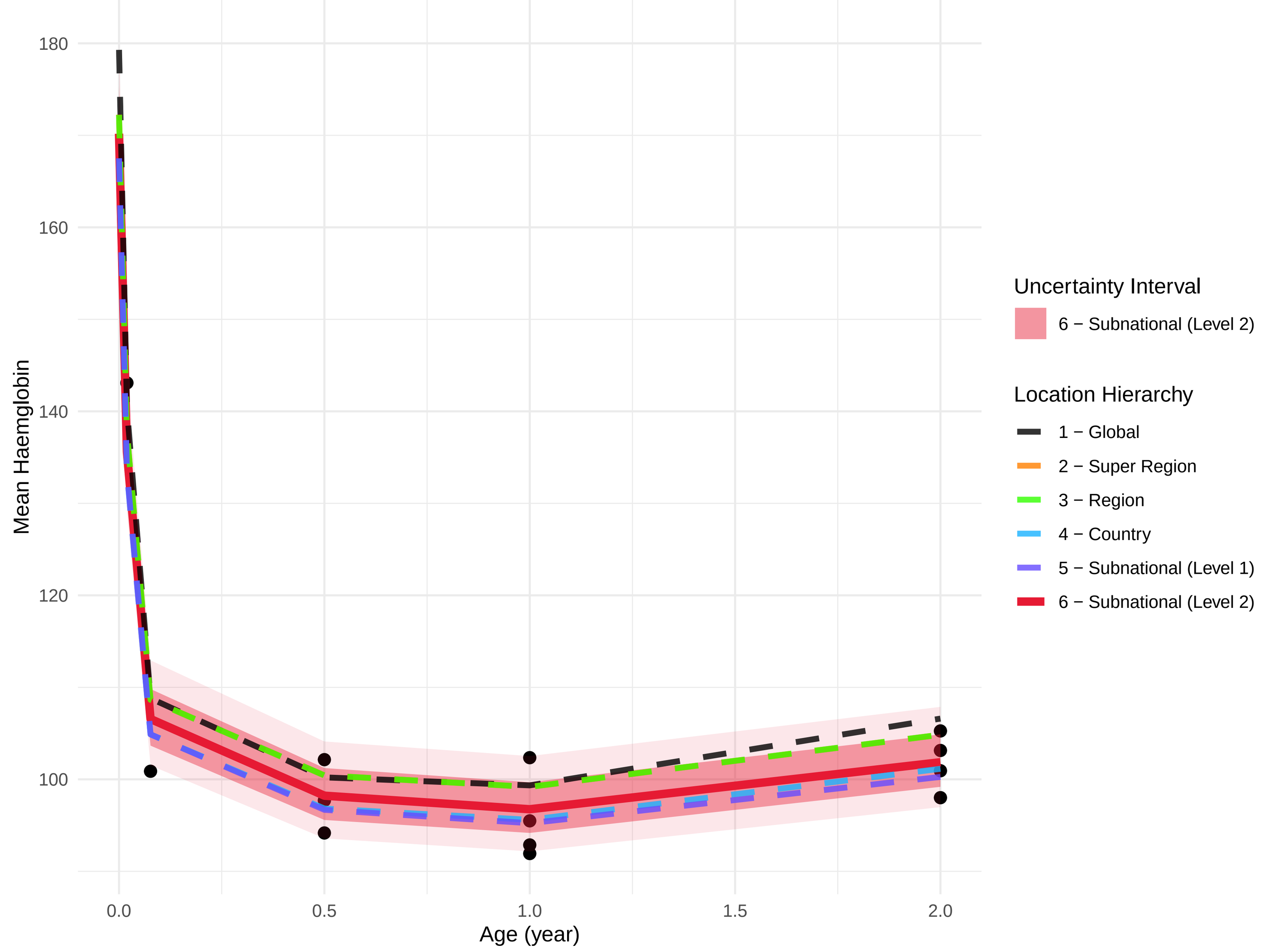

MR−BRT Age/Sex Fits − Non−pregnant Females <2 Years Old − Mean Haemglobin − Jharkhand
WHO−2024 Adjustment Method

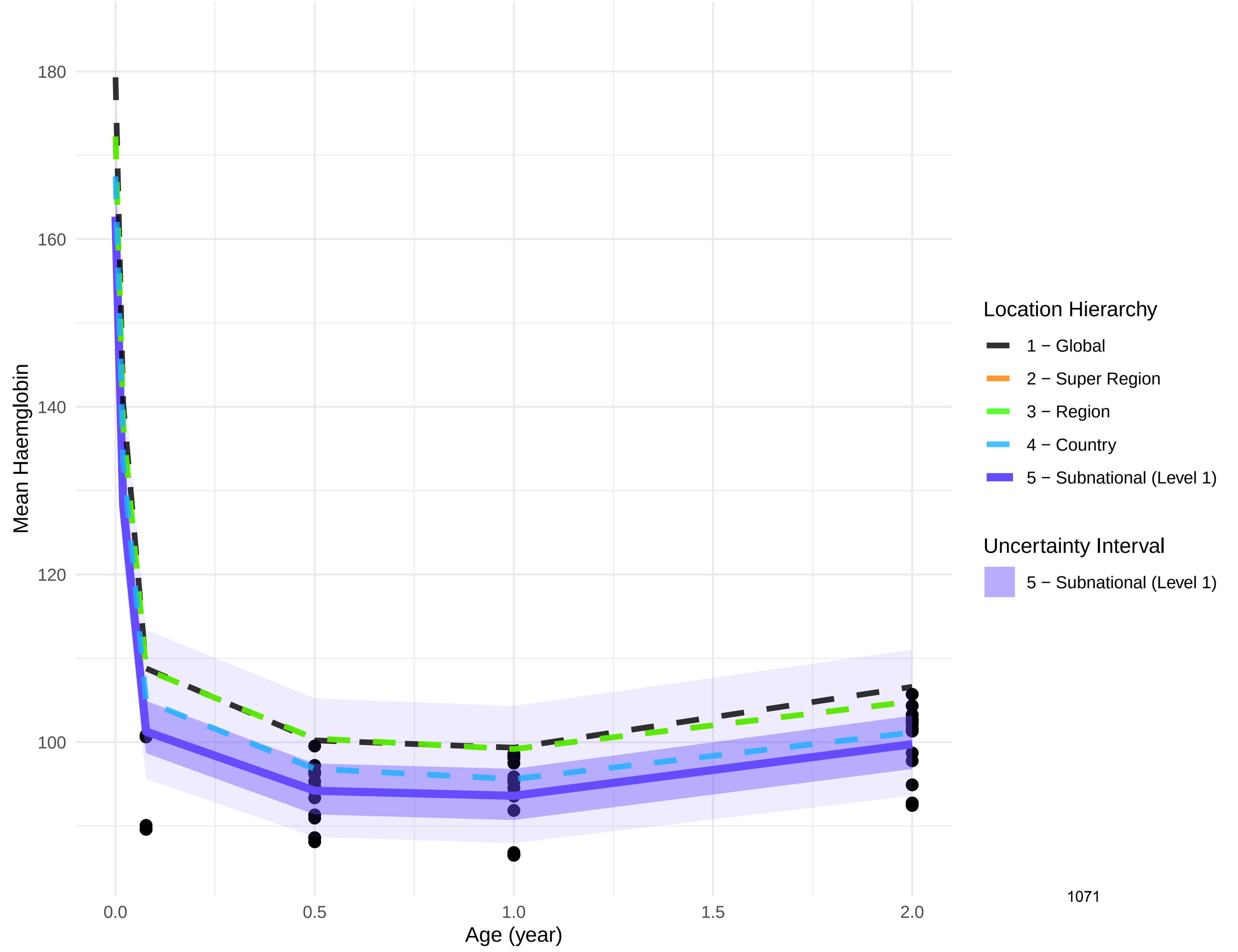

MR−BRT Age/Sex Fits − Non−pregnant Females <2 Years Old − Mean Haemglobin − Jharkhand, Rural

WHO−2024 Adjustment Method

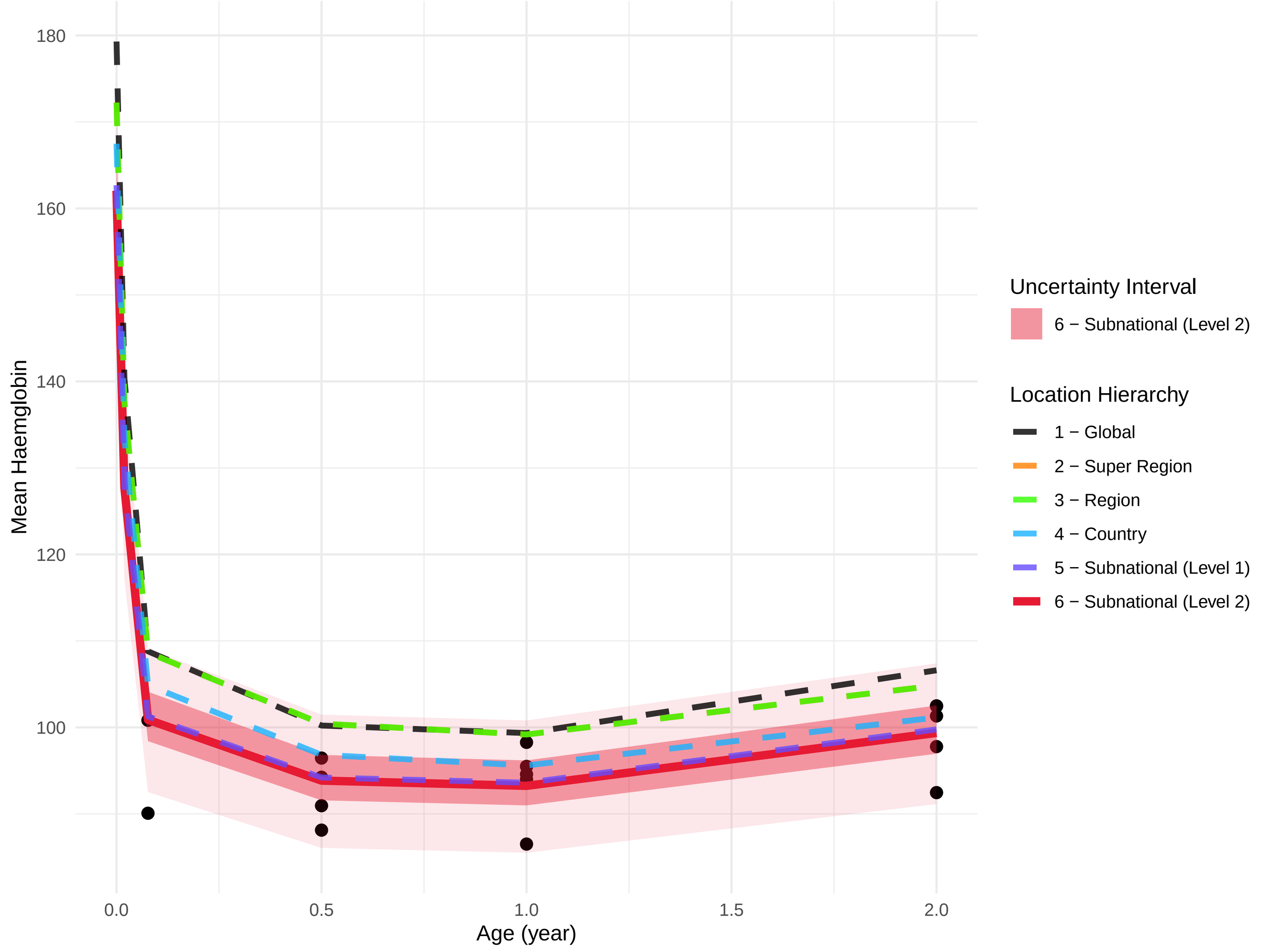

MR−BRT Age/Sex Fits − Non−pregnant Females <2 Years Old − Mean Haemglobin − Jharkhand, Urban

WHO−2024 Adjustment Method

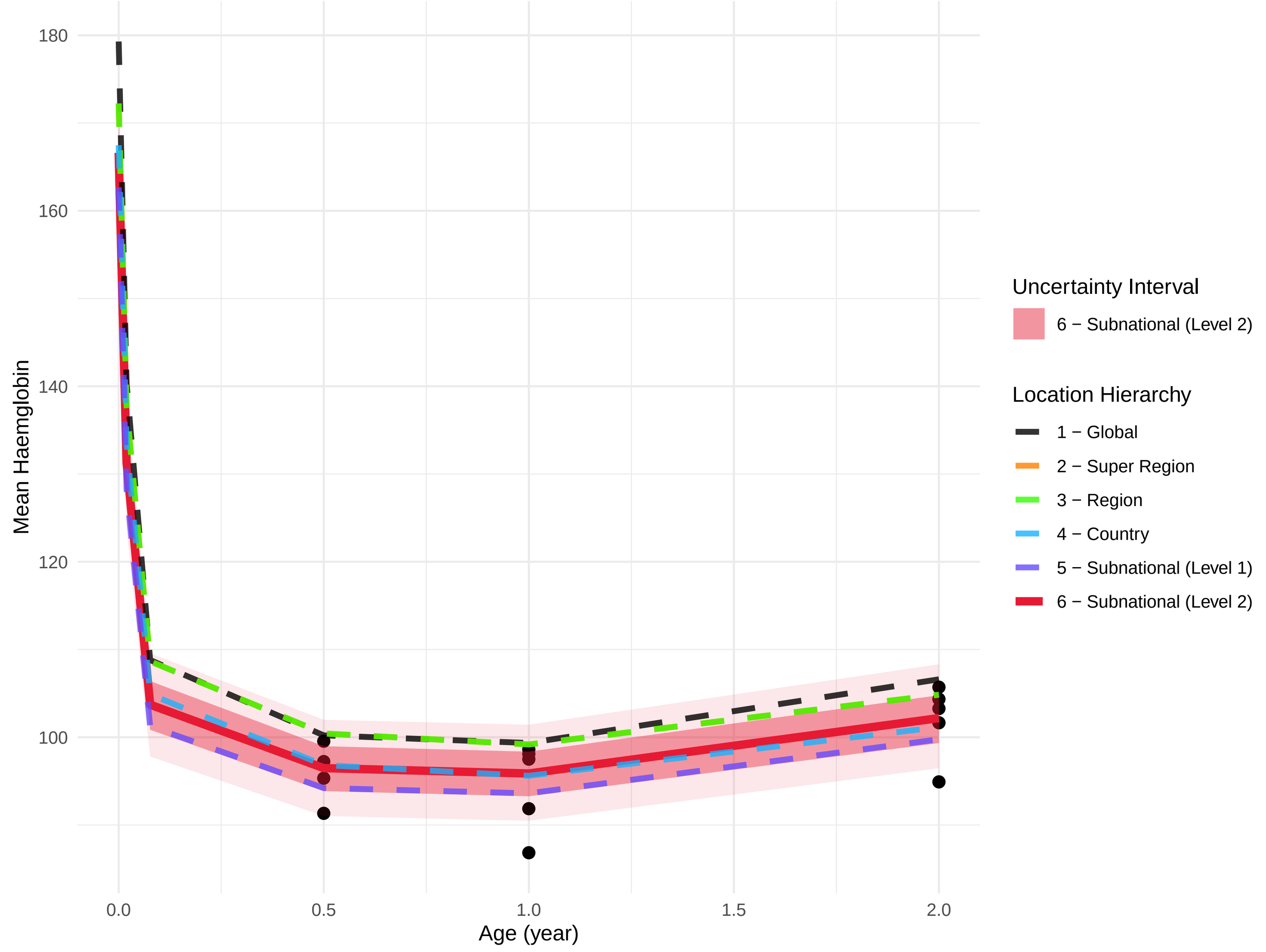

MR−BRT Age/Sex Fits − Non−pregnant Females <2 Years Old − Mean Haemglobin − Karnataka

WHO−2024 Adjustment Method

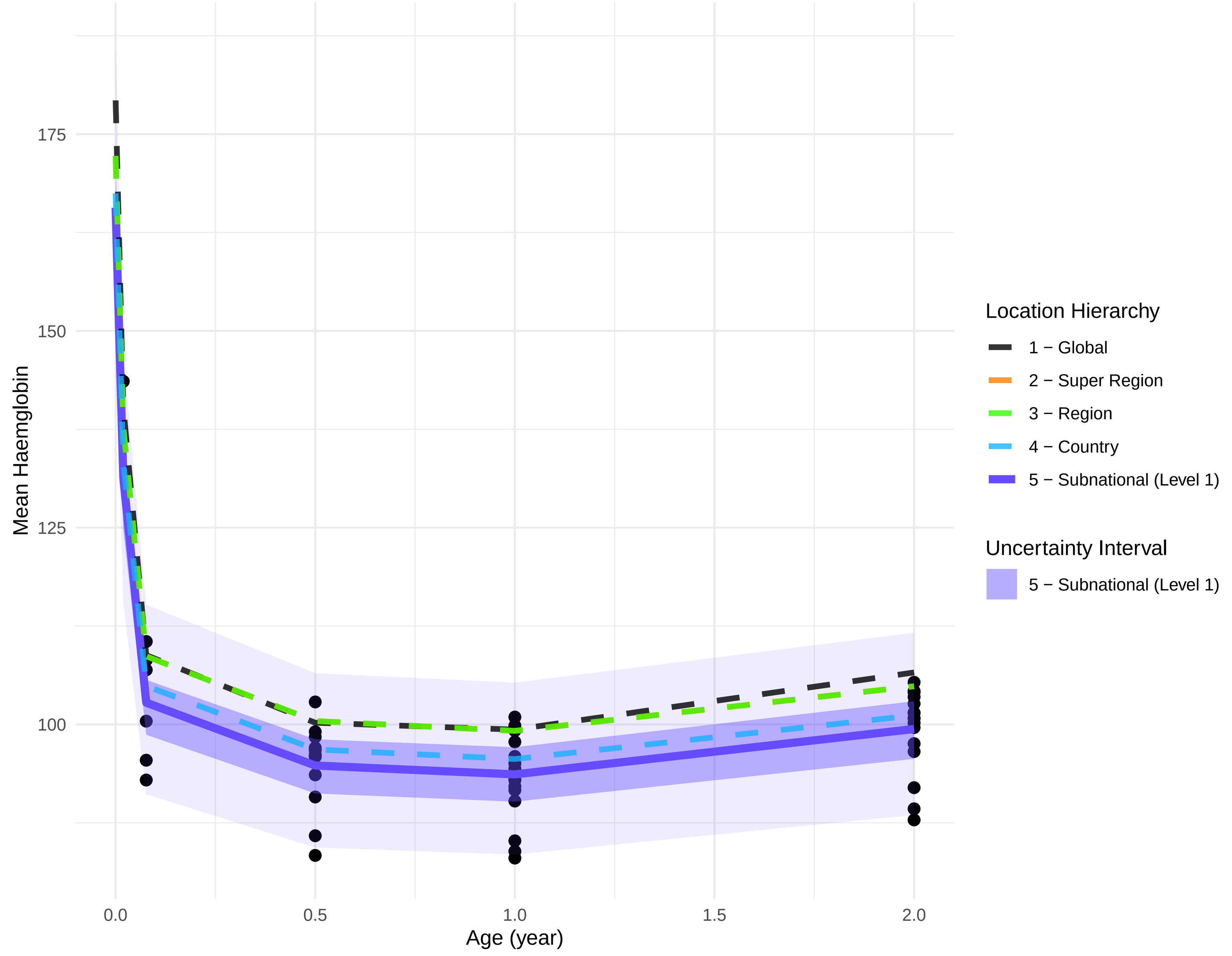

MR−BRT Age/Sex Fits − Non−pregnant Females <2 Years Old − Mean Haemglobin − Karnataka, Rural

WHO−2024 Adjustment Method

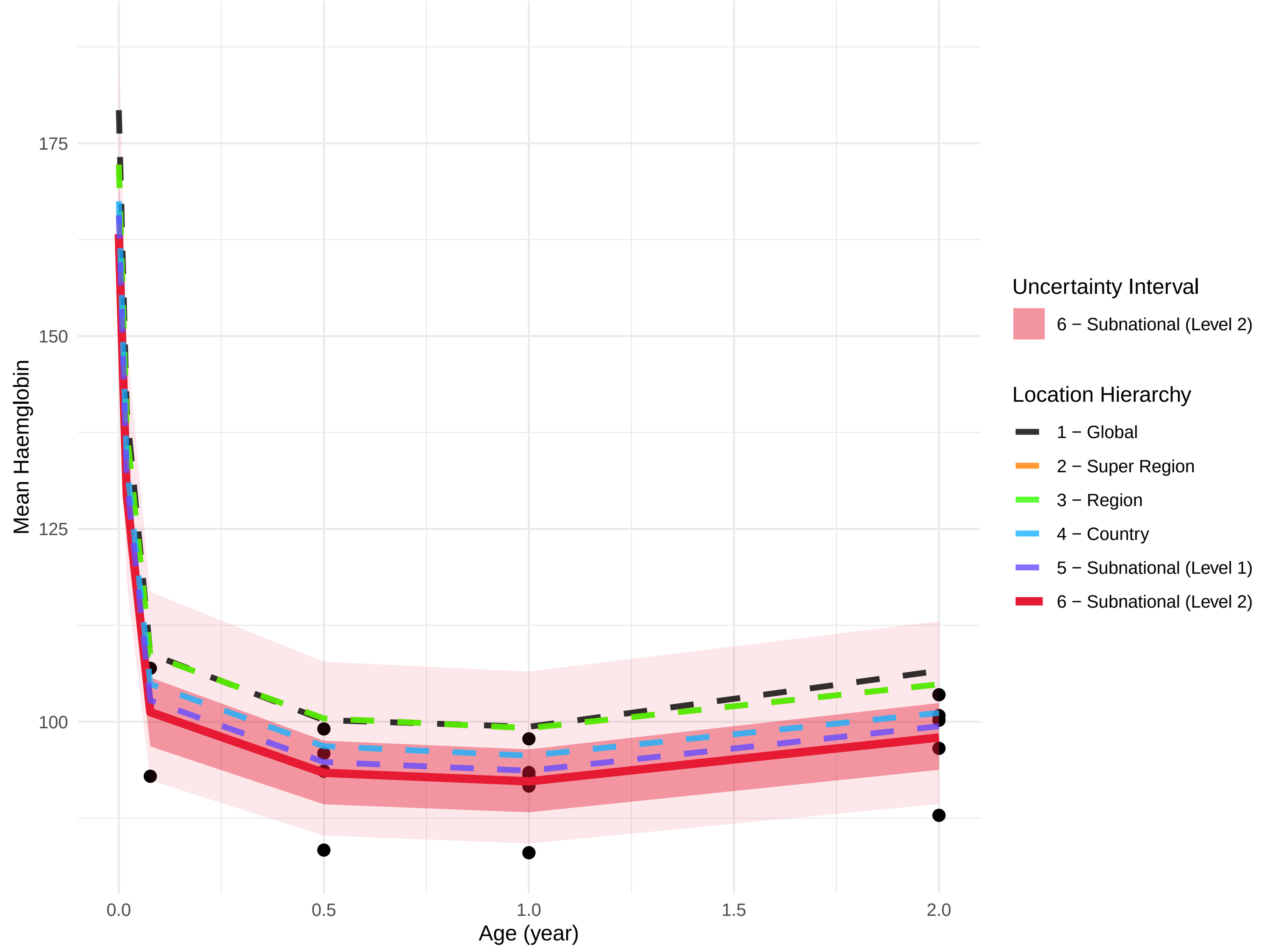

MR−BRT Age/Sex Fits − Non−pregnant Females <2 Years Old − Mean Haemglobin − Karnataka, Urban

WHO−2024 Adjustment Method

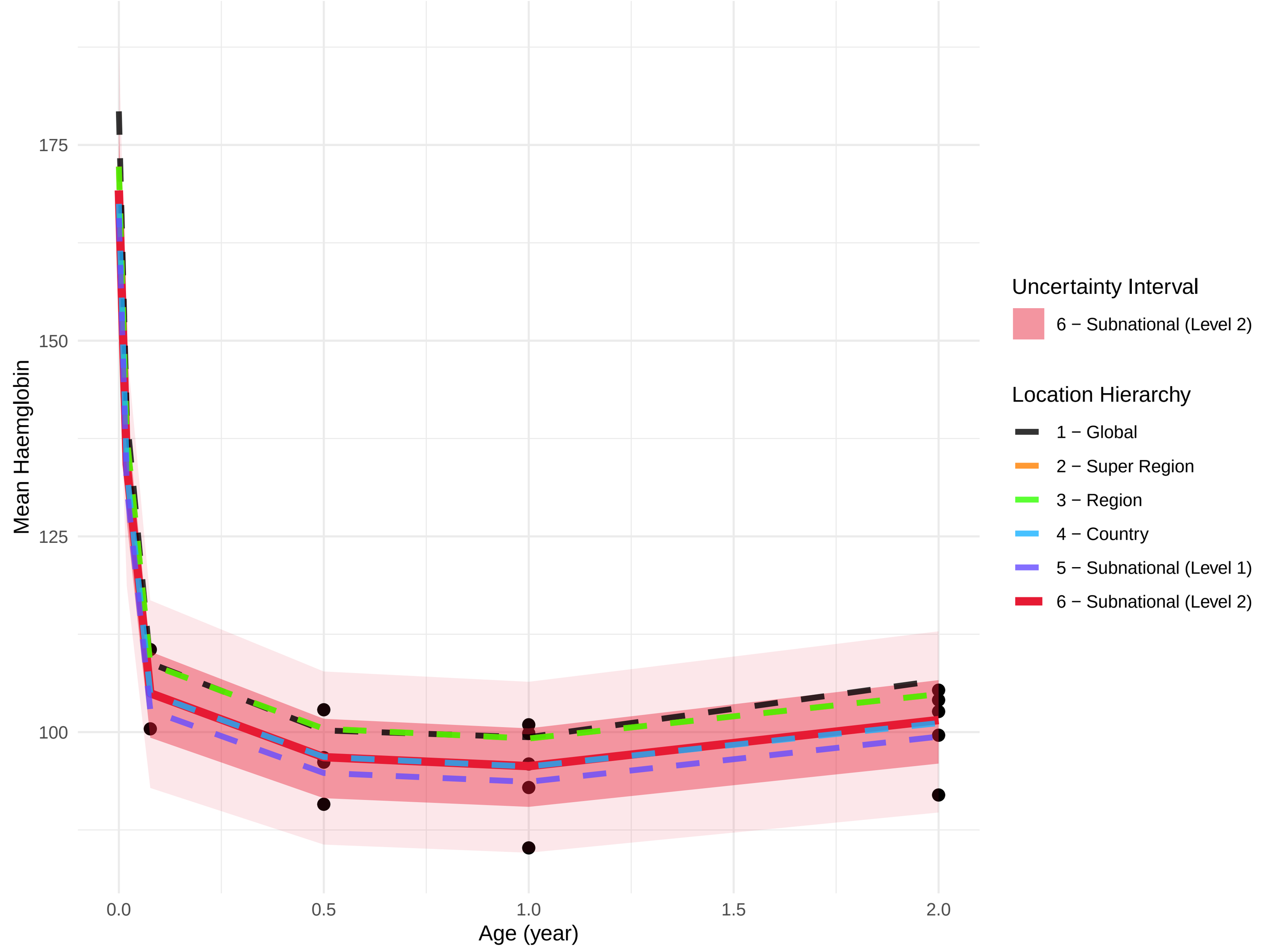

MR−BRT Age/Sex Fits − Non−pregnant Females <2 Years Old − Mean Haemglobin − Kerala

WHO−2024 Adjustment Method

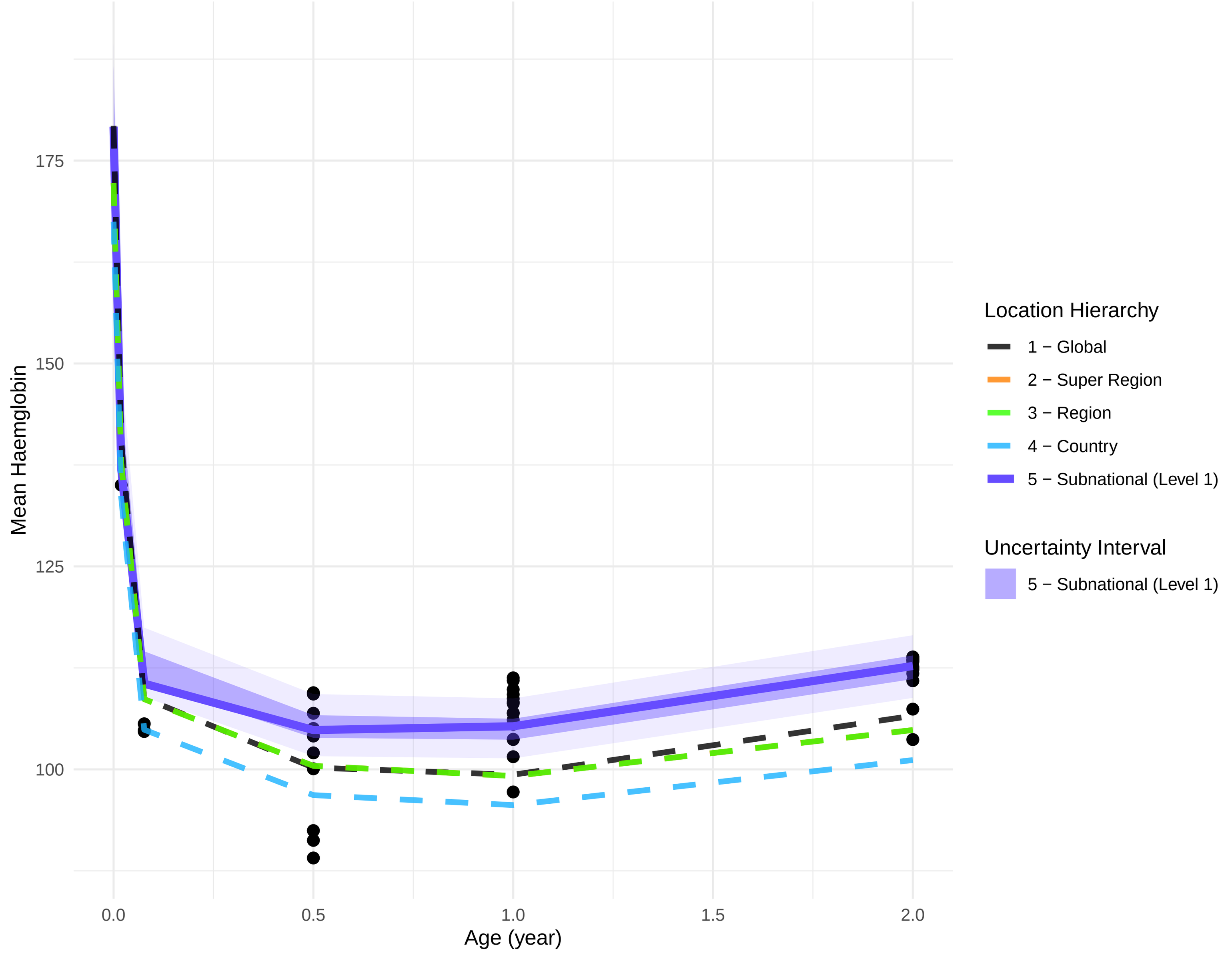

MR−BRT Age/Sex Fits − Non−pregnant Females <2 Years Old − Mean Haemglobin − Kerala, Rural

WHO−2024 Adjustment Method

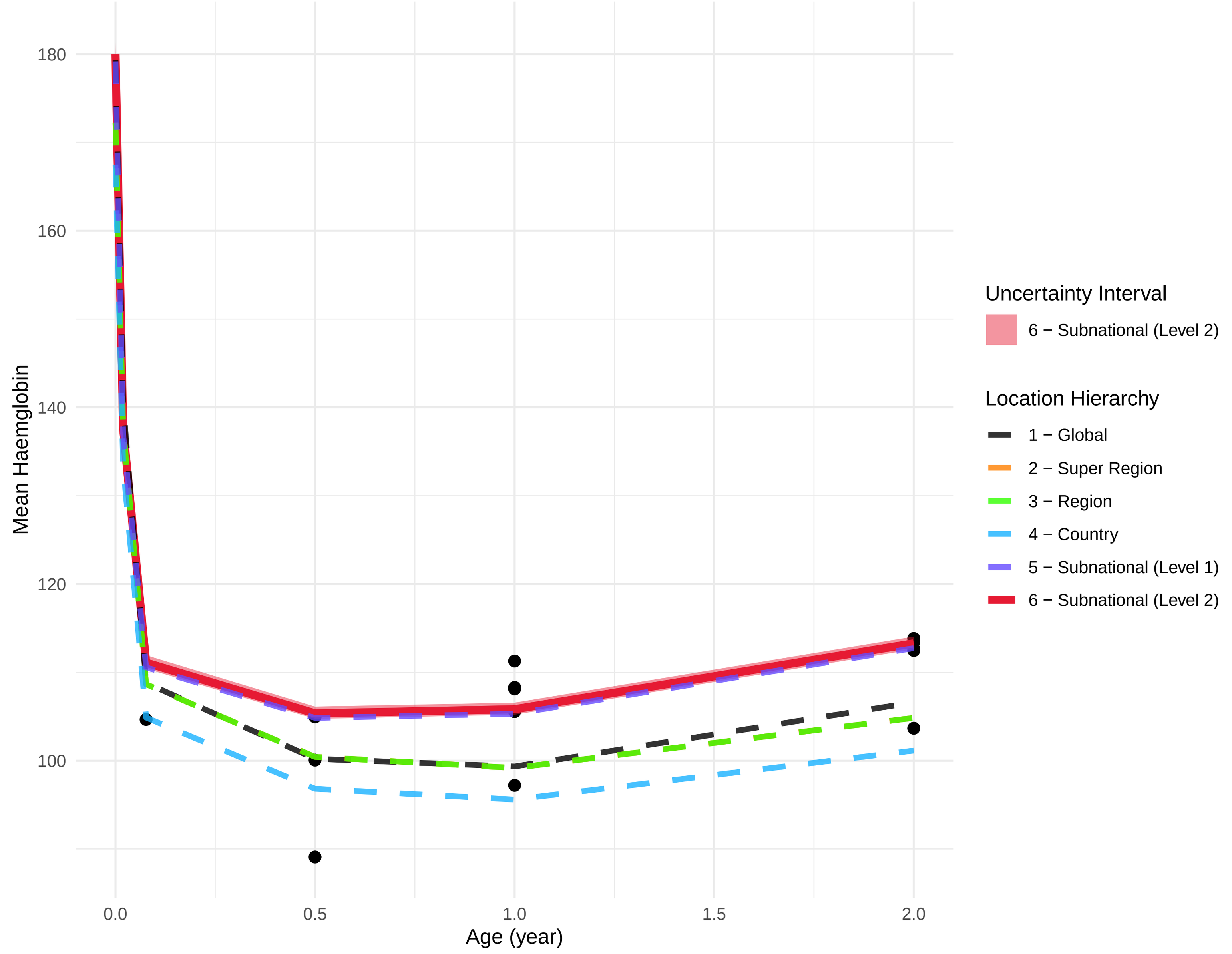

MR−BRT Age/Sex Fits − Non−pregnant Females <2 Years Old − Mean Haemglobin − Kerala, Urban

WHO−2024 Adjustment Method

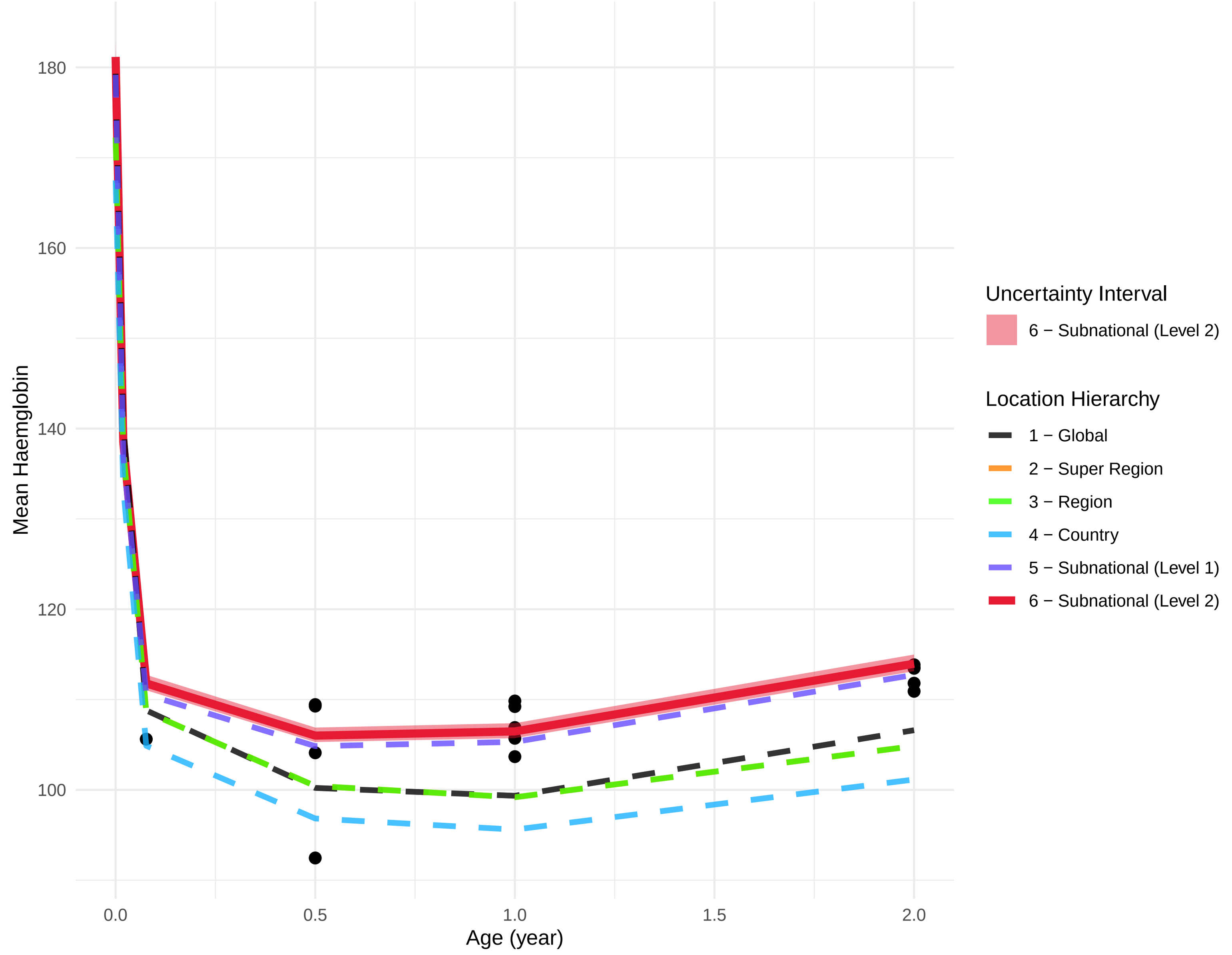

MR−BRT Age/Sex Fits − Non−pregnant Females <2 Years Old − Mean Haemglobin − Madhya Pradesh

WHO−2024 Adjustment Method

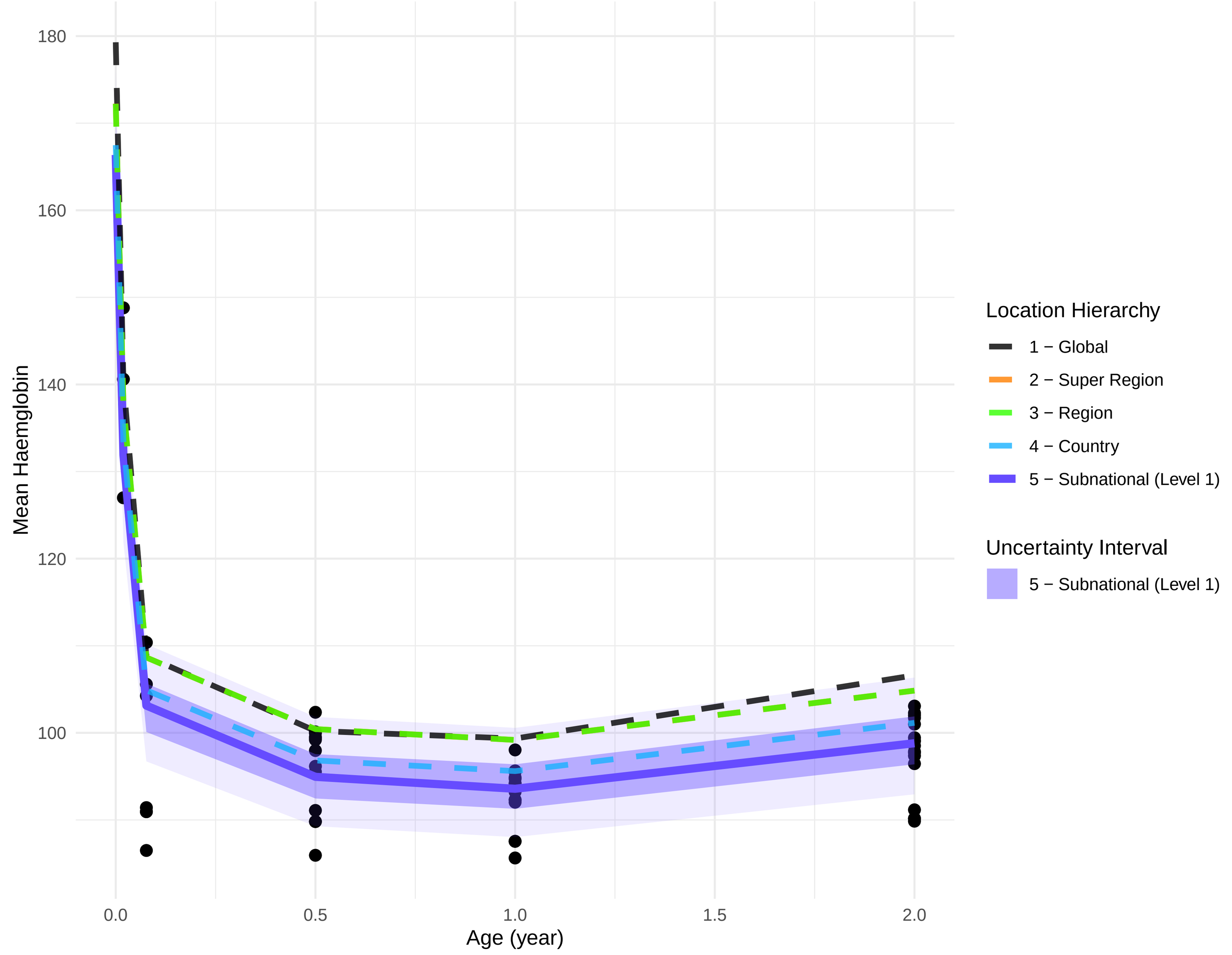

MR−BRT Age/Sex Fits − Non−pregnant Females <2 Years Old − Mean Haemglobin − Madhya Pradesh, Rural

WHO−2024 Adjustment Method

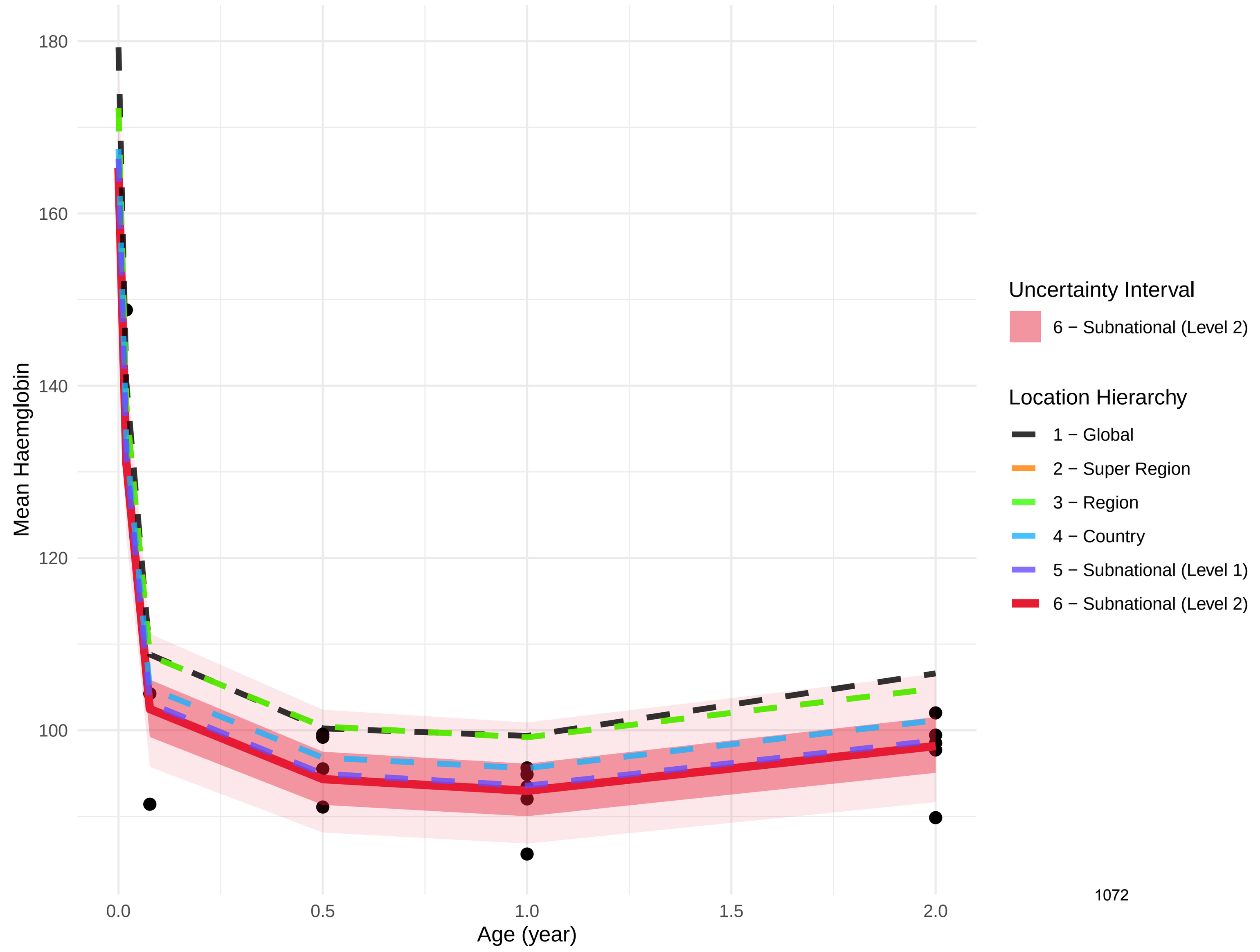

MR−BRT Age/Sex Fits − Non−pregnant Females <2 Years Old − Mean Haemglobin − Madhya Pradesh, Urban
WHO−2024 Adjustment Method

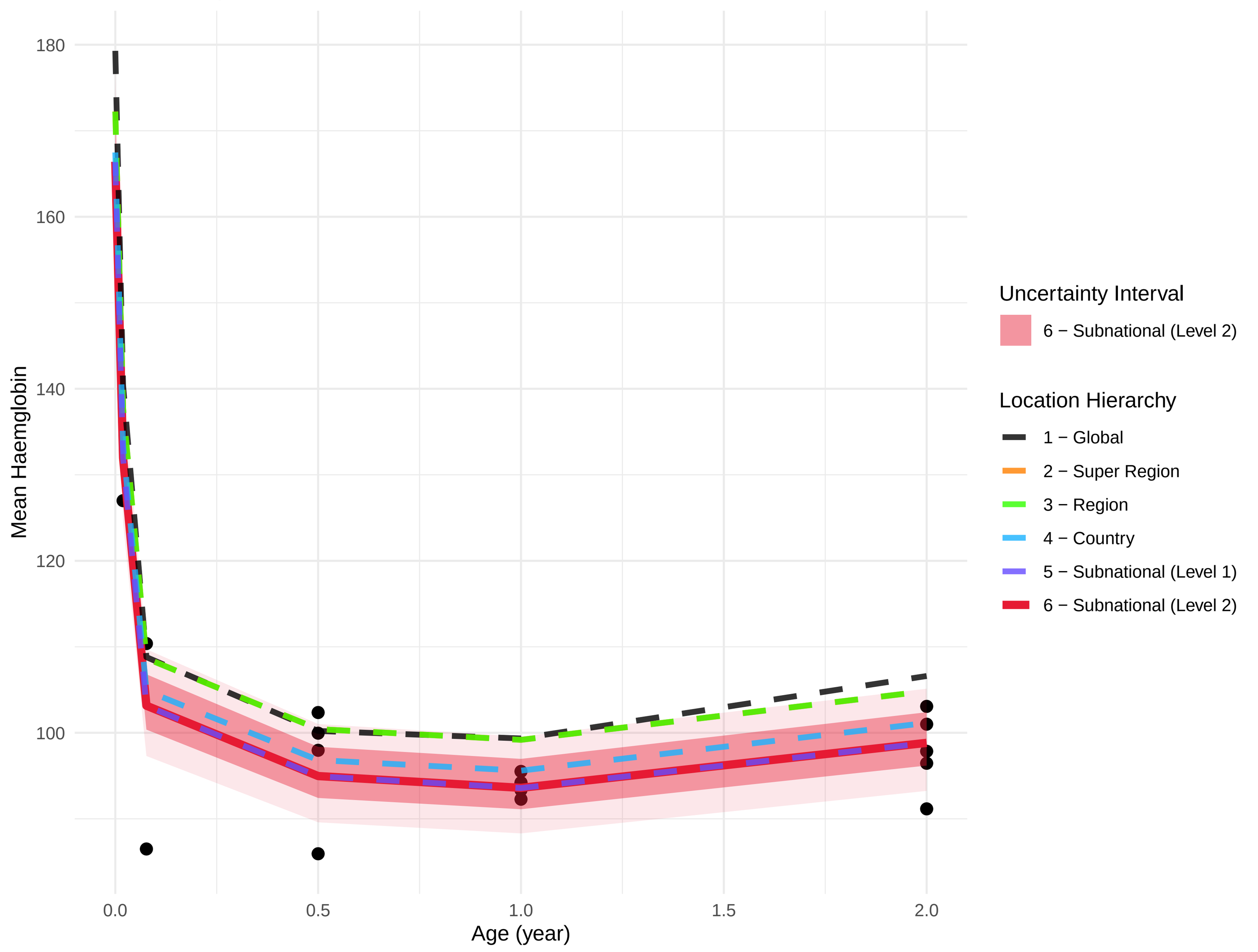

MR−BRT Age/Sex Fits − Non−pregnant Females <2 Years Old − Mean Haemglobin − Maharashtra
WHO−2024 Adjustment Method

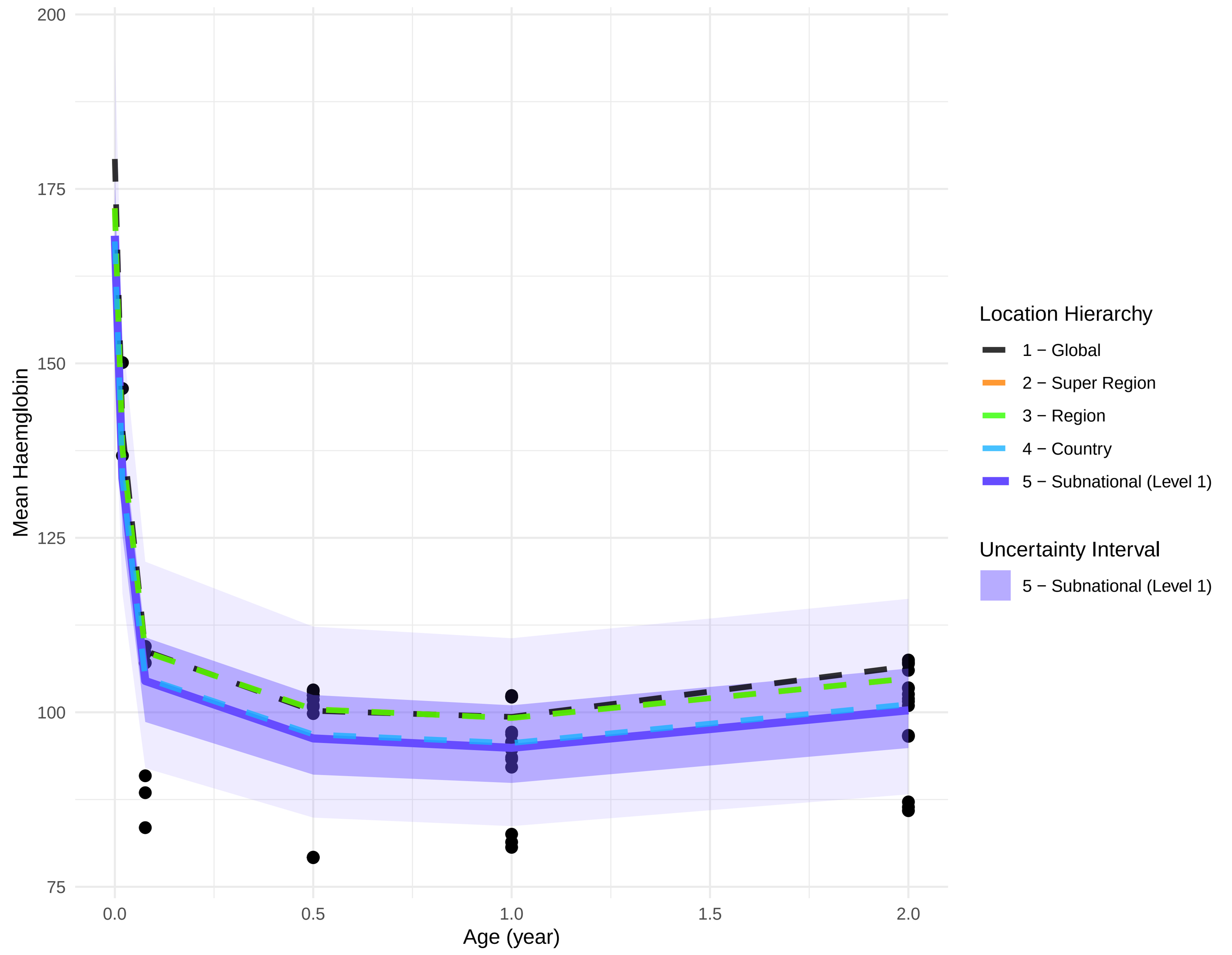

MR−BRT Age/Sex Fits − Non−pregnant Females <2 Years Old − Mean Haemglobin − Maharashtra, Rural
WHO−2024 Adjustment Method

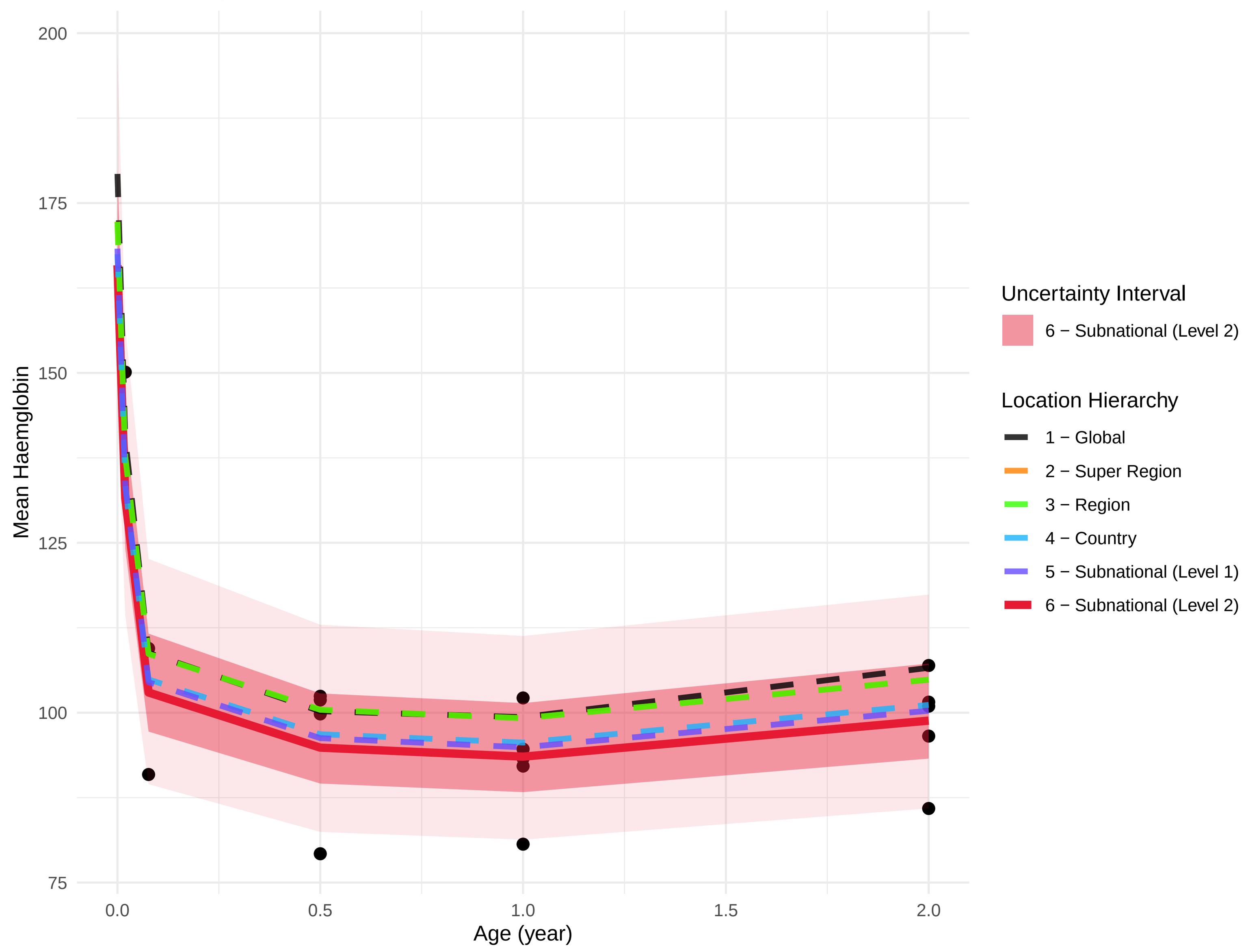

MR−BRT Age/Sex Fits − Non−pregnant Females <2 Years Old − Mean Haemglobin − Maharashtra, Urban
WHO−2024 Adjustment Method

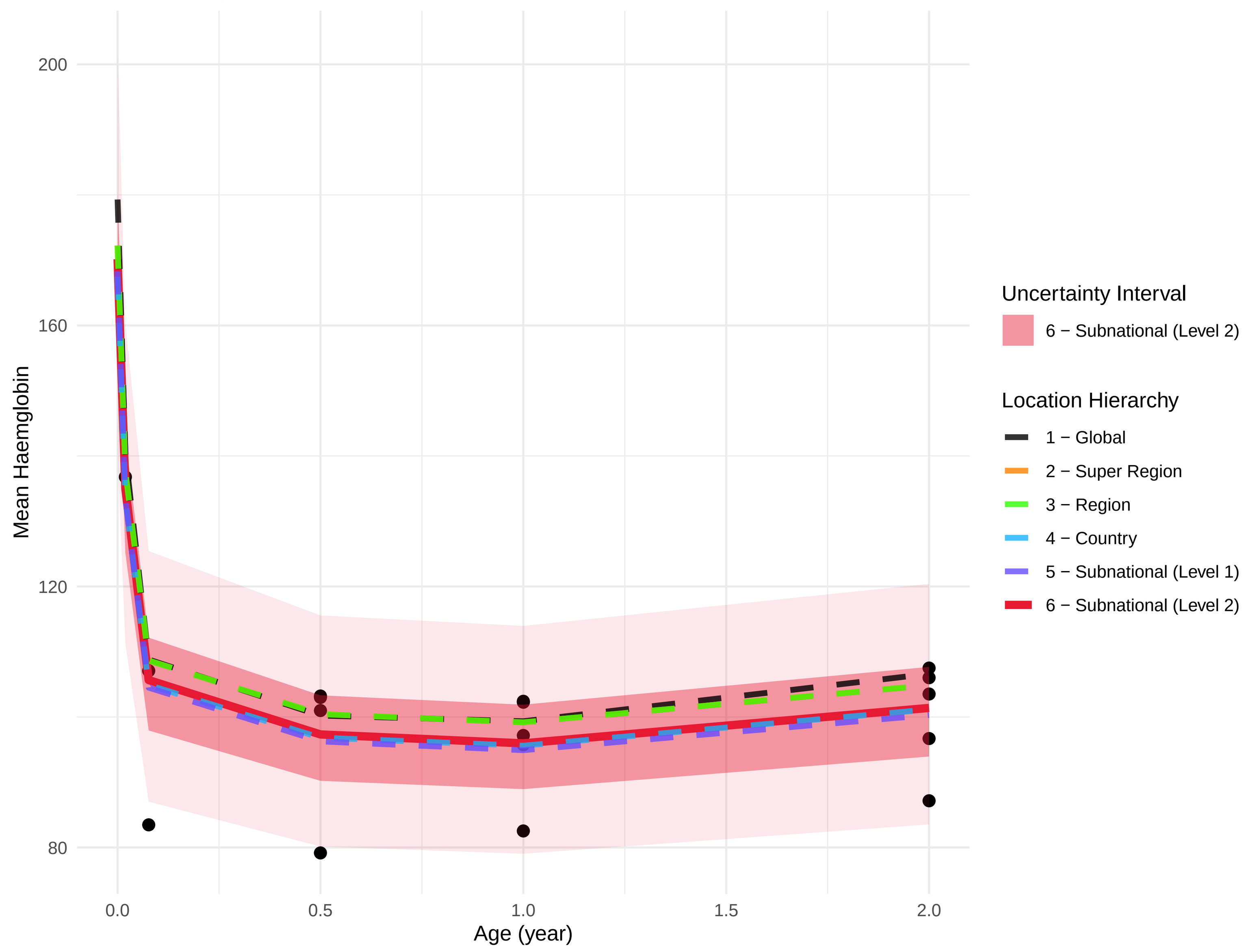

MR−BRT Age/Sex Fits − Non−pregnant Females <2 Years Old − Mean Haemglobin − Manipur
WHO−2024 Adjustment Method

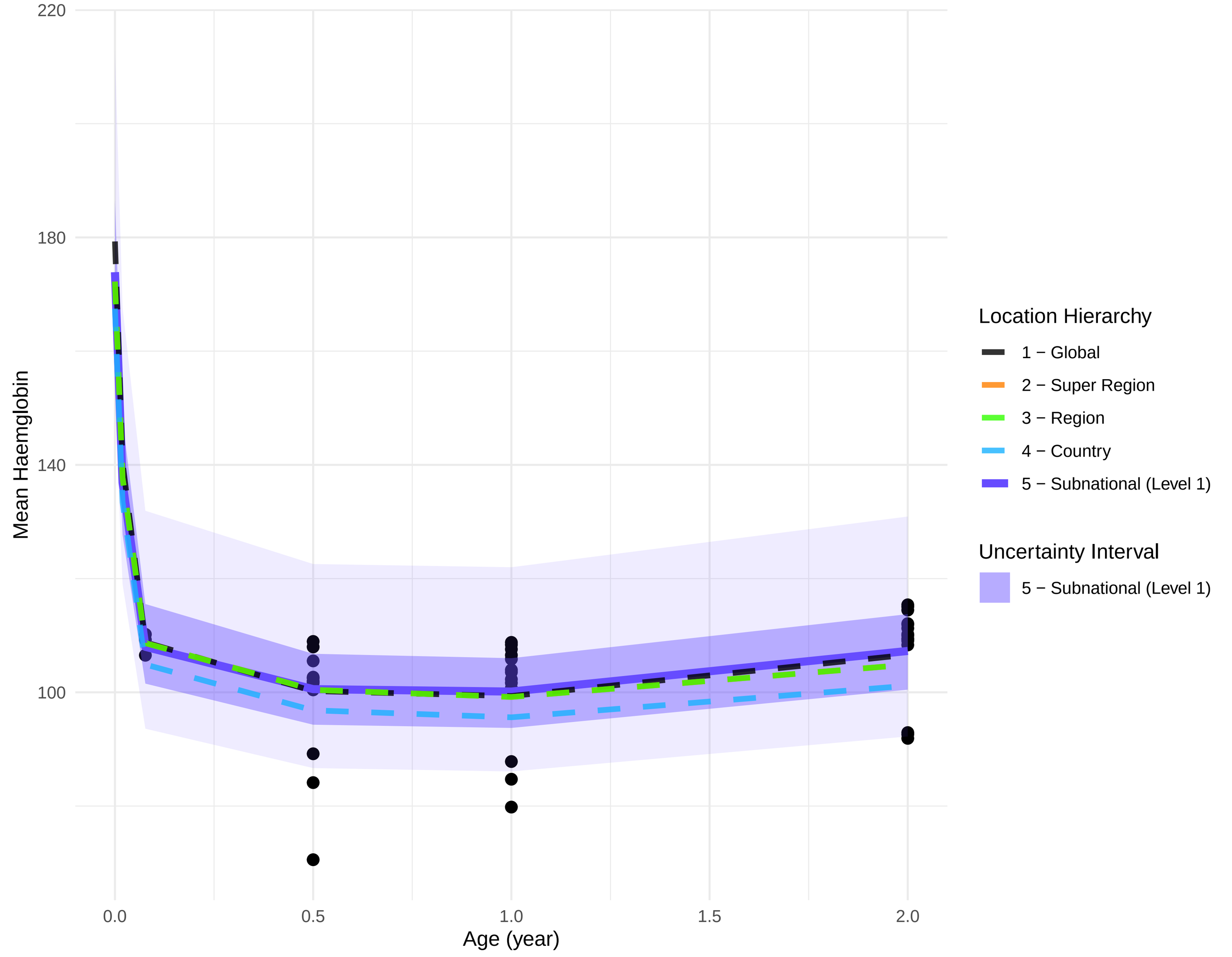

MR−BRT Age/Sex Fits − Non−pregnant Females <2 Years Old − Mean Haemglobin − Manipur, Rural
WHO−2024 Adjustment Method

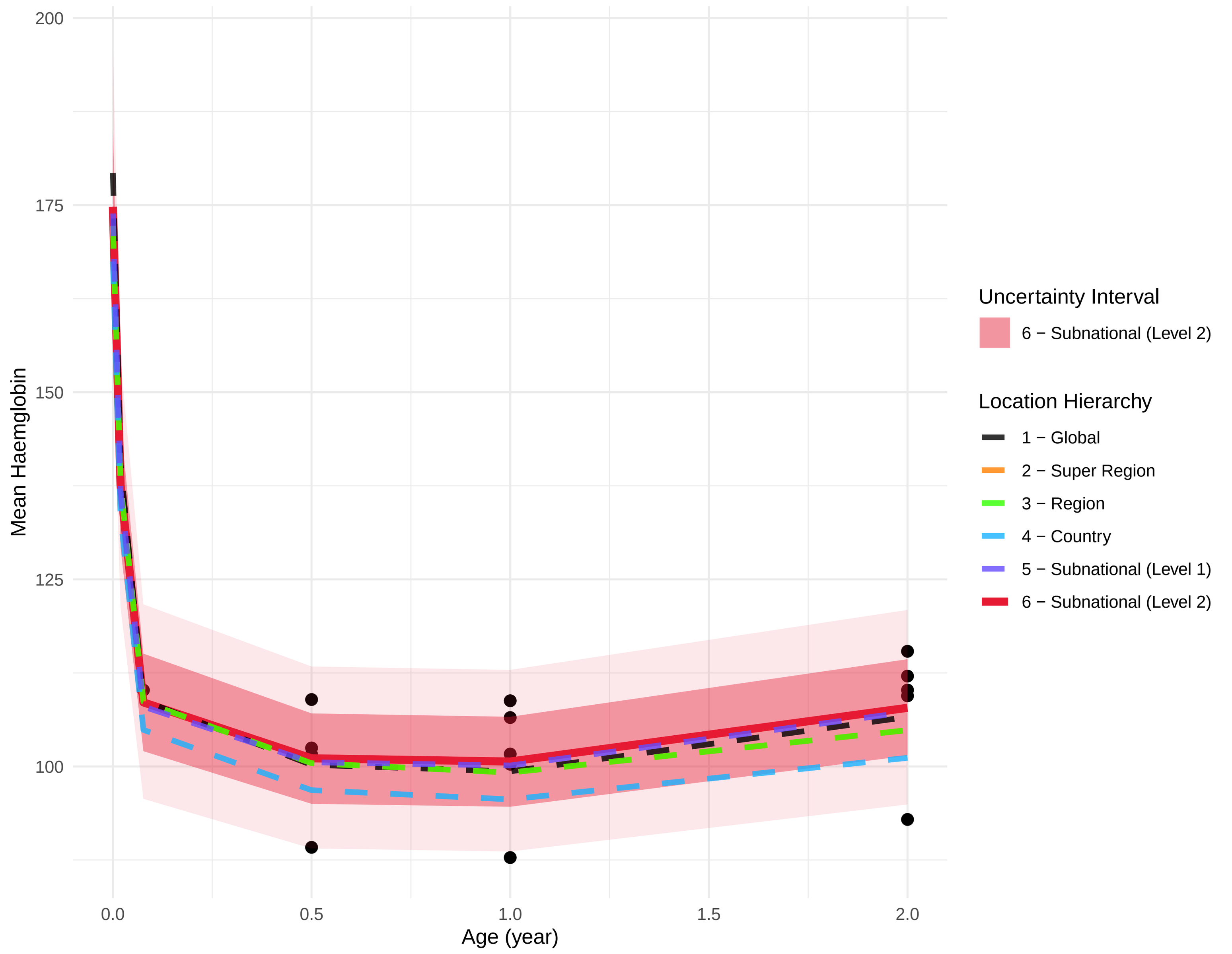

MR−BRT Age/Sex Fits − Non−pregnant Females <2 Years Old − Mean Haemglobin − Manipur, Urban
WHO−2024 Adjustment Method

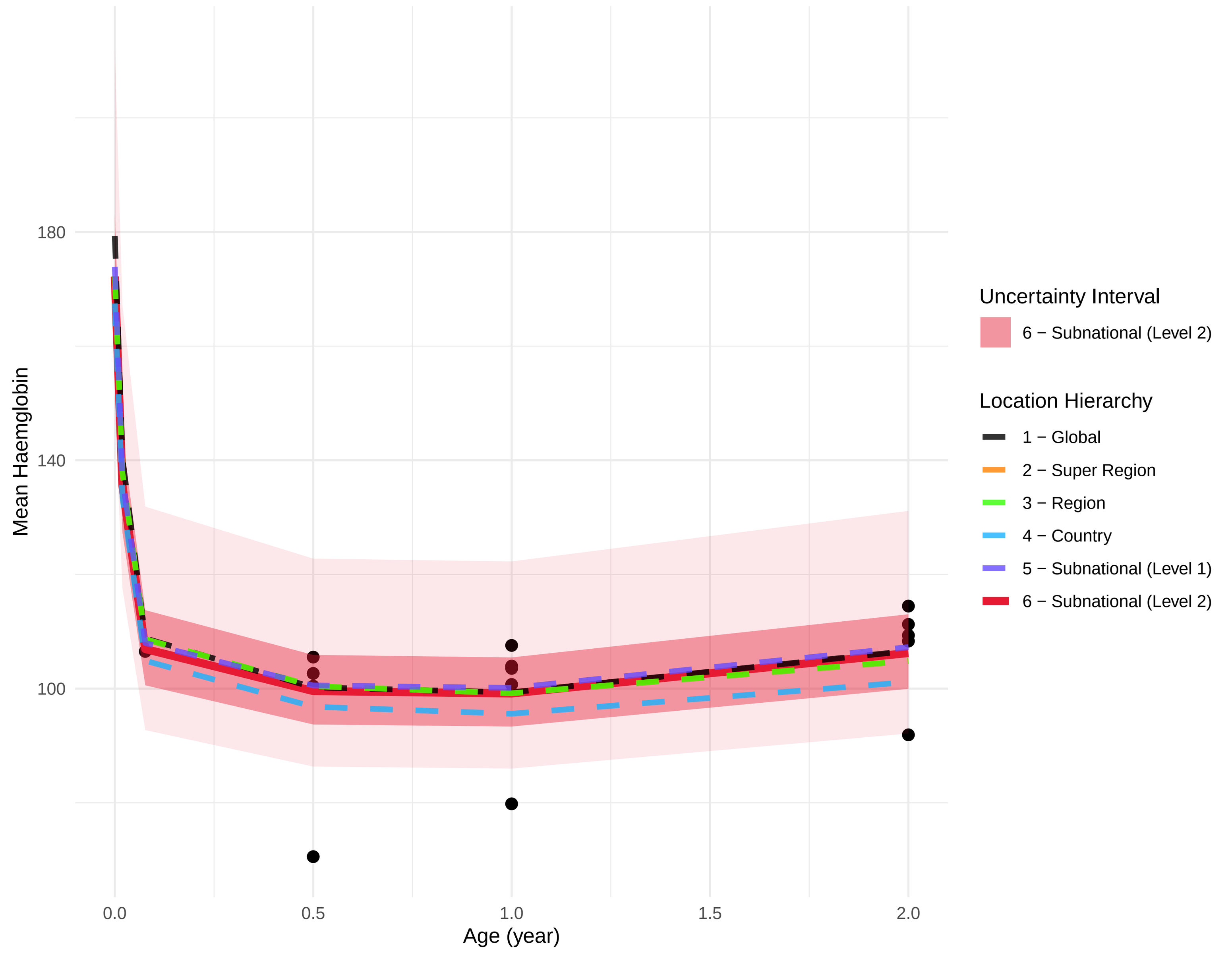

MR−BRT Age/Sex Fits − Non−pregnant Females <2 Years Old − Mean Haemglobin − Meghalaya
WHO−2024 Adjustment Method

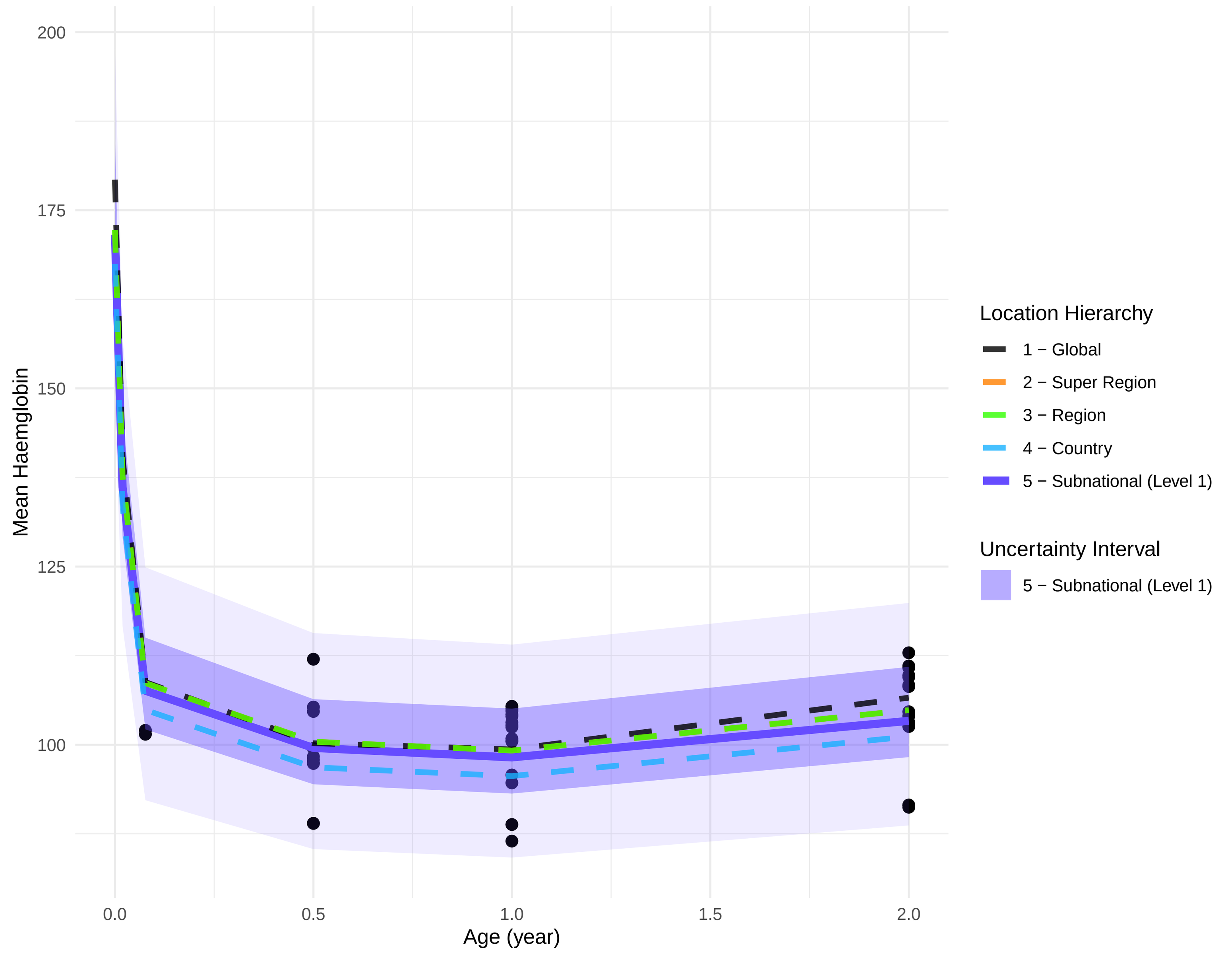

MR−BRT Age/Sex Fits − Non−pregnant Females <2 Years Old − Mean Haemglobin − Meghalaya, Rural
WHO−2024 Adjustment Method

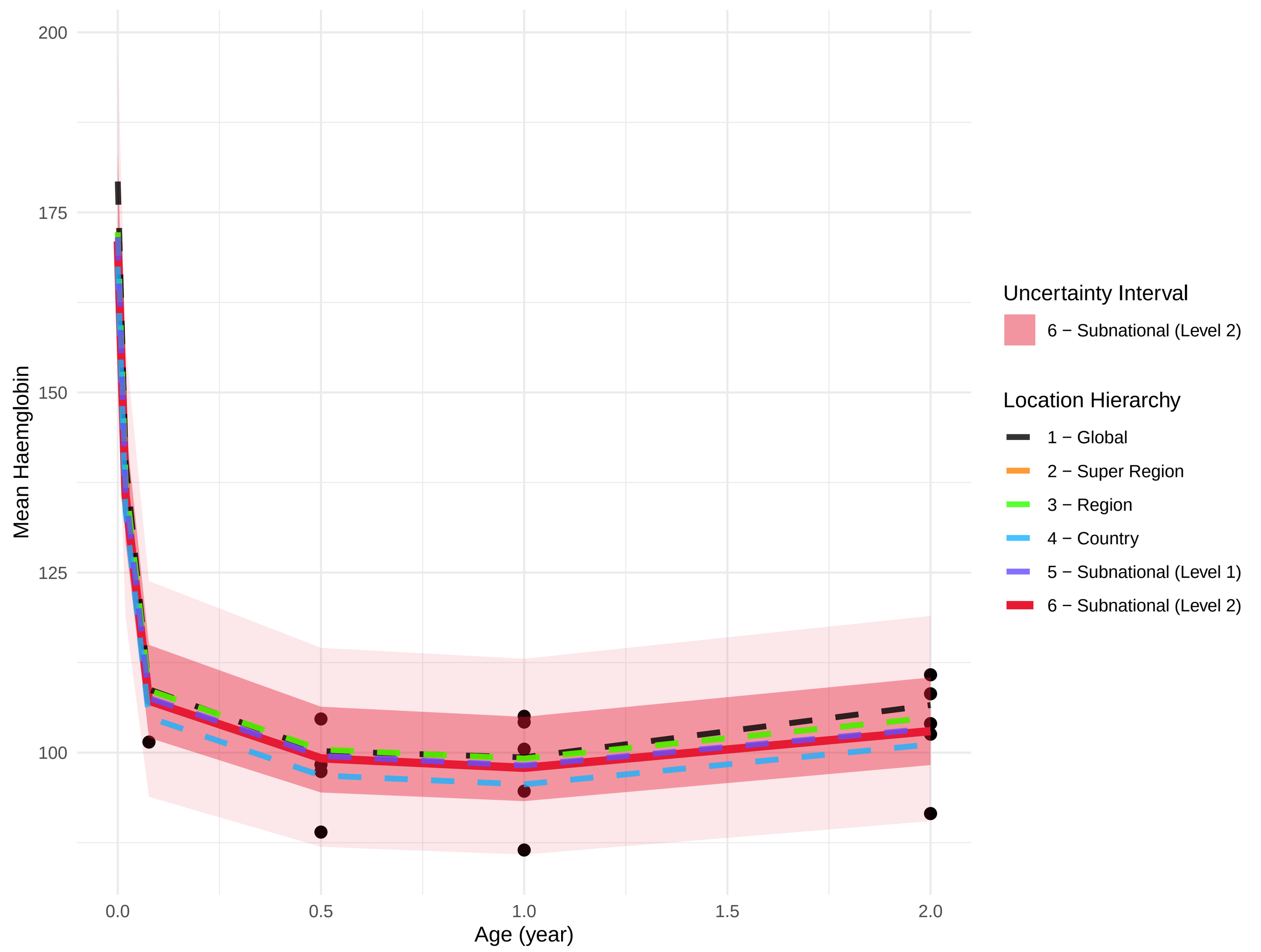

MR−BRT Age/Sex Fits − Non−pregnant Females <2 Years Old − Mean Haemglobin − Meghalaya, Urban
WHO−2024 Adjustment Method

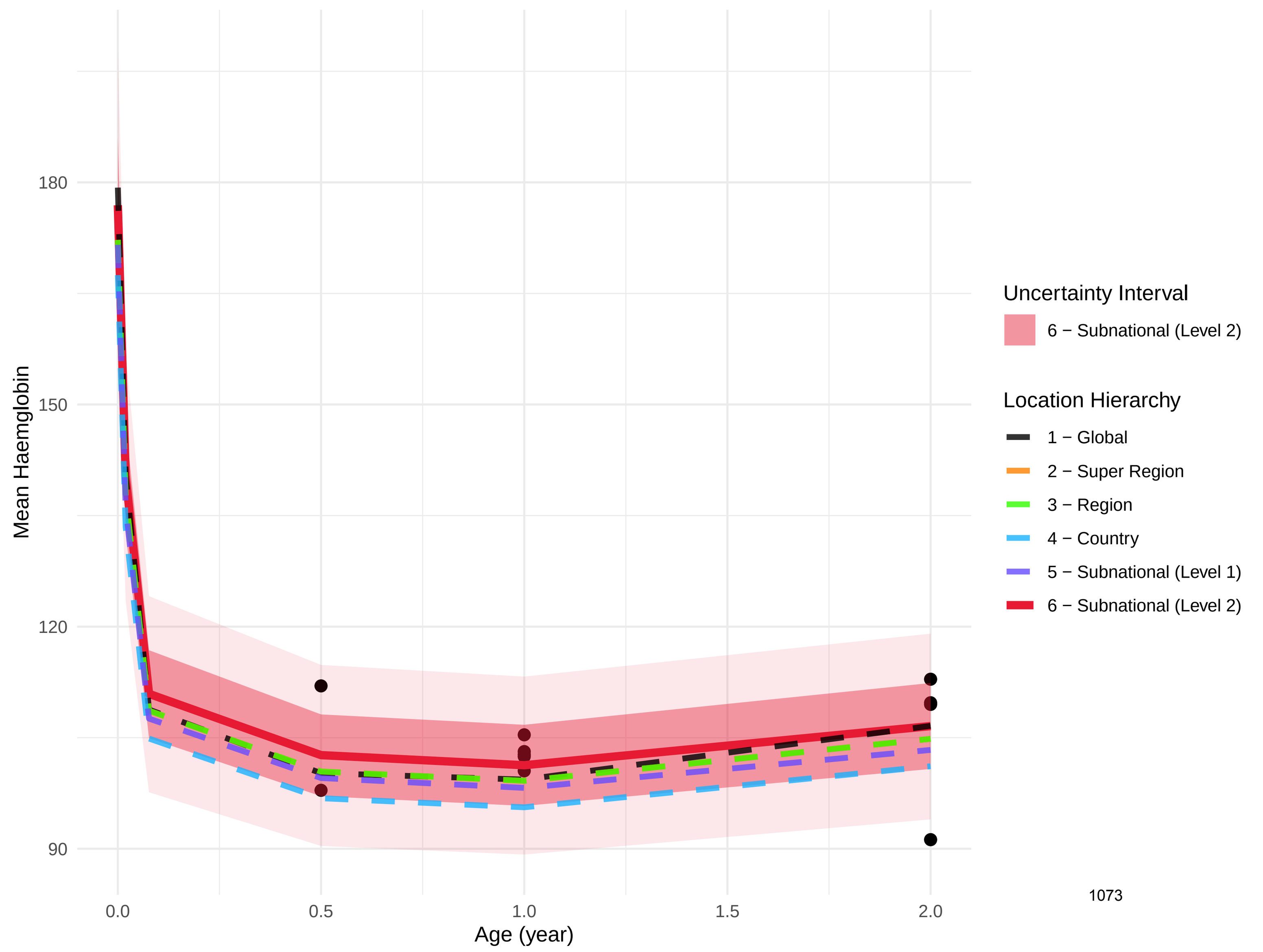

MR−BRT Age/Sex Fits − Non−pregnant Females <2 Years Old − Mean Haemglobin − Mizoram
WHO−2024 Adjustment Method

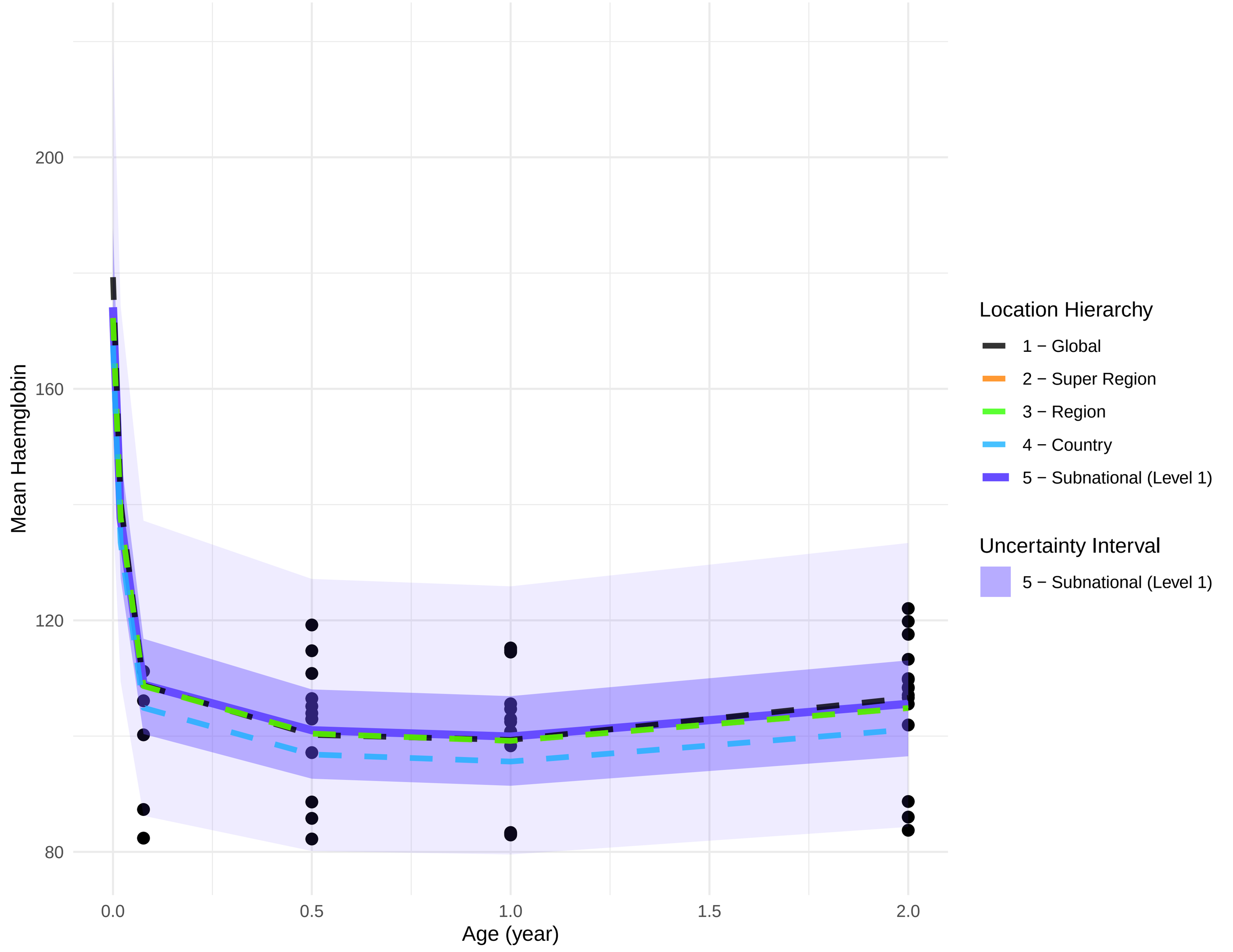

MR−BRT Age/Sex Fits − Non−pregnant Females <2 Years Old − Mean Haemglobin − Mizoram, Rural
WHO−2024 Adjustment Method

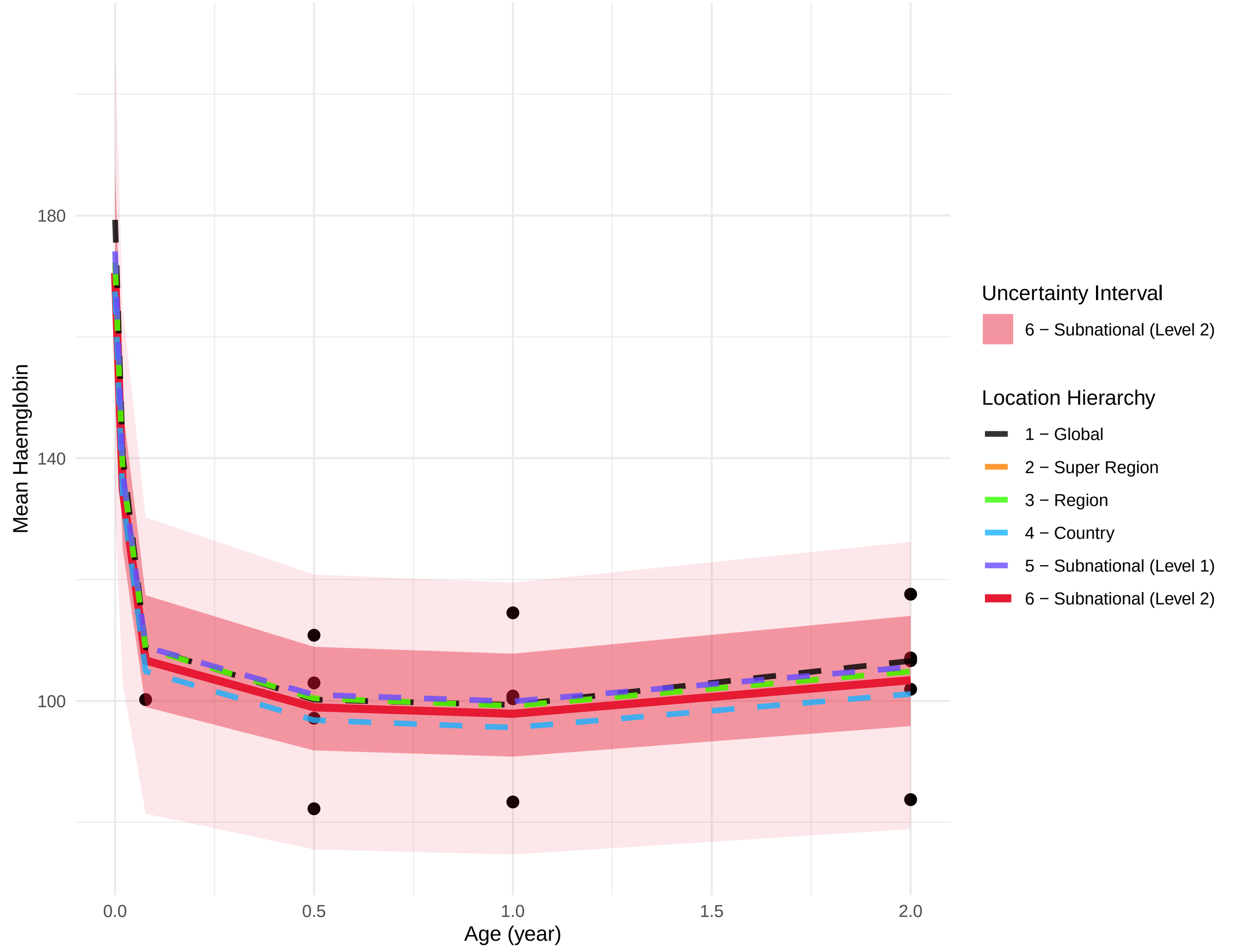

MR−BRT Age/Sex Fits − Non−pregnant Females <2 Years Old − Mean Haemglobin − Mizoram, Urban
WHO−2024 Adjustment Method

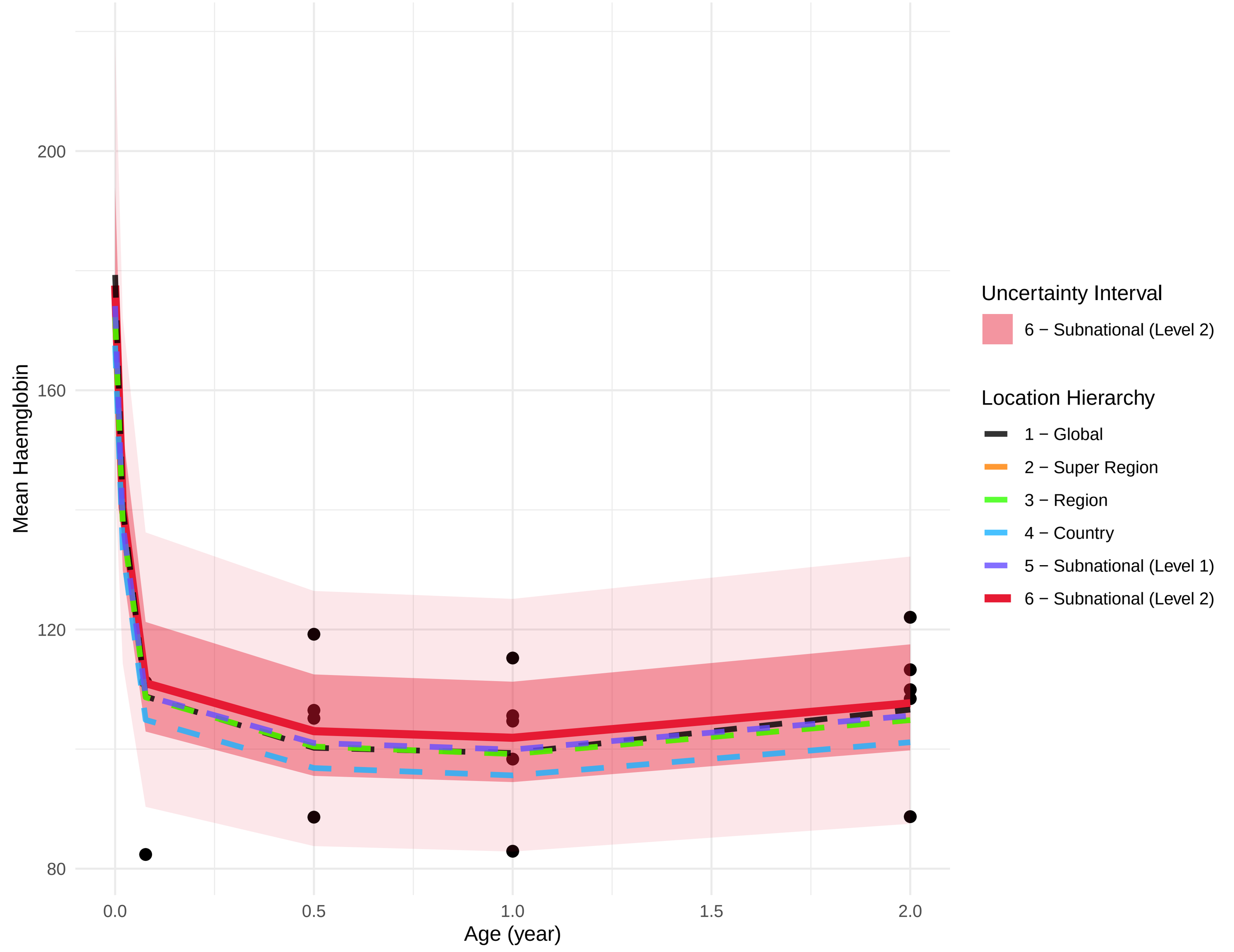

MR−BRT Age/Sex Fits − Non−pregnant Females <2 Years Old − Mean Haemglobin − Nagaland
WHO−2024 Adjustment Method

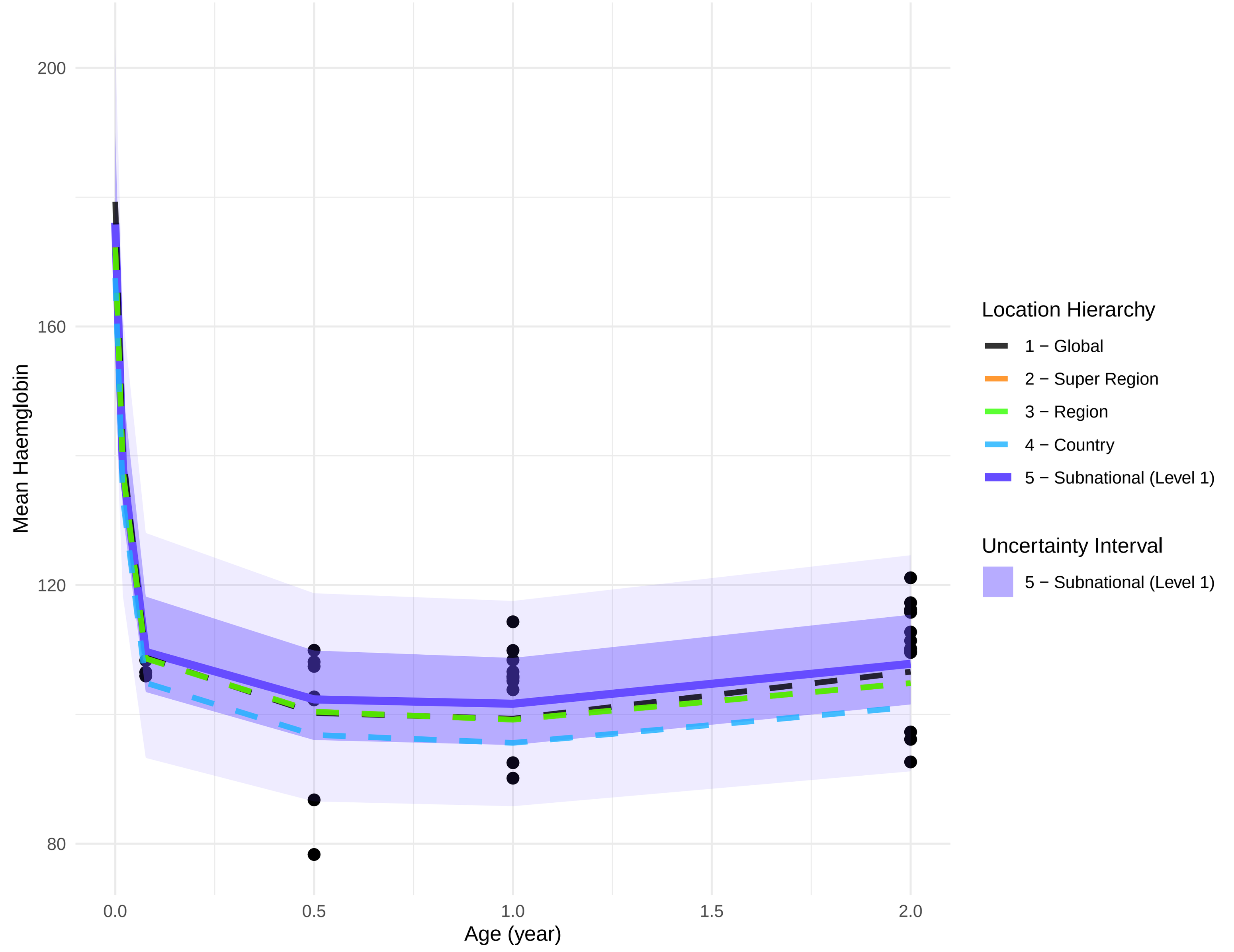

MR−BRT Age/Sex Fits − Non−pregnant Females <2 Years Old − Mean Haemglobin − Nagaland, Rural
WHO−2024 Adjustment Method

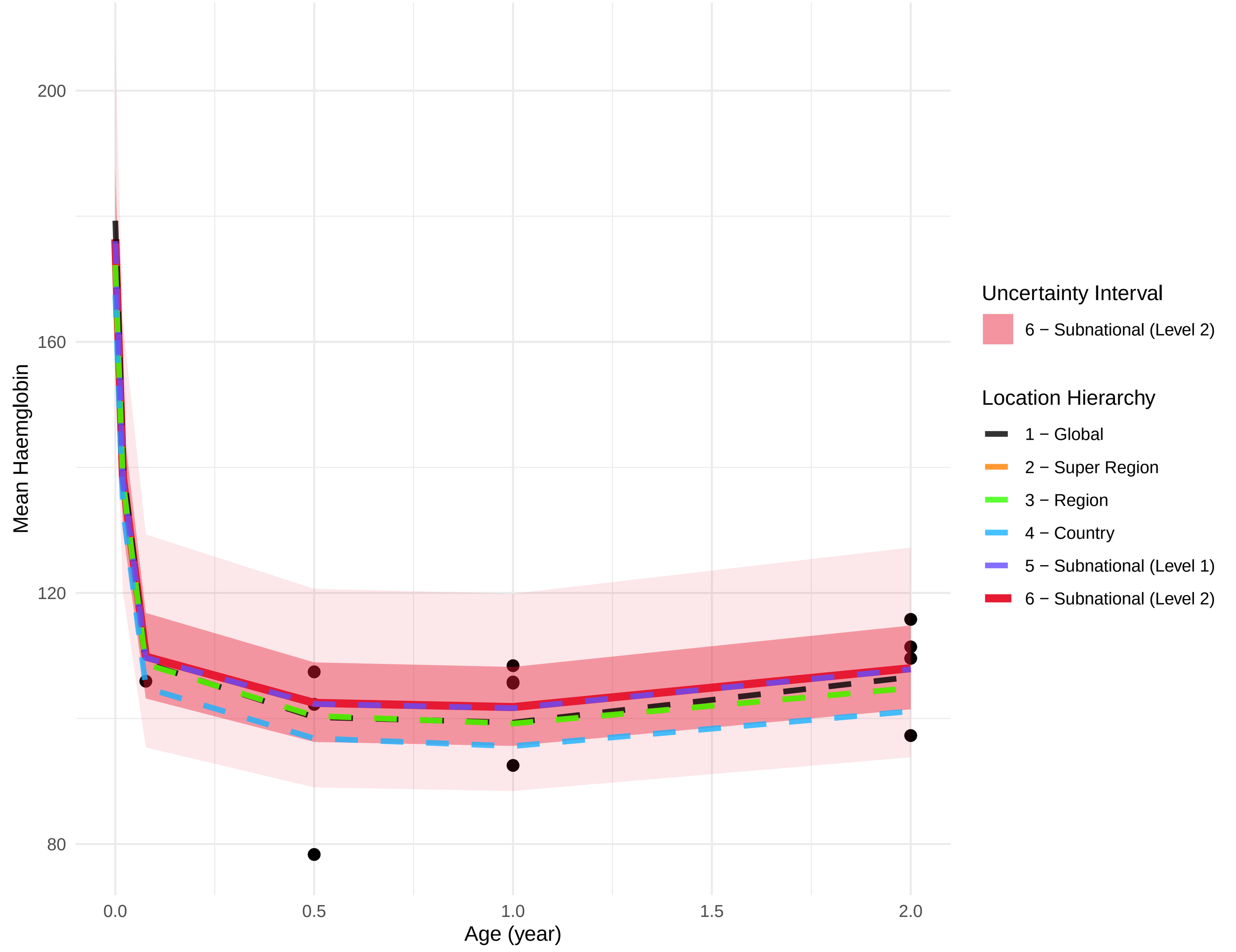

MR−BRT Age/Sex Fits − Non−pregnant Females <2 Years Old − Mean Haemglobin − Nagaland, Urban
WHO−2024 Adjustment Method

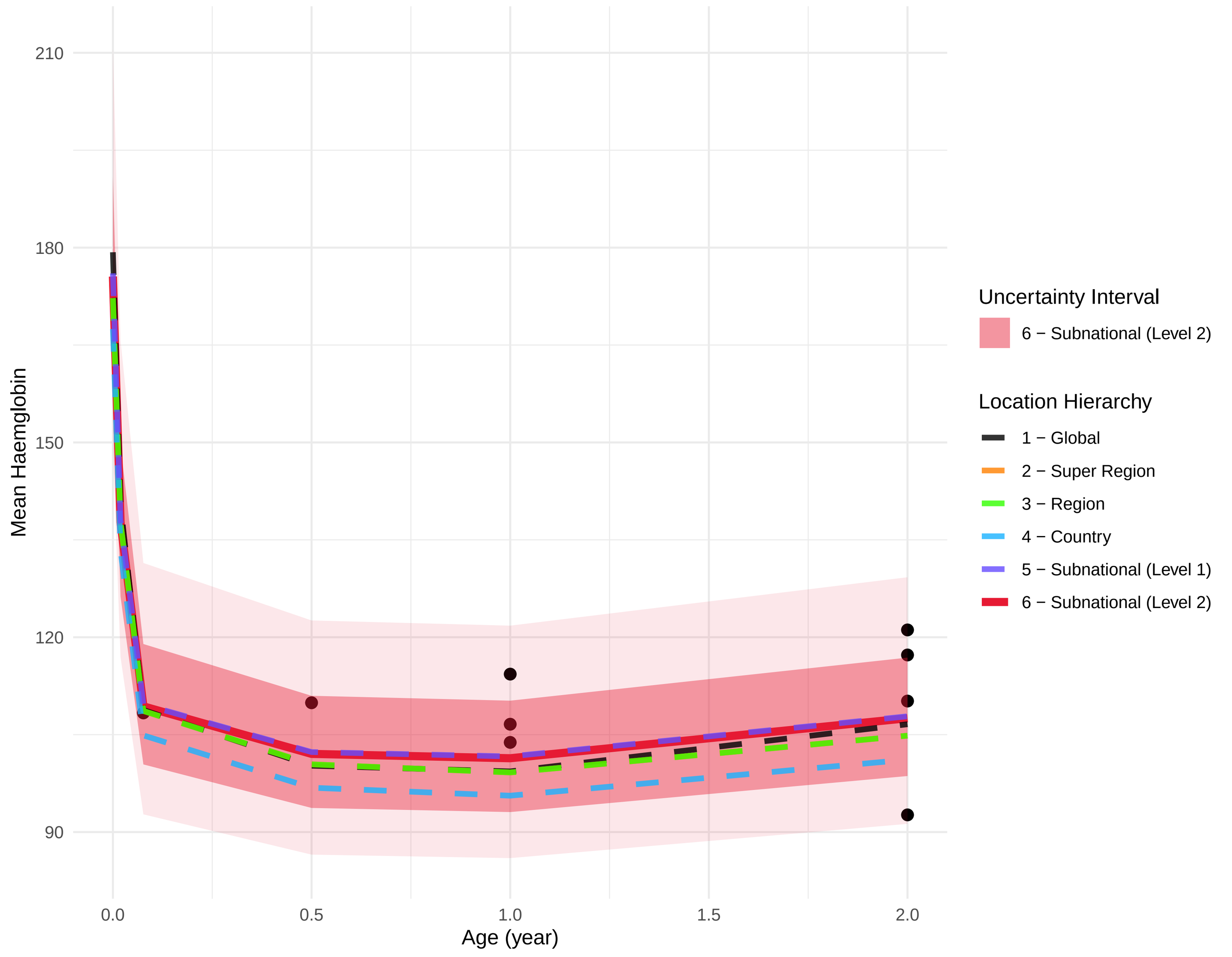

MR−BRT Age/Sex Fits − Non−pregnant Females <2 Years Old − Mean Haemglobin − Odisha
WHO−2024 Adjustment Method

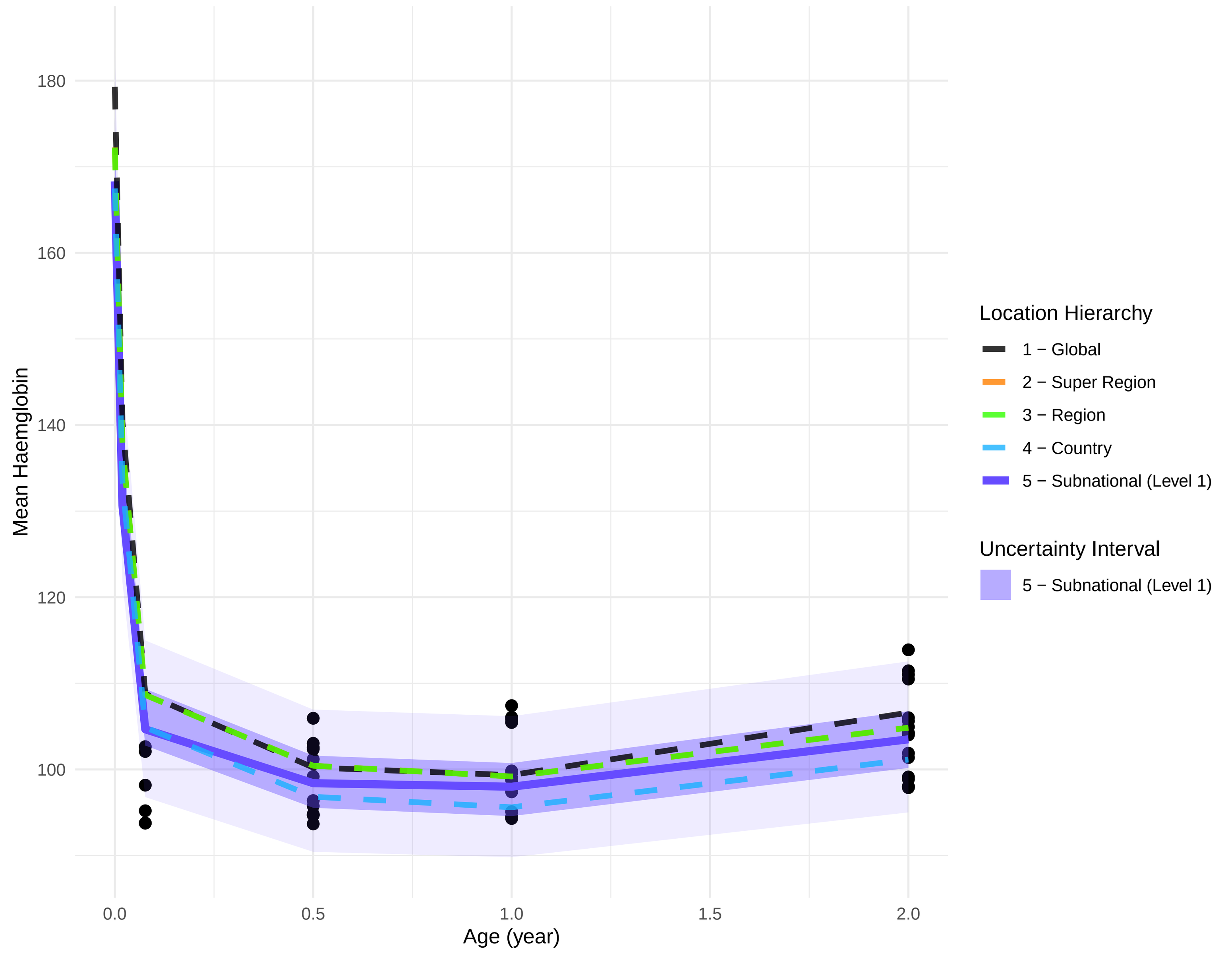

MR−BRT Age/Sex Fits − Non−pregnant Females <2 Years Old − Mean Haemglobin − Odisha, Rural
WHO−2024 Adjustment Method

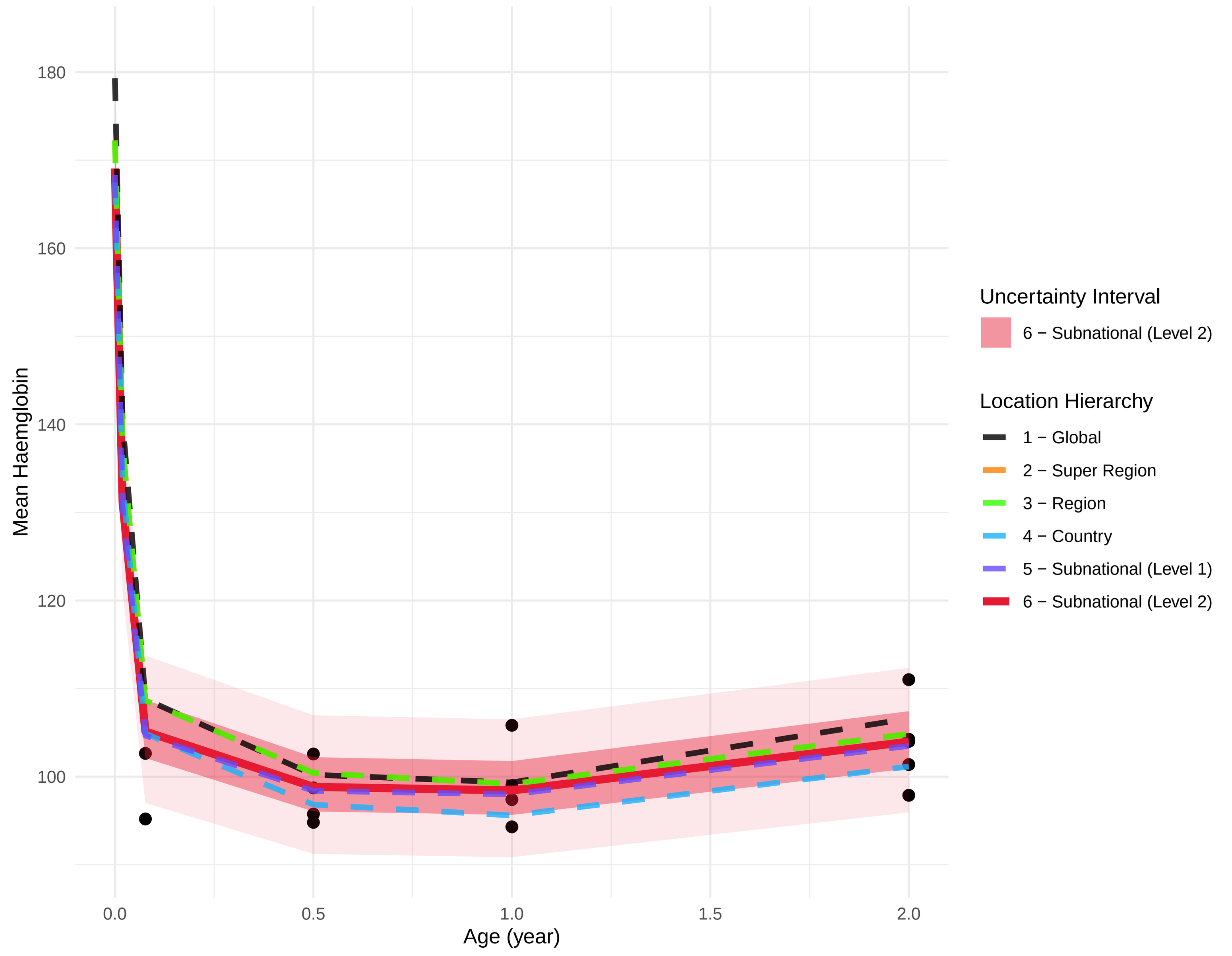

MR−BRT Age/Sex Fits − Non−pregnant Females <2 Years Old − Mean Haemglobin − Odisha, Urban
WHO−2024 Adjustment Method

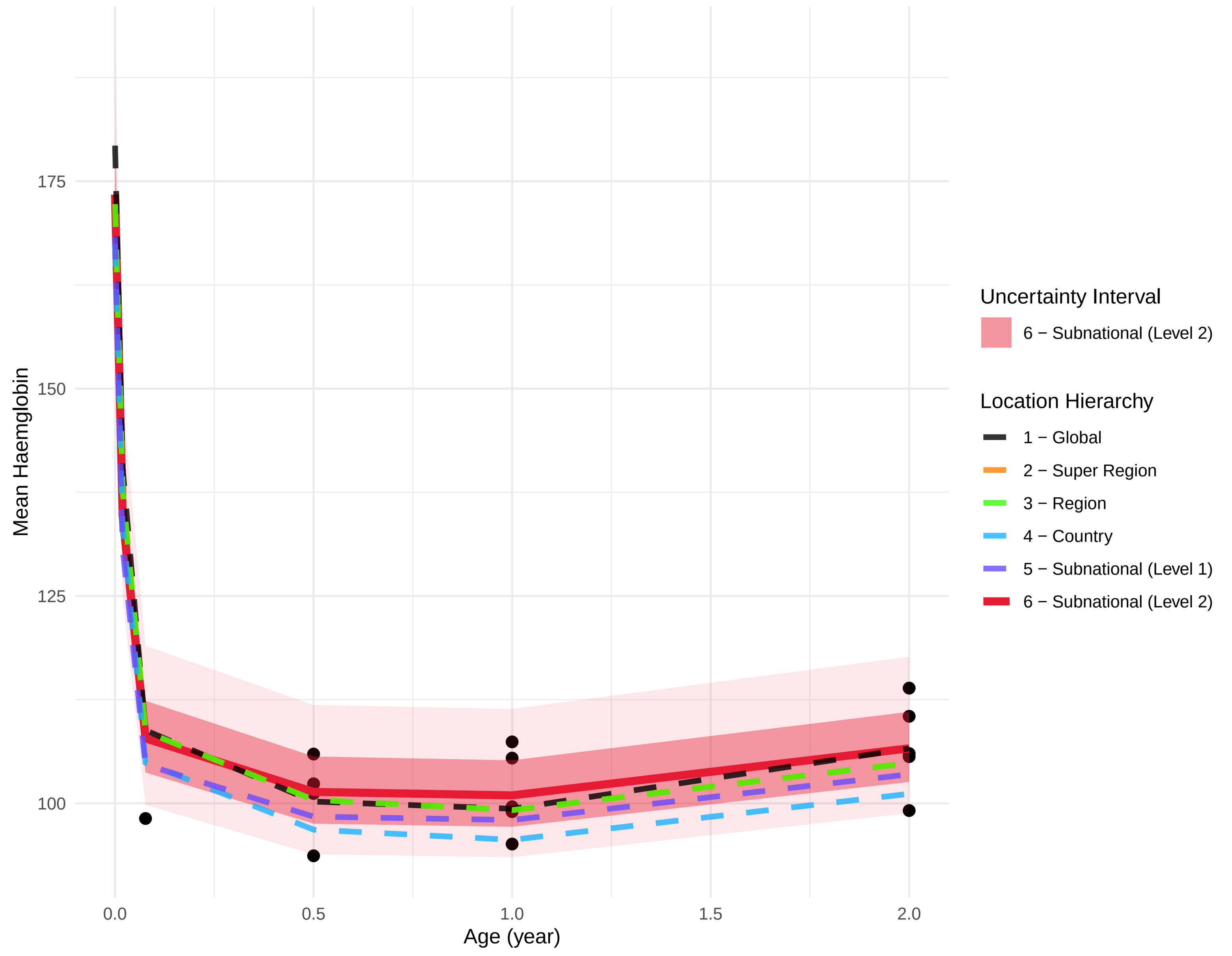

MR−BRT Age/Sex Fits − Non−pregnant Females <2 Years Old − Mean Haemglobin − Other Union Territories
WHO−2024 Adjustment Method

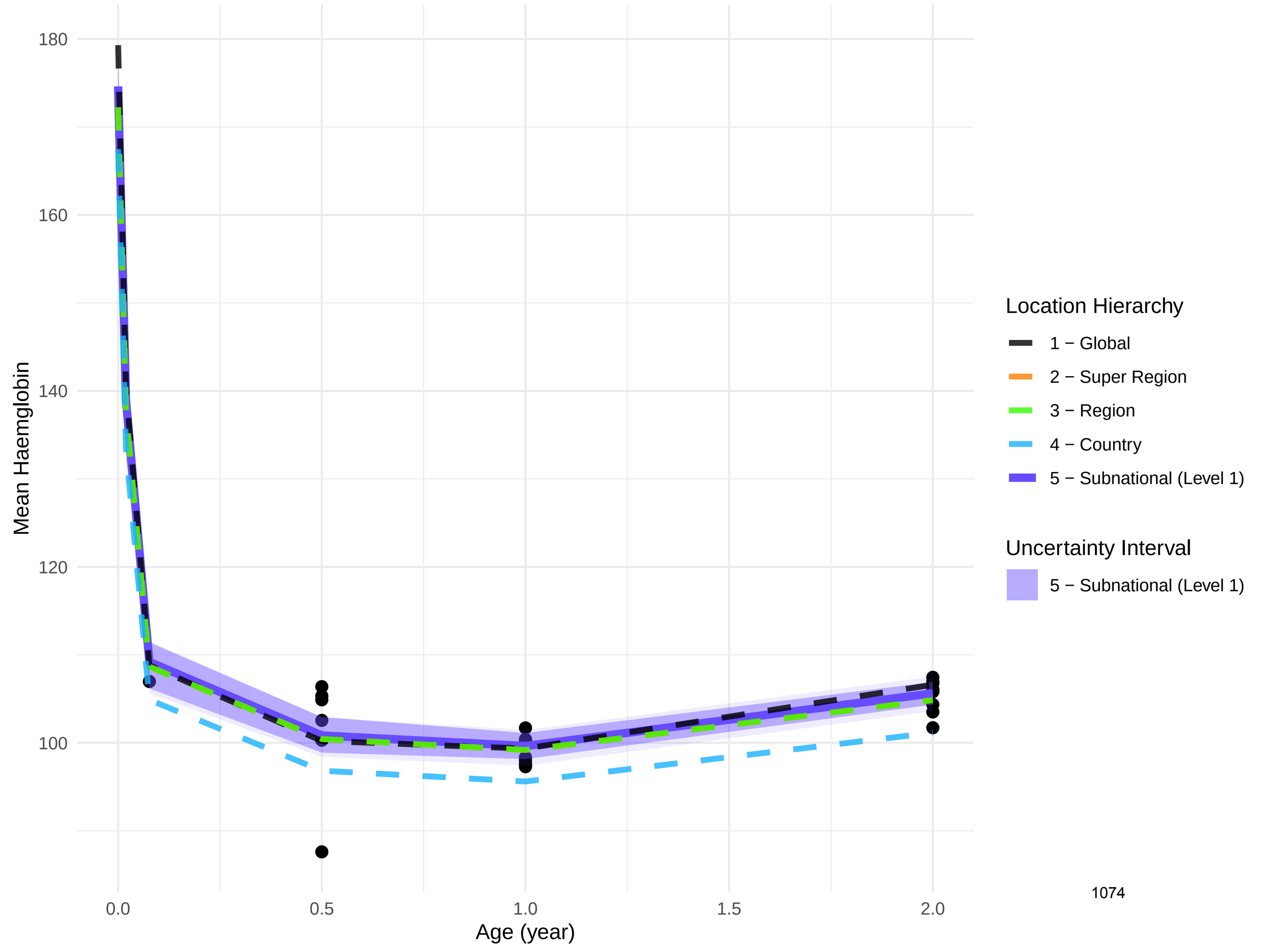

MR−BRT Age/Sex Fits − Non−pregnant Females <2 Years Old − Mean Haemglobin − Other Union Territories, Rural
WHO−2024 Adjustment Method

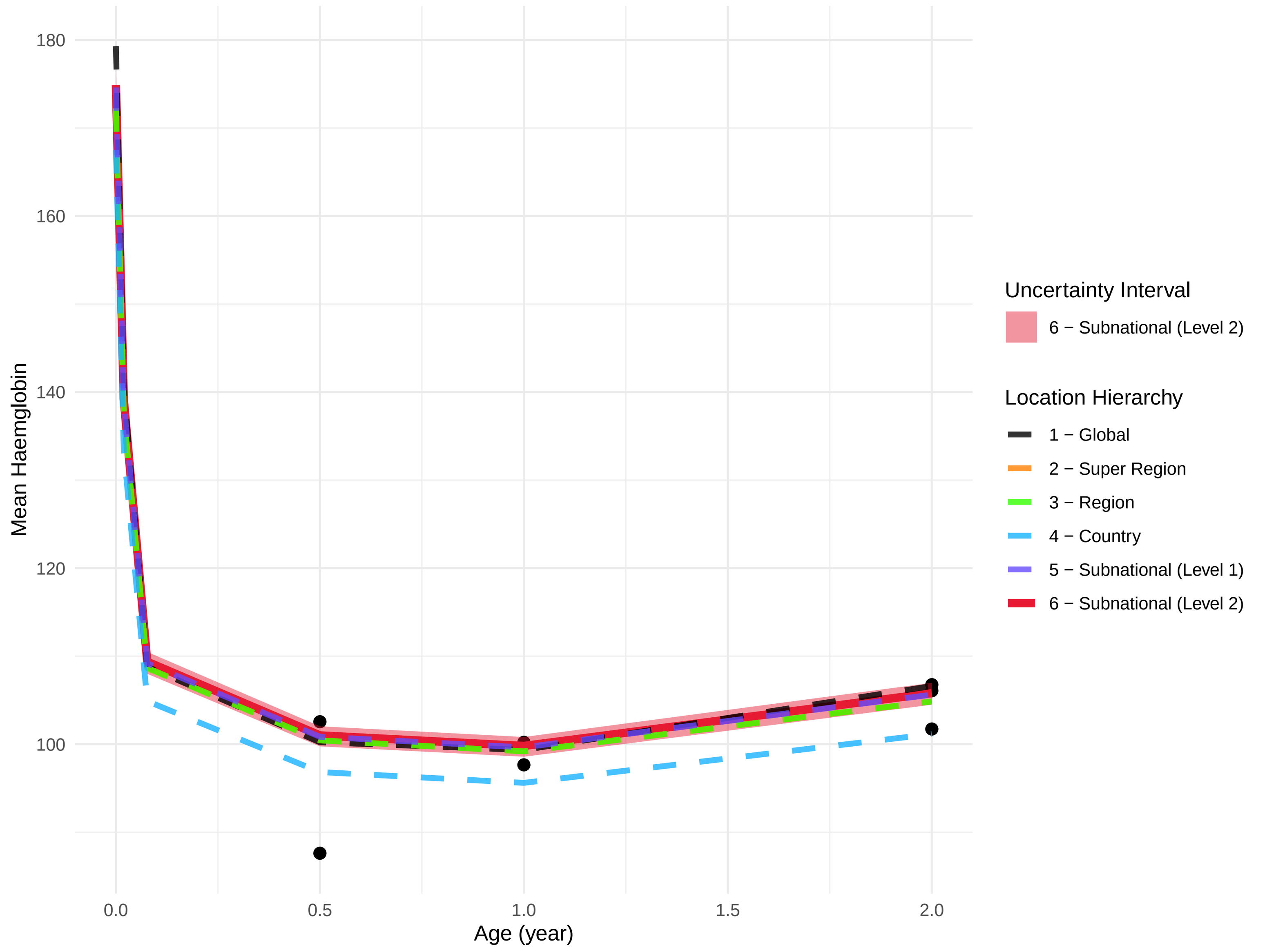

MR−BRT Age/Sex Fits − Non−pregnant Females <2 Years Old − Mean Haemglobin − Other Union Territories, Urban
WHO−2024 Adjustment Method

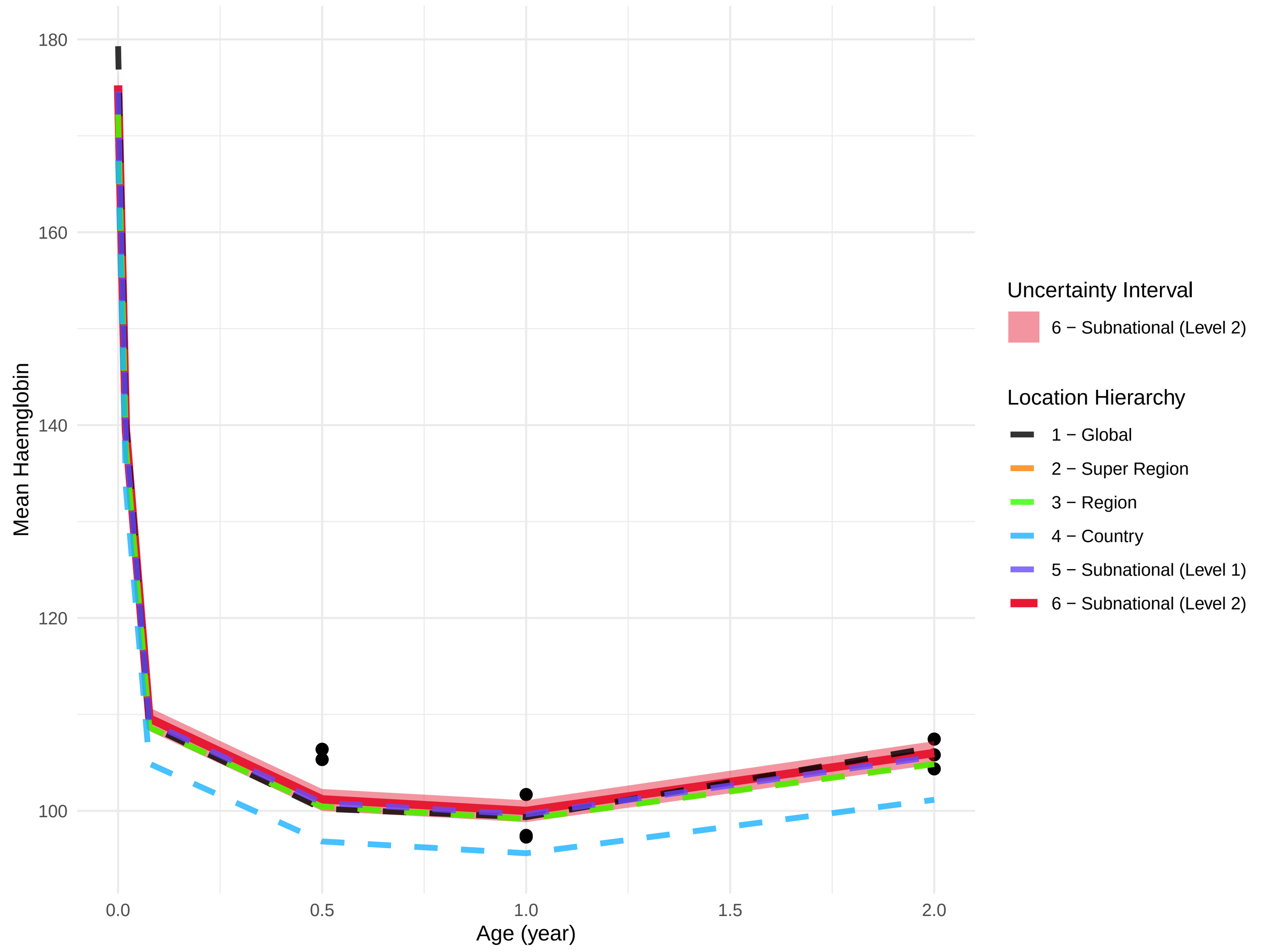

MR−BRT Age/Sex Fits − Non−pregnant Females <2 Years Old − Mean Haemglobin − Punjab
WHO−2024 Adjustment Method

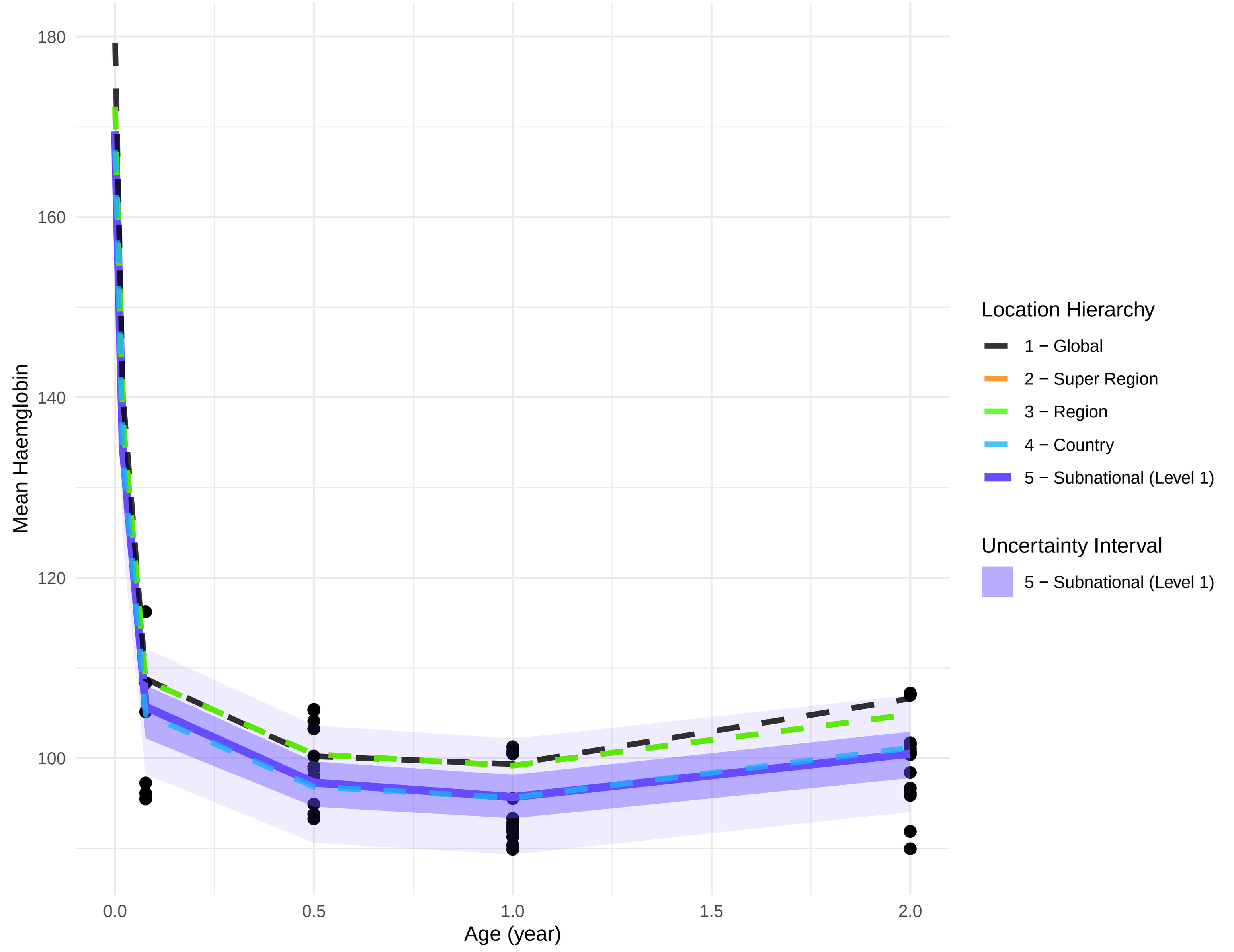

MR−BRT Age/Sex Fits − Non−pregnant Females <2 Years Old − Mean Haemglobin − Punjab, Rural
WHO−2024 Adjustment Method

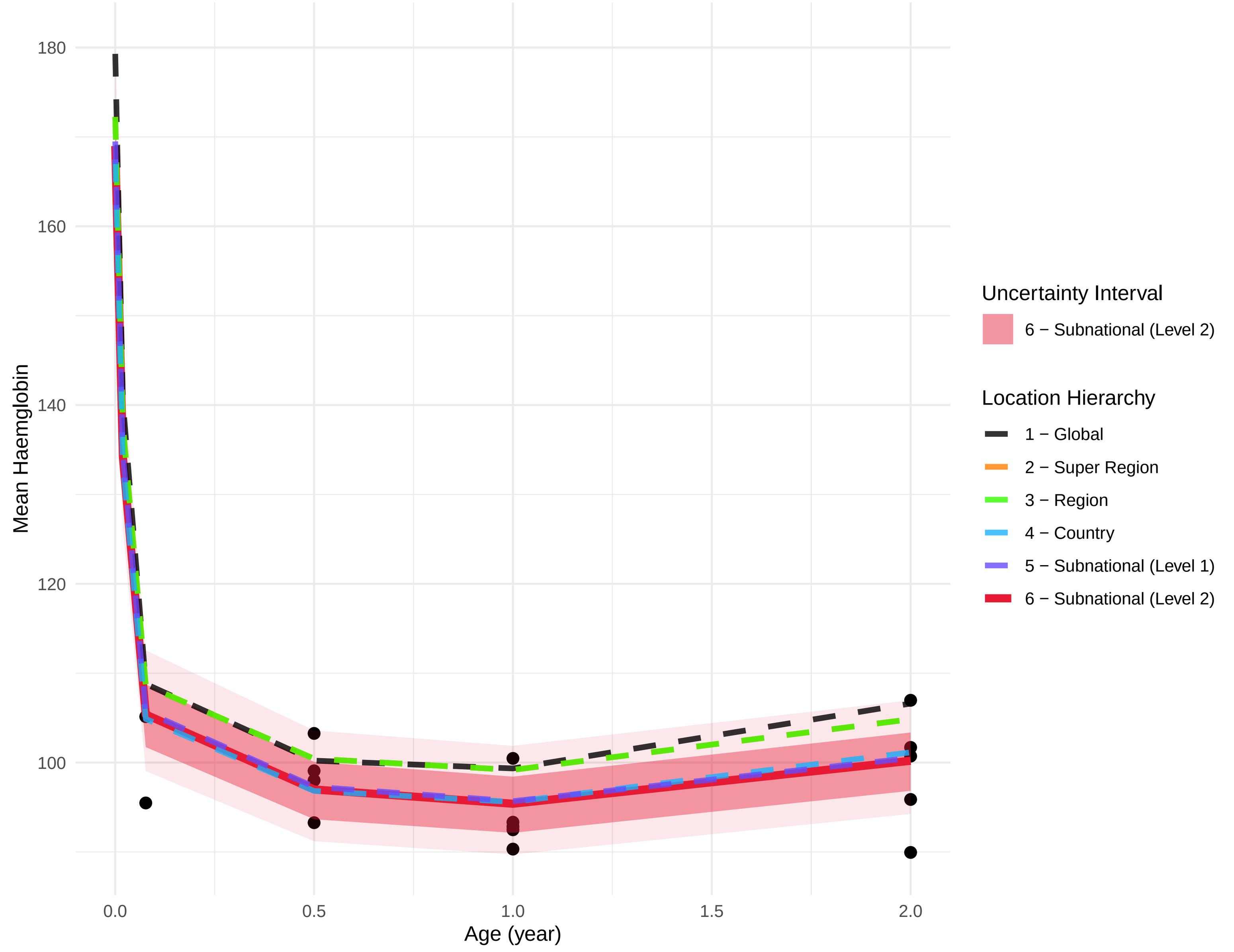

MR−BRT Age/Sex Fits − Non−pregnant Females <2 Years Old − Mean Haemglobin − Punjab, Urban
WHO−2024 Adjustment Method

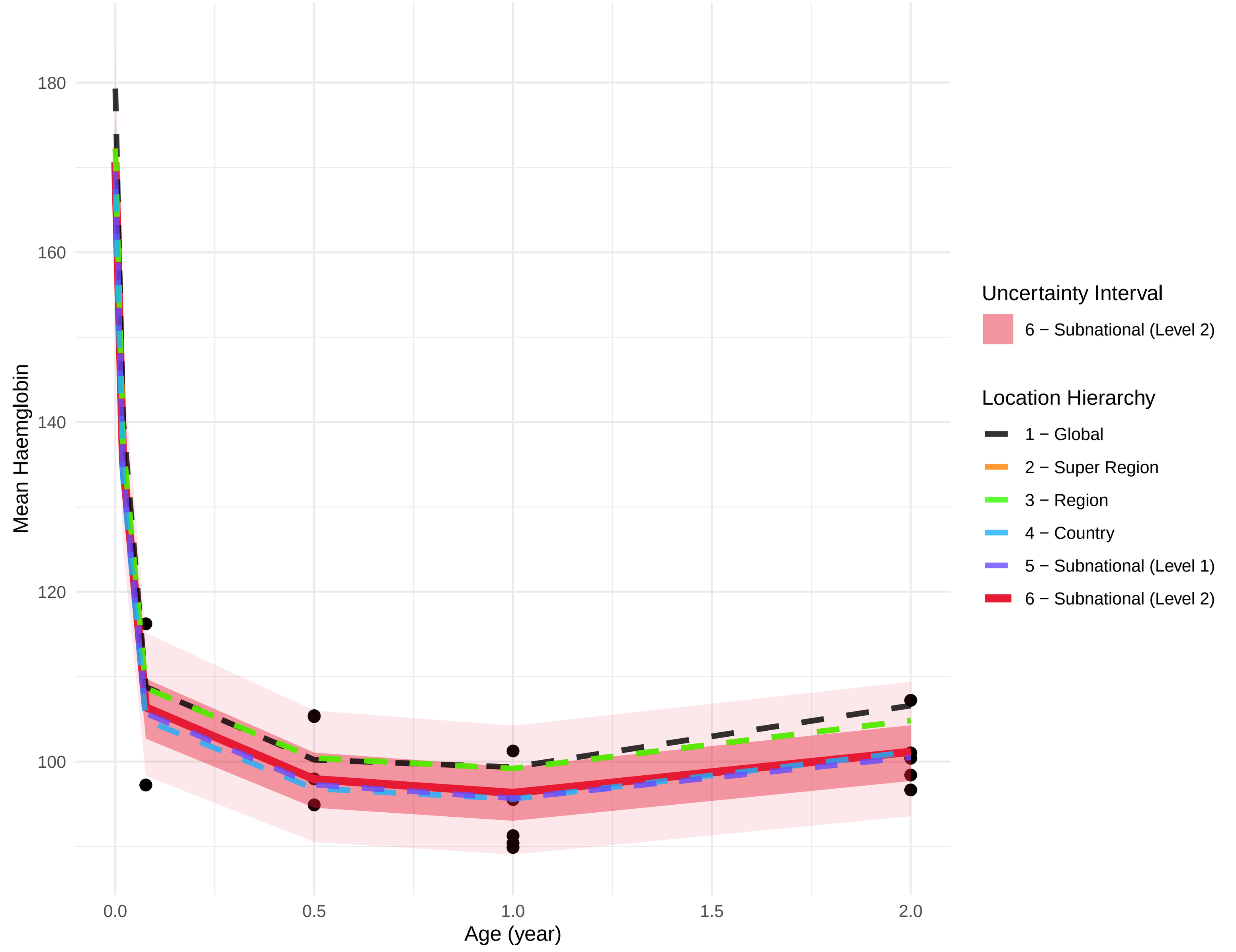

MR−BRT Age/Sex Fits − Non−pregnant Females <2 Years Old − Mean Haemglobin − Rajasthan
WHO−2024 Adjustment Method

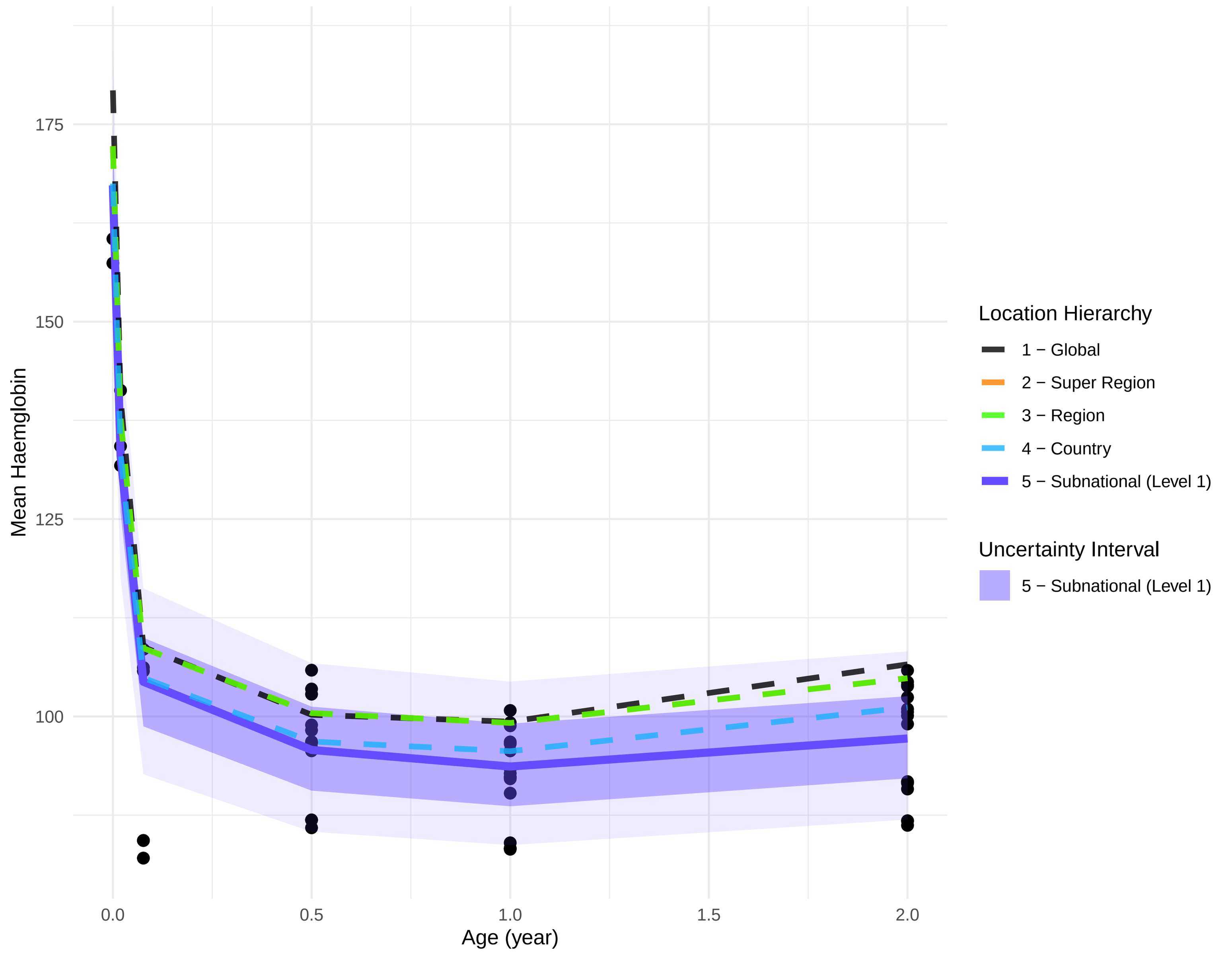

MR−BRT Age/Sex Fits − Non−pregnant Females <2 Years Old − Mean Haemglobin − Rajasthan, Rural
WHO−2024 Adjustment Method

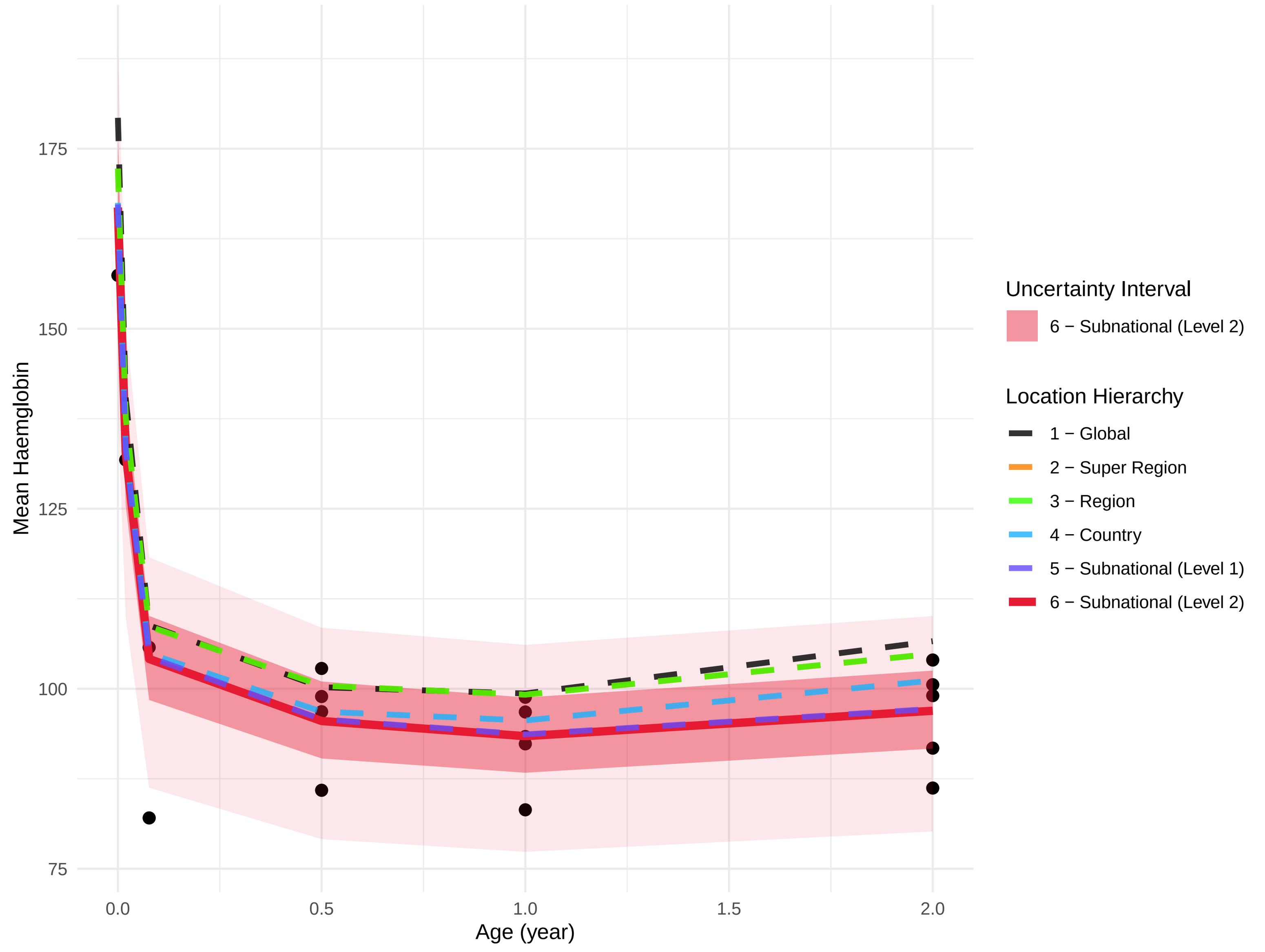

MR−BRT Age/Sex Fits − Non−pregnant Females <2 Years Old − Mean Haemglobin − Rajasthan, Urban
WHO−2024 Adjustment Method

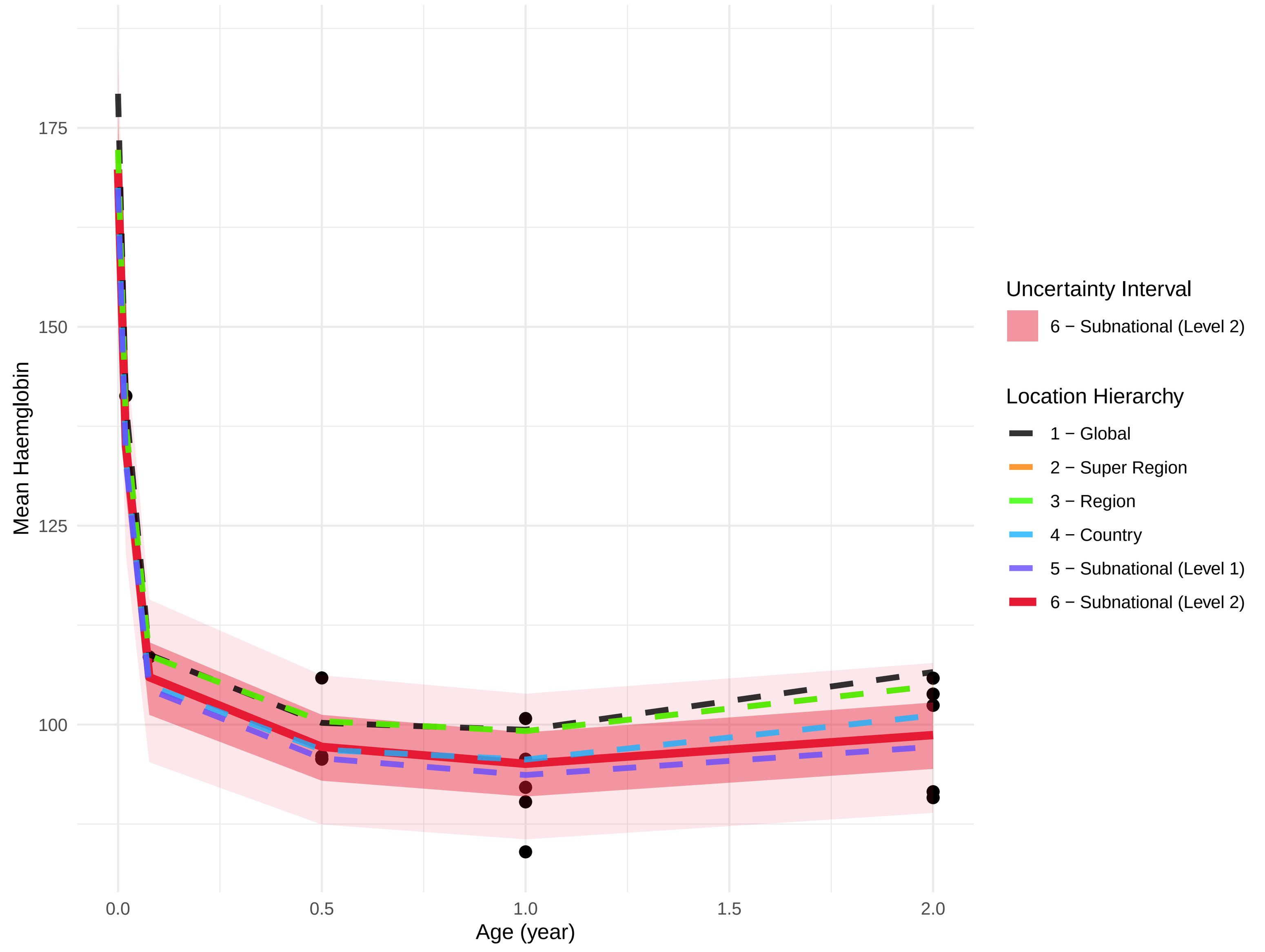

MR−BRT Age/Sex Fits − Non−pregnant Females <2 Years Old − Mean Haemglobin − Sikkim
WHO−2024 Adjustment Method

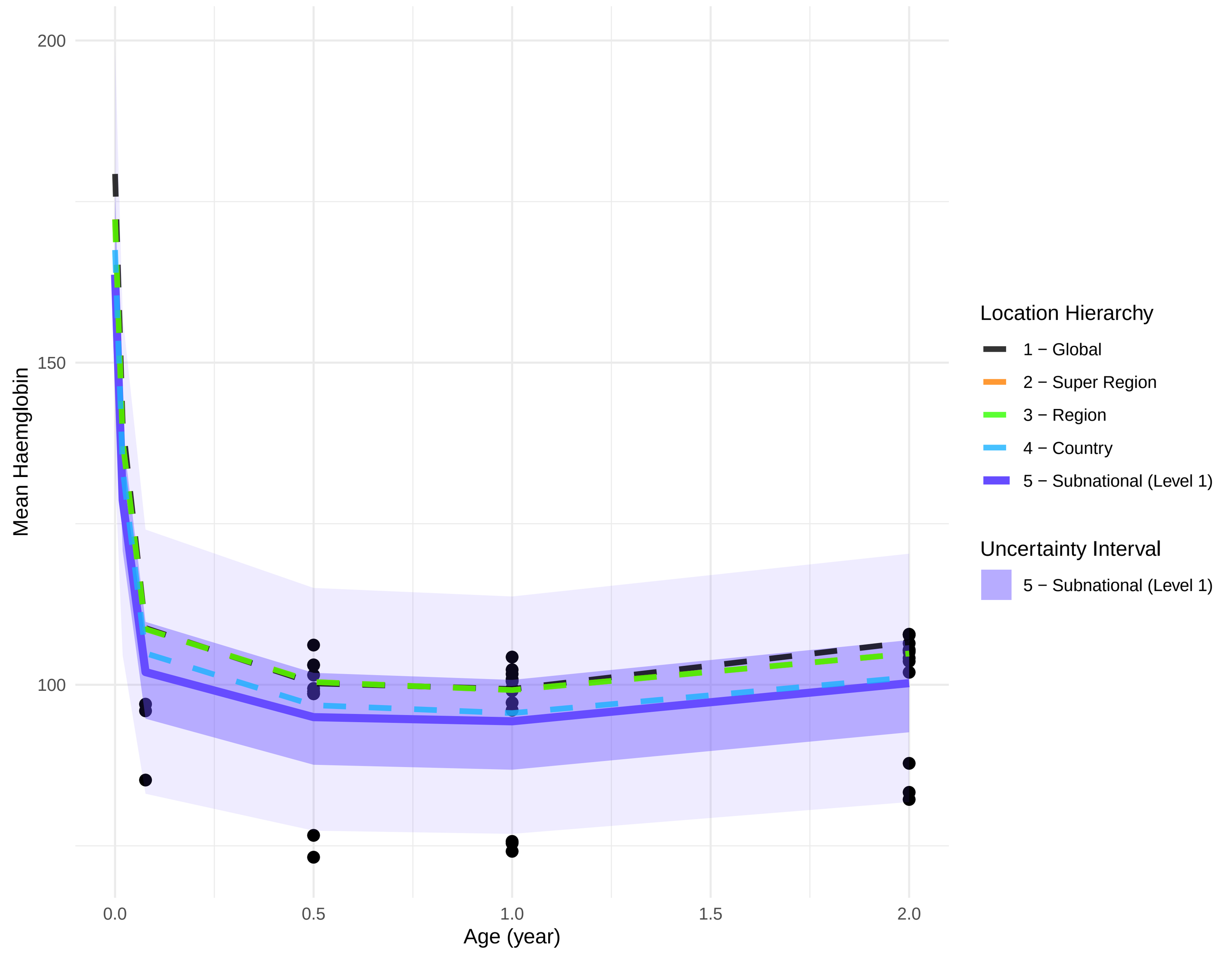

MR−BRT Age/Sex Fits − Non−pregnant Females <2 Years Old − Mean Haemglobin − Sikkim, Rural
WHO−2024 Adjustment Method

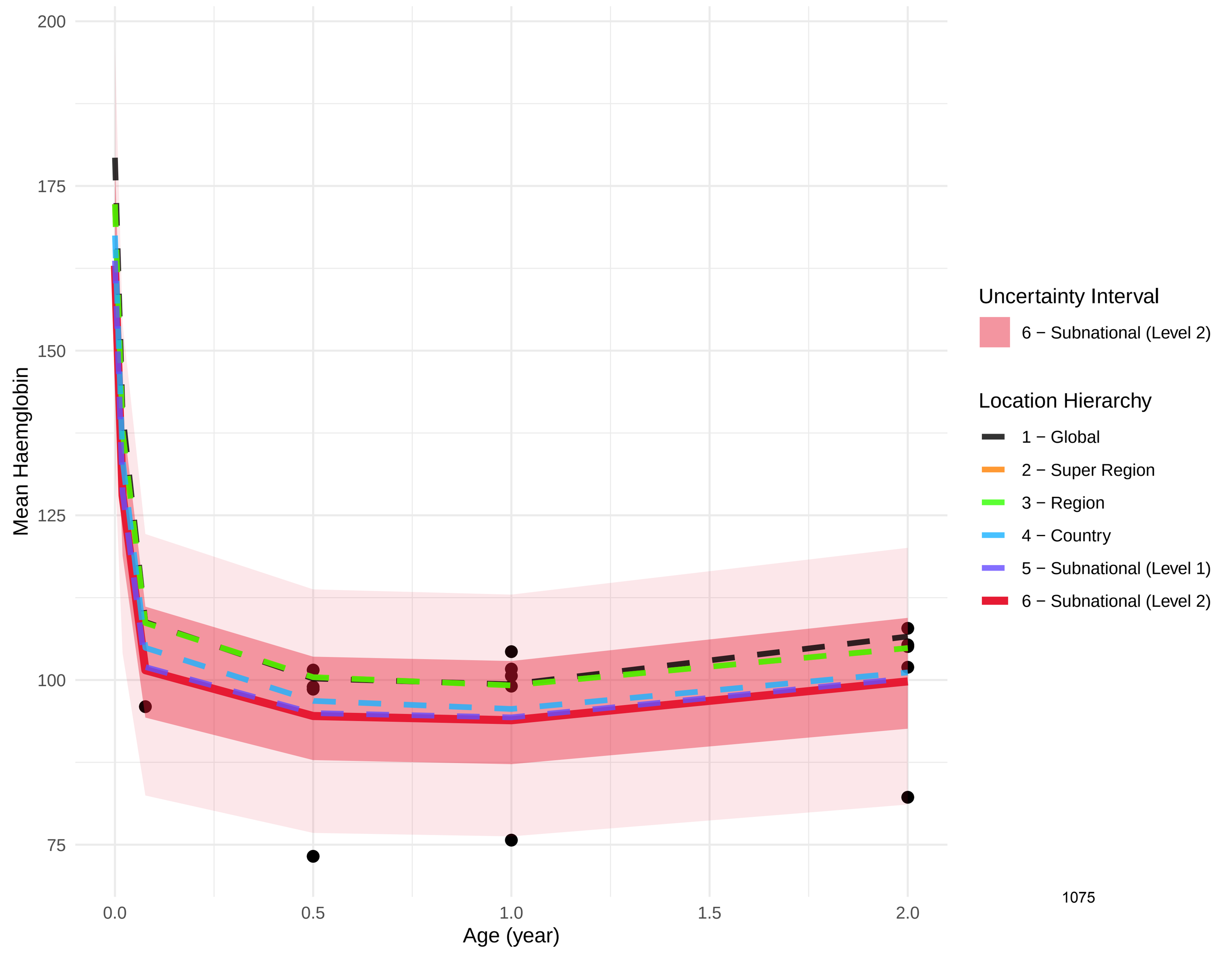

MR−BRT Age/Sex Fits − Non−pregnant Females <2 Years Old − Mean Haemglobin − Sikkim, Urban
WHO−2024 Adjustment Method

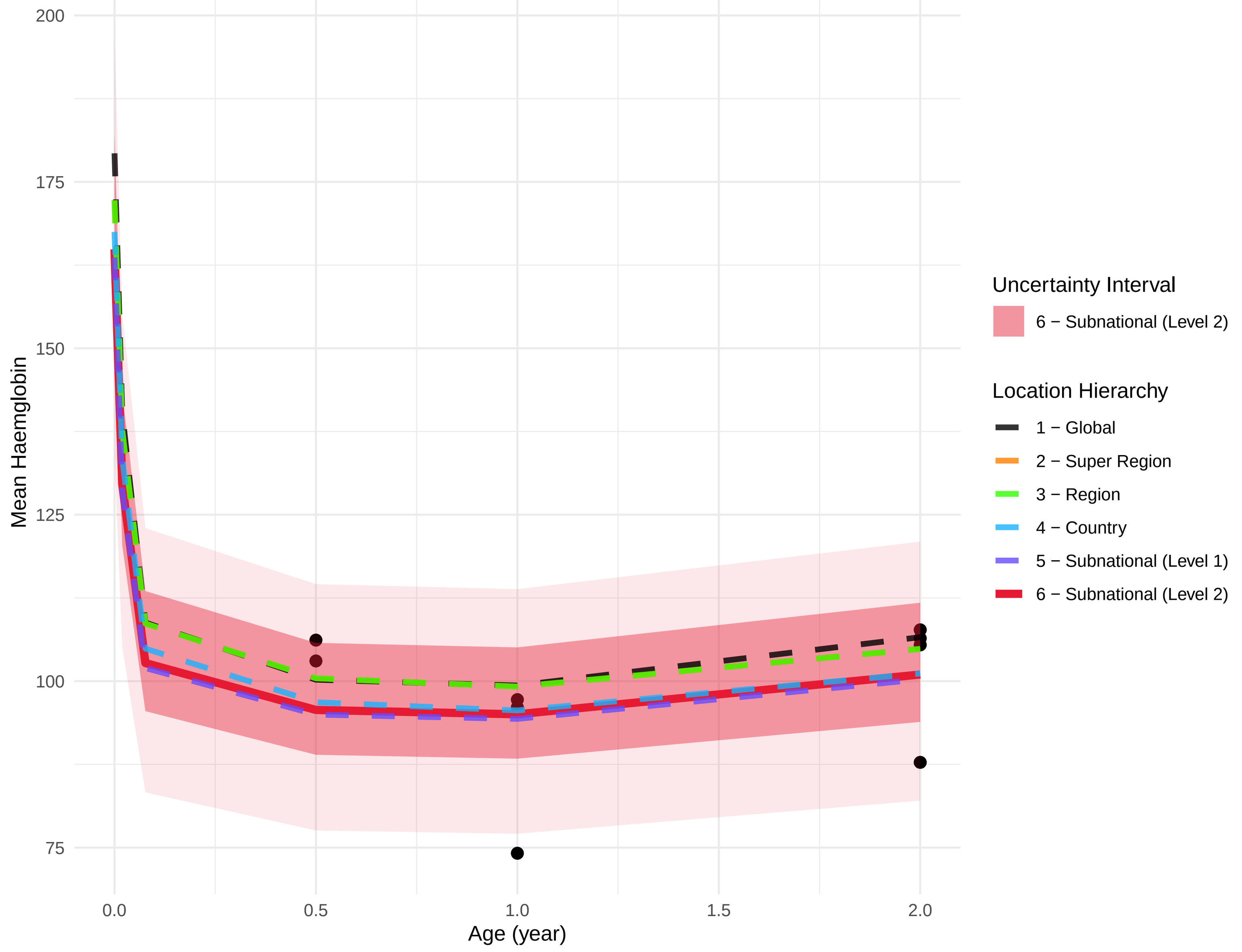

MR−BRT Age/Sex Fits − Non−pregnant Females <2 Years Old − Mean Haemglobin − Tamil Nadu
WHO−2024 Adjustment Method

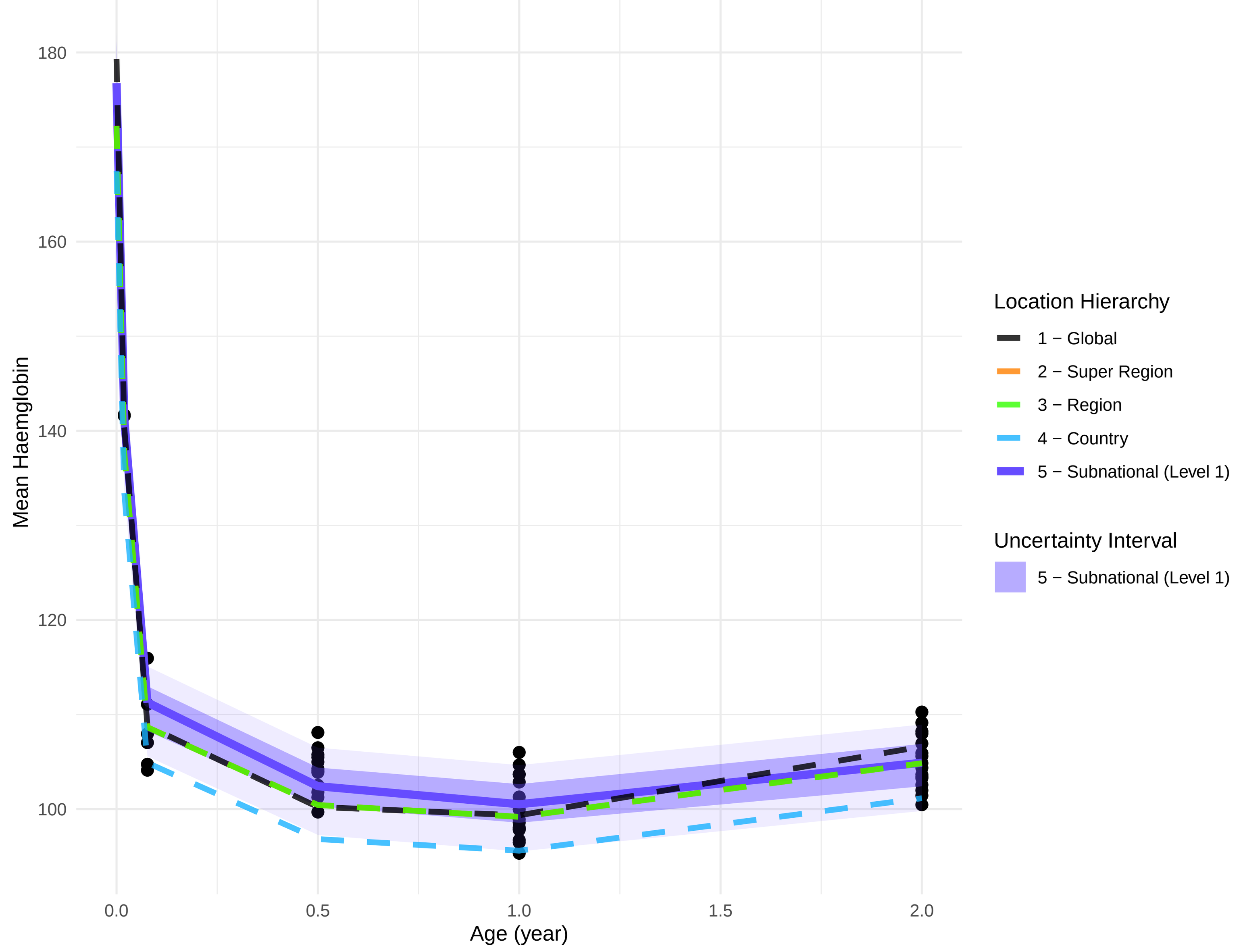

MR−BRT Age/Sex Fits − Non−pregnant Females <2 Years Old − Mean Haemglobin − Tamil Nadu, Rural
WHO−2024 Adjustment Method

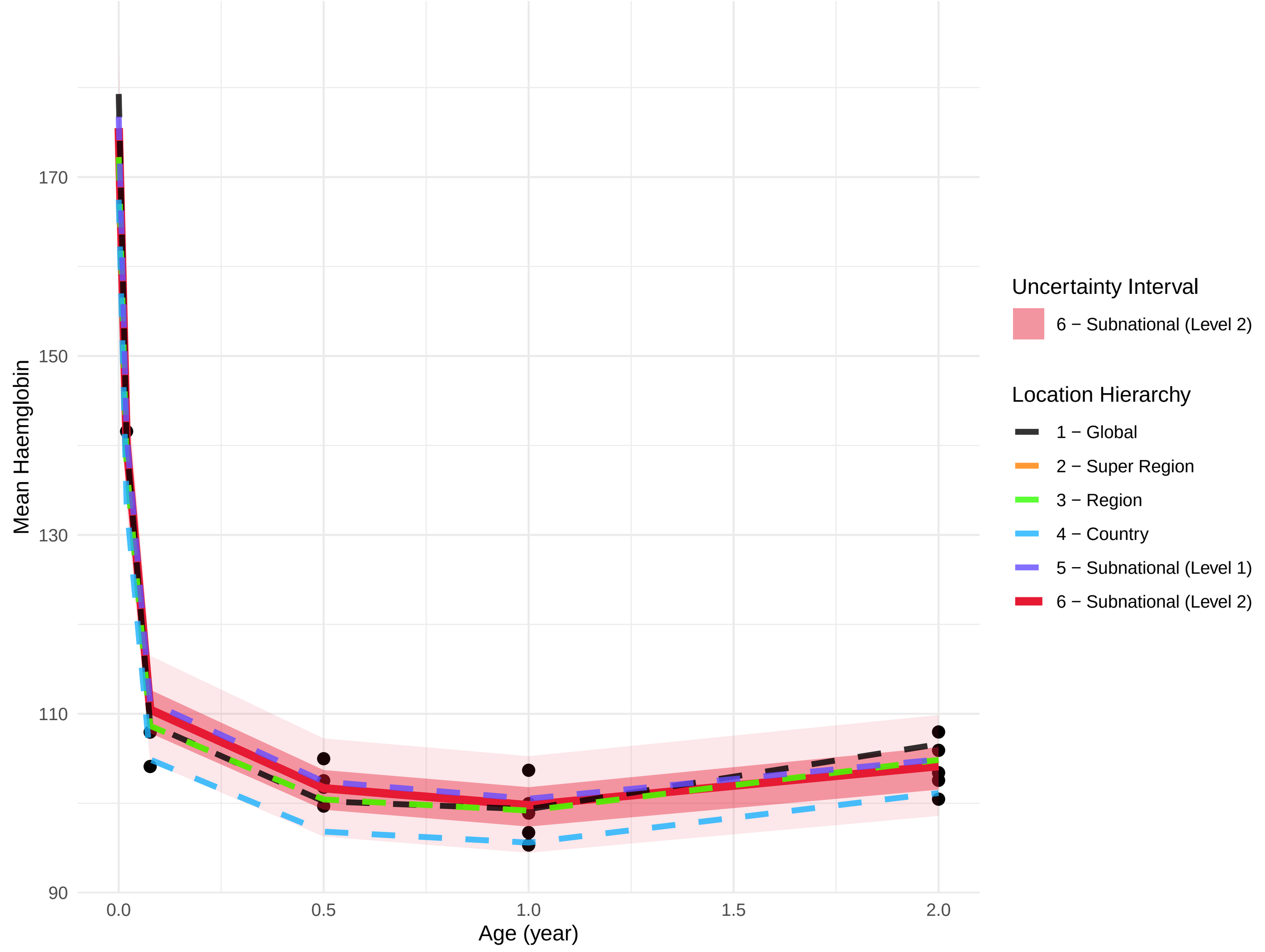

MR−BRT Age/Sex Fits − Non−pregnant Females <2 Years Old − Mean Haemglobin − Tamil Nadu, Urban
WHO−2024 Adjustment Method

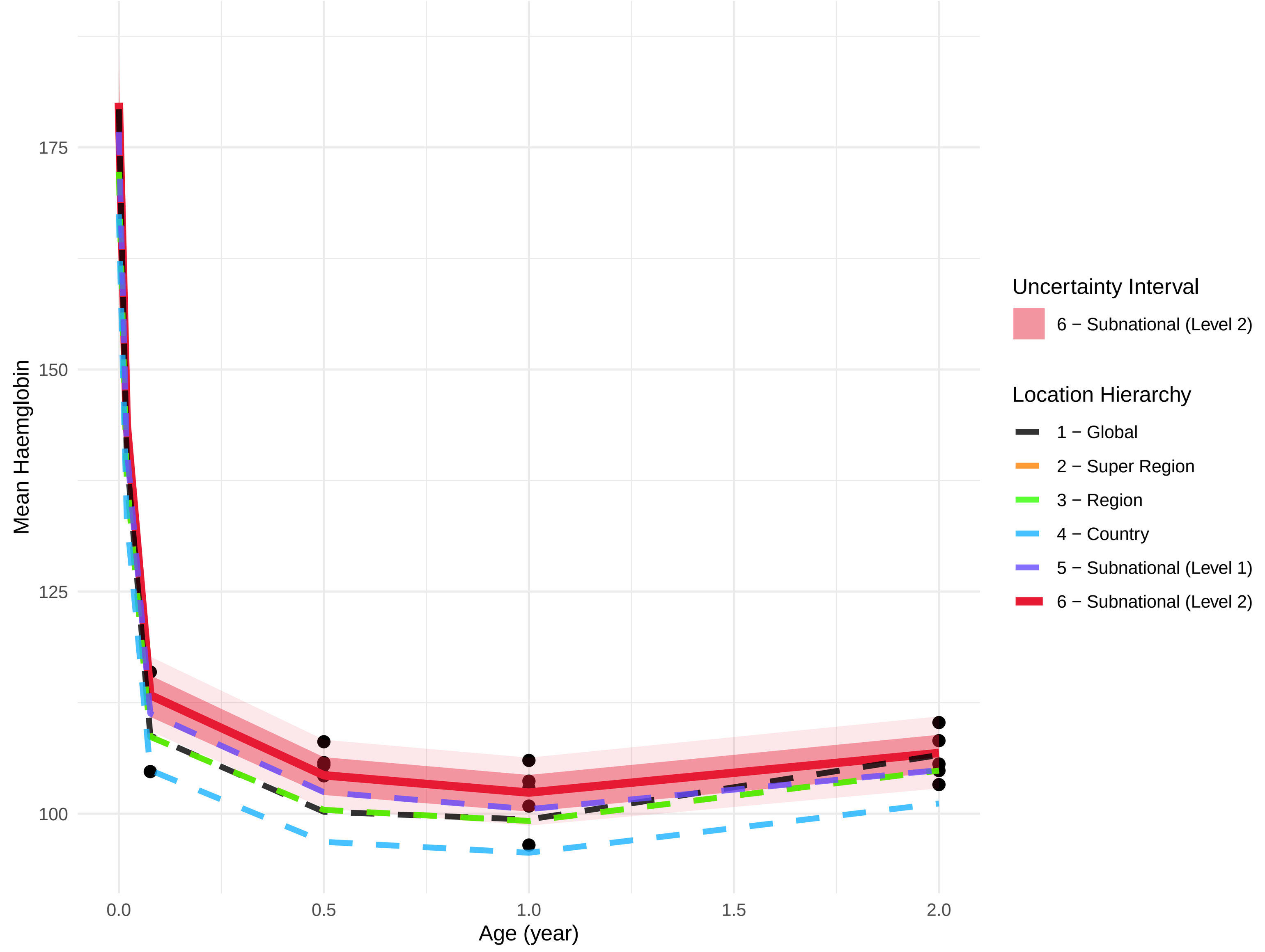

MR−BRT Age/Sex Fits − Non−pregnant Females <2 Years Old − Mean Haemglobin − Telangana
WHO−2024 Adjustment Method

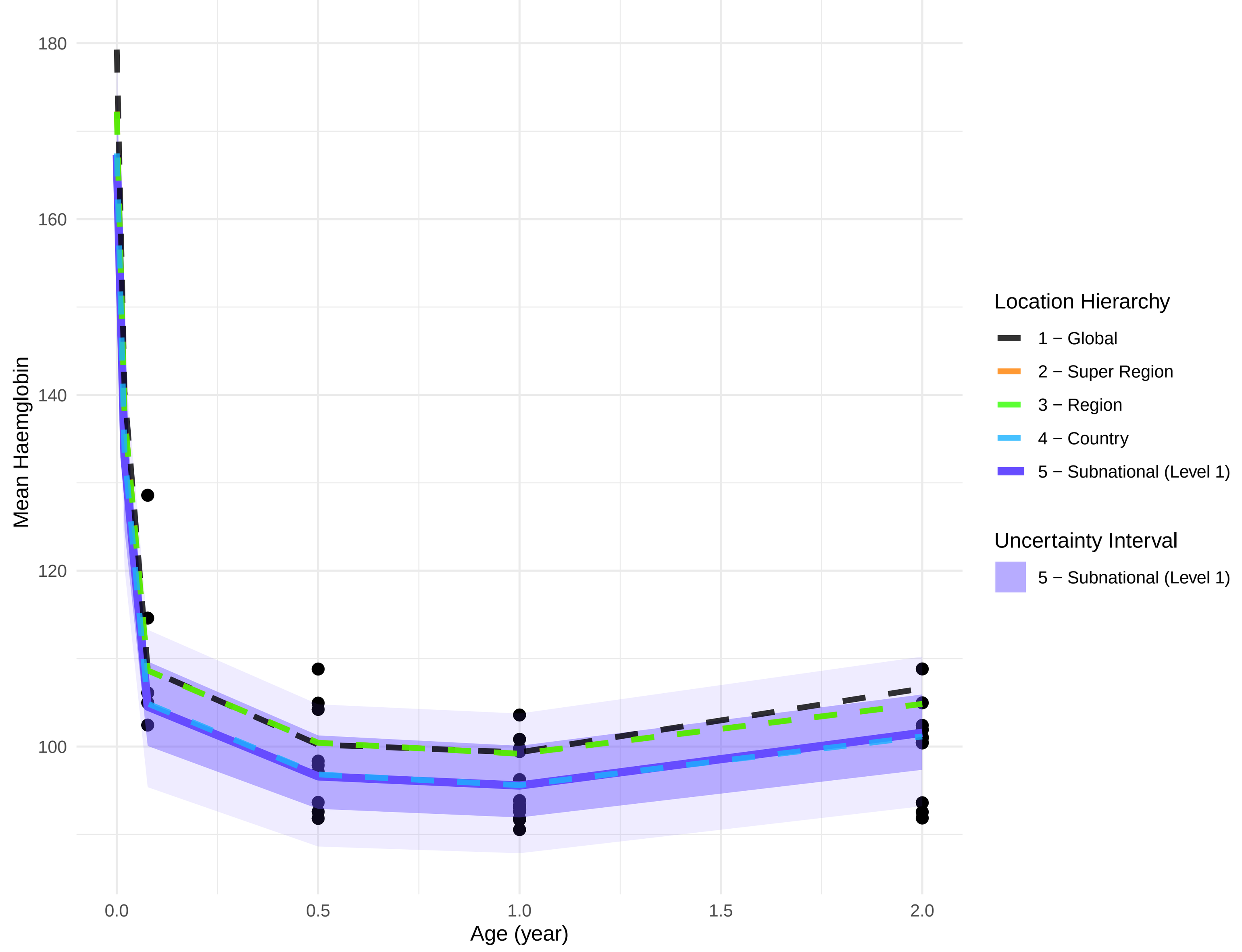

MR−BRT Age/Sex Fits − Non−pregnant Females <2 Years Old − Mean Haemglobin − Telangana, Rural
WHO−2024 Adjustment Method

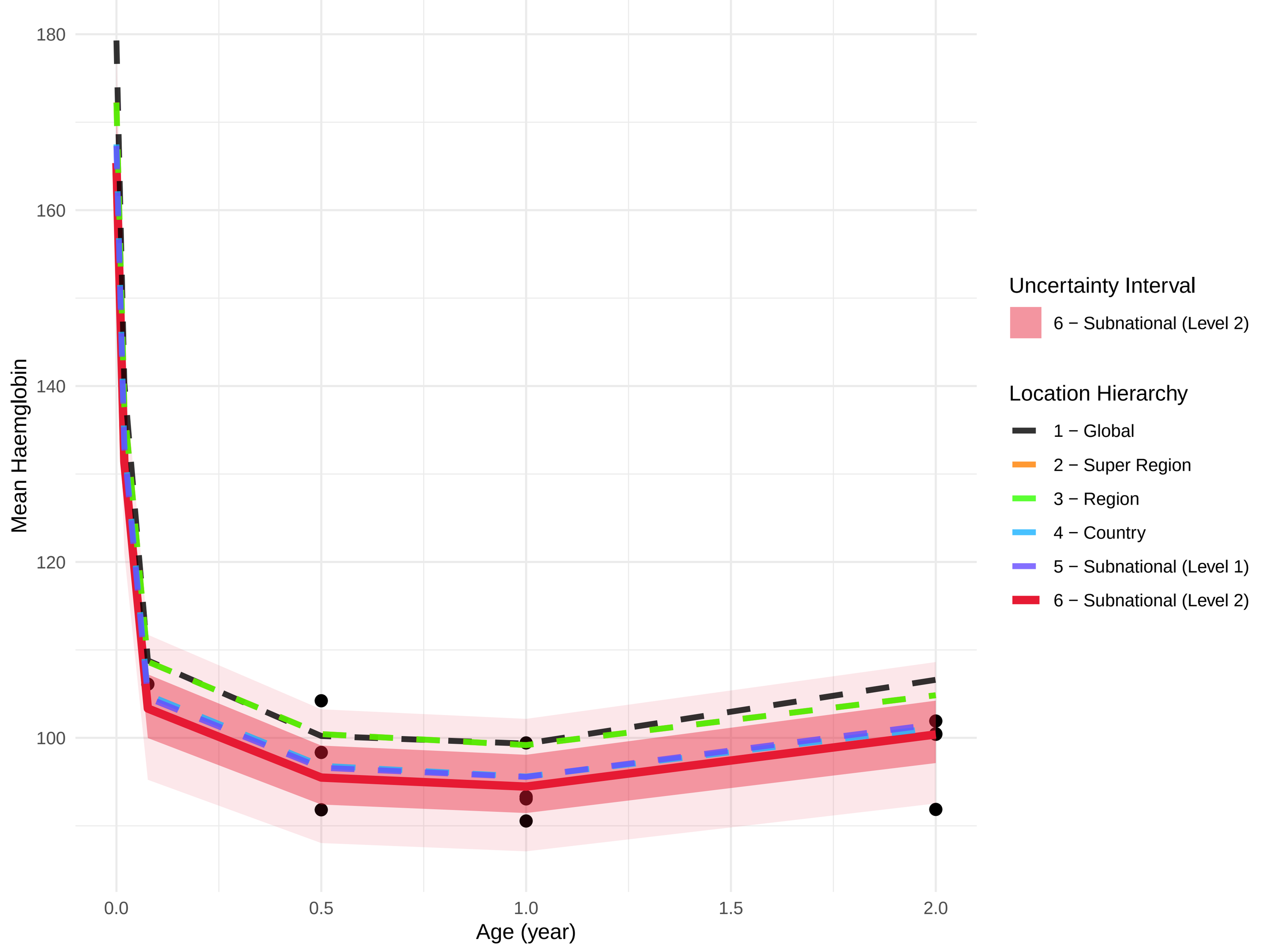

MR−BRT Age/Sex Fits − Non−pregnant Females <2 Years Old − Mean Haemglobin − Telangana, Urban
WHO−2024 Adjustment Method

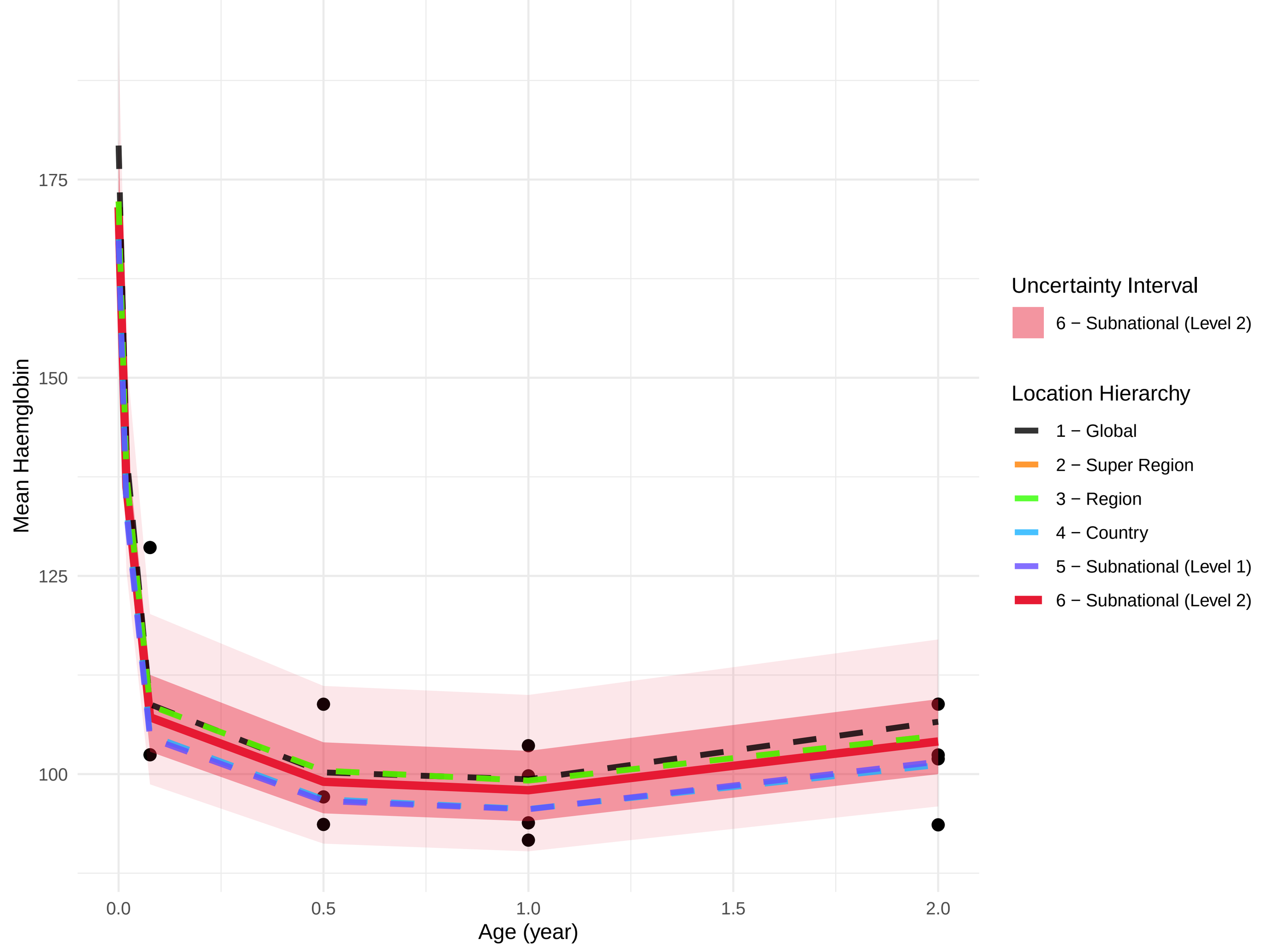

MR−BRT Age/Sex Fits − Non−pregnant Females <2 Years Old − Mean Haemglobin − Tripura
WHO−2024 Adjustment Method

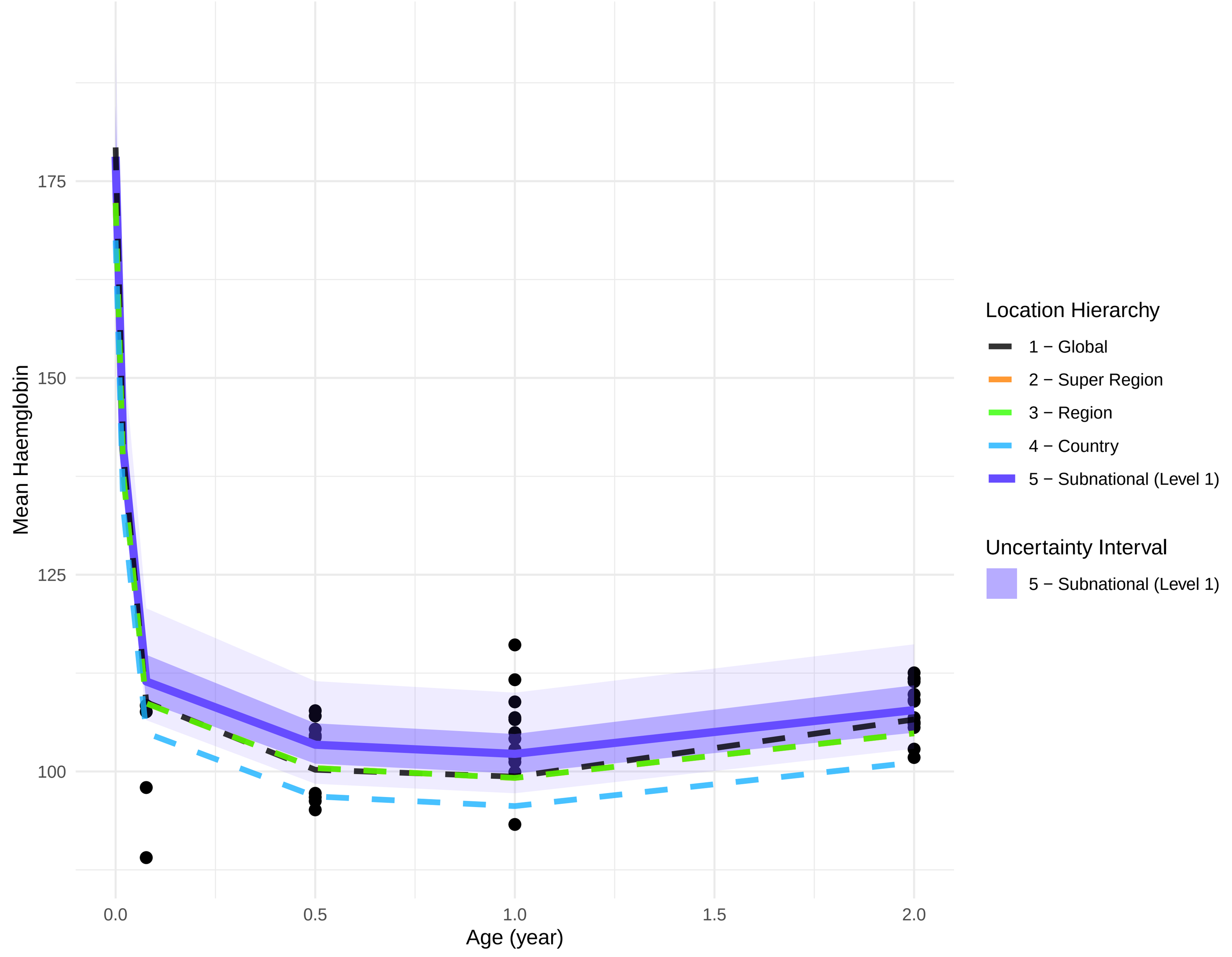

MR−BRT Age/Sex Fits − Non−pregnant Females <2 Years Old − Mean Haemglobin − Tripura, Rural
WHO−2024 Adjustment Method

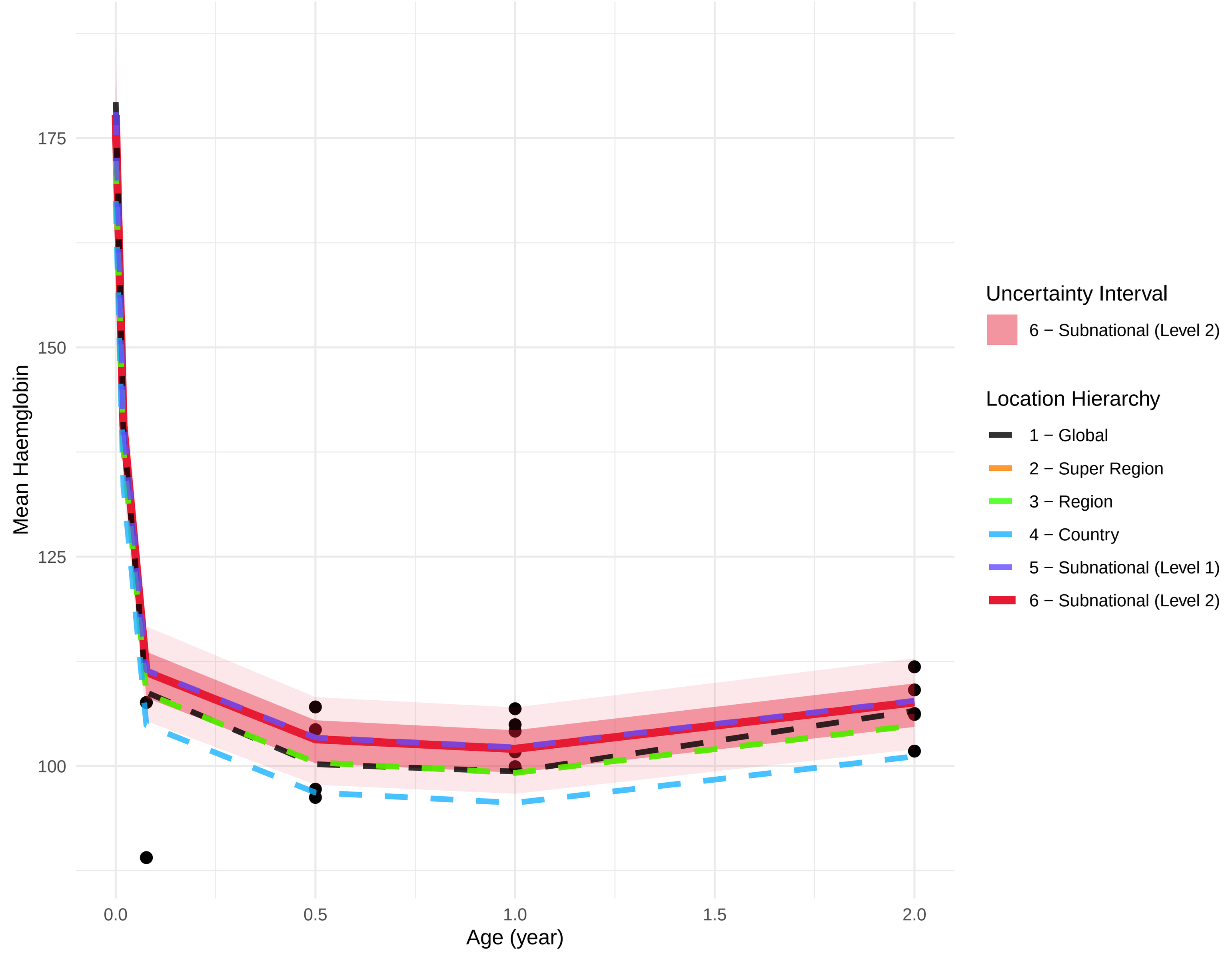

MR−BRT Age/Sex Fits − Non−pregnant Females <2 Years Old − Mean Haemglobin − Tripura, Urban
WHO−2024 Adjustment Method

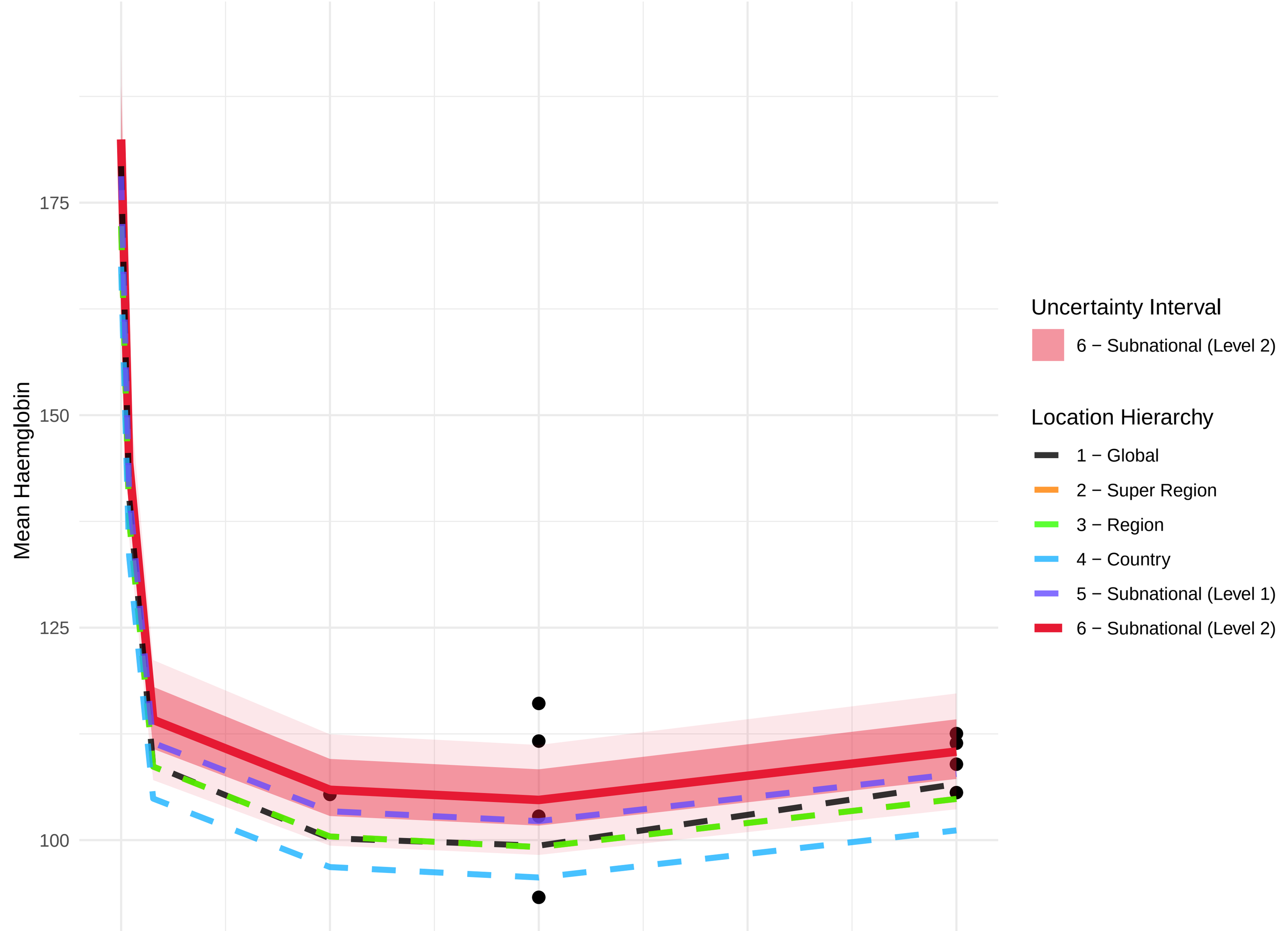

MR−BRT Age/Sex Fits − Non−pregnant Females <2 Years Old − Mean Haemglobin − Uttar Pradesh
WHO−2024 Adjustment Method

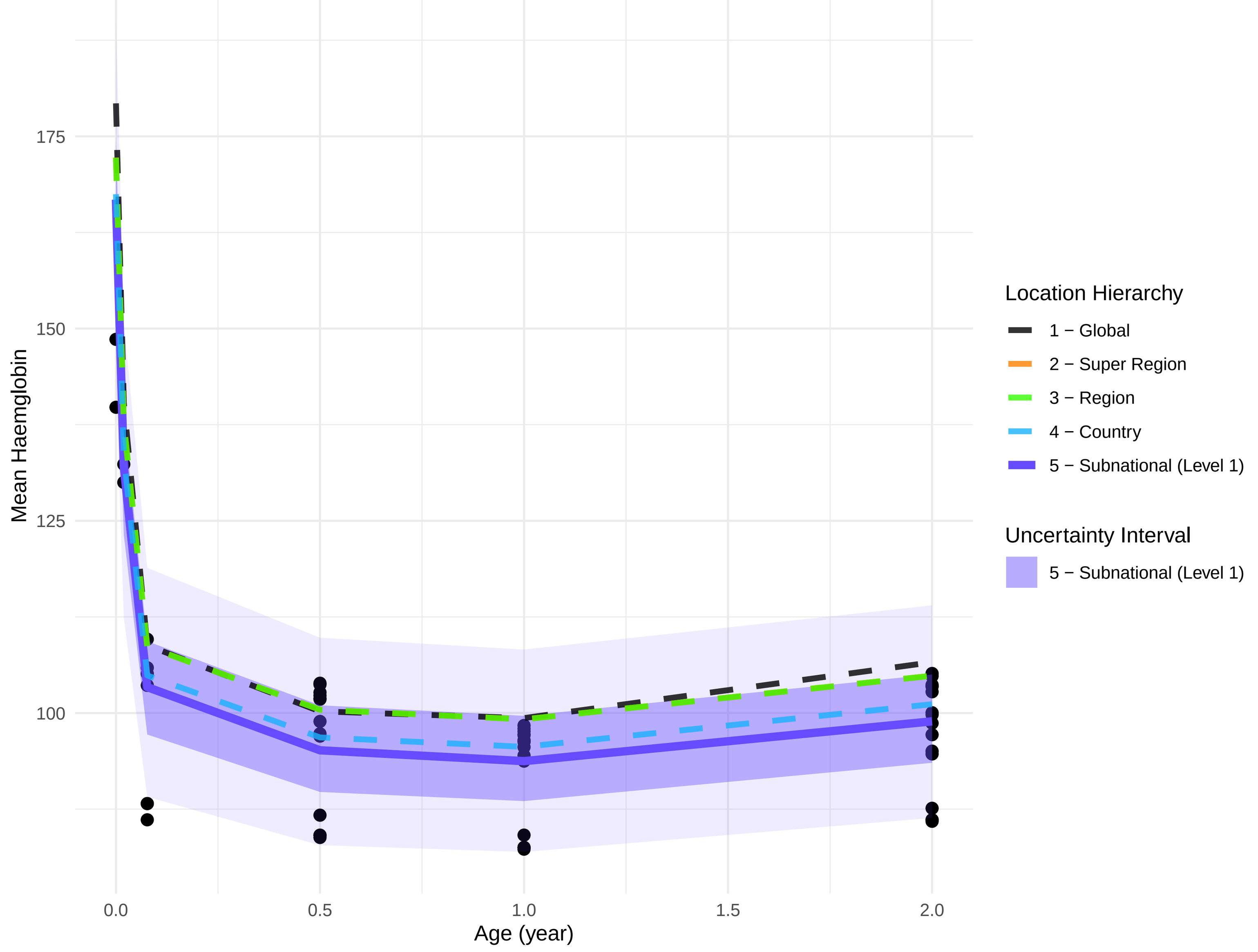

MR−BRT Age/Sex Fits − Non−pregnant Females <2 Years Old − Mean Haemglobin − Uttar Pradesh, Rural
WHO−2024 Adjustment Method

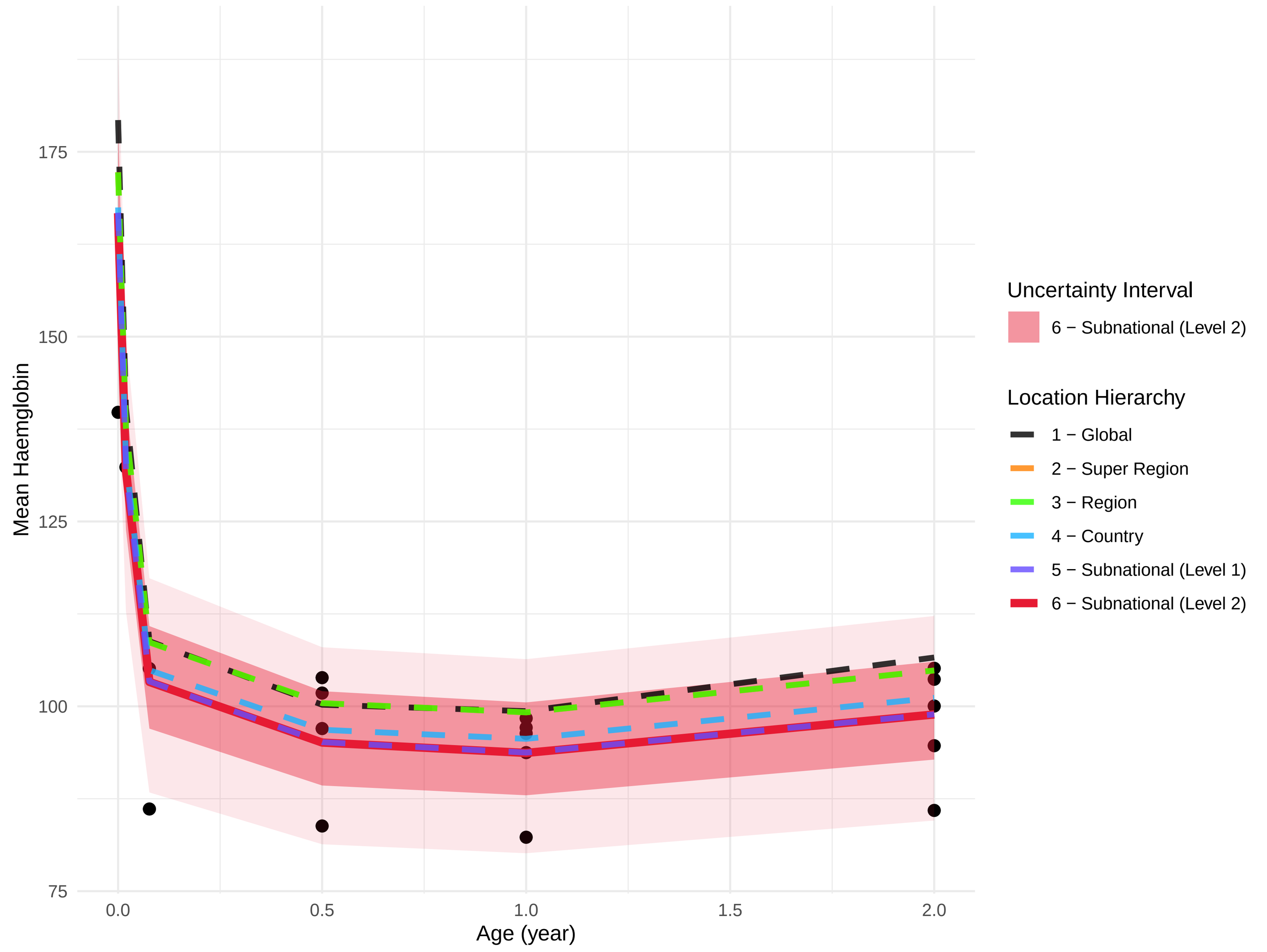

MR−BRT Age/Sex Fits − Non−pregnant Females <2 Years Old − Mean Haemglobin − Uttar Pradesh, Urban
WHO−2024 Adjustment Method

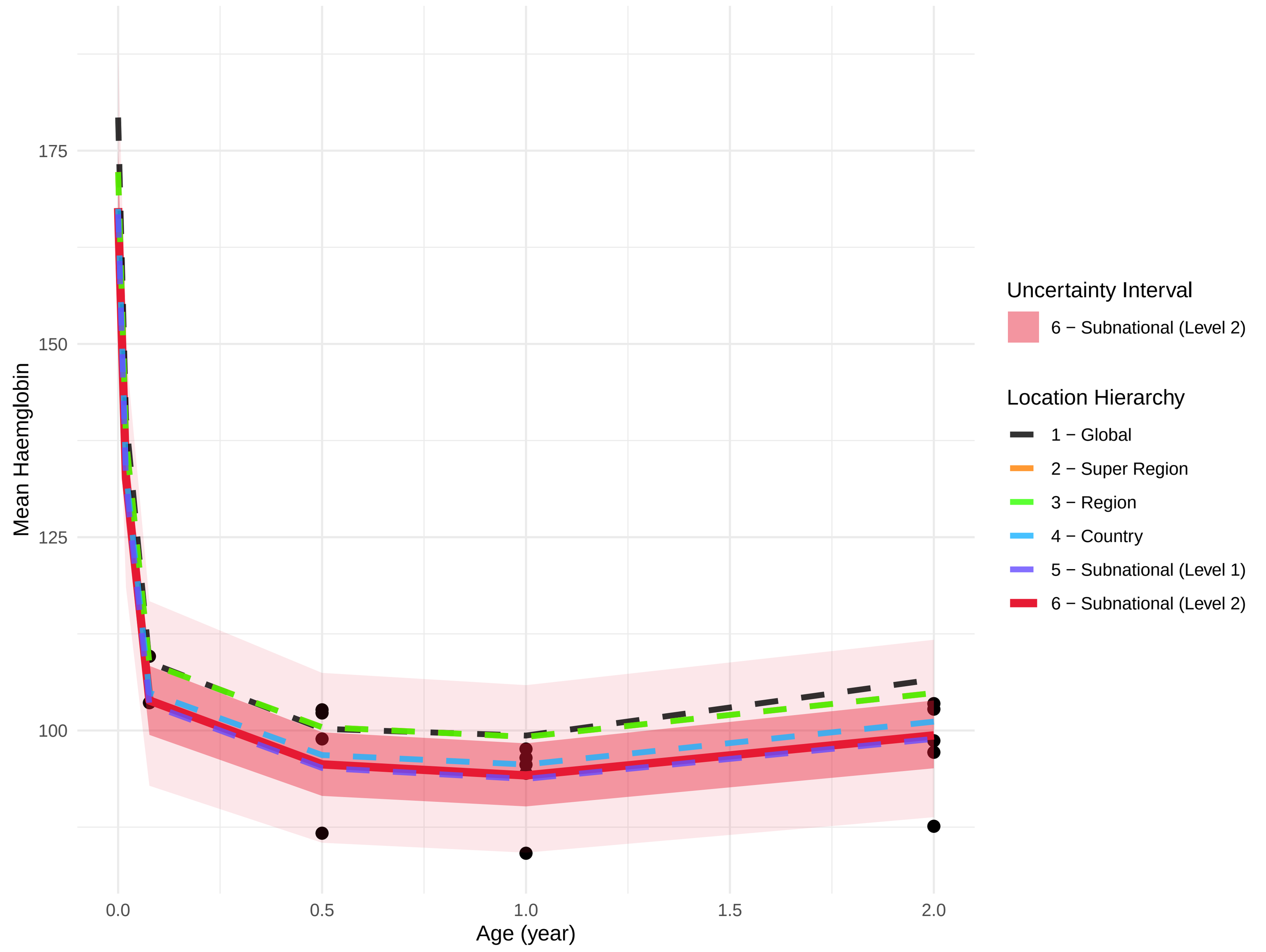

MR−BRT Age/Sex Fits − Non−pregnant Females <2 Years Old − Mean Haemglobin − Uttarakhand
WHO−2024 Adjustment Method

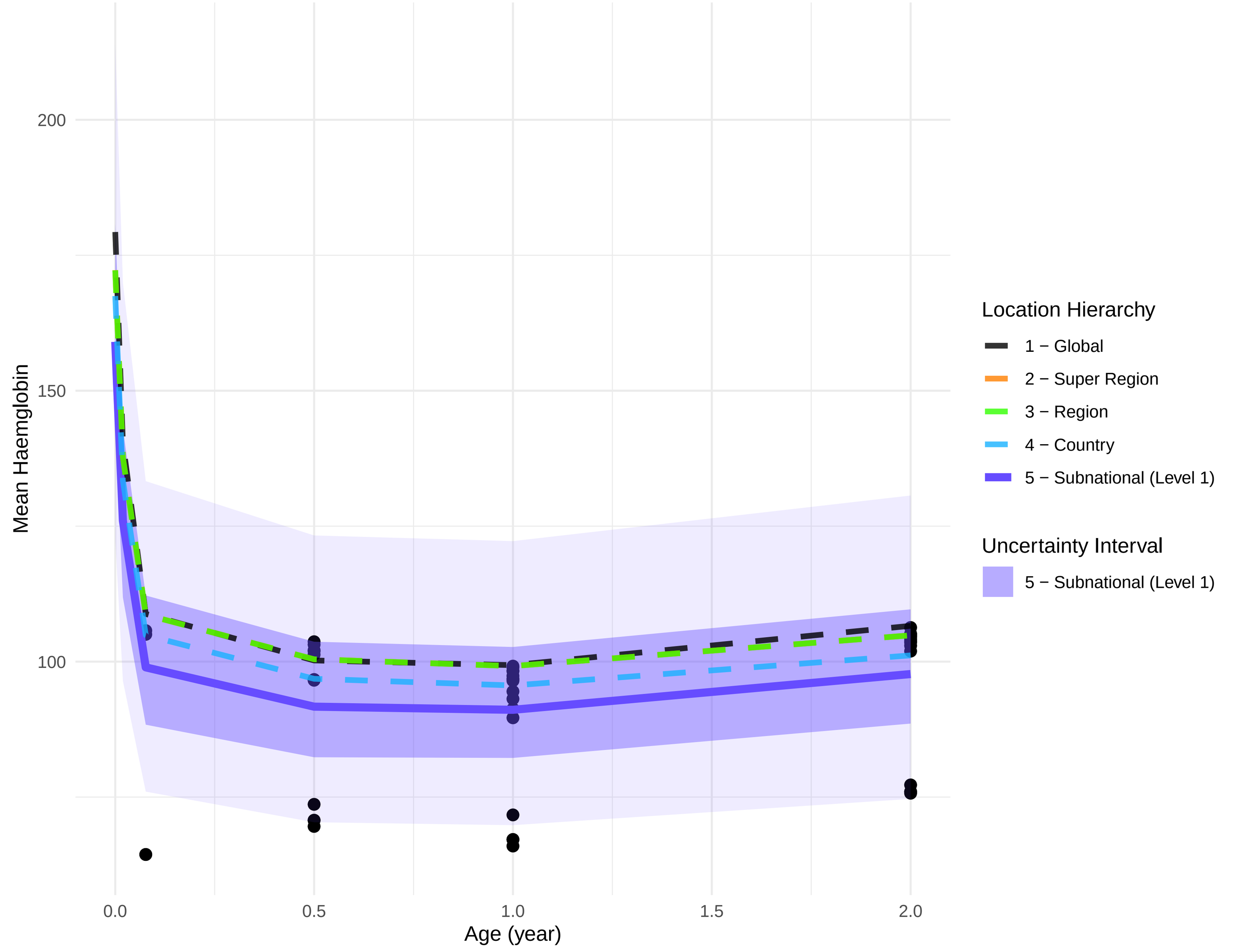

MR−BRT Age/Sex Fits − Non−pregnant Females <2 Years Old − Mean Haemglobin − Uttarakhand, Rural
WHO−2024 Adjustment Method

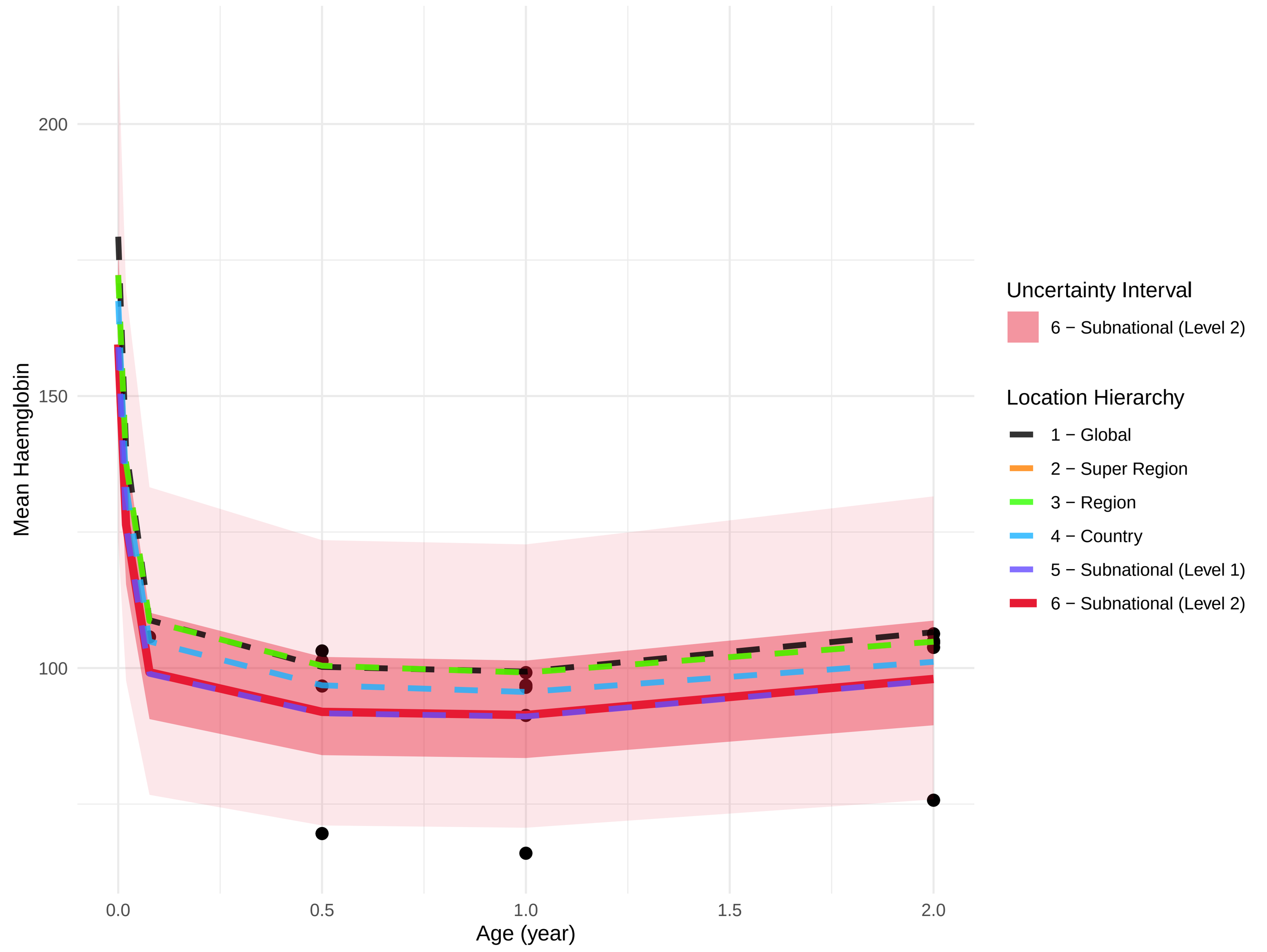

MR−BRT Age/Sex Fits − Non−pregnant Females <2 Years Old − Mean Haemglobin − Uttarakhand, Urban
WHO−2024 Adjustment Method

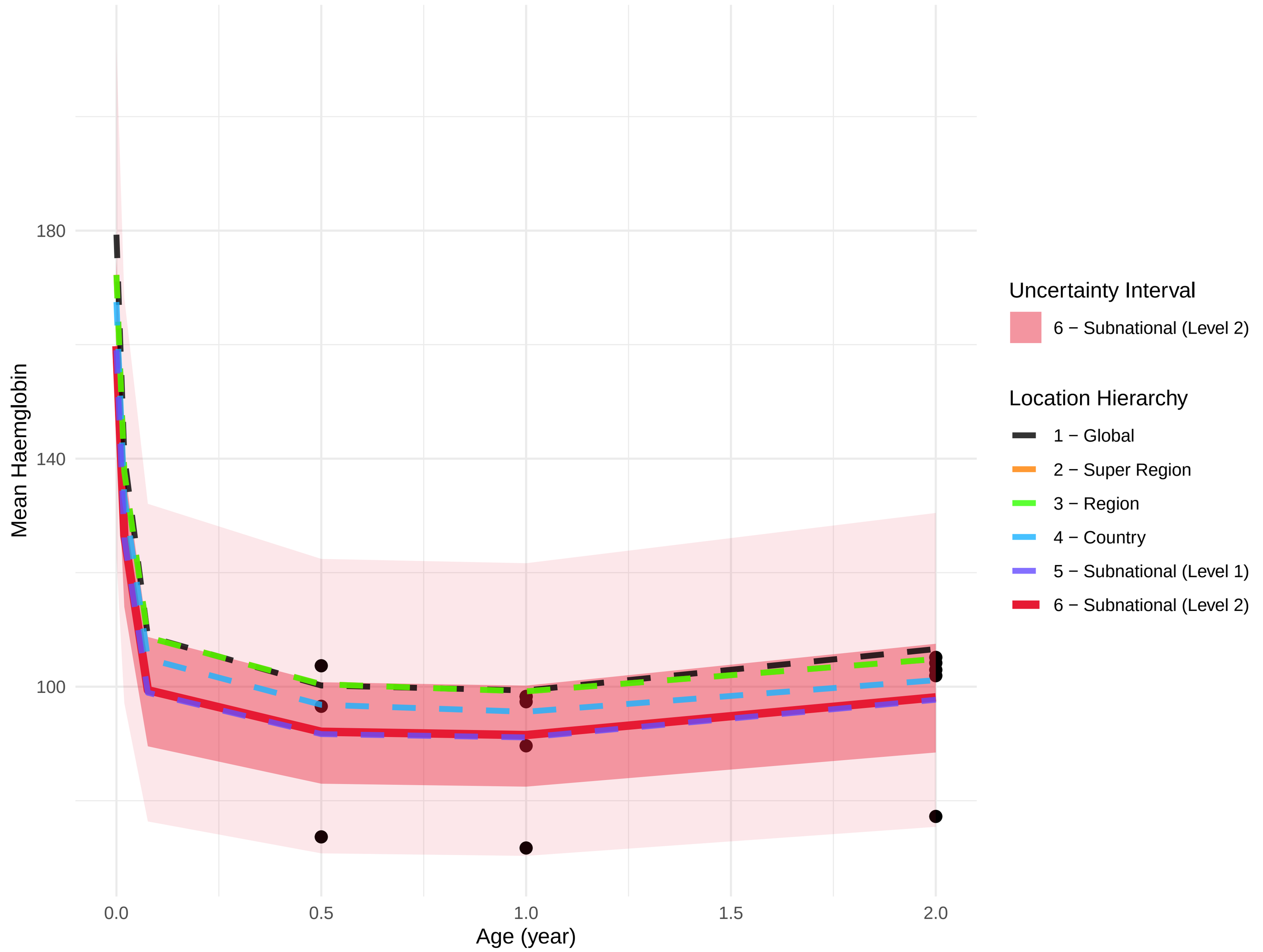

MR−BRT Age/Sex Fits − Non−pregnant Females <2 Years Old − Mean Haemglobin − West Bengal
WHO−2024 Adjustment Method

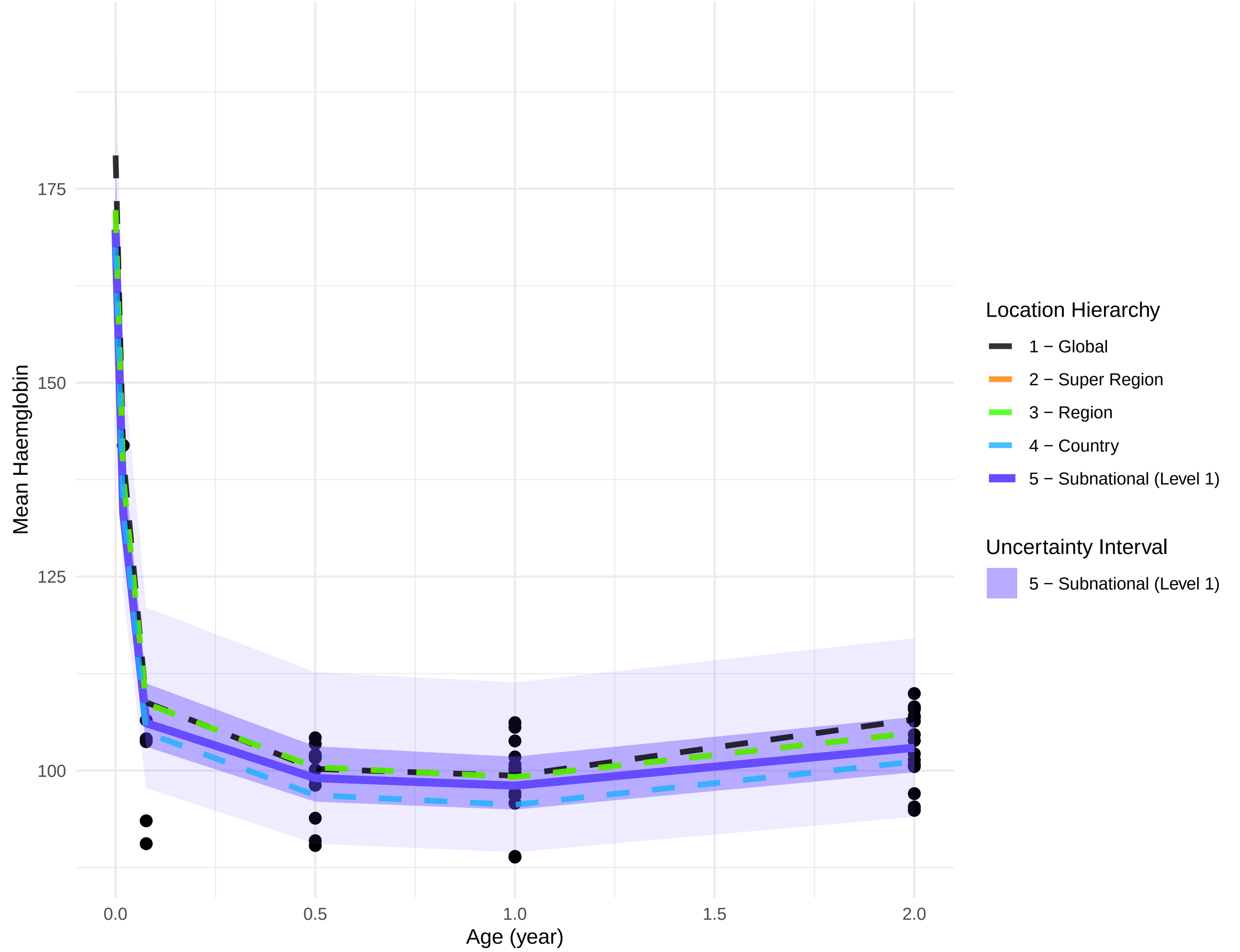

MR−BRT Age/Sex Fits − Non−pregnant Females <2 Years Old − Mean Haemglobin − West Bengal, Rural
WHO−2024 Adjustment Method

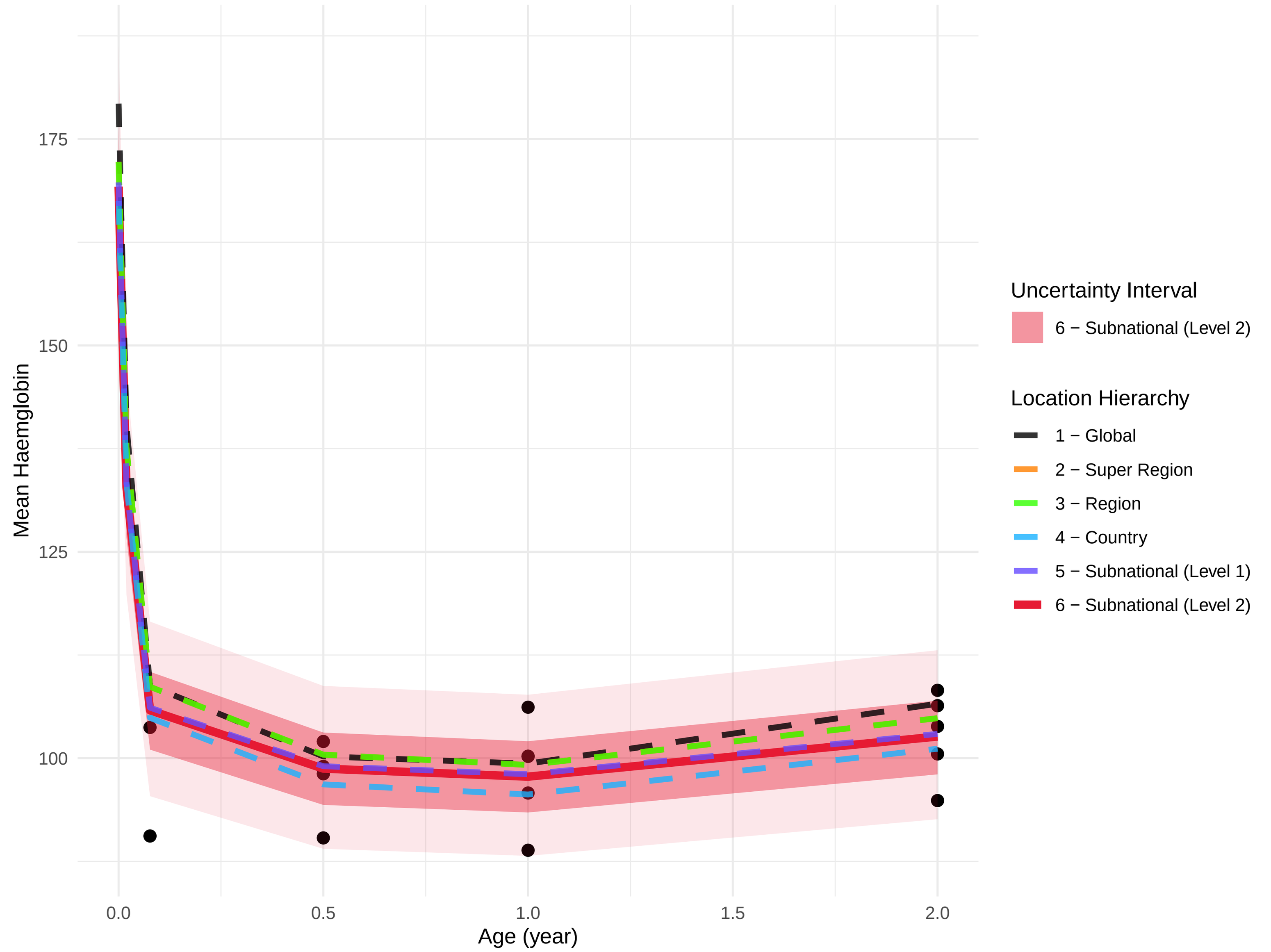

MR−BRT Age/Sex Fits − Non−pregnant Females <2 Years Old − Mean Haemglobin − West Bengal, Urban
WHO−2024 Adjustment Method

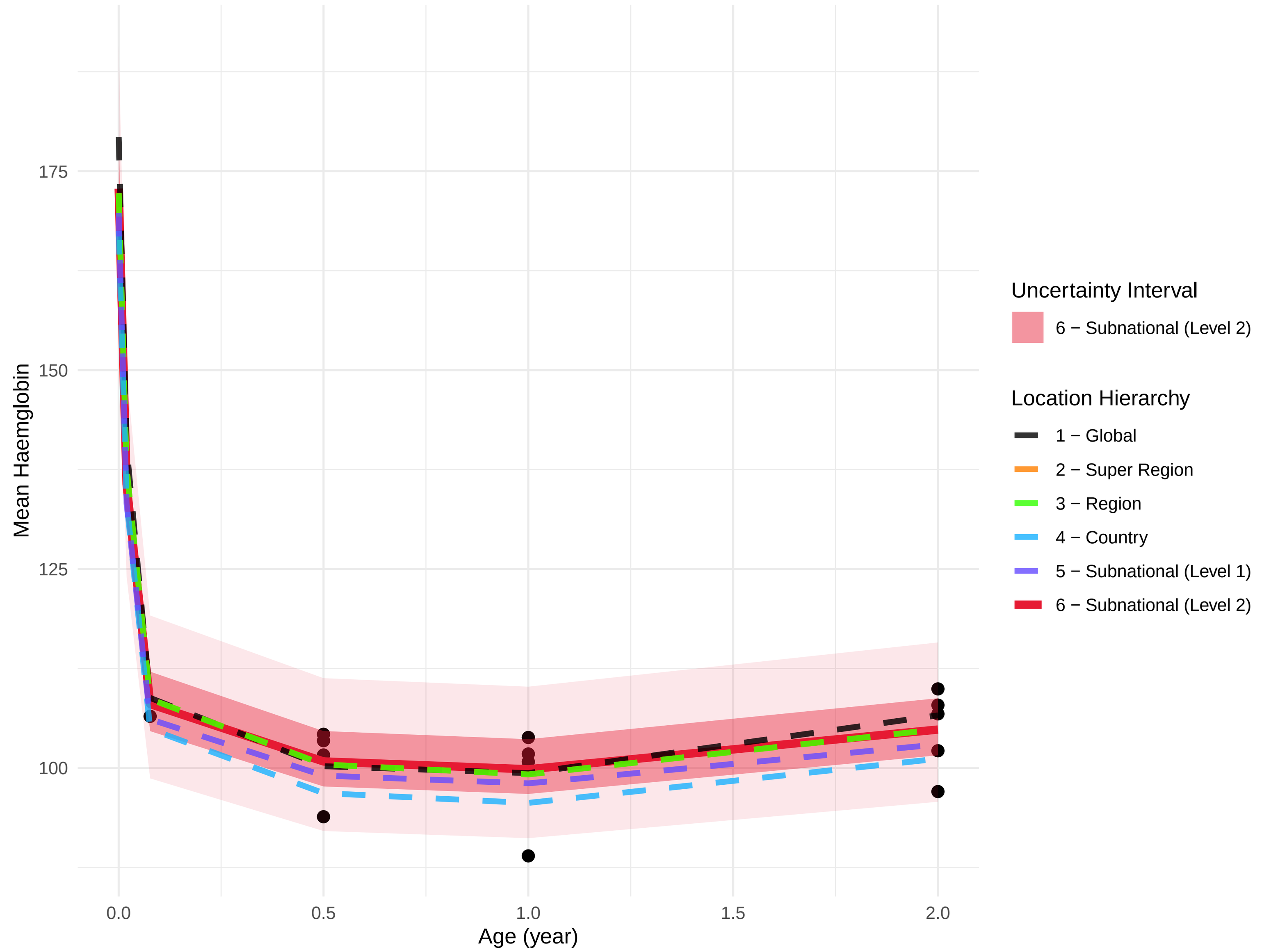

MR−BRT Age/Sex Fits − Non−pregnant Females <2 Years Old − Mean Haemglobin − Nepal
WHO−2024 Adjustment Method

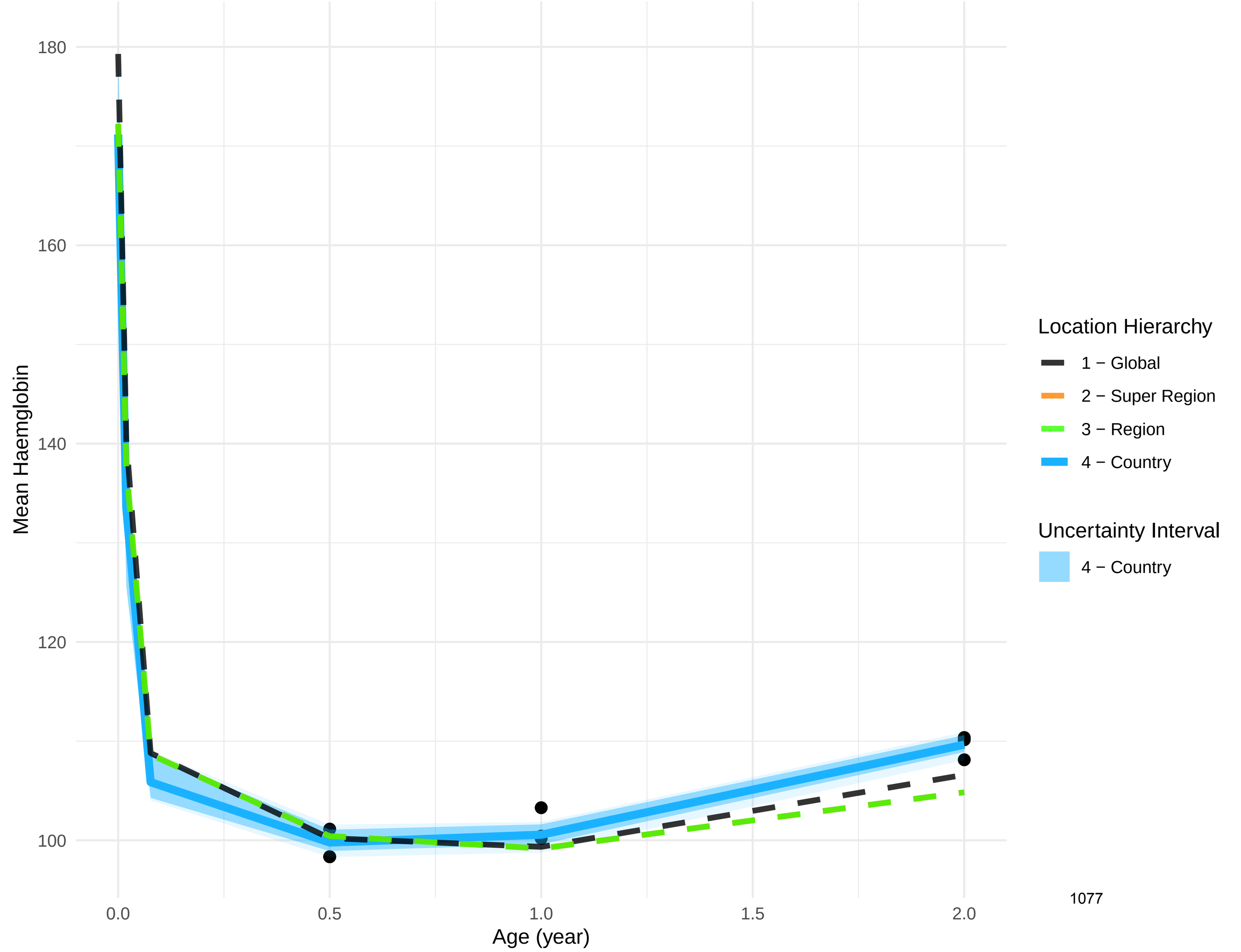

MR−BRT Age/Sex Fits − Non−pregnant Females <2 Years Old − Mean Haemglobin − Pakistan
WHO−2024 Adjustment Method

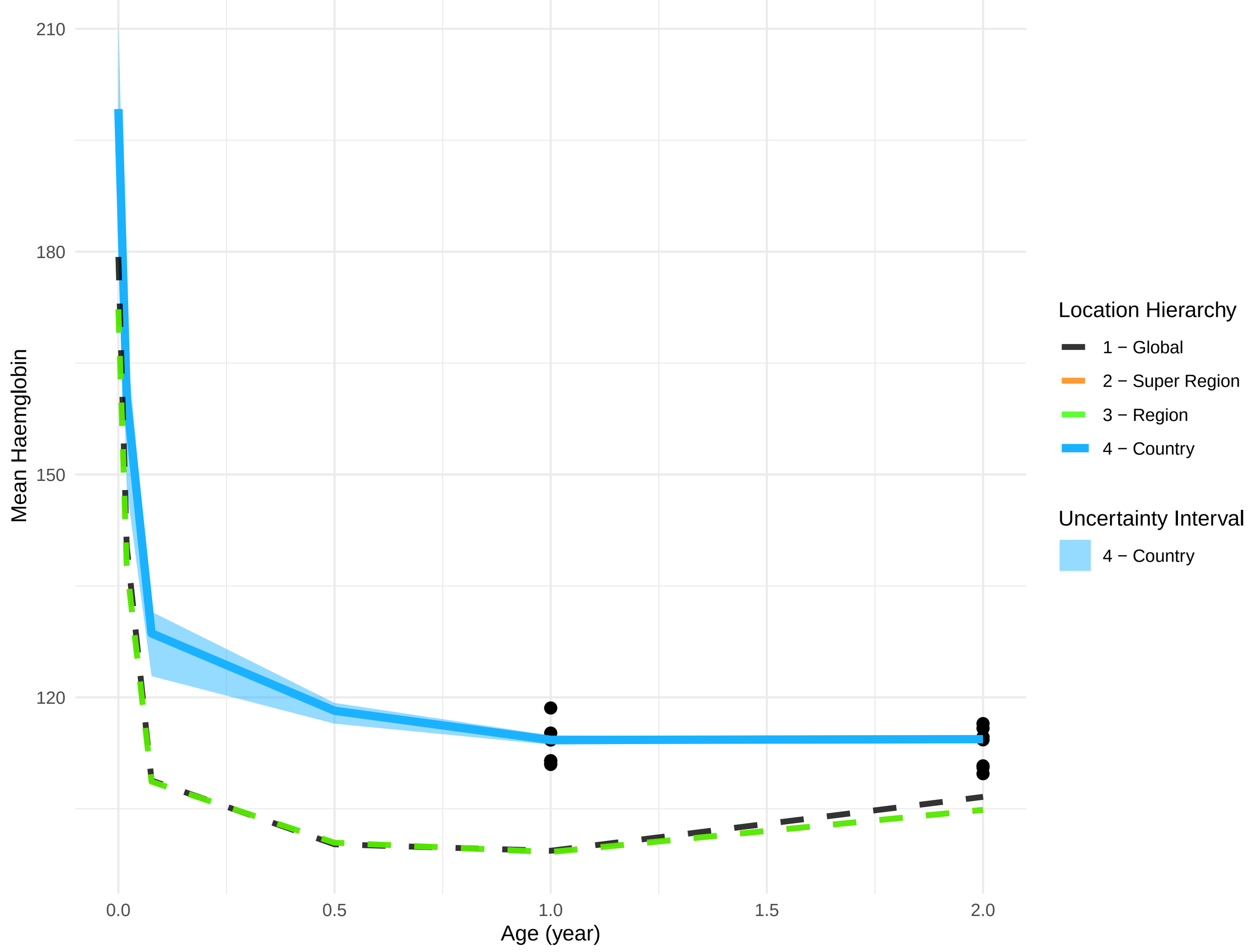

MR−BRT Age/Sex Fits − Non−pregnant Females <2 Years Old − Mean Haemglobin − Azad Jammu & Kashmir
WHO−2024 Adjustment Method

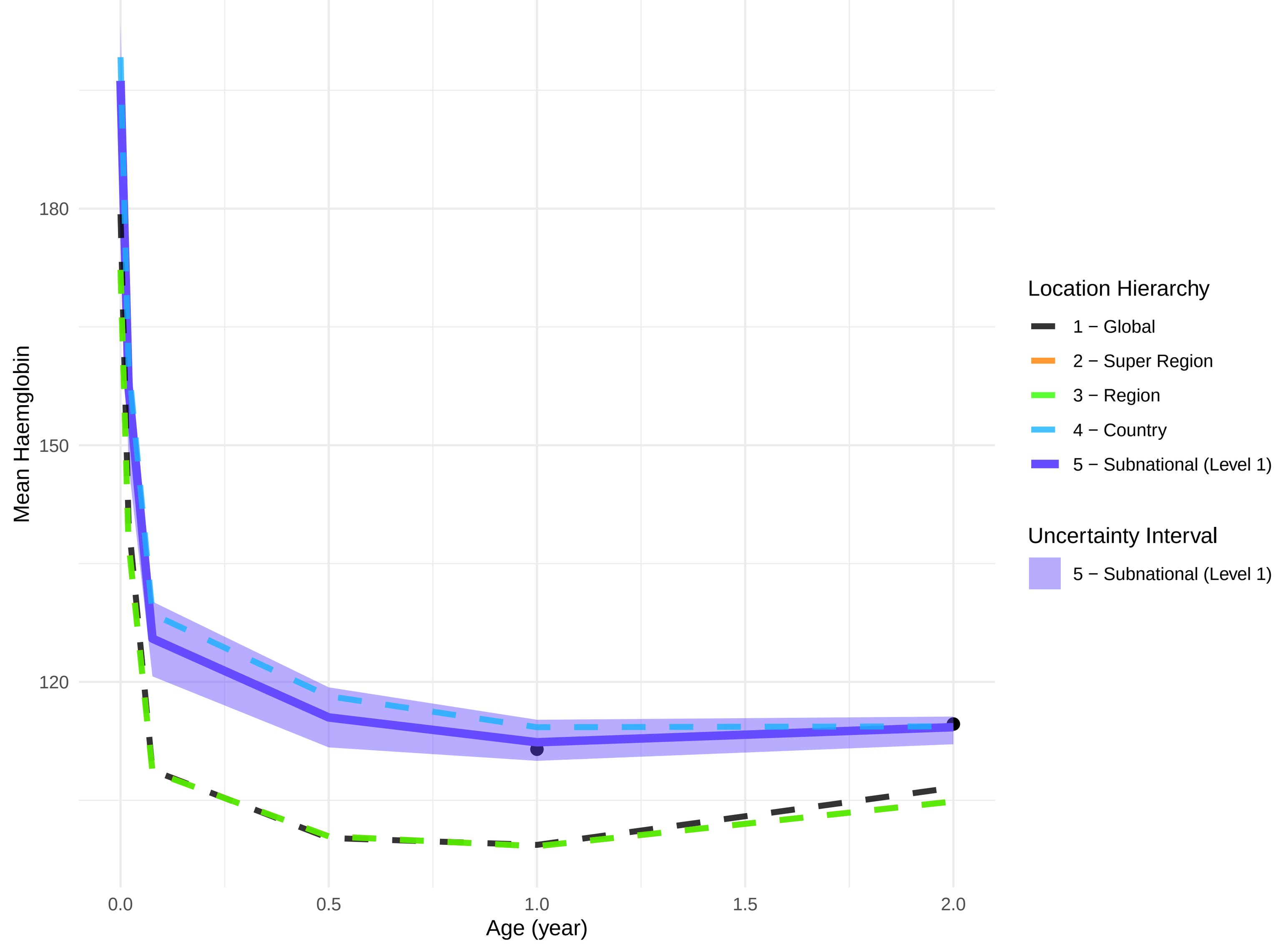

MR−BRT Age/Sex Fits − Non−pregnant Females <2 Years Old − Mean Haemglobin − Balochistan
WHO−2024 Adjustment Method

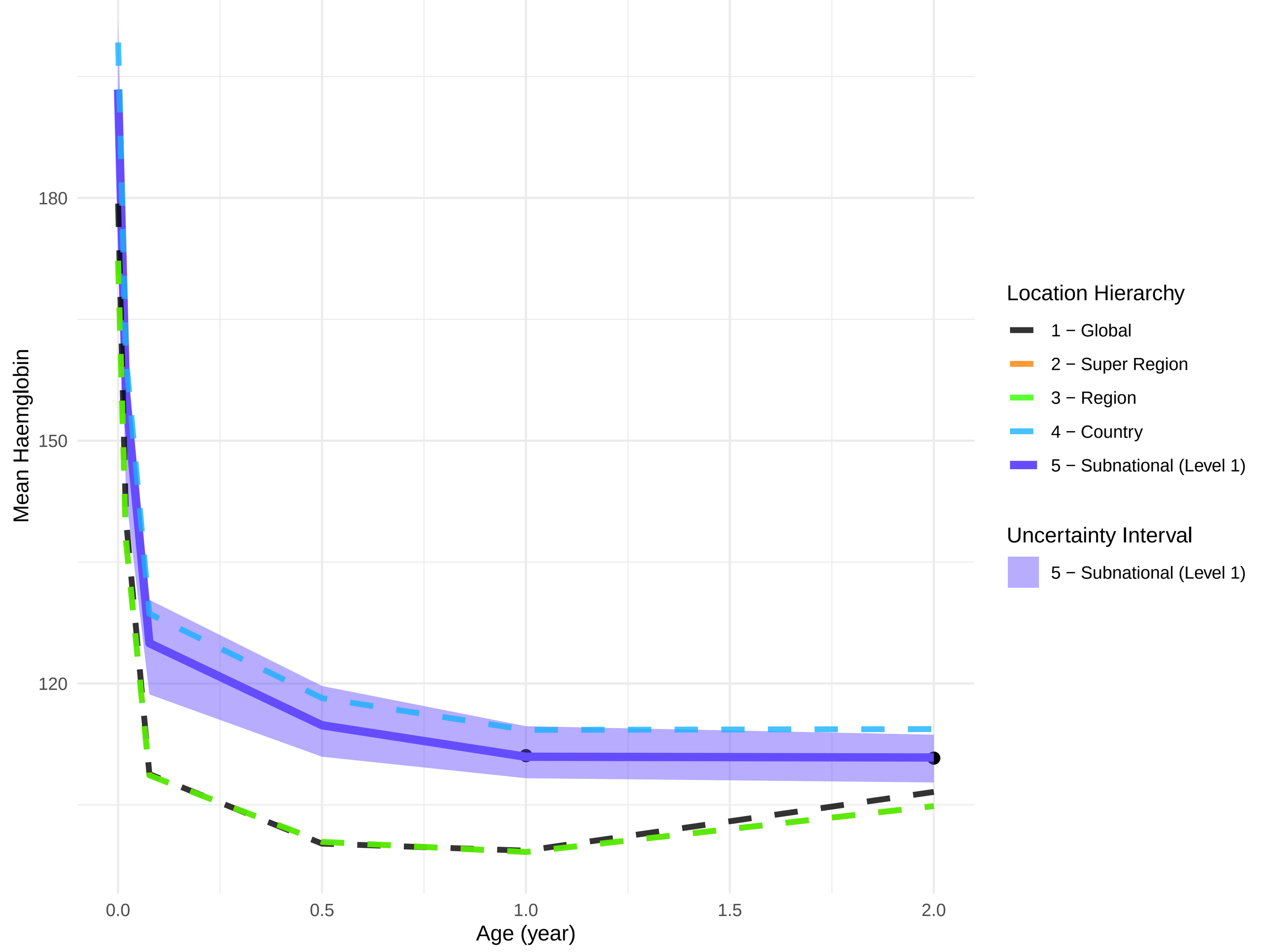

MR−BRT Age/Sex Fits − Non−pregnant Females <2 Years Old − Mean Haemglobin − Gilgit−Baltistan
WHO−2024 Adjustment Method

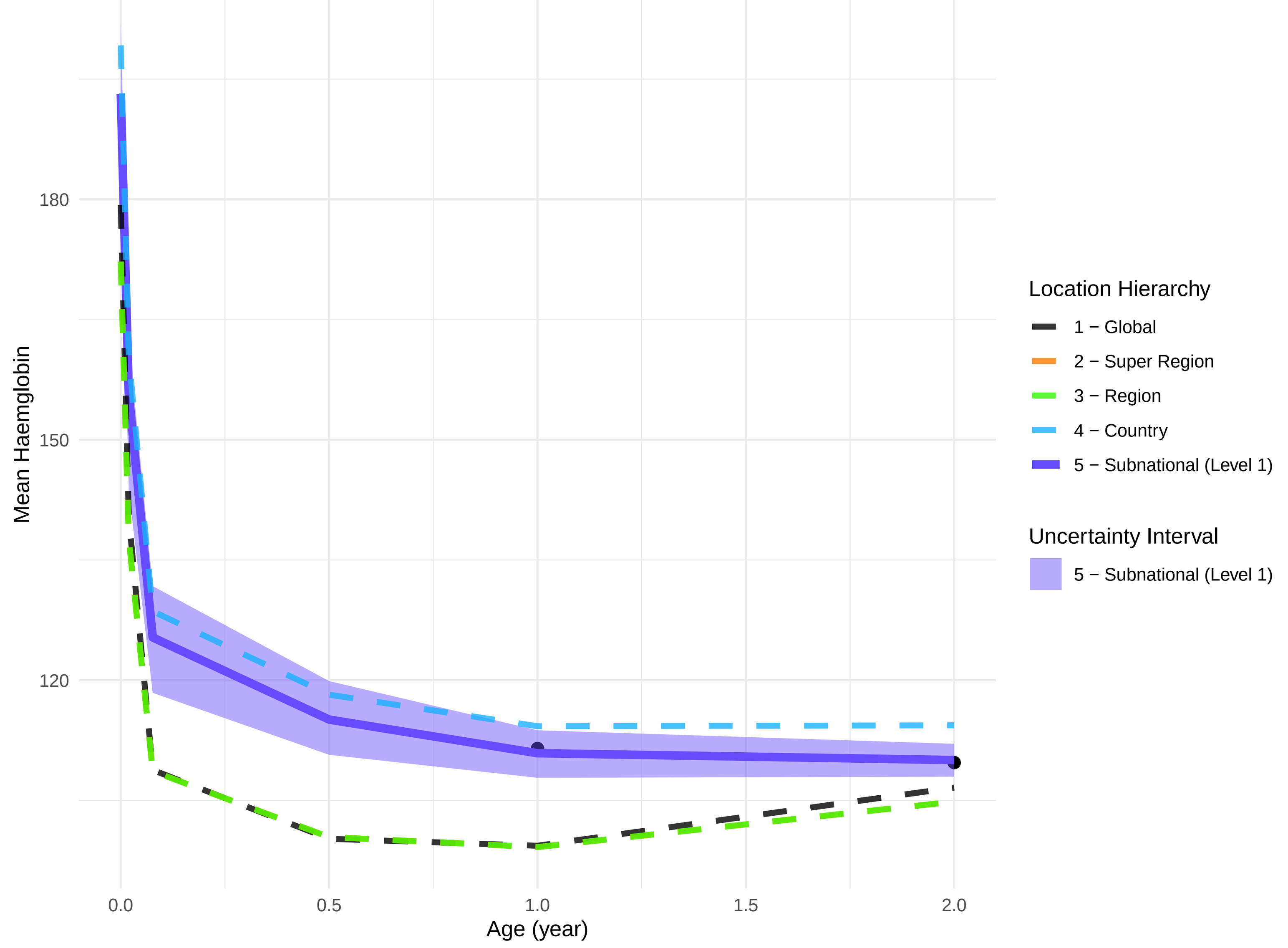

MR−BRT Age/Sex Fits − Non−pregnant Females <2 Years Old − Mean Haemglobin − Khyber Pakhtunkhwa
WHO−2024 Adjustment Method

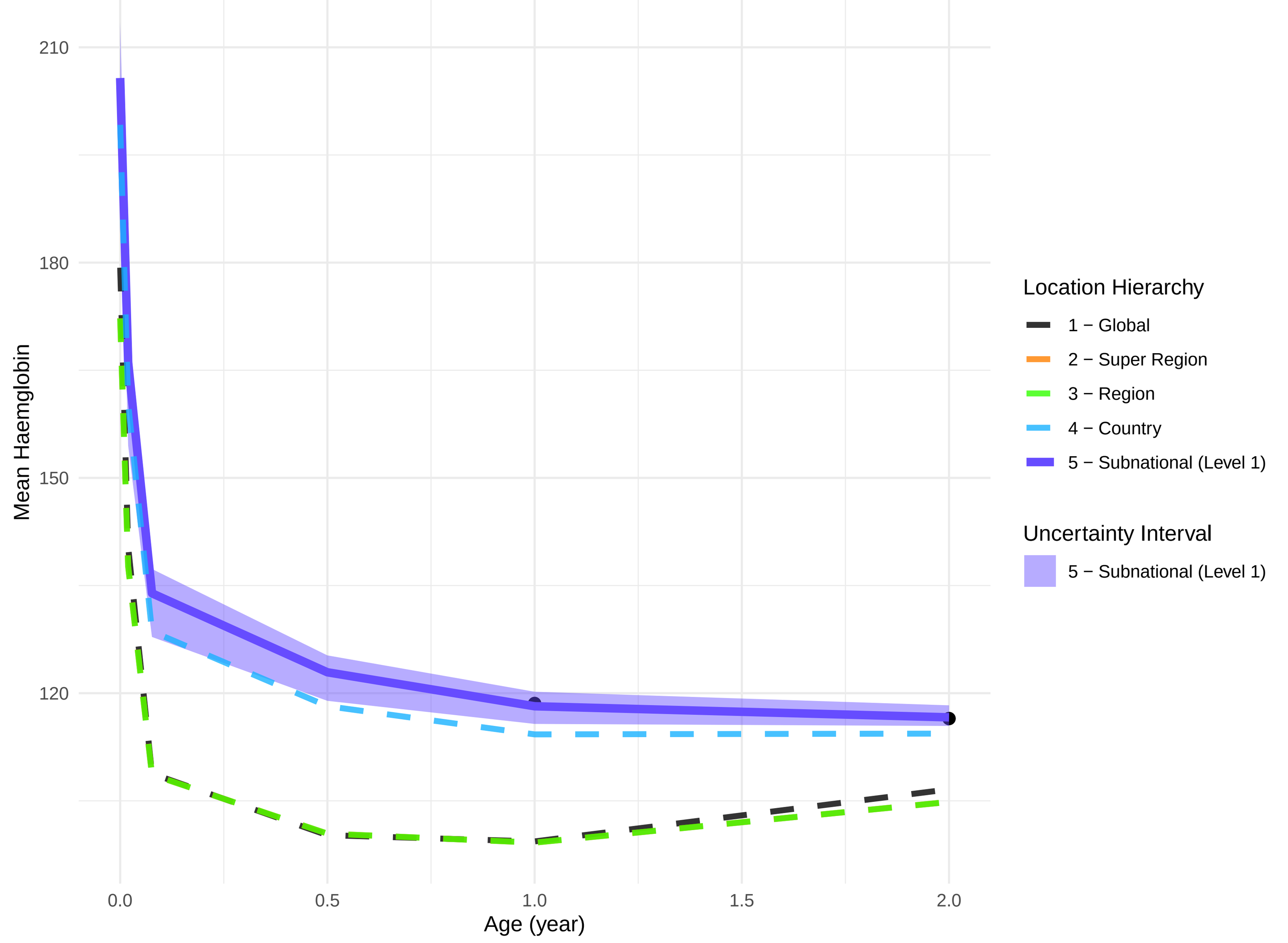

MR−BRT Age/Sex Fits − Non−pregnant Females <2 Years Old − Mean Haemglobin − Punjab
WHO−2024 Adjustment Method

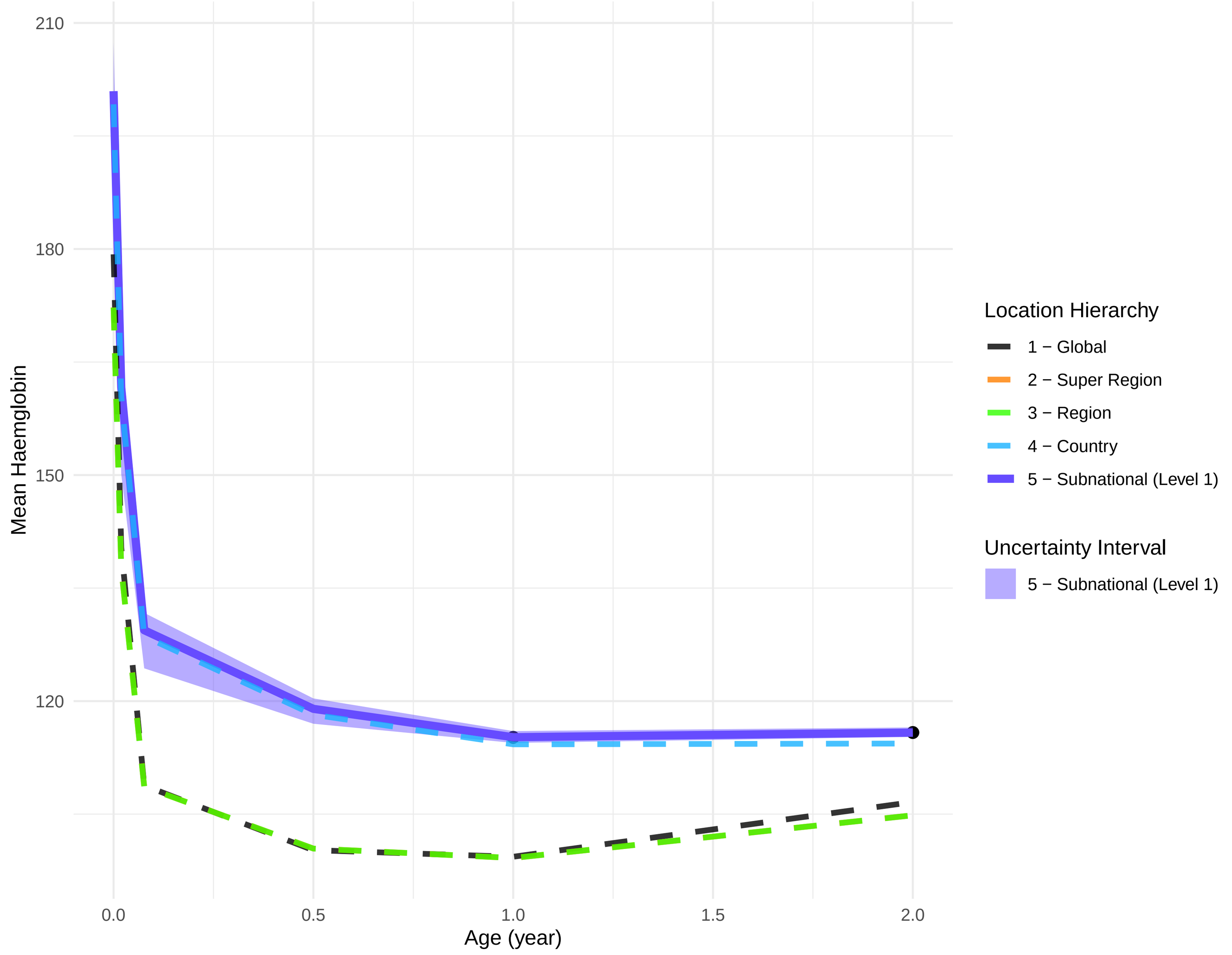

MR−BRT Age/Sex Fits − Non−pregnant Females <2 Years Old − Mean Haemglobin − Sindh
WHO−2024 Adjustment Method

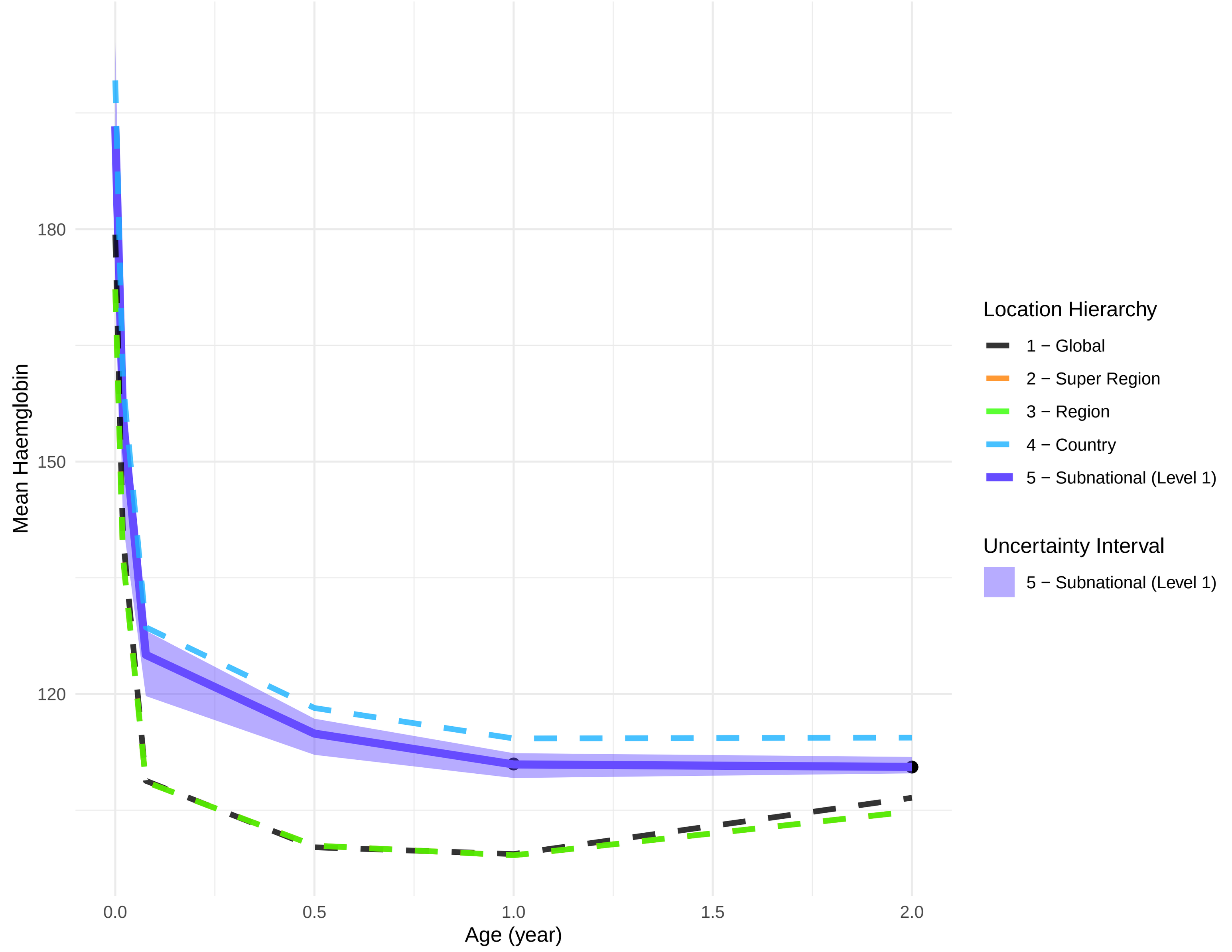

MR−BRT Age/Sex Fits − Non−pregnant Females <2 Years Old − Mean Haemglobin − Southeast Asia, East Asia, and Oceania
WHO−2024 Adjustment Method

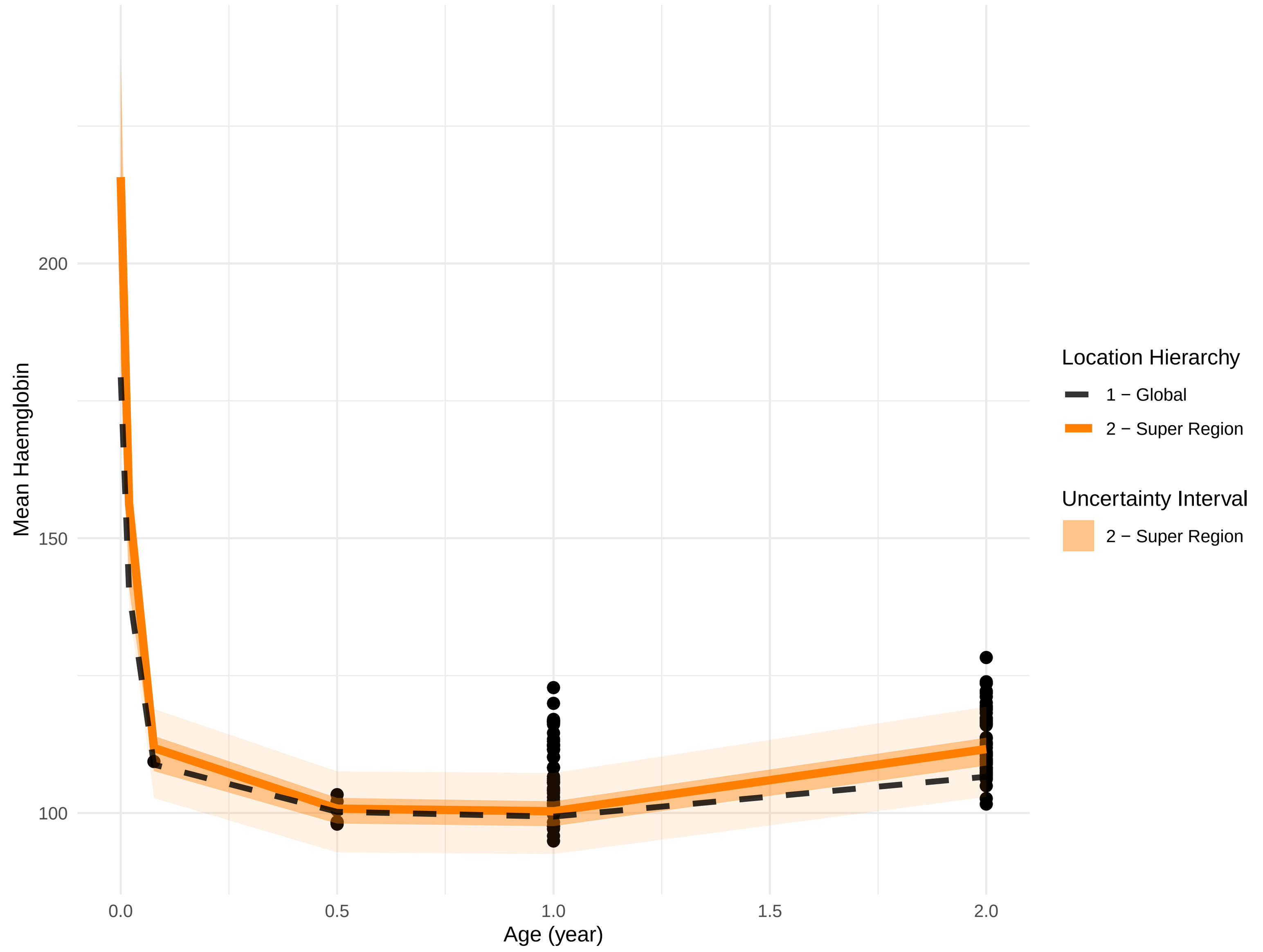

MR−BRT Age/Sex Fits − Non−pregnant Females <2 Years Old − Mean Haemglobin − Southeast Asia
WHO−2024 Adjustment Method

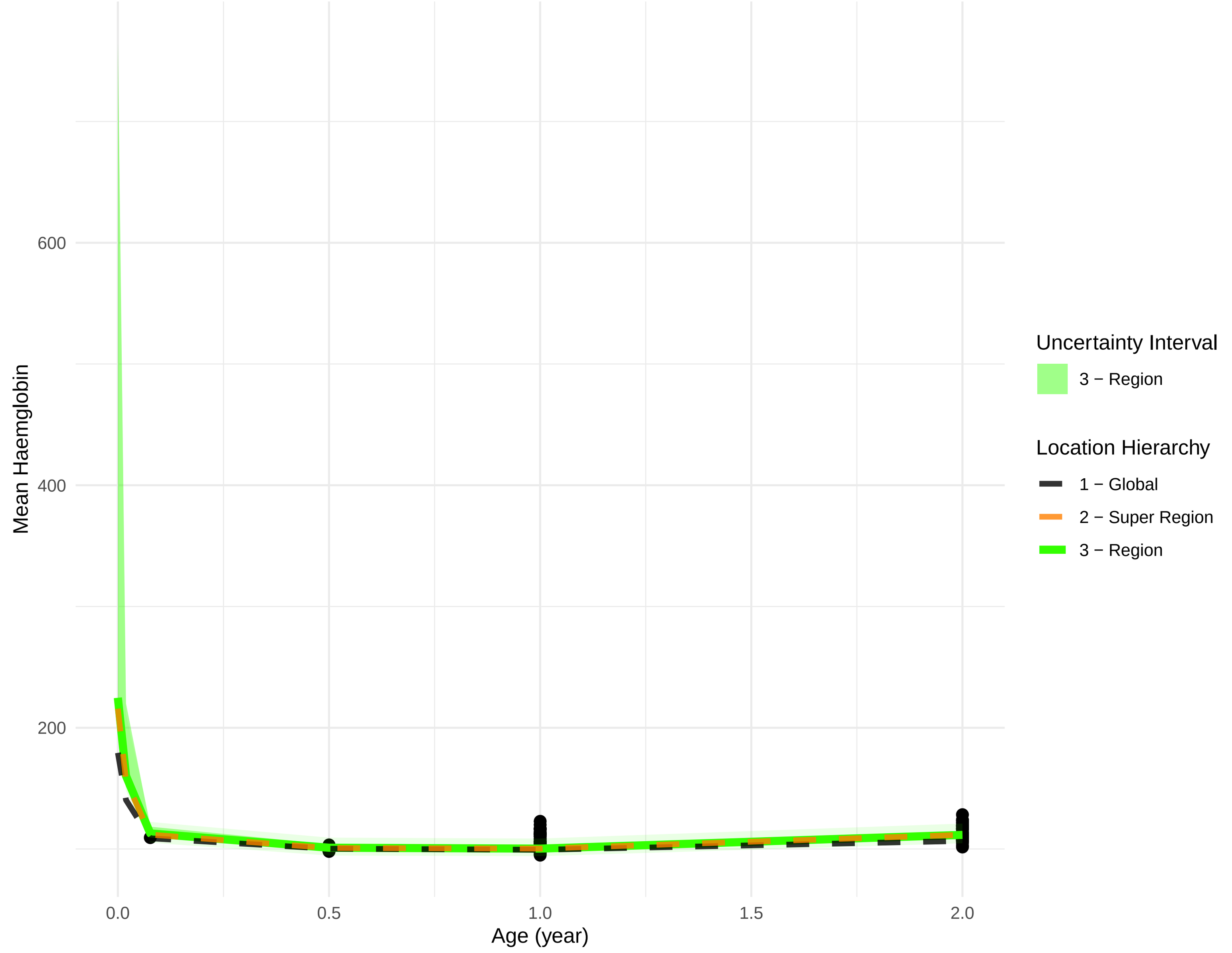

MR−BRT Age/Sex Fits − Non−pregnant Females <2 Years Old − Mean Haemglobin − Cambodia
WHO−2024 Adjustment Method

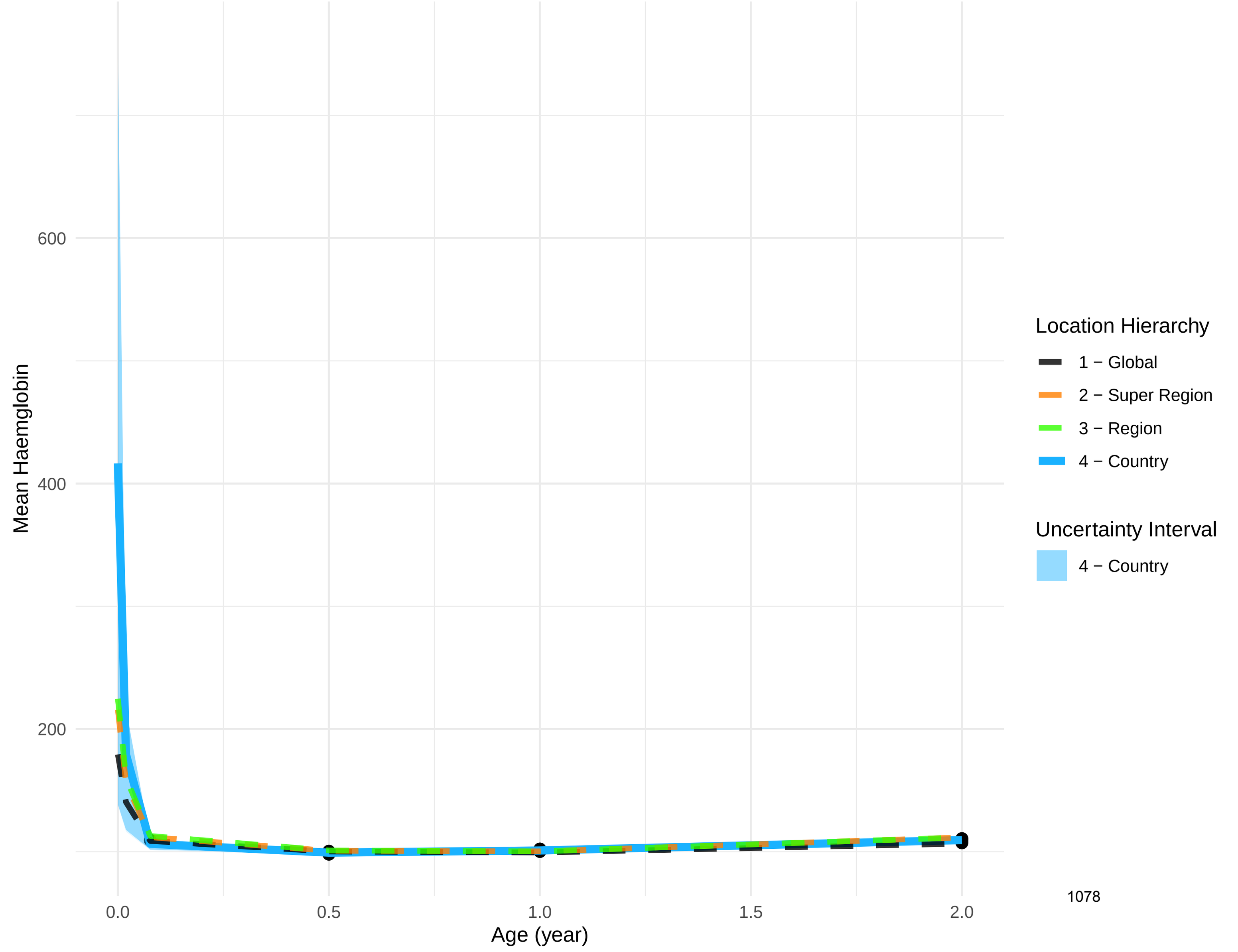

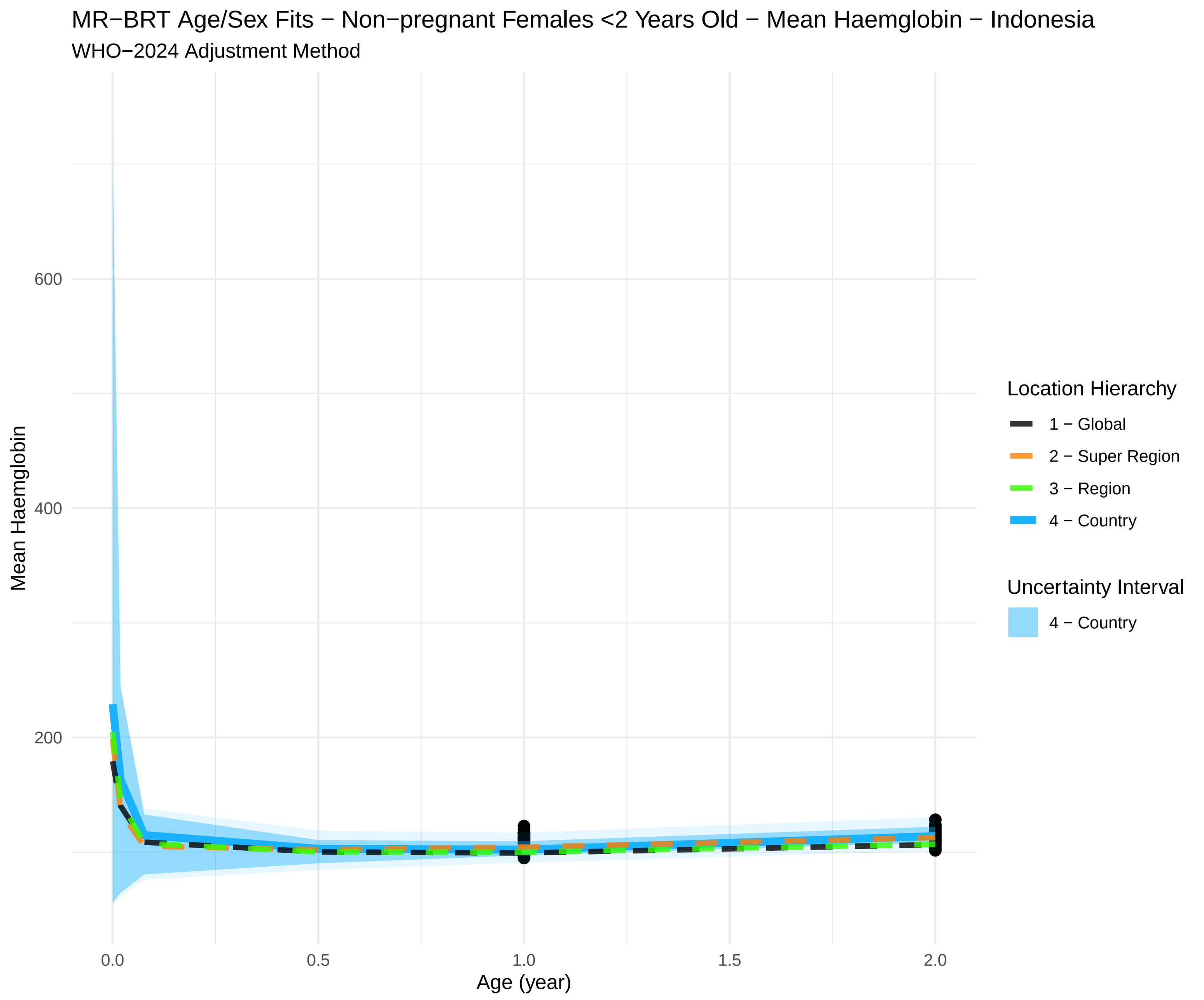
MR−BRT Age/Sex Fits − Non−pregnant Females <2 Years Old − Mean Haemglobin − Indonesia
WHO−2024 Adjustment Method
Mean Haemglobin
Age (year)
Location Hierarchy
1 − Global
2 − Super Region
3 − Region
4 − Country
Uncertainty Interval
4 − Country

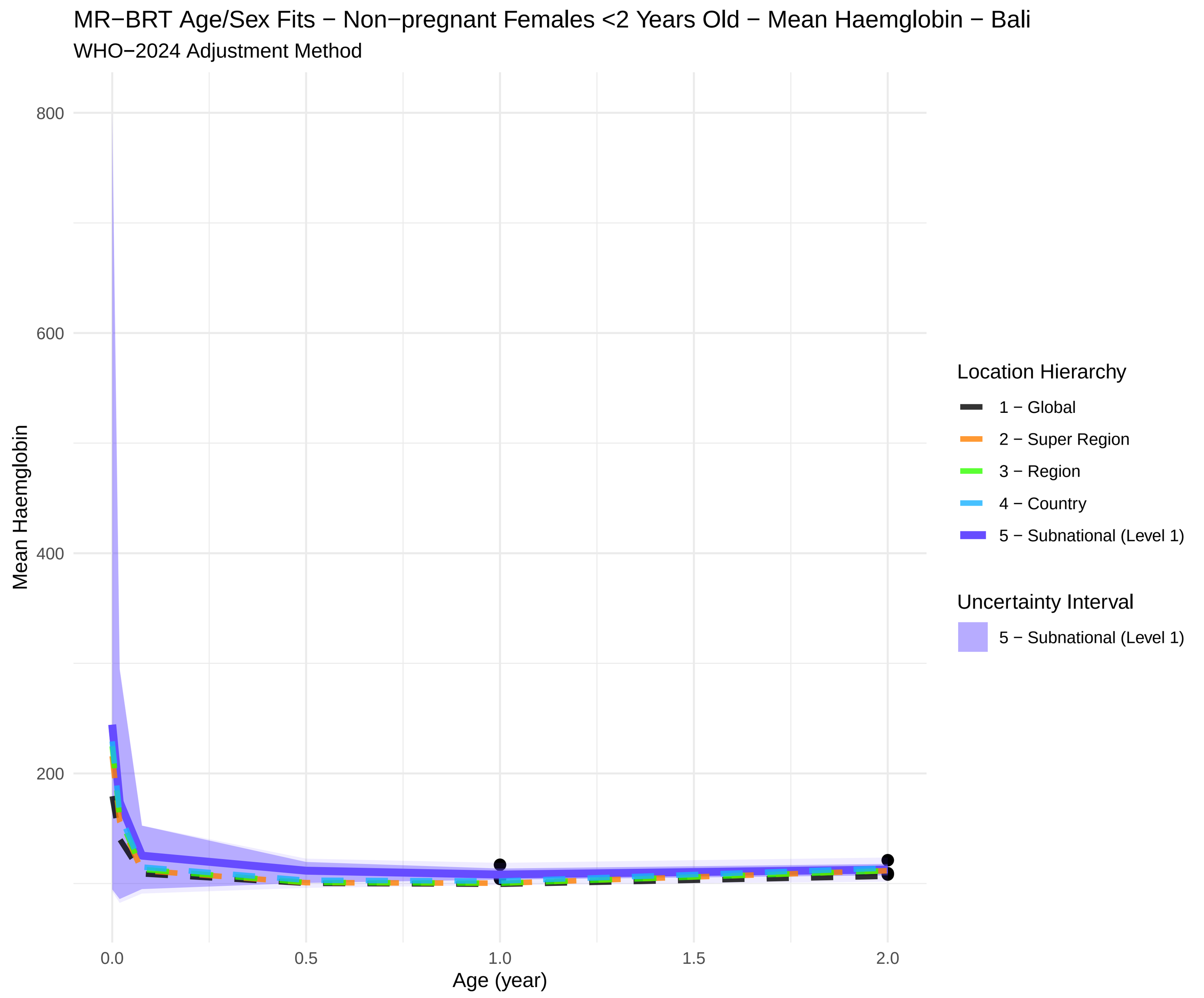
MR−BRT Age/Sex Fits − Non−pregnant Females <2 Years Old − Mean Haemglobin − Bali
WHO−2024 Adjustment Method
Mean Haemglobin
Age (year)
Location Hierarchy
1 − Global
2 − Super Region
3 − Region
4 − Country
5 − Subnational (Level 1)
Uncertainty Interval
5 − Subnational (Level 1)

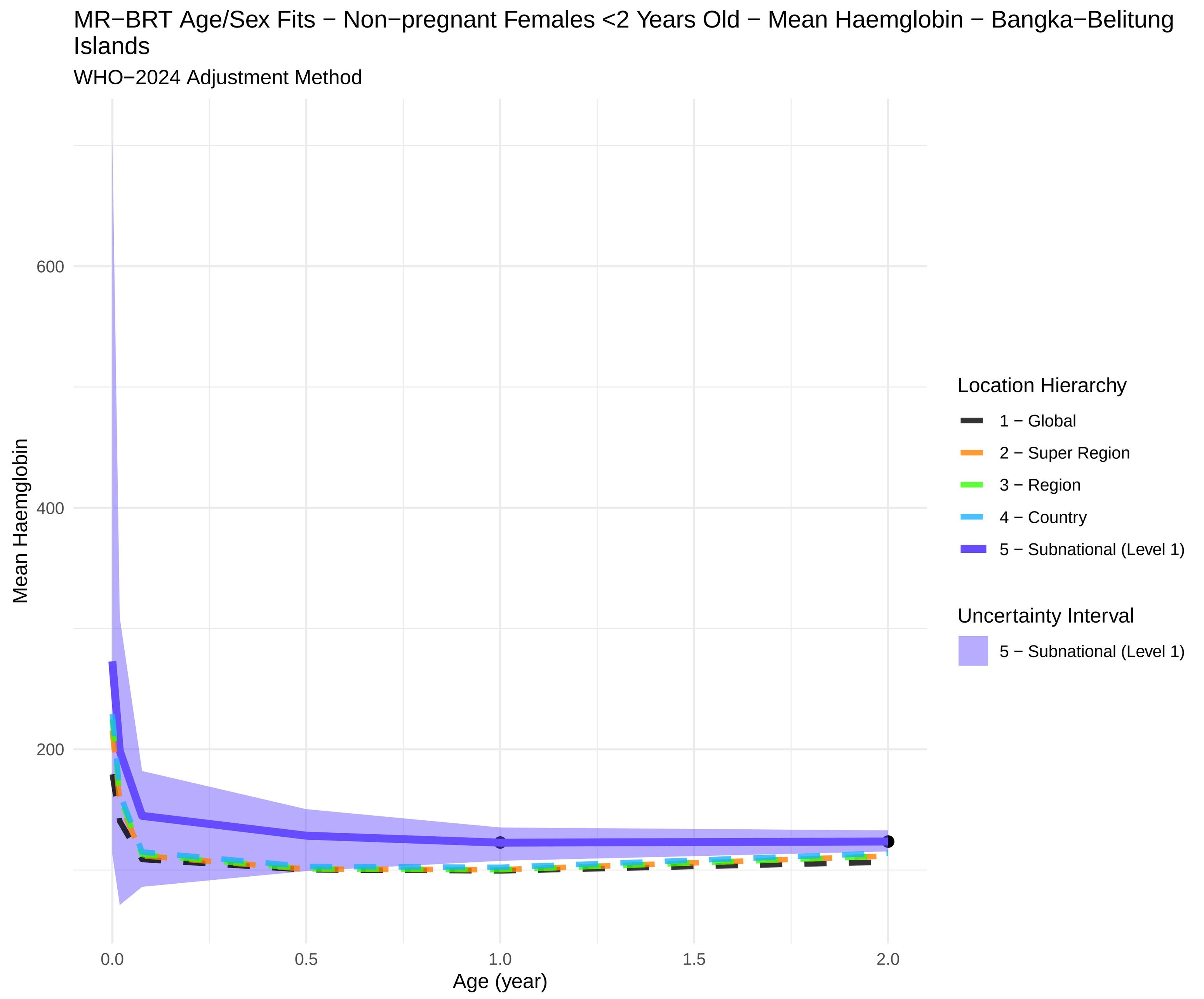
MR−BRT Age/Sex Fits − Non−pregnant Females <2 Years Old − Mean Haemglobin − Bangka−Belitung Islands
WHO−2024 Adjustment Method
Mean Haemglobin
Age (year)
Location Hierarchy
1 − Global
2 − Super Region
3 − Region
4 − Country
5 − Subnational (Level 1)
Uncertainty Interval
5 − Subnational (Level 1)

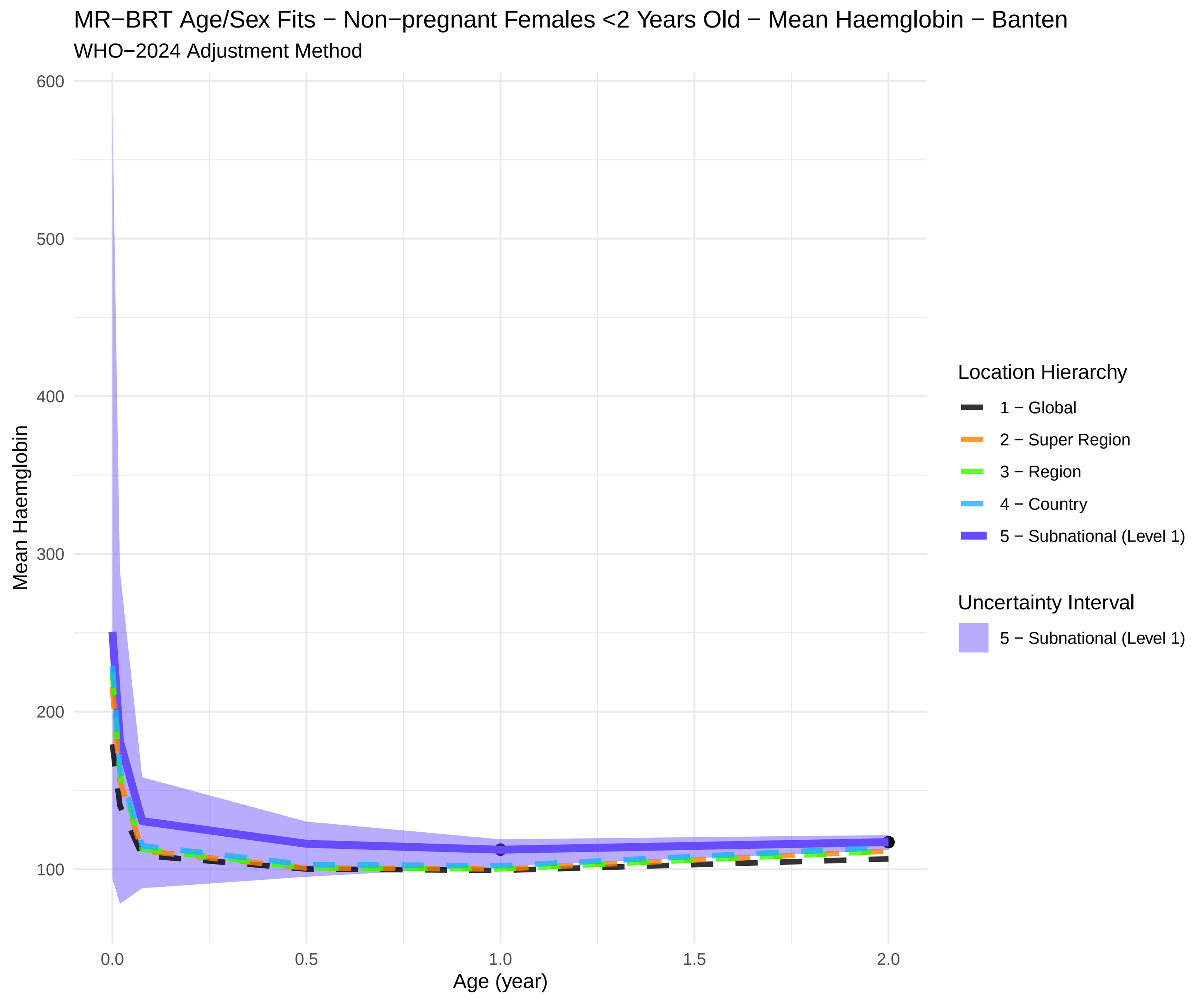
MR−BRT Age/Sex Fits − Non−pregnant Females <2 Years Old − Mean Haemglobin − Banten
WHO−2024 Adjustment Method
Mean Haemglobin
Age (year)
Location Hierarchy
1 − Global
2 − Super Region
3 − Region
4 − Country
5 − Subnational (Level 1)
Uncertainty Interval
5 − Subnational (Level 1)

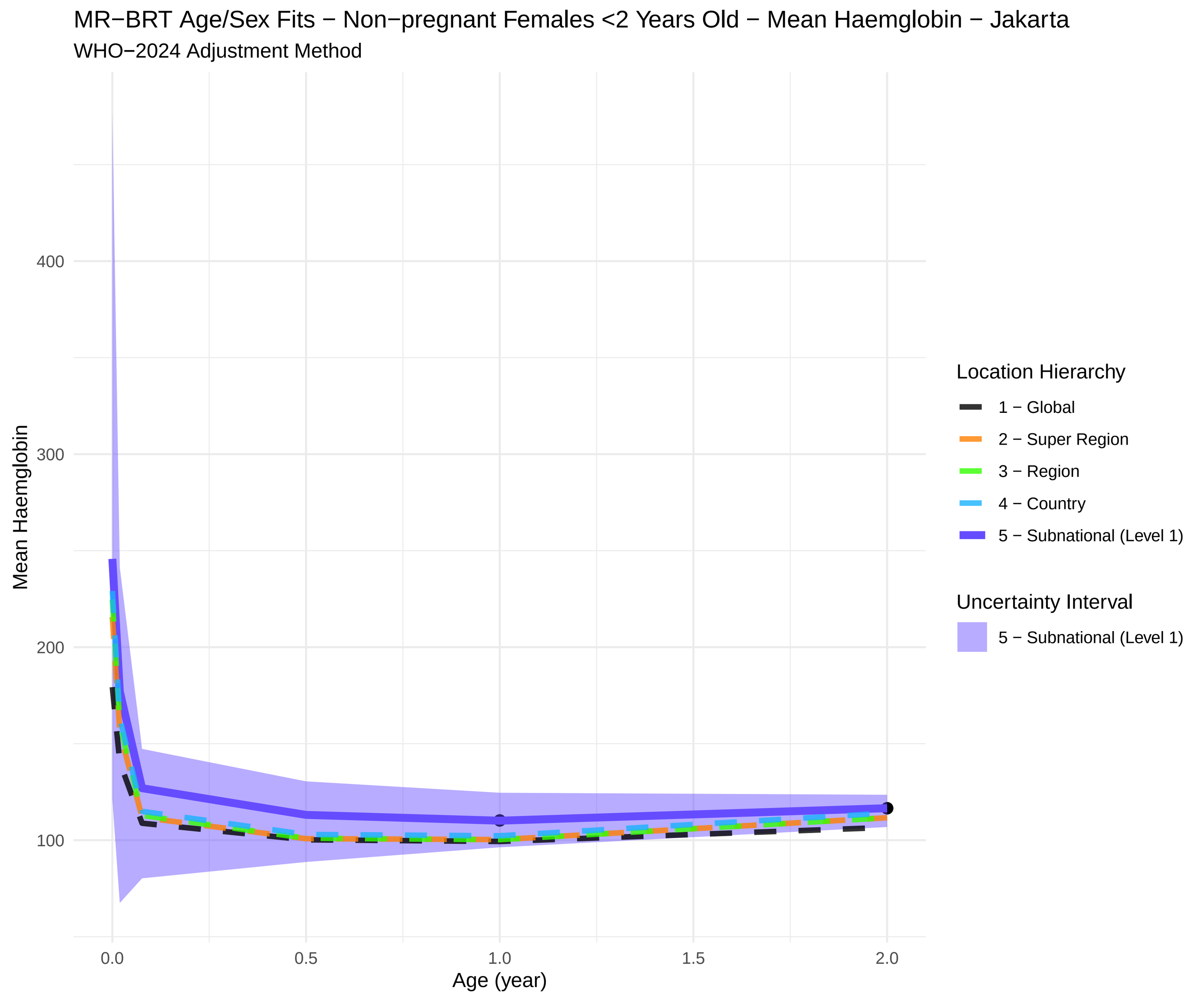
MR−BRT Age/Sex Fits − Non−pregnant Females <2 Years Old − Mean Haemglobin − Jakarta
WHO−2024 Adjustment Method
Mean Haemglobin
Age (year)
Location Hierarchy
1 − Global
2 − Super Region
3 − Region
4 − Country
5 − Subnational (Level 1)
Uncertainty Interval
5 − Subnational (Level 1)

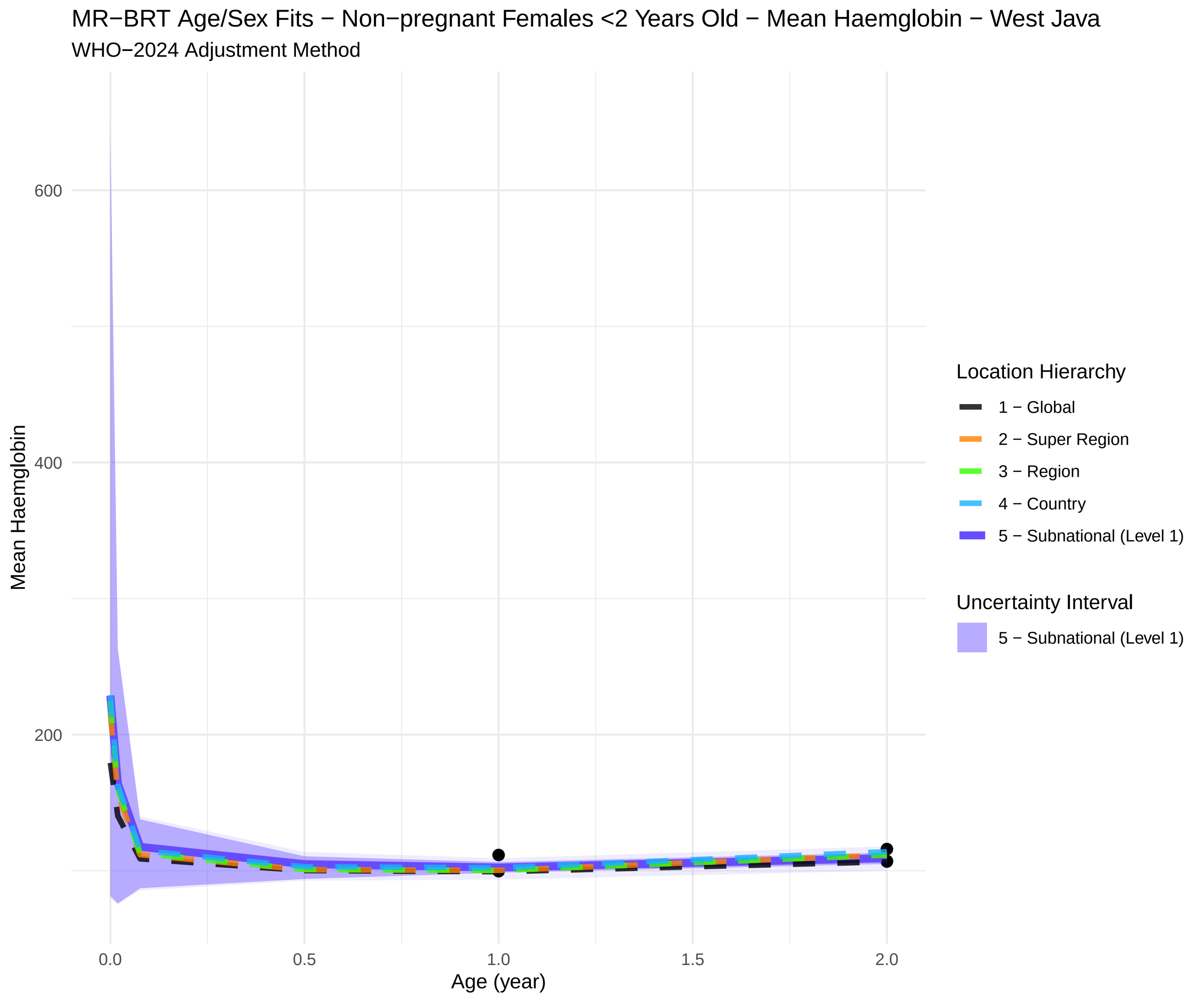
MR−BRT Age/Sex Fits − Non−pregnant Females <2 Years Old − Mean Haemglobin − West Java
WHO−2024 Adjustment Method
Mean Haemglobin
Age (year)
Location Hierarchy
1 − Global
2 − Super Region
3 − Region
4 − Country
5 − Subnational (Level 1)
Uncertainty Interval
5 − Subnational (Level 1)

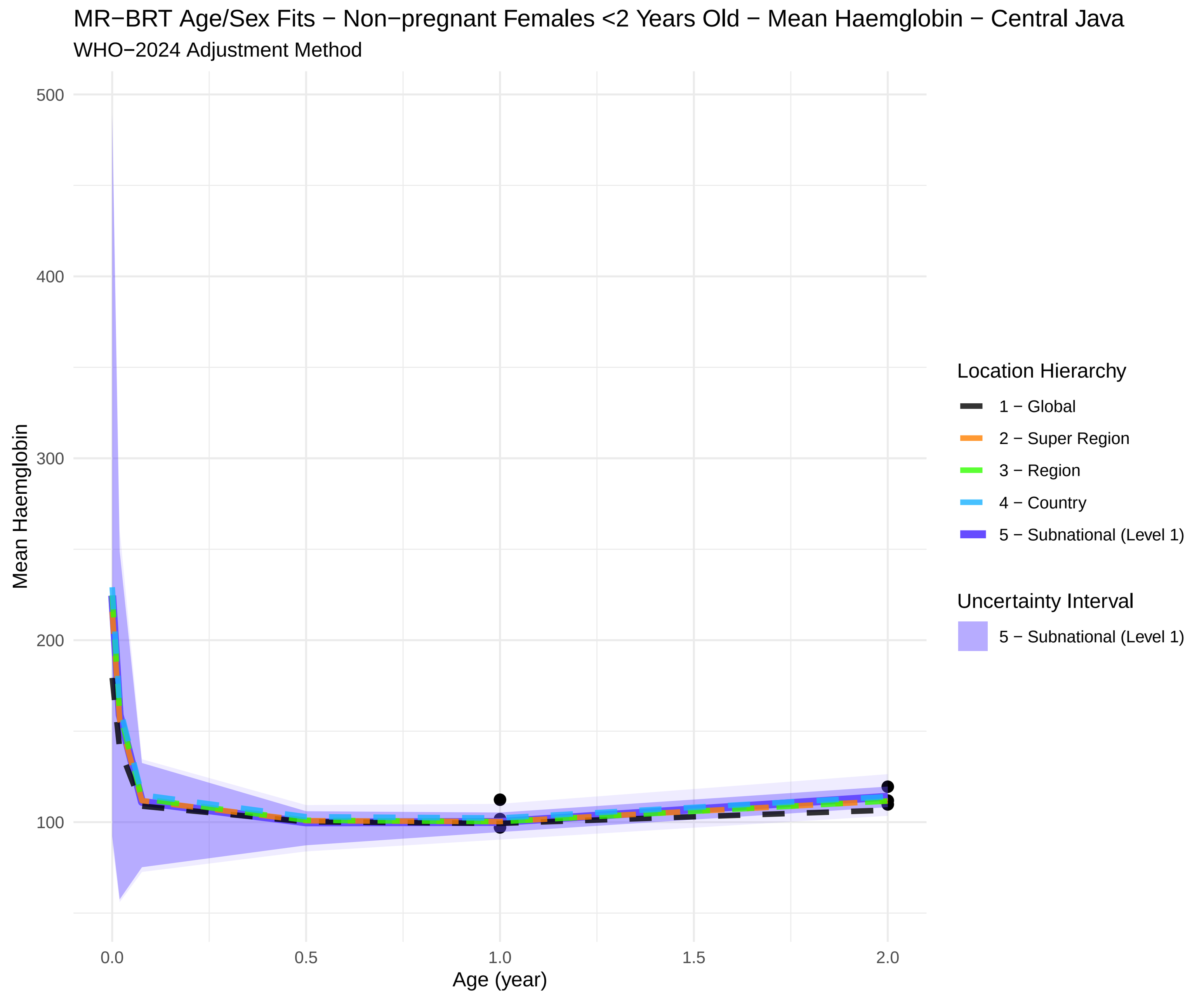
MR−BRT Age/Sex Fits − Non−pregnant Females <2 Years Old − Mean Haemglobin − Central Java
WHO−2024 Adjustment Method
Mean Haemglobin
Age (year)
Location Hierarchy
1 − Global
2 − Super Region
3 − Region
4 − Country
5 − Subnational (Level 1)
Uncertainty Interval
5 − Subnational (Level 1)

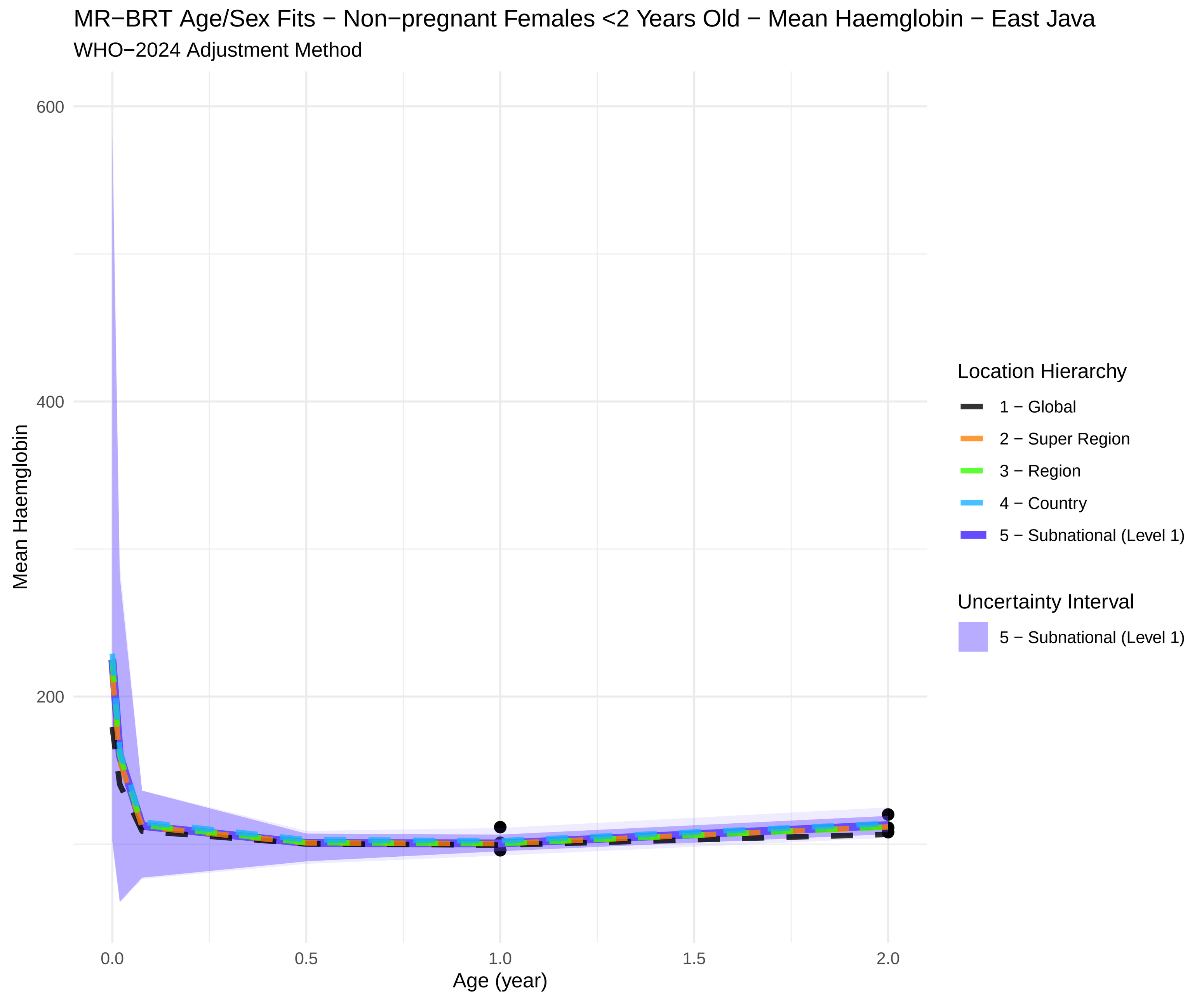
MR−BRT Age/Sex Fits − Non−pregnant Females <2 Years Old − Mean Haemglobin − East Java
WHO−2024 Adjustment Method
Mean Haemglobin
Age (year)
Location Hierarchy
1 − Global
2 − Super Region
3 − Region
4 − Country
5 − Subnational (Level 1)
Uncertainty Interval
5 − Subnational (Level 1)

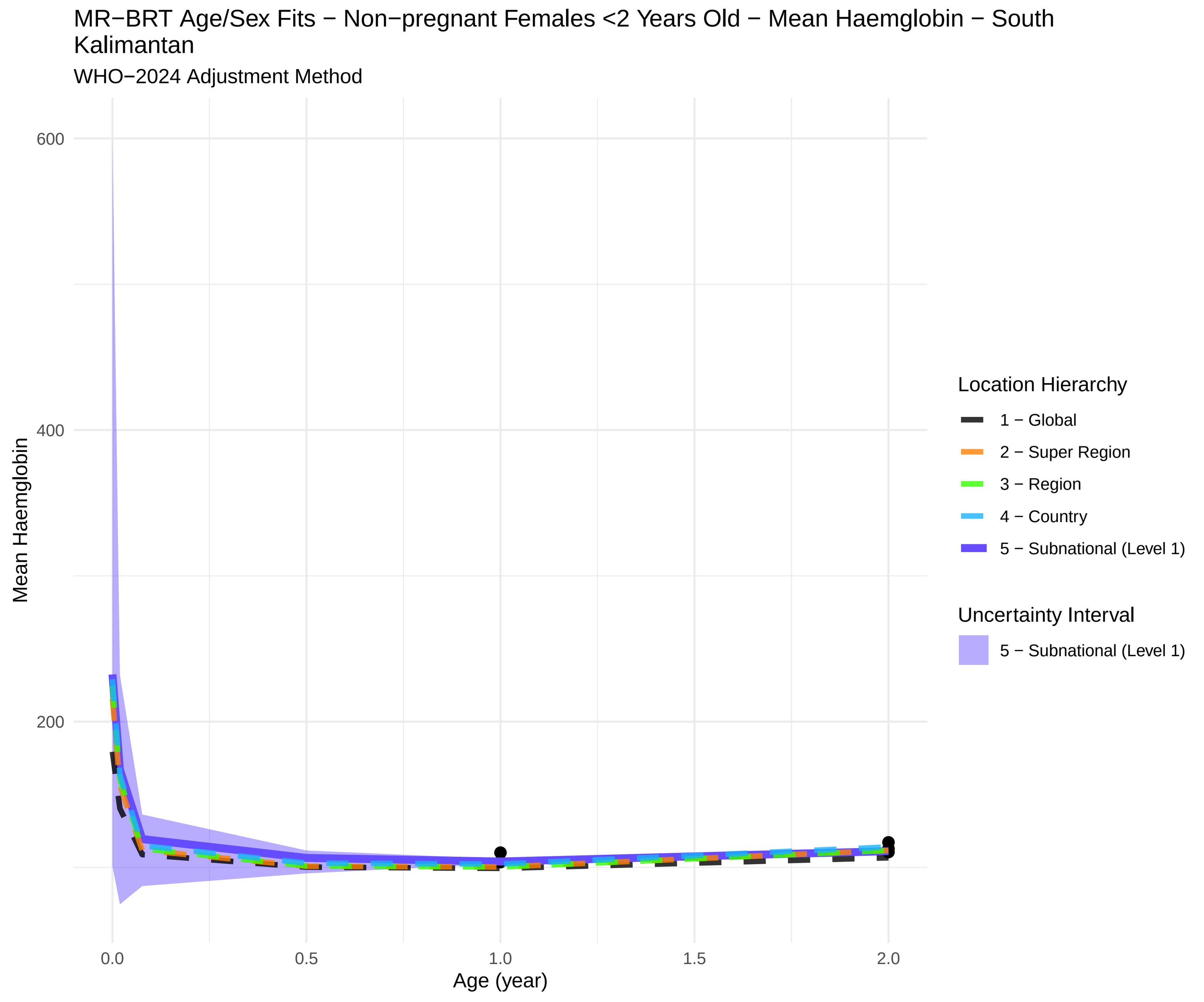
MR−BRT Age/Sex Fits − Non−pregnant Females <2 Years Old − Mean Haemglobin − South Kalimantan
WHO−2024 Adjustment Method
Mean Haemglobin
Age (year)
Location Hierarchy
1 − Global
2 − Super Region
3 − Region
4 − Country
5 − Subnational (Level 1)
Uncertainty Interval
5 − Subnational (Level 1)

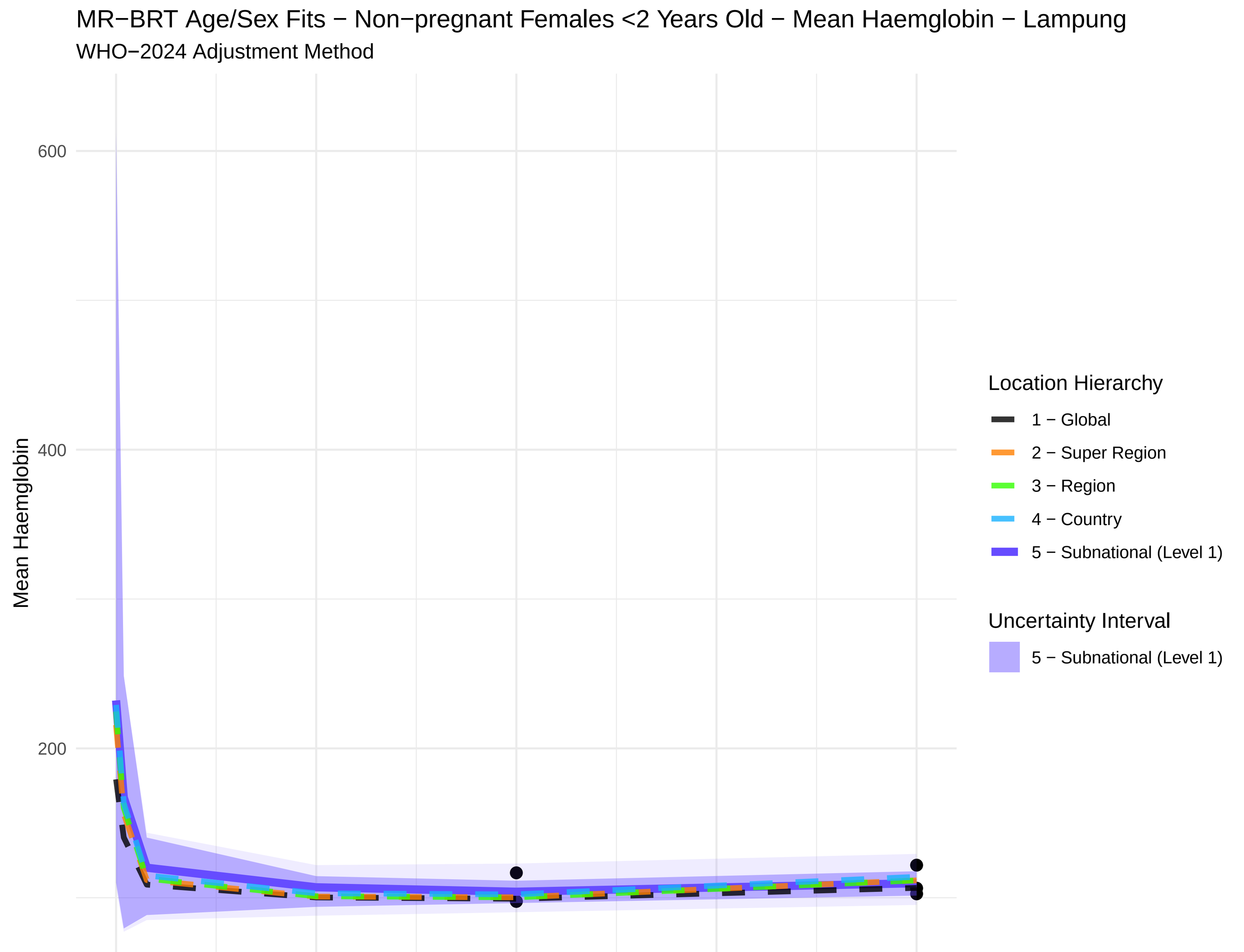
MR−BRT Age/Sex Fits − Non−pregnant Females <2 Years Old − Mean Haemglobin − Lampung
WHO−2024 Adjustment Method
Mean Haemglobin
Age (year)
Location Hierarchy
1 − Global
2 − Super Region
3 − Region
4 − Country
5 − Subnational (Level 1)
Uncertainty Interval
5 − Subnational (Level 1)

MR−BRT Age/Sex Fits − Non−pregnant Females <2 Years Old − Mean Haemglobin − West Nusa Tenggara
WHO−2024 Adjustment Method

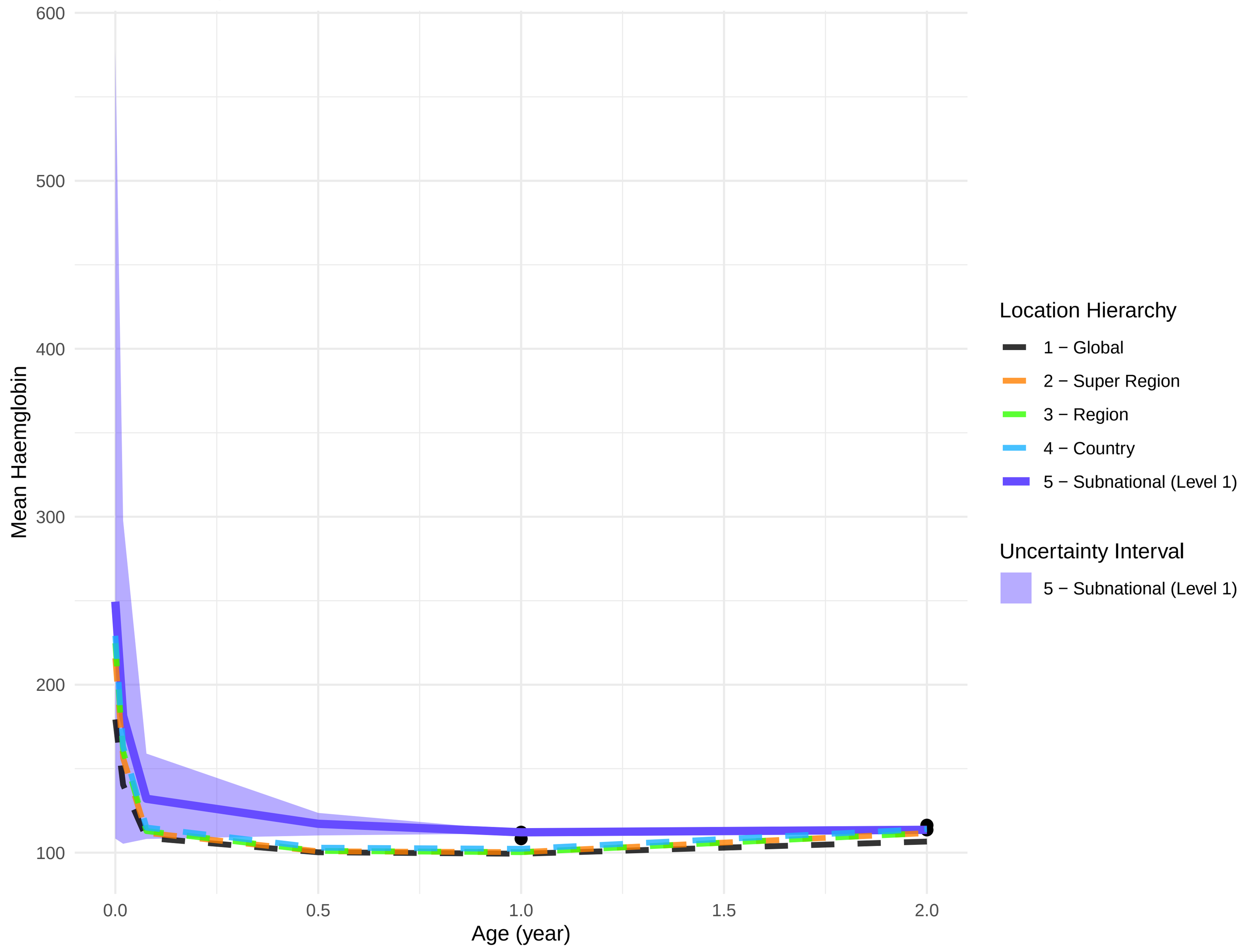

MR−BRT Age/Sex Fits − Non−pregnant Females <2 Years Old − Mean Haemglobin − Riau
WHO−2024 Adjustment Method

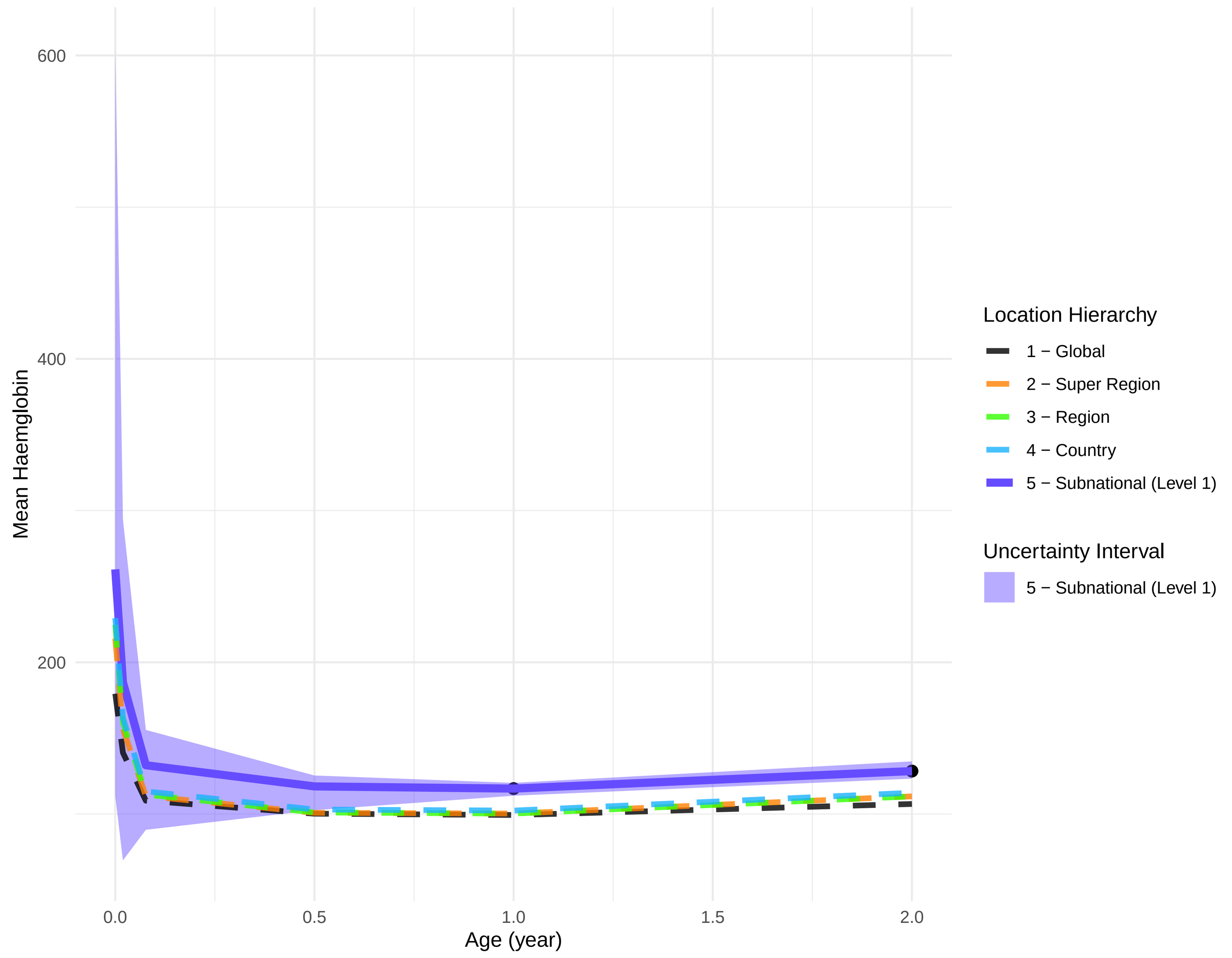

MR−BRT Age/Sex Fits − Non−pregnant Females <2 Years Old − Mean Haemglobin − West Sulawesi
WHO−2024 Adjustment Method

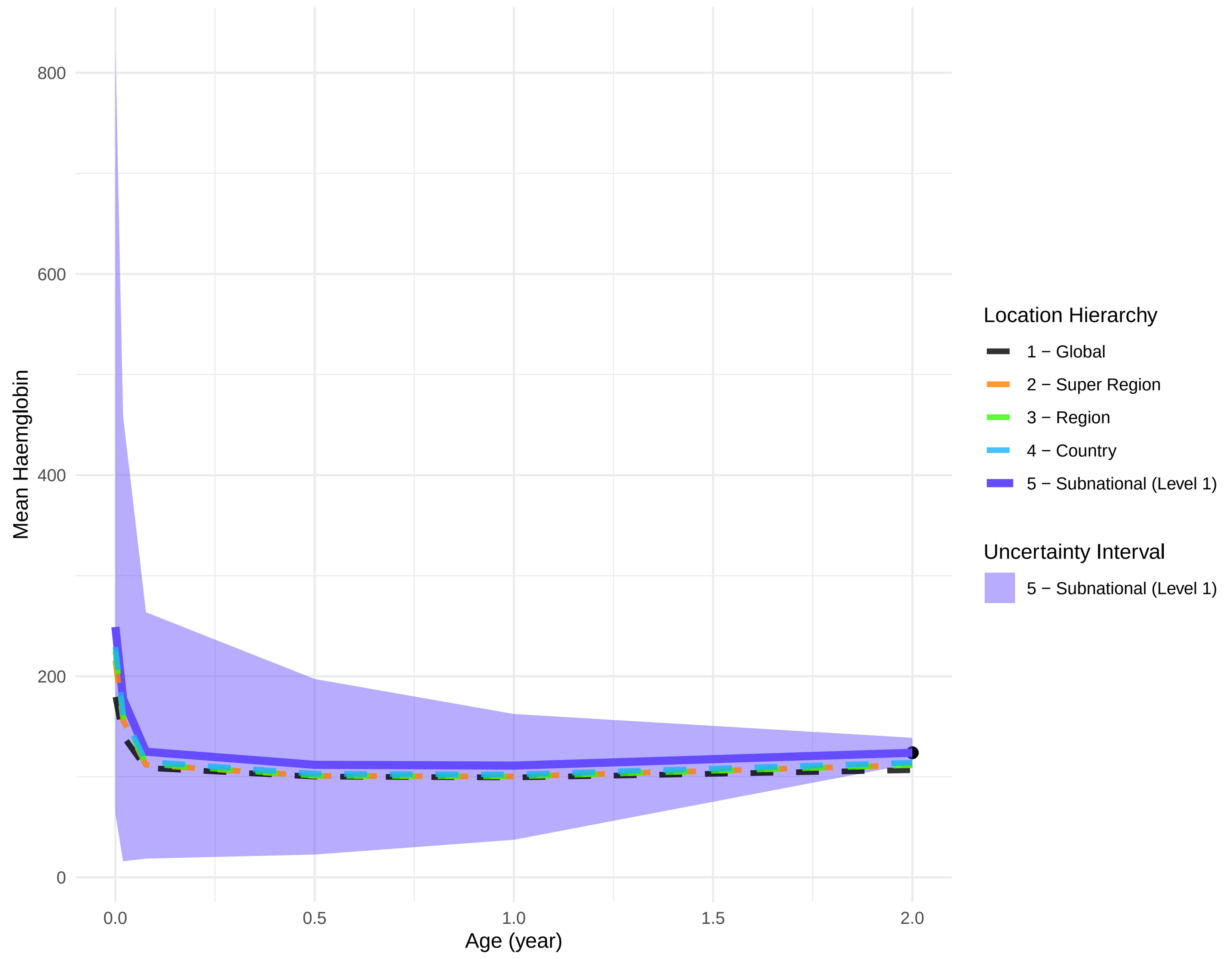

MR−BRT Age/Sex Fits − Non−pregnant Females <2 Years Old − Mean Haemglobin − South Sulawesi
WHO−2024 Adjustment Method

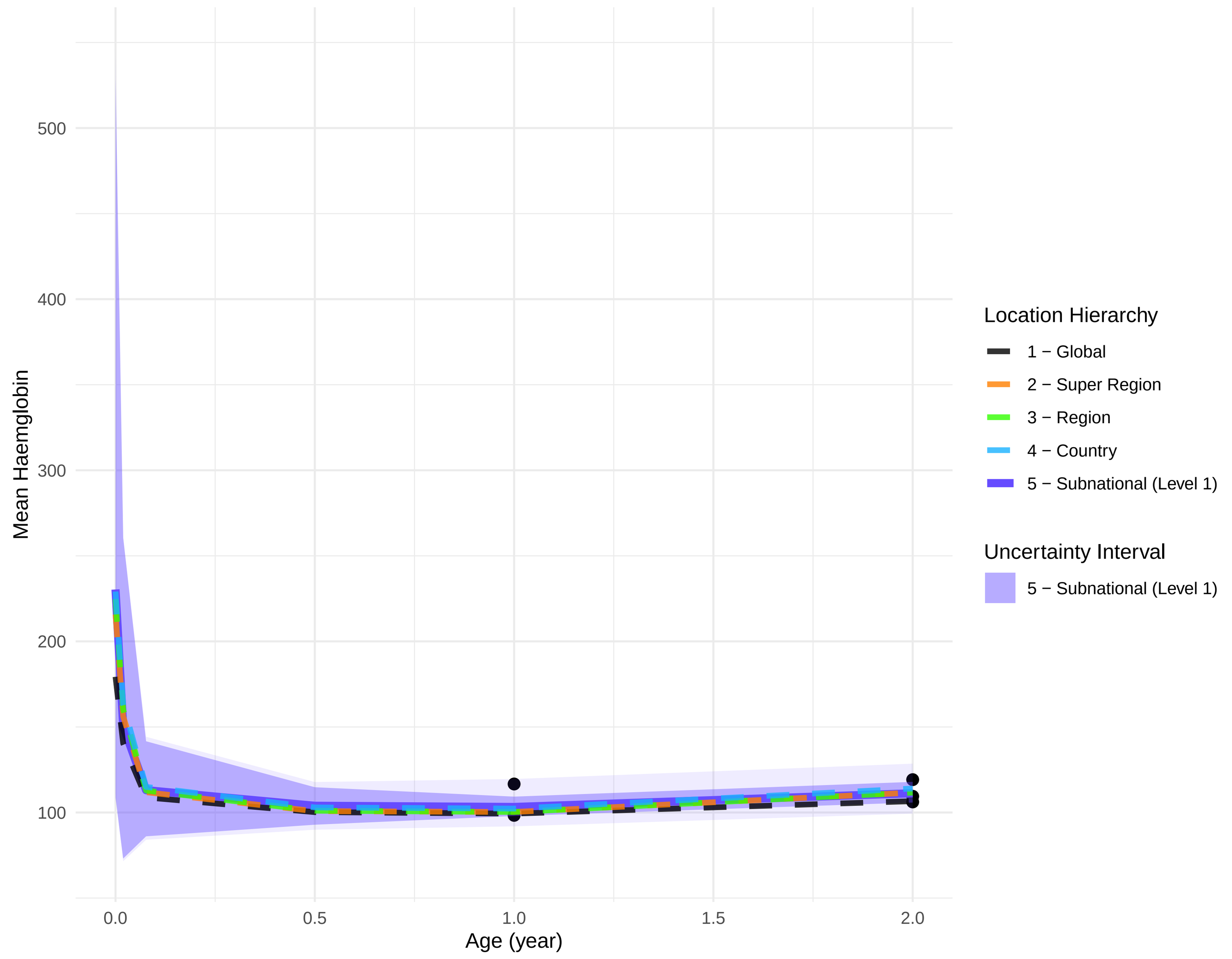

MR−BRT Age/Sex Fits − Non−pregnant Females <2 Years Old − Mean Haemglobin − West Sumatra
WHO−2024 Adjustment Method

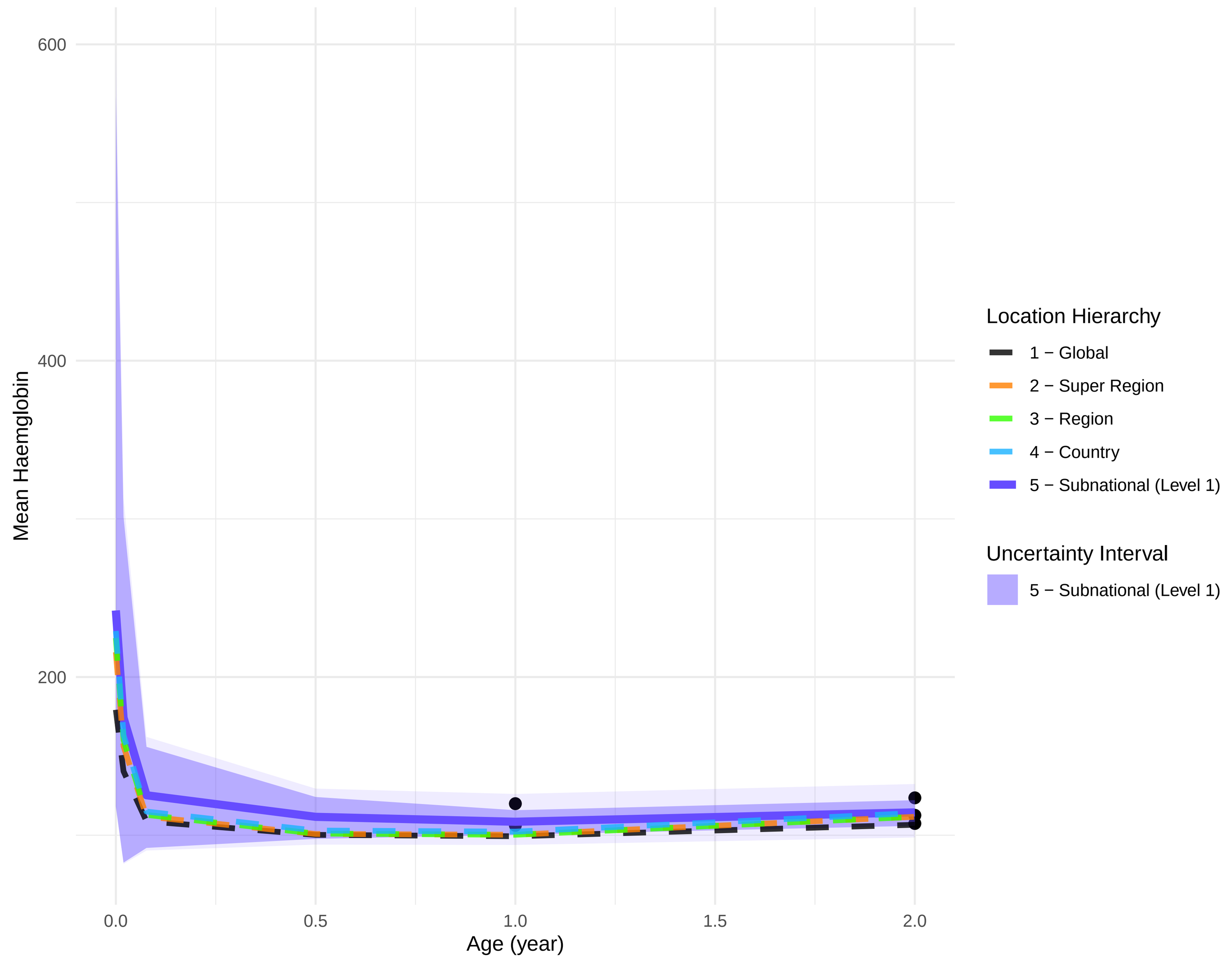

MR−BRT Age/Sex Fits − Non−pregnant Females <2 Years Old − Mean Haemglobin − South Sumatra
WHO−2024 Adjustment Method

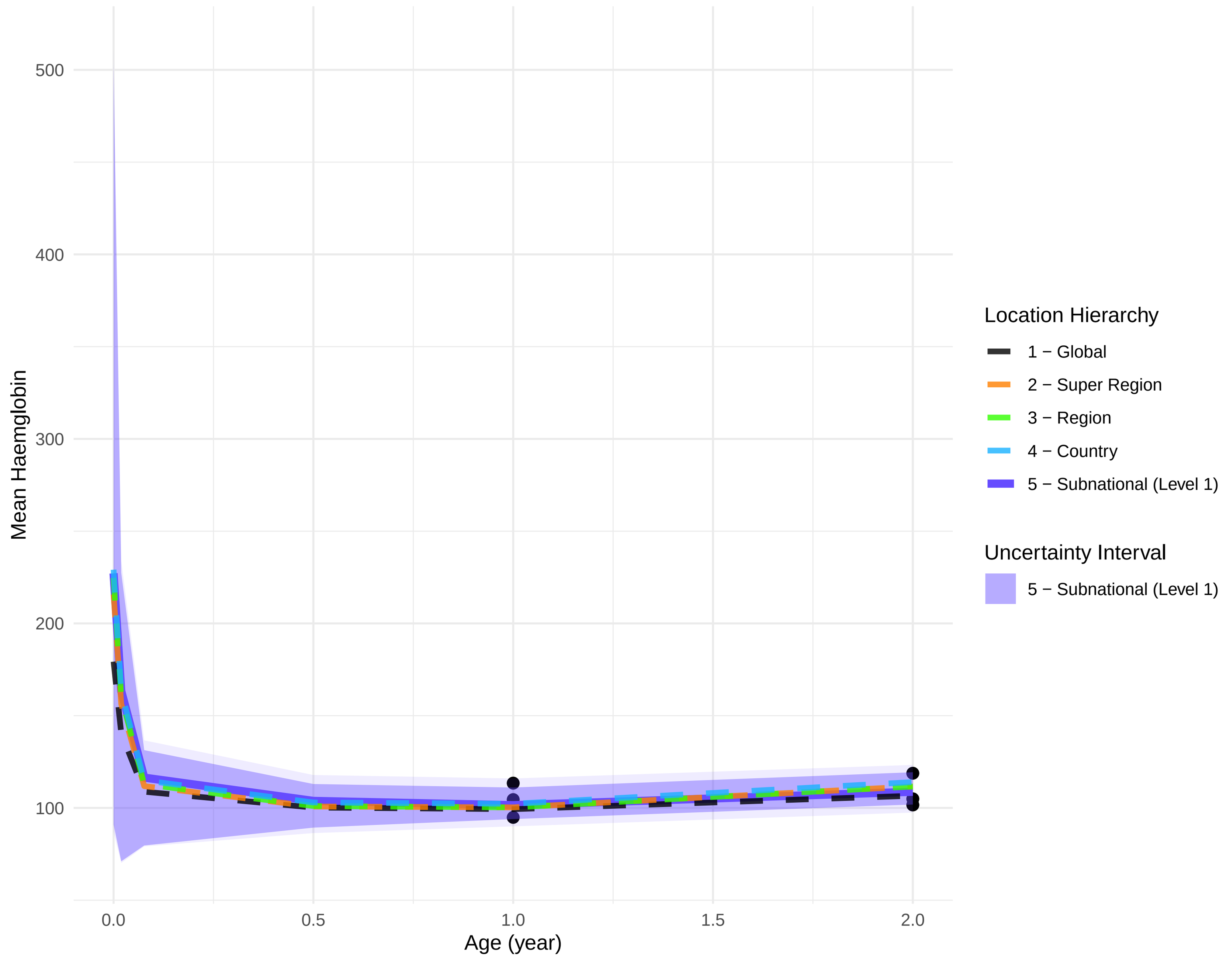

MR−BRT Age/Sex Fits − Non−pregnant Females <2 Years Old − Mean Haemglobin − North Sumatra
WHO−2024 Adjustment Method

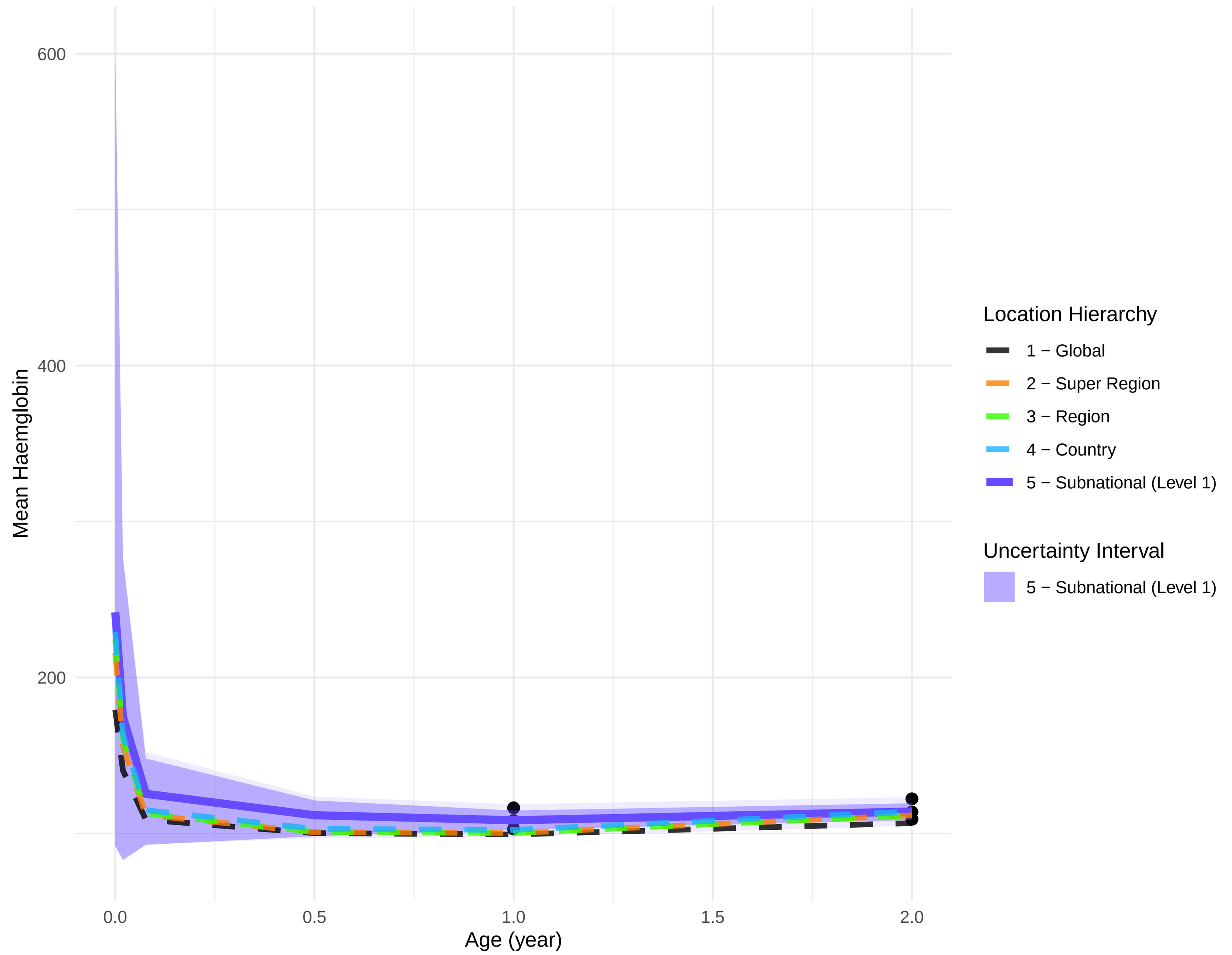

MR−BRT Age/Sex Fits − Non−pregnant Females <2 Years Old − Mean Haemglobin − Yogyakarta
WHO−2024 Adjustment Method

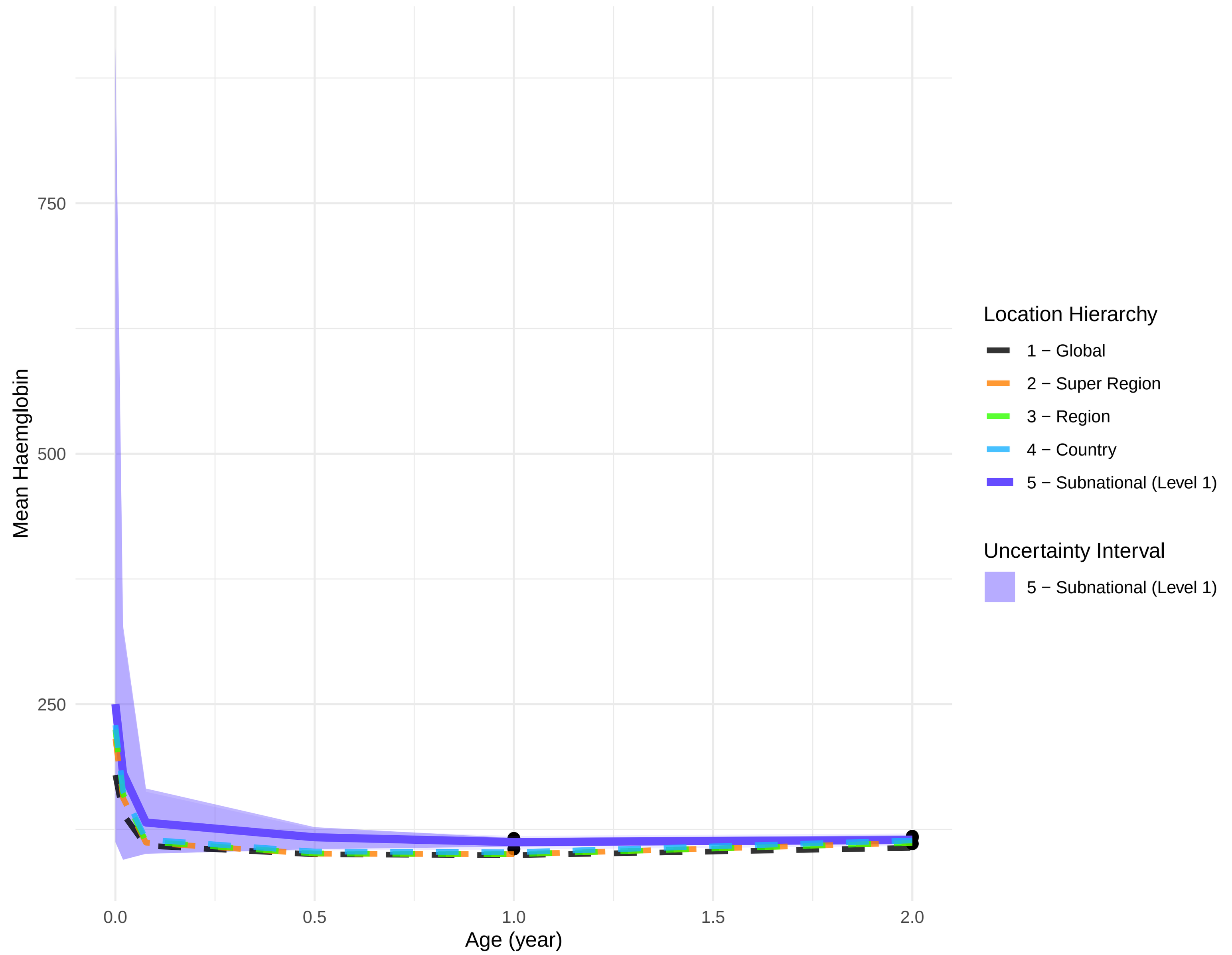

MR−BRT Age/Sex Fits − Non−pregnant Females <2 Years Old − Mean Haemglobin − Myanmar
WHO−2024 Adjustment Method

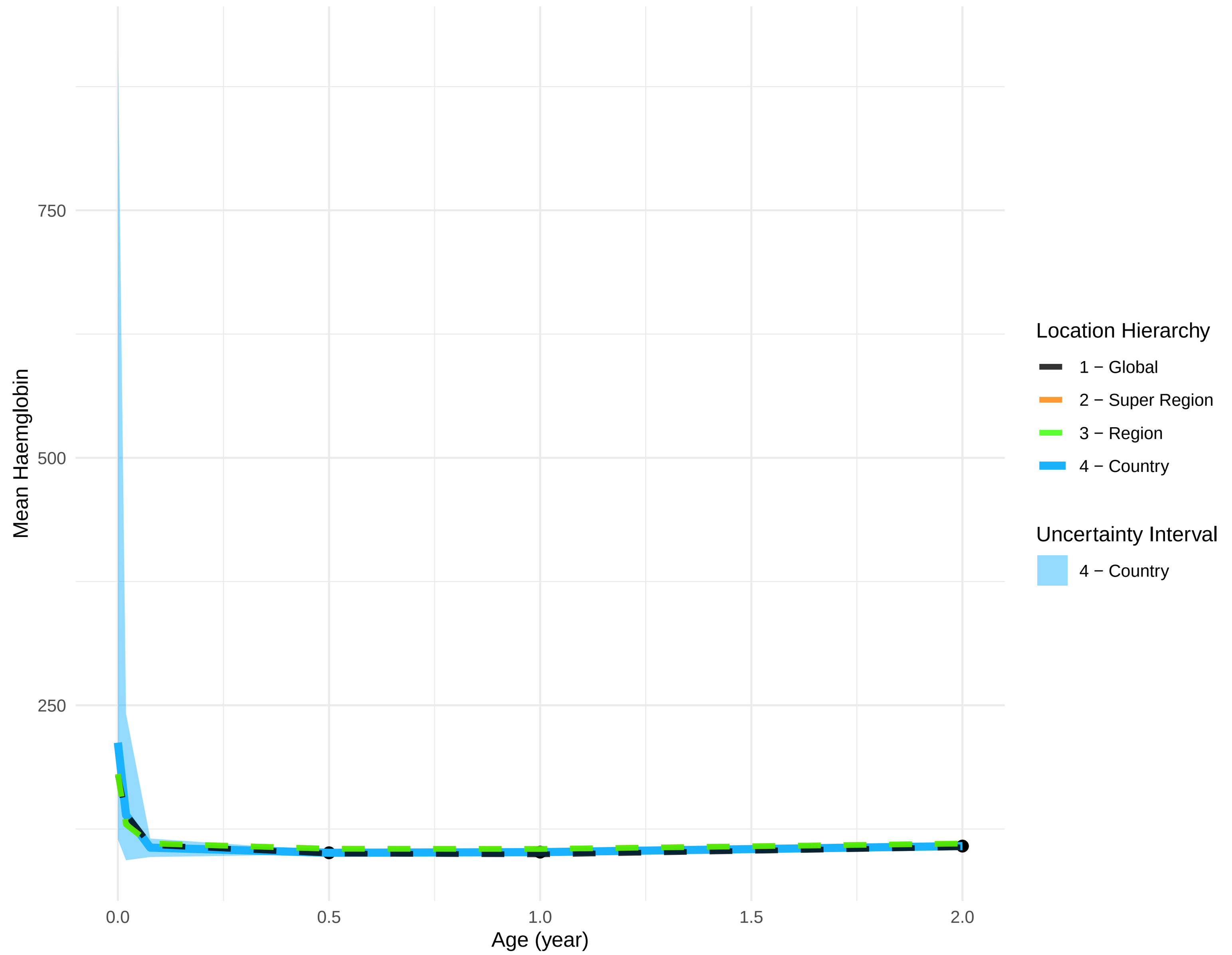

MR−BRT Age/Sex Fits − Non−pregnant Females <2 Years Old − Mean Haemglobin − Timor−Leste
WHO−2024 Adjustment Method

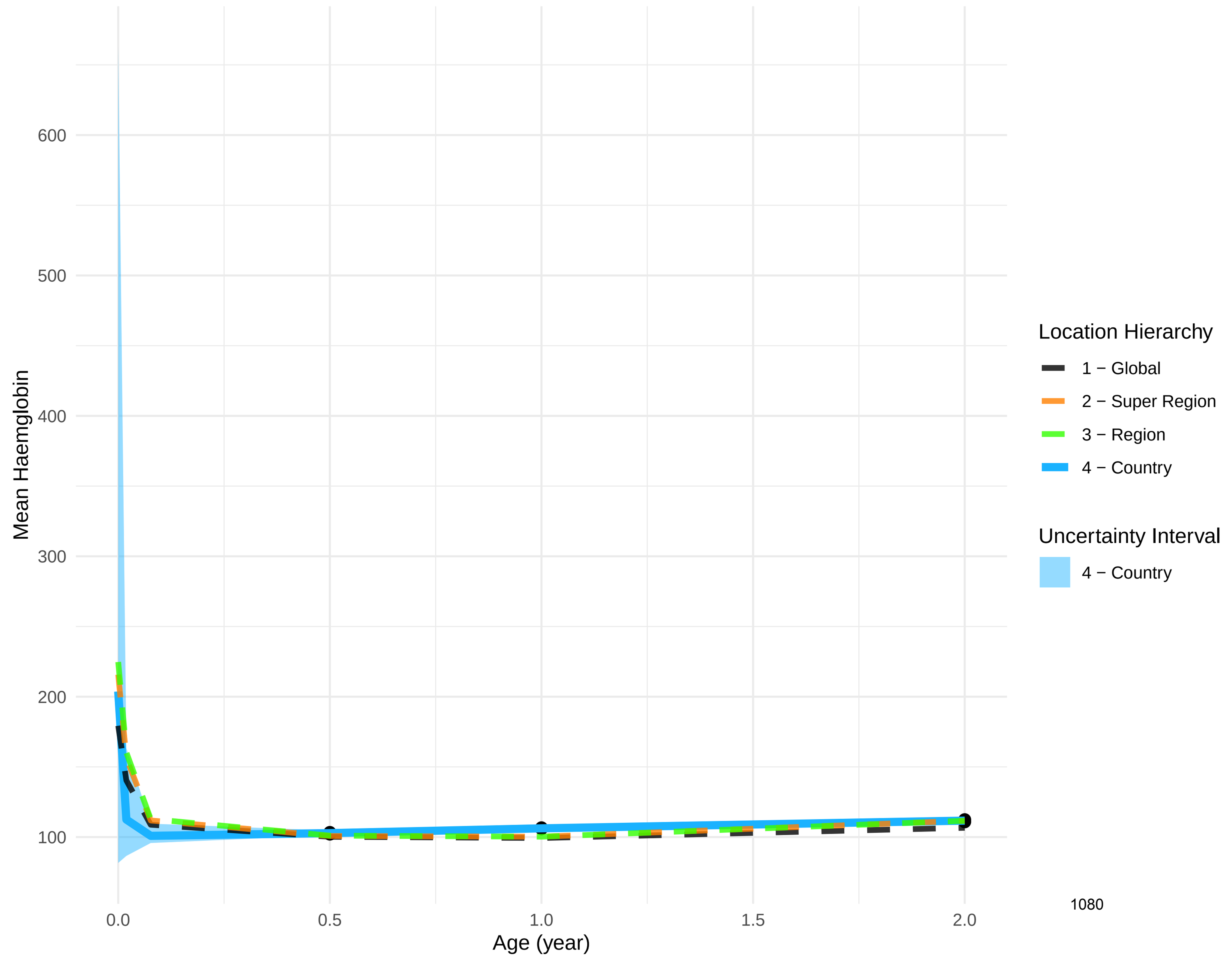

MR−BRT Age/Sex Fits − Non−pregnant Females <2 Years Old − Mean Haemglobin − Sub−Saharan Africa
WHO−2024 Adjustment Method

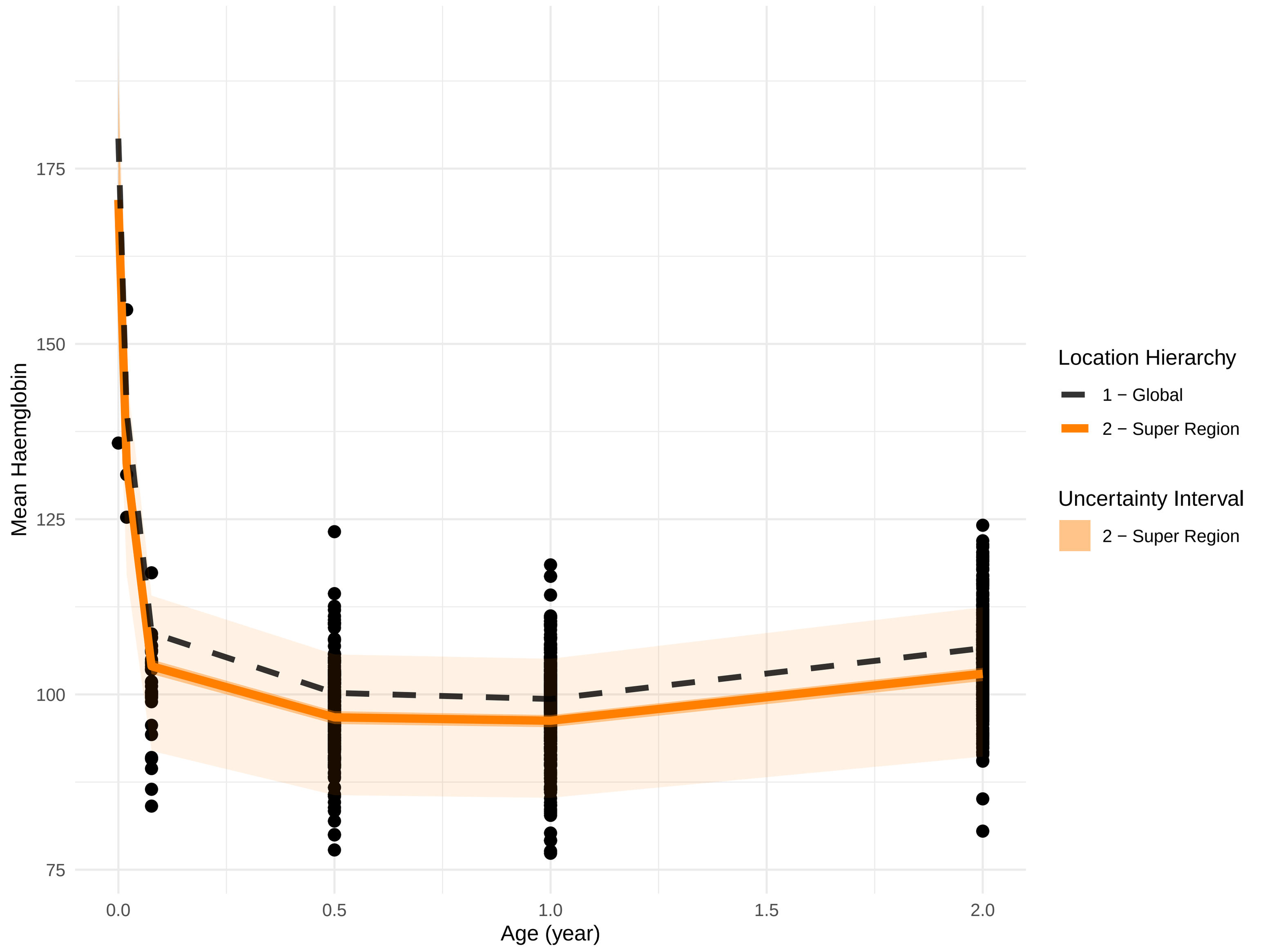

MR−BRT Age/Sex Fits − Non−pregnant Females <2 Years Old − Mean Haemglobin − Central Sub−Saharan Africa
WHO−2024 Adjustment Method

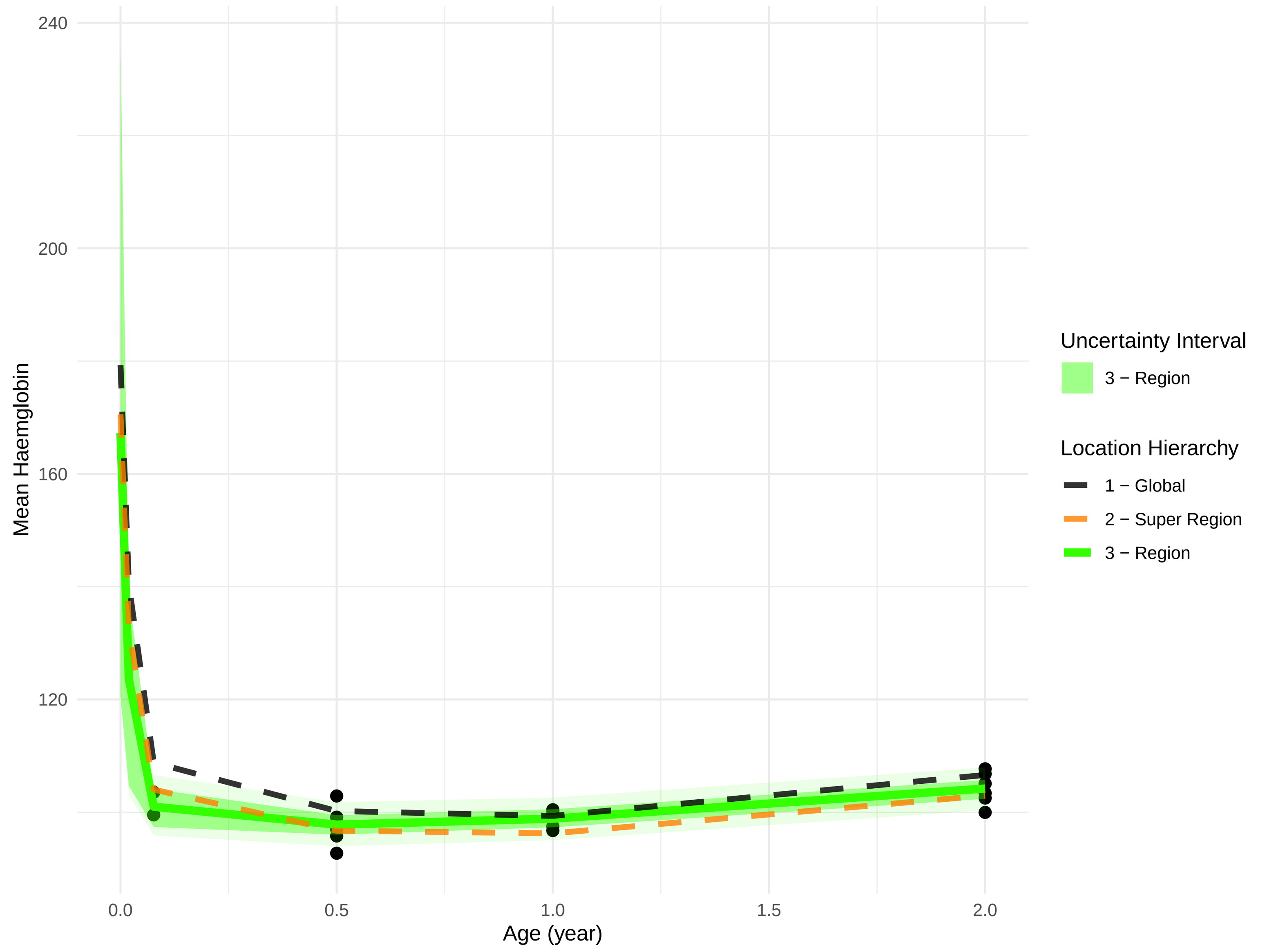

MR−BRT Age/Sex Fits − Non−pregnant Females <2 Years Old − Mean Haemglobin − Angola
WHO−2024 Adjustment Method

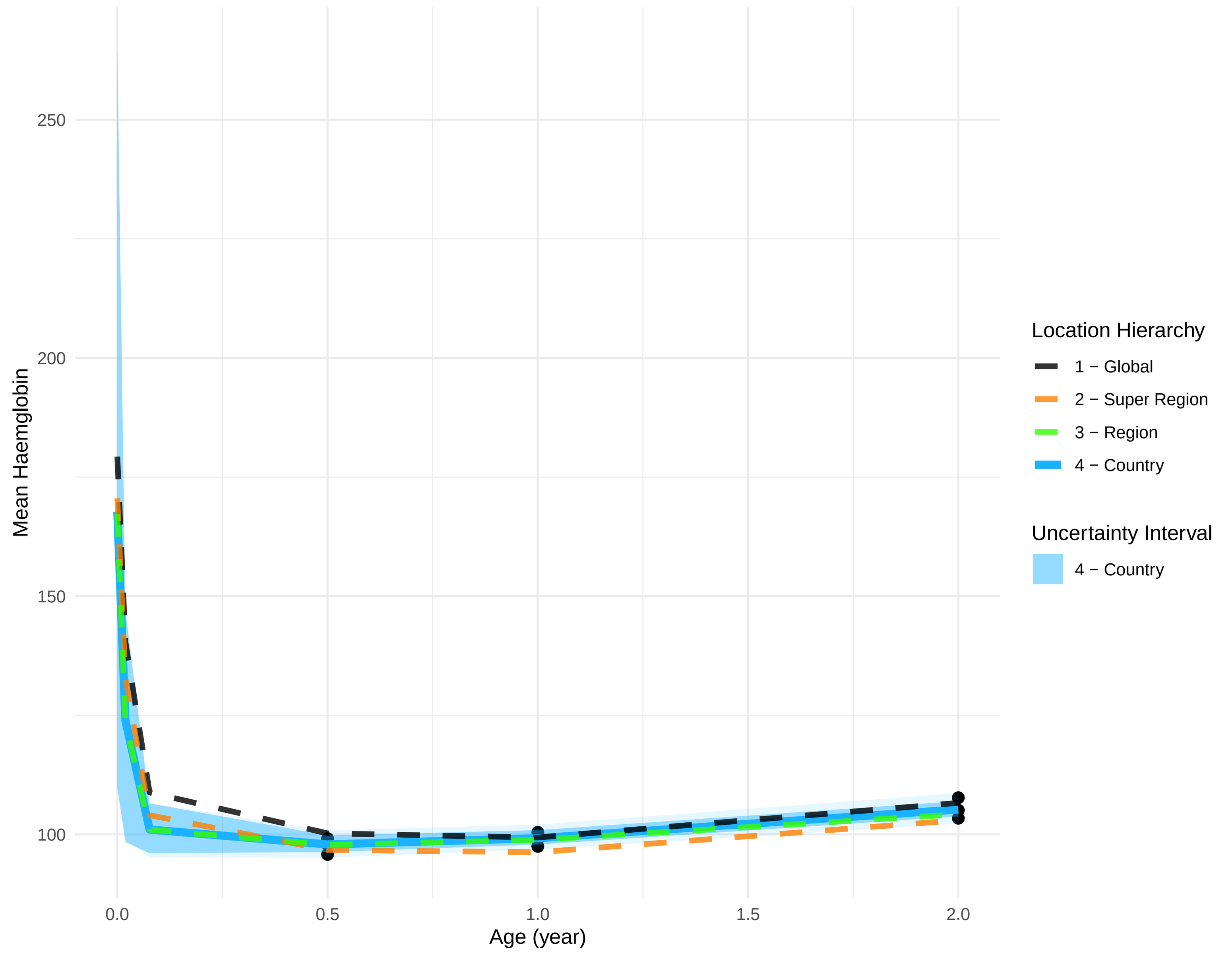

MR−BRT Age/Sex Fits − Non−pregnant Females <2 Years Old − Mean Haemglobin − Democratic Republic of the Congo
WHO−2024 Adjustment Method

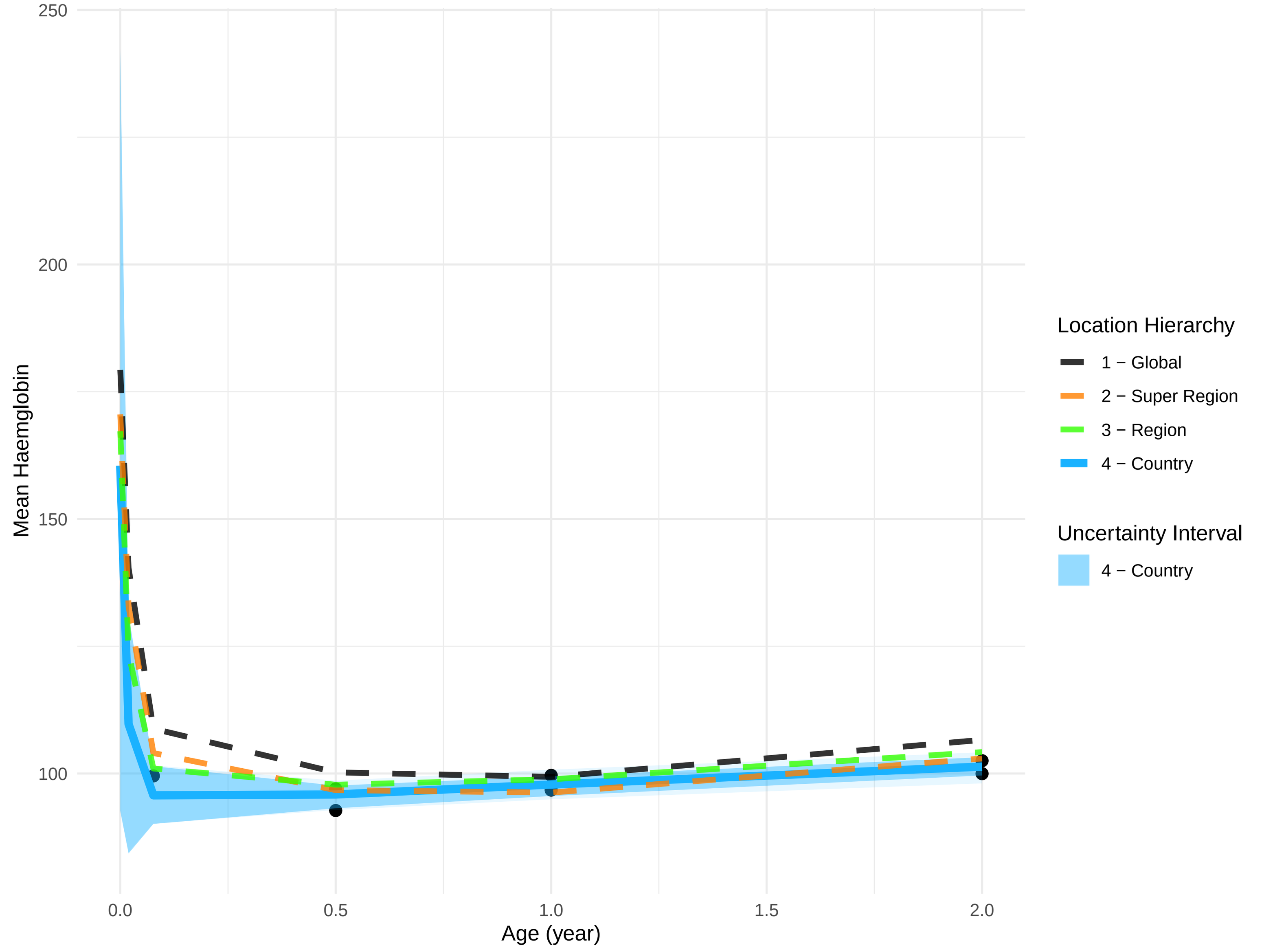

MR−BRT Age/Sex Fits − Non−pregnant Females <2 Years Old − Mean Haemglobin − Gabon
WHO−2024 Adjustment Method

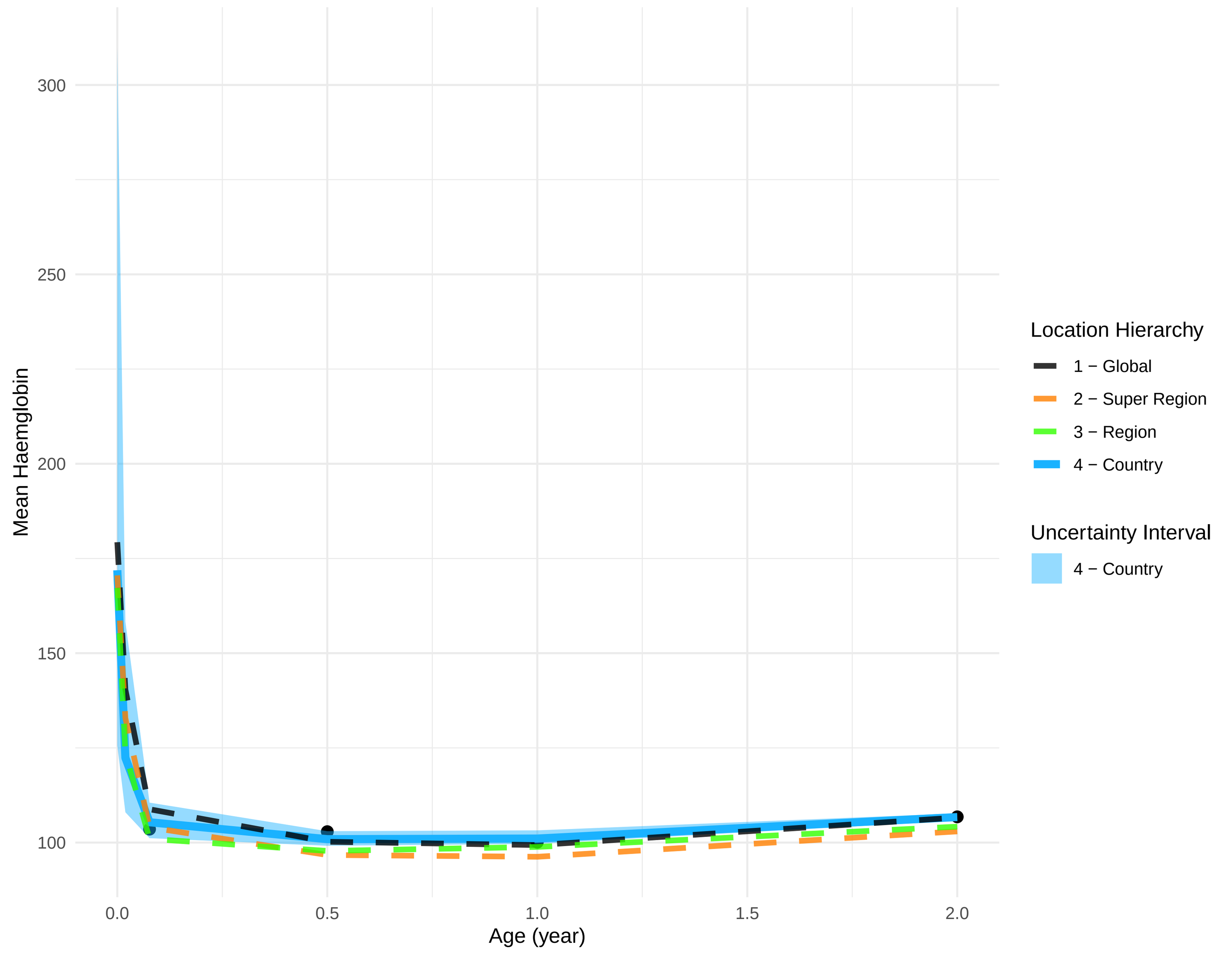

MR−BRT Age/Sex Fits − Non−pregnant Females <2 Years Old − Mean Haemglobin − Eastern Sub−Saharan Africa
WHO−2024 Adjustment Method

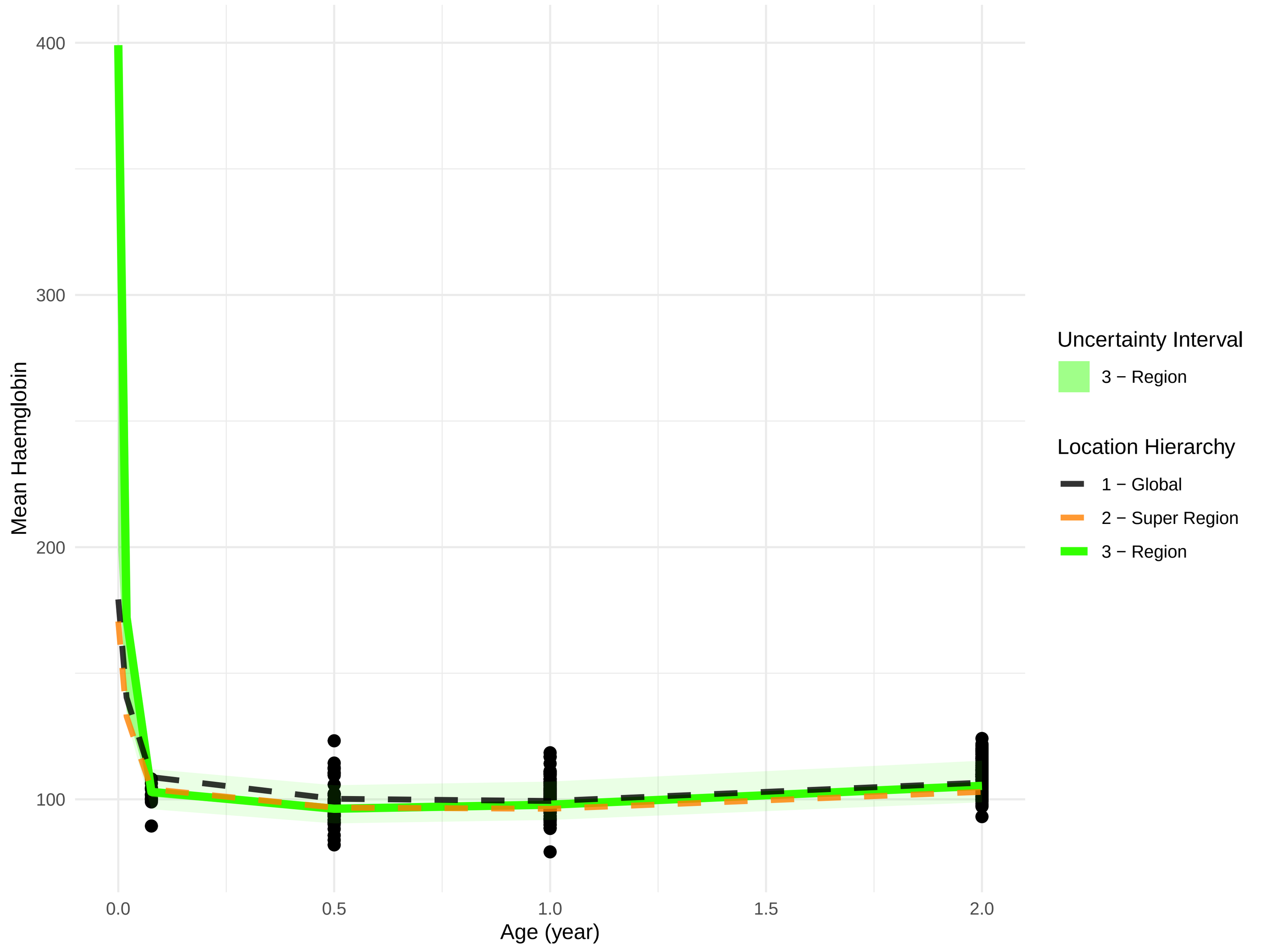

MR−BRT Age/Sex Fits − Non−pregnant Females <2 Years Old − Mean Haemglobin − Burundi
WHO−2024 Adjustment Method

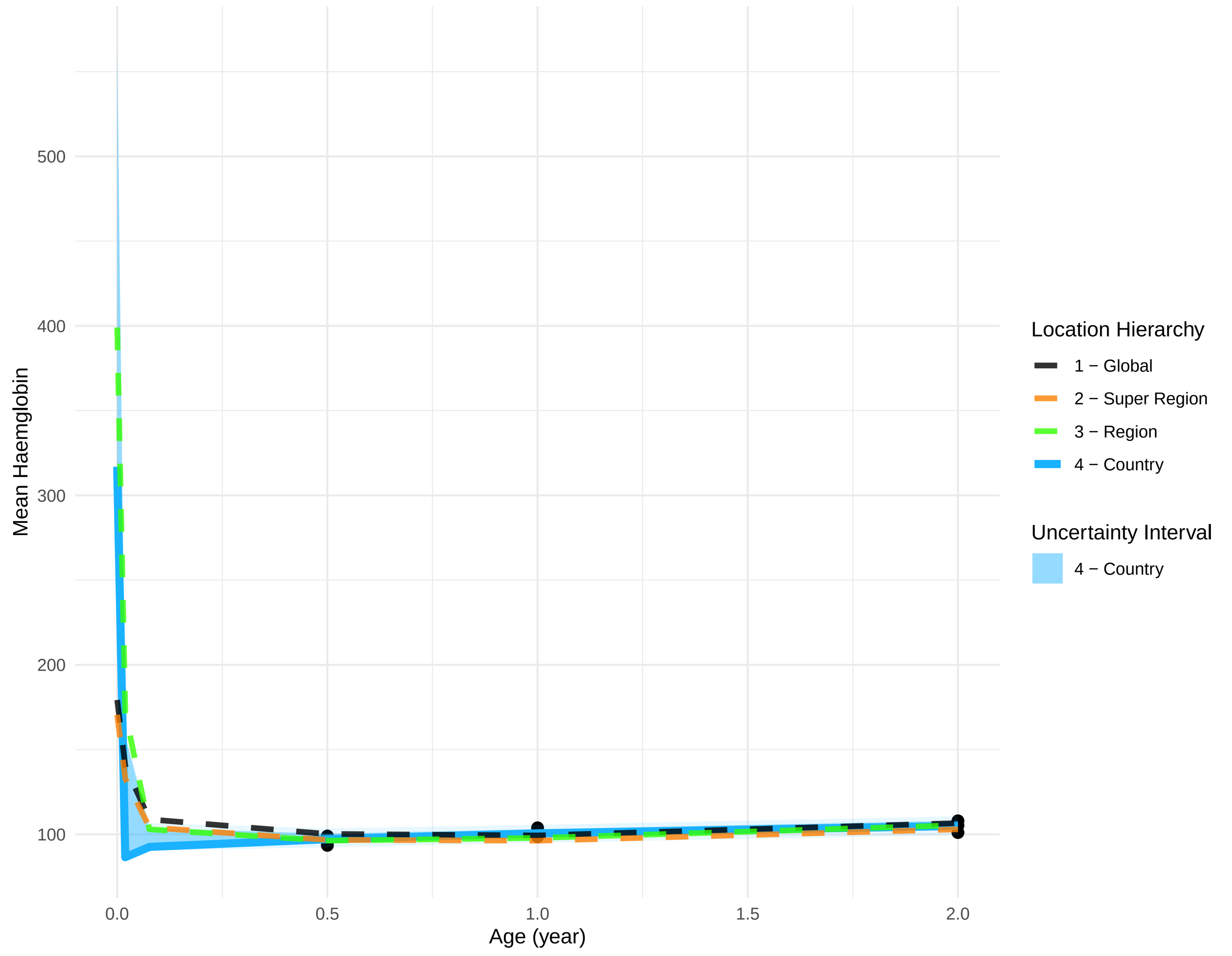

MR−BRT Age/Sex Fits − Non−pregnant Females <2 Years Old − Mean Haemglobin − Ethiopia
WHO−2024 Adjustment Method

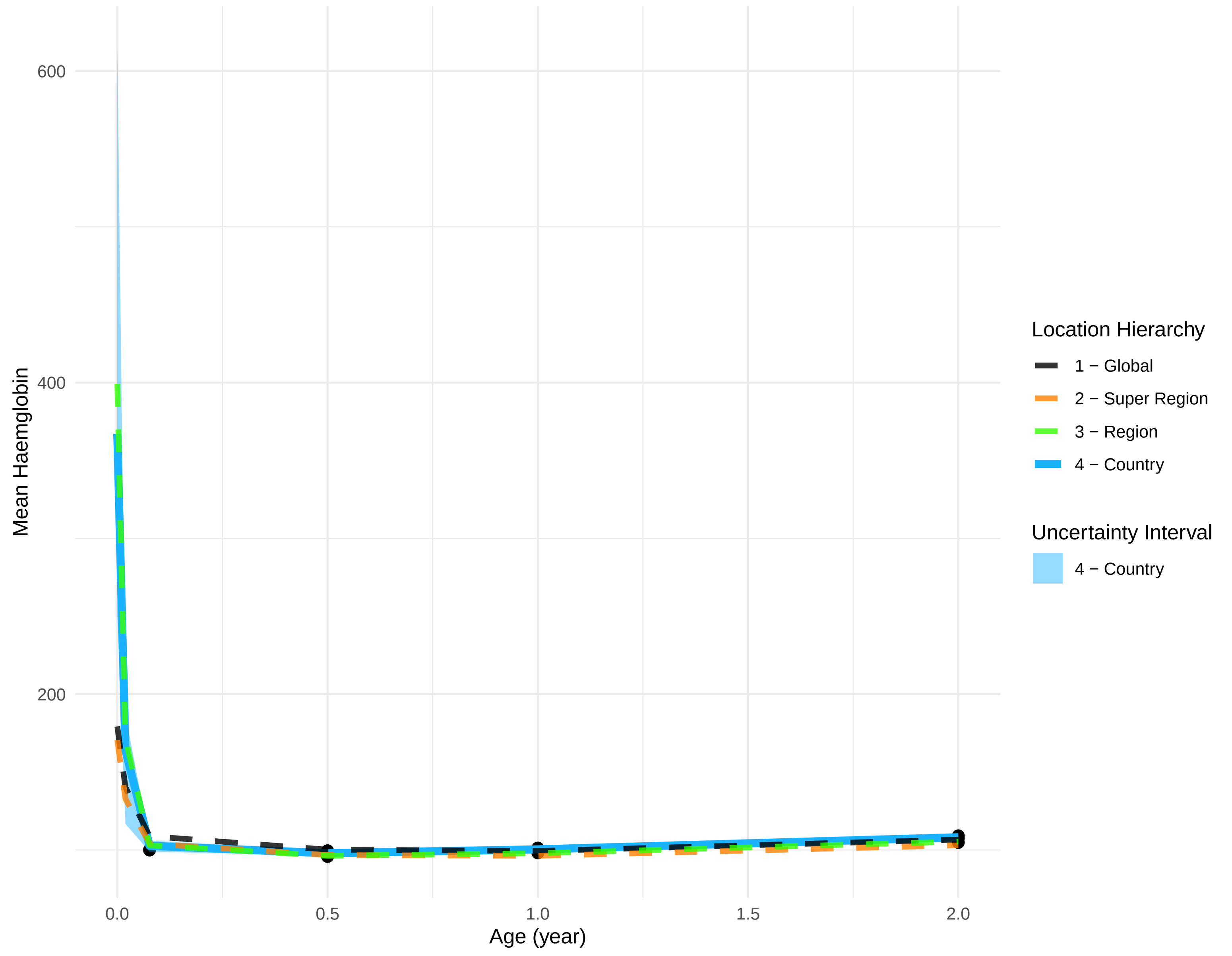

MR−BRT Age/Sex Fits − Non−pregnant Females <2 Years Old − Mean Haemglobin − Kenya
WHO−2024 Adjustment Method

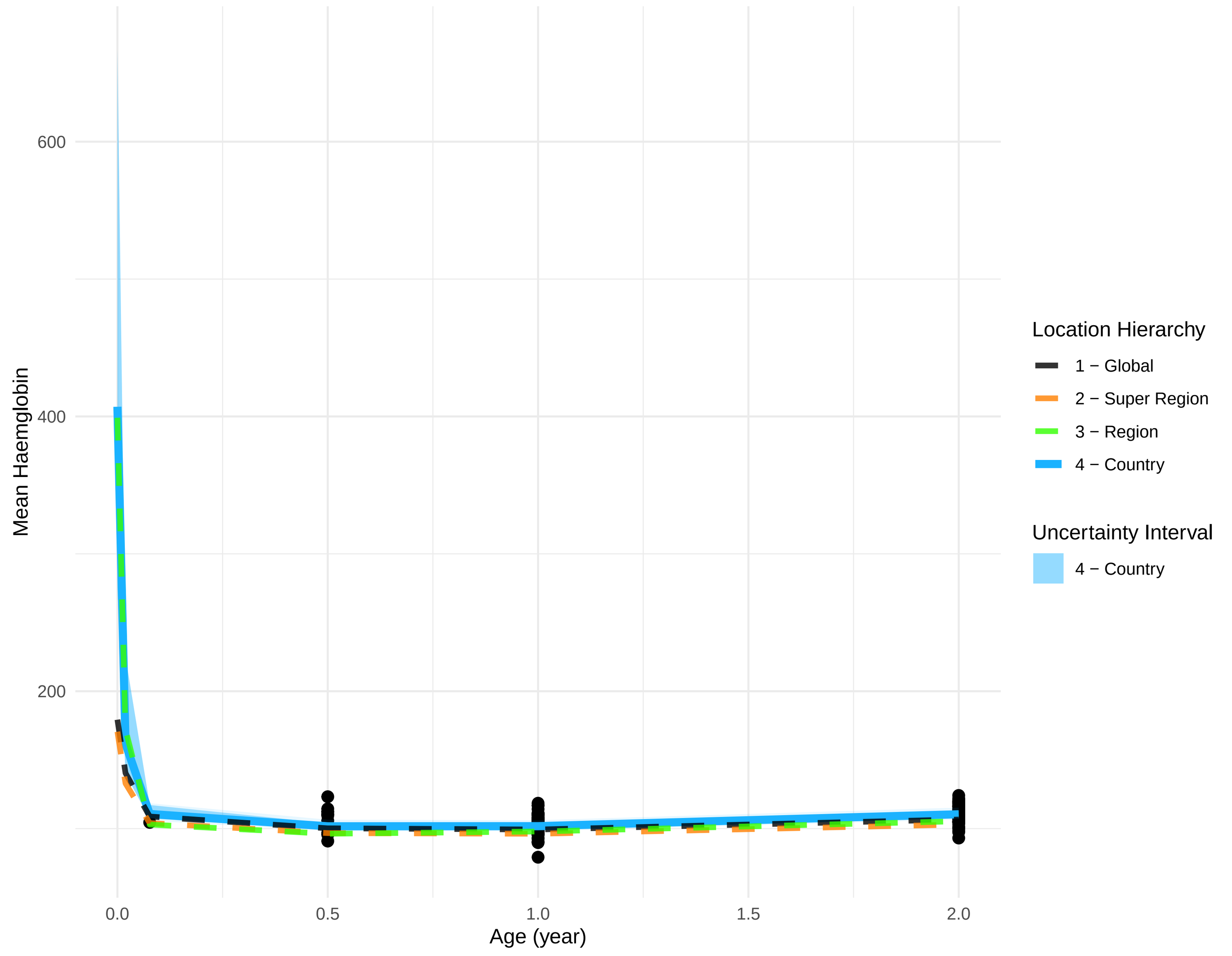

MR−BRT Age/Sex Fits − Non−pregnant Females <2 Years Old − Mean Haemglobin − Baringo
WHO−2024 Adjustment Method

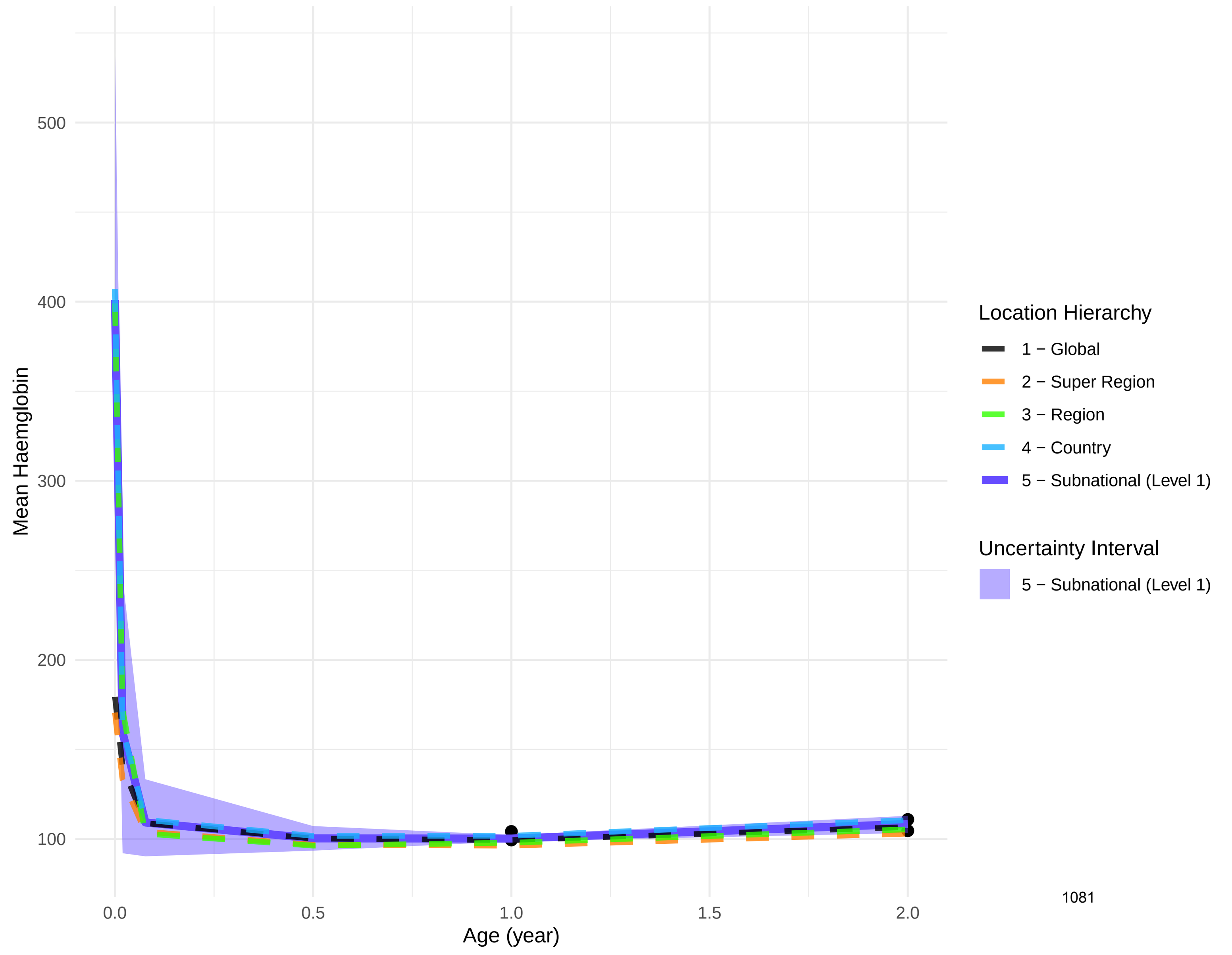

MR−BRT Age/Sex Fits − Non−pregnant Females <2 Years Old − Mean Haemglobin − Bomet
WHO−2024 Adjustment Method

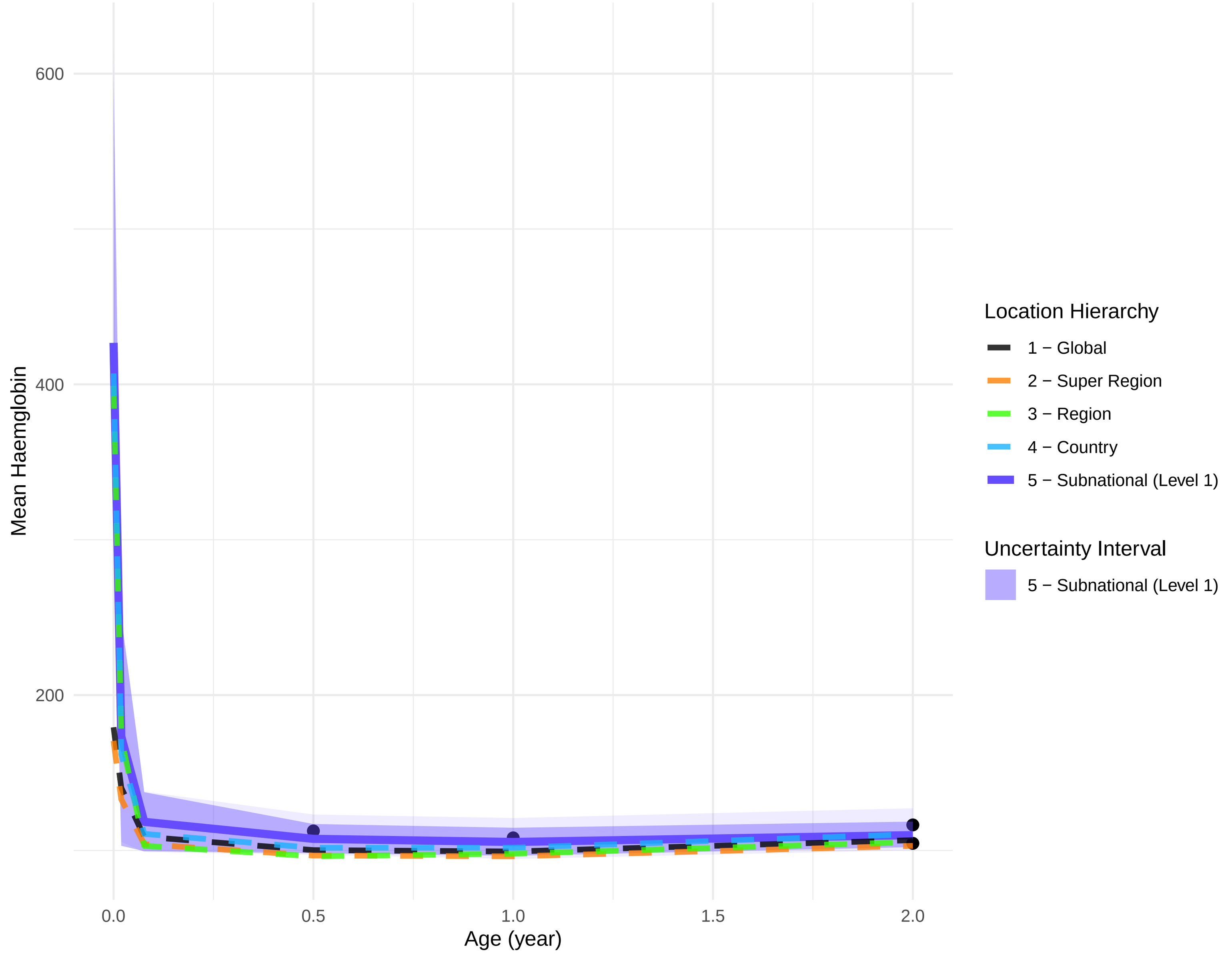

MR−BRT Age/Sex Fits − Non−pregnant Females <2 Years Old − Mean Haemglobin − Bungoma
WHO−2024 Adjustment Method

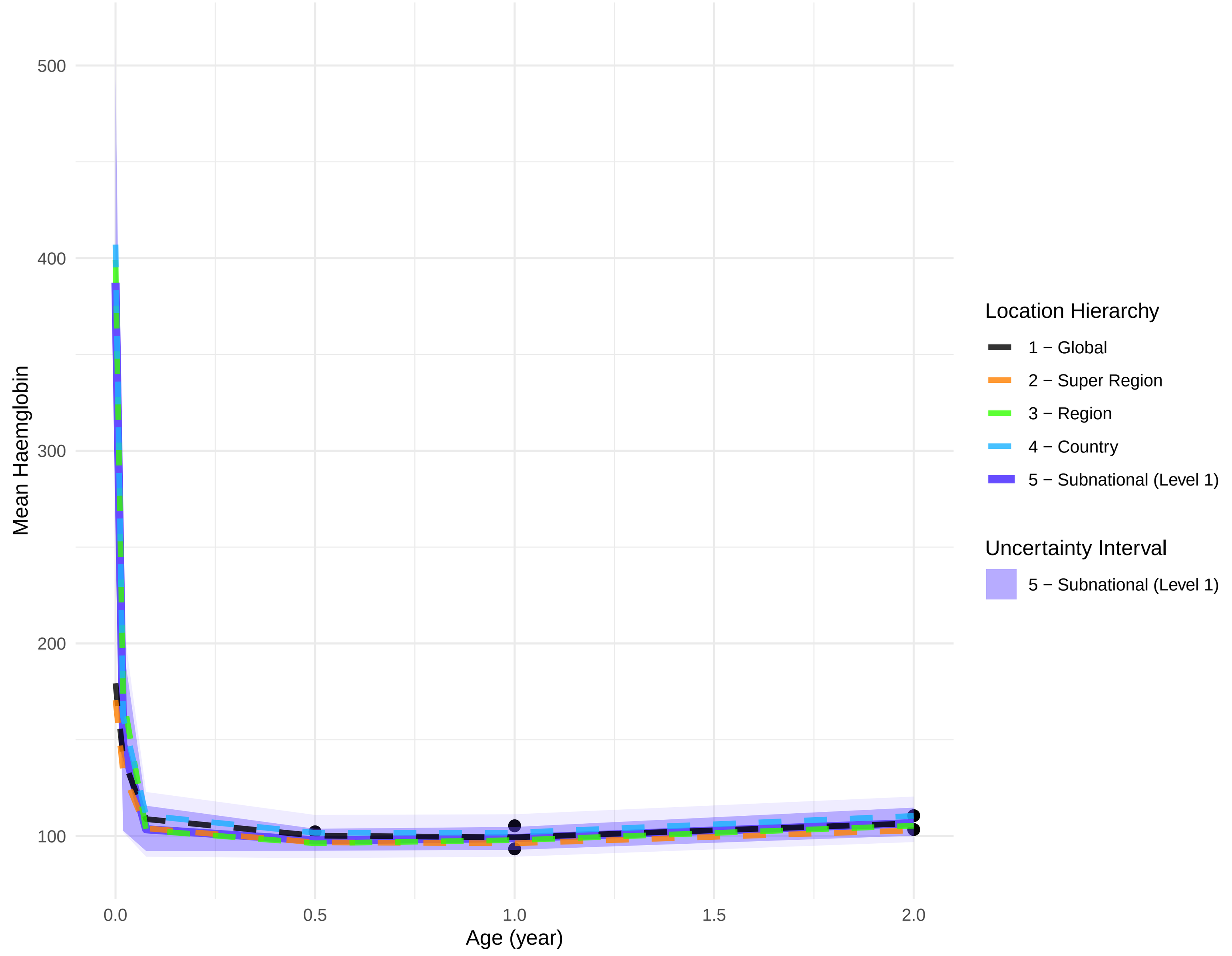

MR−BRT Age/Sex Fits − Non−pregnant Females <2 Years Old − Mean Haemglobin − Busia
WHO−2024 Adjustment Method

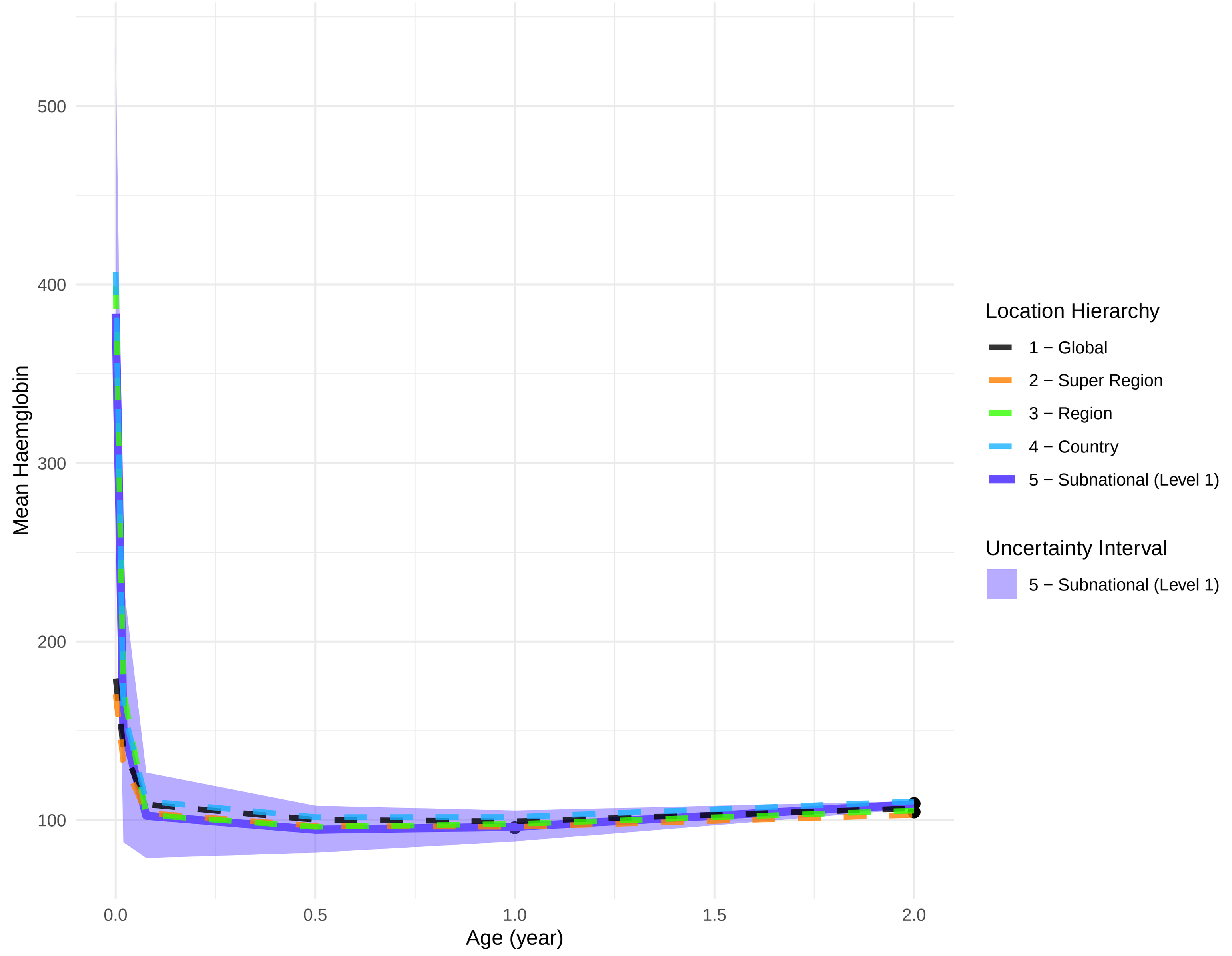

MR−BRT Age/Sex Fits − Non−pregnant Females <2 Years Old − Mean Haemglobin − Elgeyo−Marakwet
WHO−2024 Adjustment Method

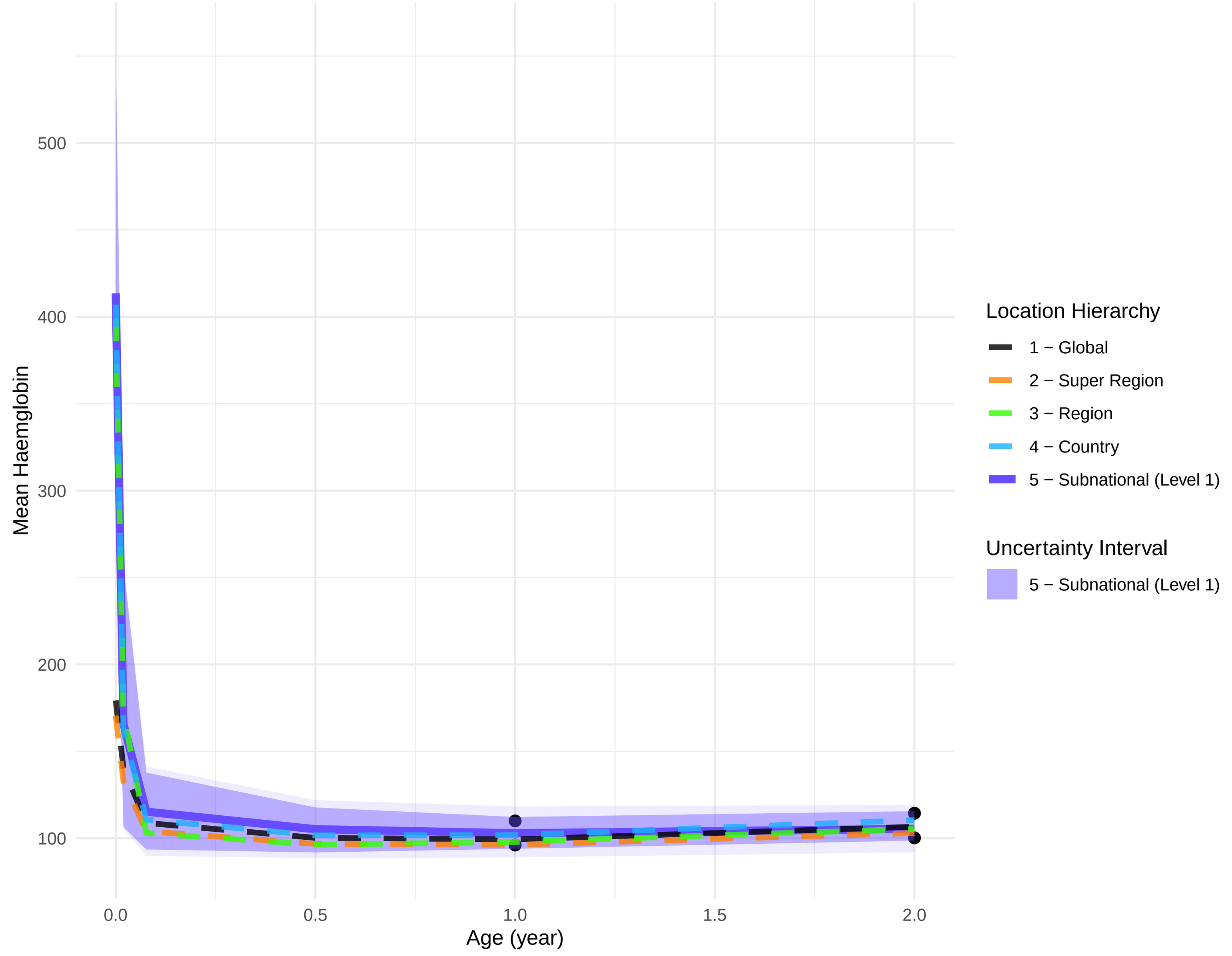

MR−BRT Age/Sex Fits − Non−pregnant Females <2 Years Old − Mean Haemglobin − Embu
WHO−2024 Adjustment Method

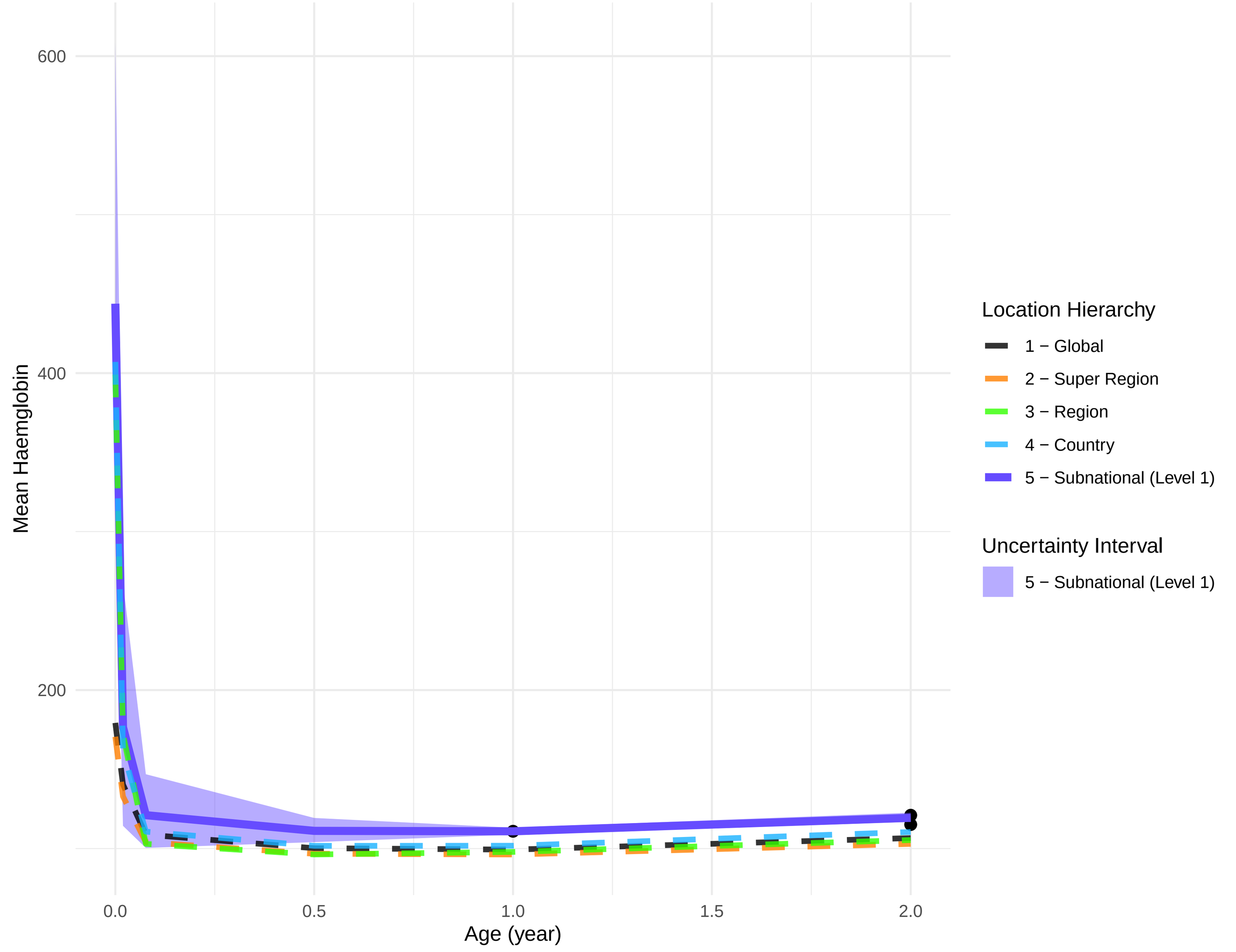

MR−BRT Age/Sex Fits − Non−pregnant Females <2 Years Old − Mean Haemglobin − Garissa
WHO−2024 Adjustment Method

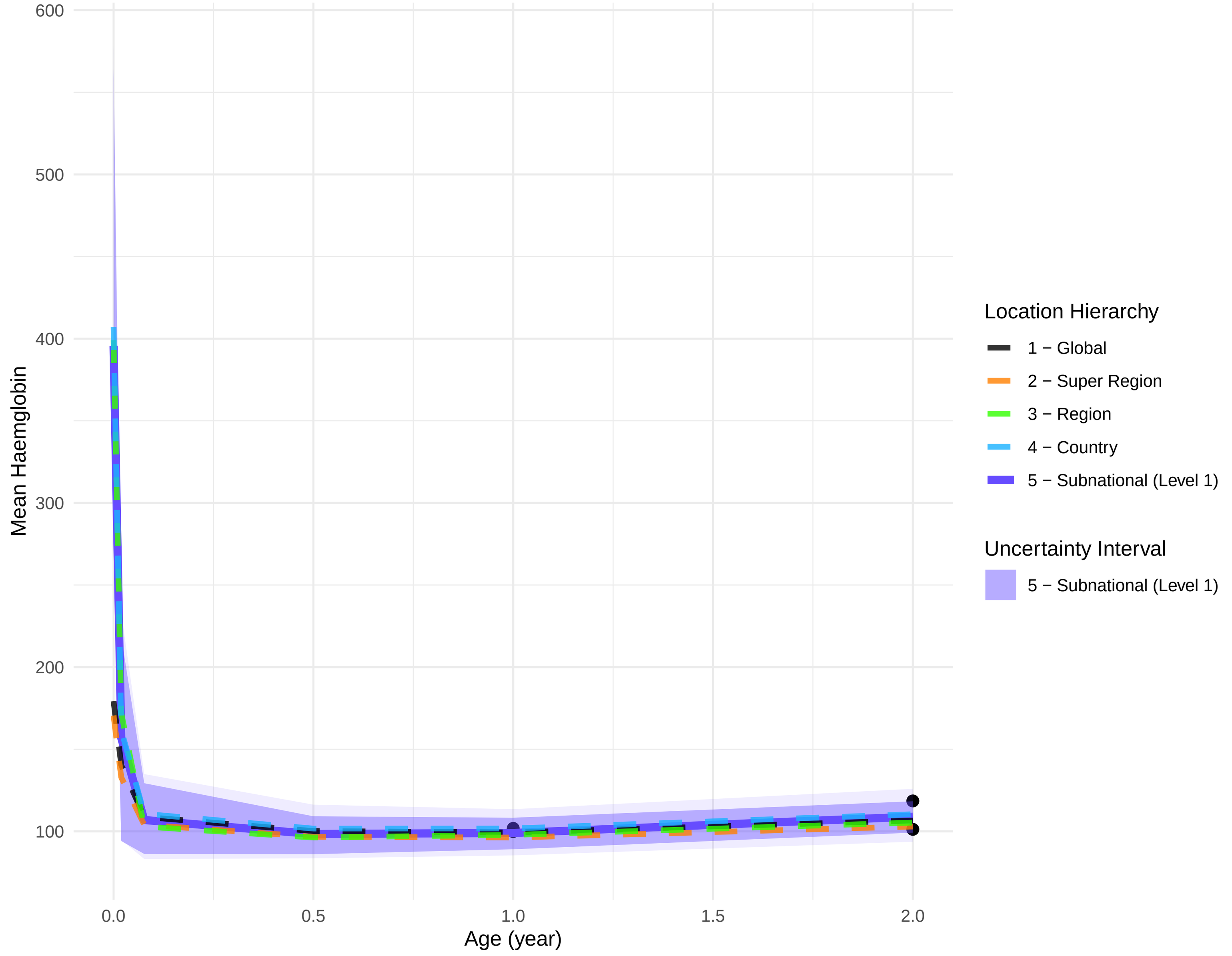

MR−BRT Age/Sex Fits − Non−pregnant Females <2 Years Old − Mean Haemglobin − Homa Bay
WHO−2024 Adjustment Method

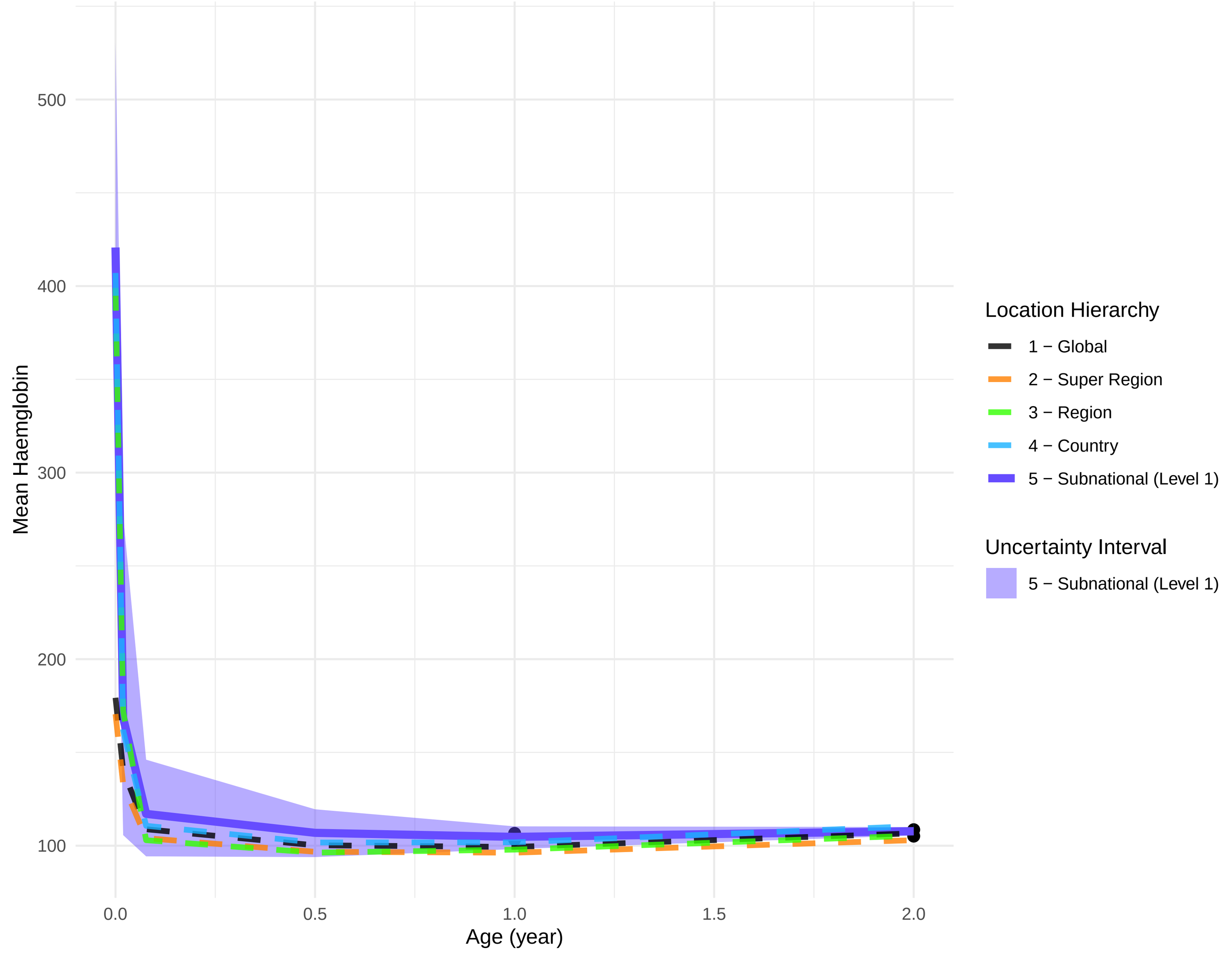

MR−BRT Age/Sex Fits − Non−pregnant Females <2 Years Old − Mean Haemglobin − Isiolo
WHO−2024 Adjustment Method

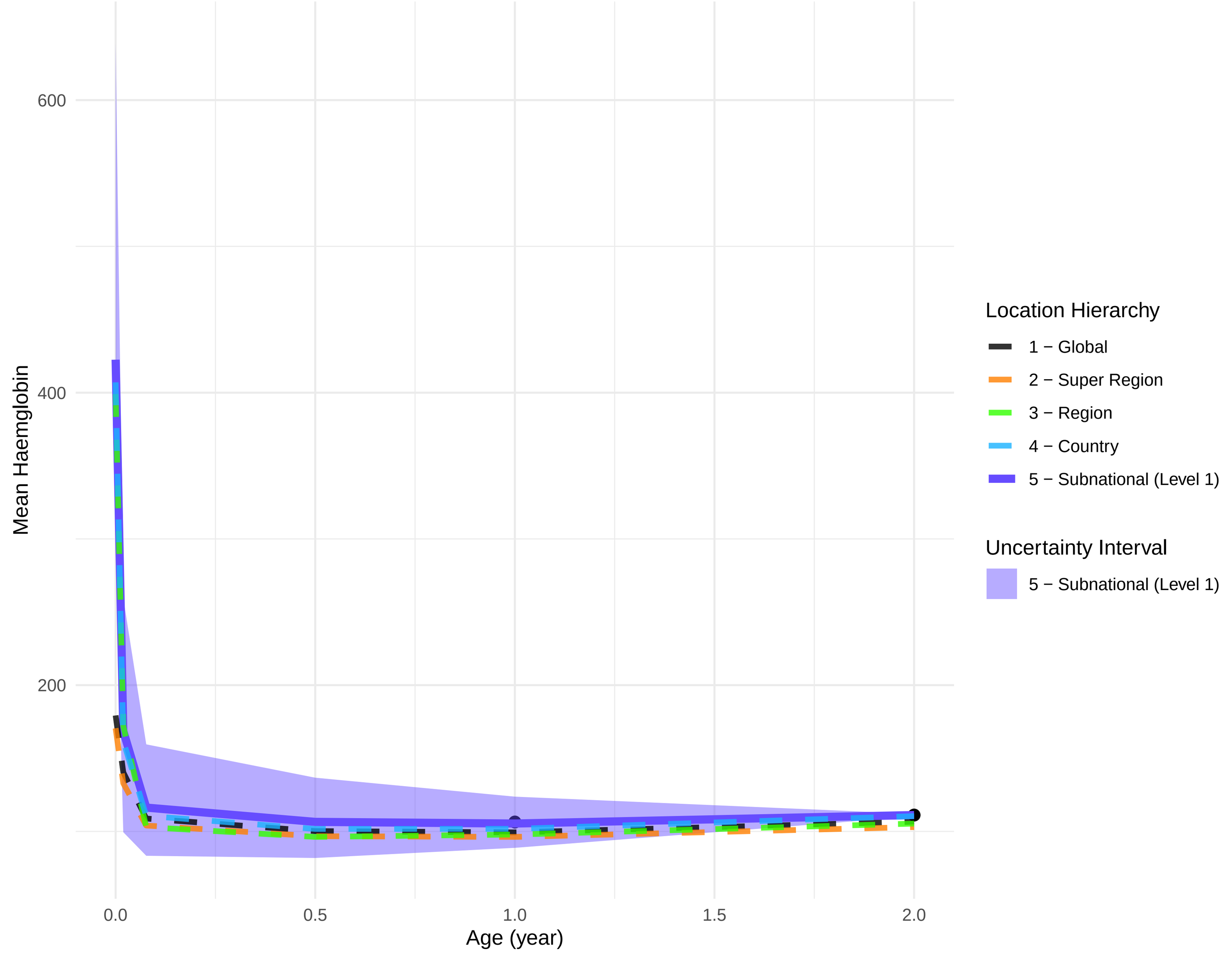

MR−BRT Age/Sex Fits − Non−pregnant Females <2 Years Old − Mean Haemglobin − Kajiado
WHO−2024 Adjustment Method

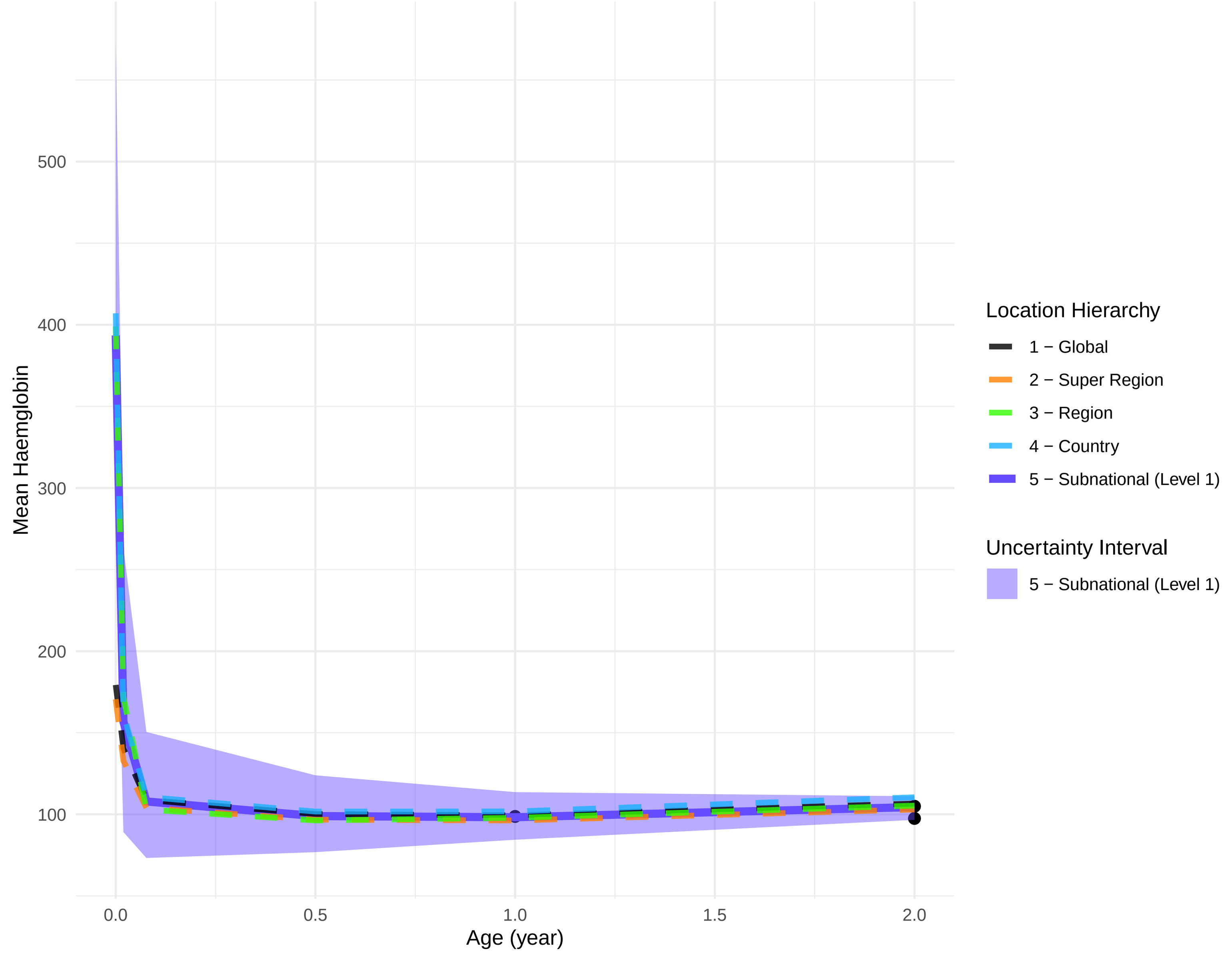

MR−BRT Age/Sex Fits − Non−pregnant Females <2 Years Old − Mean Haemglobin − Kakamega
WHO−2024 Adjustment Method

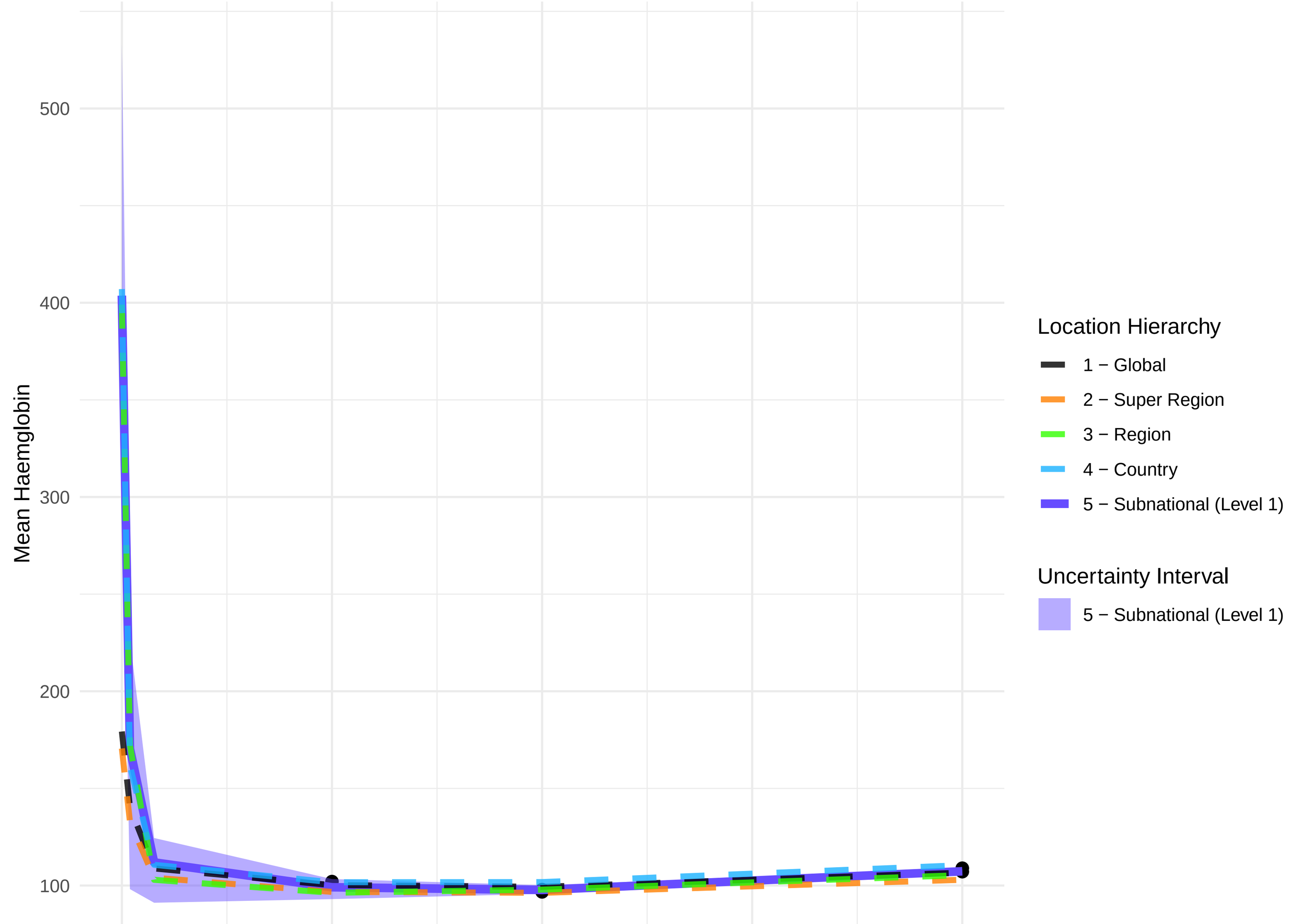

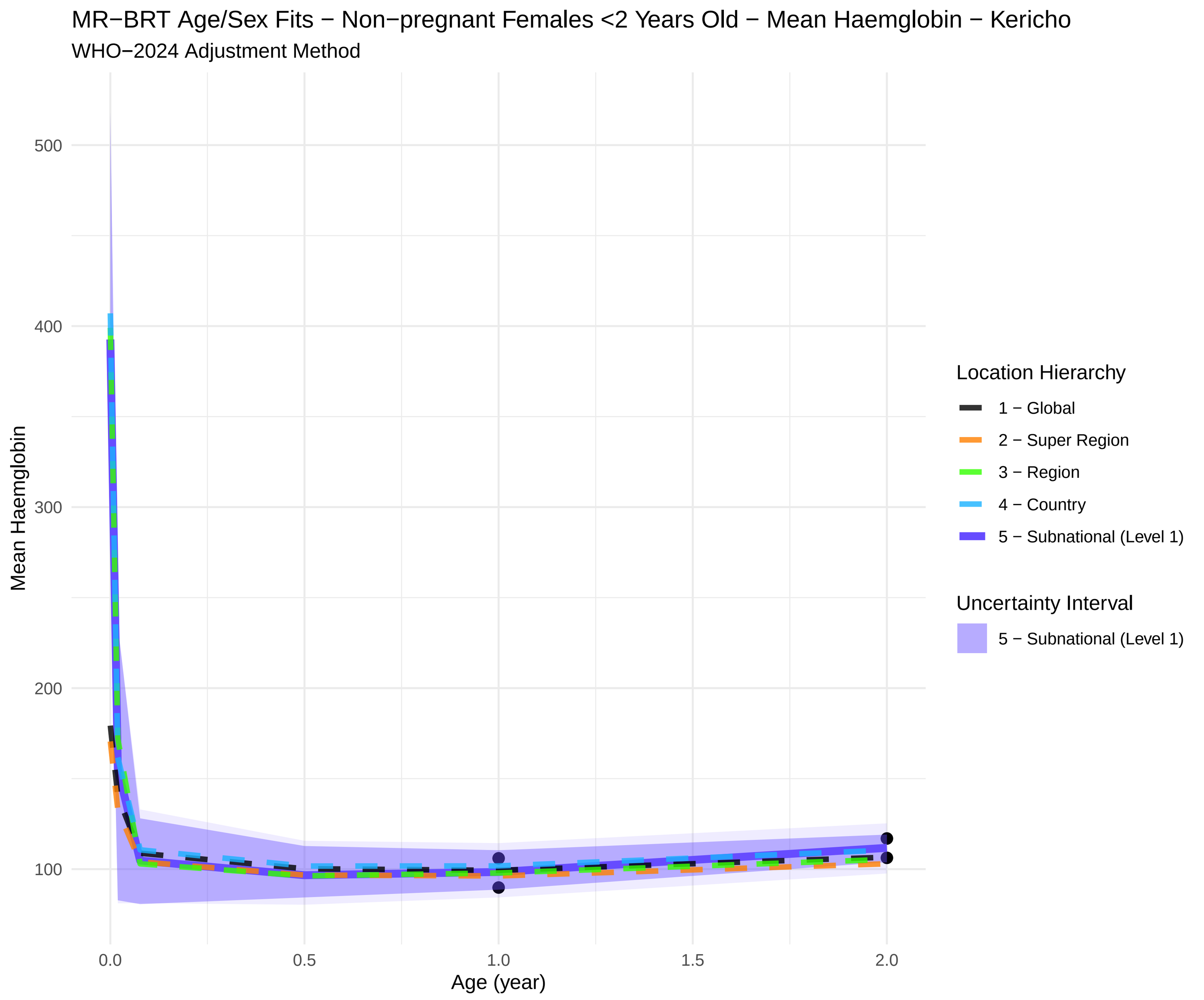
MR−BRT Age/Sex Fits − Non−pregnant Females <2 Years Old − Mean Haemglobin − Kericho
WHO−2024 Adjustment Method
Mean Haemglobin
Age (year)
Location Hierarchy
1 − Global
2 − Super Region
3 − Region
4 − Country
5 − Subnational (Level 1)
Uncertainty Interval
5 − Subnational (Level 1)

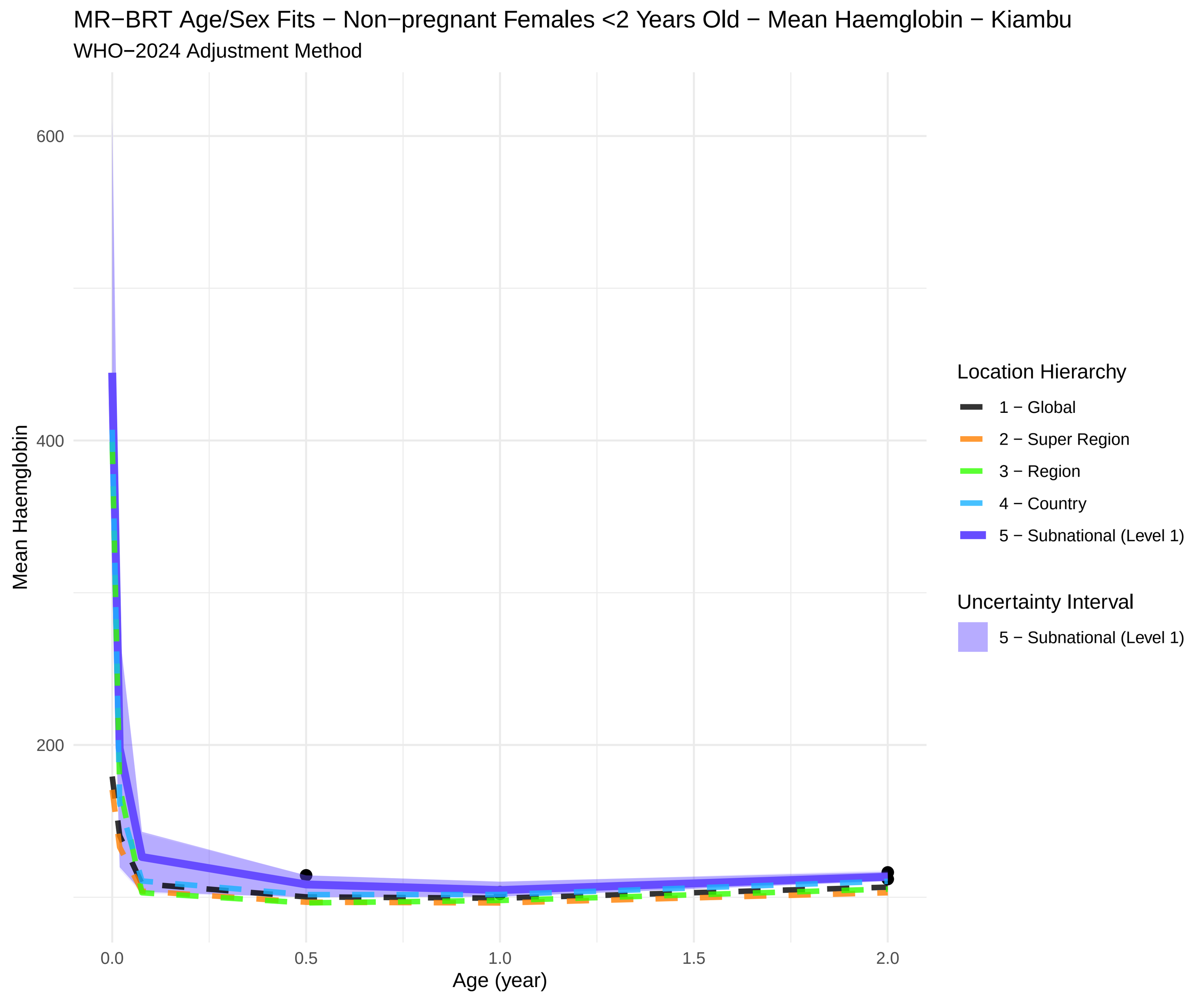
MR−BRT Age/Sex Fits − Non−pregnant Females <2 Years Old − Mean Haemglobin − Kiambu
WHO−2024 Adjustment Method
Mean Haemglobin
Age (year)
Location Hierarchy
1 − Global
2 − Super Region
3 − Region
4 − Country
5 − Subnational (Level 1)
Uncertainty Interval
5 − Subnational (Level 1)

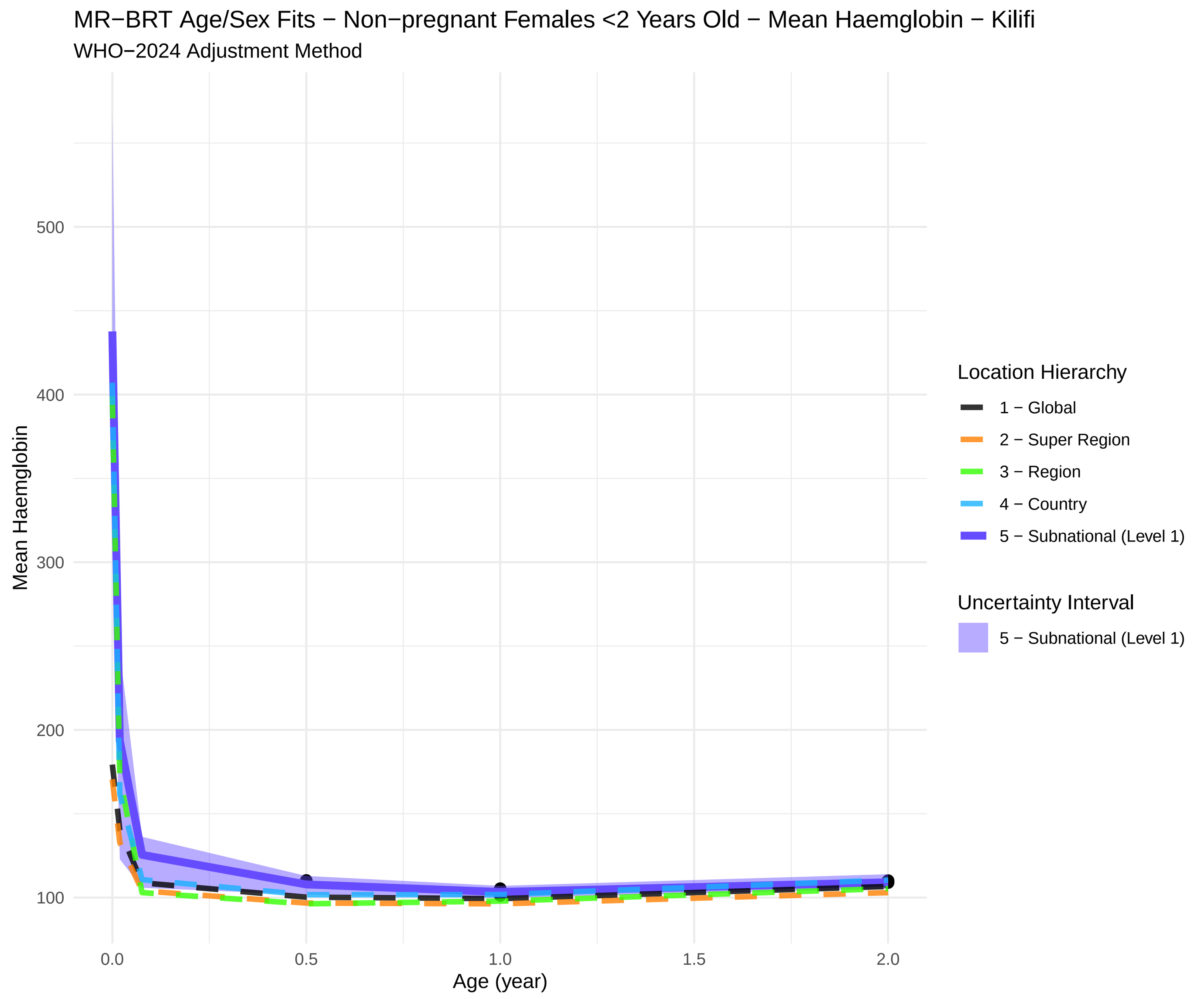
MR−BRT Age/Sex Fits − Non−pregnant Females <2 Years Old − Mean Haemglobin − Kilifi
WHO−2024 Adjustment Method
Mean Haemglobin
Age (year)
Location Hierarchy
1 − Global
2 − Super Region
3 − Region
4 − Country
5 − Subnational (Level 1)
Uncertainty Interval
5 − Subnational (Level 1)

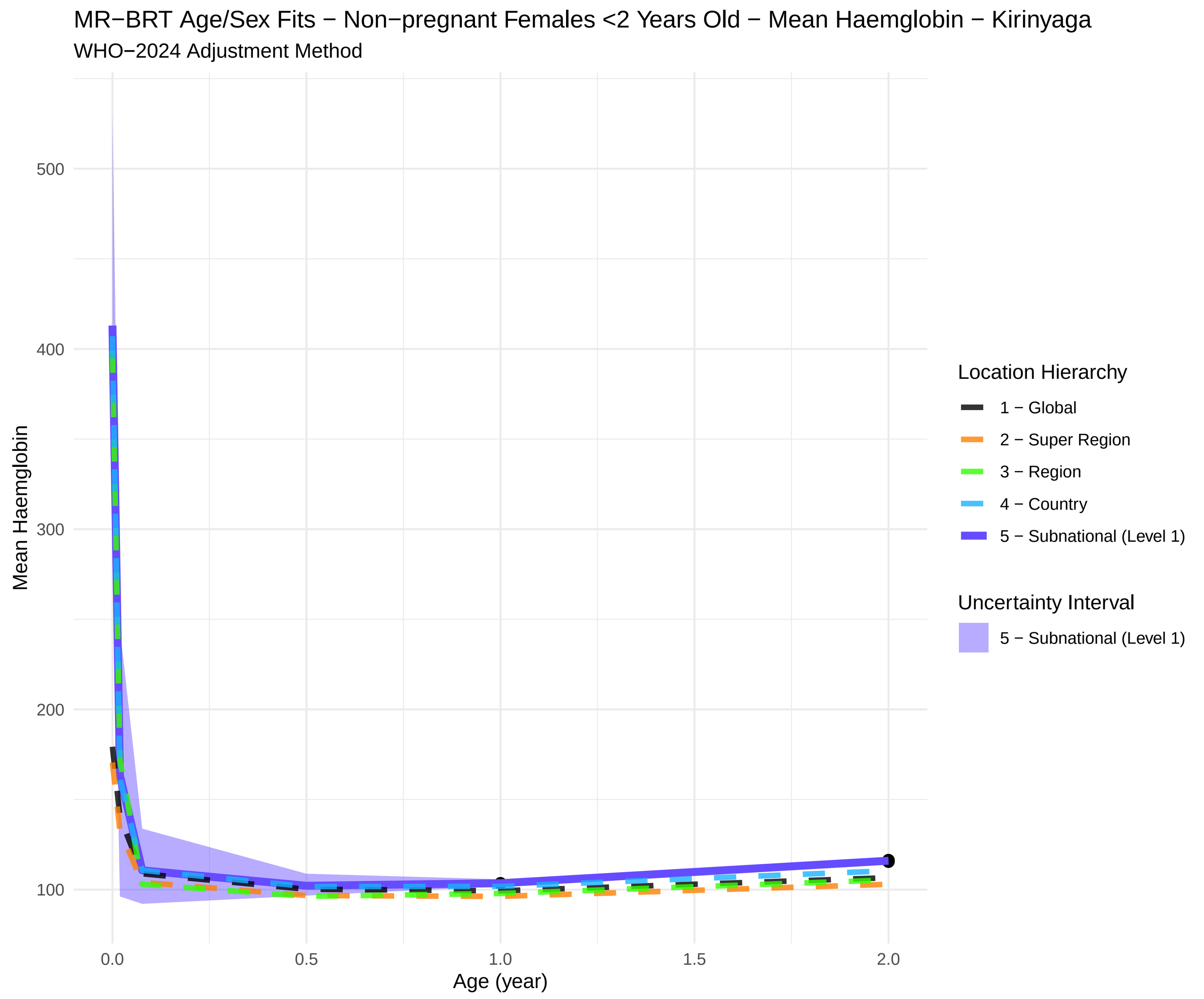
MR−BRT Age/Sex Fits − Non−pregnant Females <2 Years Old − Mean Haemglobin − Kirinyaga
WHO−2024 Adjustment Method
Mean Haemglobin
Age (year)
Location Hierarchy
1 − Global
2 − Super Region
3 − Region
4 − Country
5 − Subnational (Level 1)
Uncertainty Interval
5 − Subnational (Level 1)

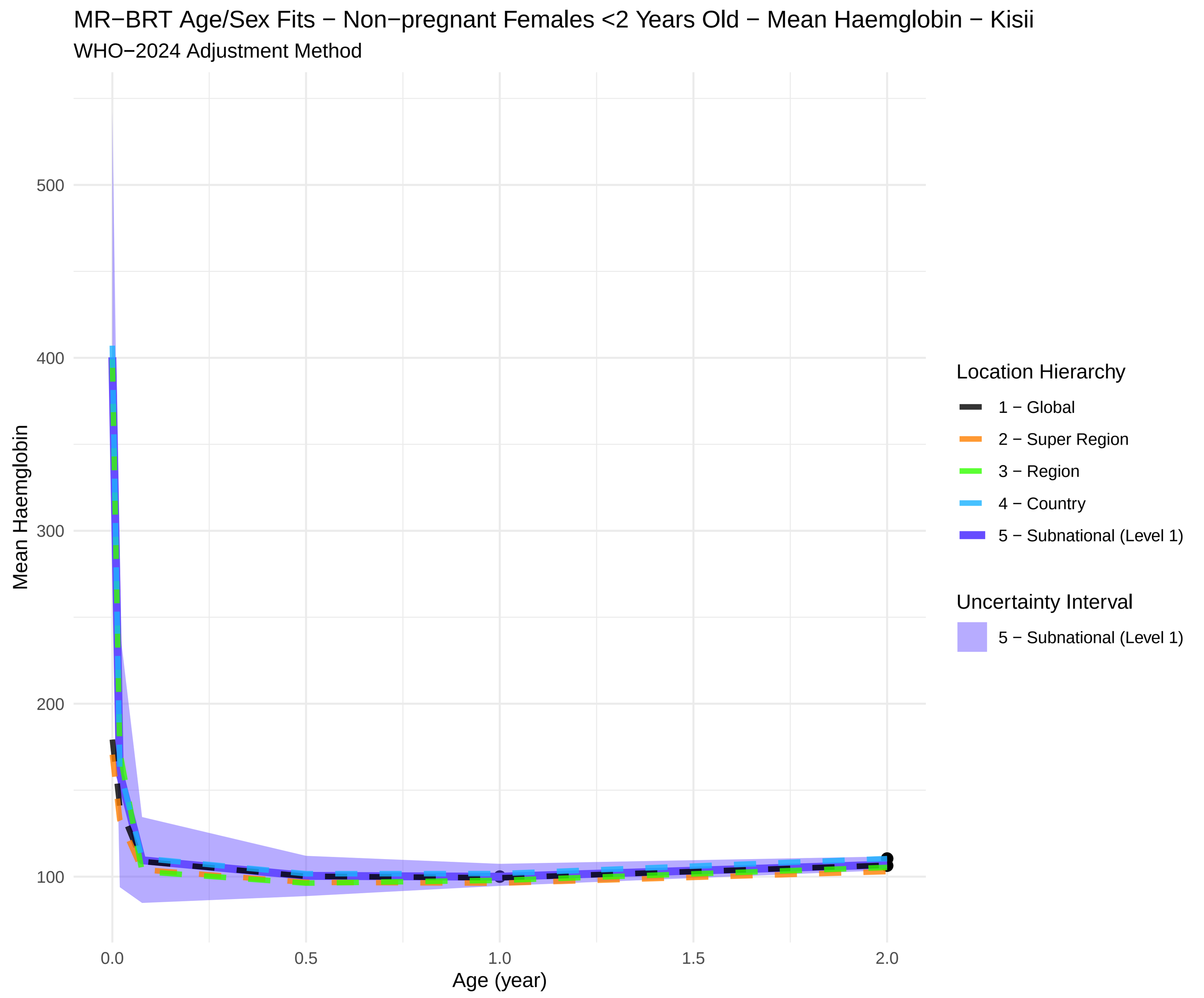
MR−BRT Age/Sex Fits − Non−pregnant Females <2 Years Old − Mean Haemglobin − Kisii
WHO−2024 Adjustment Method
Mean Haemglobin
Age (year)
Location Hierarchy
1 − Global
2 − Super Region
3 − Region
4 − Country
5 − Subnational (Level 1)
Uncertainty Interval
5 − Subnational (Level 1)

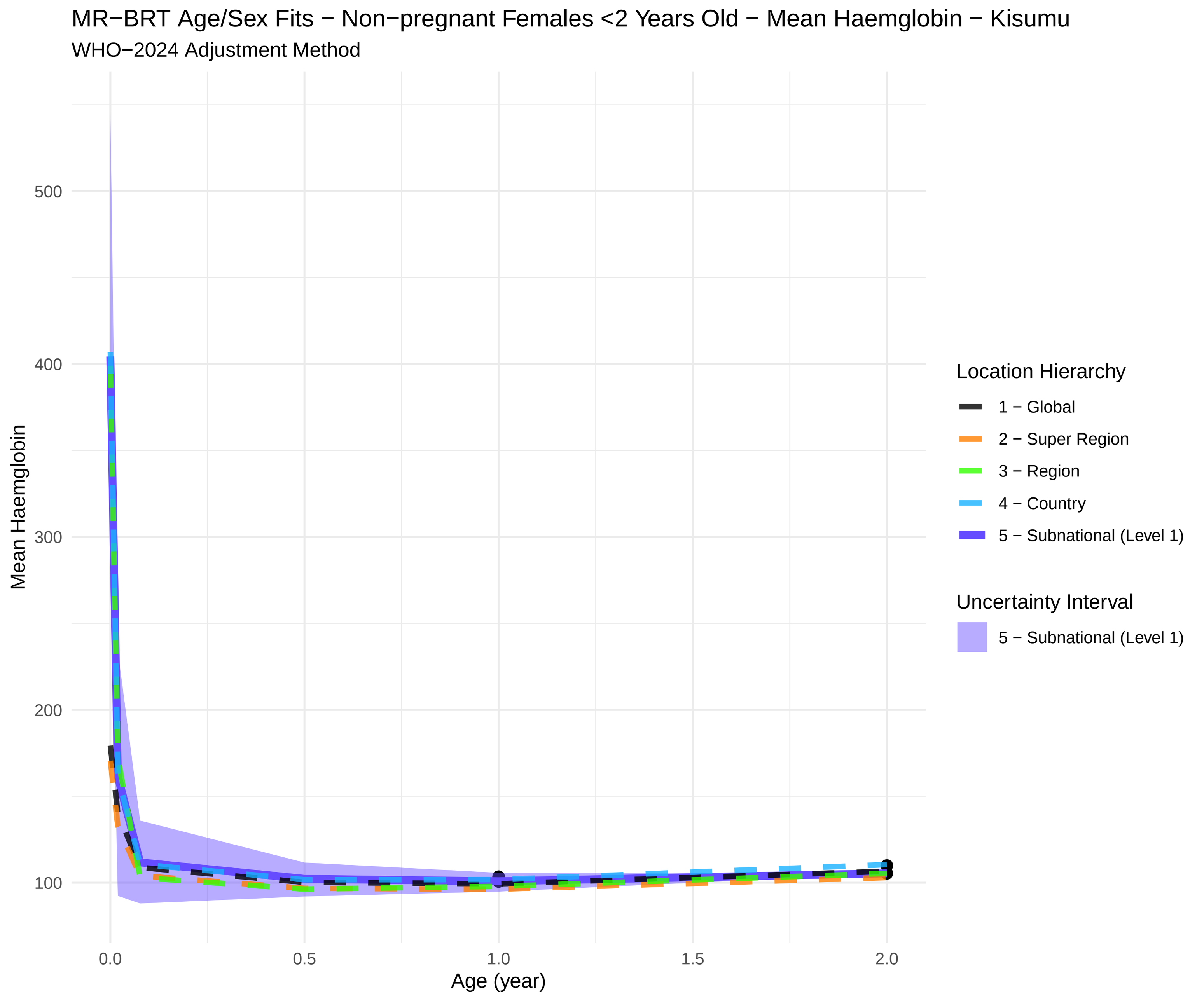
MR−BRT Age/Sex Fits − Non−pregnant Females <2 Years Old − Mean Haemglobin − Kisumu
WHO−2024 Adjustment Method
Mean Haemglobin
Age (year)
Location Hierarchy
1 − Global
2 − Super Region
3 − Region
4 − Country
5 − Subnational (Level 1)
Uncertainty Interval
5 − Subnational (Level 1)

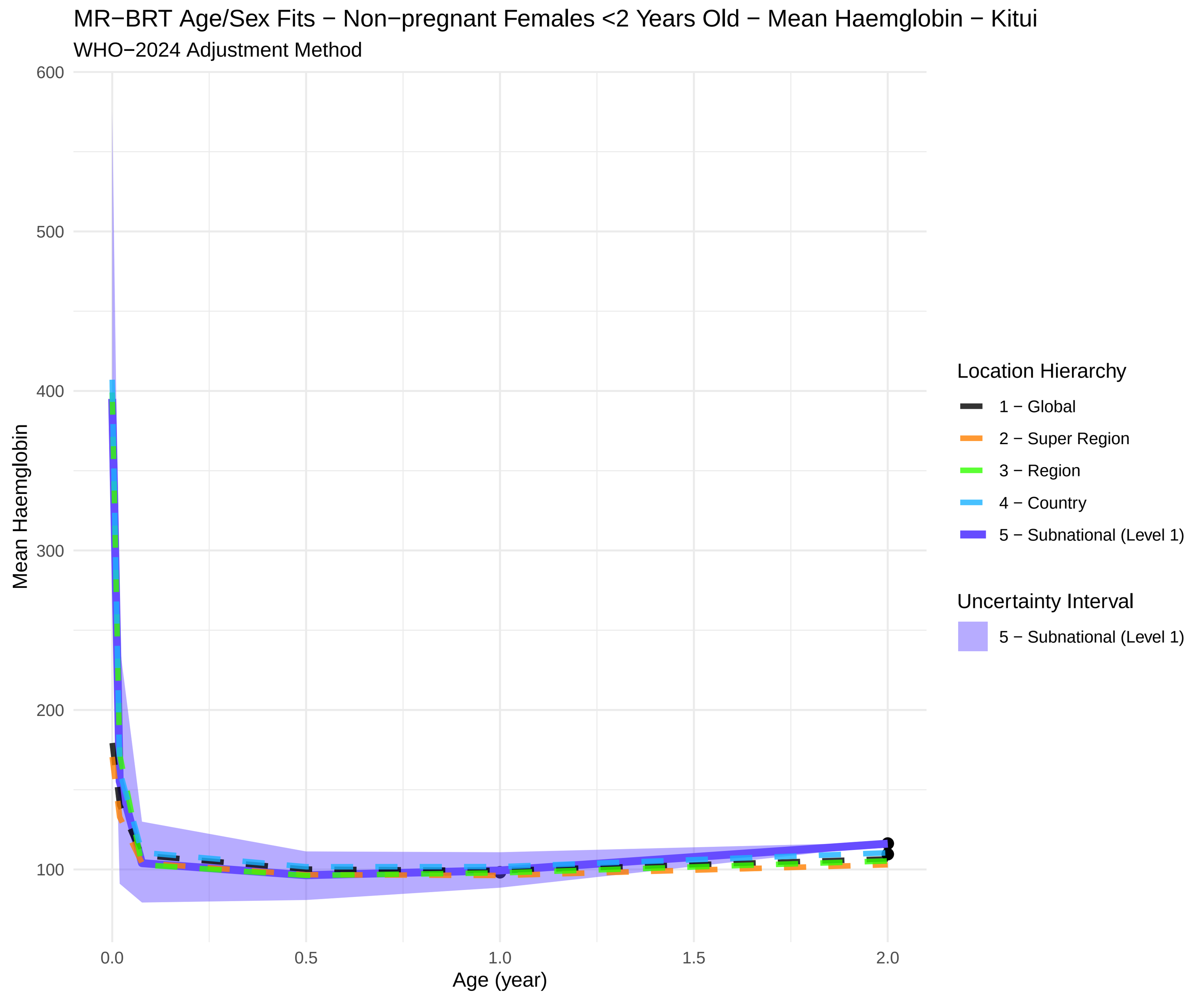
MR−BRT Age/Sex Fits − Non−pregnant Females <2 Years Old − Mean Haemglobin − Kitui
WHO−2024 Adjustment Method
Mean Haemglobin
Age (year)
Location Hierarchy
1 − Global
2 − Super Region
3 − Region
4 − Country
5 − Subnational (Level 1)
Uncertainty Interval
5 − Subnational (Level 1)

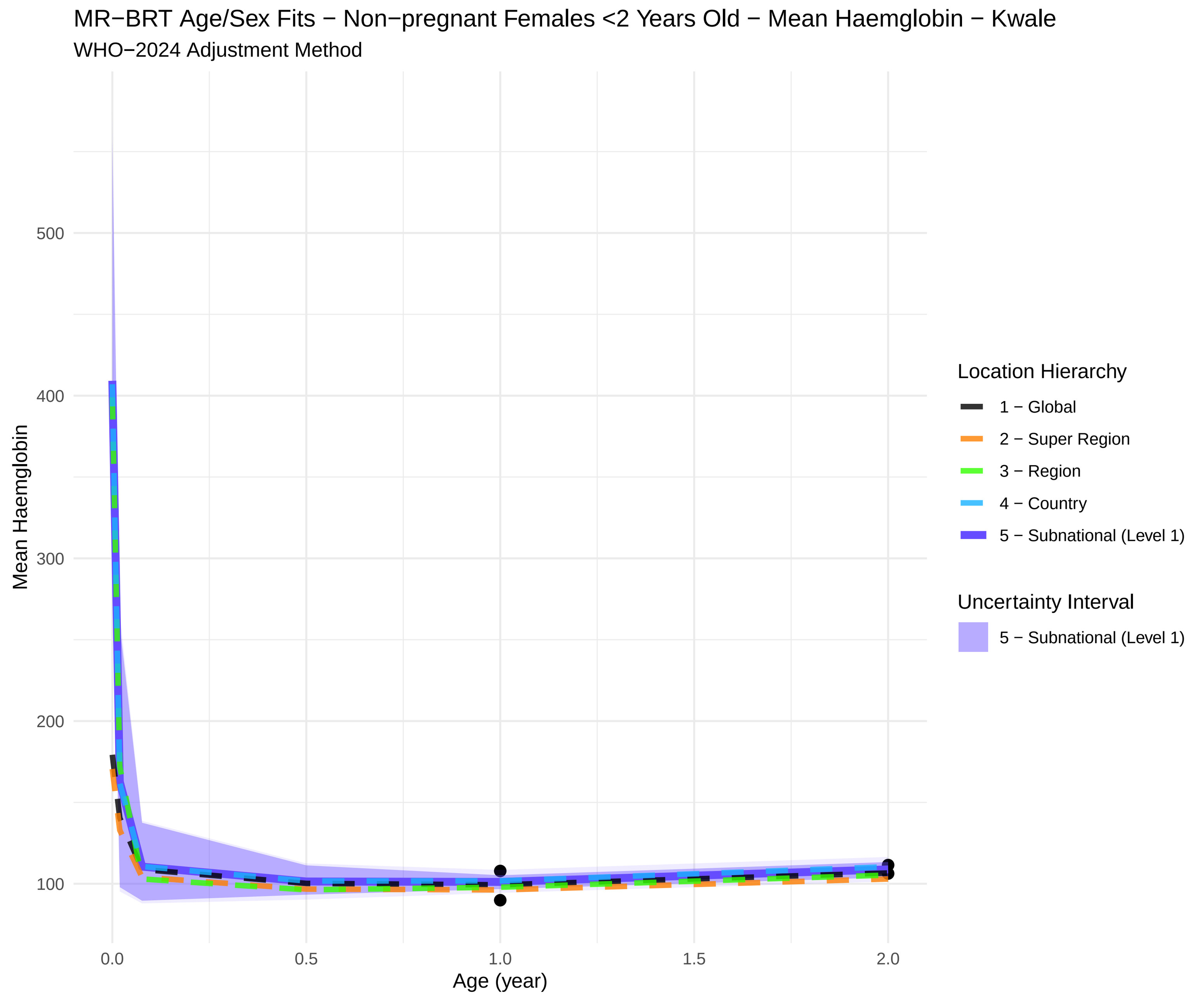
MR−BRT Age/Sex Fits − Non−pregnant Females <2 Years Old − Mean Haemglobin − Kwale
WHO−2024 Adjustment Method
Mean Haemglobin
Age (year)
Location Hierarchy
1 − Global
2 − Super Region
3 − Region
4 − Country
5 − Subnational (Level 1)
Uncertainty Interval
5 − Subnational (Level 1)

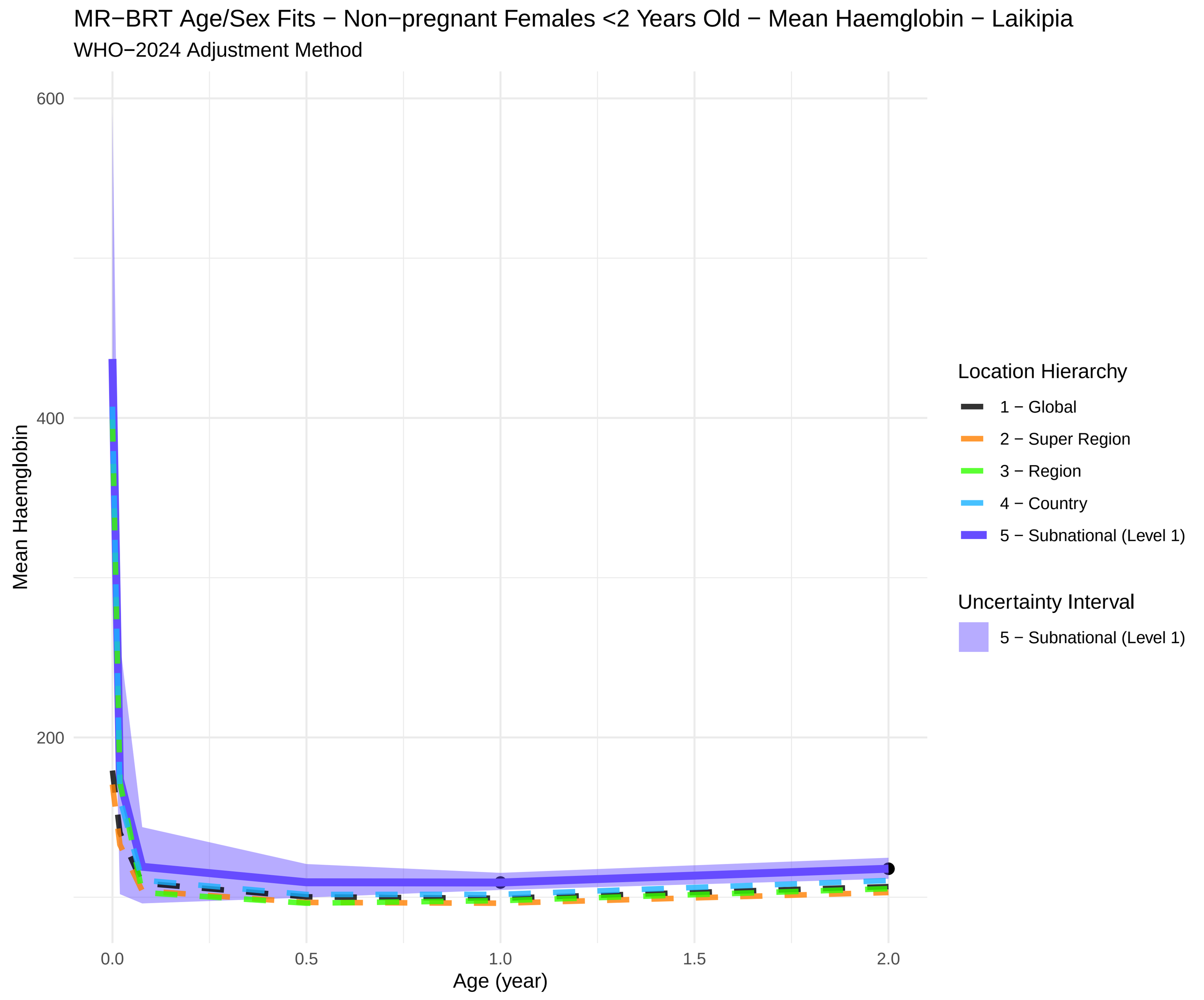
MR−BRT Age/Sex Fits − Non−pregnant Females <2 Years Old − Mean Haemglobin − Laikipia
WHO−2024 Adjustment Method
Mean Haemglobin
Age (year)
Location Hierarchy
1 − Global
2 − Super Region
3 − Region
4 − Country
5 − Subnational (Level 1)
Uncertainty Interval
5 − Subnational (Level 1)

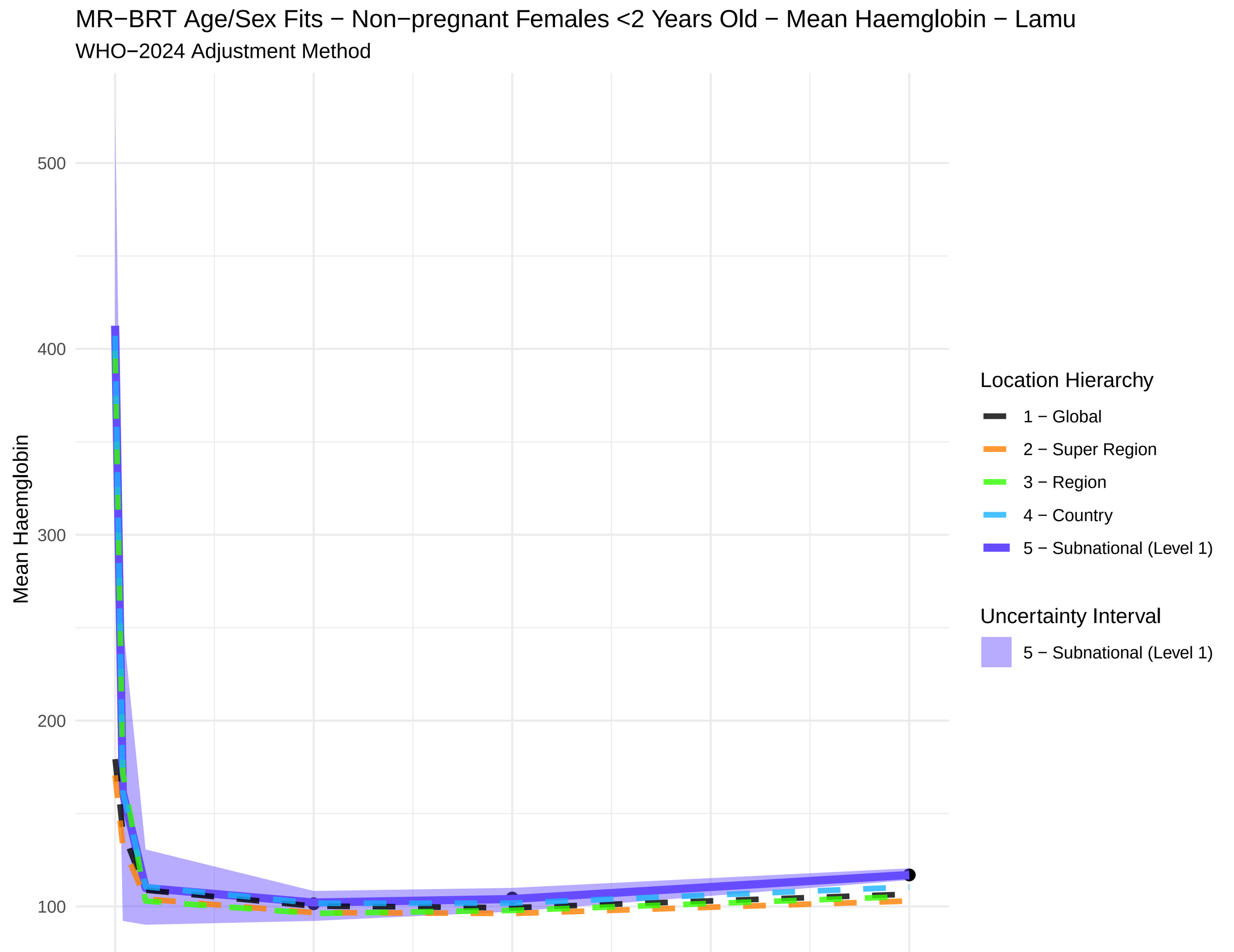
MR−BRT Age/Sex Fits − Non−pregnant Females <2 Years Old − Mean Haemglobin − Lamu
WHO−2024 Adjustment Method
Mean Haemglobin
Age (year)
Location Hierarchy
1 − Global
2 − Super Region
3 − Region
4 − Country
5 − Subnational (Level 1)
Uncertainty Interval
5 − Subnational (Level 1)

MR−BRT Age/Sex Fits − Non−pregnant Females <2 Years Old − Mean Haemglobin − Machakos
WHO−2024 Adjustment Method

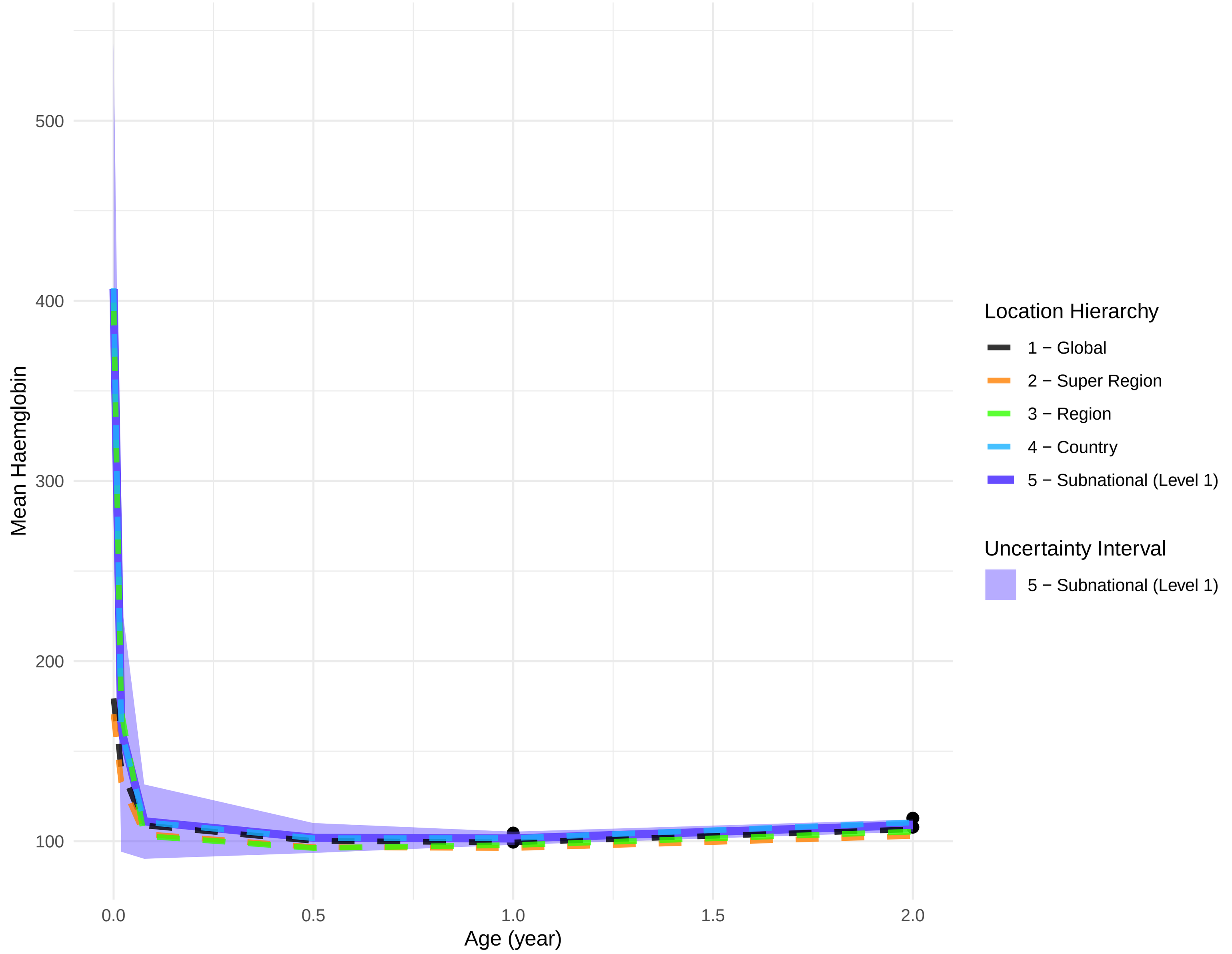

MR−BRT Age/Sex Fits − Non−pregnant Females <2 Years Old − Mean Haemglobin − Makueni
WHO−2024 Adjustment Method

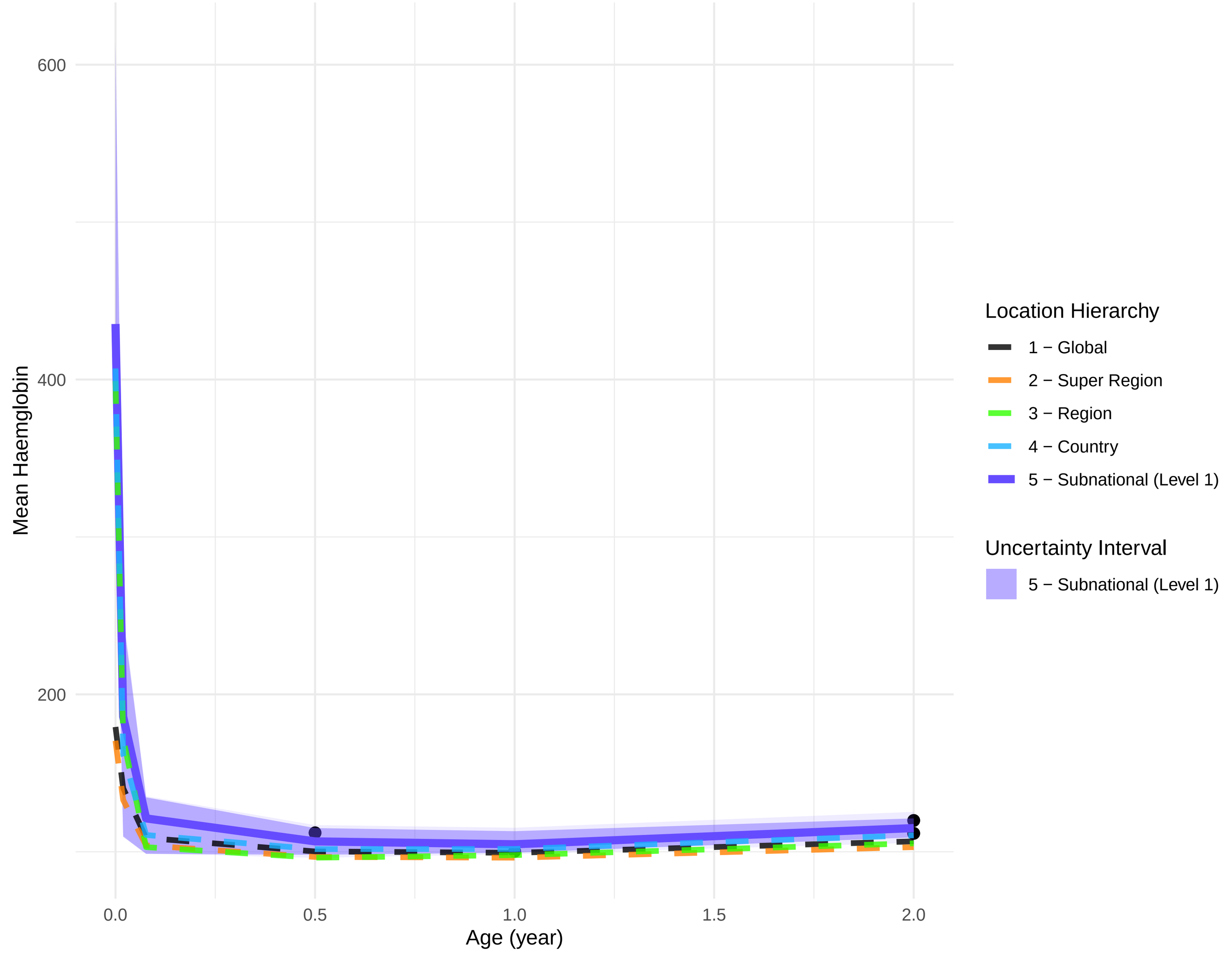

MR−BRT Age/Sex Fits − Non−pregnant Females <2 Years Old − Mean Haemglobin − Mandera
WHO−2024 Adjustment Method

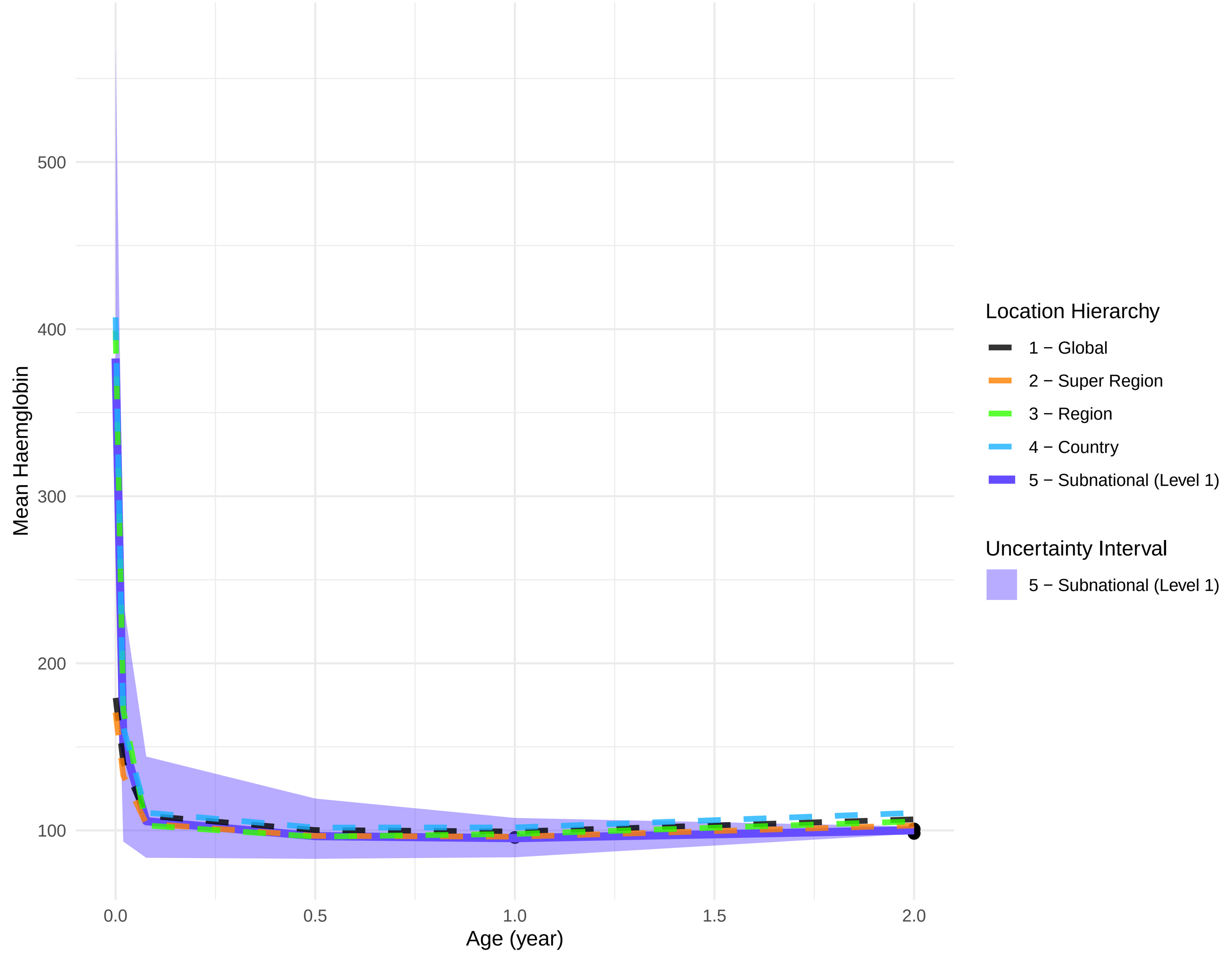

MR−BRT Age/Sex Fits − Non−pregnant Females <2 Years Old − Mean Haemglobin − Marsabit
WHO−2024 Adjustment Method

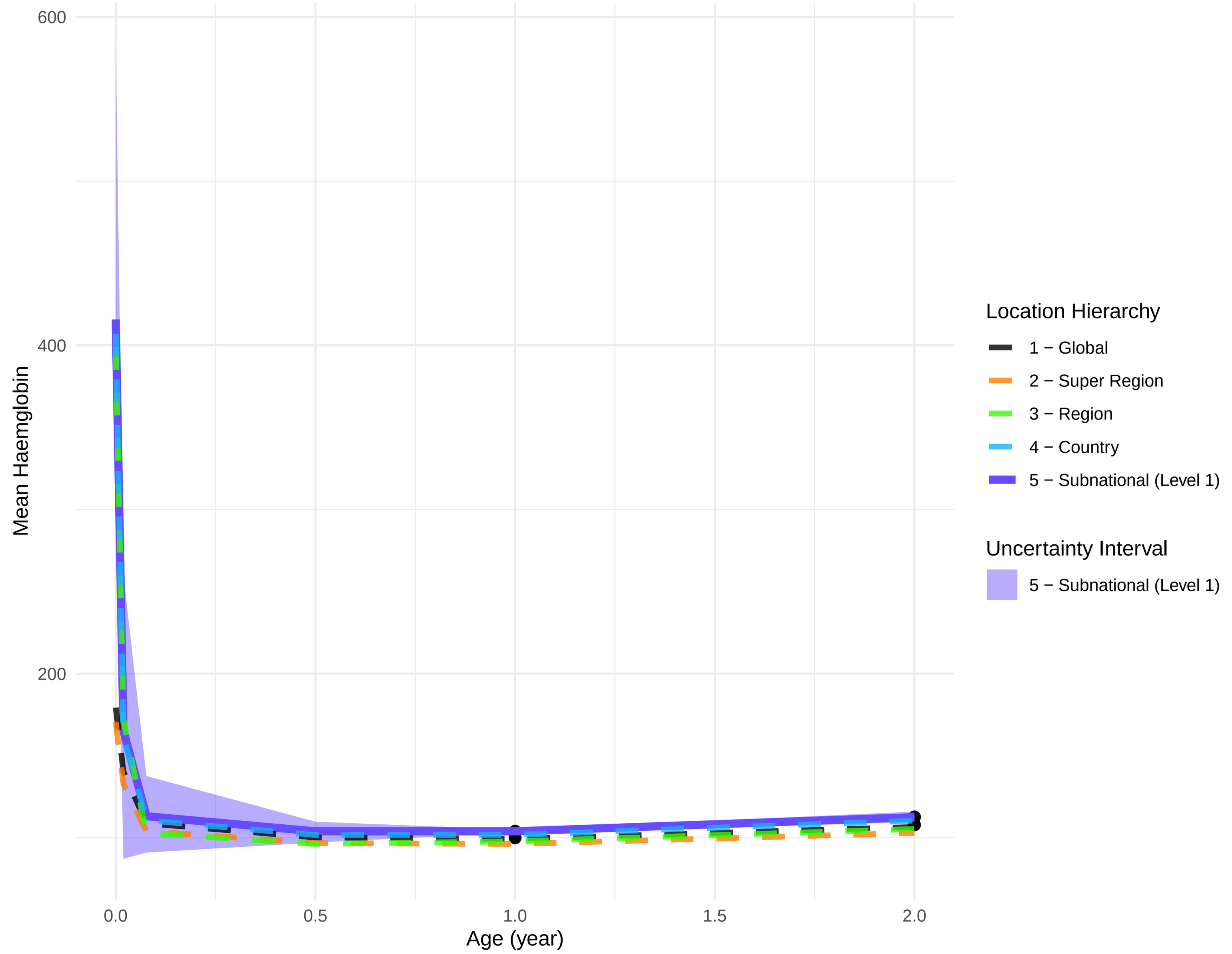

MR−BRT Age/Sex Fits − Non−pregnant Females <2 Years Old − Mean Haemglobin − Meru
WHO−2024 Adjustment Method

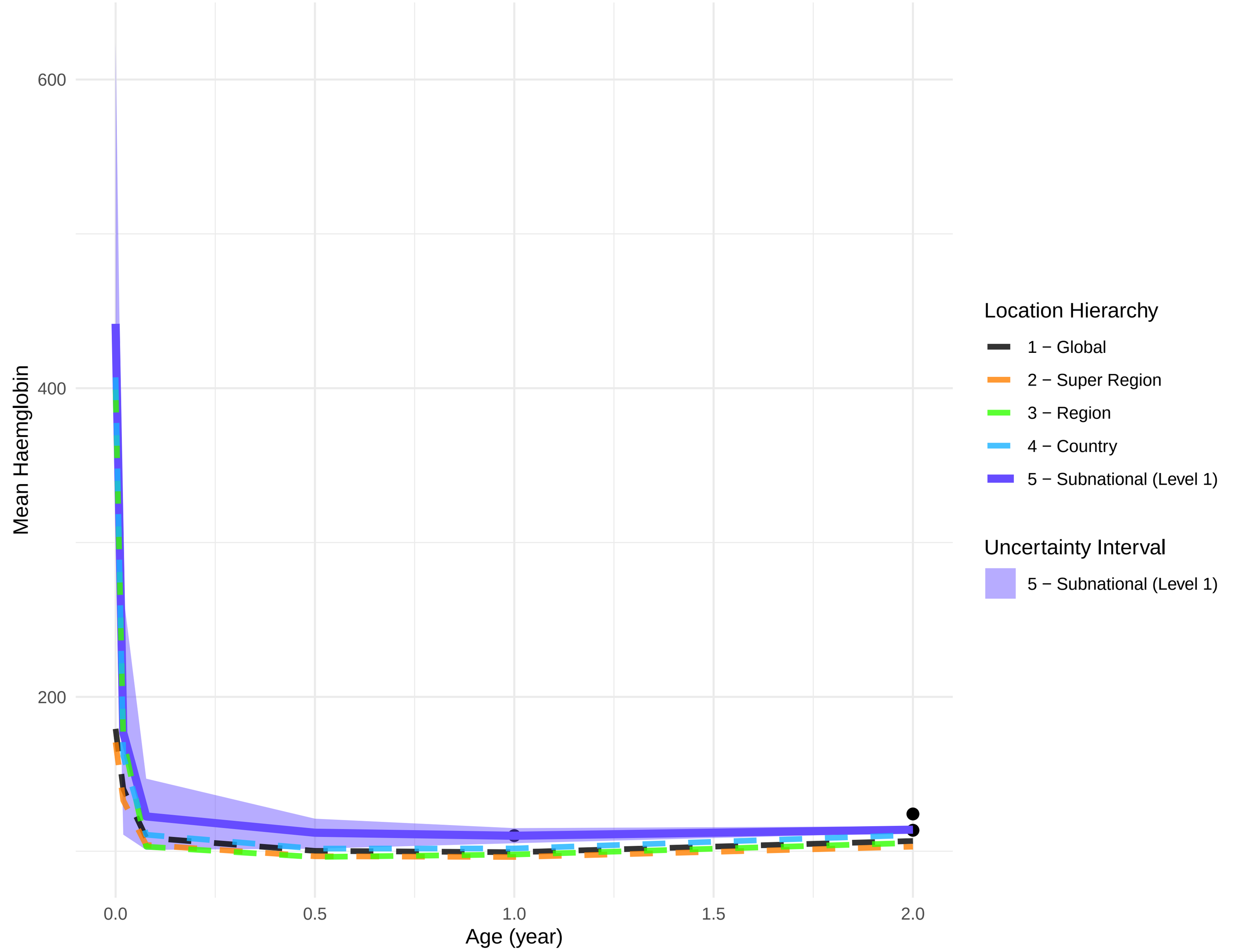

MR−BRT Age/Sex Fits − Non−pregnant Females <2 Years Old − Mean Haemglobin − Migori
WHO−2024 Adjustment Method

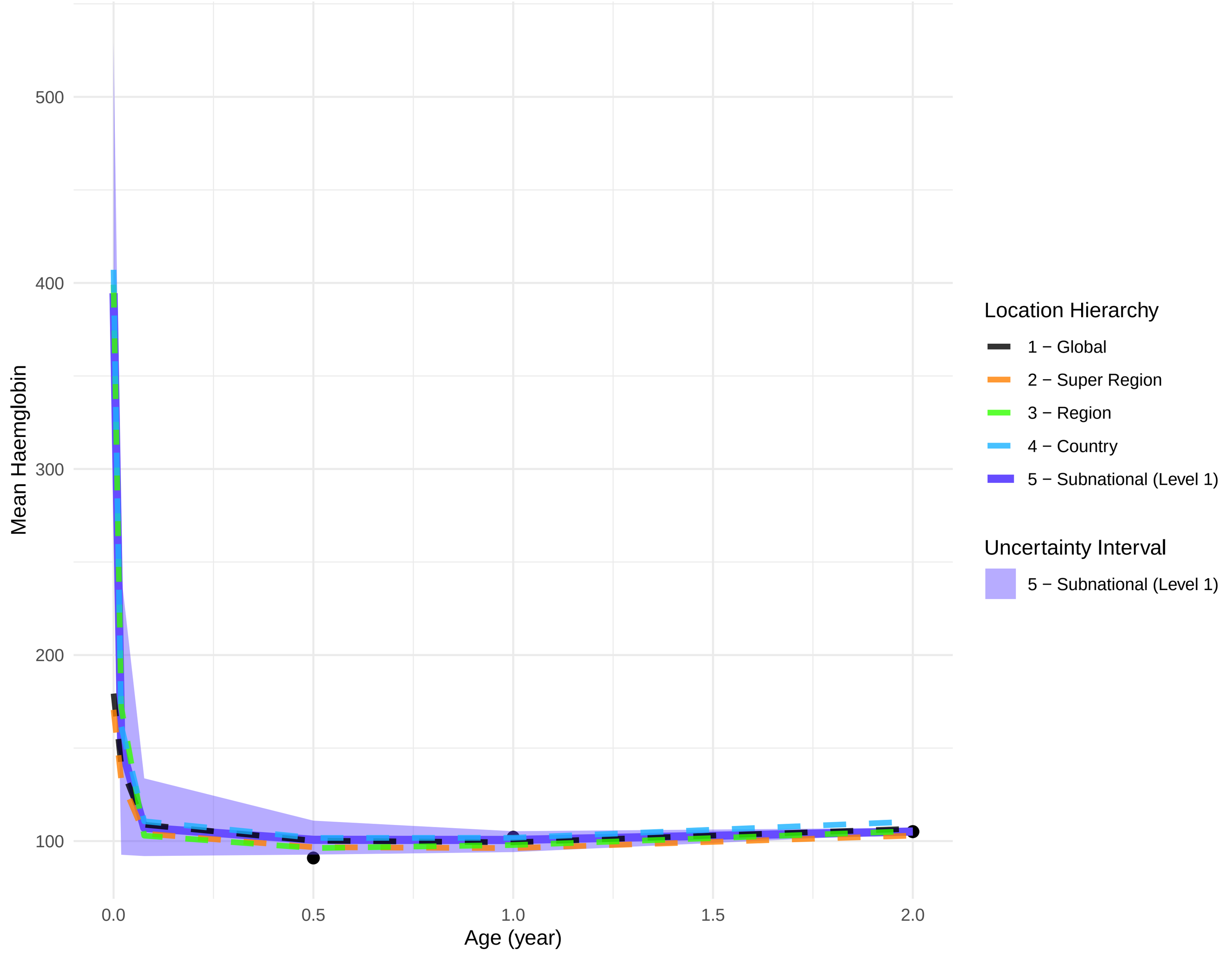

MR−BRT Age/Sex Fits − Non−pregnant Females <2 Years Old − Mean Haemglobin − Mombasa
WHO−2024 Adjustment Method

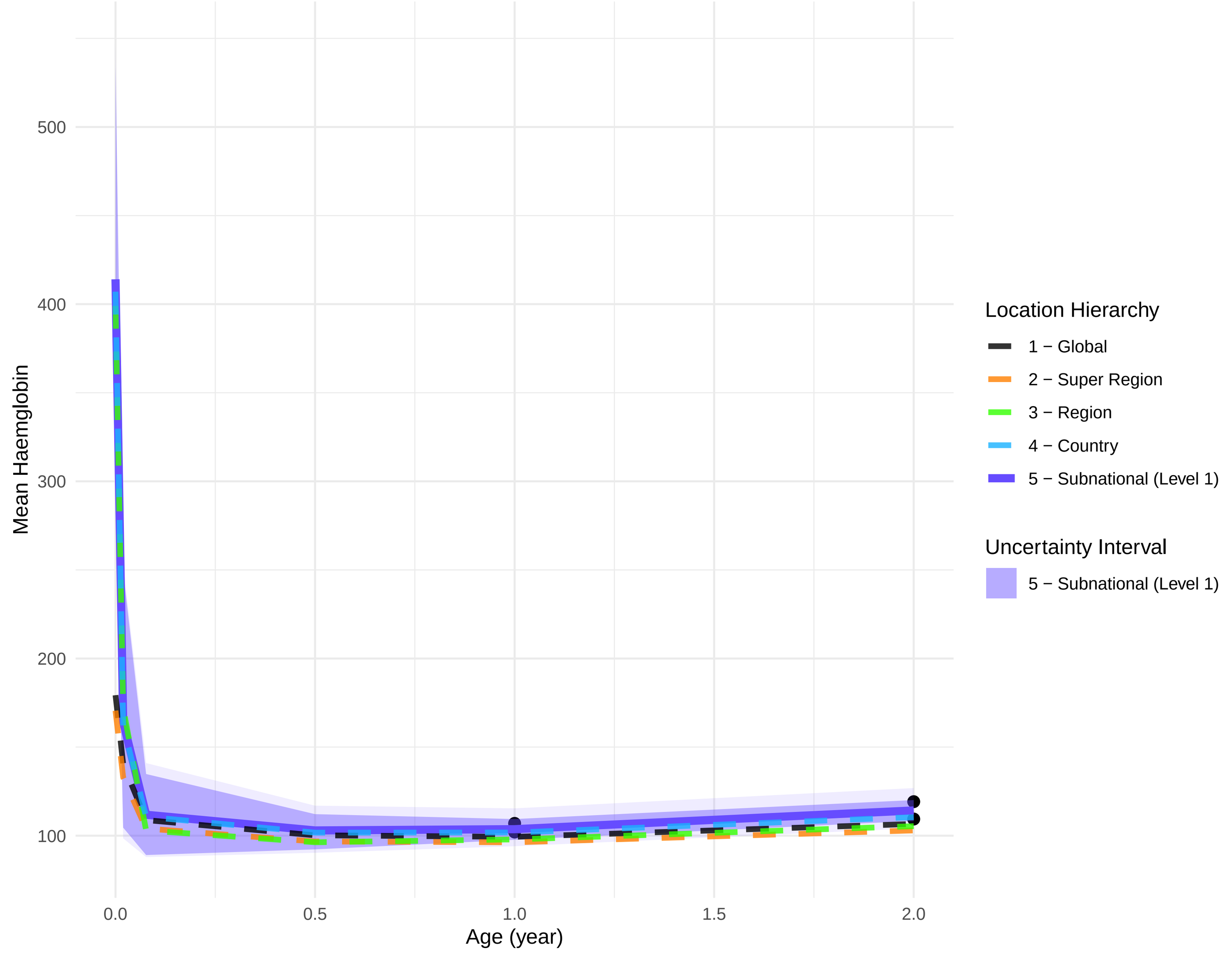

MR−BRT Age/Sex Fits − Non−pregnant Females <2 Years Old − Mean Haemglobin − Murang'a
WHO−2024 Adjustment Method

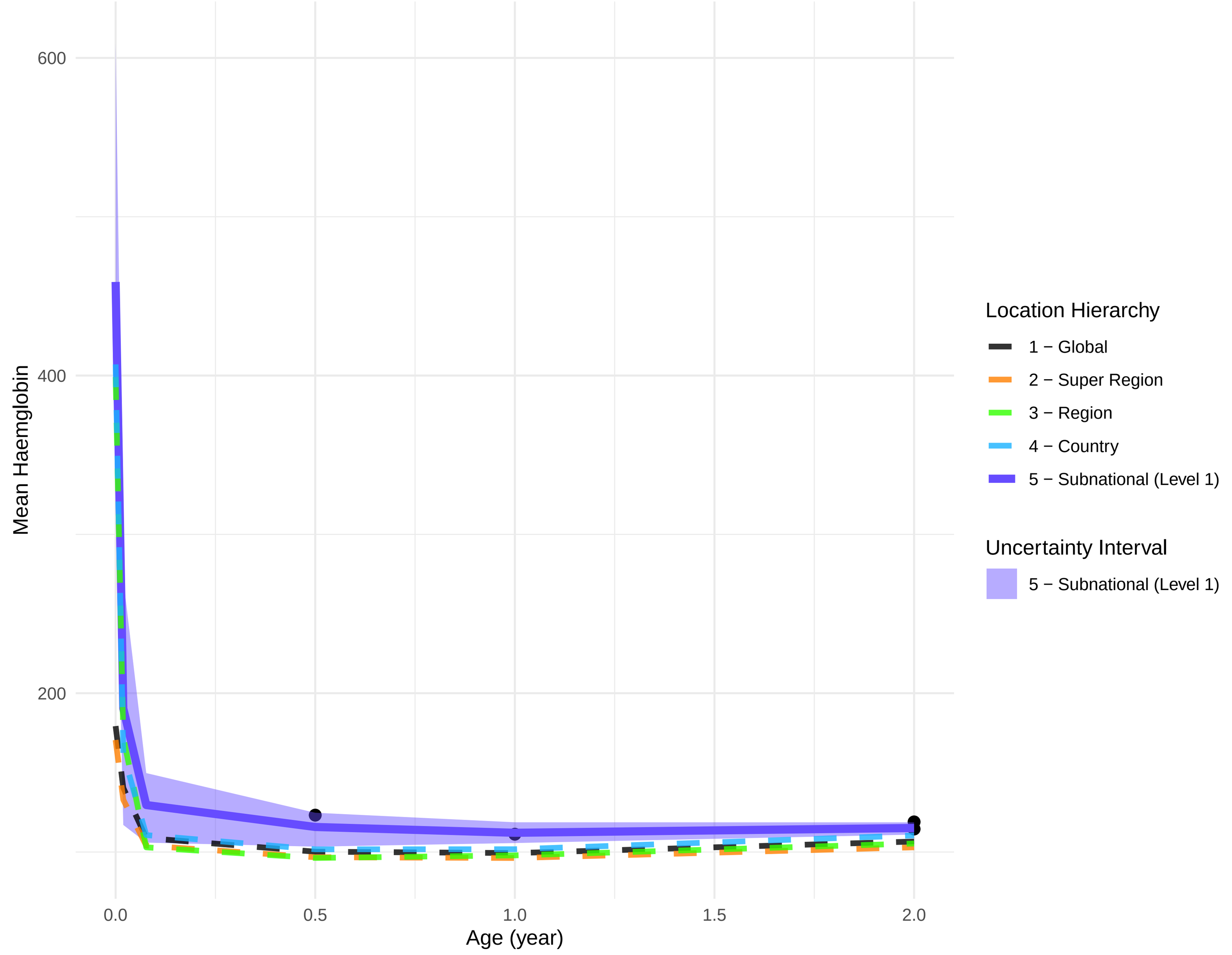

MR−BRT Age/Sex Fits − Non−pregnant Females <2 Years Old − Mean Haemglobin − Nairobi
WHO−2024 Adjustment Method

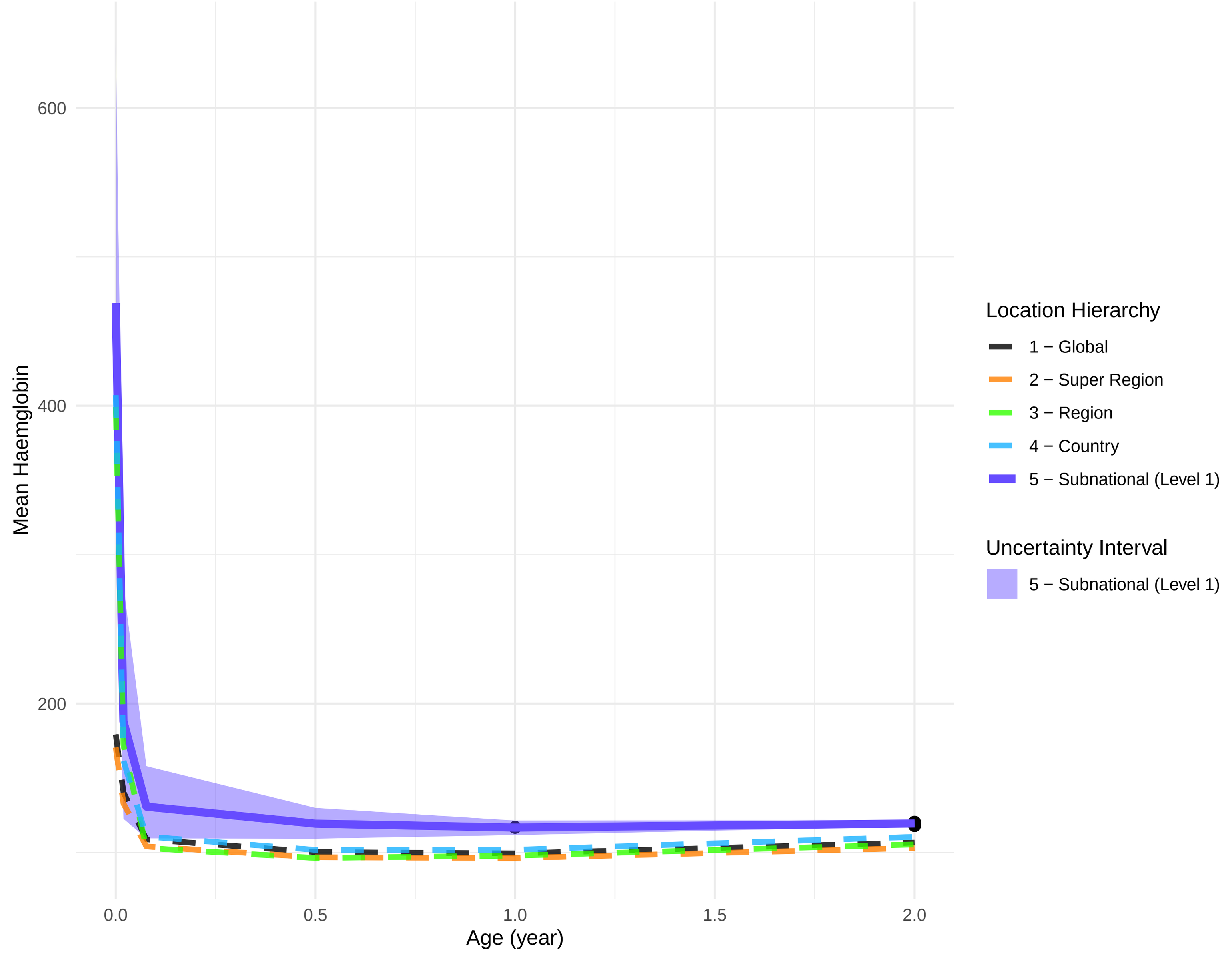

MR−BRT Age/Sex Fits − Non−pregnant Females <2 Years Old − Mean Haemglobin − Nakuru
WHO−2024 Adjustment Method

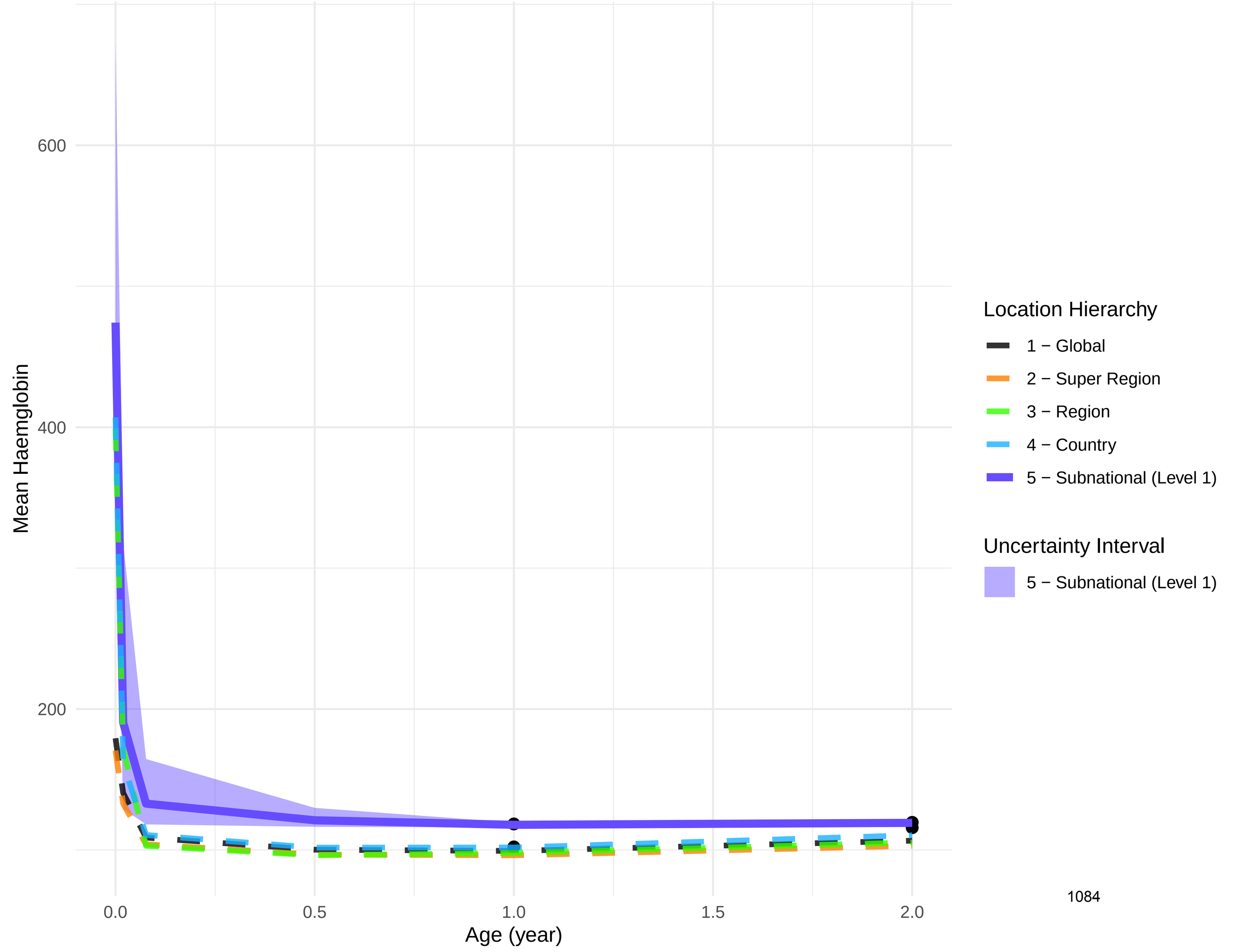

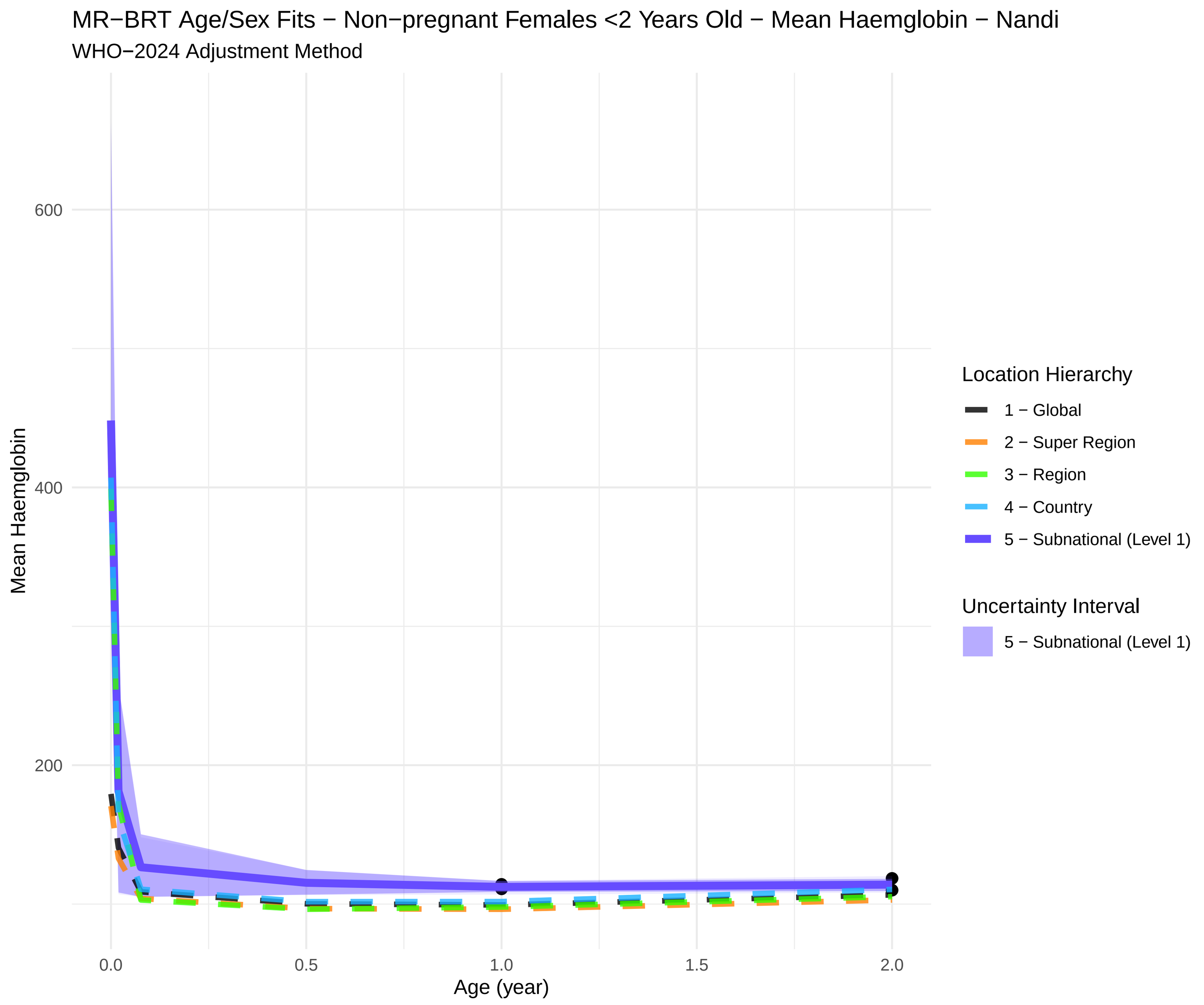
MR−BRT Age/Sex Fits − Non−pregnant Females <2 Years Old − Mean Haemglobin − Nandi
WHO−2024 Adjustment Method
Mean Haemglobin
Age (year)
Location Hierarchy
1 − Global
2 − Super Region
3 − Region
4 − Country
5 − Subnational (Level 1)
Uncertainty Interval
5 − Subnational (Level 1)

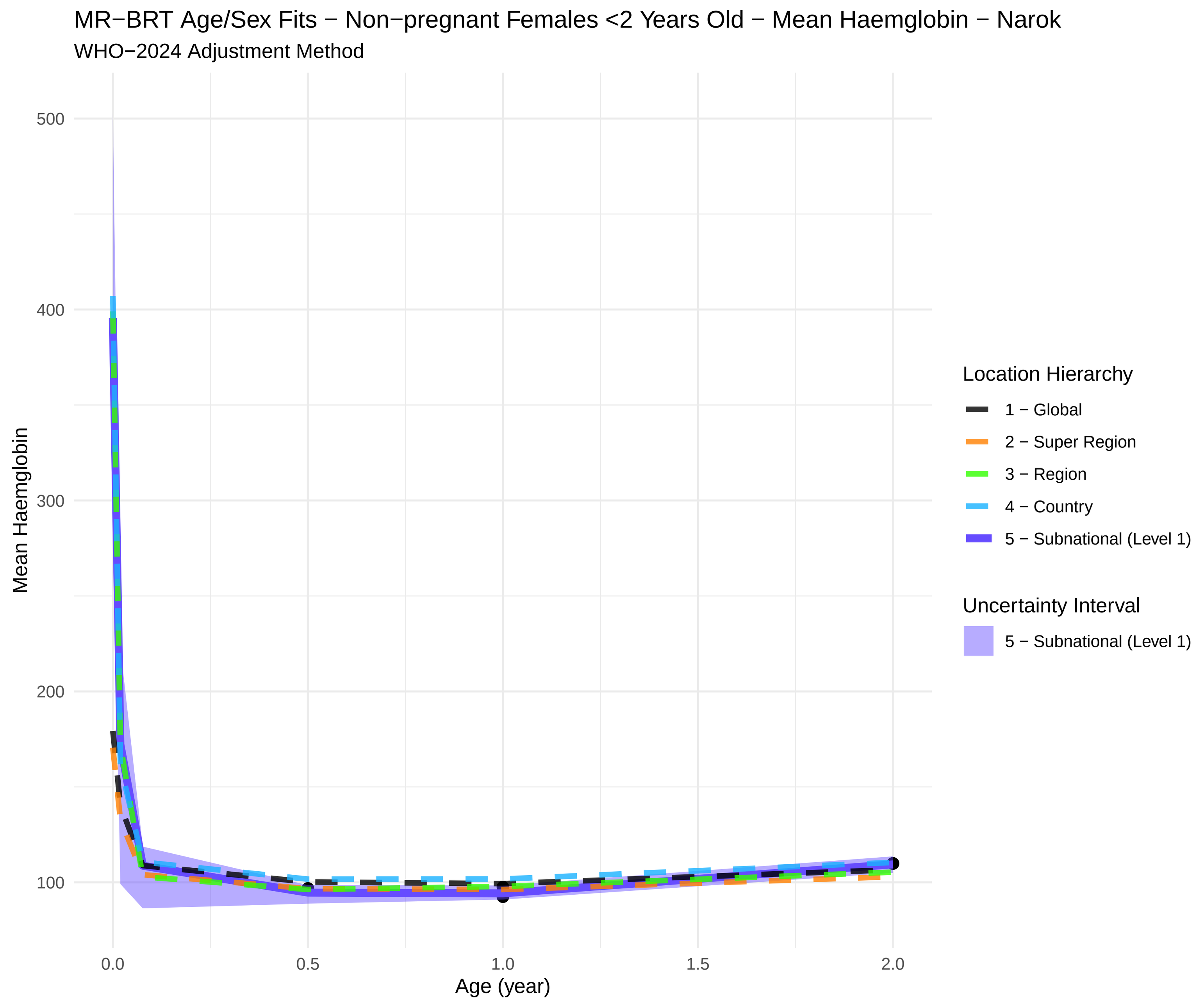
MR−BRT Age/Sex Fits − Non−pregnant Females <2 Years Old − Mean Haemglobin − Narok
WHO−2024 Adjustment Method
Mean Haemglobin
Age (year)
Location Hierarchy
1 − Global
2 − Super Region
3 − Region
4 − Country
5 − Subnational (Level 1)
Uncertainty Interval
5 − Subnational (Level 1)

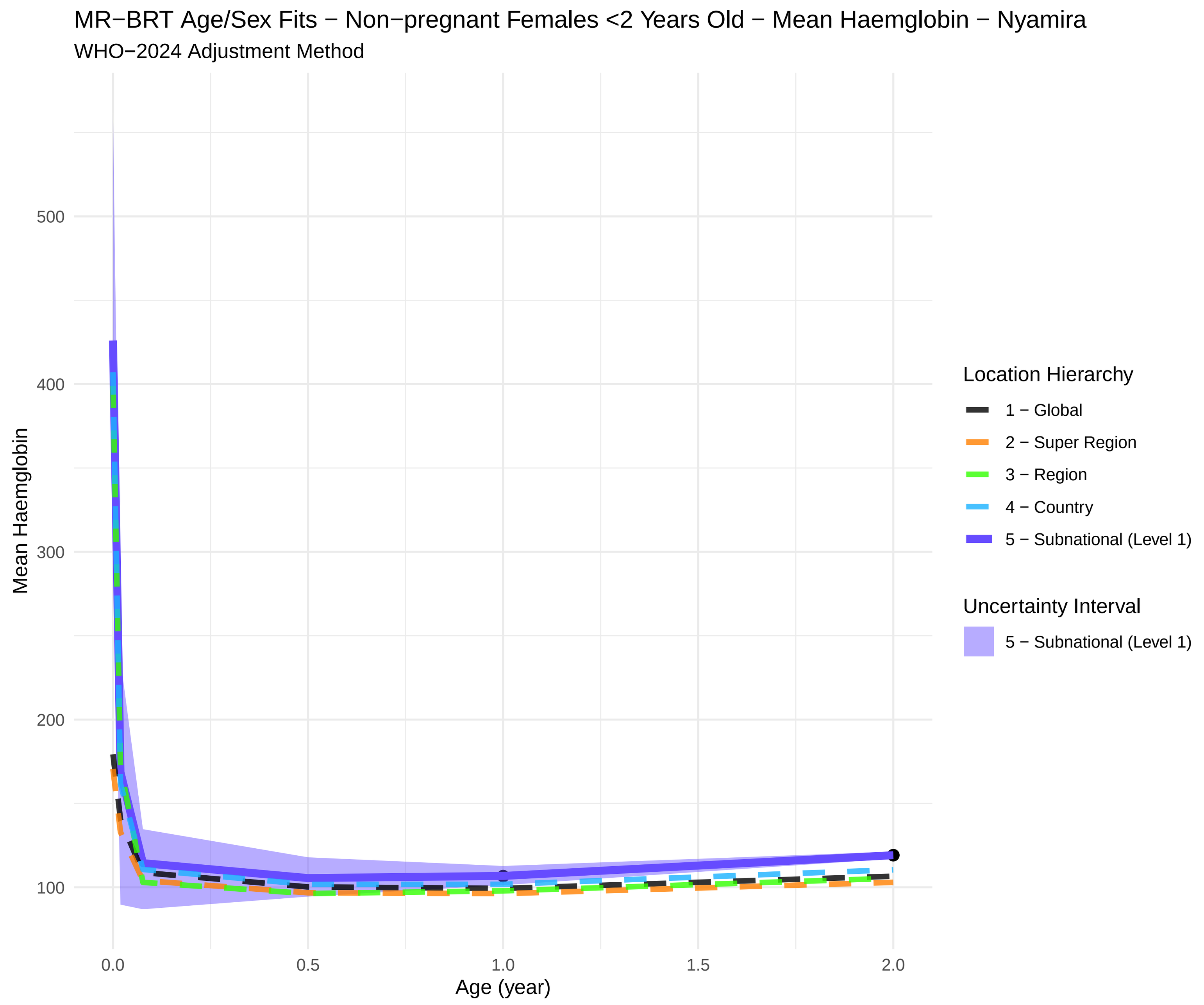
MR−BRT Age/Sex Fits − Non−pregnant Females <2 Years Old − Mean Haemglobin − Nyamira
WHO−2024 Adjustment Method
Mean Haemglobin
Age (year)
Location Hierarchy
1 − Global
2 − Super Region
3 − Region
4 − Country
5 − Subnational (Level 1)
Uncertainty Interval
5 − Subnational (Level 1)

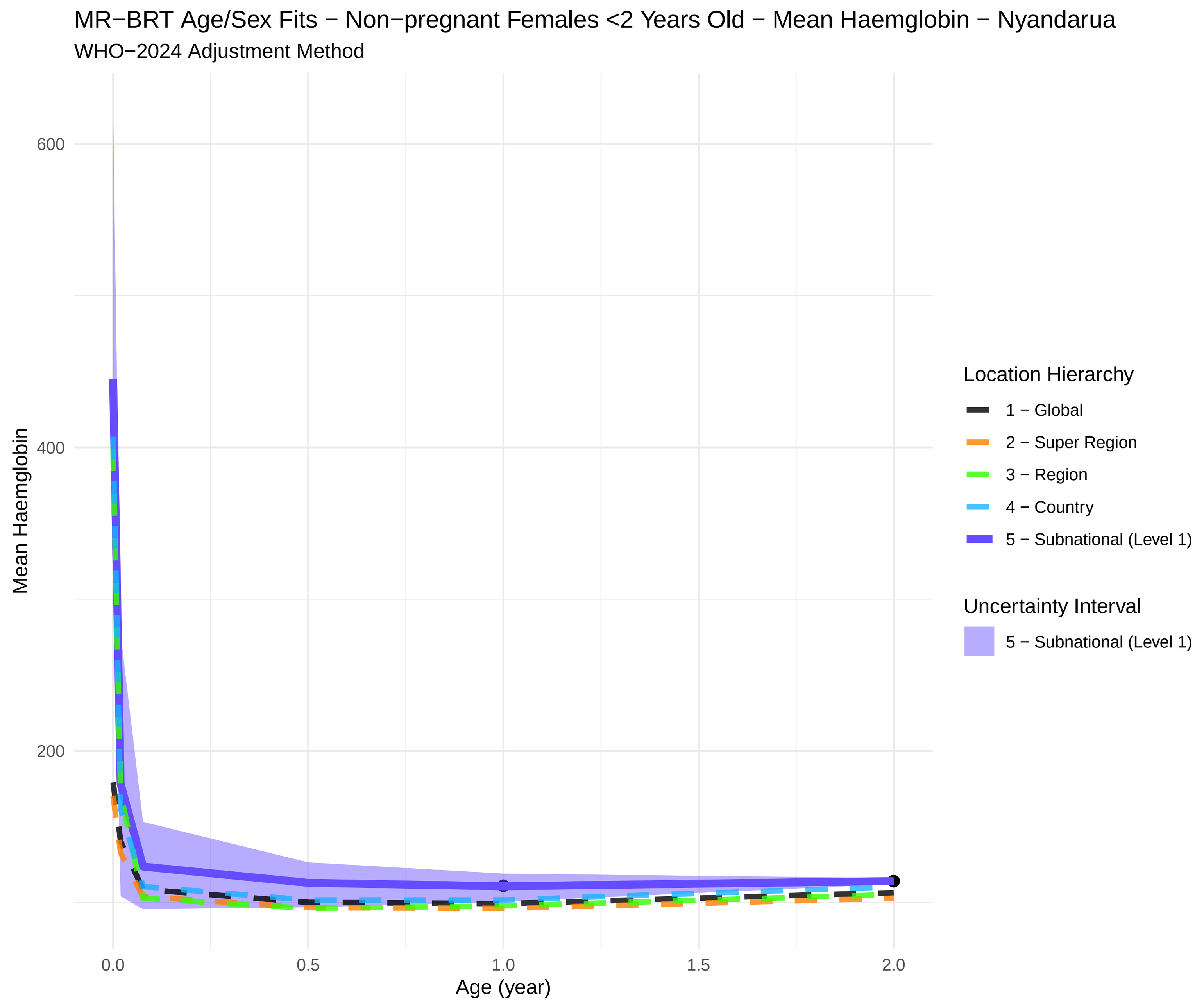
MR−BRT Age/Sex Fits − Non−pregnant Females <2 Years Old − Mean Haemglobin − Nyandarua
WHO−2024 Adjustment Method
Mean Haemglobin
Age (year)
Location Hierarchy
1 − Global
2 − Super Region
3 − Region
4 − Country
5 − Subnational (Level 1)
Uncertainty Interval
5 − Subnational (Level 1)

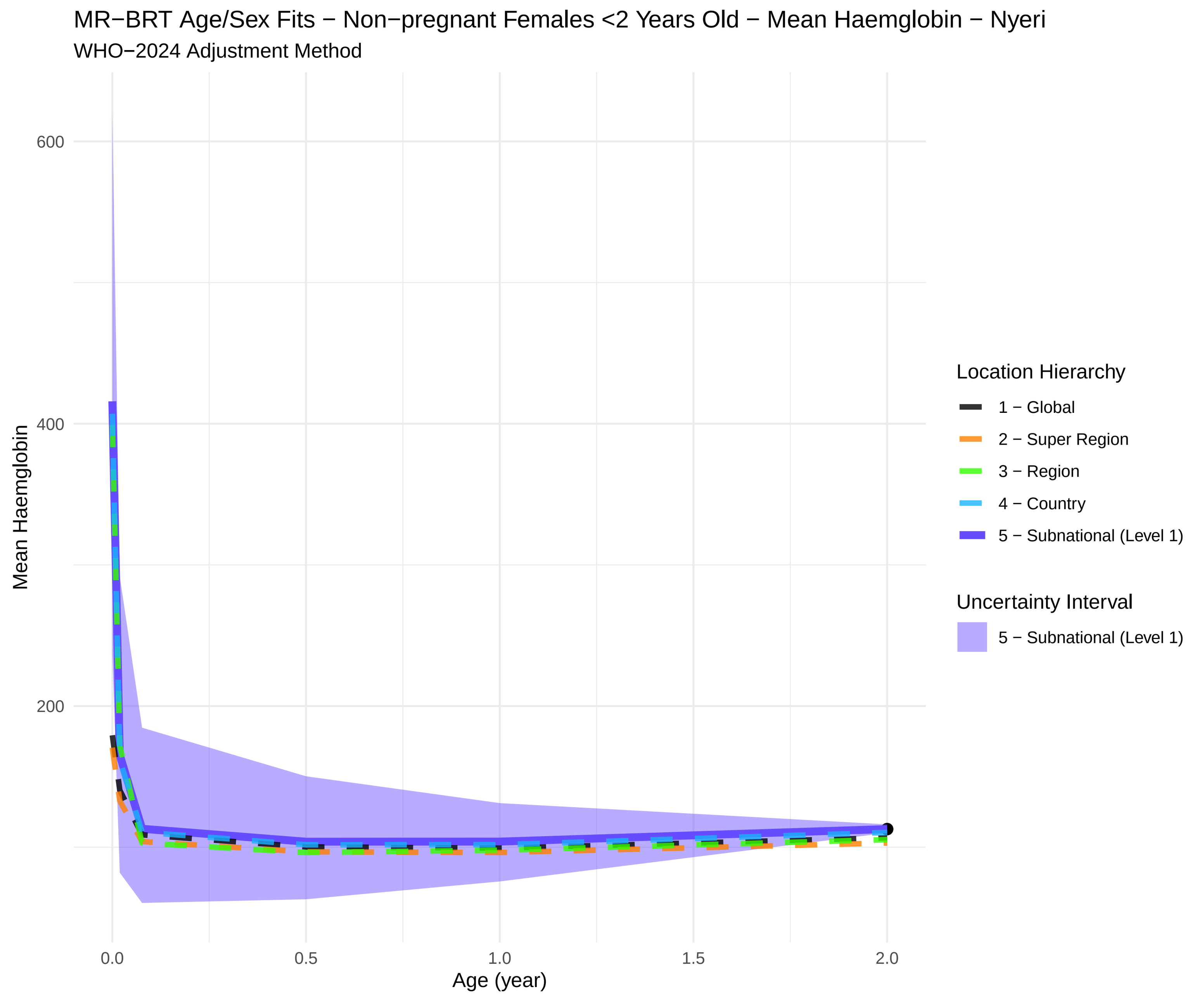
MR−BRT Age/Sex Fits − Non−pregnant Females <2 Years Old − Mean Haemglobin − Nyeri
WHO−2024 Adjustment Method
Mean Haemglobin
Age (year)
Location Hierarchy
1 − Global
2 − Super Region
3 − Region
4 − Country
5 − Subnational (Level 1)
Uncertainty Interval
5 − Subnational (Level 1)

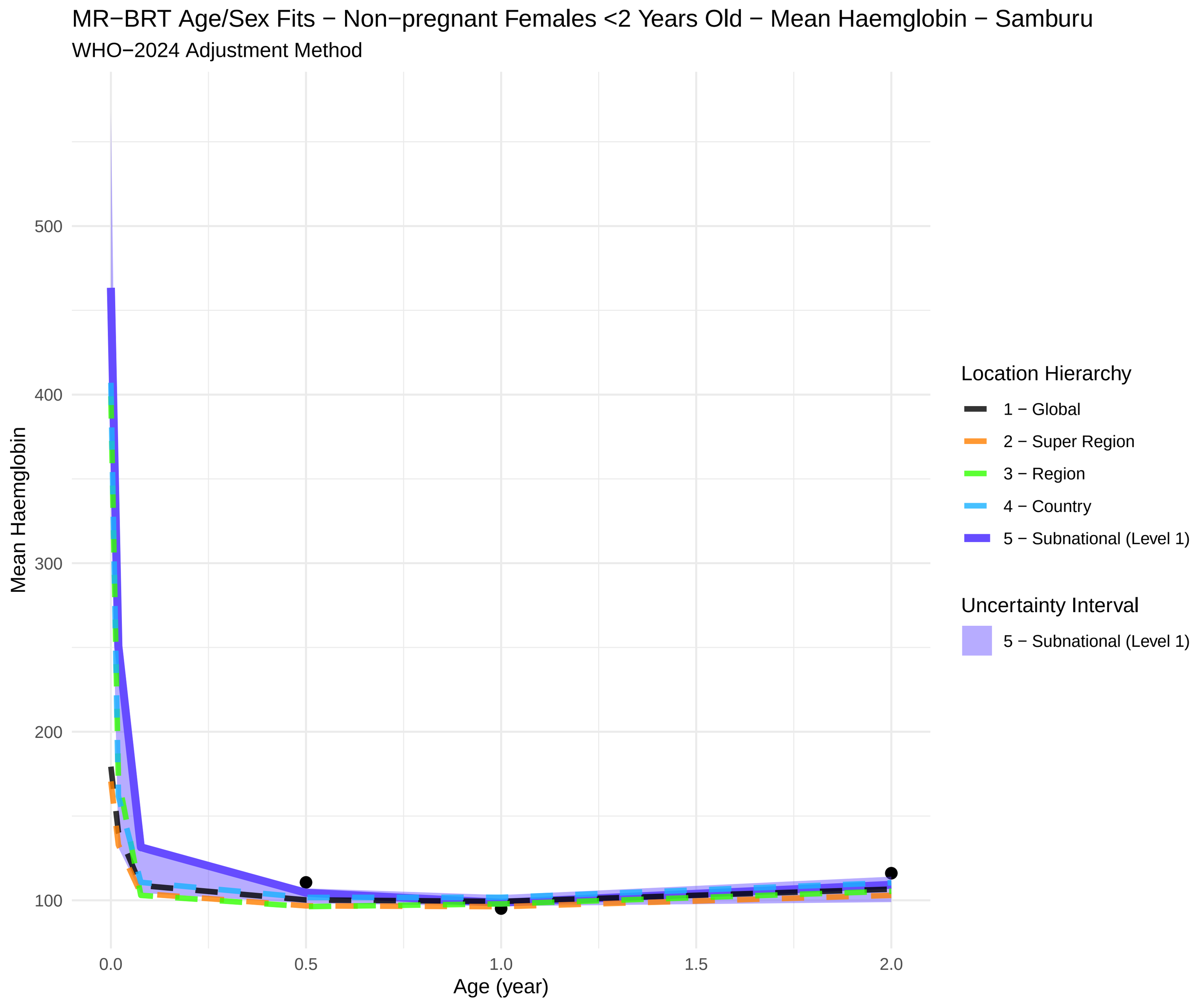
MR−BRT Age/Sex Fits − Non−pregnant Females <2 Years Old − Mean Haemglobin − Samburu
WHO−2024 Adjustment Method
Mean Haemglobin
Age (year)
Location Hierarchy
1 − Global
2 − Super Region
3 − Region
4 − Country
5 − Subnational (Level 1)
Uncertainty Interval
5 − Subnational (Level 1)

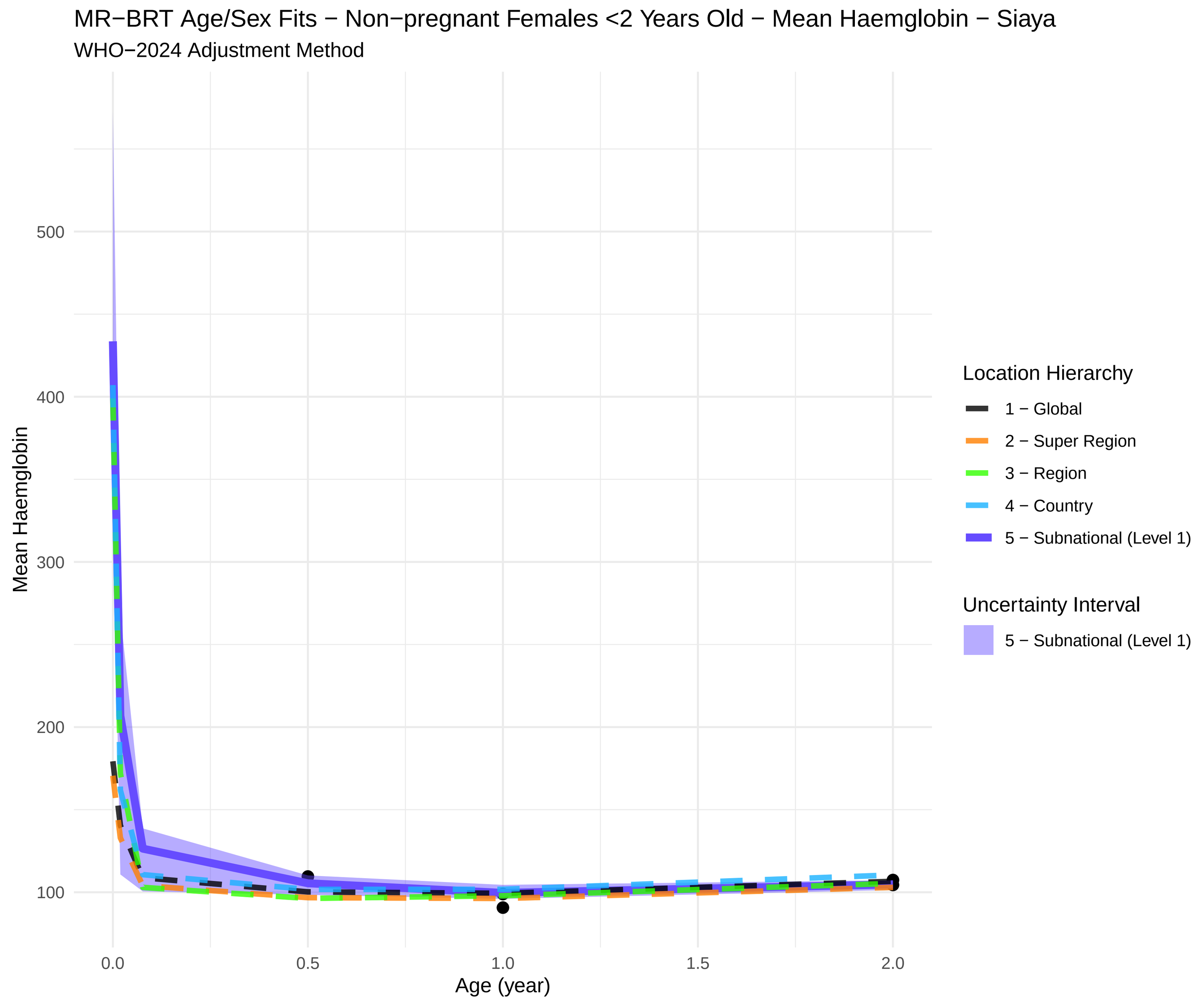
MR−BRT Age/Sex Fits − Non−pregnant Females <2 Years Old − Mean Haemglobin − Siaya
WHO−2024 Adjustment Method
Mean Haemglobin
Age (year)
Location Hierarchy
1 − Global
2 − Super Region
3 − Region
4 − Country
5 − Subnational (Level 1)
Uncertainty Interval
5 − Subnational (Level 1)

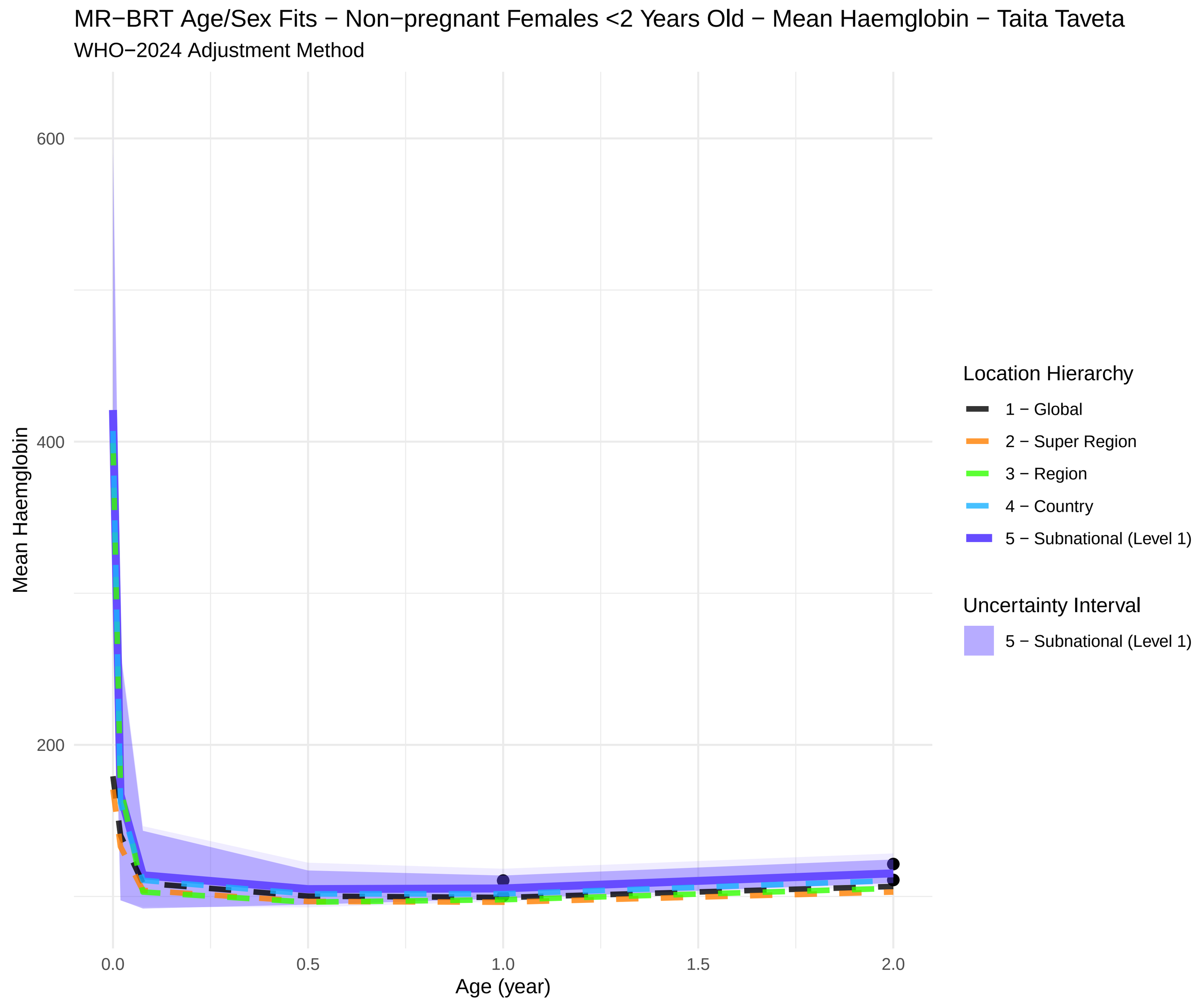
MR−BRT Age/Sex Fits − Non−pregnant Females <2 Years Old − Mean Haemglobin − Taita Taveta
WHO−2024 Adjustment Method
Mean Haemglobin
Age (year)
Location Hierarchy
1 − Global
2 − Super Region
3 − Region
4 − Country
5 − Subnational (Level 1)
Uncertainty Interval
5 − Subnational (Level 1)

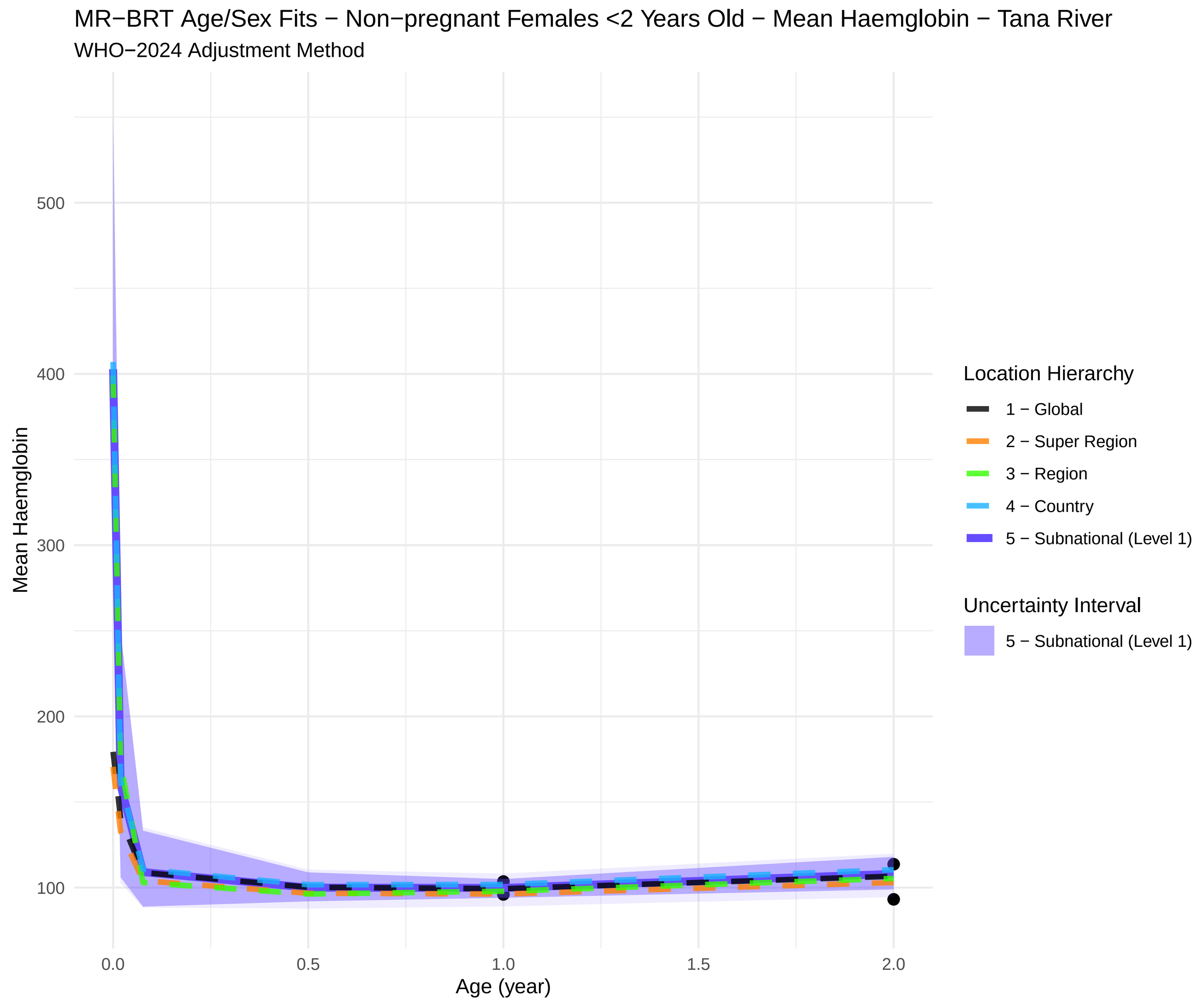
MR−BRT Age/Sex Fits − Non−pregnant Females <2 Years Old − Mean Haemglobin − Tana River
WHO−2024 Adjustment Method
Mean Haemglobin
Age (year)
Location Hierarchy
1 − Global
2 − Super Region
3 − Region
4 − Country
5 − Subnational (Level 1)
Uncertainty Interval
5 − Subnational (Level 1)

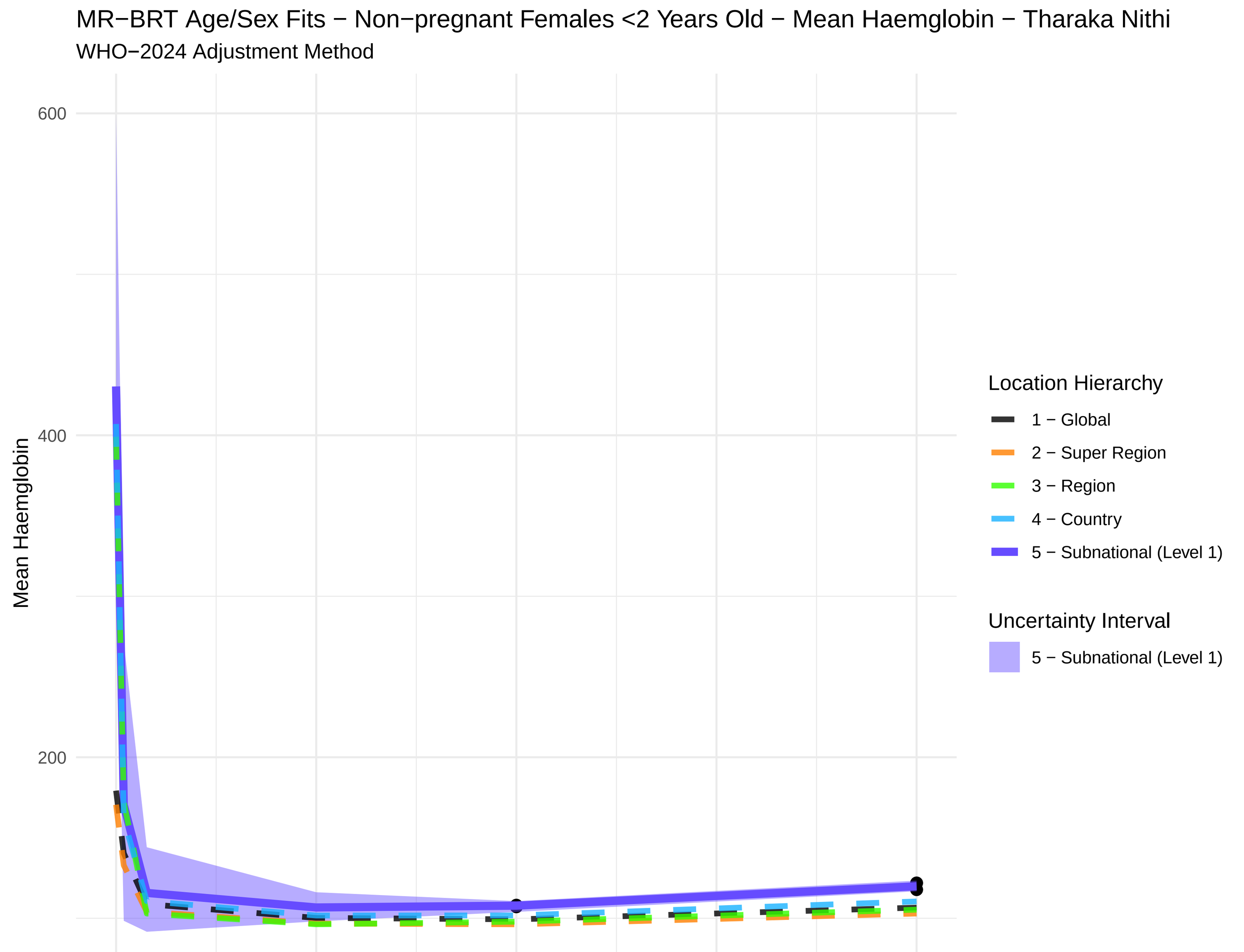
MR−BRT Age/Sex Fits − Non−pregnant Females <2 Years Old − Mean Haemglobin − Tharaka Nithi
WHO−2024 Adjustment Method
Mean Haemglobin
Age (year)
Location Hierarchy
1 − Global
2 − Super Region
3 − Region
4 − Country
5 − Subnational (Level 1)
Uncertainty Interval
5 − Subnational (Level 1)

MR−BRT Age/Sex Fits − Non−pregnant Females <2 Years Old − Mean Haemglobin − Trans Nzoia
WHO−2024 Adjustment Method

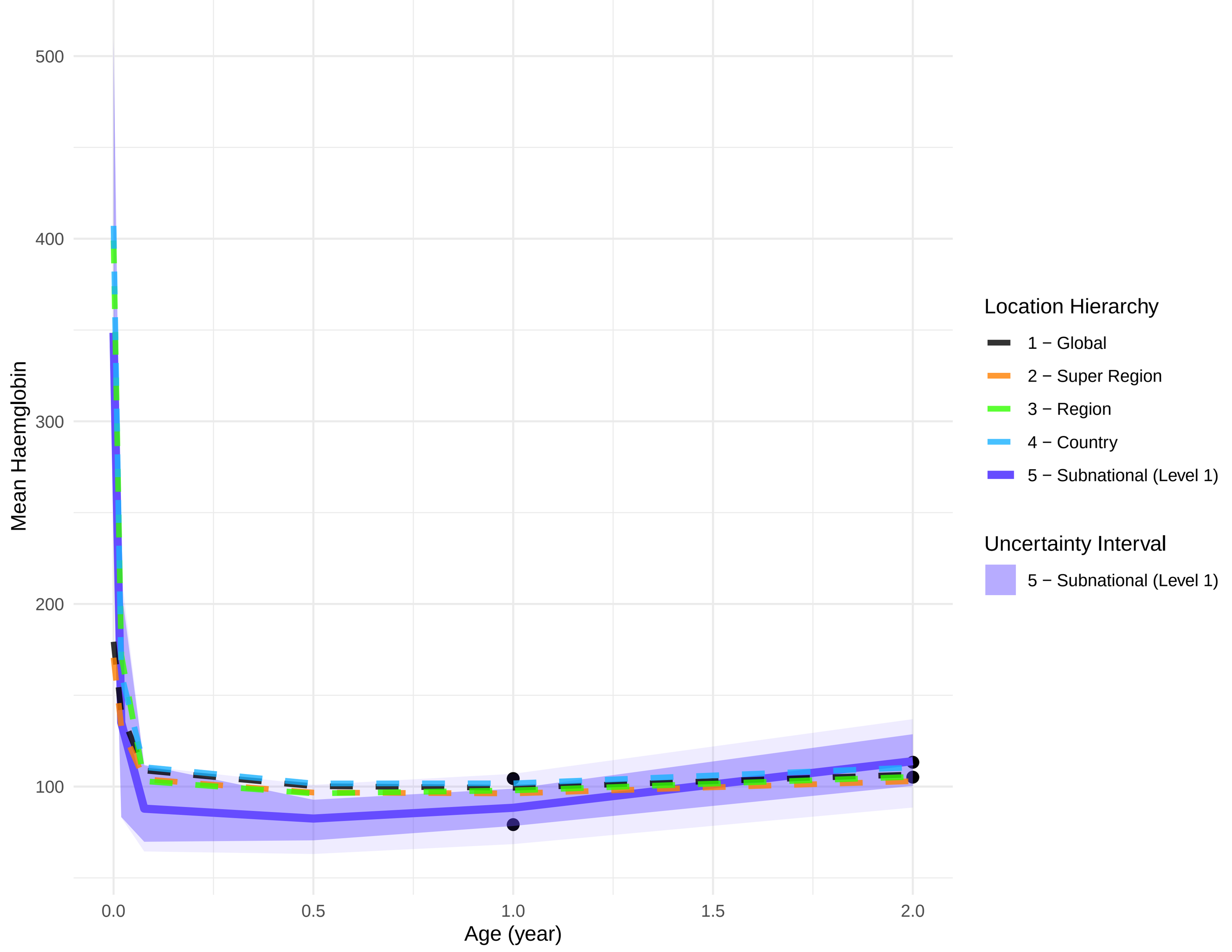

MR−BRT Age/Sex Fits − Non−pregnant Females <2 Years Old − Mean Haemglobin − Turkana
WHO−2024 Adjustment Method

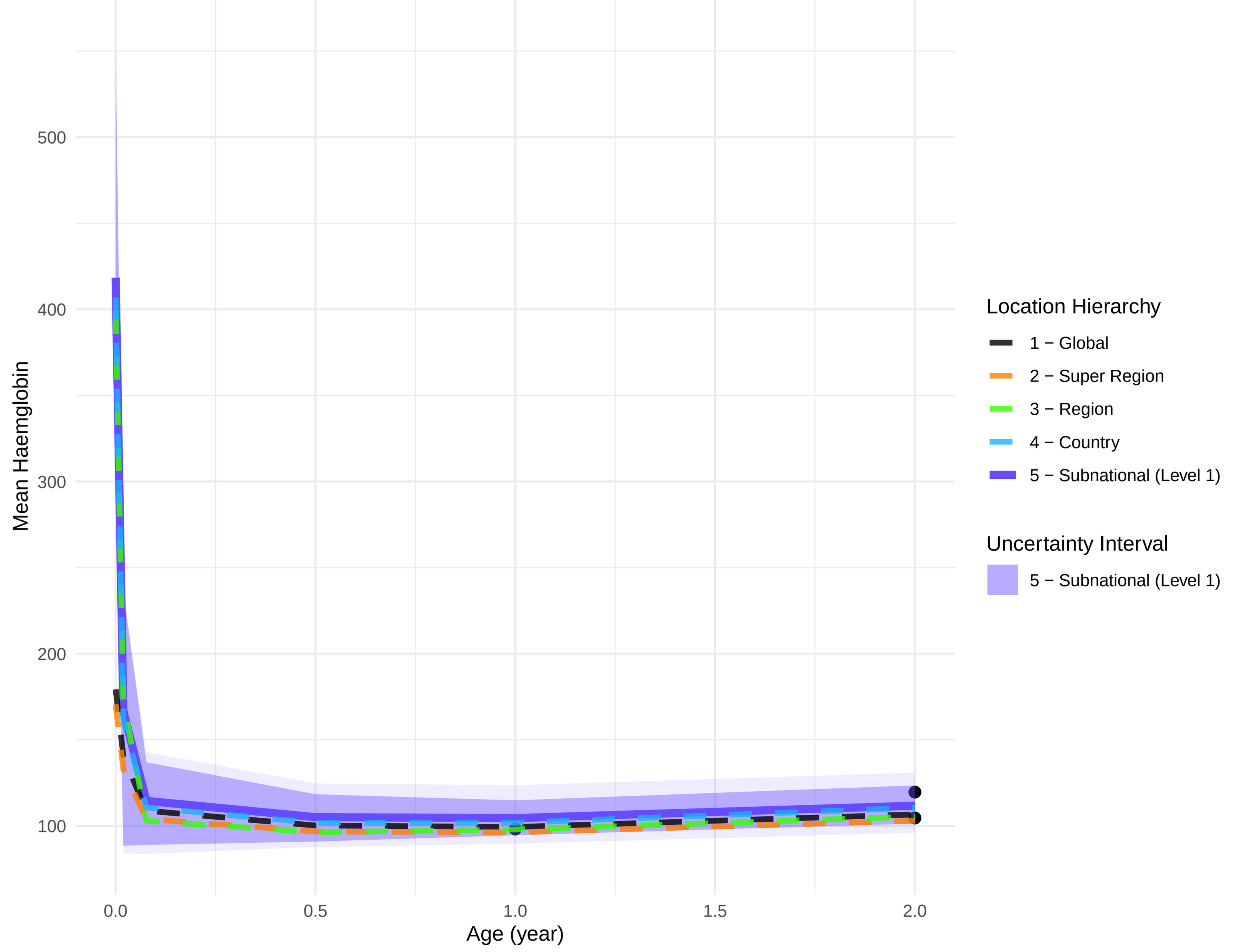

MR−BRT Age/Sex Fits − Non−pregnant Females <2 Years Old − Mean Haemglobin − Uasin Gishu
WHO−2024 Adjustment Method

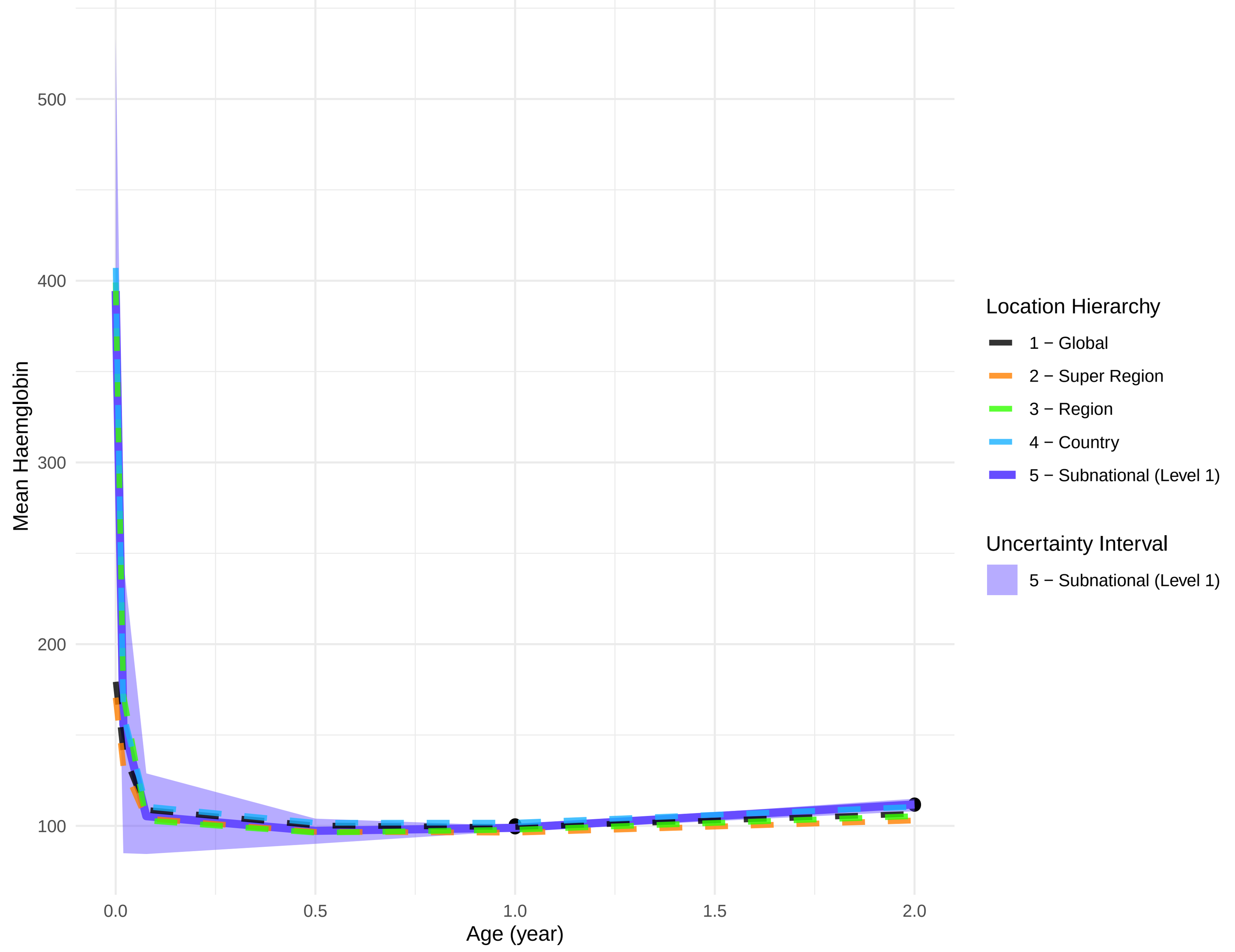

MR−BRT Age/Sex Fits − Non−pregnant Females <2 Years Old − Mean Haemglobin − Vihiga
WHO−2024 Adjustment Method

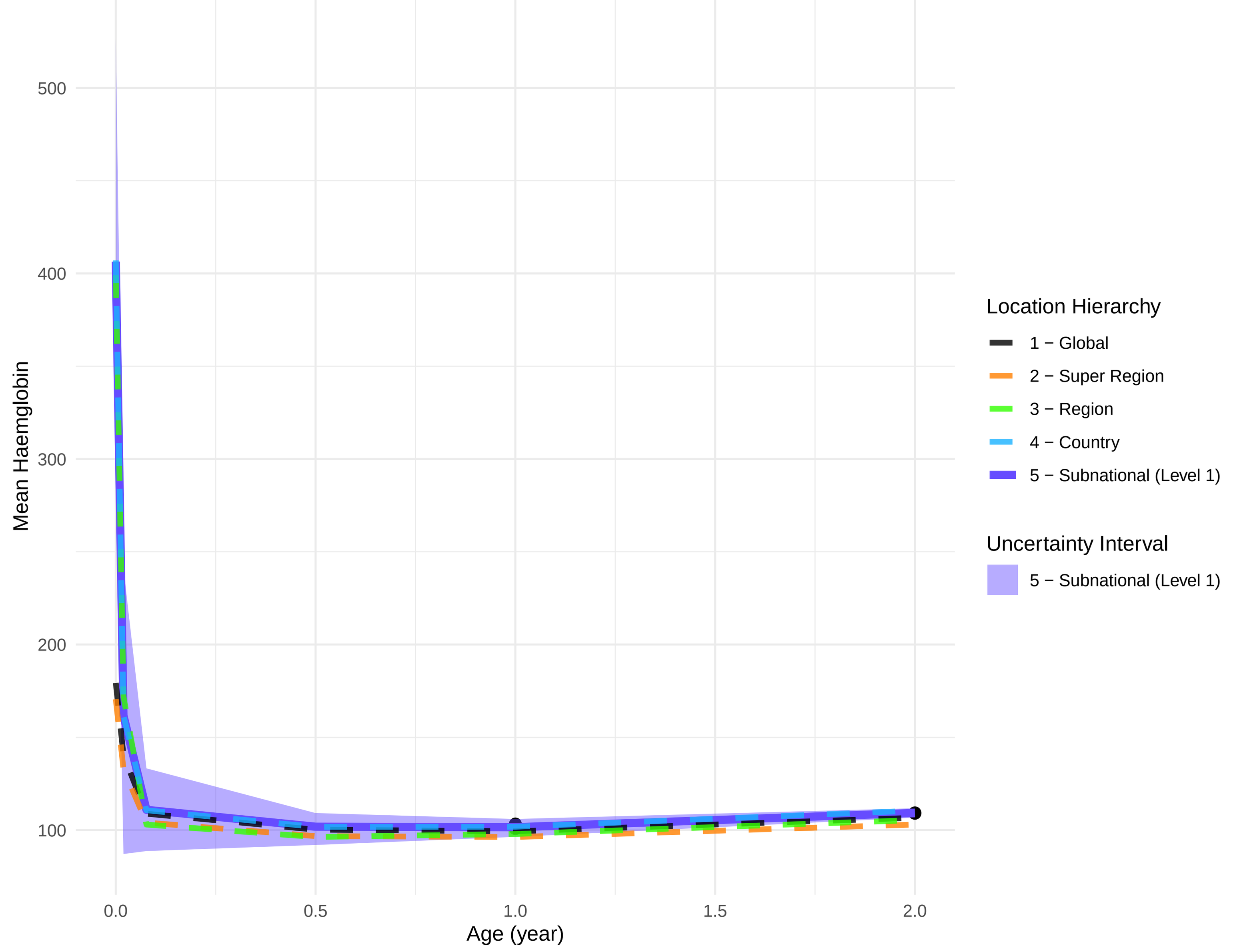

MR−BRT Age/Sex Fits − Non−pregnant Females <2 Years Old − Mean Haemglobin − Wajir
WHO−2024 Adjustment Method

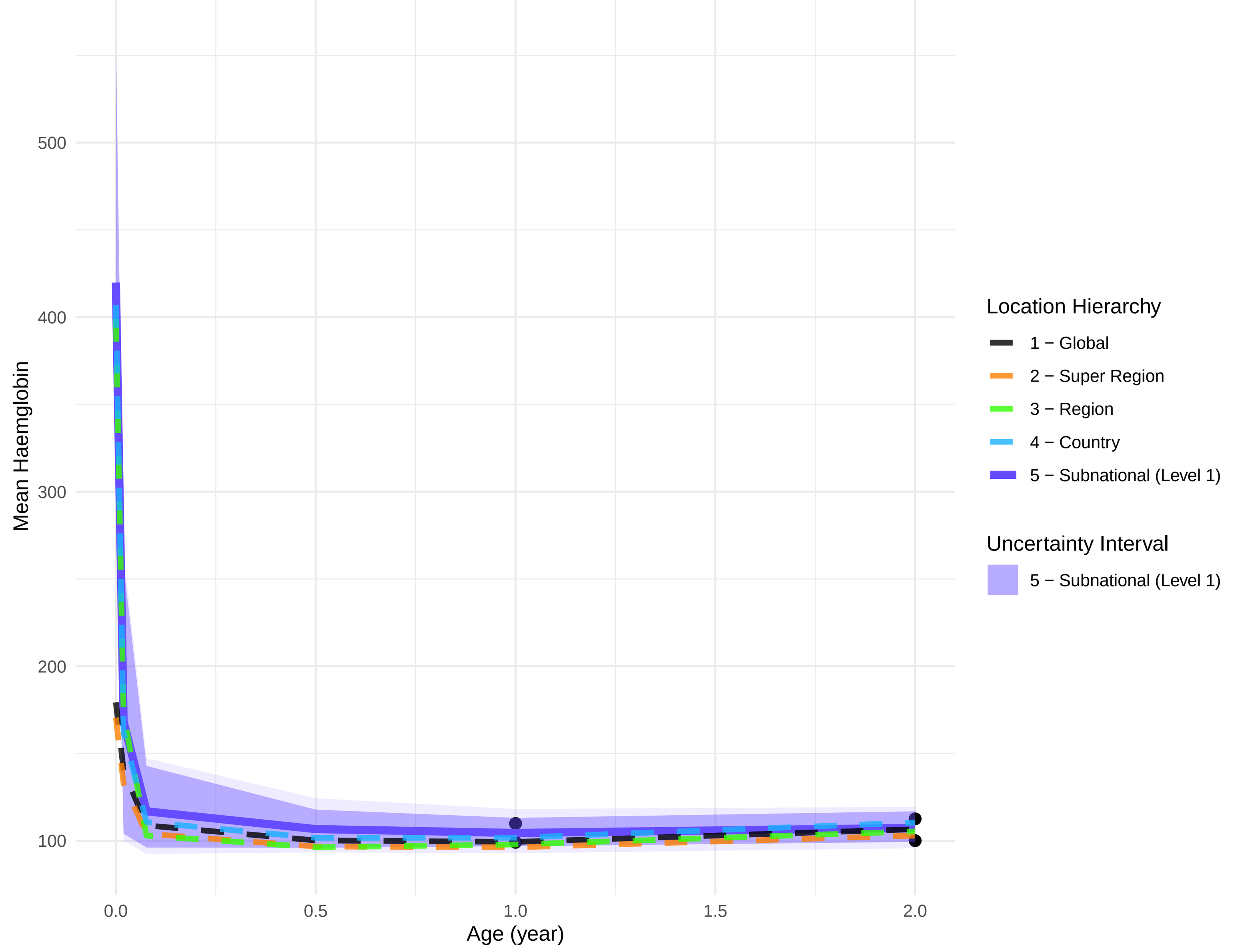

MR−BRT Age/Sex Fits − Non−pregnant Females <2 Years Old − Mean Haemglobin − West Pokot
WHO−2024 Adjustment Method

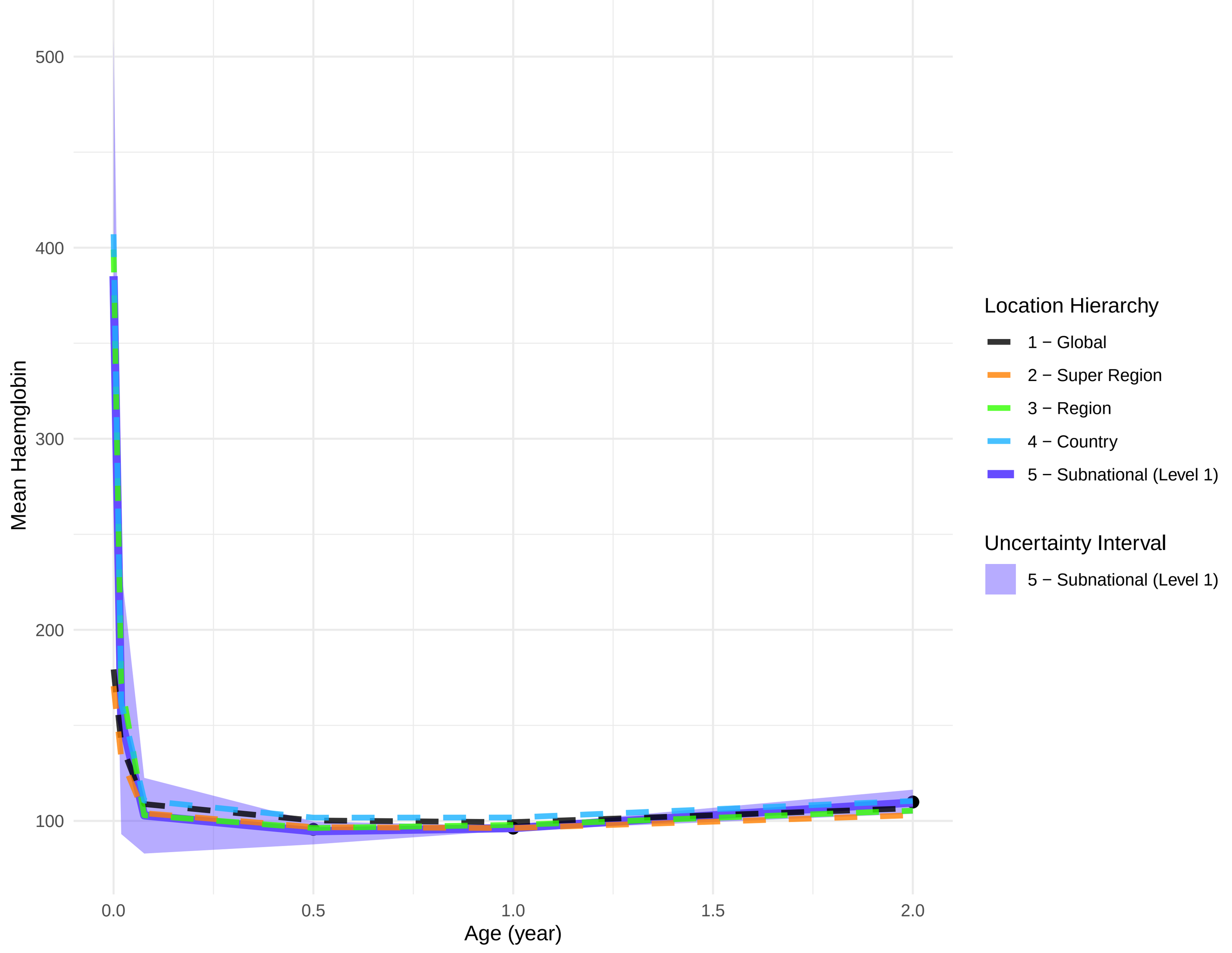

MR−BRT Age/Sex Fits − Non−pregnant Females <2 Years Old − Mean Haemglobin − Madagascar
WHO−2024 Adjustment Method

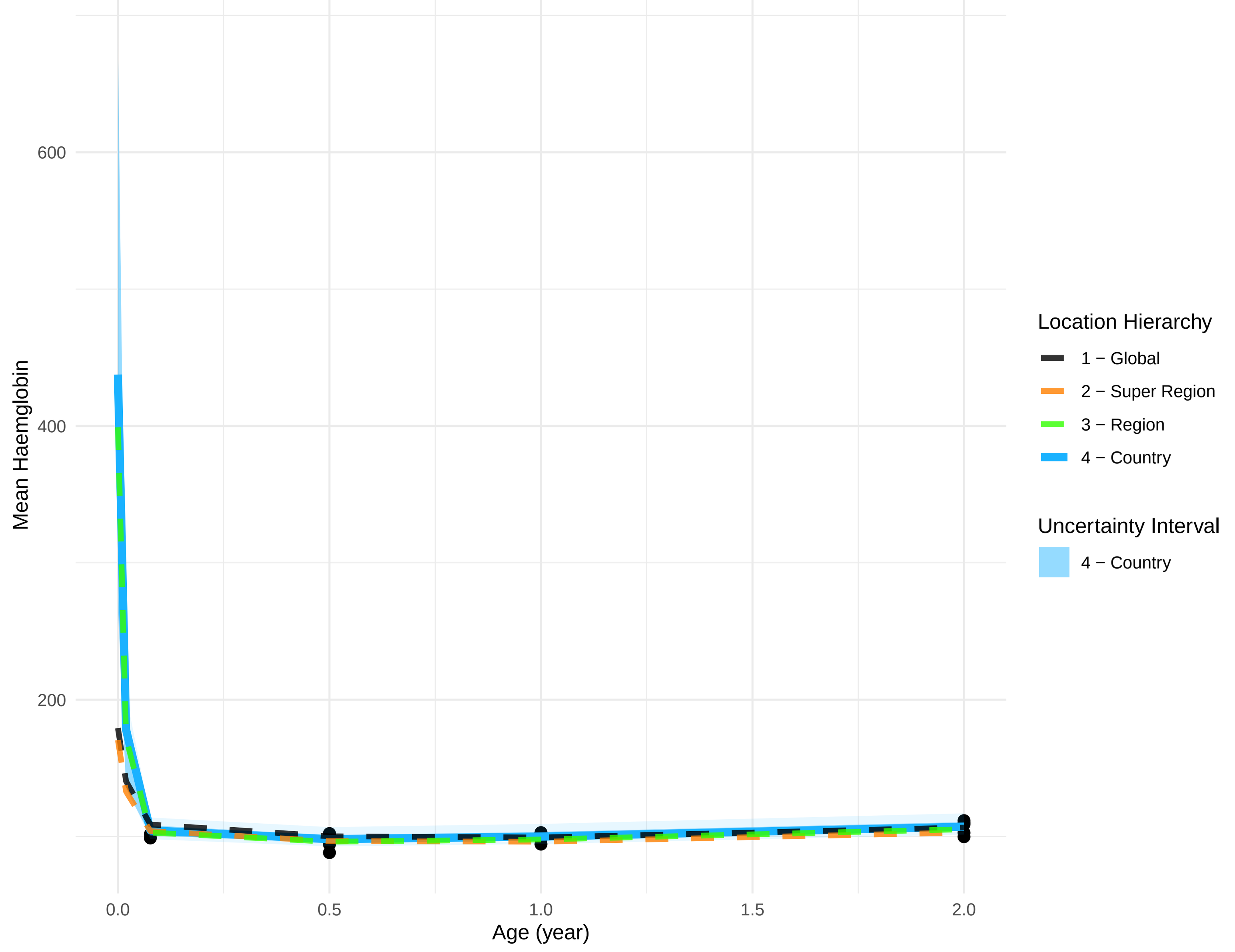

MR−BRT Age/Sex Fits − Non−pregnant Females <2 Years Old − Mean Haemglobin − Malawi
WHO−2024 Adjustment Method

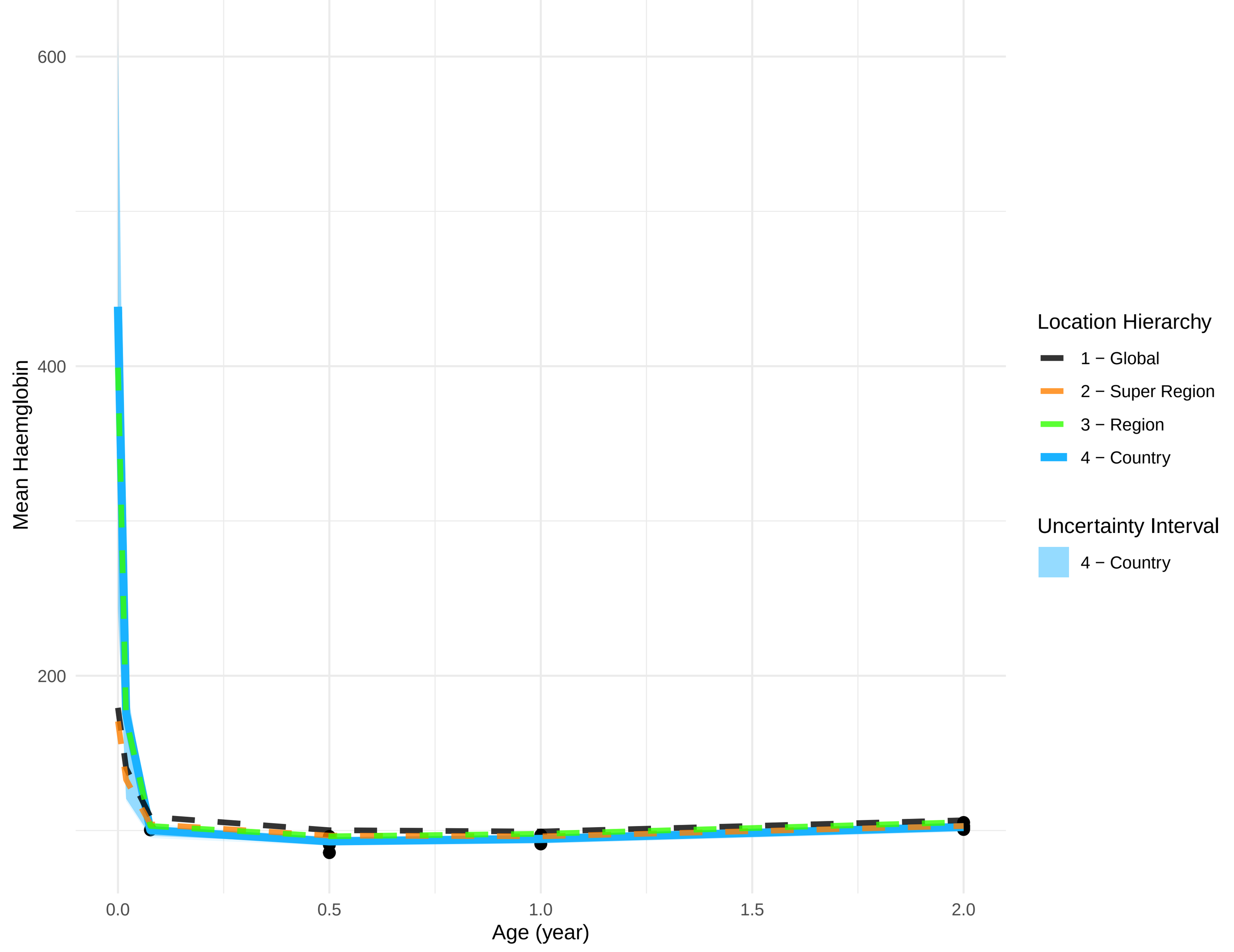

MR−BRT Age/Sex Fits − Non−pregnant Females <2 Years Old − Mean Haemglobin − Mozambique
WHO−2024 Adjustment Method

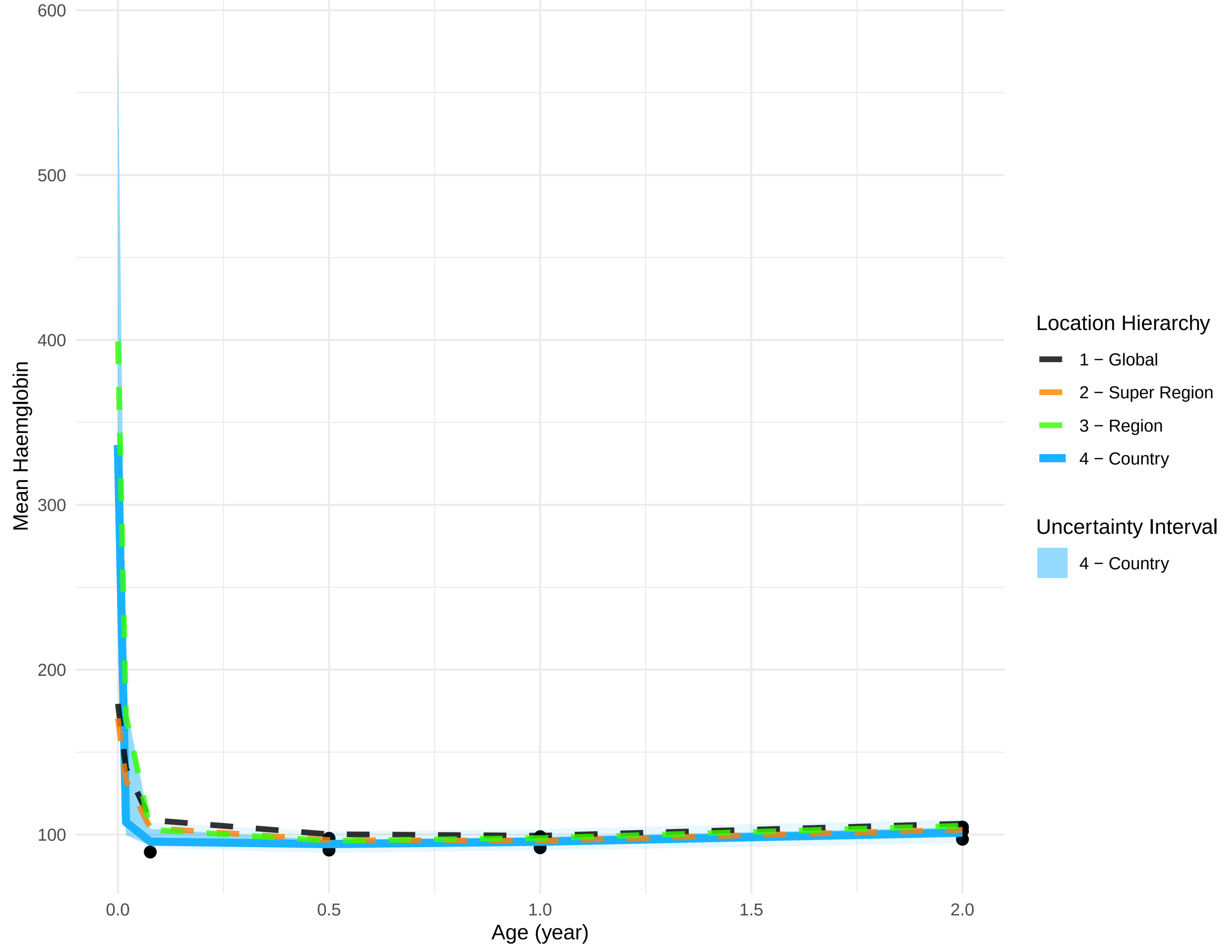

MR−BRT Age/Sex Fits − Non−pregnant Females <2 Years Old − Mean Haemglobin − Rwanda
WHO−2024 Adjustment Method

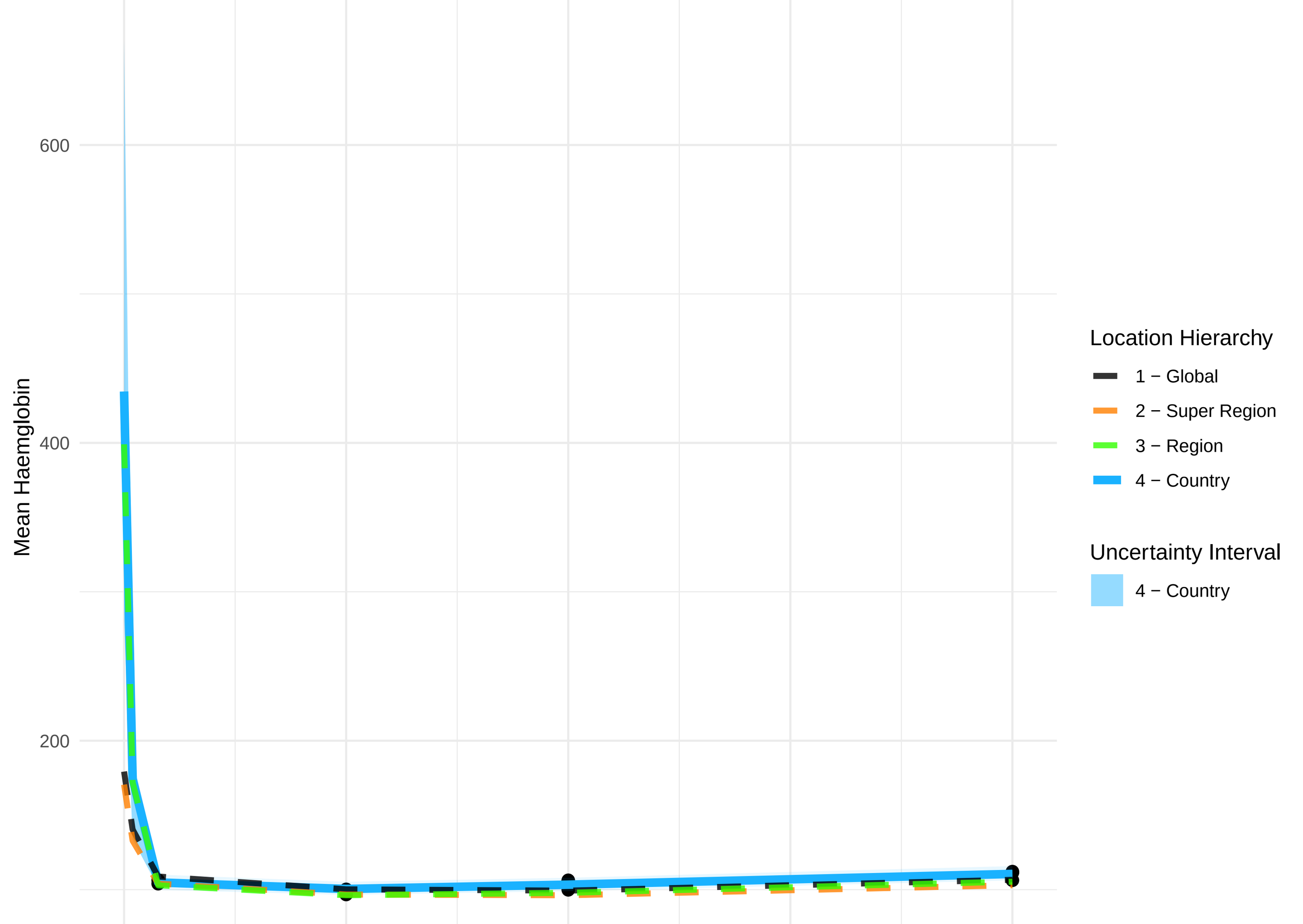

MR−BRT Age/Sex Fits − Non−pregnant Females <2 Years Old − Mean Haemglobin − Uganda
WHO−2024 Adjustment Method

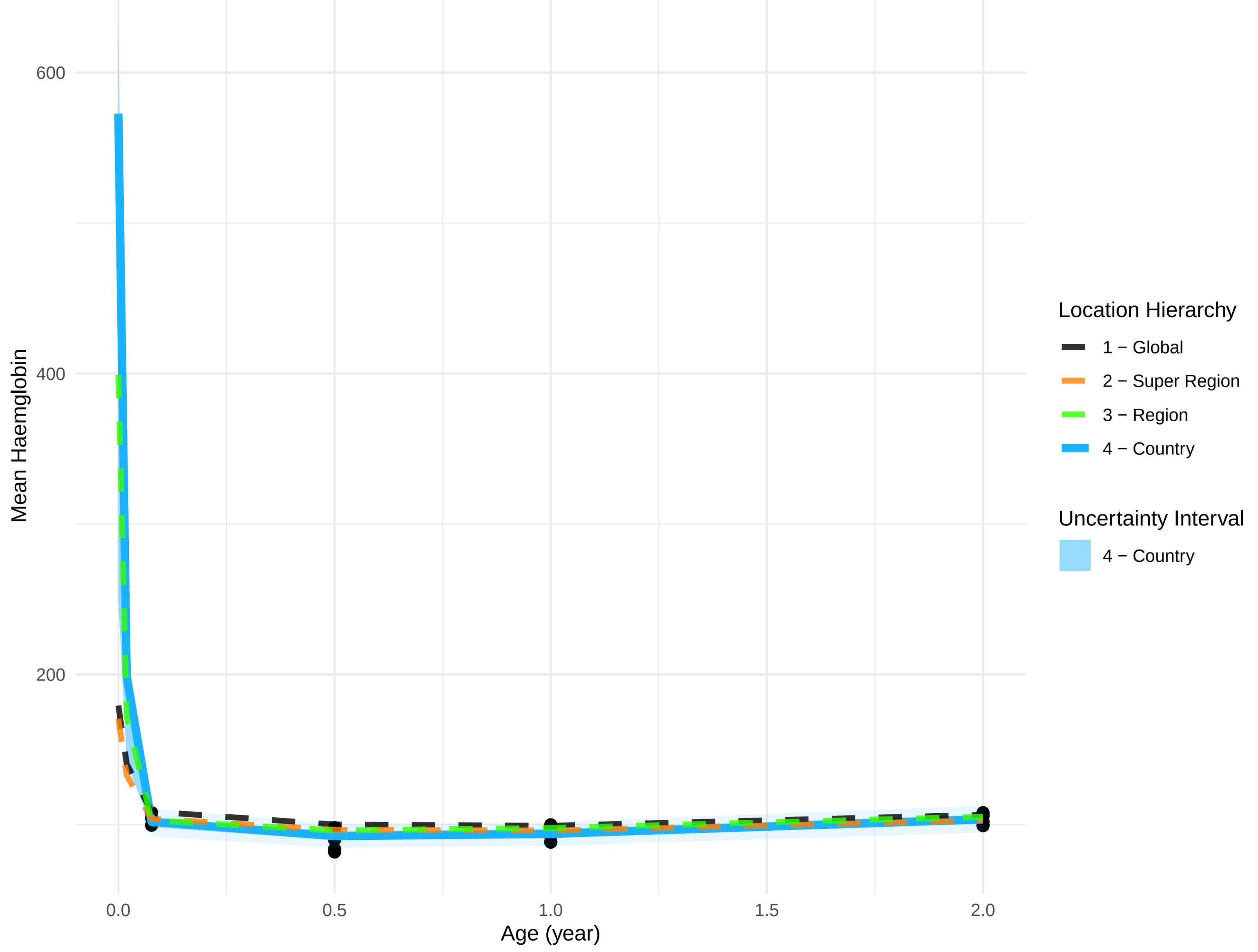

MR−BRT Age/Sex Fits − Non−pregnant Females <2 Years Old − Mean Haemglobin − United Republic of Tanzania
WHO−2024 Adjustment Method

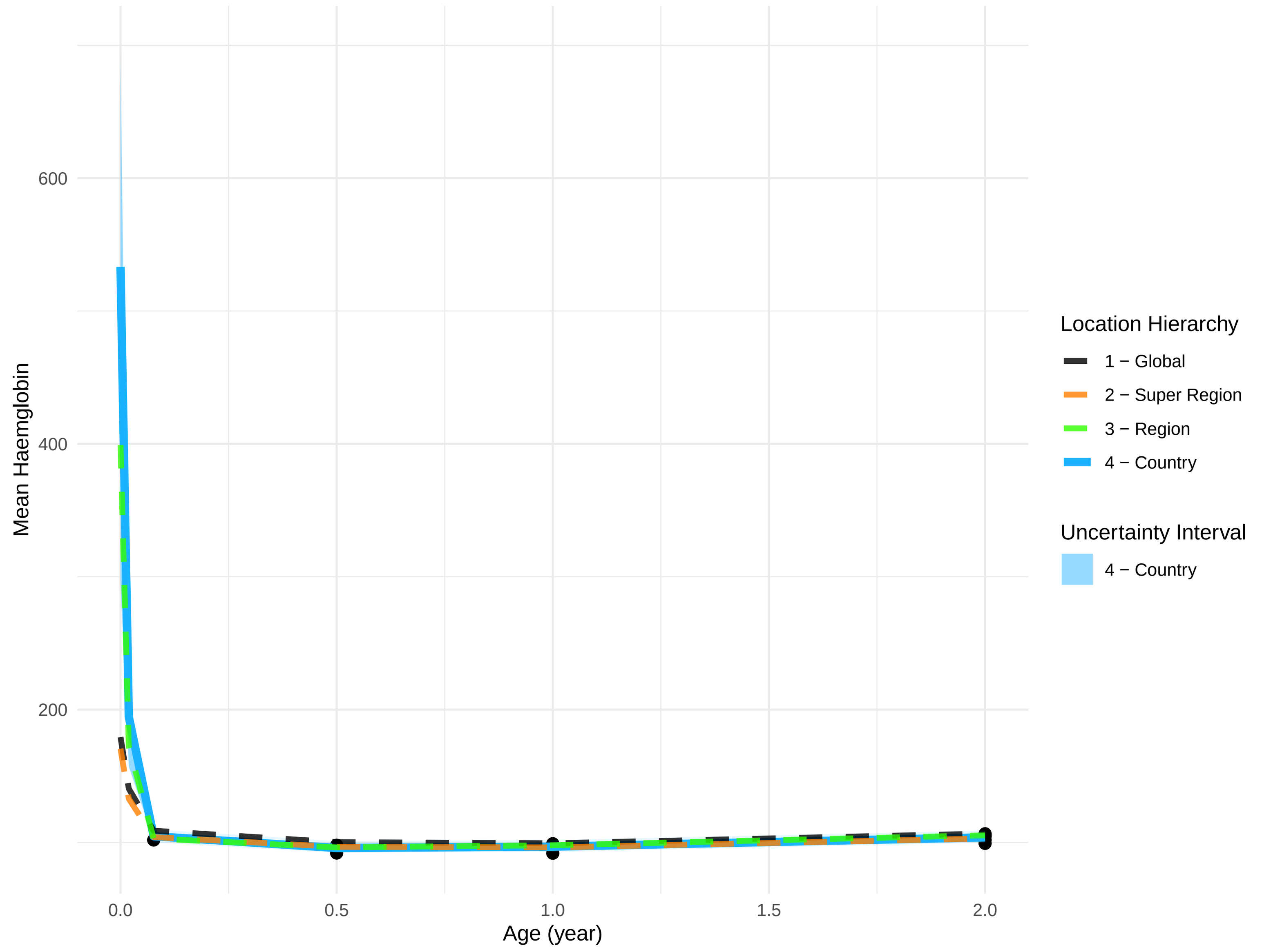

MR−BRT Age/Sex Fits − Non−pregnant Females <2 Years Old − Mean Haemglobin − Zambia
WHO−2024 Adjustment Method

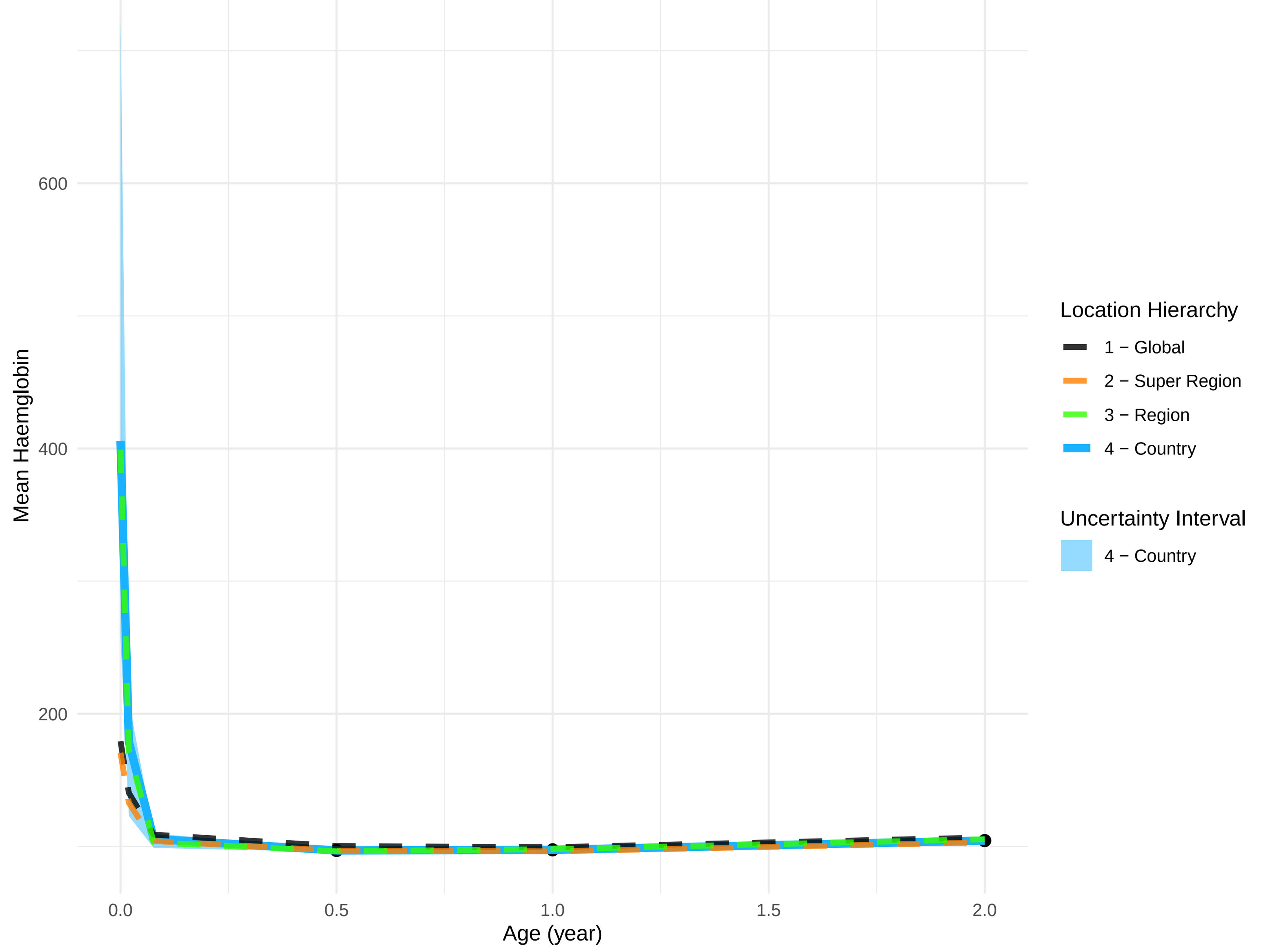

MR−BRT Age/Sex Fits − Non−pregnant Females <2 Years Old − Mean Haemglobin − Southern Sub−Saharan Africa
WHO−2024 Adjustment Method

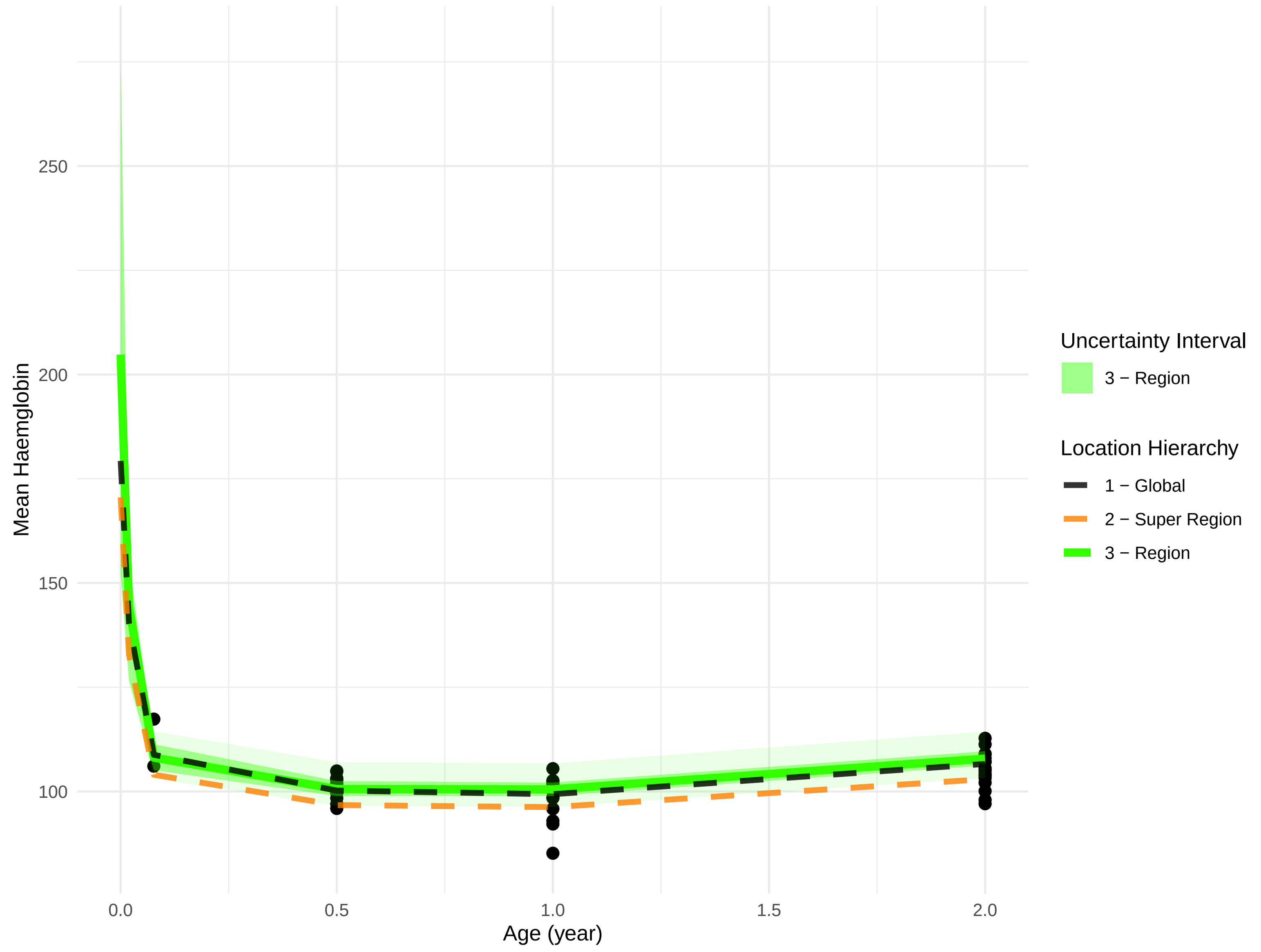

MR−BRT Age/Sex Fits − Non−pregnant Females <2 Years Old − Mean Haemglobin − Eswatini
WHO−2024 Adjustment Method

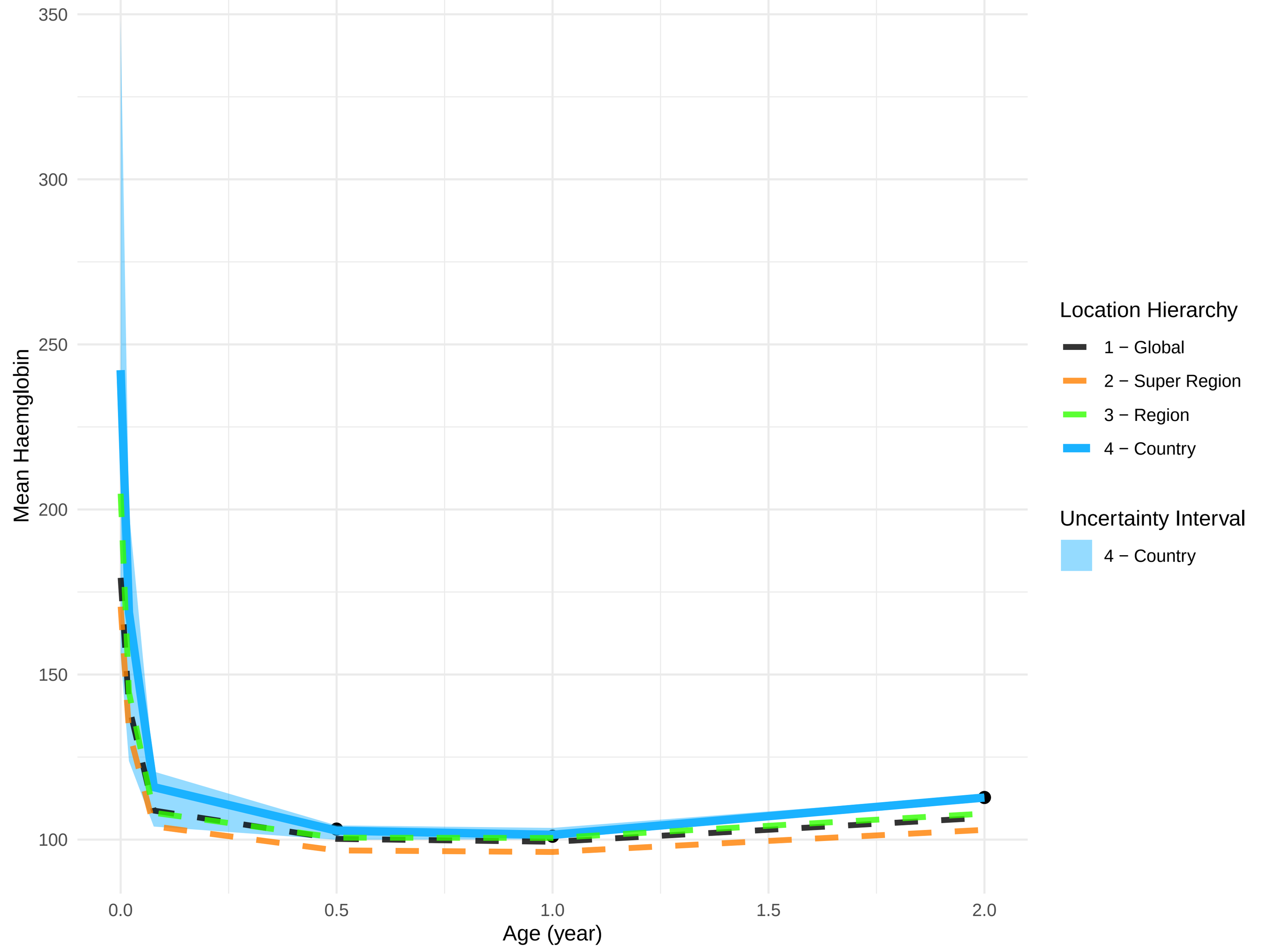

MR−BRT Age/Sex Fits − Non−pregnant Females <2 Years Old − Mean Haemglobin − Lesotho
WHO−2024 Adjustment Method

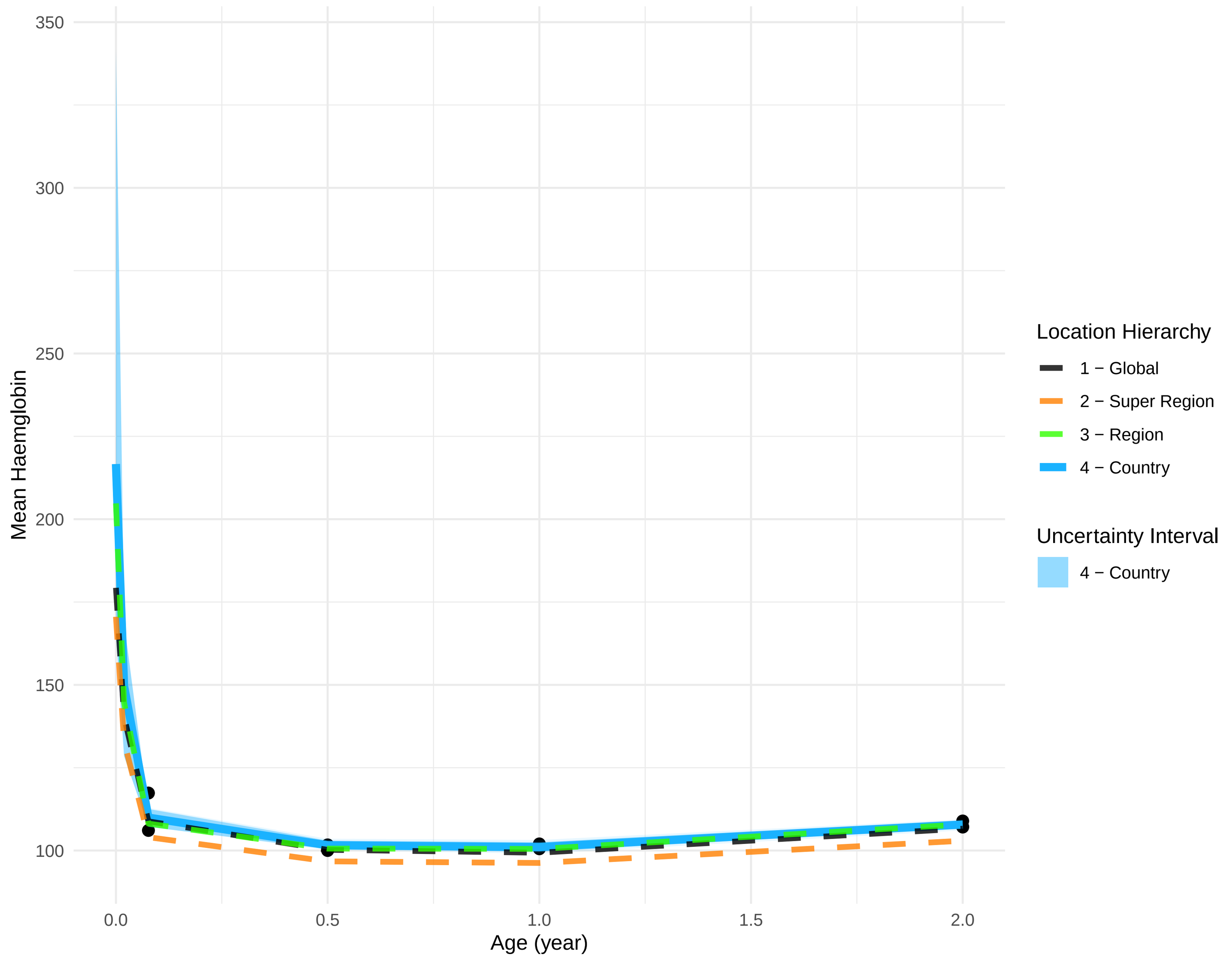

MR−BRT Age/Sex Fits − Non−pregnant Females <2 Years Old − Mean Haemglobin − Namibia
WHO−2024 Adjustment Method

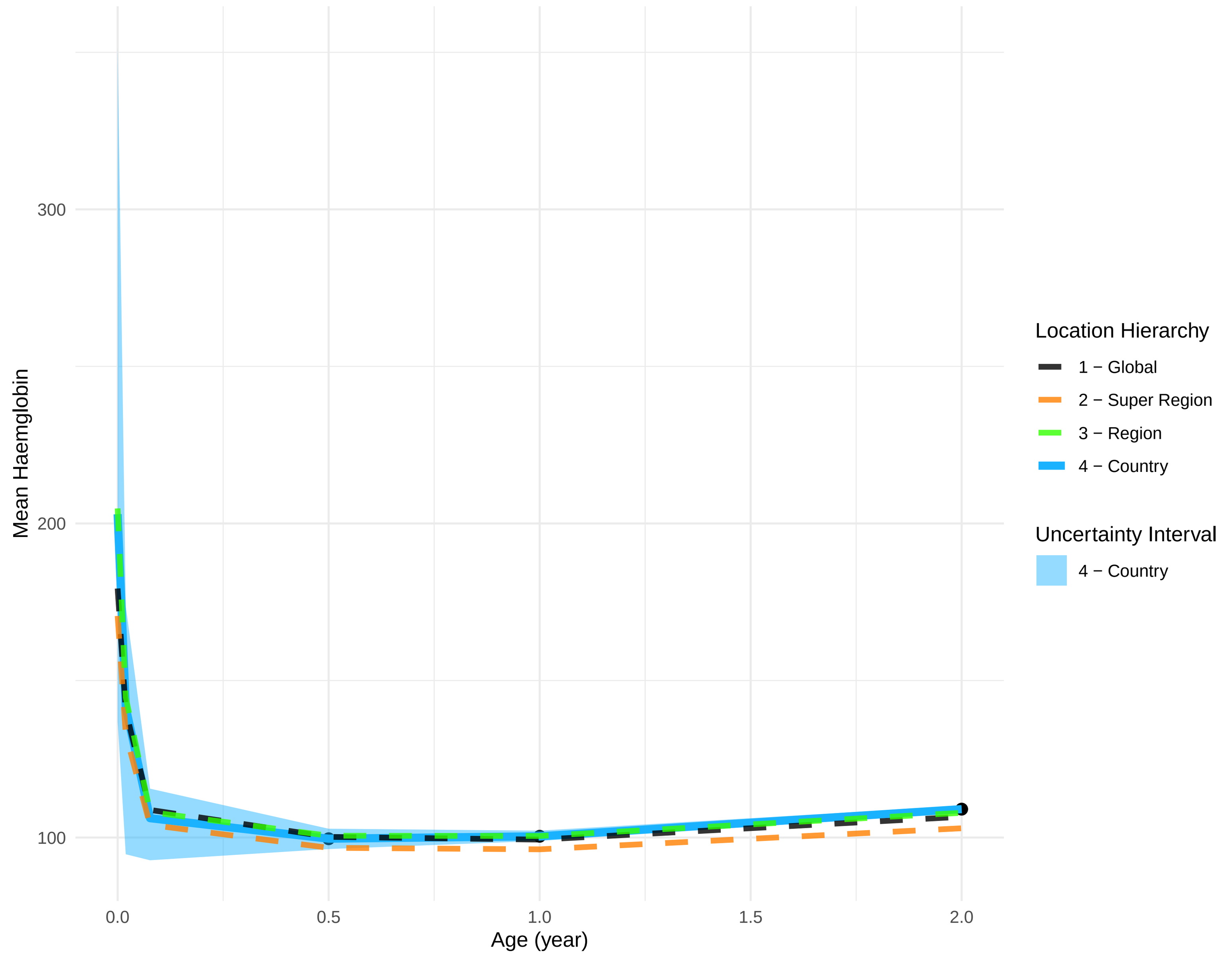

MR−BRT Age/Sex Fits − Non−pregnant Females <2 Years Old − Mean Haemglobin − South Africa
WHO−2024 Adjustment Method

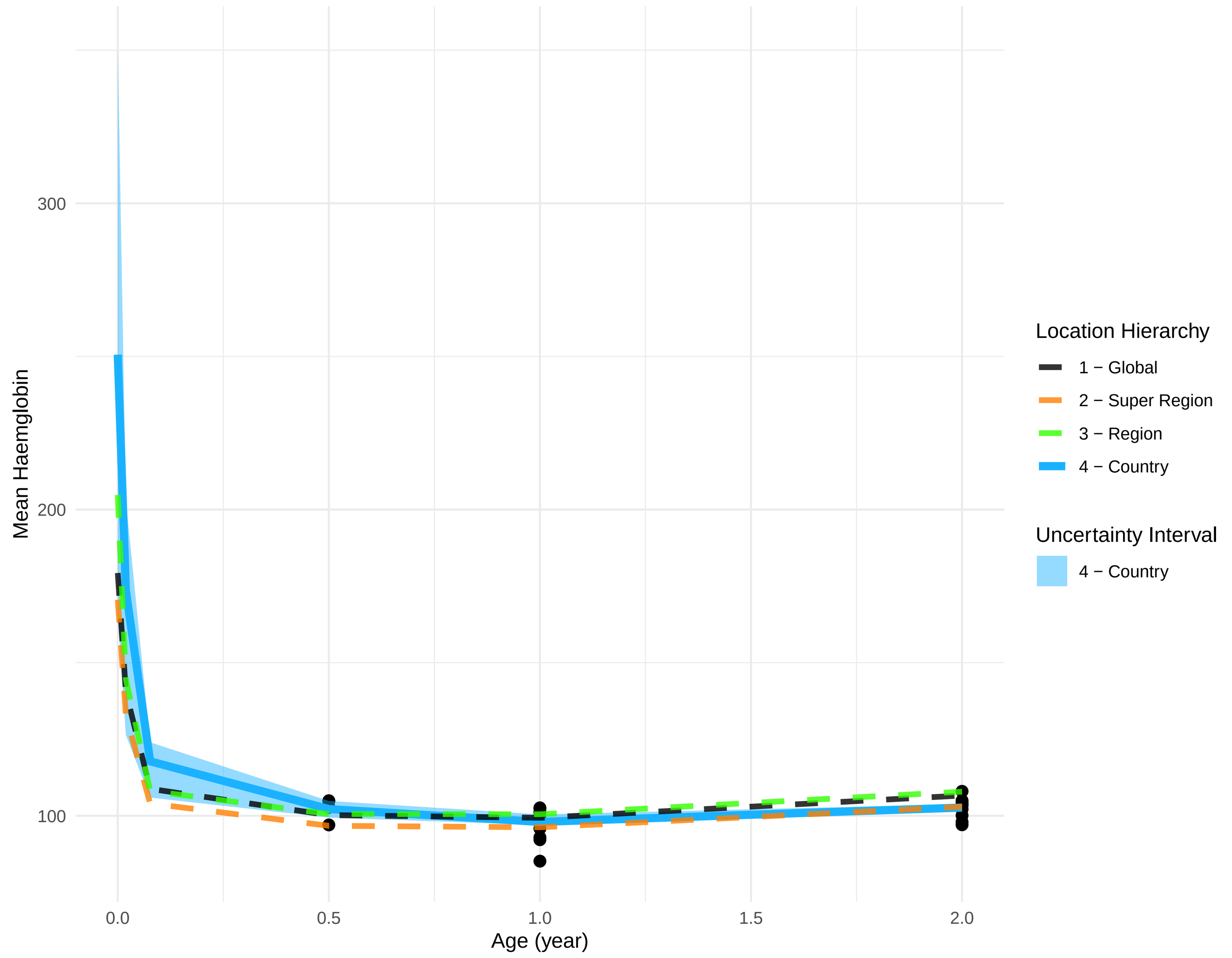

MR−BRT Age/Sex Fits − Non−pregnant Females <2 Years Old − Mean Haemglobin − Eastern Cape
WHO−2024 Adjustment Method

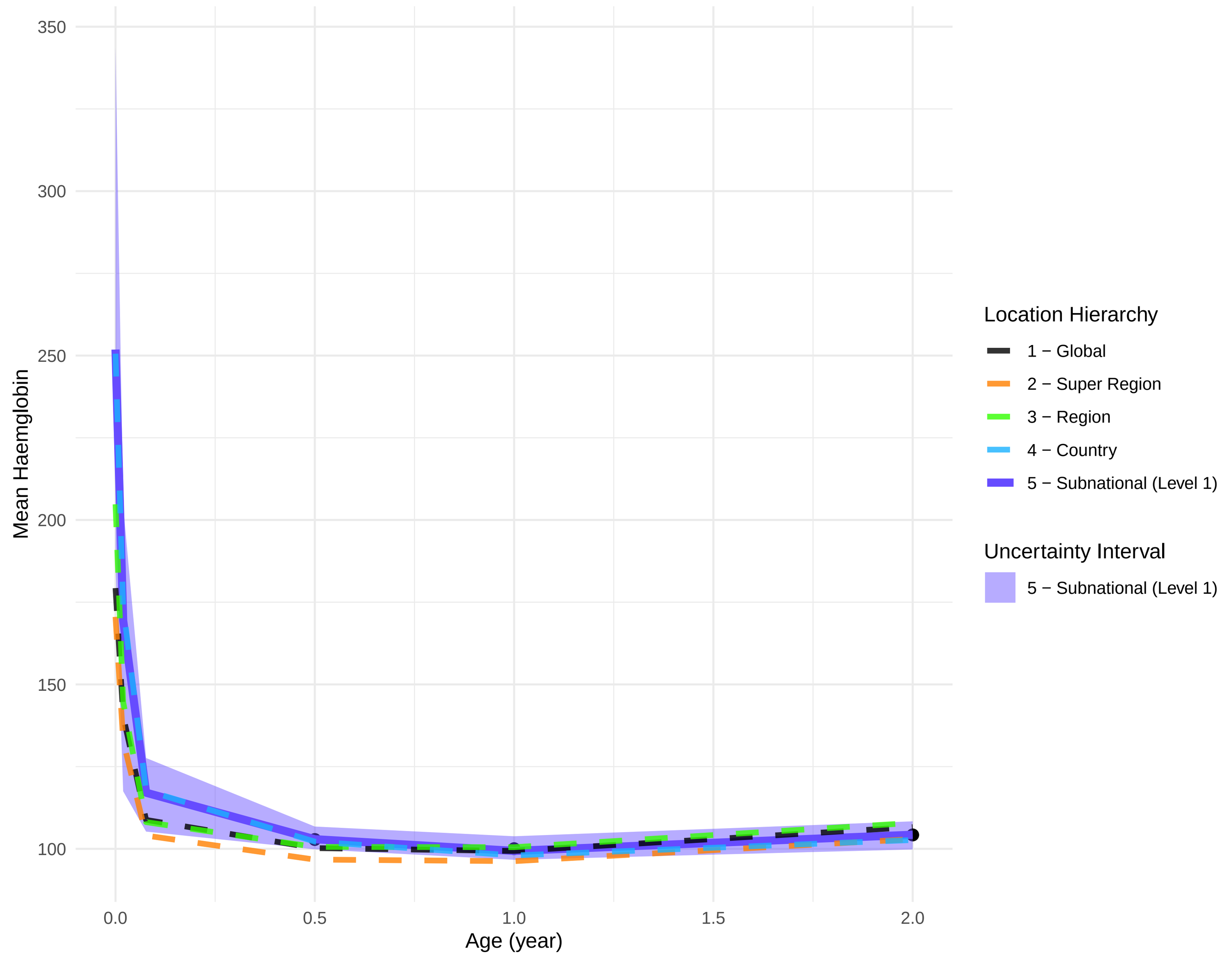

MR−BRT Age/Sex Fits − Non−pregnant Females <2 Years Old − Mean Haemglobin − Free State
WHO−2024 Adjustment Method

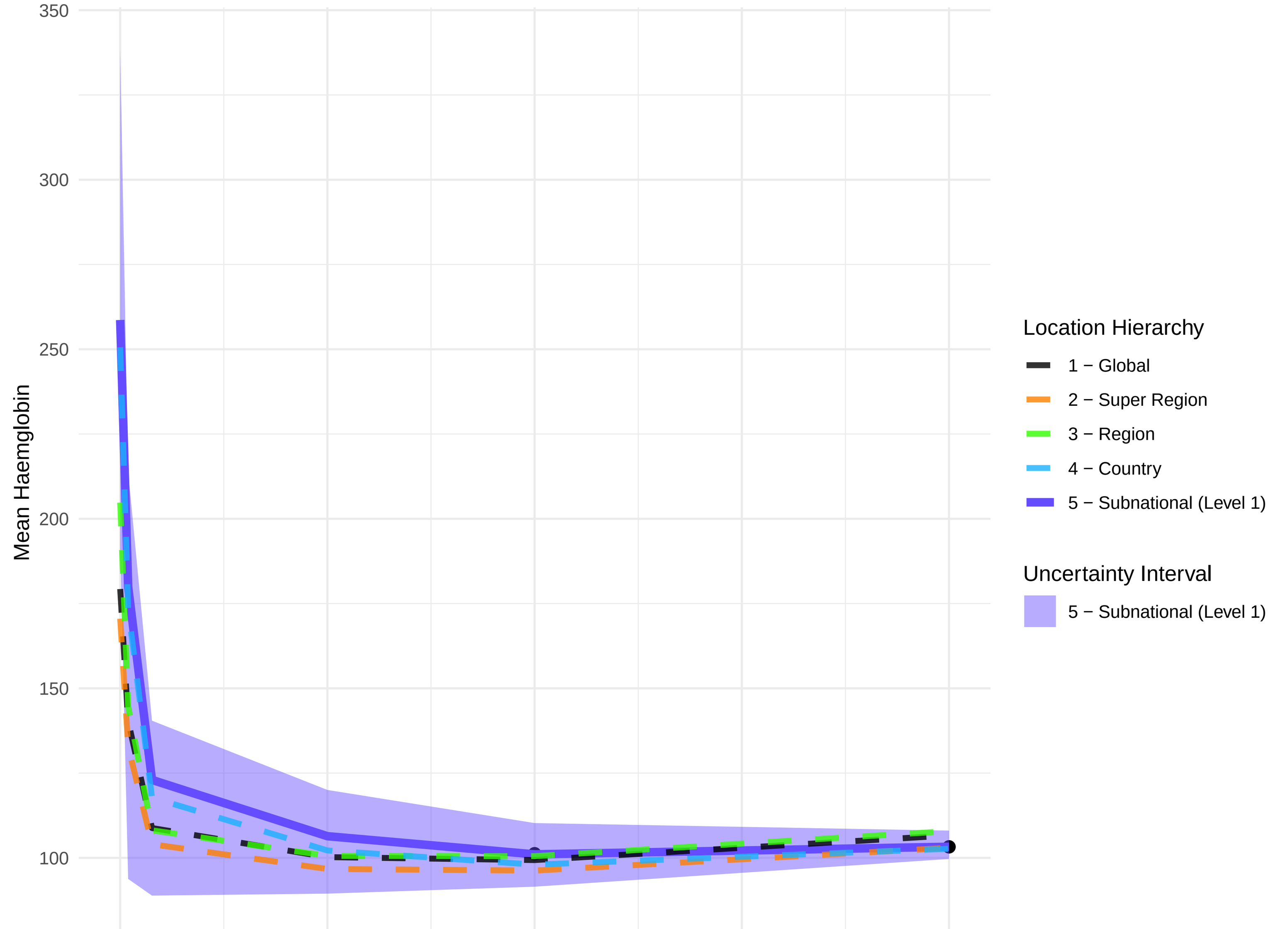

MR−BRT Age/Sex Fits − Non−pregnant Females <2 Years Old − Mean Haemglobin − Gauteng
WHO−2024 Adjustment Method

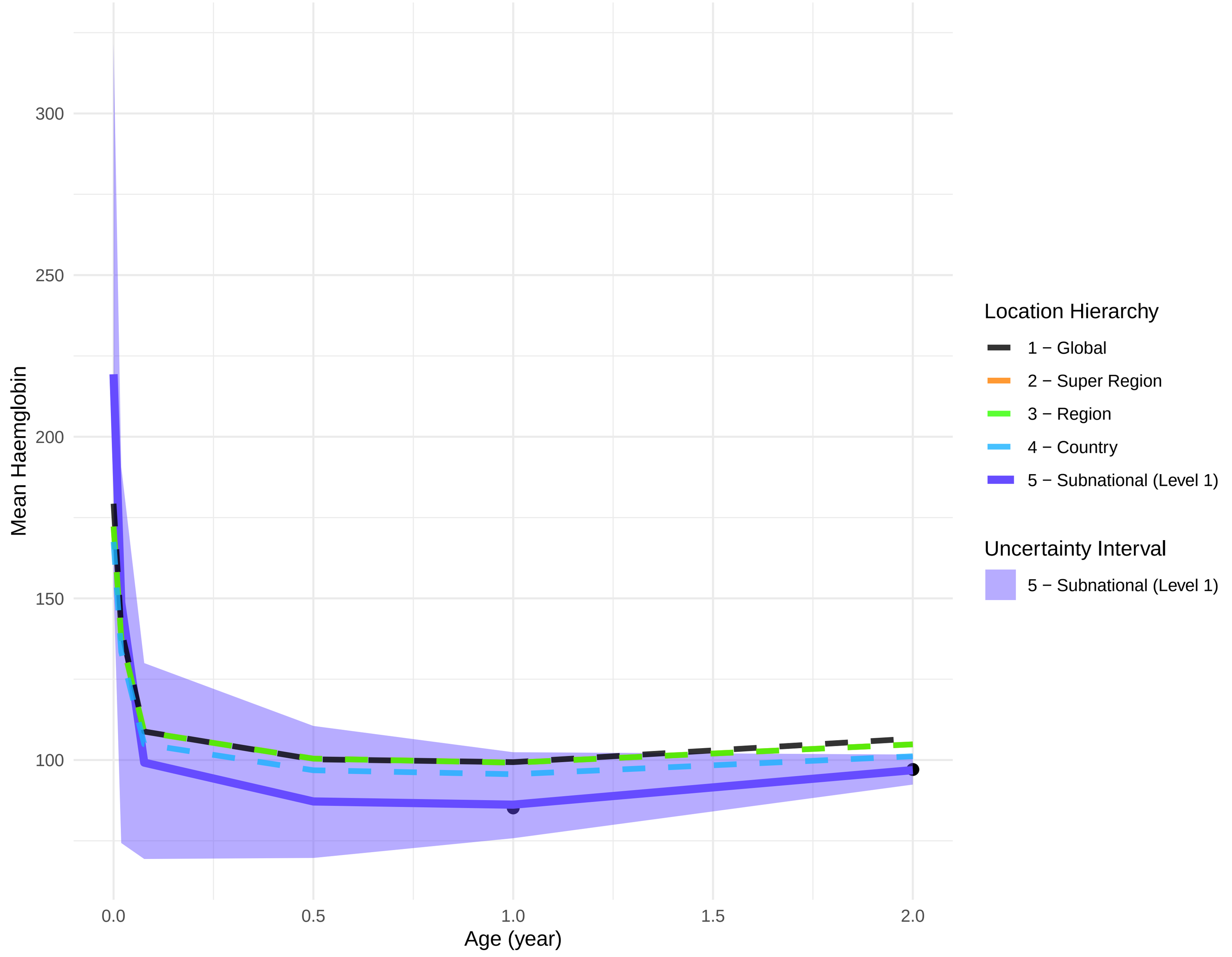

MR−BRT Age/Sex Fits − Non−pregnant Females <2 Years Old − Mean Haemglobin − KwaZulu−Natal
WHO−2024 Adjustment Method

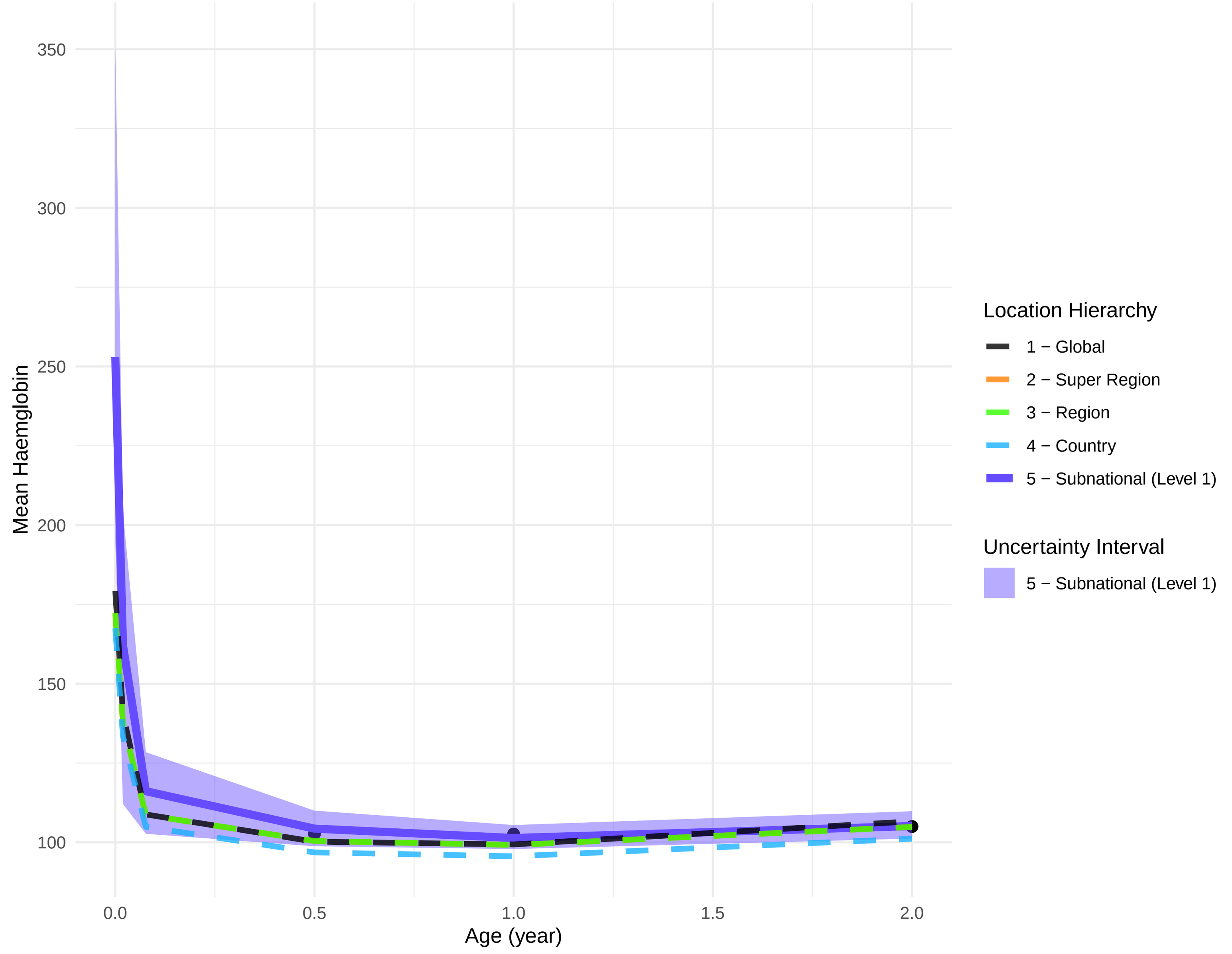

MR−BRT Age/Sex Fits − Non−pregnant Females <2 Years Old − Mean Haemglobin − Limpopo
WHO−2024 Adjustment Method

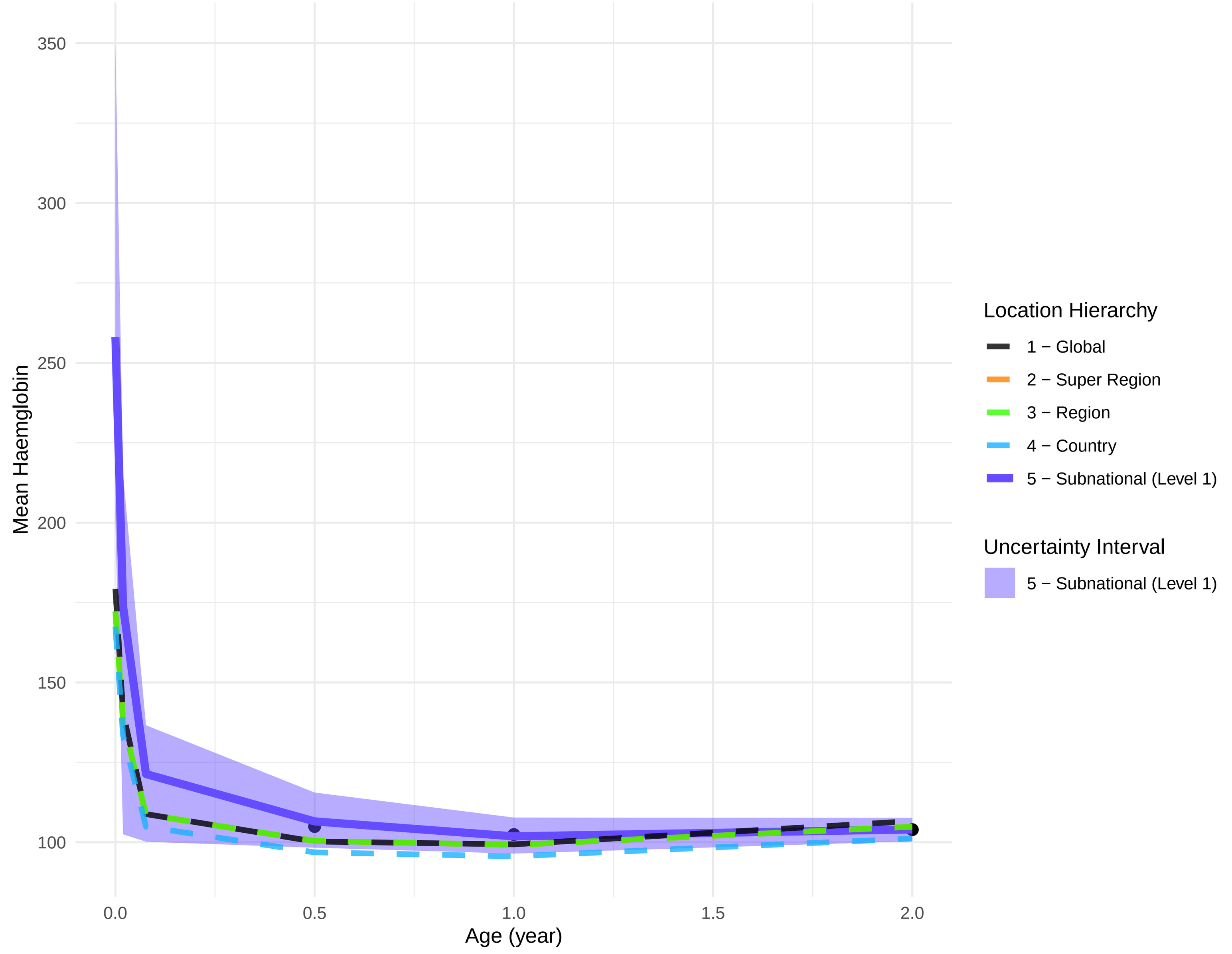

MR−BRT Age/Sex Fits − Non−pregnant Females <2 Years Old − Mean Haemglobin − Mpumalanga
WHO−2024 Adjustment Method

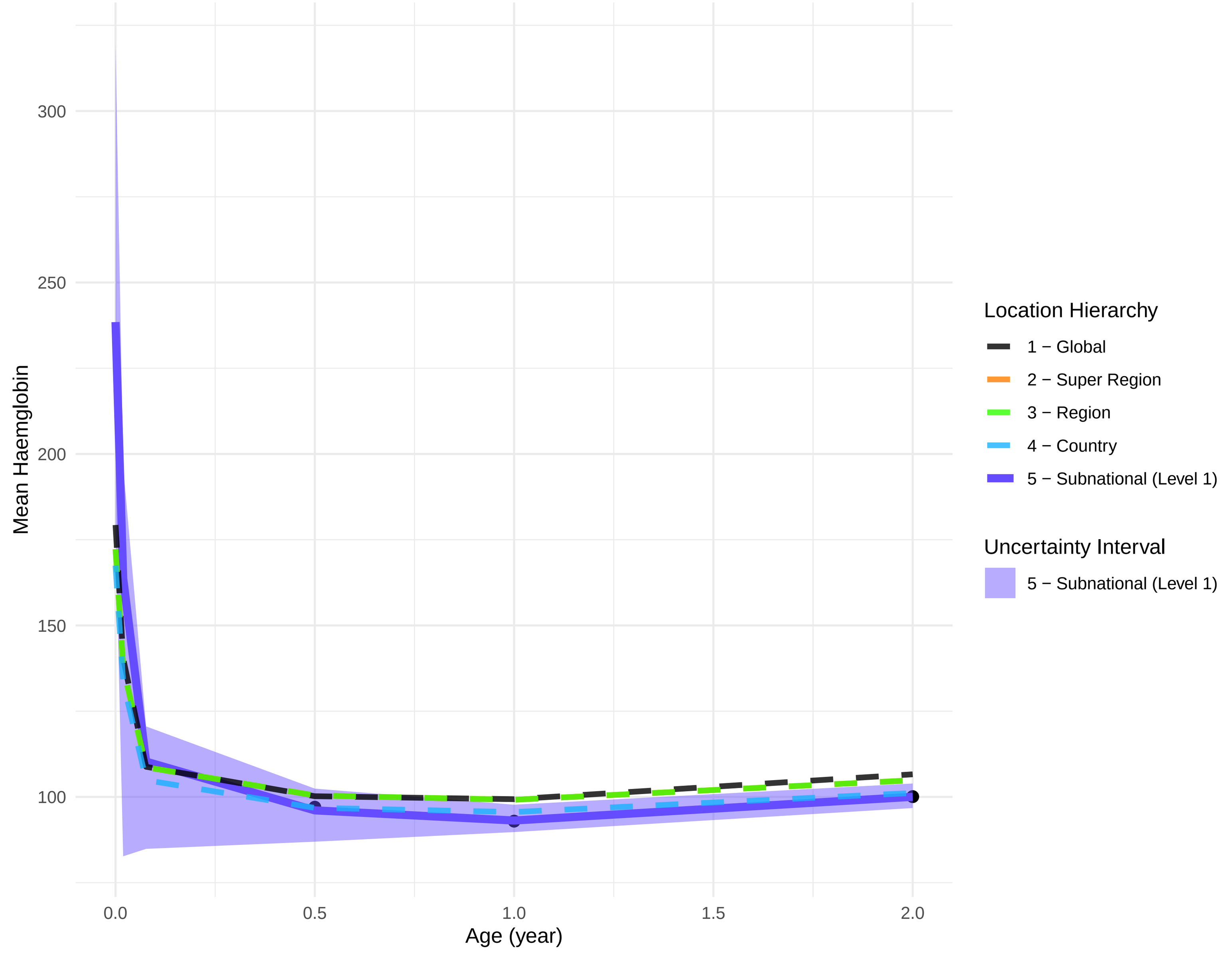

MR−BRT Age/Sex Fits − Non−pregnant Females <2 Years Old − Mean Haemglobin − North−West
WHO−2024 Adjustment Method

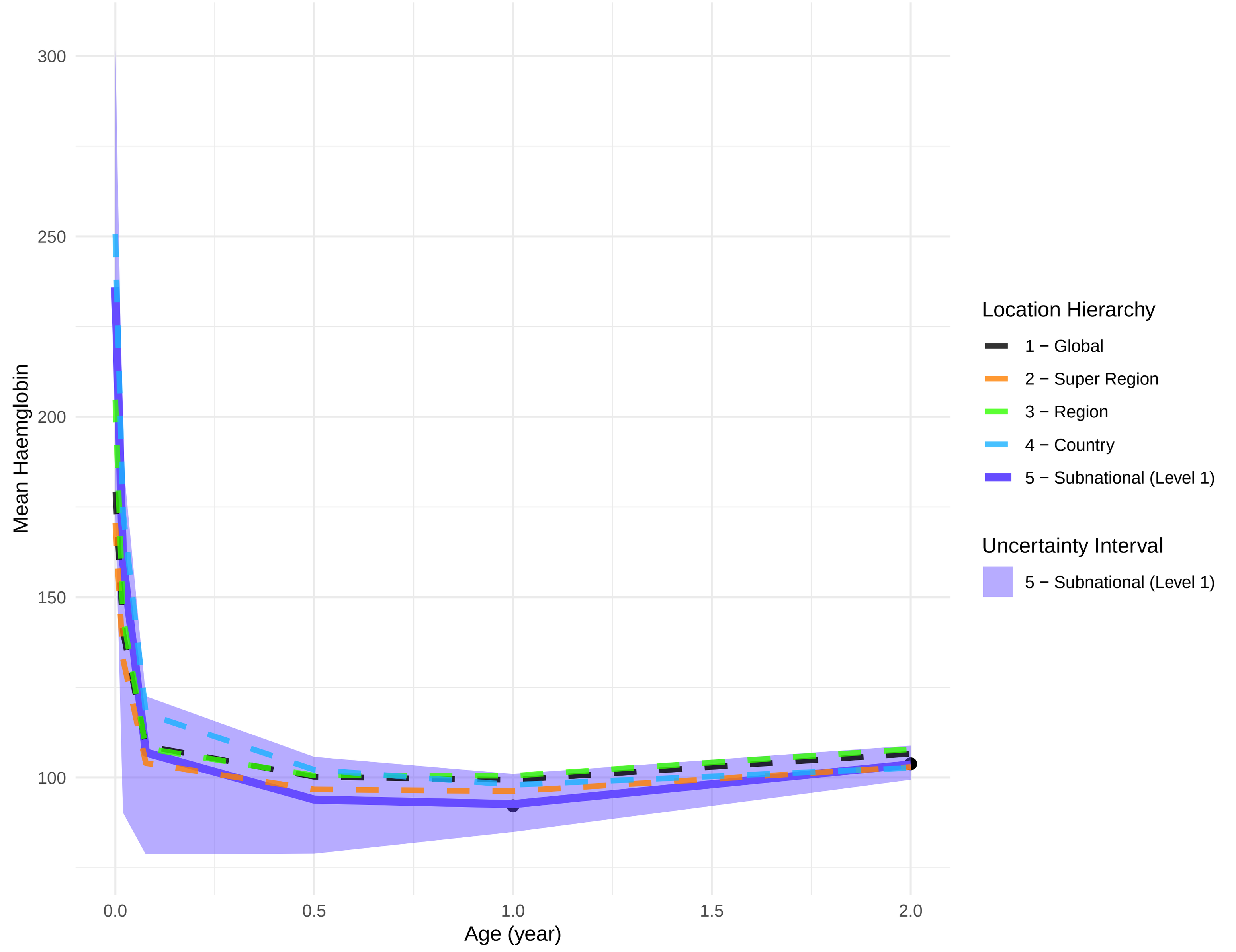

MR−BRT Age/Sex Fits − Non−pregnant Females <2 Years Old − Mean Haemglobin − Northern Cape
WHO−2024 Adjustment Method

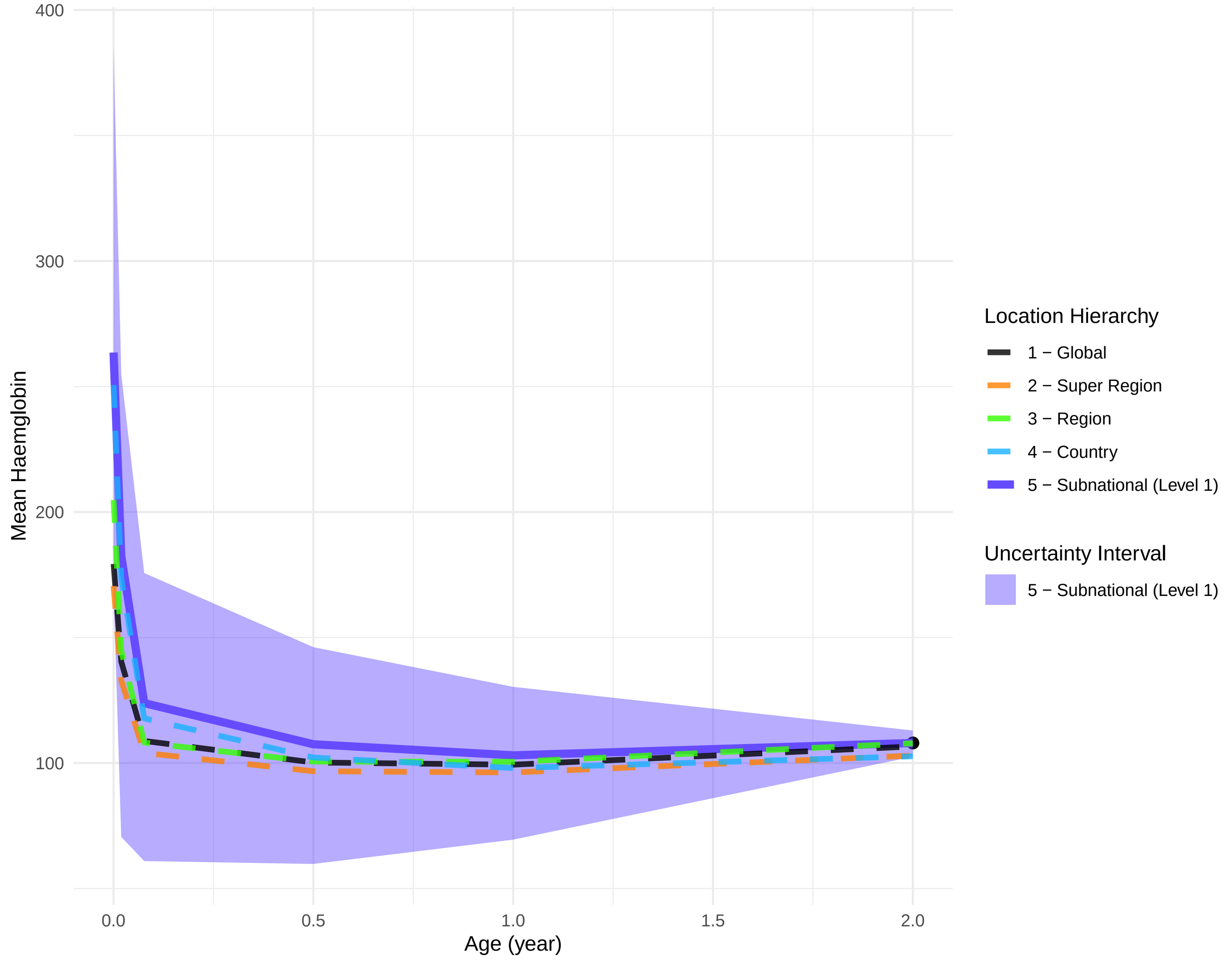

MR−BRT Age/Sex Fits − Non−pregnant Females <2 Years Old − Mean Haemglobin − Western Cape
WHO−2024 Adjustment Method

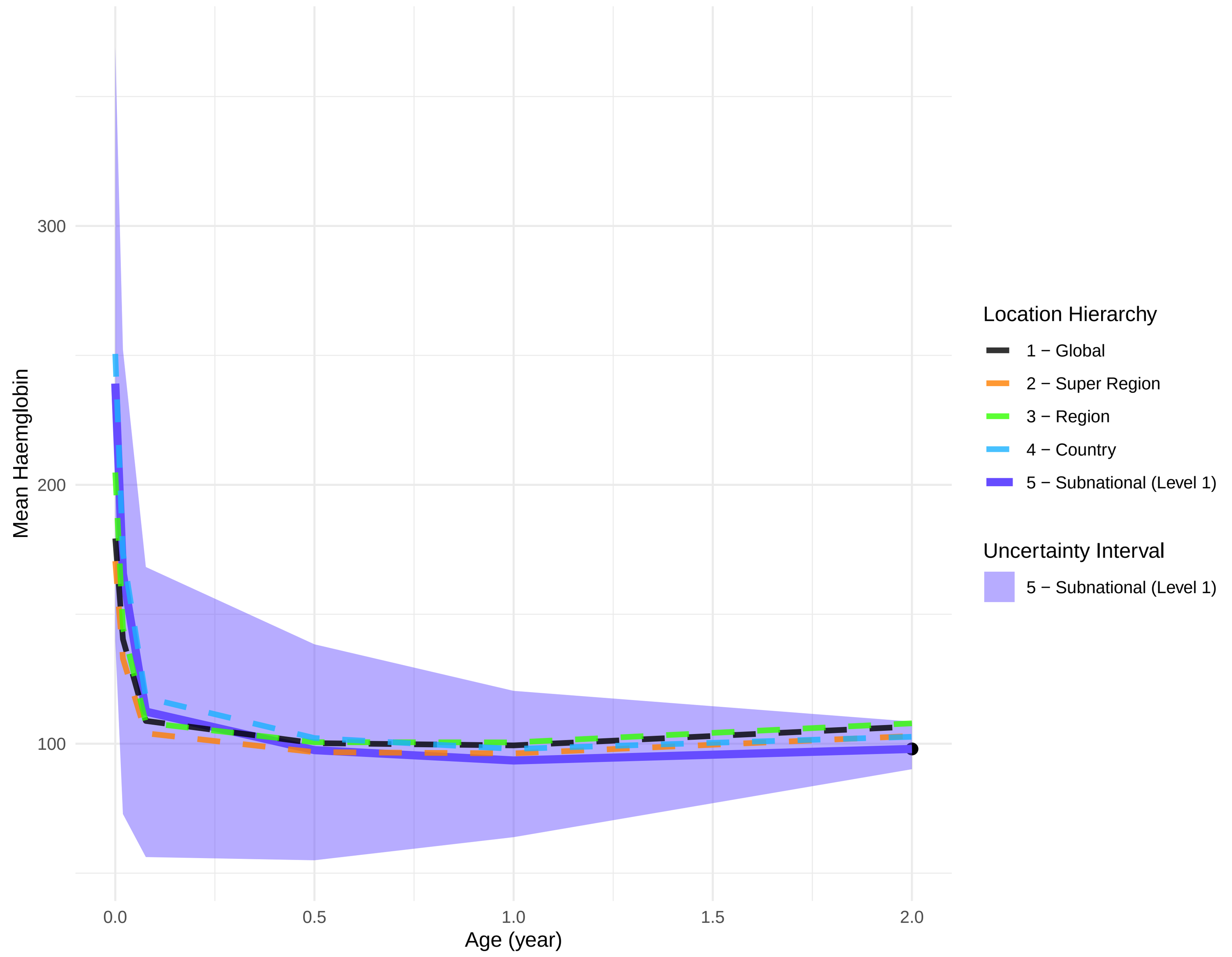

MR−BRT Age/Sex Fits − Non−pregnant Females <2 Years Old − Mean Haemglobin − Zimbabwe
WHO−2024 Adjustment Method

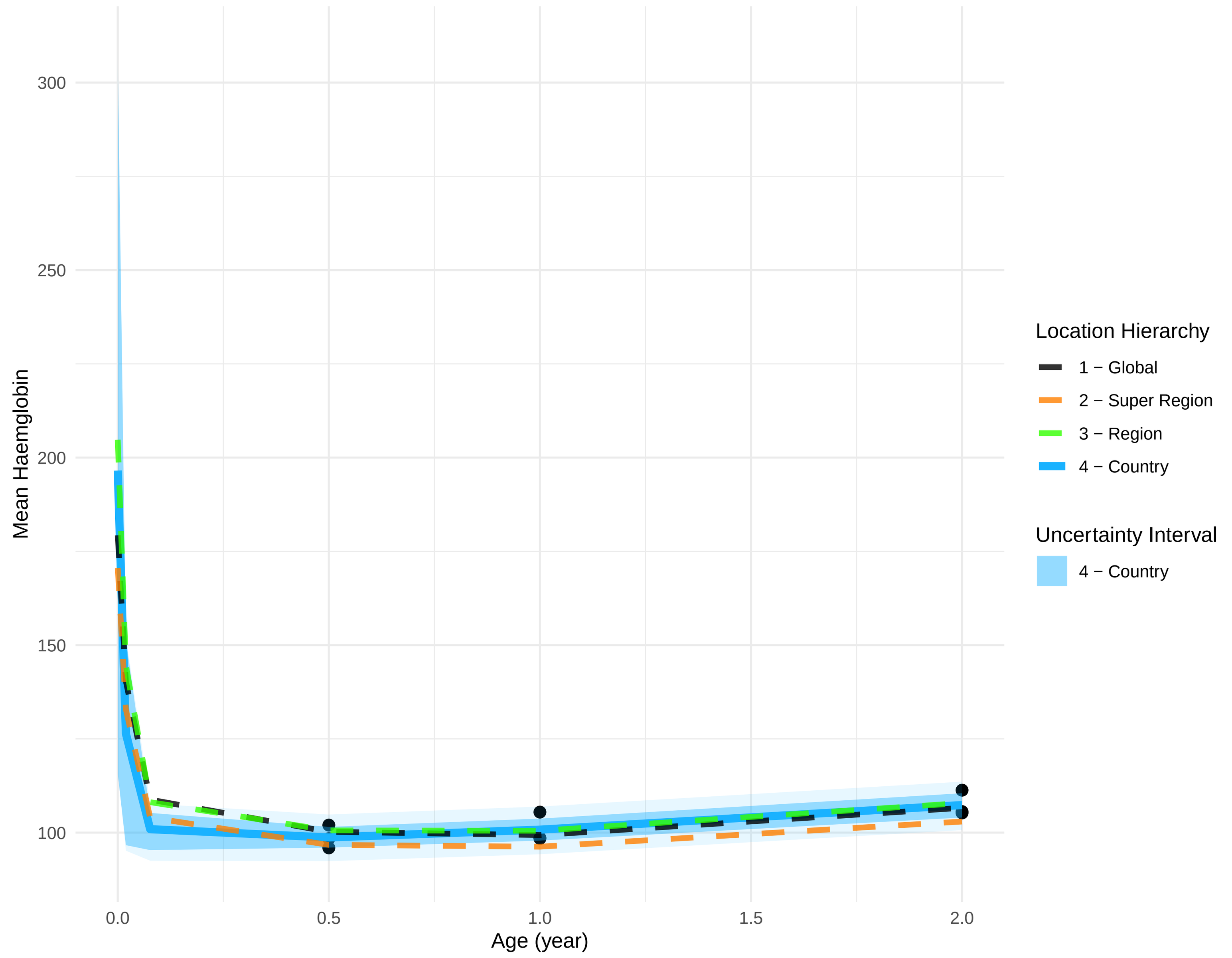

MR−BRT Age/Sex Fits − Non−pregnant Females <2 Years Old − Mean Haemglobin − Western Sub−Saharan Africa
WHO−2024 Adjustment Method

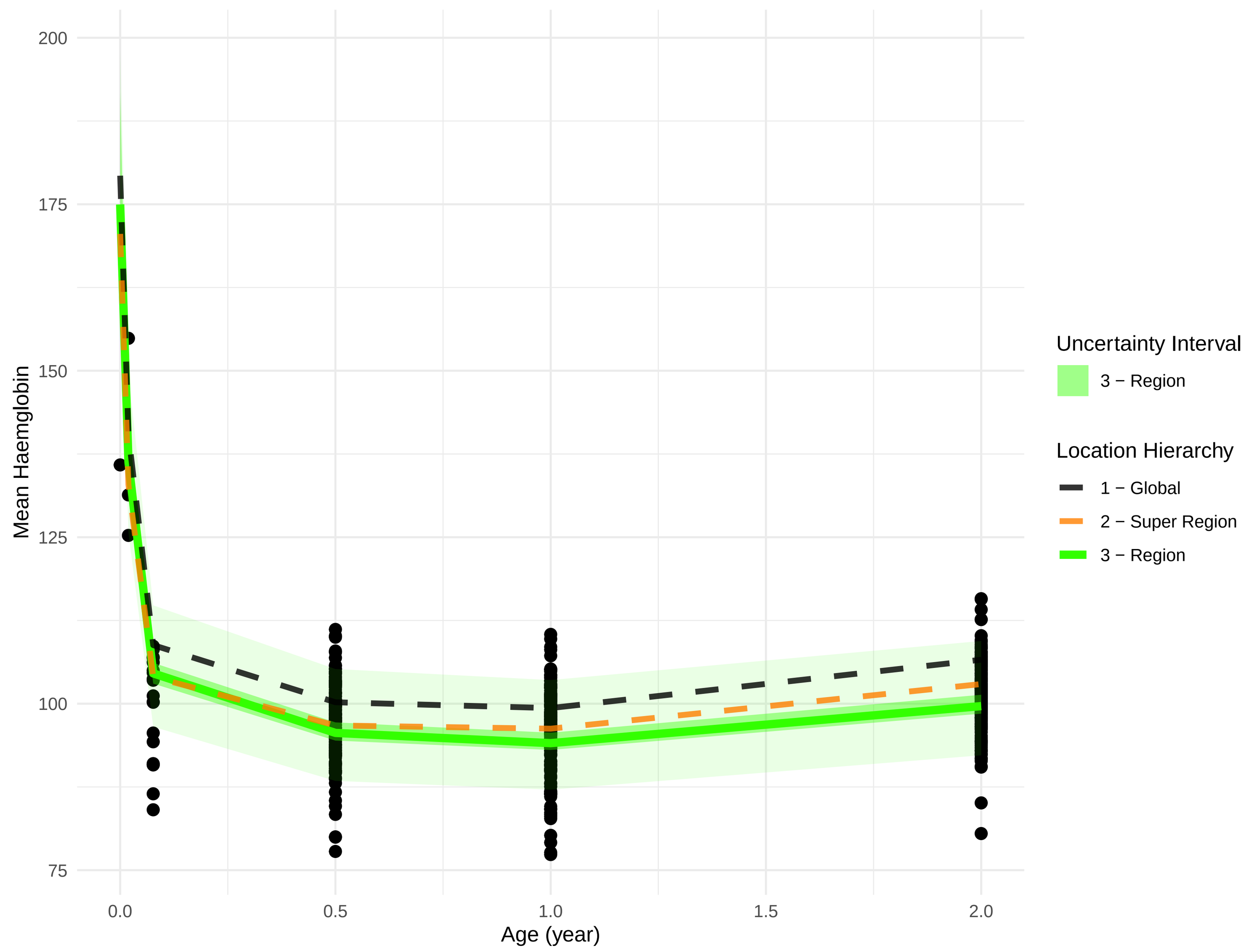

MR−BRT Age/Sex Fits − Non−pregnant Females <2 Years Old − Mean Haemglobin − Benin
WHO−2024 Adjustment Method

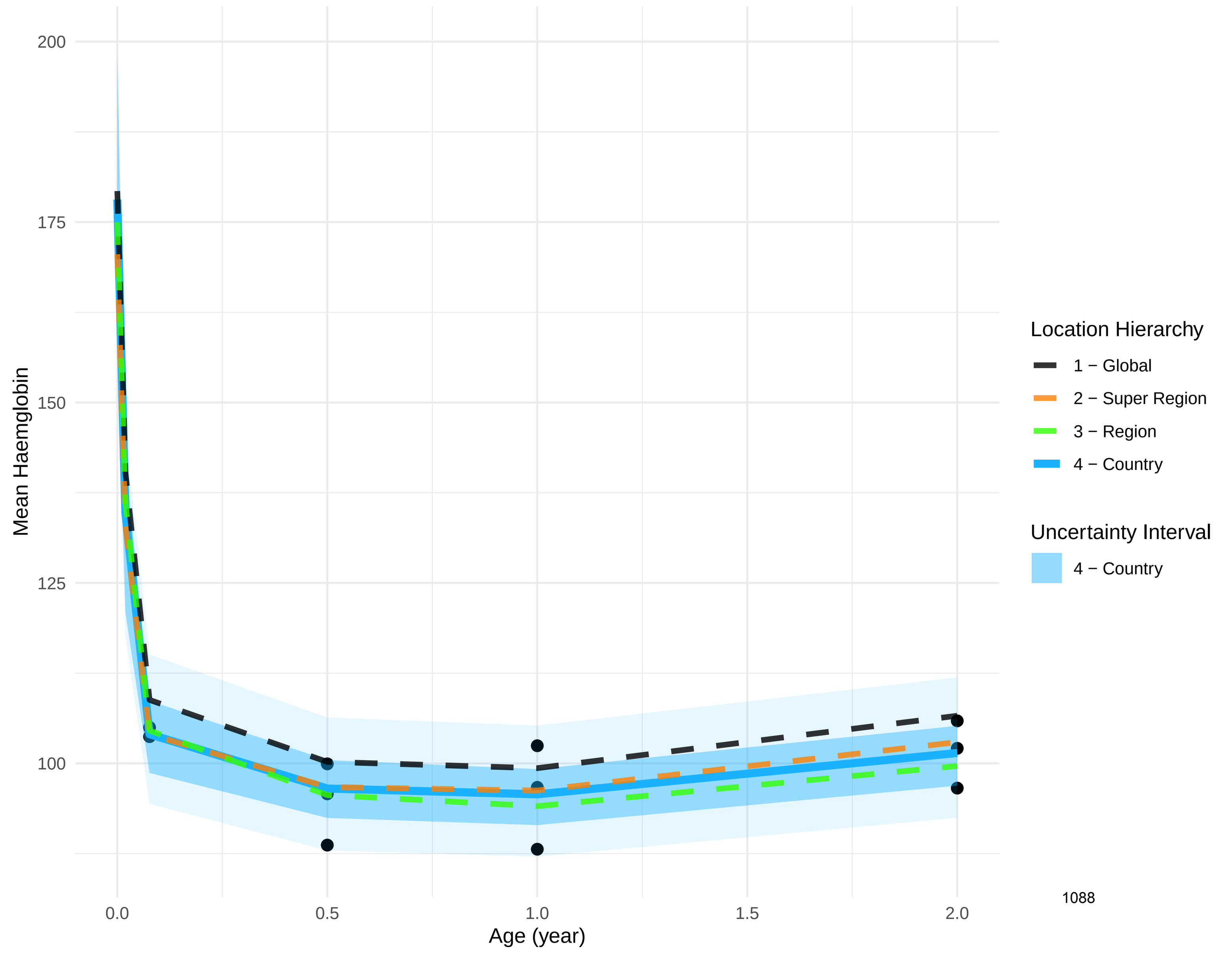

MR−BRT Age/Sex Fits − Non−pregnant Females <2 Years Old − Mean Haemglobin − Burkina Faso
WHO−2024 Adjustment Method

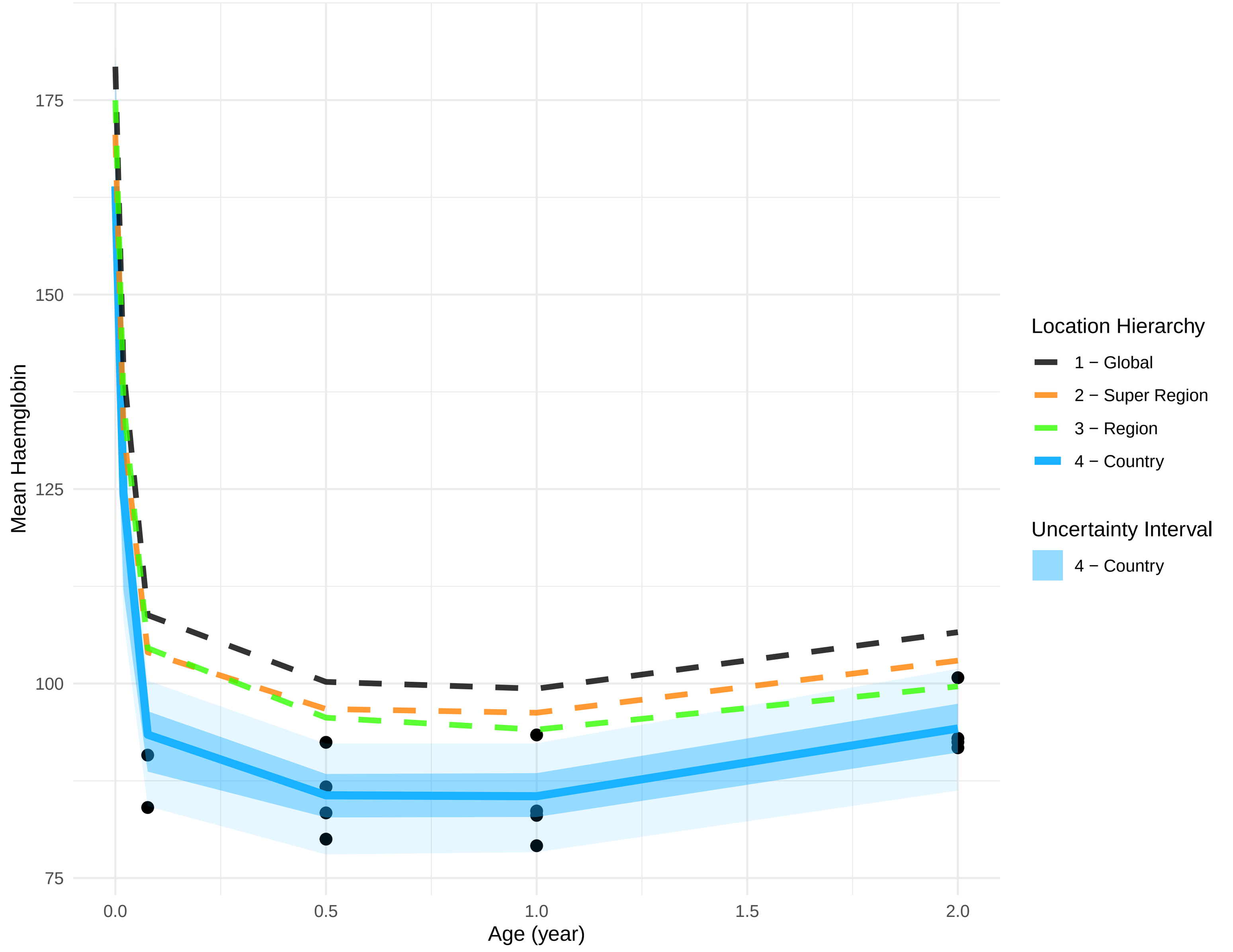

MR−BRT Age/Sex Fits − Non−pregnant Females <2 Years Old − Mean Haemglobin − Cameroon
WHO−2024 Adjustment Method

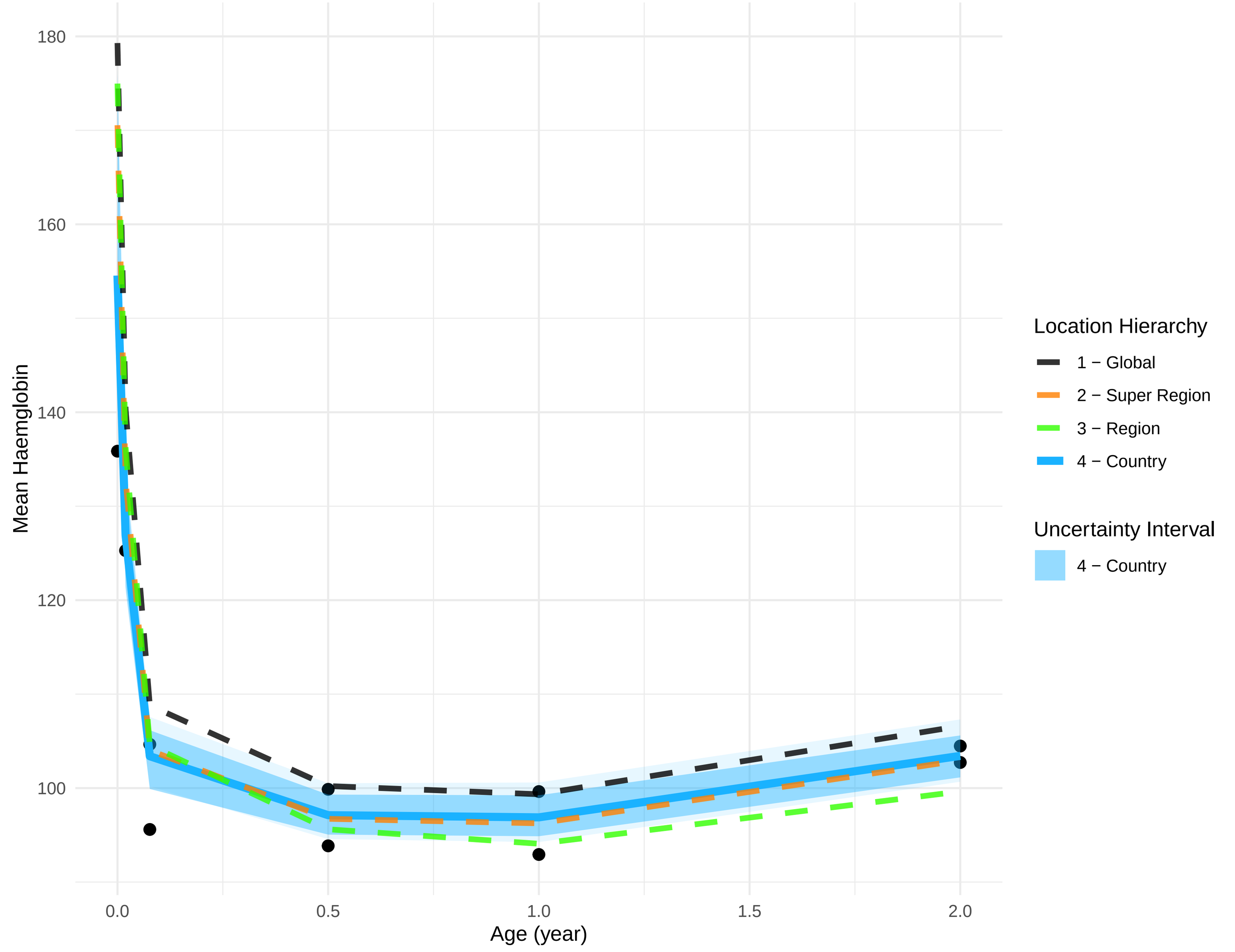

MR−BRT Age/Sex Fits − Non−pregnant Females <2 Years Old − Mean Haemglobin − Côte d'Ivoire
WHO−2024 Adjustment Method

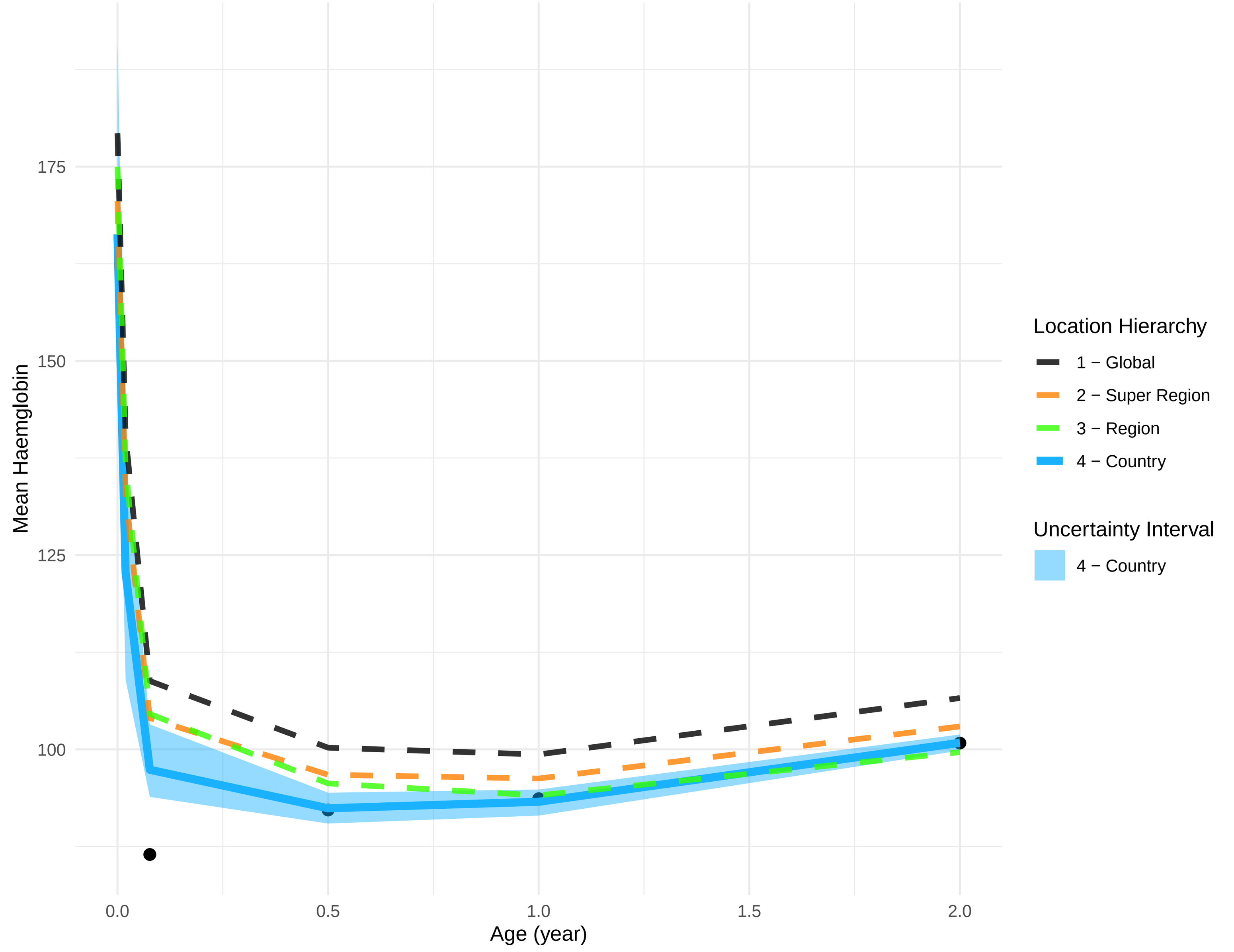

MR−BRT Age/Sex Fits − Non−pregnant Females <2 Years Old − Mean Haemglobin − Gambia
WHO−2024 Adjustment Method

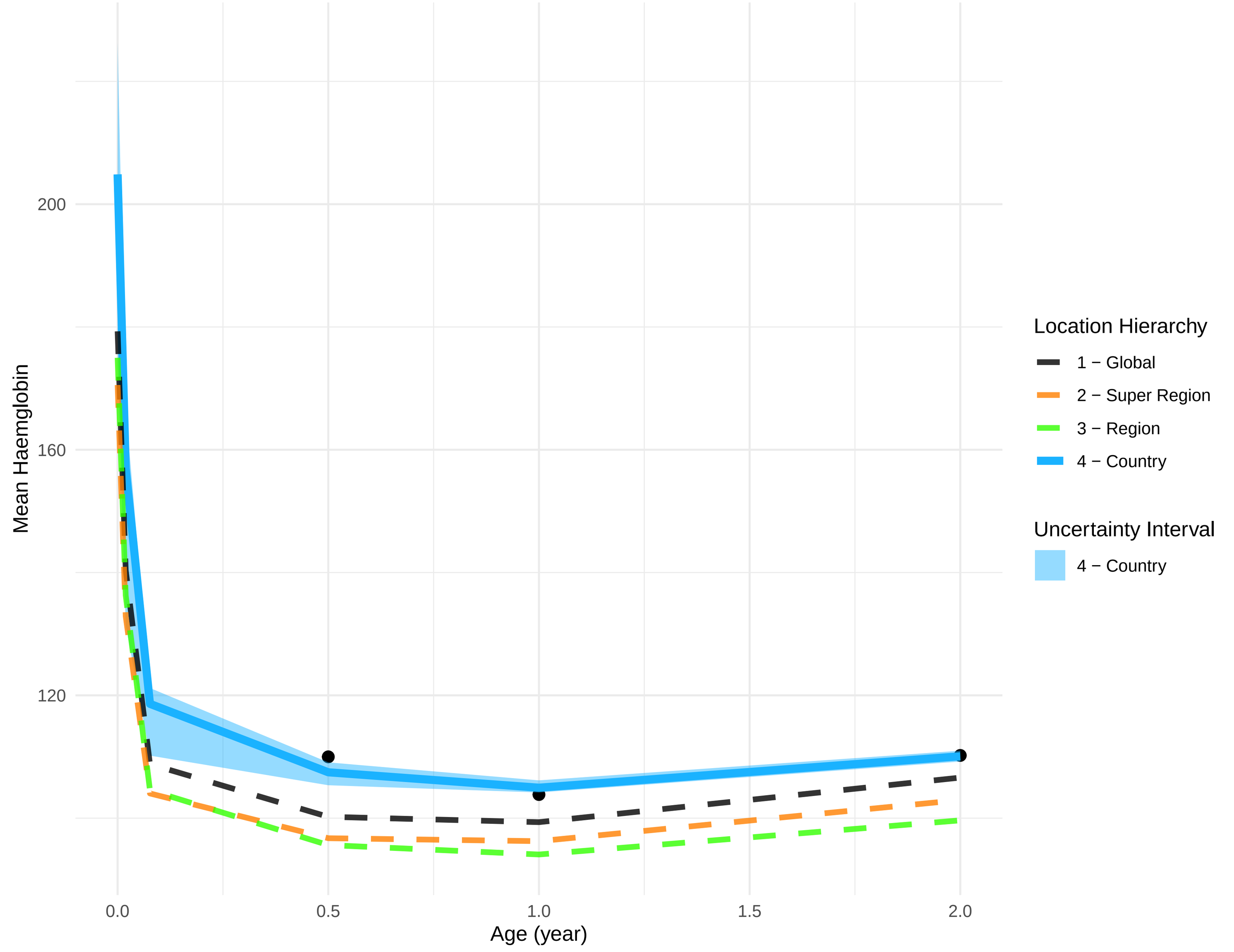

MR−BRT Age/Sex Fits − Non−pregnant Females <2 Years Old − Mean Haemglobin − Ghana
WHO−2024 Adjustment Method

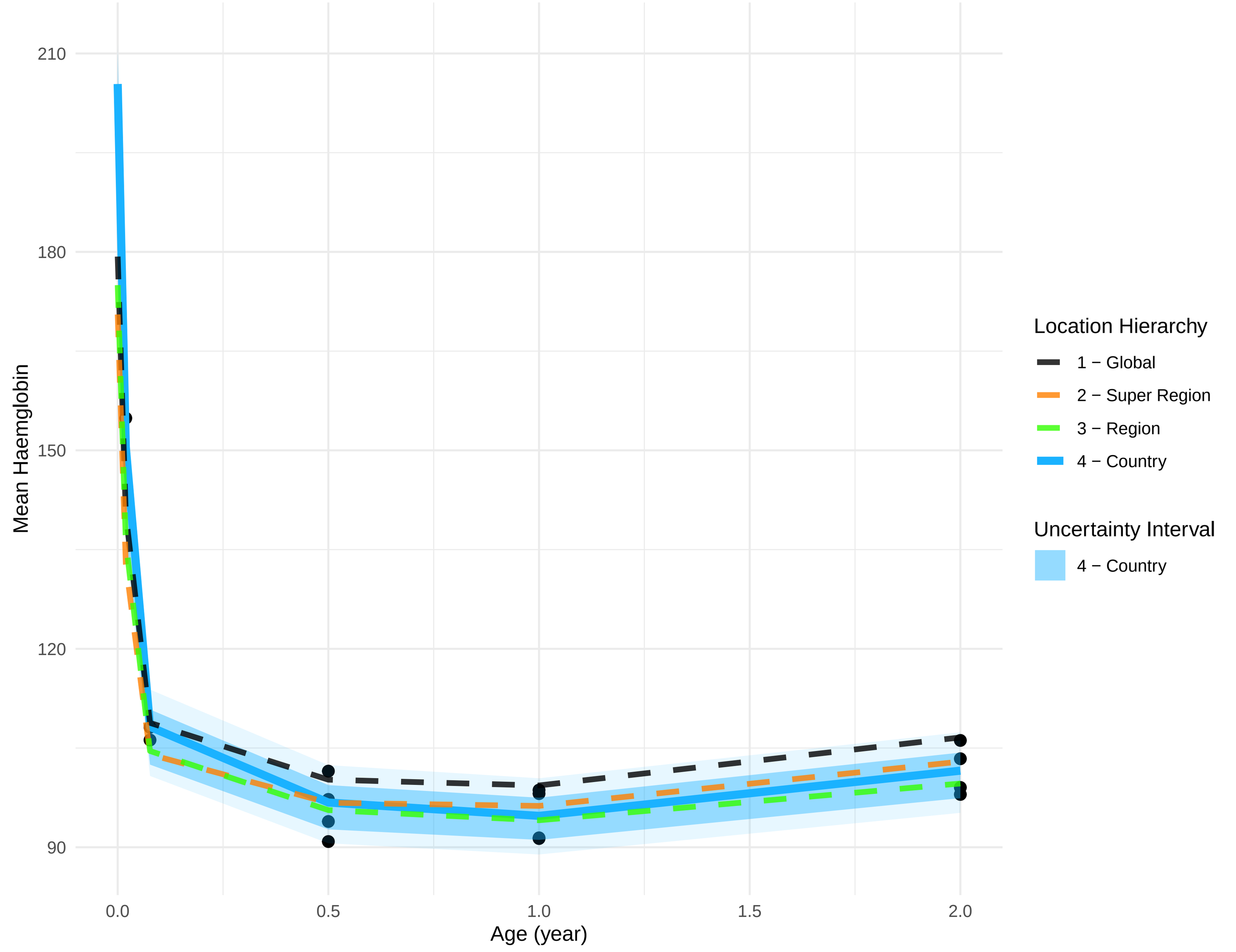

MR−BRT Age/Sex Fits − Non−pregnant Females <2 Years Old − Mean Haemglobin − Guinea
WHO−2024 Adjustment Method

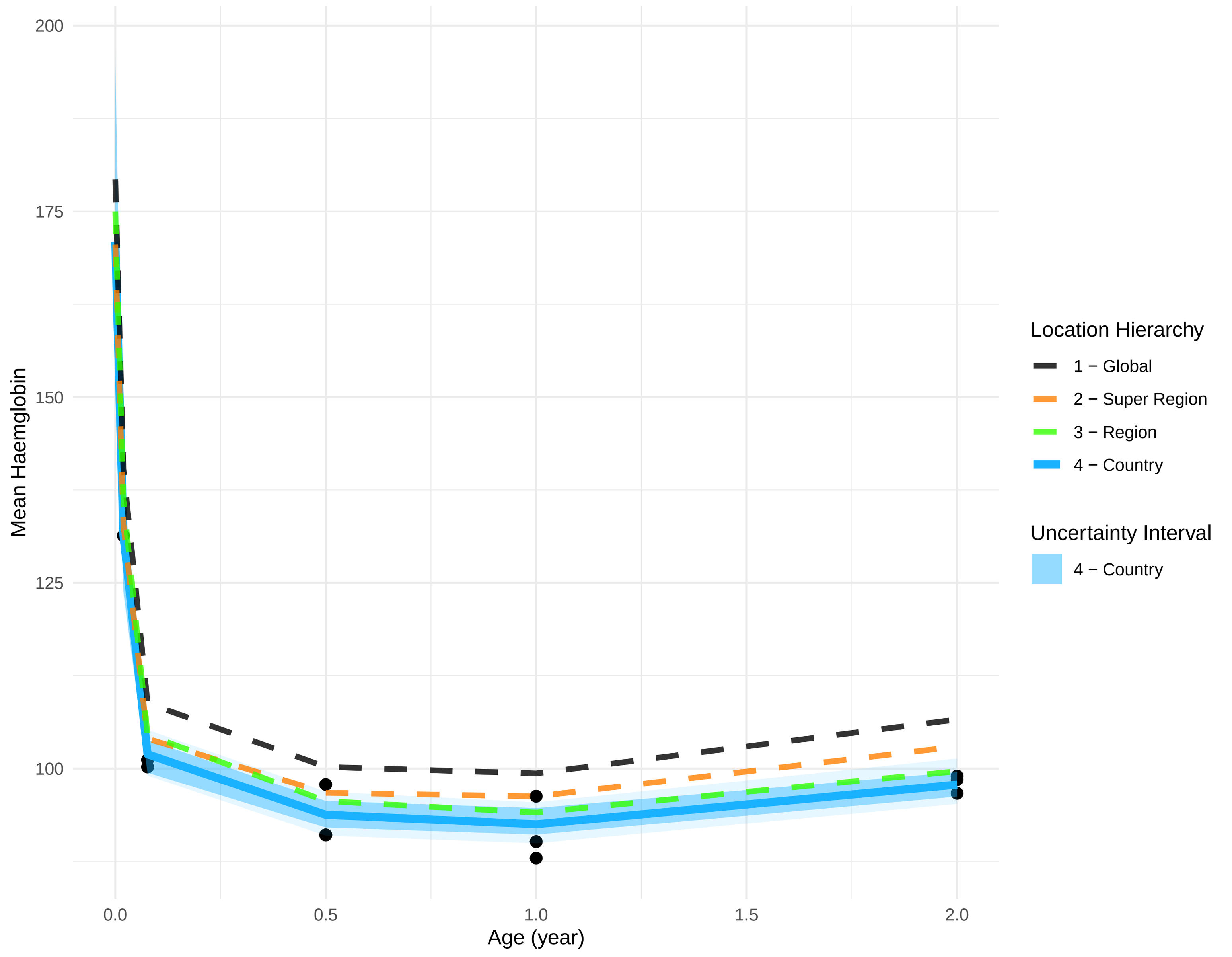

MR−BRT Age/Sex Fits − Non−pregnant Females <2 Years Old − Mean Haemglobin − Liberia
WHO−2024 Adjustment Method

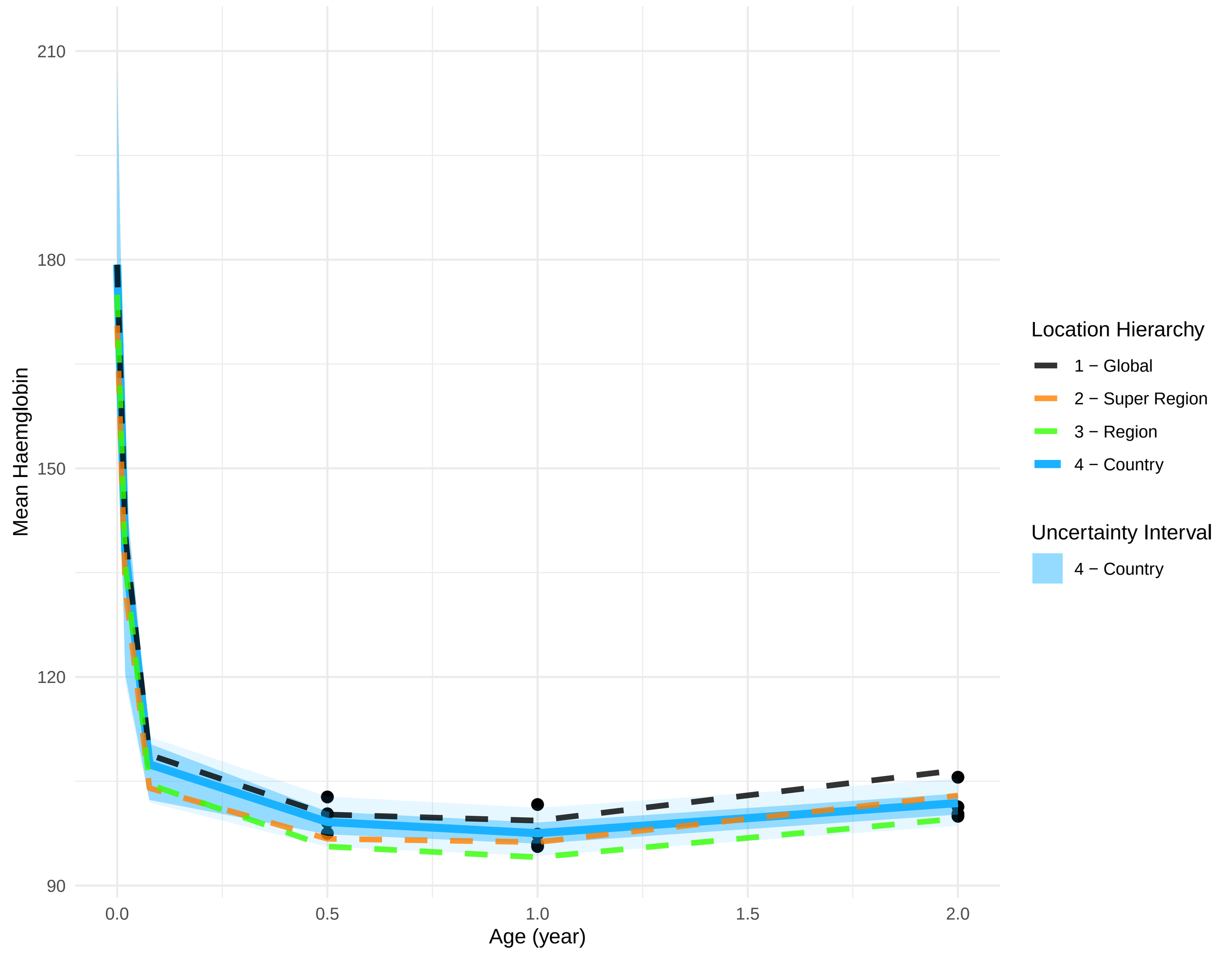

MR−BRT Age/Sex Fits − Non−pregnant Females <2 Years Old − Mean Haemglobin − Mali
WHO−2024 Adjustment Method

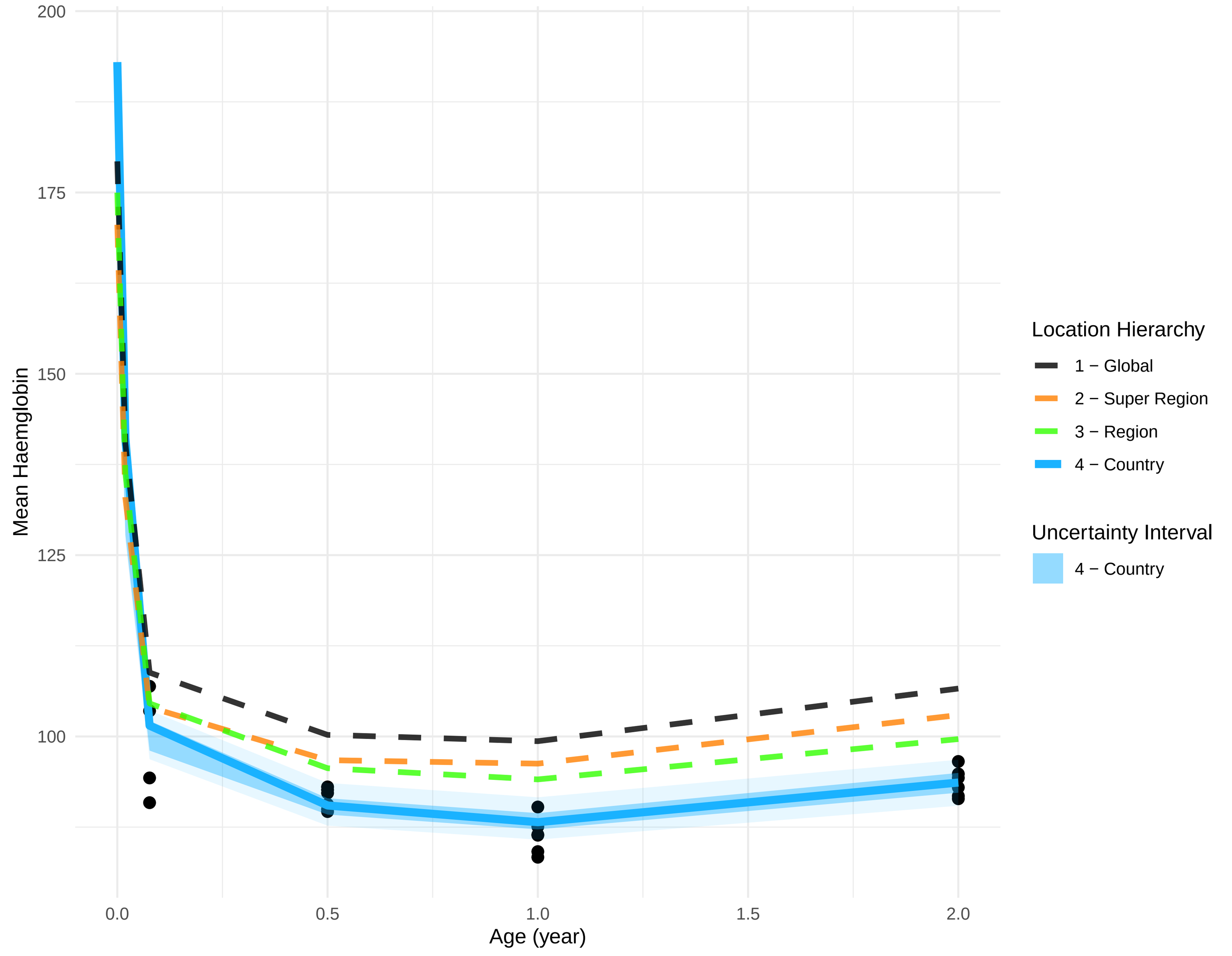

MR−BRT Age/Sex Fits − Non−pregnant Females <2 Years Old − Mean Haemglobin − Niger
WHO−2024 Adjustment Method

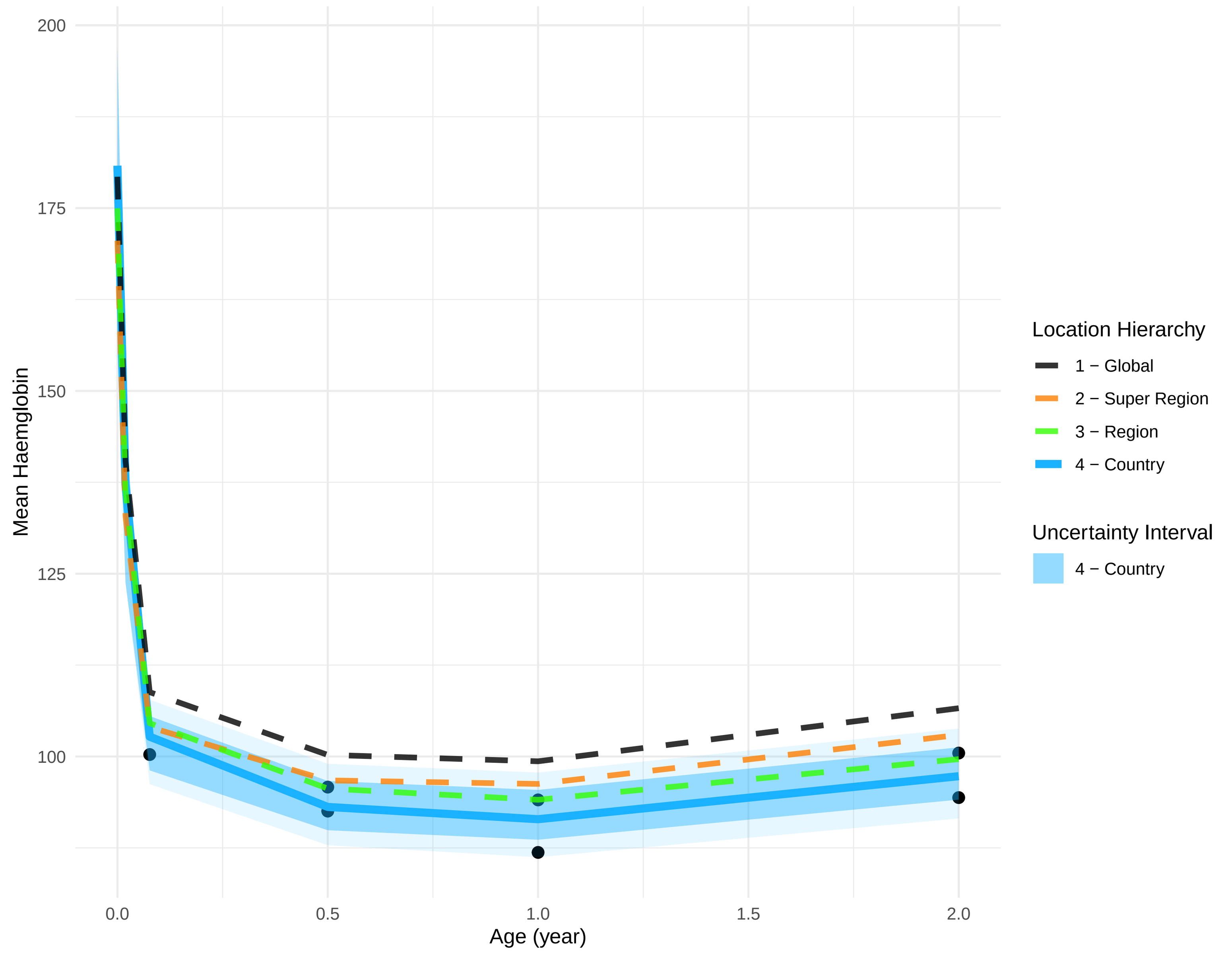

MR−BRT Age/Sex Fits − Non−pregnant Females <2 Years Old − Mean Haemglobin − Nigeria
WHO−2024 Adjustment Method

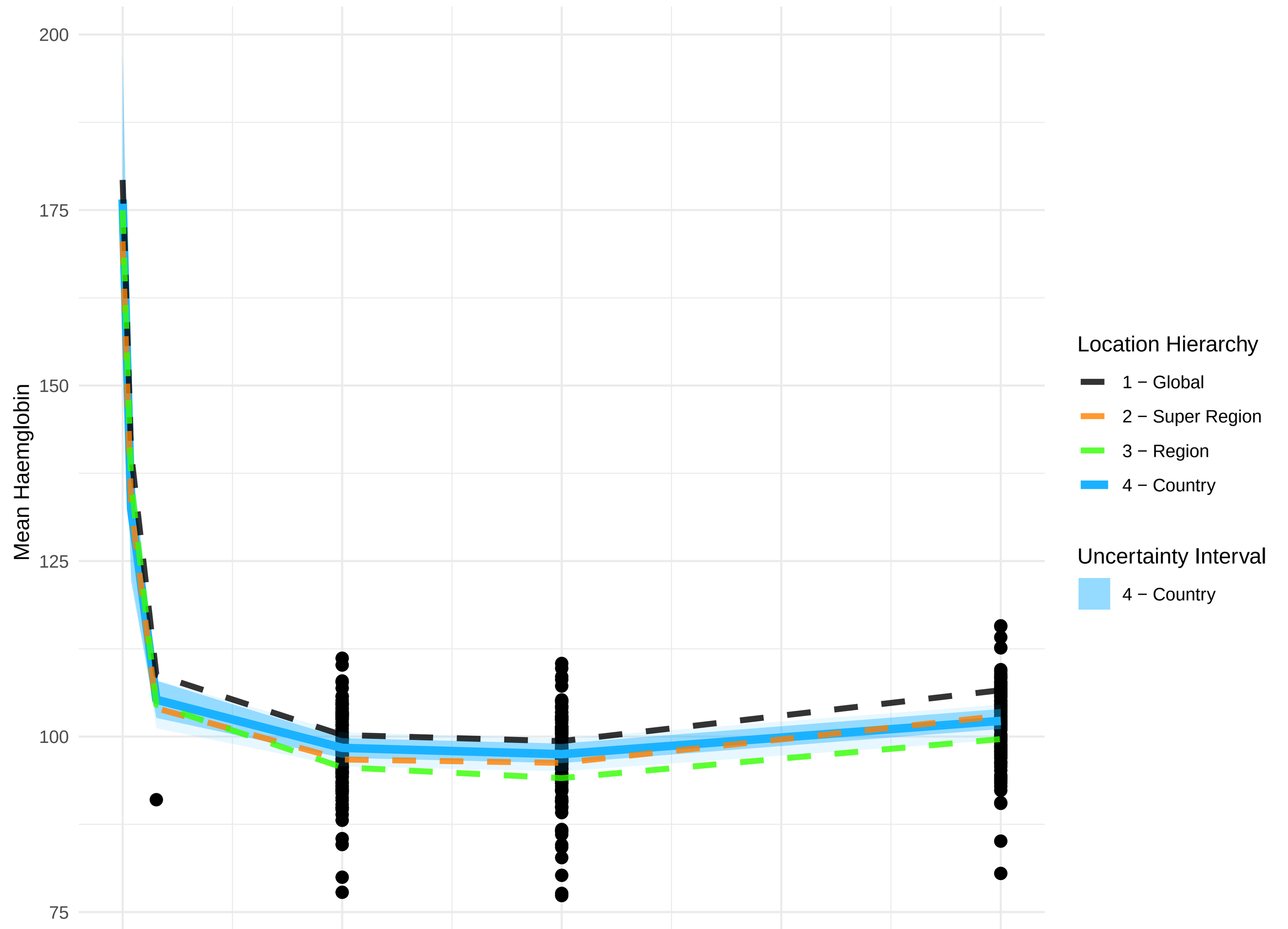

MR−BRT Age/Sex Fits − Non−pregnant Females <2 Years Old − Mean Haemglobin − Abia
WHO−2024 Adjustment Method

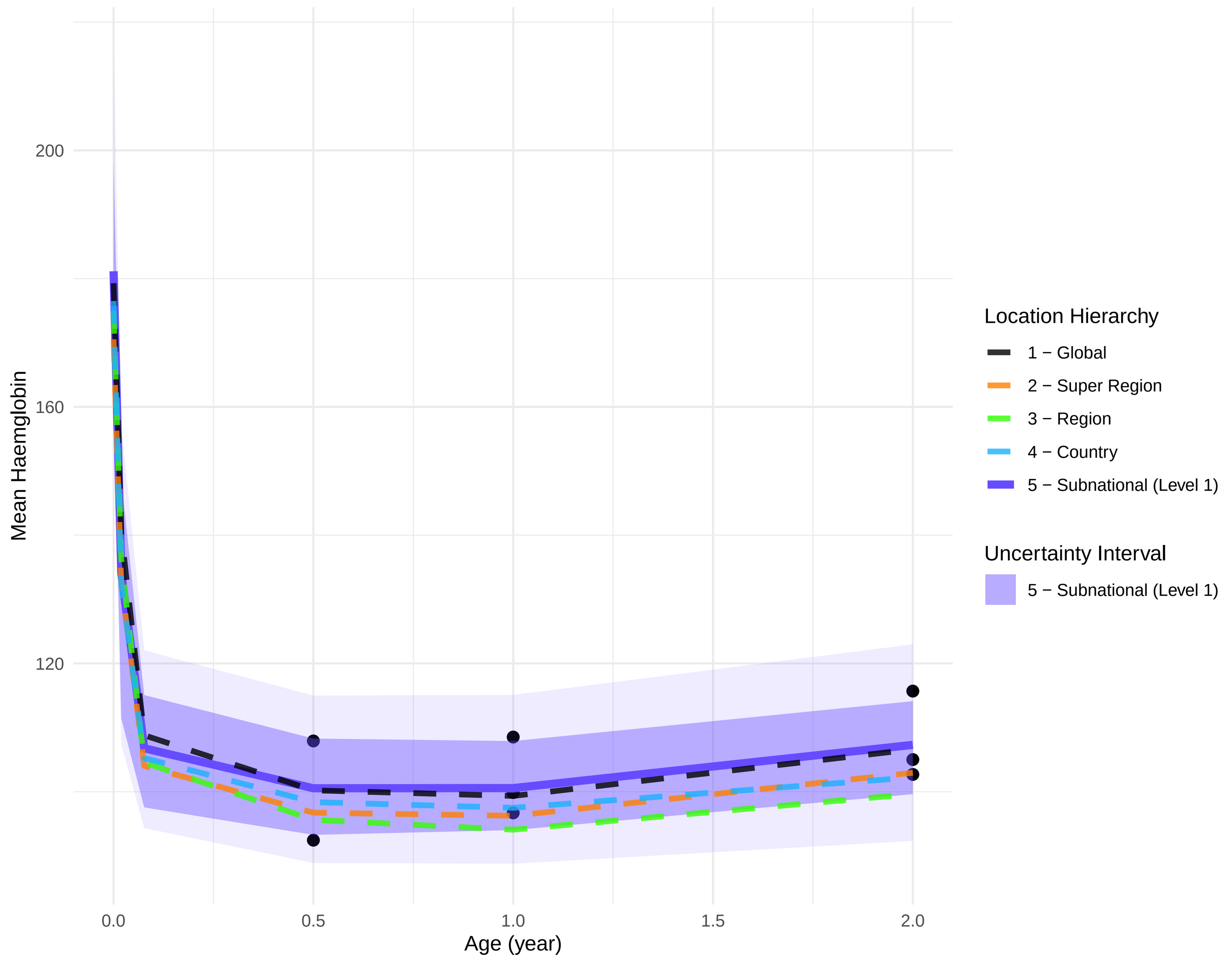

MR−BRT Age/Sex Fits − Non−pregnant Females <2 Years Old − Mean Haemglobin − Adamawa
WHO−2024 Adjustment Method

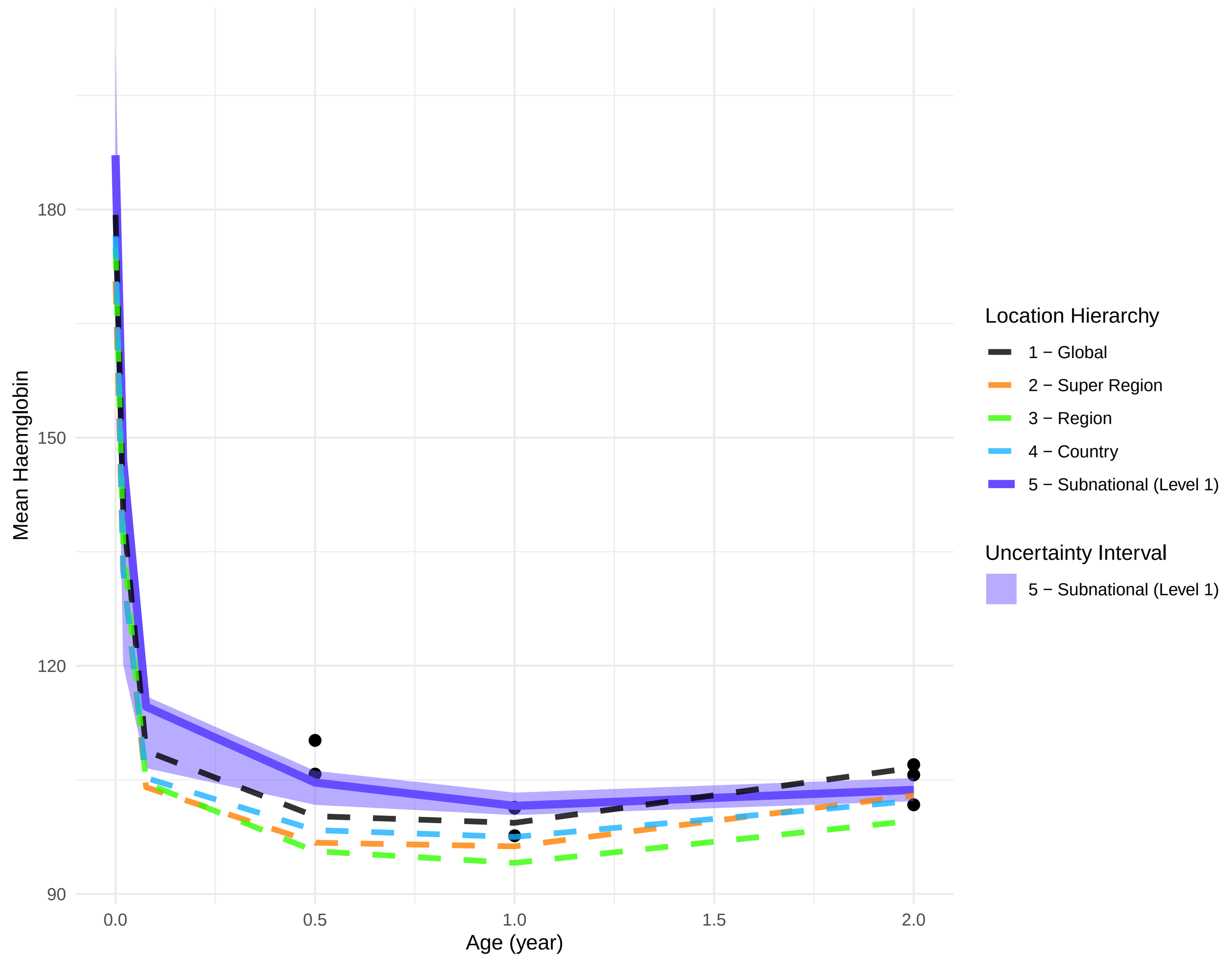

MR−BRT Age/Sex Fits − Non−pregnant Females <2 Years Old − Mean Haemglobin − Akwa Ibom
WHO−2024 Adjustment Method

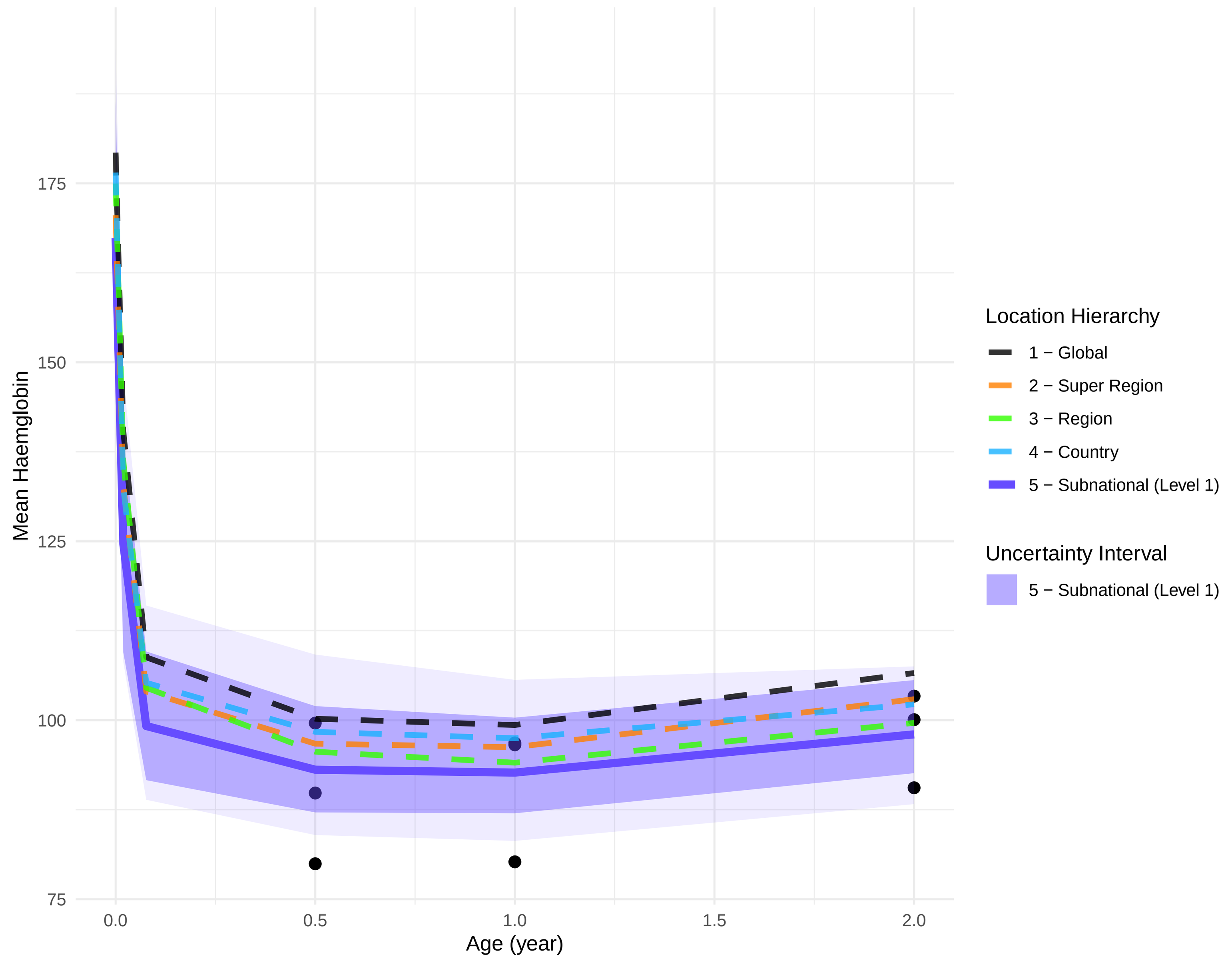

MR−BRT Age/Sex Fits − Non−pregnant Females <2 Years Old − Mean Haemglobin − Anambra
WHO−2024 Adjustment Method

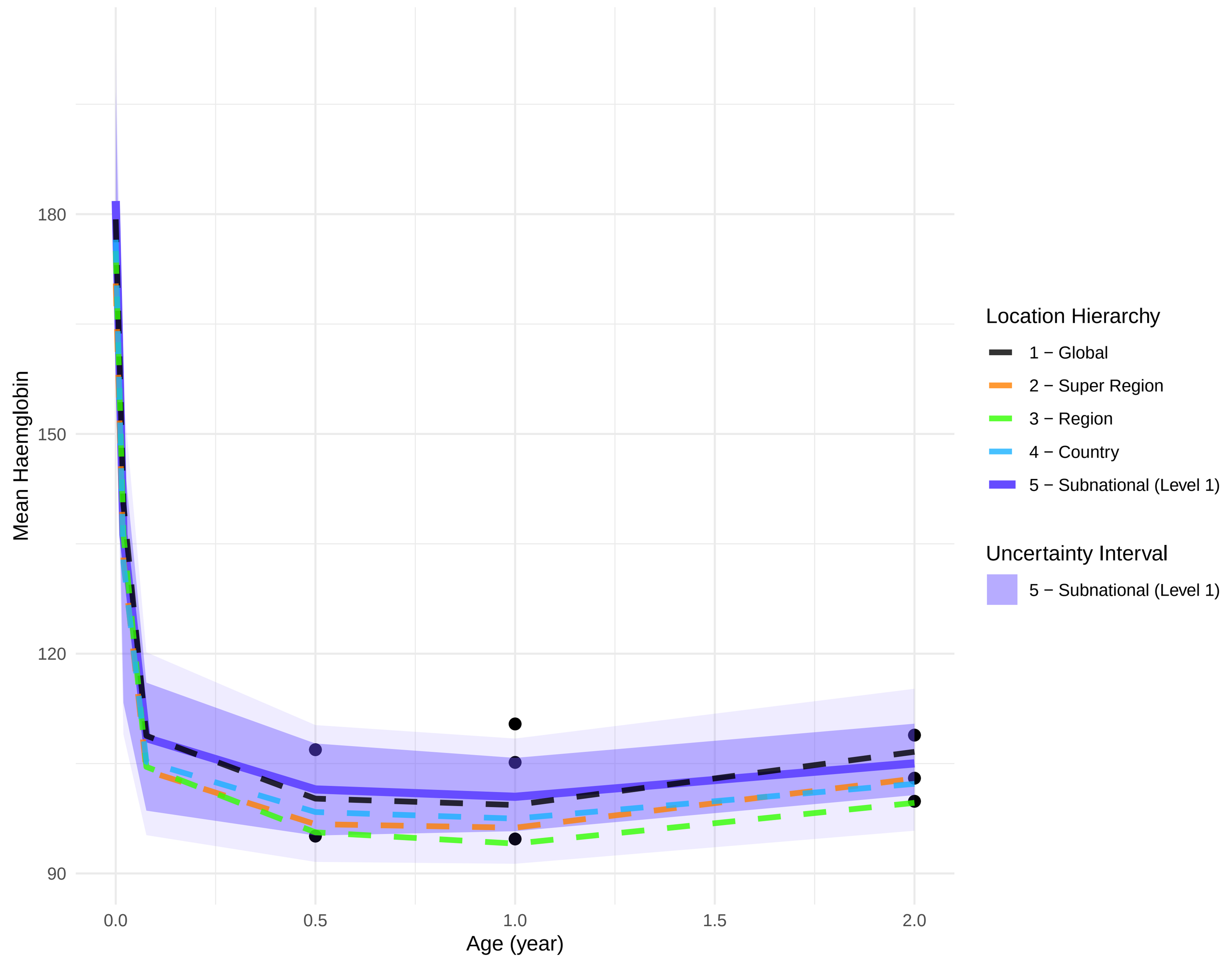

MR−BRT Age/Sex Fits − Non−pregnant Females <2 Years Old − Mean Haemglobin − Bauchi
WHO−2024 Adjustment Method

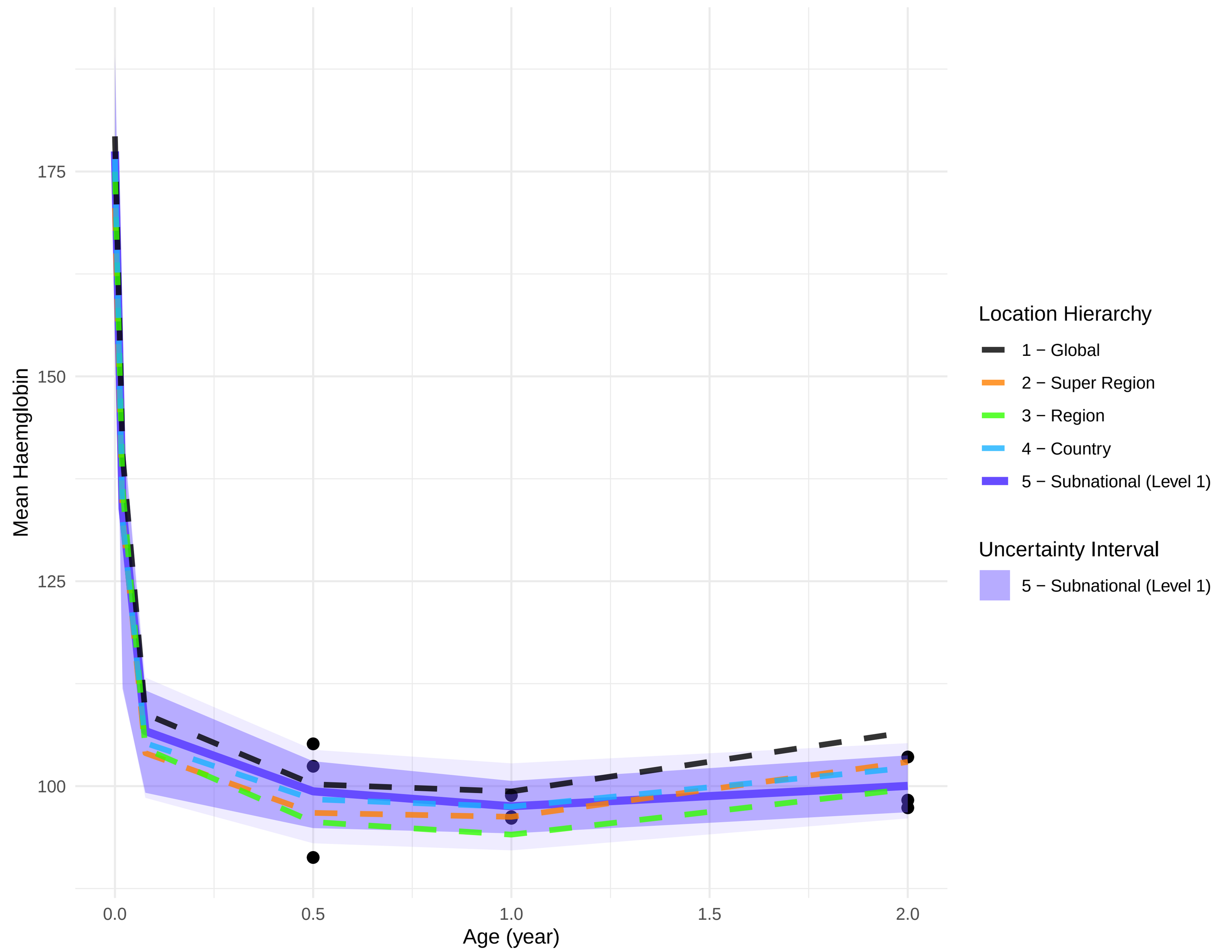

MR−BRT Age/Sex Fits − Non−pregnant Females <2 Years Old − Mean Haemglobin − Bayelsa
WHO−2024 Adjustment Method

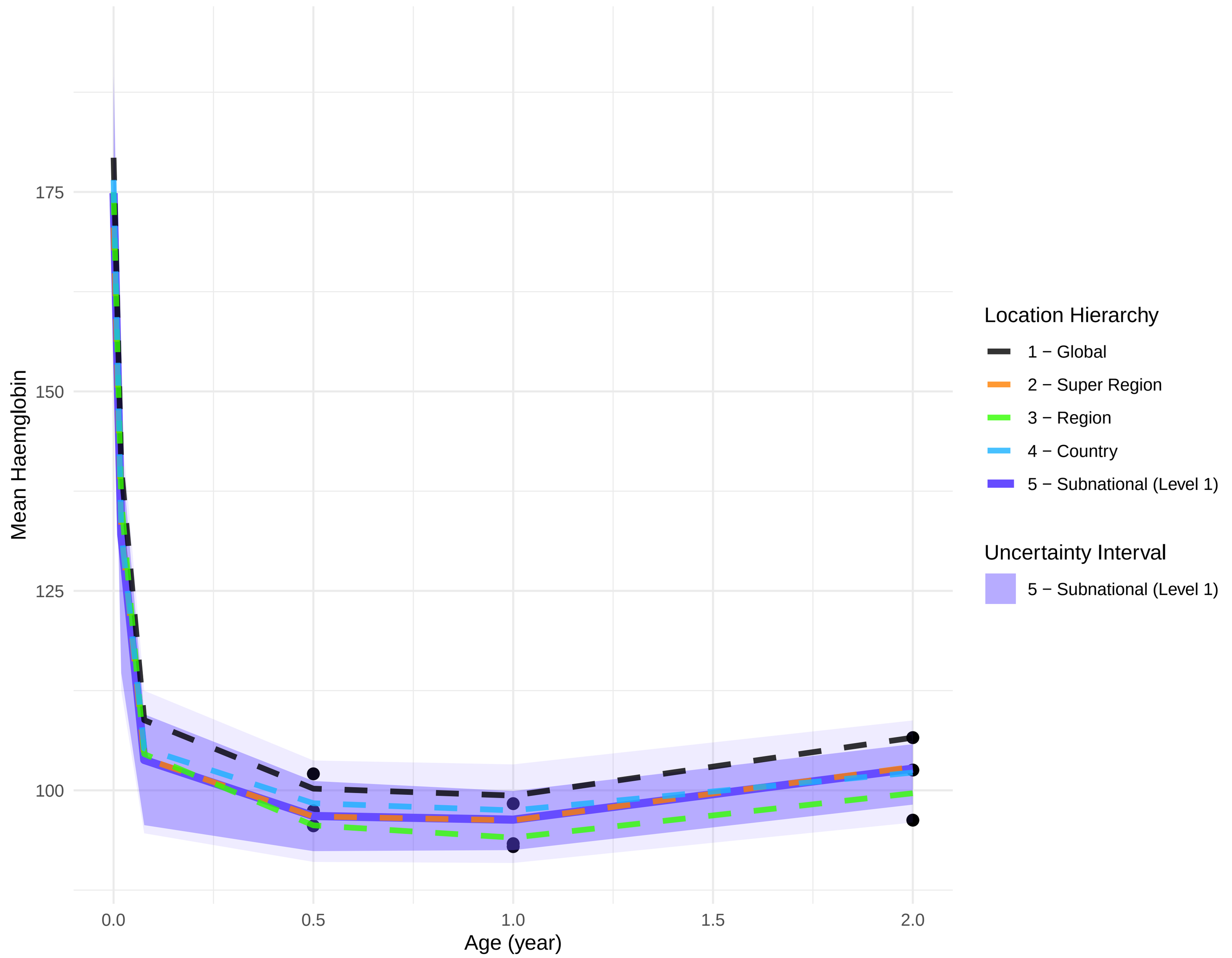

MR−BRT Age/Sex Fits − Non−pregnant Females <2 Years Old − Mean Haemglobin − Benue
WHO−2024 Adjustment Method

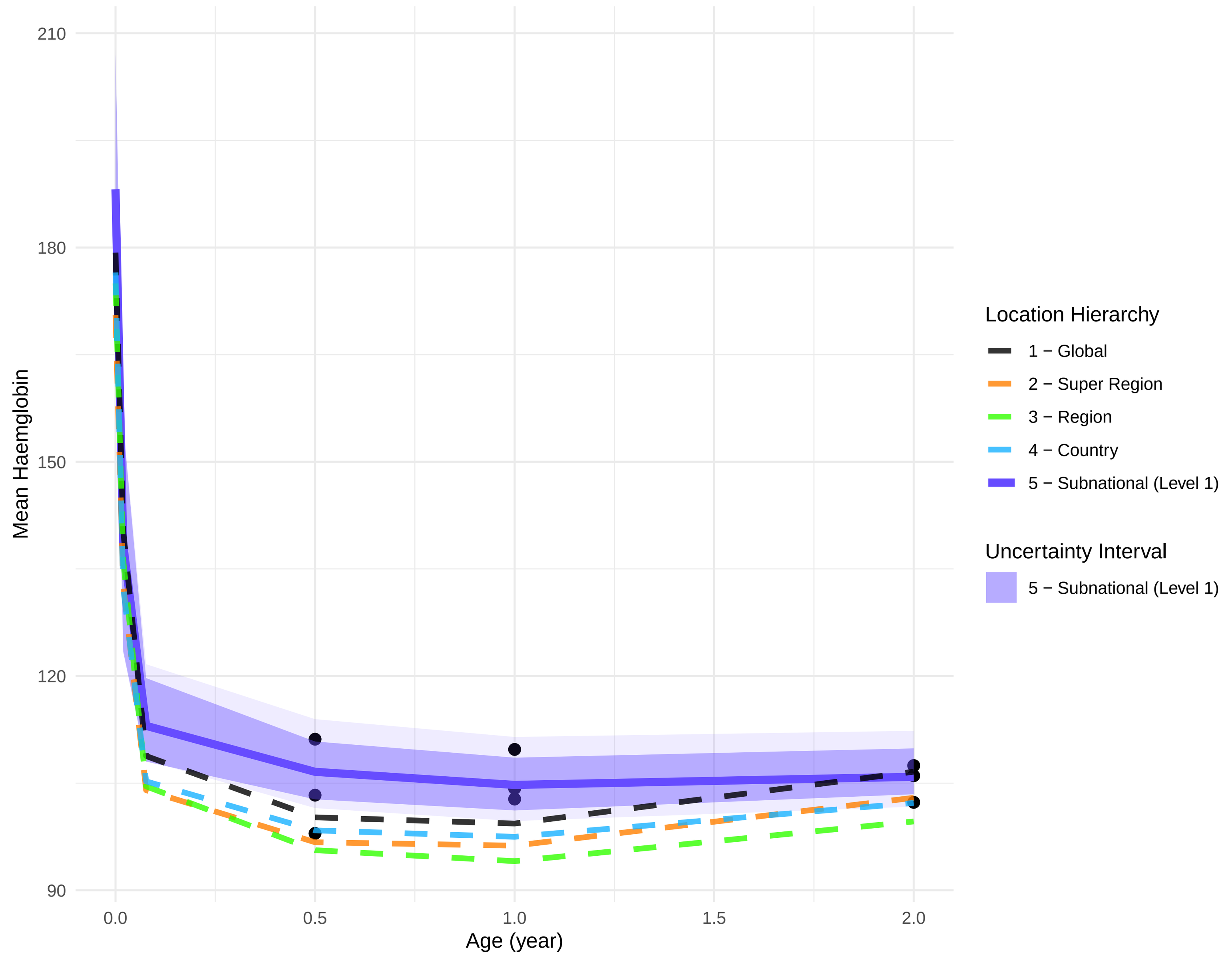

MR−BRT Age/Sex Fits − Non−pregnant Females <2 Years Old − Mean Haemglobin − Borno
WHO−2024 Adjustment Method

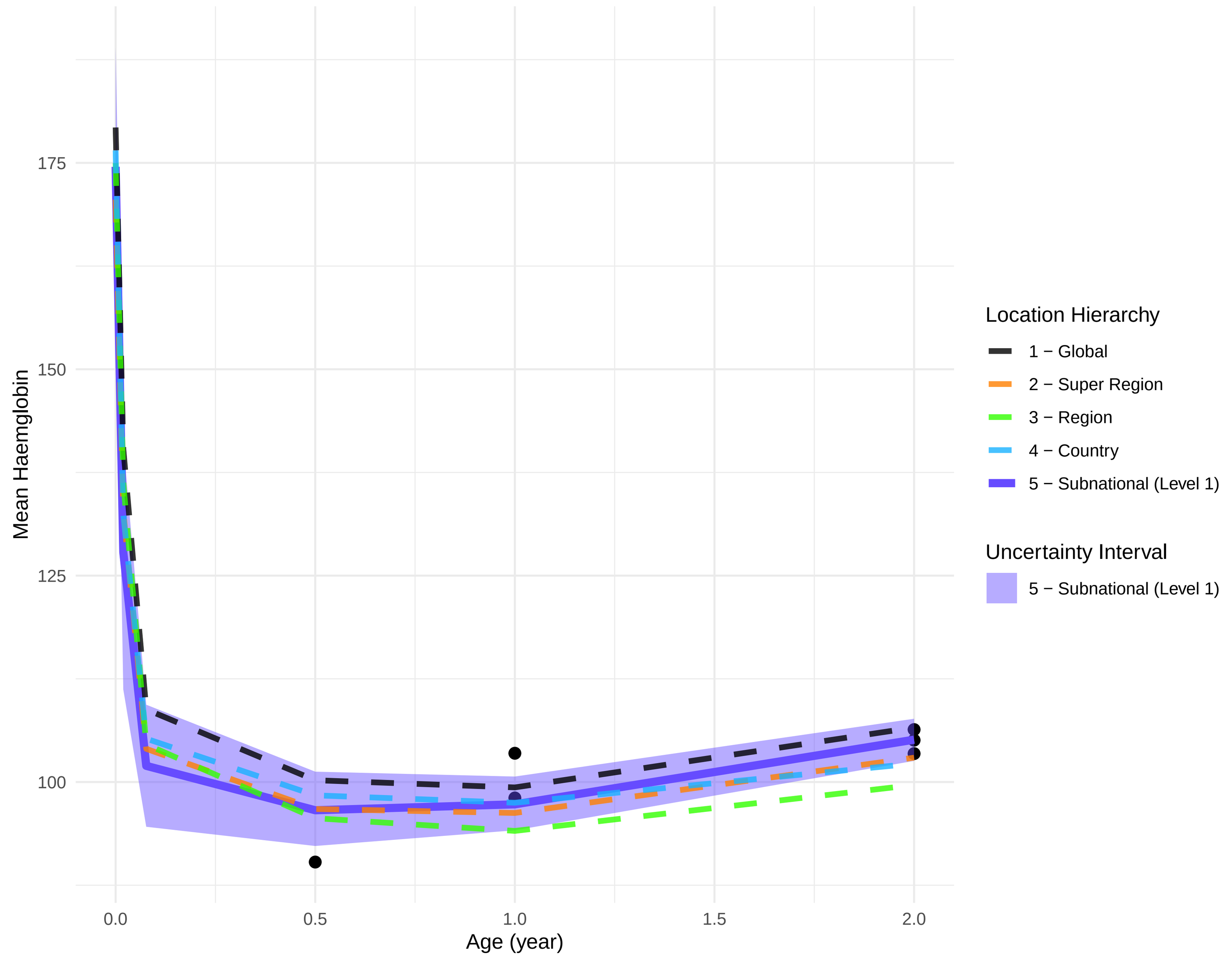

MR−BRT Age/Sex Fits − Non−pregnant Females <2 Years Old − Mean Haemglobin − Cross River
WHO−2024 Adjustment Method

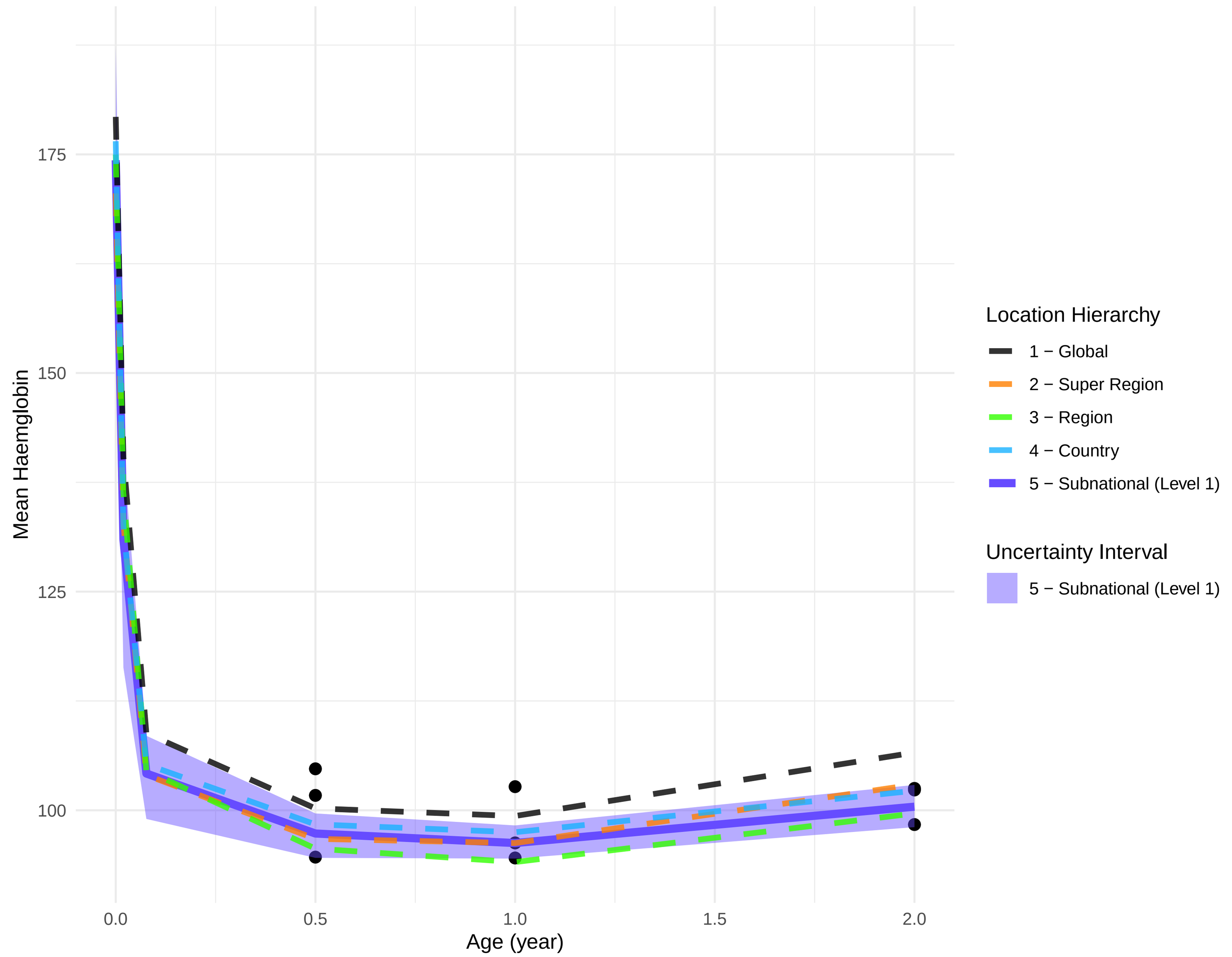

MR−BRT Age/Sex Fits − Non−pregnant Females <2 Years Old − Mean Haemglobin − Delta
WHO−2024 Adjustment Method

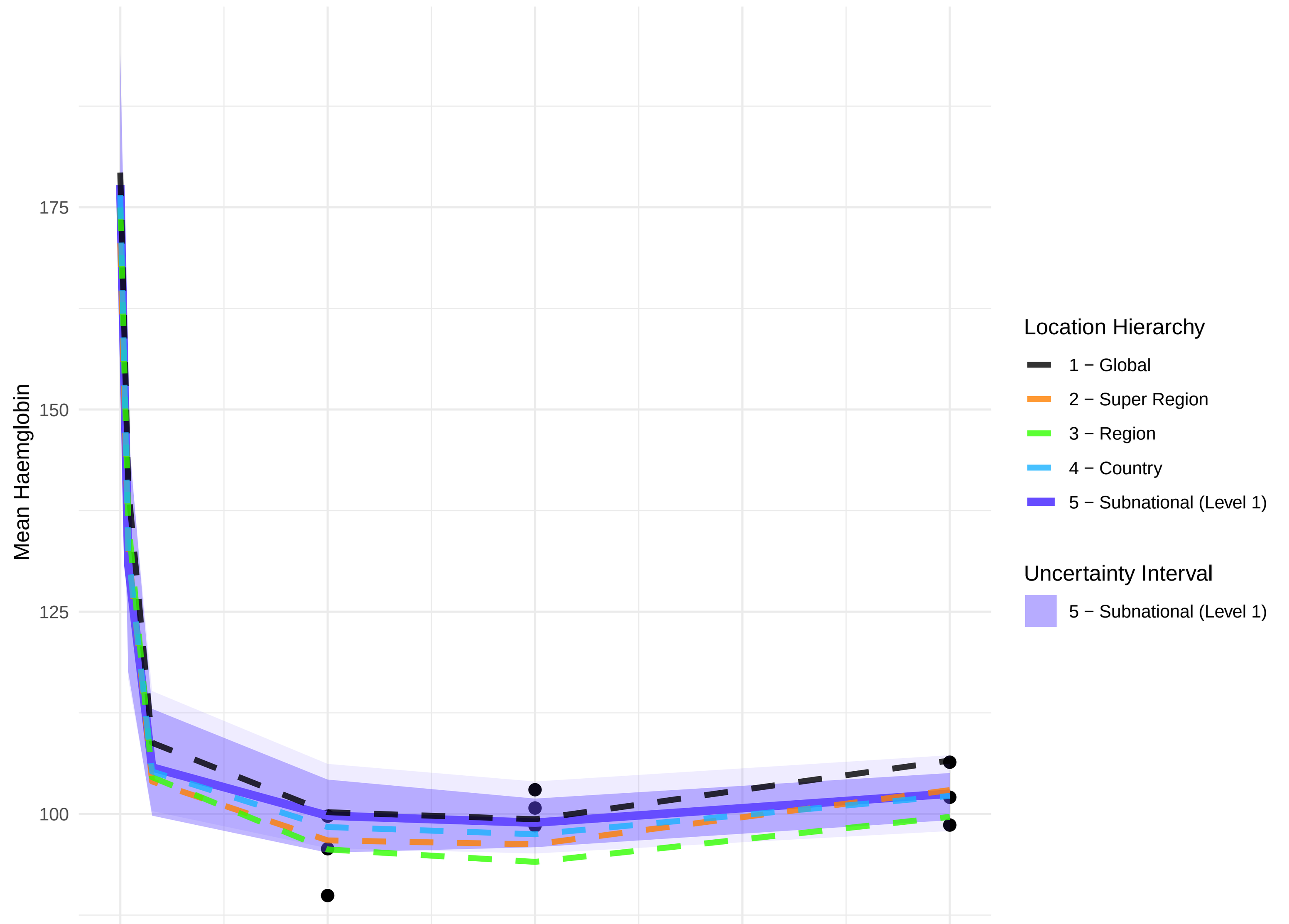

MR−BRT Age/Sex Fits − Non−pregnant Females <2 Years Old − Mean Haemglobin − Ebonyi
WHO−2024 Adjustment Method

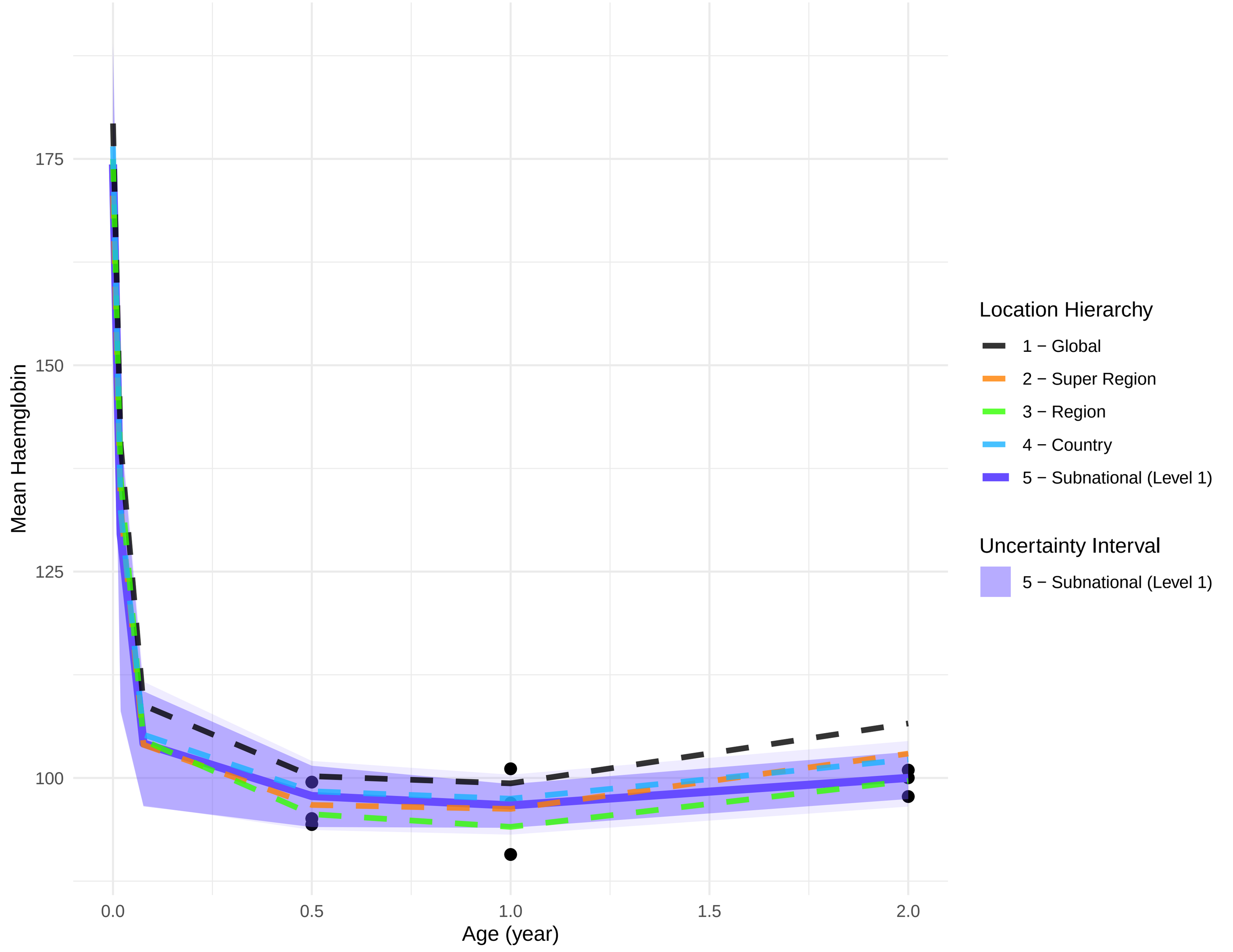

MR−BRT Age/Sex Fits − Non−pregnant Females <2 Years Old − Mean Haemglobin − Edo
WHO−2024 Adjustment Method

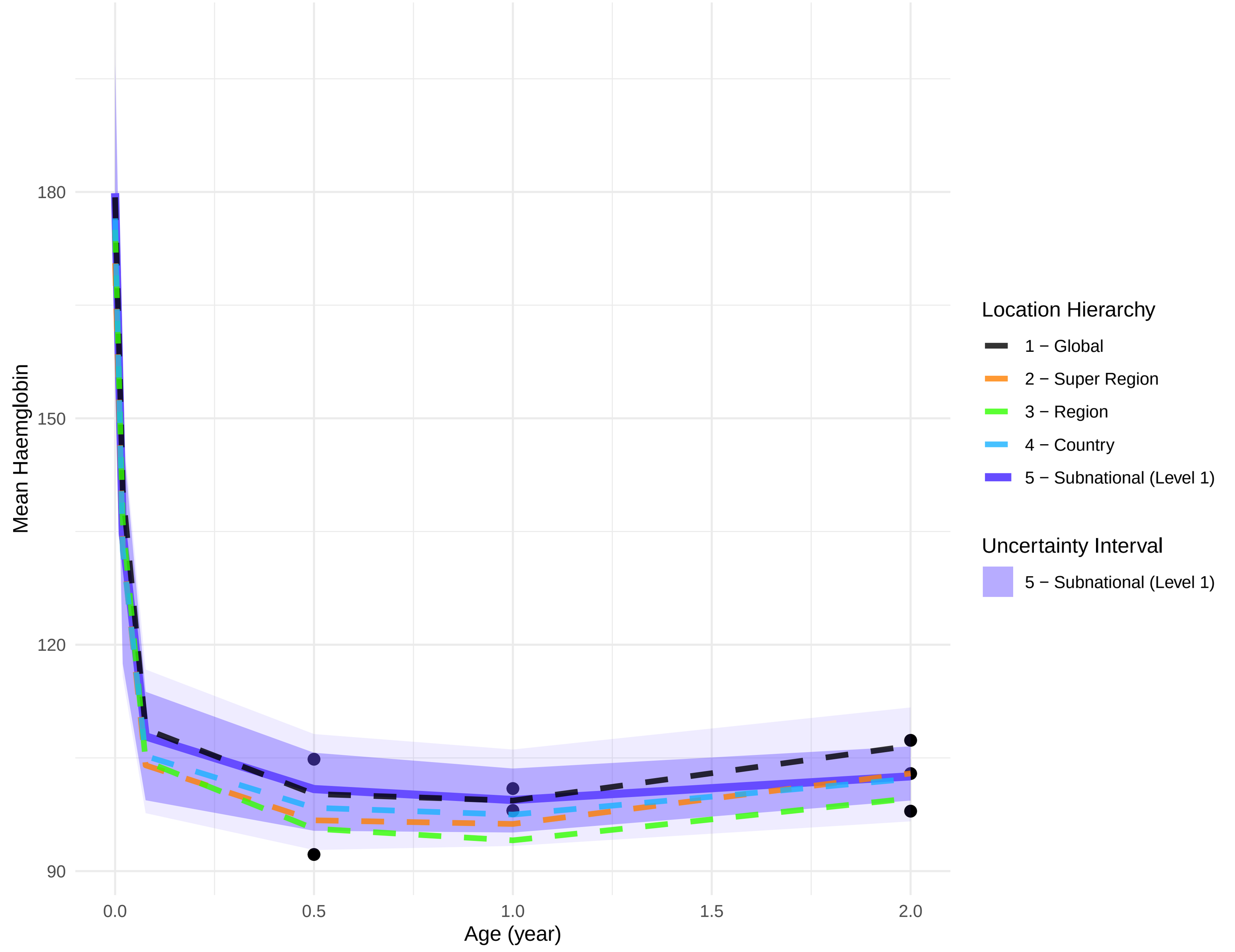

MR−BRT Age/Sex Fits − Non−pregnant Females <2 Years Old − Mean Haemglobin − Ekiti
WHO−2024 Adjustment Method

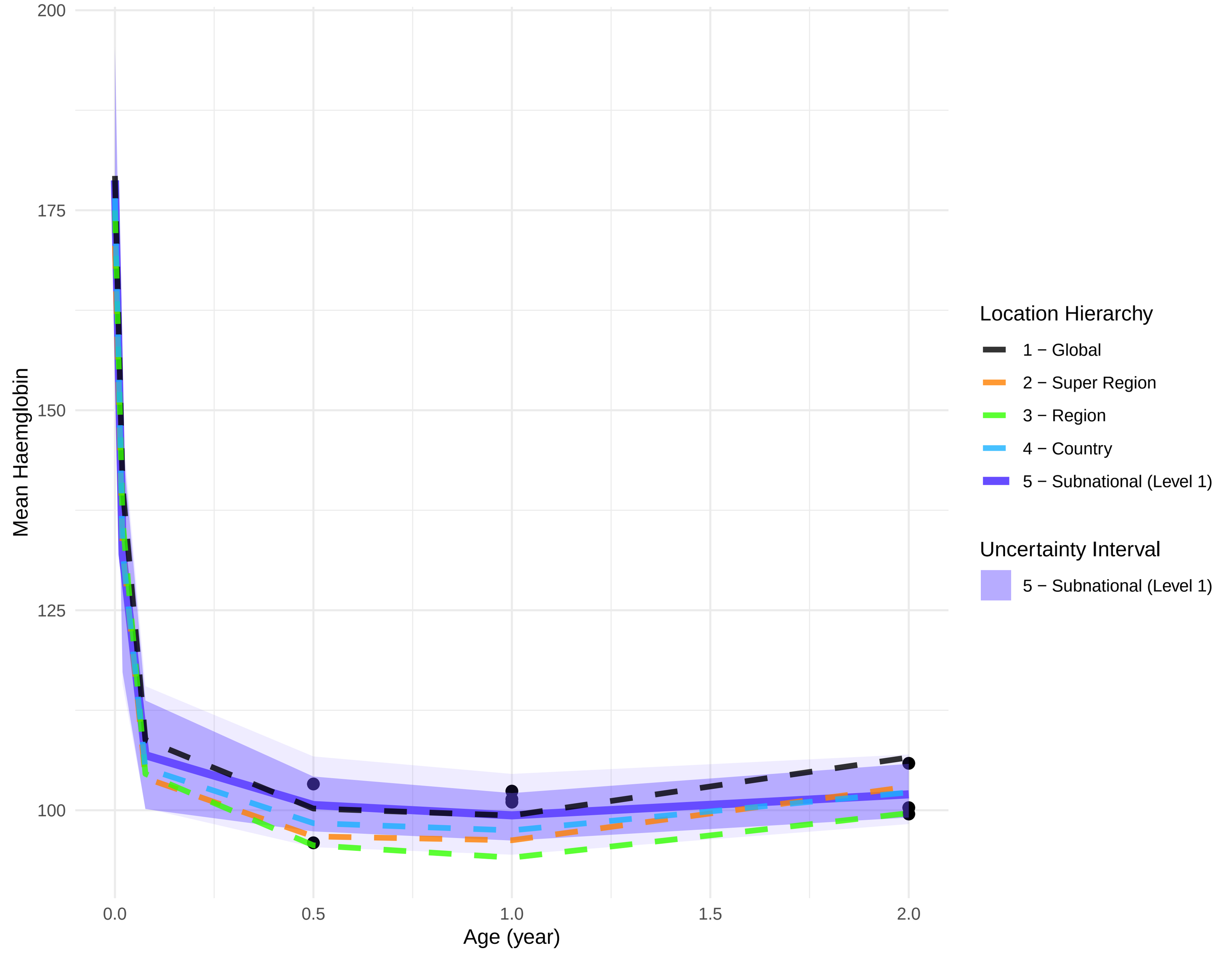

MR−BRT Age/Sex Fits − Non−pregnant Females <2 Years Old − Mean Haemglobin − Enugu
WHO−2024 Adjustment Method

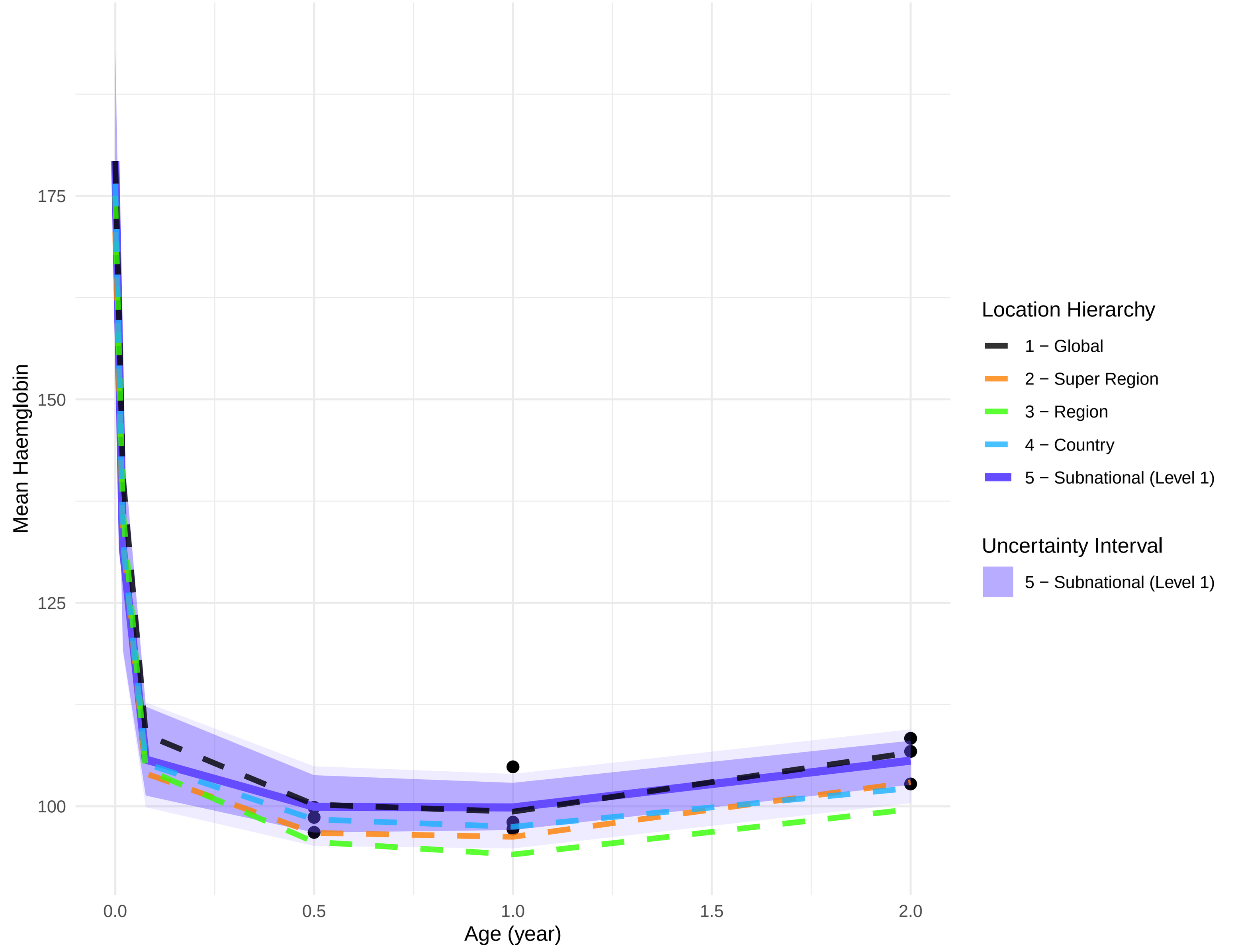

MR−BRT Age/Sex Fits − Non−pregnant Females <2 Years Old − Mean Haemglobin − FCT (Abuja)
WHO−2024 Adjustment Method

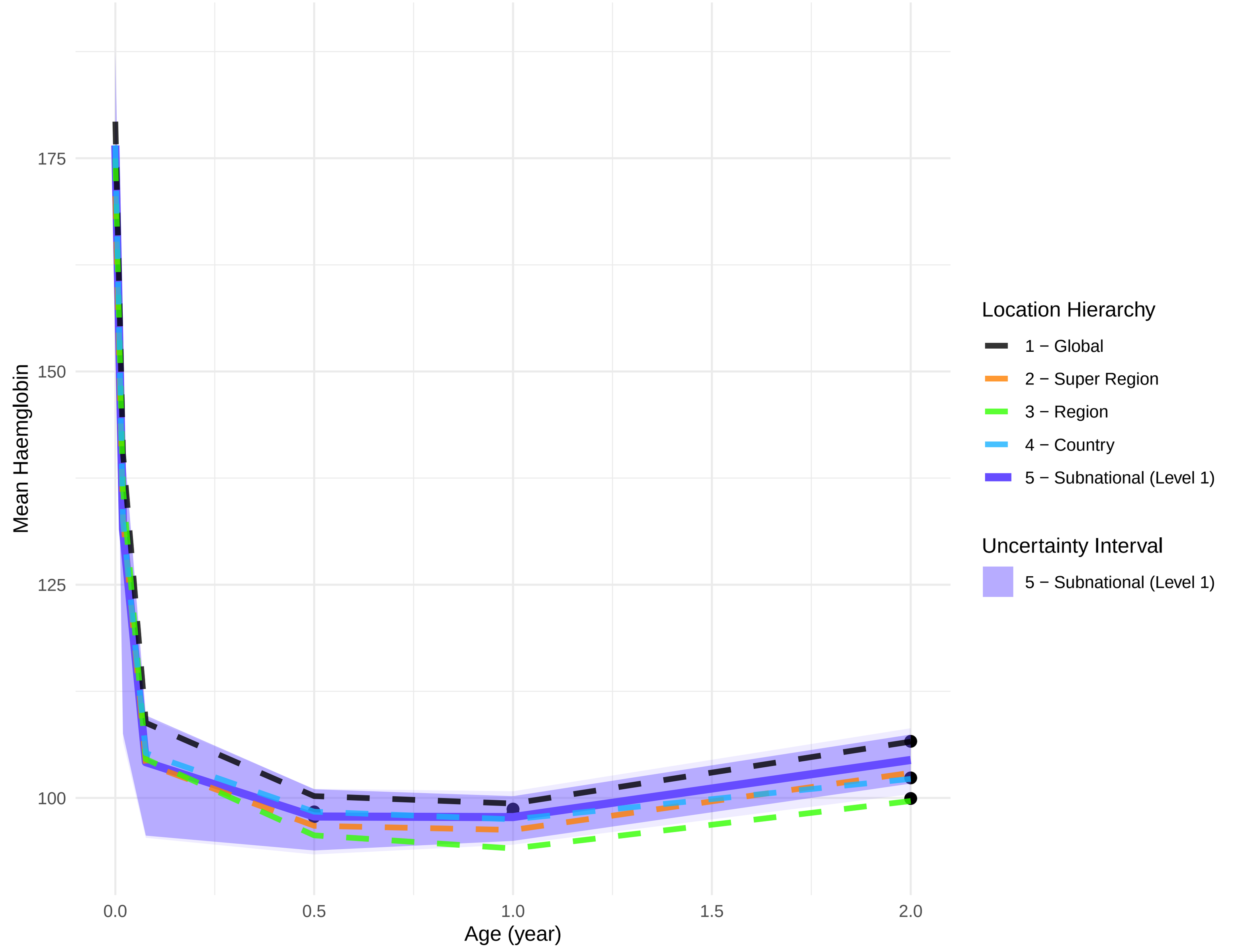

MR−BRT Age/Sex Fits − Non−pregnant Females <2 Years Old − Mean Haemglobin − Gombe
WHO−2024 Adjustment Method

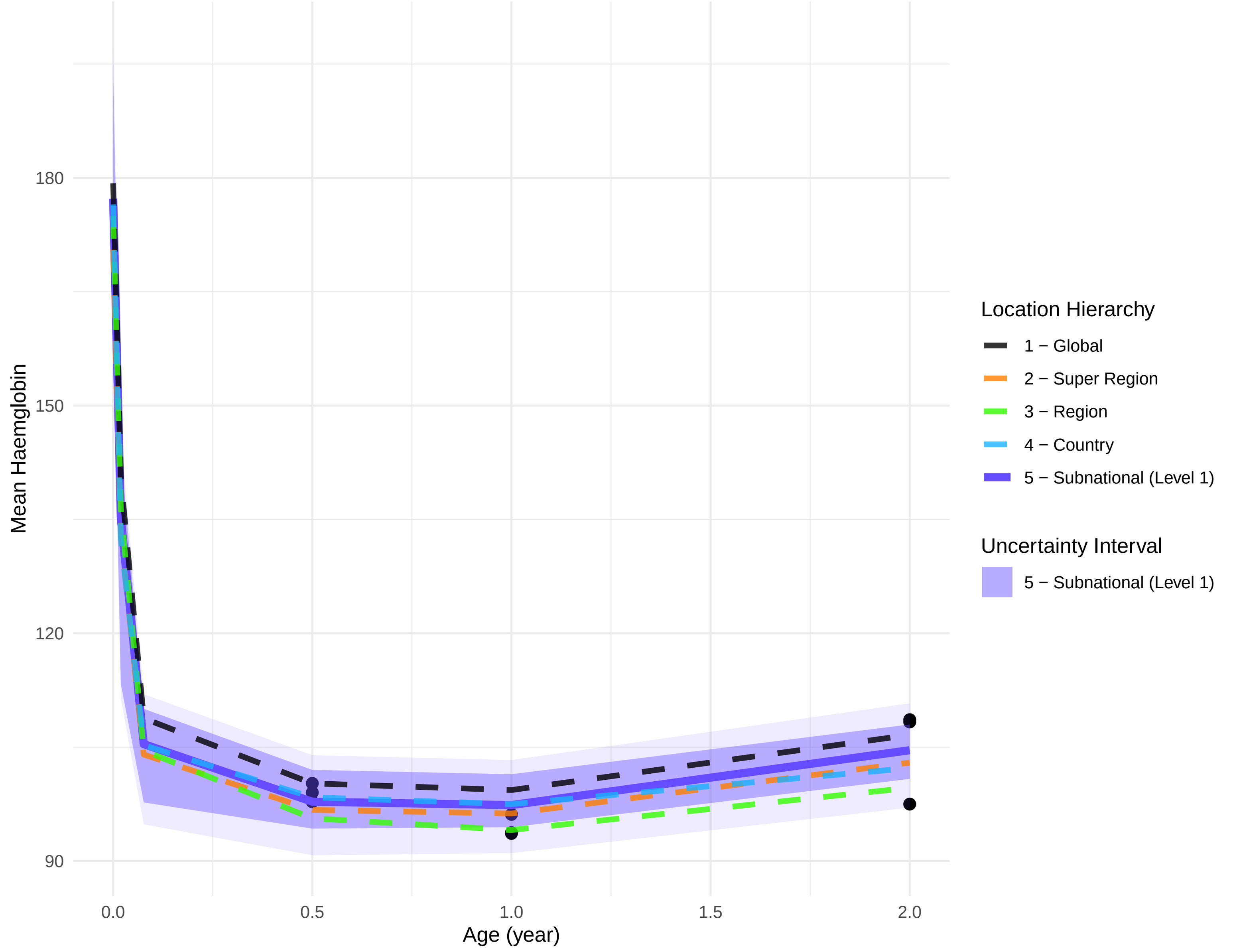

MR−BRT Age/Sex Fits − Non−pregnant Females <2 Years Old − Mean Haemglobin − Imo
WHO−2024 Adjustment Method

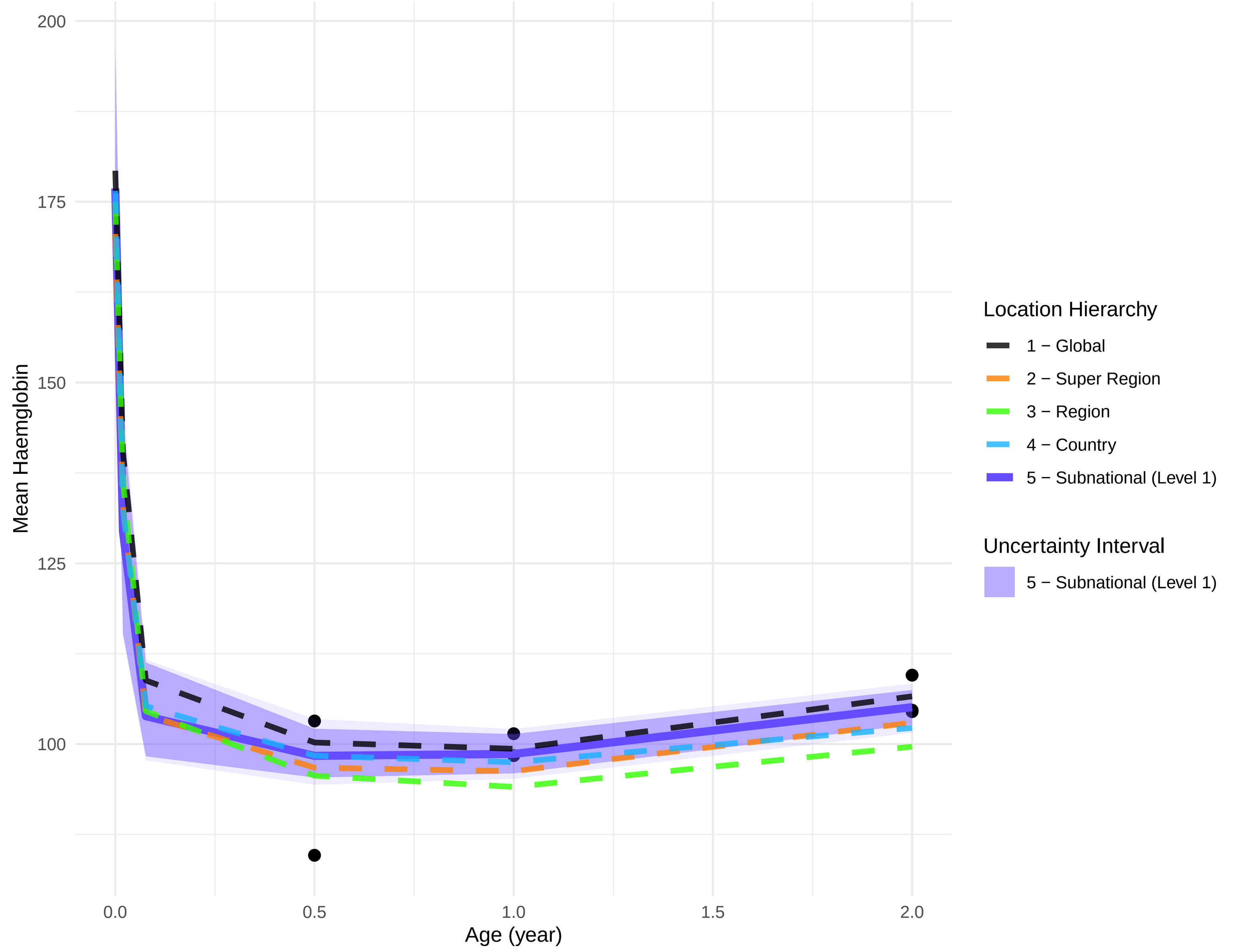

MR−BRT Age/Sex Fits − Non−pregnant Females <2 Years Old − Mean Haemglobin − Jigawa
WHO−2024 Adjustment Method

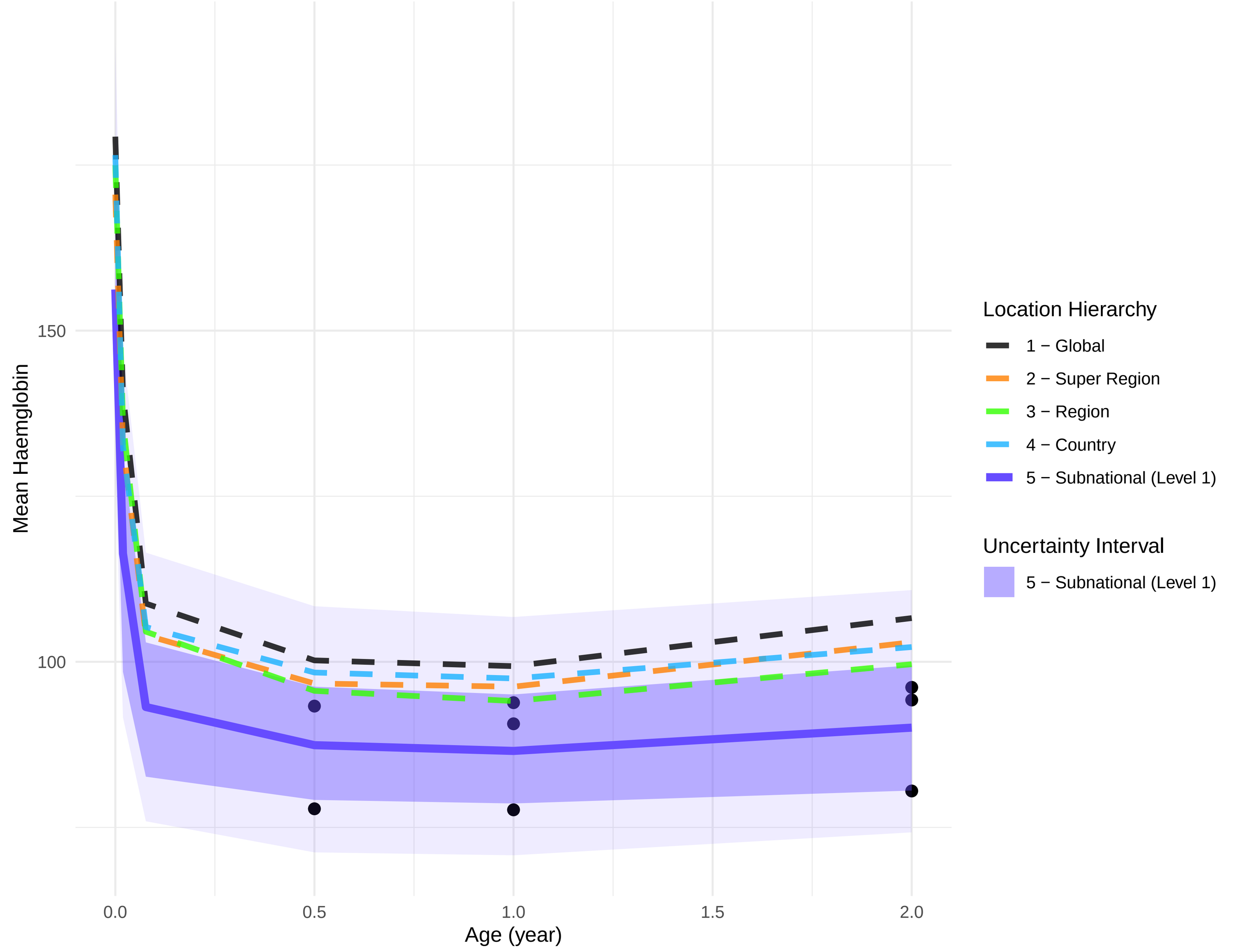

MR−BRT Age/Sex Fits − Non−pregnant Females <2 Years Old − Mean Haemglobin − Kaduna
WHO−2024 Adjustment Method

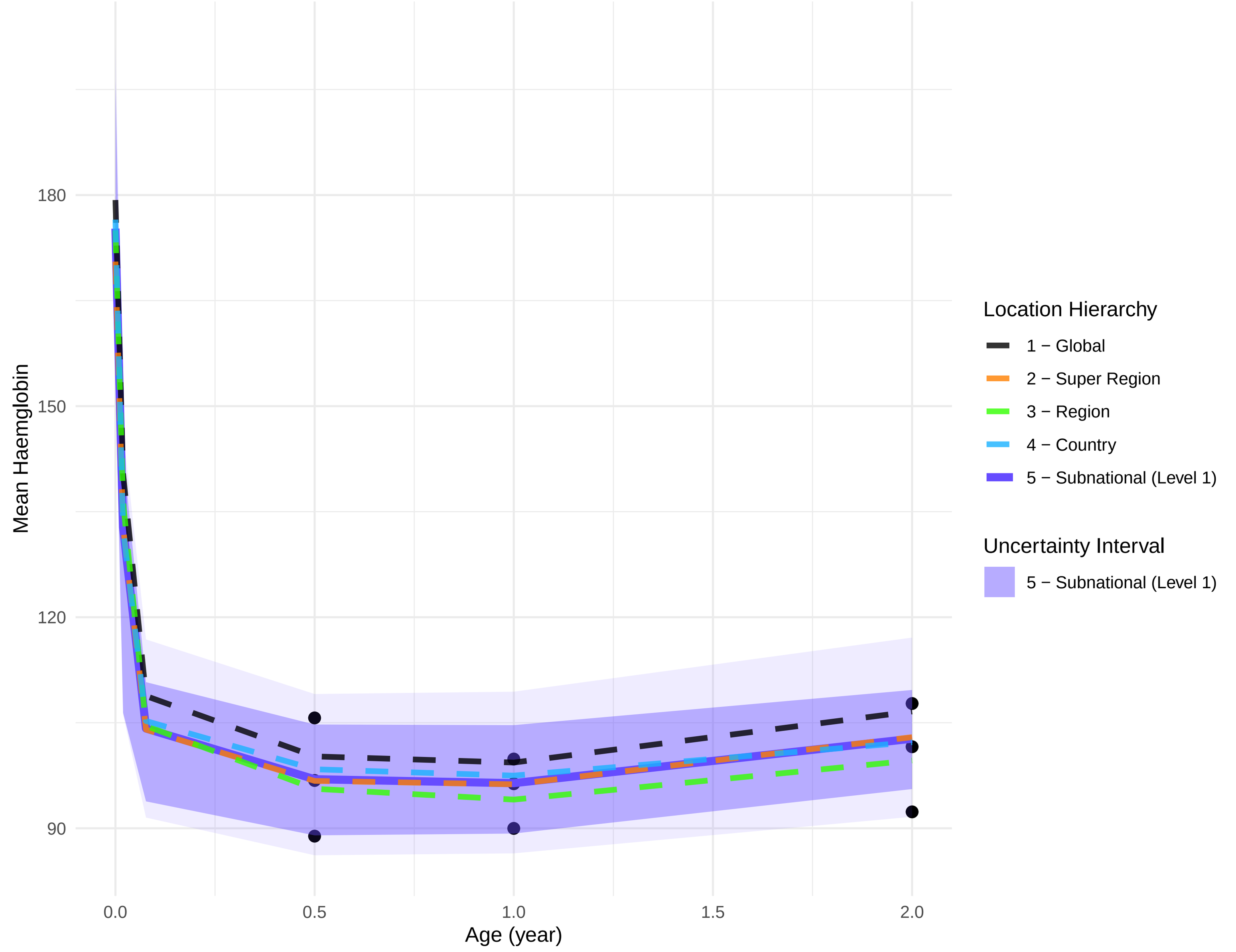

MR−BRT Age/Sex Fits − Non−pregnant Females <2 Years Old − Mean Haemglobin − Kano
WHO−2024 Adjustment Method

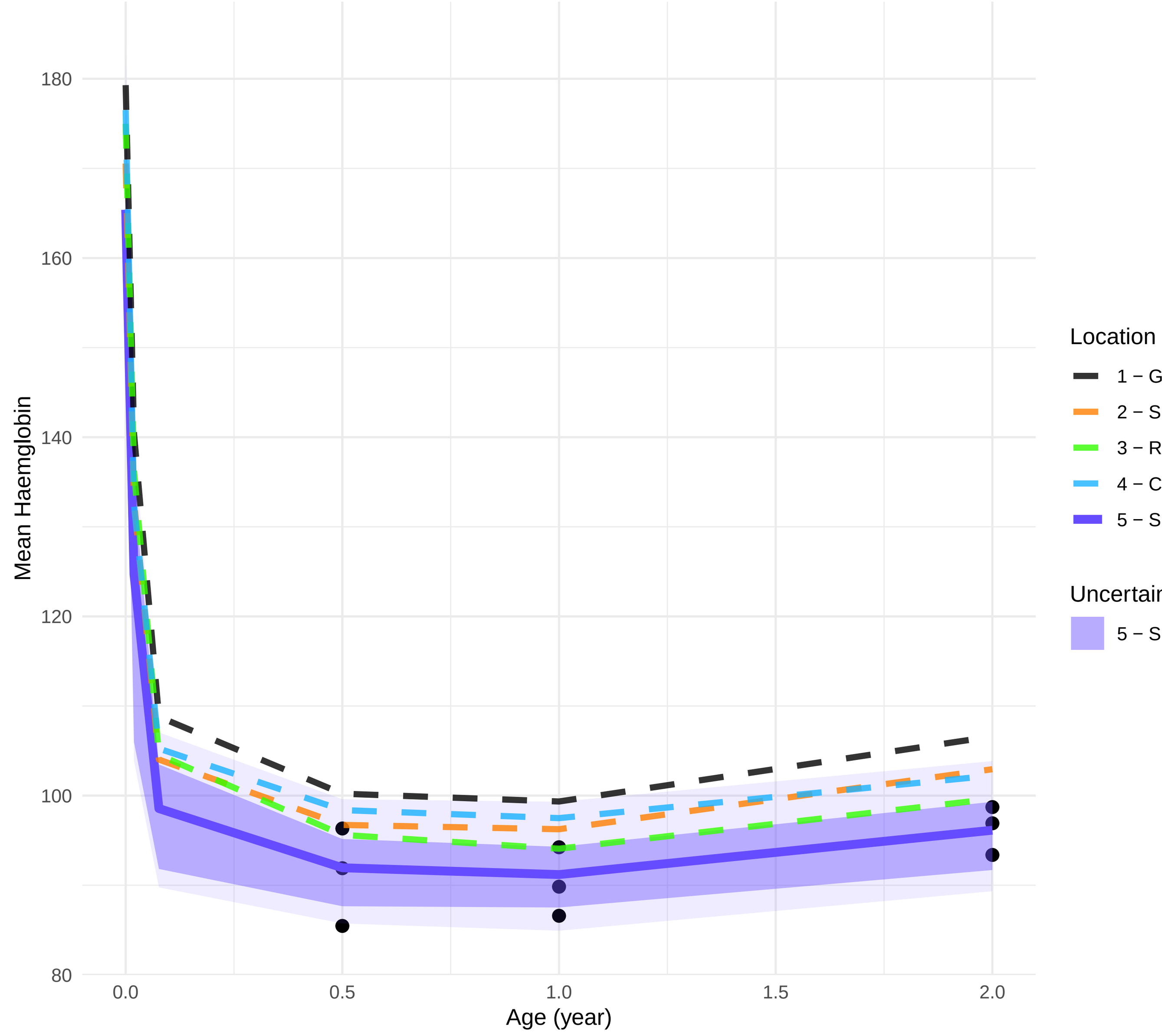

MR−BRT Age/Sex Fits − Non−pregnant Females <2 Years Old − Mean Haemglobin − Katsina
WHO−2024 Adjustment Method

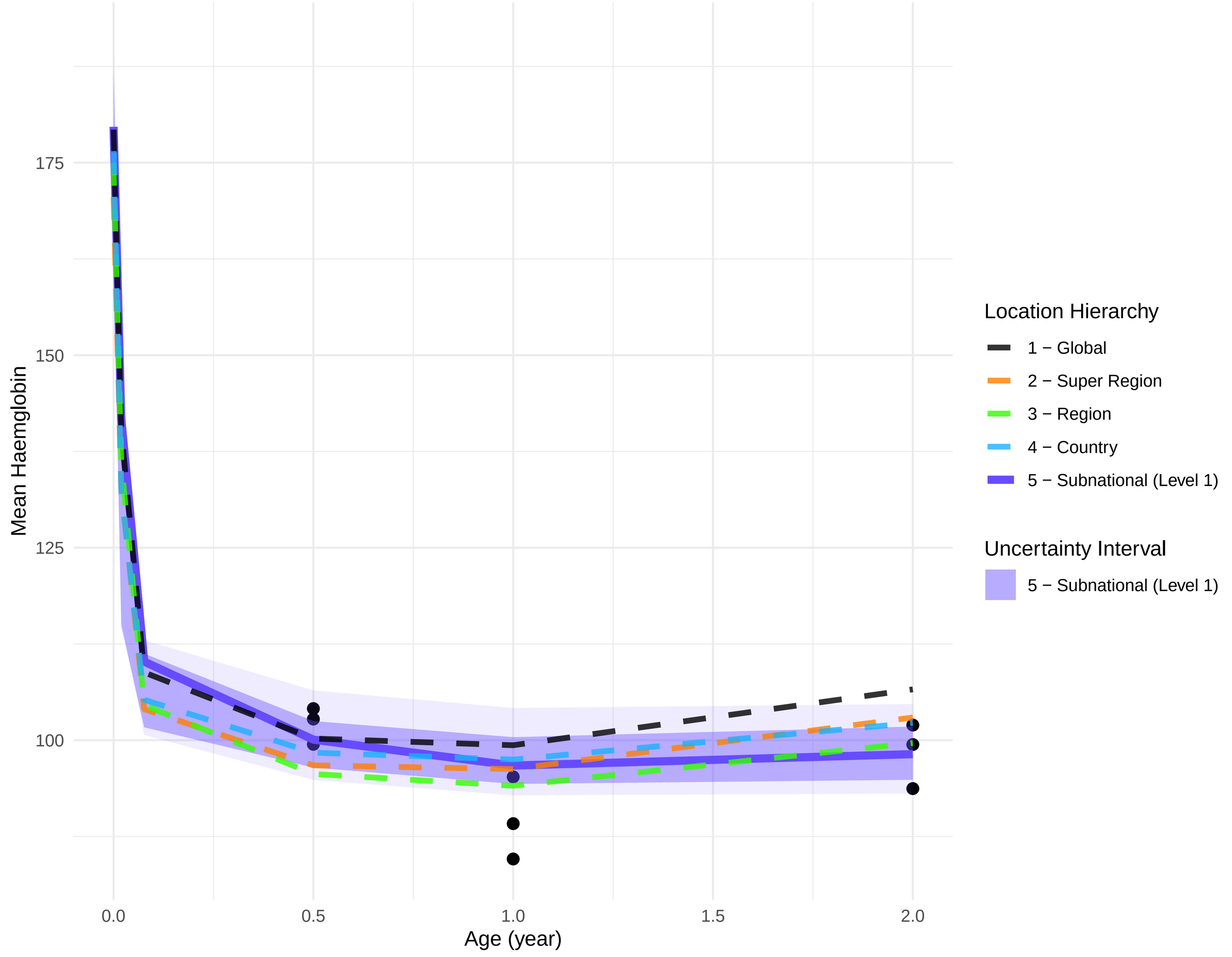

MR−BRT Age/Sex Fits − Non−pregnant Females <2 Years Old − Mean Haemglobin − Kebbi
WHO−2024 Adjustment Method

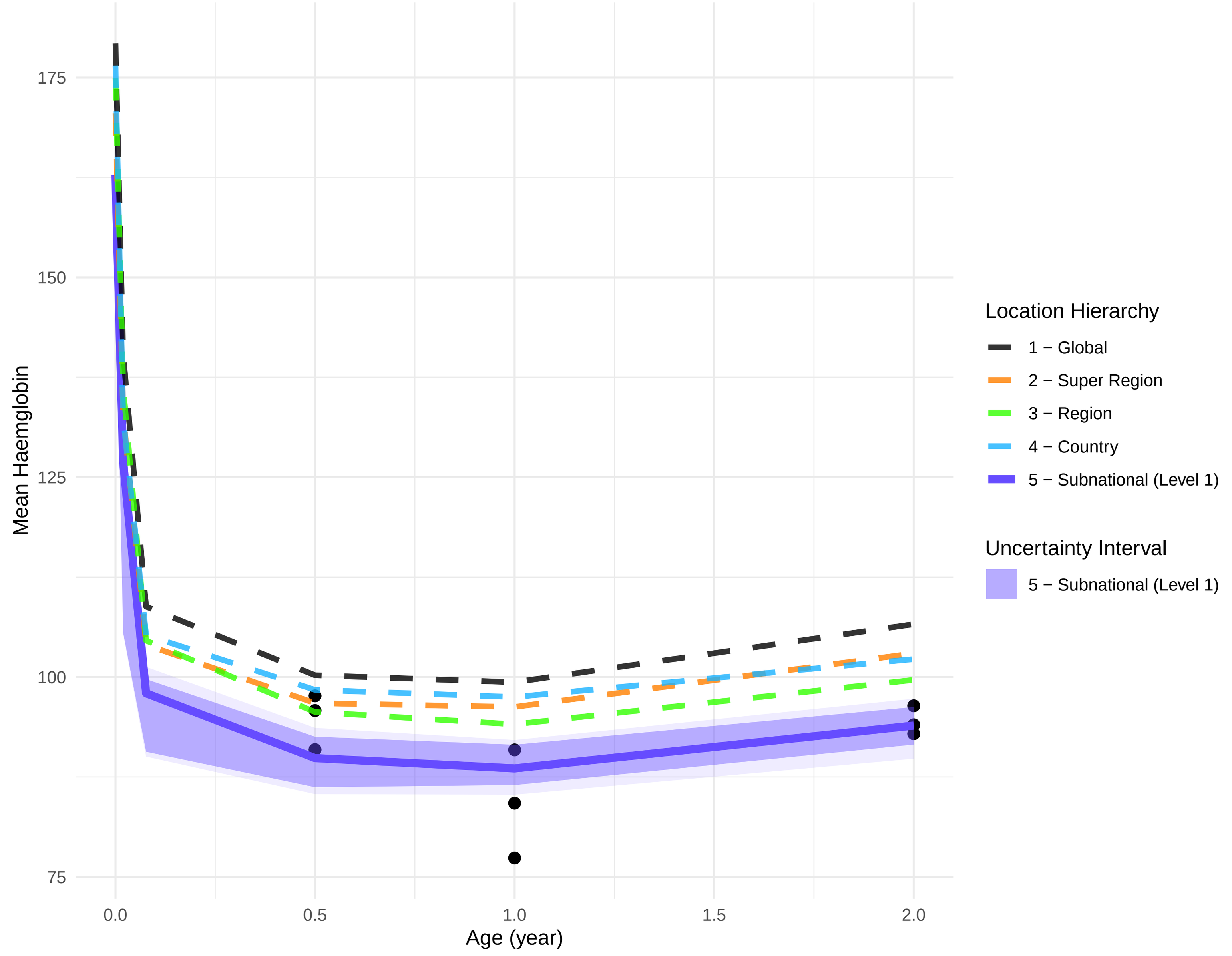

MR−BRT Age/Sex Fits − Non−pregnant Females <2 Years Old − Mean Haemglobin − Kogi
WHO−2024 Adjustment Method

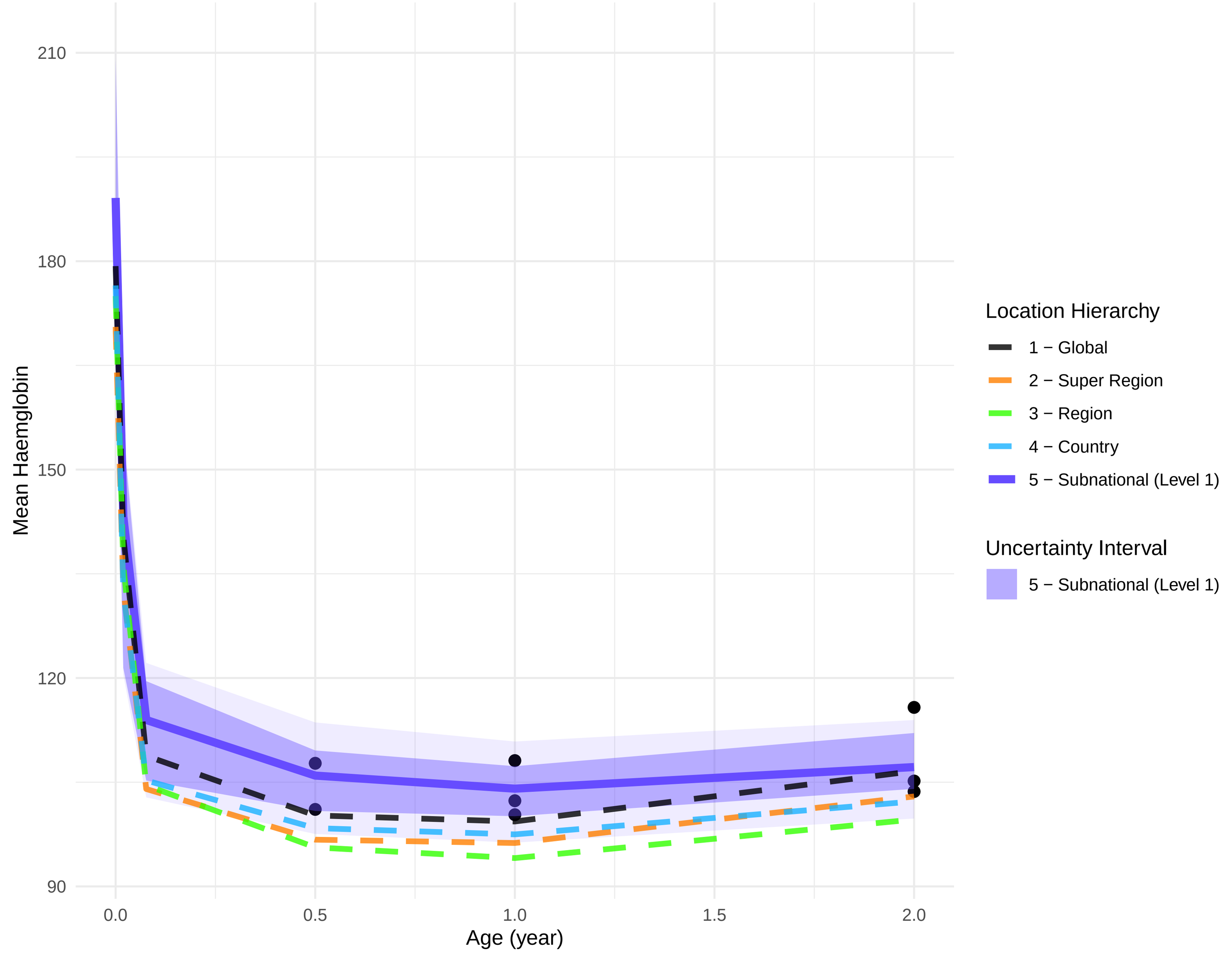

MR−BRT Age/Sex Fits − Non−pregnant Females <2 Years Old − Mean Haemglobin − Kwara
WHO−2024 Adjustment Method

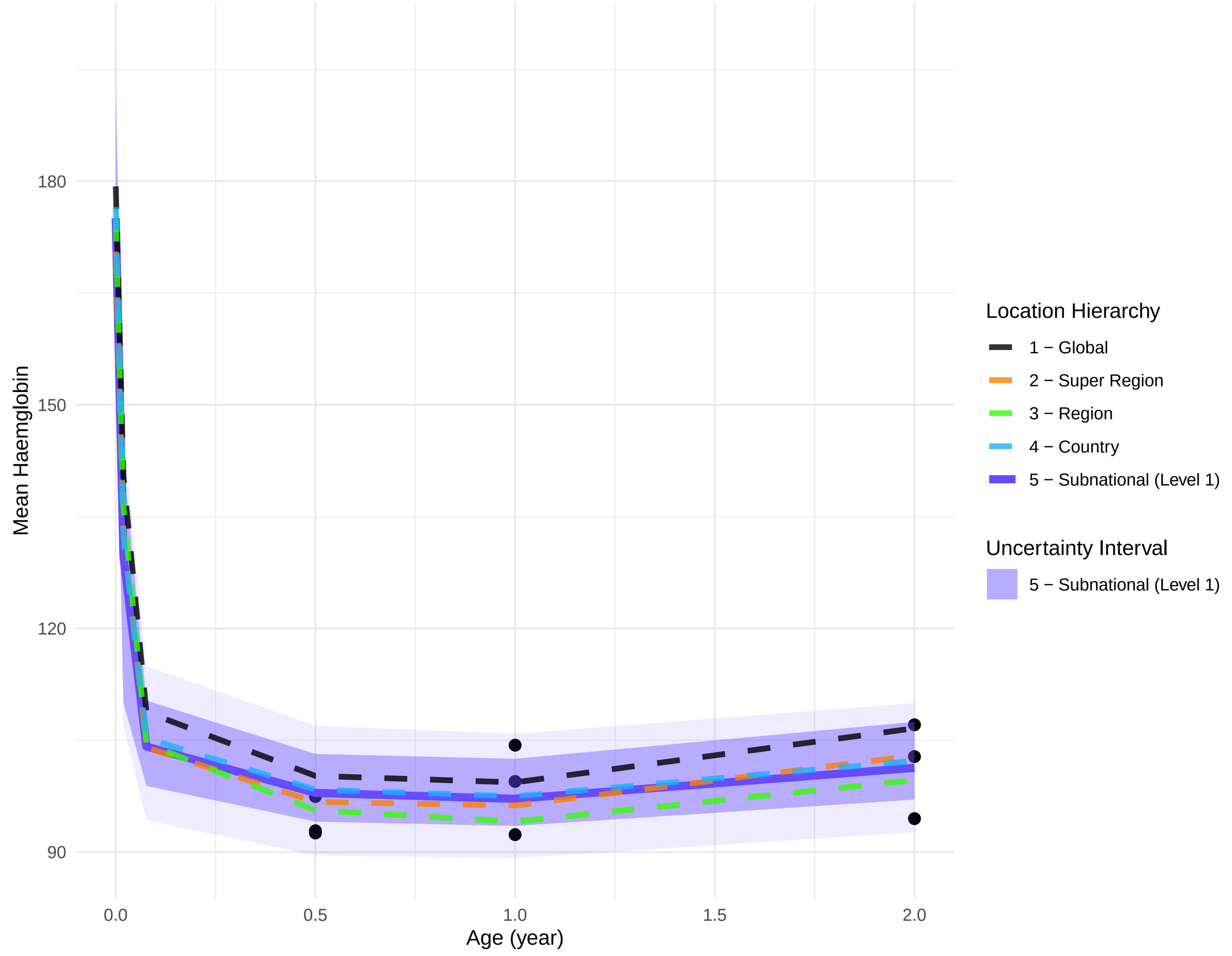

MR−BRT Age/Sex Fits − Non−pregnant Females <2 Years Old − Mean Haemglobin − Lagos
WHO−2024 Adjustment Method

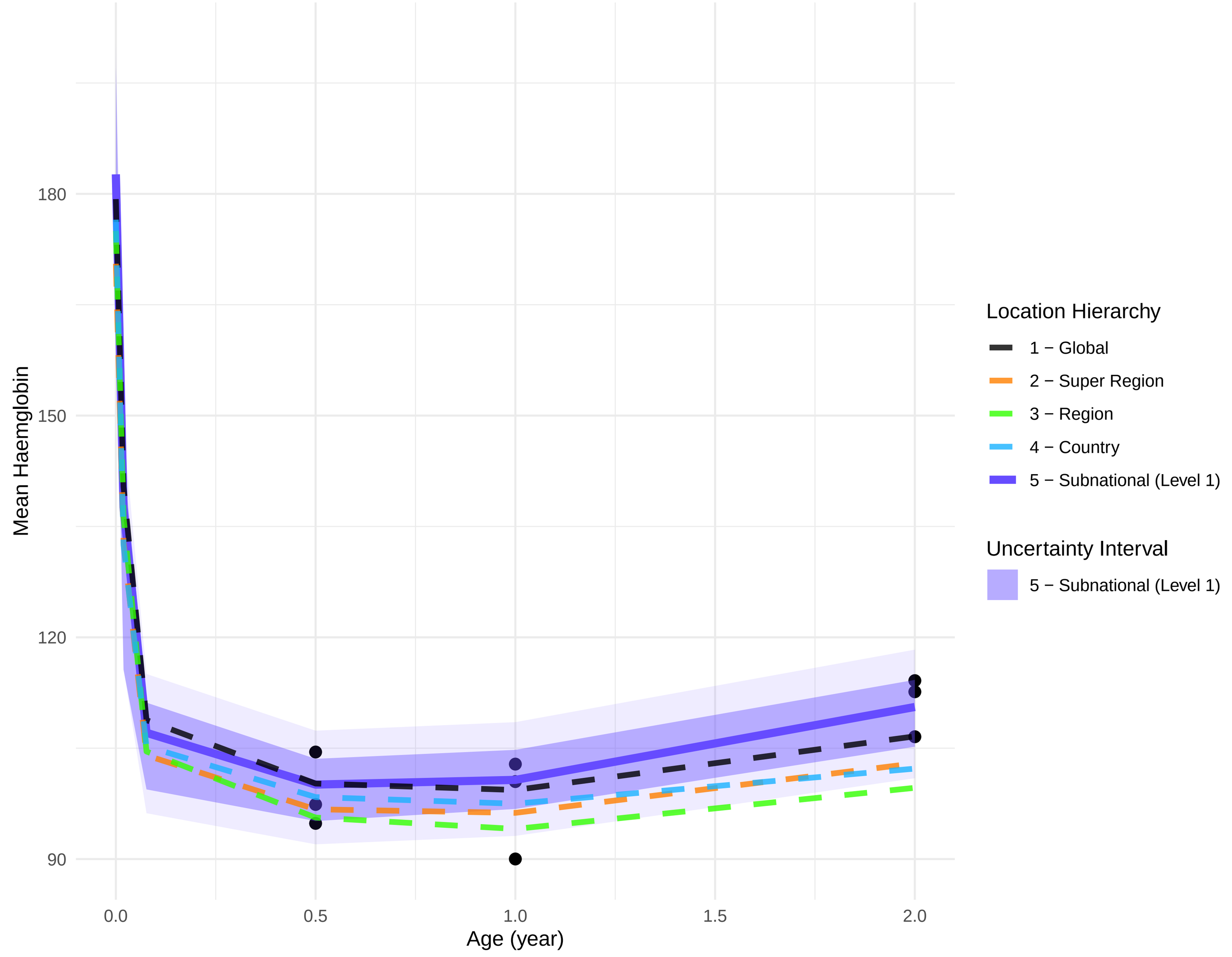

MR−BRT Age/Sex Fits − Non−pregnant Females <2 Years Old − Mean Haemglobin − Nasarawa
WHO−2024 Adjustment Method

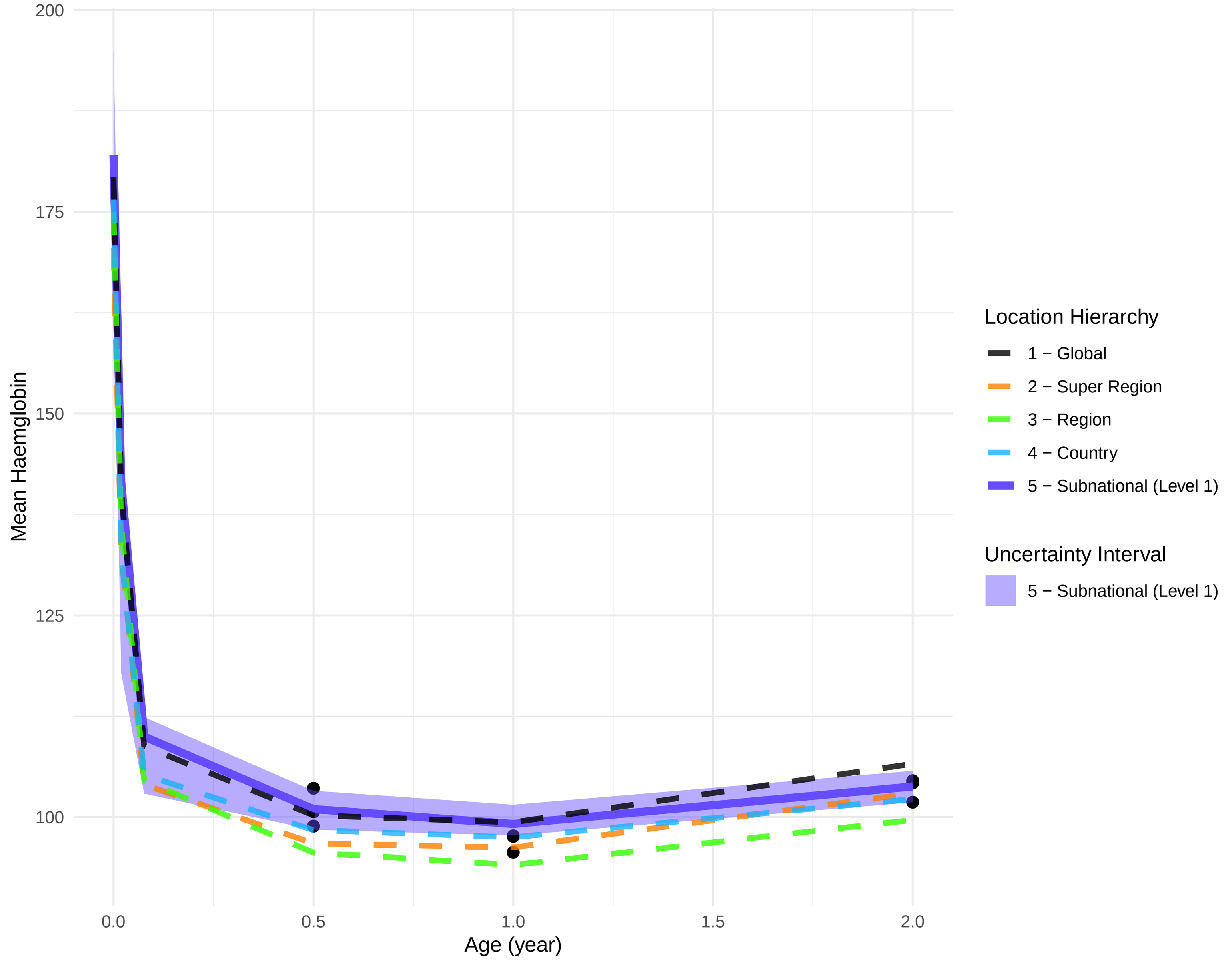

MR−BRT Age/Sex Fits − Non−pregnant Females <2 Years Old − Mean Haemglobin − Niger
WHO−2024 Adjustment Method

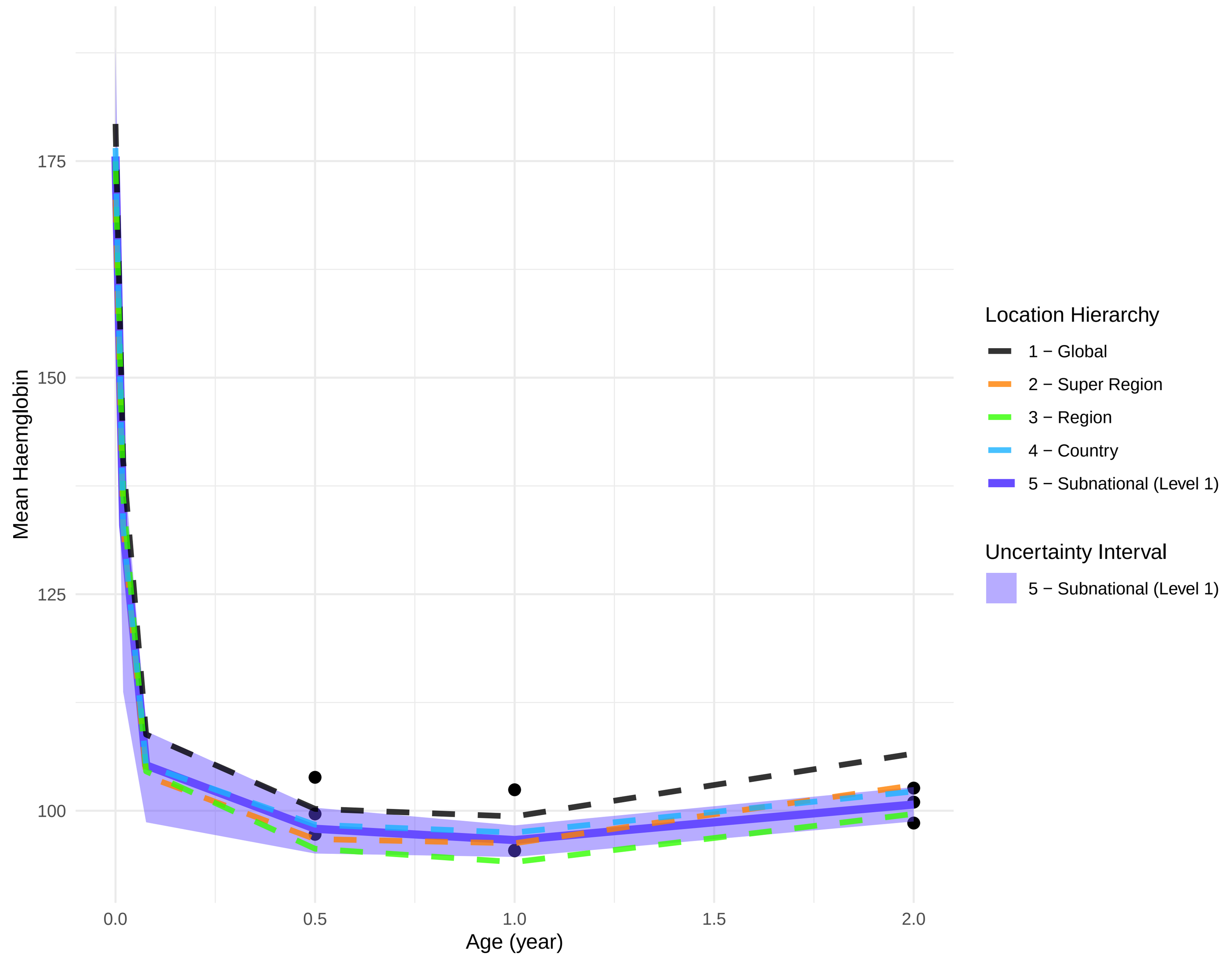

MR−BRT Age/Sex Fits − Non−pregnant Females <2 Years Old − Mean Haemglobin − Ogun
WHO−2024 Adjustment Method

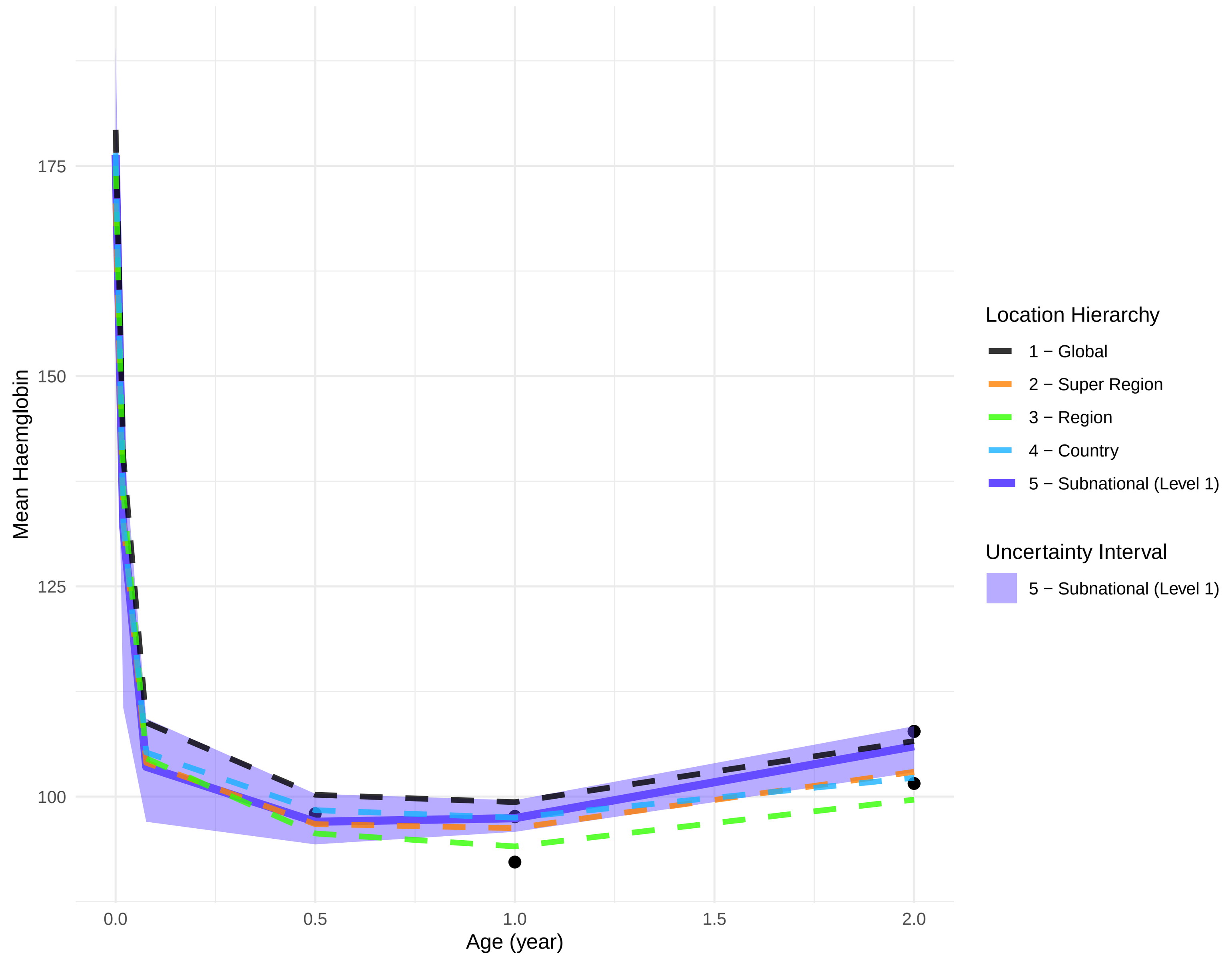

MR−BRT Age/Sex Fits − Non−pregnant Females <2 Years Old − Mean Haemglobin − Ondo
WHO−2024 Adjustment Method

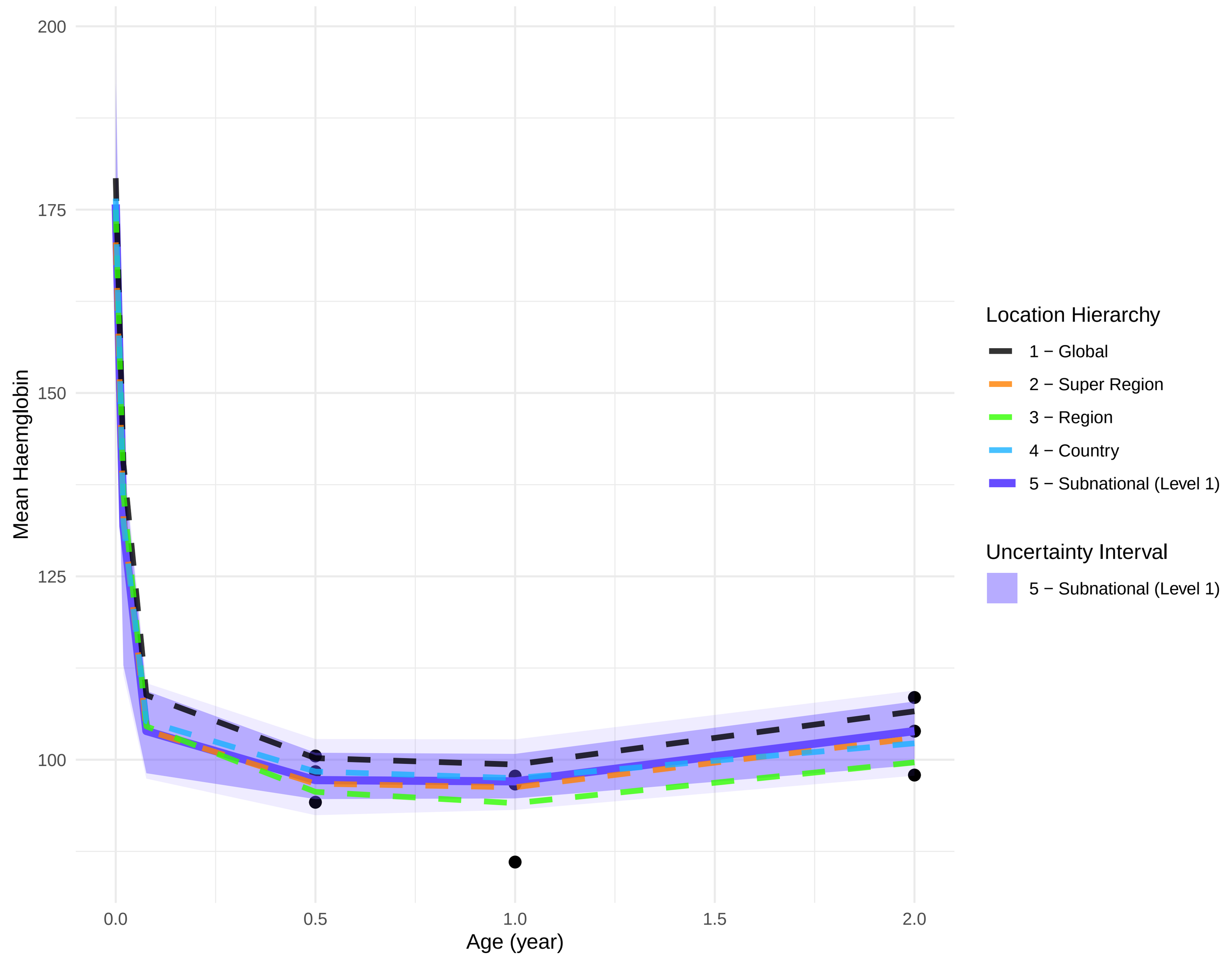

MR−BRT Age/Sex Fits − Non−pregnant Females <2 Years Old − Mean Haemglobin − Osun
WHO−2024 Adjustment Method

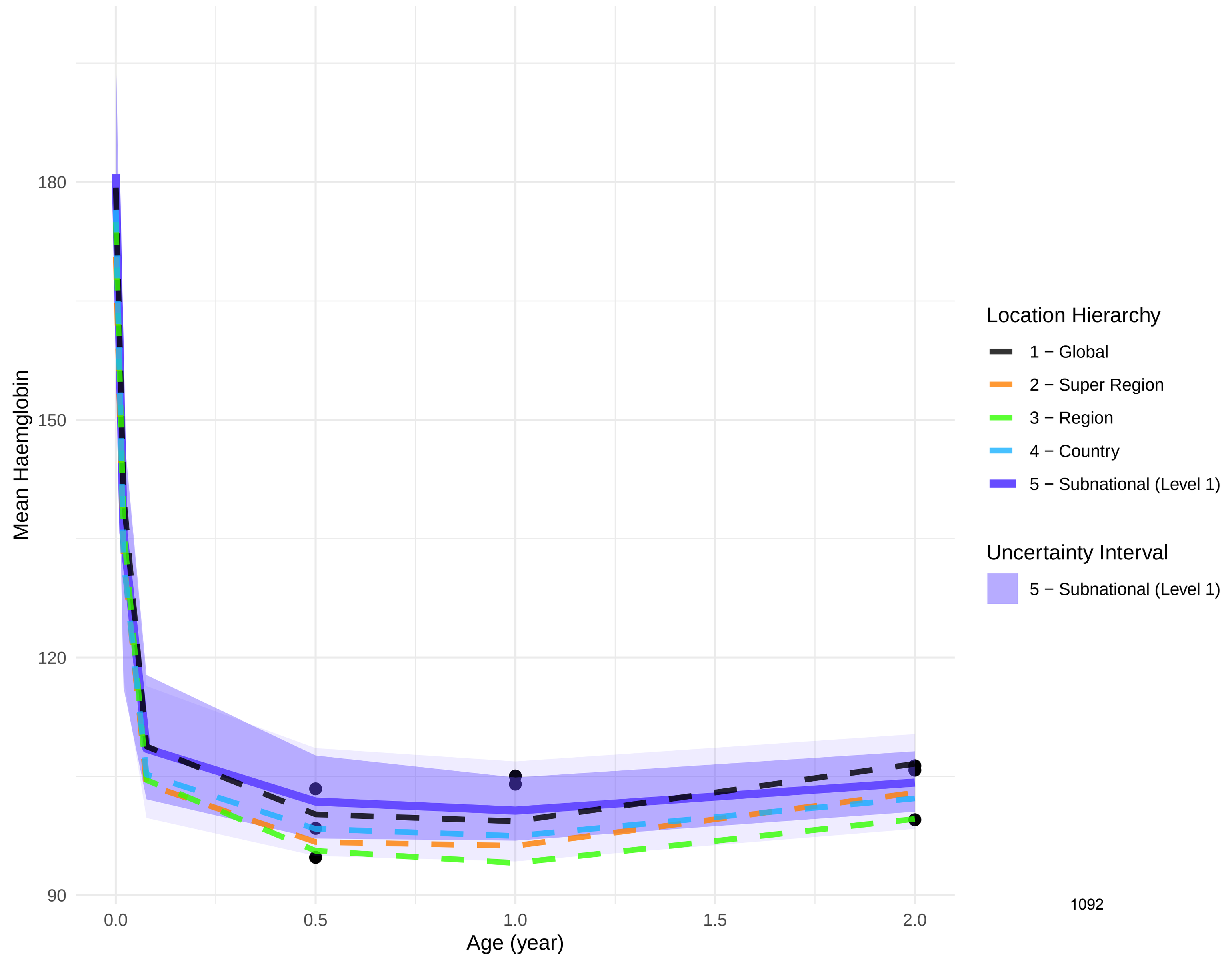

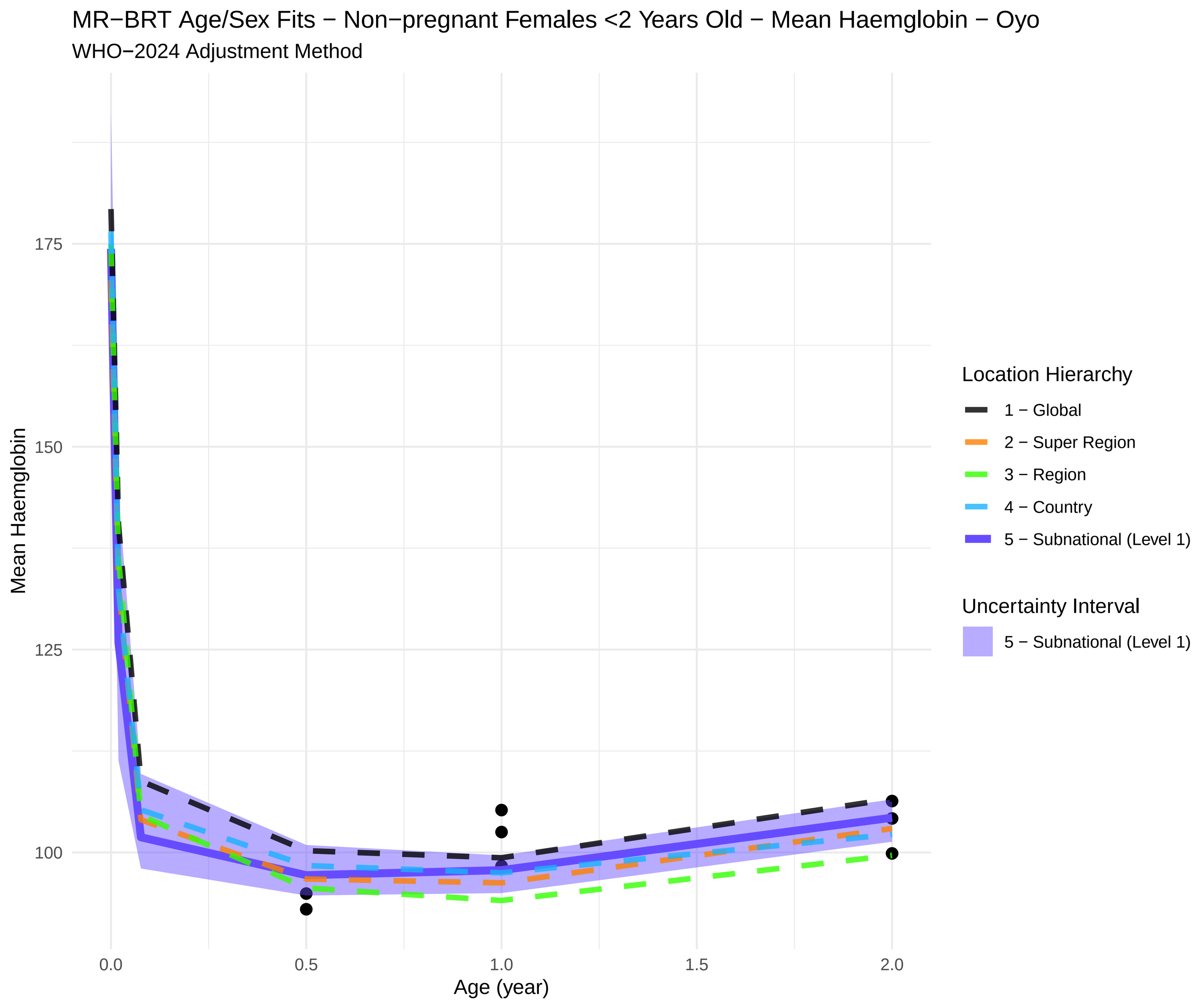
MR−BRT Age/Sex Fits − Non−pregnant Females <2 Years Old − Mean Haemglobin − Oyo
WHO−2024 Adjustment Method
Mean Haemglobin
Age (year)
Location Hierarchy
1 − Global
2 − Super Region
3 − Region
4 − Country
5 − Subnational (Level 1)
Uncertainty Interval
5 − Subnational (Level 1)

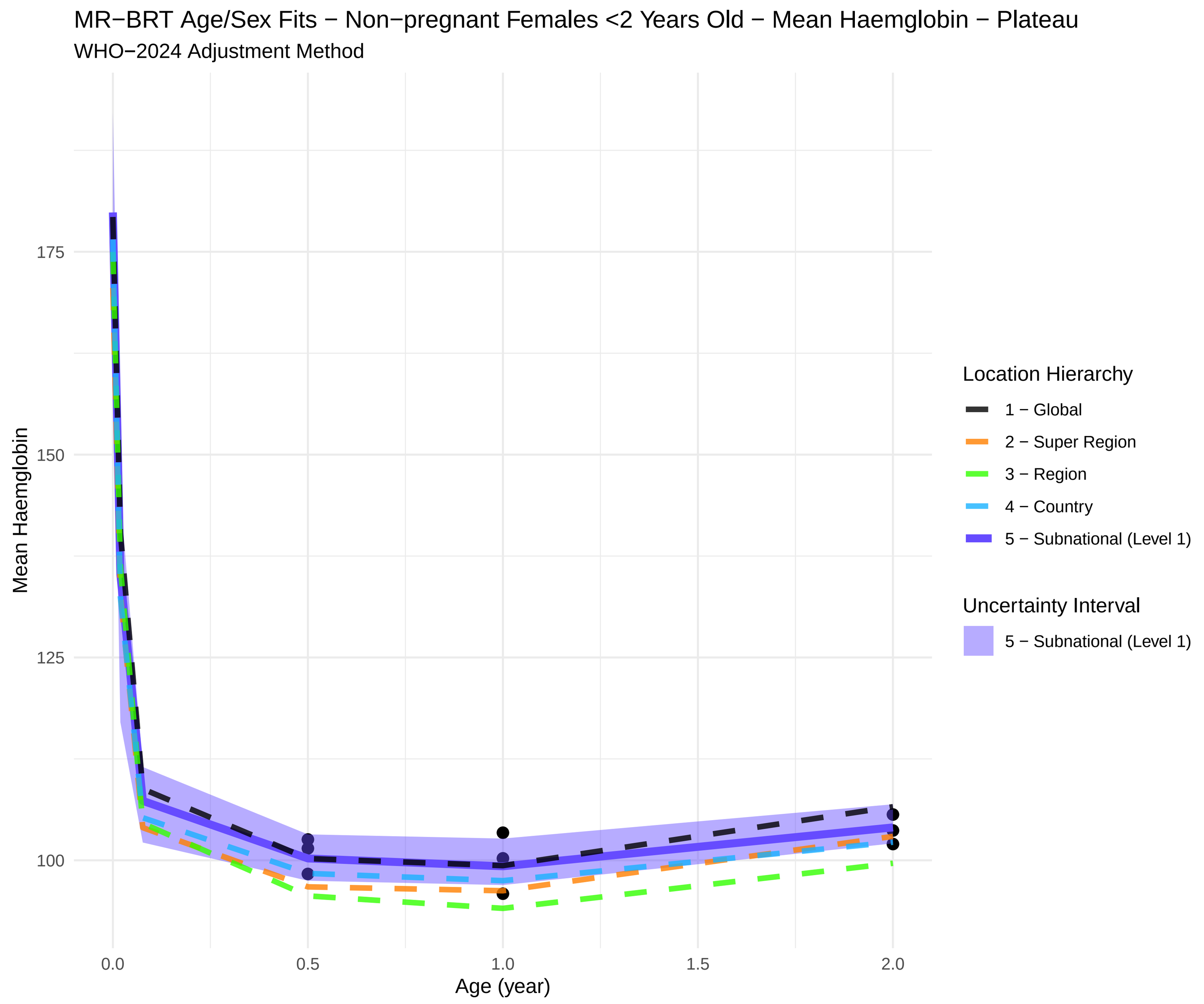
MR−BRT Age/Sex Fits − Non−pregnant Females <2 Years Old − Mean Haemglobin − Plateau
WHO−2024 Adjustment Method
Mean Haemglobin
Age (year)
Location Hierarchy
1 − Global
2 − Super Region
3 − Region
4 − Country
5 − Subnational (Level 1)
Uncertainty Interval
5 − Subnational (Level 1)

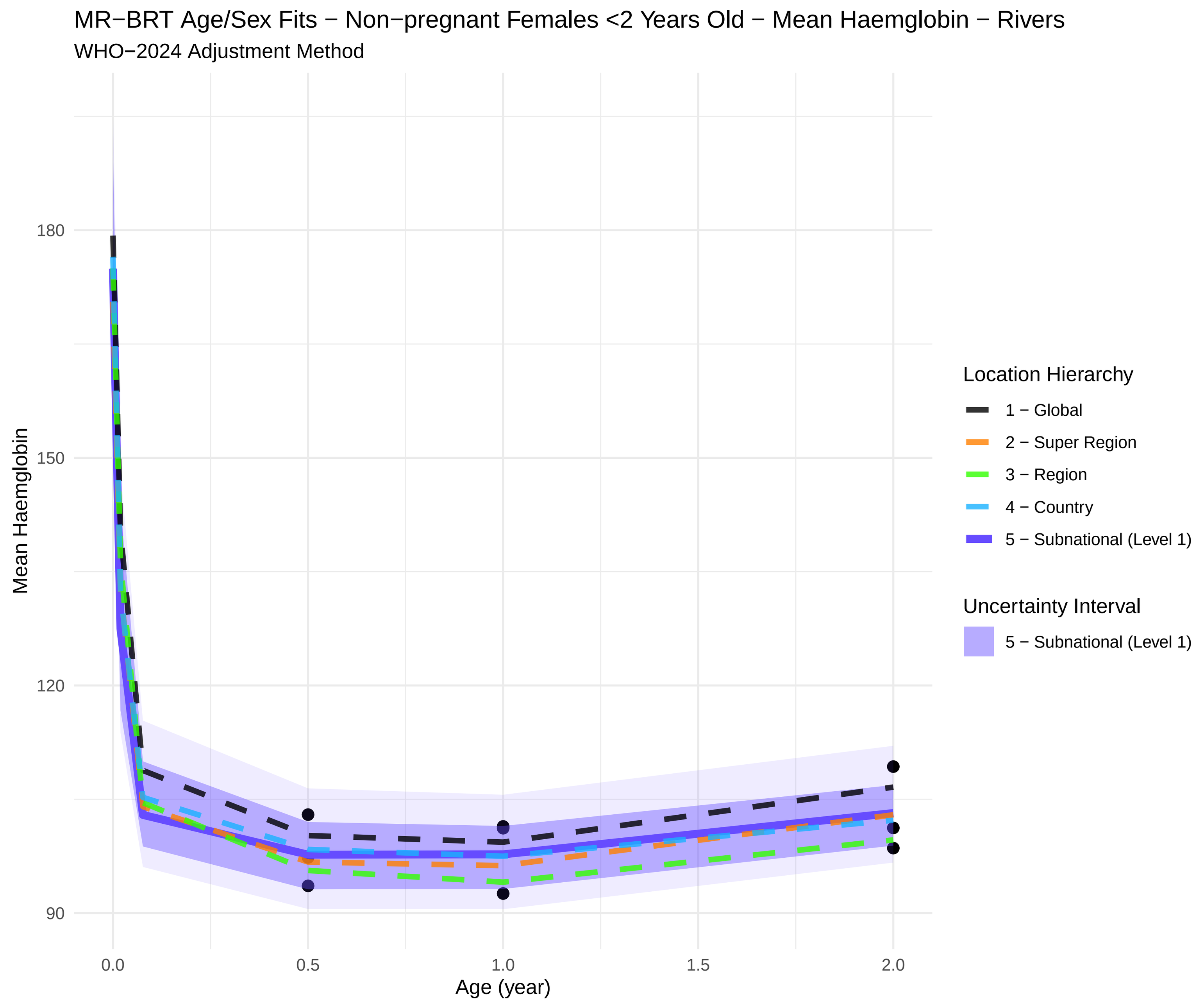
MR−BRT Age/Sex Fits − Non−pregnant Females <2 Years Old − Mean Haemglobin − Rivers
WHO−2024 Adjustment Method
Mean Haemglobin
Age (year)
Location Hierarchy
1 − Global
2 − Super Region
3 − Region
4 − Country
5 − Subnational (Level 1)
Uncertainty Interval
5 − Subnational (Level 1)

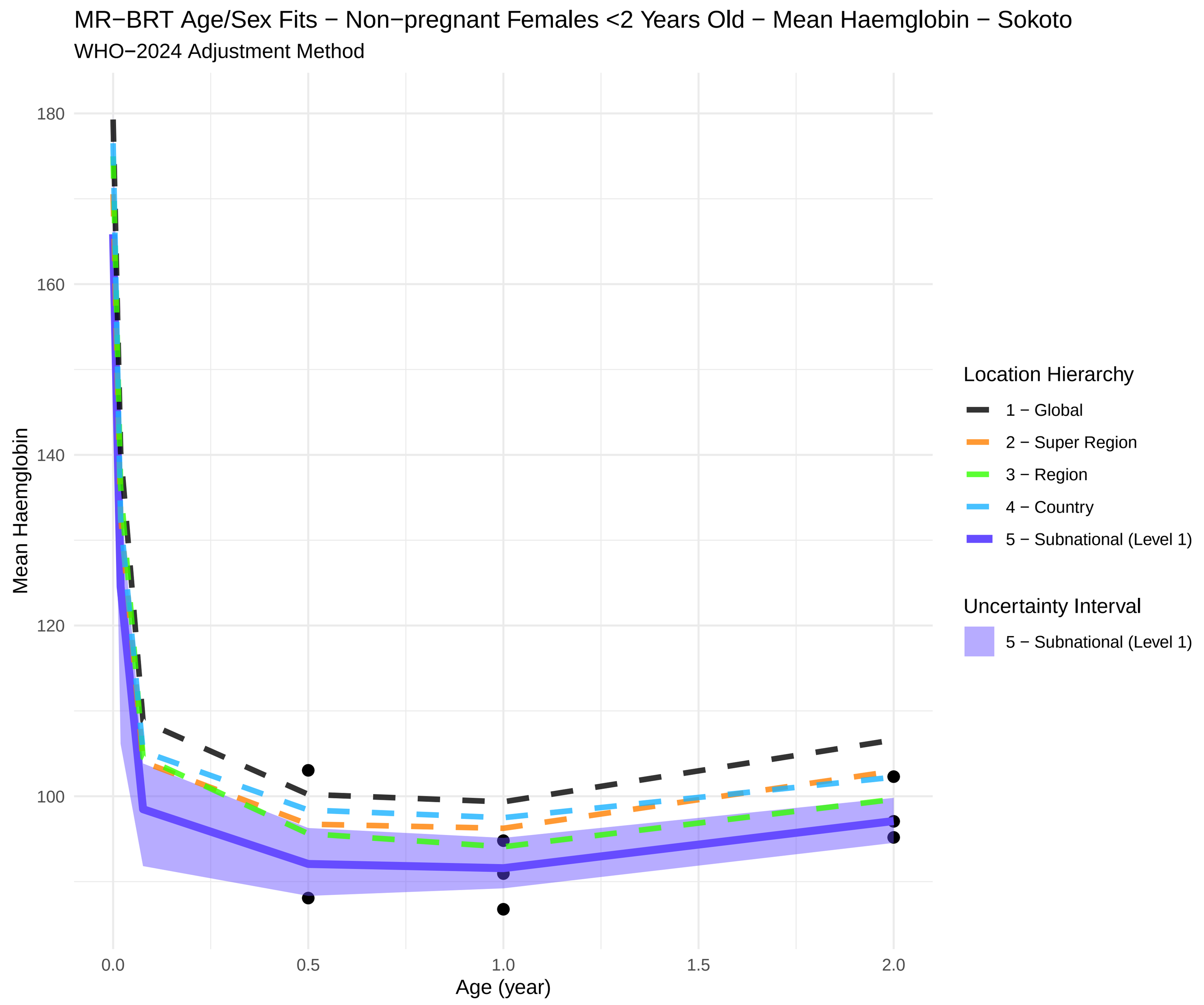
MR−BRT Age/Sex Fits − Non−pregnant Females <2 Years Old − Mean Haemglobin − Sokoto
WHO−2024 Adjustment Method
Mean Haemglobin
Age (year)
Location Hierarchy
1 − Global
2 − Super Region
3 − Region
4 − Country
5 − Subnational (Level 1)
Uncertainty Interval
5 − Subnational (Level 1)

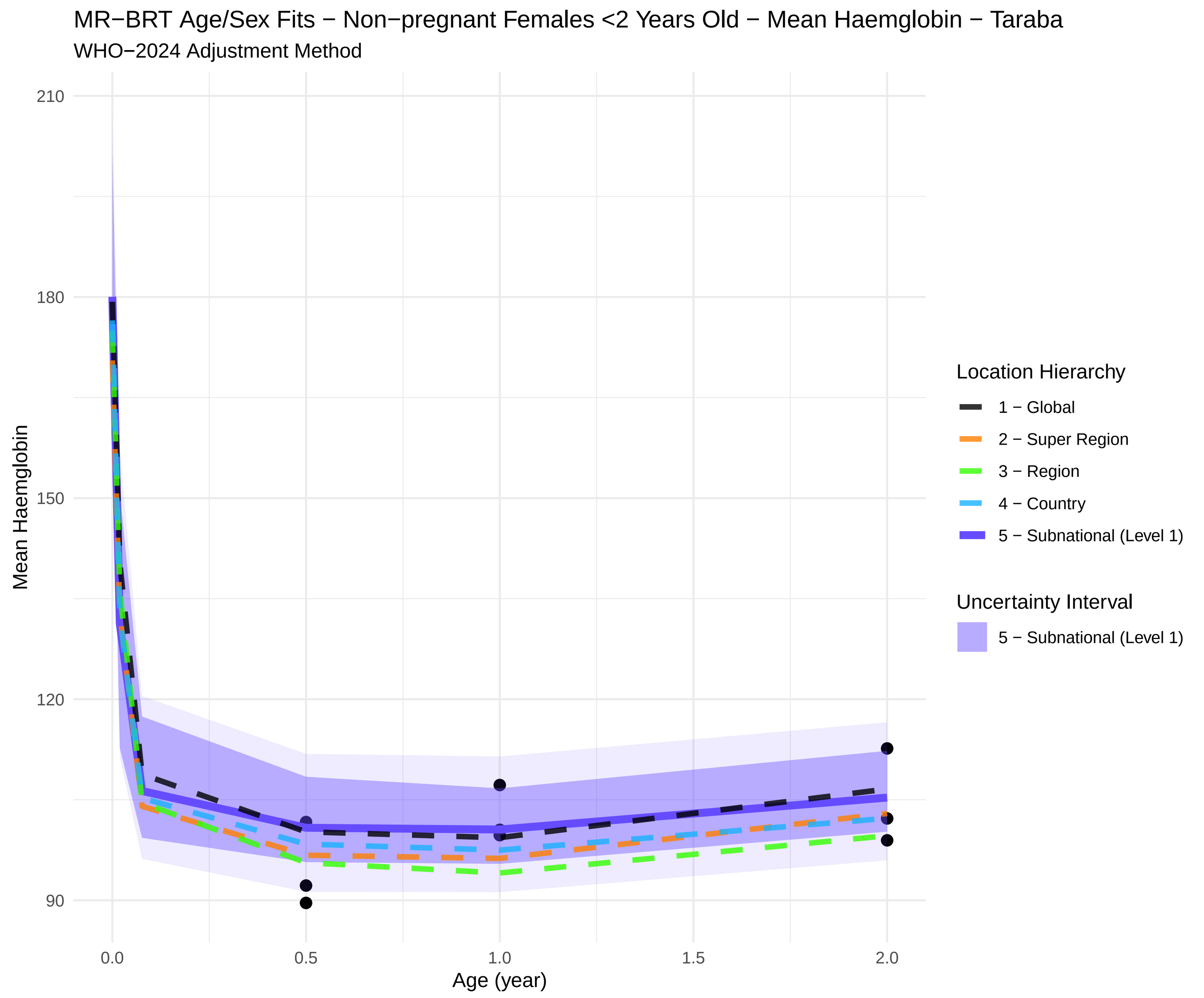
MR−BRT Age/Sex Fits − Non−pregnant Females <2 Years Old − Mean Haemglobin − Taraba
WHO−2024 Adjustment Method
Mean Haemglobin
Age (year)
Location Hierarchy
1 − Global
2 − Super Region
3 − Region
4 − Country
5 − Subnational (Level 1)
Uncertainty Interval
5 − Subnational (Level 1)

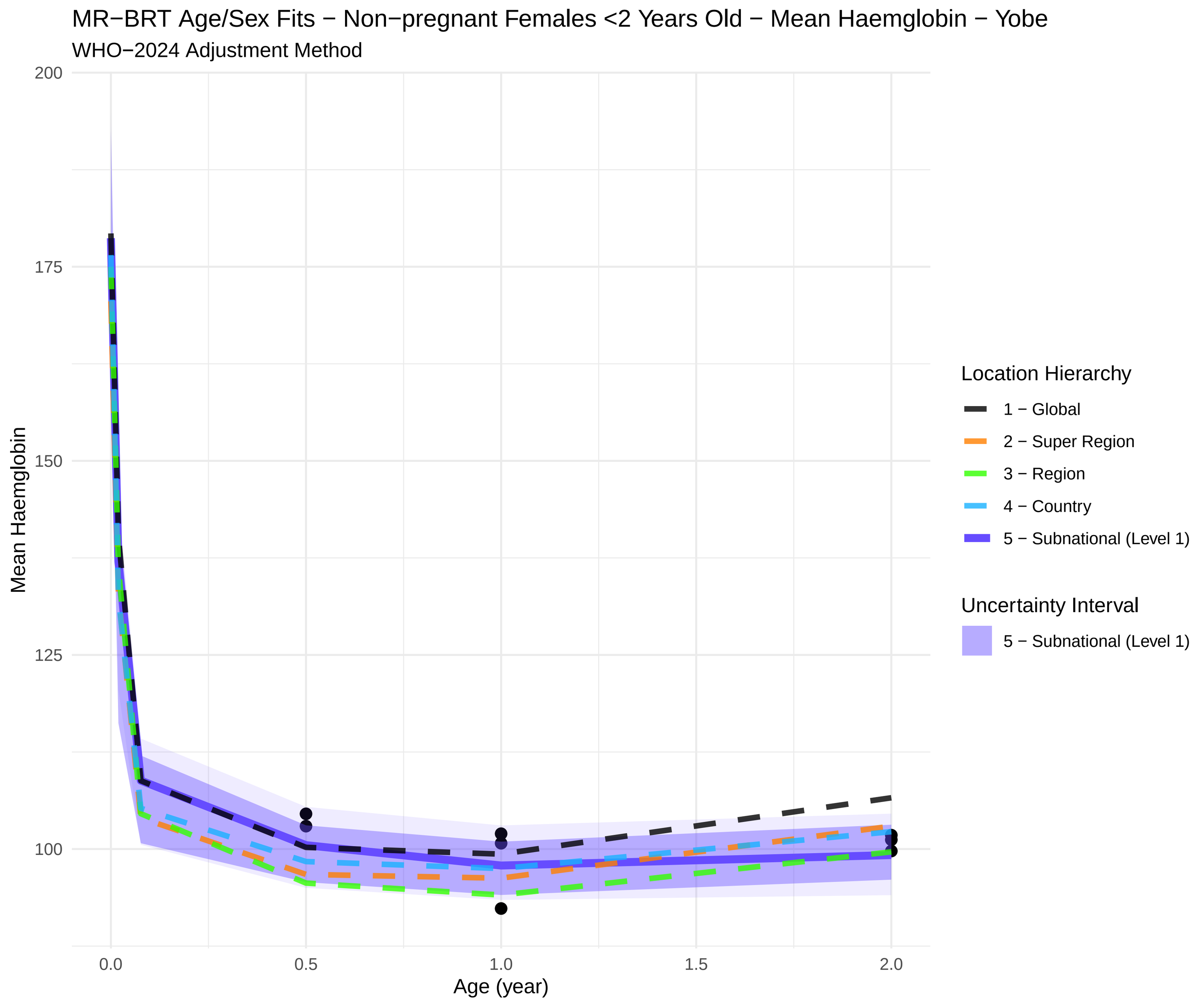
MR−BRT Age/Sex Fits − Non−pregnant Females <2 Years Old − Mean Haemglobin − Yobe
WHO−2024 Adjustment Method
Mean Haemglobin
Age (year)
Location Hierarchy
1 − Global
2 − Super Region
3 − Region
4 − Country
5 − Subnational (Level 1)
Uncertainty Interval
5 − Subnational (Level 1)

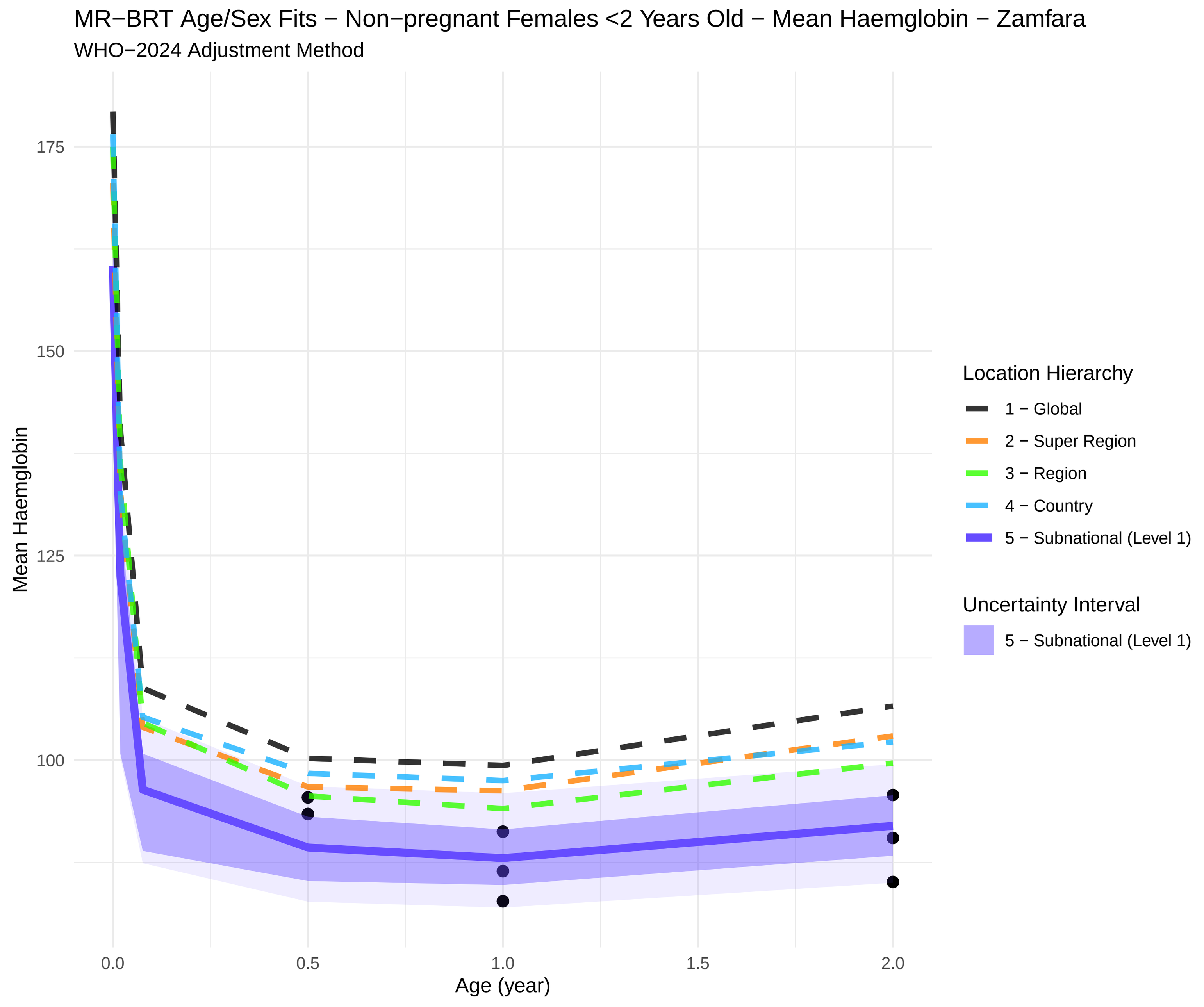
MR−BRT Age/Sex Fits − Non−pregnant Females <2 Years Old − Mean Haemglobin − Zamfara
WHO−2024 Adjustment Method
Mean Haemglobin
Age (year)
Location Hierarchy
1 − Global
2 − Super Region
3 − Region
4 − Country
5 − Subnational (Level 1)
Uncertainty Interval
5 − Subnational (Level 1)

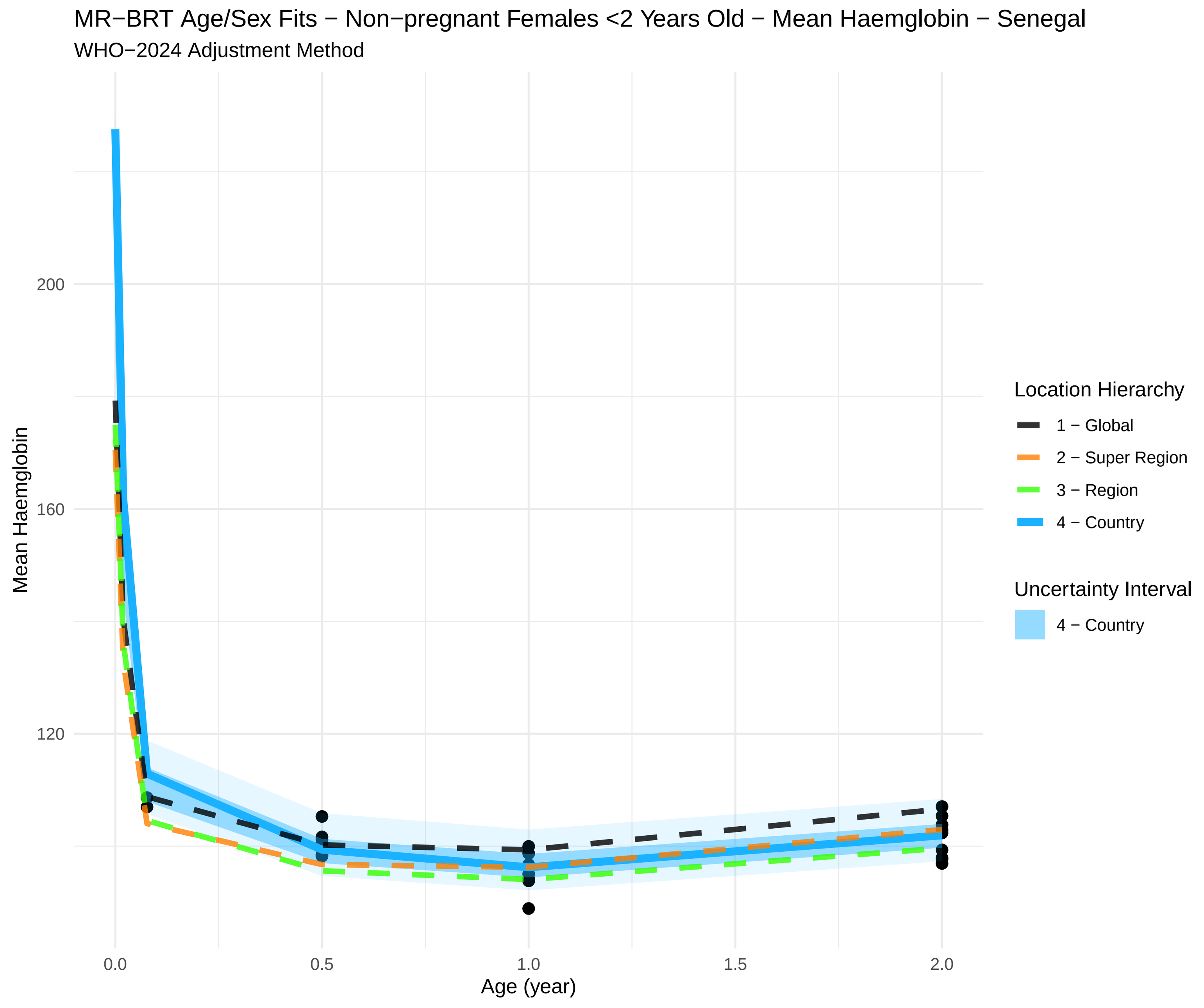
MR−BRT Age/Sex Fits − Non−pregnant Females <2 Years Old − Mean Haemglobin − Senegal
WHO−2024 Adjustment Method
Mean Haemglobin
Age (year)
Location Hierarchy
1 − Global
2 − Super Region
3 − Region
4 − Country
Uncertainty Interval
4 − Country

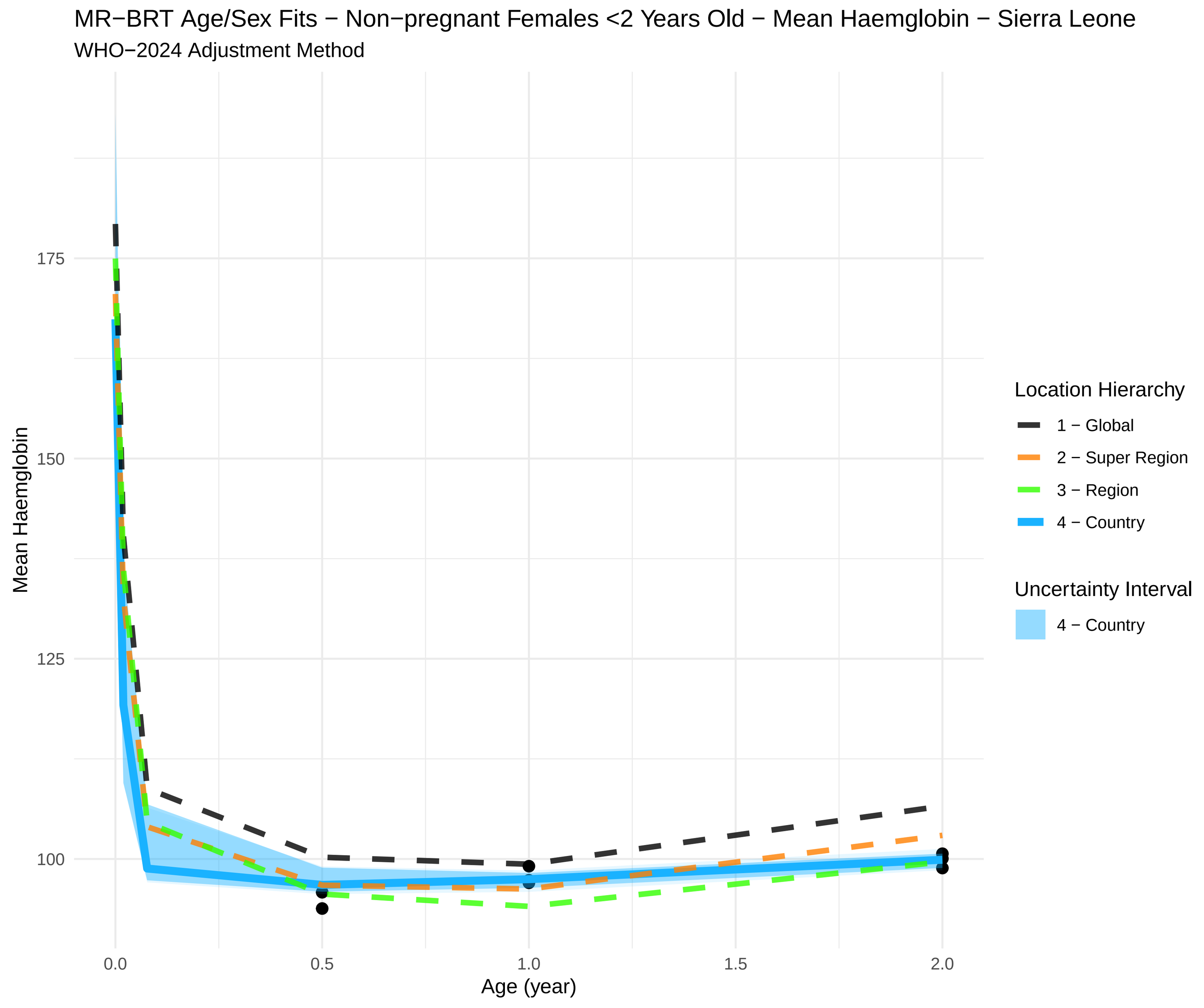
MR−BRT Age/Sex Fits − Non−pregnant Females <2 Years Old − Mean Haemglobin − Sierra Leone
WHO−2024 Adjustment Method
Mean Haemglobin
Age (year)
Location Hierarchy
1 − Global
2 − Super Region
3 − Region
4 − Country
Uncertainty Interval
4 − Country

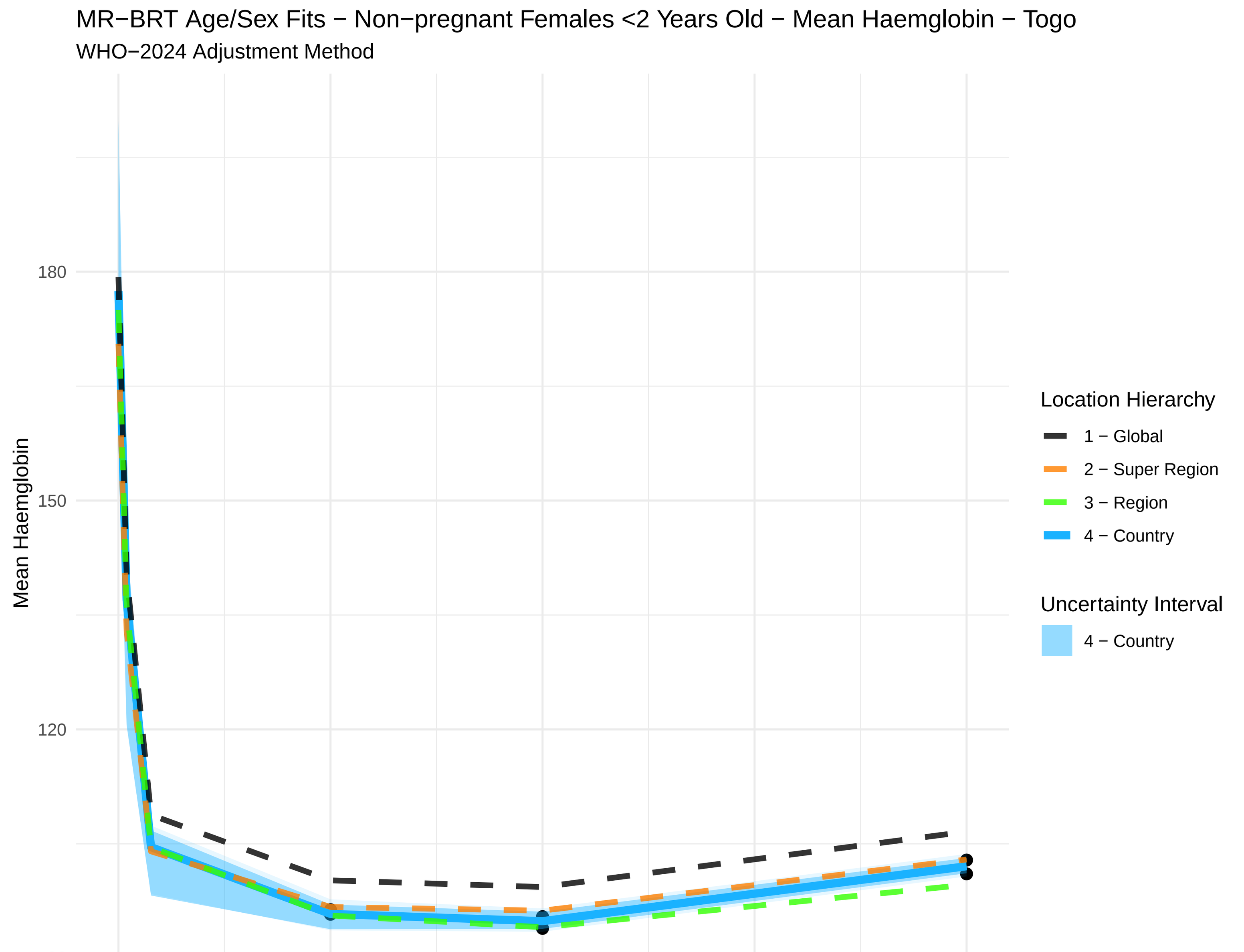
MR−BRT Age/Sex Fits − Non−pregnant Females <2 Years Old − Mean Haemglobin − Togo
WHO−2024 Adjustment Method
Mean Haemglobin
Age (year)
Location Hierarchy
1 − Global
2 − Super Region
3 − Region
4 − Country
Uncertainty Interval
4 − Country

MR−BRT Age/Sex Fits − Males <2 Years Old − Mean Haemglobin − Global
WHO−2001 Adjustment Method

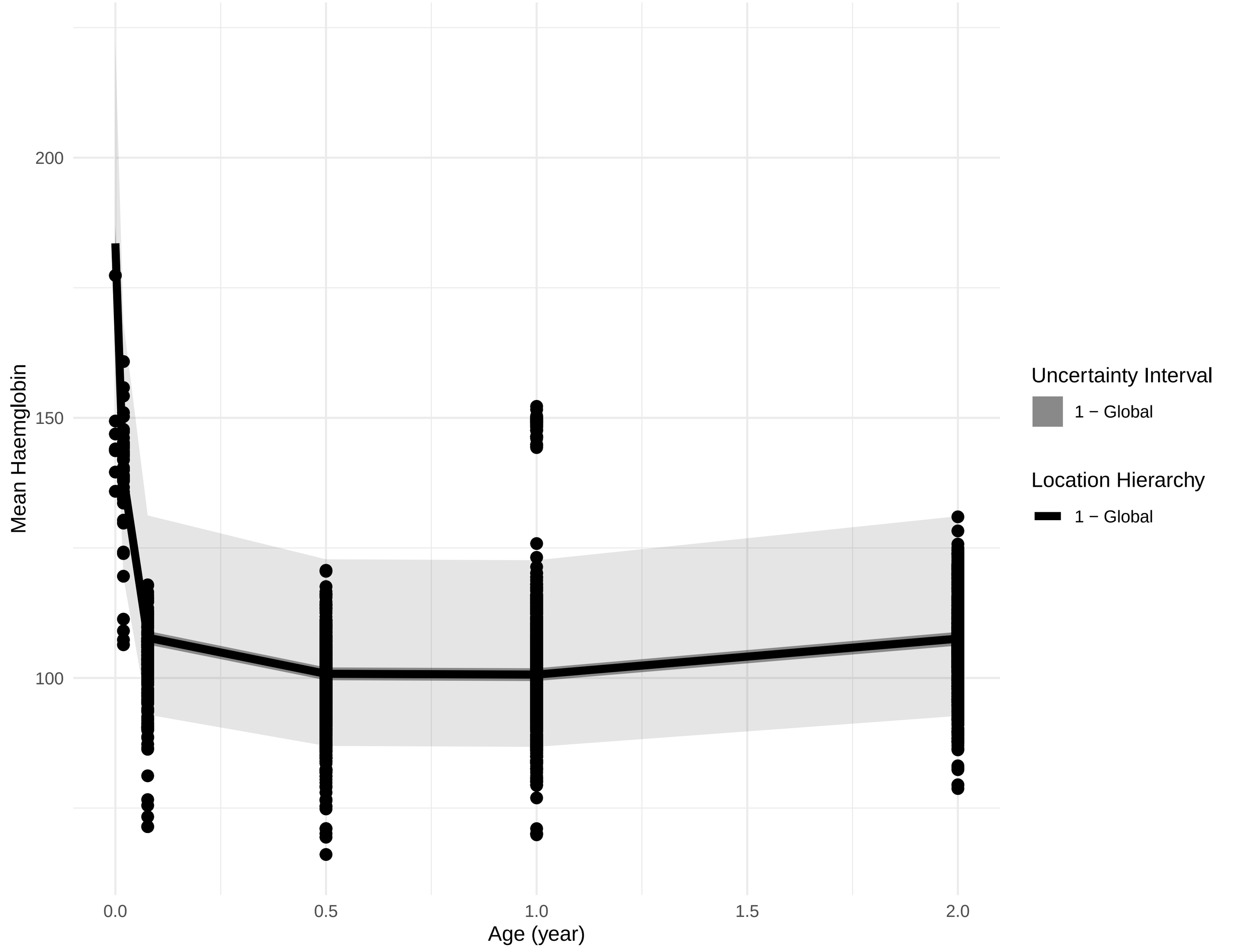

MR−BRT Age/Sex Fits − Males <2 Years Old − Mean Haemglobin − Central Europe, Eastern Europe, and Central Asia
WHO−2001 Adjustment Method

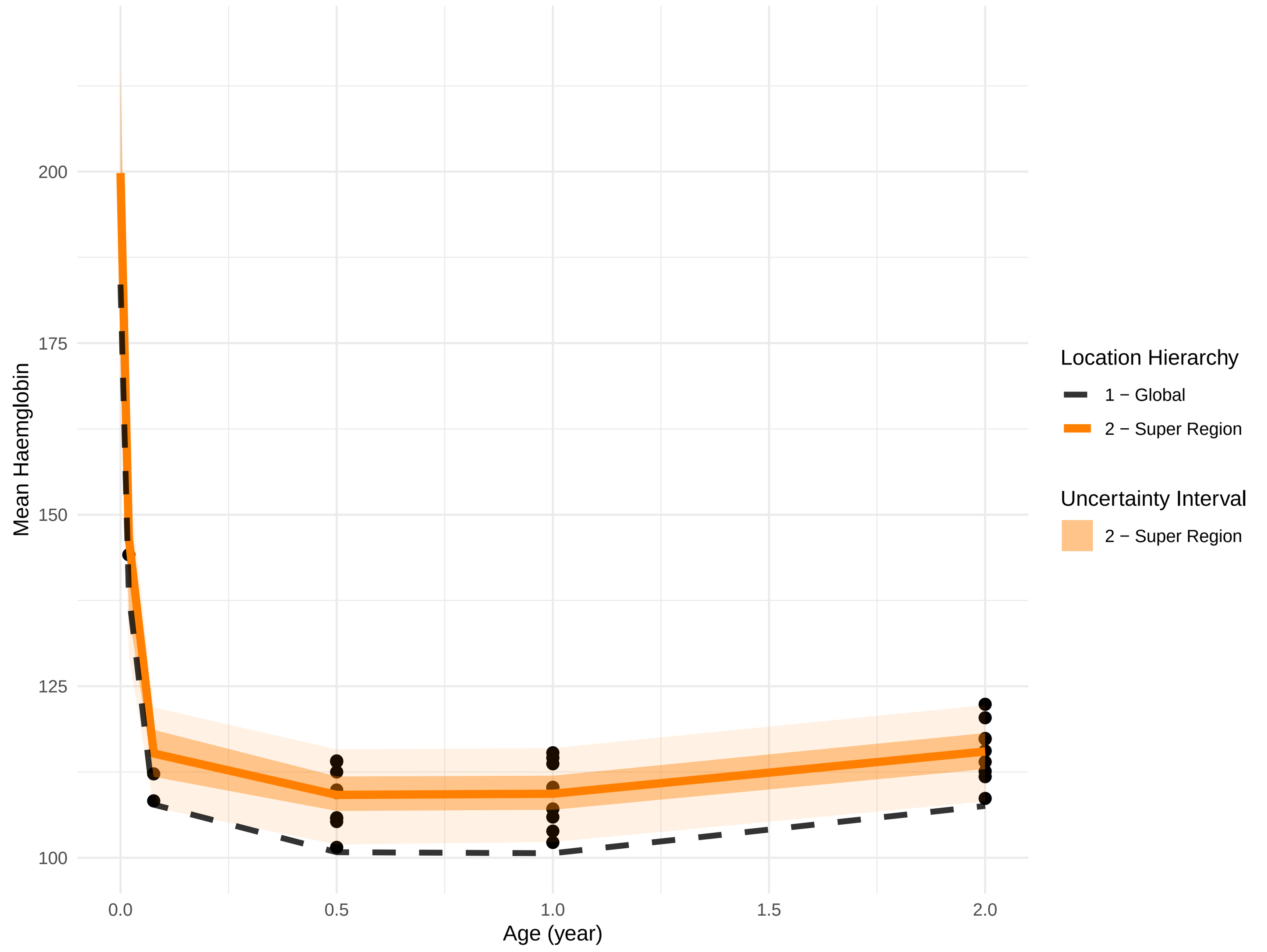

MR−BRT Age/Sex Fits − Males <2 Years Old − Mean Haemglobin − Central Asia
WHO−2001 Adjustment Method

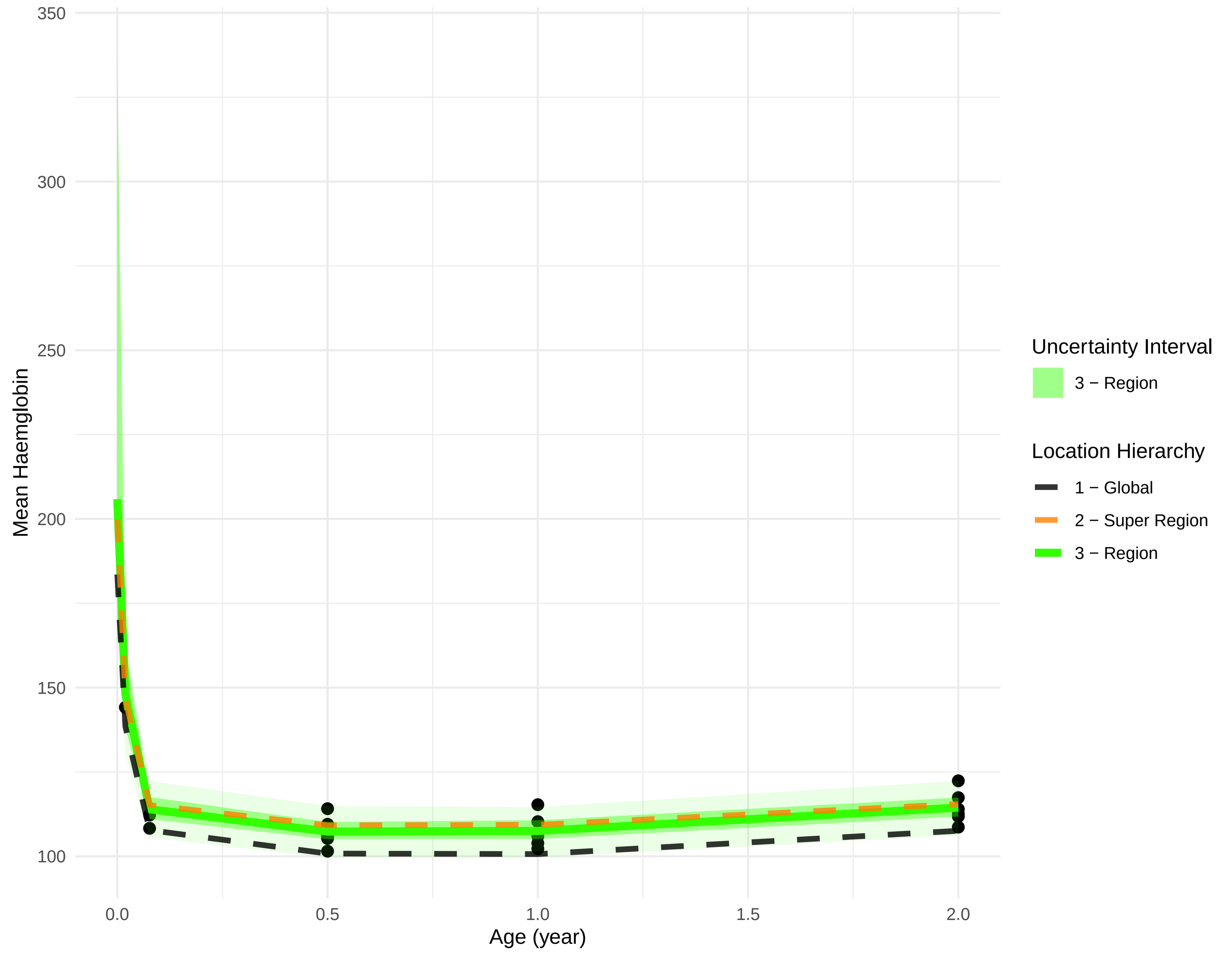

MR−BRT Age/Sex Fits − Males <2 Years Old − Mean Haemglobin − Armenia
WHO−2001 Adjustment Method

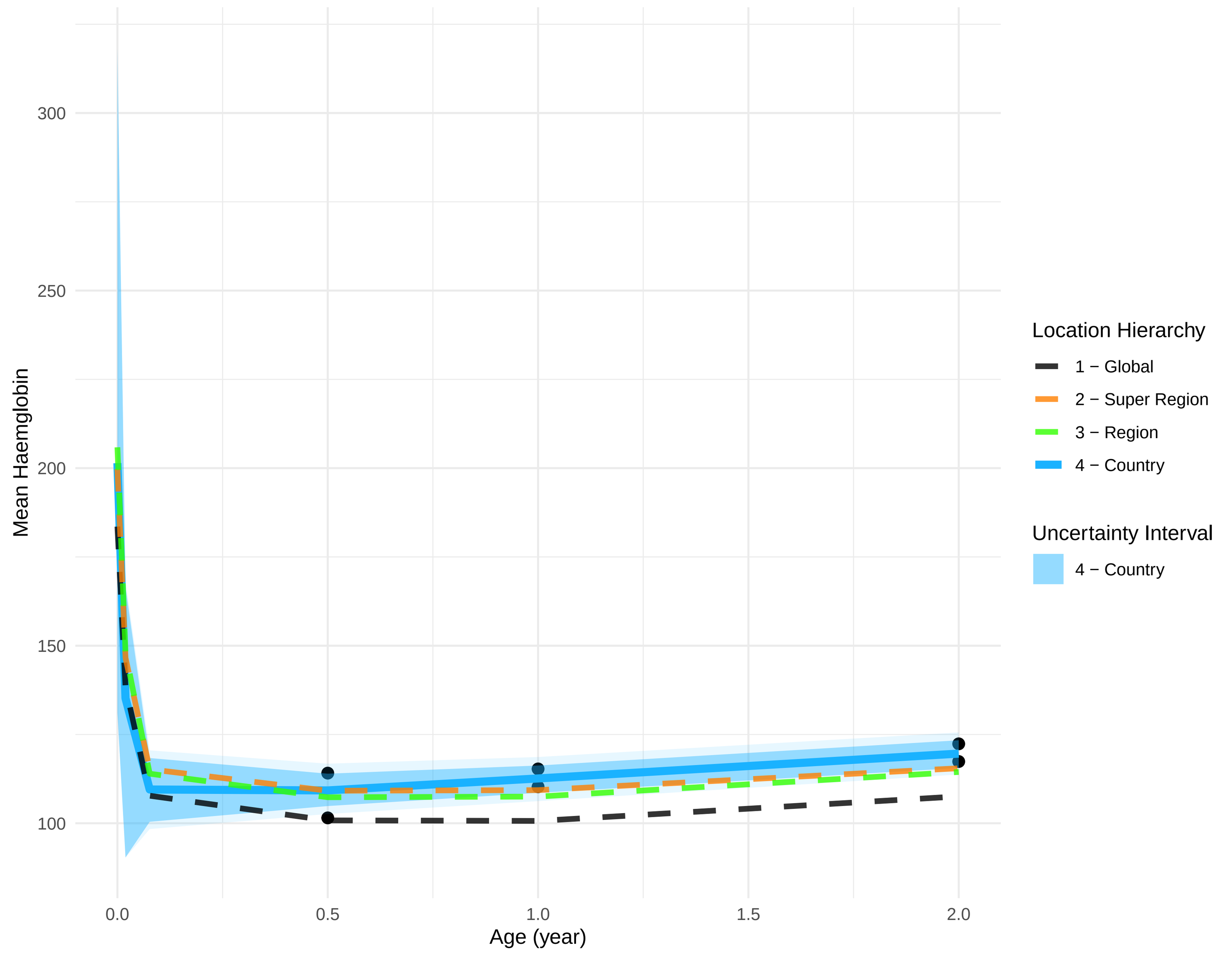

MR−BRT Age/Sex Fits − Males <2 Years Old − Mean Haemglobin − Azerbaijan
WHO−2001 Adjustment Method

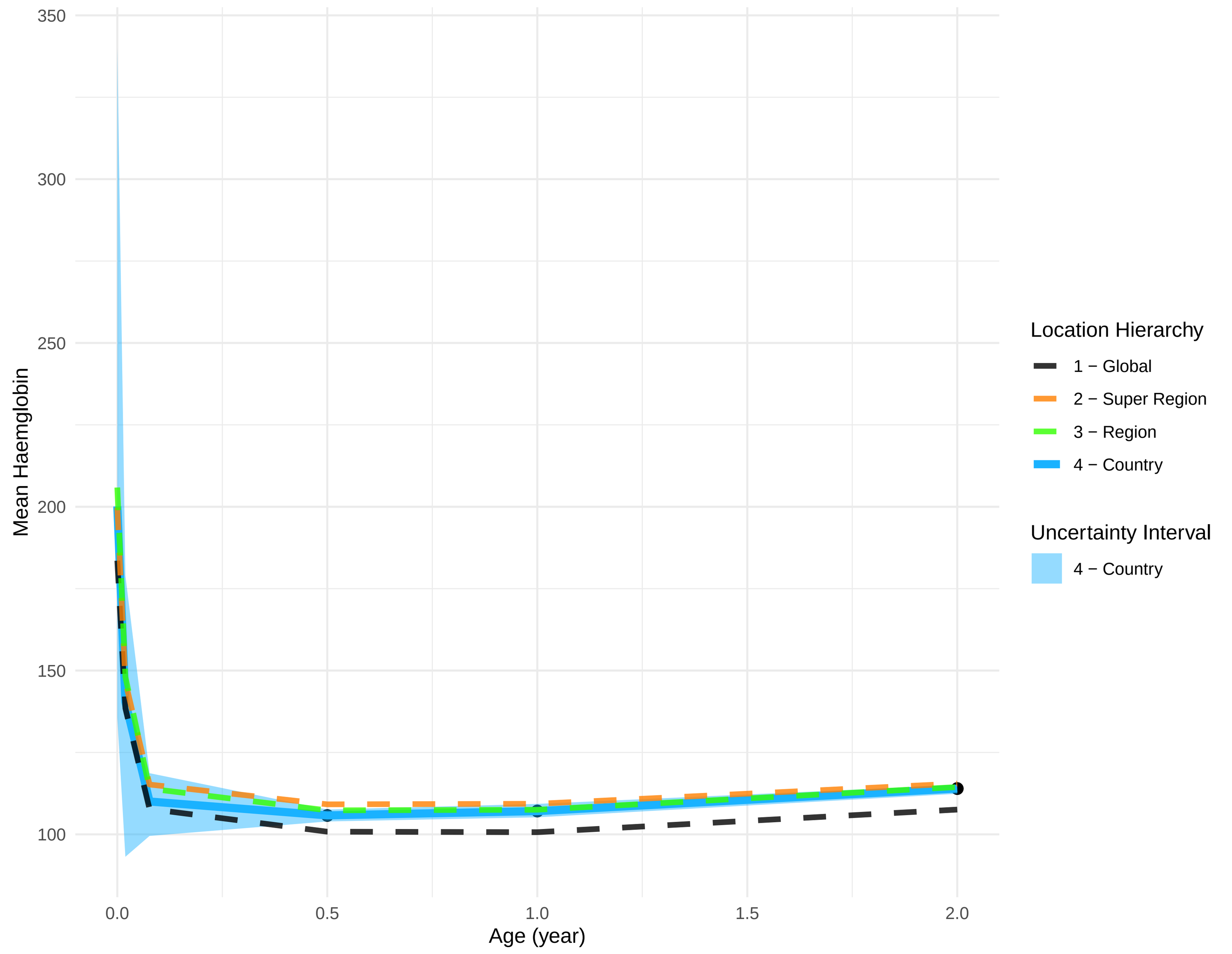

MR−BRT Age/Sex Fits − Males <2 Years Old − Mean Haemglobin − Kyrgyzstan
WHO−2001 Adjustment Method

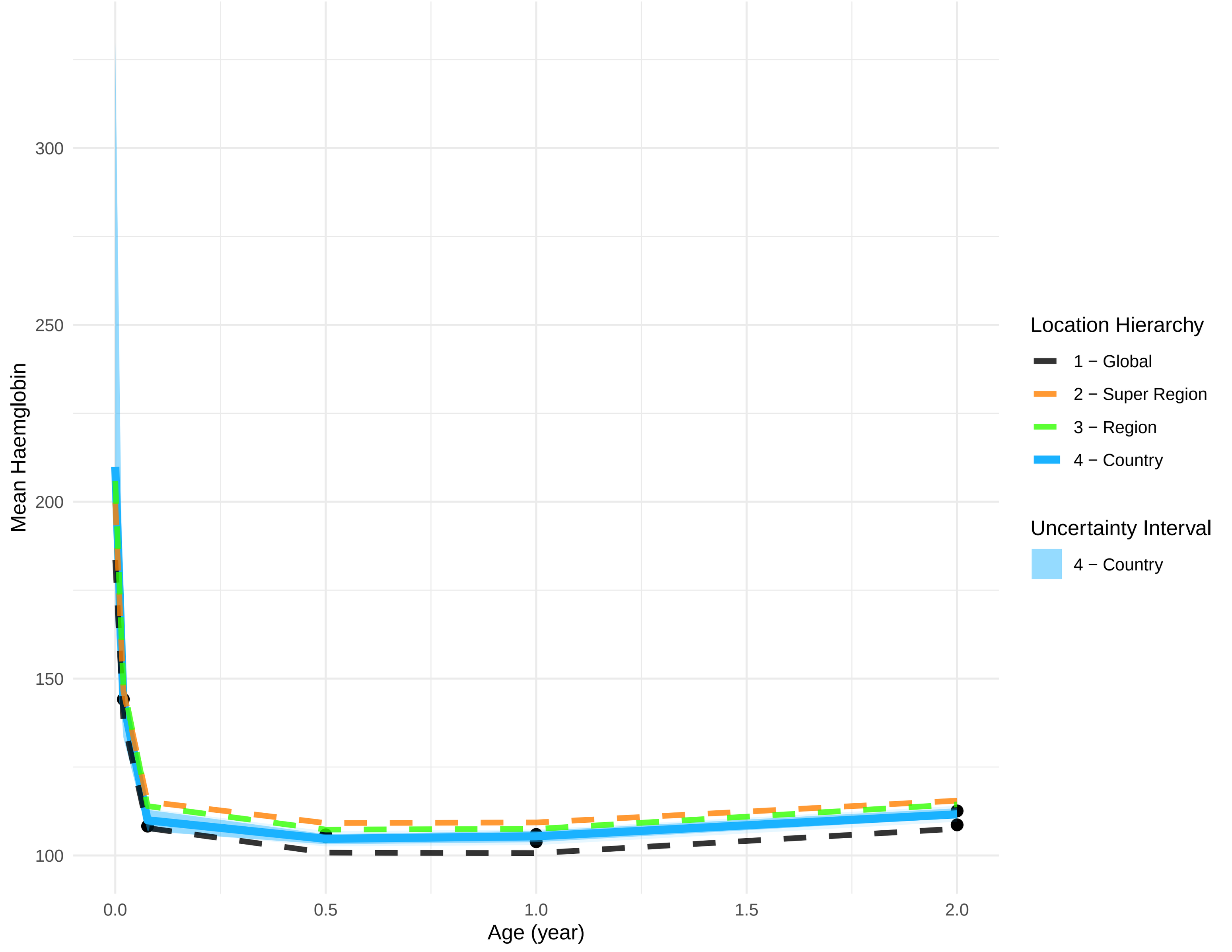

MR−BRT Age/Sex Fits − Males <2 Years Old − Mean Haemglobin − Uzbekistan
WHO−2001 Adjustment Method

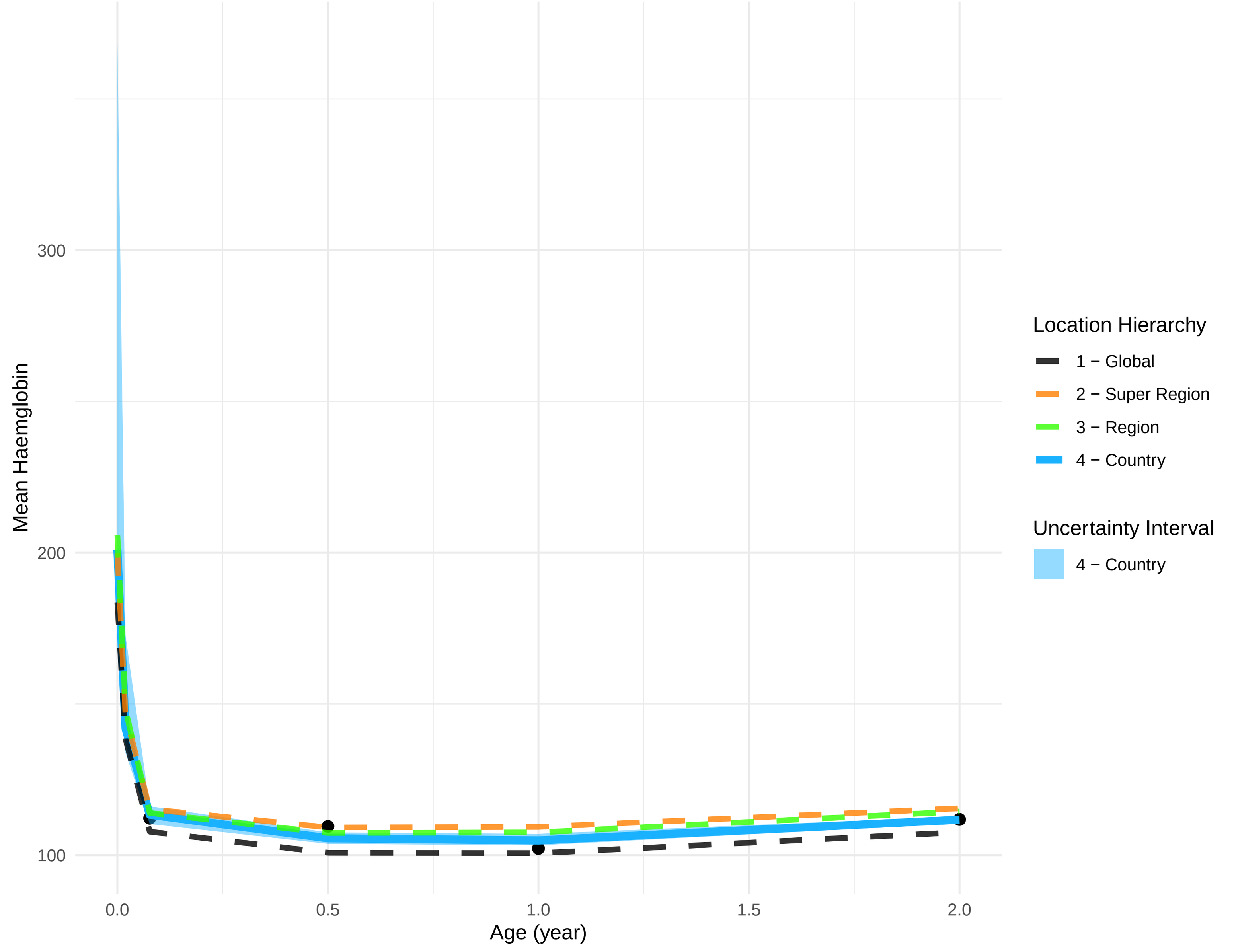

MR−BRT Age/Sex Fits − Males <2 Years Old − Mean Haemglobin − Central Europe
WHO−2001 Adjustment Method

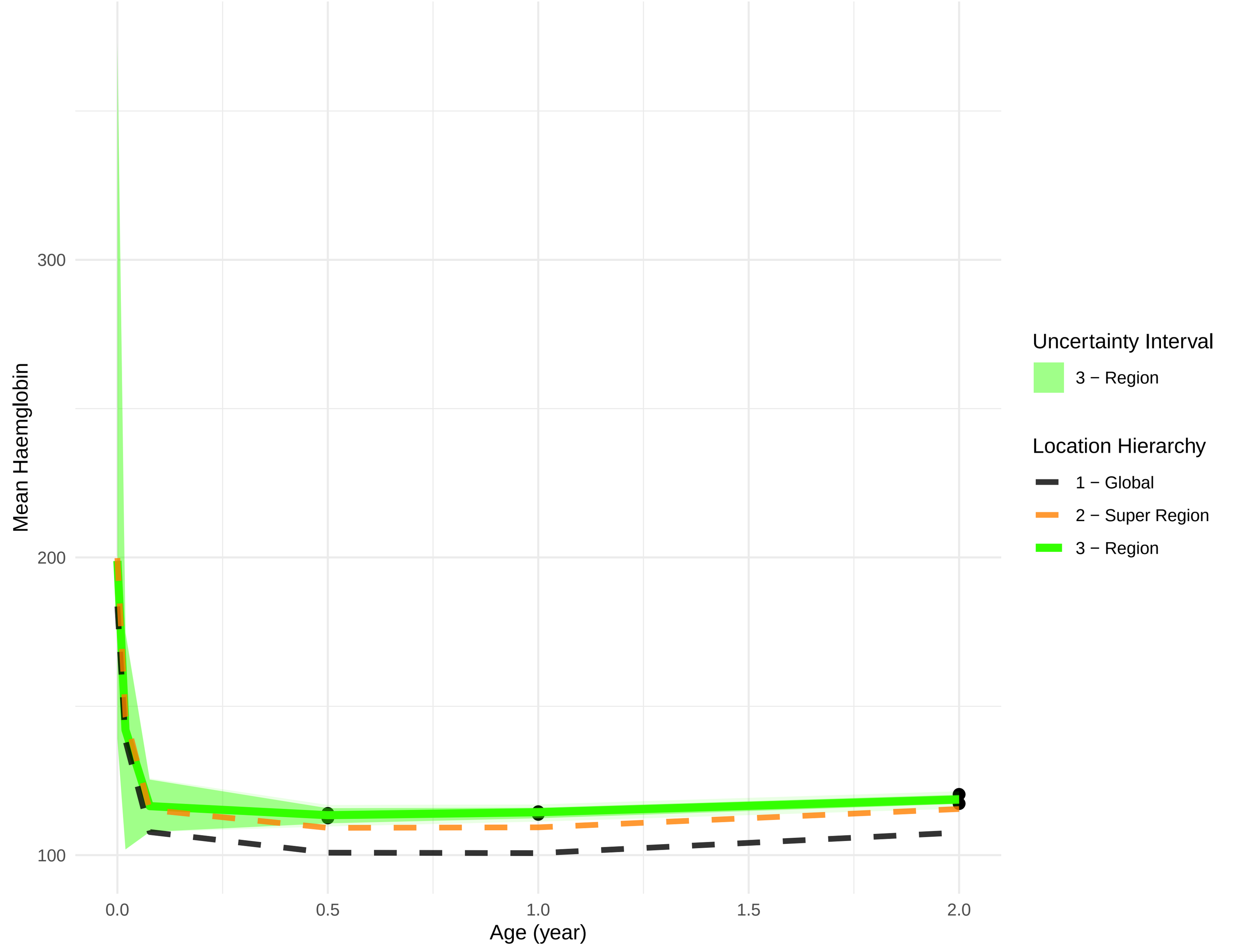

MR−BRT Age/Sex Fits − Males <2 Years Old − Mean Haemglobin − Albania
WHO−2001 Adjustment Method

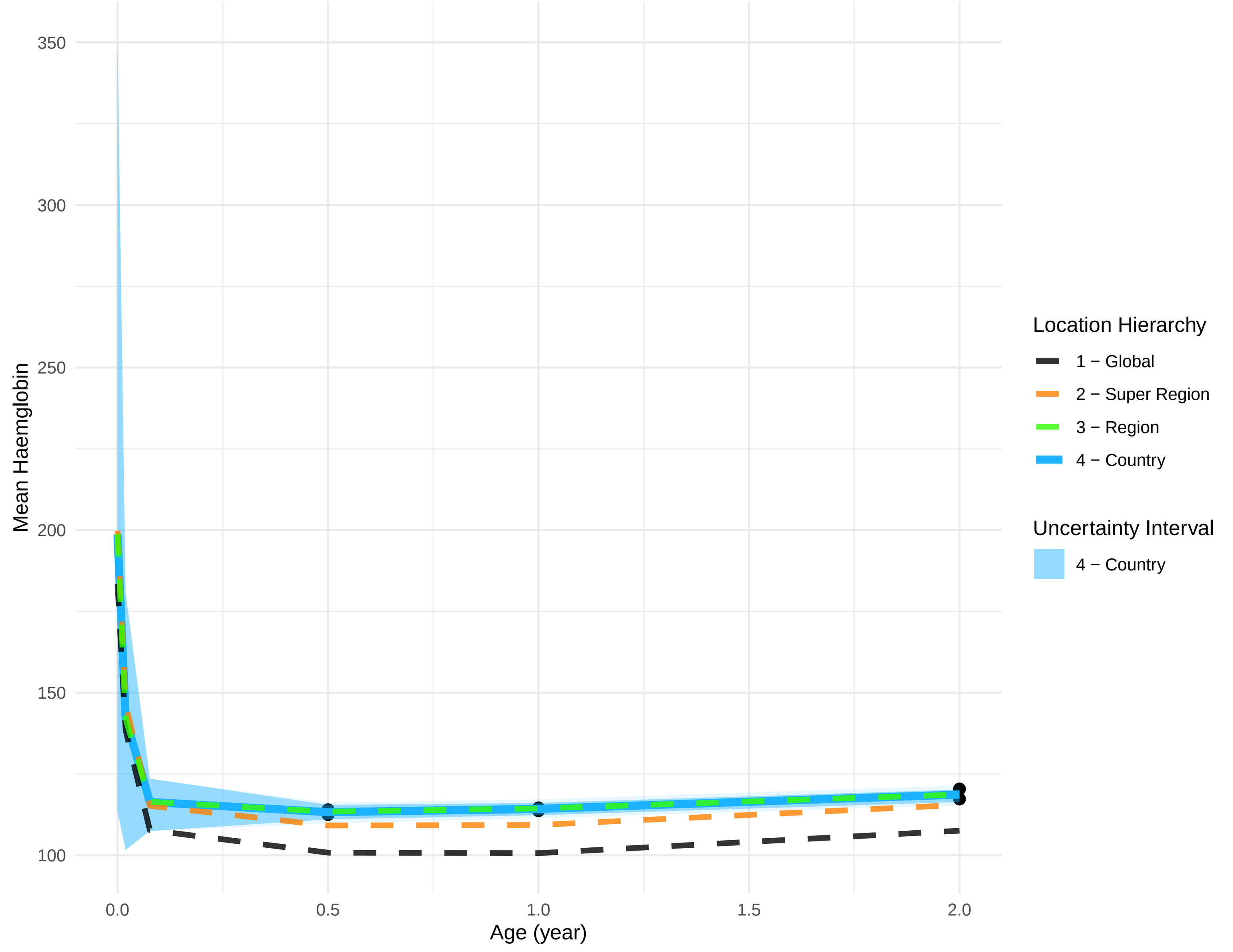

MR−BRT Age/Sex Fits − Males <2 Years Old − Mean Haemglobin − Eastern Europe
WHO−2001 Adjustment Method

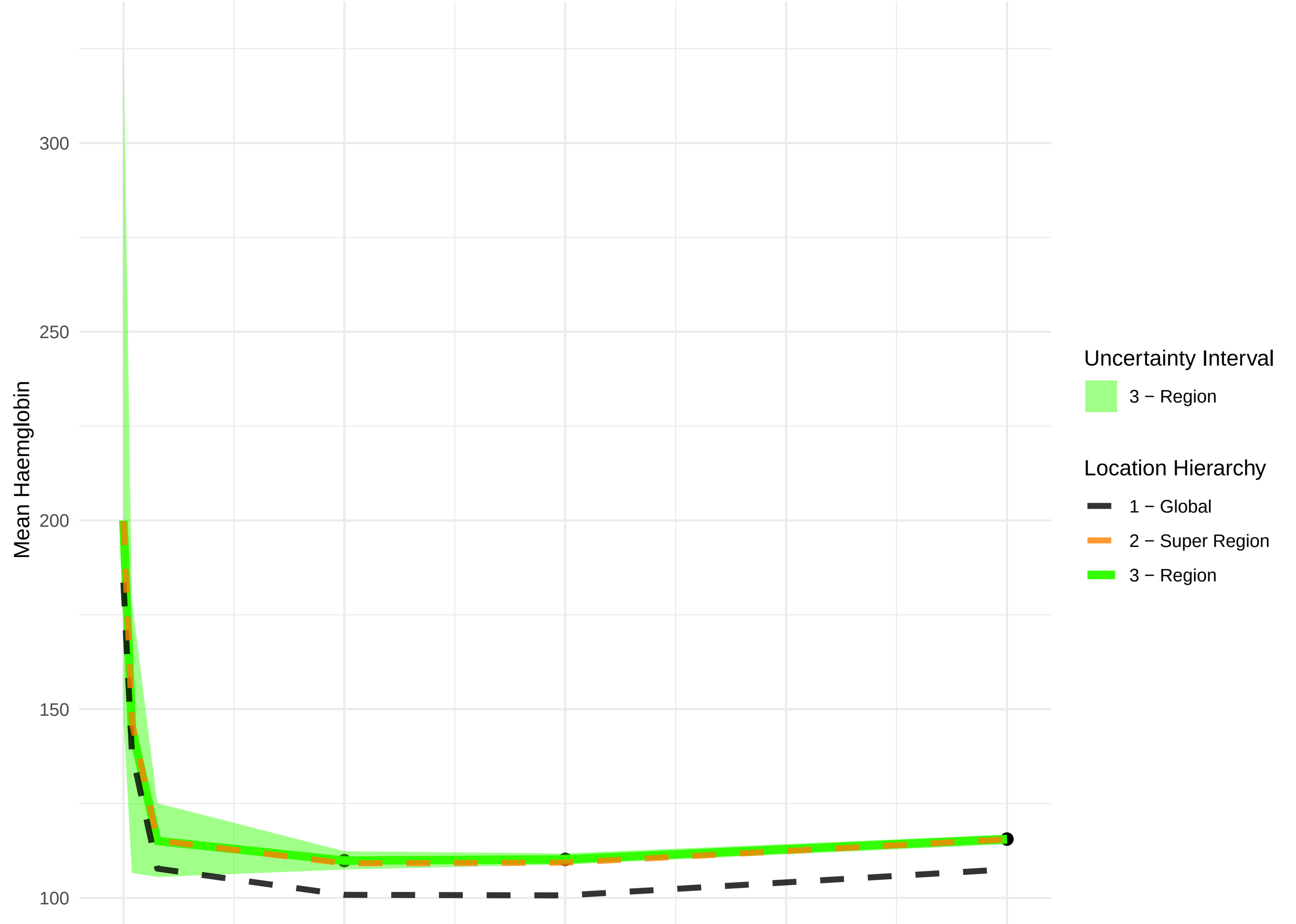

MR−BRT Age/Sex Fits − Males <2 Years Old − Mean Haemglobin − Republic of Moldova
WHO−2001 Adjustment Method

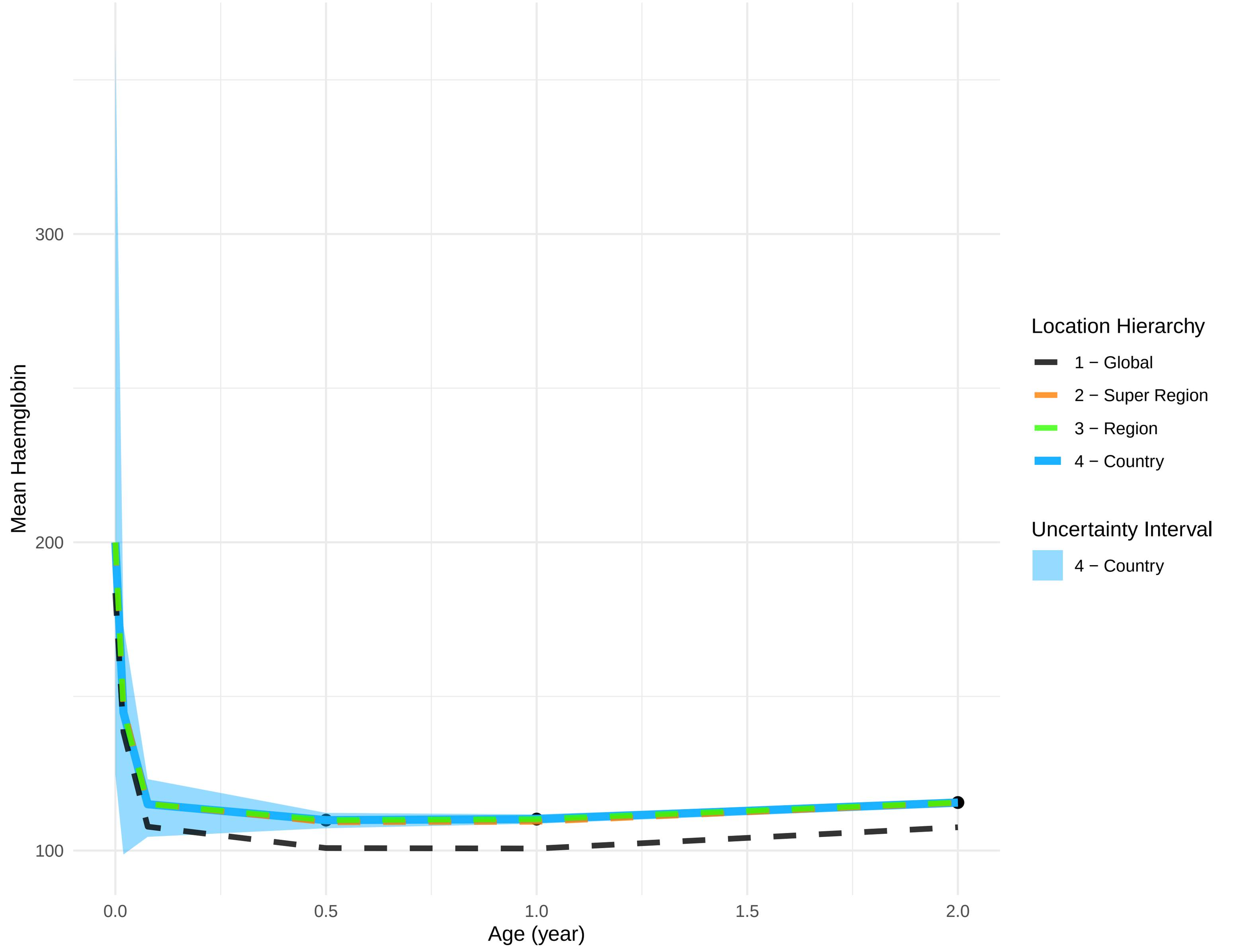

MR−BRT Age/Sex Fits − Males <2 Years Old − Mean Haemglobin − High−income
WHO−2001 Adjustment Method

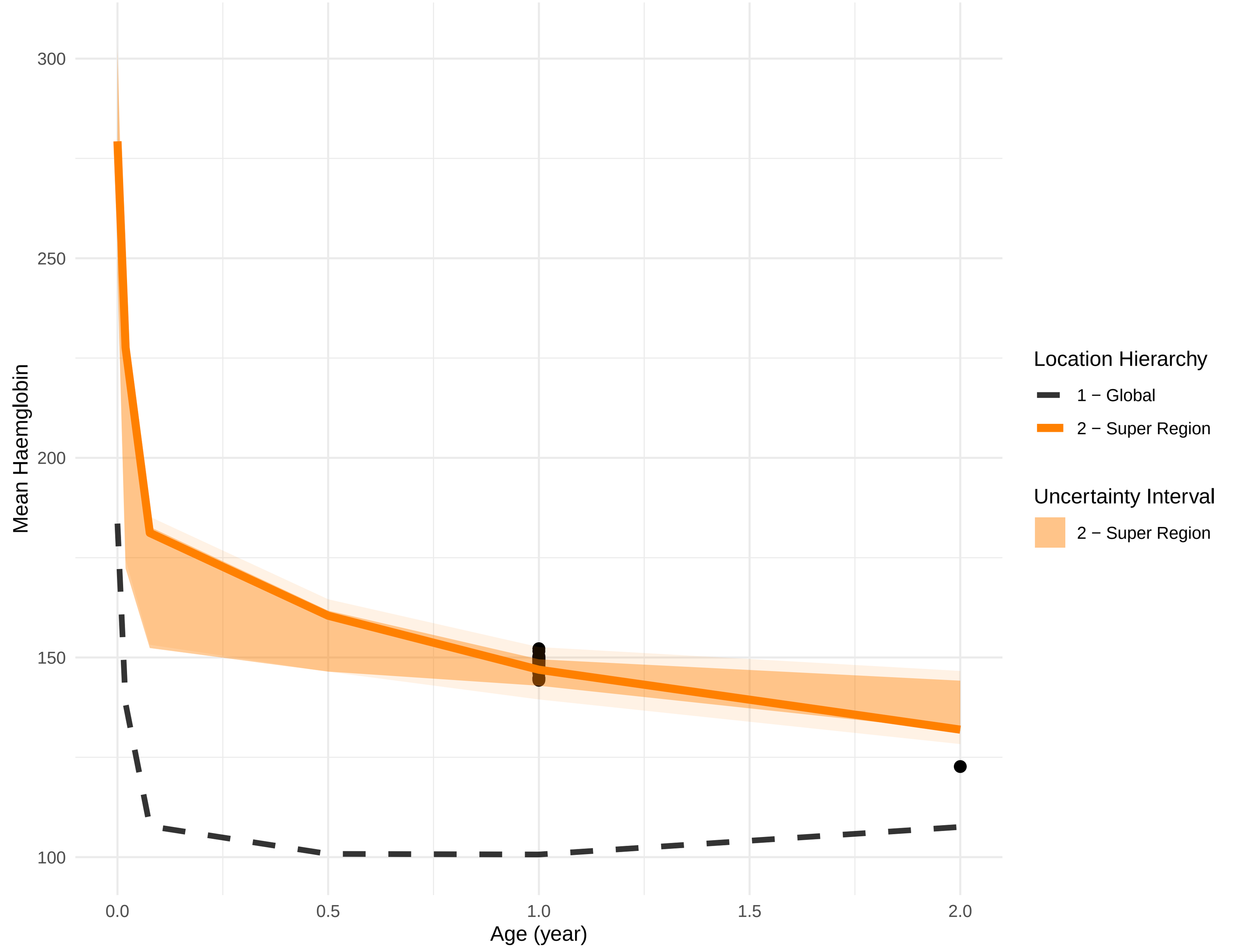

MR−BRT Age/Sex Fits − Males <2 Years Old − Mean Haemglobin − Western Europe
WHO−2001 Adjustment Method

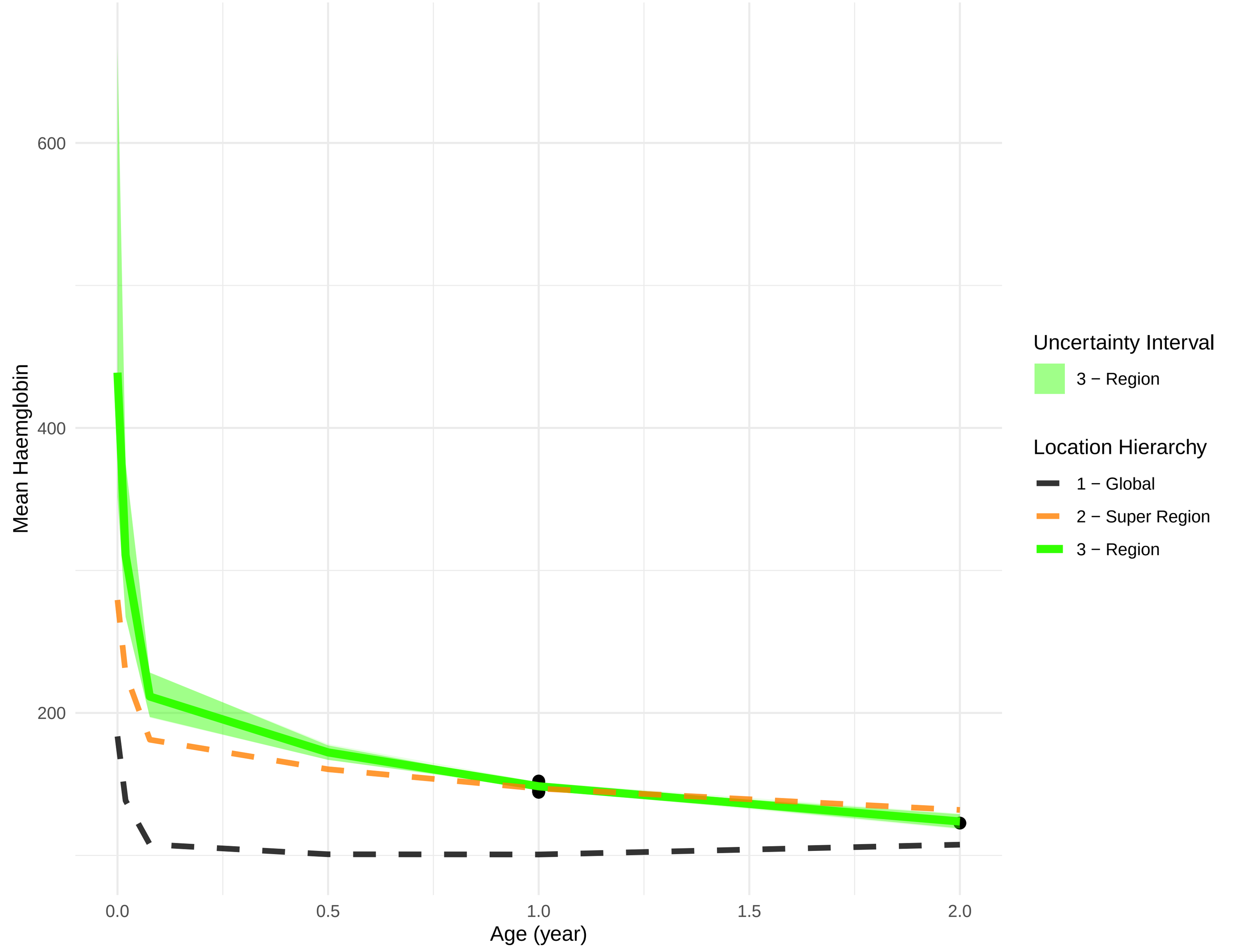

MR−BRT Age/Sex Fits − Males <2 Years Old − Mean Haemglobin − United Kingdom
WHO−2001 Adjustment Method

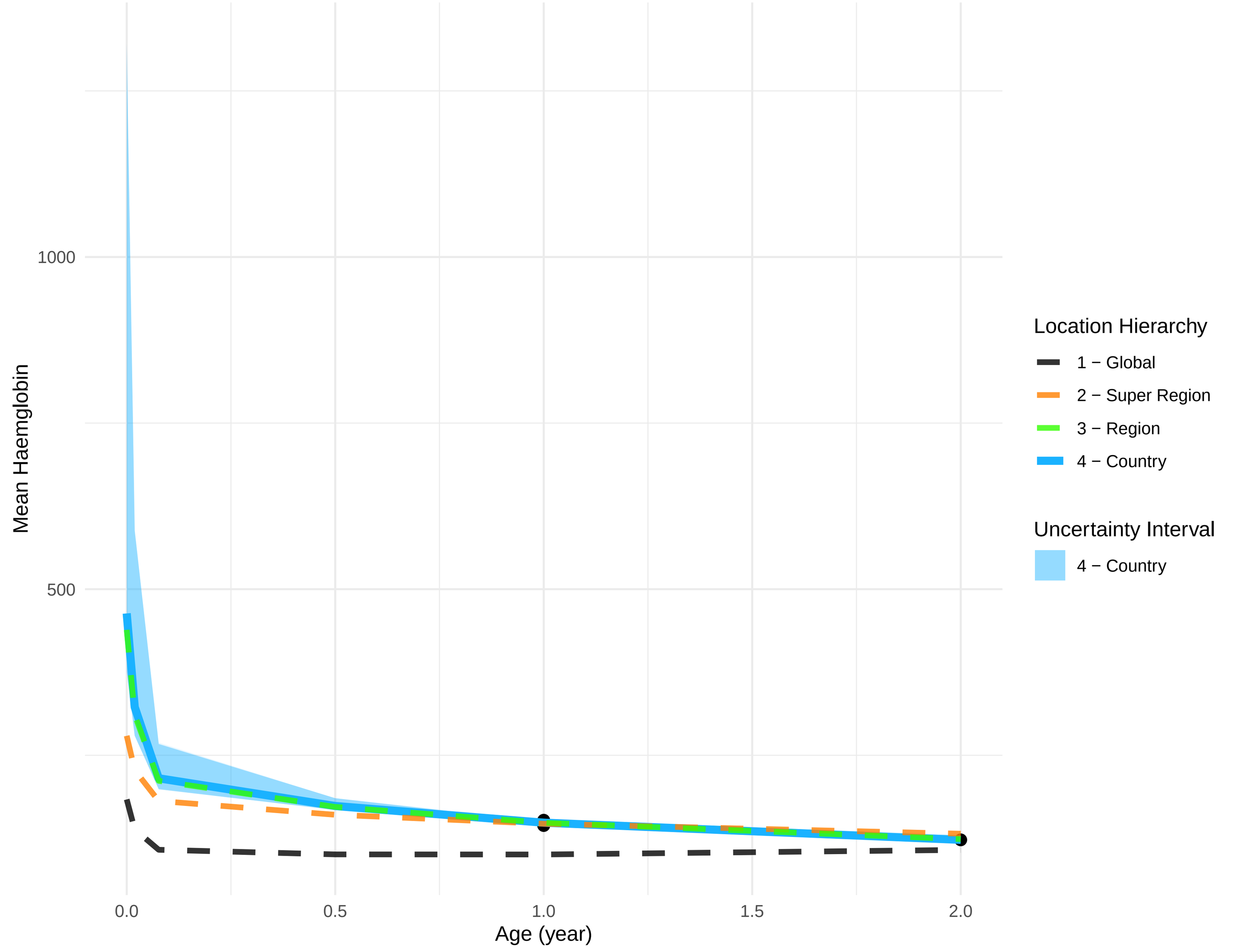

MR−BRT Age/Sex Fits − Males <2 Years Old − Mean Haemglobin − England
WHO−2001 Adjustment Method

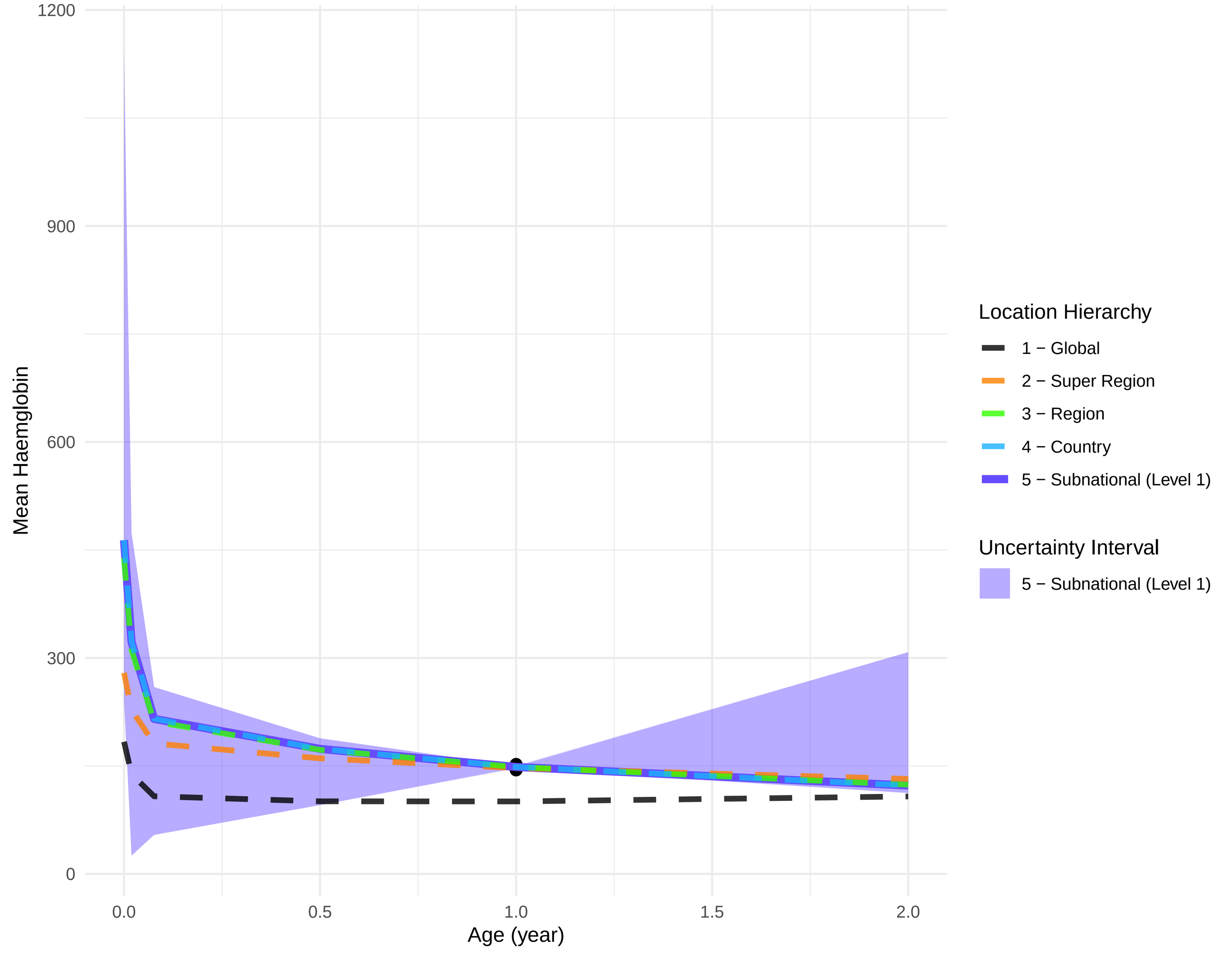

MR−BRT Age/Sex Fits − Males <2 Years Old − Mean Haemglobin − East Midlands
WHO−2001 Adjustment Method

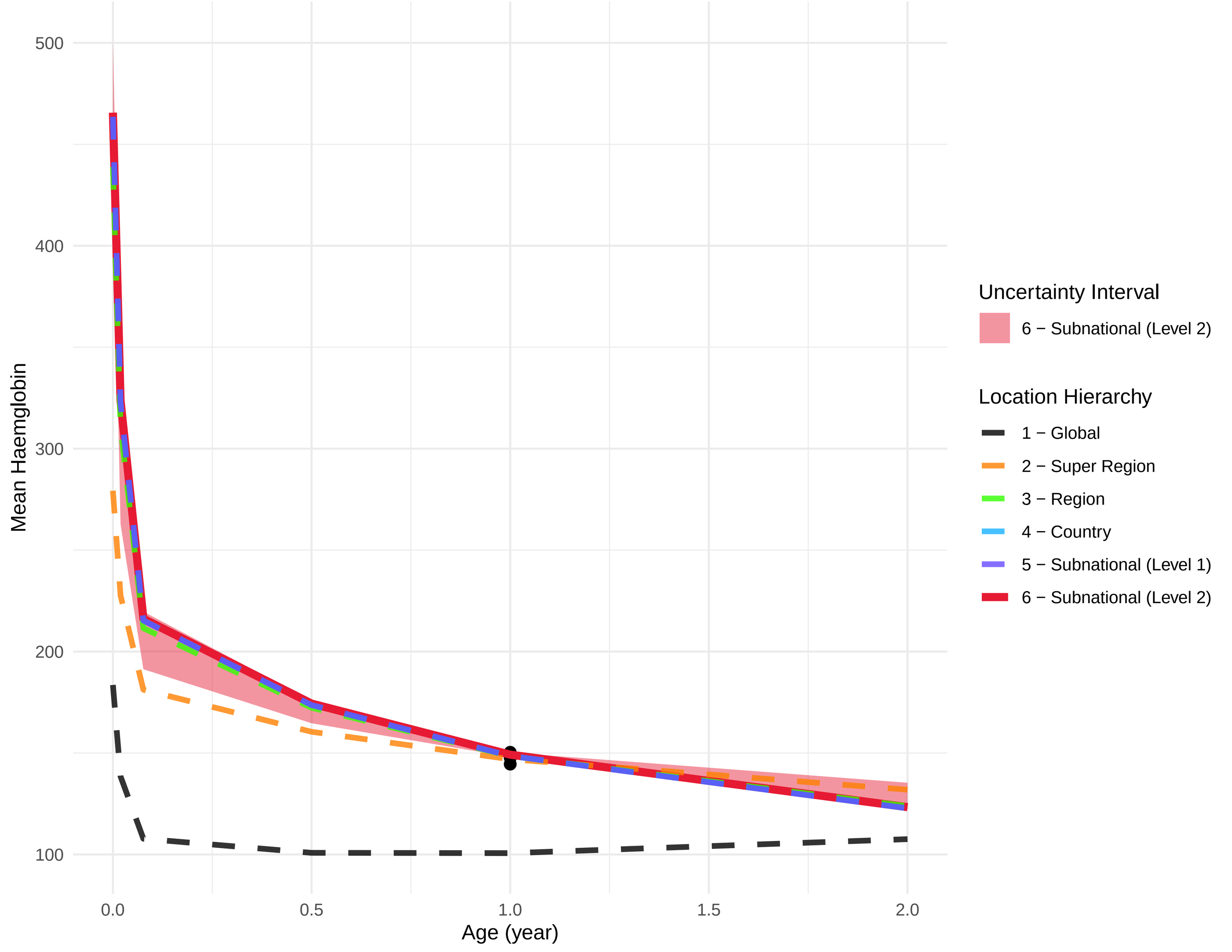

MR−BRT Age/Sex Fits − Males <2 Years Old − Mean Haemglobin − East of England
WHO−2001 Adjustment Method

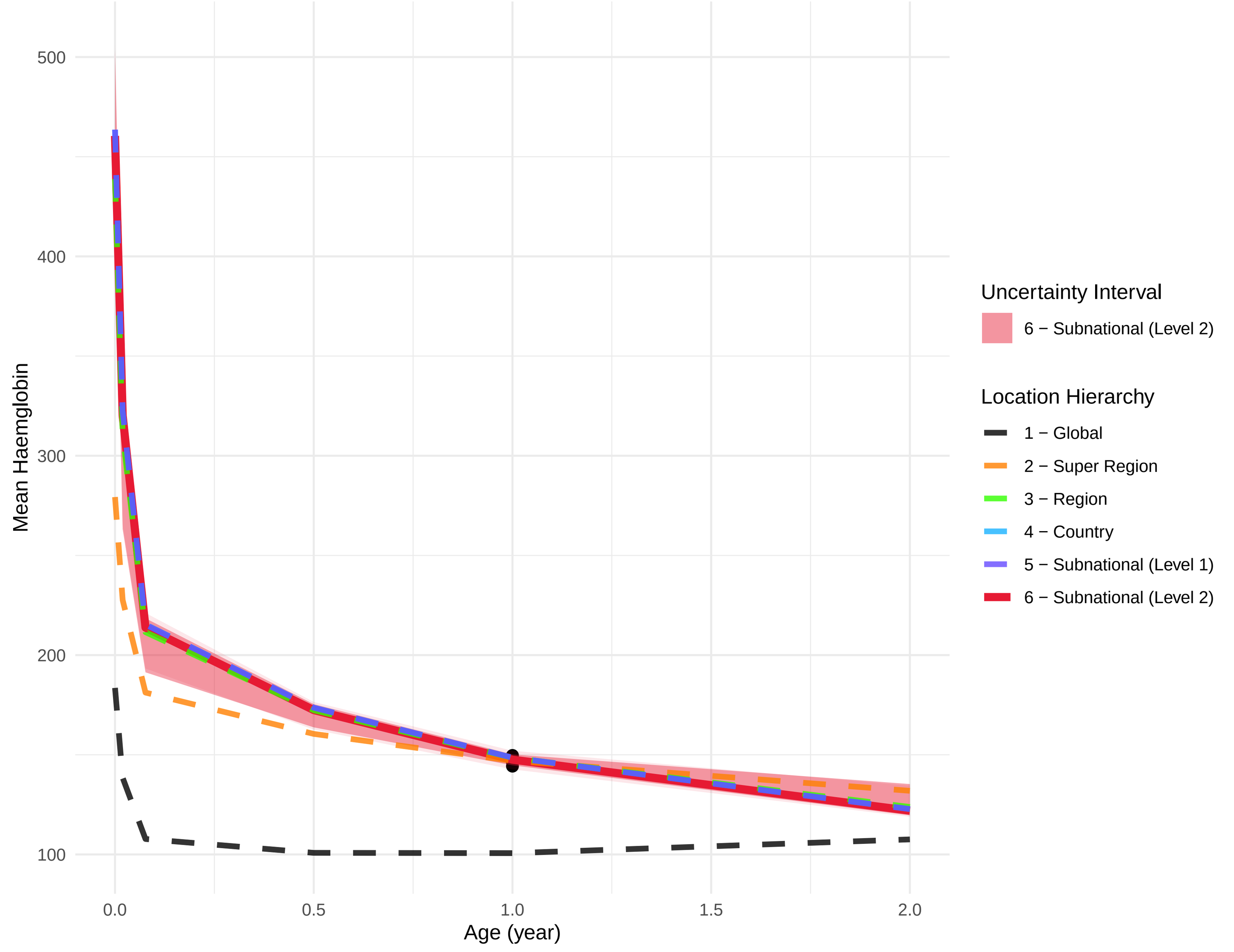

MR−BRT Age/Sex Fits − Males <2 Years Old − Mean Haemglobin − Greater London
WHO−2001 Adjustment Method

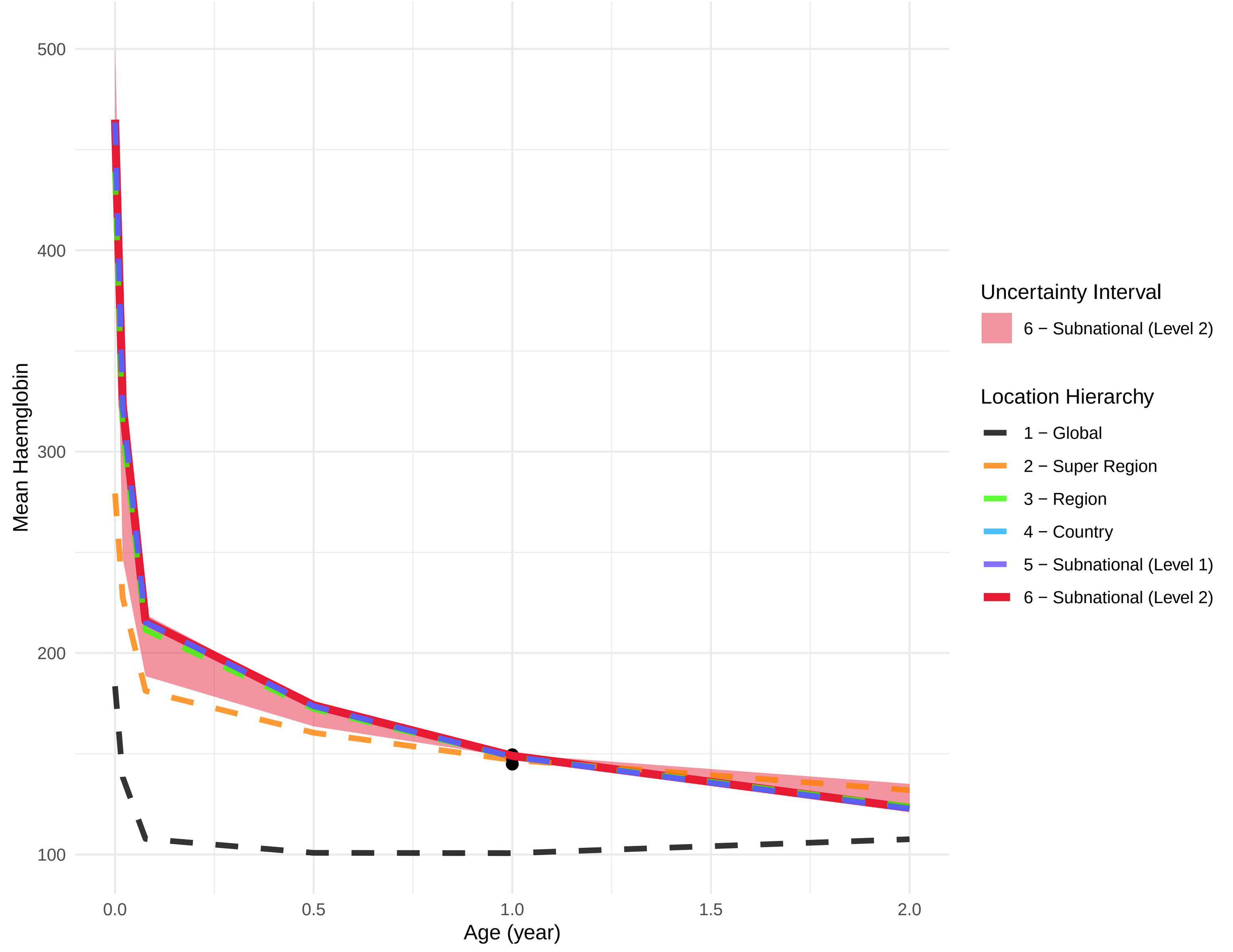

MR−BRT Age/Sex Fits − Males <2 Years Old − Mean Haemglobin − North East England
WHO−2001 Adjustment Method

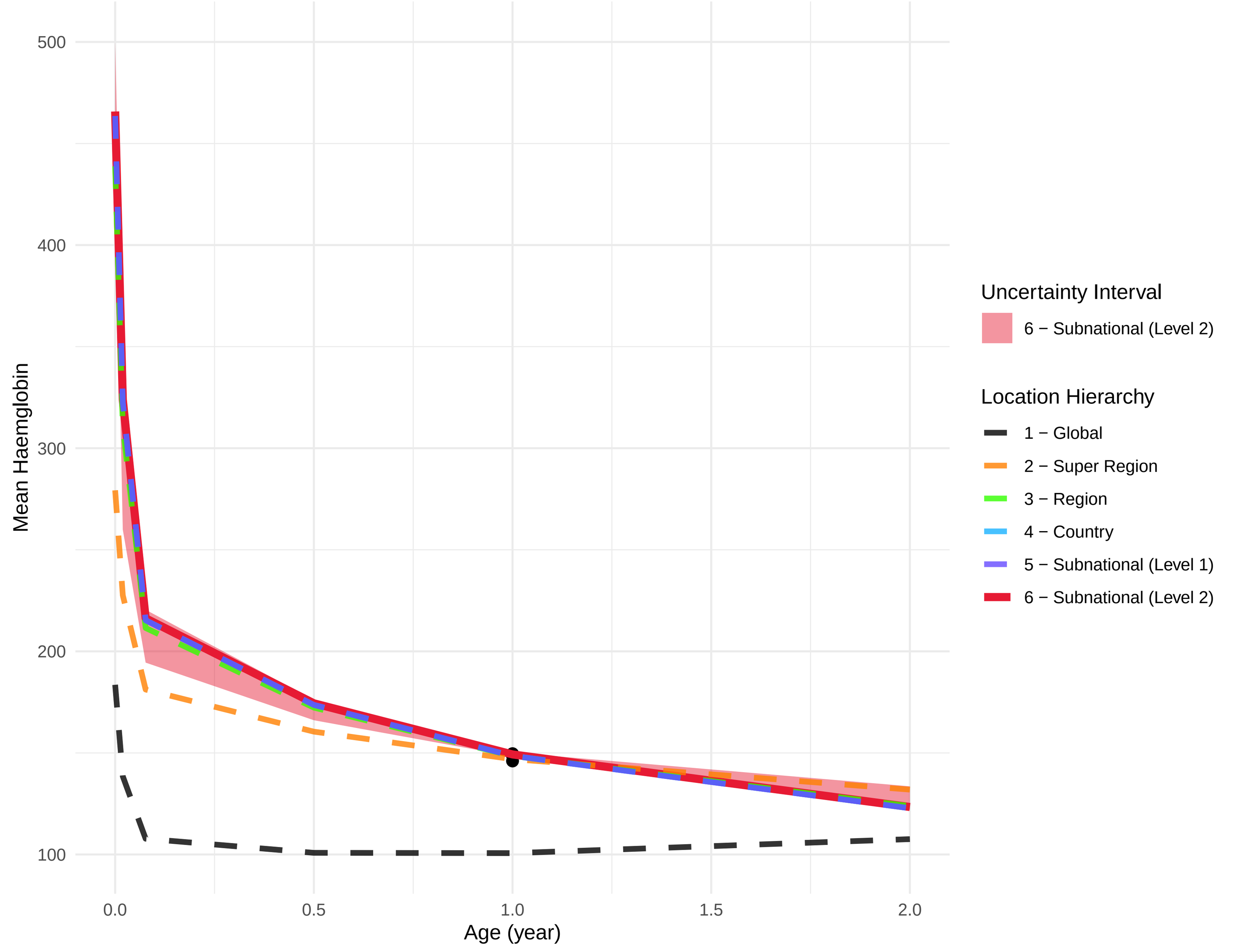

MR−BRT Age/Sex Fits − Males <2 Years Old − Mean Haemglobin − North West England
WHO−2001 Adjustment Method

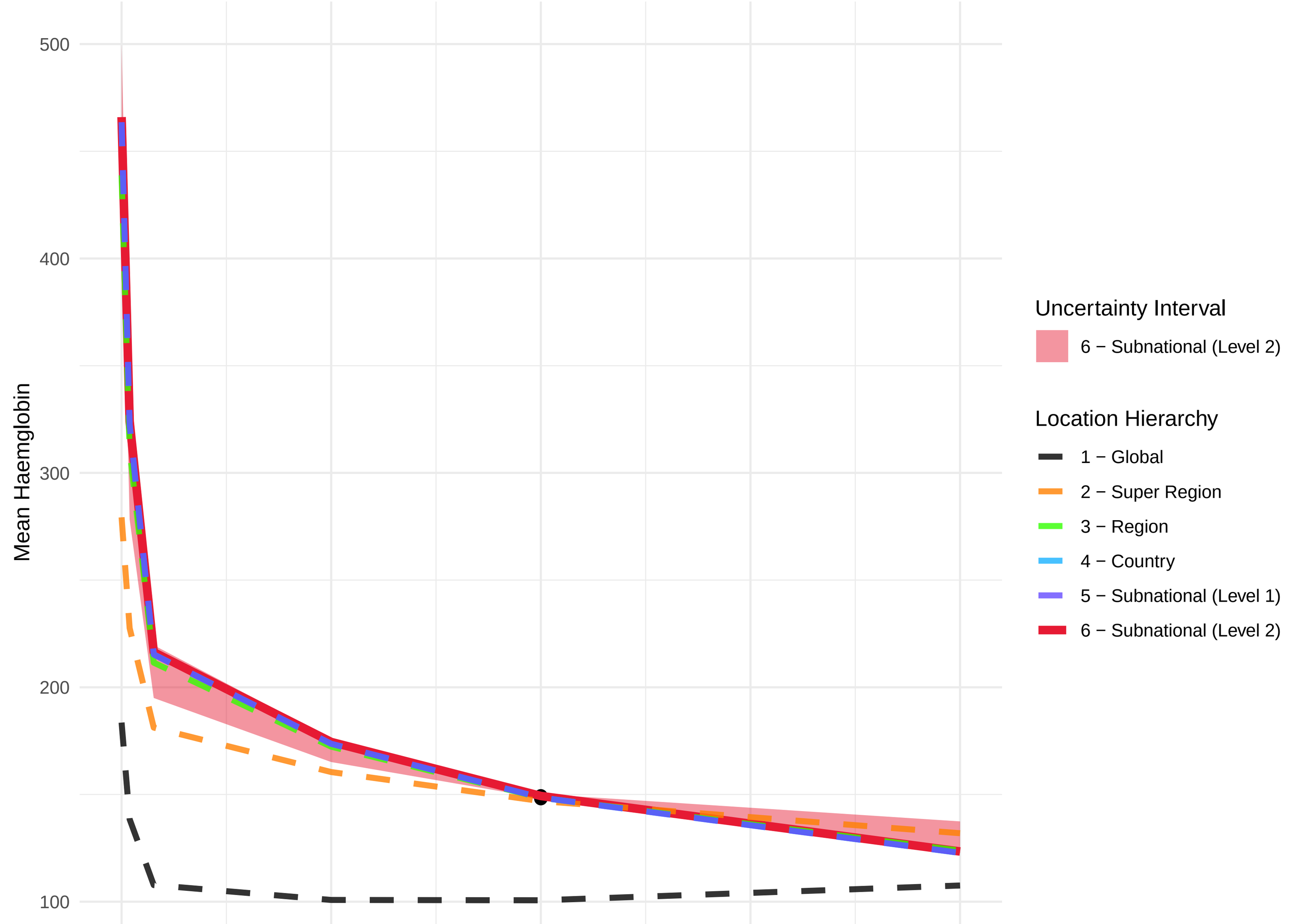

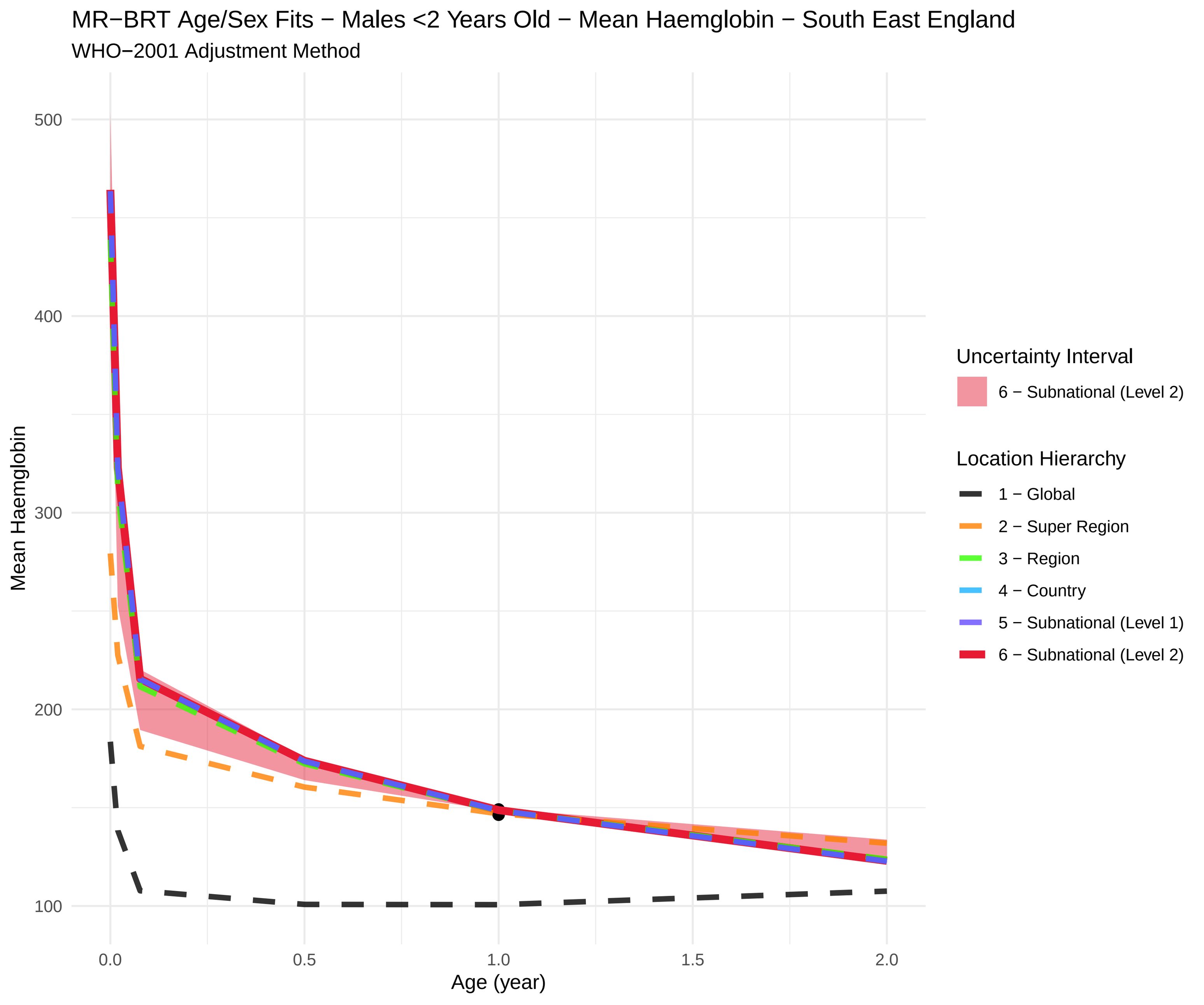
MR−BRT Age/Sex Fits − Males <2 Years Old − Mean Haemglobin − South East England
WHO−2001 Adjustment Method
Mean Haemglobin
Age (year)
Uncertainty Interval
6 − Subnational (Level 2)
Location Hierarchy
1 − Global
2 − Super Region
3 − Region
4 − Country
5 − Subnational (Level 1)
6 − Subnational (Level 2)

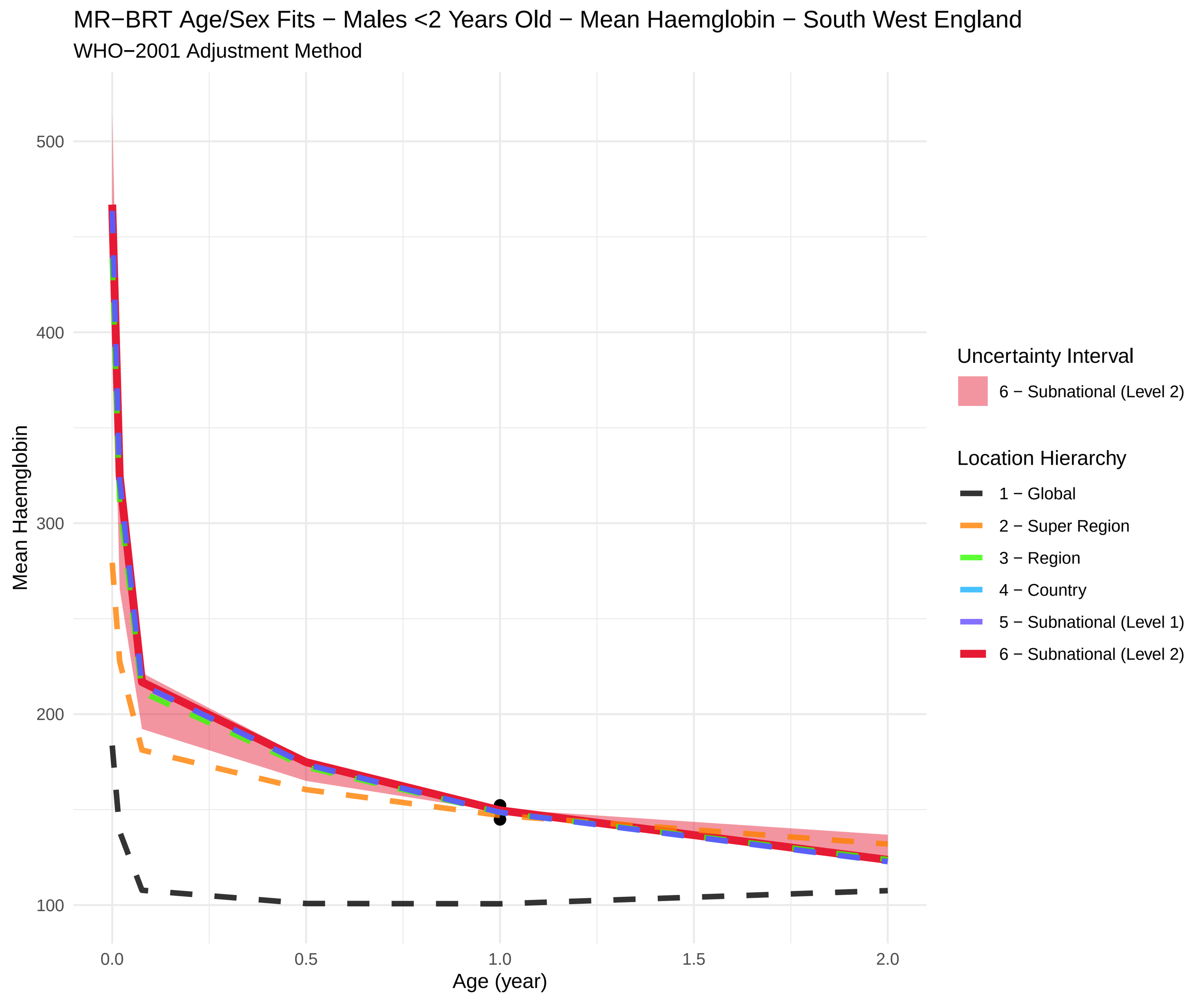
MR−BRT Age/Sex Fits − Males <2 Years Old − Mean Haemglobin − South West England
WHO−2001 Adjustment Method
Mean Haemglobin
Age (year)
Uncertainty Interval
6 − Subnational (Level 2)
Location Hierarchy
1 − Global
2 − Super Region
3 − Region
4 − Country
5 − Subnational (Level 1)
6 − Subnational (Level 2)

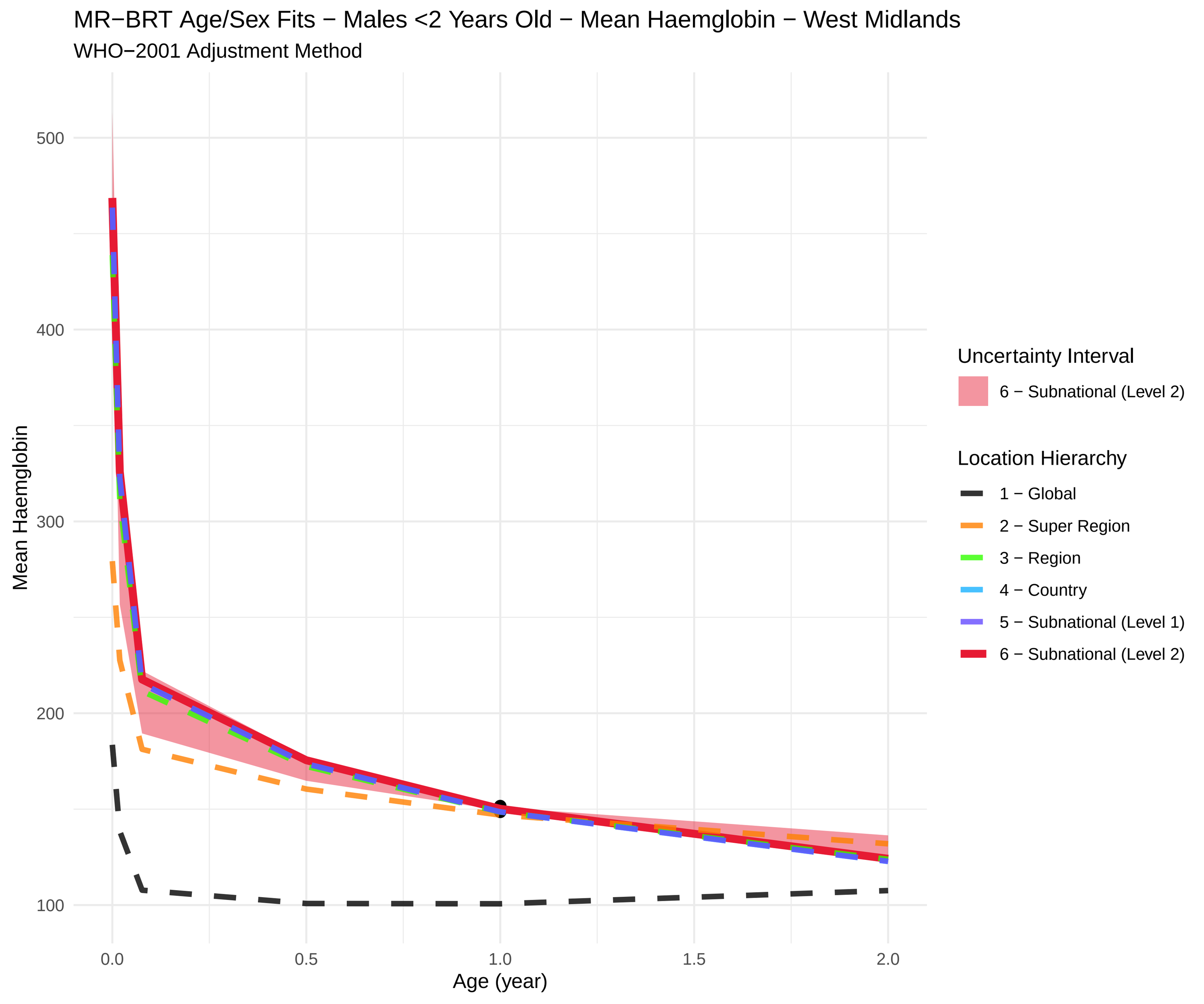
MR−BRT Age/Sex Fits − Males <2 Years Old − Mean Haemglobin − West Midlands
WHO−2001 Adjustment Method
Mean Haemglobin
Age (year)
Uncertainty Interval
6 − Subnational (Level 2)
Location Hierarchy
1 − Global
2 − Super Region
3 − Region
4 − Country
5 − Subnational (Level 1)
6 − Subnational (Level 2)

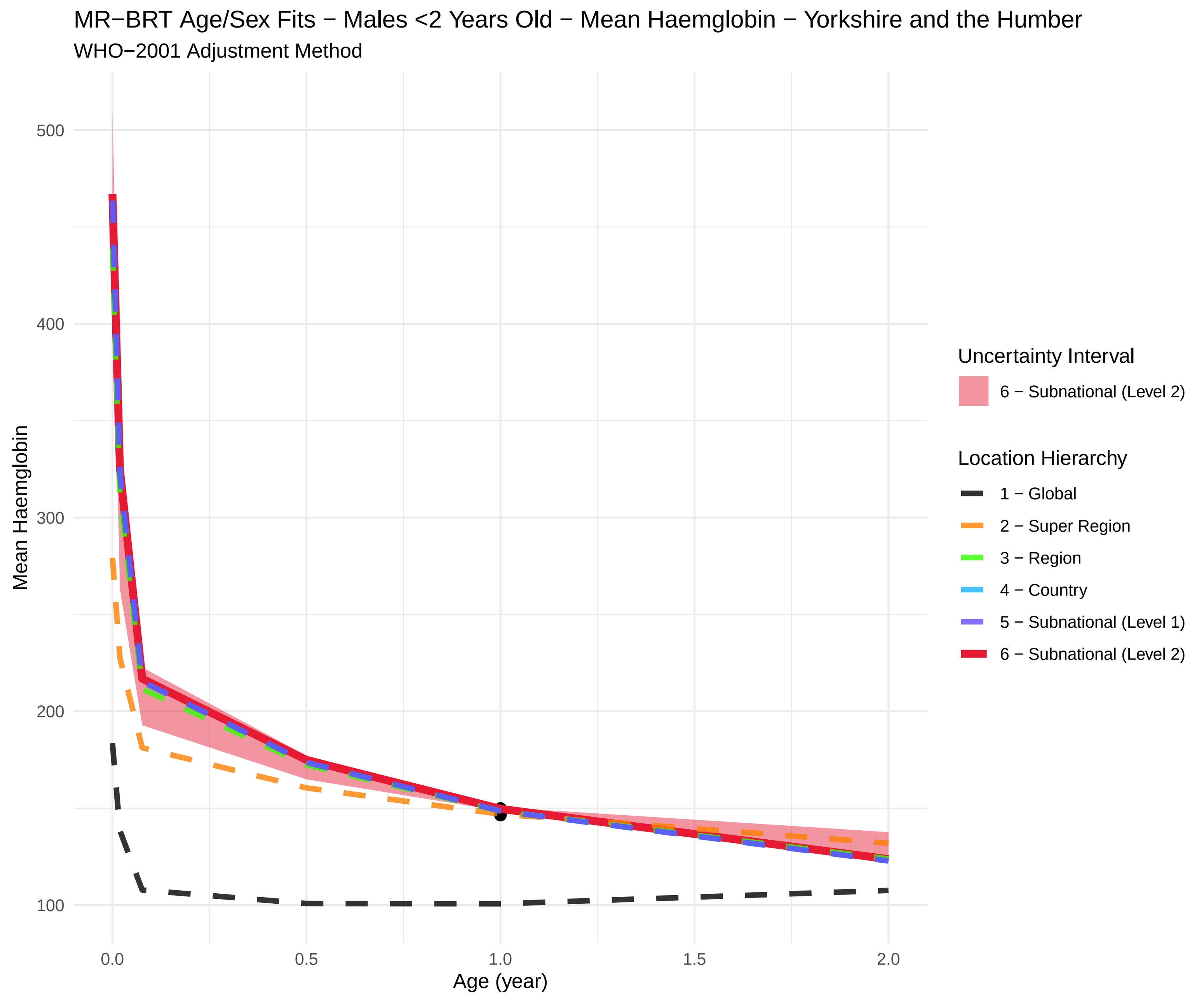
MR−BRT Age/Sex Fits − Males <2 Years Old − Mean Haemglobin − Yorkshire and the Humber
WHO−2001 Adjustment Method
Mean Haemglobin
Age (year)
Uncertainty Interval
6 − Subnational (Level 2)
Location Hierarchy
1 − Global
2 − Super Region
3 − Region
4 − Country
5 − Subnational (Level 1)
6 − Subnational (Level 2)

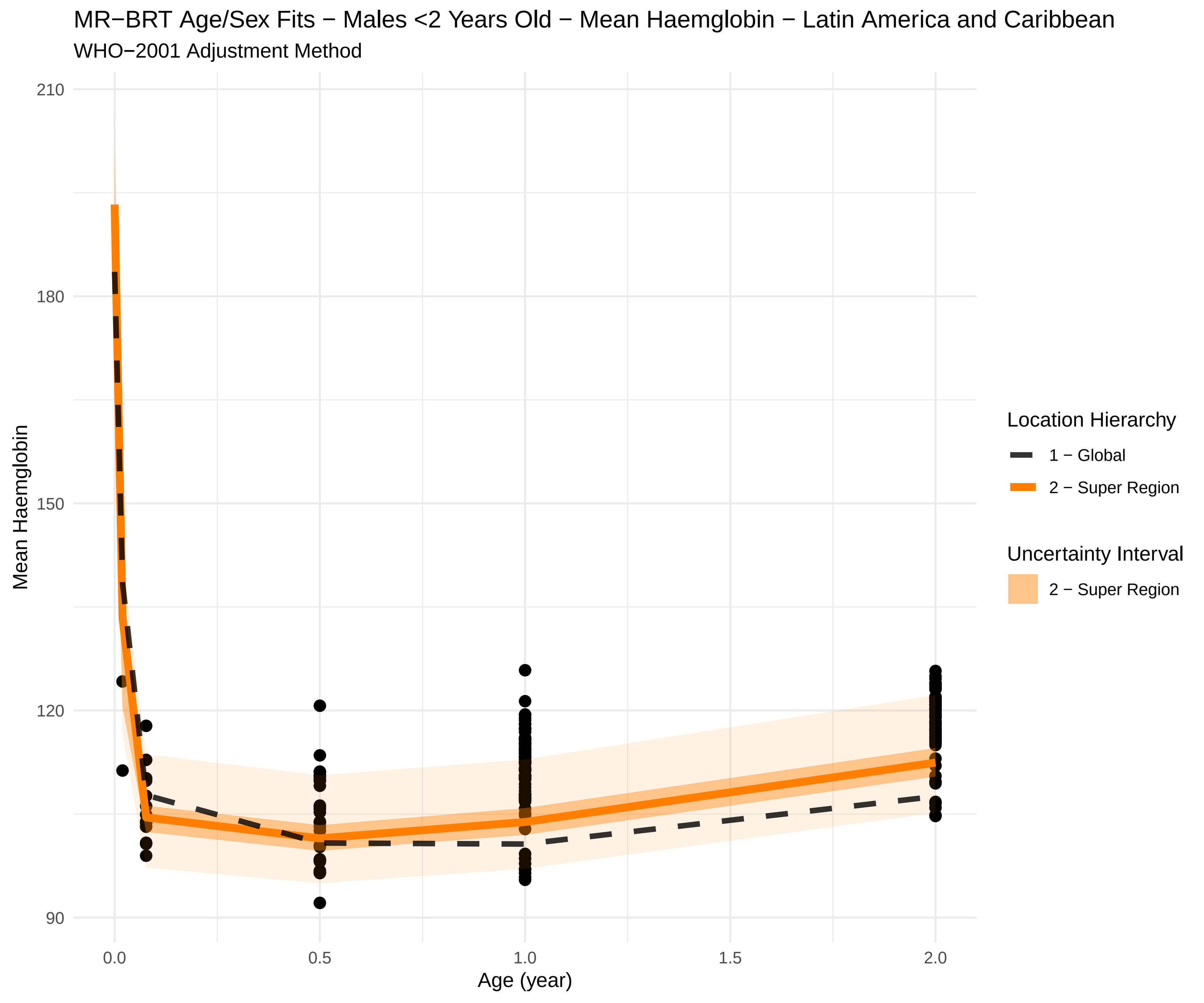
MR−BRT Age/Sex Fits − Males <2 Years Old − Mean Haemglobin − Latin America and Caribbean
WHO−2001 Adjustment Method
Mean Haemglobin
Age (year)
Location Hierarchy
1 − Global
2 − Super Region
Uncertainty Interval
2 − Super Region

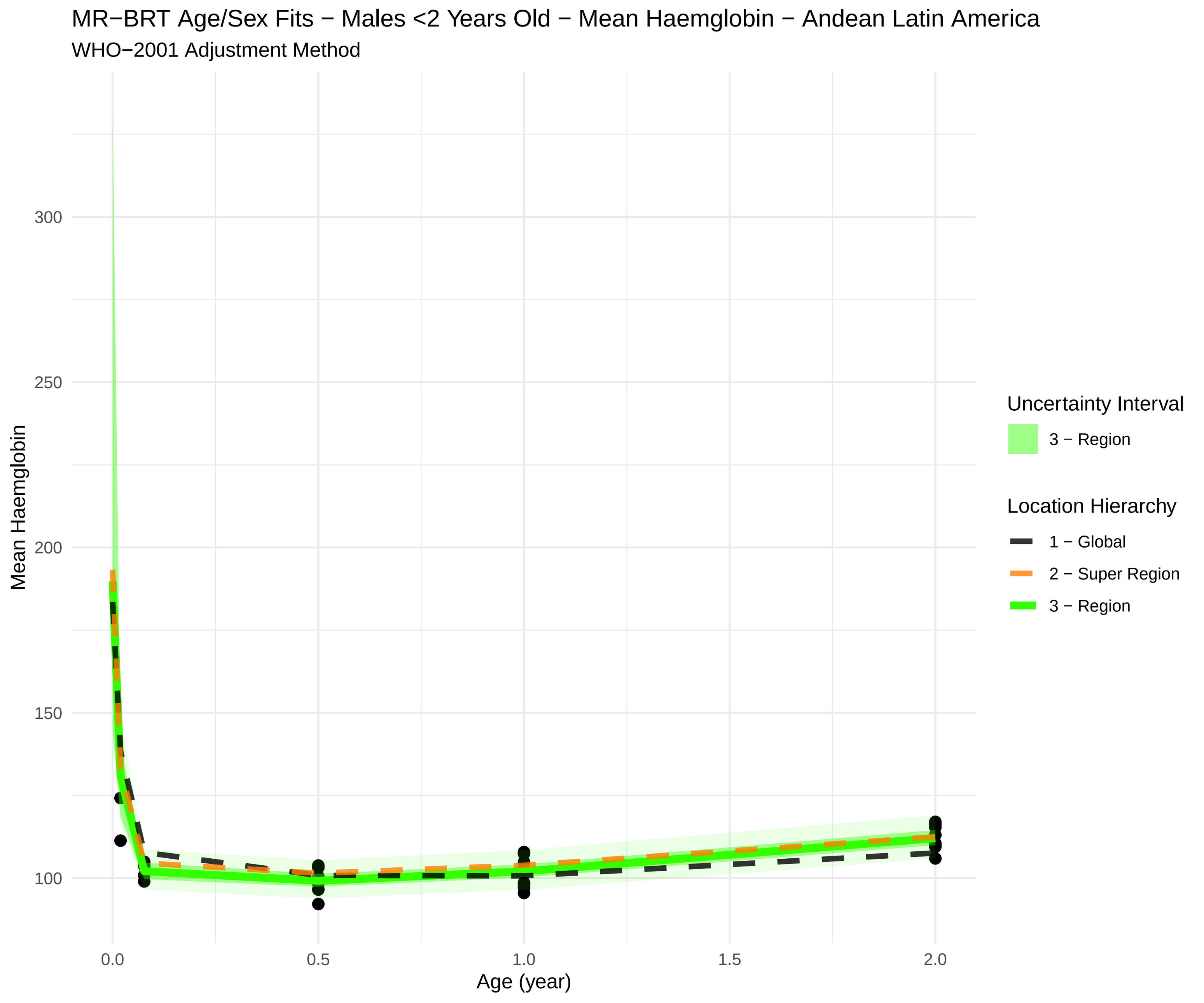
MR−BRT Age/Sex Fits − Males <2 Years Old − Mean Haemglobin − Andean Latin America
WHO−2001 Adjustment Method
Mean Haemglobin
Age (year)
Uncertainty Interval
3 − Region
Location Hierarchy
1 − Global
2 − Super Region
3 − Region

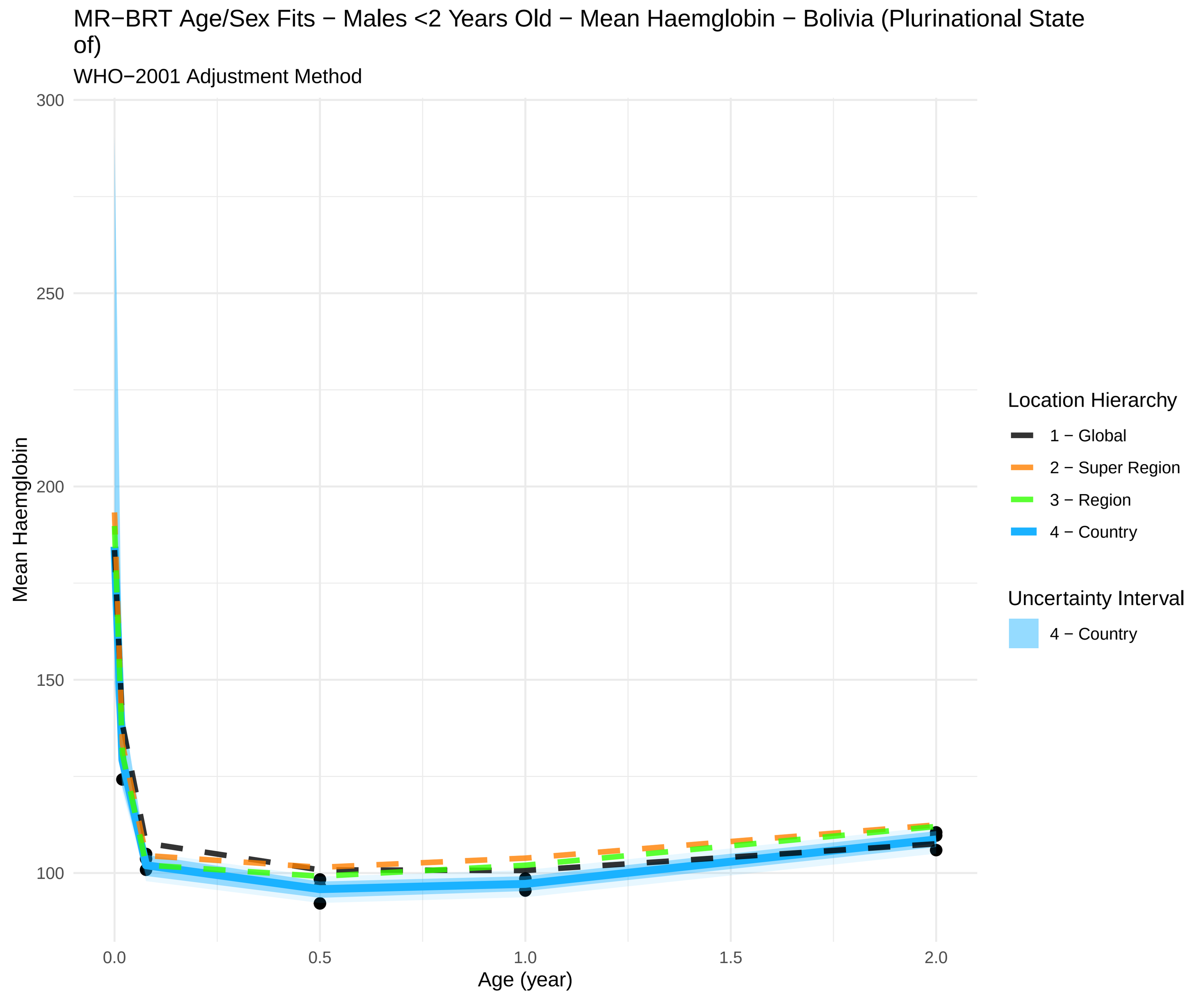
MR−BRT Age/Sex Fits − Males <2 Years Old − Mean Haemglobin − Bolivia (Plurinational State of)
WHO−2001 Adjustment Method
Mean Haemglobin
Age (year)
Location Hierarchy
1 − Global
2 − Super Region
3 − Region
4 − Country
Uncertainty Interval
4 − Country

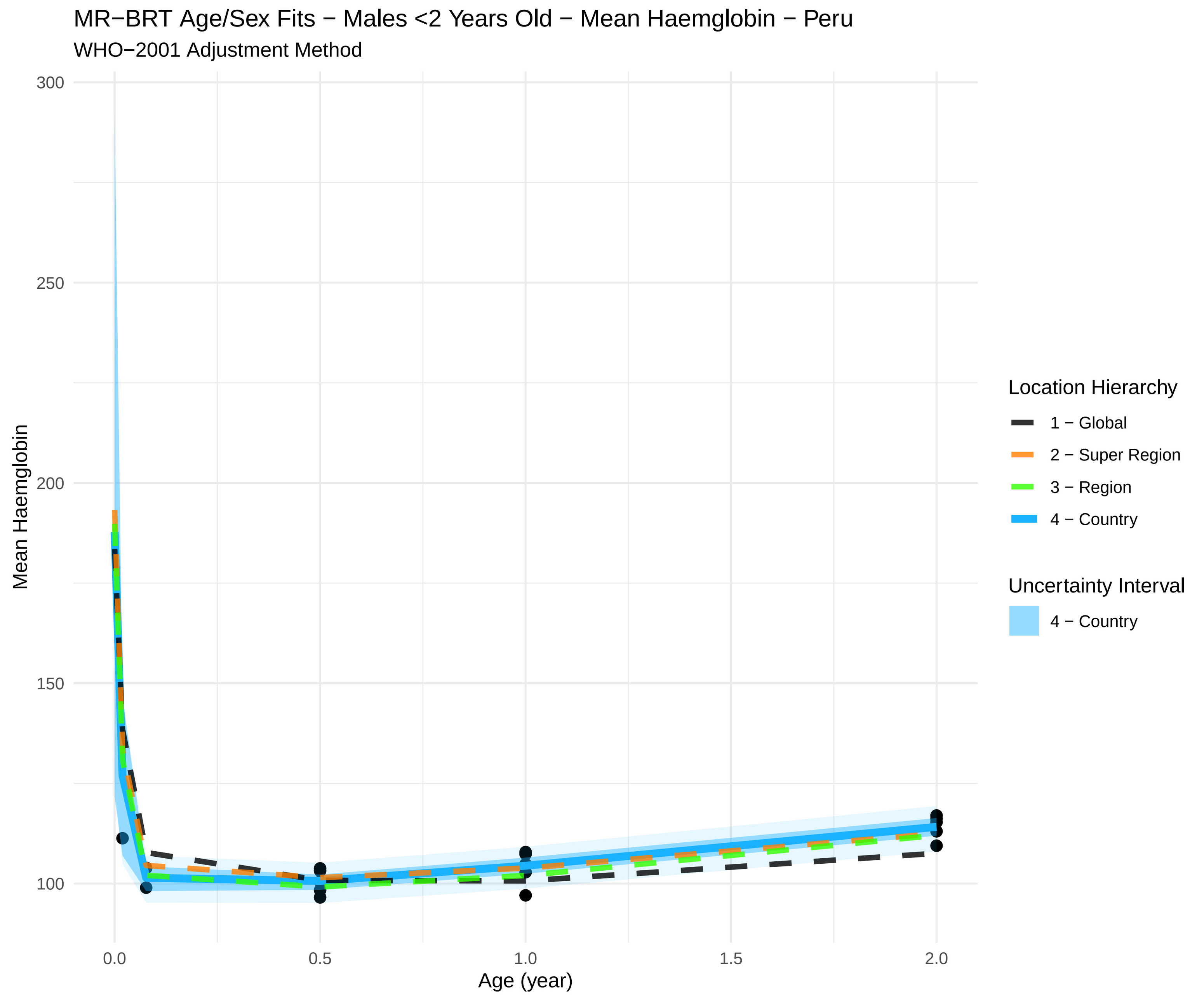
MR−BRT Age/Sex Fits − Males <2 Years Old − Mean Haemglobin − Peru
WHO−2001 Adjustment Method
Mean Haemglobin
Age (year)
Location Hierarchy
1 − Global
2 − Super Region
3 − Region
4 − Country
Uncertainty Interval
4 − Country

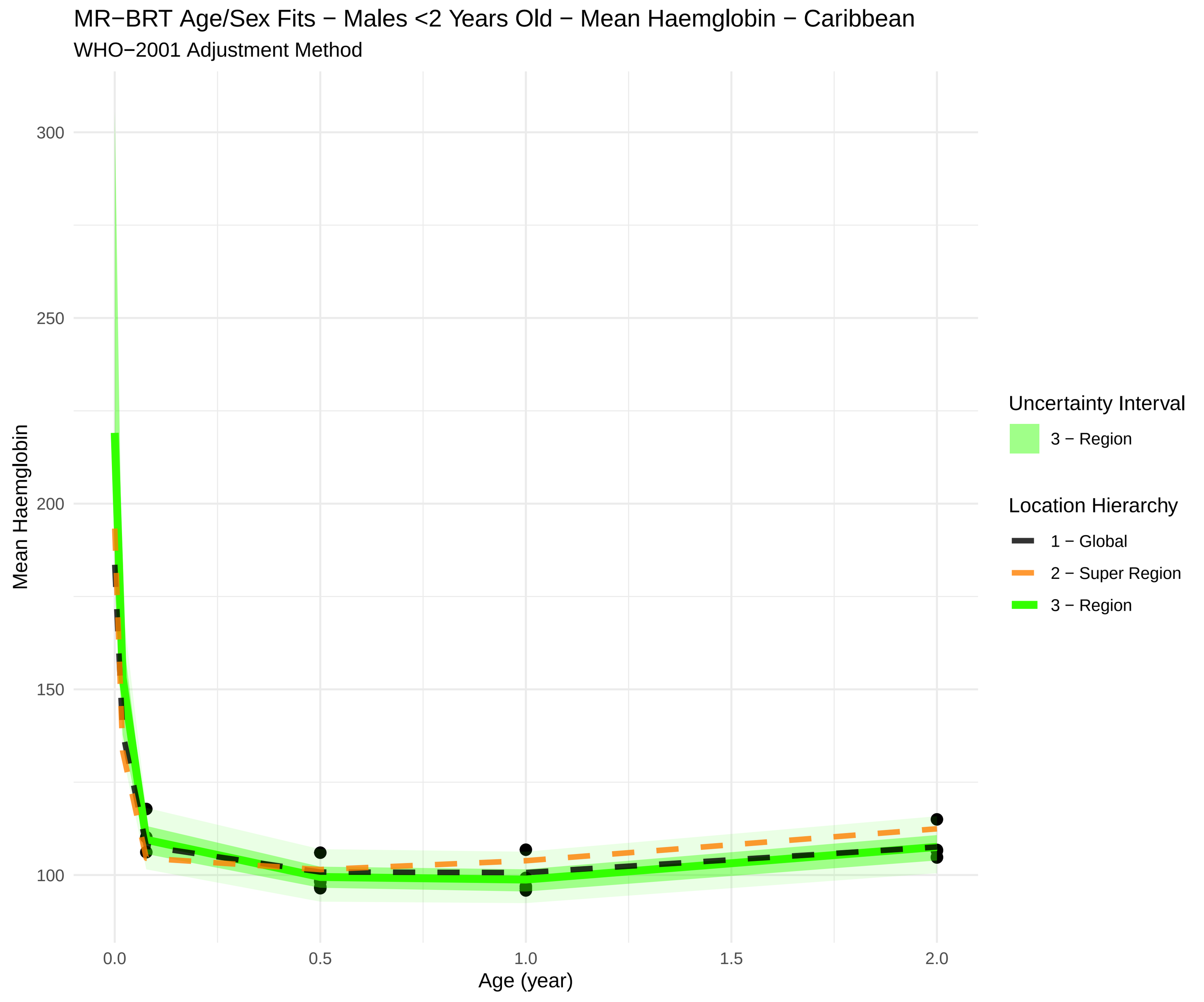
MR−BRT Age/Sex Fits − Males <2 Years Old − Mean Haemglobin − Caribbean
WHO−2001 Adjustment Method
Mean Haemglobin
Age (year)
Uncertainty Interval
3 − Region
Location Hierarchy
1 − Global
2 − Super Region
3 − Region

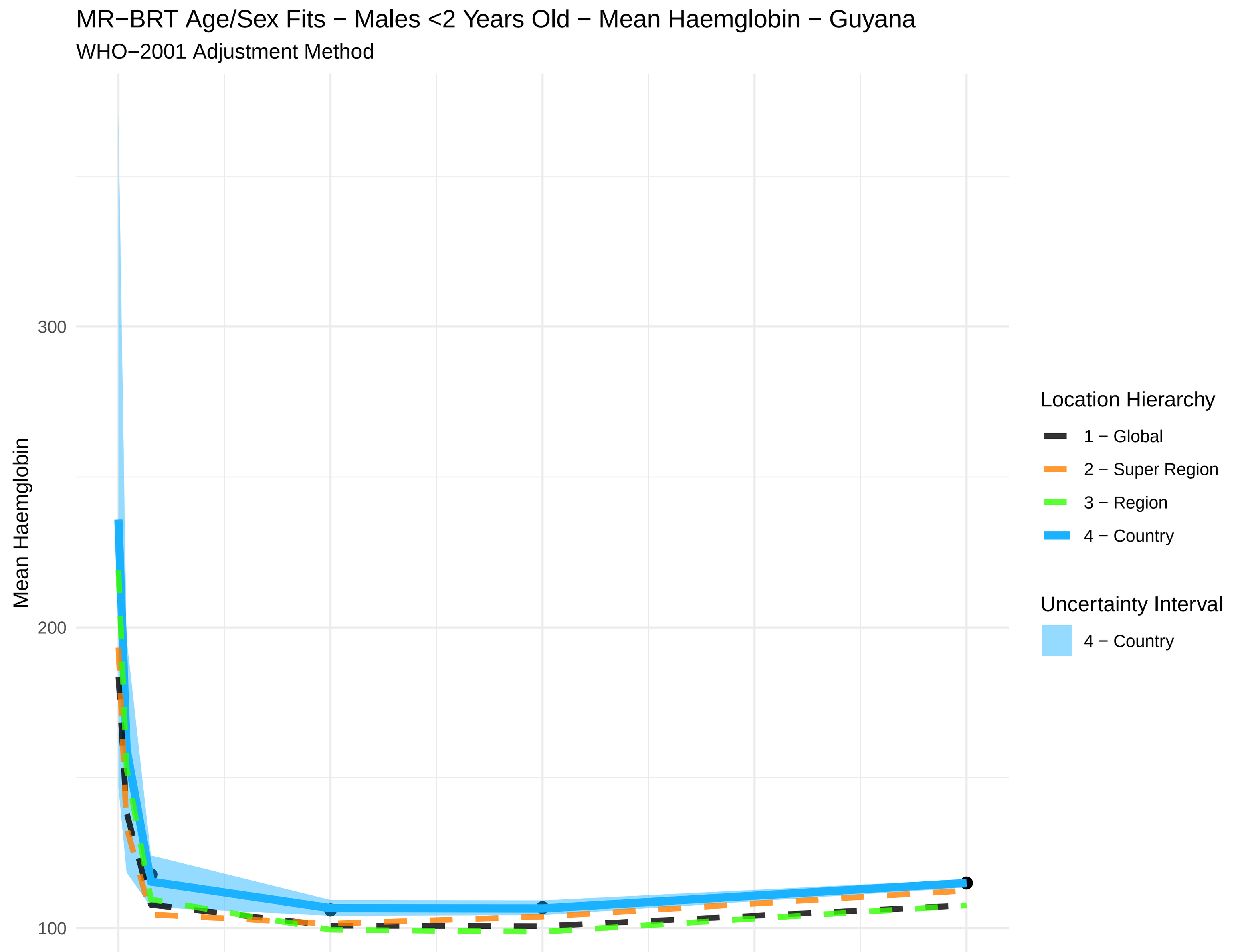
MR−BRT Age/Sex Fits − Males <2 Years Old − Mean Haemglobin − Guyana
WHO−2001 Adjustment Method
Mean Haemglobin
Age (year)
Location Hierarchy
1 − Global
2 − Super Region
3 − Region
4 − Country
Uncertainty Interval
4 − Country

MR−BRT Age/Sex Fits − Males <2 Years Old − Mean Haemglobin − Haiti
WHO−2001 Adjustment Method

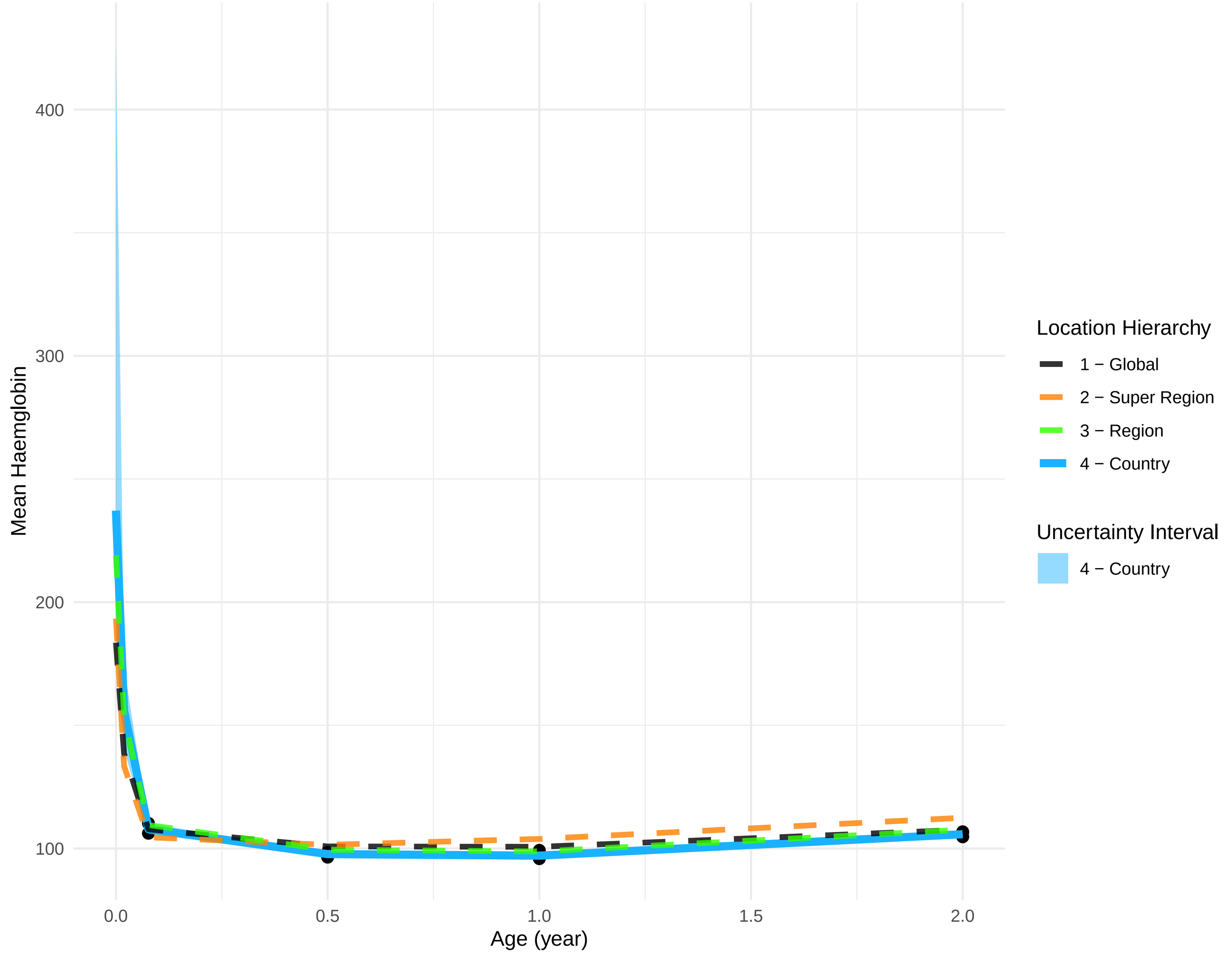

MR−BRT Age/Sex Fits − Males <2 Years Old − Mean Haemglobin − Central Latin America
WHO−2001 Adjustment Method

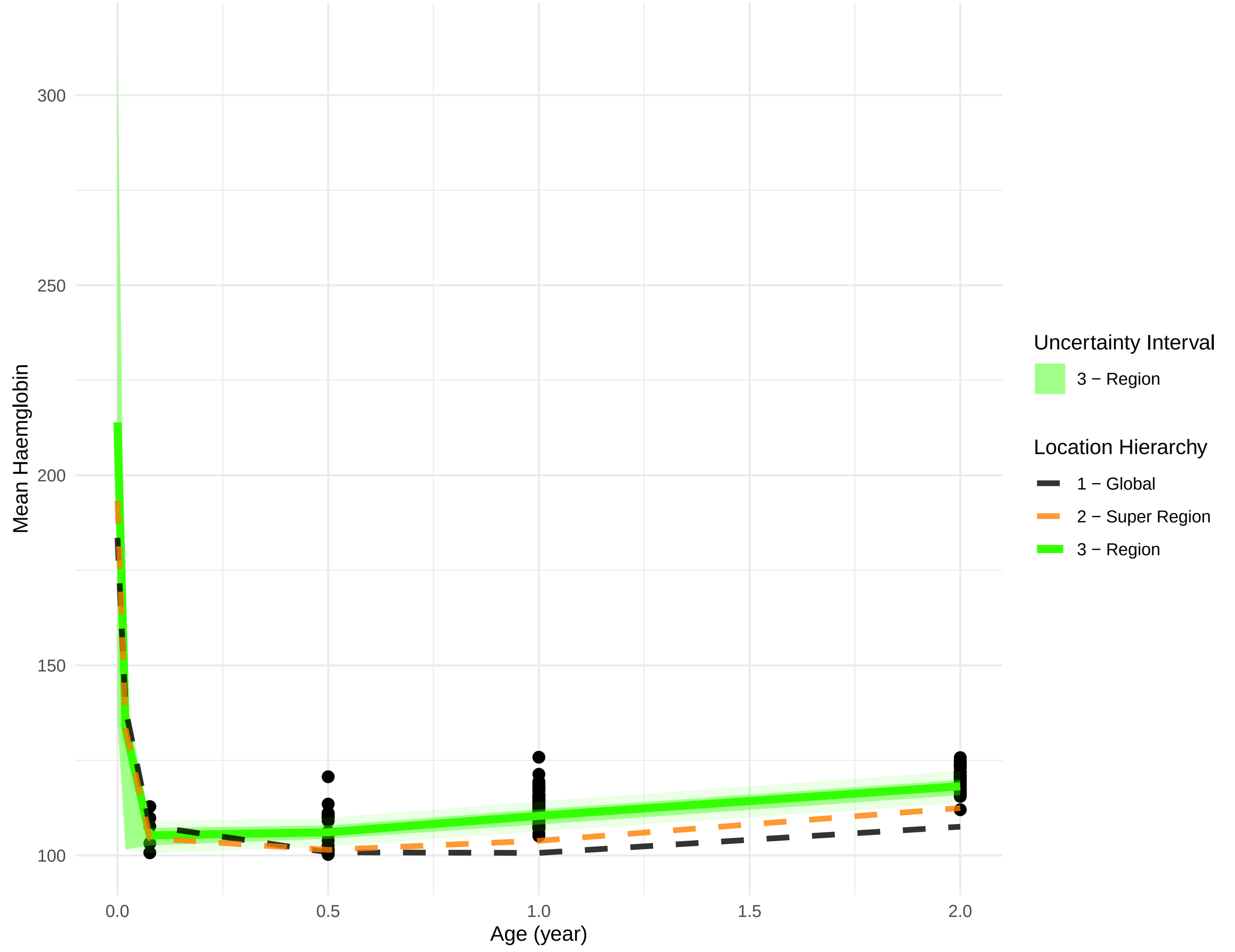

MR−BRT Age/Sex Fits − Males <2 Years Old − Mean Haemglobin − Guatemala
WHO−2001 Adjustment Method

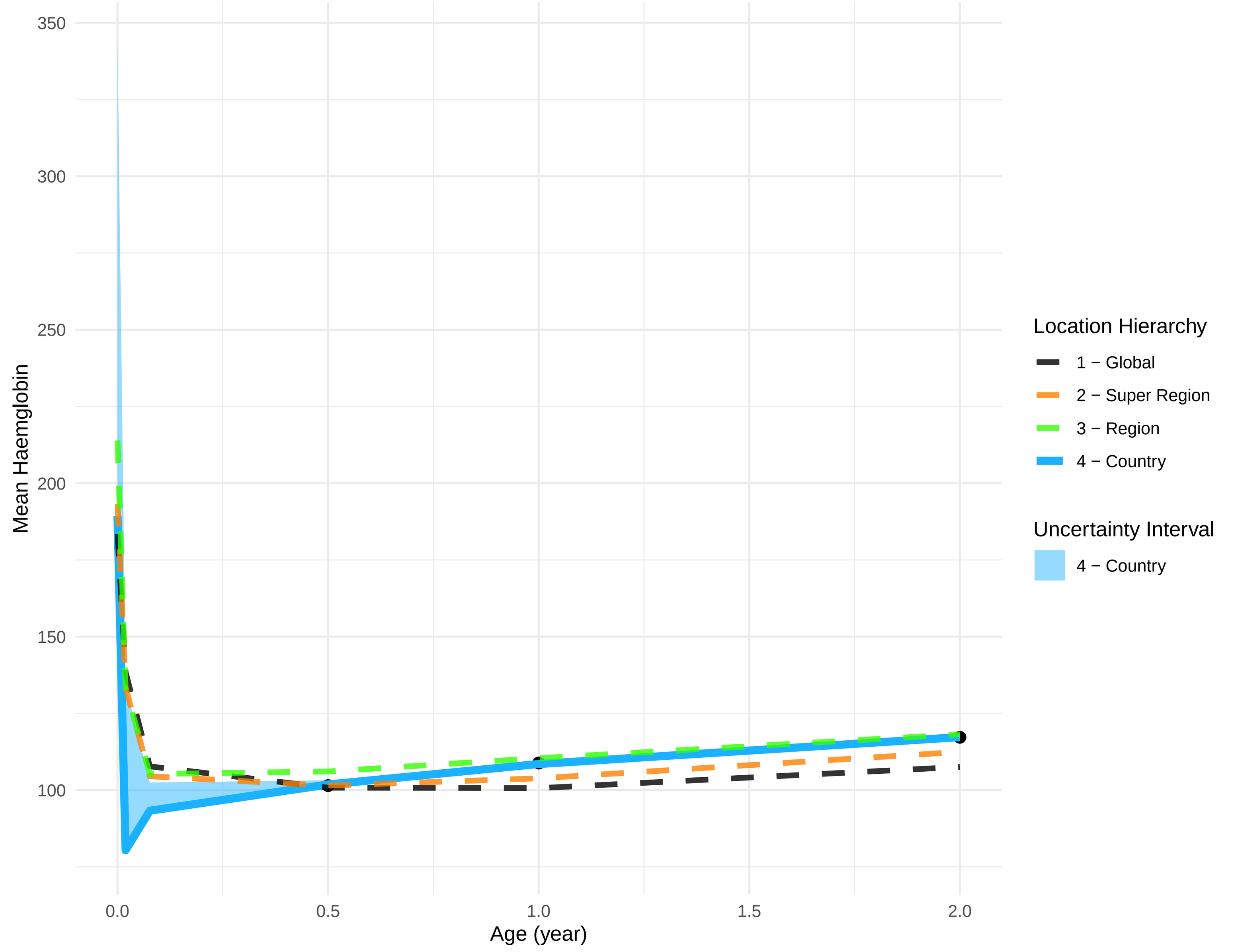

MR−BRT Age/Sex Fits − Males <2 Years Old − Mean Haemglobin − Honduras
WHO−2001 Adjustment Method

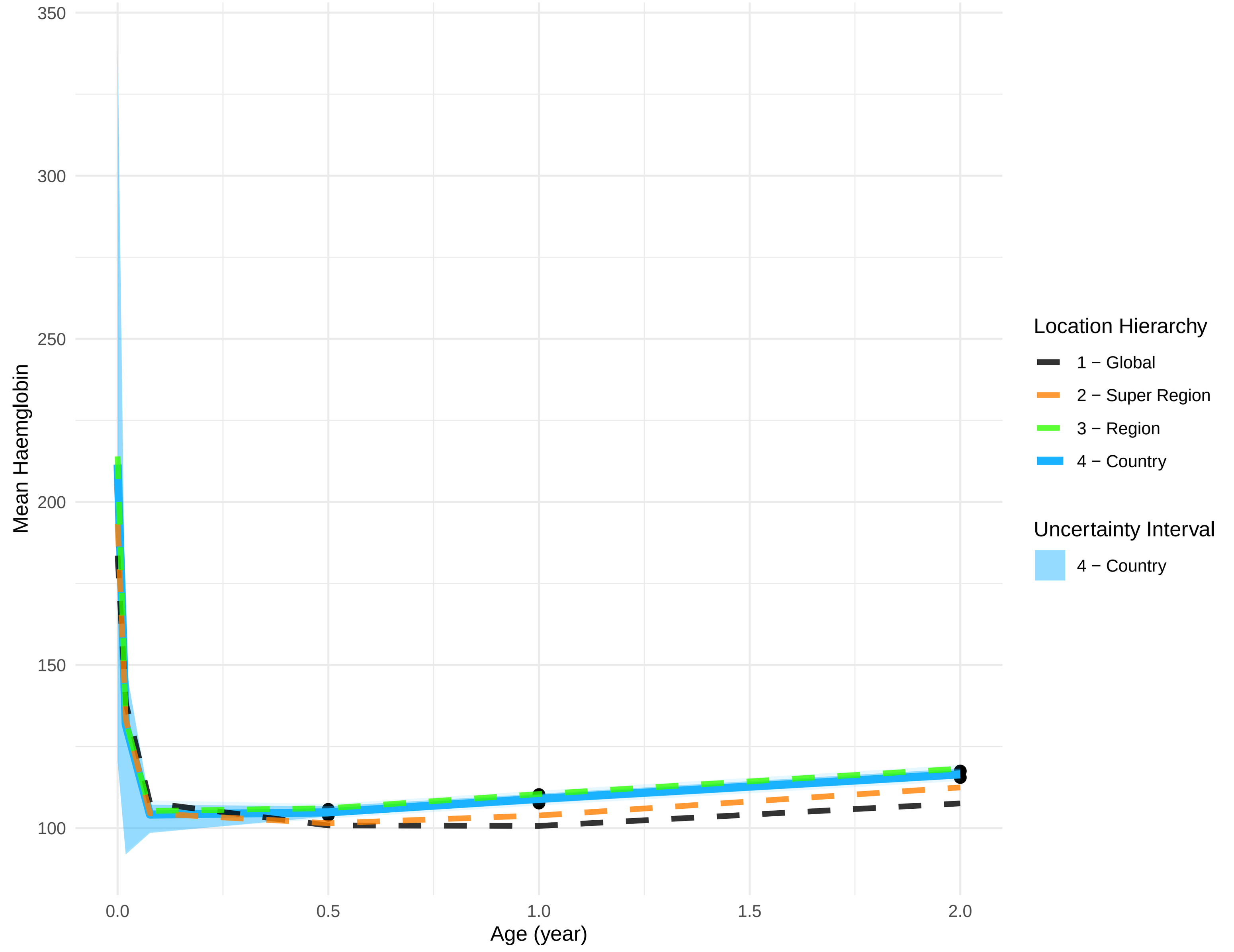

MR−BRT Age/Sex Fits − Males <2 Years Old − Mean Haemglobin − Mexico
WHO−2001 Adjustment Method

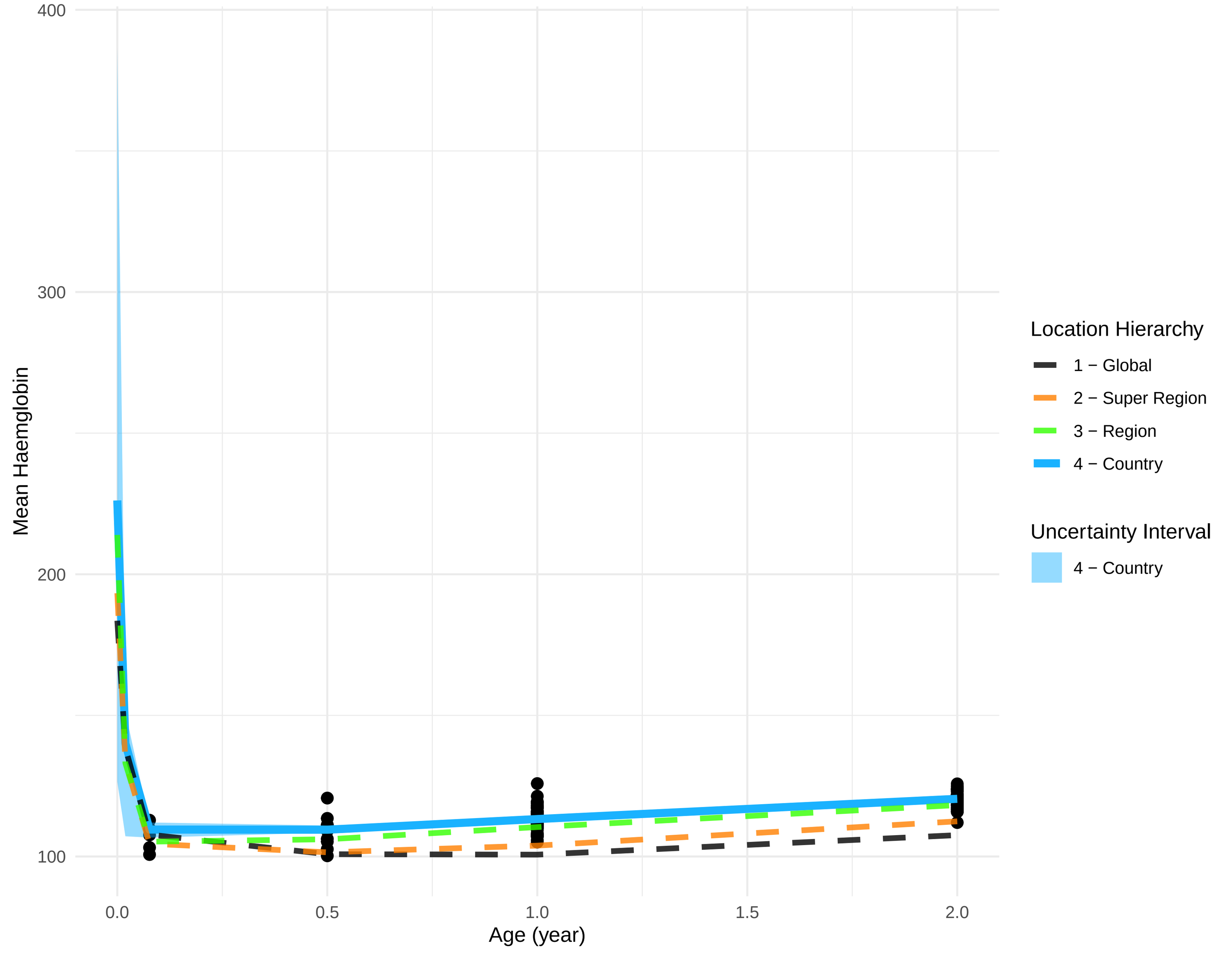

MR−BRT Age/Sex Fits − Males <2 Years Old − Mean Haemglobin − Aguascalientes
WHO−2001 Adjustment Method

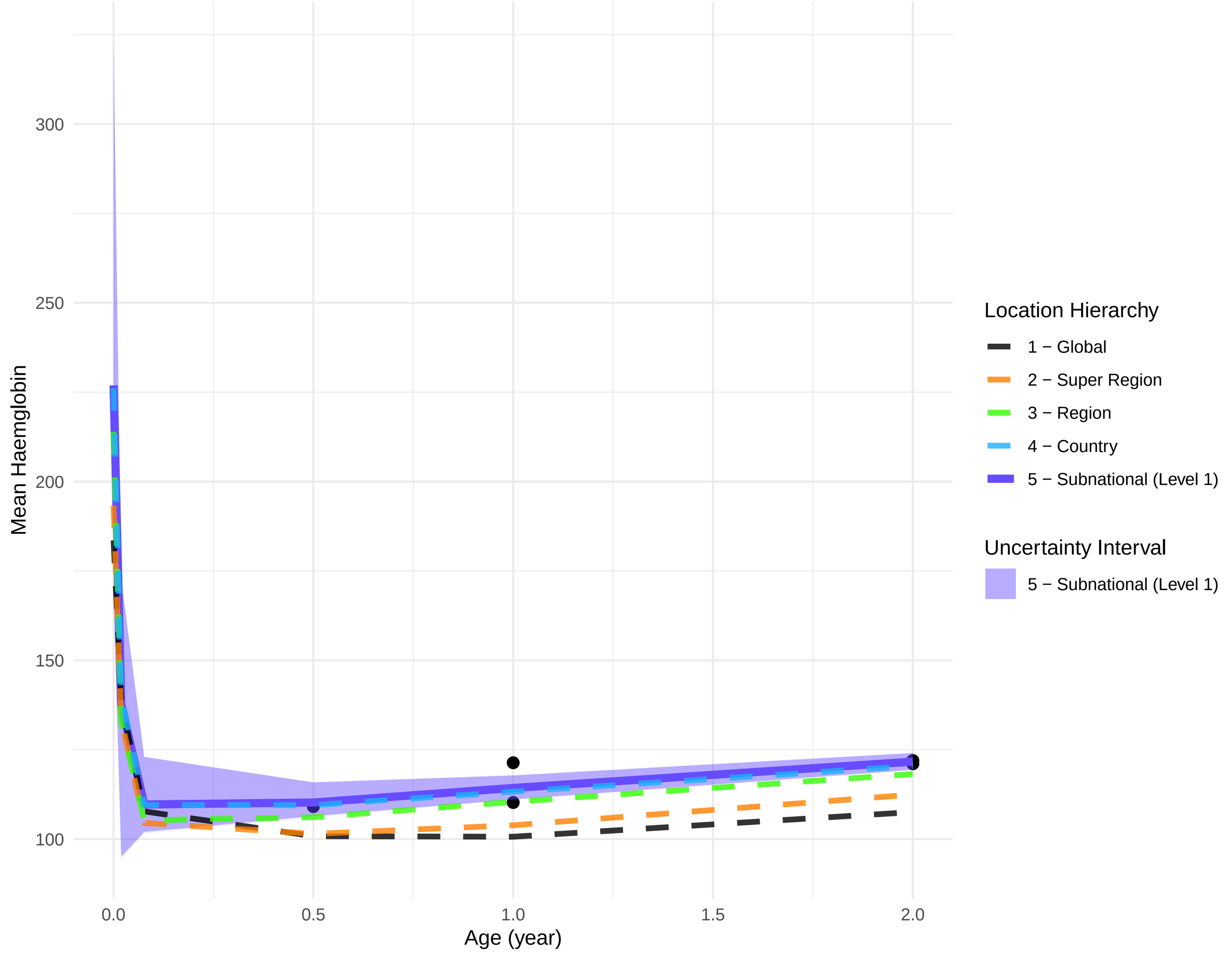

MR−BRT Age/Sex Fits − Males <2 Years Old − Mean Haemglobin − Baja California
WHO−2001 Adjustment Method

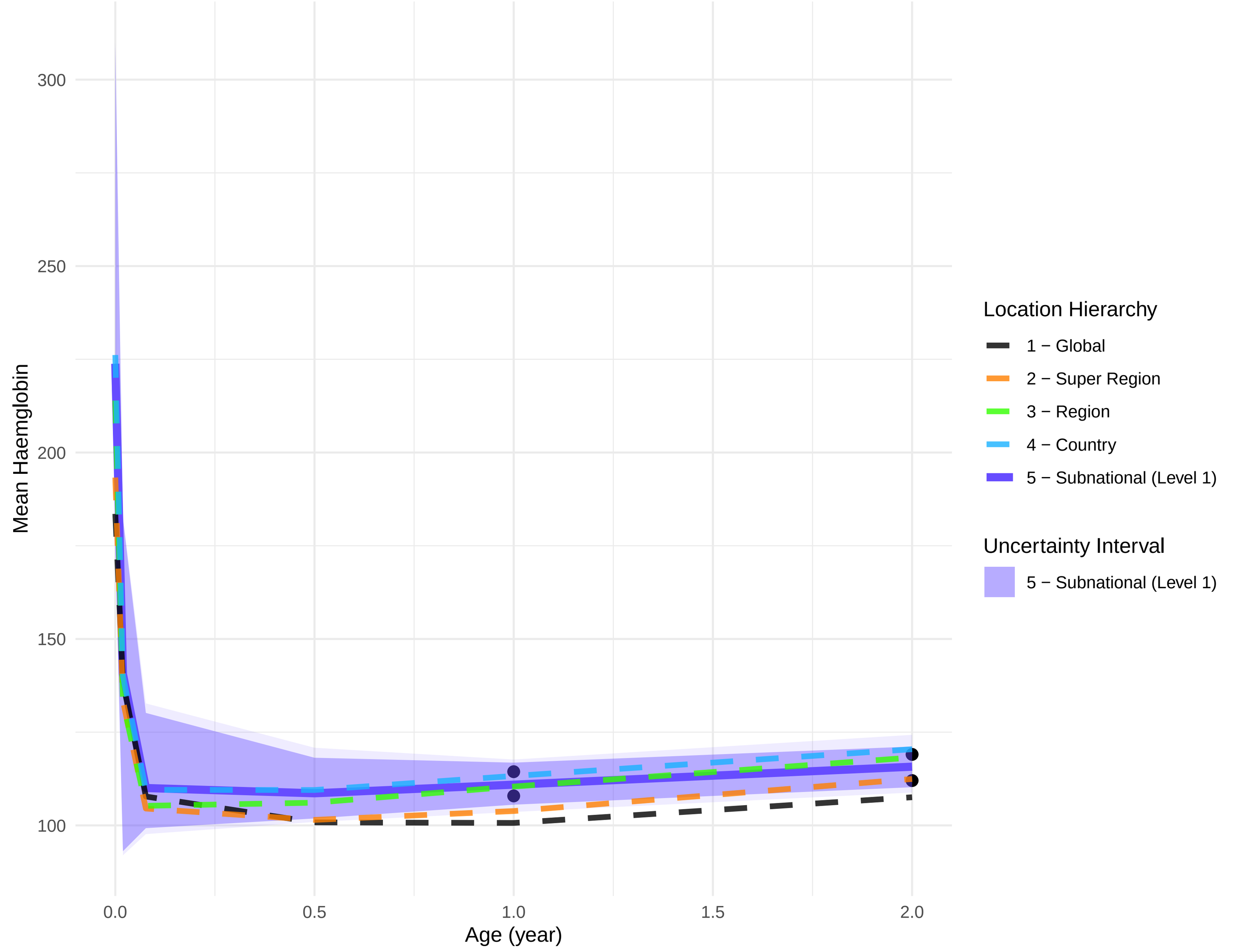

MR−BRT Age/Sex Fits − Males <2 Years Old − Mean Haemglobin − Baja California Sur
WHO−2001 Adjustment Method

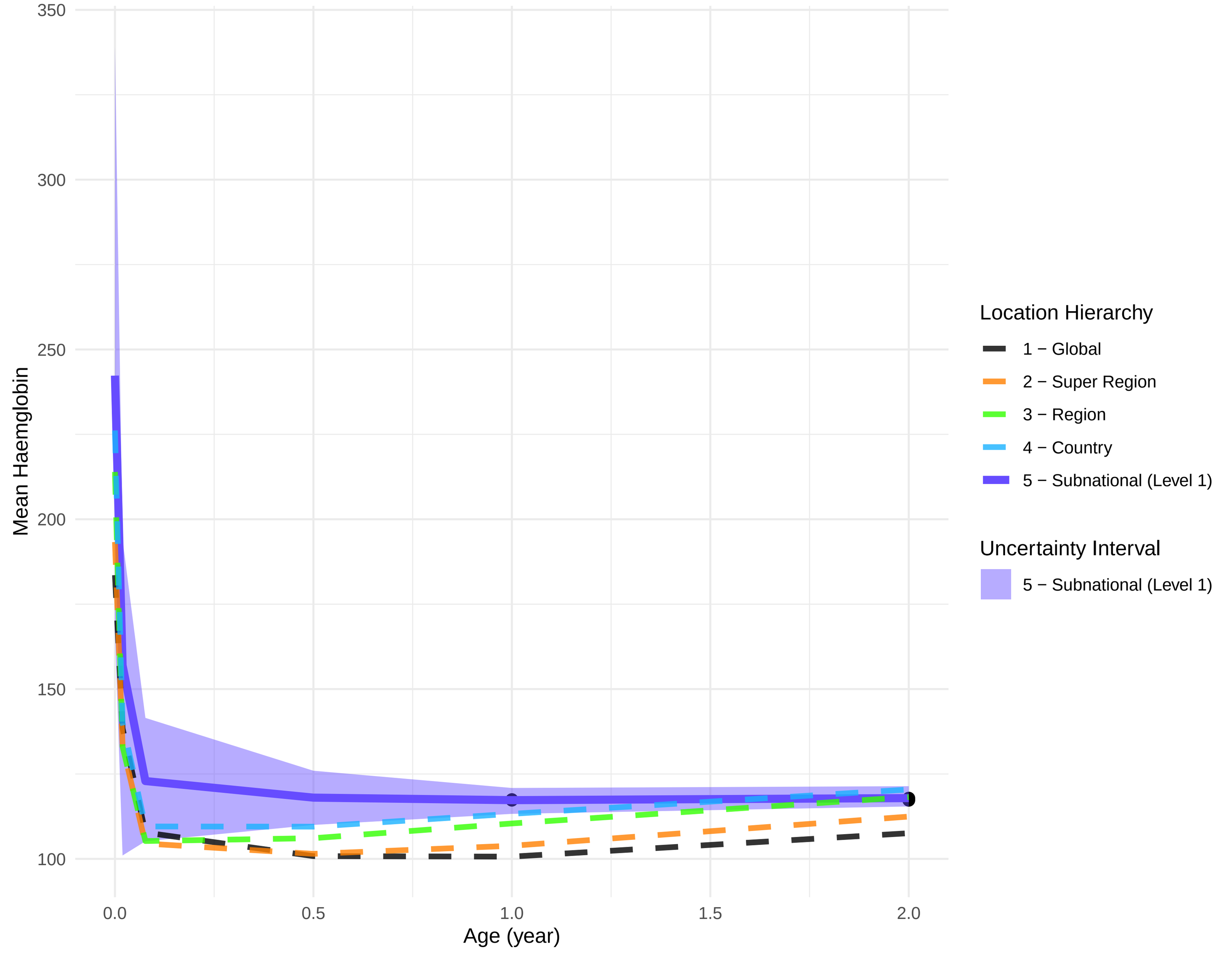

MR−BRT Age/Sex Fits − Males <2 Years Old − Mean Haemglobin − Campeche
WHO−2001 Adjustment Method

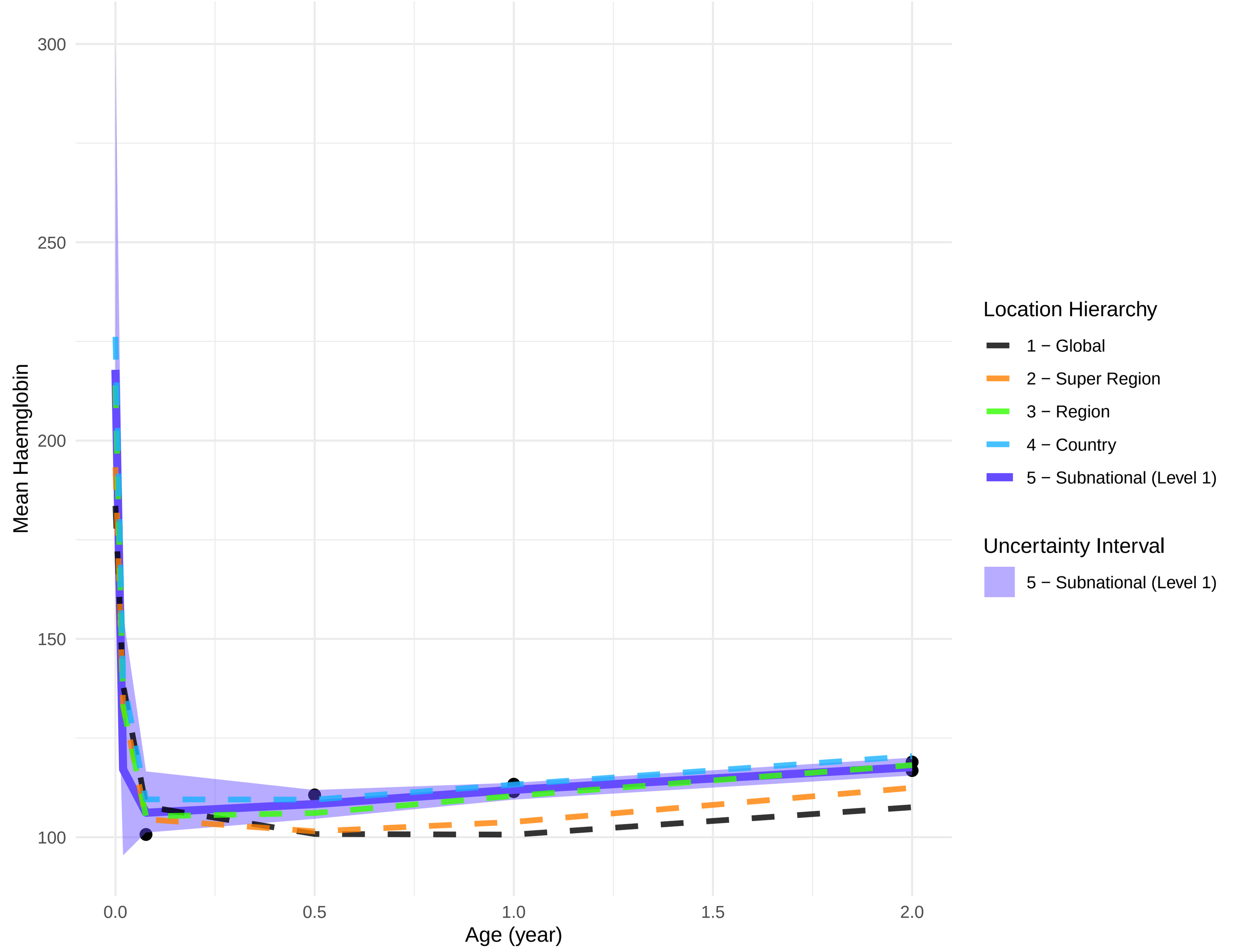

MR−BRT Age/Sex Fits − Males <2 Years Old − Mean Haemglobin − Chiapas
WHO−2001 Adjustment Method

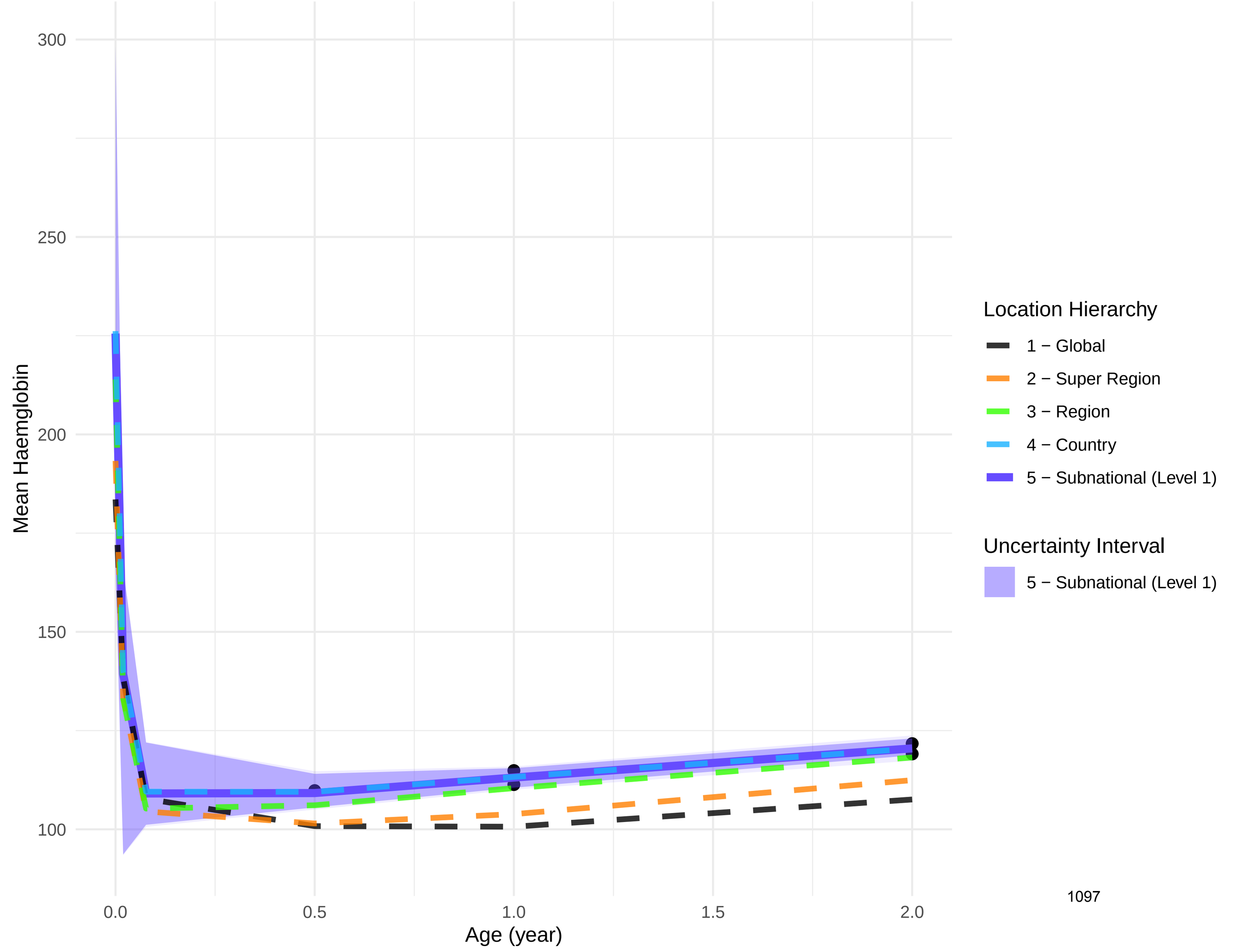

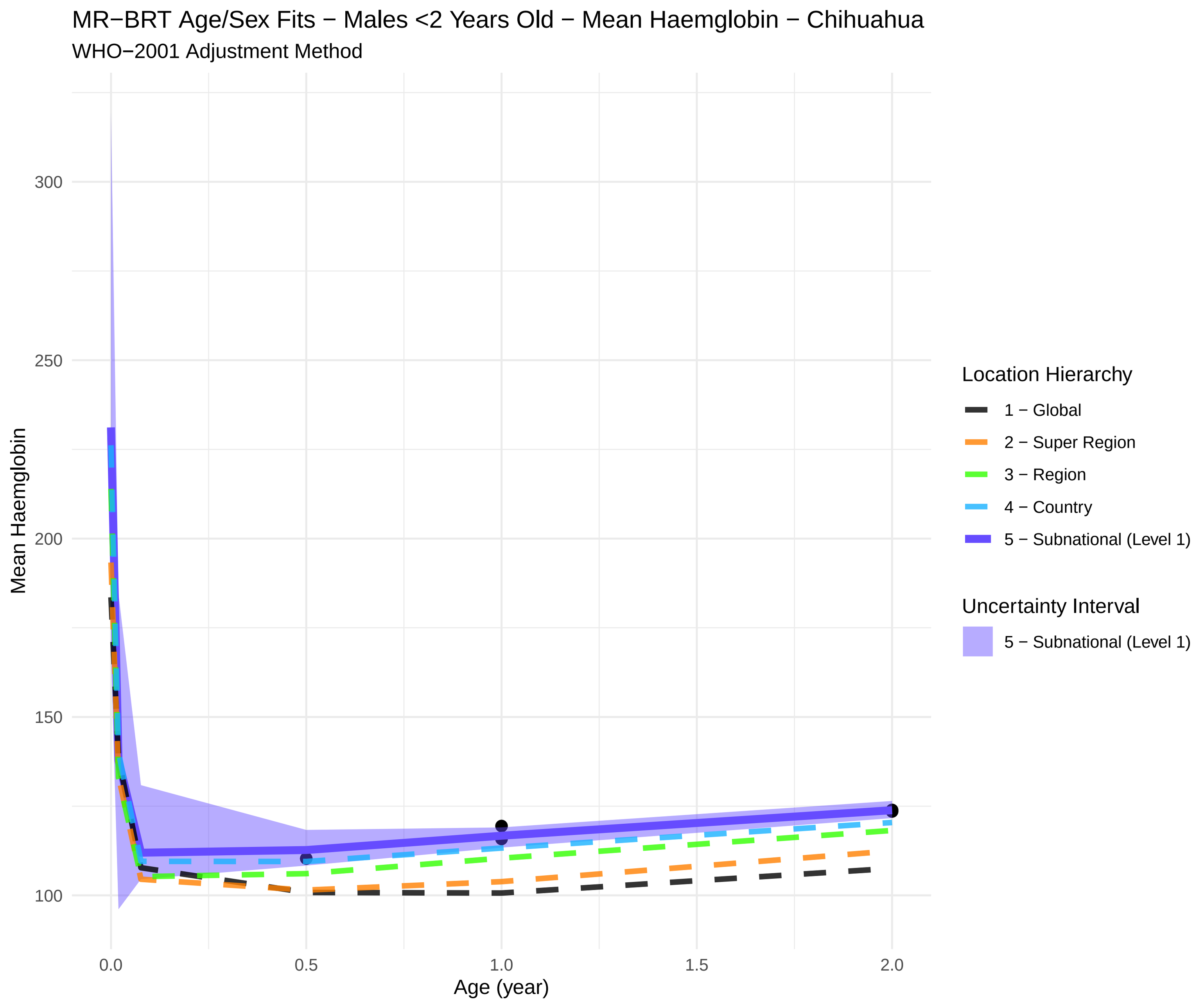
MR−BRT Age/Sex Fits − Males <2 Years Old − Mean Haemglobin − Chihuahua
WHO−2001 Adjustment Method
Mean Haemglobin
100
150
200
250
300
0.0
0.5
1.0
1.5
2.0
Age (year)
Location Hierarchy
1 − Global
2 − Super Region
3 − Region
4 − Country
5 − Subnational (Level 1)
Uncertainty Interval
5 − Subnational (Level 1)

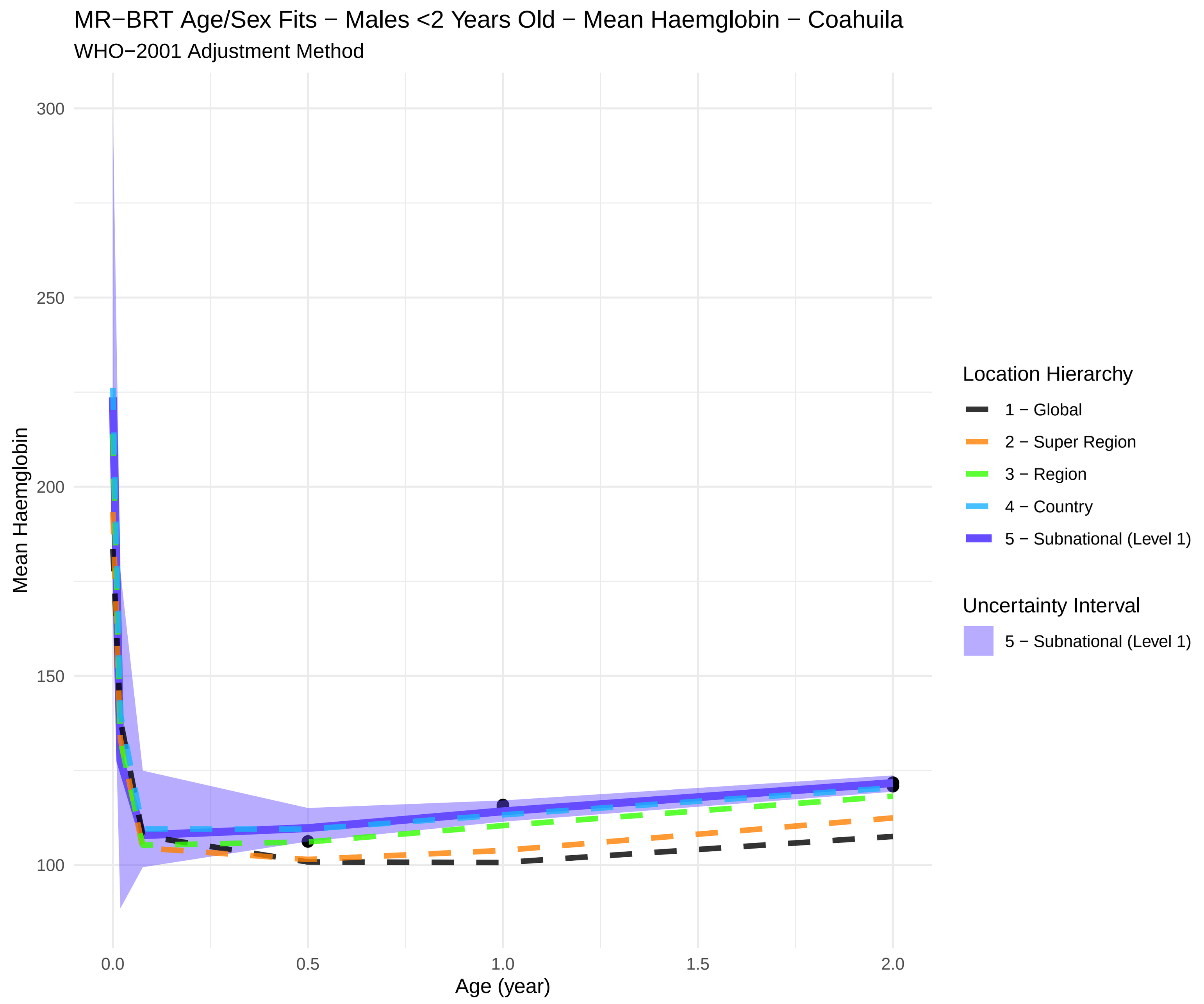
MR−BRT Age/Sex Fits − Males <2 Years Old − Mean Haemglobin − Coahuila
WHO−2001 Adjustment Method
Mean Haemglobin
100
150
200
250
300
0.0
0.5
1.0
1.5
2.0
Age (year)
Location Hierarchy
1 − Global
2 − Super Region
3 − Region
4 − Country
5 − Subnational (Level 1)
Uncertainty Interval
5 − Subnational (Level 1)

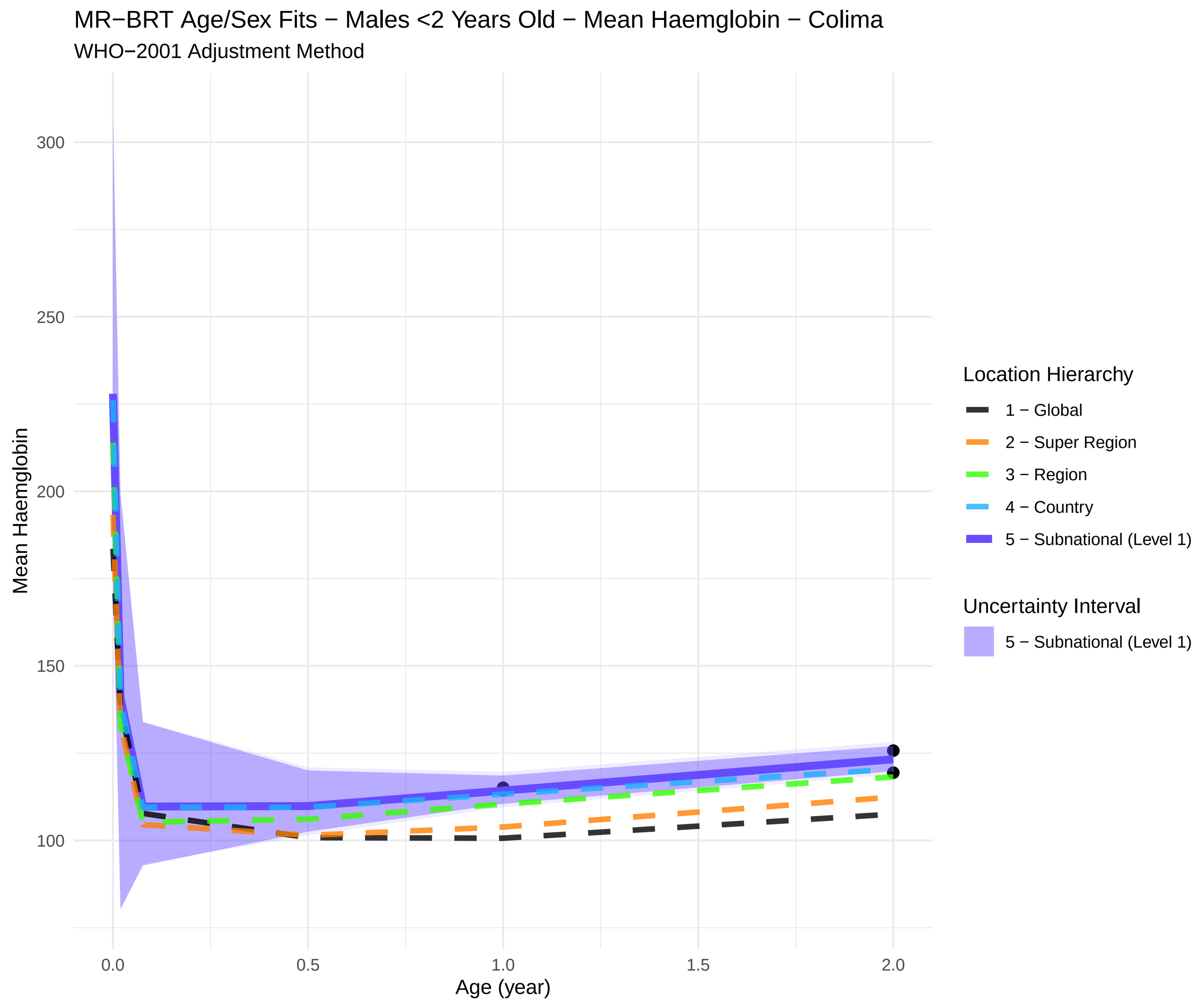
MR−BRT Age/Sex Fits − Males <2 Years Old − Mean Haemglobin − Colima
WHO−2001 Adjustment Method
Mean Haemglobin
100
150
200
250
300
0.0
0.5
1.0
1.5
2.0
Age (year)
Location Hierarchy
1 − Global
2 − Super Region
3 − Region
4 − Country
5 − Subnational (Level 1)
Uncertainty Interval
5 − Subnational (Level 1)

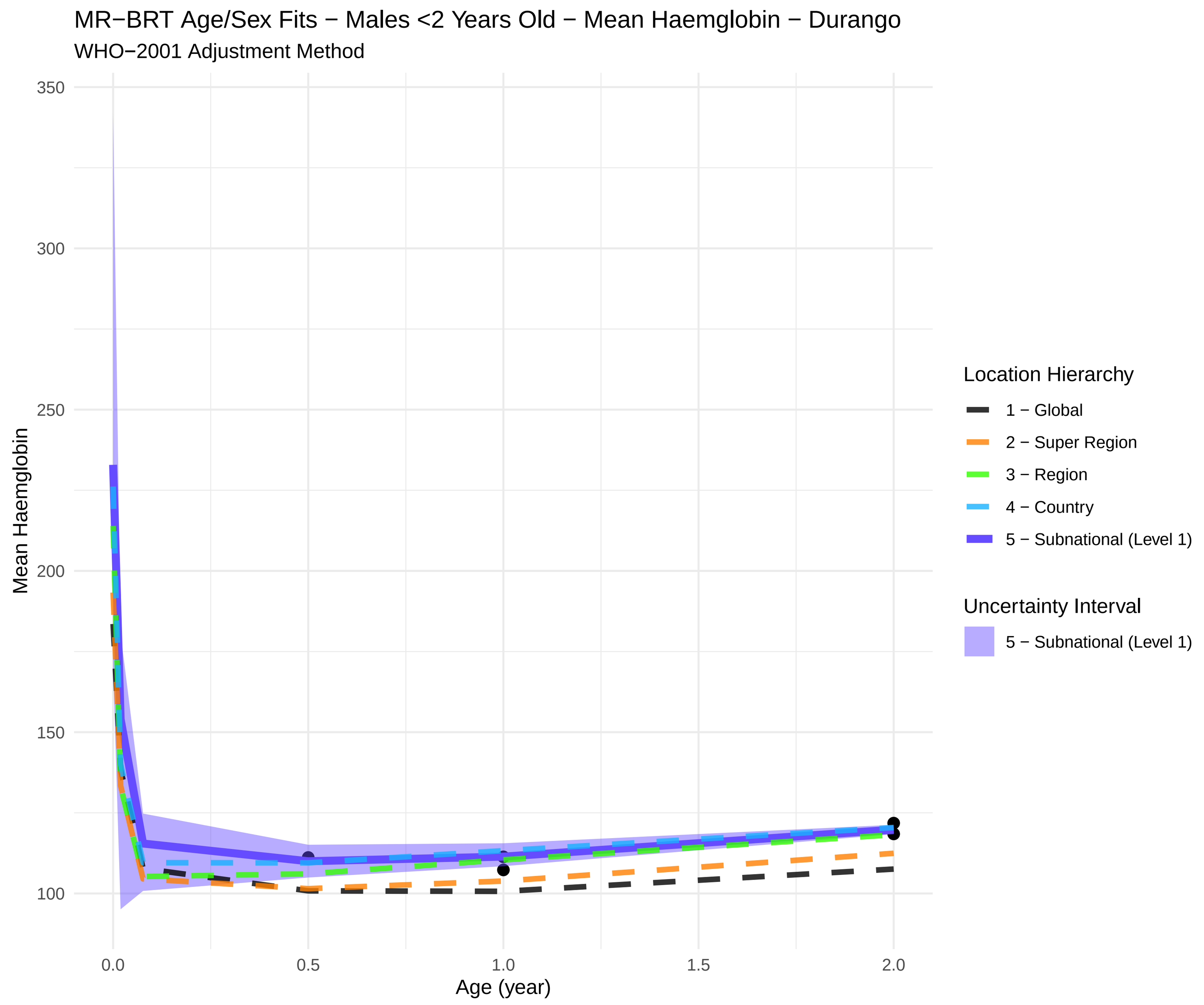
MR−BRT Age/Sex Fits − Males <2 Years Old − Mean Haemglobin − Durango
WHO−2001 Adjustment Method
Mean Haemglobin
100
150
200
250
300
350
0.0
0.5
1.0
1.5
2.0
Age (year)
Location Hierarchy
1 − Global
2 − Super Region
3 − Region
4 − Country
5 − Subnational (Level 1)
Uncertainty Interval
5 − Subnational (Level 1)

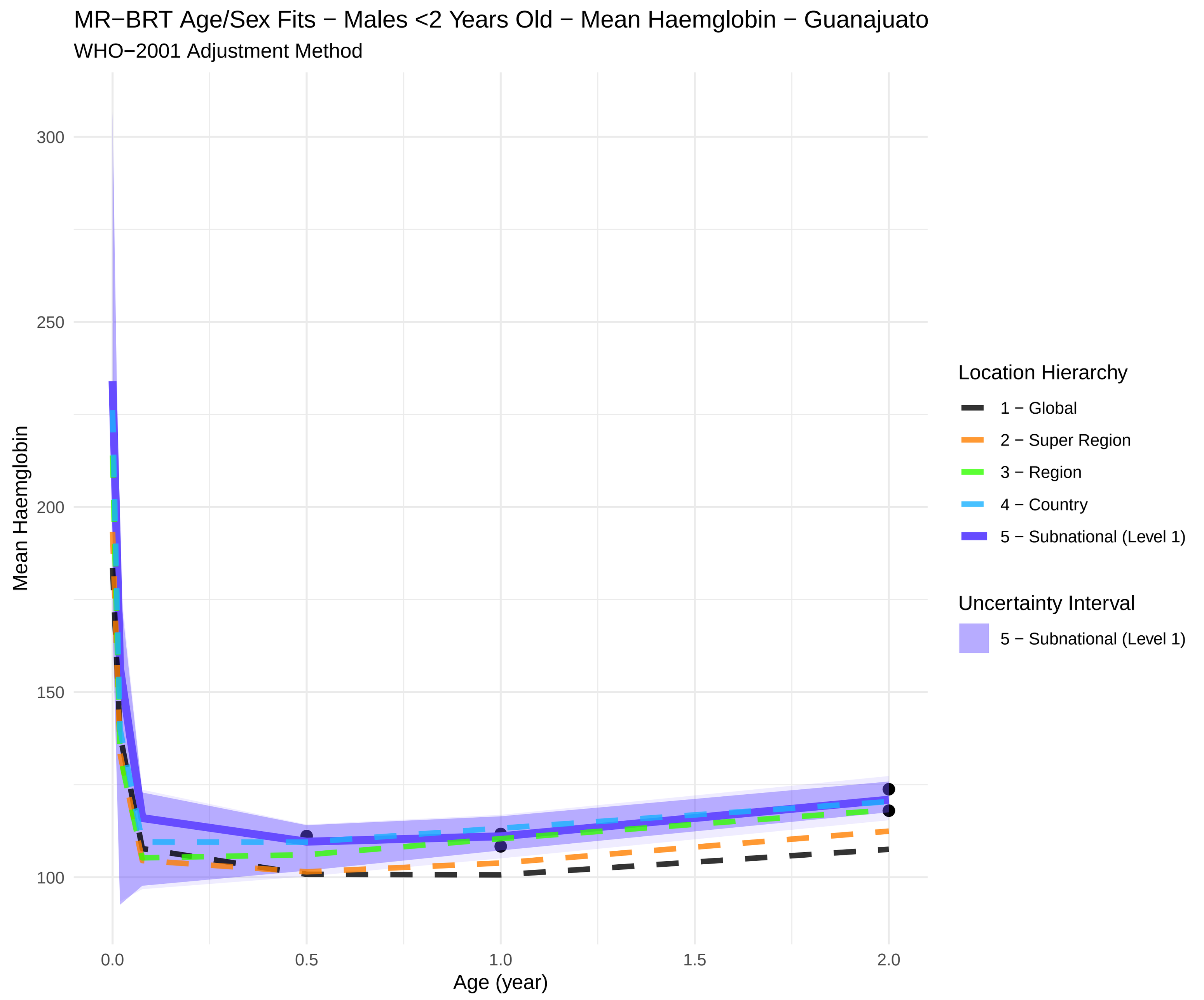
MR−BRT Age/Sex Fits − Males <2 Years Old − Mean Haemglobin − Guanajuato
WHO−2001 Adjustment Method
Mean Haemglobin
100
150
200
250
300
0.0
0.5
1.0
1.5
2.0
Age (year)
Location Hierarchy
1 − Global
2 − Super Region
3 − Region
4 − Country
5 − Subnational (Level 1)
Uncertainty Interval
5 − Subnational (Level 1)

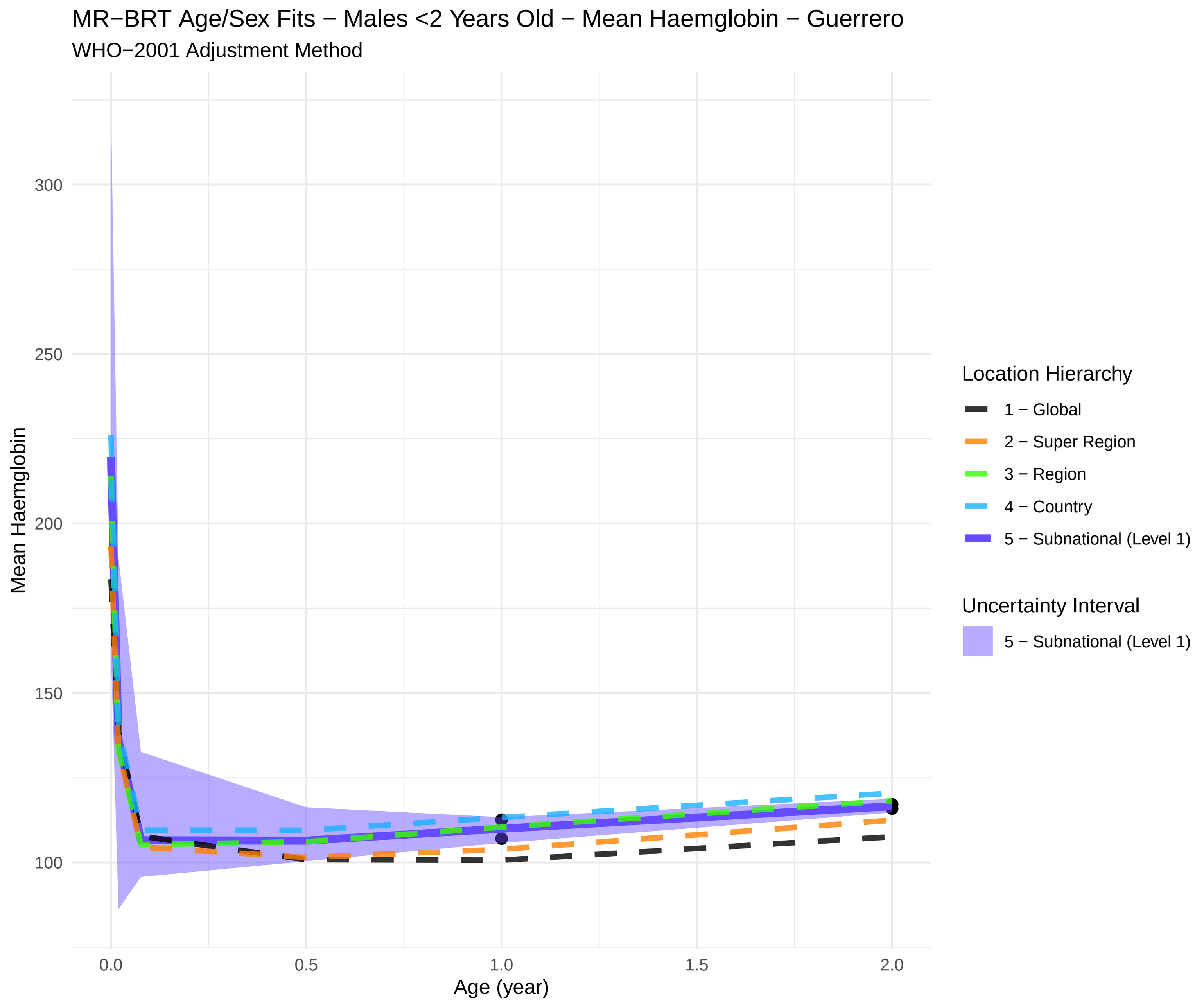
MR−BRT Age/Sex Fits − Males <2 Years Old − Mean Haemglobin − Guerrero
WHO−2001 Adjustment Method
Mean Haemglobin
100
150
200
250
300
0.0
0.5
1.0
1.5
2.0
Age (year)
Location Hierarchy
1 − Global
2 − Super Region
3 − Region
4 − Country
5 − Subnational (Level 1)
Uncertainty Interval
5 − Subnational (Level 1)

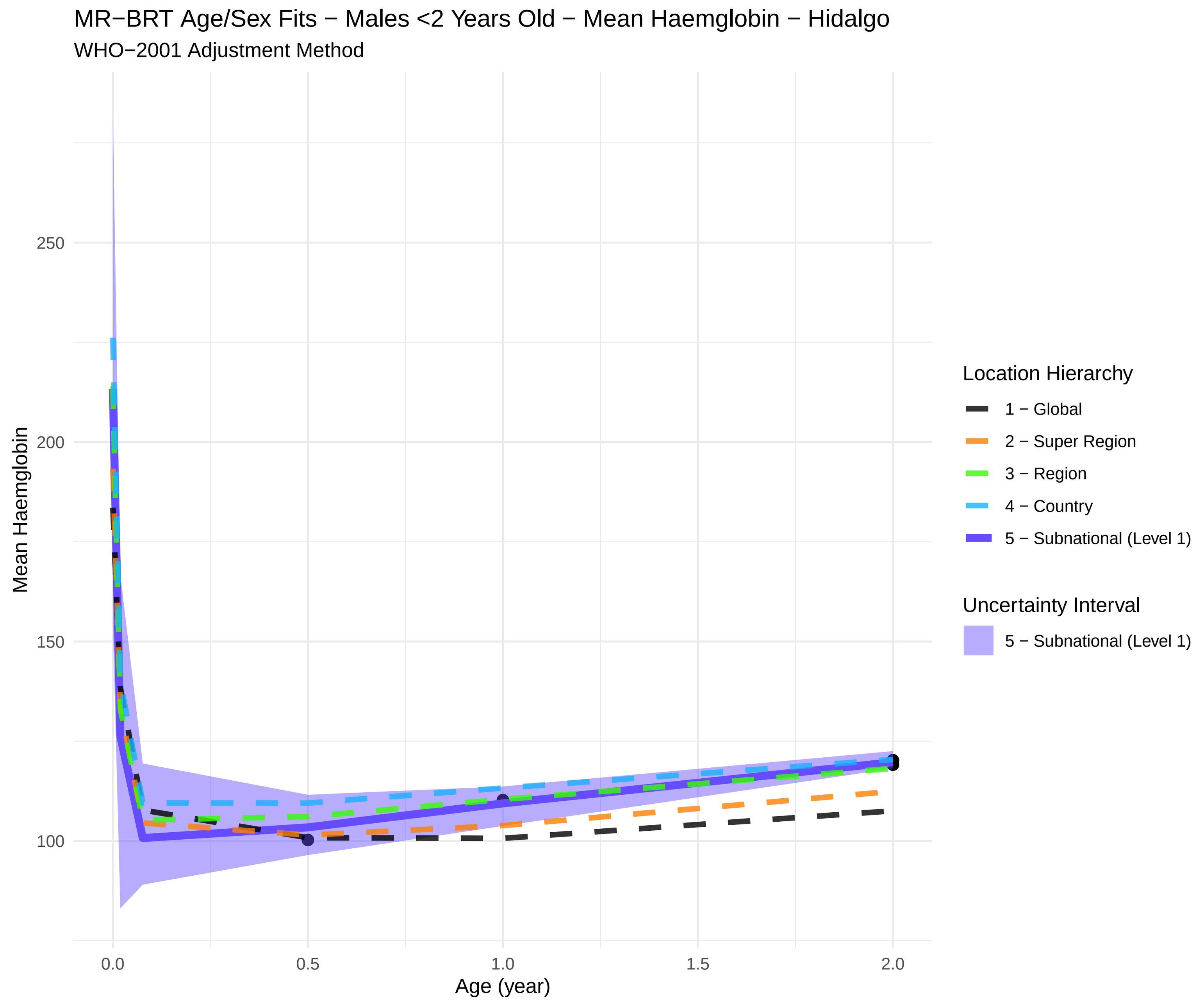
MR−BRT Age/Sex Fits − Males <2 Years Old − Mean Haemglobin − Hidalgo
WHO−2001 Adjustment Method
Mean Haemglobin
100
150
200
250
0.0
0.5
1.0
1.5
2.0
Age (year)
Location Hierarchy
1 − Global
2 − Super Region
3 − Region
4 − Country
5 − Subnational (Level 1)
Uncertainty Interval
5 − Subnational (Level 1)

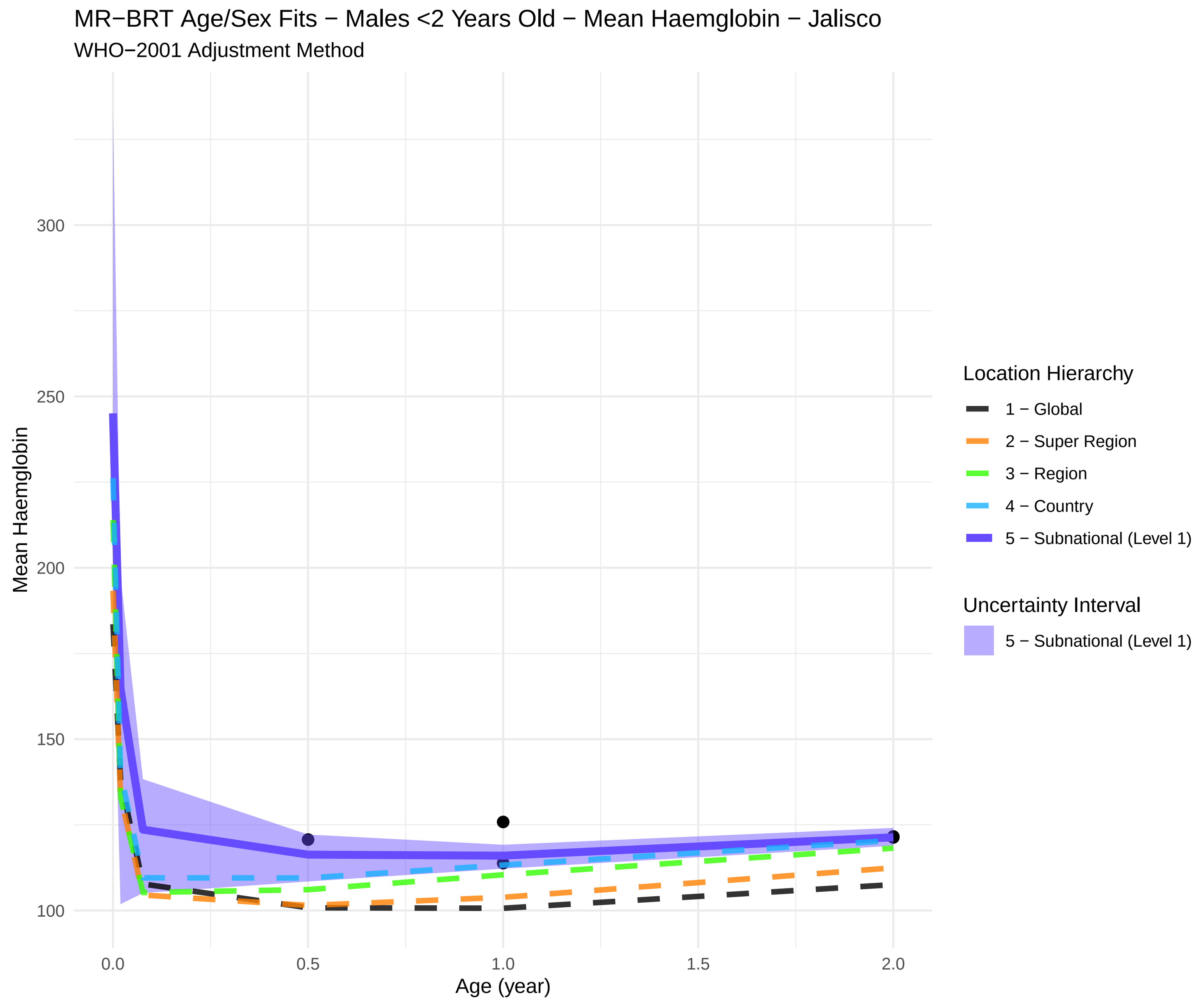
MR−BRT Age/Sex Fits − Males <2 Years Old − Mean Haemglobin − Jalisco
WHO−2001 Adjustment Method
Mean Haemglobin
100
150
200
250
300
0.0
0.5
1.0
1.5
2.0
Age (year)
Location Hierarchy
1 − Global
2 − Super Region
3 − Region
4 − Country
5 − Subnational (Level 1)
Uncertainty Interval
5 − Subnational (Level 1)

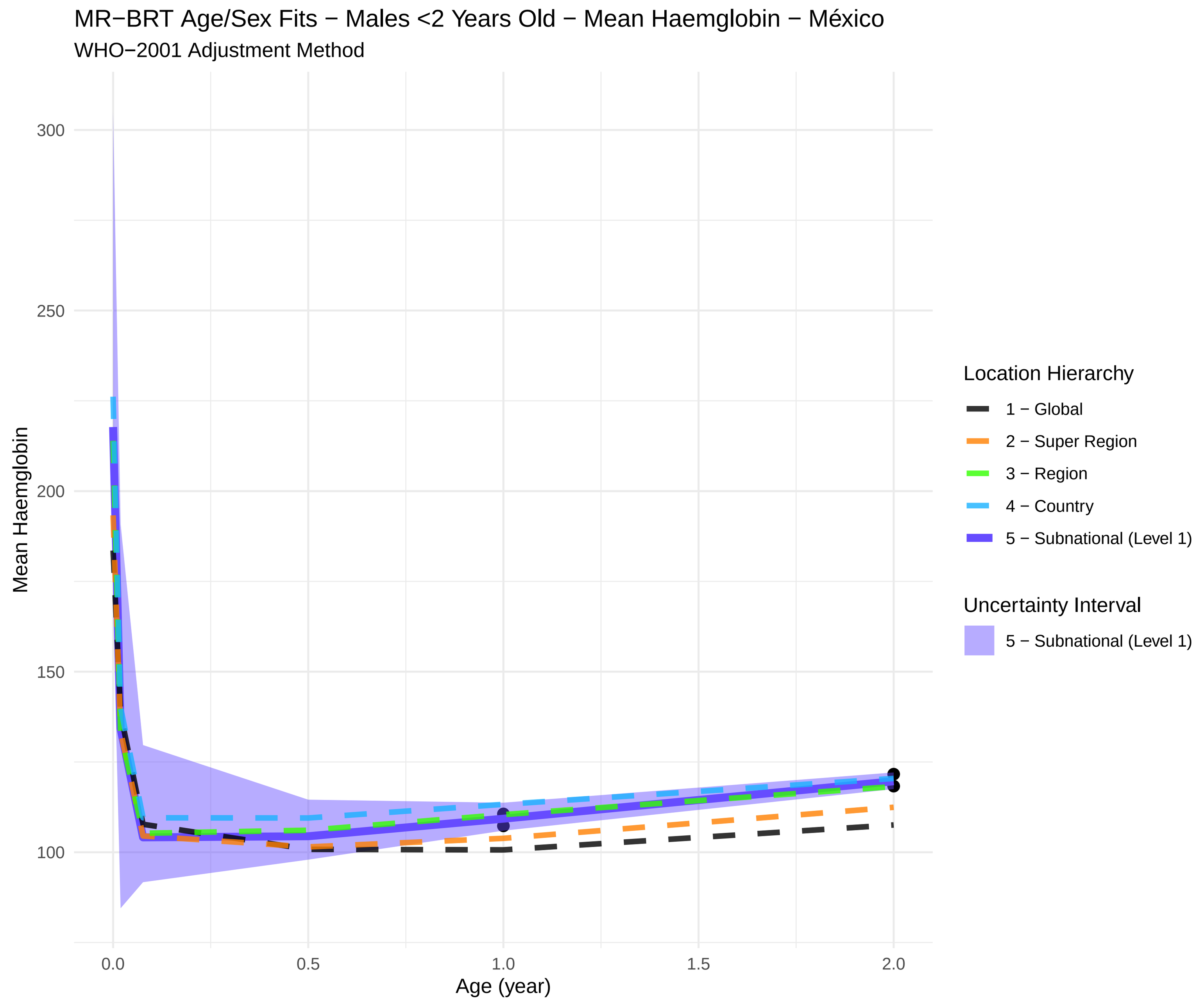
MR−BRT Age/Sex Fits − Males <2 Years Old − Mean Haemglobin − México
WHO−2001 Adjustment Method
Mean Haemglobin
100
150
200
250
300
0.0
0.5
1.0
1.5
2.0
Age (year)
Location Hierarchy
1 − Global
2 − Super Region
3 − Region
4 − Country
5 − Subnational (Level 1)
Uncertainty Interval
5 − Subnational (Level 1)

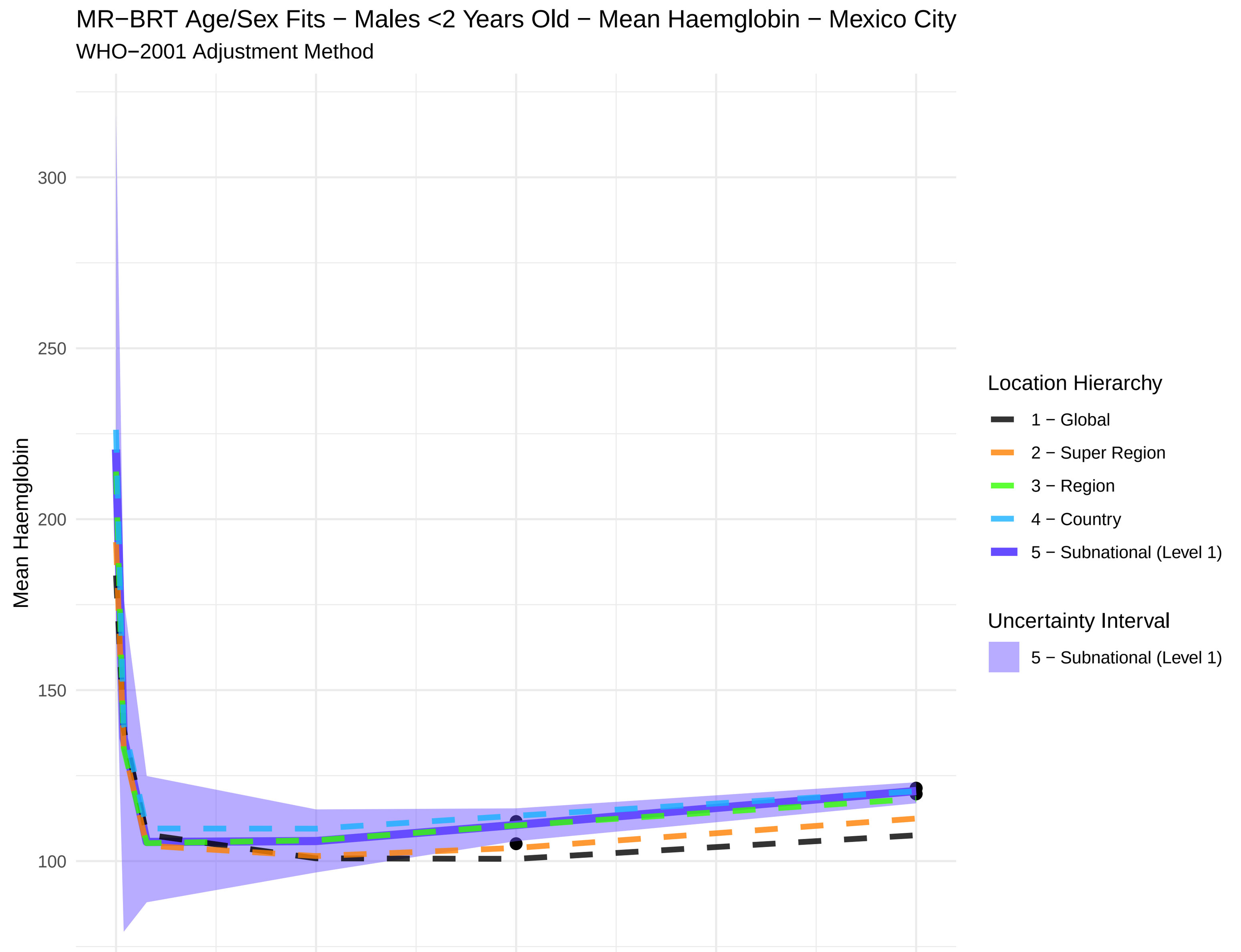
MR−BRT Age/Sex Fits − Males <2 Years Old − Mean Haemglobin − Mexico City
WHO−2001 Adjustment Method
Mean Haemglobin
100
150
200
250
300
0.0
0.5
1.0
1.5
2.0
Age (year)
Location Hierarchy
1 − Global
2 − Super Region
3 − Region
4 − Country
5 − Subnational (Level 1)
Uncertainty Interval
5 − Subnational (Level 1)

MR−BRT Age/Sex Fits − Males <2 Years Old − Mean Haemglobin − Michoacán de Ocampo
WHO−2001 Adjustment Method

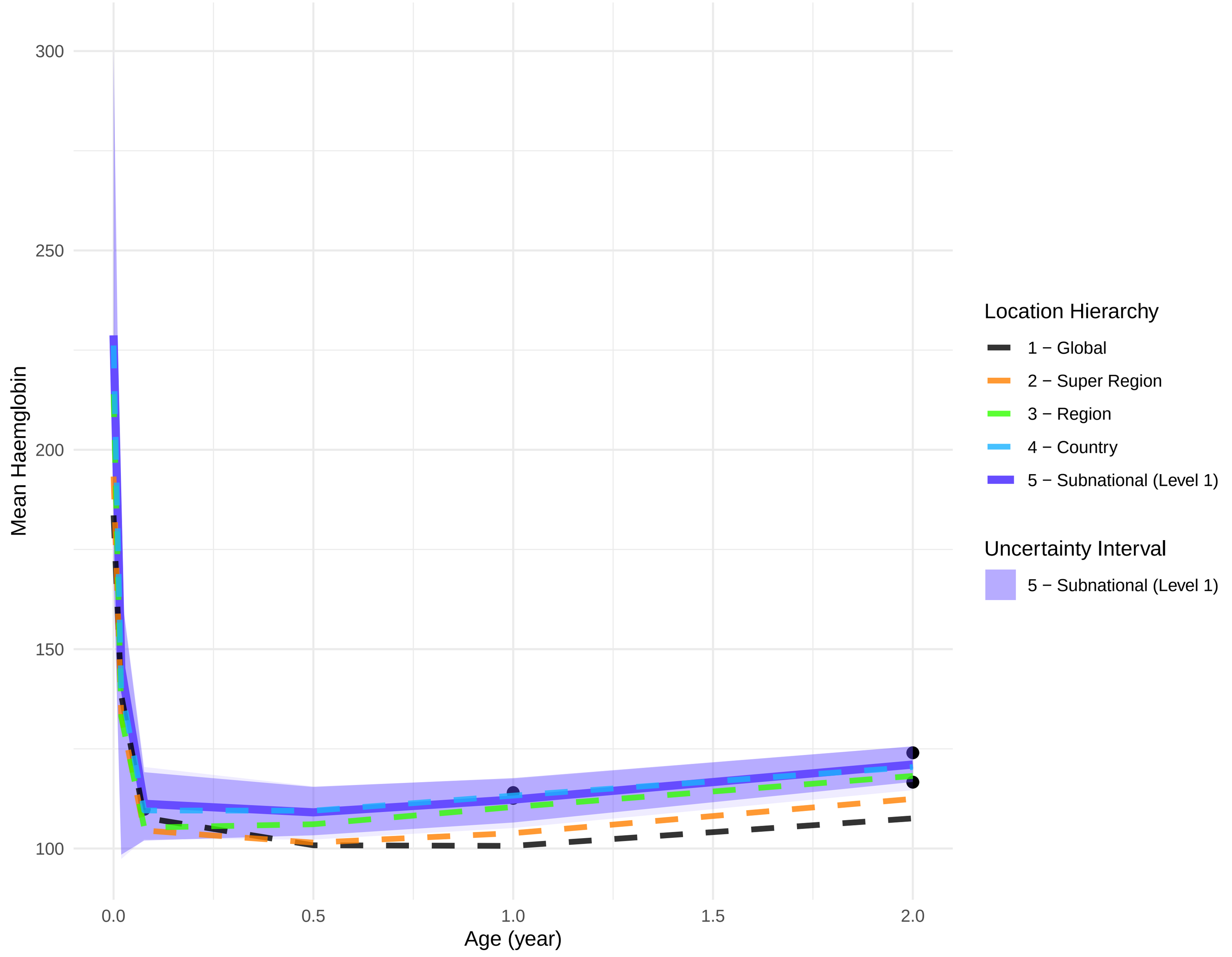

MR−BRT Age/Sex Fits − Males <2 Years Old − Mean Haemglobin − Morelos
WHO−2001 Adjustment Method

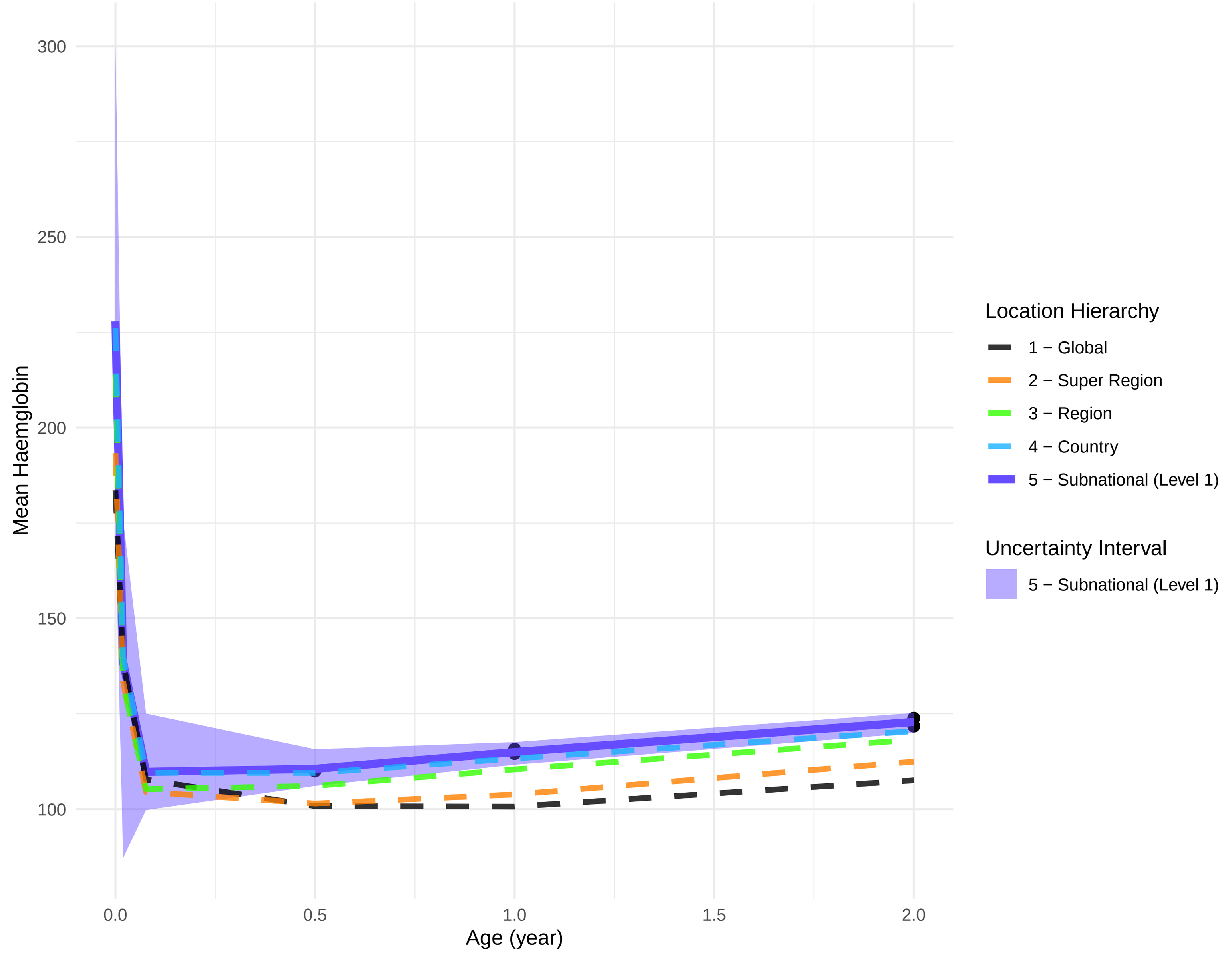

MR−BRT Age/Sex Fits − Males <2 Years Old − Mean Haemglobin − Nayarit
WHO−2001 Adjustment Method

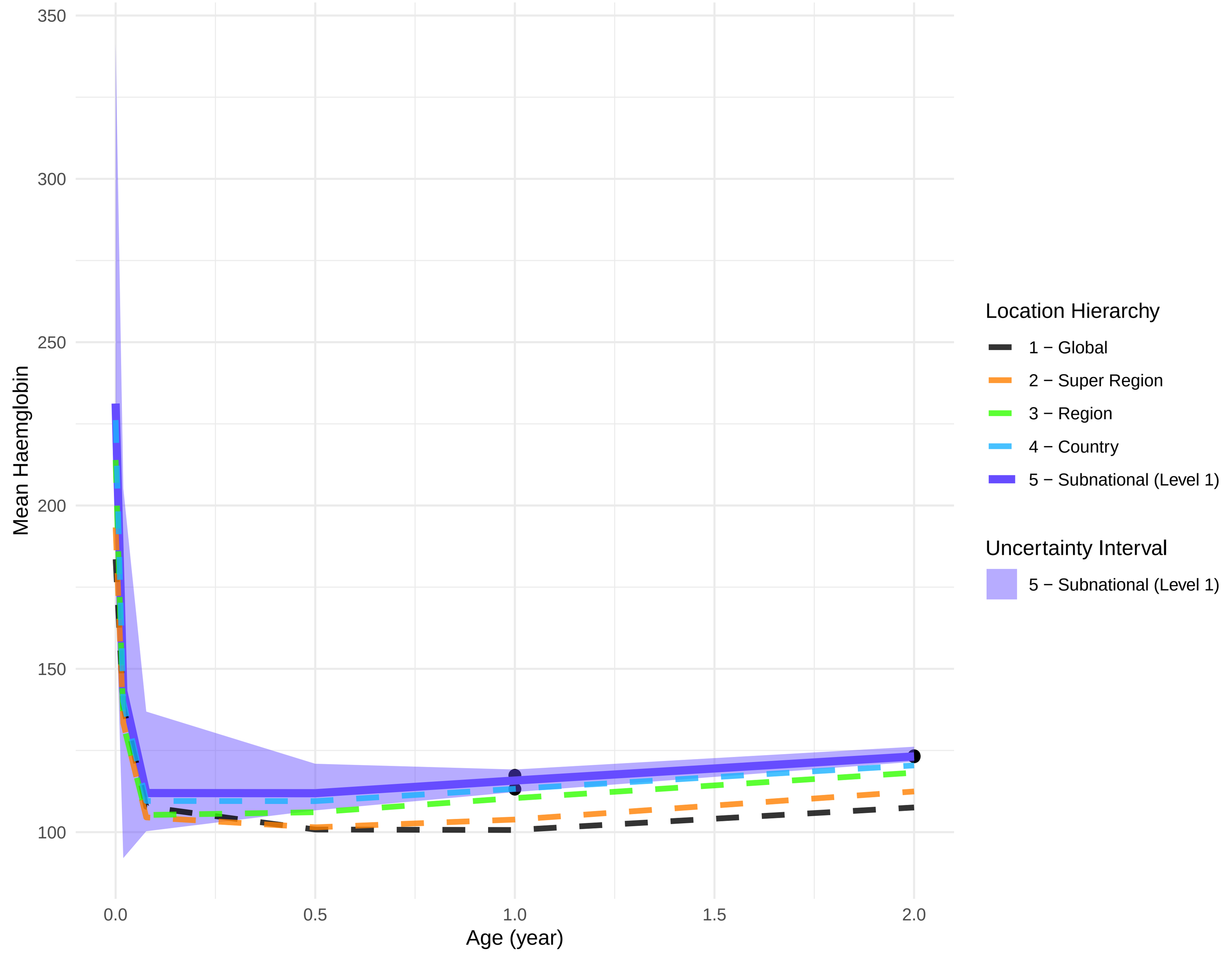

MR−BRT Age/Sex Fits − Males <2 Years Old − Mean Haemglobin − Nuevo León
WHO−2001 Adjustment Method

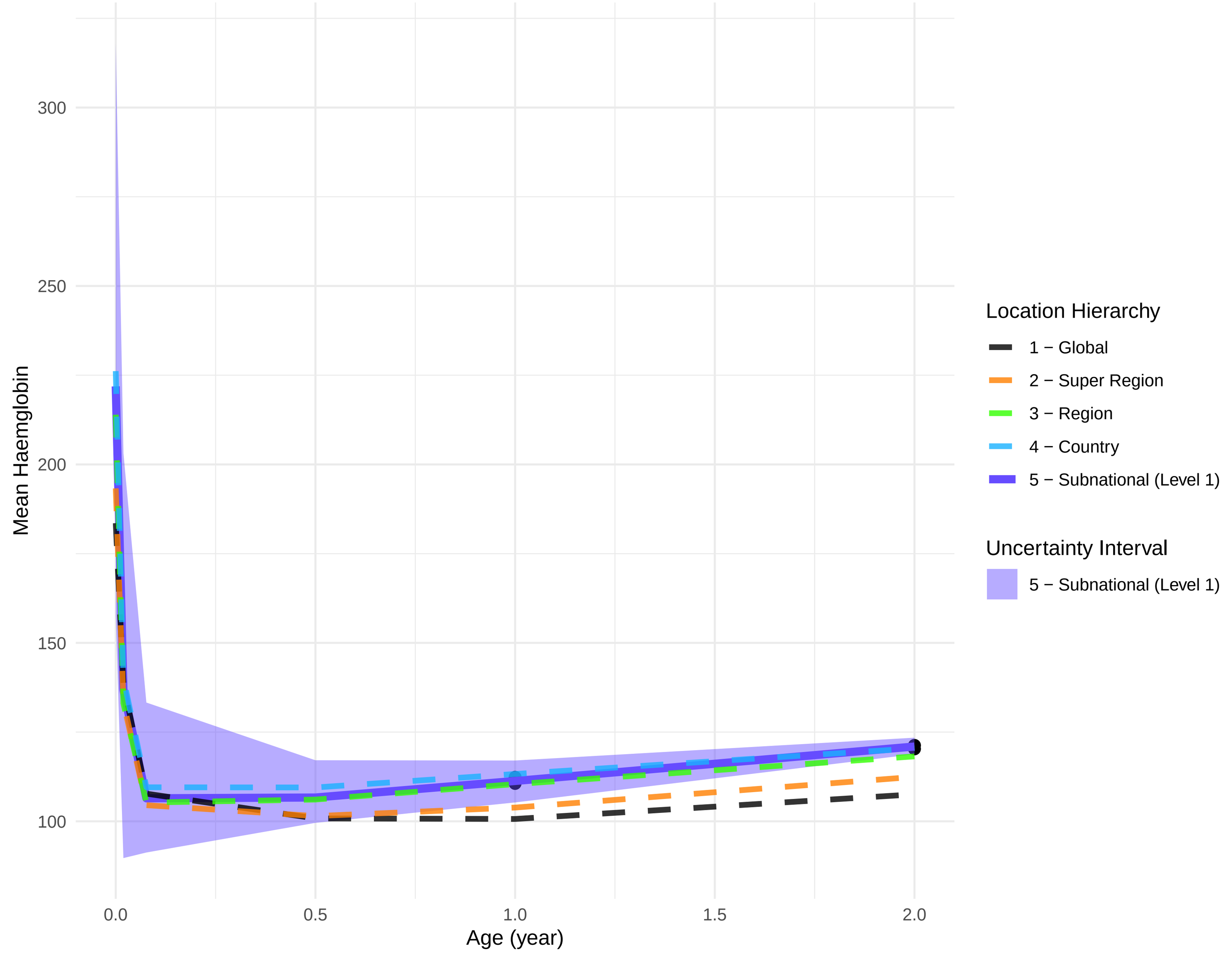

MR−BRT Age/Sex Fits − Males <2 Years Old − Mean Haemglobin − Oaxaca
WHO−2001 Adjustment Method

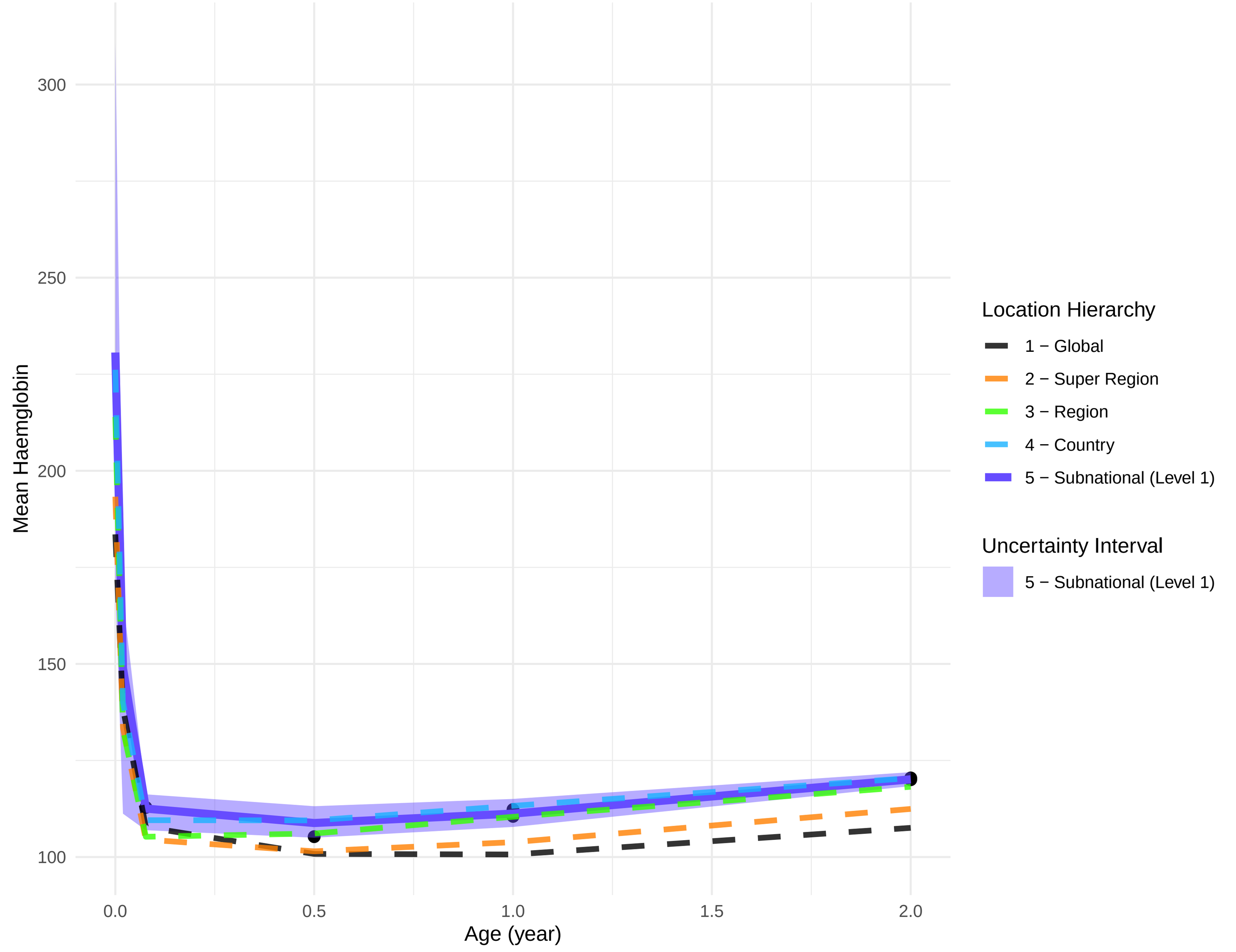

MR−BRT Age/Sex Fits − Males <2 Years Old − Mean Haemglobin − Puebla
WHO−2001 Adjustment Method

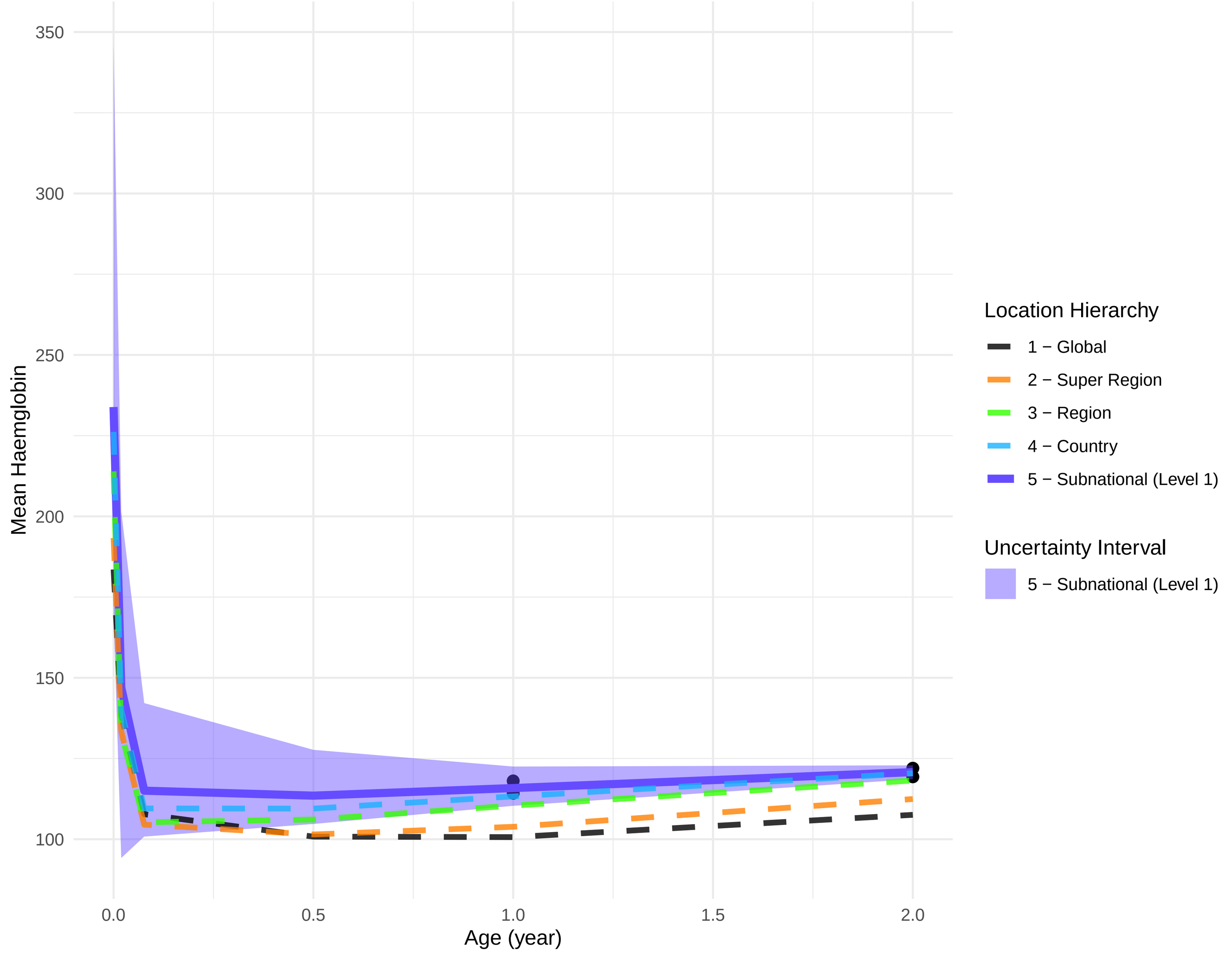

MR−BRT Age/Sex Fits − Males <2 Years Old − Mean Haemglobin − Querétaro
WHO−2001 Adjustment Method

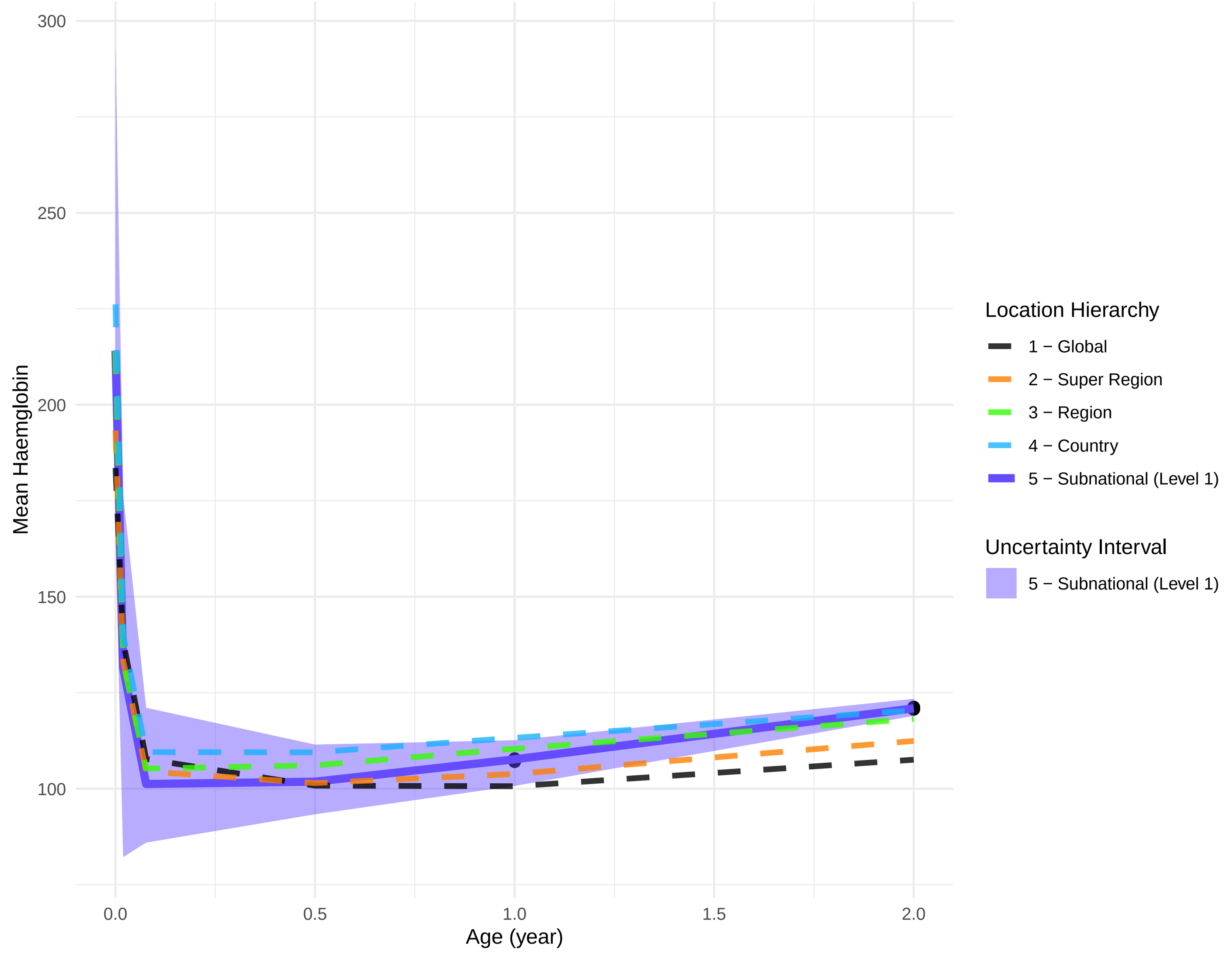

MR−BRT Age/Sex Fits − Males <2 Years Old − Mean Haemglobin − Quintana Roo
WHO−2001 Adjustment Method

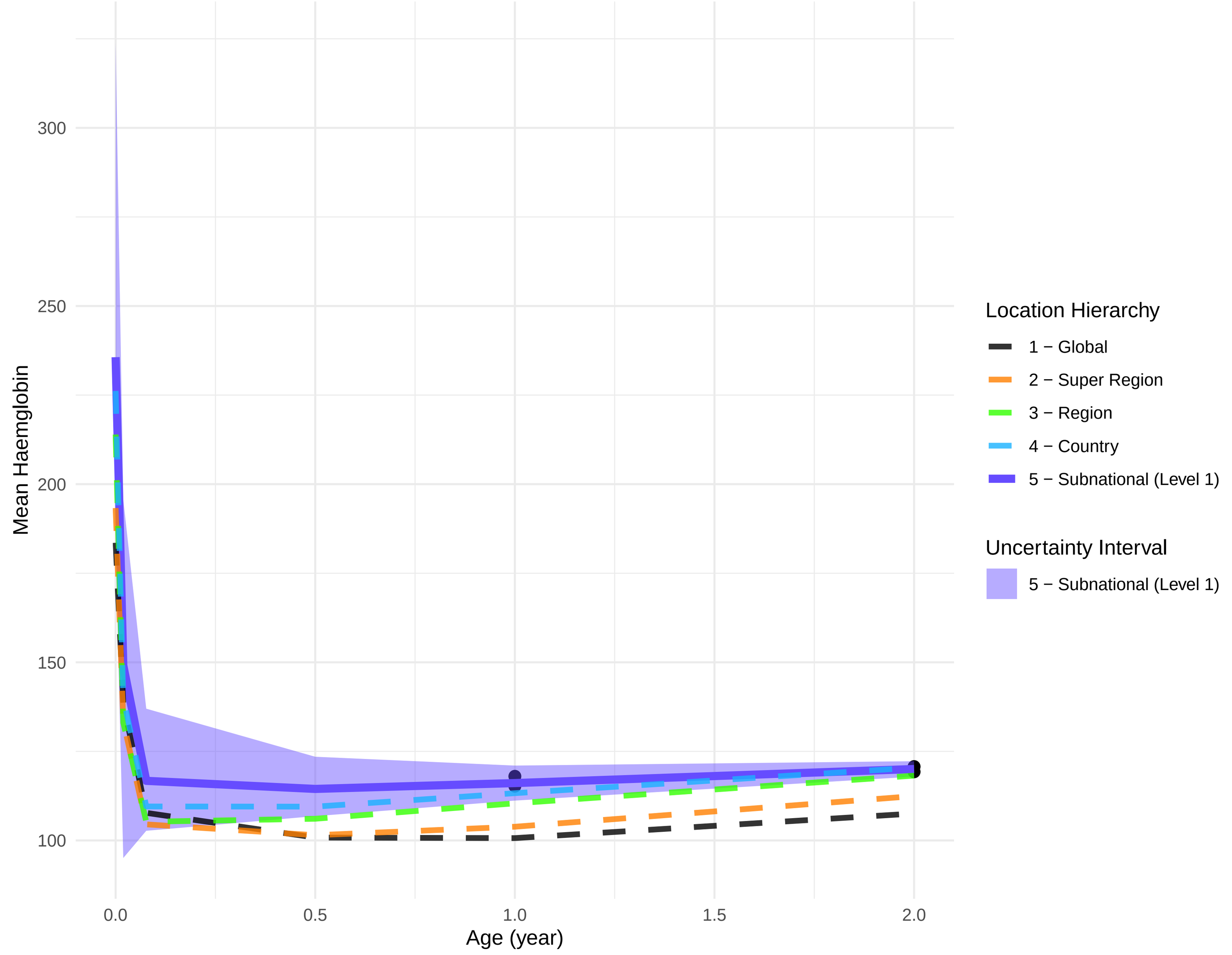

MR−BRT Age/Sex Fits − Males <2 Years Old − Mean Haemglobin − San Luis Potosí
WHO−2001 Adjustment Method

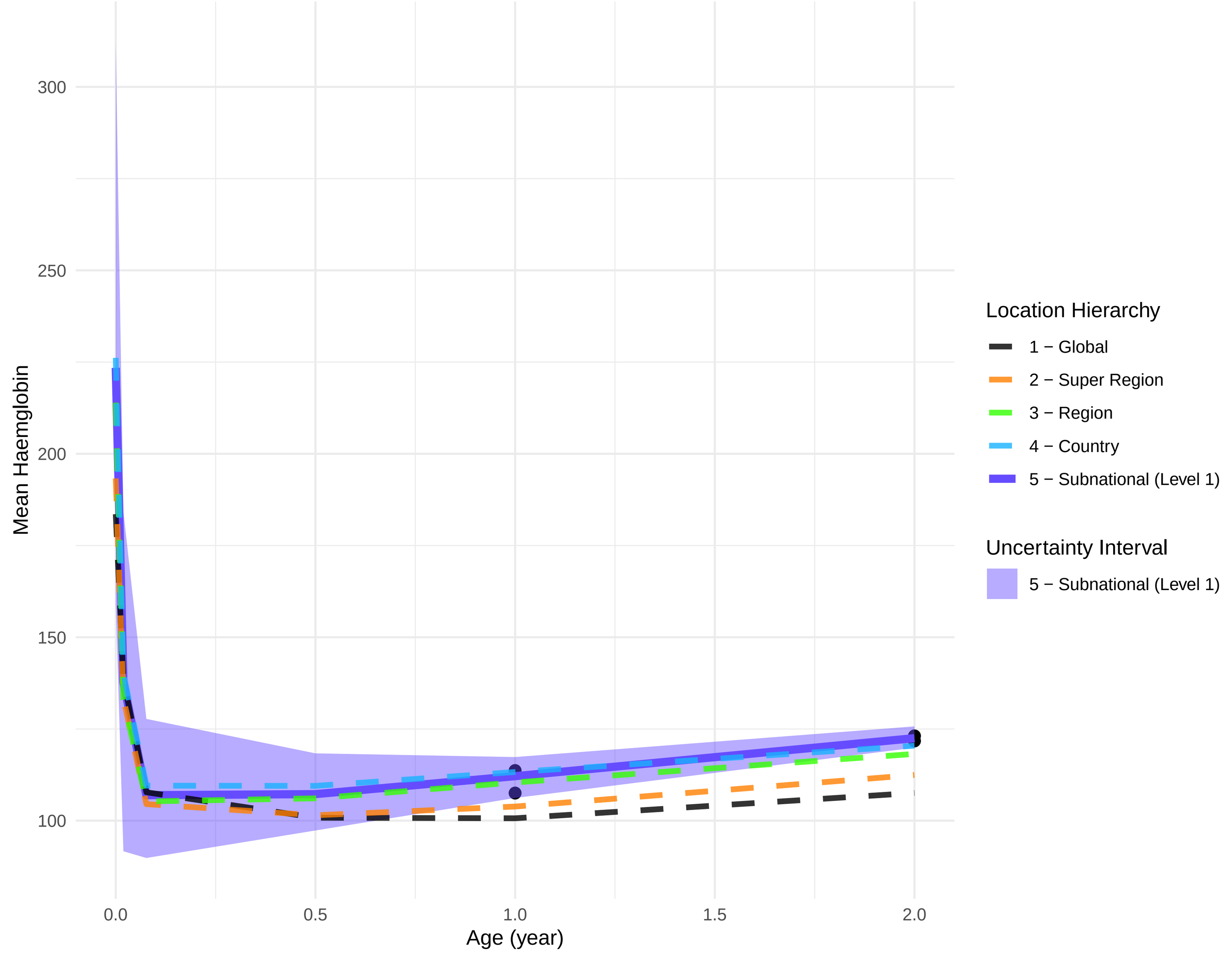

MR−BRT Age/Sex Fits − Males <2 Years Old − Mean Haemglobin − Sinaloa
WHO−2001 Adjustment Method

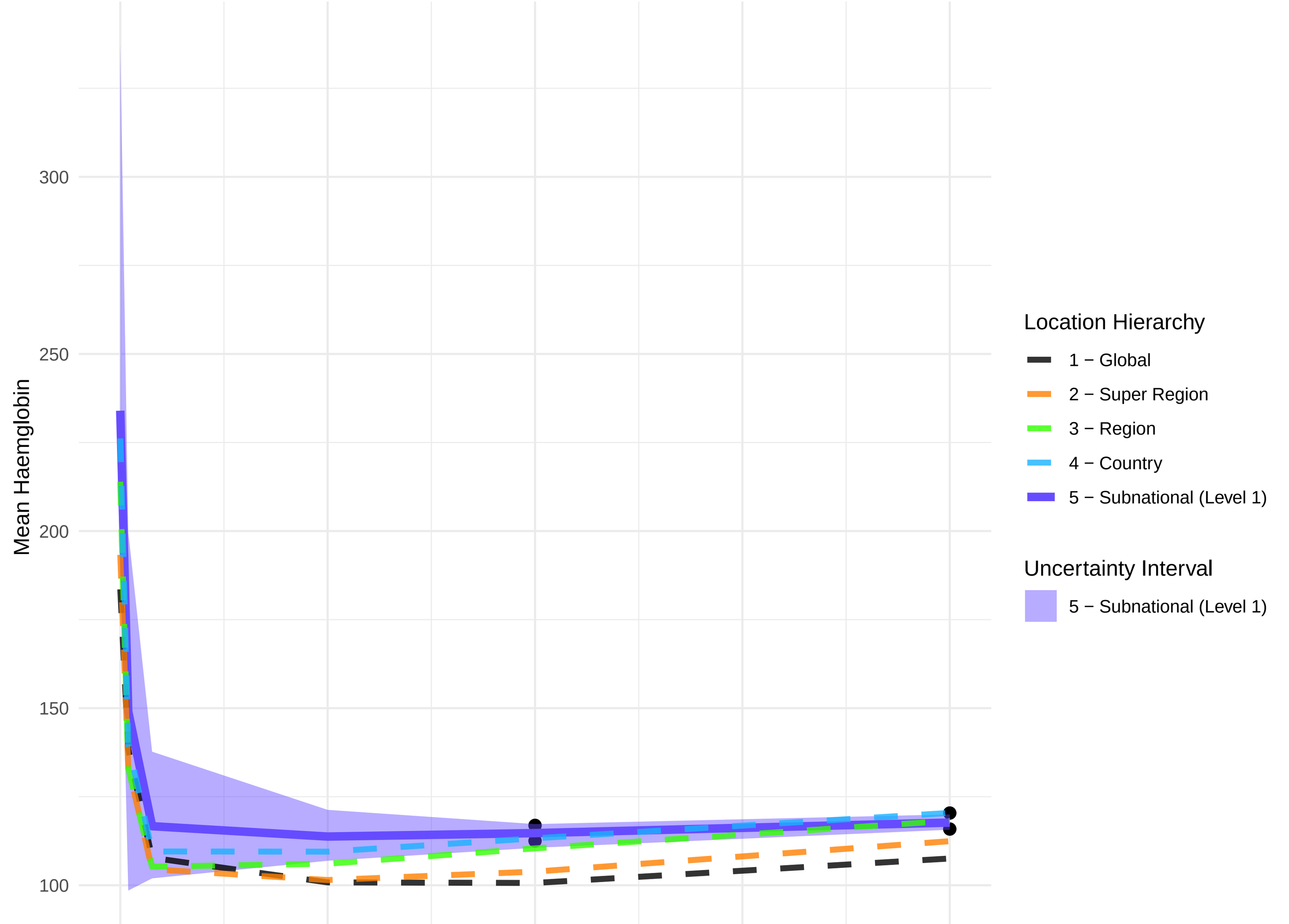

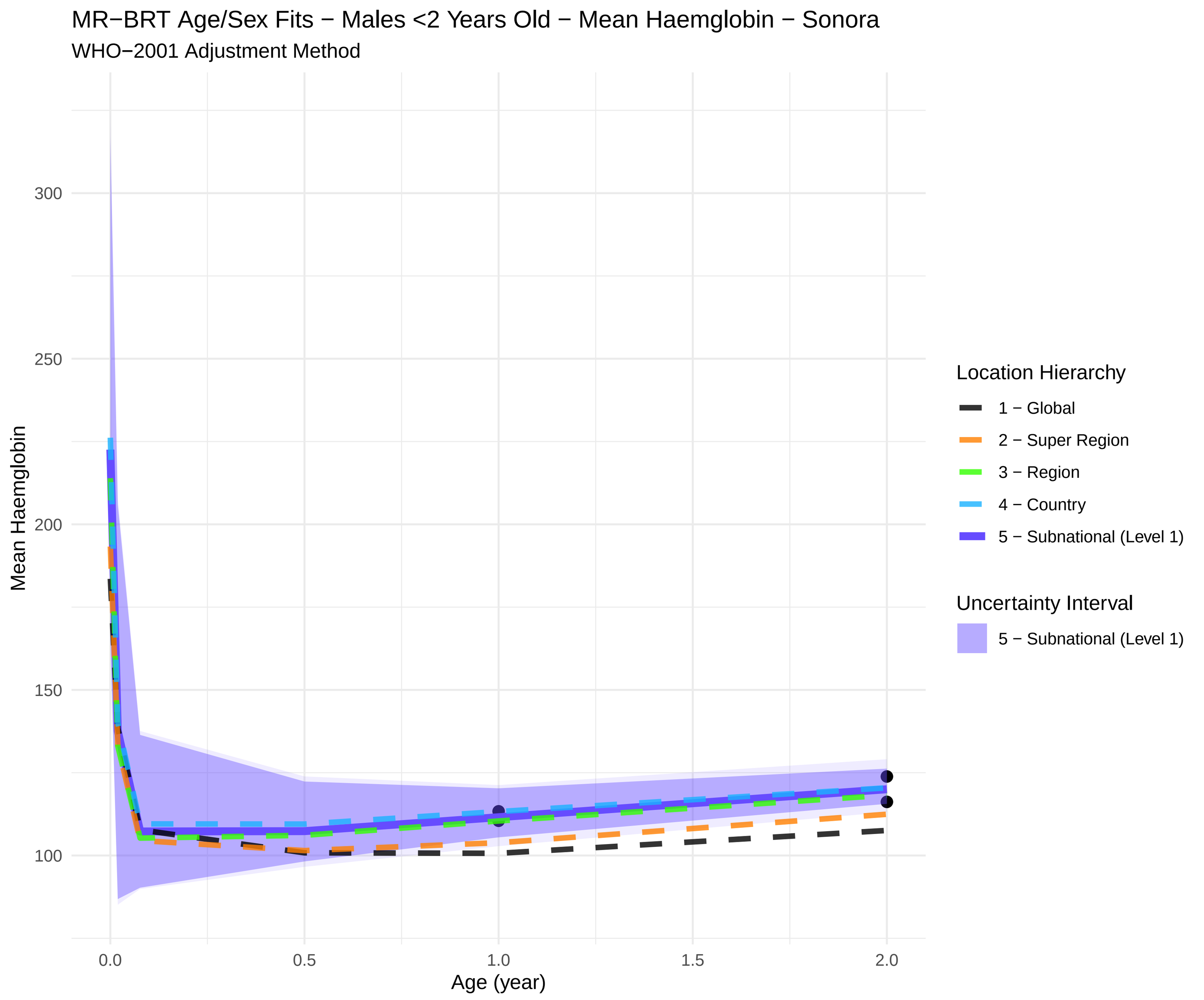
MR−BRT Age/Sex Fits − Males <2 Years Old − Mean Haemglobin − Sonora
WHO−2001 Adjustment Method
Mean Haemglobin
Age (year)
Location Hierarchy
1 − Global
2 − Super Region
3 − Region
4 − Country
5 − Subnational (Level 1)
Uncertainty Interval
5 − Subnational (Level 1)

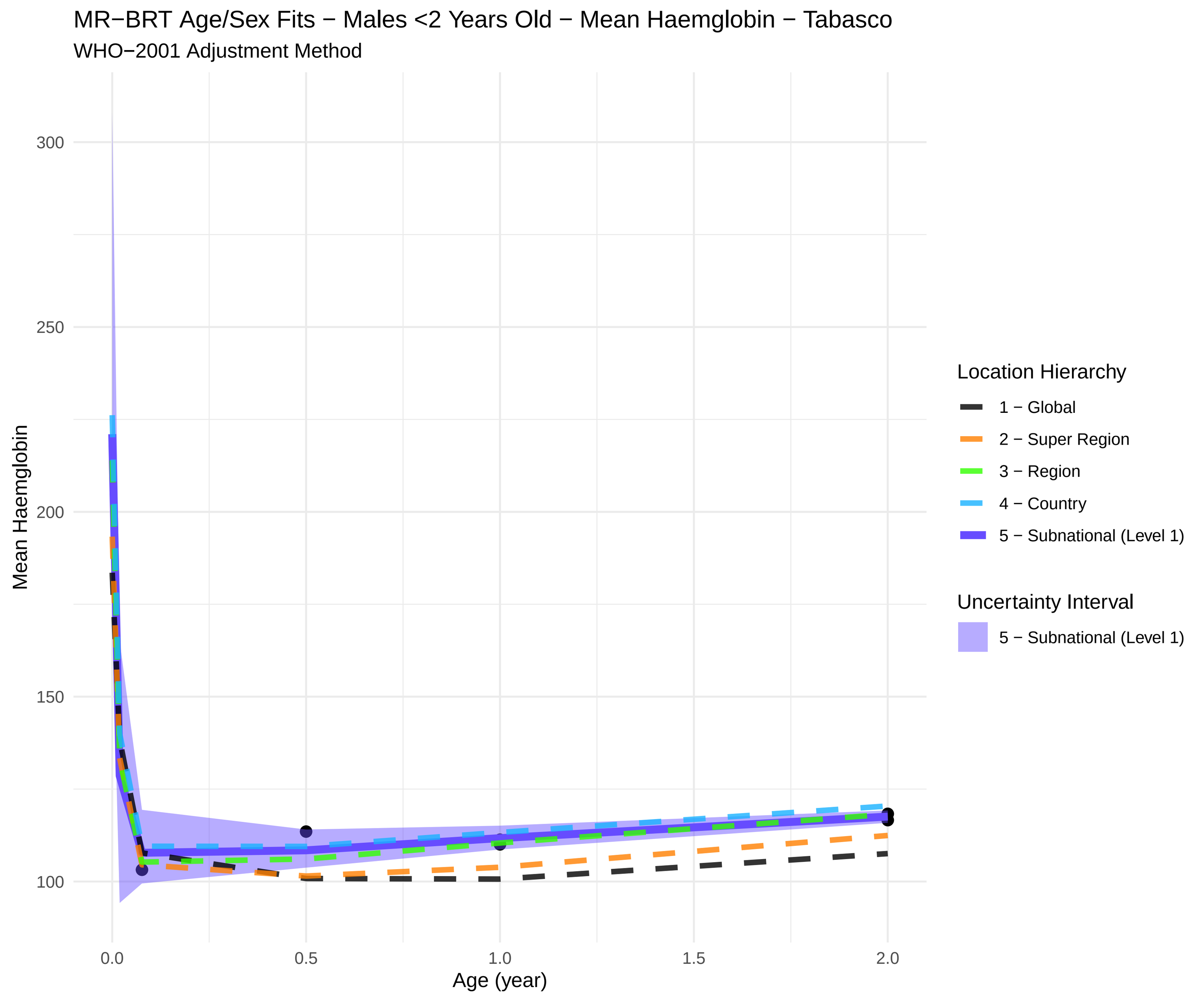
MR−BRT Age/Sex Fits − Males <2 Years Old − Mean Haemglobin − Tabasco
WHO−2001 Adjustment Method
Mean Haemglobin
Age (year)
Location Hierarchy
1 − Global
2 − Super Region
3 − Region
4 − Country
5 − Subnational (Level 1)
Uncertainty Interval
5 − Subnational (Level 1)

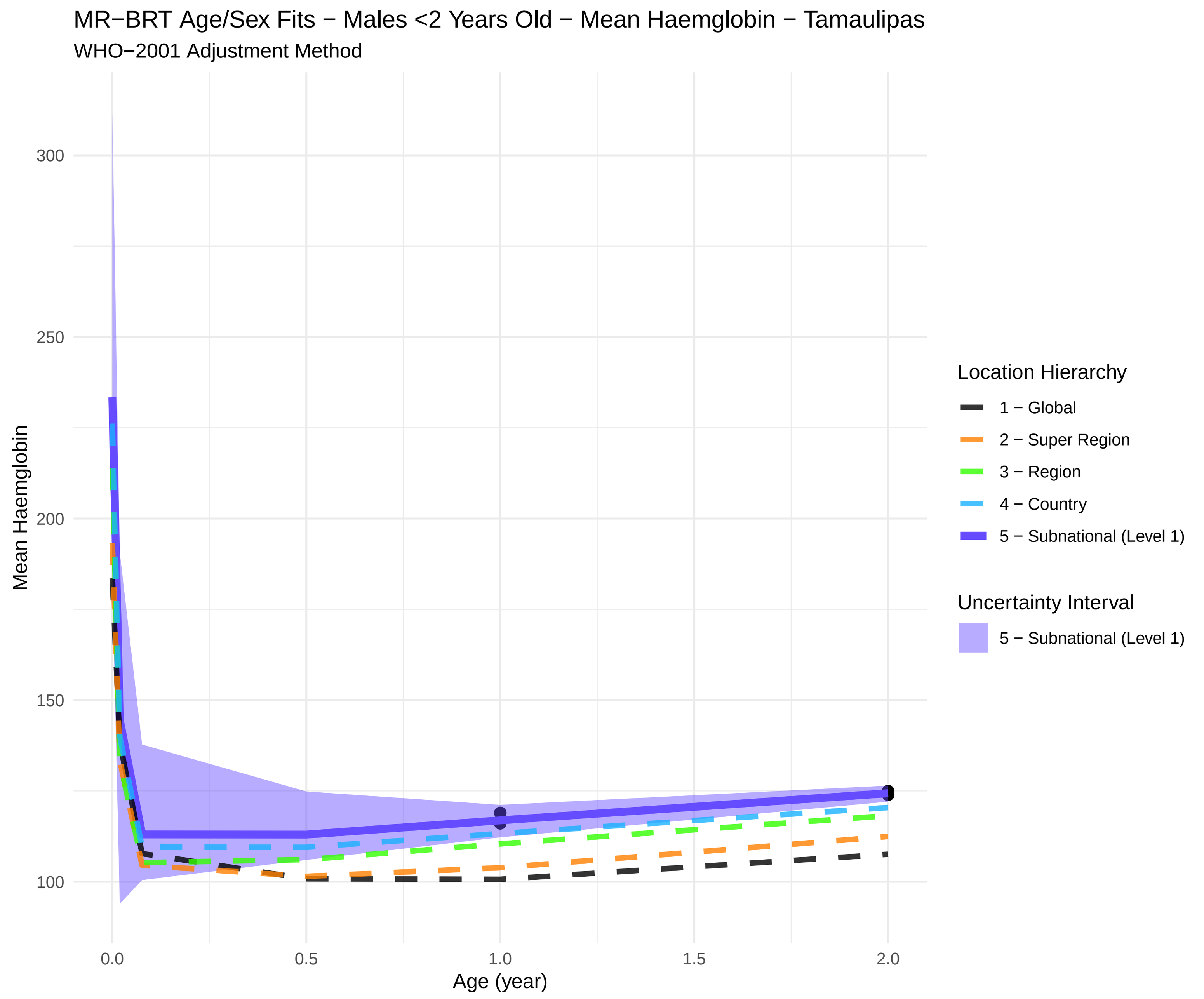
MR−BRT Age/Sex Fits − Males <2 Years Old − Mean Haemglobin − Tamaulipas
WHO−2001 Adjustment Method
Mean Haemglobin
Age (year)
Location Hierarchy
1 − Global
2 − Super Region
3 − Region
4 − Country
5 − Subnational (Level 1)
Uncertainty Interval
5 − Subnational (Level 1)

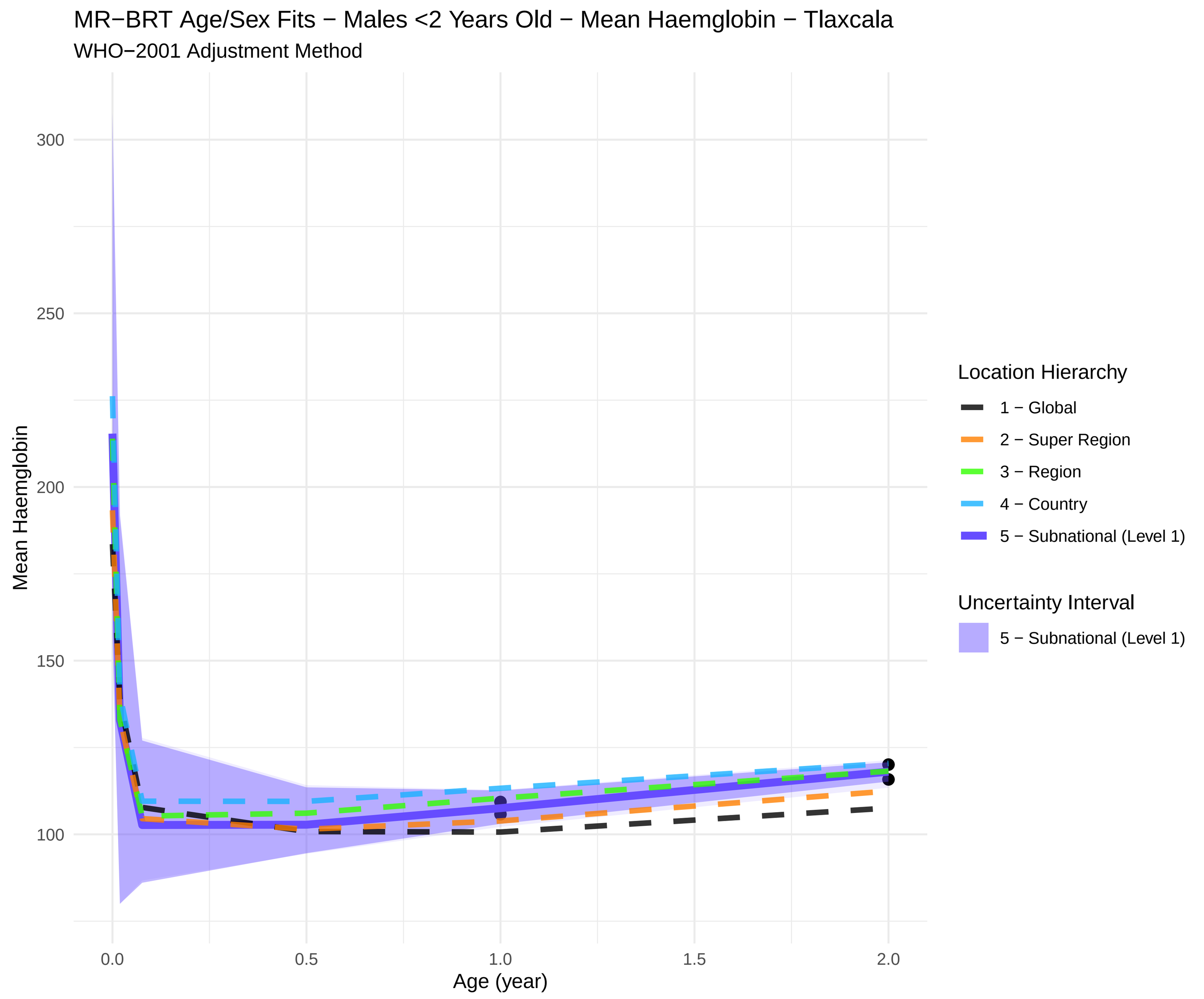
MR−BRT Age/Sex Fits − Males <2 Years Old − Mean Haemglobin − Tlaxcala
WHO−2001 Adjustment Method
Mean Haemglobin
Age (year)
Location Hierarchy
1 − Global
2 − Super Region
3 − Region
4 − Country
5 − Subnational (Level 1)
Uncertainty Interval
5 − Subnational (Level 1)

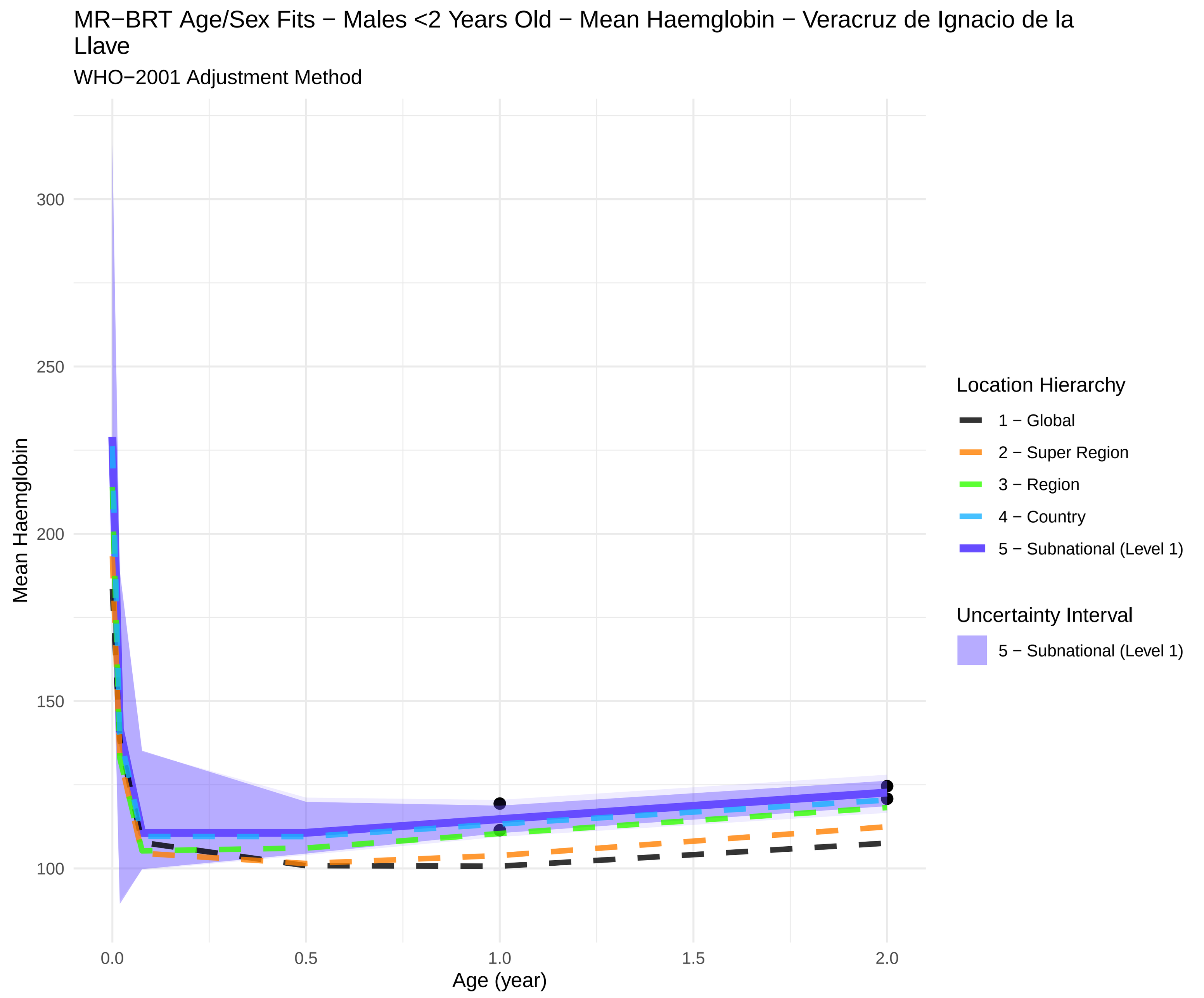
MR−BRT Age/Sex Fits − Males <2 Years Old − Mean Haemglobin − Veracruz de Ignacio de la Llave
WHO−2001 Adjustment Method
Mean Haemglobin
Age (year)
Location Hierarchy
1 − Global
2 − Super Region
3 − Region
4 − Country
5 − Subnational (Level 1)
Uncertainty Interval
5 − Subnational (Level 1)

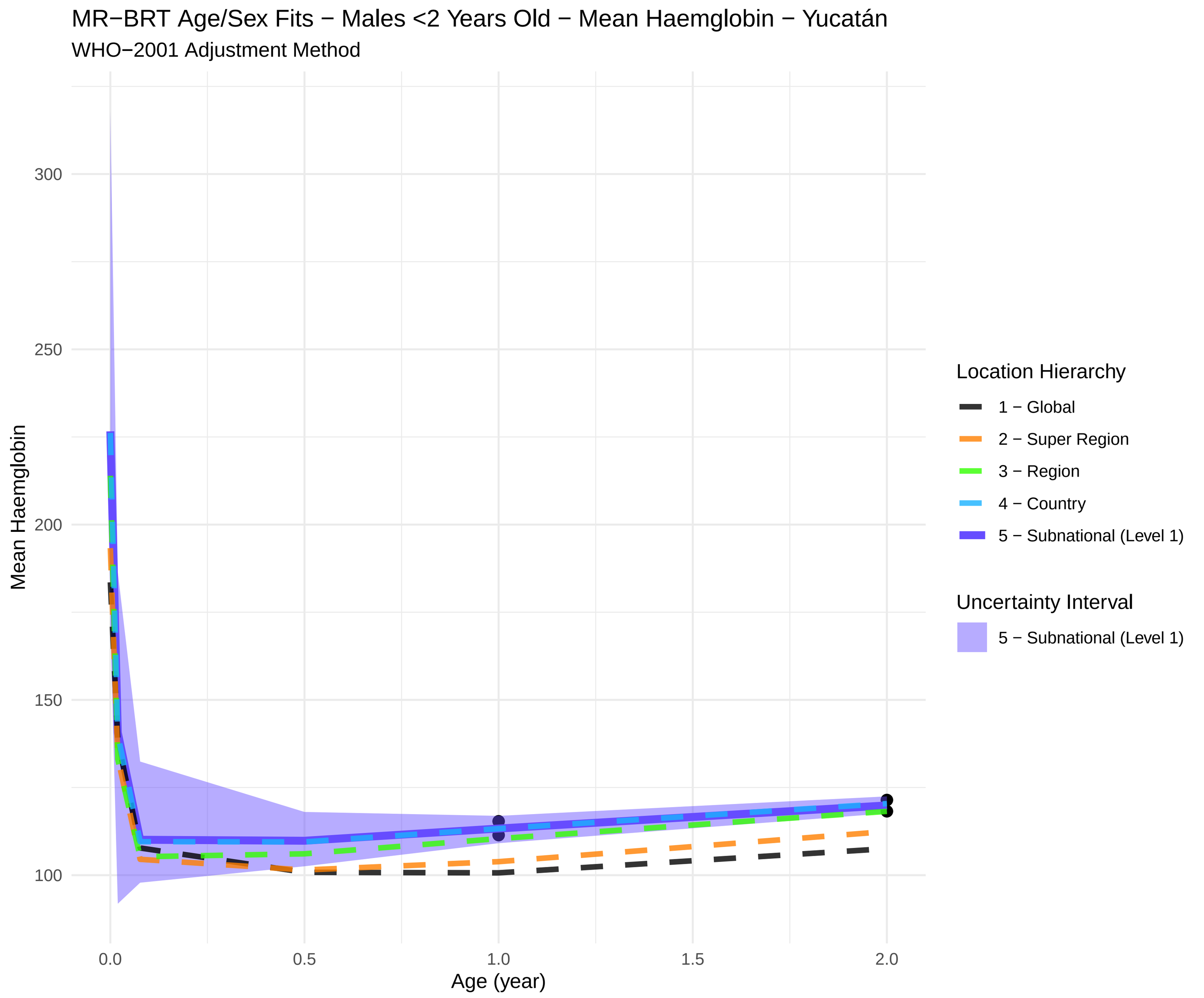
MR−BRT Age/Sex Fits − Males <2 Years Old − Mean Haemglobin − Yucatán
WHO−2001 Adjustment Method
Mean Haemglobin
Age (year)
Location Hierarchy
1 − Global
2 − Super Region
3 − Region
4 − Country
5 − Subnational (Level 1)
Uncertainty Interval
5 − Subnational (Level 1)

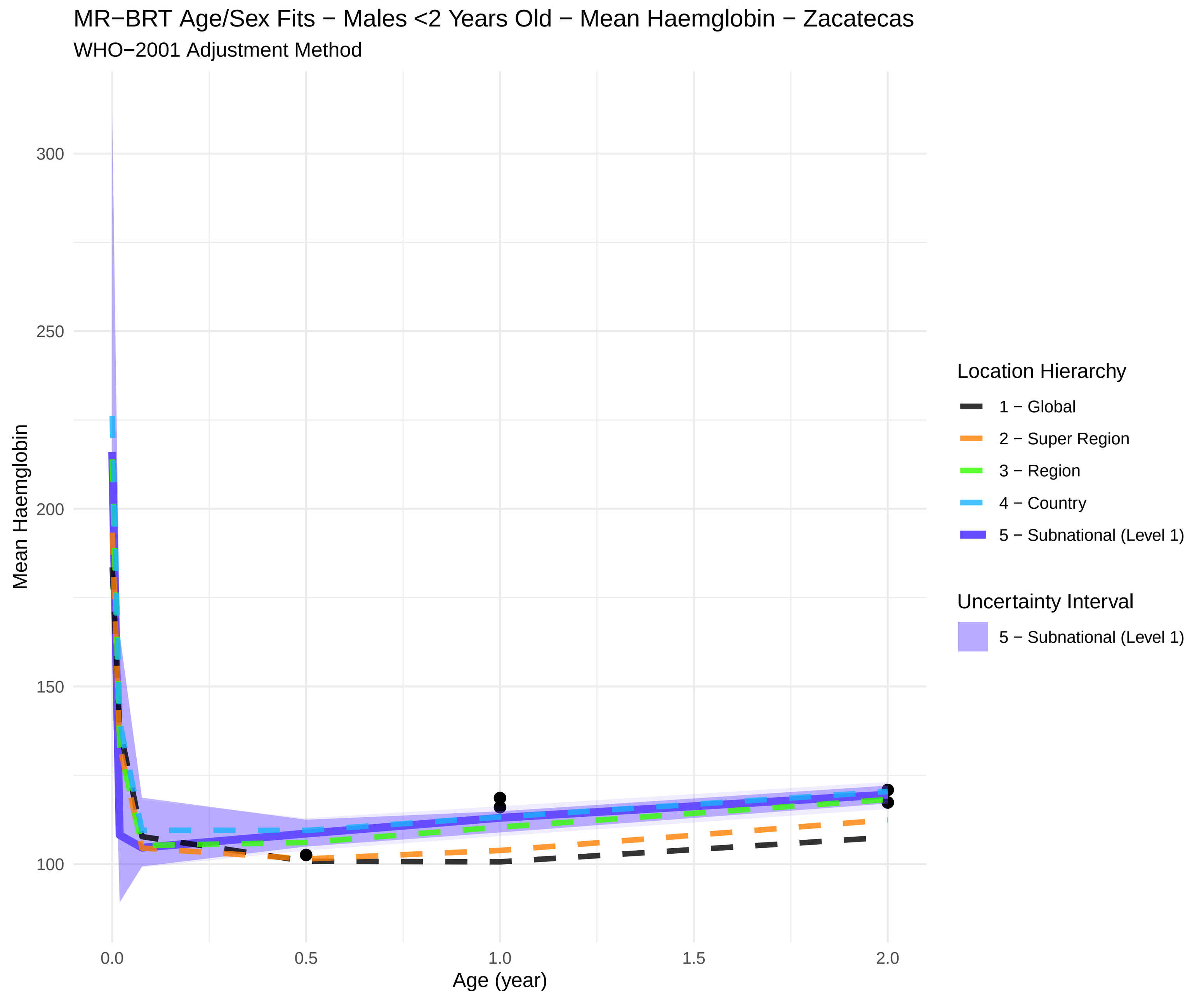
MR−BRT Age/Sex Fits − Males <2 Years Old − Mean Haemglobin − Zacatecas
WHO−2001 Adjustment Method
Mean Haemglobin
Age (year)
Location Hierarchy
1 − Global
2 − Super Region
3 − Region
4 − Country
5 − Subnational (Level 1)
Uncertainty Interval
5 − Subnational (Level 1)

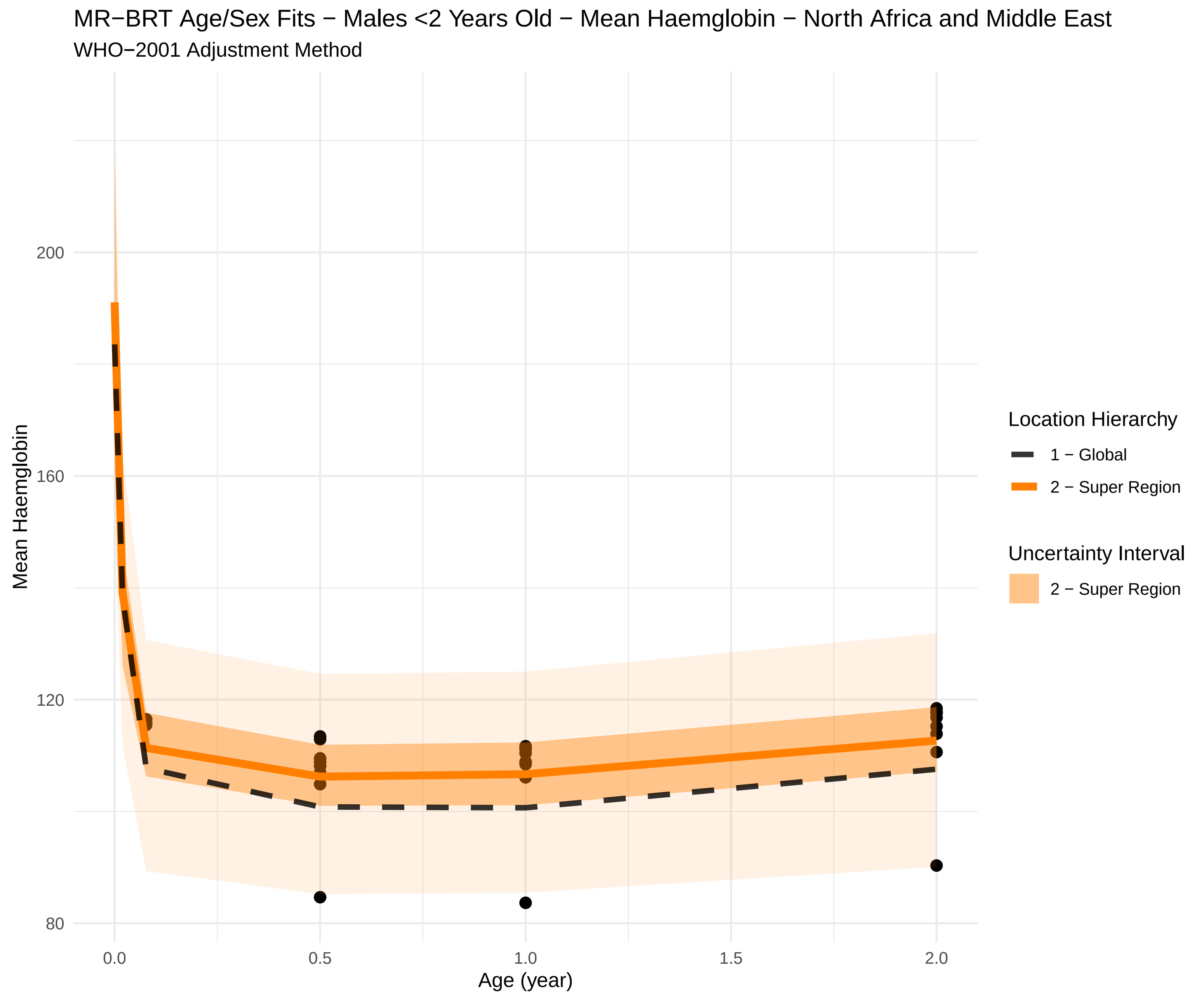
MR−BRT Age/Sex Fits − Males <2 Years Old − Mean Haemglobin − North Africa and Middle East
WHO−2001 Adjustment Method
Mean Haemglobin
Age (year)
Location Hierarchy
1 − Global
2 − Super Region
Uncertainty Interval
2 − Super Region

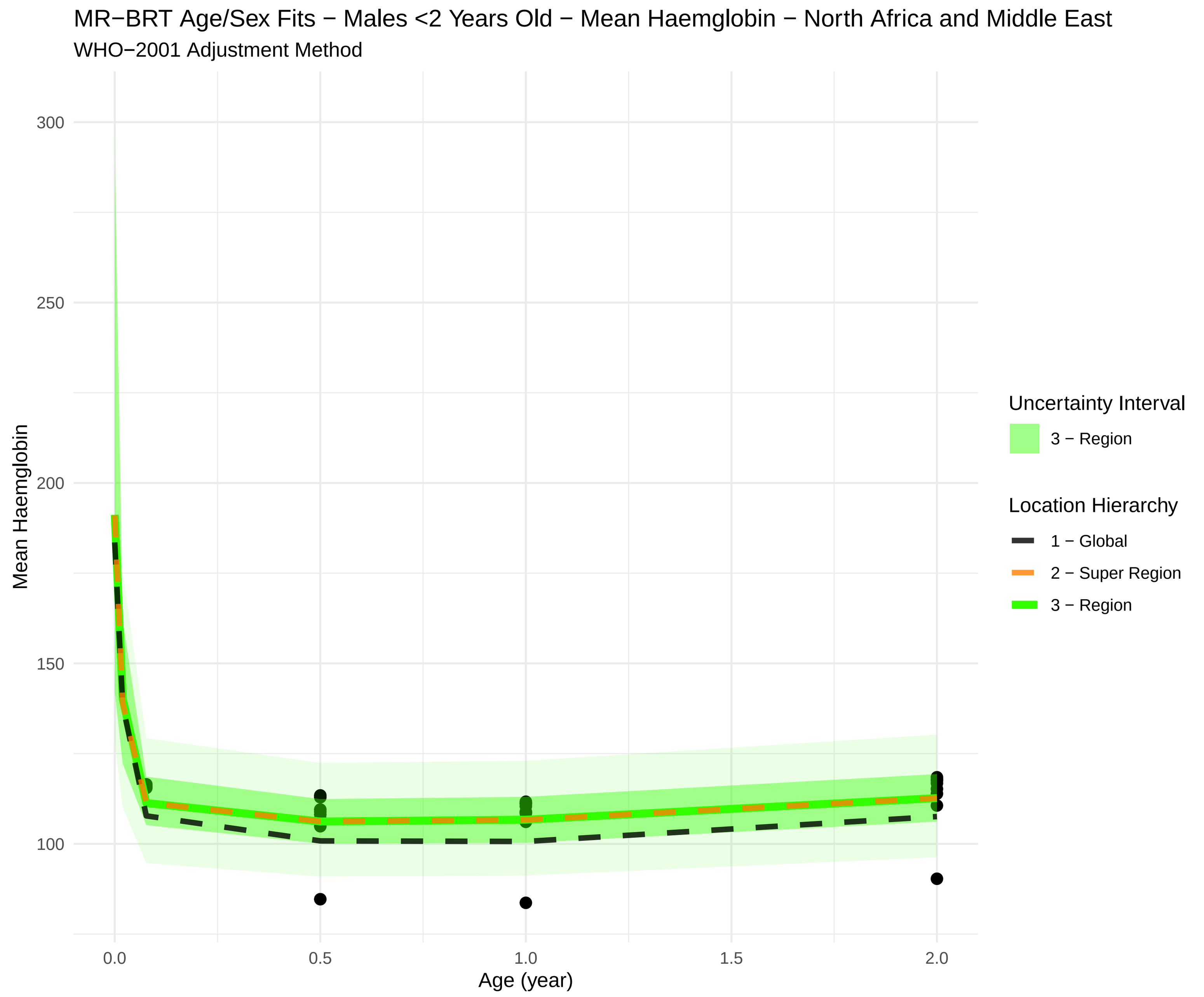
MR−BRT Age/Sex Fits − Males <2 Years Old − Mean Haemglobin − North Africa and Middle East
WHO−2001 Adjustment Method
Mean Haemglobin
Age (year)
Uncertainty Interval
3 − Region
Location Hierarchy
1 − Global
2 − Super Region
3 − Region

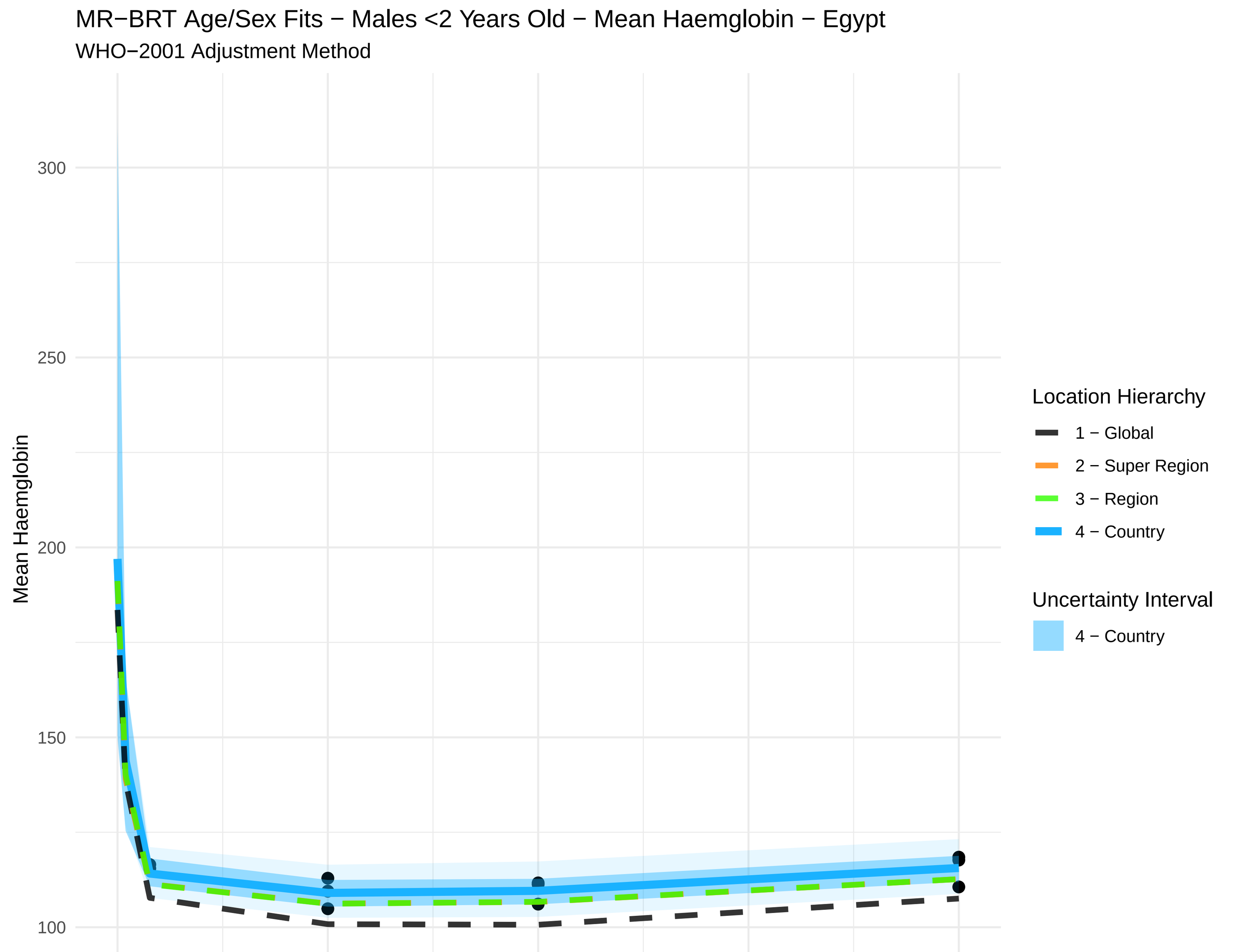
MR−BRT Age/Sex Fits − Males <2 Years Old − Mean Haemglobin − Egypt
WHO−2001 Adjustment Method
Mean Haemglobin
Age (year)
Location Hierarchy
1 − Global
2 − Super Region
3 − Region
4 − Country
Uncertainty Interval
4 − Country

MR−BRT Age/Sex Fits − Males <2 Years Old − Mean Haemoglobin − Jordan
WHO−2001 Adjustment Method

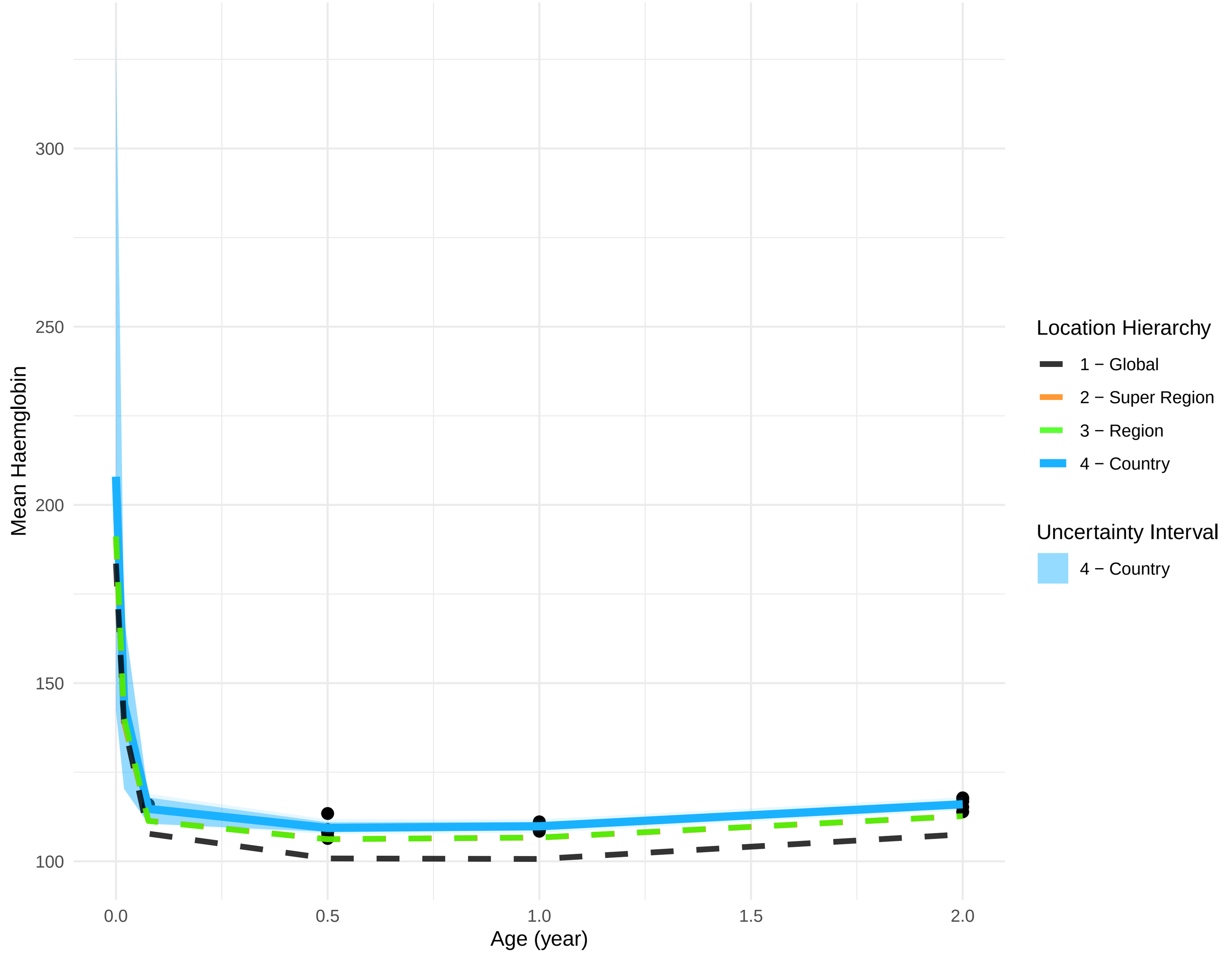

MR−BRT Age/Sex Fits − Males <2 Years Old − Mean Haemoglobin − Yemen
WHO−2001 Adjustment Method

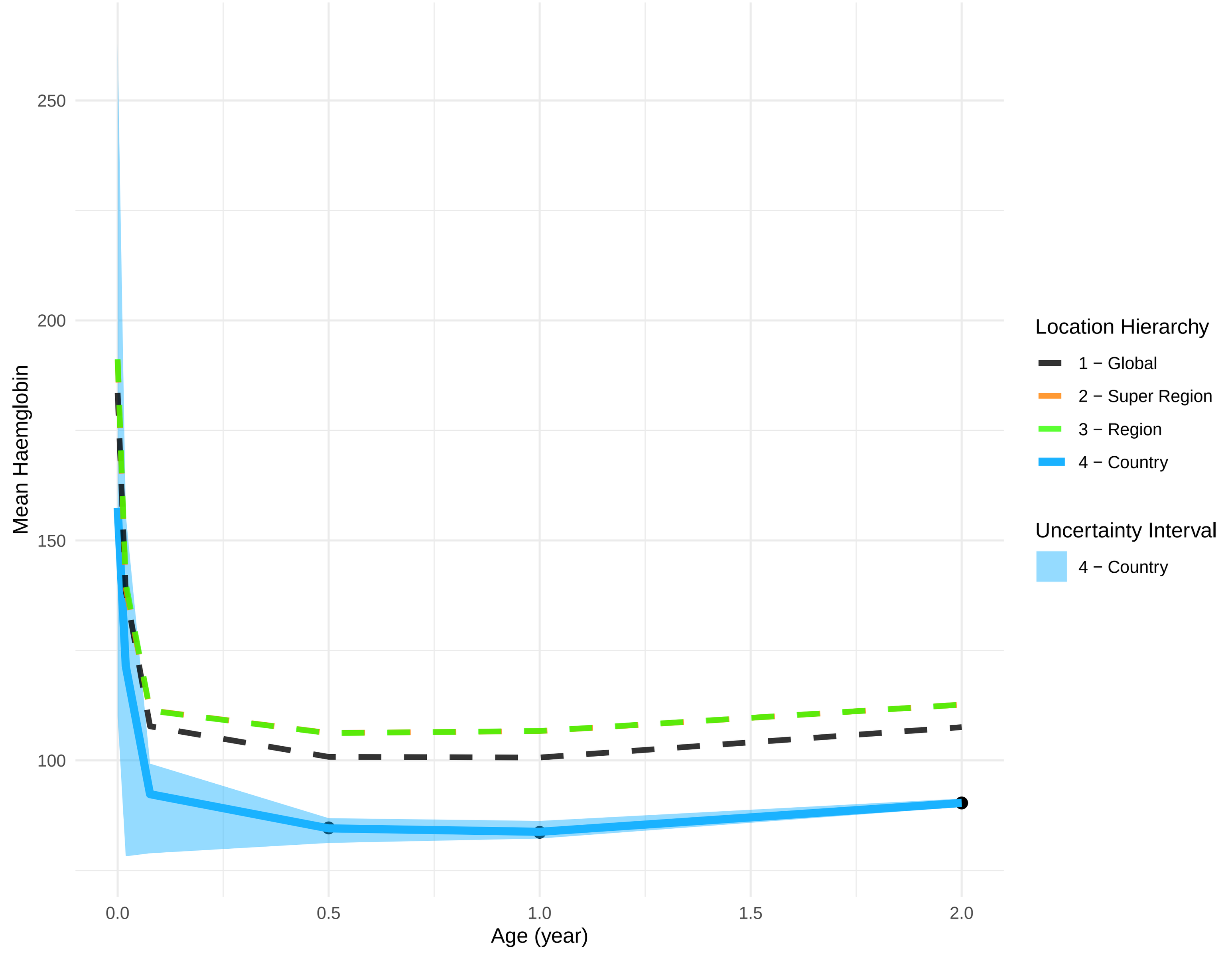

MR−BRT Age/Sex Fits − Males <2 Years Old − Mean Haemoglobin − South Asia
WHO−2001 Adjustment Method

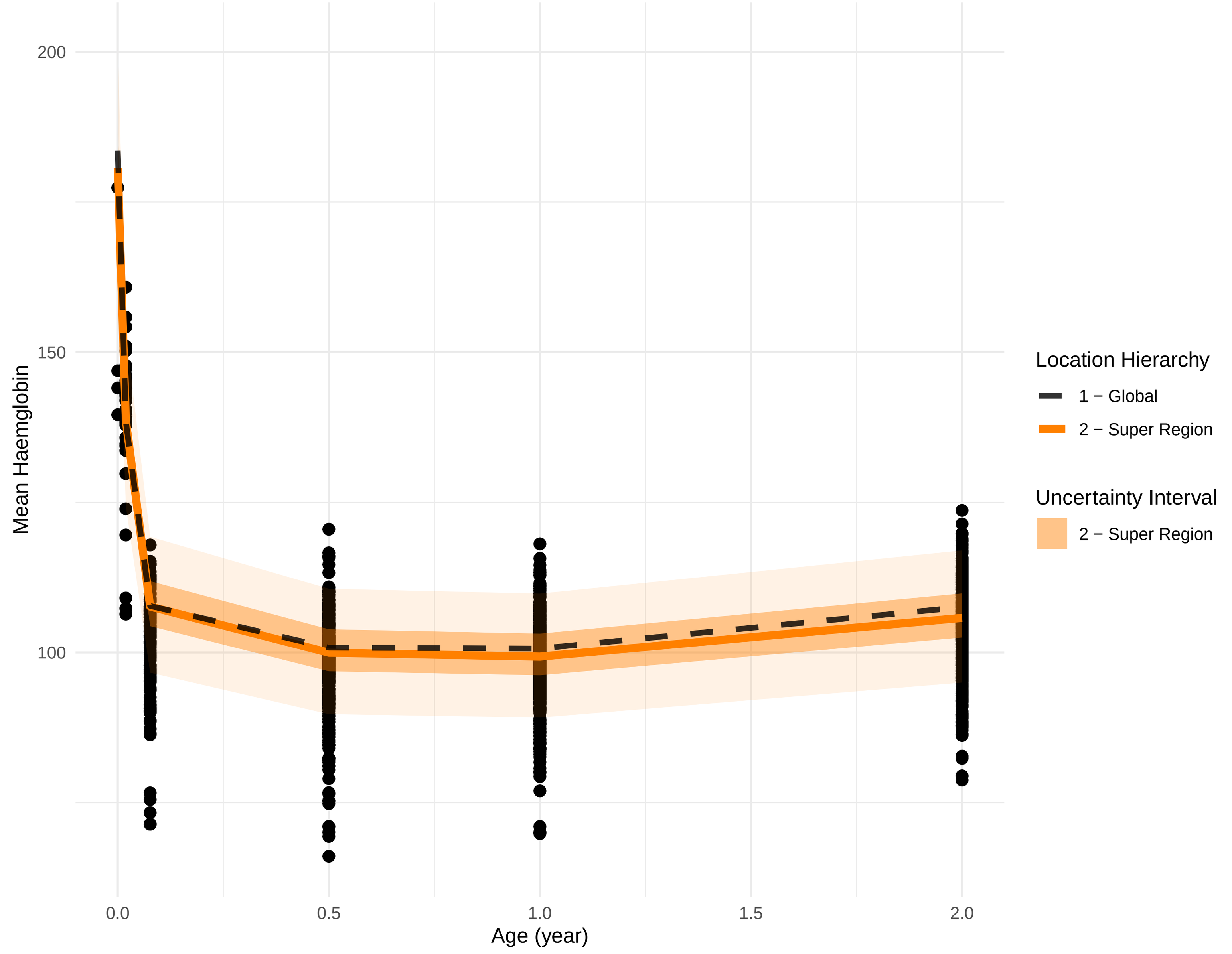

MR−BRT Age/Sex Fits − Males <2 Years Old − Mean Haemoglobin − South Asia
WHO−2001 Adjustment Method

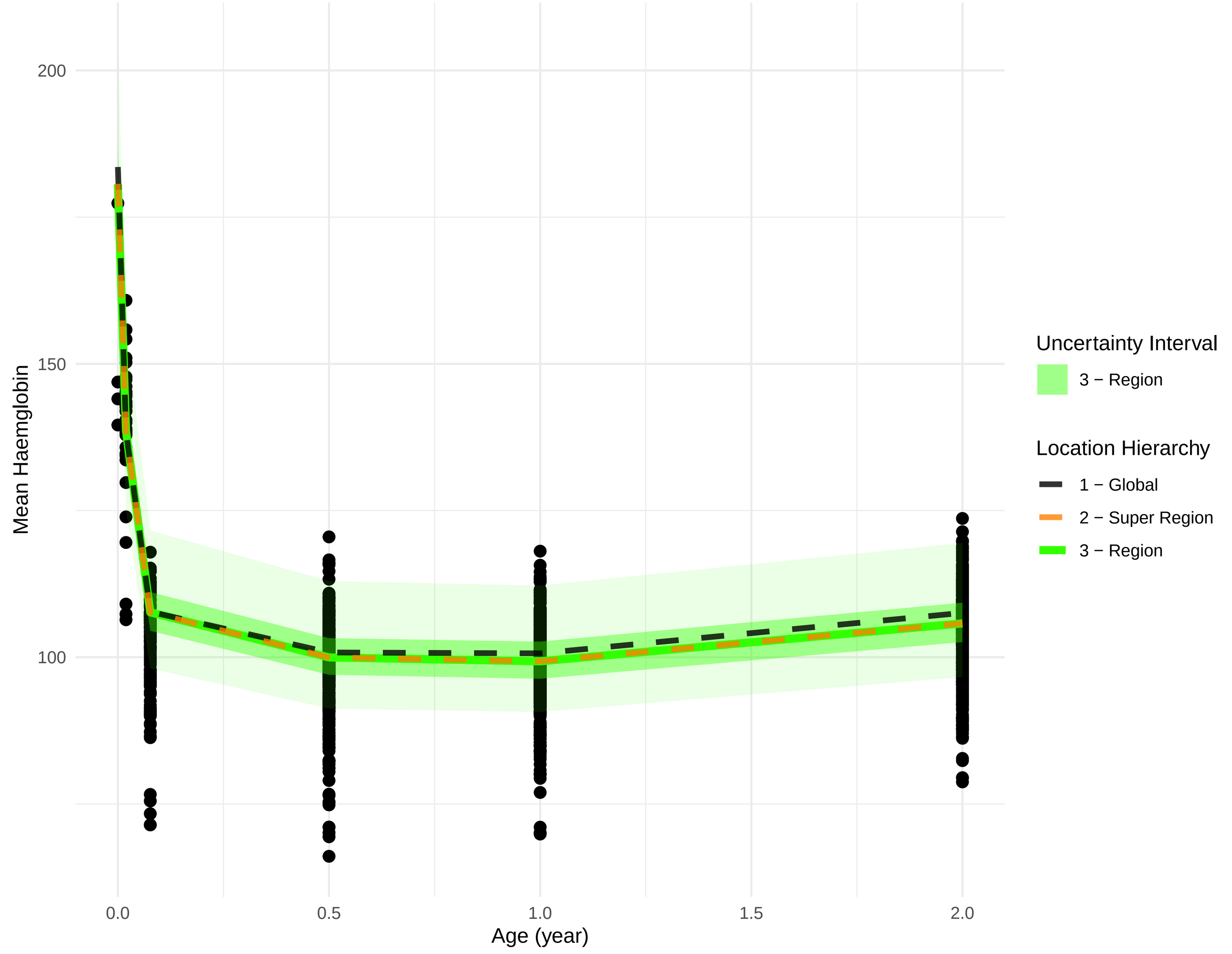

MR−BRT Age/Sex Fits − Males <2 Years Old − Mean Haemoglobin − Bangladesh
WHO−2001 Adjustment Method

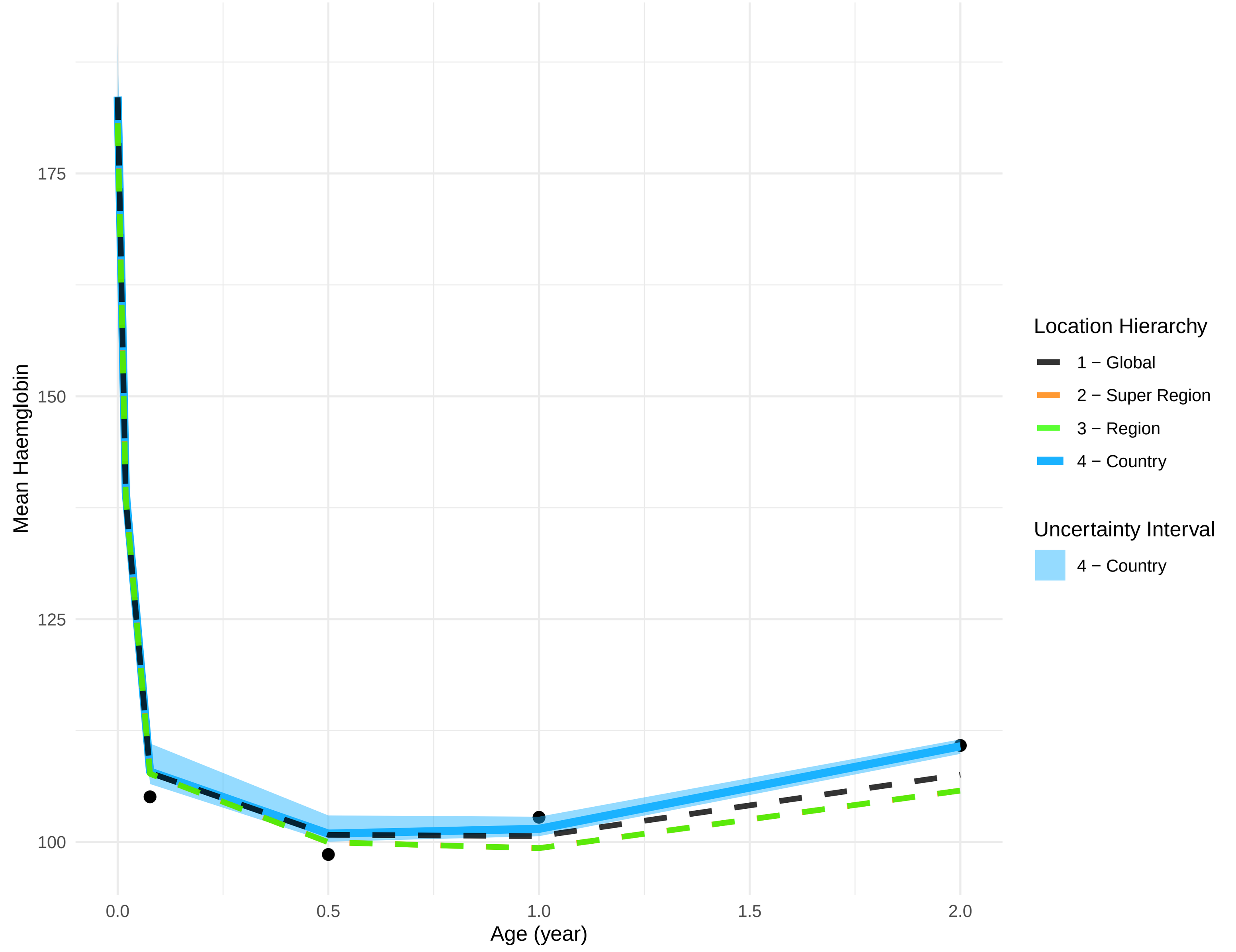

MR−BRT Age/Sex Fits − Males <2 Years Old − Mean Haemoglobin − India
WHO−2001 Adjustment Method

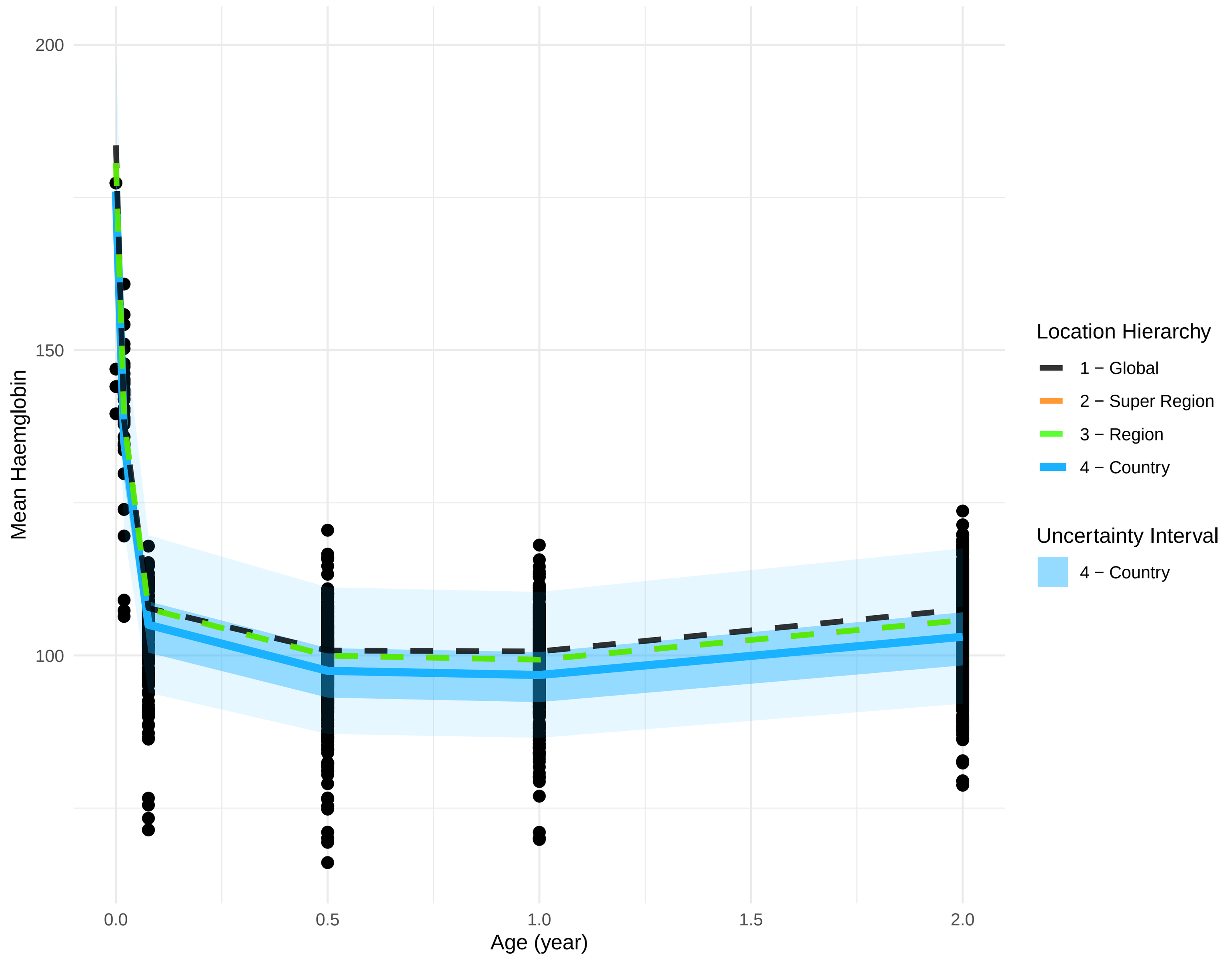

MR−BRT Age/Sex Fits − Males <2 Years Old − Mean Haemoglobin − Andhra Pradesh
WHO−2001 Adjustment Method

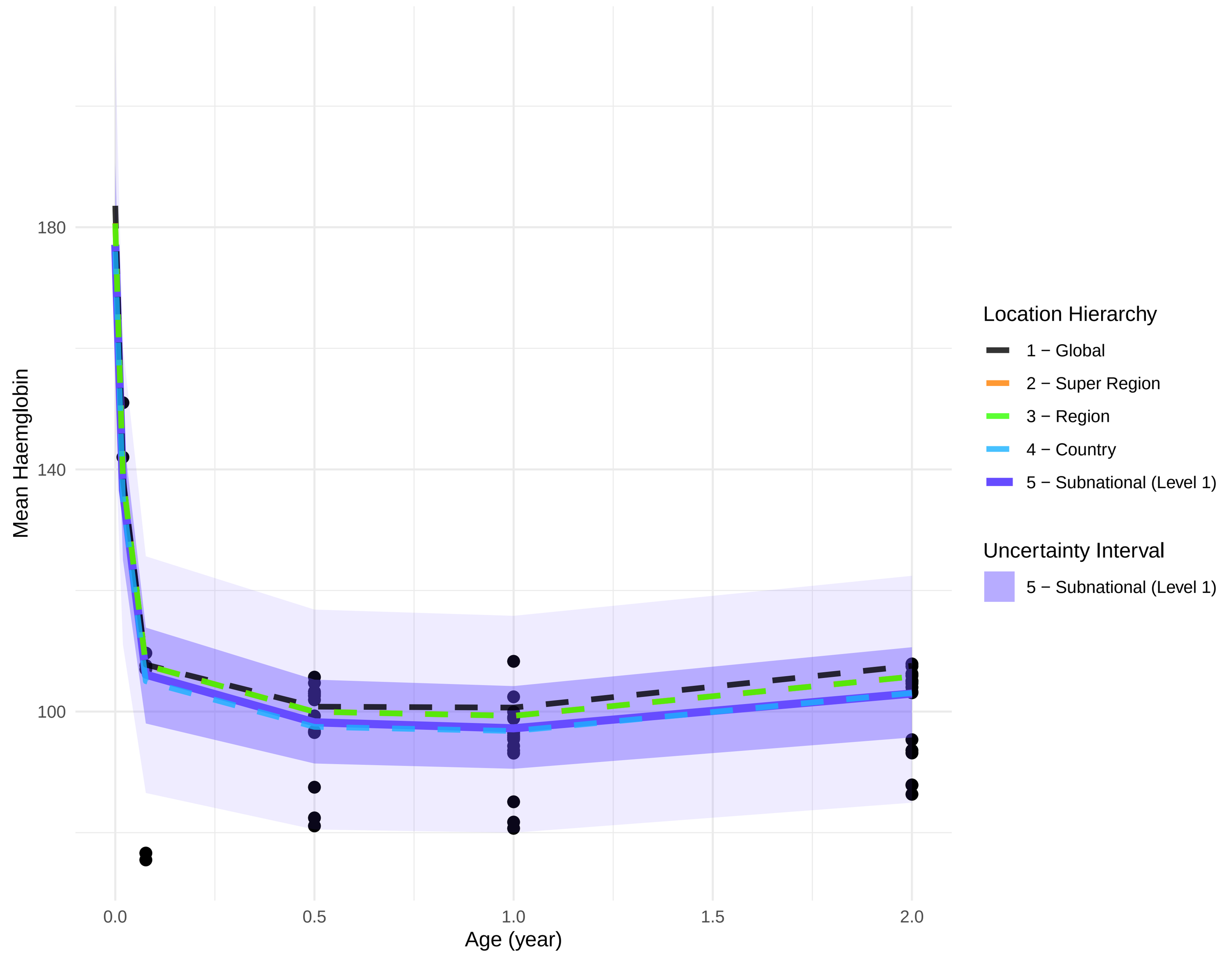

MR−BRT Age/Sex Fits − Males <2 Years Old − Mean Haemoglobin − Andhra Pradesh, Rural
WHO−2001 Adjustment Method

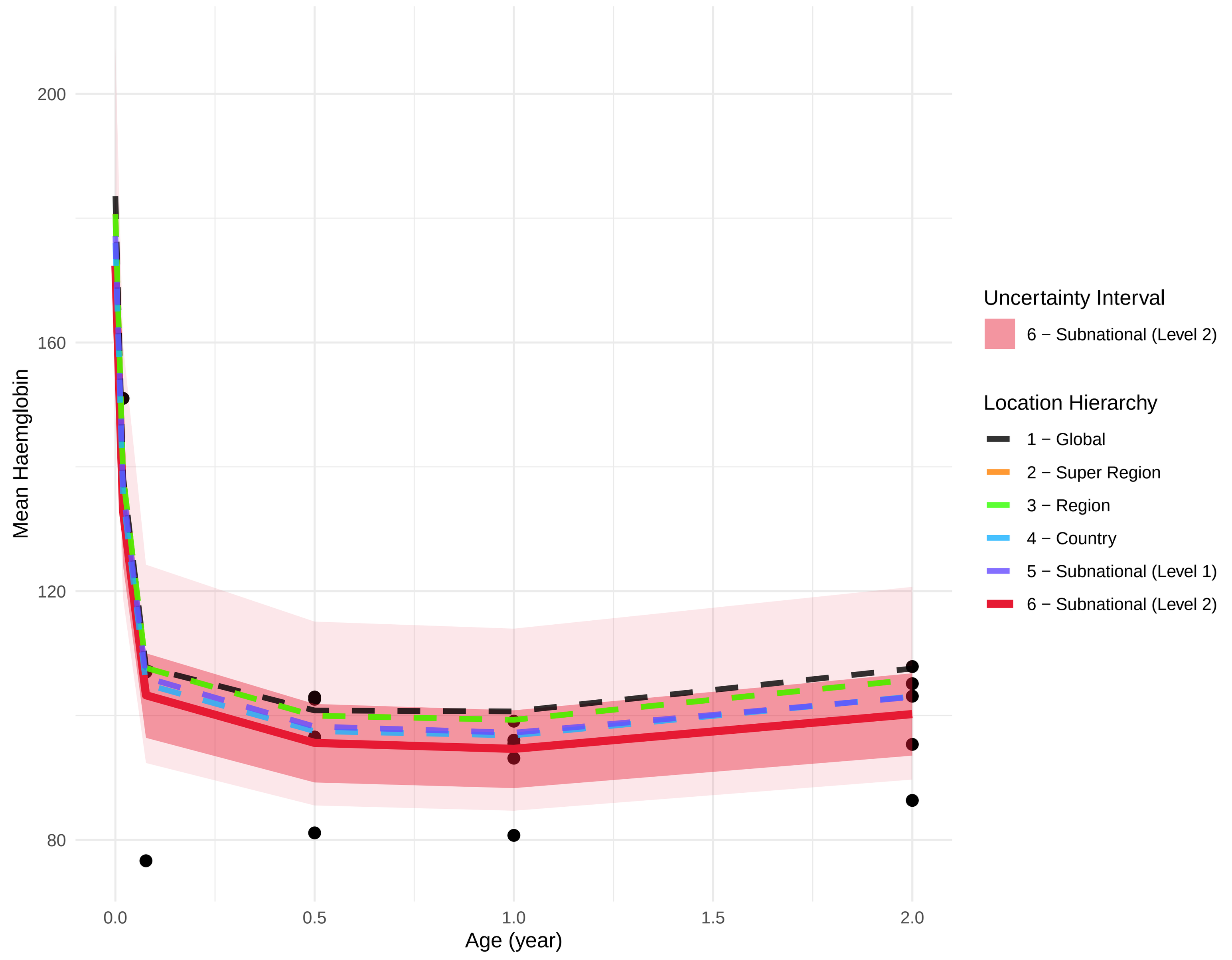

MR−BRT Age/Sex Fits − Males <2 Years Old − Mean Haemoglobin − Andhra Pradesh, Urban
WHO−2001 Adjustment Method

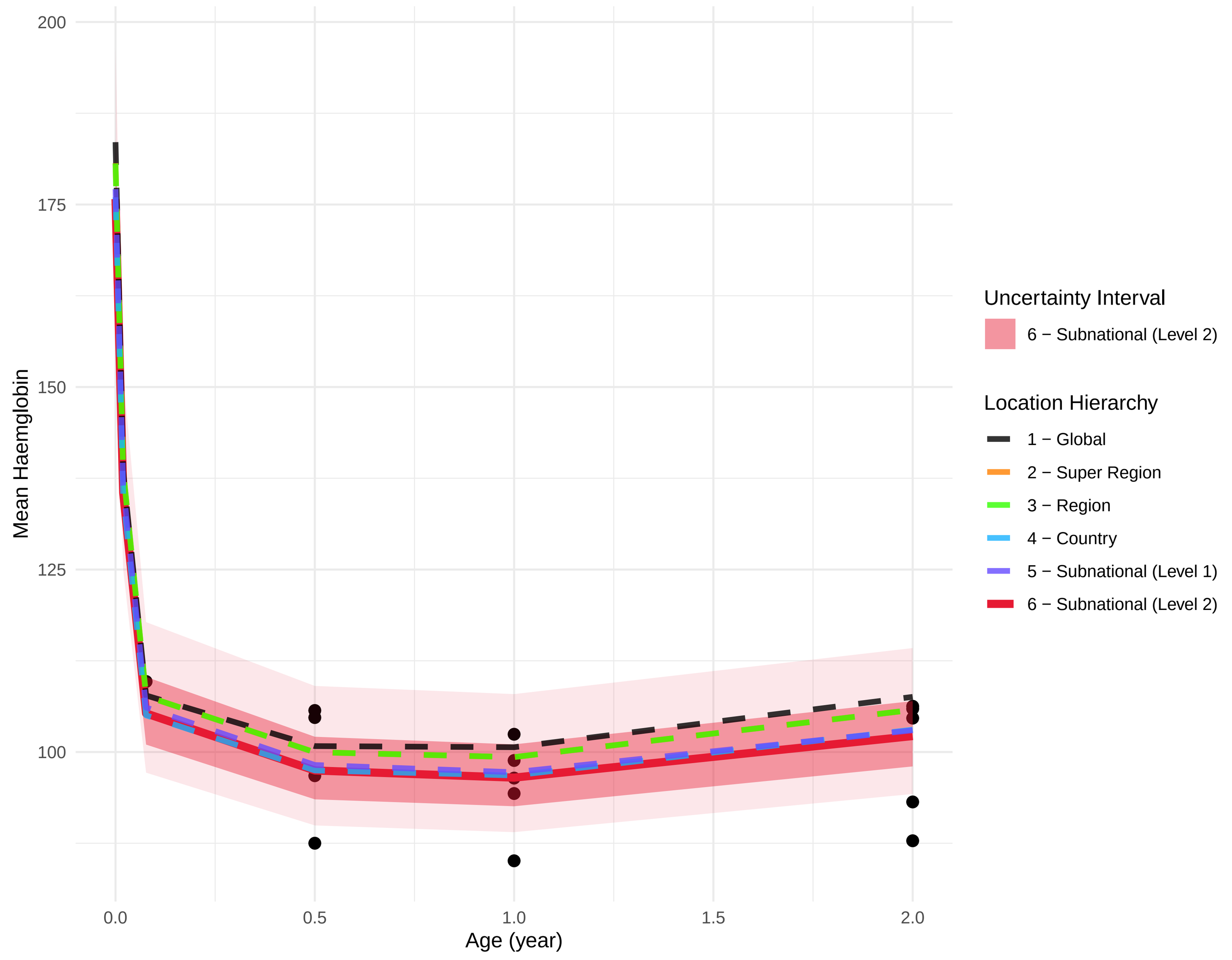

MR−BRT Age/Sex Fits − Males <2 Years Old − Mean Haemoglobin − Arunachal Pradesh
WHO−2001 Adjustment Method

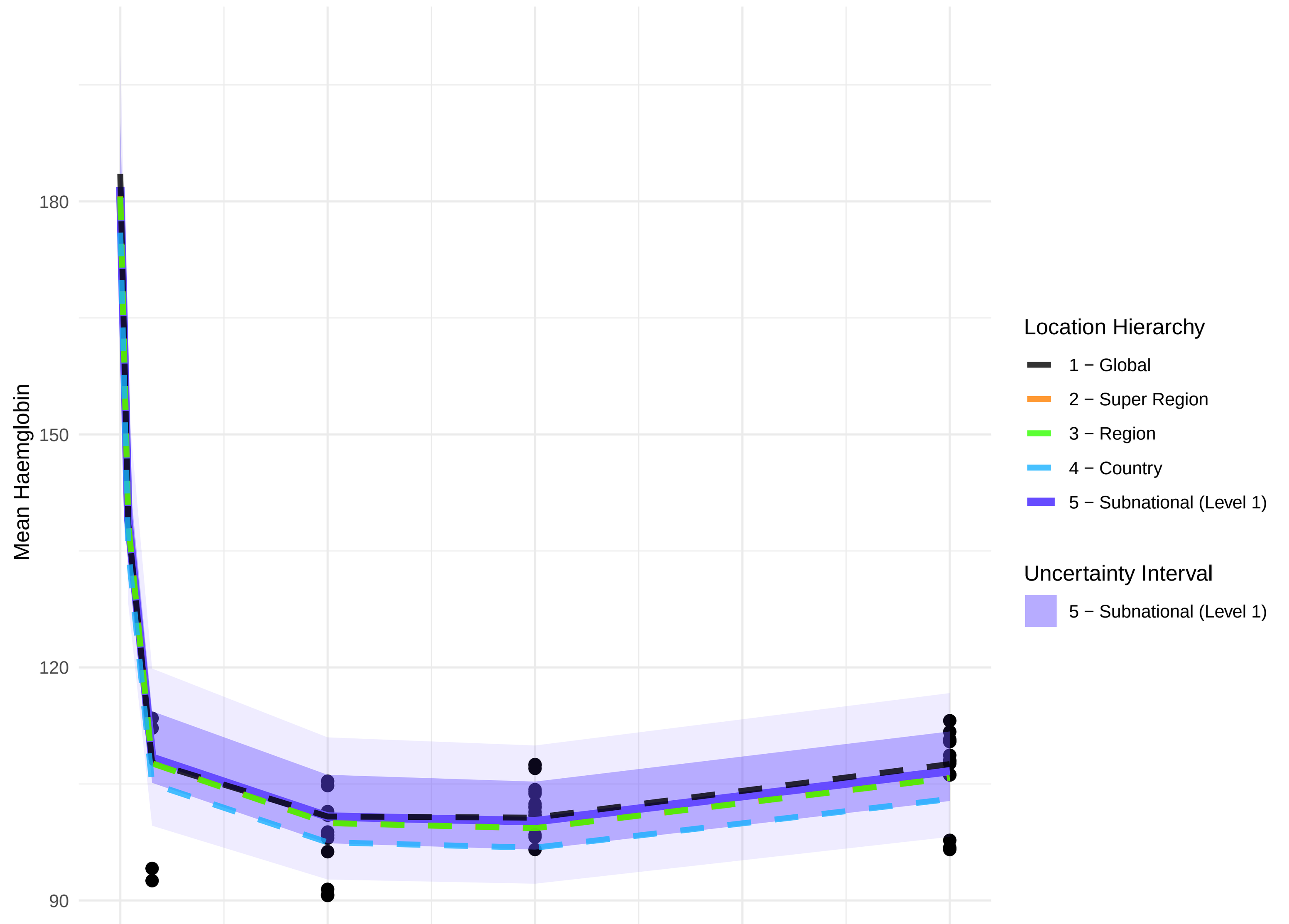

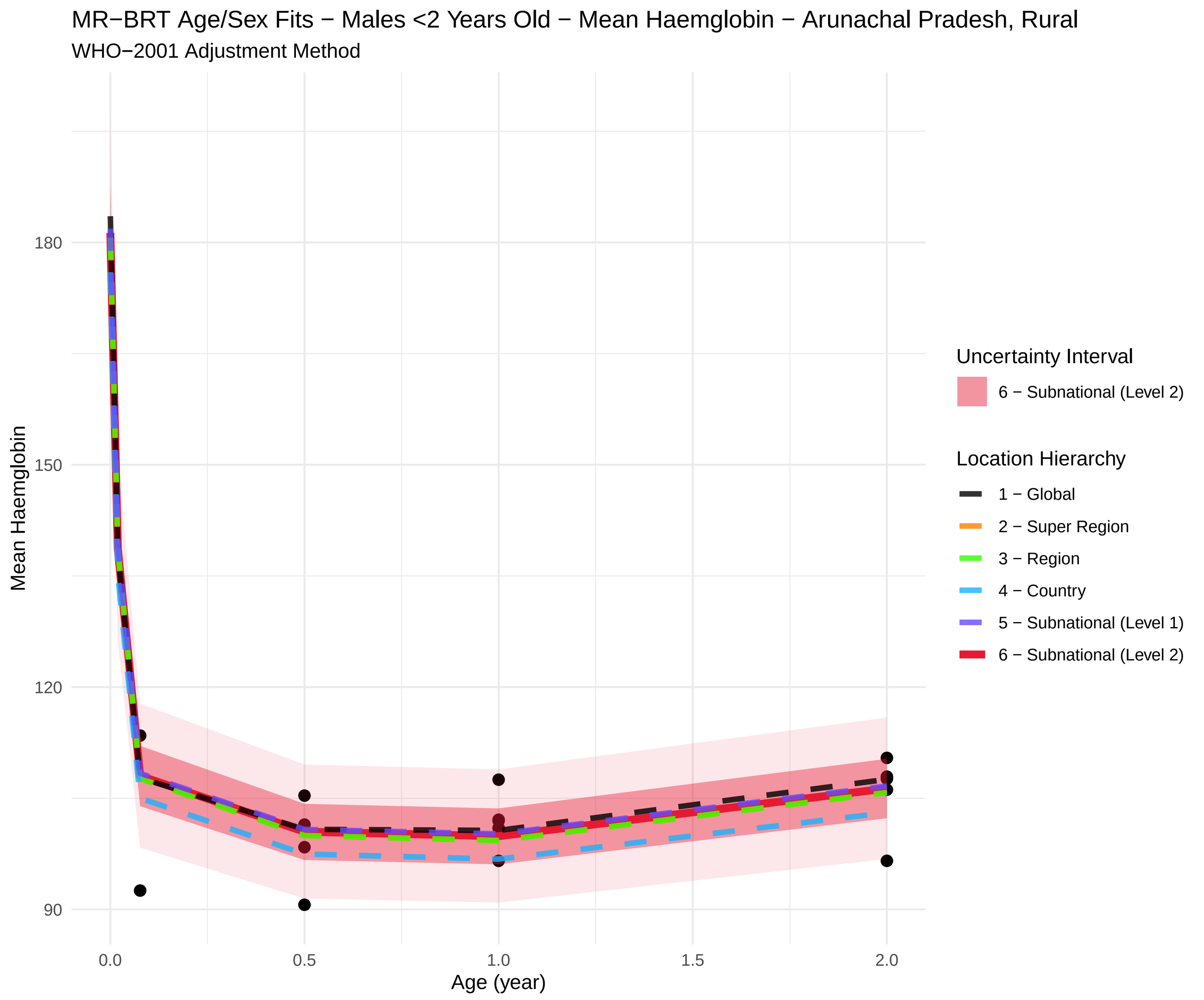
MR−BRT Age/Sex Fits − Males <2 Years Old − Mean Haemglobin − Arunachal Pradesh, Rural
WHO−2001 Adjustment Method
Mean Haemglobin
90
120
150
180
0.0
0.5
1.0
1.5
2.0
Age (year)
Uncertainty Interval
6 − Subnational (Level 2)
Location Hierarchy
1 − Global
2 − Super Region
3 − Region
4 − Country
5 − Subnational (Level 1)
6 − Subnational (Level 2)

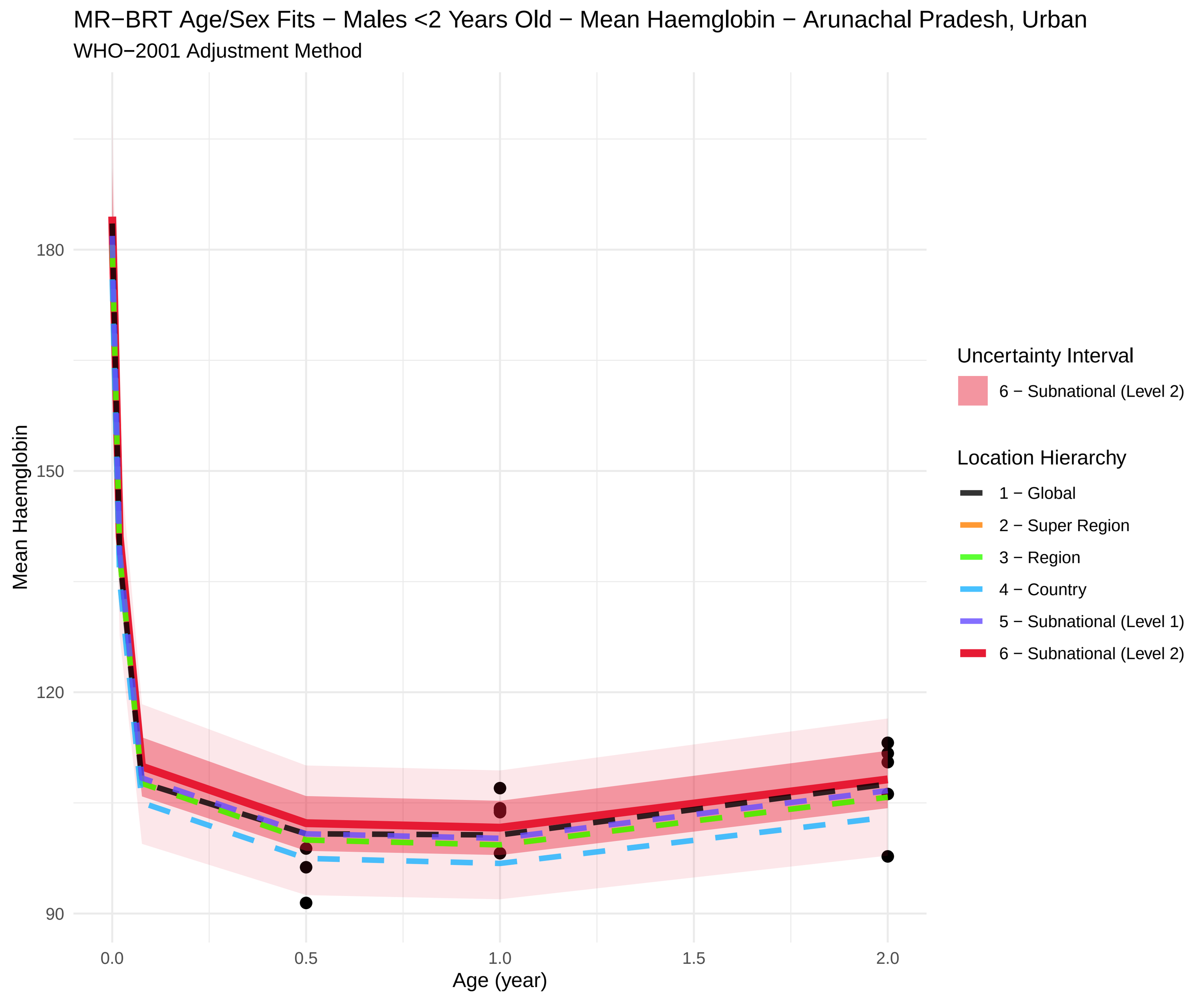
MR−BRT Age/Sex Fits − Males <2 Years Old − Mean Haemglobin − Arunachal Pradesh, Urban
WHO−2001 Adjustment Method
Mean Haemglobin
90
120
150
180
0.0
0.5
1.0
1.5
2.0
Age (year)
Uncertainty Interval
6 − Subnational (Level 2)
Location Hierarchy
1 − Global
2 − Super Region
3 − Region
4 − Country
5 − Subnational (Level 1)
6 − Subnational (Level 2)

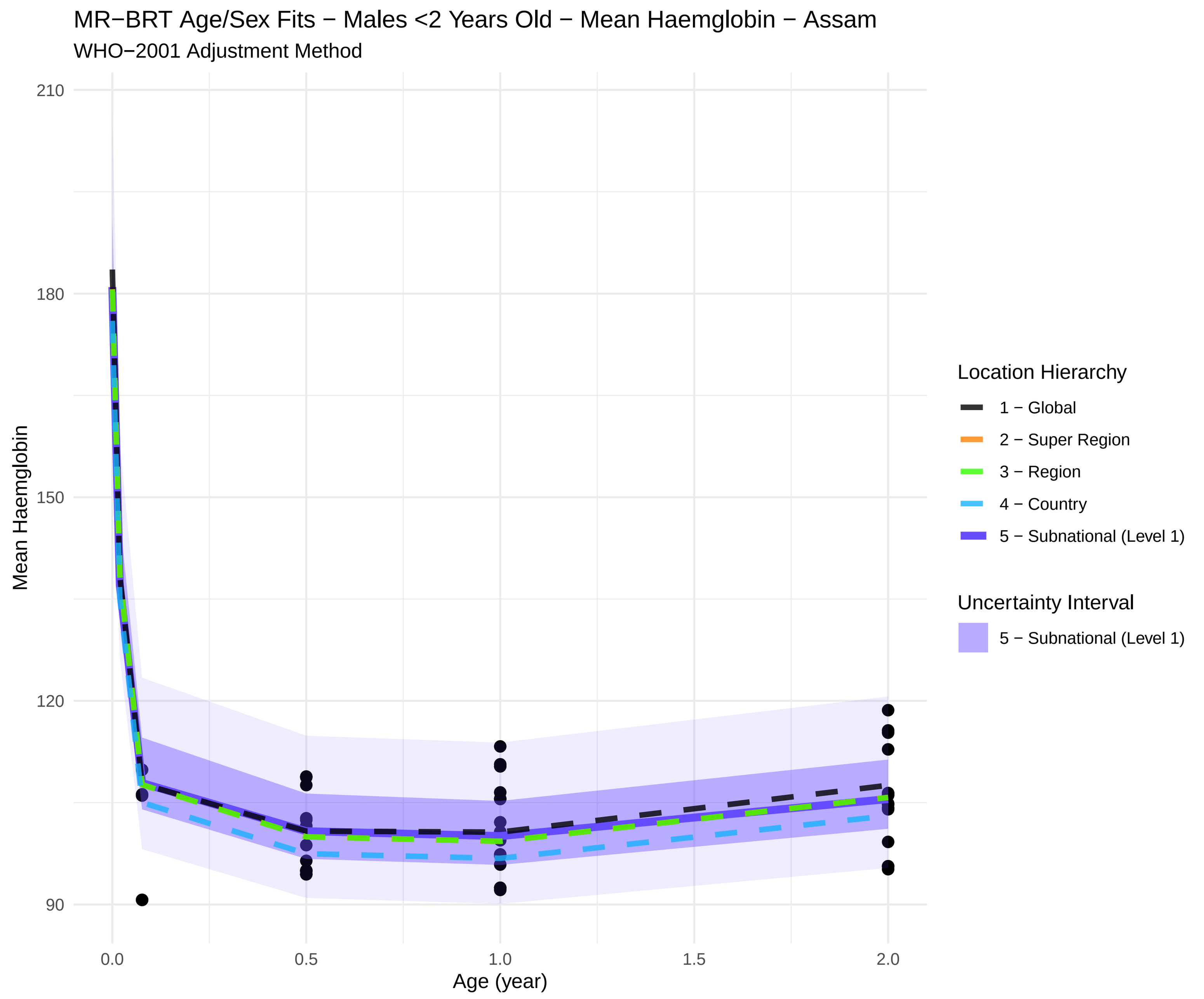
MR−BRT Age/Sex Fits − Males <2 Years Old − Mean Haemglobin − Assam
WHO−2001 Adjustment Method
Mean Haemglobin
90
120
150
180
210
0.0
0.5
1.0
1.5
2.0
Age (year)
Location Hierarchy
1 − Global
2 − Super Region
3 − Region
4 − Country
5 − Subnational (Level 1)
Uncertainty Interval
5 − Subnational (Level 1)

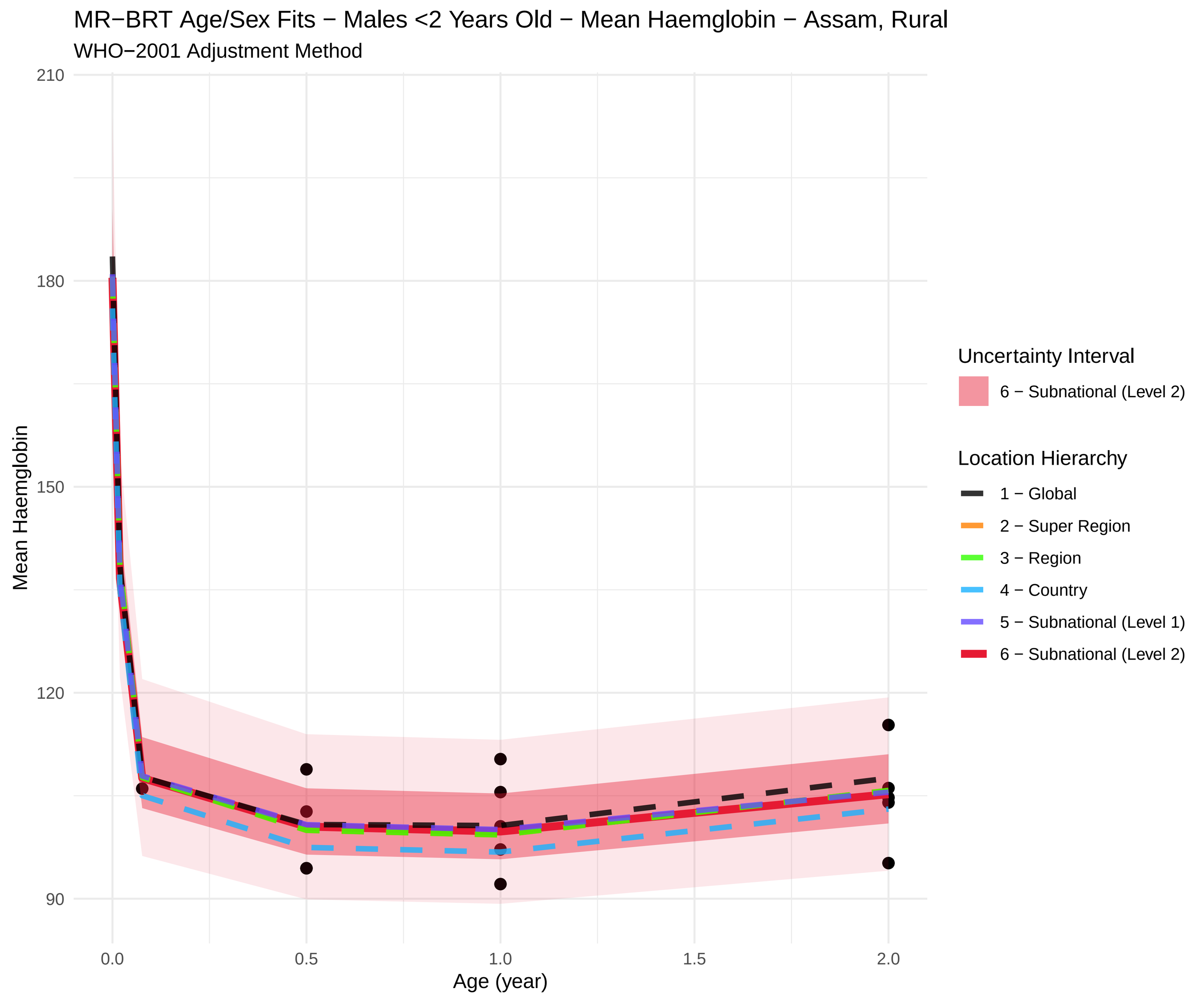
MR−BRT Age/Sex Fits − Males <2 Years Old − Mean Haemglobin − Assam, Rural
WHO−2001 Adjustment Method
Mean Haemglobin
90
120
150
180
210
0.0
0.5
1.0
1.5
2.0
Age (year)
Uncertainty Interval
6 − Subnational (Level 2)
Location Hierarchy
1 − Global
2 − Super Region
3 − Region
4 − Country
5 − Subnational (Level 1)
6 − Subnational (Level 2)

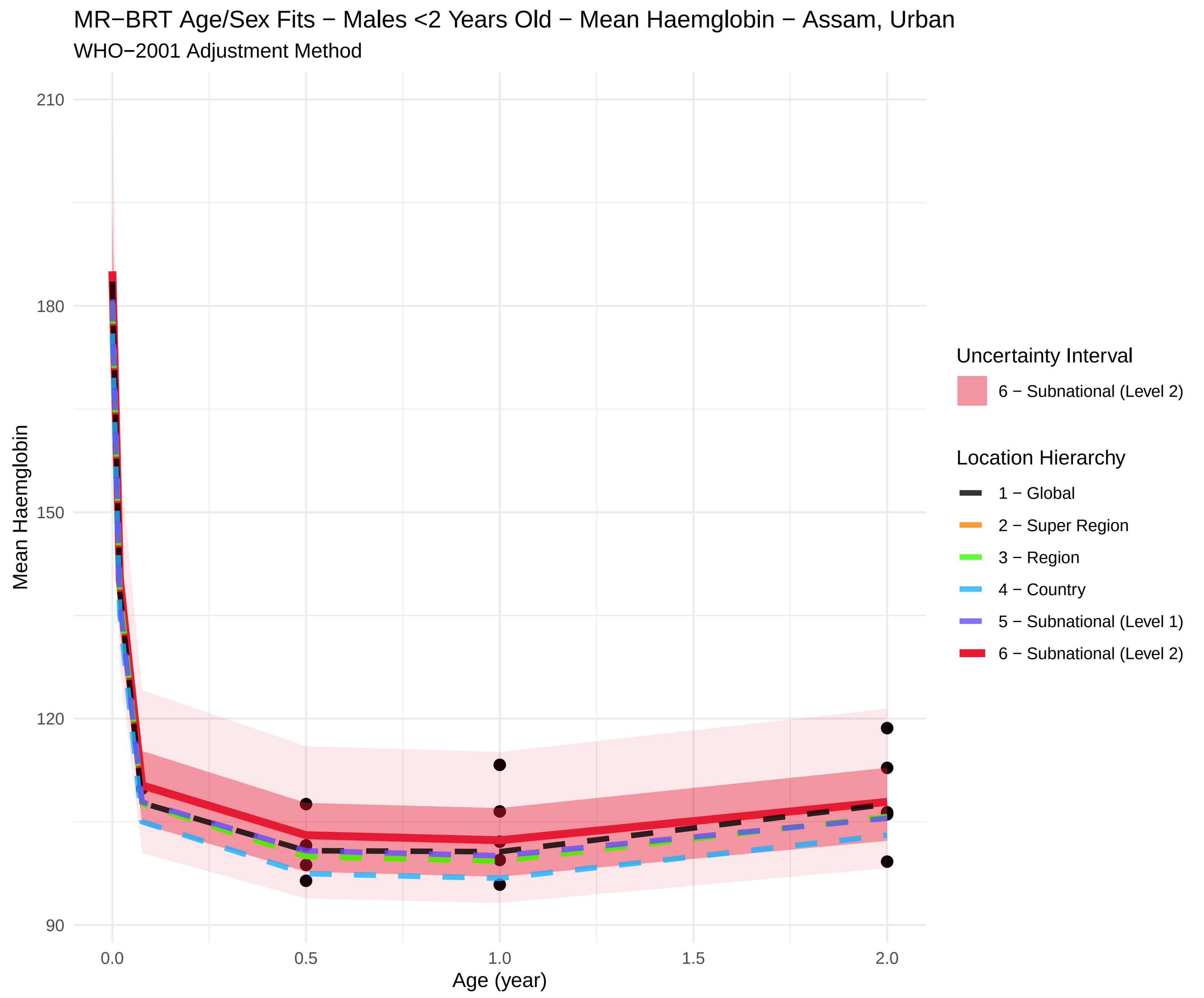
MR−BRT Age/Sex Fits − Males <2 Years Old − Mean Haemglobin − Assam, Urban
WHO−2001 Adjustment Method
Mean Haemglobin
90
120
150
180
210
0.0
0.5
1.0
1.5
2.0
Age (year)
Uncertainty Interval
6 − Subnational (Level 2)
Location Hierarchy
1 − Global
2 − Super Region
3 − Region
4 − Country
5 − Subnational (Level 1)
6 − Subnational (Level 2)

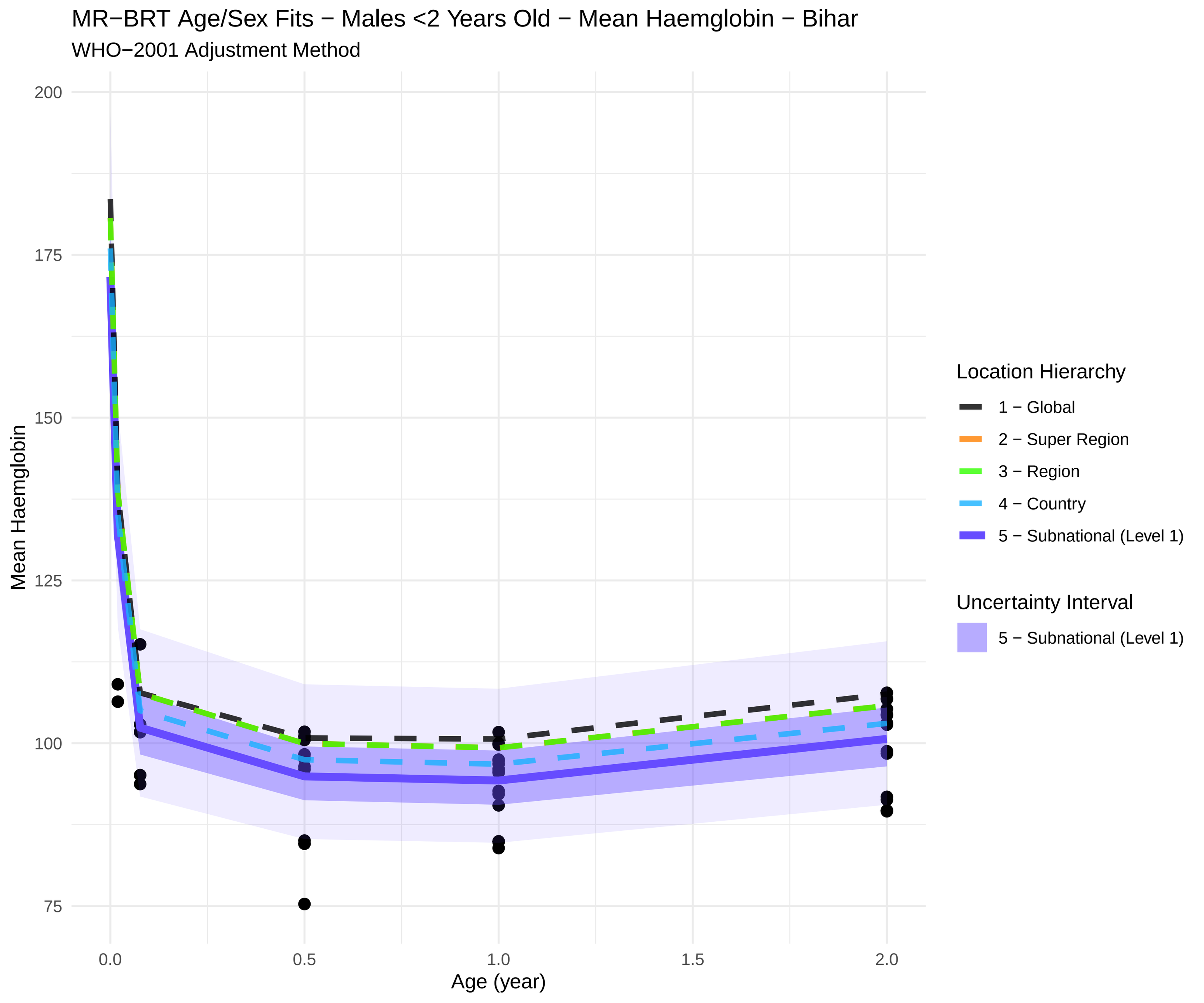
MR−BRT Age/Sex Fits − Males <2 Years Old − Mean Haemglobin − Bihar
WHO−2001 Adjustment Method
Mean Haemglobin
75
100
125
150
175
200
0.0
0.5
1.0
1.5
2.0
Age (year)
Location Hierarchy
1 − Global
2 − Super Region
3 − Region
4 − Country
5 − Subnational (Level 1)
Uncertainty Interval
5 − Subnational (Level 1)

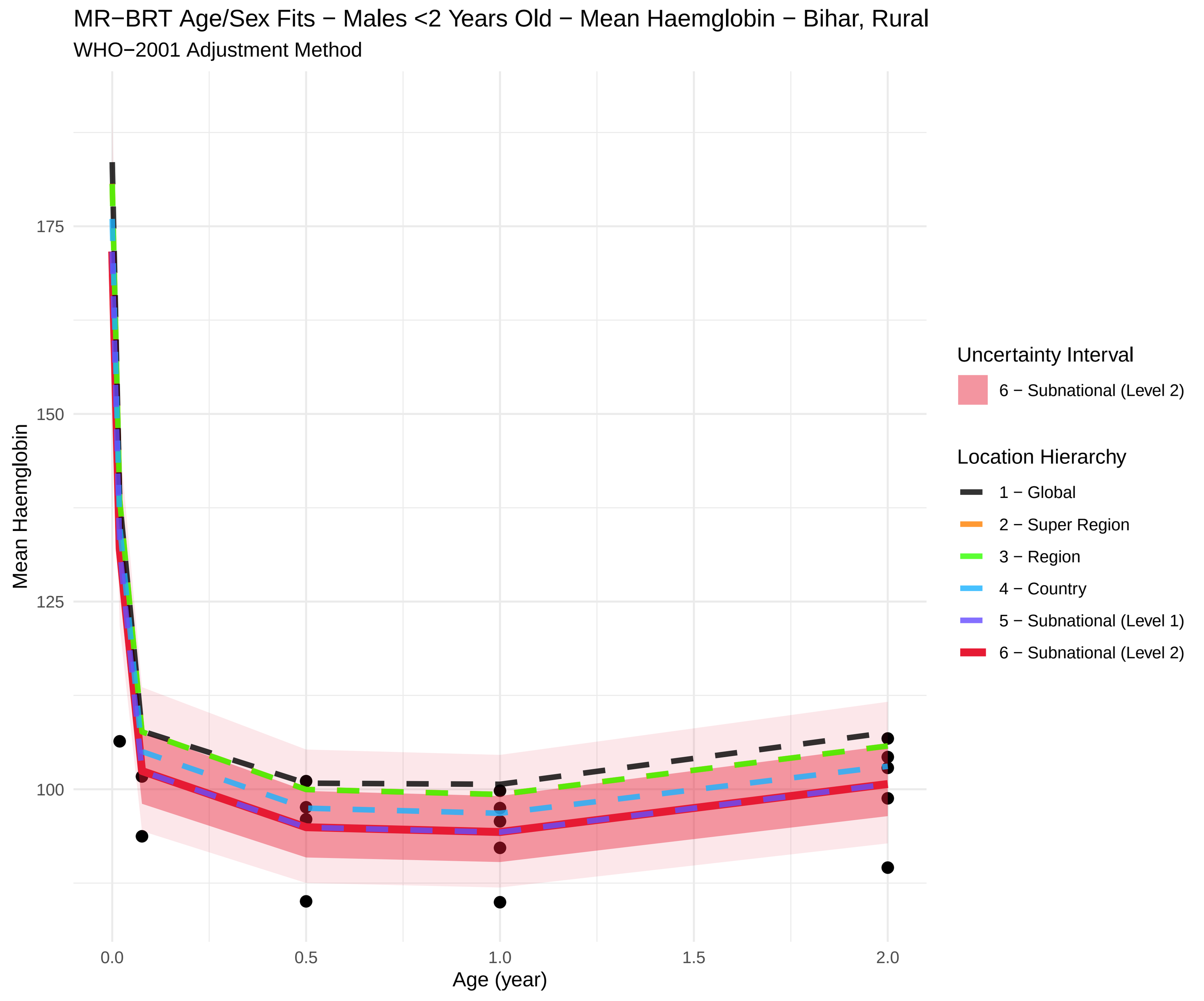
MR−BRT Age/Sex Fits − Males <2 Years Old − Mean Haemglobin − Bihar, Rural
WHO−2001 Adjustment Method
Mean Haemglobin
100
125
150
175
0.0
0.5
1.0
1.5
2.0
Age (year)
Uncertainty Interval
6 − Subnational (Level 2)
Location Hierarchy
1 − Global
2 − Super Region
3 − Region
4 − Country
5 − Subnational (Level 1)
6 − Subnational (Level 2)

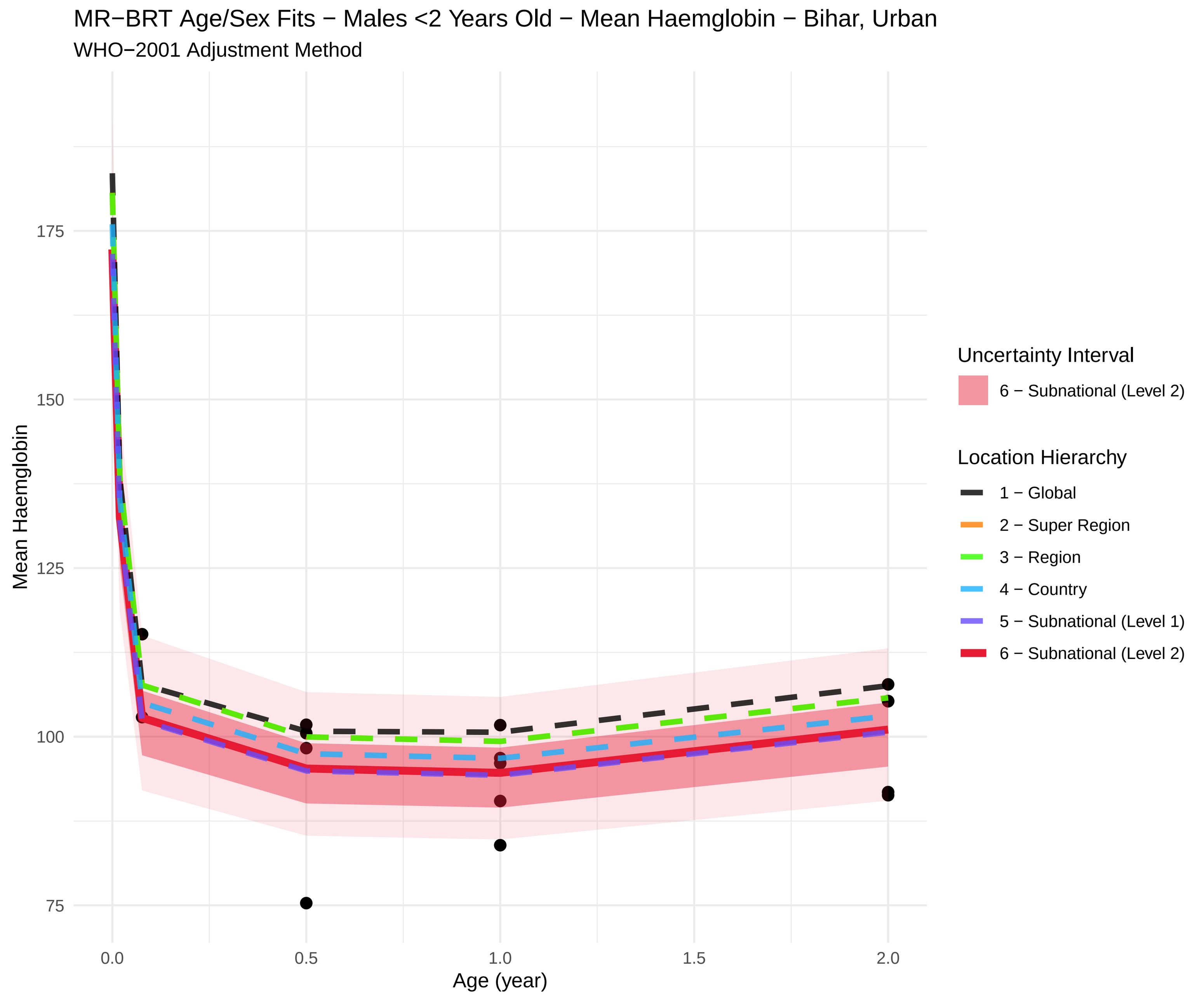
MR−BRT Age/Sex Fits − Males <2 Years Old − Mean Haemglobin − Bihar, Urban
WHO−2001 Adjustment Method
Mean Haemglobin
75
100
125
150
175
0.0
0.5
1.0
1.5
2.0
Age (year)
Uncertainty Interval
6 − Subnational (Level 2)
Location Hierarchy
1 − Global
2 − Super Region
3 − Region
4 − Country
5 − Subnational (Level 1)
6 − Subnational (Level 2)

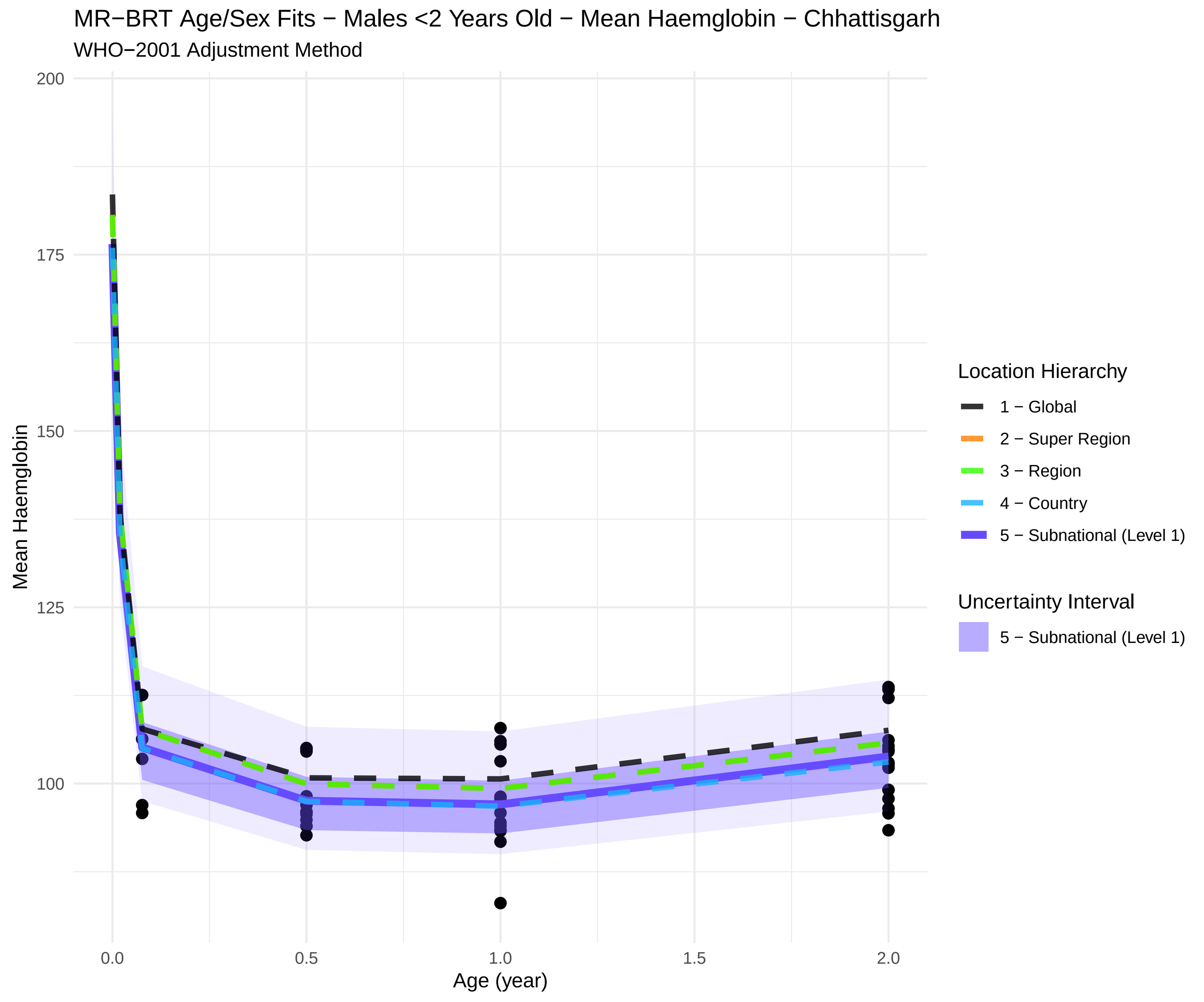
MR−BRT Age/Sex Fits − Males <2 Years Old − Mean Haemglobin − Chhattisgarh
WHO−2001 Adjustment Method
Mean Haemglobin
100
125
150
175
200
0.0
0.5
1.0
1.5
2.0
Age (year)
Location Hierarchy
1 − Global
2 − Super Region
3 − Region
4 − Country
5 − Subnational (Level 1)
Uncertainty Interval
5 − Subnational (Level 1)

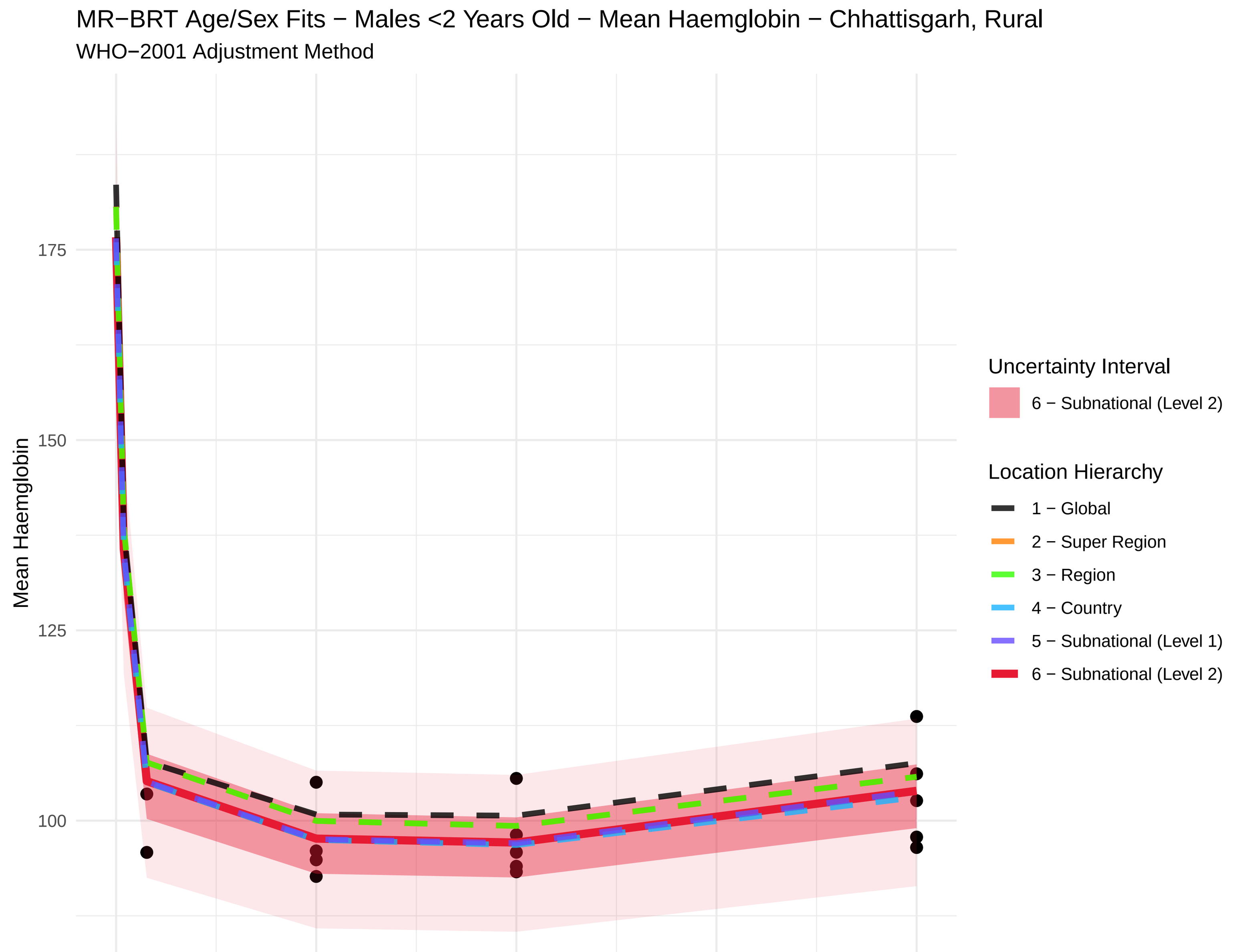
MR−BRT Age/Sex Fits − Males <2 Years Old − Mean Haemglobin − Chhattisgarh, Rural
WHO−2001 Adjustment Method
Mean Haemglobin
100
125
150
175
0.0
0.5
1.0
1.5
2.0
Age (year)
Uncertainty Interval
6 − Subnational (Level 2)
Location Hierarchy
1 − Global
2 − Super Region
3 − Region
4 − Country
5 − Subnational (Level 1)
6 − Subnational (Level 2)

MR−BRT Age/Sex Fits − Males <2 Years Old − Mean Haemglobin − Chhattisgarh, Urban
WHO−2001 Adjustment Method

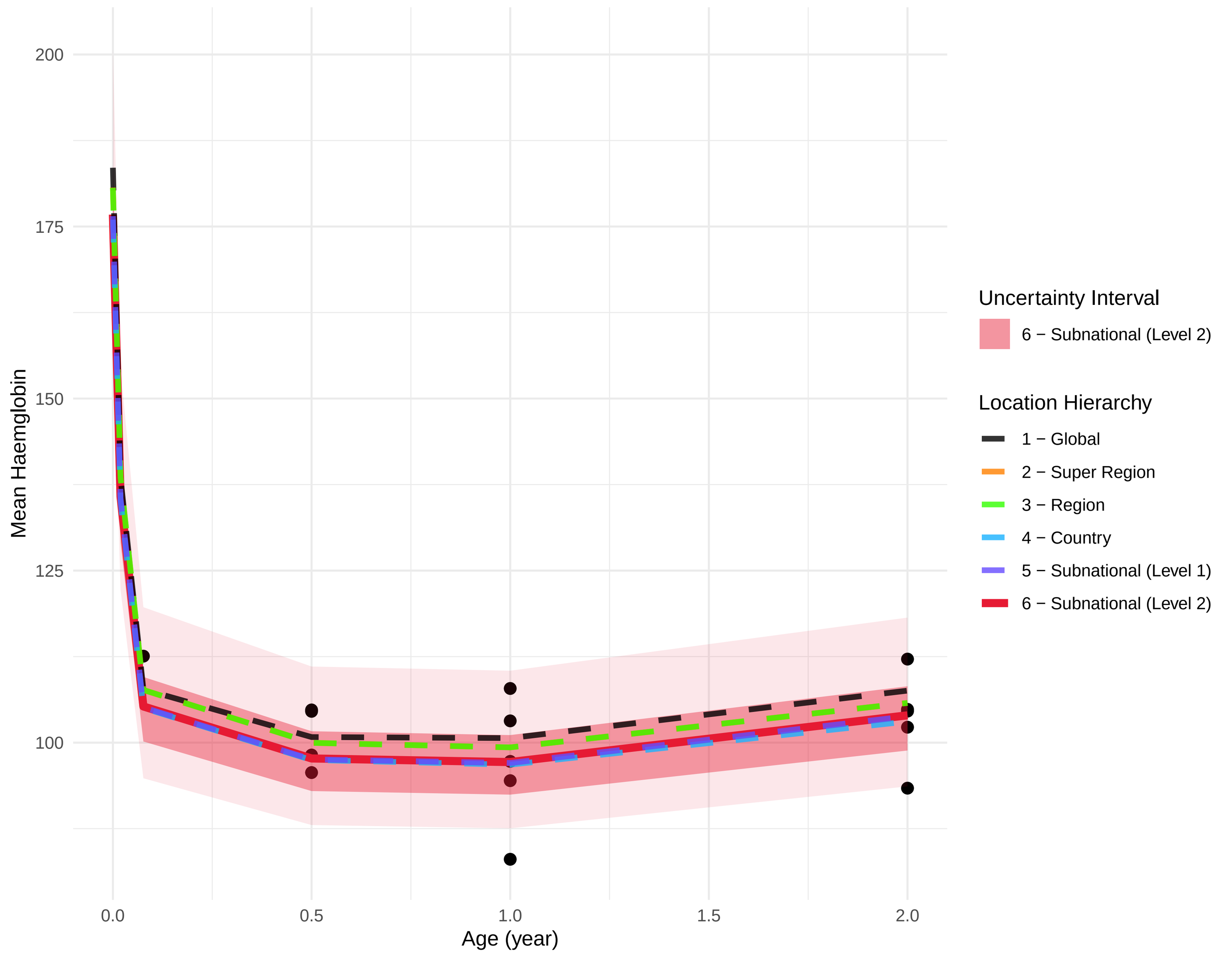

MR−BRT Age/Sex Fits − Males <2 Years Old − Mean Haemglobin − Delhi
WHO−2001 Adjustment Method

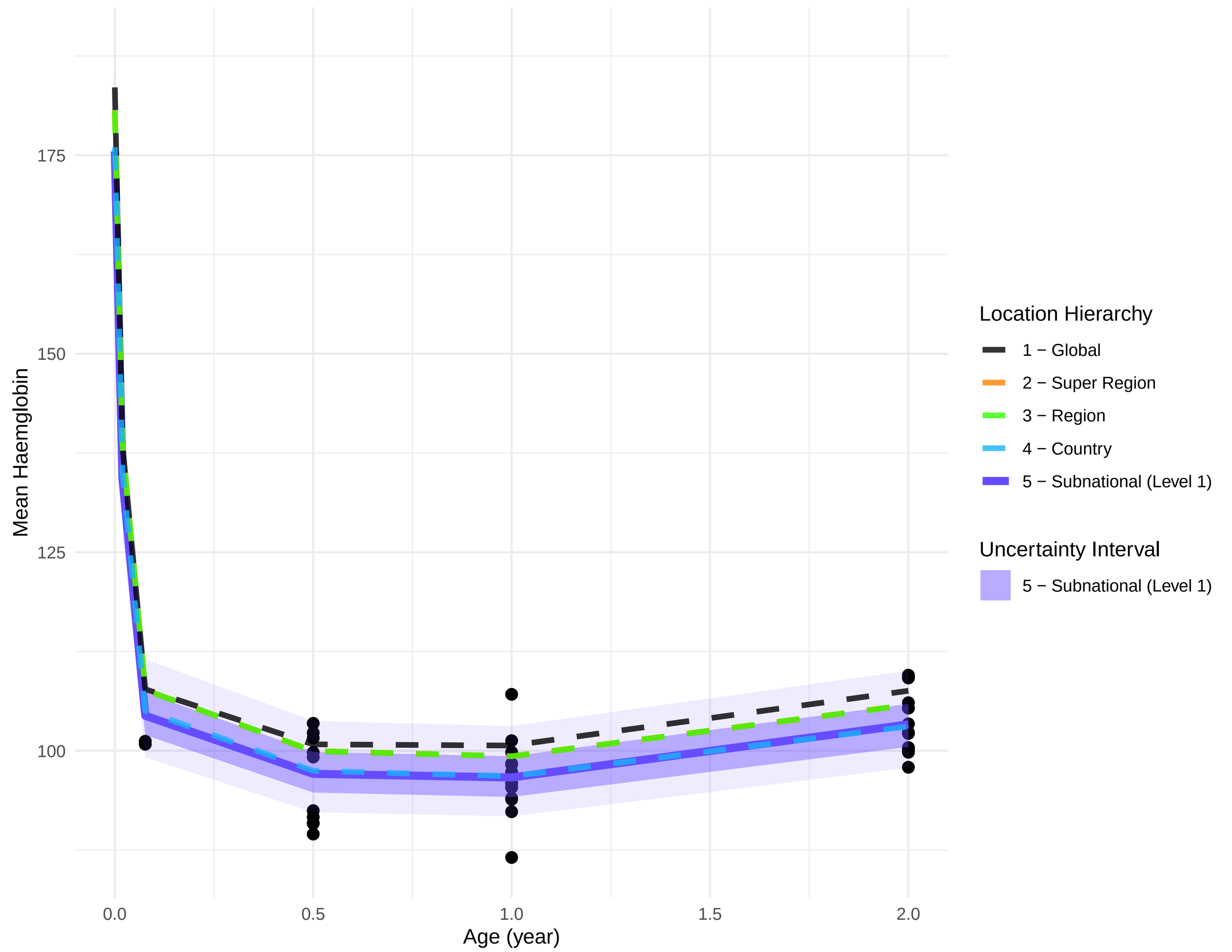

MR−BRT Age/Sex Fits − Males <2 Years Old − Mean Haemglobin − Delhi, Rural
WHO−2001 Adjustment Method

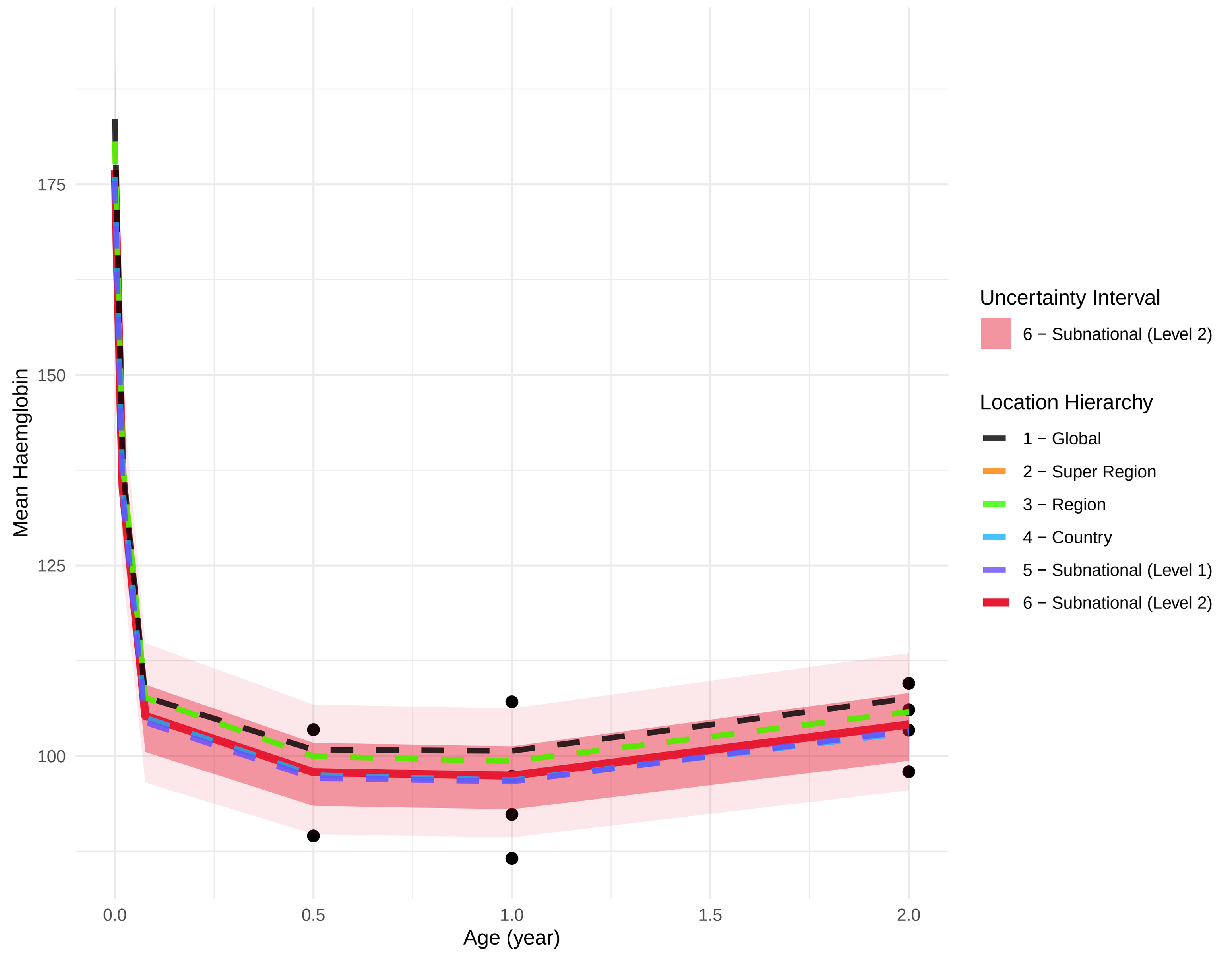

MR−BRT Age/Sex Fits − Males <2 Years Old − Mean Haemglobin − Delhi, Urban
WHO−2001 Adjustment Method

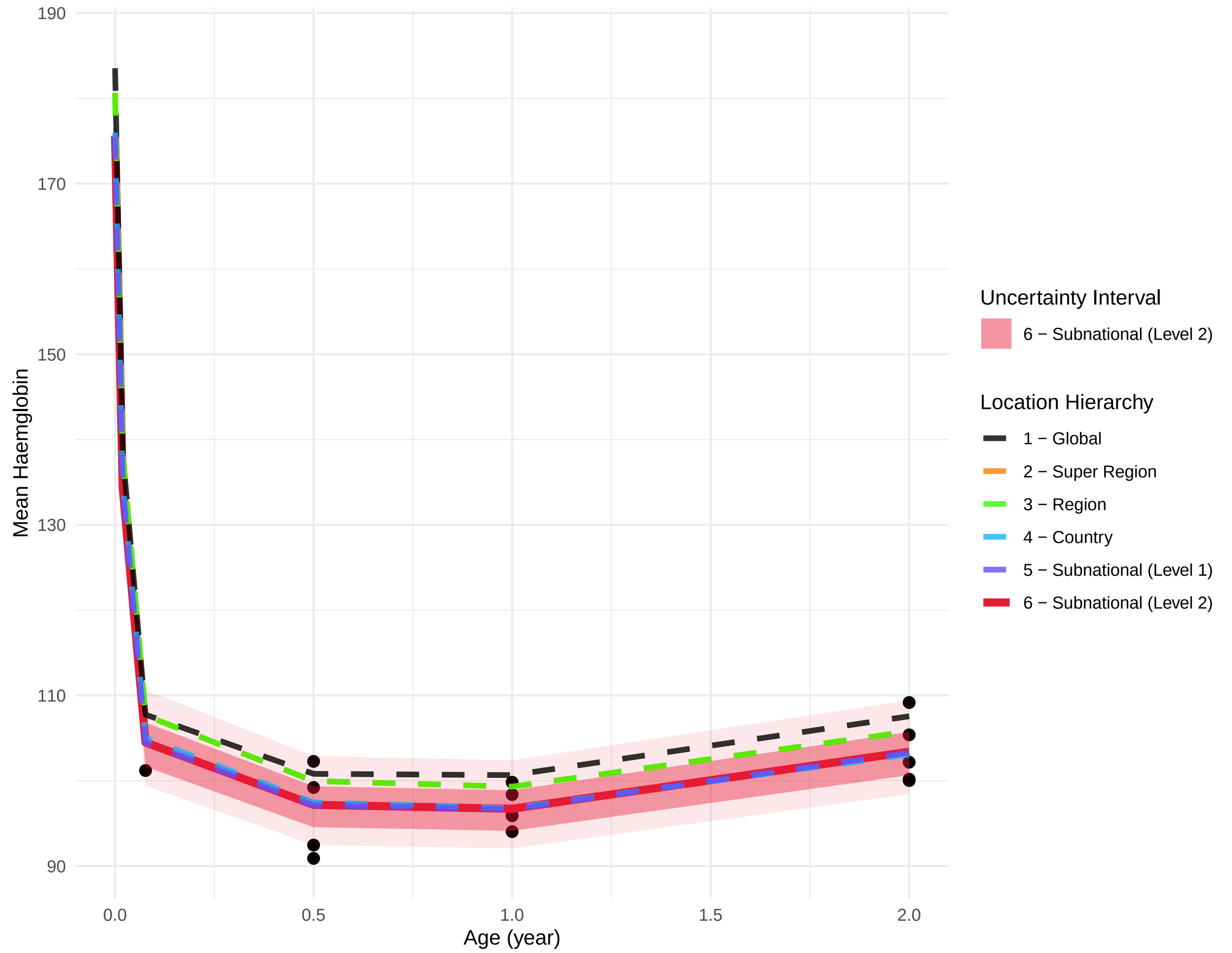

MR−BRT Age/Sex Fits − Males <2 Years Old − Mean Haemglobin − Goa
WHO−2001 Adjustment Method

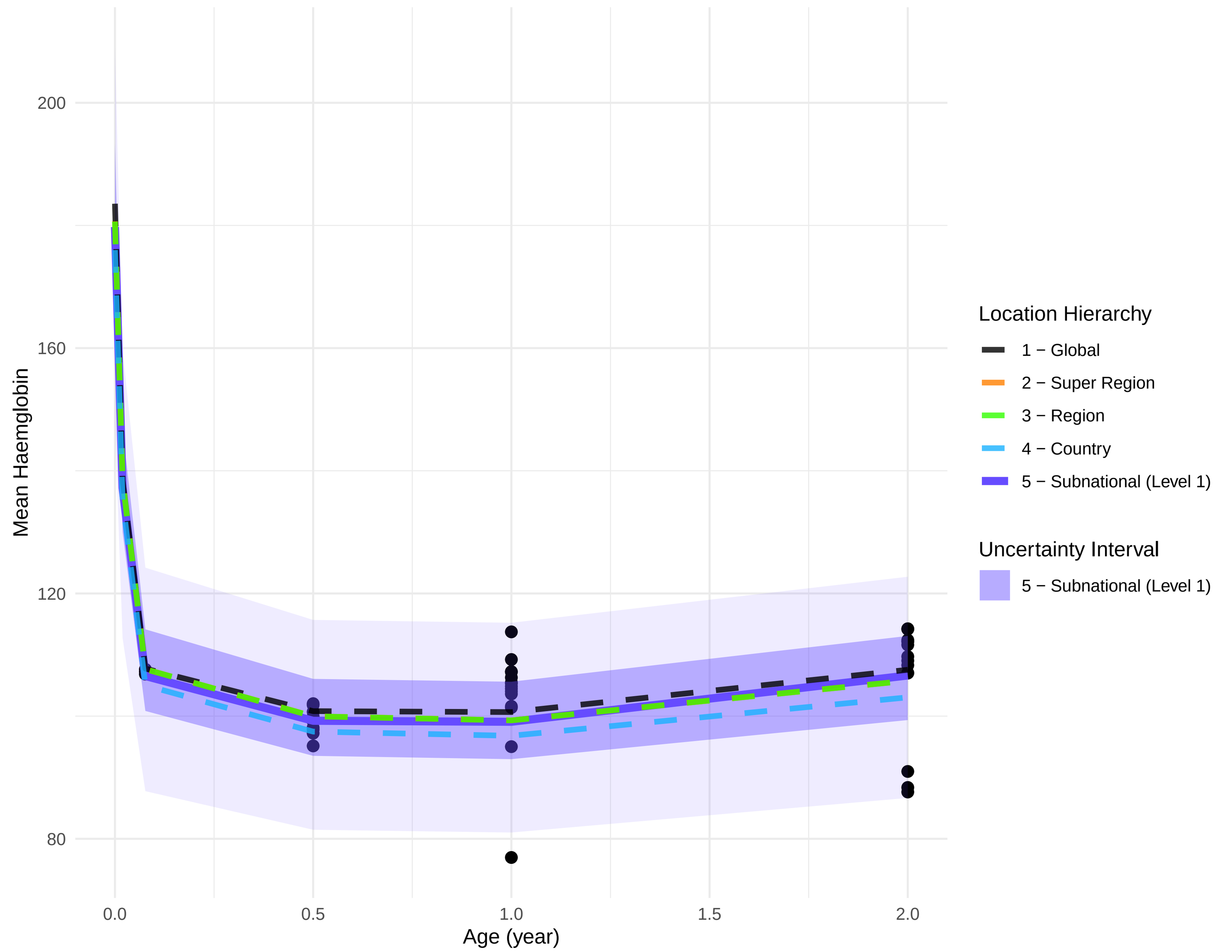

MR−BRT Age/Sex Fits − Males <2 Years Old − Mean Haemglobin − Goa, Rural
WHO−2001 Adjustment Method

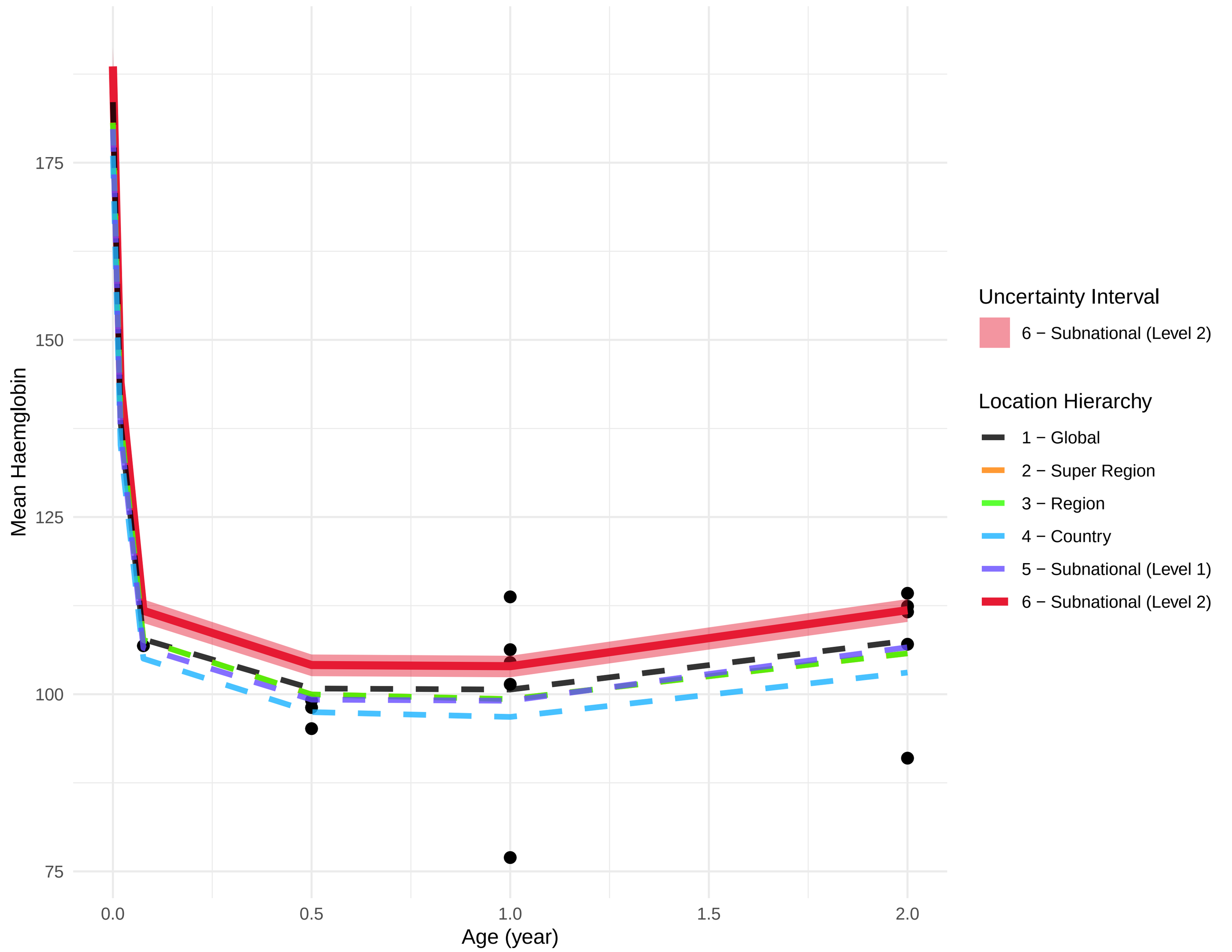

MR−BRT Age/Sex Fits − Males <2 Years Old − Mean Haemglobin − Goa, Urban
WHO−2001 Adjustment Method

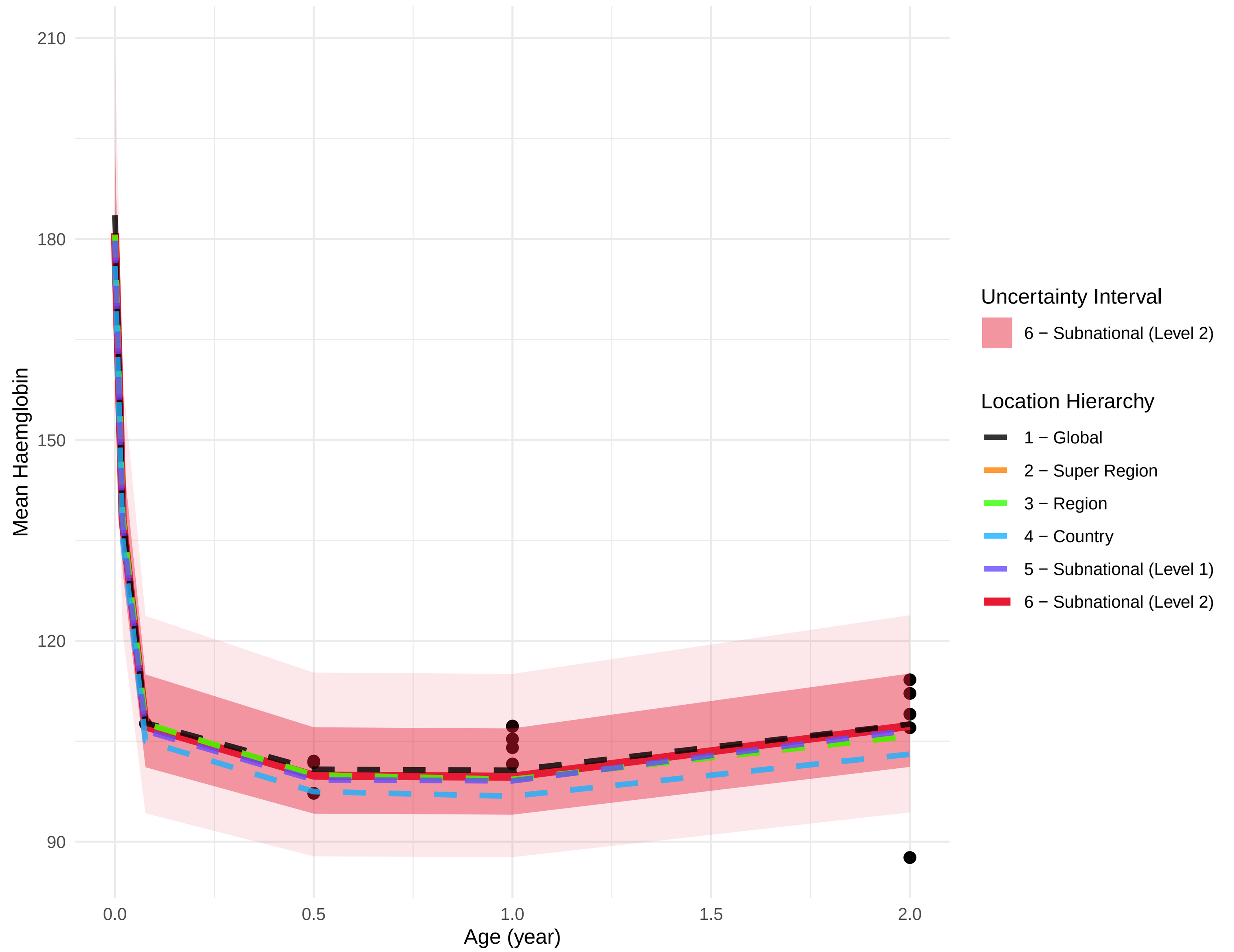

MR−BRT Age/Sex Fits − Males <2 Years Old − Mean Haemglobin − Gujarat
WHO−2001 Adjustment Method

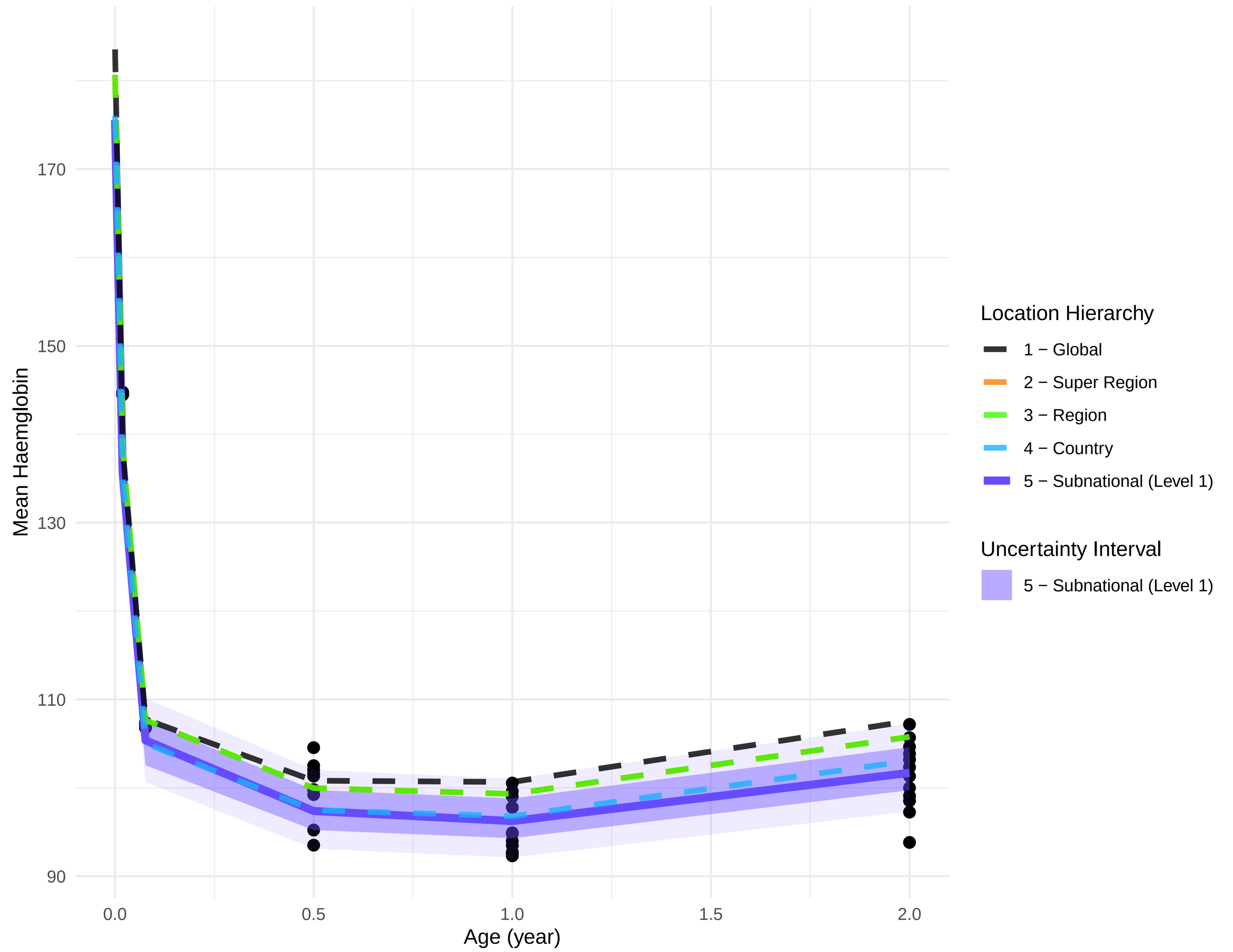

MR−BRT Age/Sex Fits − Males <2 Years Old − Mean Haemglobin − Gujarat, Rural
WHO−2001 Adjustment Method

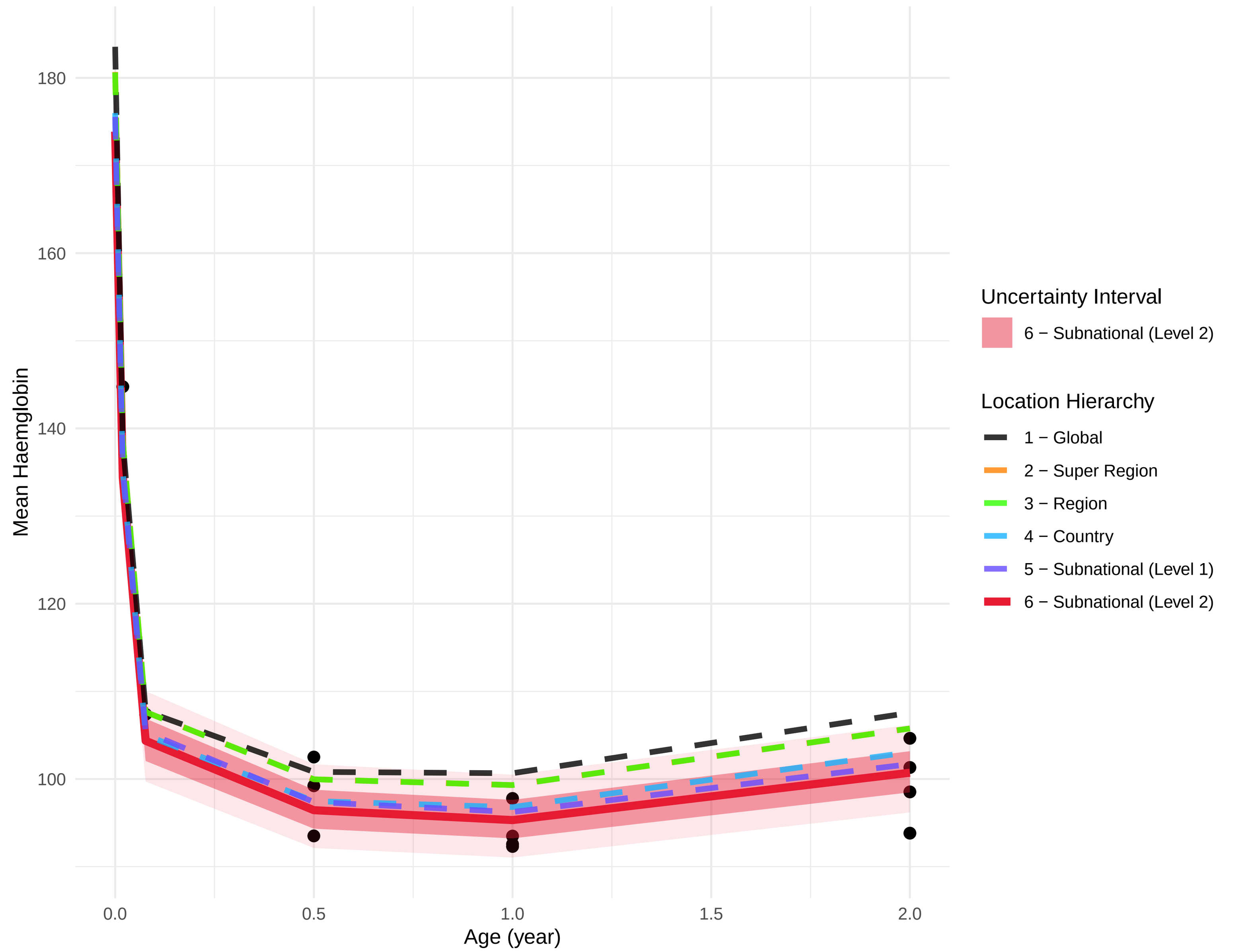

MR−BRT Age/Sex Fits − Males <2 Years Old − Mean Haemglobin − Gujarat, Urban
WHO−2001 Adjustment Method

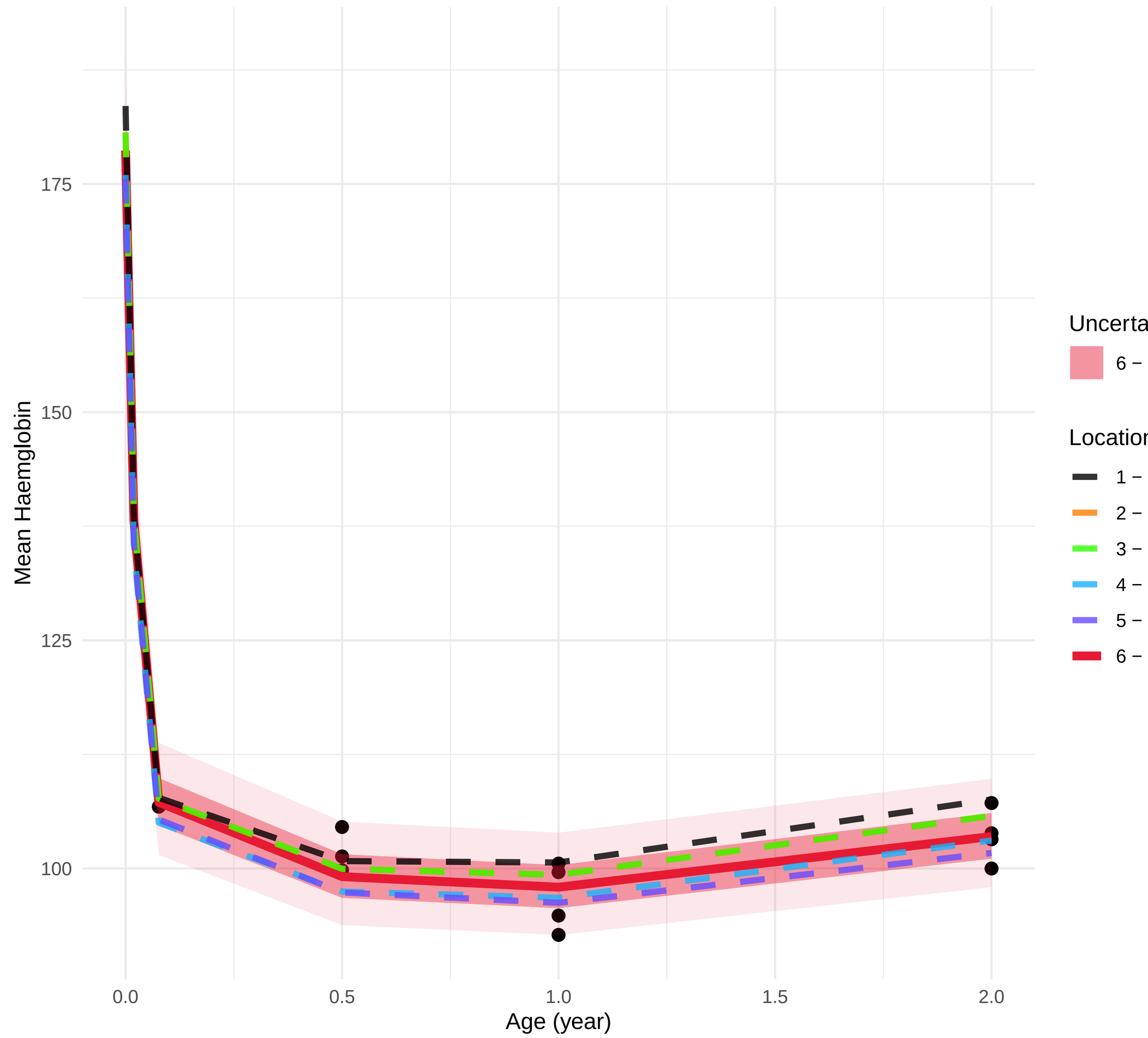

MR−BRT Age/Sex Fits − Males <2 Years Old − Mean Haemglobin − Haryana
WHO−2001 Adjustment Method

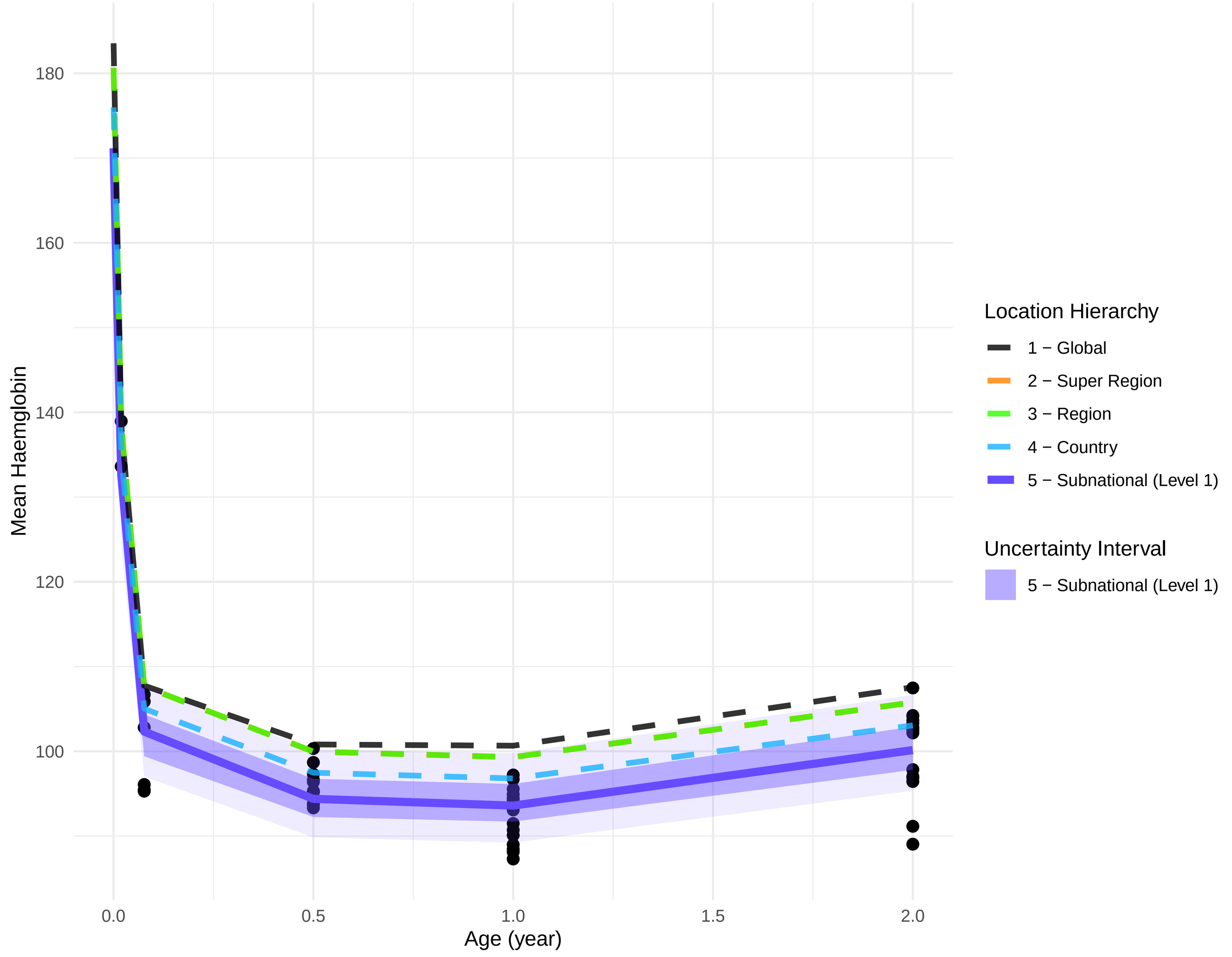

MR−BRT Age/Sex Fits − Males <2 Years Old − Mean Haemglobin − Haryana, Rural
WHO−2001 Adjustment Method

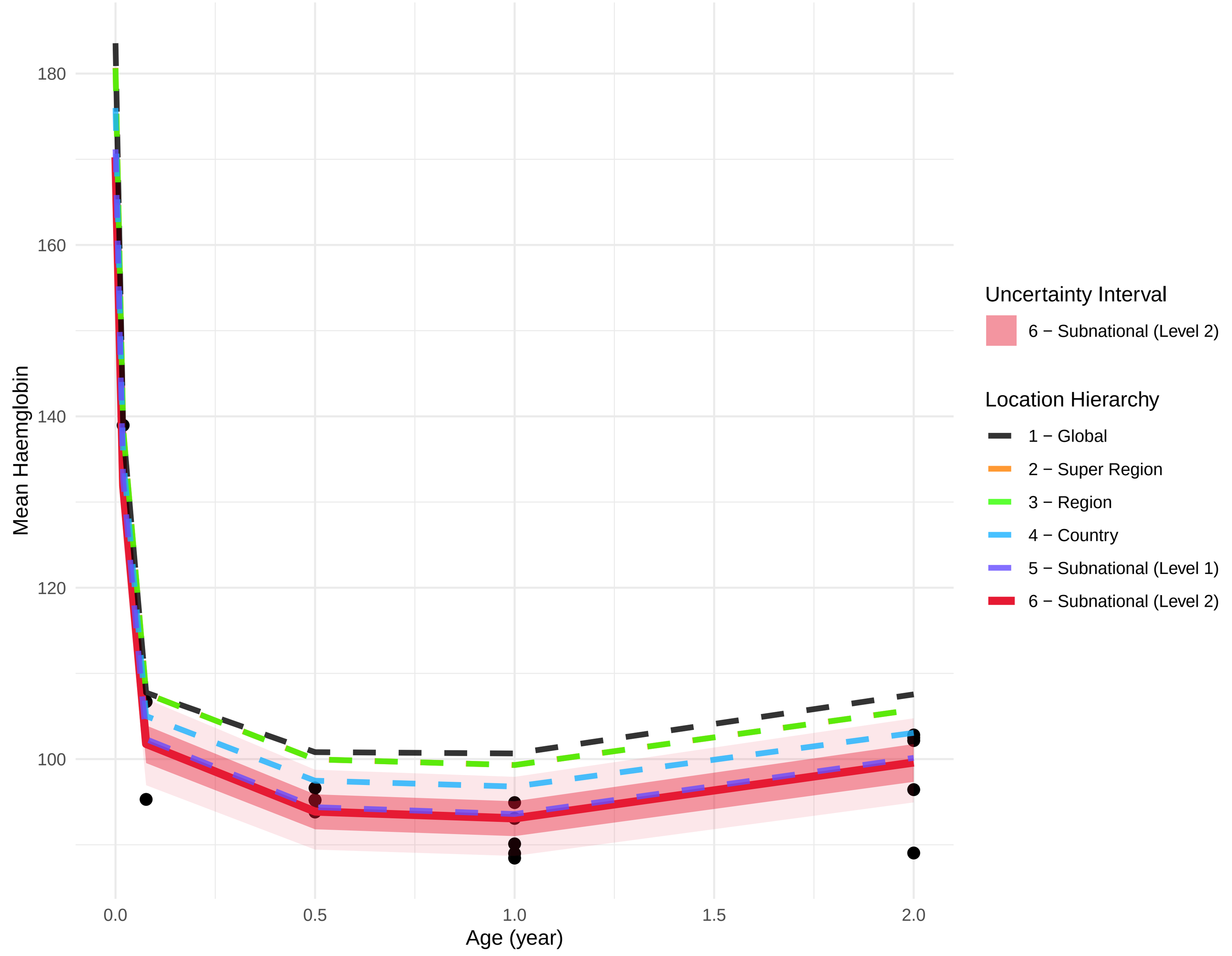

MR−BRT Age/Sex Fits − Males <2 Years Old − Mean Haemglobin − Haryana, Urban
WHO−2001 Adjustment Method

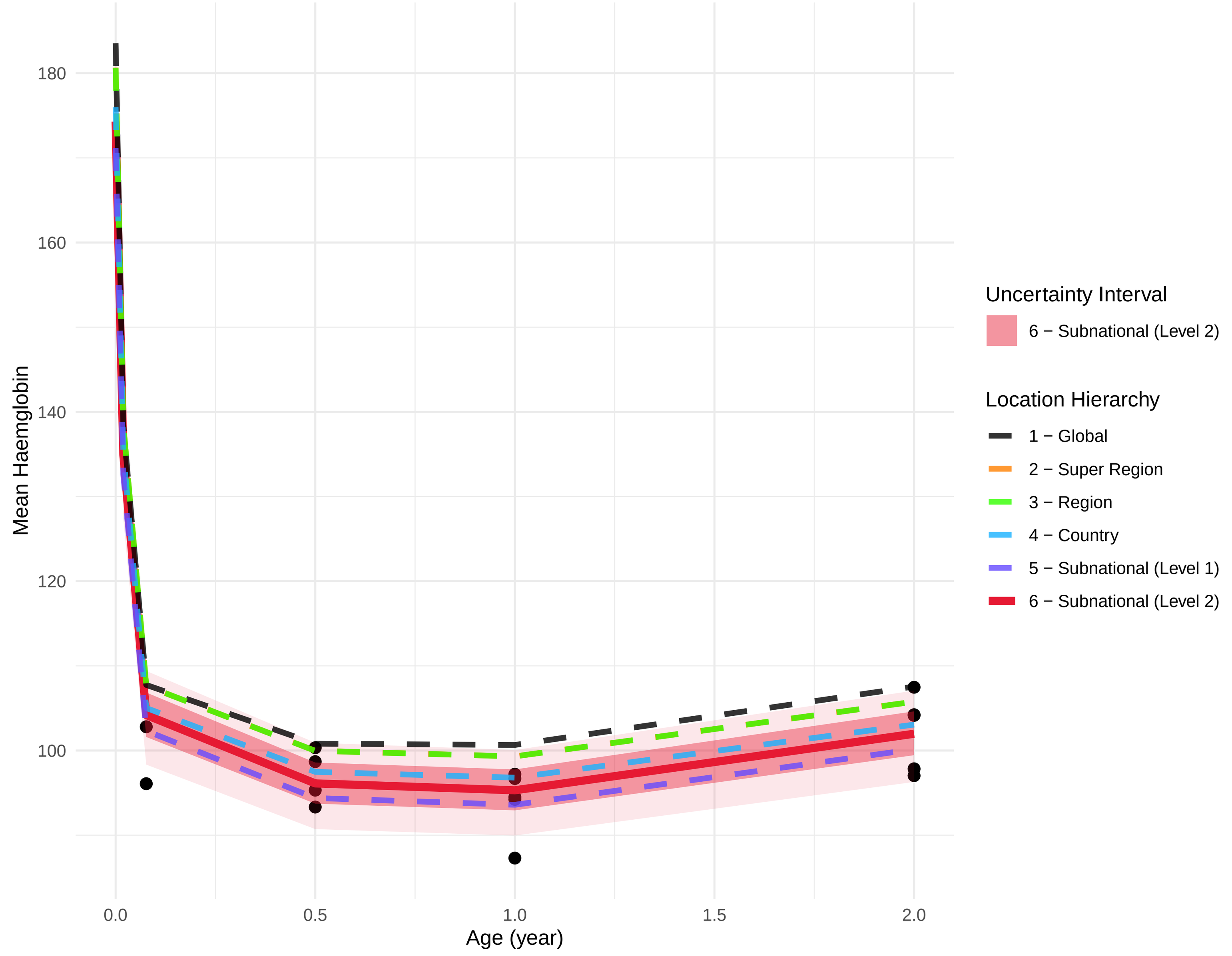

MR−BRT Age/Sex Fits − Males <2 Years Old − Mean Haemglobin − Himachal Pradesh
WHO−2001 Adjustment Method

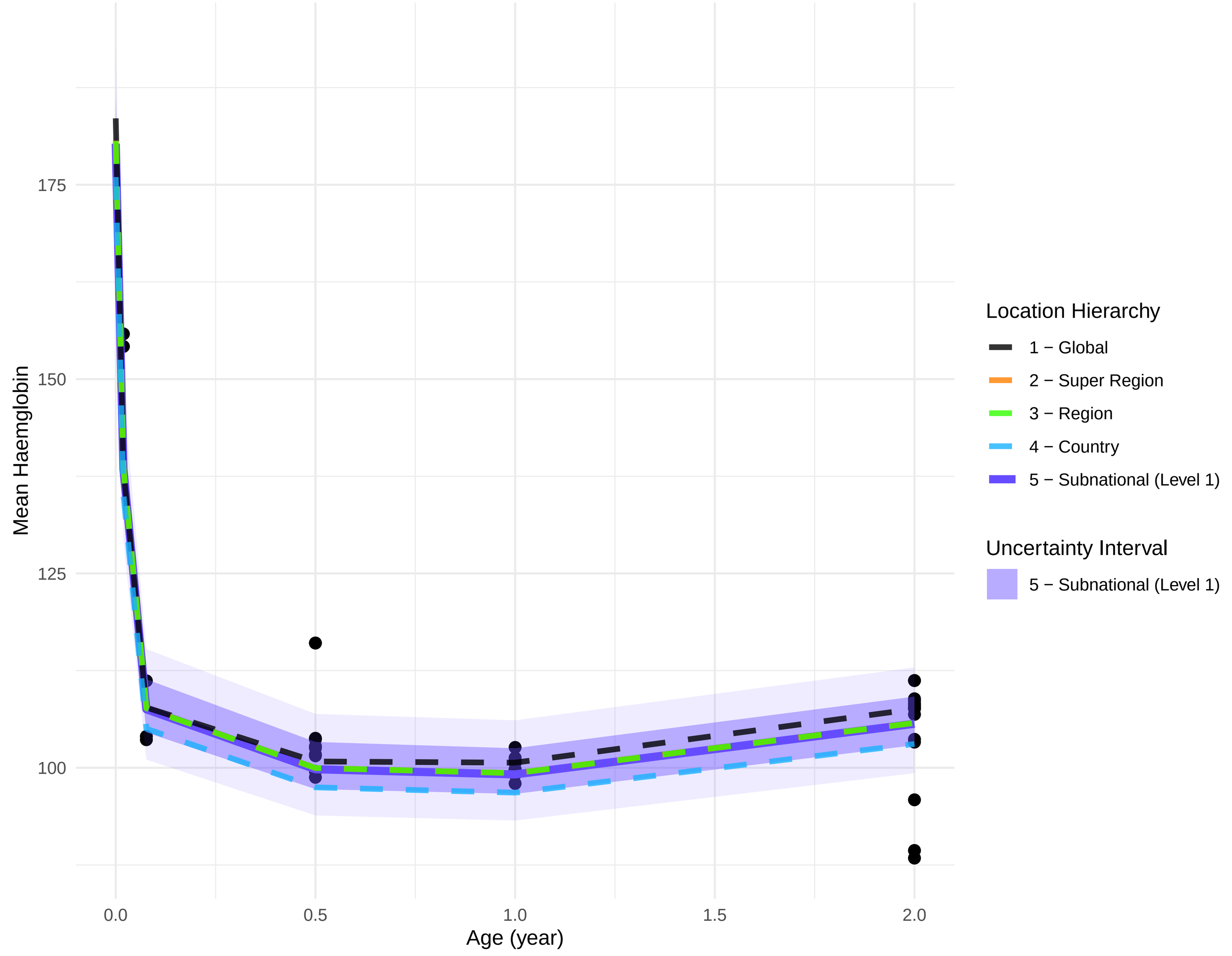

MR−BRT Age/Sex Fits − Males <2 Years Old − Mean Haemglobin − Himachal Pradesh, Rural
WHO−2001 Adjustment Method

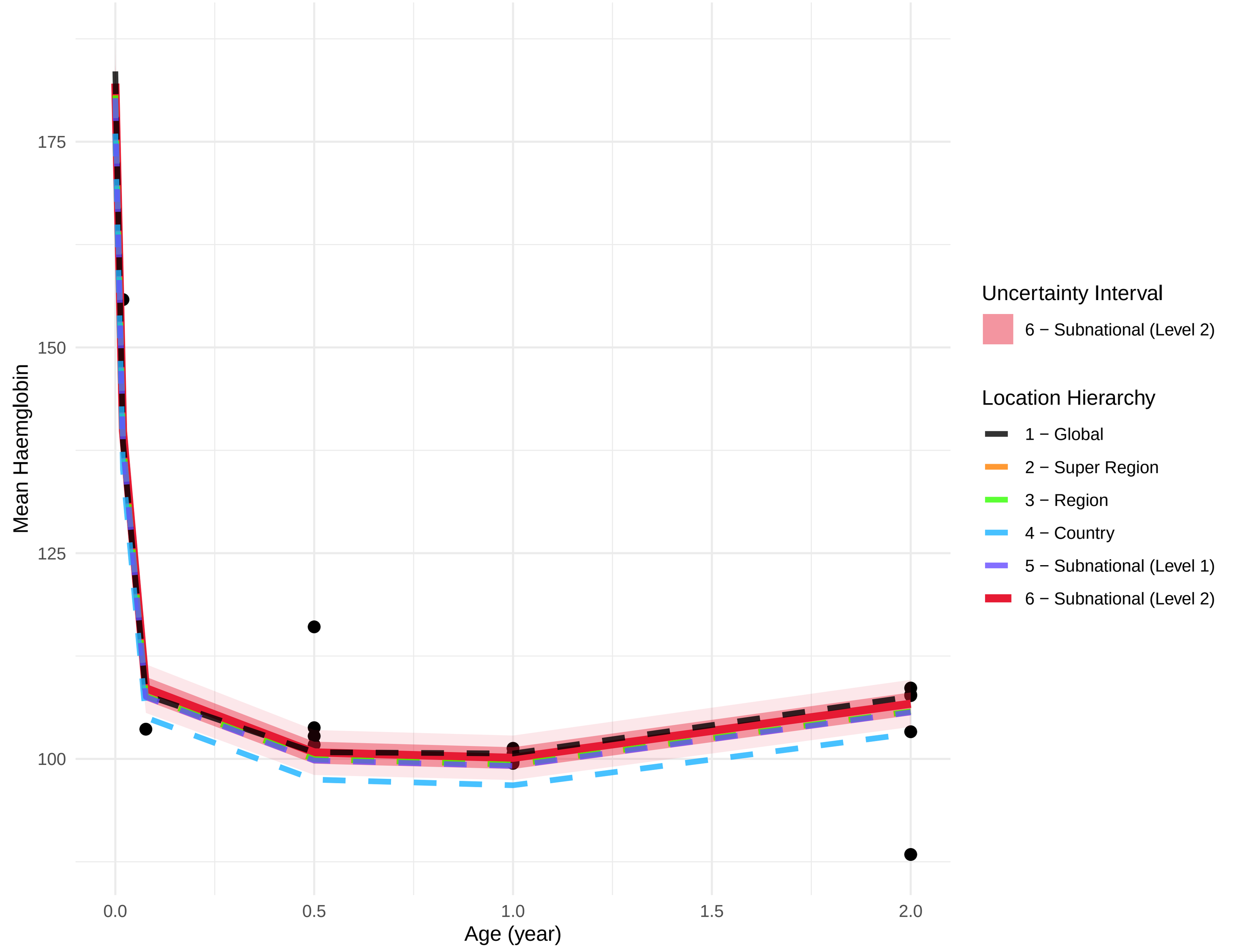

MR−BRT Age/Sex Fits − Males <2 Years Old − Mean Haemglobin − Himachal Pradesh, Urban
WHO−2001 Adjustment Method

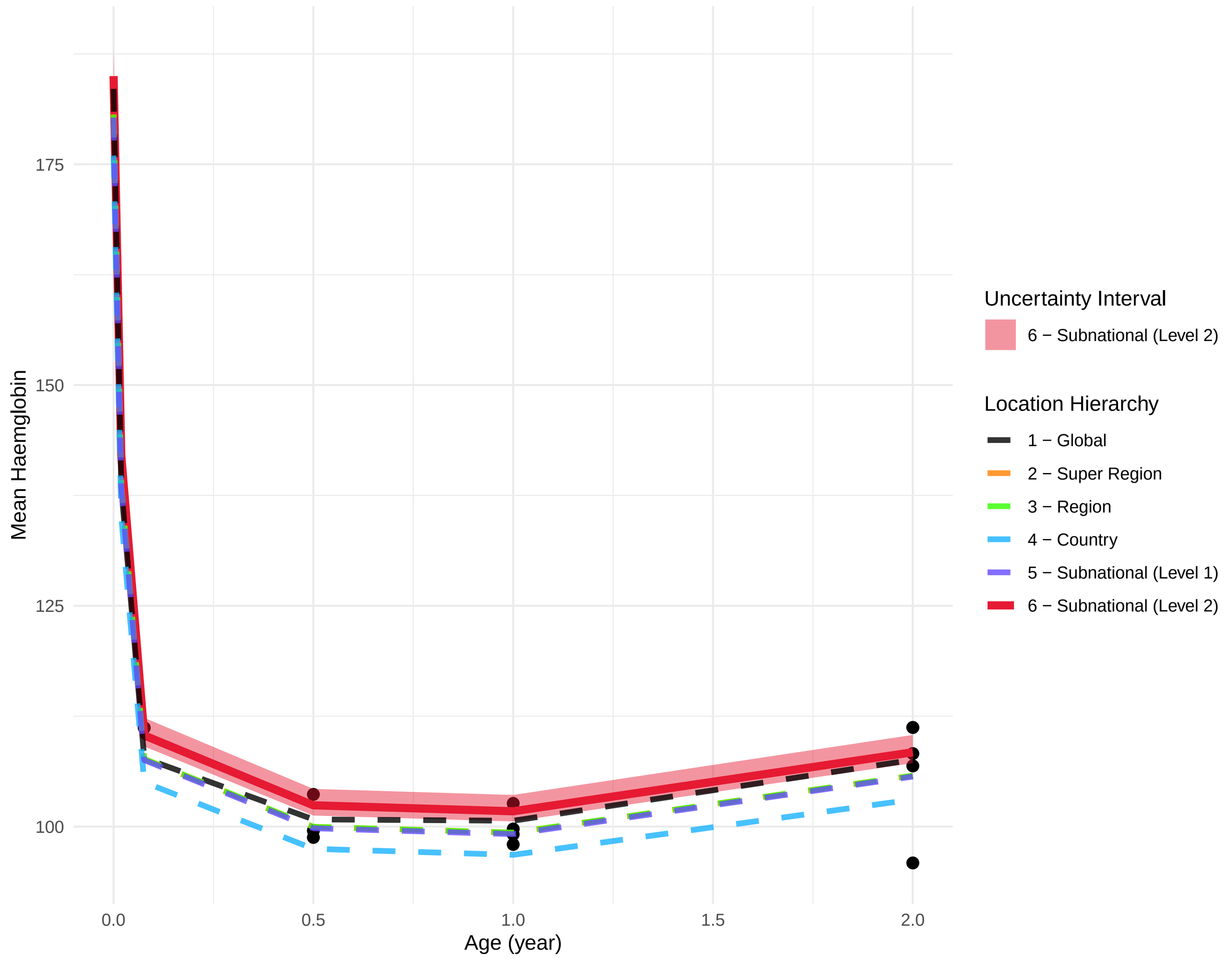

MR−BRT Age/Sex Fits − Males <2 Years Old − Mean Haemglobin − Jammu & Kashmir and Ladakh
WHO−2001 Adjustment Method

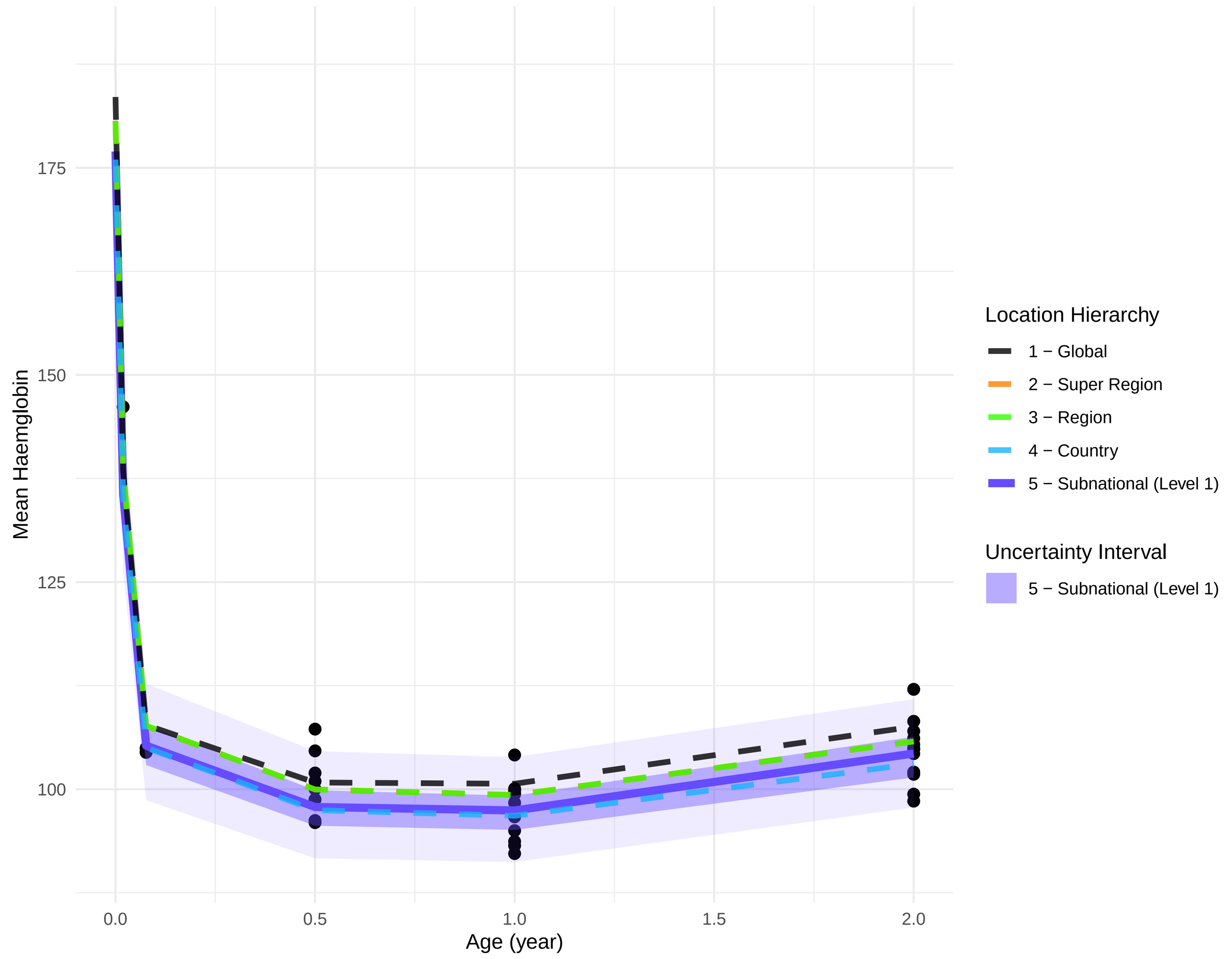

MR−BRT Age/Sex Fits − Males <2 Years Old − Mean Haemglobin − Jammu & Kashmir and Ladakh, Rural
WHO−2001 Adjustment Method

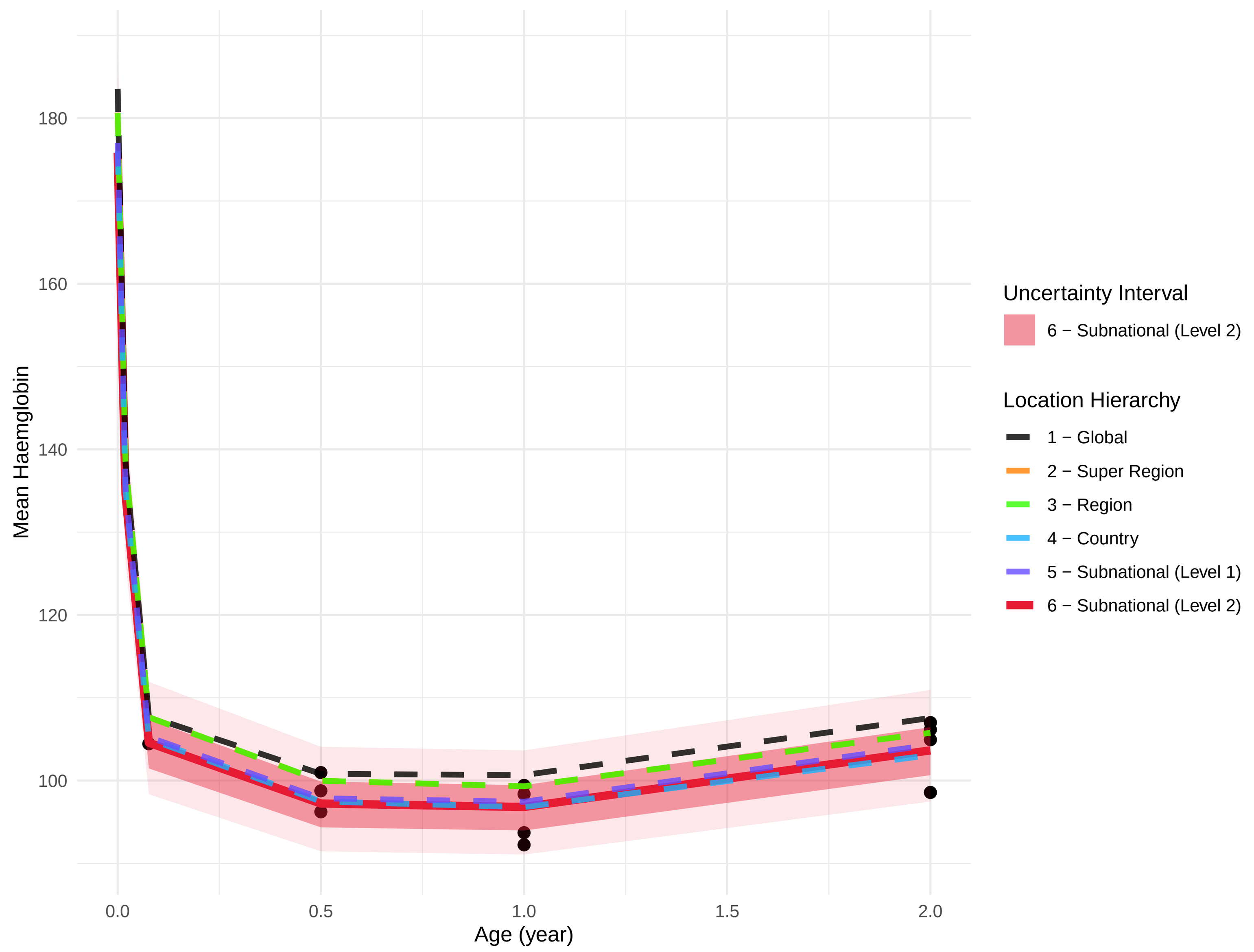

MR−BRT Age/Sex Fits − Males <2 Years Old − Mean Haemglobin − Jammu & Kashmir and Ladakh, Urban
WHO−2001 Adjustment Method

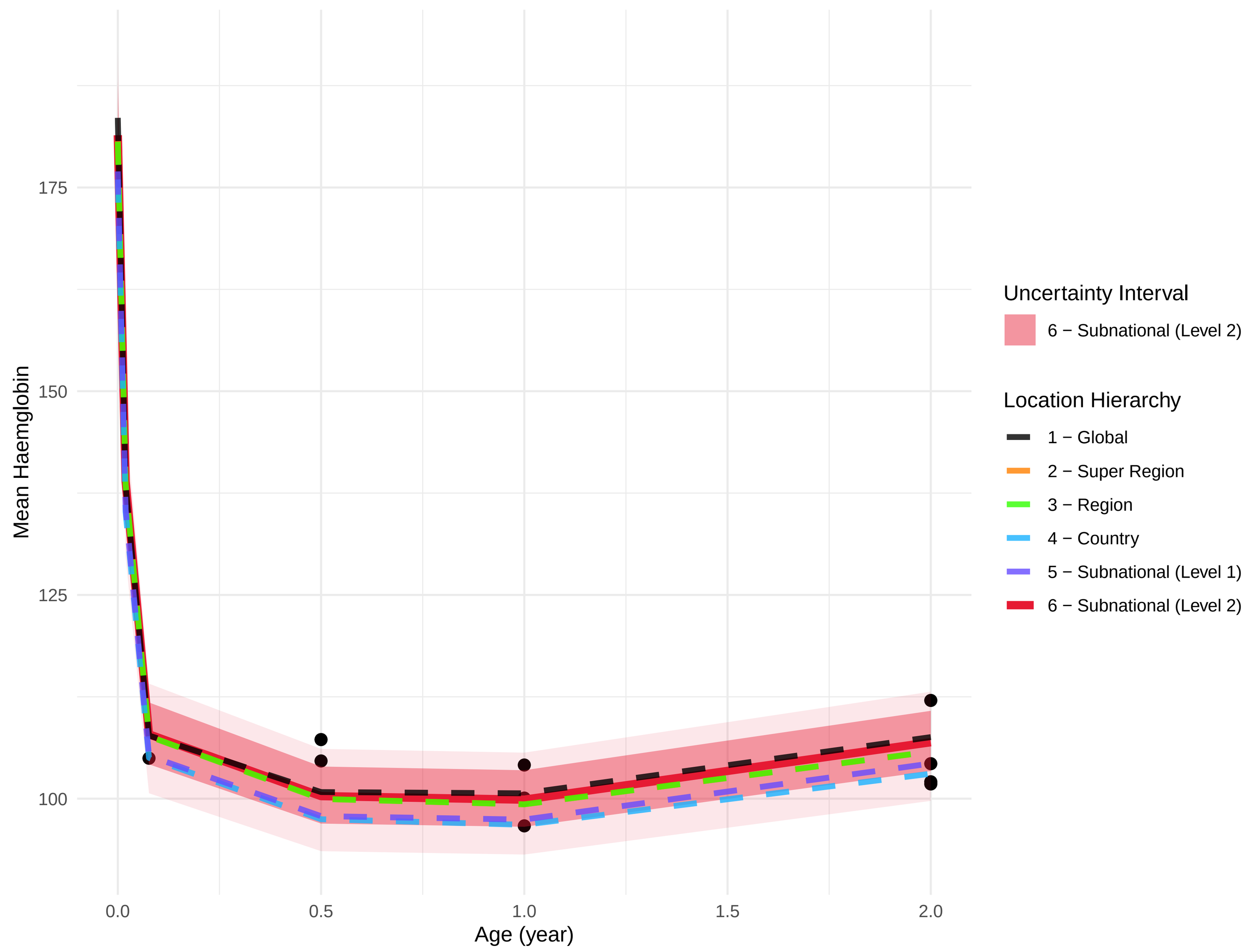

MR−BRT Age/Sex Fits − Males <2 Years Old − Mean Haemglobin − Jharkhand
WHO−2001 Adjustment Method

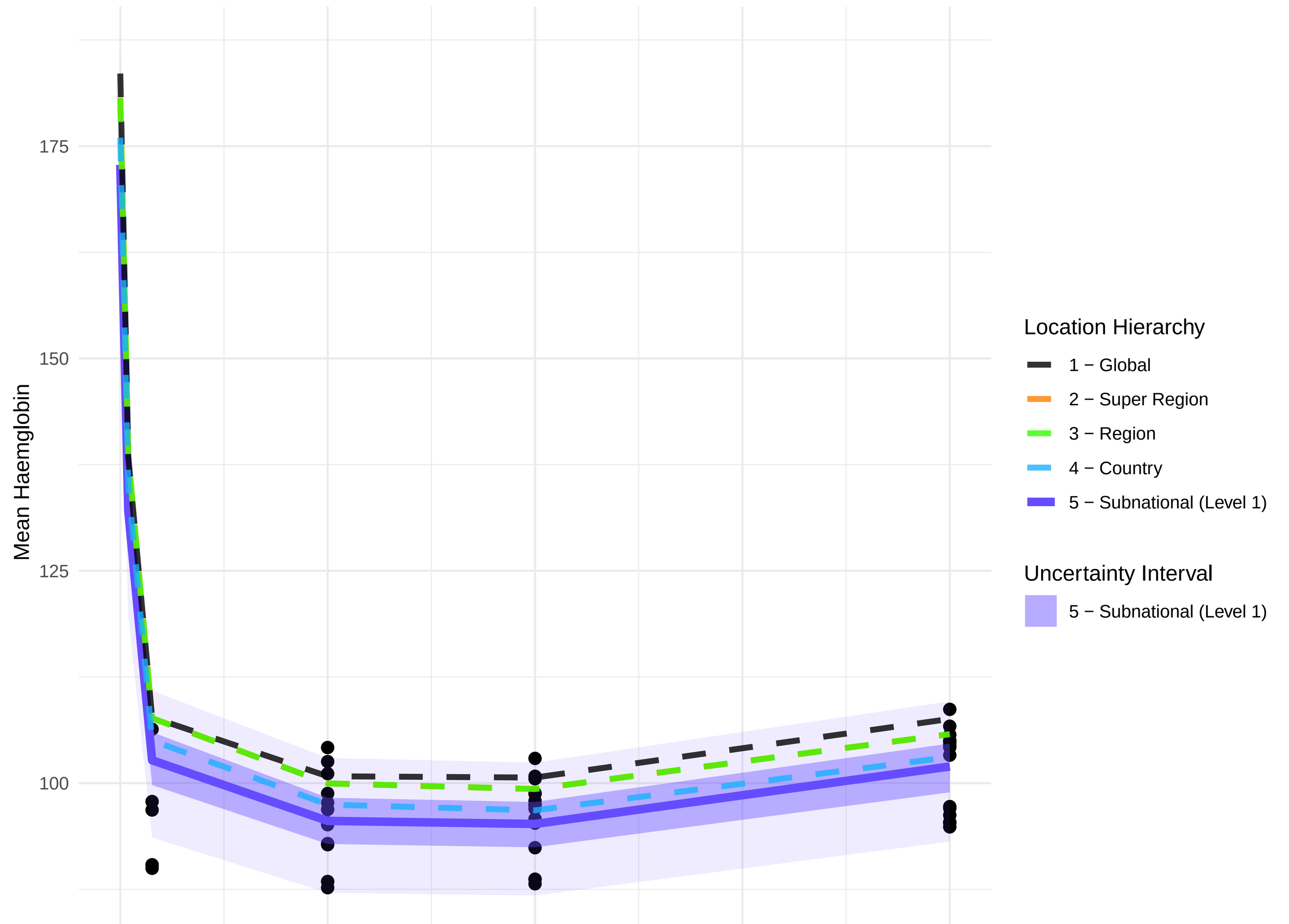

MR−BRT Age/Sex Fits − Males <2 Years Old − Mean Haemglobin − Jharkhand, Rural
WHO−2001 Adjustment Method

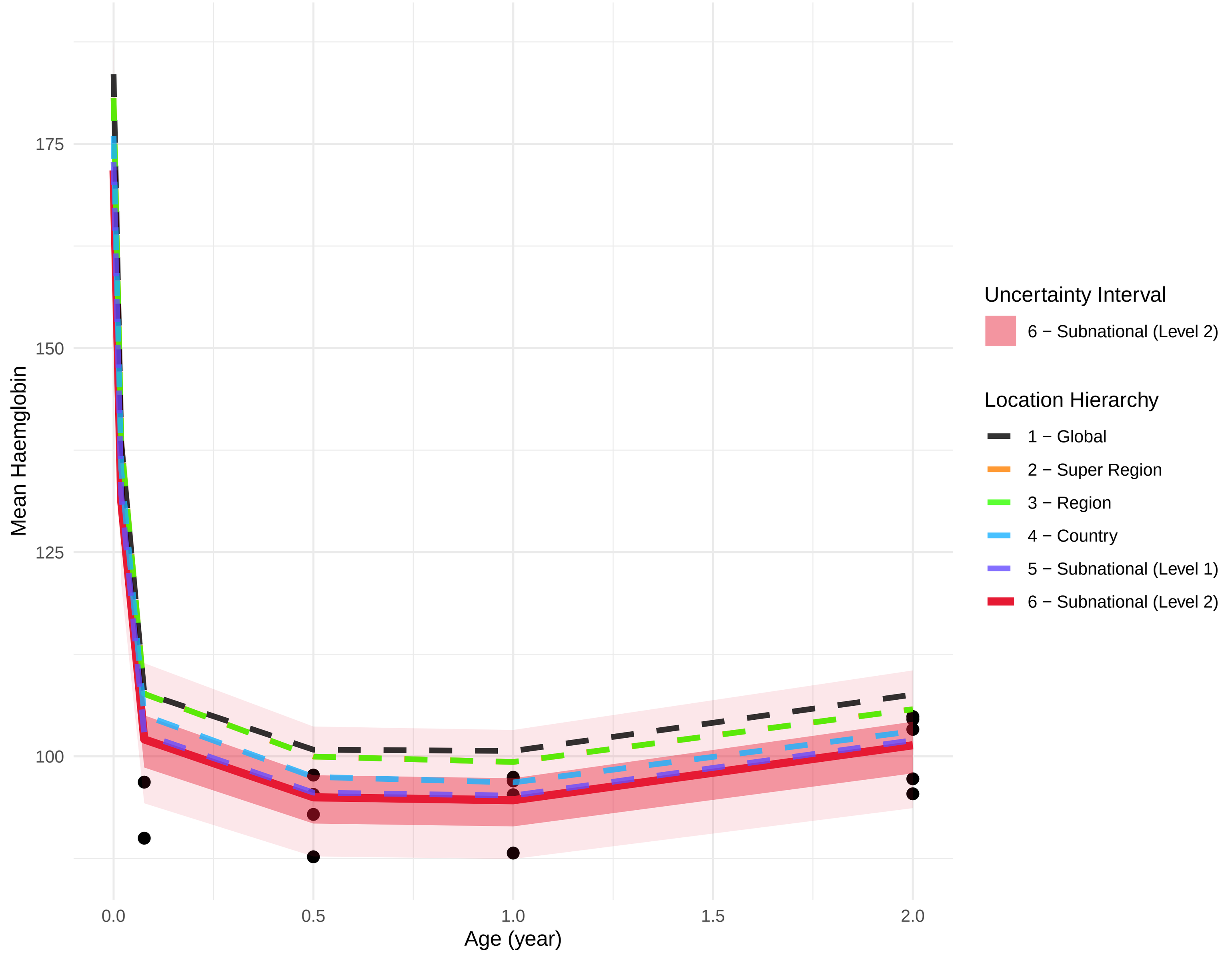

MR−BRT Age/Sex Fits − Males <2 Years Old − Mean Haemglobin − Jharkhand, Urban
WHO−2001 Adjustment Method

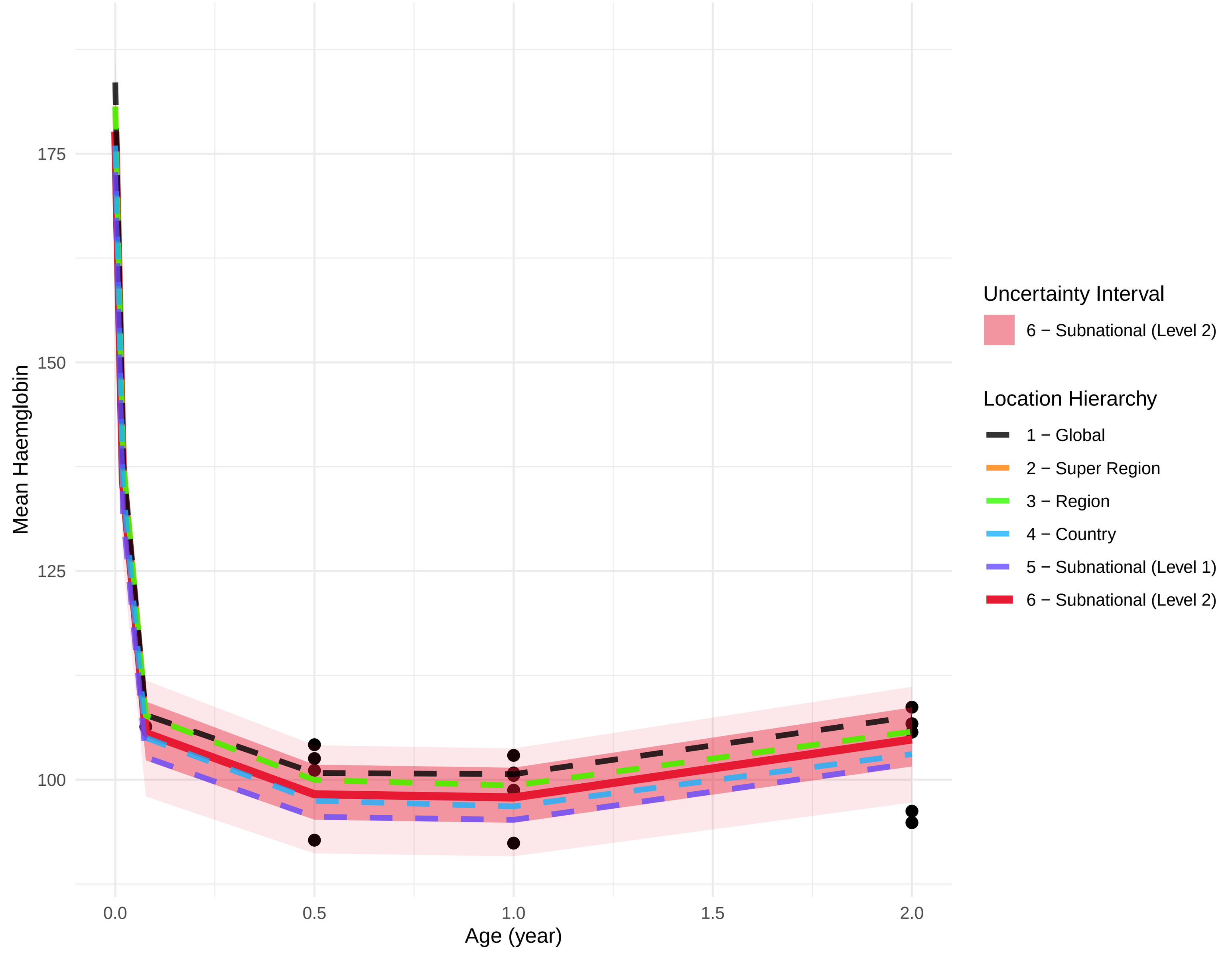

MR−BRT Age/Sex Fits − Males <2 Years Old − Mean Haemglobin − Karnataka
WHO−2001 Adjustment Method

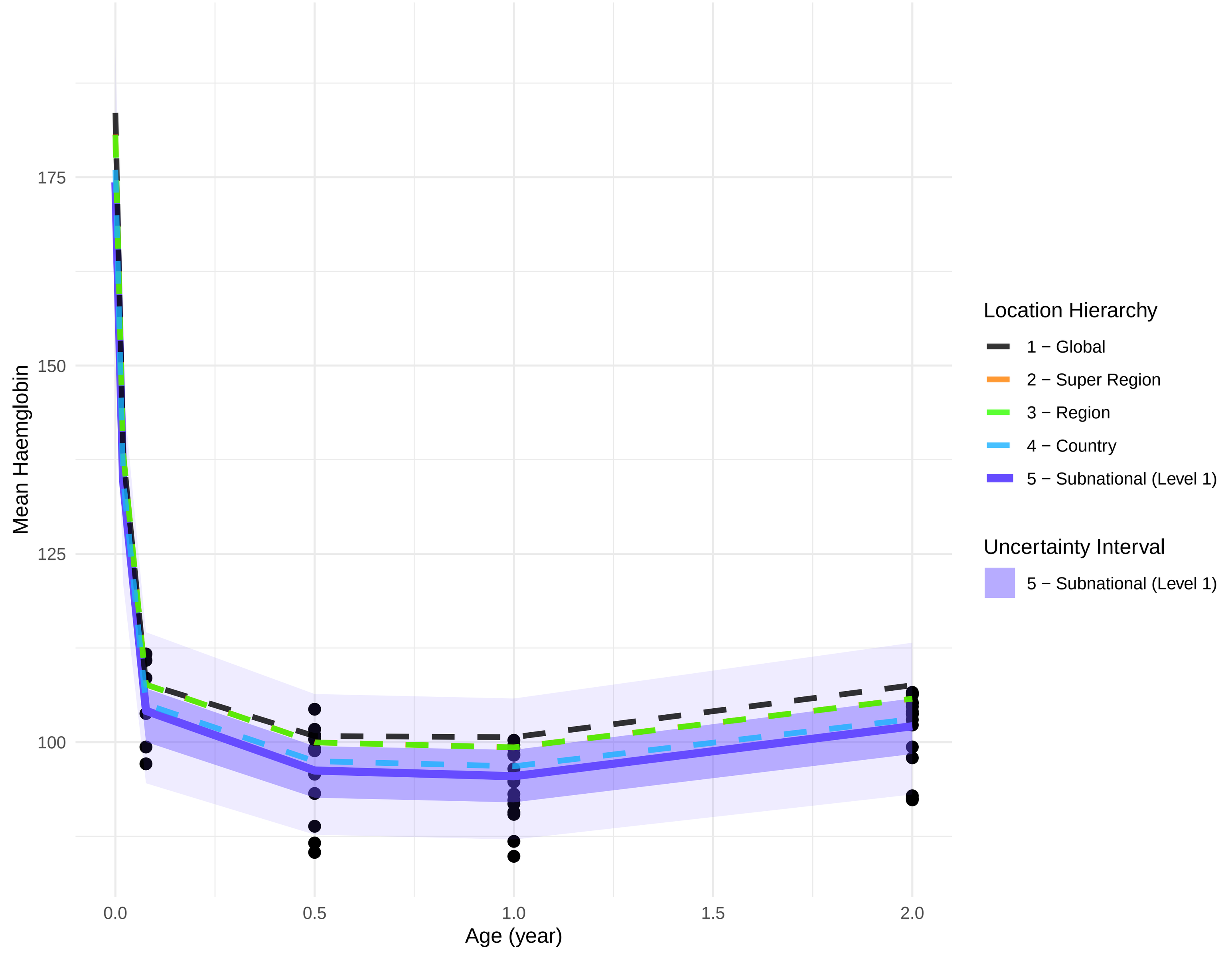

MR−BRT Age/Sex Fits − Males <2 Years Old − Mean Haemglobin − Karnataka, Rural
WHO−2001 Adjustment Method

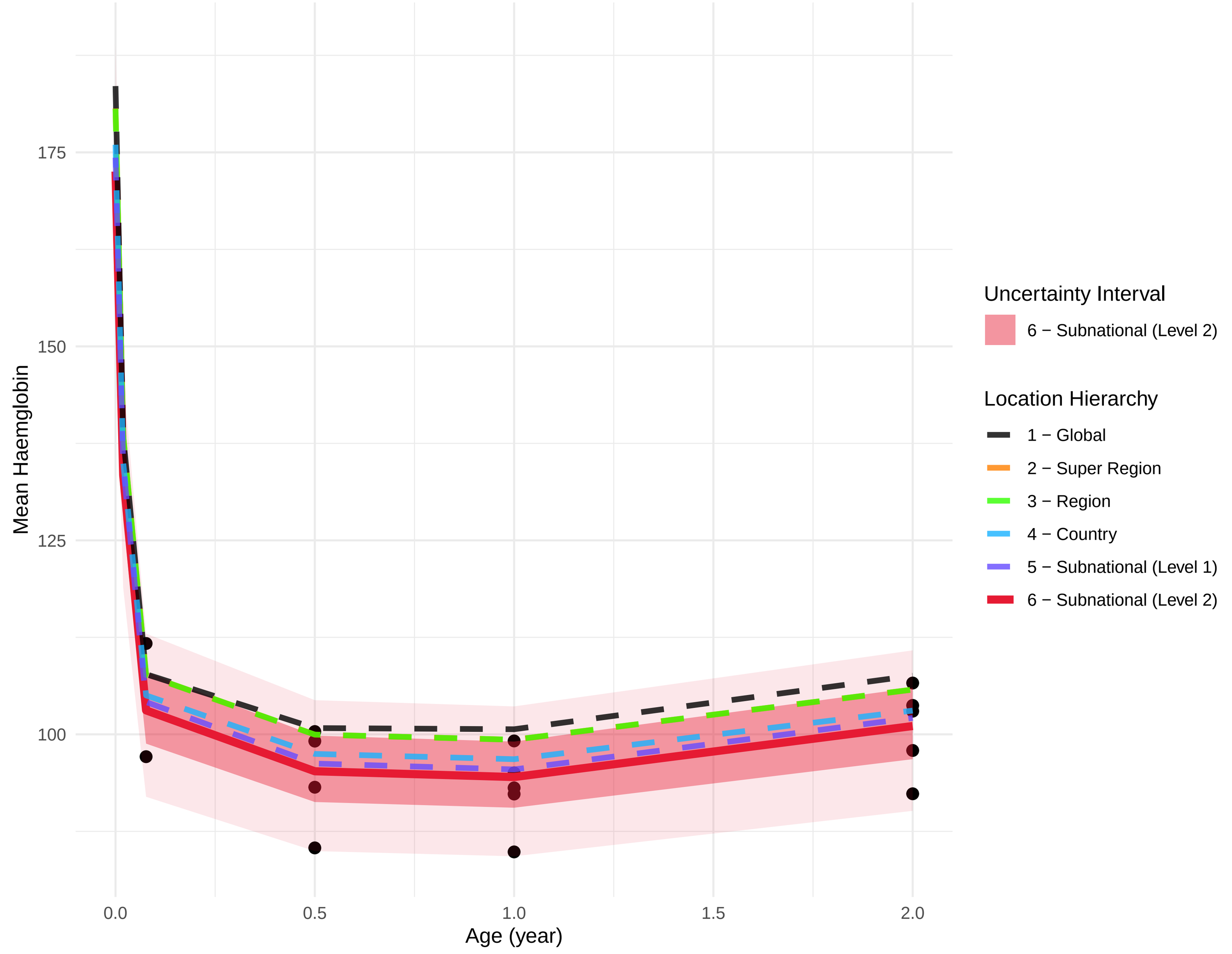

MR−BRT Age/Sex Fits − Males <2 Years Old − Mean Haemglobin − Karnataka, Urban
WHO−2001 Adjustment Method

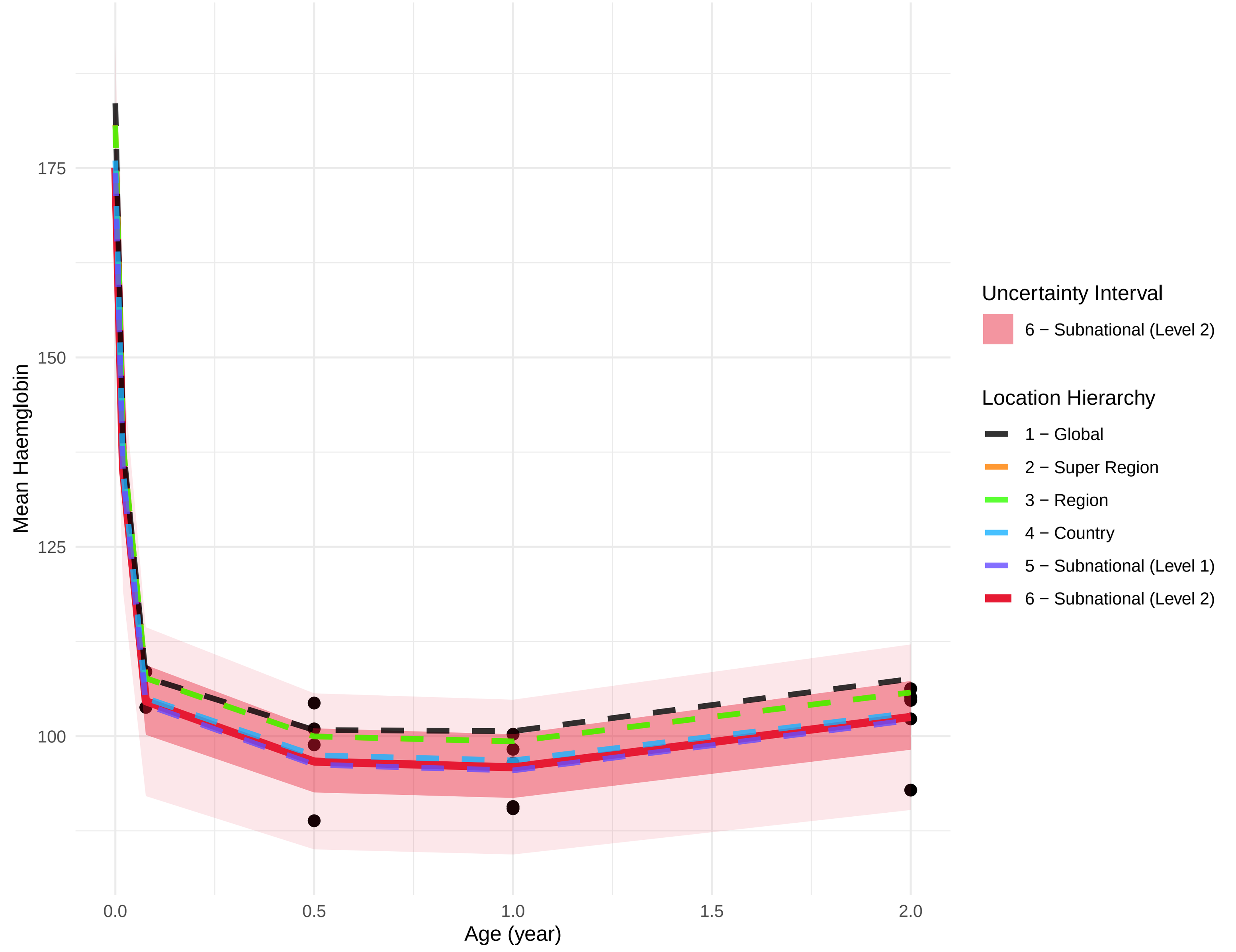

MR−BRT Age/Sex Fits − Males <2 Years Old − Mean Haemglobin − Kerala
WHO−2001 Adjustment Method

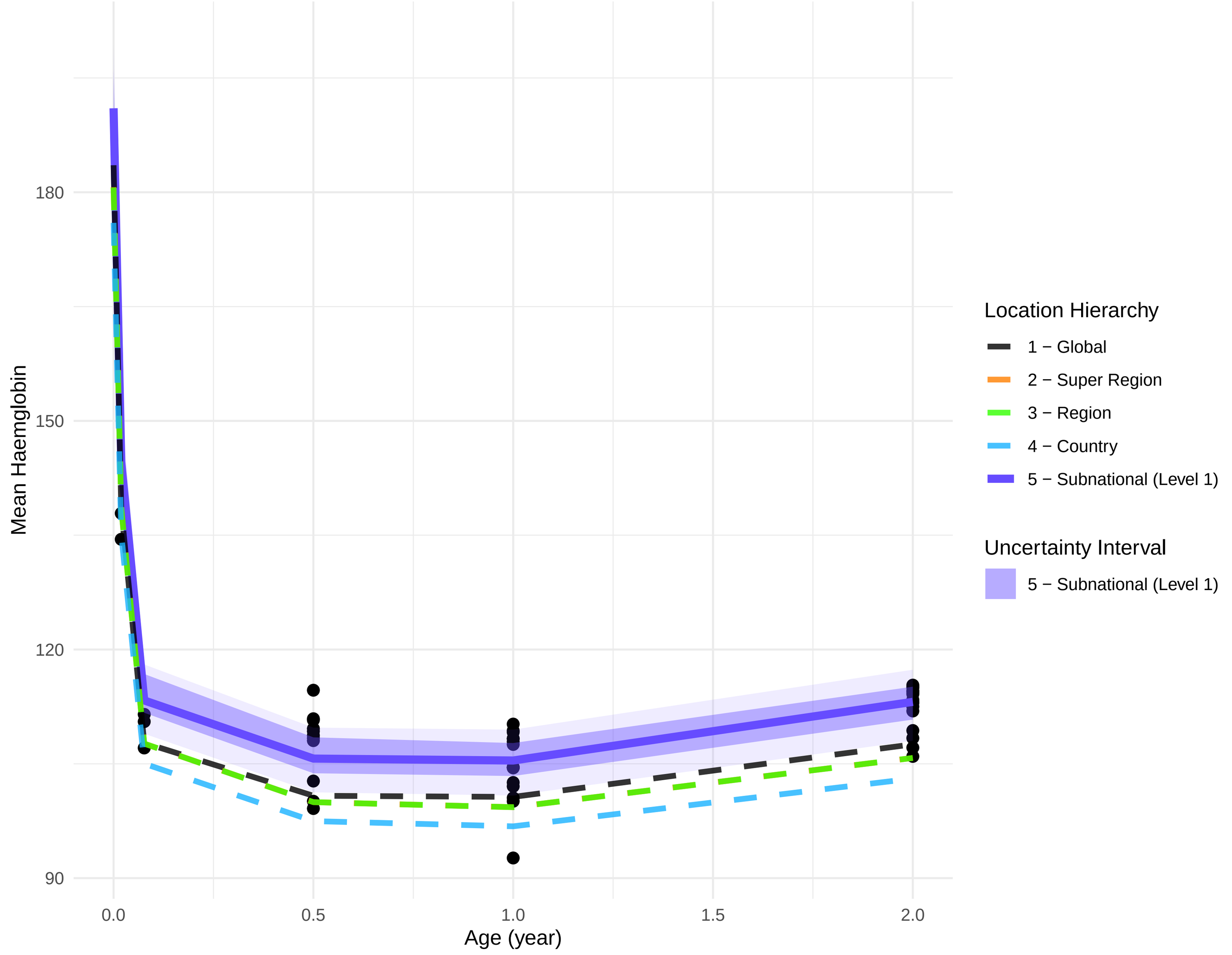

MR−BRT Age/Sex Fits − Males <2 Years Old − Mean Haemglobin − Kerala, Rural
WHO−2001 Adjustment Method

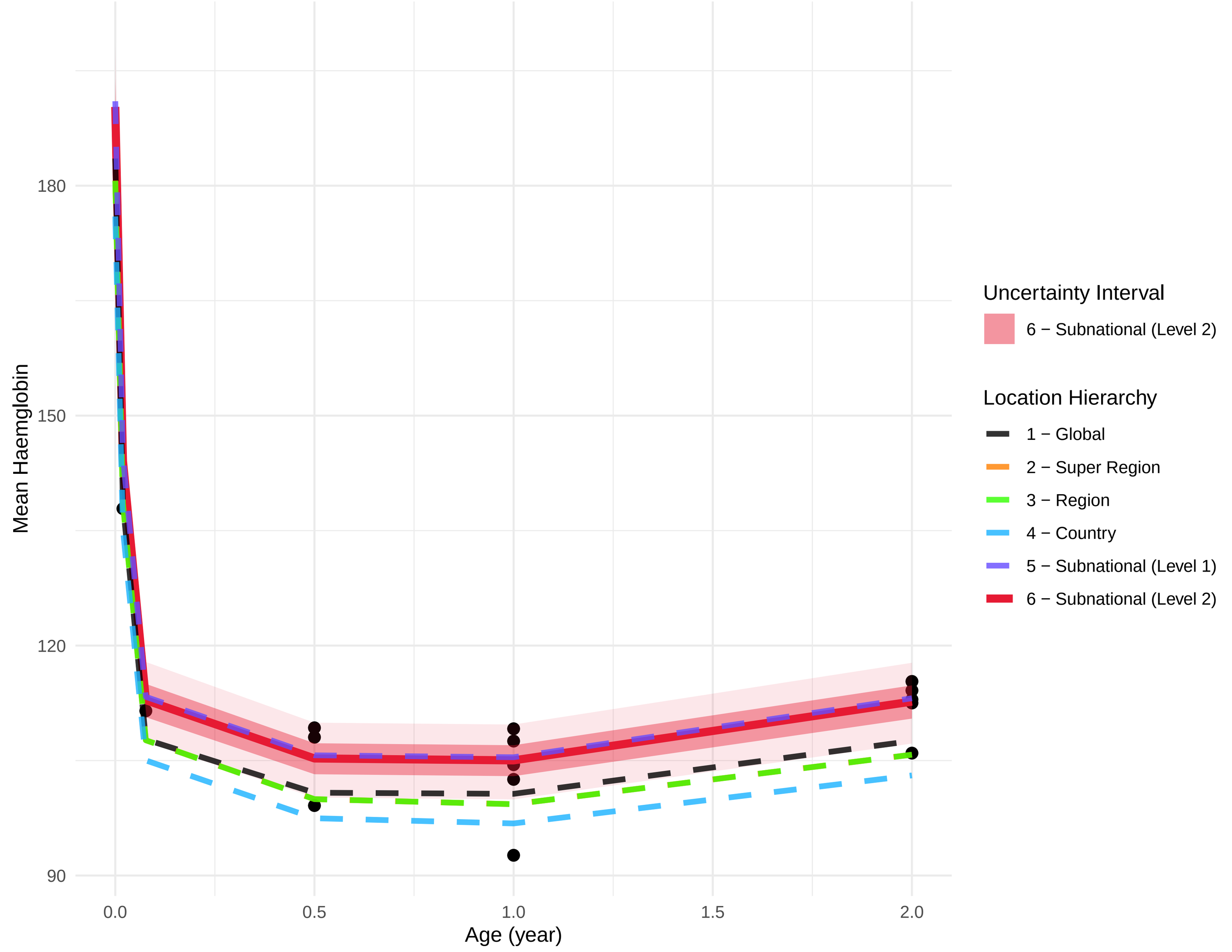

MR−BRT Age/Sex Fits − Males <2 Years Old − Mean Haemglobin − Kerala, Urban
WHO−2001 Adjustment Method

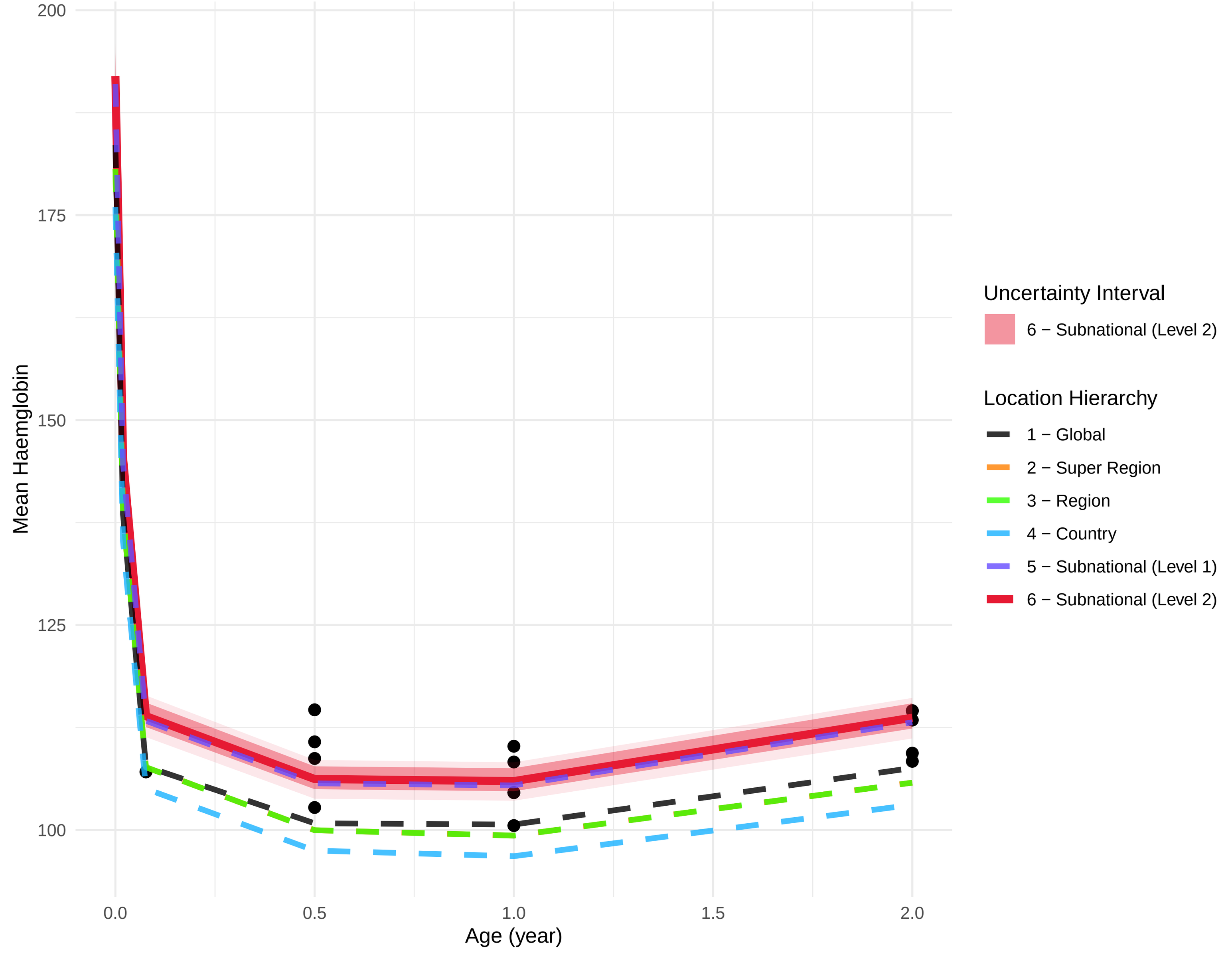

MR−BRT Age/Sex Fits − Males <2 Years Old − Mean Haemglobin − Madhya Pradesh
WHO−2001 Adjustment Method

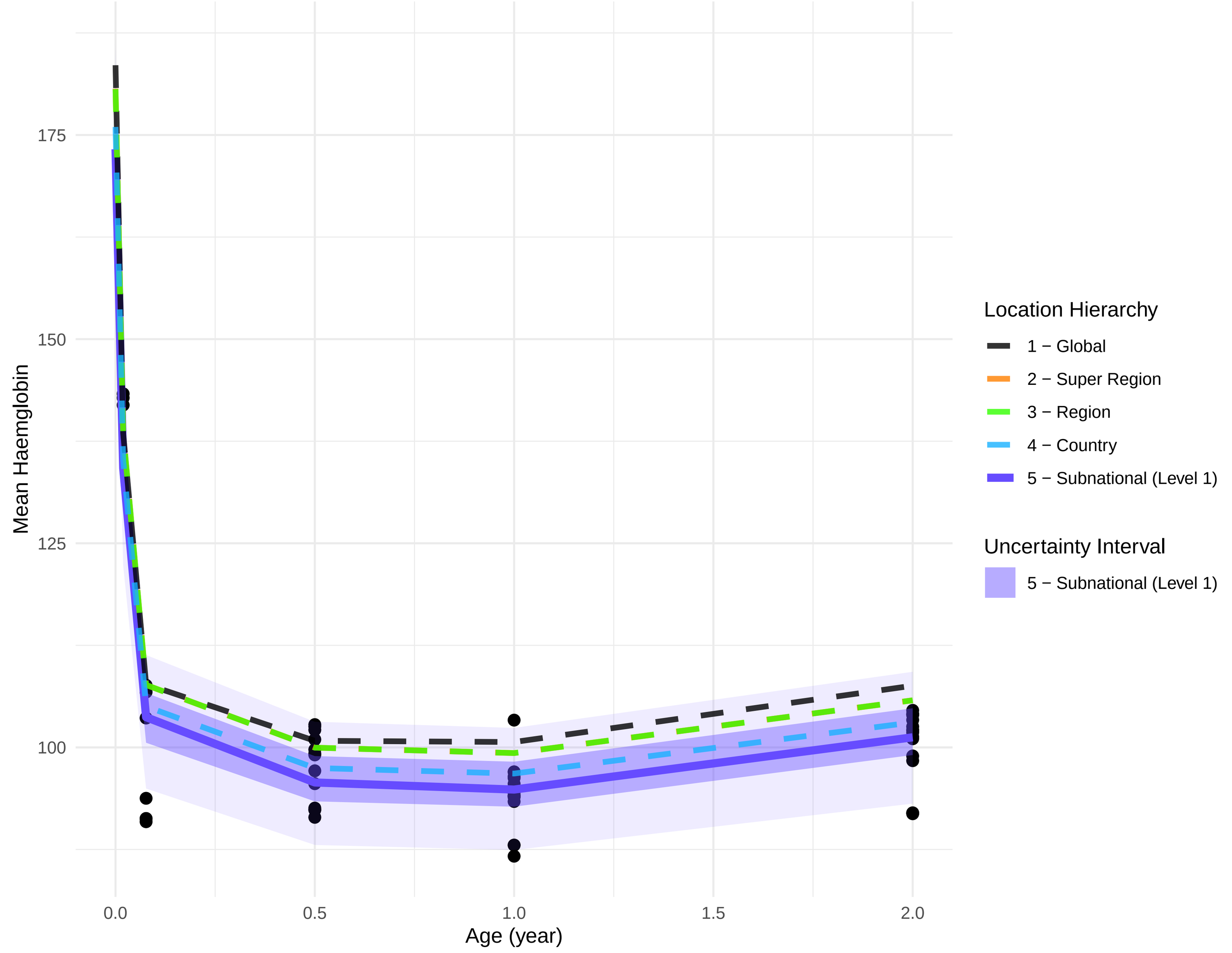

MR−BRT Age/Sex Fits − Males <2 Years Old − Mean Haemglobin − Madhya Pradesh, Rural
WHO−2001 Adjustment Method

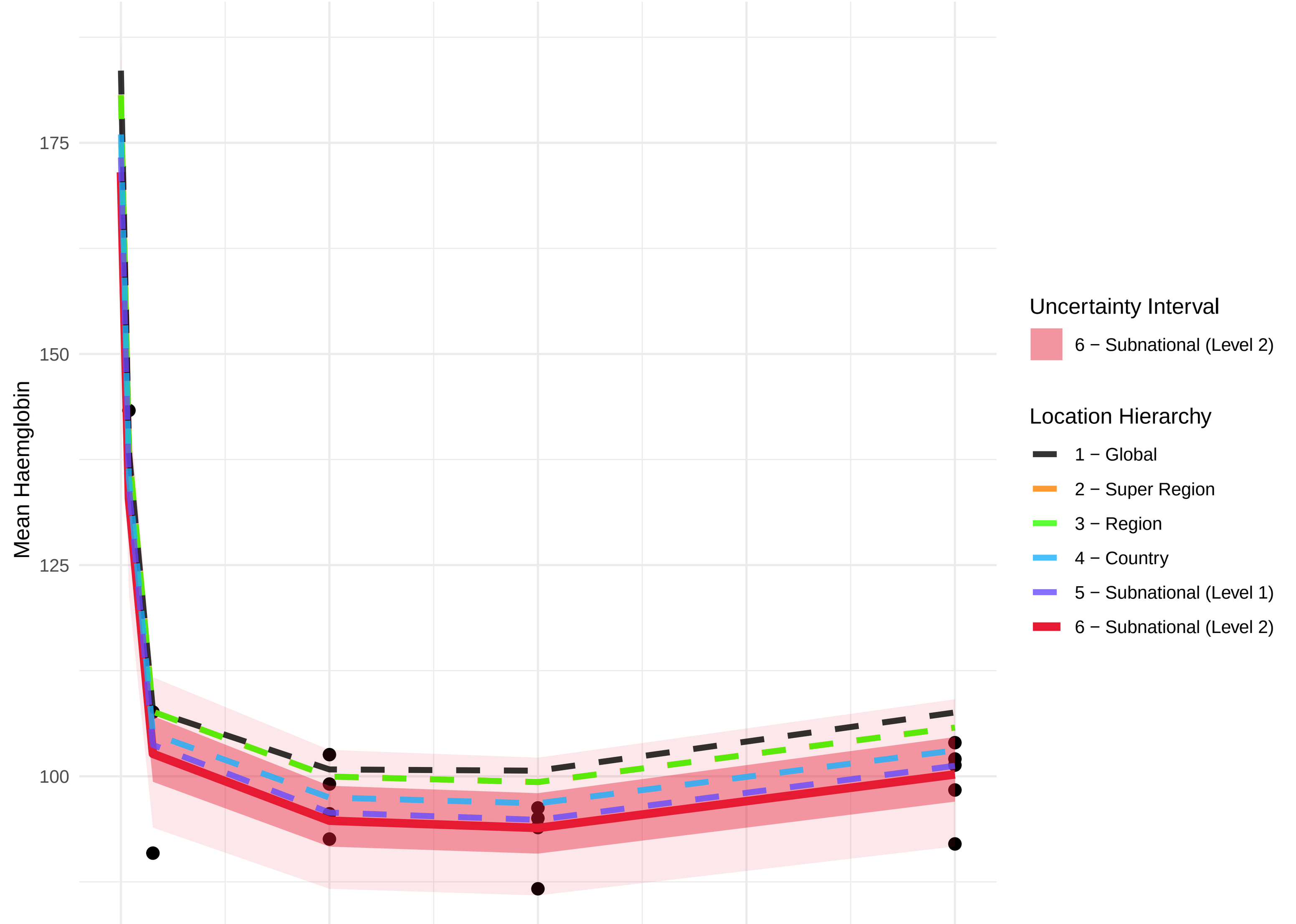

MR−BRT Age/Sex Fits − Males <2 Years Old − Mean Haemglobin − Madhya Pradesh, Urban
WHO−2001 Adjustment Method

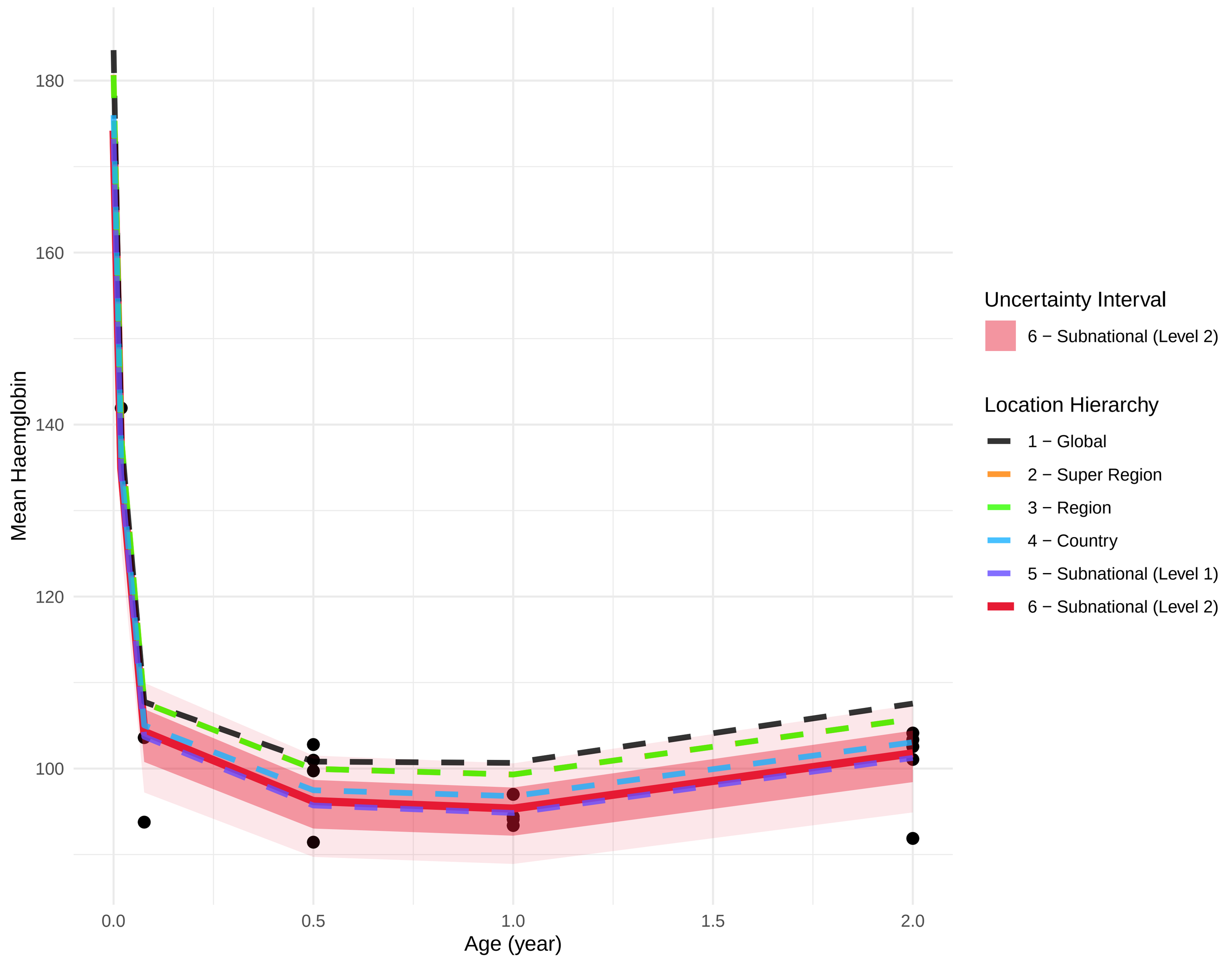

MR−BRT Age/Sex Fits − Males <2 Years Old − Mean Haemglobin − Maharashtra
WHO−2001 Adjustment Method

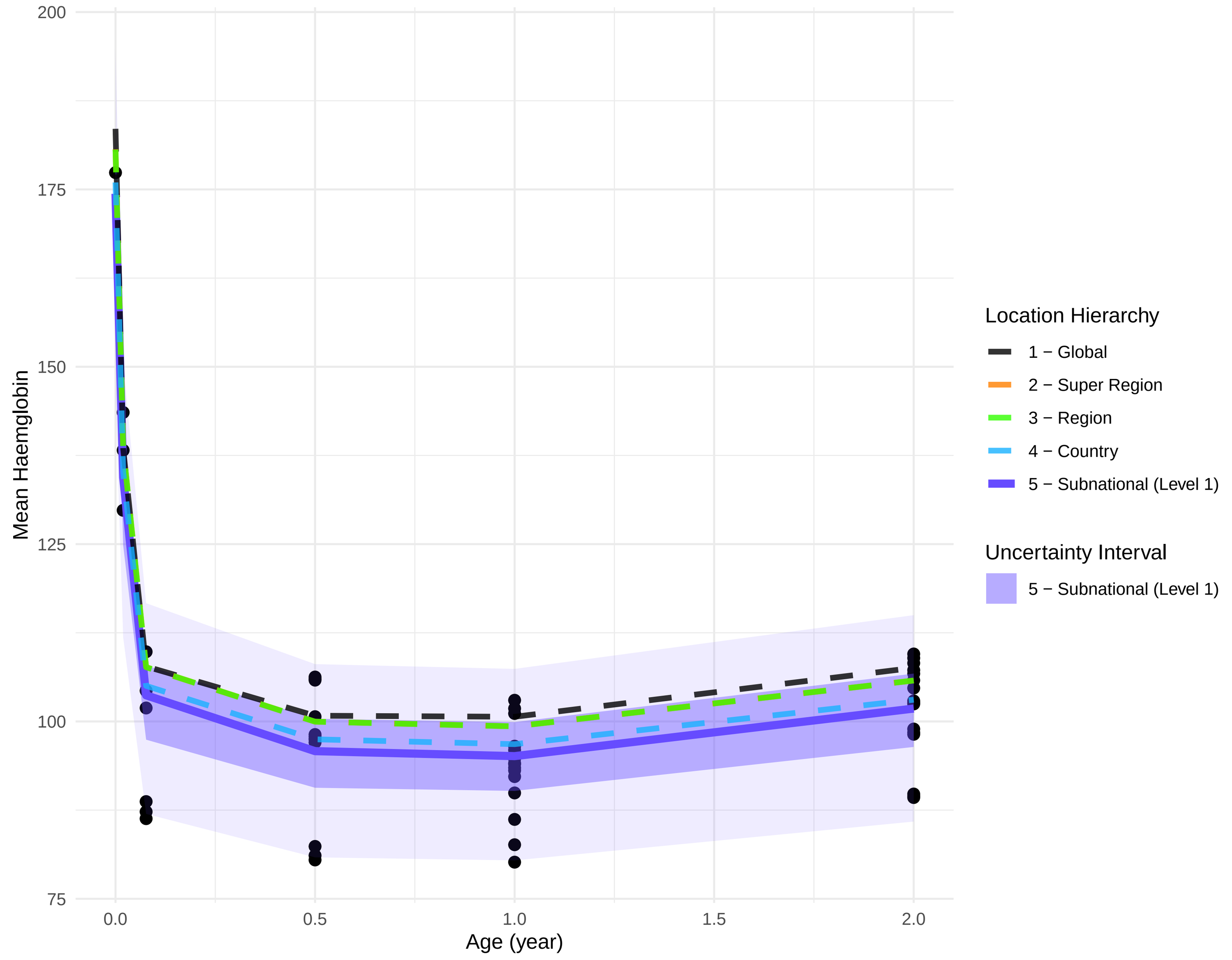

MR−BRT Age/Sex Fits − Males <2 Years Old − Mean Haemglobin − Maharashtra, Rural
WHO−2001 Adjustment Method

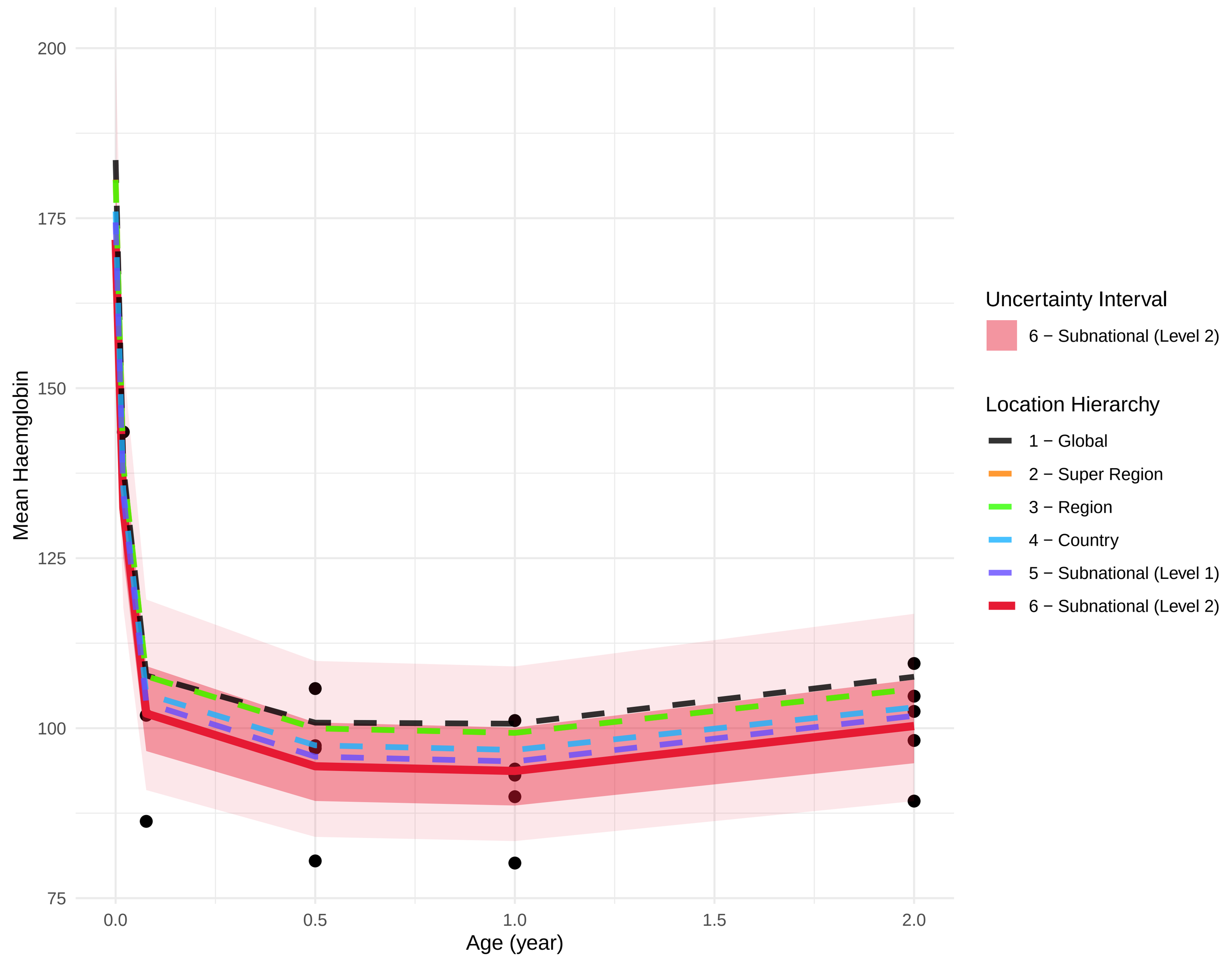

MR−BRT Age/Sex Fits − Males <2 Years Old − Mean Haemglobin − Maharashtra, Urban
WHO−2001 Adjustment Method

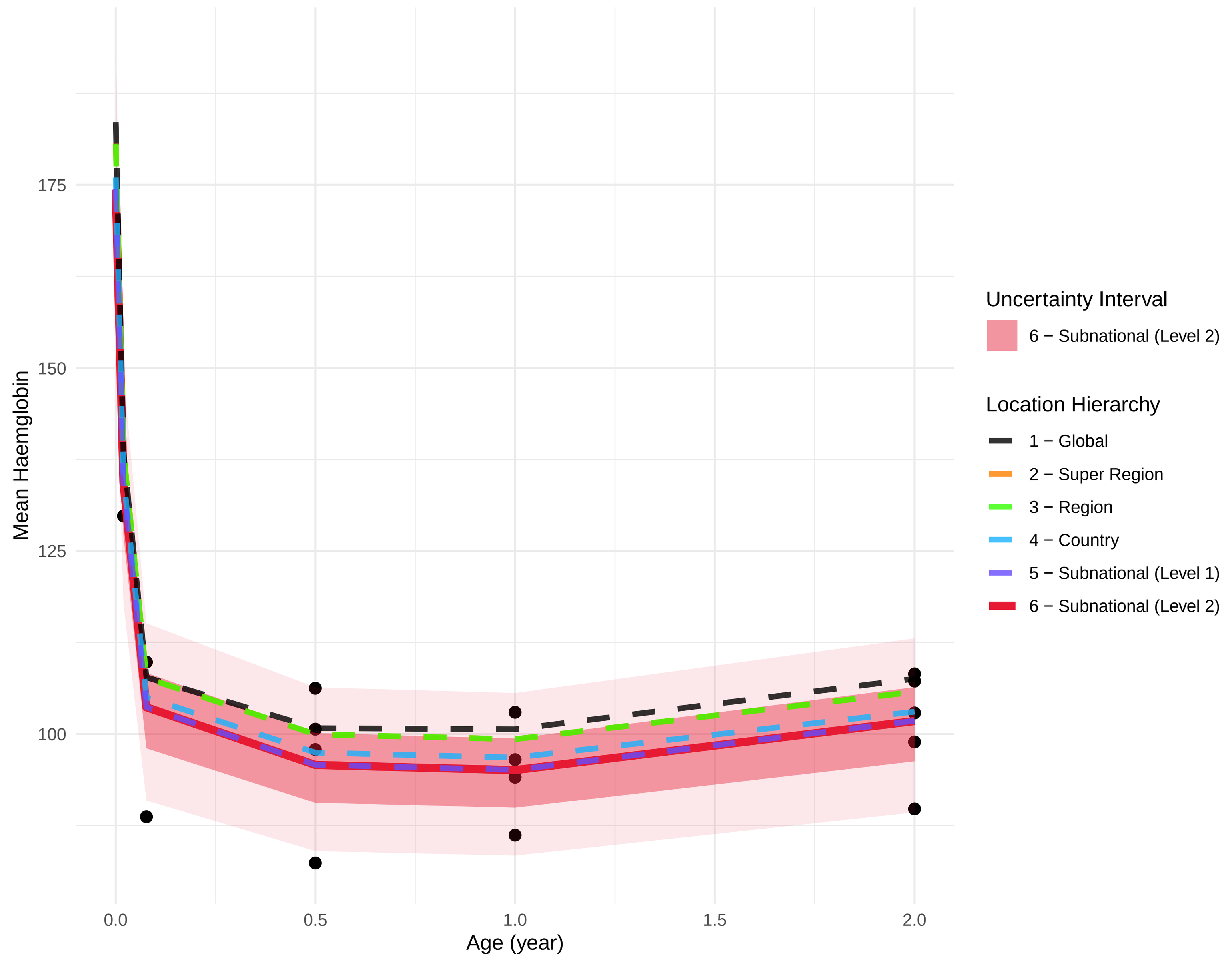

MR−BRT Age/Sex Fits − Males <2 Years Old − Mean Haemglobin − Manipur
WHO−2001 Adjustment Method

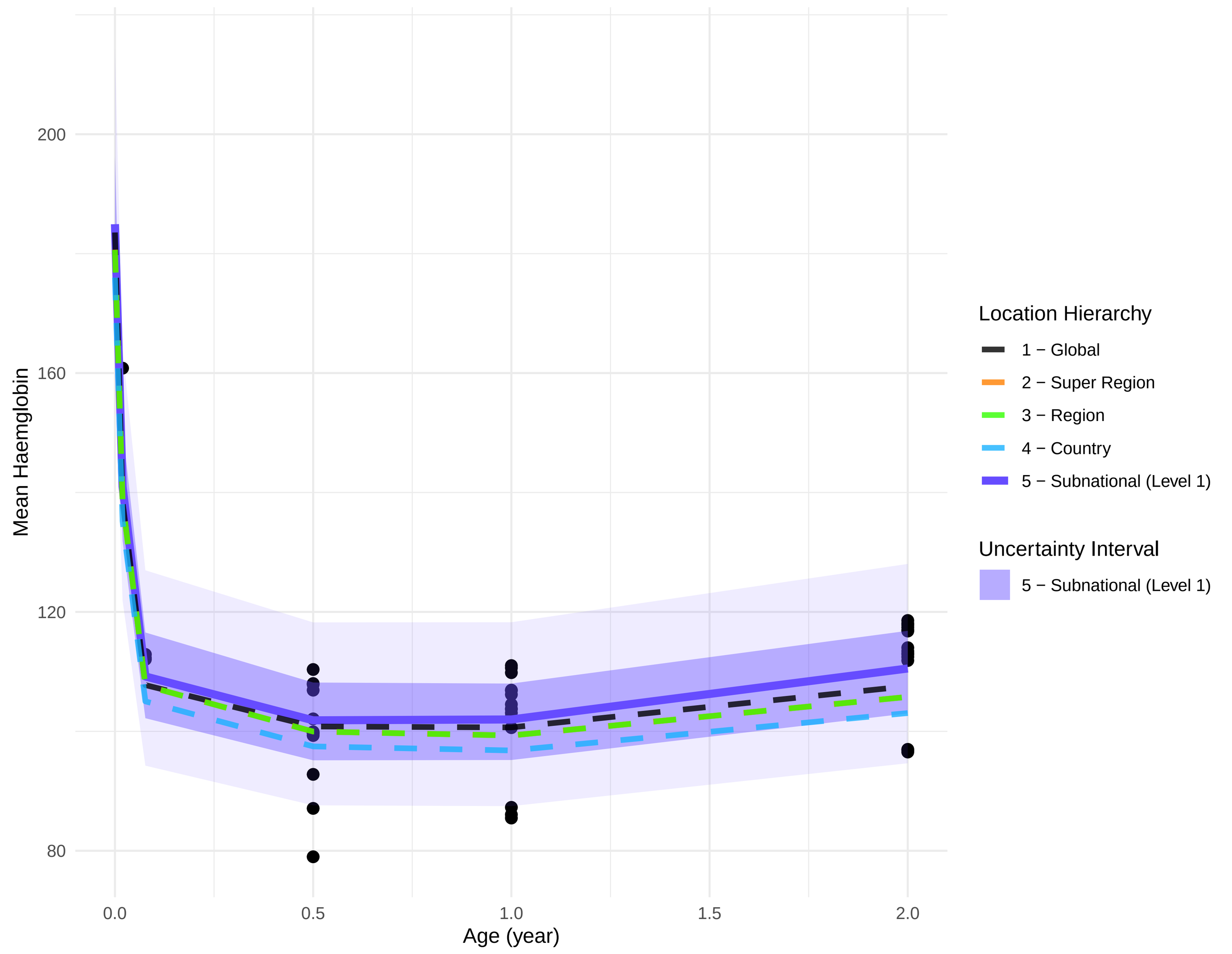

MR−BRT Age/Sex Fits − Males <2 Years Old − Mean Haemglobin − Manipur, Rural
WHO−2001 Adjustment Method

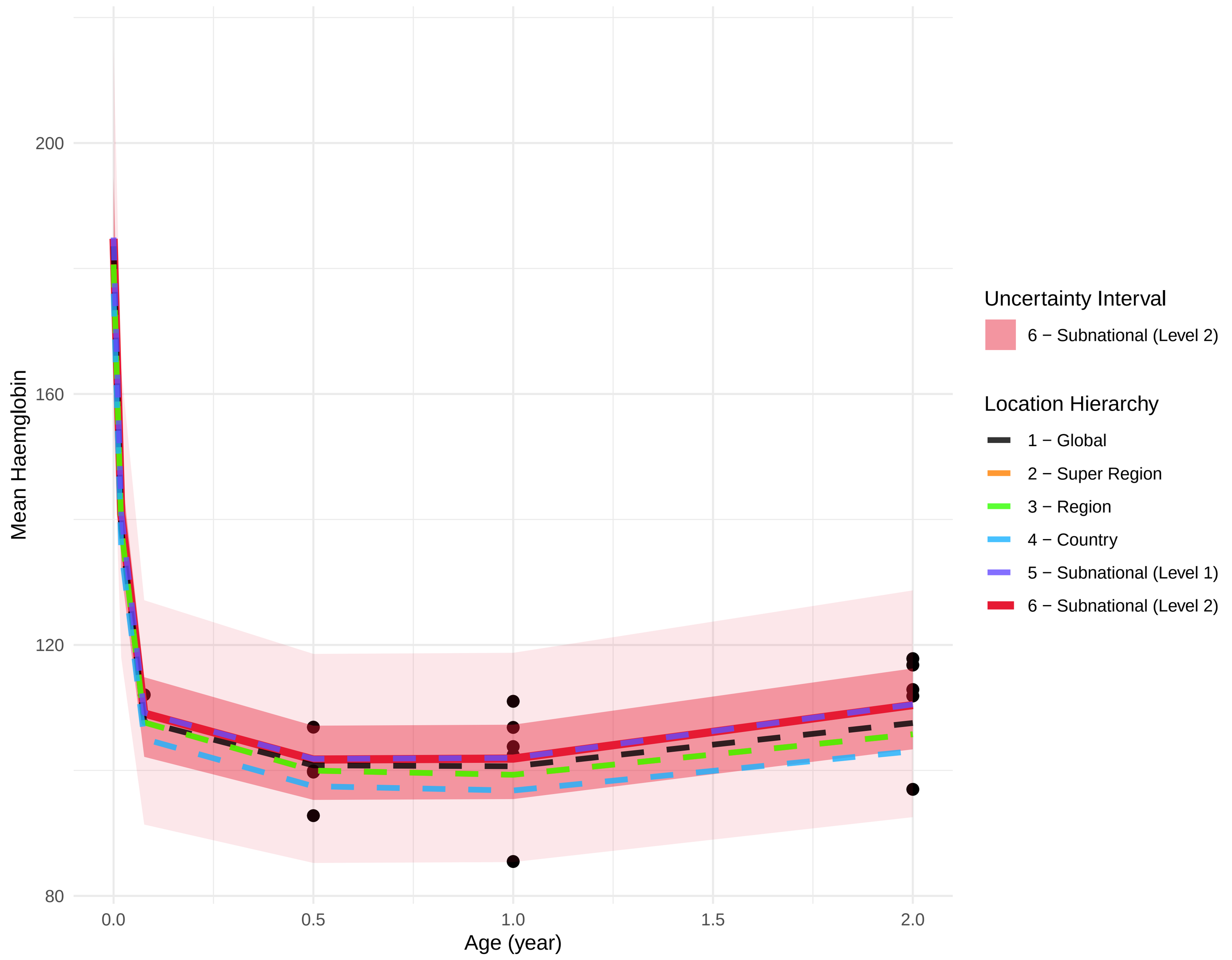

MR−BRT Age/Sex Fits − Males <2 Years Old − Mean Haemglobin − Manipur, Urban
WHO−2001 Adjustment Method

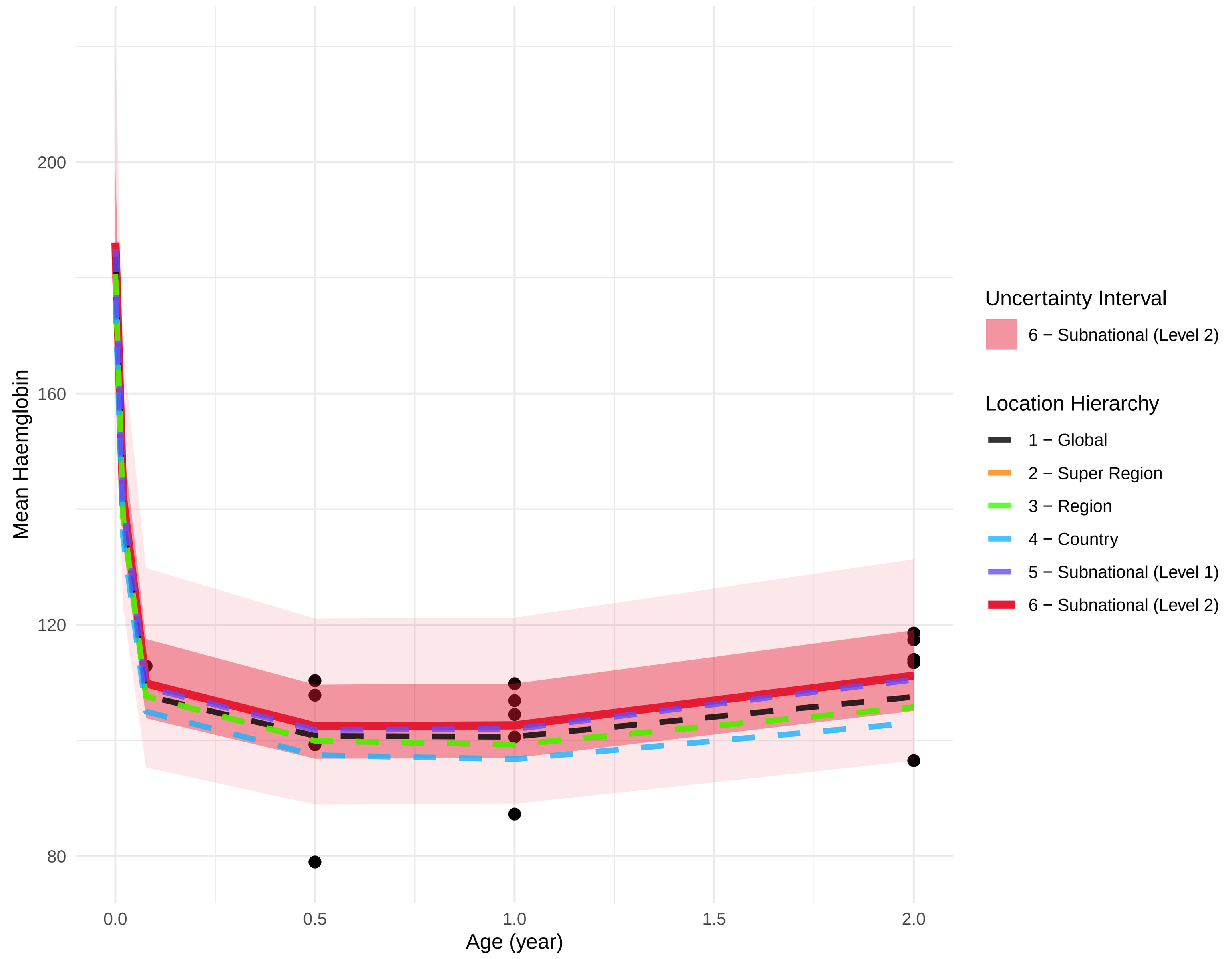

MR−BRT Age/Sex Fits − Males <2 Years Old − Mean Haemglobin − Meghalaya
WHO−2001 Adjustment Method

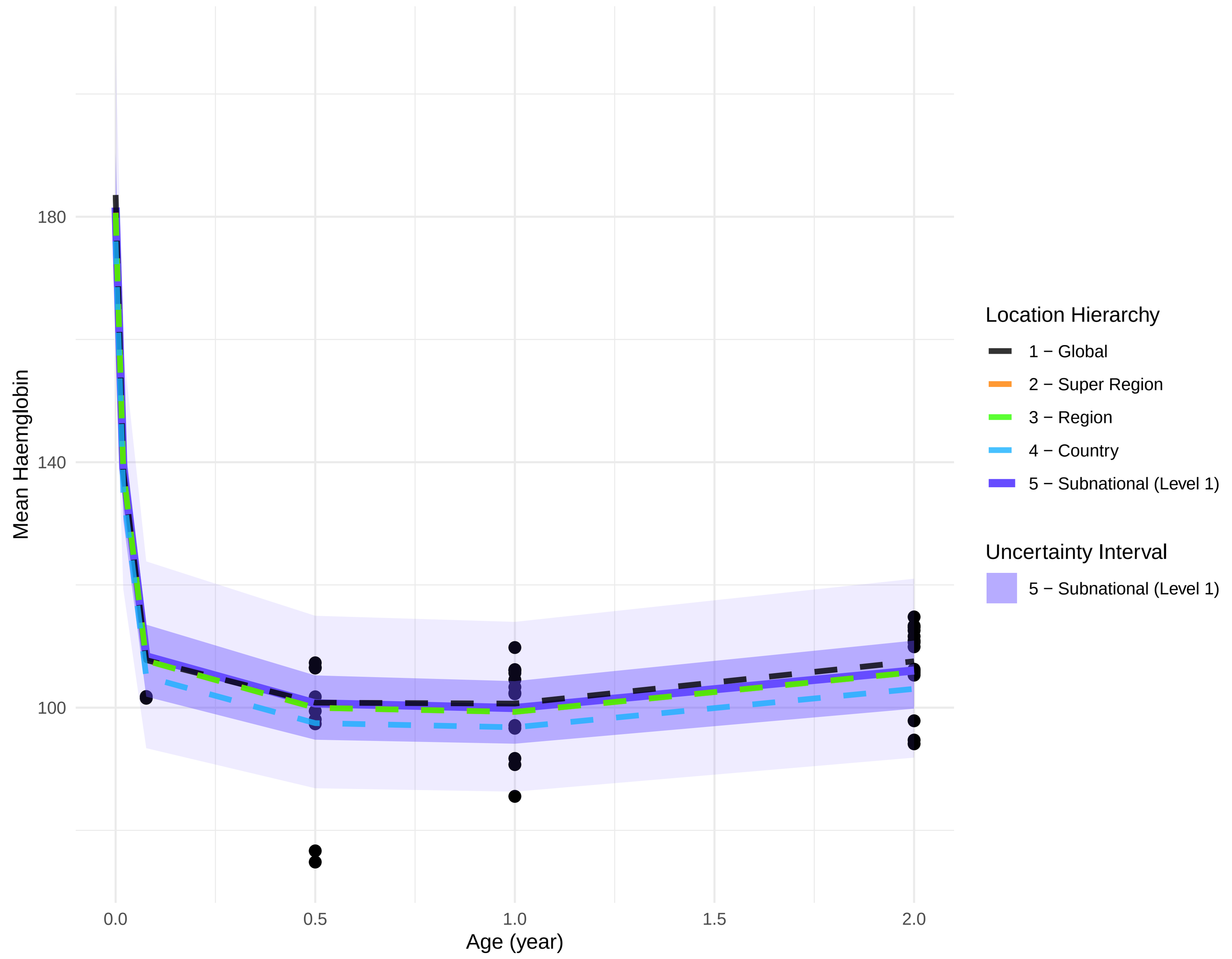

MR−BRT Age/Sex Fits − Males <2 Years Old − Mean Haemglobin − Meghalaya, Rural
WHO−2001 Adjustment Method

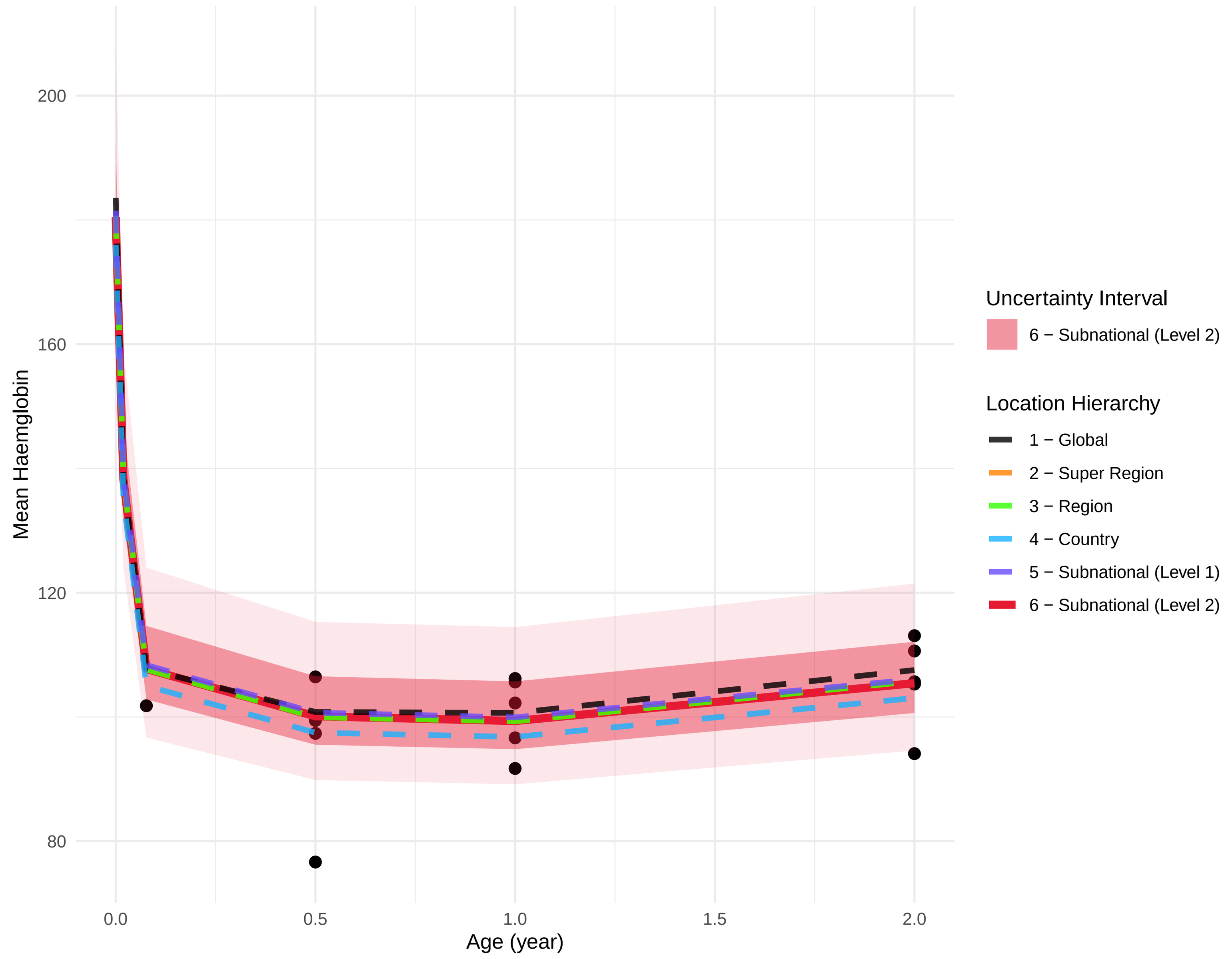

MR−BRT Age/Sex Fits − Males <2 Years Old − Mean Haemglobin − Meghalaya, Urban
WHO−2001 Adjustment Method

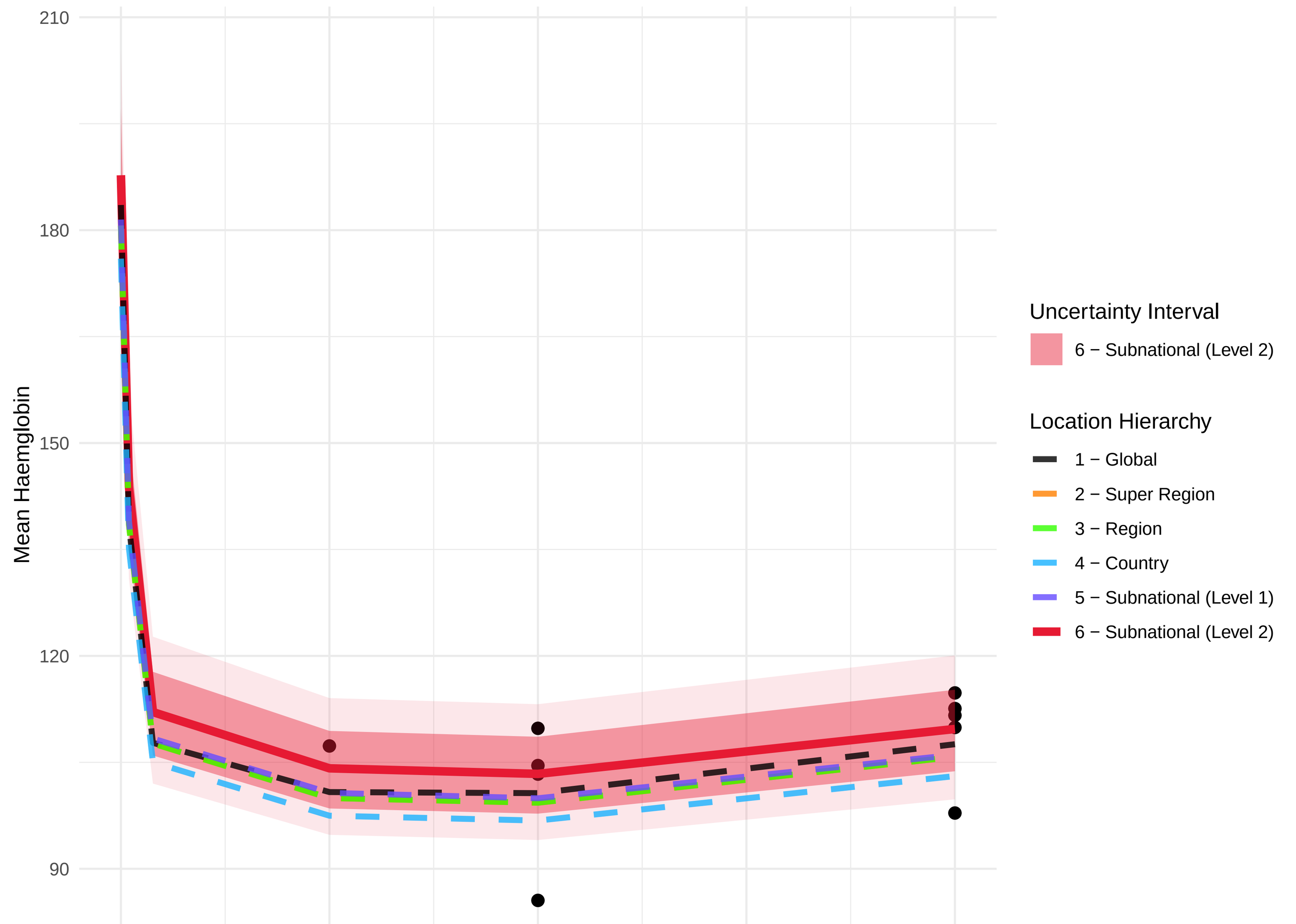

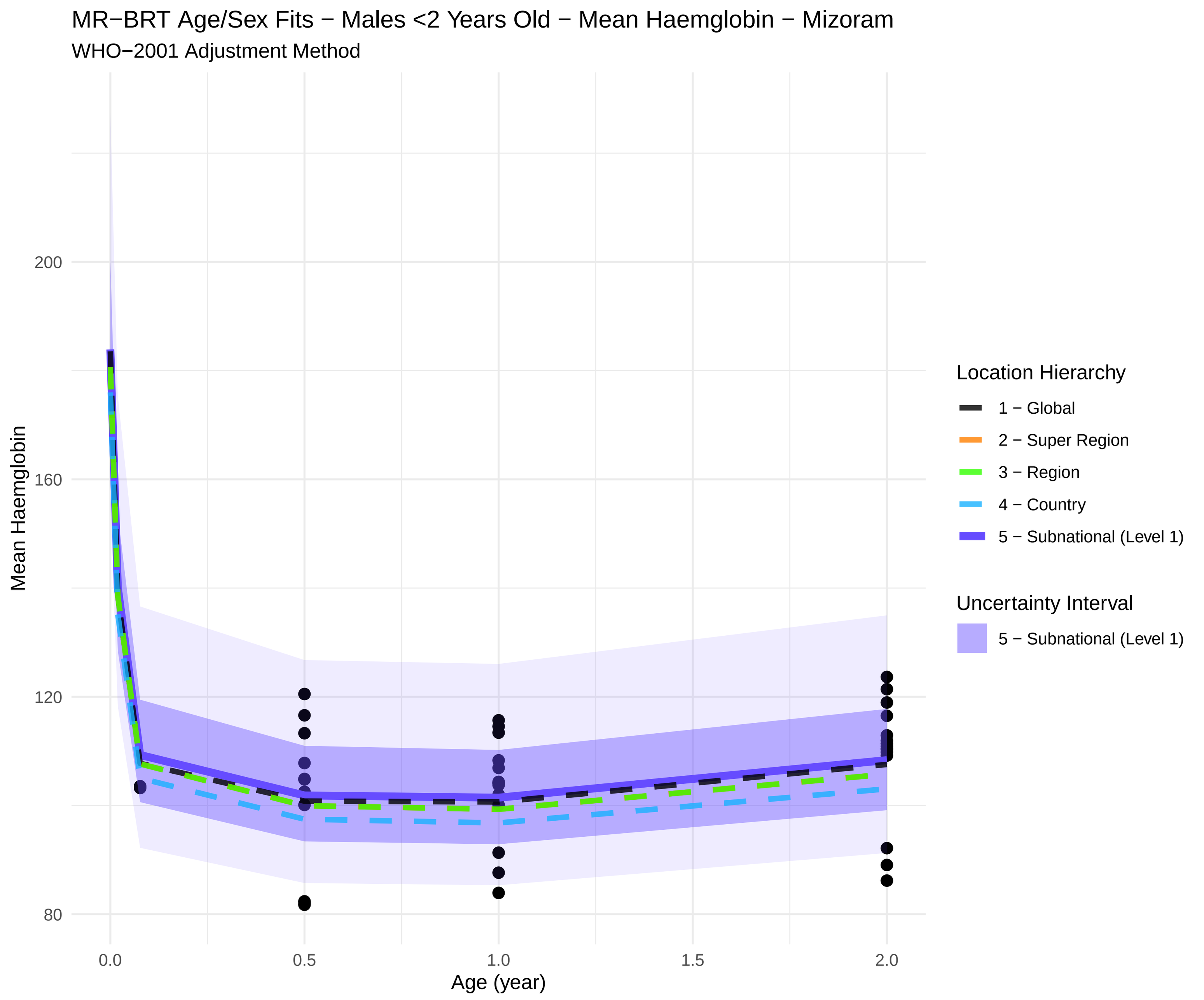
MR−BRT Age/Sex Fits − Males <2 Years Old − Mean Haemglobin − Mizoram
WHO−2001 Adjustment Method
Mean Haemglobin
Age (year)
Location Hierarchy
1 − Global
2 − Super Region
3 − Region
4 − Country
5 − Subnational (Level 1)
Uncertainty Interval
5 − Subnational (Level 1)

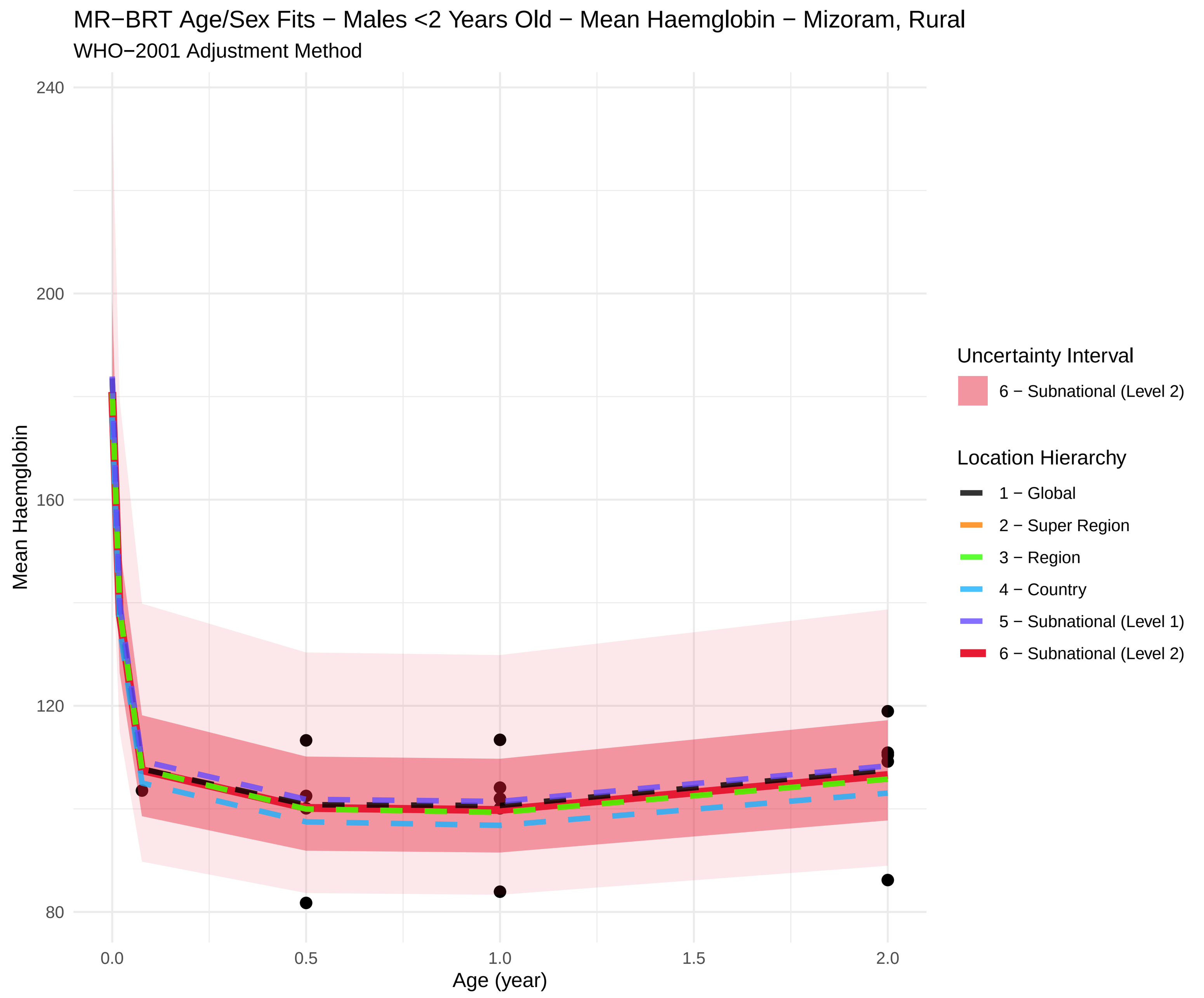
MR−BRT Age/Sex Fits − Males <2 Years Old − Mean Haemglobin − Mizoram, Rural
WHO−2001 Adjustment Method
Mean Haemglobin
Age (year)
Uncertainty Interval
6 − Subnational (Level 2)
Location Hierarchy
1 − Global
2 − Super Region
3 − Region
4 − Country
5 − Subnational (Level 1)
6 − Subnational (Level 2)

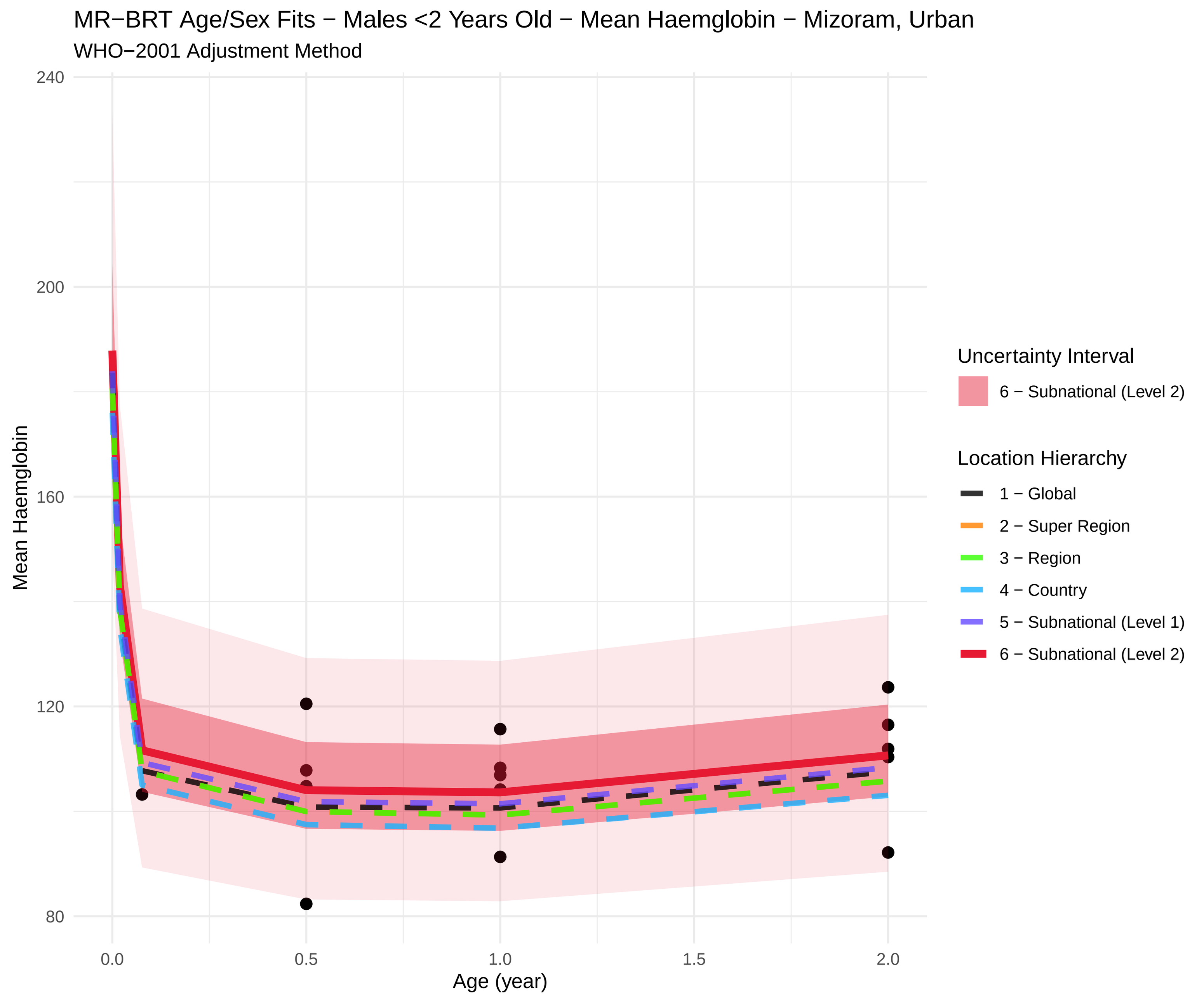
MR−BRT Age/Sex Fits − Males <2 Years Old − Mean Haemglobin − Mizoram, Urban
WHO−2001 Adjustment Method
Mean Haemglobin
Age (year)
Uncertainty Interval
6 − Subnational (Level 2)
Location Hierarchy
1 − Global
2 − Super Region
3 − Region
4 − Country
5 − Subnational (Level 1)
6 − Subnational (Level 2)

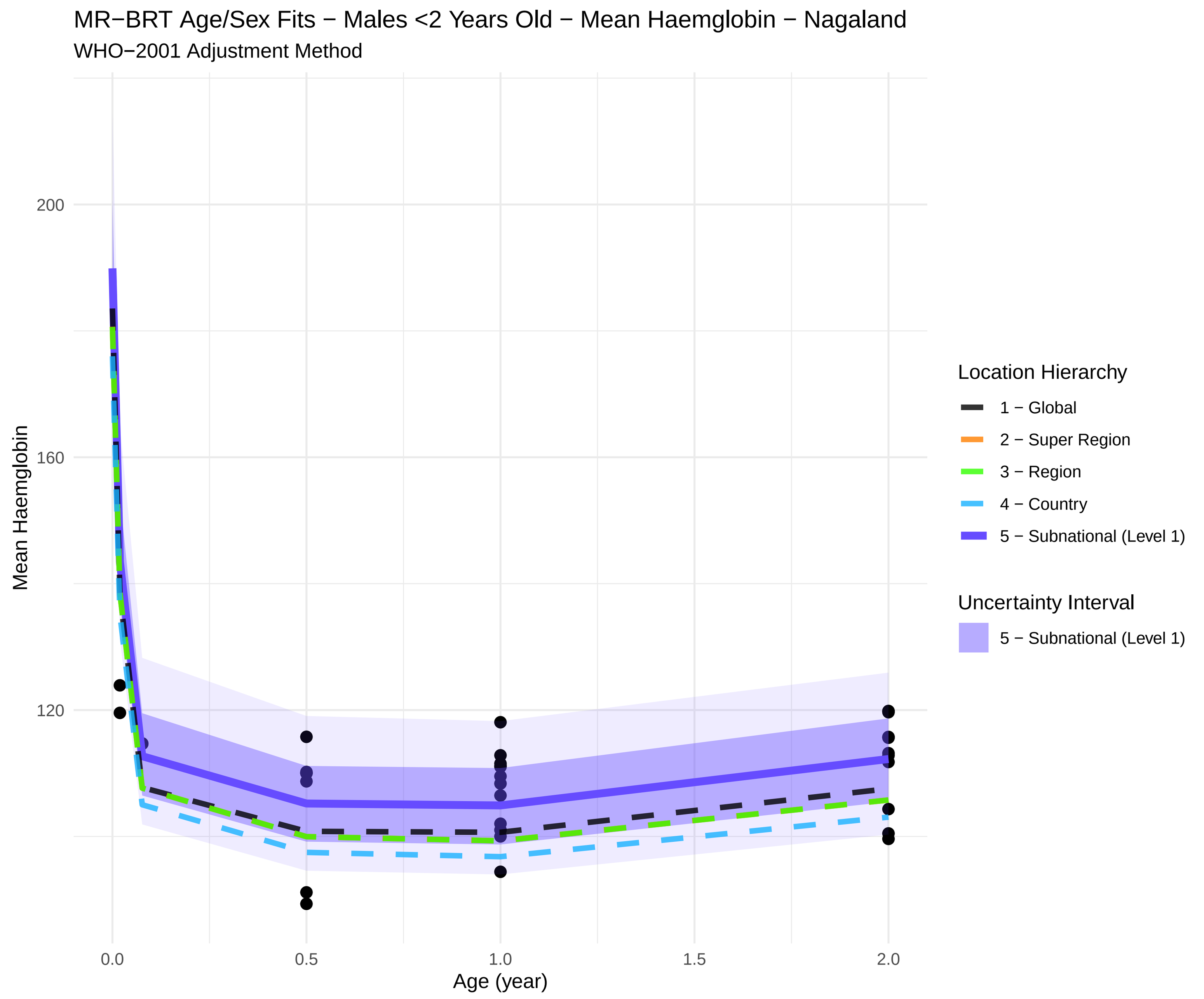
MR−BRT Age/Sex Fits − Males <2 Years Old − Mean Haemglobin − Nagaland
WHO−2001 Adjustment Method
Mean Haemglobin
Age (year)
Location Hierarchy
1 − Global
2 − Super Region
3 − Region
4 − Country
5 − Subnational (Level 1)
Uncertainty Interval
5 − Subnational (Level 1)

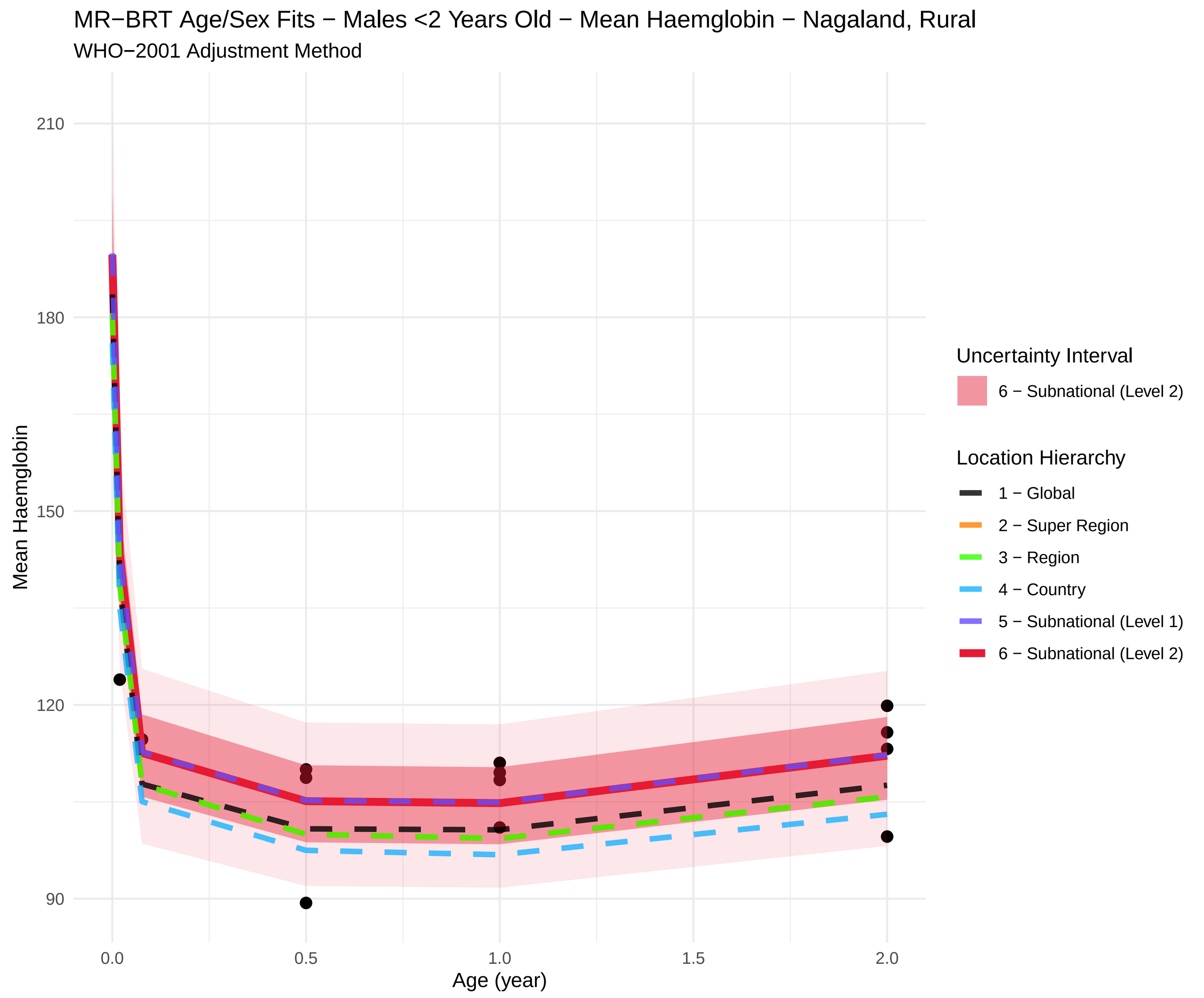
MR−BRT Age/Sex Fits − Males <2 Years Old − Mean Haemglobin − Nagaland, Rural
WHO−2001 Adjustment Method
Mean Haemglobin
Age (year)
Uncertainty Interval
6 − Subnational (Level 2)
Location Hierarchy
1 − Global
2 − Super Region
3 − Region
4 − Country
5 − Subnational (Level 1)
6 − Subnational (Level 2)

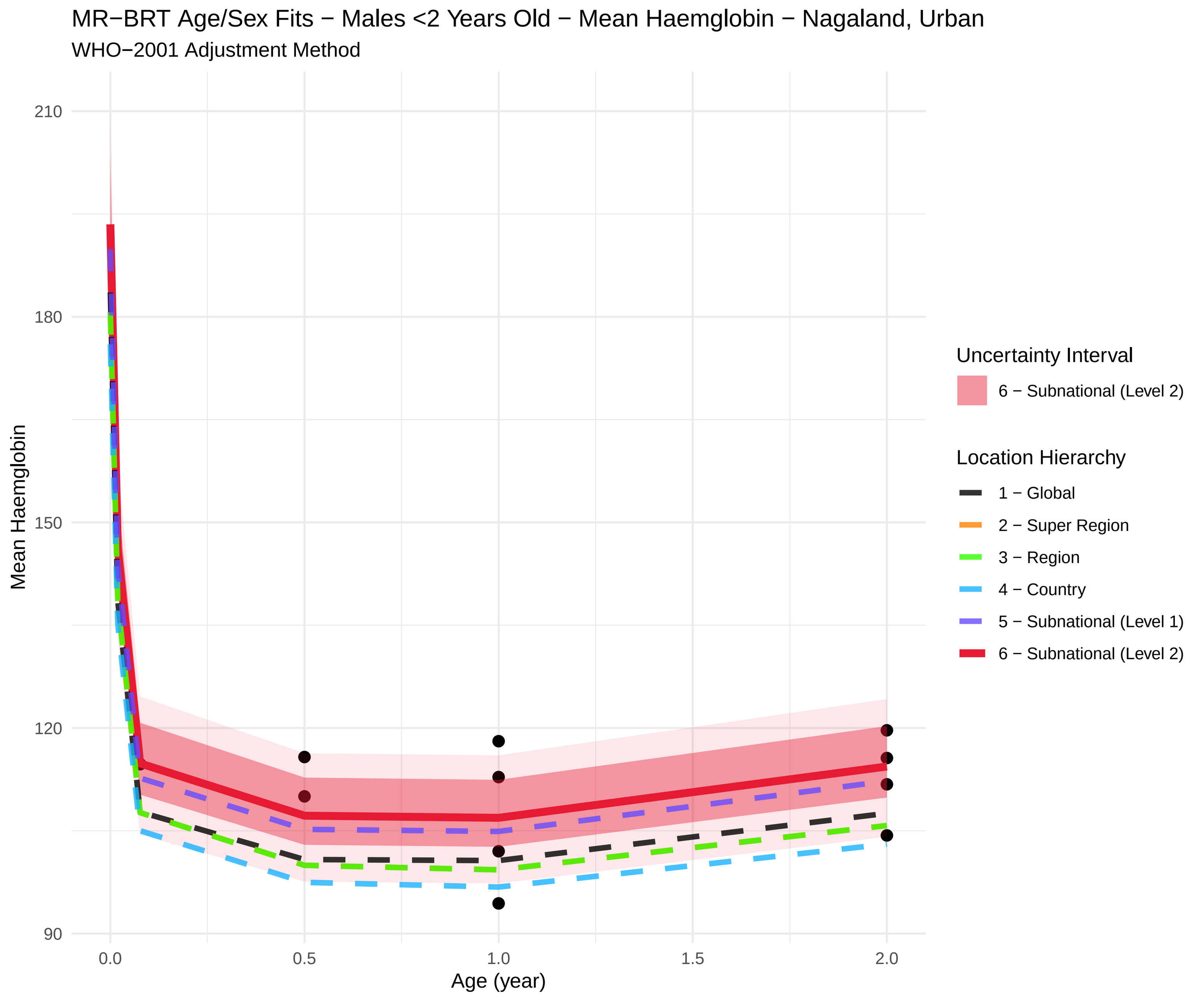
MR−BRT Age/Sex Fits − Males <2 Years Old − Mean Haemglobin − Nagaland, Urban
WHO−2001 Adjustment Method
Mean Haemglobin
Age (year)
Uncertainty Interval
6 − Subnational (Level 2)
Location Hierarchy
1 − Global
2 − Super Region
3 − Region
4 − Country
5 − Subnational (Level 1)
6 − Subnational (Level 2)

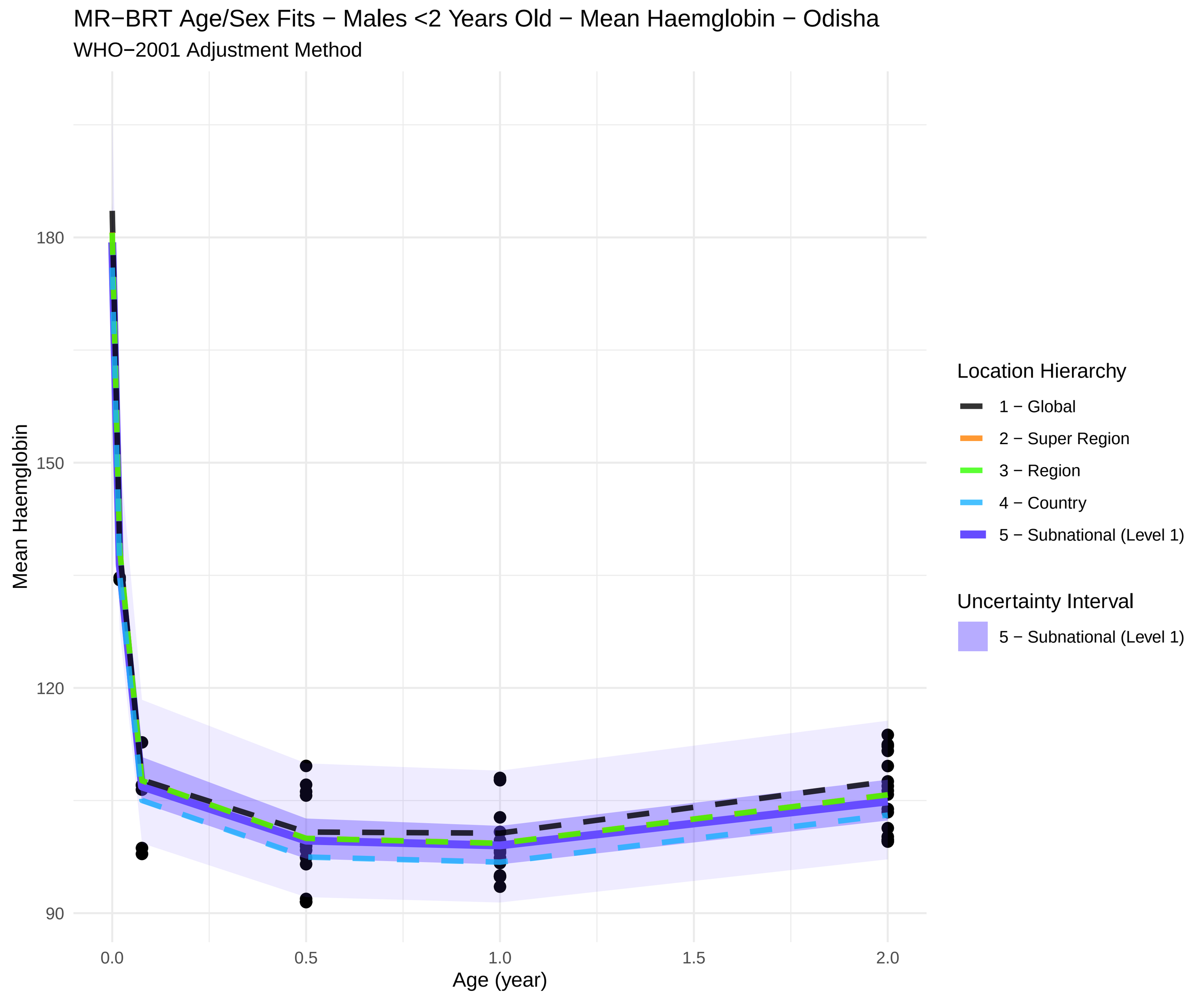
MR−BRT Age/Sex Fits − Males <2 Years Old − Mean Haemglobin − Odisha
WHO−2001 Adjustment Method
Mean Haemglobin
Age (year)
Location Hierarchy
1 − Global
2 − Super Region
3 − Region
4 − Country
5 − Subnational (Level 1)
Uncertainty Interval
5 − Subnational (Level 1)

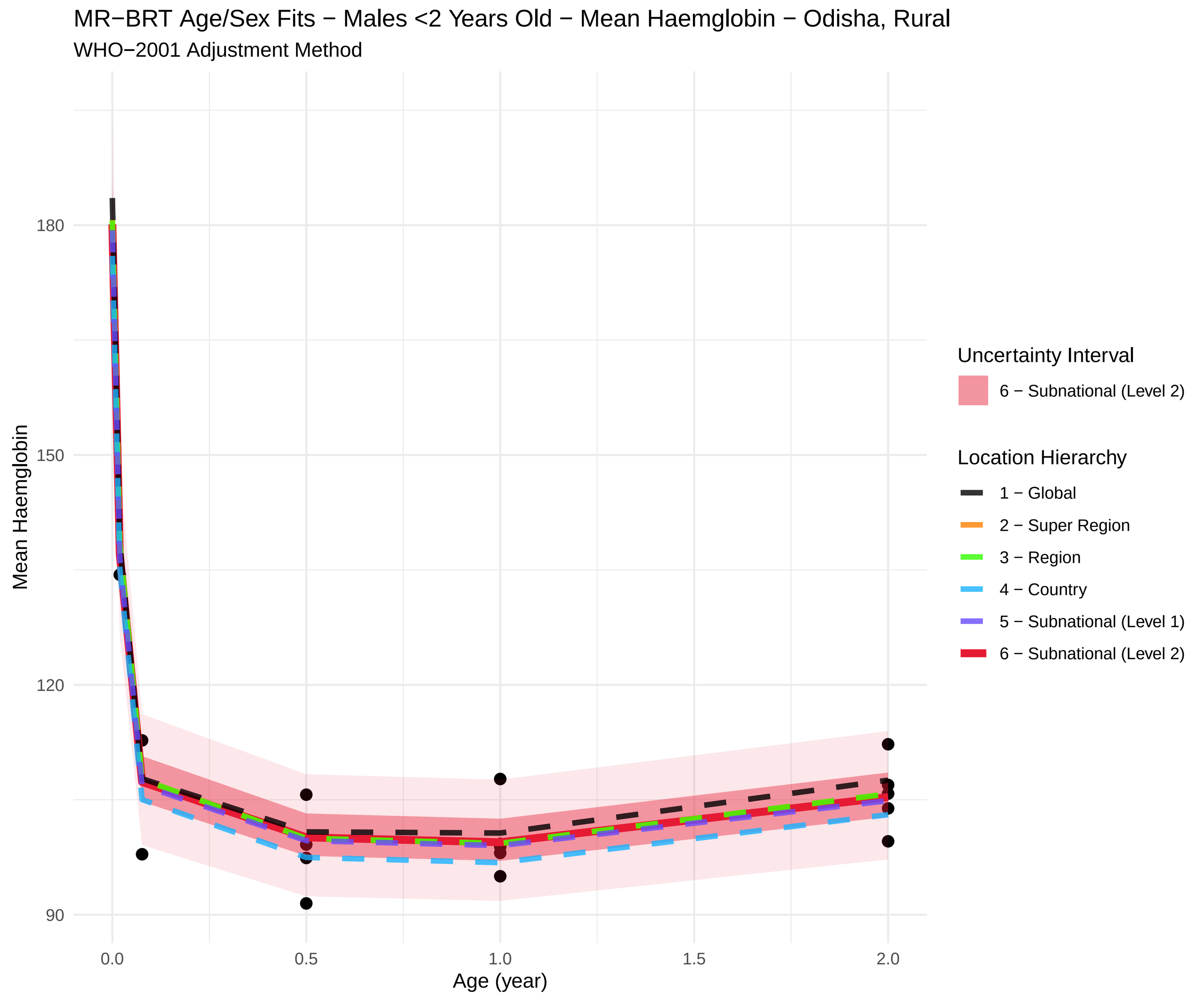
MR−BRT Age/Sex Fits − Males <2 Years Old − Mean Haemglobin − Odisha, Rural
WHO−2001 Adjustment Method
Mean Haemglobin
Age (year)
Uncertainty Interval
6 − Subnational (Level 2)
Location Hierarchy
1 − Global
2 − Super Region
3 − Region
4 − Country
5 − Subnational (Level 1)
6 − Subnational (Level 2)

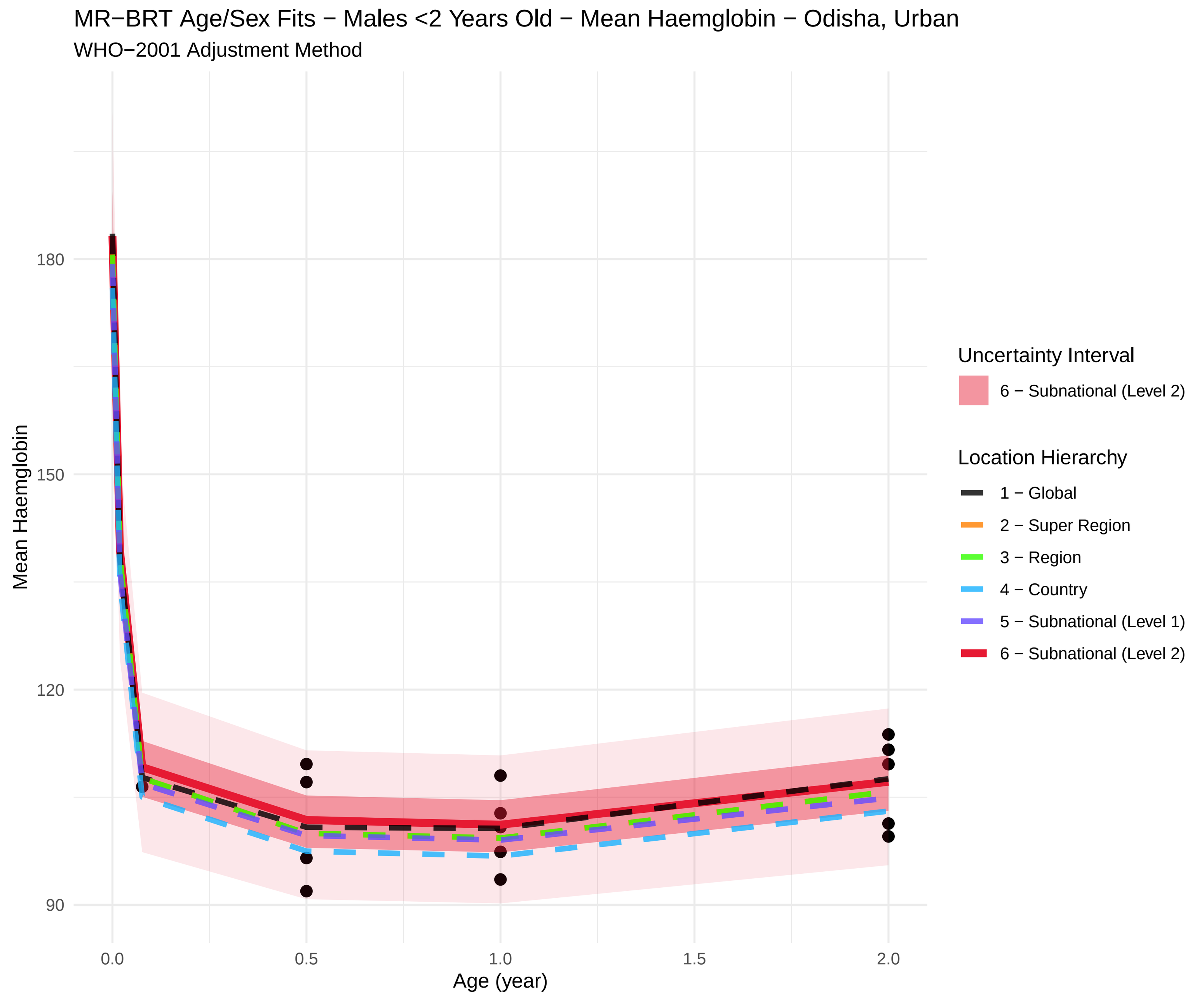
MR−BRT Age/Sex Fits − Males <2 Years Old − Mean Haemglobin − Odisha, Urban
WHO−2001 Adjustment Method
Mean Haemglobin
Age (year)
Uncertainty Interval
6 − Subnational (Level 2)
Location Hierarchy
1 − Global
2 − Super Region
3 − Region
4 − Country
5 − Subnational (Level 1)
6 − Subnational (Level 2)

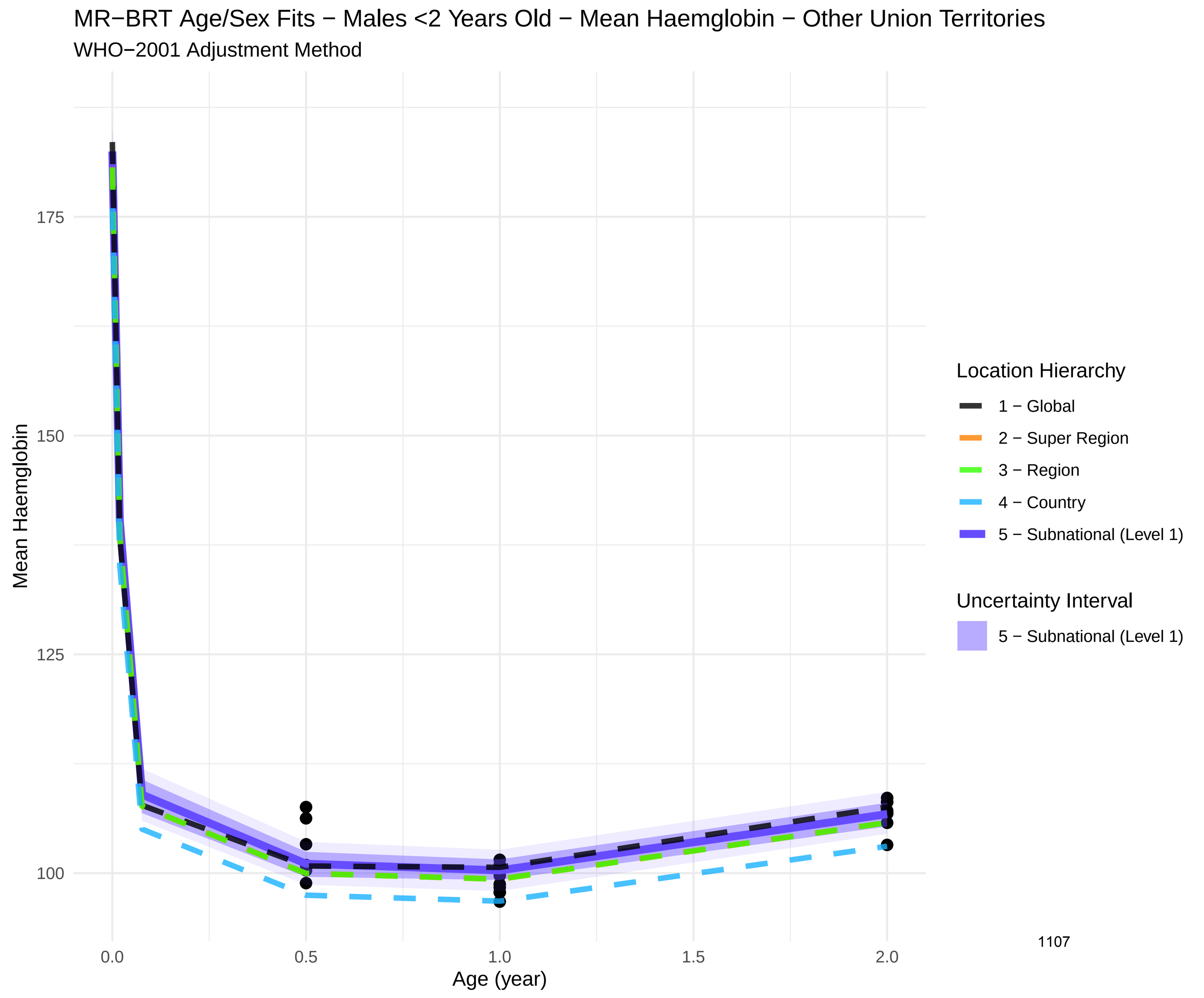
MR−BRT Age/Sex Fits − Males <2 Years Old − Mean Haemglobin − Other Union Territories
WHO−2001 Adjustment Method
Mean Haemglobin
Age (year)
Location Hierarchy
1 − Global
2 − Super Region
3 − Region
4 − Country
5 − Subnational (Level 1)
Uncertainty Interval
5 − Subnational (Level 1)

MR−BRT Age/Sex Fits − Males <2 Years Old − Mean Haemglobin − Other Union Territories, Rural
WHO−2001 Adjustment Method

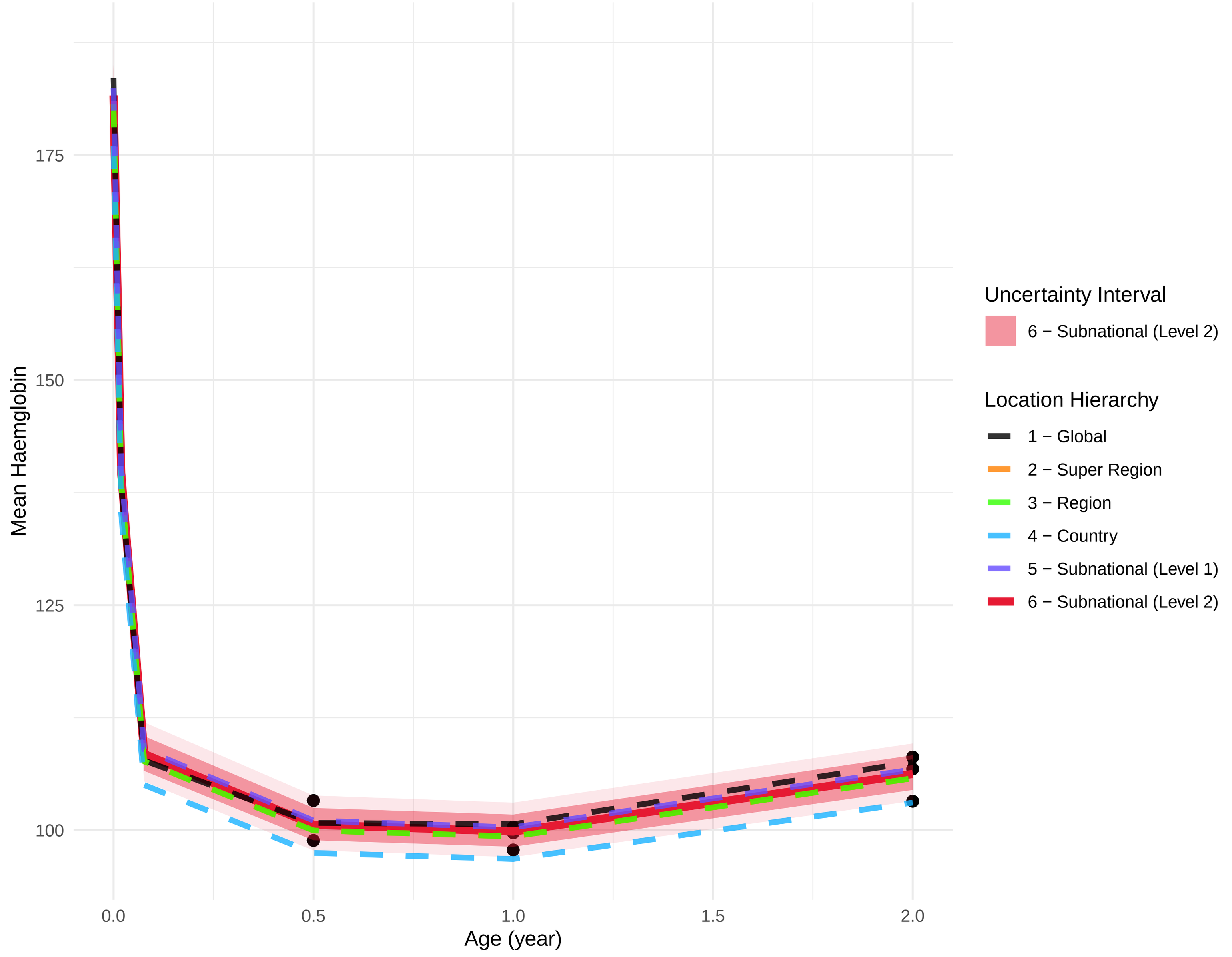

MR−BRT Age/Sex Fits − Males <2 Years Old − Mean Haemglobin − Other Union Territories, Urban
WHO−2001 Adjustment Method

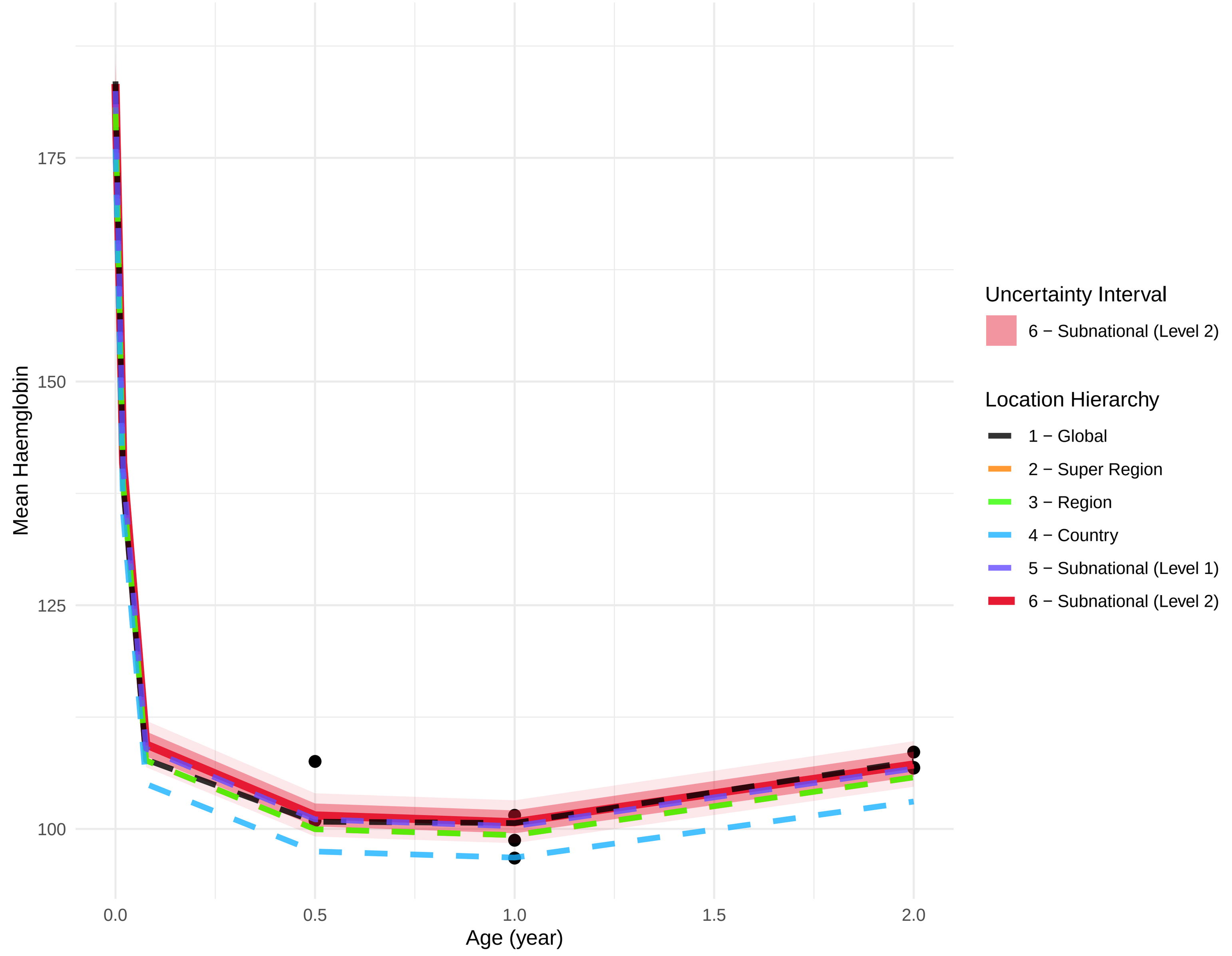

MR−BRT Age/Sex Fits − Males <2 Years Old − Mean Haemglobin − Punjab
WHO−2001 Adjustment Method

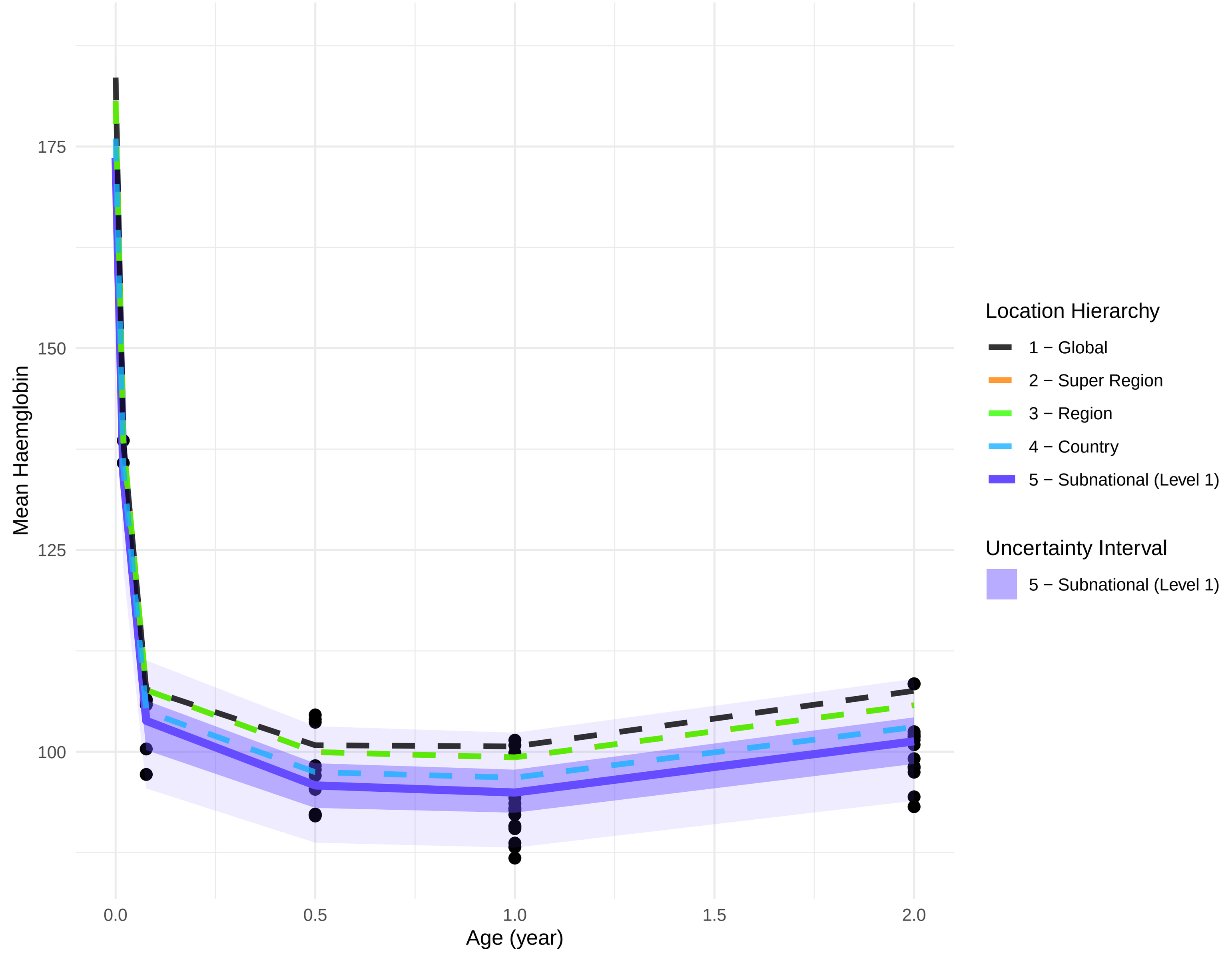

MR−BRT Age/Sex Fits − Males <2 Years Old − Mean Haemglobin − Punjab, Rural
WHO−2001 Adjustment Method

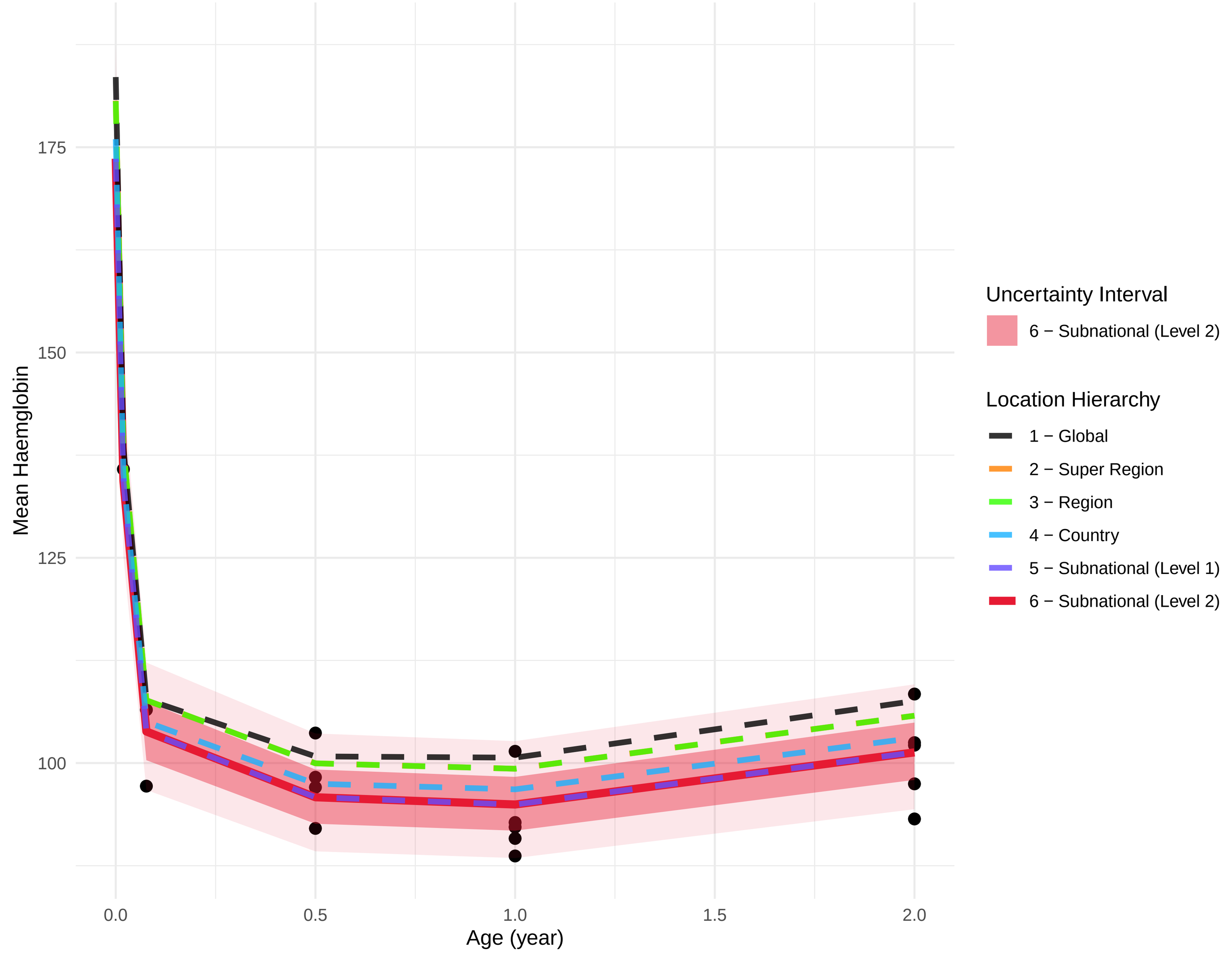

MR−BRT Age/Sex Fits − Males <2 Years Old − Mean Haemglobin − Punjab, Urban
WHO−2001 Adjustment Method

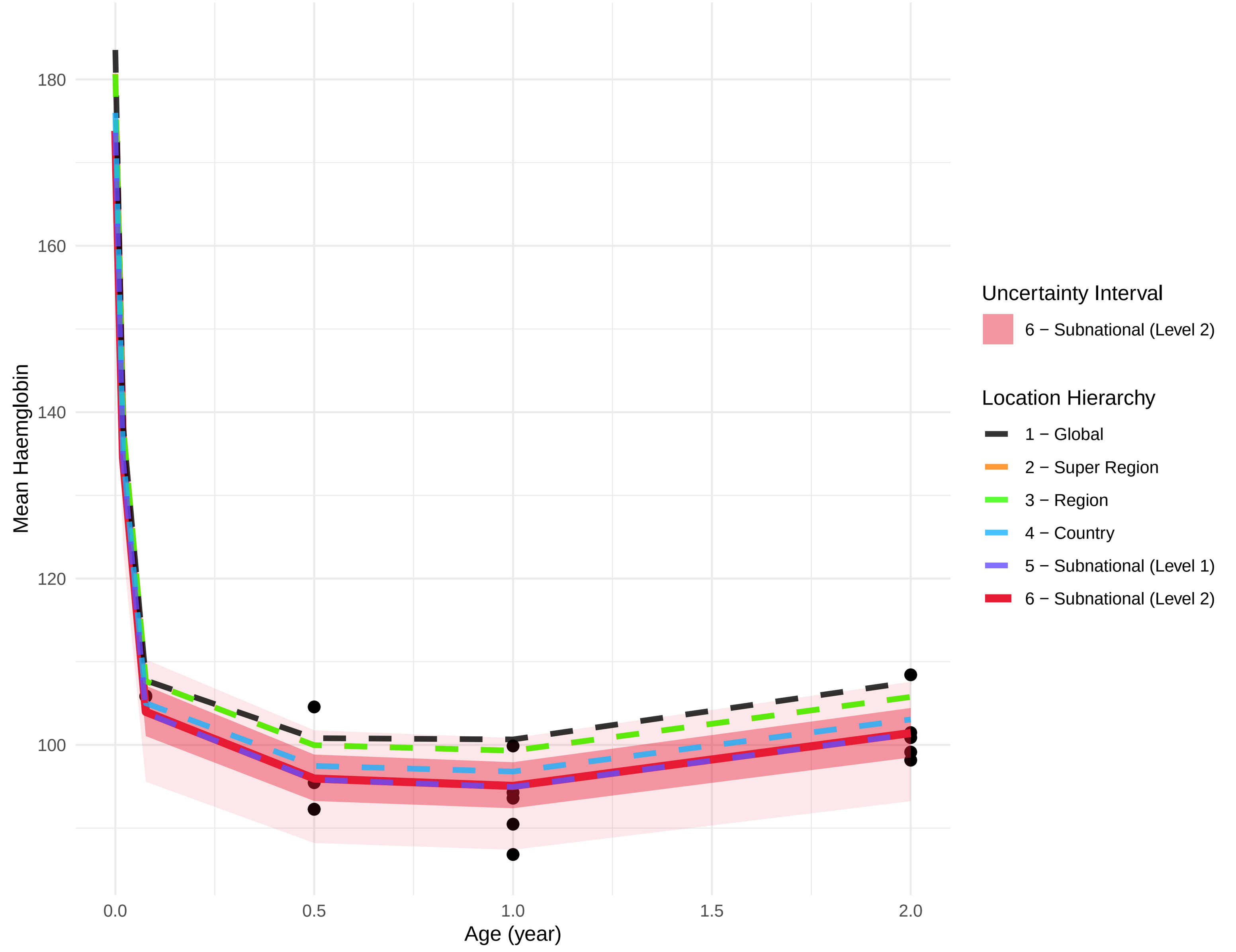

MR−BRT Age/Sex Fits − Males <2 Years Old − Mean Haemglobin − Rajasthan
WHO−2001 Adjustment Method

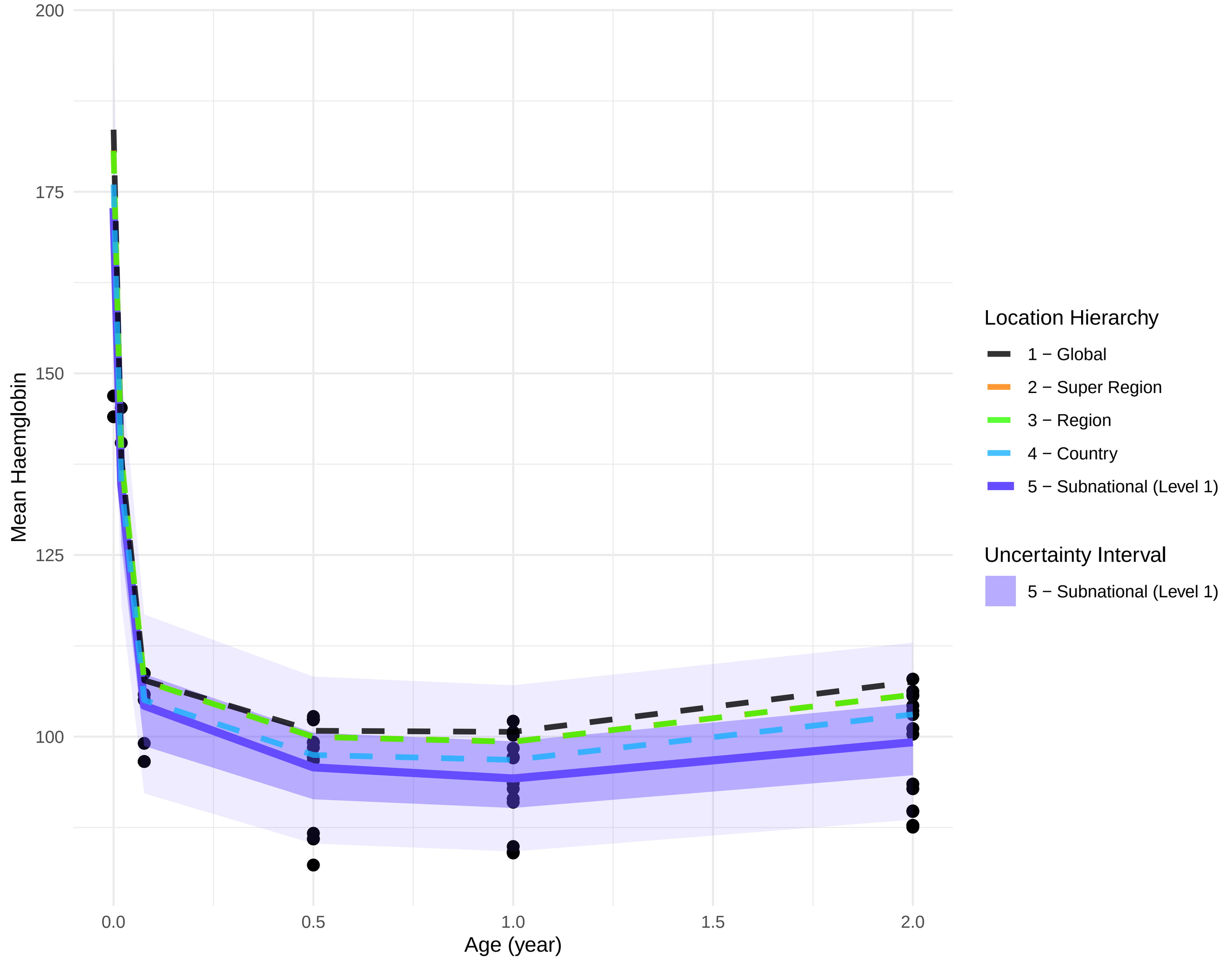

MR−BRT Age/Sex Fits − Males <2 Years Old − Mean Haemglobin − Rajasthan, Rural
WHO−2001 Adjustment Method

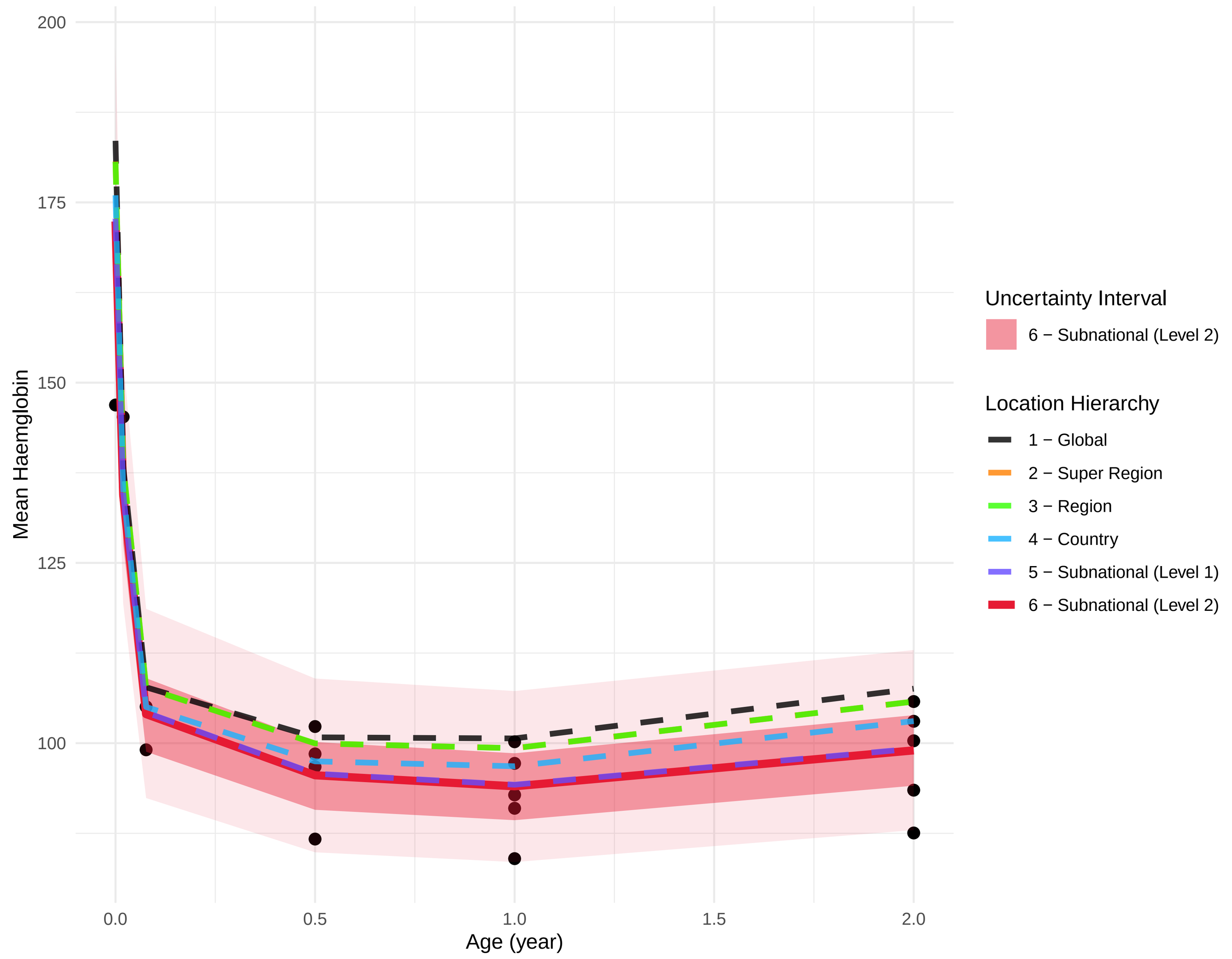

MR−BRT Age/Sex Fits − Males <2 Years Old − Mean Haemglobin − Rajasthan, Urban
WHO−2001 Adjustment Method

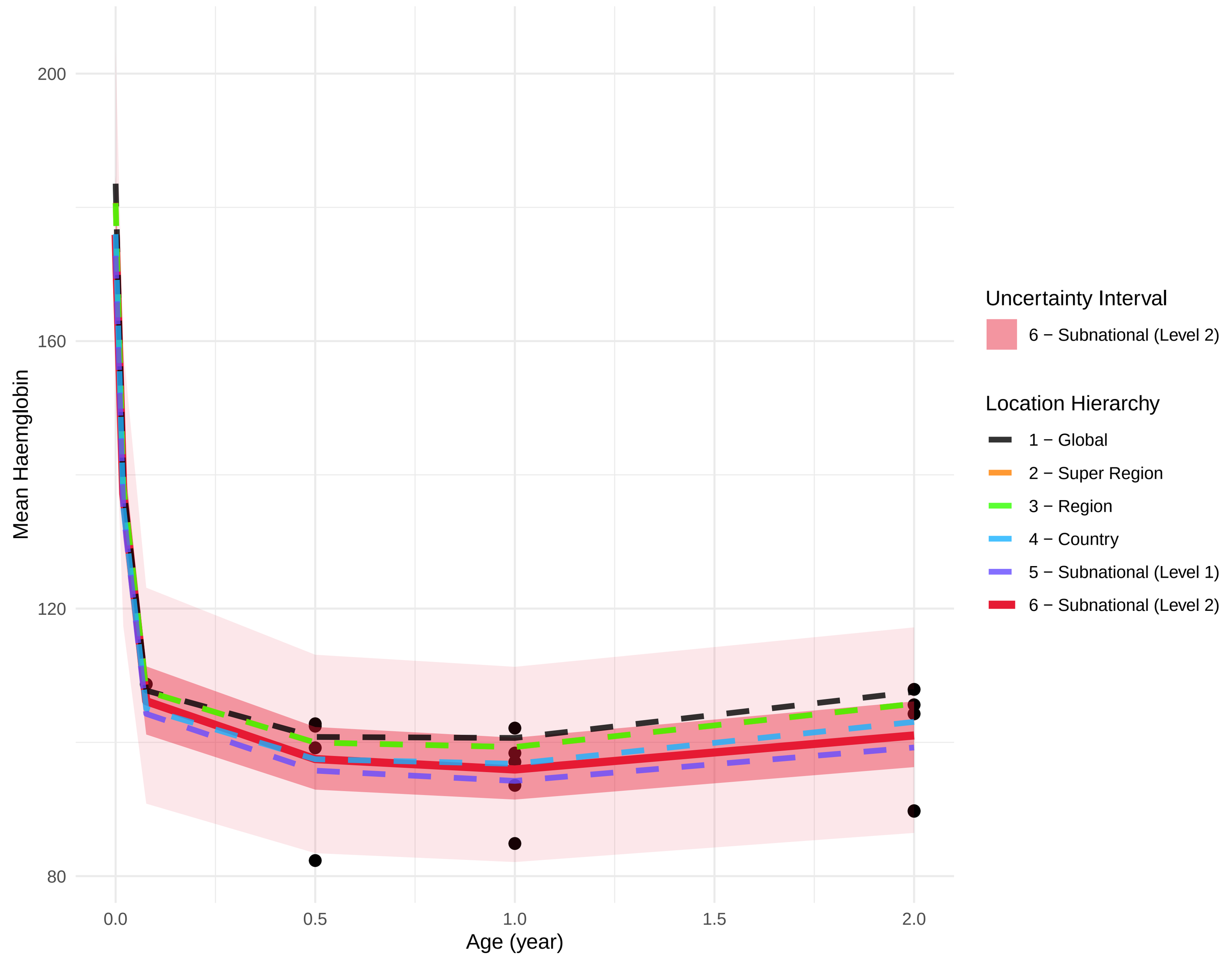

MR−BRT Age/Sex Fits − Males <2 Years Old − Mean Haemglobin − Sikkim
WHO−2001 Adjustment Method

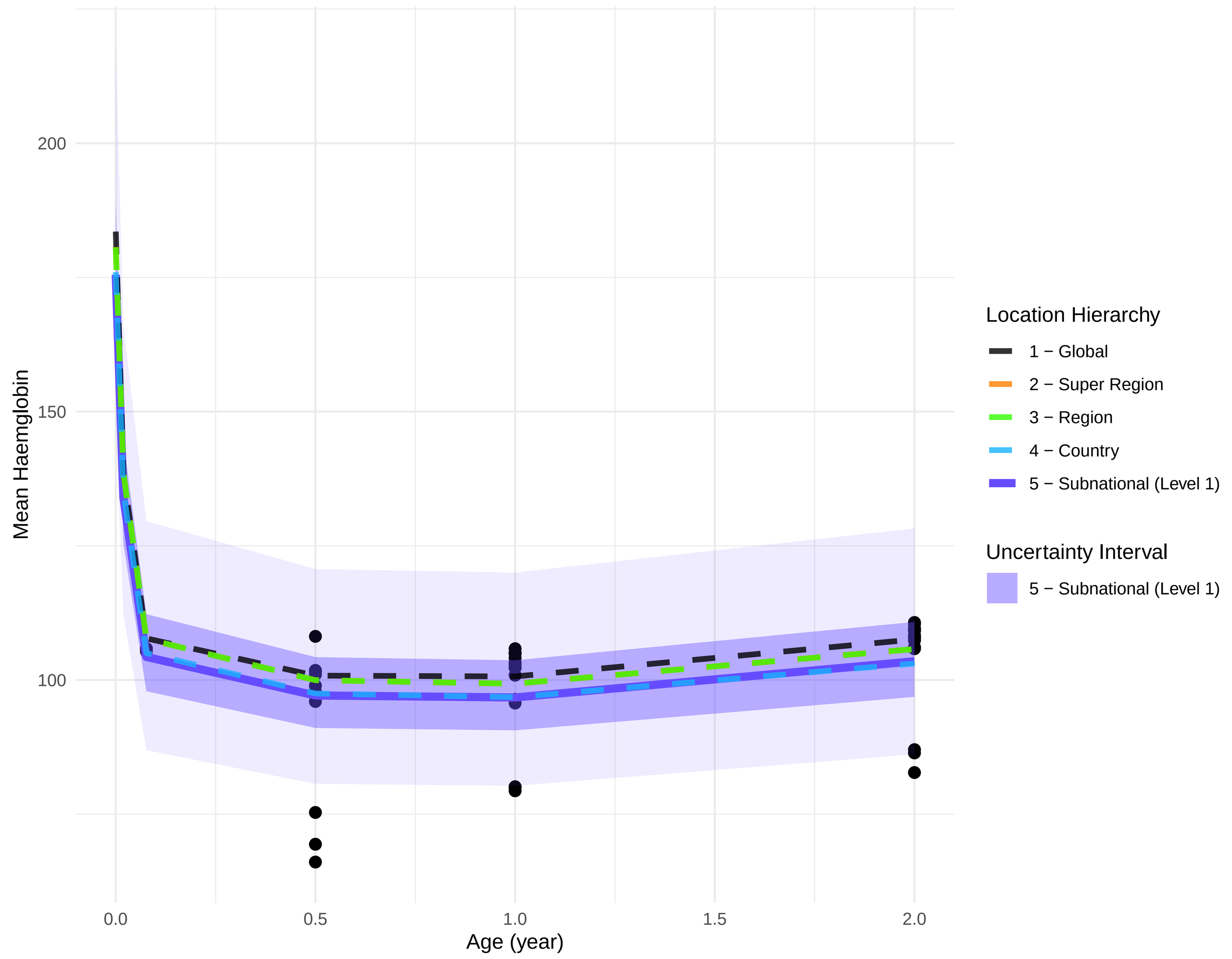

MR−BRT Age/Sex Fits − Males <2 Years Old − Mean Haemglobin − Sikkim, Rural
WHO−2001 Adjustment Method

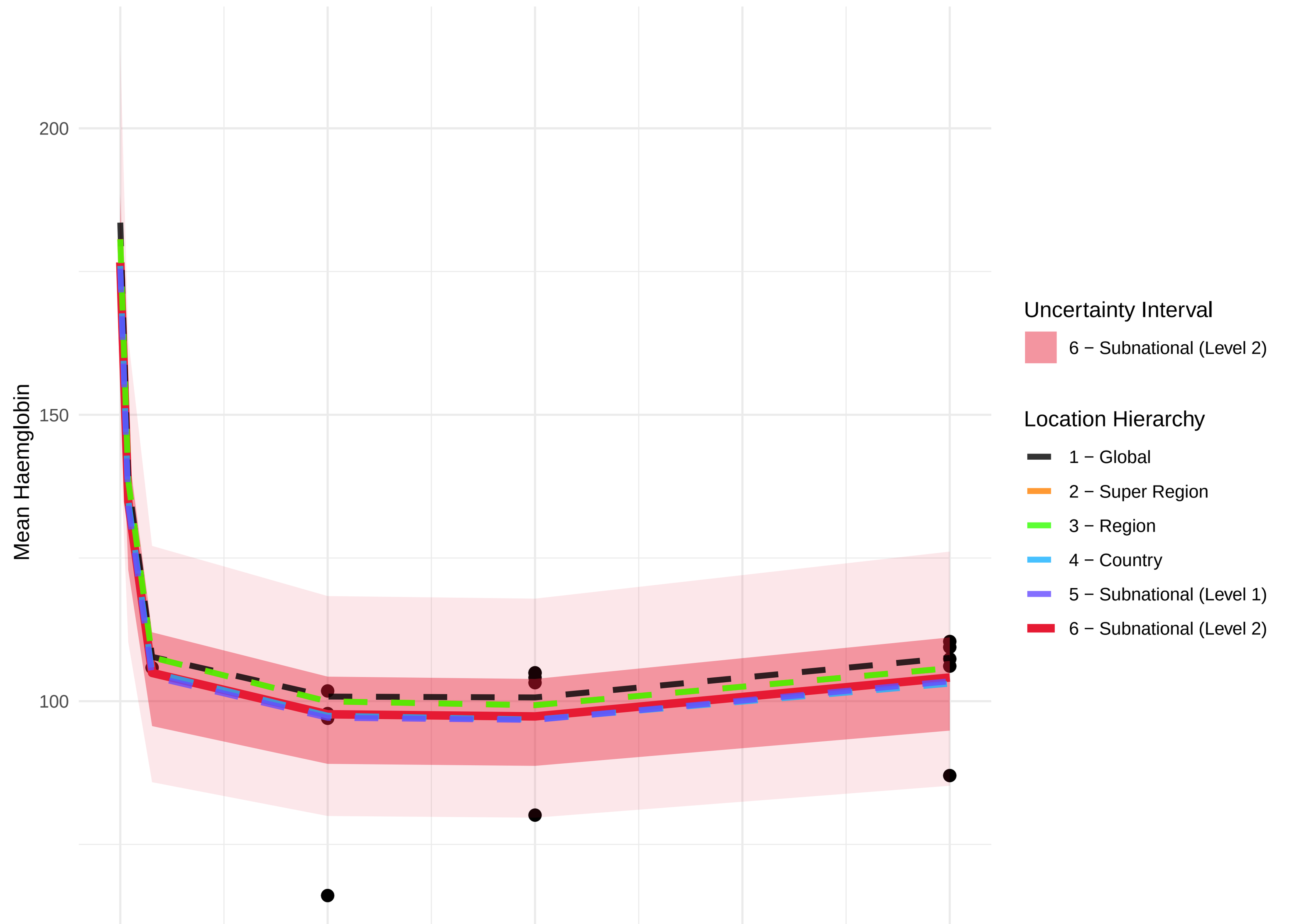

MR−BRT Age/Sex Fits − Males <2 Years Old − Mean Haemglobin − Sikkim, Urban
WHO−2001 Adjustment Method

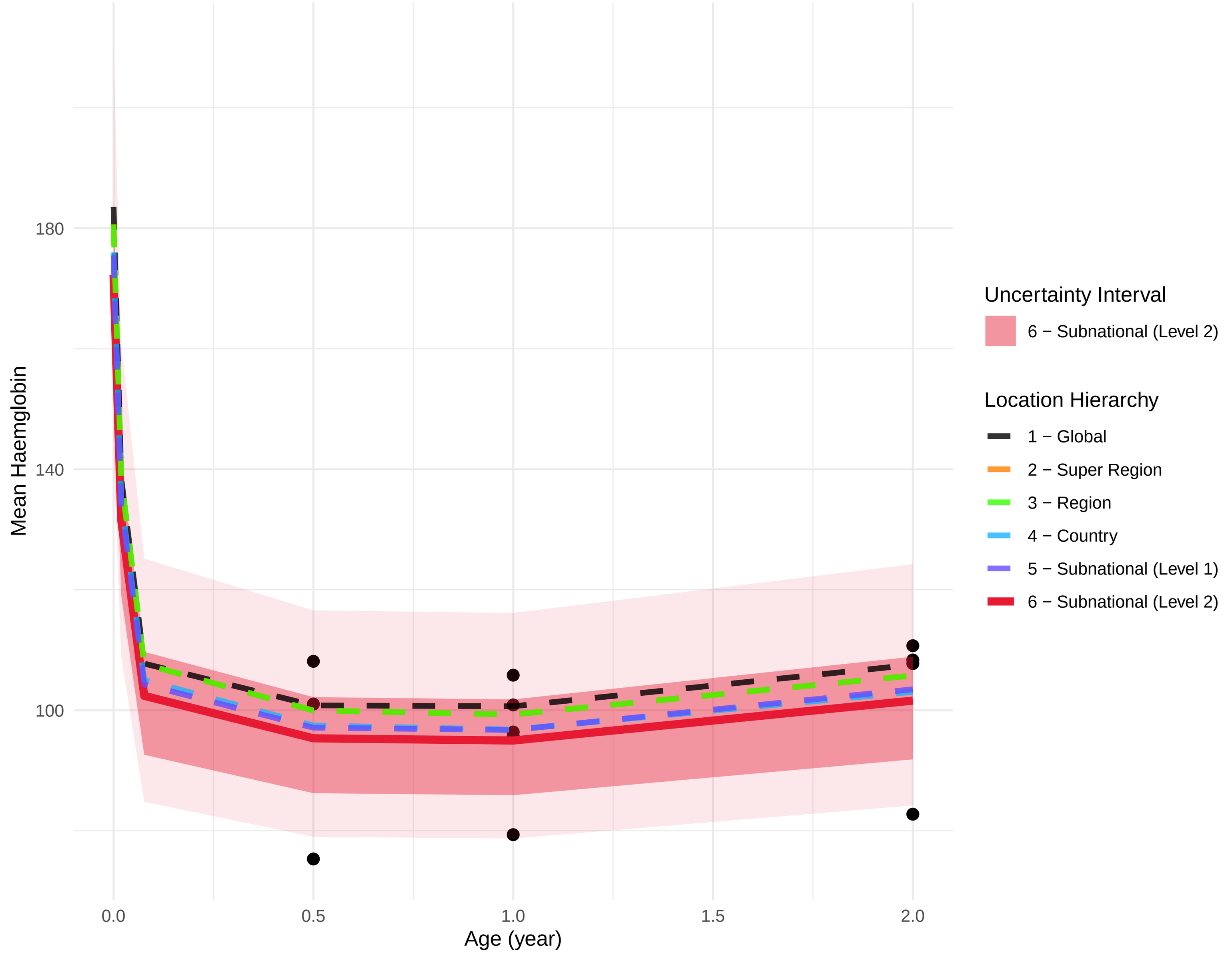

MR−BRT Age/Sex Fits − Males <2 Years Old − Mean Haemglobin − Tamil Nadu
WHO−2001 Adjustment Method

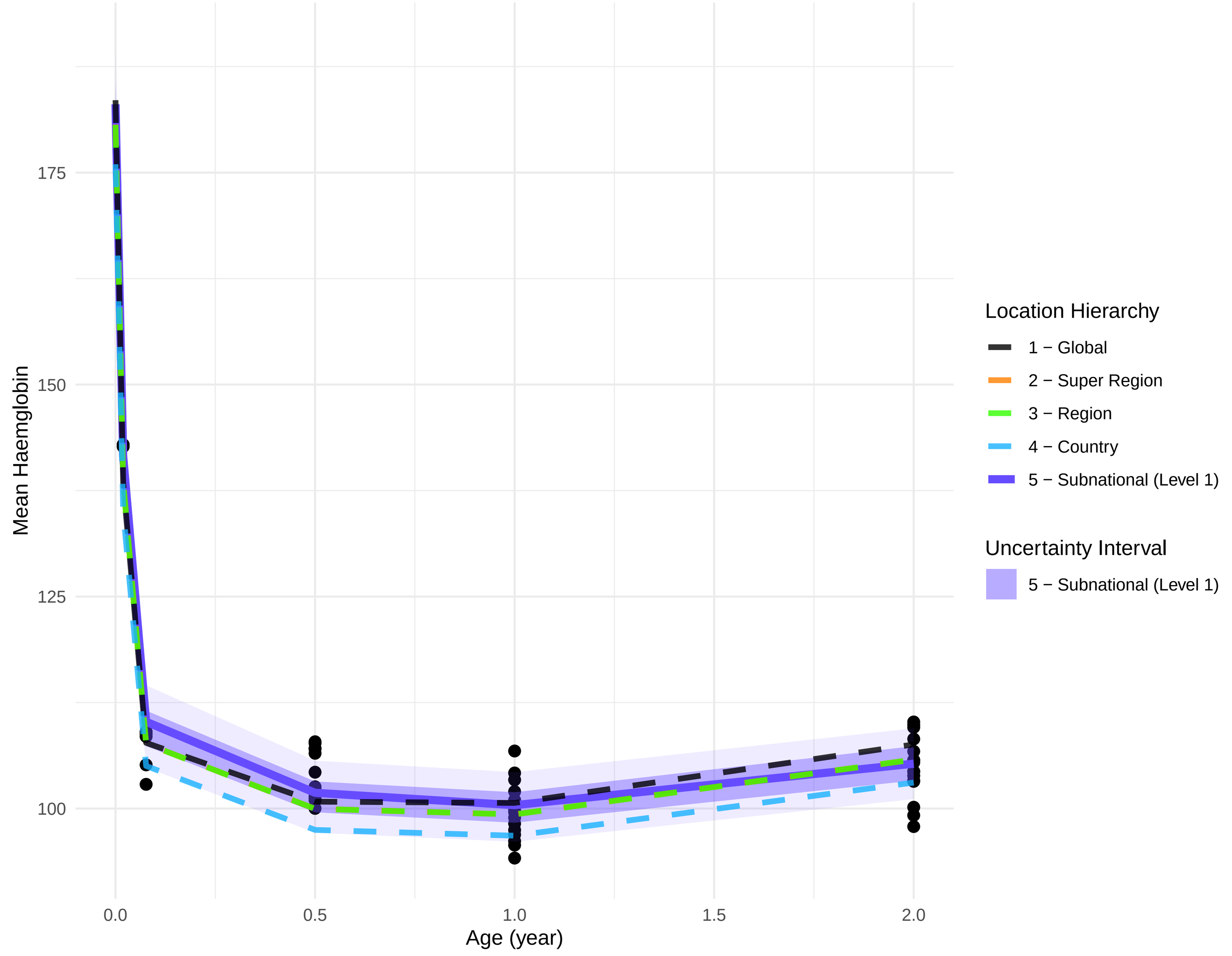

MR−BRT Age/Sex Fits − Males <2 Years Old − Mean Haemglobin − Tamil Nadu, Rural
WHO−2001 Adjustment Method

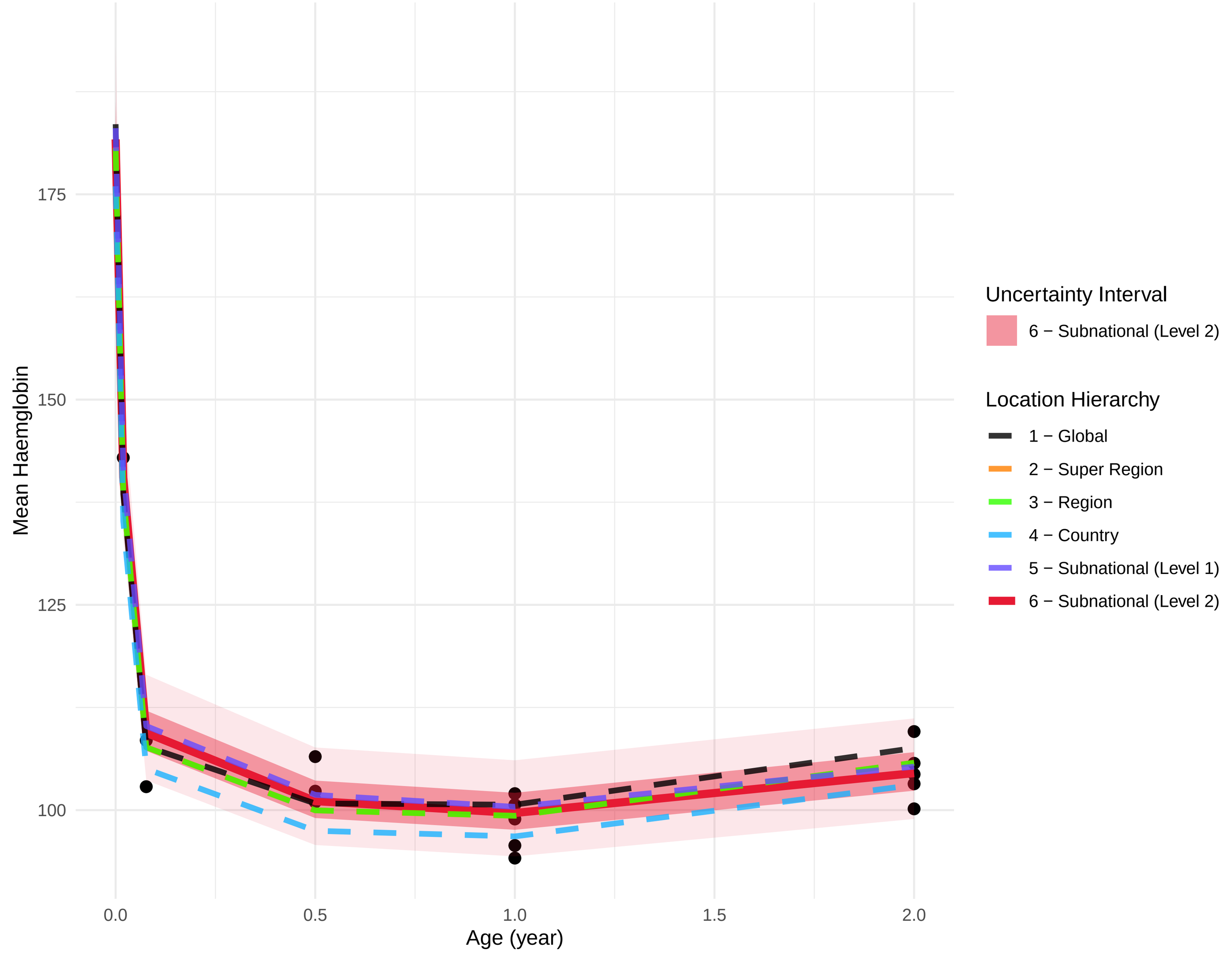

MR−BRT Age/Sex Fits − Males <2 Years Old − Mean Haemglobin − Tamil Nadu, Urban
WHO−2001 Adjustment Method

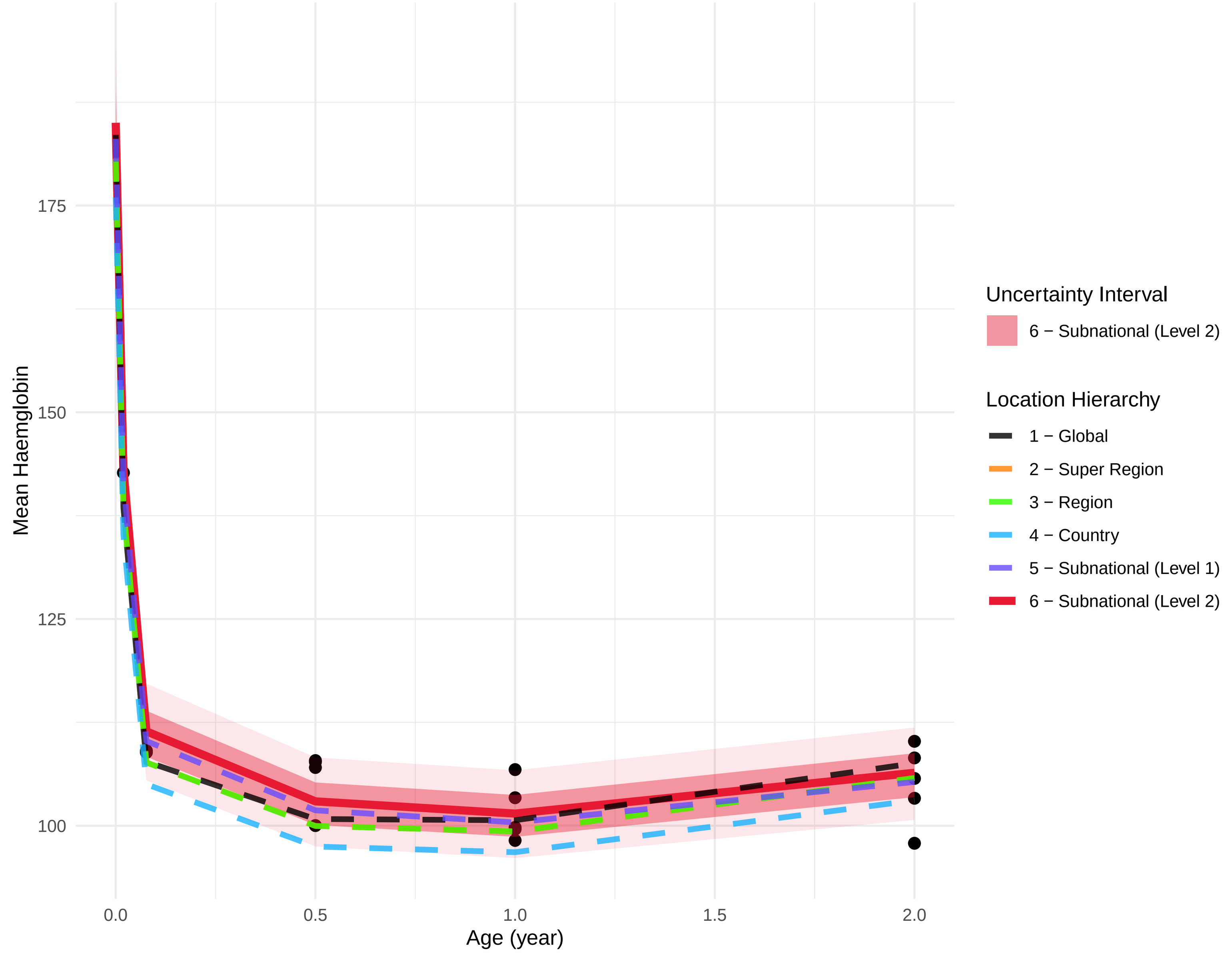

MR−BRT Age/Sex Fits − Males <2 Years Old − Mean Haemglobin − Telangana
WHO−2001 Adjustment Method

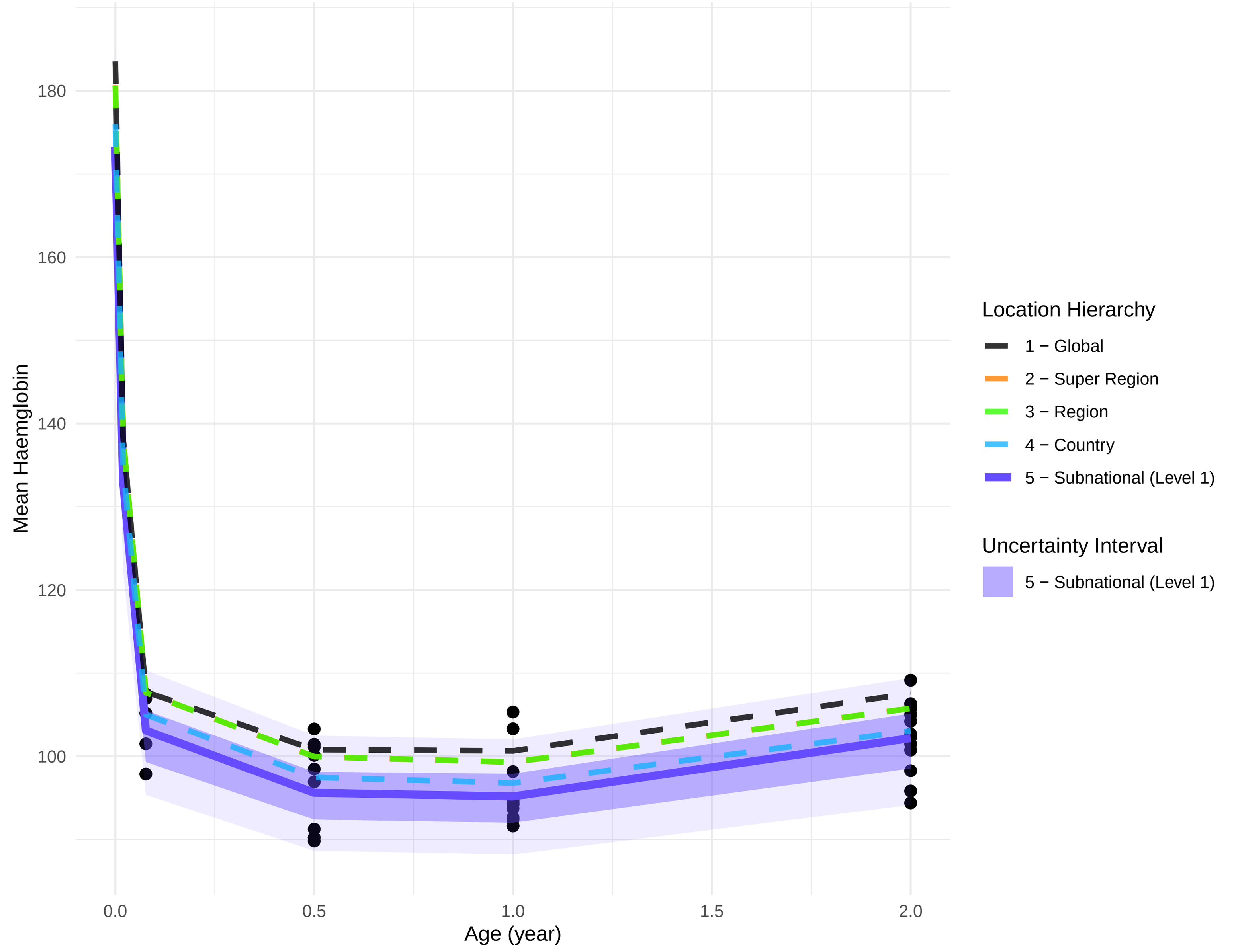

MR−BRT Age/Sex Fits − Males <2 Years Old − Mean Haemglobin − Telangana, Rural
WHO−2001 Adjustment Method

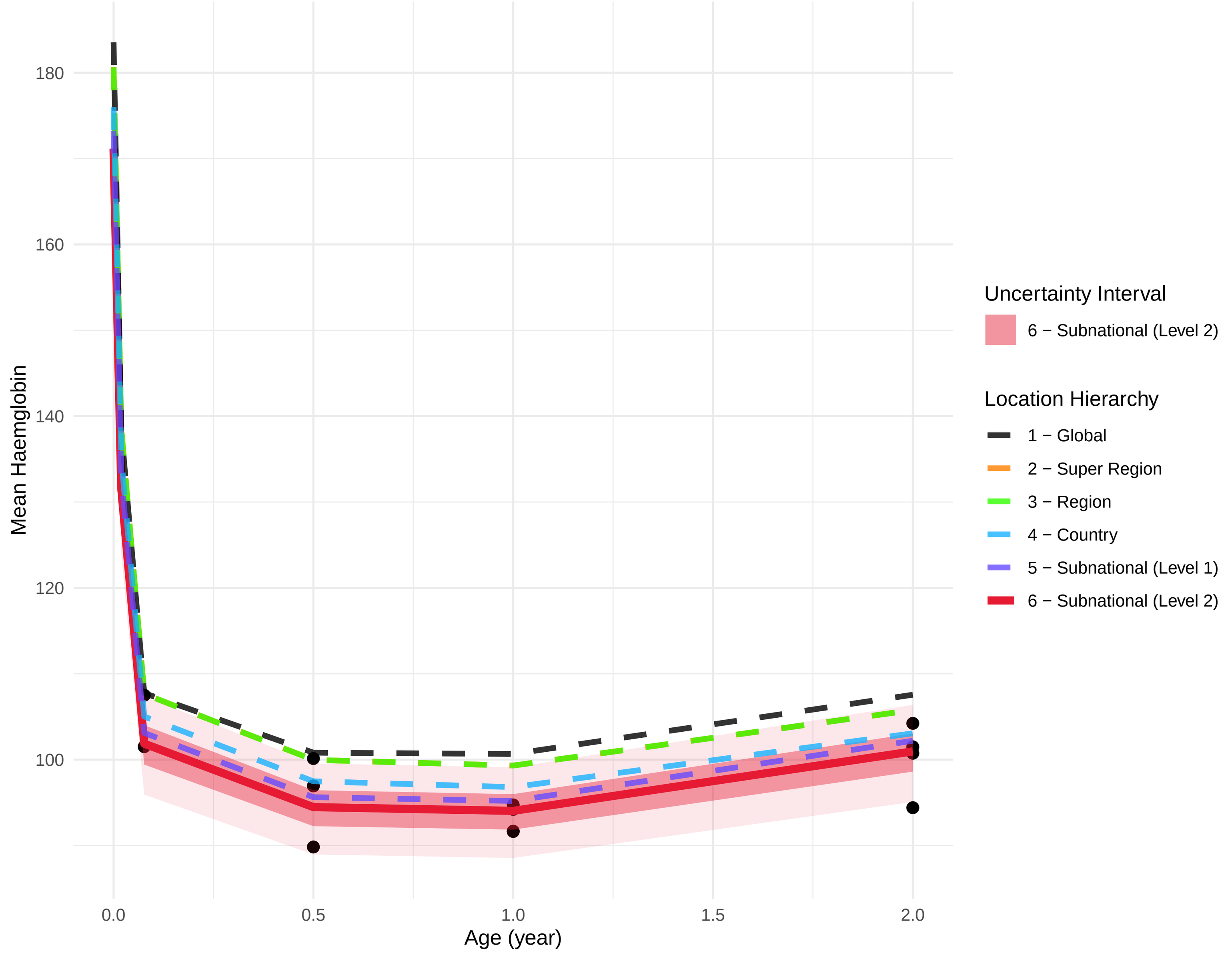

MR−BRT Age/Sex Fits − Males <2 Years Old − Mean Haemglobin − Telangana, Urban
WHO−2001 Adjustment Method

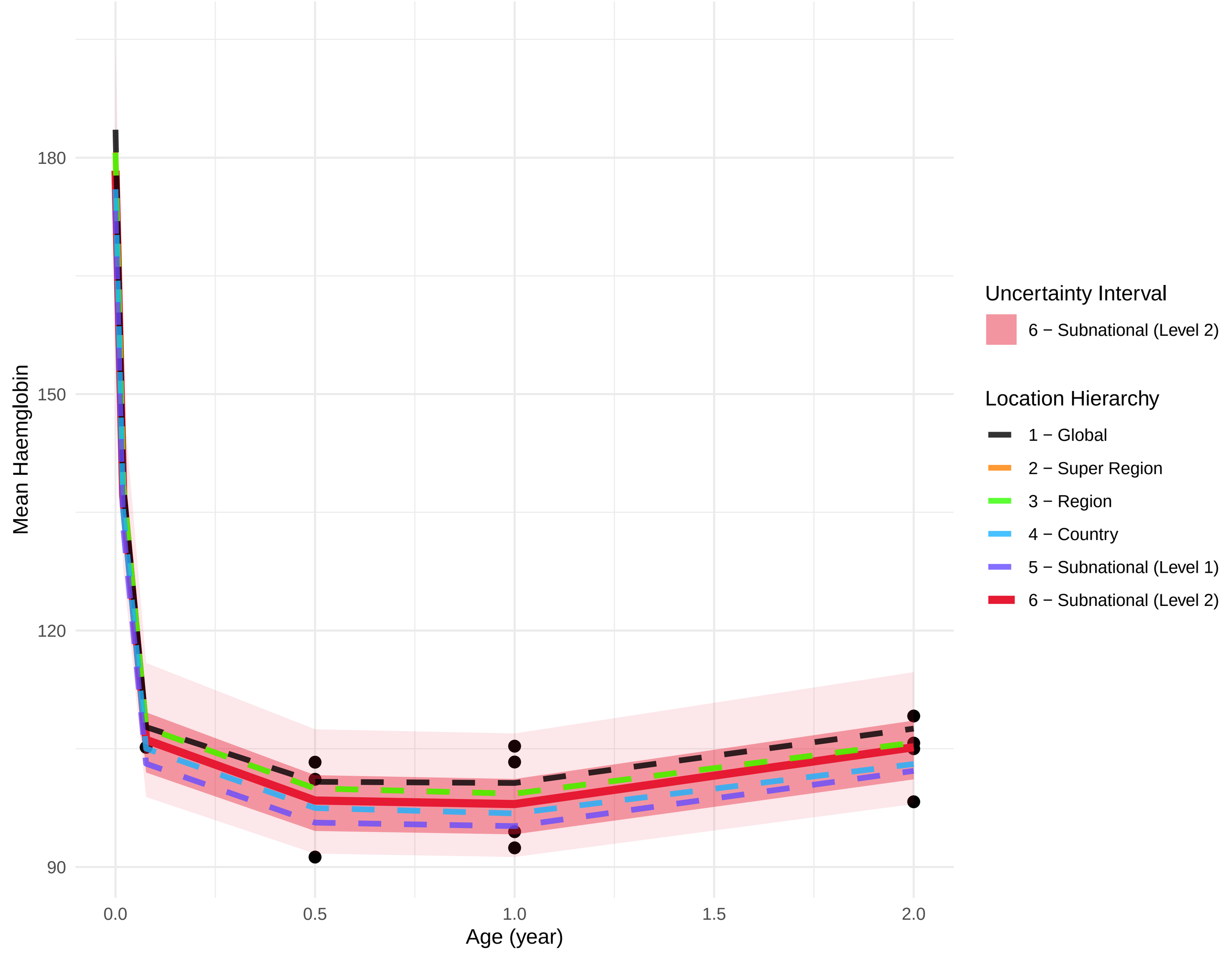

MR−BRT Age/Sex Fits − Males <2 Years Old − Mean Haemglobin − Tripura
WHO−2001 Adjustment Method

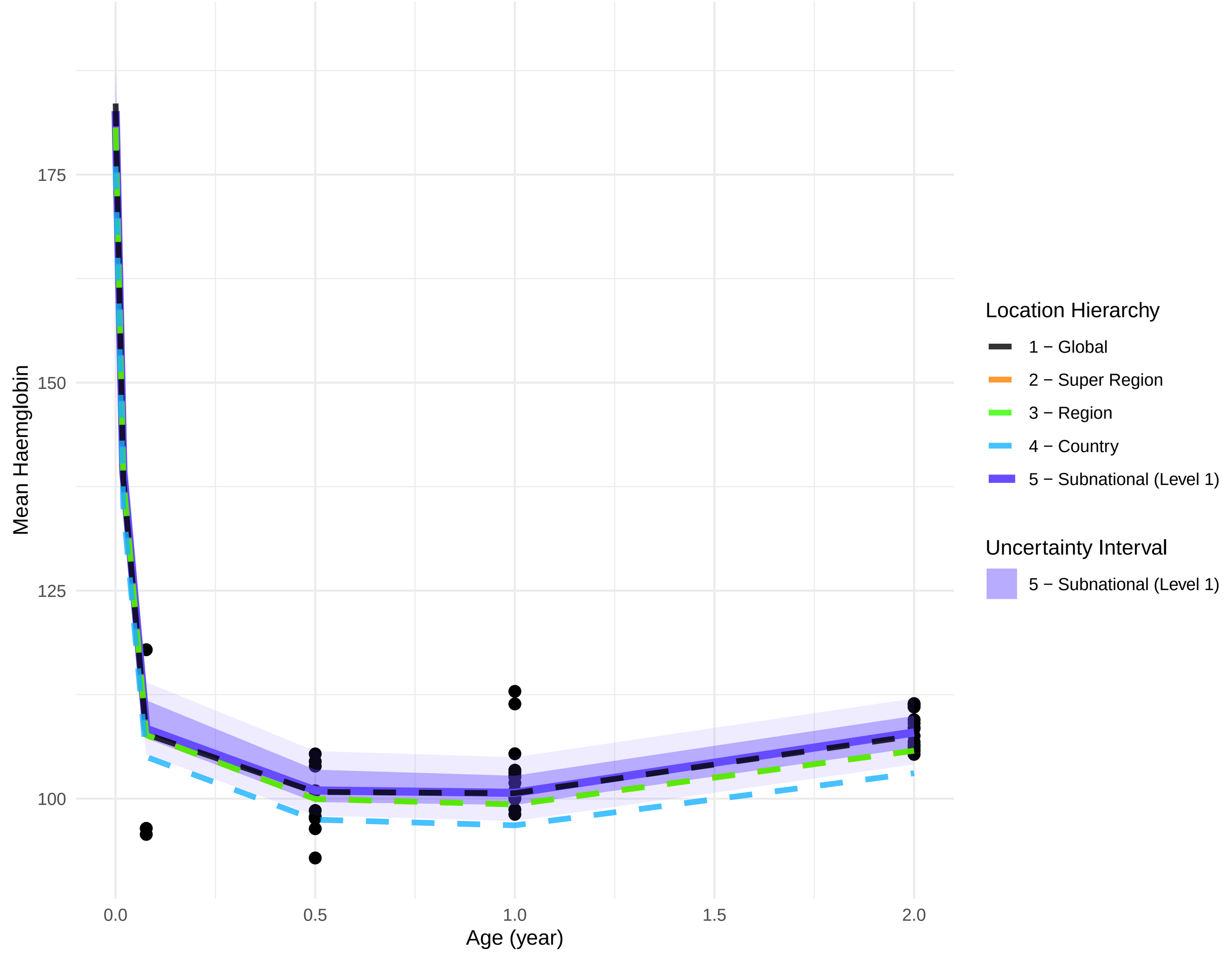

MR−BRT Age/Sex Fits − Males <2 Years Old − Mean Haemglobin − Tripura, Rural
WHO−2001 Adjustment Method

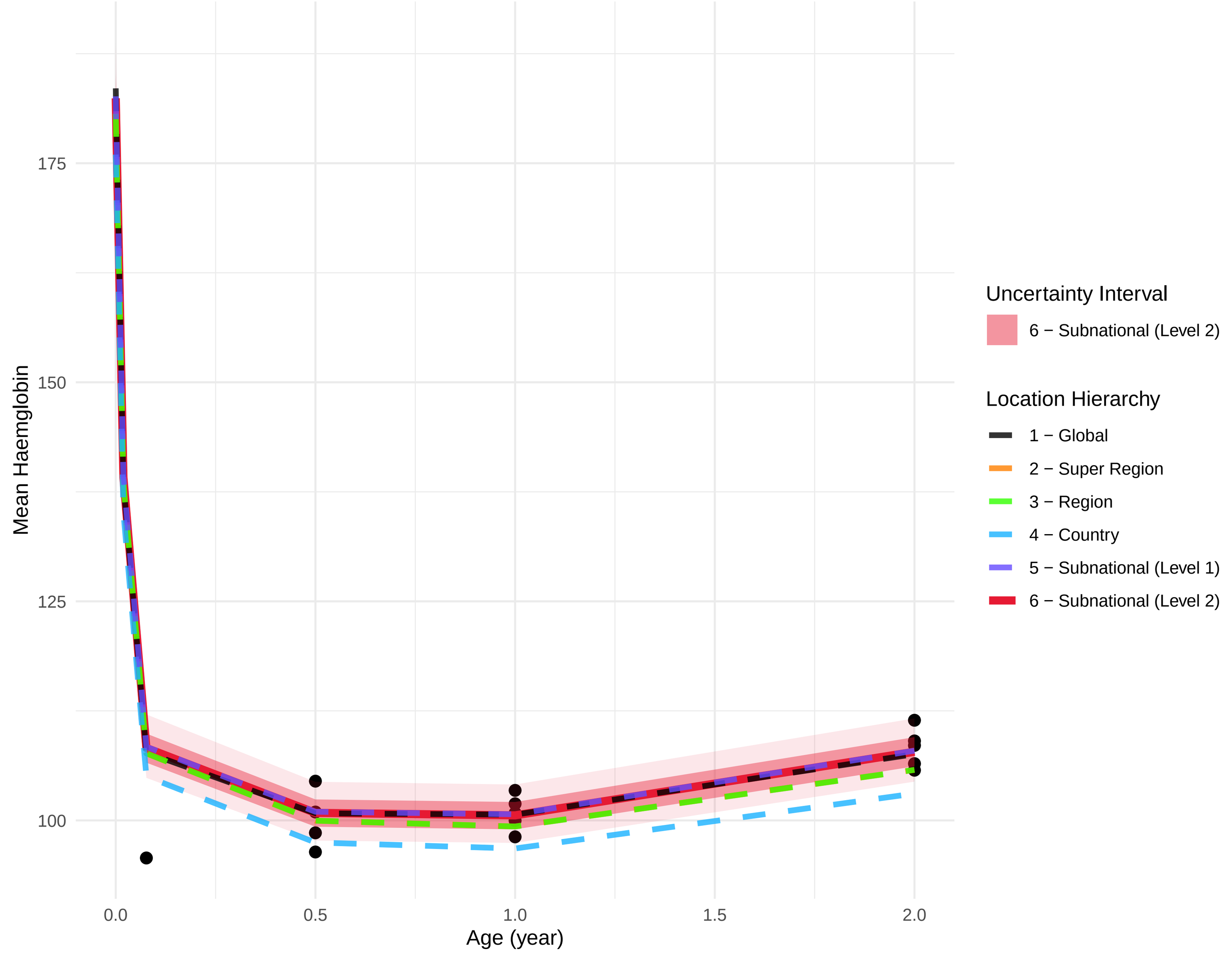

MR−BRT Age/Sex Fits − Males <2 Years Old − Mean Haemglobin − Tripura, Urban
WHO−2001 Adjustment Method

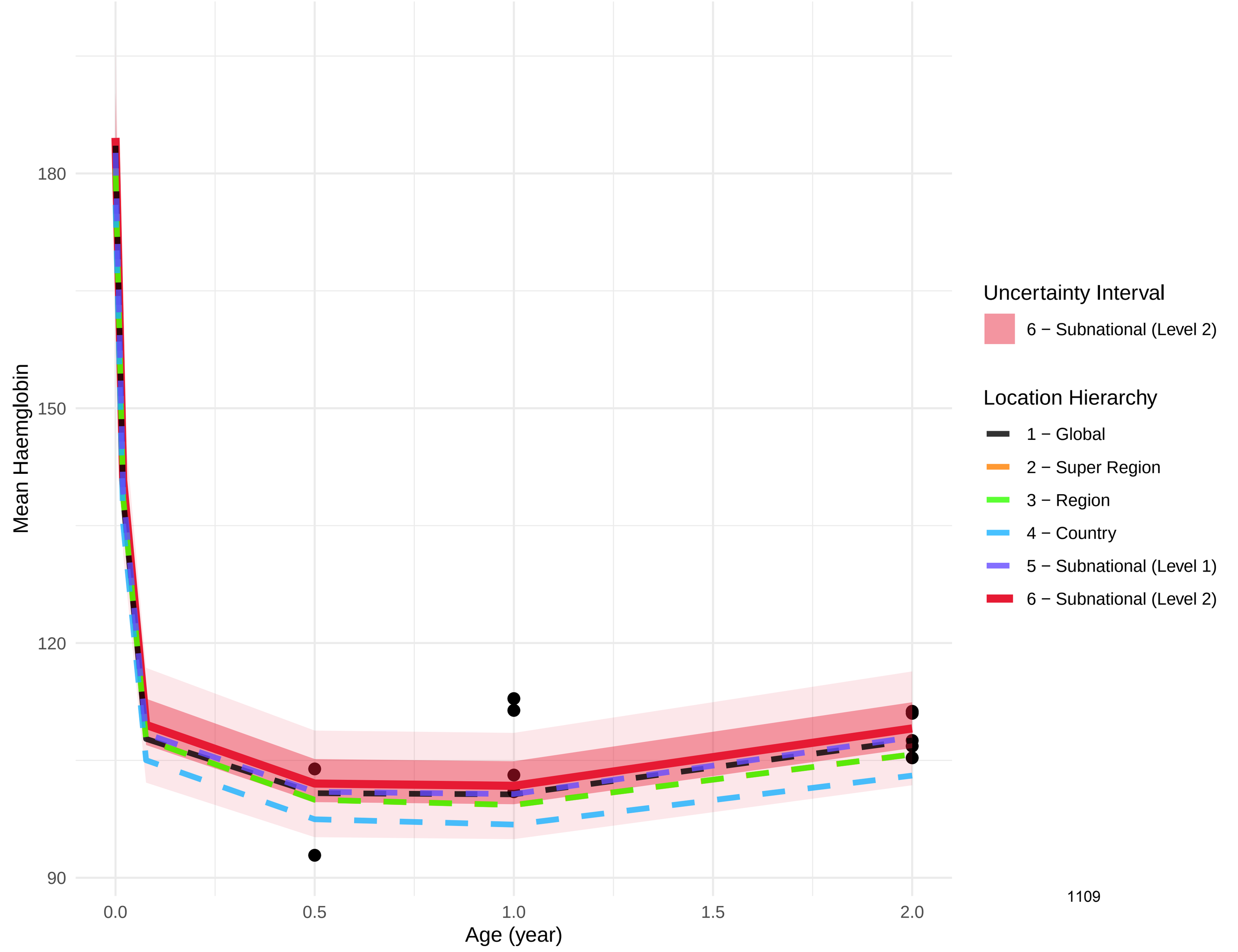

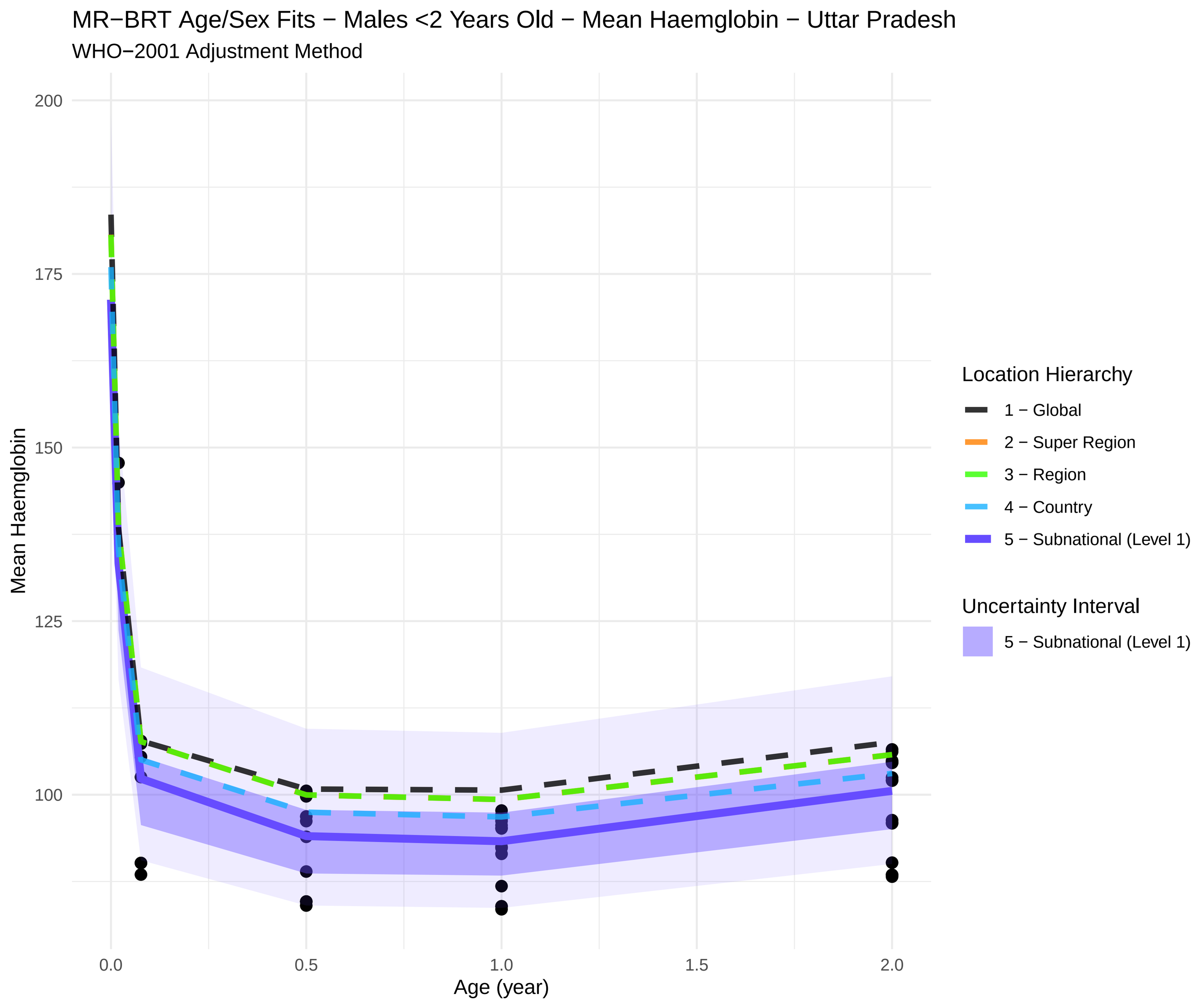
MR−BRT Age/Sex Fits − Males <2 Years Old − Mean Haemglobin − Uttar Pradesh
WHO−2001 Adjustment Method
Mean Haemglobin
Age (year)
Location Hierarchy
1 − Global
2 − Super Region
3 − Region
4 − Country
5 − Subnational (Level 1)
Uncertainty Interval
5 − Subnational (Level 1)

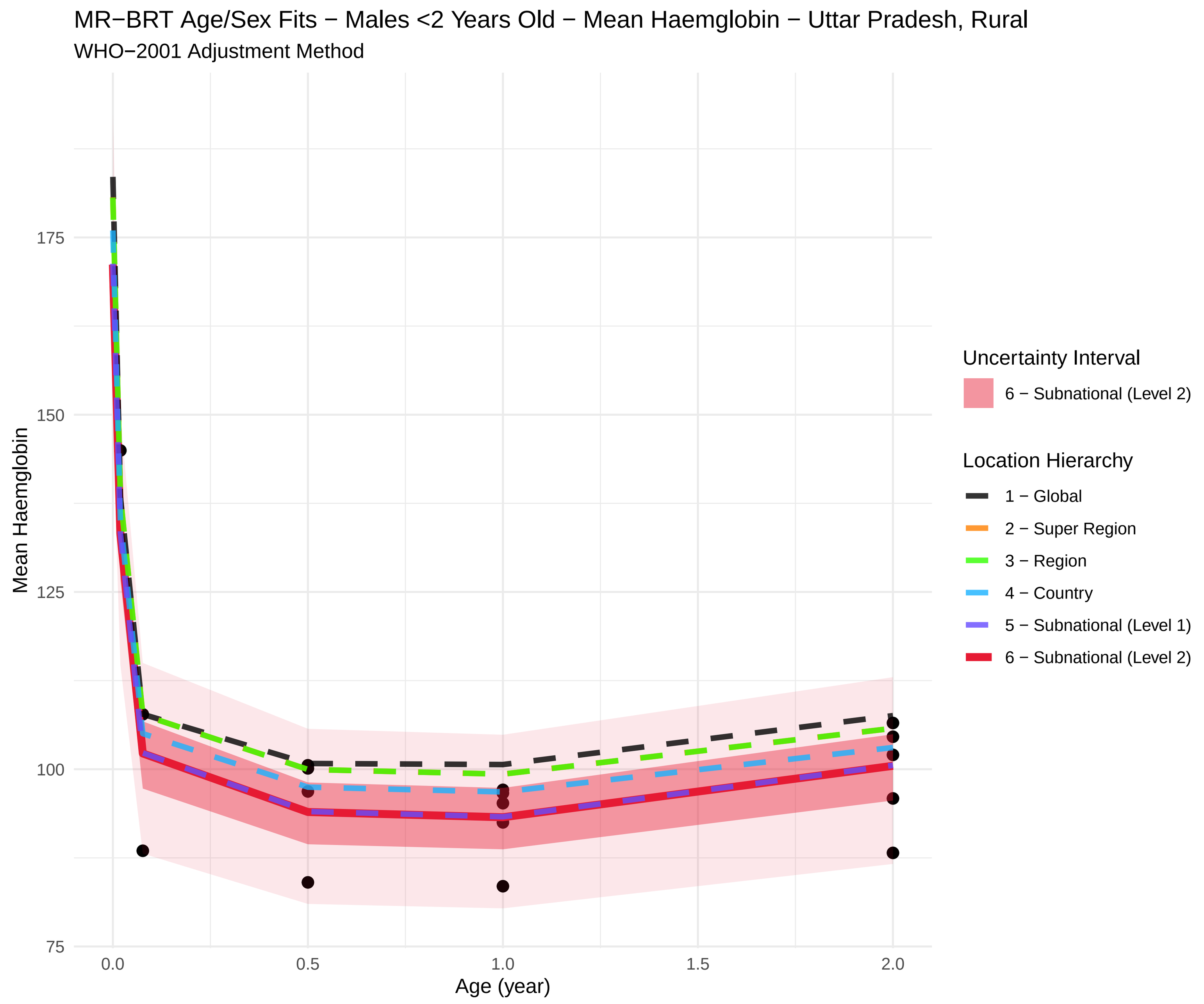
MR−BRT Age/Sex Fits − Males <2 Years Old − Mean Haemglobin − Uttar Pradesh, Rural
WHO−2001 Adjustment Method
Mean Haemglobin
Age (year)
Uncertainty Interval
6 − Subnational (Level 2)
Location Hierarchy
1 − Global
2 − Super Region
3 − Region
4 − Country
5 − Subnational (Level 1)
6 − Subnational (Level 2)

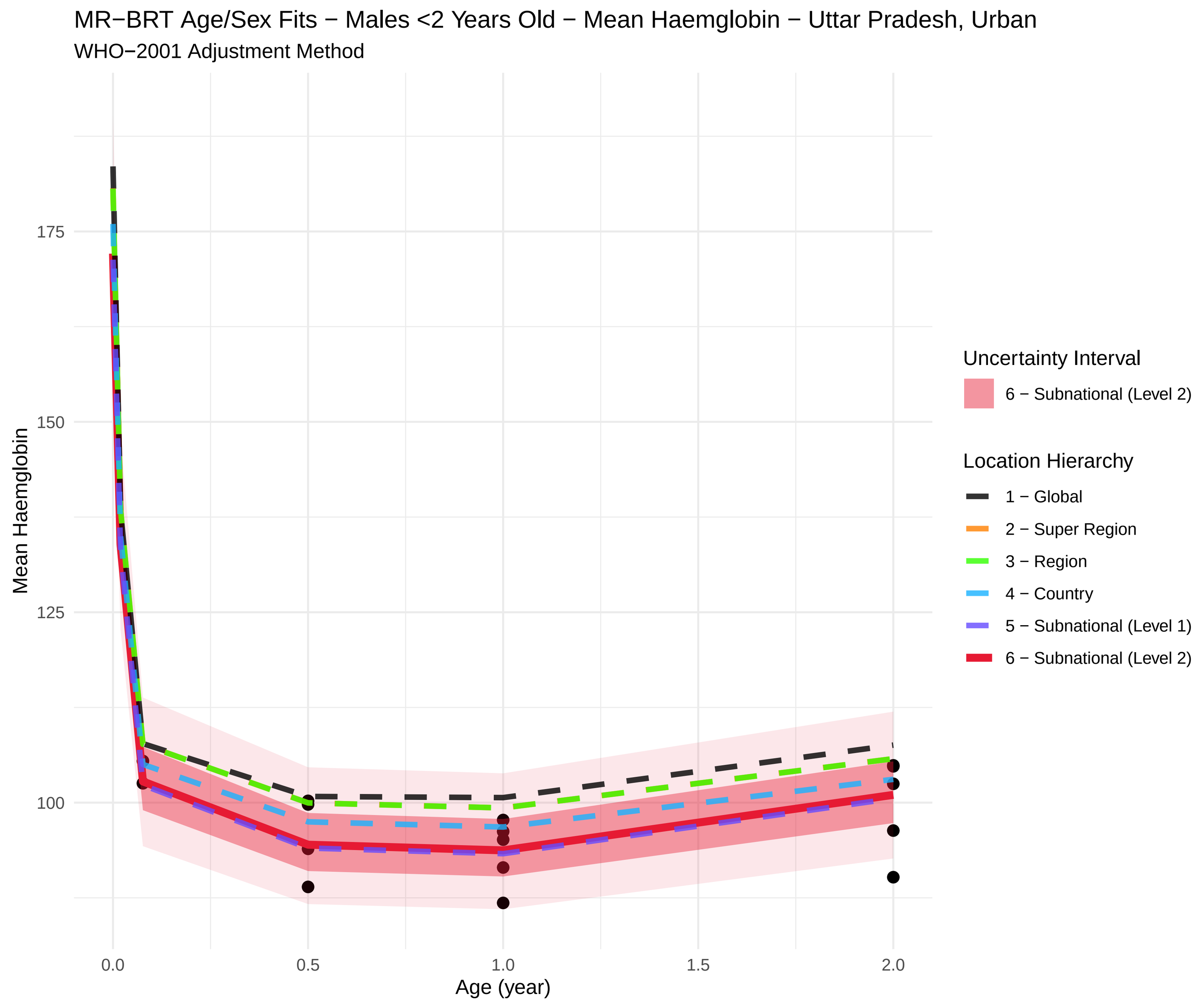
MR−BRT Age/Sex Fits − Males <2 Years Old − Mean Haemglobin − Uttar Pradesh, Urban
WHO−2001 Adjustment Method
Mean Haemglobin
Age (year)
Uncertainty Interval
6 − Subnational (Level 2)
Location Hierarchy
1 − Global
2 − Super Region
3 − Region
4 − Country
5 − Subnational (Level 1)
6 − Subnational (Level 2)

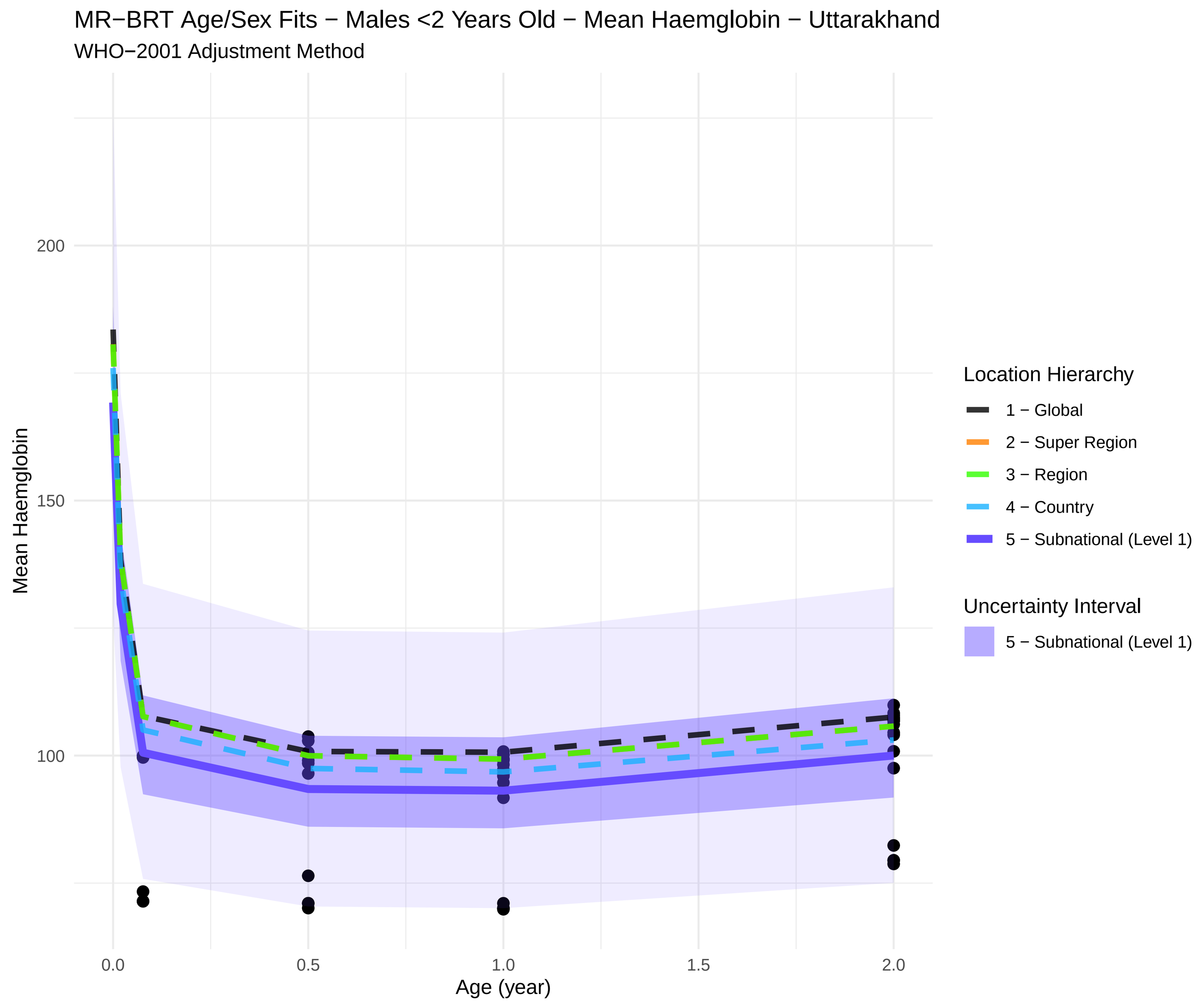
MR−BRT Age/Sex Fits − Males <2 Years Old − Mean Haemglobin − Uttarakhand
WHO−2001 Adjustment Method
Mean Haemglobin
Age (year)
Location Hierarchy
1 − Global
2 − Super Region
3 − Region
4 − Country
5 − Subnational (Level 1)
Uncertainty Interval
5 − Subnational (Level 1)

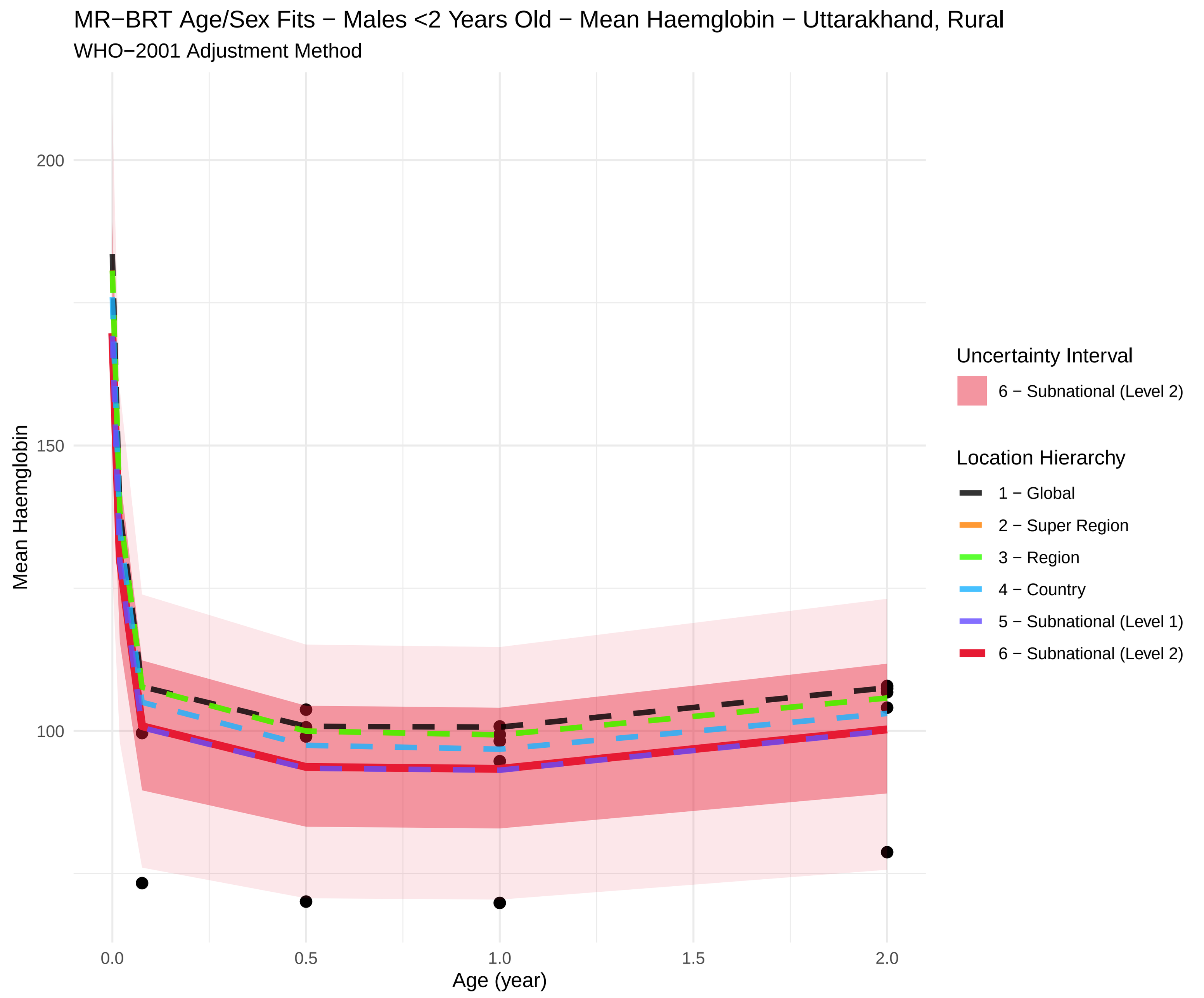
MR−BRT Age/Sex Fits − Males <2 Years Old − Mean Haemglobin − Uttarakhand, Rural
WHO−2001 Adjustment Method
Mean Haemglobin
Age (year)
Uncertainty Interval
6 − Subnational (Level 2)
Location Hierarchy
1 − Global
2 − Super Region
3 − Region
4 − Country
5 − Subnational (Level 1)
6 − Subnational (Level 2)

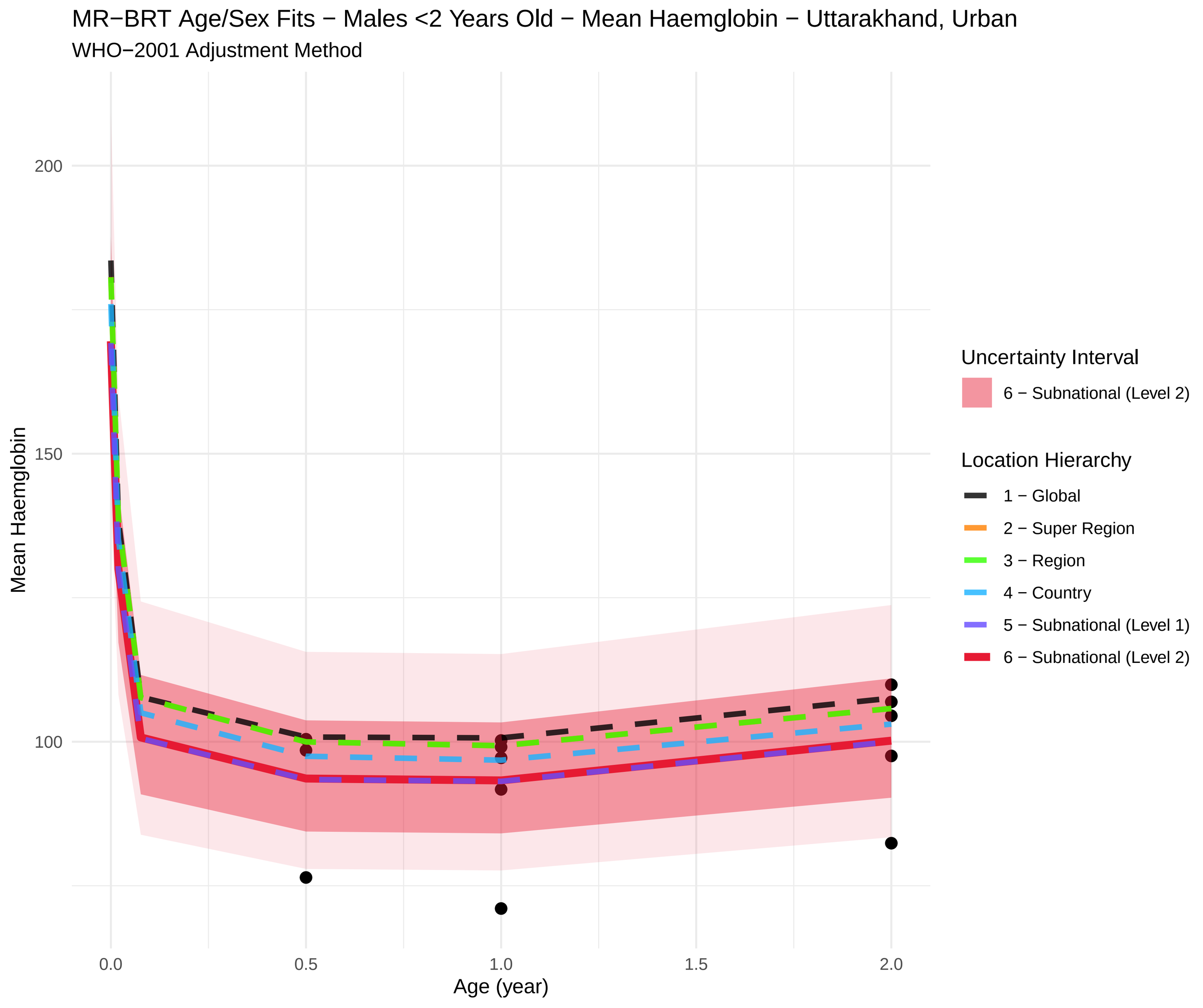
MR−BRT Age/Sex Fits − Males <2 Years Old − Mean Haemglobin − Uttarakhand, Urban
WHO−2001 Adjustment Method
Mean Haemglobin
Age (year)
Uncertainty Interval
6 − Subnational (Level 2)
Location Hierarchy
1 − Global
2 − Super Region
3 − Region
4 − Country
5 − Subnational (Level 1)
6 − Subnational (Level 2)

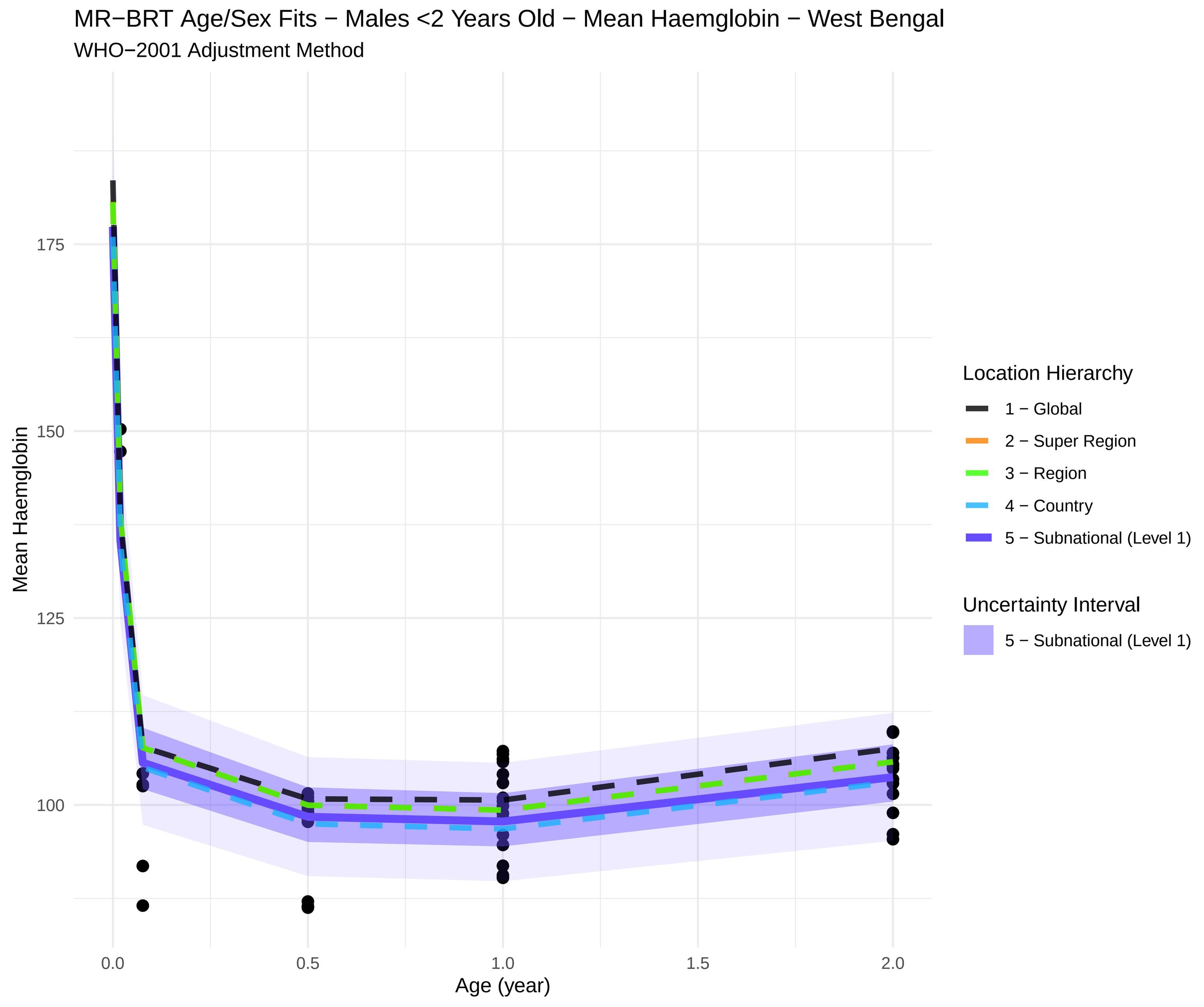
MR−BRT Age/Sex Fits − Males <2 Years Old − Mean Haemglobin − West Bengal
WHO−2001 Adjustment Method
Mean Haemglobin
Age (year)
Location Hierarchy
1 − Global
2 − Super Region
3 − Region
4 − Country
5 − Subnational (Level 1)
Uncertainty Interval
5 − Subnational (Level 1)

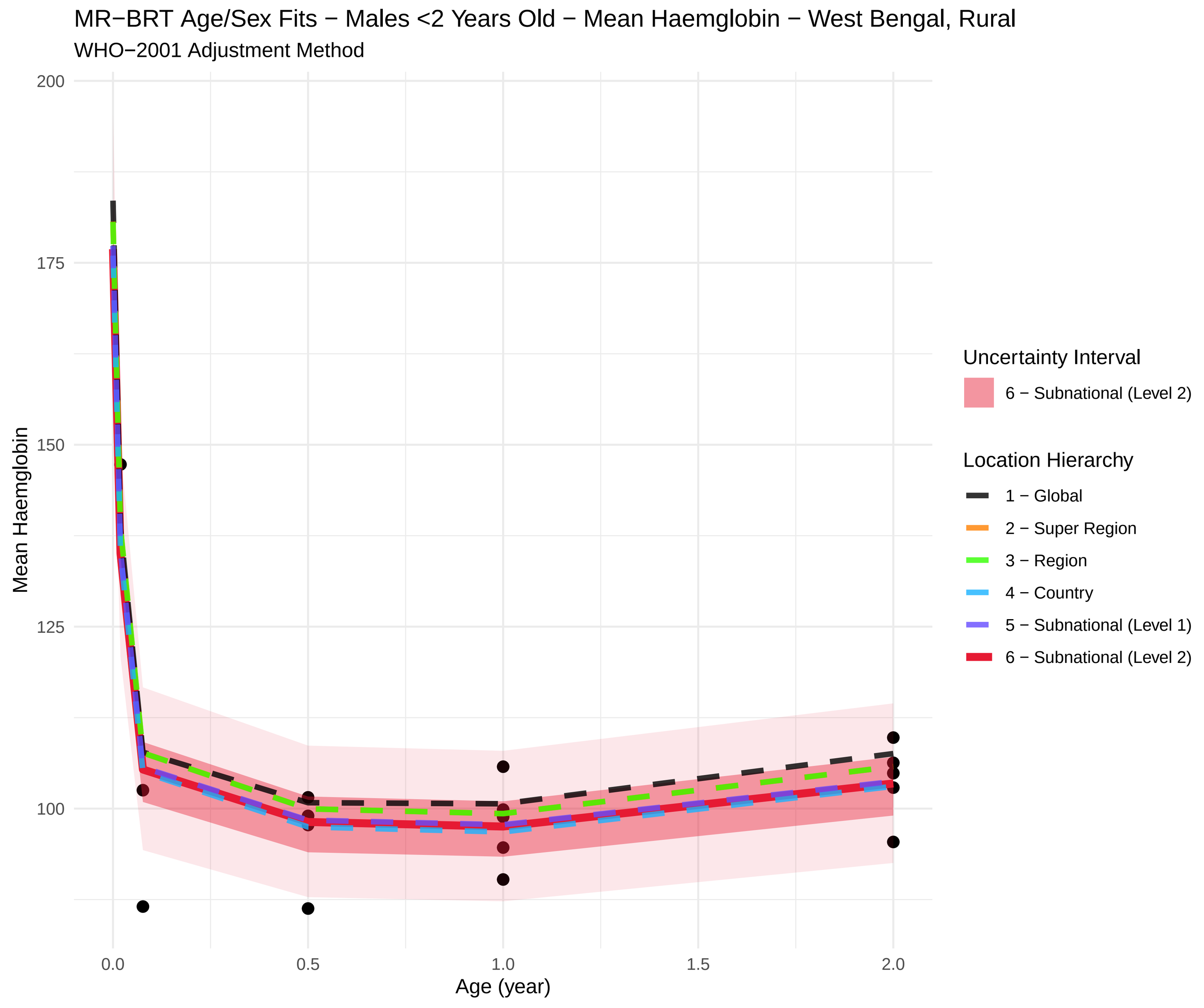
MR−BRT Age/Sex Fits − Males <2 Years Old − Mean Haemglobin − West Bengal, Rural
WHO−2001 Adjustment Method
Mean Haemglobin
Age (year)
Uncertainty Interval
6 − Subnational (Level 2)
Location Hierarchy
1 − Global
2 − Super Region
3 − Region
4 − Country
5 − Subnational (Level 1)
6 − Subnational (Level 2)

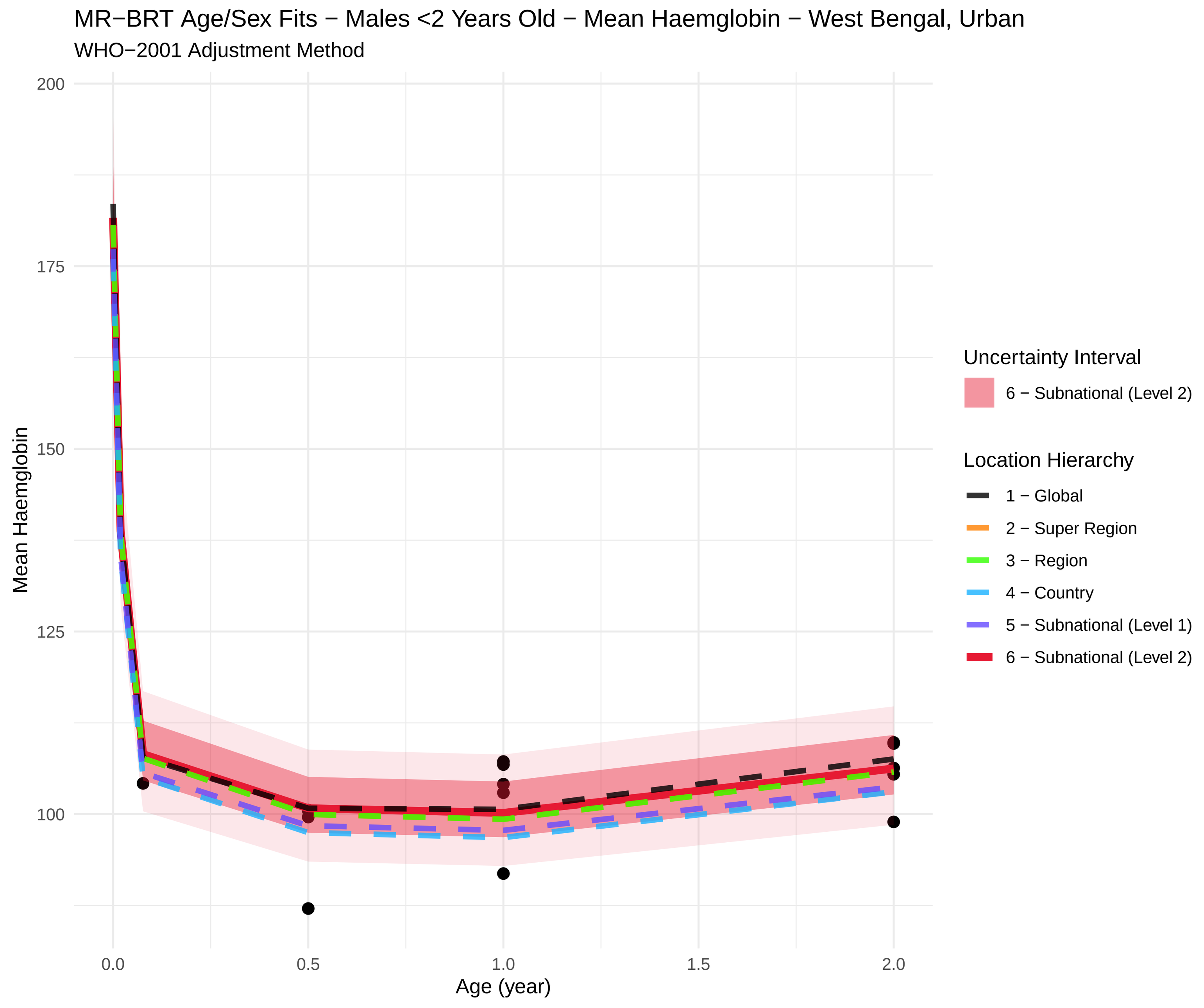
MR−BRT Age/Sex Fits − Males <2 Years Old − Mean Haemglobin − West Bengal, Urban
WHO−2001 Adjustment Method
Mean Haemglobin
Age (year)
Uncertainty Interval
6 − Subnational (Level 2)
Location Hierarchy
1 − Global
2 − Super Region
3 − Region
4 − Country
5 − Subnational (Level 1)
6 − Subnational (Level 2)

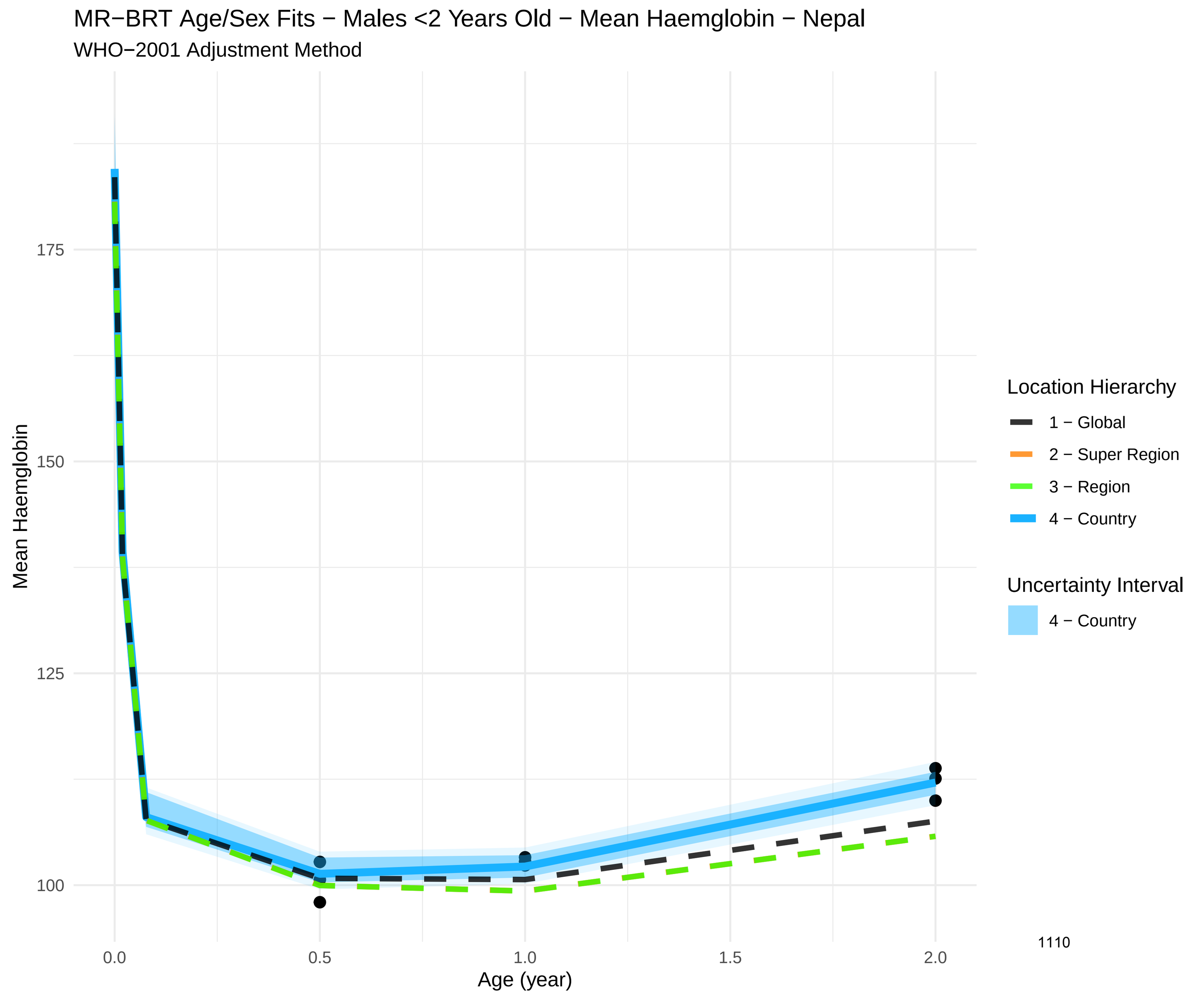
MR−BRT Age/Sex Fits − Males <2 Years Old − Mean Haemglobin − Nepal
WHO−2001 Adjustment Method
Mean Haemglobin
Age (year)
Location Hierarchy
1 − Global
2 − Super Region
3 − Region
4 − Country
Uncertainty Interval
4 − Country

MR−BRT Age/Sex Fits − Males <2 Years Old − Mean Haemglobin − Southeast Asia, East Asia, and Oceania
WHO−2001 Adjustment Method

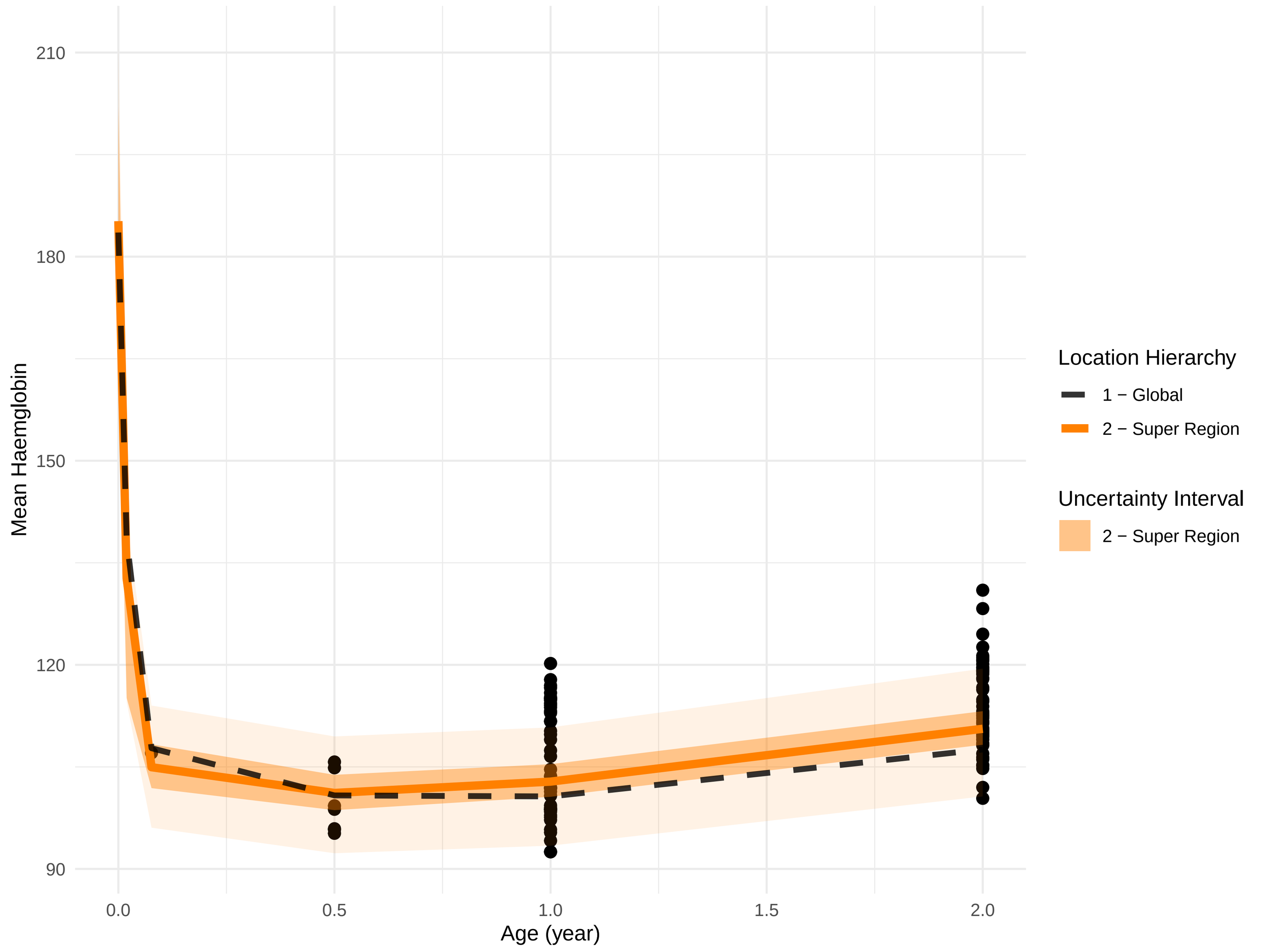

MR−BRT Age/Sex Fits − Males <2 Years Old − Mean Haemglobin − Southeast Asia
WHO−2001 Adjustment Method

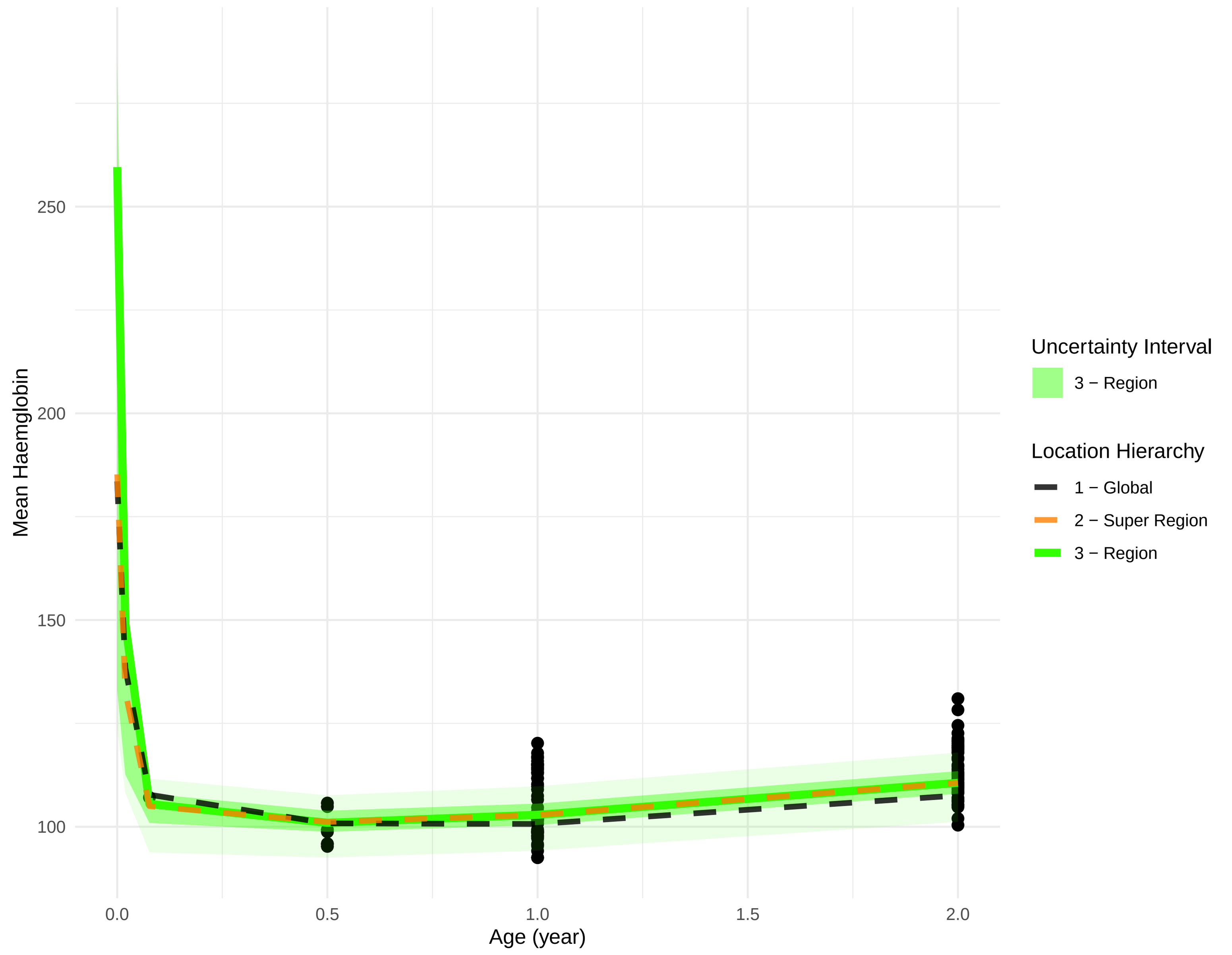

MR−BRT Age/Sex Fits − Males <2 Years Old − Mean Haemglobin − Cambodia
WHO−2001 Adjustment Method

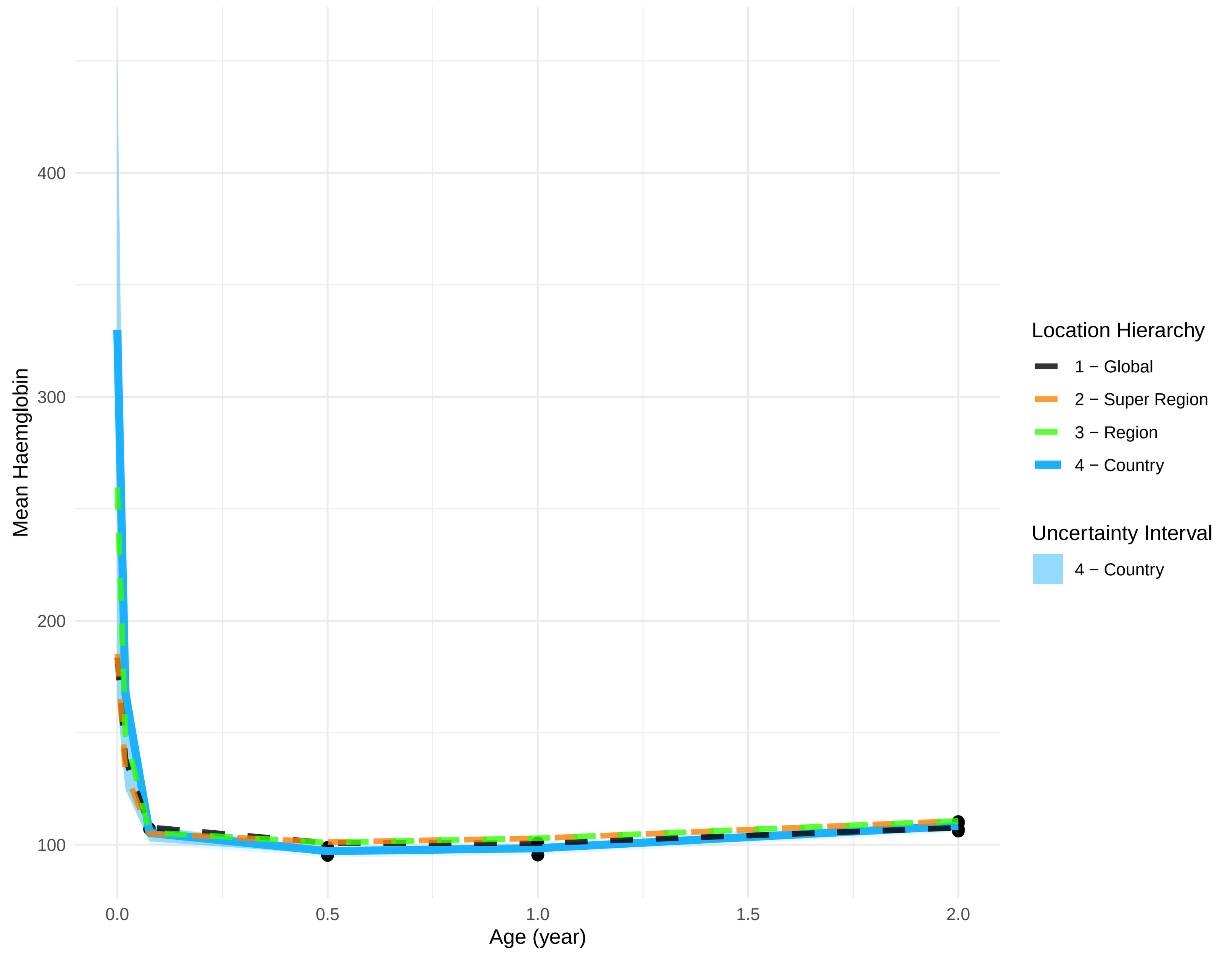

MR−BRT Age/Sex Fits − Males <2 Years Old − Mean Haemglobin − Indonesia
WHO−2001 Adjustment Method

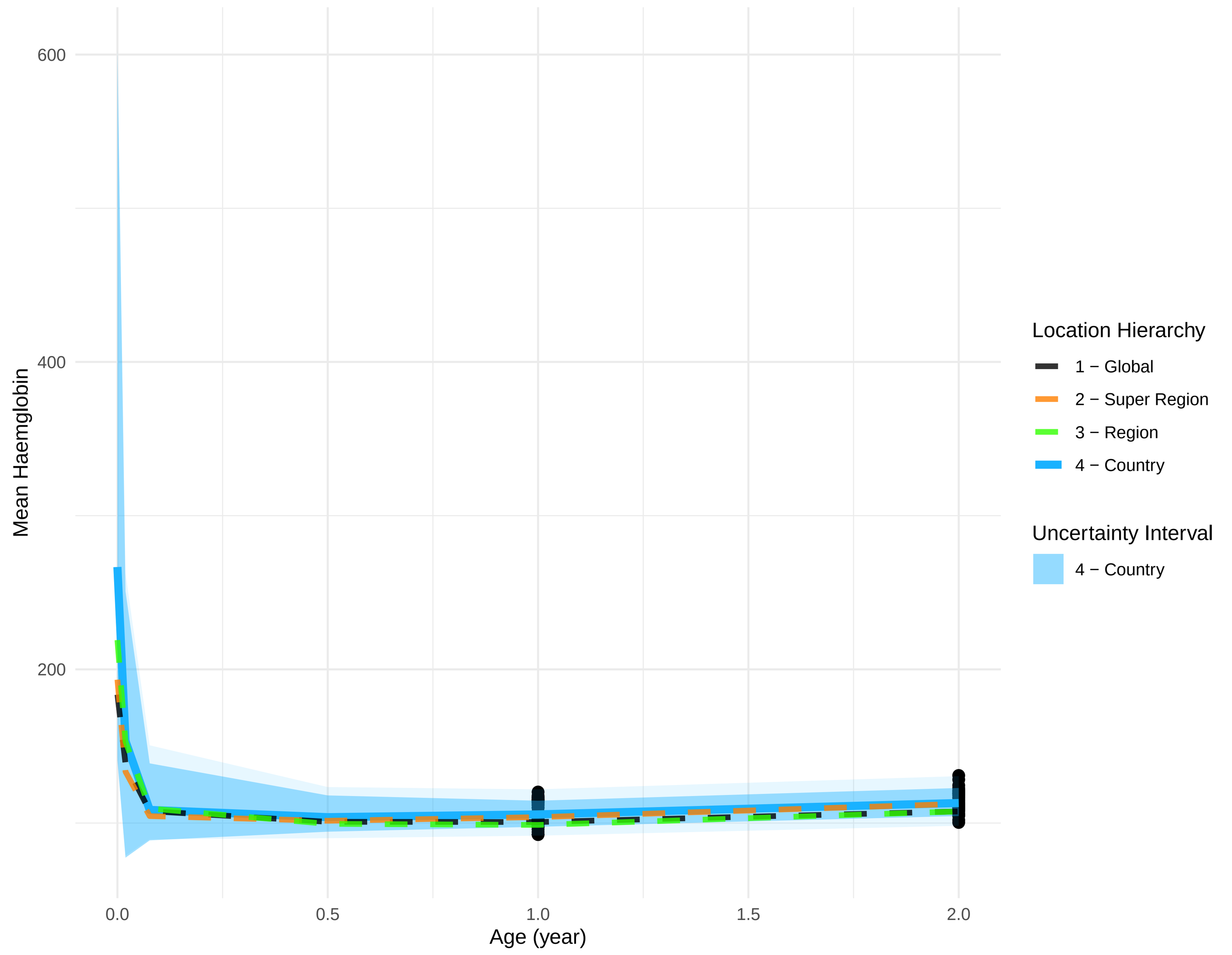

MR−BRT Age/Sex Fits − Males <2 Years Old − Mean Haemglobin − Bali
WHO−2001 Adjustment Method

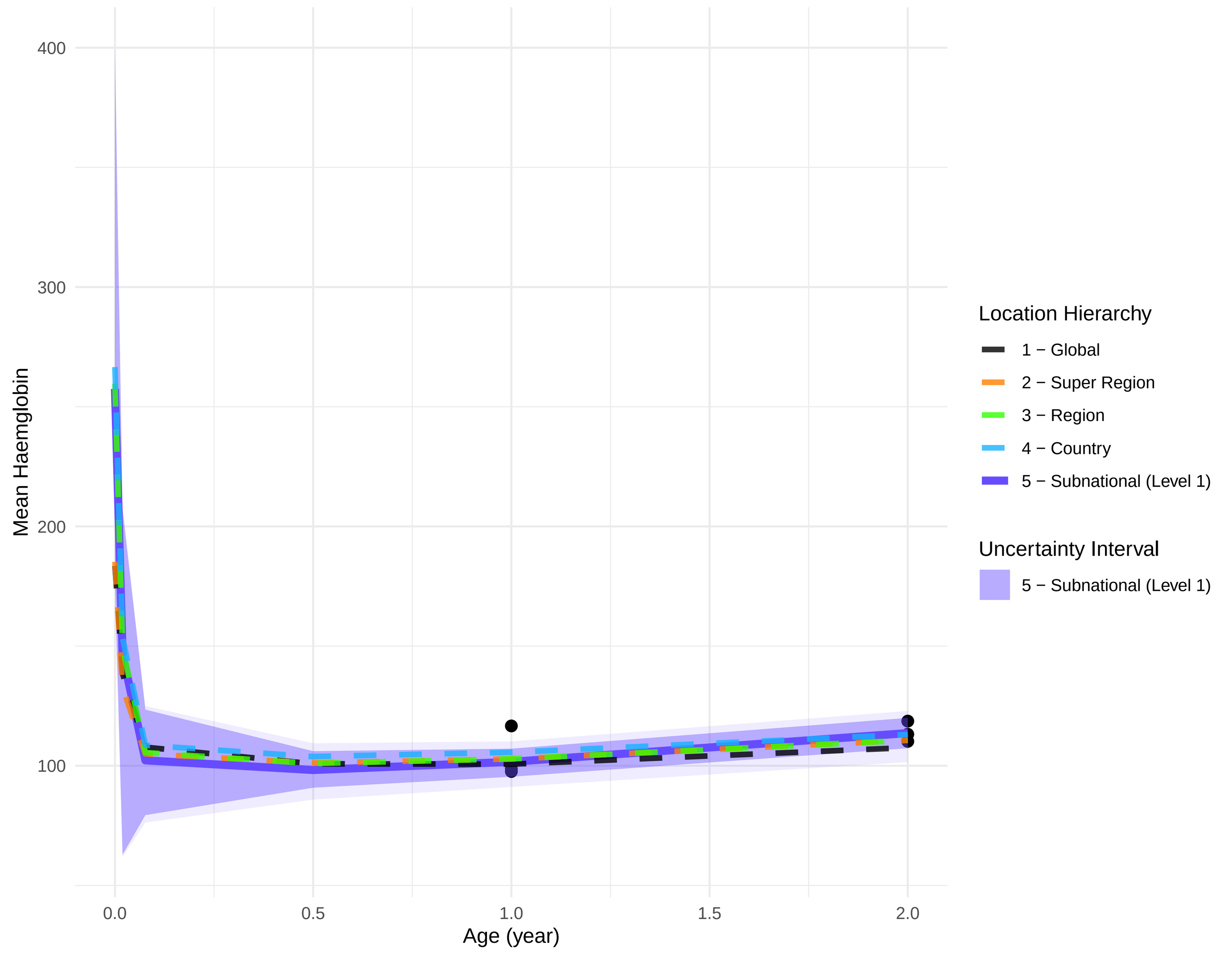

MR−BRT Age/Sex Fits − Males <2 Years Old − Mean Haemglobin − Bangka−Belitung Islands
WHO−2001 Adjustment Method

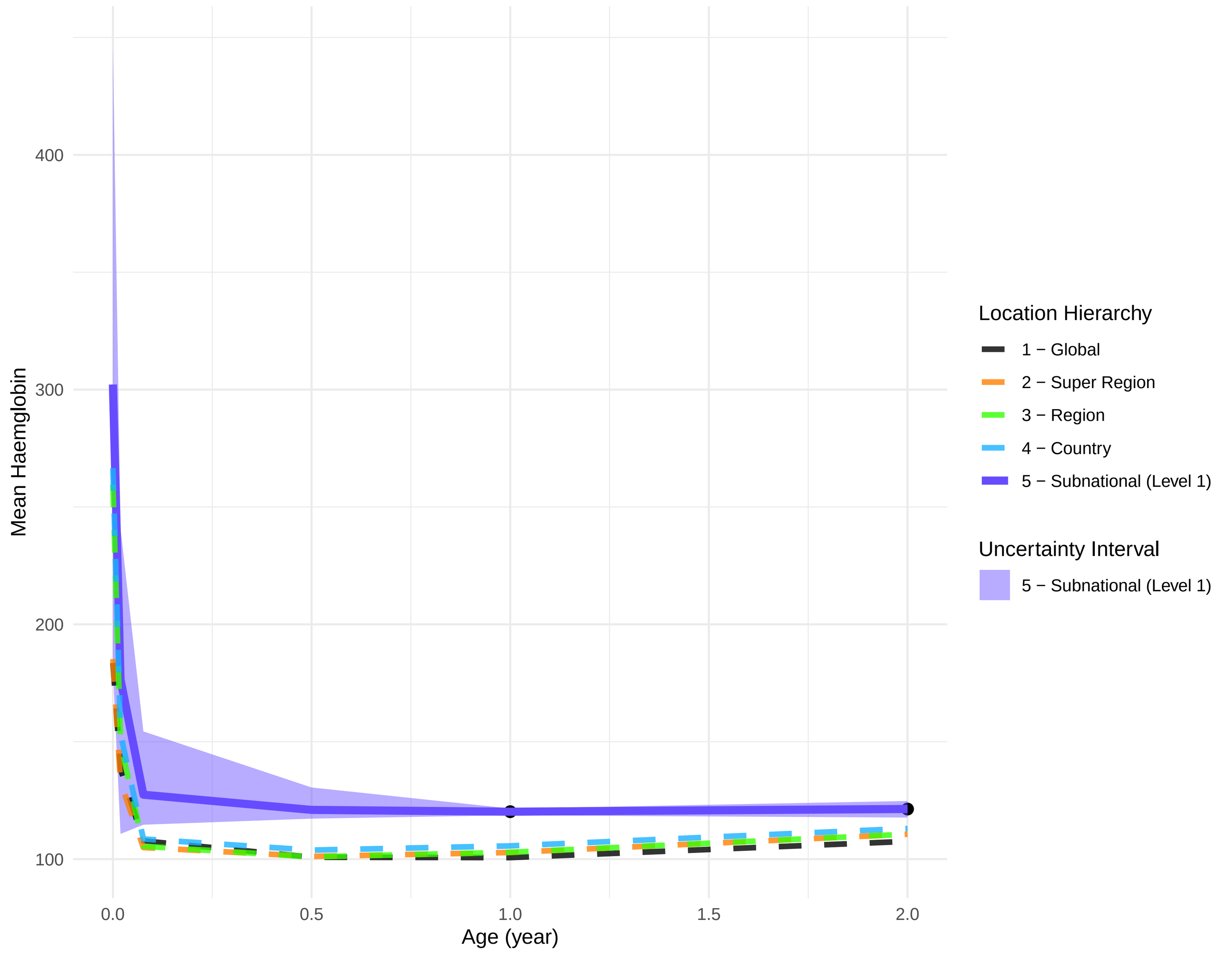

MR−BRT Age/Sex Fits − Males <2 Years Old − Mean Haemglobin − Banten
WHO−2001 Adjustment Method

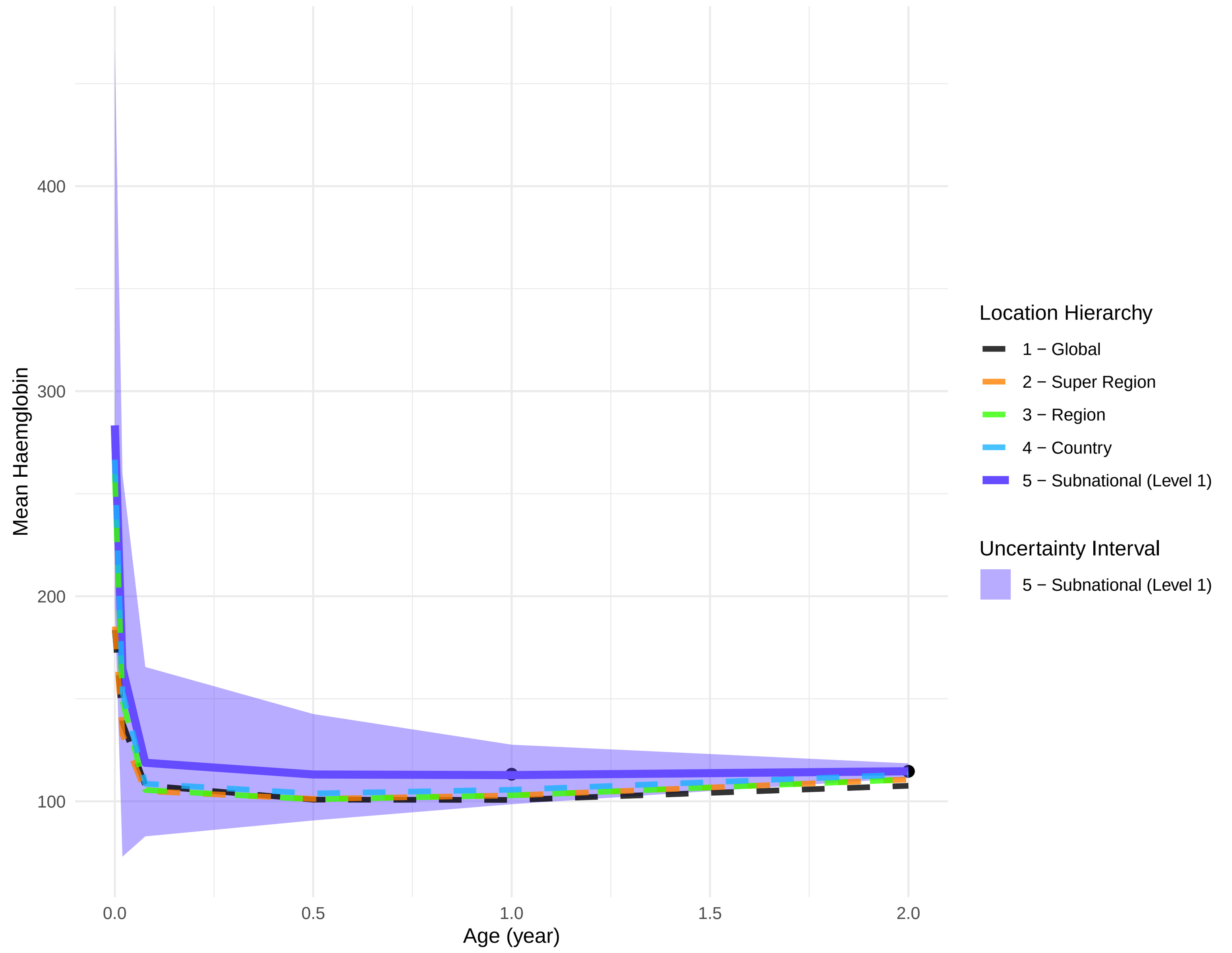

MR−BRT Age/Sex Fits − Males <2 Years Old − Mean Haemglobin − Jakarta
WHO−2001 Adjustment Method

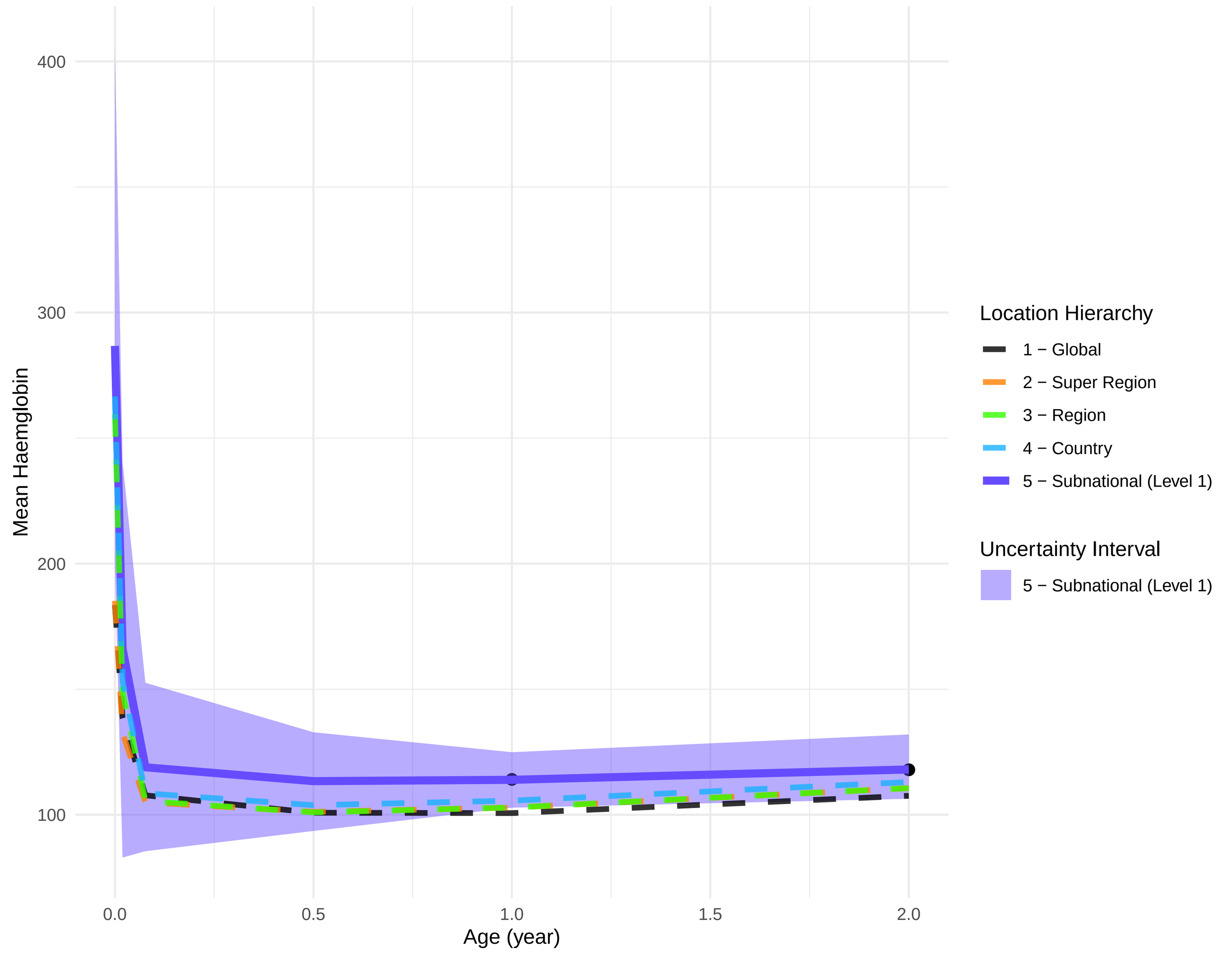

MR−BRT Age/Sex Fits − Males <2 Years Old − Mean Haemglobin − West Java
WHO−2001 Adjustment Method

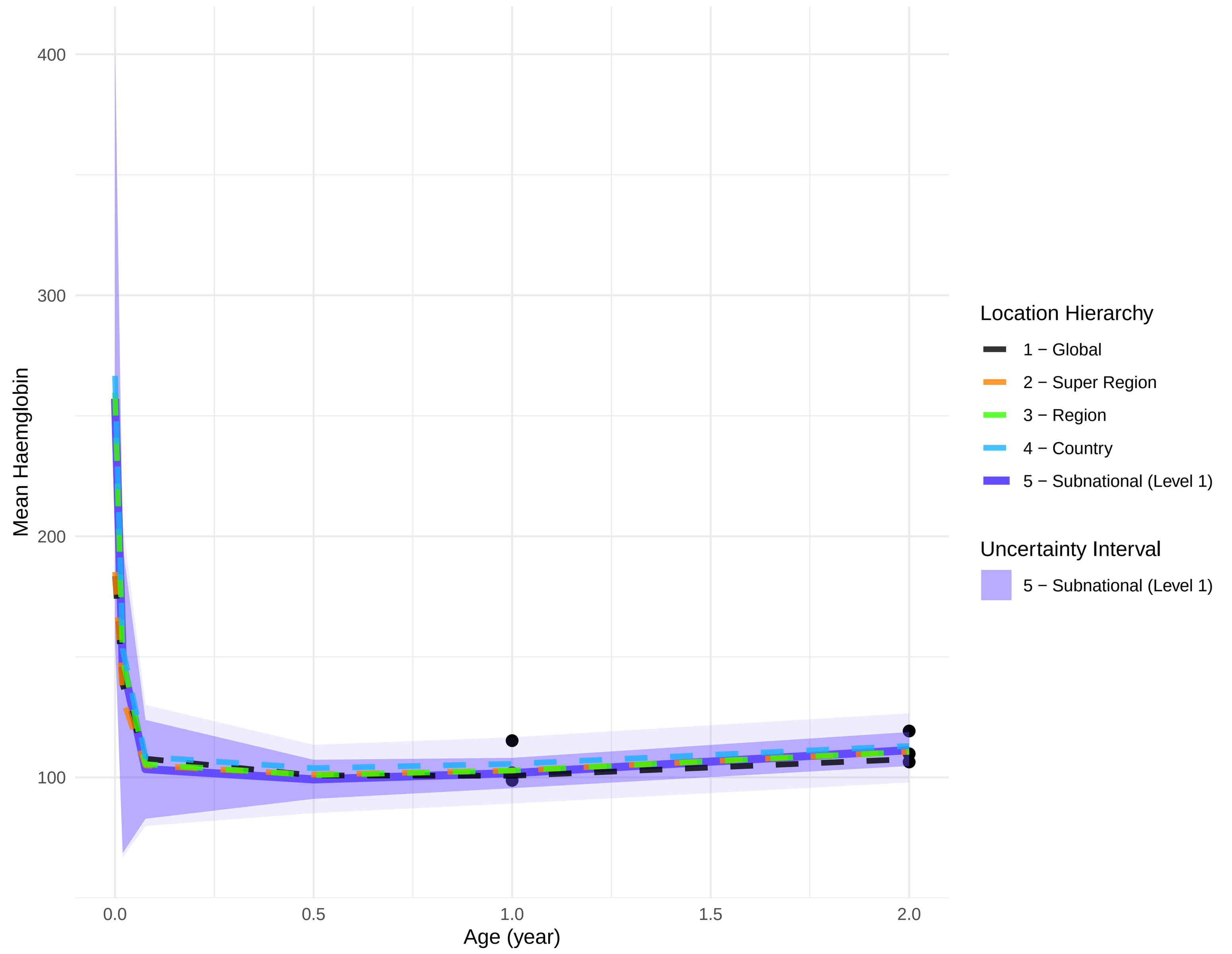

MR−BRT Age/Sex Fits − Males <2 Years Old − Mean Haemglobin − Central Java
WHO−2001 Adjustment Method

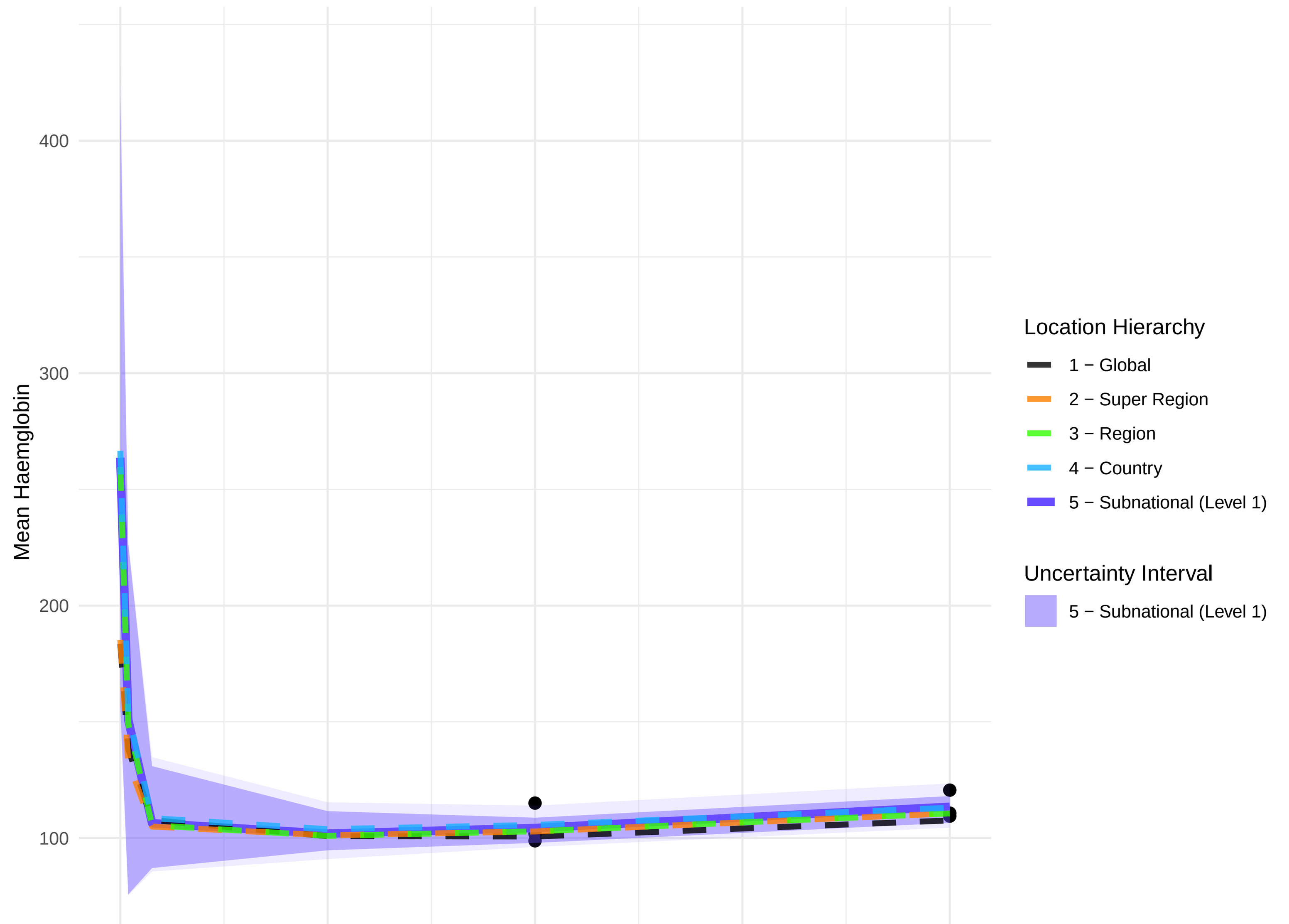

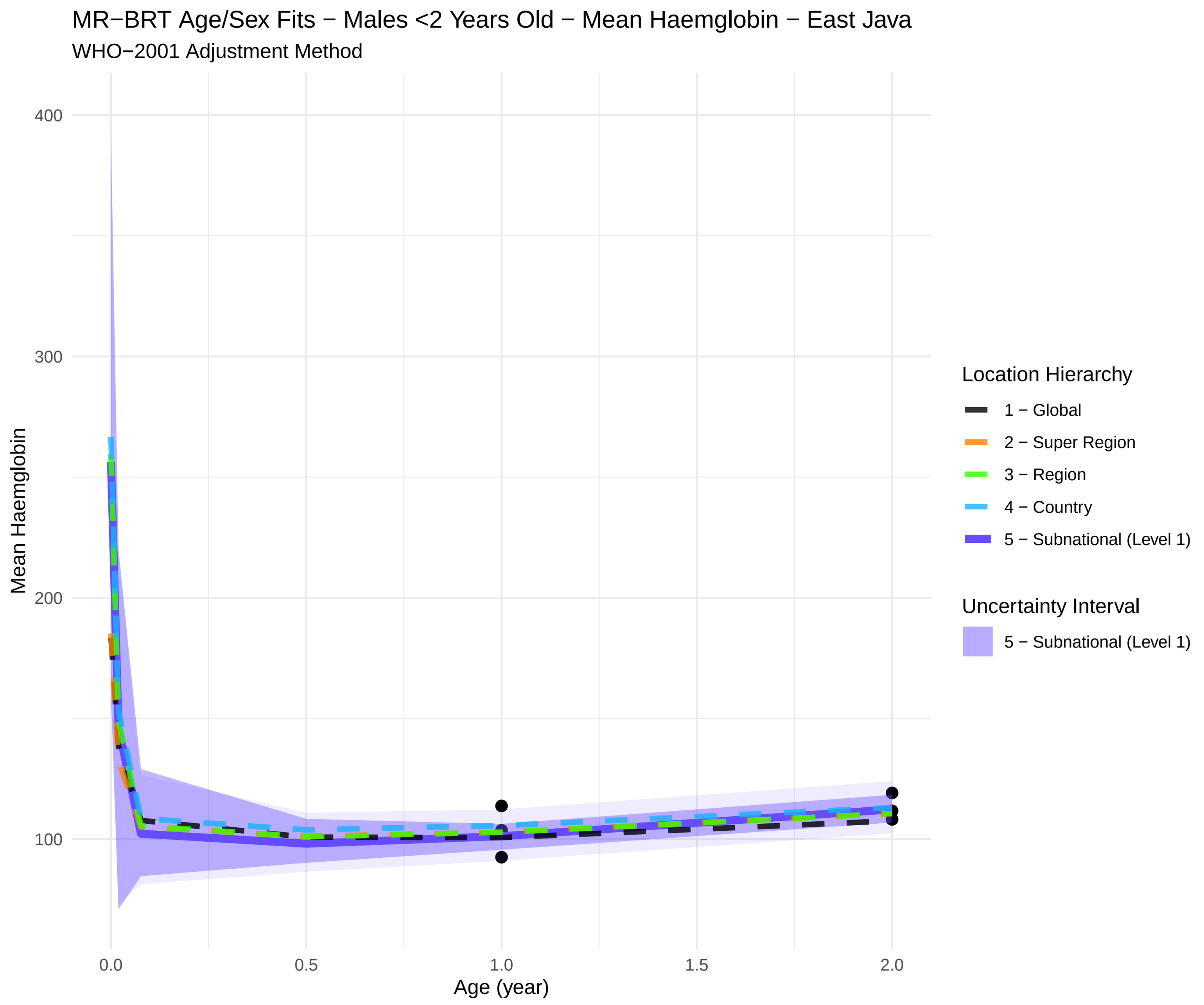
MR−BRT Age/Sex Fits − Males <2 Years Old − Mean Haemglobin − East Java
WHO−2001 Adjustment Method
Mean Haemglobin
Age (year)
Location Hierarchy
1 − Global
2 − Super Region
3 − Region
4 − Country
5 − Subnational (Level 1)
Uncertainty Interval
5 − Subnational (Level 1)

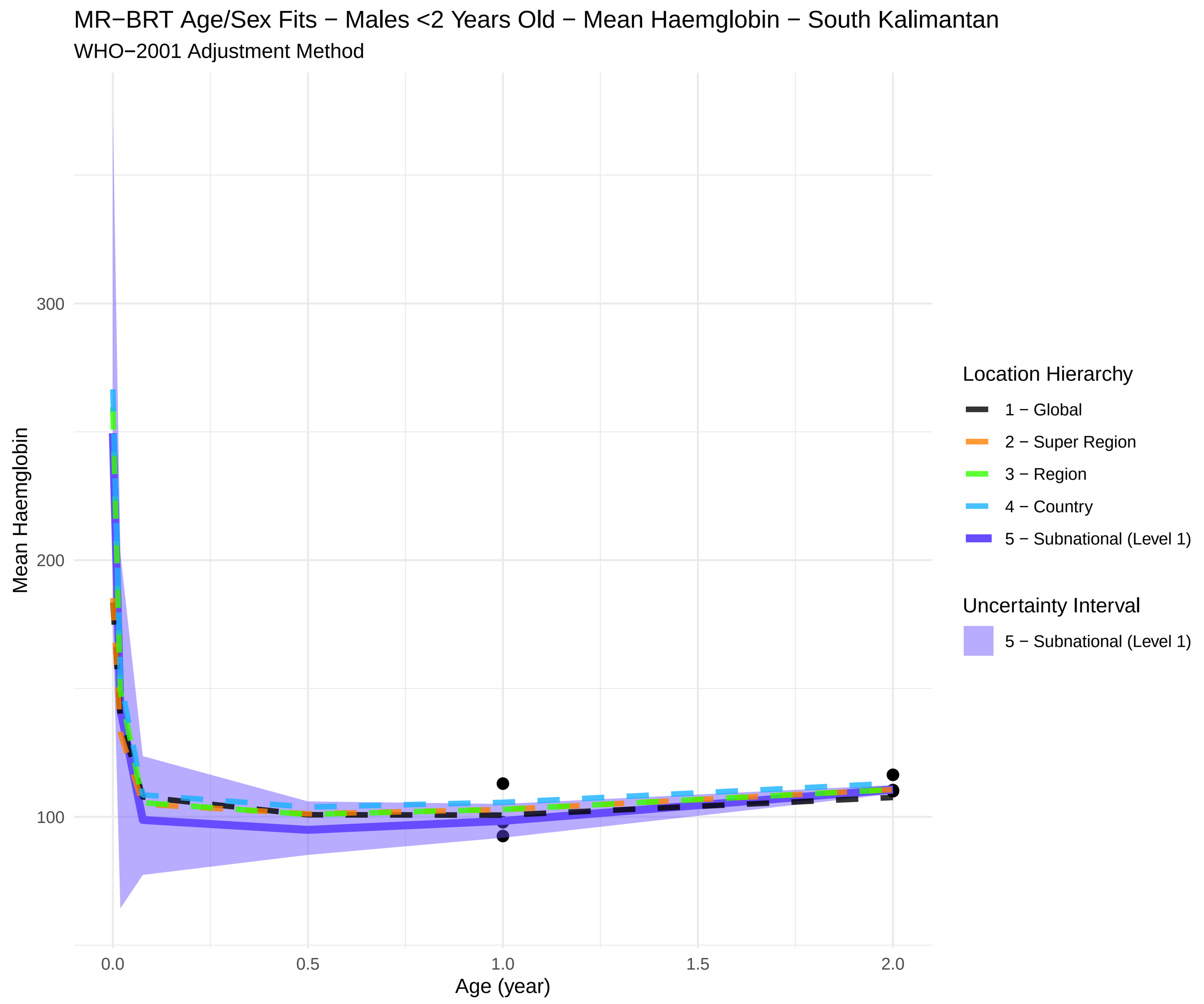
MR−BRT Age/Sex Fits − Males <2 Years Old − Mean Haemglobin − South Kalimantan
WHO−2001 Adjustment Method
Mean Haemglobin
Age (year)
Location Hierarchy
1 − Global
2 − Super Region
3 − Region
4 − Country
5 − Subnational (Level 1)
Uncertainty Interval
5 − Subnational (Level 1)

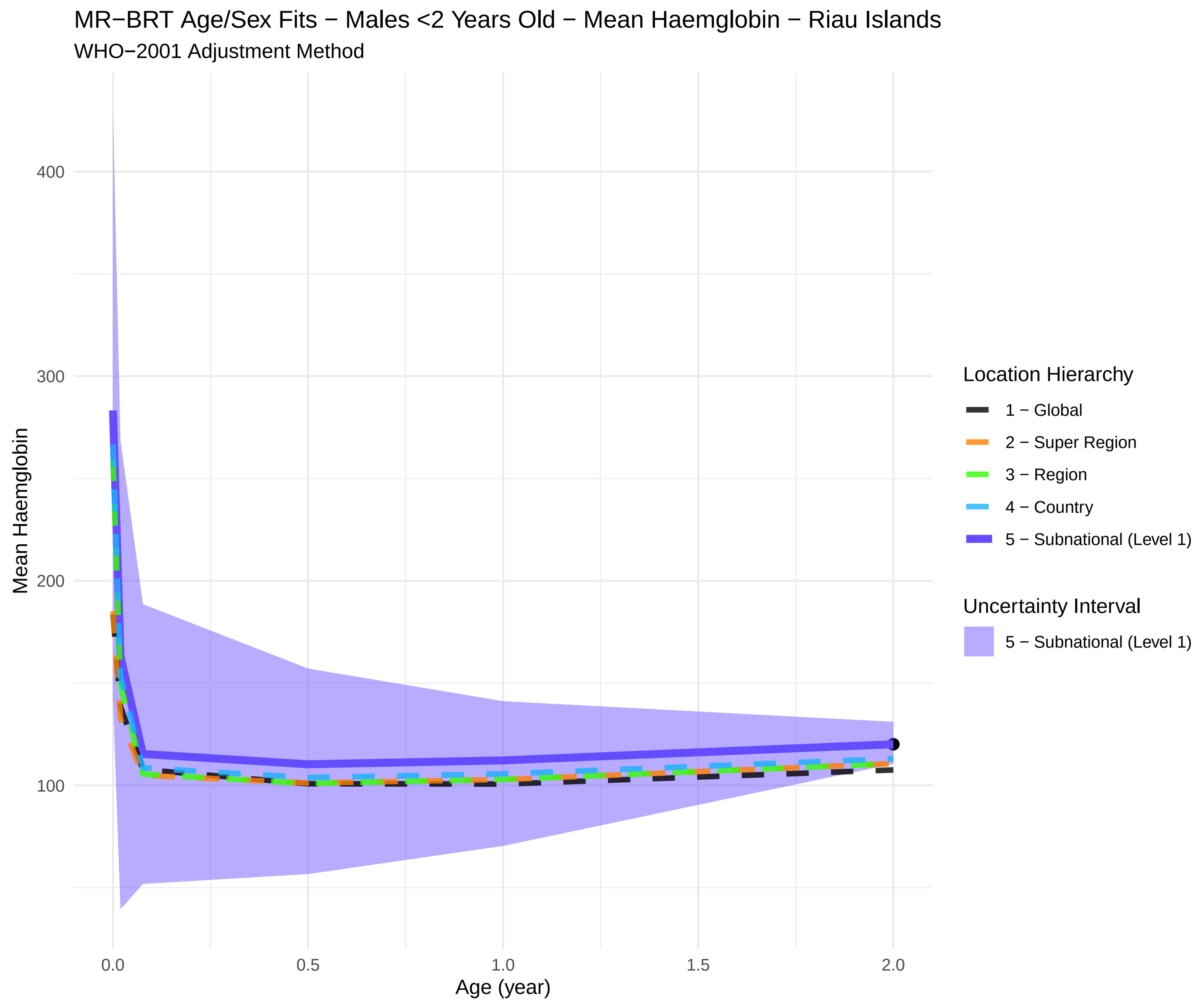
MR−BRT Age/Sex Fits − Males <2 Years Old − Mean Haemglobin − Riau Islands
WHO−2001 Adjustment Method
Mean Haemglobin
Age (year)
Location Hierarchy
1 − Global
2 − Super Region
3 − Region
4 − Country
5 − Subnational (Level 1)
Uncertainty Interval
5 − Subnational (Level 1)

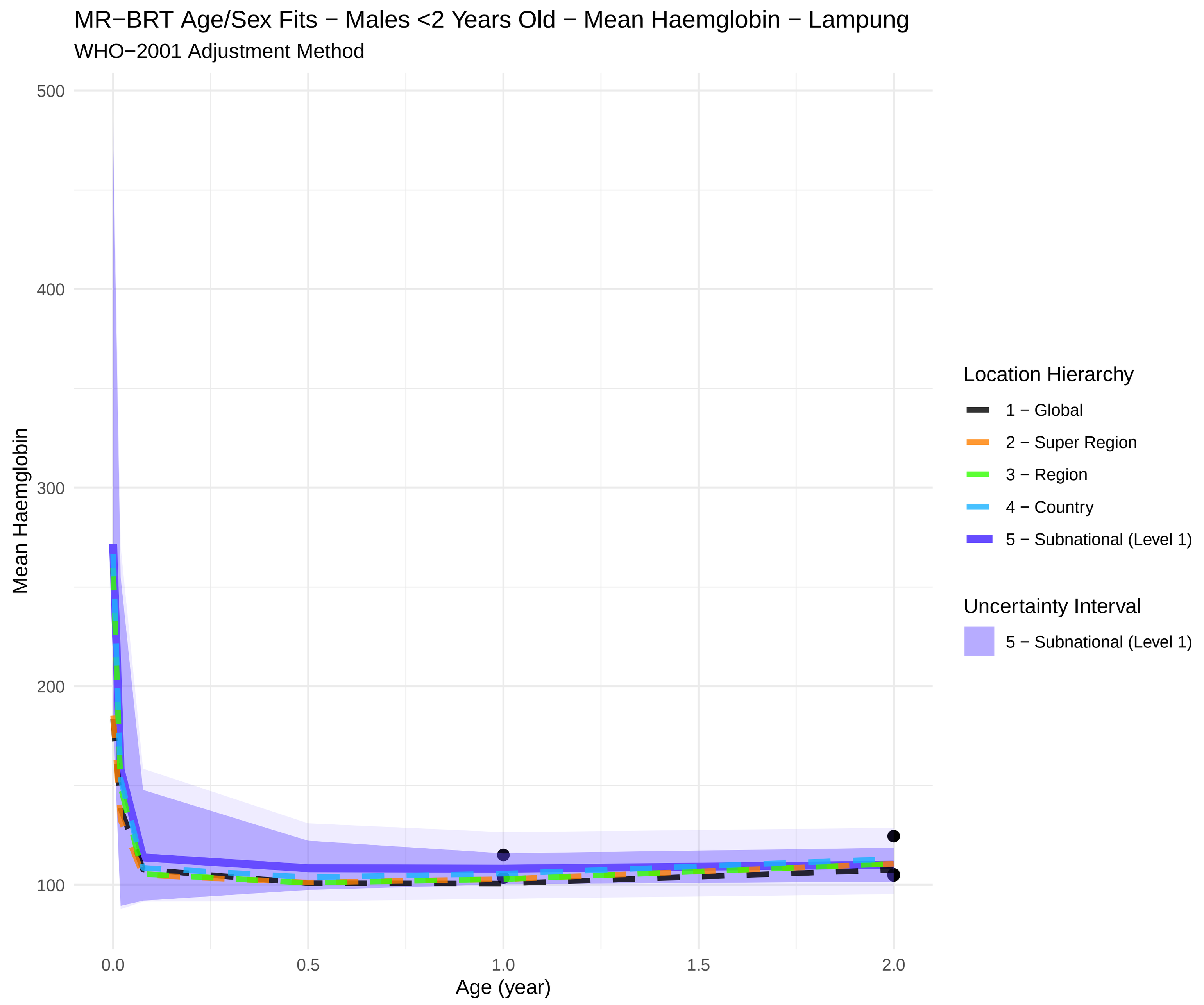
MR−BRT Age/Sex Fits − Males <2 Years Old − Mean Haemglobin − Lampung
WHO−2001 Adjustment Method
Mean Haemglobin
Age (year)
Location Hierarchy
1 − Global
2 − Super Region
3 − Region
4 − Country
5 − Subnational (Level 1)
Uncertainty Interval
5 − Subnational (Level 1)

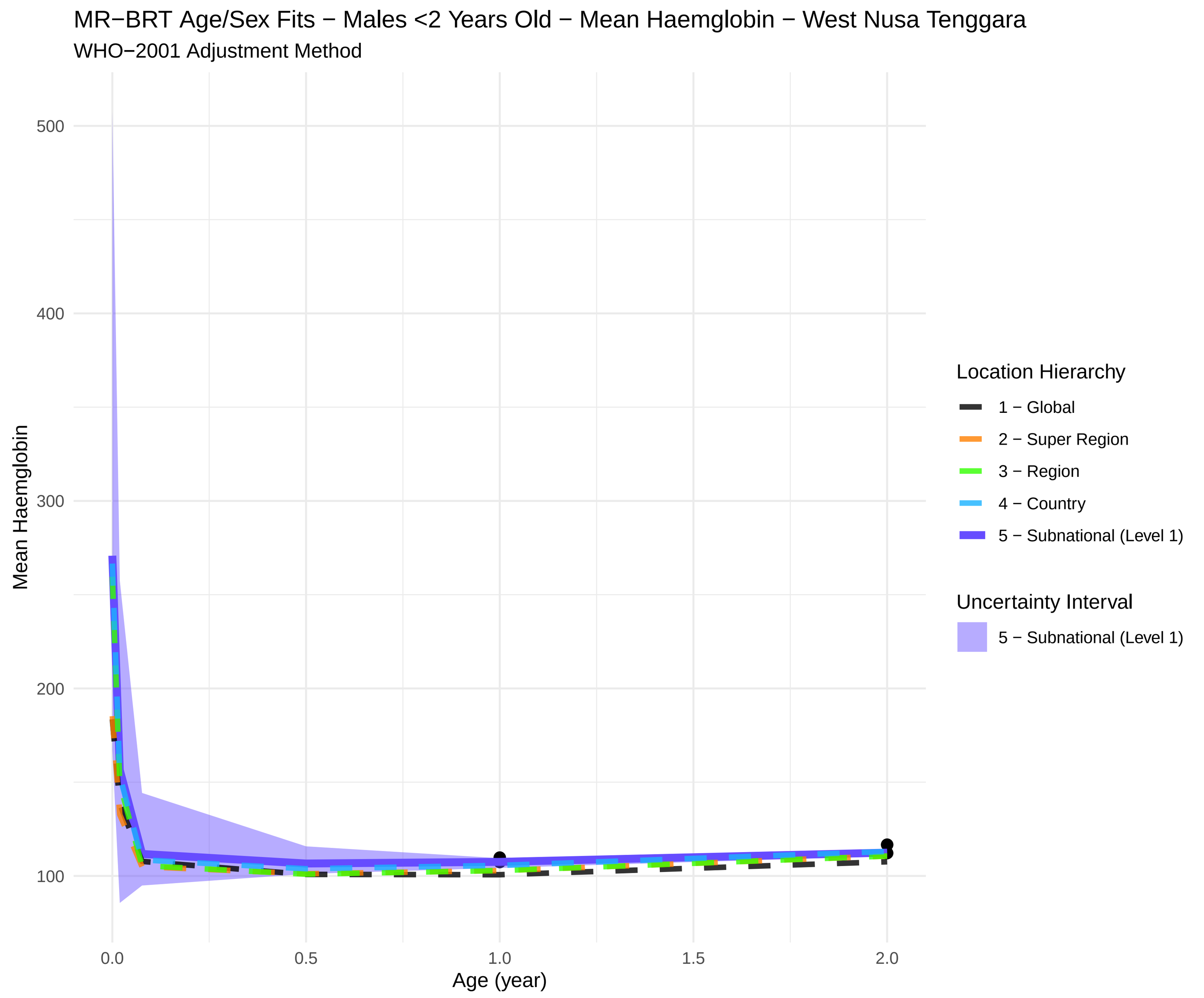
MR−BRT Age/Sex Fits − Males <2 Years Old − Mean Haemglobin − West Nusa Tenggara
WHO−2001 Adjustment Method
Mean Haemglobin
Age (year)
Location Hierarchy
1 − Global
2 − Super Region
3 − Region
4 − Country
5 − Subnational (Level 1)
Uncertainty Interval
5 − Subnational (Level 1)

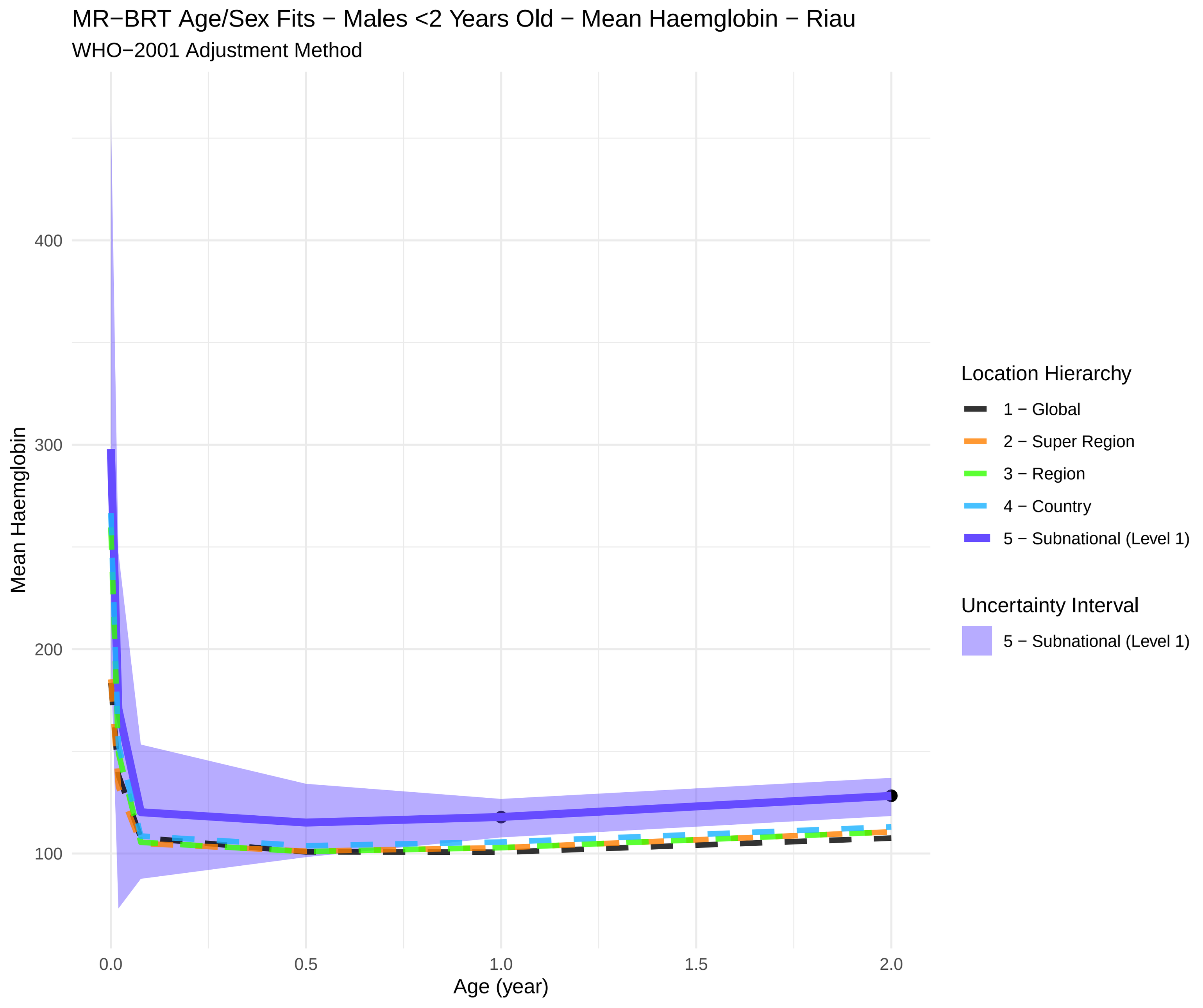
MR−BRT Age/Sex Fits − Males <2 Years Old − Mean Haemglobin − Riau
WHO−2001 Adjustment Method
Mean Haemglobin
Age (year)
Location Hierarchy
1 − Global
2 − Super Region
3 − Region
4 − Country
5 − Subnational (Level 1)
Uncertainty Interval
5 − Subnational (Level 1)

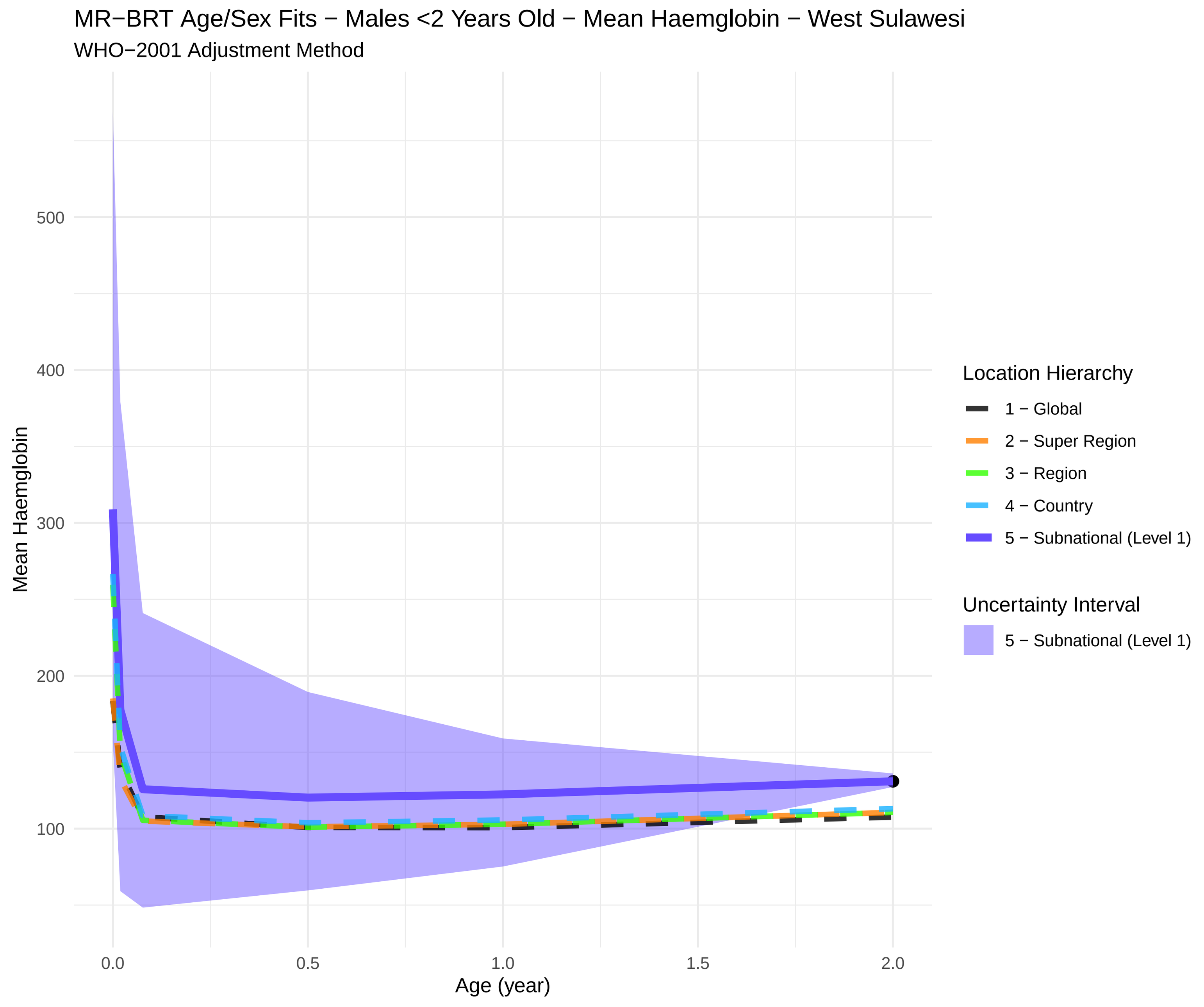
MR−BRT Age/Sex Fits − Males <2 Years Old − Mean Haemglobin − West Sulawesi
WHO−2001 Adjustment Method
Mean Haemglobin
Age (year)
Location Hierarchy
1 − Global
2 − Super Region
3 − Region
4 − Country
5 − Subnational (Level 1)
Uncertainty Interval
5 − Subnational (Level 1)

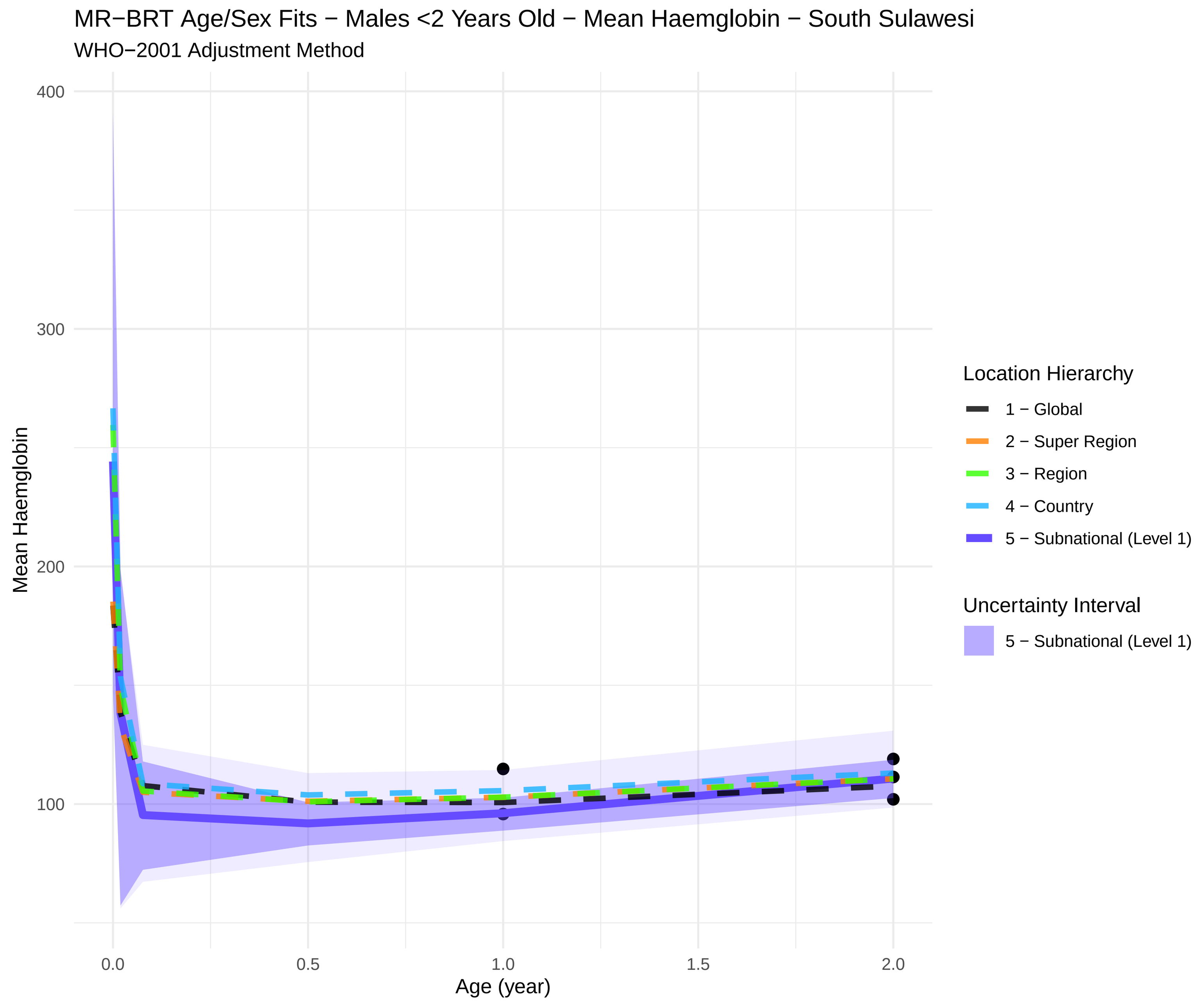
MR−BRT Age/Sex Fits − Males <2 Years Old − Mean Haemglobin − South Sulawesi
WHO−2001 Adjustment Method
Mean Haemglobin
Age (year)
Location Hierarchy
1 − Global
2 − Super Region
3 − Region
4 − Country
5 − Subnational (Level 1)
Uncertainty Interval
5 − Subnational (Level 1)

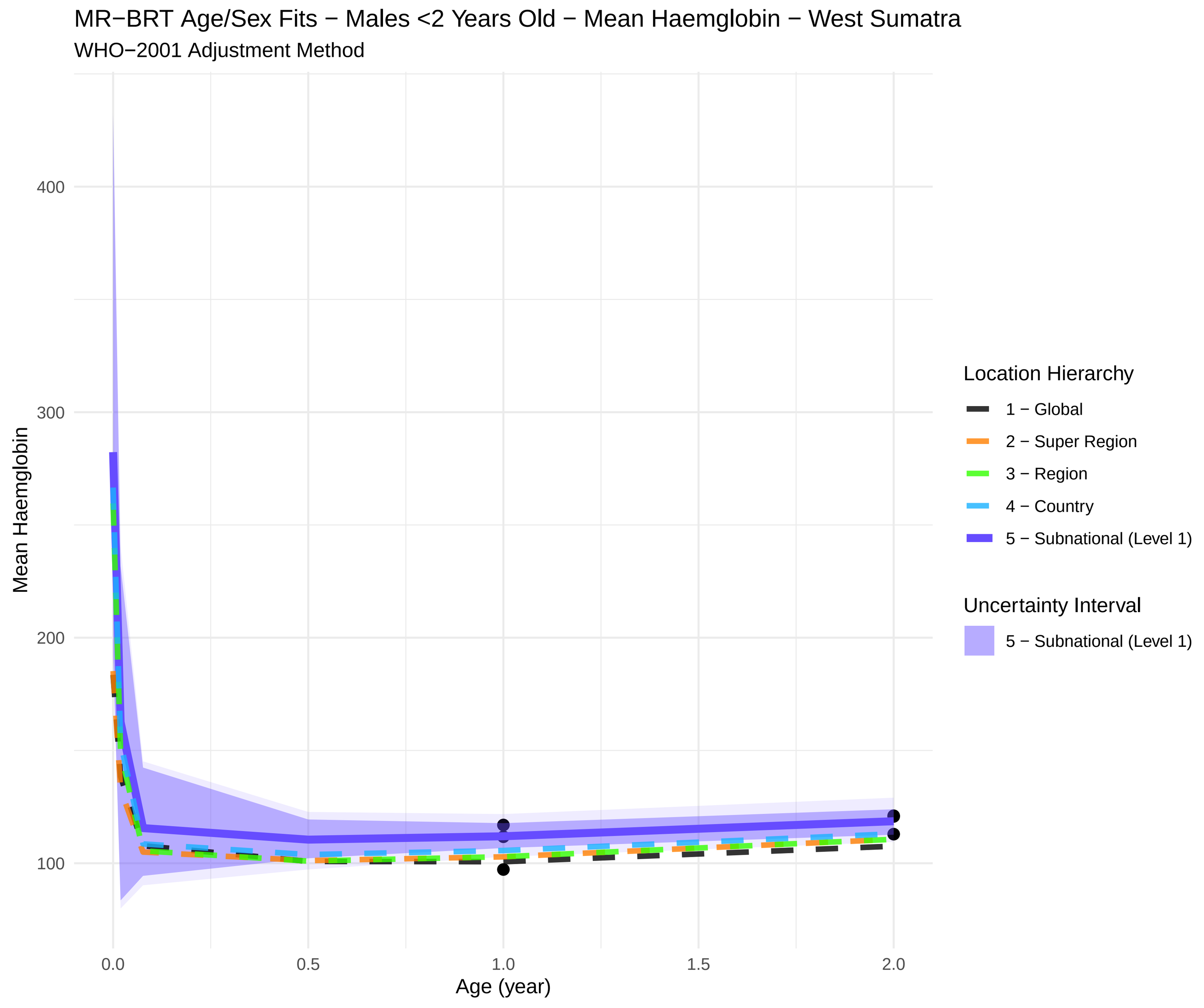
MR−BRT Age/Sex Fits − Males <2 Years Old − Mean Haemglobin − West Sumatra
WHO−2001 Adjustment Method
Mean Haemglobin
Age (year)
Location Hierarchy
1 − Global
2 − Super Region
3 − Region
4 − Country
5 − Subnational (Level 1)
Uncertainty Interval
5 − Subnational (Level 1)

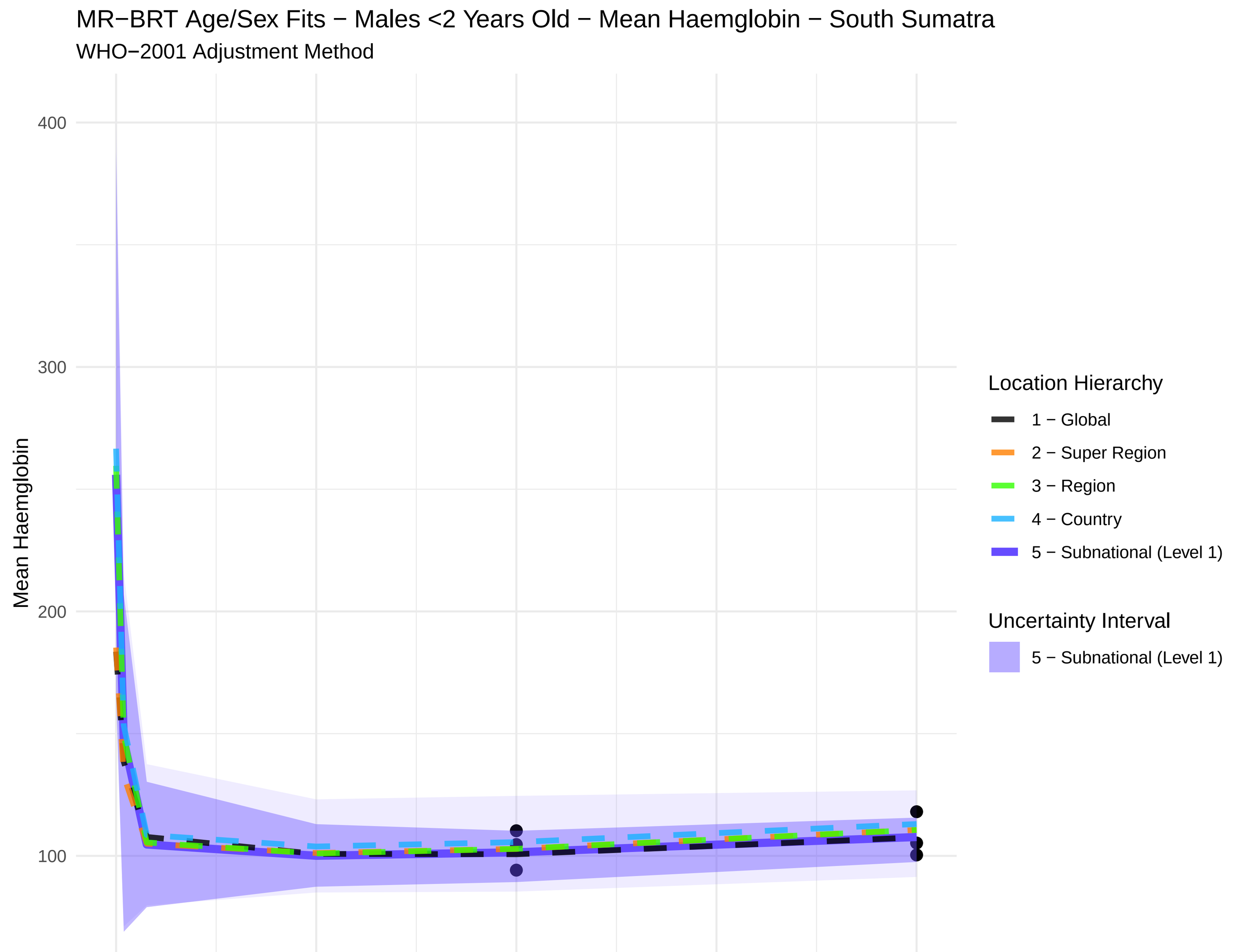
MR−BRT Age/Sex Fits − Males <2 Years Old − Mean Haemglobin − South Sumatra
WHO−2001 Adjustment Method
Mean Haemglobin
Age (year)
Location Hierarchy
1 − Global
2 − Super Region
3 − Region
4 − Country
5 − Subnational (Level 1)
Uncertainty Interval
5 − Subnational (Level 1)

MR−BRT Age/Sex Fits − Males <2 Years Old − Mean Haemglobin − North Sumatra
WHO−2001 Adjustment Method

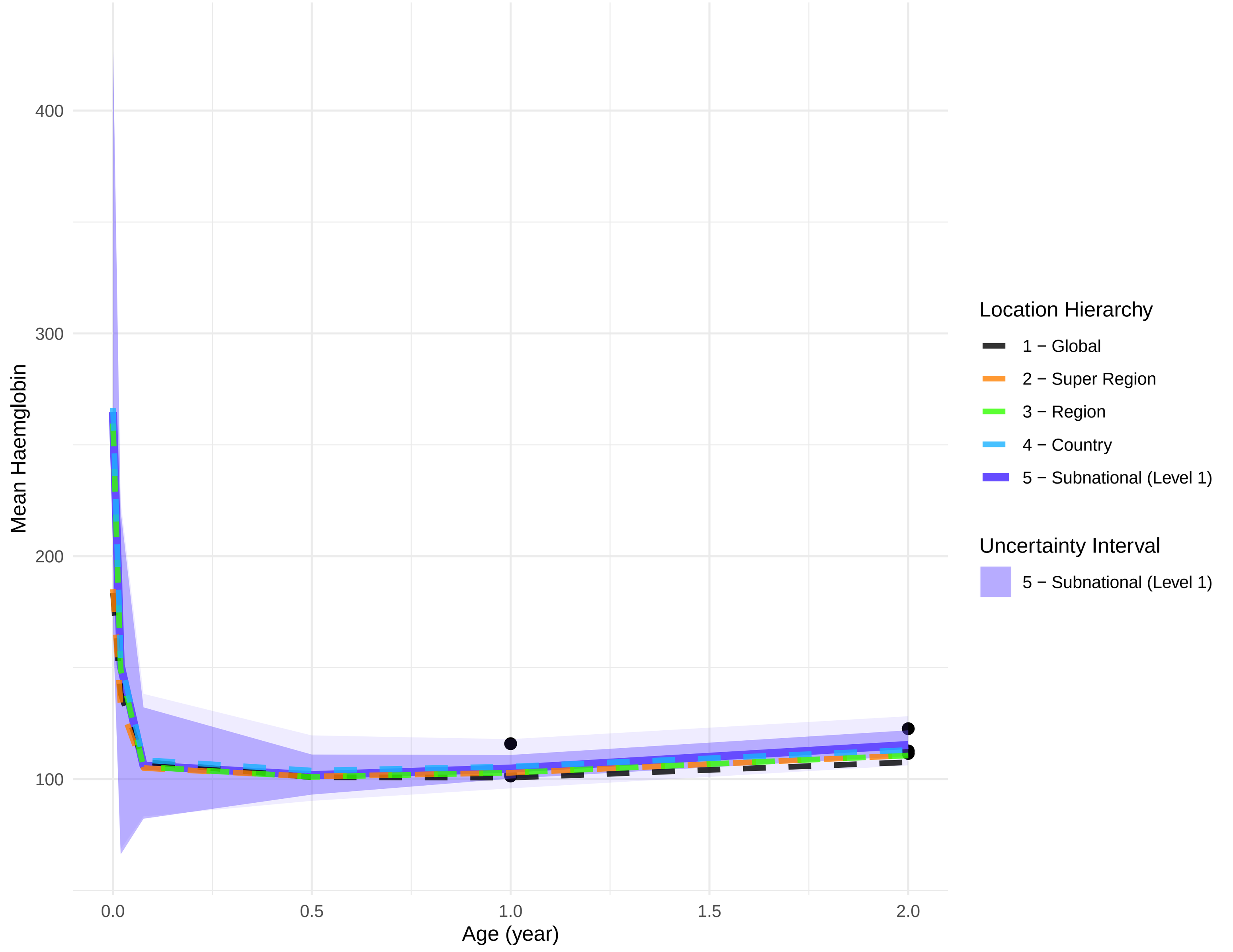

MR−BRT Age/Sex Fits − Males <2 Years Old − Mean Haemglobin − Yogyakarta
WHO−2001 Adjustment Method

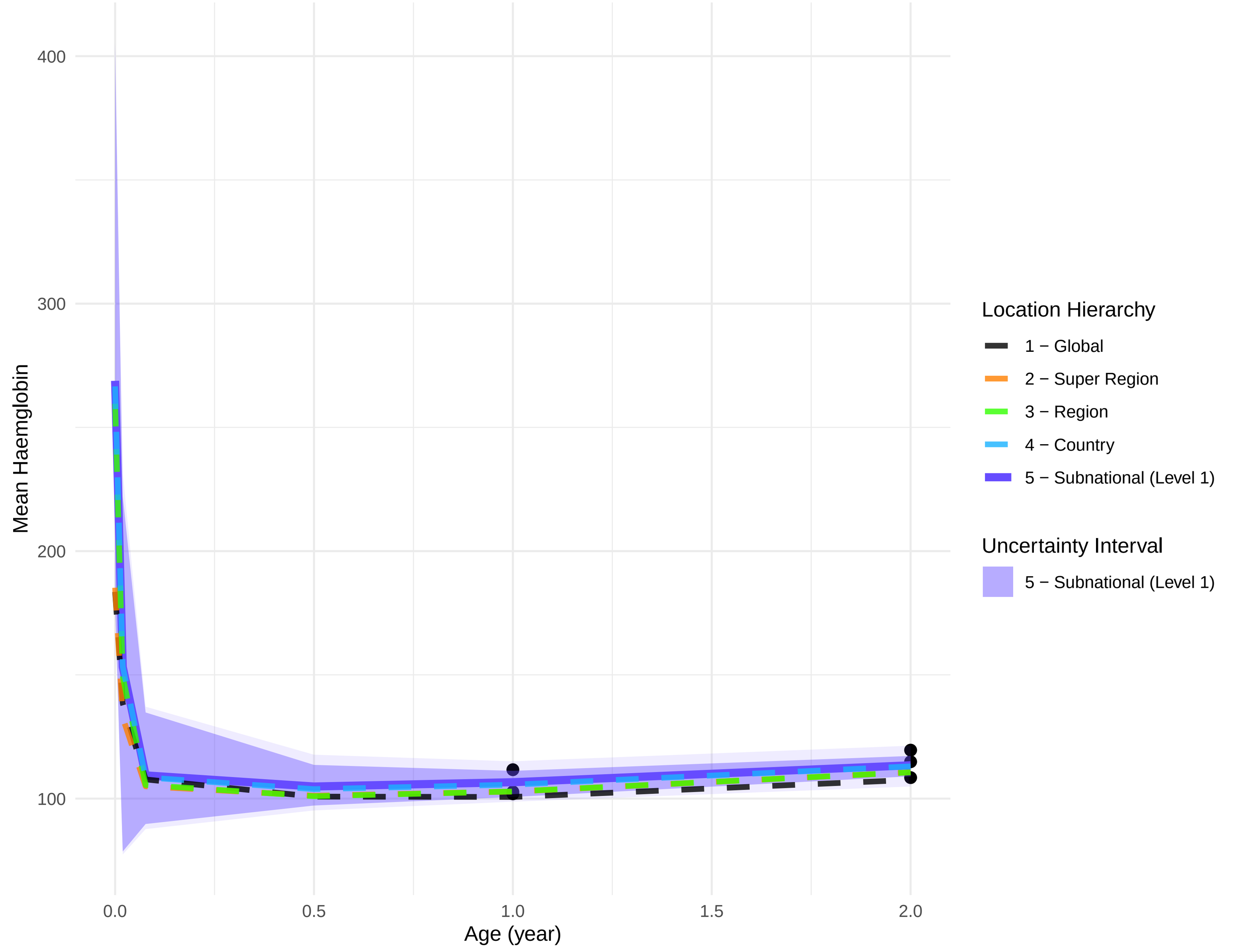

MR−BRT Age/Sex Fits − Males <2 Years Old − Mean Haemglobin − Myanmar
WHO−2001 Adjustment Method

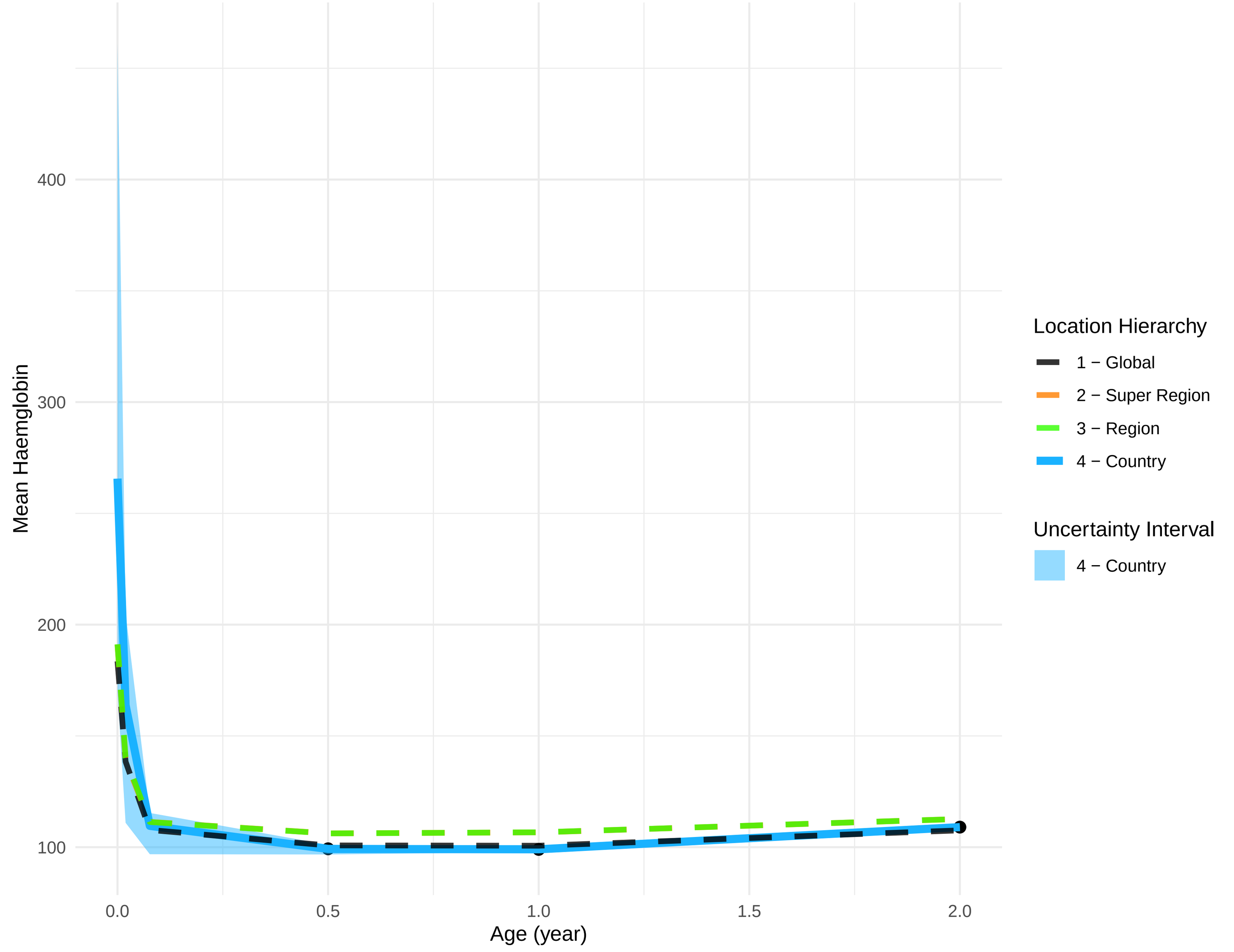

MR−BRT Age/Sex Fits − Males <2 Years Old − Mean Haemglobin − Timor−Leste
WHO−2001 Adjustment Method

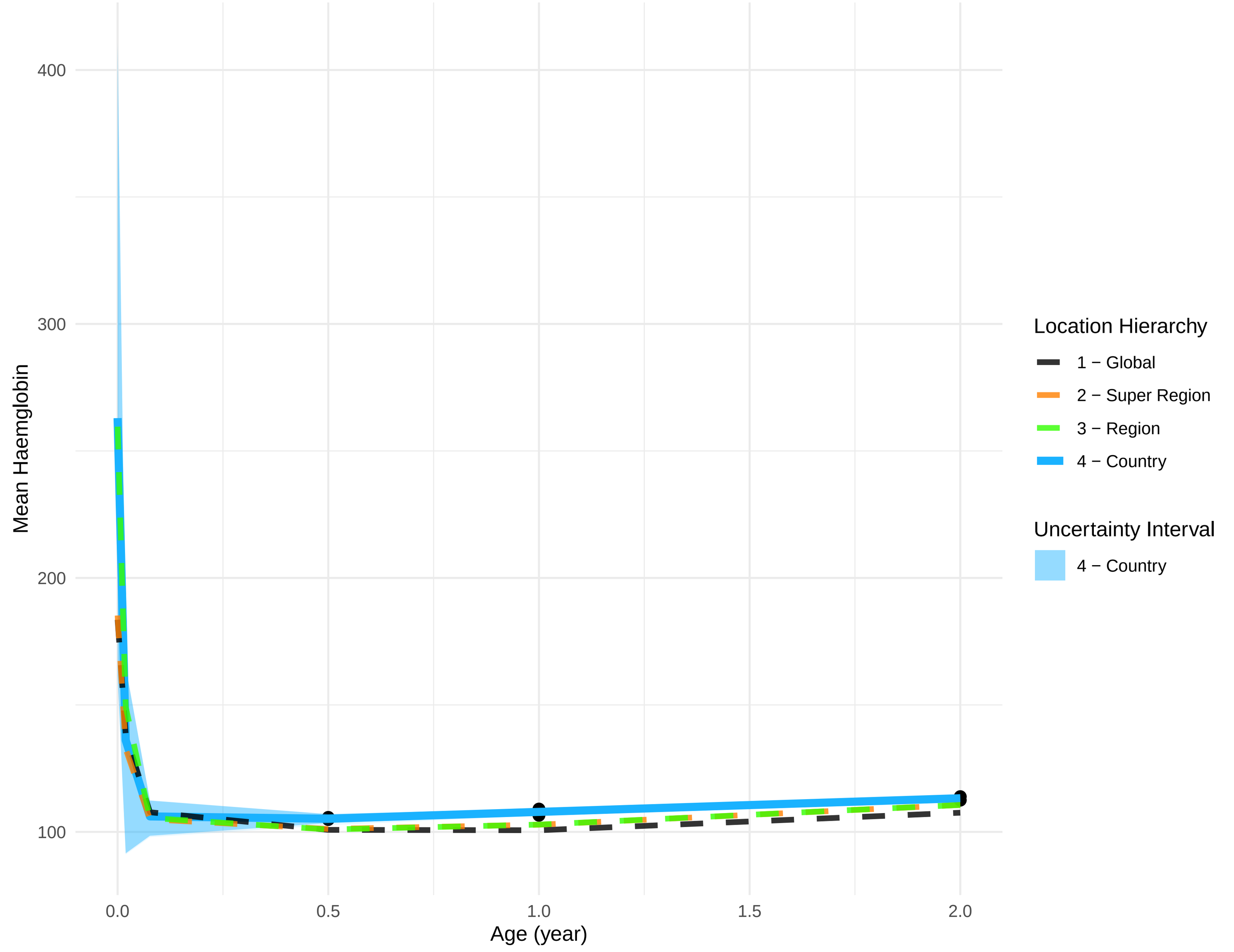

MR−BRT Age/Sex Fits − Males <2 Years Old − Mean Haemglobin − Sub−Saharan Africa
WHO−2001 Adjustment Method

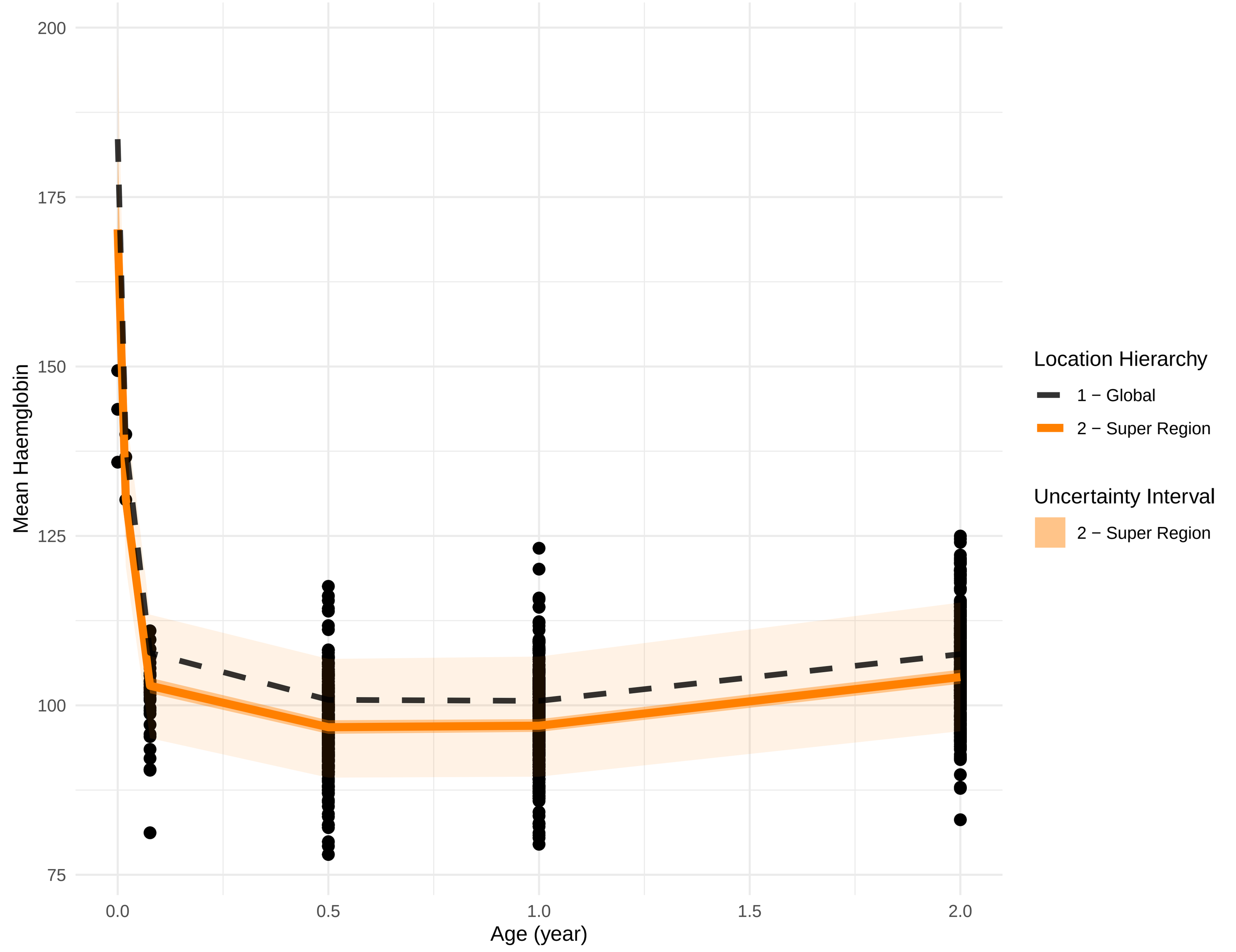

MR−BRT Age/Sex Fits − Males <2 Years Old − Mean Haemglobin − Central Sub−Saharan Africa
WHO−2001 Adjustment Method

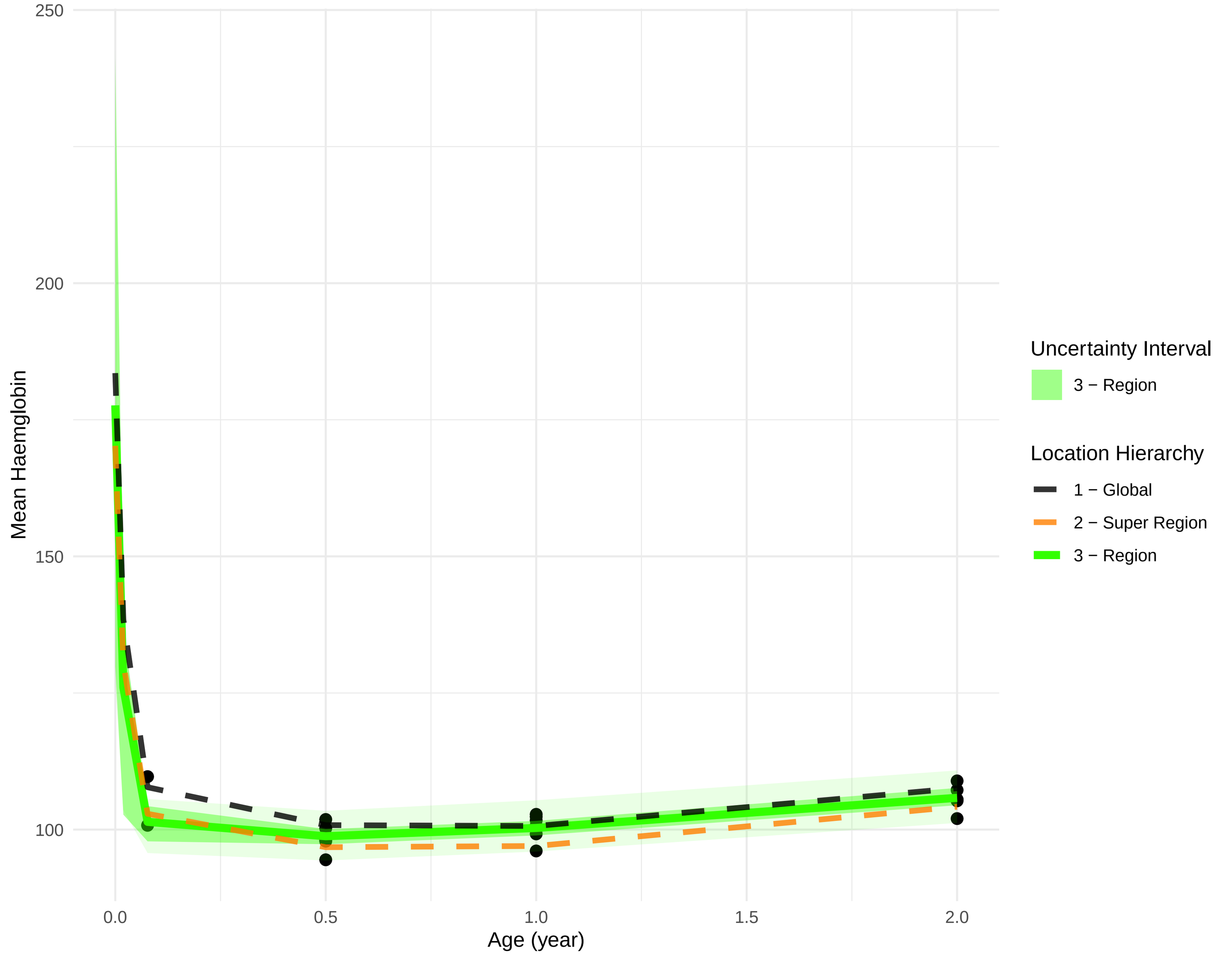

MR−BRT Age/Sex Fits − Males <2 Years Old − Mean Haemglobin − Angola
WHO−2001 Adjustment Method

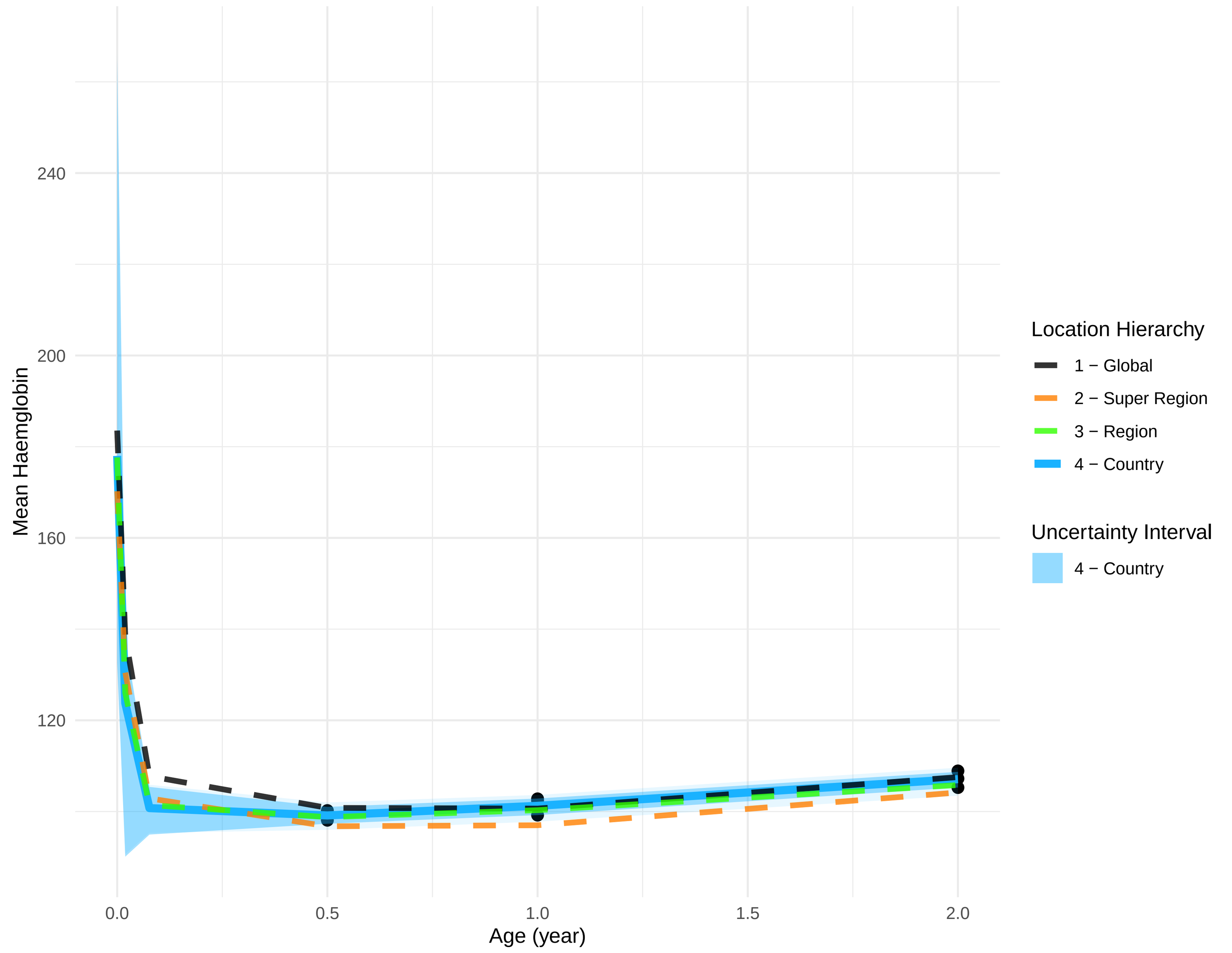

MR−BRT Age/Sex Fits − Males <2 Years Old − Mean Haemglobin − Democratic Republic of the Congo
WHO−2001 Adjustment Method

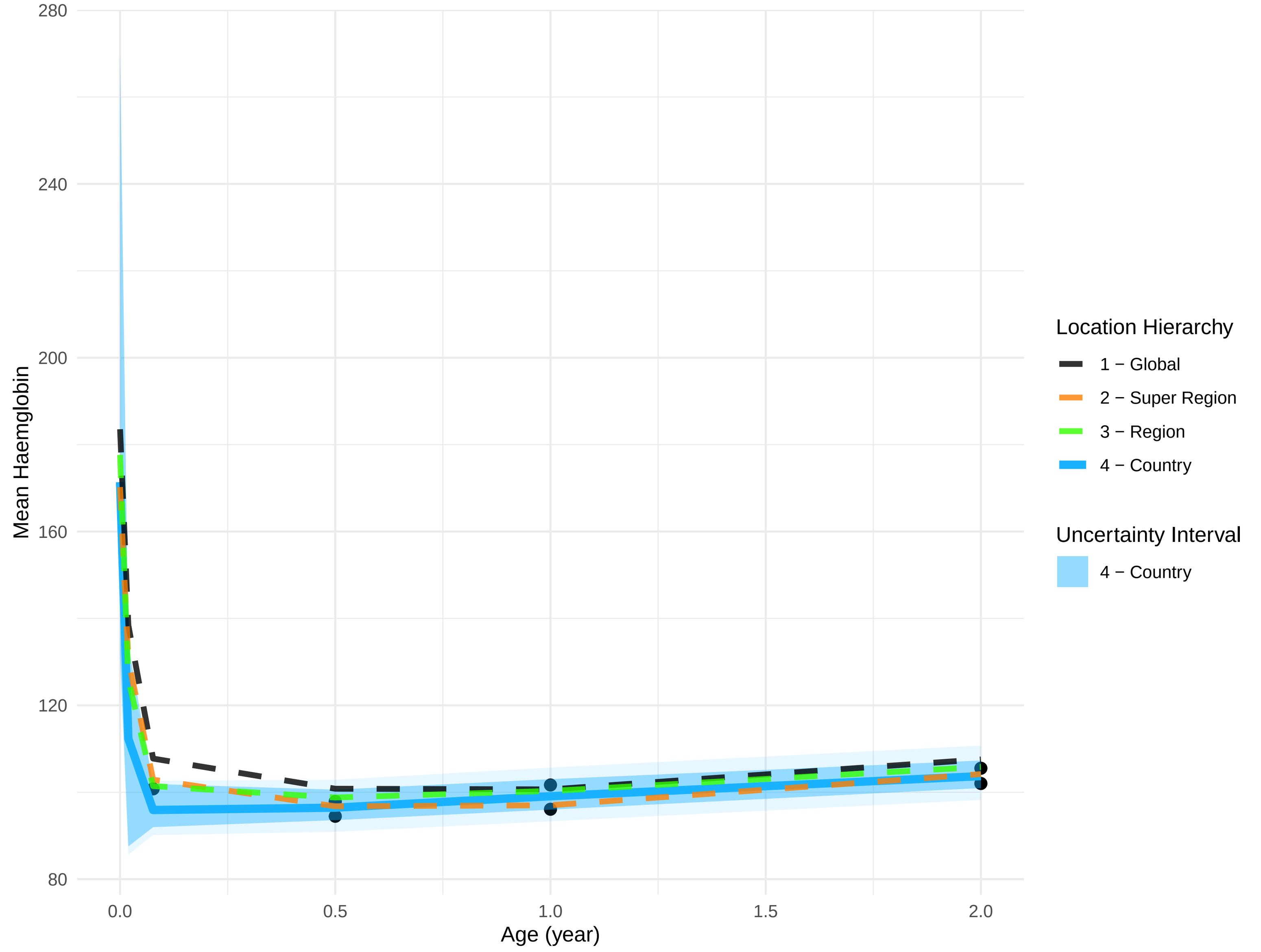

MR−BRT Age/Sex Fits − Males <2 Years Old − Mean Haemglobin − Gabon
WHO−2001 Adjustment Method

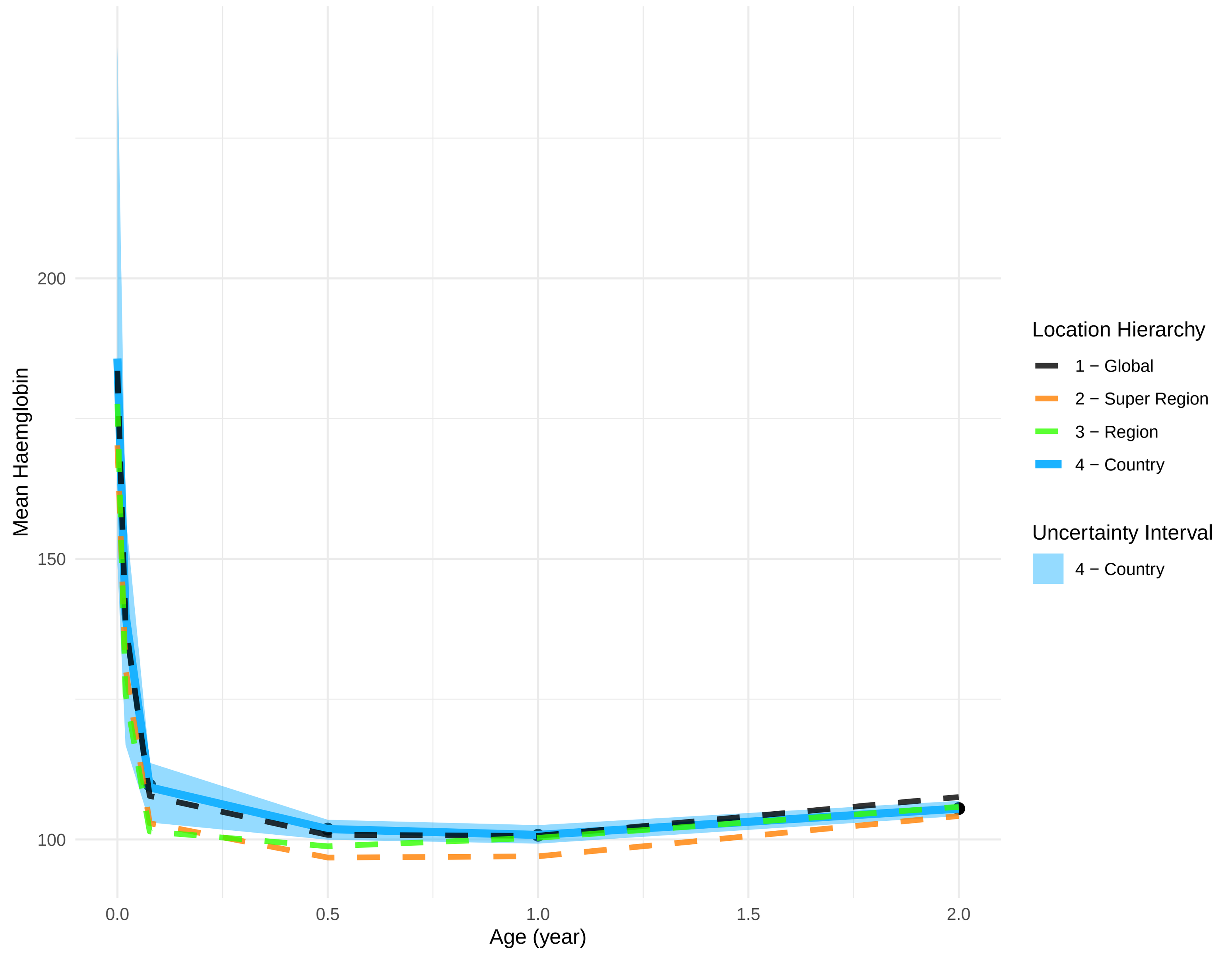

MR−BRT Age/Sex Fits − Males <2 Years Old − Mean Haemglobin − Eastern Sub−Saharan Africa
WHO−2001 Adjustment Method

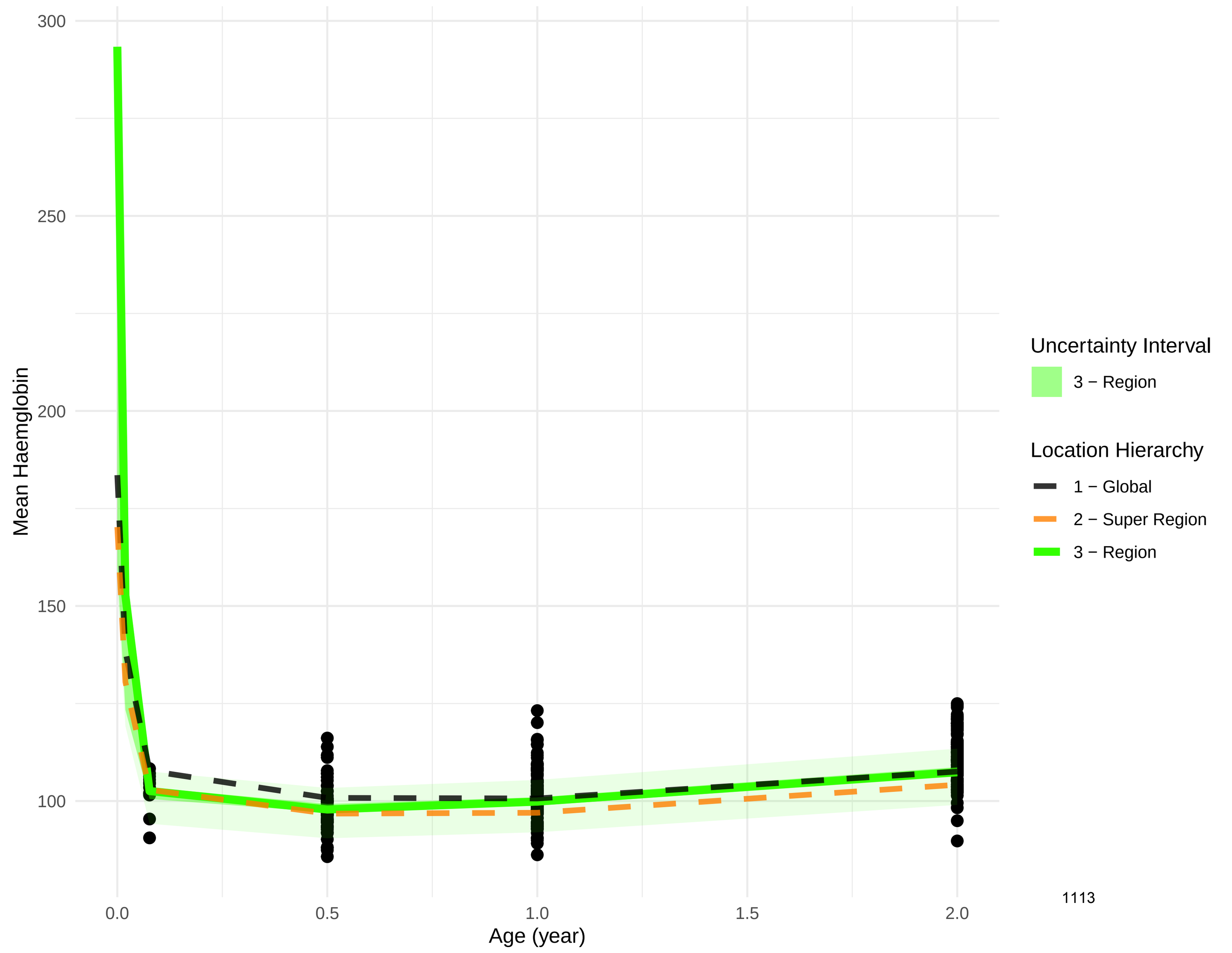

MR−BRT Age/Sex Fits − Males <2 Years Old − Mean Haemglobin − Burundi
WHO−2001 Adjustment Method

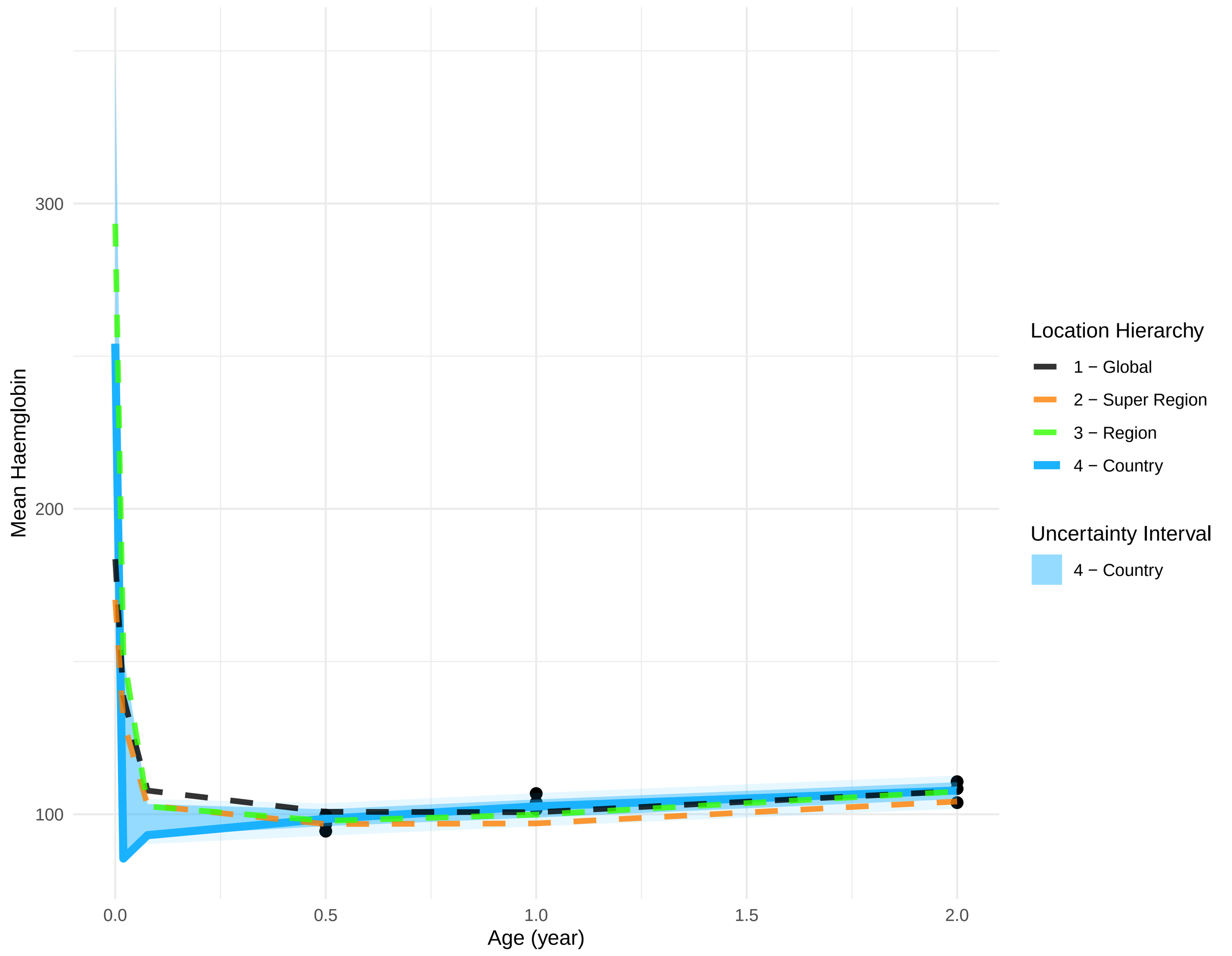

MR−BRT Age/Sex Fits − Males <2 Years Old − Mean Haemglobin − Ethiopia
WHO−2001 Adjustment Method

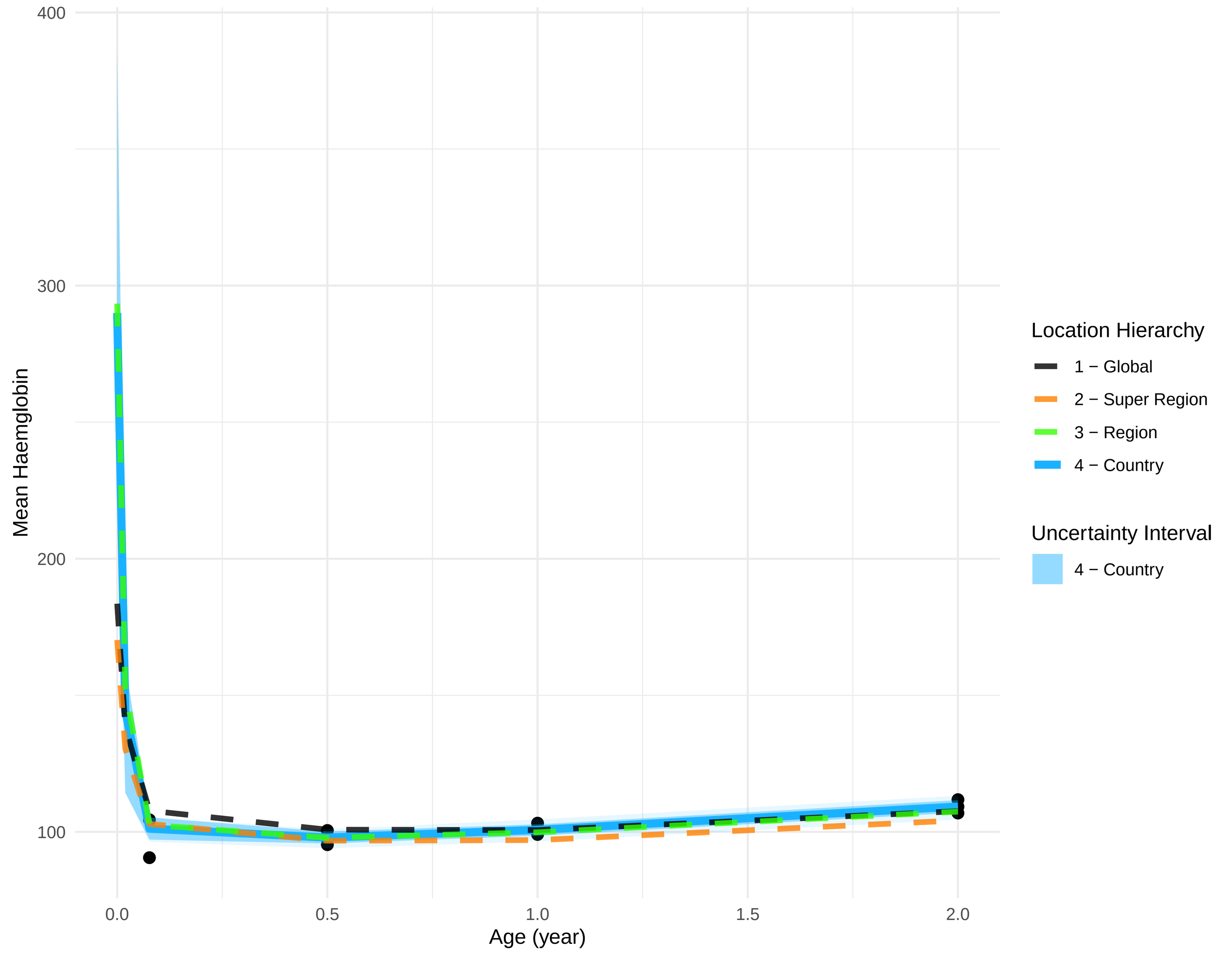

MR−BRT Age/Sex Fits − Males <2 Years Old − Mean Haemglobin − Kenya
WHO−2001 Adjustment Method

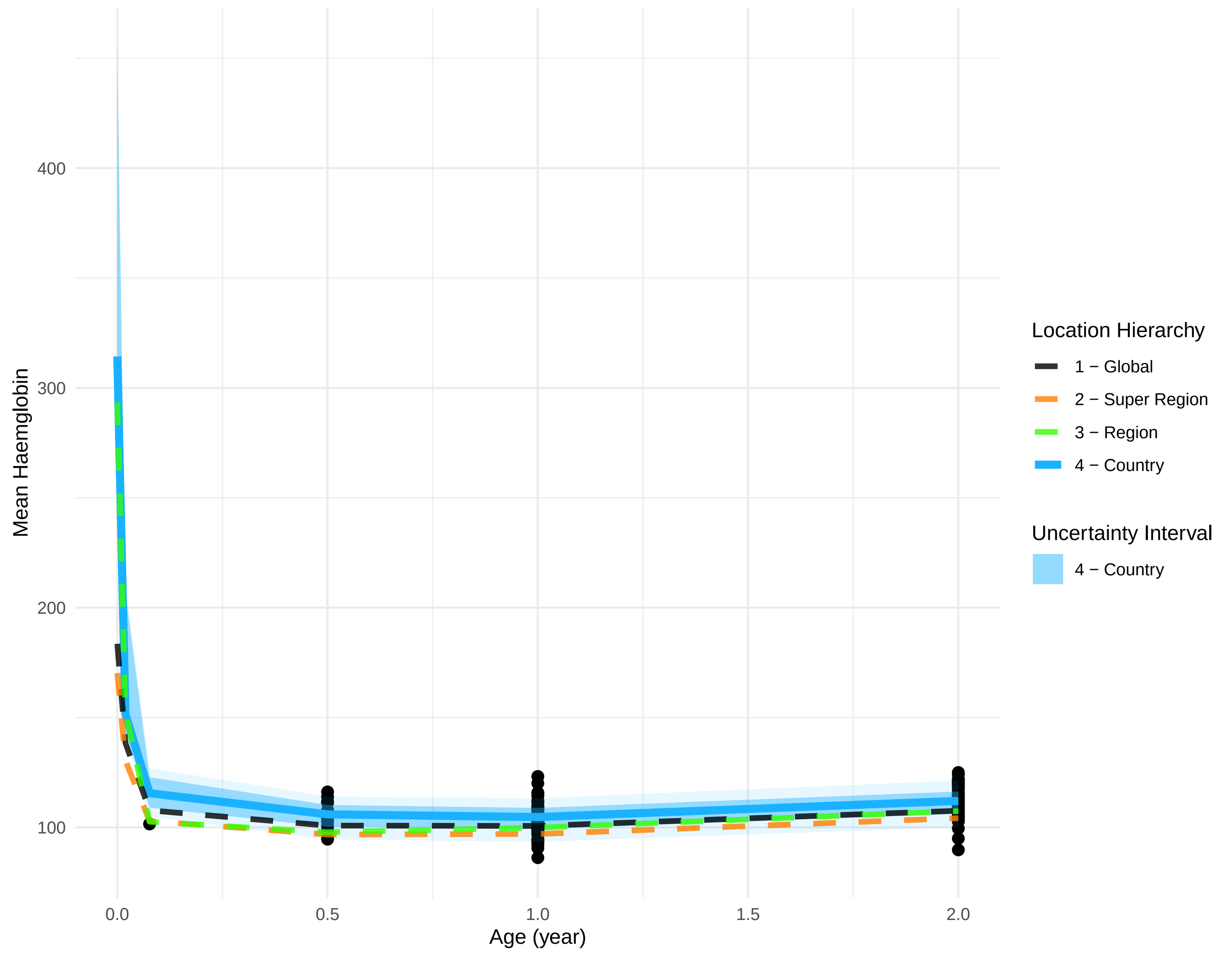

MR−BRT Age/Sex Fits − Males <2 Years Old − Mean Haemglobin − Baringo
WHO−2001 Adjustment Method

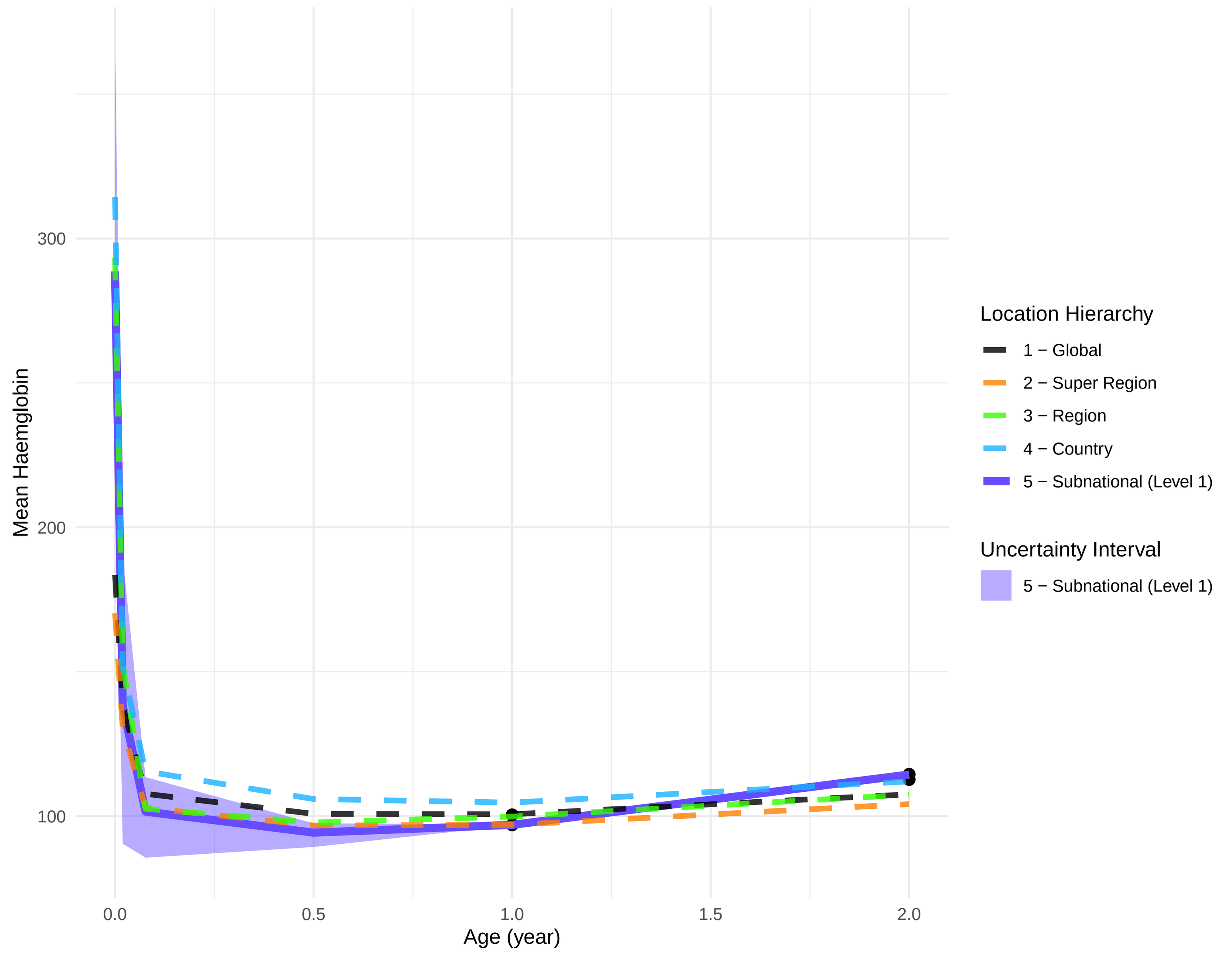

MR−BRT Age/Sex Fits − Males <2 Years Old − Mean Haemglobin − Bomet
WHO−2001 Adjustment Method

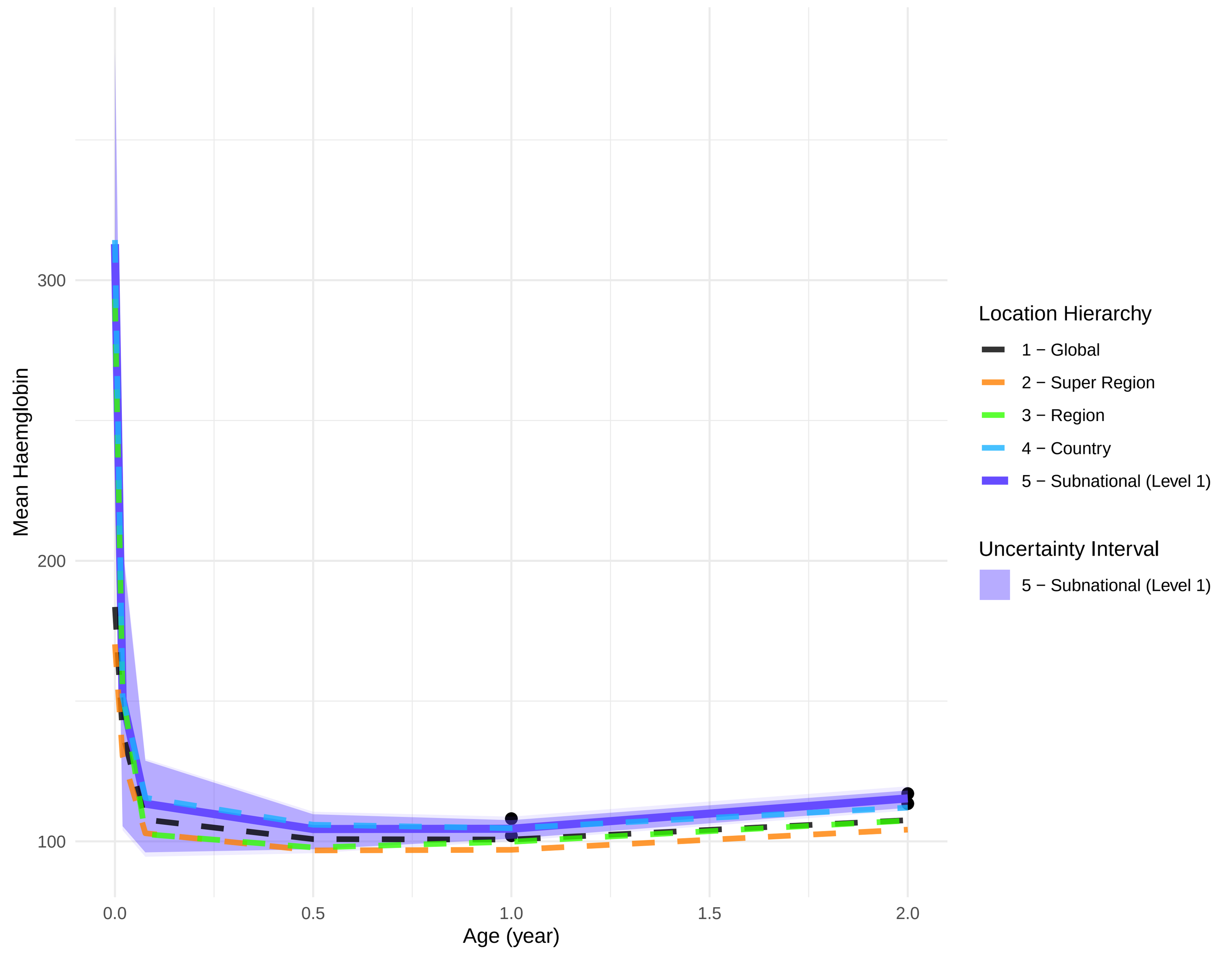

MR−BRT Age/Sex Fits − Males <2 Years Old − Mean Haemglobin − Bungoma
WHO−2001 Adjustment Method

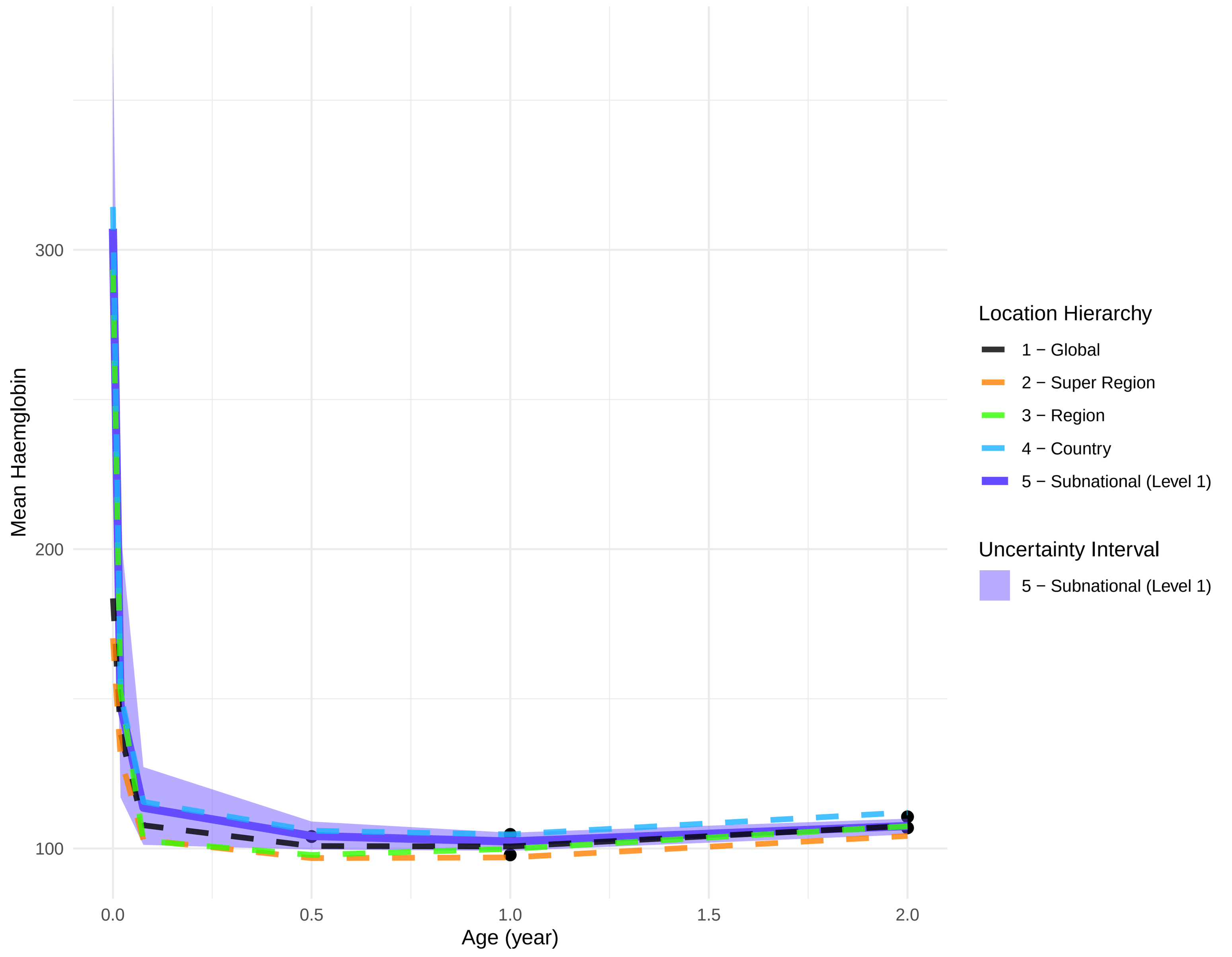

MR−BRT Age/Sex Fits − Males <2 Years Old − Mean Haemglobin − Busia
WHO−2001 Adjustment Method

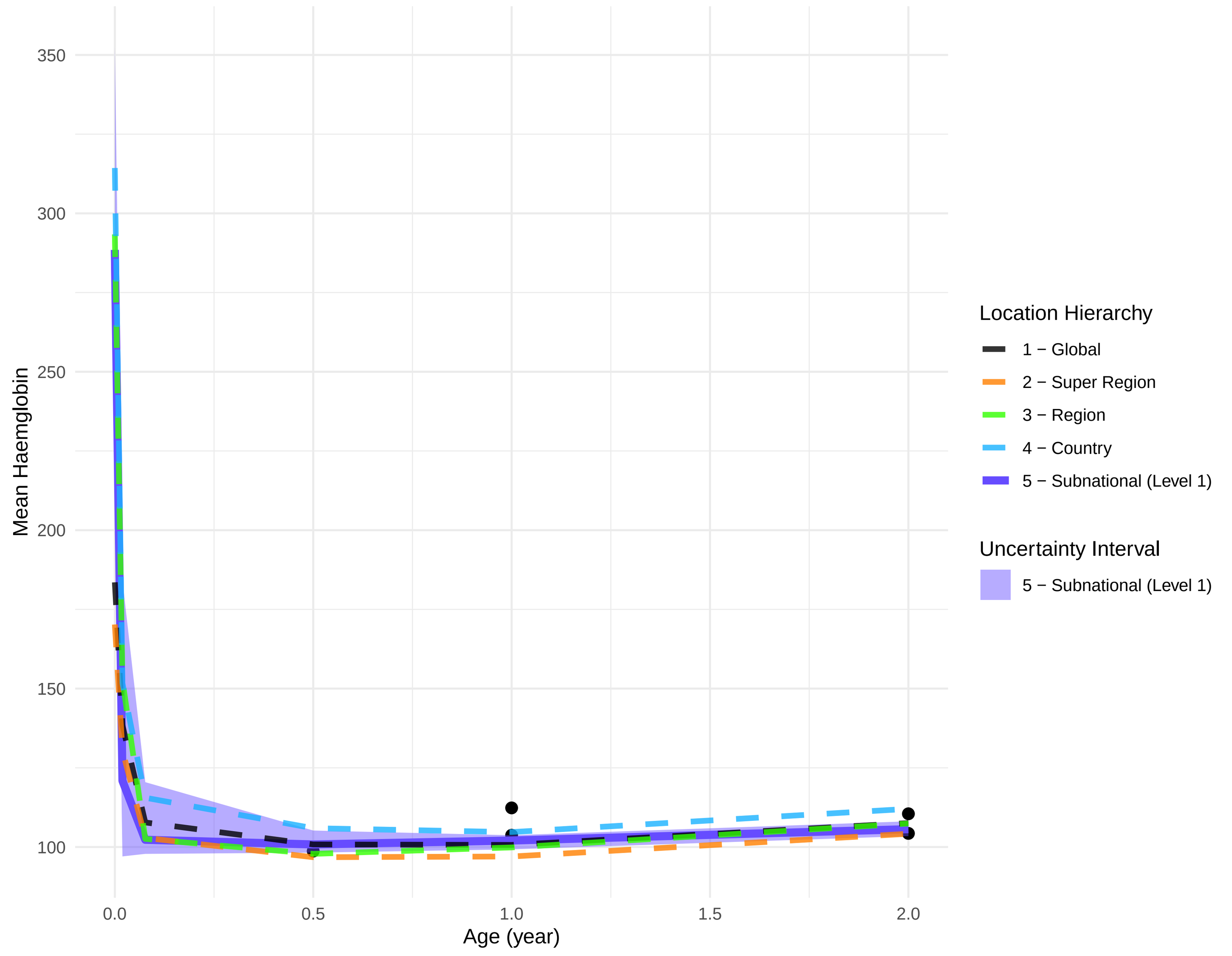

MR−BRT Age/Sex Fits − Males <2 Years Old − Mean Haemglobin − Elgeyo−Marakwet
WHO−2001 Adjustment Method

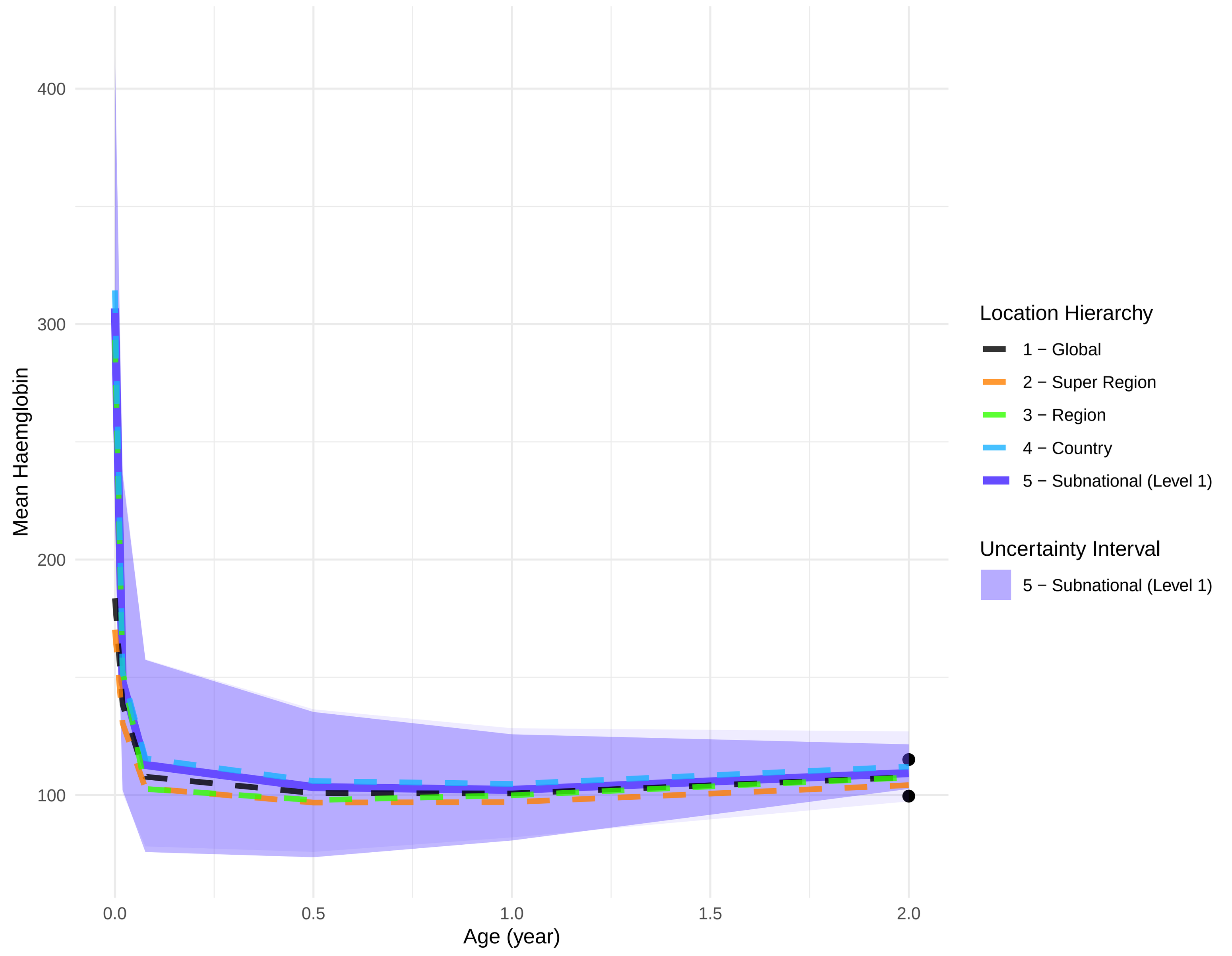

MR−BRT Age/Sex Fits − Males <2 Years Old − Mean Haemglobin − Embu
WHO−2001 Adjustment Method

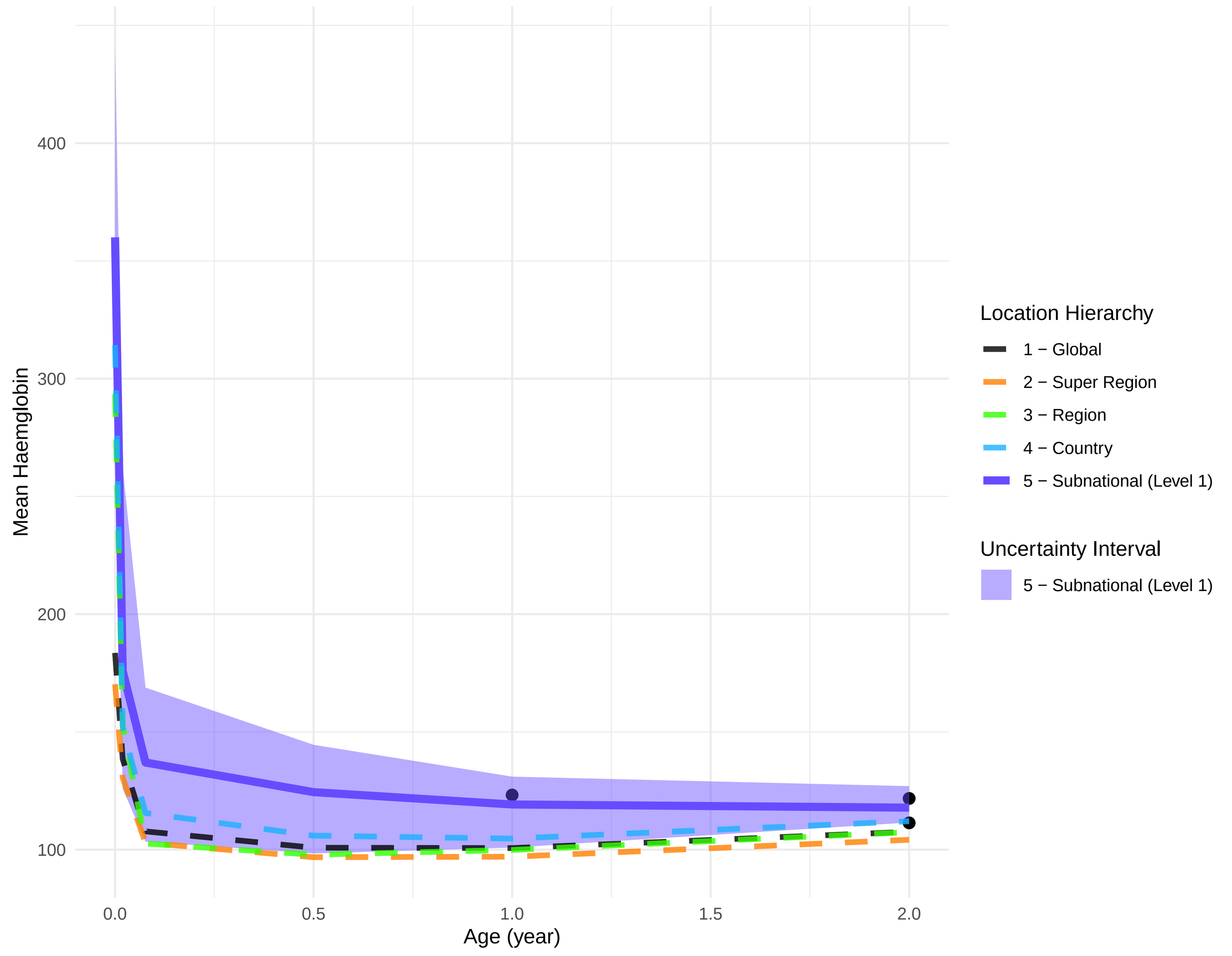

MR−BRT Age/Sex Fits − Males <2 Years Old − Mean Haemglobin − Garissa
WHO−2001 Adjustment Method

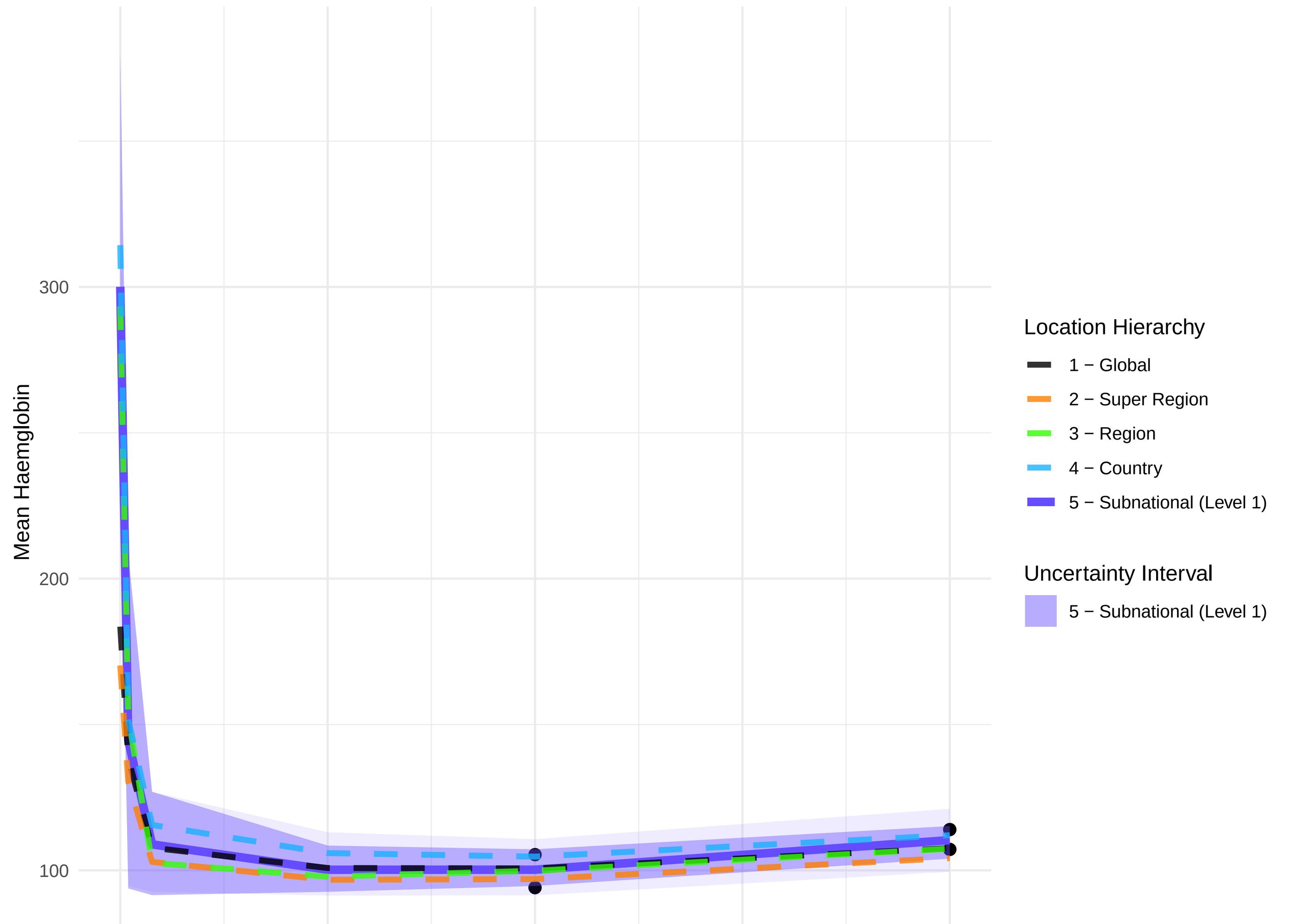

MR−BRT Age/Sex Fits − Males <2 Years Old − Mean Haemglobin − Homa Bay
WHO−2001 Adjustment Method

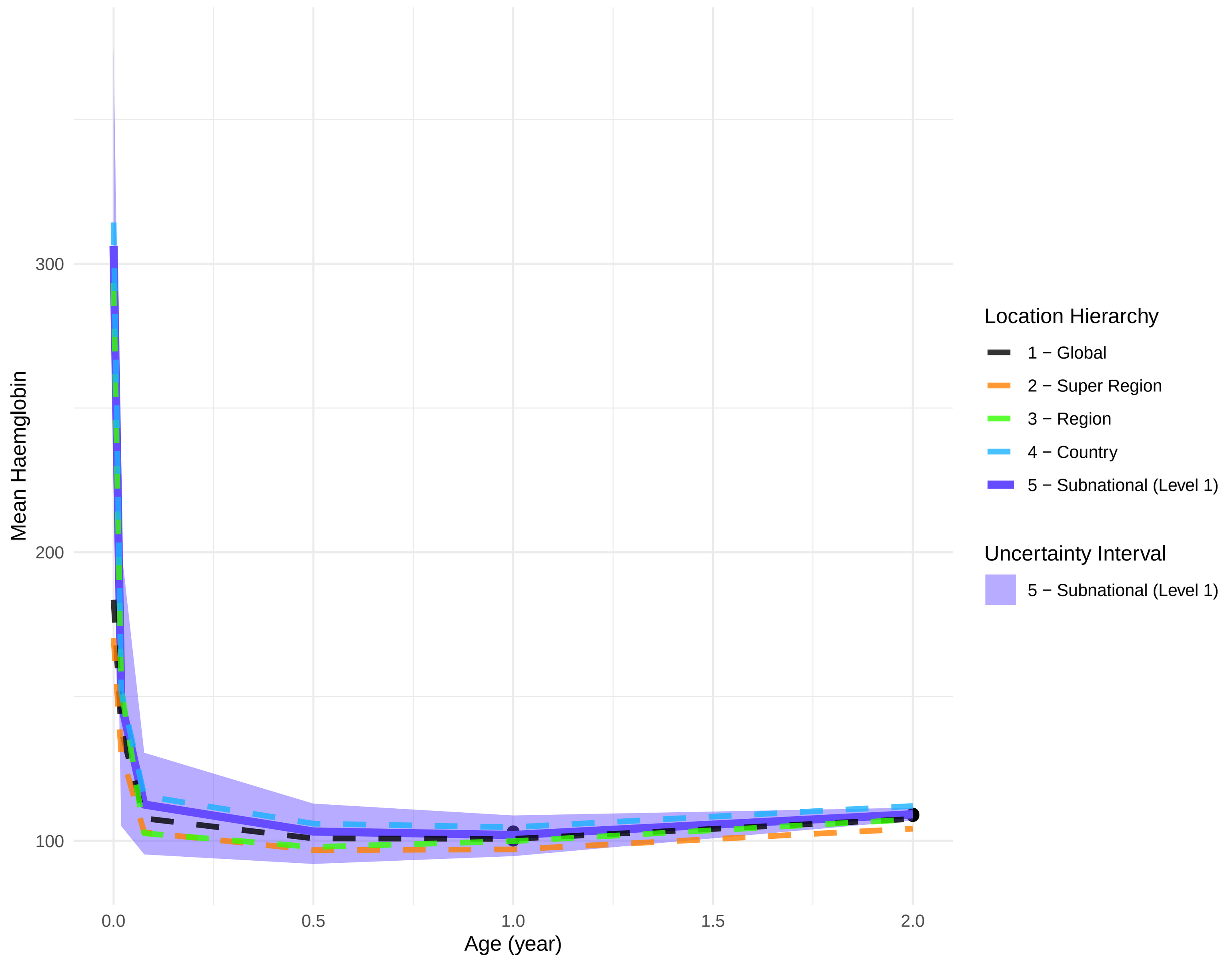

MR−BRT Age/Sex Fits − Males <2 Years Old − Mean Haemglobin − Isiolo
WHO−2001 Adjustment Method

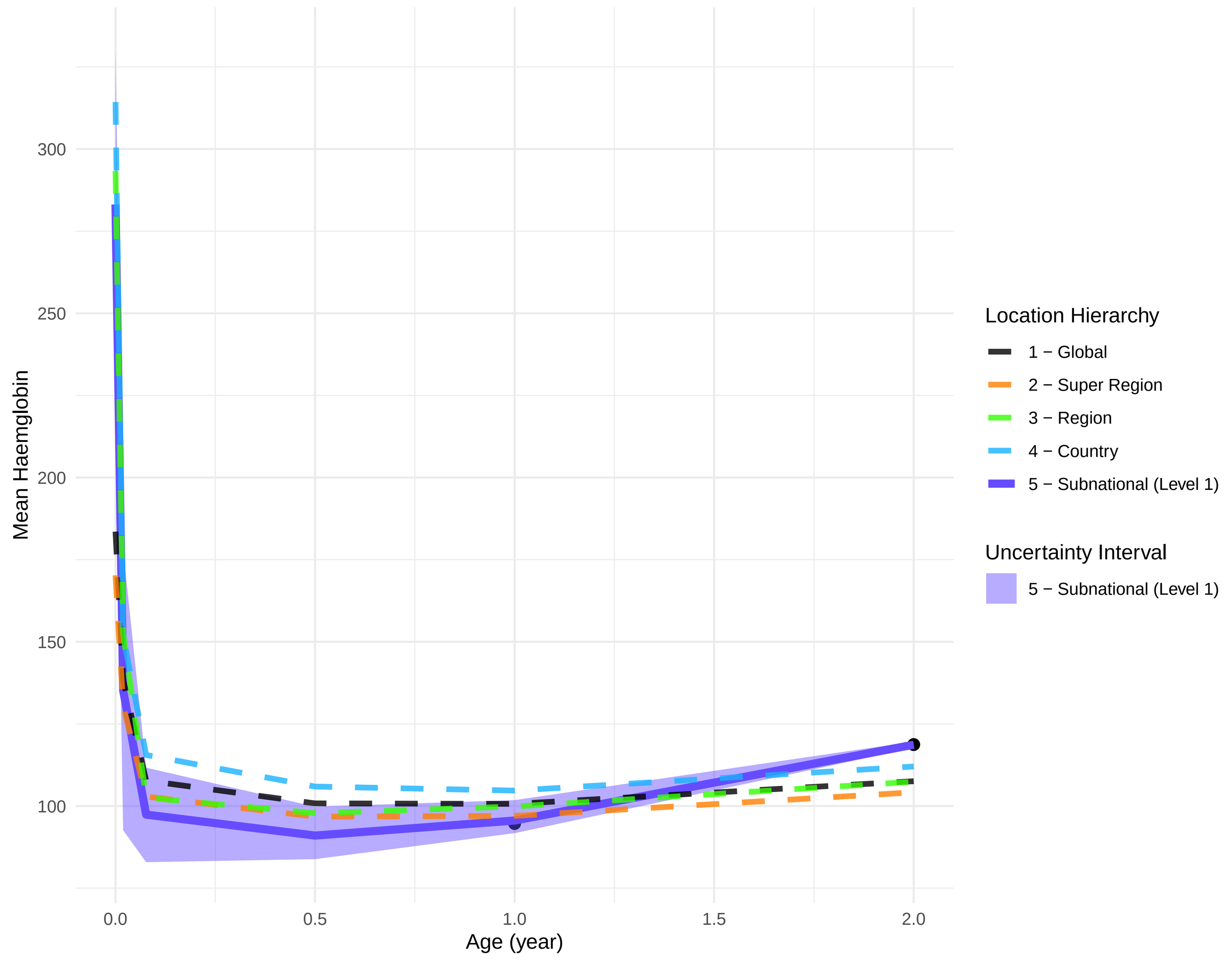

MR−BRT Age/Sex Fits − Males <2 Years Old − Mean Haemglobin − Kajiado
WHO−2001 Adjustment Method

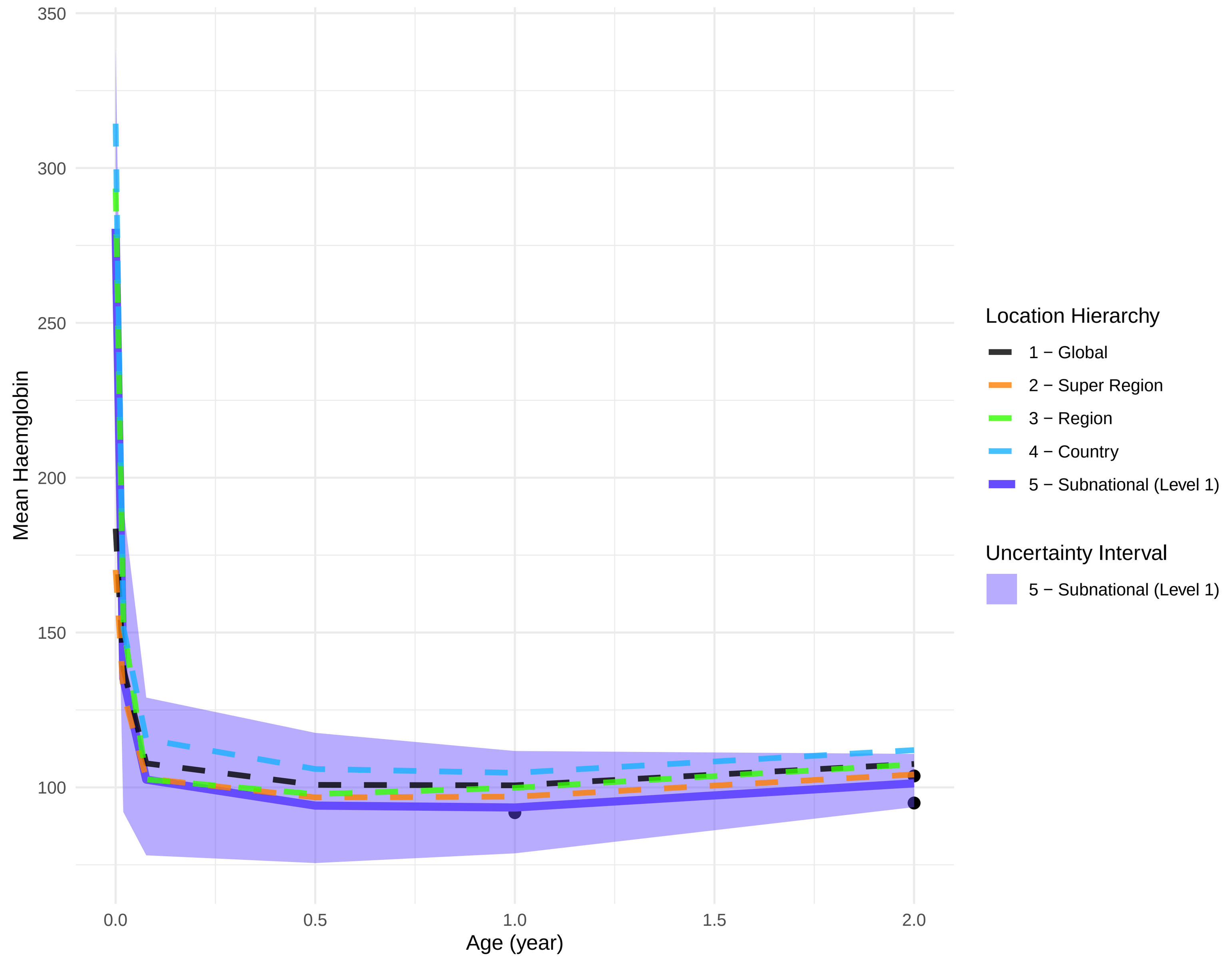

MR−BRT Age/Sex Fits − Males <2 Years Old − Mean Haemglobin − Kakamega
WHO−2001 Adjustment Method

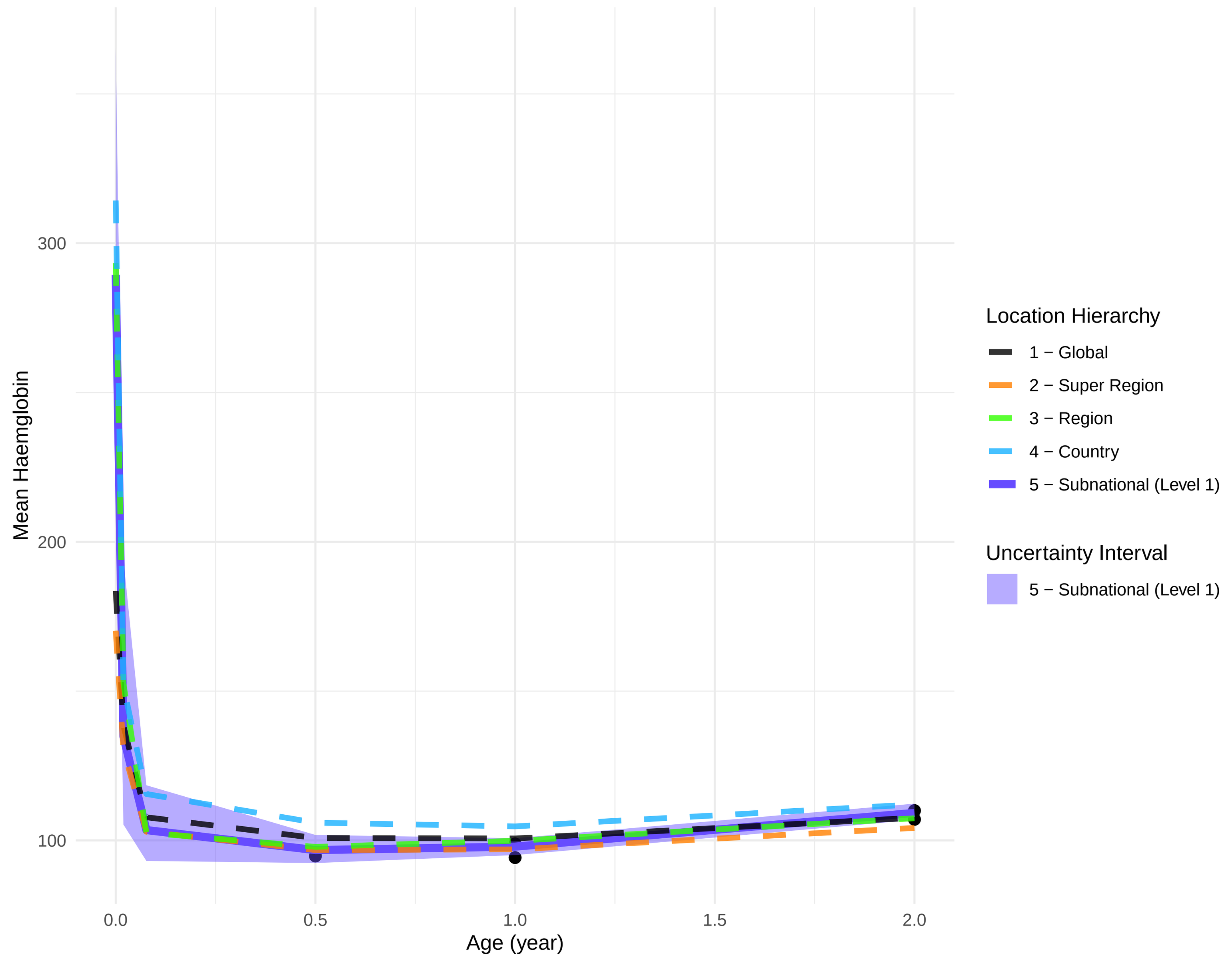

MR−BRT Age/Sex Fits − Males <2 Years Old − Mean Haemglobin − Kericho
WHO−2001 Adjustment Method

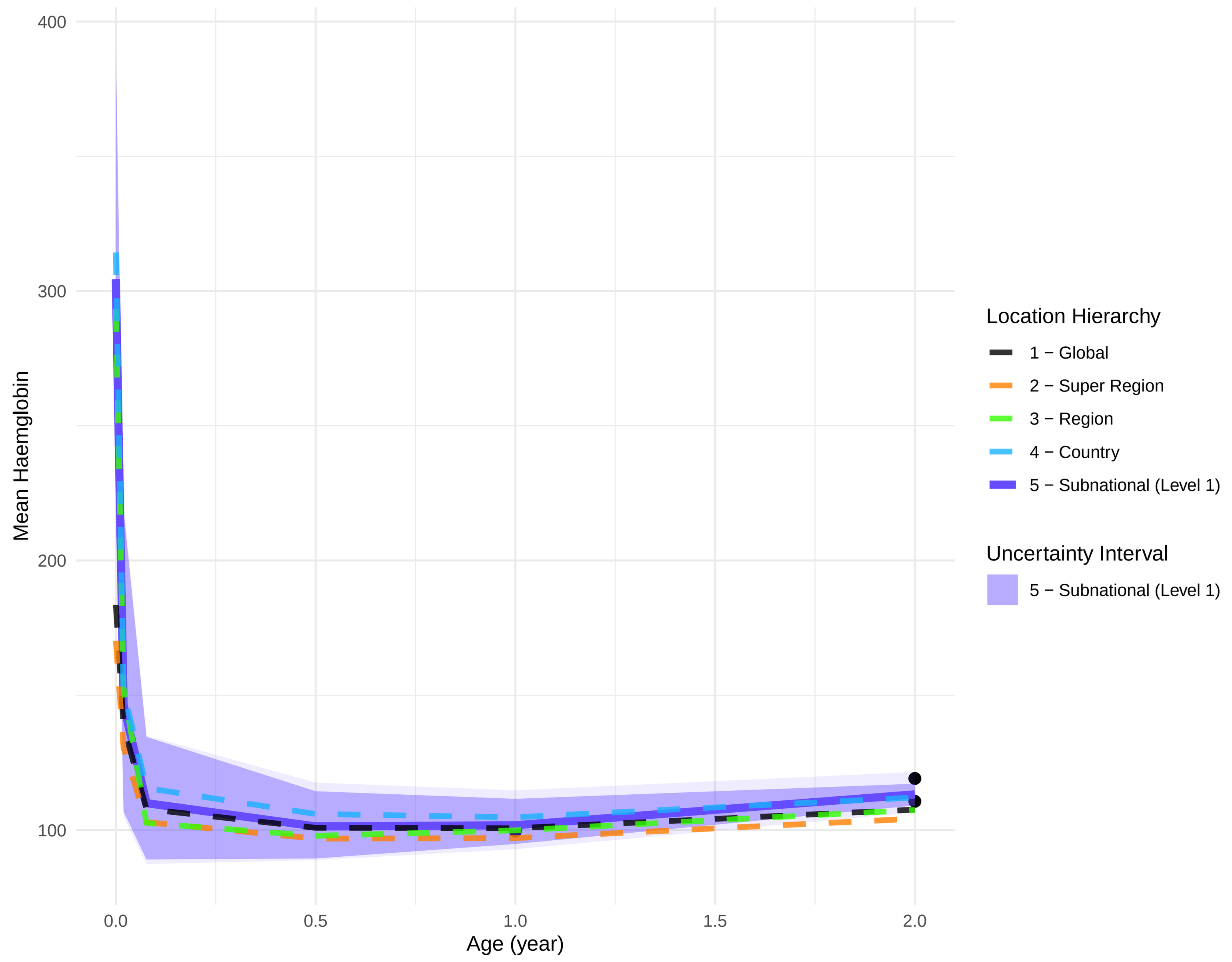

MR−BRT Age/Sex Fits − Males <2 Years Old − Mean Haemglobin − Kiambu
WHO−2001 Adjustment Method

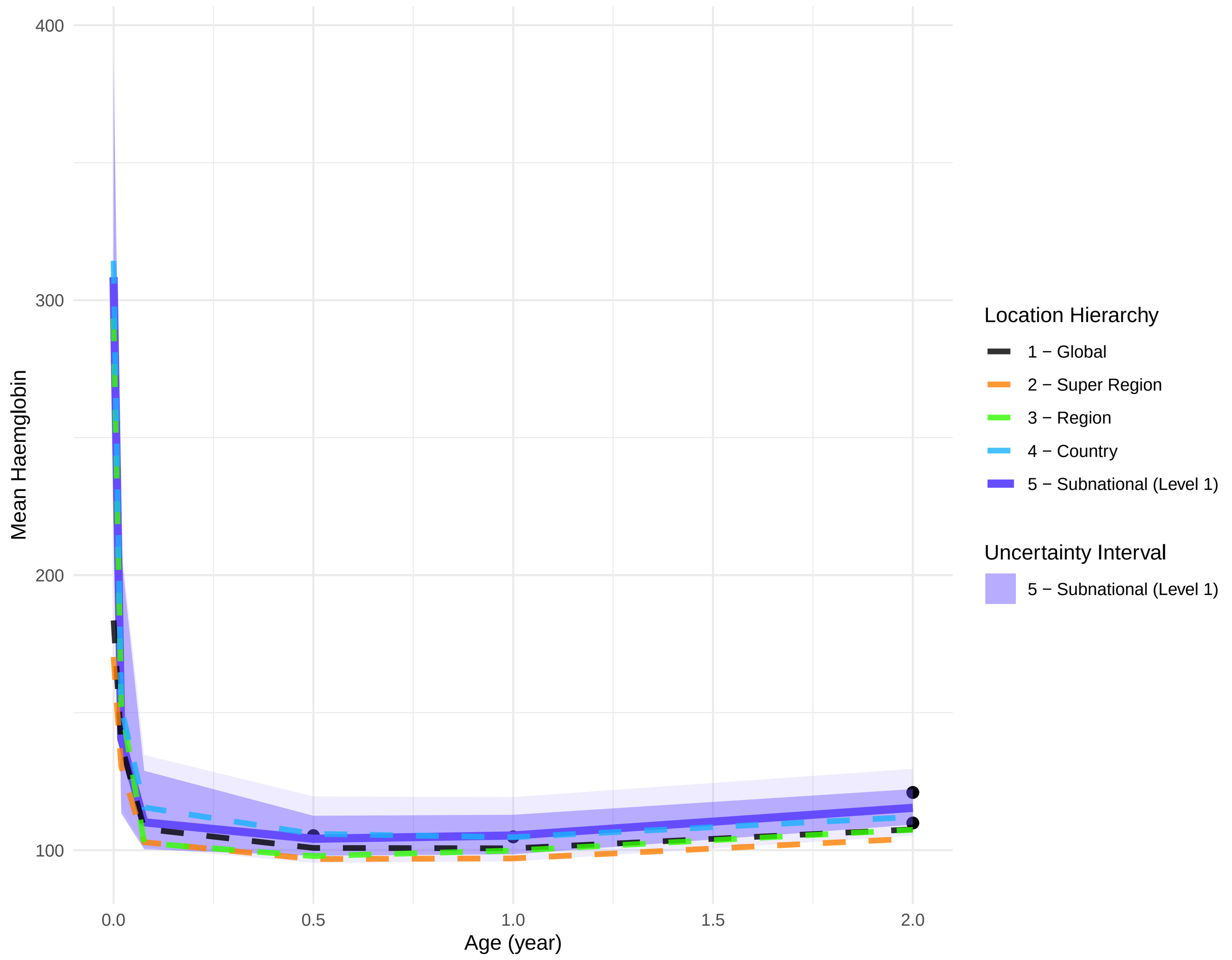

MR−BRT Age/Sex Fits − Males <2 Years Old − Mean Haemglobin − Kilifi
WHO−2001 Adjustment Method

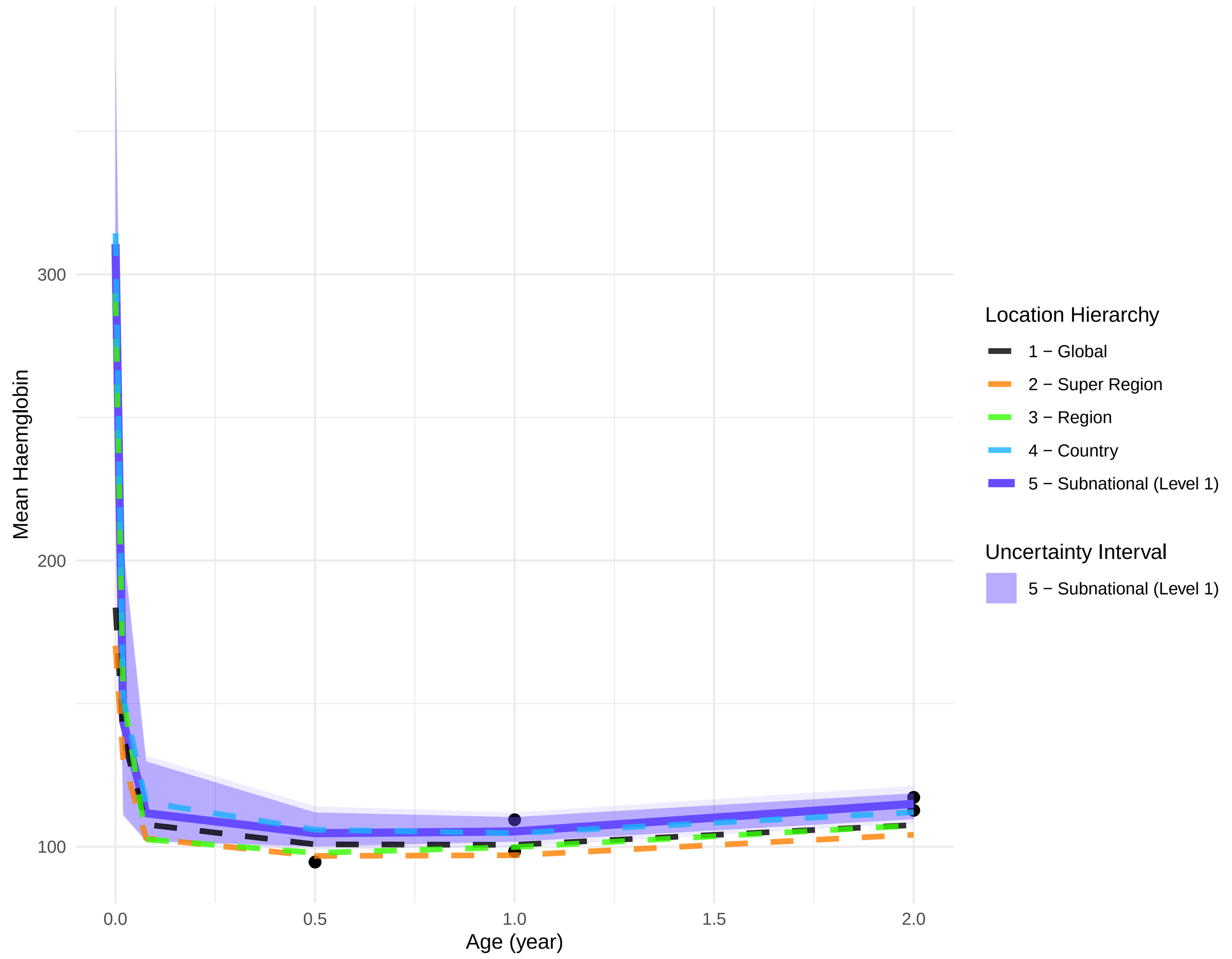

MR−BRT Age/Sex Fits − Males <2 Years Old − Mean Haemglobin − Kirinyaga
WHO−2001 Adjustment Method

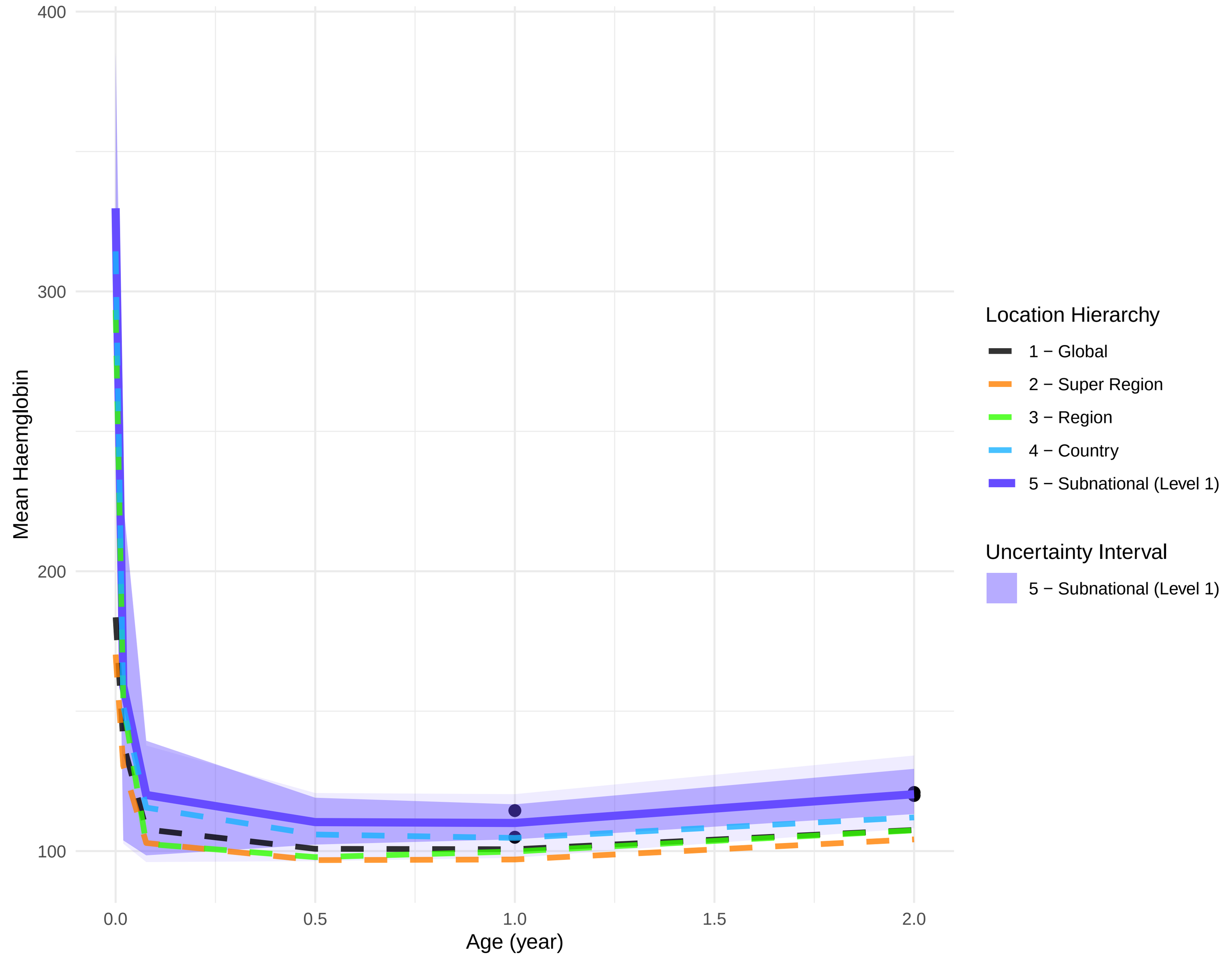

MR−BRT Age/Sex Fits − Males <2 Years Old − Mean Haemglobin − Kisii
WHO−2001 Adjustment Method

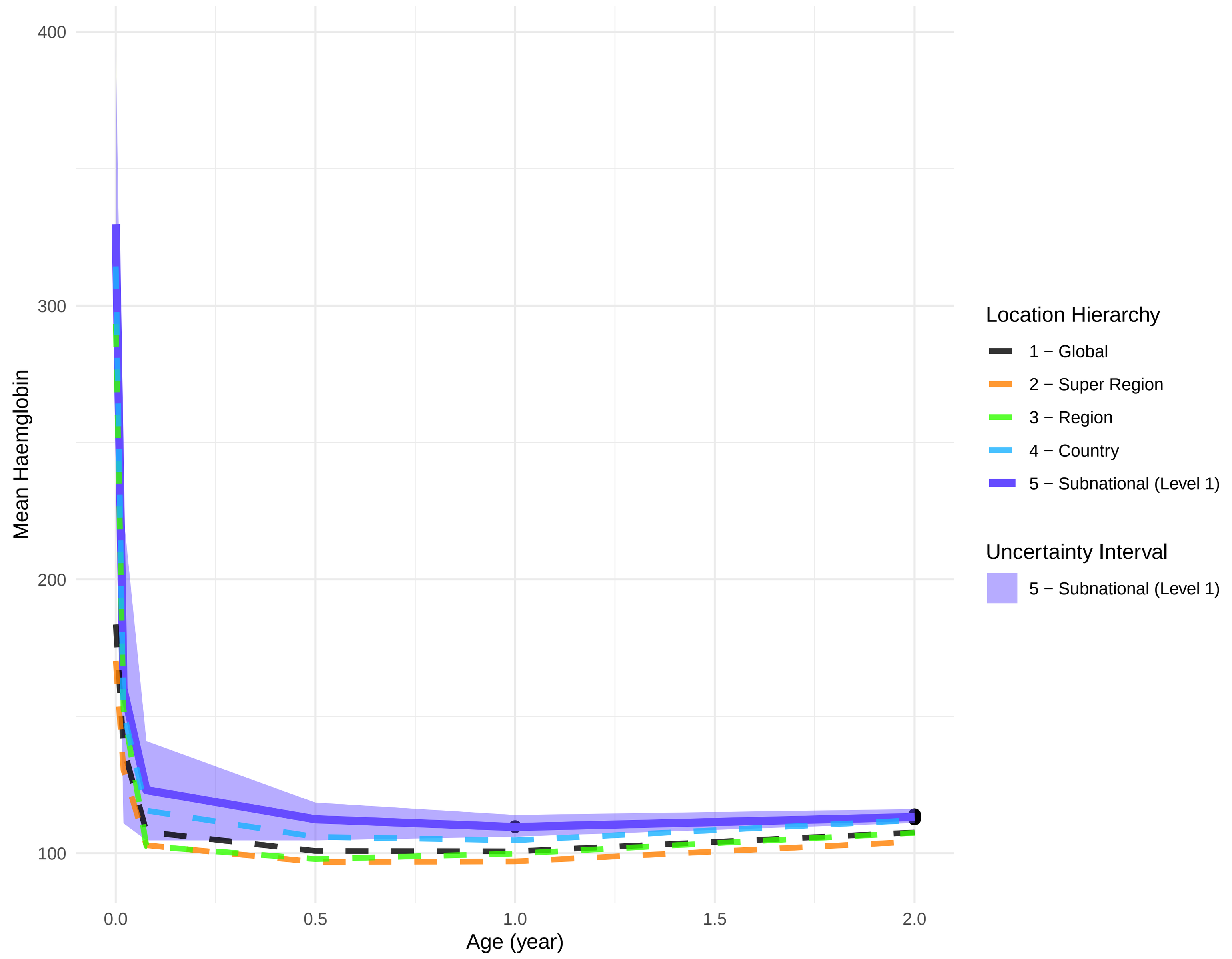

MR−BRT Age/Sex Fits − Males <2 Years Old − Mean Haemglobin − Kisumu
WHO−2001 Adjustment Method

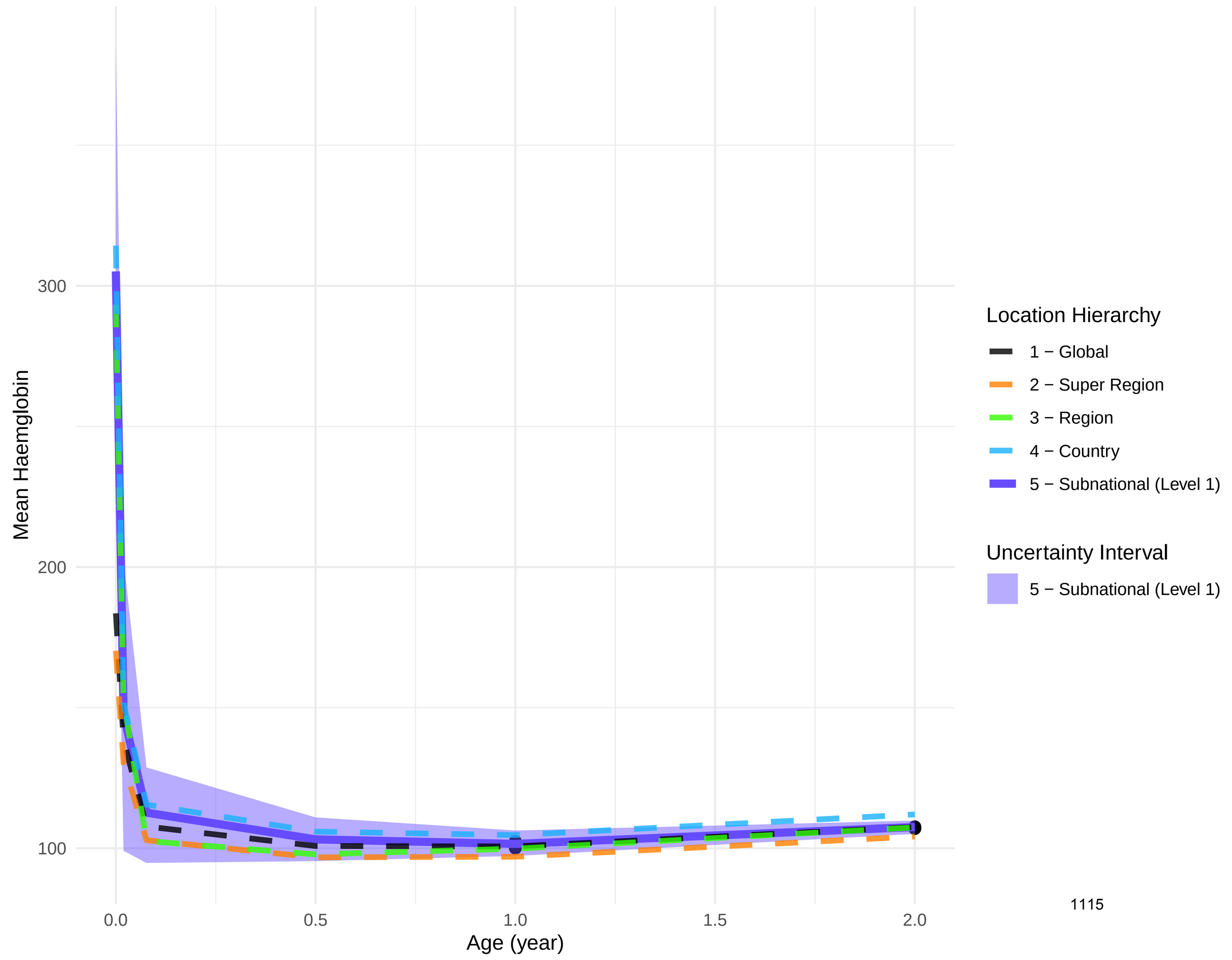

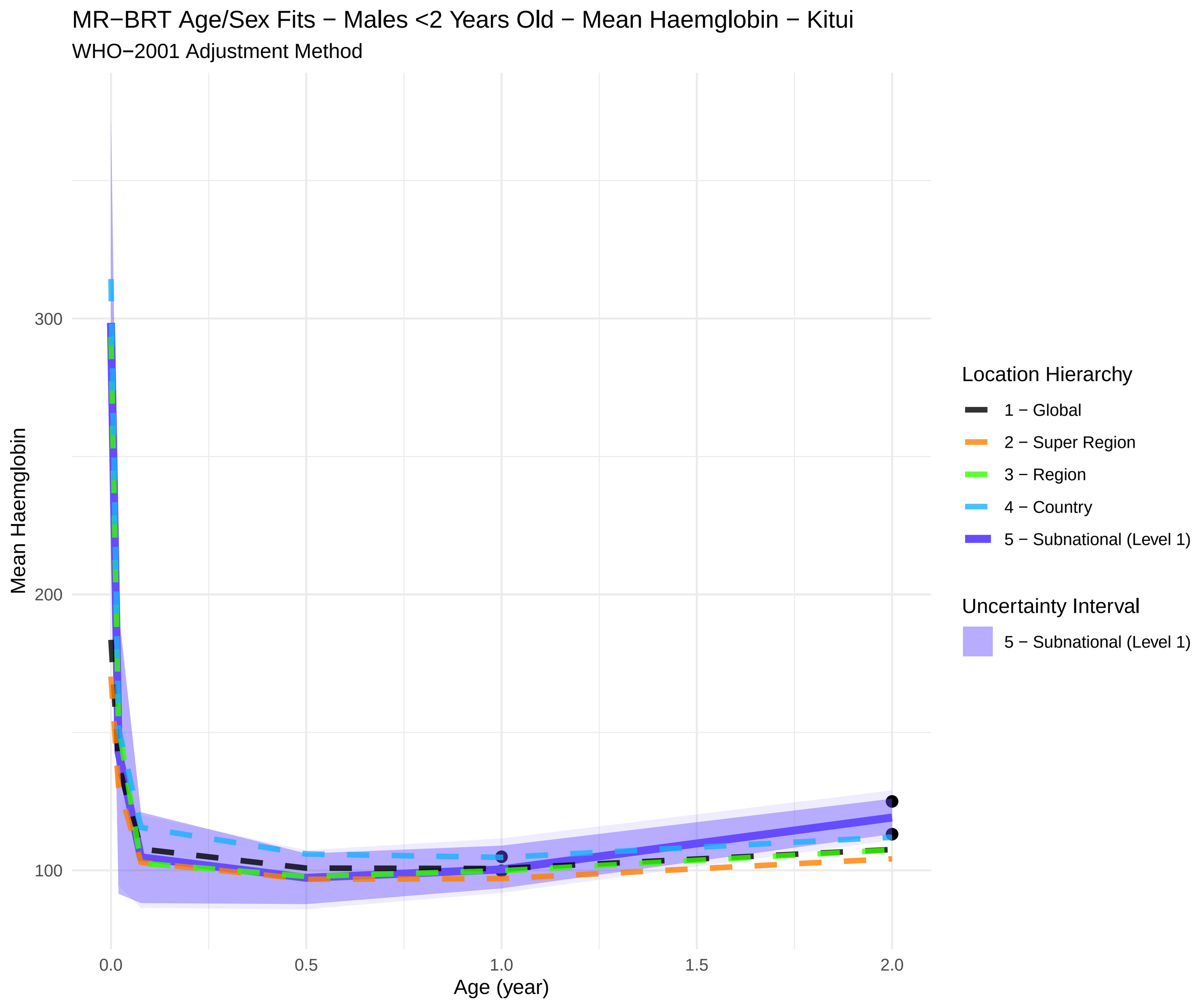
MR−BRT Age/Sex Fits − Males <2 Years Old − Mean Haemglobin − Kitui
WHO−2001 Adjustment Method
Mean Haemglobin
Age (year)
Location Hierarchy
1 − Global
2 − Super Region
3 − Region
4 − Country
5 − Subnational (Level 1)
Uncertainty Interval
5 − Subnational (Level 1)

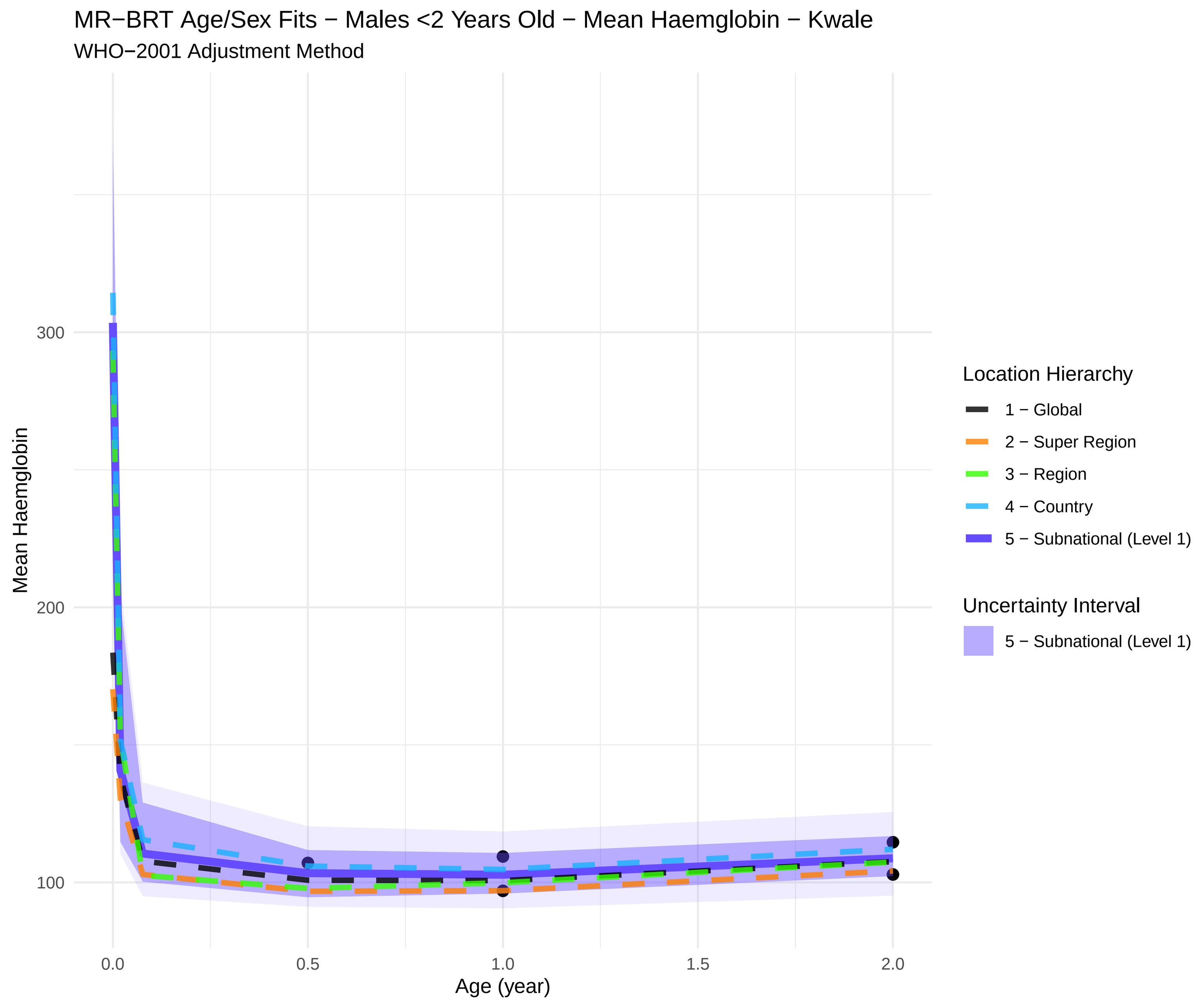
MR−BRT Age/Sex Fits − Males <2 Years Old − Mean Haemglobin − Kwale
WHO−2001 Adjustment Method
Mean Haemglobin
Age (year)
Location Hierarchy
1 − Global
2 − Super Region
3 − Region
4 − Country
5 − Subnational (Level 1)
Uncertainty Interval
5 − Subnational (Level 1)

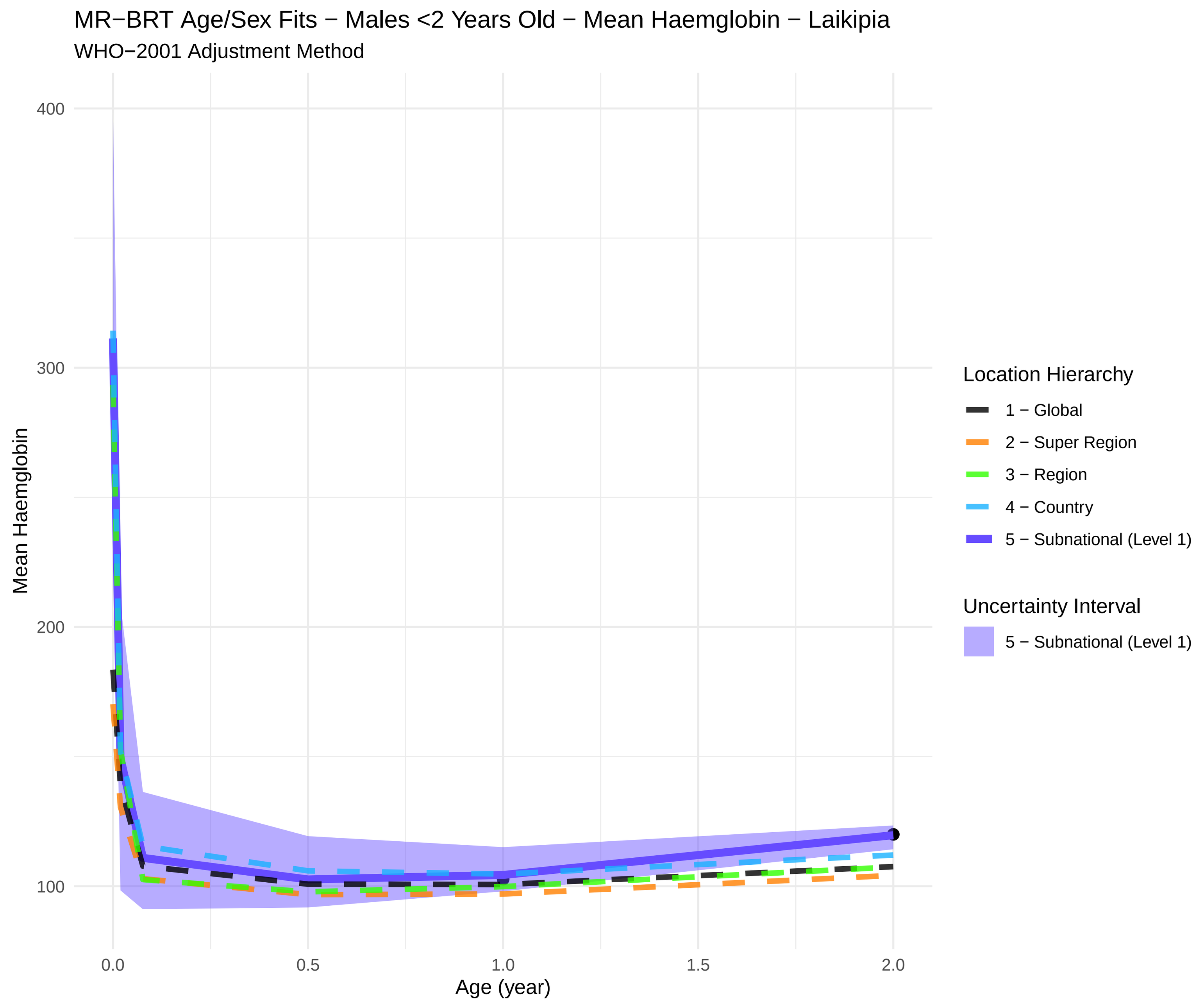
MR−BRT Age/Sex Fits − Males <2 Years Old − Mean Haemglobin − Laikipia
WHO−2001 Adjustment Method
Mean Haemglobin
Age (year)
Location Hierarchy
1 − Global
2 − Super Region
3 − Region
4 − Country
5 − Subnational (Level 1)
Uncertainty Interval
5 − Subnational (Level 1)

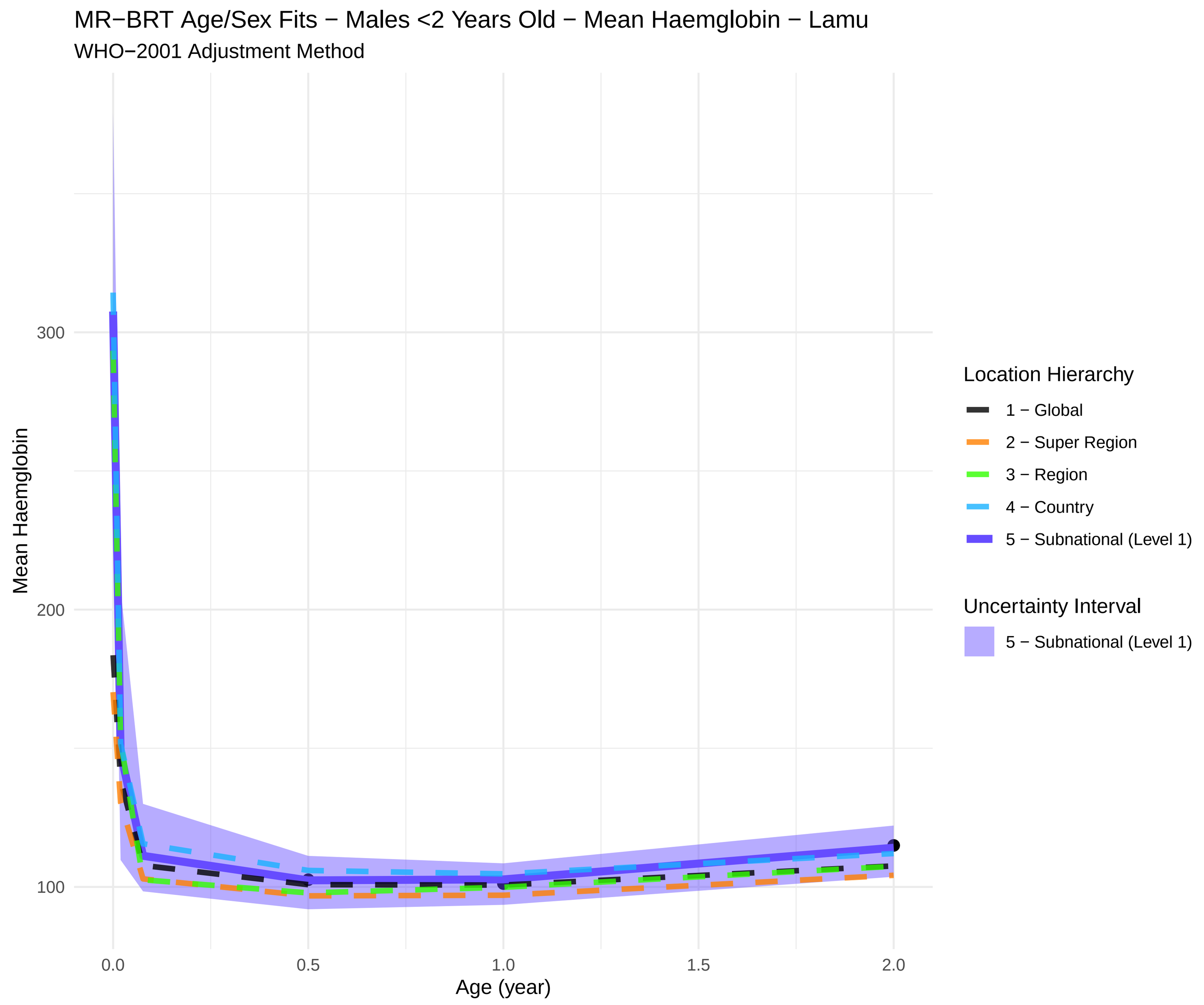
MR−BRT Age/Sex Fits − Males <2 Years Old − Mean Haemglobin − Lamu
WHO−2001 Adjustment Method
Mean Haemglobin
Age (year)
Location Hierarchy
1 − Global
2 − Super Region
3 − Region
4 − Country
5 − Subnational (Level 1)
Uncertainty Interval
5 − Subnational (Level 1)

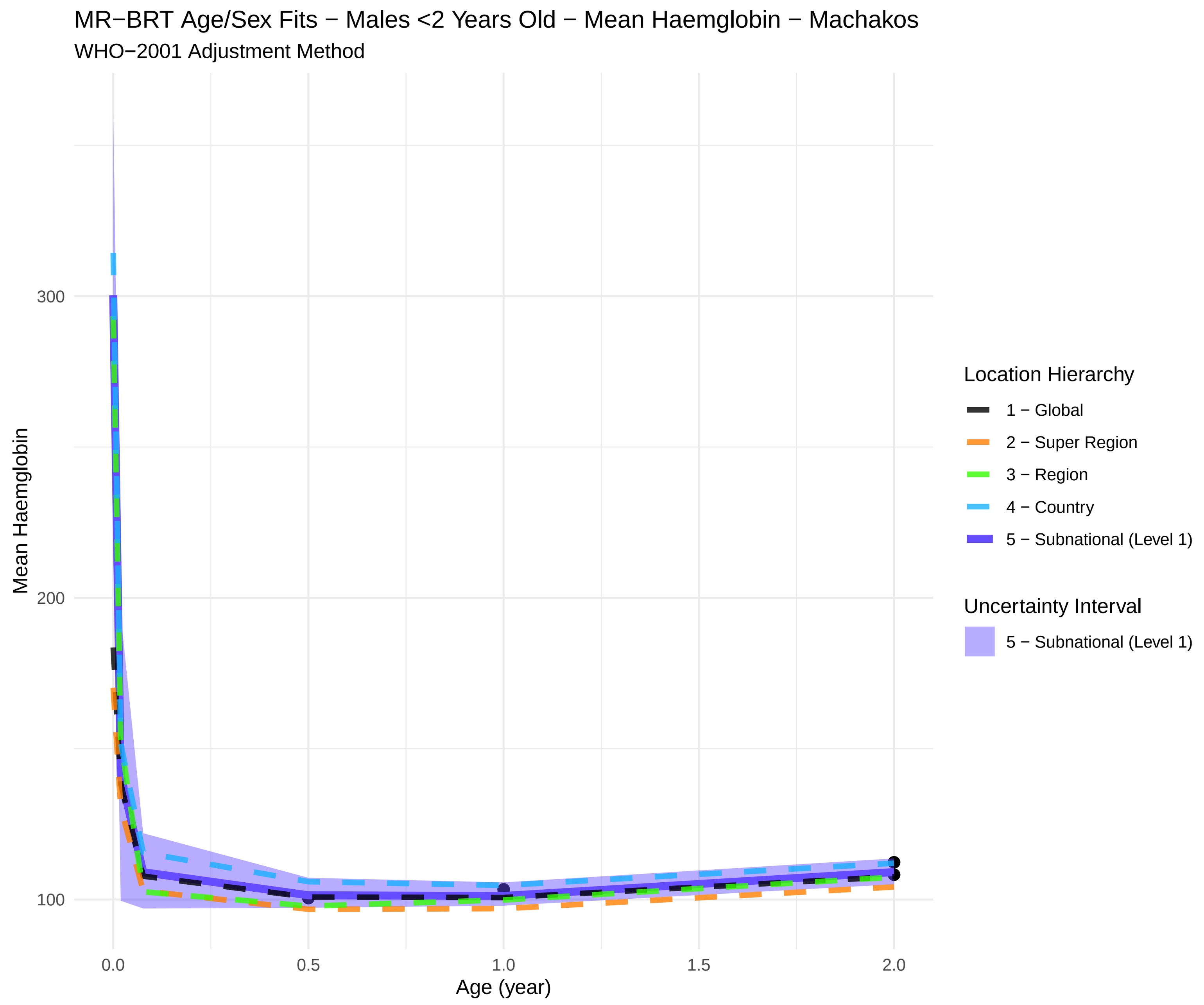
MR−BRT Age/Sex Fits − Males <2 Years Old − Mean Haemglobin − Machakos
WHO−2001 Adjustment Method
Mean Haemglobin
Age (year)
Location Hierarchy
1 − Global
2 − Super Region
3 − Region
4 − Country
5 − Subnational (Level 1)
Uncertainty Interval
5 − Subnational (Level 1)

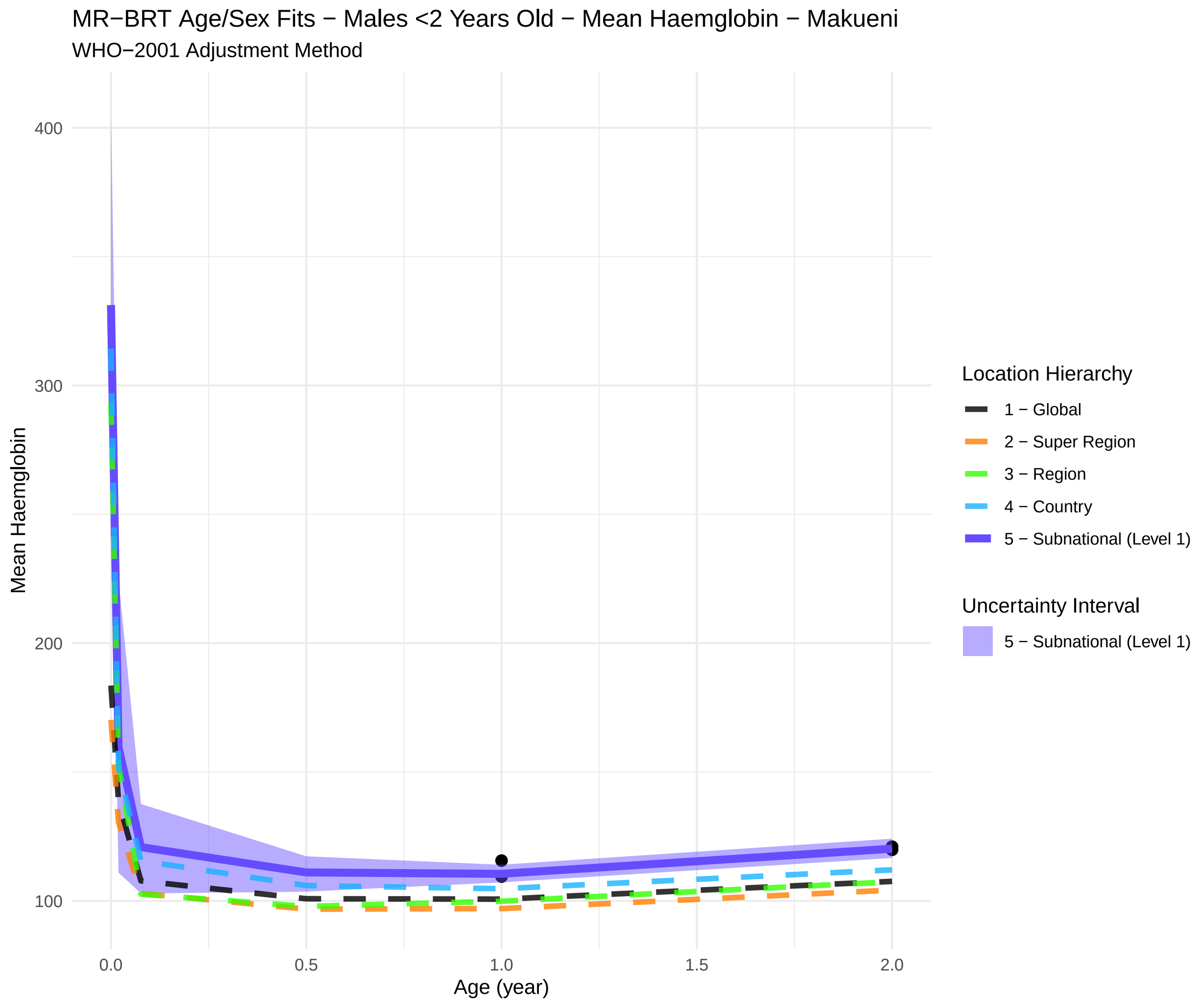
MR−BRT Age/Sex Fits − Males <2 Years Old − Mean Haemglobin − Makueni
WHO−2001 Adjustment Method
Mean Haemglobin
Age (year)
Location Hierarchy
1 − Global
2 − Super Region
3 − Region
4 − Country
5 − Subnational (Level 1)
Uncertainty Interval
5 − Subnational (Level 1)

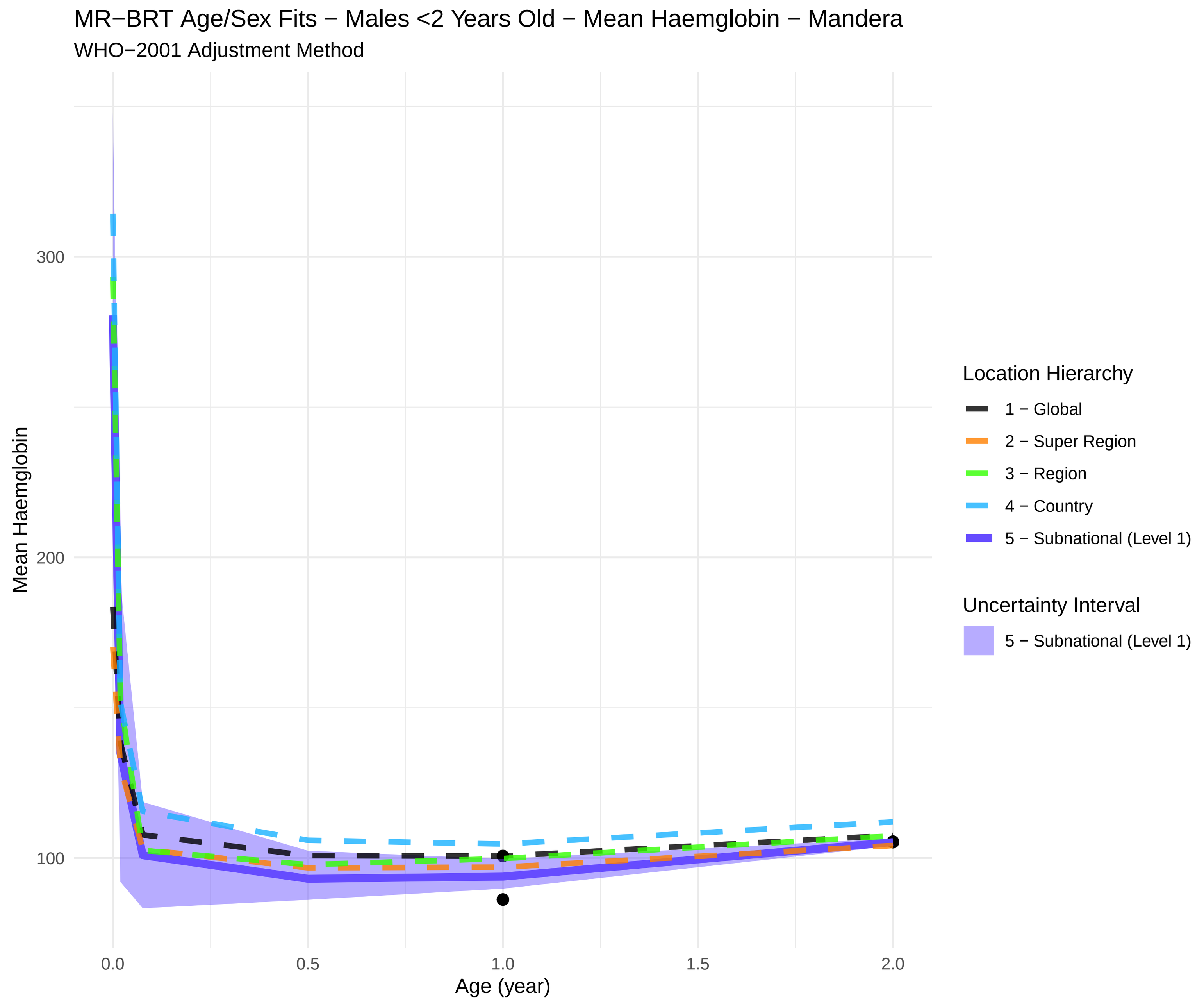
MR−BRT Age/Sex Fits − Males <2 Years Old − Mean Haemglobin − Mandera
WHO−2001 Adjustment Method
Mean Haemglobin
Age (year)
Location Hierarchy
1 − Global
2 − Super Region
3 − Region
4 − Country
5 − Subnational (Level 1)
Uncertainty Interval
5 − Subnational (Level 1)

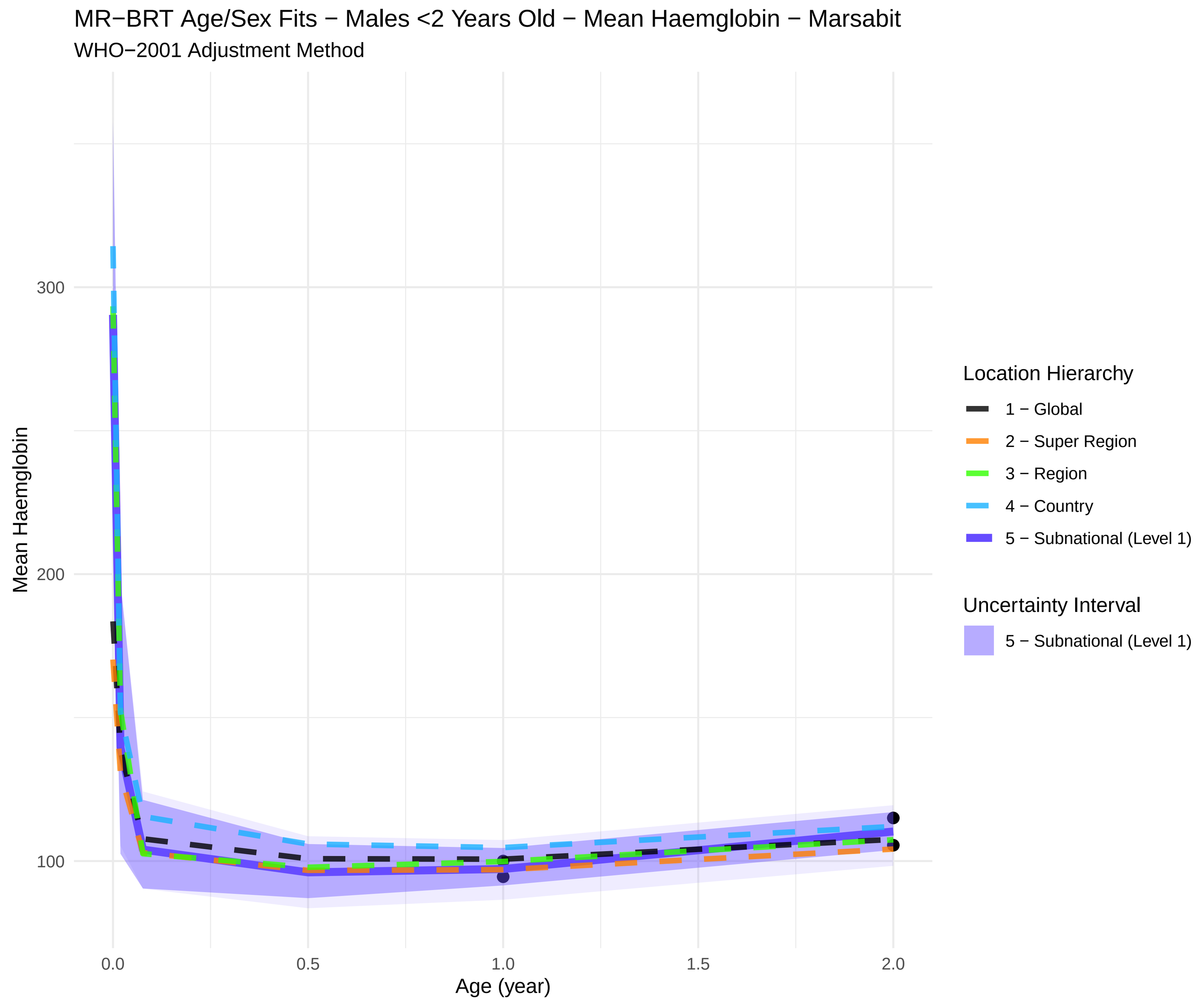
MR−BRT Age/Sex Fits − Males <2 Years Old − Mean Haemglobin − Marsabit
WHO−2001 Adjustment Method
Mean Haemglobin
Age (year)
Location Hierarchy
1 − Global
2 − Super Region
3 − Region
4 − Country
5 − Subnational (Level 1)
Uncertainty Interval
5 − Subnational (Level 1)

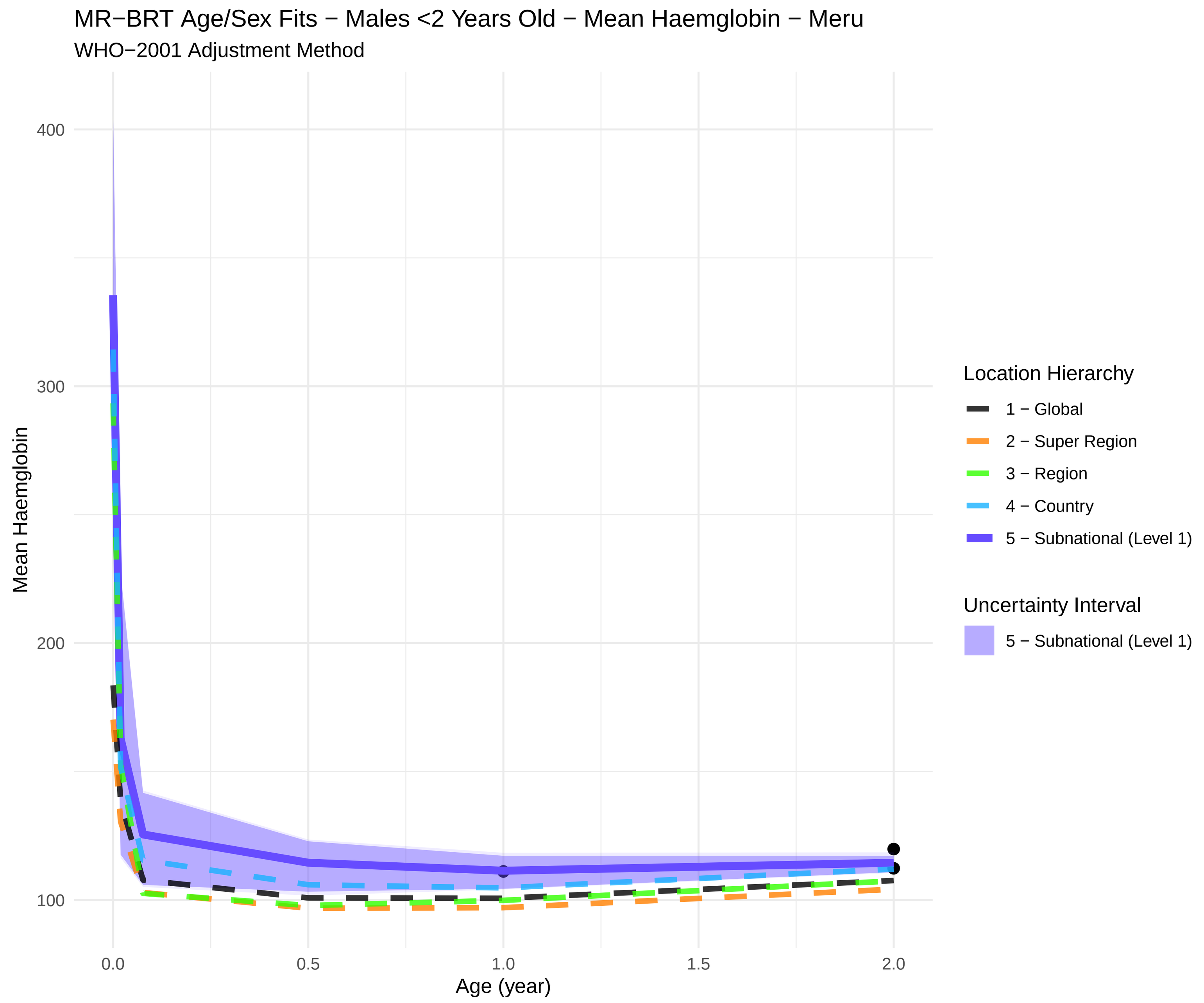
MR−BRT Age/Sex Fits − Males <2 Years Old − Mean Haemglobin − Meru
WHO−2001 Adjustment Method
Mean Haemglobin
Age (year)
Location Hierarchy
1 − Global
2 − Super Region
3 − Region
4 − Country
5 − Subnational (Level 1)
Uncertainty Interval
5 − Subnational (Level 1)

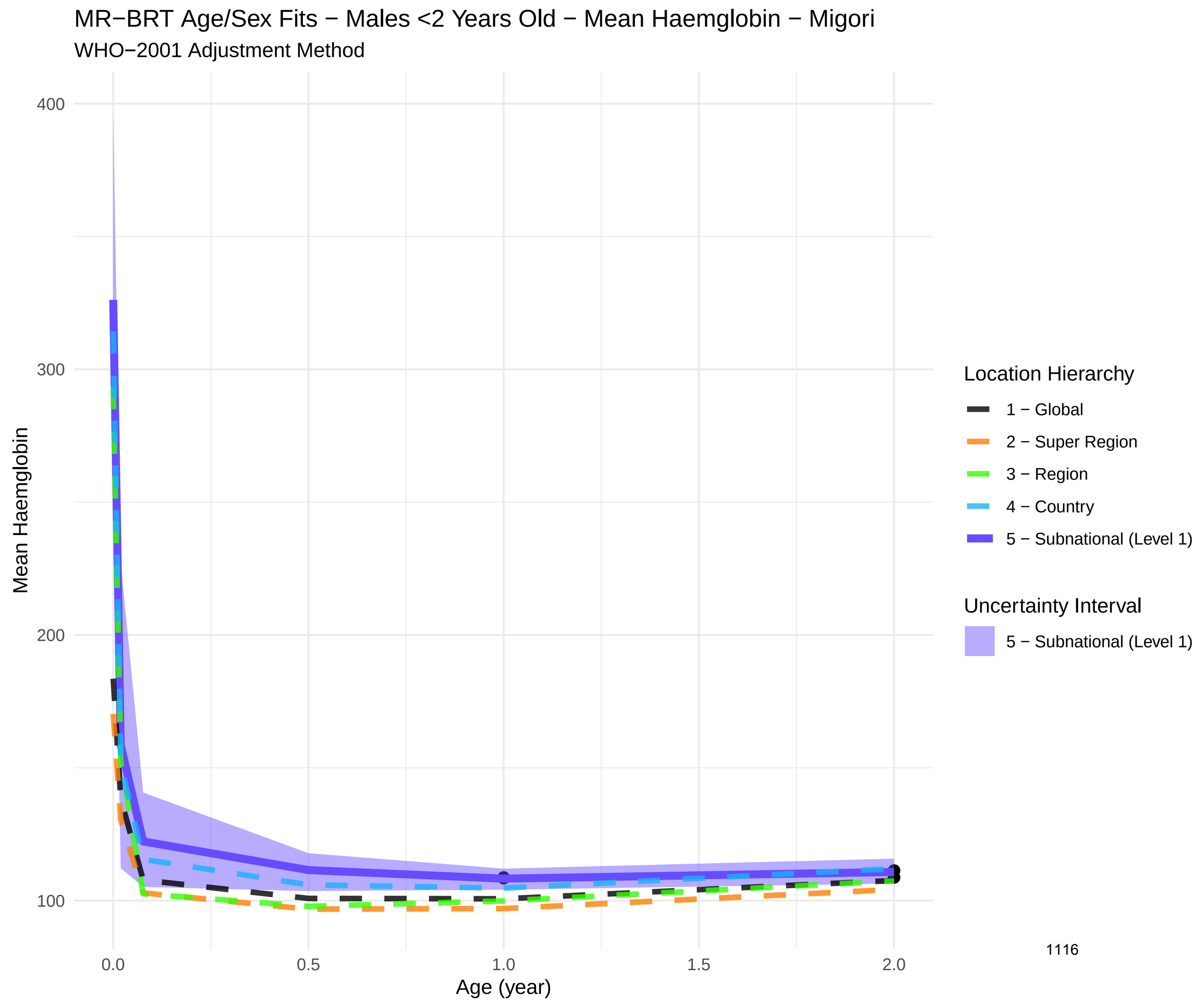
MR−BRT Age/Sex Fits − Males <2 Years Old − Mean Haemglobin − Migori
WHO−2001 Adjustment Method
Mean Haemglobin
Age (year)
Location Hierarchy
1 − Global
2 − Super Region
3 − Region
4 − Country
5 − Subnational (Level 1)
Uncertainty Interval
5 − Subnational (Level 1)

MR−BRT Age/Sex Fits − Males <2 Years Old − Mean Haemglobin − Mombasa
WHO−2001 Adjustment Method

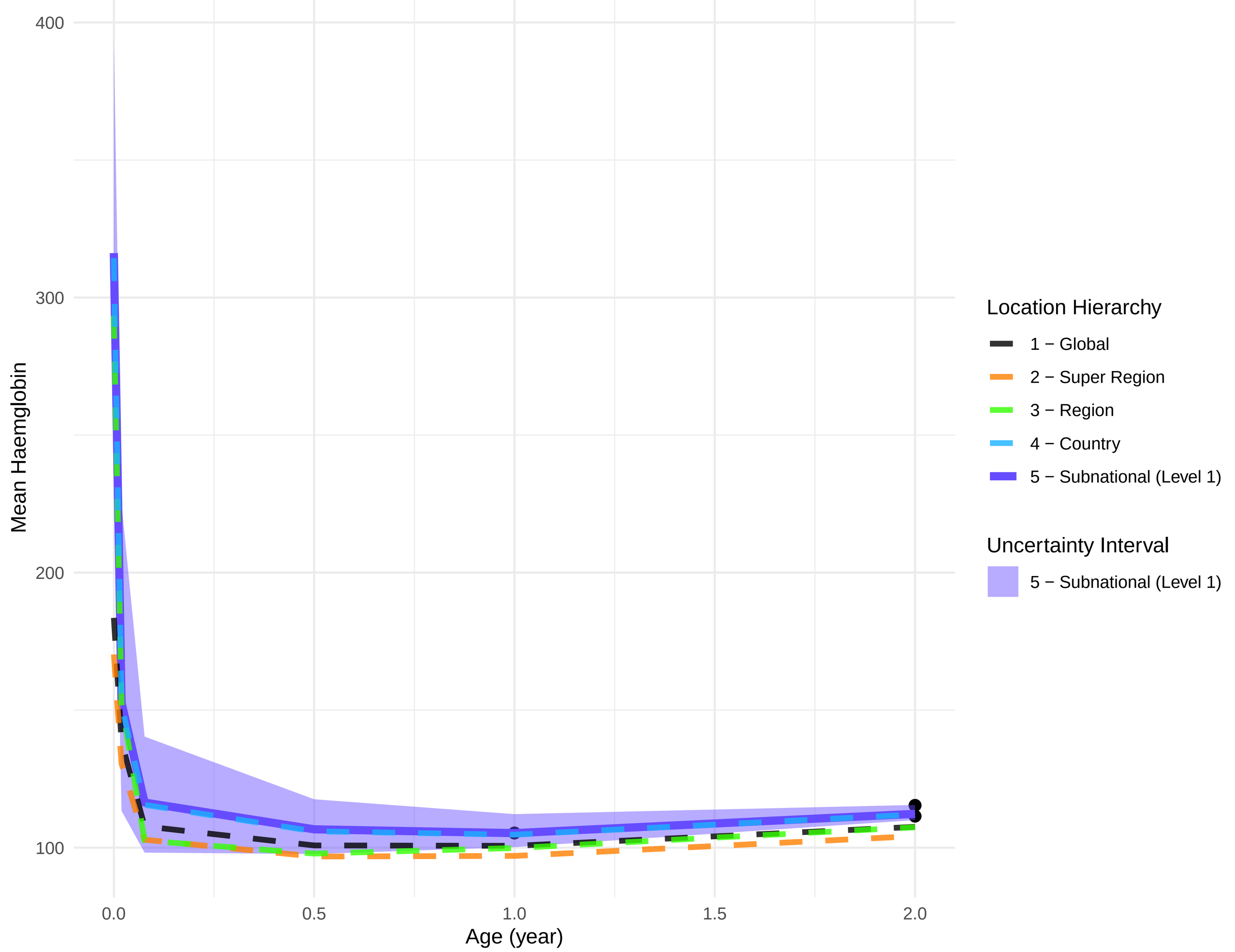

MR−BRT Age/Sex Fits − Males <2 Years Old − Mean Haemglobin − Murang'a
WHO−2001 Adjustment Method

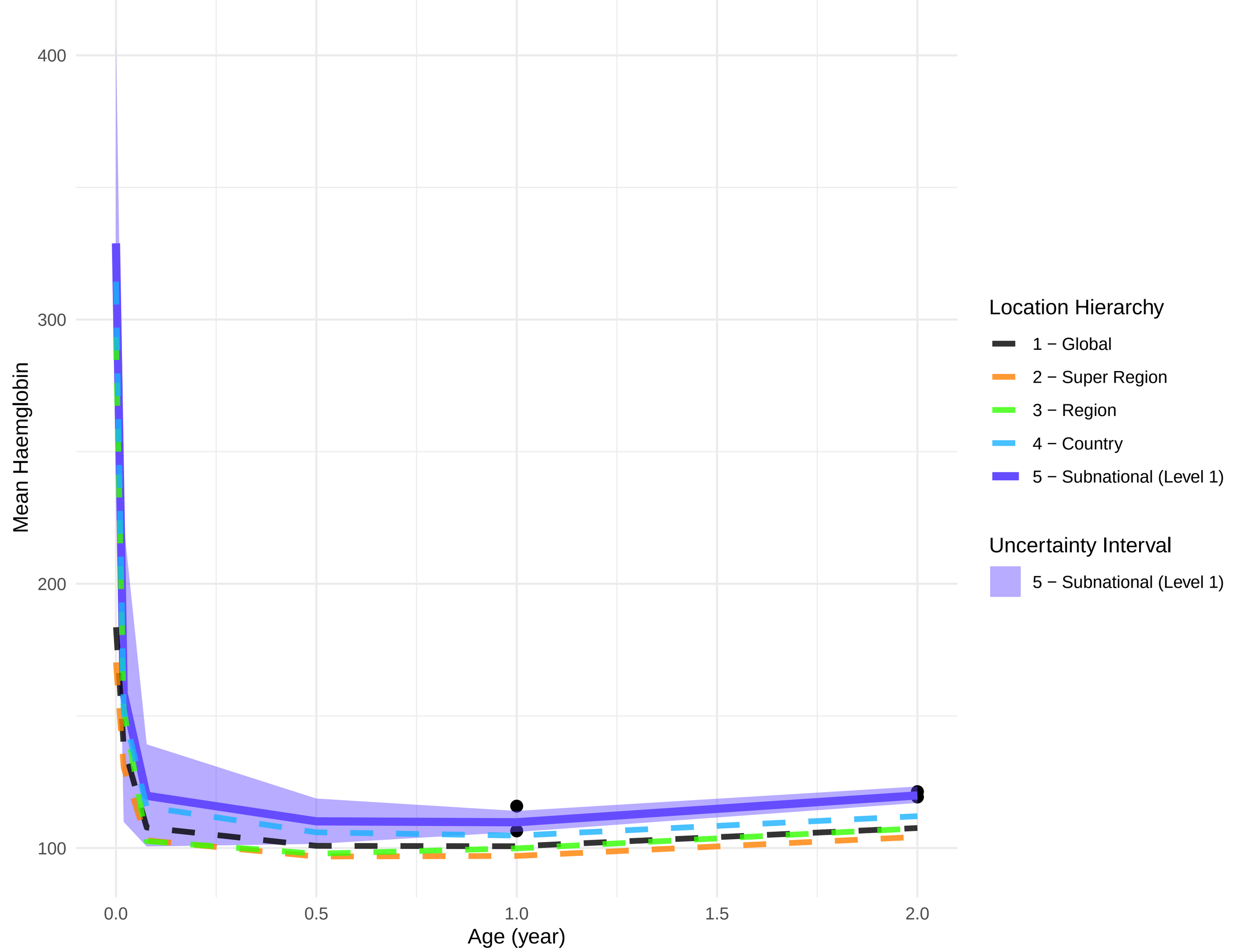

MR−BRT Age/Sex Fits − Males <2 Years Old − Mean Haemglobin − Nairobi
WHO−2001 Adjustment Method

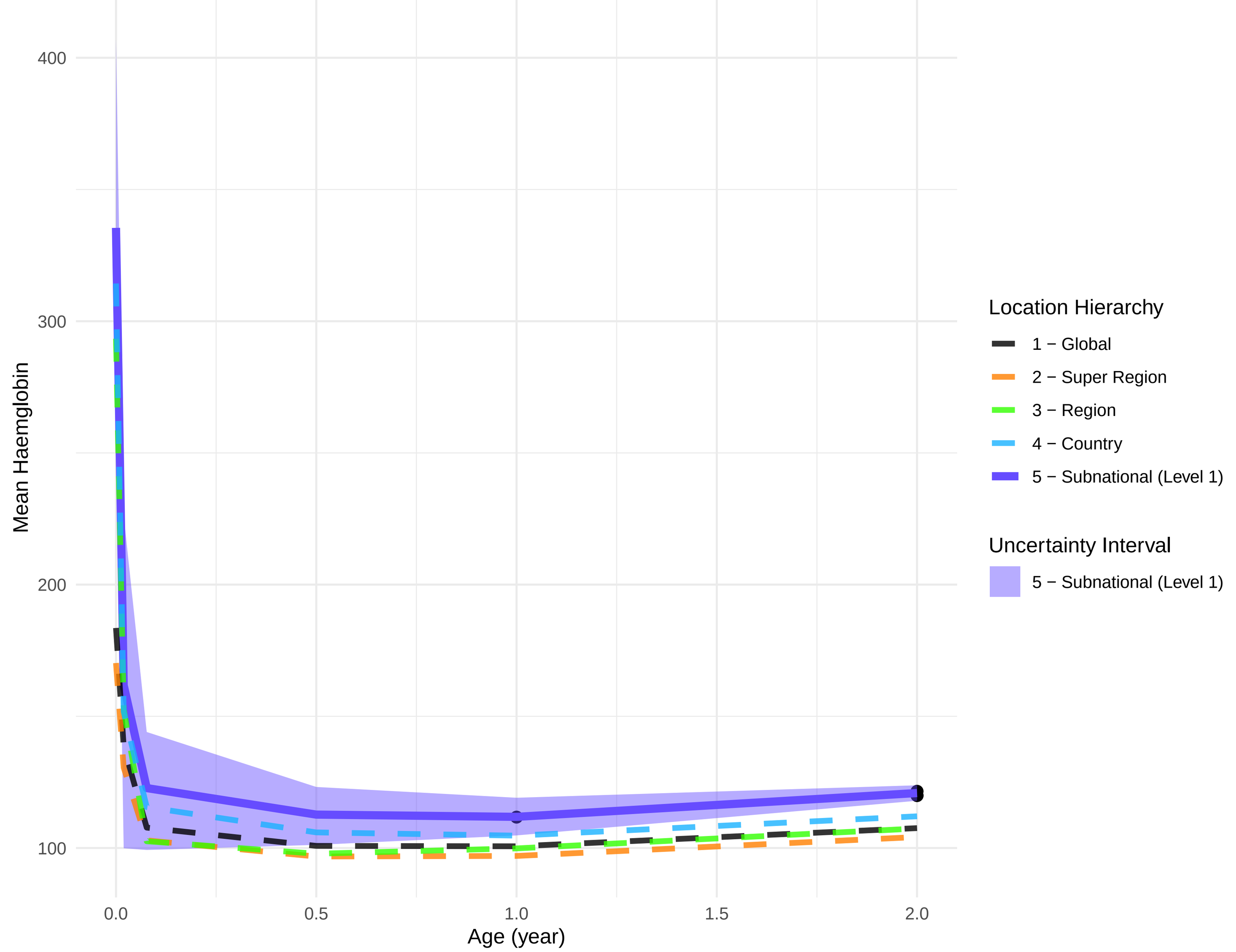

MR−BRT Age/Sex Fits − Males <2 Years Old − Mean Haemglobin − Nakuru
WHO−2001 Adjustment Method

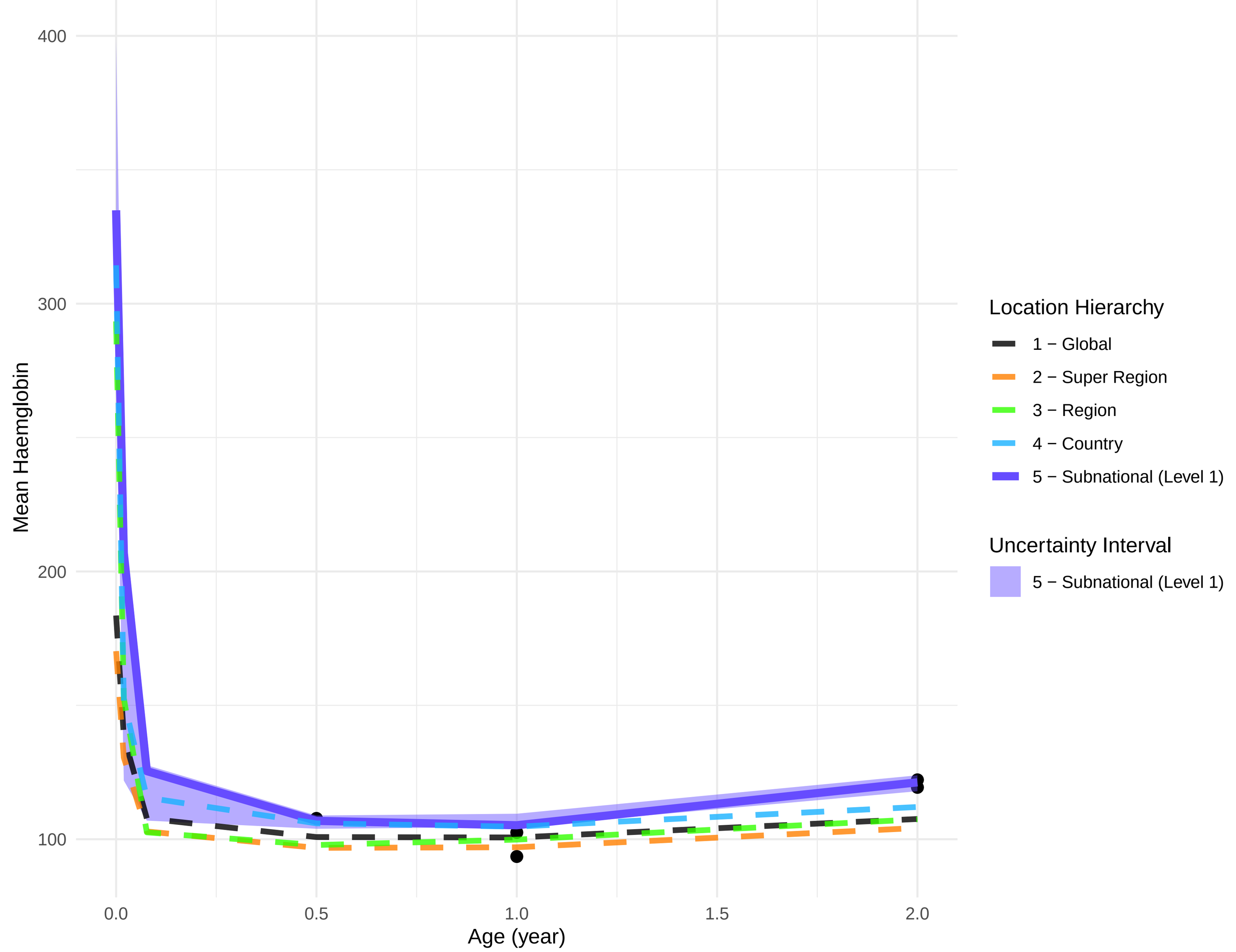

MR−BRT Age/Sex Fits − Males <2 Years Old − Mean Haemglobin − Nandi
WHO−2001 Adjustment Method

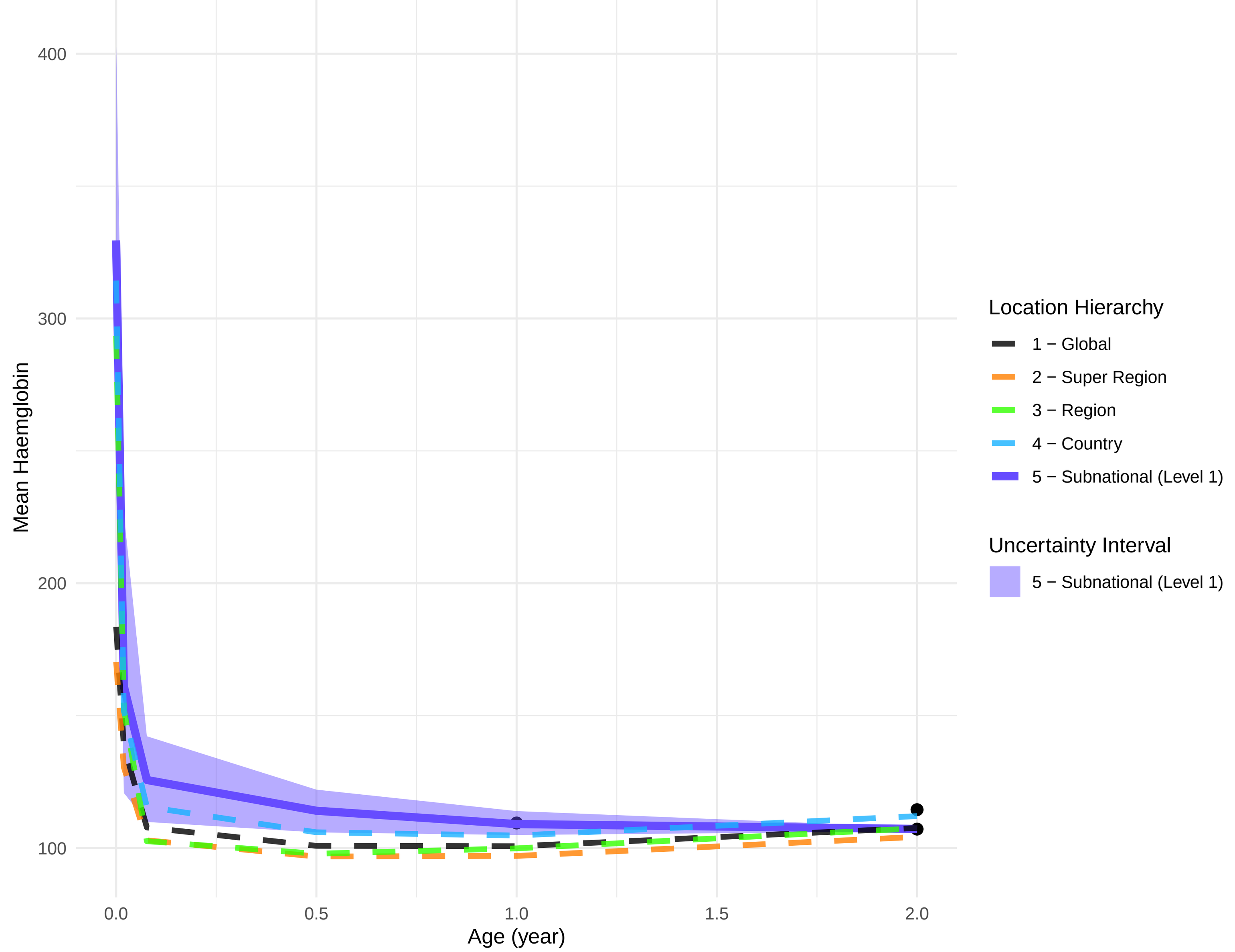

MR−BRT Age/Sex Fits − Males <2 Years Old − Mean Haemglobin − Narok
WHO−2001 Adjustment Method

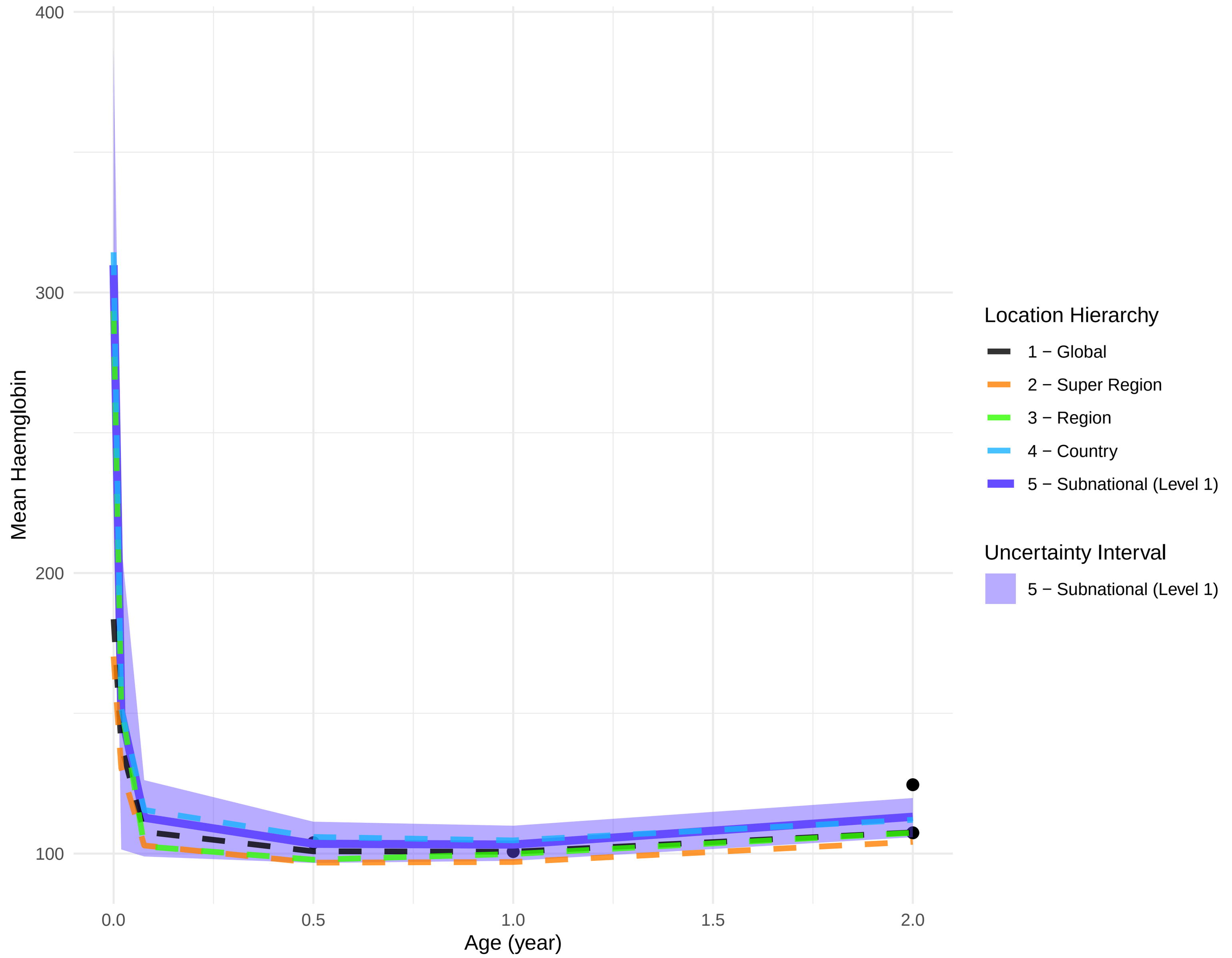

MR−BRT Age/Sex Fits − Males <2 Years Old − Mean Haemglobin − Nyamira
WHO−2001 Adjustment Method

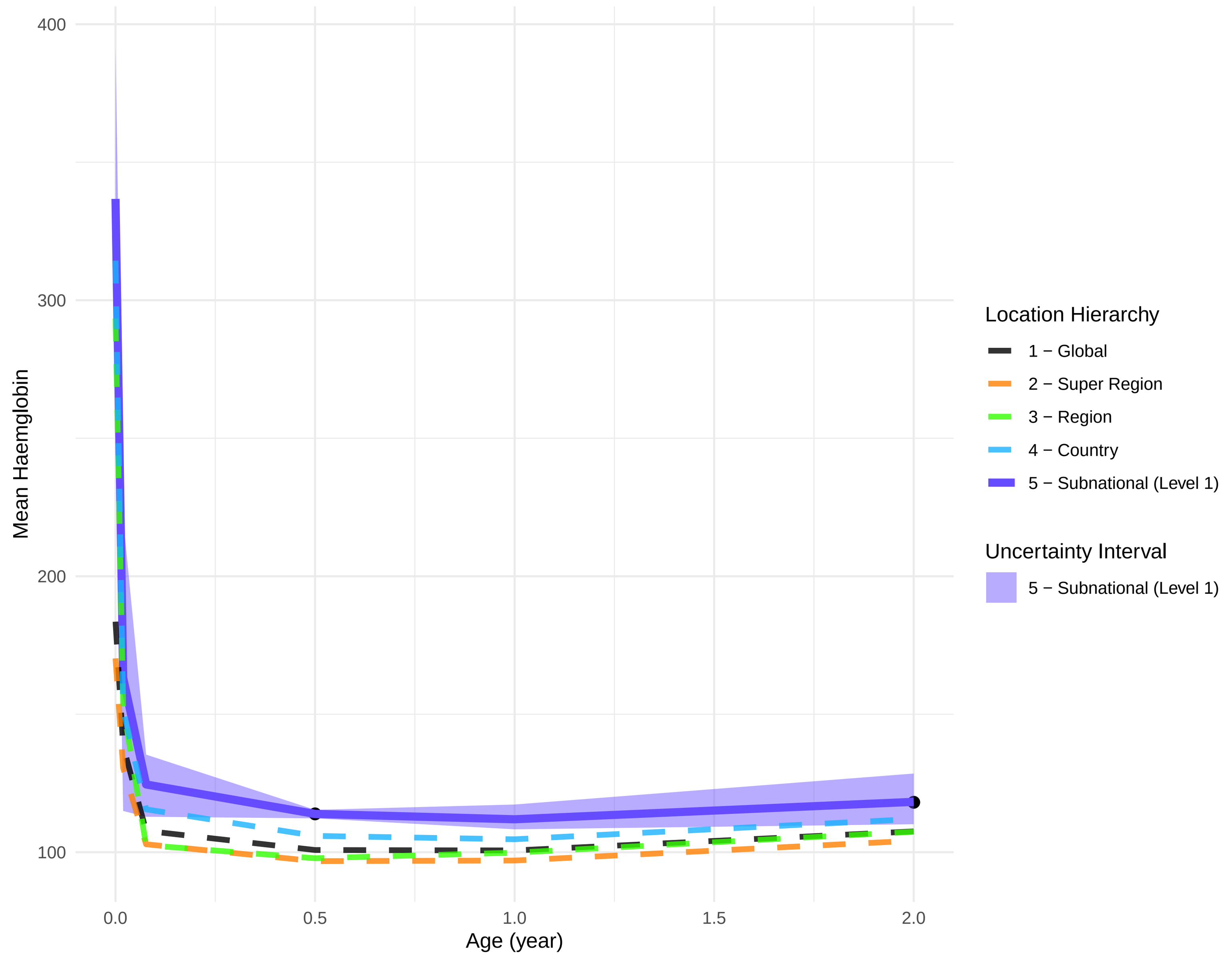

MR−BRT Age/Sex Fits − Males <2 Years Old − Mean Haemglobin − Nyandarua
WHO−2001 Adjustment Method

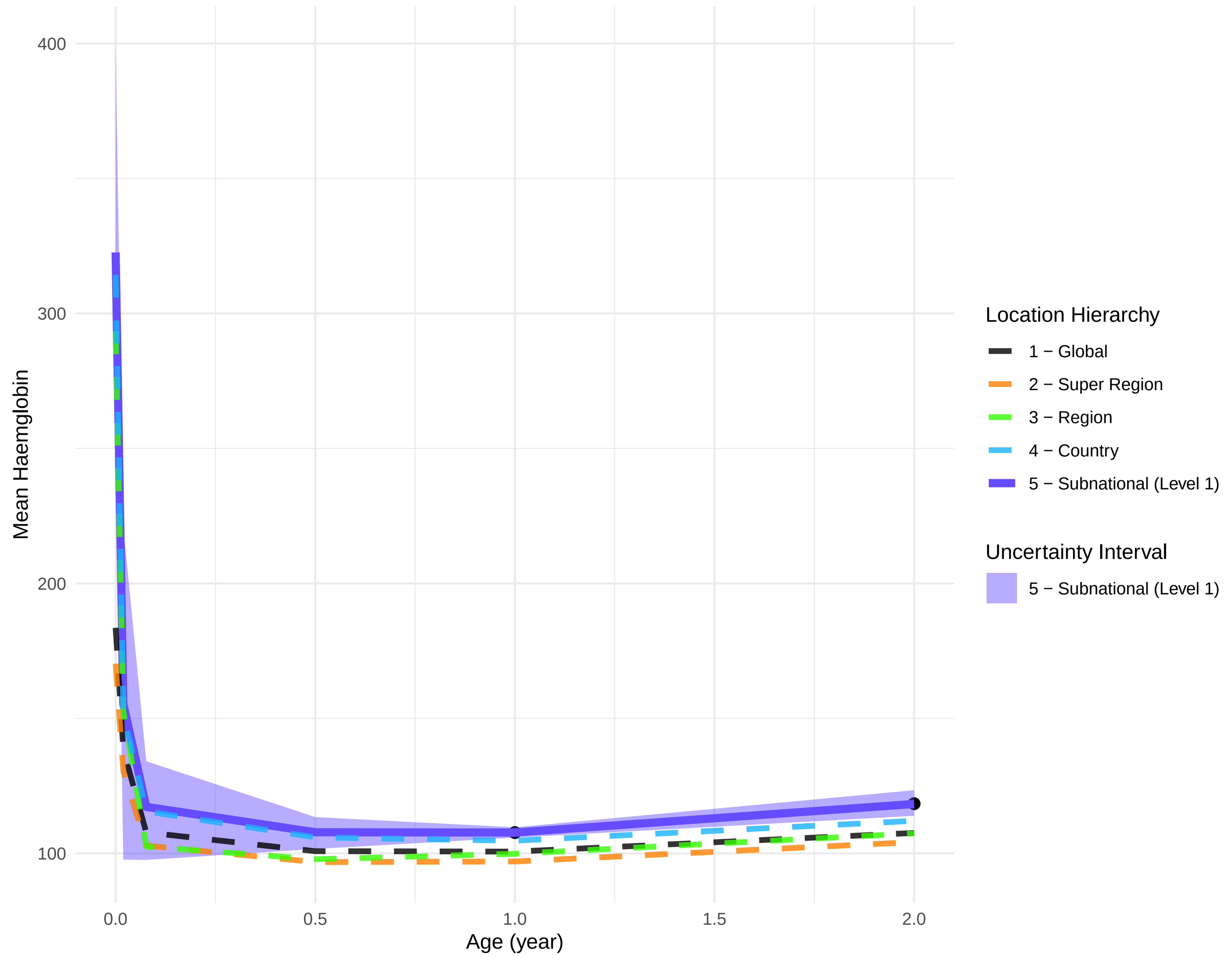

MR−BRT Age/Sex Fits − Males <2 Years Old − Mean Haemglobin − Nyeri
WHO−2001 Adjustment Method

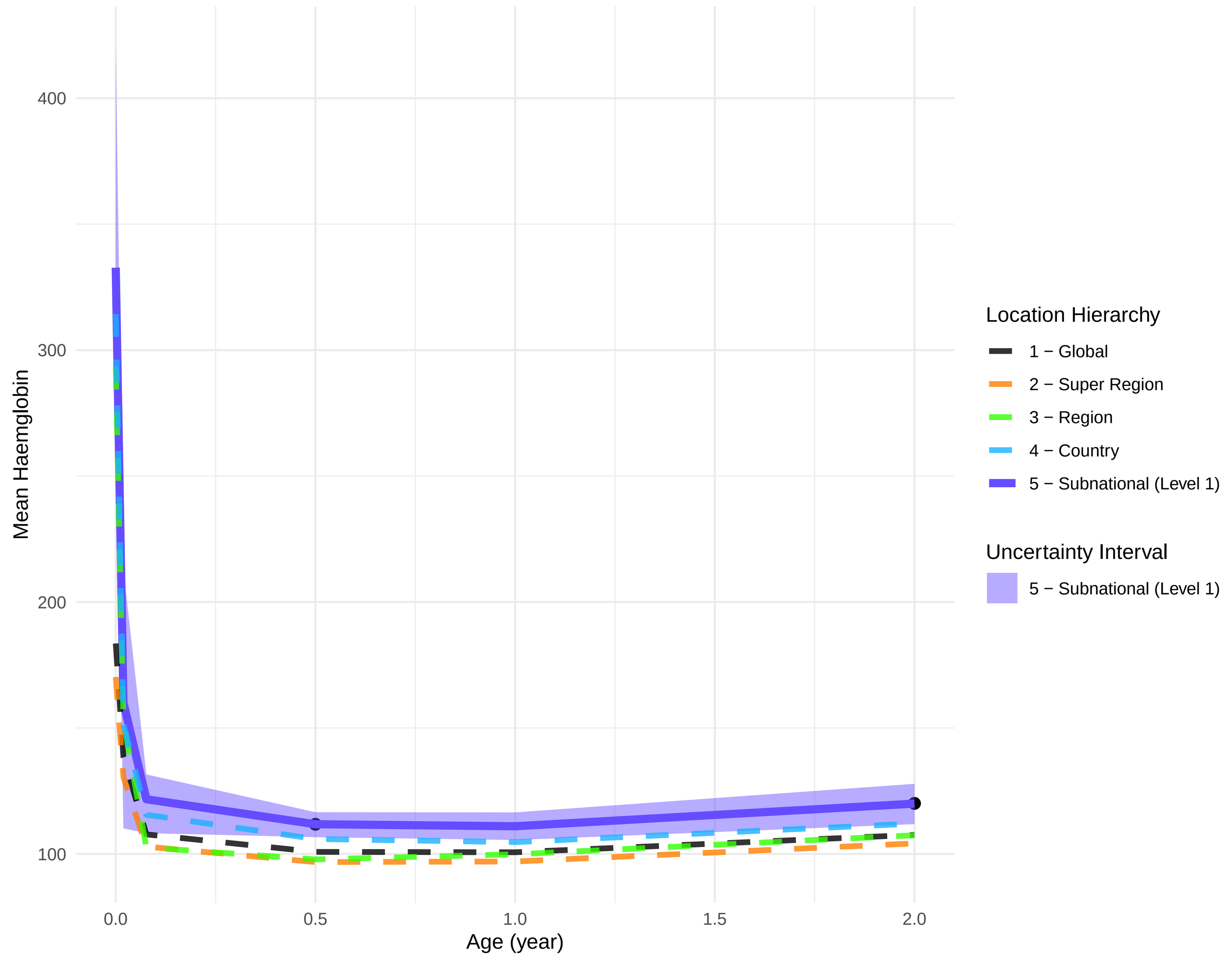

MR−BRT Age/Sex Fits − Males <2 Years Old − Mean Haemglobin − Samburu
WHO−2001 Adjustment Method

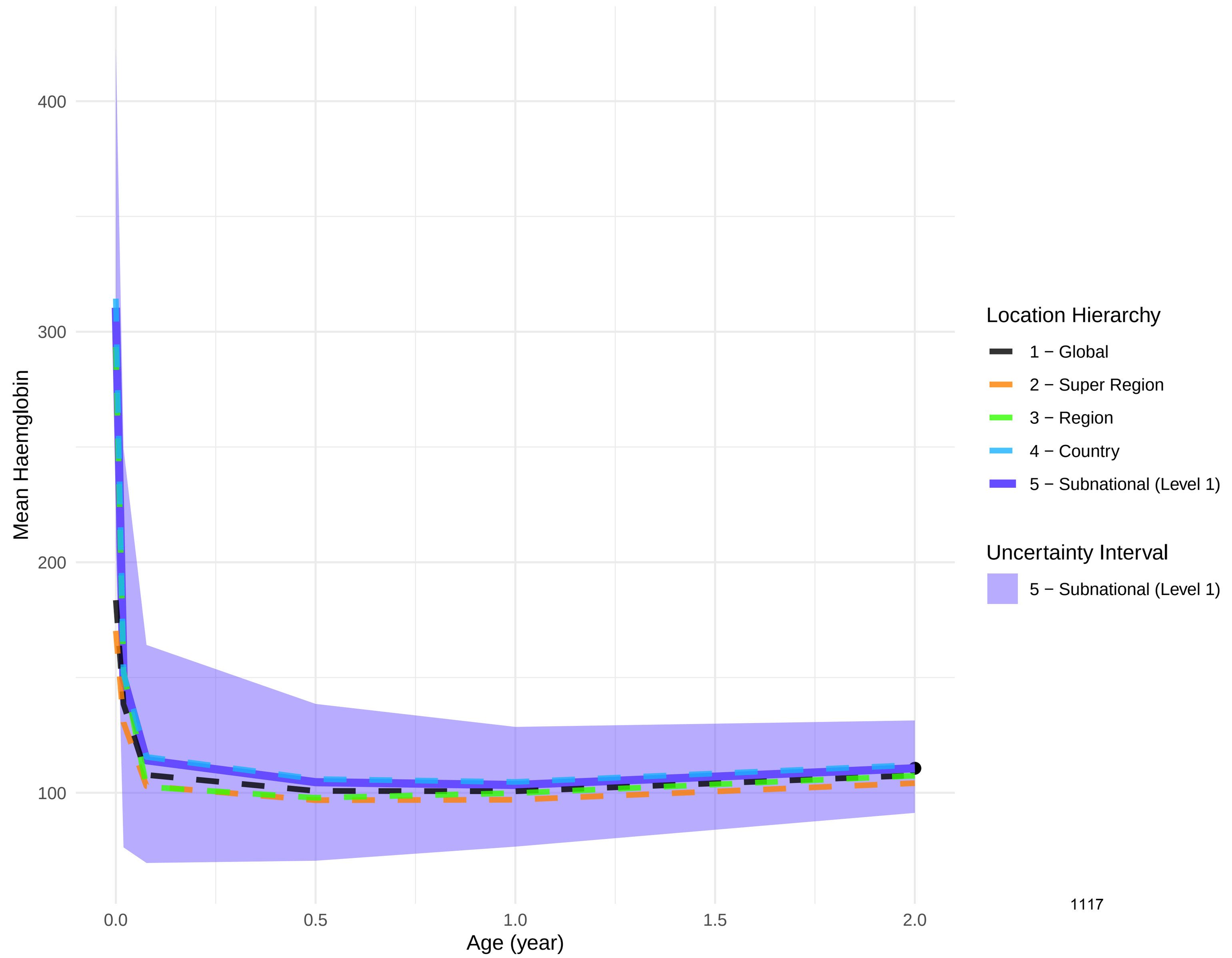

MR−BRT Age/Sex Fits − Males <2 Years Old − Mean Haemglobin − Siaya
WHO−2001 Adjustment Method

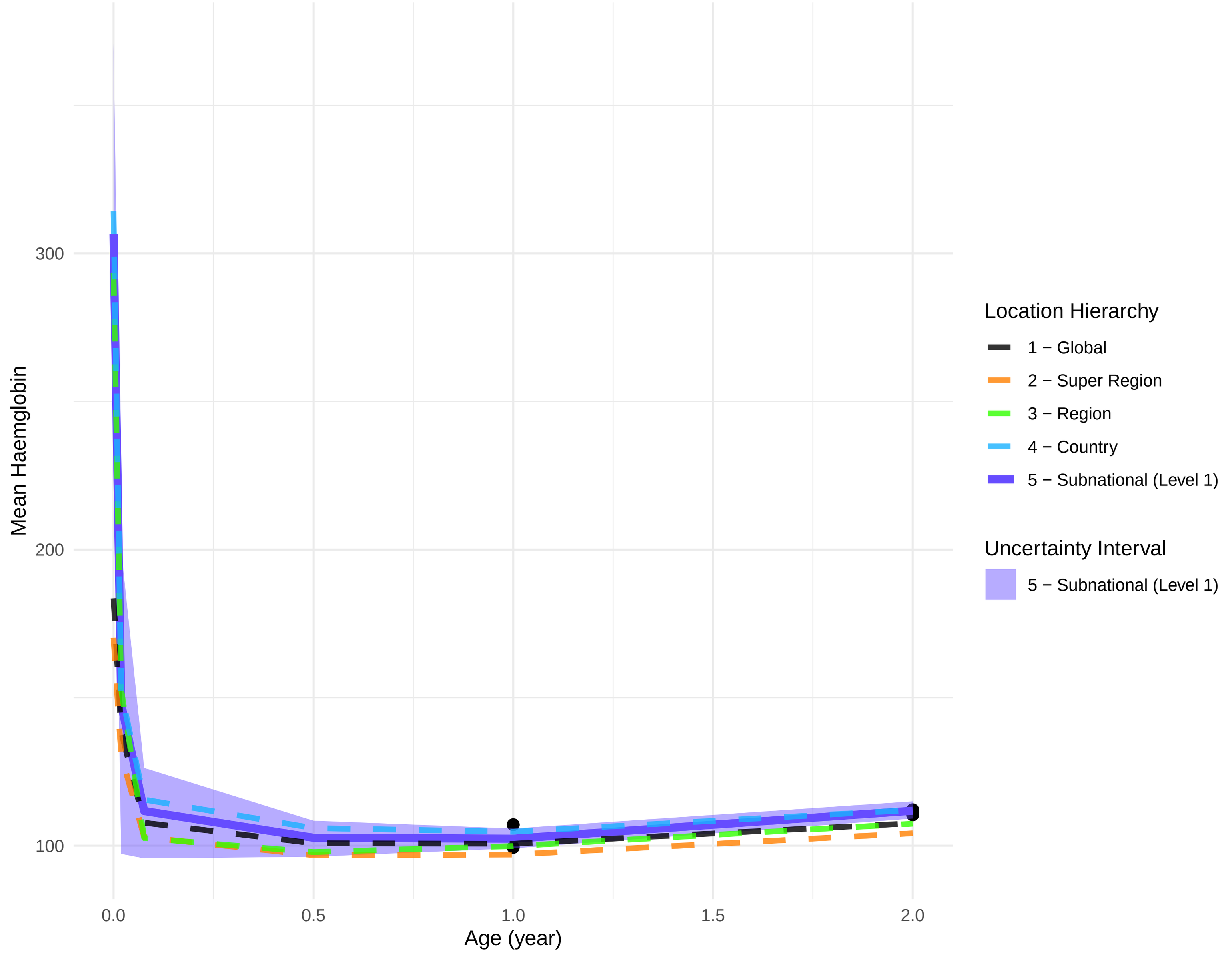

MR−BRT Age/Sex Fits − Males <2 Years Old − Mean Haemglobin − Taita Taveta
WHO−2001 Adjustment Method

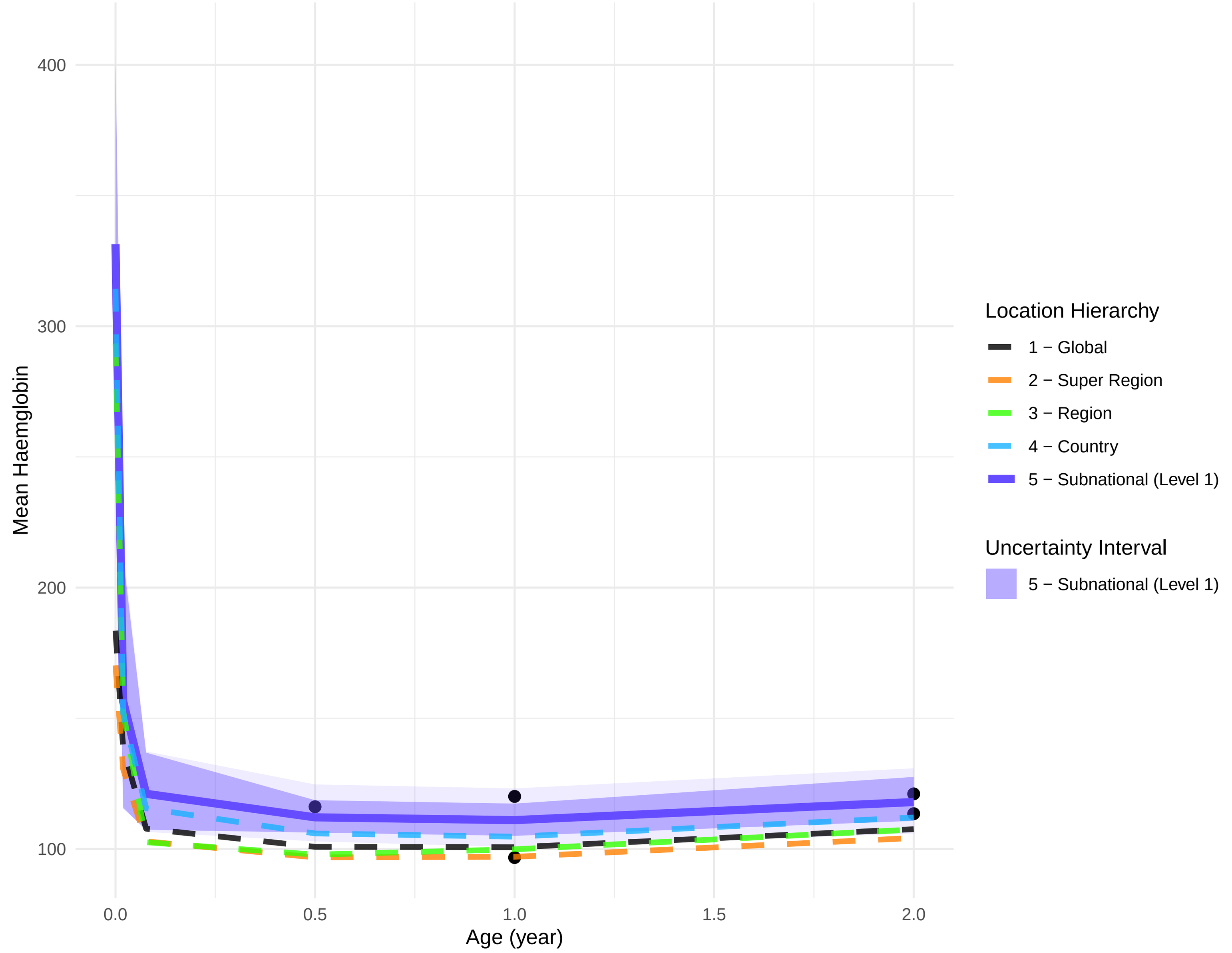

MR−BRT Age/Sex Fits − Males <2 Years Old − Mean Haemglobin − Tana River
WHO−2001 Adjustment Method

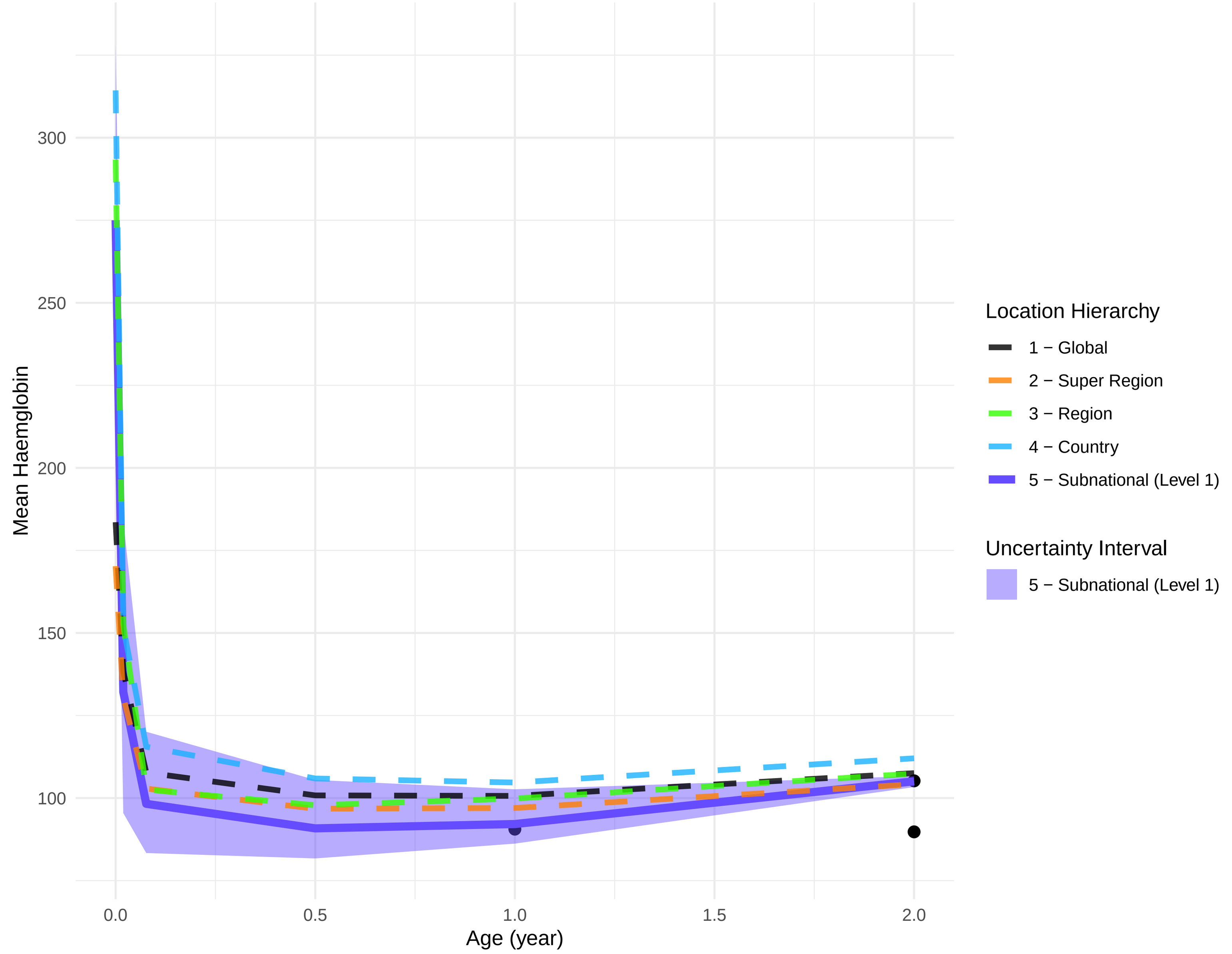

MR−BRT Age/Sex Fits − Males <2 Years Old − Mean Haemglobin − Tharaka Nithi
WHO−2001 Adjustment Method

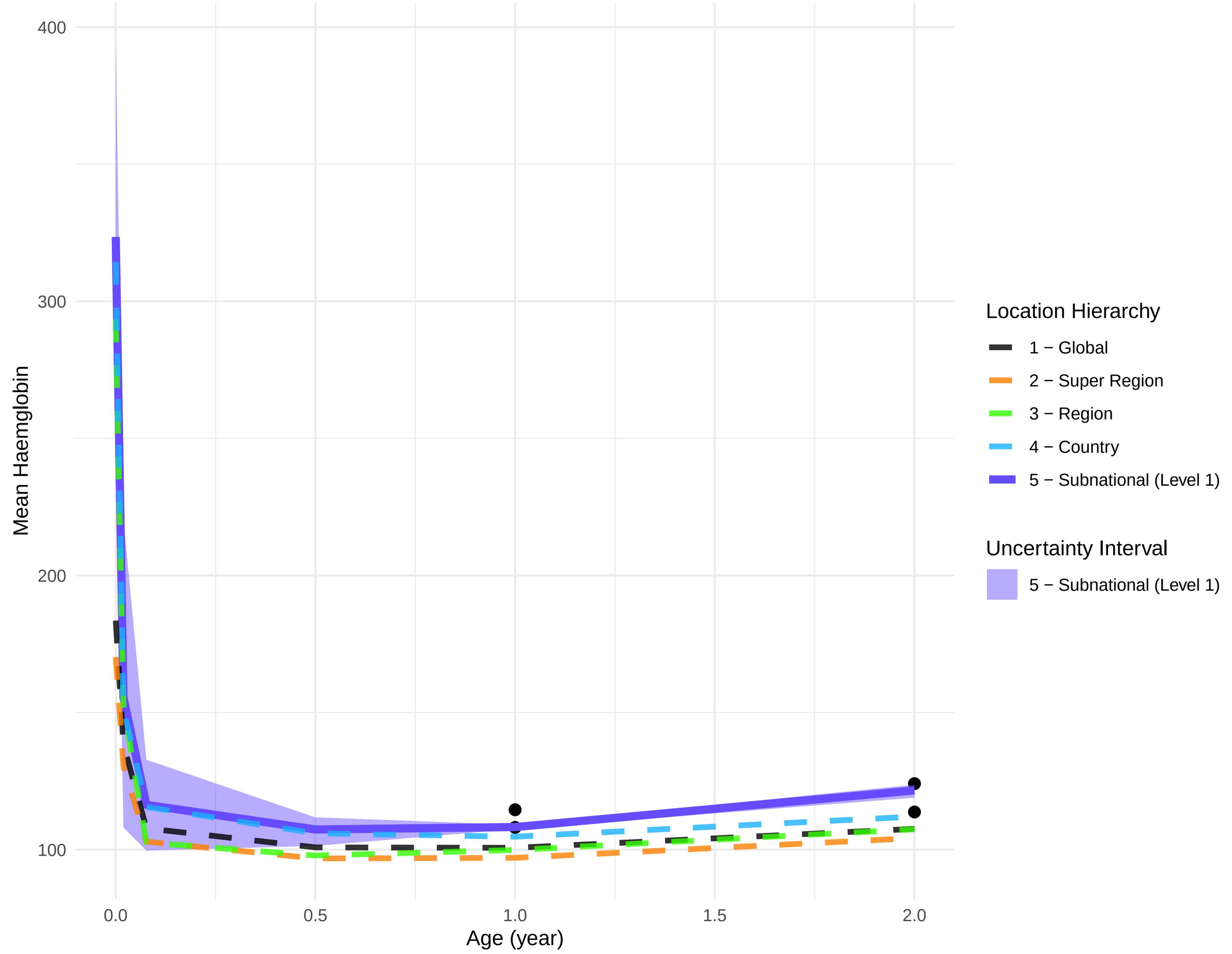

MR−BRT Age/Sex Fits − Males <2 Years Old − Mean Haemglobin − Trans Nzoia
WHO−2001 Adjustment Method

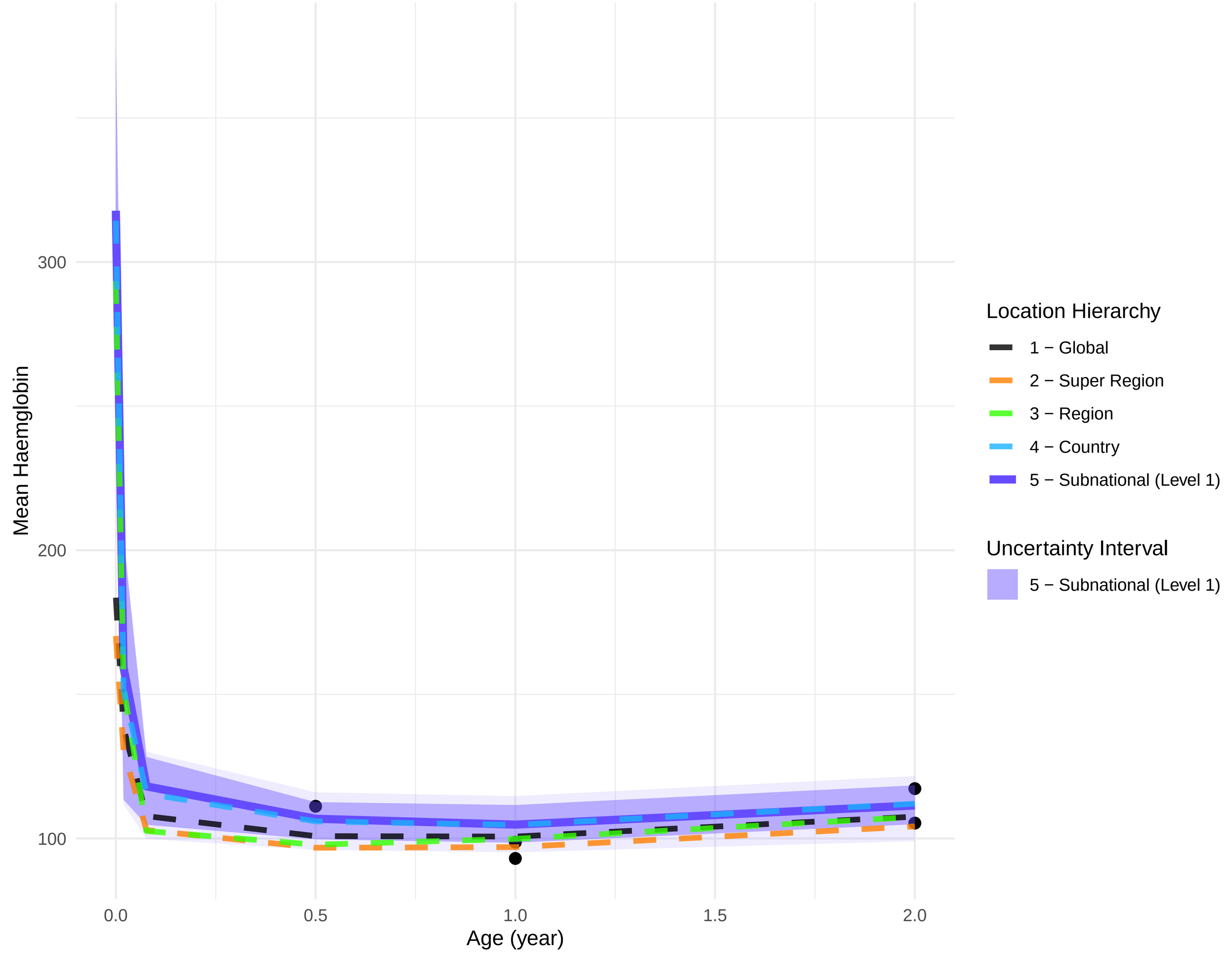

MR−BRT Age/Sex Fits − Males <2 Years Old − Mean Haemglobin − Turkana
WHO−2001 Adjustment Method

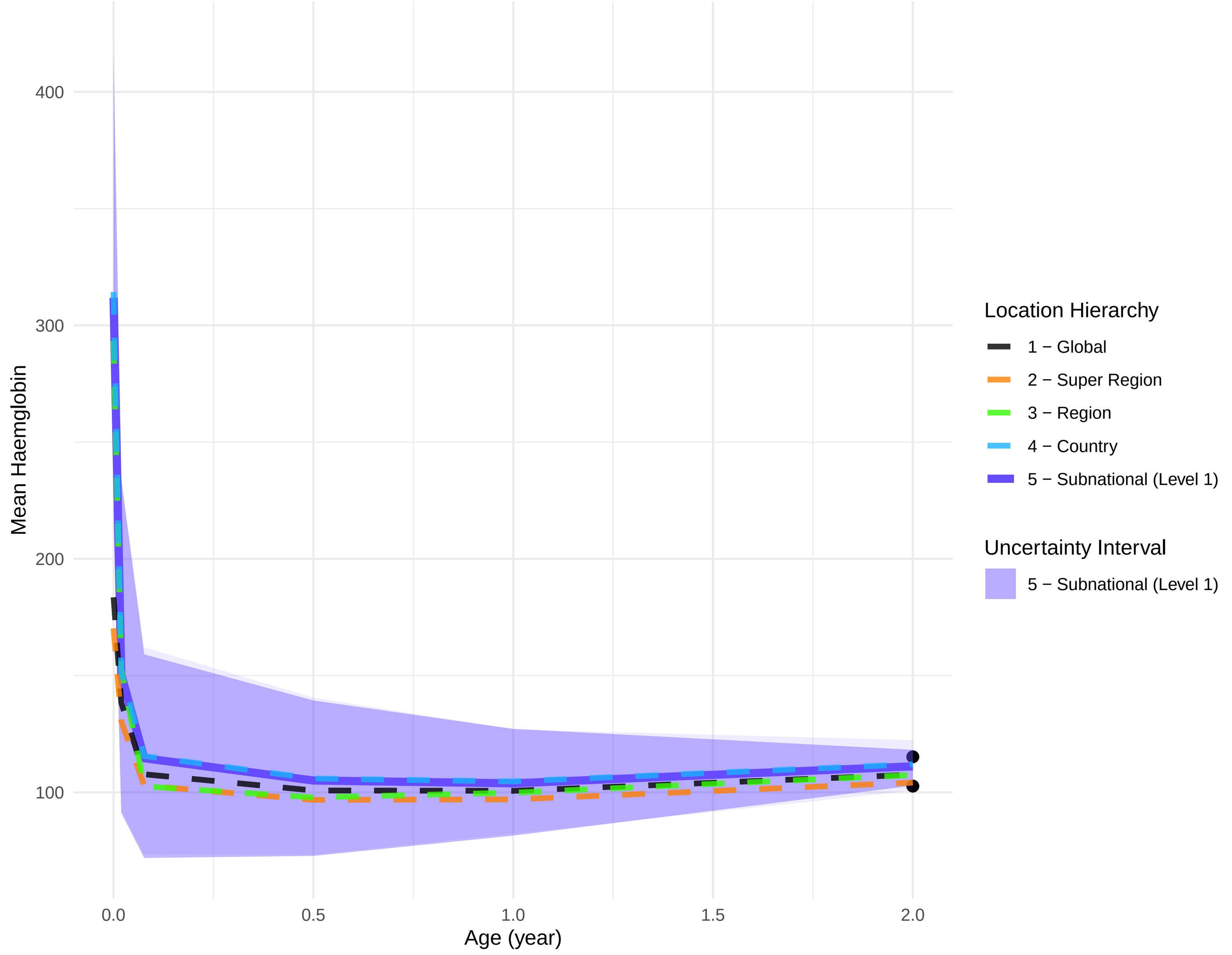

MR−BRT Age/Sex Fits − Males <2 Years Old − Mean Haemglobin − Uasin Gishu
WHO−2001 Adjustment Method

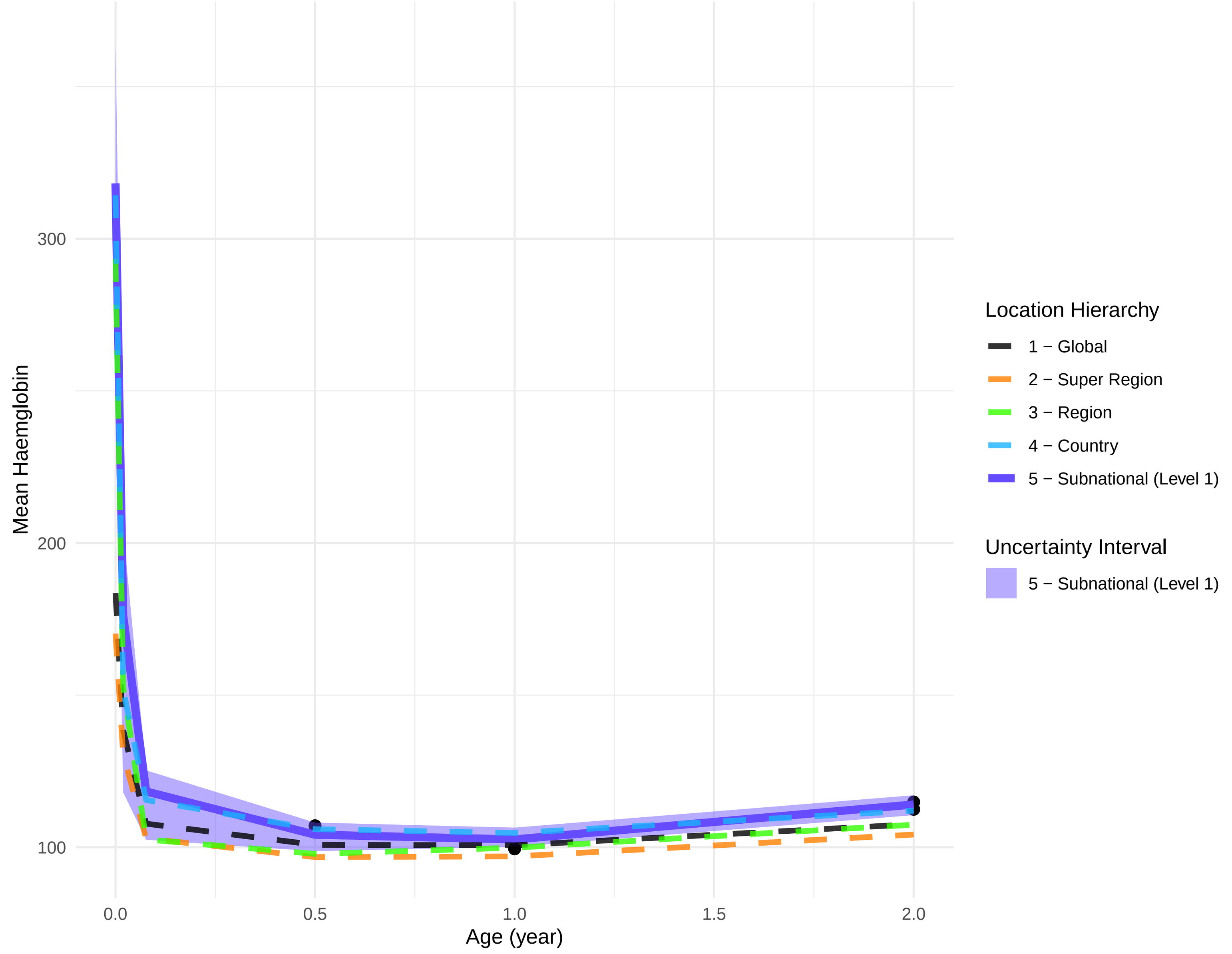

MR−BRT Age/Sex Fits − Males <2 Years Old − Mean Haemglobin − Vihiga
WHO−2001 Adjustment Method

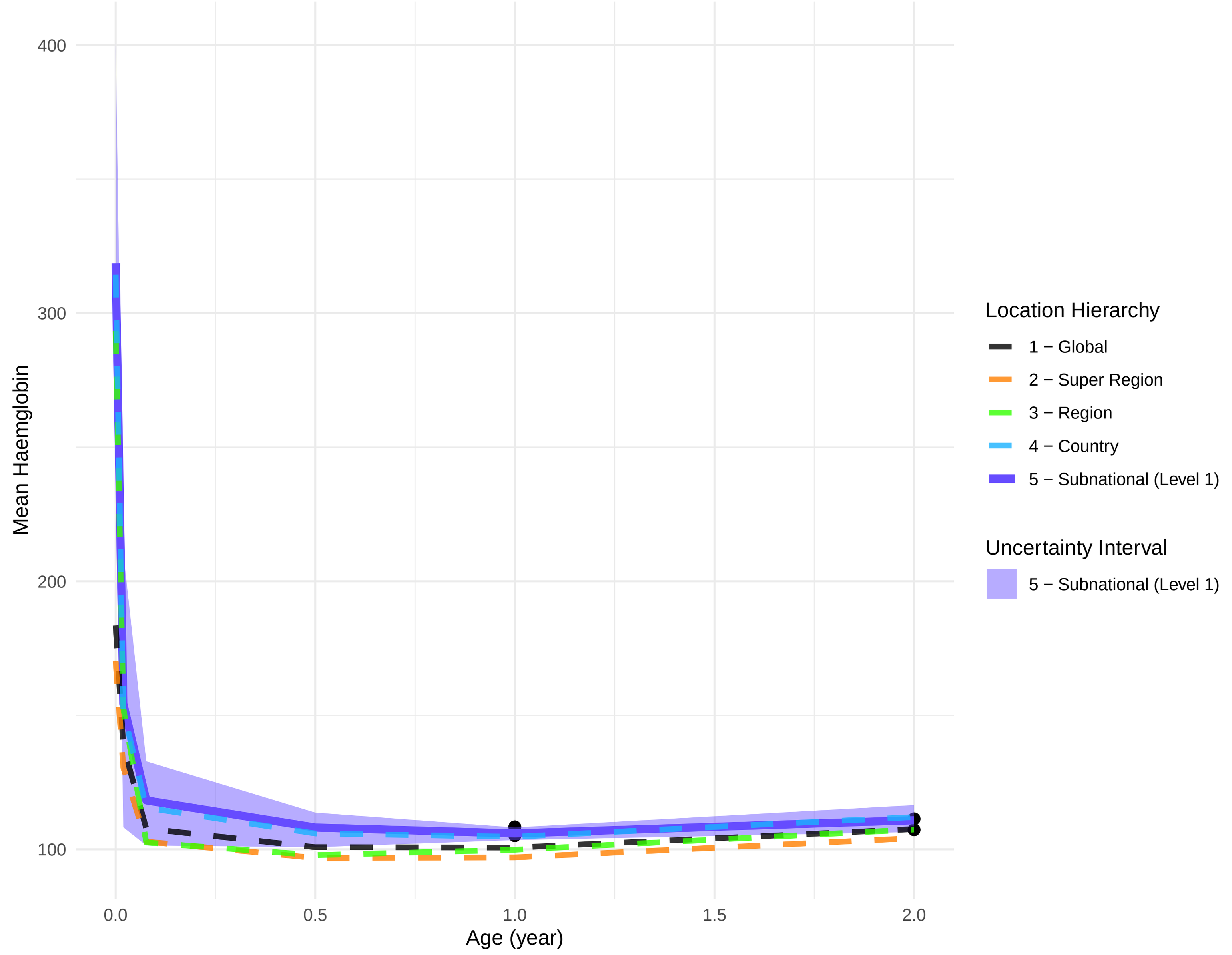

MR−BRT Age/Sex Fits − Males <2 Years Old − Mean Haemglobin − Wajir
WHO−2001 Adjustment Method

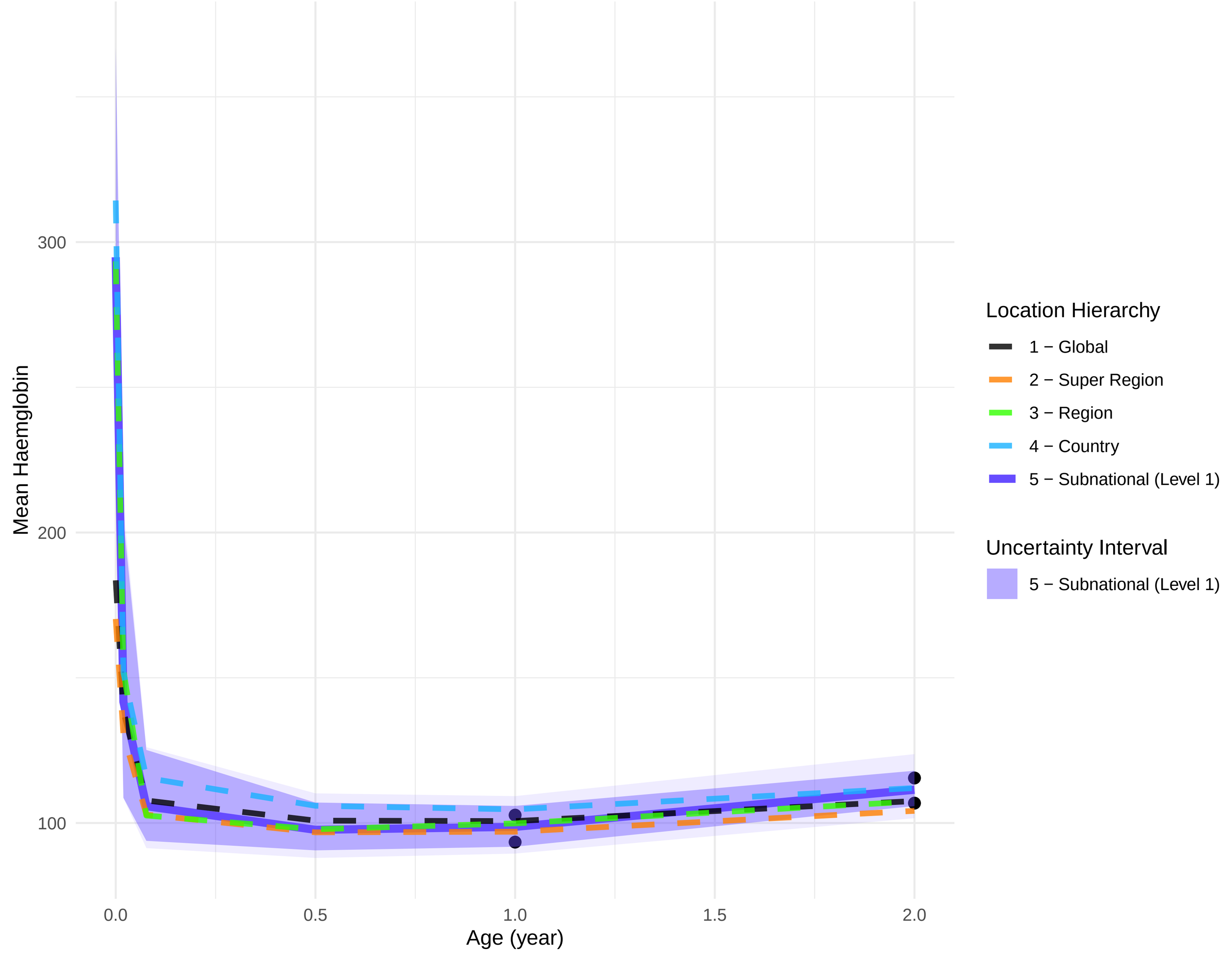

MR−BRT Age/Sex Fits − Males <2 Years Old − Mean Haemglobin − West Pokot
WHO−2001 Adjustment Method

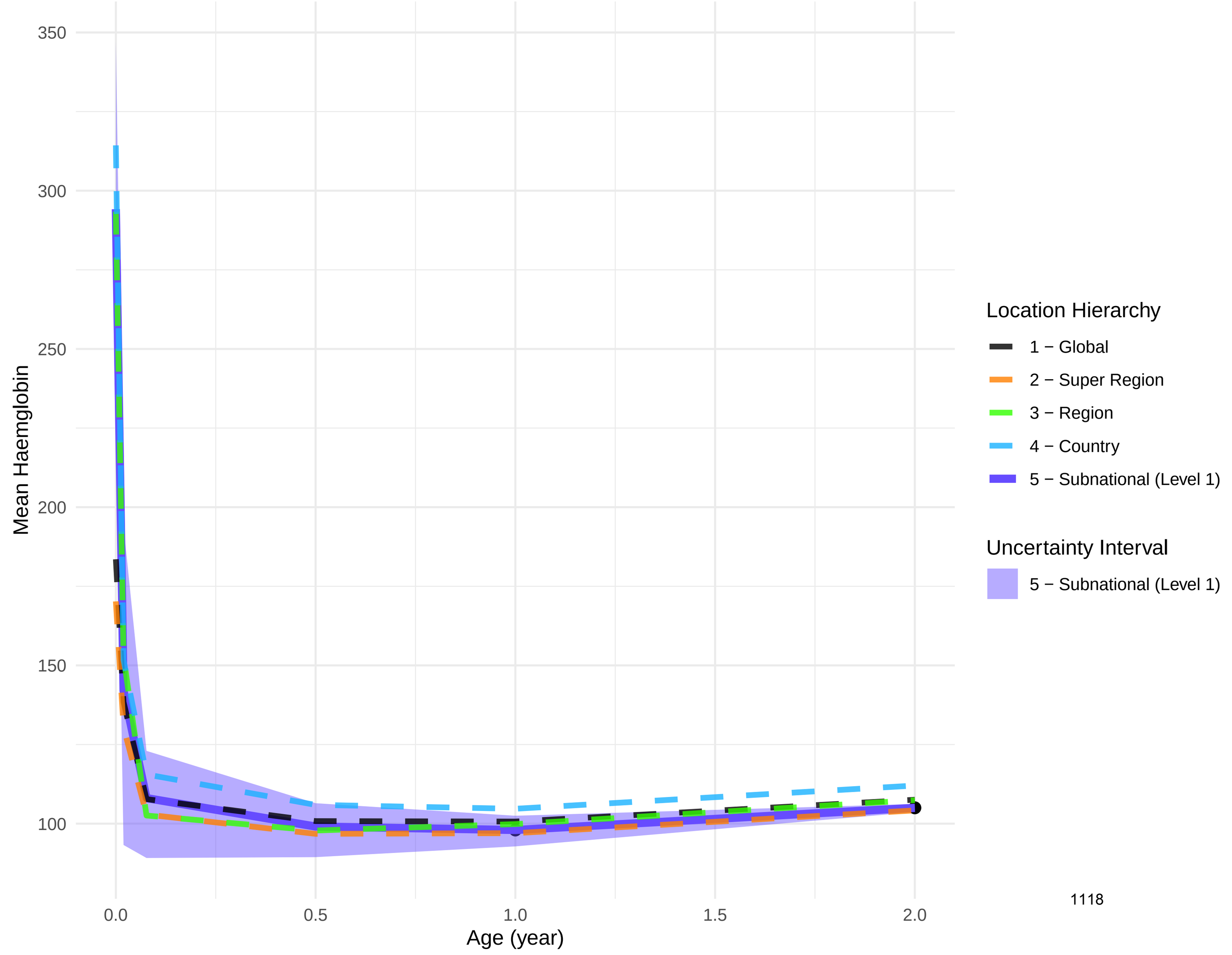

MR−BRT Age/Sex Fits − Males <2 Years Old − Mean Haemglobin − Madagascar
WHO−2001 Adjustment Method

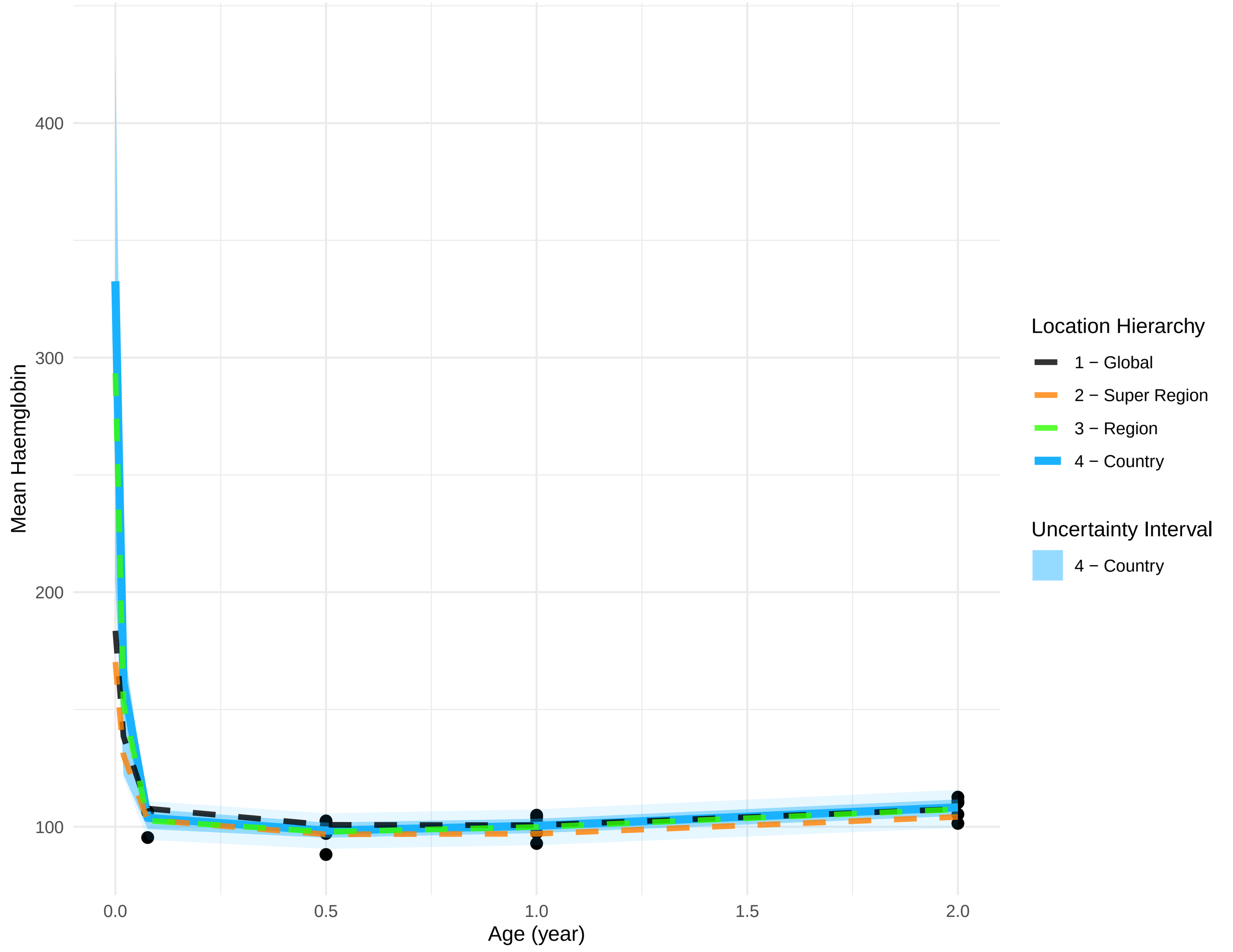

MR−BRT Age/Sex Fits − Males <2 Years Old − Mean Haemglobin − Malawi
WHO−2001 Adjustment Method

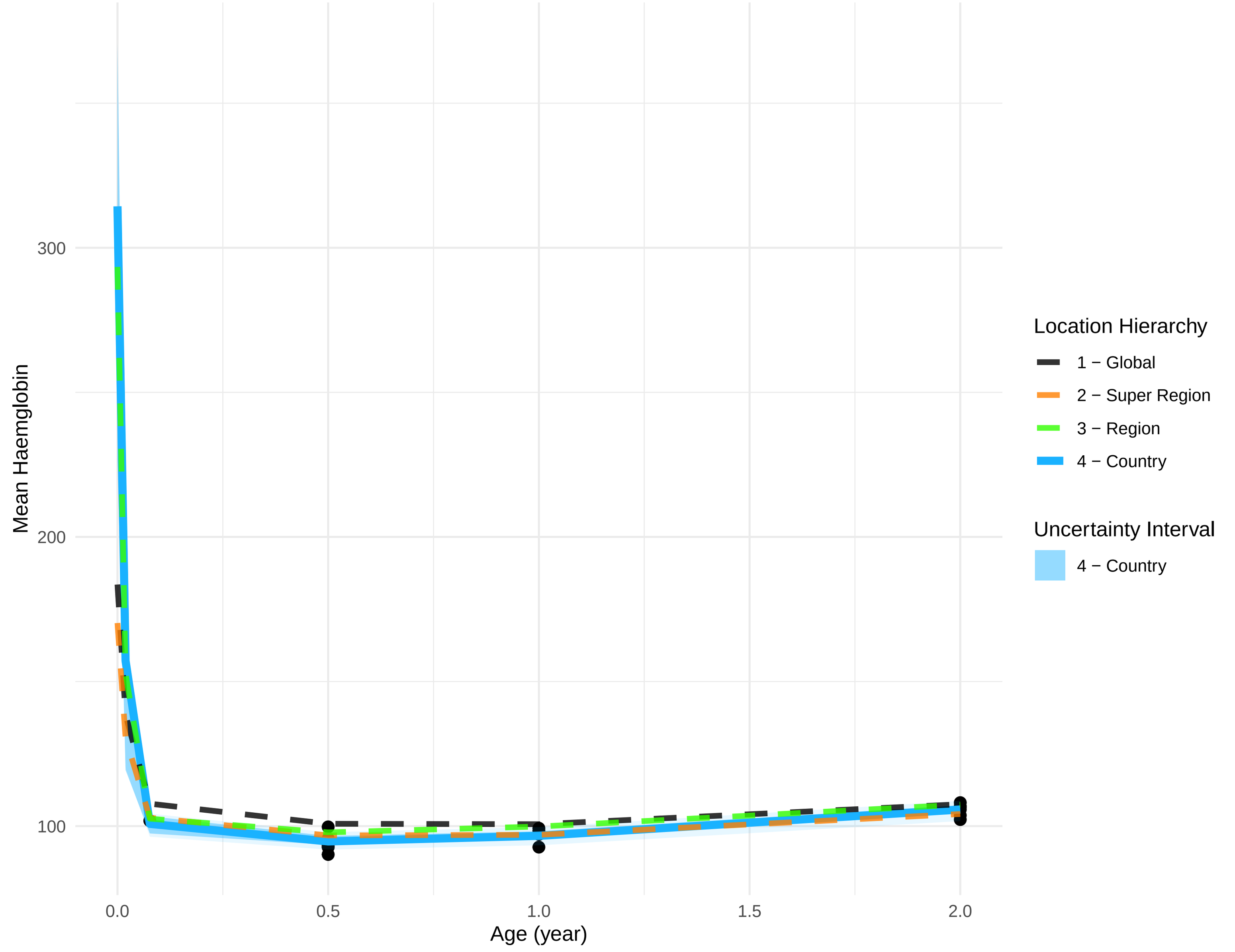

MR−BRT Age/Sex Fits − Males <2 Years Old − Mean Haemglobin − Mozambique
WHO−2001 Adjustment Method

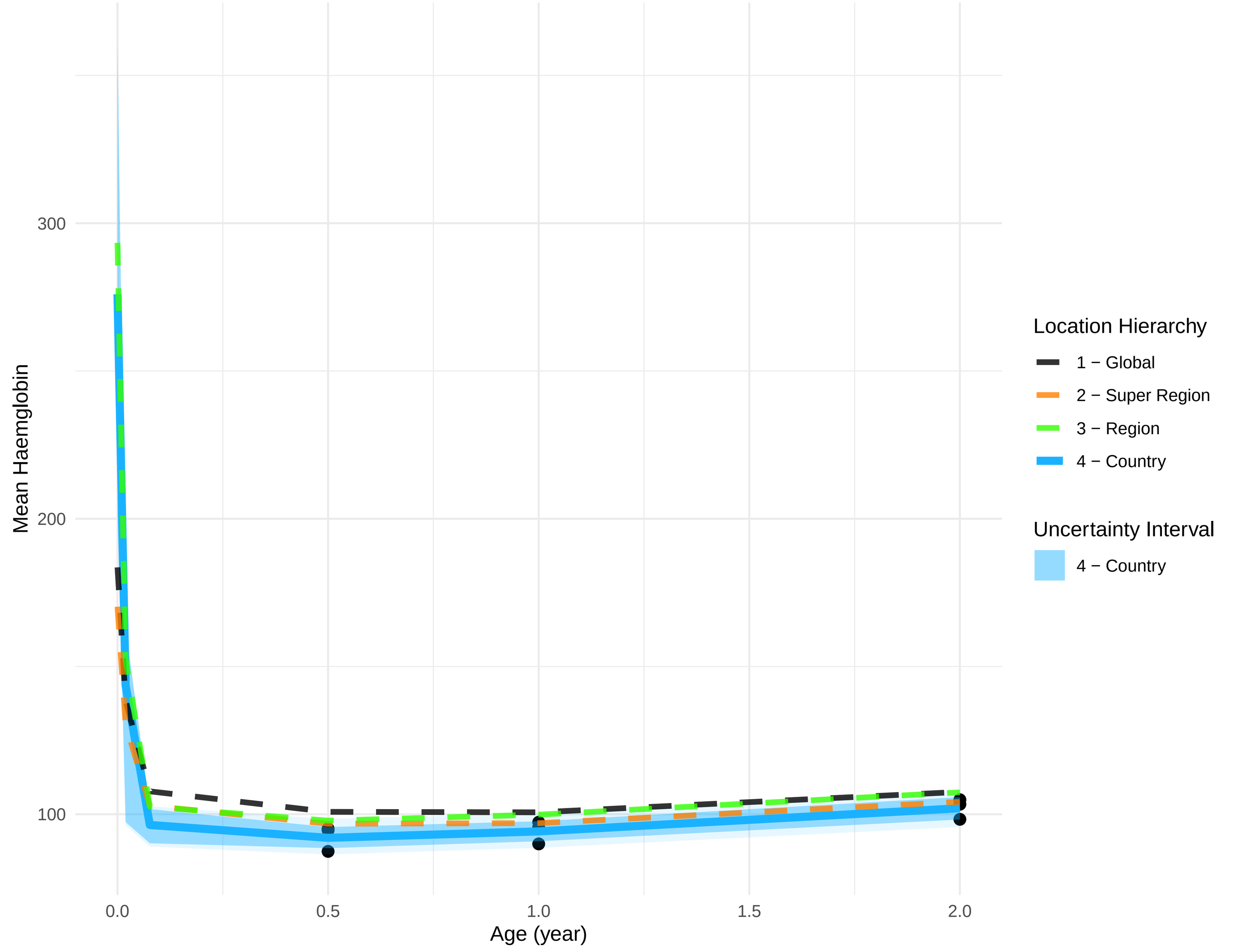

MR−BRT Age/Sex Fits − Males <2 Years Old − Mean Haemglobin − Rwanda
WHO−2001 Adjustment Method

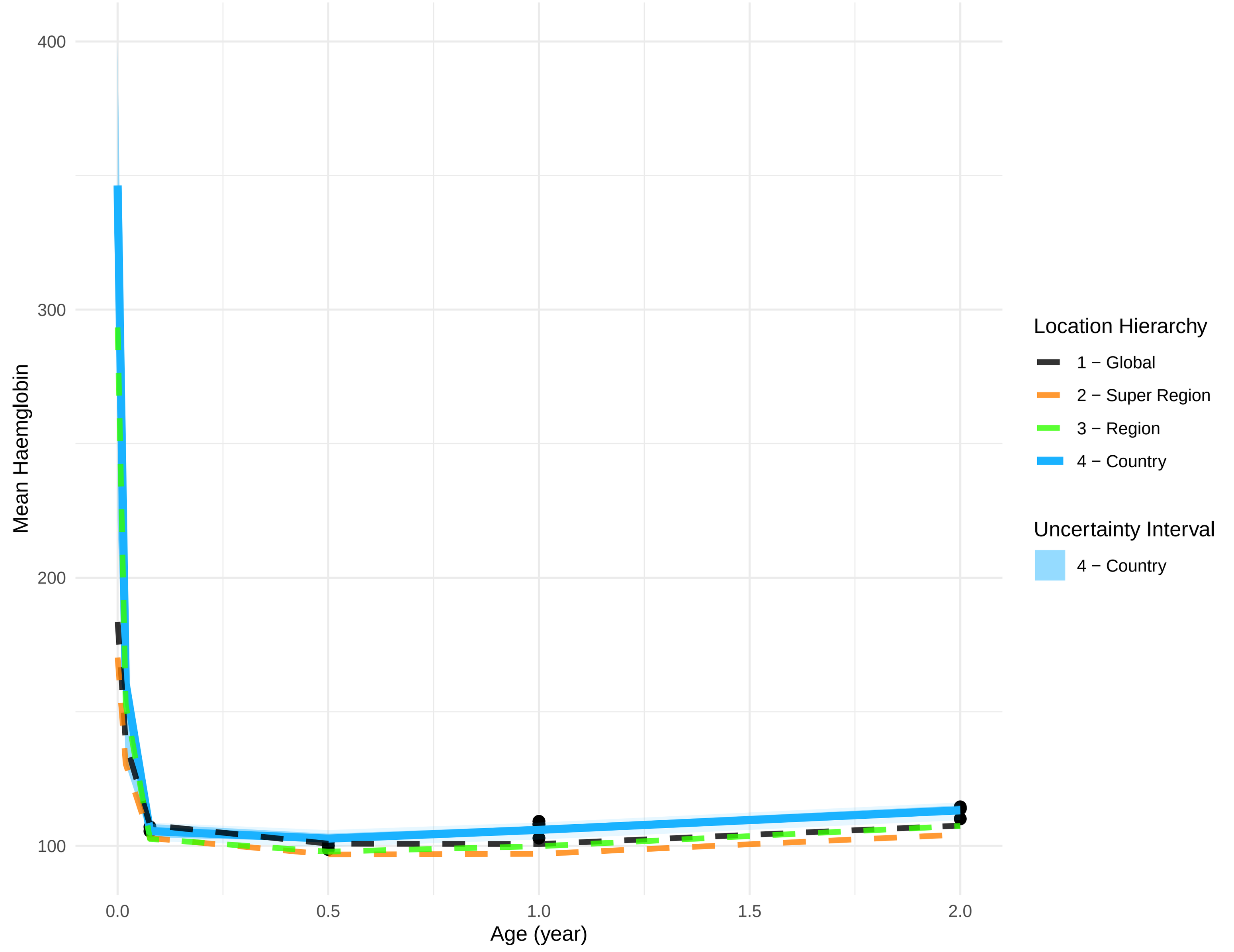

MR−BRT Age/Sex Fits − Males <2 Years Old − Mean Haemglobin − Uganda
WHO−2001 Adjustment Method

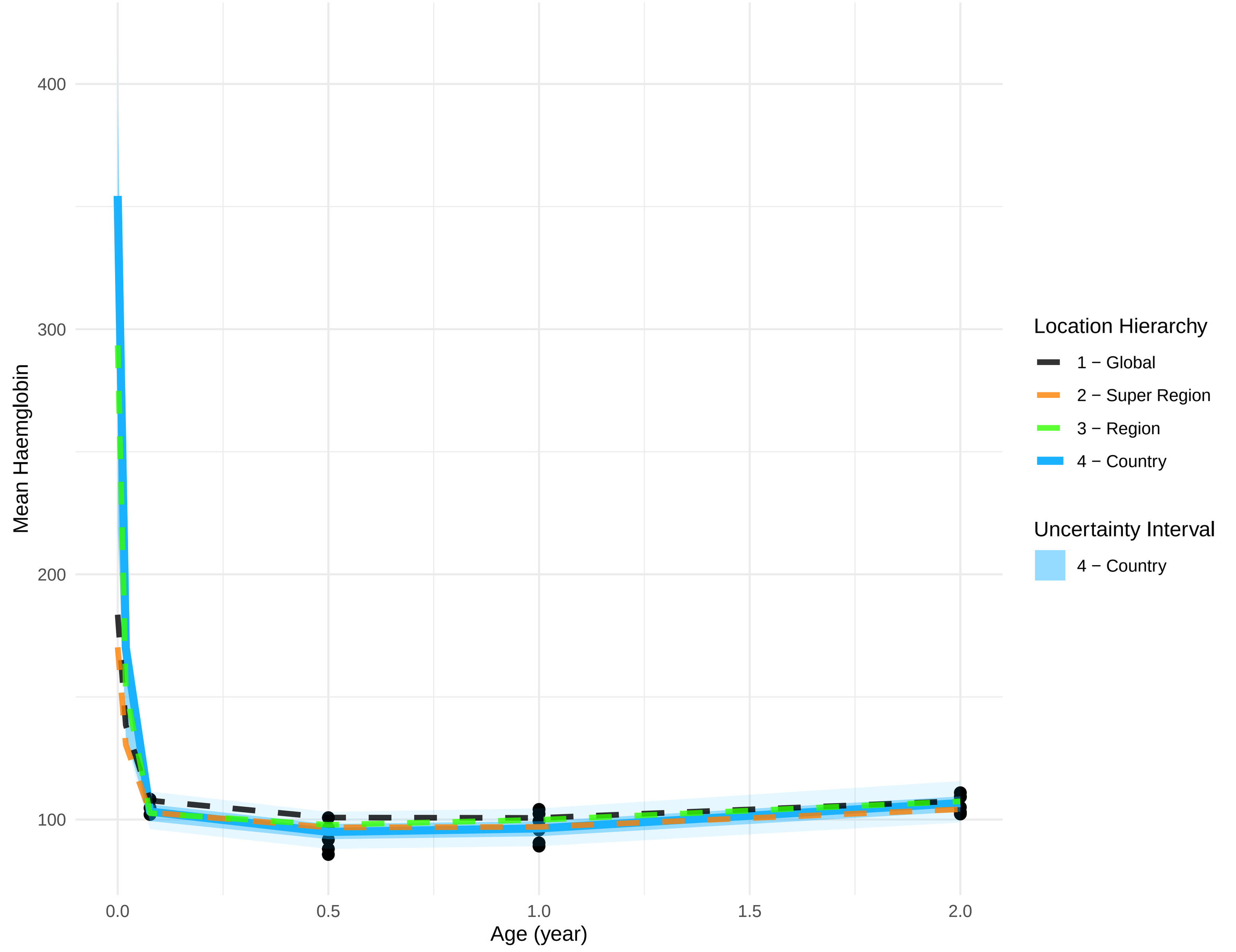

MR−BRT Age/Sex Fits − Males <2 Years Old − Mean Haemglobin − United Republic of Tanzania
WHO−2001 Adjustment Method

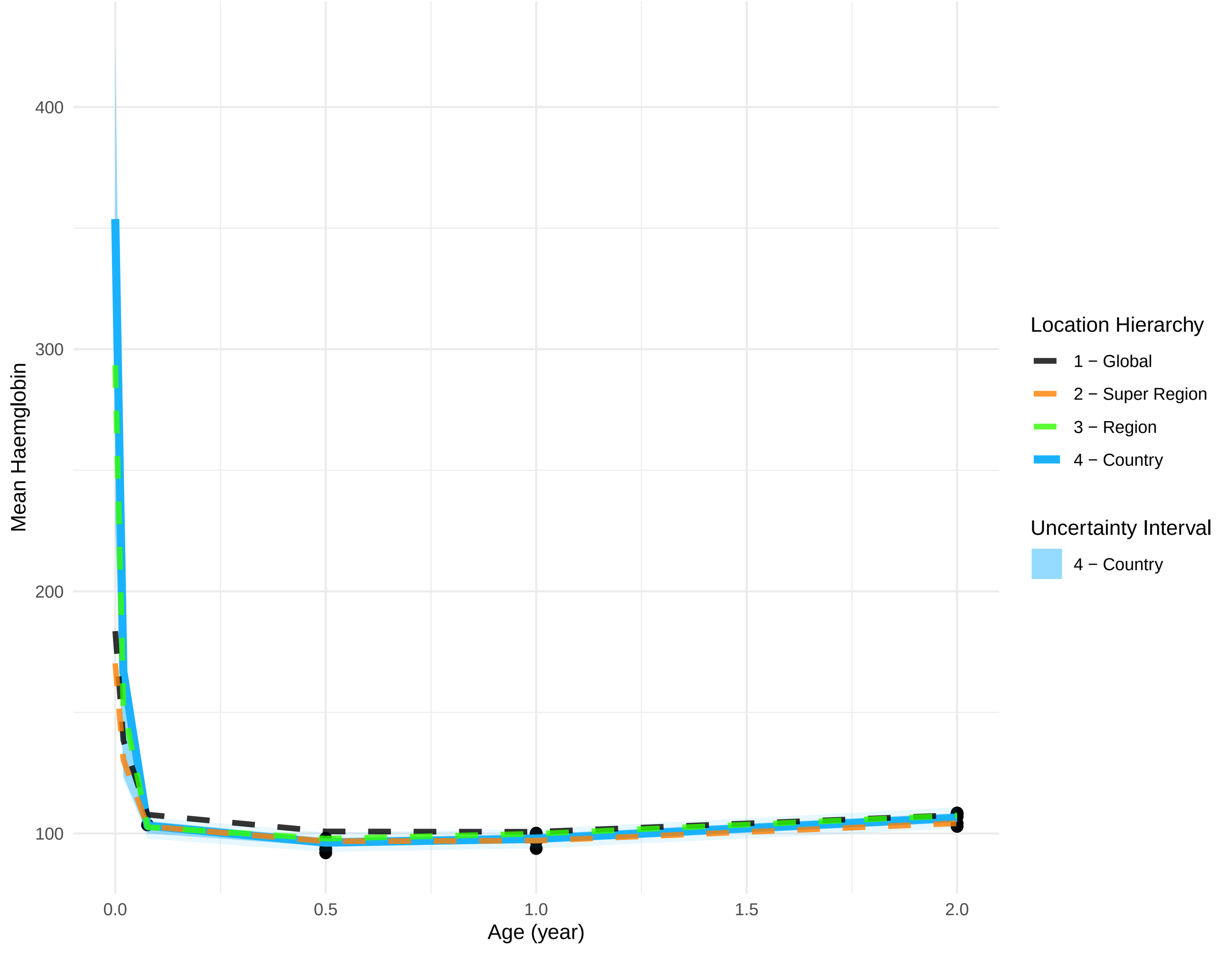

MR−BRT Age/Sex Fits − Males <2 Years Old − Mean Haemglobin − Zambia
WHO−2001 Adjustment Method

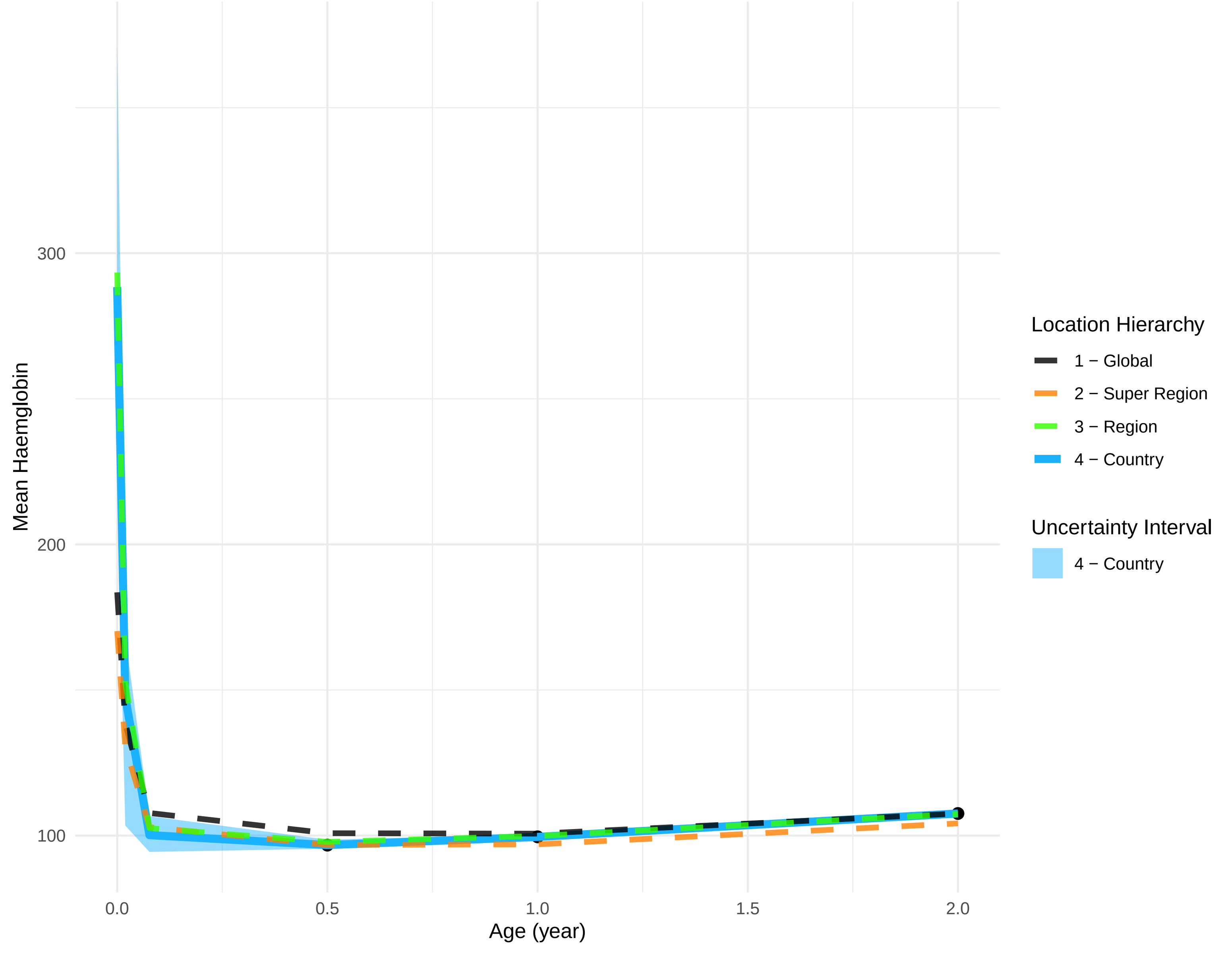

MR−BRT Age/Sex Fits − Males <2 Years Old − Mean Haemglobin − Southern Sub−Saharan Africa
WHO−2001 Adjustment Method

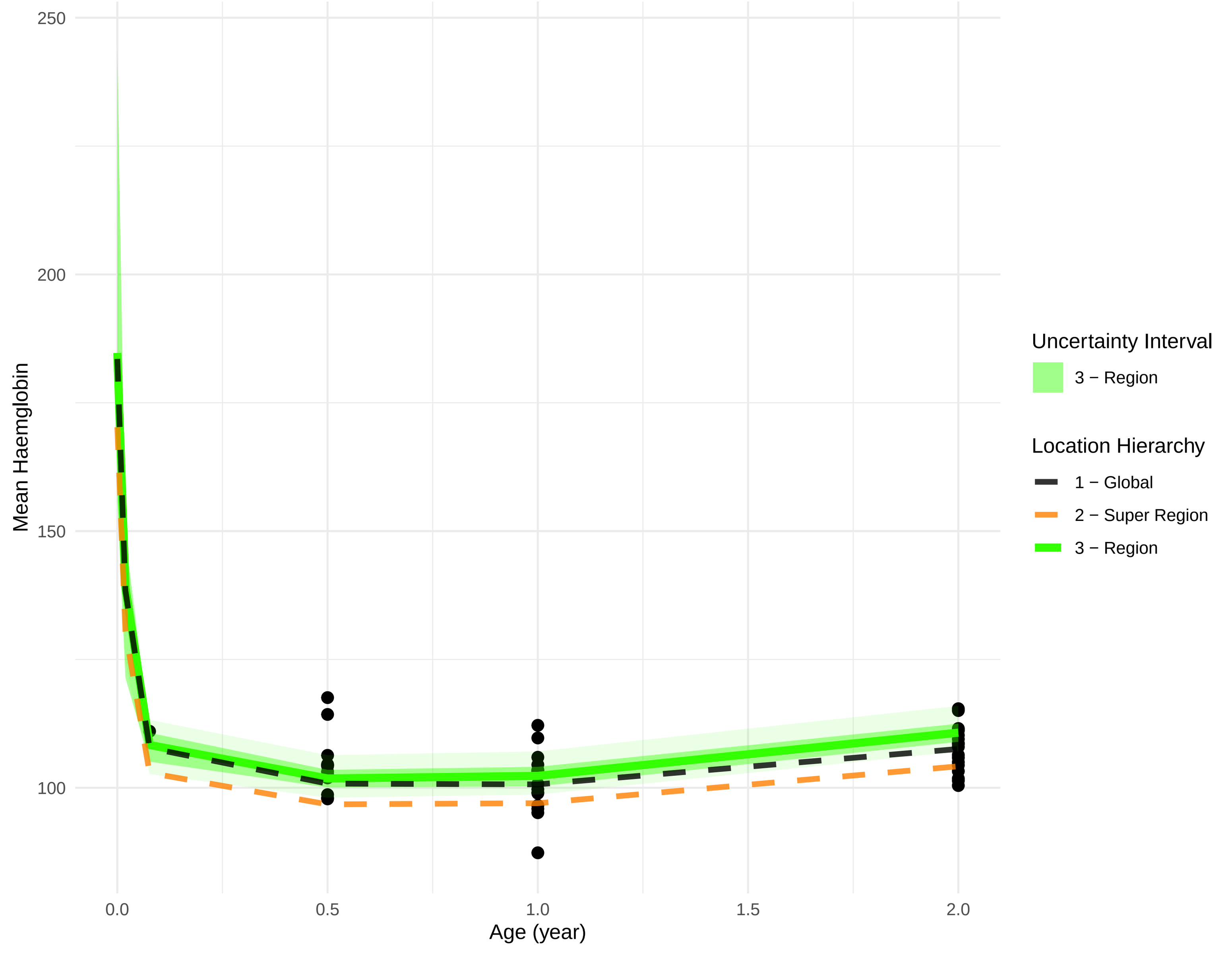

MR−BRT Age/Sex Fits − Males <2 Years Old − Mean Haemglobin − Eswatini
WHO−2001 Adjustment Method

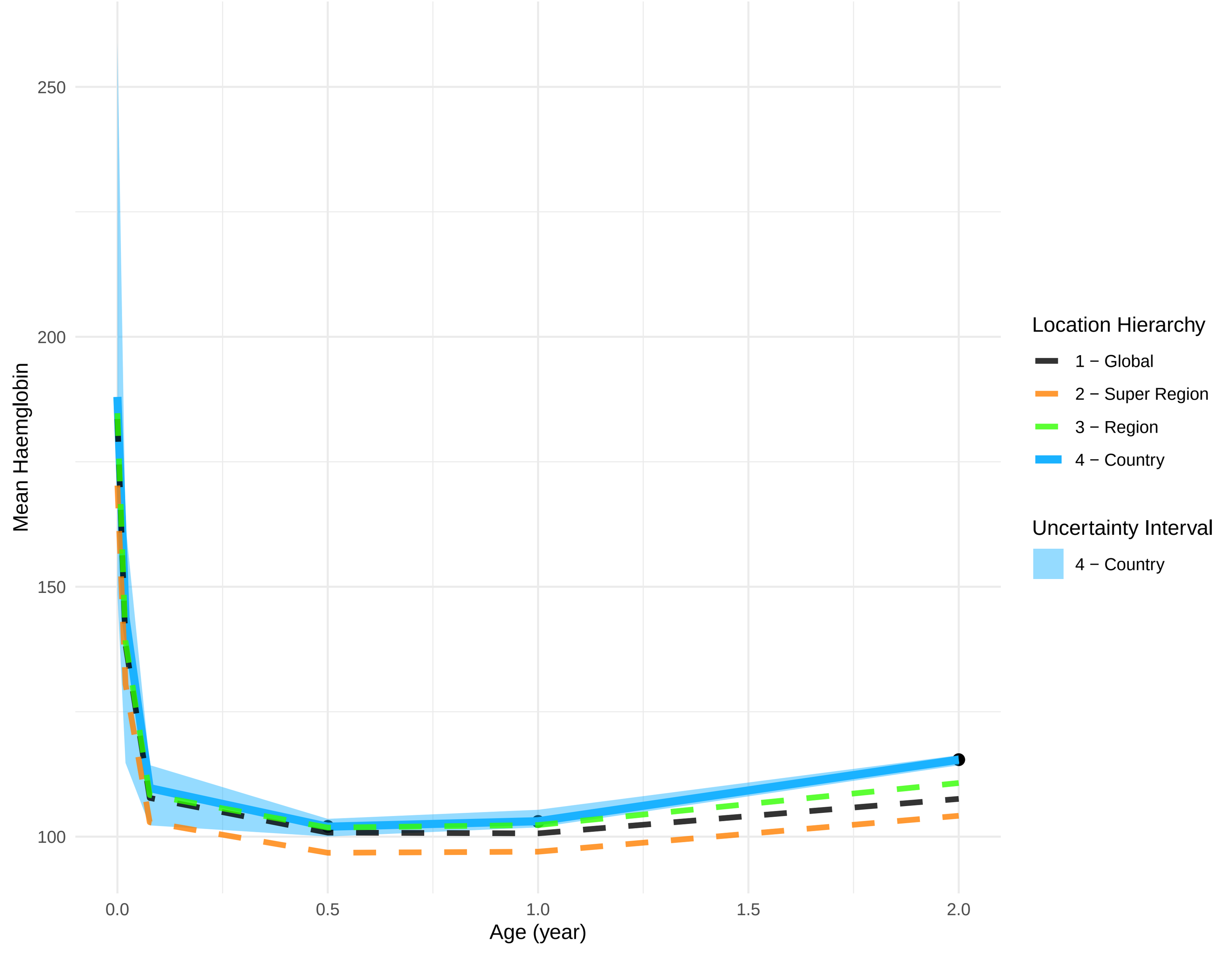

MR−BRT Age/Sex Fits − Males <2 Years Old − Mean Haemglobin − Lesotho
WHO−2001 Adjustment Method

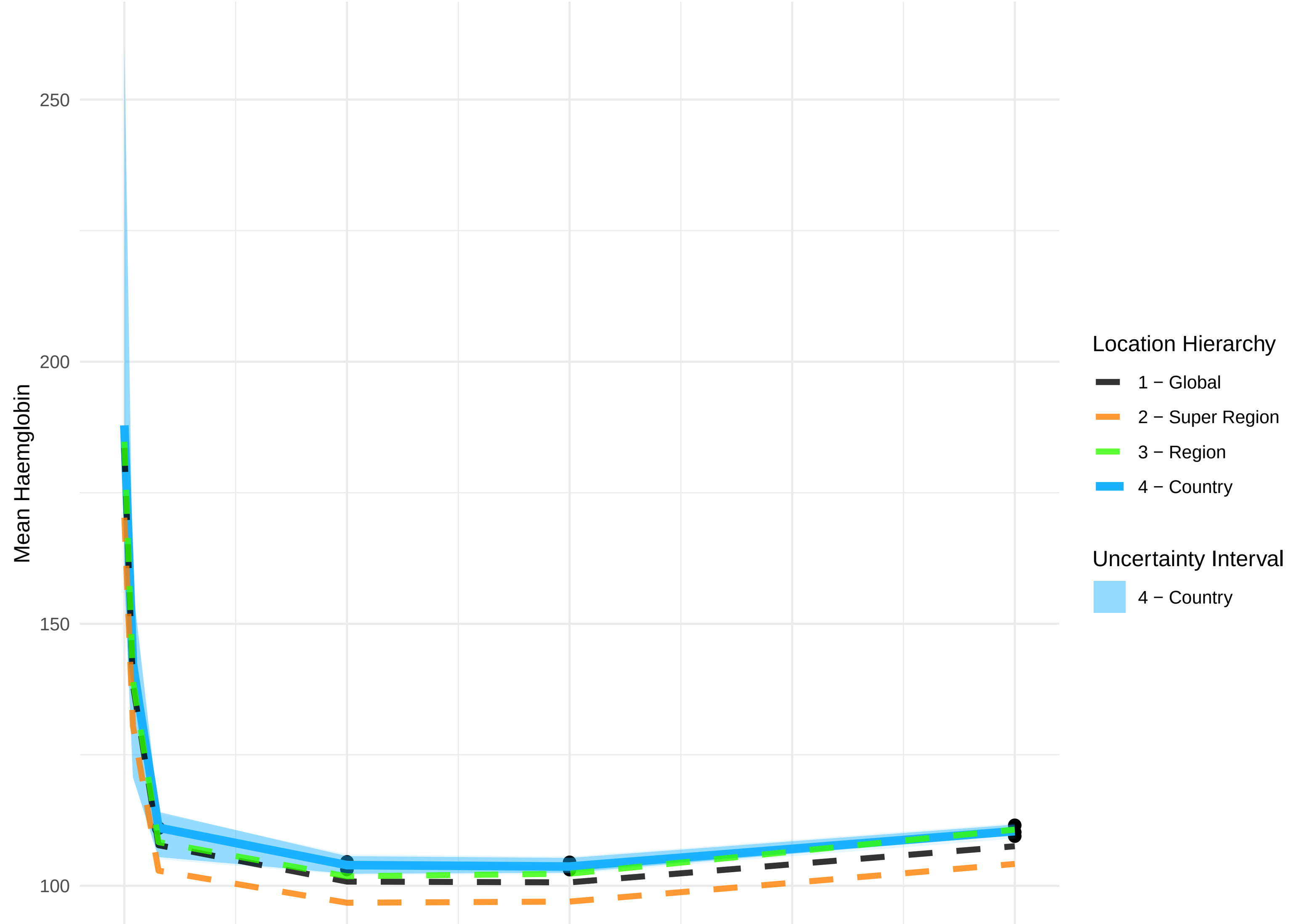

MR−BRT Age/Sex Fits − Males <2 Years Old − Mean Haemglobin − Namibia
WHO−2001 Adjustment Method

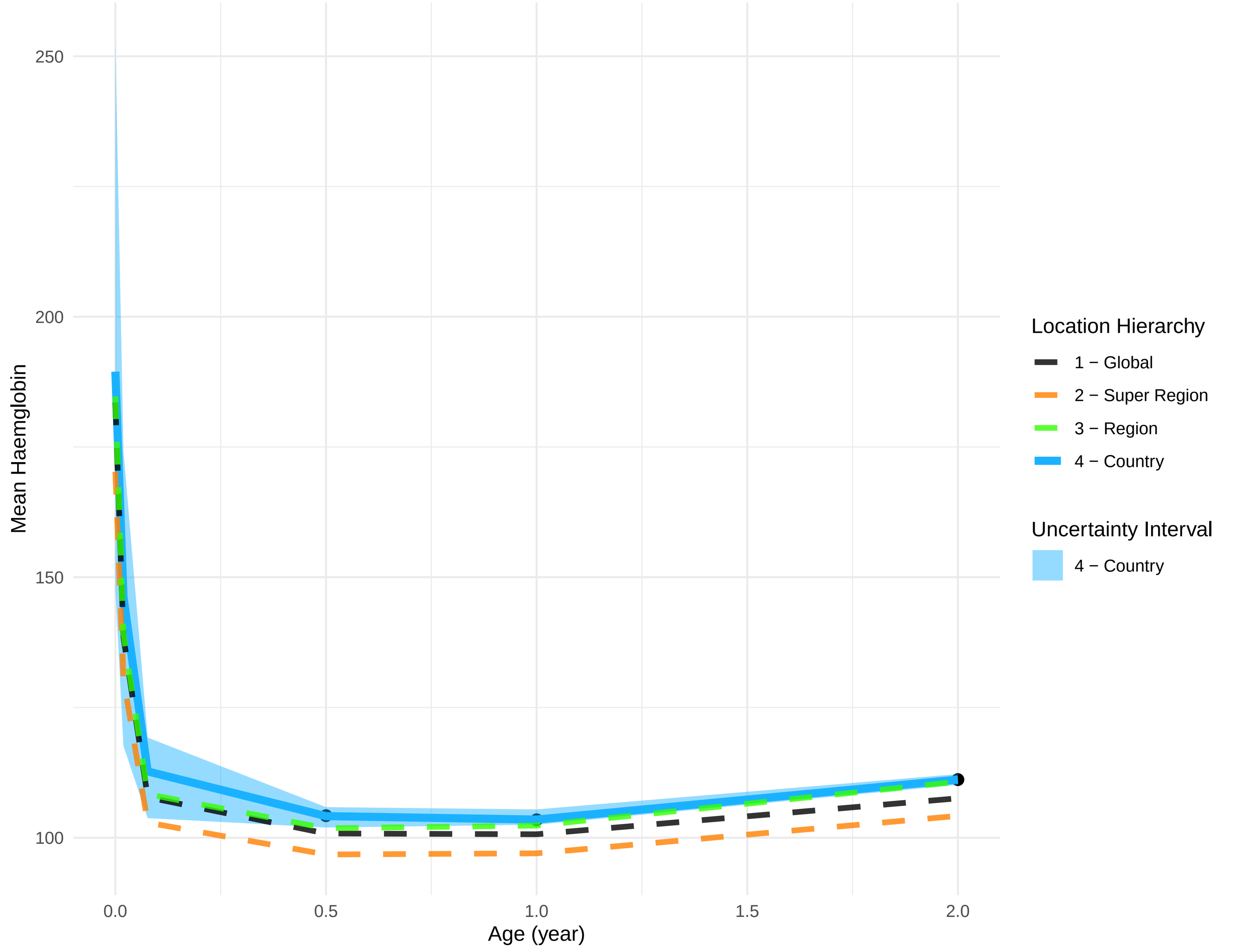

MR−BRT Age/Sex Fits − Males <2 Years Old − Mean Haemglobin − South Africa
WHO−2001 Adjustment Method

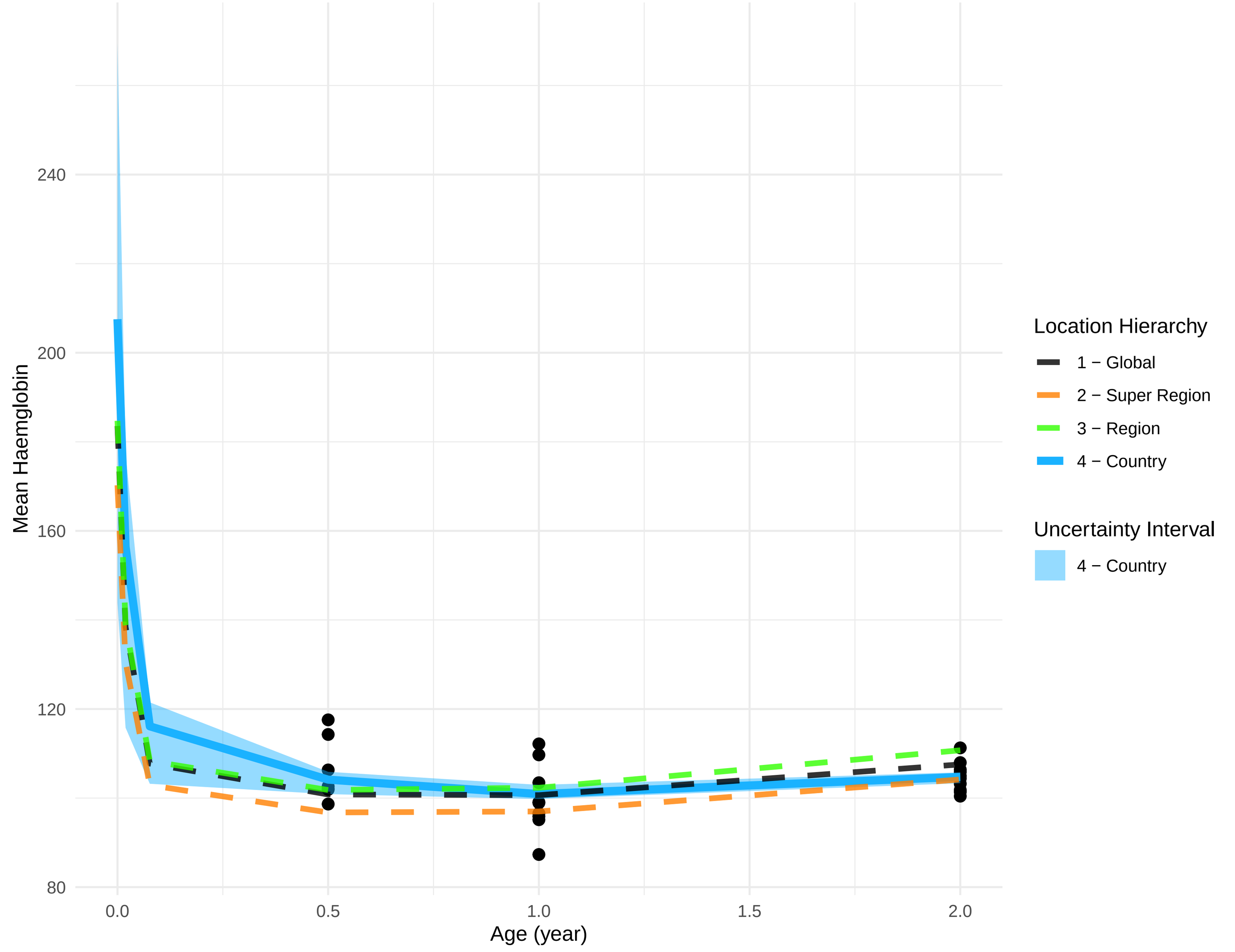

MR−BRT Age/Sex Fits − Males <2 Years Old − Mean Haemglobin − Eastern Cape
WHO−2001 Adjustment Method

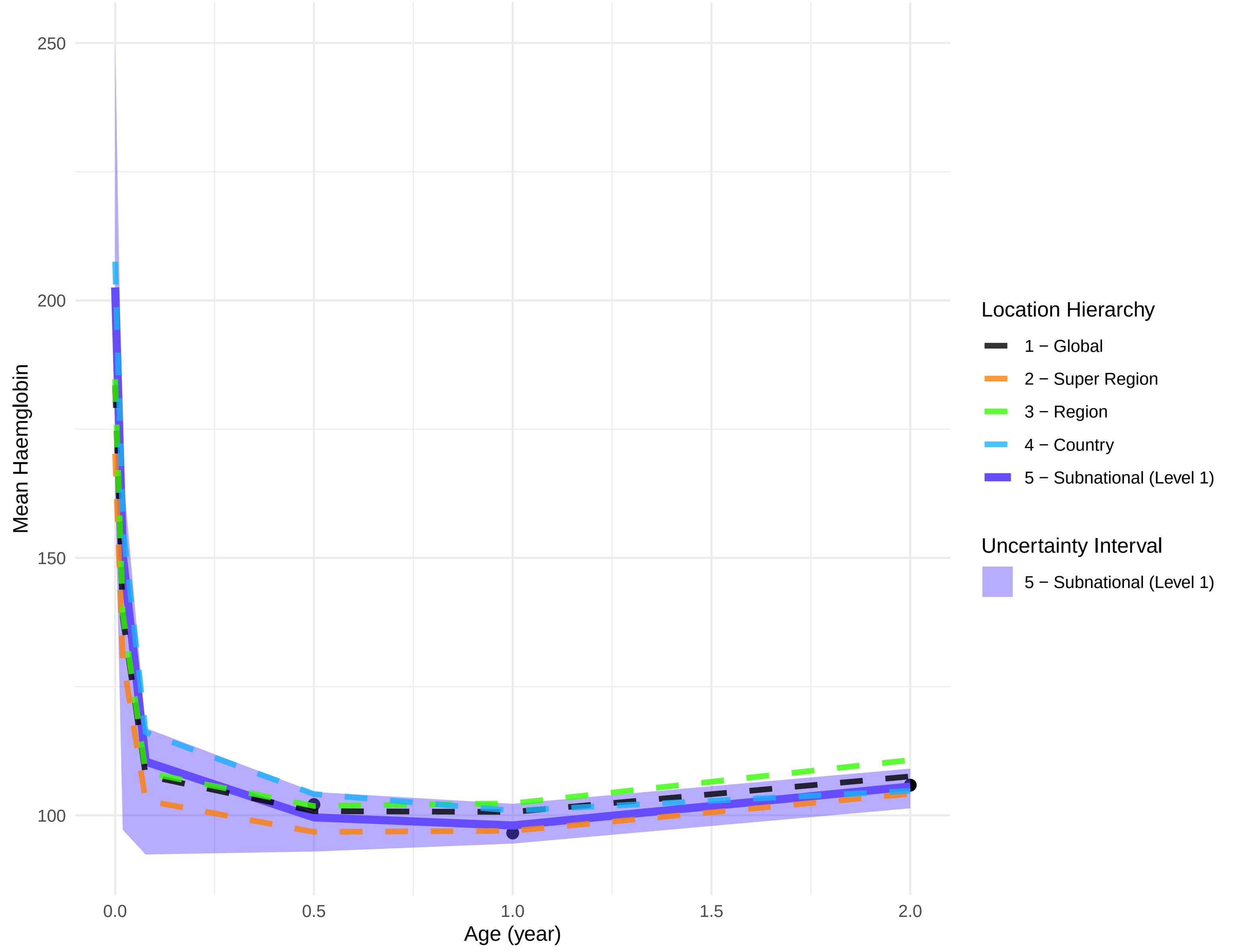

MR−BRT Age/Sex Fits − Males <2 Years Old − Mean Haemglobin − Free State
WHO−2001 Adjustment Method

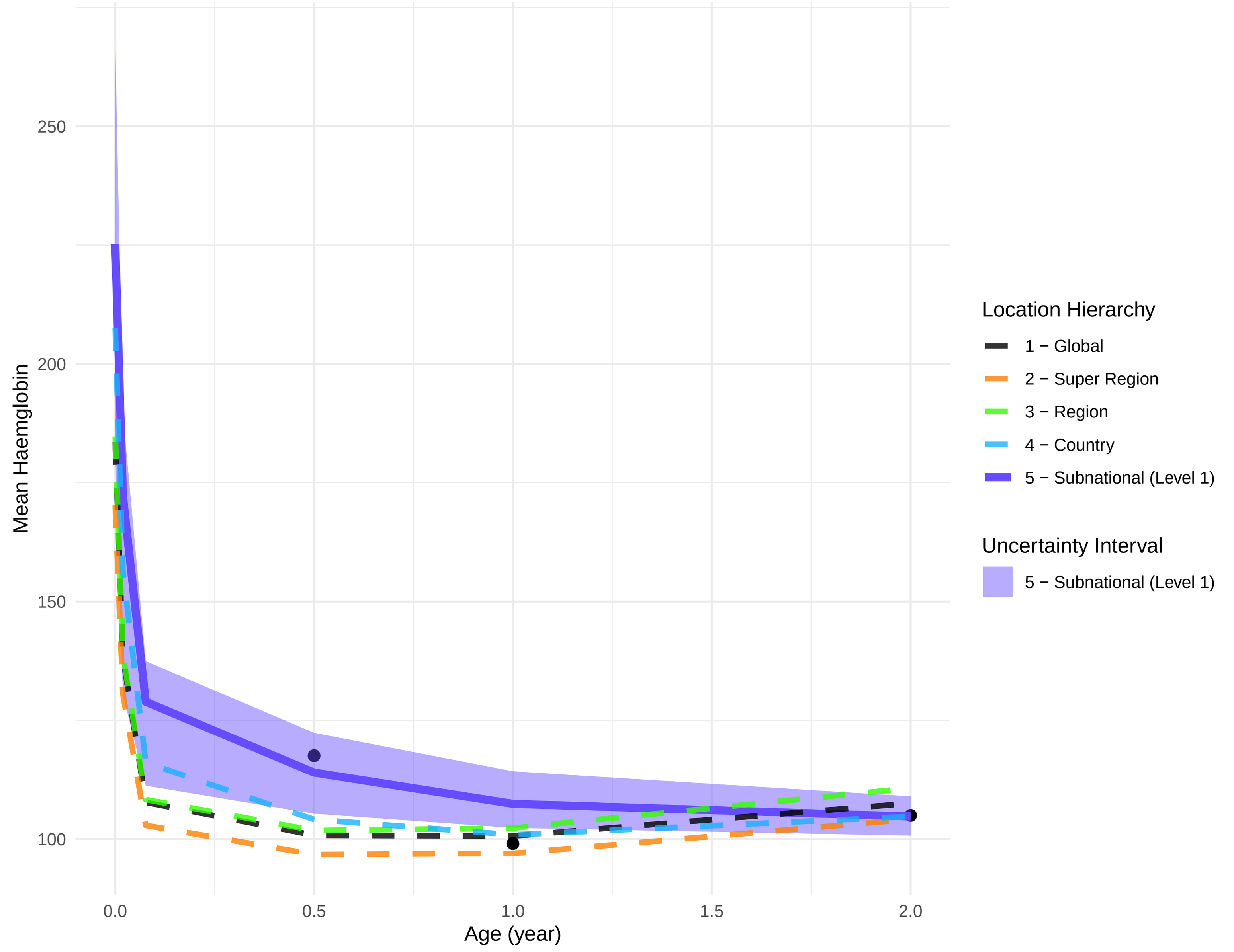

MR−BRT Age/Sex Fits − Males <2 Years Old − Mean Haemglobin − Gauteng
WHO−2001 Adjustment Method

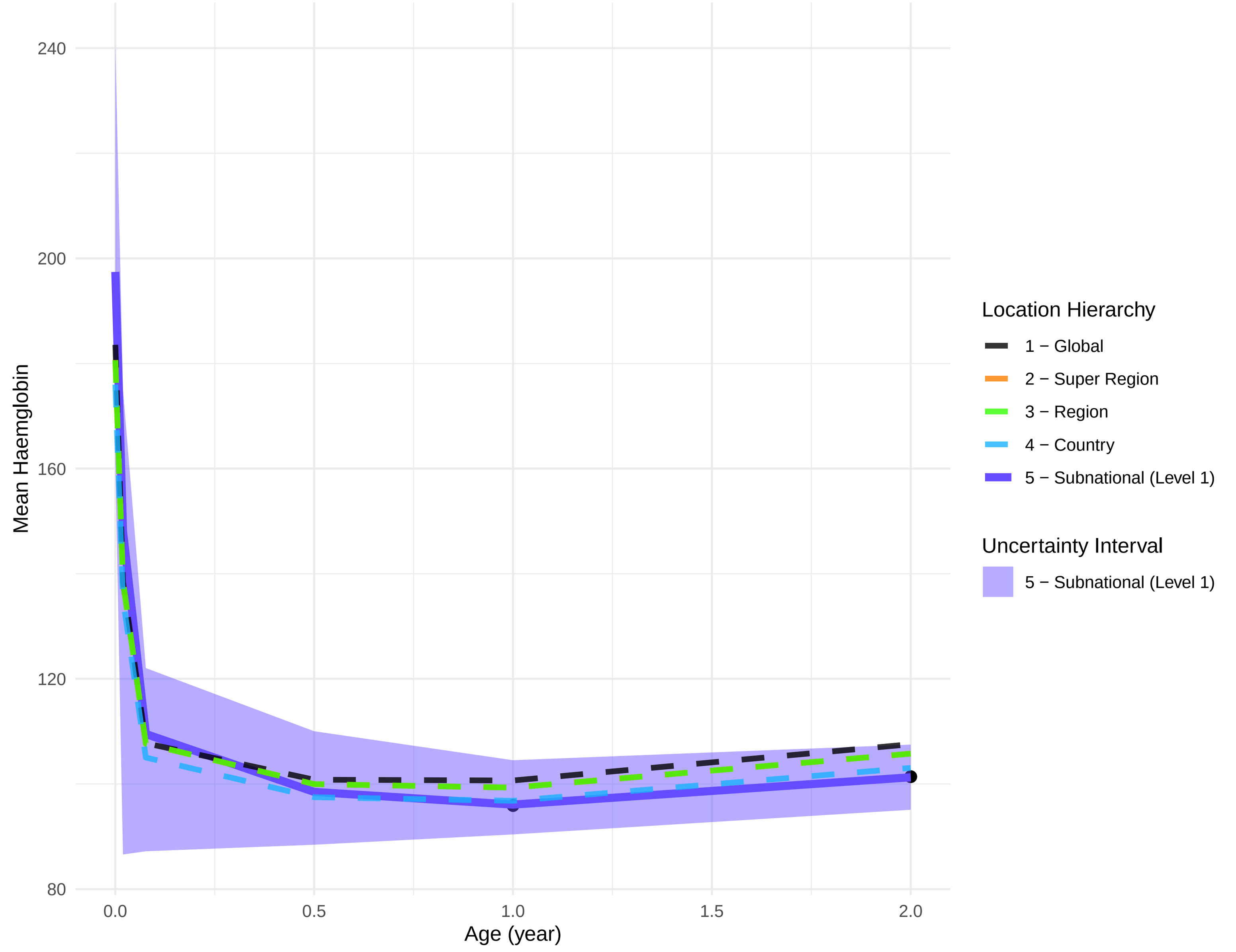

MR−BRT Age/Sex Fits − Males <2 Years Old − Mean Haemglobin − KwaZulu−Natal
WHO−2001 Adjustment Method

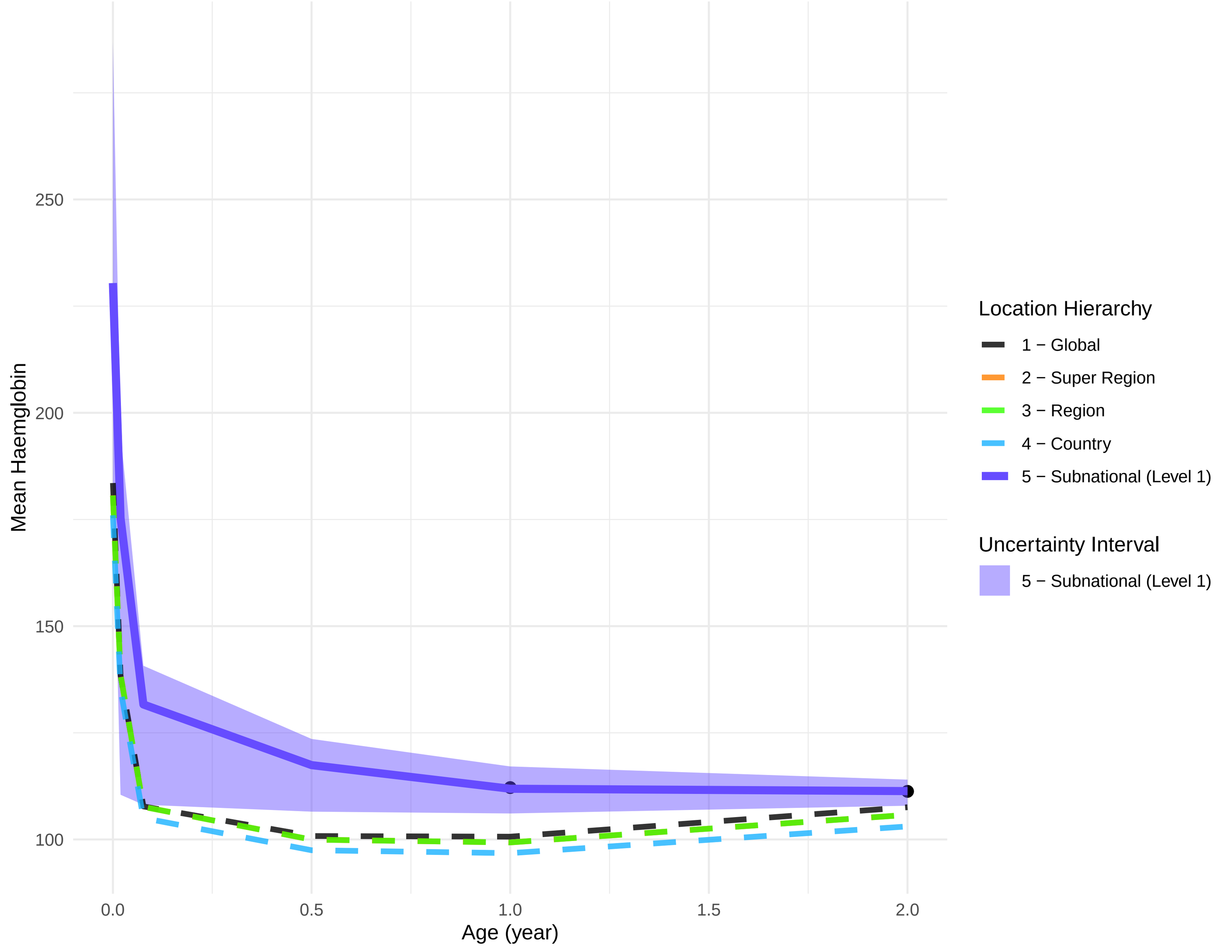

MR−BRT Age/Sex Fits − Males <2 Years Old − Mean Haemglobin − Limpopo
WHO−2001 Adjustment Method

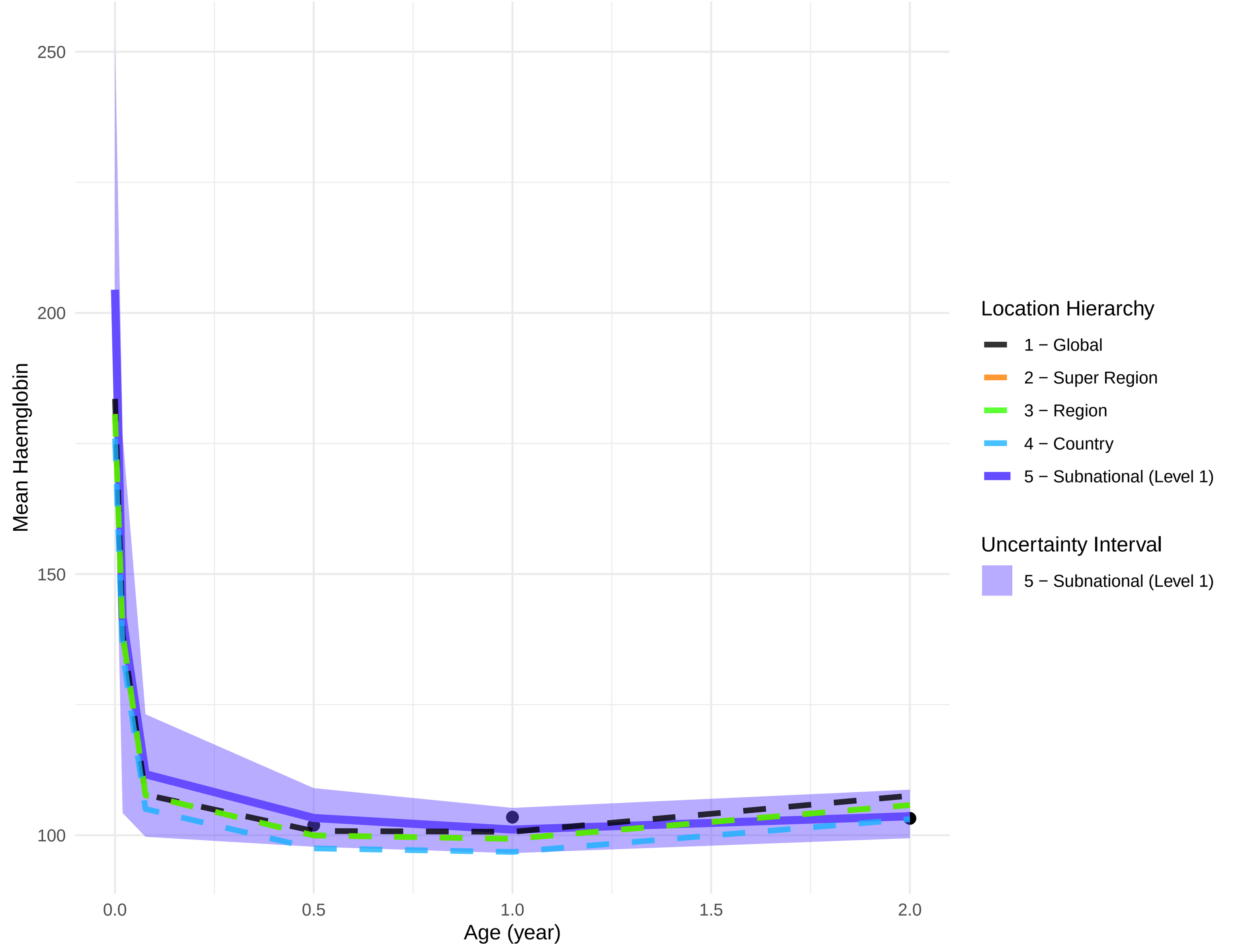

MR−BRT Age/Sex Fits − Males <2 Years Old − Mean Haemglobin − Mpumalanga
WHO−2001 Adjustment Method

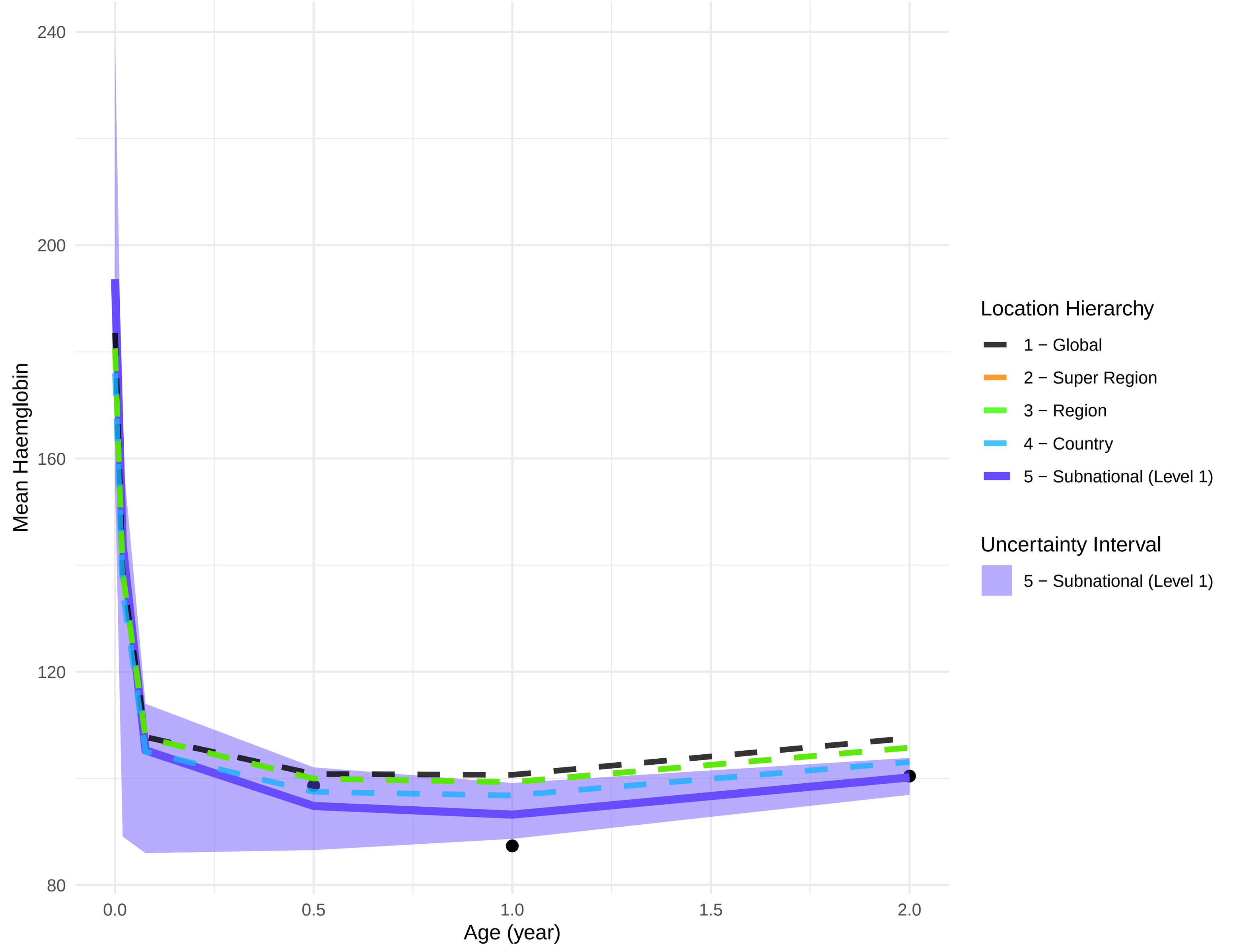

MR−BRT Age/Sex Fits − Males <2 Years Old − Mean Haemglobin − North−West
WHO−2001 Adjustment Method

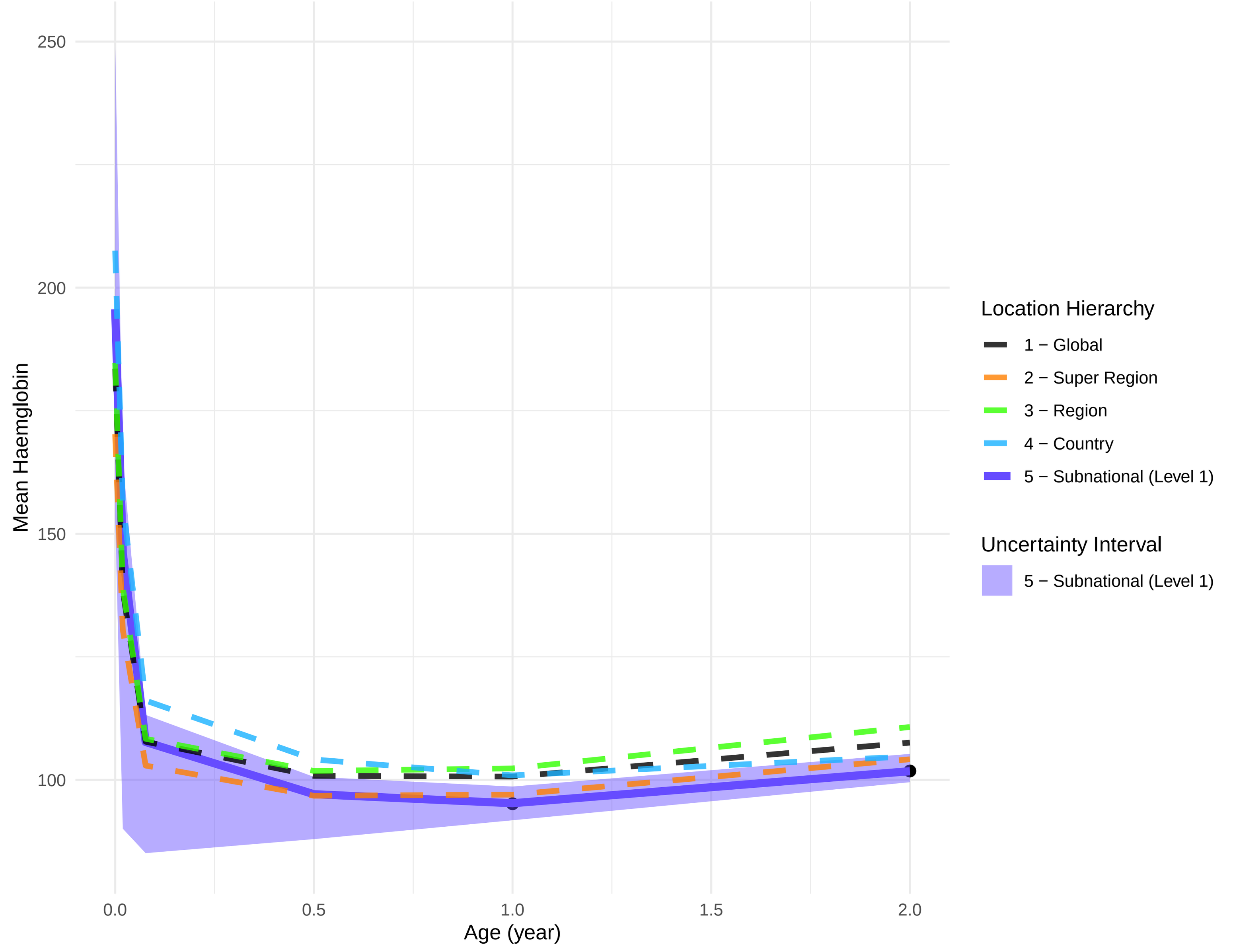

MR−BRT Age/Sex Fits − Males <2 Years Old − Mean Haemglobin − Northern Cape
WHO−2001 Adjustment Method

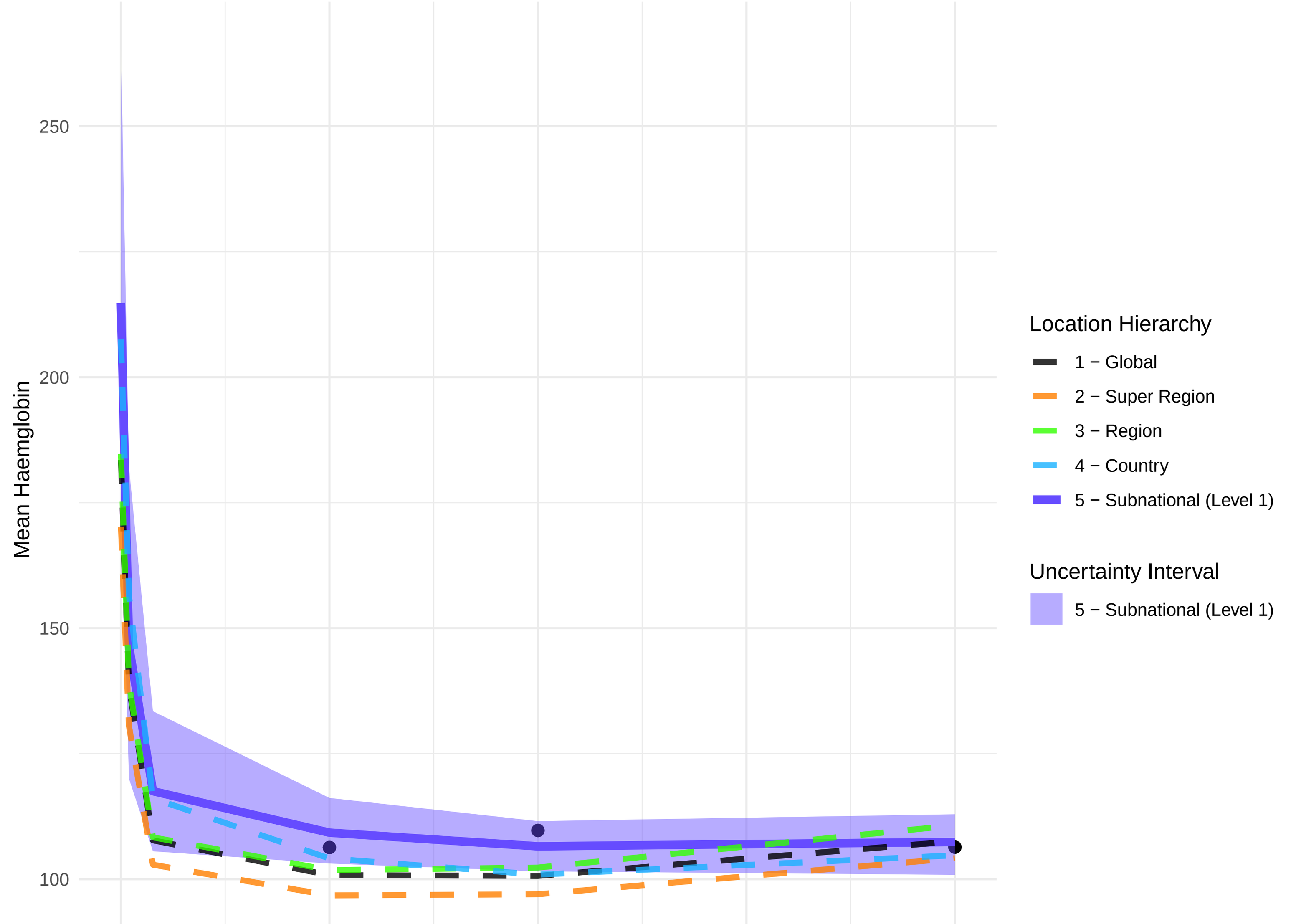

MR−BRT Age/Sex Fits − Males <2 Years Old − Mean Haemglobin − Western Cape
WHO−2001 Adjustment Method

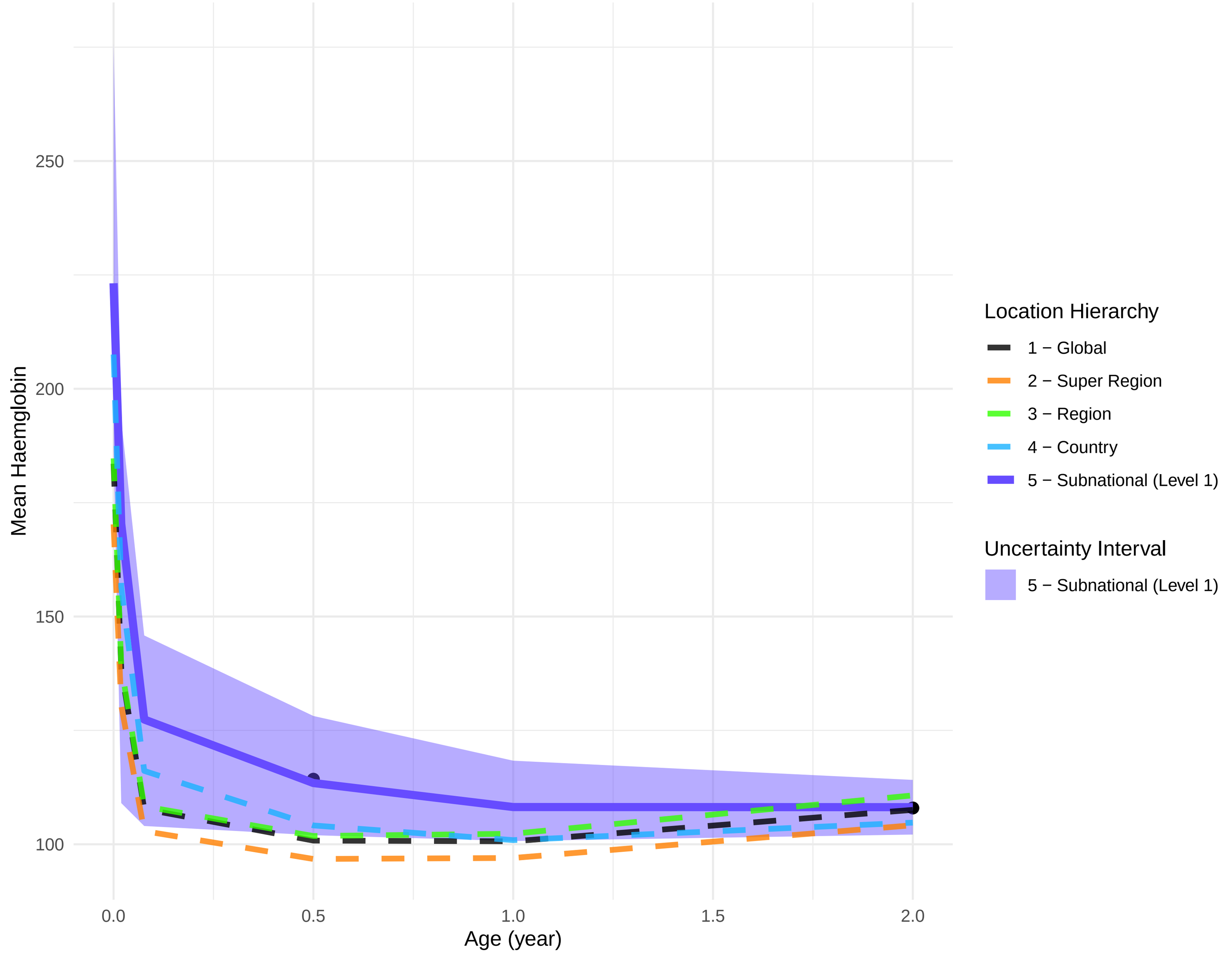

MR−BRT Age/Sex Fits − Males <2 Years Old − Mean Haemglobin − Zimbabwe
WHO−2001 Adjustment Method

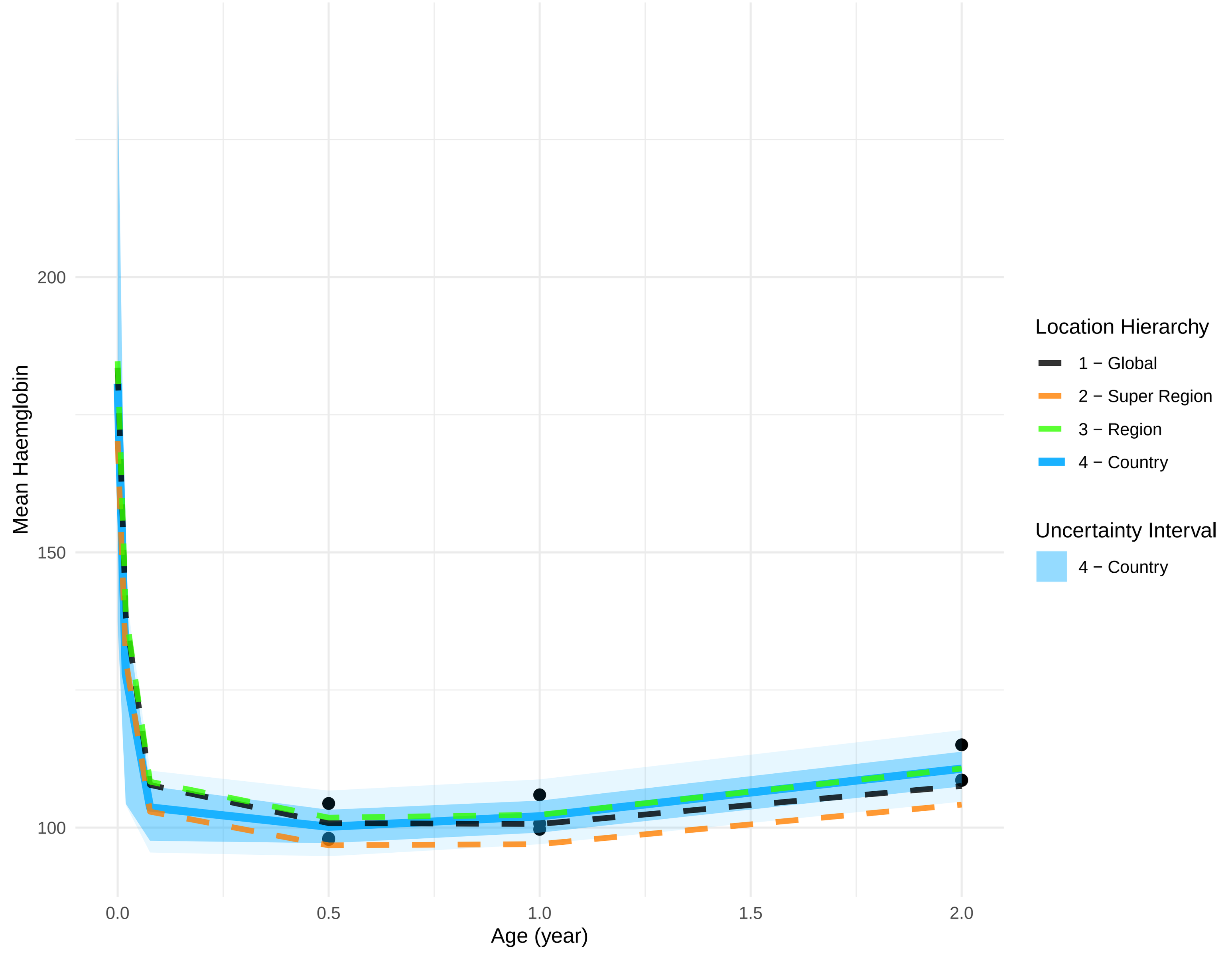

MR−BRT Age/Sex Fits − Males <2 Years Old − Mean Haemglobin − Western Sub−Saharan Africa
WHO−2001 Adjustment Method

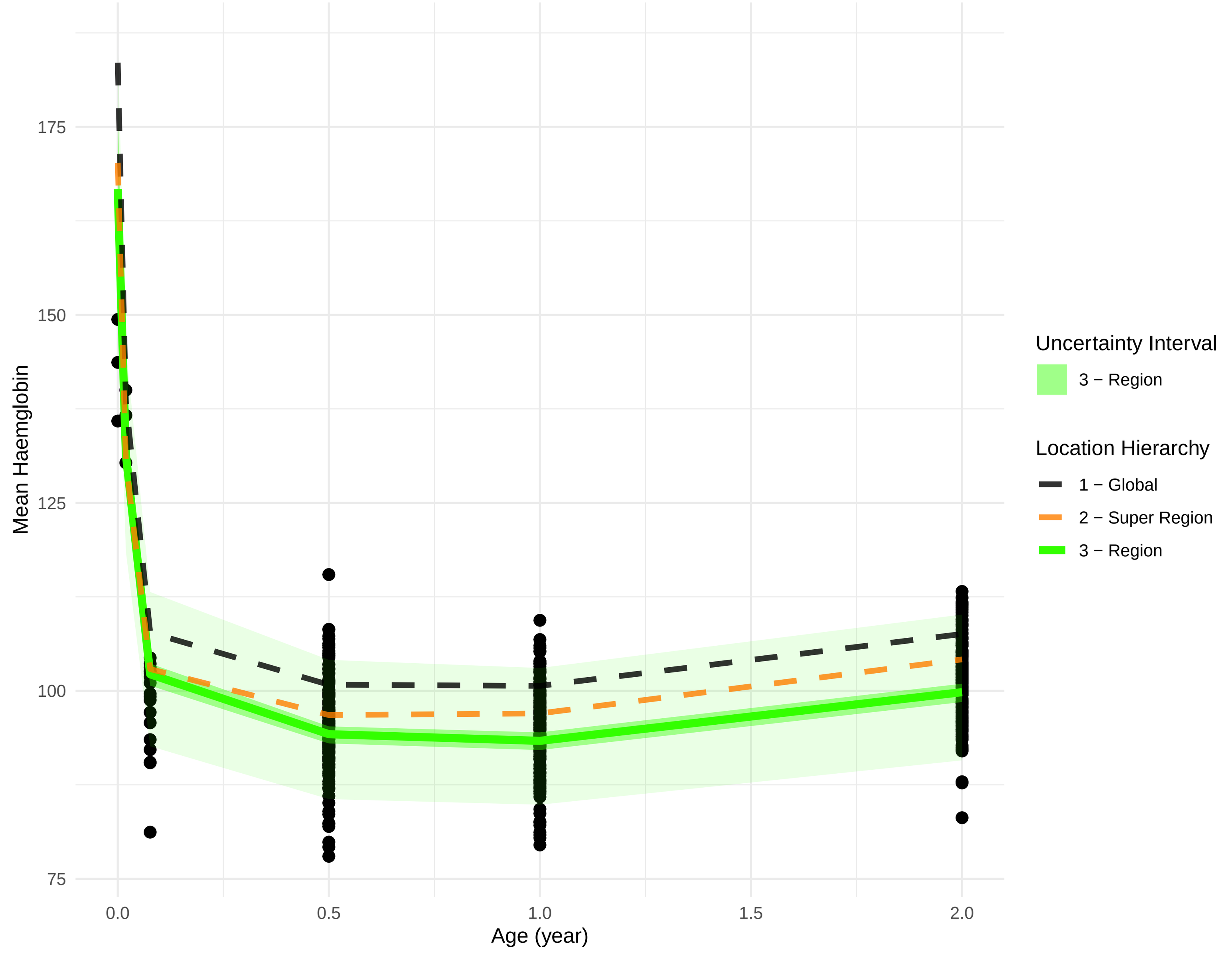

MR−BRT Age/Sex Fits − Males <2 Years Old − Mean Haemglobin − Benin
WHO−2001 Adjustment Method

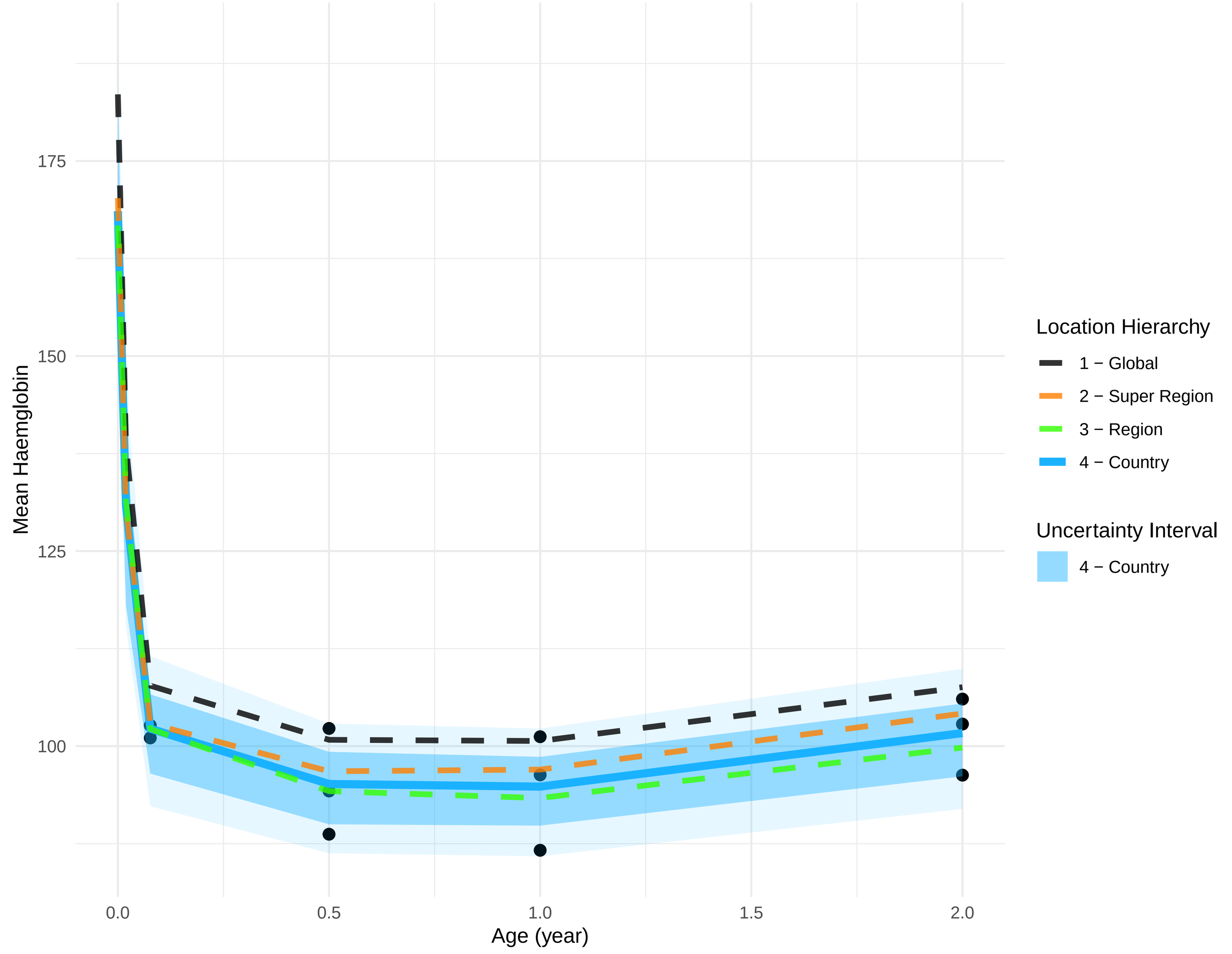

MR−BRT Age/Sex Fits − Males <2 Years Old − Mean Haemglobin − Burkina Faso
WHO−2001 Adjustment Method

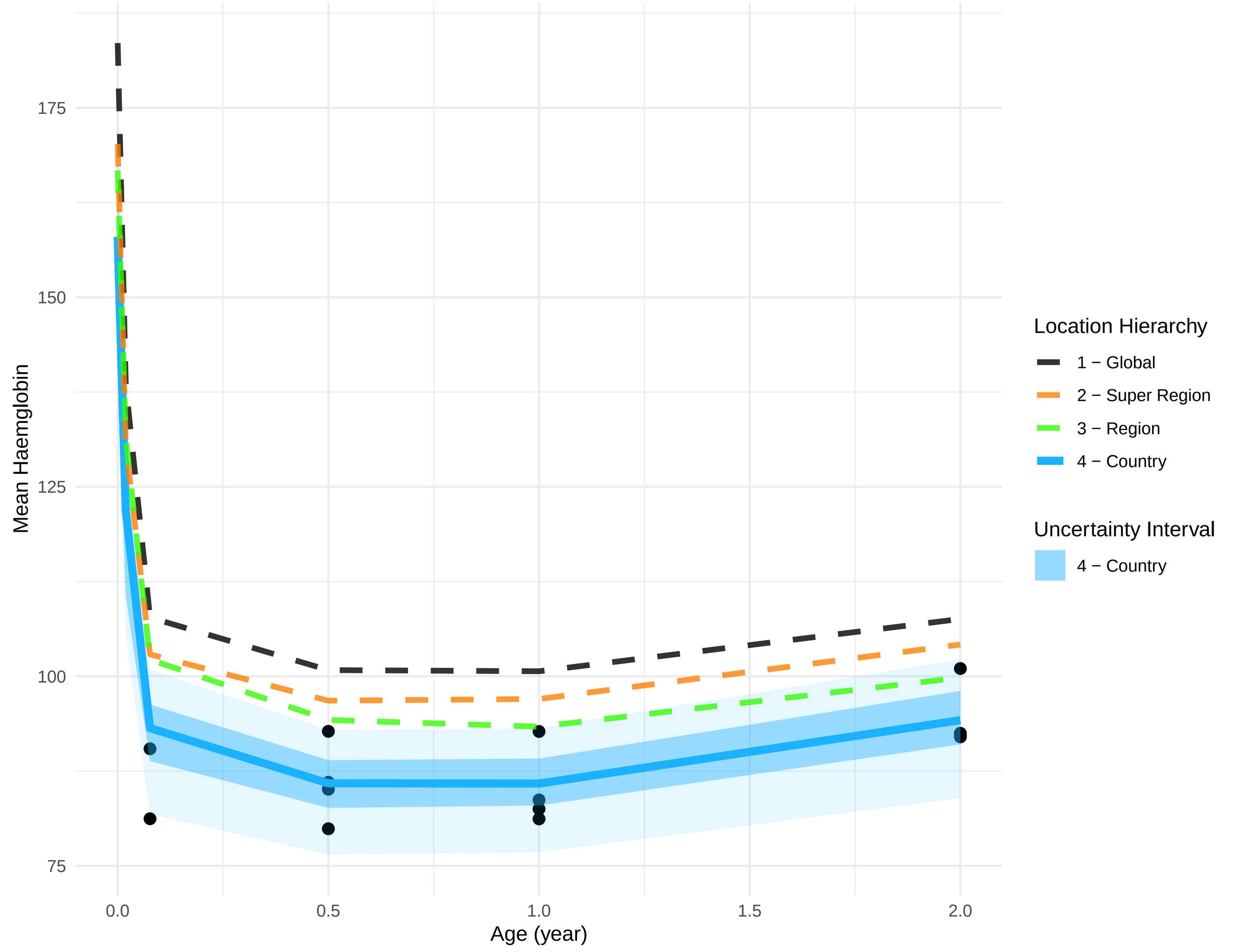

MR−BRT Age/Sex Fits − Males <2 Years Old − Mean Haemglobin − Cameroon
WHO−2001 Adjustment Method

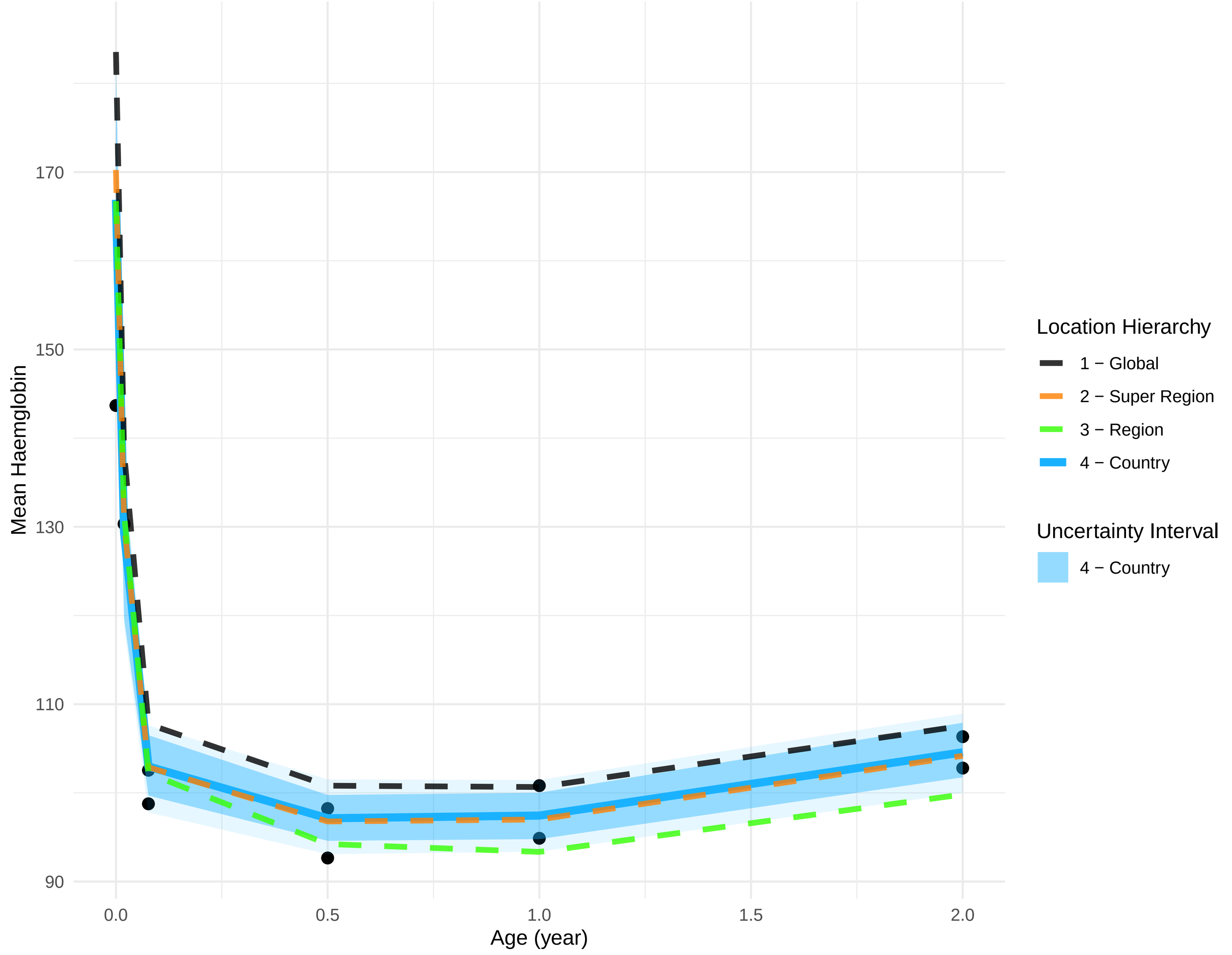

MR−BRT Age/Sex Fits − Males <2 Years Old − Mean Haemglobin − Côte d'Ivoire
WHO−2001 Adjustment Method

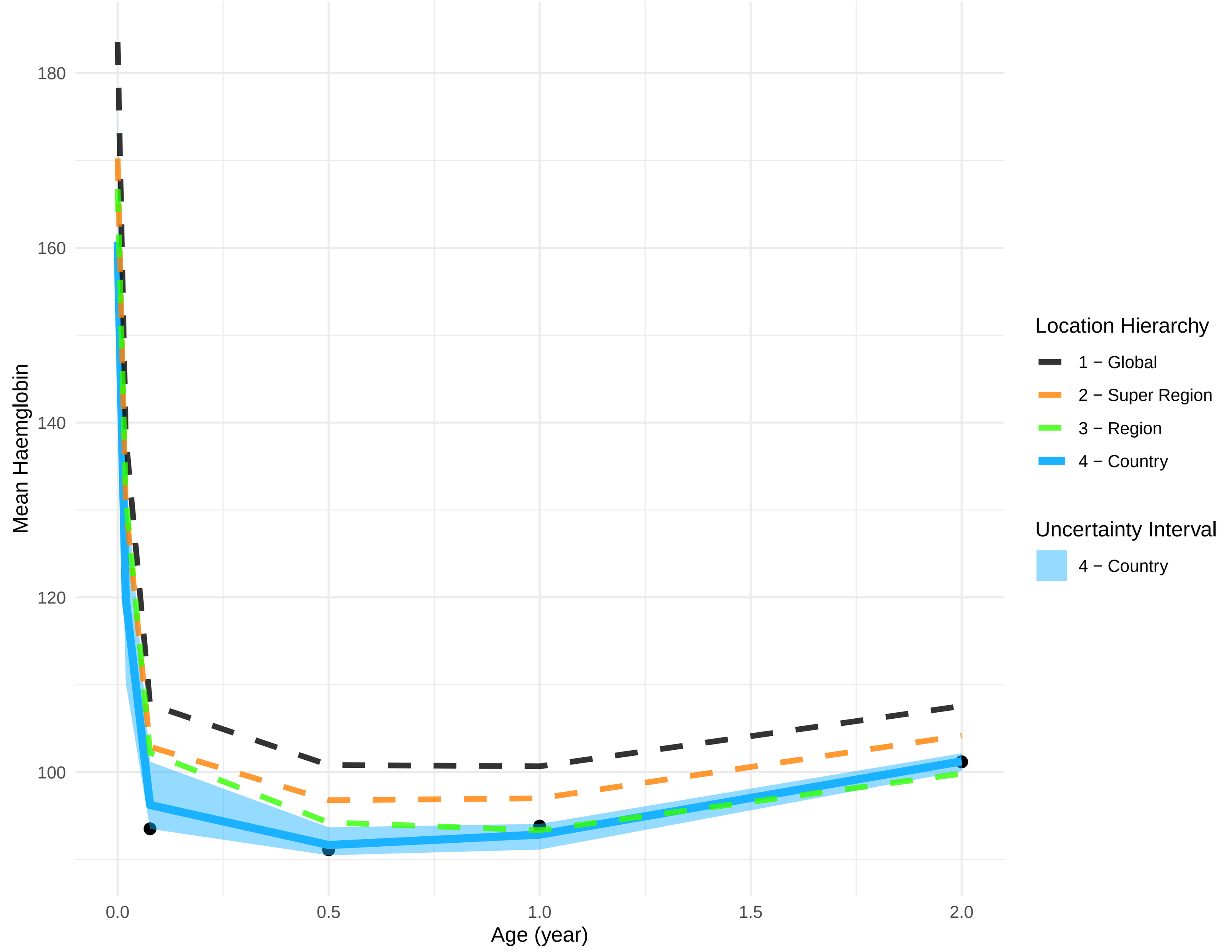

MR−BRT Age/Sex Fits − Males <2 Years Old − Mean Haemglobin − Gambia
WHO−2001 Adjustment Method

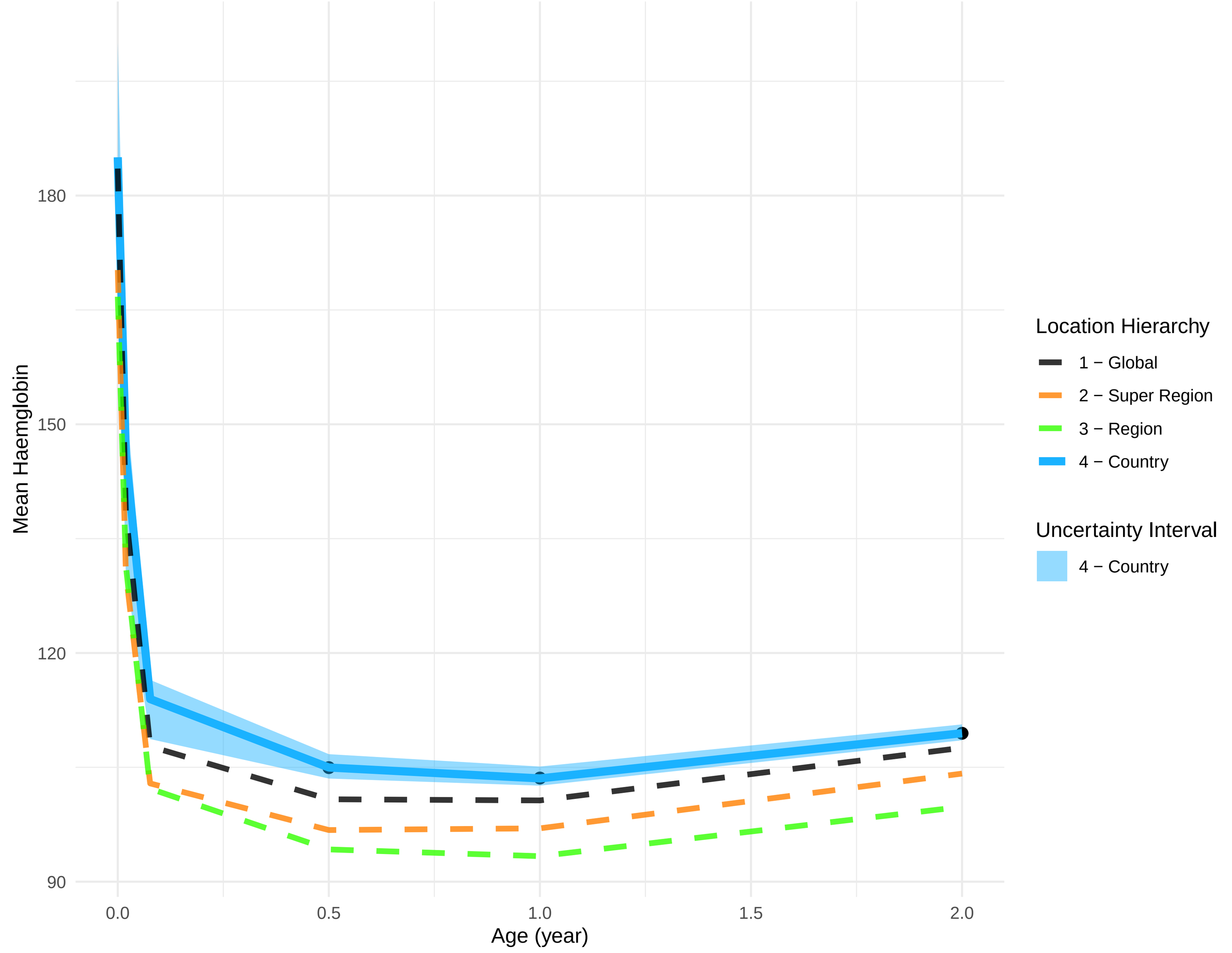

MR−BRT Age/Sex Fits − Males <2 Years Old − Mean Haemglobin − Ghana
WHO−2001 Adjustment Method

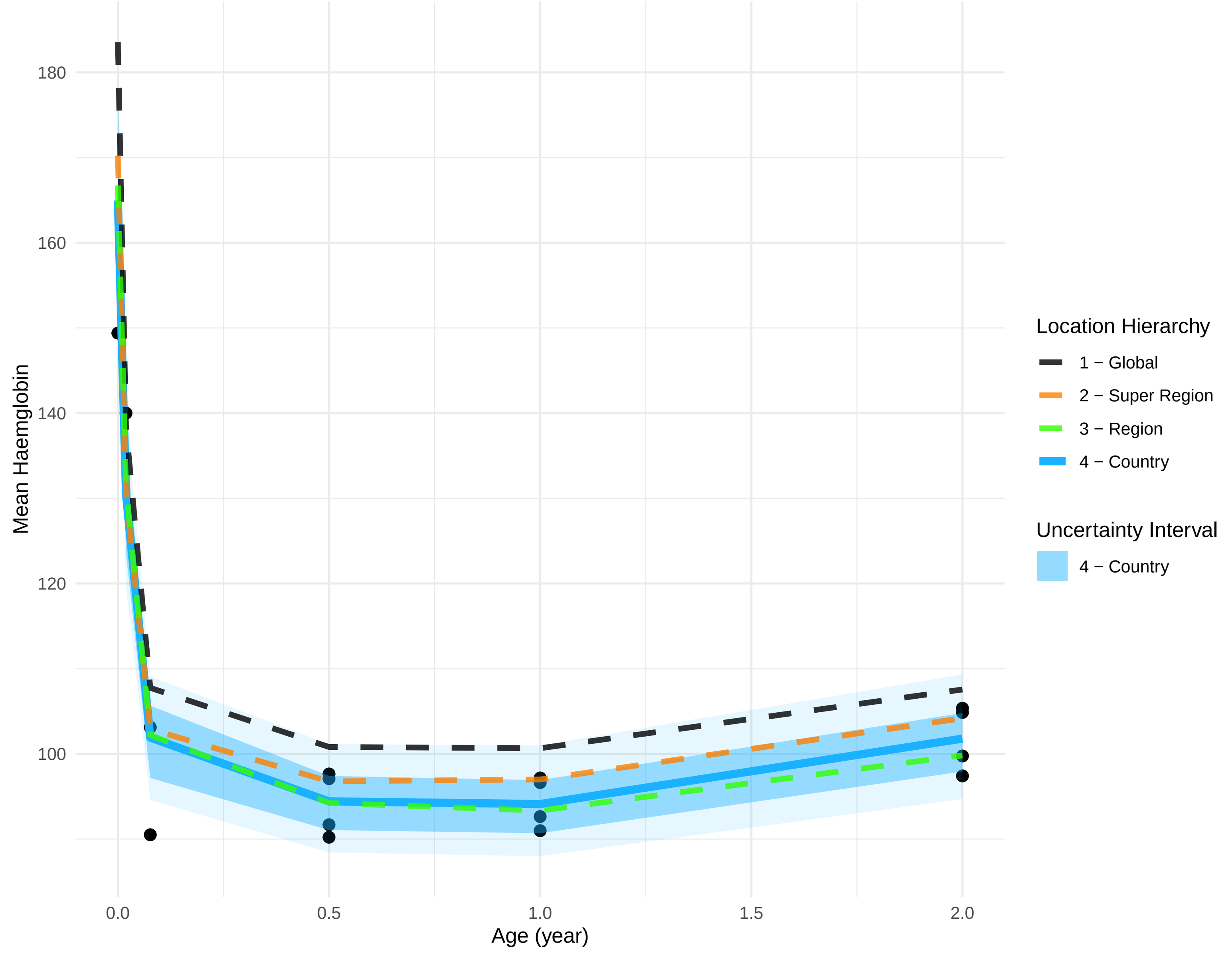

MR−BRT Age/Sex Fits − Males <2 Years Old − Mean Haemglobin − Guinea
WHO−2001 Adjustment Method

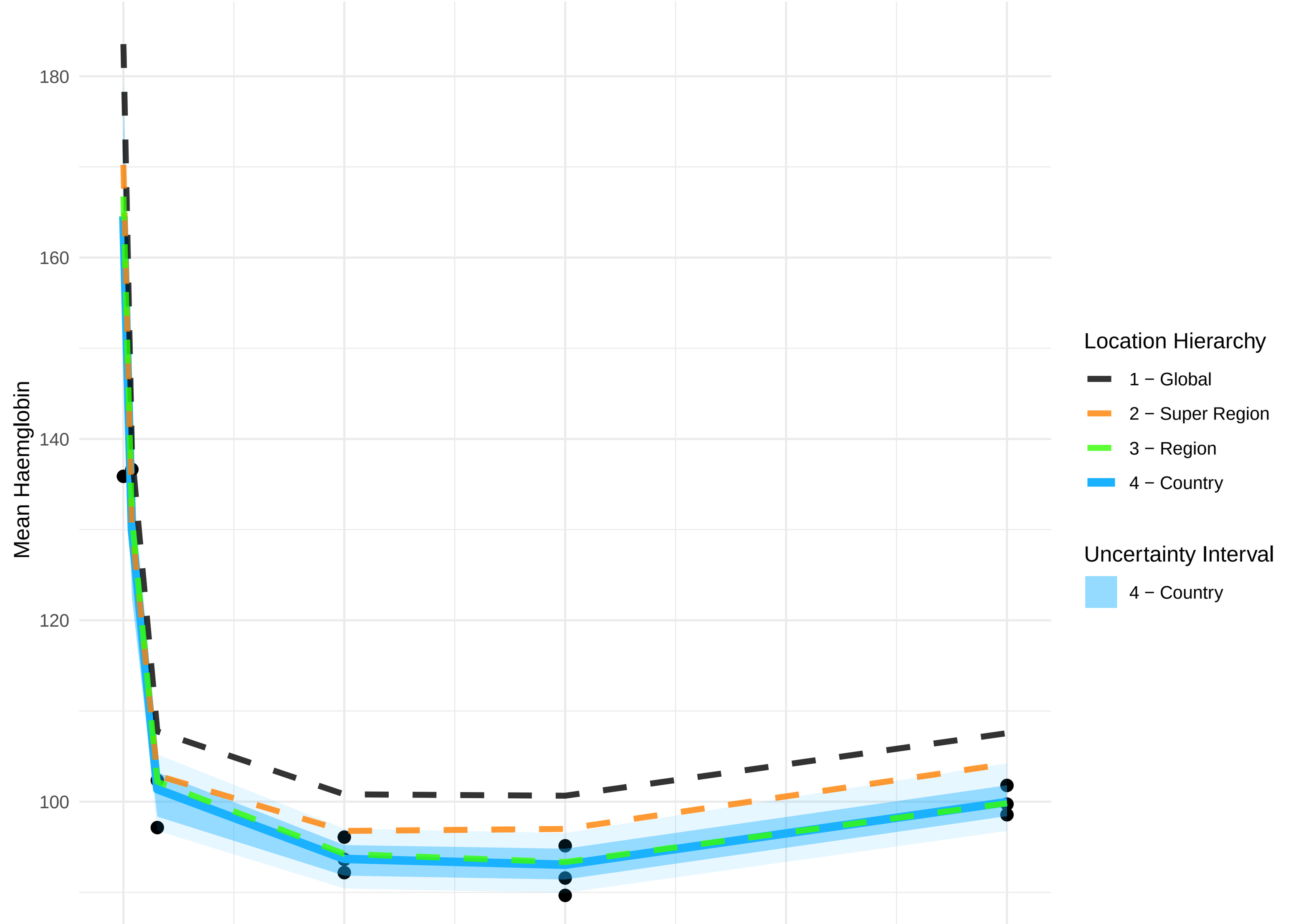

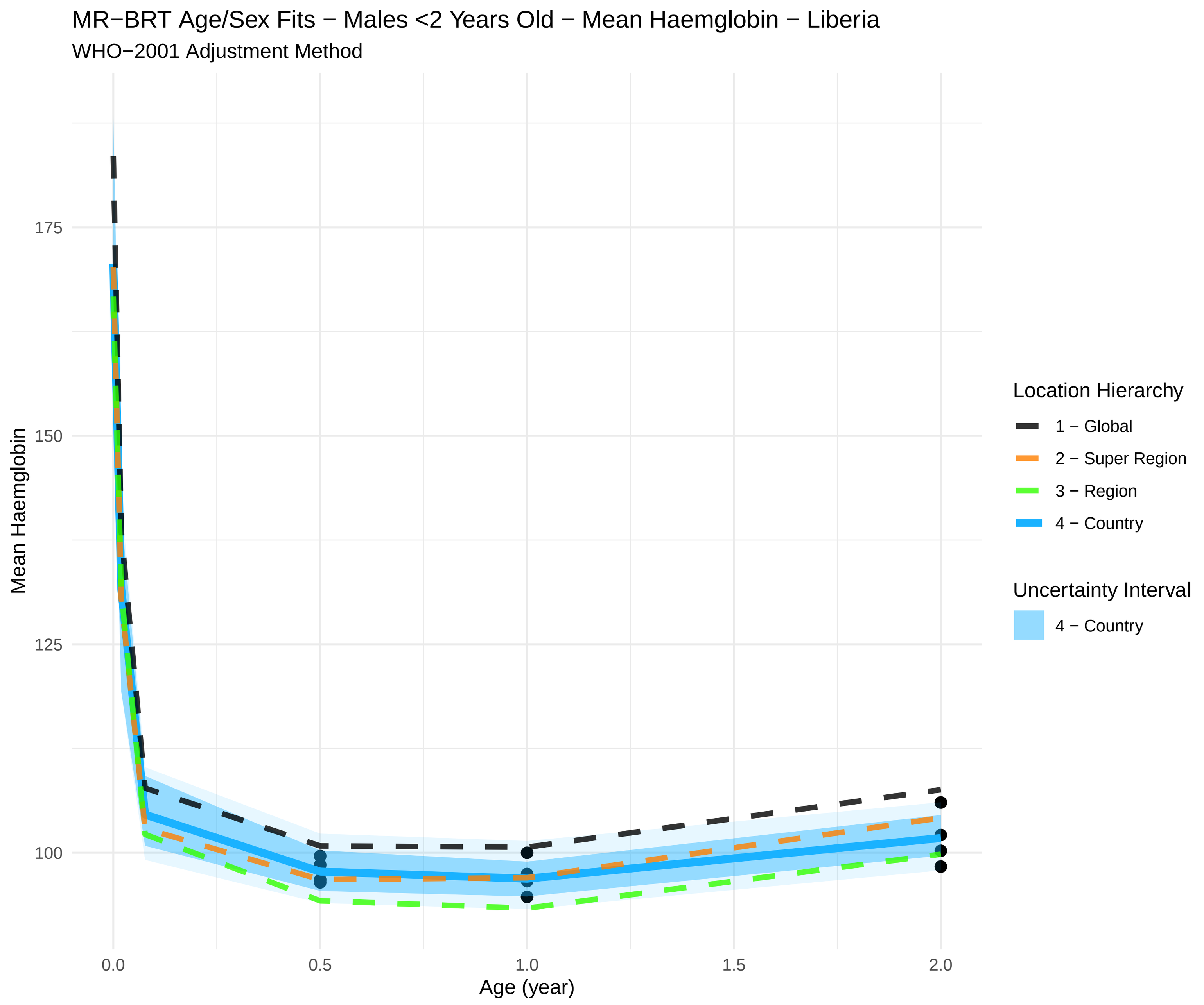
MR−BRT Age/Sex Fits − Males <2 Years Old − Mean Haemglobin − Liberia
WHO−2001 Adjustment Method
Mean Haemglobin
Age (year)
Location Hierarchy
1 − Global
2 − Super Region
3 − Region
4 − Country
Uncertainty Interval
4 − Country

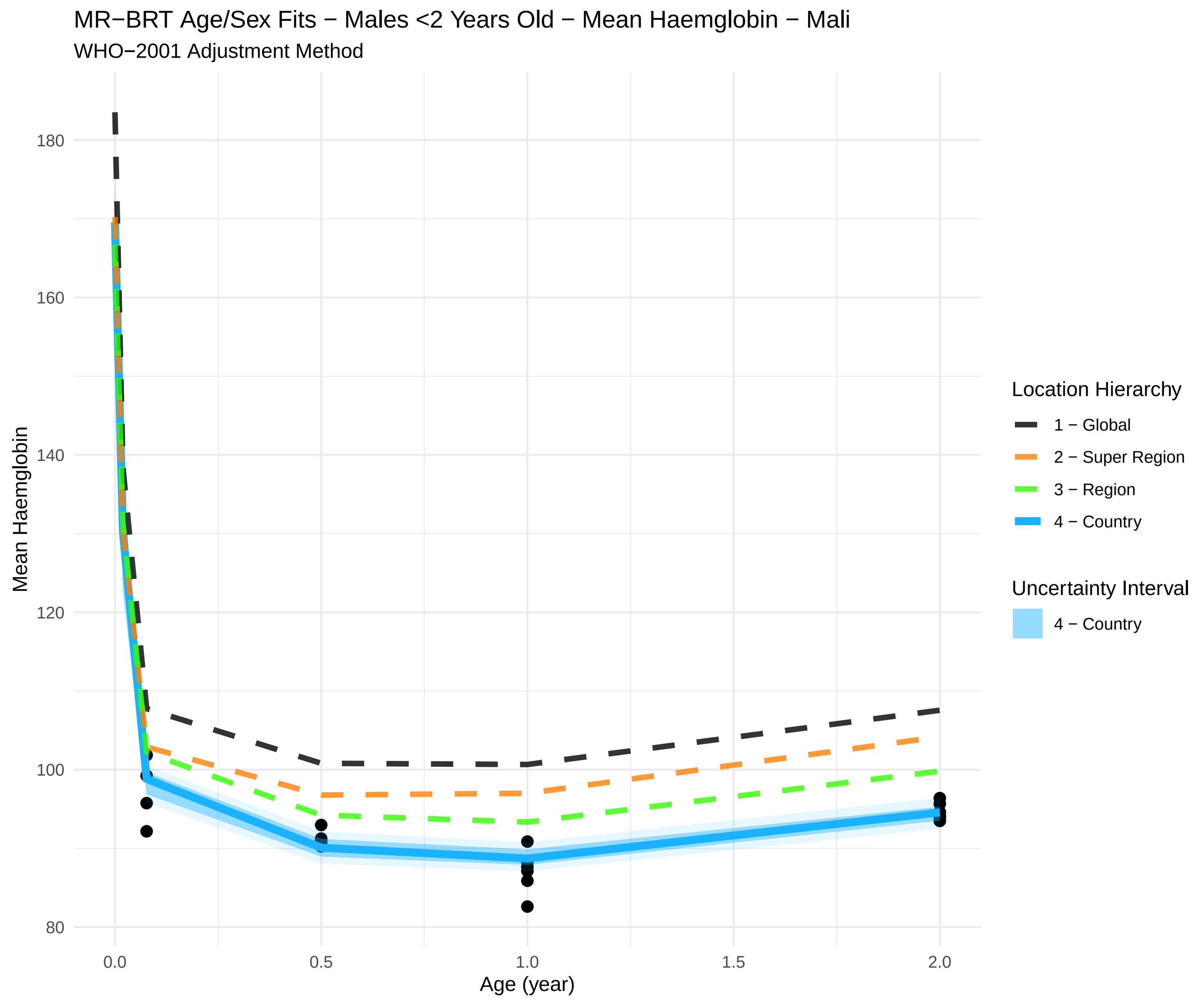
MR−BRT Age/Sex Fits − Males <2 Years Old − Mean Haemglobin − Mali
WHO−2001 Adjustment Method
Mean Haemglobin
Age (year)
Location Hierarchy
1 − Global
2 − Super Region
3 − Region
4 − Country
Uncertainty Interval
4 − Country

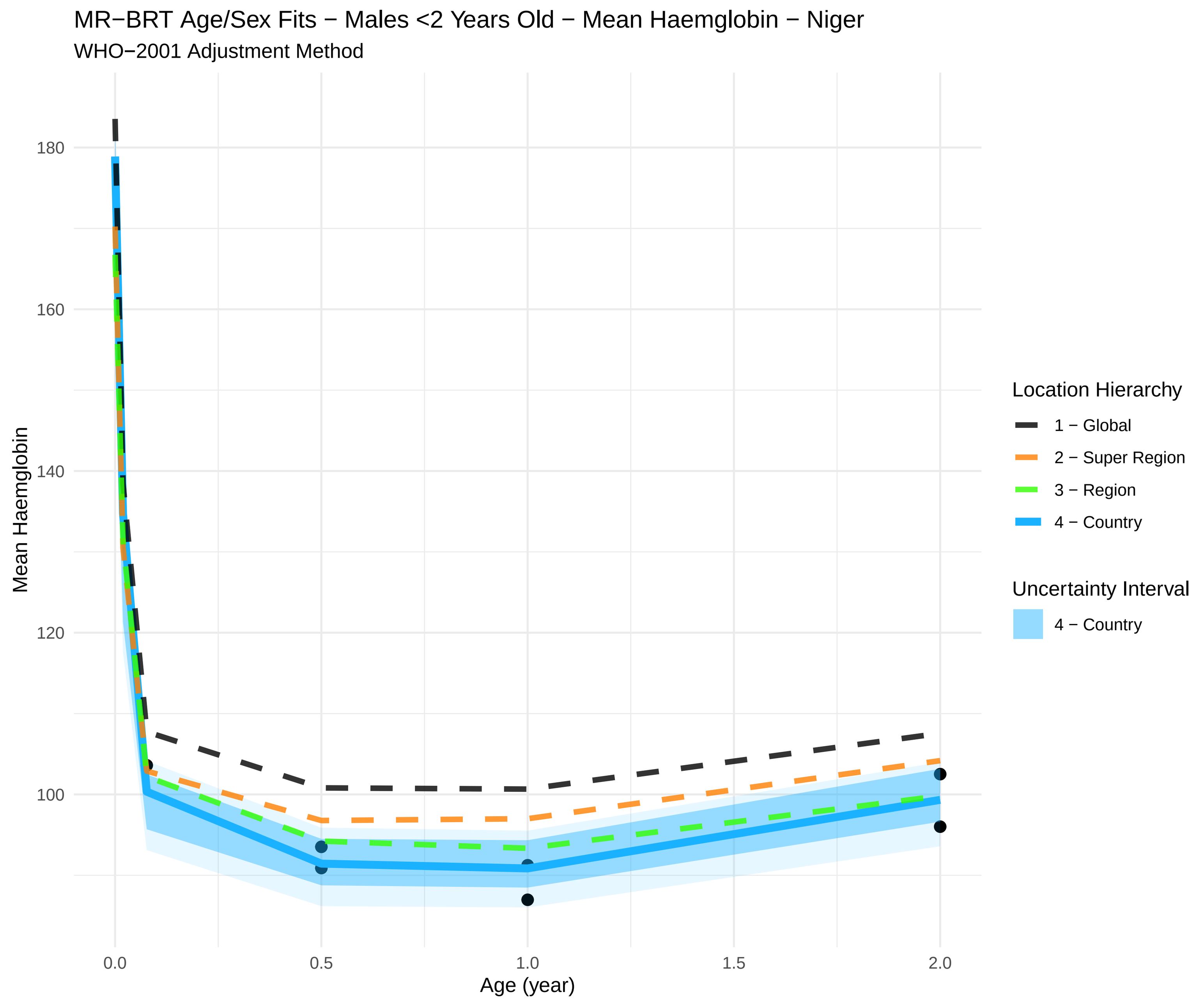
MR−BRT Age/Sex Fits − Males <2 Years Old − Mean Haemglobin − Niger
WHO−2001 Adjustment Method
Mean Haemglobin
Age (year)
Location Hierarchy
1 − Global
2 − Super Region
3 − Region
4 − Country
Uncertainty Interval
4 − Country

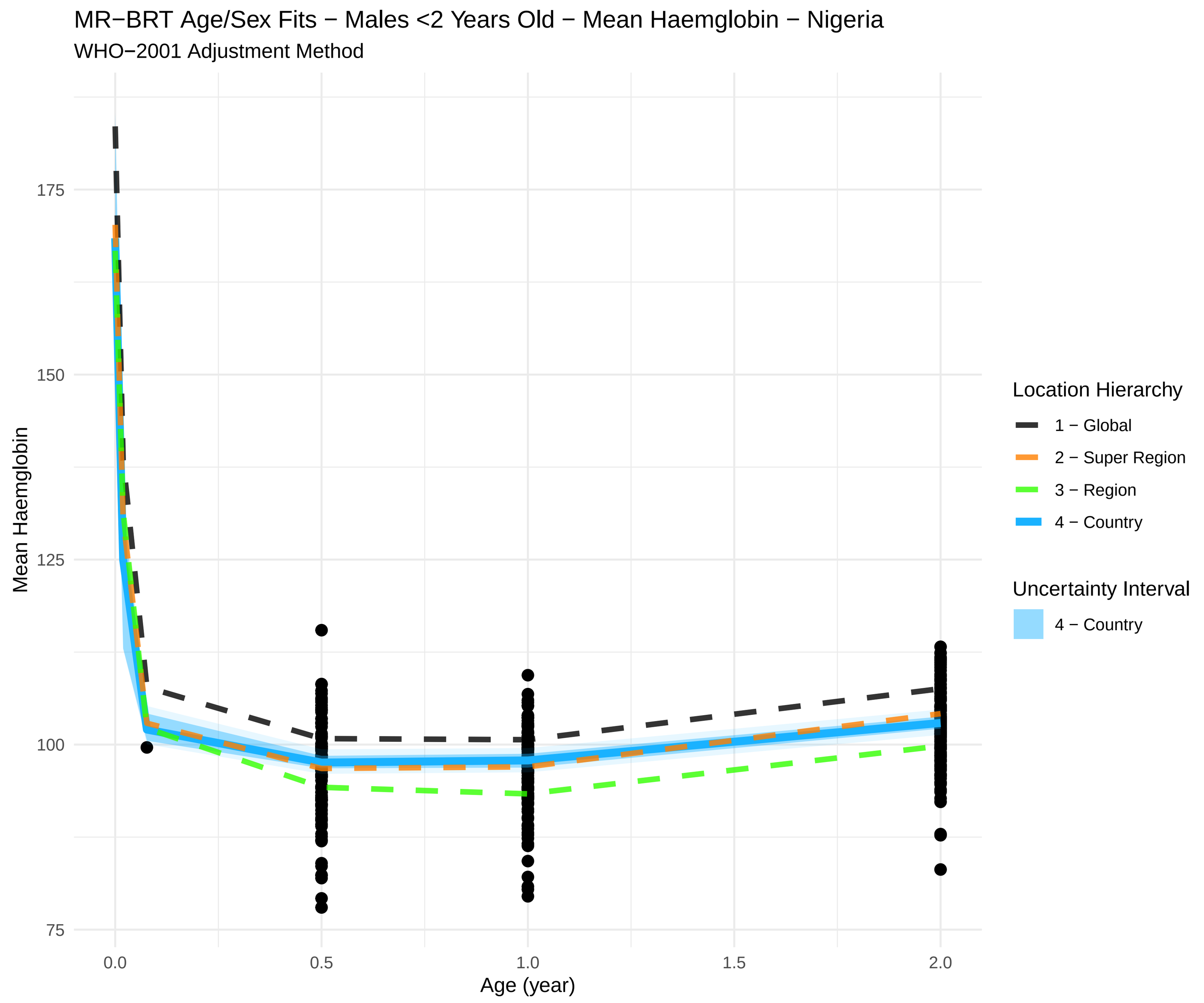
MR−BRT Age/Sex Fits − Males <2 Years Old − Mean Haemglobin − Nigeria
WHO−2001 Adjustment Method
Mean Haemglobin
Age (year)
Location Hierarchy
1 − Global
2 − Super Region
3 − Region
4 − Country
Uncertainty Interval
4 − Country

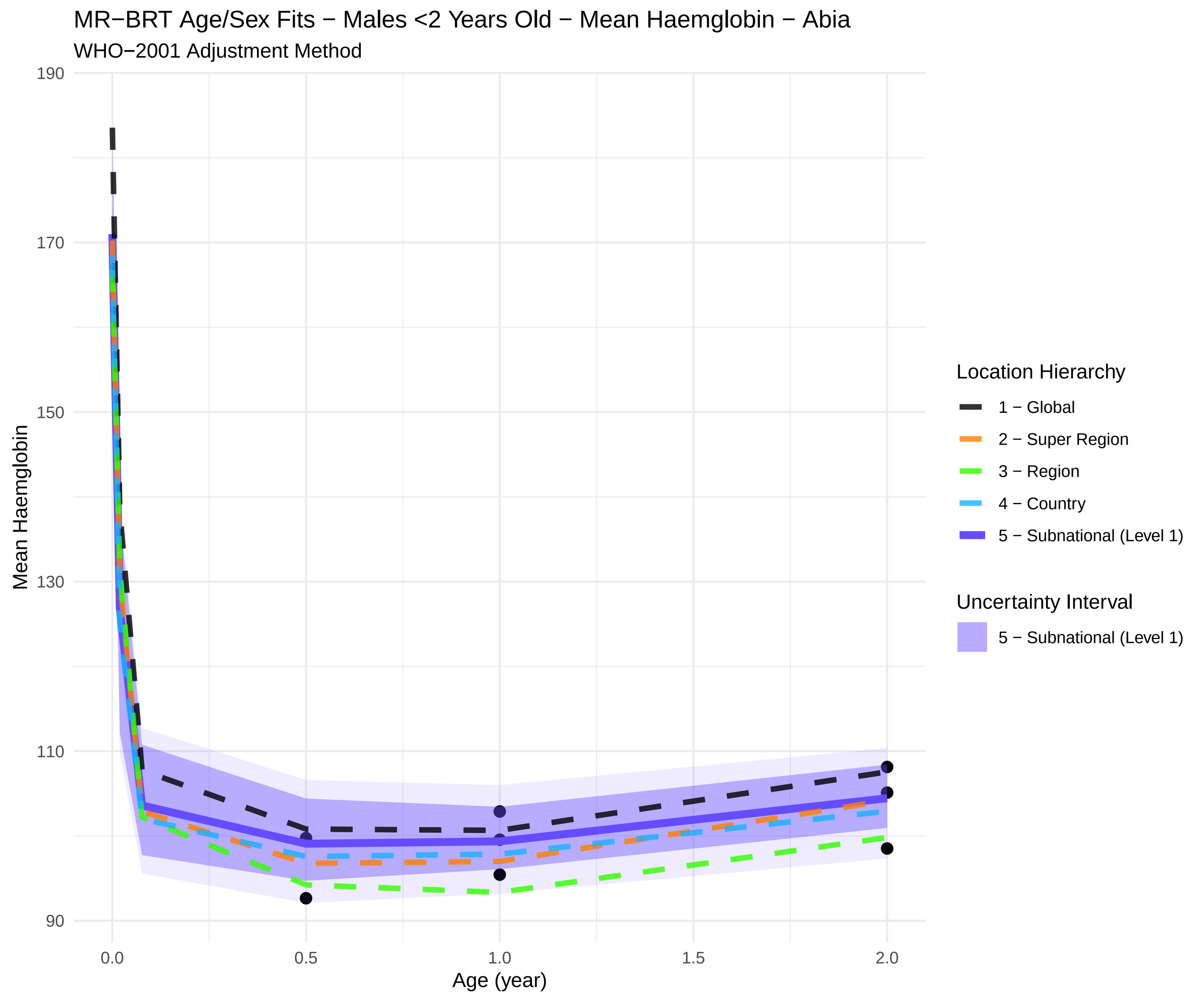
MR−BRT Age/Sex Fits − Males <2 Years Old − Mean Haemglobin − Abia
WHO−2001 Adjustment Method
Mean Haemglobin
Age (year)
Location Hierarchy
1 − Global
2 − Super Region
3 − Region
4 − Country
5 − Subnational (Level 1)
Uncertainty Interval
5 − Subnational (Level 1)

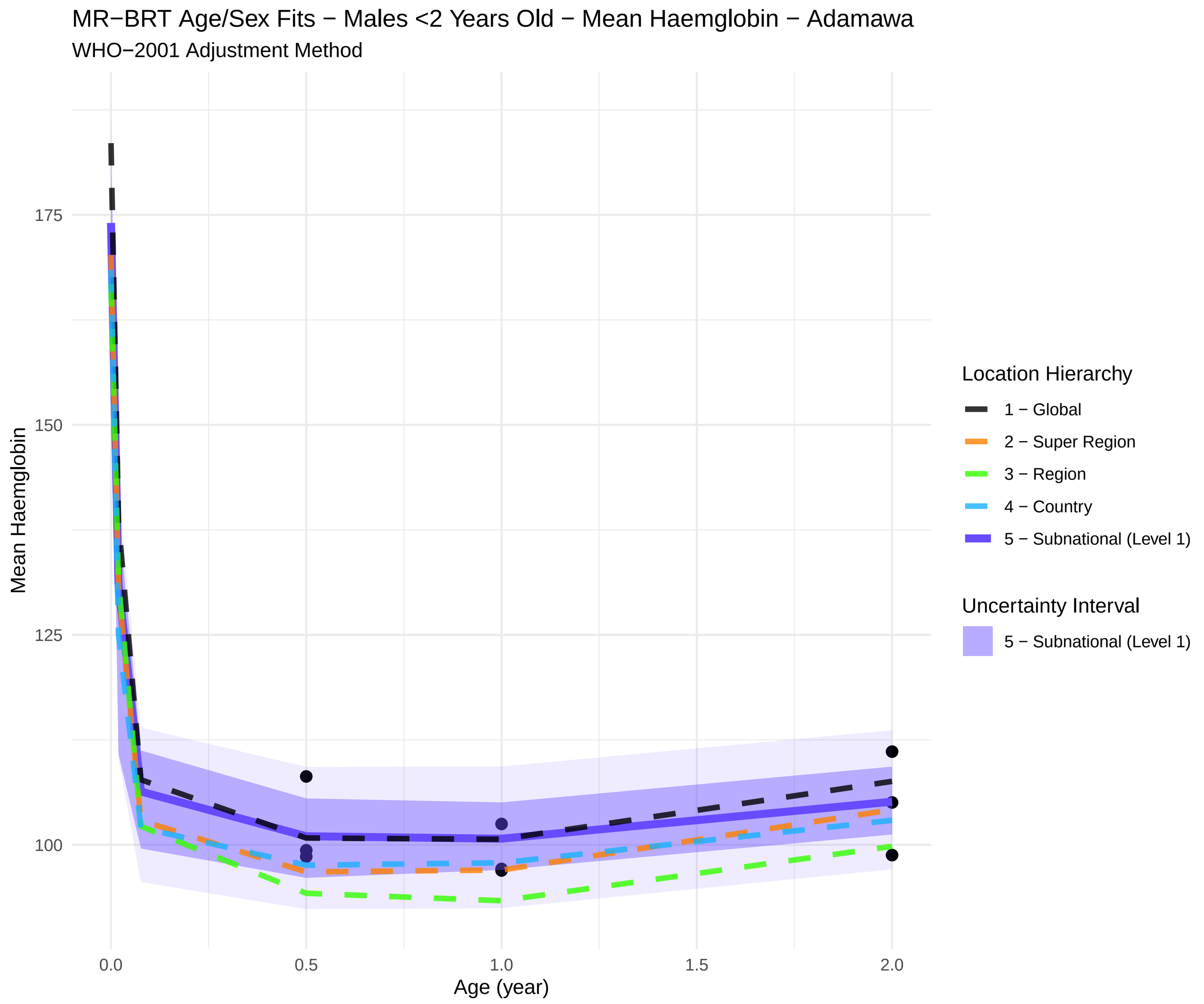
MR−BRT Age/Sex Fits − Males <2 Years Old − Mean Haemglobin − Adamawa
WHO−2001 Adjustment Method
Mean Haemglobin
Age (year)
Location Hierarchy
1 − Global
2 − Super Region
3 − Region
4 − Country
5 − Subnational (Level 1)
Uncertainty Interval
5 − Subnational (Level 1)

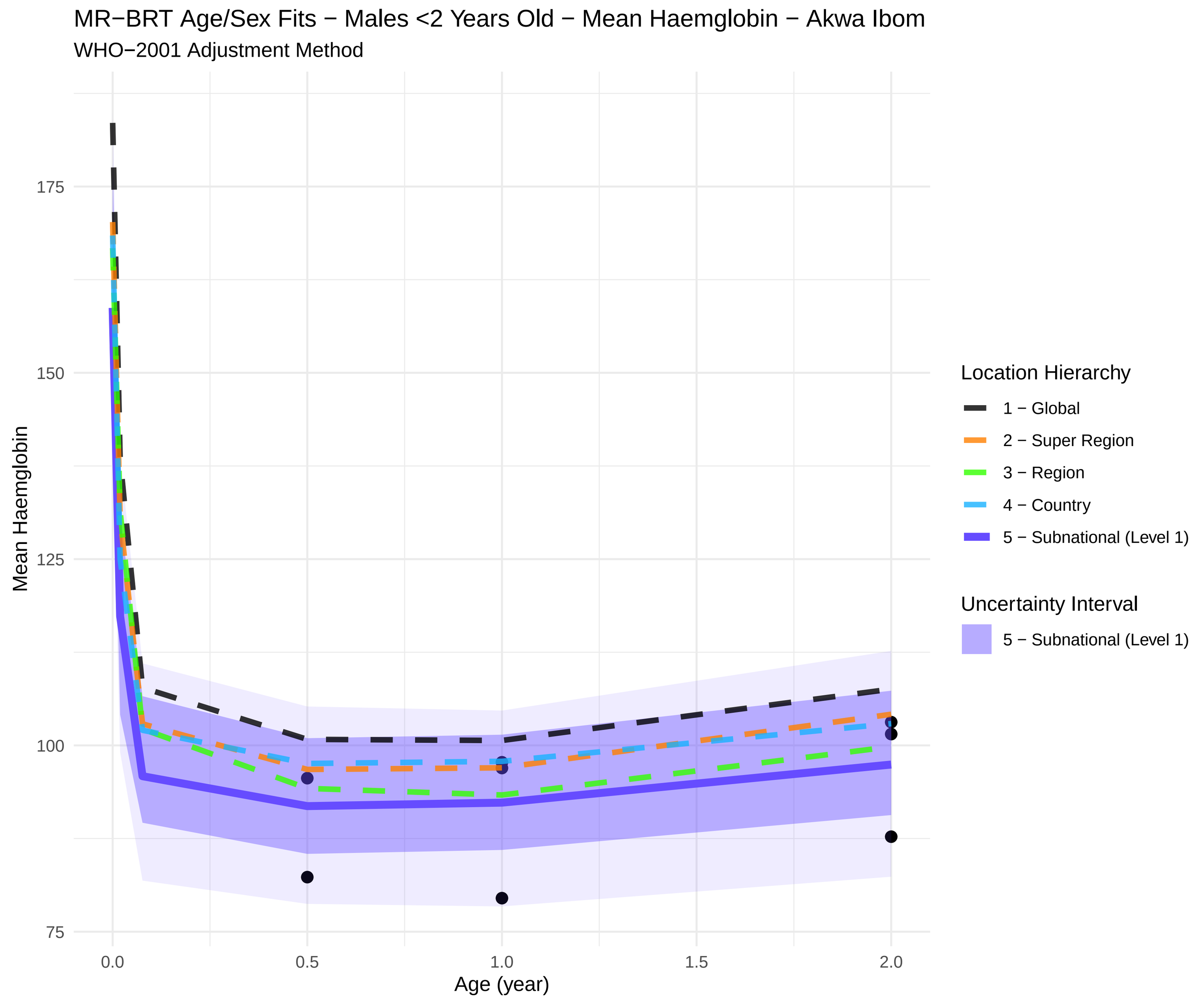
MR−BRT Age/Sex Fits − Males <2 Years Old − Mean Haemglobin − Akwa Ibom
WHO−2001 Adjustment Method
Mean Haemglobin
Age (year)
Location Hierarchy
1 − Global
2 − Super Region
3 − Region
4 − Country
5 − Subnational (Level 1)
Uncertainty Interval
5 − Subnational (Level 1)

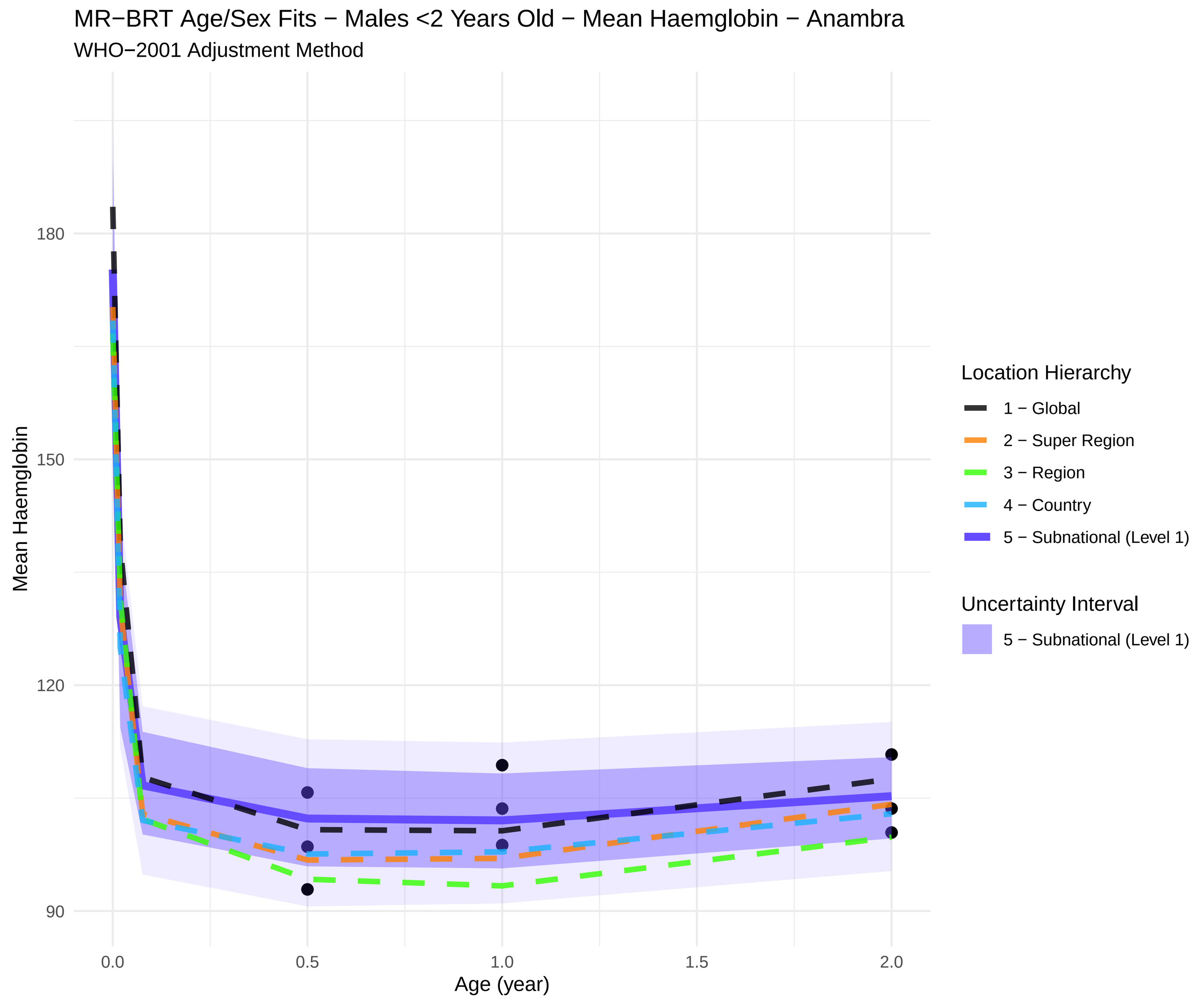
MR−BRT Age/Sex Fits − Males <2 Years Old − Mean Haemglobin − Anambra
WHO−2001 Adjustment Method
Mean Haemglobin
Age (year)
Location Hierarchy
1 − Global
2 − Super Region
3 − Region
4 − Country
5 − Subnational (Level 1)
Uncertainty Interval
5 − Subnational (Level 1)

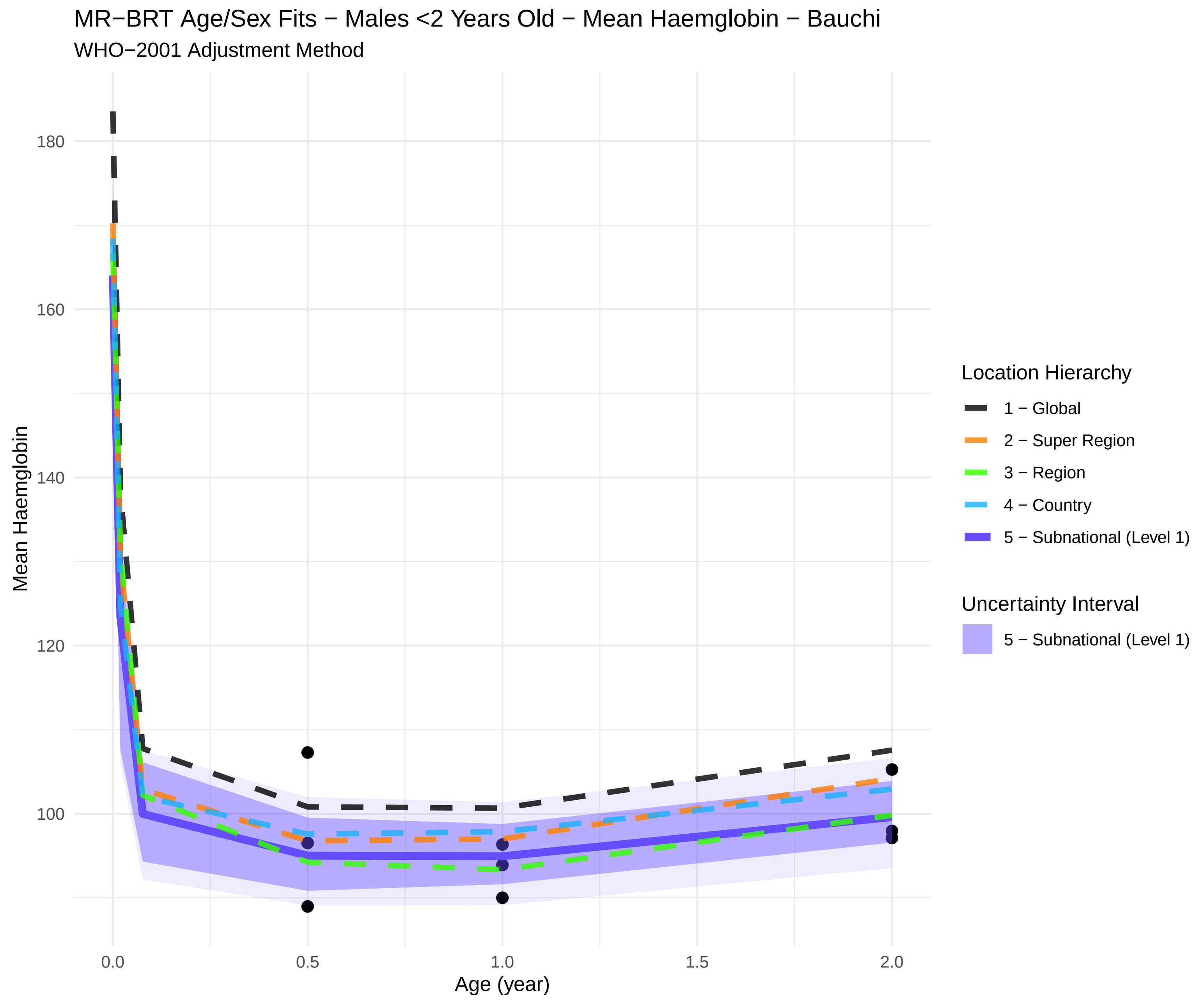
MR−BRT Age/Sex Fits − Males <2 Years Old − Mean Haemglobin − Bauchi
WHO−2001 Adjustment Method
Mean Haemglobin
Age (year)
Location Hierarchy
1 − Global
2 − Super Region
3 − Region
4 − Country
5 − Subnational (Level 1)
Uncertainty Interval
5 − Subnational (Level 1)

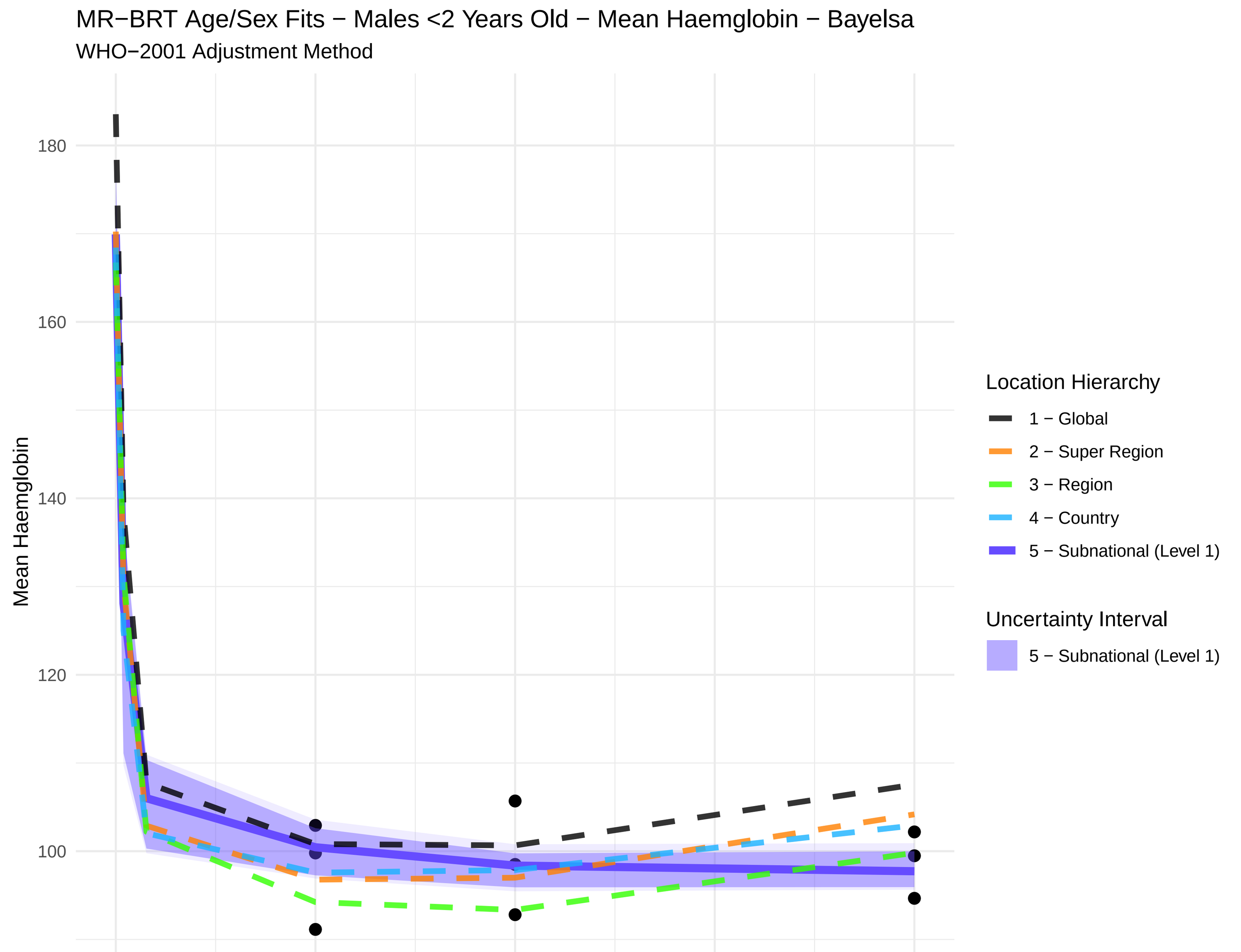
MR−BRT Age/Sex Fits − Males <2 Years Old − Mean Haemglobin − Bayelsa
WHO−2001 Adjustment Method
Mean Haemglobin
Age (year)
Location Hierarchy
1 − Global
2 − Super Region
3 − Region
4 − Country
5 − Subnational (Level 1)
Uncertainty Interval
5 − Subnational (Level 1)

MR−BRT Age/Sex Fits − Males <2 Years Old − Mean Haemglobin − Benue
WHO−2001 Adjustment Method

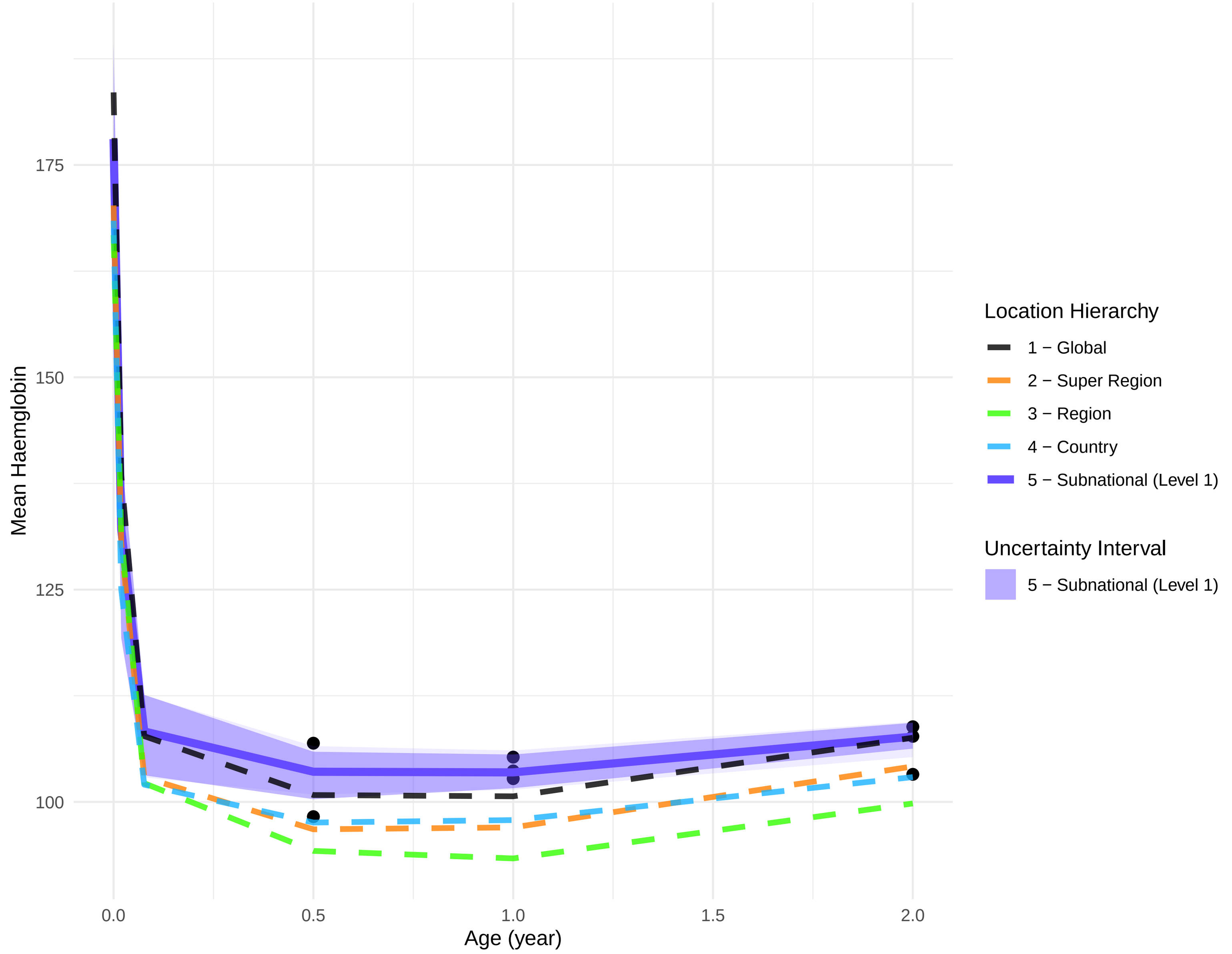

MR−BRT Age/Sex Fits − Males <2 Years Old − Mean Haemglobin − Borno
WHO−2001 Adjustment Method

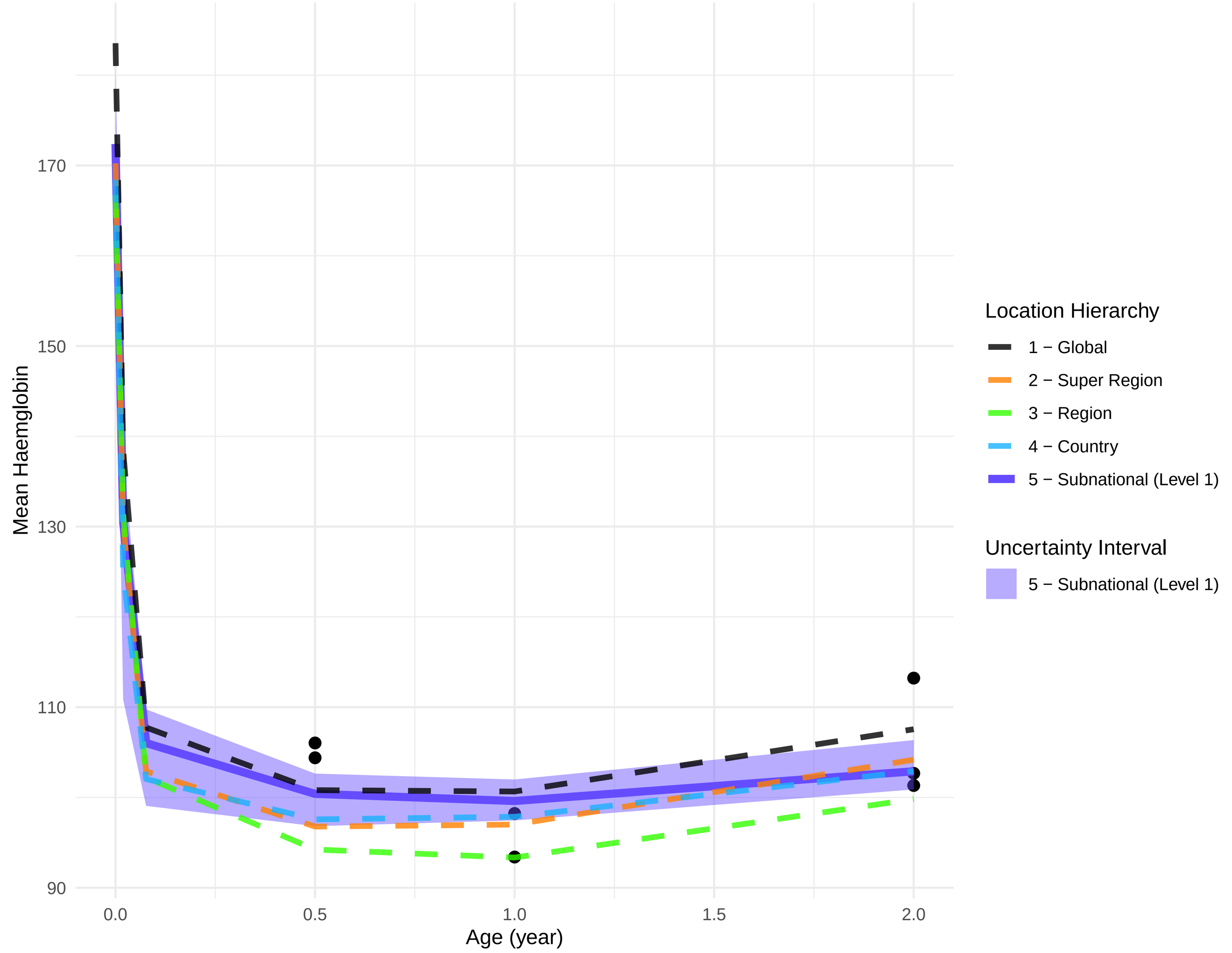

MR−BRT Age/Sex Fits − Males <2 Years Old − Mean Haemglobin − Cross River
WHO−2001 Adjustment Method

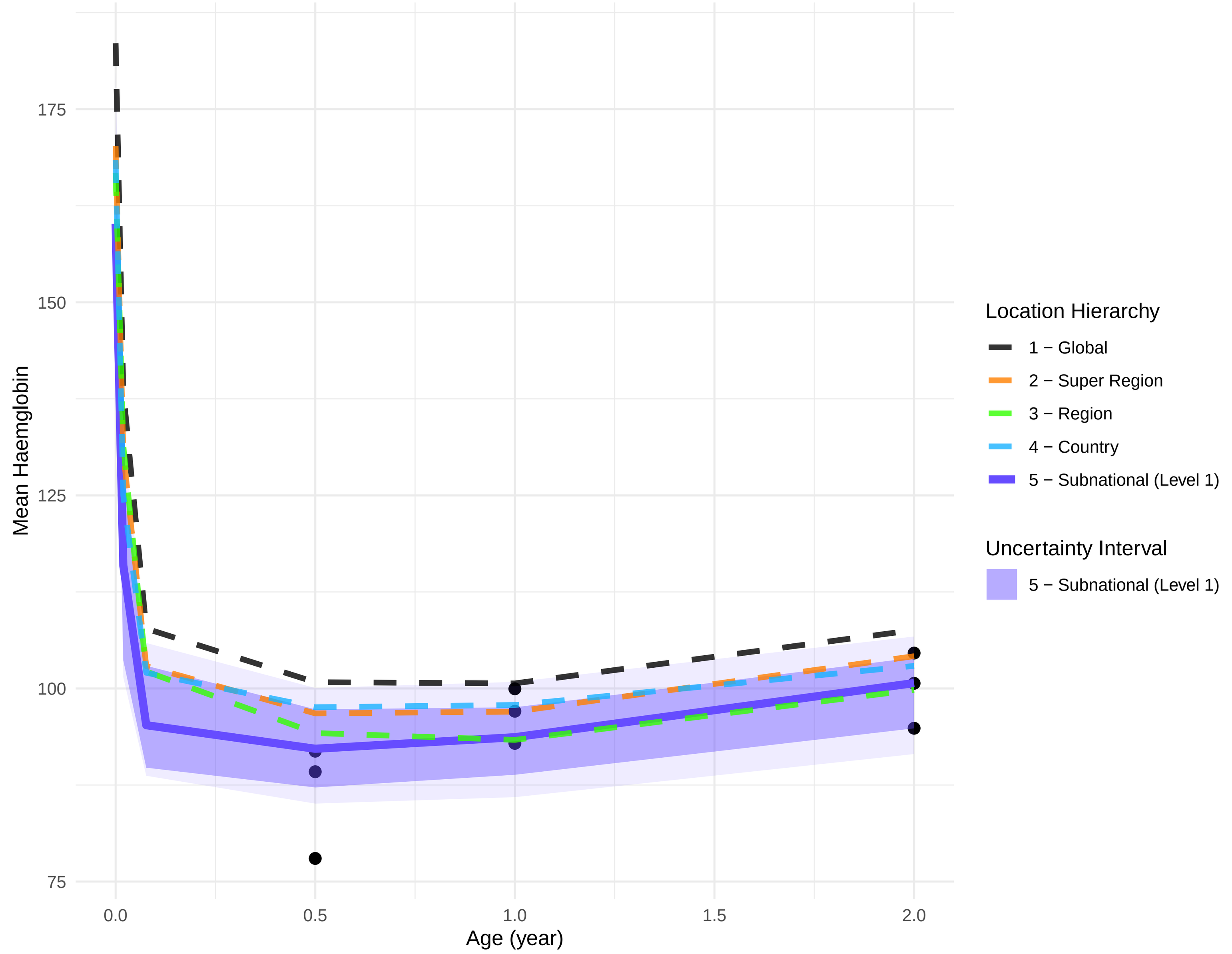

MR−BRT Age/Sex Fits − Males <2 Years Old − Mean Haemglobin − Delta
WHO−2001 Adjustment Method

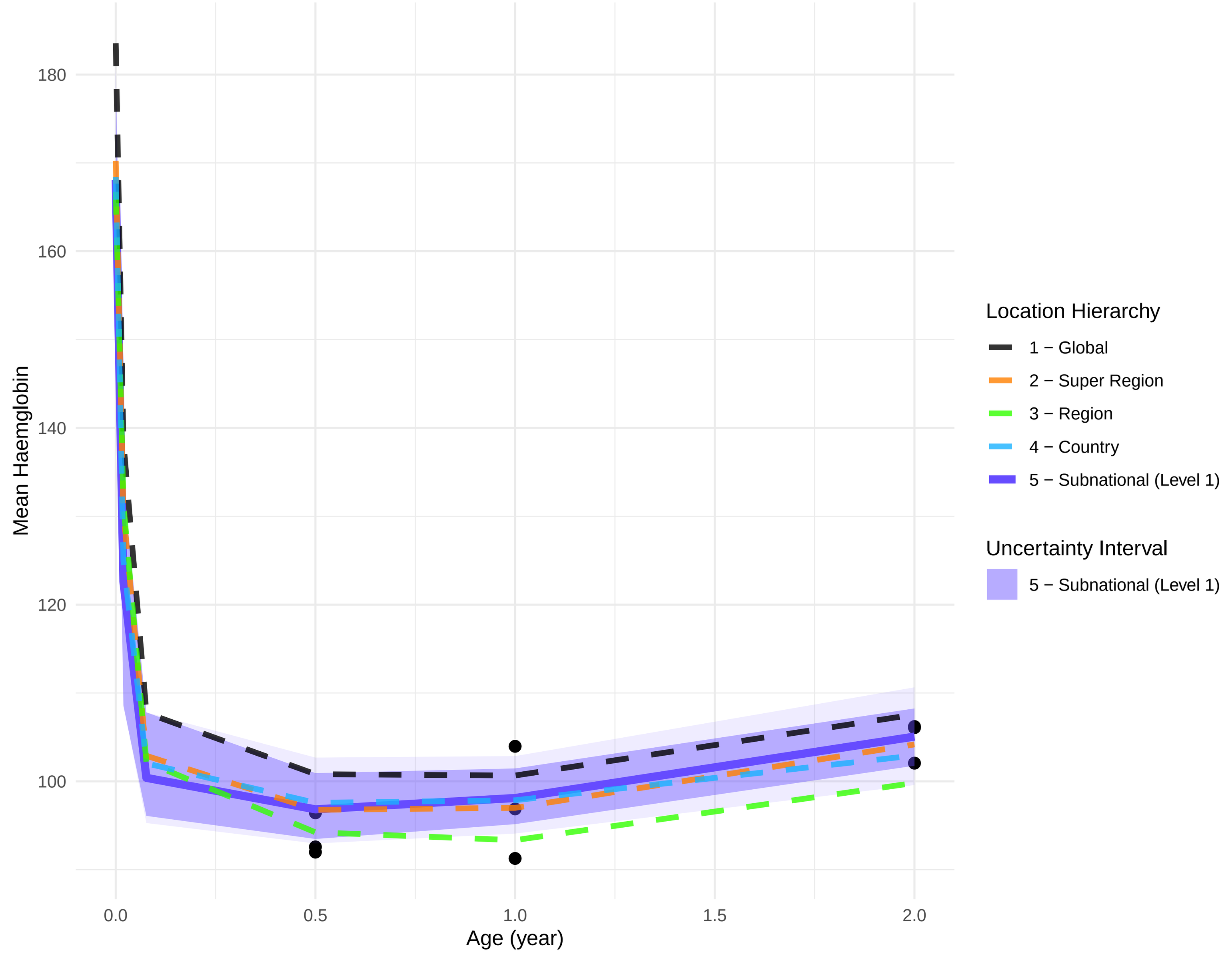

MR−BRT Age/Sex Fits − Males <2 Years Old − Mean Haemglobin − Ebonyi
WHO−2001 Adjustment Method

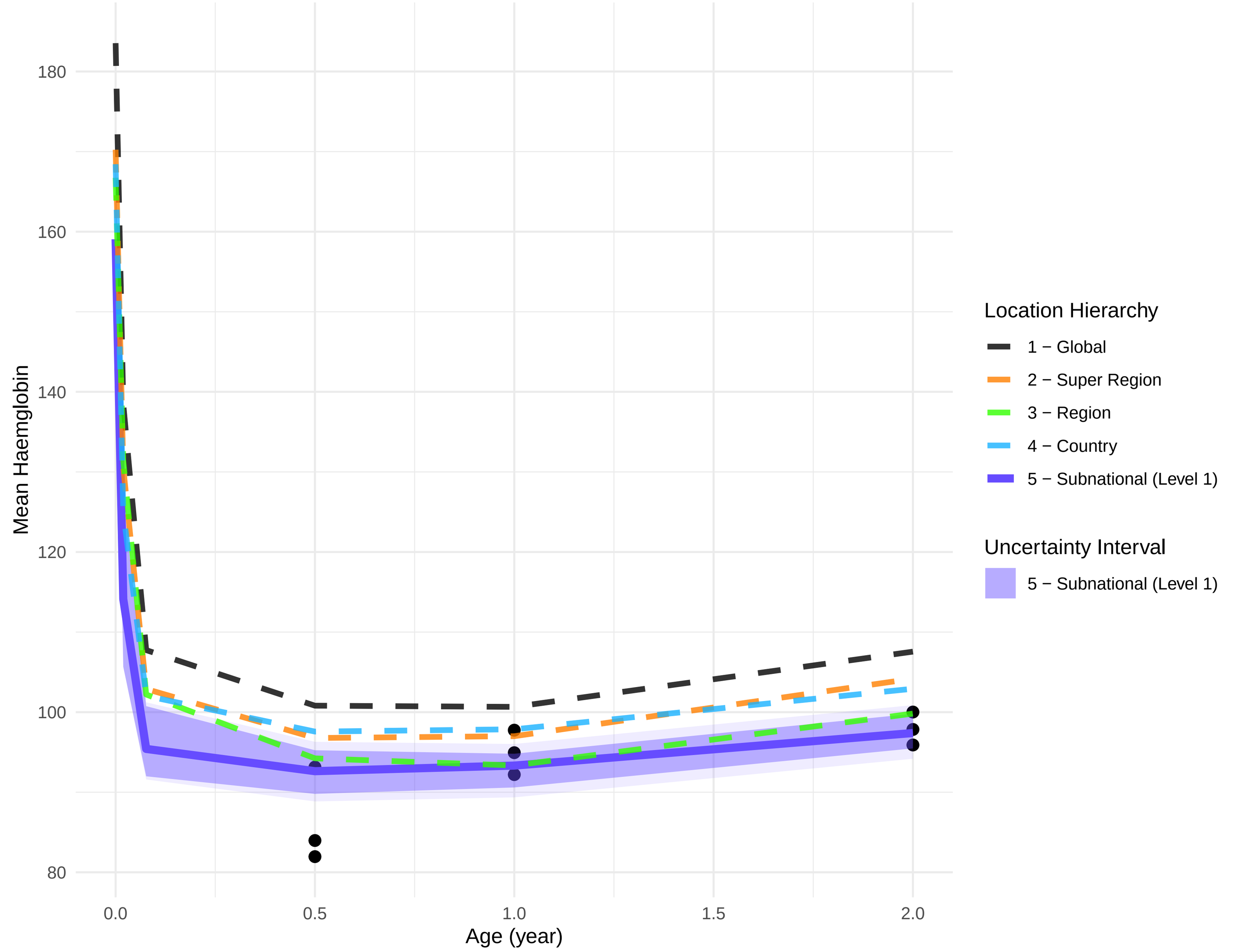

MR−BRT Age/Sex Fits − Males <2 Years Old − Mean Haemglobin − Edo
WHO−2001 Adjustment Method

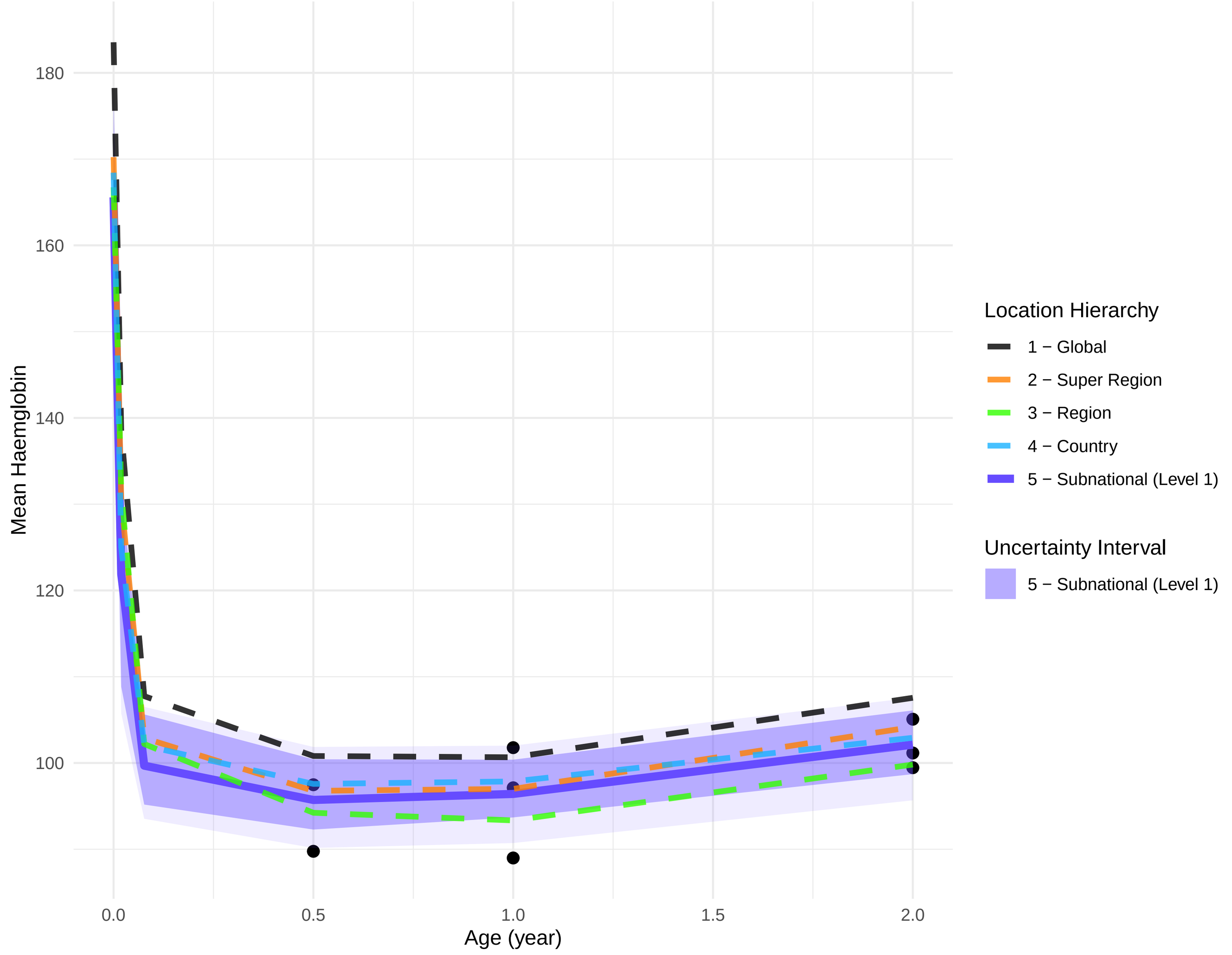

MR−BRT Age/Sex Fits − Males <2 Years Old − Mean Haemglobin − Ekiti
WHO−2001 Adjustment Method

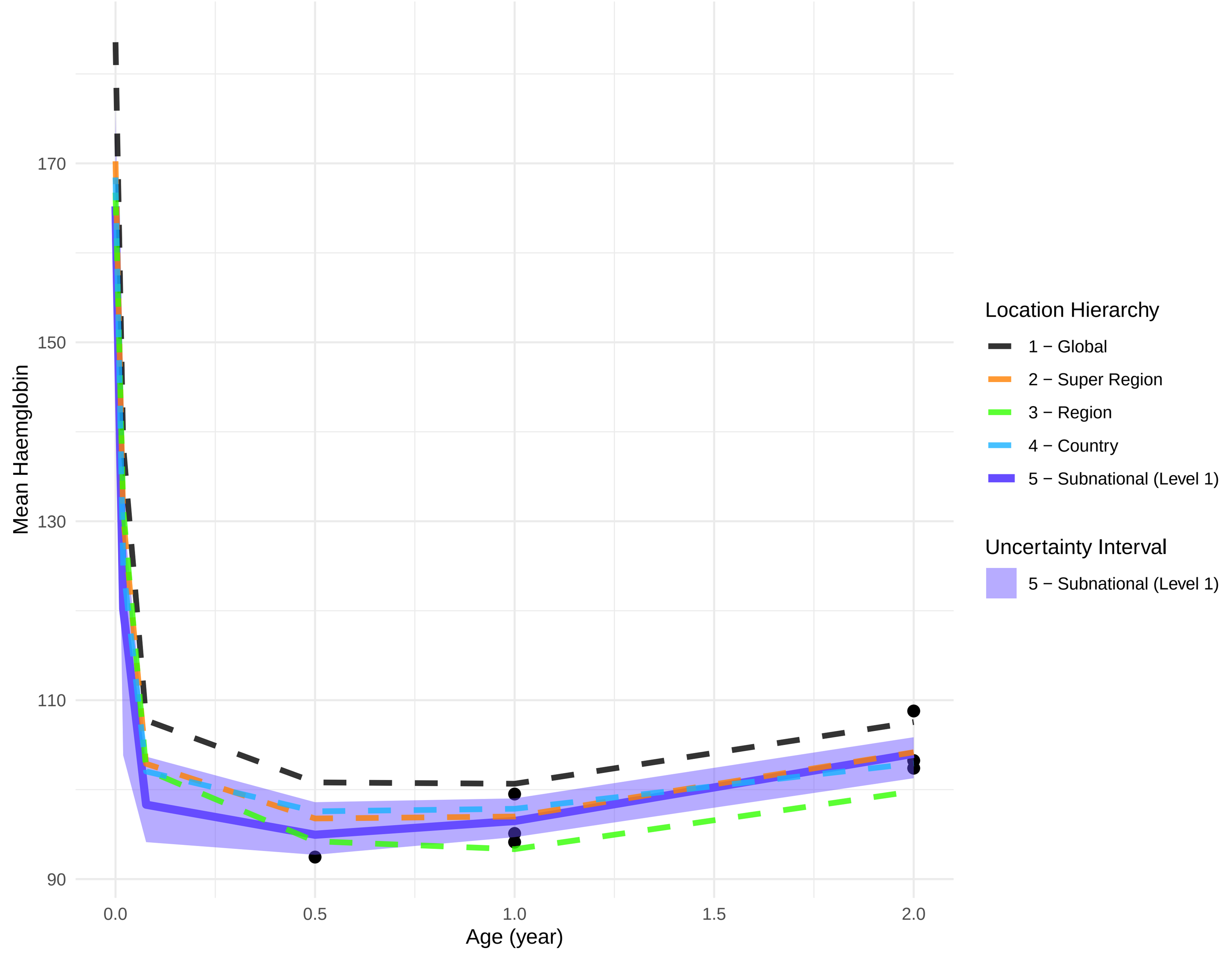

MR−BRT Age/Sex Fits − Males <2 Years Old − Mean Haemglobin − Enugu
WHO−2001 Adjustment Method

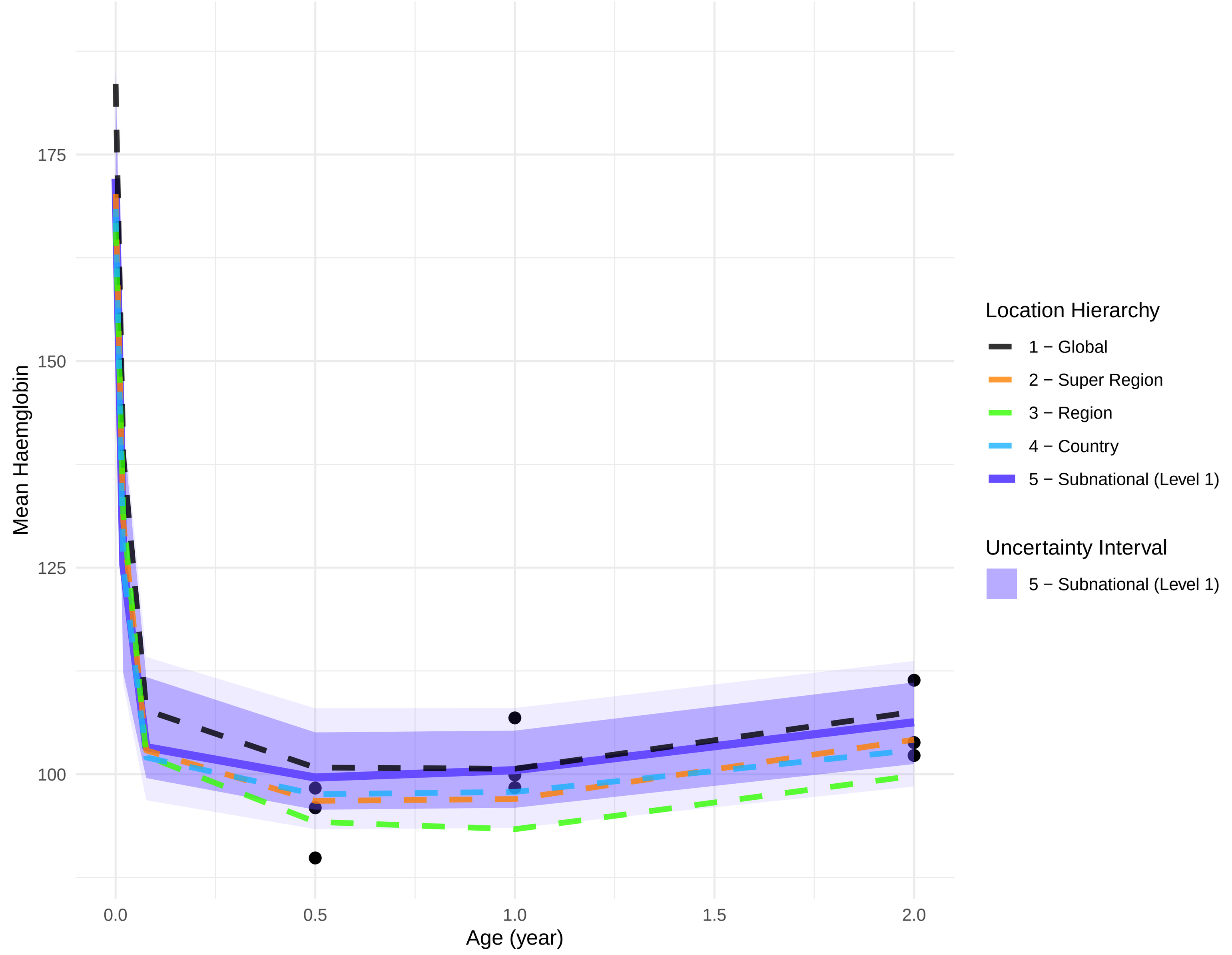

MR−BRT Age/Sex Fits − Males <2 Years Old − Mean Haemglobin − FCT (Abuja)
WHO−2001 Adjustment Method

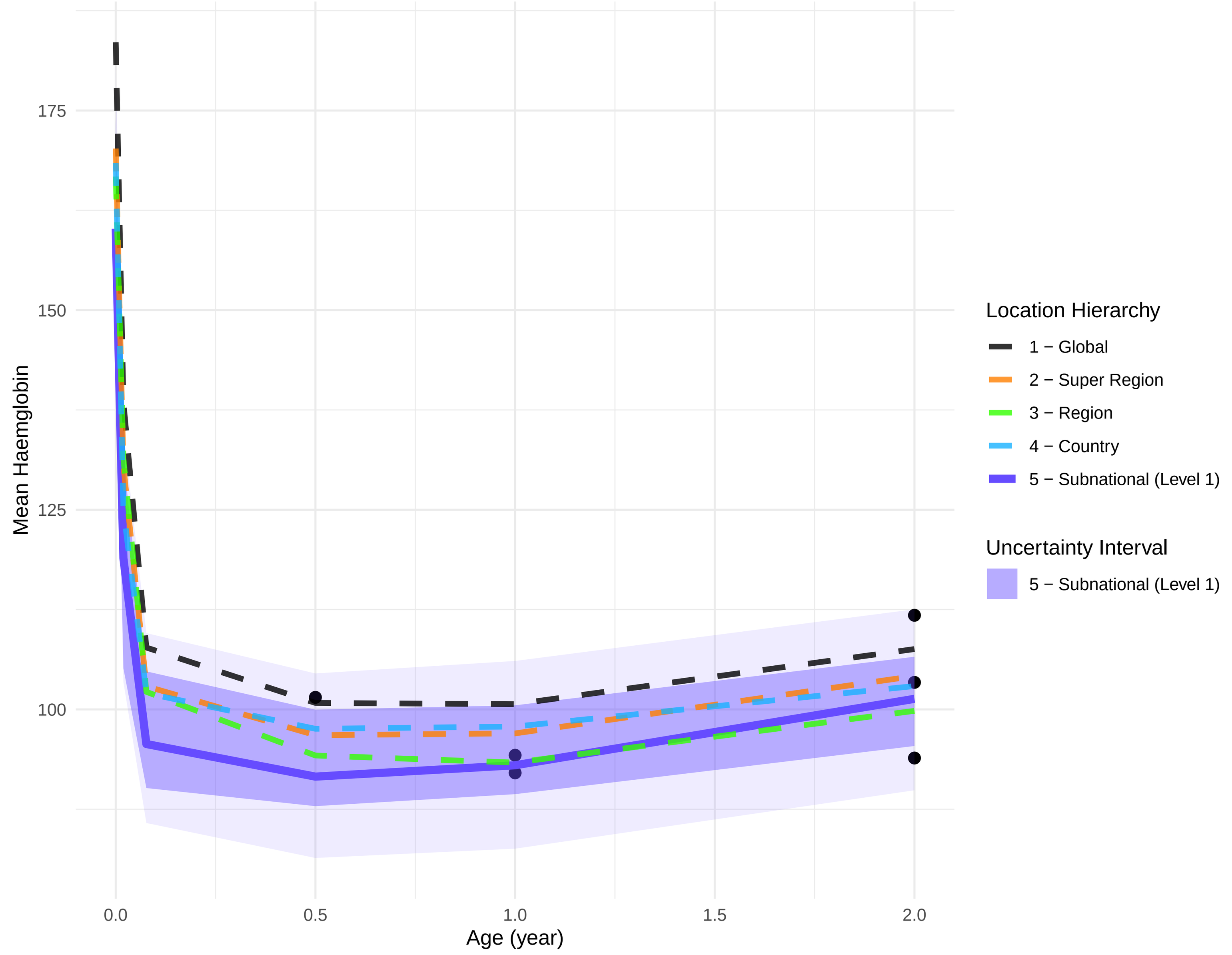

MR−BRT Age/Sex Fits − Males <2 Years Old − Mean Haemglobin − Gombe
WHO−2001 Adjustment Method

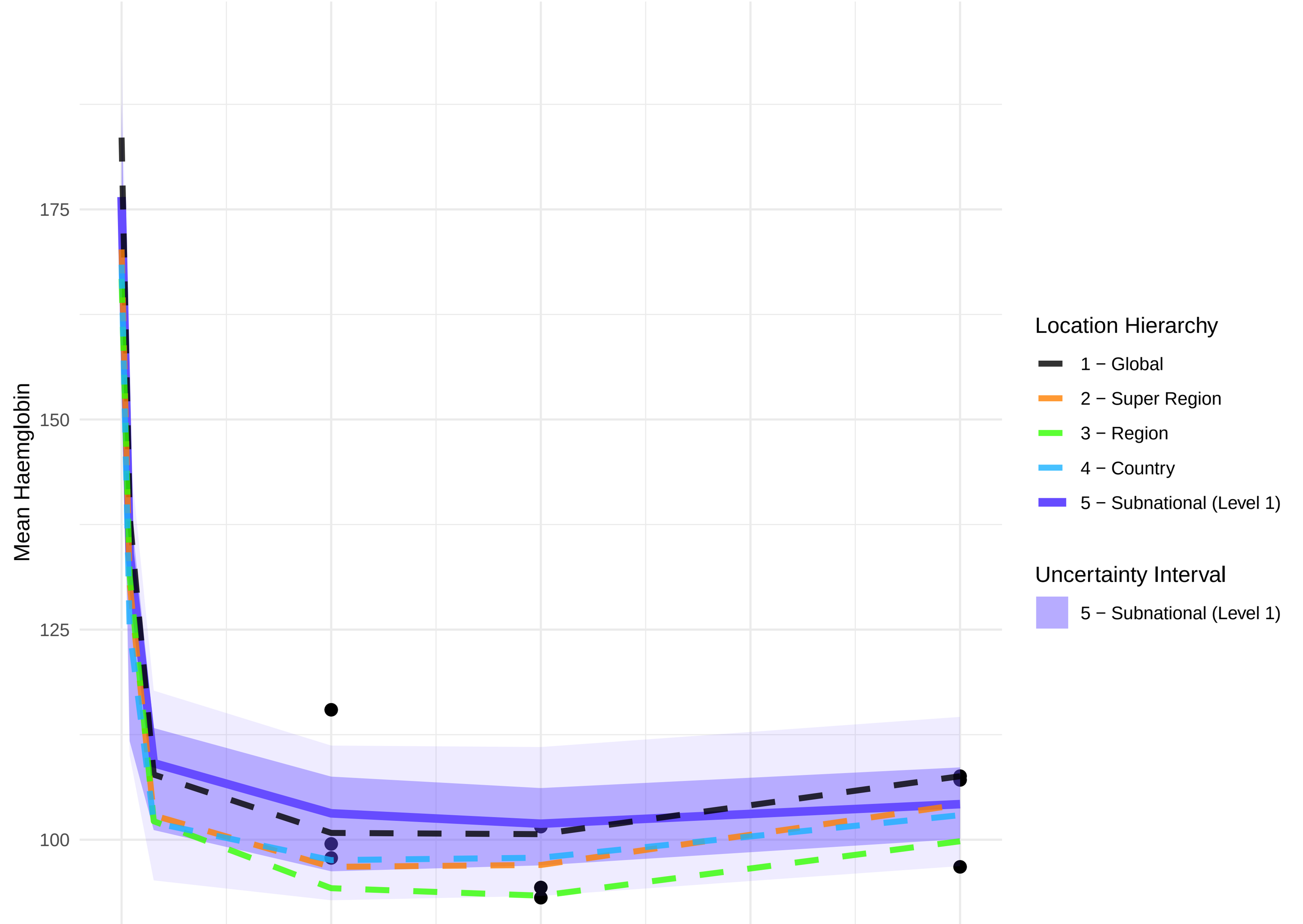

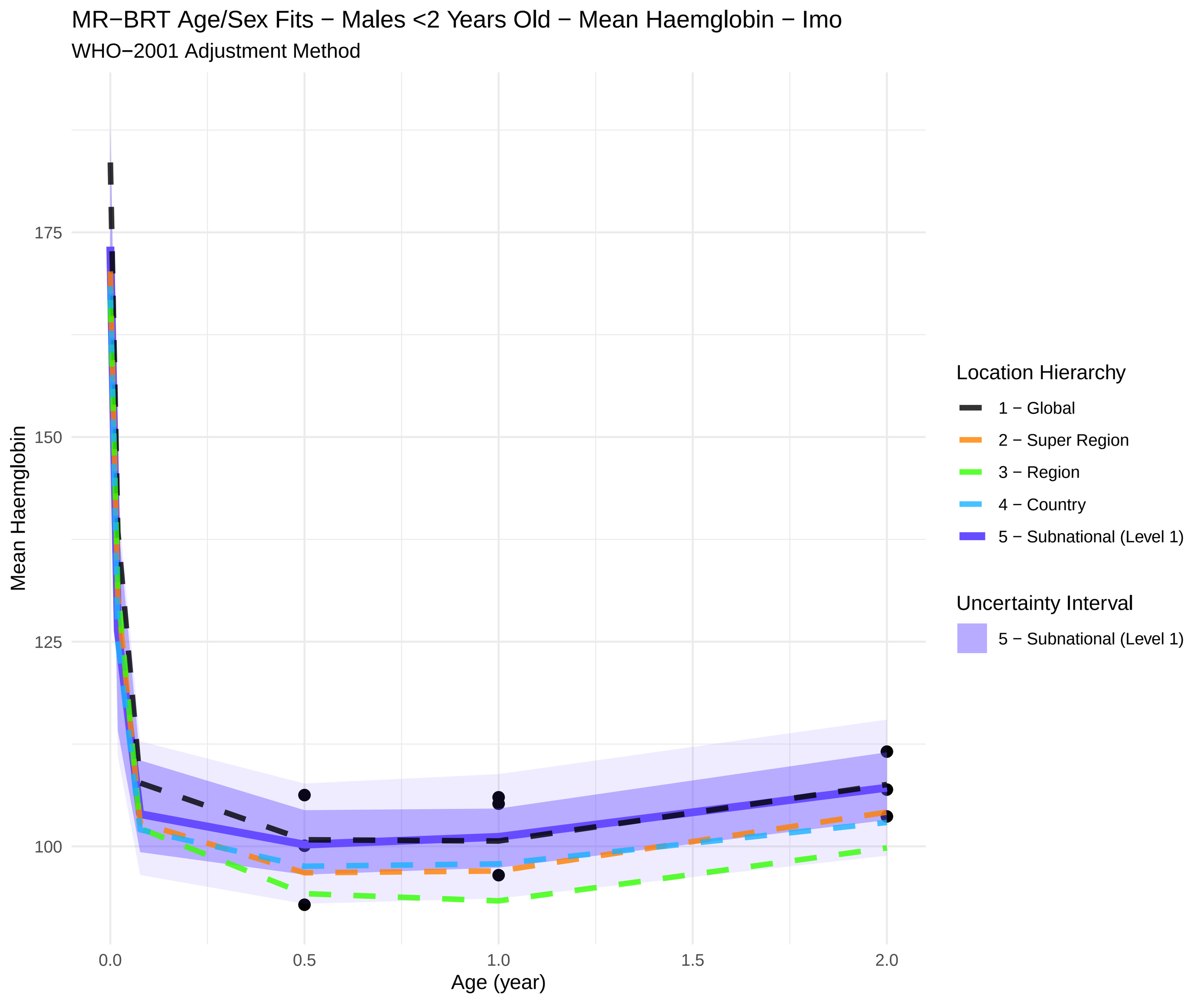
MR−BRT Age/Sex Fits − Males <2 Years Old − Mean Haemglobin − Imo
WHO−2001 Adjustment Method
Mean Haemglobin
100
125
150
175
0.0
0.5
1.0
1.5
2.0
Age (year)
Location Hierarchy
1 − Global
2 − Super Region
3 − Region
4 − Country
5 − Subnational (Level 1)
Uncertainty Interval
5 − Subnational (Level 1)

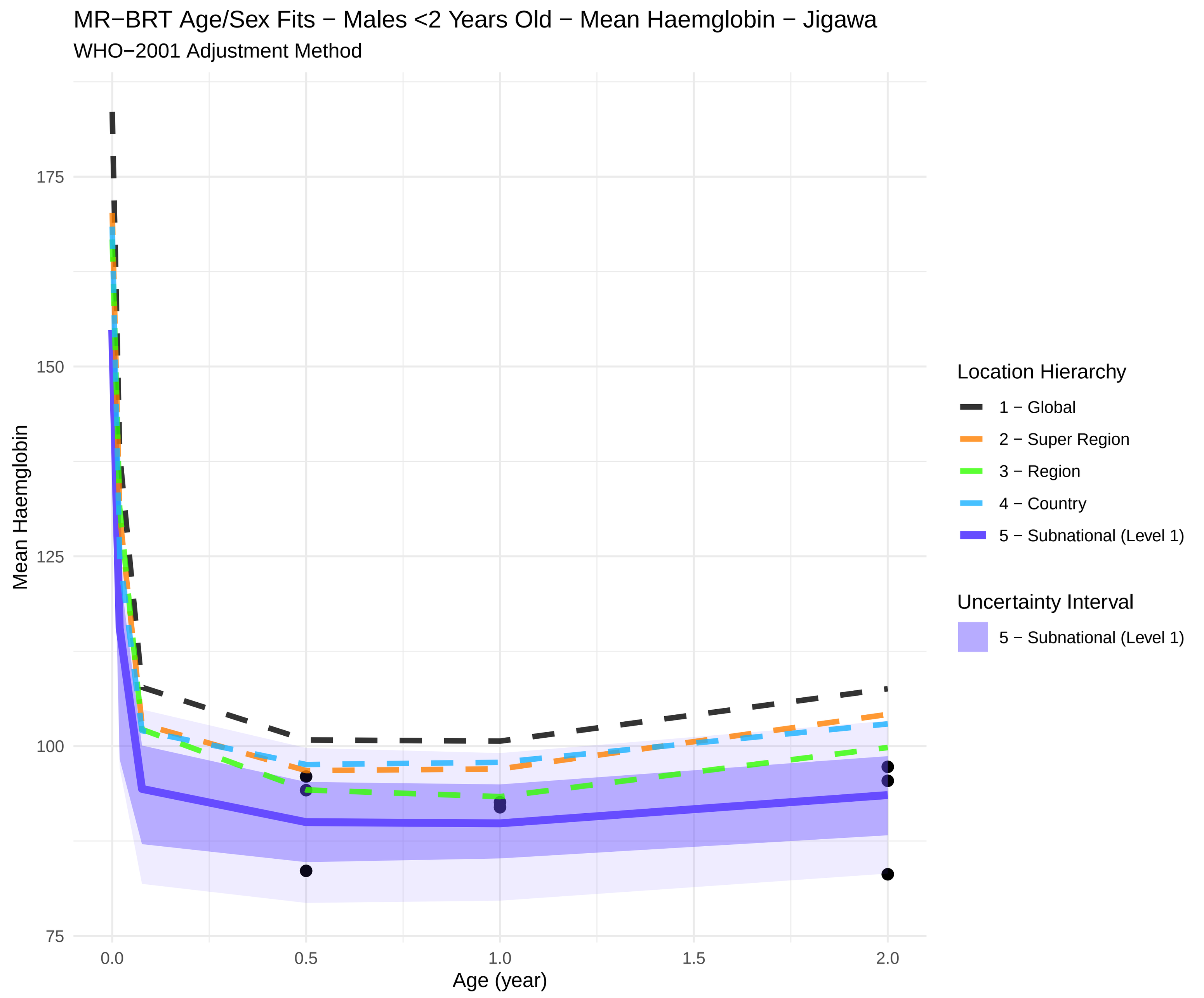
MR−BRT Age/Sex Fits − Males <2 Years Old − Mean Haemglobin − Jigawa
WHO−2001 Adjustment Method
Mean Haemglobin
75
100
125
150
175
0.0
0.5
1.0
1.5
2.0
Age (year)
Location Hierarchy
1 − Global
2 − Super Region
3 − Region
4 − Country
5 − Subnational (Level 1)
Uncertainty Interval
5 − Subnational (Level 1)

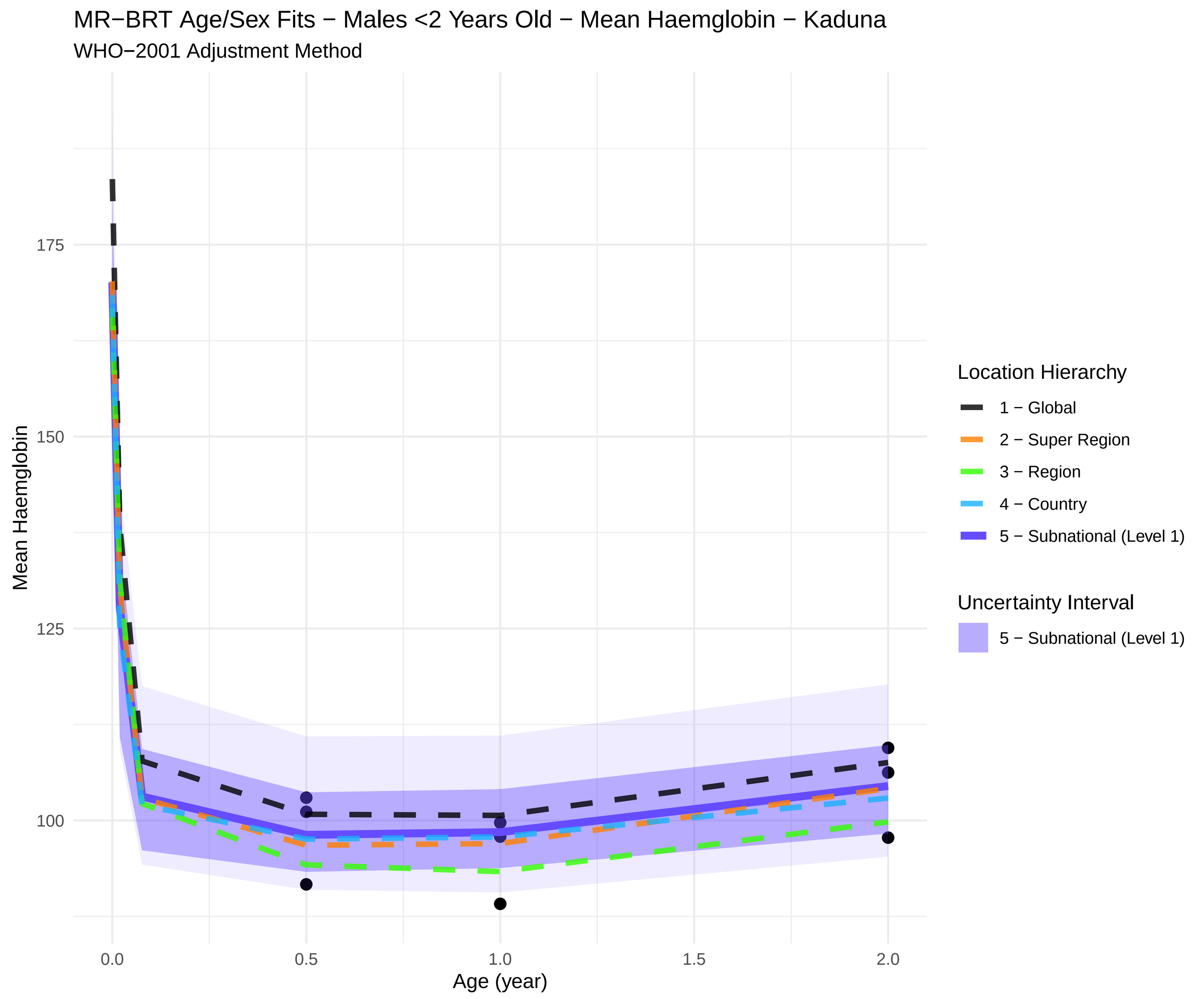
MR−BRT Age/Sex Fits − Males <2 Years Old − Mean Haemglobin − Kaduna
WHO−2001 Adjustment Method
Mean Haemglobin
100
125
150
175
0.0
0.5
1.0
1.5
2.0
Age (year)
Location Hierarchy
1 − Global
2 − Super Region
3 − Region
4 − Country
5 − Subnational (Level 1)
Uncertainty Interval
5 − Subnational (Level 1)

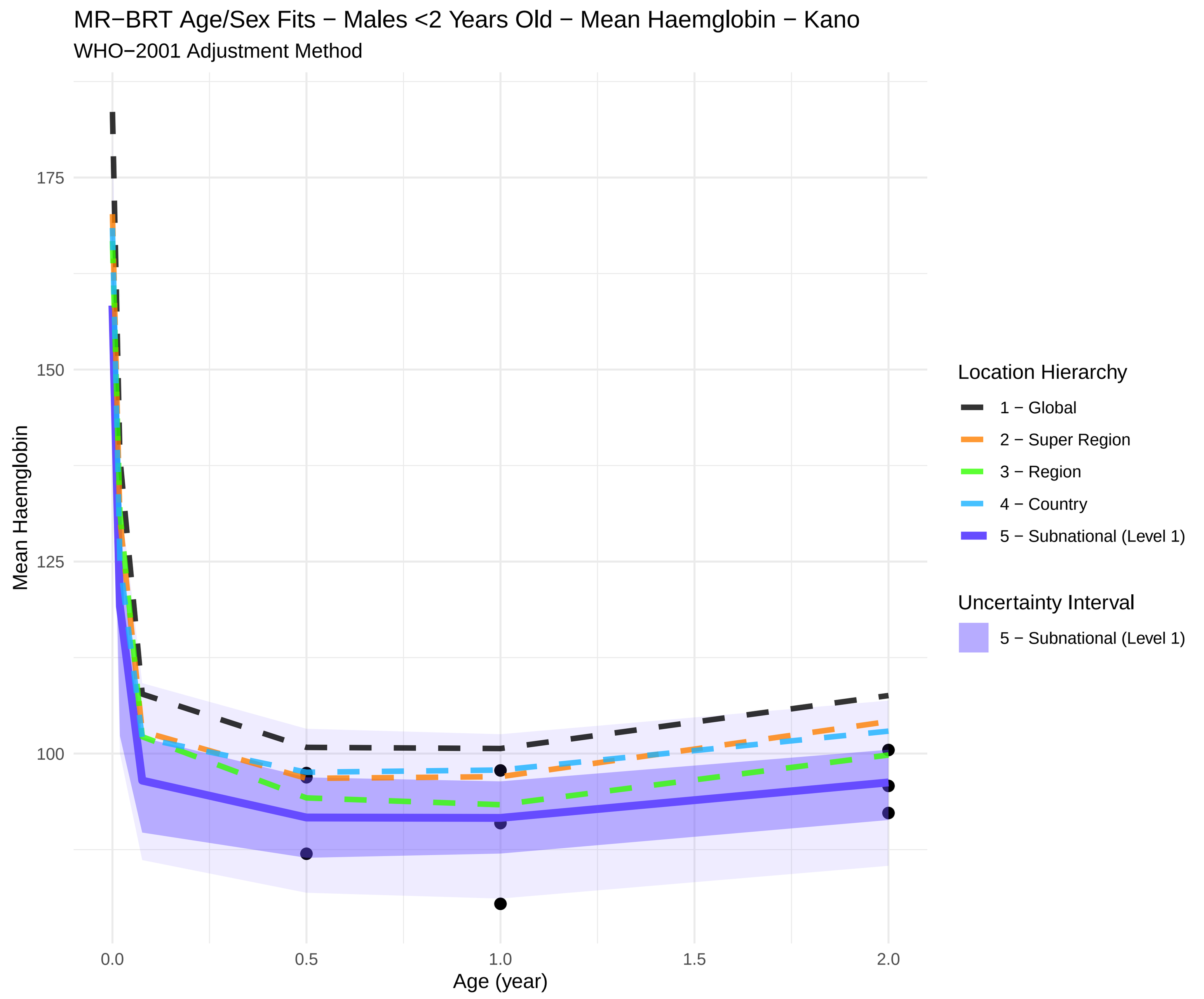
MR−BRT Age/Sex Fits − Males <2 Years Old − Mean Haemglobin − Kano
WHO−2001 Adjustment Method
Mean Haemglobin
100
125
150
175
0.0
0.5
1.0
1.5
2.0
Age (year)
Location Hierarchy
1 − Global
2 − Super Region
3 − Region
4 − Country
5 − Subnational (Level 1)
Uncertainty Interval
5 − Subnational (Level 1)

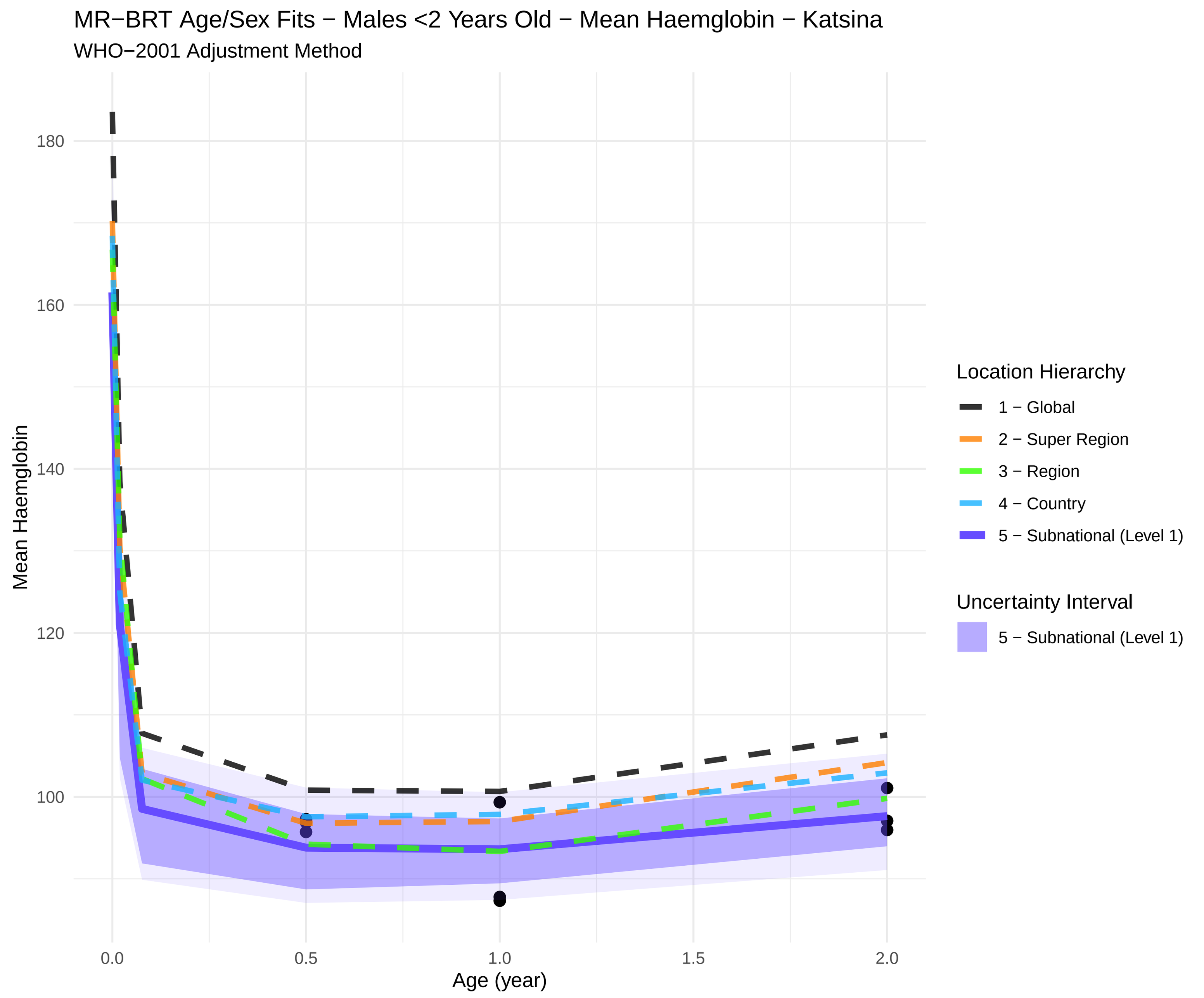
MR−BRT Age/Sex Fits − Males <2 Years Old − Mean Haemglobin − Katsina
WHO−2001 Adjustment Method
Mean Haemglobin
100
120
140
160
180
0.0
0.5
1.0
1.5
2.0
Age (year)
Location Hierarchy
1 − Global
2 − Super Region
3 − Region
4 − Country
5 − Subnational (Level 1)
Uncertainty Interval
5 − Subnational (Level 1)

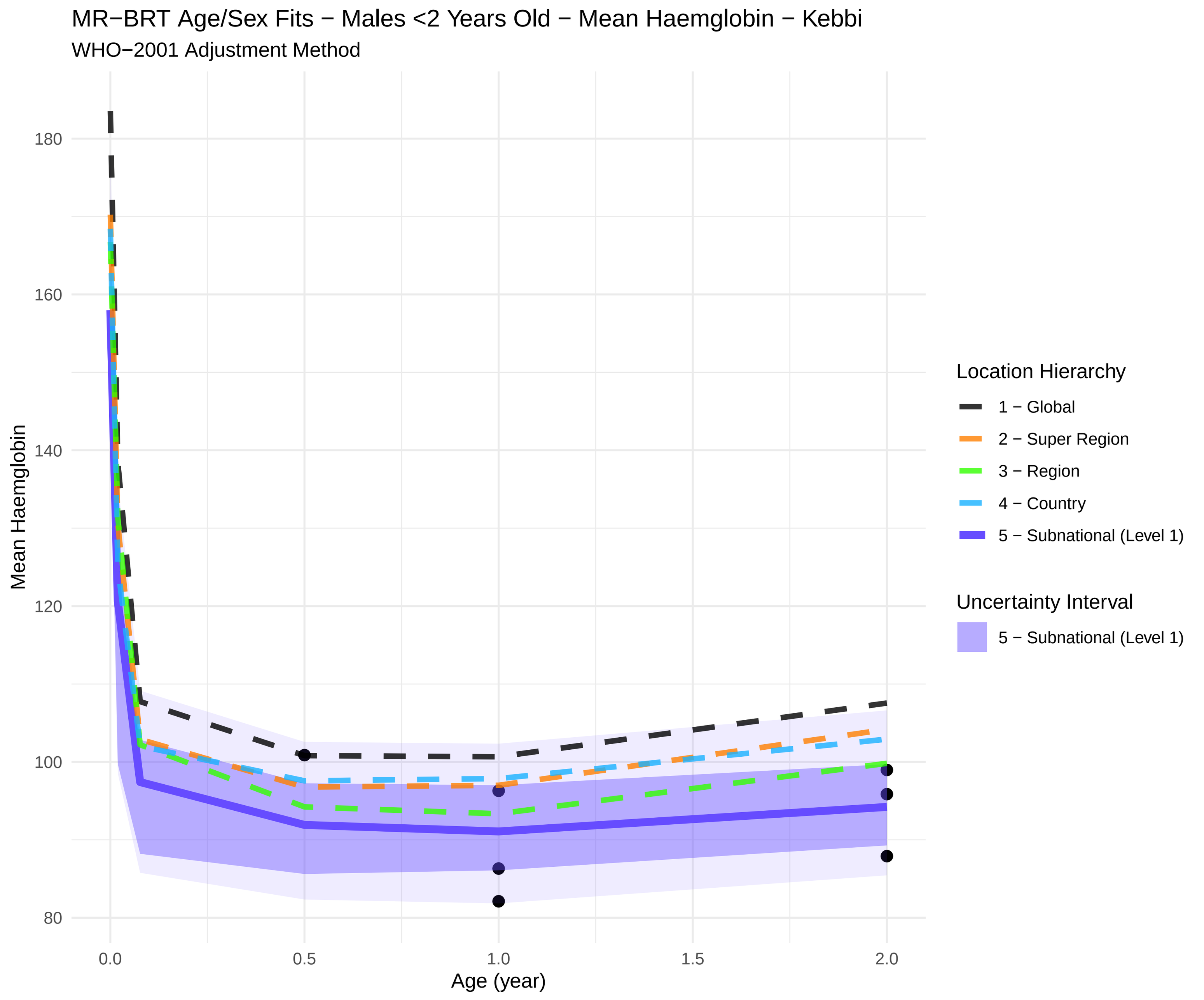
MR−BRT Age/Sex Fits − Males <2 Years Old − Mean Haemglobin − Kebbi
WHO−2001 Adjustment Method
Mean Haemglobin
80
100
120
140
160
180
0.0
0.5
1.0
1.5
2.0
Age (year)
Location Hierarchy
1 − Global
2 − Super Region
3 − Region
4 − Country
5 − Subnational (Level 1)
Uncertainty Interval
5 − Subnational (Level 1)

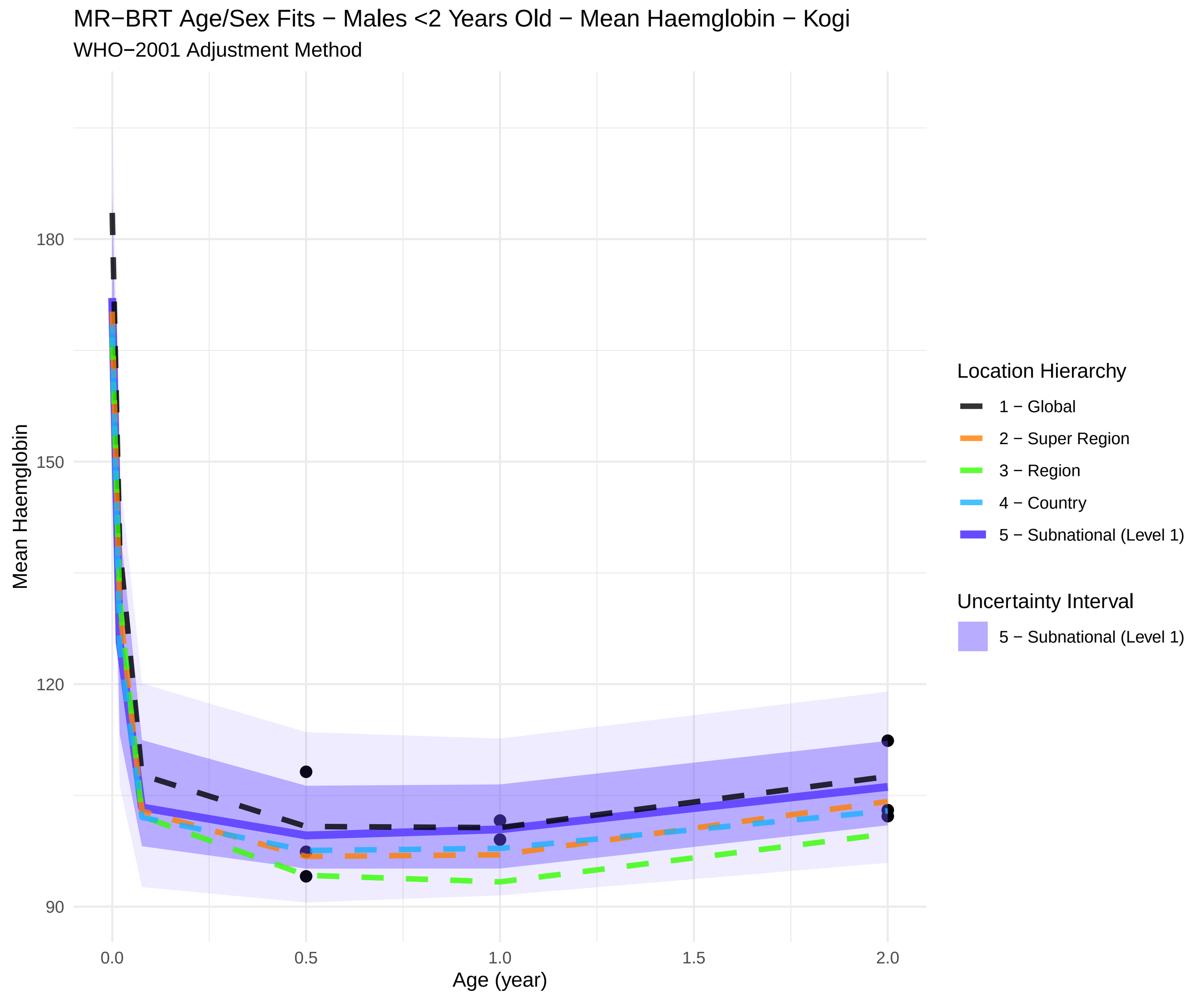
MR−BRT Age/Sex Fits − Males <2 Years Old − Mean Haemglobin − Kogi
WHO−2001 Adjustment Method
Mean Haemglobin
90
120
150
180
0.0
0.5
1.0
1.5
2.0
Age (year)
Location Hierarchy
1 − Global
2 − Super Region
3 − Region
4 − Country
5 − Subnational (Level 1)
Uncertainty Interval
5 − Subnational (Level 1)

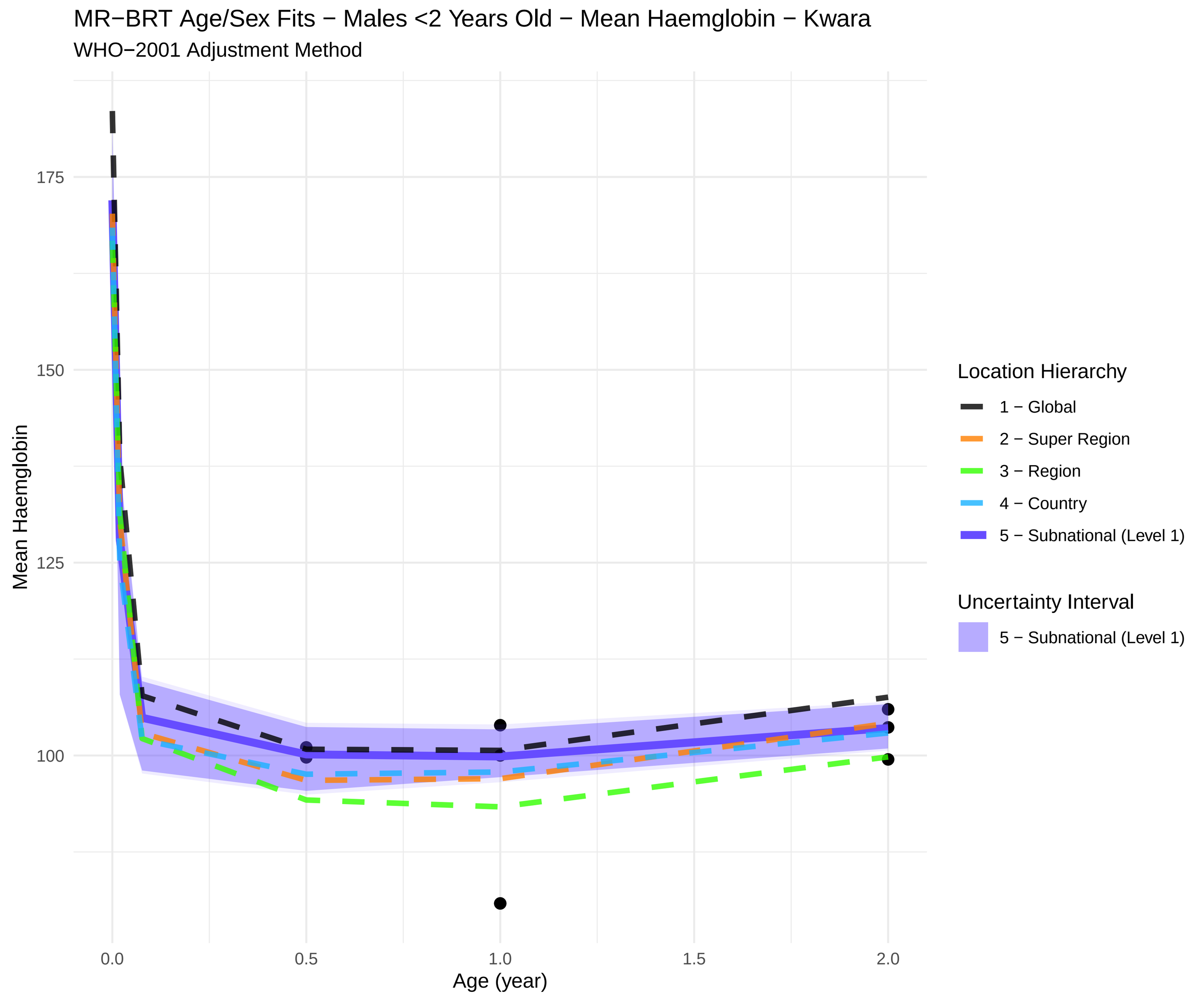
MR−BRT Age/Sex Fits − Males <2 Years Old − Mean Haemglobin − Kwara
WHO−2001 Adjustment Method
Mean Haemglobin
100
125
150
175
0.0
0.5
1.0
1.5
2.0
Age (year)
Location Hierarchy
1 − Global
2 − Super Region
3 − Region
4 − Country
5 − Subnational (Level 1)
Uncertainty Interval
5 − Subnational (Level 1)

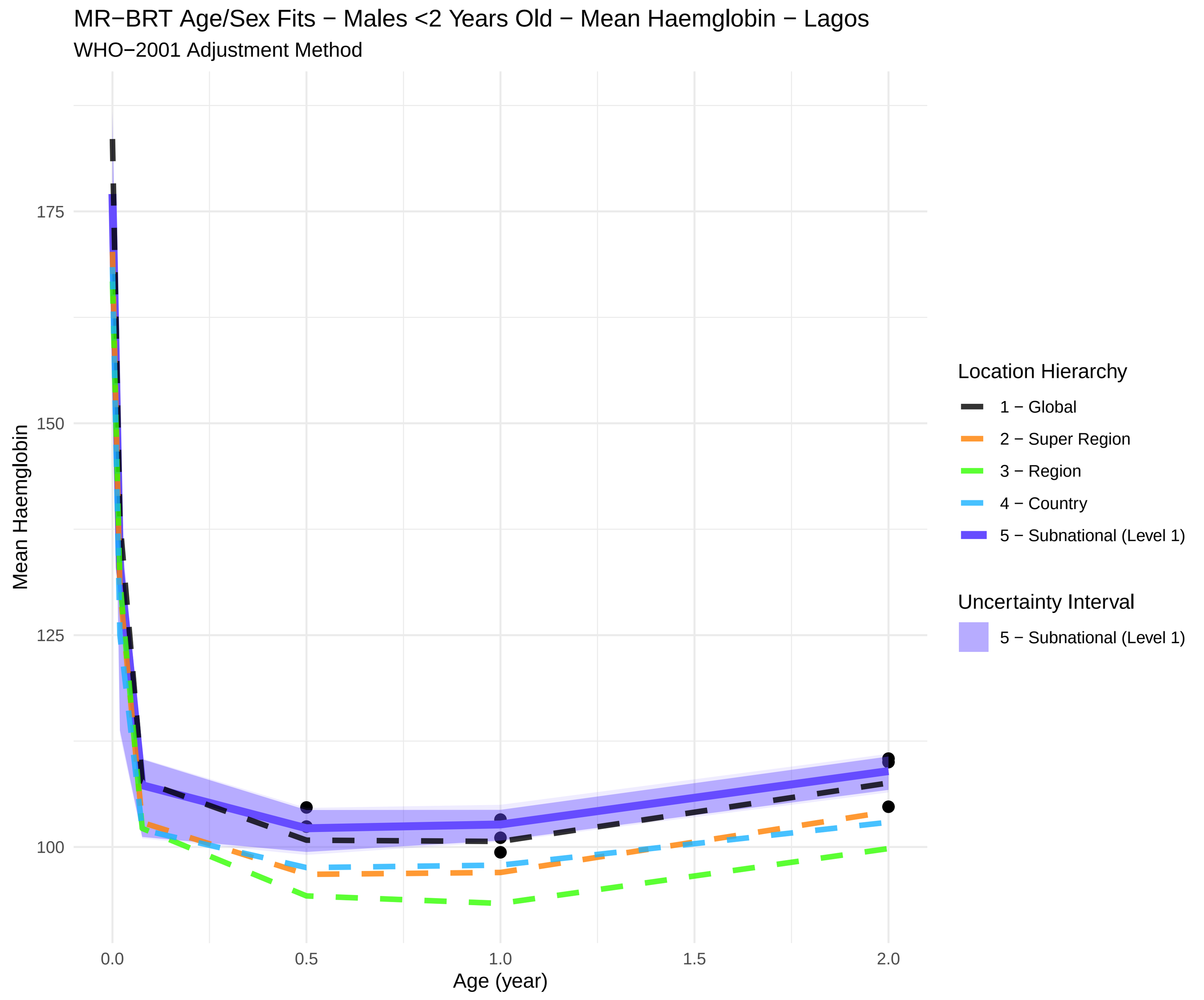
MR−BRT Age/Sex Fits − Males <2 Years Old − Mean Haemglobin − Lagos
WHO−2001 Adjustment Method
Mean Haemglobin
100
125
150
175
0.0
0.5
1.0
1.5
2.0
Age (year)
Location Hierarchy
1 − Global
2 − Super Region
3 − Region
4 − Country
5 − Subnational (Level 1)
Uncertainty Interval
5 − Subnational (Level 1)

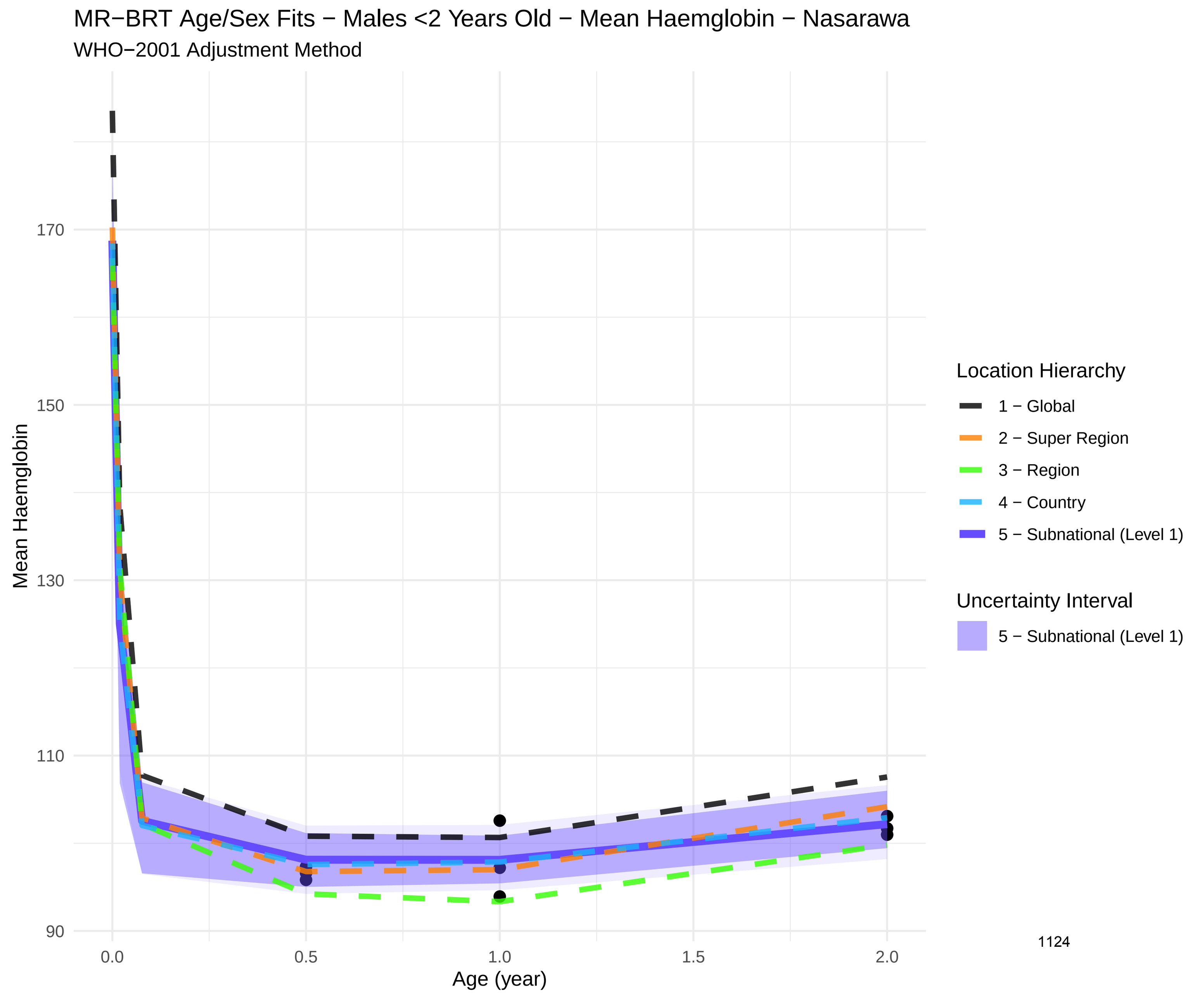
MR−BRT Age/Sex Fits − Males <2 Years Old − Mean Haemglobin − Nasarawa
WHO−2001 Adjustment Method
Mean Haemglobin
90
110
130
150
170
0.0
0.5
1.0
1.5
2.0
Age (year)
Location Hierarchy
1 − Global
2 − Super Region
3 − Region
4 − Country
5 − Subnational (Level 1)
Uncertainty Interval
5 − Subnational (Level 1)

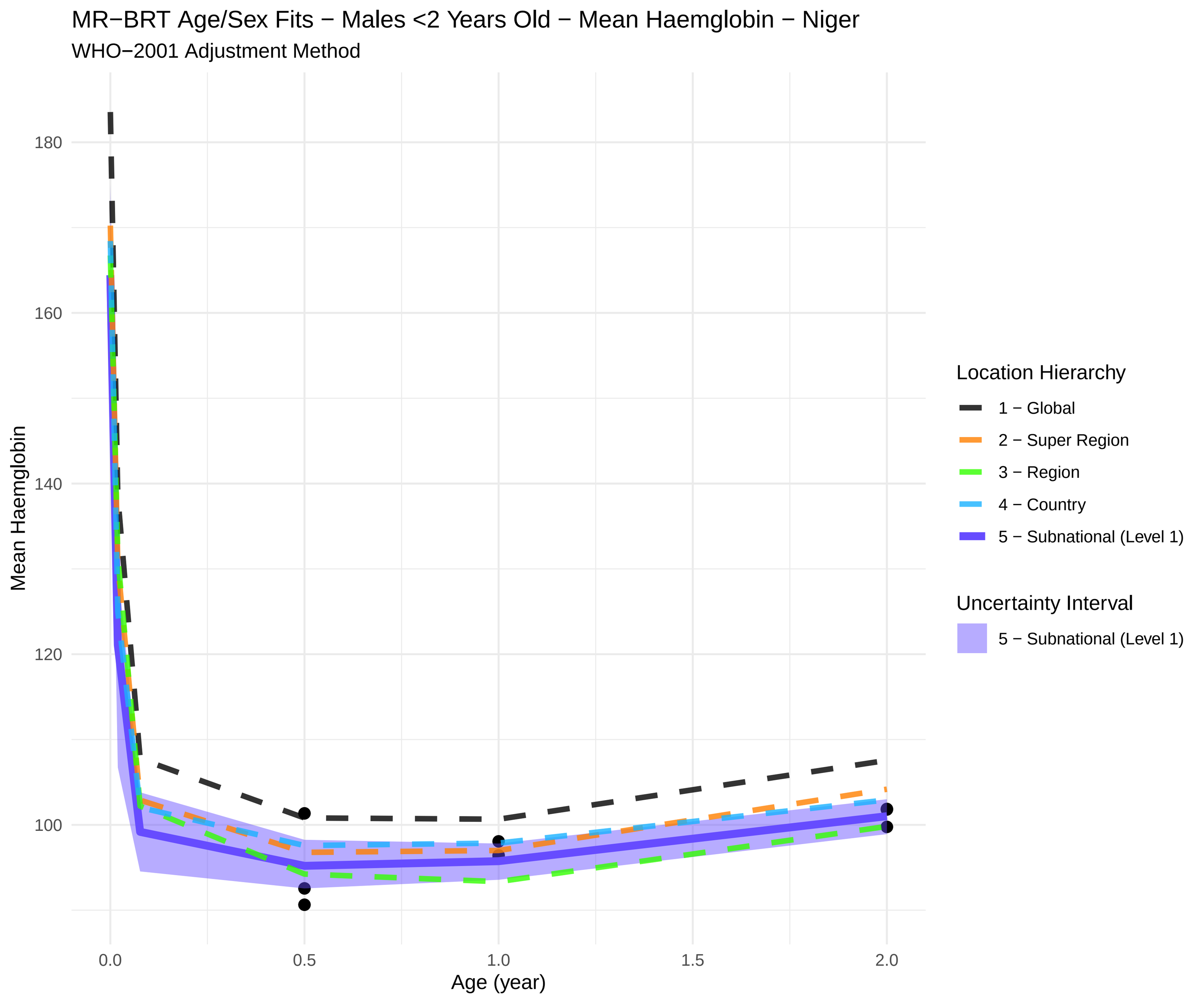
MR−BRT Age/Sex Fits − Males <2 Years Old − Mean Haemglobin − Niger
WHO−2001 Adjustment Method
Mean Haemglobin
Age (year)
Location Hierarchy
1 − Global
2 − Super Region
3 − Region
4 − Country
5 − Subnational (Level 1)
Uncertainty Interval
5 − Subnational (Level 1)

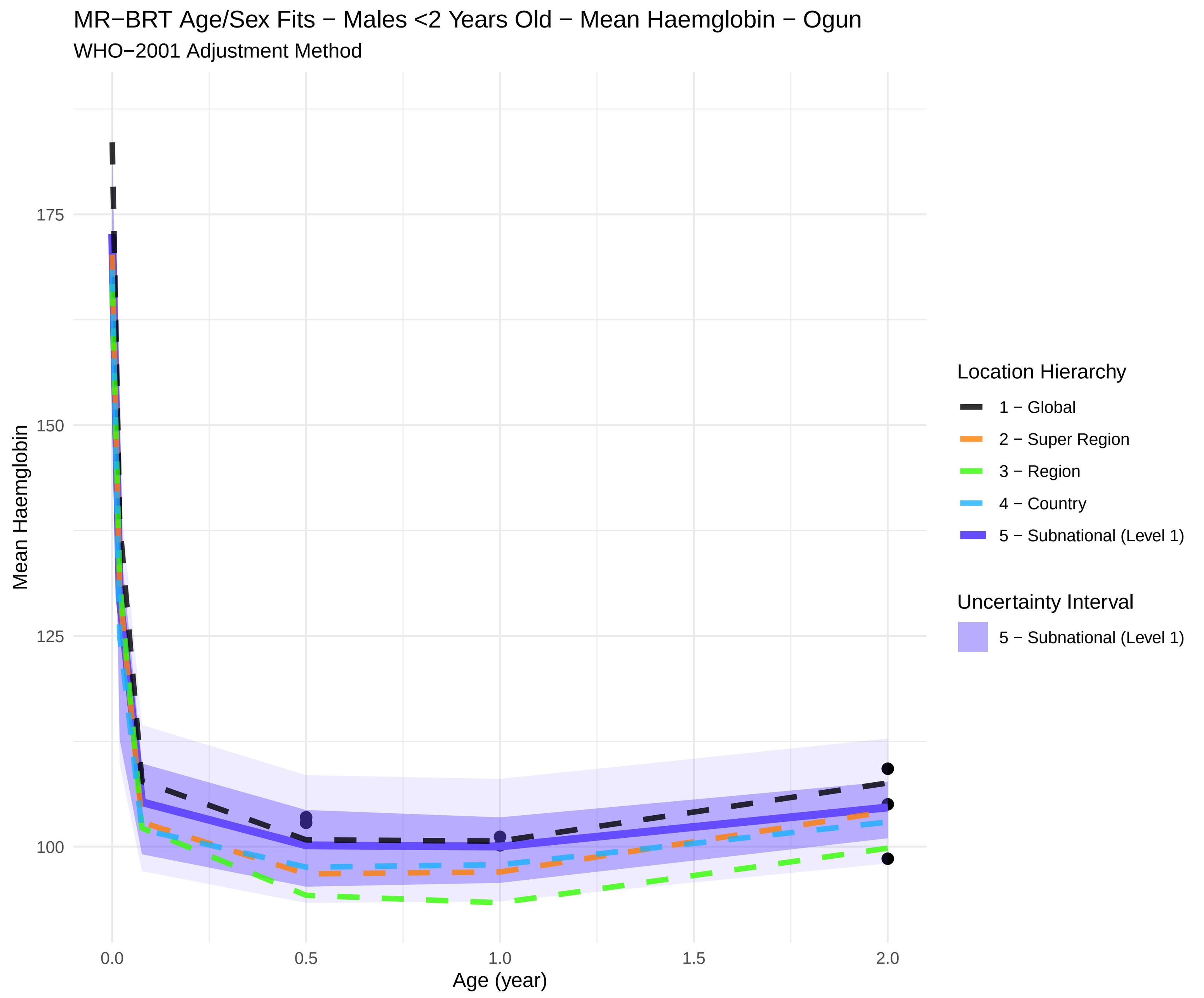
MR−BRT Age/Sex Fits − Males <2 Years Old − Mean Haemglobin − Ogun
WHO−2001 Adjustment Method
Mean Haemglobin
Age (year)
Location Hierarchy
1 − Global
2 − Super Region
3 − Region
4 − Country
5 − Subnational (Level 1)
Uncertainty Interval
5 − Subnational (Level 1)

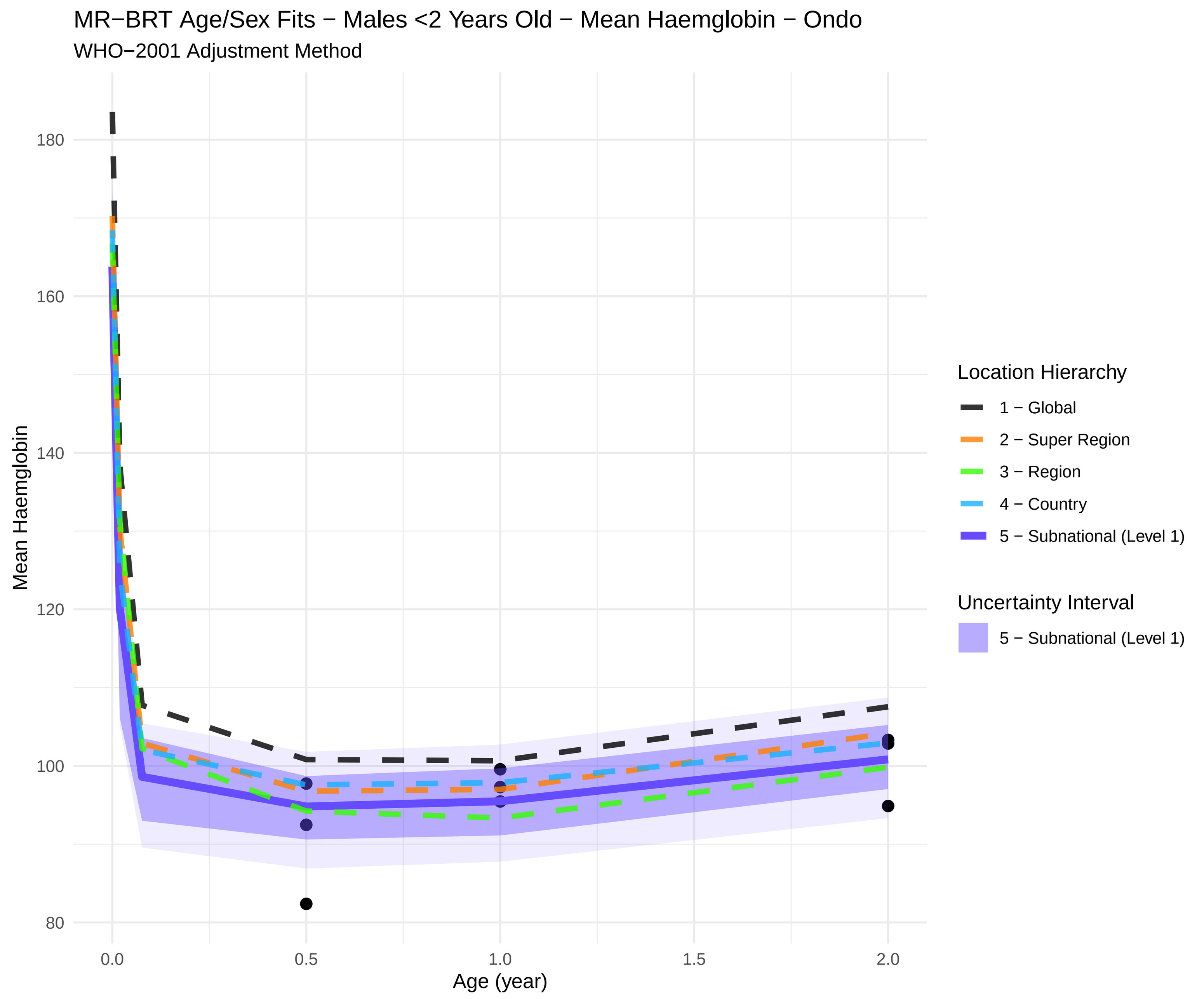
MR−BRT Age/Sex Fits − Males <2 Years Old − Mean Haemglobin − Ondo
WHO−2001 Adjustment Method
Mean Haemglobin
Age (year)
Location Hierarchy
1 − Global
2 − Super Region
3 − Region
4 − Country
5 − Subnational (Level 1)
Uncertainty Interval
5 − Subnational (Level 1)

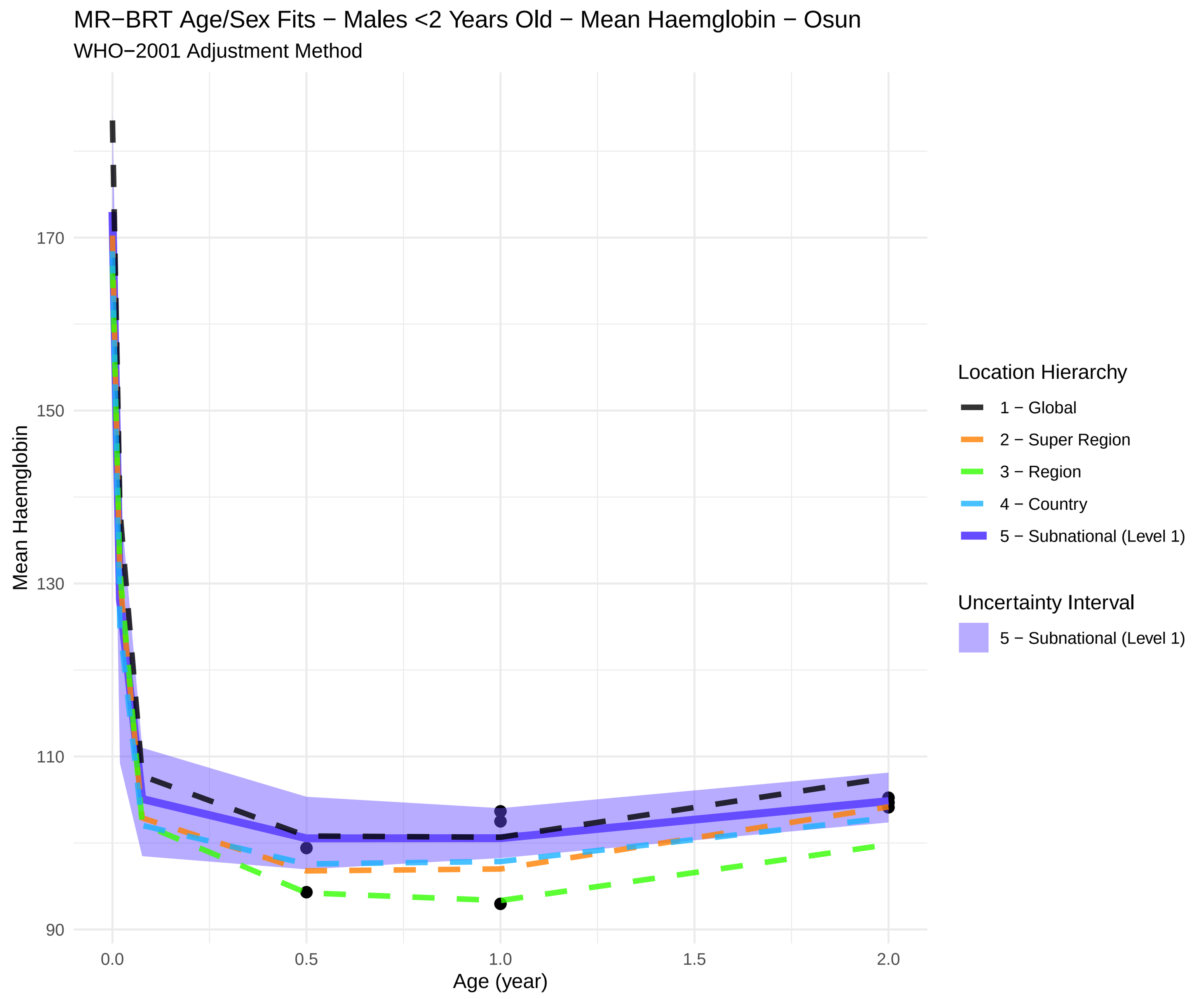
MR−BRT Age/Sex Fits − Males <2 Years Old − Mean Haemglobin − Osun
WHO−2001 Adjustment Method
Mean Haemglobin
Age (year)
Location Hierarchy
1 − Global
2 − Super Region
3 − Region
4 − Country
5 − Subnational (Level 1)
Uncertainty Interval
5 − Subnational (Level 1)

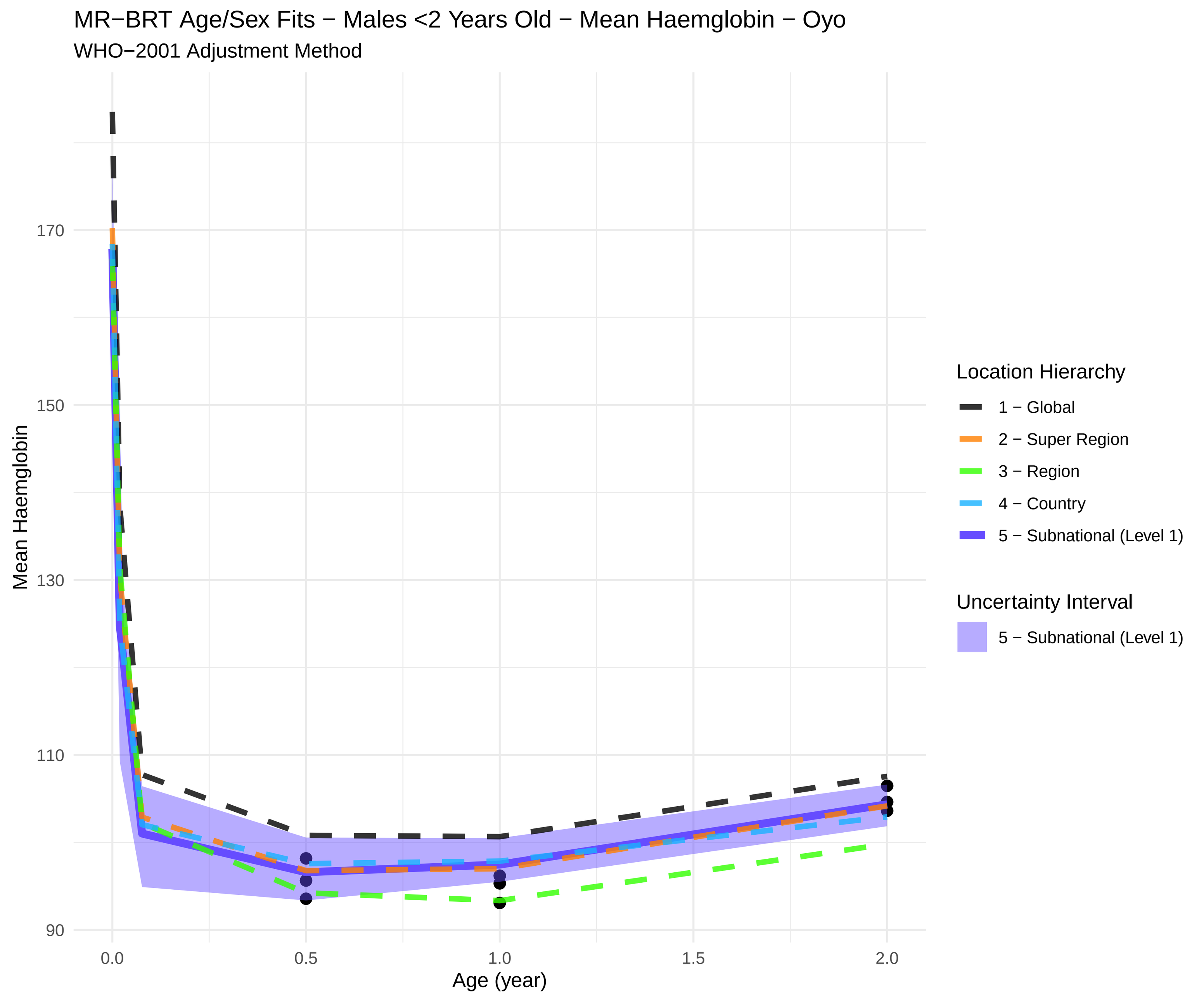
MR−BRT Age/Sex Fits − Males <2 Years Old − Mean Haemglobin − Oyo
WHO−2001 Adjustment Method
Mean Haemglobin
Age (year)
Location Hierarchy
1 − Global
2 − Super Region
3 − Region
4 − Country
5 − Subnational (Level 1)
Uncertainty Interval
5 − Subnational (Level 1)

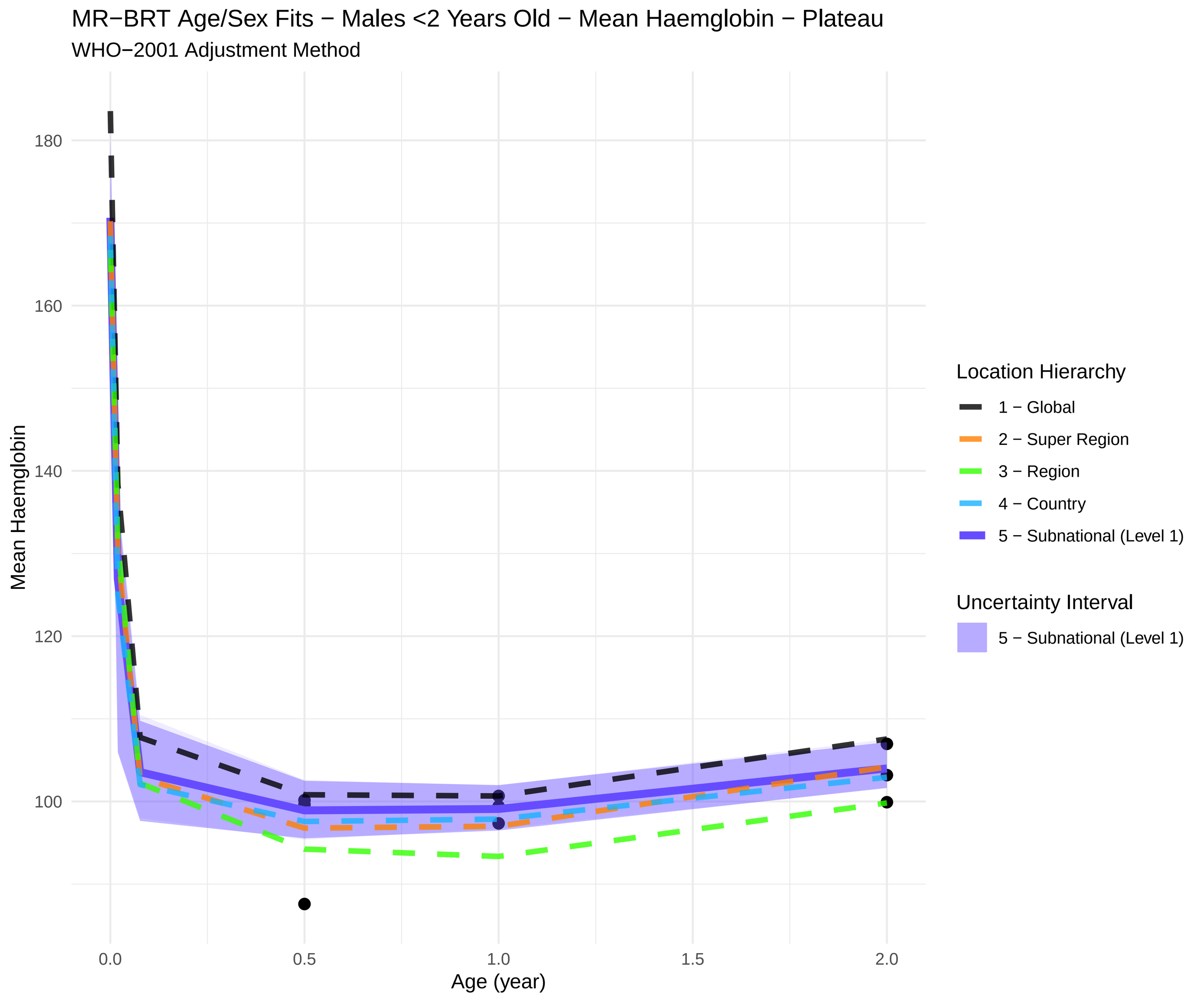
MR−BRT Age/Sex Fits − Males <2 Years Old − Mean Haemglobin − Plateau
WHO−2001 Adjustment Method
Mean Haemglobin
Age (year)
Location Hierarchy
1 − Global
2 − Super Region
3 − Region
4 − Country
5 − Subnational (Level 1)
Uncertainty Interval
5 − Subnational (Level 1)

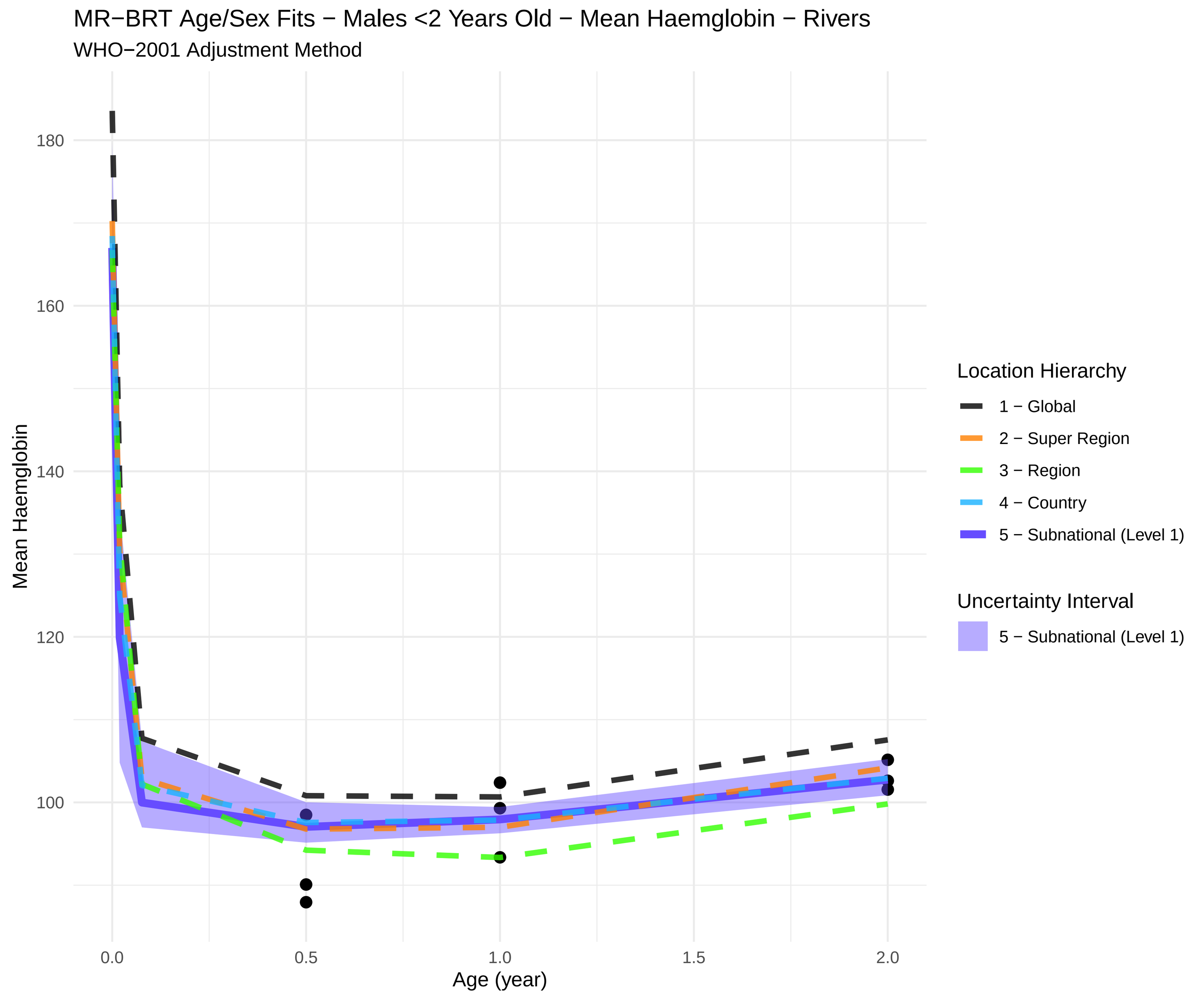
MR−BRT Age/Sex Fits − Males <2 Years Old − Mean Haemglobin − Rivers
WHO−2001 Adjustment Method
Mean Haemglobin
Age (year)
Location Hierarchy
1 − Global
2 − Super Region
3 − Region
4 − Country
5 − Subnational (Level 1)
Uncertainty Interval
5 − Subnational (Level 1)

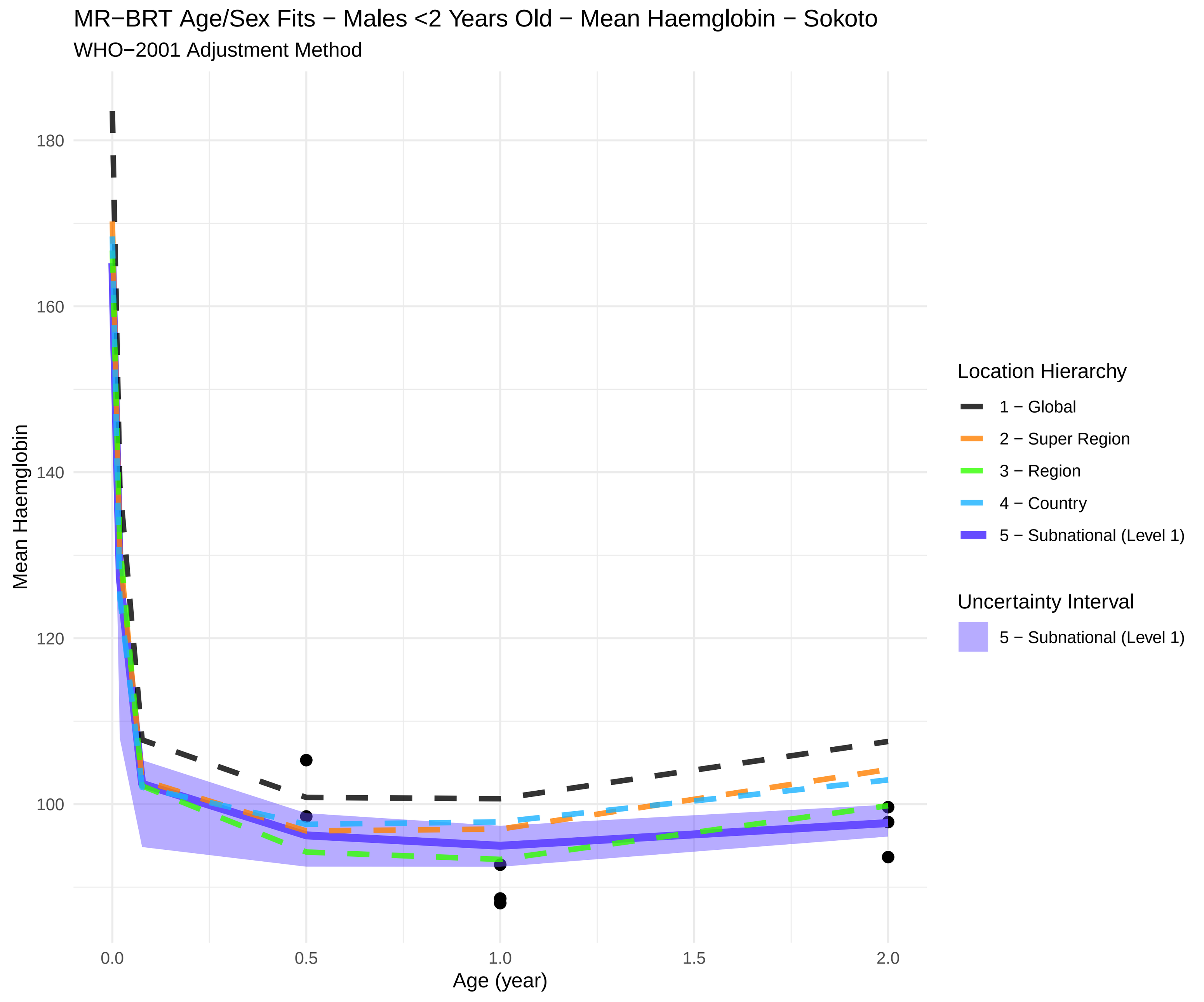
MR−BRT Age/Sex Fits − Males <2 Years Old − Mean Haemglobin − Sokoto
WHO−2001 Adjustment Method
Mean Haemglobin
Age (year)
Location Hierarchy
1 − Global
2 − Super Region
3 − Region
4 − Country
5 − Subnational (Level 1)
Uncertainty Interval
5 − Subnational (Level 1)

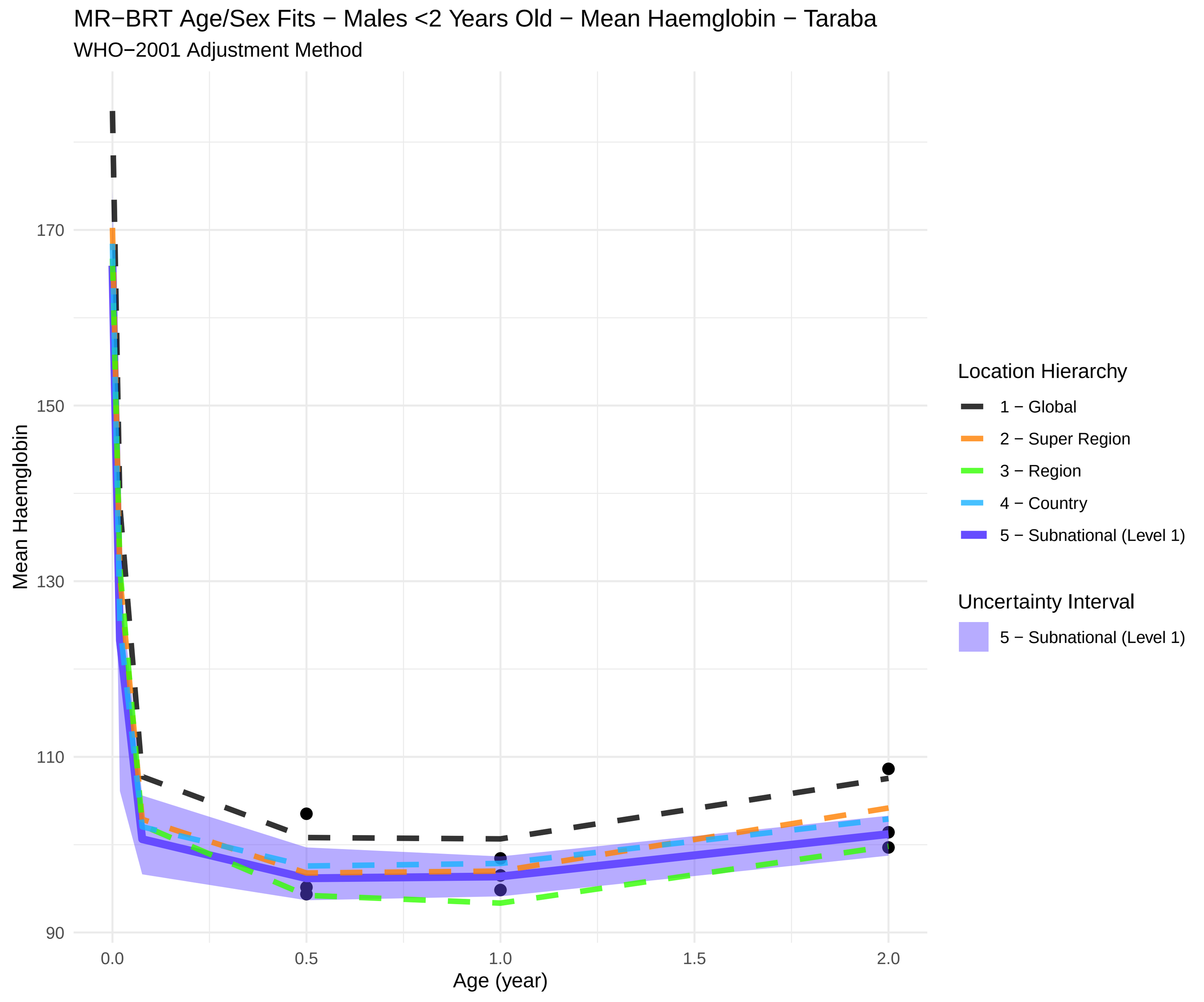
MR−BRT Age/Sex Fits − Males <2 Years Old − Mean Haemglobin − Taraba
WHO−2001 Adjustment Method
Mean Haemglobin
Age (year)
Location Hierarchy
1 − Global
2 − Super Region
3 − Region
4 − Country
5 − Subnational (Level 1)
Uncertainty Interval
5 − Subnational (Level 1)

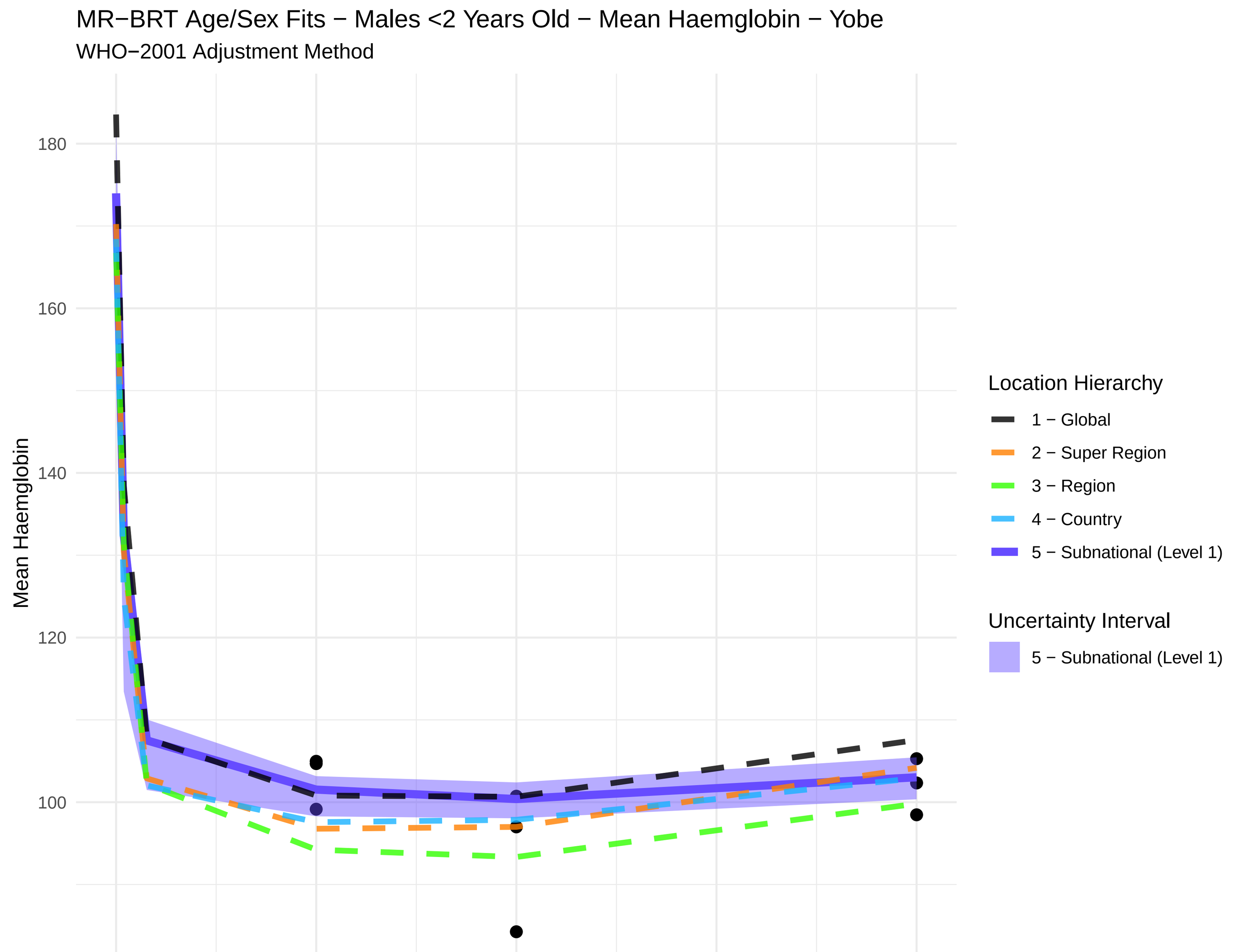
MR−BRT Age/Sex Fits − Males <2 Years Old − Mean Haemglobin − Yobe
WHO−2001 Adjustment Method
Mean Haemglobin
Age (year)
Location Hierarchy
1 − Global
2 − Super Region
3 − Region
4 − Country
5 − Subnational (Level 1)
Uncertainty Interval
5 − Subnational (Level 1)

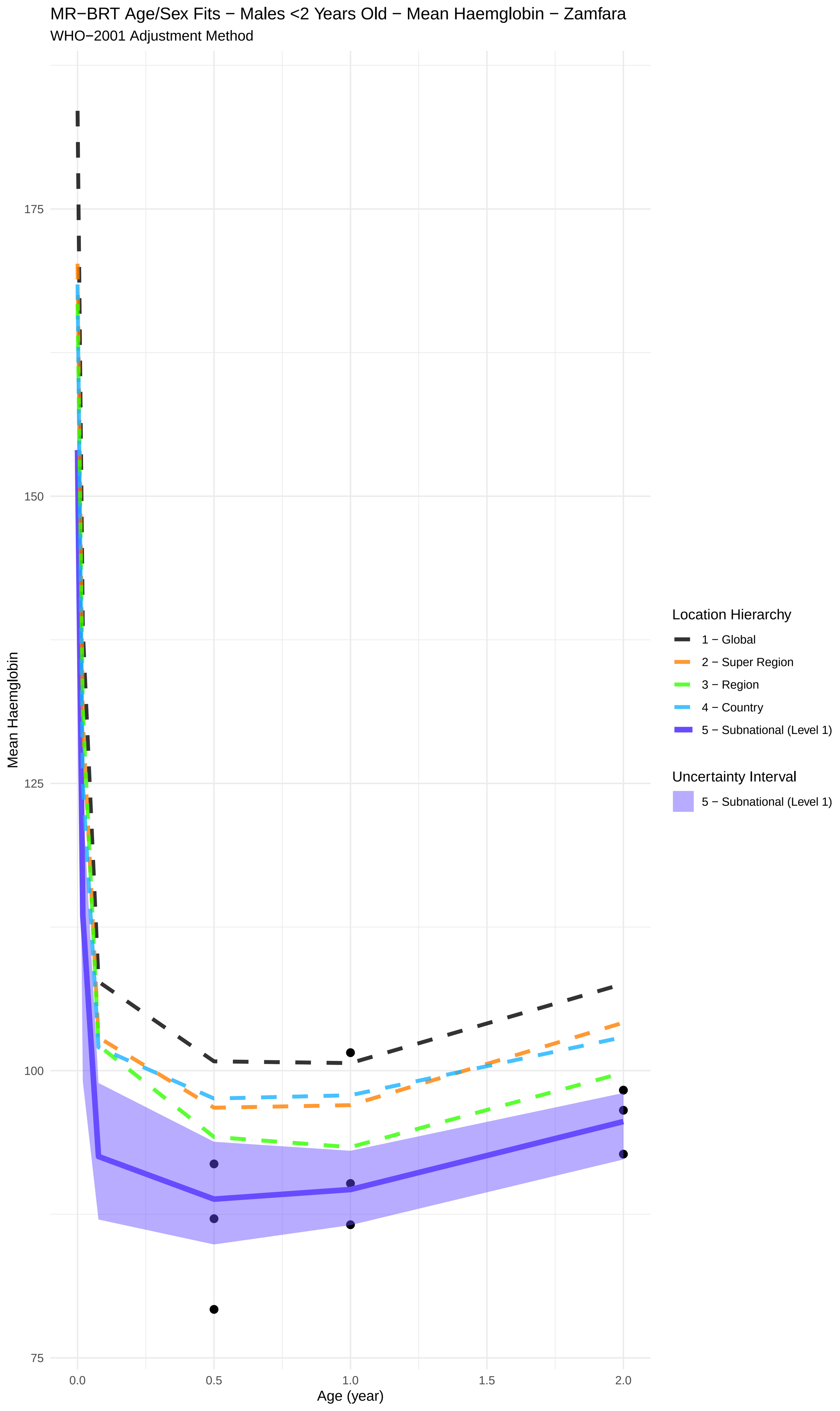

MR−BRT Age/Sex Fits − Males <2 Years Old − Mean Haemglobin − Zamfara
WHO−2001 Adjustment Method
Mean Haemglobin
Age (year)
Location Hierarchy
1 − Global
2 − Super Region
3 − Region
4 − Country
5 − Subnational (Level 1)
Uncertainty Interval
5 − Subnational (Level 1)

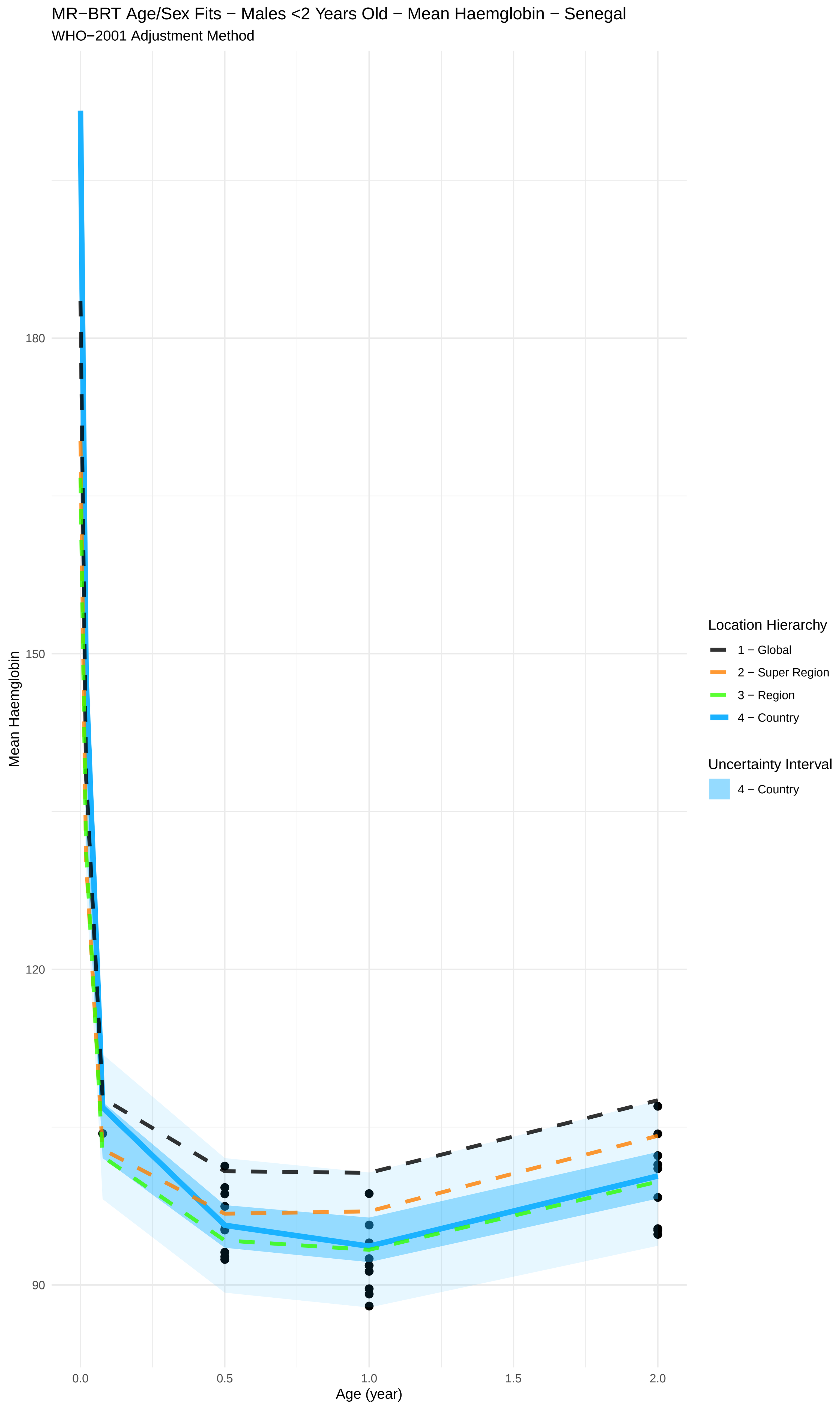

MR−BRT Age/Sex Fits − Males <2 Years Old − Mean Haemglobin − Senegal
WHO−2001 Adjustment Method
Mean Haemglobin
Age (year)
Location Hierarchy
1 − Global
2 − Super Region
3 − Region
4 − Country
Uncertainty Interval
4 − Country

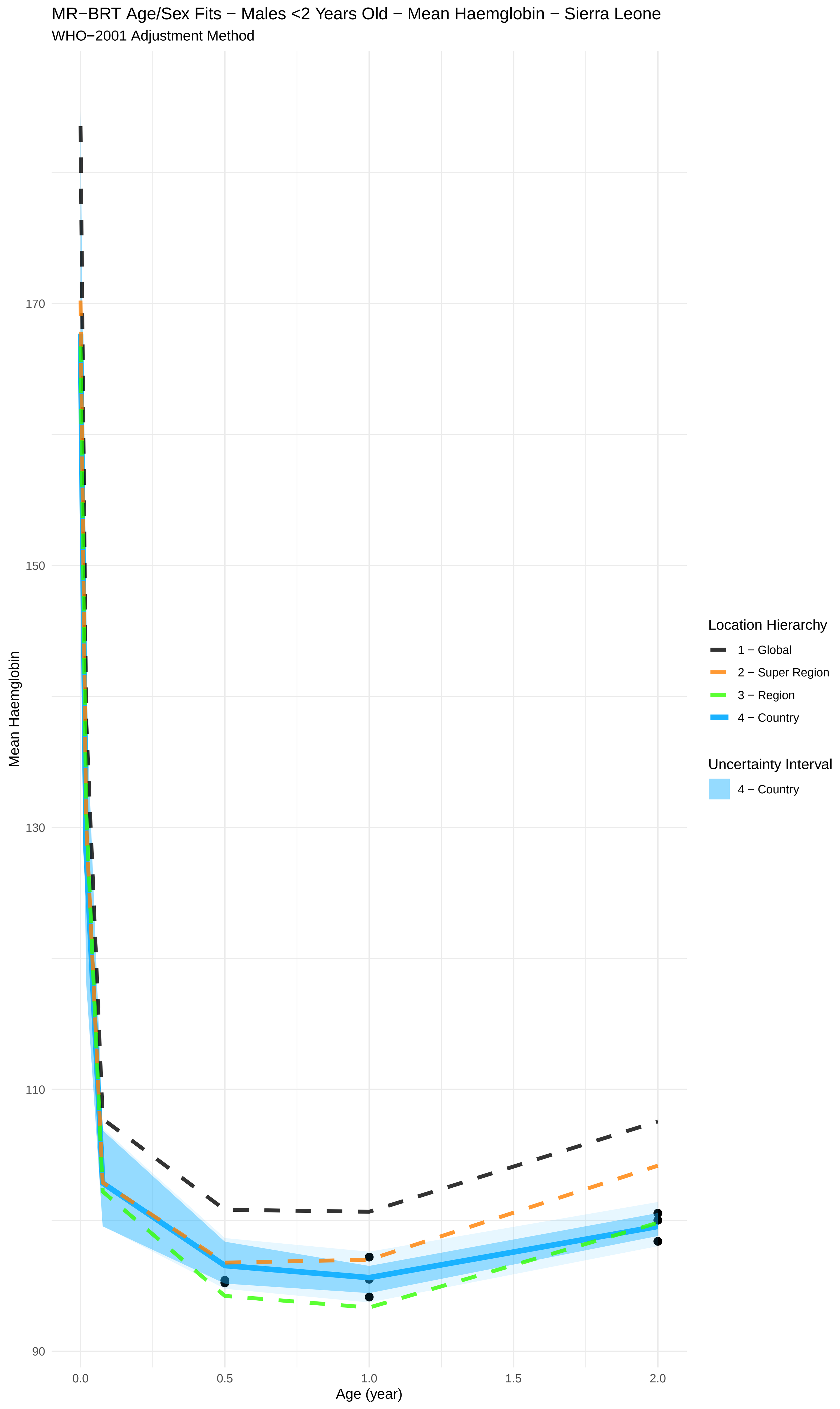

MR−BRT Age/Sex Fits − Males <2 Years Old − Mean Haemglobin − Sierra Leone
WHO−2001 Adjustment Method
Mean Haemglobin
Age (year)
Location Hierarchy
1 − Global
2 − Super Region
3 − Region
4 − Country
Uncertainty Interval
4 − Country

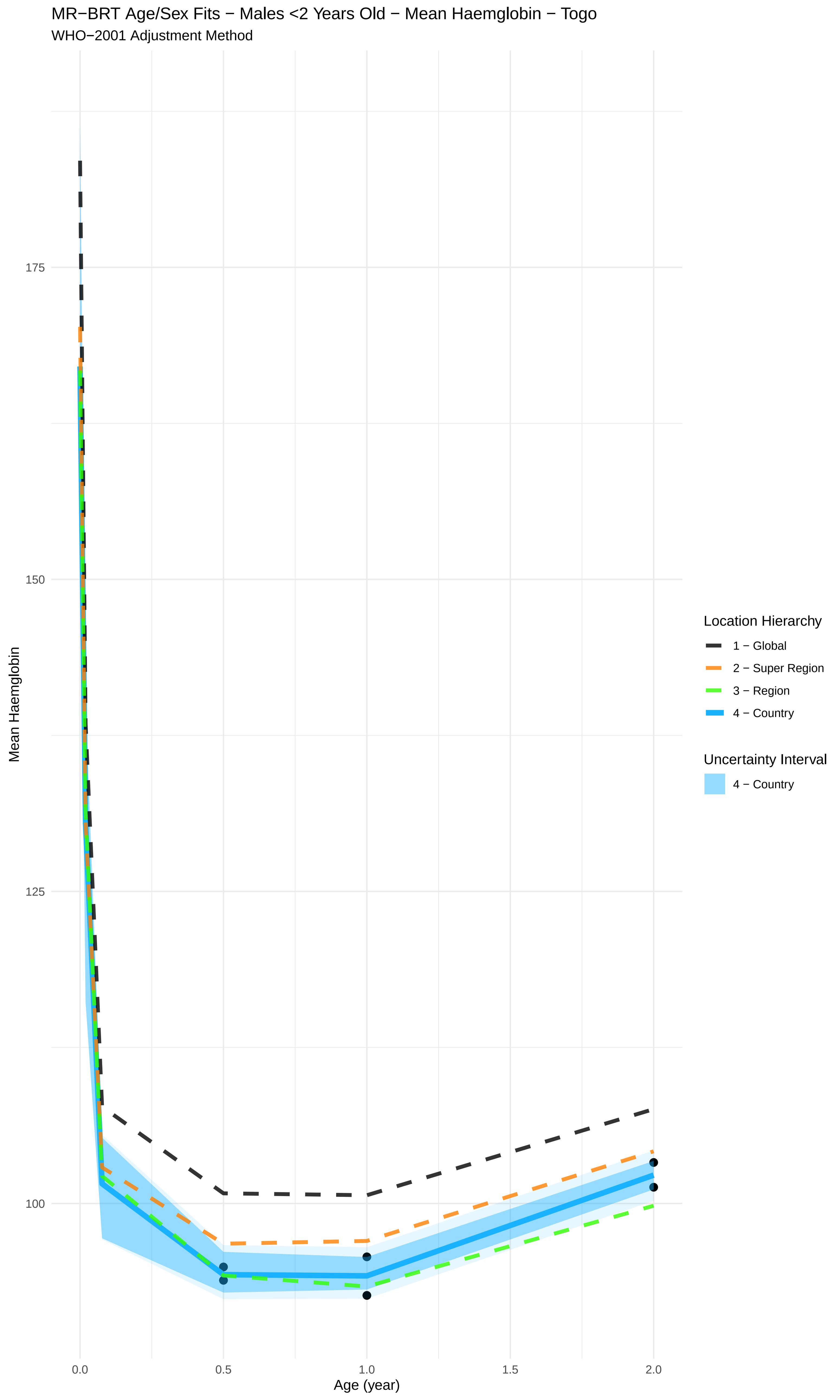

MR−BRT Age/Sex Fits − Males <2 Years Old − Mean Haemglobin − Togo
WHO−2001 Adjustment Method
Mean Haemglobin
Age (year)
Location Hierarchy
1 − Global
2 − Super Region
3 − Region
4 − Country
Uncertainty Interval
4 − Country

MR−BRT Age/Sex Fits − Non−pregnant Females <2 Years Old − Mean Haemglobin − Global
WHO−2001 Adjustment Method

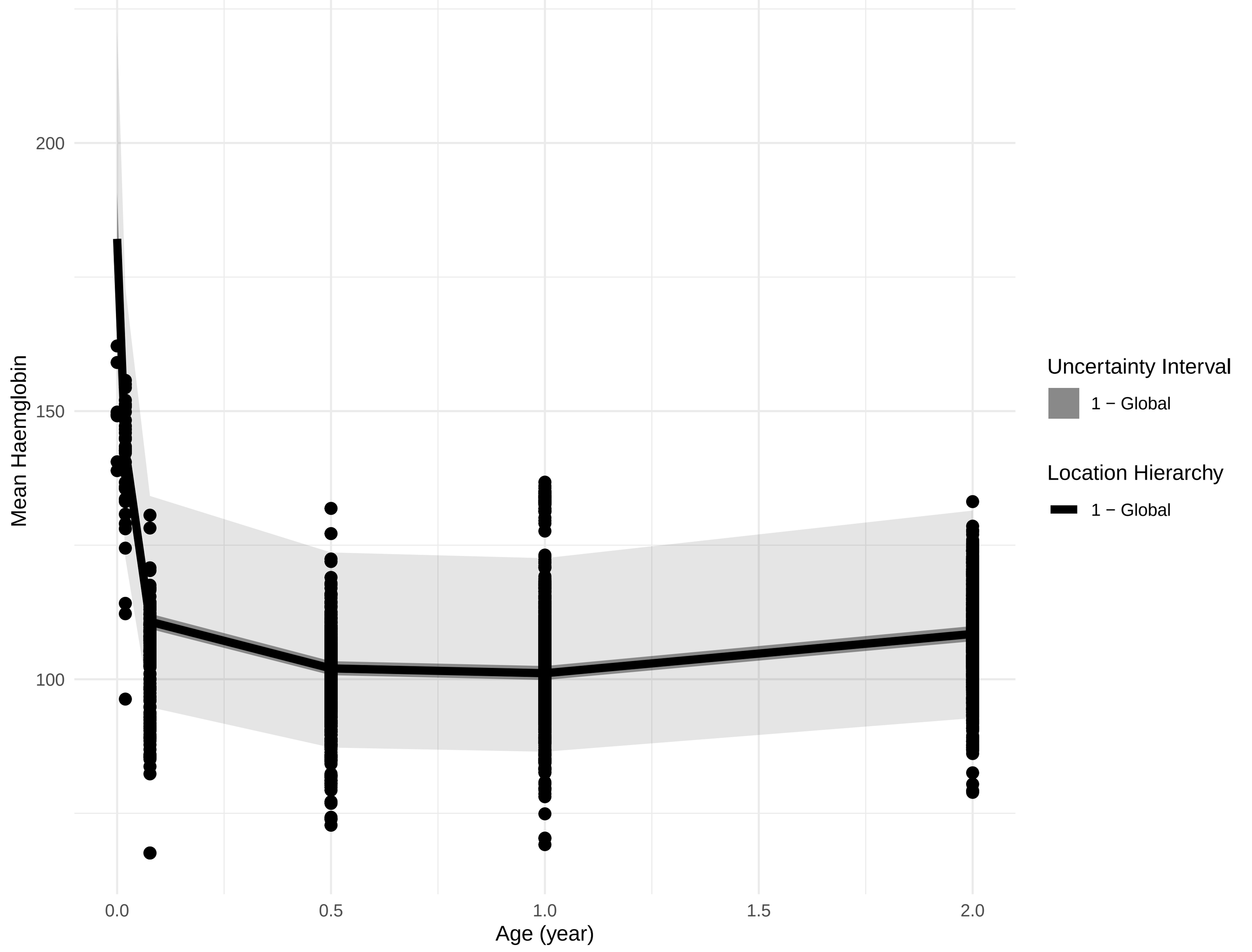

MR−BRT Age/Sex Fits − Non−pregnant Females <2 Years Old − Mean Haemglobin − Central Europe, Eastern Europe, and Central Asia
WHO−2001 Adjustment Method

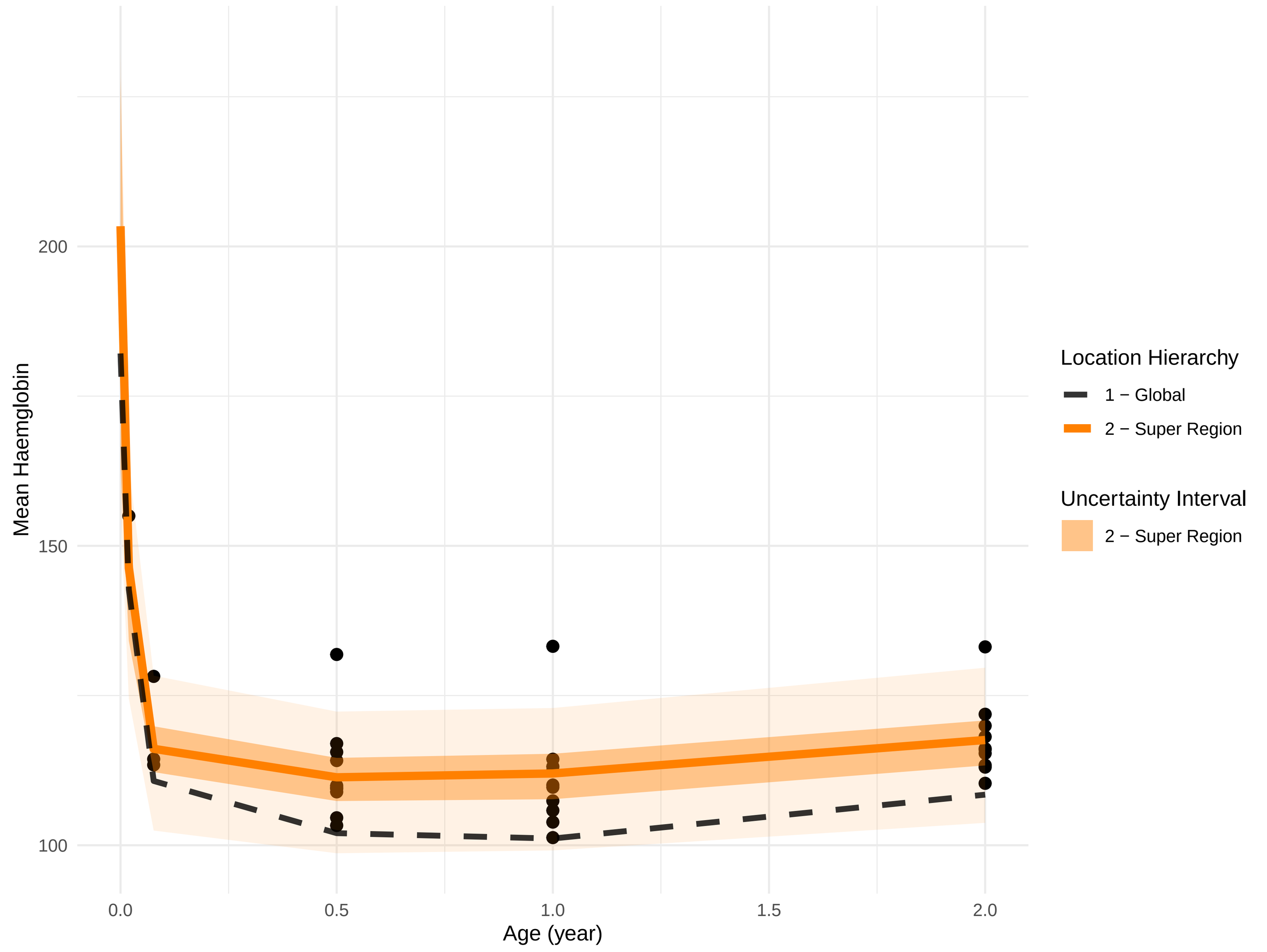

MR−BRT Age/Sex Fits − Non−pregnant Females <2 Years Old − Mean Haemglobin − Central Asia
WHO−2001 Adjustment Method

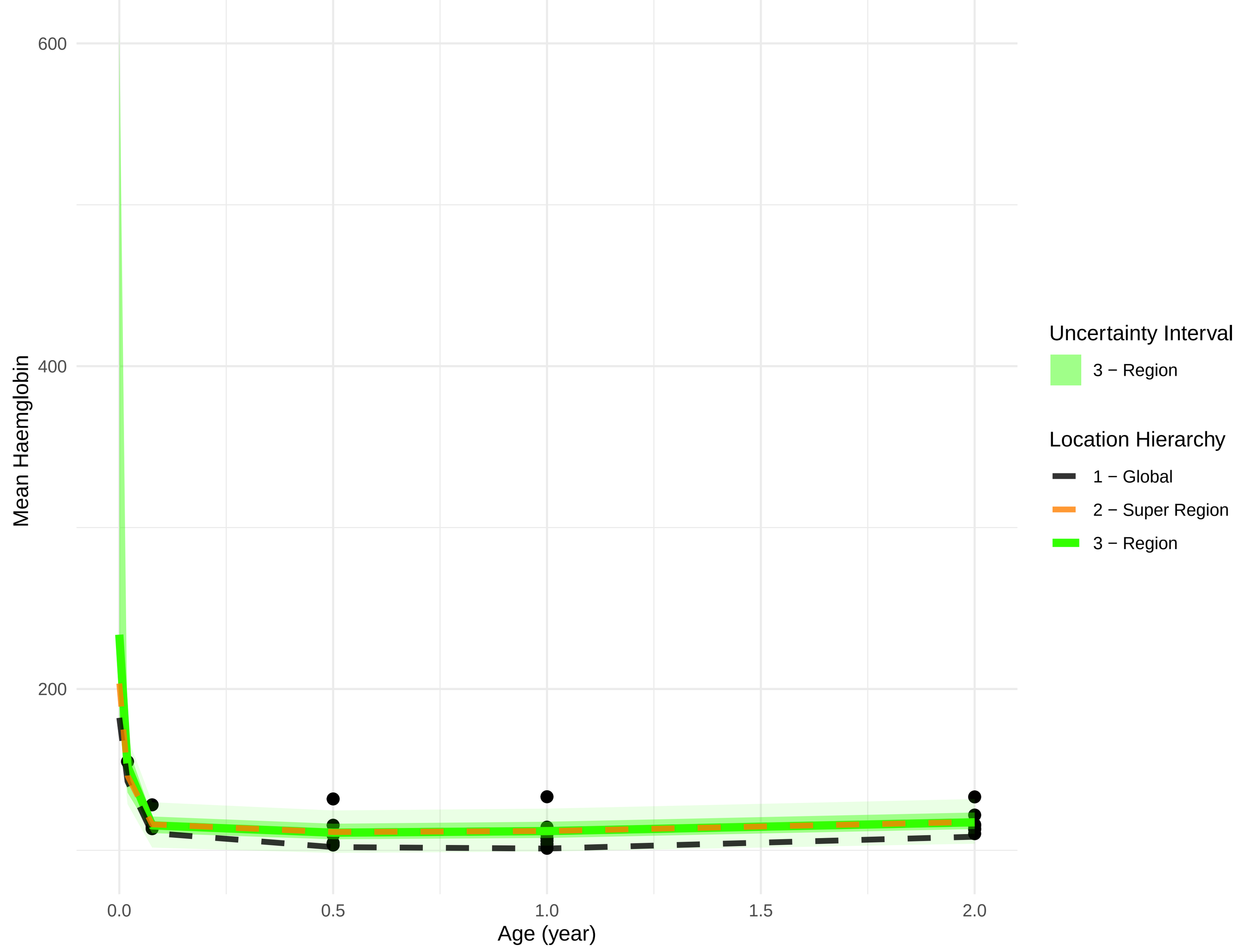

MR−BRT Age/Sex Fits − Non−pregnant Females <2 Years Old − Mean Haemglobin − Armenia
WHO−2001 Adjustment Method

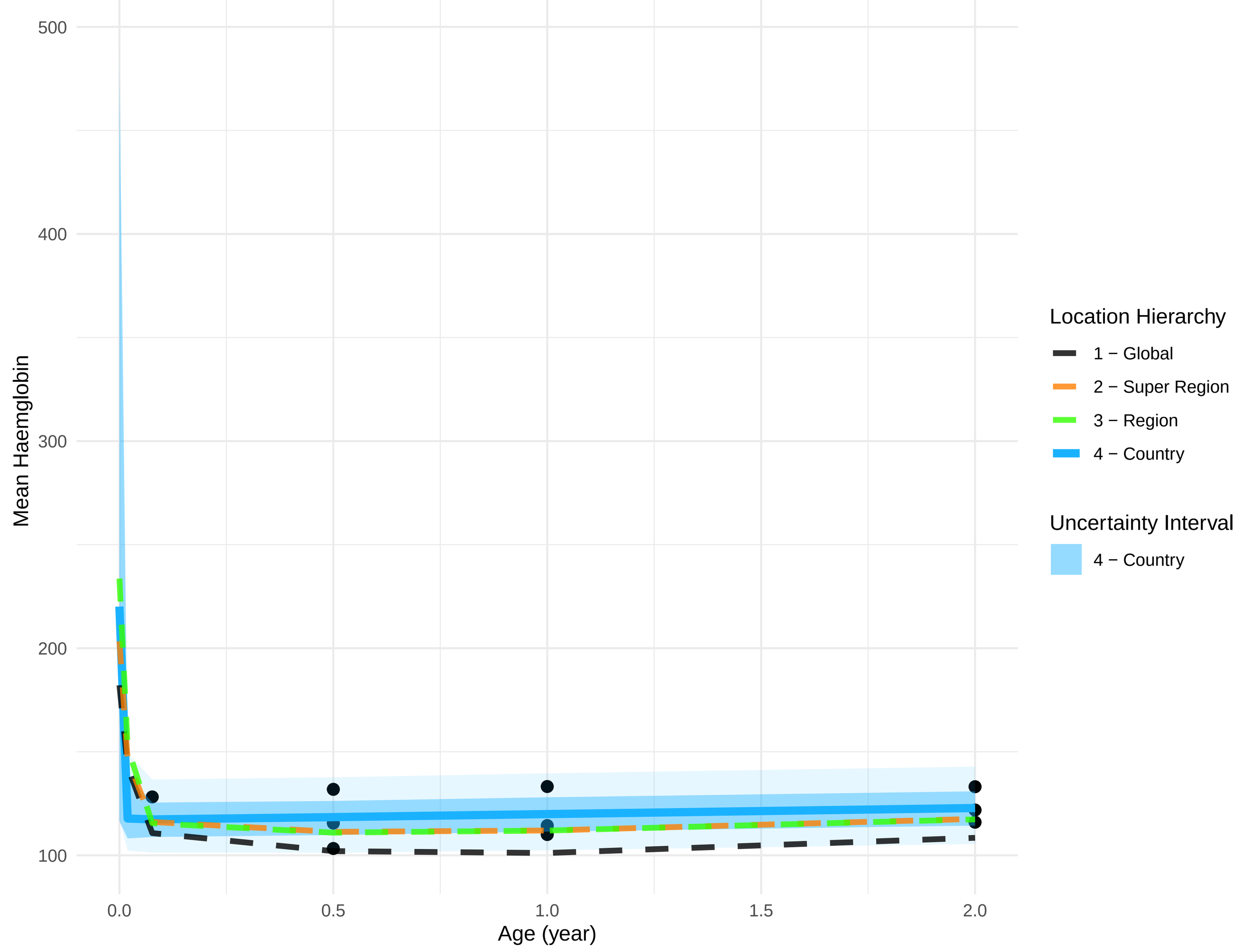

MR−BRT Age/Sex Fits − Non−pregnant Females <2 Years Old − Mean Haemglobin − Azerbaijan
WHO−2001 Adjustment Method

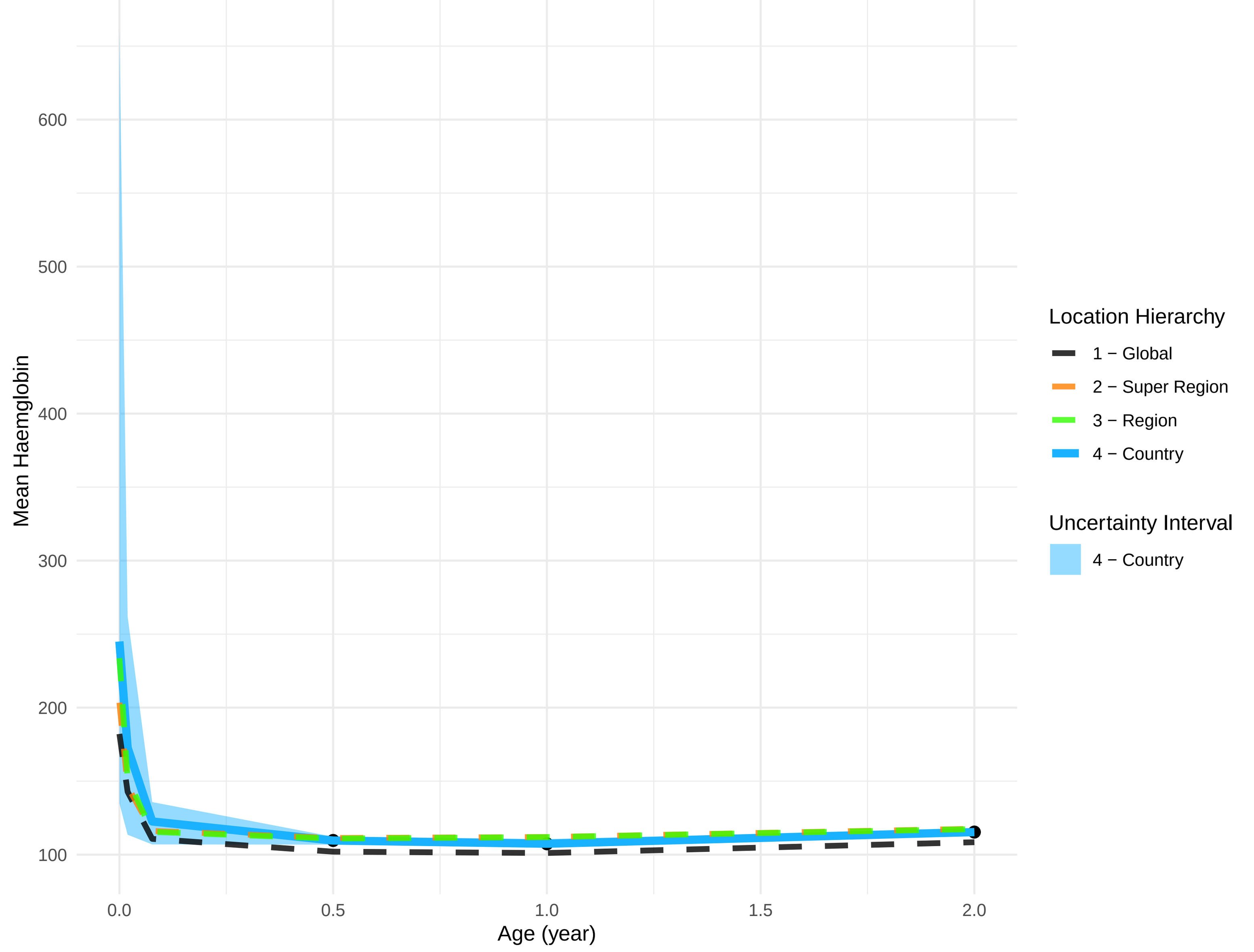

MR−BRT Age/Sex Fits − Non−pregnant Females <2 Years Old − Mean Haemglobin − Kyrgyzstan
WHO−2001 Adjustment Method

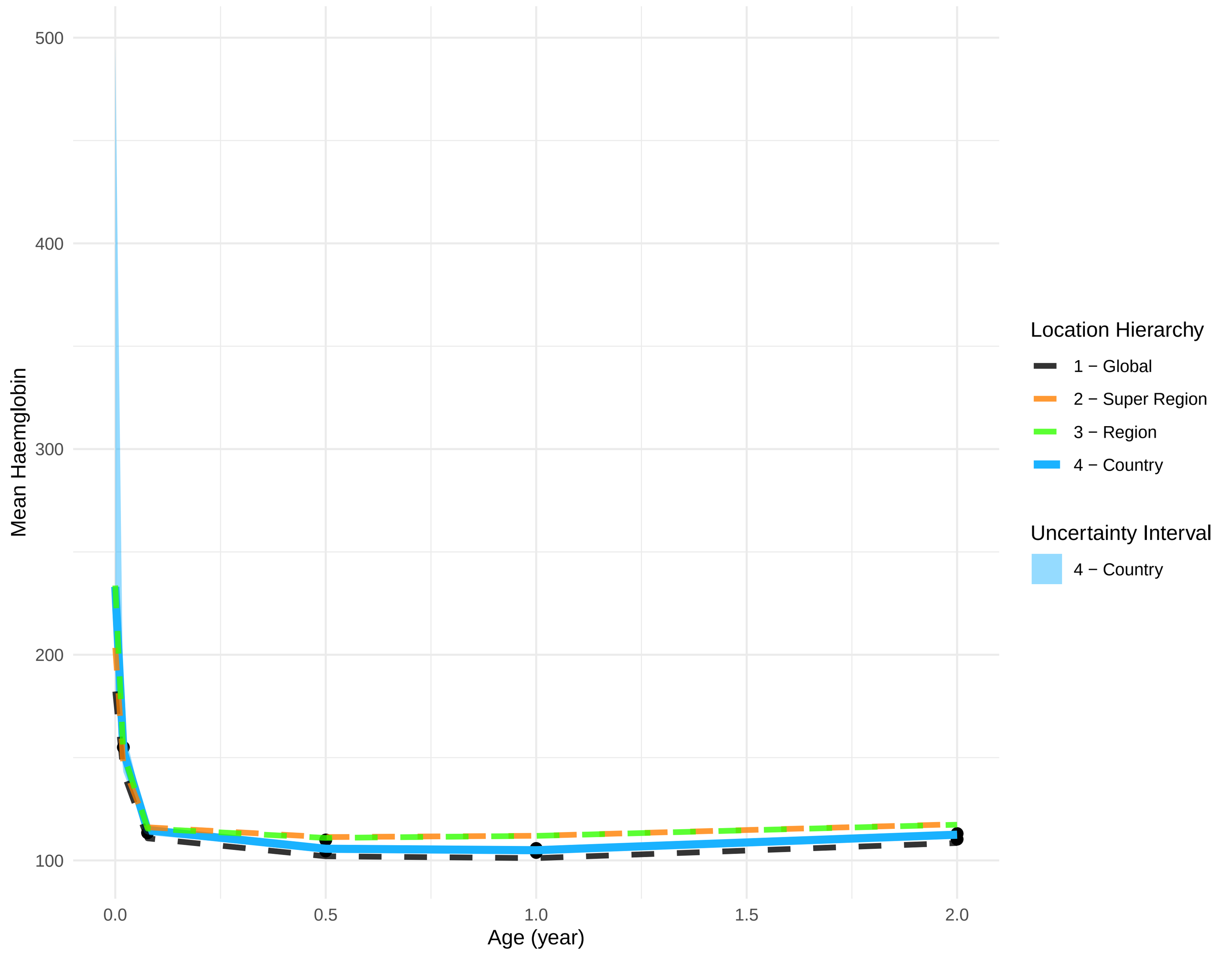

MR−BRT Age/Sex Fits − Non−pregnant Females <2 Years Old − Mean Haemglobin − Uzbekistan
WHO−2001 Adjustment Method

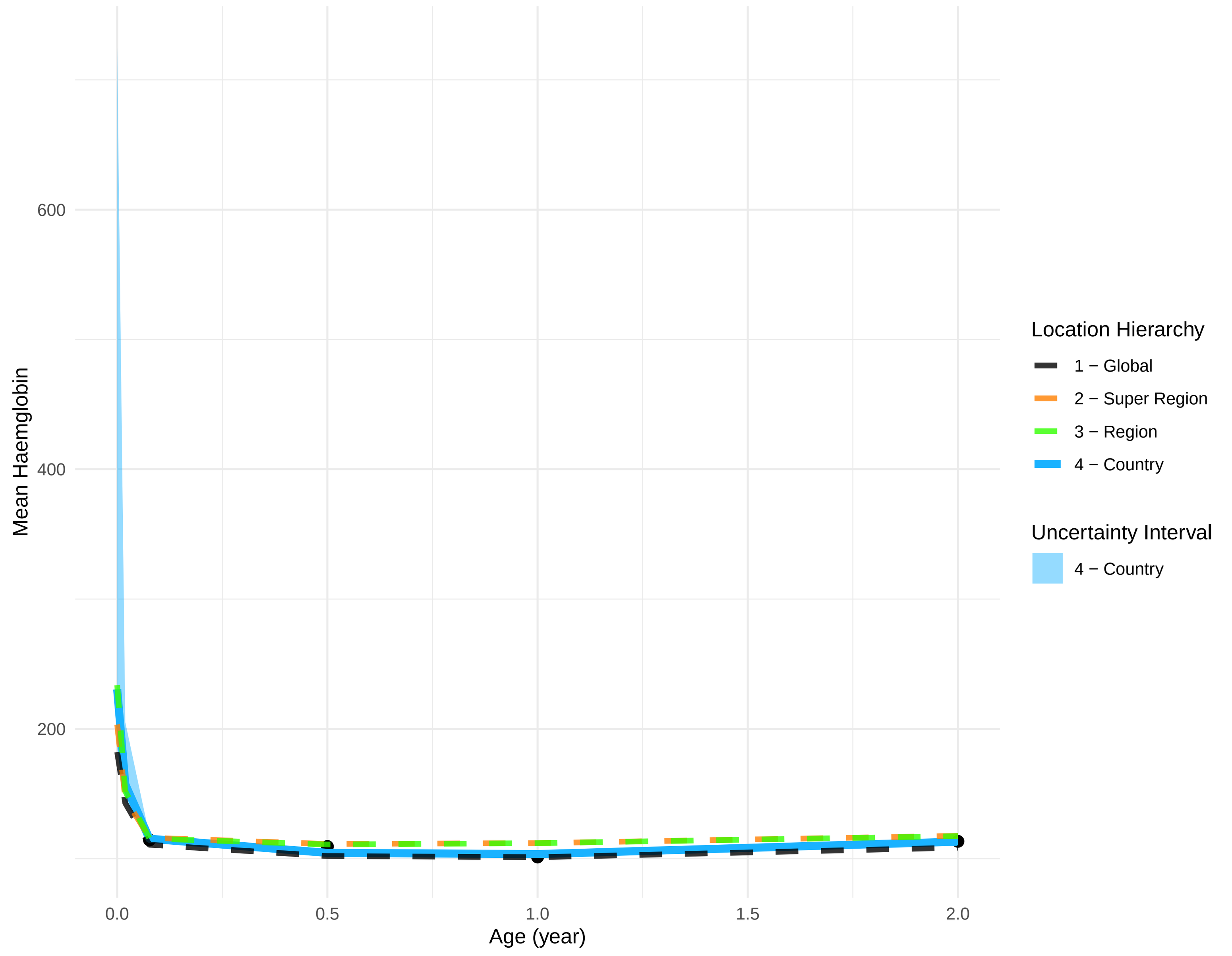

MR−BRT Age/Sex Fits − Non−pregnant Females <2 Years Old − Mean Haemglobin − Central Europe
WHO−2001 Adjustment Method

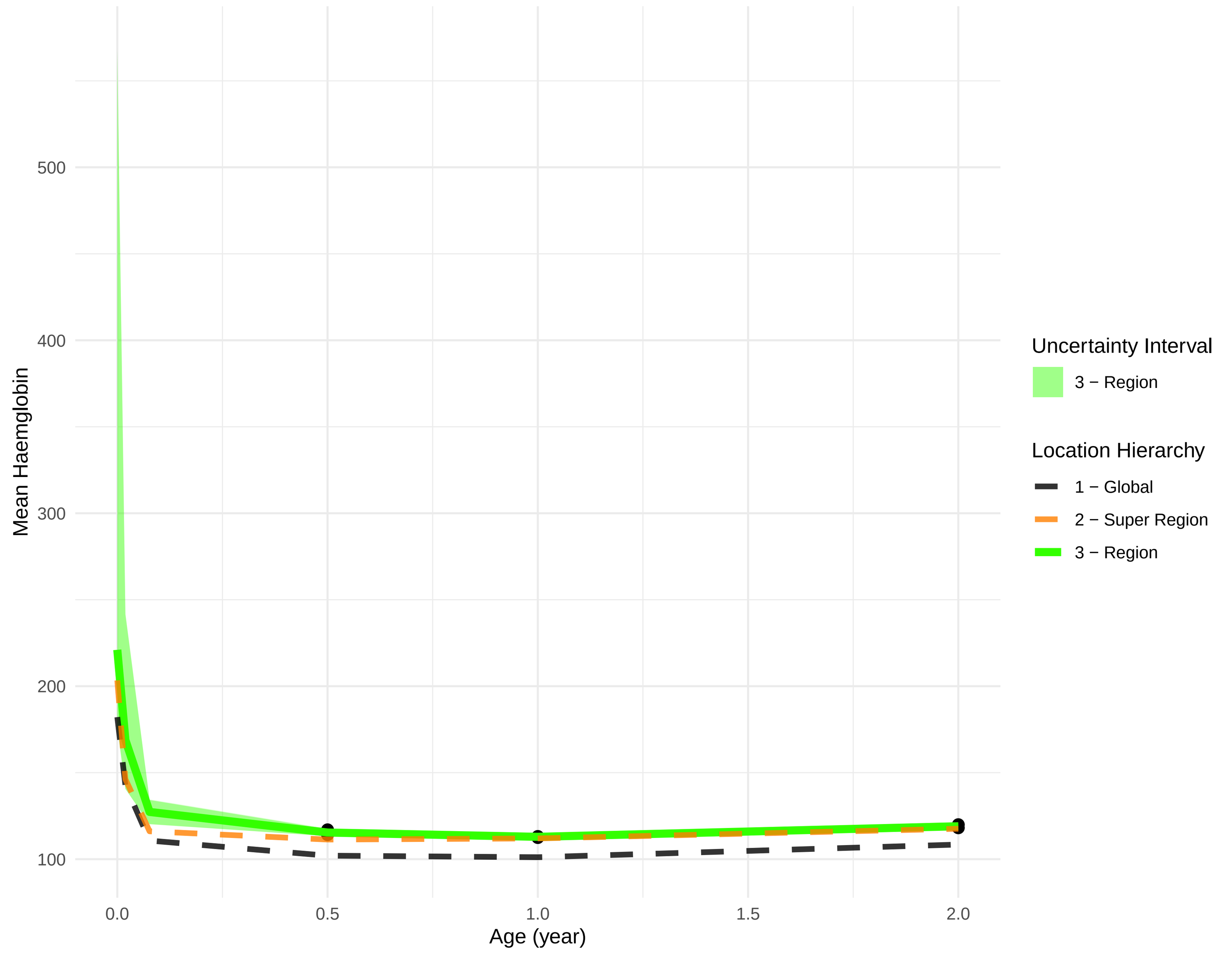

MR−BRT Age/Sex Fits − Non−pregnant Females <2 Years Old − Mean Haemglobin − Albania
WHO−2001 Adjustment Method

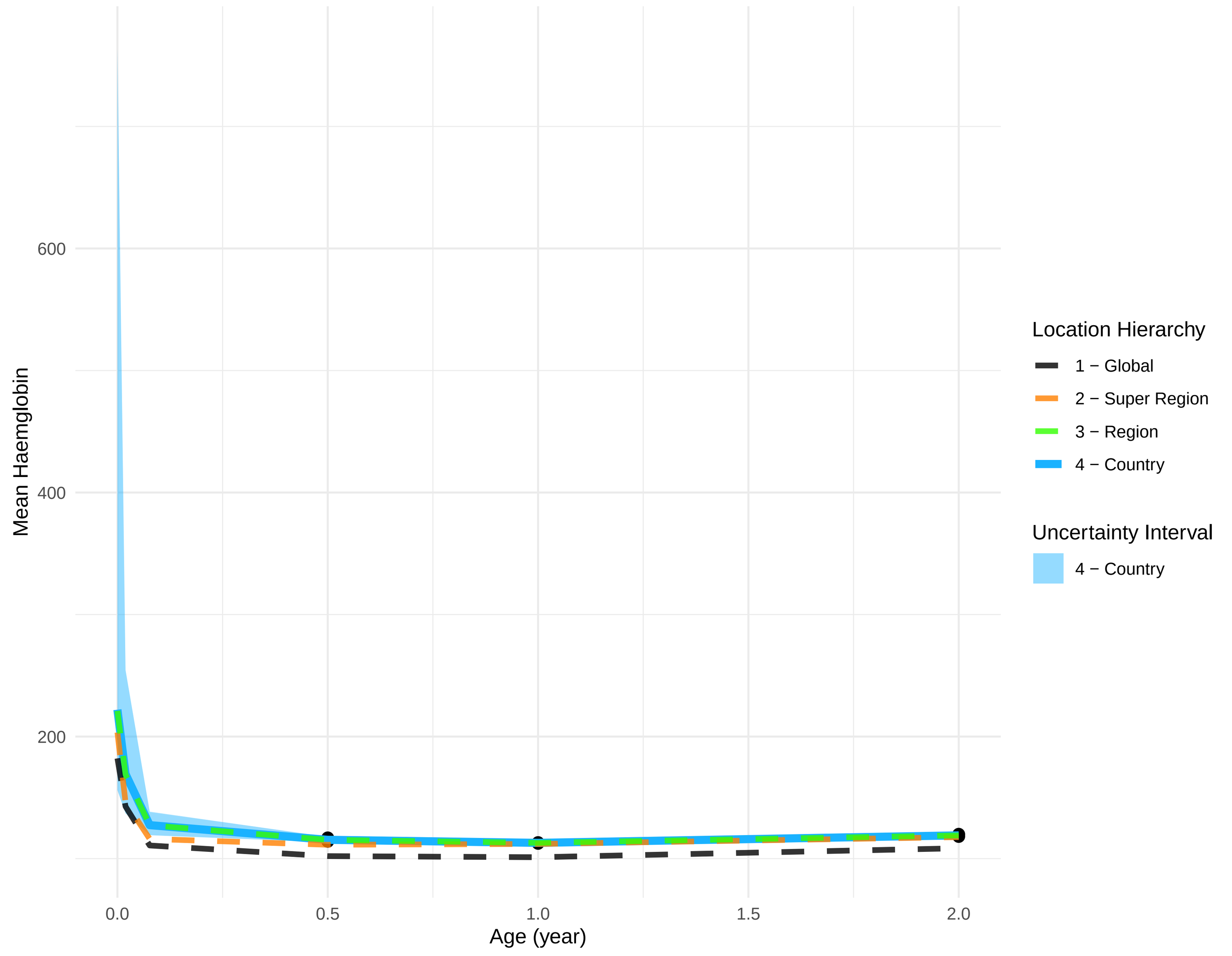

MR−BRT Age/Sex Fits − Non−pregnant Females <2 Years Old − Mean Haemglobin − Eastern Europe
WHO−2001 Adjustment Method

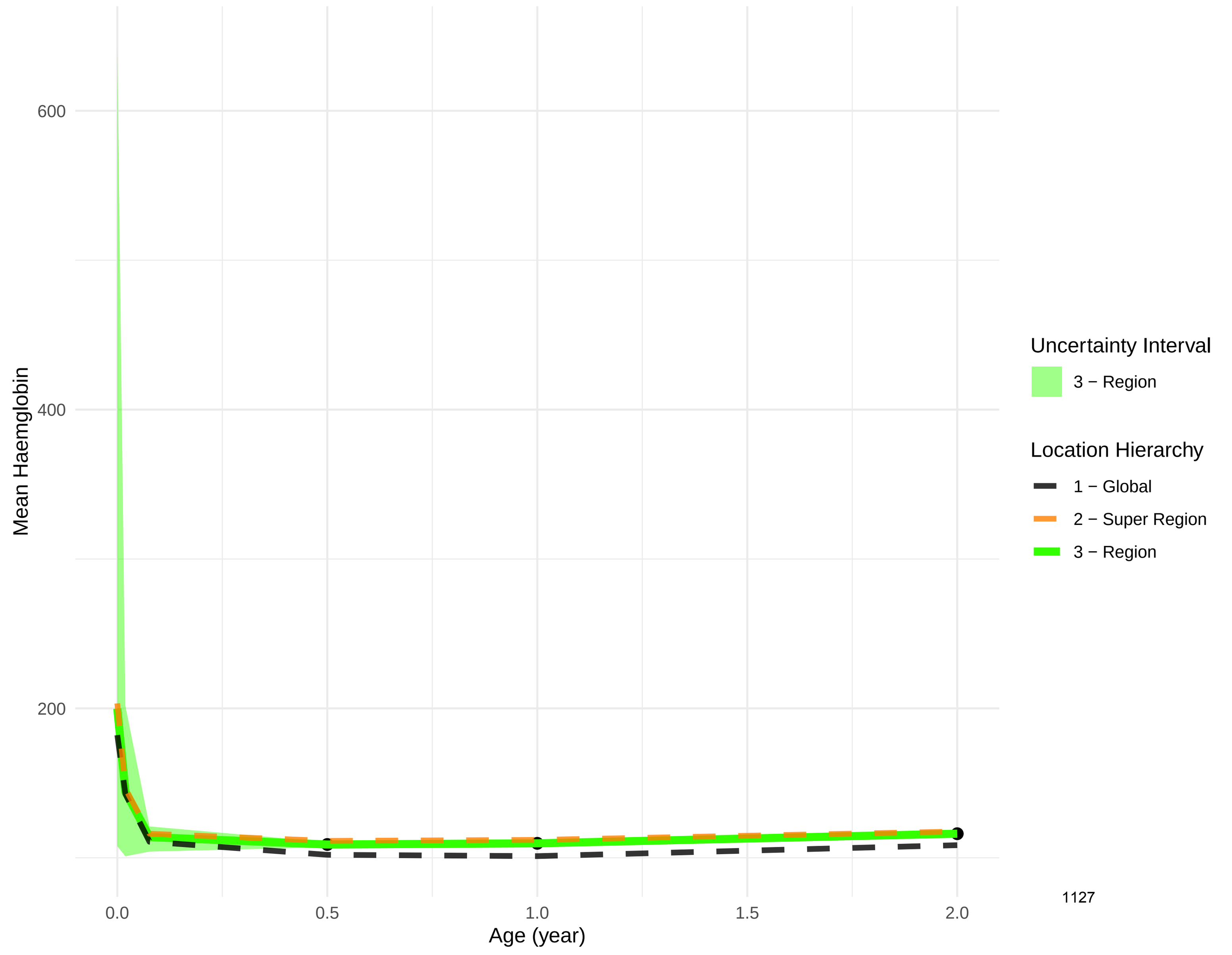

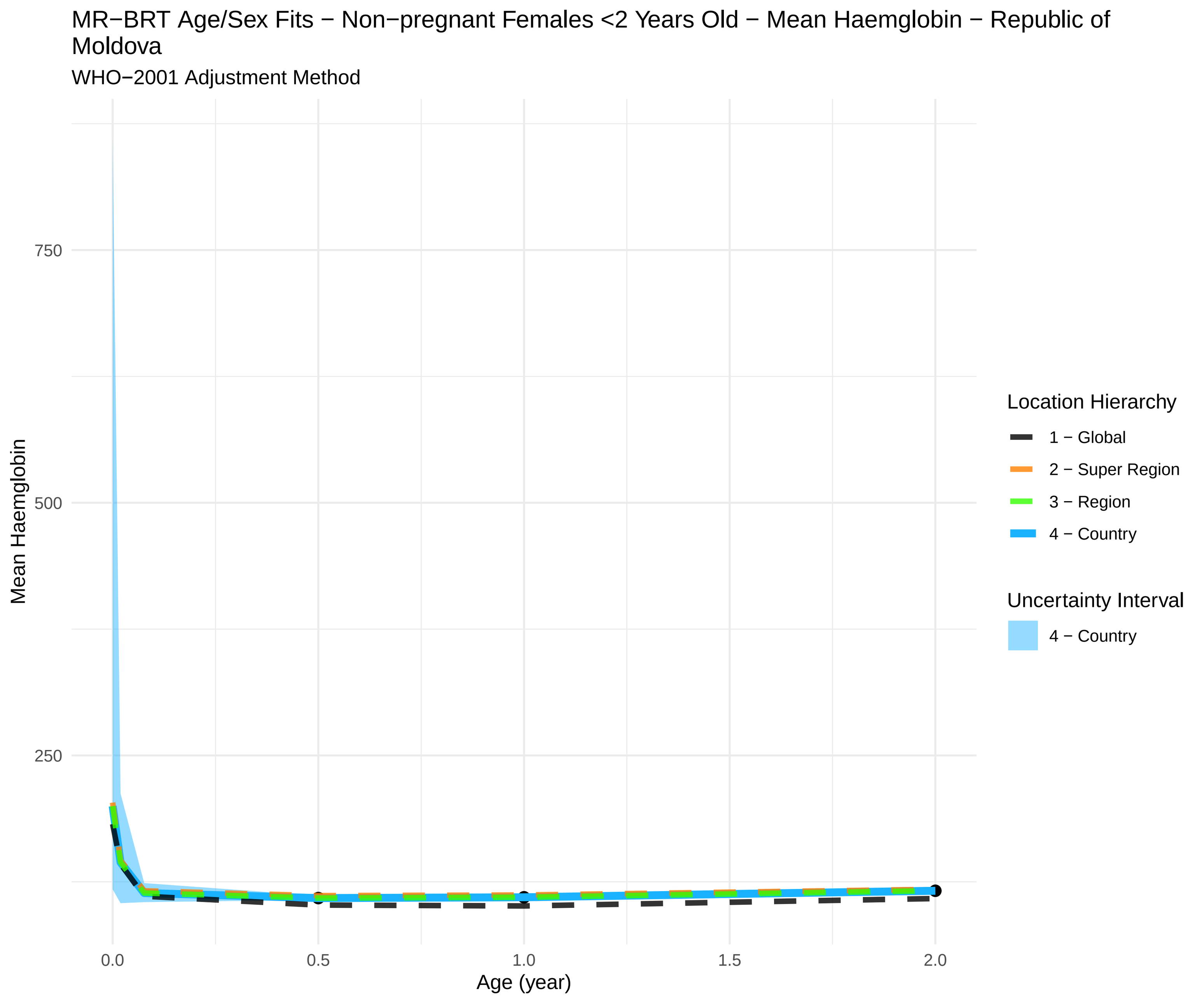
MR−BRT Age/Sex Fits − Non-pregnant Females <2 Years Old − Mean Haemglobin − Republic of Moldova
WHO−2001 Adjustment Method
Mean Haemglobin
Age (year)
Location Hierarchy
1 − Global
2 − Super Region
3 − Region
4 − Country
Uncertainty Interval
4 − Country

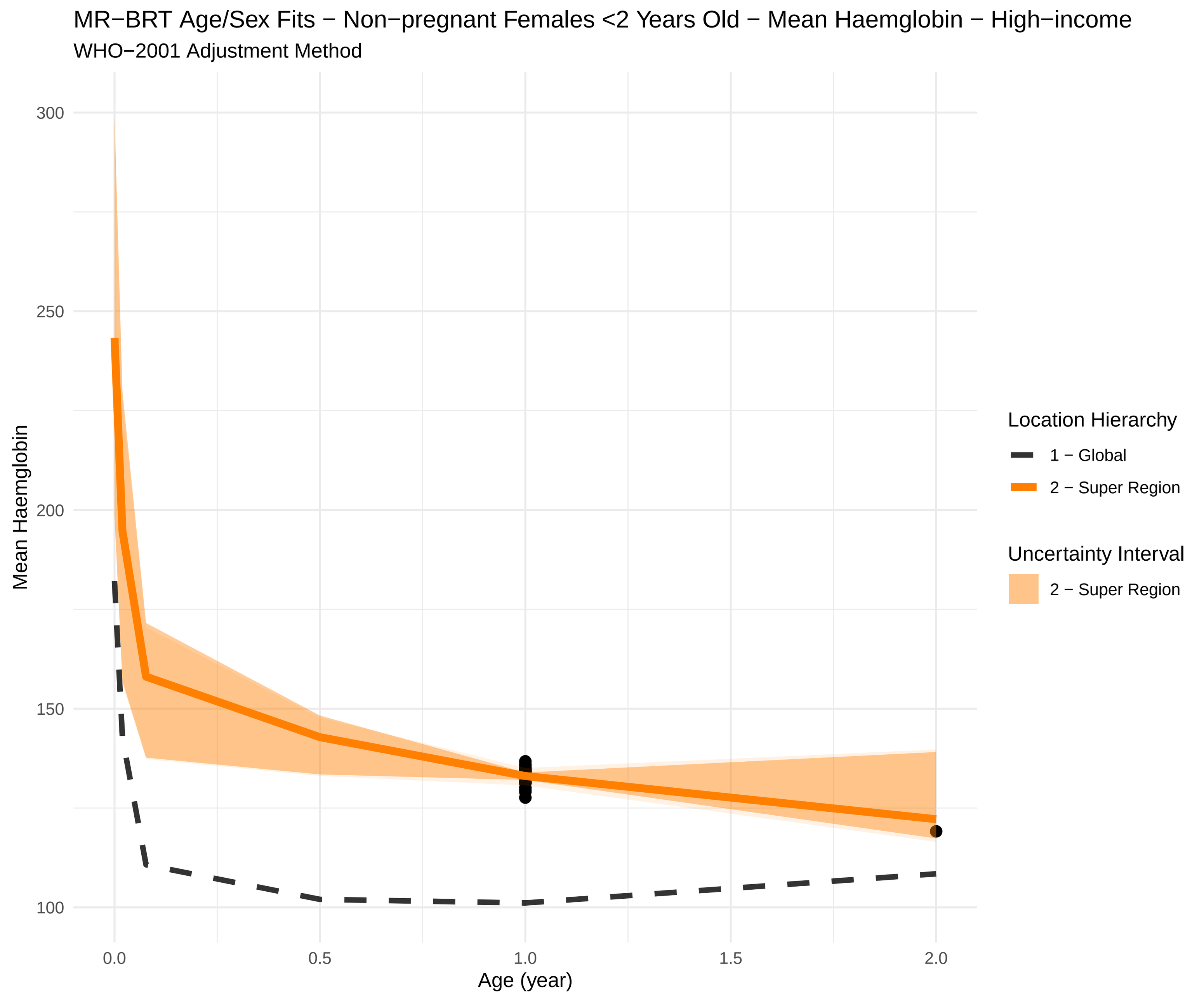
MR−BRT Age/Sex Fits − Non-pregnant Females <2 Years Old − Mean Haemglobin − High−income
WHO−2001 Adjustment Method
Mean Haemglobin
Age (year)
Location Hierarchy
1 − Global
2 − Super Region
Uncertainty Interval
2 − Super Region

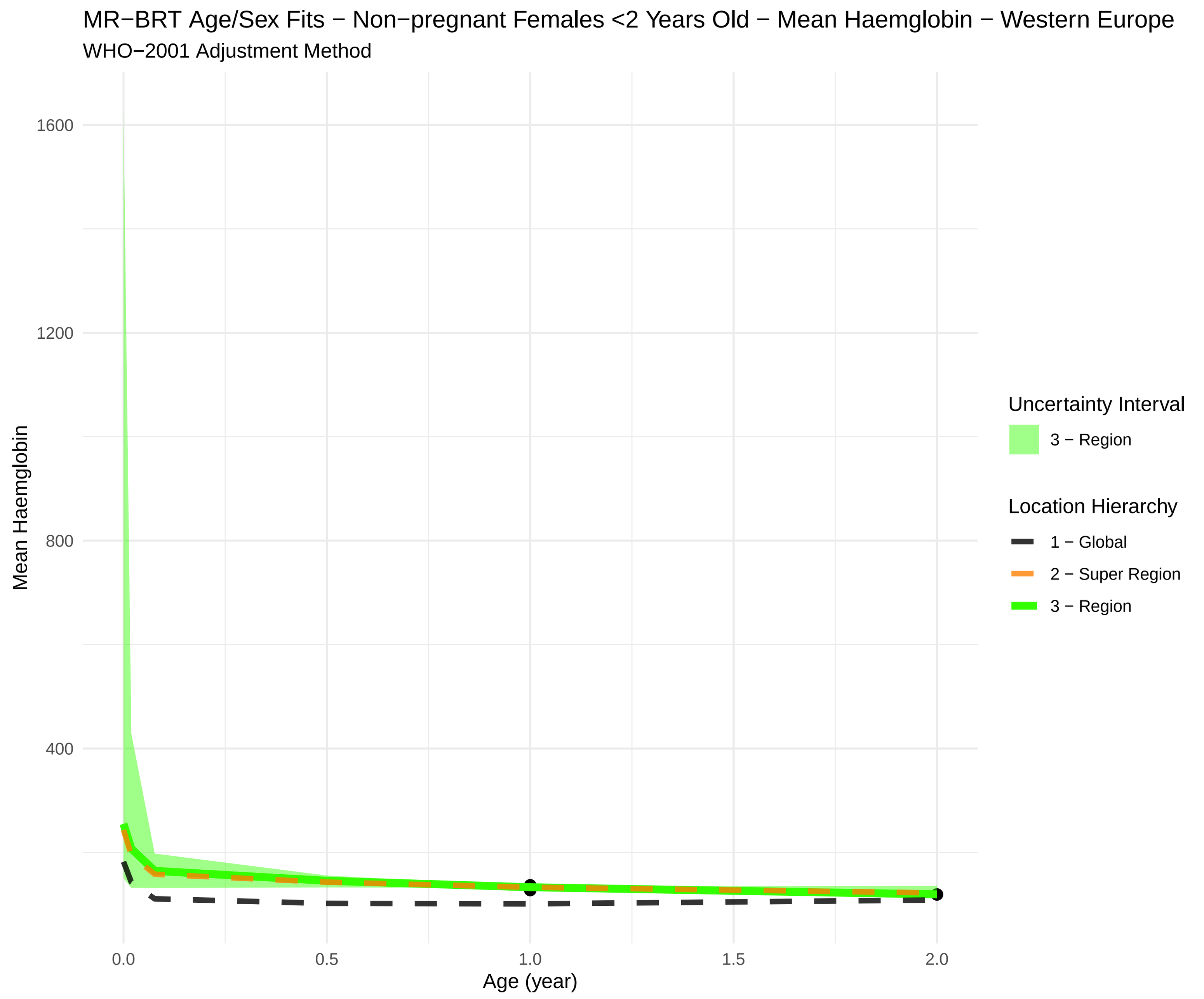
MR−BRT Age/Sex Fits − Non-pregnant Females <2 Years Old − Mean Haemglobin − Western Europe
WHO−2001 Adjustment Method
Mean Haemglobin
Age (year)
Uncertainty Interval
3 − Region
Location Hierarchy
1 − Global
2 − Super Region
3 − Region

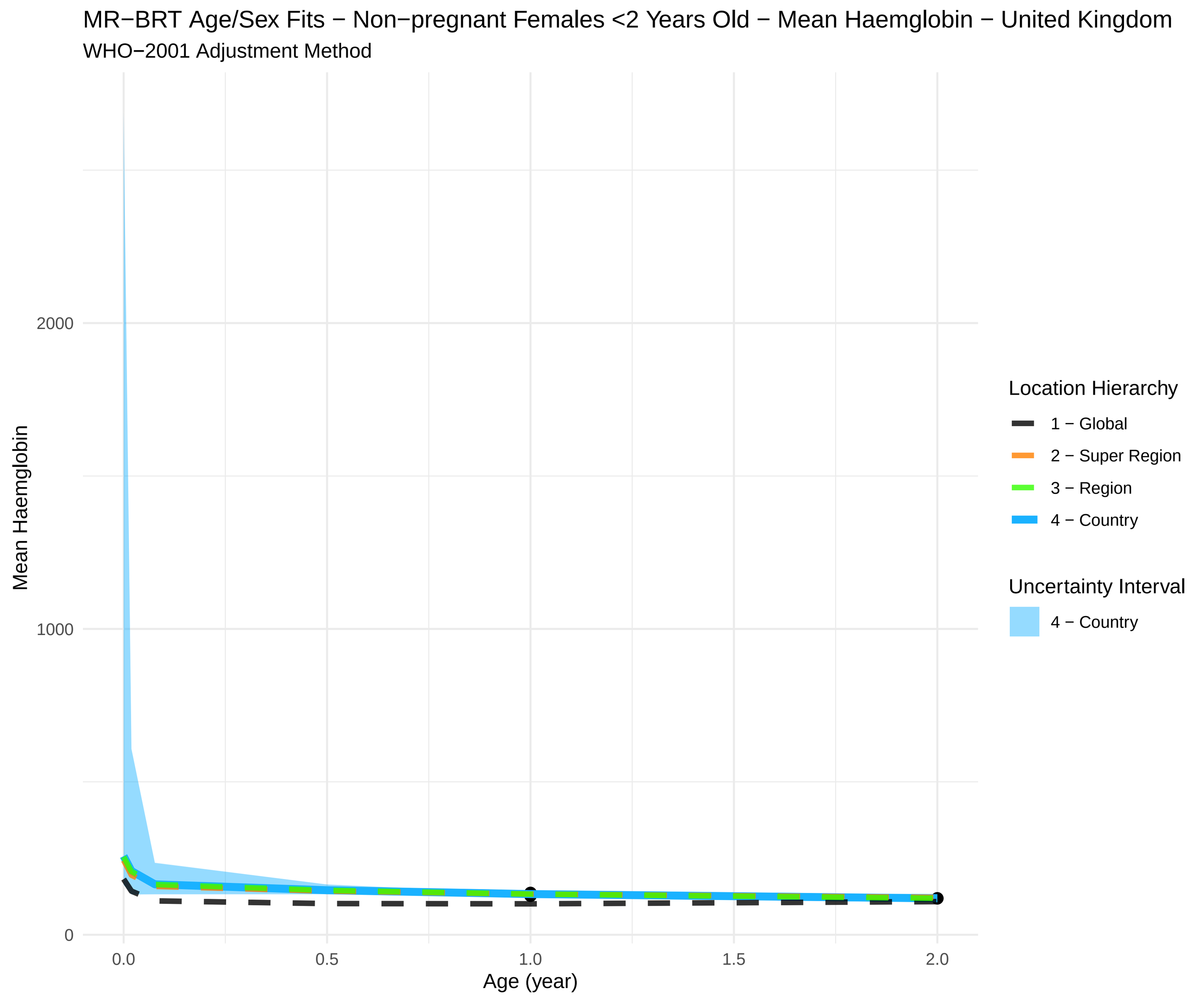
MR−BRT Age/Sex Fits − Non-pregnant Females <2 Years Old − Mean Haemglobin − United Kingdom
WHO−2001 Adjustment Method
Mean Haemglobin
Age (year)
Location Hierarchy
1 − Global
2 − Super Region
3 − Region
4 − Country
Uncertainty Interval
4 − Country

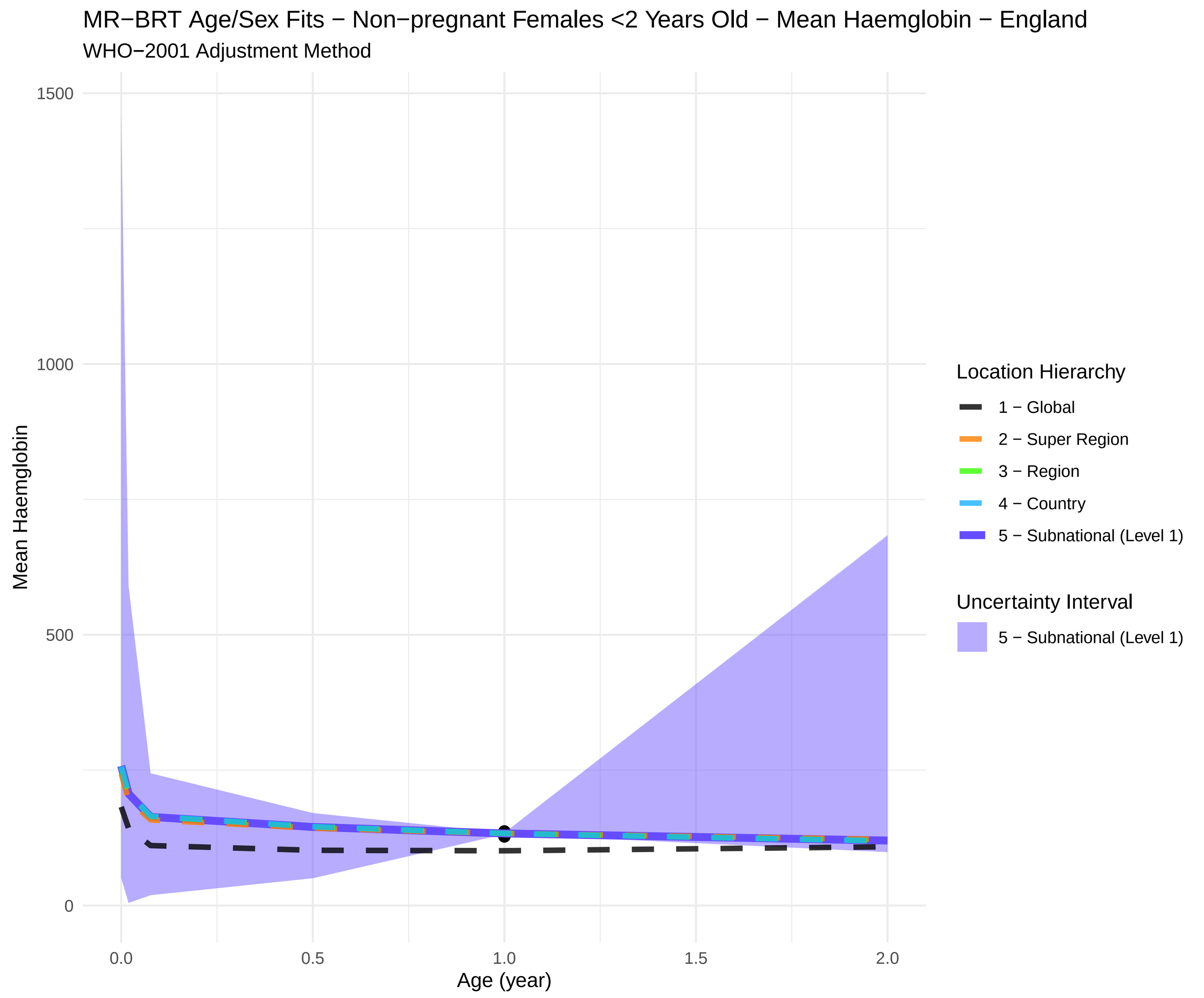
MR−BRT Age/Sex Fits − Non-pregnant Females <2 Years Old − Mean Haemglobin − England
WHO−2001 Adjustment Method
Mean Haemglobin
Age (year)
Location Hierarchy
1 − Global
2 − Super Region
3 − Region
4 − Country
5 − Subnational (Level 1)
Uncertainty Interval
5 − Subnational (Level 1)

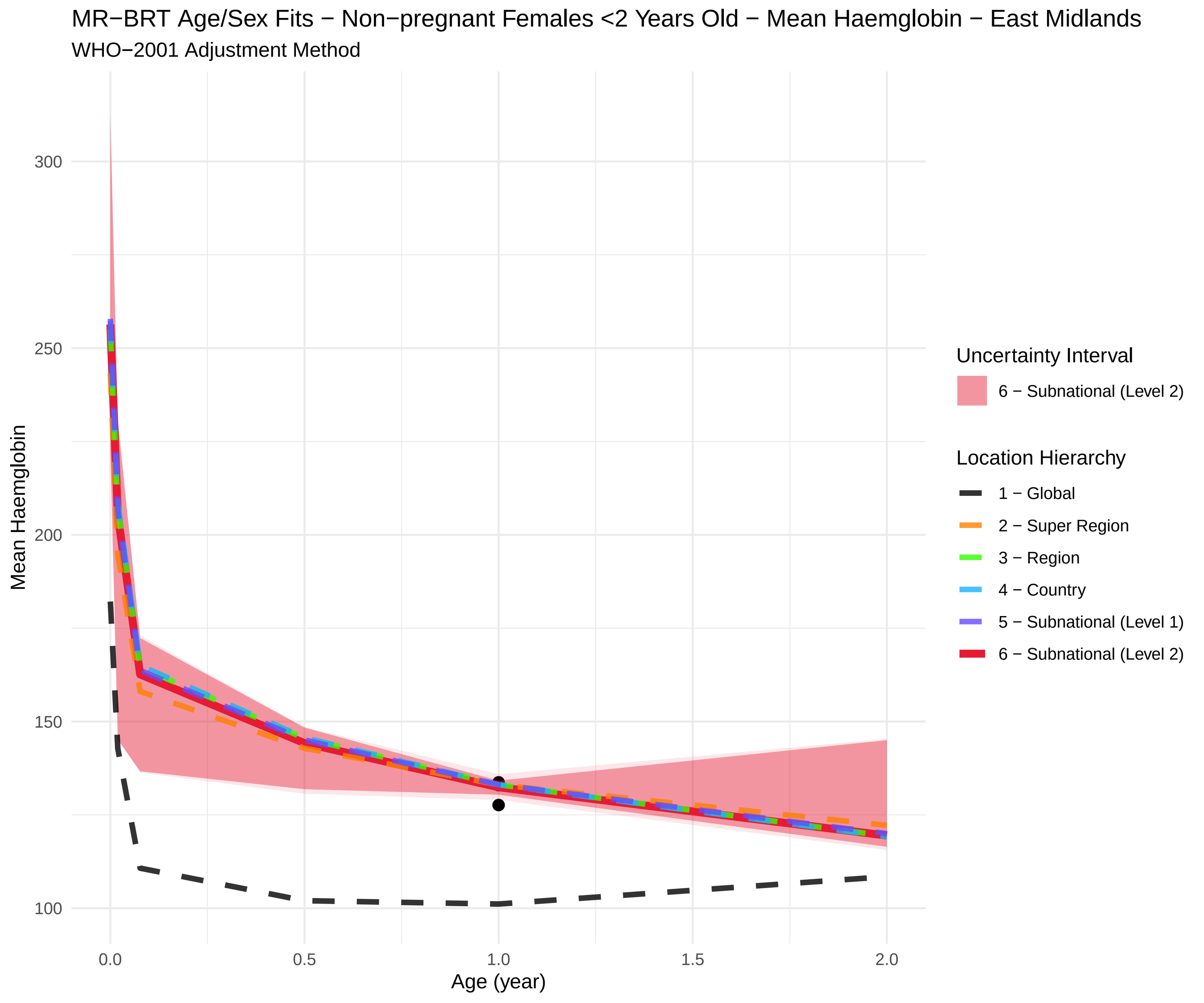
MR−BRT Age/Sex Fits − Non-pregnant Females <2 Years Old − Mean Haemglobin − East Midlands
WHO−2001 Adjustment Method
Mean Haemglobin
Age (year)
Uncertainty Interval
6 − Subnational (Level 2)
Location Hierarchy
1 − Global
2 − Super Region
3 − Region
4 − Country
5 − Subnational (Level 1)
6 − Subnational (Level 2)

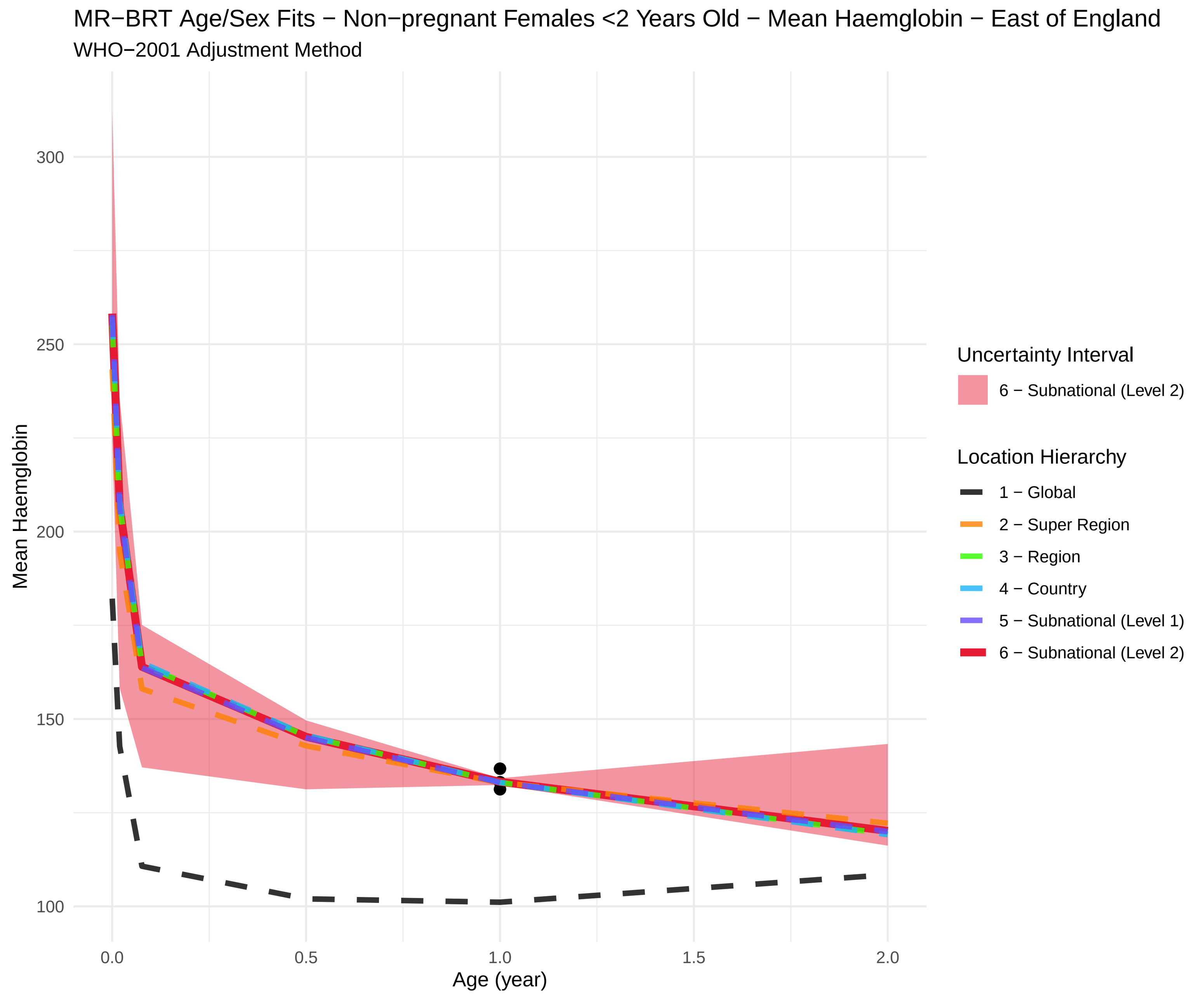
MR−BRT Age/Sex Fits − Non-pregnant Females <2 Years Old − Mean Haemglobin − East of England
WHO−2001 Adjustment Method
Mean Haemglobin
Age (year)
Uncertainty Interval
6 − Subnational (Level 2)
Location Hierarchy
1 − Global
2 − Super Region
3 − Region
4 − Country
5 − Subnational (Level 1)
6 − Subnational (Level 2)

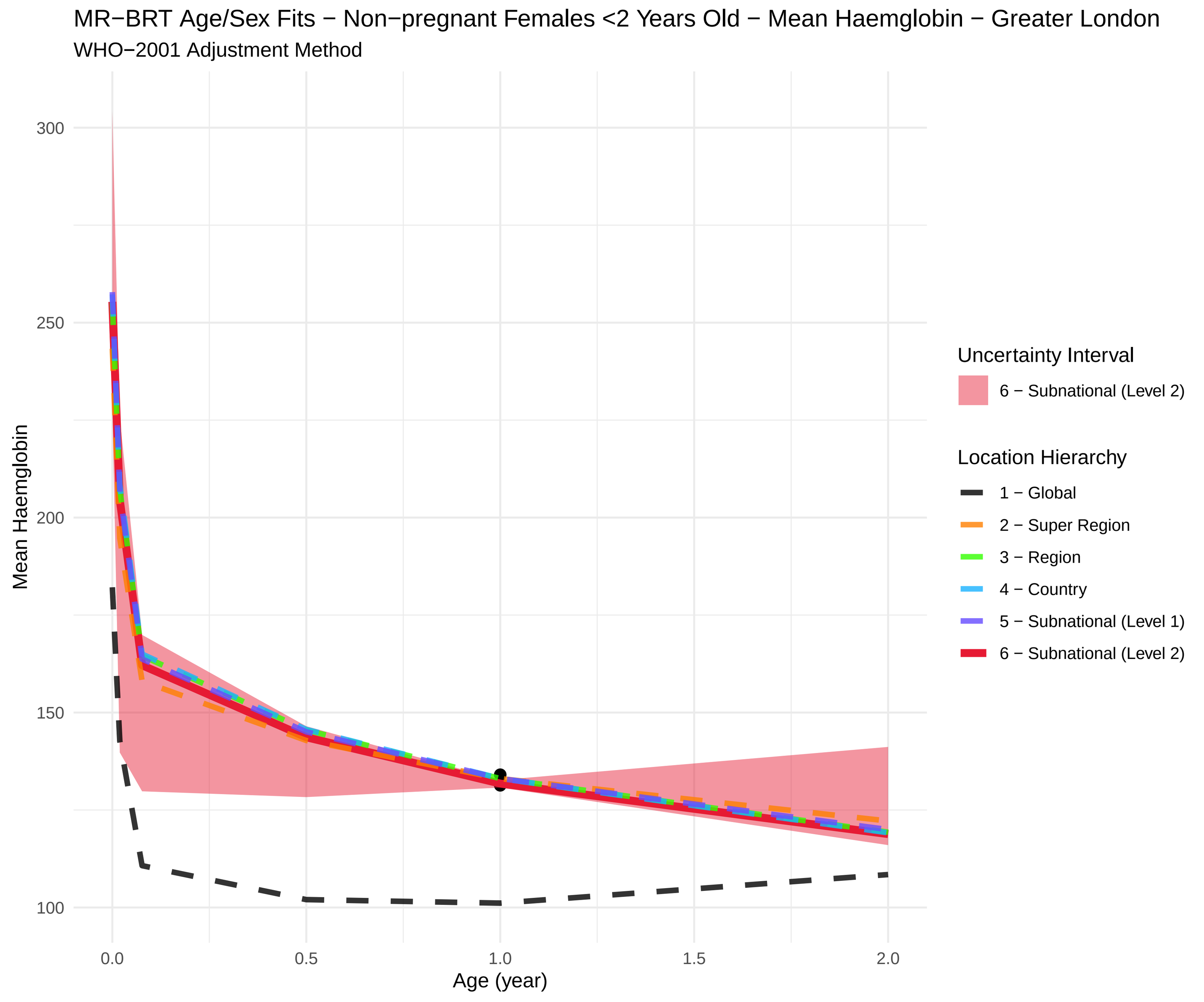
MR−BRT Age/Sex Fits − Non-pregnant Females <2 Years Old − Mean Haemglobin − Greater London
WHO−2001 Adjustment Method
Mean Haemglobin
Age (year)
Uncertainty Interval
6 − Subnational (Level 2)
Location Hierarchy
1 − Global
2 − Super Region
3 − Region
4 − Country
5 − Subnational (Level 1)
6 − Subnational (Level 2)

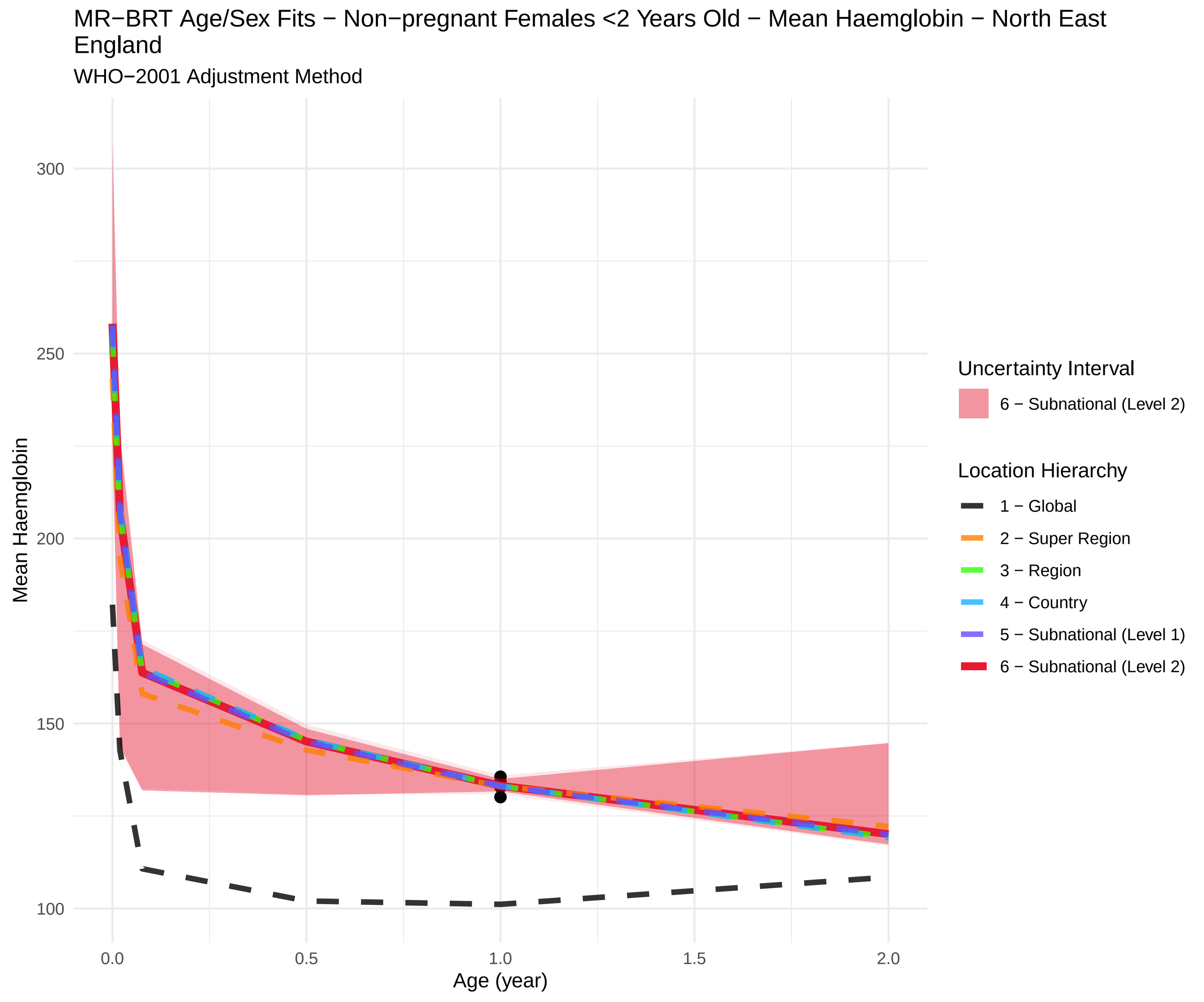
MR−BRT Age/Sex Fits − Non-pregnant Females <2 Years Old − Mean Haemglobin − North East England
WHO−2001 Adjustment Method
Mean Haemglobin
Age (year)
Uncertainty Interval
6 − Subnational (Level 2)
Location Hierarchy
1 − Global
2 − Super Region
3 − Region
4 − Country
5 − Subnational (Level 1)
6 − Subnational (Level 2)

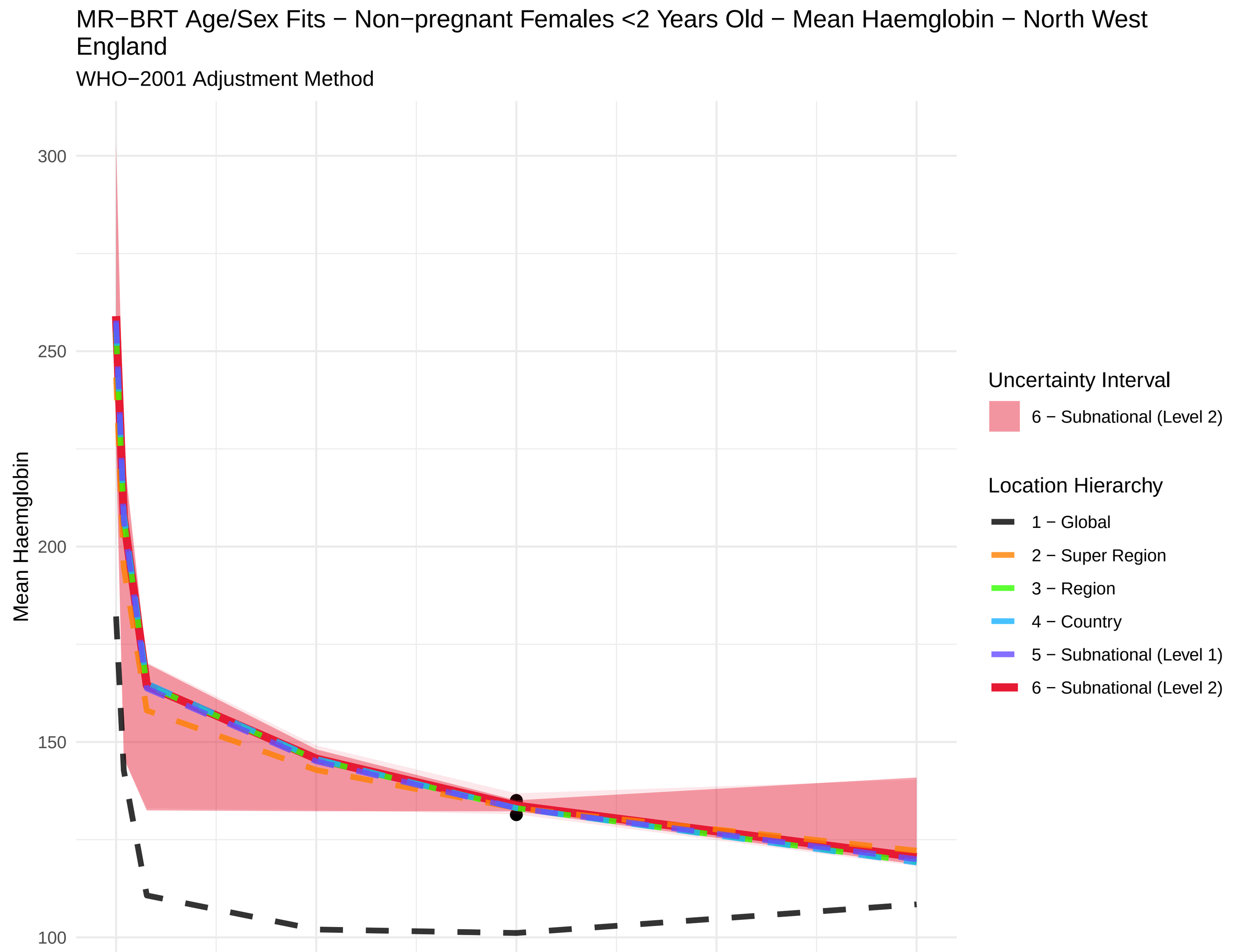
MR−BRT Age/Sex Fits − Non-pregnant Females <2 Years Old − Mean Haemglobin − North West England
WHO−2001 Adjustment Method
Mean Haemglobin
Age (year)
Uncertainty Interval
6 − Subnational (Level 2)
Location Hierarchy
1 − Global
2 − Super Region
3 − Region
4 − Country
5 − Subnational (Level 1)
6 − Subnational (Level 2)

MR−BRT Age/Sex Fits − Non−pregnant Females <2 Years Old − Mean Haemglobin − South East England
WHO−2001 Adjustment Method

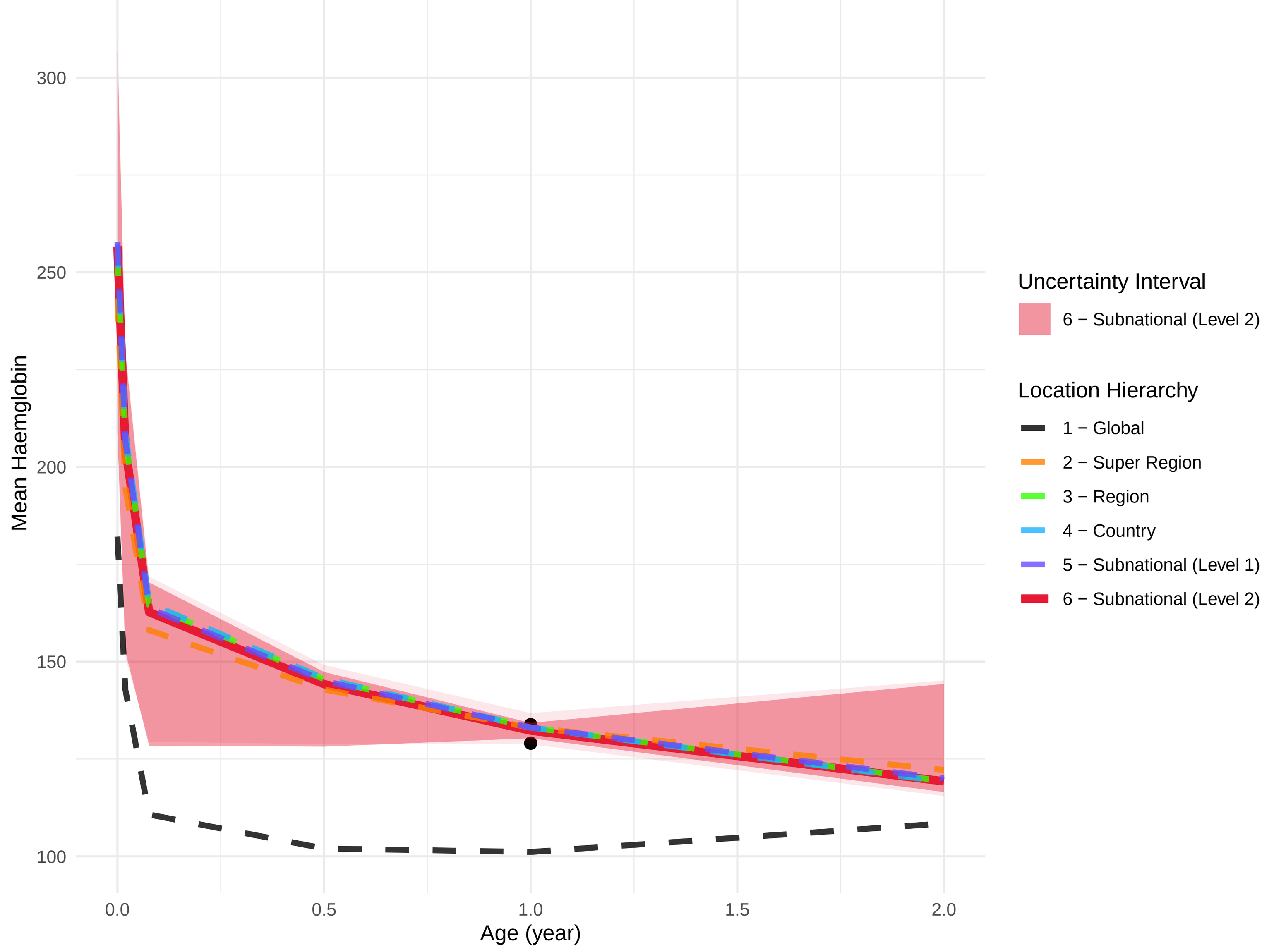

MR−BRT Age/Sex Fits − Non−pregnant Females <2 Years Old − Mean Haemglobin − South West England
WHO−2001 Adjustment Method

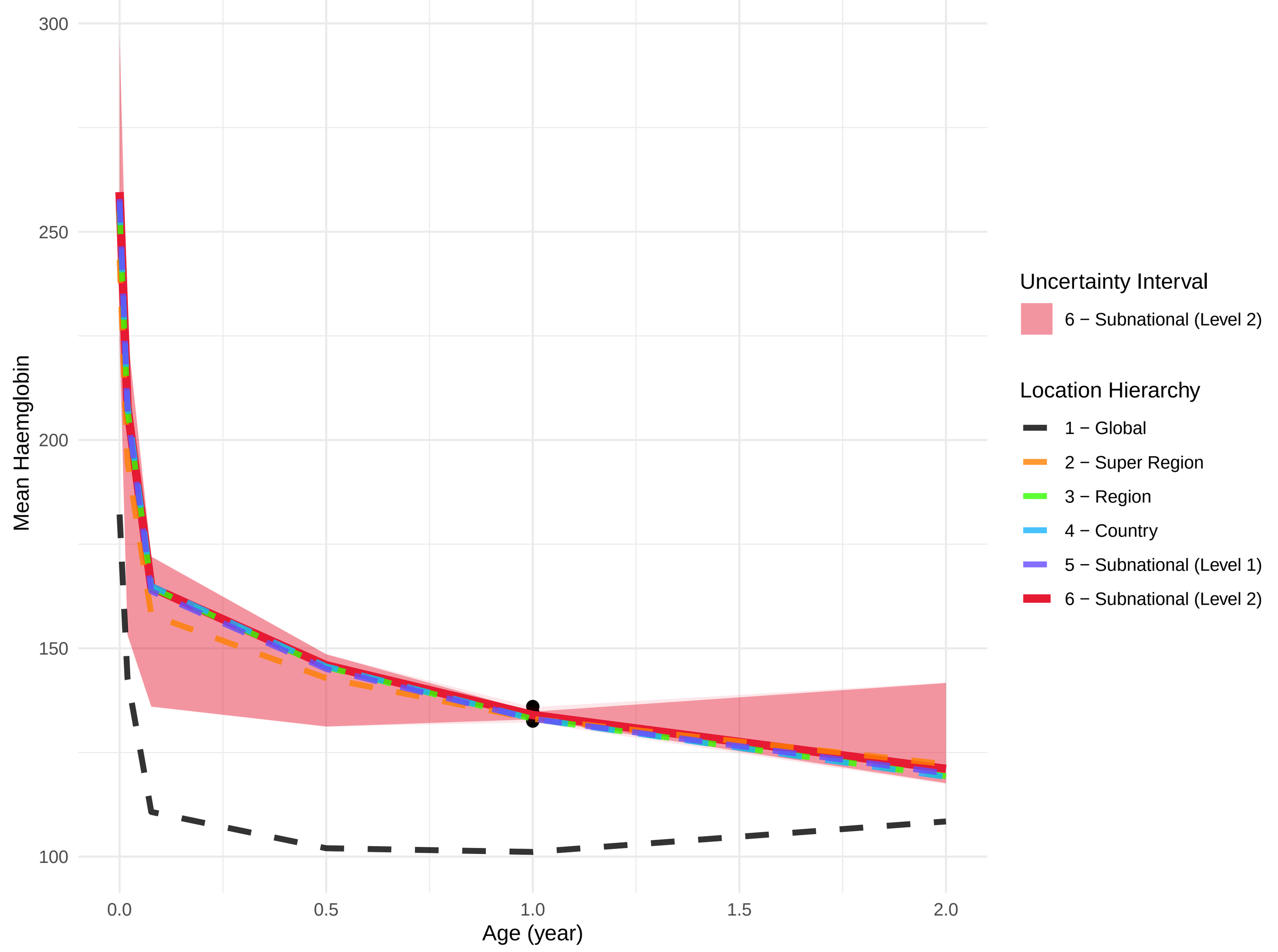

MR−BRT Age/Sex Fits − Non−pregnant Females <2 Years Old − Mean Haemglobin − West Midlands
WHO−2001 Adjustment Method

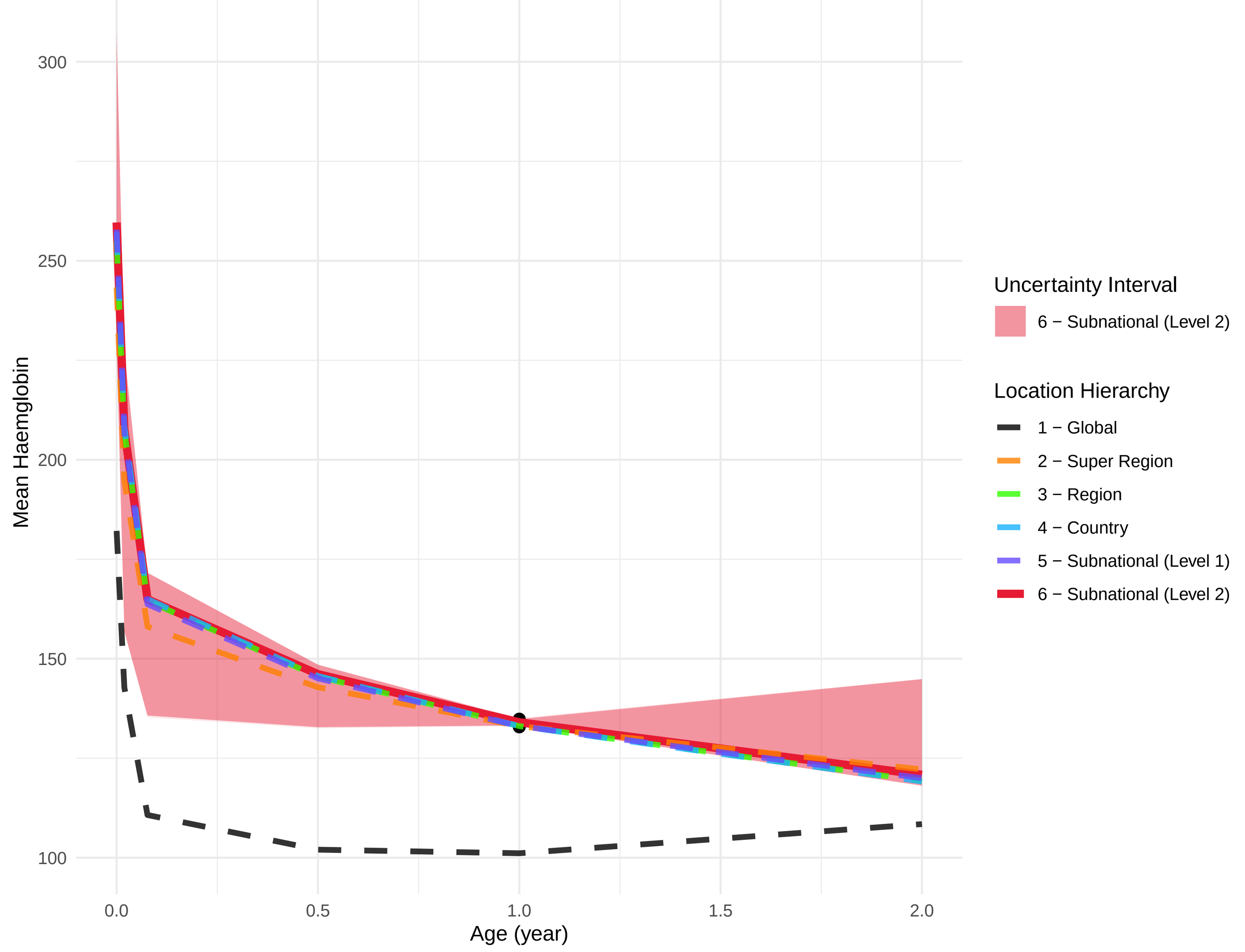

MR−BRT Age/Sex Fits − Non−pregnant Females <2 Years Old − Mean Haemglobin − Yorkshire and the Humber
WHO−2001 Adjustment Method

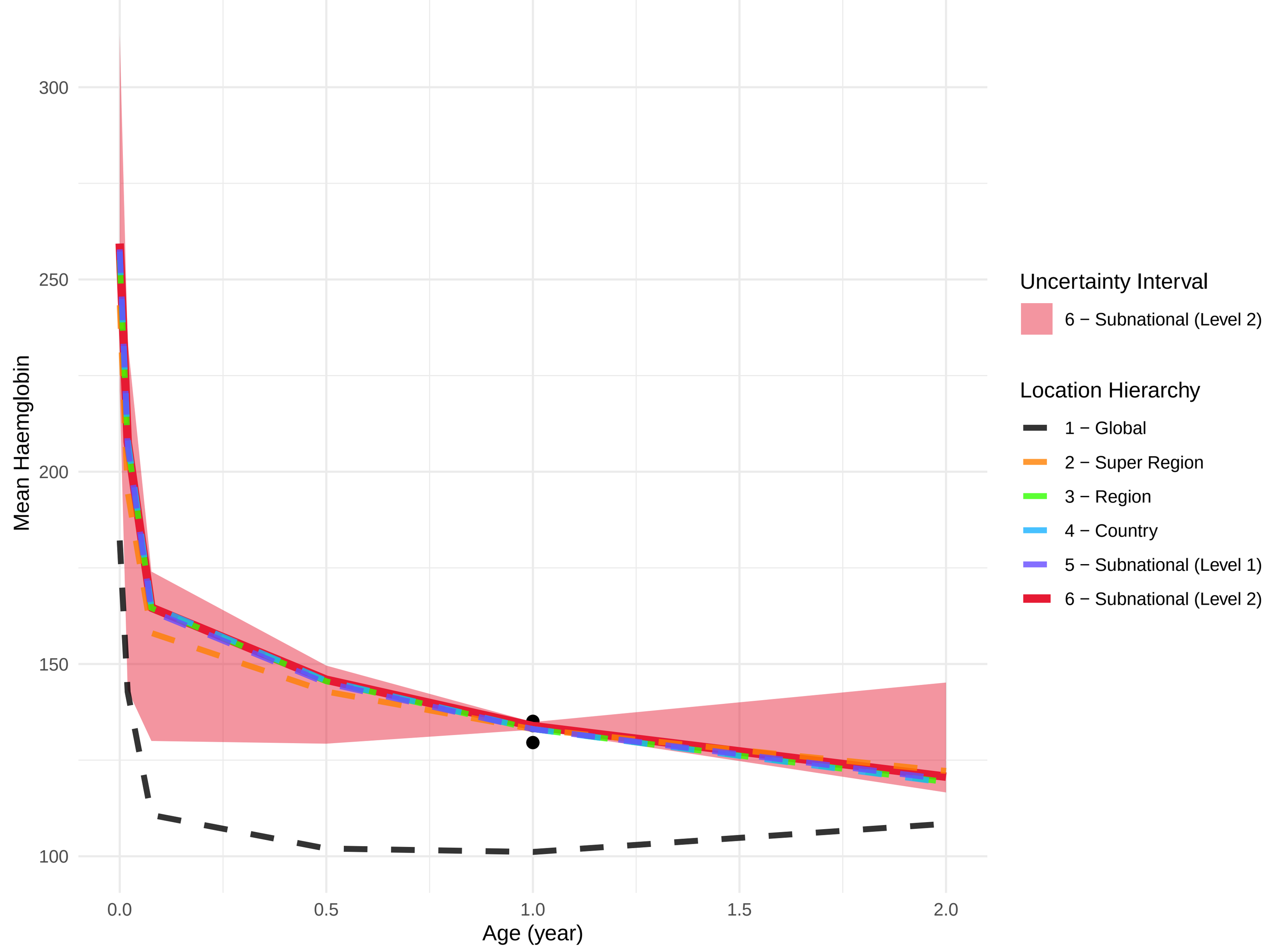

MR−BRT Age/Sex Fits − Non−pregnant Females <2 Years Old − Mean Haemglobin − Latin America and Caribbean
WHO−2001 Adjustment Method

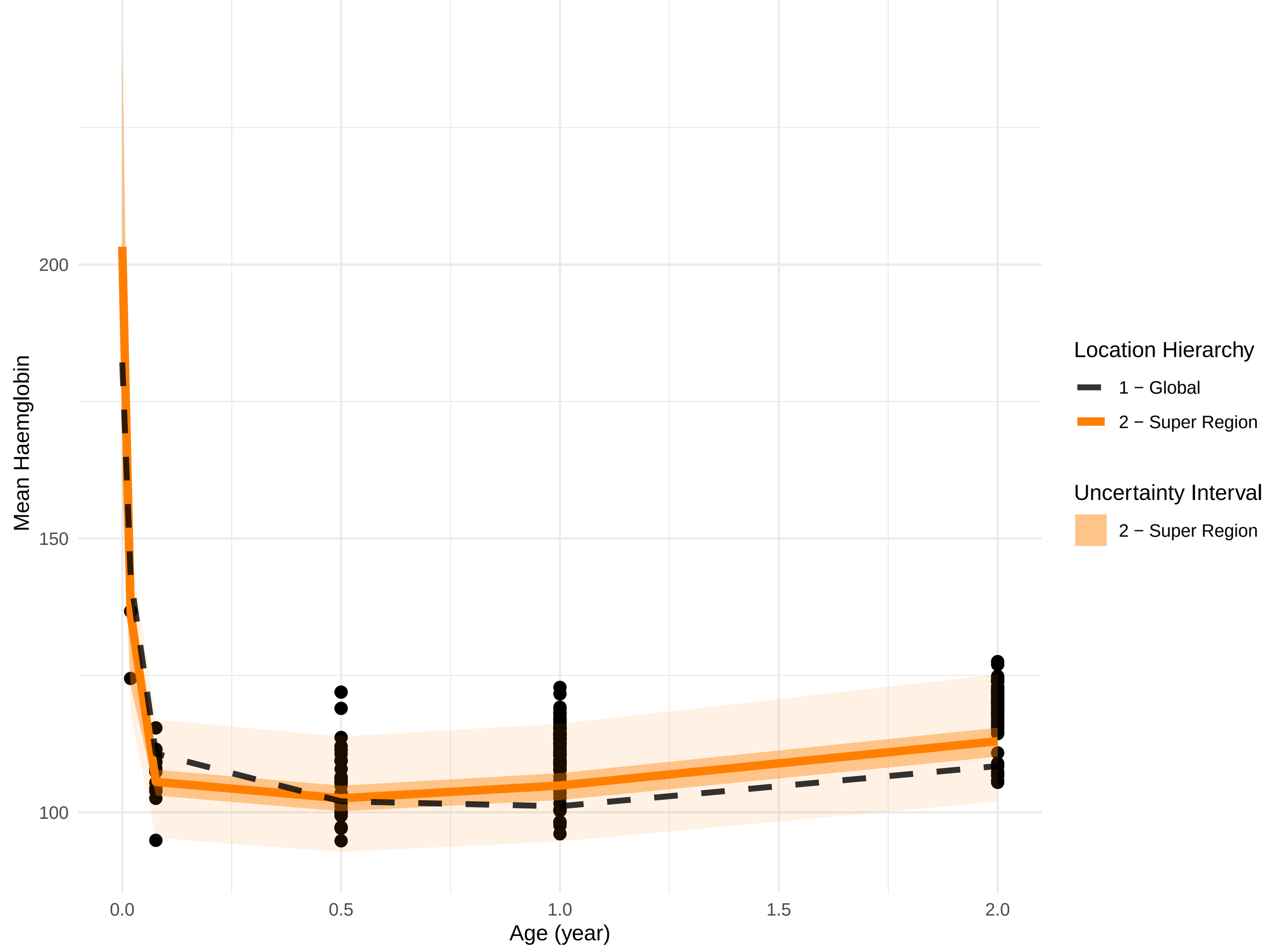

MR−BRT Age/Sex Fits − Non−pregnant Females <2 Years Old − Mean Haemglobin − Andean Latin America
WHO−2001 Adjustment Method

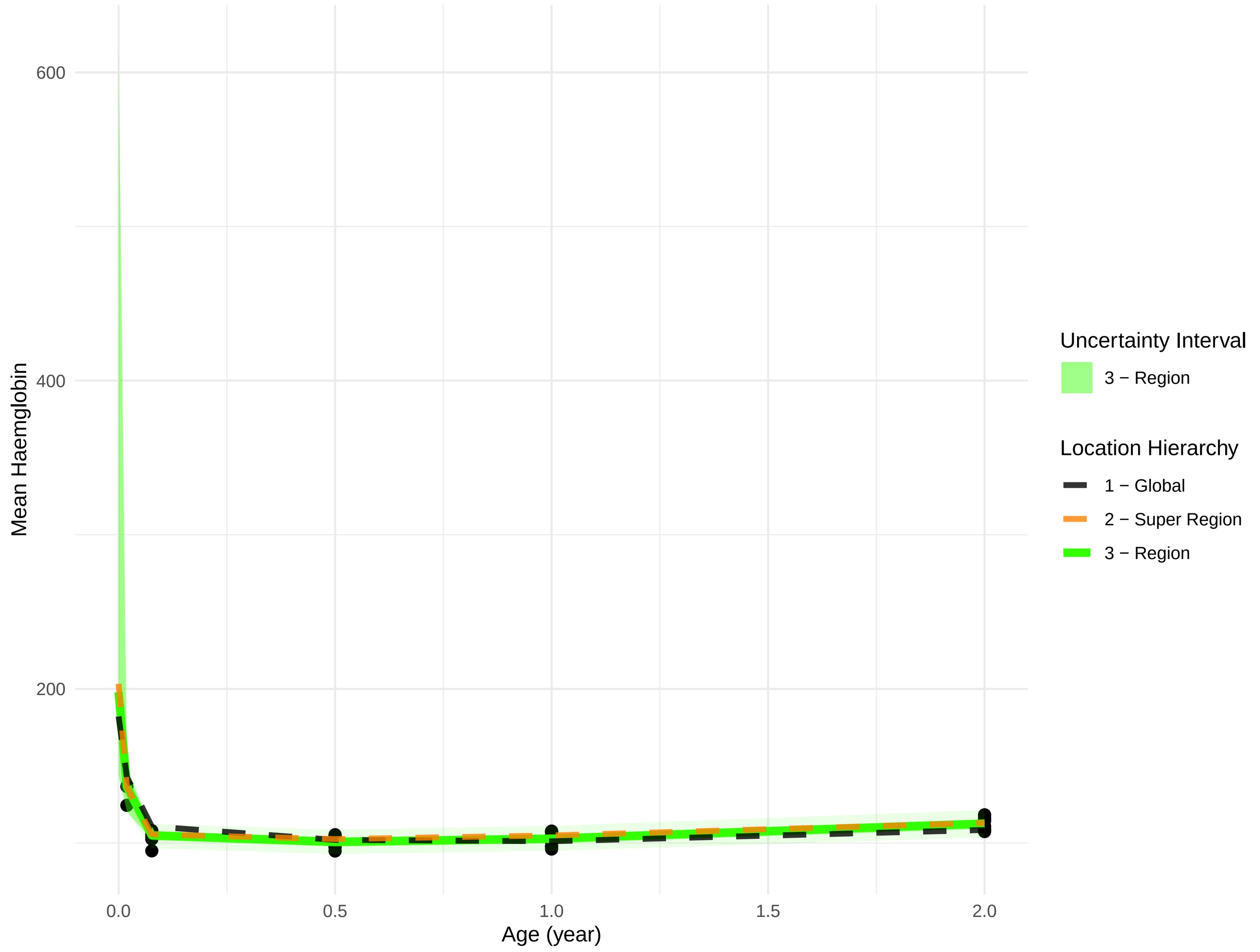

MR−BRT Age/Sex Fits − Non−pregnant Females <2 Years Old − Mean Haemglobin − Bolivia (Plurinational State of)
WHO−2001 Adjustment Method

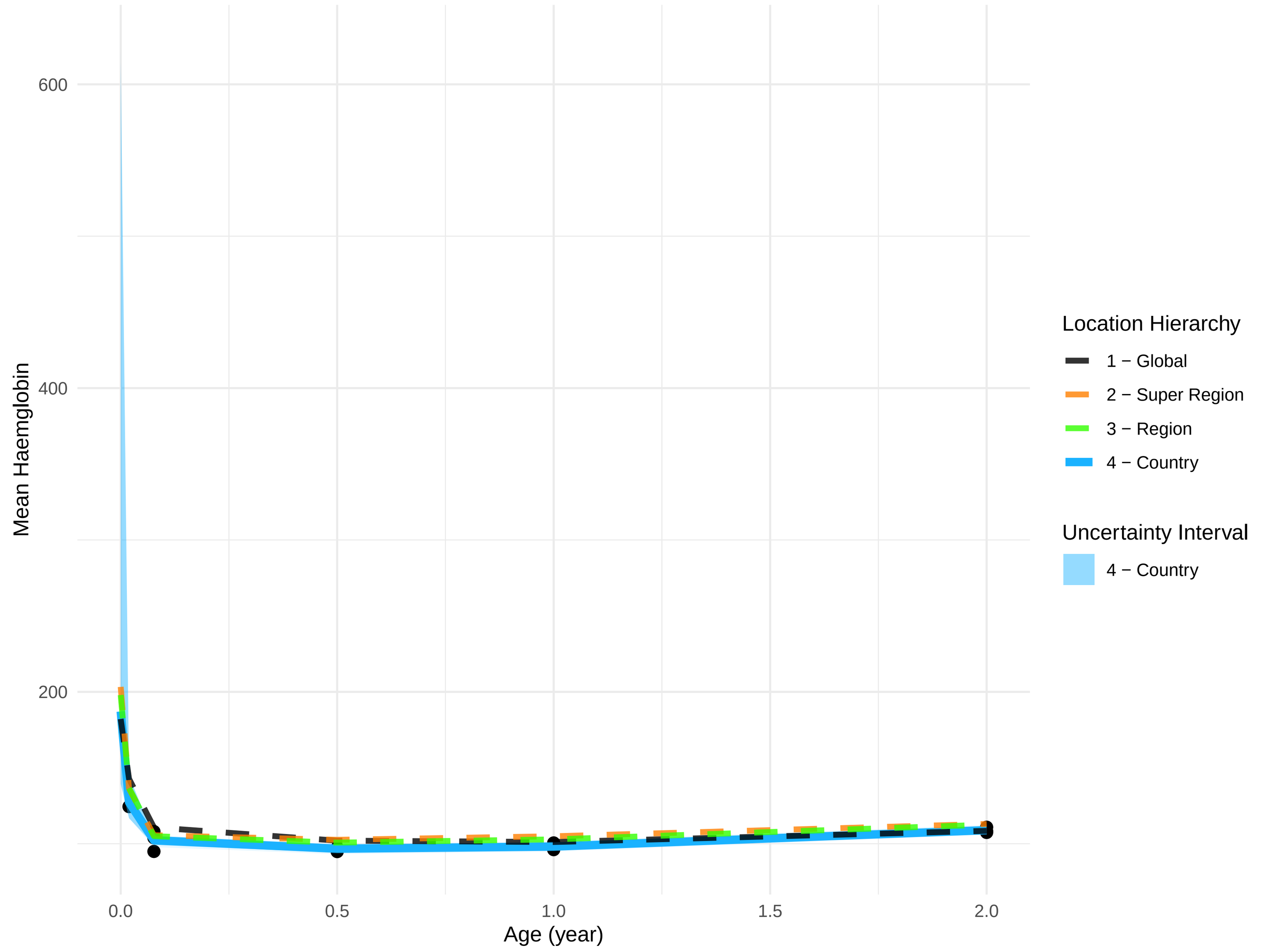

MR−BRT Age/Sex Fits − Non−pregnant Females <2 Years Old − Mean Haemglobin − Peru
WHO−2001 Adjustment Method

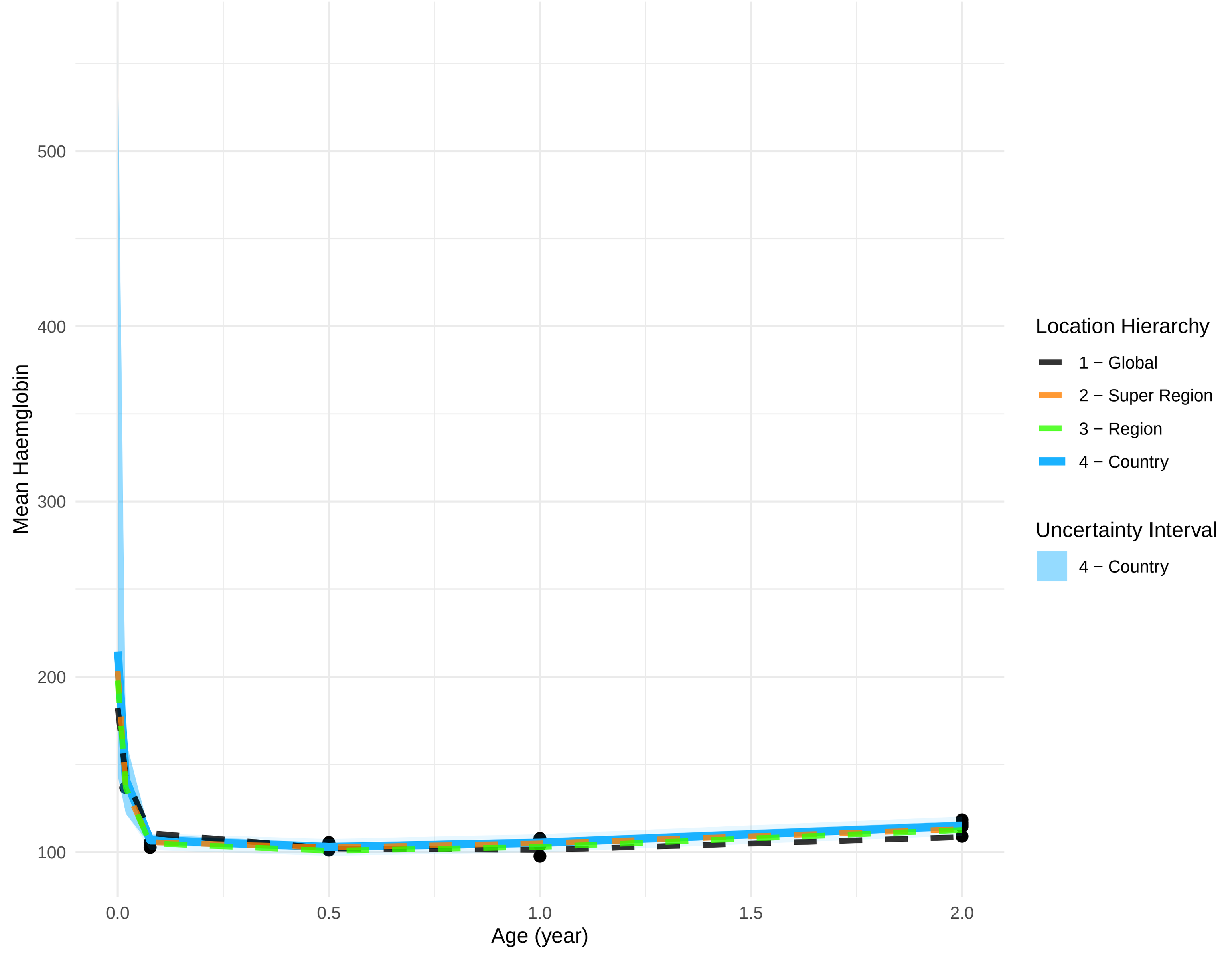

MR−BRT Age/Sex Fits − Non−pregnant Females <2 Years Old − Mean Haemglobin − Caribbean
WHO−2001 Adjustment Method

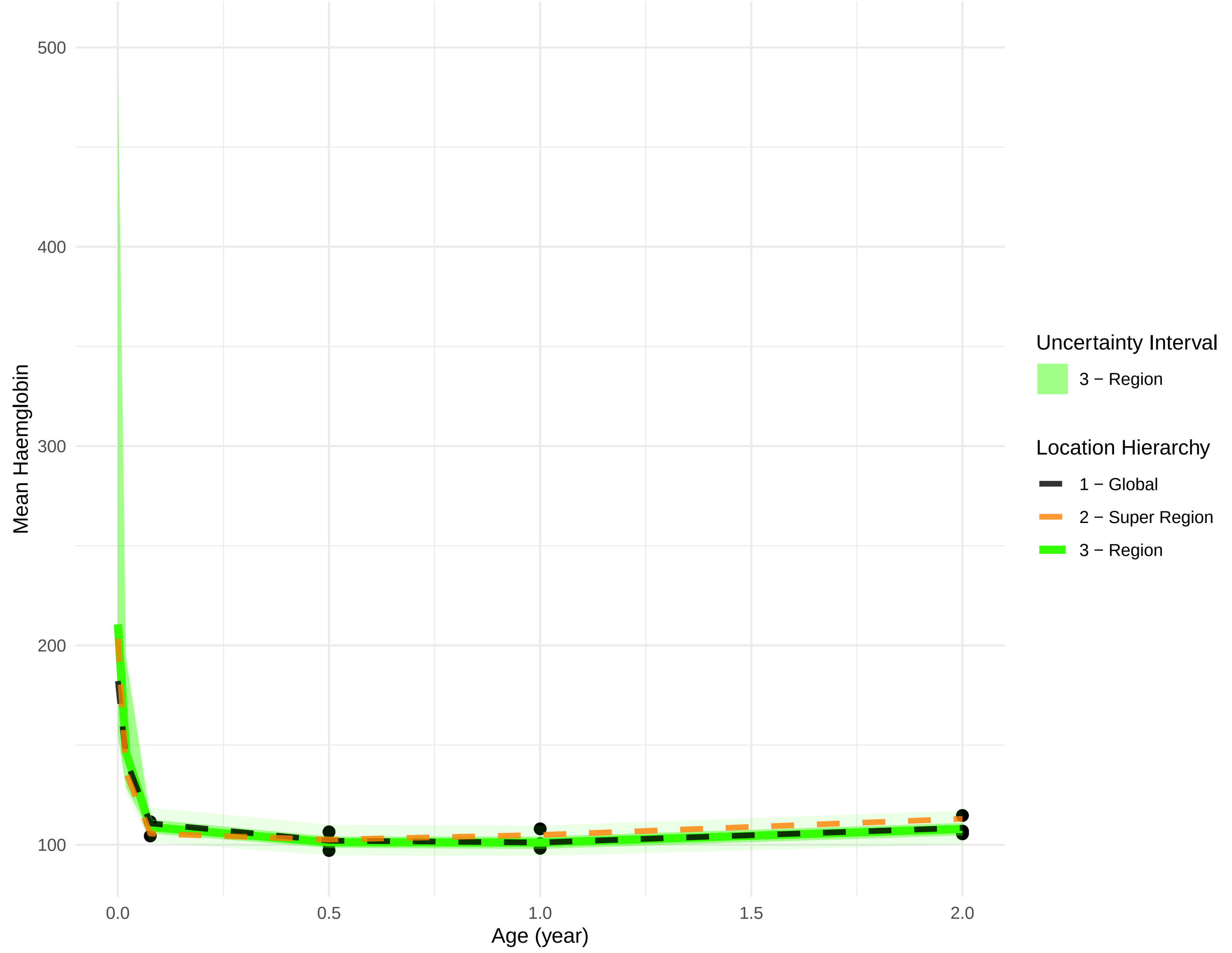

MR−BRT Age/Sex Fits − Non−pregnant Females <2 Years Old − Mean Haemglobin − Guyana
WHO−2001 Adjustment Method

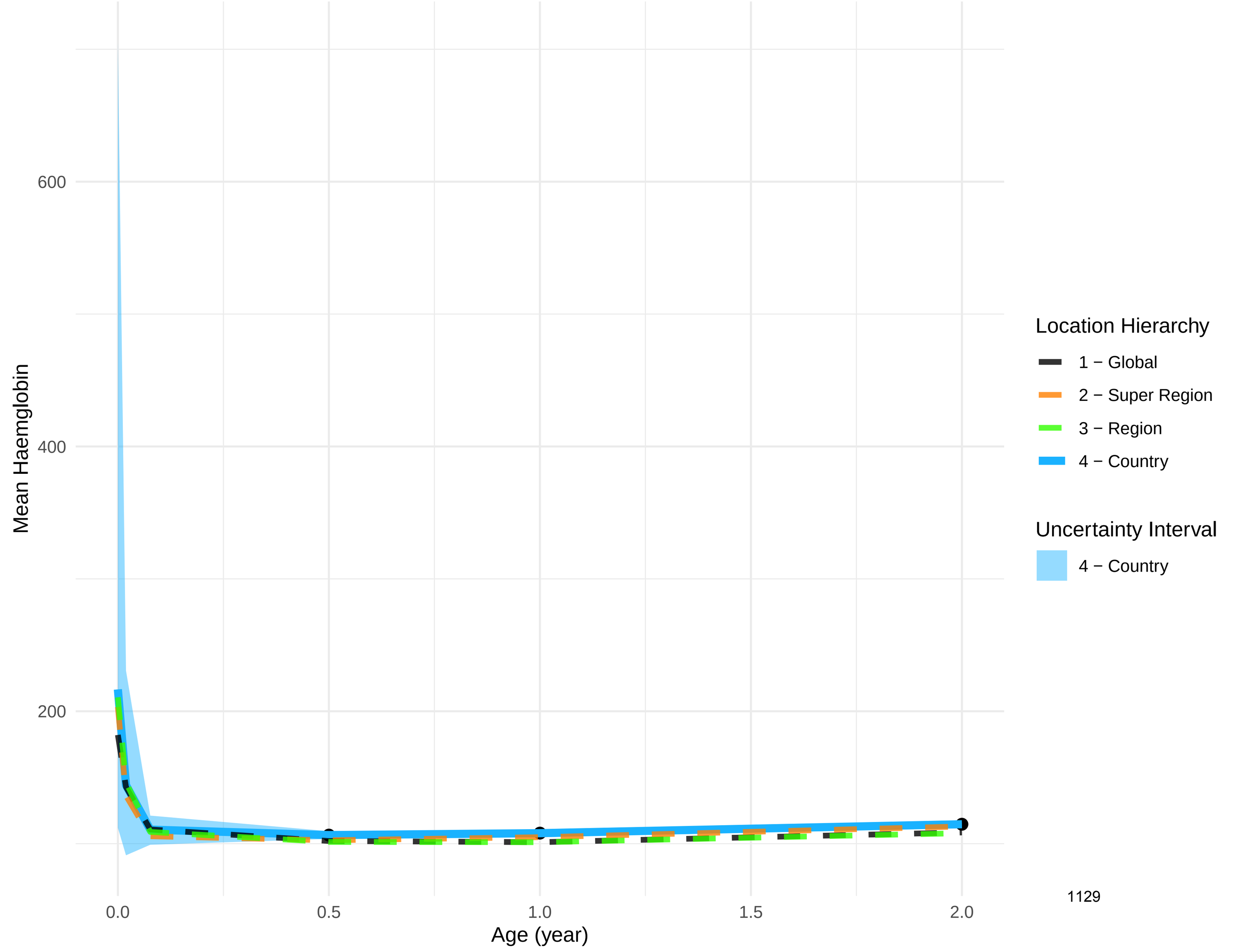

MR−BRT Age/Sex Fits − Non−pregnant Females <2 Years Old − Mean Haemglobin − Haiti
WHO−2001 Adjustment Method

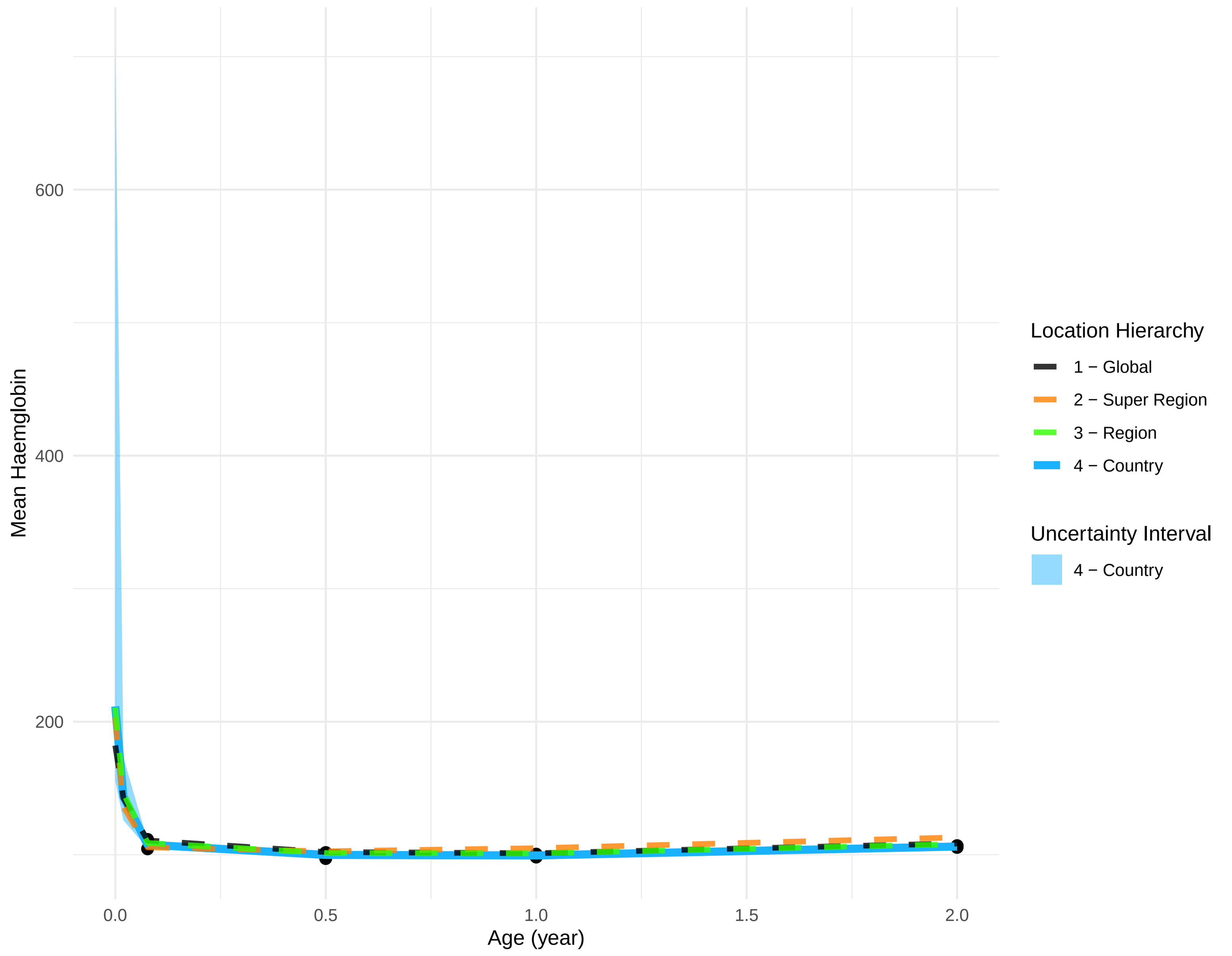

MR−BRT Age/Sex Fits − Non−pregnant Females <2 Years Old − Mean Haemglobin − Central Latin America
WHO−2001 Adjustment Method

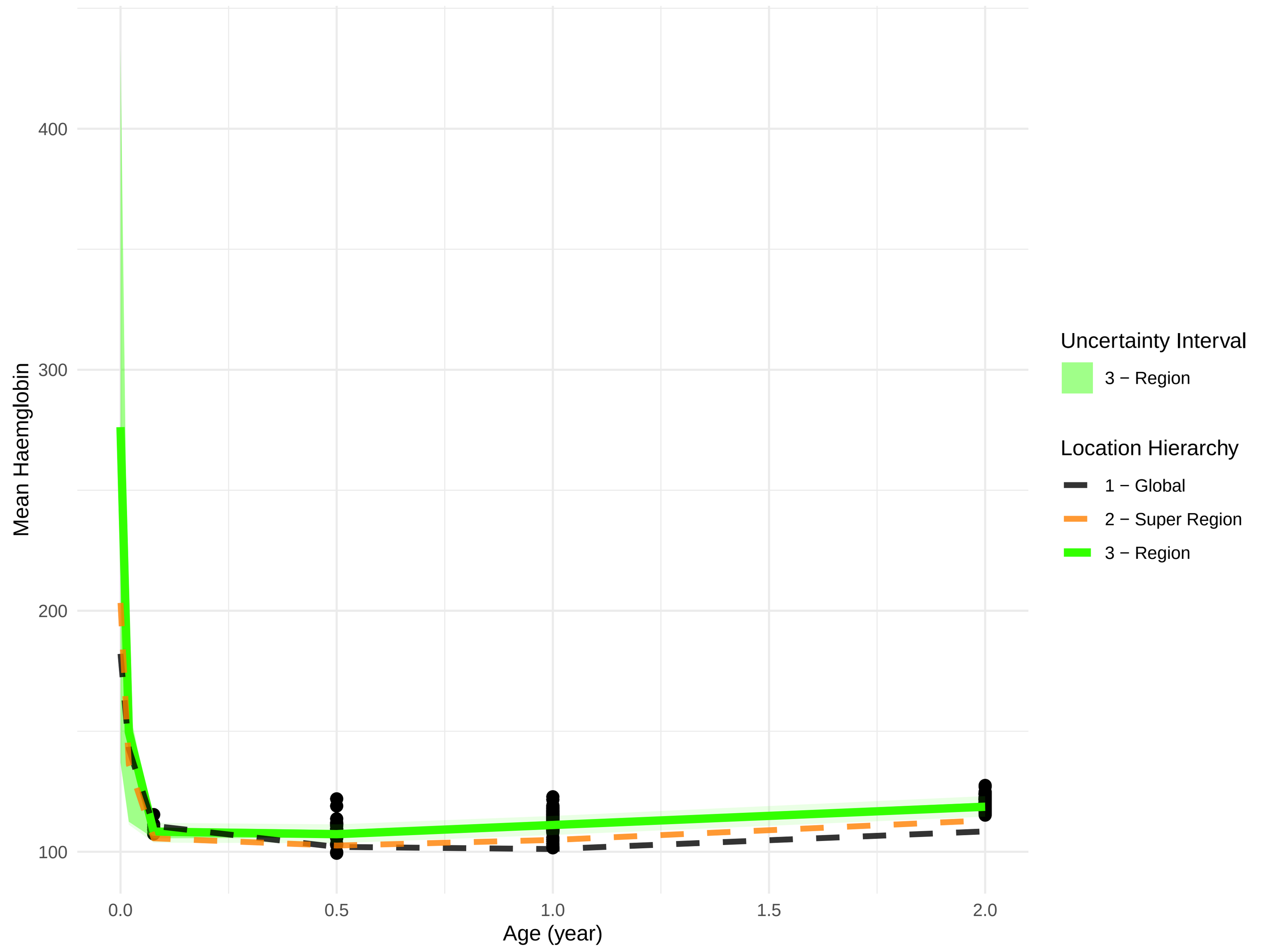

MR−BRT Age/Sex Fits − Non−pregnant Females <2 Years Old − Mean Haemglobin − Guatemala
WHO−2001 Adjustment Method

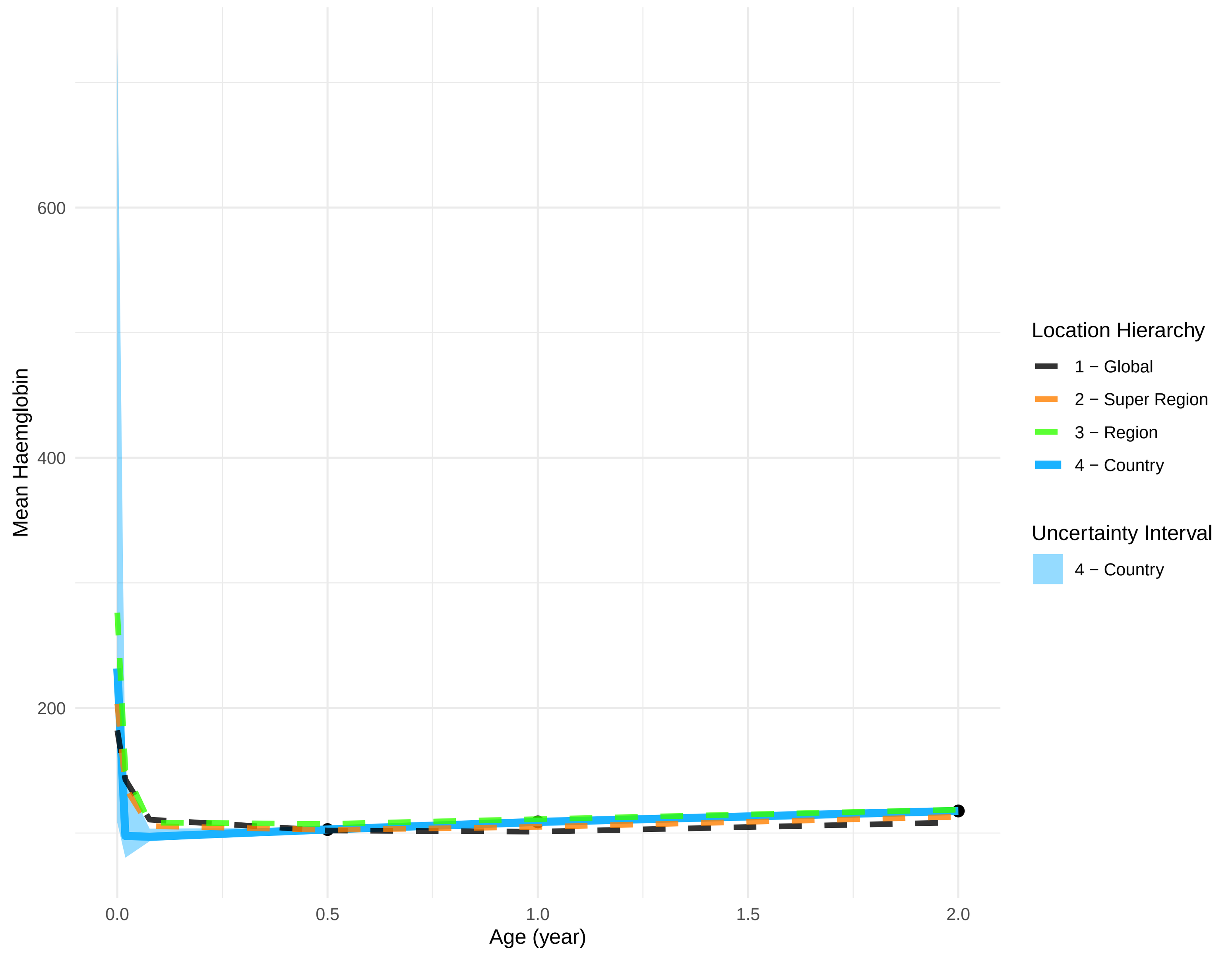

MR−BRT Age/Sex Fits − Non−pregnant Females <2 Years Old − Mean Haemglobin − Honduras
WHO−2001 Adjustment Method

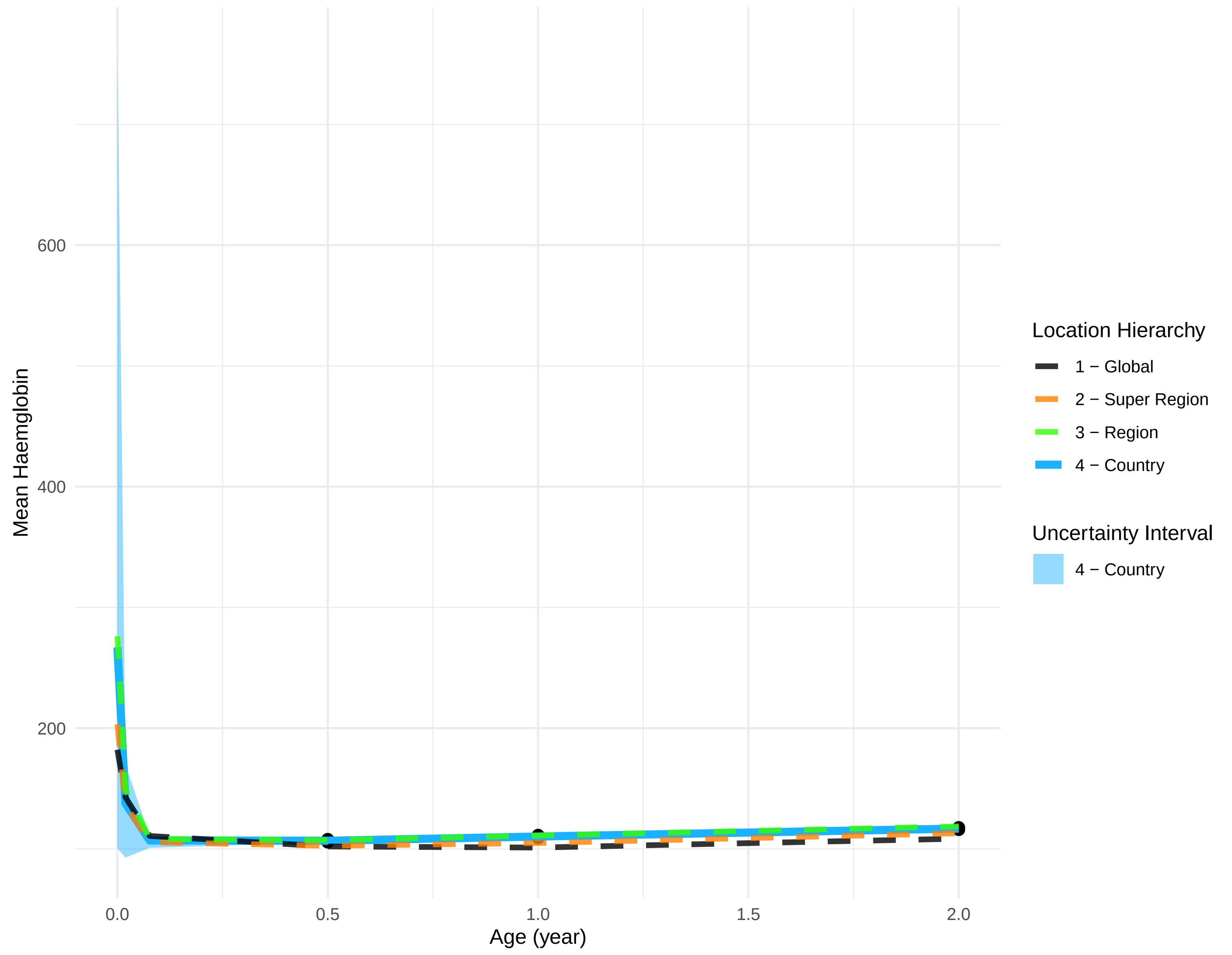

MR−BRT Age/Sex Fits − Non−pregnant Females <2 Years Old − Mean Haemglobin − Mexico
WHO−2001 Adjustment Method

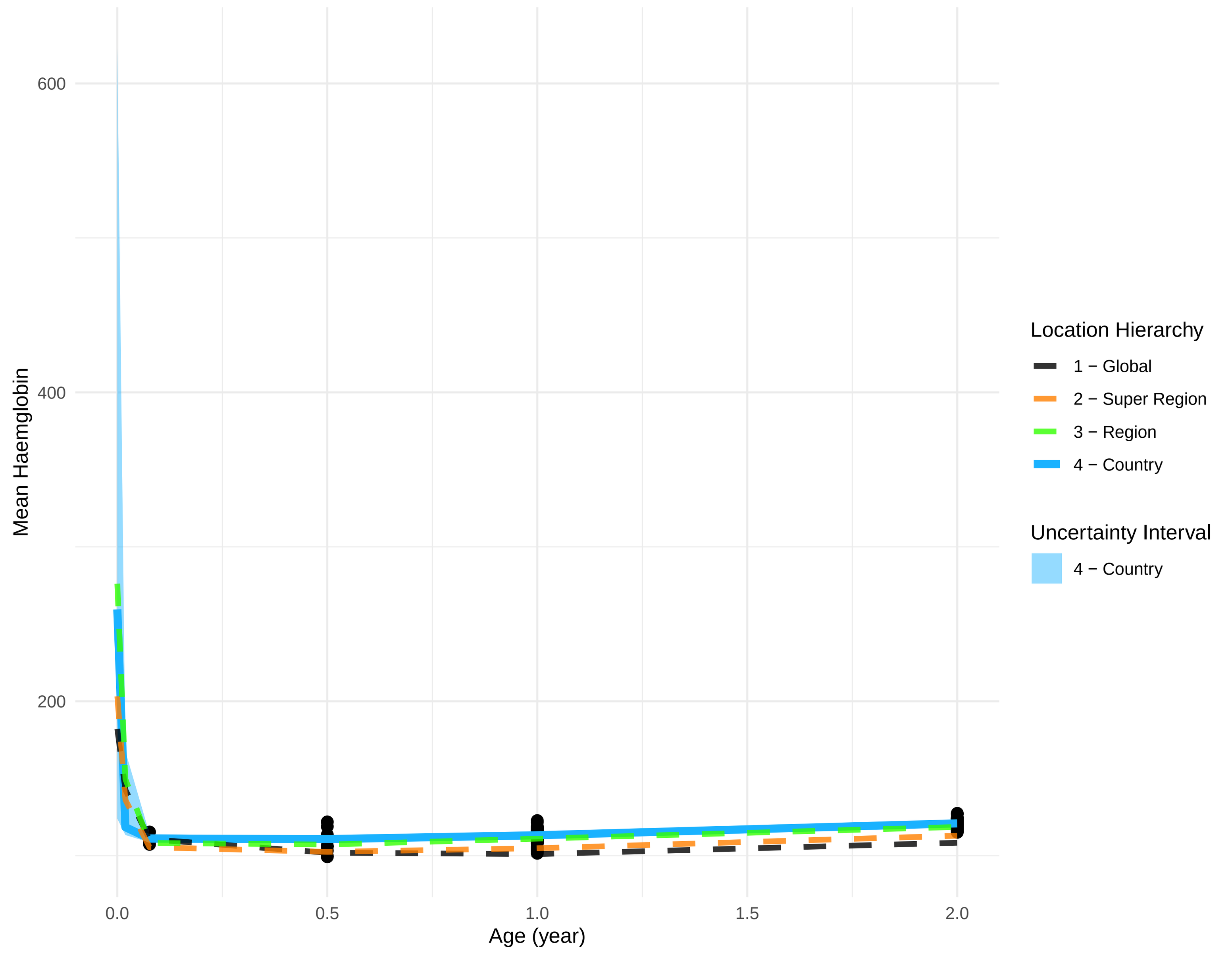

MR−BRT Age/Sex Fits − Non−pregnant Females <2 Years Old − Mean Haemglobin − Aguascalientes
WHO−2001 Adjustment Method

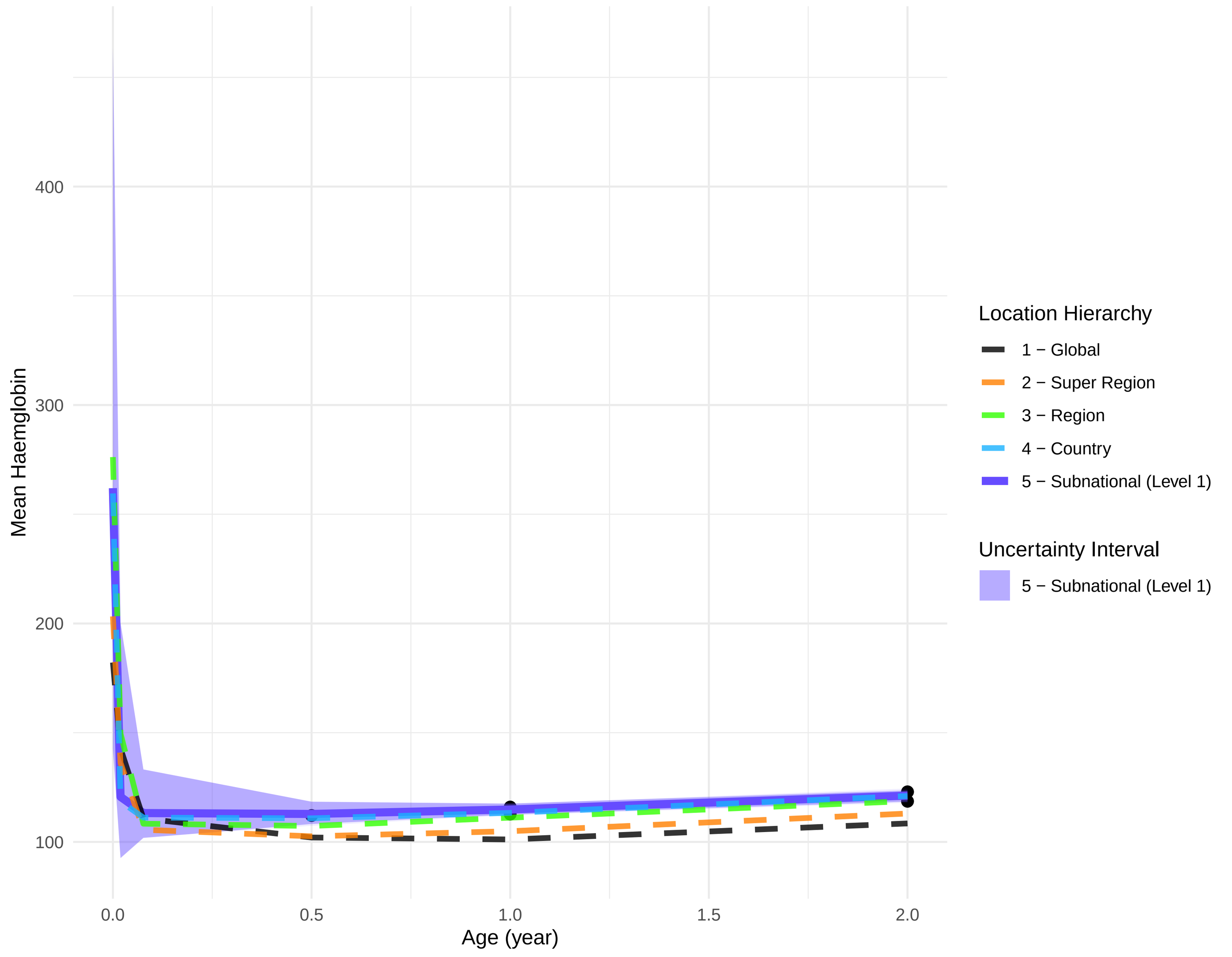

MR−BRT Age/Sex Fits − Non−pregnant Females <2 Years Old − Mean Haemglobin − Baja California
WHO−2001 Adjustment Method

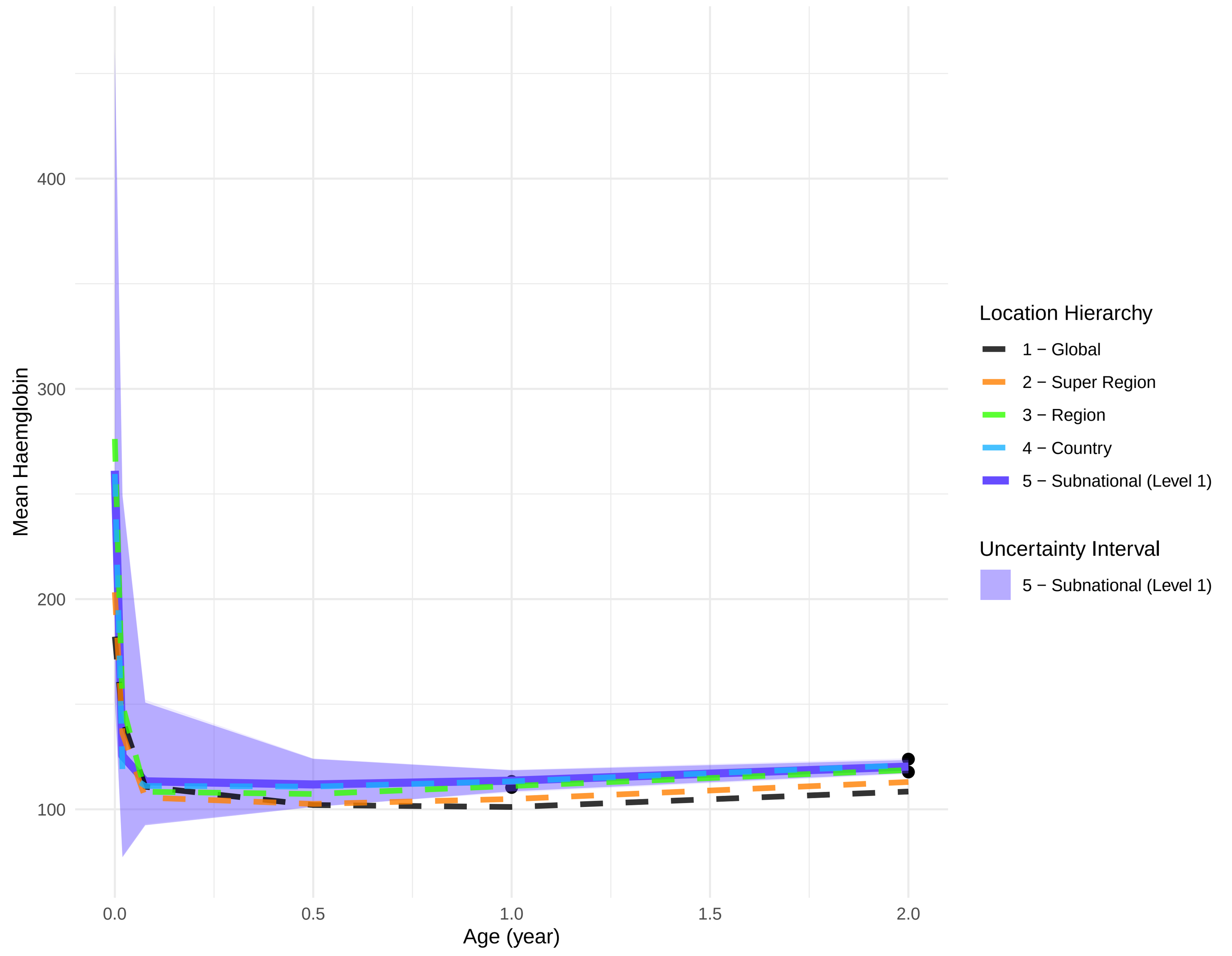

MR−BRT Age/Sex Fits − Non−pregnant Females <2 Years Old − Mean Haemglobin − Baja California Sur
WHO−2001 Adjustment Method

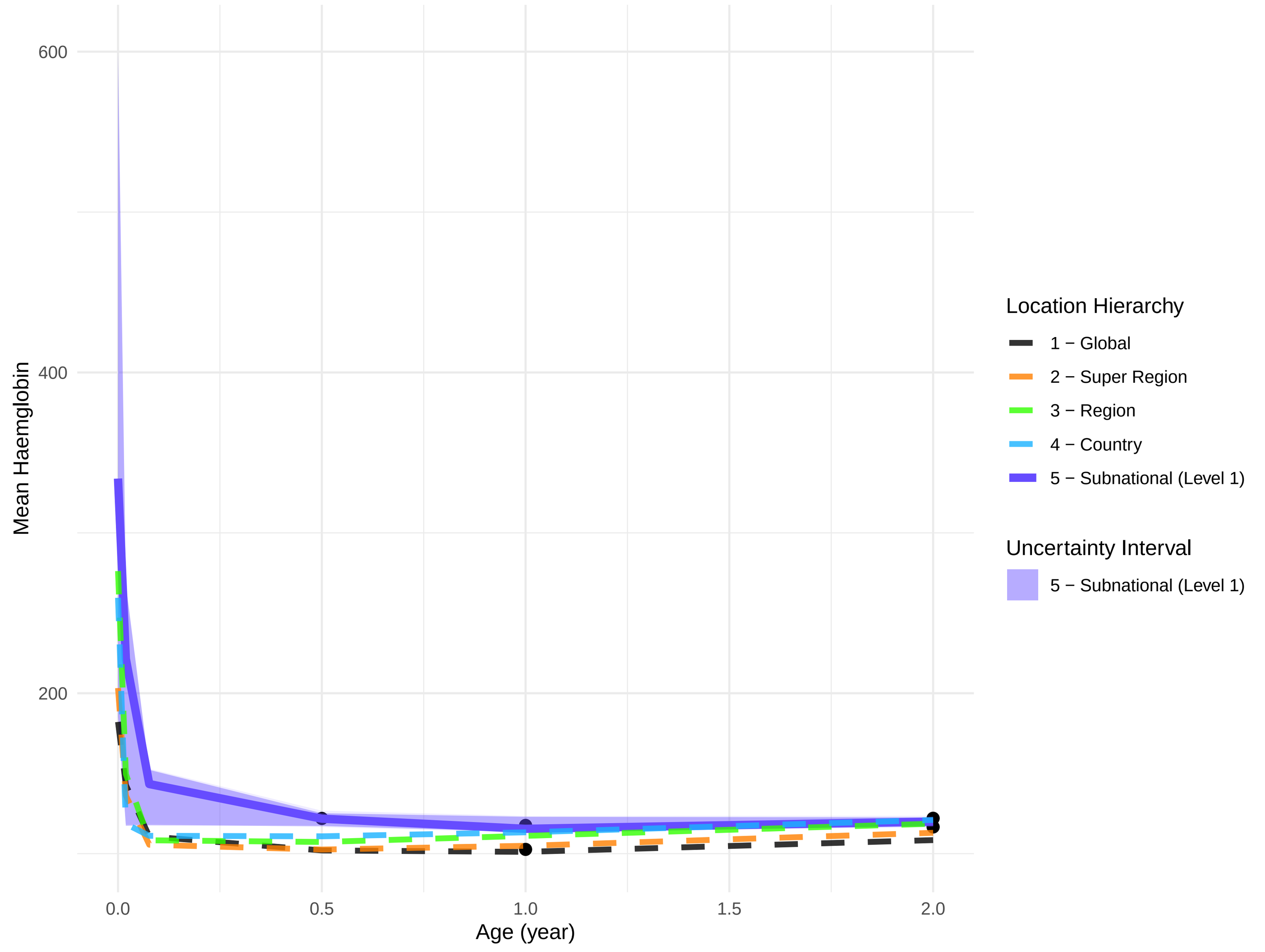

MR−BRT Age/Sex Fits − Non−pregnant Females <2 Years Old − Mean Haemglobin − Campeche
WHO−2001 Adjustment Method

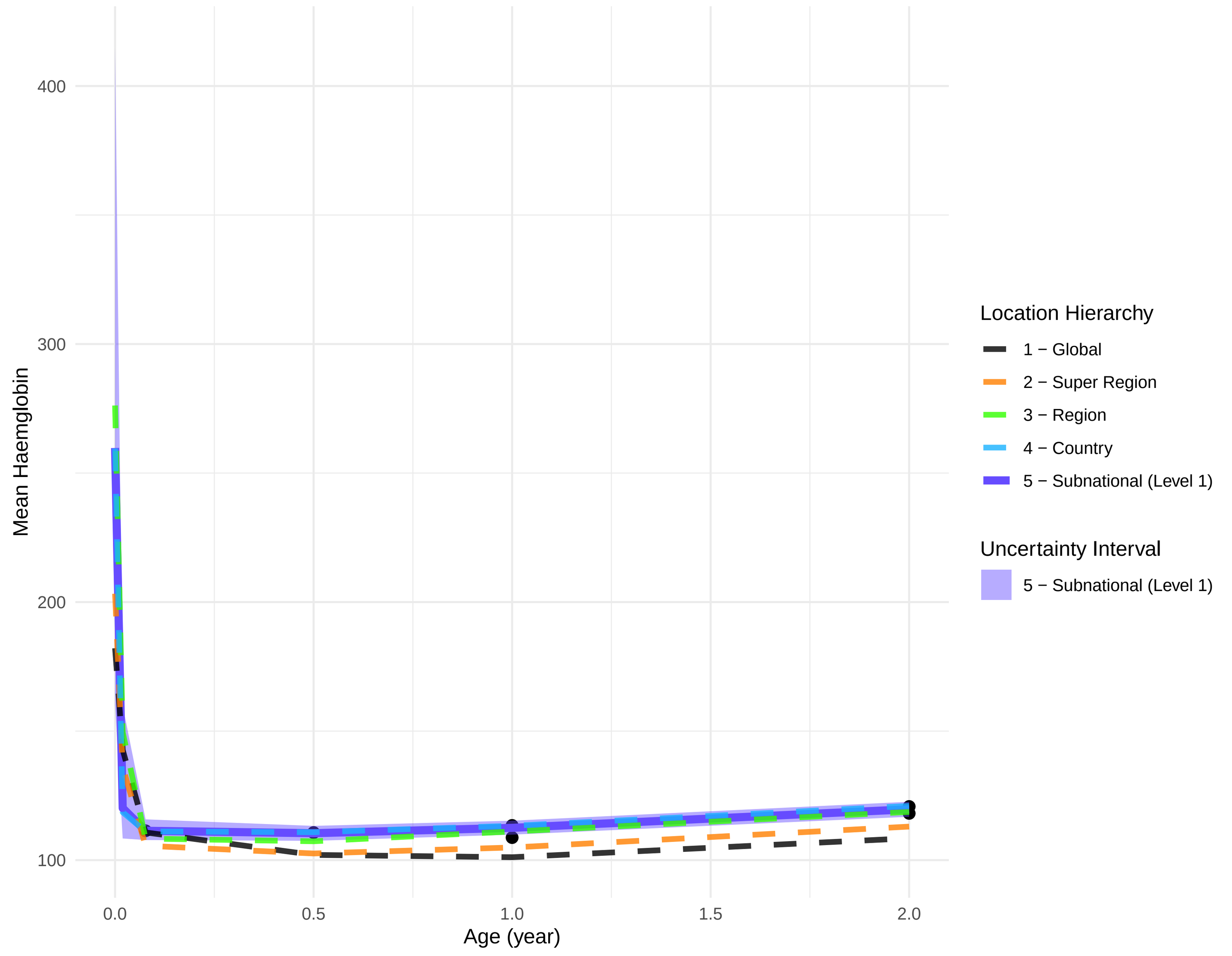

MR−BRT Age/Sex Fits − Non−pregnant Females <2 Years Old − Mean Haemglobin − Chiapas
WHO−2001 Adjustment Method

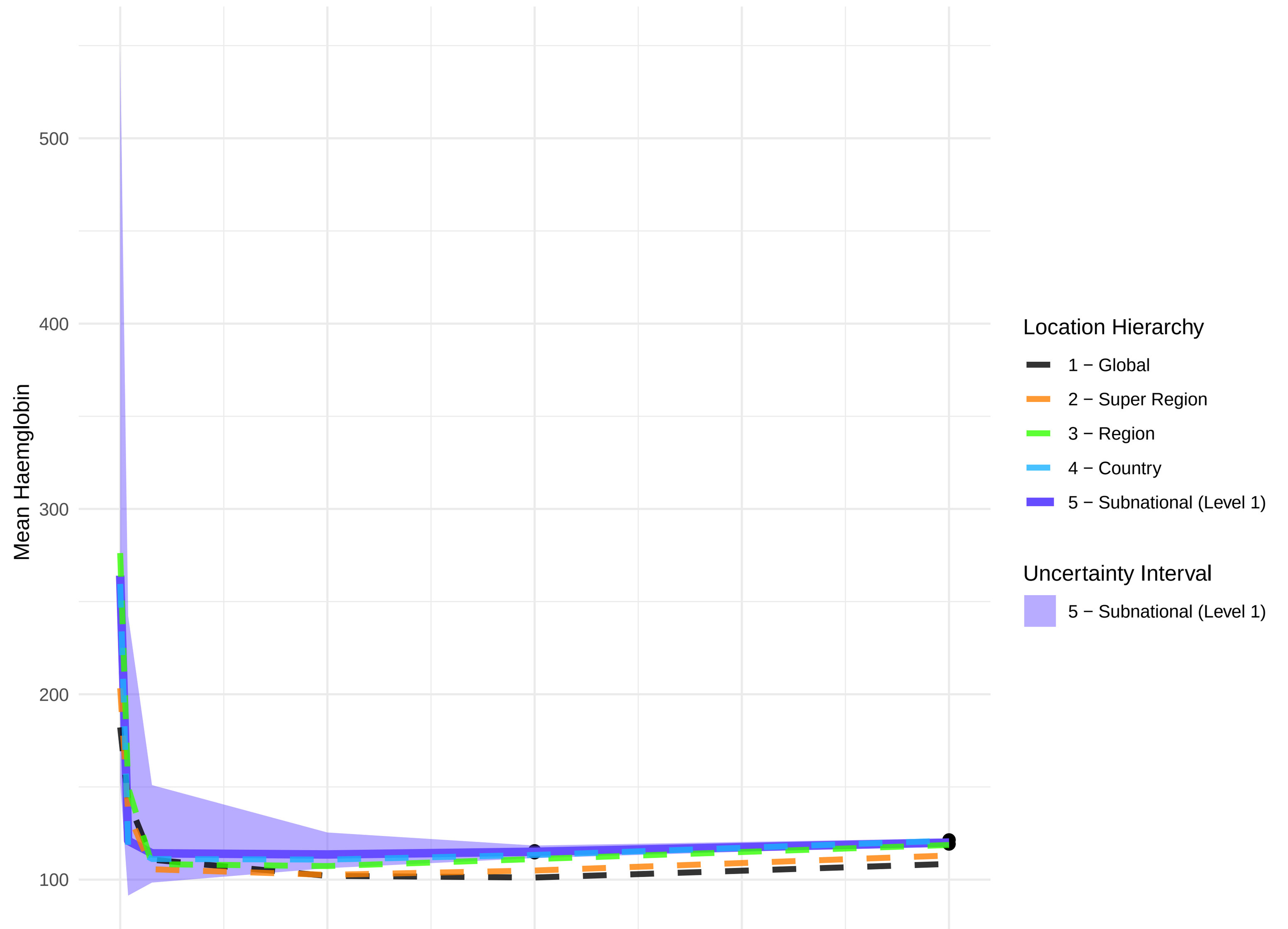

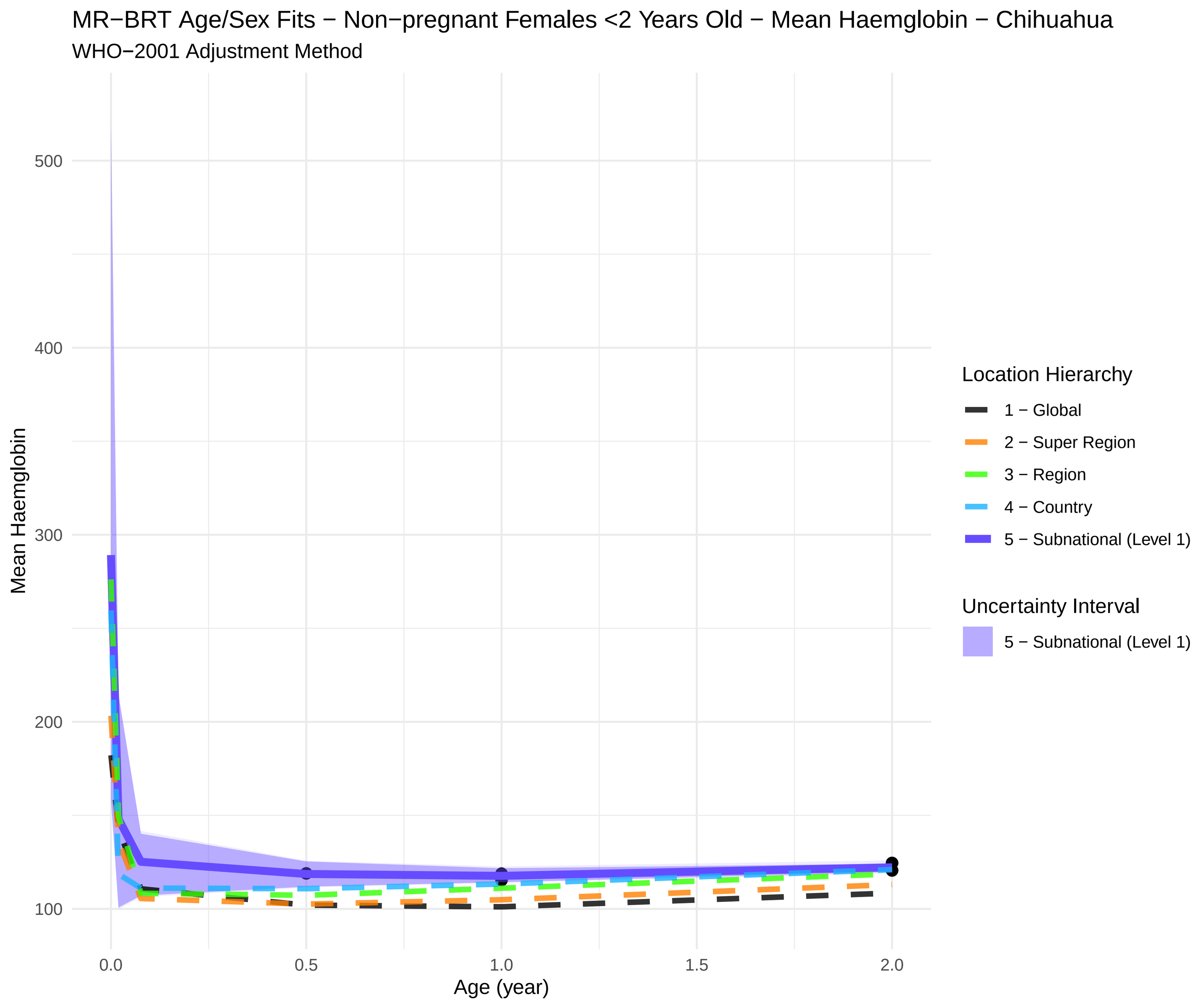
MR−BRT Age/Sex Fits − Non−pregnant Females <2 Years Old − Mean Haemglobin − Chihuahua
WHO−2001 Adjustment Method
Mean Haemglobin
Age (year)
Location Hierarchy
1 − Global
2 − Super Region
3 − Region
4 − Country
5 − Subnational (Level 1)
Uncertainty Interval
5 − Subnational (Level 1)

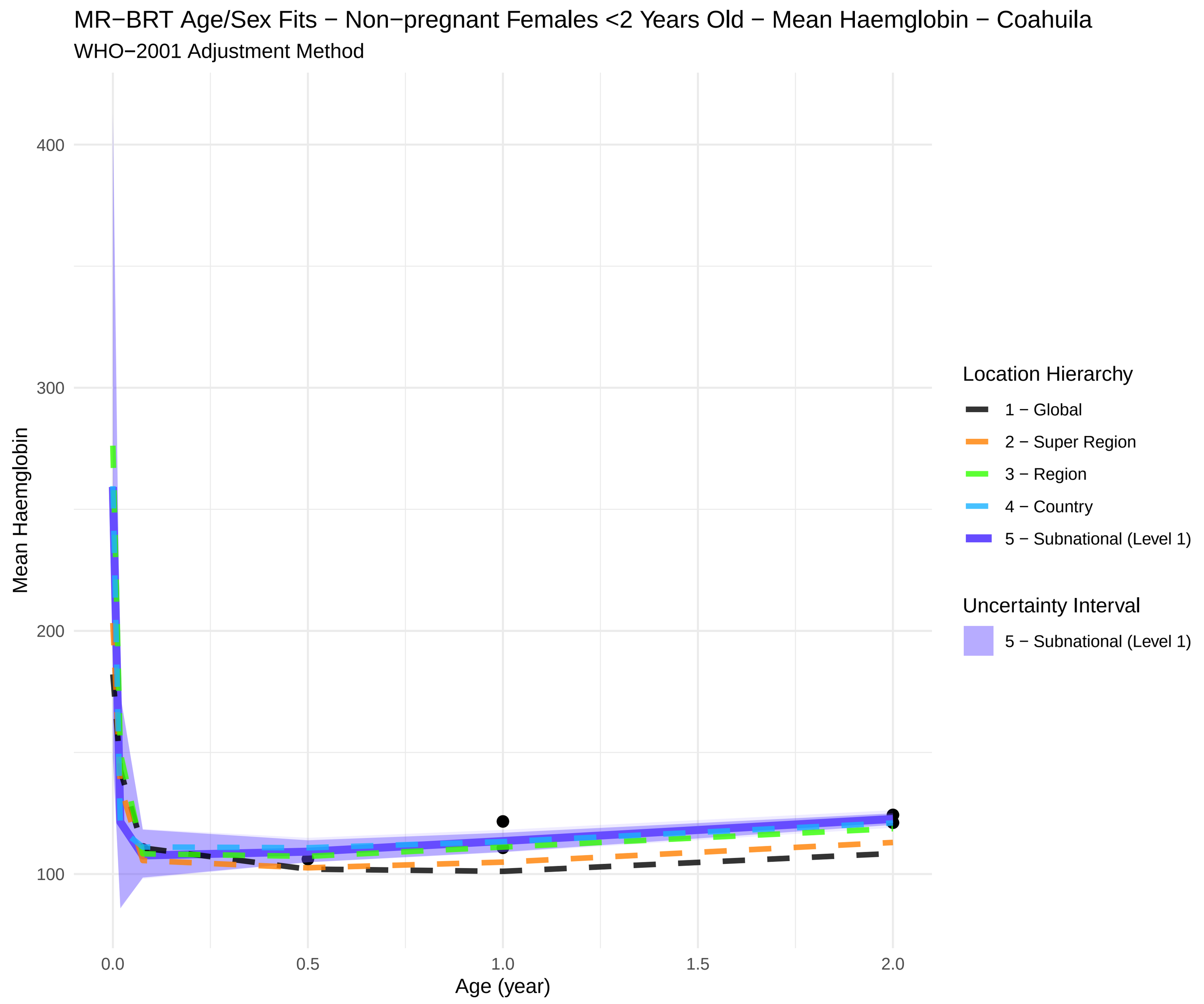
MR−BRT Age/Sex Fits − Non−pregnant Females <2 Years Old − Mean Haemglobin − Coahuila
WHO−2001 Adjustment Method
Mean Haemglobin
Age (year)
Location Hierarchy
1 − Global
2 − Super Region
3 − Region
4 − Country
5 − Subnational (Level 1)
Uncertainty Interval
5 − Subnational (Level 1)

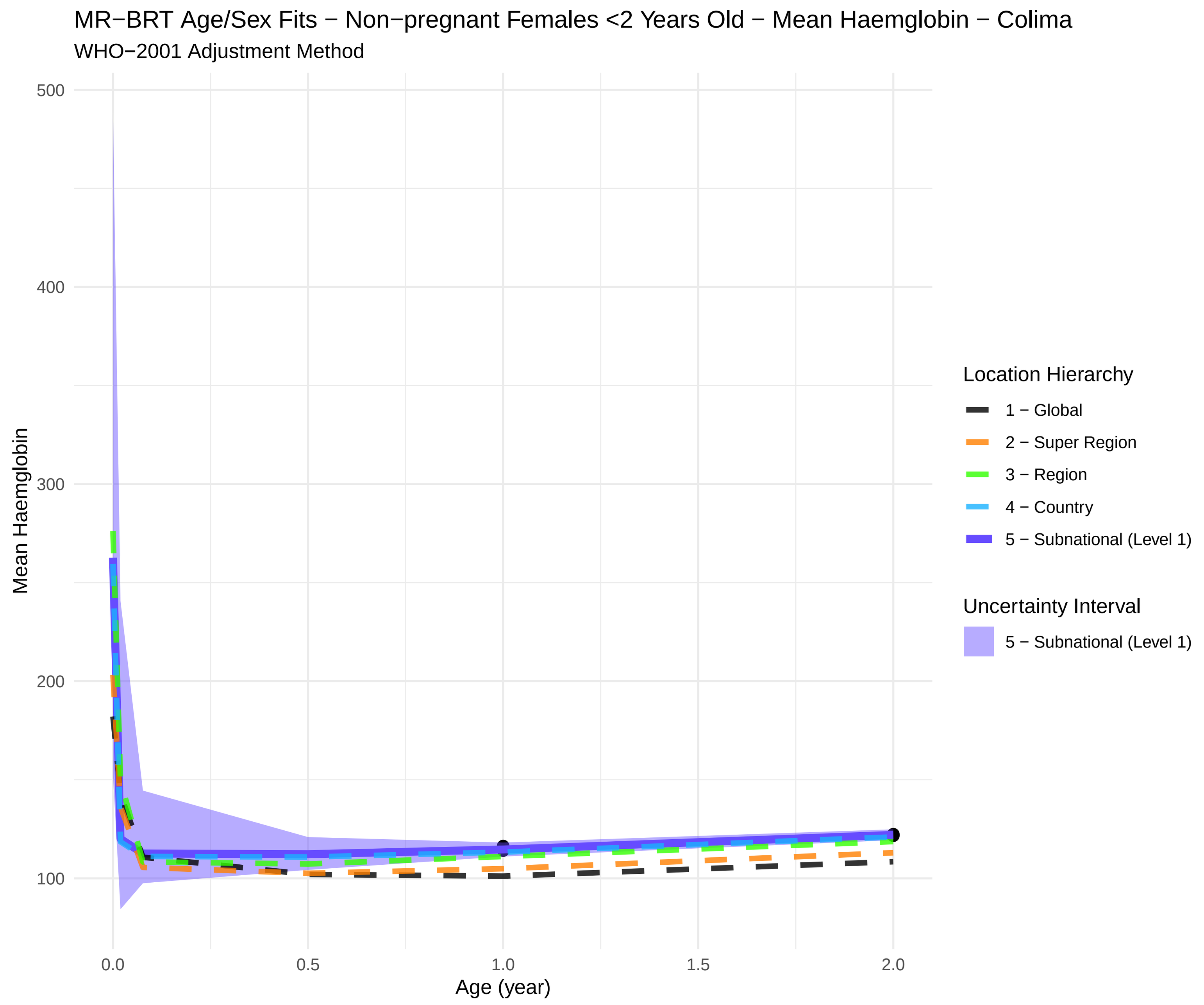
MR−BRT Age/Sex Fits − Non−pregnant Females <2 Years Old − Mean Haemglobin − Colima
WHO−2001 Adjustment Method
Mean Haemglobin
Age (year)
Location Hierarchy
1 − Global
2 − Super Region
3 − Region
4 − Country
5 − Subnational (Level 1)
Uncertainty Interval
5 − Subnational (Level 1)

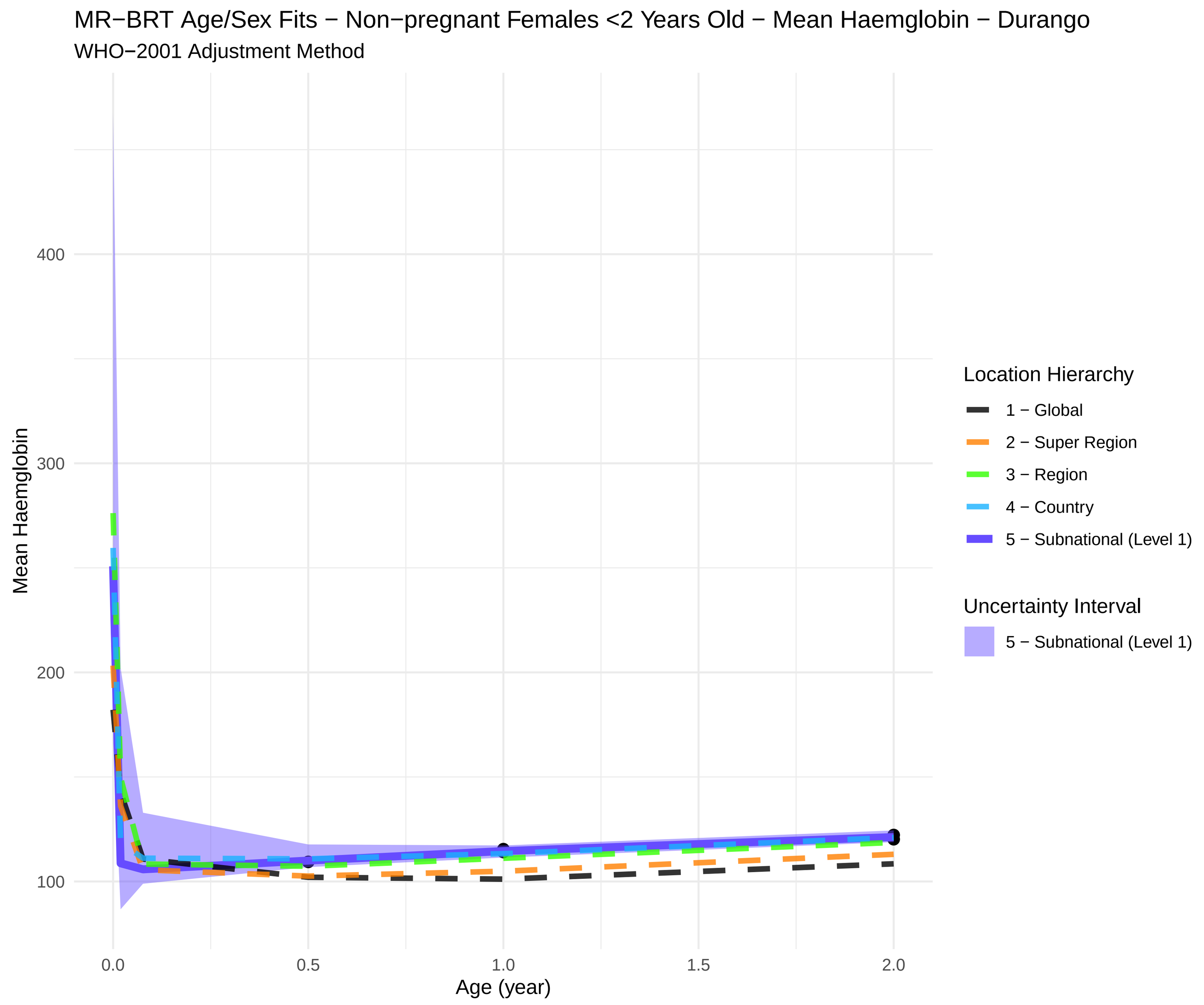
MR−BRT Age/Sex Fits − Non−pregnant Females <2 Years Old − Mean Haemglobin − Durango
WHO−2001 Adjustment Method
Mean Haemglobin
Age (year)
Location Hierarchy
1 − Global
2 − Super Region
3 − Region
4 − Country
5 − Subnational (Level 1)
Uncertainty Interval
5 − Subnational (Level 1)

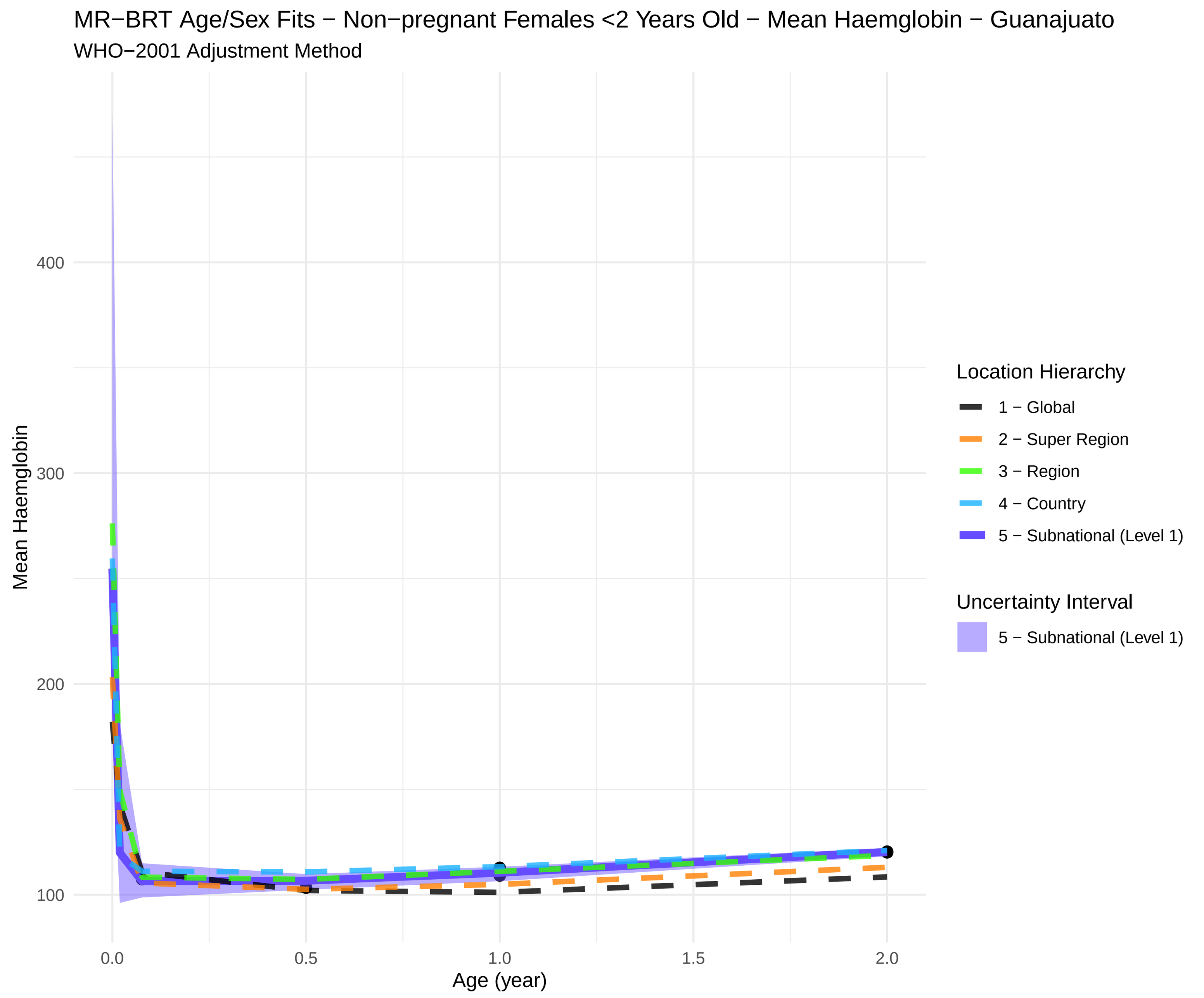
MR−BRT Age/Sex Fits − Non−pregnant Females <2 Years Old − Mean Haemglobin − Guanajuato
WHO−2001 Adjustment Method
Mean Haemglobin
Age (year)
Location Hierarchy
1 − Global
2 − Super Region
3 − Region
4 − Country
5 − Subnational (Level 1)
Uncertainty Interval
5 − Subnational (Level 1)

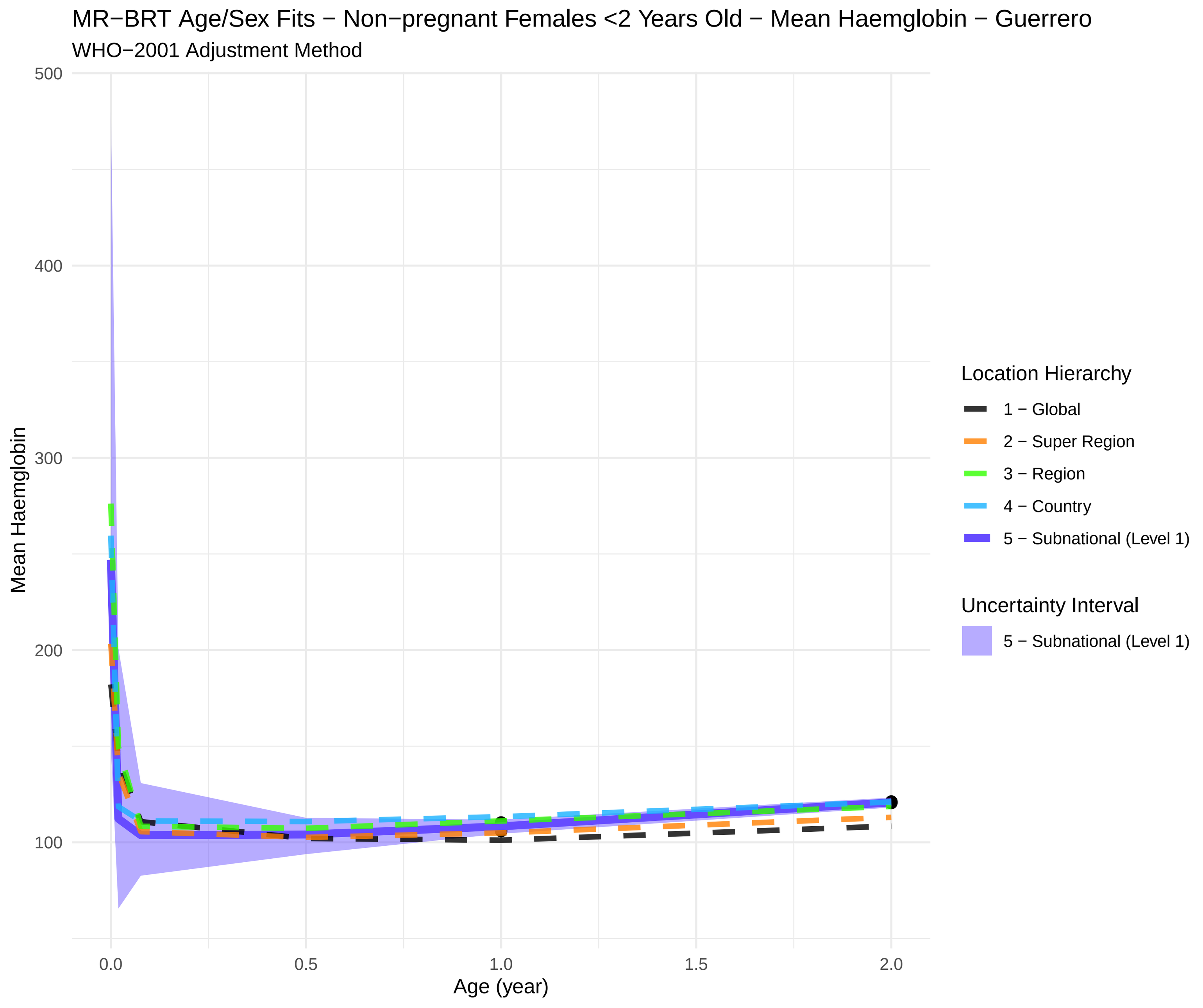
MR−BRT Age/Sex Fits − Non−pregnant Females <2 Years Old − Mean Haemglobin − Guerrero
WHO−2001 Adjustment Method
Mean Haemglobin
Age (year)
Location Hierarchy
1 − Global
2 − Super Region
3 − Region
4 − Country
5 − Subnational (Level 1)
Uncertainty Interval
5 − Subnational (Level 1)

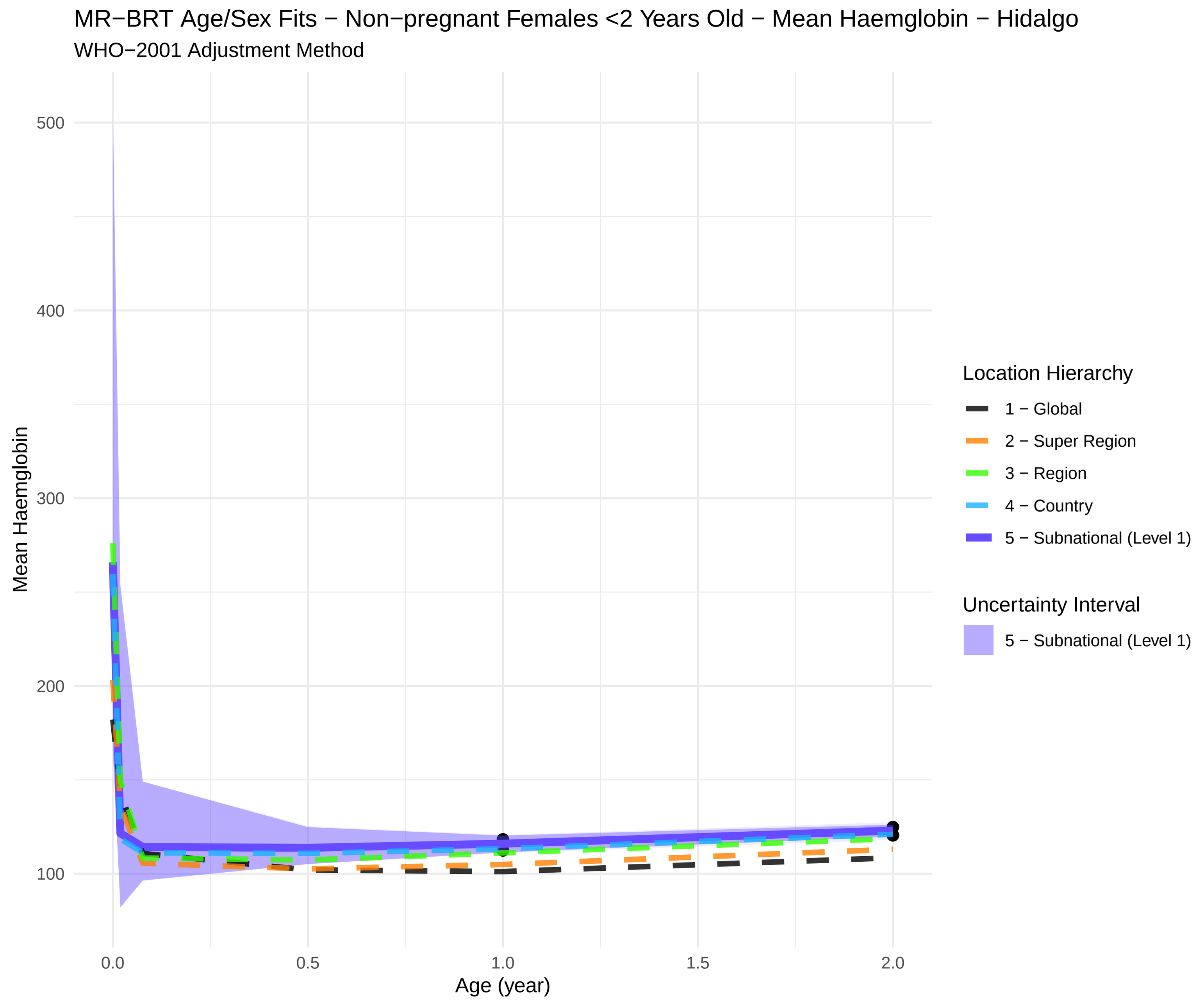
MR−BRT Age/Sex Fits − Non−pregnant Females <2 Years Old − Mean Haemglobin − Hidalgo
WHO−2001 Adjustment Method
Mean Haemglobin
Age (year)
Location Hierarchy
1 − Global
2 − Super Region
3 − Region
4 − Country
5 − Subnational (Level 1)
Uncertainty Interval
5 − Subnational (Level 1)

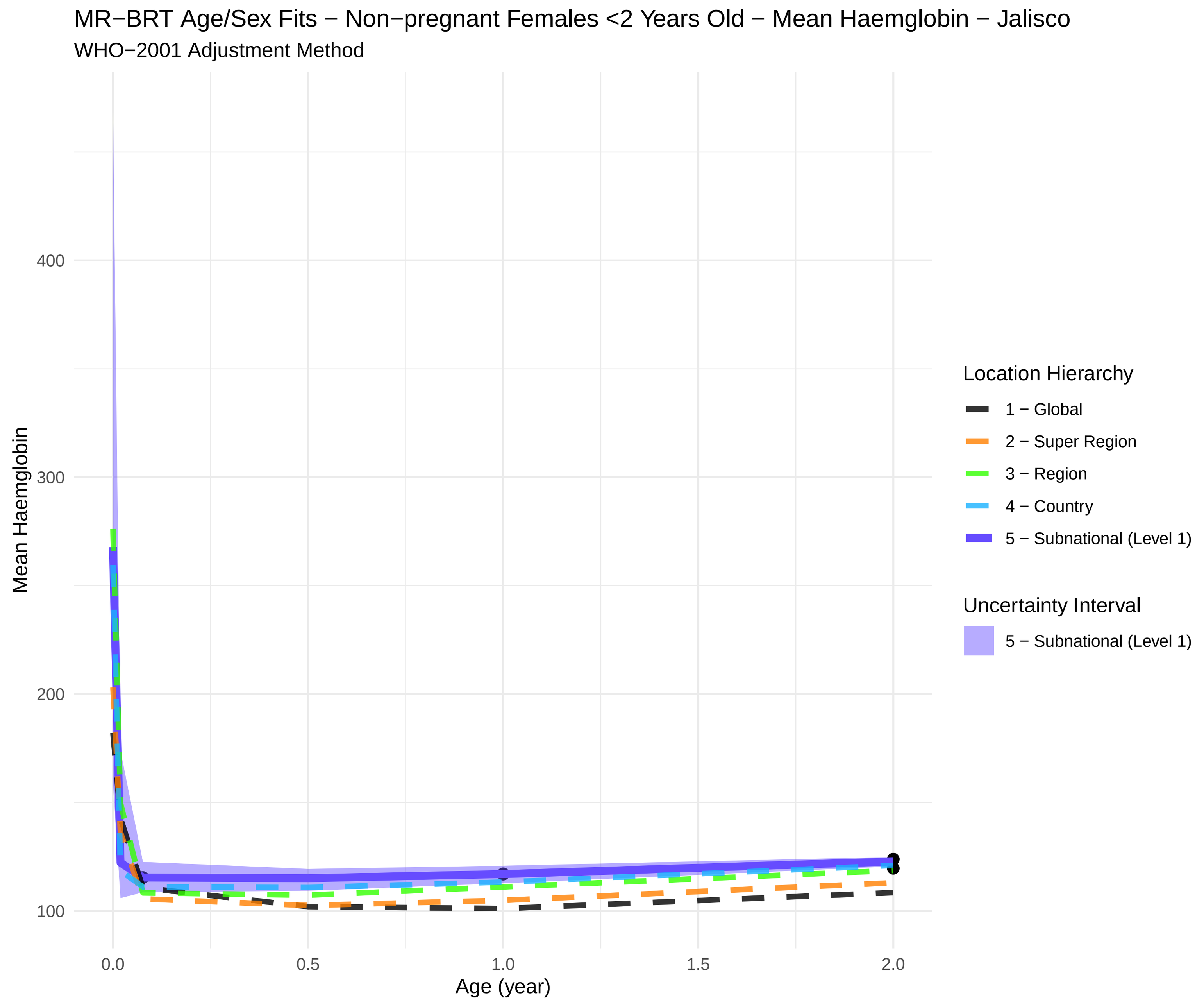
MR−BRT Age/Sex Fits − Non−pregnant Females <2 Years Old − Mean Haemglobin − Jalisco
WHO−2001 Adjustment Method
Mean Haemglobin
Age (year)
Location Hierarchy
1 − Global
2 − Super Region
3 − Region
4 − Country
5 − Subnational (Level 1)
Uncertainty Interval
5 − Subnational (Level 1)

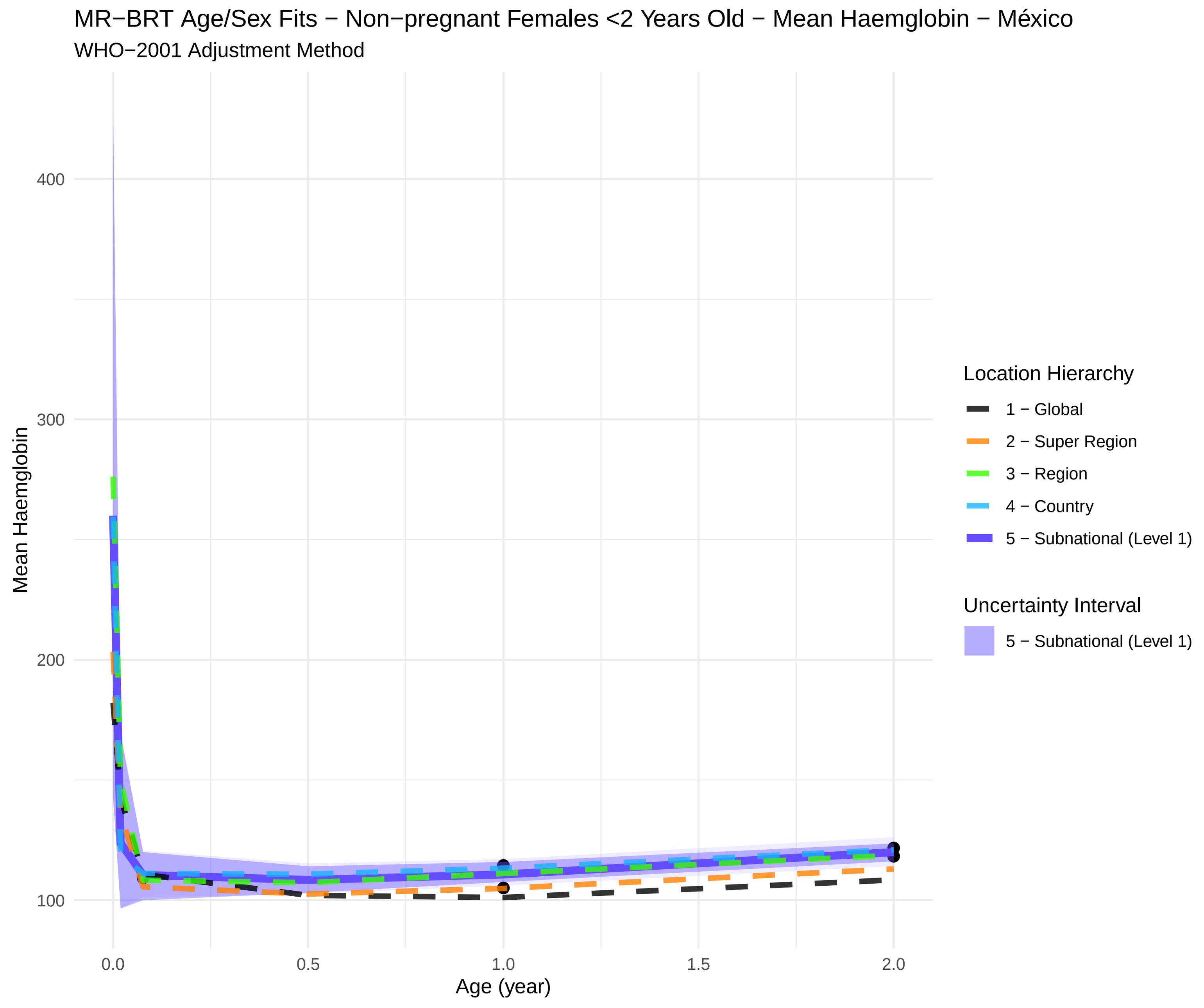
MR−BRT Age/Sex Fits − Non−pregnant Females <2 Years Old − Mean Haemglobin − México
WHO−2001 Adjustment Method
Mean Haemglobin
Age (year)
Location Hierarchy
1 − Global
2 − Super Region
3 − Region
4 − Country
5 − Subnational (Level 1)
Uncertainty Interval
5 − Subnational (Level 1)

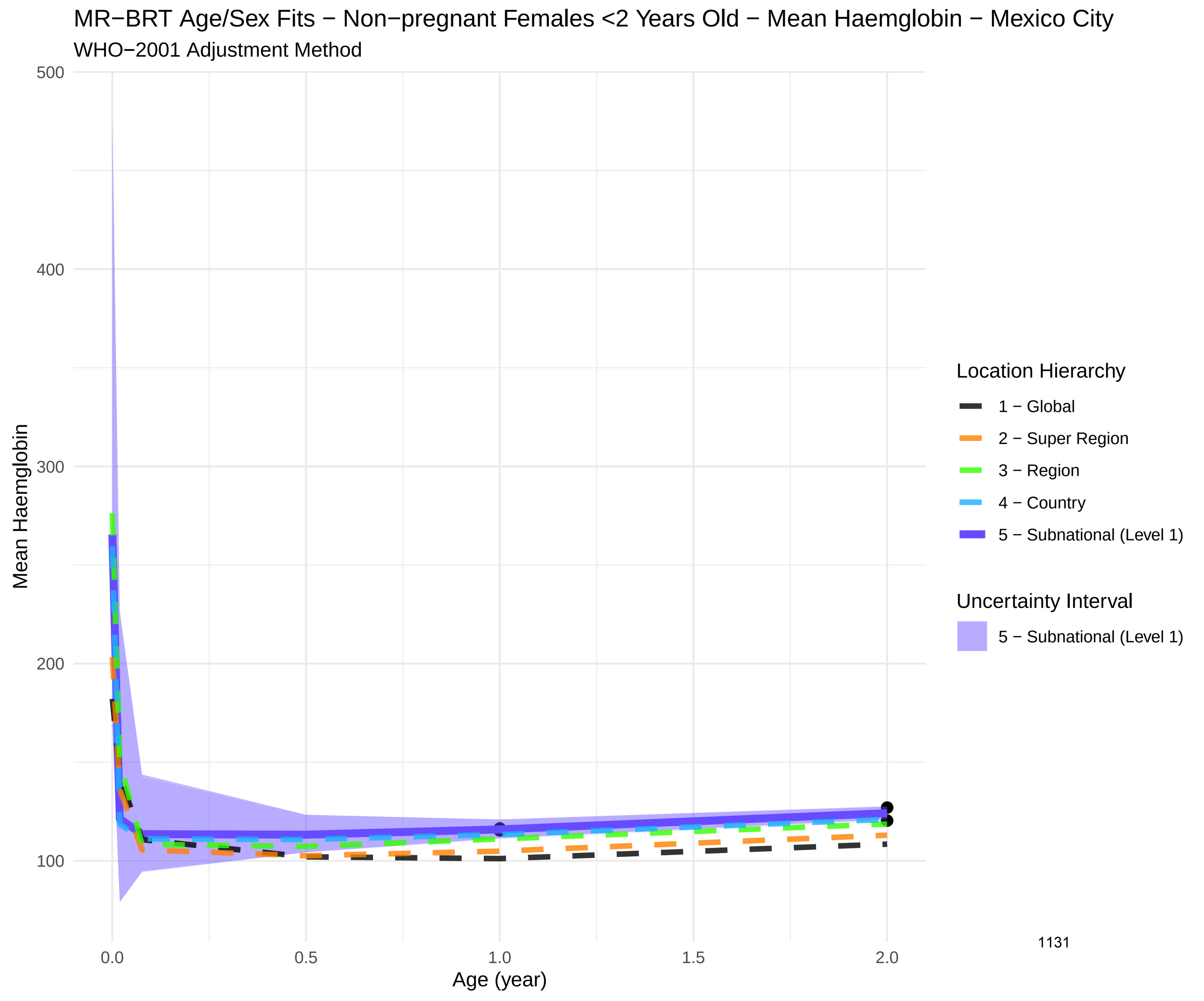
MR−BRT Age/Sex Fits − Non−pregnant Females <2 Years Old − Mean Haemglobin − Mexico City
WHO−2001 Adjustment Method
Mean Haemglobin
Age (year)
Location Hierarchy
1 − Global
2 − Super Region
3 − Region
4 − Country
5 − Subnational (Level 1)
Uncertainty Interval
5 − Subnational (Level 1)

MR−BRT Age/Sex Fits − Non−pregnant Females <2 Years Old − Mean Haemglobin − Michoacán de Ocampo
WHO−2001 Adjustment Method

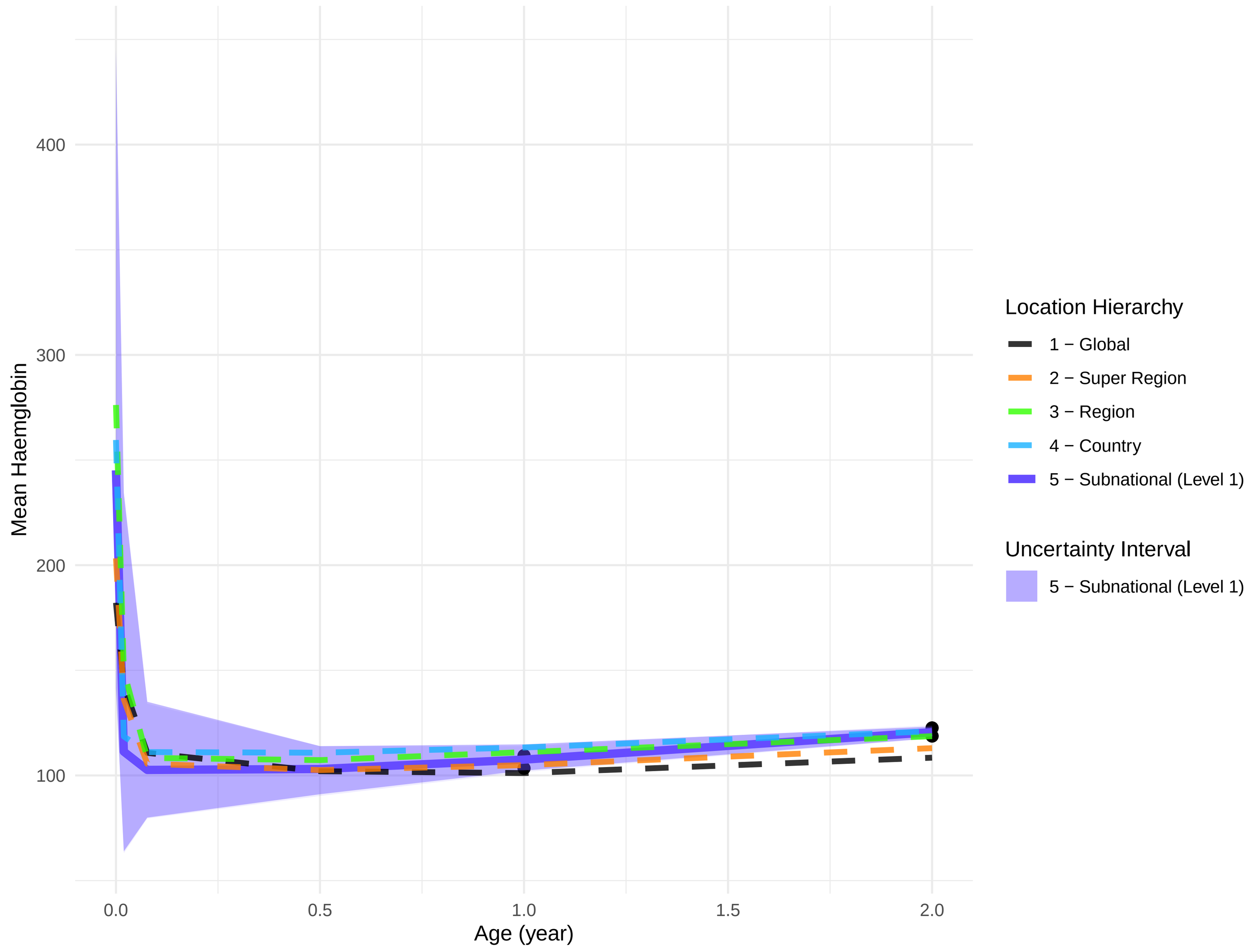

MR−BRT Age/Sex Fits − Non−pregnant Females <2 Years Old − Mean Haemglobin − Morelos
WHO−2001 Adjustment Method

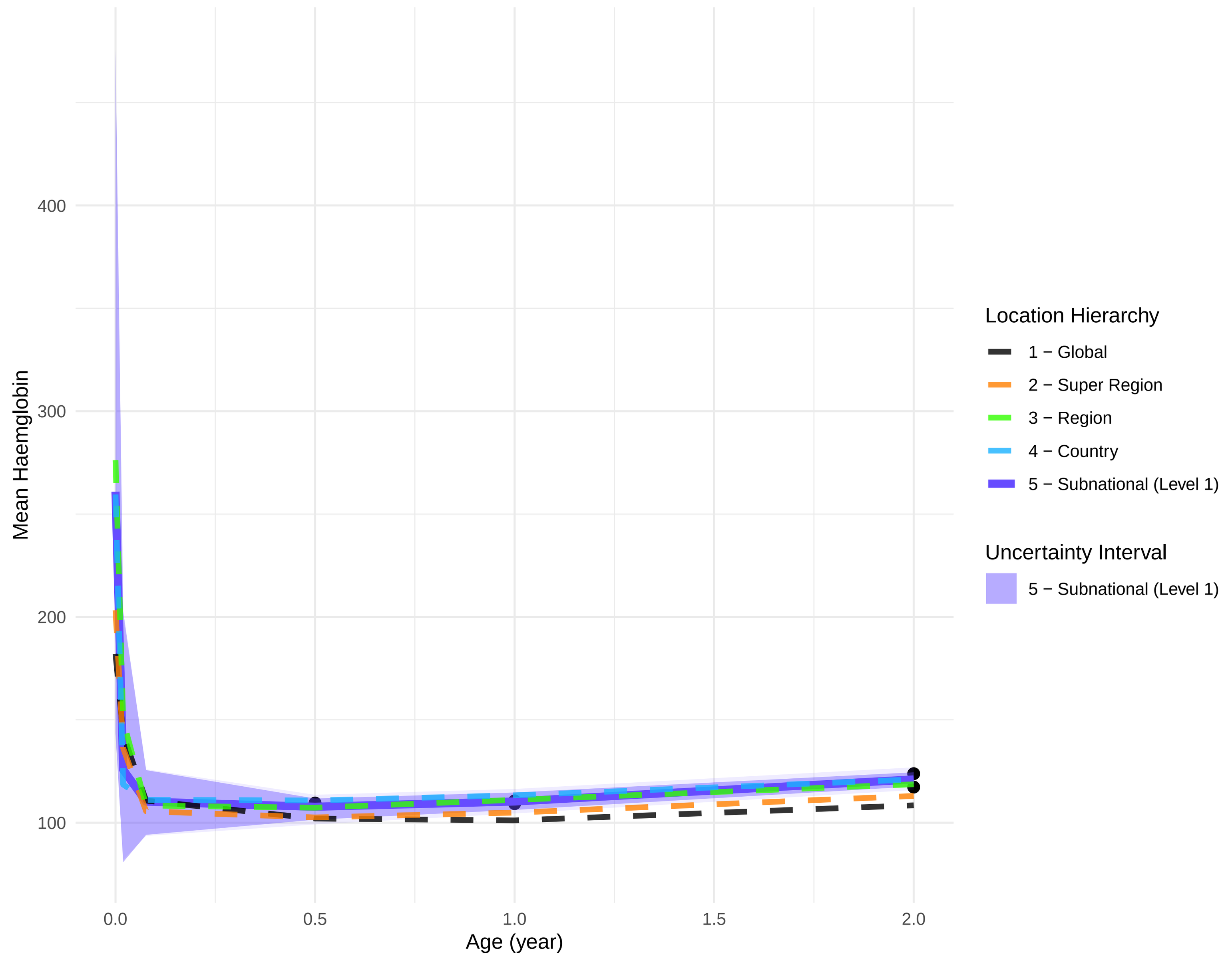

MR−BRT Age/Sex Fits − Non−pregnant Females <2 Years Old − Mean Haemglobin − Nayarit
WHO−2001 Adjustment Method

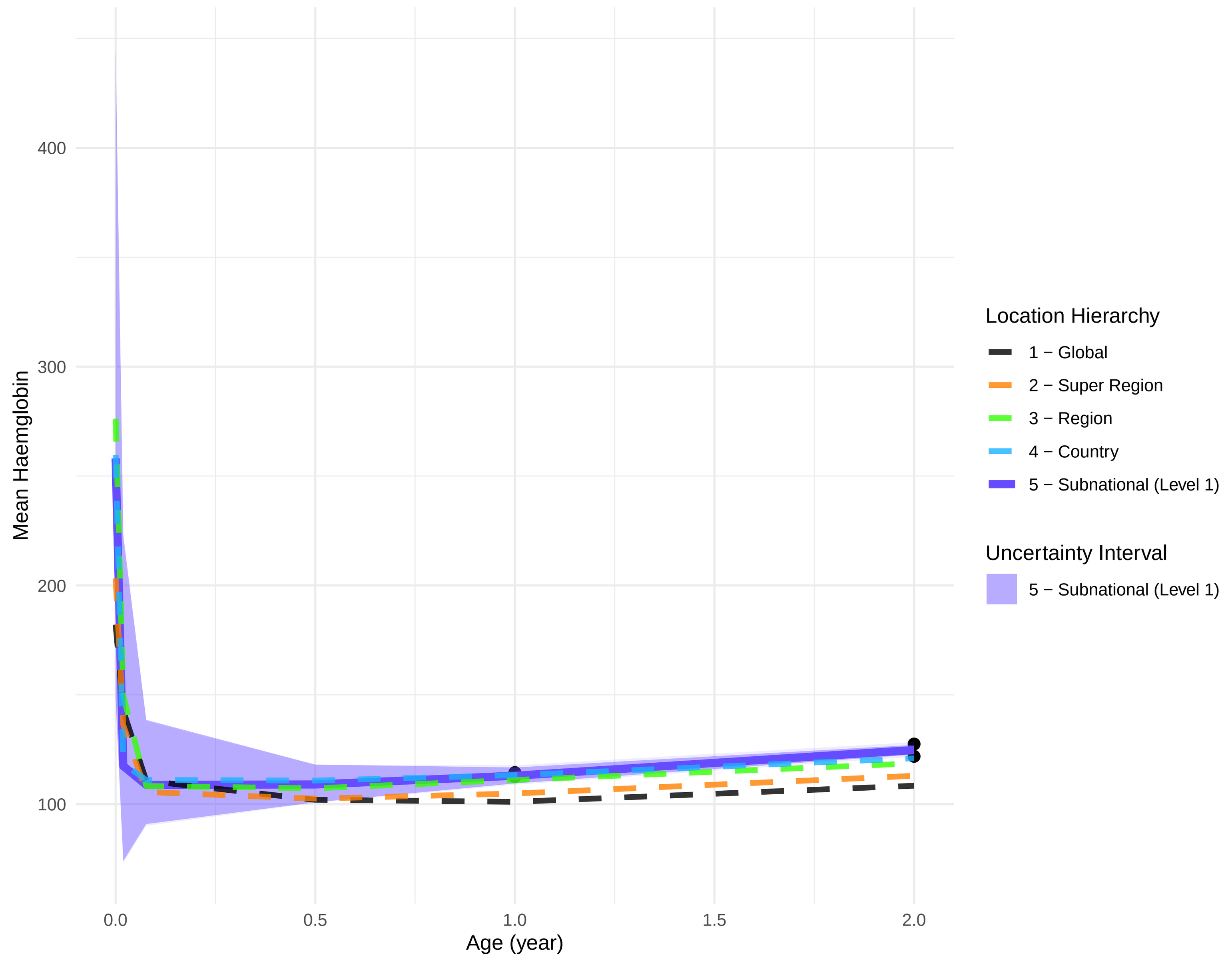

MR−BRT Age/Sex Fits − Non−pregnant Females <2 Years Old − Mean Haemglobin − Nuevo León
WHO−2001 Adjustment Method

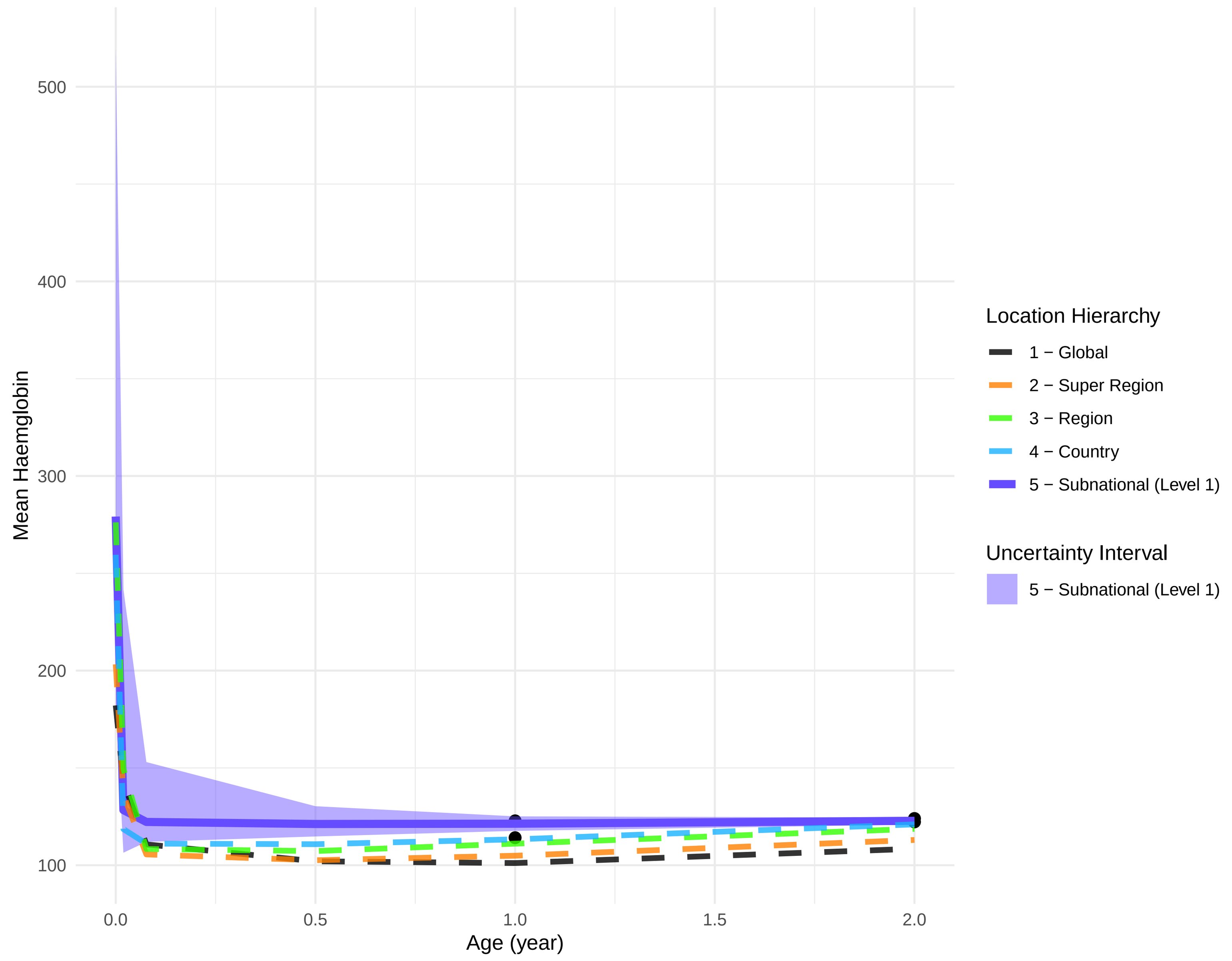

MR−BRT Age/Sex Fits − Non−pregnant Females <2 Years Old − Mean Haemglobin − Oaxaca
WHO−2001 Adjustment Method

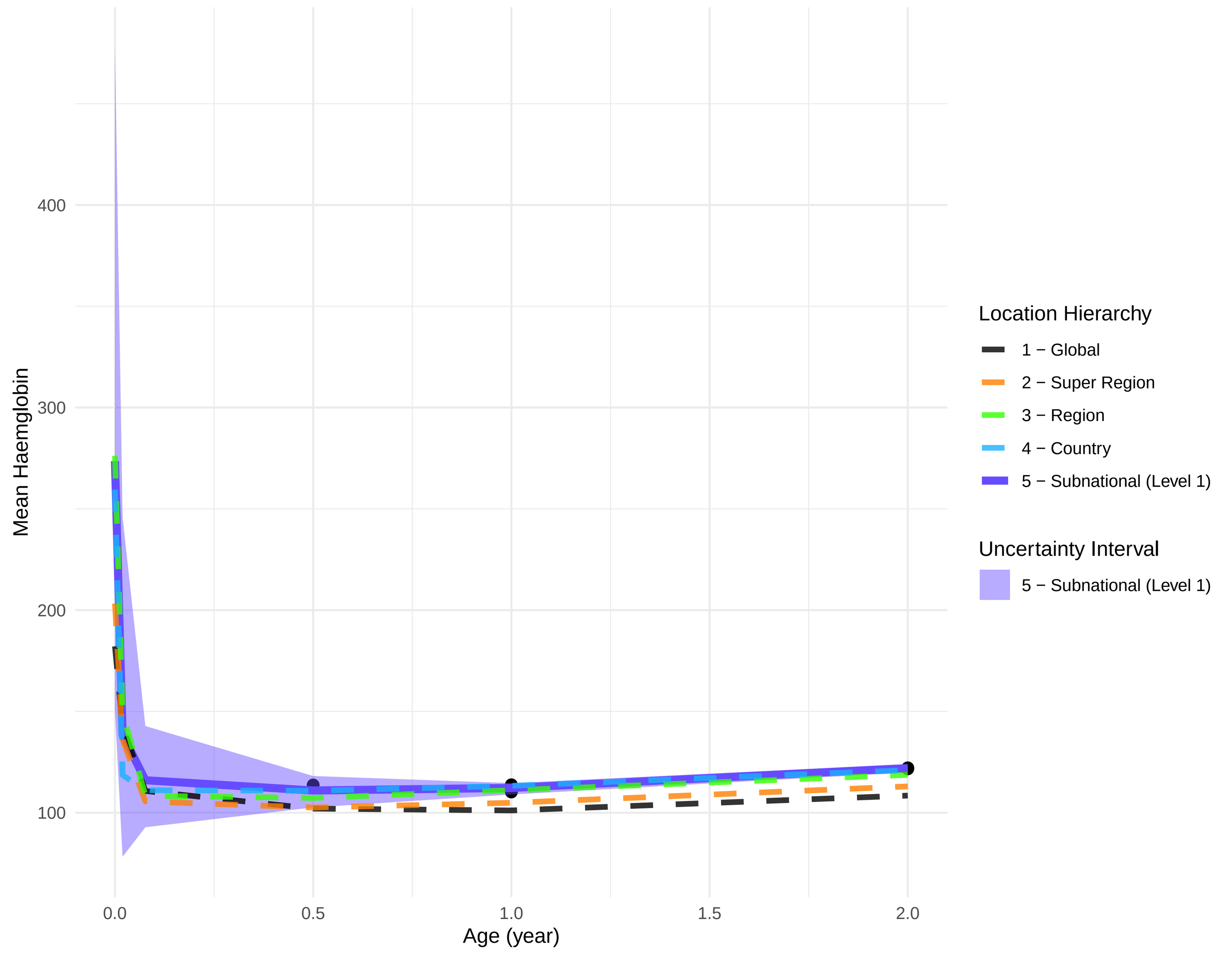

MR−BRT Age/Sex Fits − Non−pregnant Females <2 Years Old − Mean Haemglobin − Puebla
WHO−2001 Adjustment Method

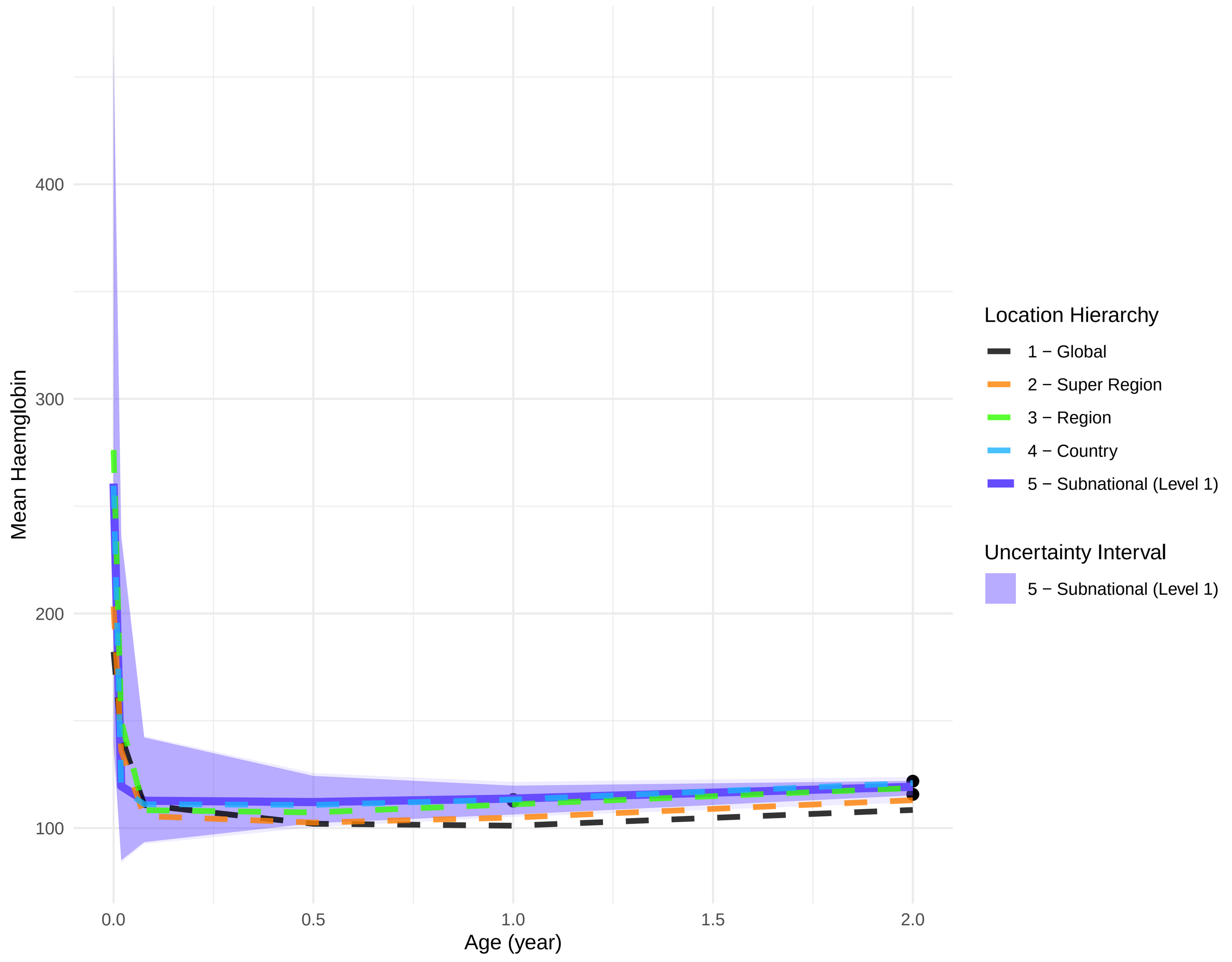

MR−BRT Age/Sex Fits − Non−pregnant Females <2 Years Old − Mean Haemglobin − Querétaro
WHO−2001 Adjustment Method

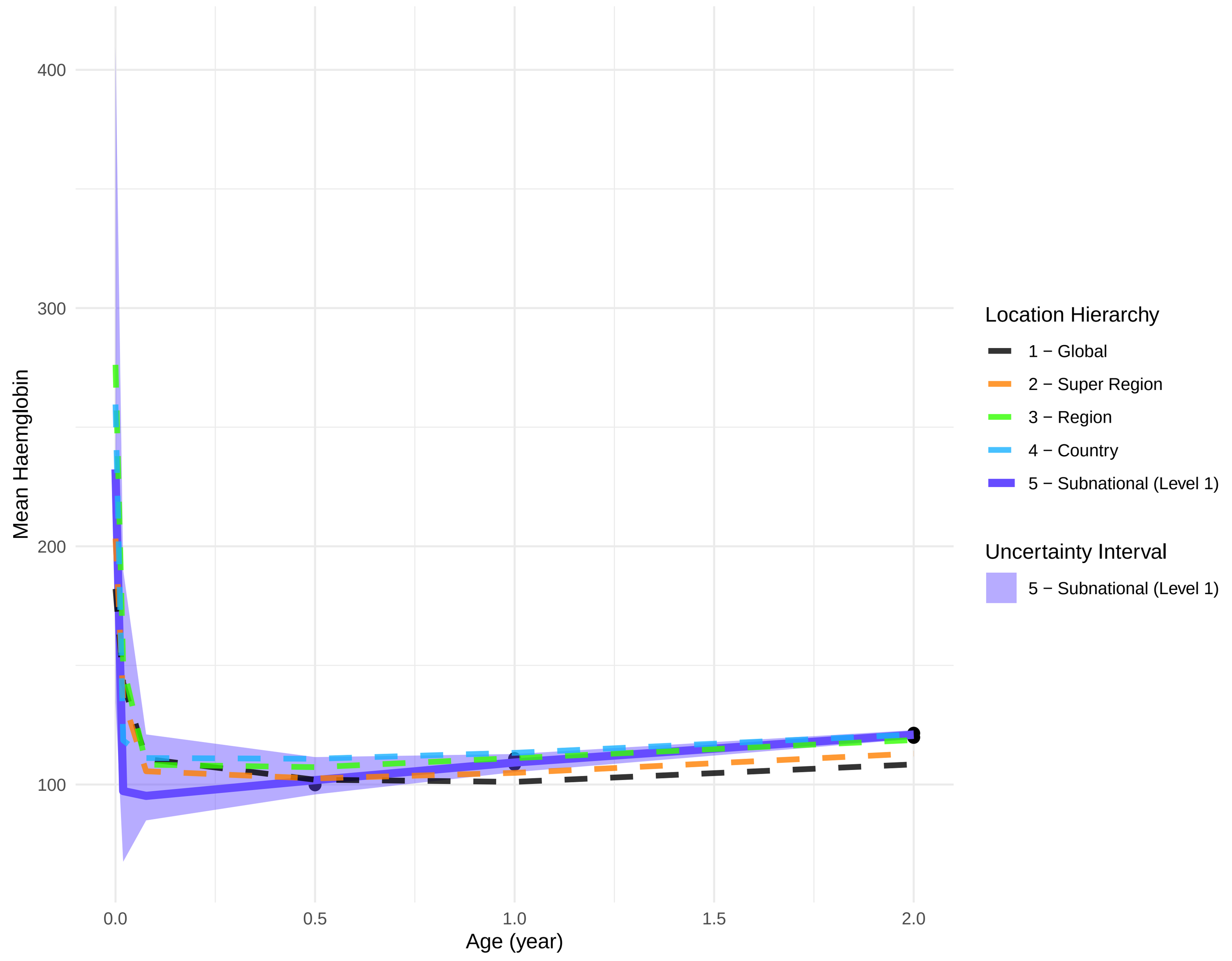

MR−BRT Age/Sex Fits − Non−pregnant Females <2 Years Old − Mean Haemglobin − Quintana Roo
WHO−2001 Adjustment Method

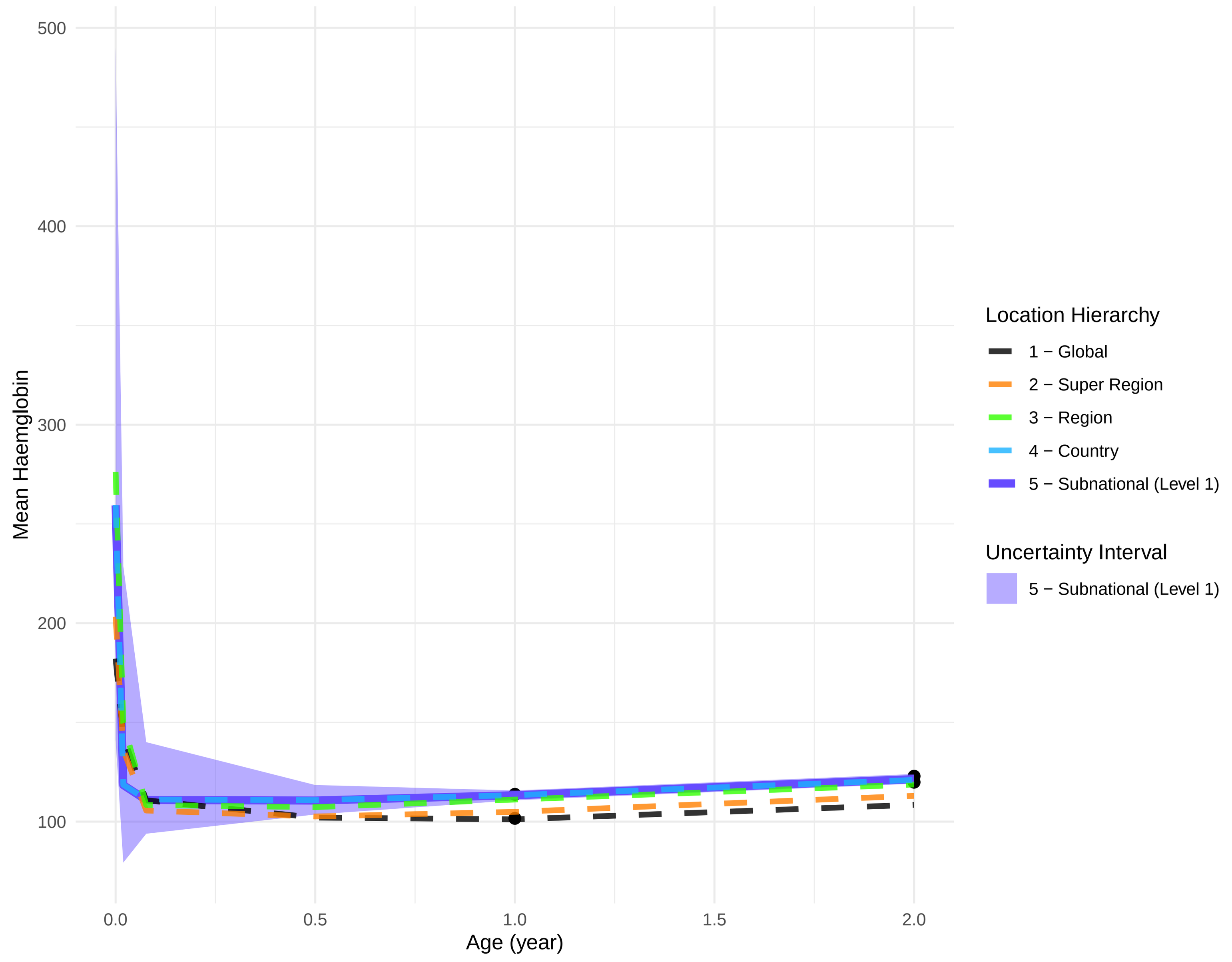

MR−BRT Age/Sex Fits − Non−pregnant Females <2 Years Old − Mean Haemglobin − San Luis Potosí
WHO−2001 Adjustment Method

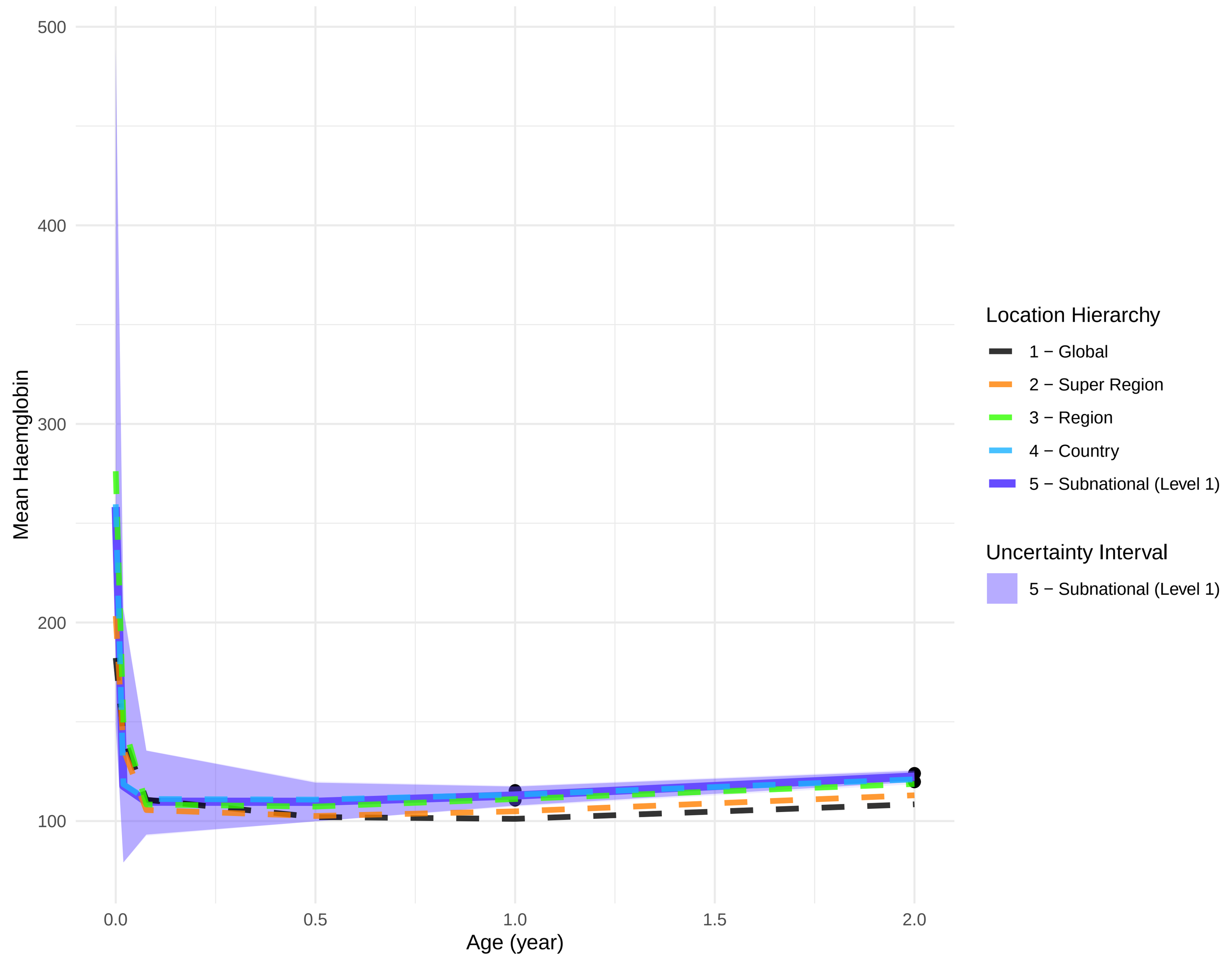

MR−BRT Age/Sex Fits − Non−pregnant Females <2 Years Old − Mean Haemglobin − Sinaloa
WHO−2001 Adjustment Method

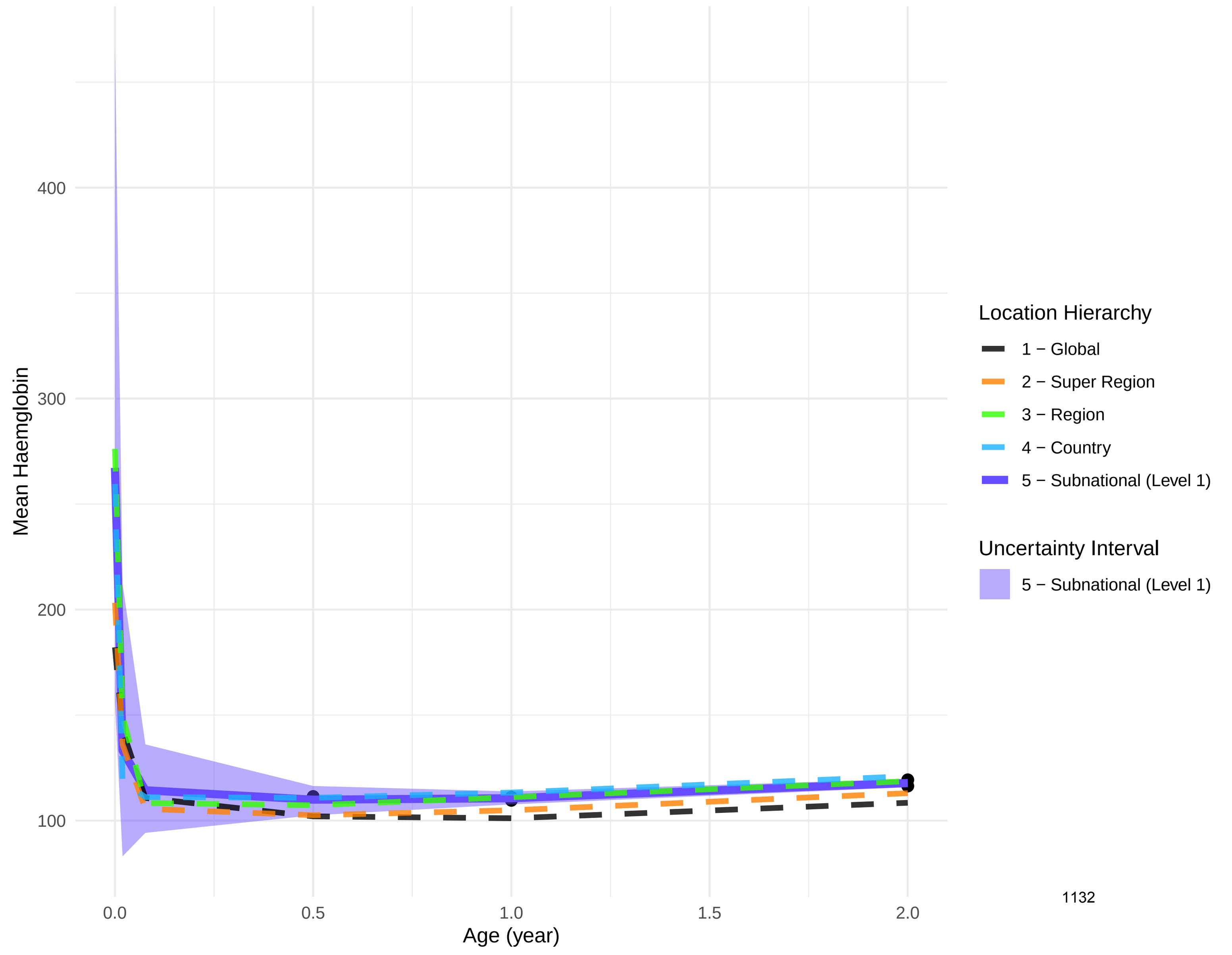

MR−BRT Age/Sex Fits − Non−pregnant Females <2 Years Old − Mean Haemglobin − Sonora
WHO−2001 Adjustment Method

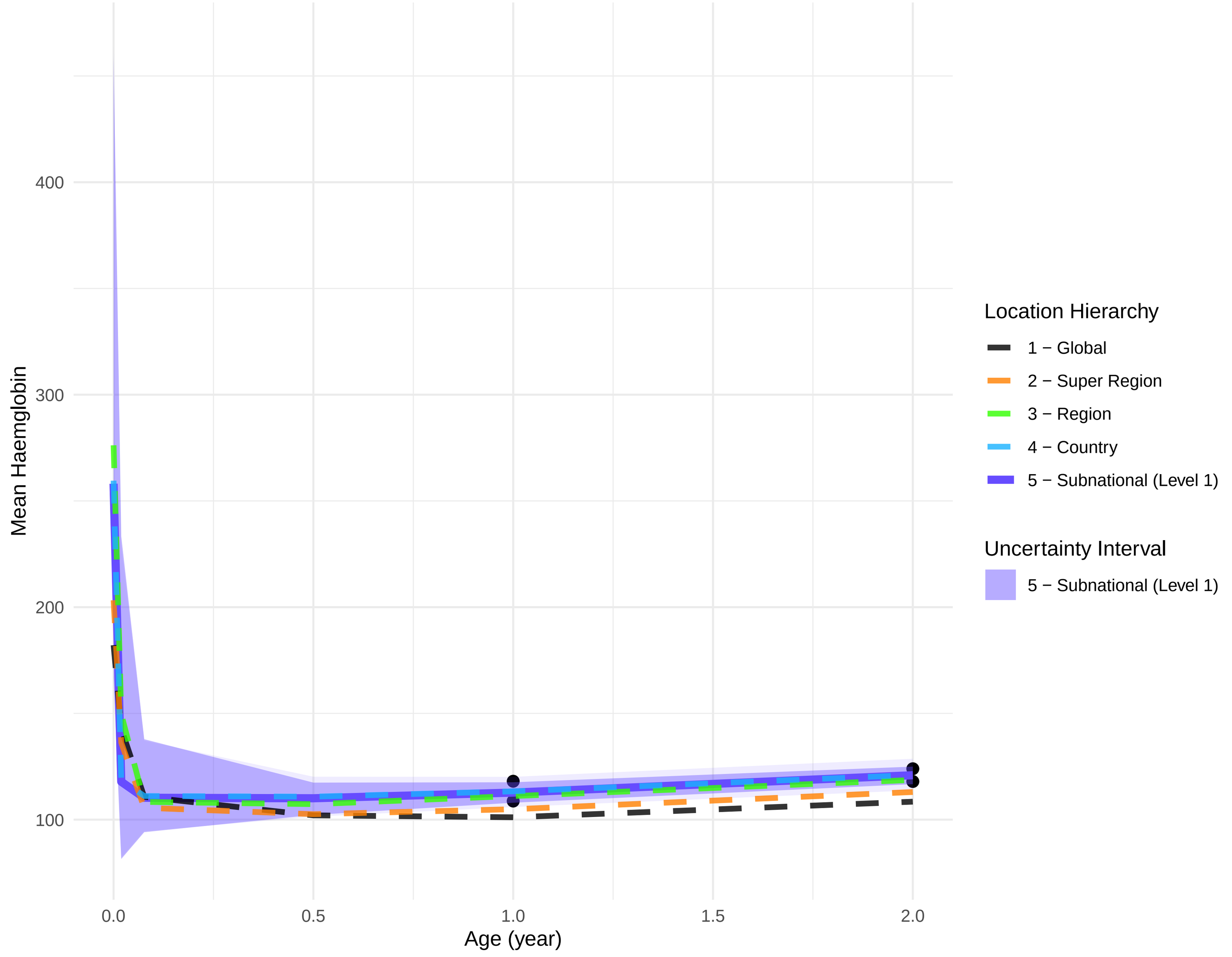

MR−BRT Age/Sex Fits − Non−pregnant Females <2 Years Old − Mean Haemglobin − Tabasco
WHO−2001 Adjustment Method

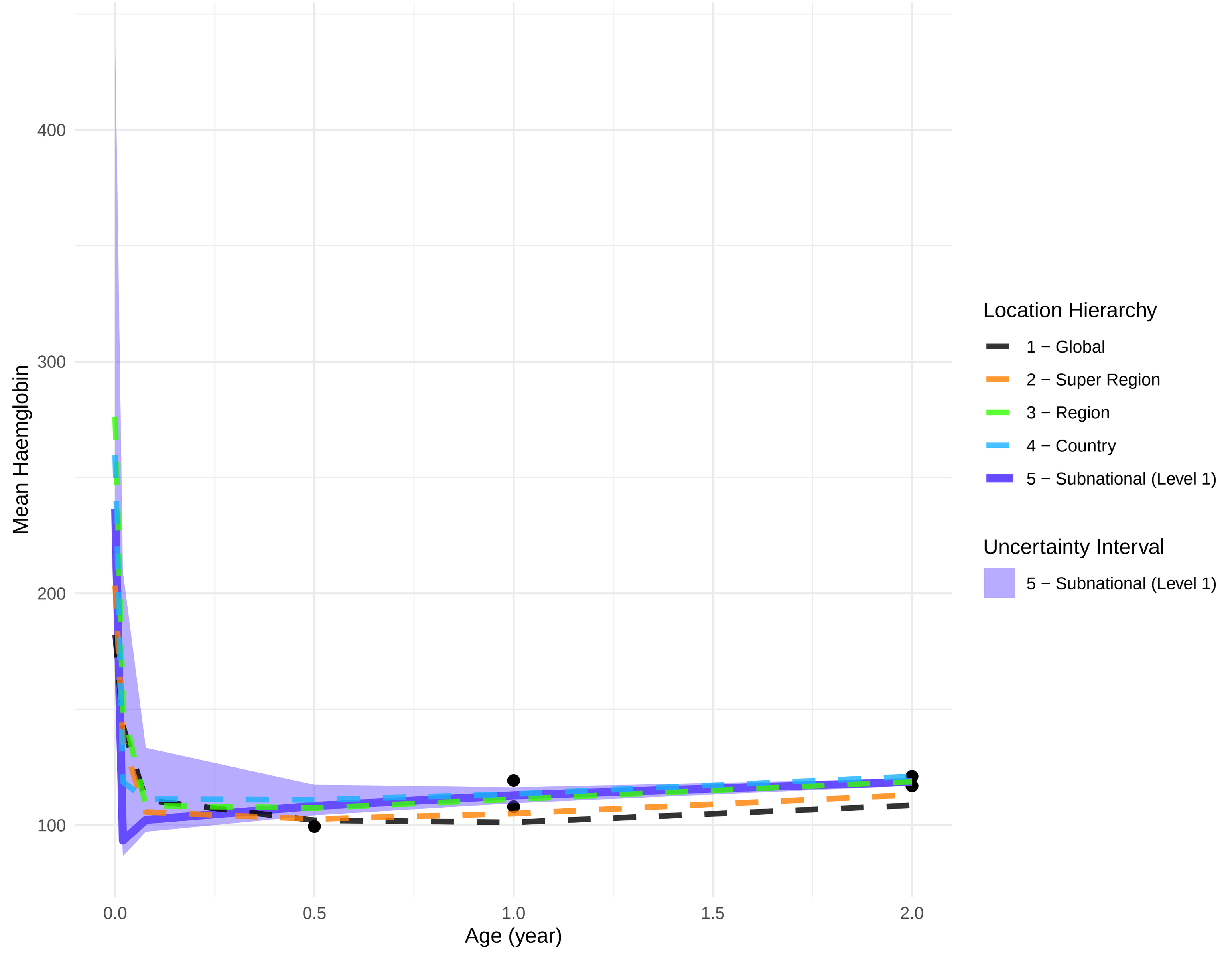

MR−BRT Age/Sex Fits − Non−pregnant Females <2 Years Old − Mean Haemglobin − Tamaulipas
WHO−2001 Adjustment Method

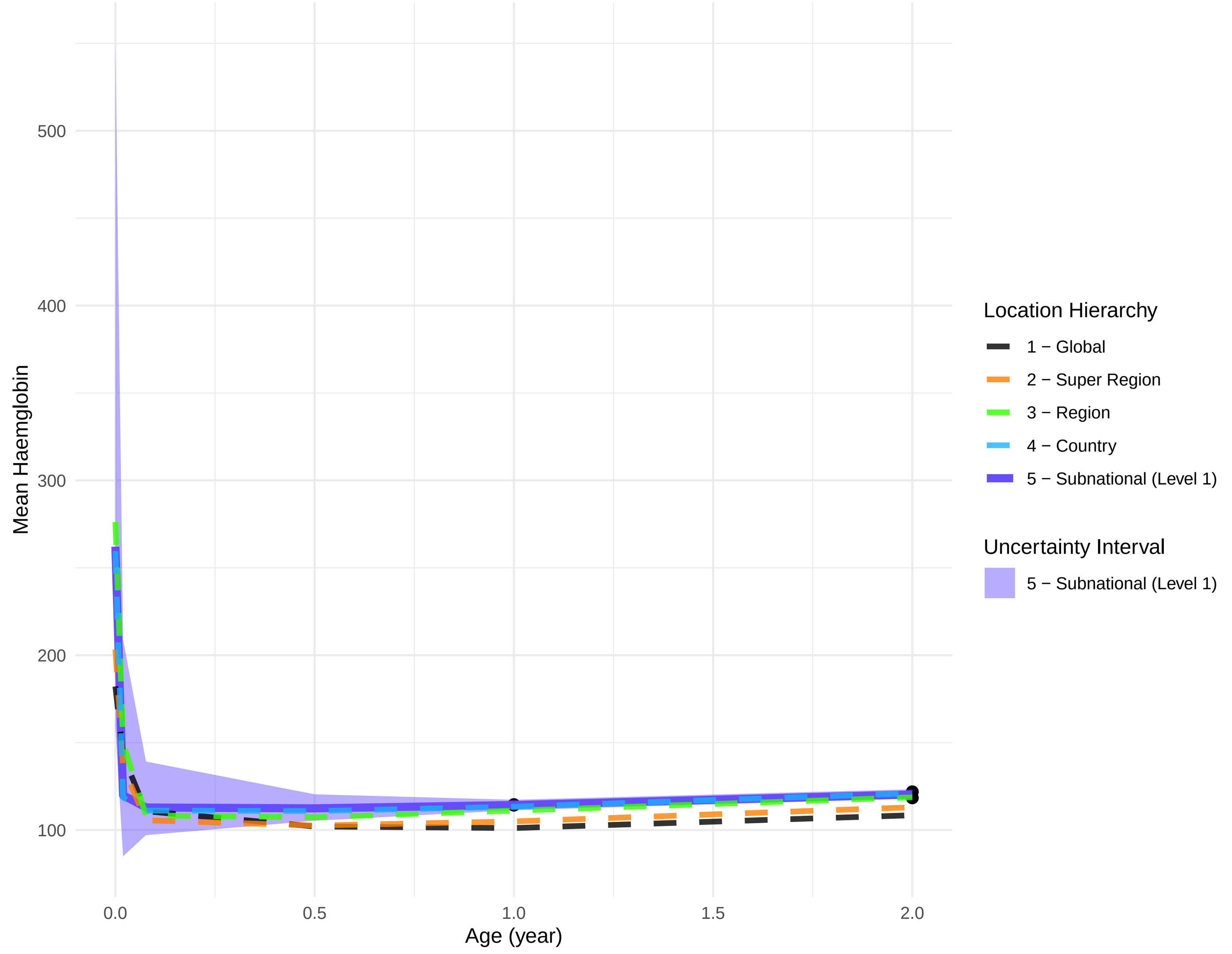

MR−BRT Age/Sex Fits − Non−pregnant Females <2 Years Old − Mean Haemglobin − Tlaxcala
WHO−2001 Adjustment Method

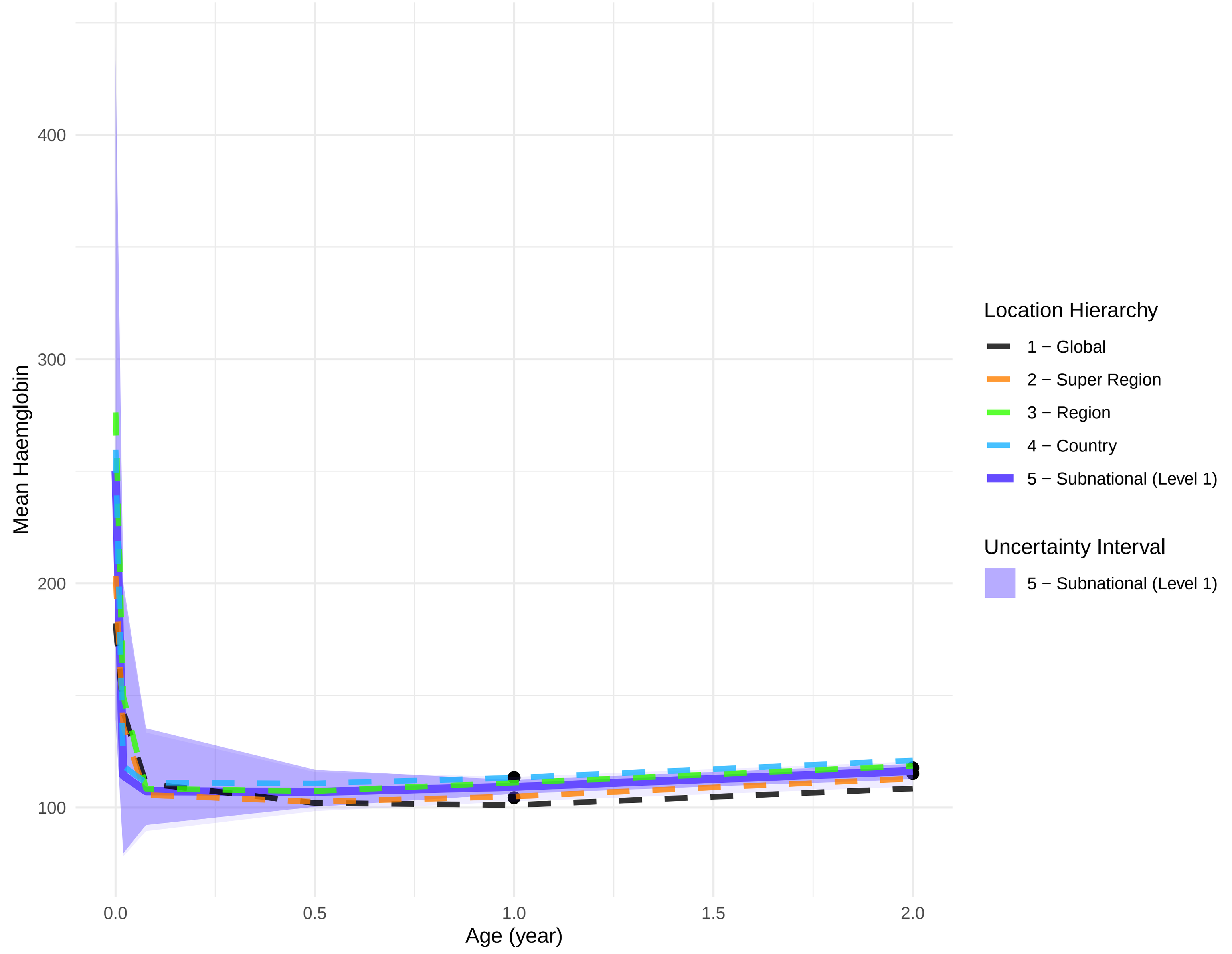

MR−BRT Age/Sex Fits − Non−pregnant Females <2 Years Old − Mean Haemglobin − Veracruz de Ignacio de la Llave
WHO−2001 Adjustment Method

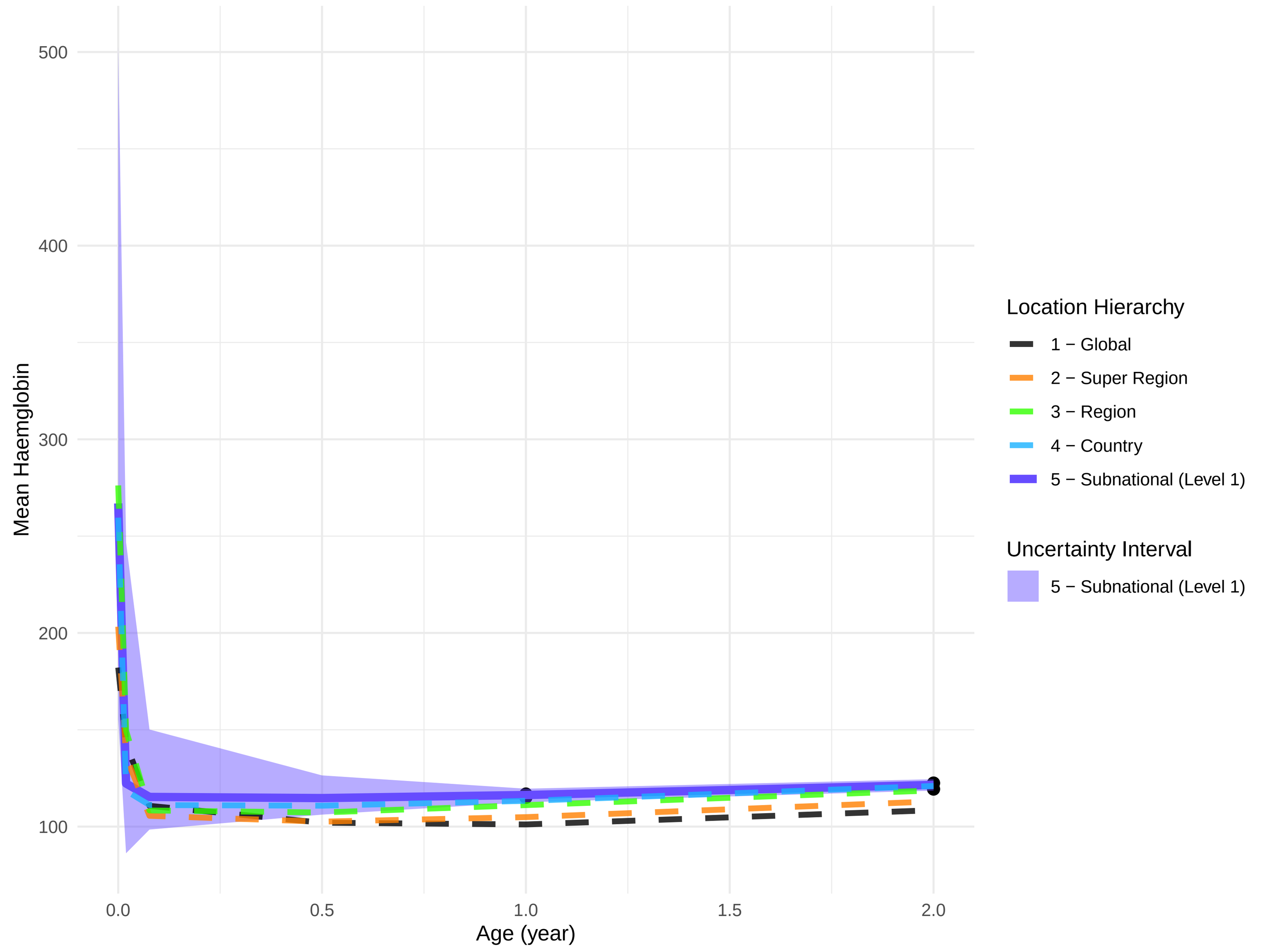

MR−BRT Age/Sex Fits − Non−pregnant Females <2 Years Old − Mean Haemglobin − Yucatán
WHO−2001 Adjustment Method

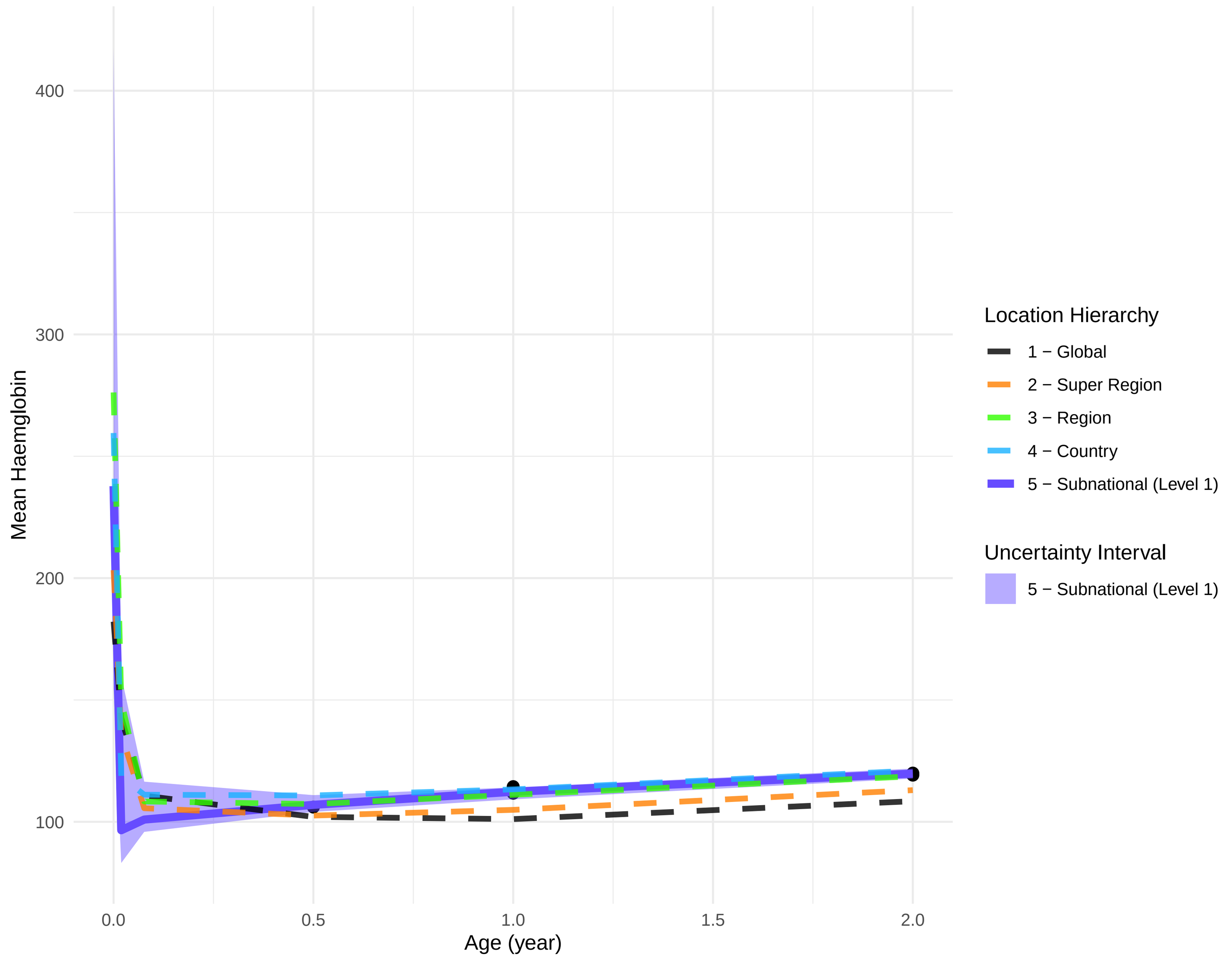

MR−BRT Age/Sex Fits − Non−pregnant Females <2 Years Old − Mean Haemglobin − Zacatecas
WHO−2001 Adjustment Method

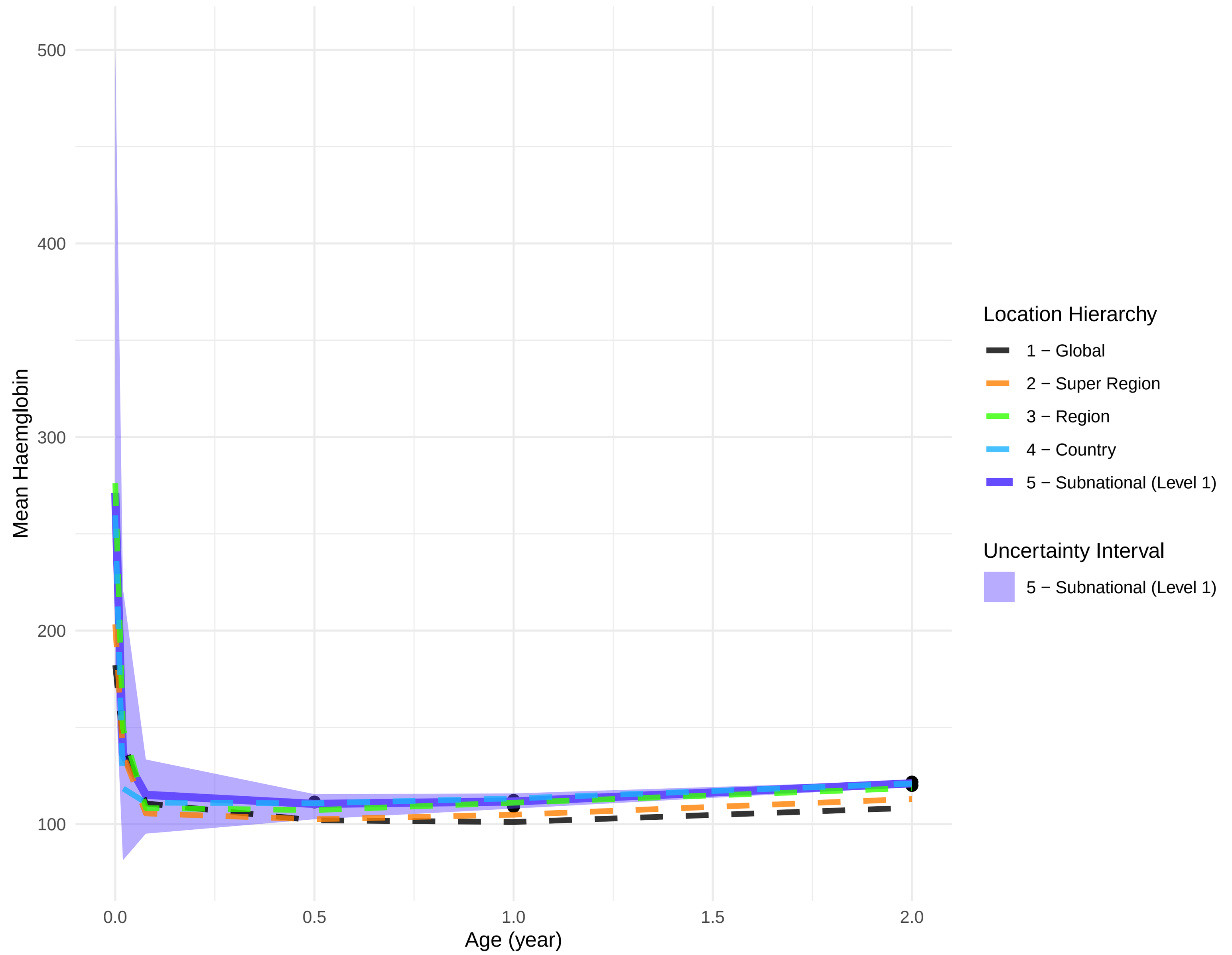

MR−BRT Age/Sex Fits − Non−pregnant Females <2 Years Old − Mean Haemglobin − North Africa and Middle East
WHO−2001 Adjustment Method

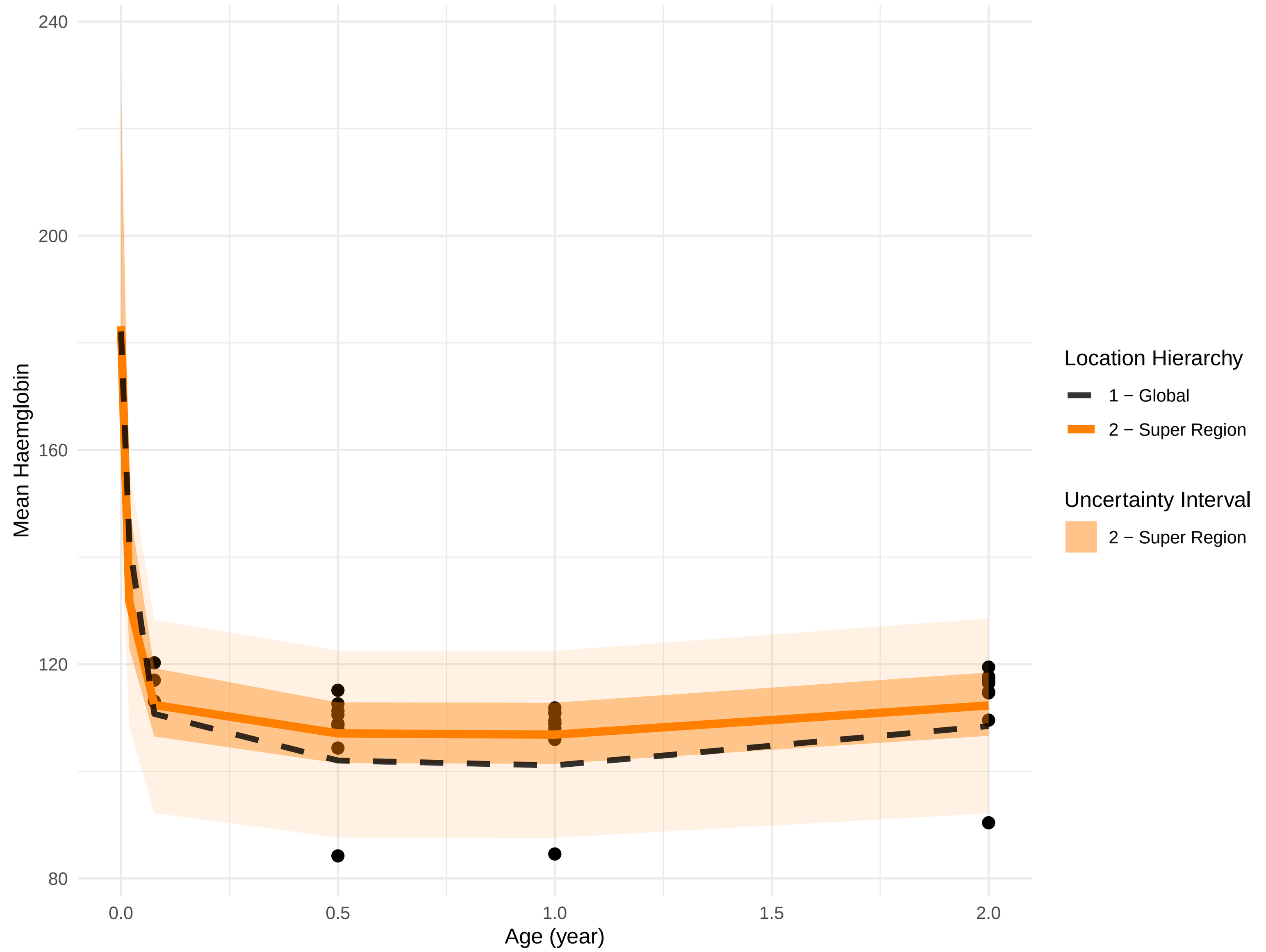

MR−BRT Age/Sex Fits − Non−pregnant Females <2 Years Old − Mean Haemglobin − North Africa and Middle East
WHO−2001 Adjustment Method

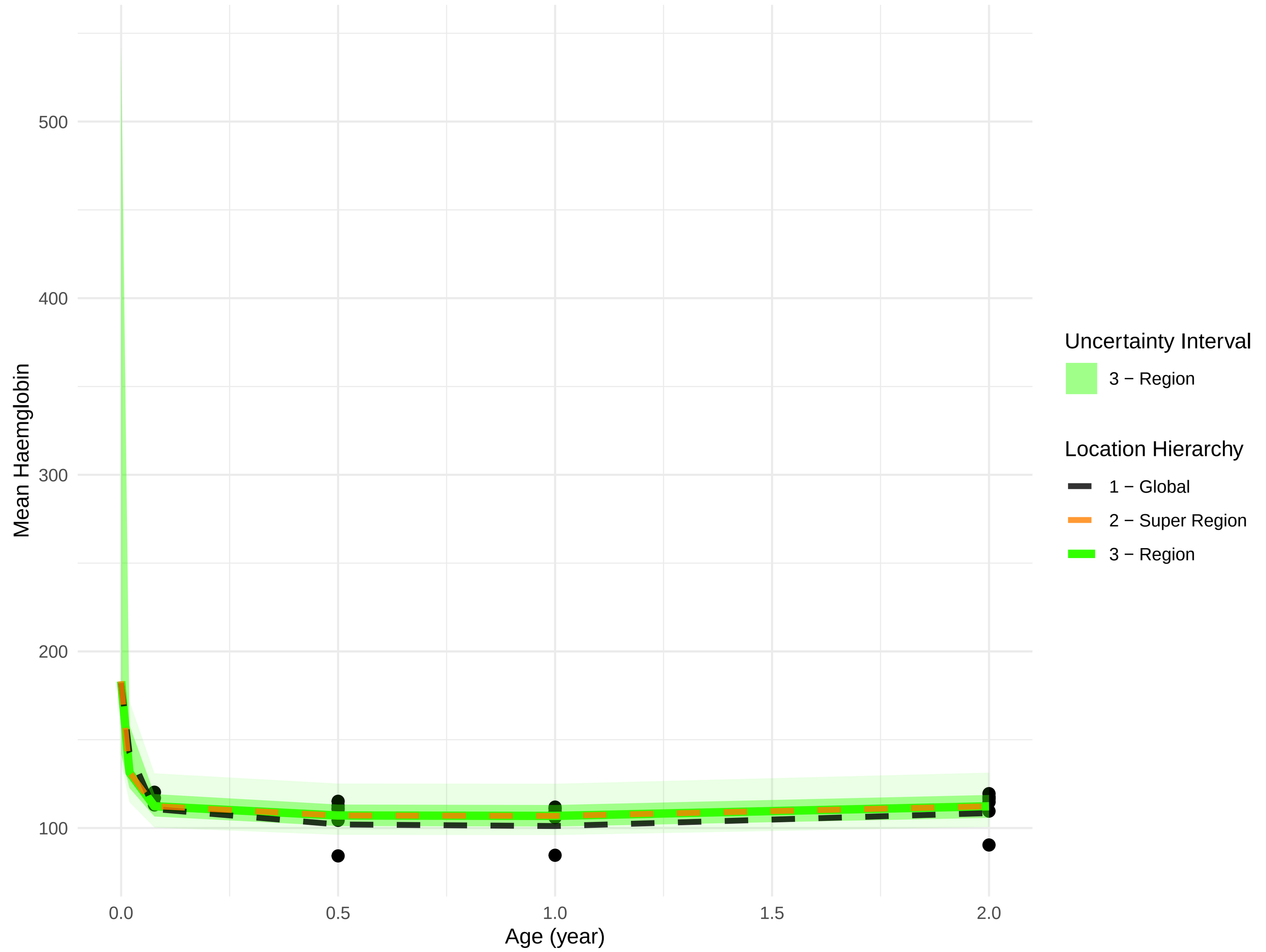

MR−BRT Age/Sex Fits − Non−pregnant Females <2 Years Old − Mean Haemglobin − Egypt
WHO−2001 Adjustment Method

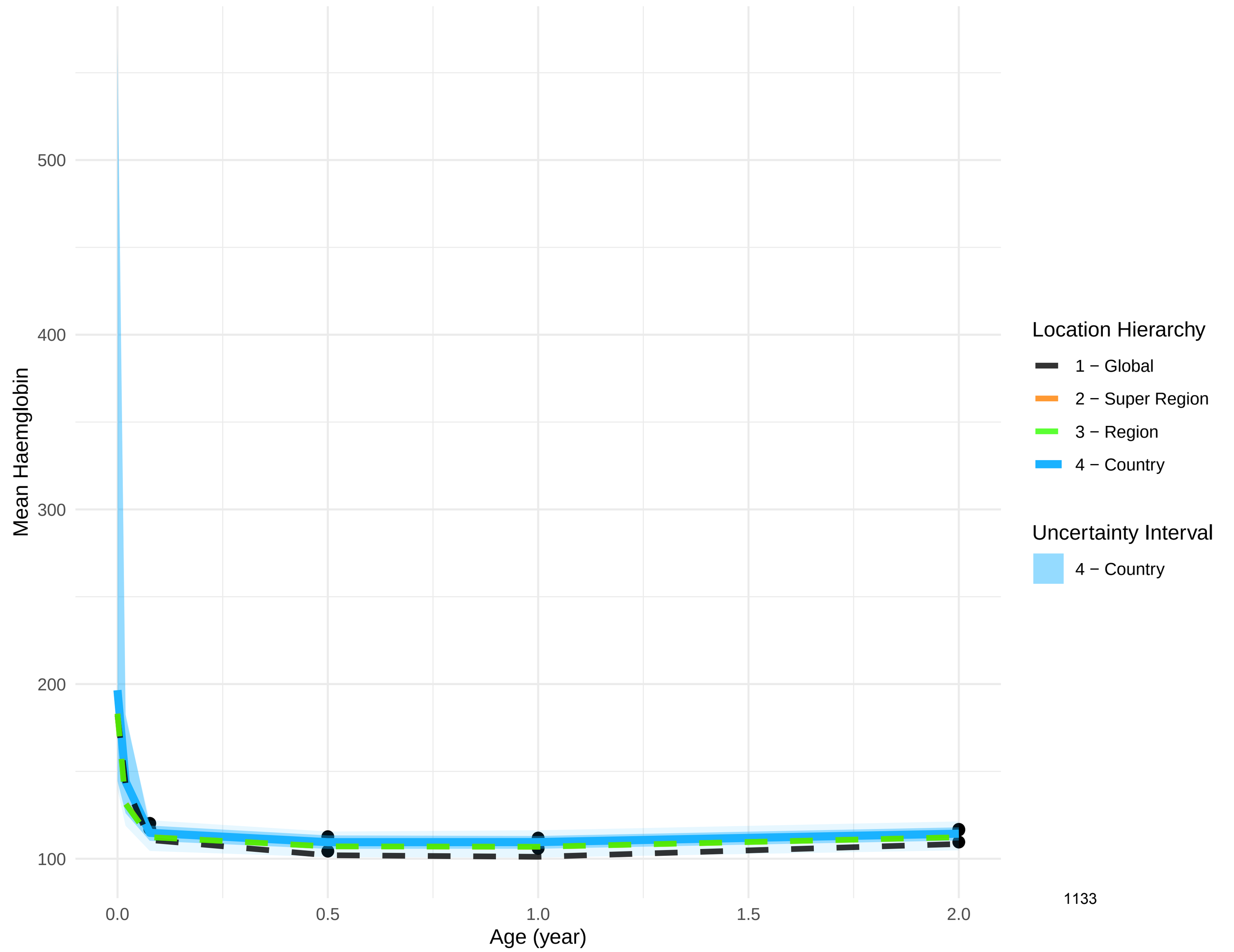

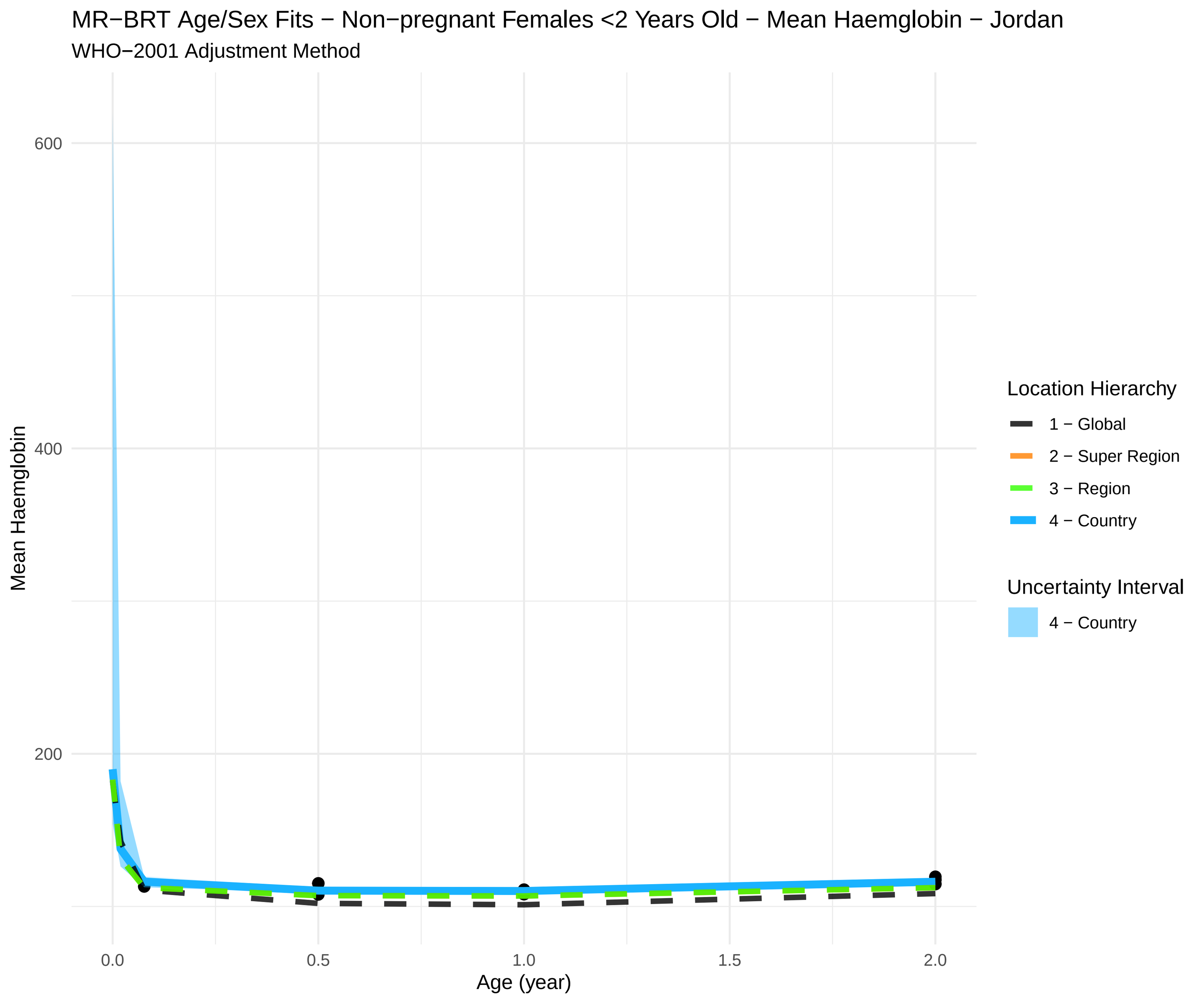
MR−BRT Age/Sex Fits − Non−pregnant Females <2 Years Old − Mean Haemglobin − Jordan
WHO−2001 Adjustment Method
Mean Haemglobin
Age (year)
Location Hierarchy
1 − Global
2 − Super Region
3 − Region
4 − Country
Uncertainty Interval
4 − Country

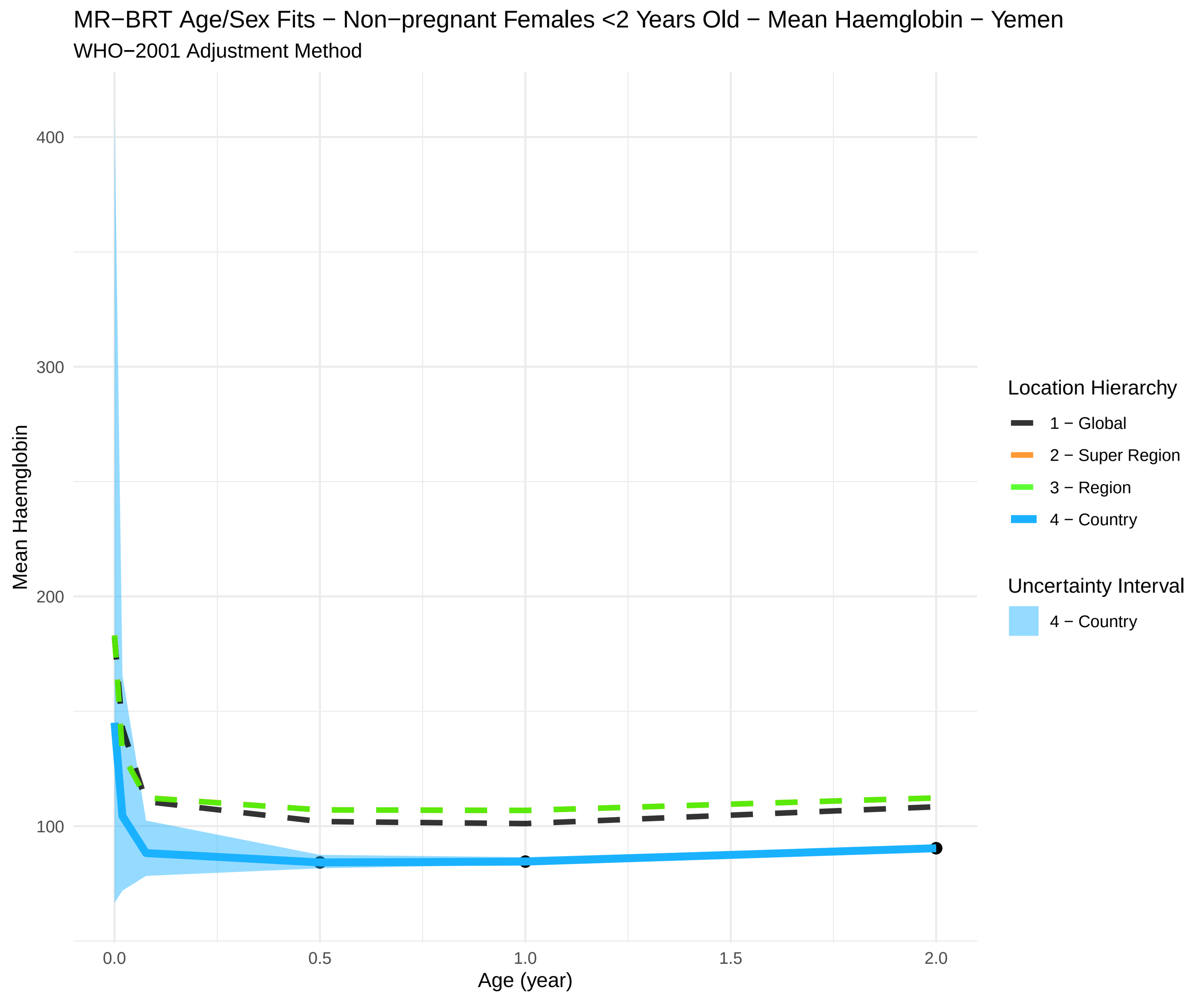
MR−BRT Age/Sex Fits − Non−pregnant Females <2 Years Old − Mean Haemglobin − Yemen
WHO−2001 Adjustment Method
Mean Haemglobin
Age (year)
Location Hierarchy
1 − Global
2 − Super Region
3 − Region
4 − Country
Uncertainty Interval
4 − Country

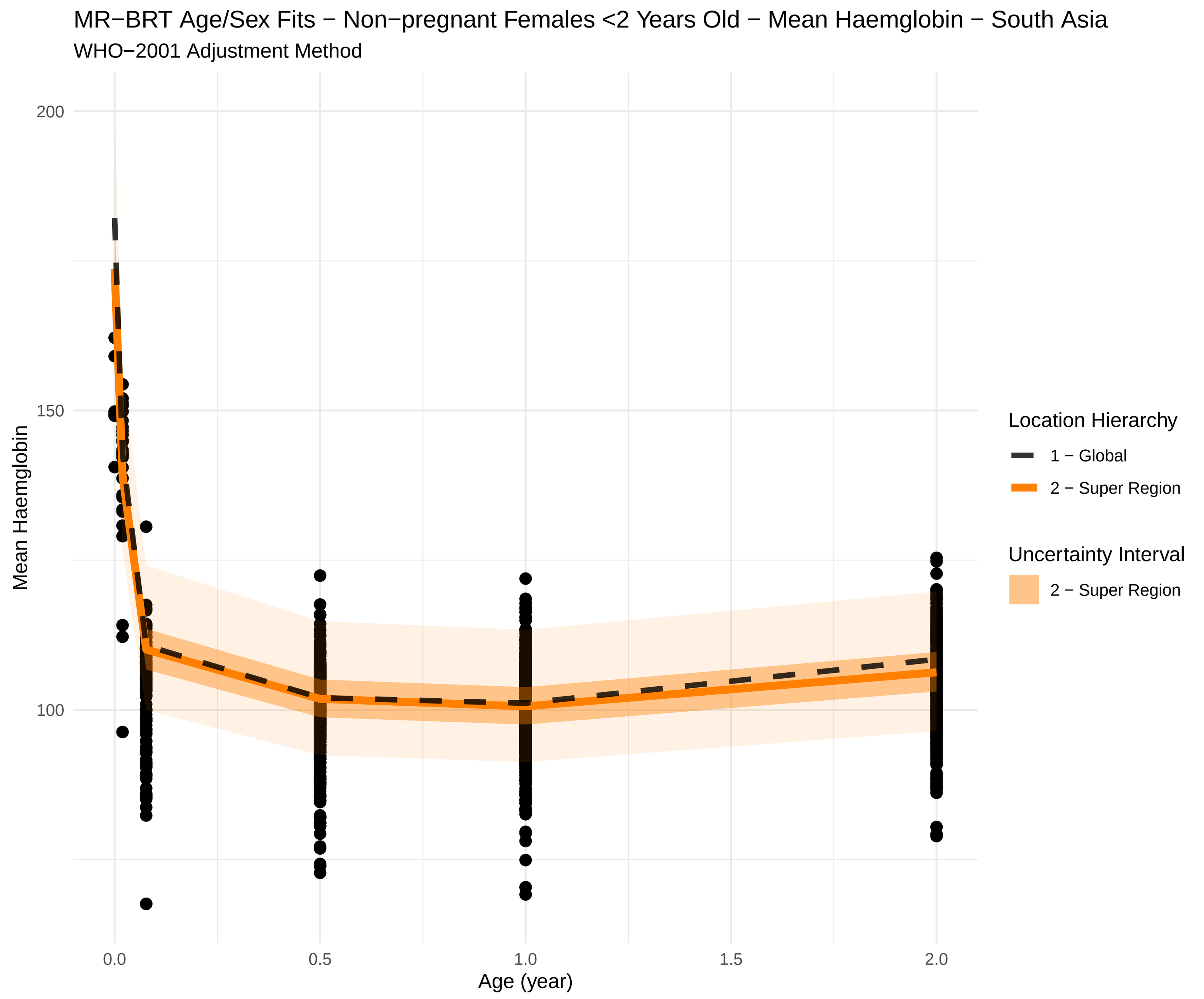
MR−BRT Age/Sex Fits − Non−pregnant Females <2 Years Old − Mean Haemglobin − South Asia
WHO−2001 Adjustment Method
Mean Haemglobin
Age (year)
Location Hierarchy
1 − Global
2 − Super Region
Uncertainty Interval
2 − Super Region

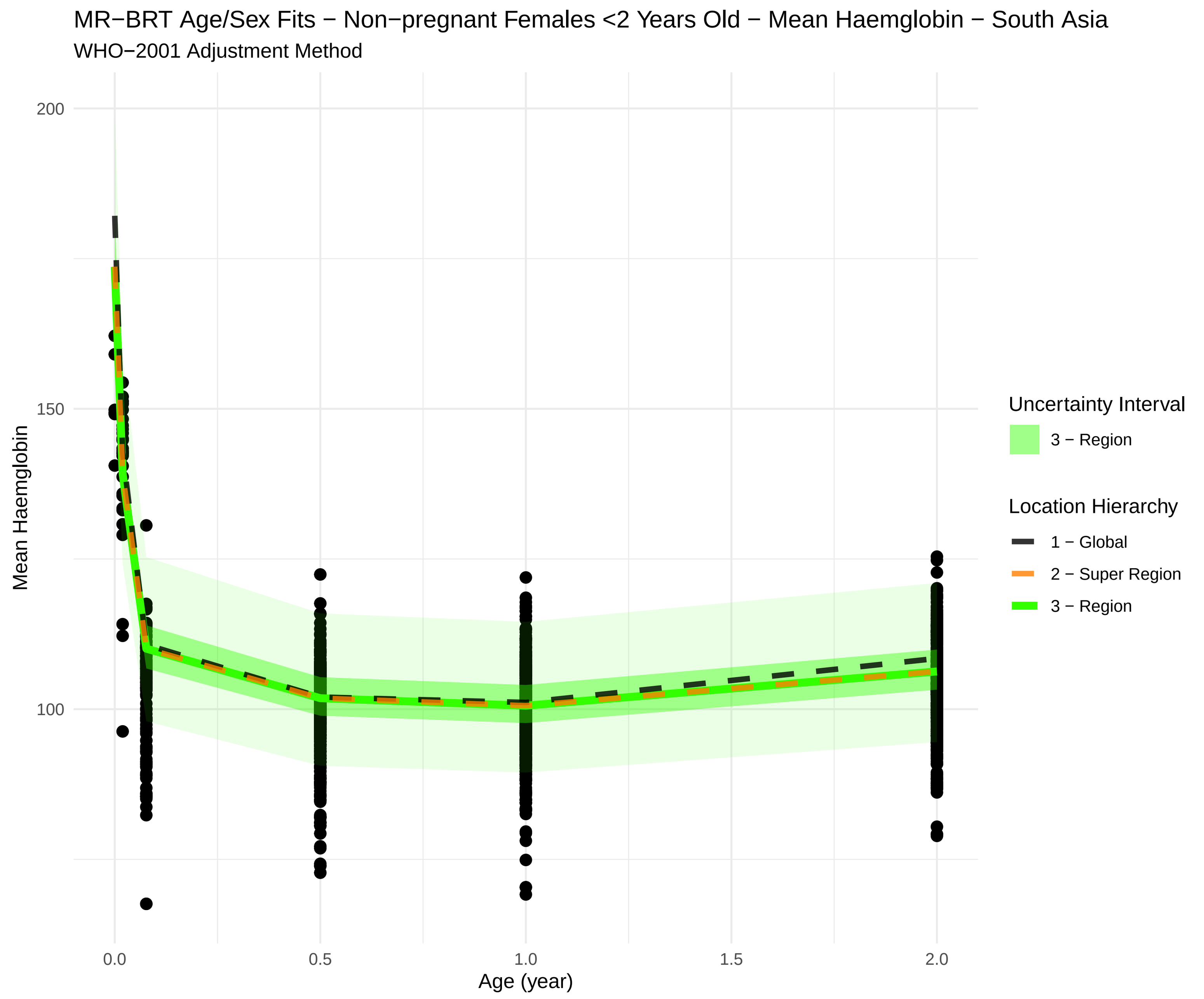
MR−BRT Age/Sex Fits − Non−pregnant Females <2 Years Old − Mean Haemglobin − South Asia
WHO−2001 Adjustment Method
Mean Haemglobin
Age (year)
Uncertainty Interval
3 − Region
Location Hierarchy
1 − Global
2 − Super Region
3 − Region

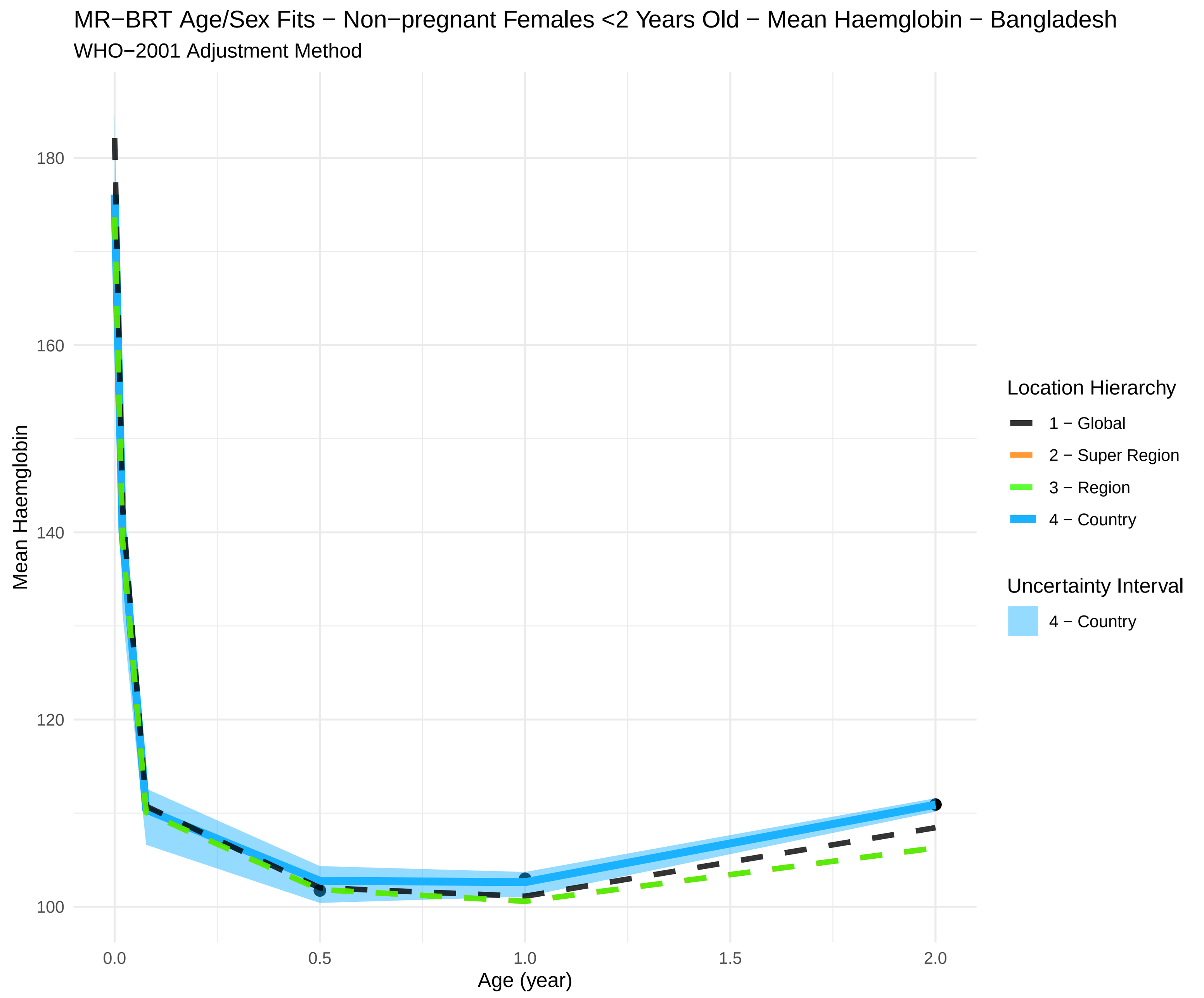
MR−BRT Age/Sex Fits − Non−pregnant Females <2 Years Old − Mean Haemglobin − Bangladesh
WHO−2001 Adjustment Method
Mean Haemglobin
Age (year)
Location Hierarchy
1 − Global
2 − Super Region
3 − Region
4 − Country
Uncertainty Interval
4 − Country

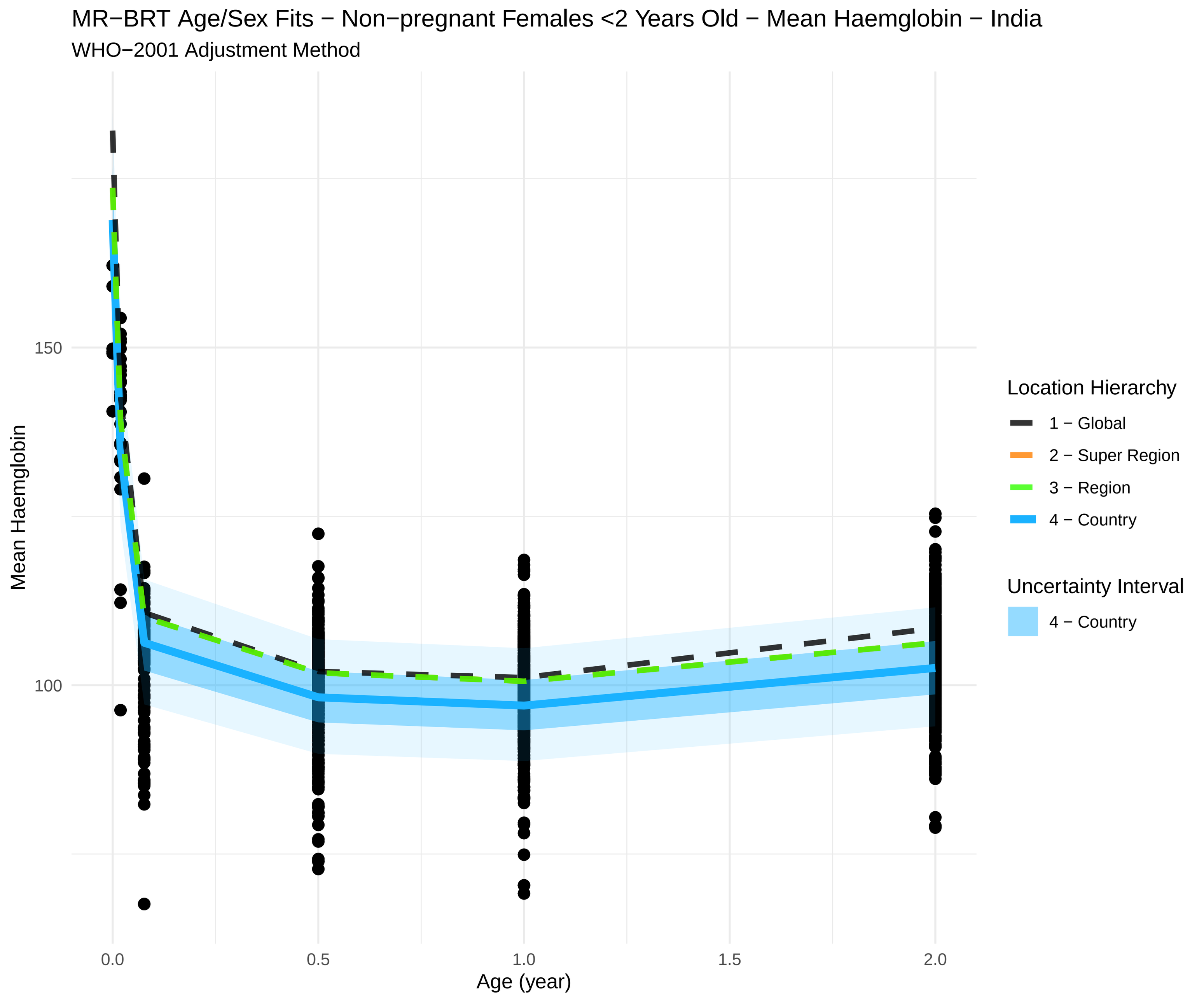
MR−BRT Age/Sex Fits − Non−pregnant Females <2 Years Old − Mean Haemglobin − India
WHO−2001 Adjustment Method
Mean Haemglobin
Age (year)
Location Hierarchy
1 − Global
2 − Super Region
3 − Region
4 − Country
Uncertainty Interval
4 − Country

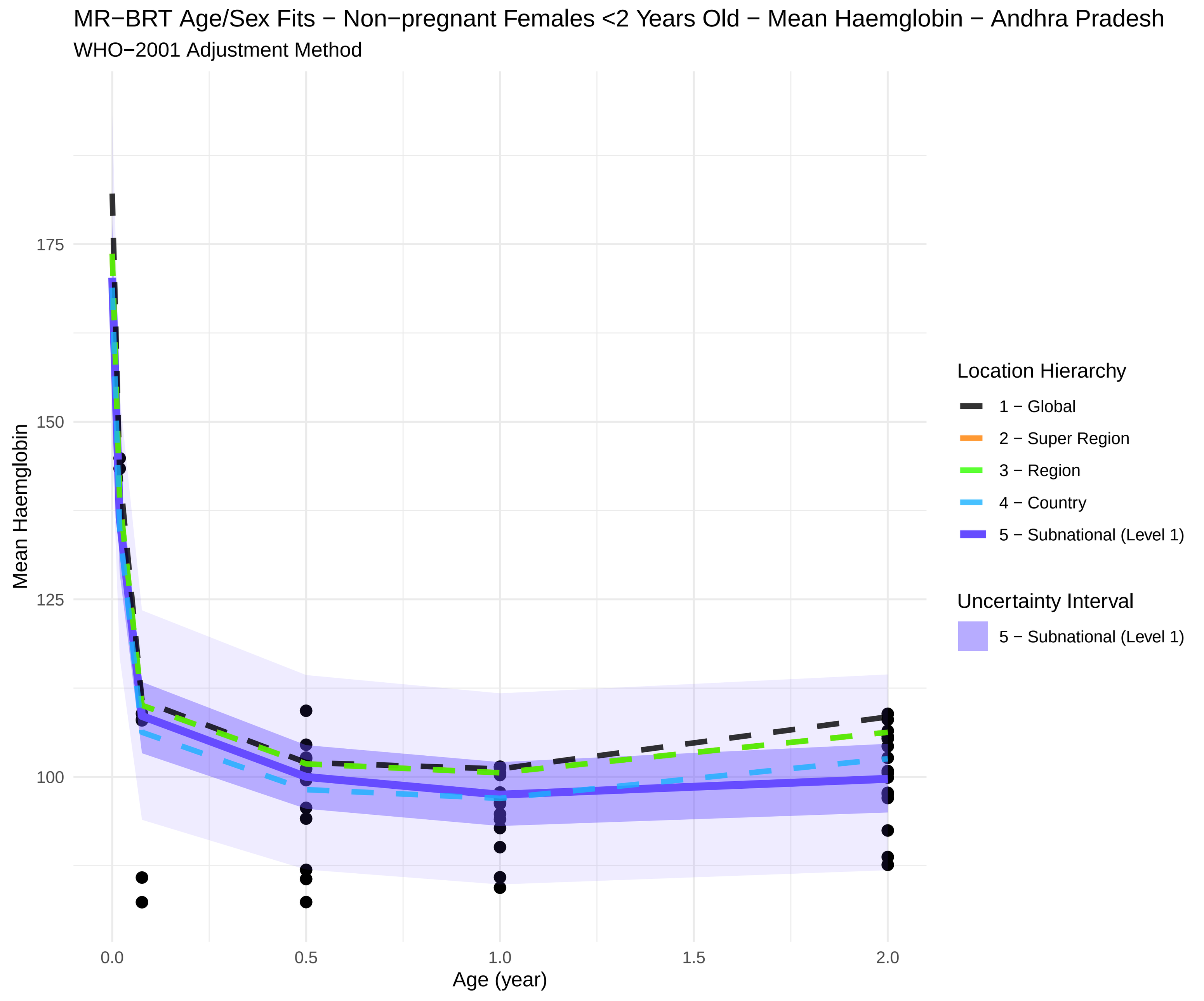
MR−BRT Age/Sex Fits − Non−pregnant Females <2 Years Old − Mean Haemglobin − Andhra Pradesh
WHO−2001 Adjustment Method
Mean Haemglobin
Age (year)
Location Hierarchy
1 − Global
2 − Super Region
3 − Region
4 − Country
5 − Subnational (Level 1)
Uncertainty Interval
5 − Subnational (Level 1)

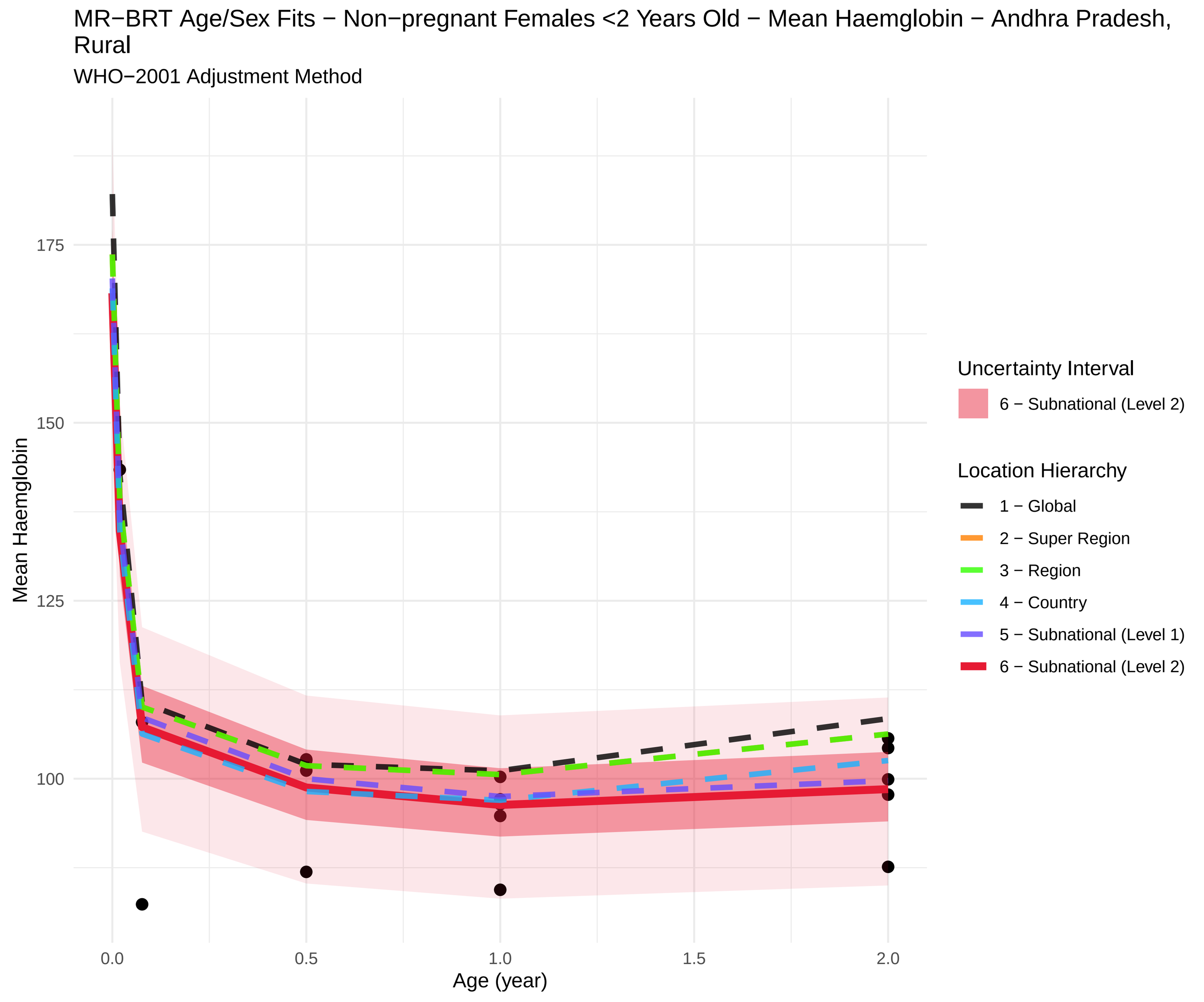
MR−BRT Age/Sex Fits − Non−pregnant Females <2 Years Old − Mean Haemglobin − Andhra Pradesh, Rural
WHO−2001 Adjustment Method
Mean Haemglobin
Age (year)
Uncertainty Interval
6 − Subnational (Level 2)
Location Hierarchy
1 − Global
2 − Super Region
3 − Region
4 − Country
5 − Subnational (Level 1)
6 − Subnational (Level 2)

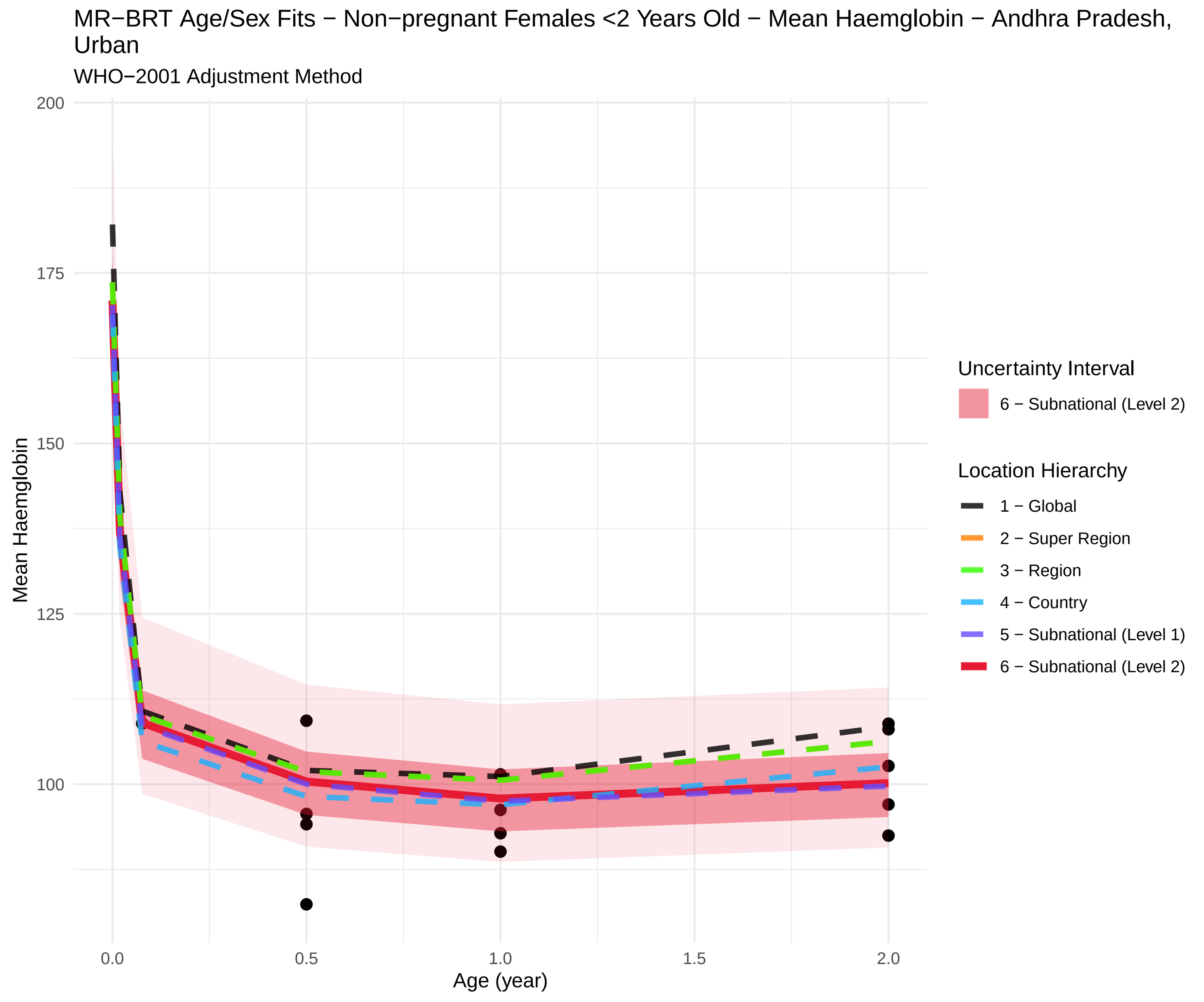
MR−BRT Age/Sex Fits − Non−pregnant Females <2 Years Old − Mean Haemglobin − Andhra Pradesh, Urban
WHO−2001 Adjustment Method
Mean Haemglobin
Age (year)
Uncertainty Interval
6 − Subnational (Level 2)
Location Hierarchy
1 − Global
2 − Super Region
3 − Region
4 − Country
5 − Subnational (Level 1)
6 − Subnational (Level 2)

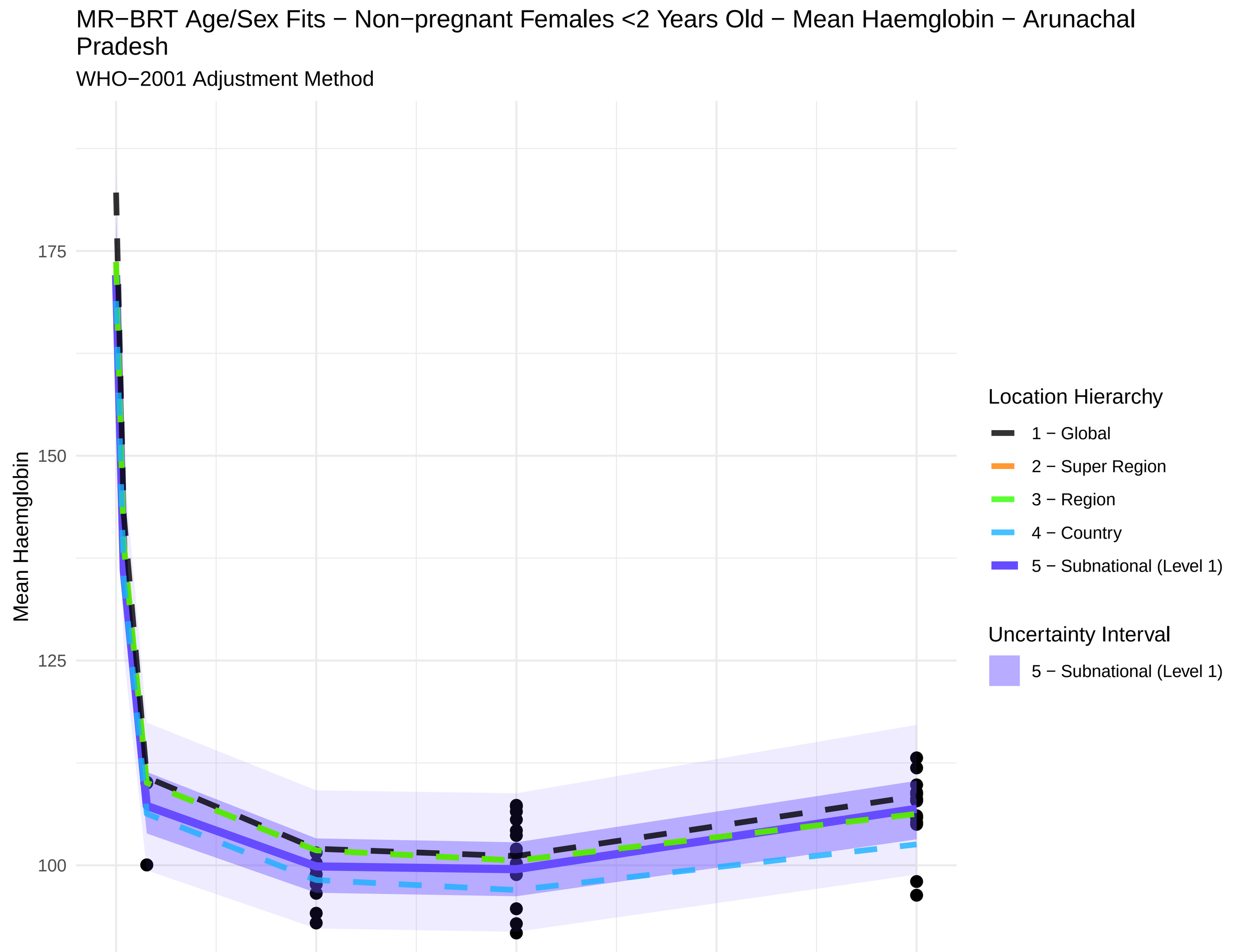
MR−BRT Age/Sex Fits − Non−pregnant Females <2 Years Old − Mean Haemglobin − Arunachal Pradesh
WHO−2001 Adjustment Method
Mean Haemglobin
Age (year)
Location Hierarchy
1 − Global
2 − Super Region
3 − Region
4 − Country
5 − Subnational (Level 1)
Uncertainty Interval
5 − Subnational (Level 1)

MR−BRT Age/Sex Fits − Non−pregnant Females <2 Years Old − Mean Haemglobin − Arunachal Pradesh, Rural
WHO−2001 Adjustment Method

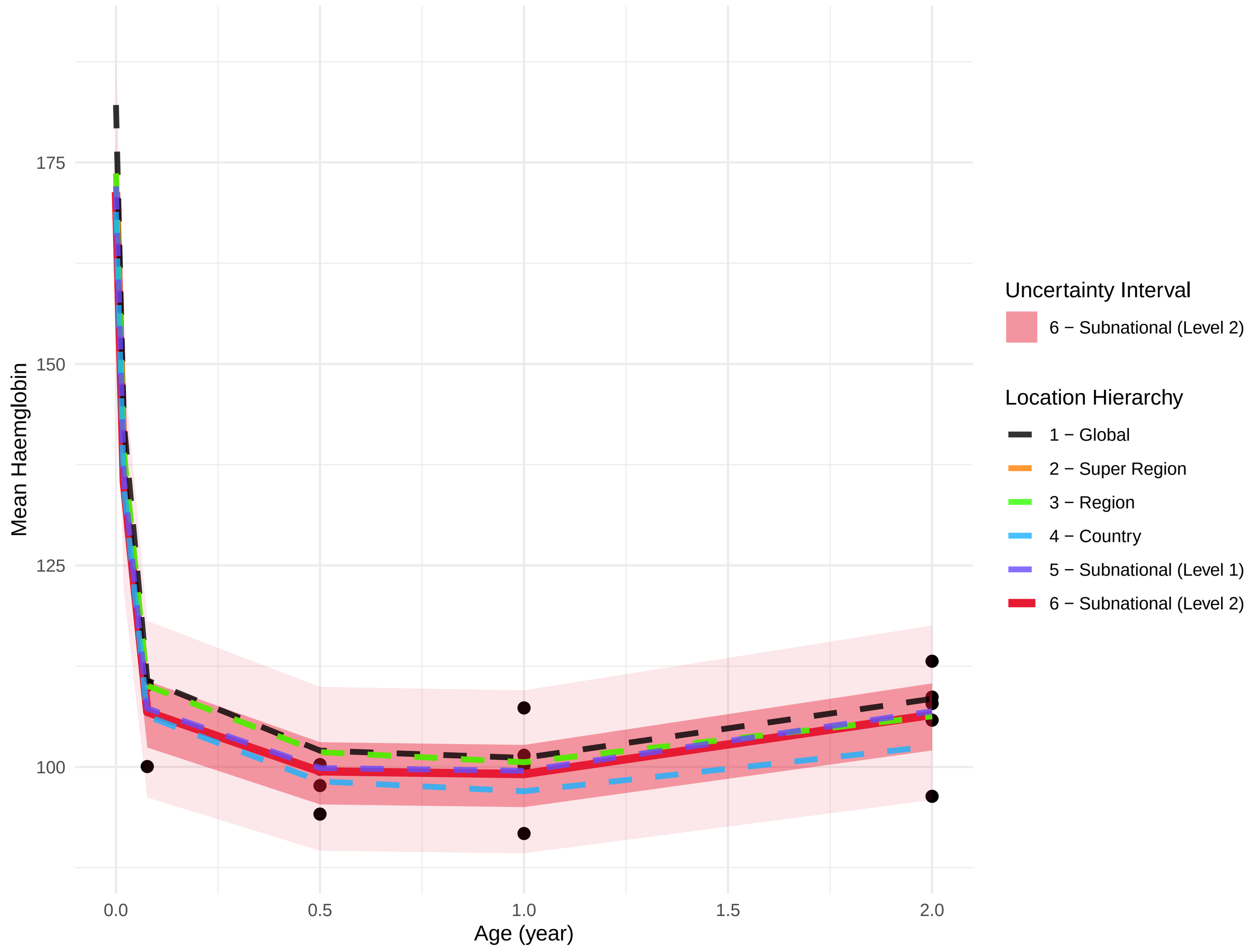

MR−BRT Age/Sex Fits − Non−pregnant Females <2 Years Old − Mean Haemglobin − Arunachal Pradesh, Urban
WHO−2001 Adjustment Method

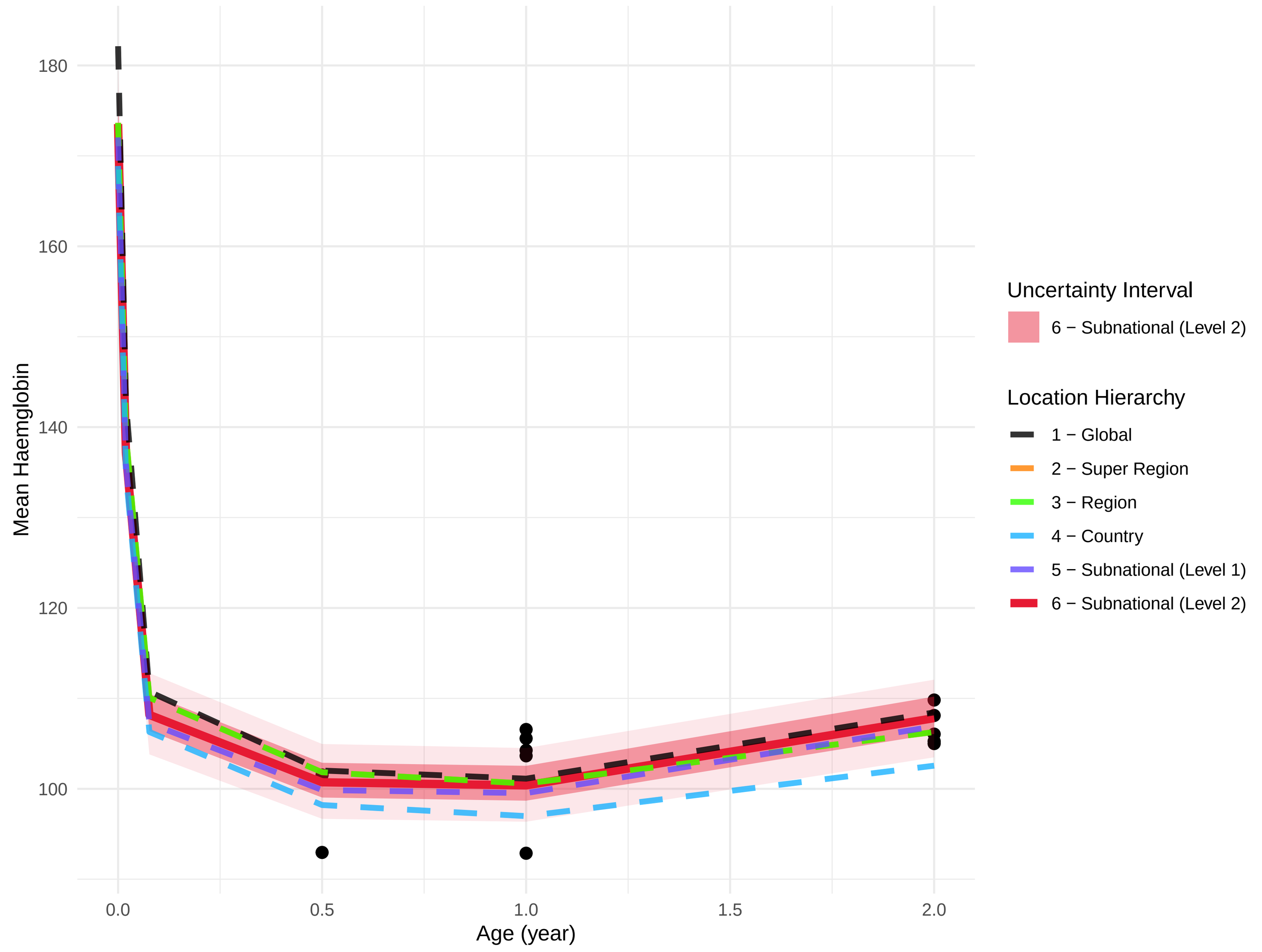

MR−BRT Age/Sex Fits − Non−pregnant Females <2 Years Old − Mean Haemglobin − Assam
WHO−2001 Adjustment Method

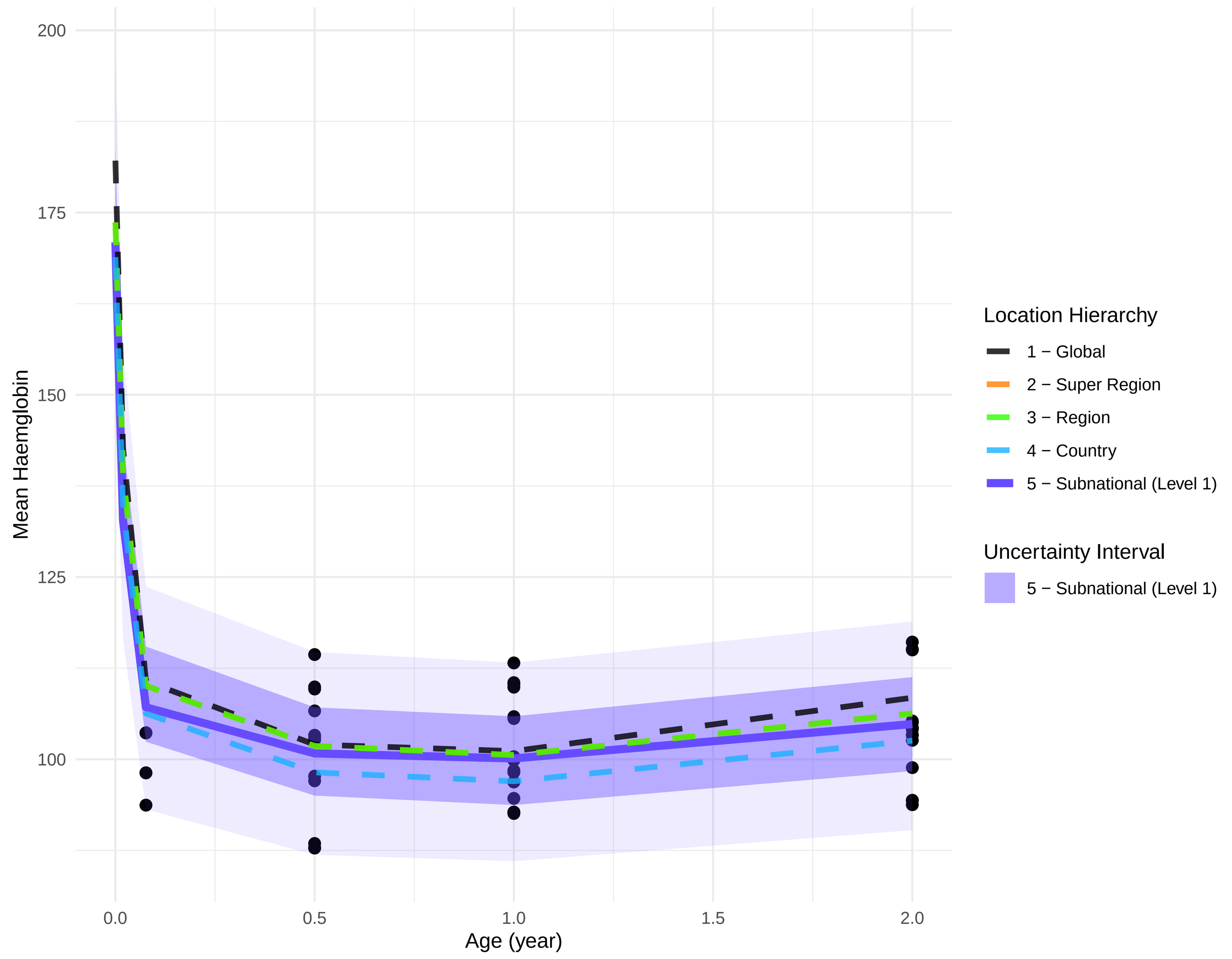

MR−BRT Age/Sex Fits − Non−pregnant Females <2 Years Old − Mean Haemglobin − Assam, Rural
WHO−2001 Adjustment Method

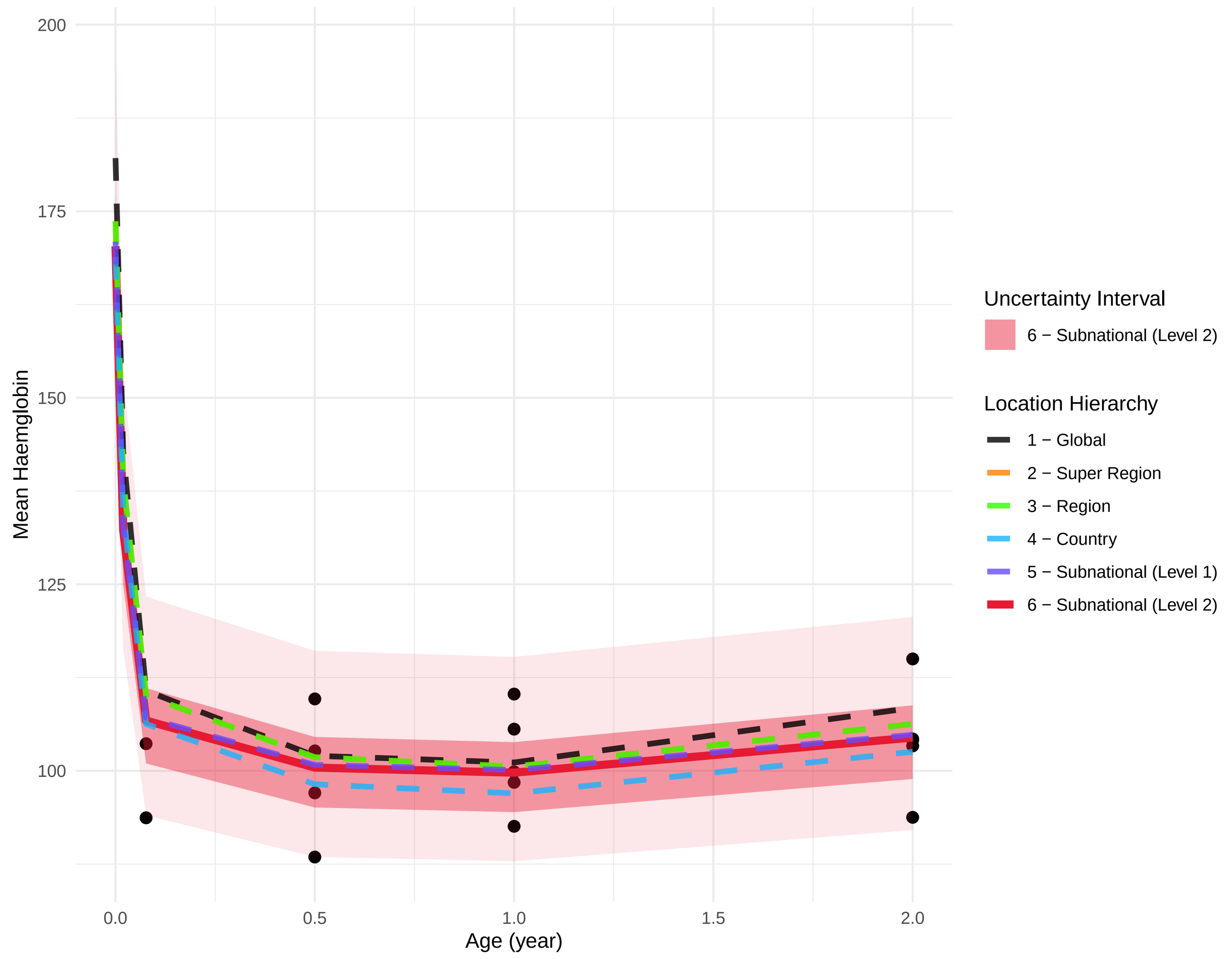

MR−BRT Age/Sex Fits − Non−pregnant Females <2 Years Old − Mean Haemglobin − Assam, Urban
WHO−2001 Adjustment Method

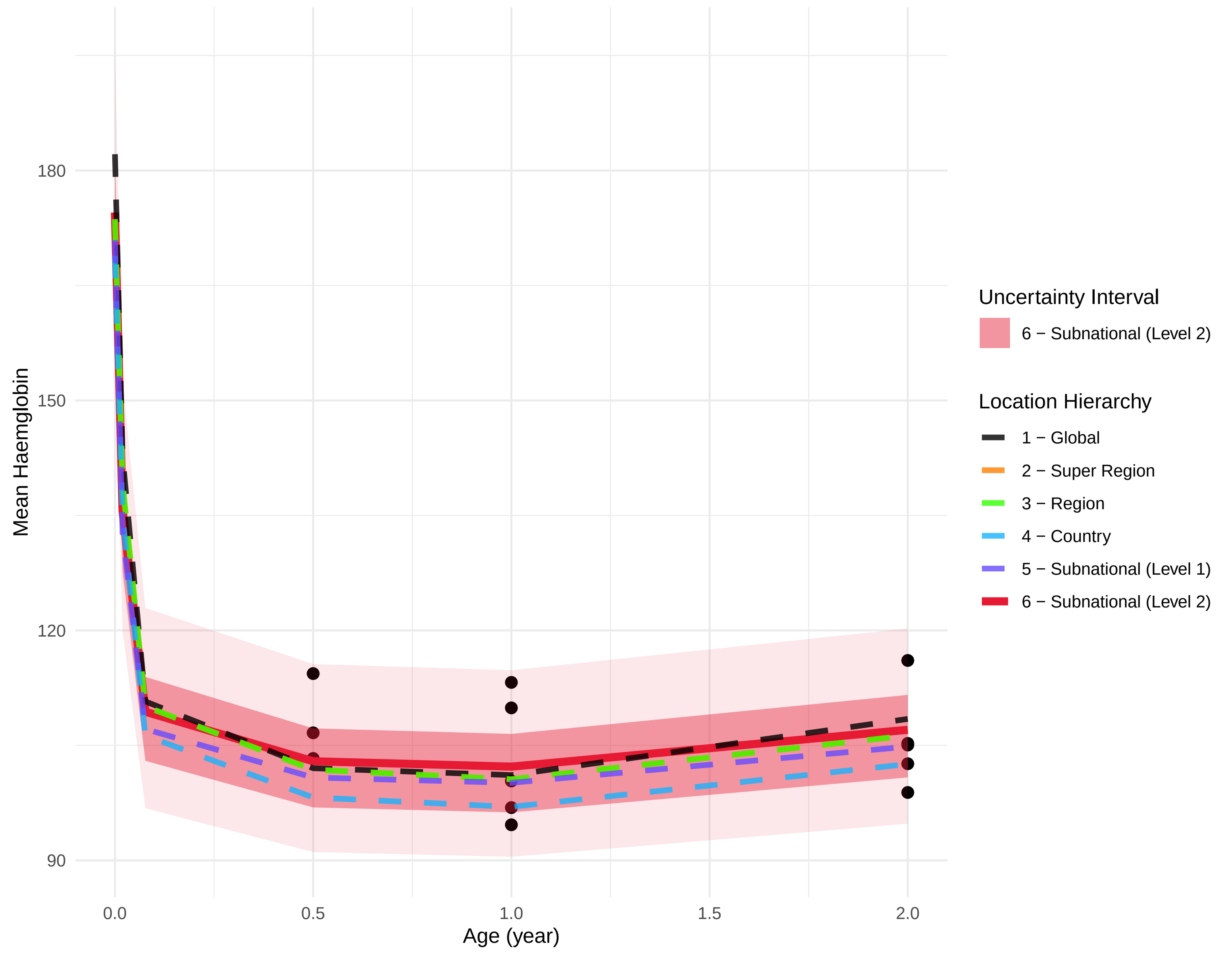

MR−BRT Age/Sex Fits − Non−pregnant Females <2 Years Old − Mean Haemglobin − Bihar
WHO−2001 Adjustment Method

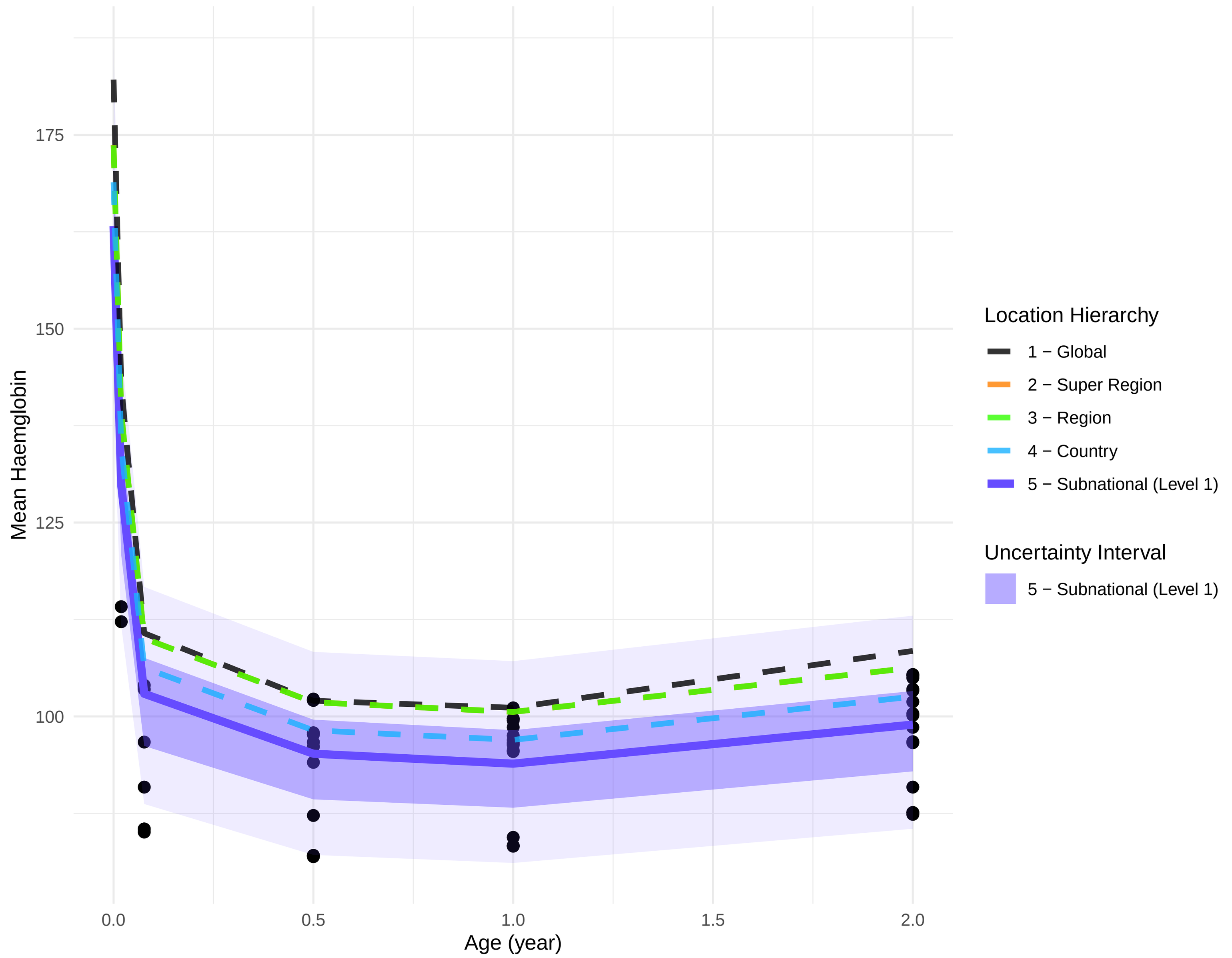

MR−BRT Age/Sex Fits − Non−pregnant Females <2 Years Old − Mean Haemglobin − Bihar, Rural
WHO−2001 Adjustment Method

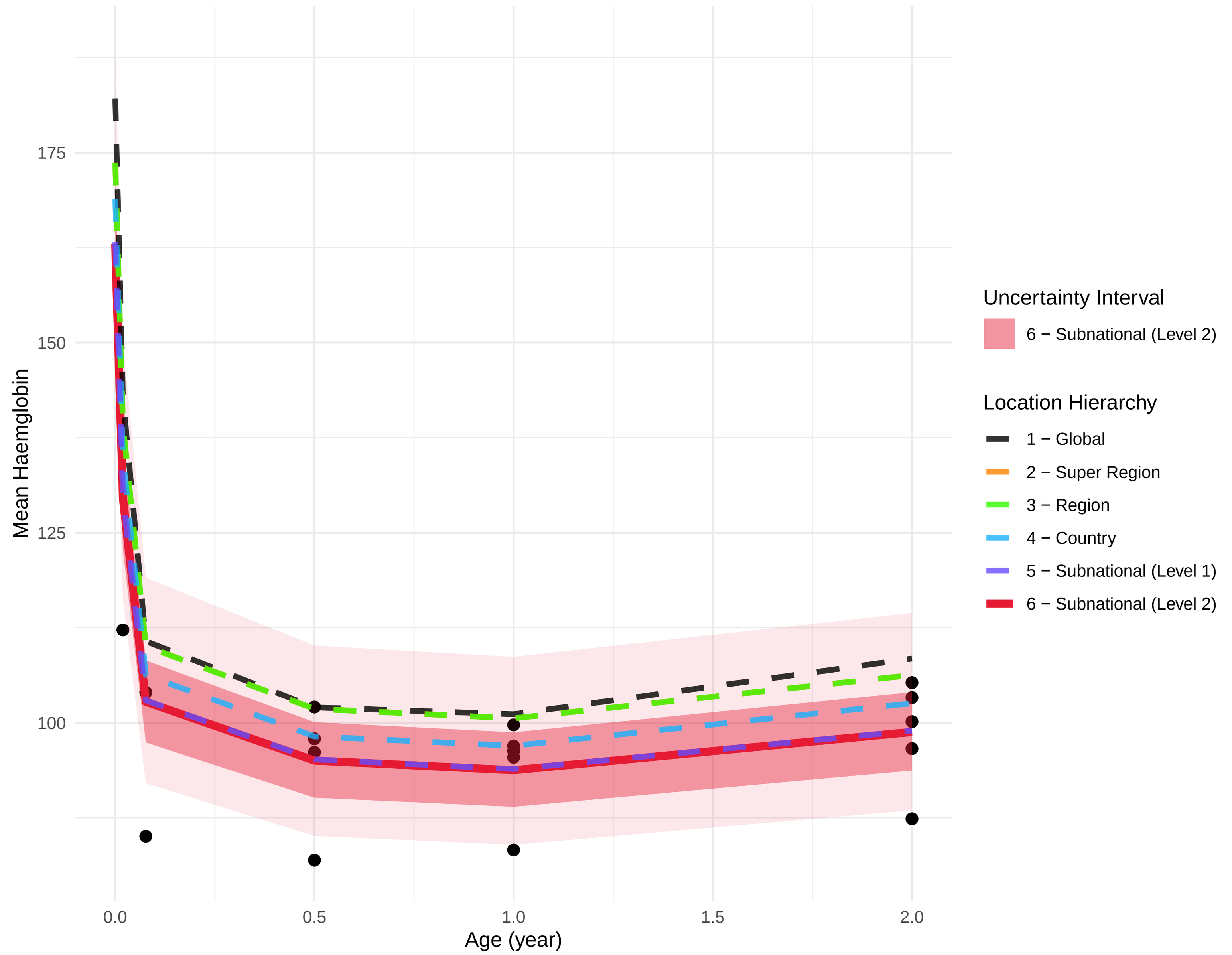

MR−BRT Age/Sex Fits − Non−pregnant Females <2 Years Old − Mean Haemglobin − Bihar, Urban
WHO−2001 Adjustment Method

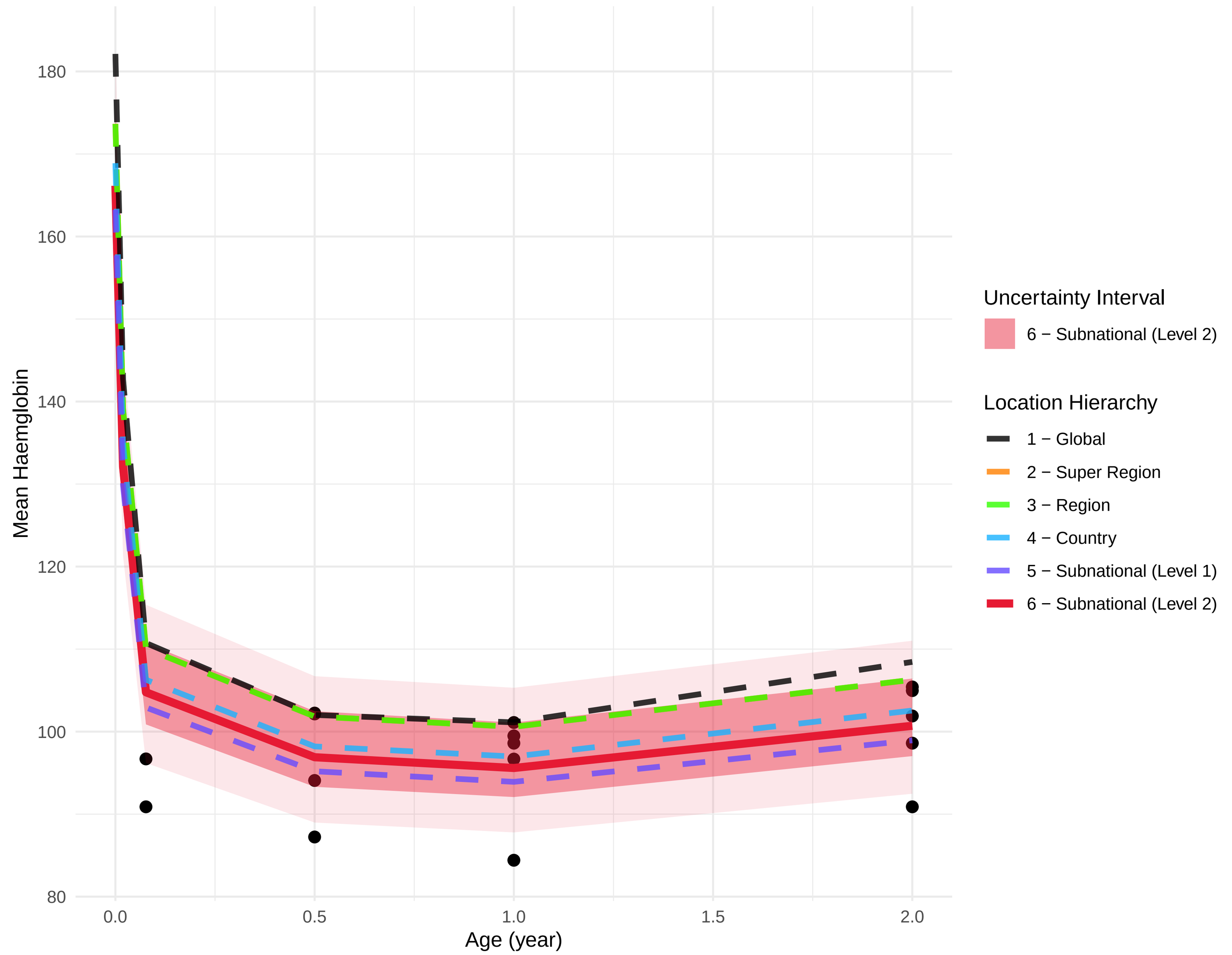

MR−BRT Age/Sex Fits − Non−pregnant Females <2 Years Old − Mean Haemglobin − Chhattisgarh
WHO−2001 Adjustment Method

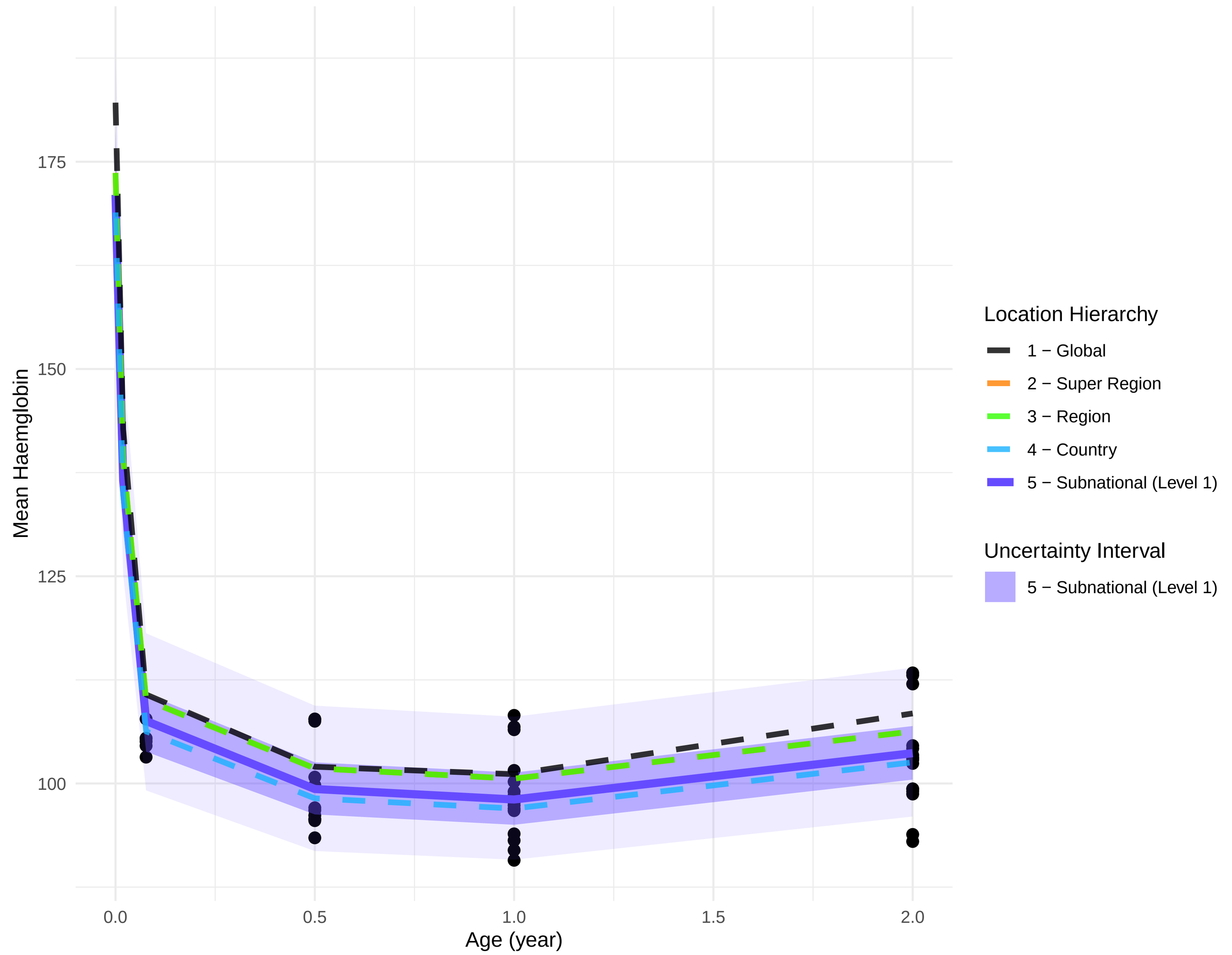

MR−BRT Age/Sex Fits − Non−pregnant Females <2 Years Old − Mean Haemglobin − Chhattisgarh, Rural
WHO−2001 Adjustment Method

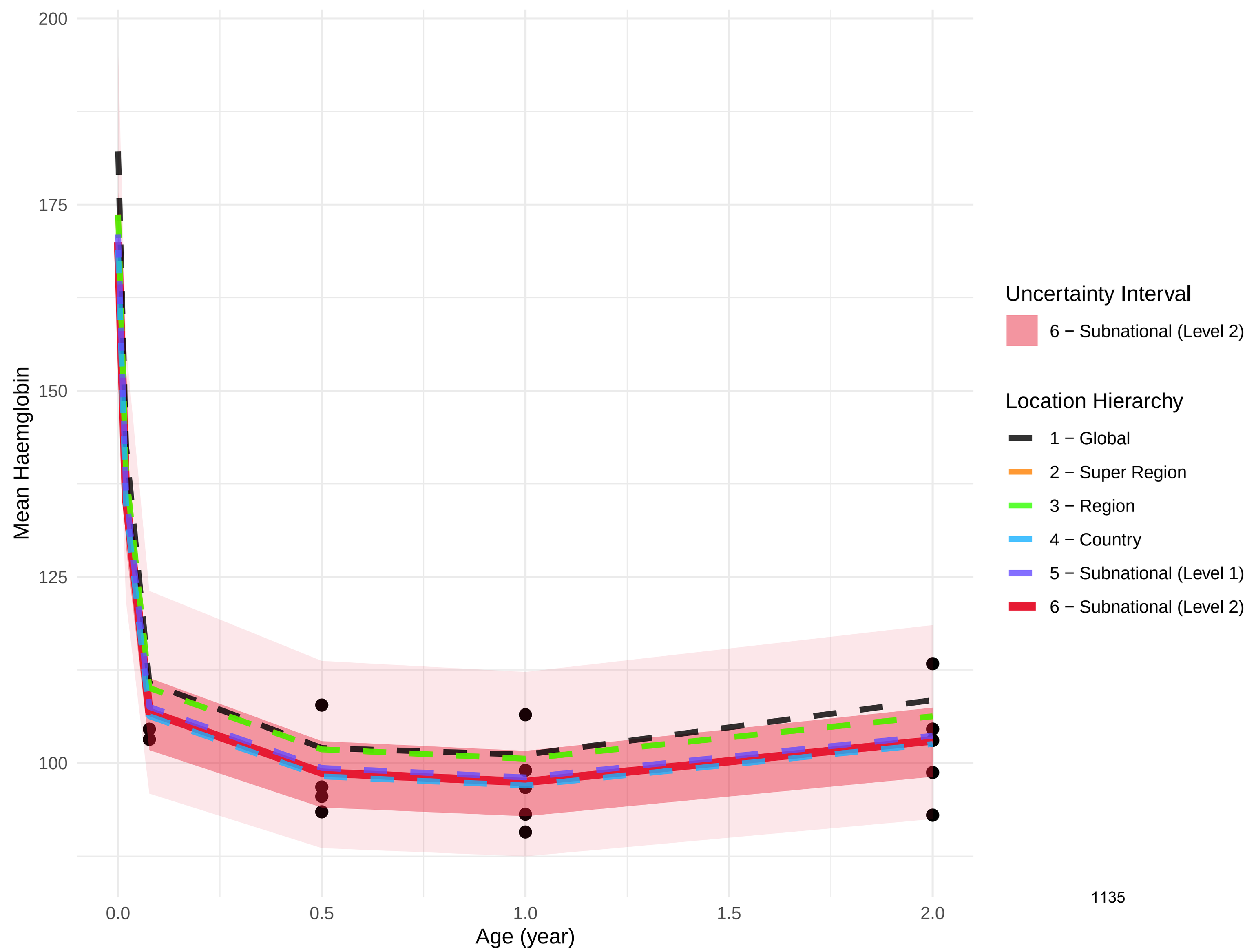

MR−BRT Age/Sex Fits − Non−pregnant Females <2 Years Old − Mean Haemglobin − Chhattisgarh, Urban
WHO−2001 Adjustment Method

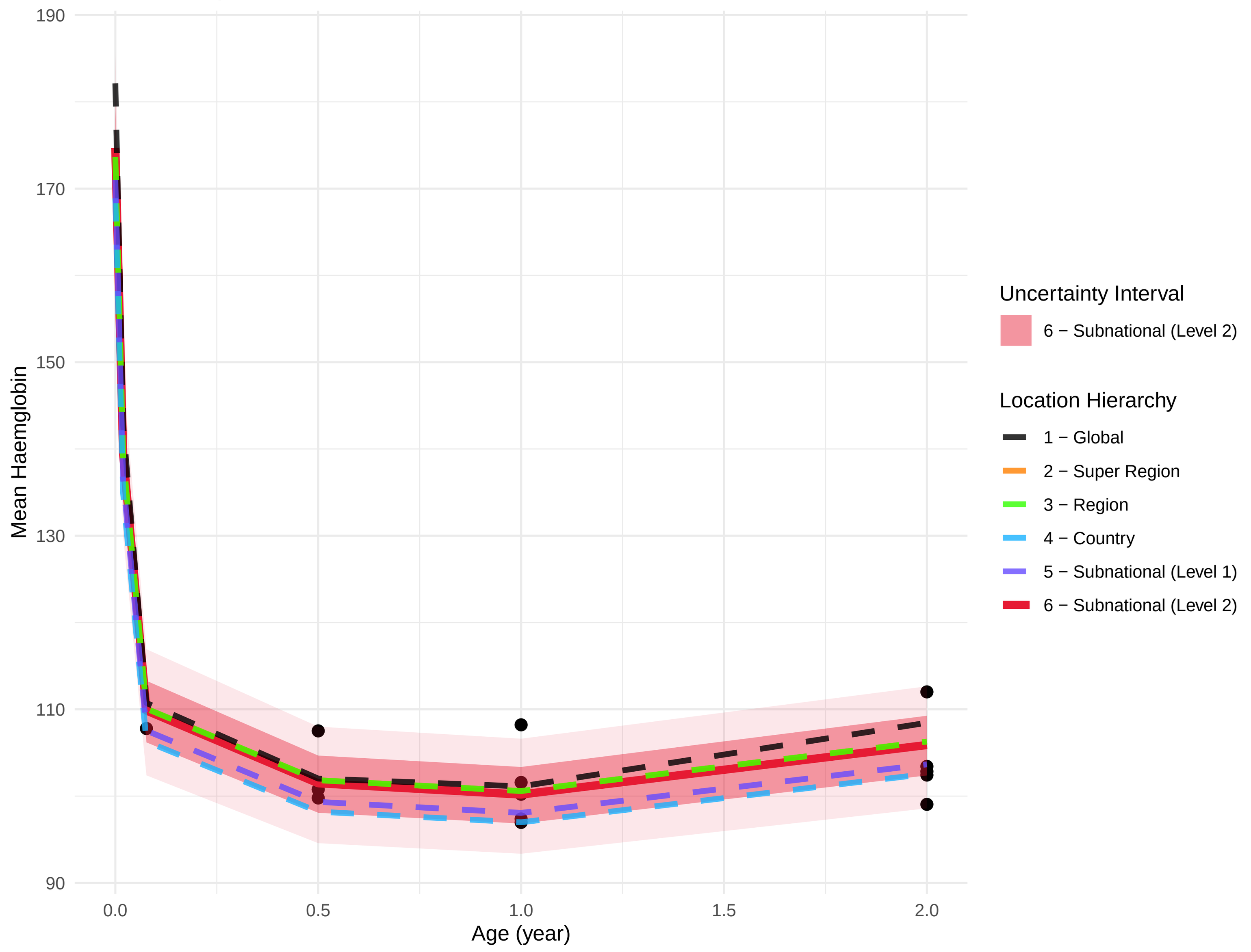

MR−BRT Age/Sex Fits − Non−pregnant Females <2 Years Old − Mean Haemglobin − Delhi
WHO−2001 Adjustment Method

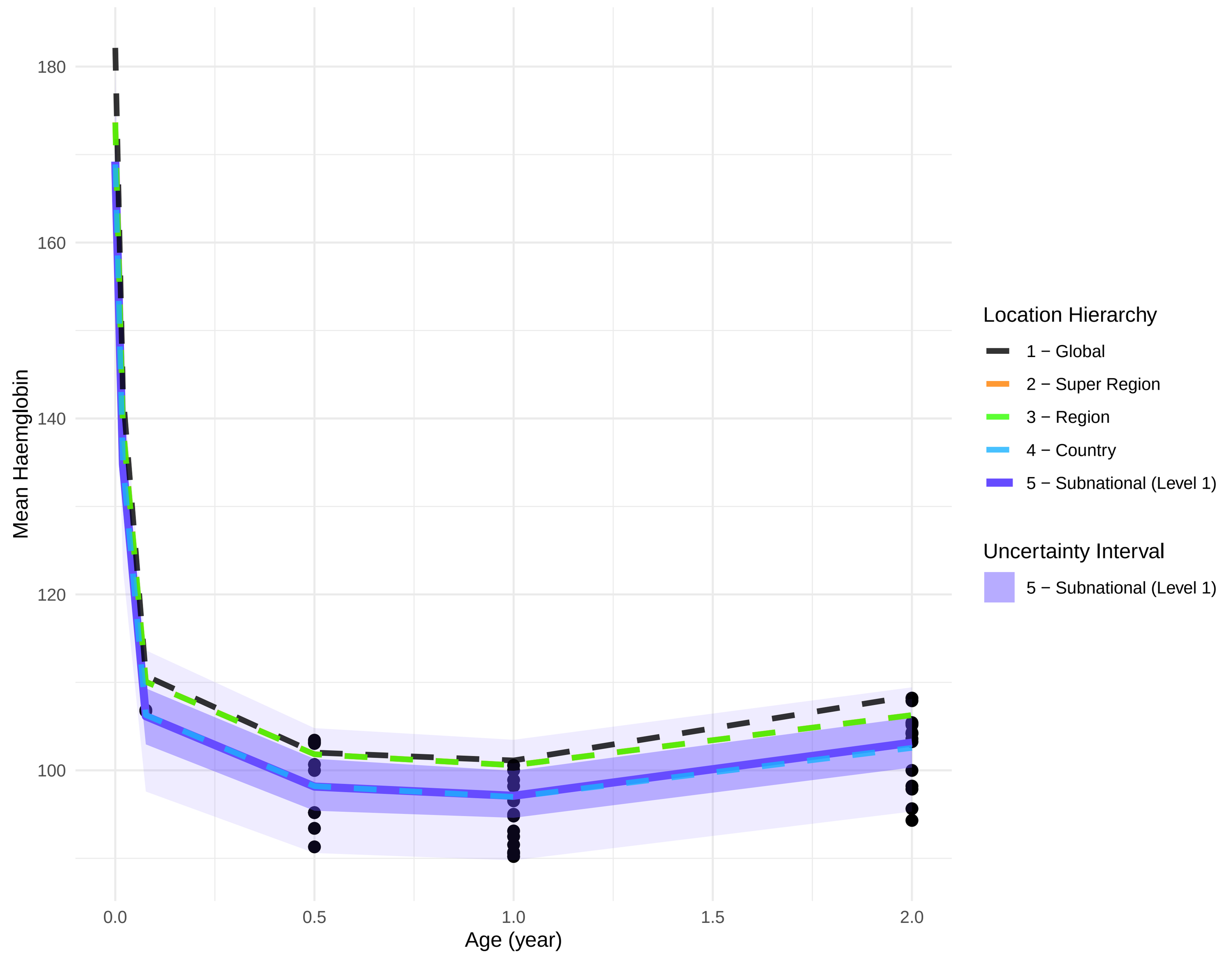

MR−BRT Age/Sex Fits − Non−pregnant Females <2 Years Old − Mean Haemglobin − Delhi, Rural
WHO−2001 Adjustment Method

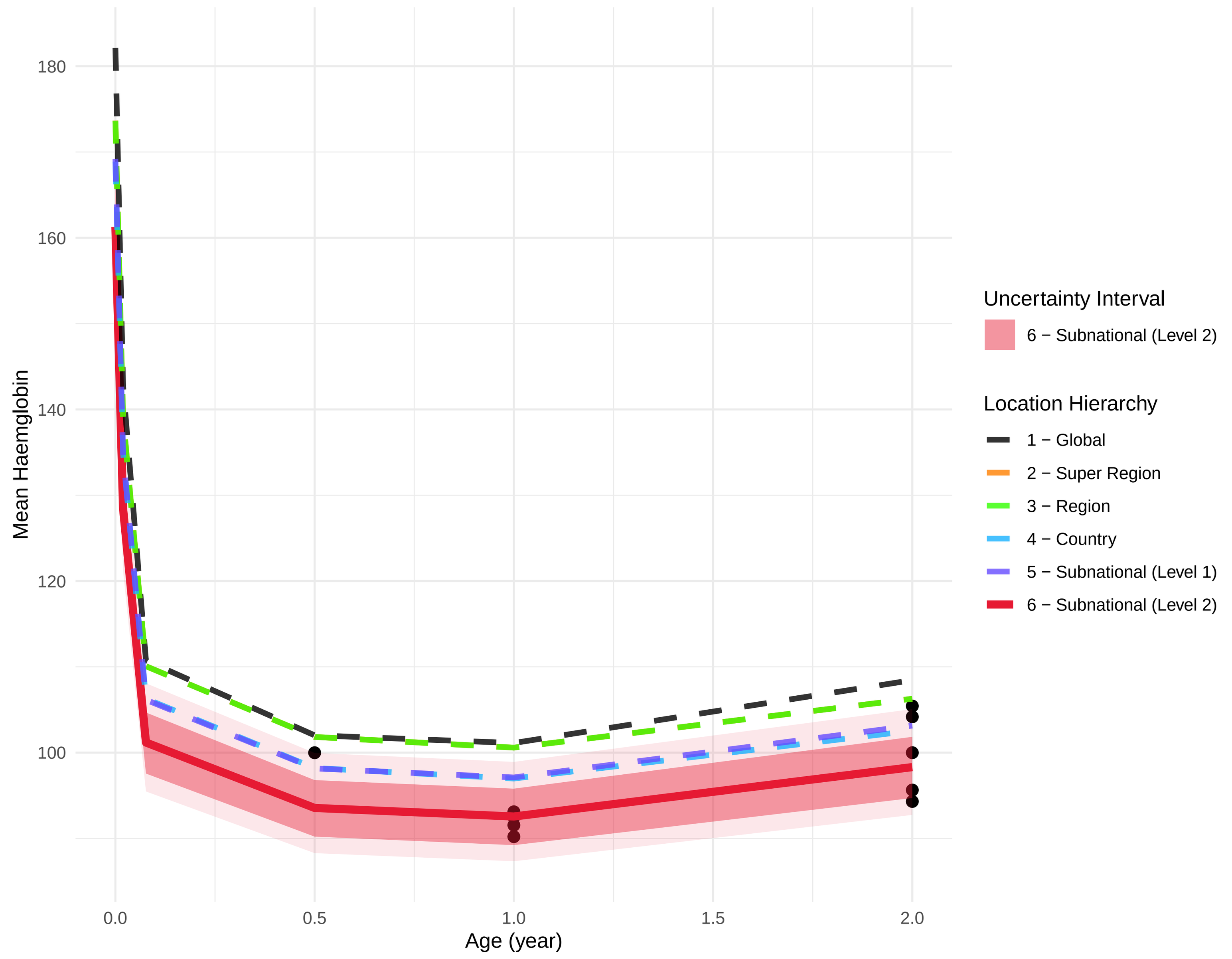

MR−BRT Age/Sex Fits − Non−pregnant Females <2 Years Old − Mean Haemglobin − Delhi, Urban
WHO−2001 Adjustment Method

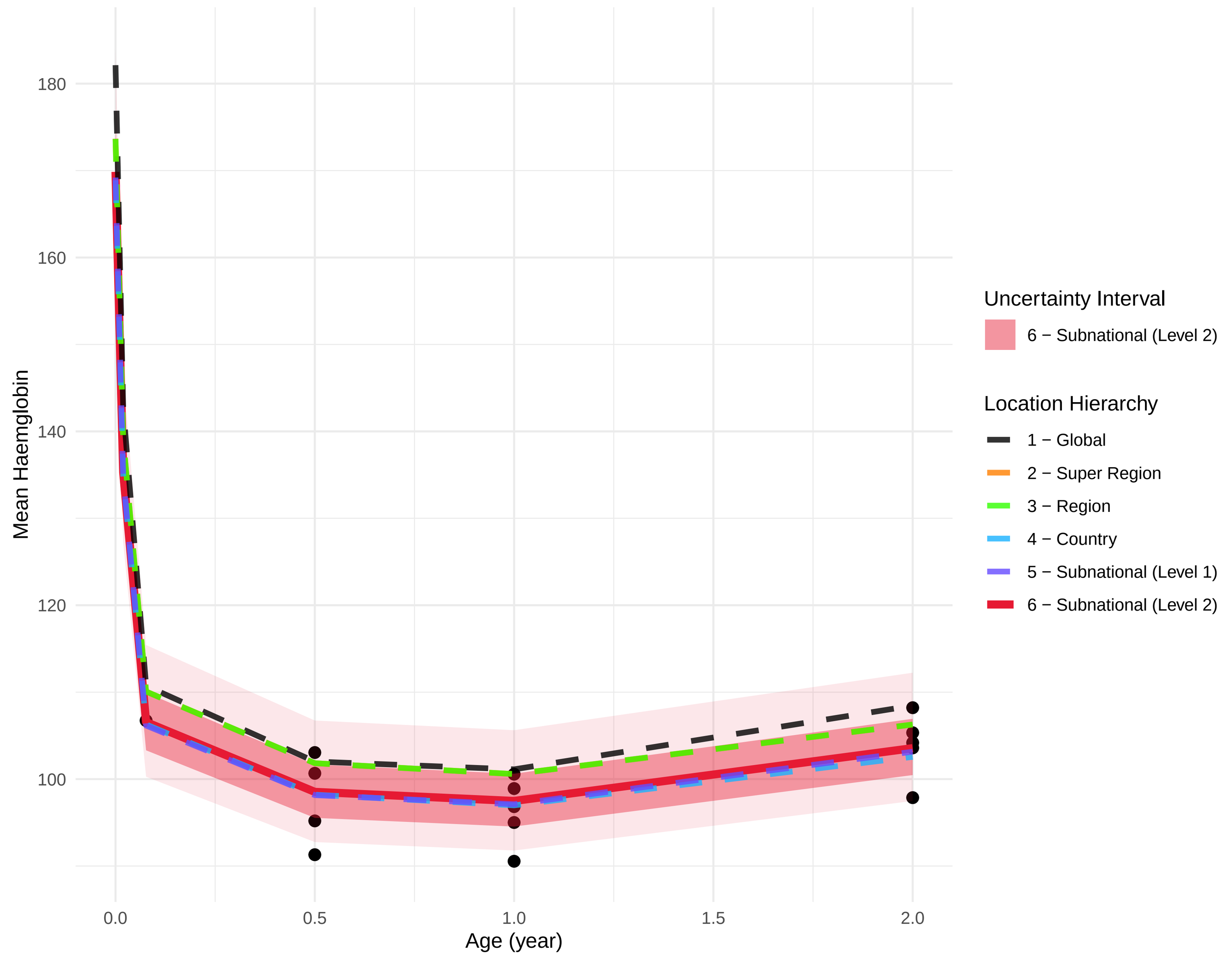

MR−BRT Age/Sex Fits − Non−pregnant Females <2 Years Old − Mean Haemglobin − Goa
WHO−2001 Adjustment Method

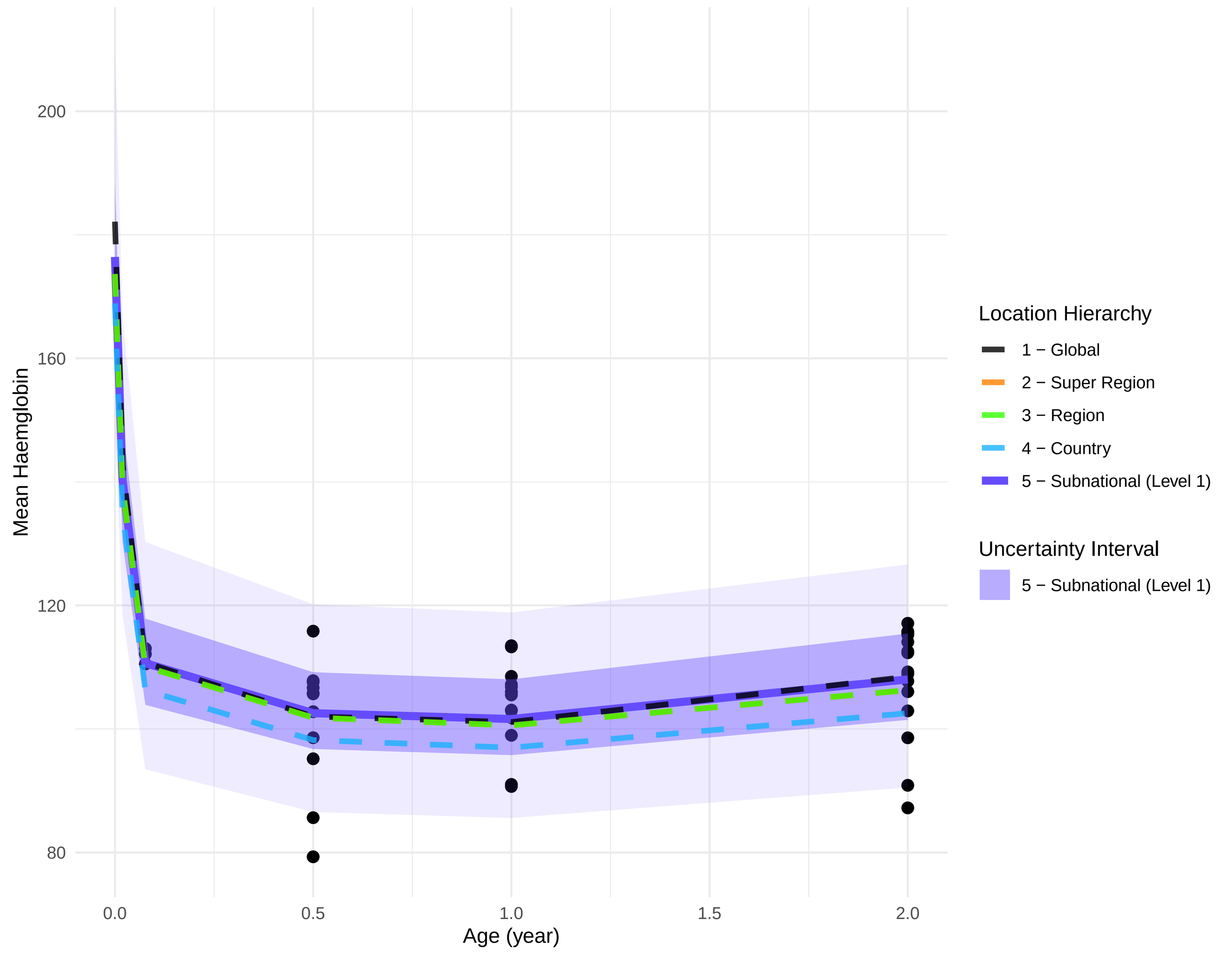

MR−BRT Age/Sex Fits − Non−pregnant Females <2 Years Old − Mean Haemglobin − Goa, Rural
WHO−2001 Adjustment Method

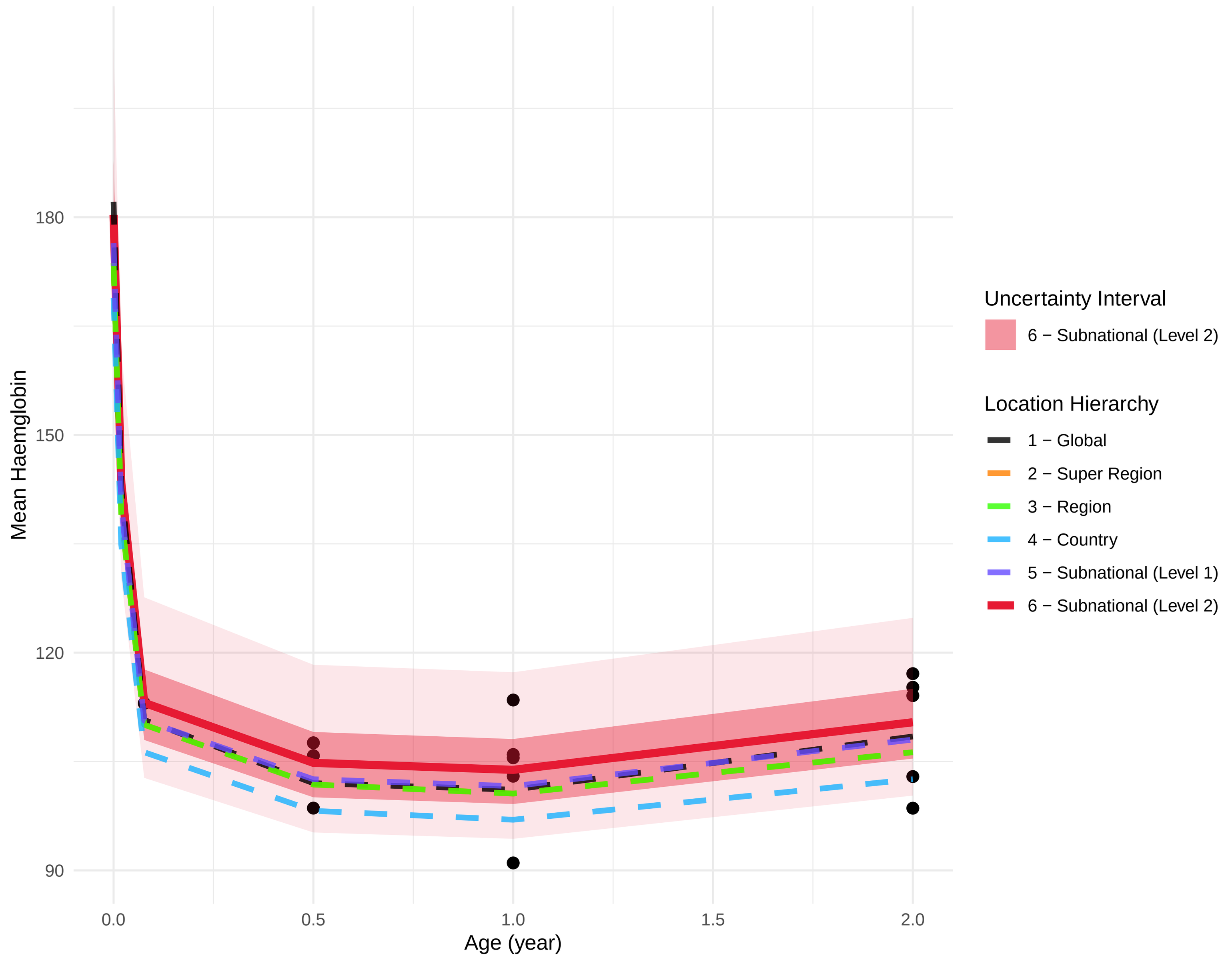

MR−BRT Age/Sex Fits − Non−pregnant Females <2 Years Old − Mean Haemglobin − Goa, Urban
WHO−2001 Adjustment Method

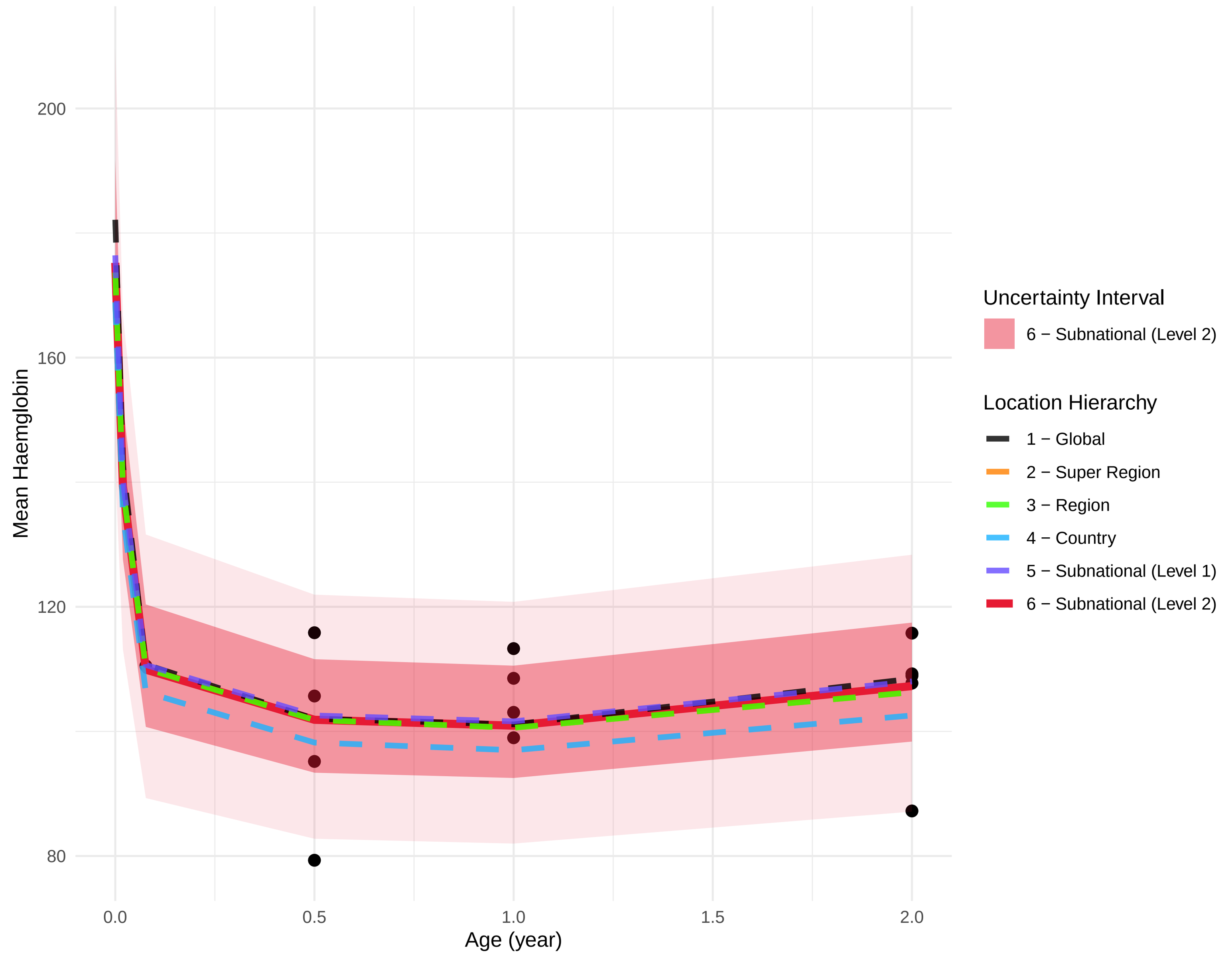

MR−BRT Age/Sex Fits − Non−pregnant Females <2 Years Old − Mean Haemglobin − Gujarat
WHO−2001 Adjustment Method

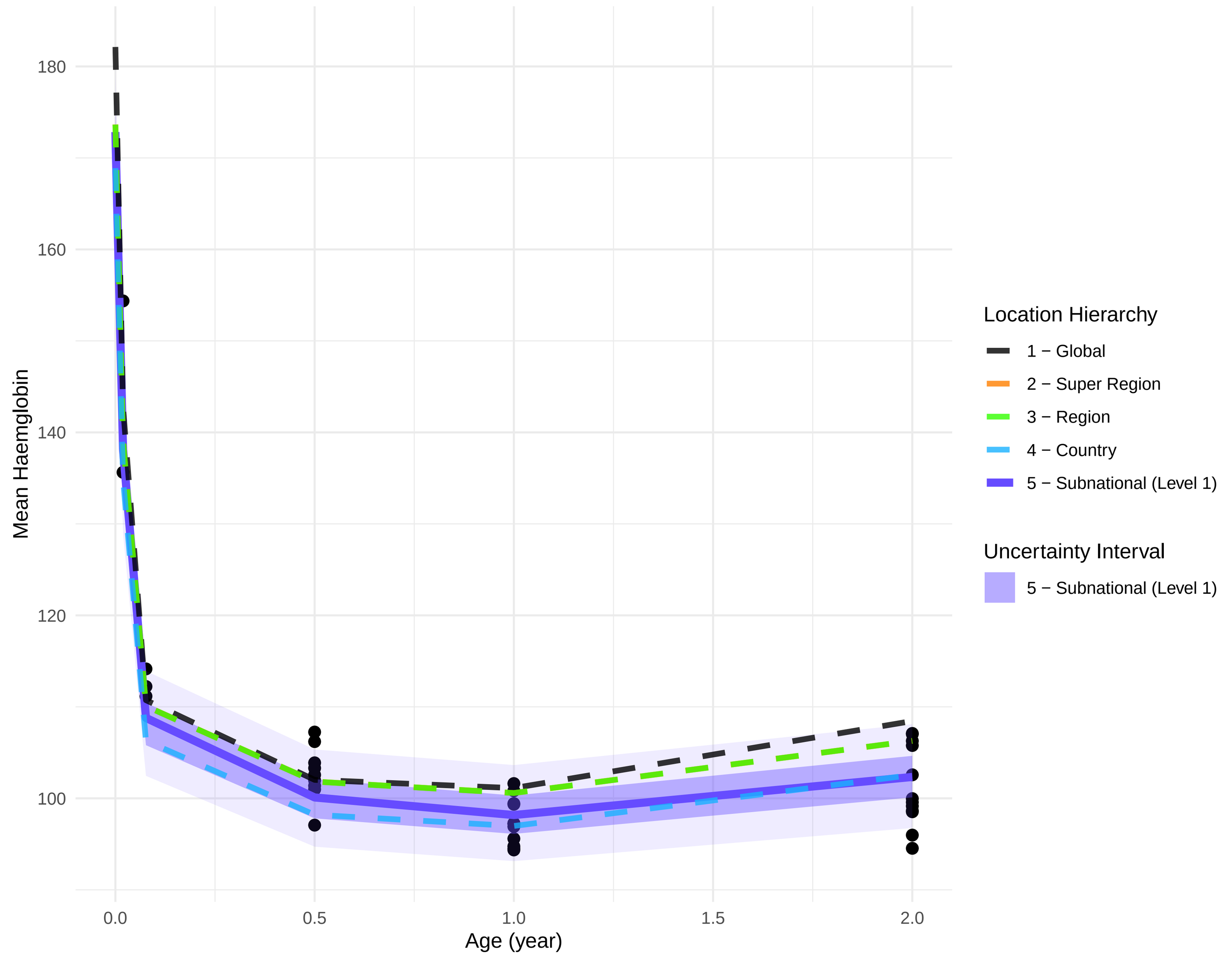

MR−BRT Age/Sex Fits − Non−pregnant Females <2 Years Old − Mean Haemglobin − Gujarat, Rural
WHO−2001 Adjustment Method

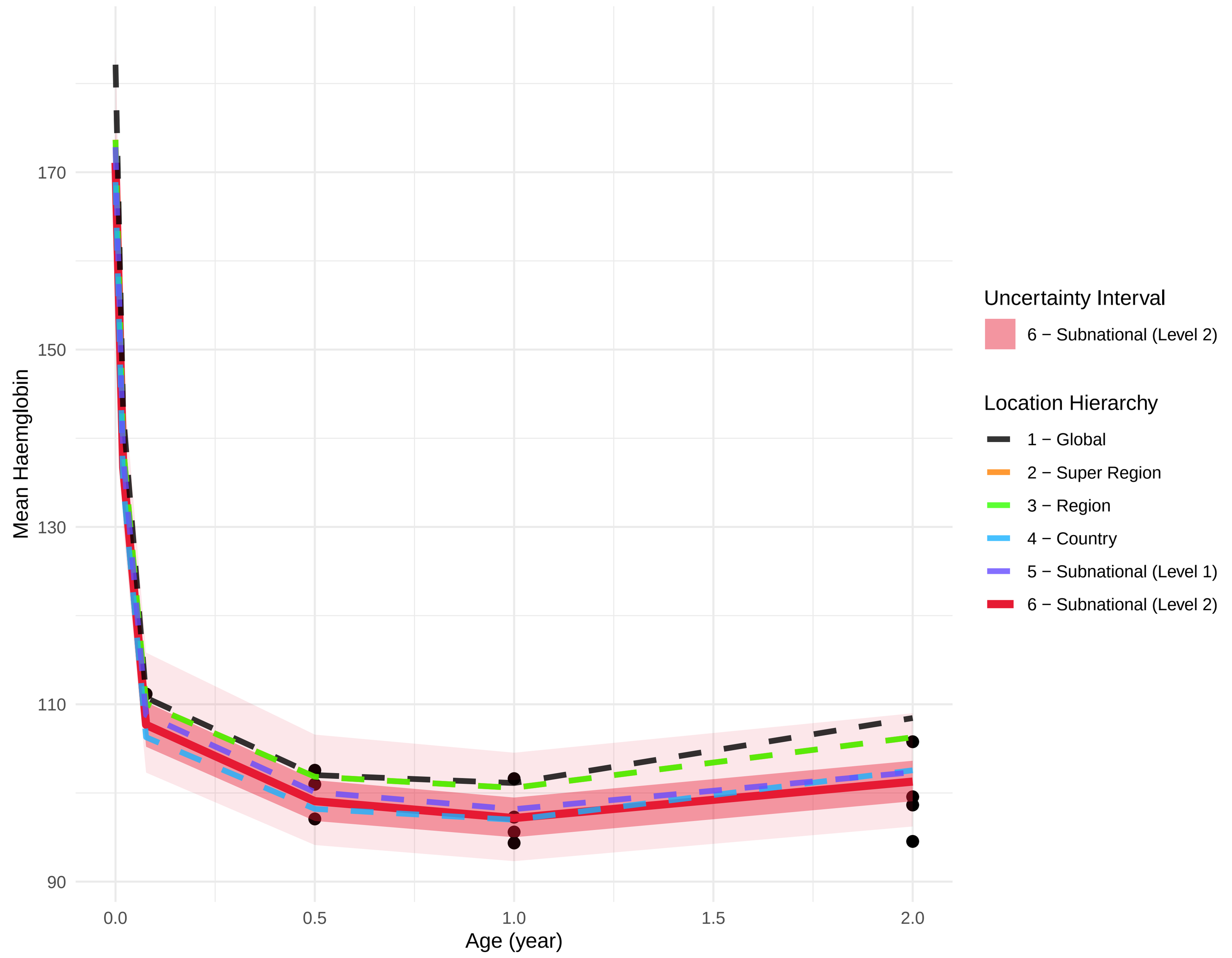

MR−BRT Age/Sex Fits − Non−pregnant Females <2 Years Old − Mean Haemglobin − Gujarat, Urban
WHO−2001 Adjustment Method

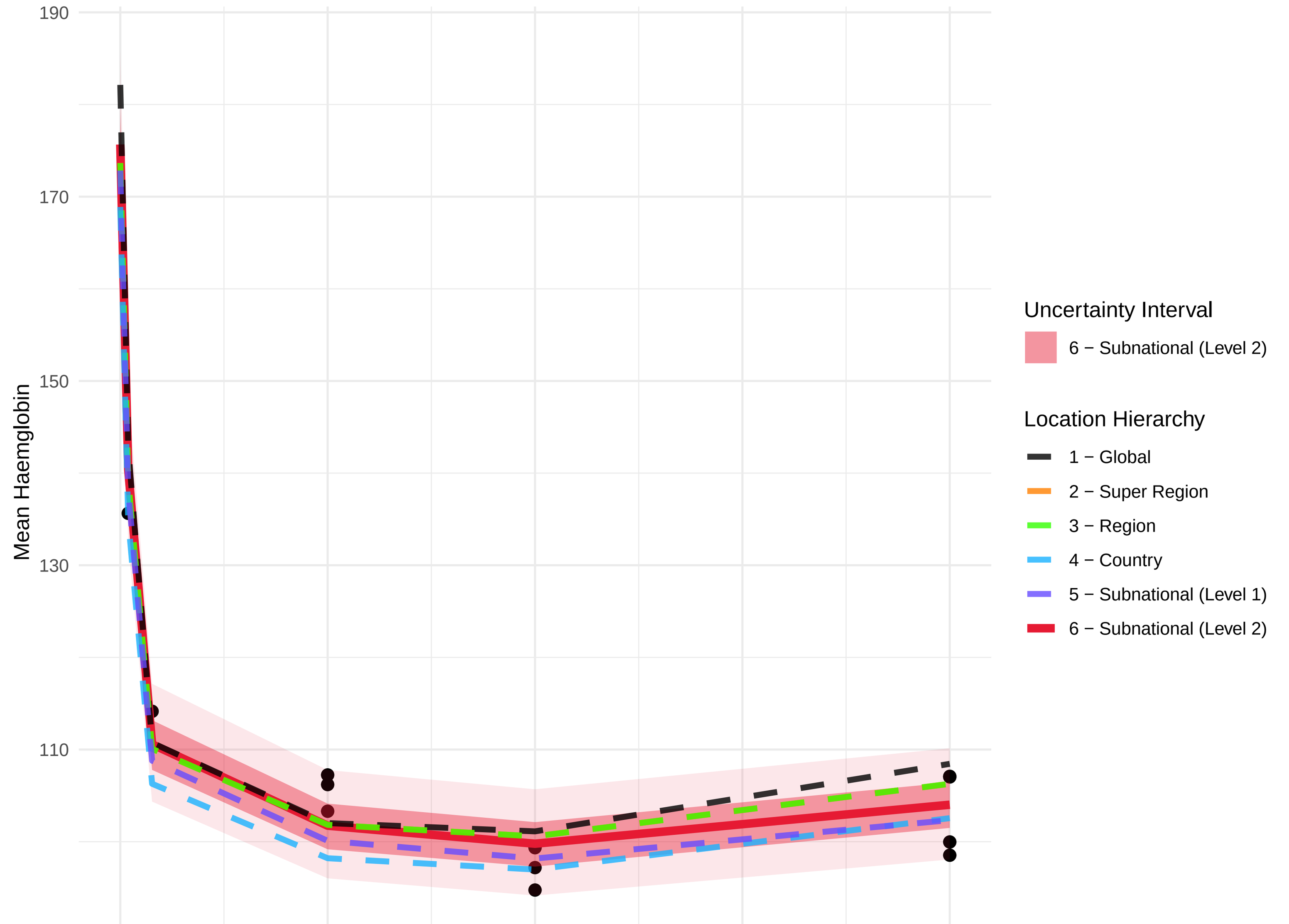

MR−BRT Age/Sex Fits − Non−pregnant Females <2 Years Old − Mean Haemglobin − Haryana
WHO−2001 Adjustment Method

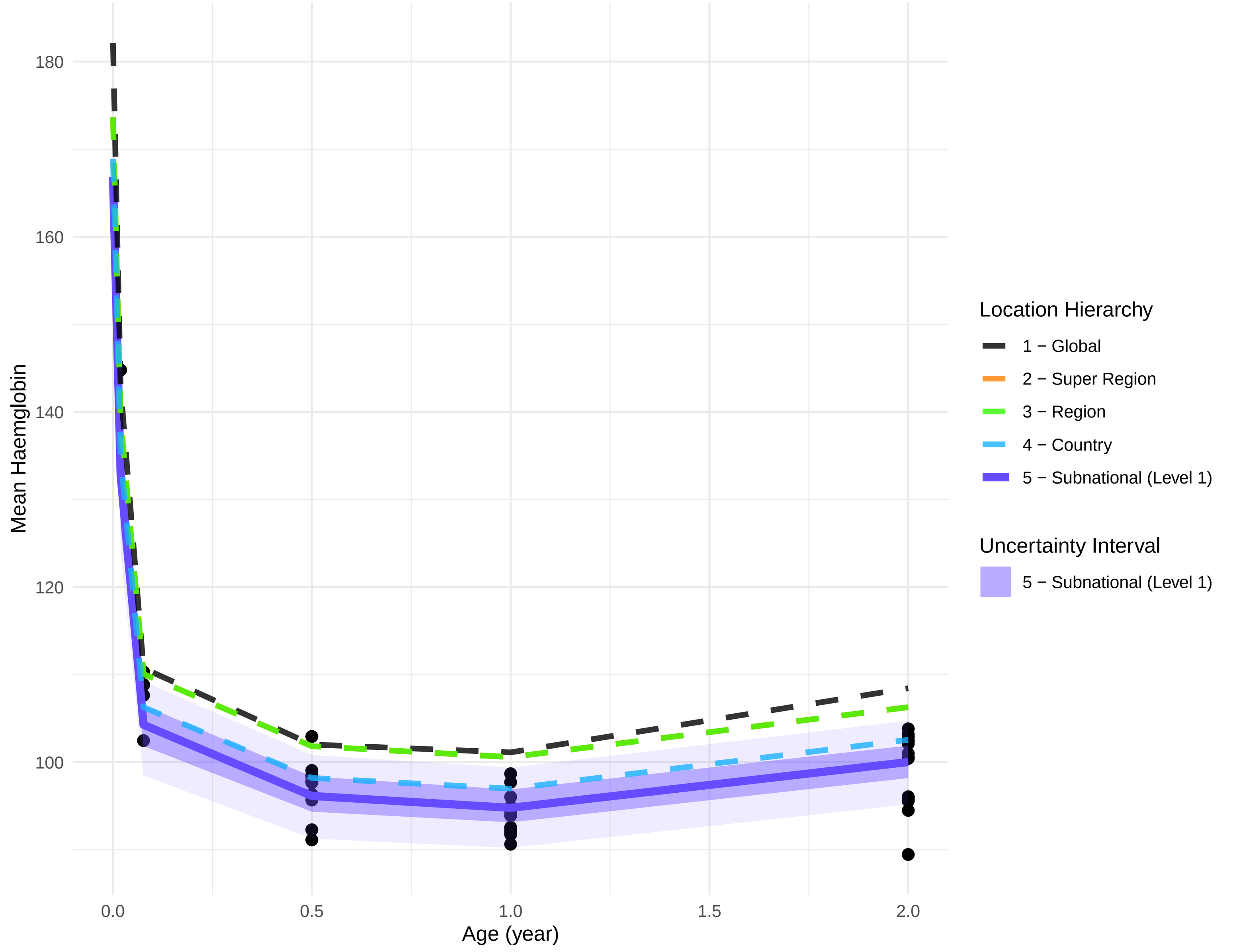

MR−BRT Age/Sex Fits − Non−pregnant Females <2 Years Old − Mean Haemglobin − Haryana, Rural
WHO−2001 Adjustment Method

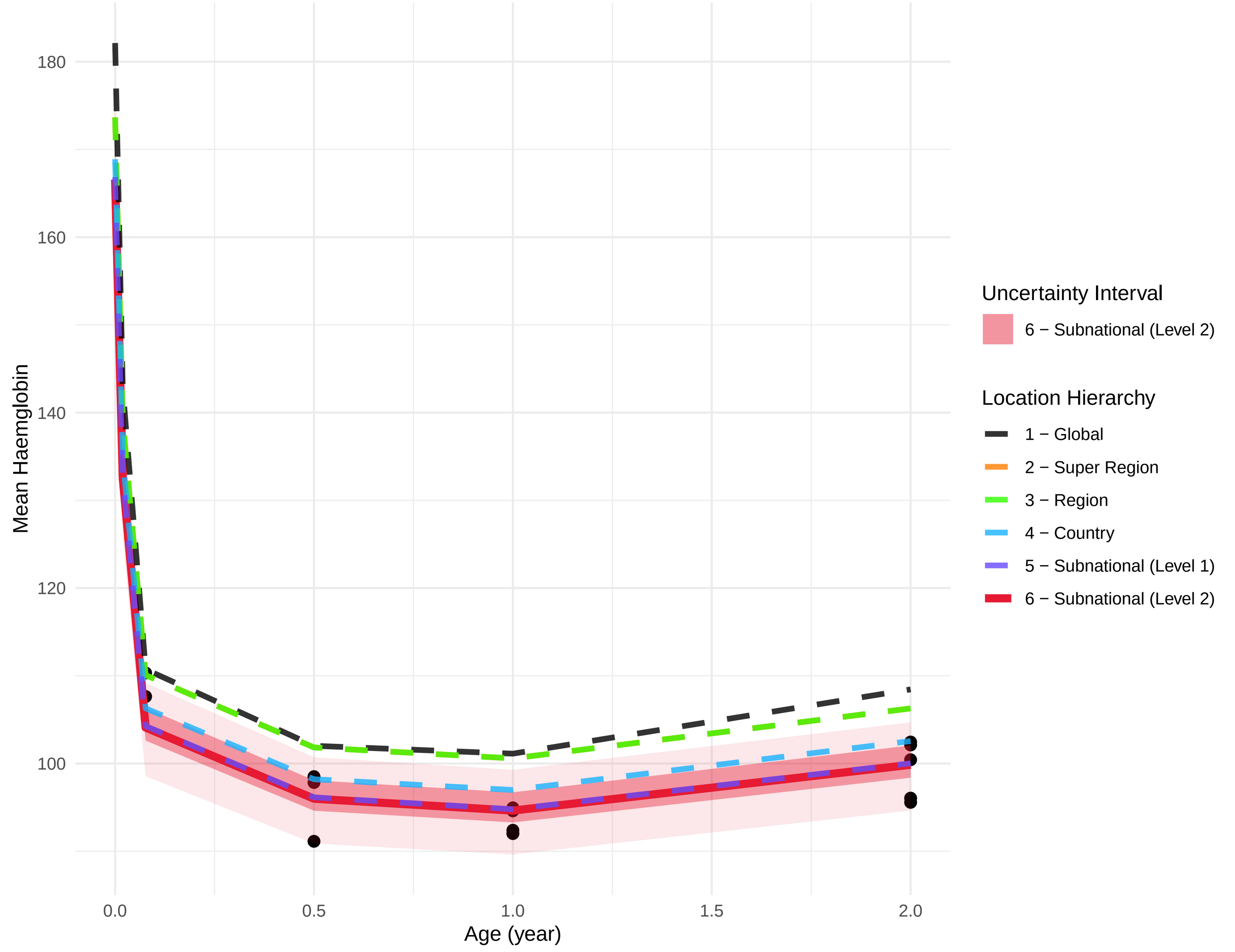

MR−BRT Age/Sex Fits − Non−pregnant Females <2 Years Old − Mean Haemglobin − Haryana, Urban
WHO−2001 Adjustment Method

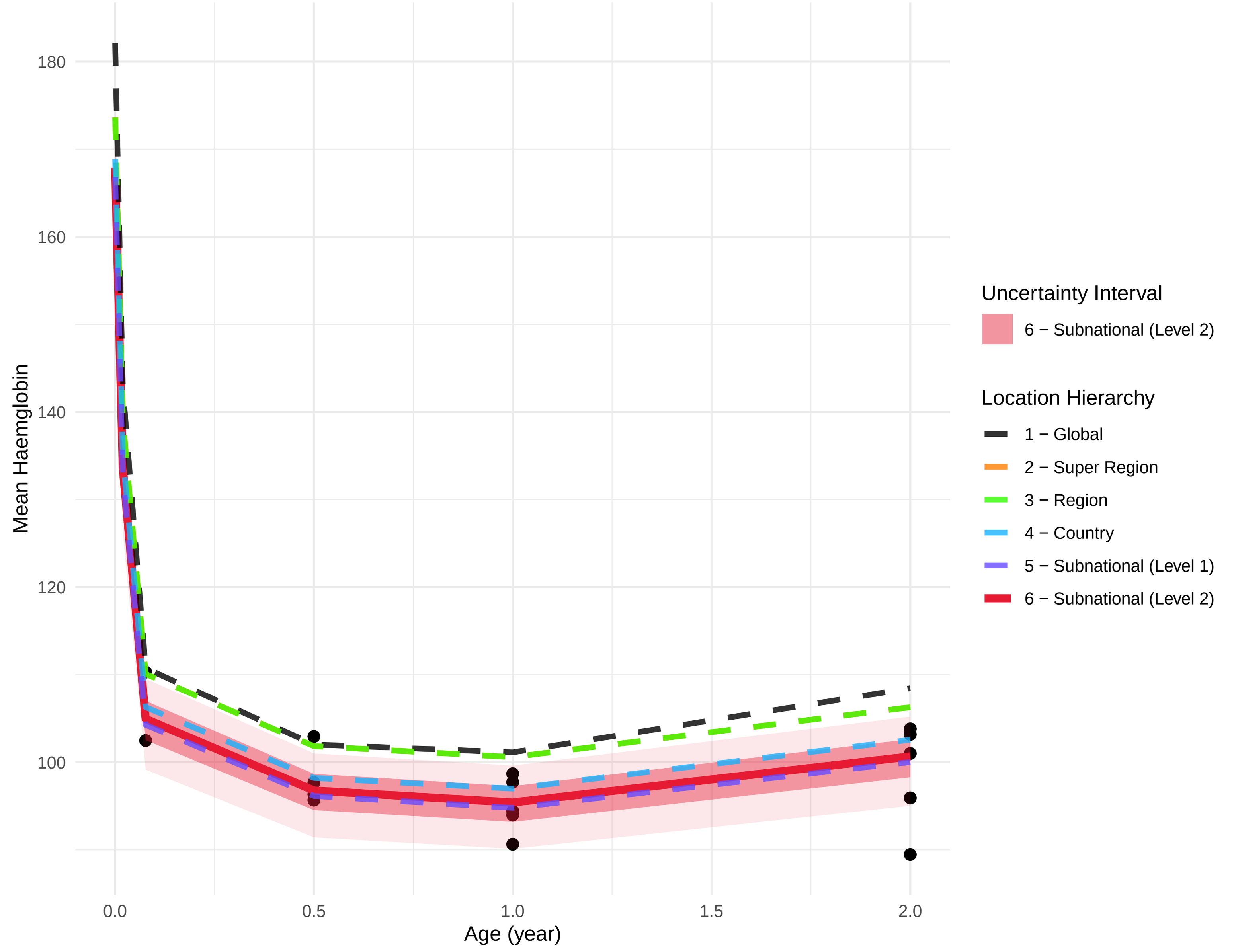

MR−BRT Age/Sex Fits − Non−pregnant Females <2 Years Old − Mean Haemglobin − Himachal Pradesh
WHO−2001 Adjustment Method

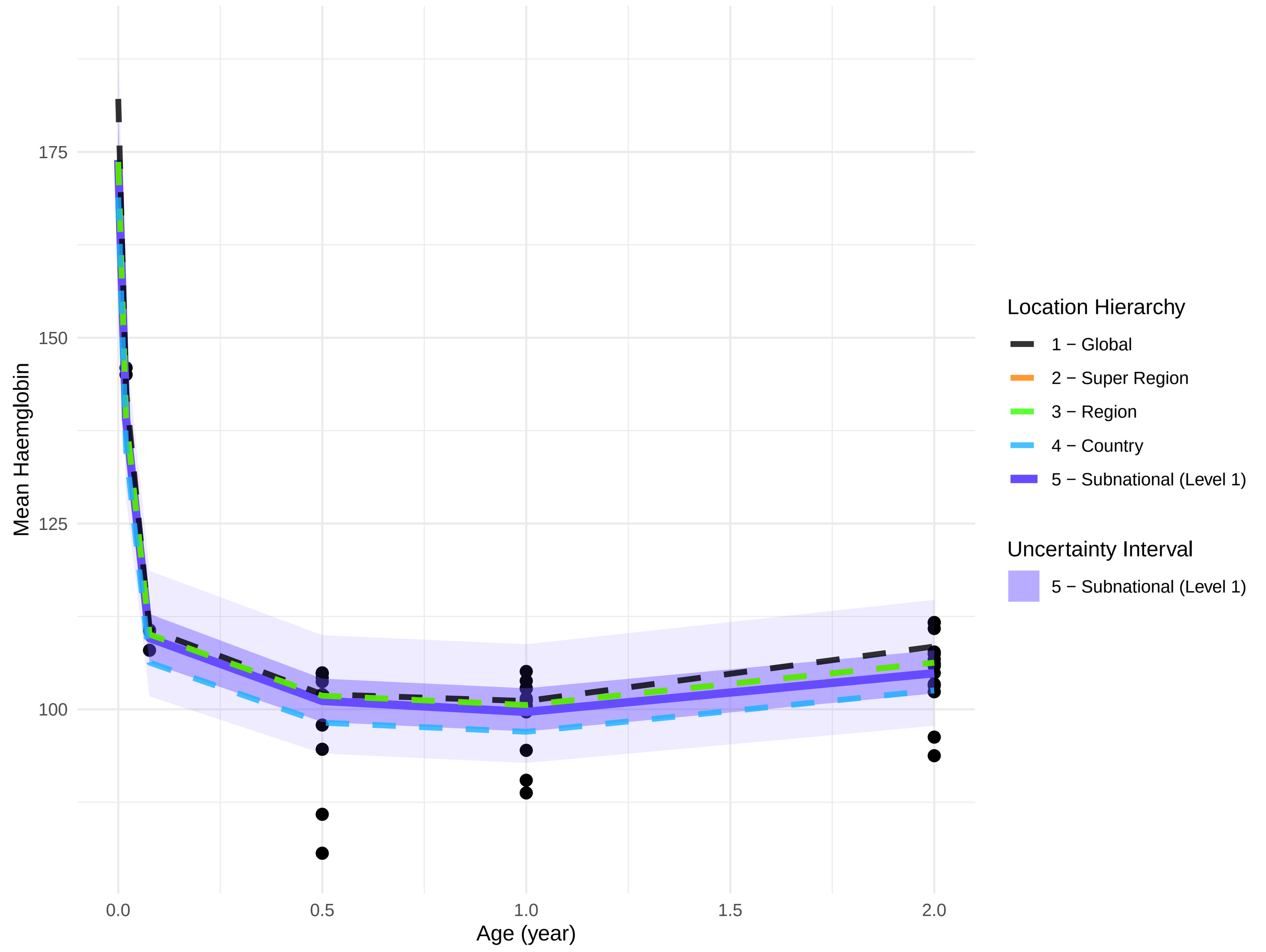

MR−BRT Age/Sex Fits − Non−pregnant Females <2 Years Old − Mean Haemglobin − Himachal Pradesh, Rural
WHO−2001 Adjustment Method

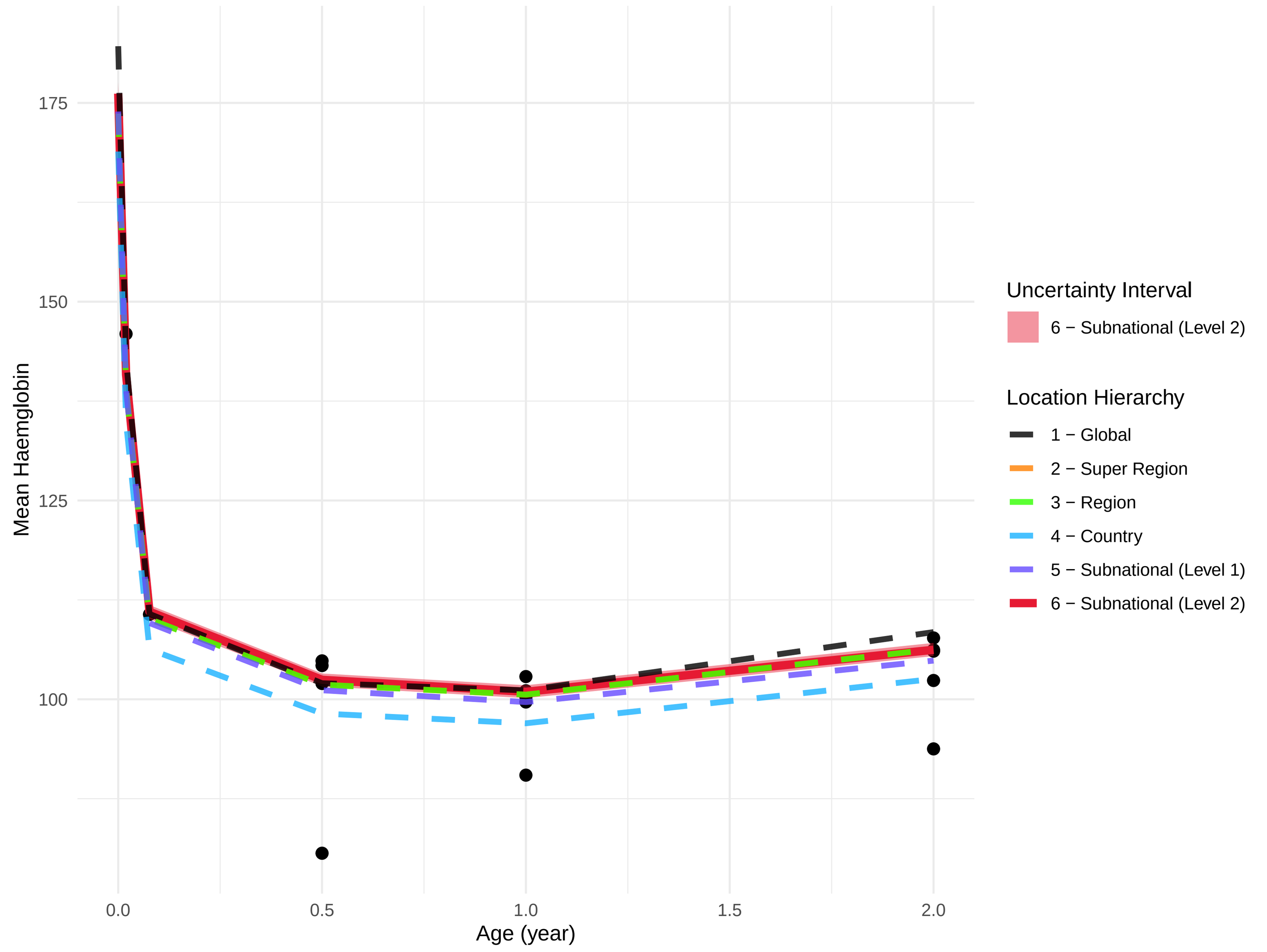

MR−BRT Age/Sex Fits − Non−pregnant Females <2 Years Old − Mean Haemglobin − Himachal Pradesh, Urban
WHO−2001 Adjustment Method

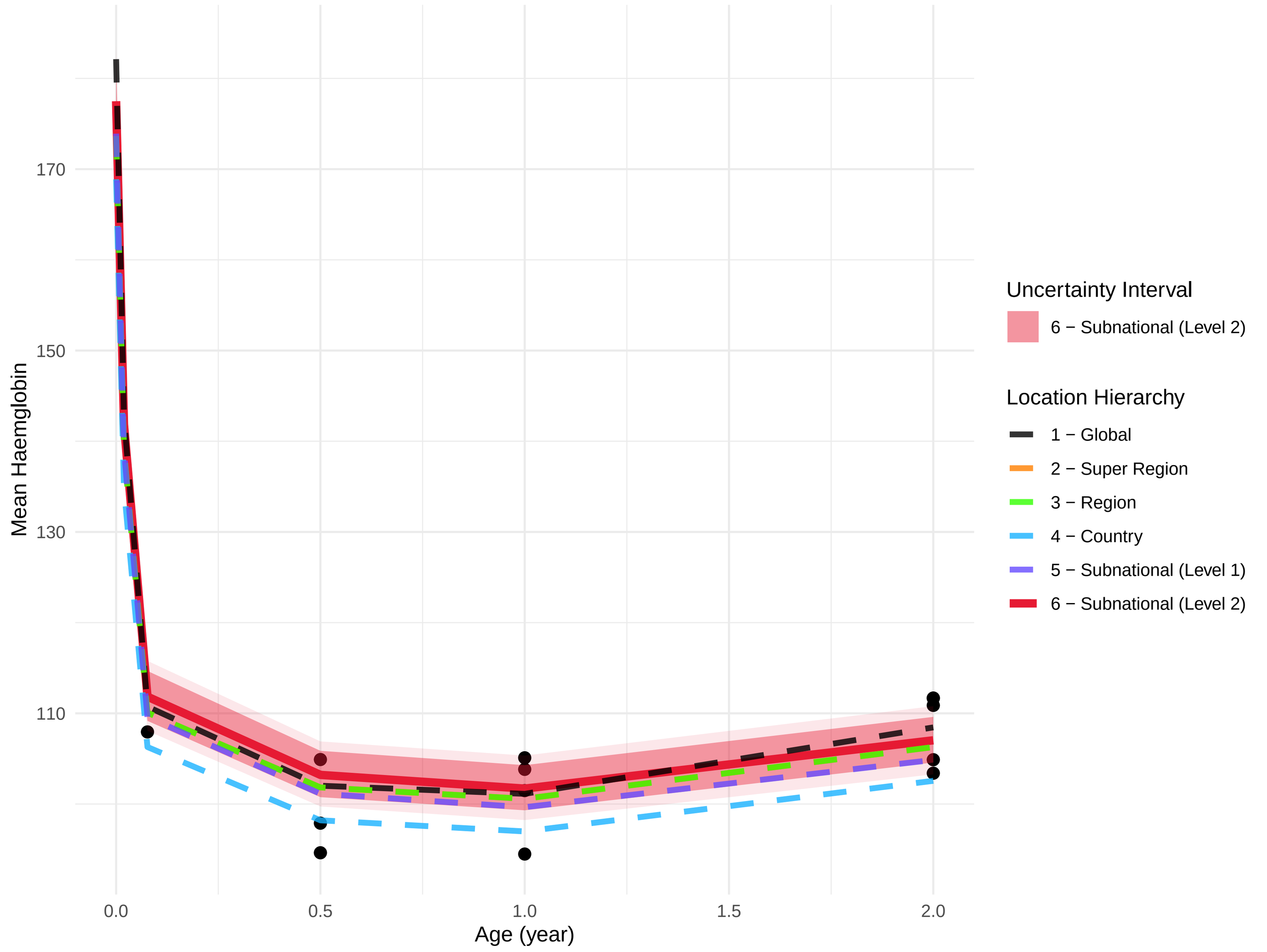

MR−BRT Age/Sex Fits − Non−pregnant Females <2 Years Old − Mean Haemglobin − Jammu & Kashmir and Ladakh
WHO−2001 Adjustment Method

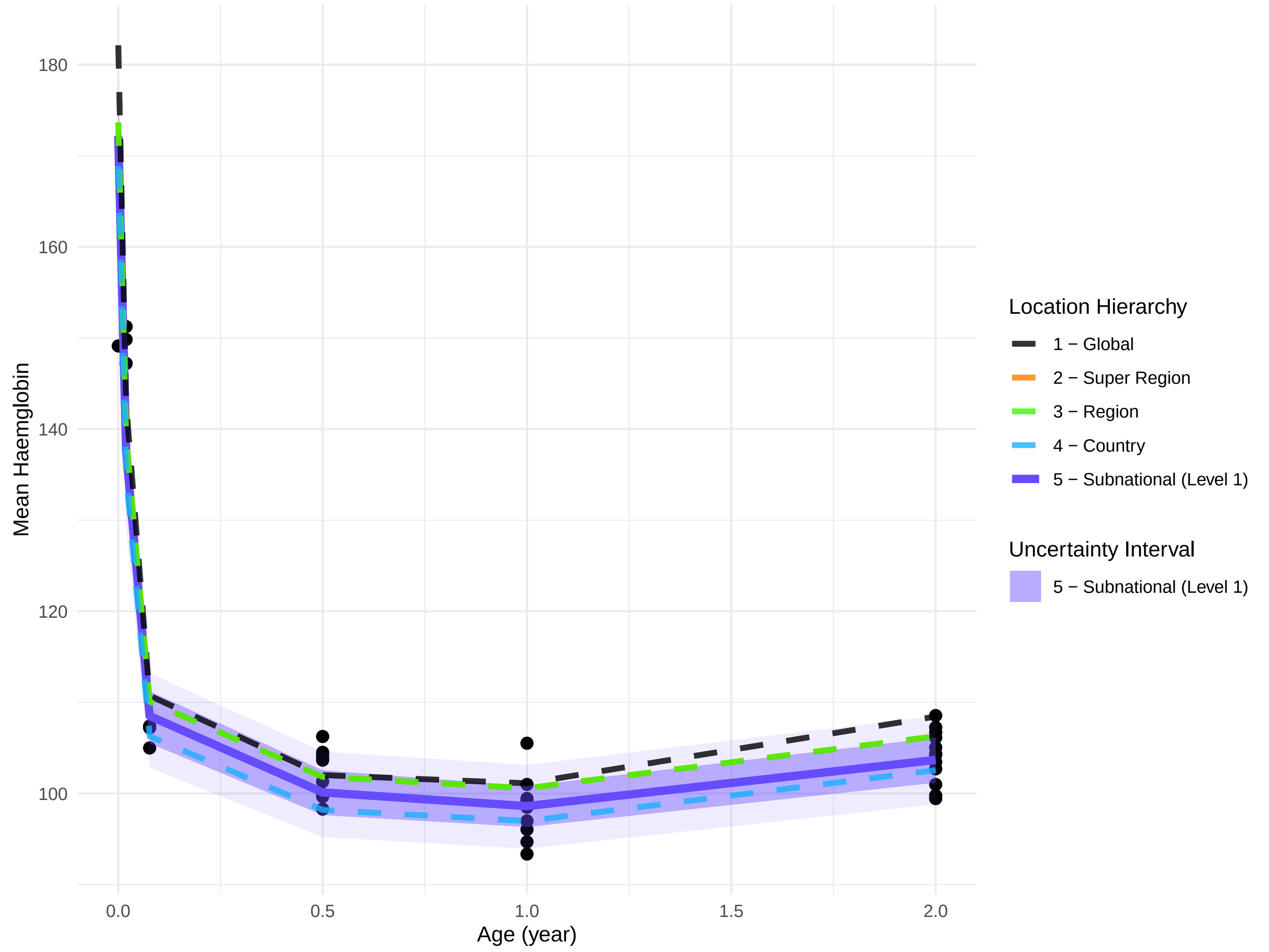

MR−BRT Age/Sex Fits − Non−pregnant Females <2 Years Old − Mean Haemglobin − Jammu & Kashmir and Ladakh, Rural
WHO−2001 Adjustment Method

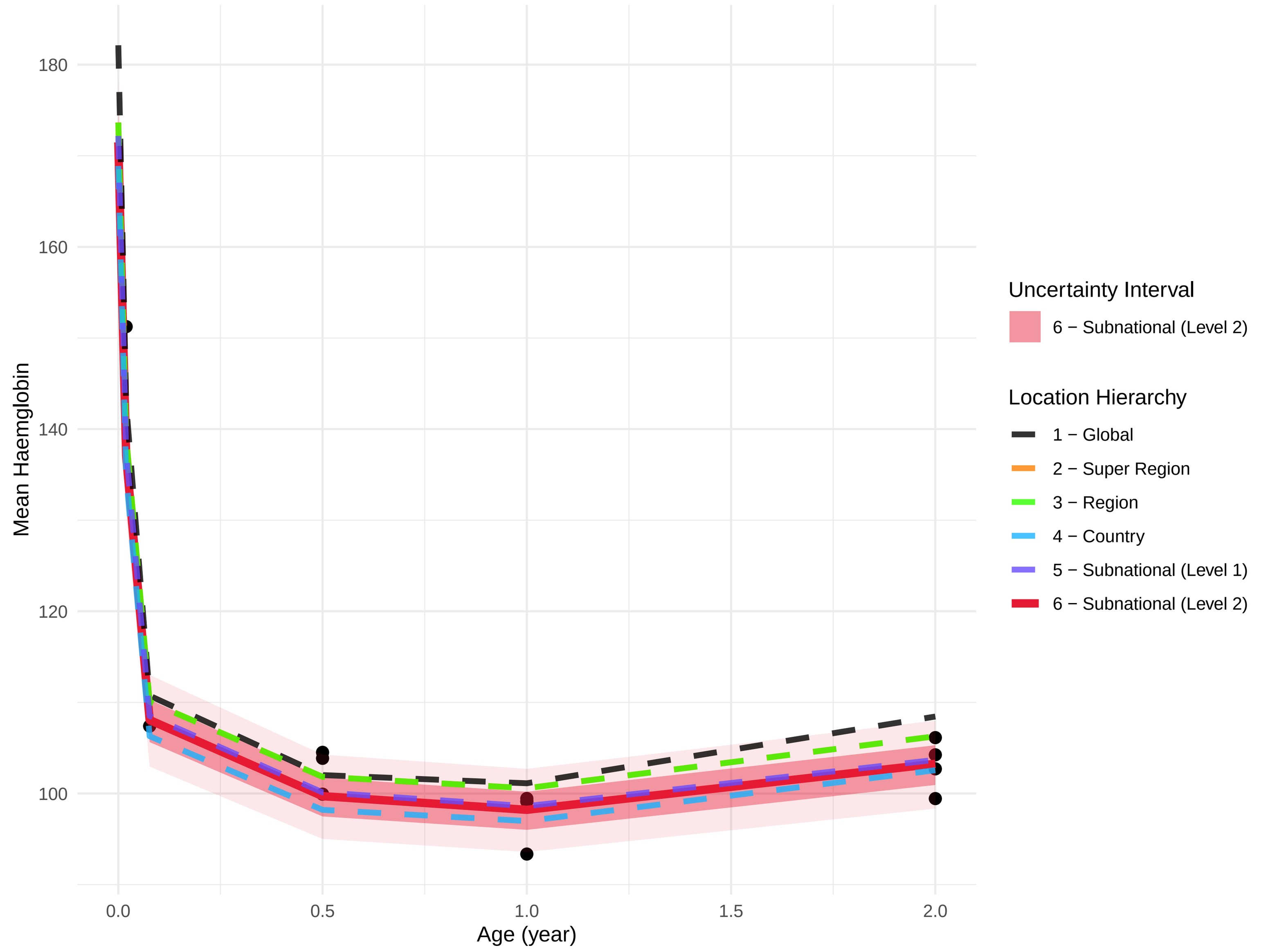

MR−BRT Age/Sex Fits − Non−pregnant Females <2 Years Old − Mean Haemglobin − Jammu & Kashmir and Ladakh, Urban
WHO−2001 Adjustment Method

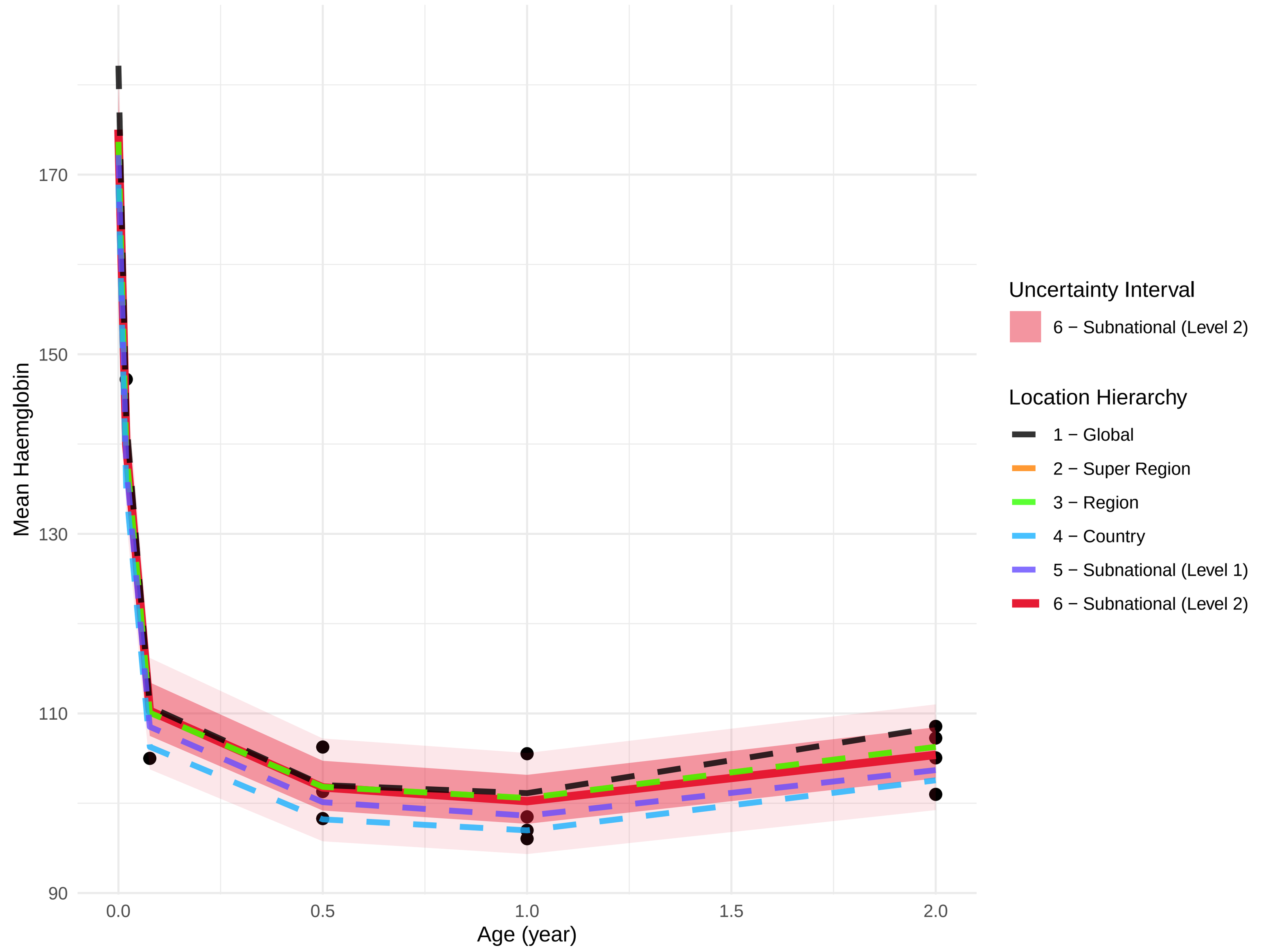

MR−BRT Age/Sex Fits − Non−pregnant Females <2 Years Old − Mean Haemglobin − Jharkhand
WHO−2001 Adjustment Method

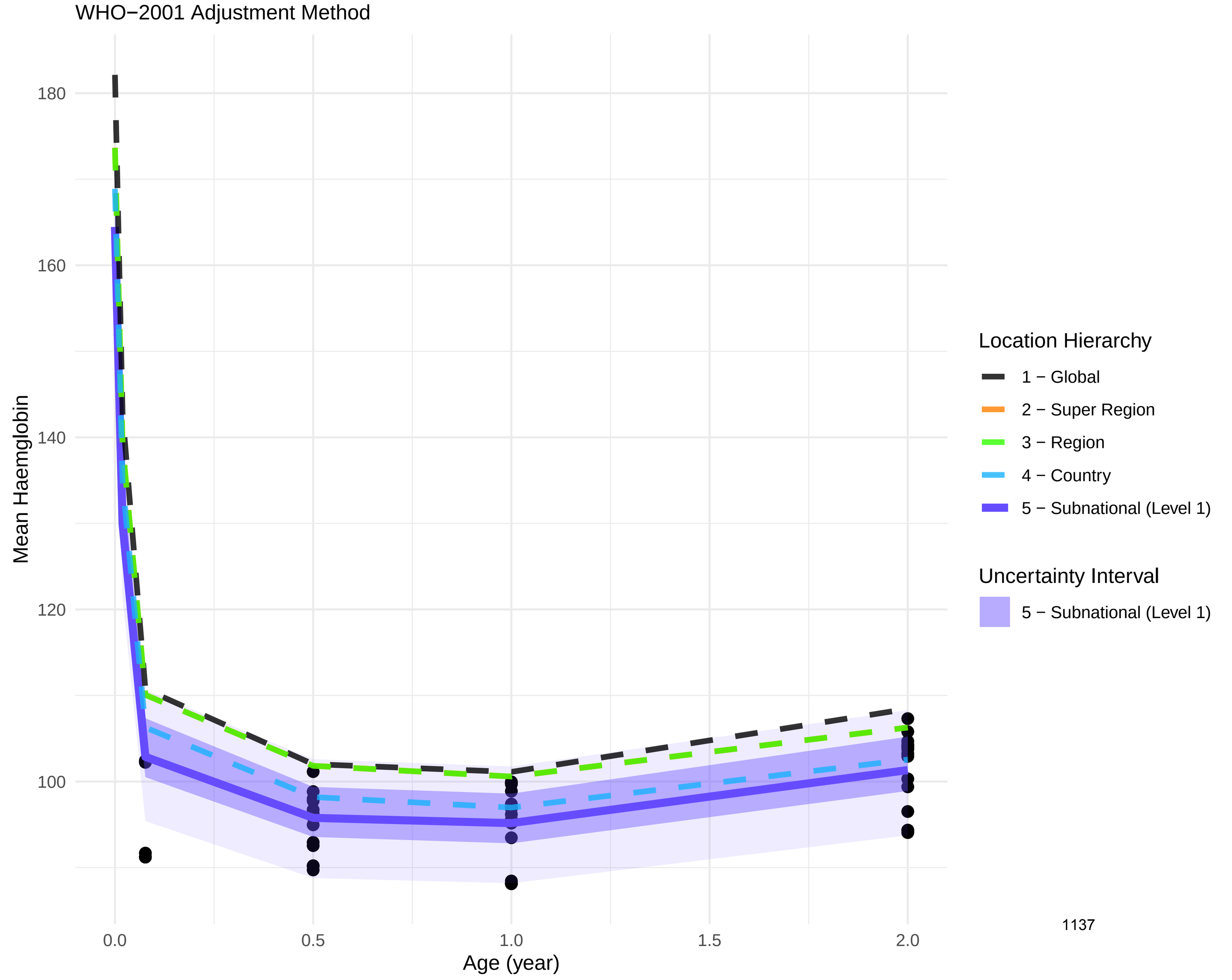

MR−BRT Age/Sex Fits − Non-pregnant Females <2 Years Old − Mean Haemglobin − Jharkhand, Rural
WHO−2001 Adjustment Method

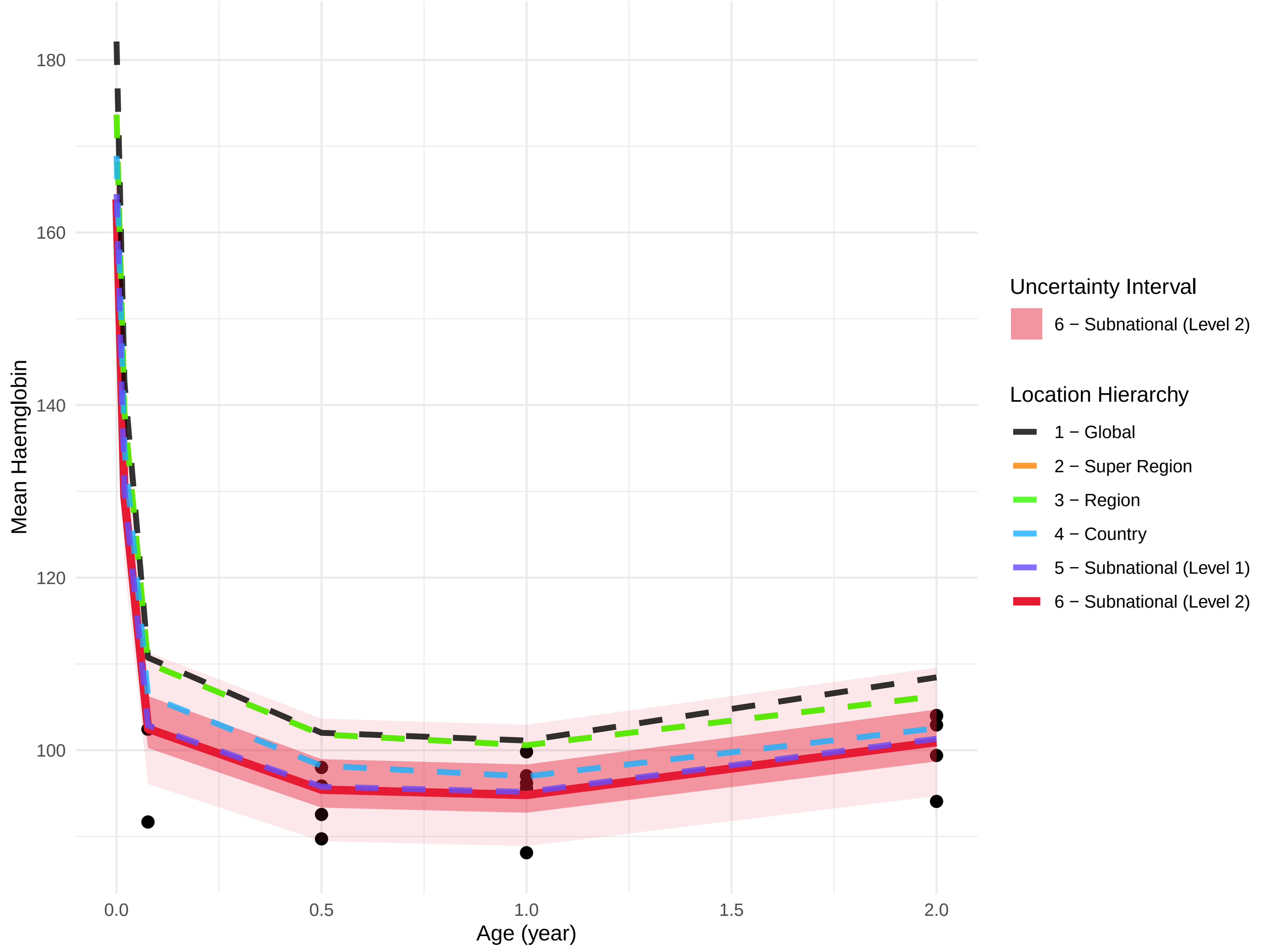

MR−BRT Age/Sex Fits − Non-pregnant Females <2 Years Old − Mean Haemglobin − Jharkhand, Urban
WHO−2001 Adjustment Method

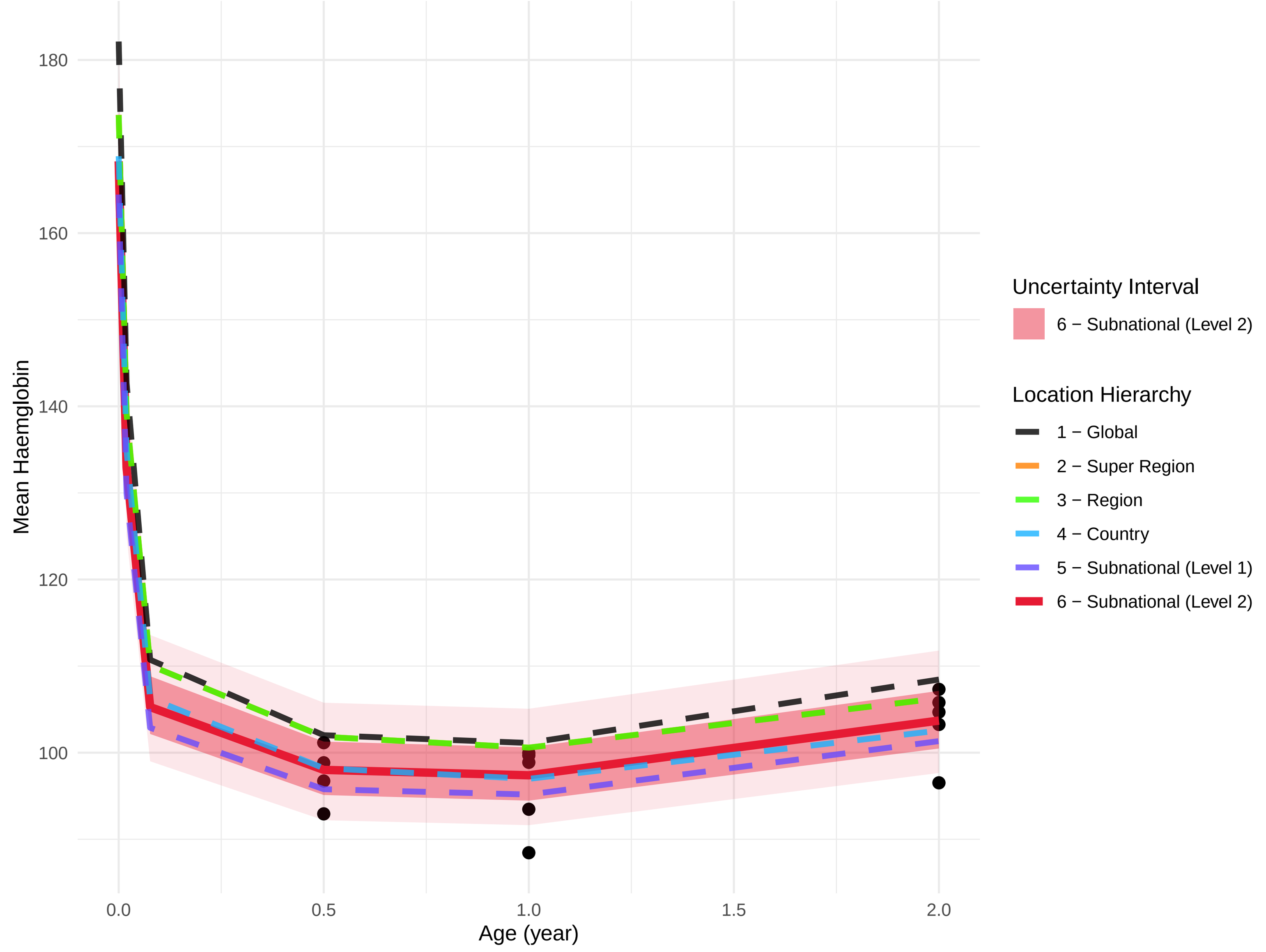

MR−BRT Age/Sex Fits − Non-pregnant Females <2 Years Old − Mean Haemglobin − Karnataka
WHO−2001 Adjustment Method

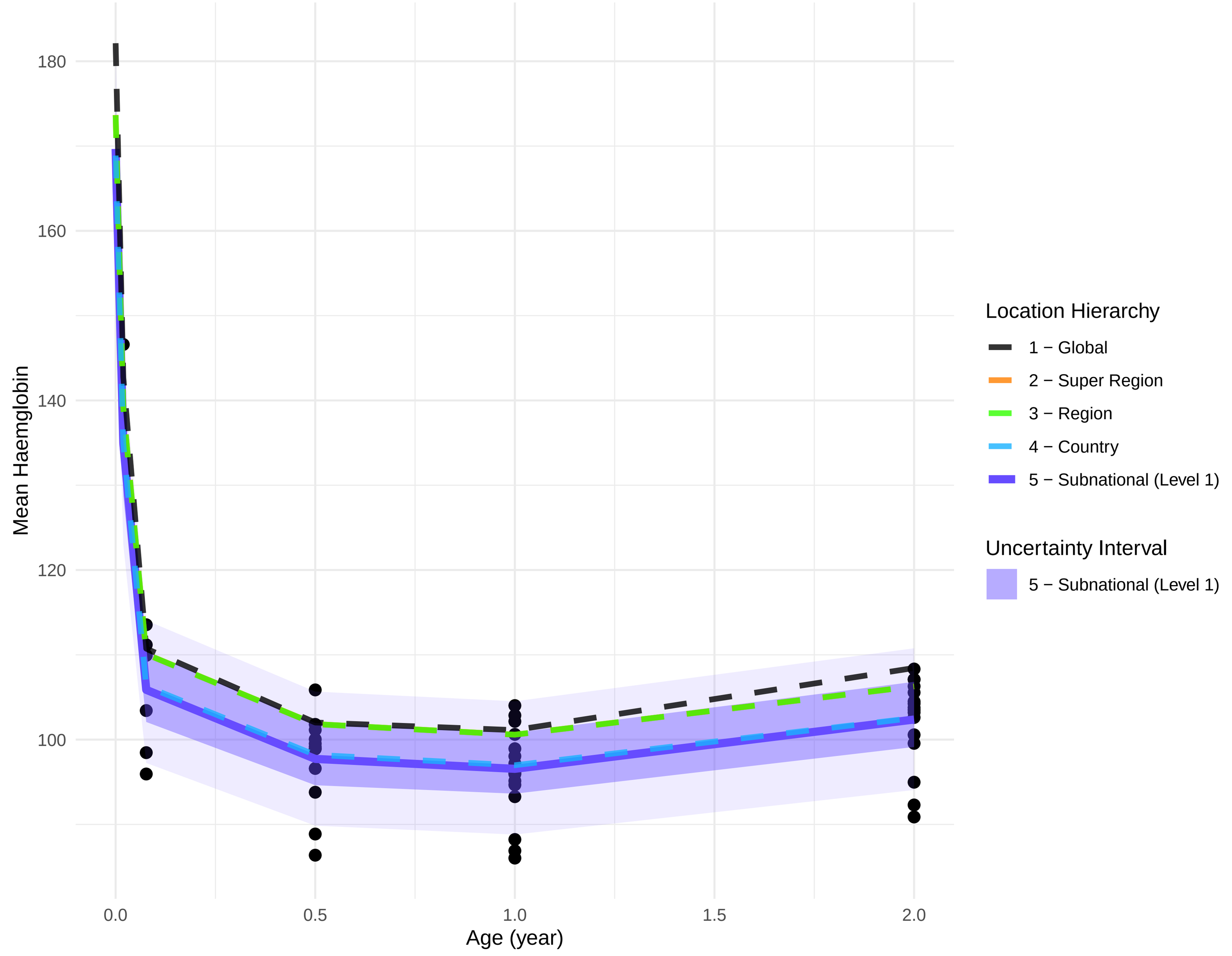

MR−BRT Age/Sex Fits − Non-pregnant Females <2 Years Old − Mean Haemglobin − Karnataka, Rural
WHO−2001 Adjustment Method

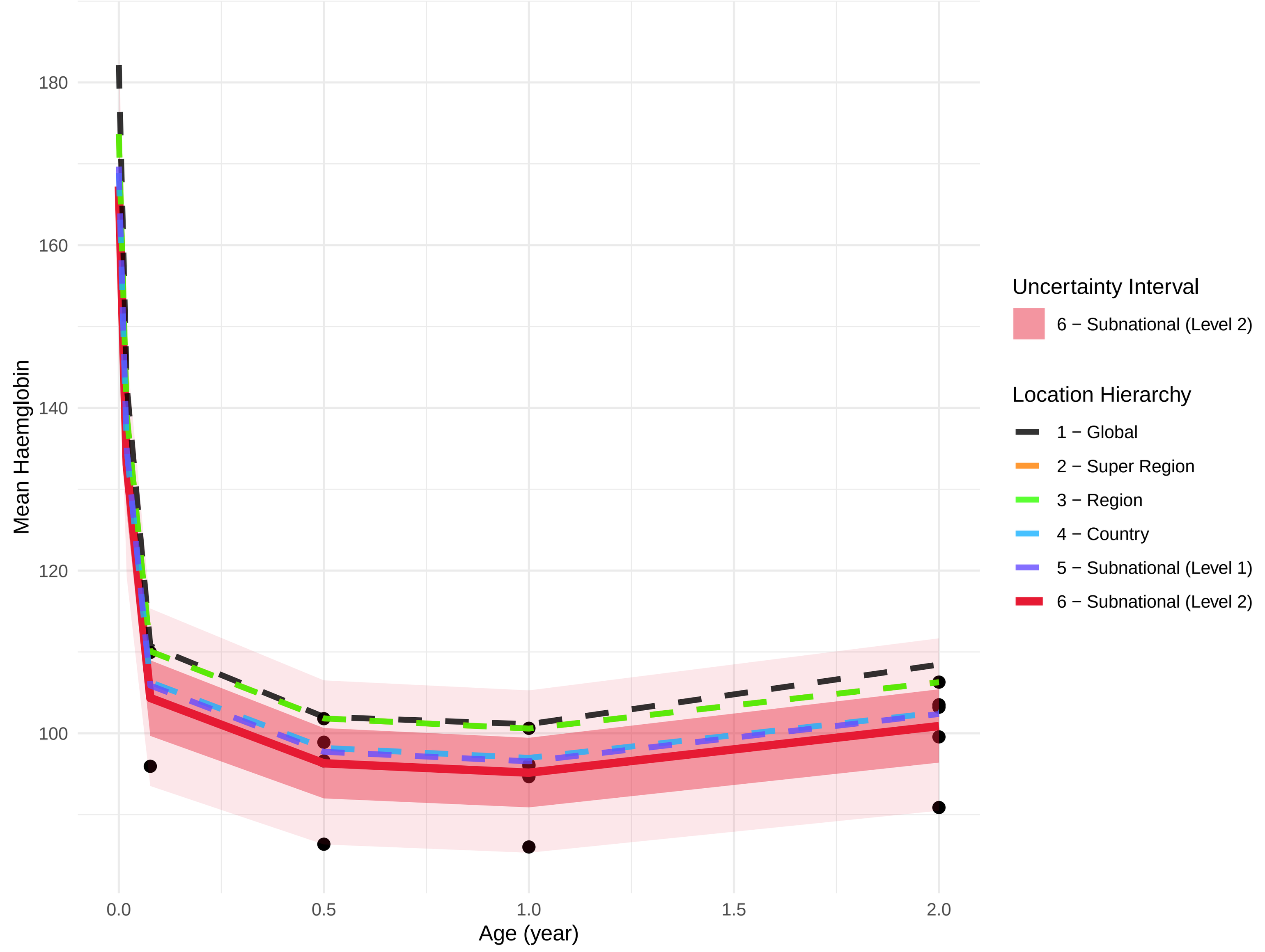

MR−BRT Age/Sex Fits − Non-pregnant Females <2 Years Old − Mean Haemglobin − Karnataka, Urban
WHO−2001 Adjustment Method

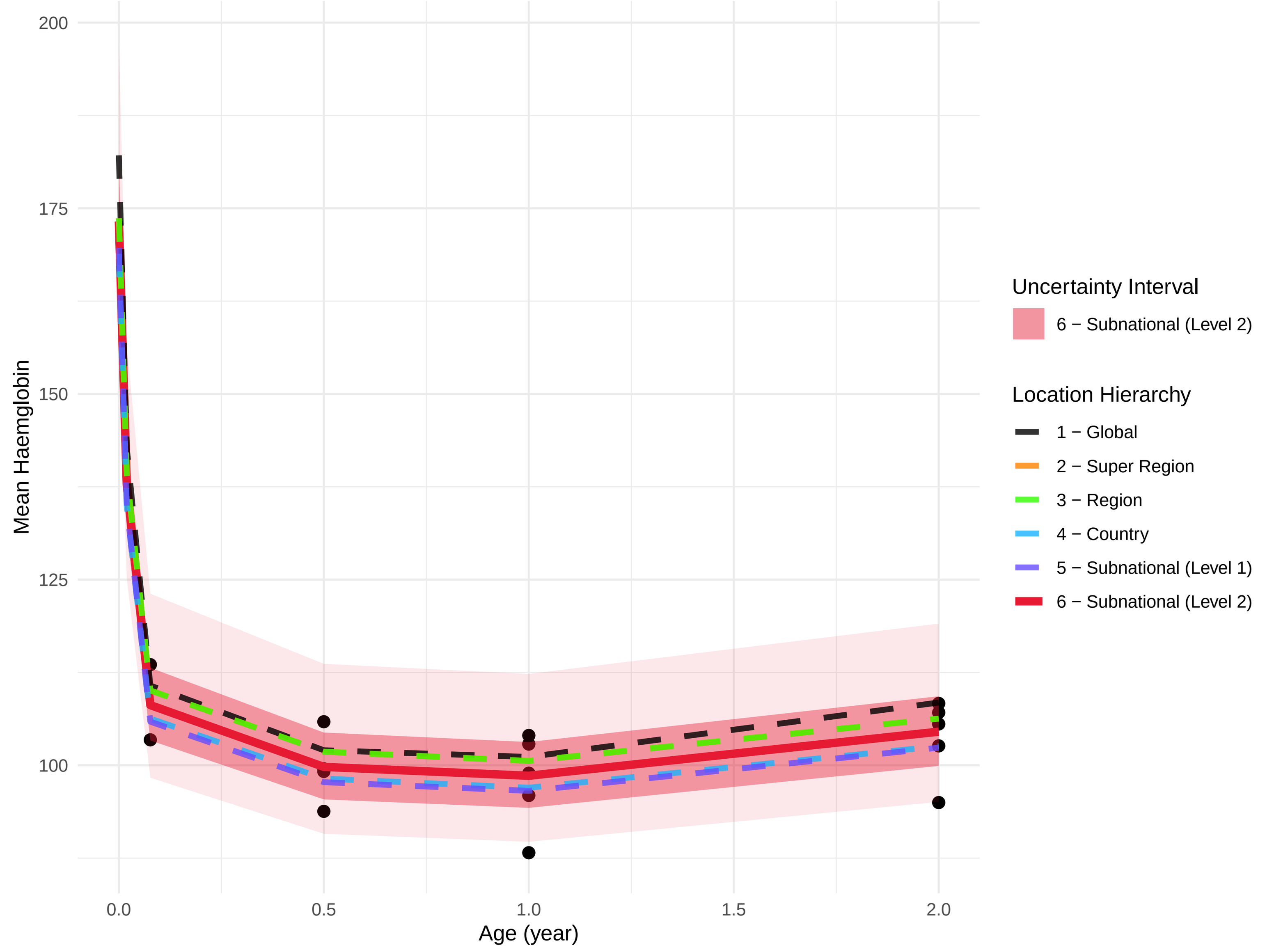

MR−BRT Age/Sex Fits − Non-pregnant Females <2 Years Old − Mean Haemglobin − Kerala
WHO−2001 Adjustment Method

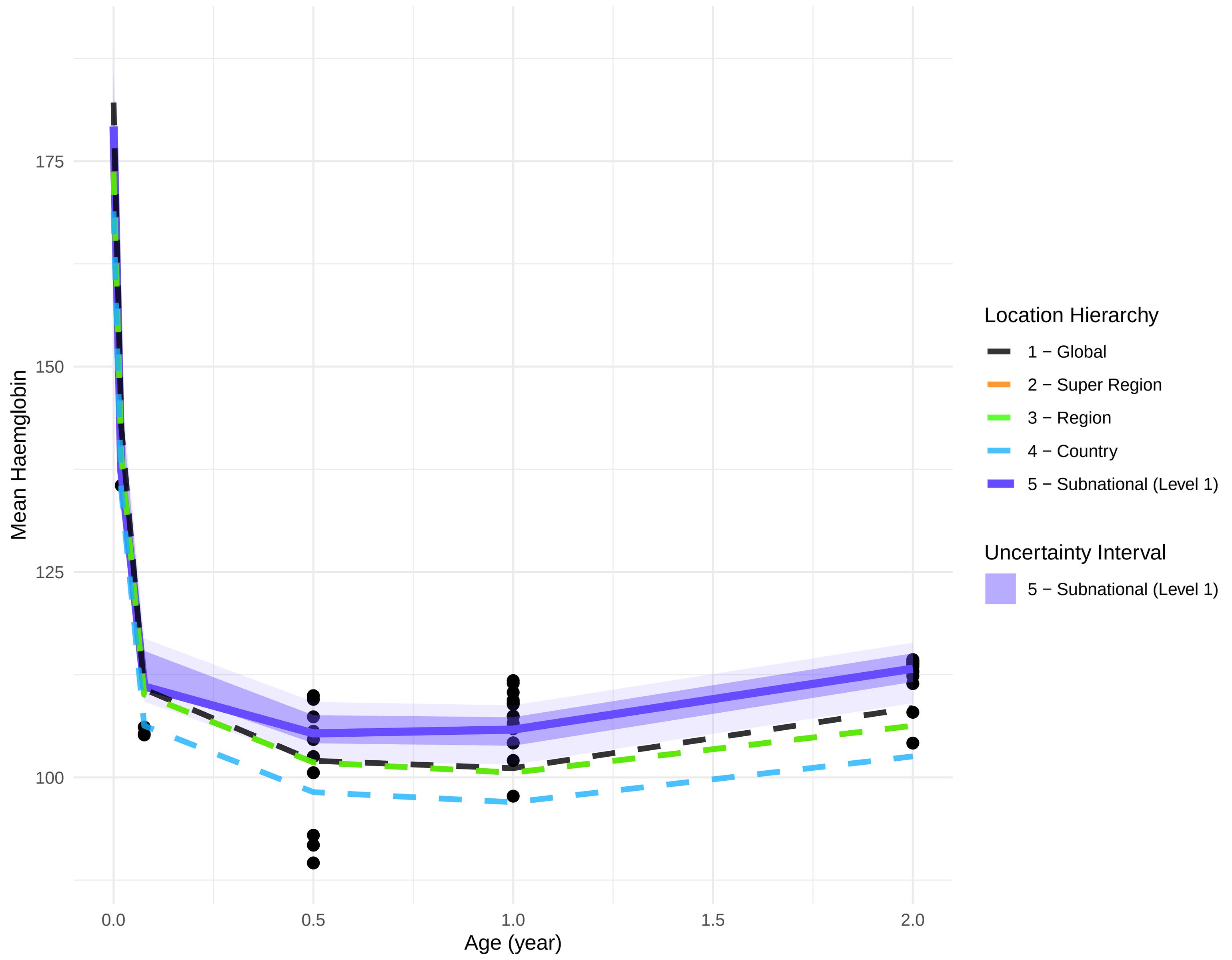

MR−BRT Age/Sex Fits − Non-pregnant Females <2 Years Old − Mean Haemglobin − Kerala, Rural
WHO−2001 Adjustment Method

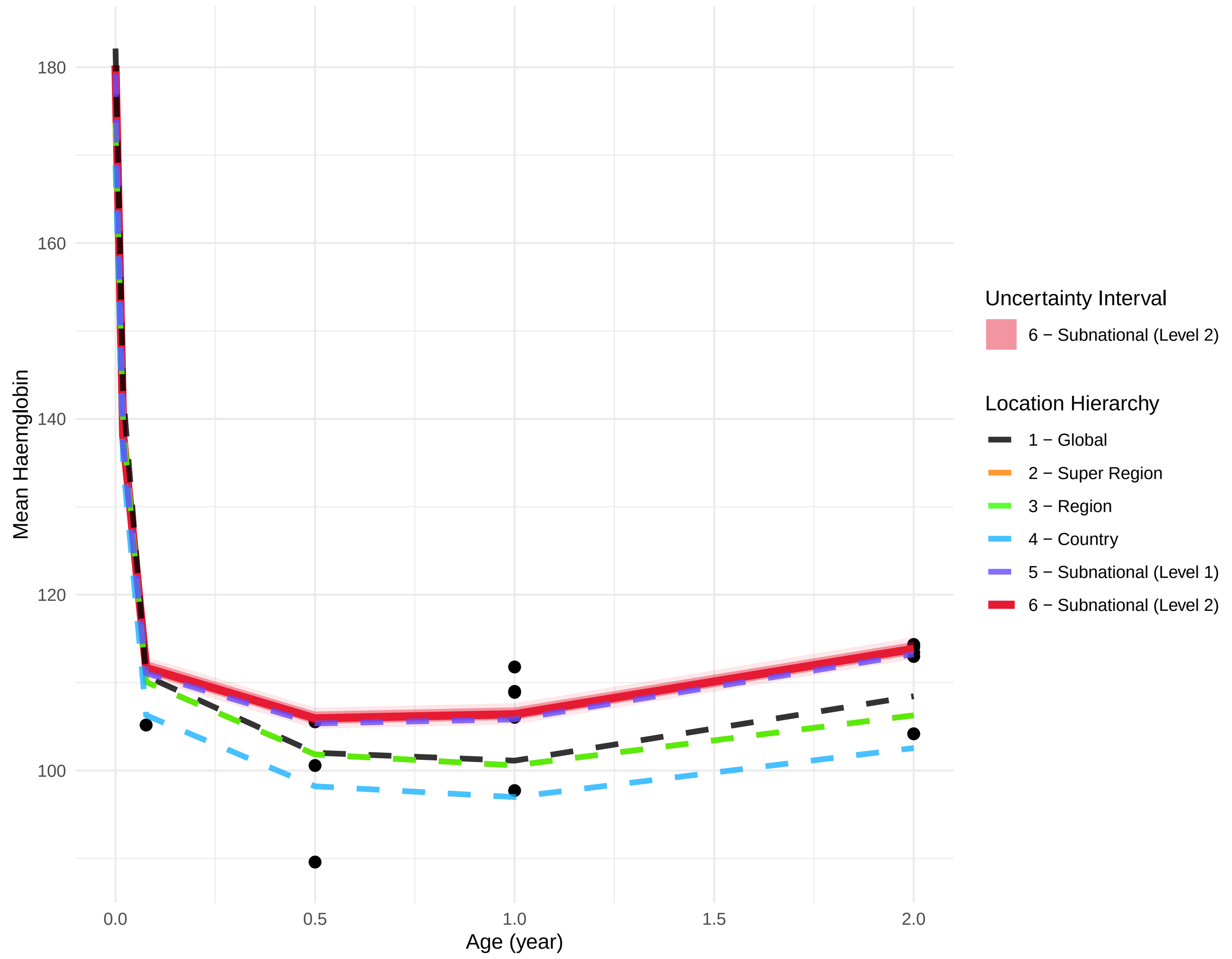

MR−BRT Age/Sex Fits − Non-pregnant Females <2 Years Old − Mean Haemglobin − Kerala, Urban
WHO−2001 Adjustment Method

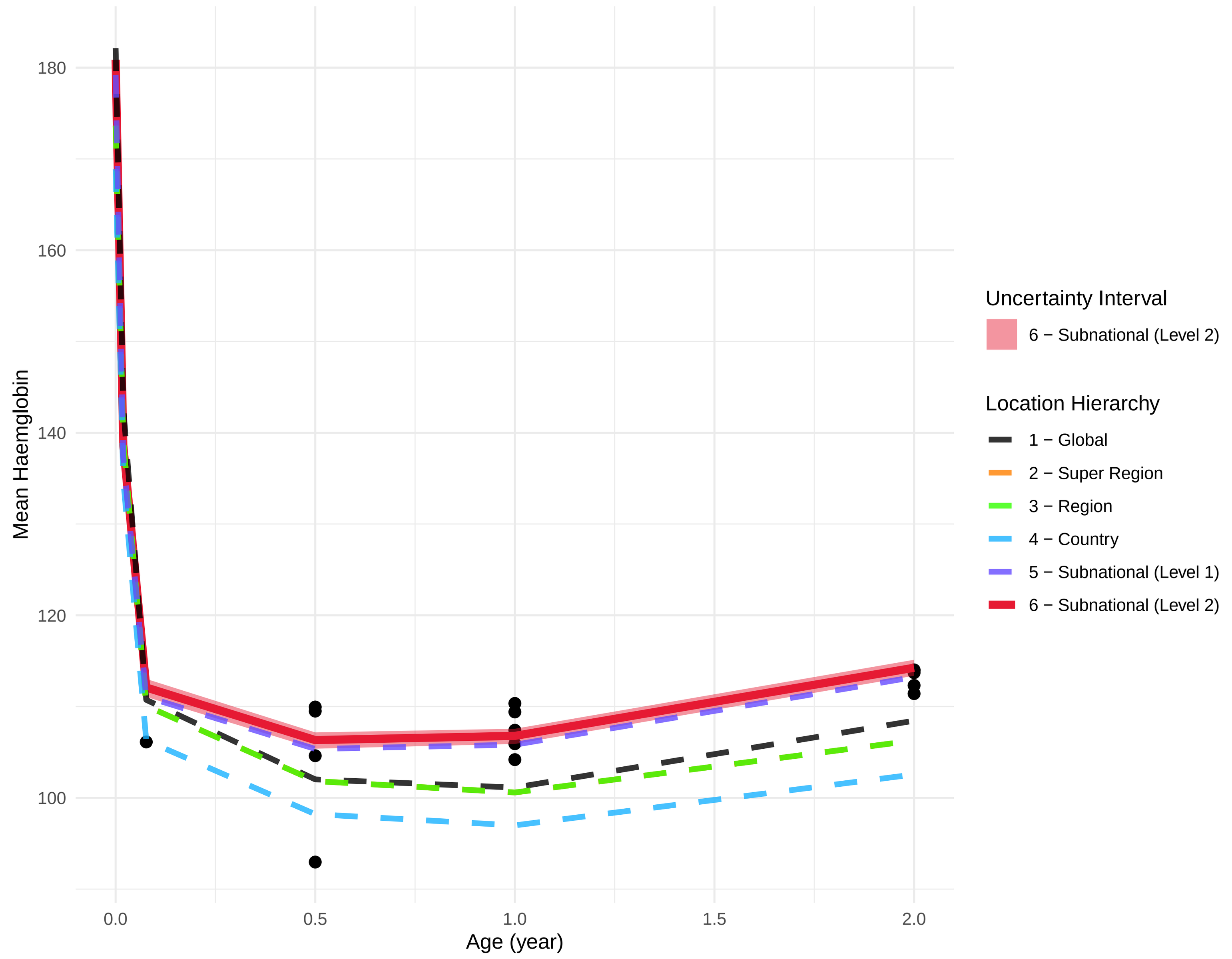

MR−BRT Age/Sex Fits − Non-pregnant Females <2 Years Old − Mean Haemglobin − Madhya Pradesh
WHO−2001 Adjustment Method

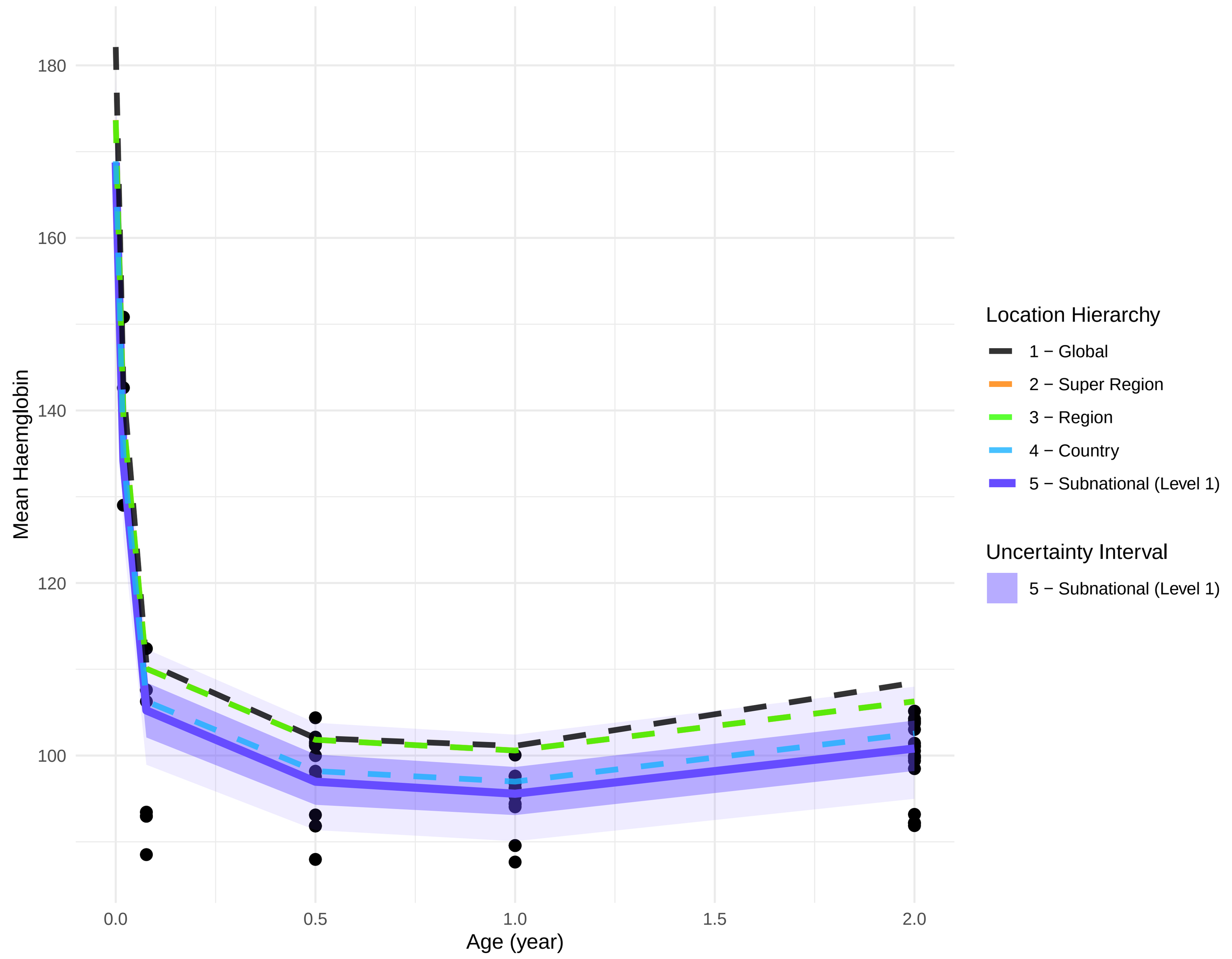

MR−BRT Age/Sex Fits − Non-pregnant Females <2 Years Old − Mean Haemglobin − Madhya Pradesh, Rural
WHO−2001 Adjustment Method

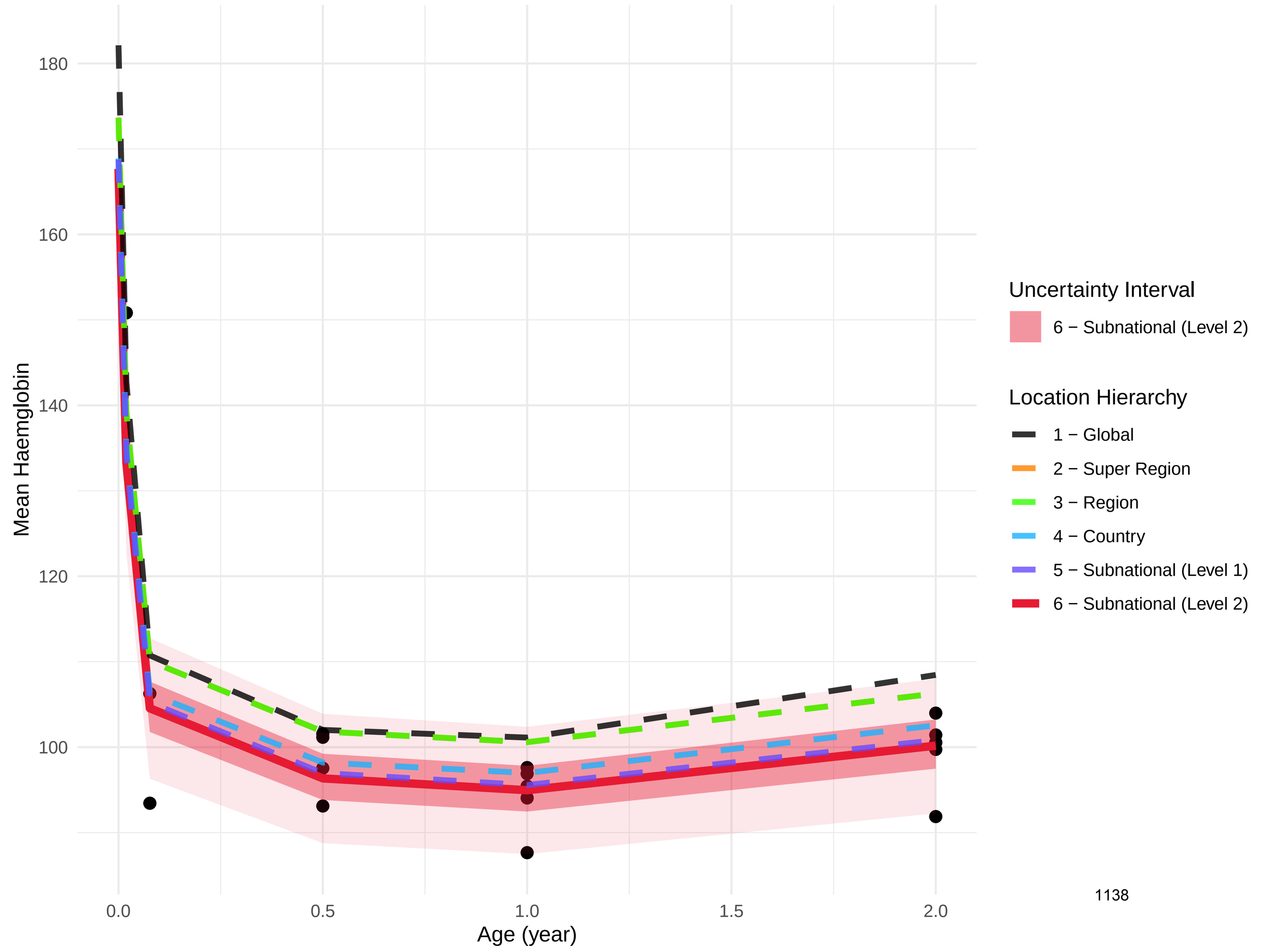

MR−BRT Age/Sex Fits − Non−pregnant Females <2 Years Old − Mean Haemglobin − Madhya Pradesh, Urban
WHO−2001 Adjustment Method

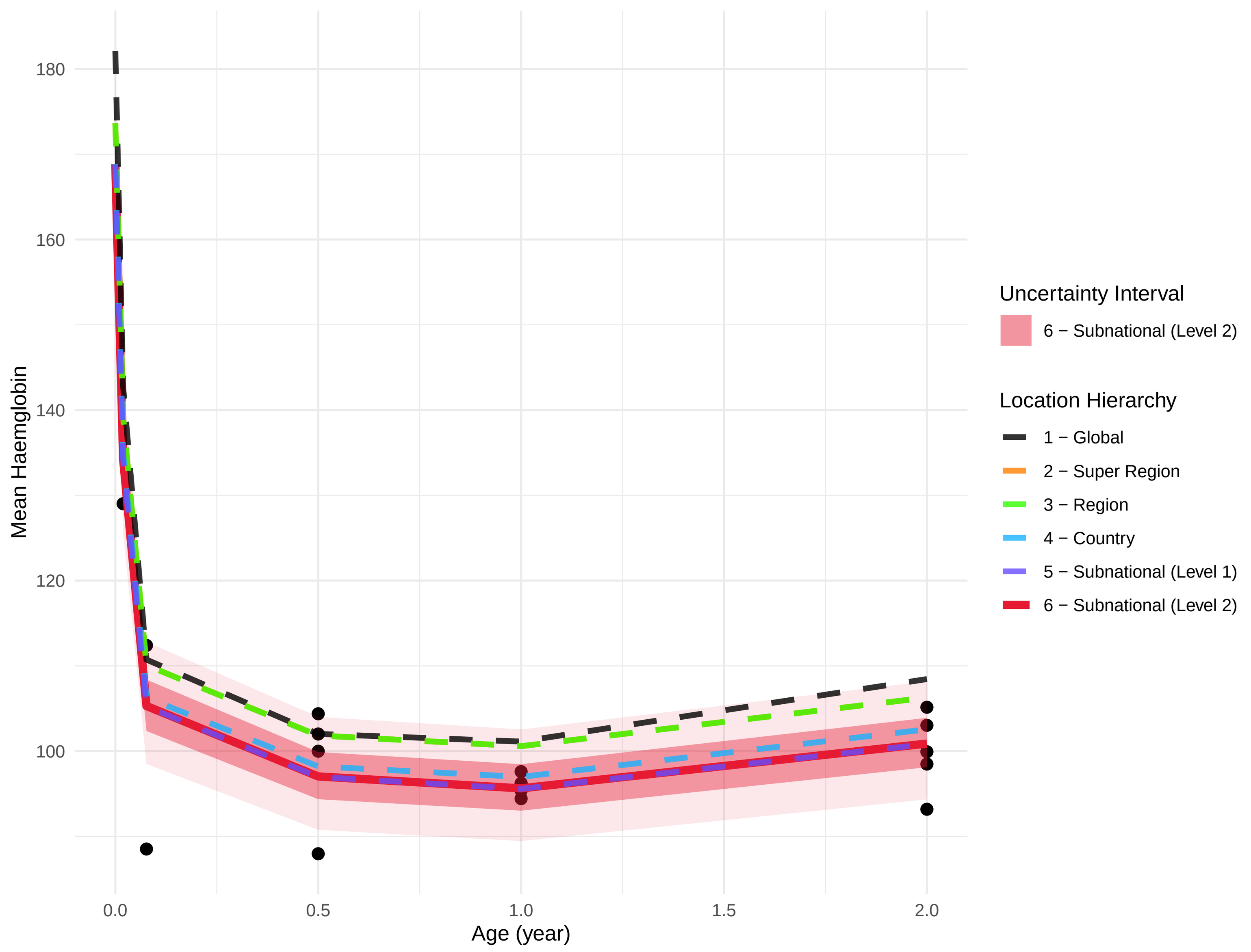

MR−BRT Age/Sex Fits − Non−pregnant Females <2 Years Old − Mean Haemglobin − Maharashtra
WHO−2001 Adjustment Method

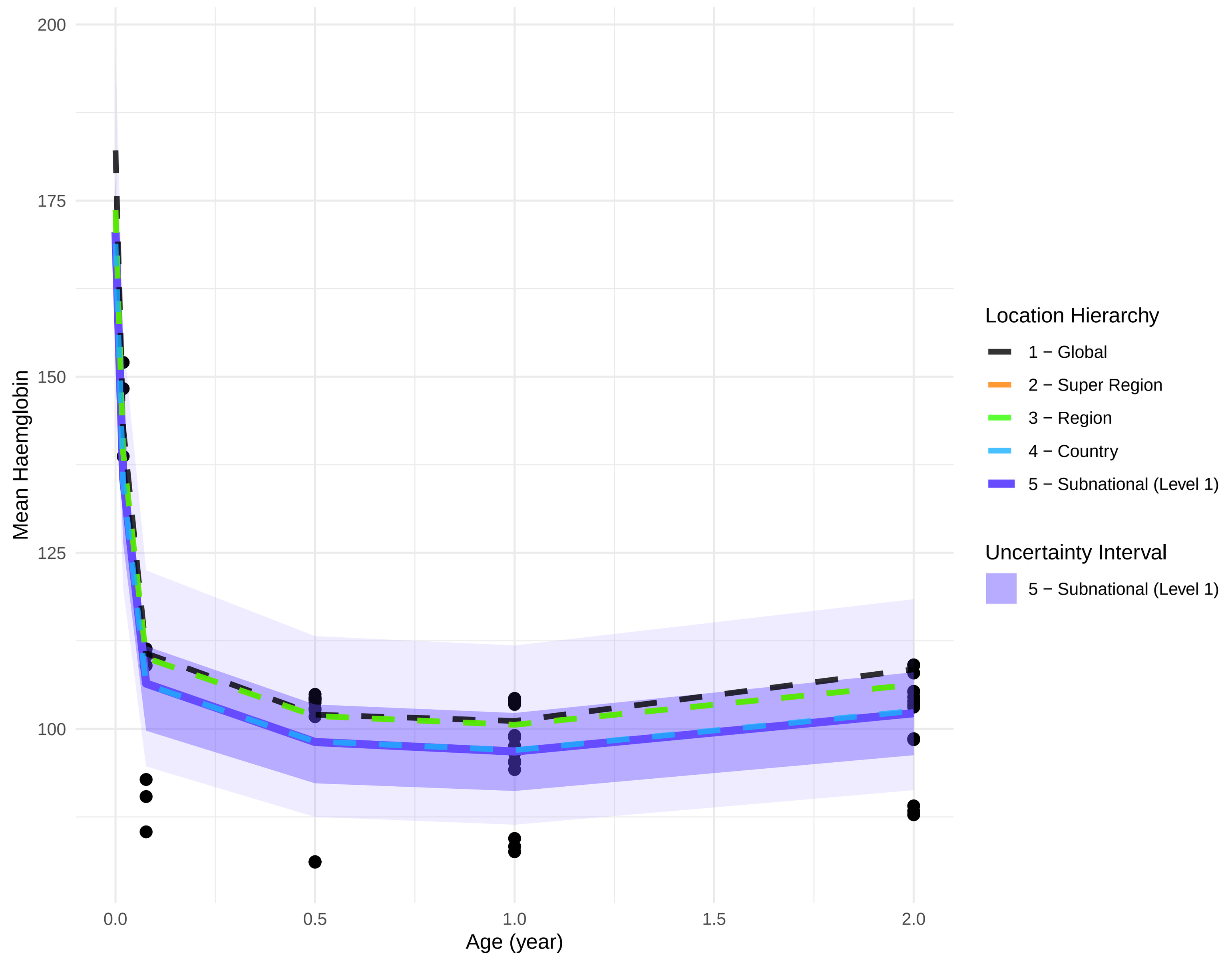

MR−BRT Age/Sex Fits − Non−pregnant Females <2 Years Old − Mean Haemglobin − Maharashtra, Rural
WHO−2001 Adjustment Method

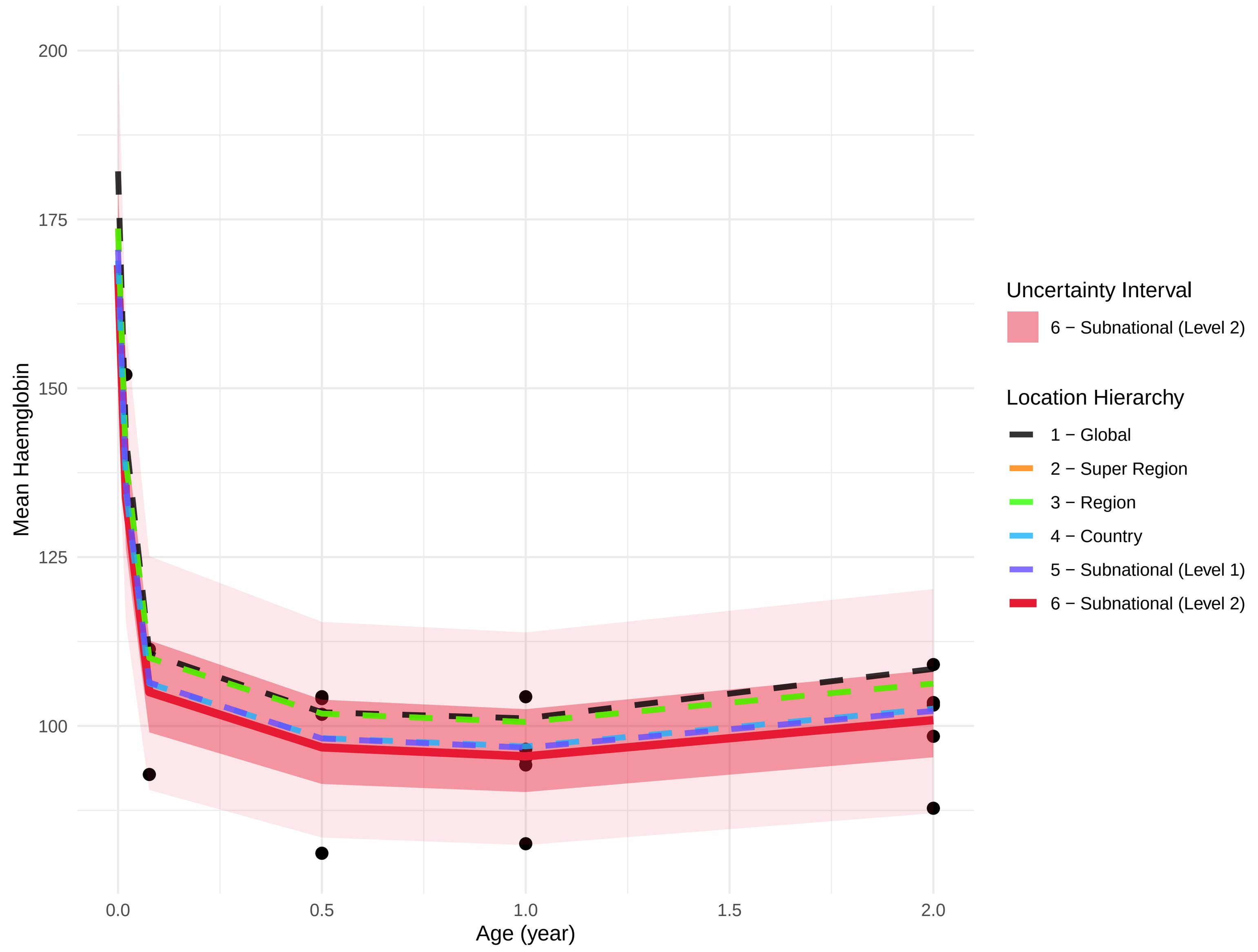

MR−BRT Age/Sex Fits − Non−pregnant Females <2 Years Old − Mean Haemglobin − Maharashtra, Urban
WHO−2001 Adjustment Method

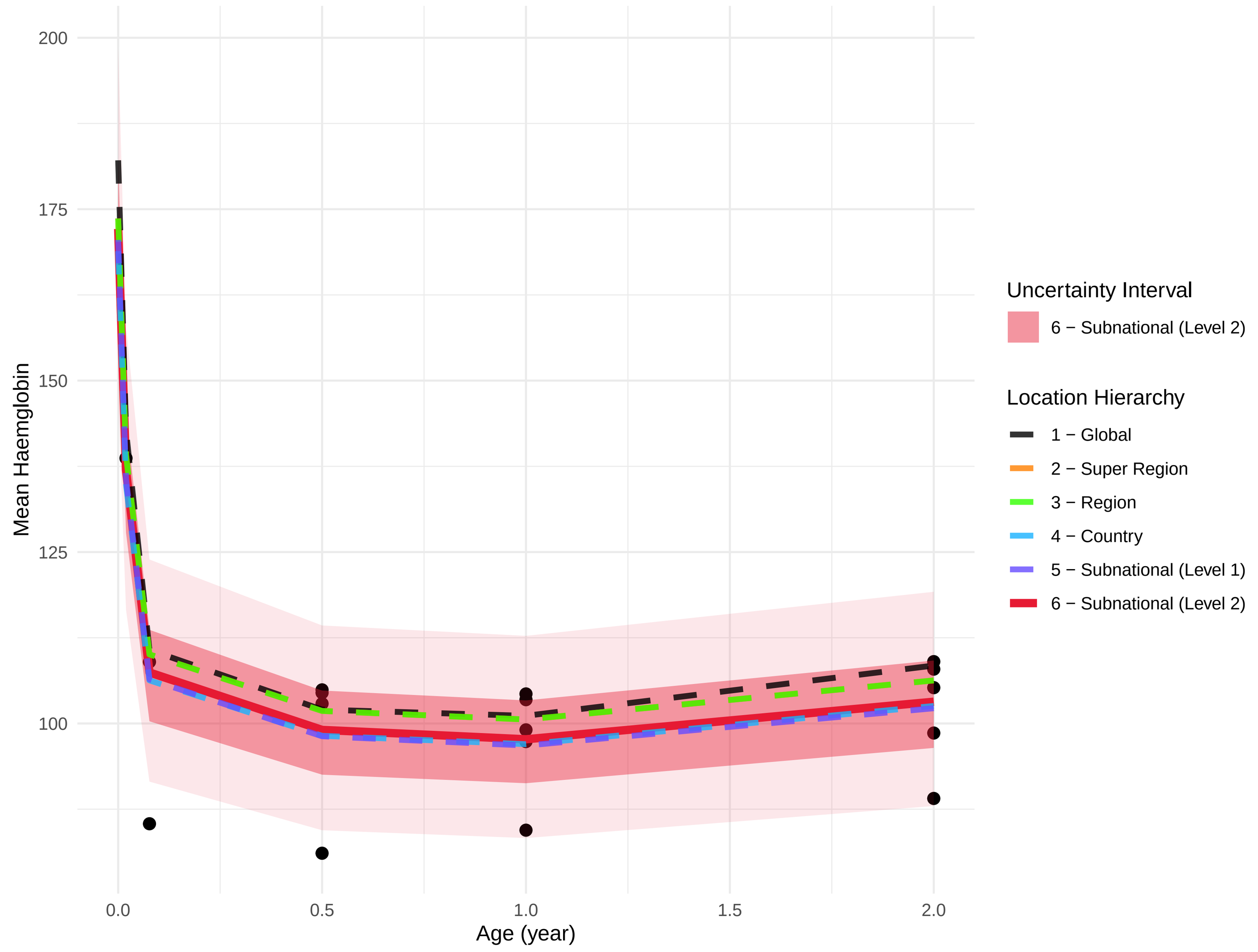

MR−BRT Age/Sex Fits − Non−pregnant Females <2 Years Old − Mean Haemglobin − Manipur
WHO−2001 Adjustment Method

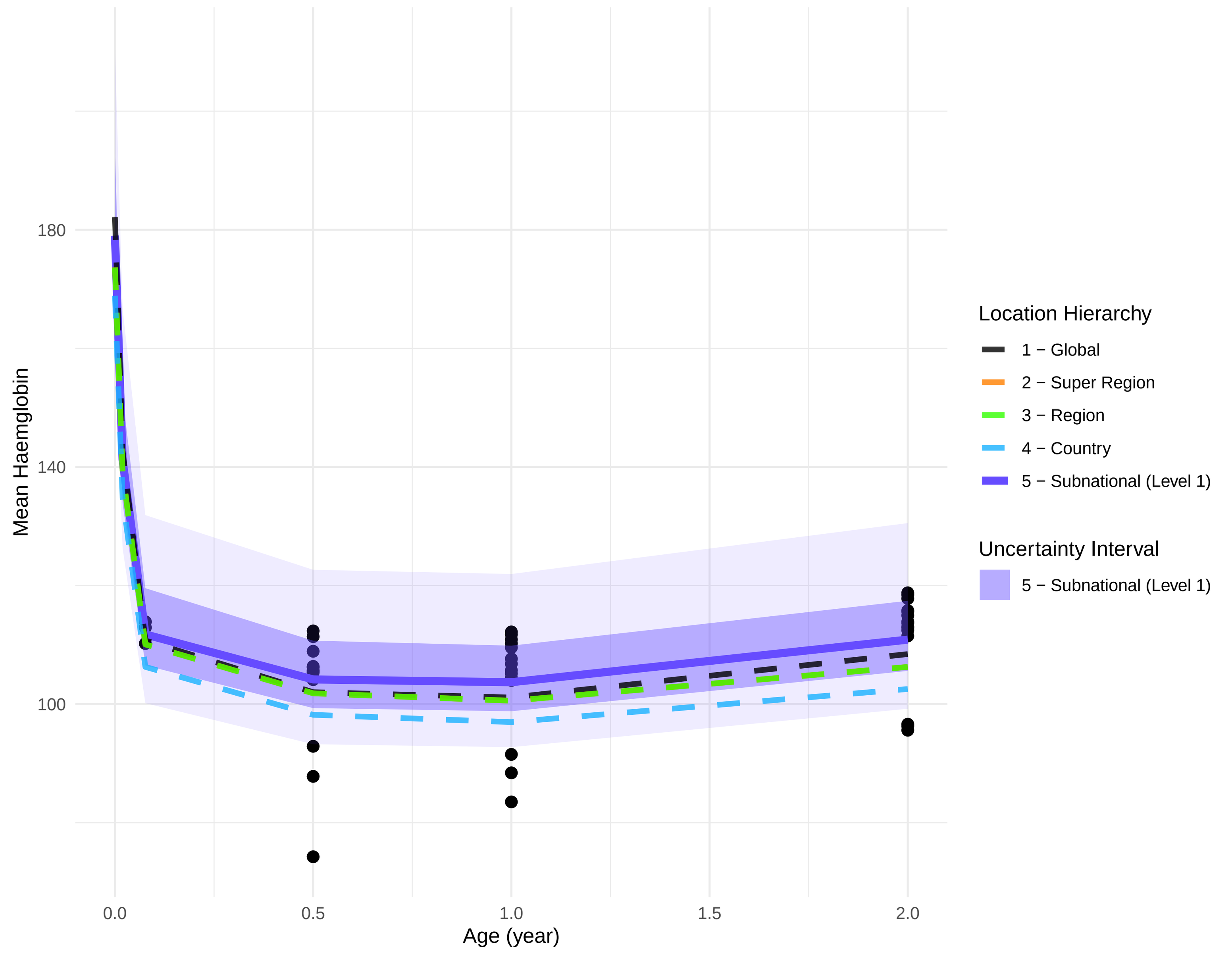

MR−BRT Age/Sex Fits − Non−pregnant Females <2 Years Old − Mean Haemglobin − Manipur, Rural
WHO−2001 Adjustment Method

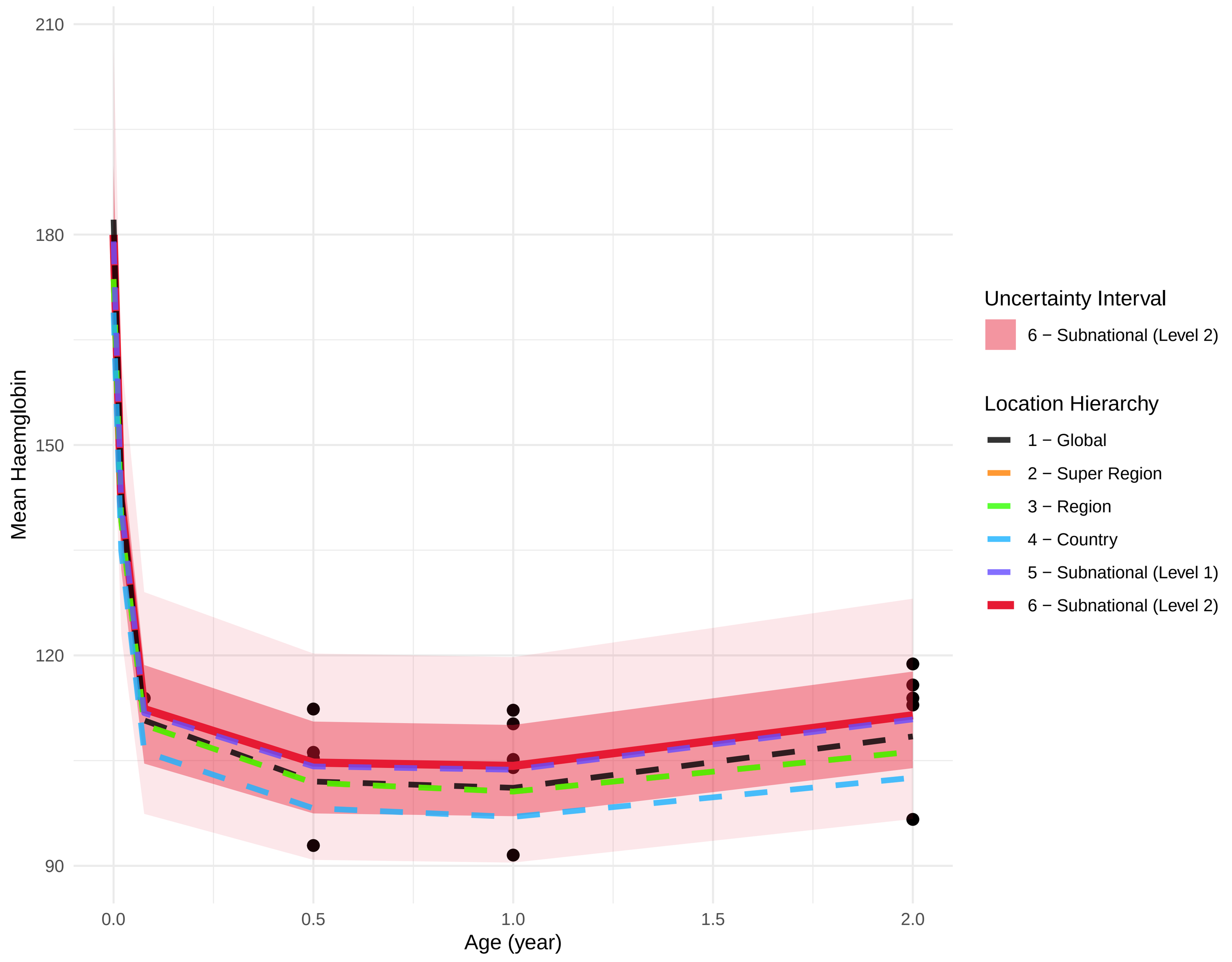

MR−BRT Age/Sex Fits − Non−pregnant Females <2 Years Old − Mean Haemglobin − Manipur, Urban
WHO−2001 Adjustment Method

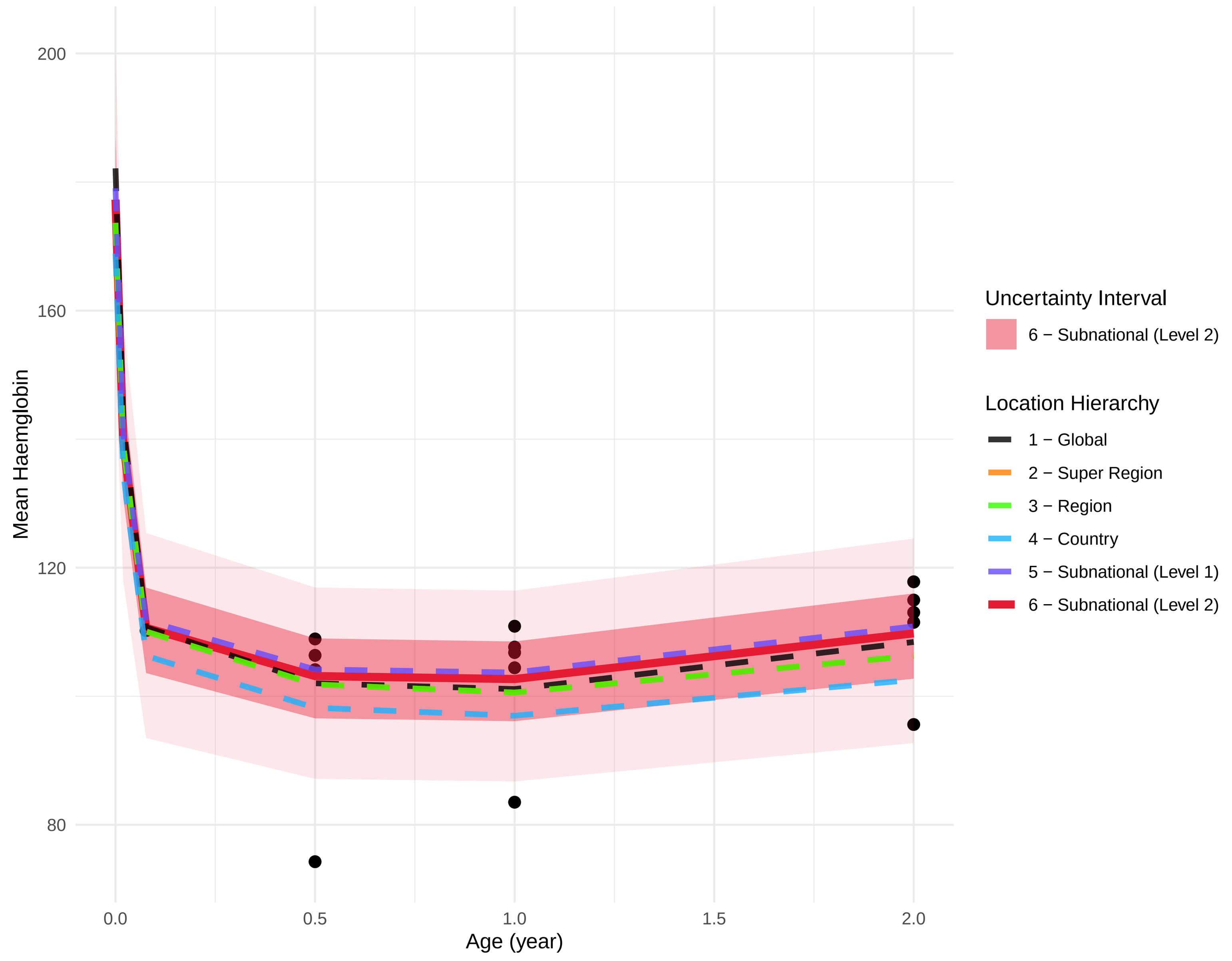

MR−BRT Age/Sex Fits − Non−pregnant Females <2 Years Old − Mean Haemglobin − Meghalaya
WHO−2001 Adjustment Method

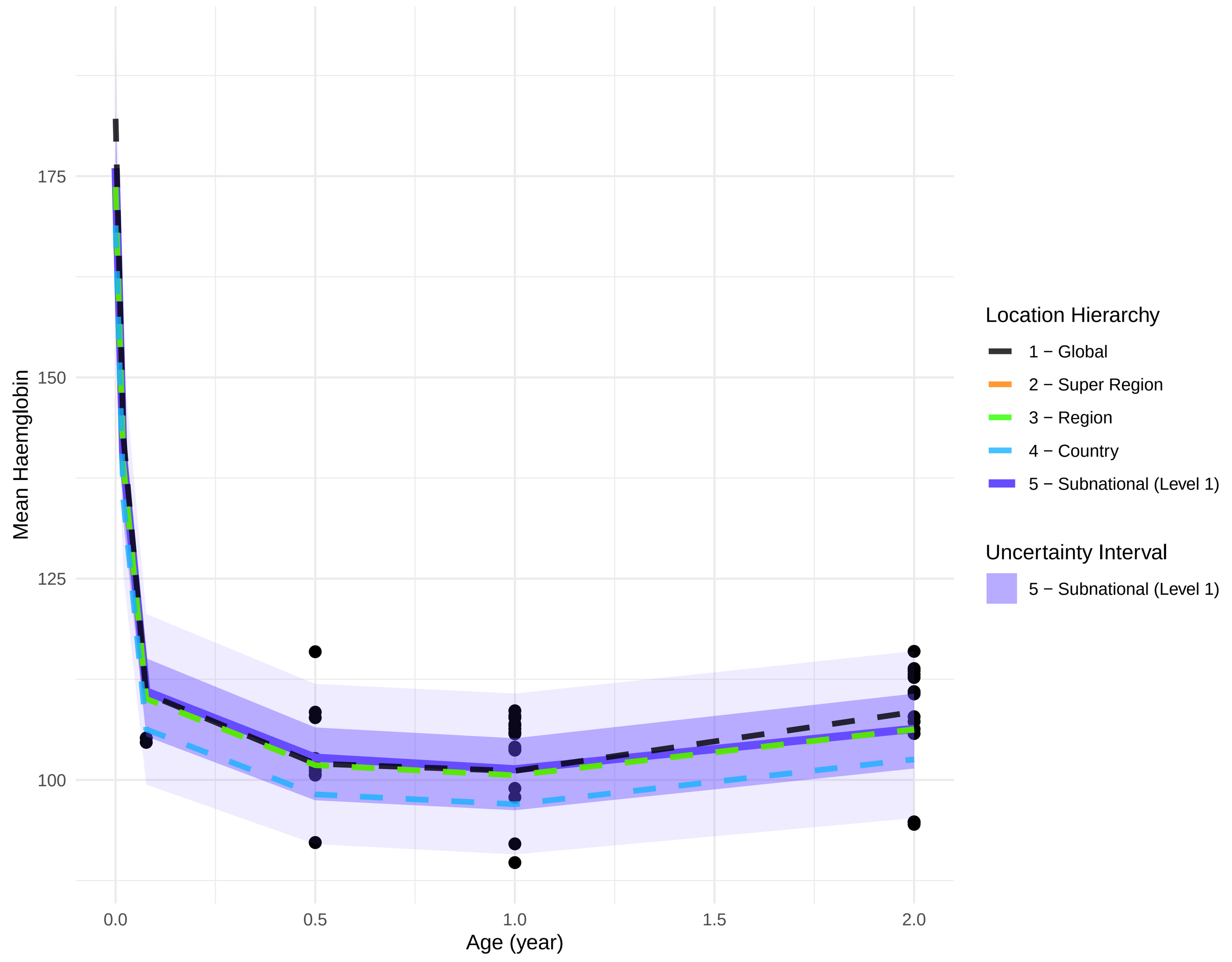

MR−BRT Age/Sex Fits − Non−pregnant Females <2 Years Old − Mean Haemglobin − Meghalaya, Rural
WHO−2001 Adjustment Method

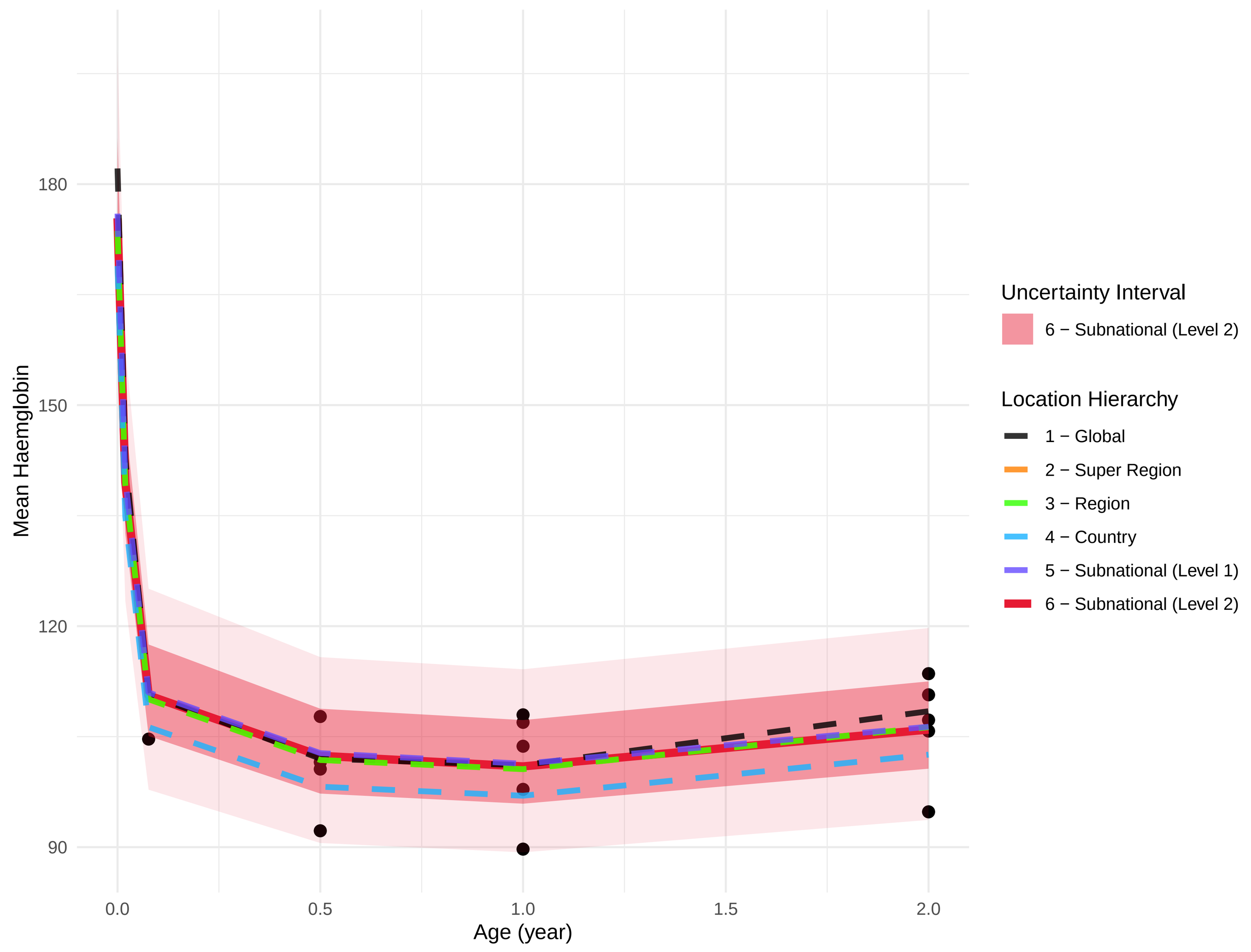

MR−BRT Age/Sex Fits − Non−pregnant Females <2 Years Old − Mean Haemglobin − Meghalaya, Urban
WHO−2001 Adjustment Method

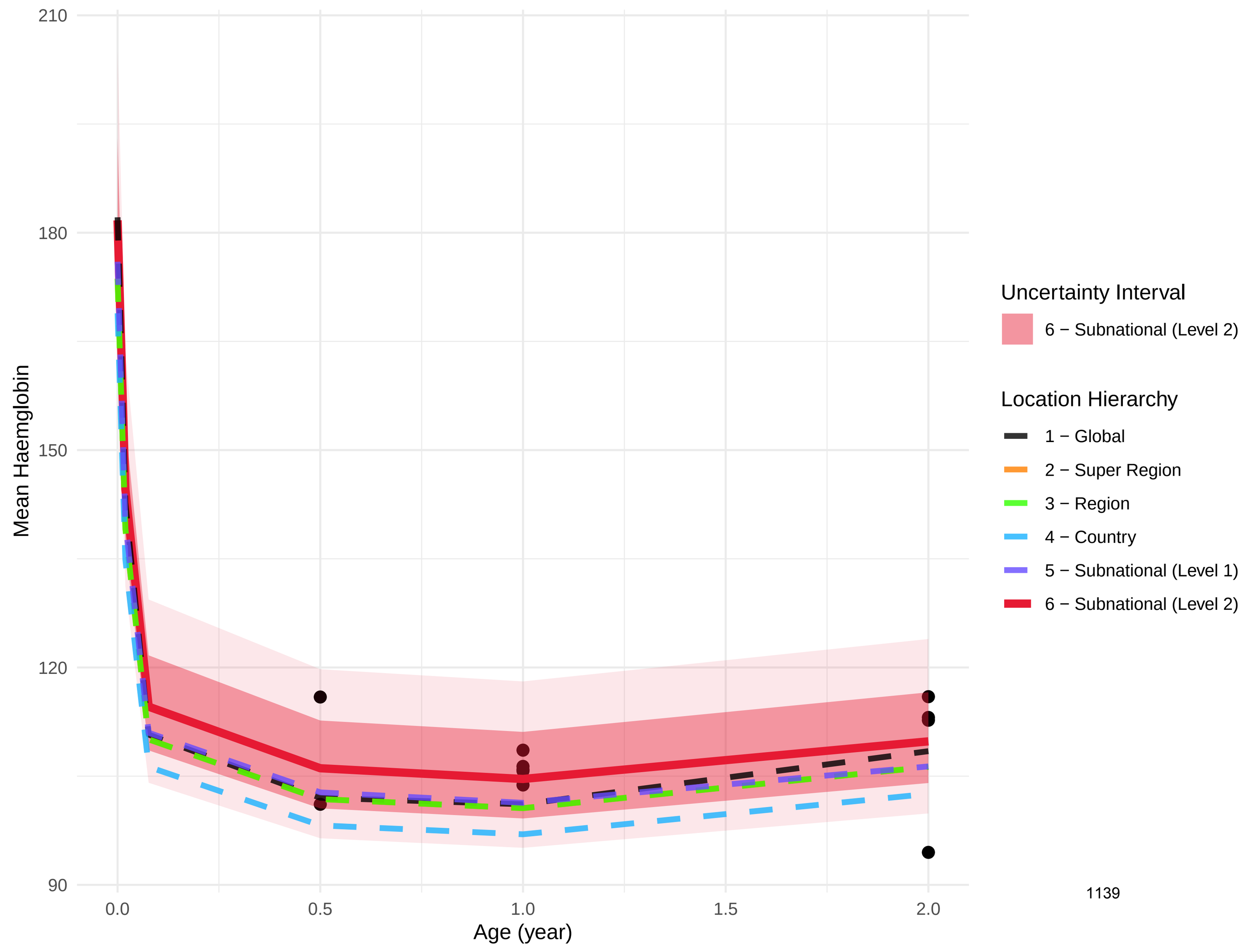

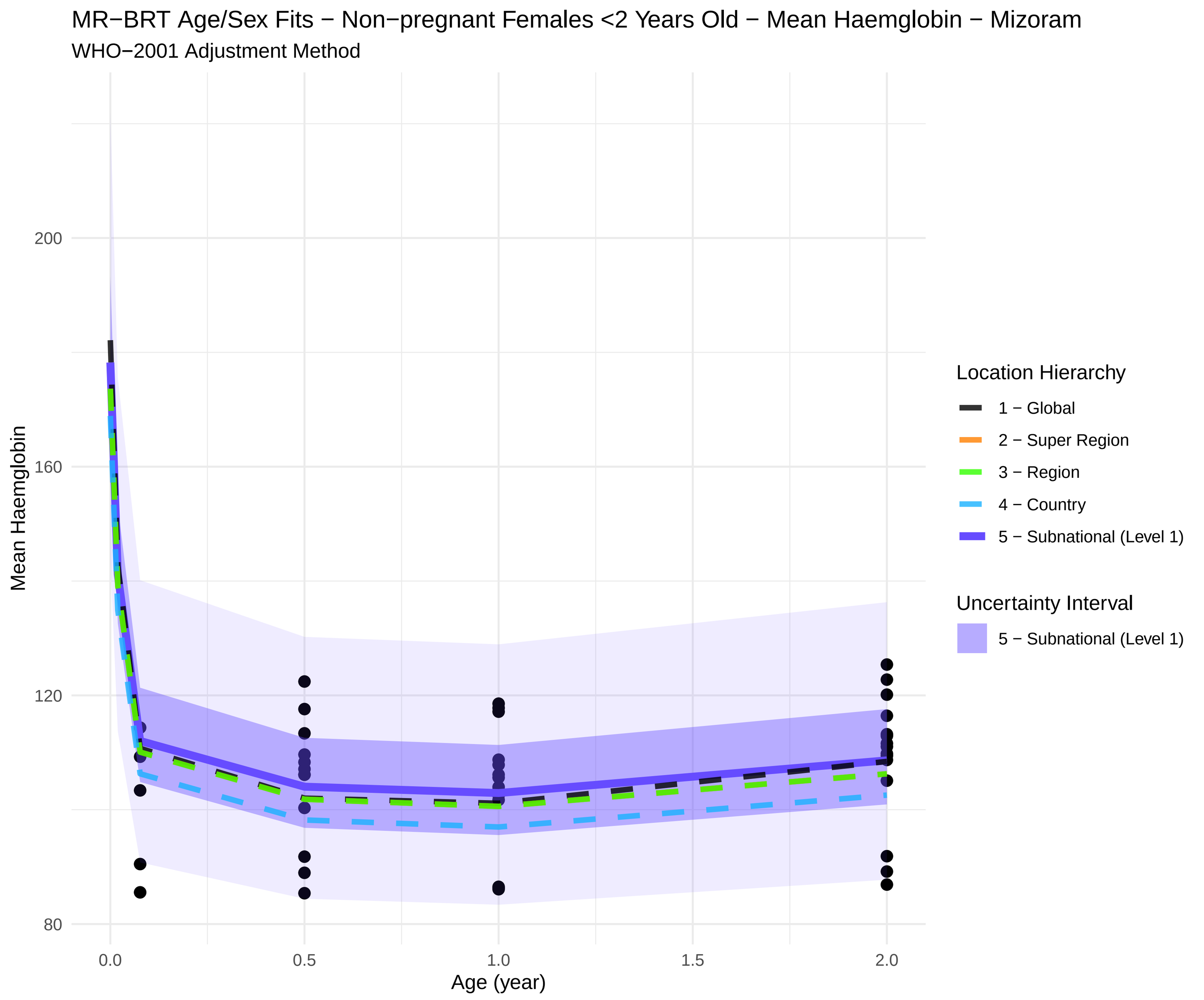
MR−BRT Age/Sex Fits − Non−pregnant Females <2 Years Old − Mean Haemglobin − Mizoram
WHO−2001 Adjustment Method
Mean Haemglobin
200
160
120
80
0.0
0.5
1.0
1.5
2.0
Age (year)
Location Hierarchy
1 − Global
2 − Super Region
3 − Region
4 − Country
5 − Subnational (Level 1)
Uncertainty Interval
5 − Subnational (Level 1)

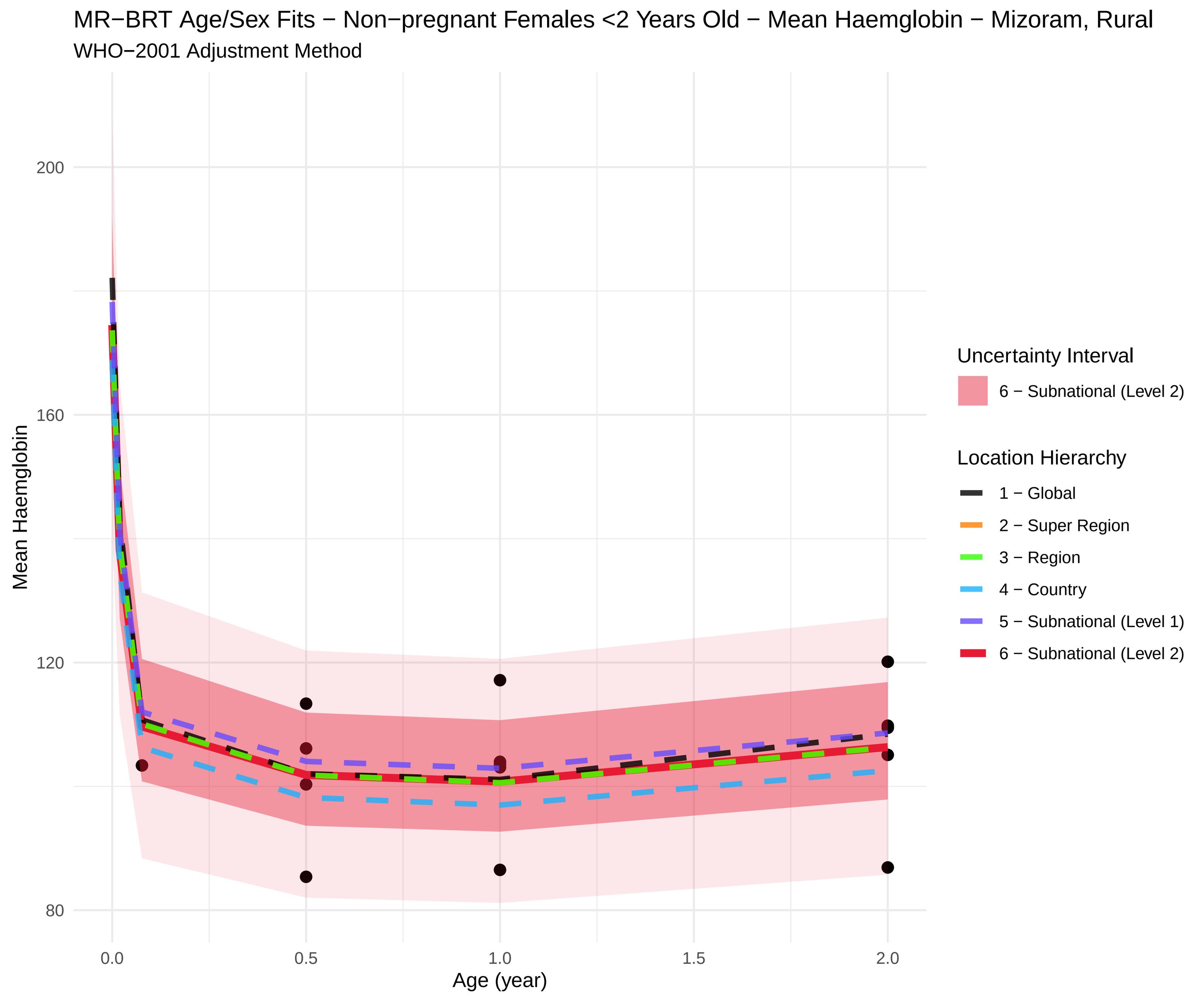
MR−BRT Age/Sex Fits − Non−pregnant Females <2 Years Old − Mean Haemglobin − Mizoram, Rural
WHO−2001 Adjustment Method
Mean Haemglobin
200
160
120
80
0.0
0.5
1.0
1.5
2.0
Age (year)
Uncertainty Interval
6 − Subnational (Level 2)
Location Hierarchy
1 − Global
2 − Super Region
3 − Region
4 − Country
5 − Subnational (Level 1)
6 − Subnational (Level 2)

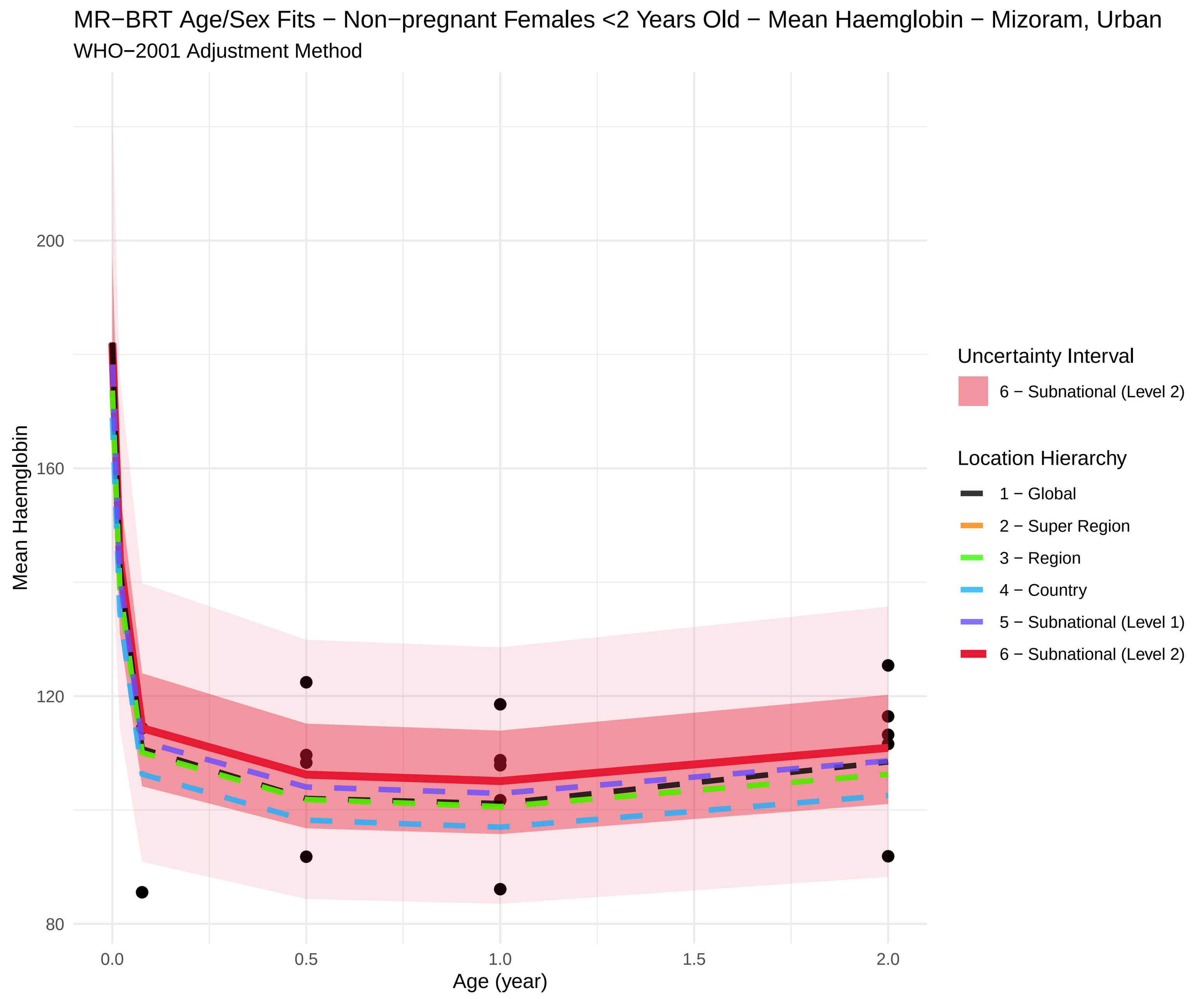
MR−BRT Age/Sex Fits − Non−pregnant Females <2 Years Old − Mean Haemglobin − Mizoram, Urban
WHO−2001 Adjustment Method
Mean Haemglobin
200
160
120
80
0.0
0.5
1.0
1.5
2.0
Age (year)
Uncertainty Interval
6 − Subnational (Level 2)
Location Hierarchy
1 − Global
2 − Super Region
3 − Region
4 − Country
5 − Subnational (Level 1)
6 − Subnational (Level 2)

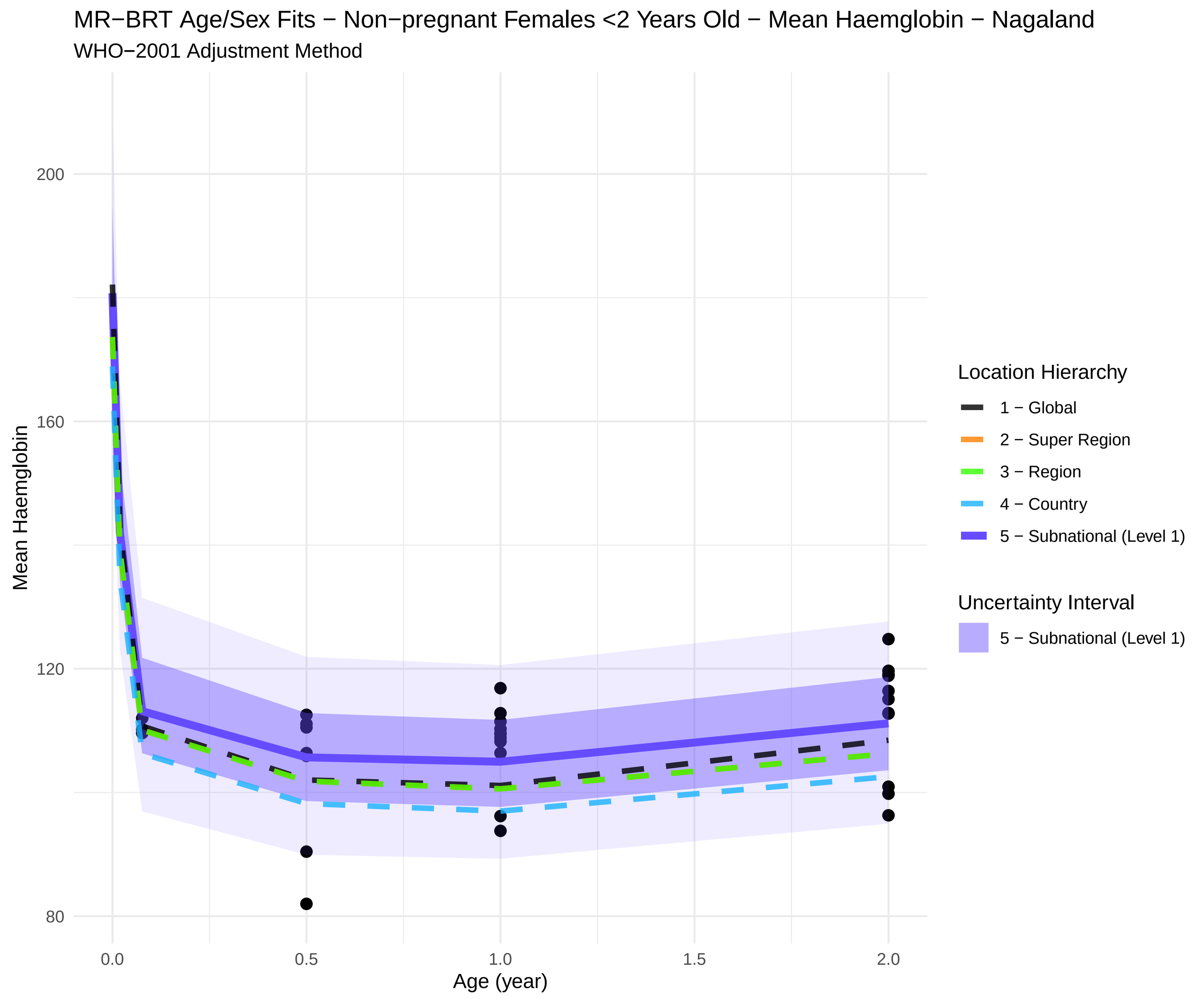
MR−BRT Age/Sex Fits − Non−pregnant Females <2 Years Old − Mean Haemglobin − Nagaland
WHO−2001 Adjustment Method
Mean Haemglobin
200
160
120
80
0.0
0.5
1.0
1.5
2.0
Age (year)
Location Hierarchy
1 − Global
2 − Super Region
3 − Region
4 − Country
5 − Subnational (Level 1)
Uncertainty Interval
5 − Subnational (Level 1)

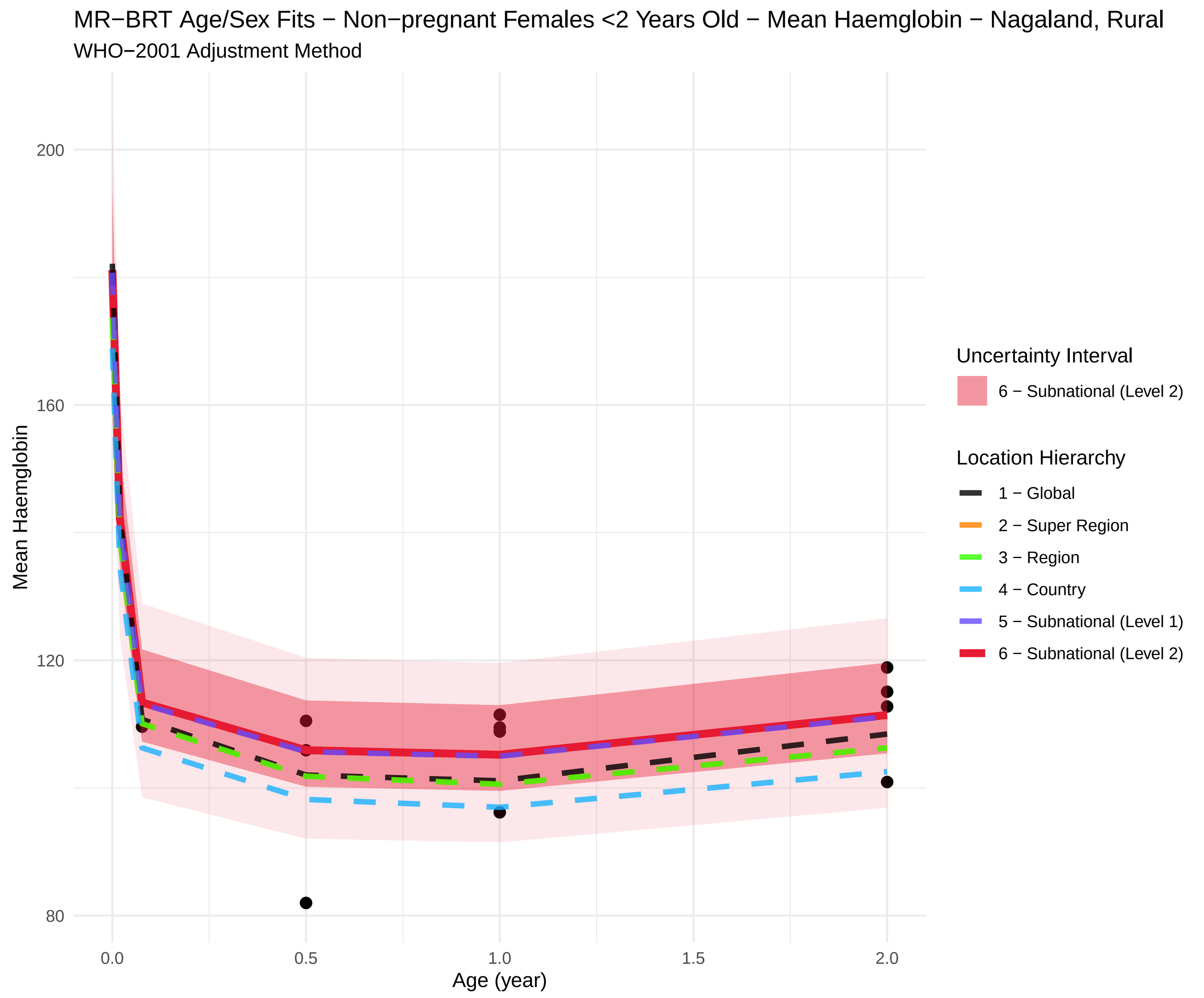
MR−BRT Age/Sex Fits − Non−pregnant Females <2 Years Old − Mean Haemglobin − Nagaland, Rural
WHO−2001 Adjustment Method
Mean Haemglobin
200
160
120
80
0.0
0.5
1.0
1.5
2.0
Age (year)
Uncertainty Interval
6 − Subnational (Level 2)
Location Hierarchy
1 − Global
2 − Super Region
3 − Region
4 − Country
5 − Subnational (Level 1)
6 − Subnational (Level 2)

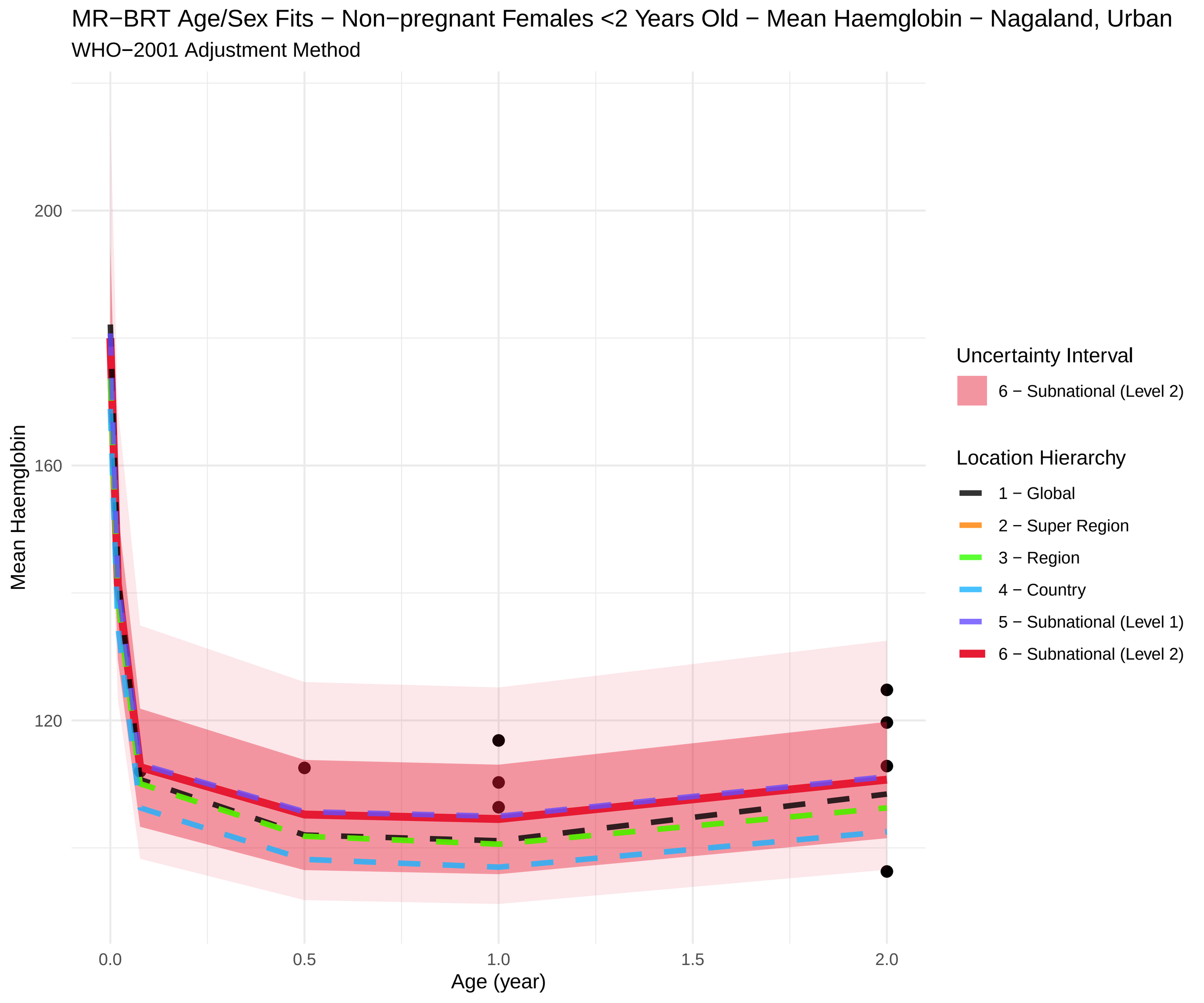
MR−BRT Age/Sex Fits − Non−pregnant Females <2 Years Old − Mean Haemglobin − Nagaland, Urban
WHO−2001 Adjustment Method
Mean Haemglobin
200
160
120
0.0
0.5
1.0
1.5
2.0
Age (year)
Uncertainty Interval
6 − Subnational (Level 2)
Location Hierarchy
1 − Global
2 − Super Region
3 − Region
4 − Country
5 − Subnational (Level 1)
6 − Subnational (Level 2)

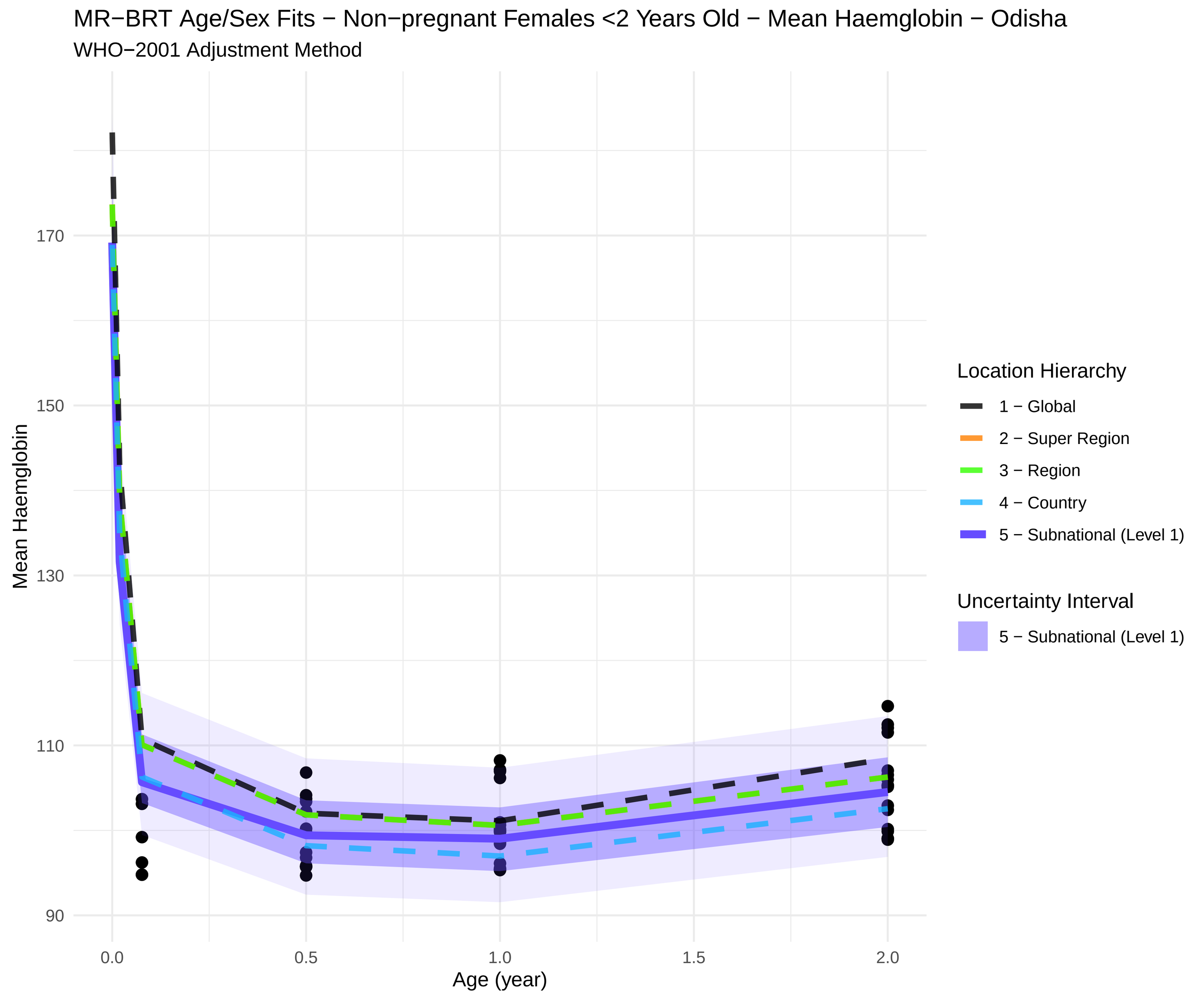
MR−BRT Age/Sex Fits − Non−pregnant Females <2 Years Old − Mean Haemglobin − Odisha
WHO−2001 Adjustment Method
Mean Haemglobin
170
150
130
110
90
0.0
0.5
1.0
1.5
2.0
Age (year)
Location Hierarchy
1 − Global
2 − Super Region
3 − Region
4 − Country
5 − Subnational (Level 1)
Uncertainty Interval
5 − Subnational (Level 1)

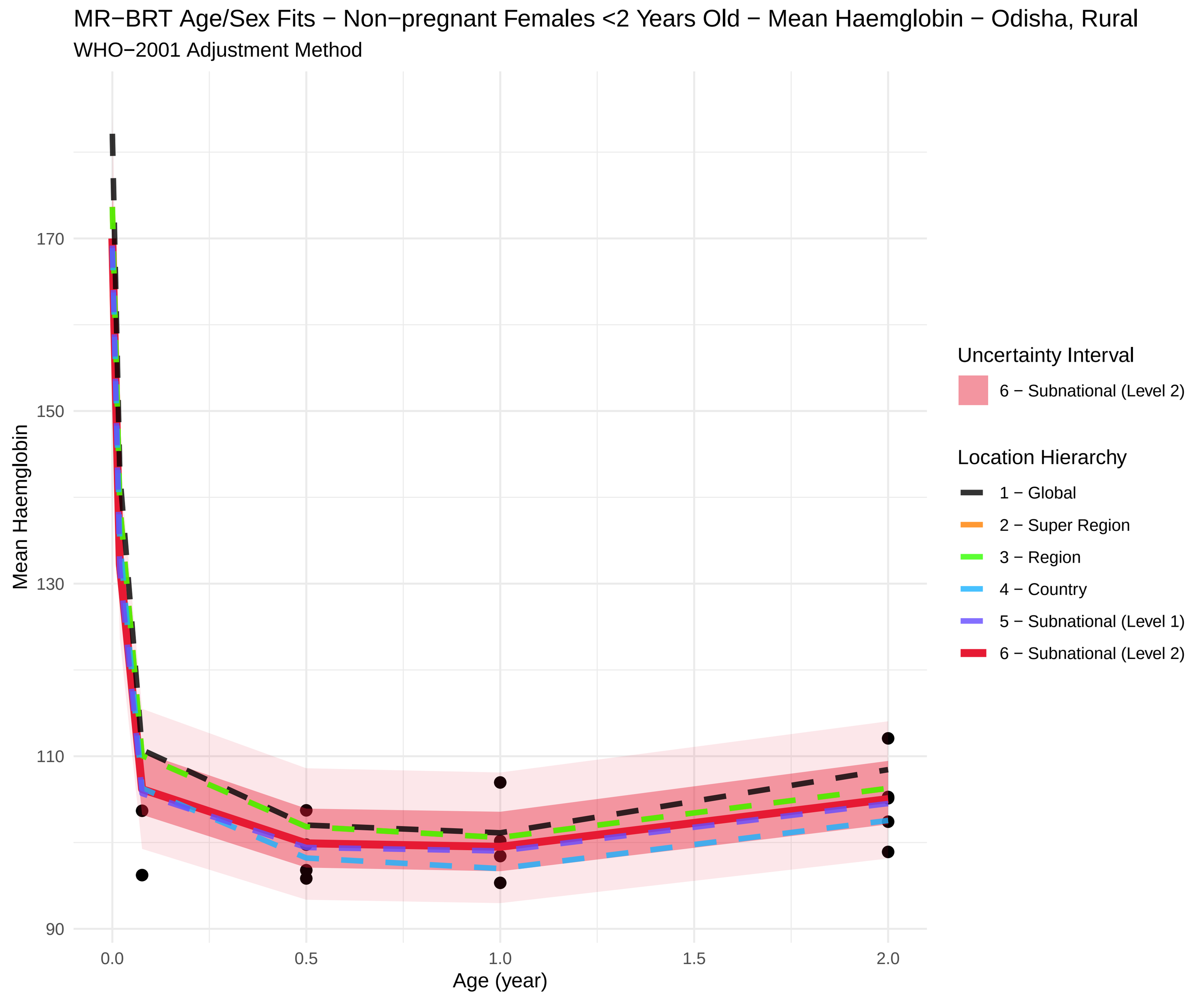
MR−BRT Age/Sex Fits − Non−pregnant Females <2 Years Old − Mean Haemglobin − Odisha, Rural
WHO−2001 Adjustment Method
Mean Haemglobin
170
150
130
110
90
0.0
0.5
1.0
1.5
2.0
Age (year)
Uncertainty Interval
6 − Subnational (Level 2)
Location Hierarchy
1 − Global
2 − Super Region
3 − Region
4 − Country
5 − Subnational (Level 1)
6 − Subnational (Level 2)

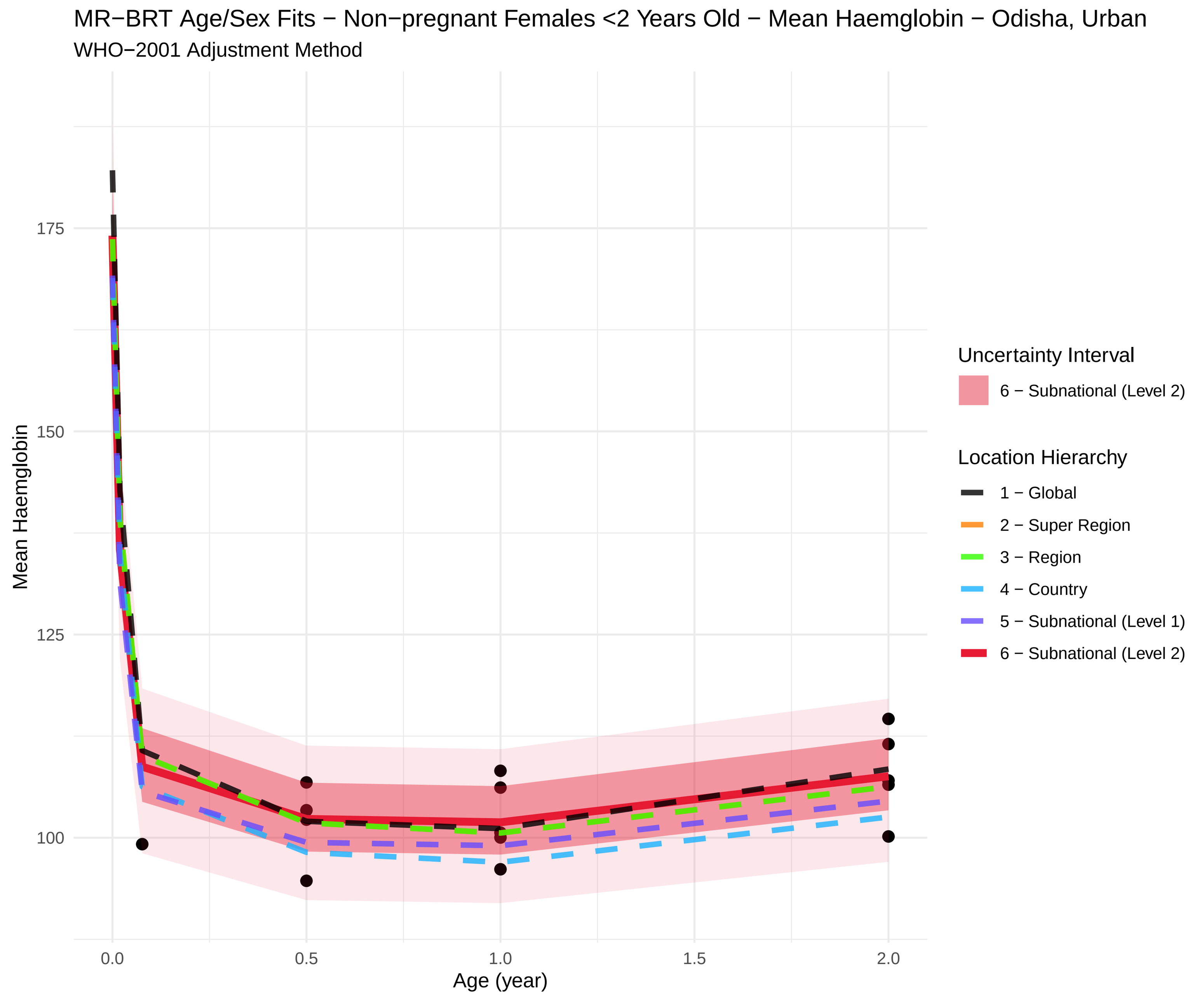
MR−BRT Age/Sex Fits − Non−pregnant Females <2 Years Old − Mean Haemglobin − Odisha, Urban
WHO−2001 Adjustment Method
Mean Haemglobin
175
150
125
100
0.0
0.5
1.0
1.5
2.0
Age (year)
Uncertainty Interval
6 − Subnational (Level 2)
Location Hierarchy
1 − Global
2 − Super Region
3 − Region
4 − Country
5 − Subnational (Level 1)
6 − Subnational (Level 2)

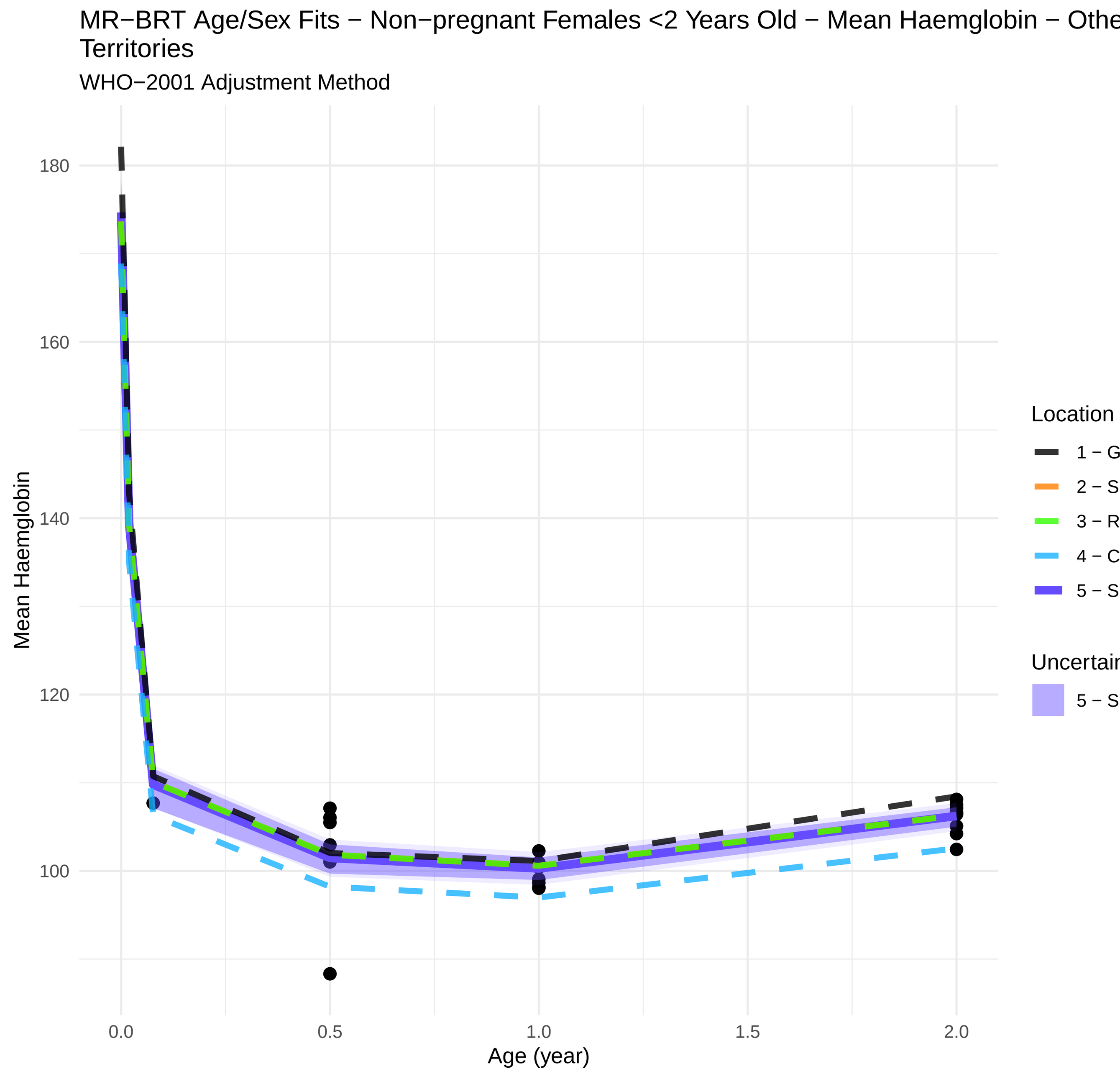
MR−BRT Age/Sex Fits − Non−pregnant Females <2 Years Old − Mean Haemglobin − Other Union Territories
WHO−2001 Adjustment Method
Mean Haemglobin
180
160
140
120
100
0.0
0.5
1.0
1.5
2.0
Age (year)
Location Hierarchy
1 − Global
2 − Super Region
3 − Region
4 − Country
5 − Subnational (Level 1)
Uncertainty Interval
5 − Subnational (Level 1)

MR−BRT Age/Sex Fits − Non-pregnant Females <2 Years Old − Mean Haemglobin − Other Union Territories, Rural
WHO−2001 Adjustment Method

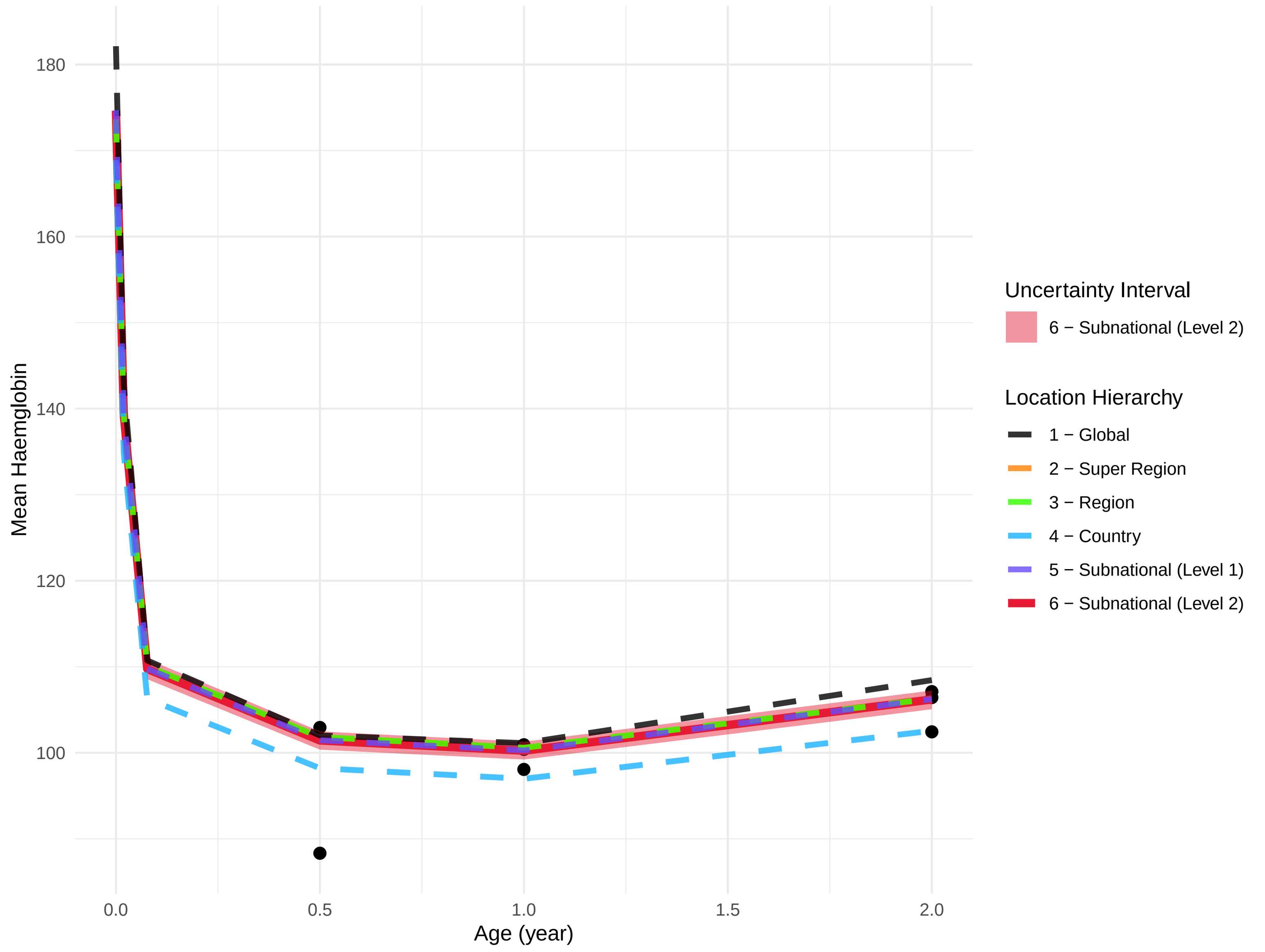

MR−BRT Age/Sex Fits − Non-pregnant Females <2 Years Old − Mean Haemglobin − Other Union Territories, Urban
WHO−2001 Adjustment Method

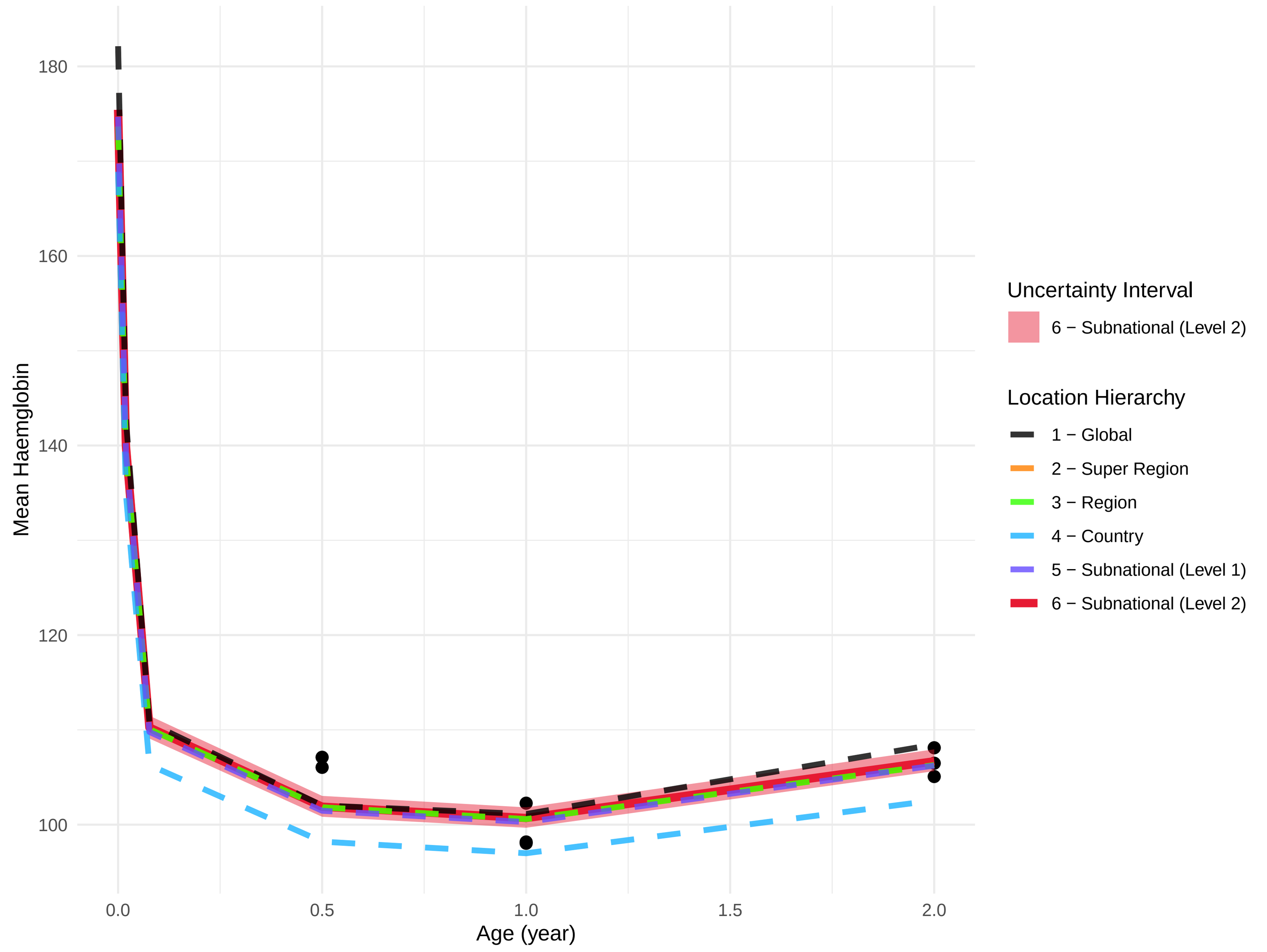

MR−BRT Age/Sex Fits − Non-pregnant Females <2 Years Old − Mean Haemglobin − Punjab
WHO−2001 Adjustment Method

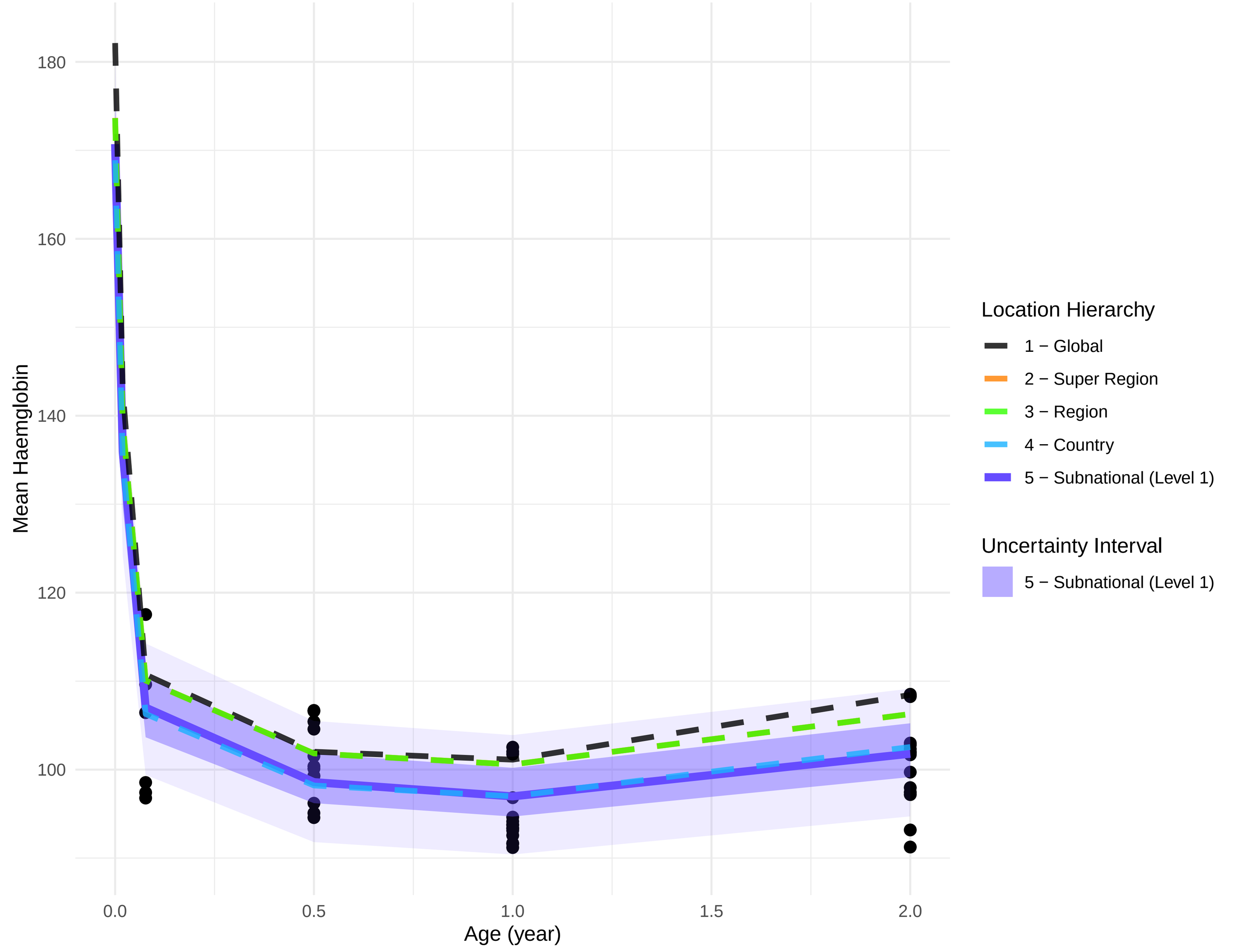

MR−BRT Age/Sex Fits − Non-pregnant Females <2 Years Old − Mean Haemglobin − Punjab, Rural
WHO−2001 Adjustment Method

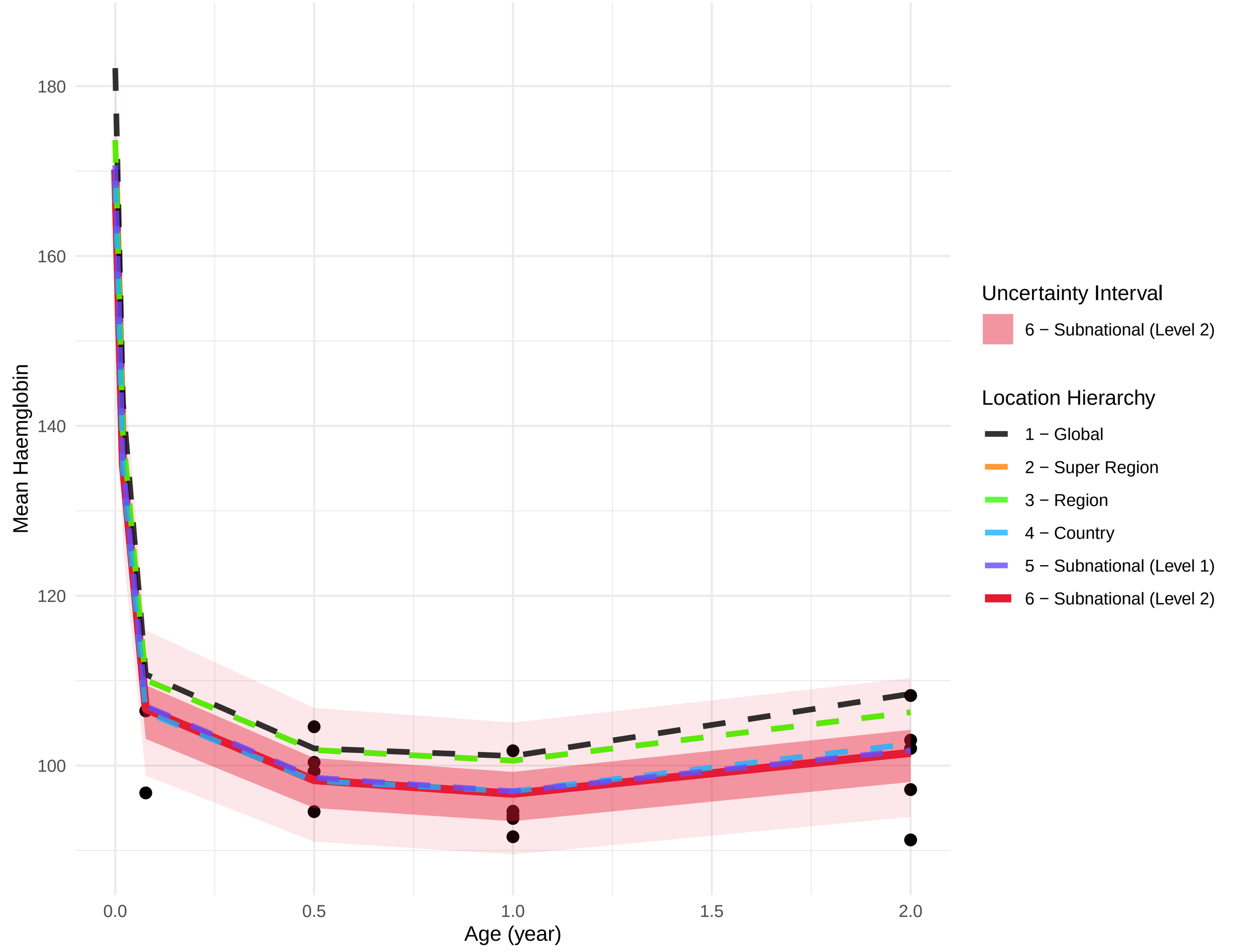

MR−BRT Age/Sex Fits − Non-pregnant Females <2 Years Old − Mean Haemglobin − Punjab, Urban
WHO−2001 Adjustment Method

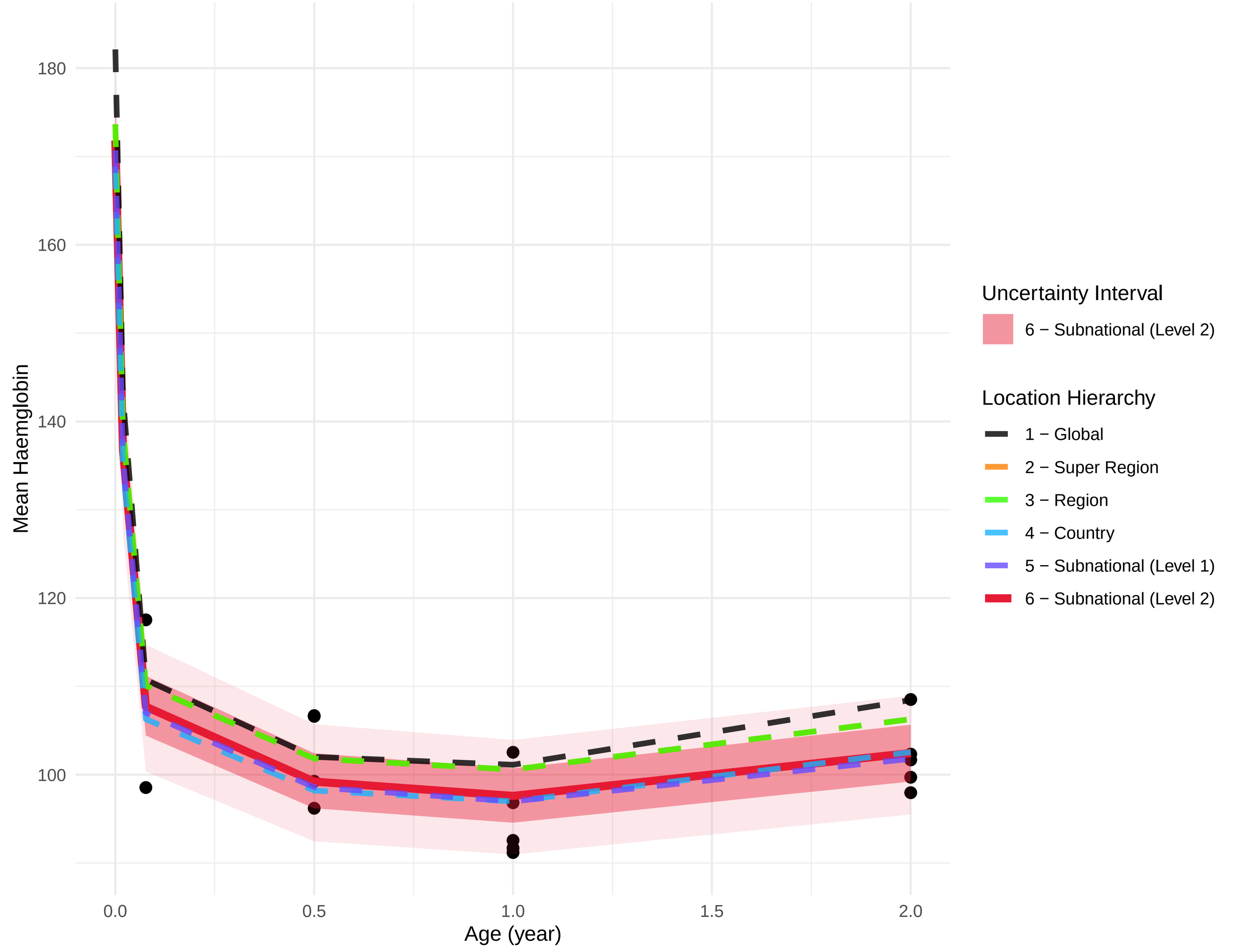

MR−BRT Age/Sex Fits − Non-pregnant Females <2 Years Old − Mean Haemglobin − Rajasthan
WHO−2001 Adjustment Method

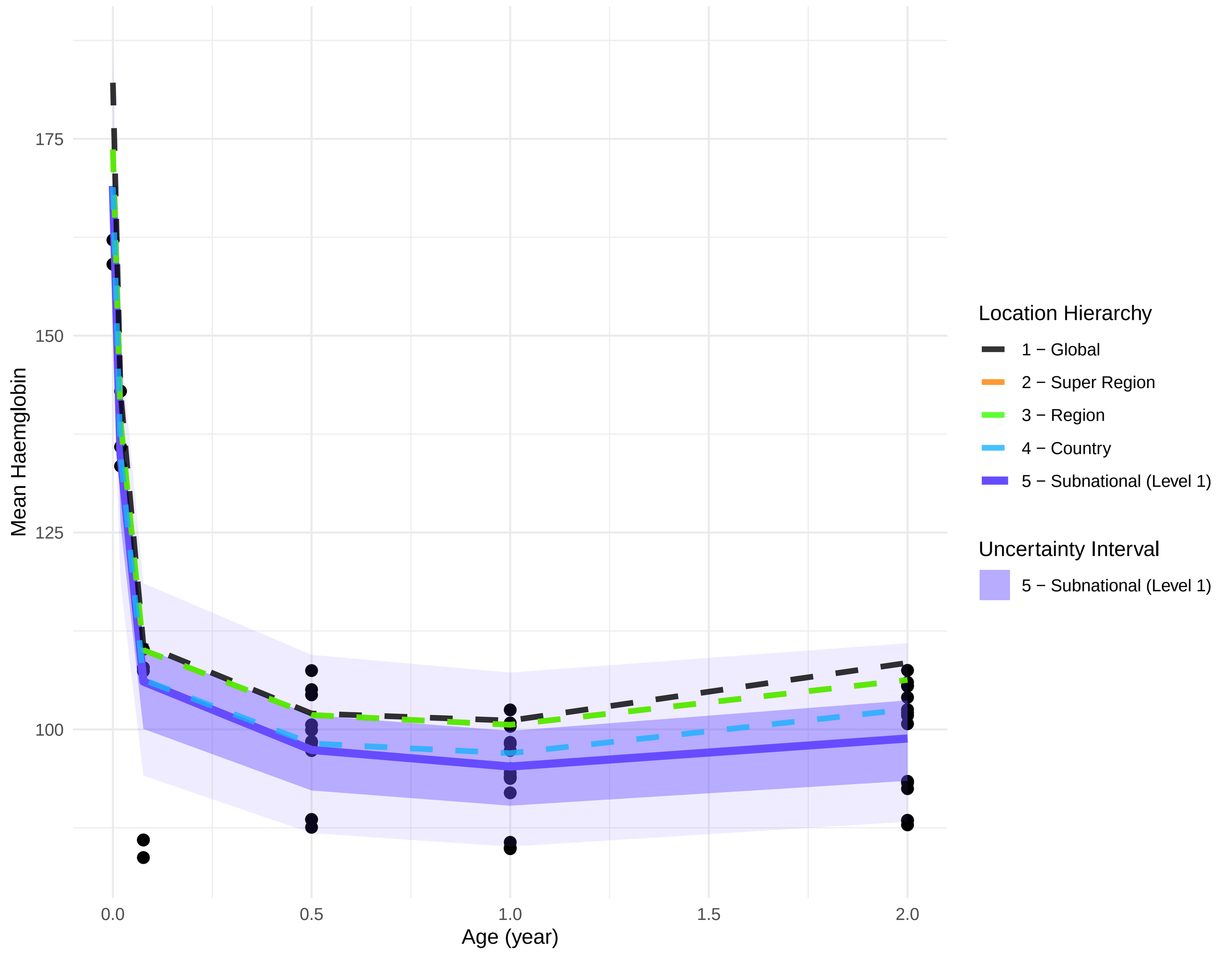

MR−BRT Age/Sex Fits − Non-pregnant Females <2 Years Old − Mean Haemglobin − Rajasthan, Rural
WHO−2001 Adjustment Method

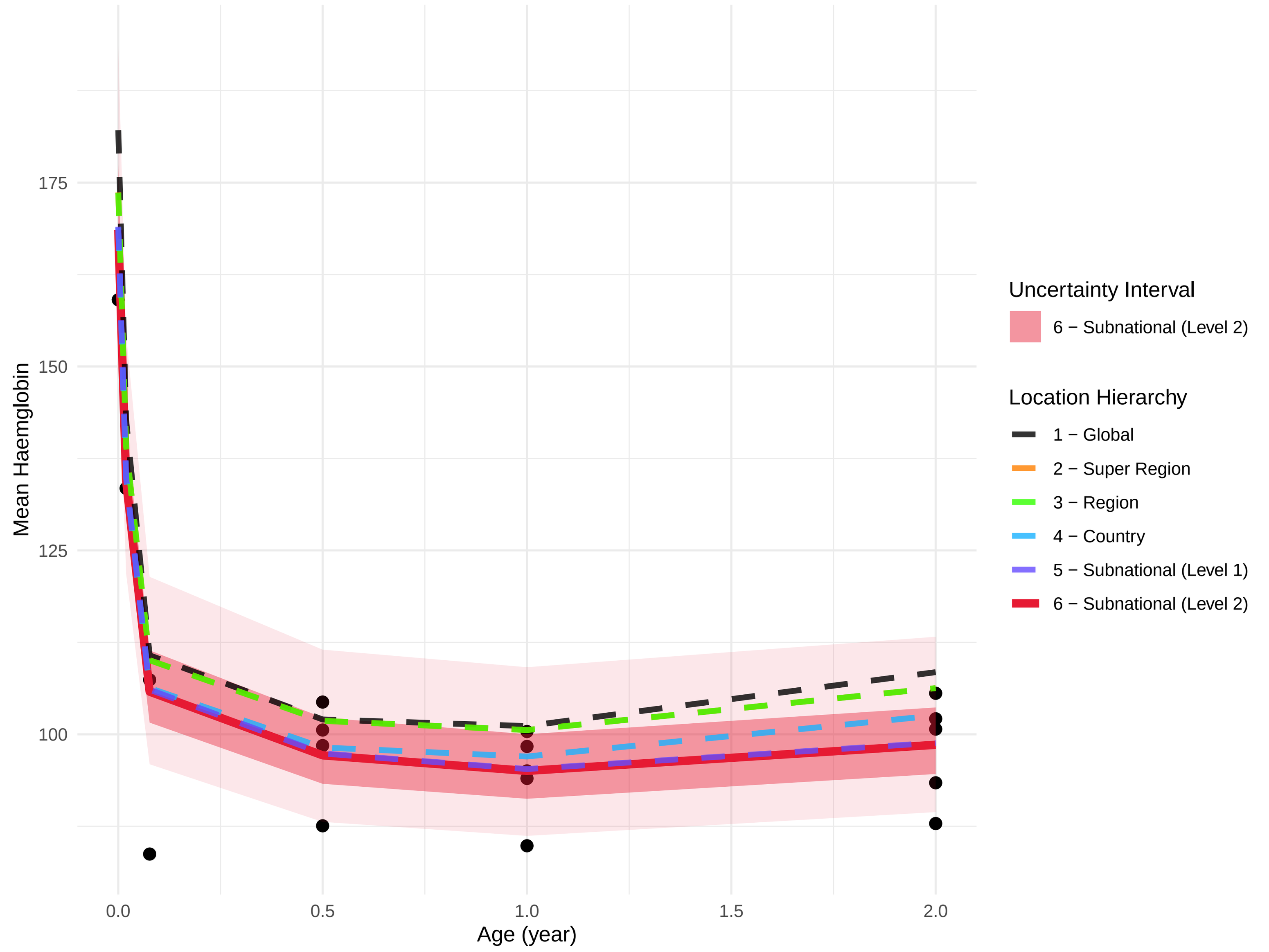

MR−BRT Age/Sex Fits − Non-pregnant Females <2 Years Old − Mean Haemglobin − Rajasthan, Urban
WHO−2001 Adjustment Method

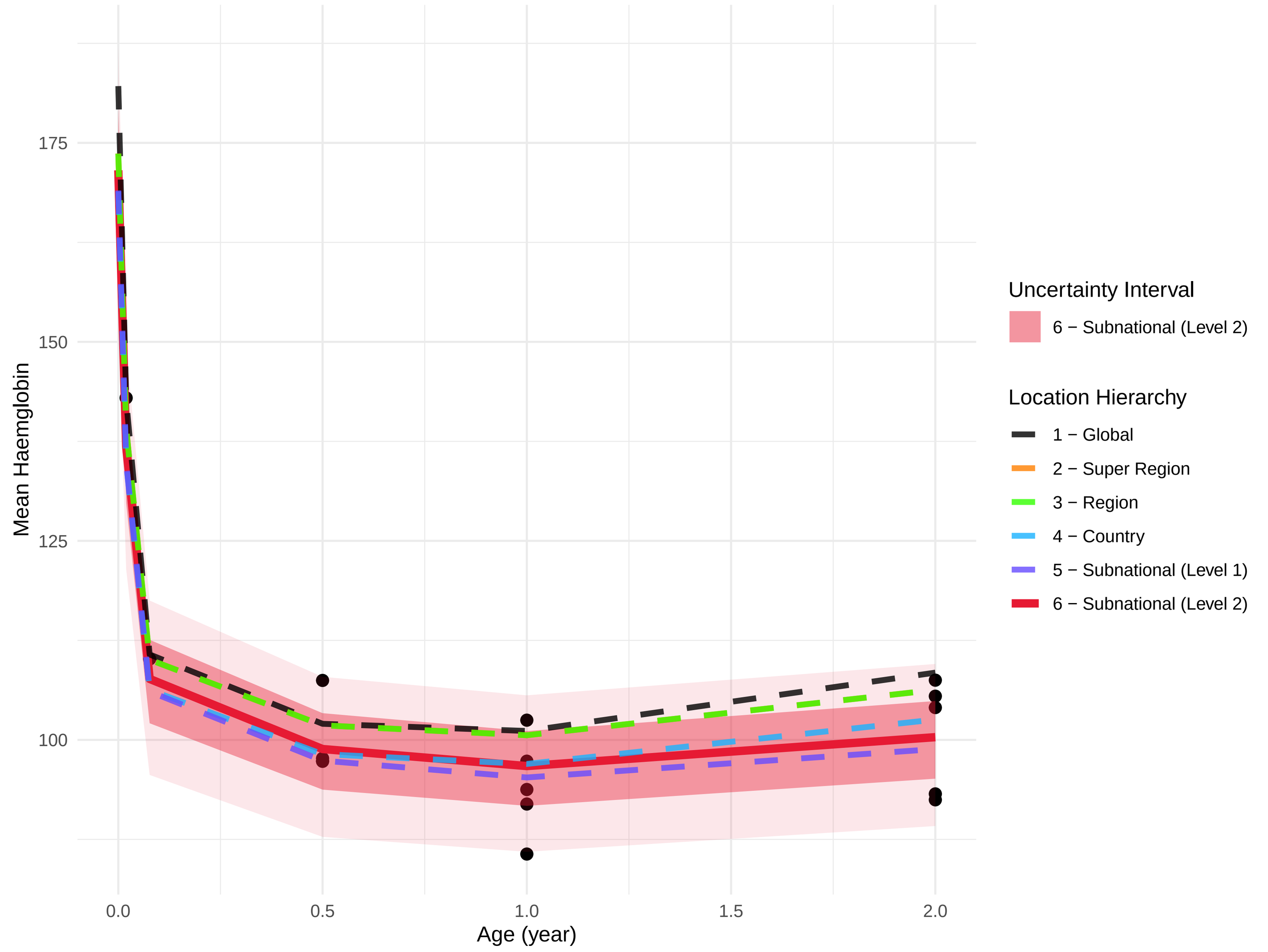

MR−BRT Age/Sex Fits − Non-pregnant Females <2 Years Old − Mean Haemglobin − Sikkim
WHO−2001 Adjustment Method

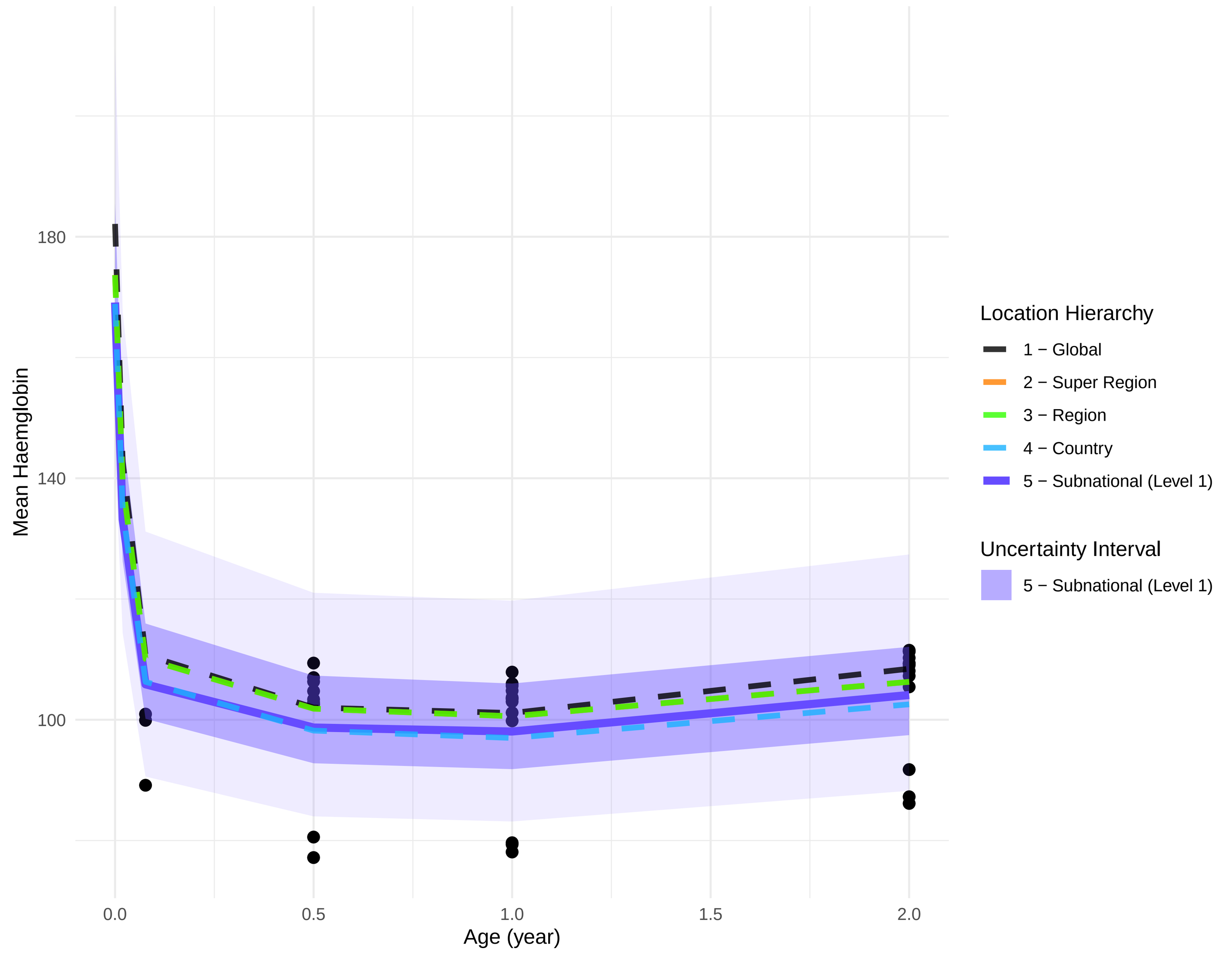

MR−BRT Age/Sex Fits − Non-pregnant Females <2 Years Old − Mean Haemglobin − Sikkim, Rural
WHO−2001 Adjustment Method

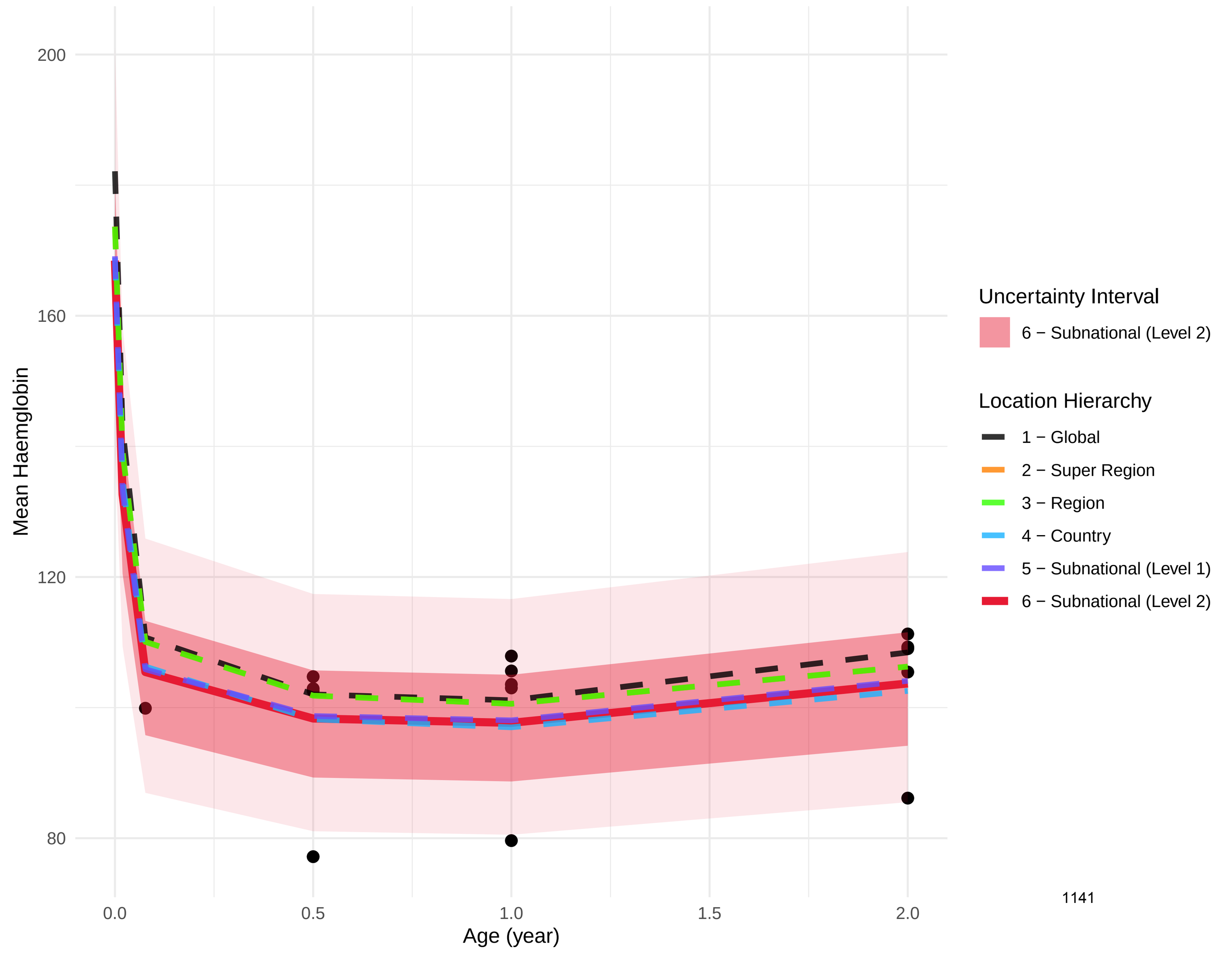

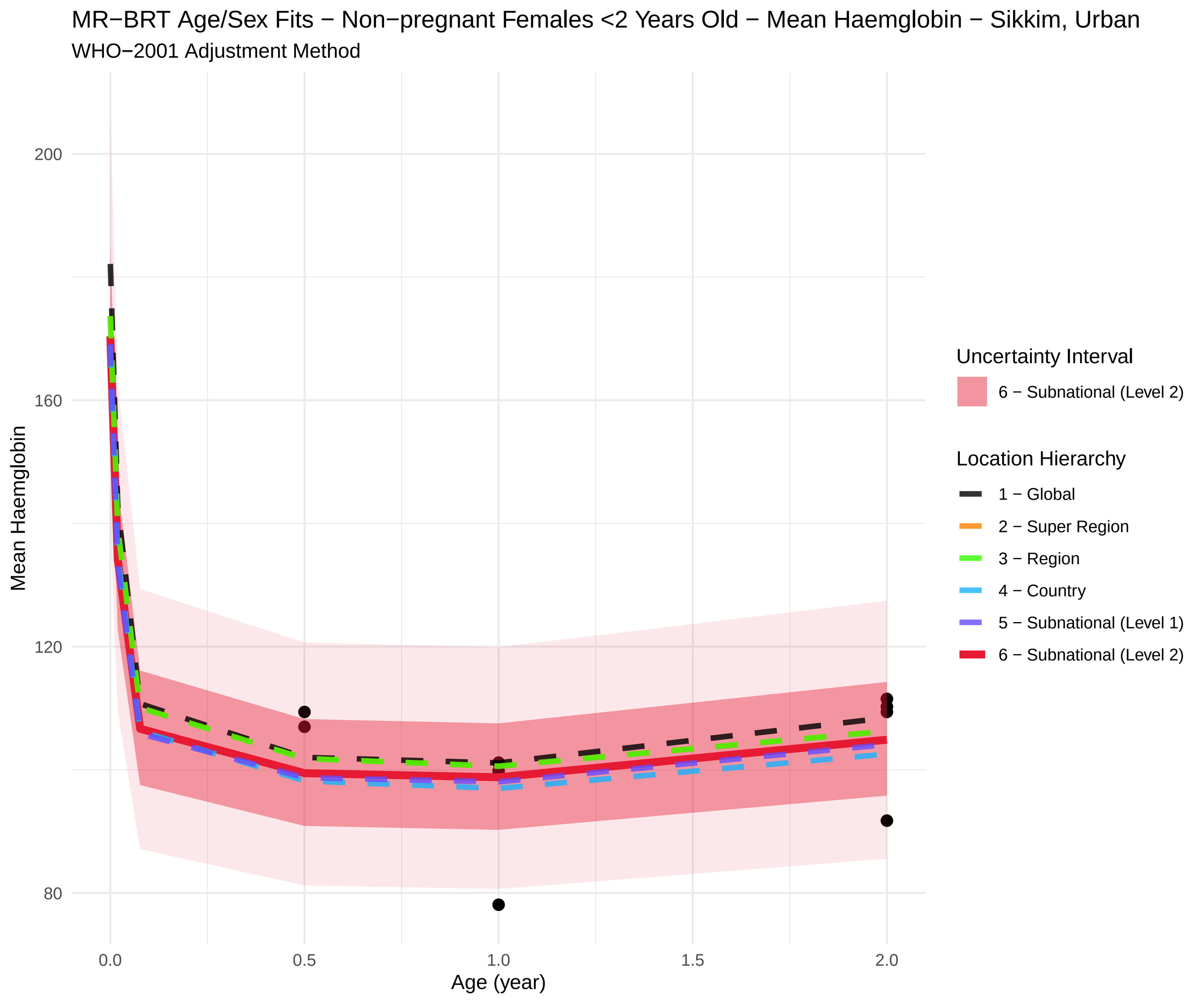
MR−BRT Age/Sex Fits − Non−pregnant Females <2 Years Old − Mean Haemglobin − Sikkim, Urban
WHO−2001 Adjustment Method
Mean Haemglobin
Age (year)
Uncertainty Interval
6 − Subnational (Level 2)
Location Hierarchy
1 − Global
2 − Super Region
3 − Region
4 − Country
5 − Subnational (Level 1)
6 − Subnational (Level 2)

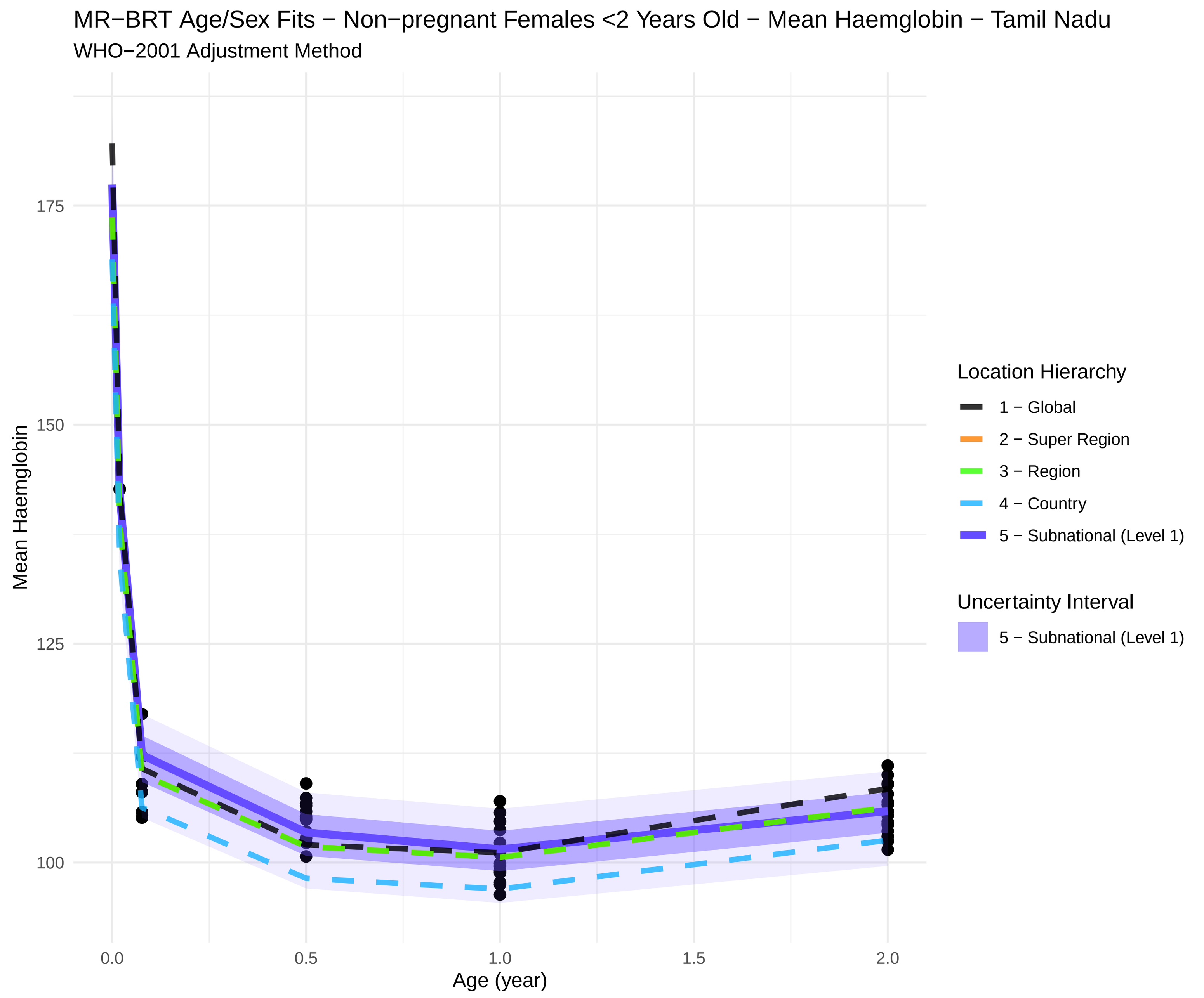
MR−BRT Age/Sex Fits − Non−pregnant Females <2 Years Old − Mean Haemglobin − Tamil Nadu
WHO−2001 Adjustment Method
Mean Haemglobin
Age (year)
Location Hierarchy
1 − Global
2 − Super Region
3 − Region
4 − Country
5 − Subnational (Level 1)
Uncertainty Interval
5 − Subnational (Level 1)

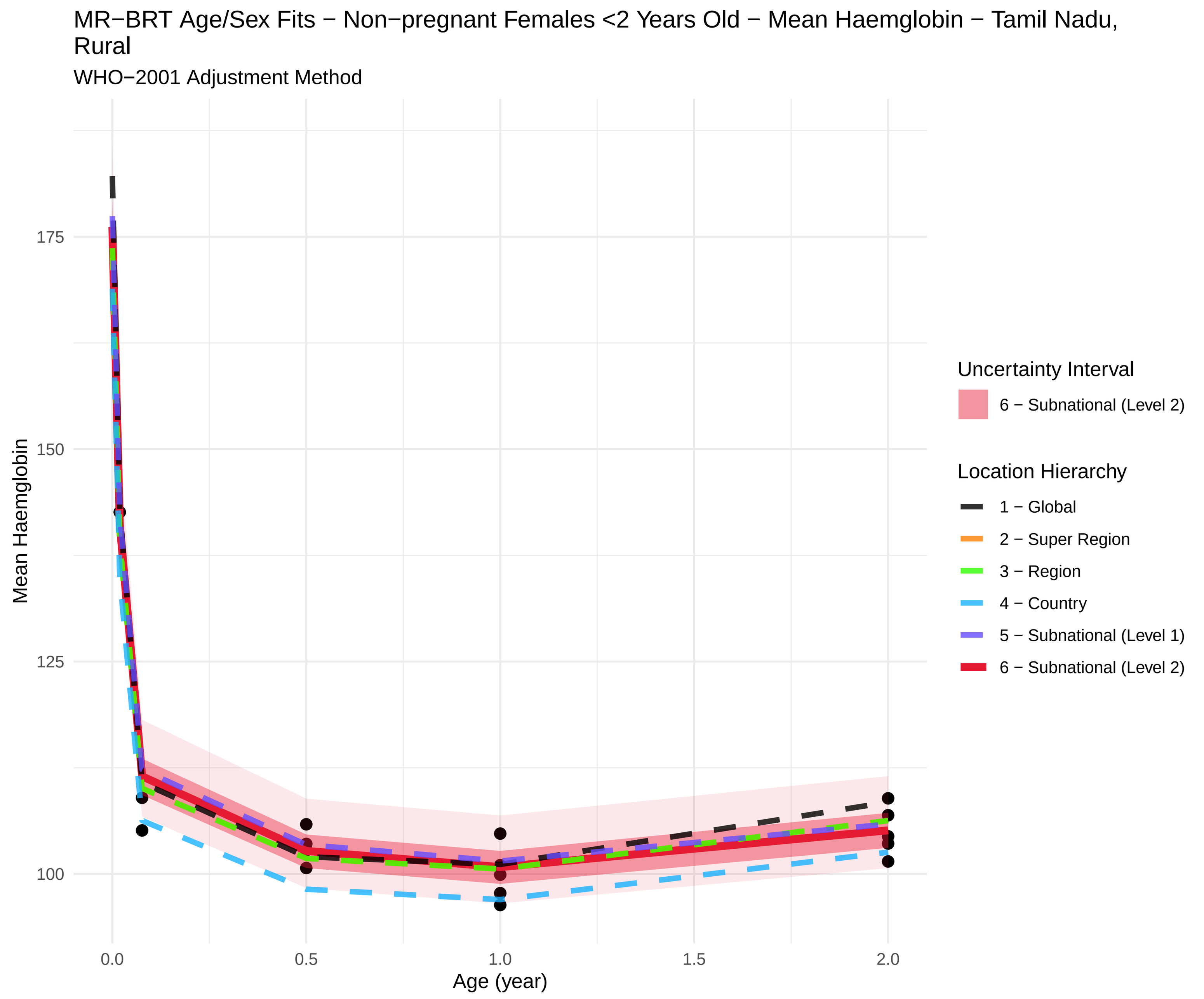
MR−BRT Age/Sex Fits − Non−pregnant Females <2 Years Old − Mean Haemglobin − Tamil Nadu, Rural
WHO−2001 Adjustment Method
Mean Haemglobin
Age (year)
Uncertainty Interval
6 − Subnational (Level 2)
Location Hierarchy
1 − Global
2 − Super Region
3 − Region
4 − Country
5 − Subnational (Level 1)
6 − Subnational (Level 2)

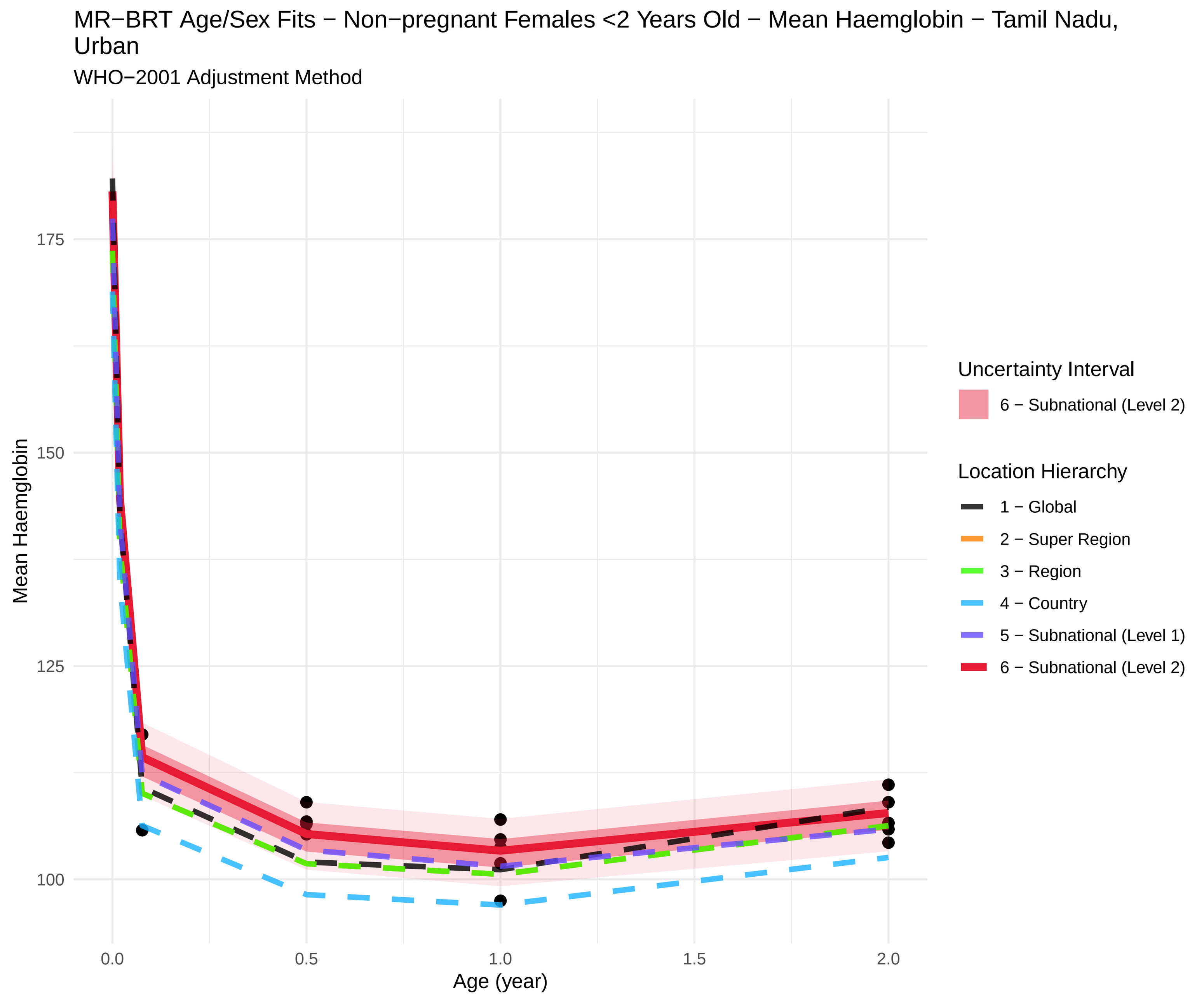
MR−BRT Age/Sex Fits − Non−pregnant Females <2 Years Old − Mean Haemglobin − Tamil Nadu, Urban
WHO−2001 Adjustment Method
Mean Haemglobin
Age (year)
Uncertainty Interval
6 − Subnational (Level 2)
Location Hierarchy
1 − Global
2 − Super Region
3 − Region
4 − Country
5 − Subnational (Level 1)
6 − Subnational (Level 2)

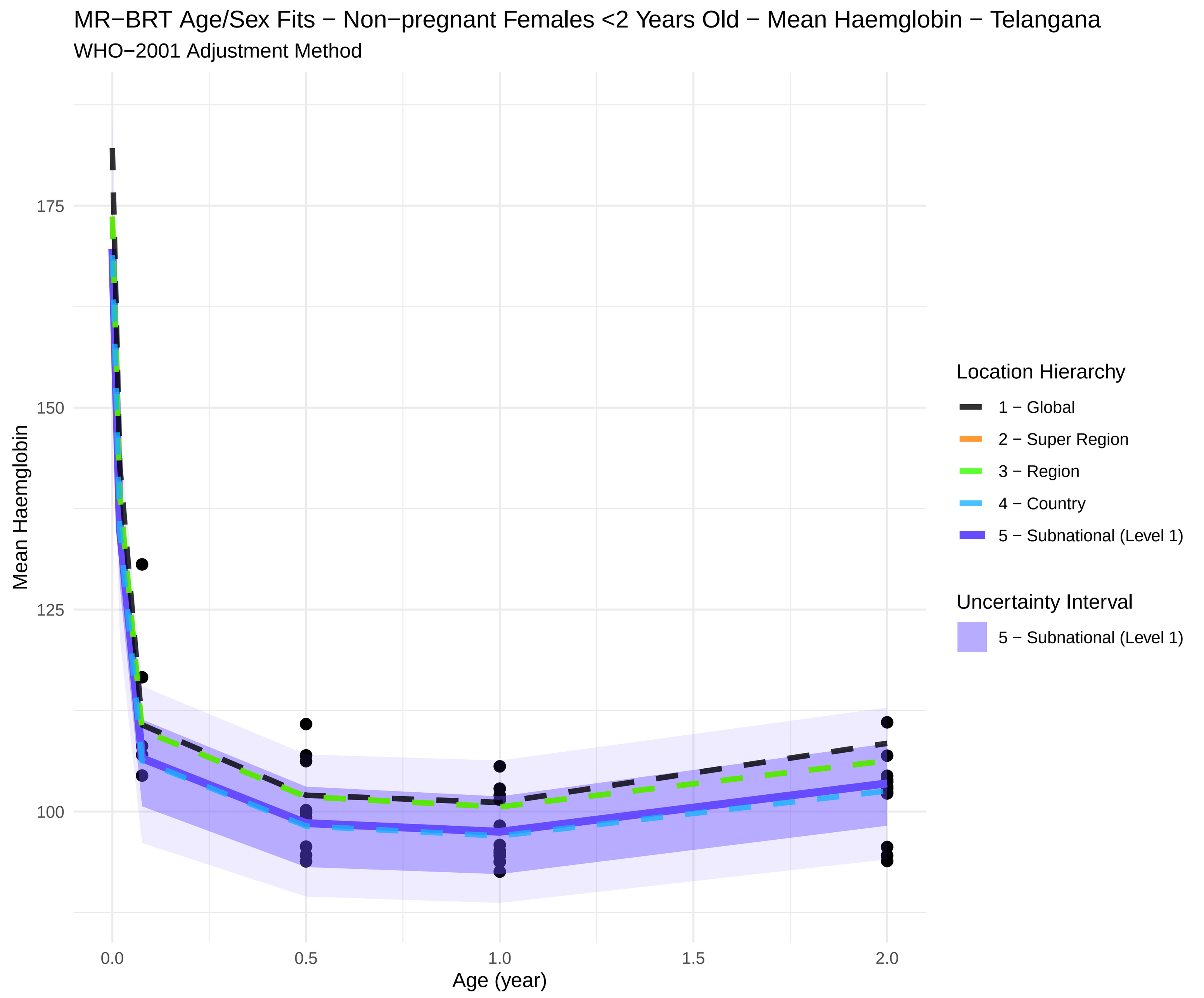
MR−BRT Age/Sex Fits − Non−pregnant Females <2 Years Old − Mean Haemglobin − Telangana
WHO−2001 Adjustment Method
Mean Haemglobin
Age (year)
Location Hierarchy
1 − Global
2 − Super Region
3 − Region
4 − Country
5 − Subnational (Level 1)
Uncertainty Interval
5 − Subnational (Level 1)

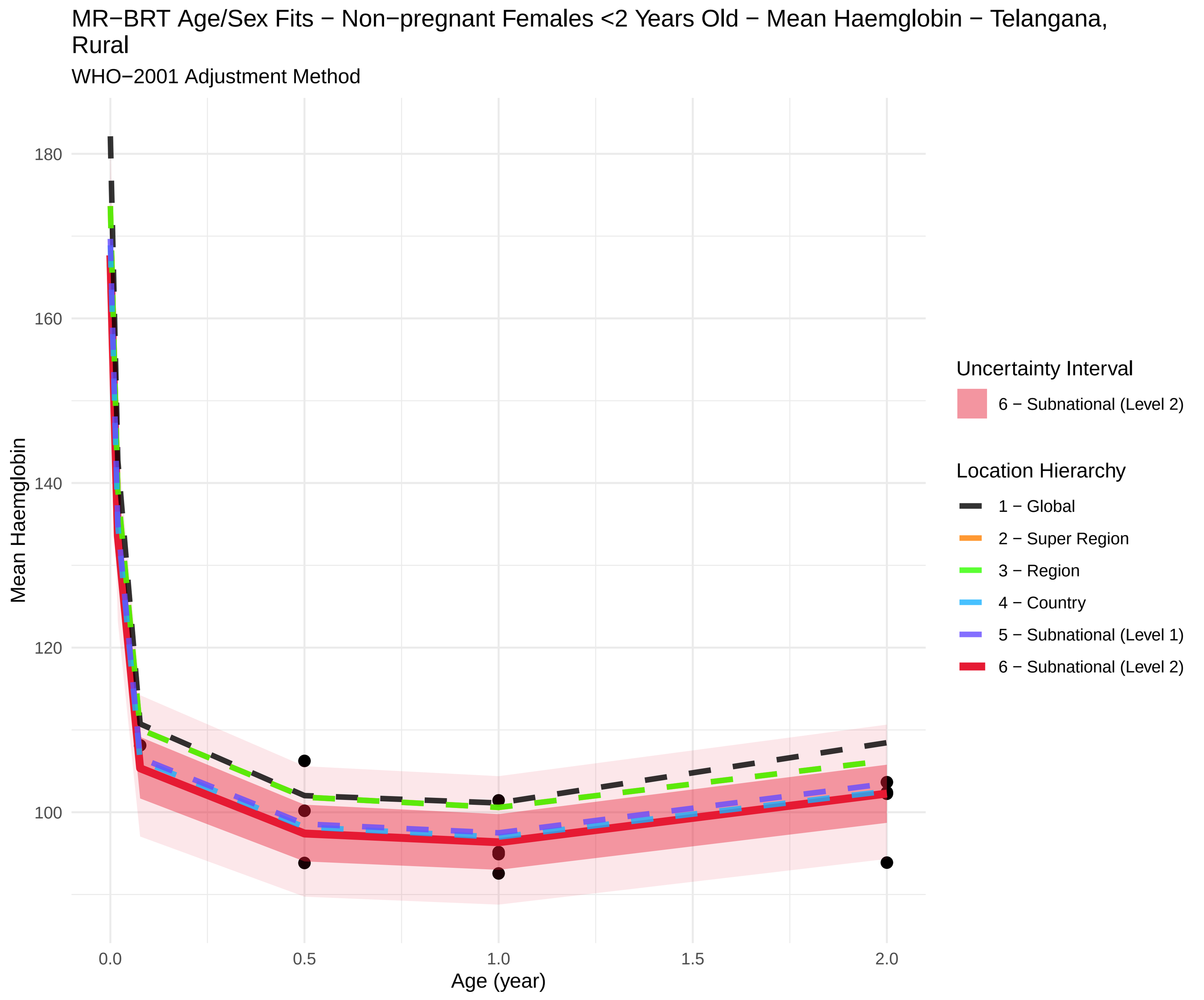
MR−BRT Age/Sex Fits − Non−pregnant Females <2 Years Old − Mean Haemglobin − Telangana, Rural
WHO−2001 Adjustment Method
Mean Haemglobin
Age (year)
Uncertainty Interval
6 − Subnational (Level 2)
Location Hierarchy
1 − Global
2 − Super Region
3 − Region
4 − Country
5 − Subnational (Level 1)
6 − Subnational (Level 2)

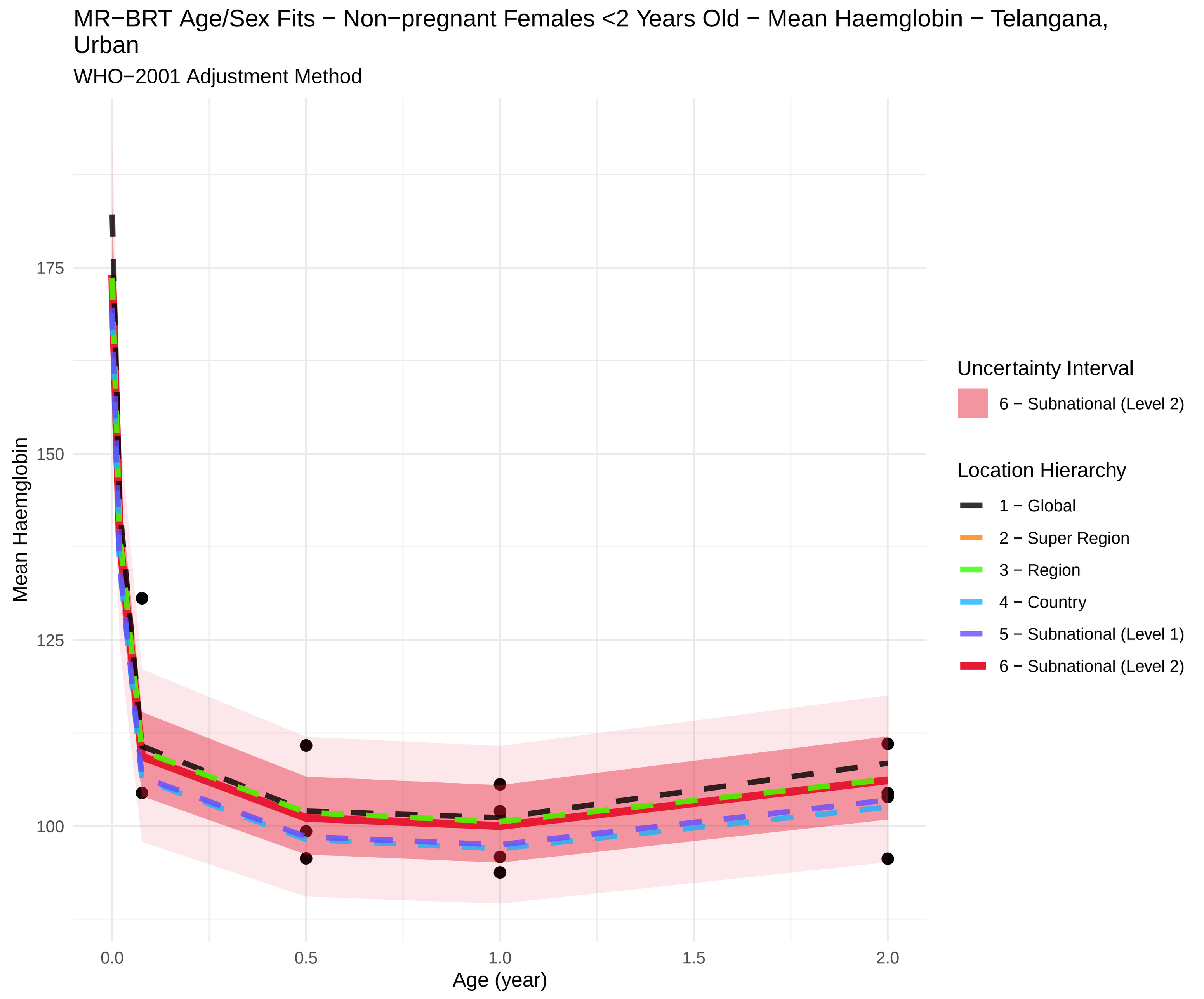
MR−BRT Age/Sex Fits − Non−pregnant Females <2 Years Old − Mean Haemglobin − Telangana, Urban
WHO−2001 Adjustment Method
Mean Haemglobin
Age (year)
Uncertainty Interval
6 − Subnational (Level 2)
Location Hierarchy
1 − Global
2 − Super Region
3 − Region
4 − Country
5 − Subnational (Level 1)
6 − Subnational (Level 2)

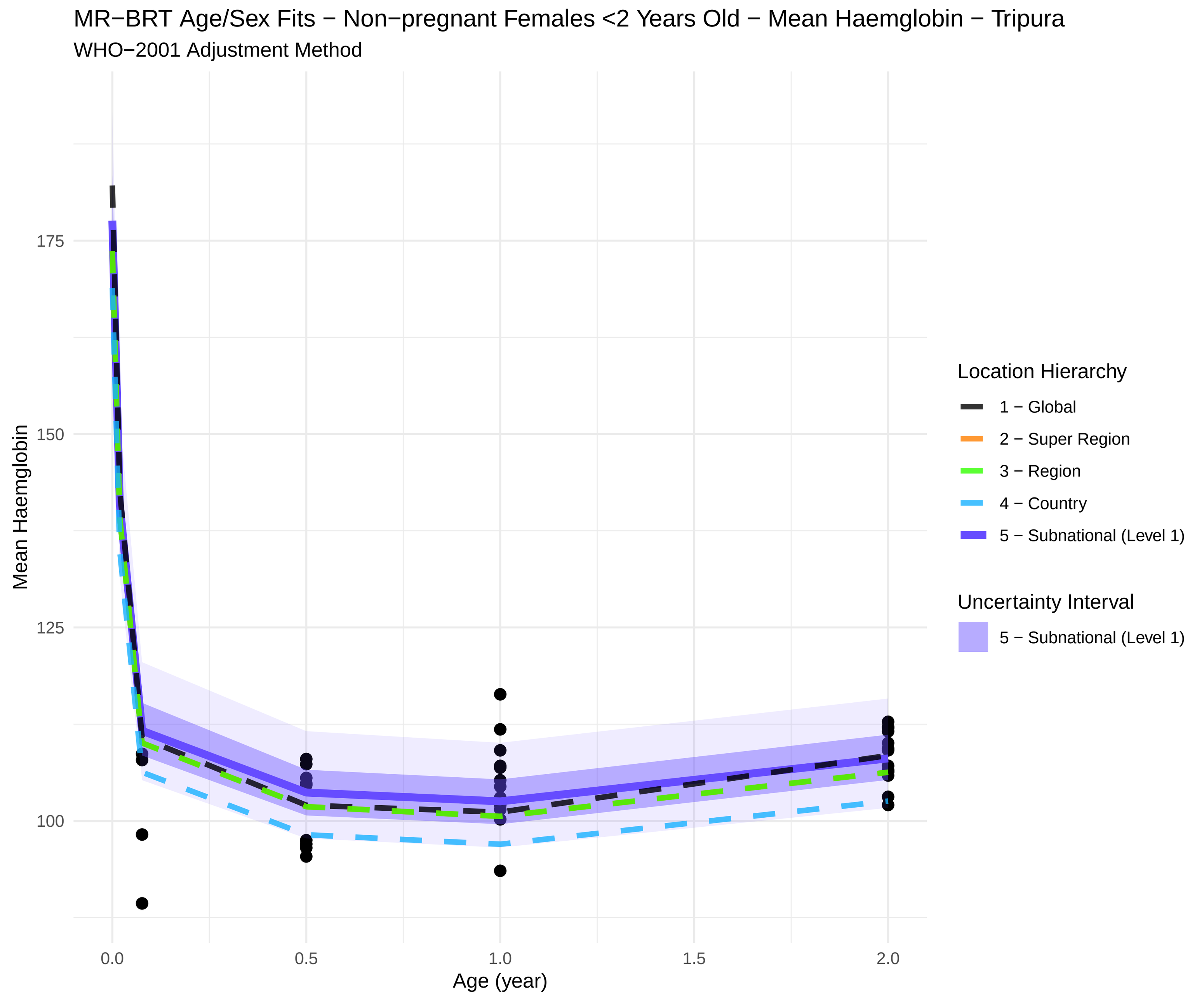
MR−BRT Age/Sex Fits − Non−pregnant Females <2 Years Old − Mean Haemglobin − Tripura
WHO−2001 Adjustment Method
Mean Haemglobin
Age (year)
Location Hierarchy
1 − Global
2 − Super Region
3 − Region
4 − Country
5 − Subnational (Level 1)
Uncertainty Interval
5 − Subnational (Level 1)

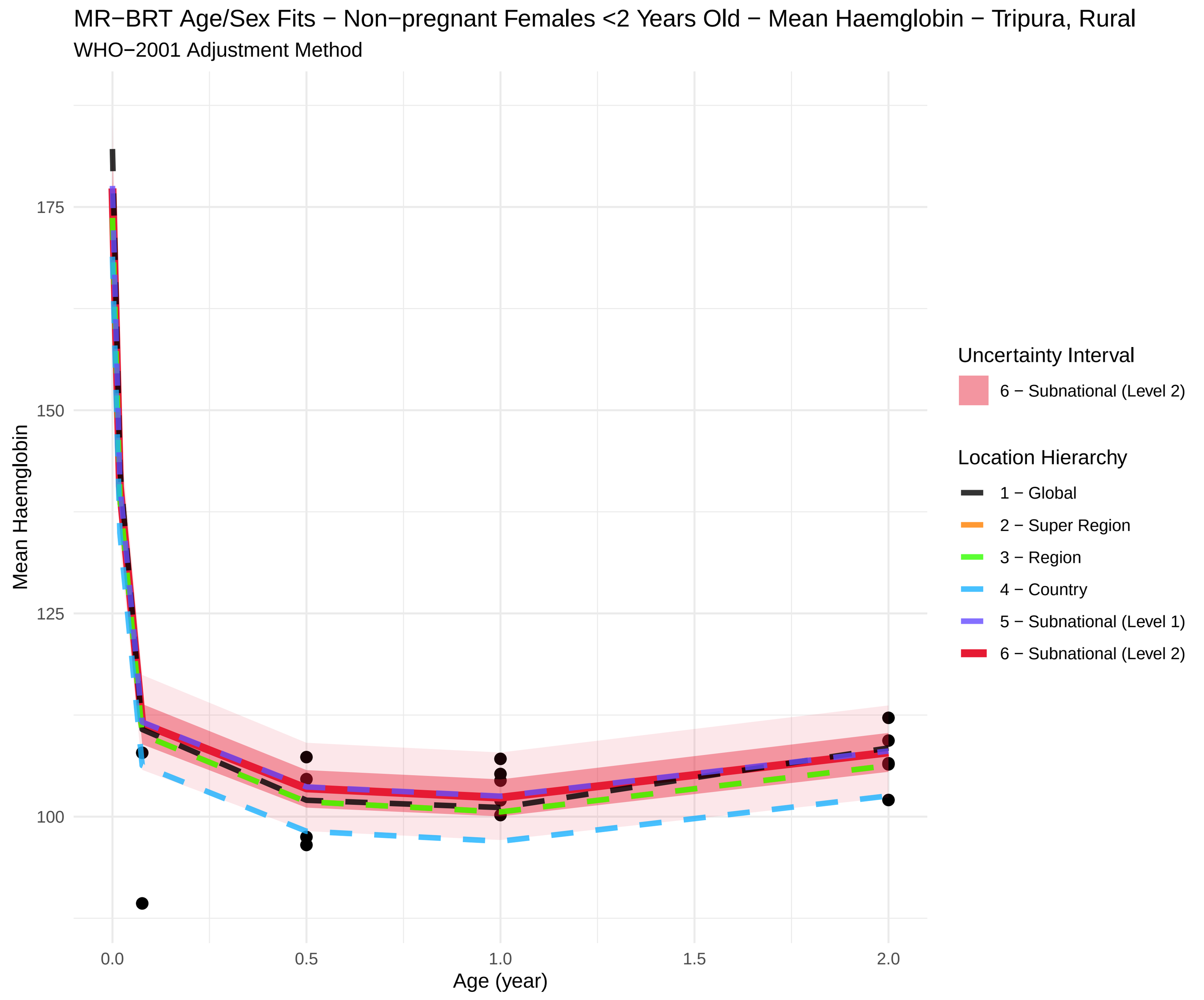
MR−BRT Age/Sex Fits − Non−pregnant Females <2 Years Old − Mean Haemglobin − Tripura, Rural
WHO−2001 Adjustment Method
Mean Haemglobin
Age (year)
Uncertainty Interval
6 − Subnational (Level 2)
Location Hierarchy
1 − Global
2 − Super Region
3 − Region
4 − Country
5 − Subnational (Level 1)
6 − Subnational (Level 2)

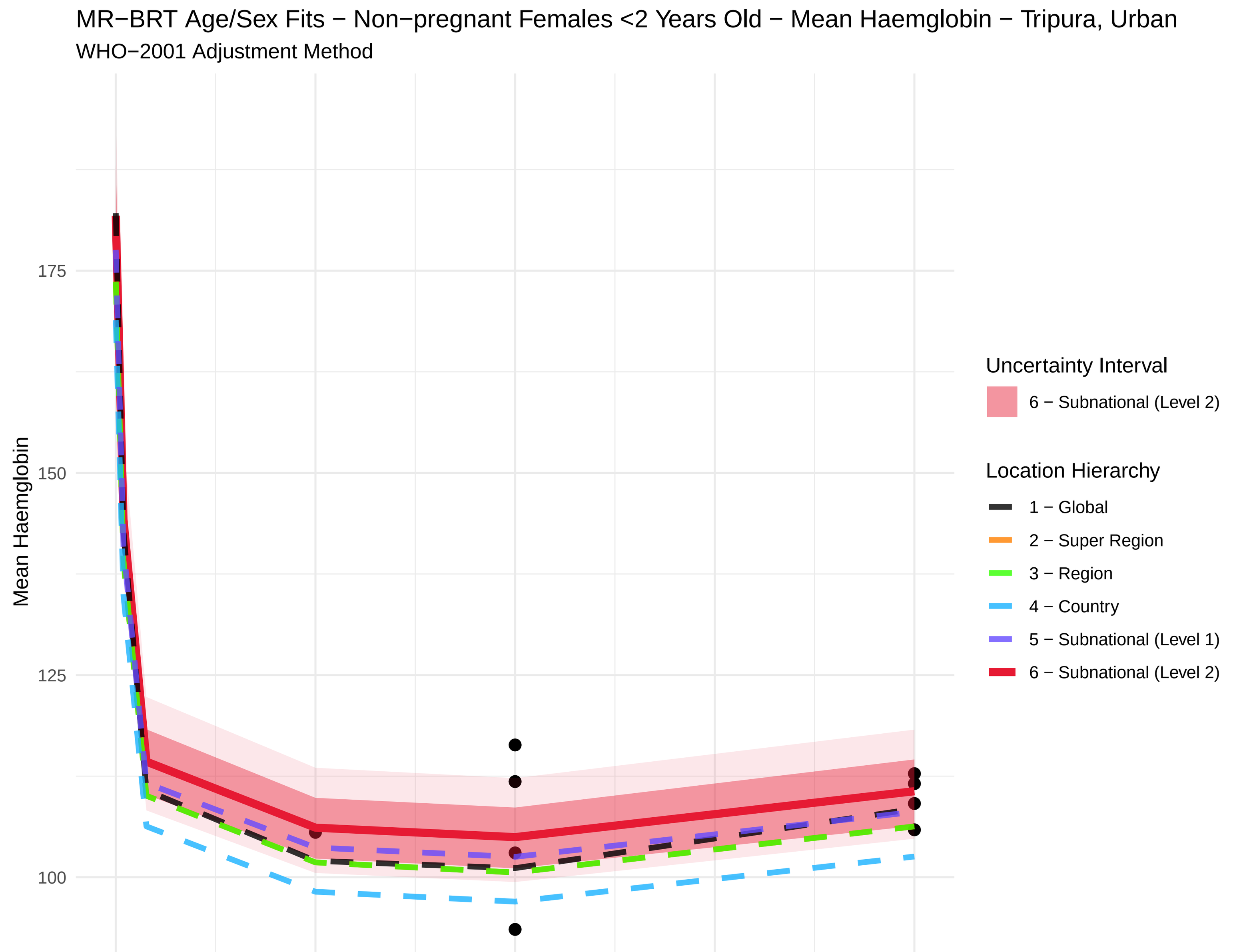
MR−BRT Age/Sex Fits − Non−pregnant Females <2 Years Old − Mean Haemglobin − Tripura, Urban
WHO−2001 Adjustment Method
Mean Haemglobin
Age (year)
Uncertainty Interval
6 − Subnational (Level 2)
Location Hierarchy
1 − Global
2 − Super Region
3 − Region
4 − Country
5 − Subnational (Level 1)
6 − Subnational (Level 2)

MR−BRT Age/Sex Fits − Non−pregnant Females <2 Years Old − Mean Haemglobin − Uttar Pradesh
WHO−2001 Adjustment Method

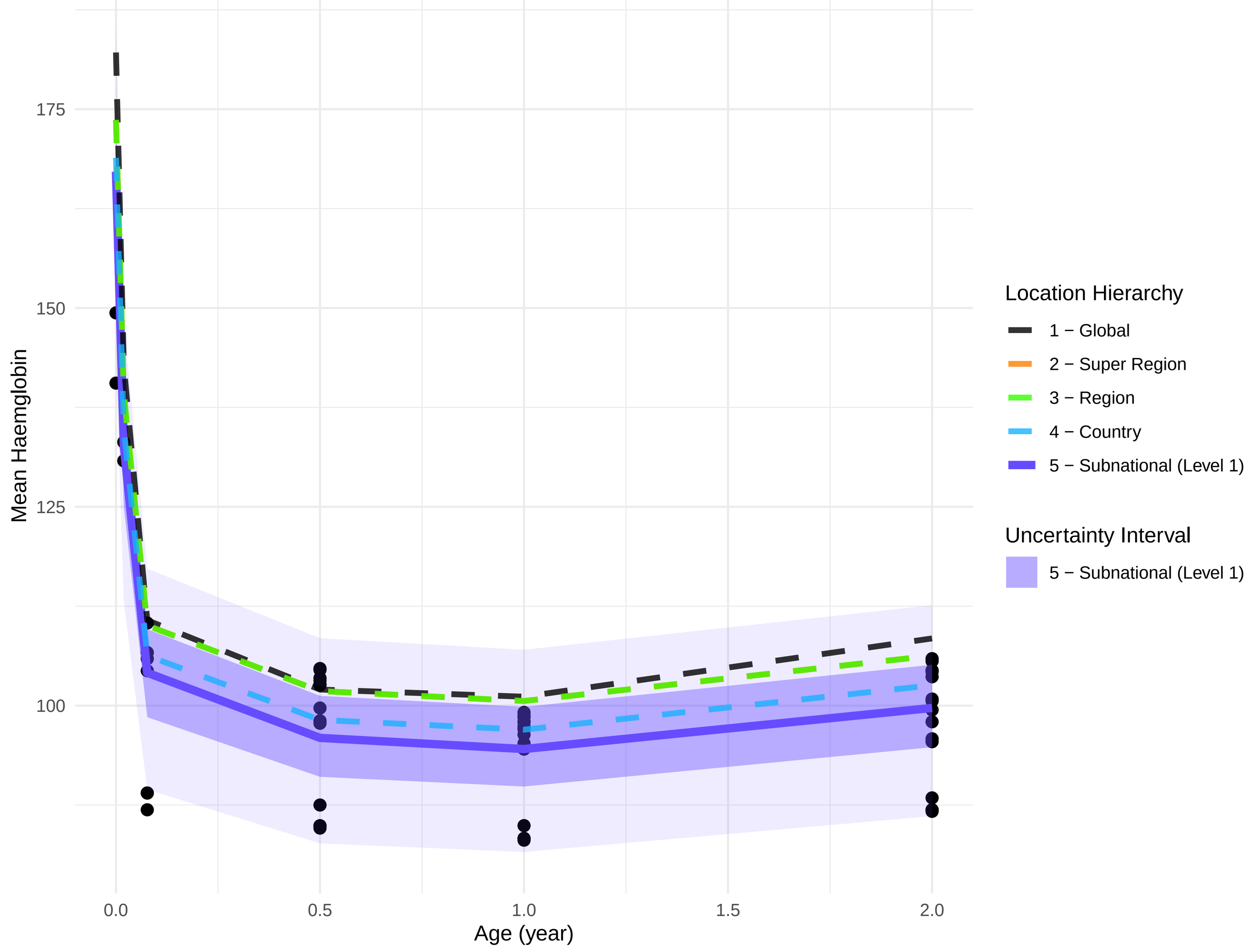

MR−BRT Age/Sex Fits − Non−pregnant Females <2 Years Old − Mean Haemglobin − Uttar Pradesh, Rural
WHO−2001 Adjustment Method

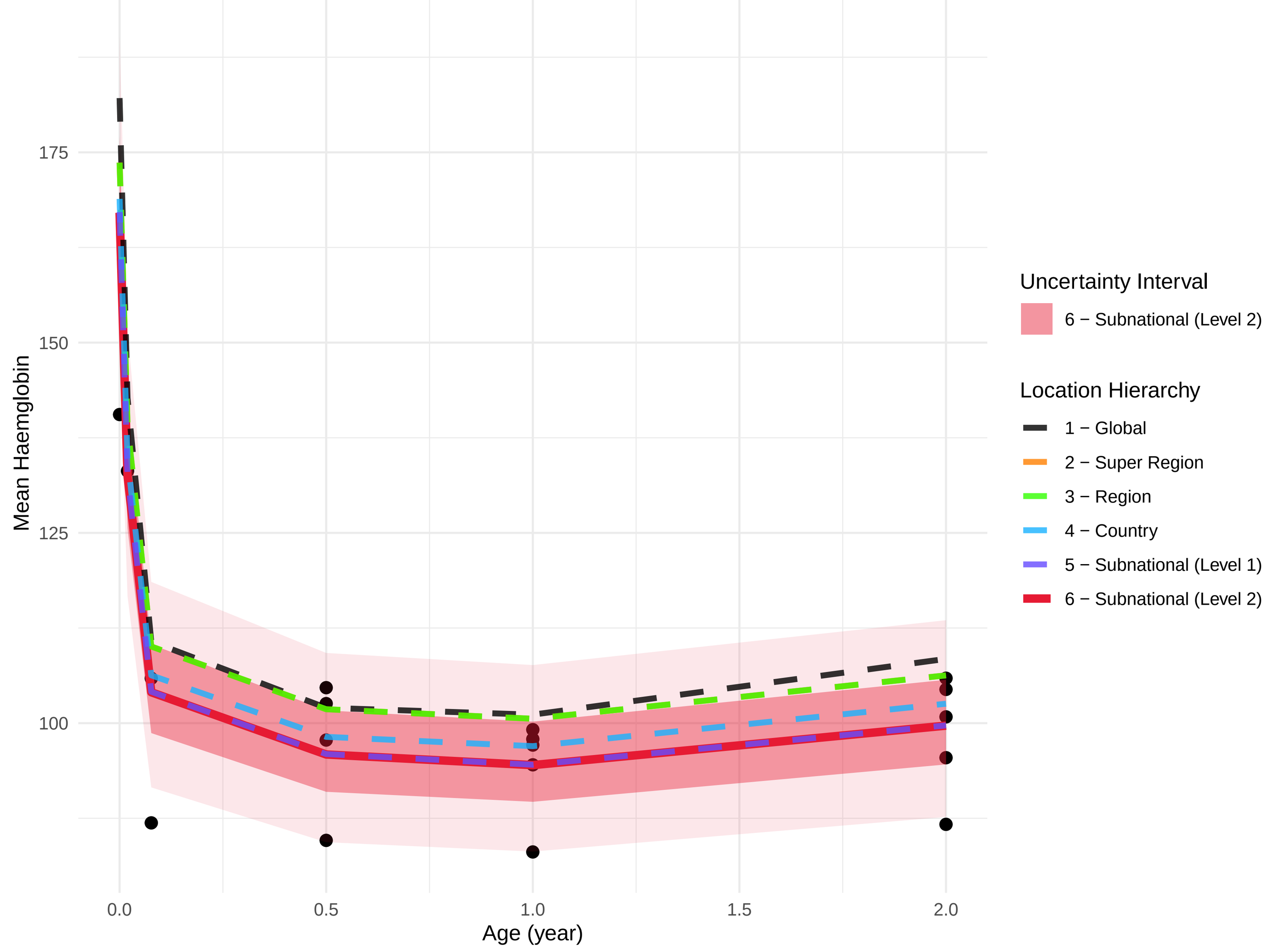

MR−BRT Age/Sex Fits − Non−pregnant Females <2 Years Old − Mean Haemglobin − Uttar Pradesh, Urban
WHO−2001 Adjustment Method

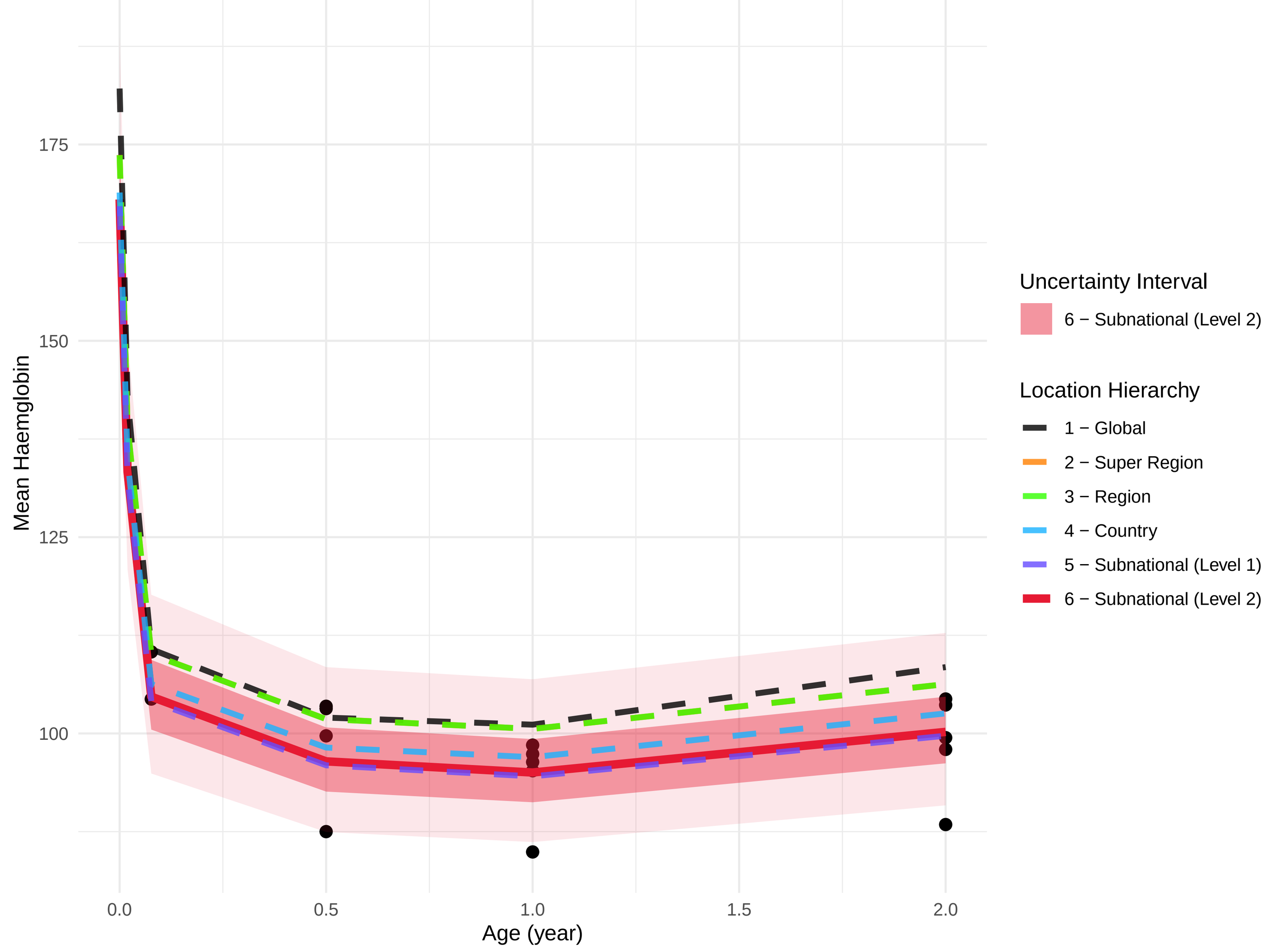

MR−BRT Age/Sex Fits − Non−pregnant Females <2 Years Old − Mean Haemglobin − Uttarakhand
WHO−2001 Adjustment Method

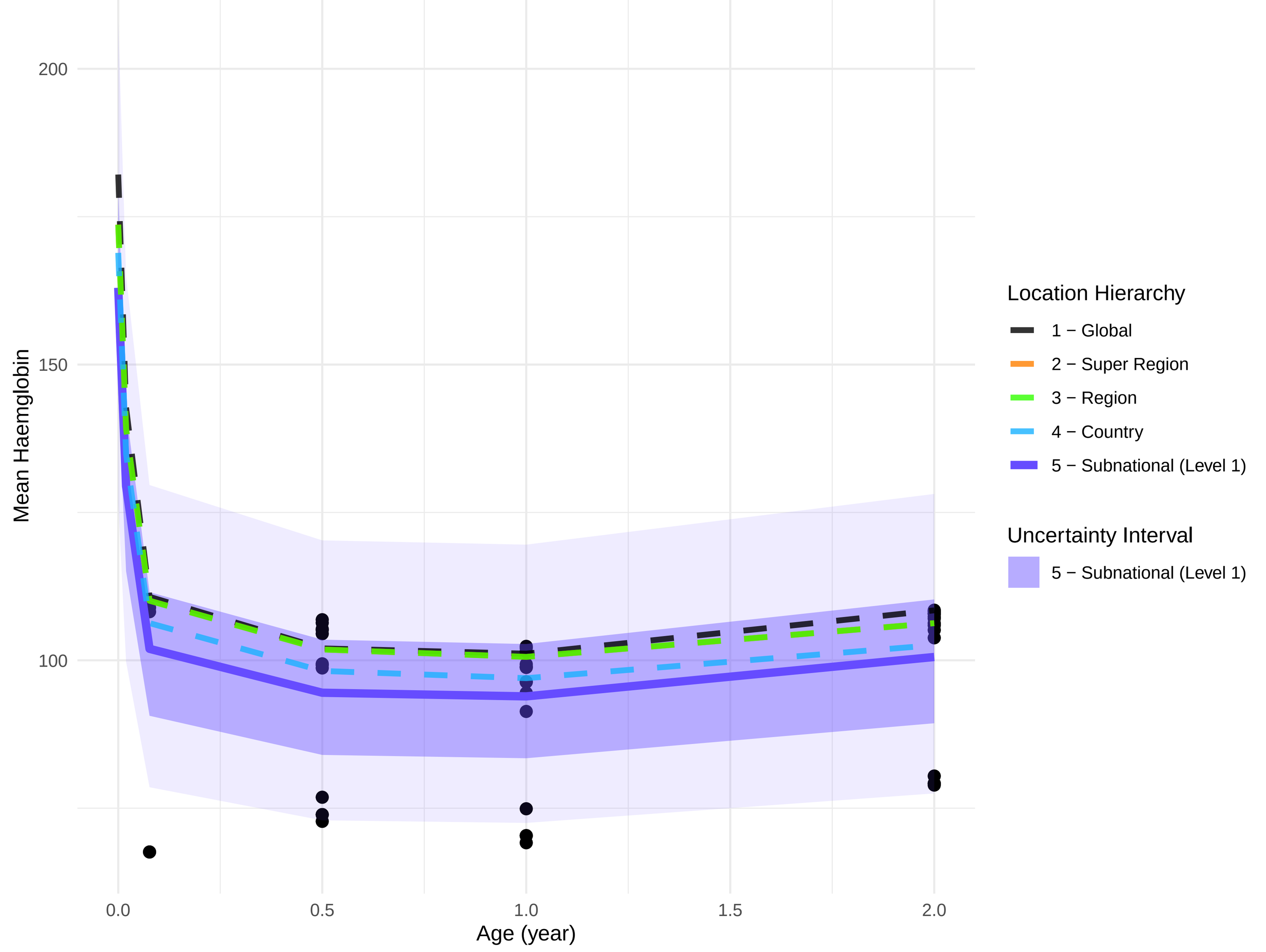

MR−BRT Age/Sex Fits − Non−pregnant Females <2 Years Old − Mean Haemglobin − Uttarakhand, Rural
WHO−2001 Adjustment Method

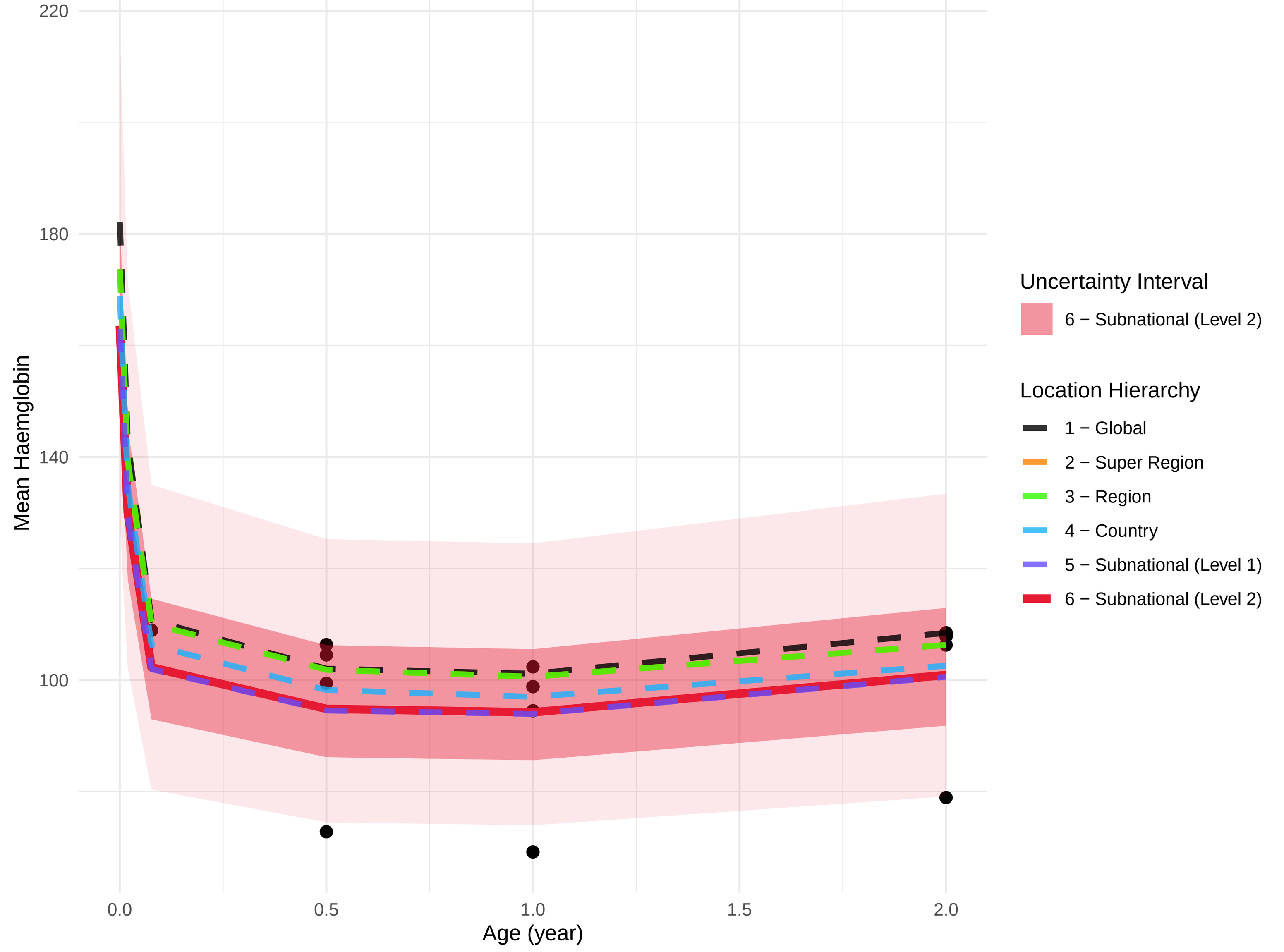

MR−BRT Age/Sex Fits − Non−pregnant Females <2 Years Old − Mean Haemglobin − Uttarakhand, Urban
WHO−2001 Adjustment Method

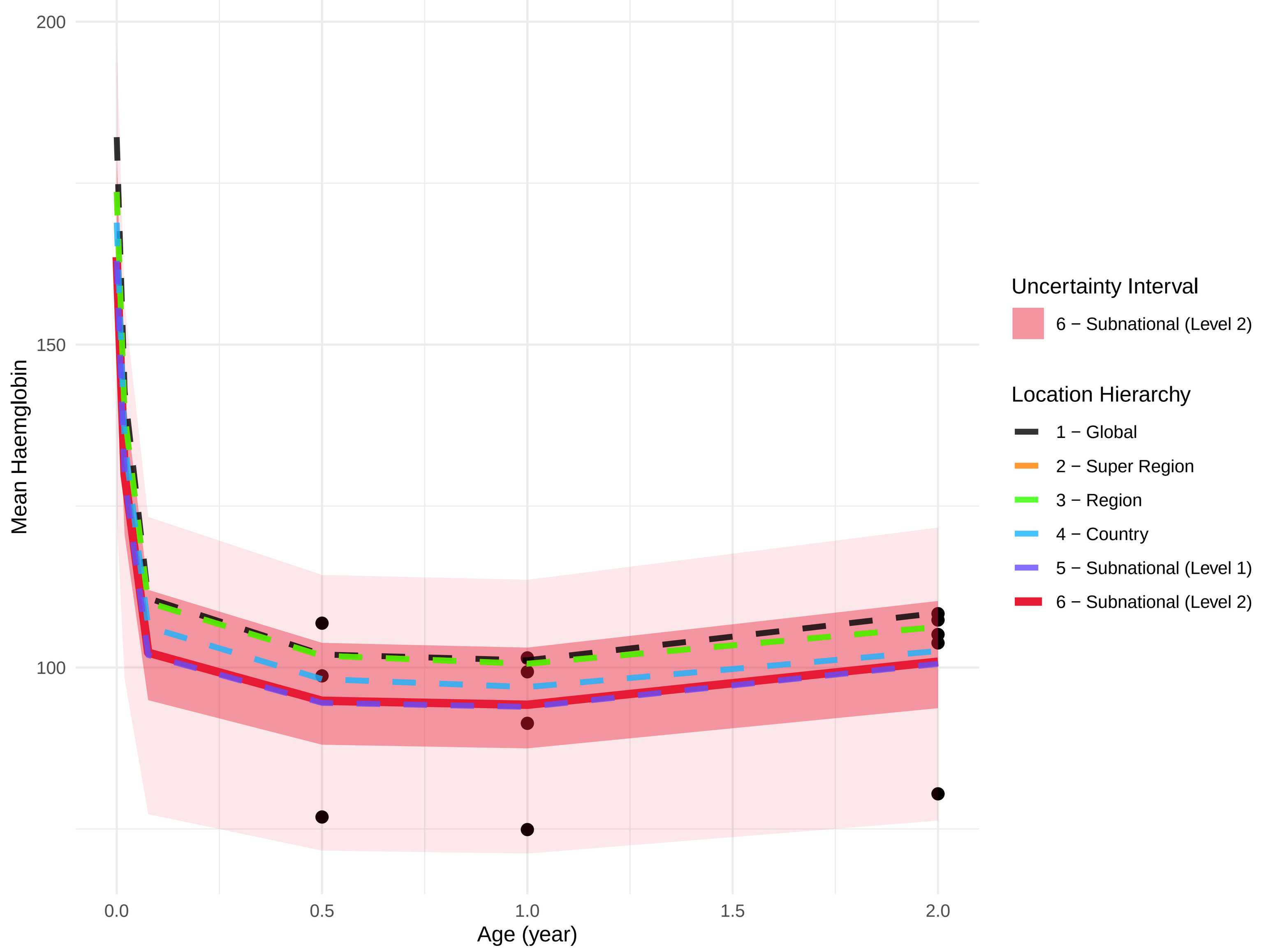

MR−BRT Age/Sex Fits − Non−pregnant Females <2 Years Old − Mean Haemglobin − West Bengal
WHO−2001 Adjustment Method

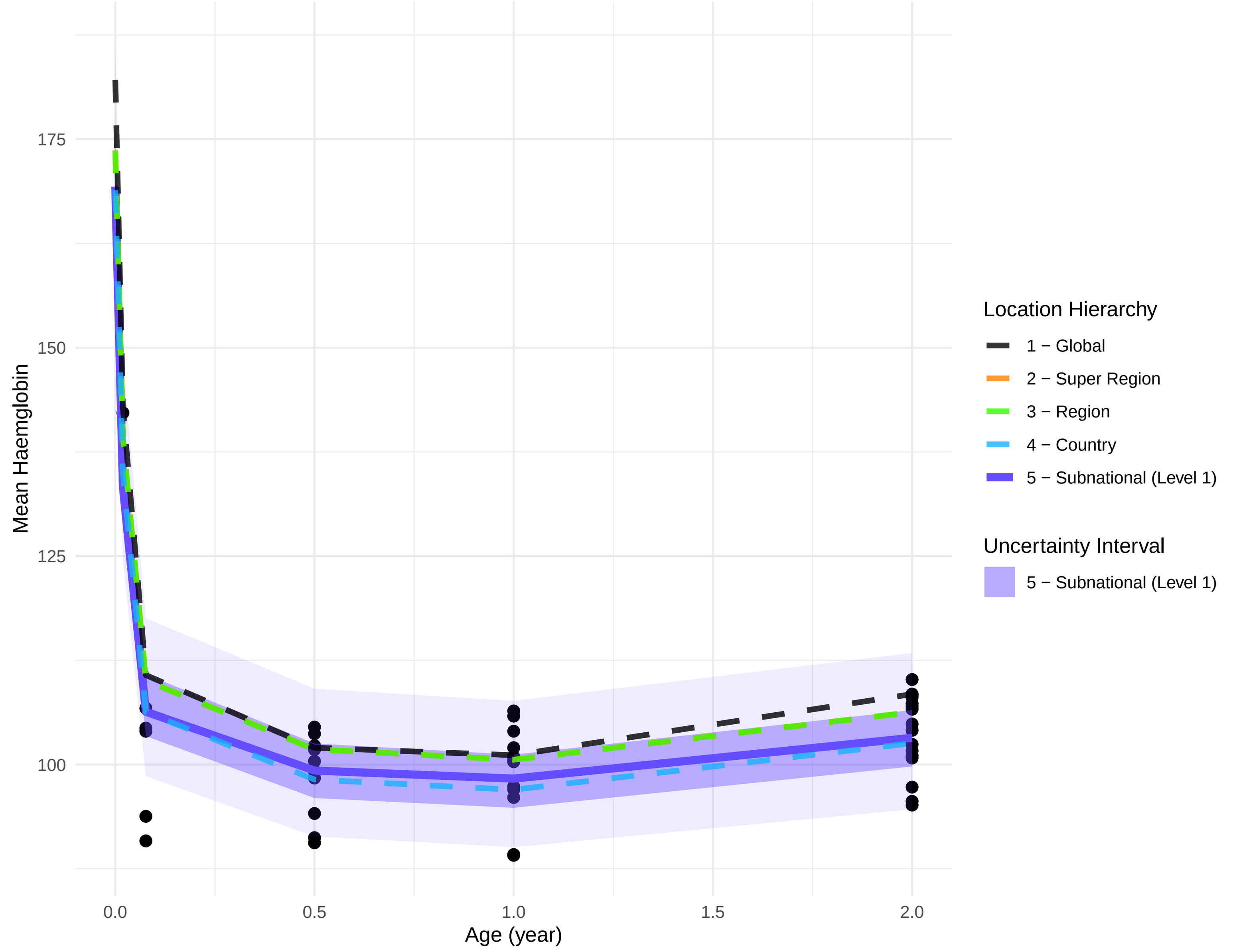

MR−BRT Age/Sex Fits − Non−pregnant Females <2 Years Old − Mean Haemglobin − West Bengal, Rural
WHO−2001 Adjustment Method

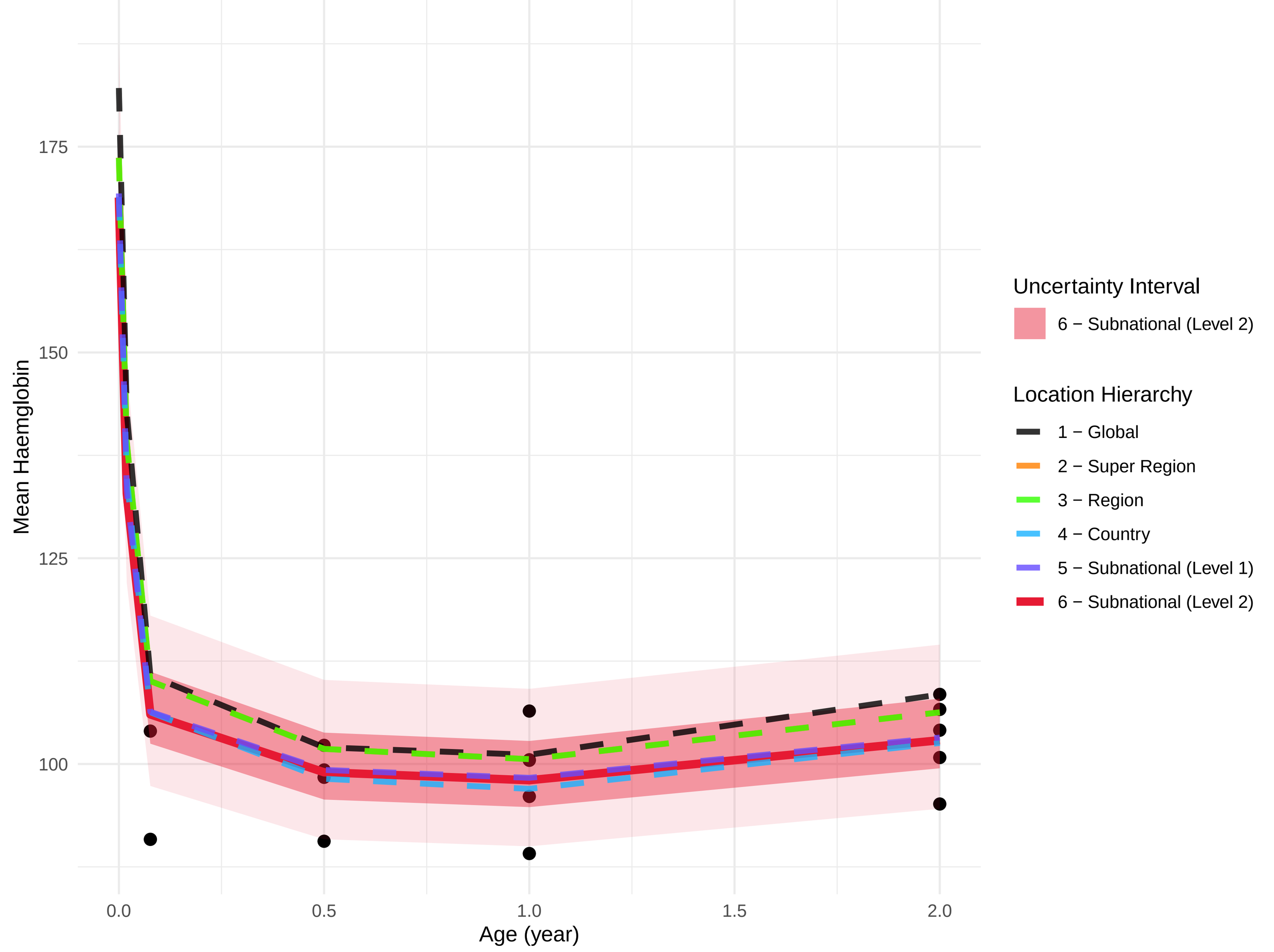

MR−BRT Age/Sex Fits − Non−pregnant Females <2 Years Old − Mean Haemglobin − West Bengal, Urban
WHO−2001 Adjustment Method

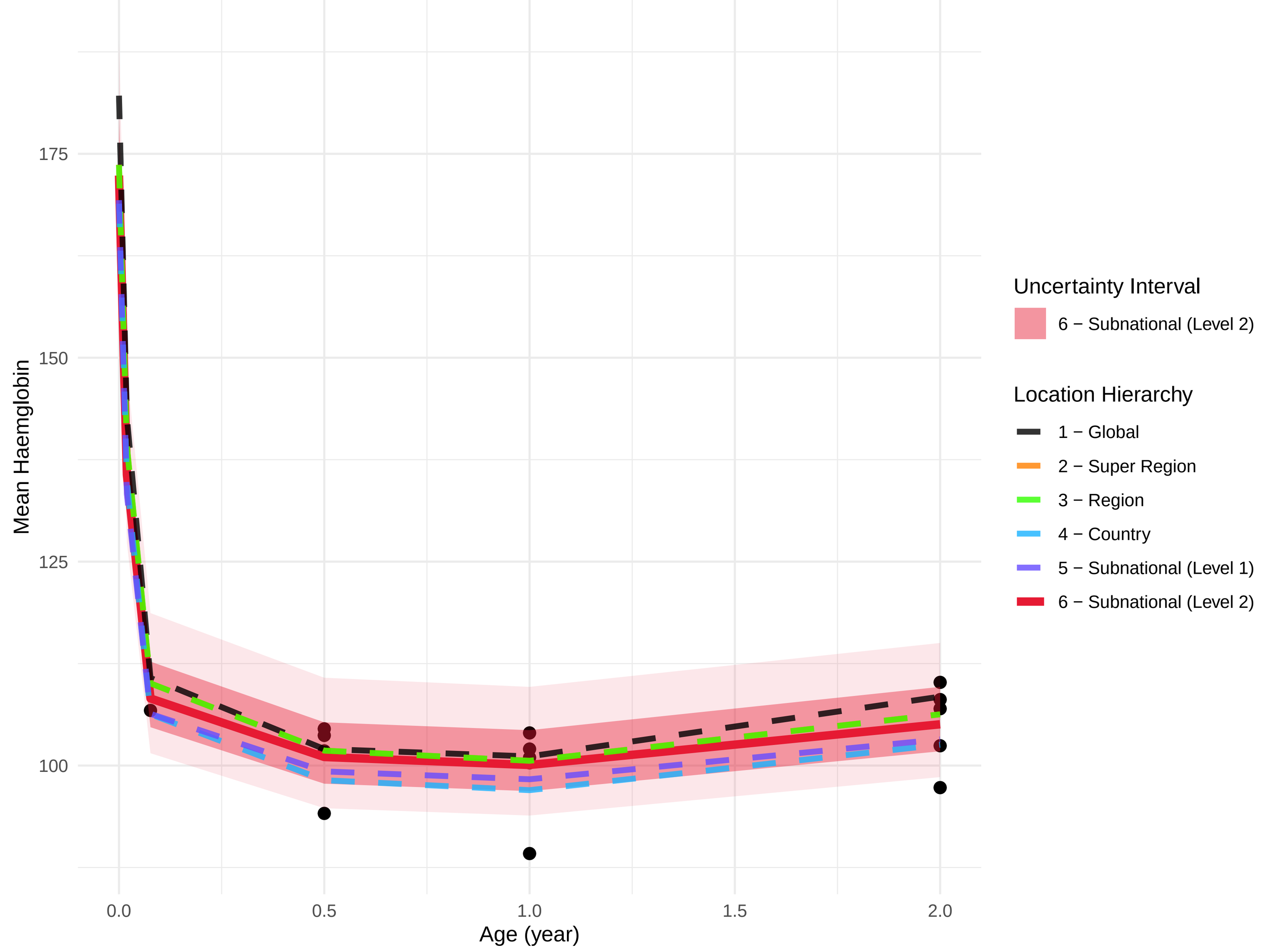

MR−BRT Age/Sex Fits − Non−pregnant Females <2 Years Old − Mean Haemglobin − Nepal
WHO−2001 Adjustment Method

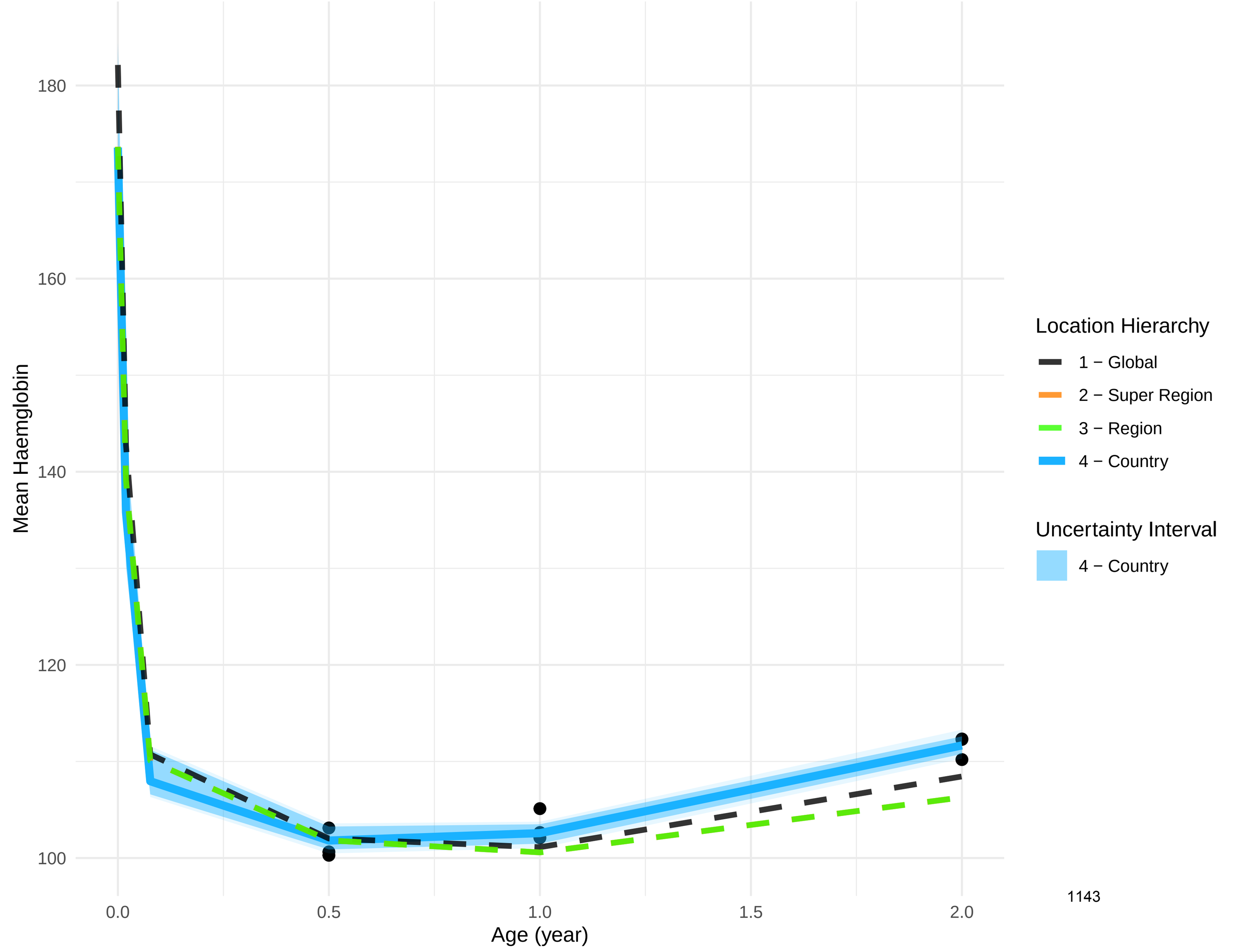

MR−BRT Age/Sex Fits − Non−pregnant Females <2 Years Old − Mean Haemglobin − Pakistan
WHO−2001 Adjustment Method

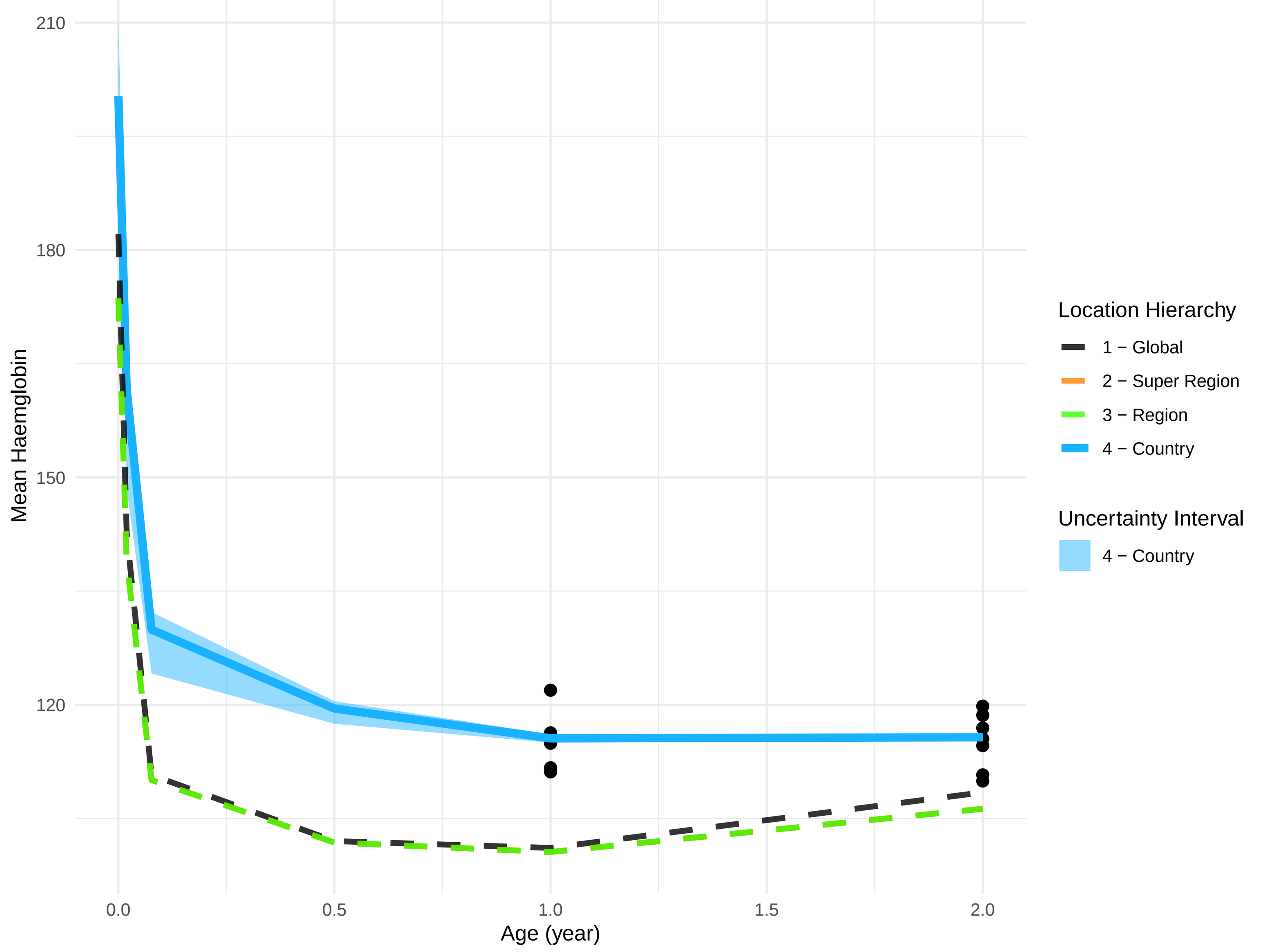

MR−BRT Age/Sex Fits − Non−pregnant Females <2 Years Old − Mean Haemglobin − Azad Jammu & Kashmir
WHO−2001 Adjustment Method

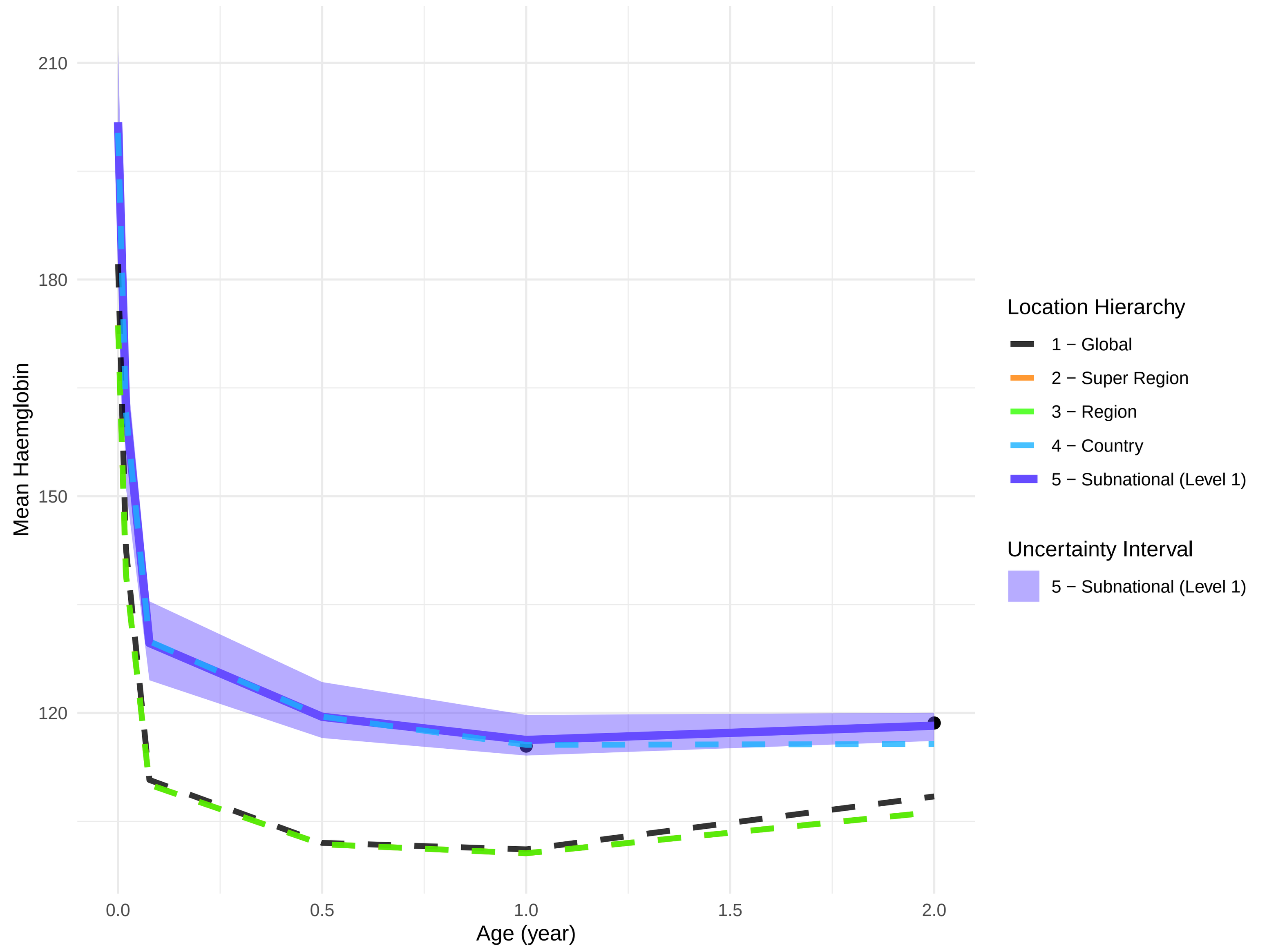

MR−BRT Age/Sex Fits − Non−pregnant Females <2 Years Old − Mean Haemglobin − Balochistan
WHO−2001 Adjustment Method

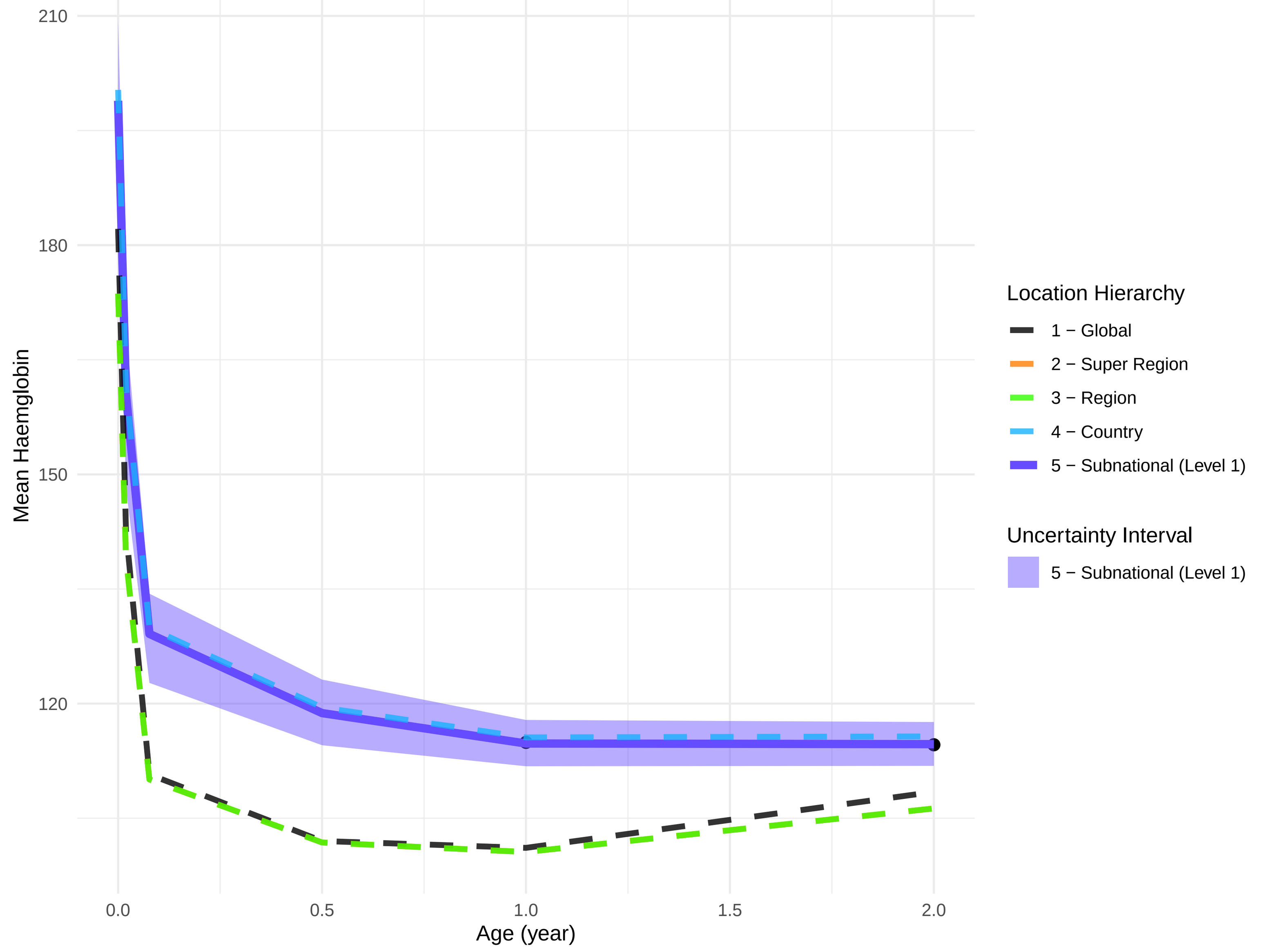

MR−BRT Age/Sex Fits − Non−pregnant Females <2 Years Old − Mean Haemglobin − Gilgit−Baltistan
WHO−2001 Adjustment Method

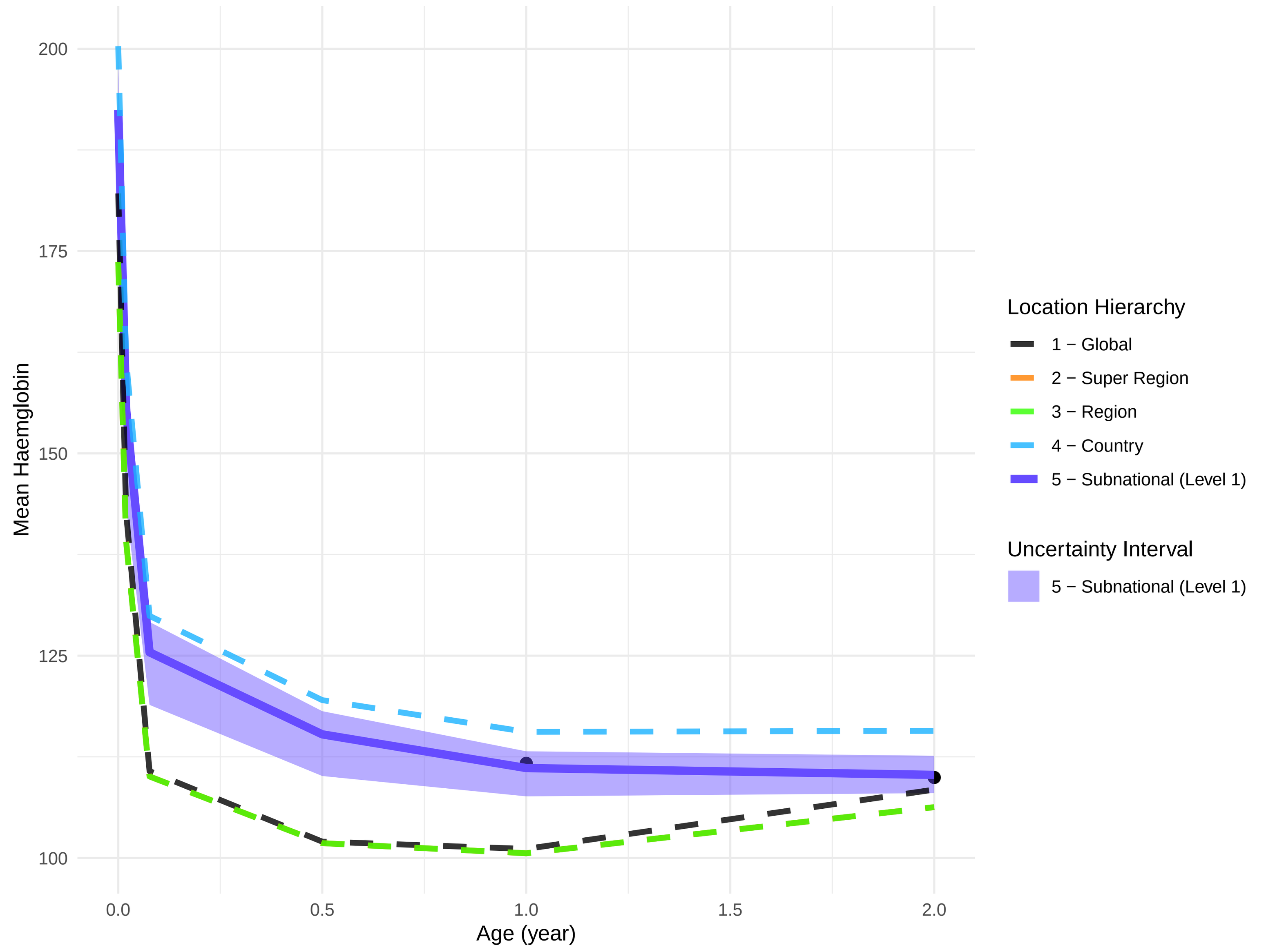

MR−BRT Age/Sex Fits − Non−pregnant Females <2 Years Old − Mean Haemglobin − Khyber Pakhtunkhwa
WHO−2001 Adjustment Method

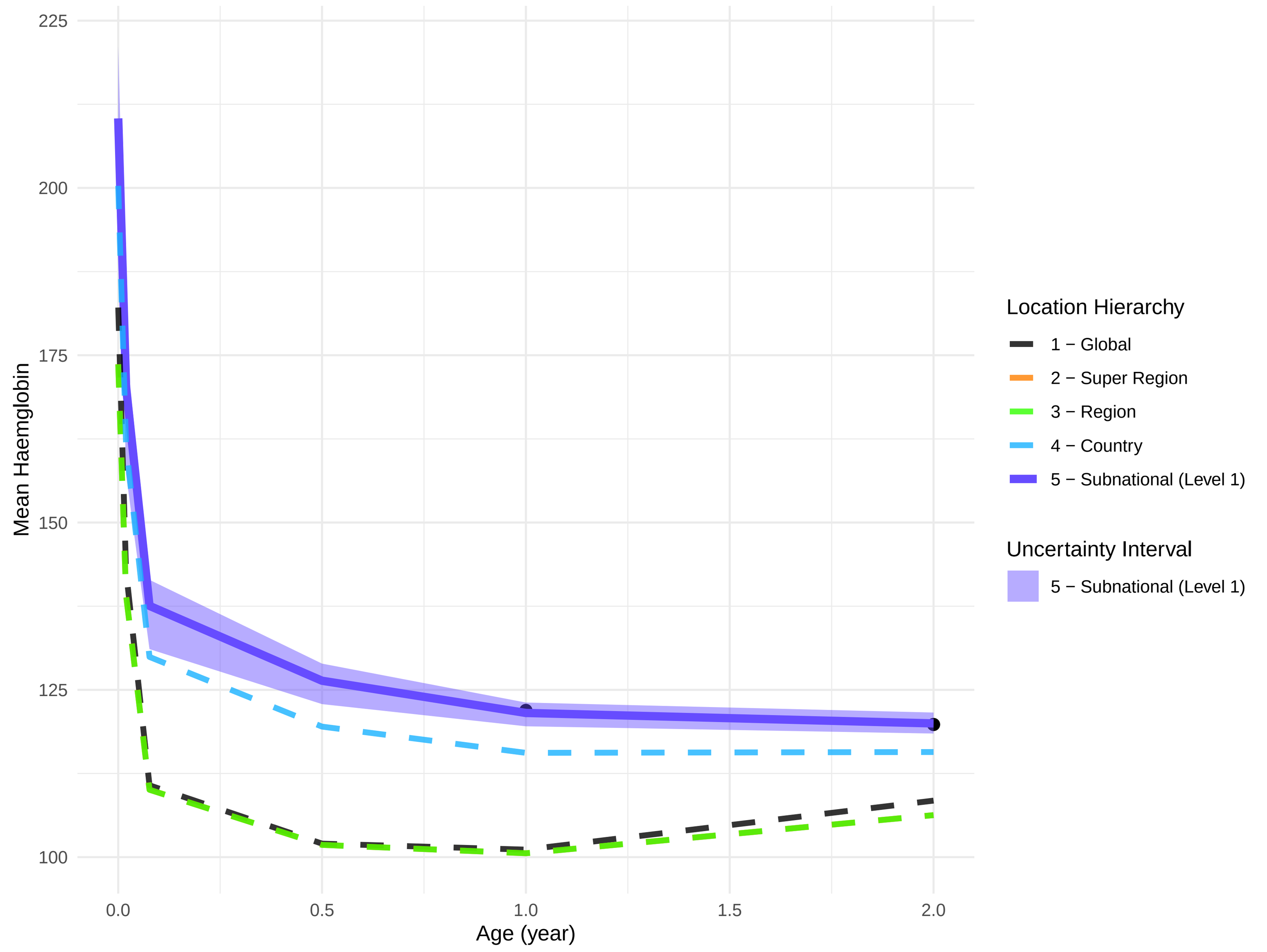

MR−BRT Age/Sex Fits − Non−pregnant Females <2 Years Old − Mean Haemglobin − Punjab
WHO−2001 Adjustment Method

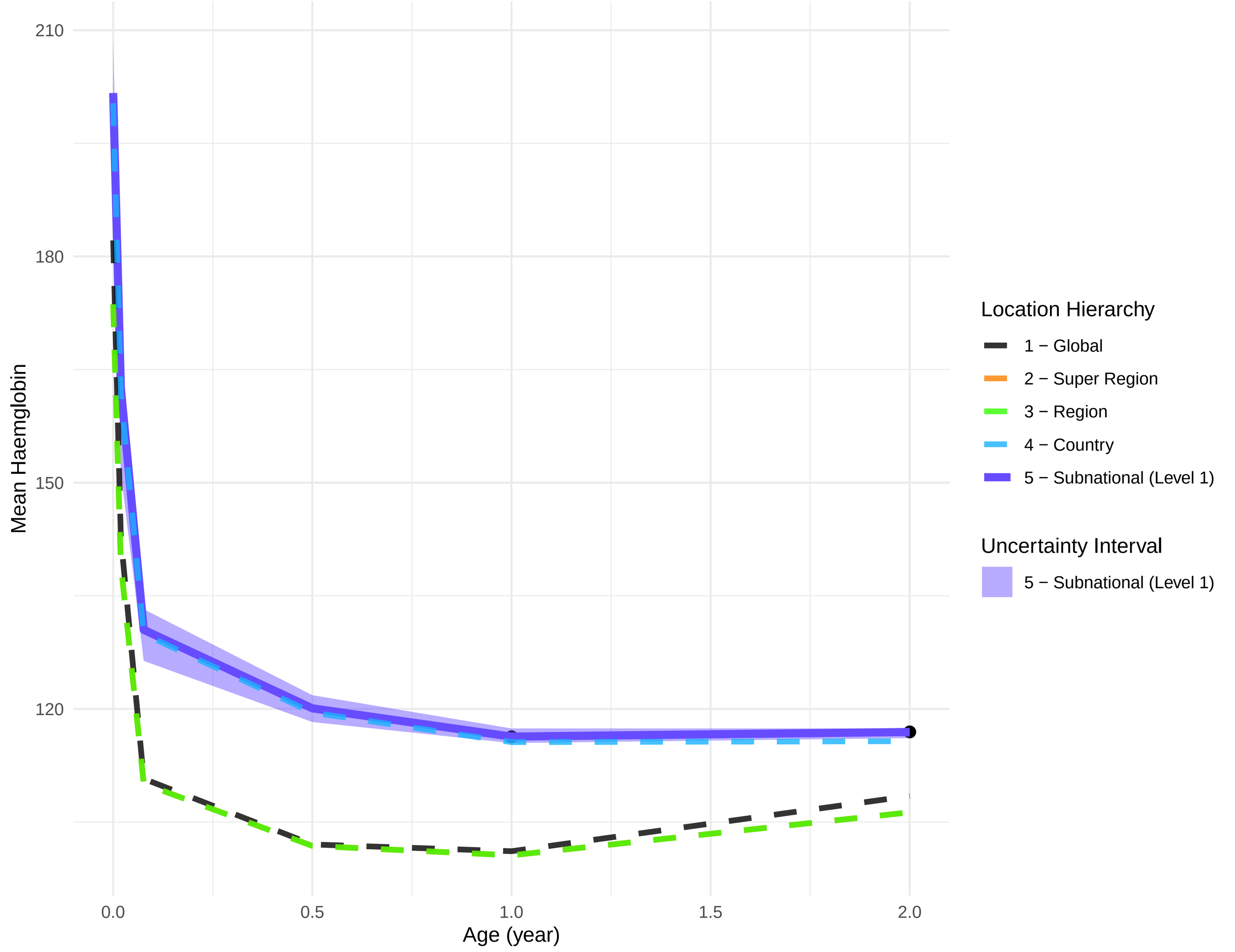

MR−BRT Age/Sex Fits − Non−pregnant Females <2 Years Old − Mean Haemglobin − Sindh
WHO−2001 Adjustment Method

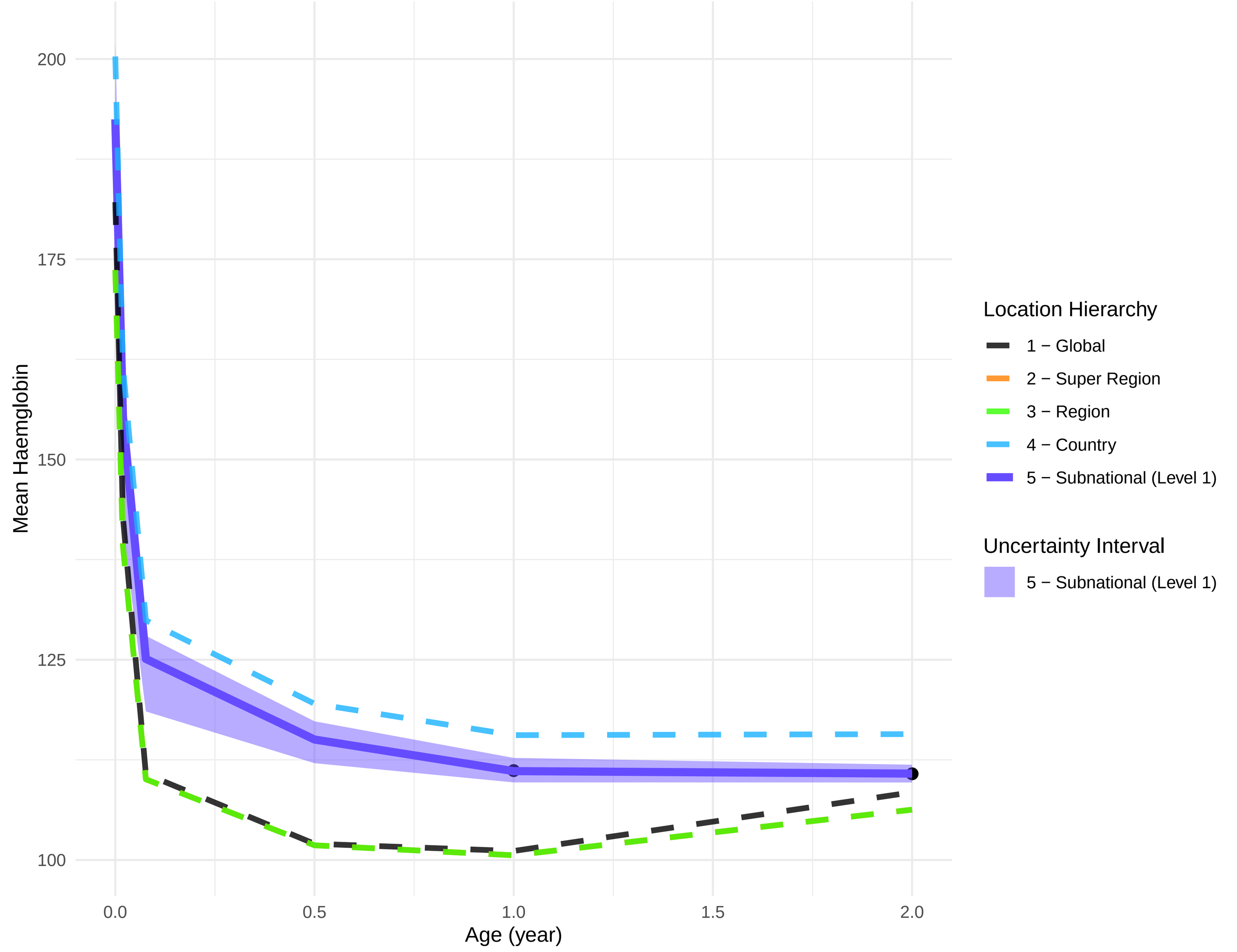

MR−BRT Age/Sex Fits − Non−pregnant Females <2 Years Old − Mean Haemglobin − Southeast Asia, East Asia, and Oceania
WHO−2001 Adjustment Method

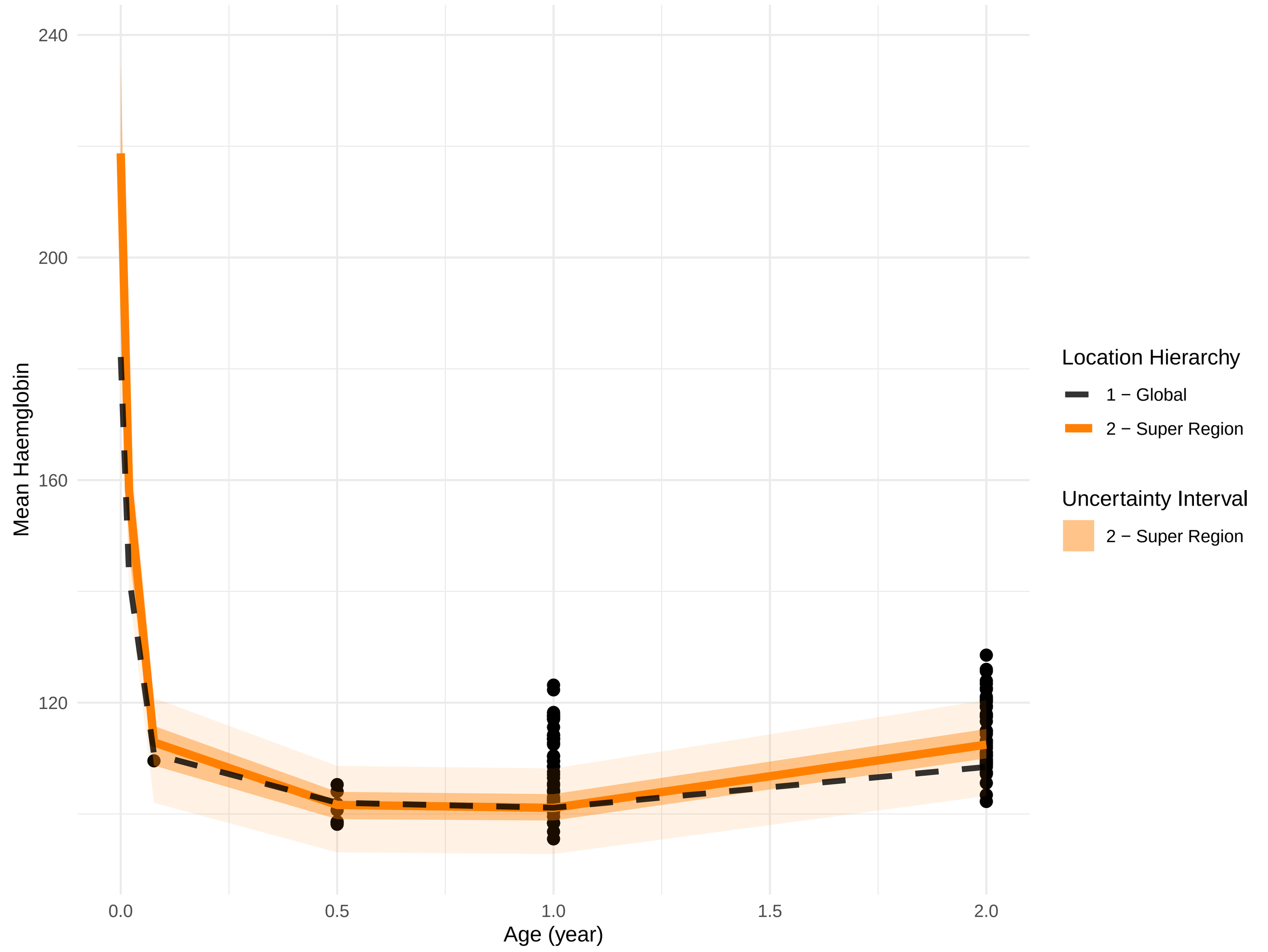

MR−BRT Age/Sex Fits − Non−pregnant Females <2 Years Old − Mean Haemglobin − Southeast Asia
WHO−2001 Adjustment Method

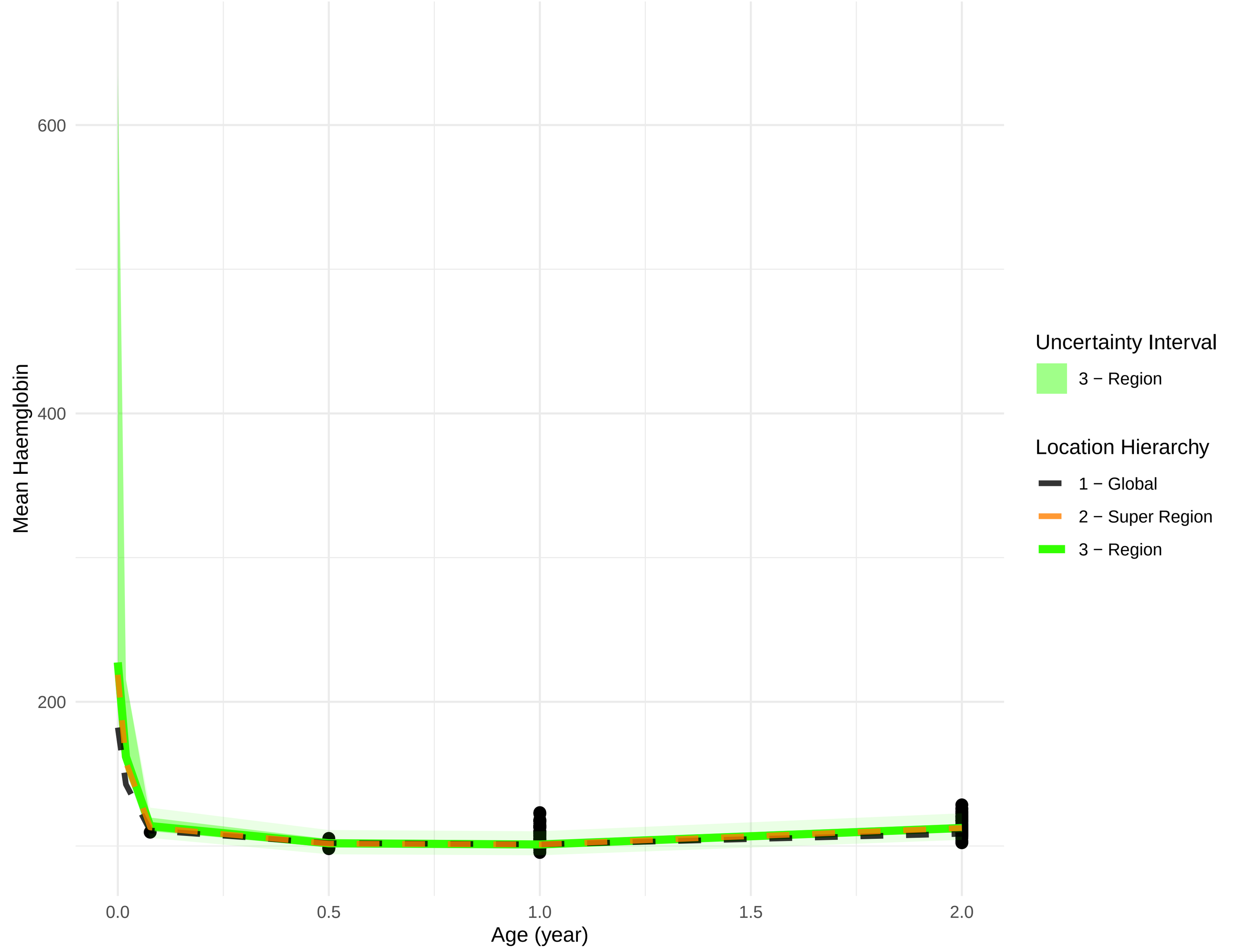

MR−BRT Age/Sex Fits − Non−pregnant Females <2 Years Old − Mean Haemglobin − Cambodia
WHO−2001 Adjustment Method

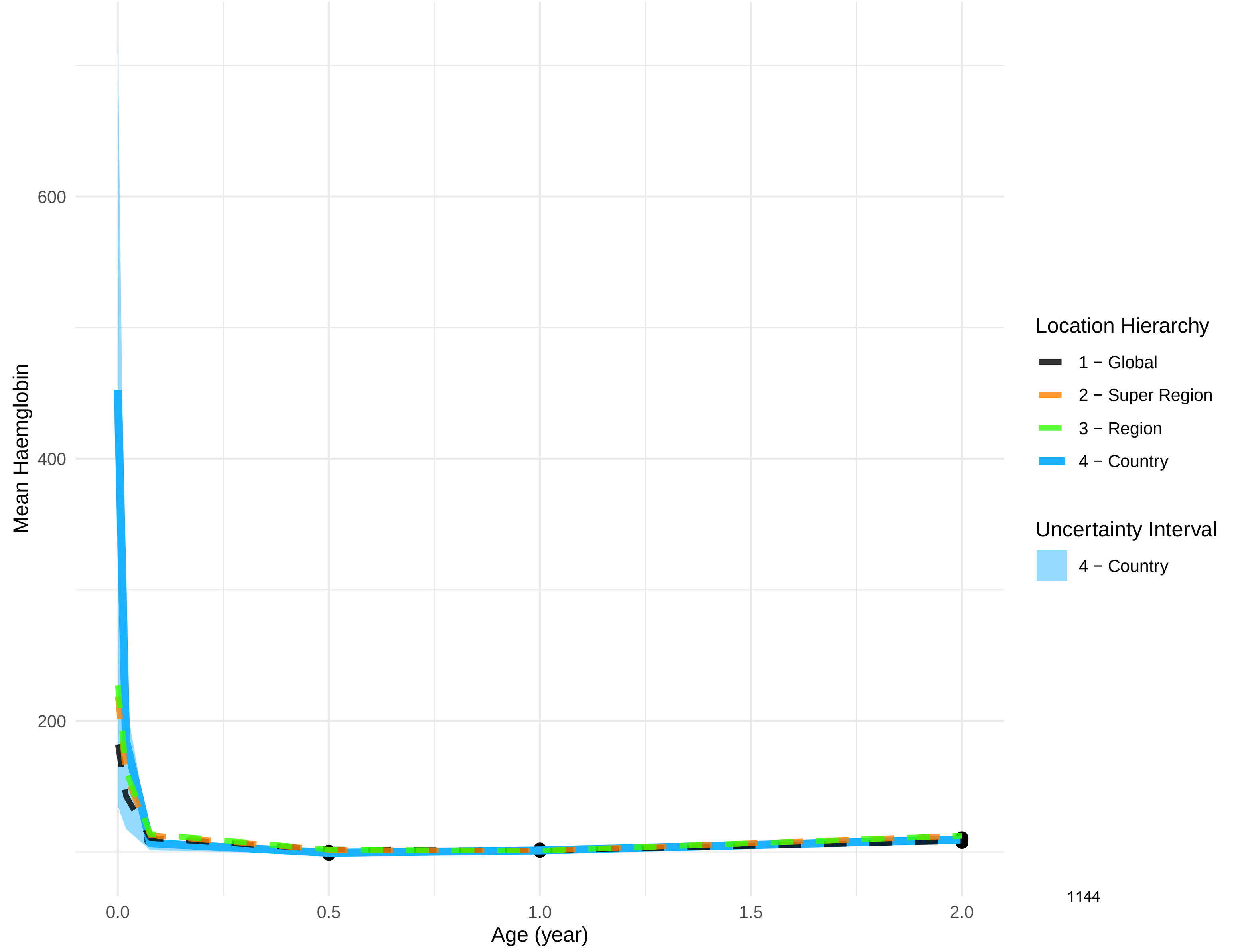

MR−BRT Age/Sex Fits − Non−pregnant Females <2 Years Old − Mean Haemglobin − Indonesia
WHO−2001 Adjustment Method

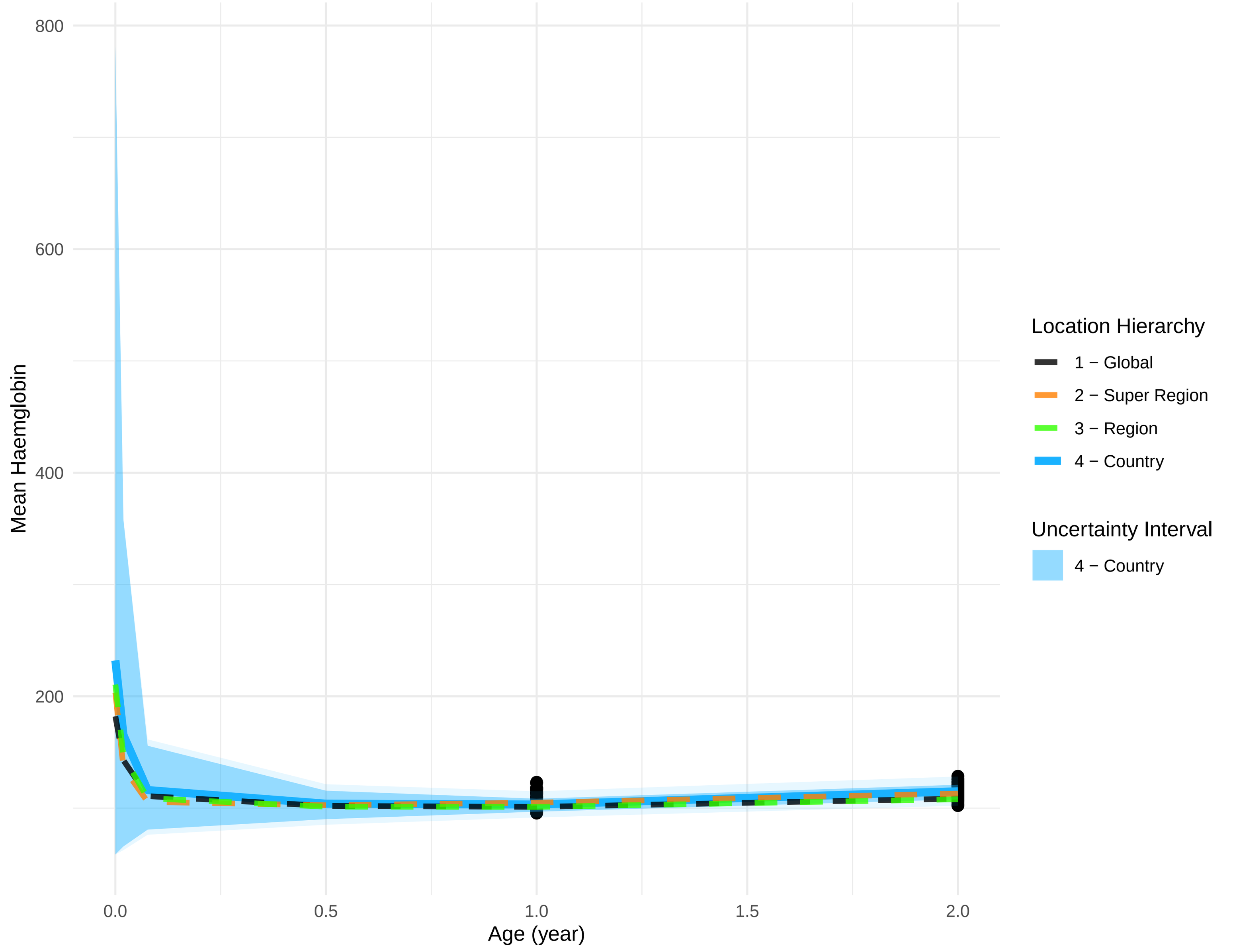

MR−BRT Age/Sex Fits − Non−pregnant Females <2 Years Old − Mean Haemglobin − Bali
WHO−2001 Adjustment Method

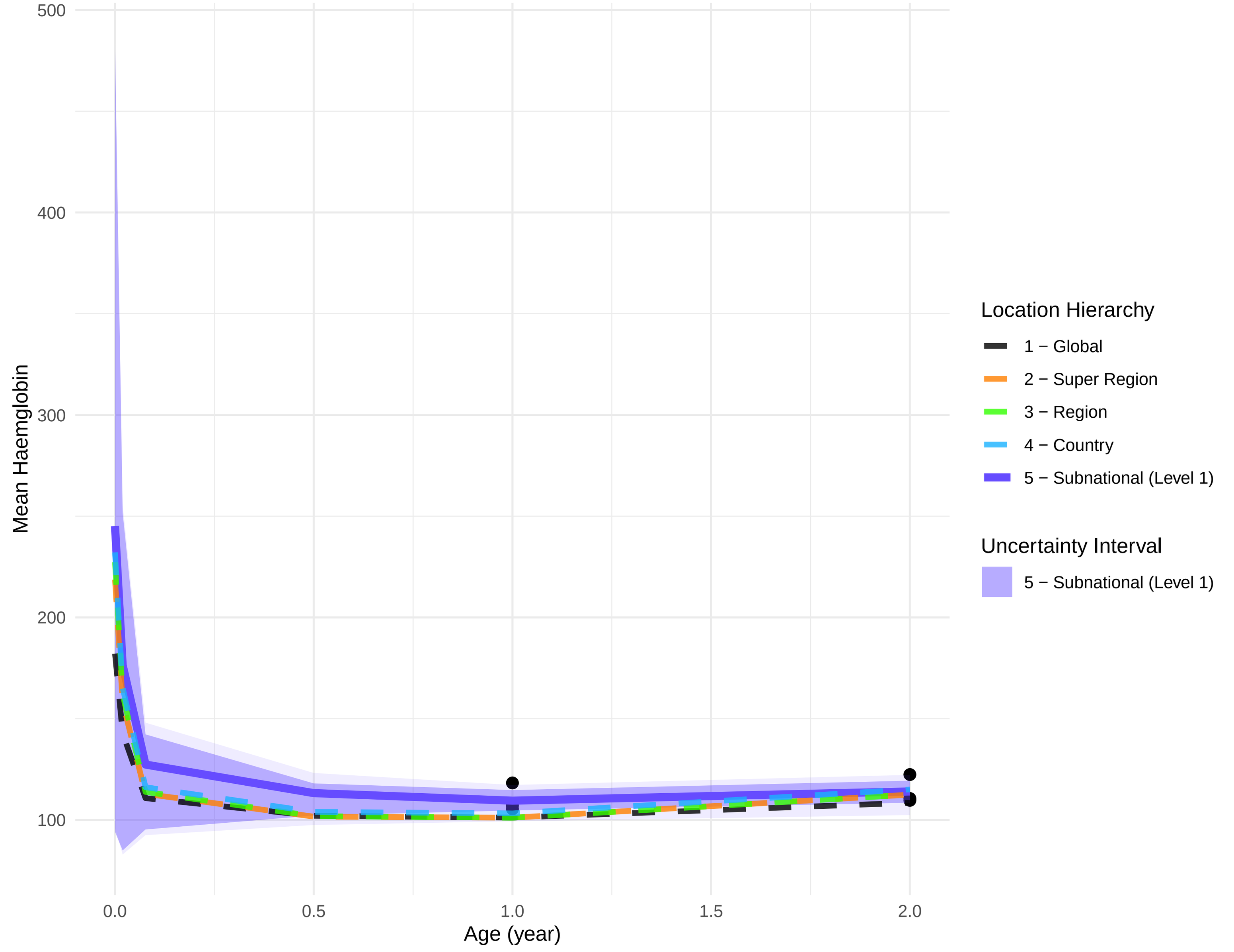

MR−BRT Age/Sex Fits − Non−pregnant Females <2 Years Old − Mean Haemglobin − Bangka−Belitung Islands
WHO−2001 Adjustment Method

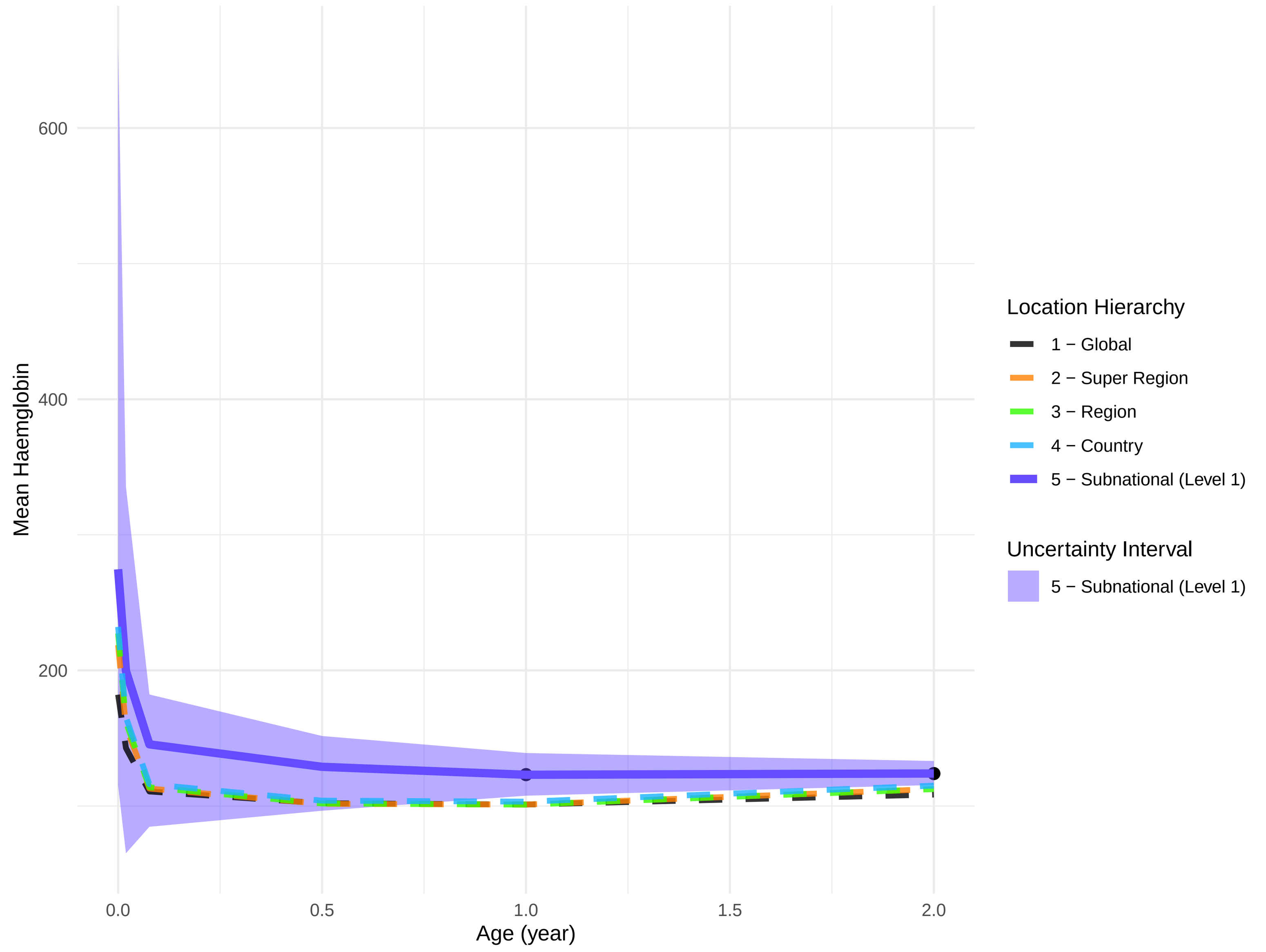

MR−BRT Age/Sex Fits − Non−pregnant Females <2 Years Old − Mean Haemglobin − Banten
WHO−2001 Adjustment Method

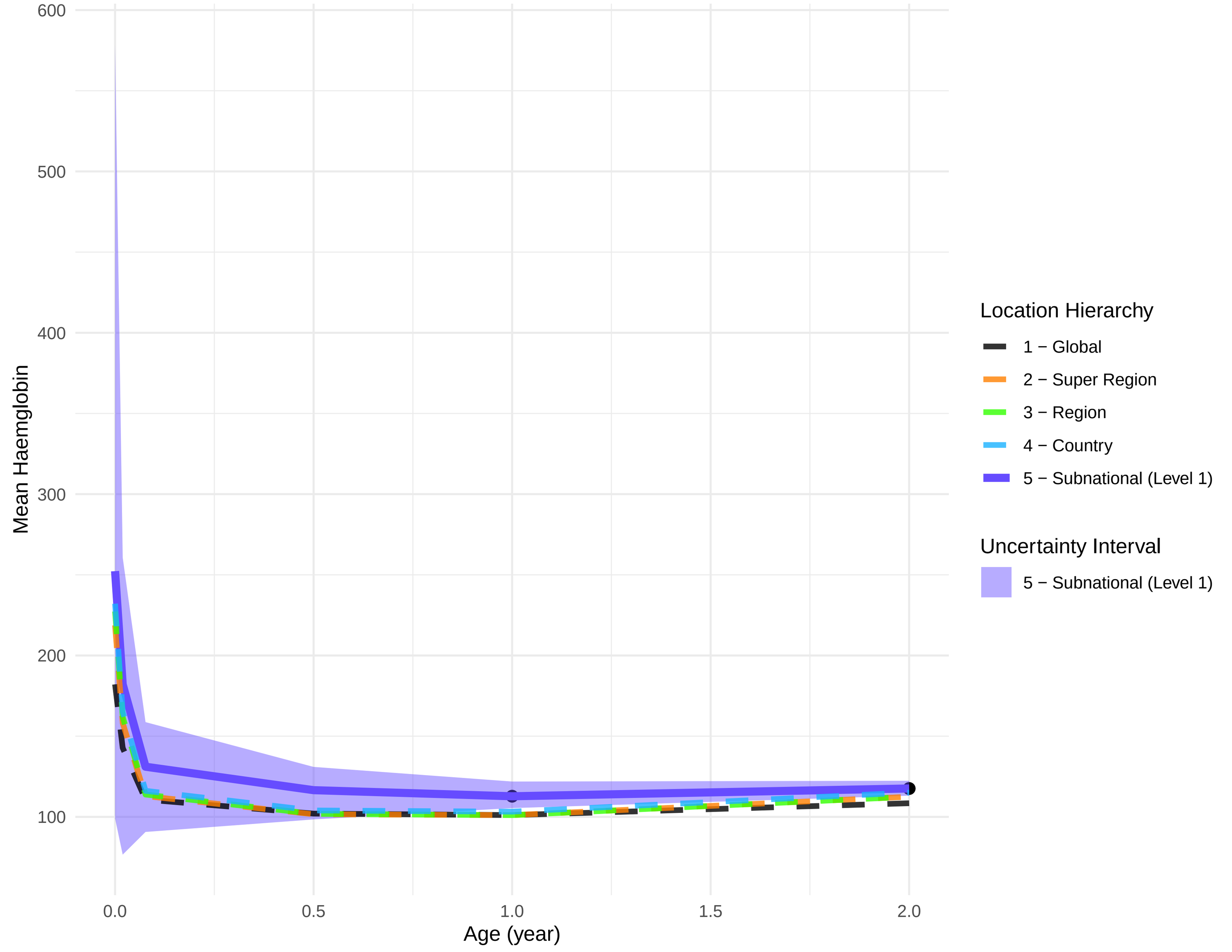

MR−BRT Age/Sex Fits − Non−pregnant Females <2 Years Old − Mean Haemglobin − Jakarta
WHO−2001 Adjustment Method

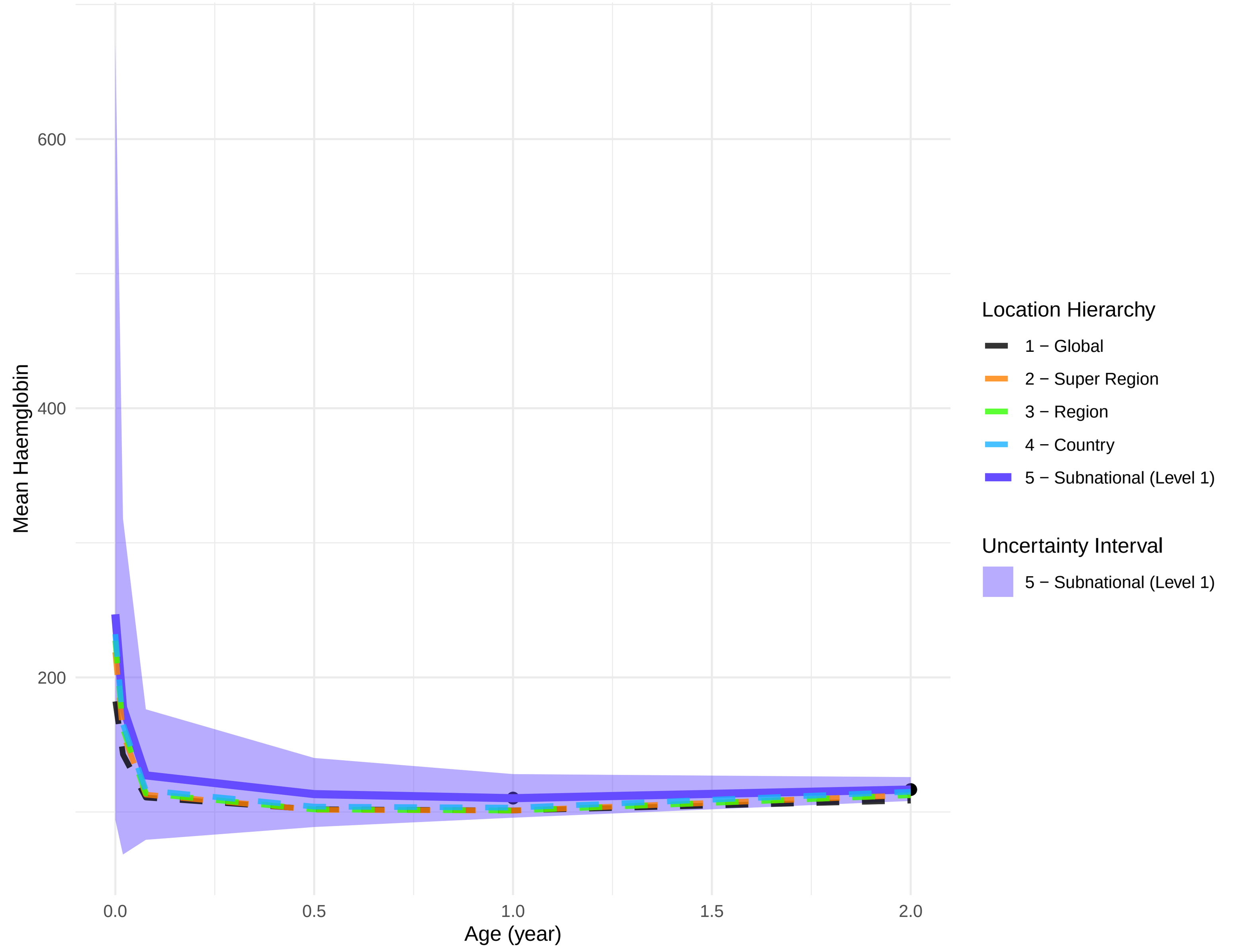

MR−BRT Age/Sex Fits − Non−pregnant Females <2 Years Old − Mean Haemglobin − West Java
WHO−2001 Adjustment Method

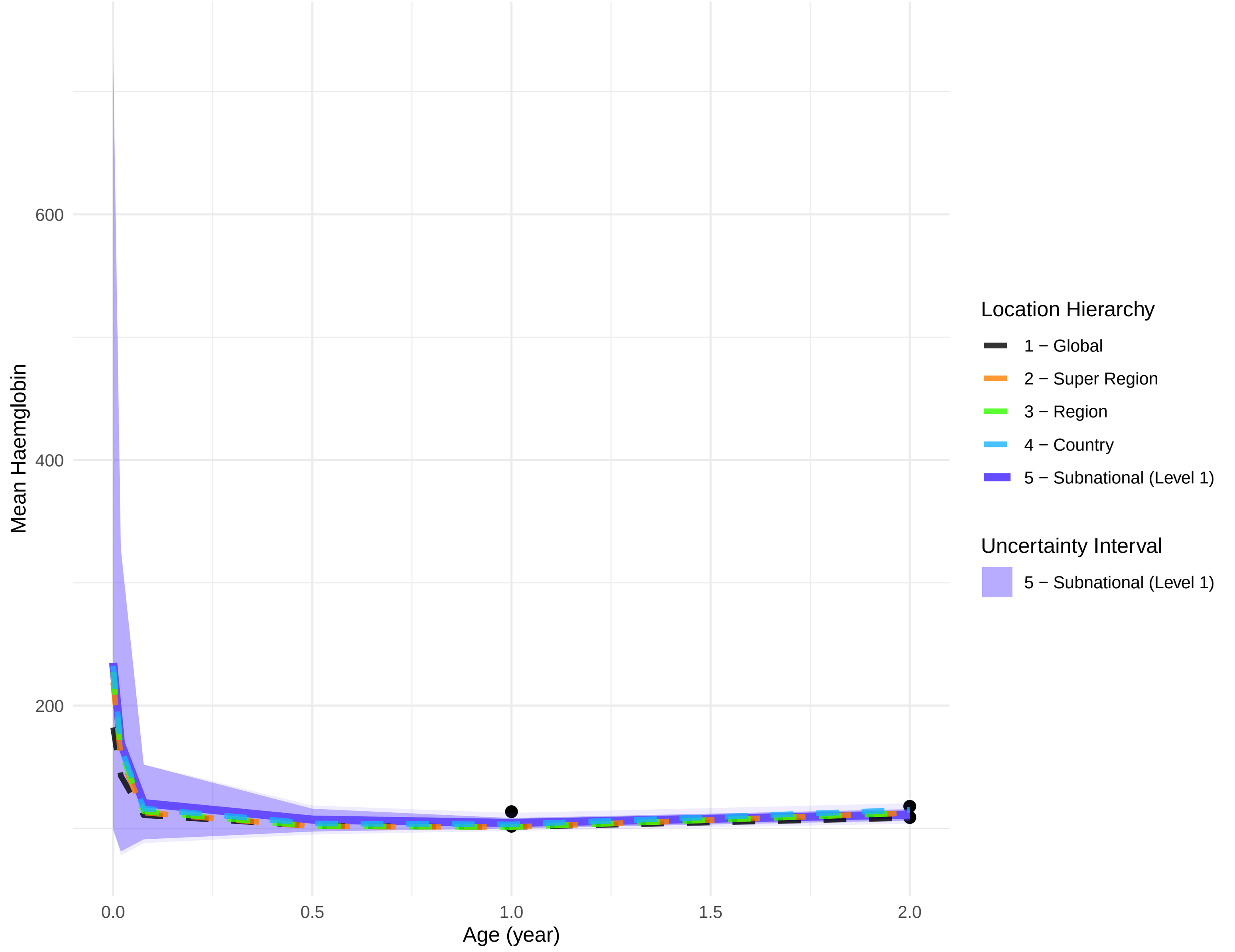

MR−BRT Age/Sex Fits − Non−pregnant Females <2 Years Old − Mean Haemglobin − Central Java
WHO−2001 Adjustment Method

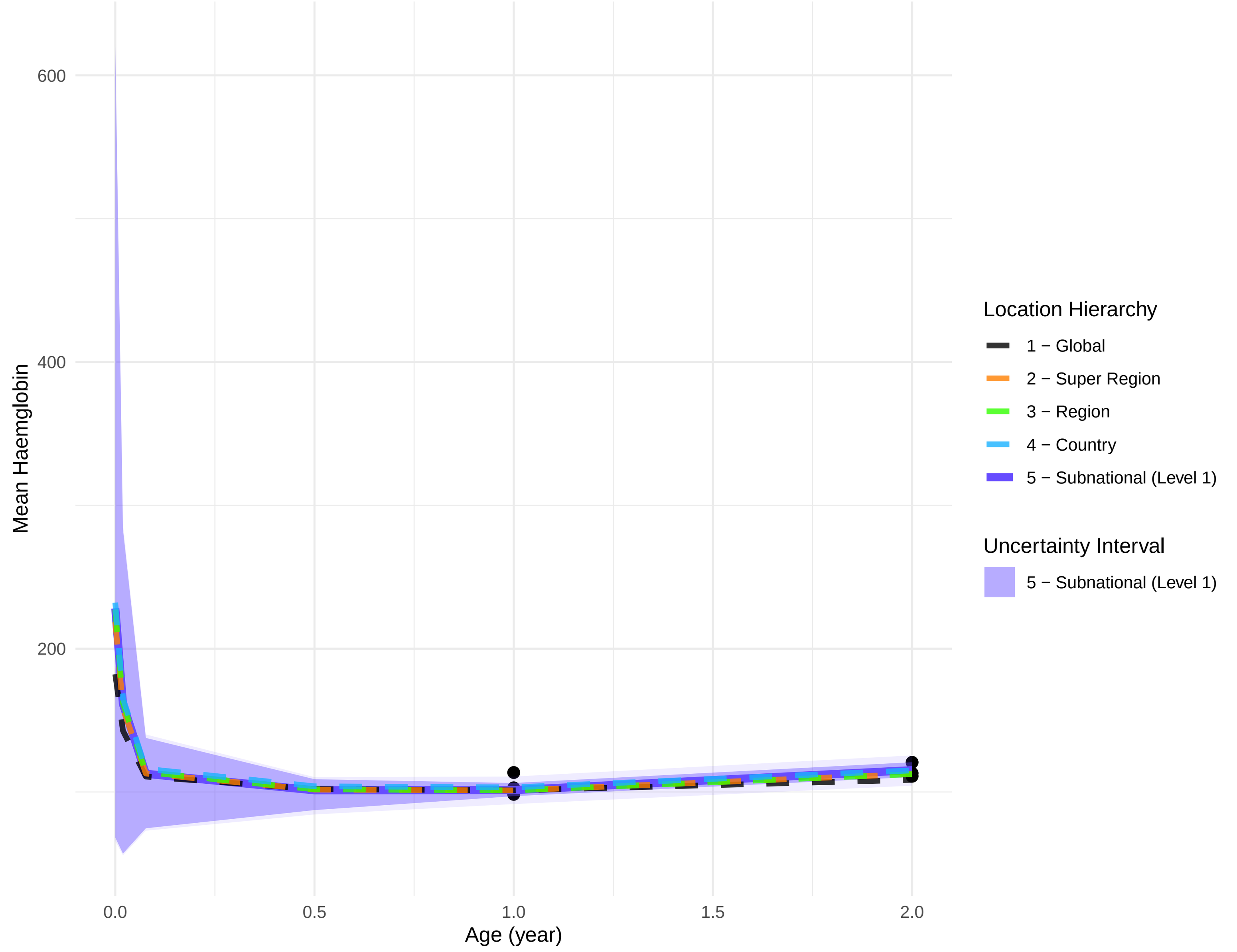

MR−BRT Age/Sex Fits − Non−pregnant Females <2 Years Old − Mean Haemglobin − East Java
WHO−2001 Adjustment Method

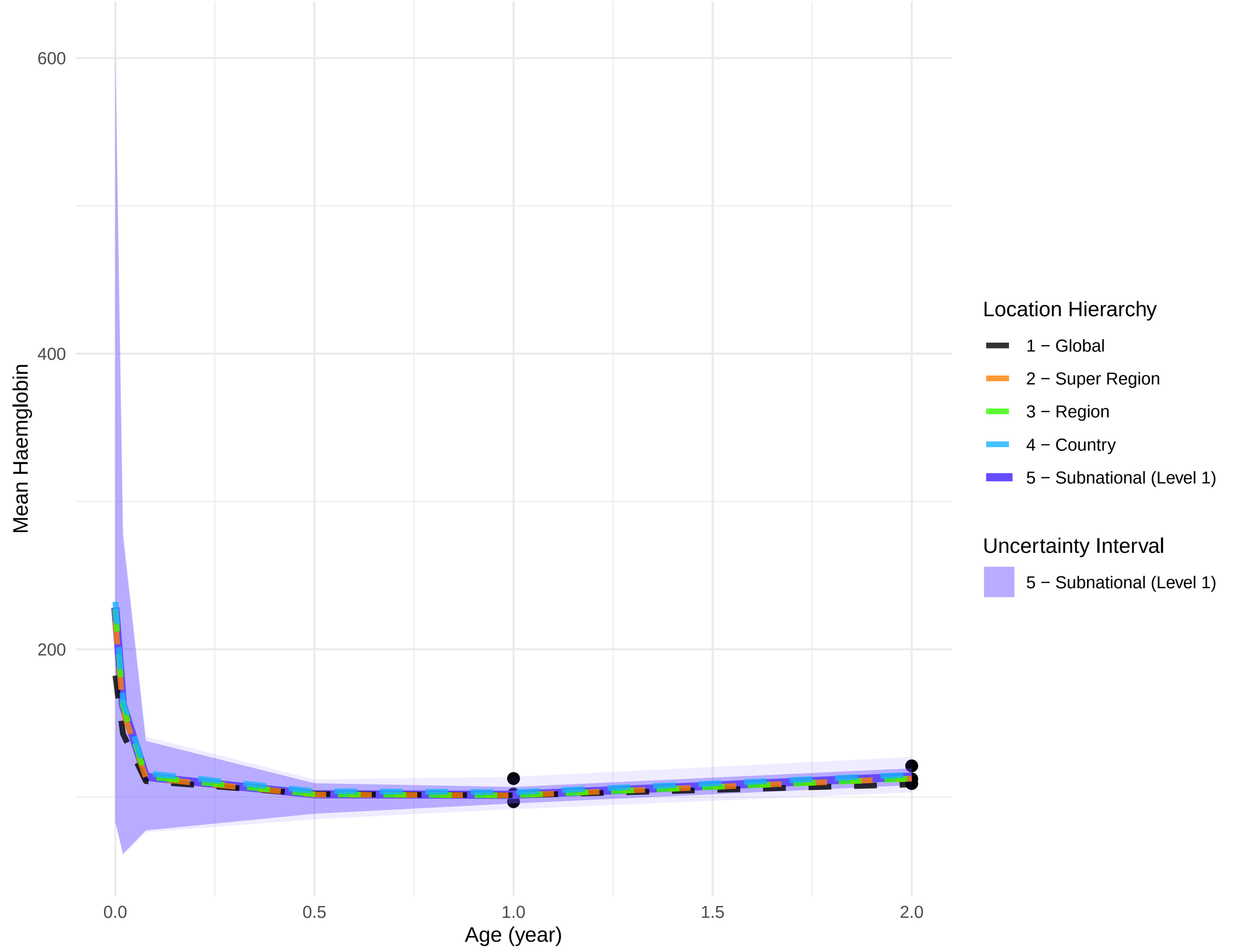

MR−BRT Age/Sex Fits − Non−pregnant Females <2 Years Old − Mean Haemglobin − South Kalimantan
WHO−2001 Adjustment Method

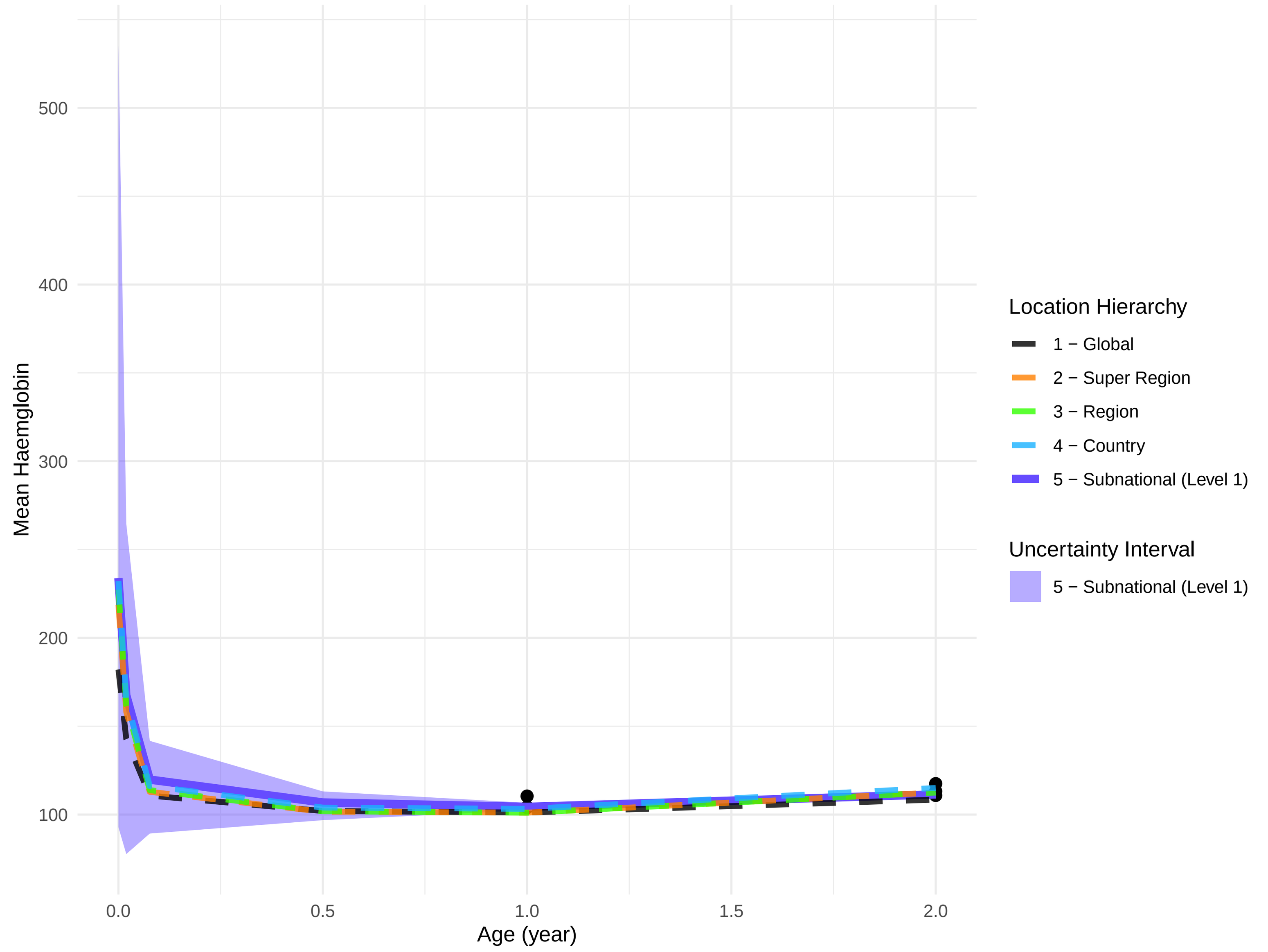

MR−BRT Age/Sex Fits − Non−pregnant Females <2 Years Old − Mean Haemglobin − Lampung
WHO−2001 Adjustment Method

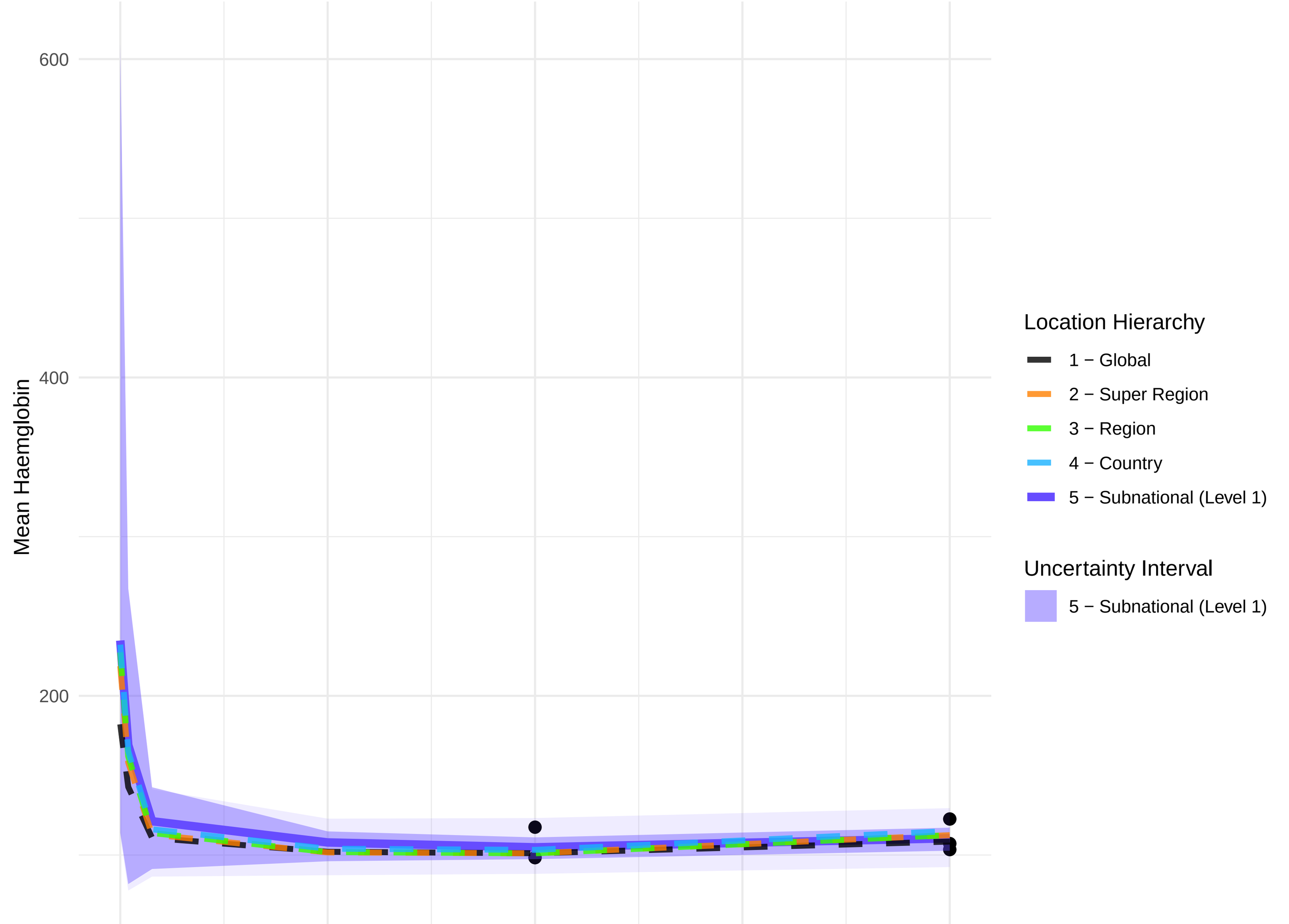

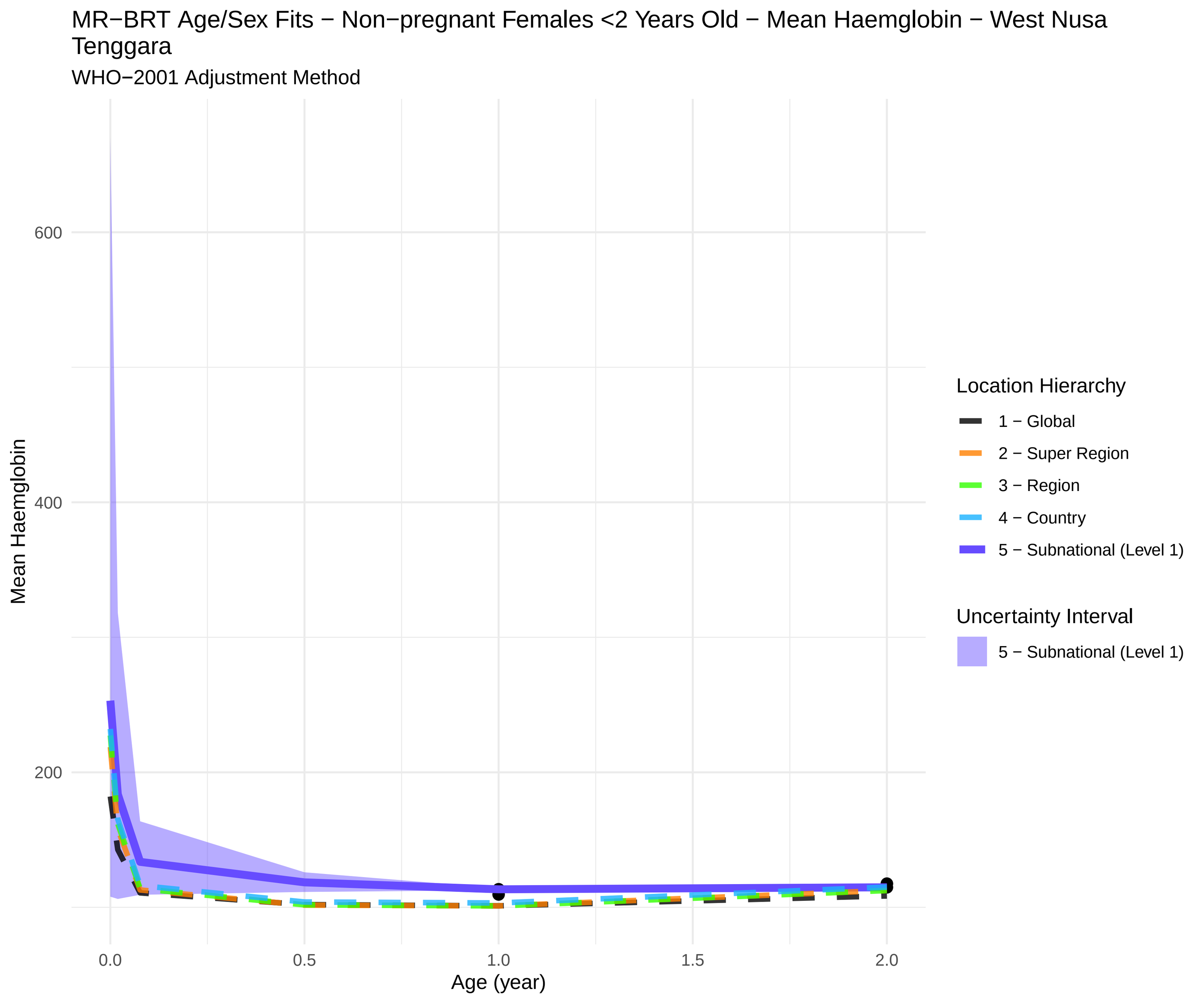
MR−BRT Age/Sex Fits − Non−pregnant Females <2 Years Old − Mean Haemglobin − West Nusa Tenggara
WHO−2001 Adjustment Method
Mean Haemglobin
Age (year)
Location Hierarchy
1 − Global
2 − Super Region
3 − Region
4 − Country
5 − Subnational (Level 1)
Uncertainty Interval
5 − Subnational (Level 1)

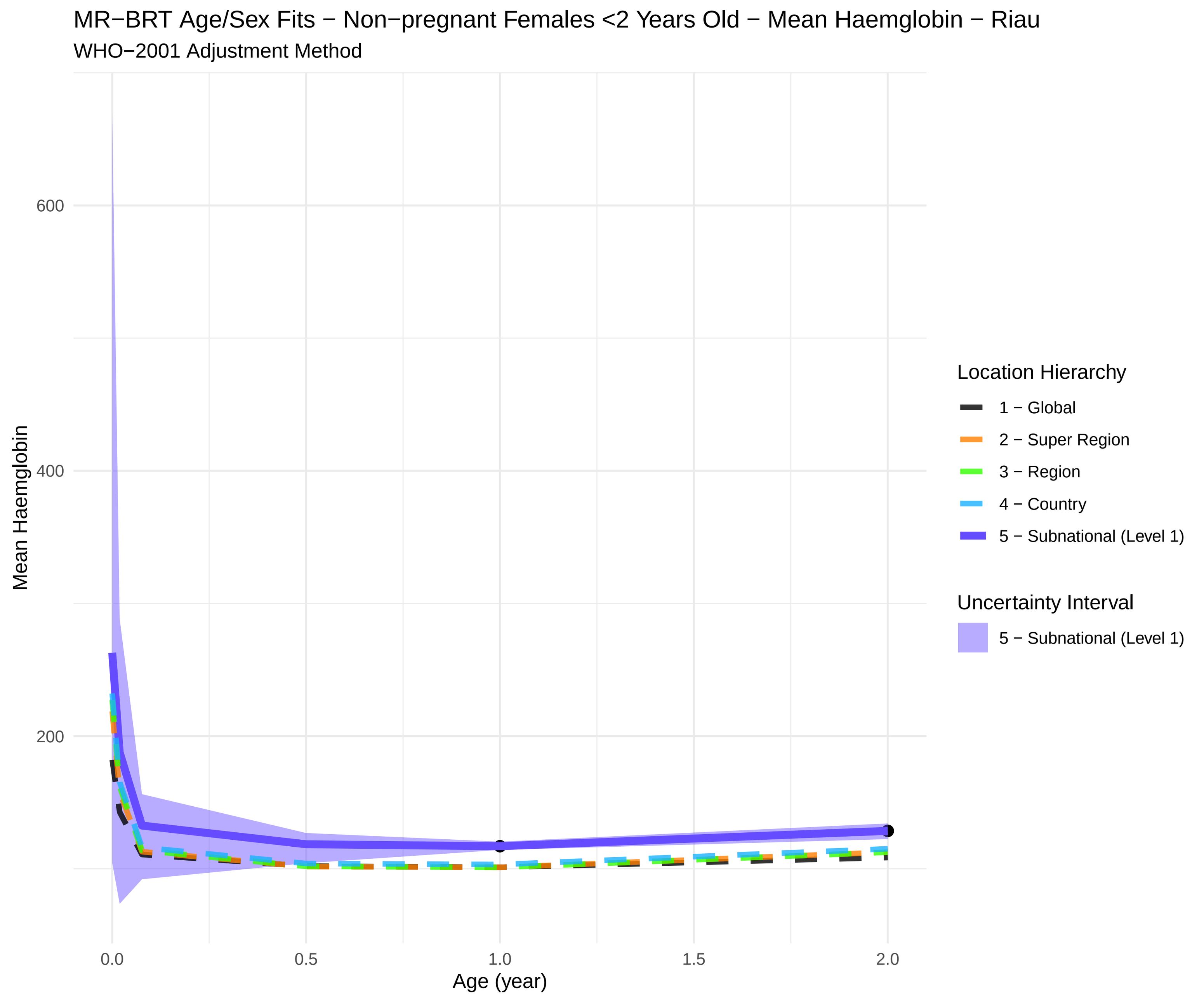
MR−BRT Age/Sex Fits − Non−pregnant Females <2 Years Old − Mean Haemglobin − Riau
WHO−2001 Adjustment Method
Mean Haemglobin
Age (year)
Location Hierarchy
1 − Global
2 − Super Region
3 − Region
4 − Country
5 − Subnational (Level 1)
Uncertainty Interval
5 − Subnational (Level 1)

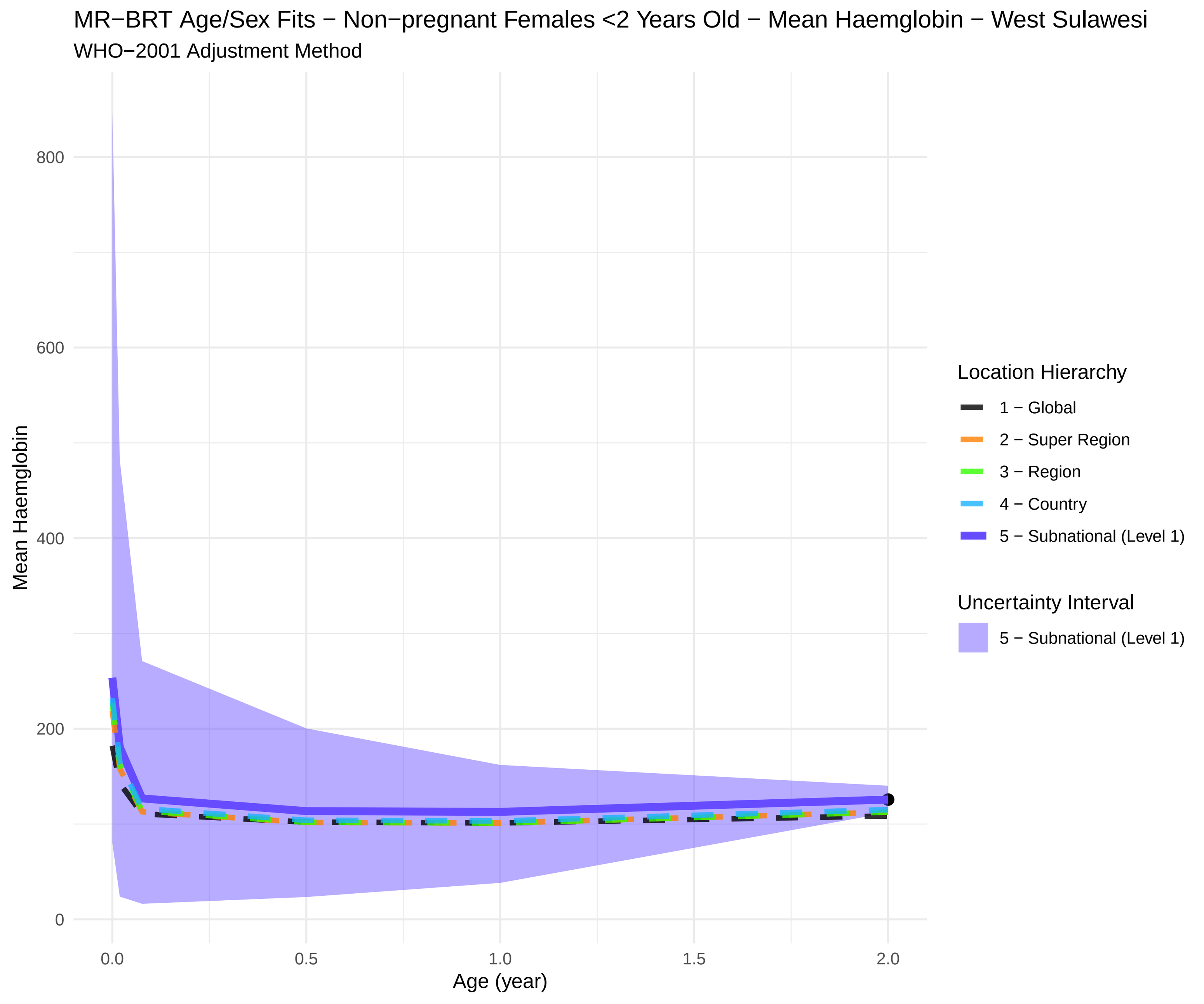
MR−BRT Age/Sex Fits − Non−pregnant Females <2 Years Old − Mean Haemglobin − West Sulawesi
WHO−2001 Adjustment Method
Mean Haemglobin
Age (year)
Location Hierarchy
1 − Global
2 − Super Region
3 − Region
4 − Country
5 − Subnational (Level 1)
Uncertainty Interval
5 − Subnational (Level 1)

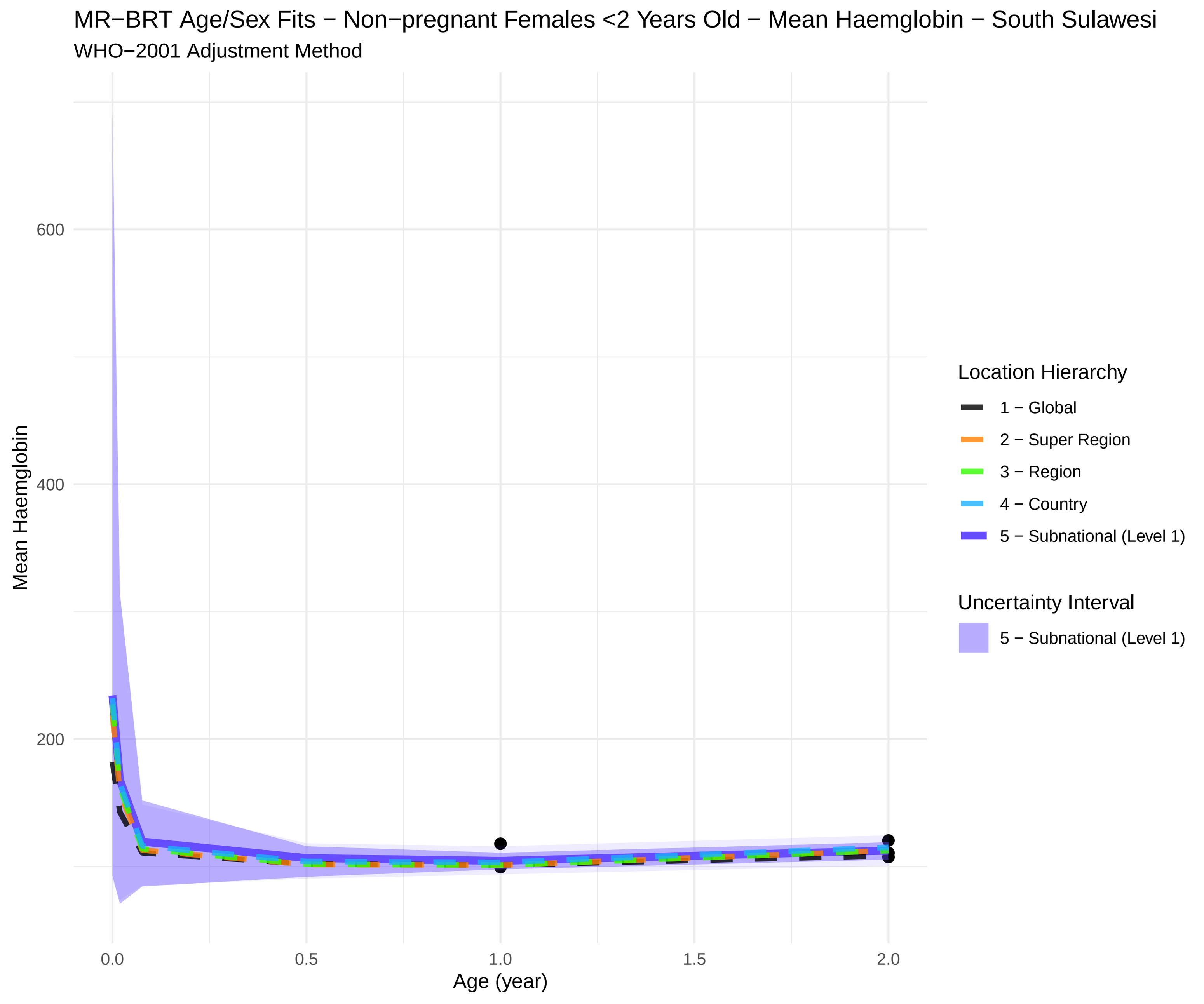
MR−BRT Age/Sex Fits − Non−pregnant Females <2 Years Old − Mean Haemglobin − South Sulawesi
WHO−2001 Adjustment Method
Mean Haemglobin
Age (year)
Location Hierarchy
1 − Global
2 − Super Region
3 − Region
4 − Country
5 − Subnational (Level 1)
Uncertainty Interval
5 − Subnational (Level 1)

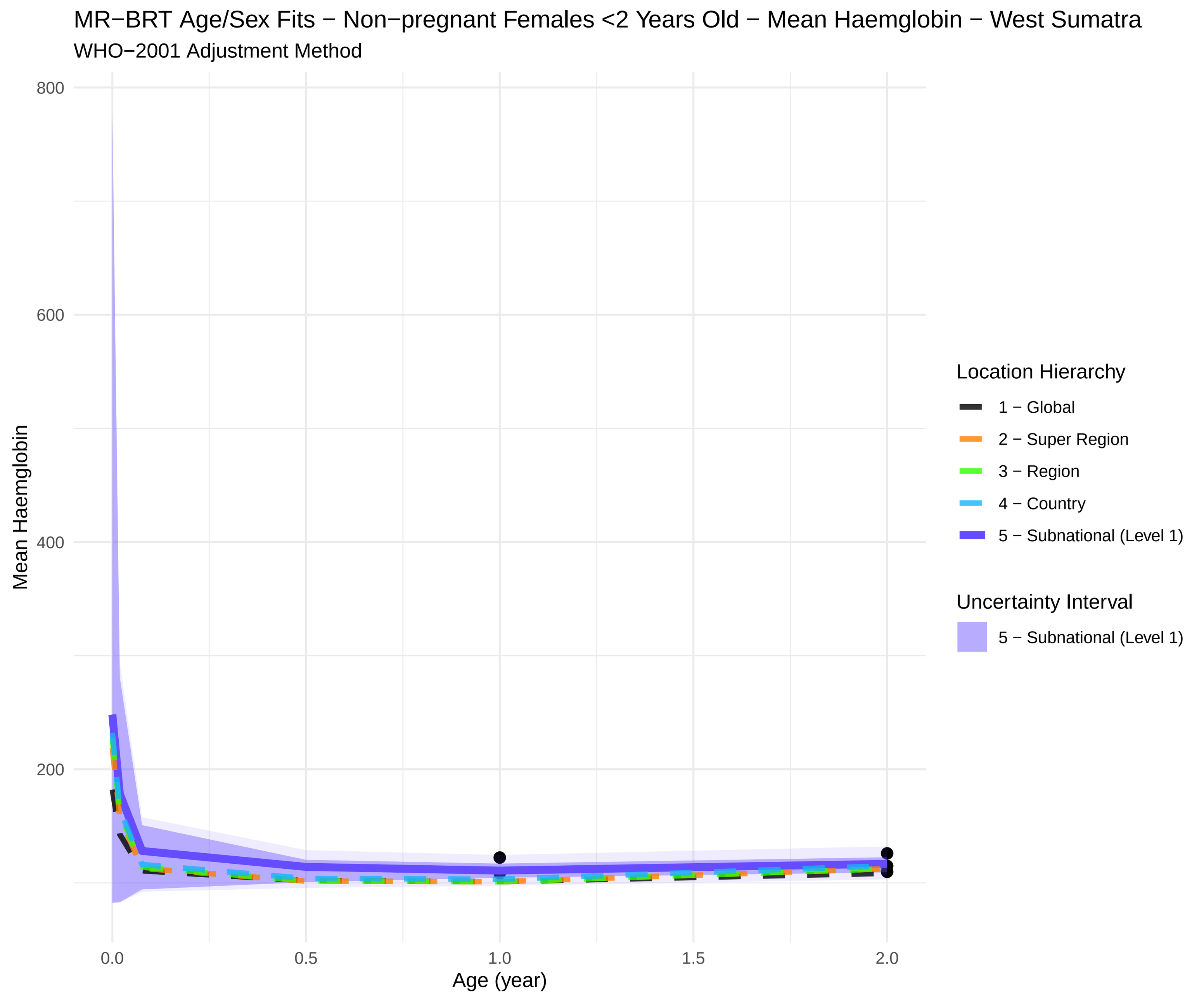
MR−BRT Age/Sex Fits − Non−pregnant Females <2 Years Old − Mean Haemglobin − West Sumatra
WHO−2001 Adjustment Method
Mean Haemglobin
Age (year)
Location Hierarchy
1 − Global
2 − Super Region
3 − Region
4 − Country
5 − Subnational (Level 1)
Uncertainty Interval
5 − Subnational (Level 1)

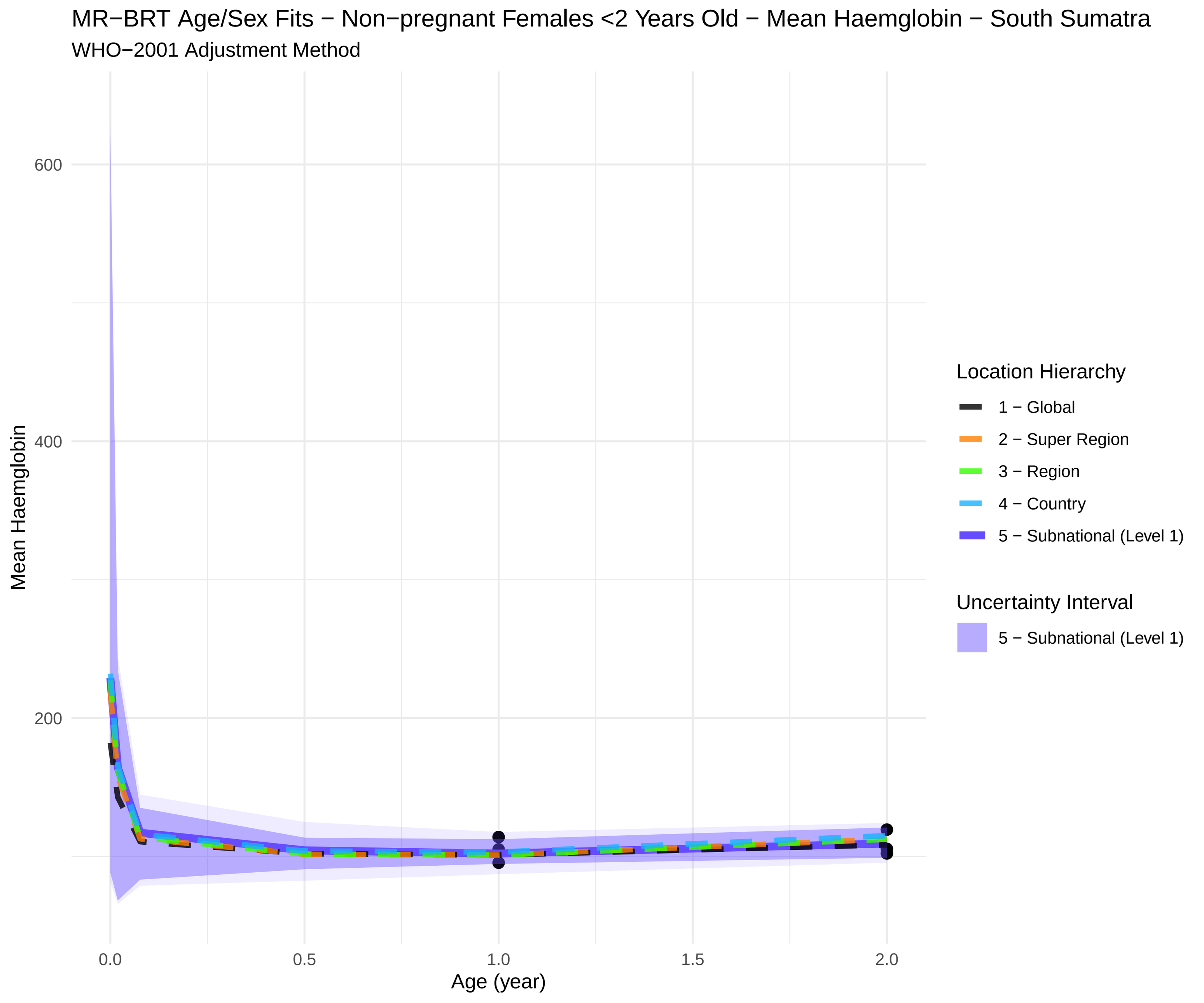
MR−BRT Age/Sex Fits − Non−pregnant Females <2 Years Old − Mean Haemglobin − South Sumatra
WHO−2001 Adjustment Method
Mean Haemglobin
Age (year)
Location Hierarchy
1 − Global
2 − Super Region
3 − Region
4 − Country
5 − Subnational (Level 1)
Uncertainty Interval
5 − Subnational (Level 1)

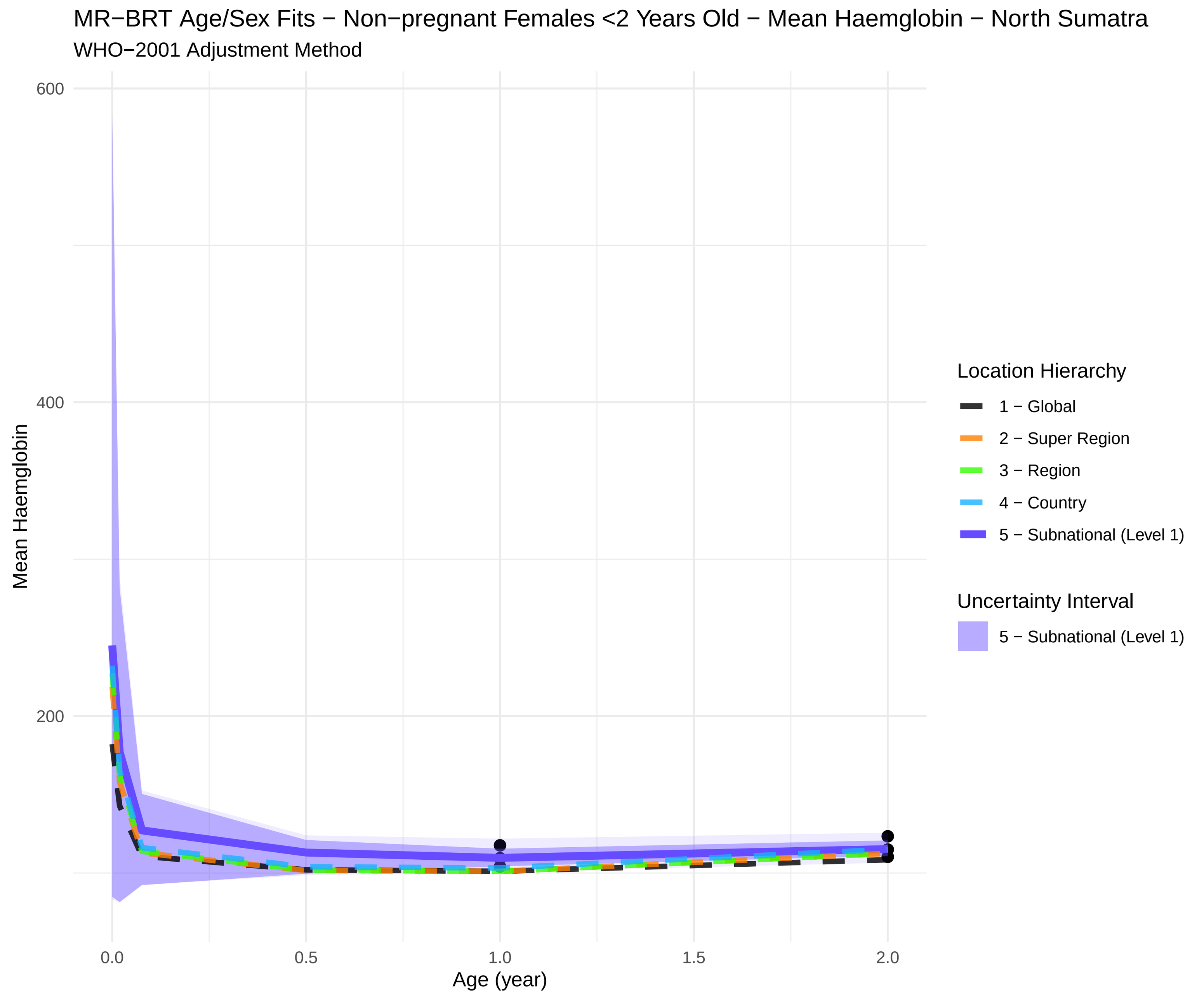
MR−BRT Age/Sex Fits − Non−pregnant Females <2 Years Old − Mean Haemglobin − North Sumatra
WHO−2001 Adjustment Method
Mean Haemglobin
Age (year)
Location Hierarchy
1 − Global
2 − Super Region
3 − Region
4 − Country
5 − Subnational (Level 1)
Uncertainty Interval
5 − Subnational (Level 1)

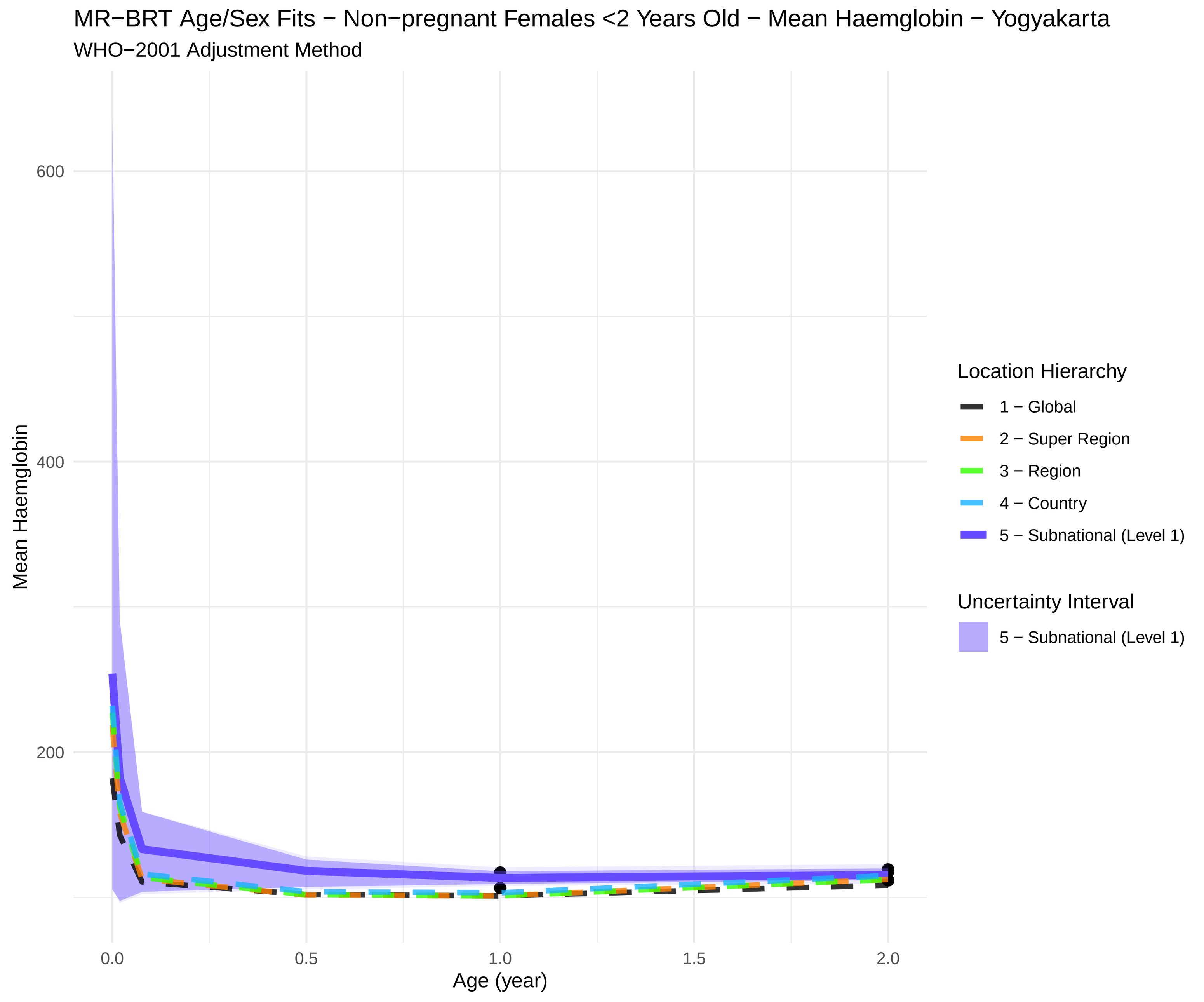
MR−BRT Age/Sex Fits − Non−pregnant Females <2 Years Old − Mean Haemglobin − Yogyakarta
WHO−2001 Adjustment Method
Mean Haemglobin
Age (year)
Location Hierarchy
1 − Global
2 − Super Region
3 − Region
4 − Country
5 − Subnational (Level 1)
Uncertainty Interval
5 − Subnational (Level 1)

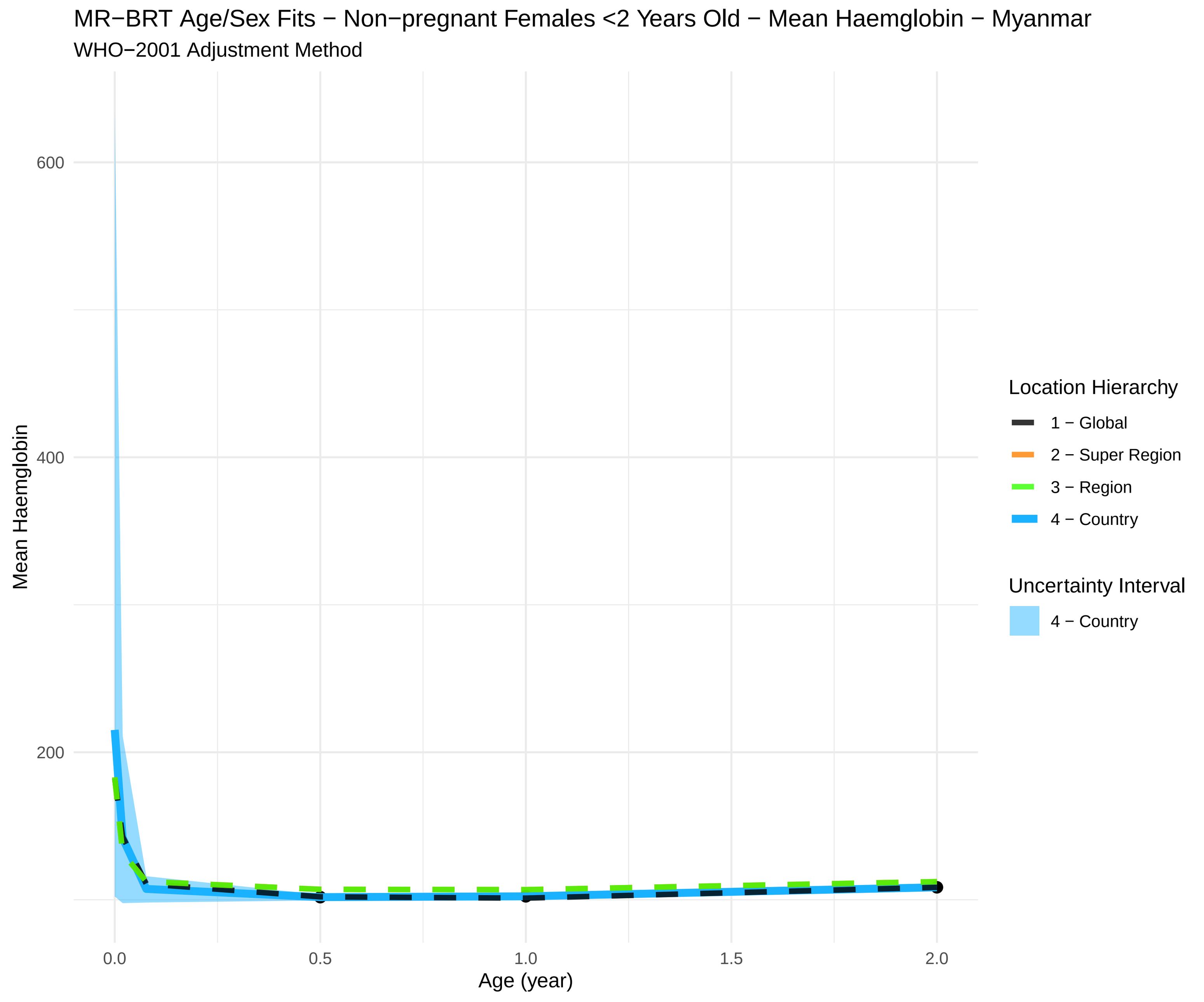
MR−BRT Age/Sex Fits − Non−pregnant Females <2 Years Old − Mean Haemglobin − Myanmar
WHO−2001 Adjustment Method
Mean Haemglobin
Age (year)
Location Hierarchy
1 − Global
2 − Super Region
3 − Region
4 − Country
Uncertainty Interval
4 − Country

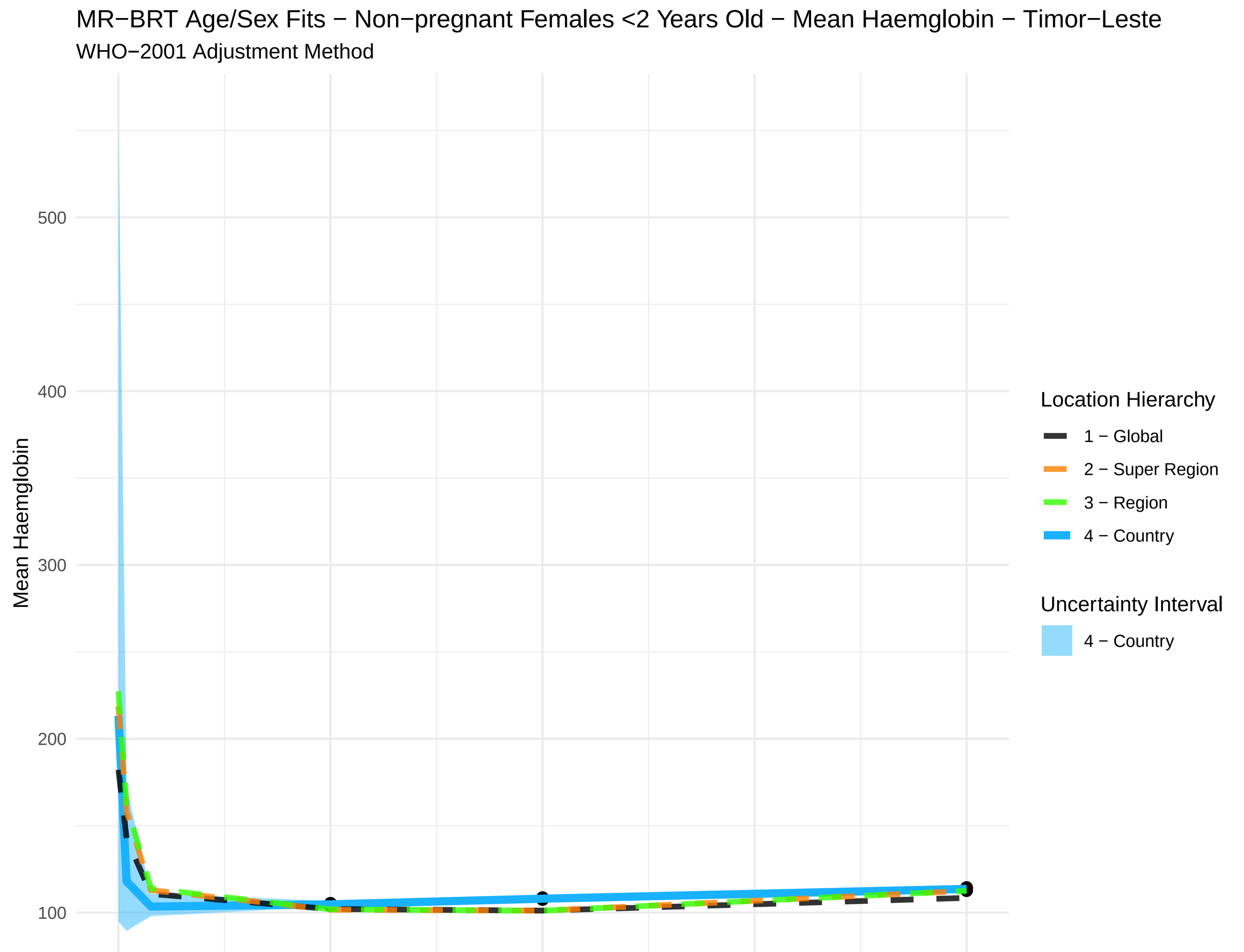
MR−BRT Age/Sex Fits − Non−pregnant Females <2 Years Old − Mean Haemglobin − Timor−Leste
WHO−2001 Adjustment Method
Mean Haemglobin
Age (year)
Location Hierarchy
1 − Global
2 − Super Region
3 − Region
4 − Country
Uncertainty Interval
4 − Country

MR−BRT Age/Sex Fits − Non−pregnant Females <2 Years Old − Mean Haemglobin − Sub−Saharan Africa

WHO−2001 Adjustment Method

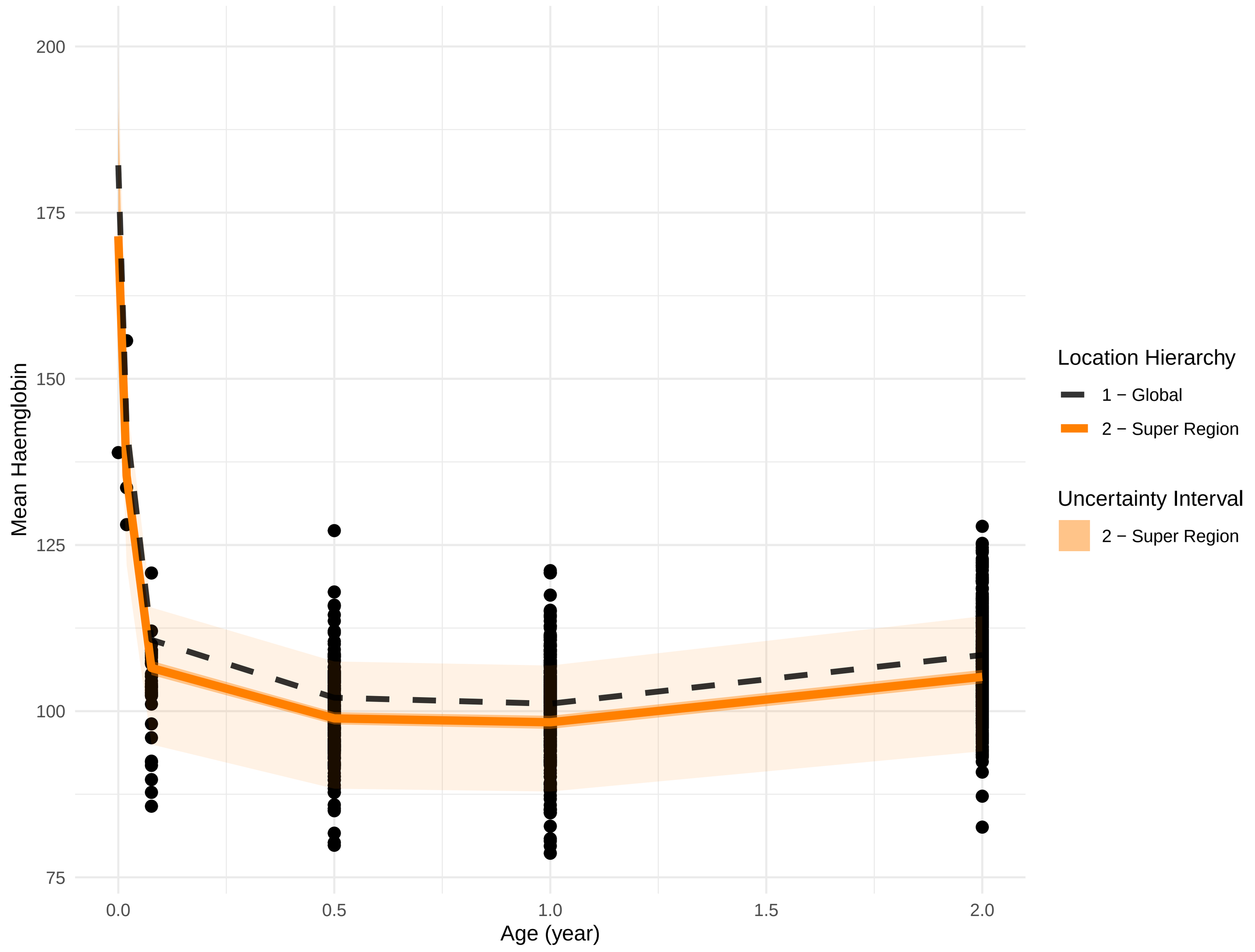

MR−BRT Age/Sex Fits − Non−pregnant Females <2 Years Old − Mean Haemglobin − Central Sub−Saharan Africa

WHO−2001 Adjustment Method

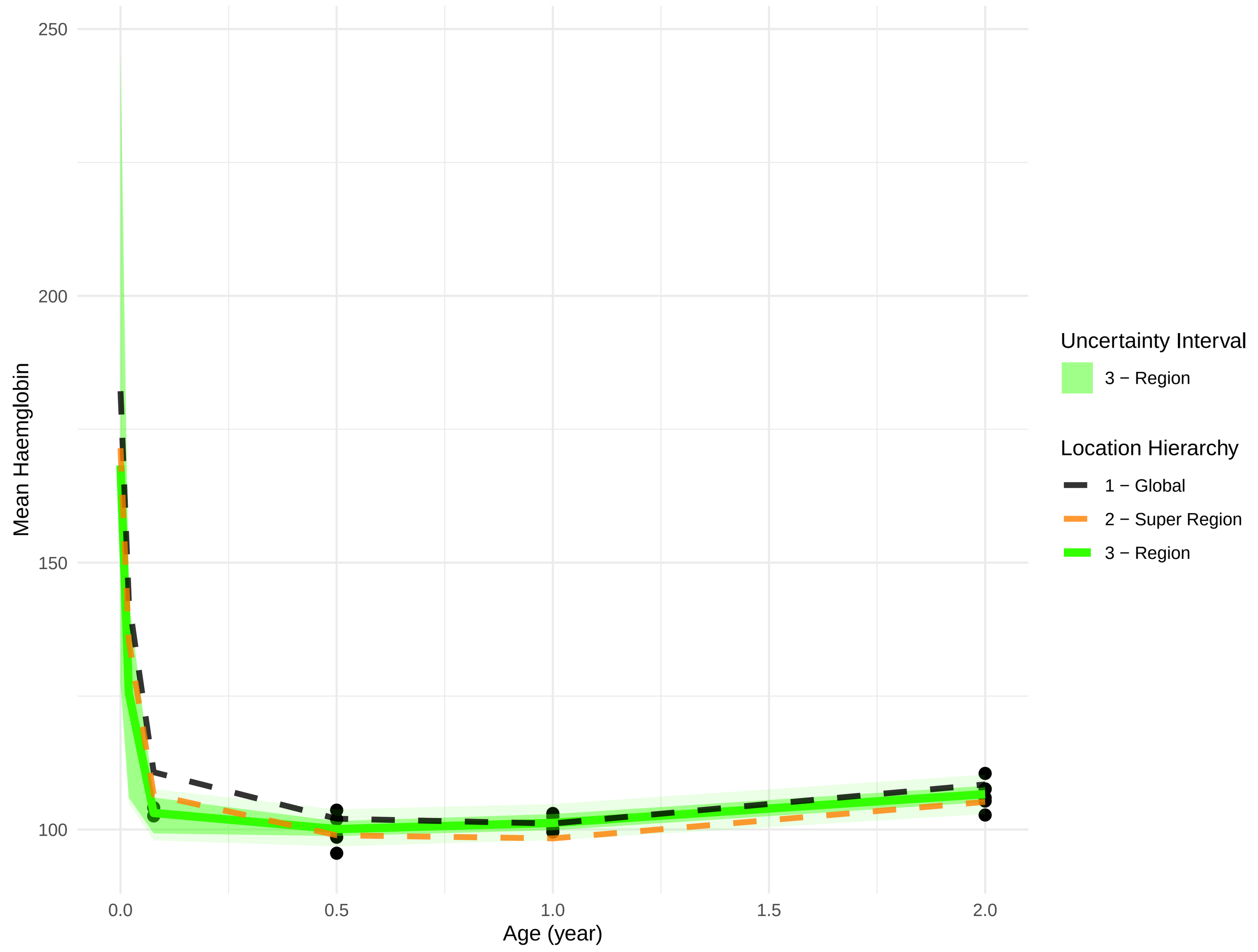

MR−BRT Age/Sex Fits − Non−pregnant Females <2 Years Old − Mean Haemglobin − Angola

WHO−2001 Adjustment Method

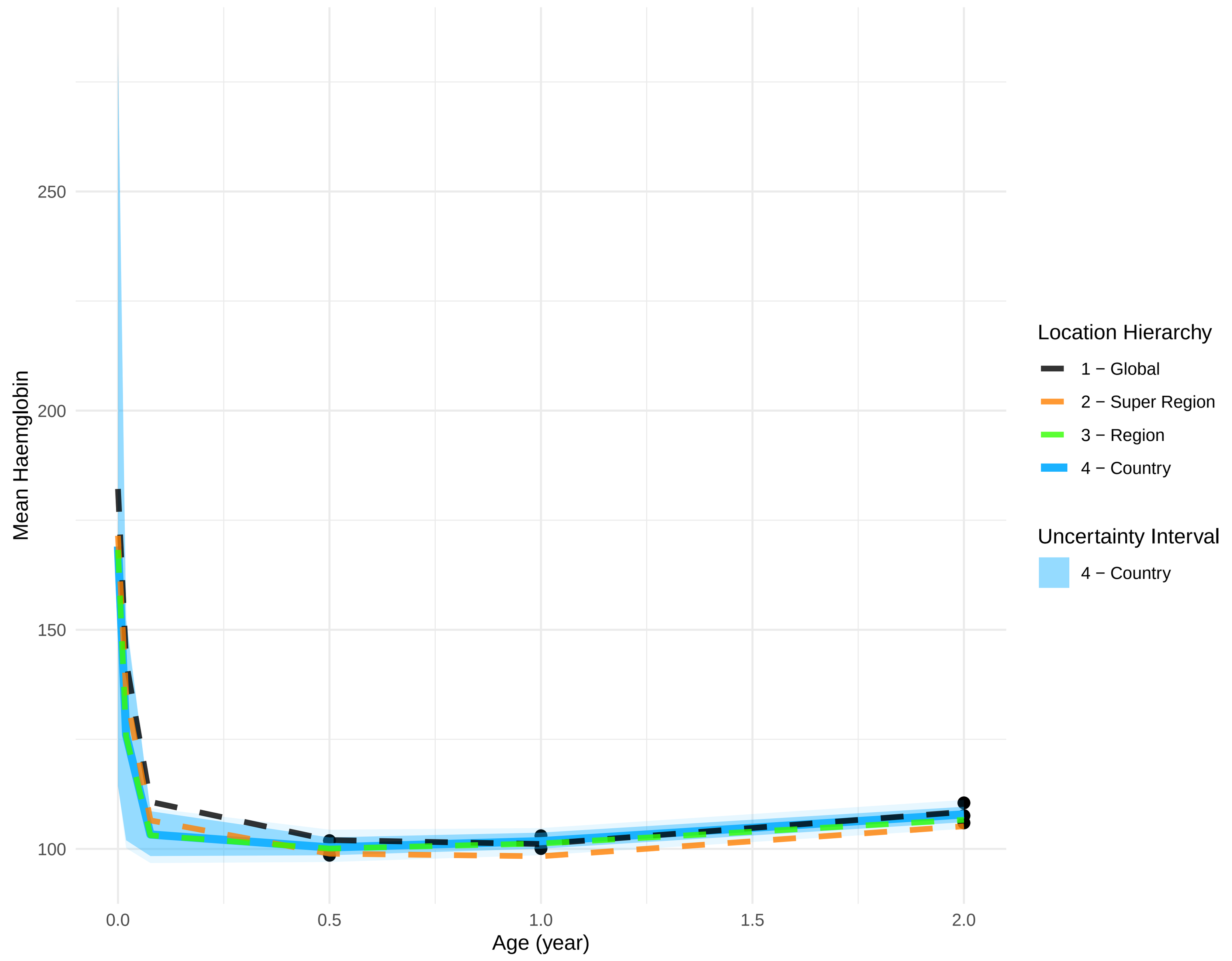

MR−BRT Age/Sex Fits − Non−pregnant Females <2 Years Old − Mean Haemglobin − Democratic Republic of the Congo

WHO−2001 Adjustment Method

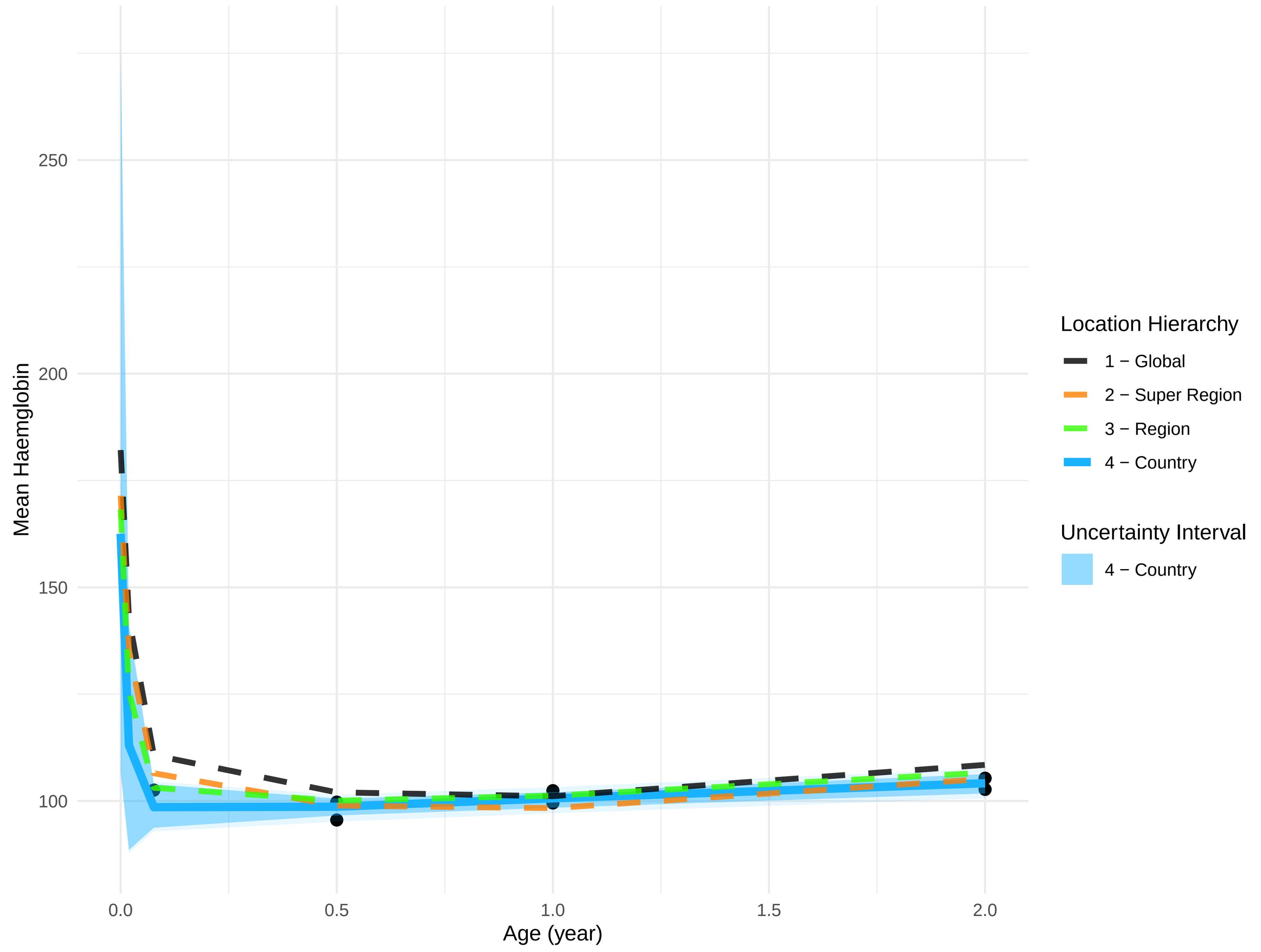

MR−BRT Age/Sex Fits − Non−pregnant Females <2 Years Old − Mean Haemglobin − Gabon

WHO−2001 Adjustment Method

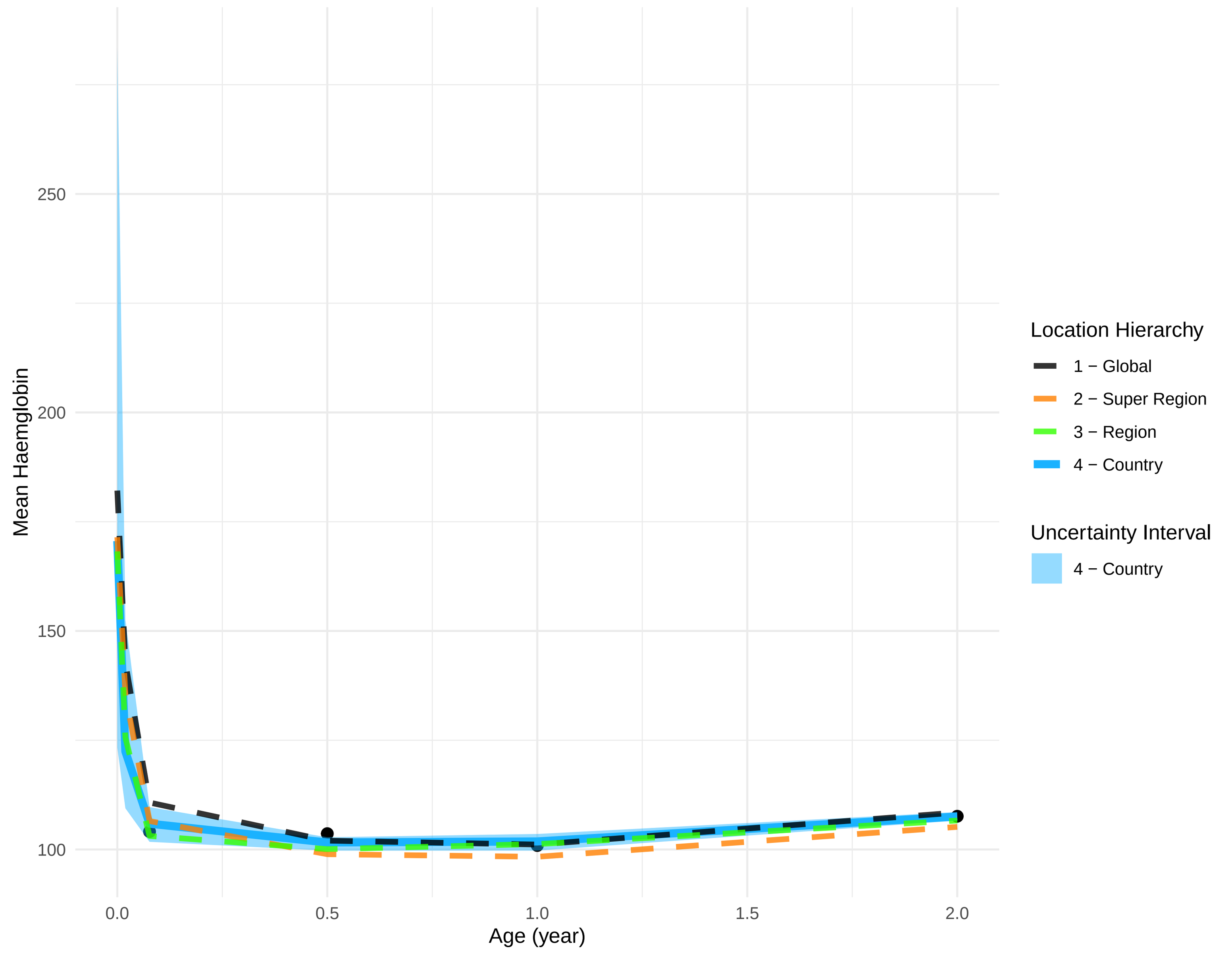

MR−BRT Age/Sex Fits − Non−pregnant Females <2 Years Old − Mean Haemglobin − Eastern Sub−Saharan Africa

WHO−2001 Adjustment Method

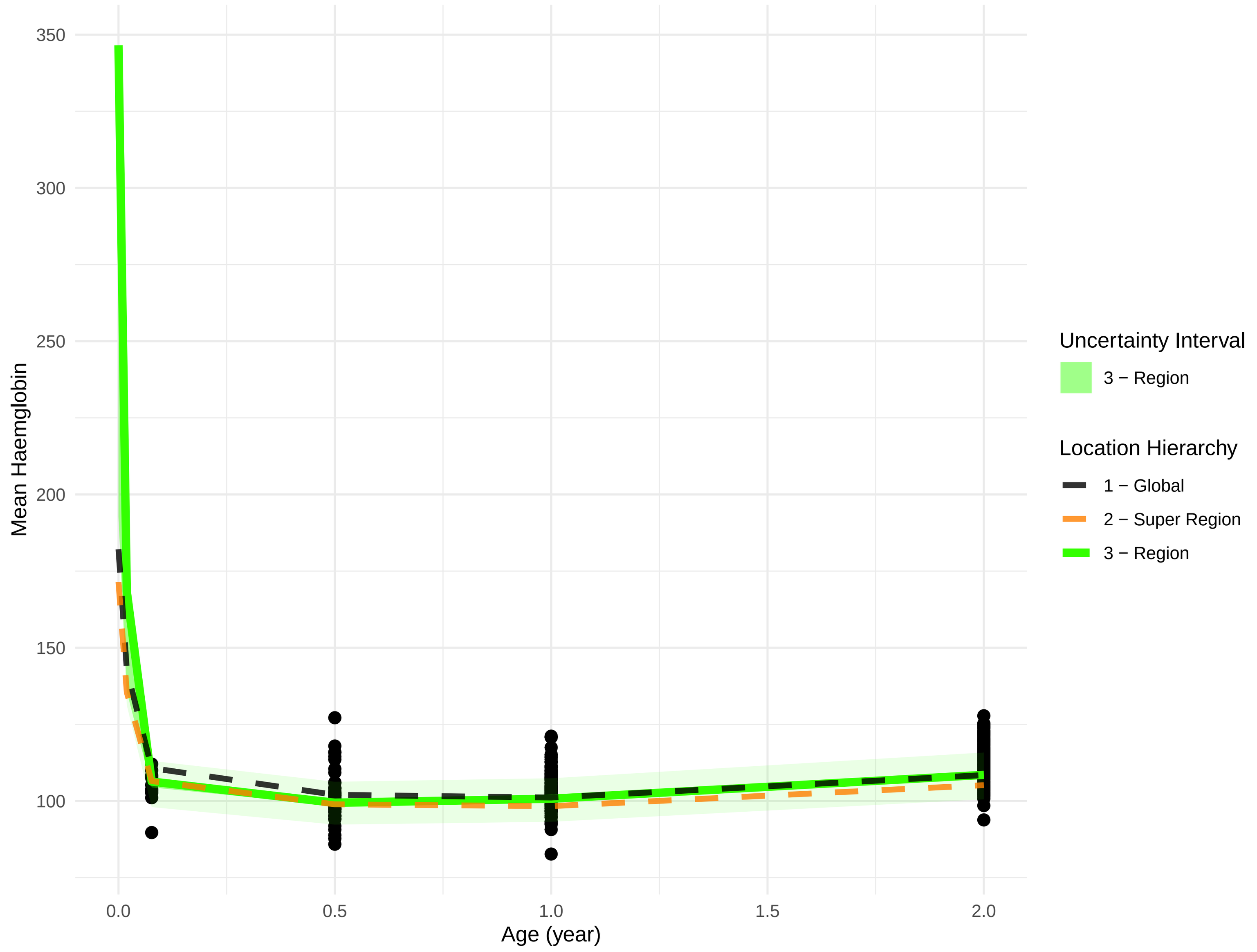

MR−BRT Age/Sex Fits − Non−pregnant Females <2 Years Old − Mean Haemglobin − Burundi

WHO−2001 Adjustment Method

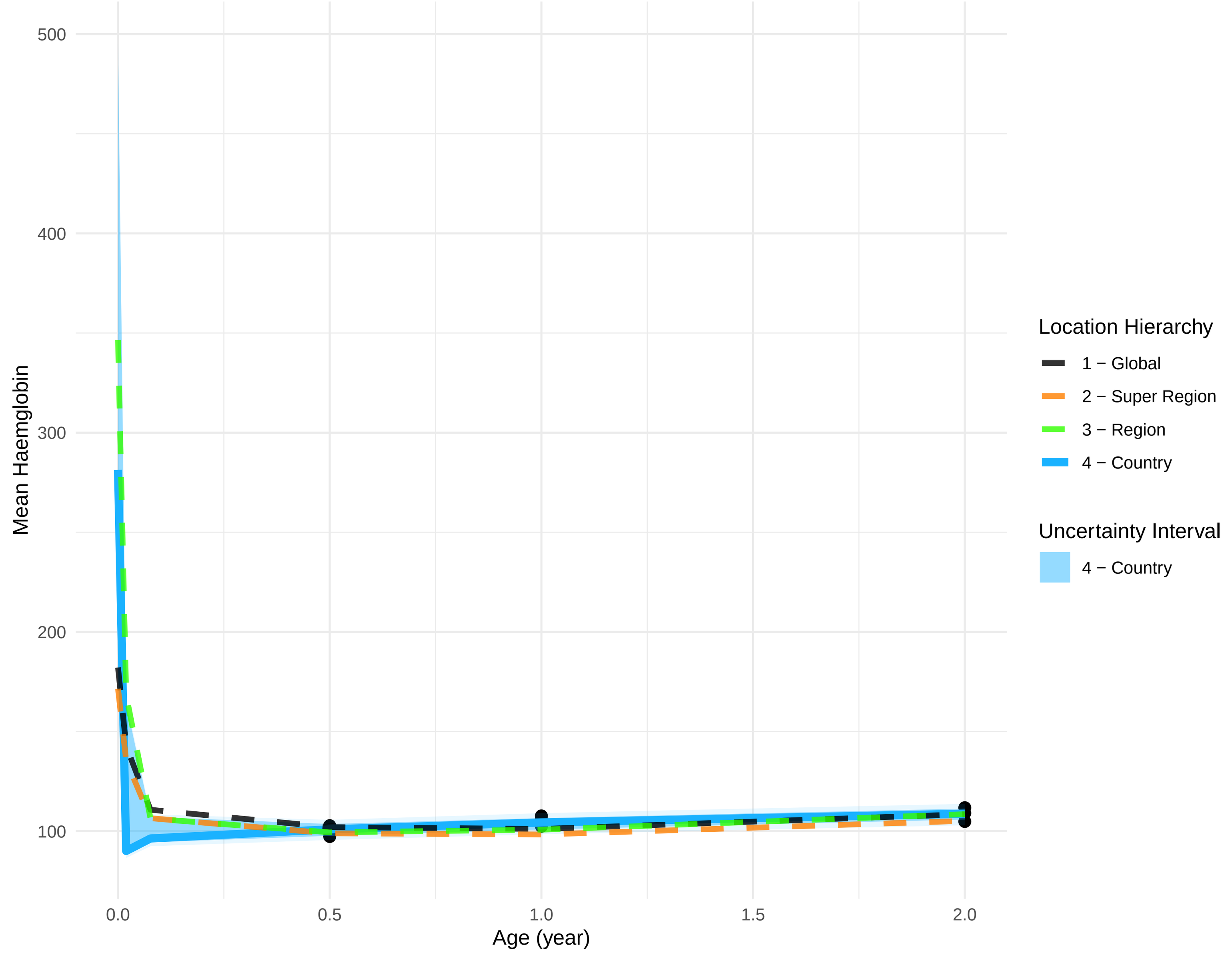

MR−BRT Age/Sex Fits − Non−pregnant Females <2 Years Old − Mean Haemglobin − Ethiopia

WHO−2001 Adjustment Method

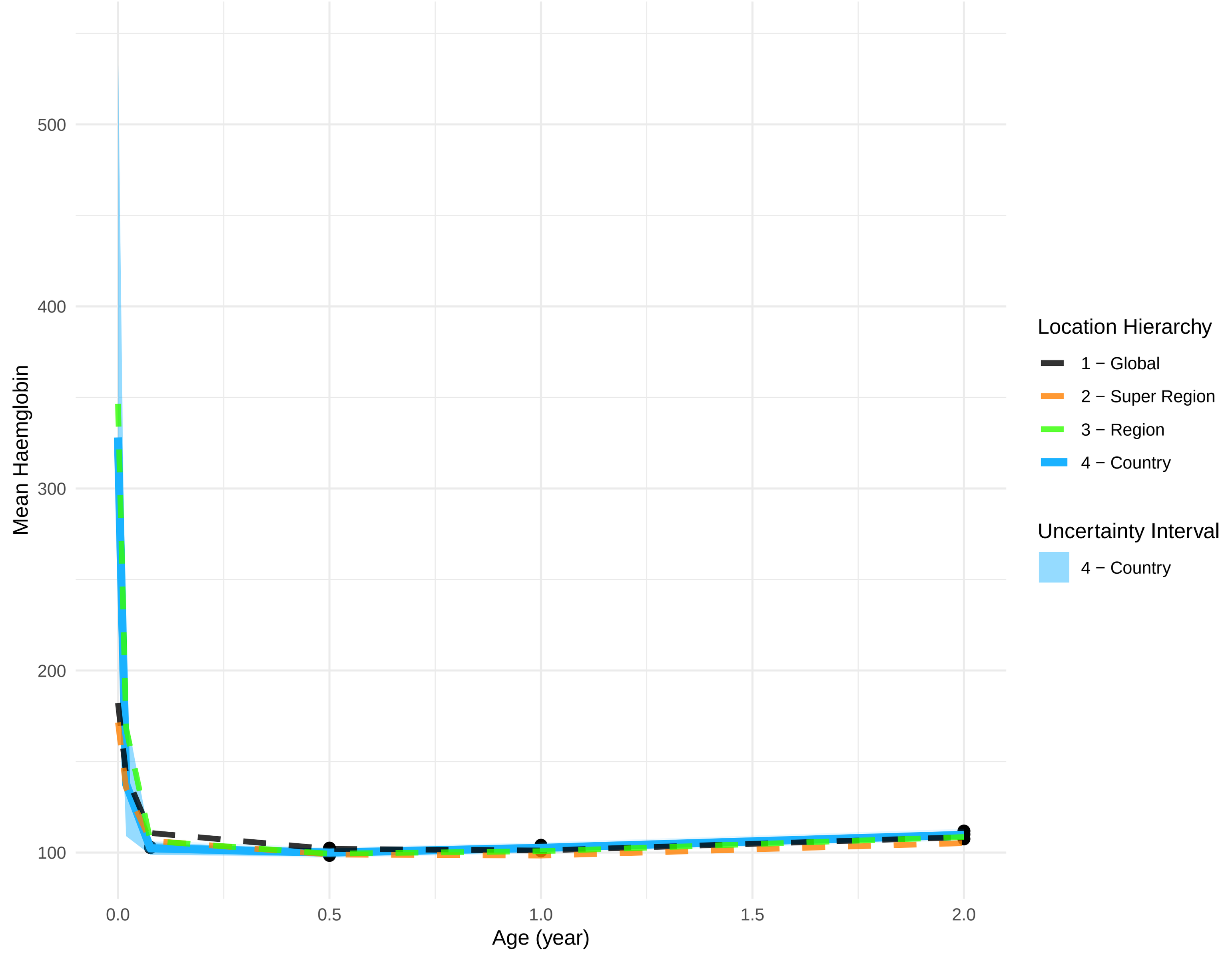

MR−BRT Age/Sex Fits − Non−pregnant Females <2 Years Old − Mean Haemglobin − Kenya

WHO−2001 Adjustment Method

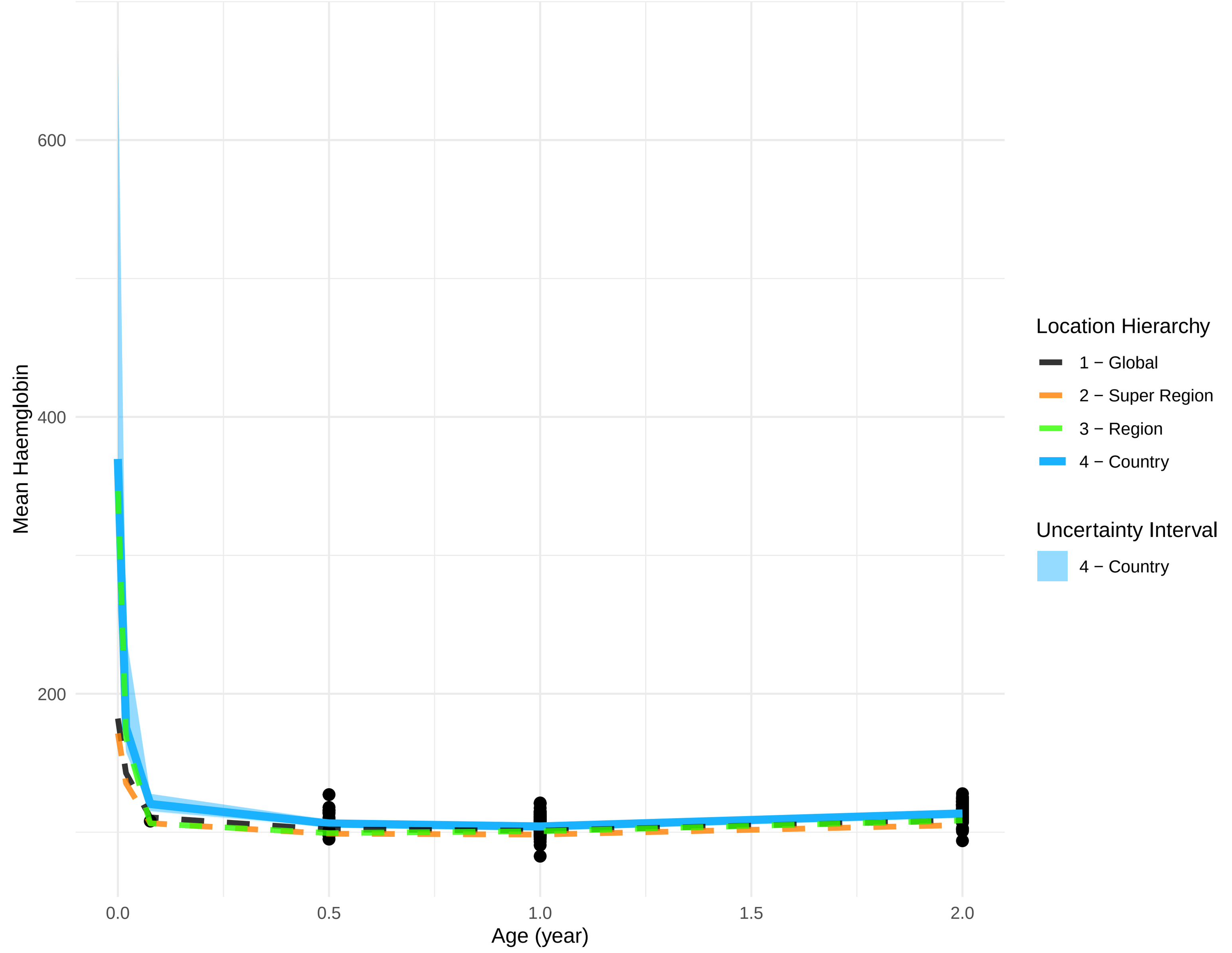

MR−BRT Age/Sex Fits − Non−pregnant Females <2 Years Old − Mean Haemglobin − Baringo

WHO−2001 Adjustment Method

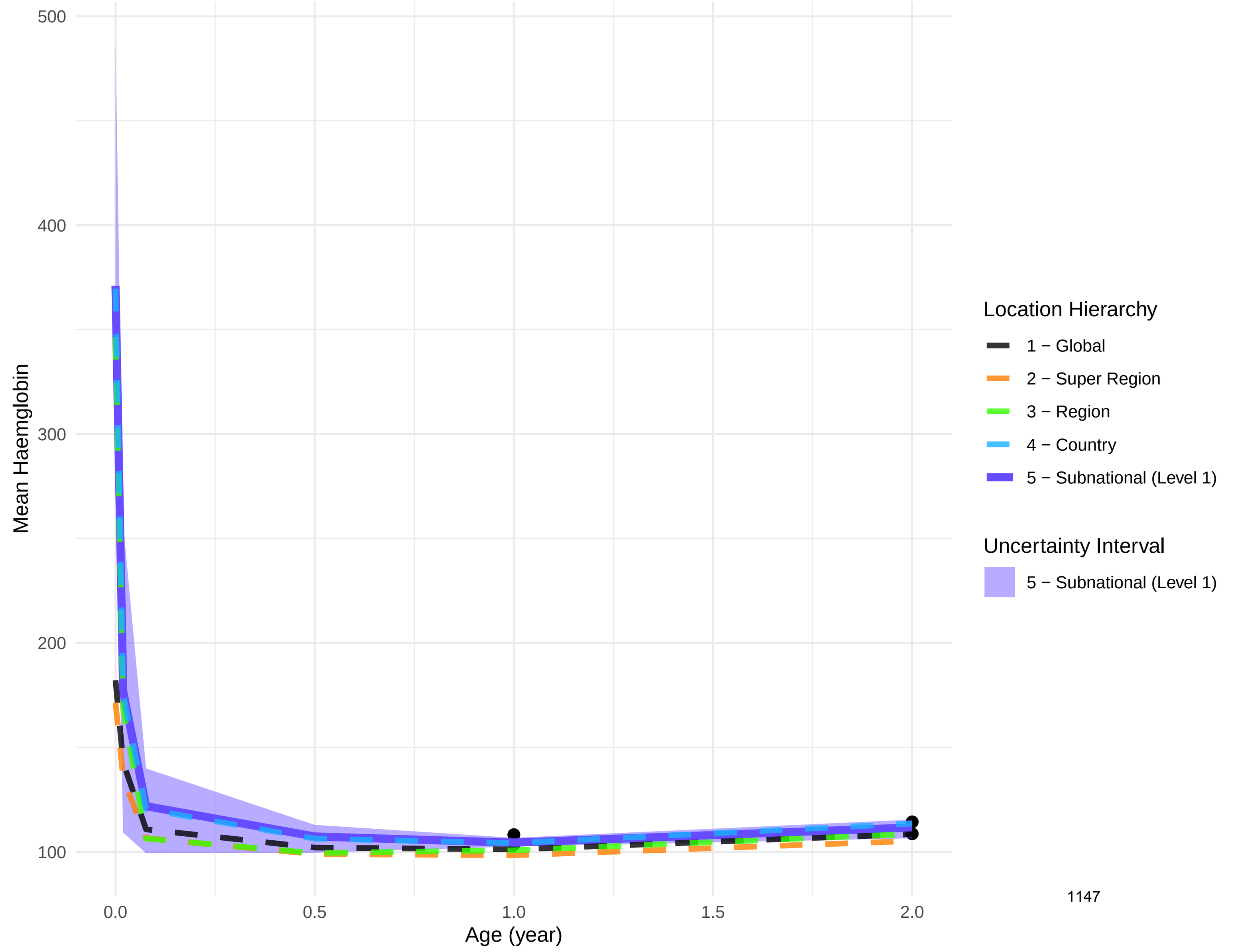

MR−BRT Age/Sex Fits − Non−pregnant Females <2 Years Old − Mean Haemglobin − Bomet
WHO−2001 Adjustment Method

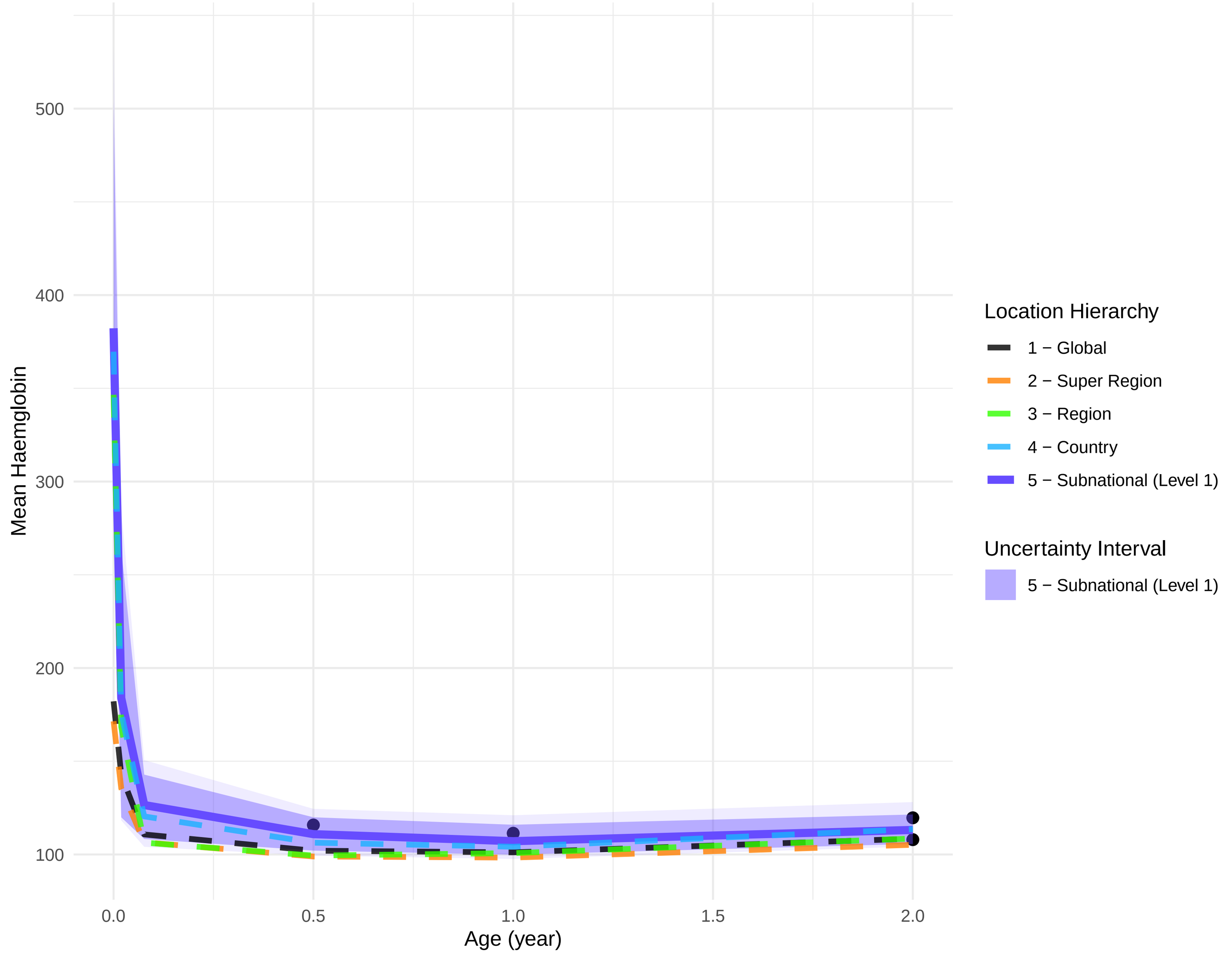

MR−BRT Age/Sex Fits − Non−pregnant Females <2 Years Old − Mean Haemglobin − Bungoma
WHO−2001 Adjustment Method

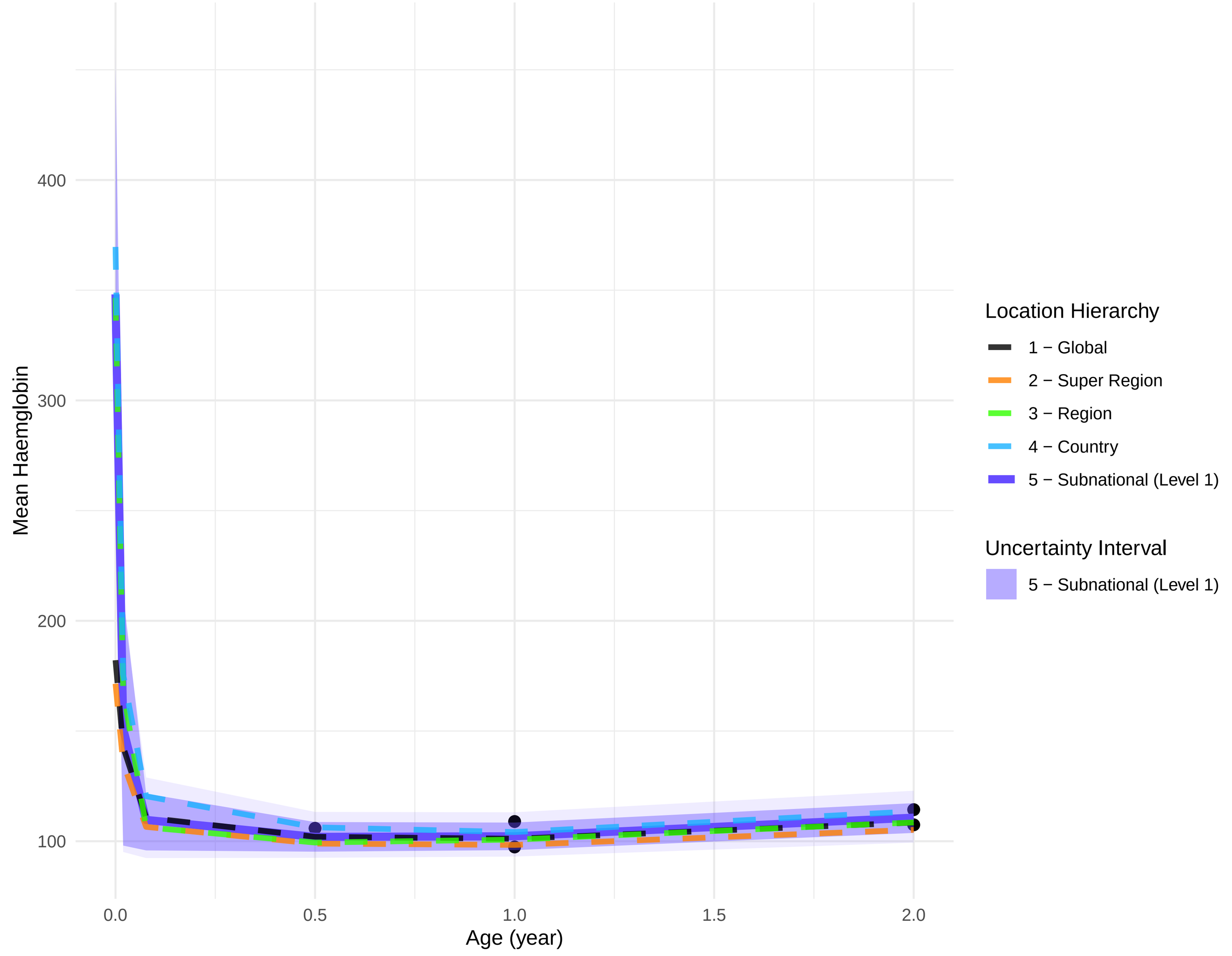

MR−BRT Age/Sex Fits − Non−pregnant Females <2 Years Old − Mean Haemglobin − Busia
WHO−2001 Adjustment Method

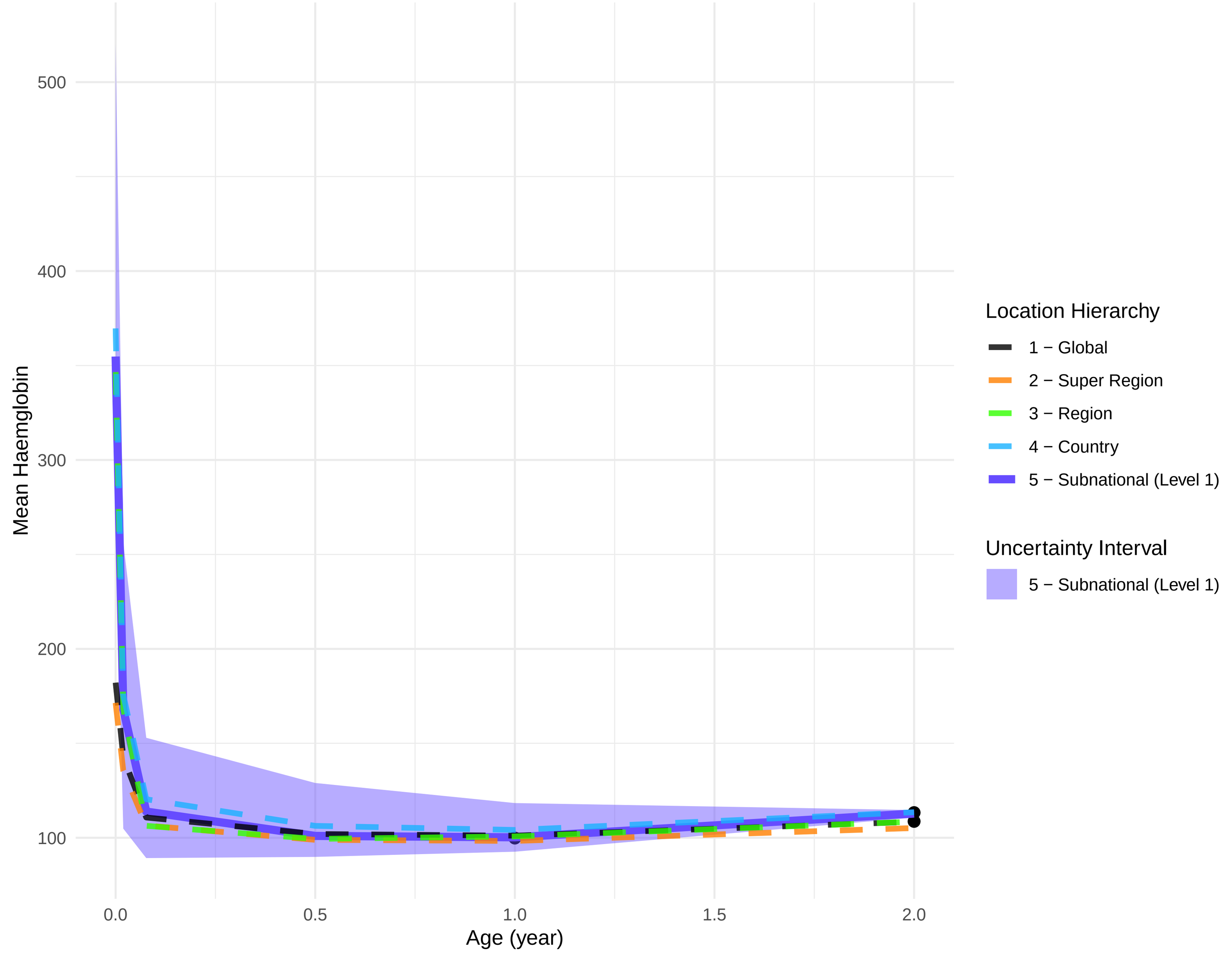

MR−BRT Age/Sex Fits − Non−pregnant Females <2 Years Old − Mean Haemglobin − Elgeyo−Marakwet
WHO−2001 Adjustment Method

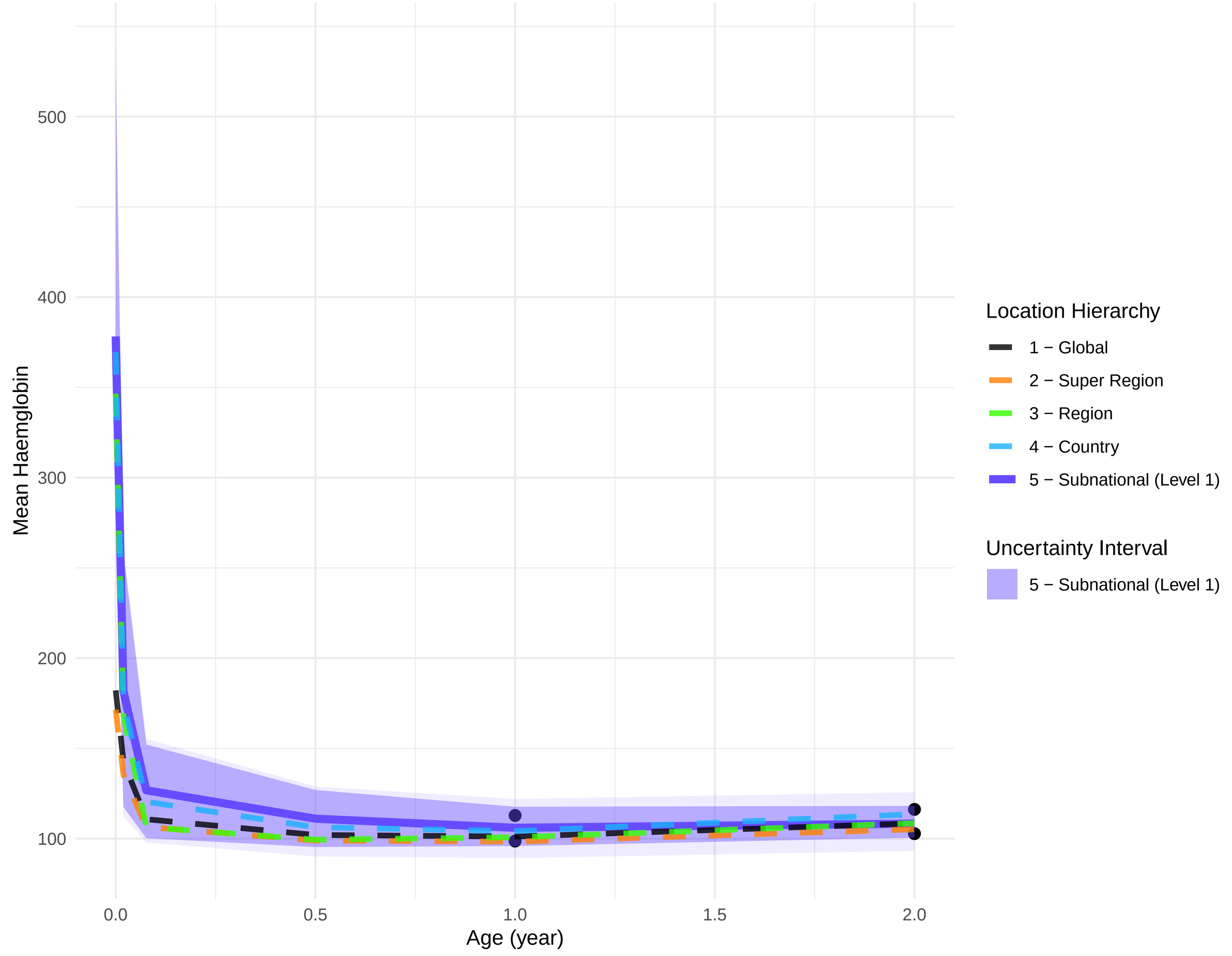

MR−BRT Age/Sex Fits − Non−pregnant Females <2 Years Old − Mean Haemglobin − Embu
WHO−2001 Adjustment Method

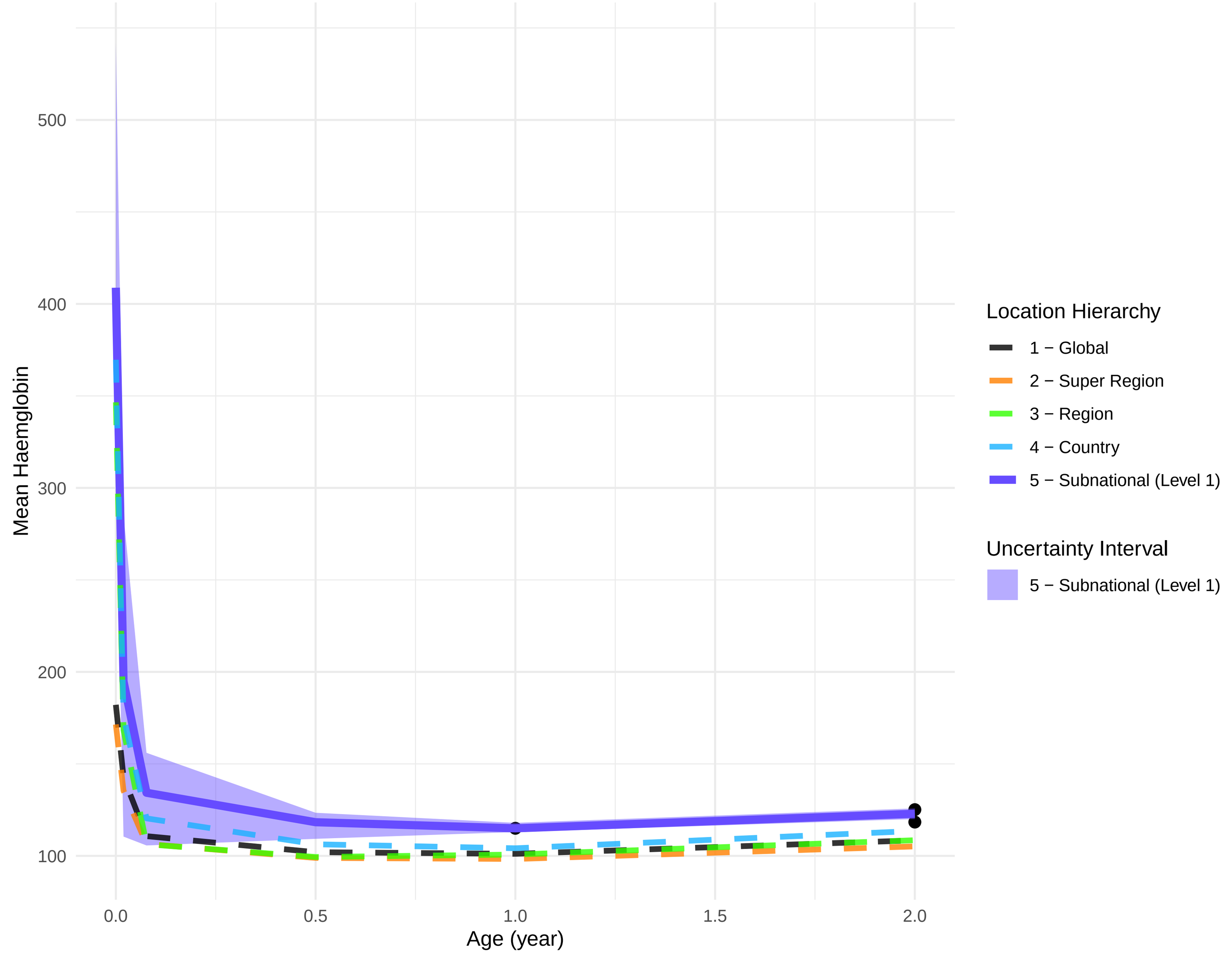

MR−BRT Age/Sex Fits − Non−pregnant Females <2 Years Old − Mean Haemglobin − Garissa
WHO−2001 Adjustment Method

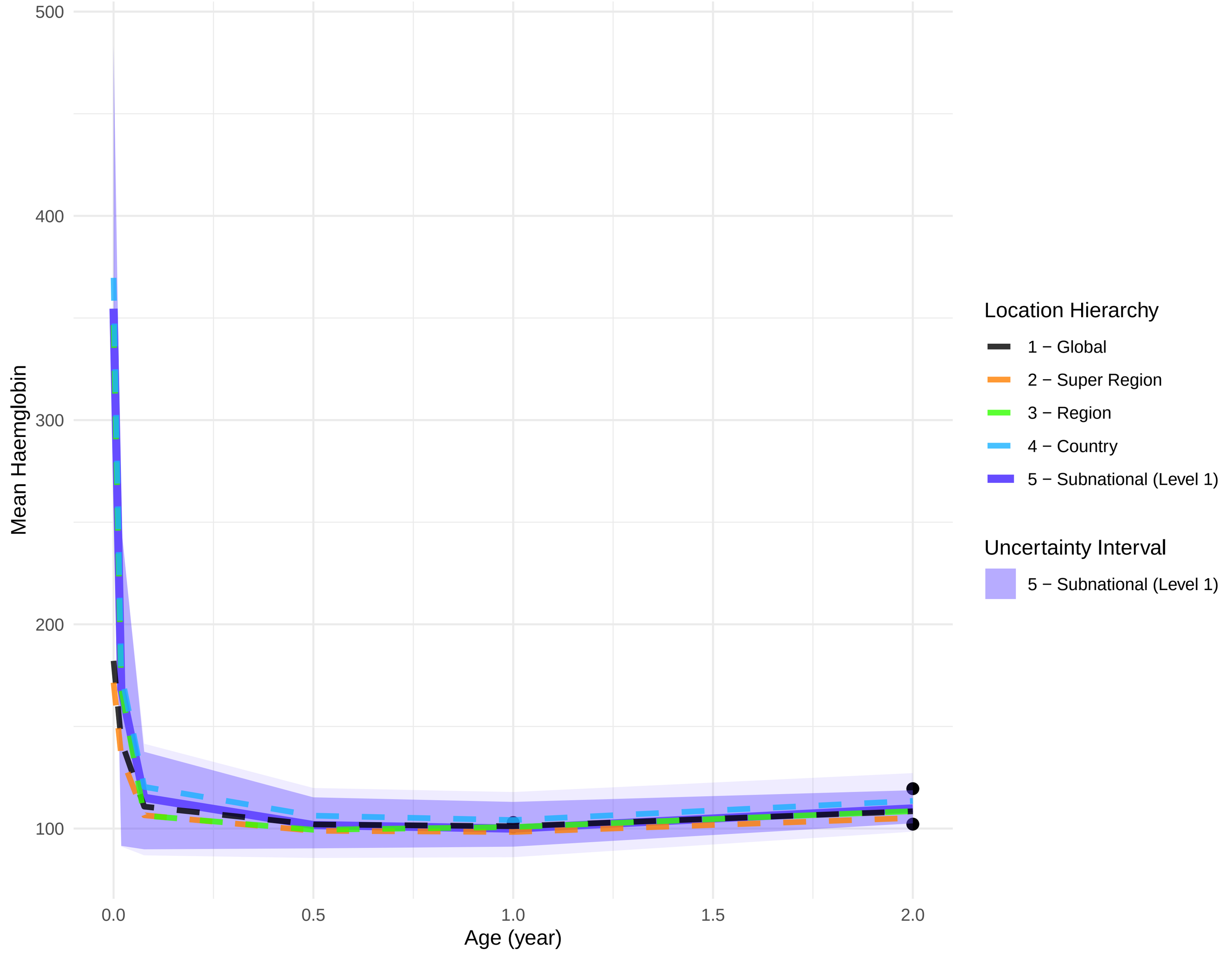

MR−BRT Age/Sex Fits − Non−pregnant Females <2 Years Old − Mean Haemglobin − Homa Bay
WHO−2001 Adjustment Method

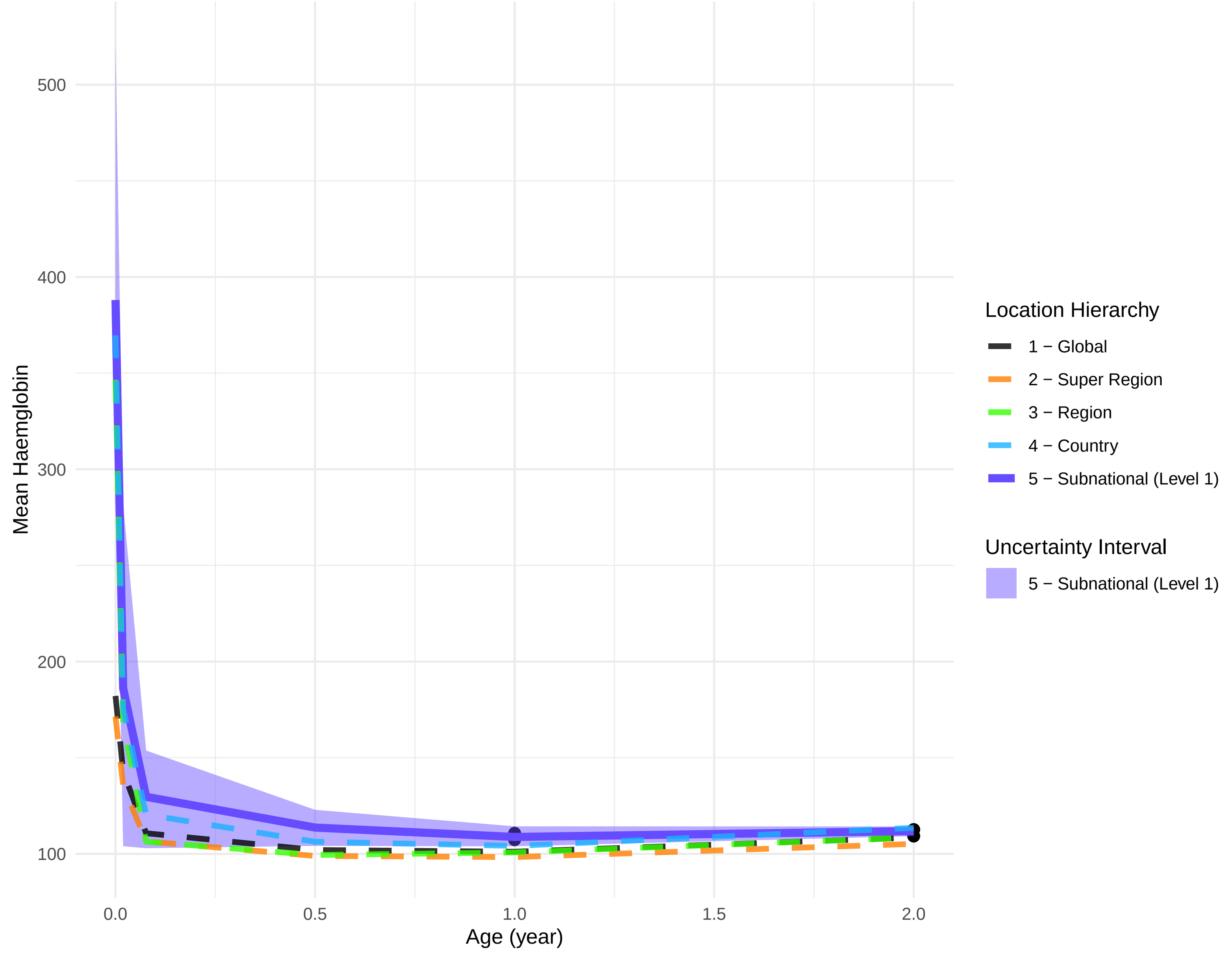

MR−BRT Age/Sex Fits − Non−pregnant Females <2 Years Old − Mean Haemglobin − Isiolo
WHO−2001 Adjustment Method

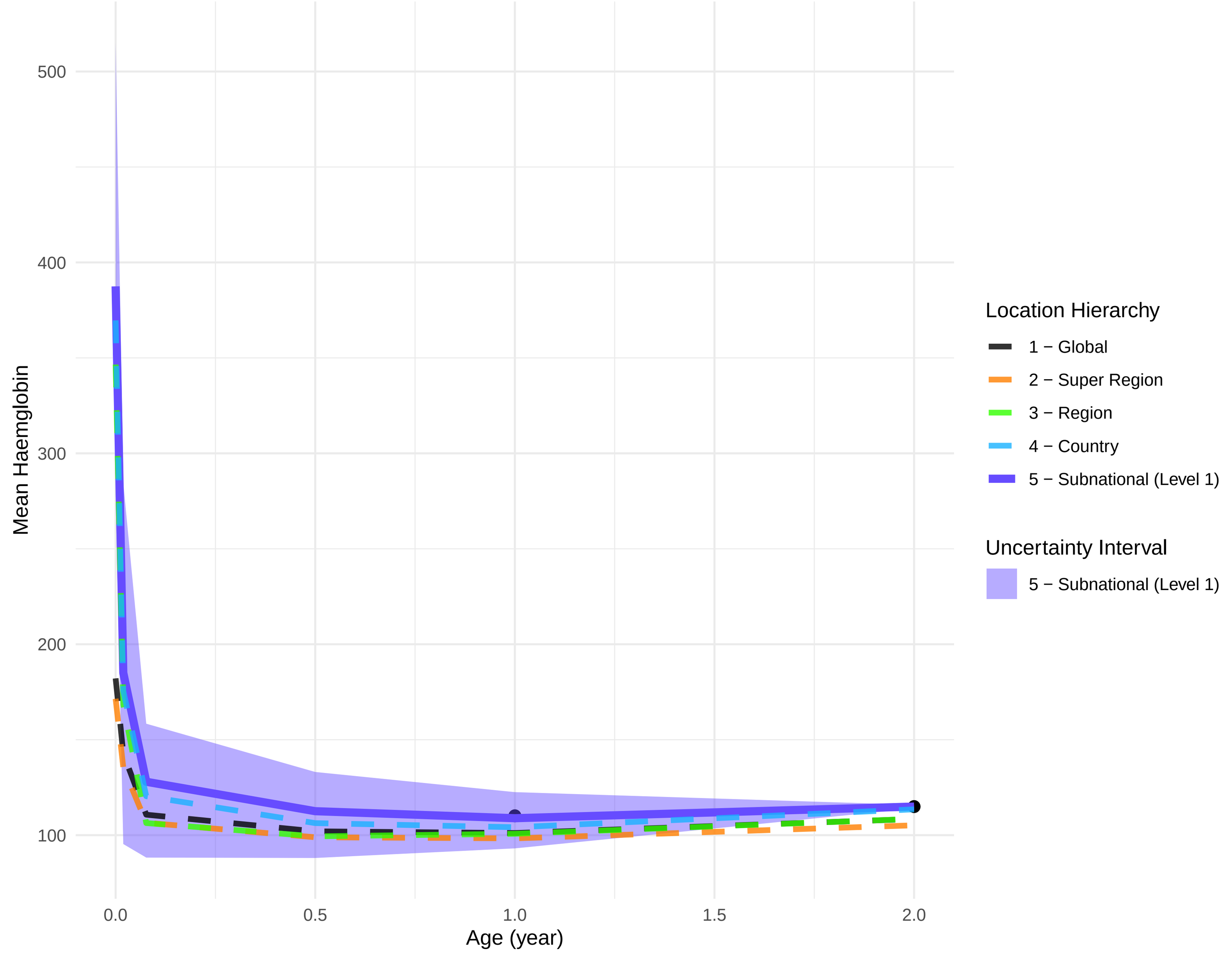

MR−BRT Age/Sex Fits − Non−pregnant Females <2 Years Old − Mean Haemglobin − Kajiado
WHO−2001 Adjustment Method

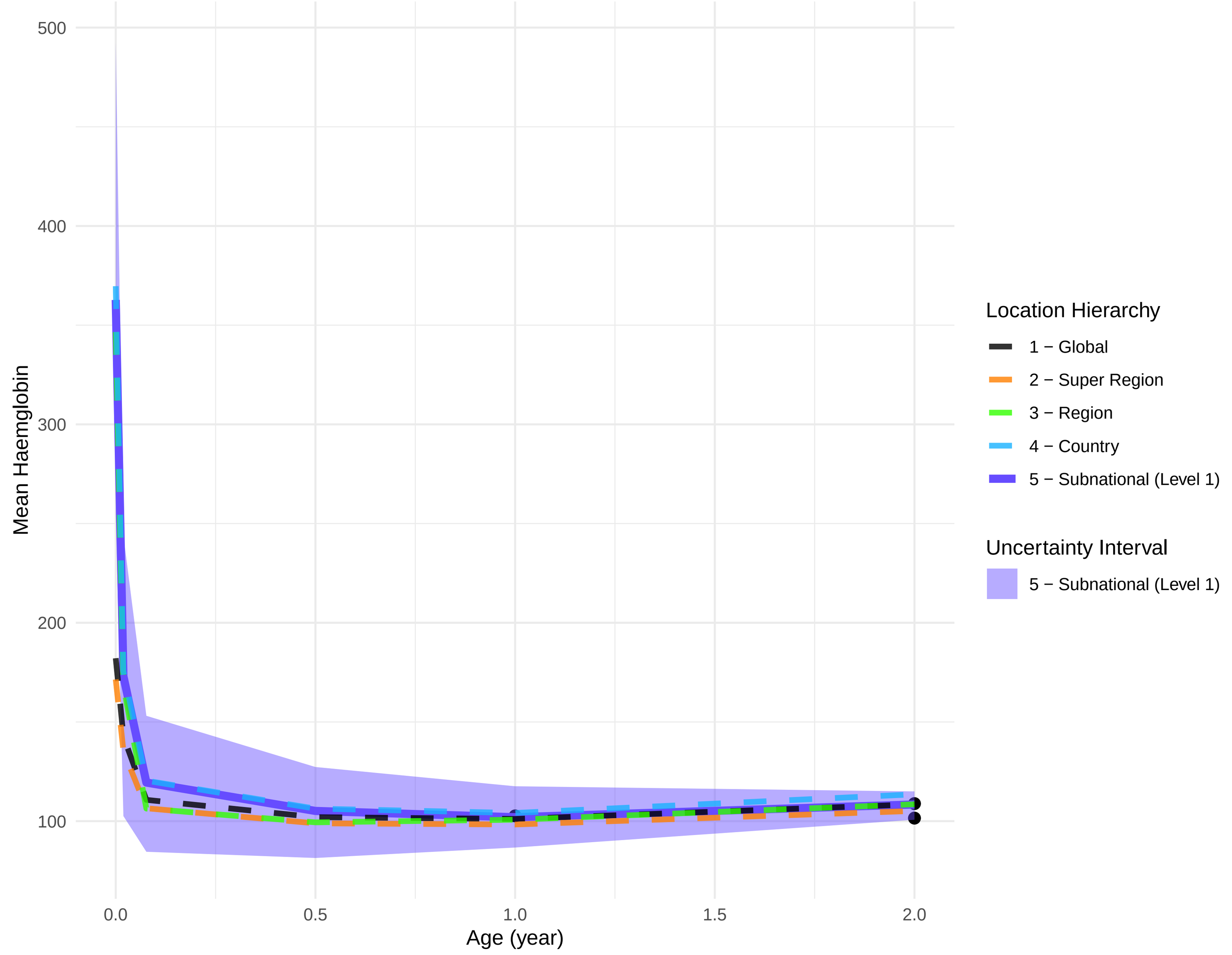

MR−BRT Age/Sex Fits − Non−pregnant Females <2 Years Old − Mean Haemglobin − Kakamega
WHO−2001 Adjustment Method

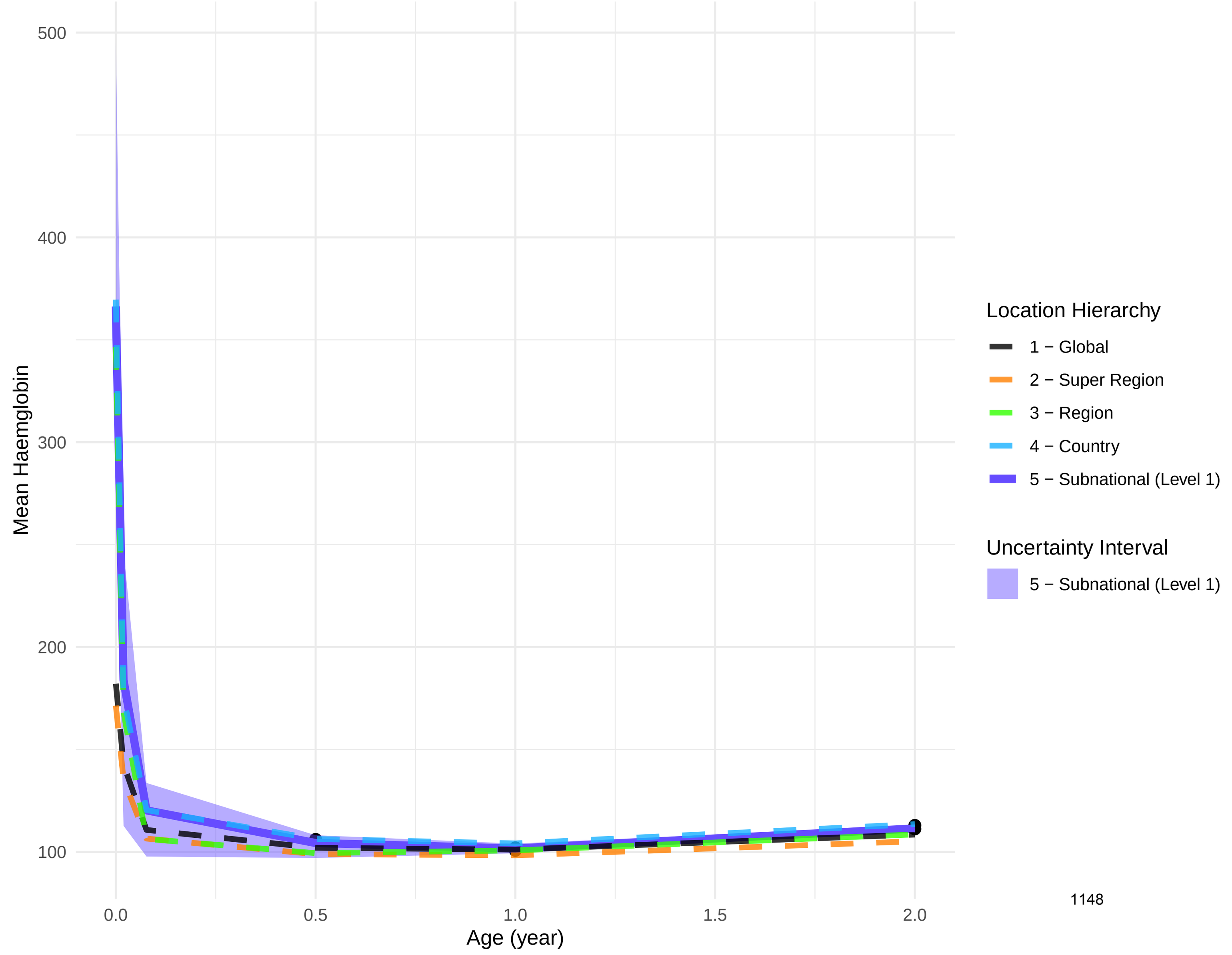

MR−BRT Age/Sex Fits − Non−pregnant Females <2 Years Old − Mean Haemglobin − Kericho
WHO−2001 Adjustment Method

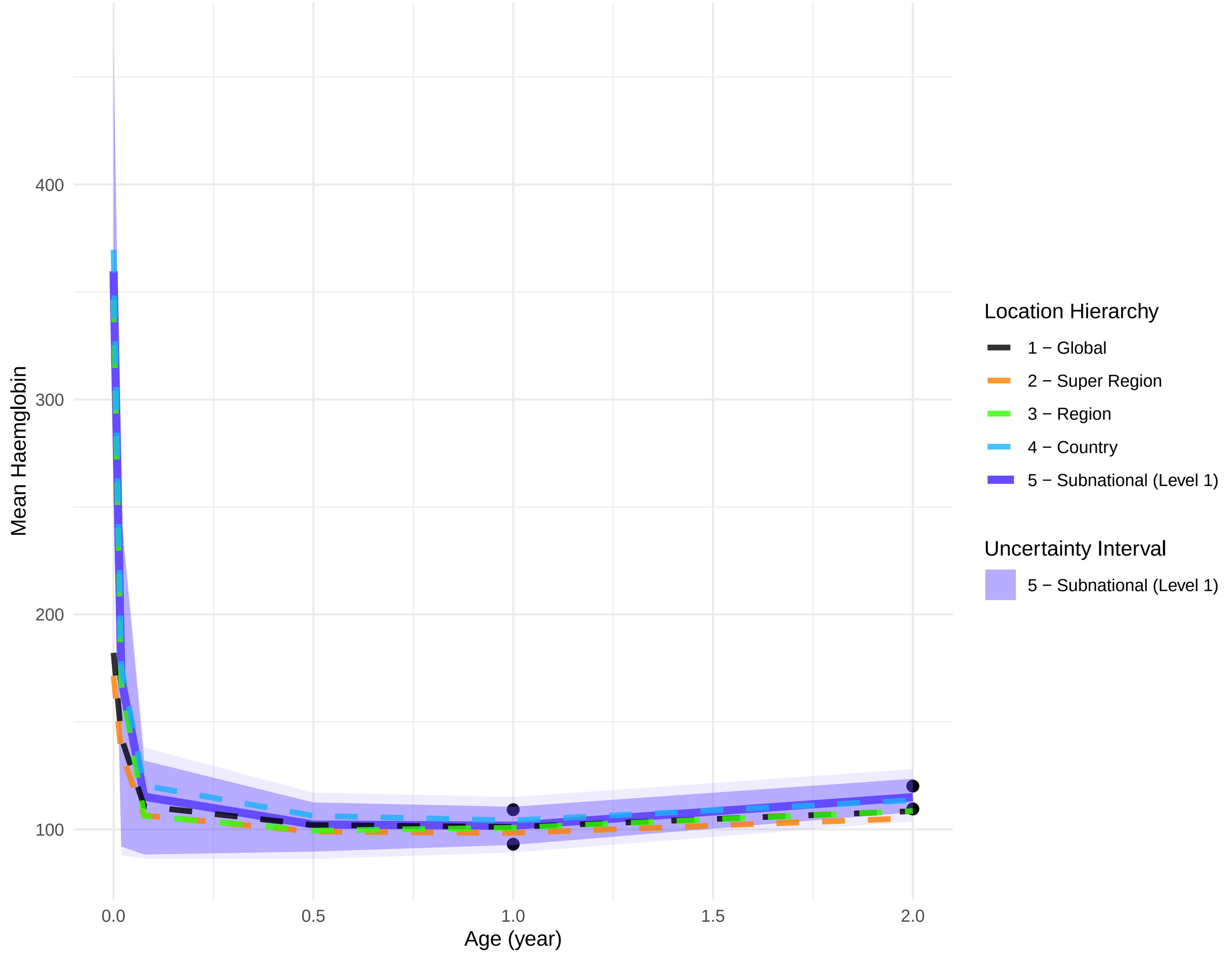

MR−BRT Age/Sex Fits − Non−pregnant Females <2 Years Old − Mean Haemglobin − Kiambu
WHO−2001 Adjustment Method

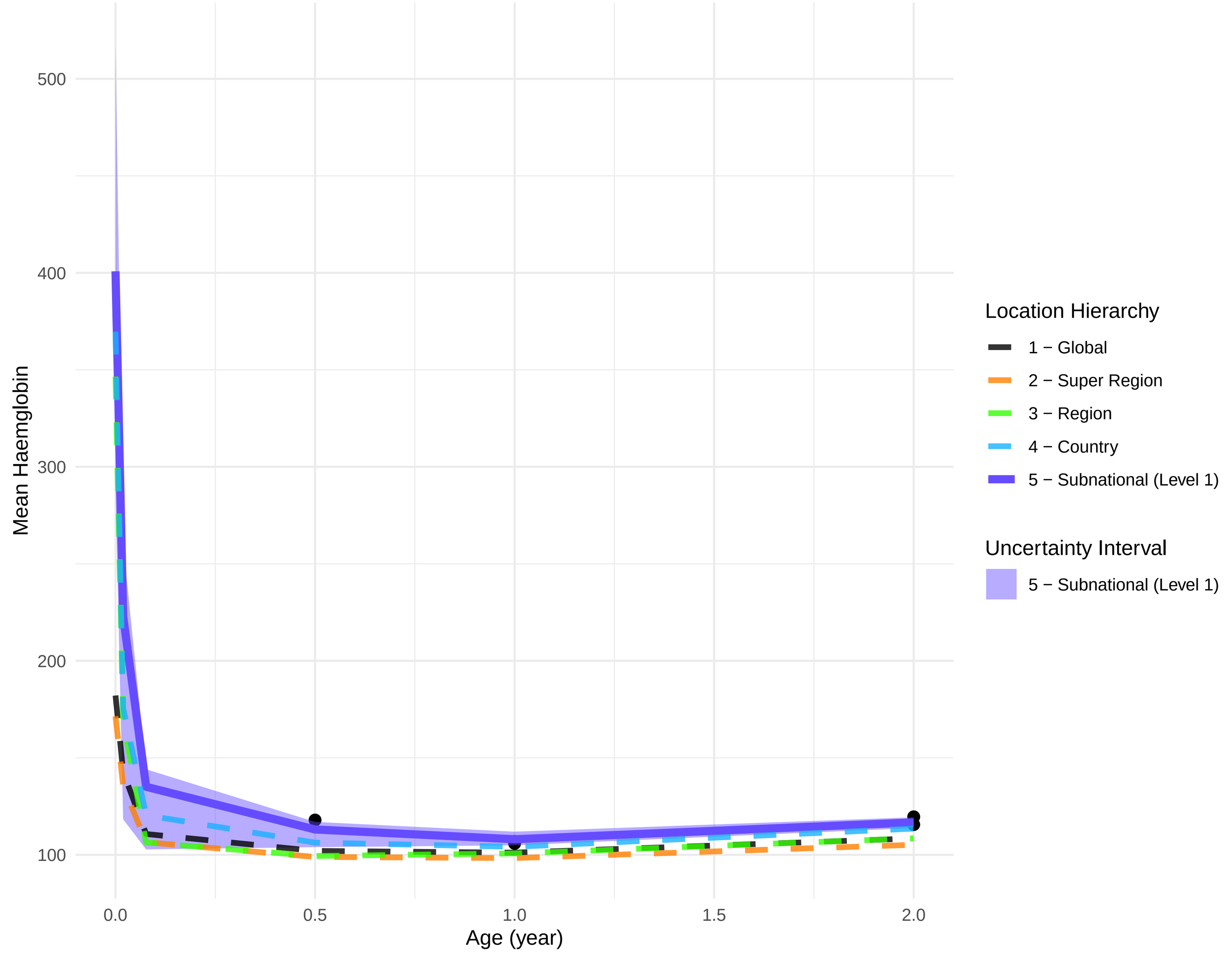

MR−BRT Age/Sex Fits − Non−pregnant Females <2 Years Old − Mean Haemglobin − Kilifi
WHO−2001 Adjustment Method

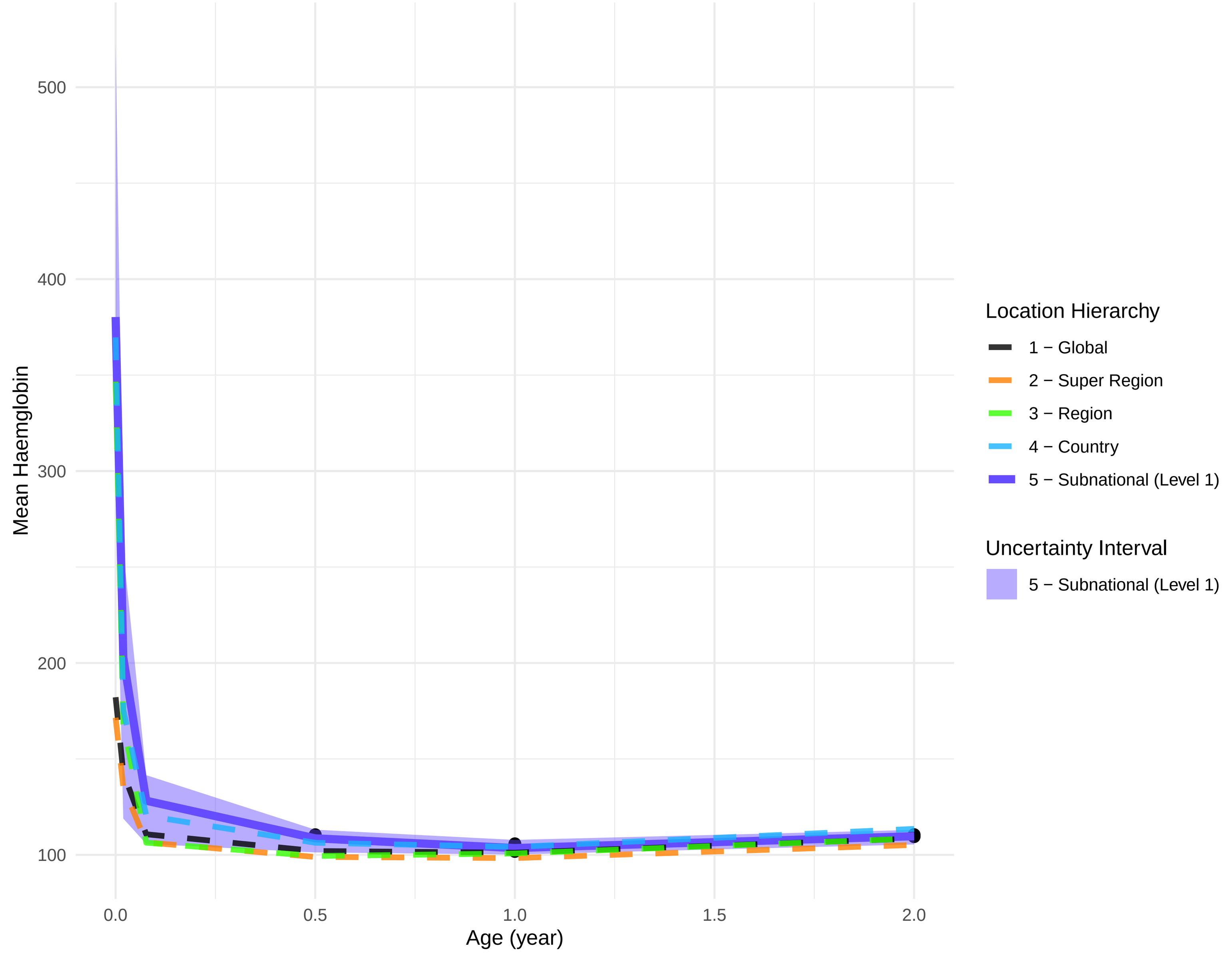

MR−BRT Age/Sex Fits − Non−pregnant Females <2 Years Old − Mean Haemglobin − Kirinyaga
WHO−2001 Adjustment Method

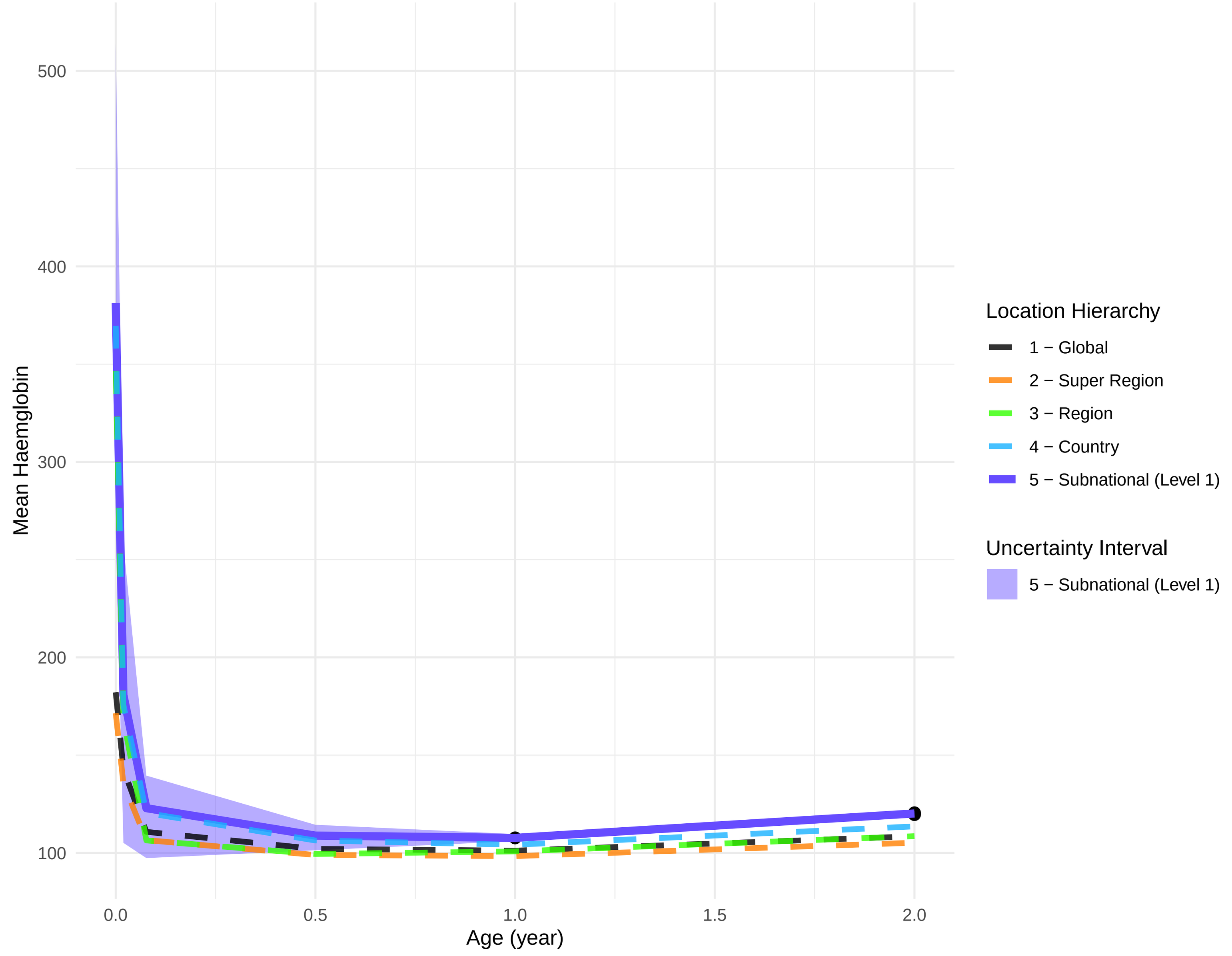

MR−BRT Age/Sex Fits − Non−pregnant Females <2 Years Old − Mean Haemglobin − Kisii
WHO−2001 Adjustment Method

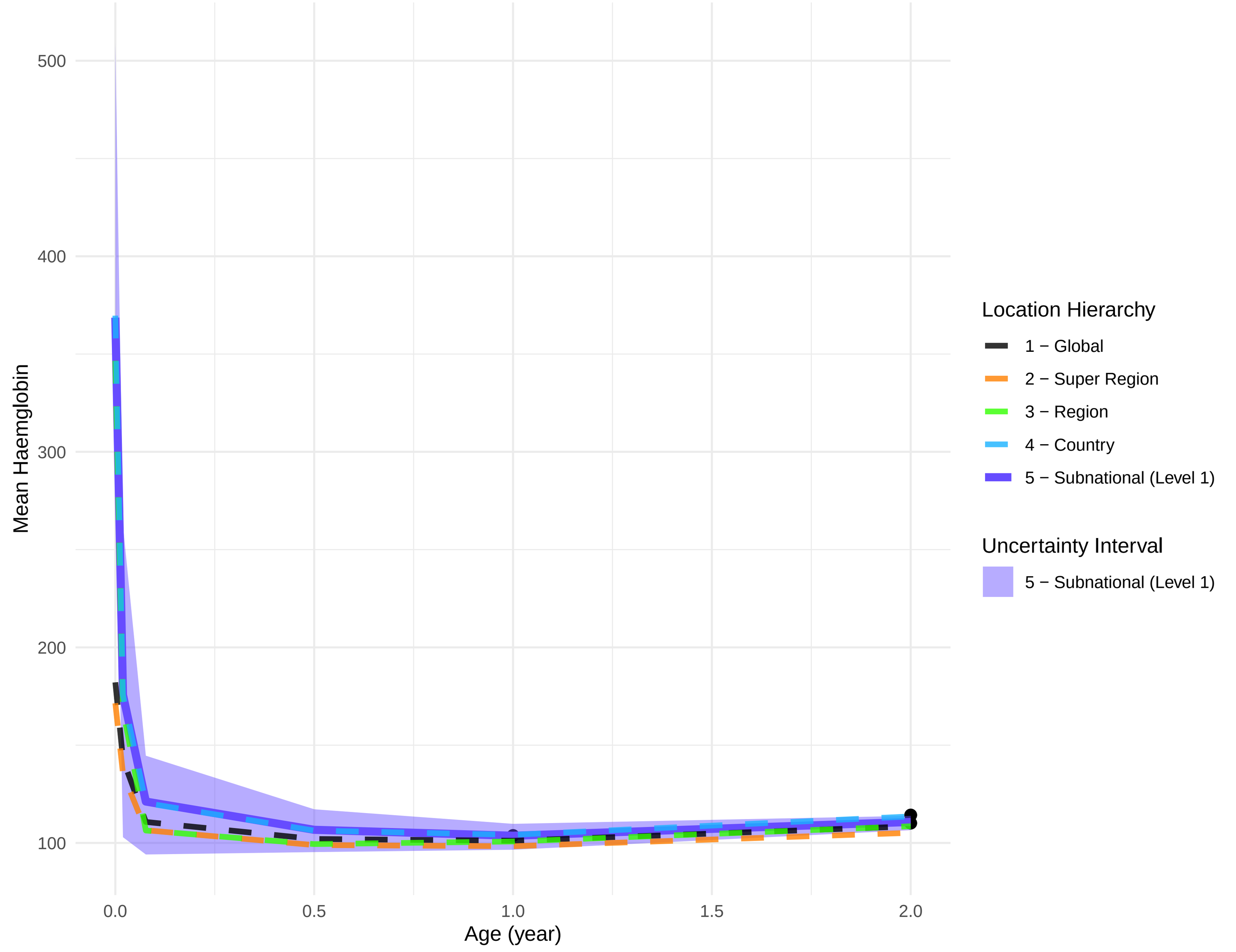

MR−BRT Age/Sex Fits − Non−pregnant Females <2 Years Old − Mean Haemglobin − Kisumu
WHO−2001 Adjustment Method

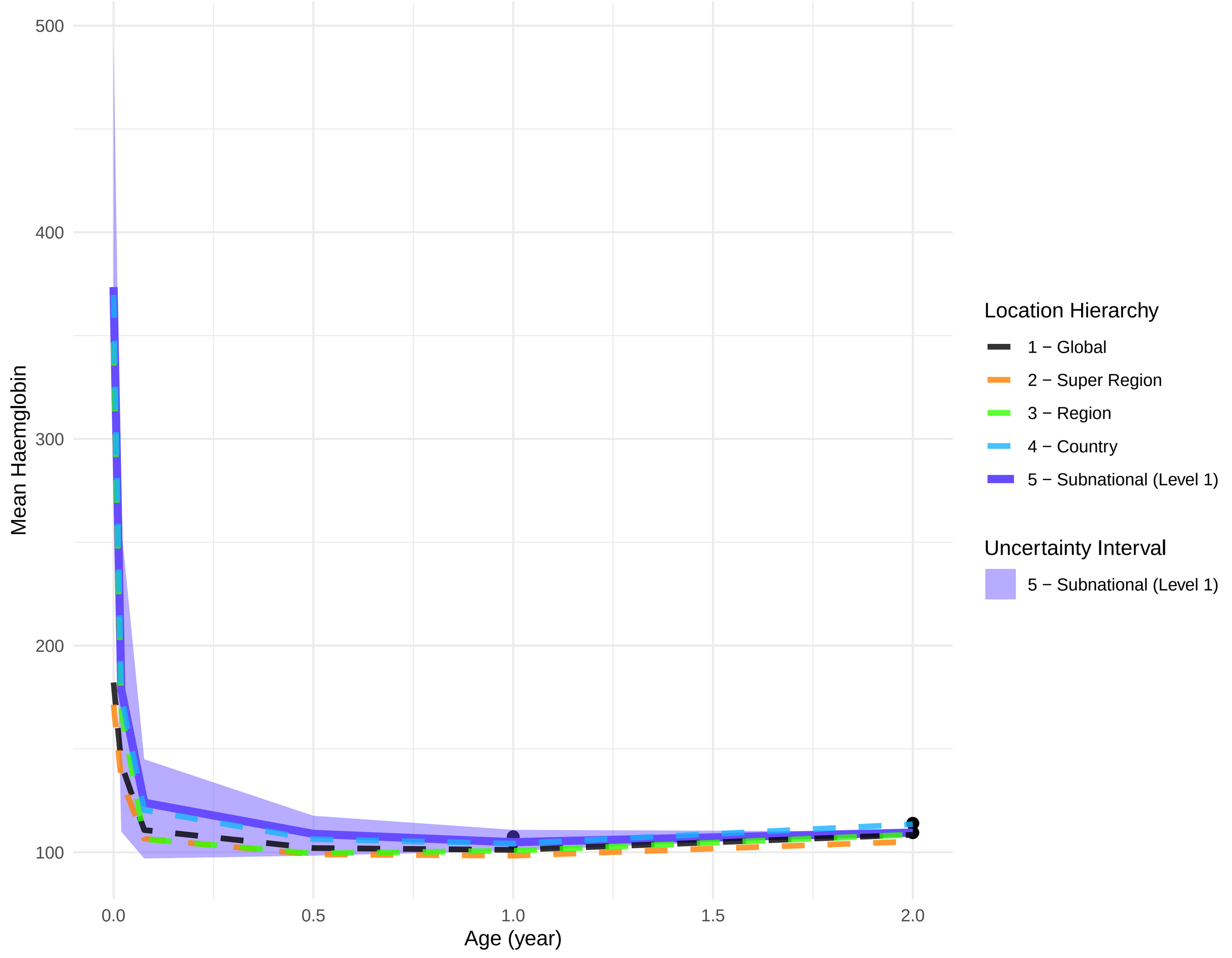

MR−BRT Age/Sex Fits − Non−pregnant Females <2 Years Old − Mean Haemglobin − Kitui
WHO−2001 Adjustment Method

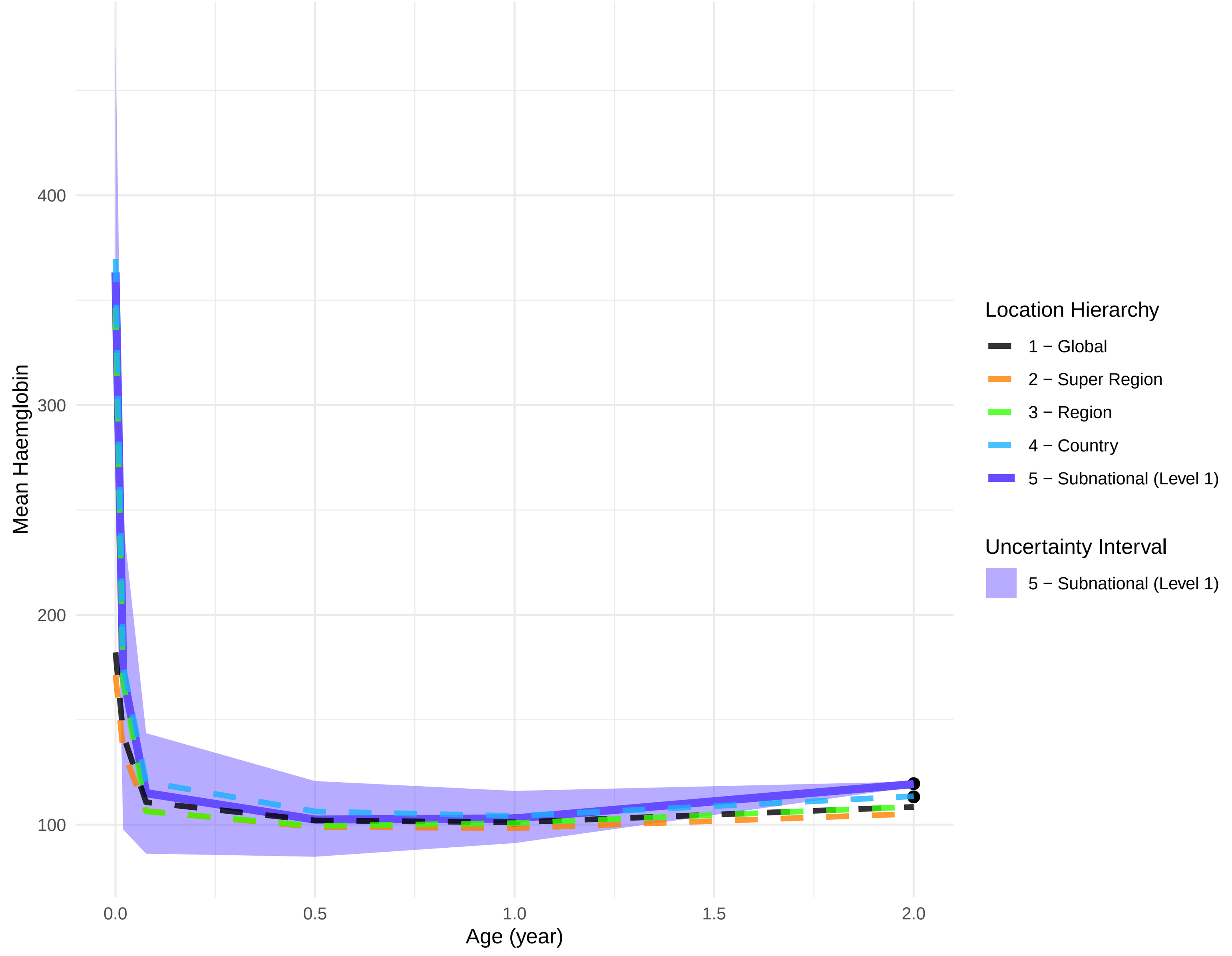

MR−BRT Age/Sex Fits − Non−pregnant Females <2 Years Old − Mean Haemglobin − Kwale
WHO−2001 Adjustment Method

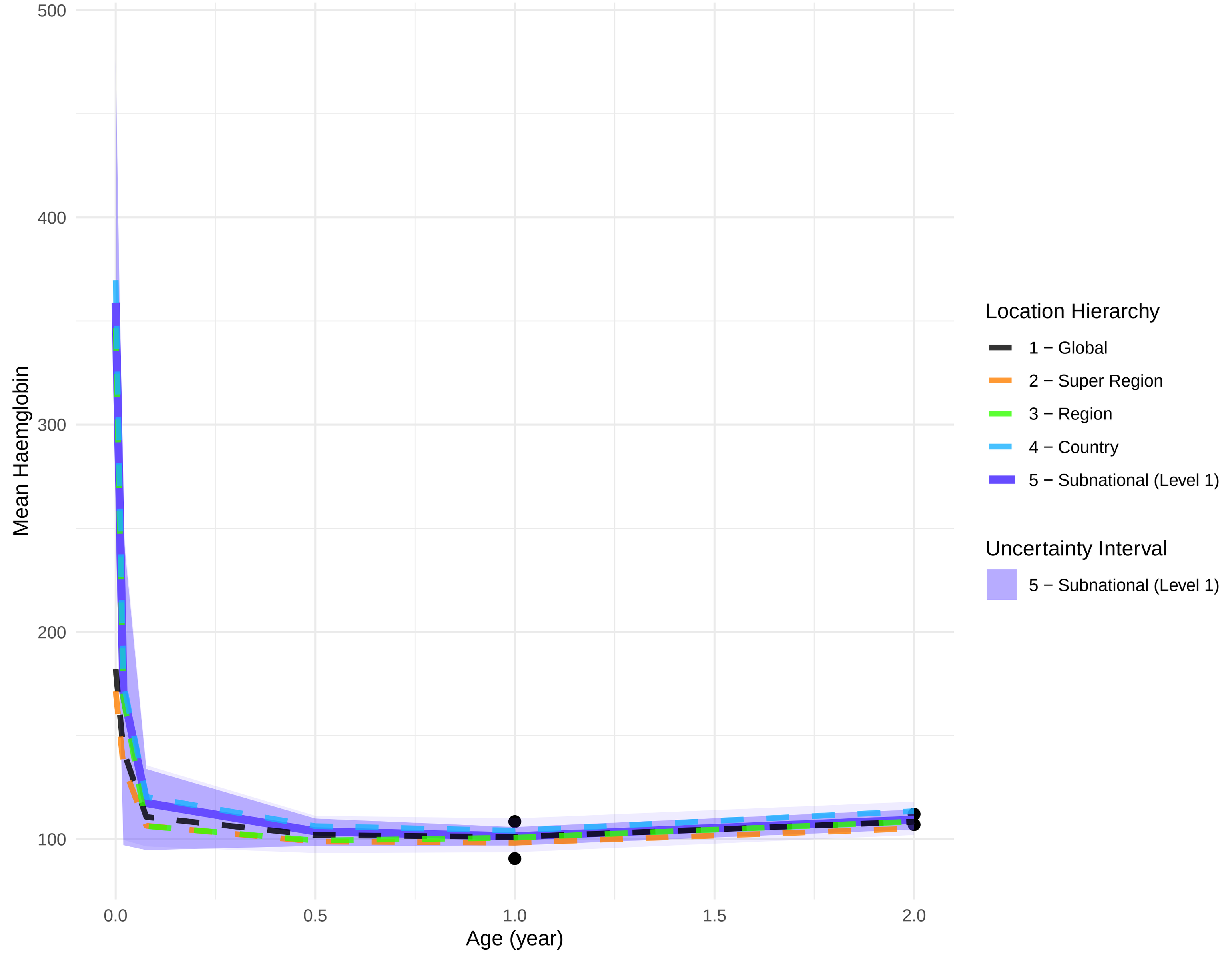

MR−BRT Age/Sex Fits − Non−pregnant Females <2 Years Old − Mean Haemglobin − Laikipia
WHO−2001 Adjustment Method

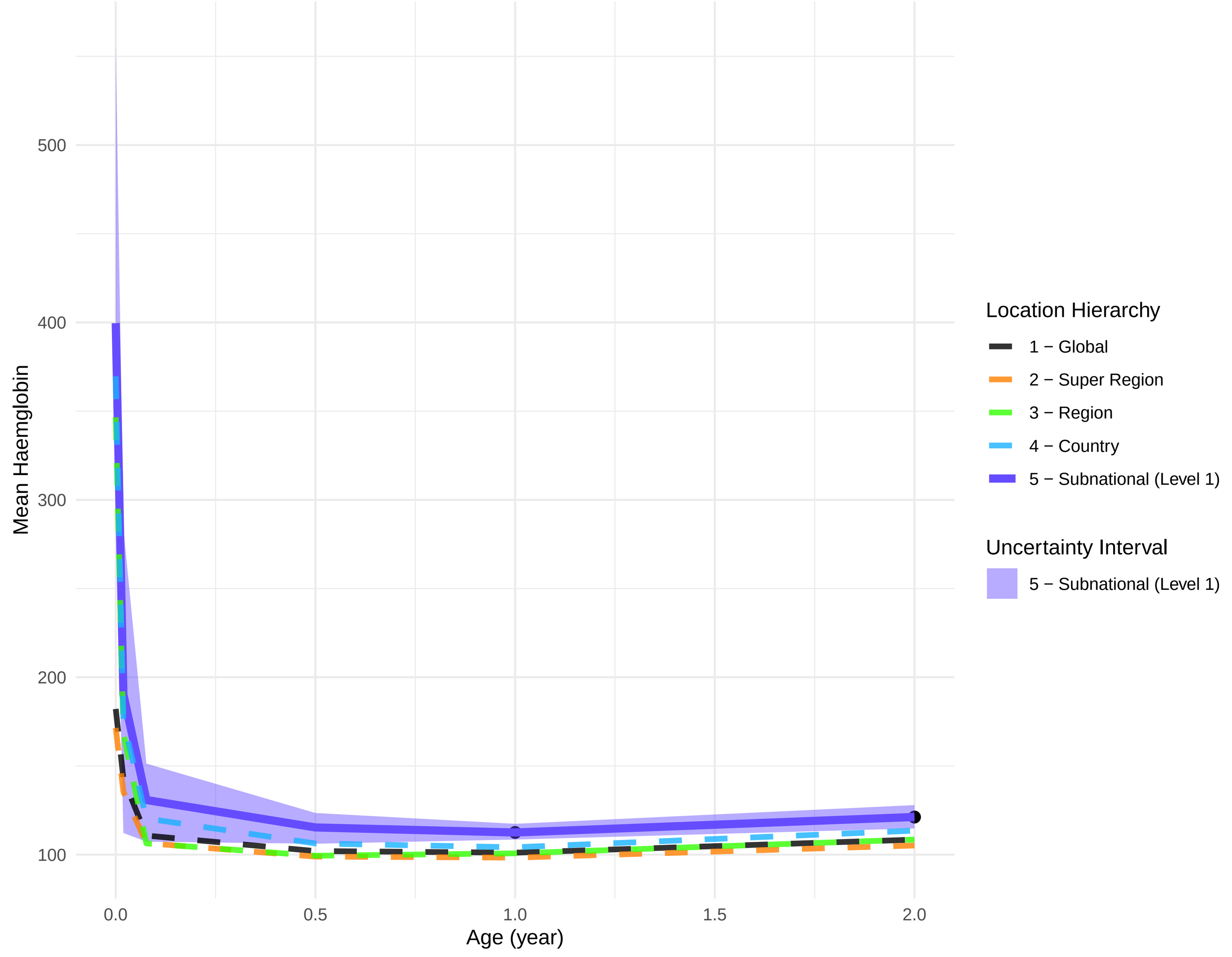

MR−BRT Age/Sex Fits − Non−pregnant Females <2 Years Old − Mean Haemglobin − Lamu
WHO−2001 Adjustment Method

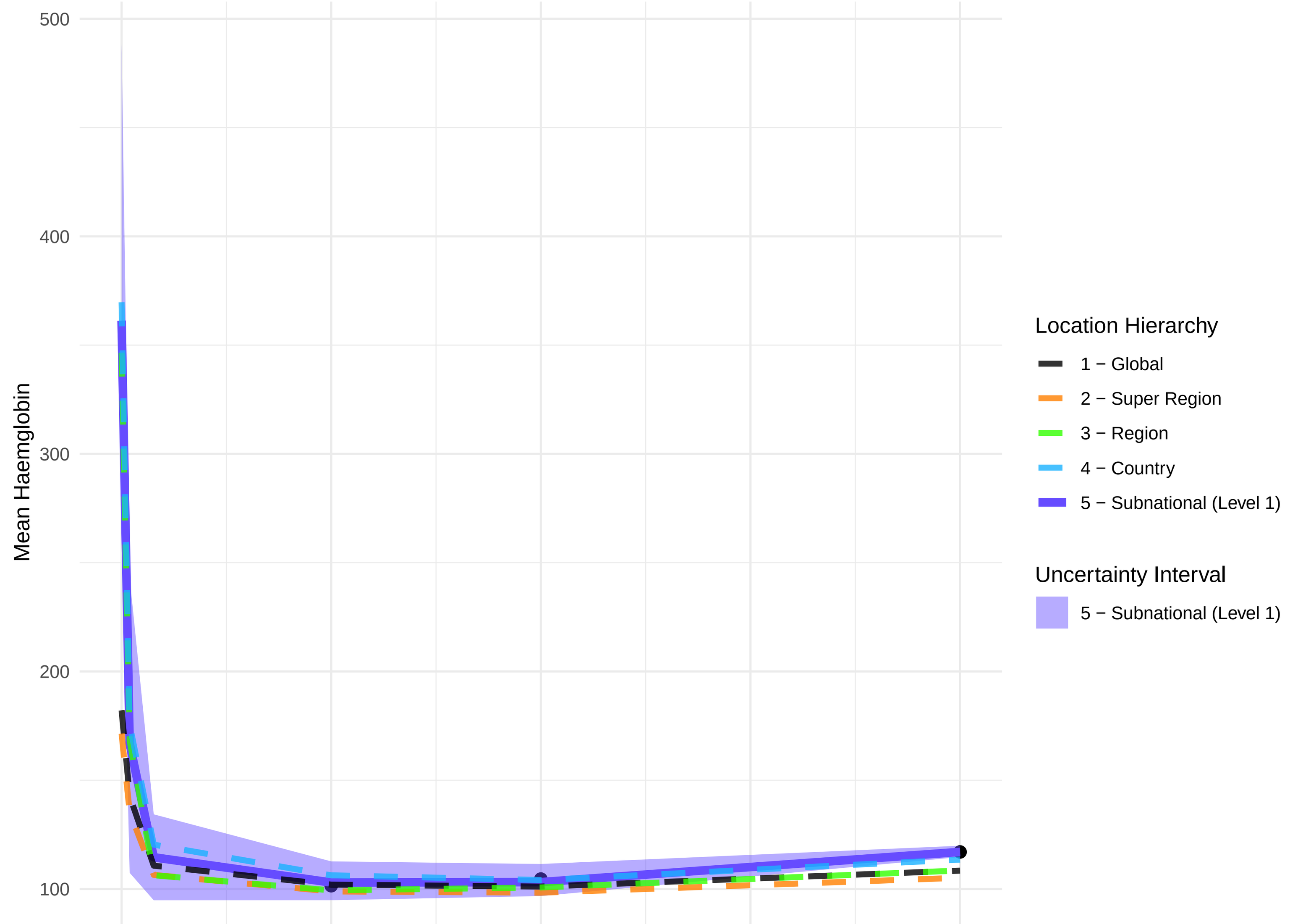

MR−BRT Age/Sex Fits − Non−pregnant Females <2 Years Old − Mean Haemglobin − Machakos
WHO−2001 Adjustment Method

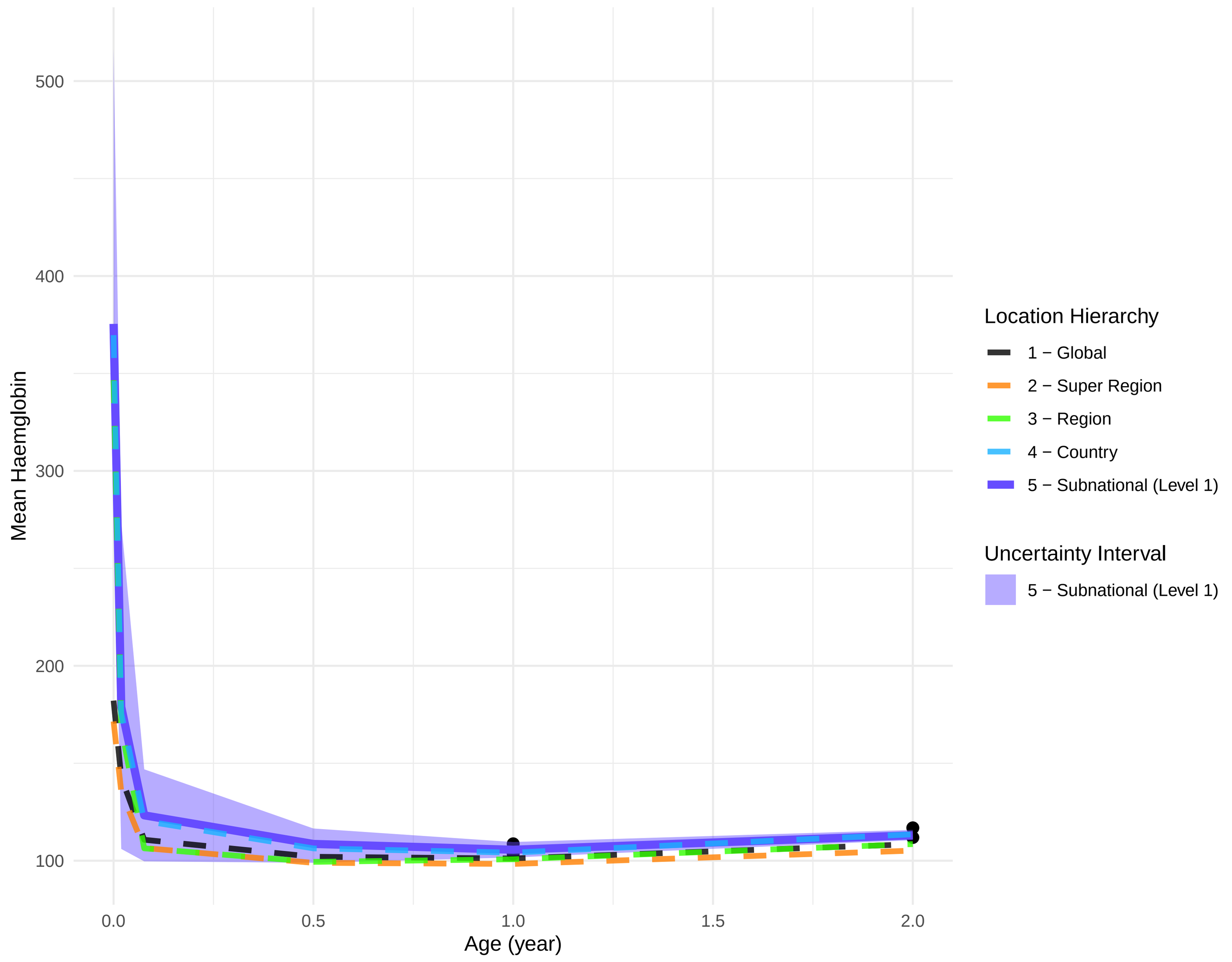

MR−BRT Age/Sex Fits − Non−pregnant Females <2 Years Old − Mean Haemglobin − Makueni
WHO−2001 Adjustment Method

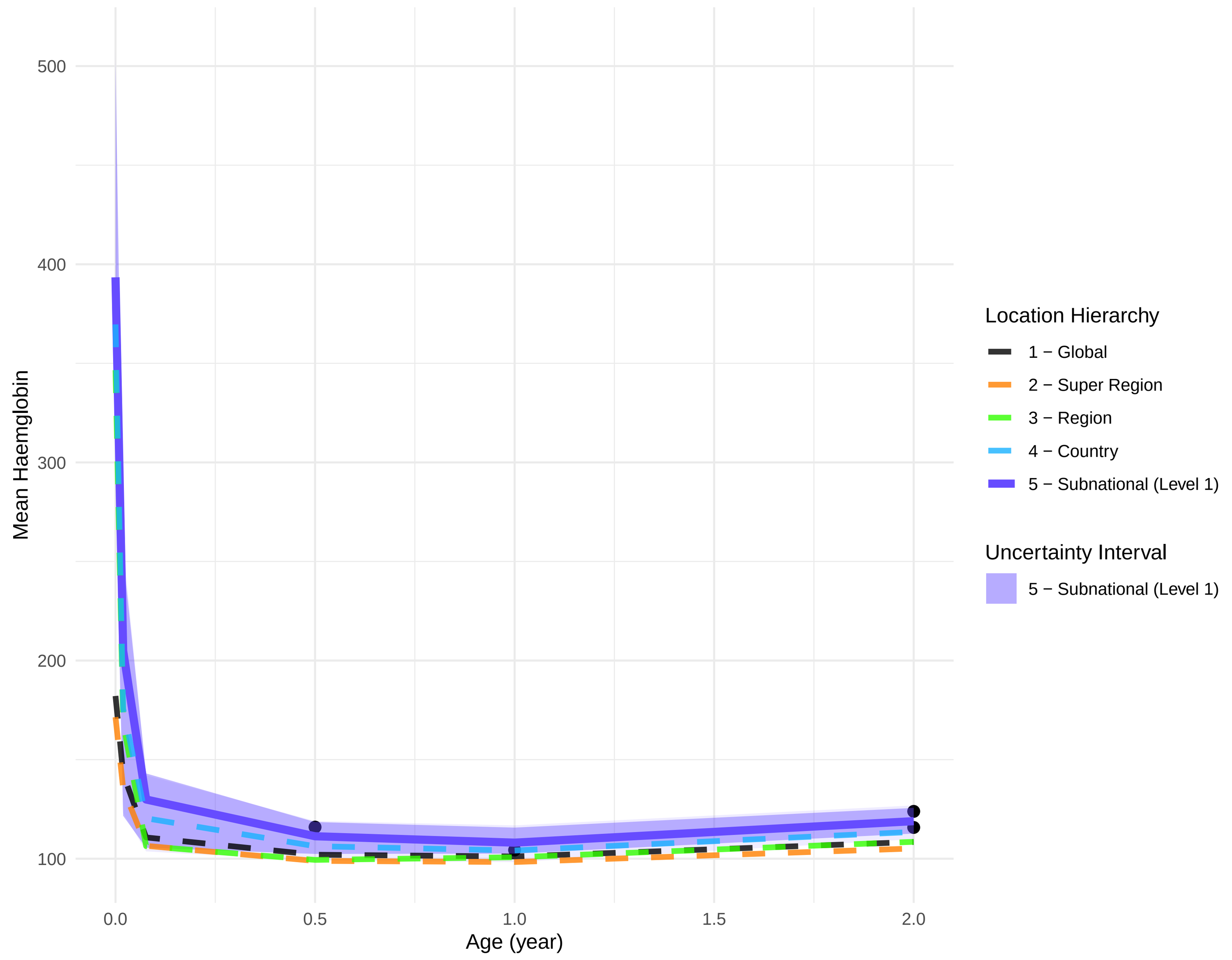

MR−BRT Age/Sex Fits − Non−pregnant Females <2 Years Old − Mean Haemglobin − Mandera
WHO−2001 Adjustment Method

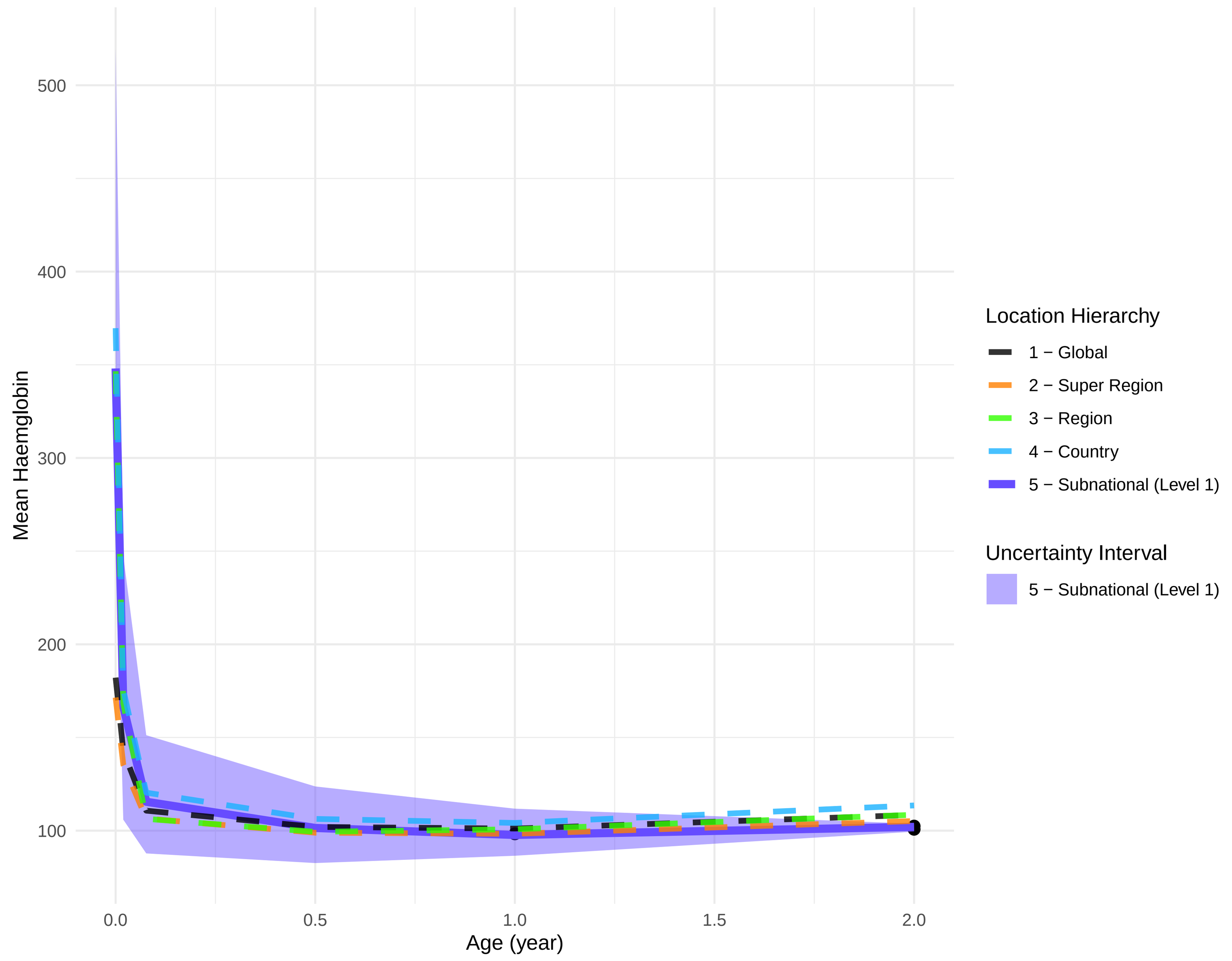

MR−BRT Age/Sex Fits − Non−pregnant Females <2 Years Old − Mean Haemglobin − Marsabit
WHO−2001 Adjustment Method

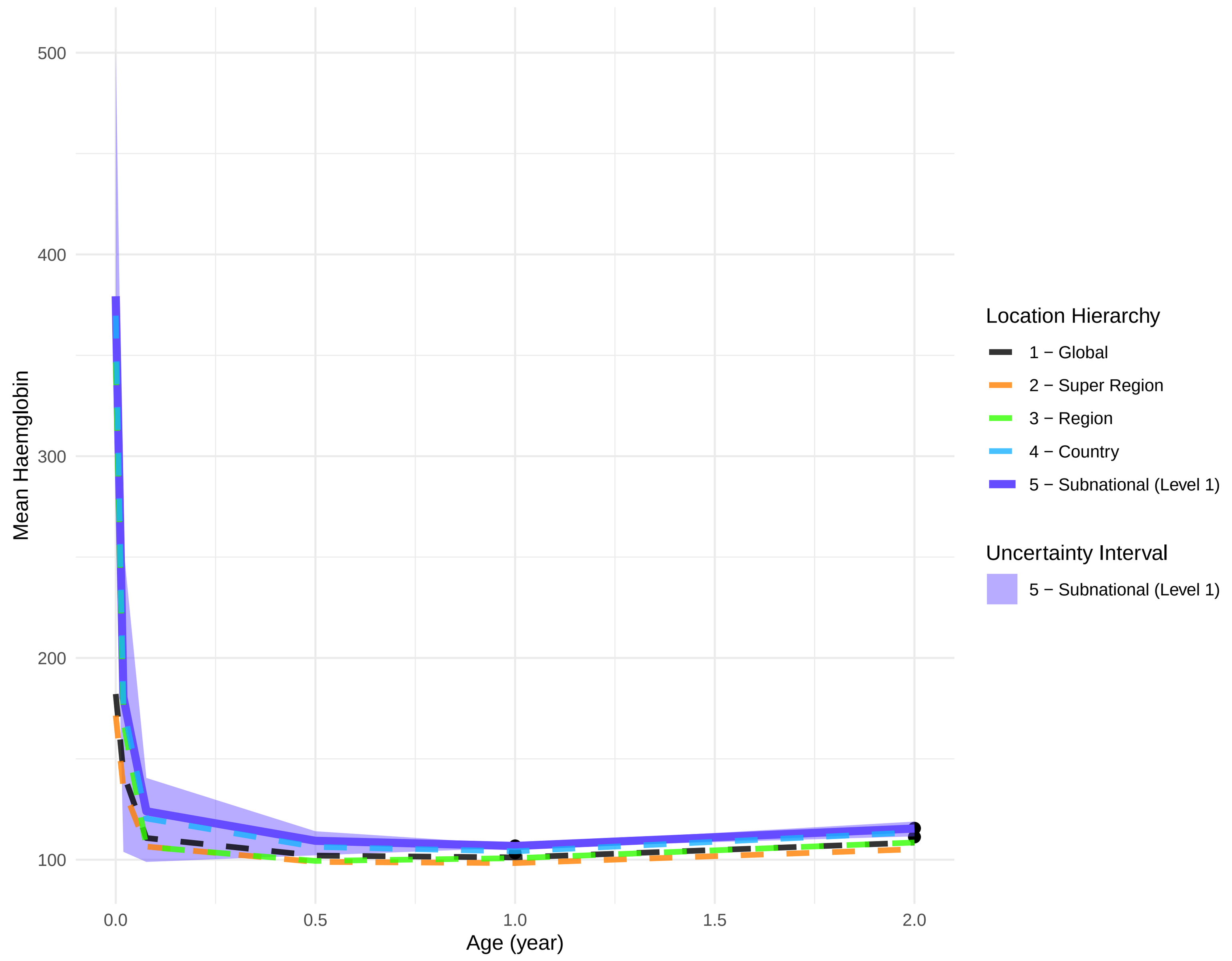

MR−BRT Age/Sex Fits − Non−pregnant Females <2 Years Old − Mean Haemglobin − Meru
WHO−2001 Adjustment Method

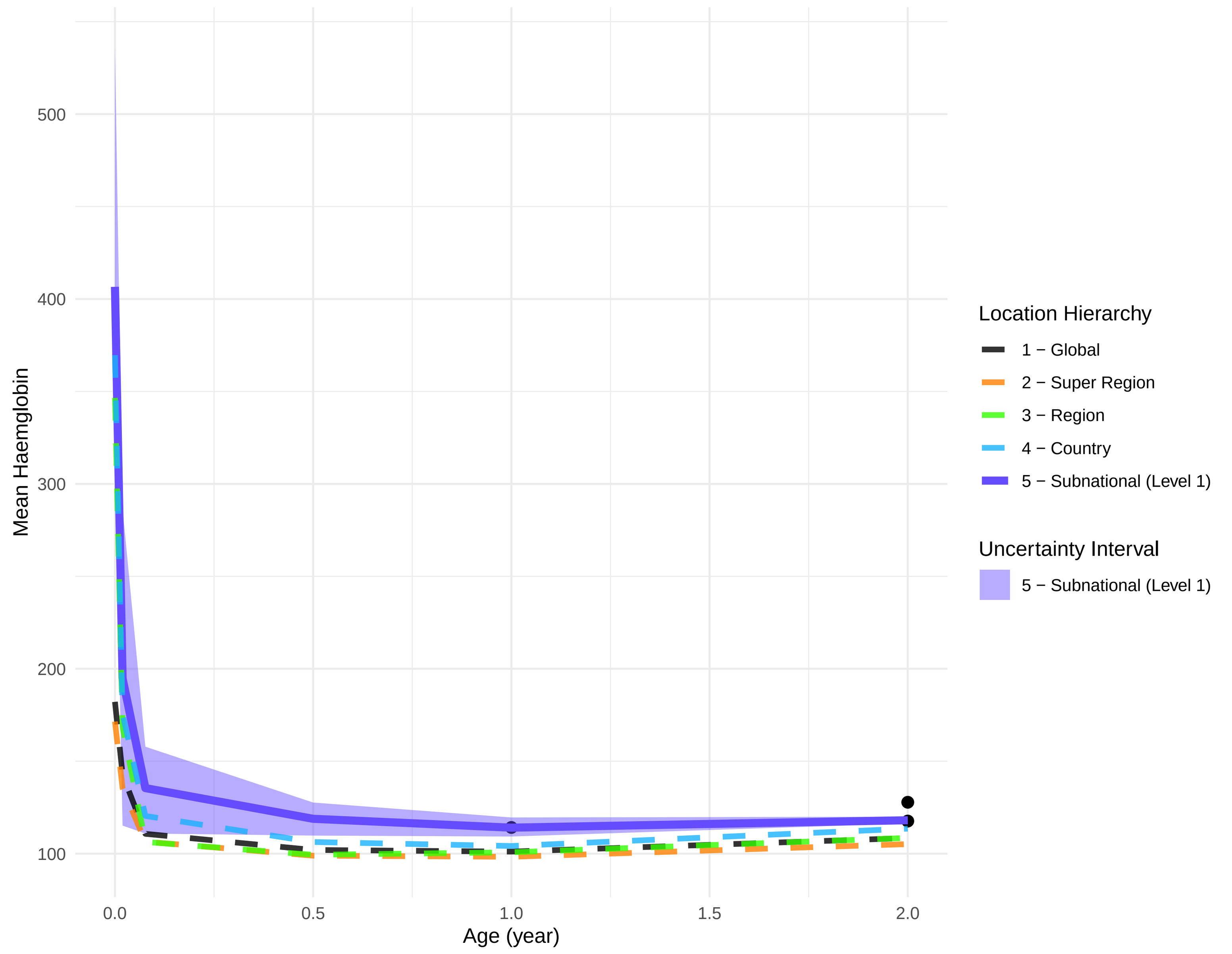

MR−BRT Age/Sex Fits − Non−pregnant Females <2 Years Old − Mean Haemglobin − Migori
WHO−2001 Adjustment Method

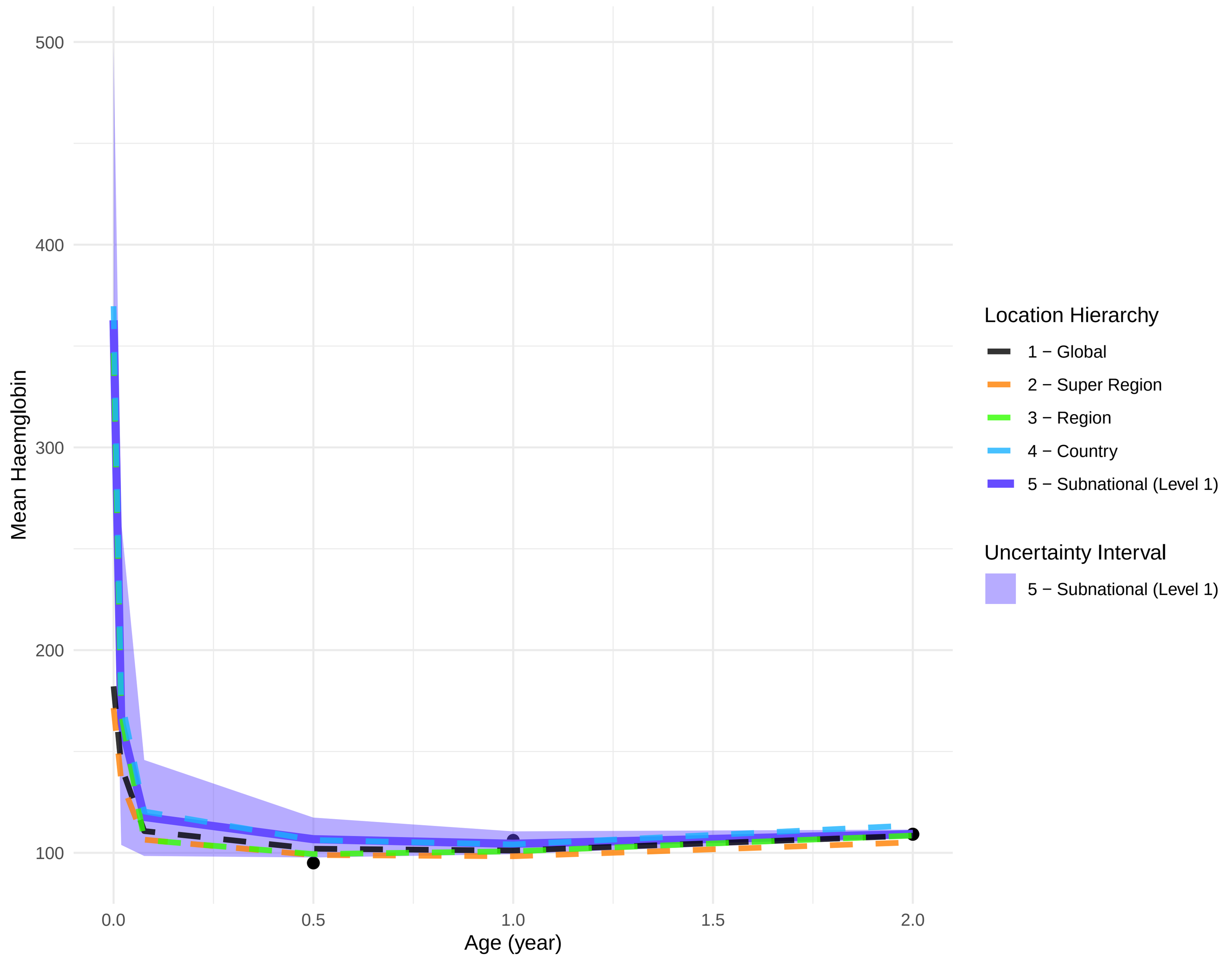

MR−BRT Age/Sex Fits − Non−pregnant Females <2 Years Old − Mean Haemglobin − Mombasa
WHO−2001 Adjustment Method

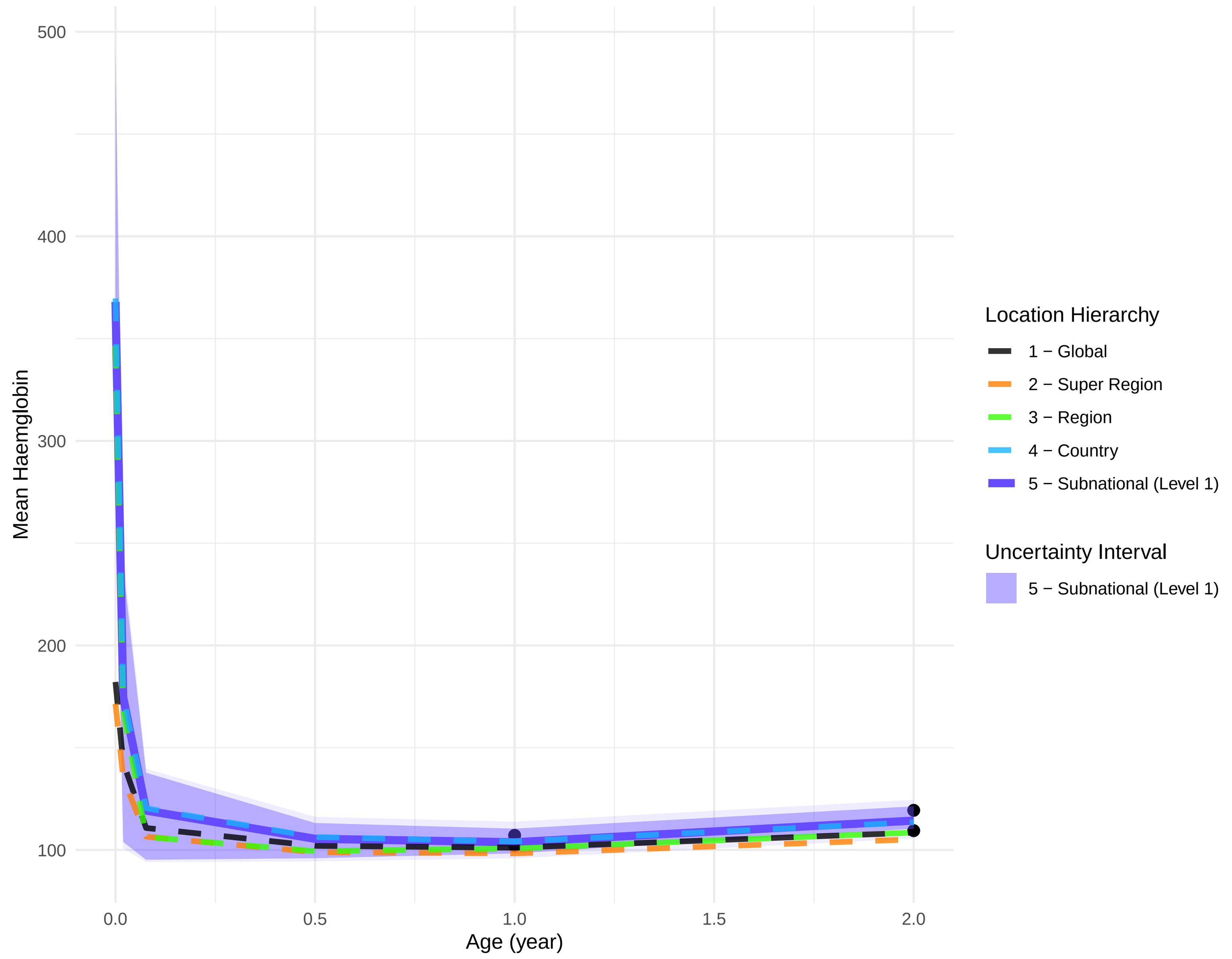

MR−BRT Age/Sex Fits − Non−pregnant Females <2 Years Old − Mean Haemglobin − Murang'a
WHO−2001 Adjustment Method

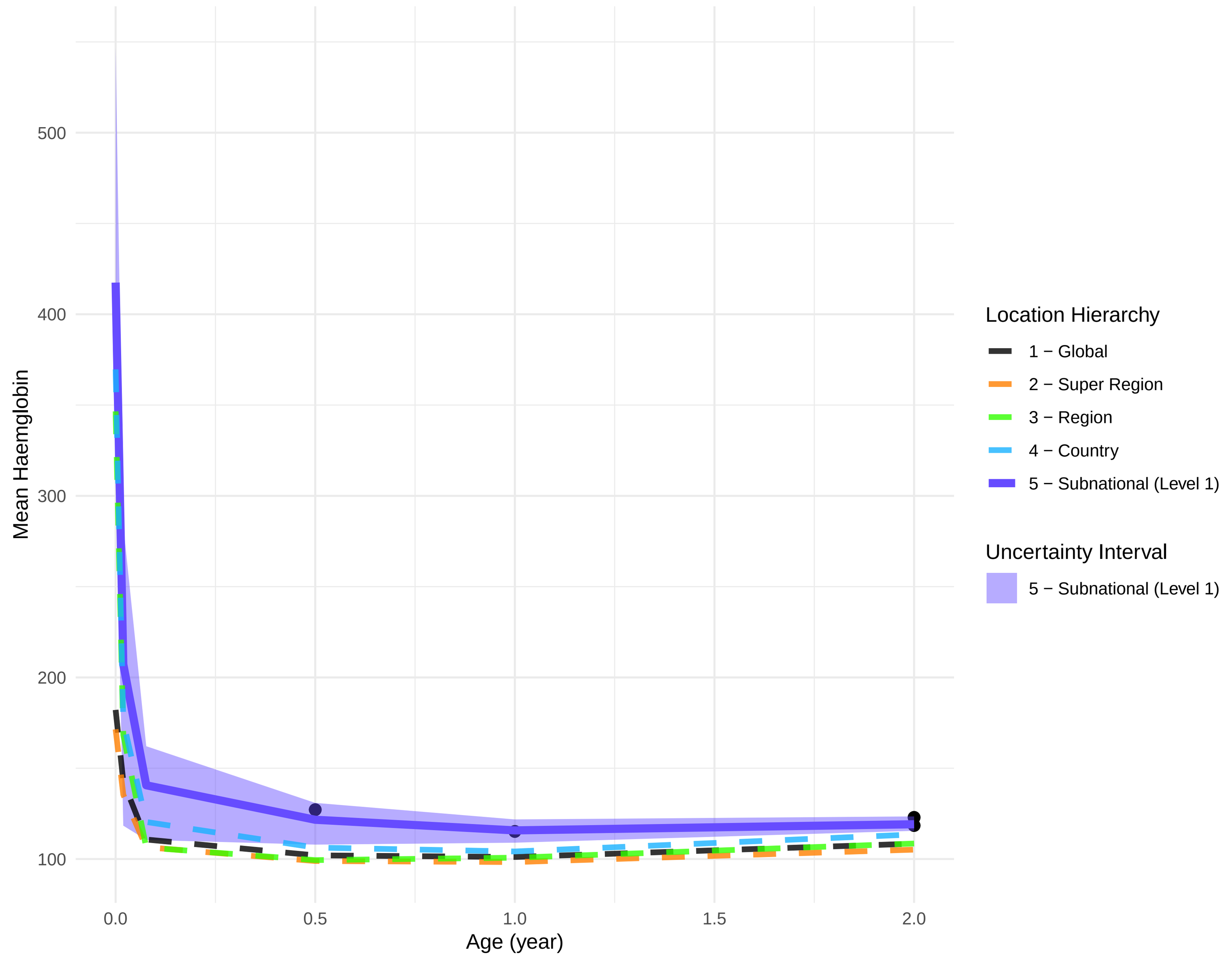

MR−BRT Age/Sex Fits − Non−pregnant Females <2 Years Old − Mean Haemglobin − Nairobi
WHO−2001 Adjustment Method

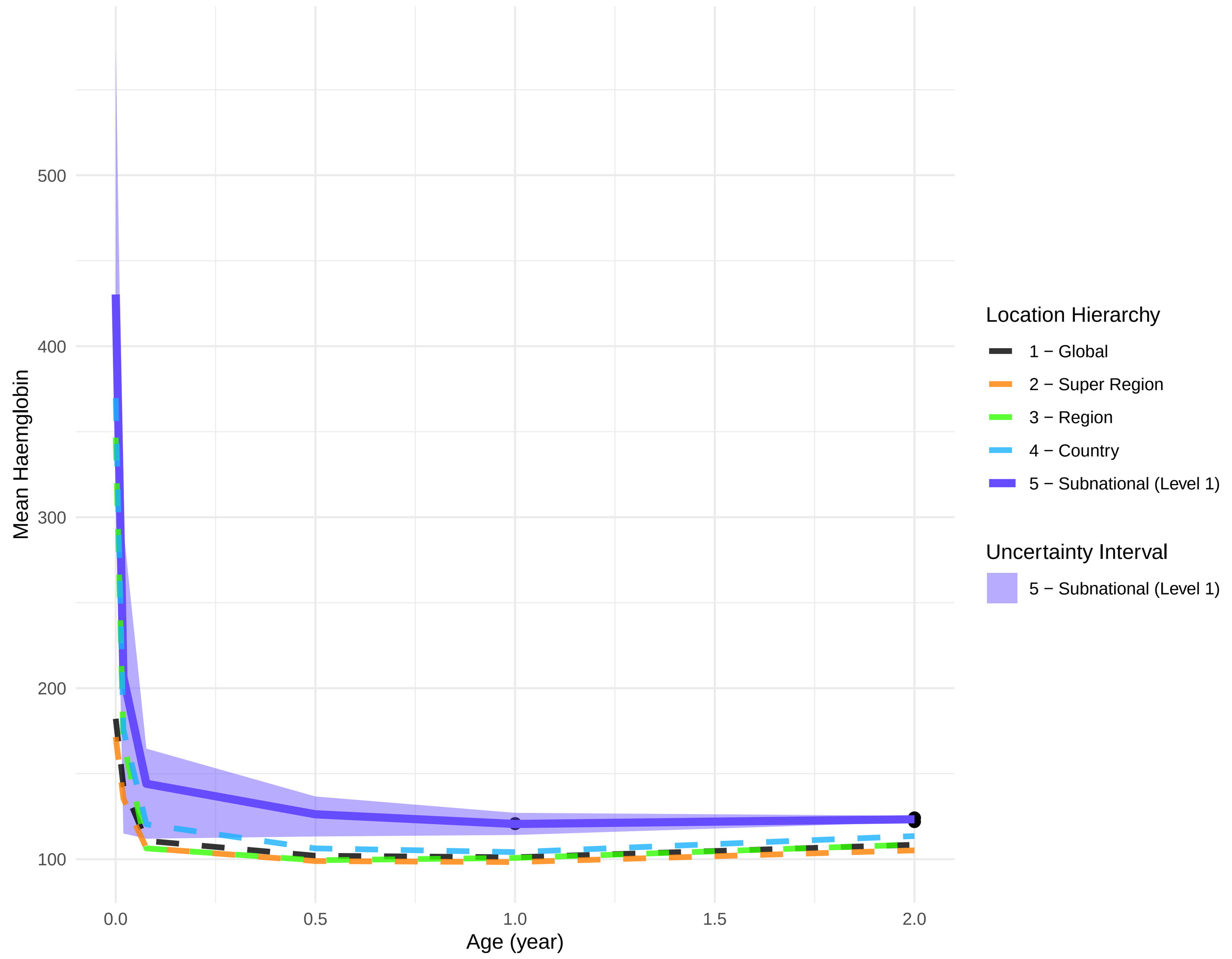

MR−BRT Age/Sex Fits − Non−pregnant Females <2 Years Old − Mean Haemglobin − Nakuru
WHO−2001 Adjustment Method

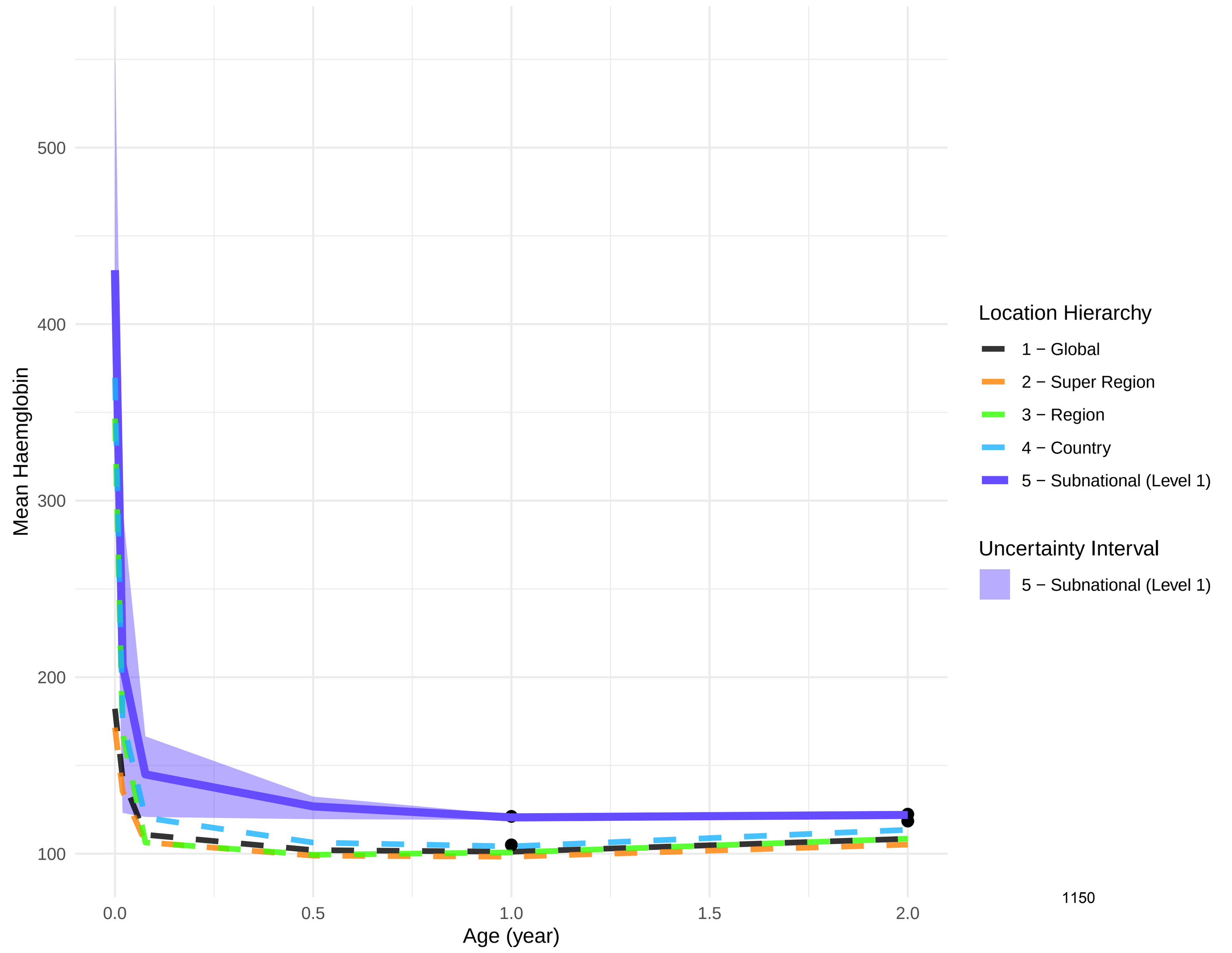

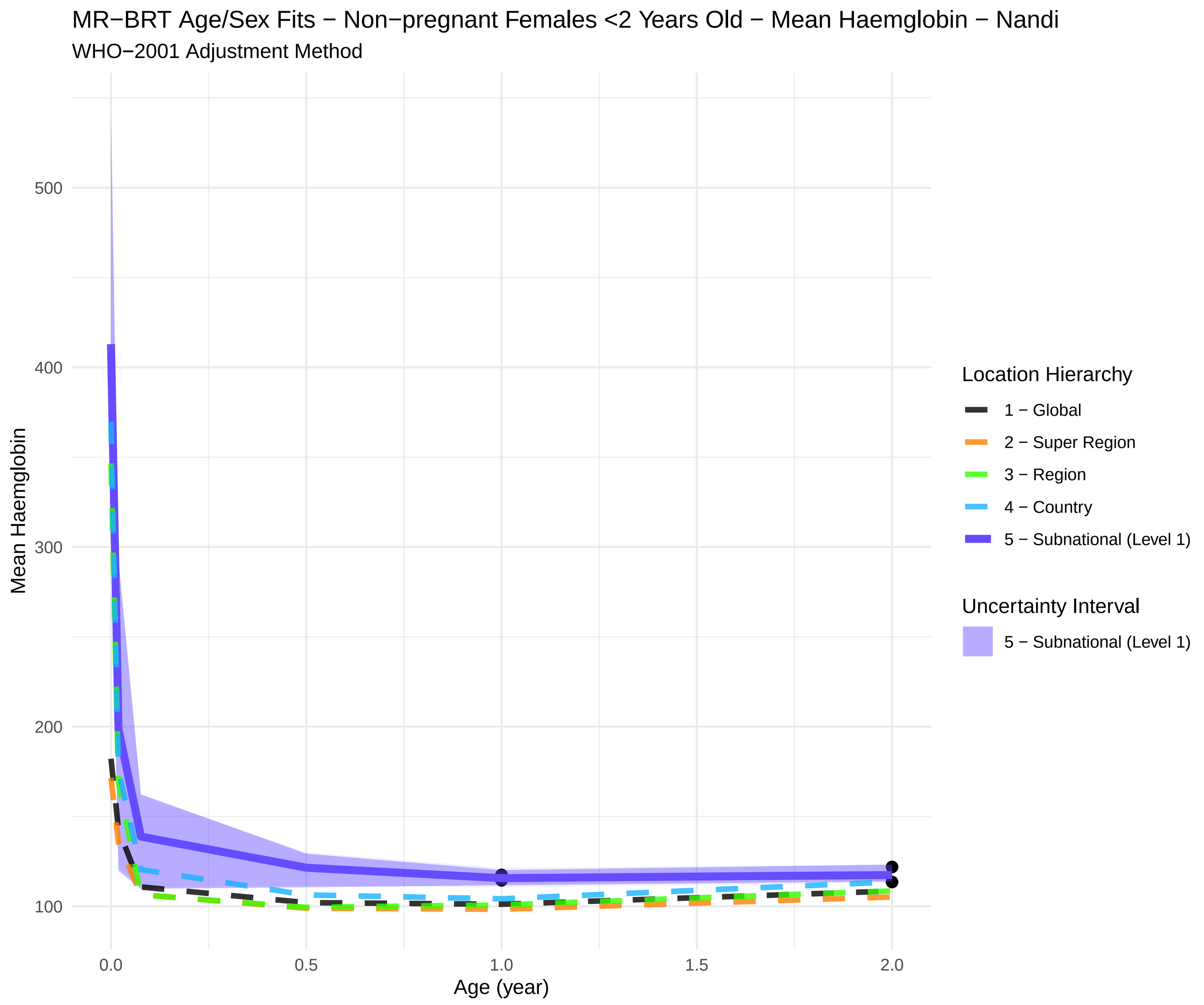
MR−BRT Age/Sex Fits − Non−pregnant Females <2 Years Old − Mean Haemglobin − Nandi
WHO−2001 Adjustment Method
Mean Haemglobin
Age (year)
Location Hierarchy
1 − Global
2 − Super Region
3 − Region
4 − Country
5 − Subnational (Level 1)
Uncertainty Interval
5 − Subnational (Level 1)

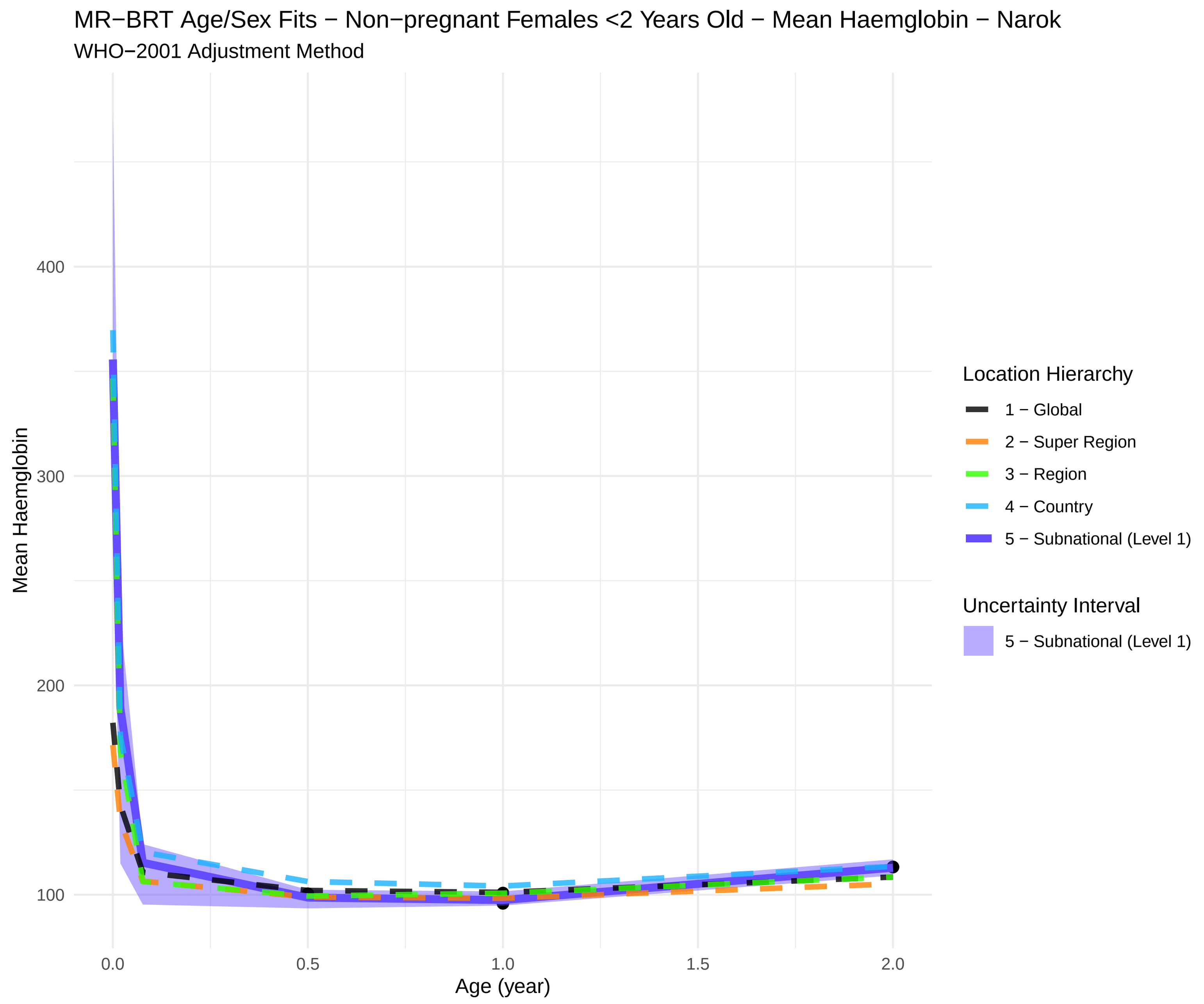
MR−BRT Age/Sex Fits − Non−pregnant Females <2 Years Old − Mean Haemglobin − Narok
WHO−2001 Adjustment Method
Mean Haemglobin
Age (year)
Location Hierarchy
1 − Global
2 − Super Region
3 − Region
4 − Country
5 − Subnational (Level 1)
Uncertainty Interval
5 − Subnational (Level 1)

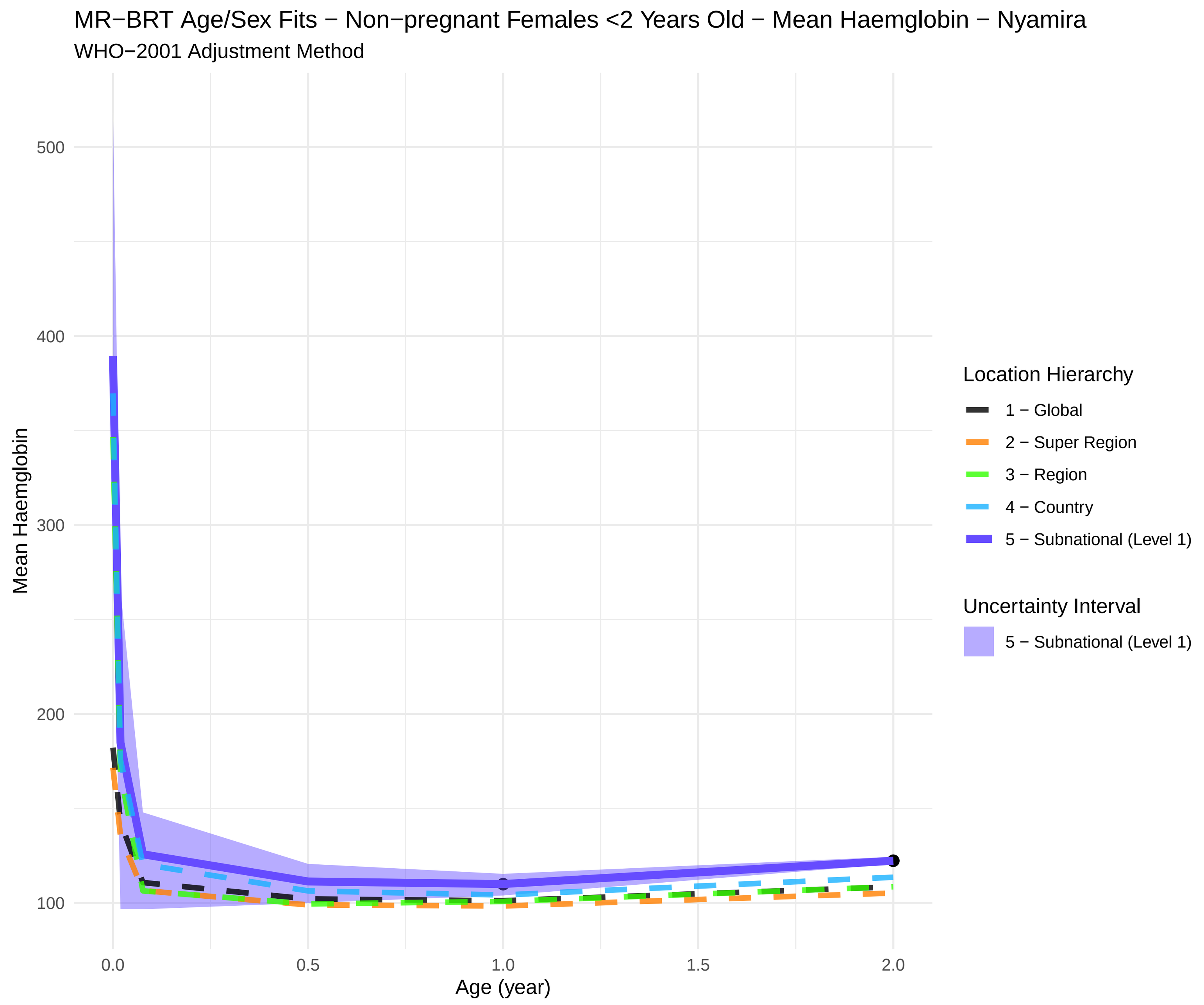
MR−BRT Age/Sex Fits − Non−pregnant Females <2 Years Old − Mean Haemglobin − Nyamira
WHO−2001 Adjustment Method
Mean Haemglobin
Age (year)
Location Hierarchy
1 − Global
2 − Super Region
3 − Region
4 − Country
5 − Subnational (Level 1)
Uncertainty Interval
5 − Subnational (Level 1)

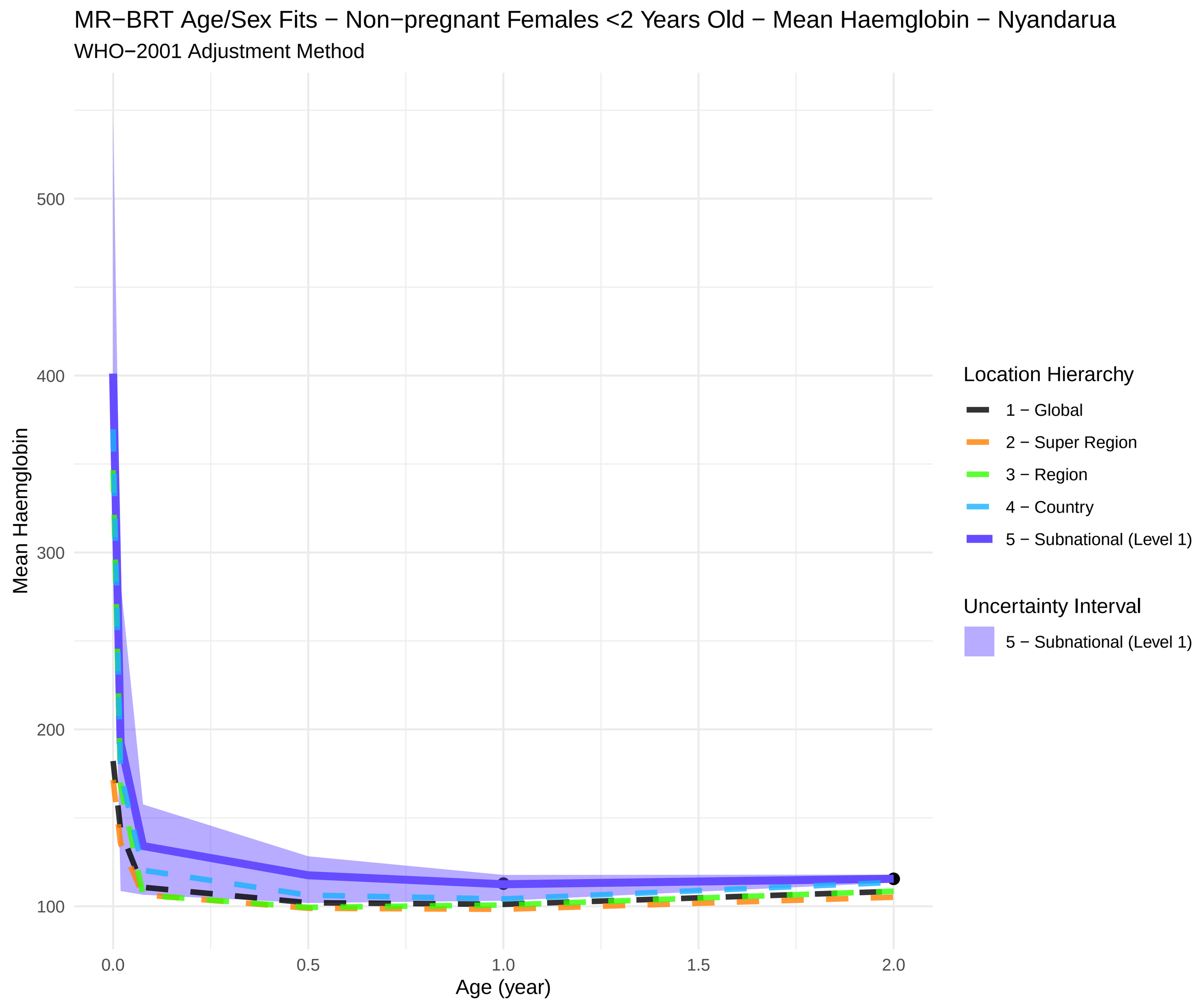
MR−BRT Age/Sex Fits − Non−pregnant Females <2 Years Old − Mean Haemglobin − Nyandarua
WHO−2001 Adjustment Method
Mean Haemglobin
Age (year)
Location Hierarchy
1 − Global
2 − Super Region
3 − Region
4 − Country
5 − Subnational (Level 1)
Uncertainty Interval
5 − Subnational (Level 1)

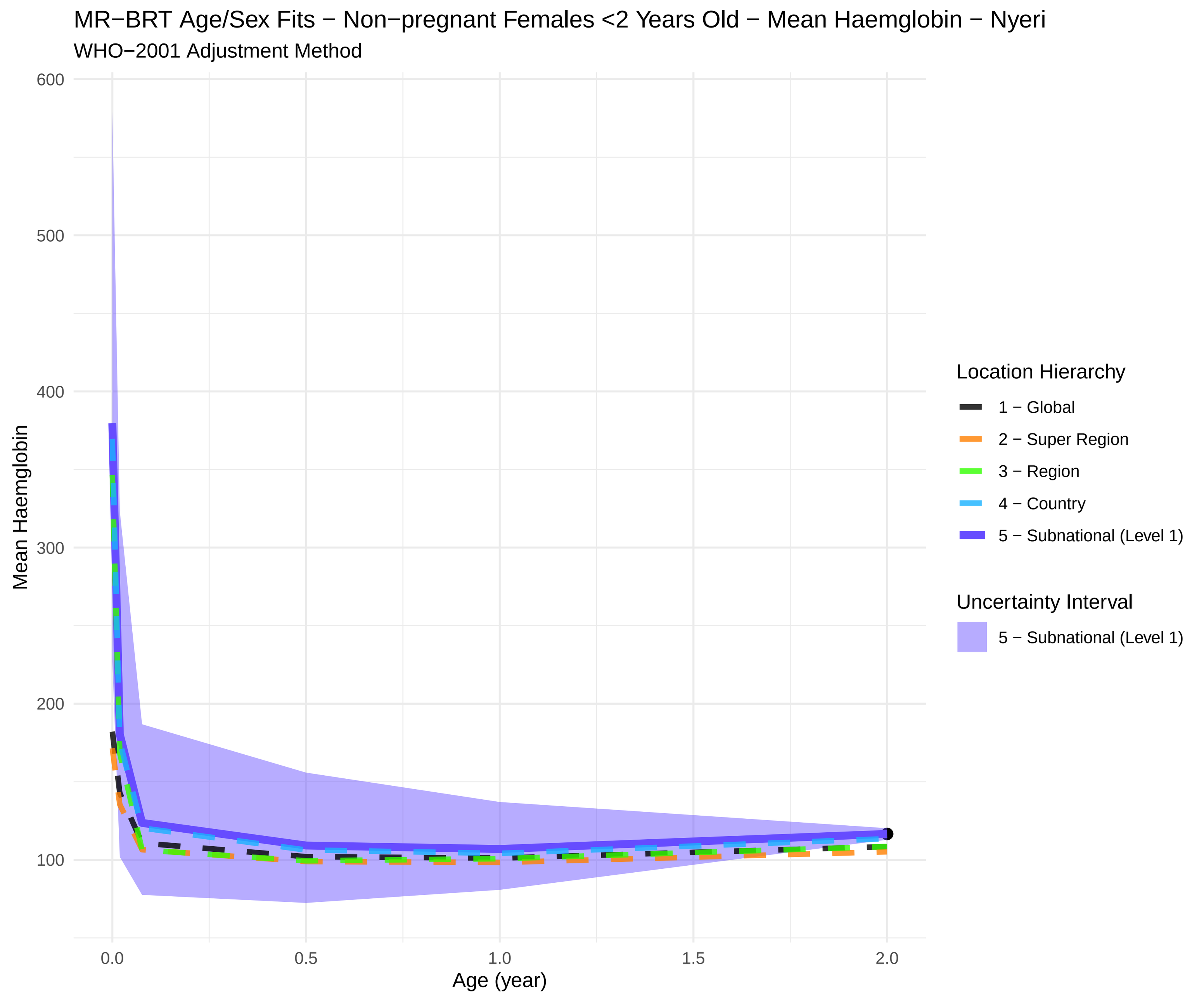
MR−BRT Age/Sex Fits − Non−pregnant Females <2 Years Old − Mean Haemglobin − Nyeri
WHO−2001 Adjustment Method
Mean Haemglobin
Age (year)
Location Hierarchy
1 − Global
2 − Super Region
3 − Region
4 − Country
5 − Subnational (Level 1)
Uncertainty Interval
5 − Subnational (Level 1)

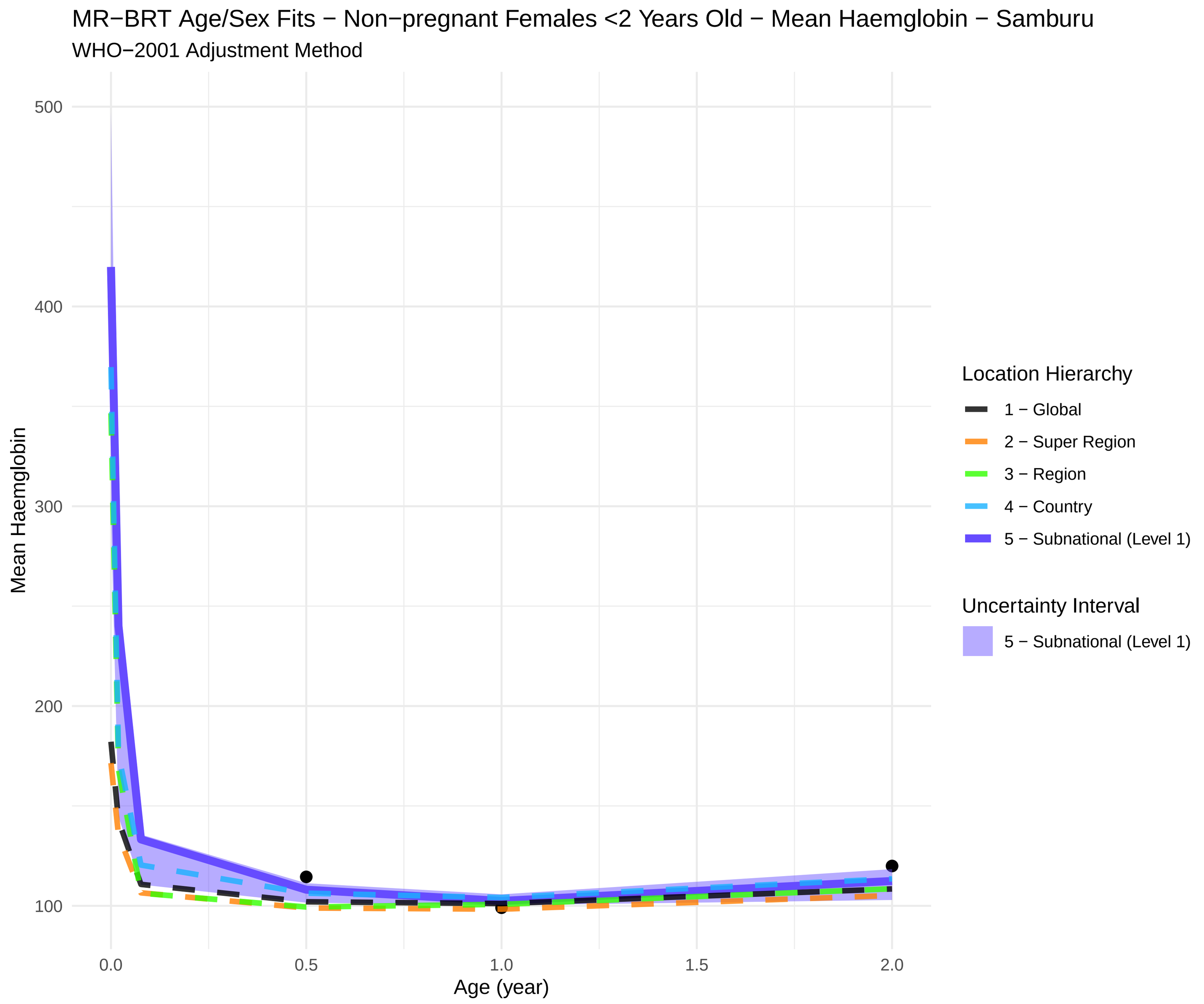
MR−BRT Age/Sex Fits − Non−pregnant Females <2 Years Old − Mean Haemglobin − Samburu
WHO−2001 Adjustment Method
Mean Haemglobin
Age (year)
Location Hierarchy
1 − Global
2 − Super Region
3 − Region
4 − Country
5 − Subnational (Level 1)
Uncertainty Interval
5 − Subnational (Level 1)

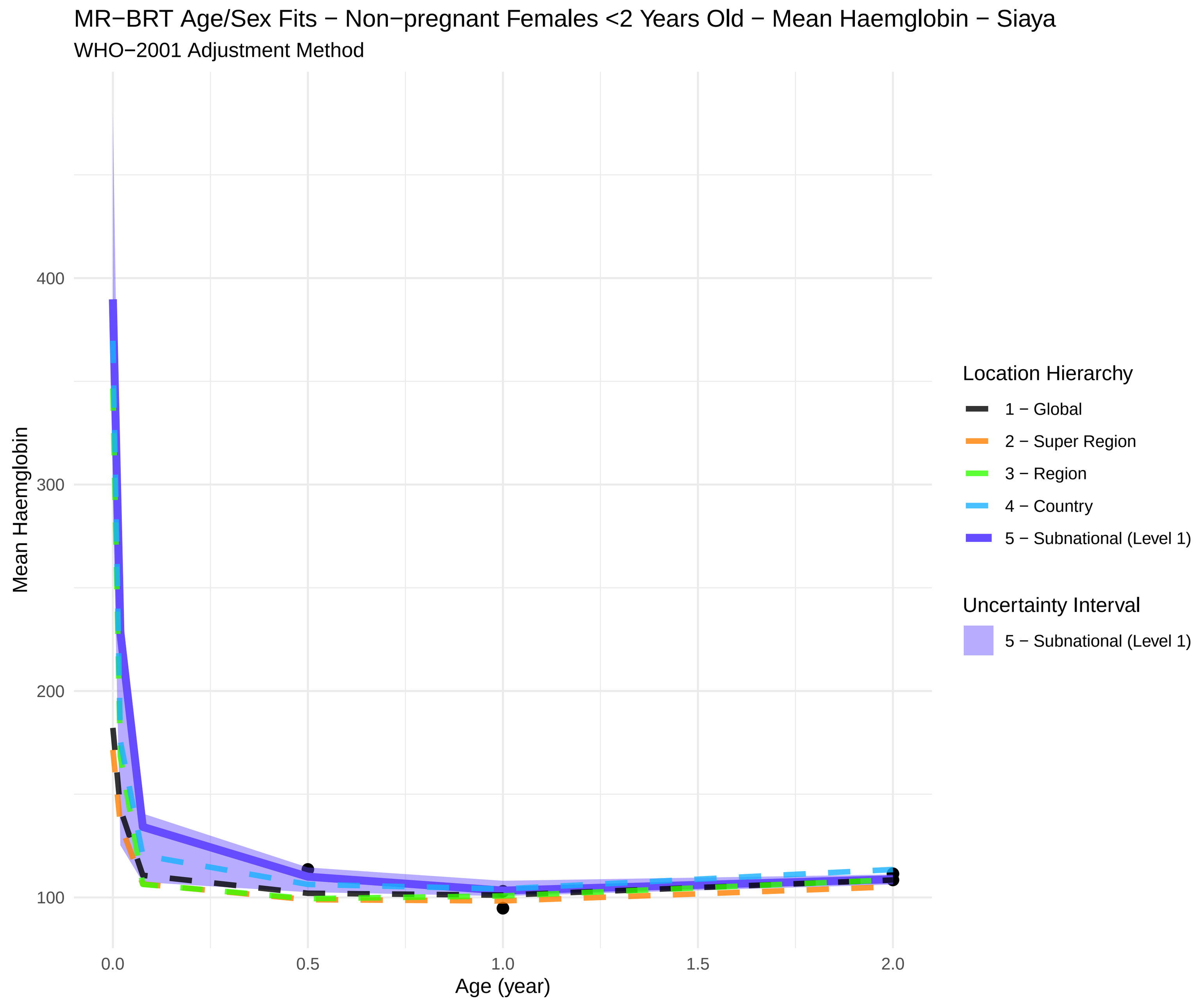
MR−BRT Age/Sex Fits − Non−pregnant Females <2 Years Old − Mean Haemglobin − Siaya
WHO−2001 Adjustment Method
Mean Haemglobin
Age (year)
Location Hierarchy
1 − Global
2 − Super Region
3 − Region
4 − Country
5 − Subnational (Level 1)
Uncertainty Interval
5 − Subnational (Level 1)

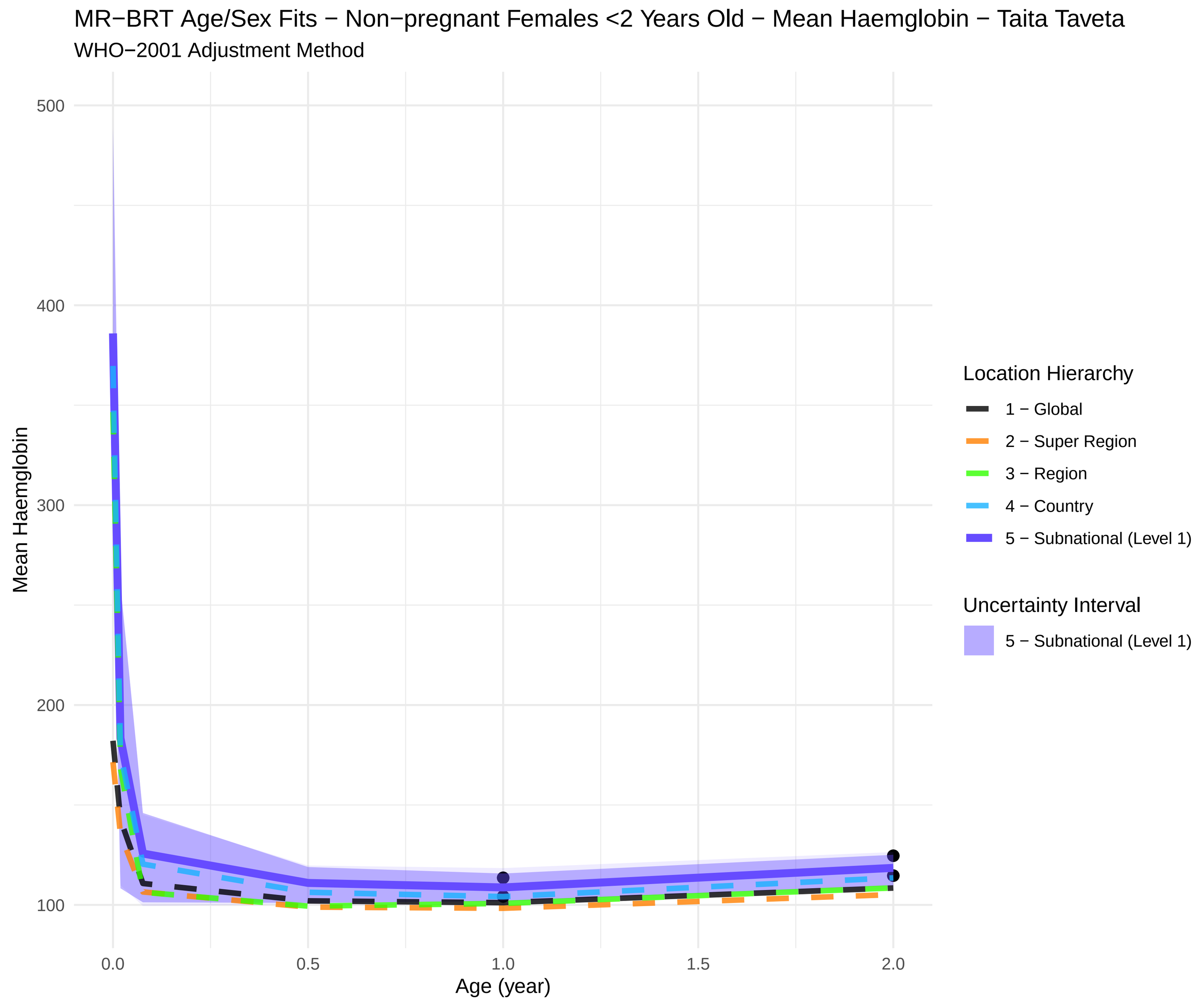
MR−BRT Age/Sex Fits − Non−pregnant Females <2 Years Old − Mean Haemglobin − Taita Taveta
WHO−2001 Adjustment Method
Mean Haemglobin
Age (year)
Location Hierarchy
1 − Global
2 − Super Region
3 − Region
4 − Country
5 − Subnational (Level 1)
Uncertainty Interval
5 − Subnational (Level 1)

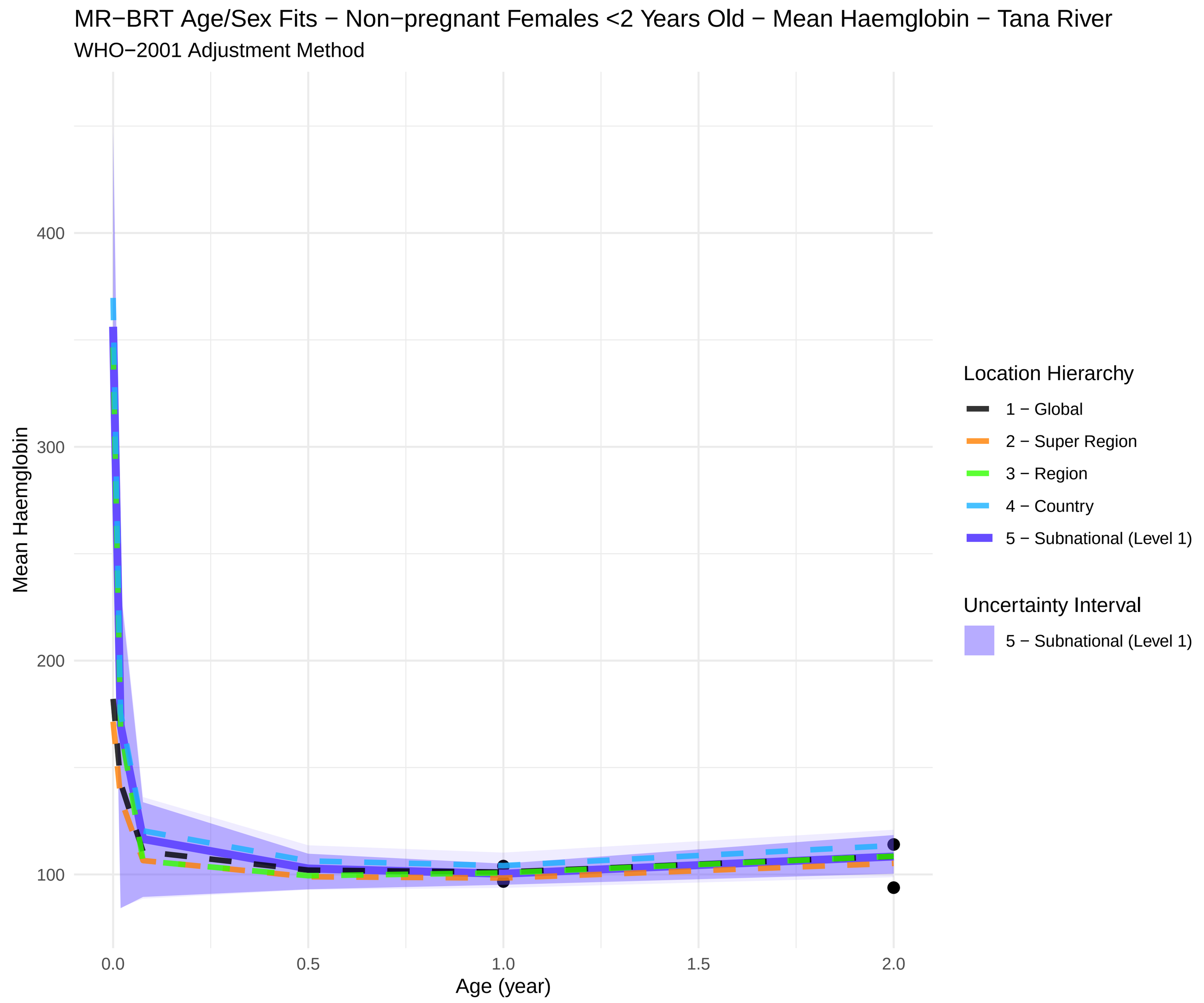
MR−BRT Age/Sex Fits − Non−pregnant Females <2 Years Old − Mean Haemglobin − Tana River
WHO−2001 Adjustment Method
Mean Haemglobin
Age (year)
Location Hierarchy
1 − Global
2 − Super Region
3 − Region
4 − Country
5 − Subnational (Level 1)
Uncertainty Interval
5 − Subnational (Level 1)

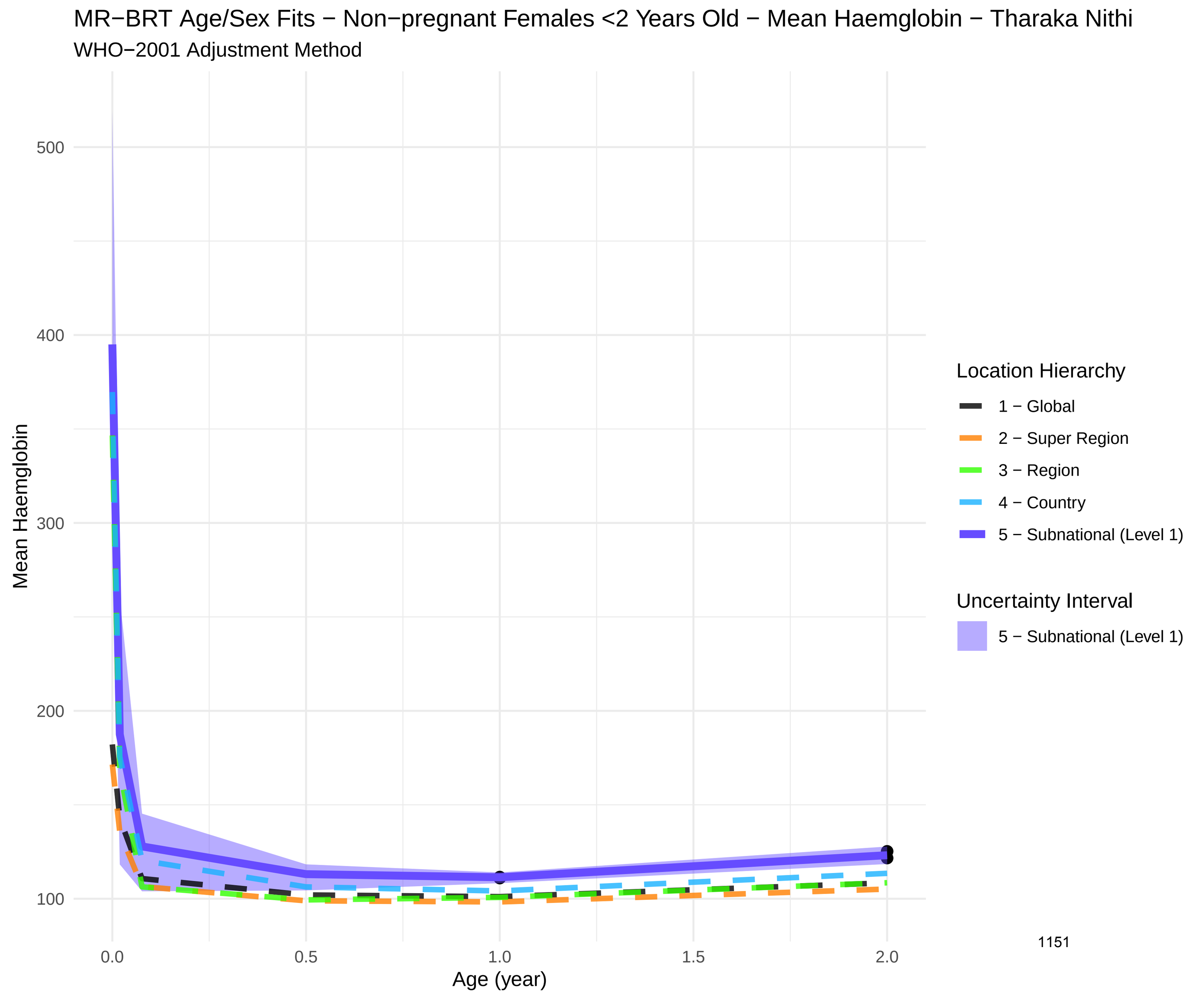
MR−BRT Age/Sex Fits − Non−pregnant Females <2 Years Old − Mean Haemglobin − Tharaka Nithi
WHO−2001 Adjustment Method
Mean Haemglobin
Age (year)
Location Hierarchy
1 − Global
2 − Super Region
3 − Region
4 − Country
5 − Subnational (Level 1)
Uncertainty Interval
5 − Subnational (Level 1)

MR−BRT Age/Sex Fits − Non−pregnant Females <2 Years Old − Mean Haemglobin − Trans Nzoia
WHO−2001 Adjustment Method

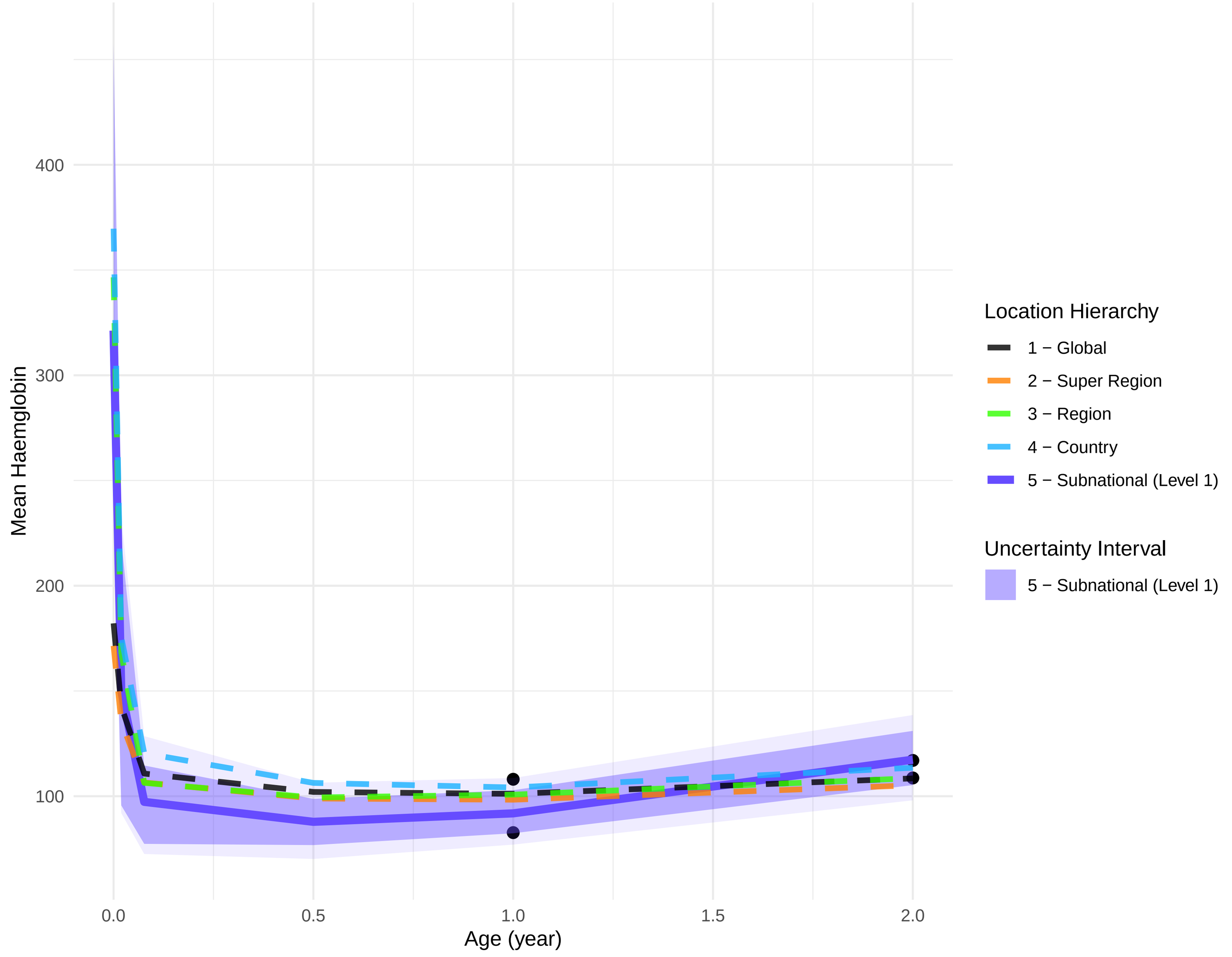

MR−BRT Age/Sex Fits − Non−pregnant Females <2 Years Old − Mean Haemglobin − Turkana
WHO−2001 Adjustment Method

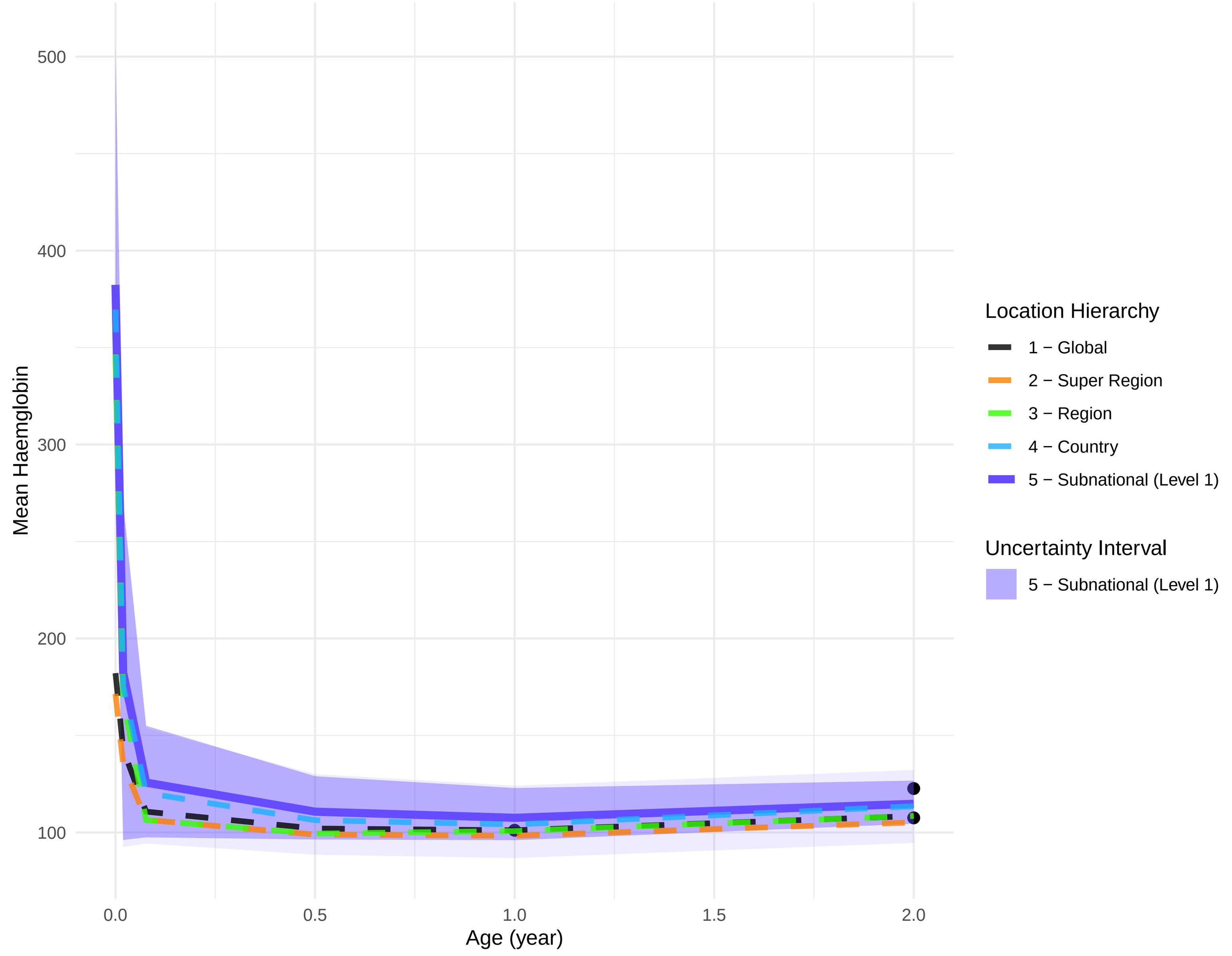

MR−BRT Age/Sex Fits − Non−pregnant Females <2 Years Old − Mean Haemglobin − Uasin Gishu
WHO−2001 Adjustment Method

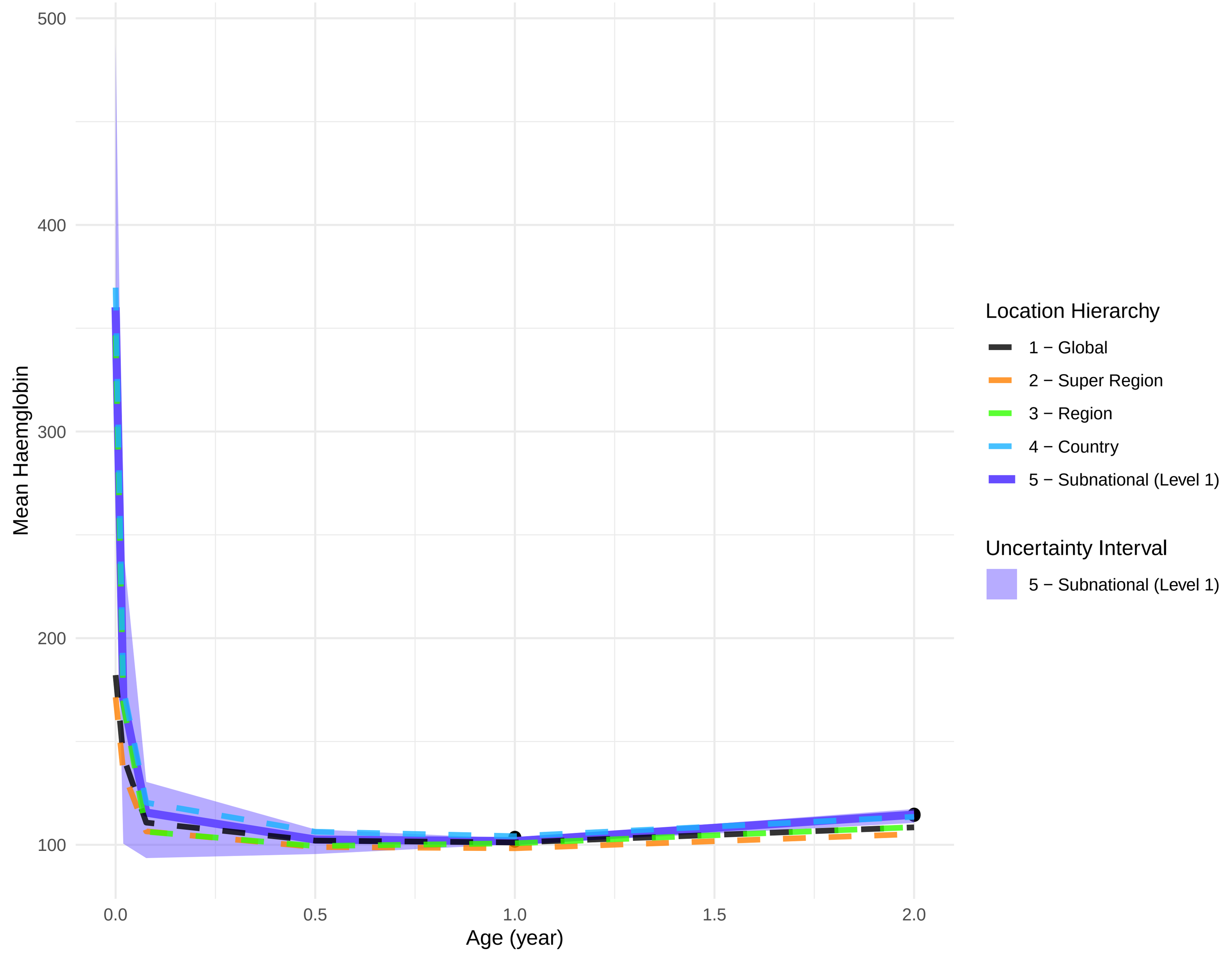

MR−BRT Age/Sex Fits − Non−pregnant Females <2 Years Old − Mean Haemglobin − Vihiga
WHO−2001 Adjustment Method

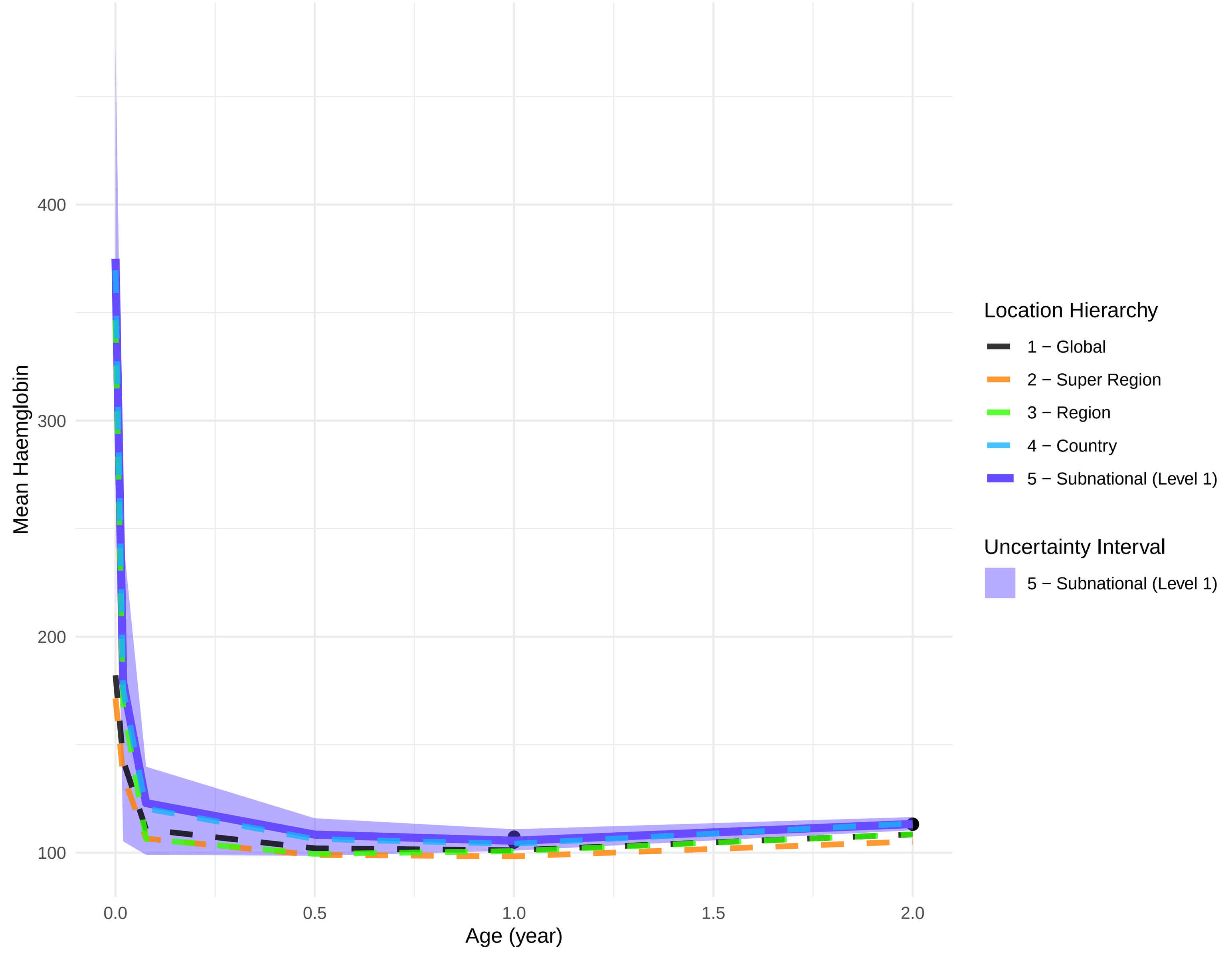

MR−BRT Age/Sex Fits − Non−pregnant Females <2 Years Old − Mean Haemglobin − Wajir
WHO−2001 Adjustment Method

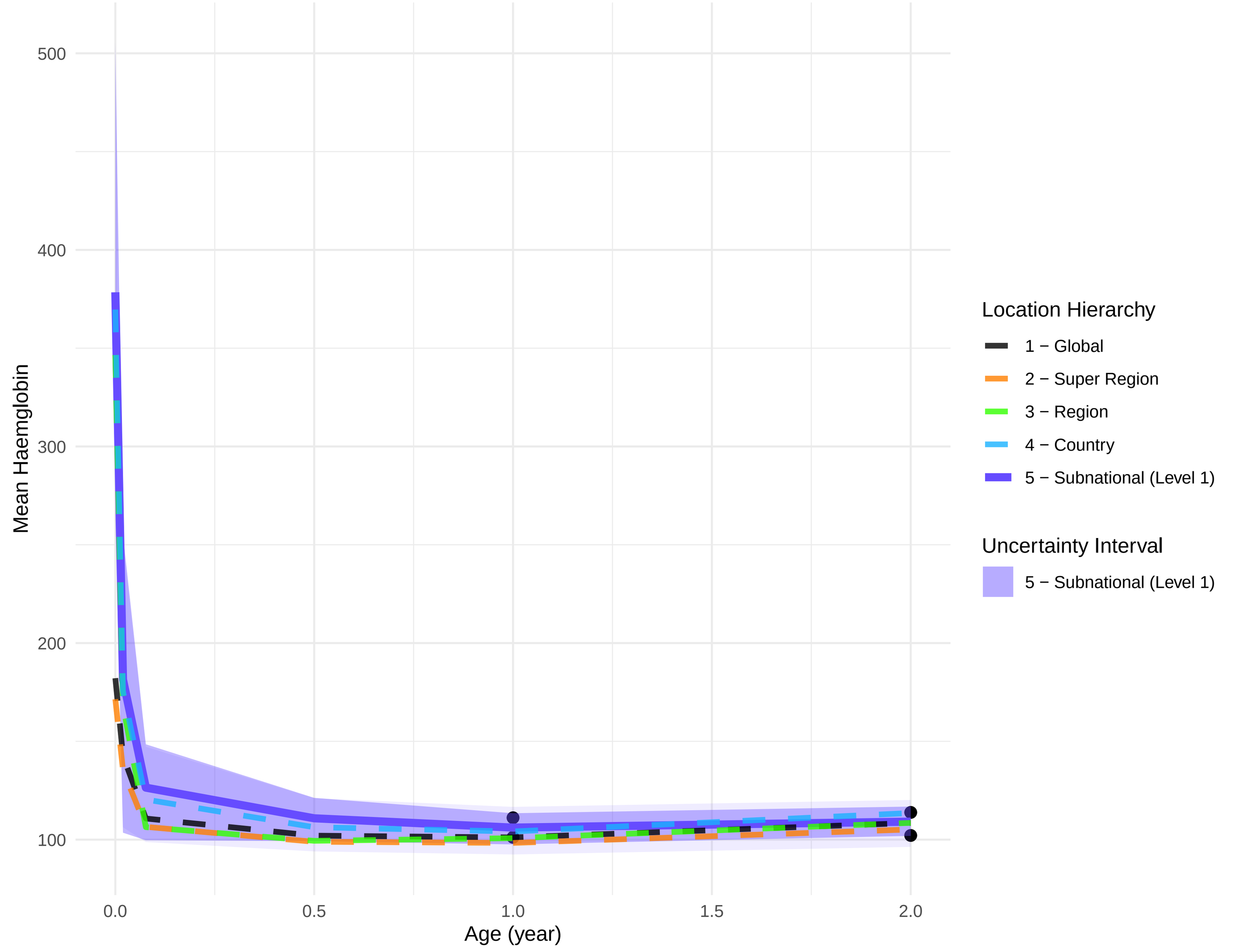

MR−BRT Age/Sex Fits − Non−pregnant Females <2 Years Old − Mean Haemglobin − West Pokot
WHO−2001 Adjustment Method

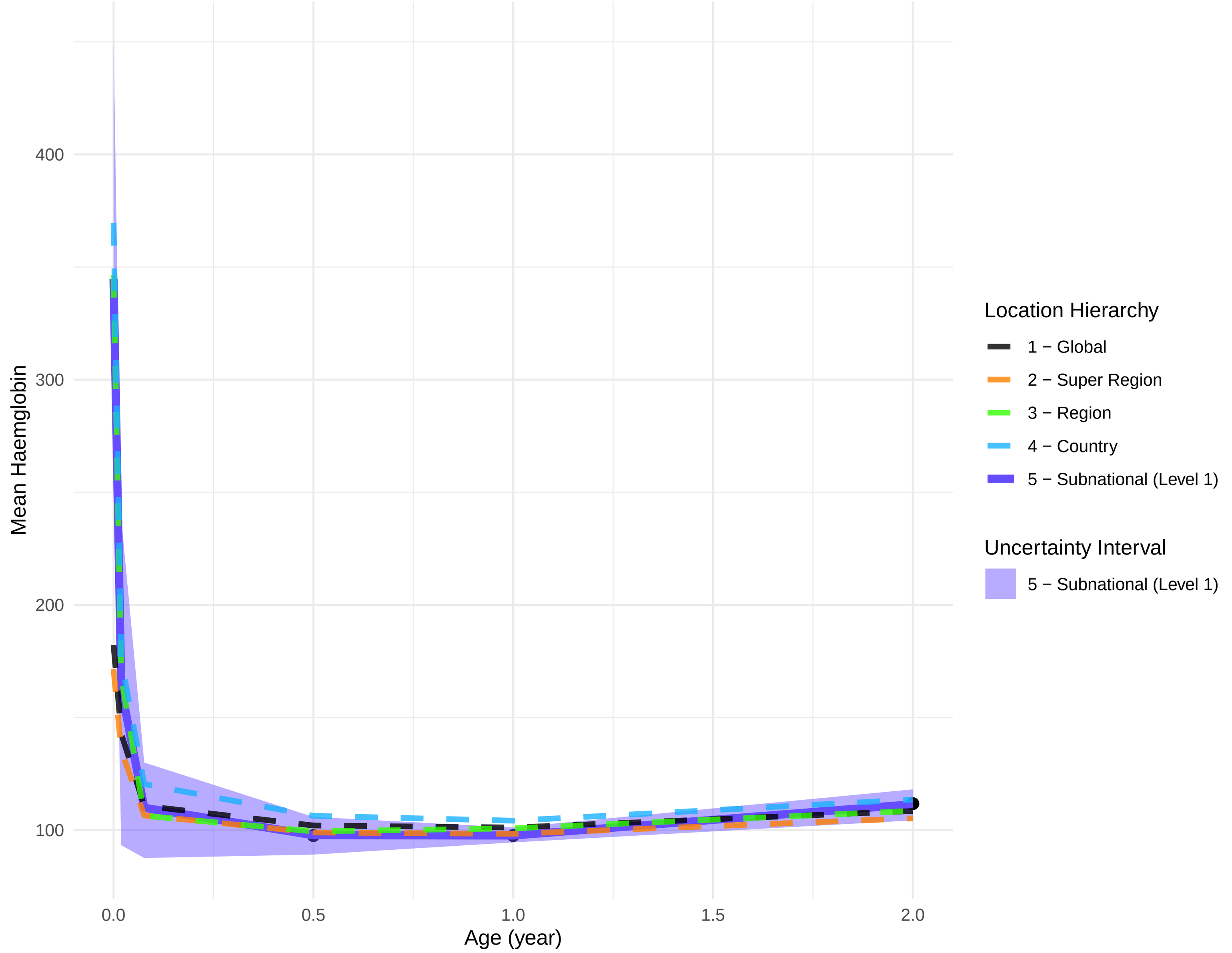

MR−BRT Age/Sex Fits − Non−pregnant Females <2 Years Old − Mean Haemglobin − Madagascar
WHO−2001 Adjustment Method

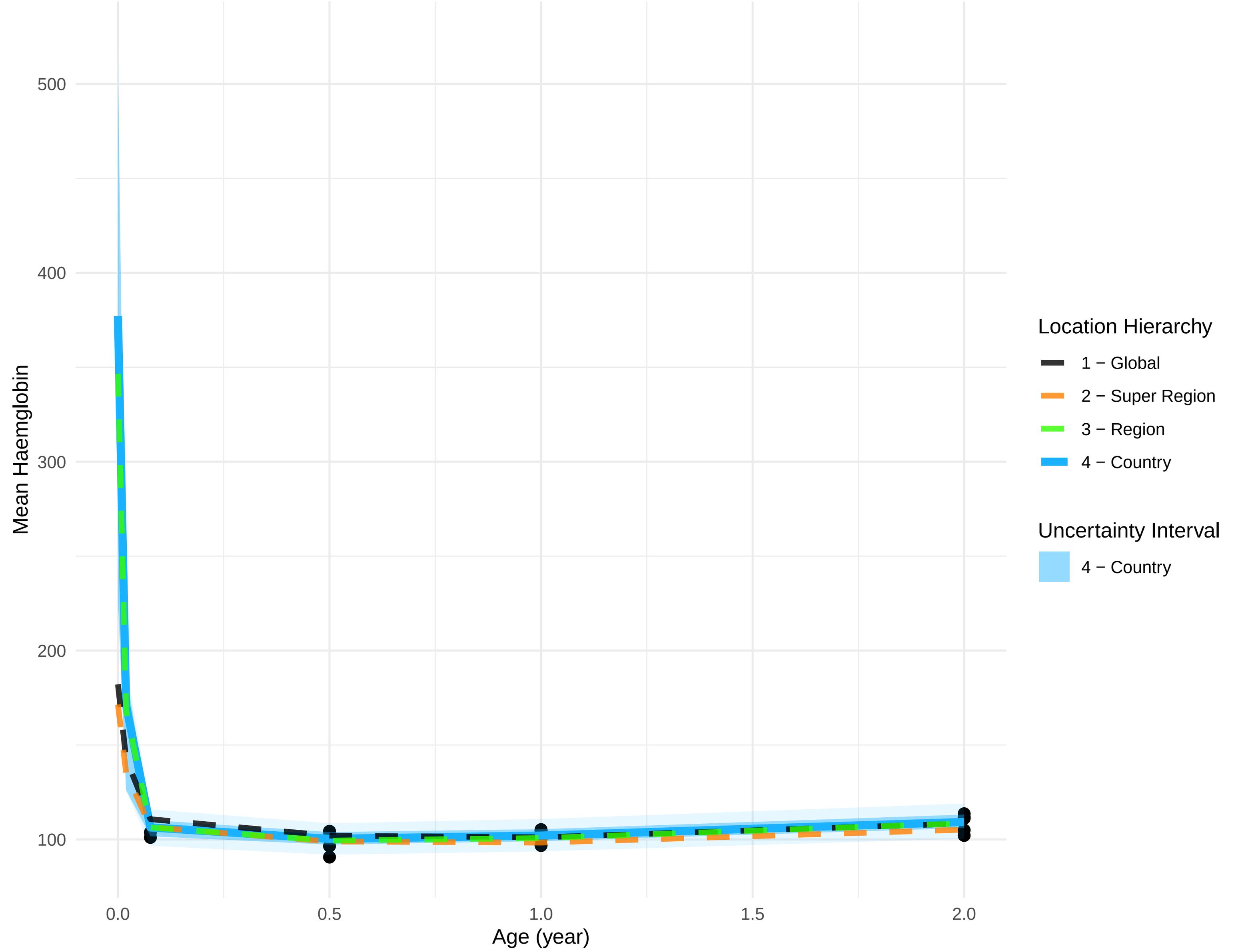

MR−BRT Age/Sex Fits − Non−pregnant Females <2 Years Old − Mean Haemglobin − Malawi
WHO−2001 Adjustment Method

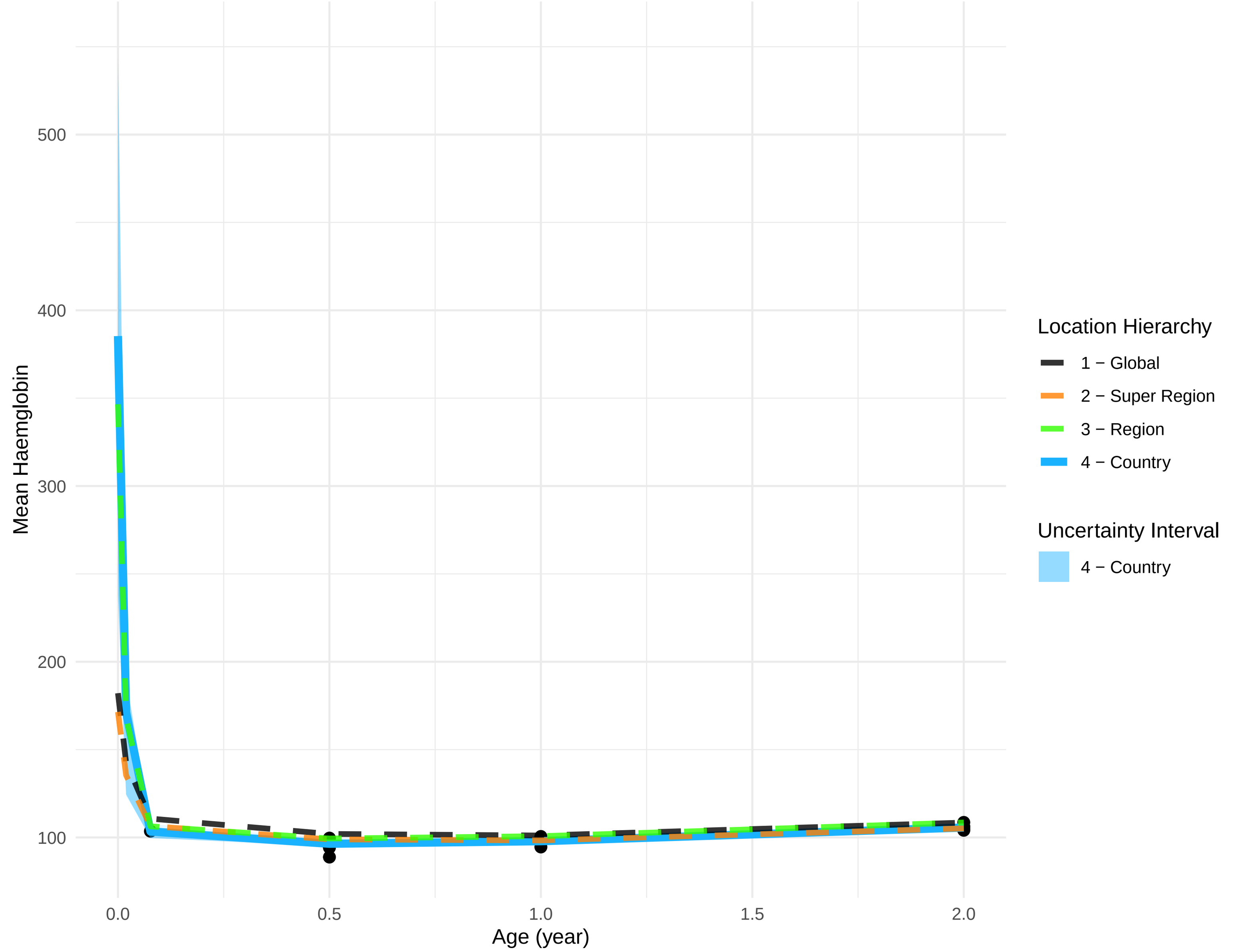

MR−BRT Age/Sex Fits − Non−pregnant Females <2 Years Old − Mean Haemglobin − Mozambique
WHO−2001 Adjustment Method

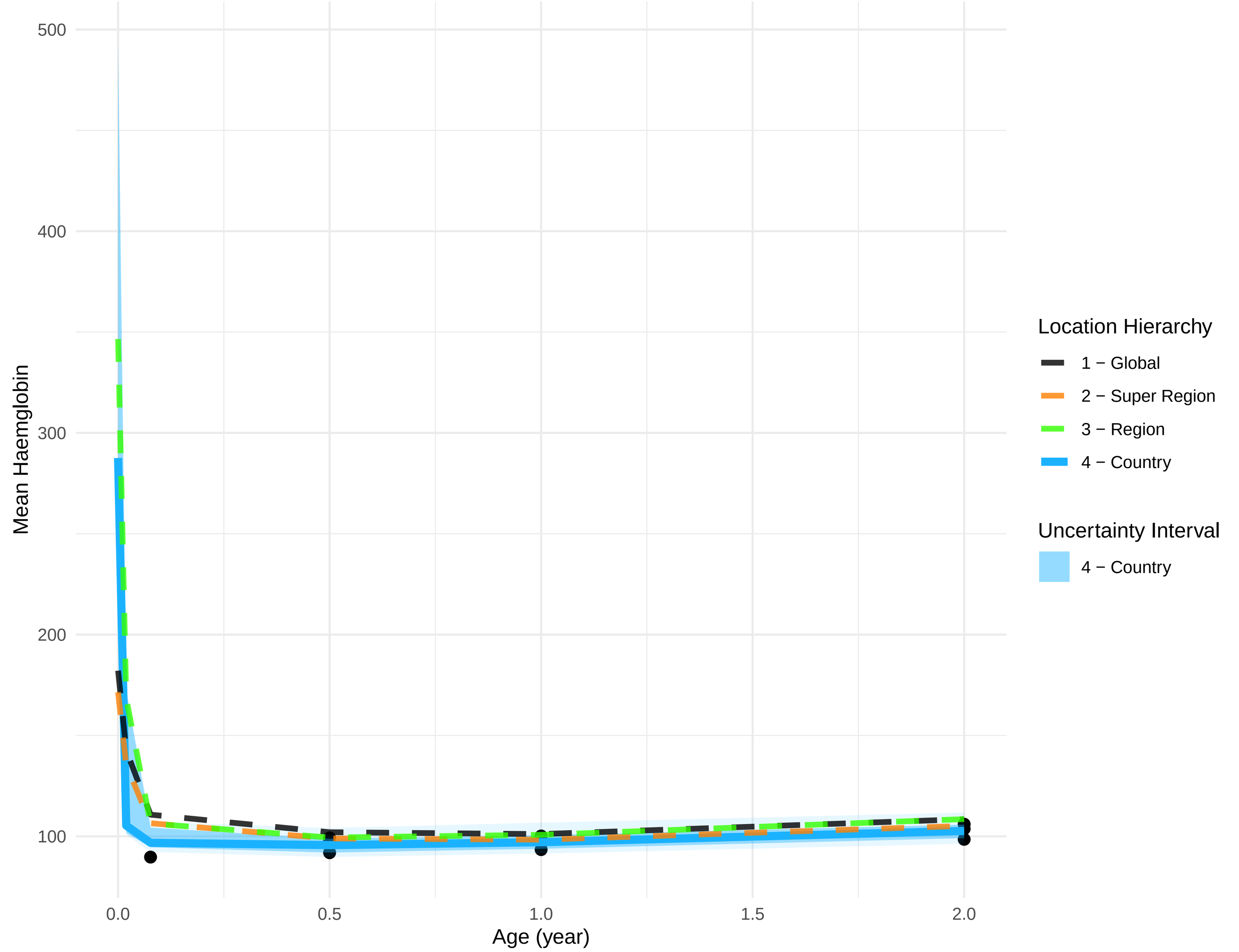

MR−BRT Age/Sex Fits − Non−pregnant Females <2 Years Old − Mean Haemglobin − Rwanda
WHO−2001 Adjustment Method

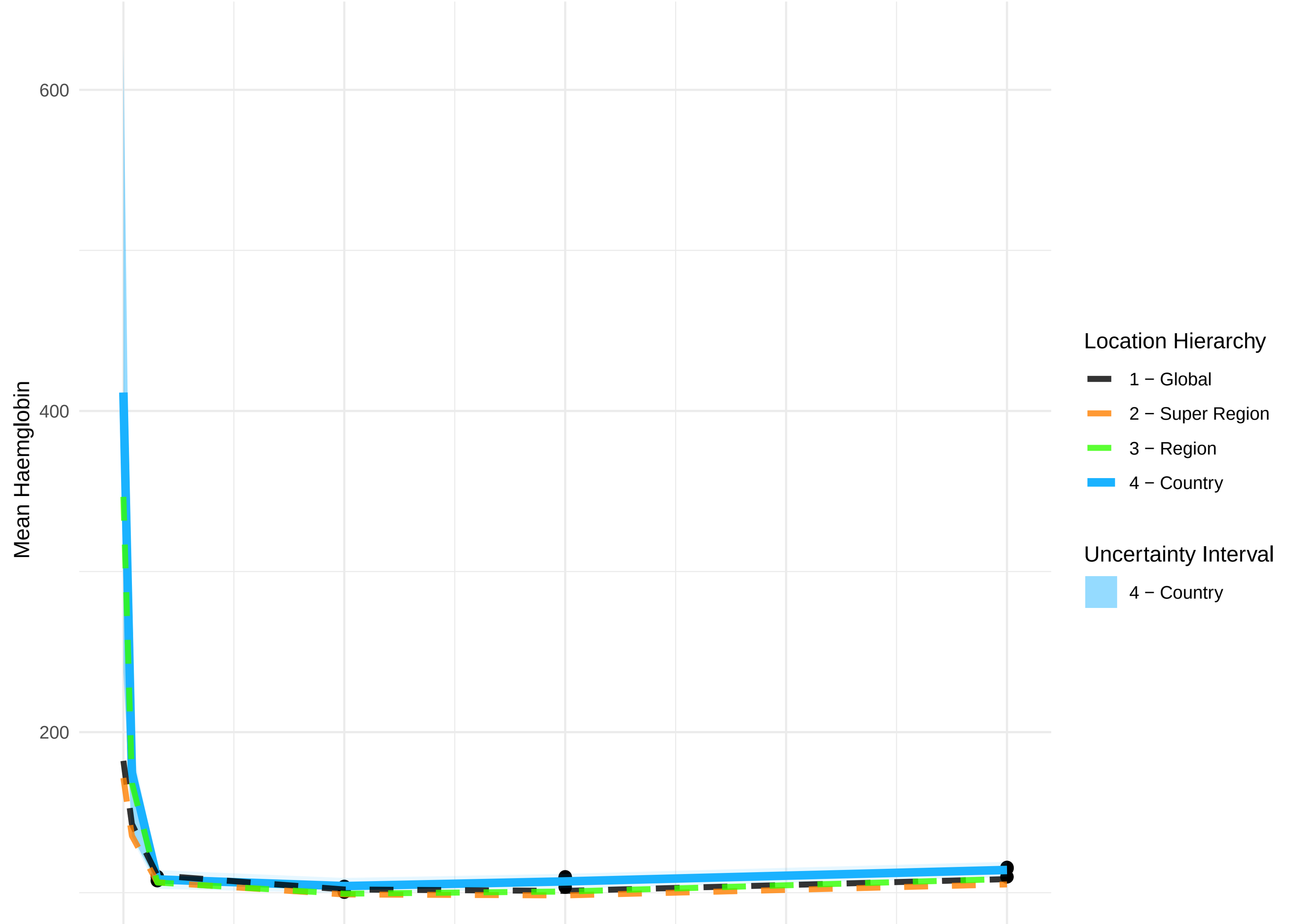

MR−BRT Age/Sex Fits − Non−pregnant Females <2 Years Old − Mean Haemglobin − Uganda
WHO−2001 Adjustment Method

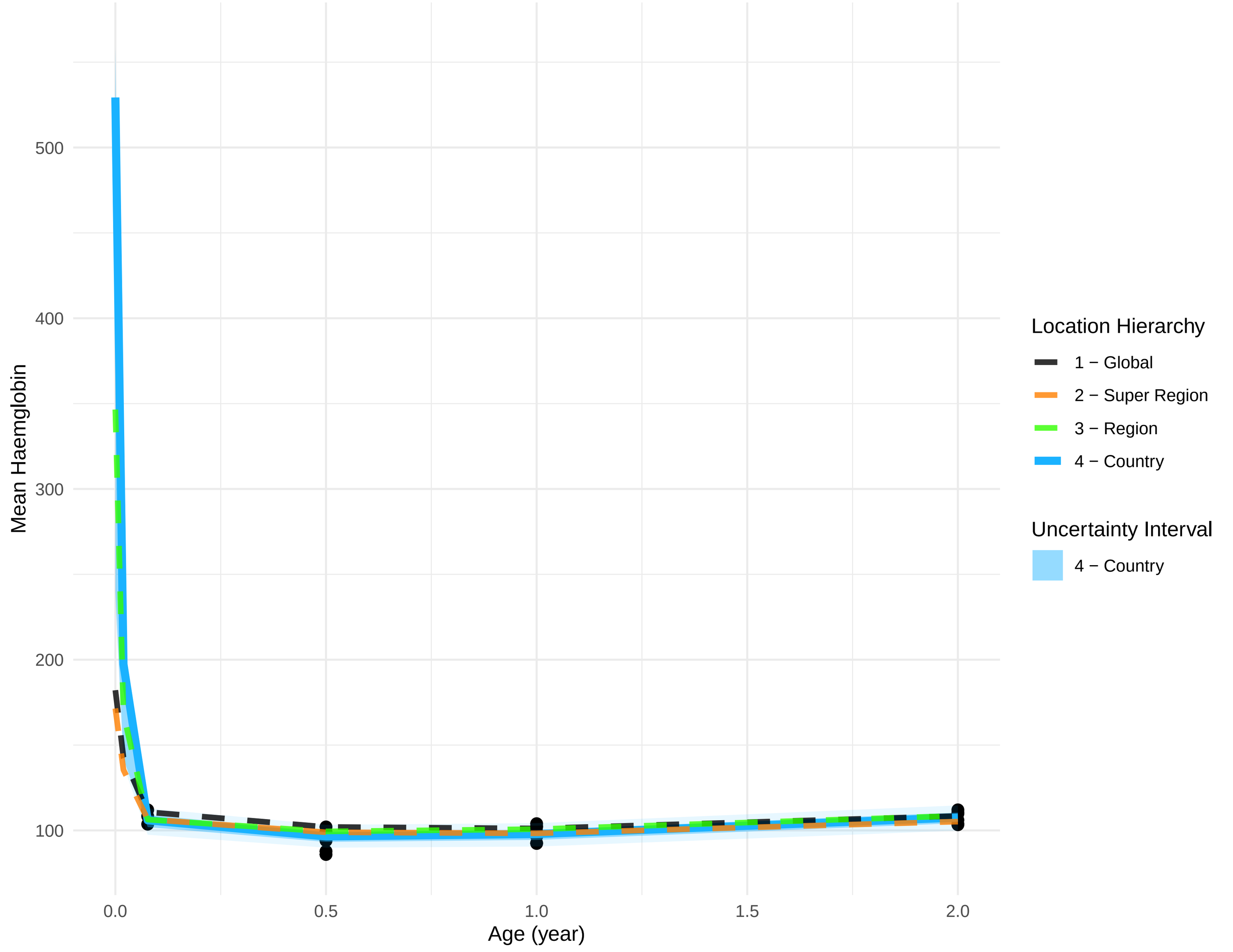

MR−BRT Age/Sex Fits − Non−pregnant Females <2 Years Old − Mean Haemglobin − United Republic of Tanzania
WHO−2001 Adjustment Method

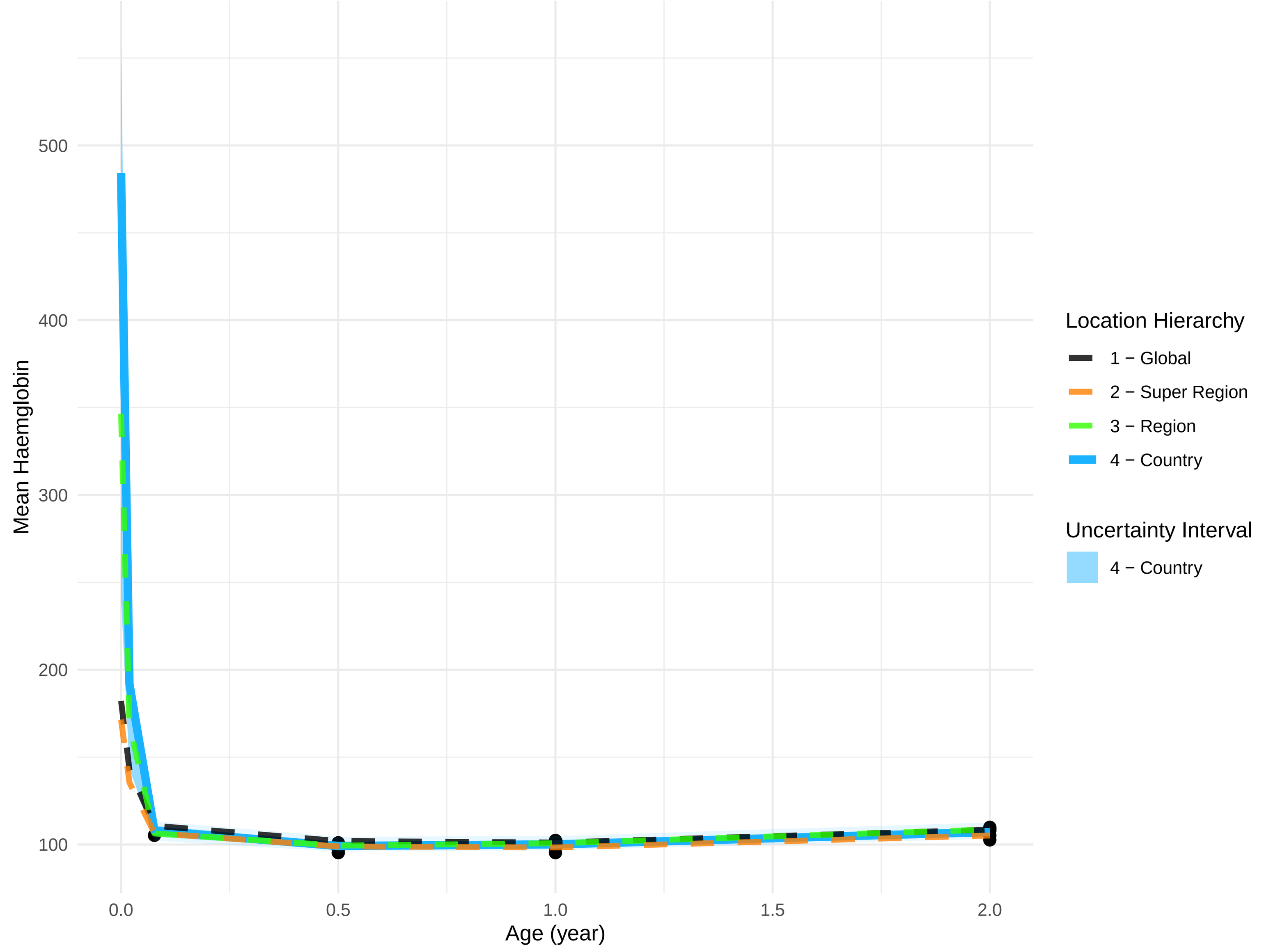

MR−BRT Age/Sex Fits − Non−pregnant Females <2 Years Old − Mean Haemglobin − Zambia
WHO−2001 Adjustment Method

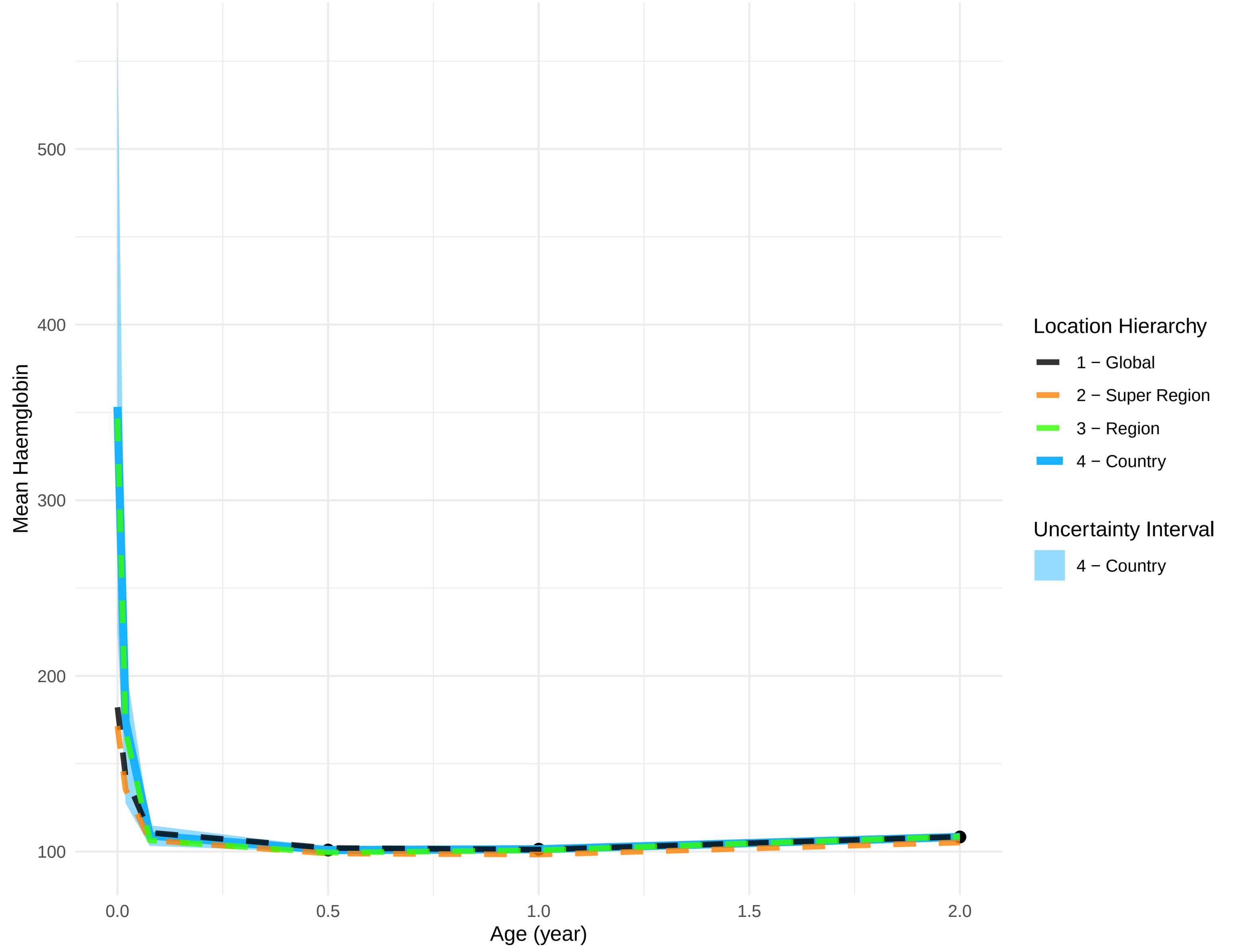

MR−BRT Age/Sex Fits − Non−pregnant Females <2 Years Old − Mean Haemglobin − Southern Sub−Saharan Africa
WHO−2001 Adjustment Method

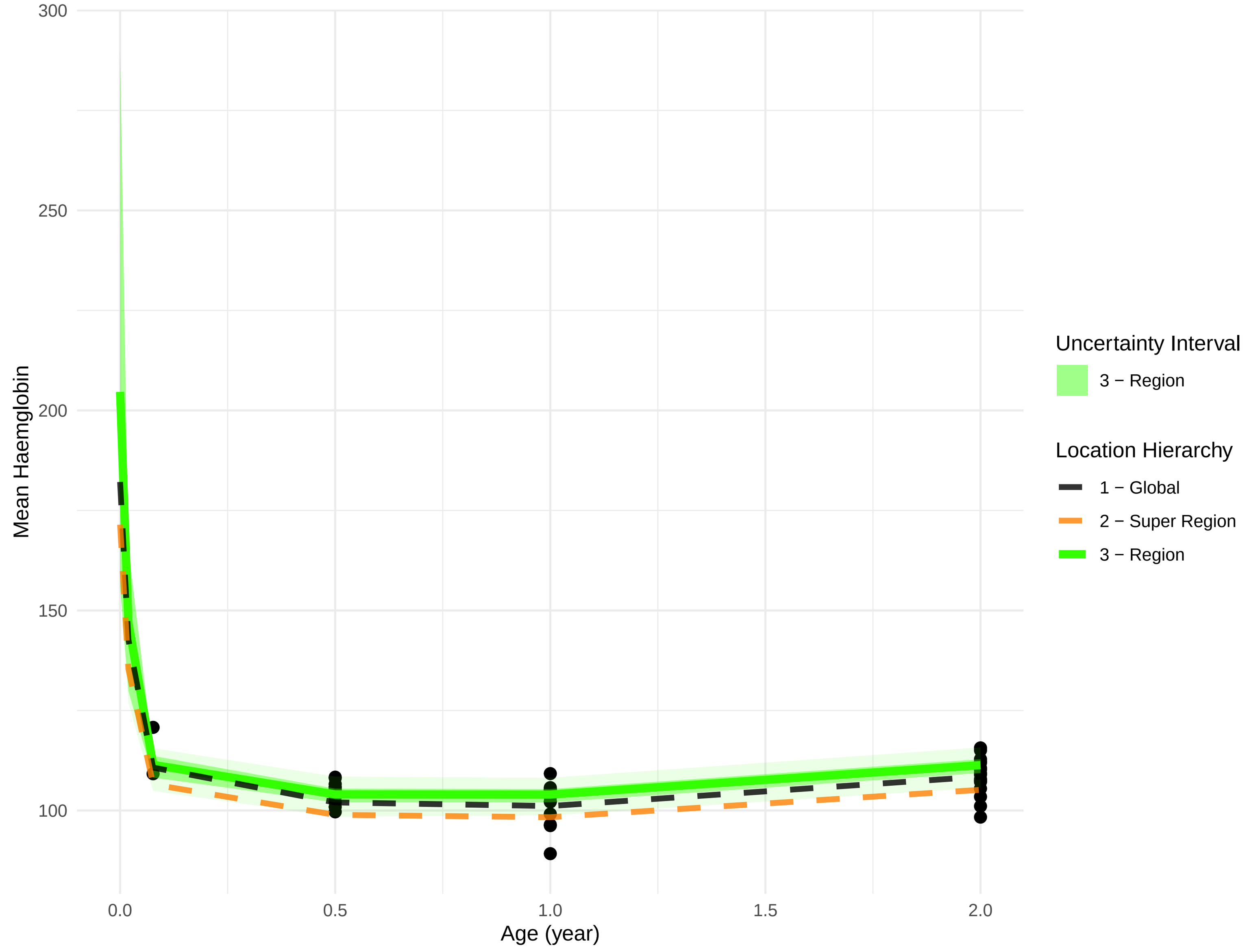

MR−BRT Age/Sex Fits − Non−pregnant Females <2 Years Old − Mean Haemglobin − Eswatini
WHO−2001 Adjustment Method

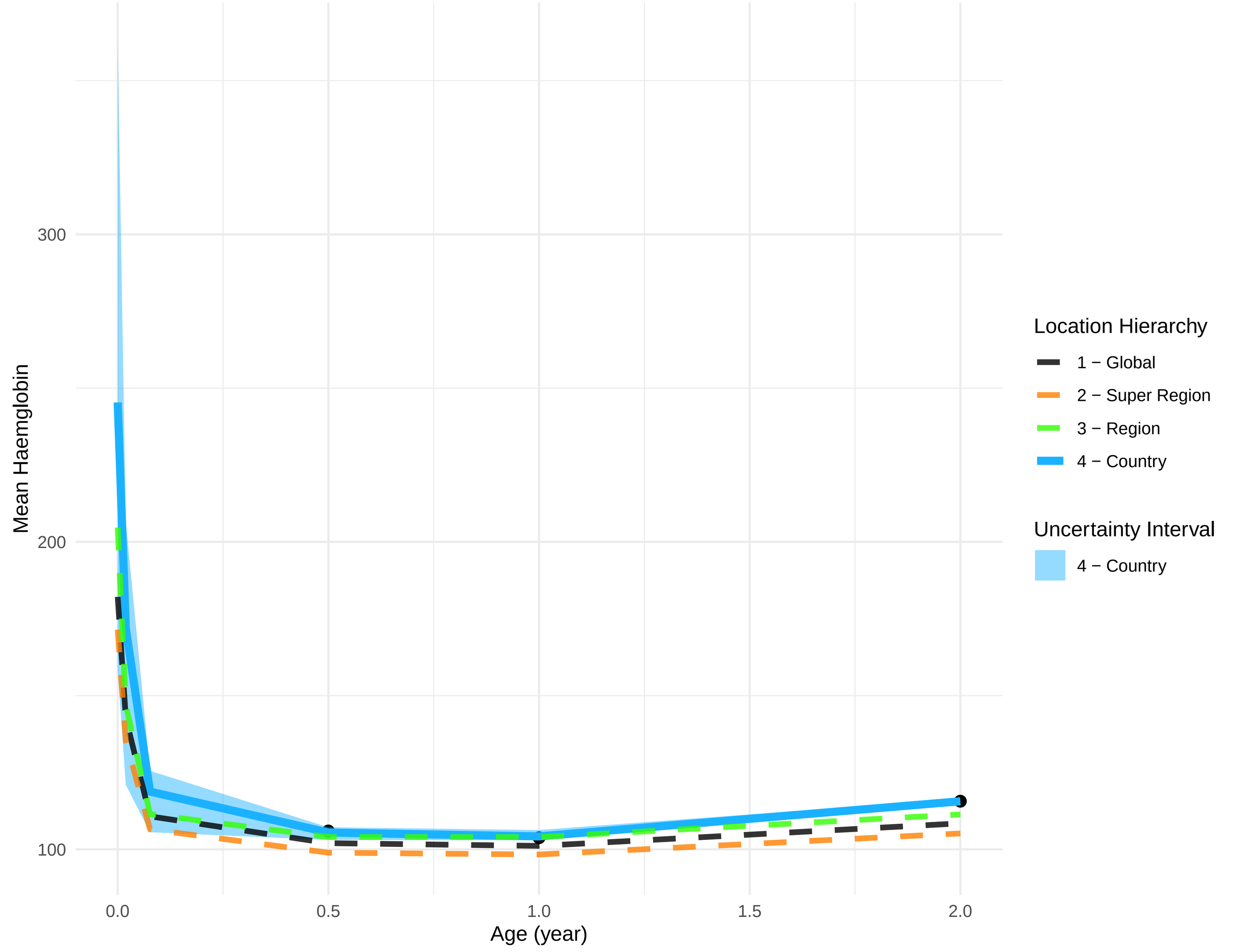

MR−BRT Age/Sex Fits − Non−pregnant Females <2 Years Old − Mean Haemglobin − Lesotho
WHO−2001 Adjustment Method

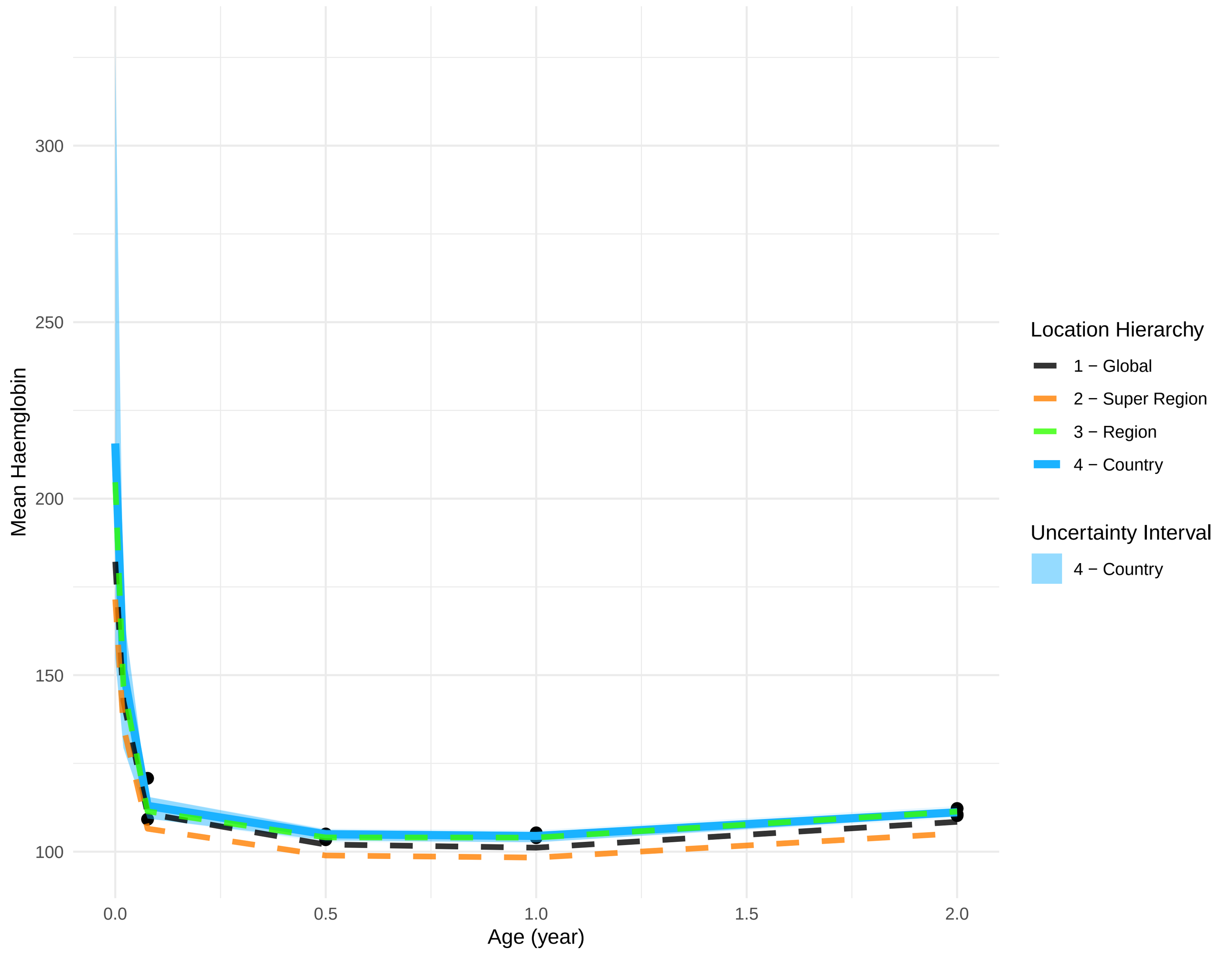

MR−BRT Age/Sex Fits − Non−pregnant Females <2 Years Old − Mean Haemglobin − Namibia
WHO−2001 Adjustment Method

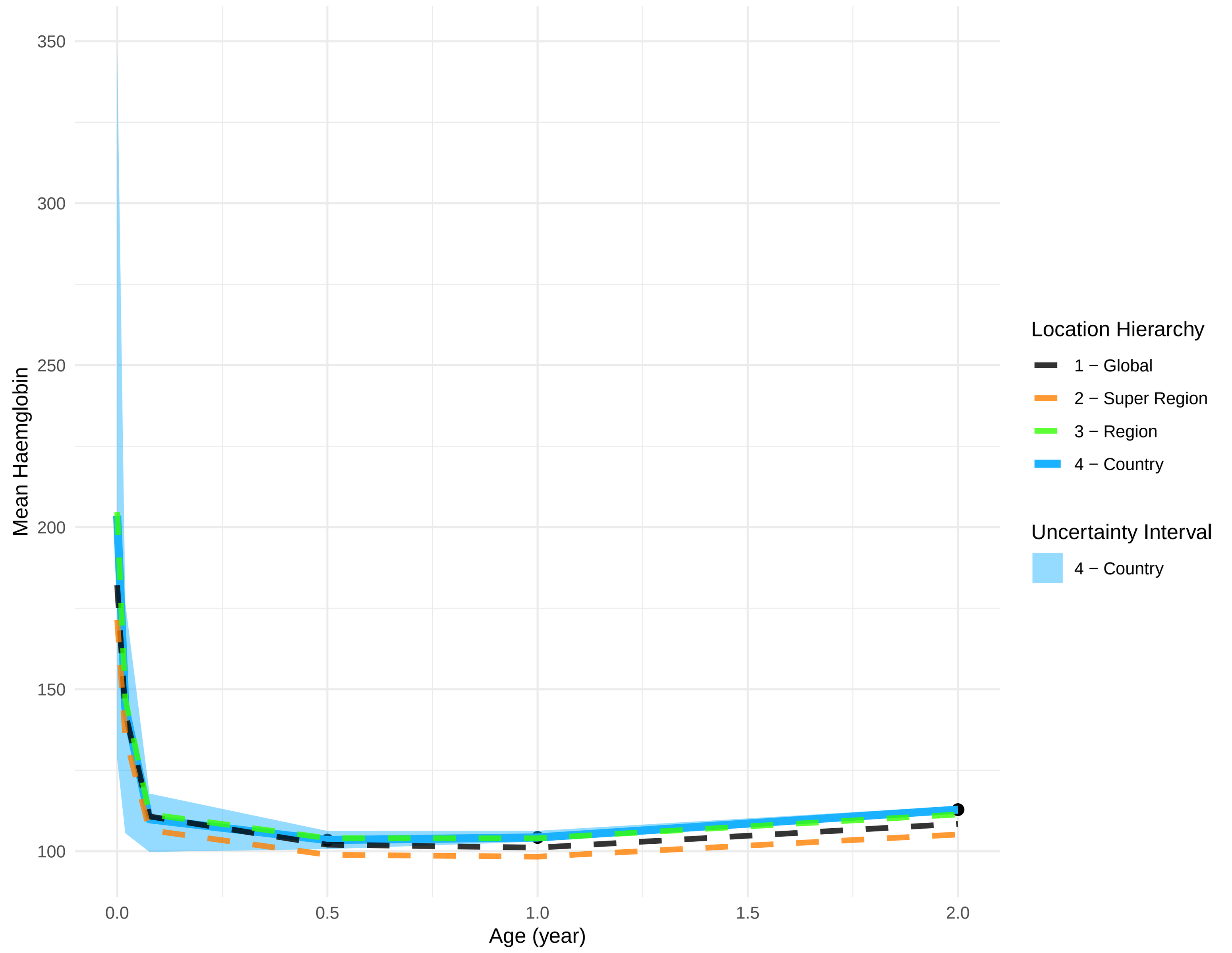

MR−BRT Age/Sex Fits − Non−pregnant Females <2 Years Old − Mean Haemglobin − South Africa
WHO−2001 Adjustment Method

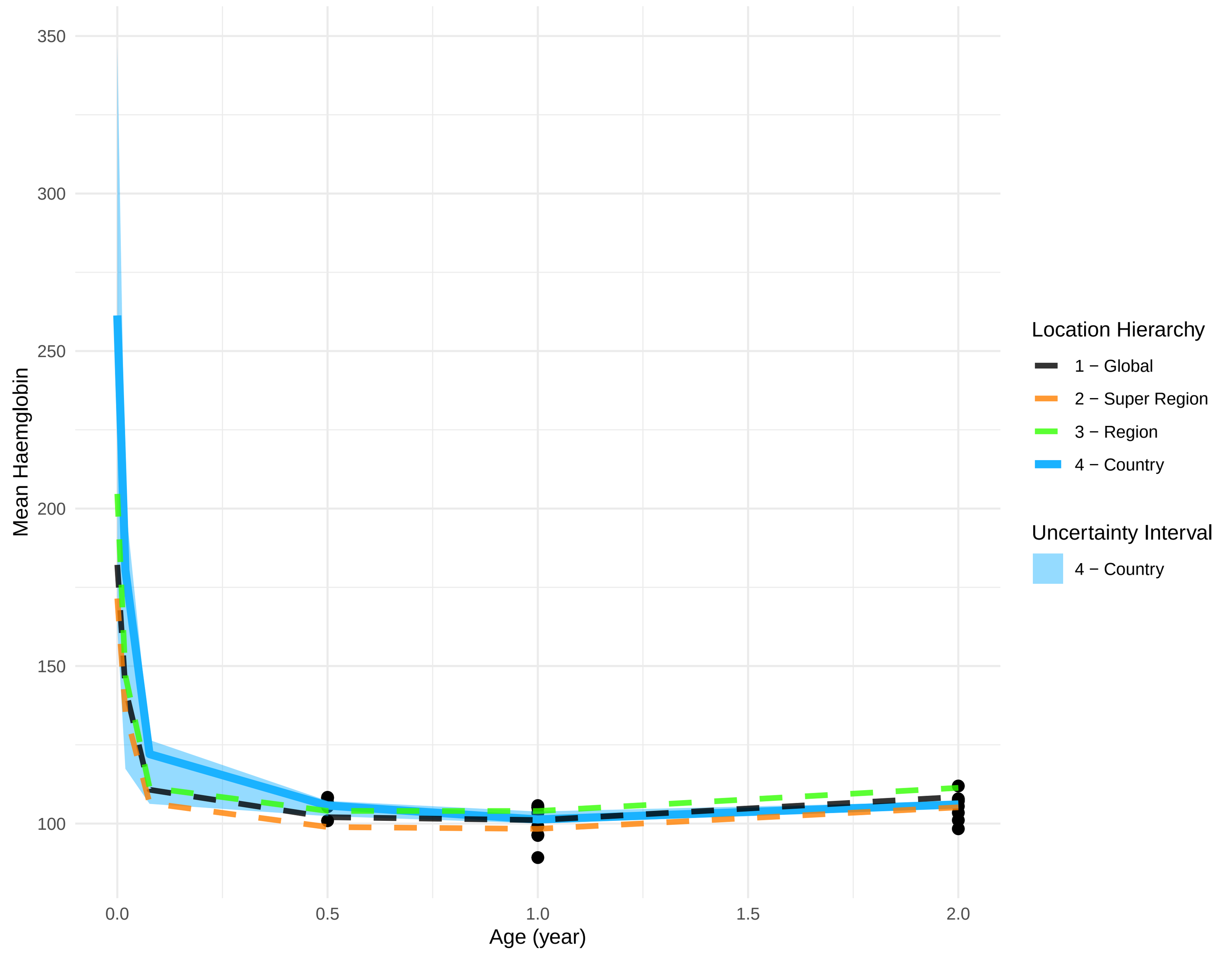

MR−BRT Age/Sex Fits − Non−pregnant Females <2 Years Old − Mean Haemglobin − Eastern Cape
WHO−2001 Adjustment Method

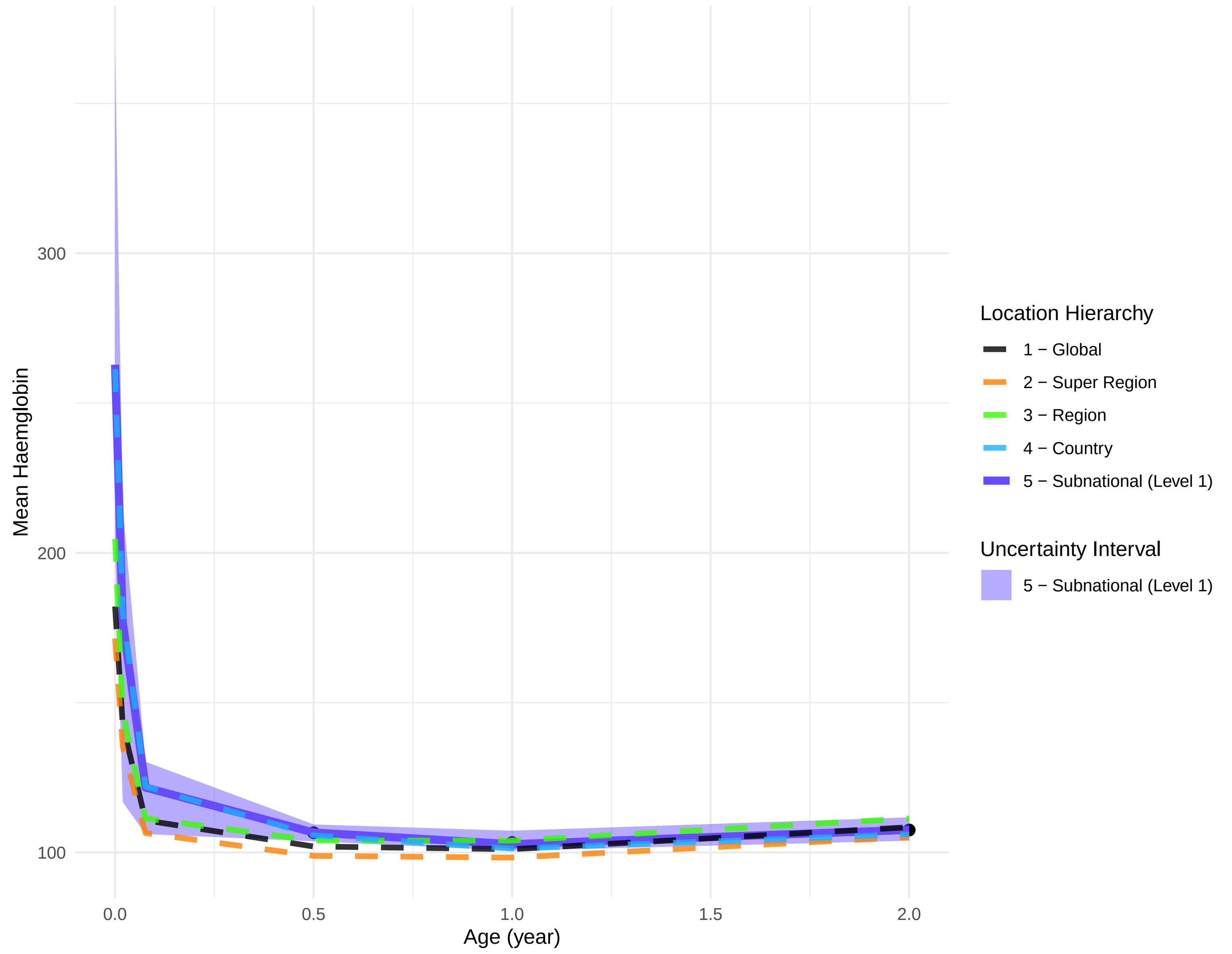

MR−BRT Age/Sex Fits − Non−pregnant Females <2 Years Old − Mean Haemglobin − Free State
WHO−2001 Adjustment Method

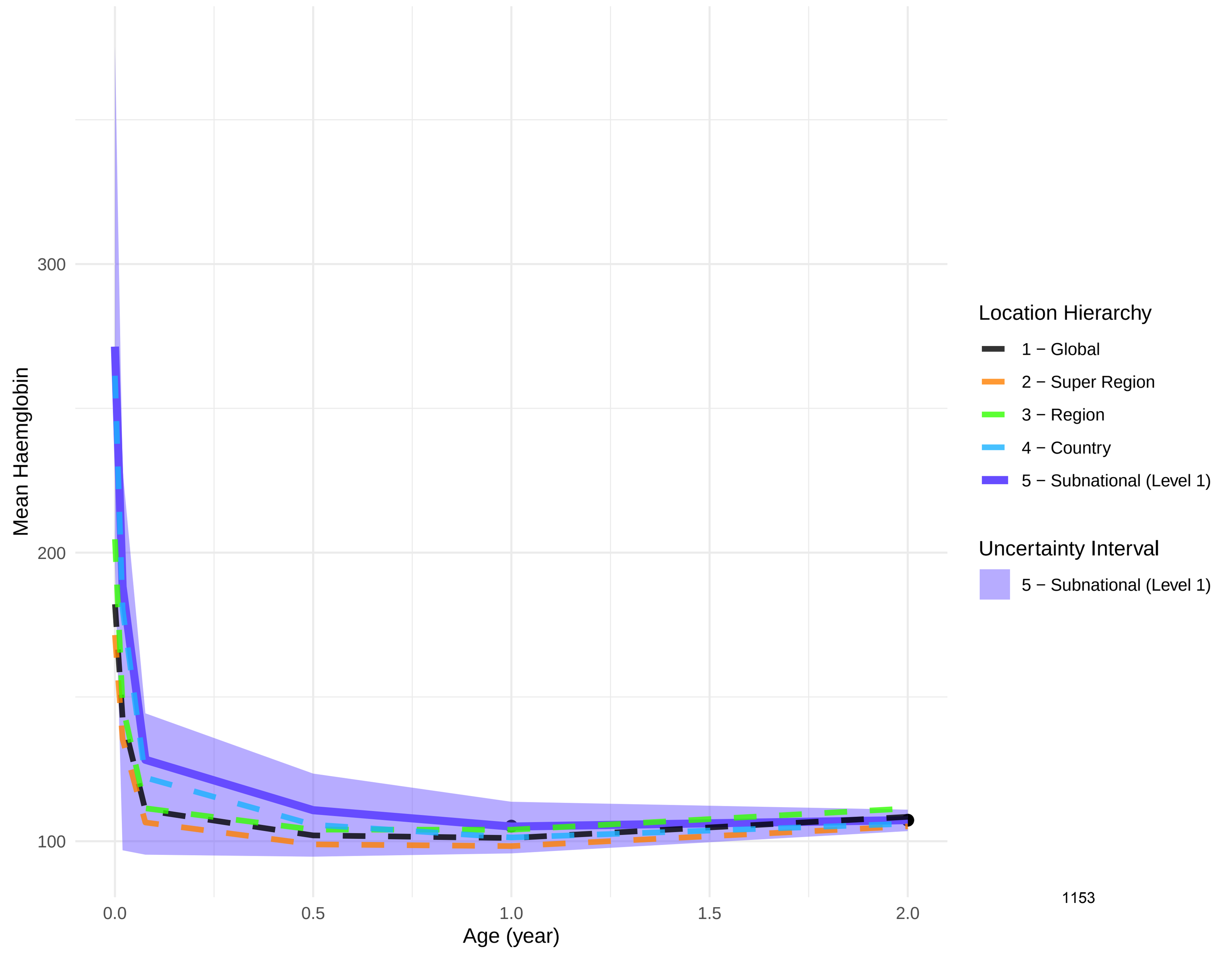

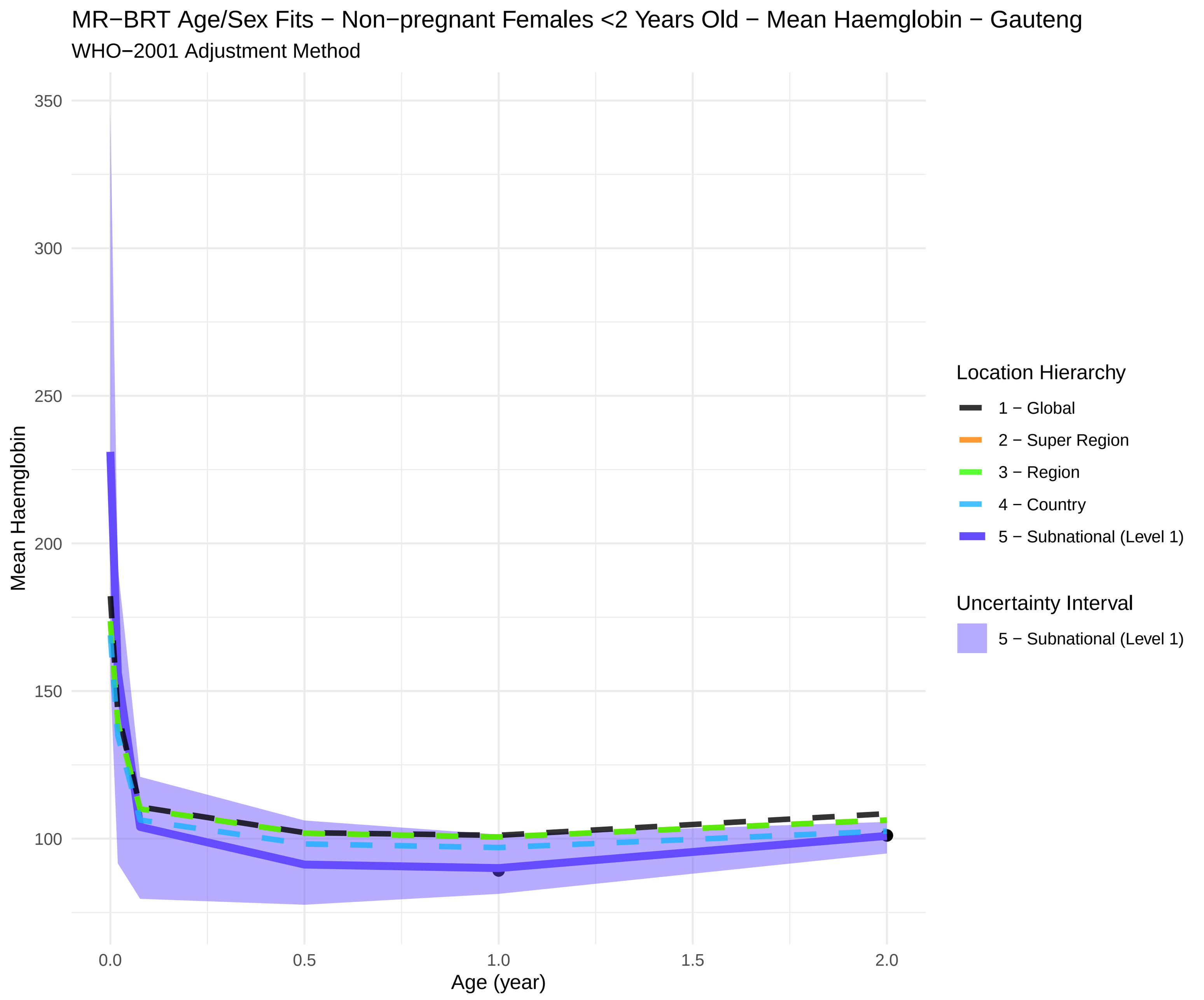
MR−BRT Age/Sex Fits − Non−pregnant Females <2 Years Old − Mean Haemglobin − Gauteng
WHO−2001 Adjustment Method
Mean Haemglobin
Age (year)
Location Hierarchy
1 − Global
2 − Super Region
3 − Region
4 − Country
5 − Subnational (Level 1)
Uncertainty Interval
5 − Subnational (Level 1)

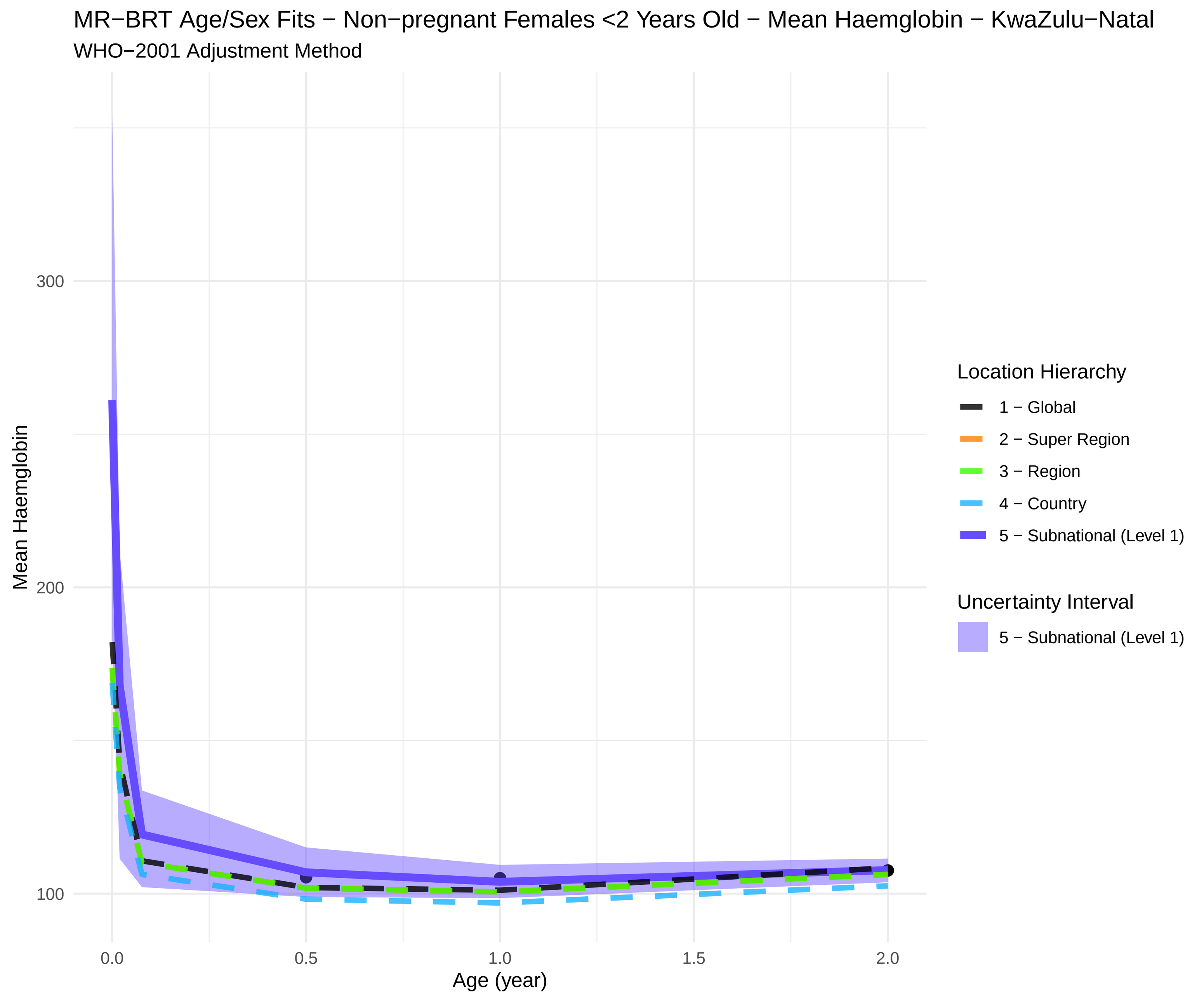
MR−BRT Age/Sex Fits − Non−pregnant Females <2 Years Old − Mean Haemglobin − KwaZulu−Natal
WHO−2001 Adjustment Method
Mean Haemglobin
Age (year)
Location Hierarchy
1 − Global
2 − Super Region
3 − Region
4 − Country
5 − Subnational (Level 1)
Uncertainty Interval
5 − Subnational (Level 1)

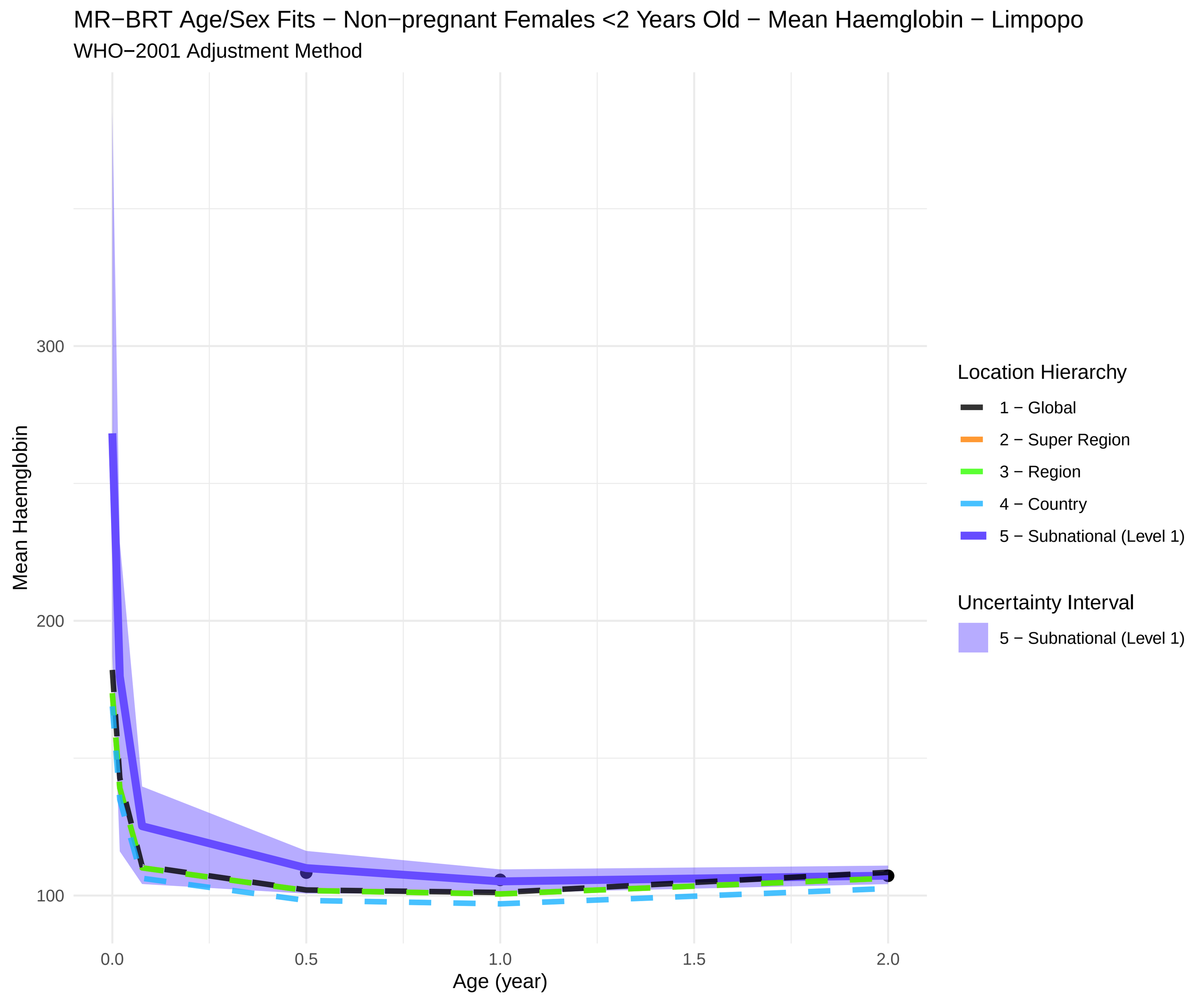
MR−BRT Age/Sex Fits − Non−pregnant Females <2 Years Old − Mean Haemglobin − Limpopo
WHO−2001 Adjustment Method
Mean Haemglobin
Age (year)
Location Hierarchy
1 − Global
2 − Super Region
3 − Region
4 − Country
5 − Subnational (Level 1)
Uncertainty Interval
5 − Subnational (Level 1)

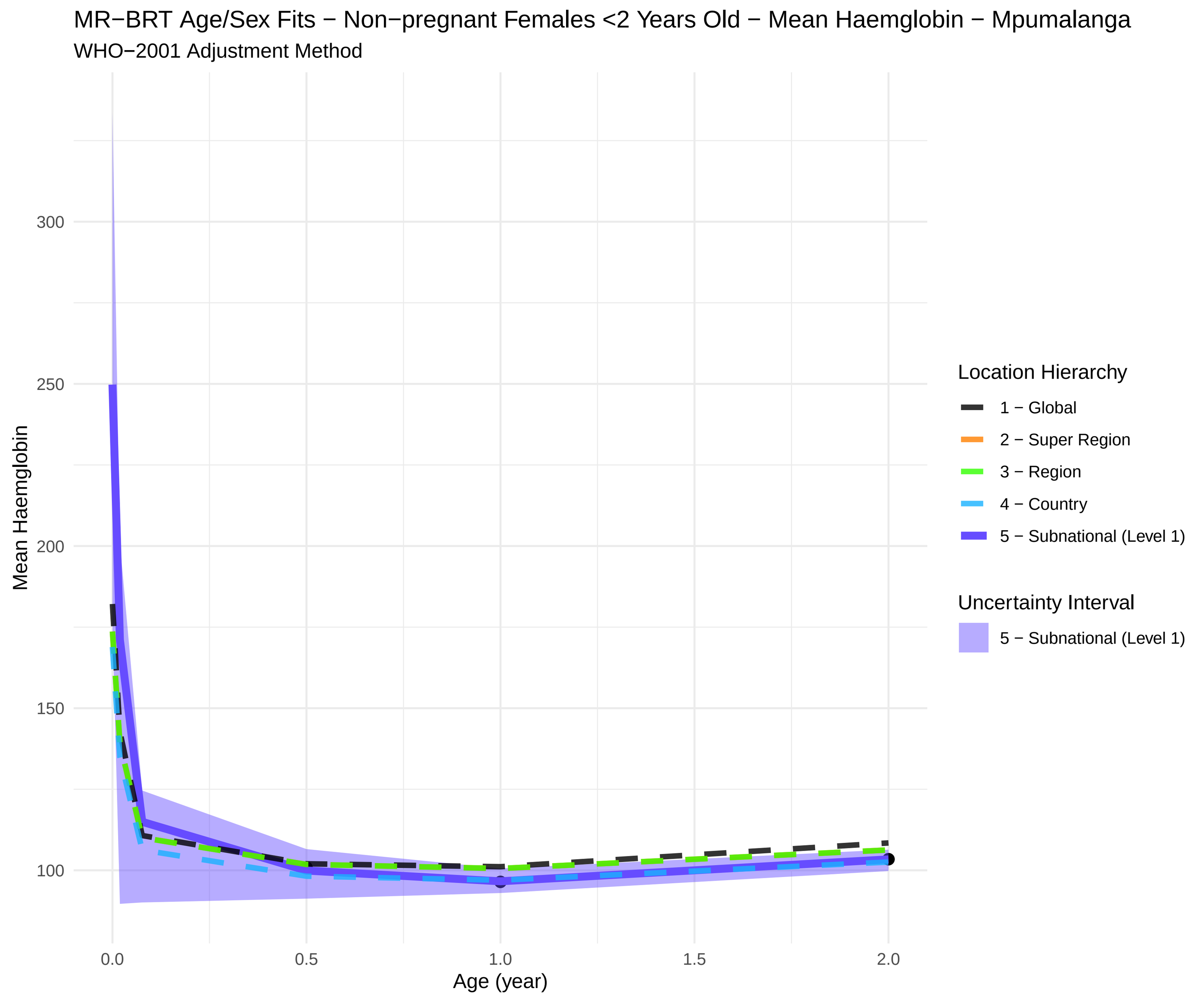
MR−BRT Age/Sex Fits − Non−pregnant Females <2 Years Old − Mean Haemglobin − Mpumalanga
WHO−2001 Adjustment Method
Mean Haemglobin
Age (year)
Location Hierarchy
1 − Global
2 − Super Region
3 − Region
4 − Country
5 − Subnational (Level 1)
Uncertainty Interval
5 − Subnational (Level 1)

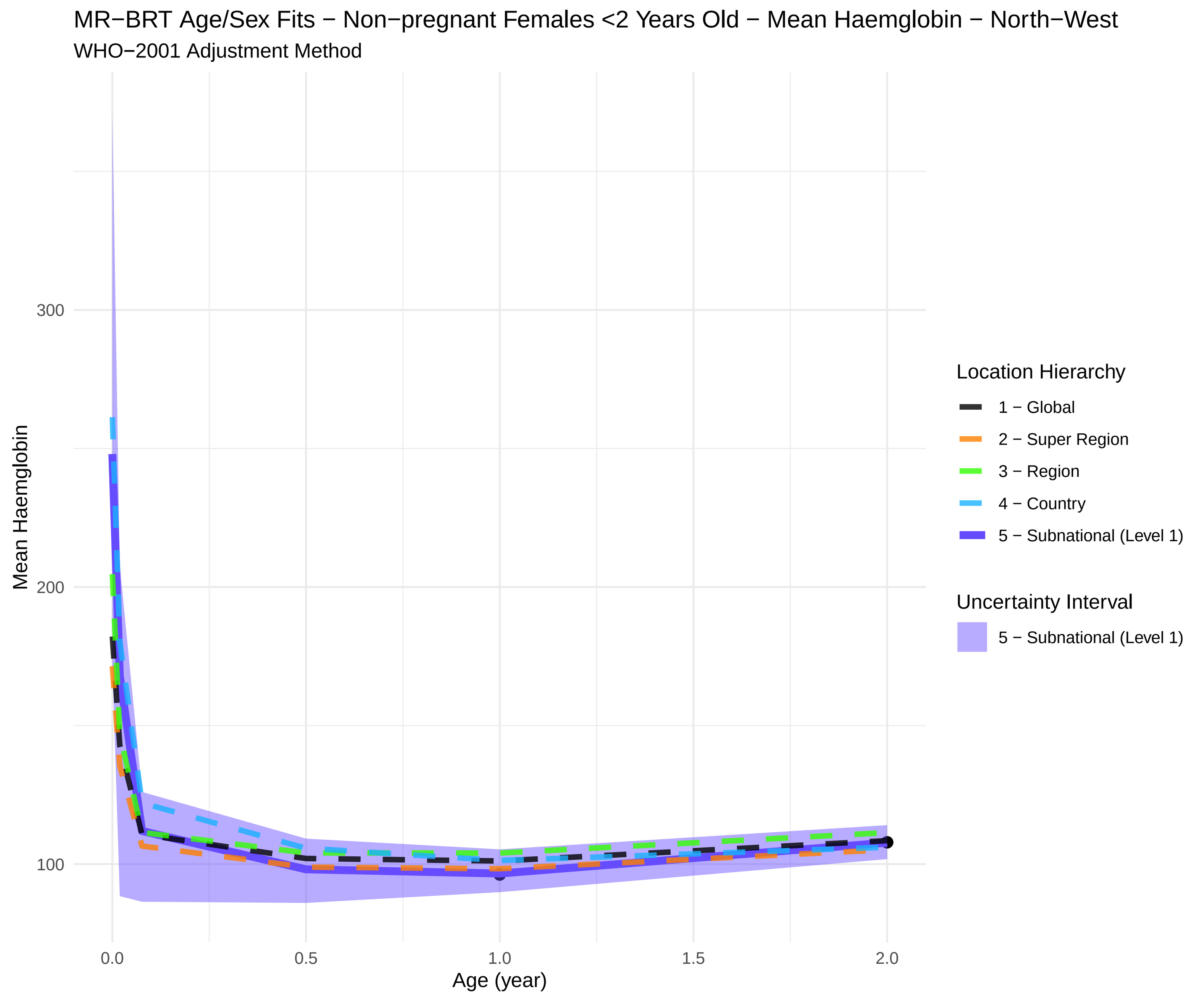
MR−BRT Age/Sex Fits − Non−pregnant Females <2 Years Old − Mean Haemglobin − North−West
WHO−2001 Adjustment Method
Mean Haemglobin
Age (year)
Location Hierarchy
1 − Global
2 − Super Region
3 − Region
4 − Country
5 − Subnational (Level 1)
Uncertainty Interval
5 − Subnational (Level 1)

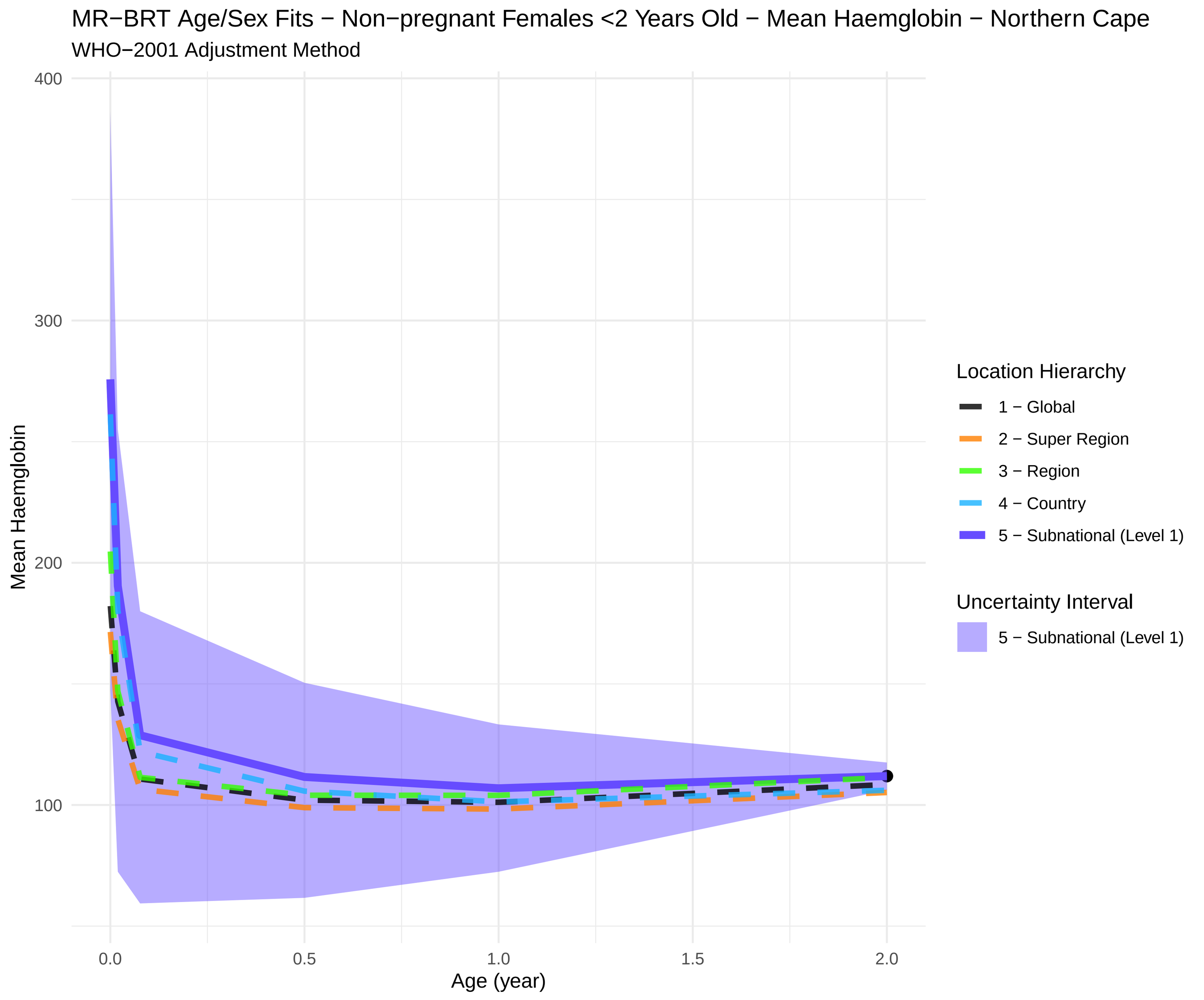
MR−BRT Age/Sex Fits − Non−pregnant Females <2 Years Old − Mean Haemglobin − Northern Cape
WHO−2001 Adjustment Method
Mean Haemglobin
Age (year)
Location Hierarchy
1 − Global
2 − Super Region
3 − Region
4 − Country
5 − Subnational (Level 1)
Uncertainty Interval
5 − Subnational (Level 1)

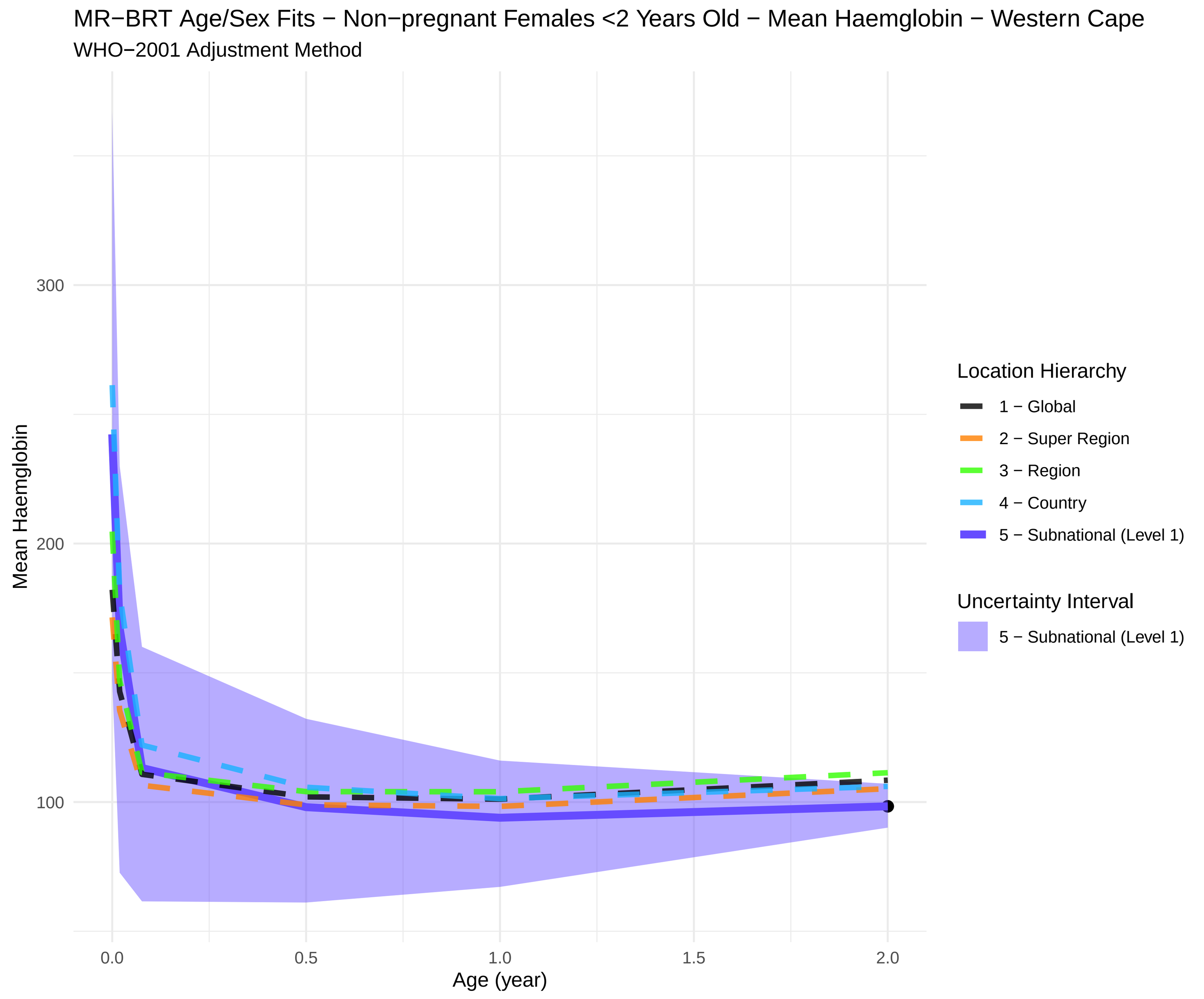
MR−BRT Age/Sex Fits − Non−pregnant Females <2 Years Old − Mean Haemglobin − Western Cape
WHO−2001 Adjustment Method
Mean Haemglobin
Age (year)
Location Hierarchy
1 − Global
2 − Super Region
3 − Region
4 − Country
5 − Subnational (Level 1)
Uncertainty Interval
5 − Subnational (Level 1)

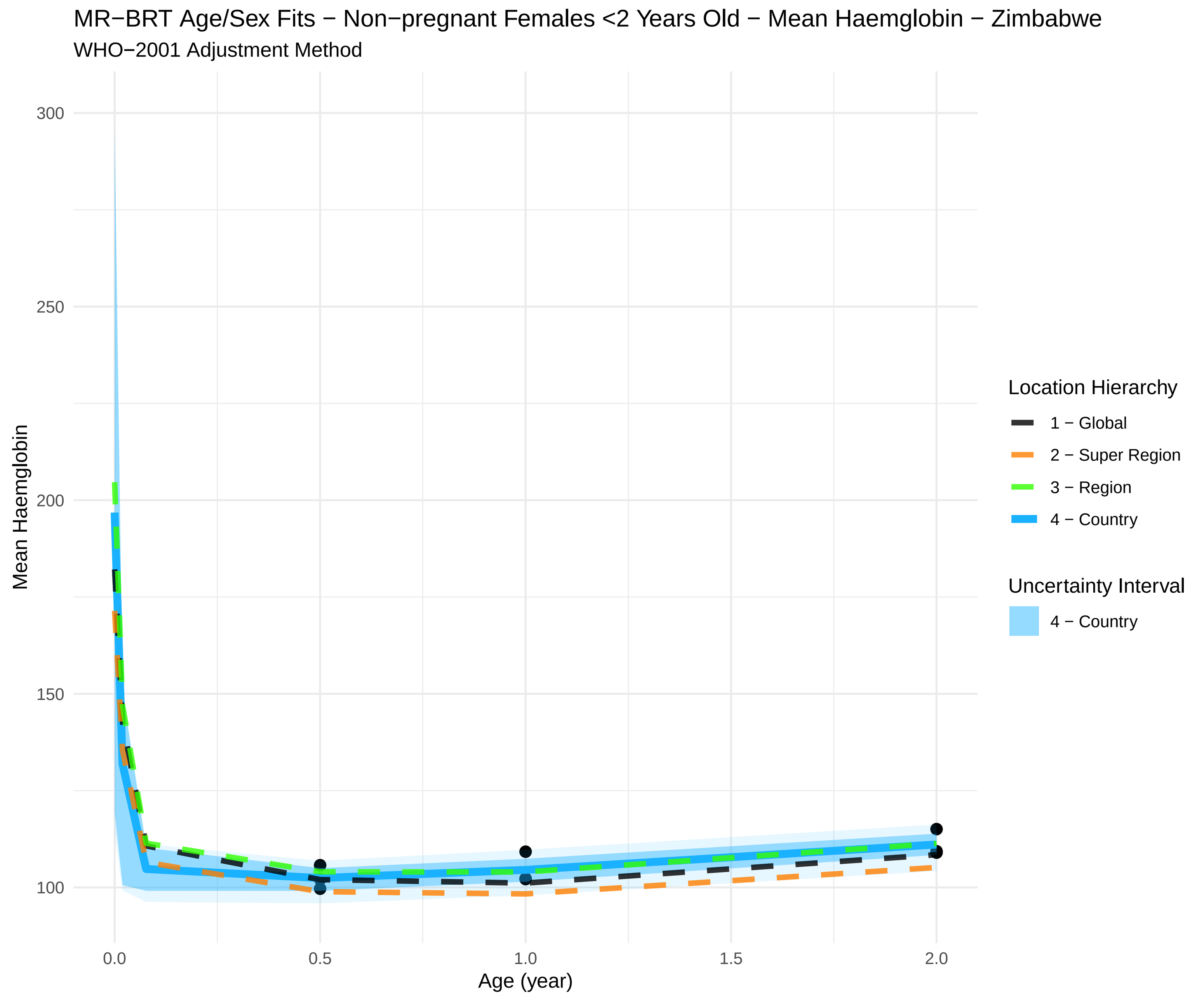
MR−BRT Age/Sex Fits − Non−pregnant Females <2 Years Old − Mean Haemglobin − Zimbabwe
WHO−2001 Adjustment Method
Mean Haemglobin
Age (year)
Location Hierarchy
1 − Global
2 − Super Region
3 − Region
4 − Country
Uncertainty Interval
4 − Country

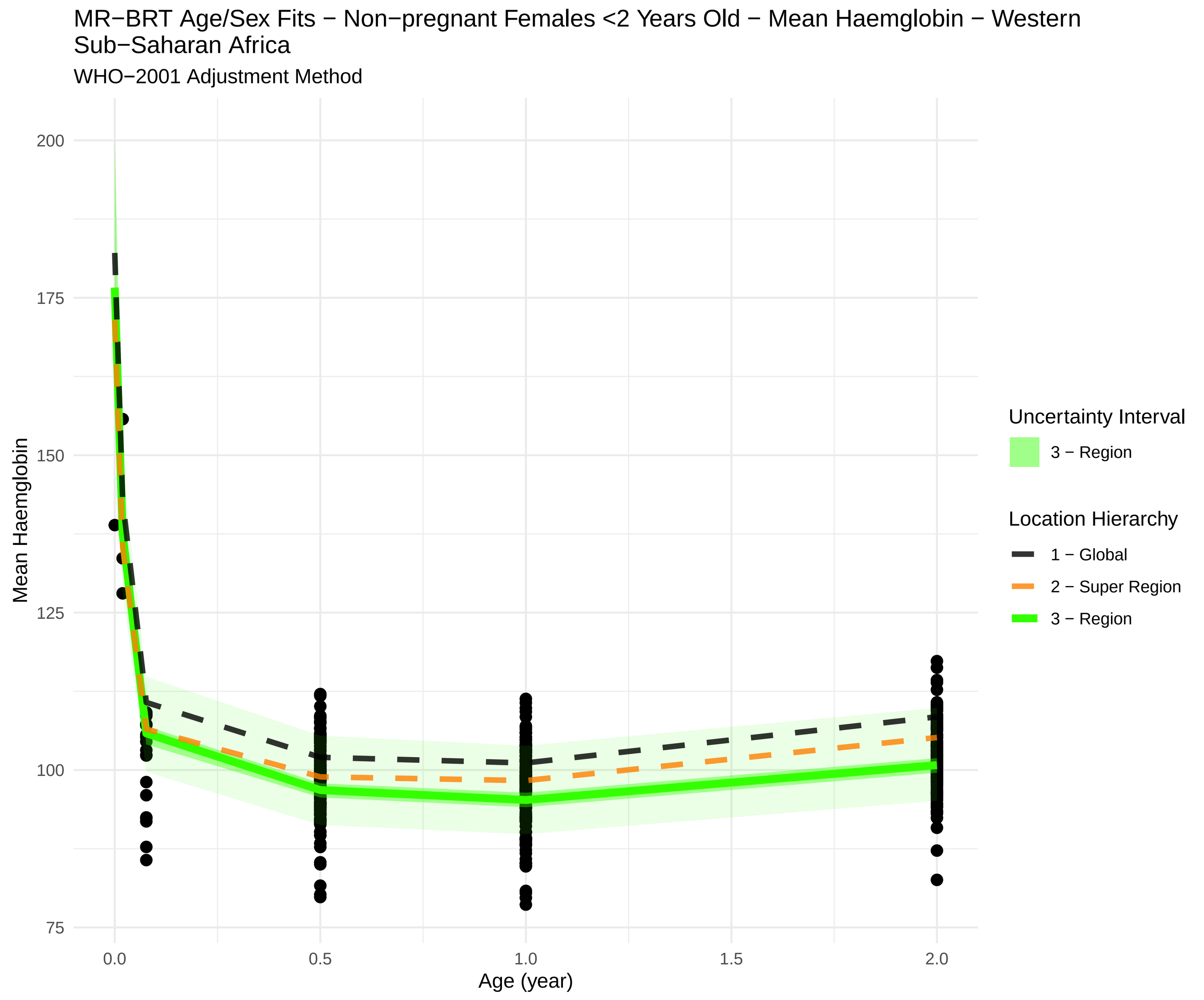
MR−BRT Age/Sex Fits − Non−pregnant Females <2 Years Old − Mean Haemglobin − Western Sub−Saharan Africa
WHO−2001 Adjustment Method
Mean Haemglobin
Age (year)
Uncertainty Interval
3 − Region
Location Hierarchy
1 − Global
2 − Super Region
3 − Region

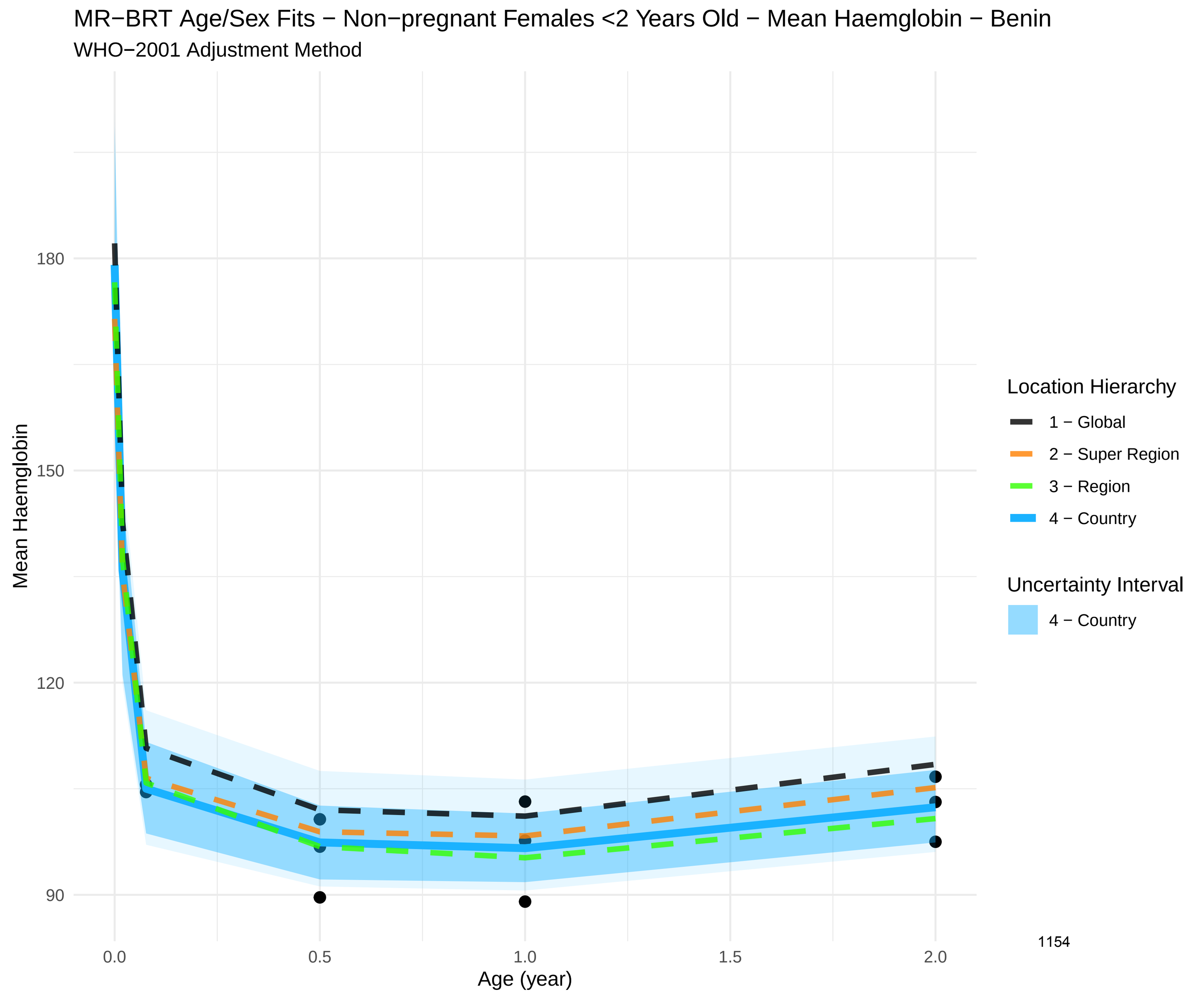
MR−BRT Age/Sex Fits − Non−pregnant Females <2 Years Old − Mean Haemglobin − Benin
WHO−2001 Adjustment Method
Mean Haemglobin
Age (year)
Location Hierarchy
1 − Global
2 − Super Region
3 − Region
4 − Country
Uncertainty Interval
4 − Country

MR−BRT Age/Sex Fits − Non-pregnant Females <2 Years Old − Mean Haemglobin − Burkina Faso
WHO−2001 Adjustment Method

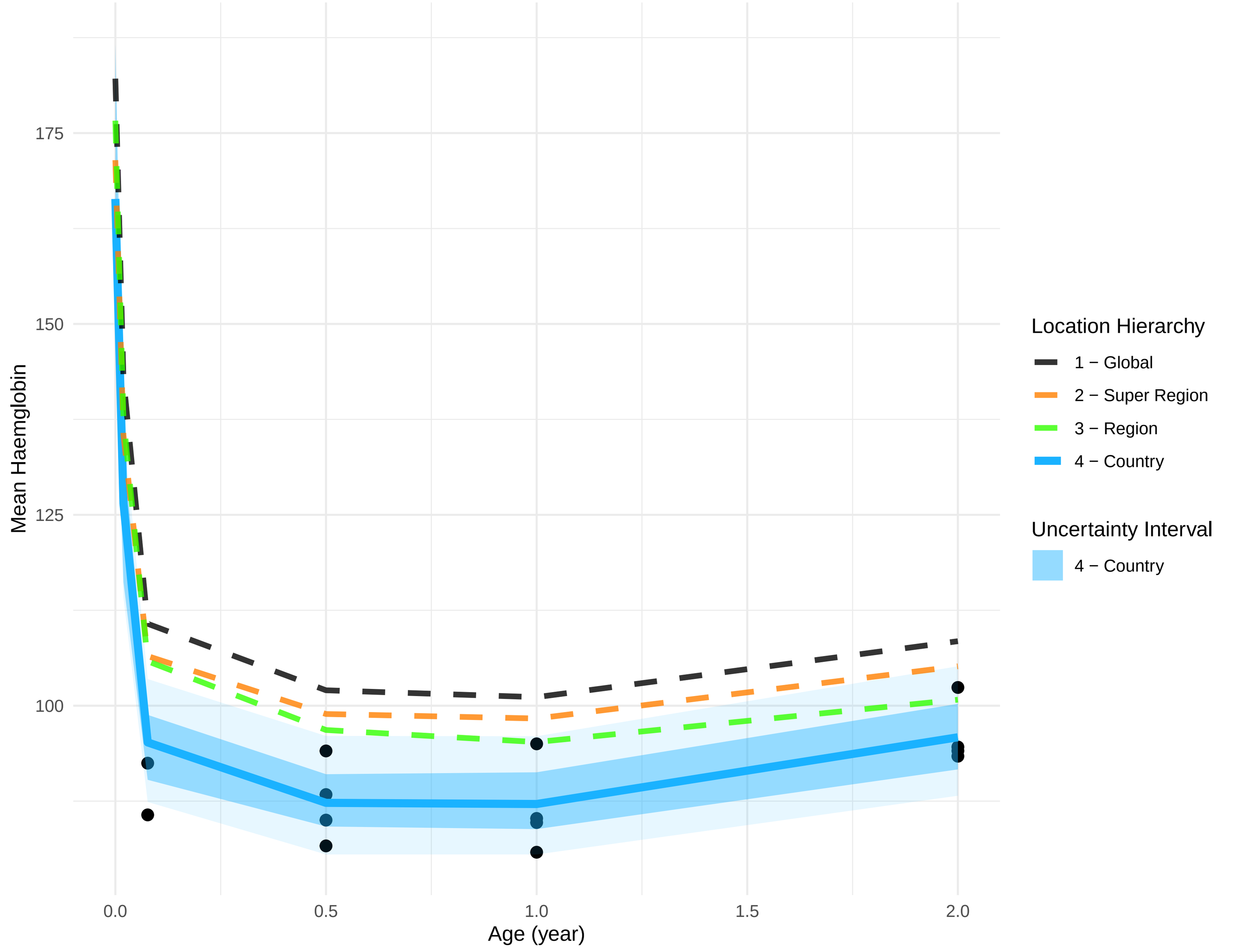

MR−BRT Age/Sex Fits − Non-pregnant Females <2 Years Old − Mean Haemglobin − Cameroon
WHO−2001 Adjustment Method

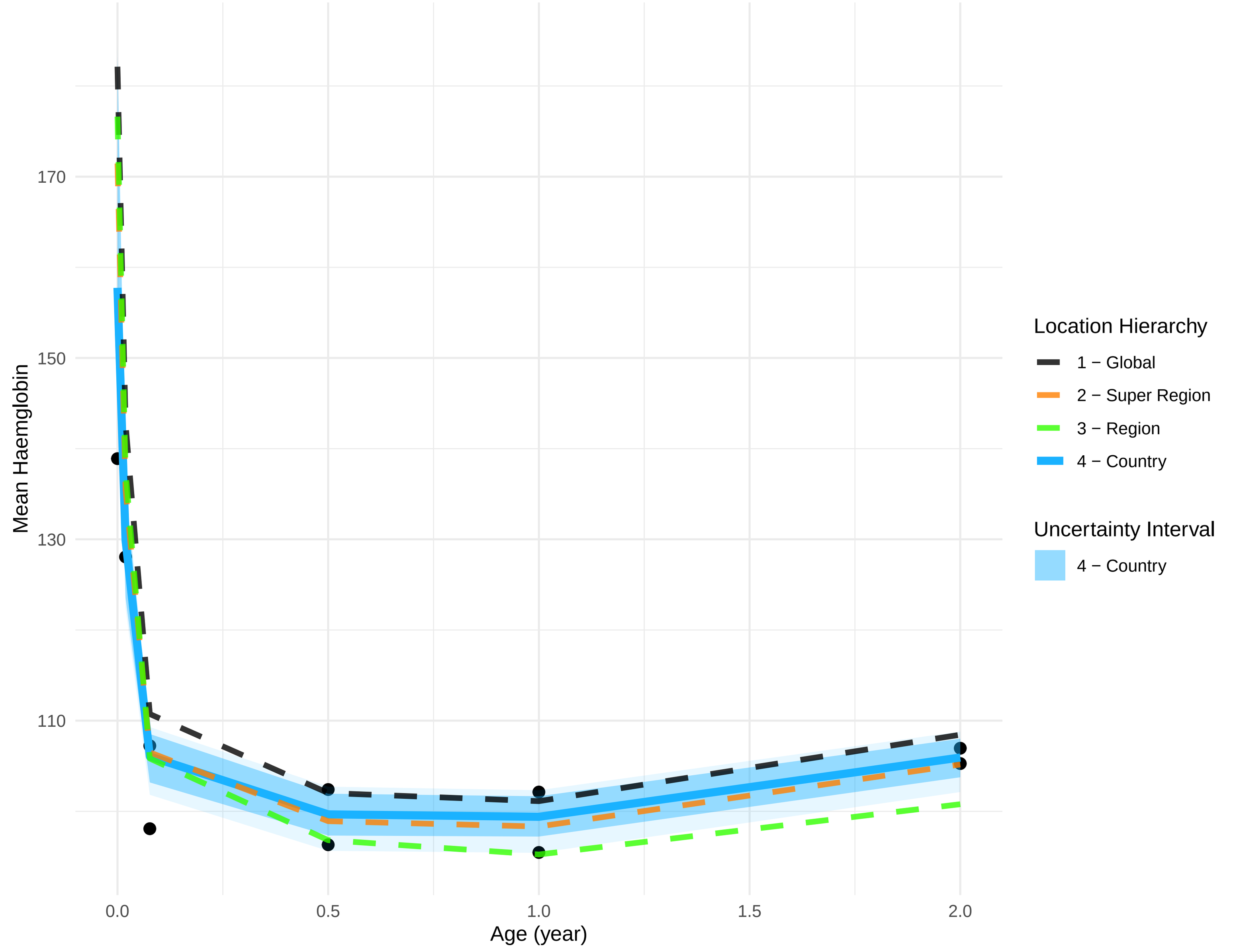

MR−BRT Age/Sex Fits − Non-pregnant Females <2 Years Old − Mean Haemglobin − Côte d'Ivoire
WHO−2001 Adjustment Method

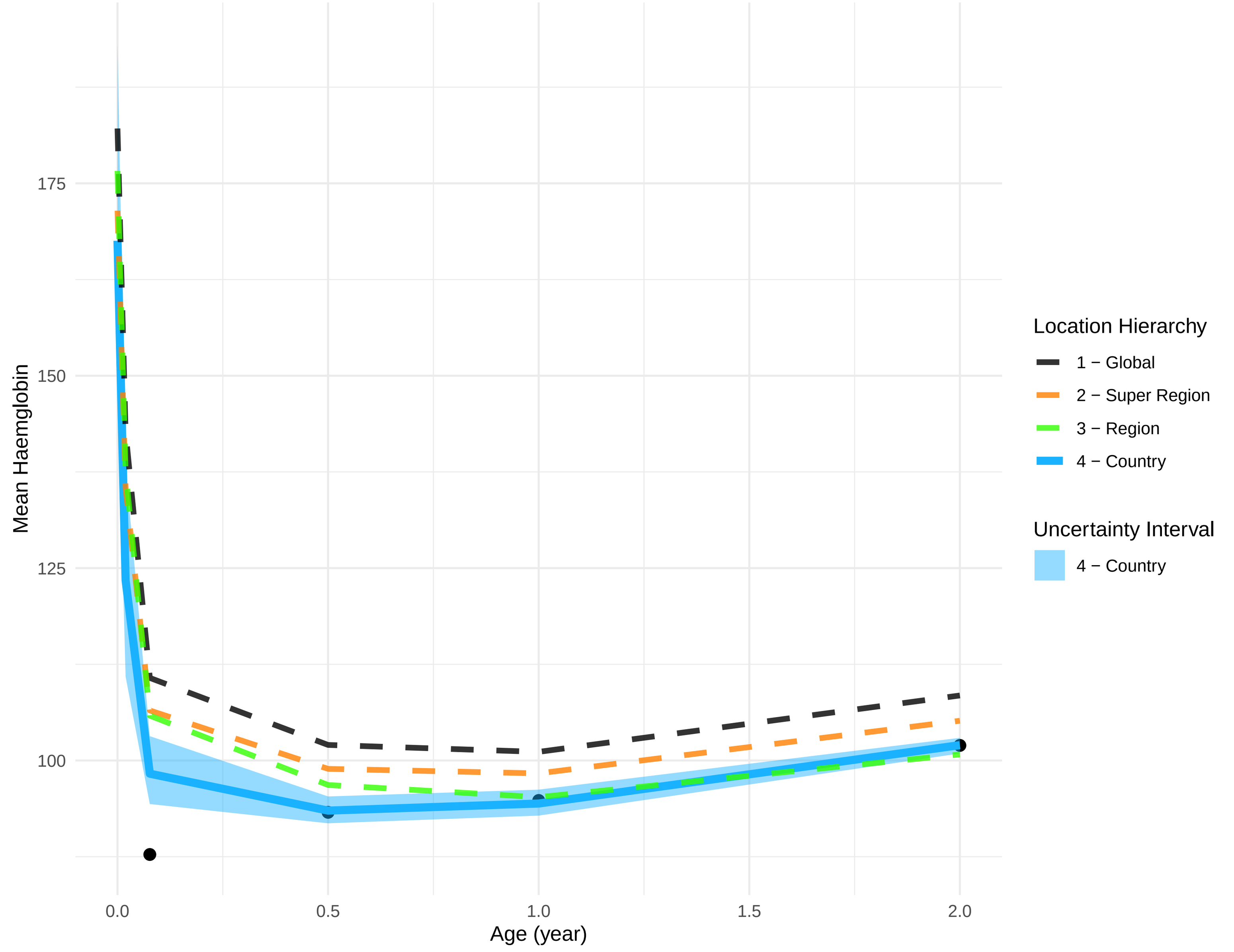

MR−BRT Age/Sex Fits − Non-pregnant Females <2 Years Old − Mean Haemglobin − Gambia
WHO−2001 Adjustment Method

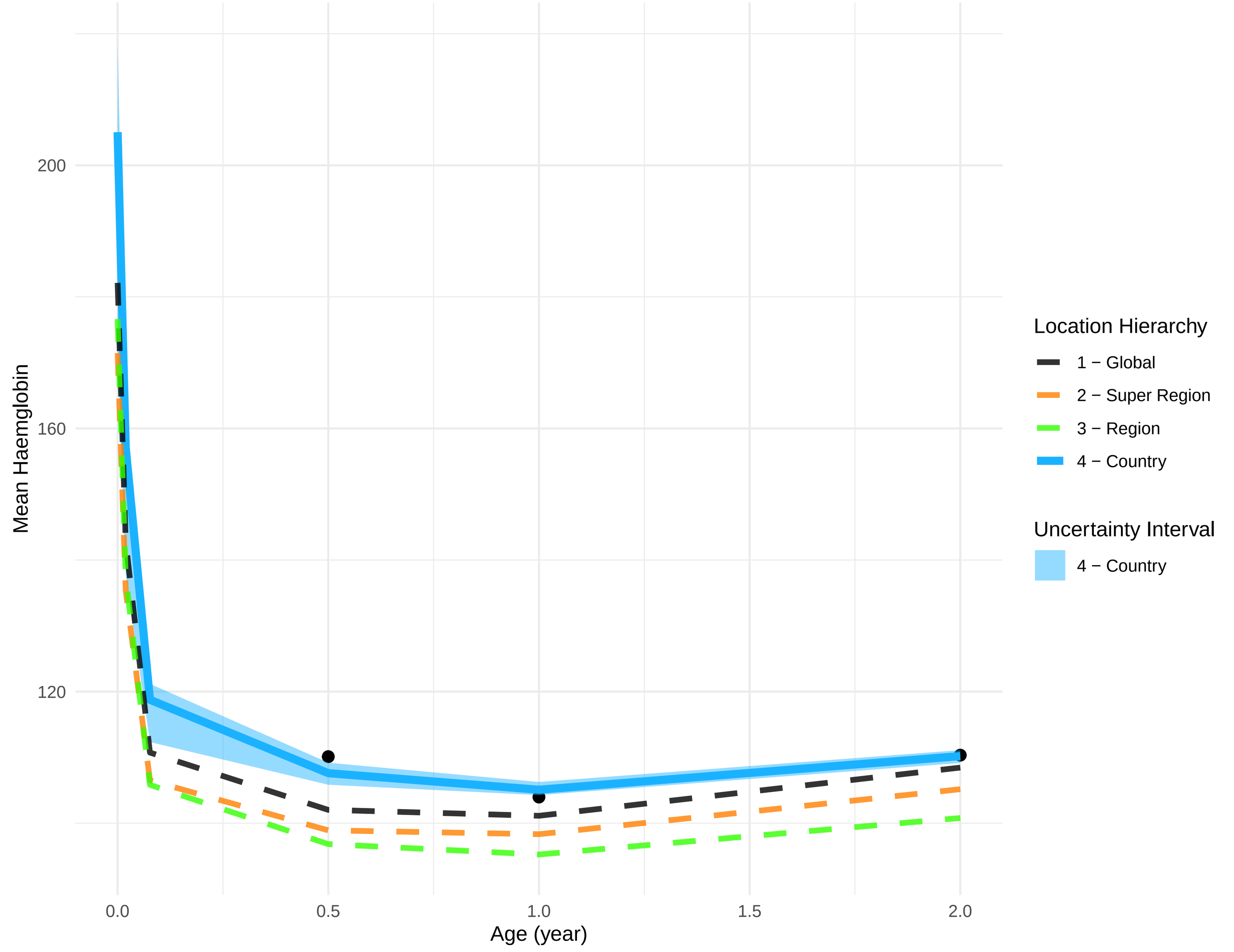

MR−BRT Age/Sex Fits − Non-pregnant Females <2 Years Old − Mean Haemglobin − Ghana
WHO−2001 Adjustment Method

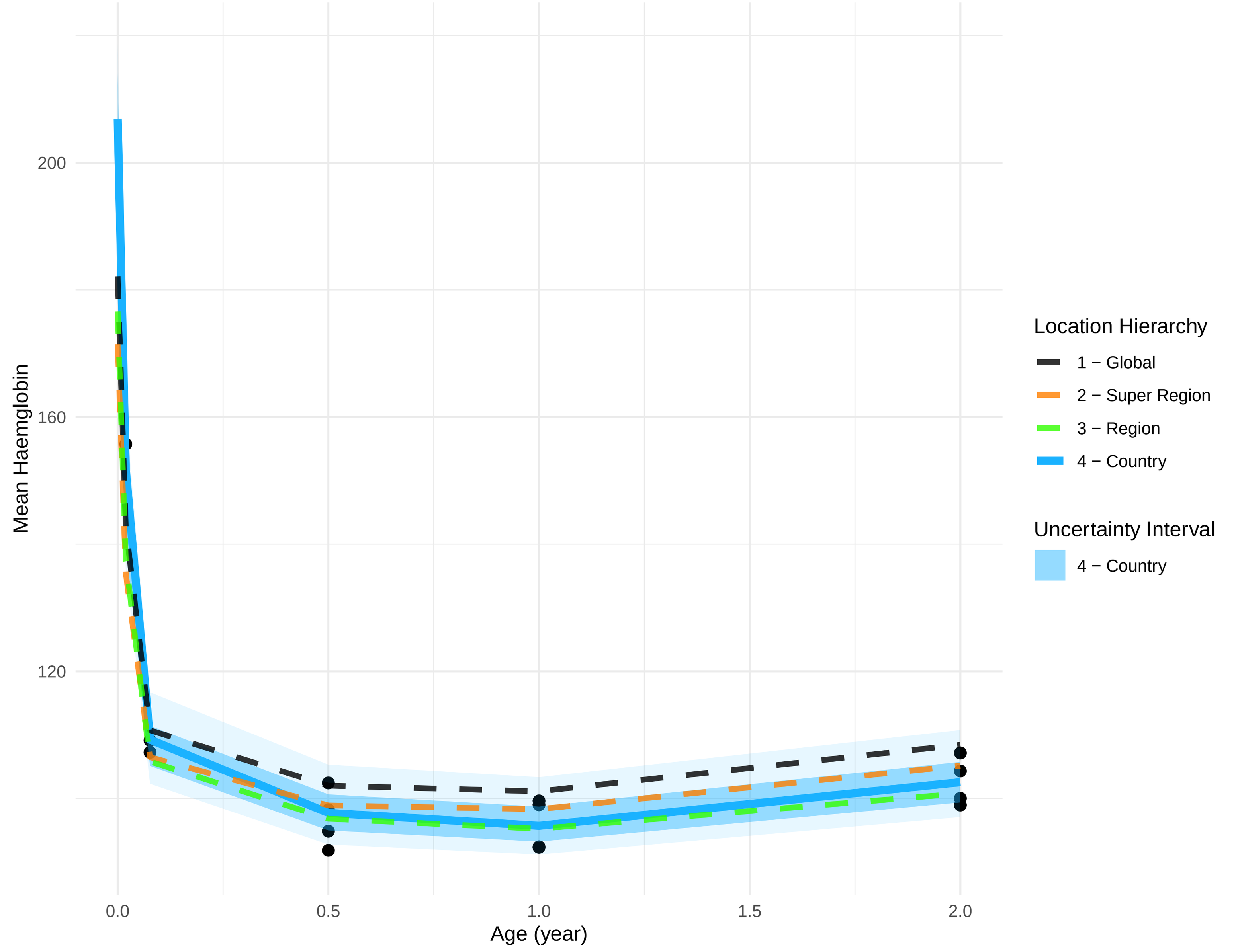

MR−BRT Age/Sex Fits − Non-pregnant Females <2 Years Old − Mean Haemglobin − Guinea
WHO−2001 Adjustment Method

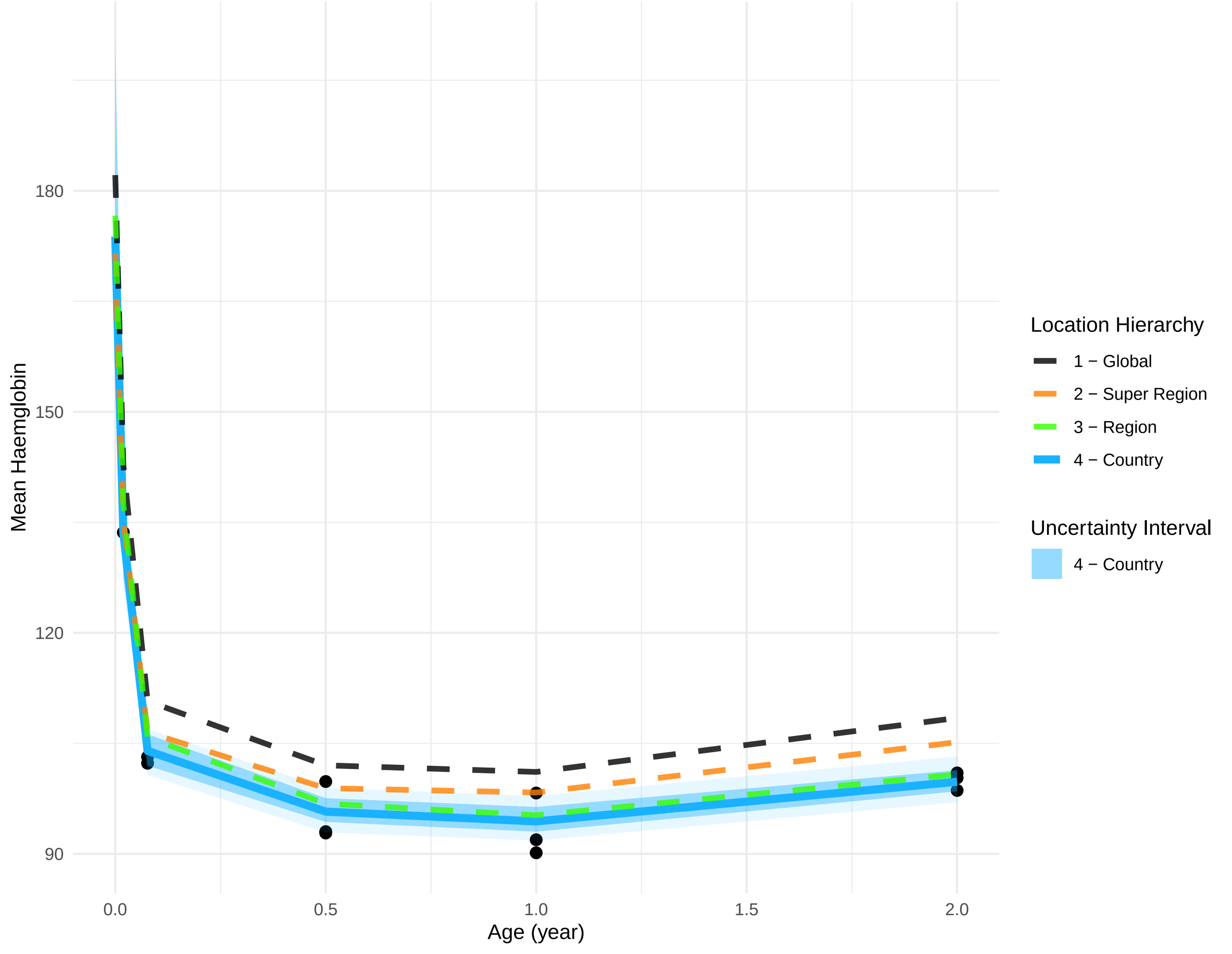

MR−BRT Age/Sex Fits − Non-pregnant Females <2 Years Old − Mean Haemglobin − Liberia
WHO−2001 Adjustment Method

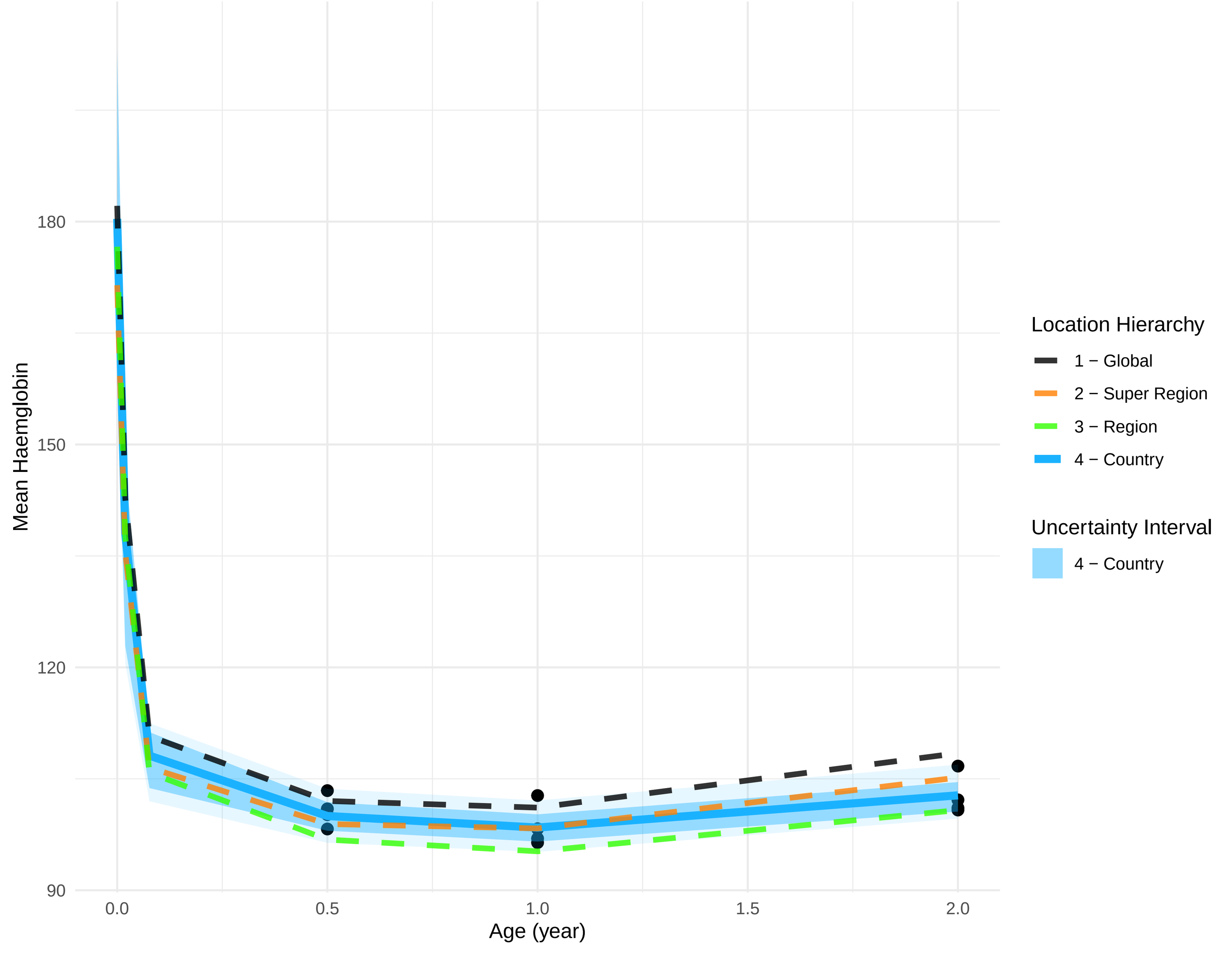

MR−BRT Age/Sex Fits − Non-pregnant Females <2 Years Old − Mean Haemglobin − Mali
WHO−2001 Adjustment Method

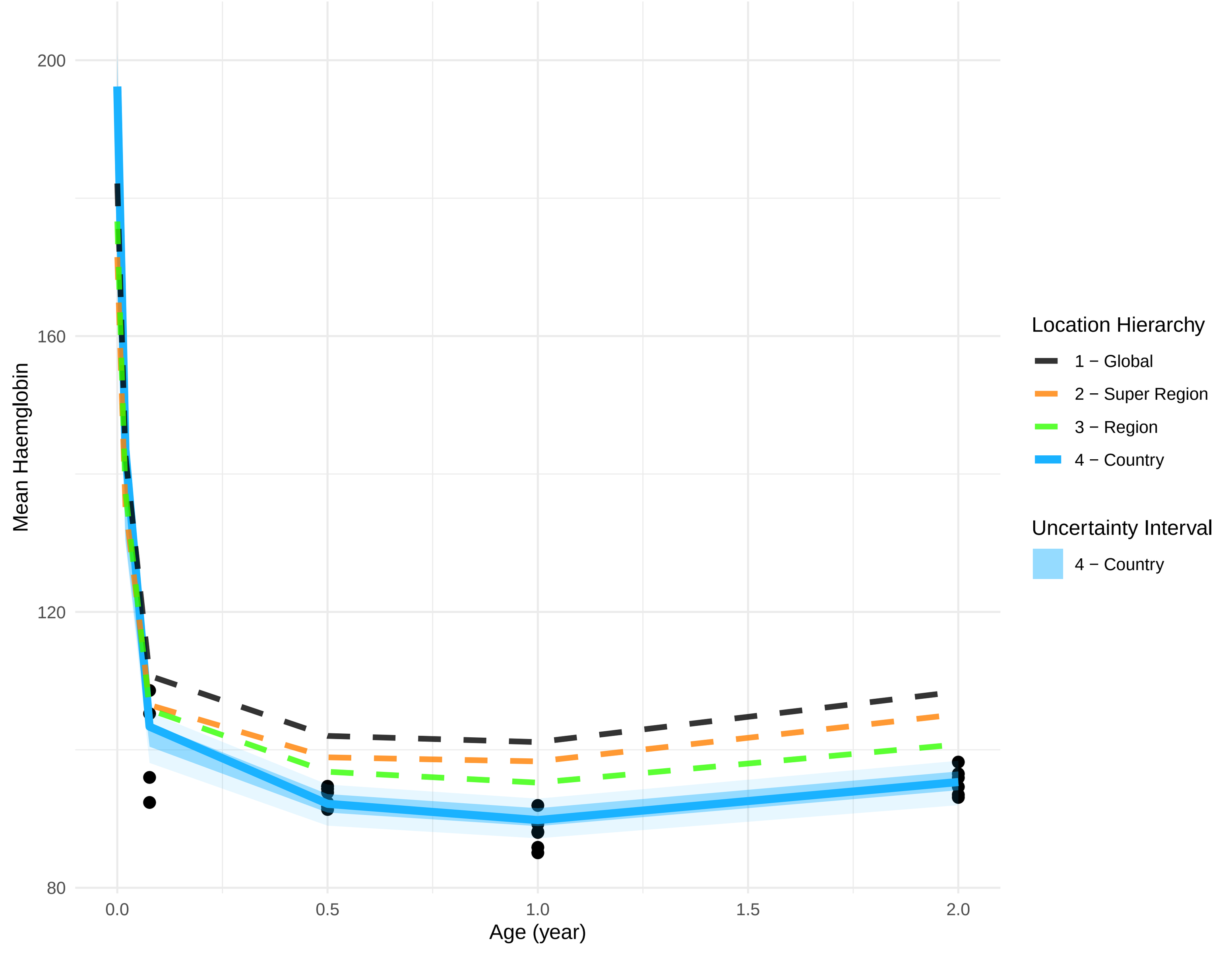

MR−BRT Age/Sex Fits − Non-pregnant Females <2 Years Old − Mean Haemglobin − Niger
WHO−2001 Adjustment Method

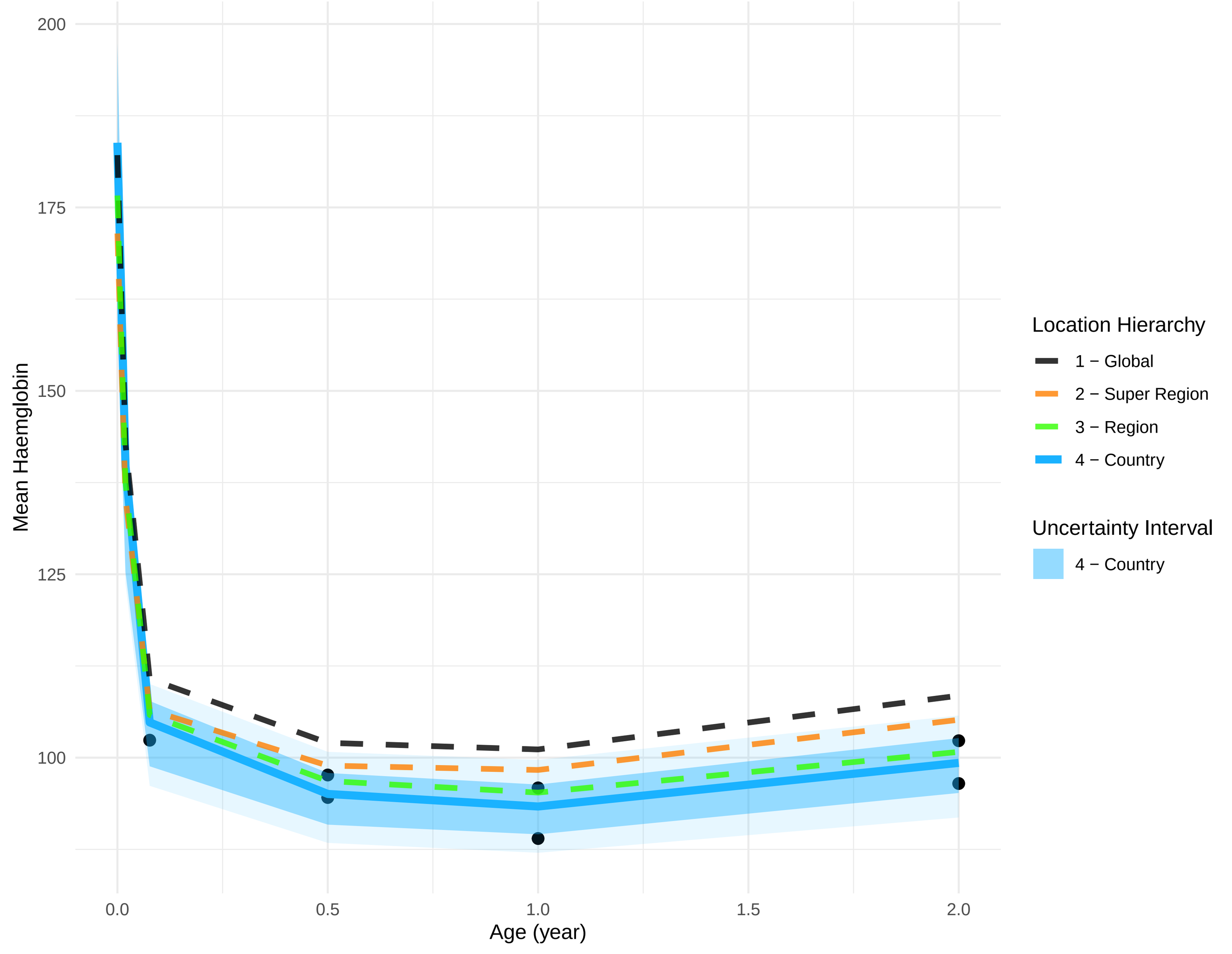

MR−BRT Age/Sex Fits − Non-pregnant Females <2 Years Old − Mean Haemglobin − Nigeria
WHO−2001 Adjustment Method

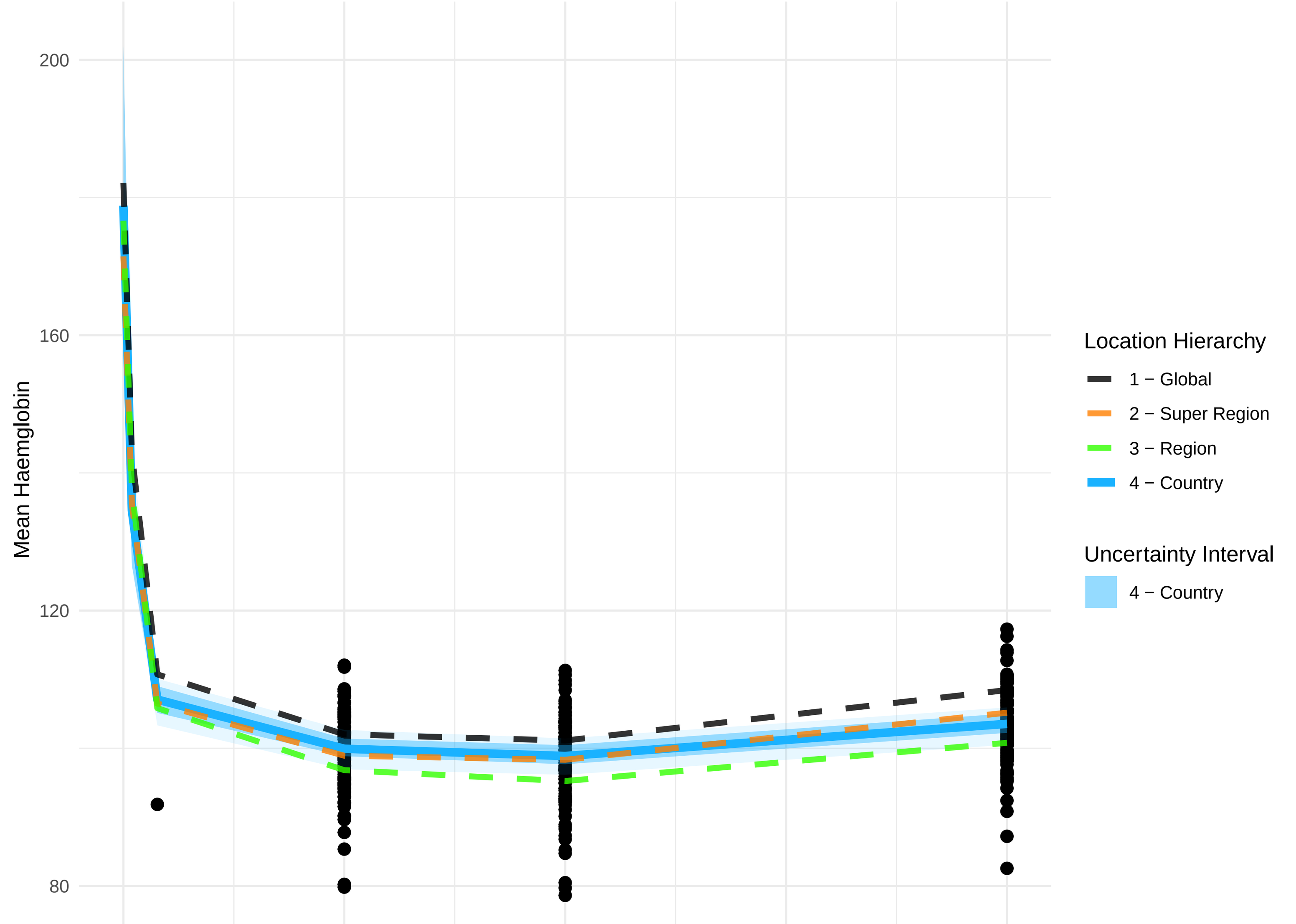

MR−BRT Age/Sex Fits − Non−pregnant Females <2 Years Old − Mean Haemglobin − Abia
WHO−2001 Adjustment Method

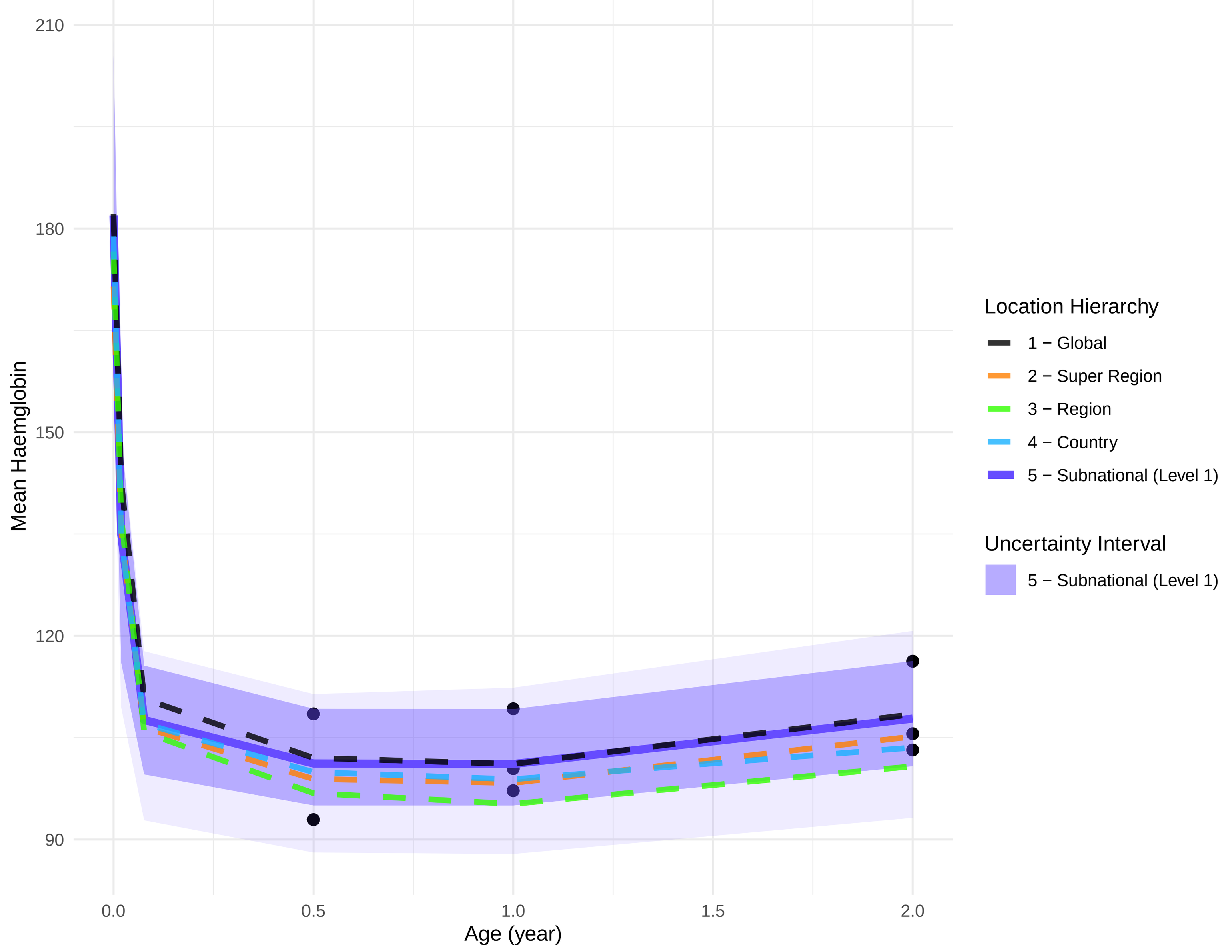

MR−BRT Age/Sex Fits − Non−pregnant Females <2 Years Old − Mean Haemglobin − Adamawa
WHO−2001 Adjustment Method

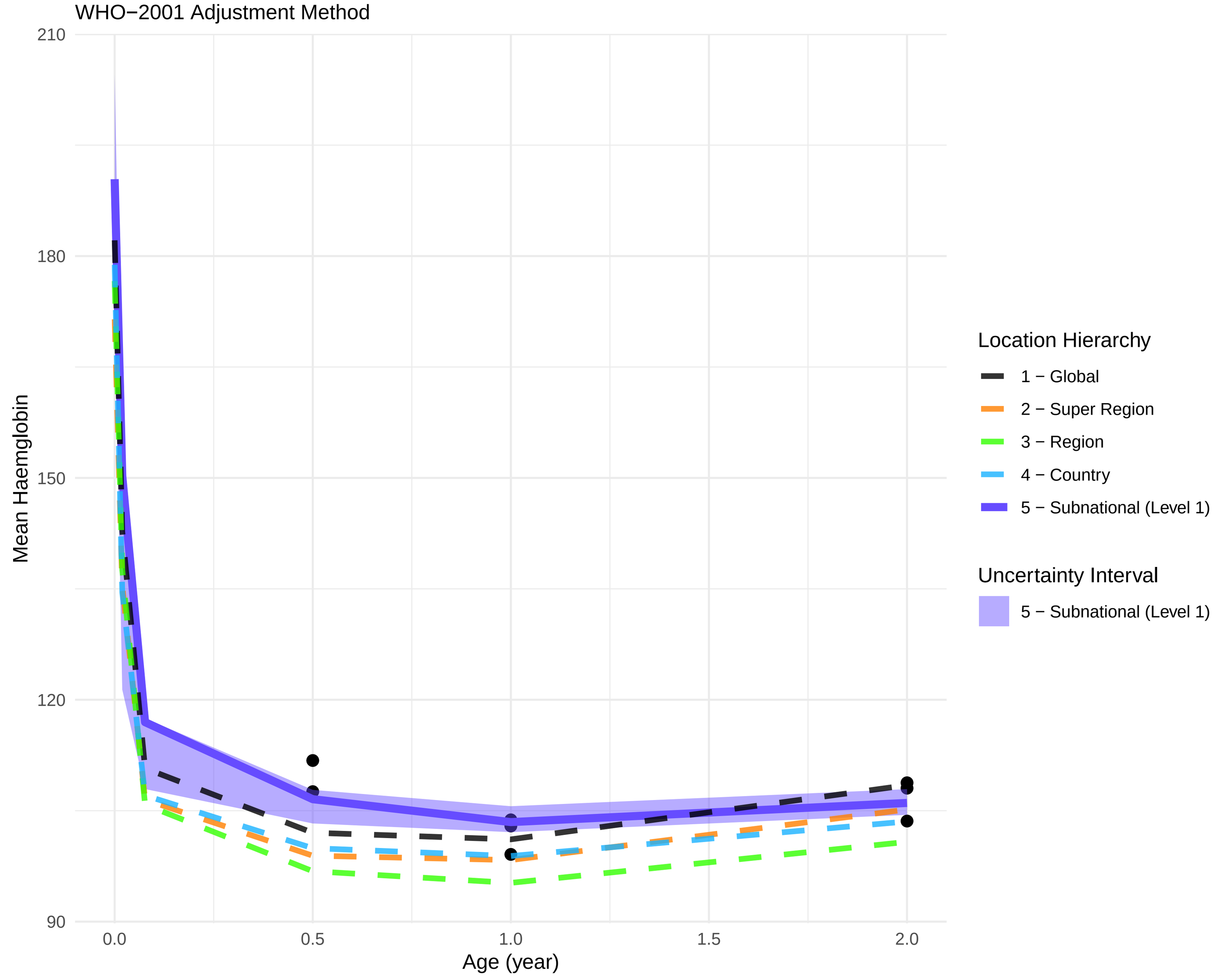

MR−BRT Age/Sex Fits − Non−pregnant Females <2 Years Old − Mean Haemglobin − Akwa Ibom
WHO−2001 Adjustment Method

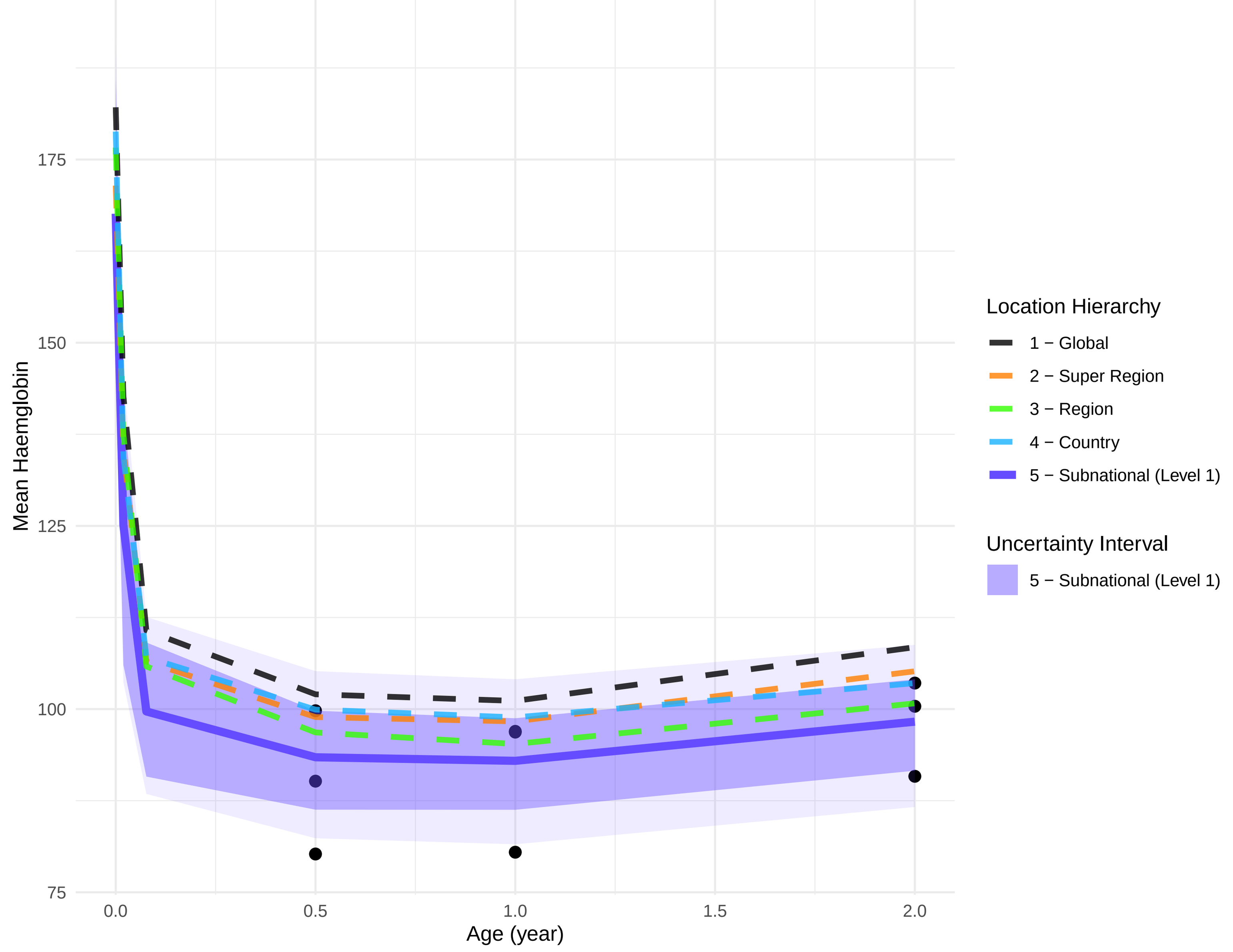

MR−BRT Age/Sex Fits − Non−pregnant Females <2 Years Old − Mean Haemglobin − Anambra
WHO−2001 Adjustment Method

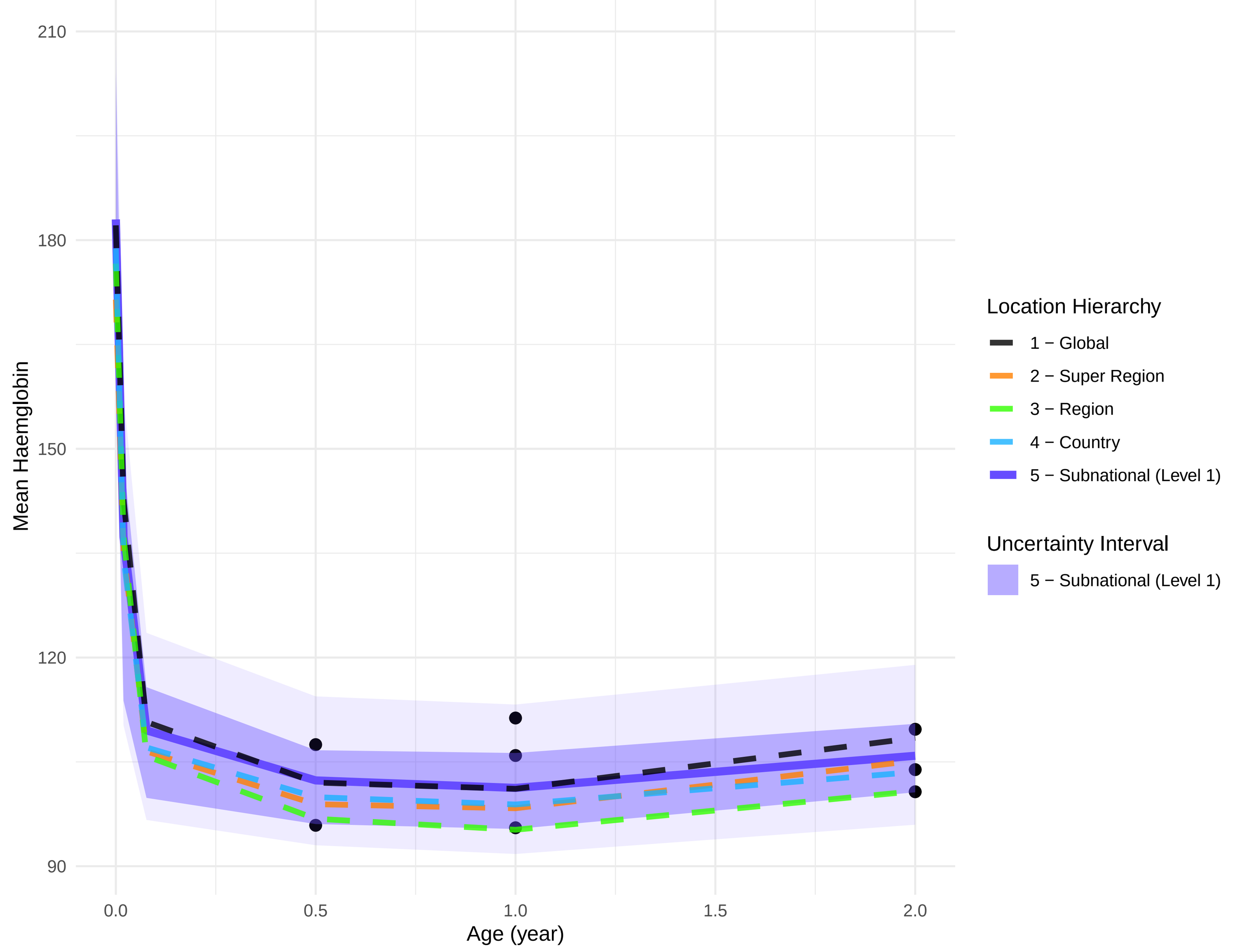

MR−BRT Age/Sex Fits − Non−pregnant Females <2 Years Old − Mean Haemglobin − Bauchi
WHO−2001 Adjustment Method

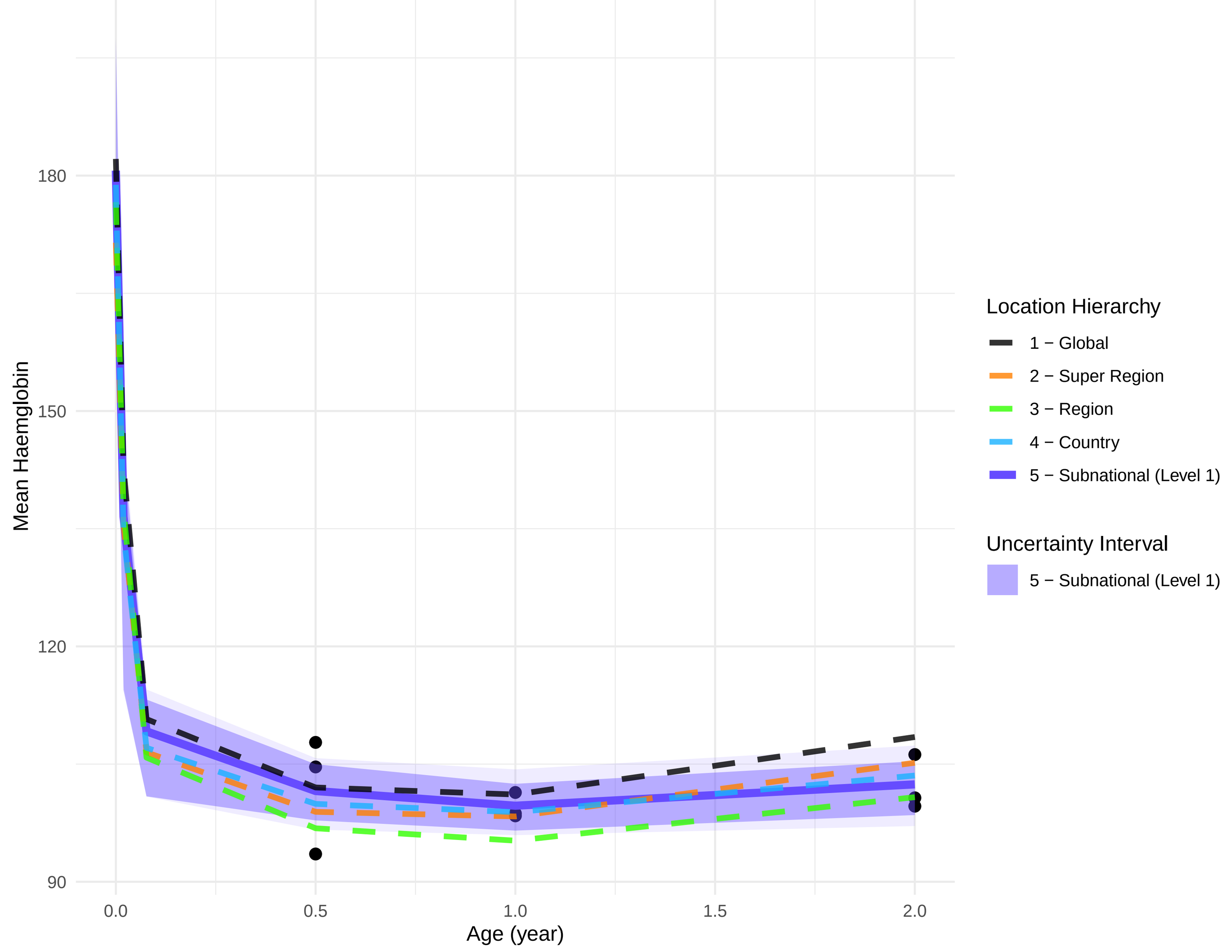

MR−BRT Age/Sex Fits − Non−pregnant Females <2 Years Old − Mean Haemglobin − Bayelsa
WHO−2001 Adjustment Method

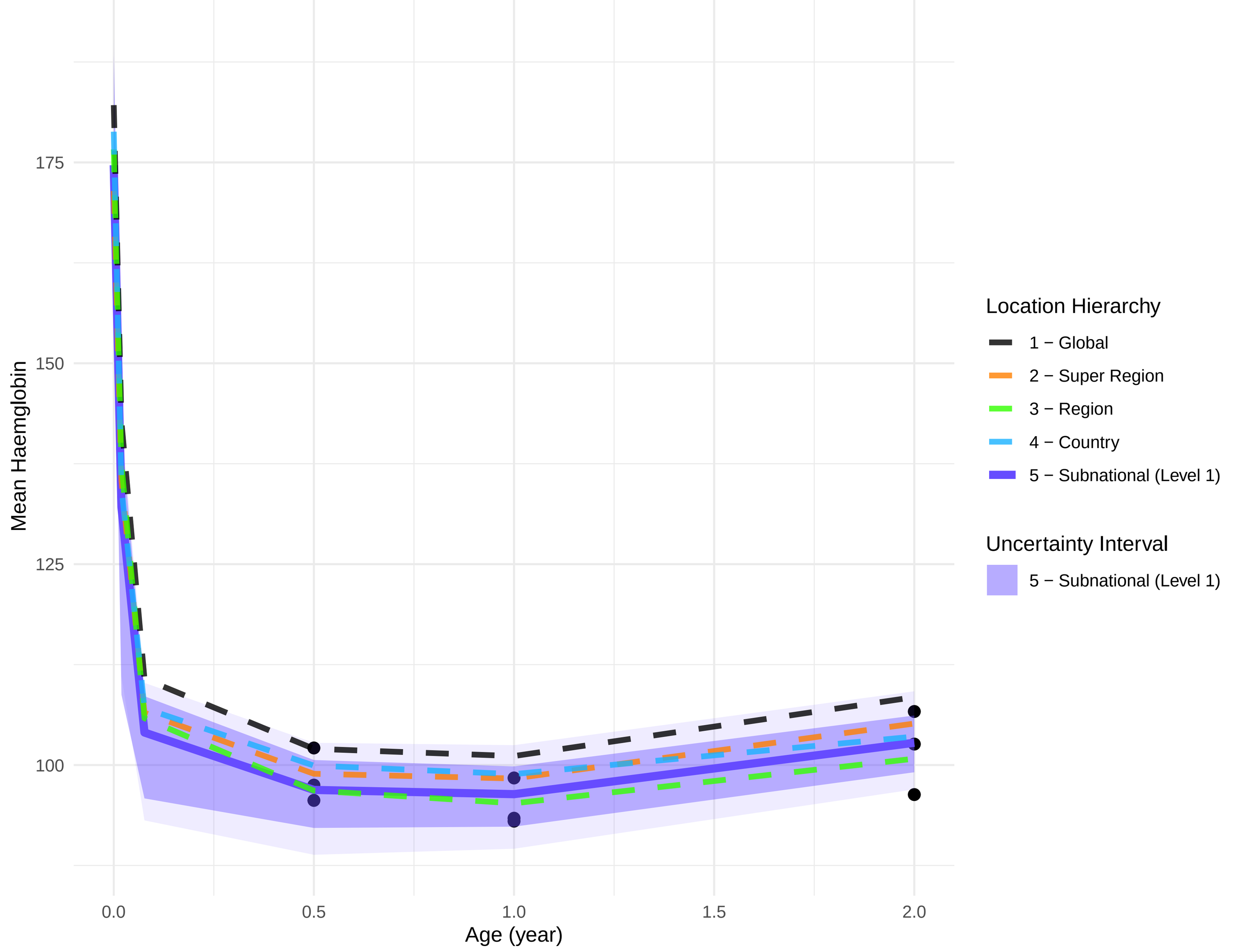

MR−BRT Age/Sex Fits − Non−pregnant Females <2 Years Old − Mean Haemglobin − Benue
WHO−2001 Adjustment Method

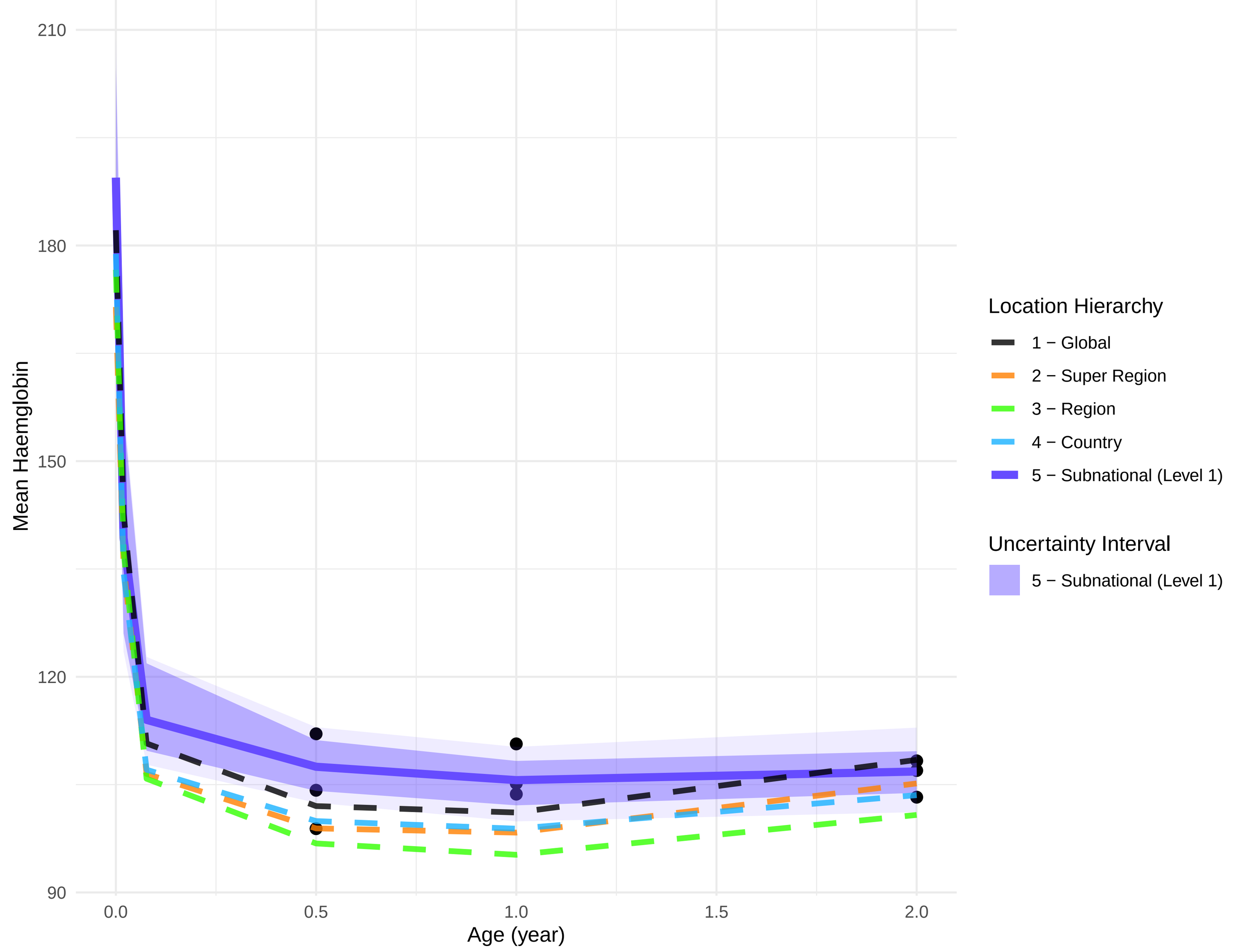

MR−BRT Age/Sex Fits − Non−pregnant Females <2 Years Old − Mean Haemglobin − Borno
WHO−2001 Adjustment Method

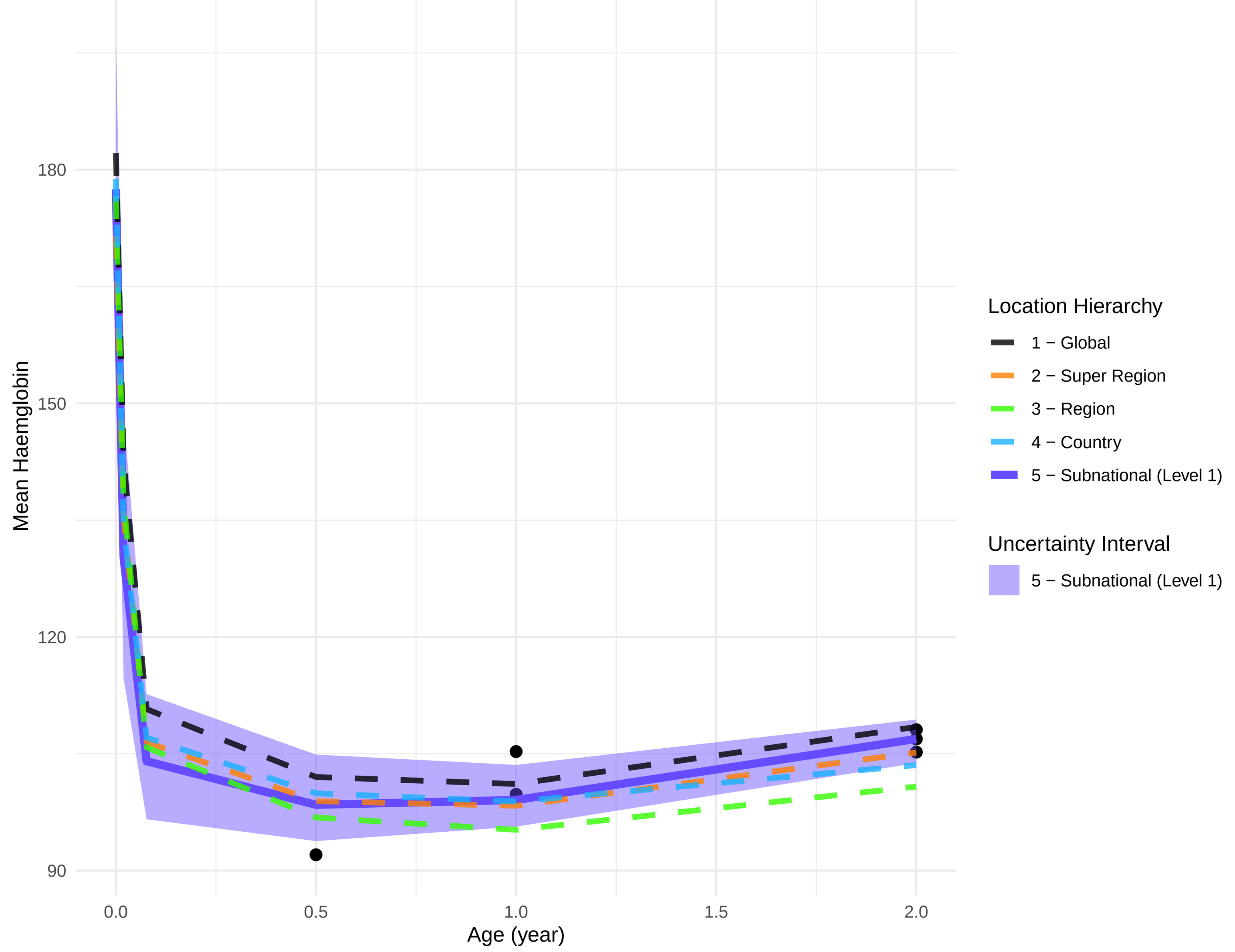

MR−BRT Age/Sex Fits − Non−pregnant Females <2 Years Old − Mean Haemglobin − Cross River
WHO−2001 Adjustment Method

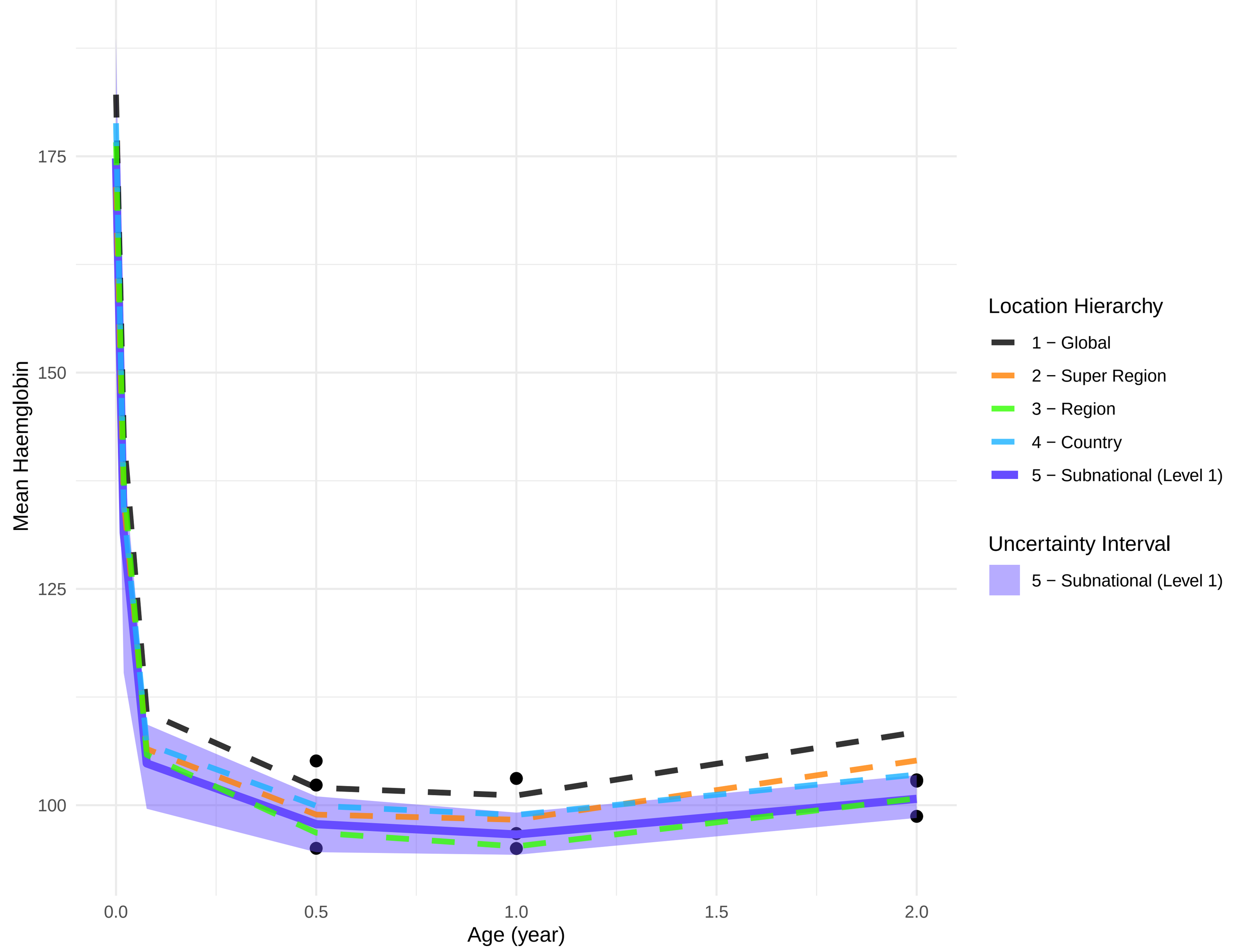

MR−BRT Age/Sex Fits − Non−pregnant Females <2 Years Old − Mean Haemglobin − Delta
WHO−2001 Adjustment Method

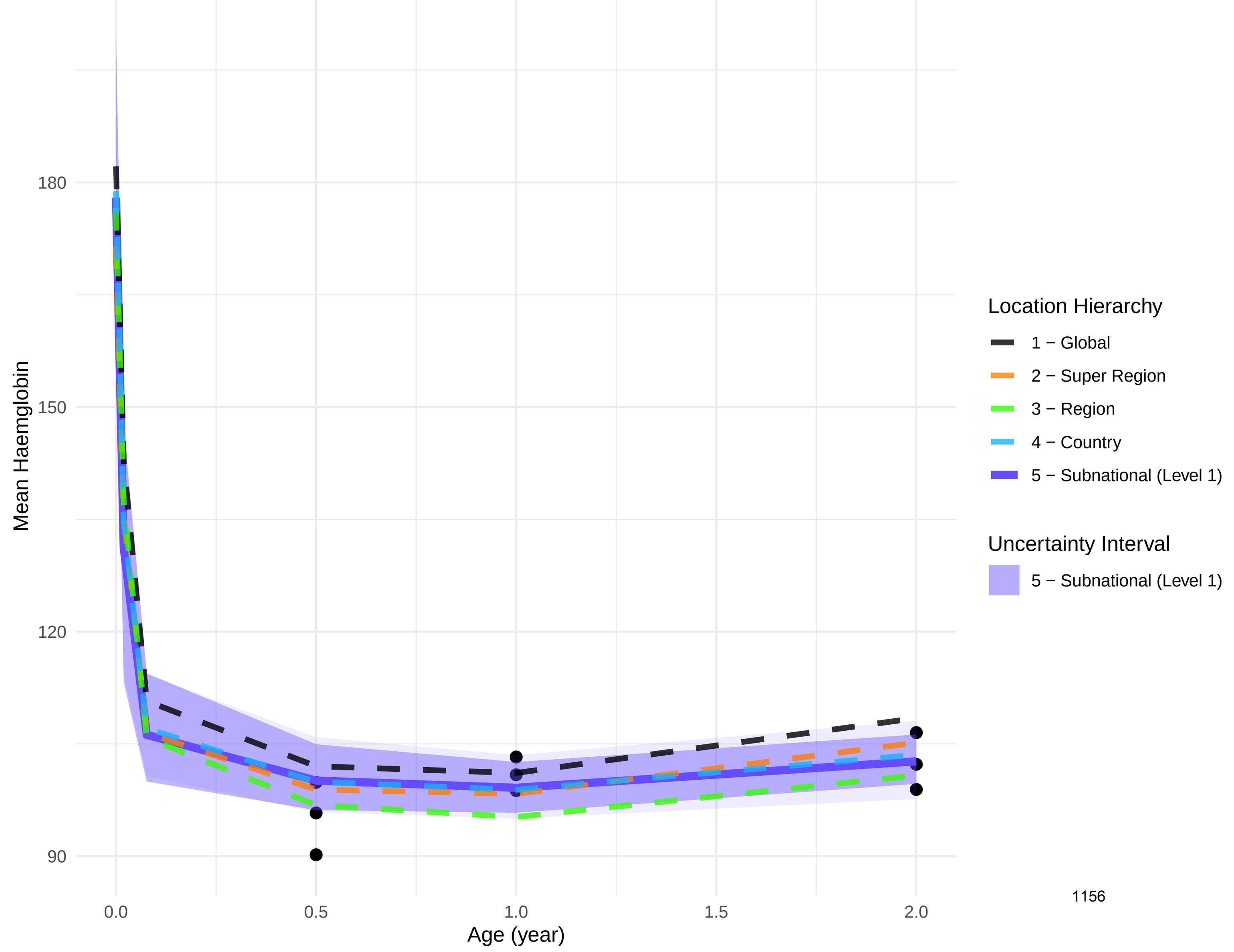

MR−BRT Age/Sex Fits − Non−pregnant Females <2 Years Old − Mean Haemglobin − Ebonyi
WHO−2001 Adjustment Method

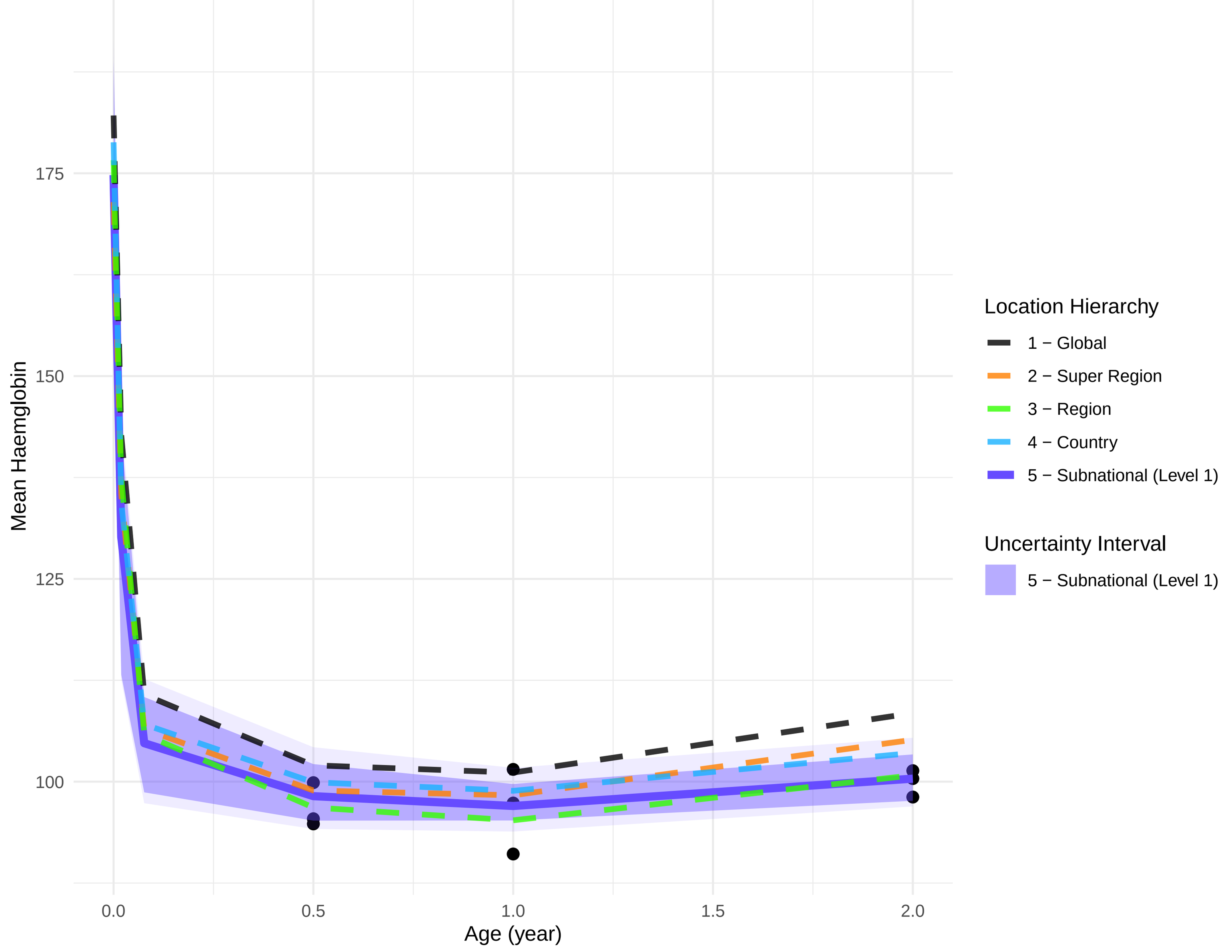

MR−BRT Age/Sex Fits − Non−pregnant Females <2 Years Old − Mean Haemglobin − Edo
WHO−2001 Adjustment Method

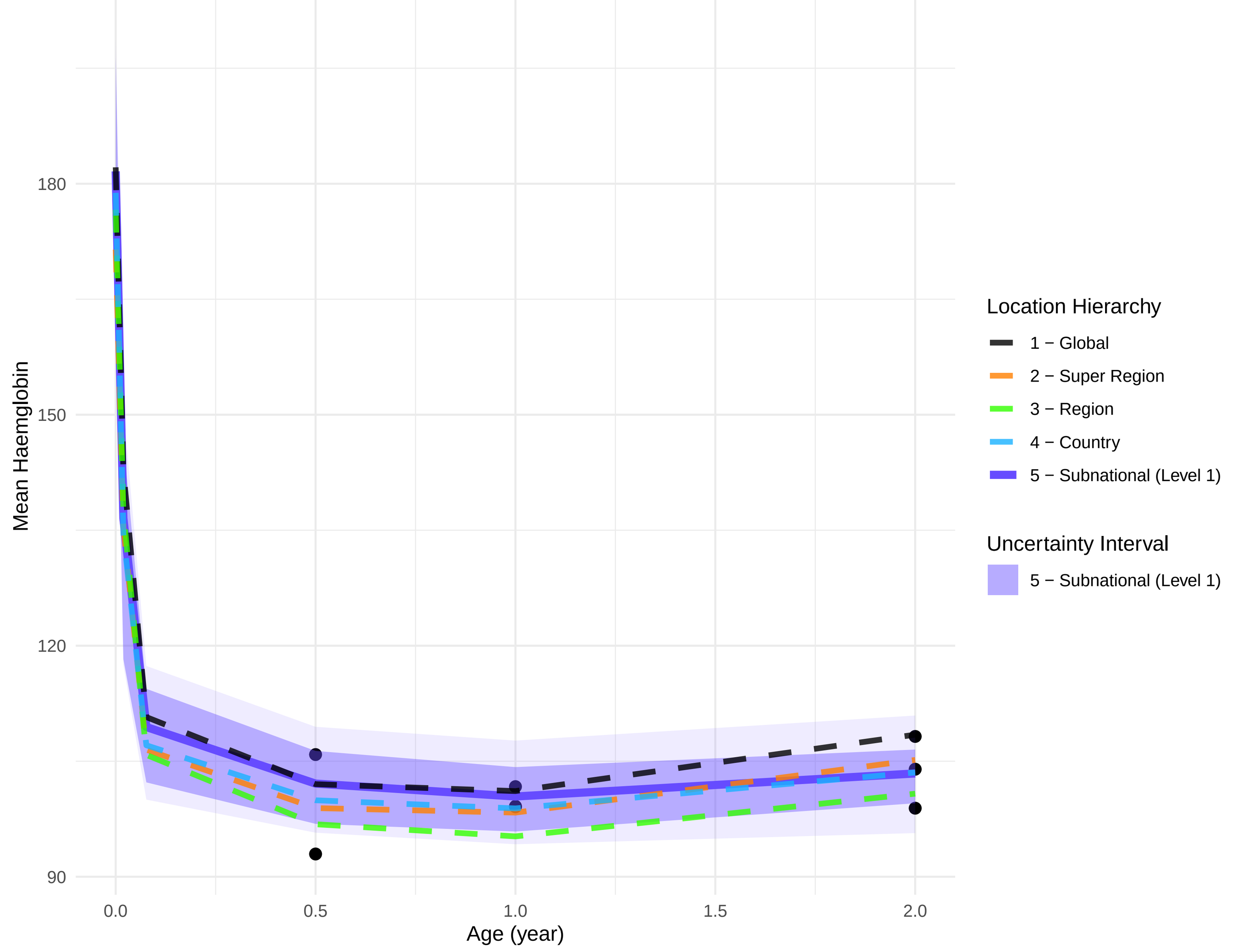

MR−BRT Age/Sex Fits − Non−pregnant Females <2 Years Old − Mean Haemglobin − Ekiti
WHO−2001 Adjustment Method

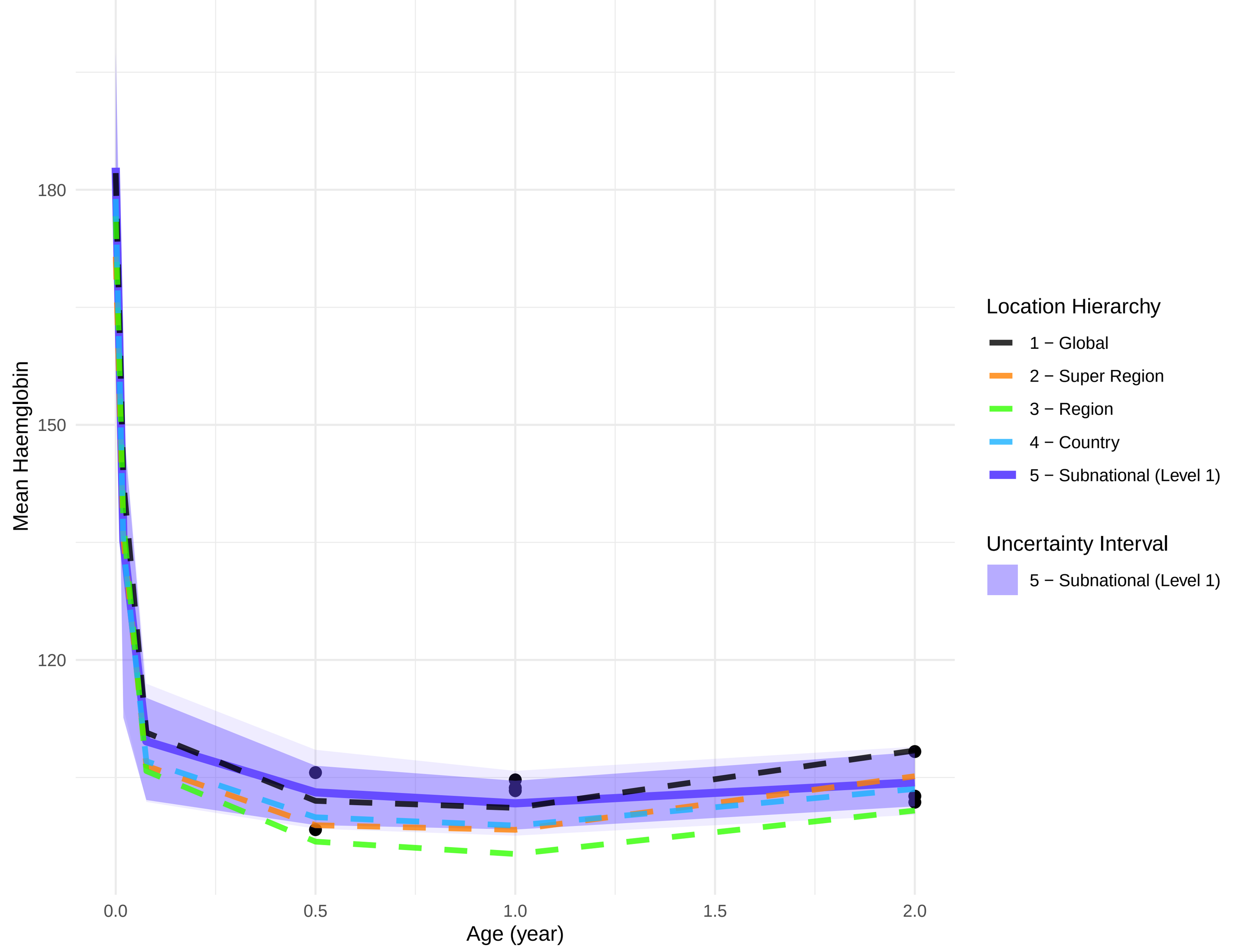

MR−BRT Age/Sex Fits − Non−pregnant Females <2 Years Old − Mean Haemglobin − Enugu
WHO−2001 Adjustment Method

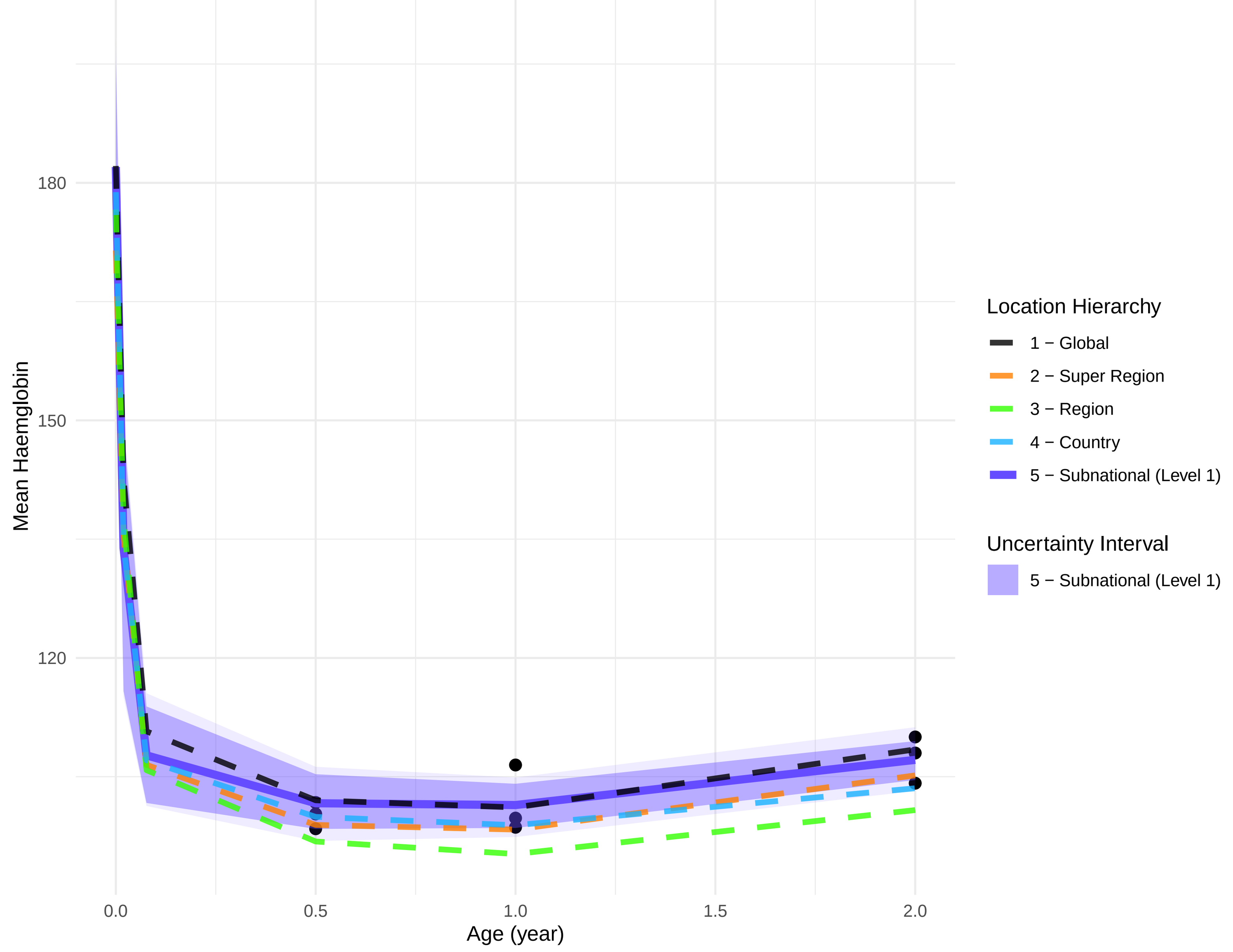

MR−BRT Age/Sex Fits − Non−pregnant Females <2 Years Old − Mean Haemglobin − FCT (Abuja)
WHO−2001 Adjustment Method

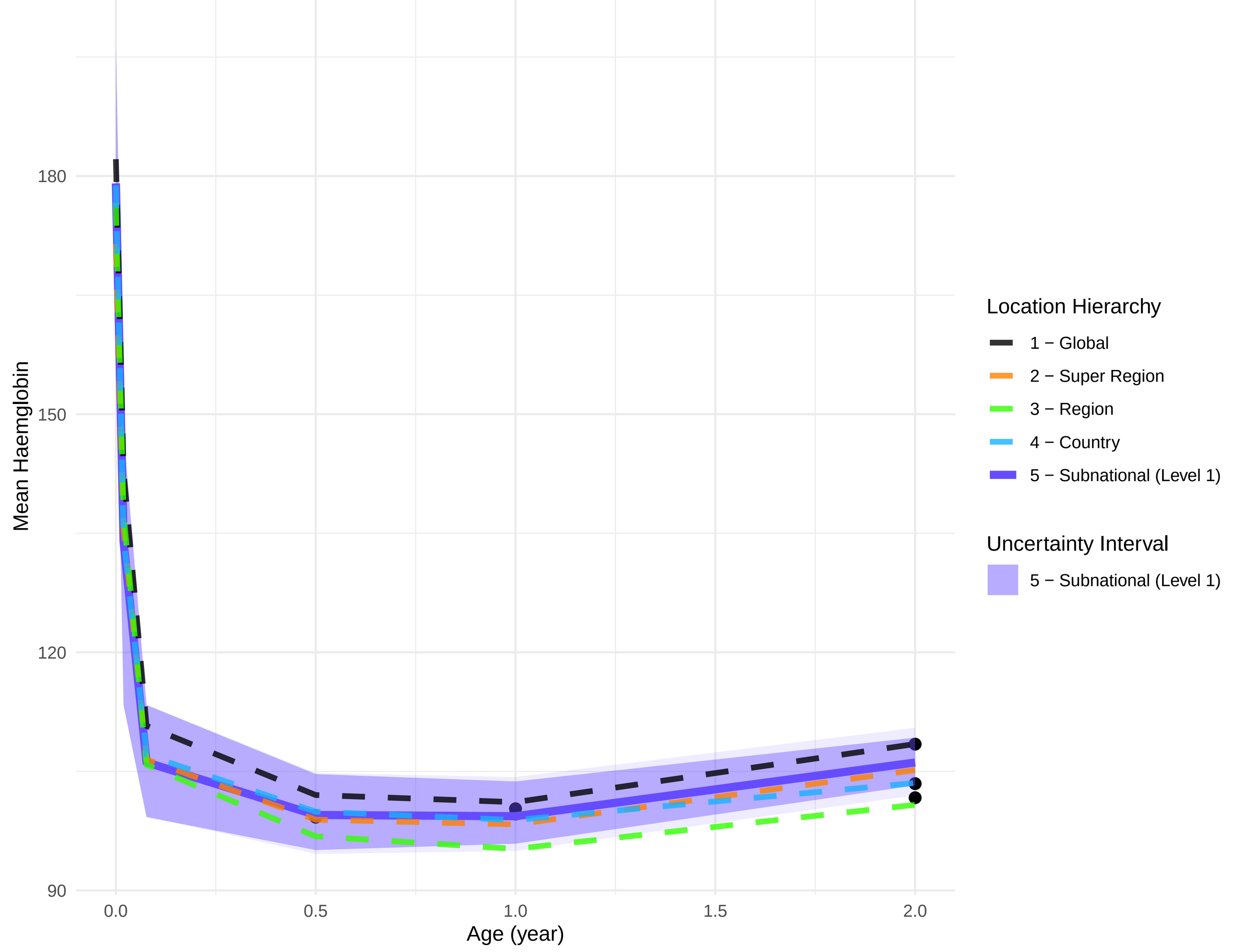

MR−BRT Age/Sex Fits − Non−pregnant Females <2 Years Old − Mean Haemglobin − Gombe
WHO−2001 Adjustment Method

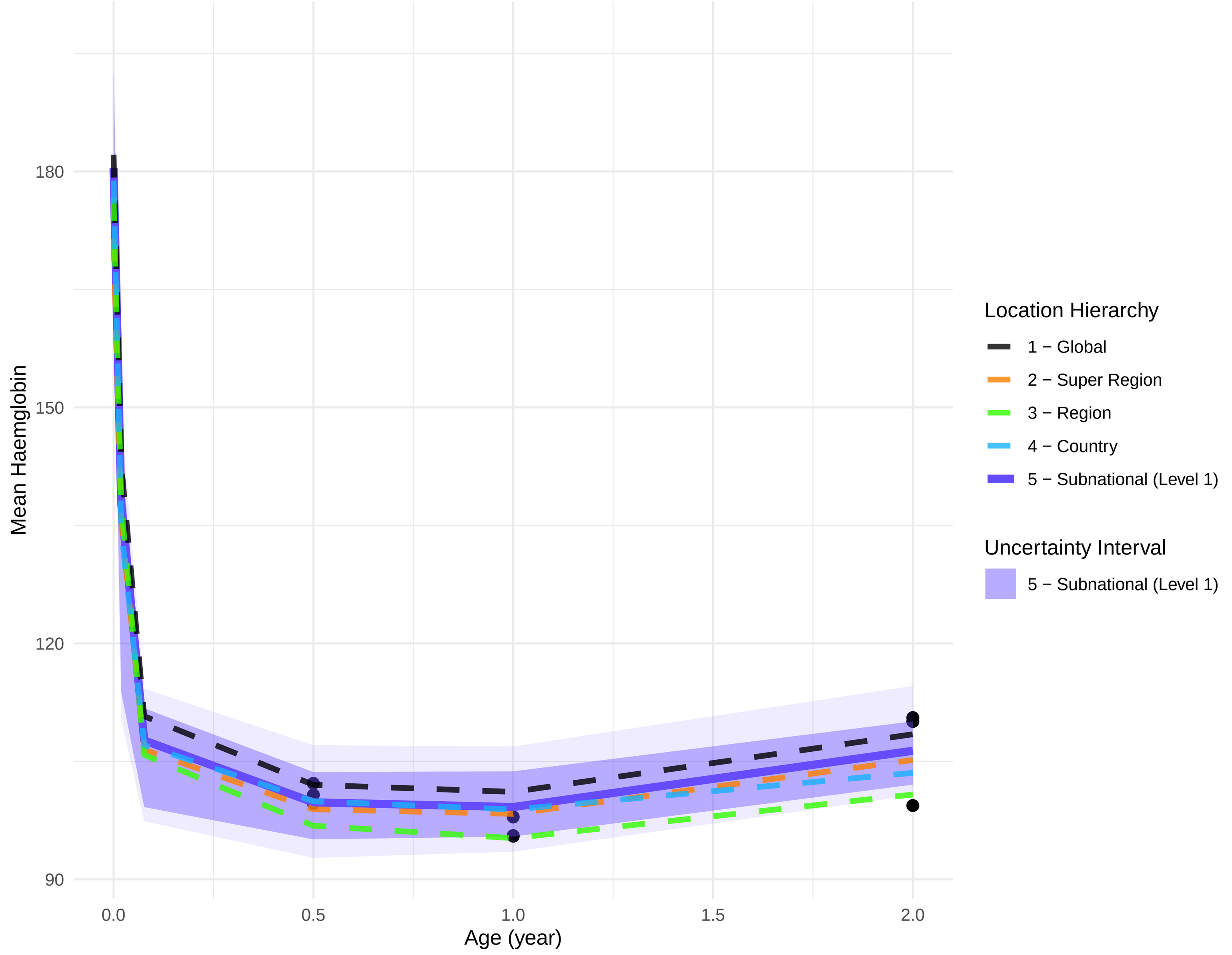

MR−BRT Age/Sex Fits − Non−pregnant Females <2 Years Old − Mean Haemglobin − Imo
WHO−2001 Adjustment Method

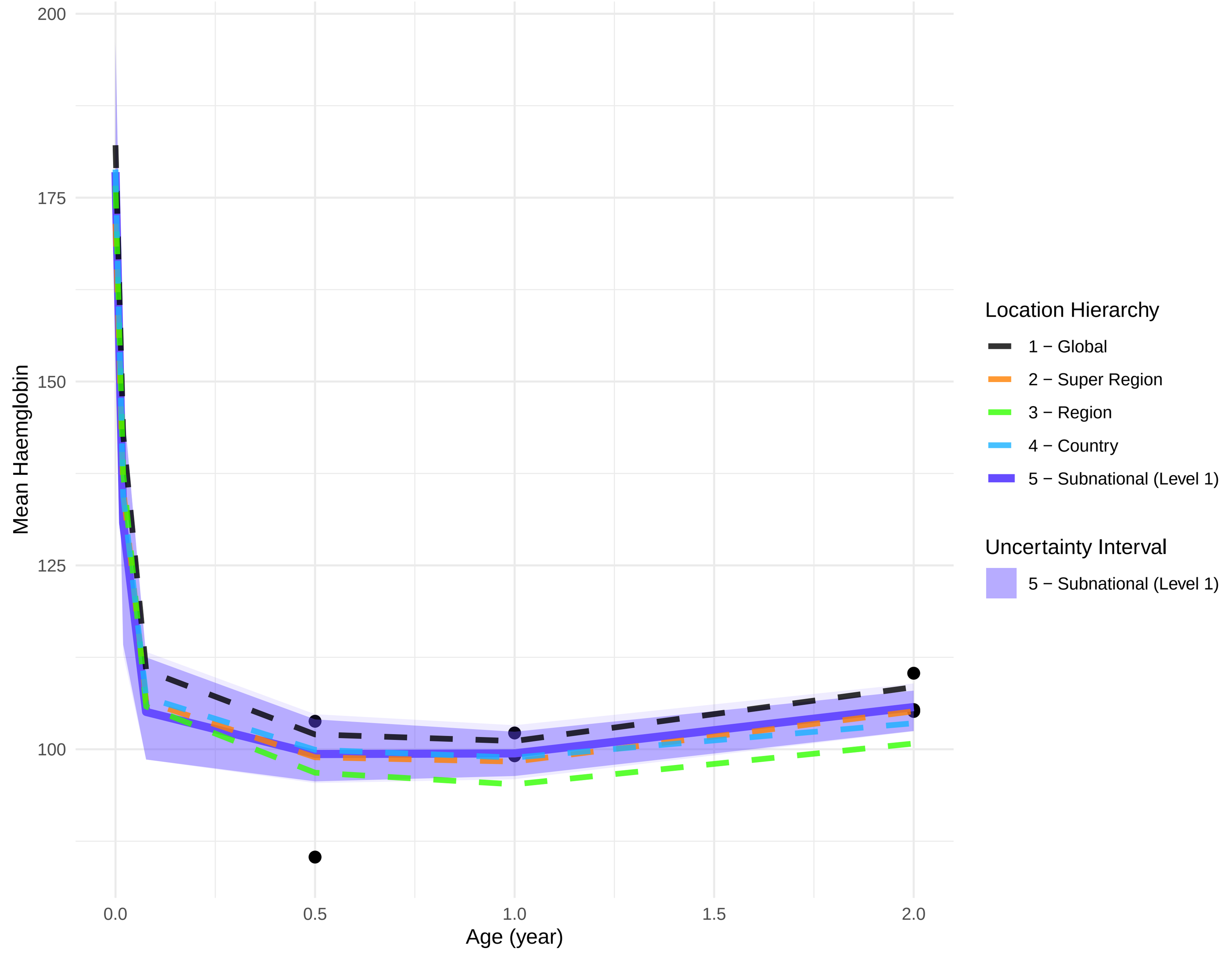

MR−BRT Age/Sex Fits − Non−pregnant Females <2 Years Old − Mean Haemglobin − Jigawa
WHO−2001 Adjustment Method

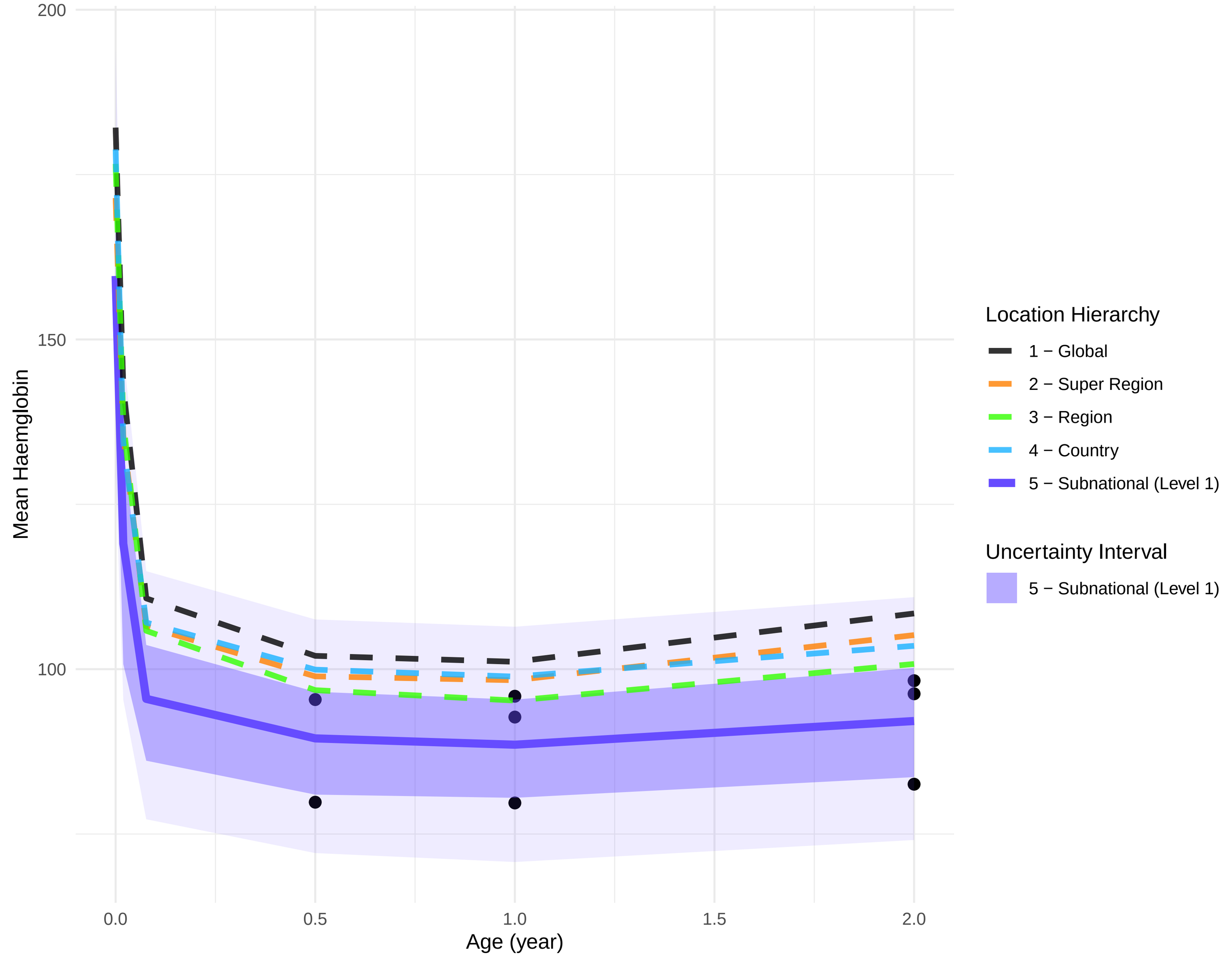

MR−BRT Age/Sex Fits − Non−pregnant Females <2 Years Old − Mean Haemglobin − Kaduna
WHO−2001 Adjustment Method

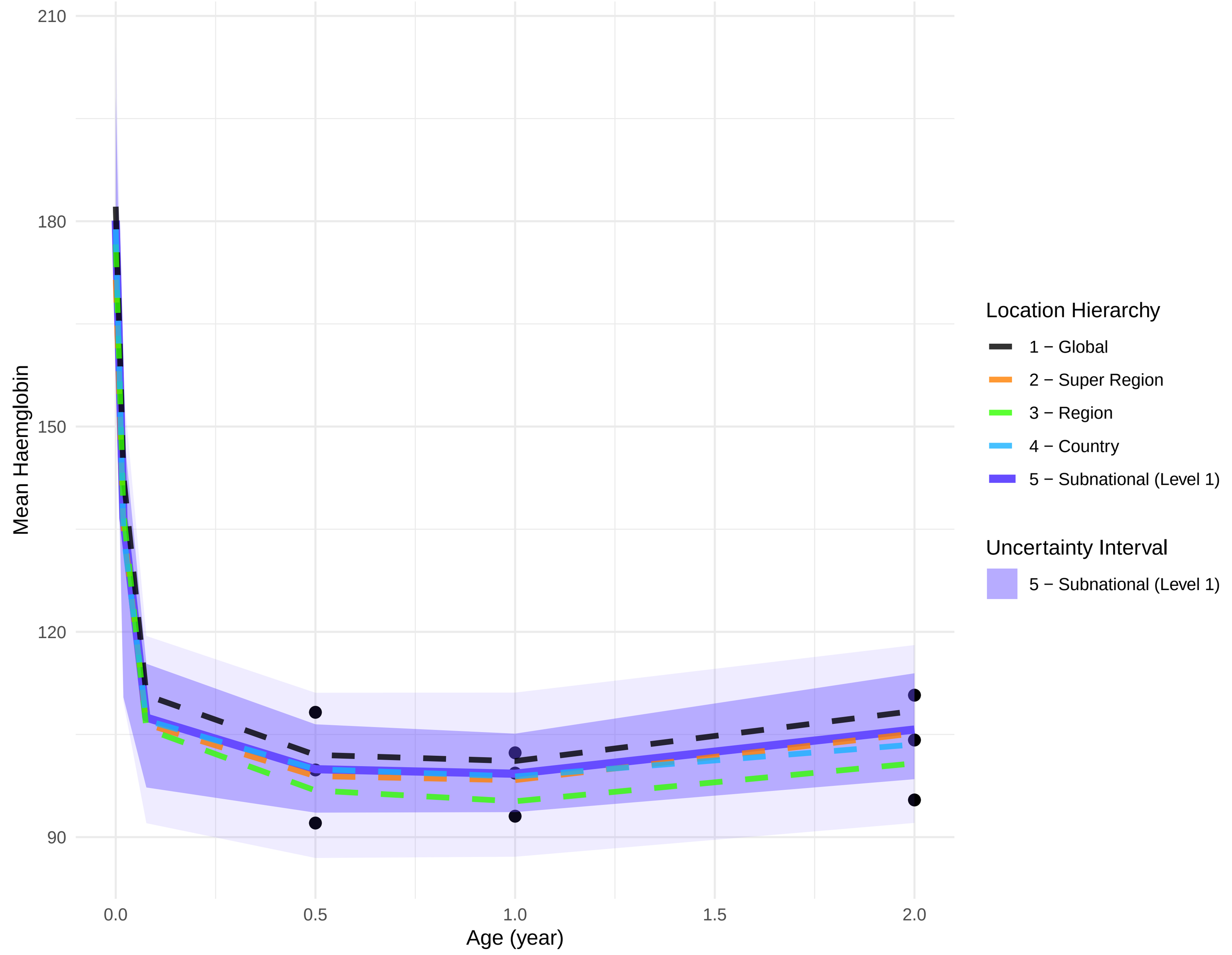

MR−BRT Age/Sex Fits − Non−pregnant Females <2 Years Old − Mean Haemglobin − Kano
WHO−2001 Adjustment Method

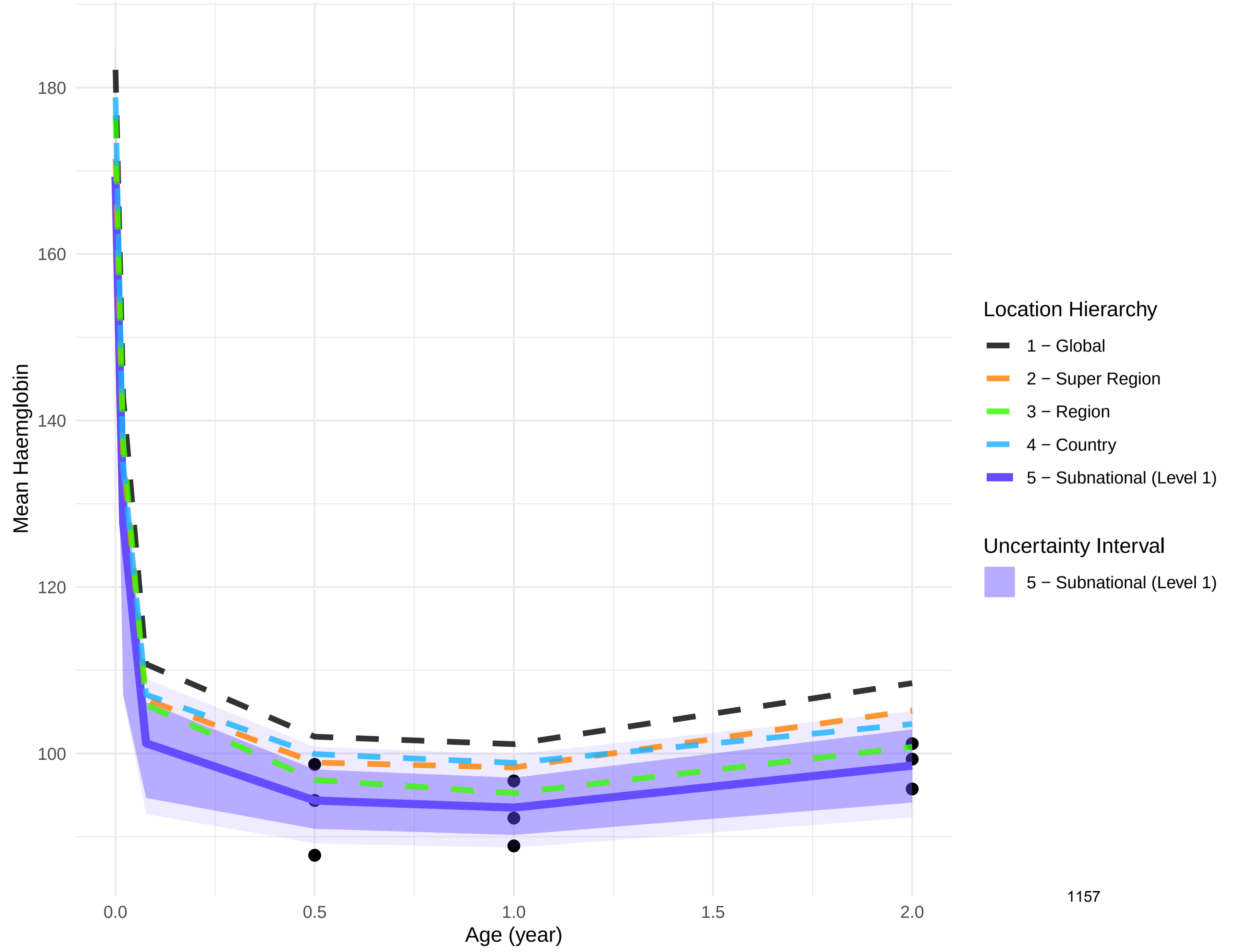

MR−BRT Age/Sex Fits − Non−pregnant Females <2 Years Old − Mean Haemglobin − Katsina
WHO−2001 Adjustment Method

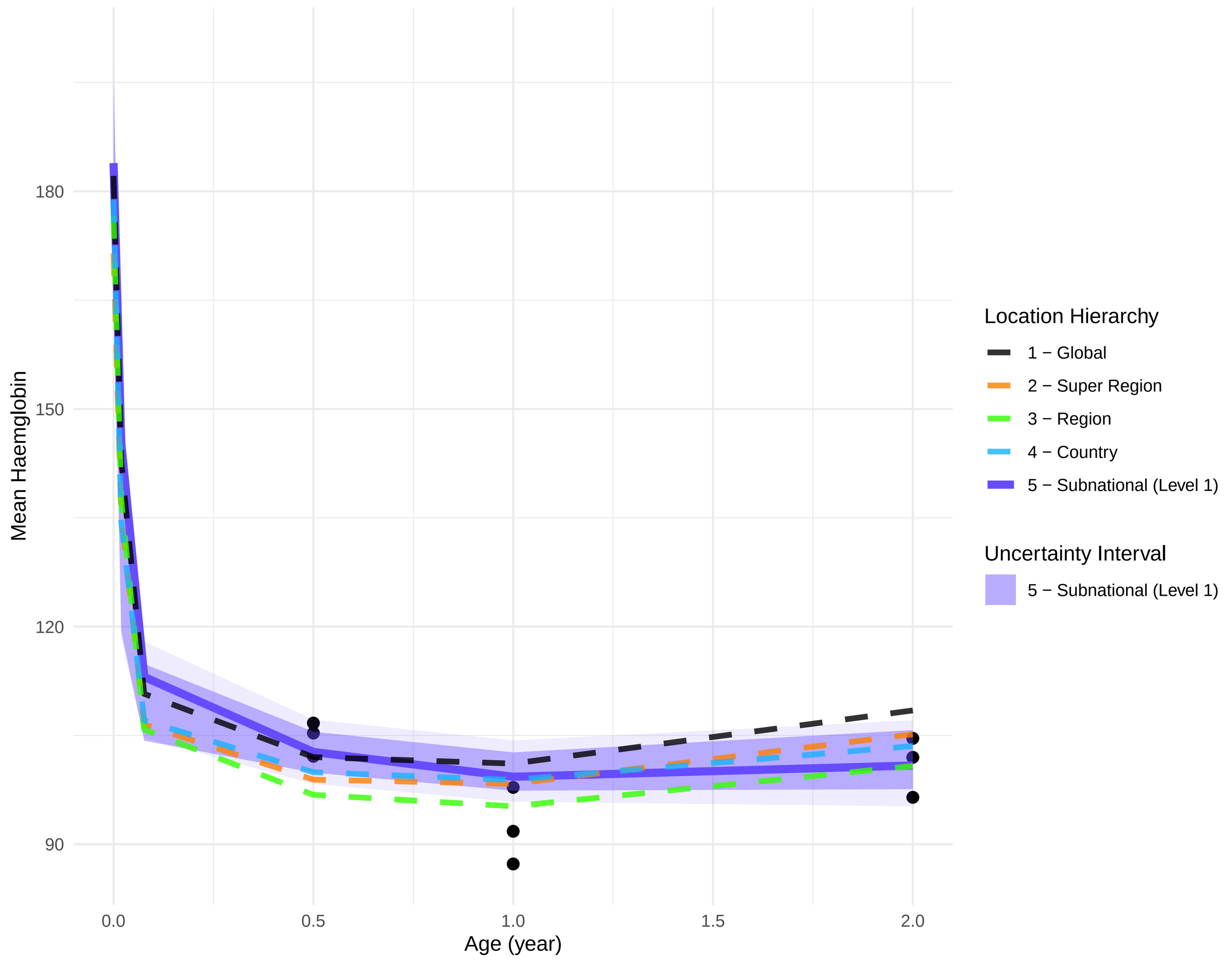

MR−BRT Age/Sex Fits − Non−pregnant Females <2 Years Old − Mean Haemglobin − Kebbi
WHO−2001 Adjustment Method

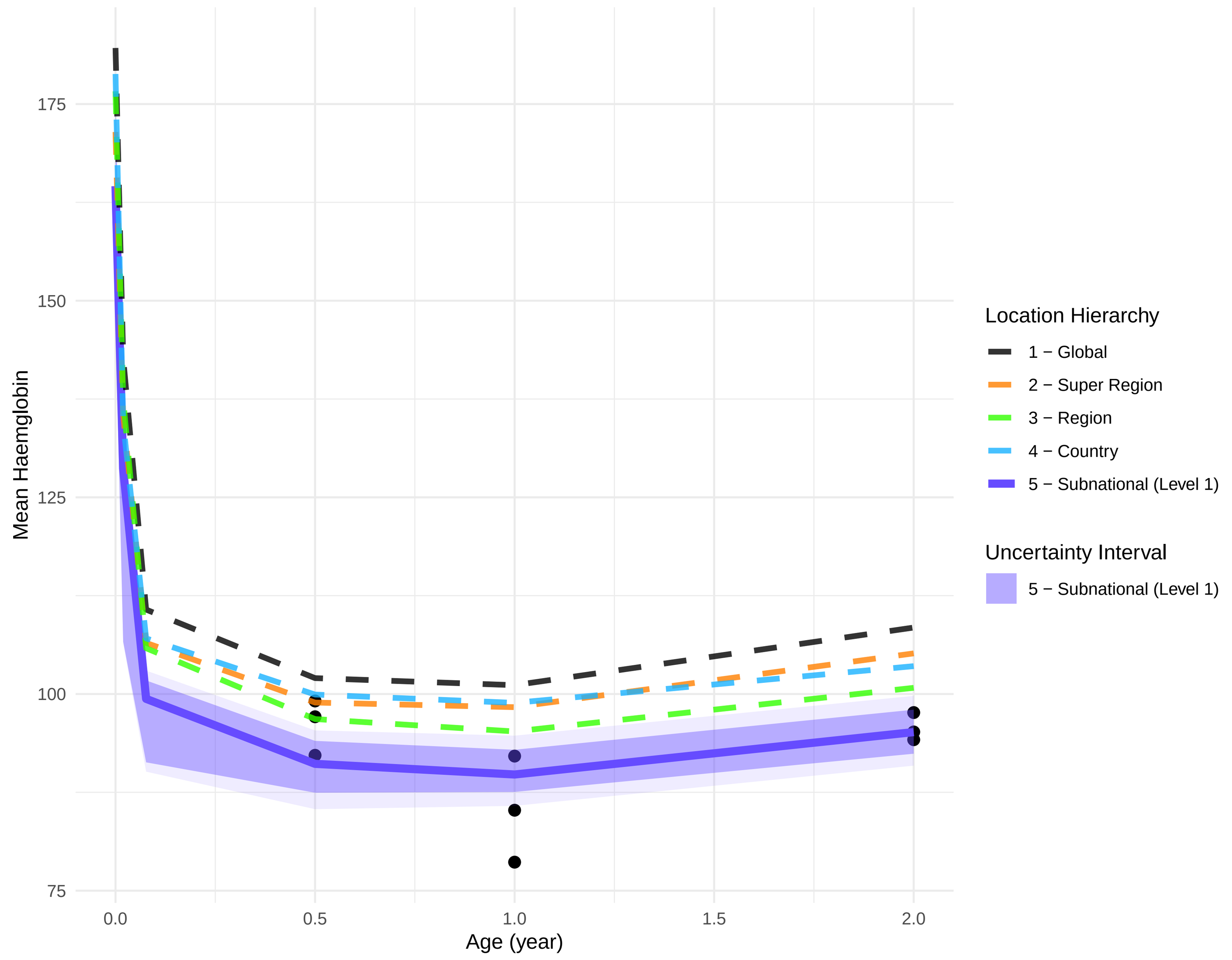

MR−BRT Age/Sex Fits − Non−pregnant Females <2 Years Old − Mean Haemglobin − Kogi
WHO−2001 Adjustment Method

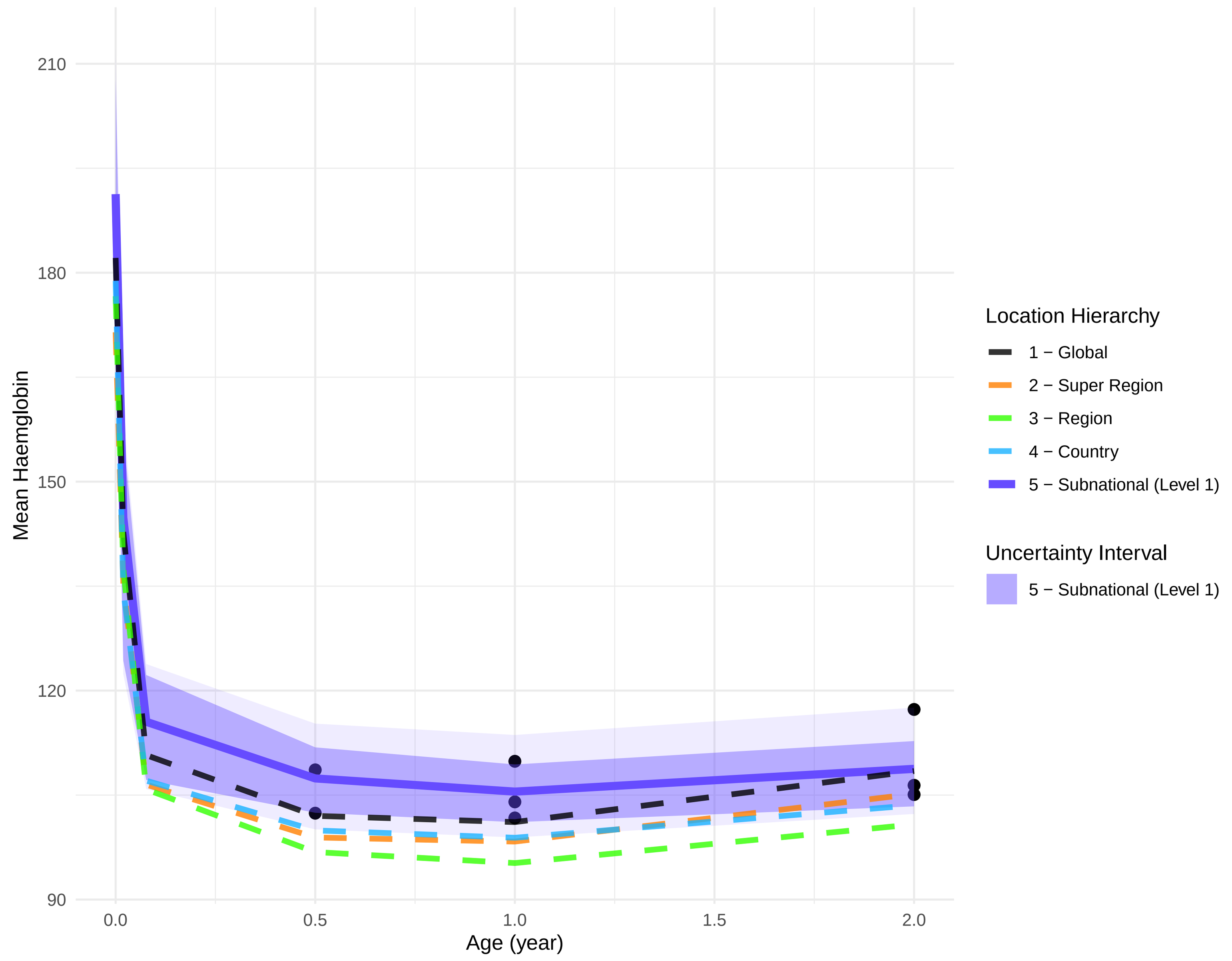

MR−BRT Age/Sex Fits − Non−pregnant Females <2 Years Old − Mean Haemglobin − Kwara
WHO−2001 Adjustment Method

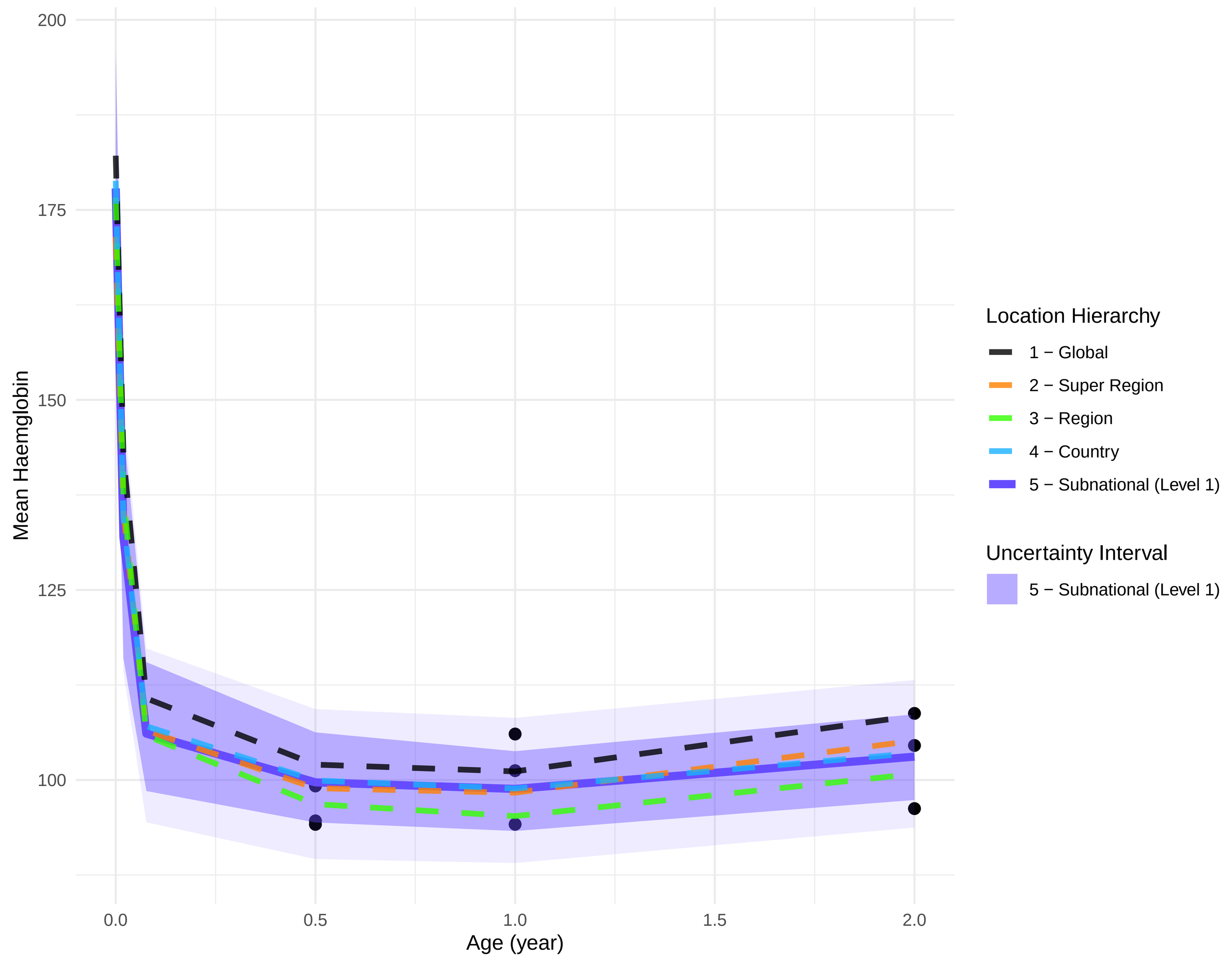

MR−BRT Age/Sex Fits − Non−pregnant Females <2 Years Old − Mean Haemglobin − Lagos
WHO−2001 Adjustment Method

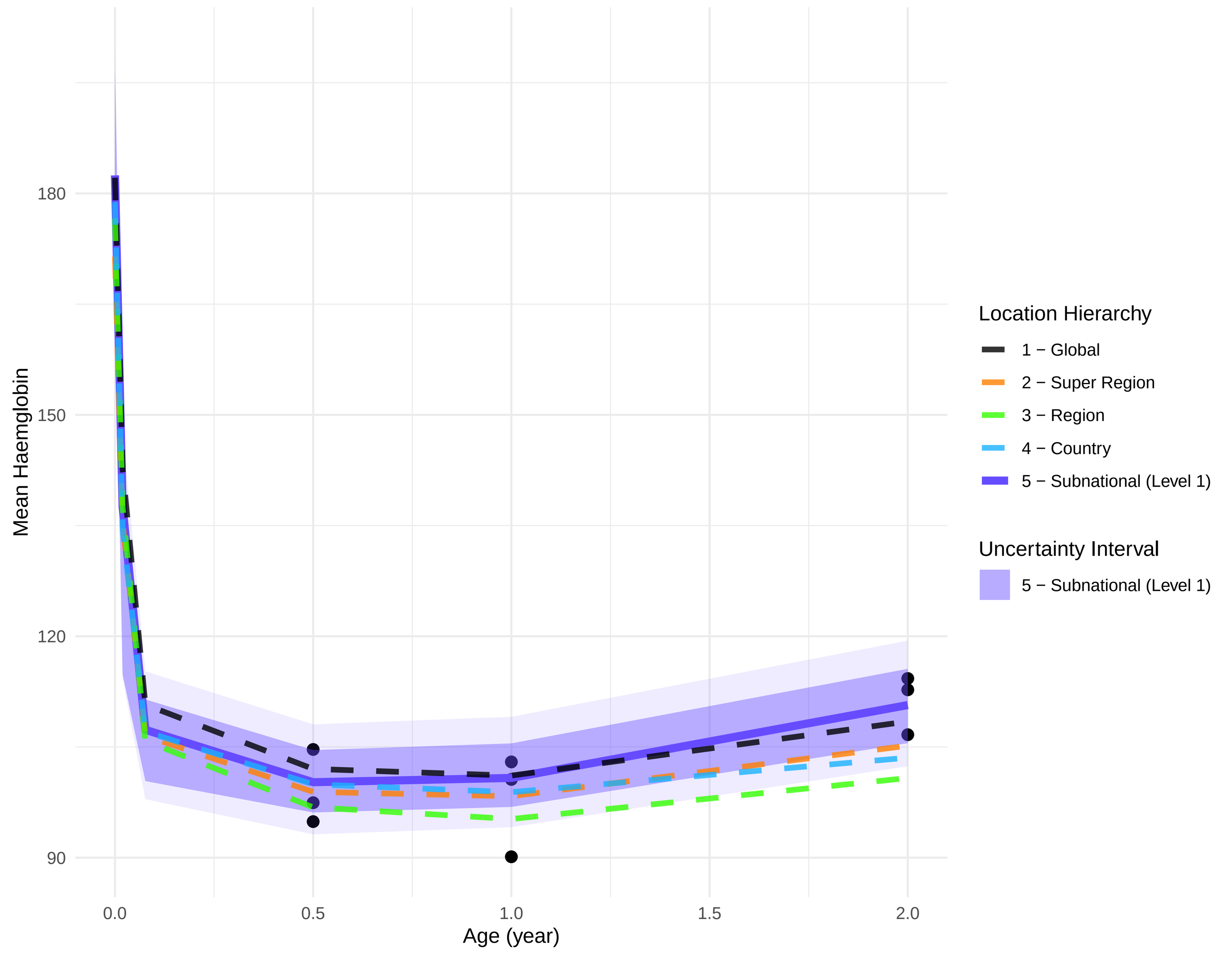

MR−BRT Age/Sex Fits − Non−pregnant Females <2 Years Old − Mean Haemglobin − Nasarawa
WHO−2001 Adjustment Method

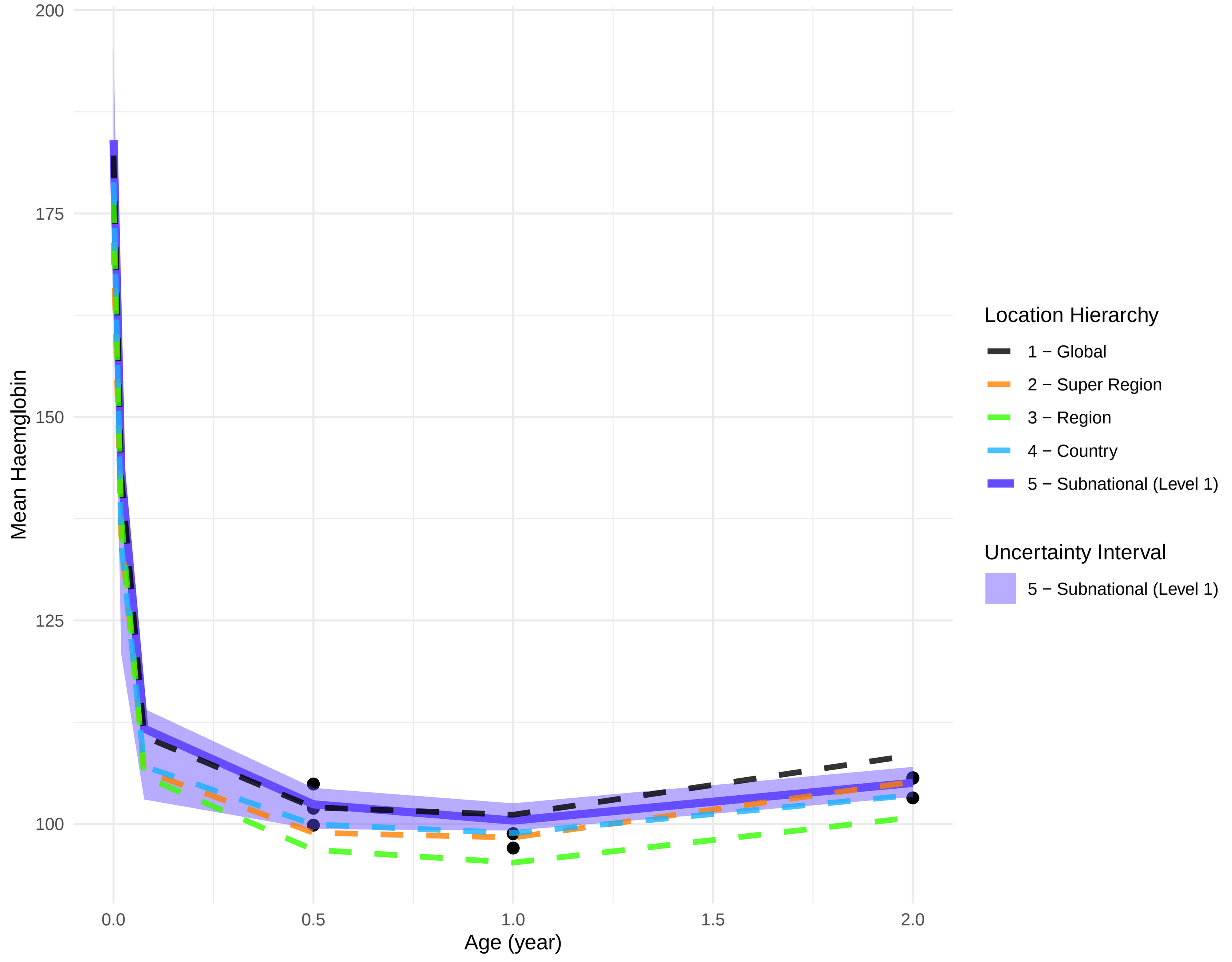

MR−BRT Age/Sex Fits − Non−pregnant Females <2 Years Old − Mean Haemglobin − Niger
WHO−2001 Adjustment Method

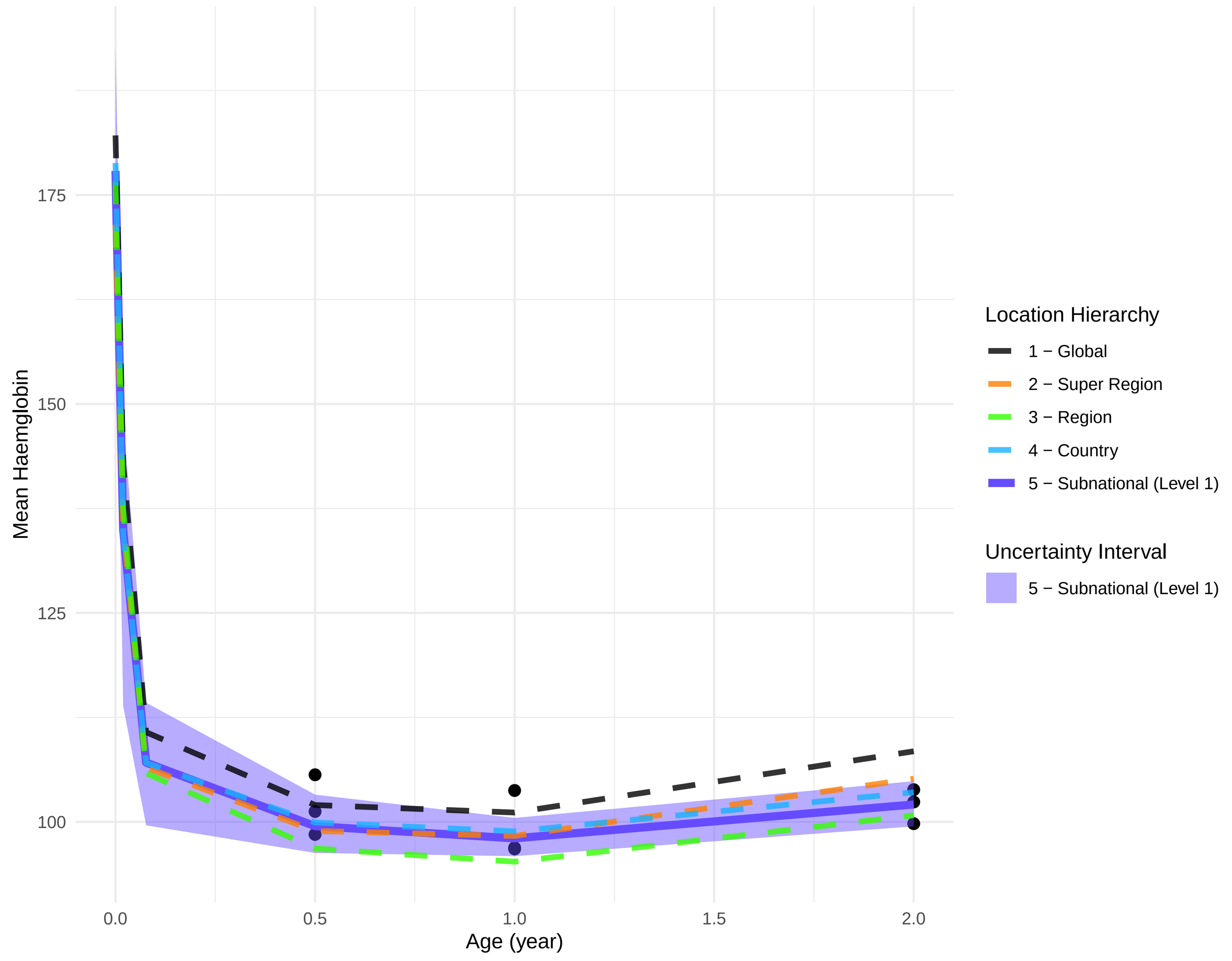

MR−BRT Age/Sex Fits − Non−pregnant Females <2 Years Old − Mean Haemglobin − Ogun
WHO−2001 Adjustment Method

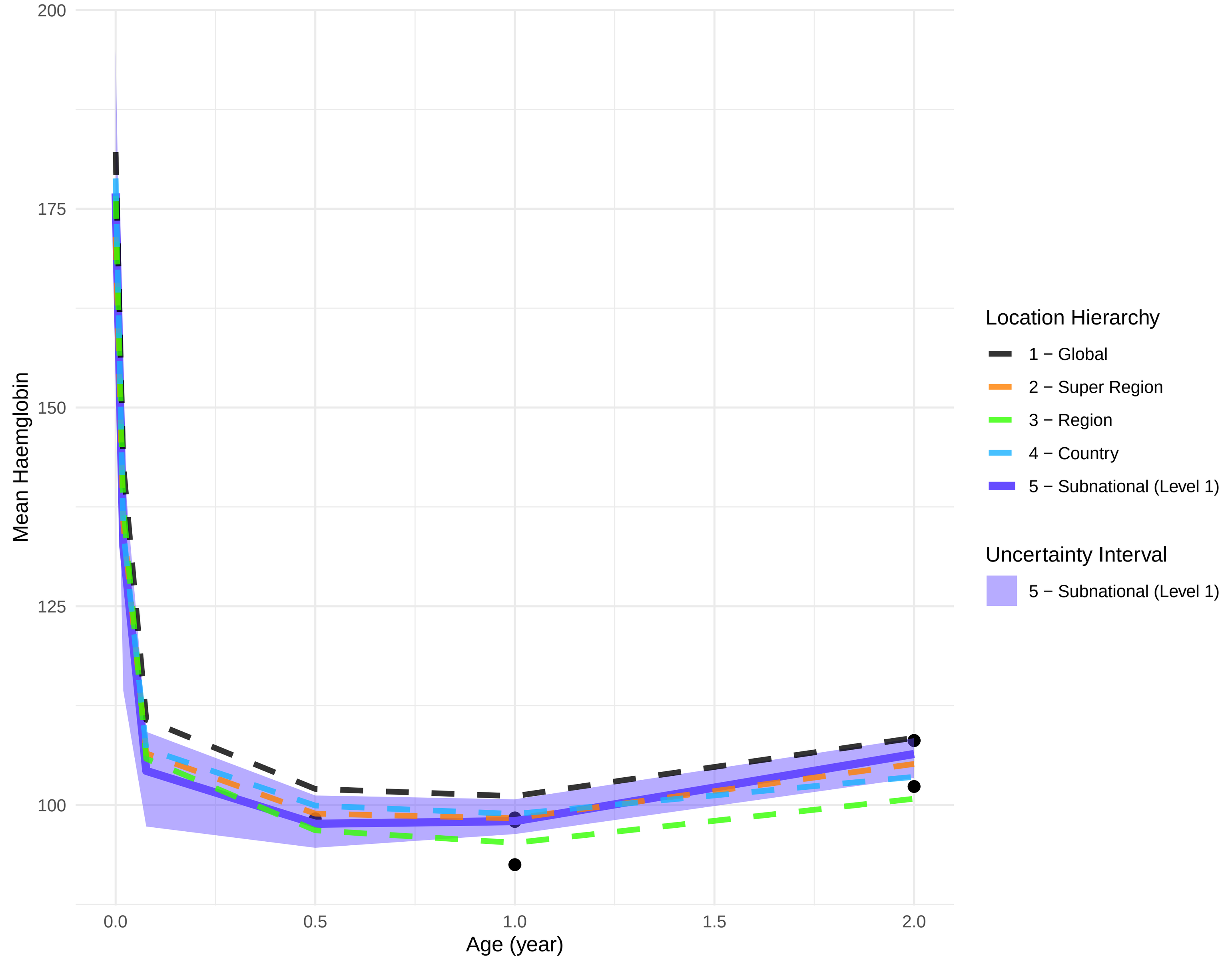

MR−BRT Age/Sex Fits − Non−pregnant Females <2 Years Old − Mean Haemglobin − Ondo
WHO−2001 Adjustment Method

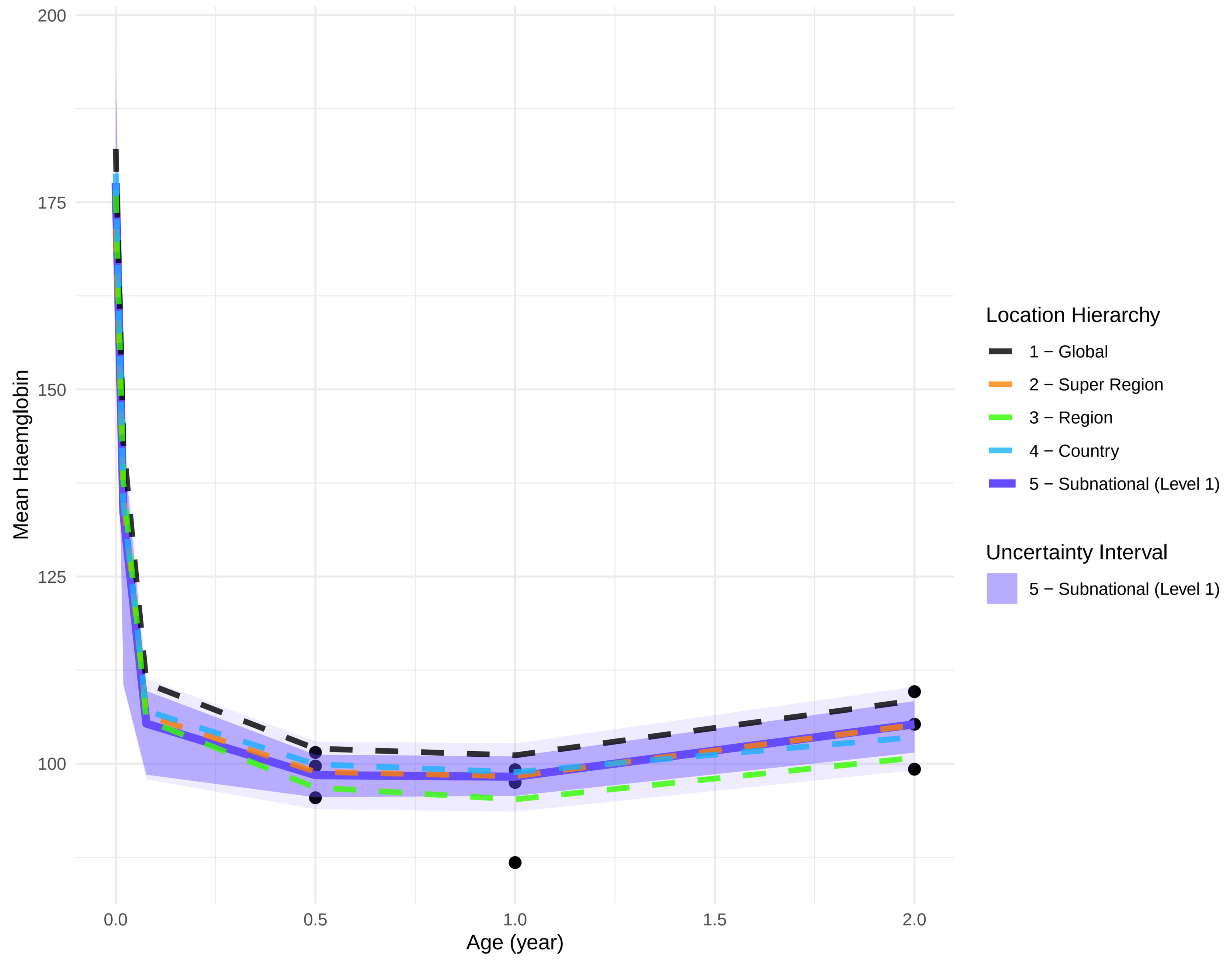

MR−BRT Age/Sex Fits − Non−pregnant Females <2 Years Old − Mean Haemglobin − Osun
WHO−2001 Adjustment Method

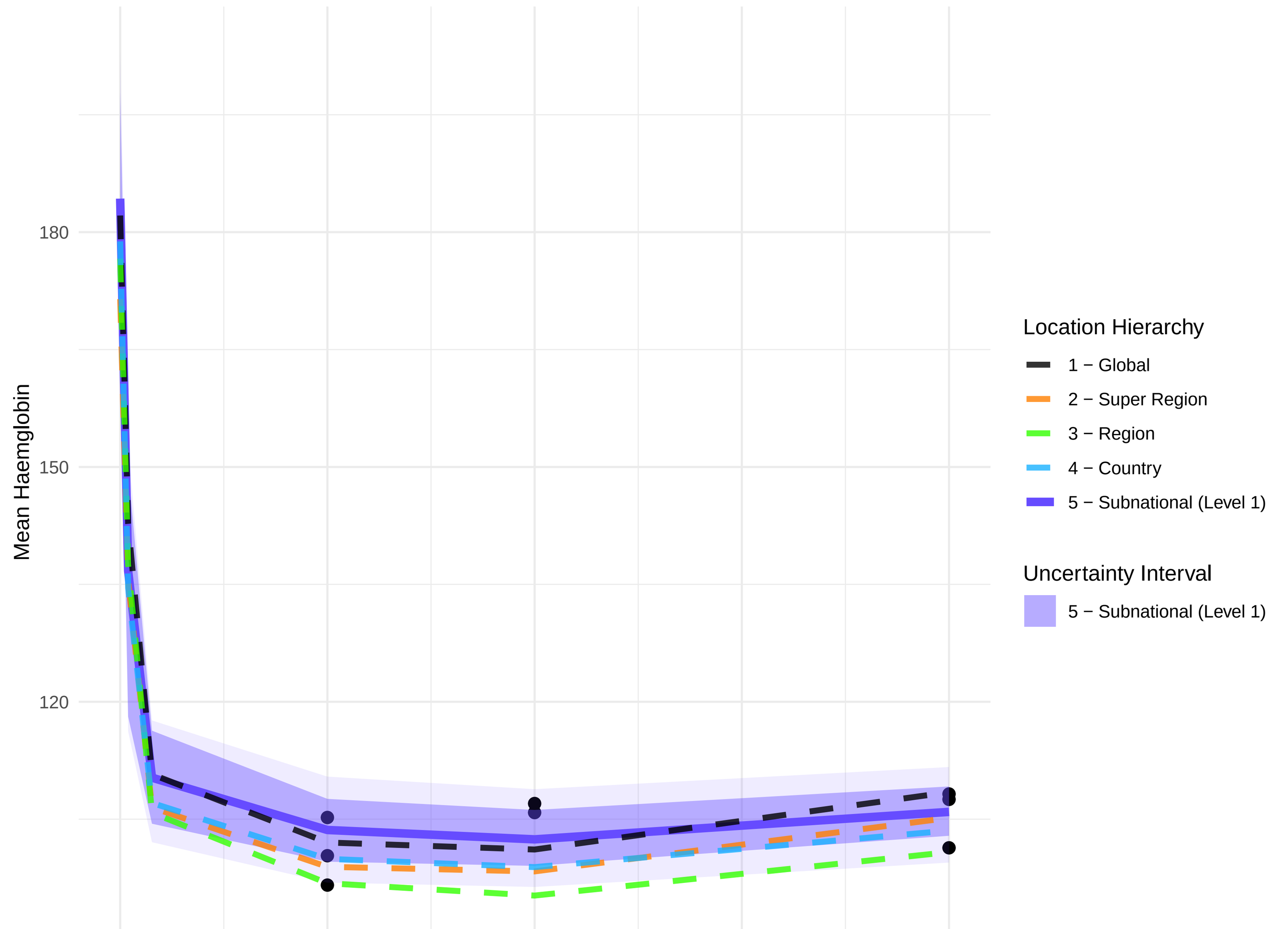

MR−BRT Age/Sex Fits − Non-pregnant Females <2 Years Old − Mean Haemglobin − Oyo
WHO−2001 Adjustment Method

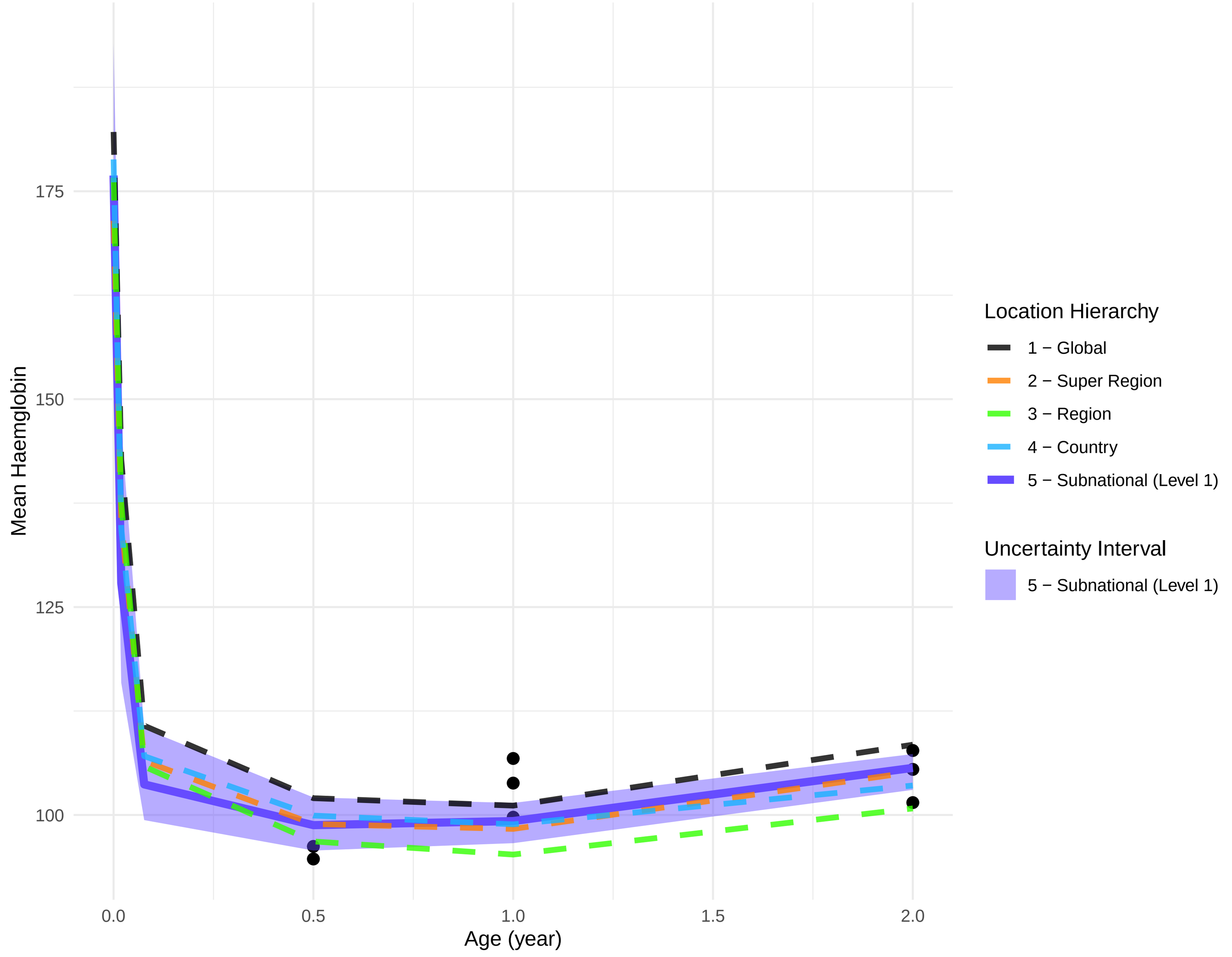

MR−BRT Age/Sex Fits − Non-pregnant Females <2 Years Old − Mean Haemglobin − Plateau
WHO−2001 Adjustment Method

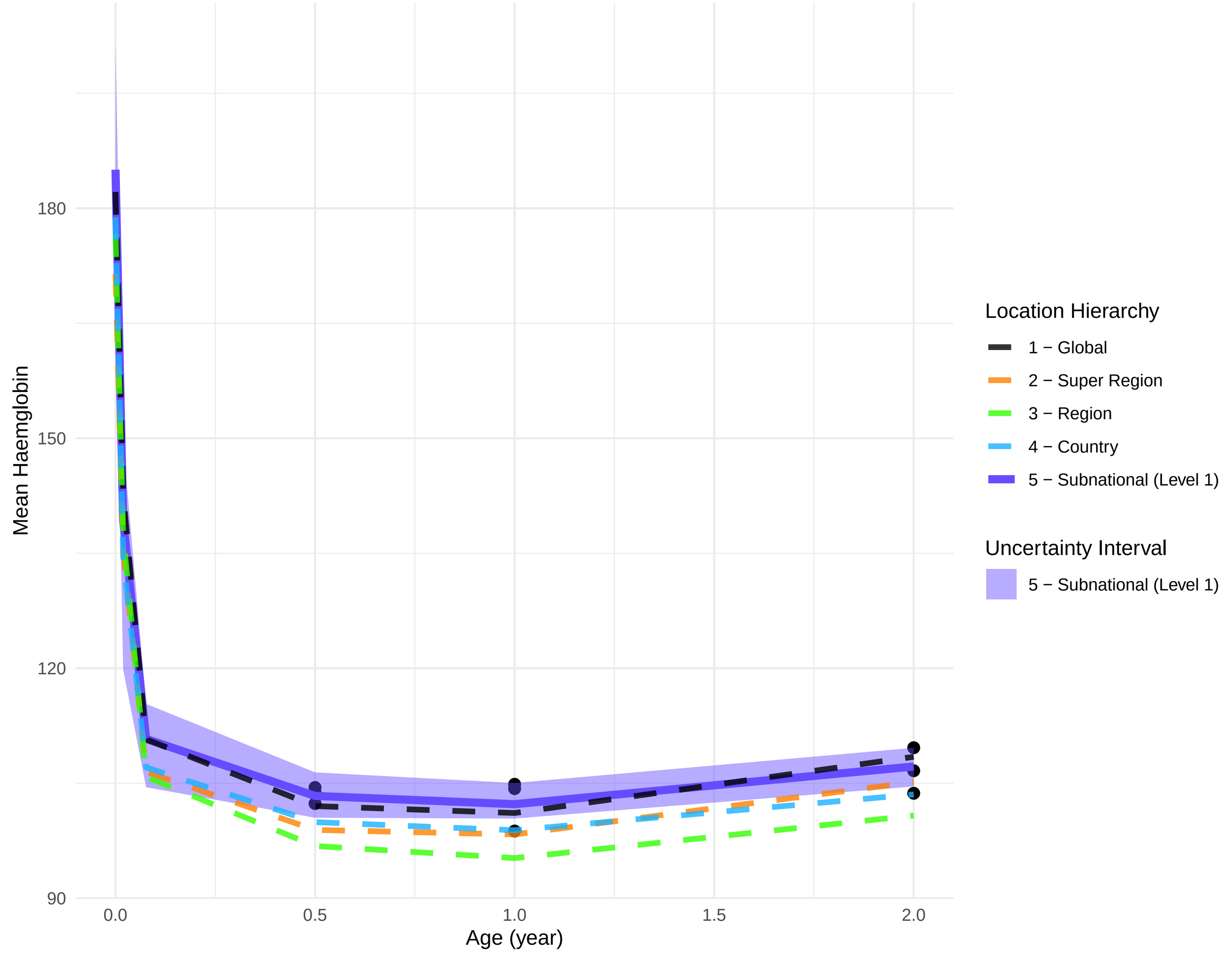

MR−BRT Age/Sex Fits − Non-pregnant Females <2 Years Old − Mean Haemglobin − Rivers
WHO−2001 Adjustment Method

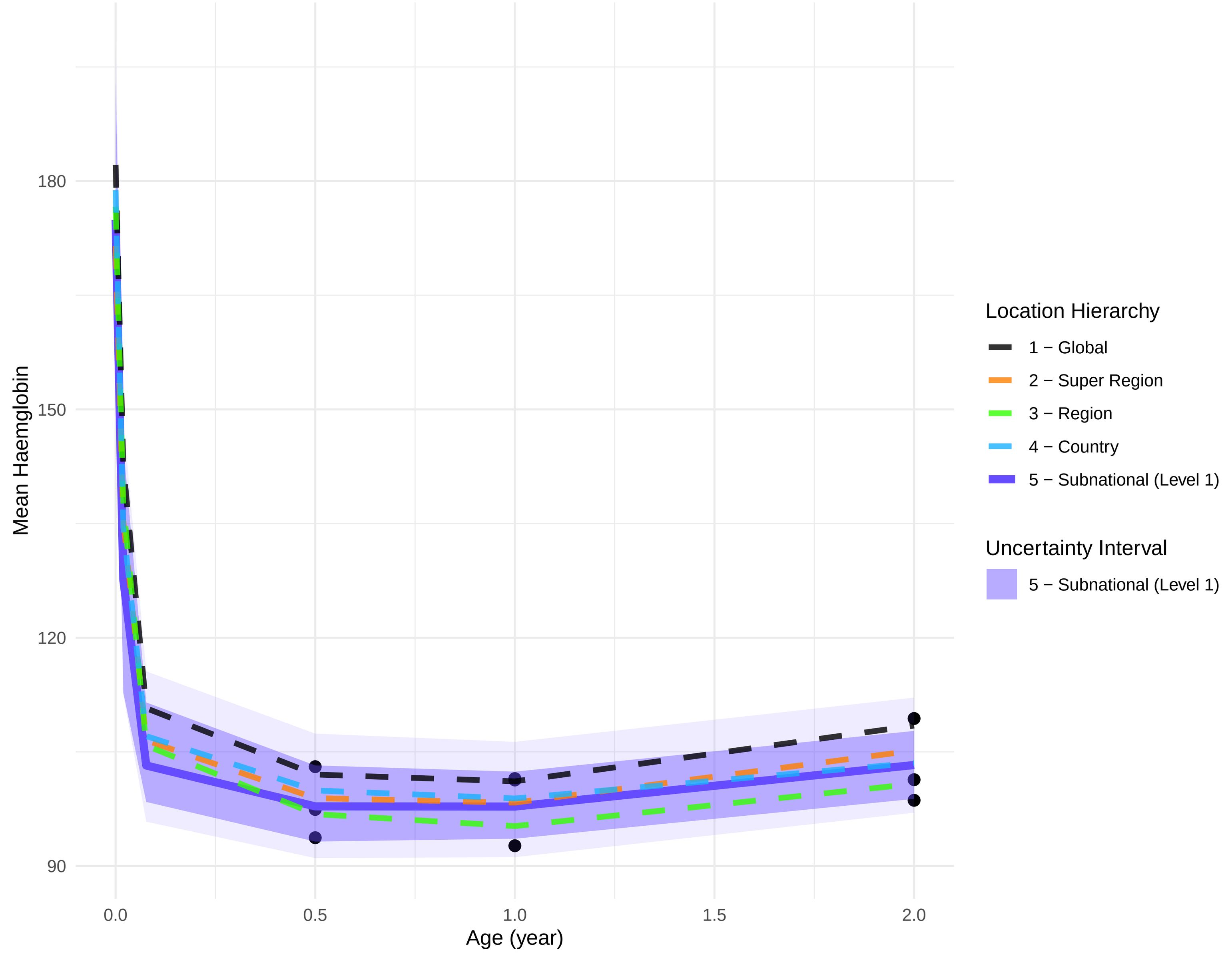

MR−BRT Age/Sex Fits − Non-pregnant Females <2 Years Old − Mean Haemglobin − Sokoto
WHO−2001 Adjustment Method

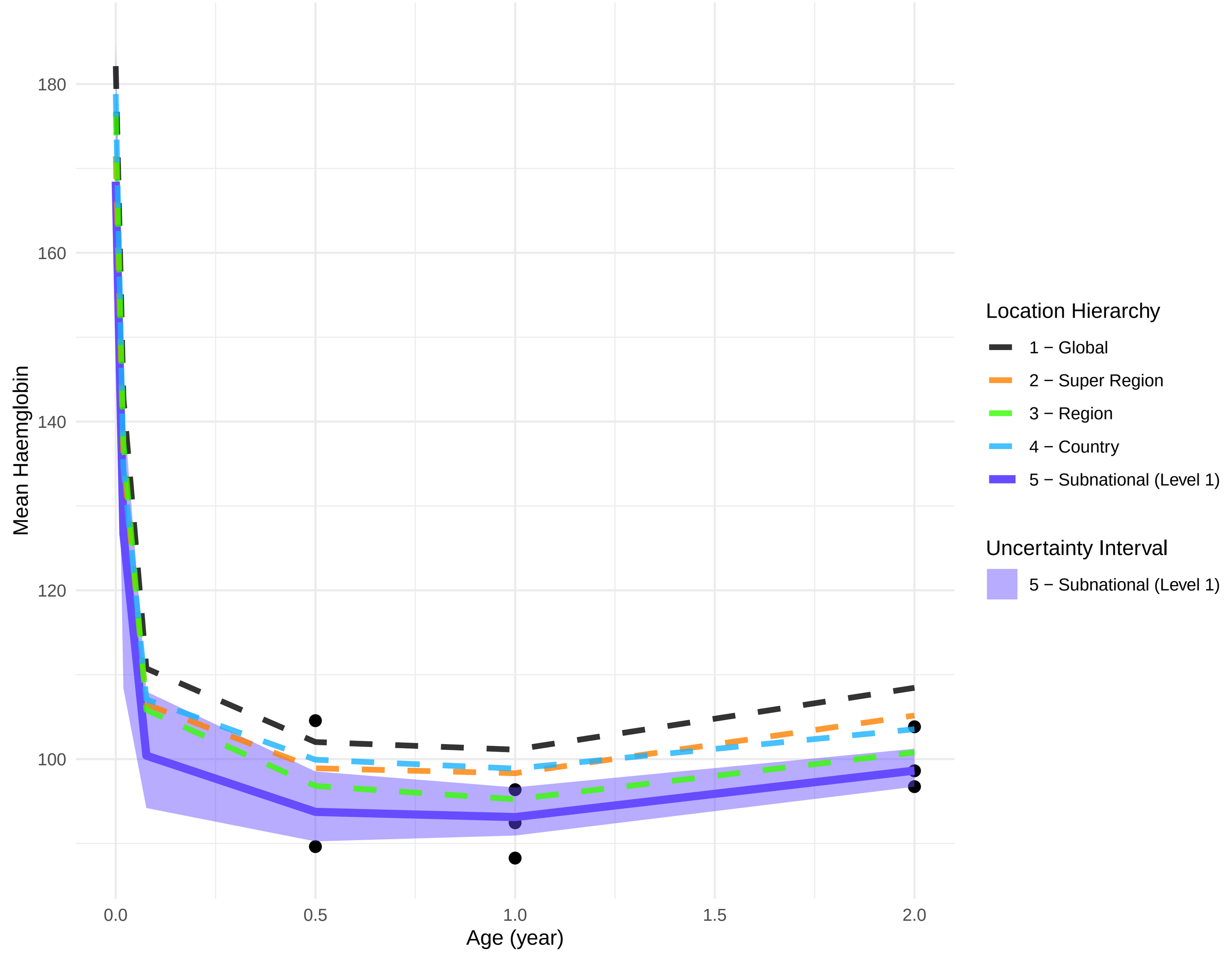

MR−BRT Age/Sex Fits − Non-pregnant Females <2 Years Old − Mean Haemglobin − Taraba
WHO−2001 Adjustment Method

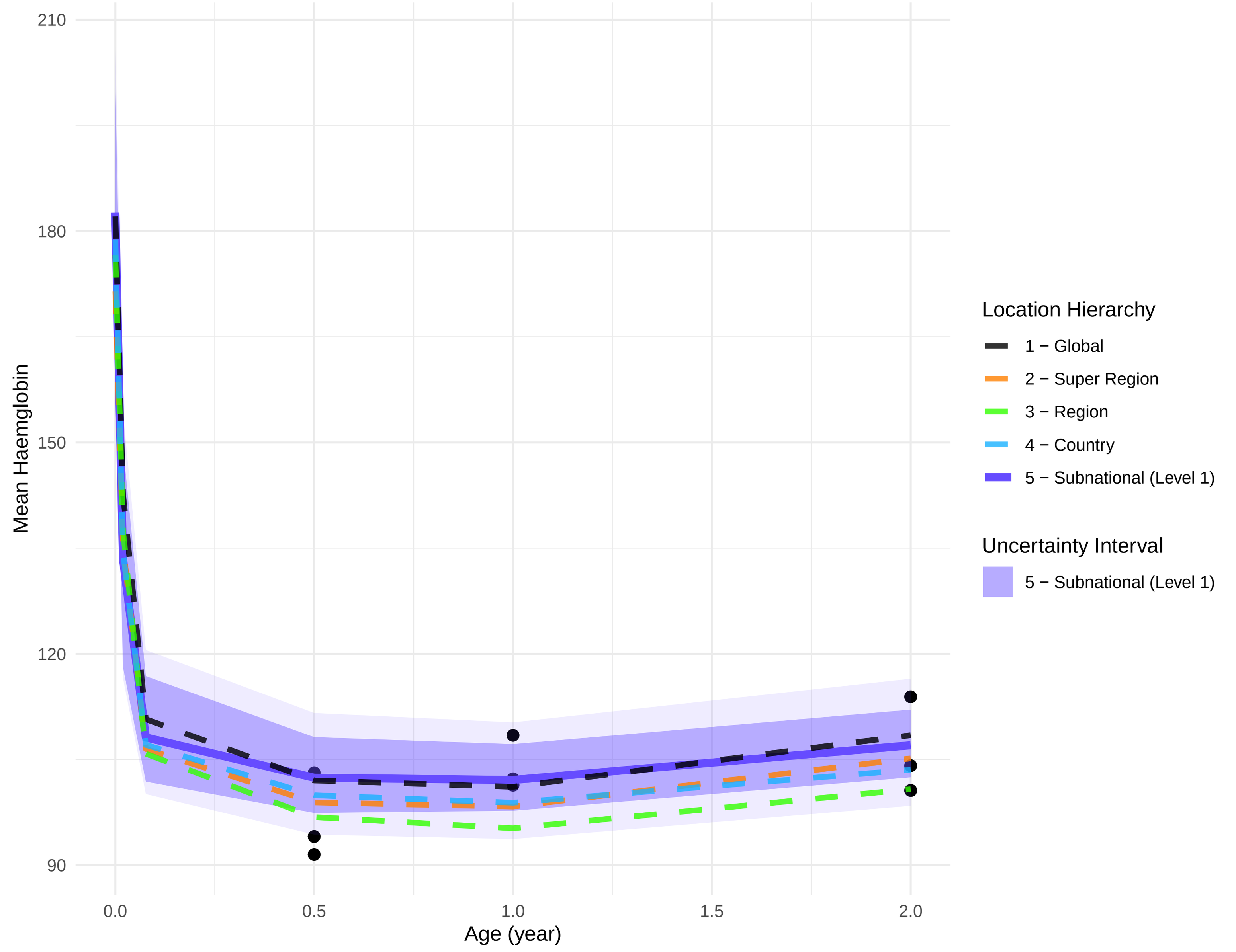

MR−BRT Age/Sex Fits − Non-pregnant Females <2 Years Old − Mean Haemglobin − Yobe
WHO−2001 Adjustment Method

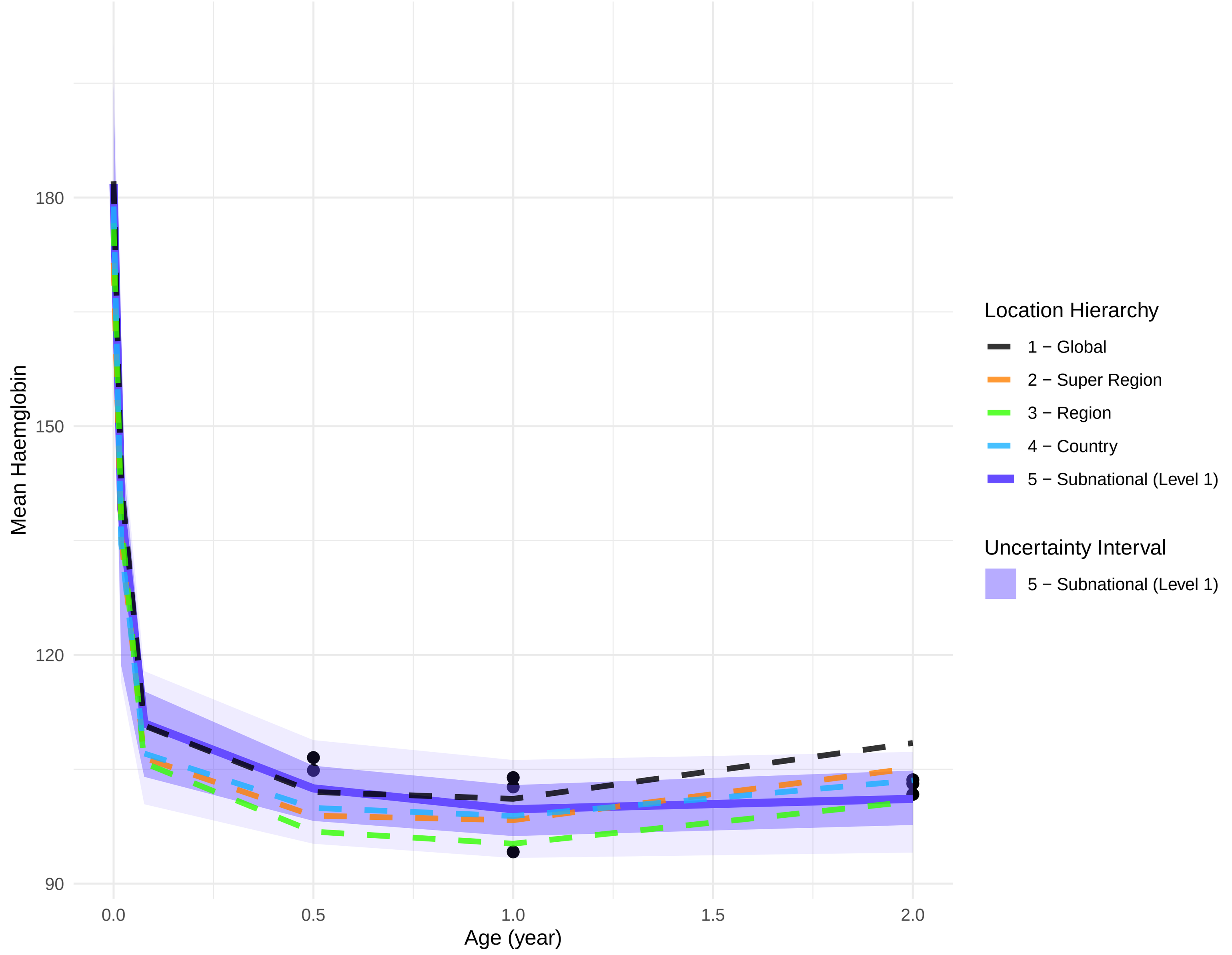

MR−BRT Age/Sex Fits − Non-pregnant Females <2 Years Old − Mean Haemglobin − Zamfara
WHO−2001 Adjustment Method

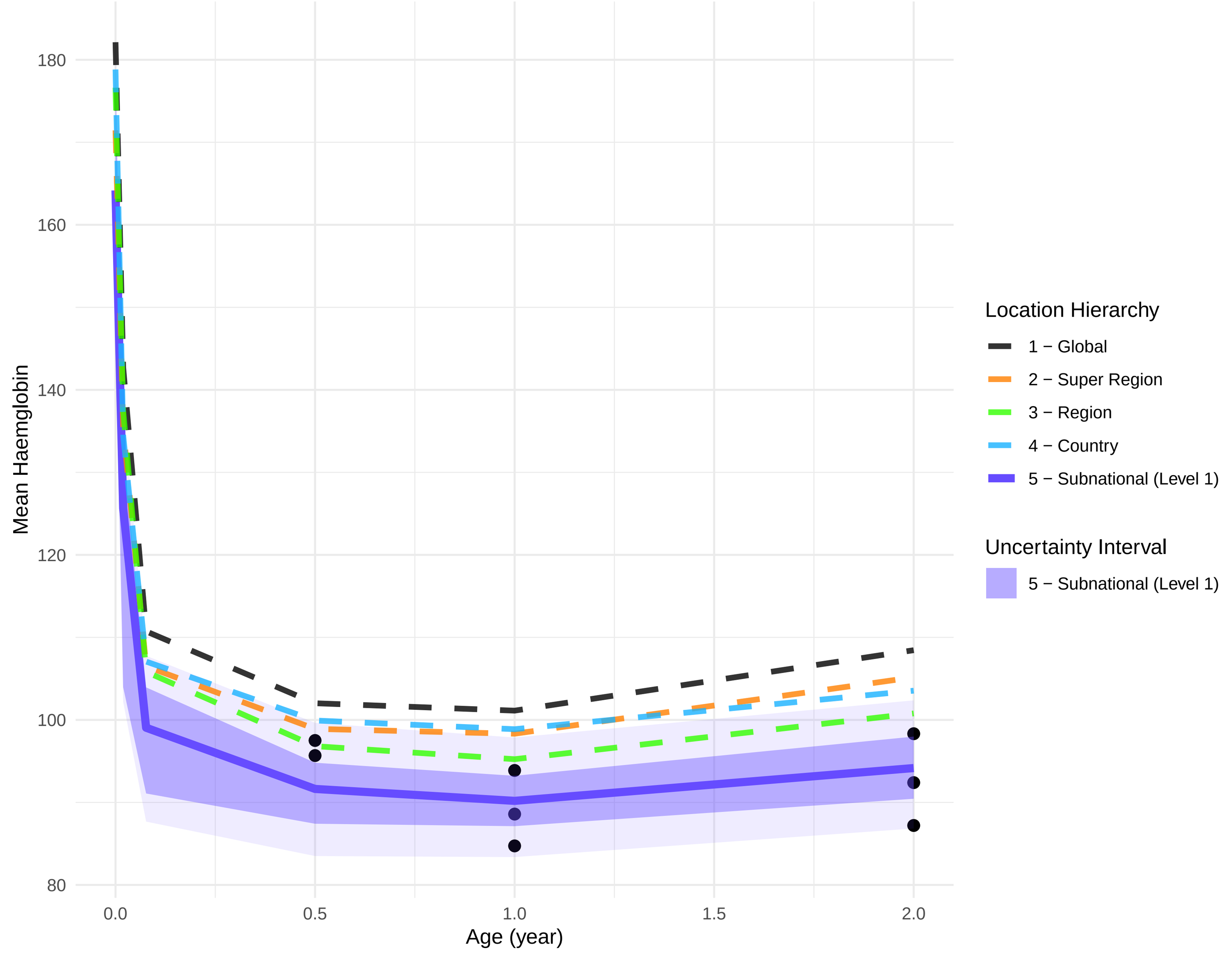

MR−BRT Age/Sex Fits − Non-pregnant Females <2 Years Old − Mean Haemglobin − Senegal
WHO−2001 Adjustment Method

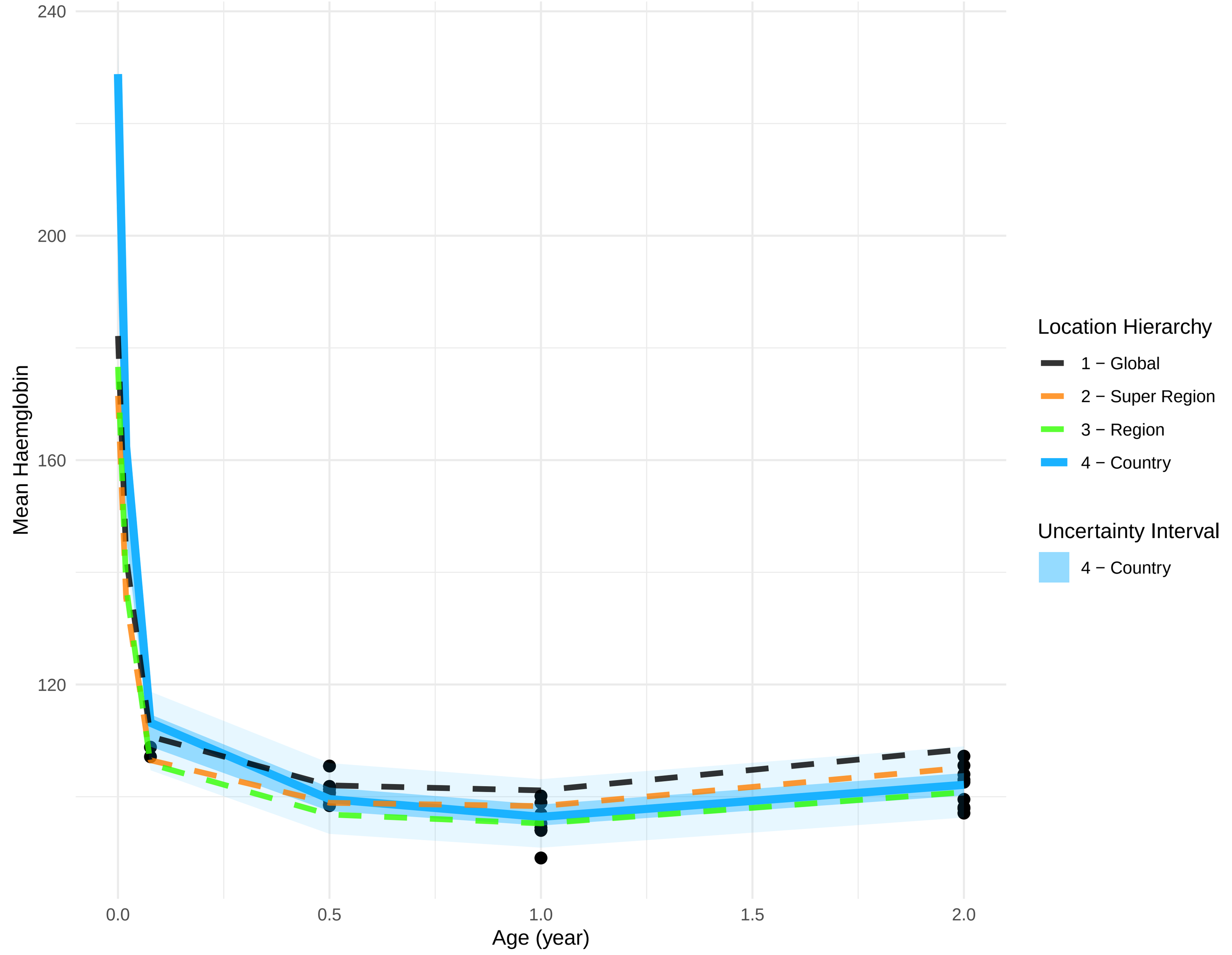

MR−BRT Age/Sex Fits − Non-pregnant Females <2 Years Old − Mean Haemglobin − Sierra Leone
WHO−2001 Adjustment Method

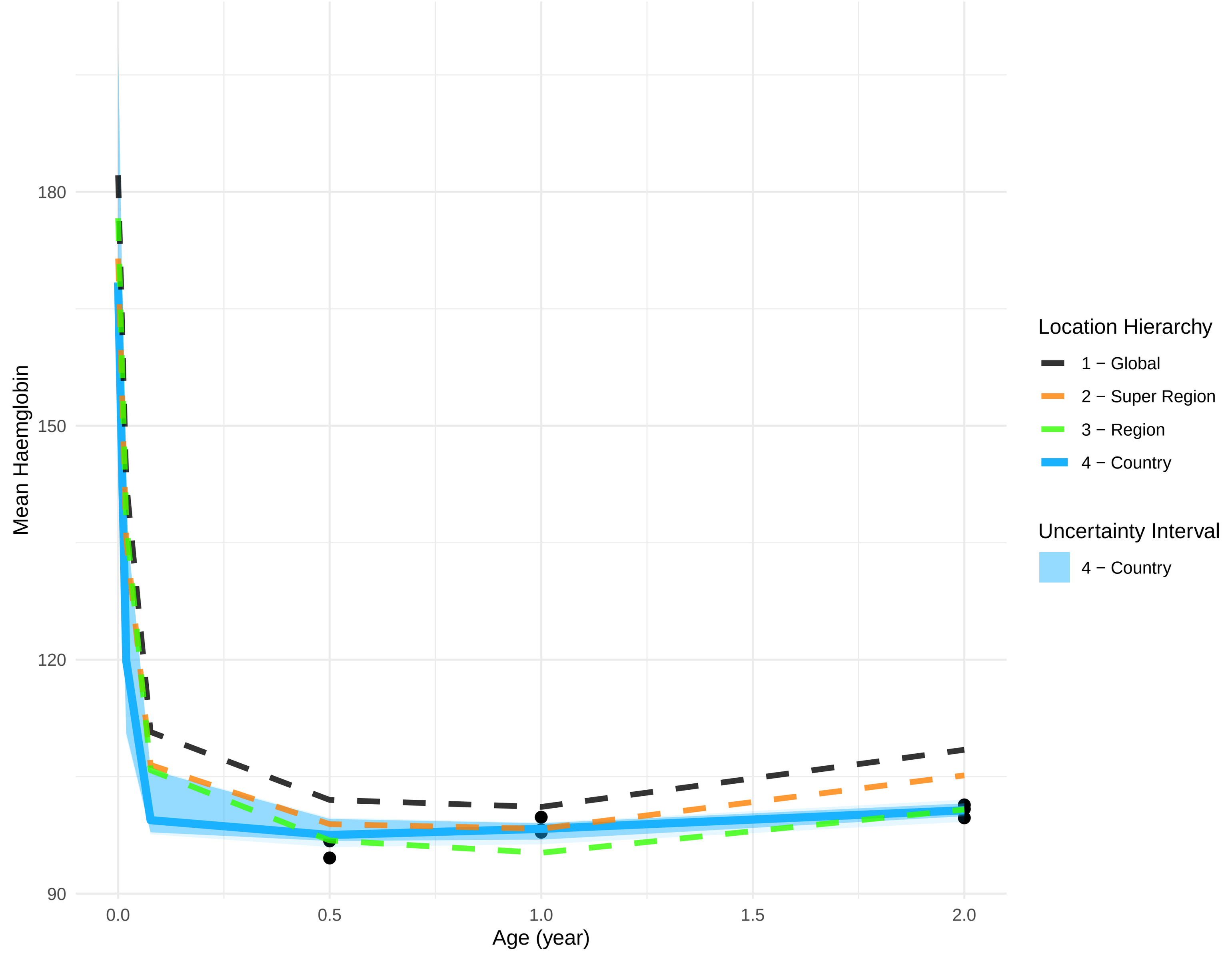

MR−BRT Age/Sex Fits − Non-pregnant Females <2 Years Old − Mean Haemglobin − Togo
WHO−2001 Adjustment Method

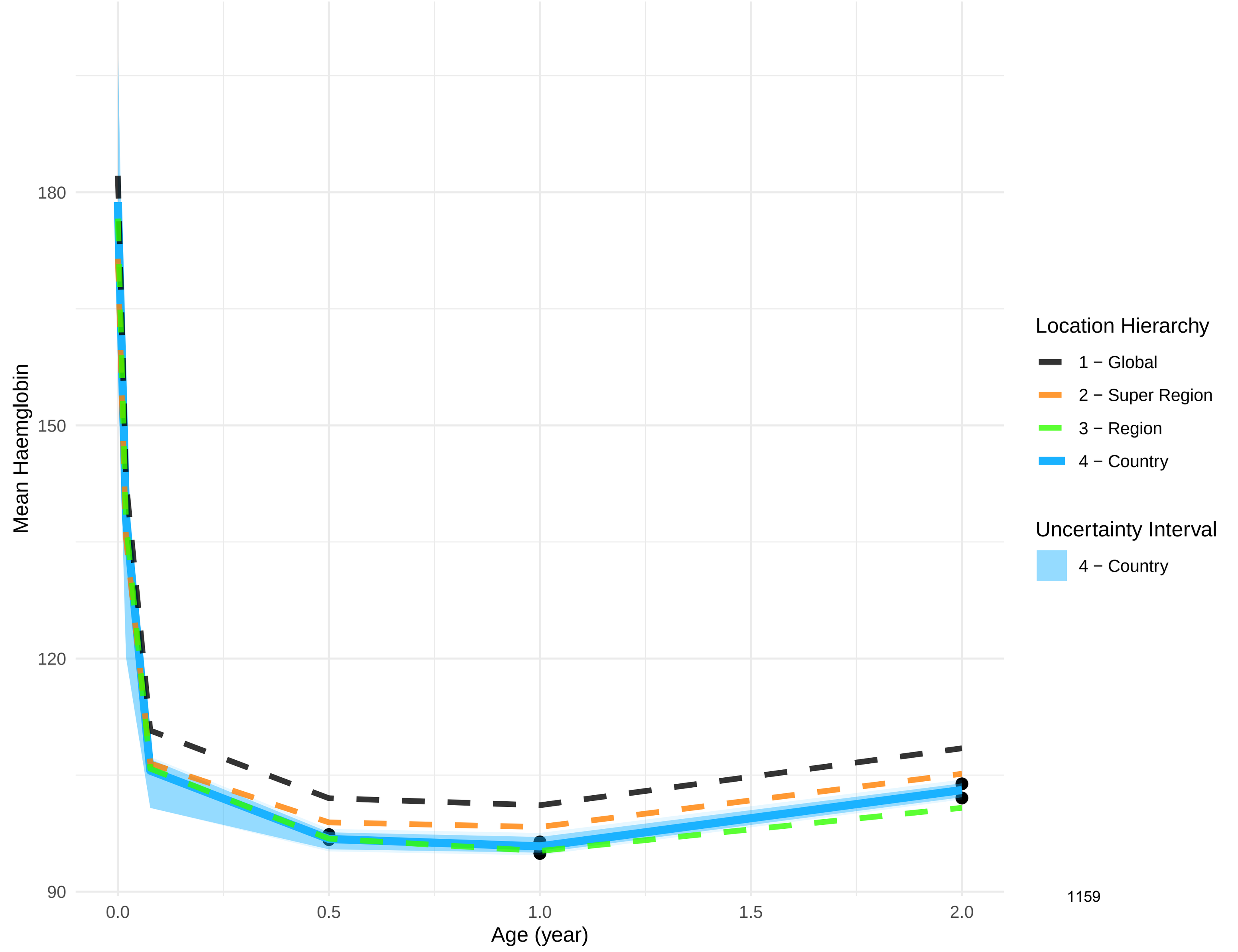

Section 5: MR-BRT measure-to-measure modelled estimates

MR-BRT modelled trends displaying the relationship of mean haemoglobin and total, moderate plus severe, and severe anaemia status. The plots are broken up into the age and sex bins modelled, which are: 1) children under 5 years old, both sexes; 2) children 5 to 14 years old, both sexes; 3) males 15 years and older; and 4) non-pregnant females 15 years and older. The points indicate the input data used in the model, where the size of the point signifies how heavily the given input point influenced the model (which was derived by taking the inverse of the standard error associated with the input point). The vertical dashed lines indicate the frequency knots assigned by the model and used in predicting the spline. The solid line represents the estimated spline for various relationships: either mean haemoglobin as a predictor for total, moderate plus severe, and severe anaemia, or total anaemia prevalence or moderate plus severe anaemia prevalence as a predictor for mean haemoglobin. Each combination was modelled using the input data set adjusted with the WHO-2001 and WHO-2024 elevation adjustment methods, resulting in a total of 10 plots for each age and sex combination.

The spline estimated in each model was utilized to impute missing measures from tabulated input data sources. This approach enabled us to generate estimated values for mean haemoglobin as well as total, moderate plus severe, and severe anaemia prevalence for each combination of age, sex, location, year, and survey present in our input data. By doing so, we ensured internal consistency across the input data to be used in the ST-GPR model.

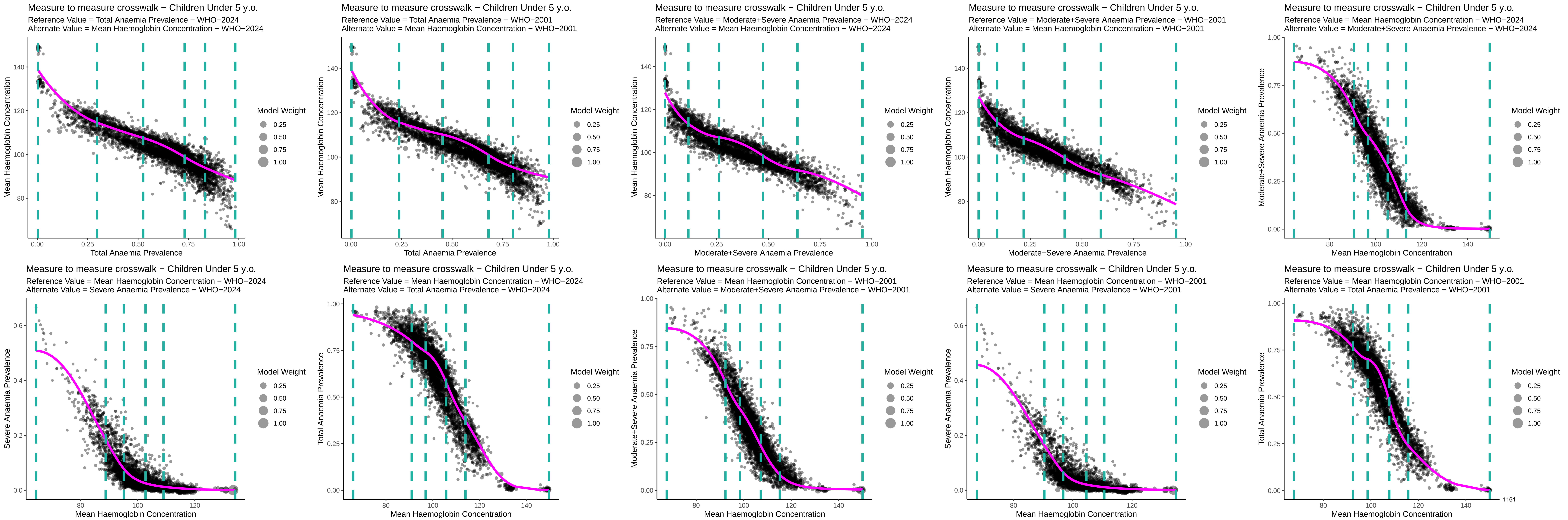
Measure to measure crosswalk − Children Under 5 y.o.
Reference Value = Total Anaemia Prevalence − WHO−2024
Alternate Value = Mean Haemoglobin Concentration − WHO−2024
Mean Haemoglobin Concentration
Total Anaemia Prevalence
Model Weight
0.25
0.50
0.75
1.00
Measure to measure crosswalk − Children Under 5 y.o.
Reference Value = Total Anaemia Prevalence − WHO−2001
Alternate Value = Mean Haemoglobin Concentration − WHO−2001
Mean Haemoglobin Concentration
Total Anaemia Prevalence
Measure to measure crosswalk − Children Under 5 y.o.
Reference Value = Moderate+Severe Anaemia Prevalence − WHO−2024
Alternate Value = Mean Haemoglobin Concentration − WHO−2024
Mean Haemoglobin Concentration
Moderate+Severe Anaemia Prevalence
Measure to measure crosswalk − Children Under 5 y.o.
Reference Value = Moderate+Severe Anaemia Prevalence − WHO−2001
Alternate Value = Mean Haemoglobin Concentration − WHO−2001
Mean Haemoglobin Concentration
Moderate+Severe Anaemia Prevalence
Measure to measure crosswalk − Children Under 5 y.o.
Reference Value = Mean Haemoglobin Concentration − WHO−2024
Alternate Value = Moderate+Severe Anaemia Prevalence − WHO−2024
Moderate+Severe Anaemia Prevalence
Mean Haemoglobin Concentration
Measure to measure crosswalk − Children Under 5 y.o.
Reference Value = Mean Haemoglobin Concentration − WHO−2024
Alternate Value = Severe Anaemia Prevalence − WHO−2024
Severe Anaemia Prevalence
Mean Haemoglobin Concentration
Measure to measure crosswalk − Children Under 5 y.o.
Reference Value = Mean Haemoglobin Concentration − WHO−2024
Alternate Value = Total Anaemia Prevalence − WHO−2024
Total Anaemia Prevalence
Mean Haemoglobin Concentration
Measure to measure crosswalk − Children Under 5 y.o.
Reference Value = Mean Haemoglobin Concentration − WHO−2001
Alternate Value = Moderate+Severe Anaemia Prevalence − WHO−2001
Moderate+Severe Anaemia Prevalence
Mean Haemoglobin Concentration
Measure to measure crosswalk − Children Under 5 y.o.
Reference Value = Mean Haemoglobin Concentration − WHO−2001
Alternate Value = Severe Anaemia Prevalence − WHO−2001
Severe Anaemia Prevalence
Mean Haemoglobin Concentration
Measure to measure crosswalk − Children Under 5 y.o.
Reference Value = Mean Haemoglobin Concentration − WHO−2001
Alternate Value = Total Anaemia Prevalence − WHO−2001
Total Anaemia Prevalence
Mean Haemoglobin Concentration

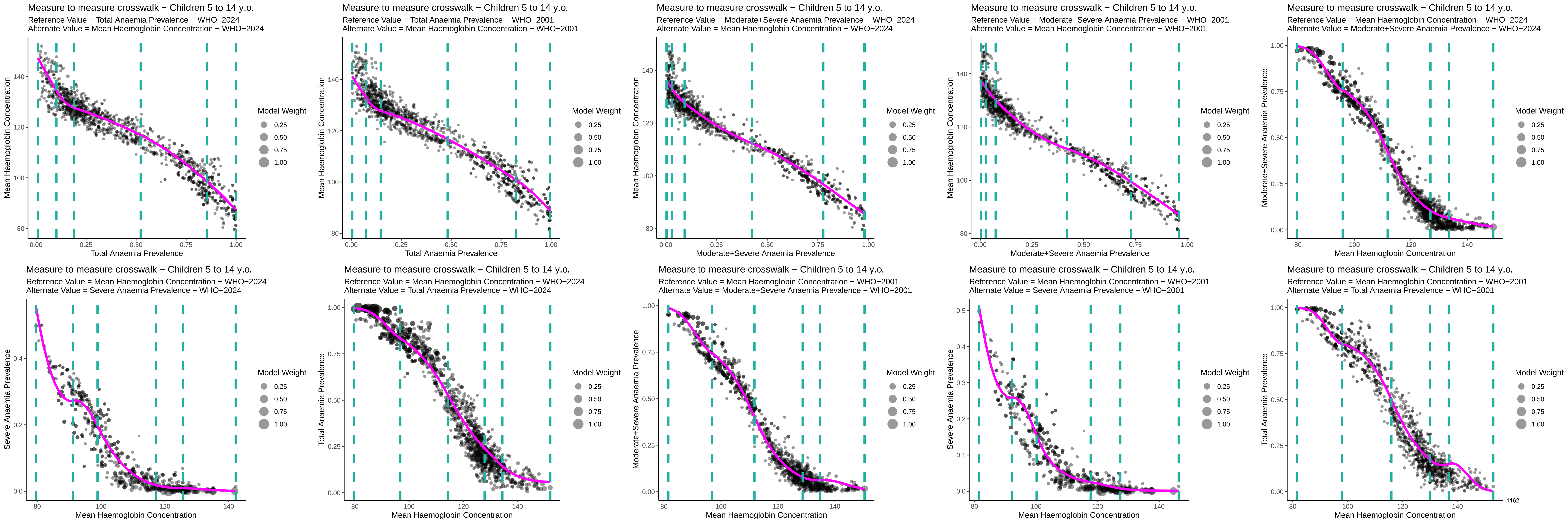

Measure to measure crosswalk − Children 5 to 14 y.o.
Reference Value = Total Anaemia Prevalence − WHO−2024
Alternate Value = Mean Haemoglobin Concentration − WHO−2024
Mean Haemoglobin Concentration
Total Anaemia Prevalence
Model Weight
0.25
0.50
0.75
1.00
Measure to measure crosswalk − Children 5 to 14 y.o.
Reference Value = Total Anaemia Prevalence − WHO−2001
Alternate Value = Mean Haemoglobin Concentration − WHO−2001
Mean Haemoglobin Concentration
Total Anaemia Prevalence
Model Weight
Measure to measure crosswalk − Children 5 to 14 y.o.
Reference Value = Moderate+Severe Anaemia Prevalence − WHO−2024
Alternate Value = Mean Haemoglobin Concentration − WHO−2024
Mean Haemoglobin Concentration
Moderate+Severe Anaemia Prevalence
Model Weight
Measure to measure crosswalk − Children 5 to 14 y.o.
Reference Value = Moderate+Severe Anaemia Prevalence − WHO−2001
Alternate Value = Mean Haemoglobin Concentration − WHO−2001
Mean Haemoglobin Concentration
Moderate+Severe Anaemia Prevalence
Model Weight
Measure to measure crosswalk − Children 5 to 14 y.o.
Reference Value = Mean Haemoglobin Concentration − WHO−2024
Alternate Value = Moderate+Severe Anaemia Prevalence − WHO−2024
Moderate+Severe Anaemia Prevalence
Mean Haemoglobin Concentration
Model Weight
Measure to measure crosswalk − Children 5 to 14 y.o.
Reference Value = Mean Haemoglobin Concentration − WHO−2024
Alternate Value = Severe Anaemia Prevalence − WHO−2024
Severe Anaemia Prevalence
Mean Haemoglobin Concentration
Model Weight
Measure to measure crosswalk − Children 5 to 14 y.o.
Reference Value = Mean Haemoglobin Concentration − WHO−2024
Alternate Value = Total Anaemia Prevalence − WHO−2024
Total Anaemia Prevalence
Mean Haemoglobin Concentration
Model Weight
Measure to measure crosswalk − Children 5 to 14 y.o.
Reference Value = Mean Haemoglobin Concentration − WHO−2001
Alternate Value = Moderate+Severe Anaemia Prevalence − WHO−2001
Moderate+Severe Anaemia Prevalence
Mean Haemoglobin Concentration
Model Weight
Measure to measure crosswalk − Children 5 to 14 y.o.
Reference Value = Mean Haemoglobin Concentration − WHO−2001
Alternate Value = Severe Anaemia Prevalence − WHO−2001
Severe Anaemia Prevalence
Mean Haemoglobin Concentration
Model Weight
Measure to measure crosswalk − Children 5 to 14 y.o.
Reference Value = Mean Haemoglobin Concentration − WHO−2001
Alternate Value = Total Anaemia Prevalence − WHO−2001
Total Anaemia Prevalence
Mean Haemoglobin Concentration
Model Weight

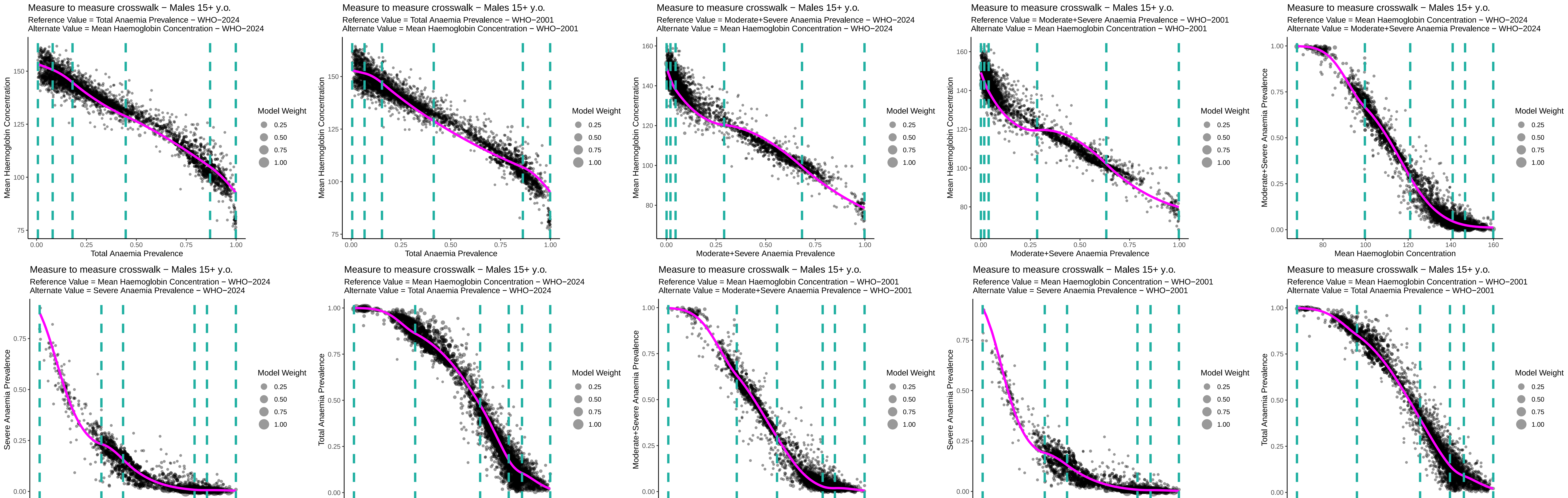
Measure to measure crosswalk − Males 15+ y.o.
Reference Value = Total Anaemia Prevalence − WHO−2024
Alternate Value = Mean Haemoglobin Concentration − WHO−2024
Mean Haemoglobin Concentration
Total Anaemia Prevalence
Model Weight
0.25
0.50
0.75
1.00
Measure to measure crosswalk − Males 15+ y.o.
Reference Value = Total Anaemia Prevalence − WHO−2001
Alternate Value = Mean Haemoglobin Concentration − WHO−2001
Measure to measure crosswalk − Males 15+ y.o.
Reference Value = Moderate+Severe Anaemia Prevalence − WHO−2024
Alternate Value = Mean Haemoglobin Concentration − WHO−2024
Moderate+Severe Anaemia Prevalence
Measure to measure crosswalk − Males 15+ y.o.
Reference Value = Moderate+Severe Anaemia Prevalence − WHO−2001
Alternate Value = Mean Haemoglobin Concentration − WHO−2001
Measure to measure crosswalk − Males 15+ y.o.
Reference Value = Mean Haemoglobin Concentration − WHO−2024
Alternate Value = Moderate+Severe Anaemia Prevalence − WHO−2024
Measure to measure crosswalk − Males 15+ y.o.
Reference Value = Mean Haemoglobin Concentration − WHO−2024
Alternate Value = Severe Anaemia Prevalence − WHO−2024
Severe Anaemia Prevalence
Measure to measure crosswalk − Males 15+ y.o.
Reference Value = Mean Haemoglobin Concentration − WHO−2024
Alternate Value = Total Anaemia Prevalence − WHO−2024
Measure to measure crosswalk − Males 15+ y.o.
Reference Value = Mean Haemoglobin Concentration − WHO−2001
Alternate Value = Moderate+Severe Anaemia Prevalence − WHO−2001
Measure to measure crosswalk − Males 15+ y.o.
Reference Value = Mean Haemoglobin Concentration − WHO−2001
Alternate Value = Severe Anaemia Prevalence − WHO−2001
Measure to measure crosswalk − Males 15+ y.o.
Reference Value = Mean Haemoglobin Concentration − WHO−2001
Alternate Value = Total Anaemia Prevalence − WHO−2001

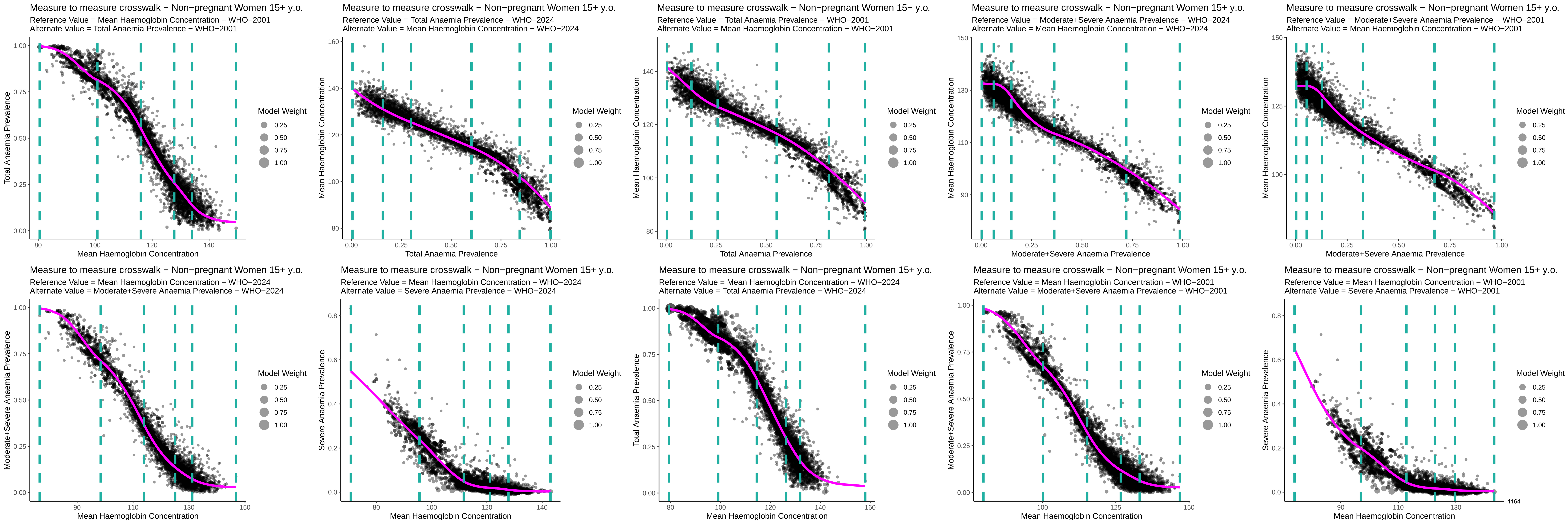

Measure to measure crosswalk − Non−pregnant Women 15+ y.o.
Reference Value = Mean Haemoglobin Concentration − WHO−2001
Alternate Value = Total Anaemia Prevalence − WHO−2001
Total Anaemia Prevalence
Mean Haemoglobin Concentration
Model Weight
0.25
0.50
0.75
1.00
Measure to measure crosswalk − Non−pregnant Women 15+ y.o.
Reference Value = Total Anaemia Prevalence − WHO−2024
Alternate Value = Mean Haemoglobin Concentration − WHO−2024
Mean Haemoglobin Concentration
Total Anaemia Prevalence
Model Weight
Measure to measure crosswalk − Non−pregnant Women 15+ y.o.
Reference Value = Total Anaemia Prevalence − WHO−2001
Alternate Value = Mean Haemoglobin Concentration − WHO−2001
Mean Haemoglobin Concentration
Total Anaemia Prevalence
Model Weight
Measure to measure crosswalk − Non−pregnant Women 15+ y.o.
Reference Value = Moderate+Severe Anaemia Prevalence − WHO−2024
Alternate Value = Mean Haemoglobin Concentration − WHO−2024
Mean Haemoglobin Concentration
Moderate+Severe Anaemia Prevalence
Model Weight
Measure to measure crosswalk − Non−pregnant Women 15+ y.o.
Reference Value = Moderate+Severe Anaemia Prevalence − WHO−2001
Alternate Value = Mean Haemoglobin Concentration − WHO−2001
Mean Haemoglobin Concentration
Moderate+Severe Anaemia Prevalence
Model Weight
Measure to measure crosswalk − Non−pregnant Women 15+ y.o.
Reference Value = Mean Haemoglobin Concentration − WHO−2024
Alternate Value = Moderate+Severe Anaemia Prevalence − WHO−2024
Moderate+Severe Anaemia Prevalence
Mean Haemoglobin Concentration
Model Weight
Measure to measure crosswalk − Non−pregnant Women 15+ y.o.
Reference Value = Mean Haemoglobin Concentration − WHO−2024
Alternate Value = Severe Anaemia Prevalence − WHO−2024
Severe Anaemia Prevalence
Mean Haemoglobin Concentration
Model Weight
Measure to measure crosswalk − Non−pregnant Women 15+ y.o.
Reference Value = Mean Haemoglobin Concentration − WHO−2024
Alternate Value = Total Anaemia Prevalence − WHO−2024
Total Anaemia Prevalence
Mean Haemoglobin Concentration
Model Weight
Measure to measure crosswalk − Non−pregnant Women 15+ y.o.
Reference Value = Mean Haemoglobin Concentration − WHO−2001
Alternate Value = Moderate+Severe Anaemia Prevalence − WHO−2001
Moderate+Severe Anaemia Prevalence
Mean Haemoglobin Concentration
Model Weight
Measure to measure crosswalk − Non−pregnant Women 15+ y.o.
Reference Value = Mean Haemoglobin Concentration − WHO−2001
Alternate Value = Severe Anaemia Prevalence − WHO−2001
Severe Anaemia Prevalence
Mean Haemoglobin Concentration
Model Weight

Section 6: MR-BRT modelled elevation adjustment crosswalk betas

MR-BRT modelled betas used to crosswalk total, moderate plus severe, and severe anaemia input data point from the WHO-2001 adjustment envelope to WHO-2024 adjustment envelope, or vice versa. The predicted betas are expressed in logit space and are applied to the logit-transformed reference anaemia prevalence value. This is done by adding the corresponding set of betas based on the input point's age, sex, elevation of residence, and anaemia prevalence, as determined by the reported elevation adjustment method for that input point. This set of crosswalk models are used to ensure each input data source is present in the WHO-2001 and WHO-2024 elevation adjustment anaemia envelopes, therefore creating internal consistency between the two sets of elevation adjustment ST-GPR models.

Model	Data input	Reference or alternative case definition	Gamma	Beta coefficient, logit difference (95% UI)*
**Total Anaemia**	Non-pregnant WRA<br>0% to 20% ref. anaemia prevalence<br>No elevation	Ref	0.00040	---
	Children under 5 y.o.	Alt		0.055<br>(-0.031 to 0.13)
	Children 5-14 y.o.	Alt		0.017<br>(-0.068 to 0.099)
	Males 15+ y.o.	Alt		-0.018<br>(-0.097 to 0.058)
	Pregnant WRA	Alt		0.008<br>(-0.076 to 0.093)
	1 to 400 metres	Alt		0.21<br>(0.18 to 0.24)
	401 to 800 metres	Alt		0.32<br>(0.28 to 0.36)
	801 to 1,200 metres	Alt		0.51<br>(0.47 to 0.55)
	1,201 to 1,600 metres	Alt		0.52<br>(0.49 to 0.57)
	1,601 to 2,000 metres	Alt		0.44<br>(0.4 to 0.48)
	2,001 to 2,400 metres	Alt		0.29<br>(0.24 to 0.33)
	2,401 to 2,800 metres	Alt		0.13<br>(0.067 to 0.18)
	2,801 to 3,200 metres	Alt		-0.34<br>(-0.43 to -0.26)
	3,201 to 3,600 metres	Alt		-0.5<br>(-0.65 to -0.36)
	3,601 to 4,000 metres	Alt		-0.96<br>(-1.1 to -0.85)
	4,001 to 4,400 metres	Alt		-1.2<br>(-1.4 to -0.97)
	4,401 to 4,800 metres	Alt		-2<br>(-2.8 to -1.2)
	20% to 40% ref. anaemia prev.	Alt		-0.033<br>(-0.11 to 0.04)
	40% to 60% ref. anaemia prev.	Alt		-0.037<br>(-0.11 to 0.033)
	60% to 80% ref. anaemia prev.	Alt		-0.052<br>(-0.13 to 0.022)
	80% to 100% ref. anaemia prev.	Alt		-0.096<br>(-0.18 to -0.017)

Model	Data input	Reference or alternative case definition	Gamma	Beta coefficient, logit difference (95% UI)*
Moderate + Severe Anaemia	Non-pregnant WRA<br>0% to 20% ref. anaemia prevalence<br>No elevation	Ref	0.0027	---
	Children under 5 y.o.	Alt		-0.13<br>(-0.33 to 0.080)
	Children 5-14 y.o.	Alt		-0.10<br>(-0.31 to 0.089)
	Males 15+ y.o.	Alt		-0.23<br>(-0.43 to -0.027)
	Pregnant WRA	Alt		-0.16<br>(-0.34 to 0.046)
	1 to 400 metres	Alt		0.16<br>(0.13 to 0.20)
	401 to 800 metres	Alt		0.28<br>(0.24 to 0.37)
	801 to 1,200 metres	Alt		0.41<br>(0.36 to 0.45)
	1,201 to 1,600 metres	Alt		0.39<br>(0.33 to 0.44)
	1,601 to 2,000 metres	Alt		0.37<br>(0.32 to 0.43)
	2,001 to 2,400 metres	Alt		0.28<br>(0.22 to 0.35)
	2,401 to 2,800 metres	Alt		0.12<br>(0.037 to 0.22)
	2,801 to 3,200 metres	Alt		-0.16<br>(-0.27 to -0.039)
	3,201 to 3,600 metres	Alt		-0.52<br>(-0.72 to -0.36)
	3,601 to 4,000 metres	Alt		-0.89<br>(-1 to -0.73)
	4,001 to 4,400 metres	Alt		-1.1<br>(-1.4 to -0.87)
	4,401 to 4,800 metres	Alt		-1.9<br>(-2.9 to -0.69)
	20% to 40% ref. anaemia prev.	Alt		-0.20<br>(-0.40 to -0.0014)
	40% to 60% ref. anaemia prev.	Alt		-0.20<br>(-0.37 to 0.00079)
	60% to 80% ref. anaemia prev.	Alt		-0.26<br>(-0.47 to -0.063)
	80% to 100% ref. anaemia prev.	Alt		-0.33<br>(-0.54 to -0.14)

Model	Data input	Reference or alternative case definition	Gamma	Beta coefficient, logit difference (95% UI)*
Severe Anaemia	Non-pregnant WRA<br>0% to 20% ref. anaemia prevalence<br>No elevation	Ref	0.00040	---
	Children under 5 y.o.	Alt		-0.032<br>(-0.47 to 0.40)
	Children 5-14 y.o.	Alt		-0.092<br>(-0.55 to 0.36)
	Males 15+ y.o.	Alt		-0.034<br>(-0.50 to 0.38)
	Pregnant WRA	Alt		-0.037<br>(-0.49 to 0.39)
	1 to 400 metres	Alt		0.14<br>(0.11 to 0.17)
	401 to 800 metres	Alt		0.24<br>(0.17 to 0.30)
	801 to 1,200 metres	Alt		0.34<br>(0.25 to 0.41)
	1,201 to 1,600 metres	Alt		0.36<br>(0.28 to 0.44)
	1,601 to 2,000 metres	Alt		0.29<br>(0.19 to 0.39)
	2,001 to 2,400 metres	Alt		0.18<br>(0.053 to 0.31)
	2,401 to 2,800 metres	Alt		0.13<br>(-0.0034 to 0.28)
	2,801 to 3,200 metres	Alt		-0.097<br>(-0.33 to 0.13)
	3,201 to 3,600 metres	Alt		-0.36<br>(-0.70 to -0.024)
	3,601 to 4,000 metres	Alt		-0.89<br>(-1.1 to -0.63)
	4,001 to 4,400 metres	Alt		-0.81<br>(-1.3 to -0.33)
	20% to 40% ref. anaemia prev.	Alt		-0.12<br>(-0.57 to 0.30)
	40% to 60% ref. anaemia prev.	Alt		-0.11<br>(-0.59 to 0.40)
	60% to 80% ref. anaemia prev.	Alt		-0.19<br>(-1.0 to 0.60)

Section 7: Post-ensemble modelled estimates for the WHO-2001 and WHO-2024 envelopes

Mean haemoglobin and total, moderate plus severe, and severe anaemia prevalence estimates for all age, sex, location, and year combinations produced in our modelling pipeline. Each page is associated with a location, where males and females (both pregnant and non-pregnant) are shown on the left- and right-hand side of each page, respectively. Each plot is faceted by modelled age group, and each plot displays years along the x-axis. The top row of plots displays modelled mean haemoglobin estimates, and the bottom row of plots displays modelled total (blue), moderate plus severe (yellow), and severe (red) anaemia prevalence. The solid line in each plot is associated with modelled estimates from the WHO-2024 elevation adjustment anaemia envelope and the dashed line is associated with the WHO-2001 elevation adjustment envelope. The 95% uncertainty interval (shown by the lightly shaded band in each plot) is associated with the WHO-2024 elevation adjustment anaemia envelope only. Input data is plotted (when present) and is colour coded by MR-BRT adjustment method used in the mean haemoglobin plots and is encoded by shape in the anaemia prevalence plots.

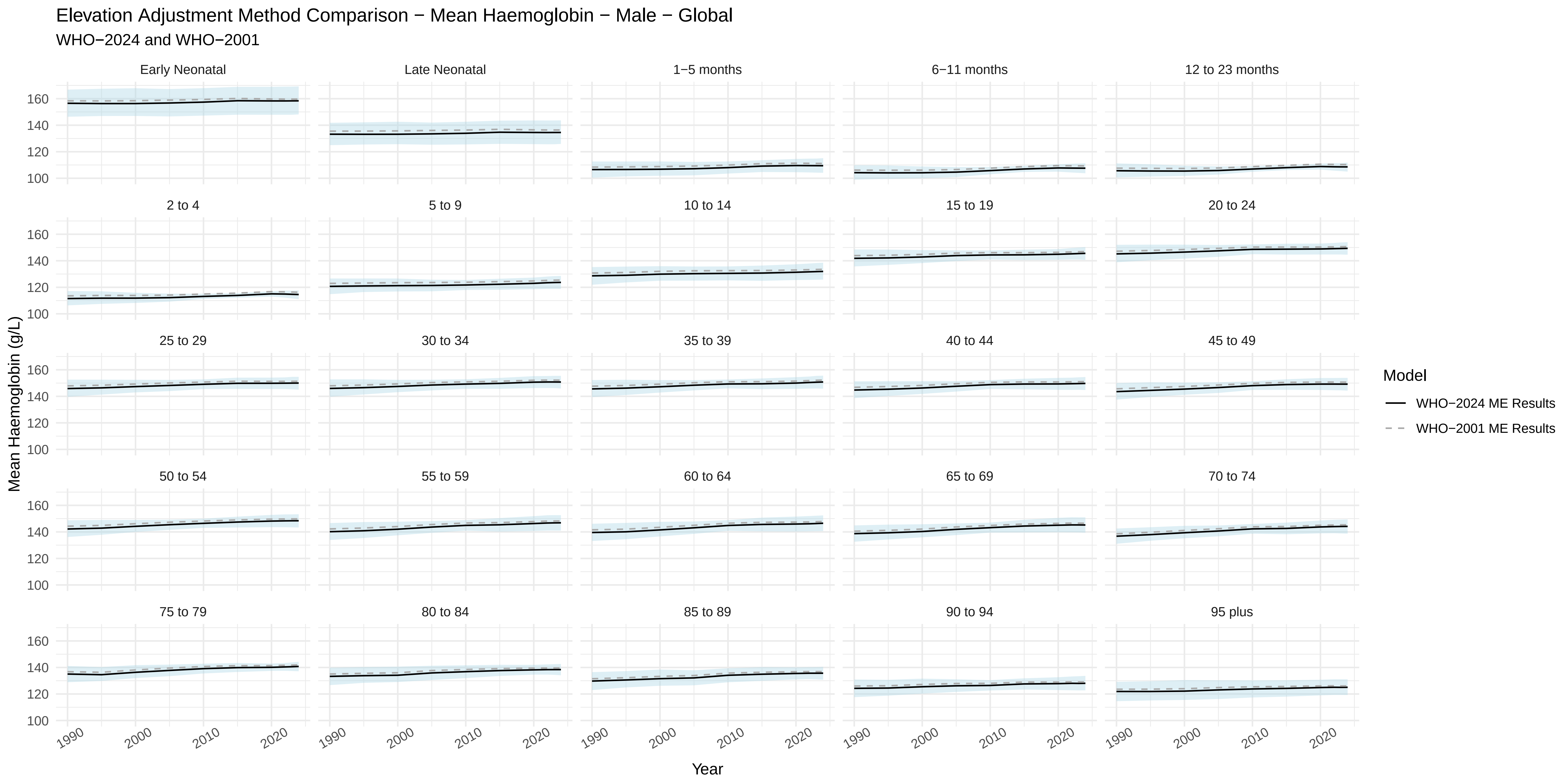
Elevation Adjustment Method Comparison − Mean Haemoglobin − Male − Global
WHO−2024 and WHO−2001
Early Neonatal
Late Neonatal
1−5 months
6−11 months
12 to 23 months
2 to 4
5 to 9
10 to 14
15 to 19
20 to 24
25 to 29
30 to 34
35 to 39
40 to 44
45 to 49
50 to 54
55 to 59
60 to 64
65 to 69
70 to 74
75 to 79
80 to 84
85 to 89
90 to 94
95 plus
Mean Haemoglobin (g/L)
Year
Model
WHO−2024 ME Results
WHO−2001 ME Results

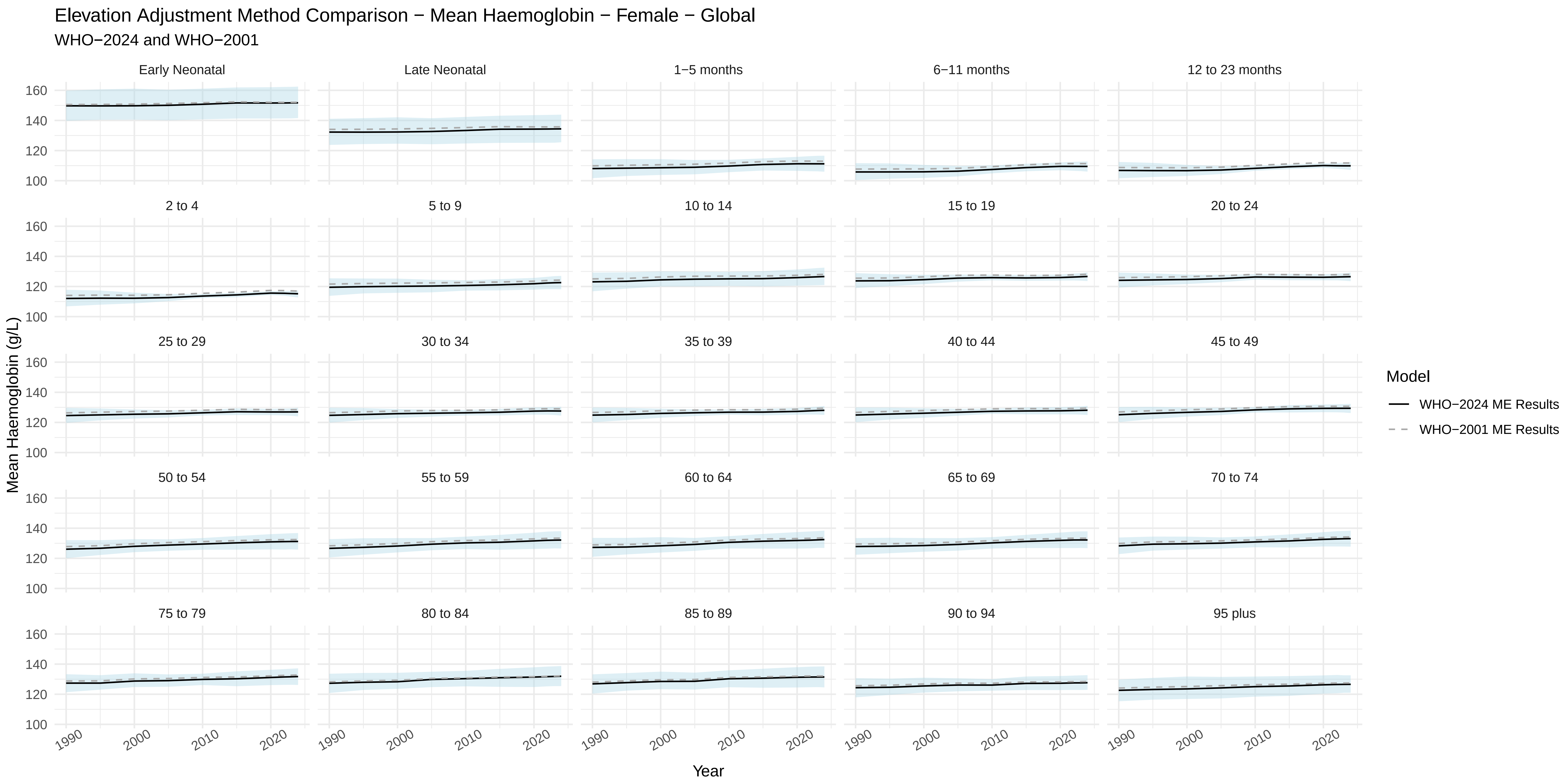
Elevation Adjustment Method Comparison − Mean Haemoglobin − Female − Global
WHO−2024 and WHO−2001
Early Neonatal
Late Neonatal
1−5 months
6−11 months
12 to 23 months
2 to 4
5 to 9
10 to 14
15 to 19
20 to 24
25 to 29
30 to 34
35 to 39
40 to 44
45 to 49
50 to 54
55 to 59
60 to 64
65 to 69
70 to 74
75 to 79
80 to 84
85 to 89
90 to 94
95 plus
Mean Haemoglobin (g/L)
Year
Model
WHO−2024 ME Results
WHO−2001 ME Results

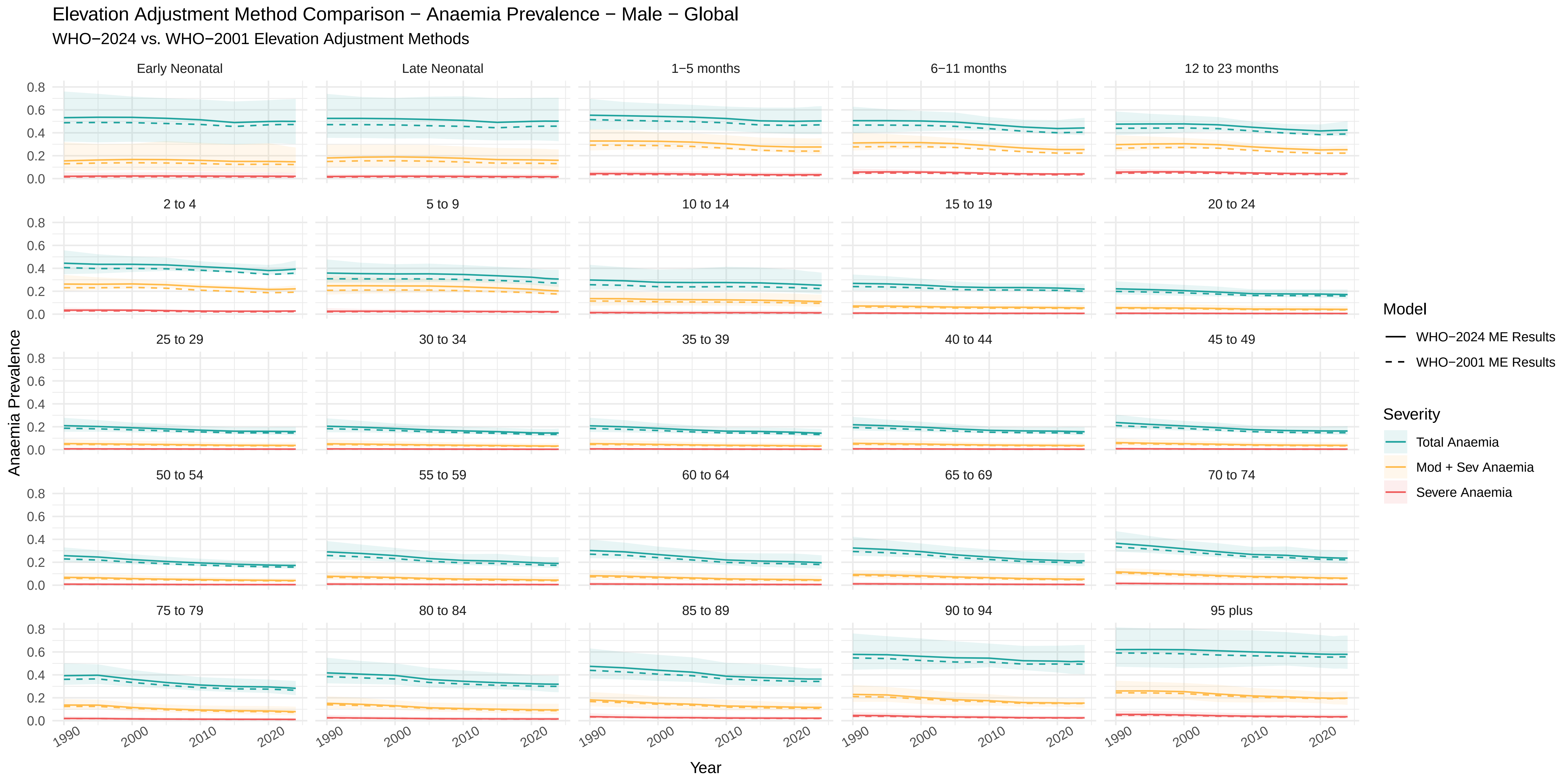
Elevation Adjustment Method Comparison − Anaemia Prevalence − Male − Global
WHO−2024 vs. WHO−2001 Elevation Adjustment Methods
Early Neonatal
Late Neonatal
1−5 months
6−11 months
12 to 23 months
2 to 4
5 to 9
10 to 14
15 to 19
20 to 24
25 to 29
30 to 34
35 to 39
40 to 44
45 to 49
50 to 54
55 to 59
60 to 64
65 to 69
70 to 74
75 to 79
80 to 84
85 to 89
90 to 94
95 plus
Anaemia Prevalence
Year
Model
WHO−2024 ME Results
WHO−2001 ME Results
Severity
Total Anaemia
Mod + Sev Anaemia
Severe Anaemia

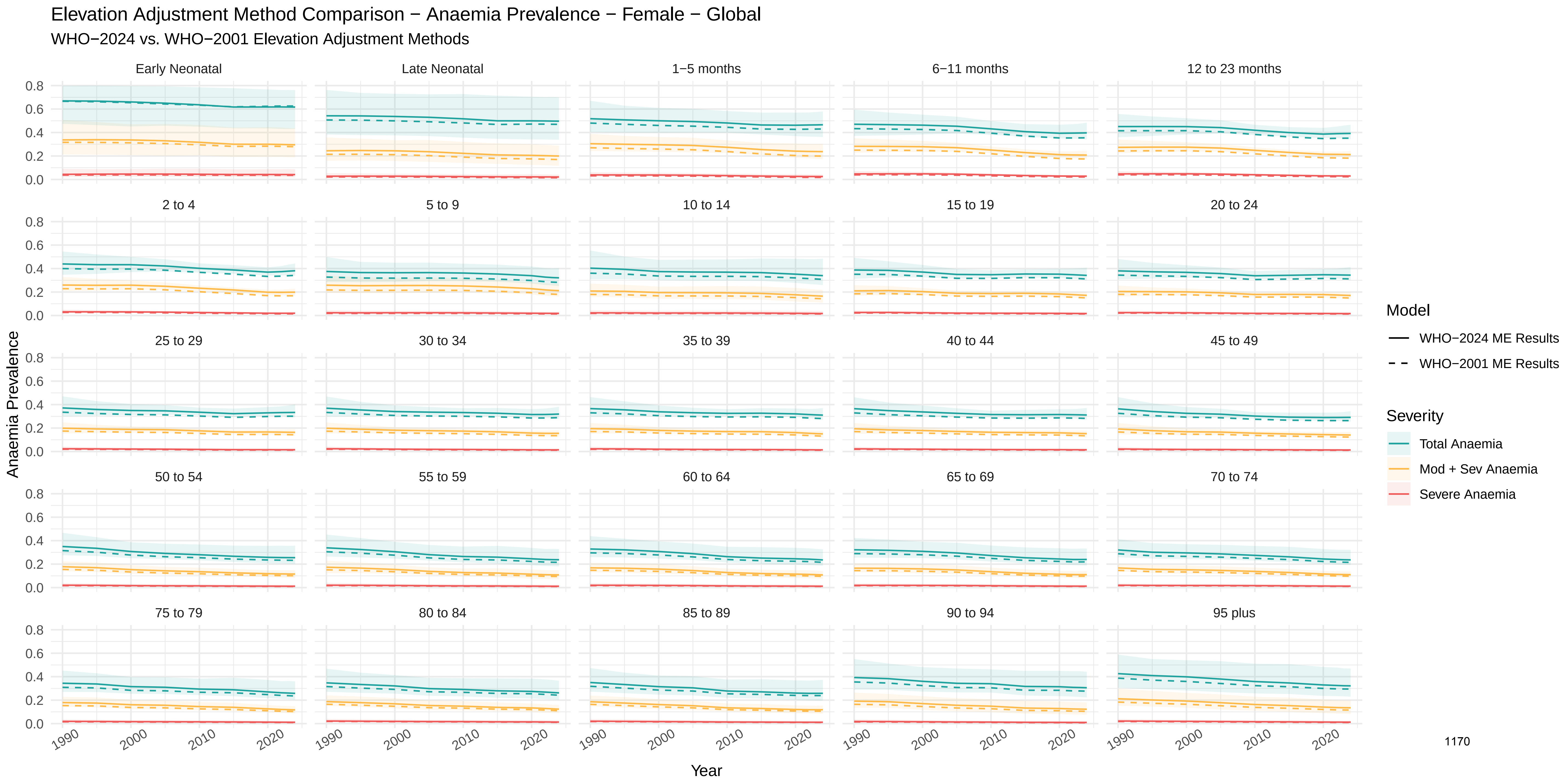
Elevation Adjustment Method Comparison − Anaemia Prevalence − Female − Global
WHO−2024 vs. WHO−2001 Elevation Adjustment Methods
Early Neonatal
Late Neonatal
1−5 months
6−11 months
12 to 23 months
2 to 4
5 to 9
10 to 14
15 to 19
20 to 24
25 to 29
30 to 34
35 to 39
40 to 44
45 to 49
50 to 54
55 to 59
60 to 64
65 to 69
70 to 74
75 to 79
80 to 84
85 to 89
90 to 94
95 plus
Anaemia Prevalence
Year
Model
WHO−2024 ME Results
WHO−2001 ME Results
Severity
Total Anaemia
Mod + Sev Anaemia
Severe Anaemia

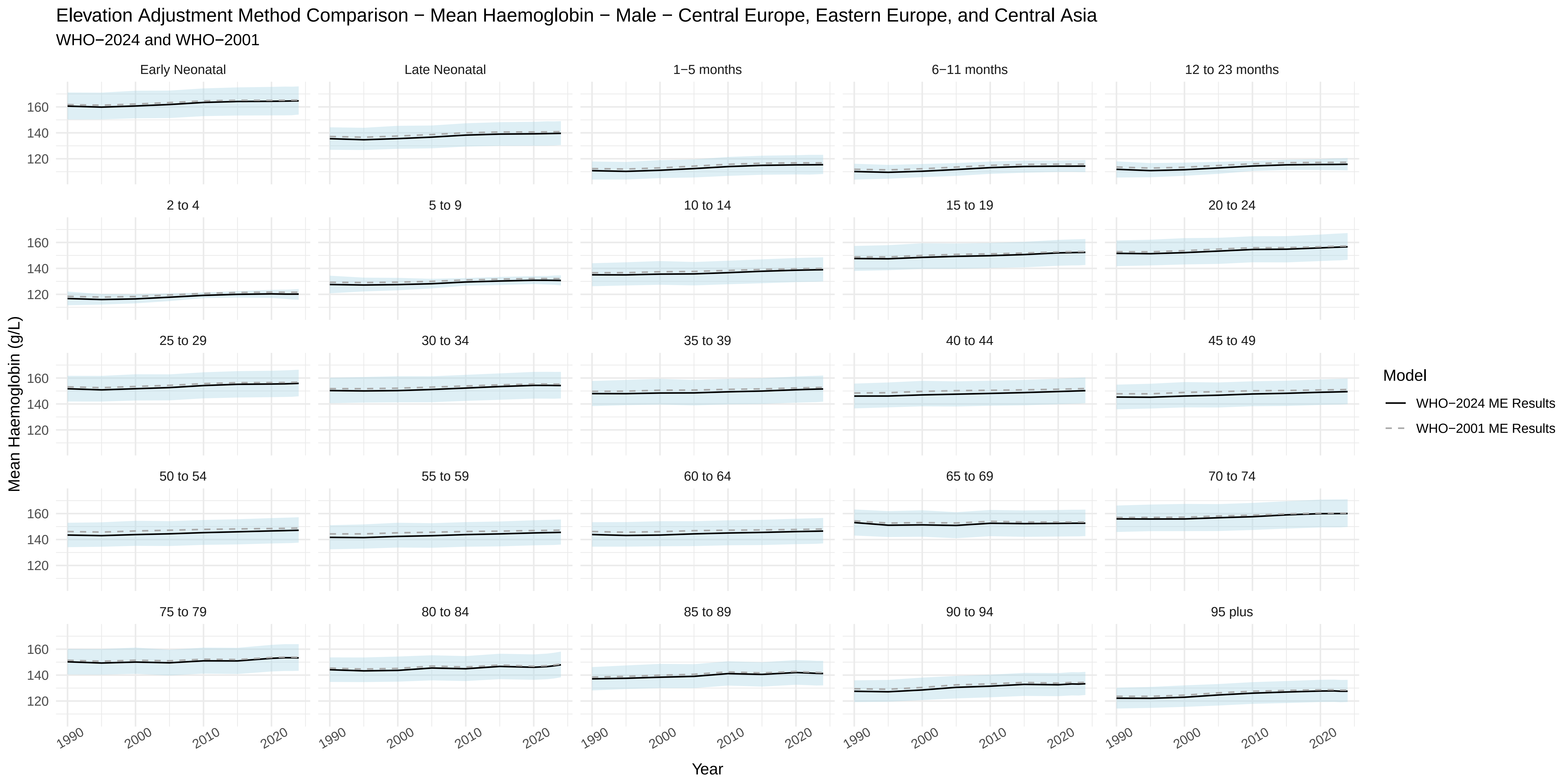
Elevation Adjustment Method Comparison − Mean Haemoglobin − Male − Central Europe, Eastern Europe, and Central Asia
WHO−2024 and WHO−2001
Early Neonatal
Late Neonatal
1−5 months
6−11 months
12 to 23 months
2 to 4
5 to 9
10 to 14
15 to 19
20 to 24
25 to 29
30 to 34
35 to 39
40 to 44
45 to 49
50 to 54
55 to 59
60 to 64
65 to 69
70 to 74
75 to 79
80 to 84
85 to 89
90 to 94
95 plus
Mean Haemoglobin (g/L)
Year
Model
WHO−2024 ME Results
WHO−2001 ME Results

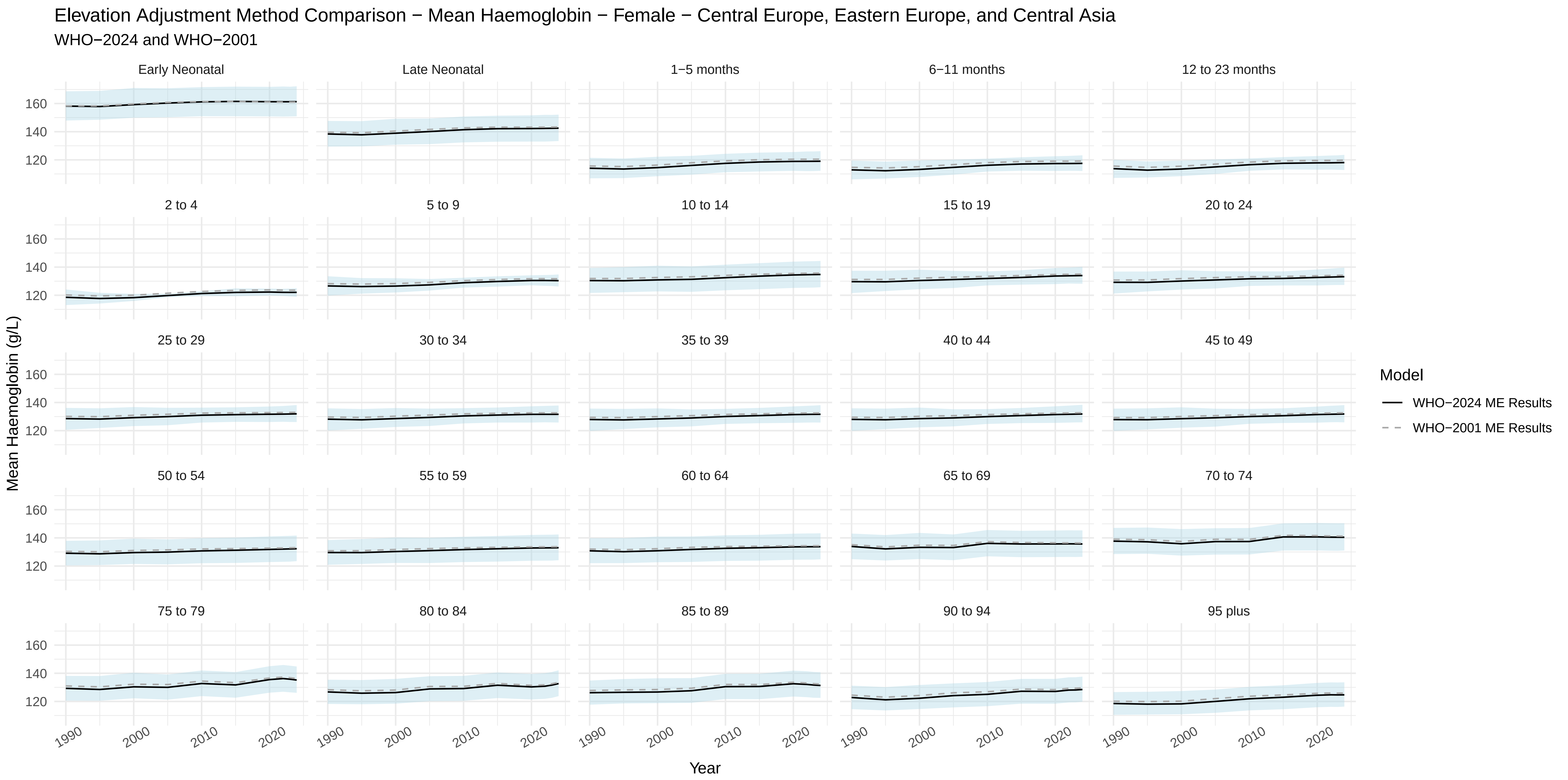
Elevation Adjustment Method Comparison − Mean Haemoglobin − Female − Central Europe, Eastern Europe, and Central Asia
WHO−2024 and WHO−2001
Early Neonatal
Late Neonatal
1−5 months
6−11 months
12 to 23 months
2 to 4
5 to 9
10 to 14
15 to 19
20 to 24
25 to 29
30 to 34
35 to 39
40 to 44
45 to 49
50 to 54
55 to 59
60 to 64
65 to 69
70 to 74
75 to 79
80 to 84
85 to 89
90 to 94
95 plus
Mean Haemoglobin (g/L)
Year
Model
WHO−2024 ME Results
WHO−2001 ME Results

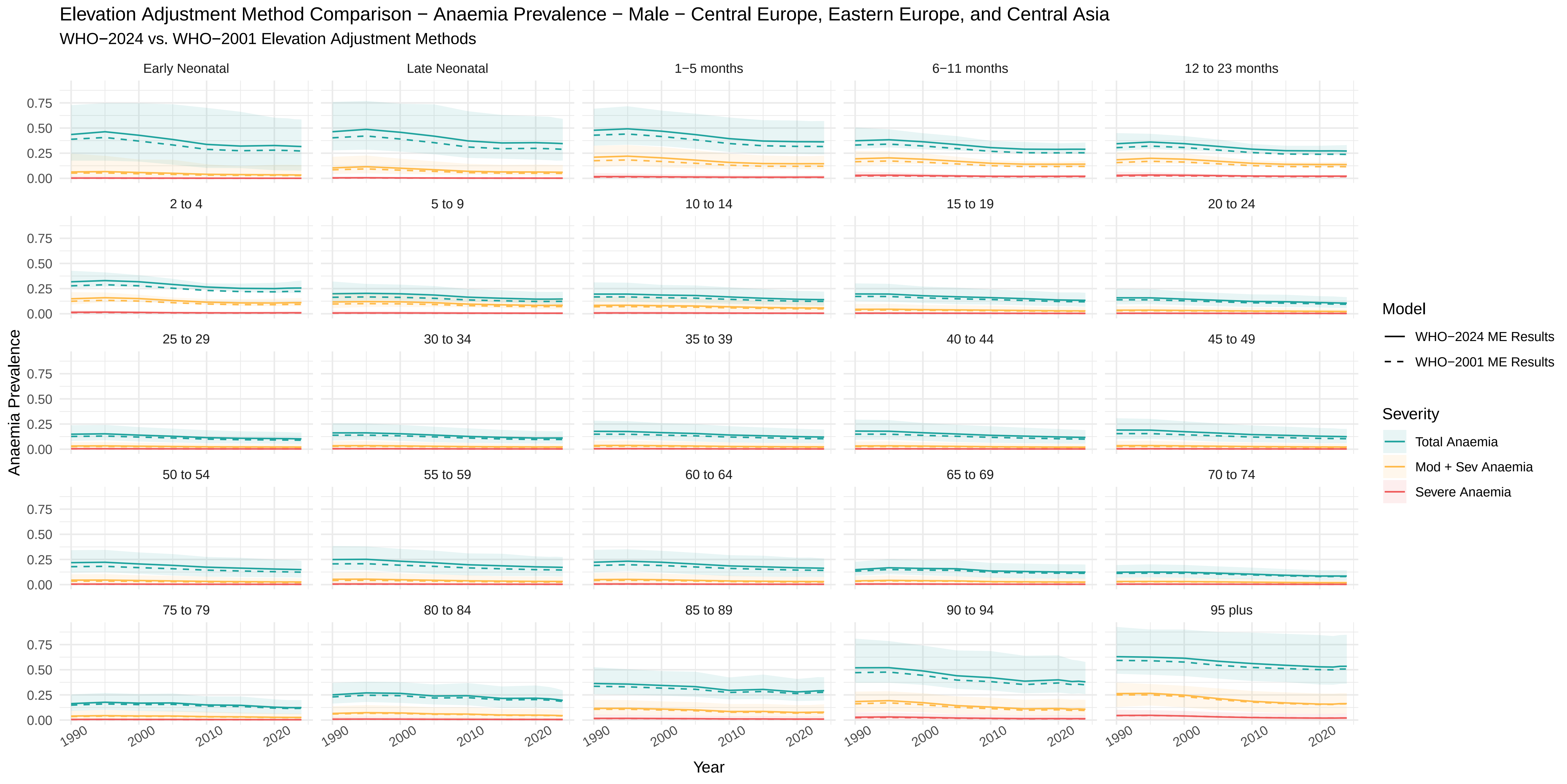
Elevation Adjustment Method Comparison − Anaemia Prevalence − Male − Central Europe, Eastern Europe, and Central Asia
WHO−2024 vs. WHO−2001 Elevation Adjustment Methods
Early Neonatal
Late Neonatal
1−5 months
6−11 months
12 to 23 months
2 to 4
5 to 9
10 to 14
15 to 19
20 to 24
25 to 29
30 to 34
35 to 39
40 to 44
45 to 49
50 to 54
55 to 59
60 to 64
65 to 69
70 to 74
75 to 79
80 to 84
85 to 89
90 to 94
95 plus
Anaemia Prevalence
Year
Model
WHO−2024 ME Results
WHO−2001 ME Results
Severity
Total Anaemia
Mod + Sev Anaemia
Severe Anaemia

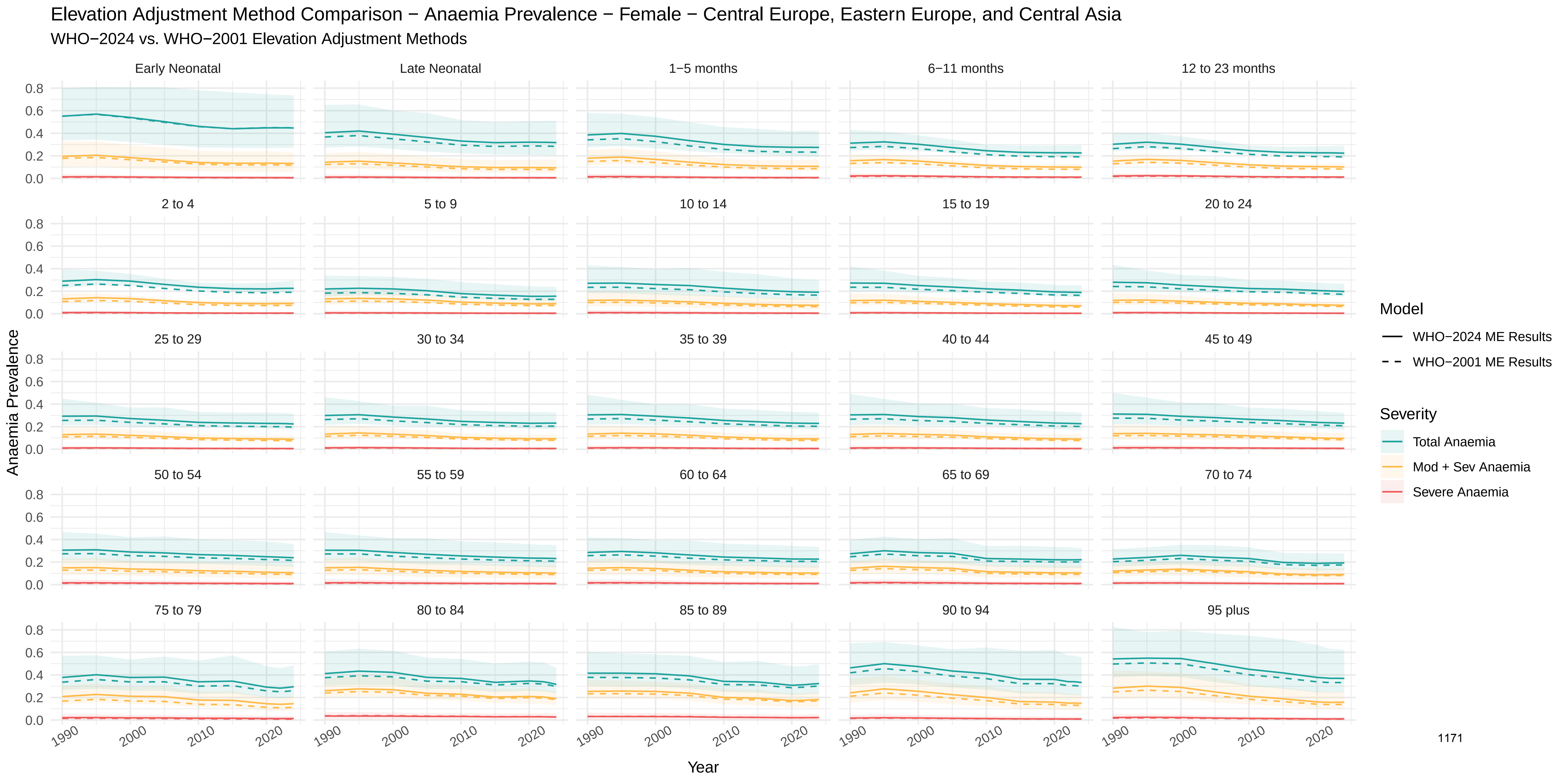
Elevation Adjustment Method Comparison − Anaemia Prevalence − Female − Central Europe, Eastern Europe, and Central Asia
WHO−2024 vs. WHO−2001 Elevation Adjustment Methods
Early Neonatal
Late Neonatal
1−5 months
6−11 months
12 to 23 months
2 to 4
5 to 9
10 to 14
15 to 19
20 to 24
25 to 29
30 to 34
35 to 39
40 to 44
45 to 49
50 to 54
55 to 59
60 to 64
65 to 69
70 to 74
75 to 79
80 to 84
85 to 89
90 to 94
95 plus
Anaemia Prevalence
Year
Model
WHO−2024 ME Results
WHO−2001 ME Results
Severity
Total Anaemia
Mod + Sev Anaemia
Severe Anaemia

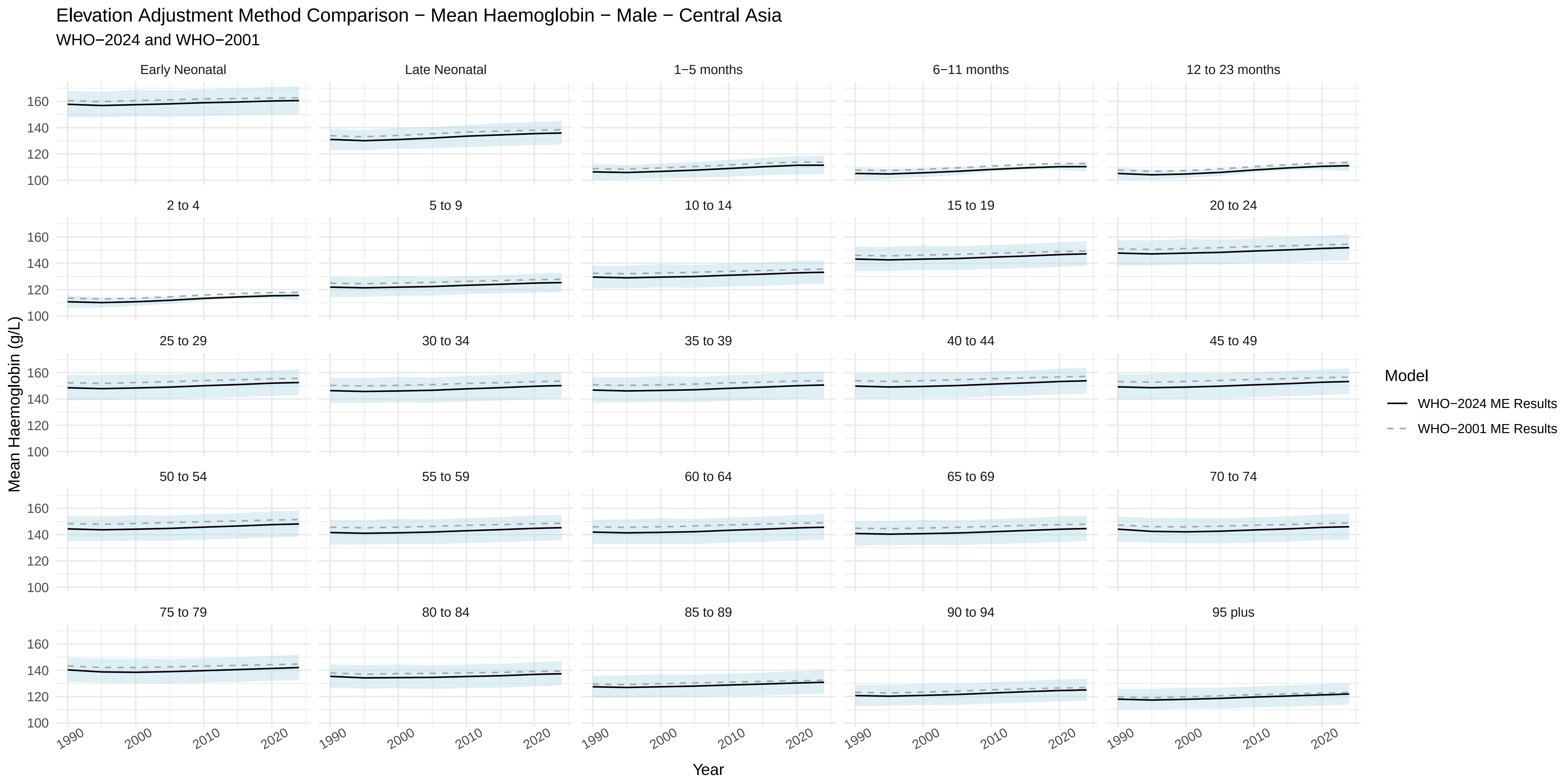
Elevation Adjustment Method Comparison − Mean Haemoglobin − Male − Central Asia
WHO−2024 and WHO−2001
Early Neonatal
Late Neonatal
1−5 months
6−11 months
12 to 23 months
2 to 4
5 to 9
10 to 14
15 to 19
20 to 24
25 to 29
30 to 34
35 to 39
40 to 44
45 to 49
50 to 54
55 to 59
60 to 64
65 to 69
70 to 74
75 to 79
80 to 84
85 to 89
90 to 94
95 plus
Mean Haemoglobin (g/L)
Year
Model
WHO−2024 ME Results
WHO−2001 ME Results

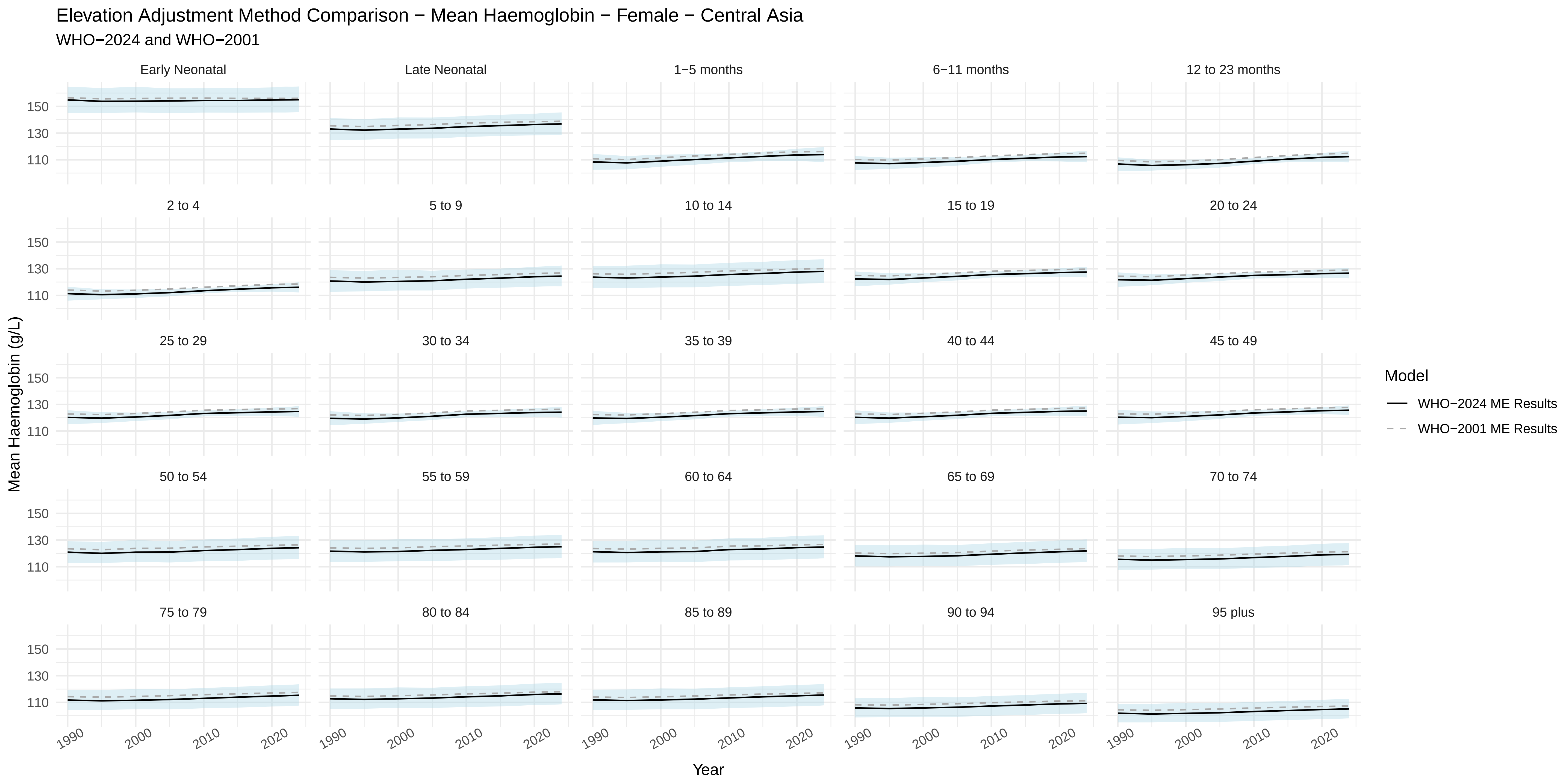
Elevation Adjustment Method Comparison − Mean Haemoglobin − Female − Central Asia
WHO−2024 and WHO−2001
Early Neonatal
Late Neonatal
1−5 months
6−11 months
12 to 23 months
2 to 4
5 to 9
10 to 14
15 to 19
20 to 24
25 to 29
30 to 34
35 to 39
40 to 44
45 to 49
50 to 54
55 to 59
60 to 64
65 to 69
70 to 74
75 to 79
80 to 84
85 to 89
90 to 94
95 plus
Mean Haemoglobin (g/L)
Year
Model
WHO−2024 ME Results
WHO−2001 ME Results

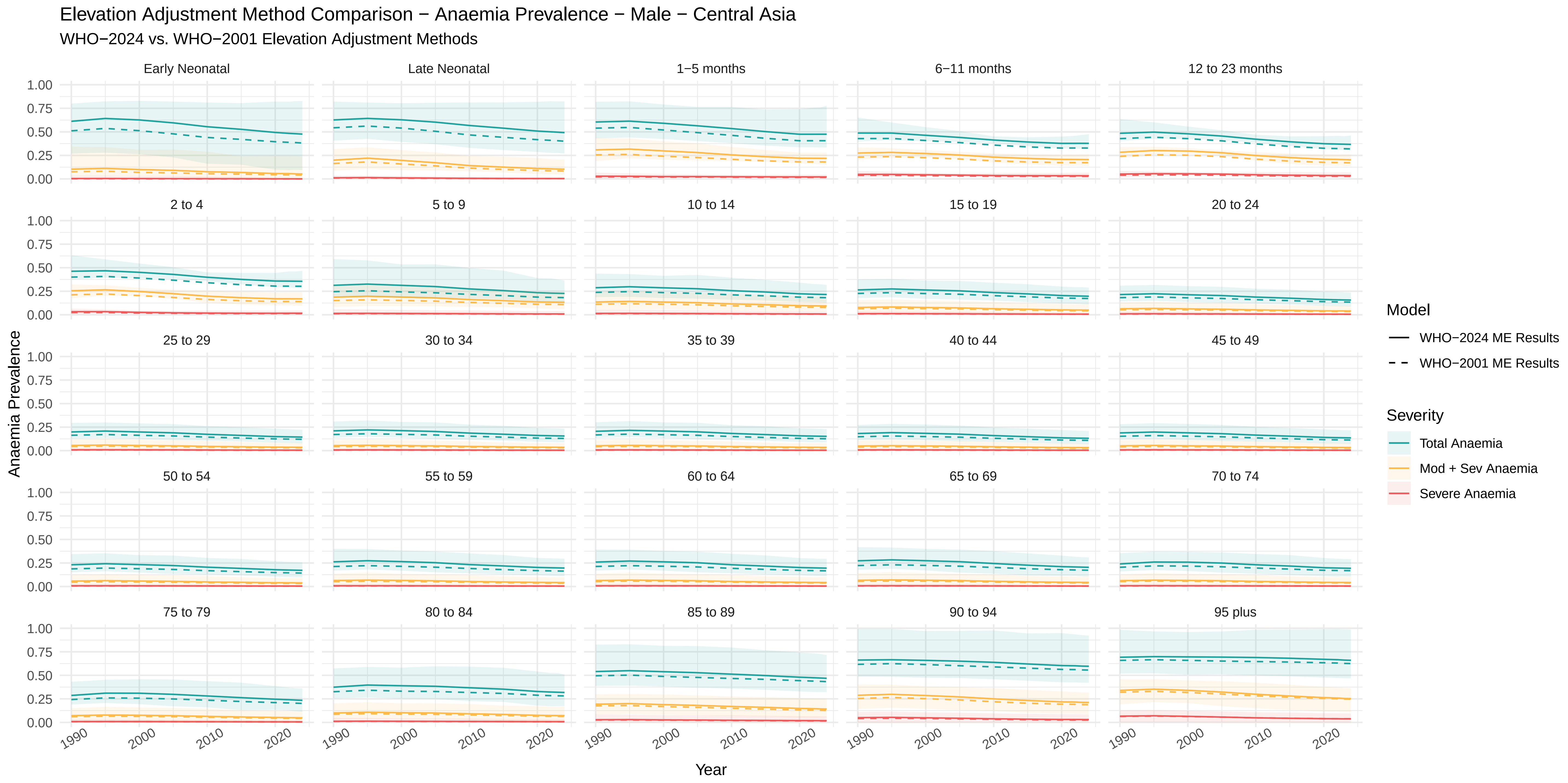
Elevation Adjustment Method Comparison − Anaemia Prevalence − Male − Central Asia
WHO−2024 vs. WHO−2001 Elevation Adjustment Methods
Early Neonatal
Late Neonatal
1−5 months
6−11 months
12 to 23 months
2 to 4
5 to 9
10 to 14
15 to 19
20 to 24
25 to 29
30 to 34
35 to 39
40 to 44
45 to 49
50 to 54
55 to 59
60 to 64
65 to 69
70 to 74
75 to 79
80 to 84
85 to 89
90 to 94
95 plus
Anaemia Prevalence
Year
Model
WHO−2024 ME Results
WHO−2001 ME Results
Severity
Total Anaemia
Mod + Sev Anaemia
Severe Anaemia

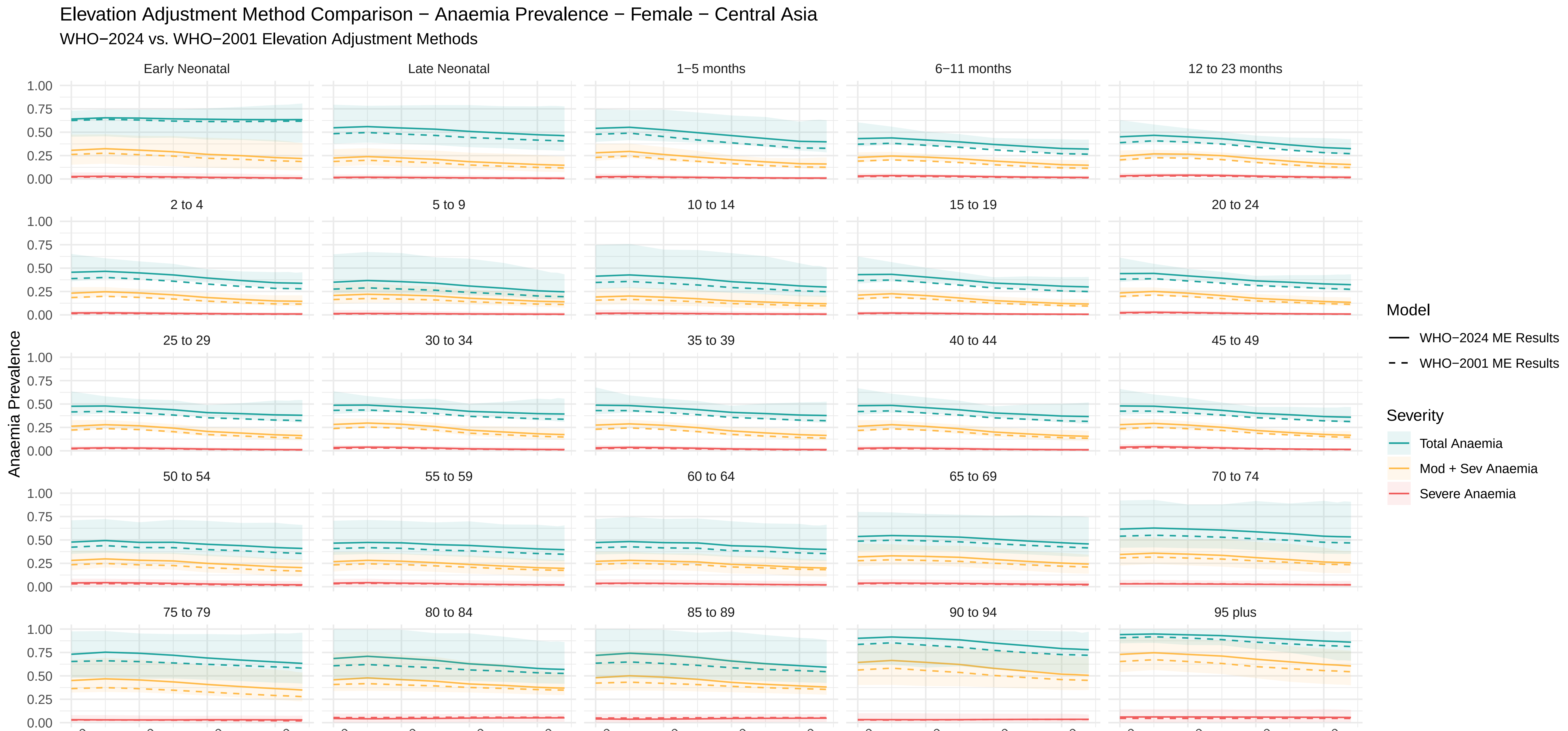
Elevation Adjustment Method Comparison − Anaemia Prevalence − Female − Central Asia
WHO−2024 vs. WHO−2001 Elevation Adjustment Methods
Early Neonatal
Late Neonatal
1−5 months
6−11 months
12 to 23 months
2 to 4
5 to 9
10 to 14
15 to 19
20 to 24
25 to 29
30 to 34
35 to 39
40 to 44
45 to 49
50 to 54
55 to 59
60 to 64
65 to 69
70 to 74
75 to 79
80 to 84
85 to 89
90 to 94
95 plus
Anaemia Prevalence
Year
Model
WHO−2024 ME Results
WHO−2001 ME Results
Severity
Total Anaemia
Mod + Sev Anaemia
Severe Anaemia

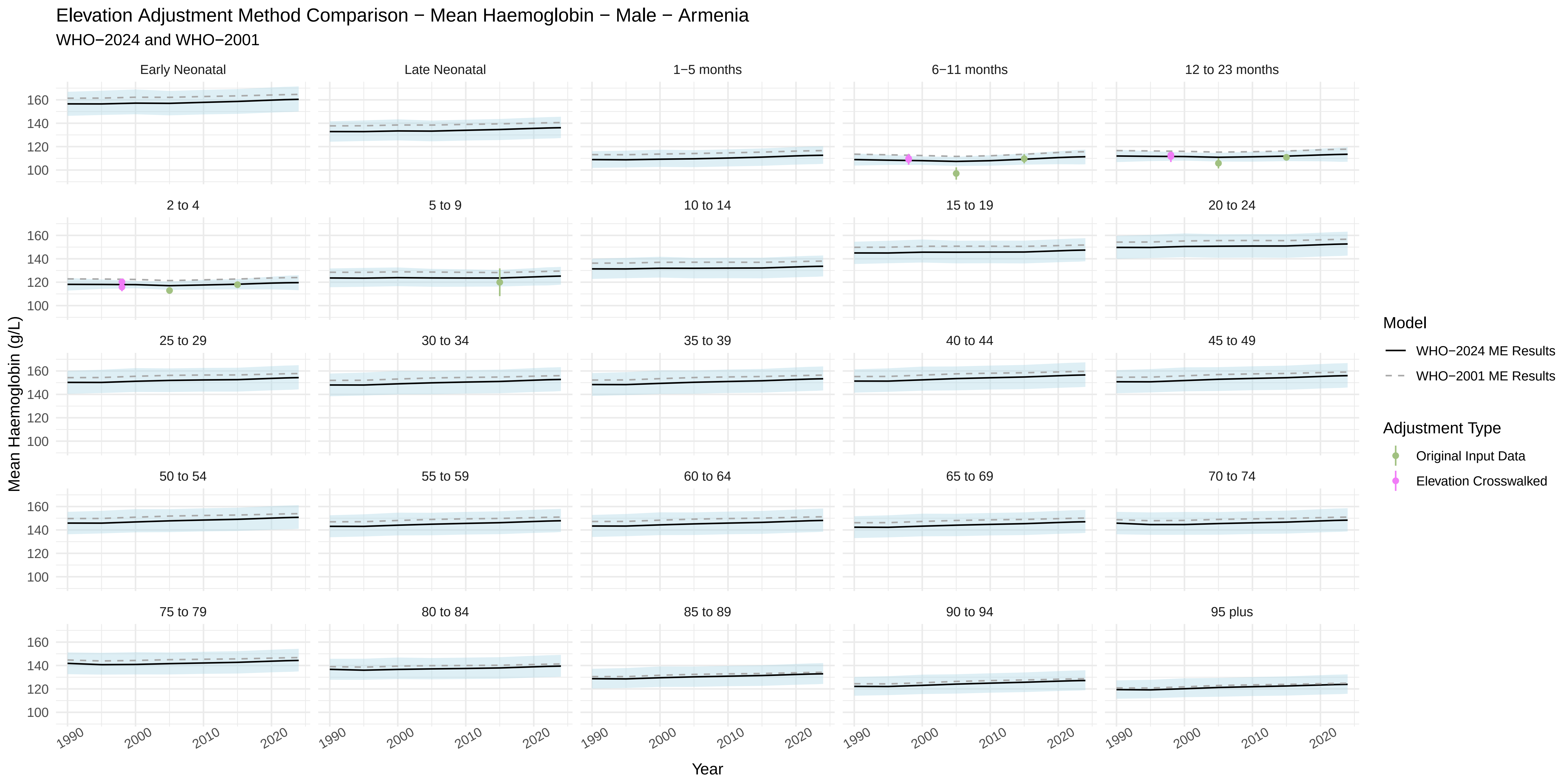
Elevation Adjustment Method Comparison − Mean Haemoglobin − Male − Armenia
WHO−2024 and WHO−2001
Early Neonatal
Late Neonatal
1−5 months
6−11 months
12 to 23 months
2 to 4
5 to 9
10 to 14
15 to 19
20 to 24
25 to 29
30 to 34
35 to 39
40 to 44
45 to 49
50 to 54
55 to 59
60 to 64
65 to 69
70 to 74
75 to 79
80 to 84
85 to 89
90 to 94
95 plus
Mean Haemoglobin (g/L)
Year
Model
WHO−2024 ME Results
WHO−2001 ME Results
Adjustment Type
Original Input Data
Elevation Crosswalked

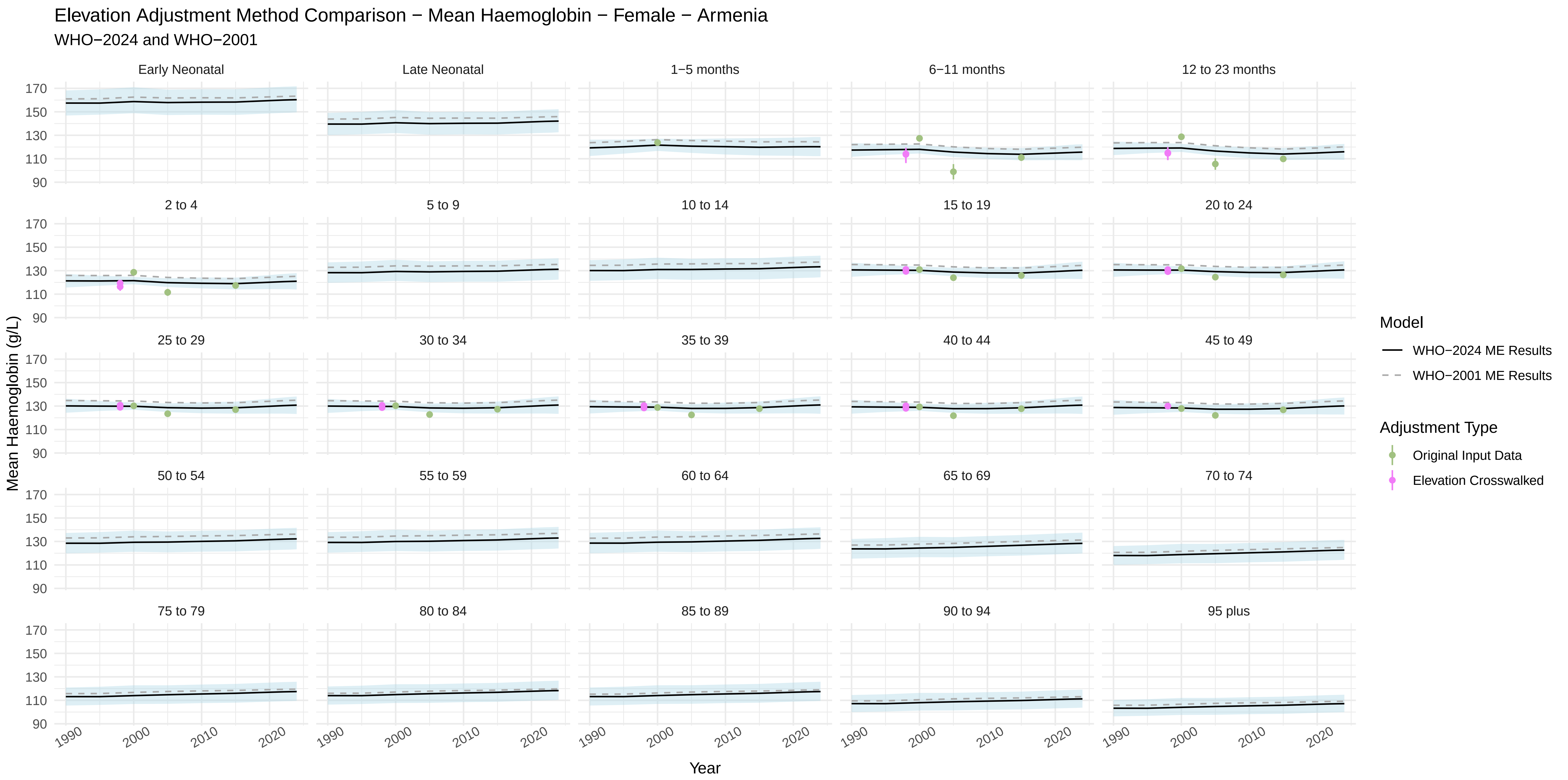
Elevation Adjustment Method Comparison − Mean Haemoglobin − Female − Armenia
WHO−2024 and WHO−2001
Early Neonatal
Late Neonatal
1−5 months
6−11 months
12 to 23 months
2 to 4
5 to 9
10 to 14
15 to 19
20 to 24
25 to 29
30 to 34
35 to 39
40 to 44
45 to 49
50 to 54
55 to 59
60 to 64
65 to 69
70 to 74
75 to 79
80 to 84
85 to 89
90 to 94
95 plus
Mean Haemoglobin (g/L)
Year
Model
WHO−2024 ME Results
WHO−2001 ME Results
Adjustment Type
Original Input Data
Elevation Crosswalked

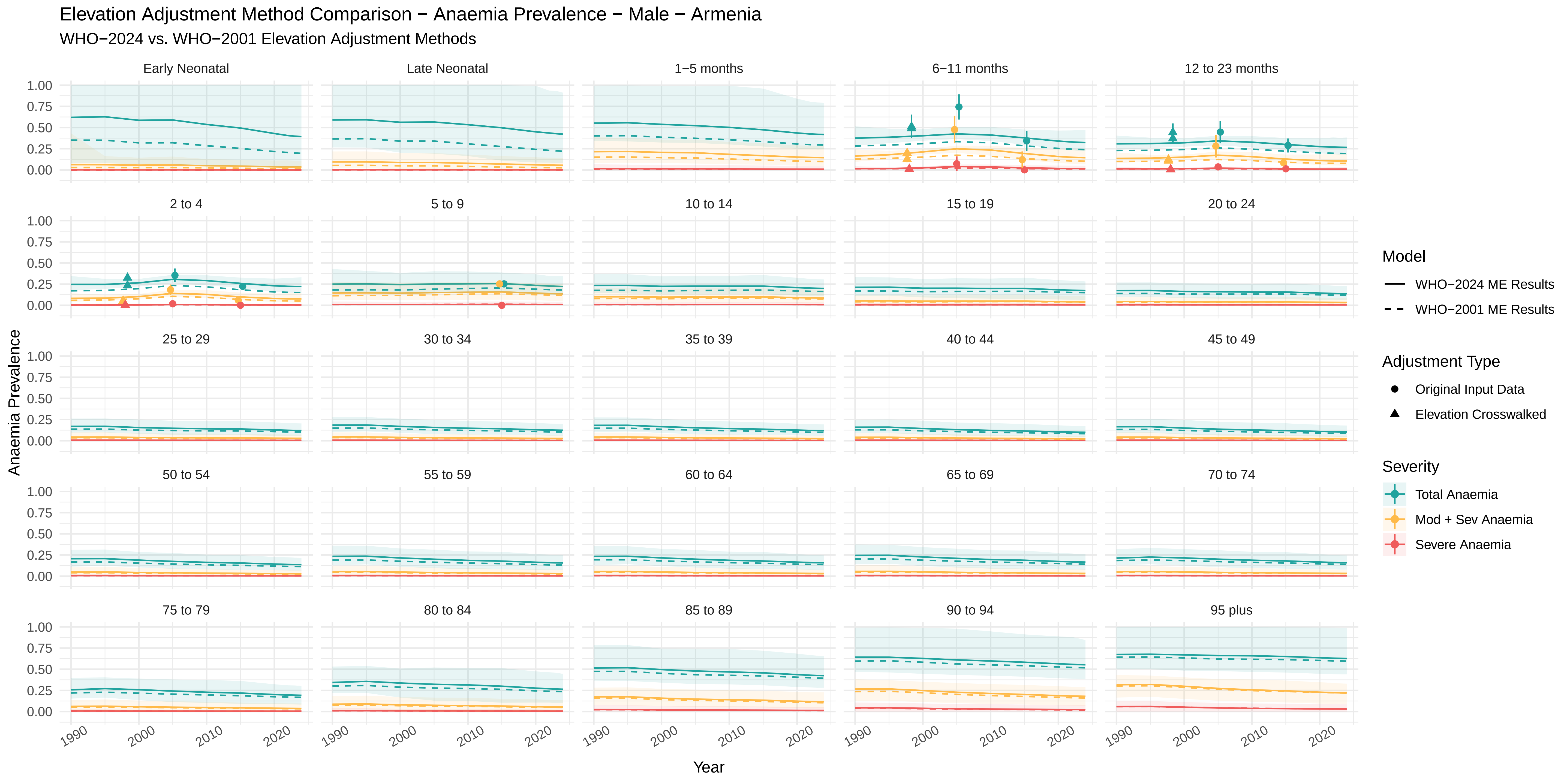
Elevation Adjustment Method Comparison − Anaemia Prevalence − Male − Armenia
WHO−2024 vs. WHO−2001 Elevation Adjustment Methods
Early Neonatal
Late Neonatal
1−5 months
6−11 months
12 to 23 months
2 to 4
5 to 9
10 to 14
15 to 19
20 to 24
25 to 29
30 to 34
35 to 39
40 to 44
45 to 49
50 to 54
55 to 59
60 to 64
65 to 69
70 to 74
75 to 79
80 to 84
85 to 89
90 to 94
95 plus
Anaemia Prevalence
Year
Model
WHO−2024 ME Results
WHO−2001 ME Results
Adjustment Type
Original Input Data
Elevation Crosswalked
Severity
Total Anaemia
Mod + Sev Anaemia
Severe Anaemia

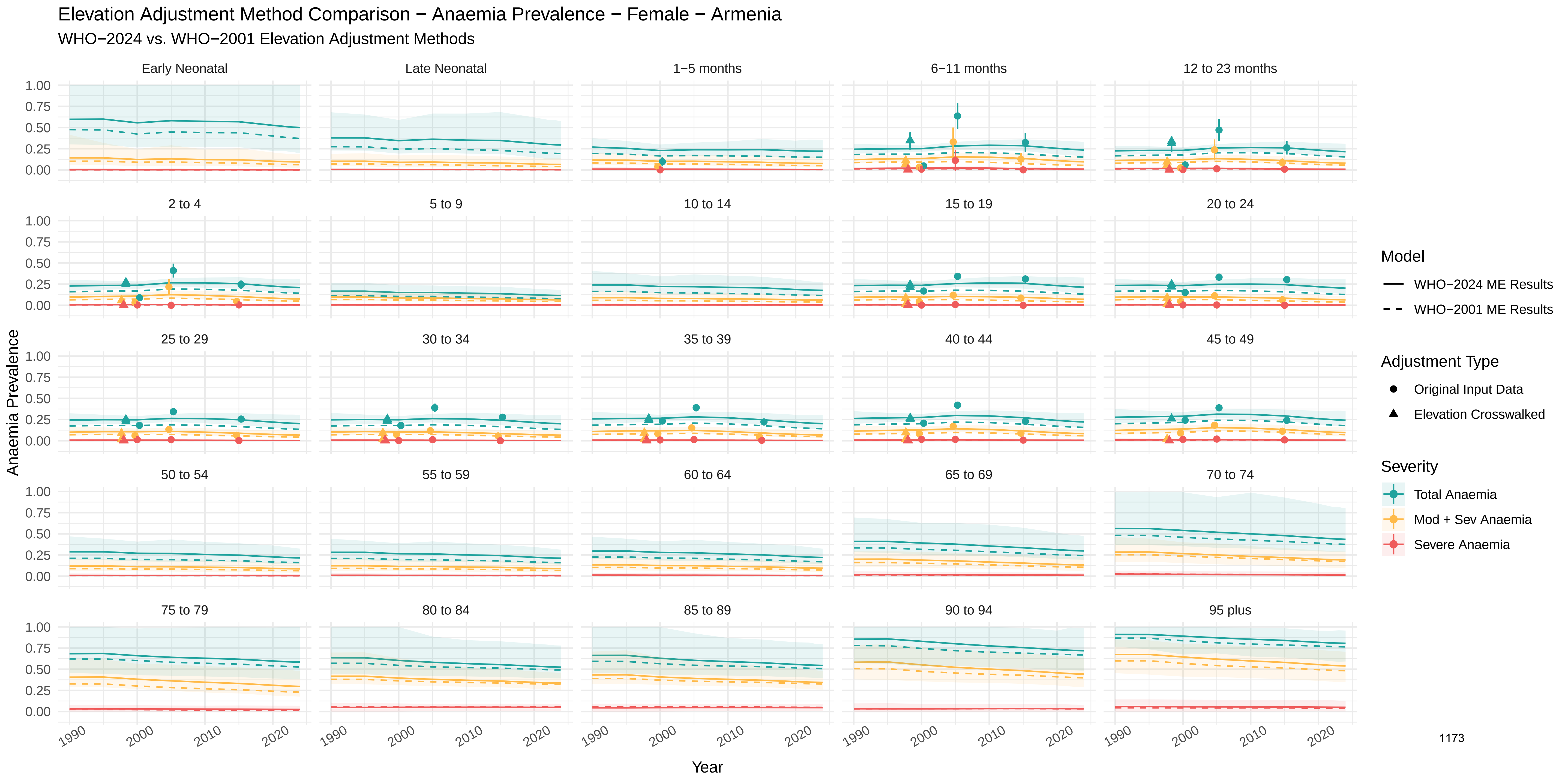
Elevation Adjustment Method Comparison − Anaemia Prevalence − Female − Armenia
WHO−2024 vs. WHO−2001 Elevation Adjustment Methods
Early Neonatal
Late Neonatal
1−5 months
6−11 months
12 to 23 months
2 to 4
5 to 9
10 to 14
15 to 19
20 to 24
25 to 29
30 to 34
35 to 39
40 to 44
45 to 49
50 to 54
55 to 59
60 to 64
65 to 69
70 to 74
75 to 79
80 to 84
85 to 89
90 to 94
95 plus
Anaemia Prevalence
Year
Model
WHO−2024 ME Results
WHO−2001 ME Results
Adjustment Type
Original Input Data
Elevation Crosswalked
Severity
Total Anaemia
Mod + Sev Anaemia
Severe Anaemia

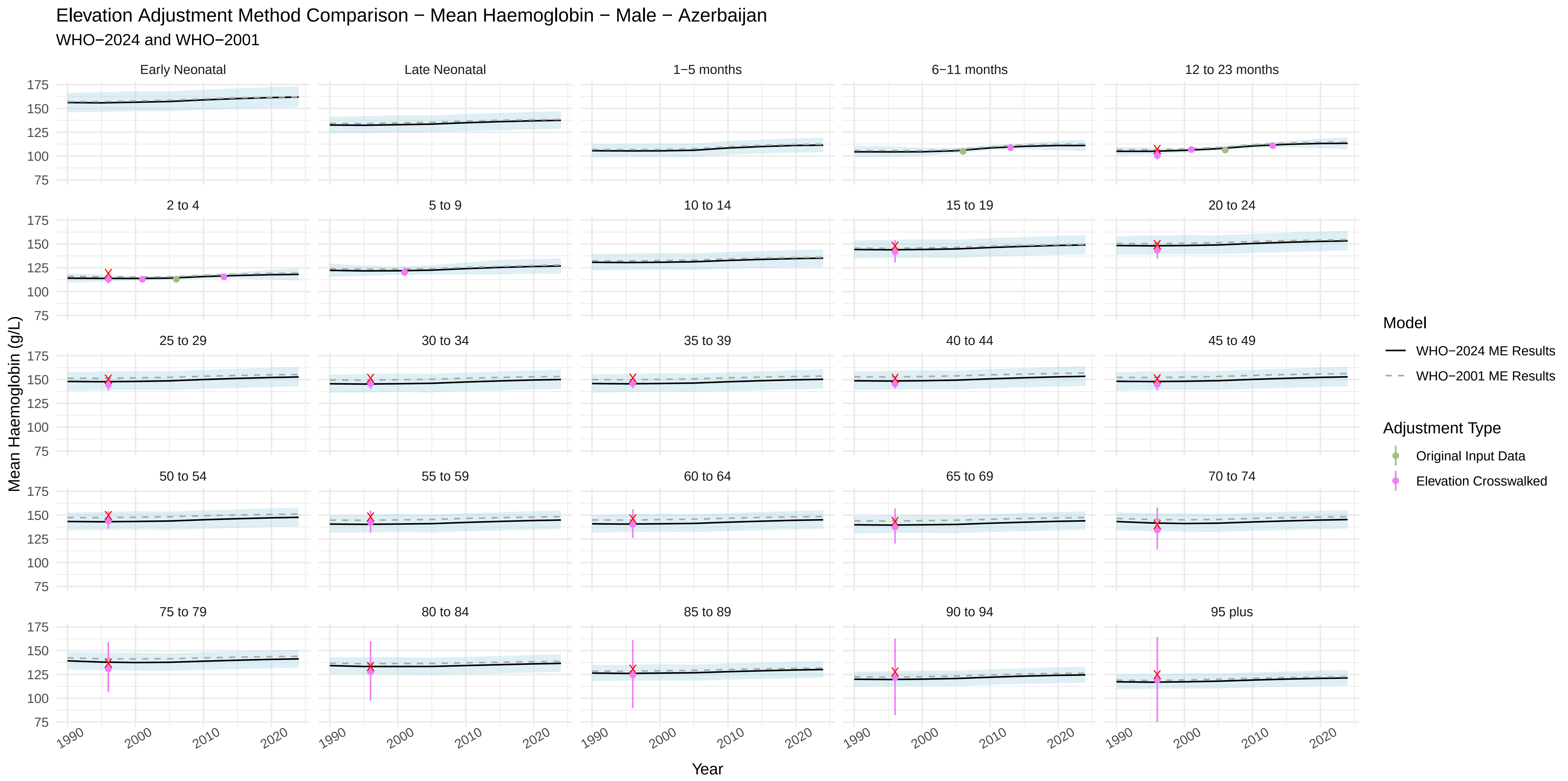
Elevation Adjustment Method Comparison − Mean Haemoglobin − Male − Azerbaijan
WHO−2024 and WHO−2001
Early Neonatal
Late Neonatal
1−5 months
6−11 months
12 to 23 months
2 to 4
5 to 9
10 to 14
15 to 19
20 to 24
25 to 29
30 to 34
35 to 39
40 to 44
45 to 49
50 to 54
55 to 59
60 to 64
65 to 69
70 to 74
75 to 79
80 to 84
85 to 89
90 to 94
95 plus
Mean Haemoglobin (g/L)
Year
Model
WHO−2024 ME Results
WHO−2001 ME Results
Adjustment Type
Original Input Data
Elevation Crosswalked

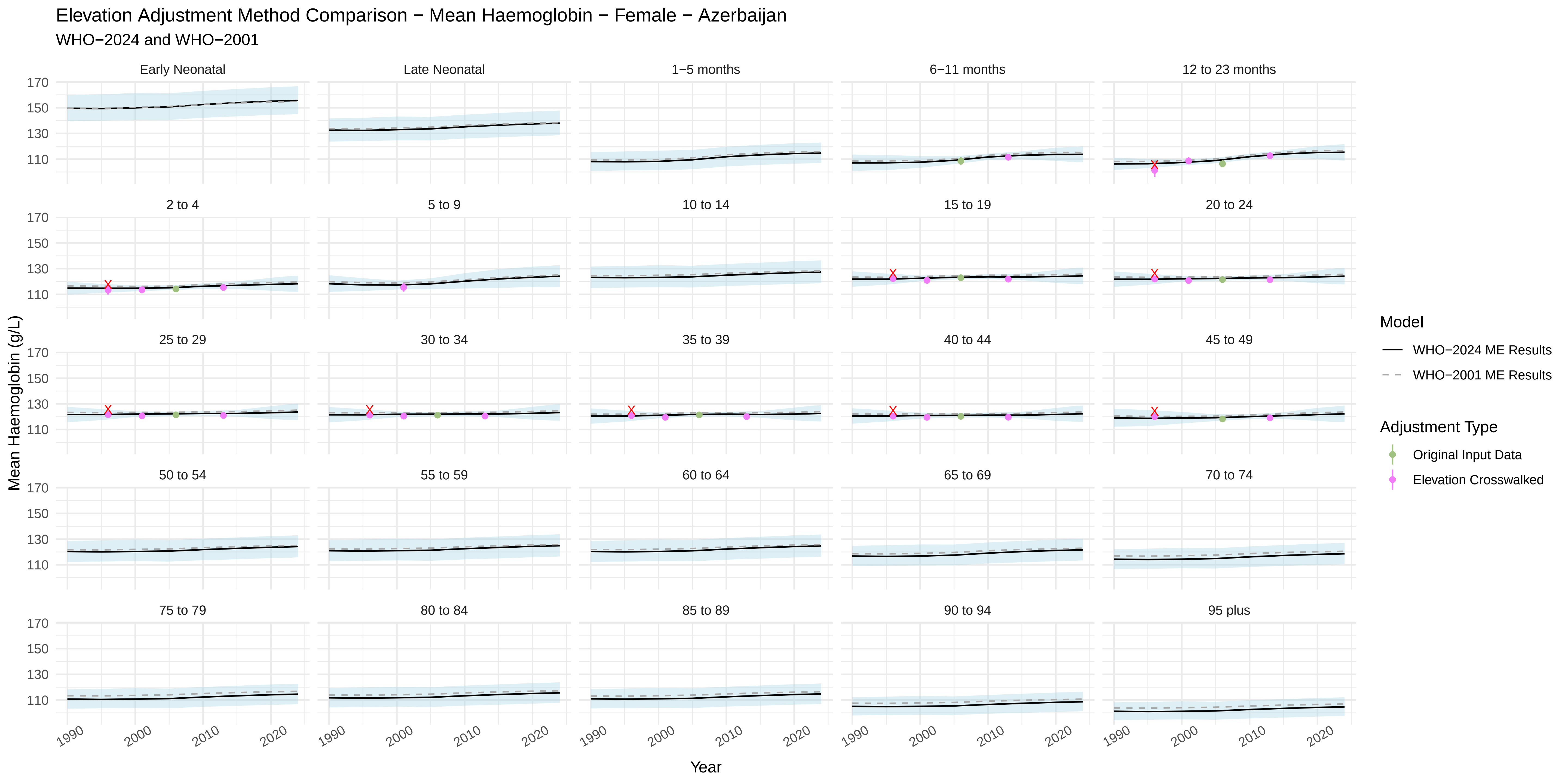
Elevation Adjustment Method Comparison − Mean Haemoglobin − Female − Azerbaijan
WHO−2024 and WHO−2001
Early Neonatal
Late Neonatal
1−5 months
6−11 months
12 to 23 months
2 to 4
5 to 9
10 to 14
15 to 19
20 to 24
25 to 29
30 to 34
35 to 39
40 to 44
45 to 49
50 to 54
55 to 59
60 to 64
65 to 69
70 to 74
75 to 79
80 to 84
85 to 89
90 to 94
95 plus
Mean Haemoglobin (g/L)
Year
Model
WHO−2024 ME Results
WHO−2001 ME Results
Adjustment Type
Original Input Data
Elevation Crosswalked

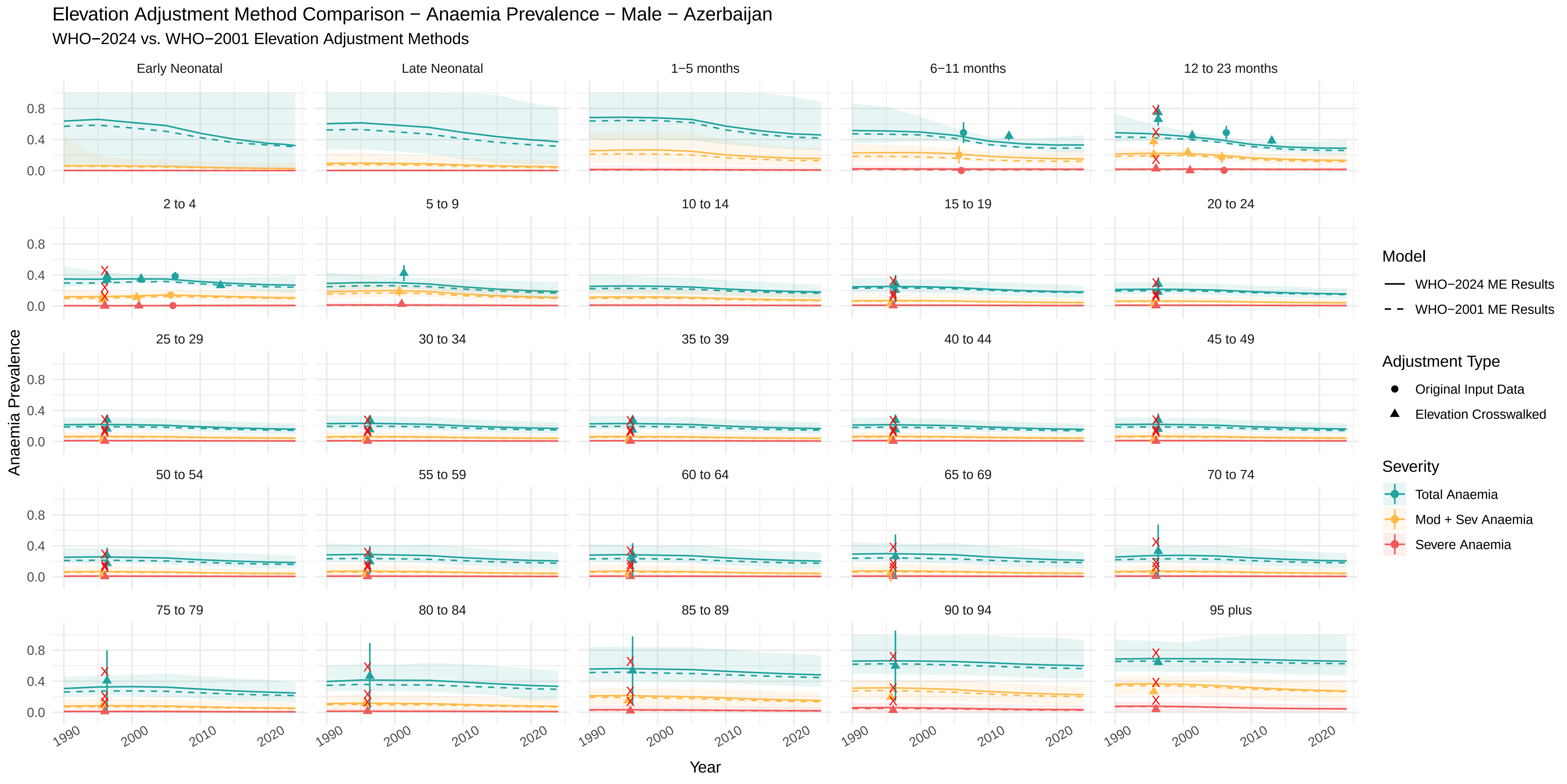
Elevation Adjustment Method Comparison − Anaemia Prevalence − Male − Azerbaijan
WHO−2024 vs. WHO−2001 Elevation Adjustment Methods
Early Neonatal
Late Neonatal
1−5 months
6−11 months
12 to 23 months
2 to 4
5 to 9
10 to 14
15 to 19
20 to 24
25 to 29
30 to 34
35 to 39
40 to 44
45 to 49
50 to 54
55 to 59
60 to 64
65 to 69
70 to 74
75 to 79
80 to 84
85 to 89
90 to 94
95 plus
Anaemia Prevalence
Year
Model
WHO−2024 ME Results
WHO−2001 ME Results
Adjustment Type
Original Input Data
Elevation Crosswalked
Severity
Total Anaemia
Mod + Sev Anaemia
Severe Anaemia

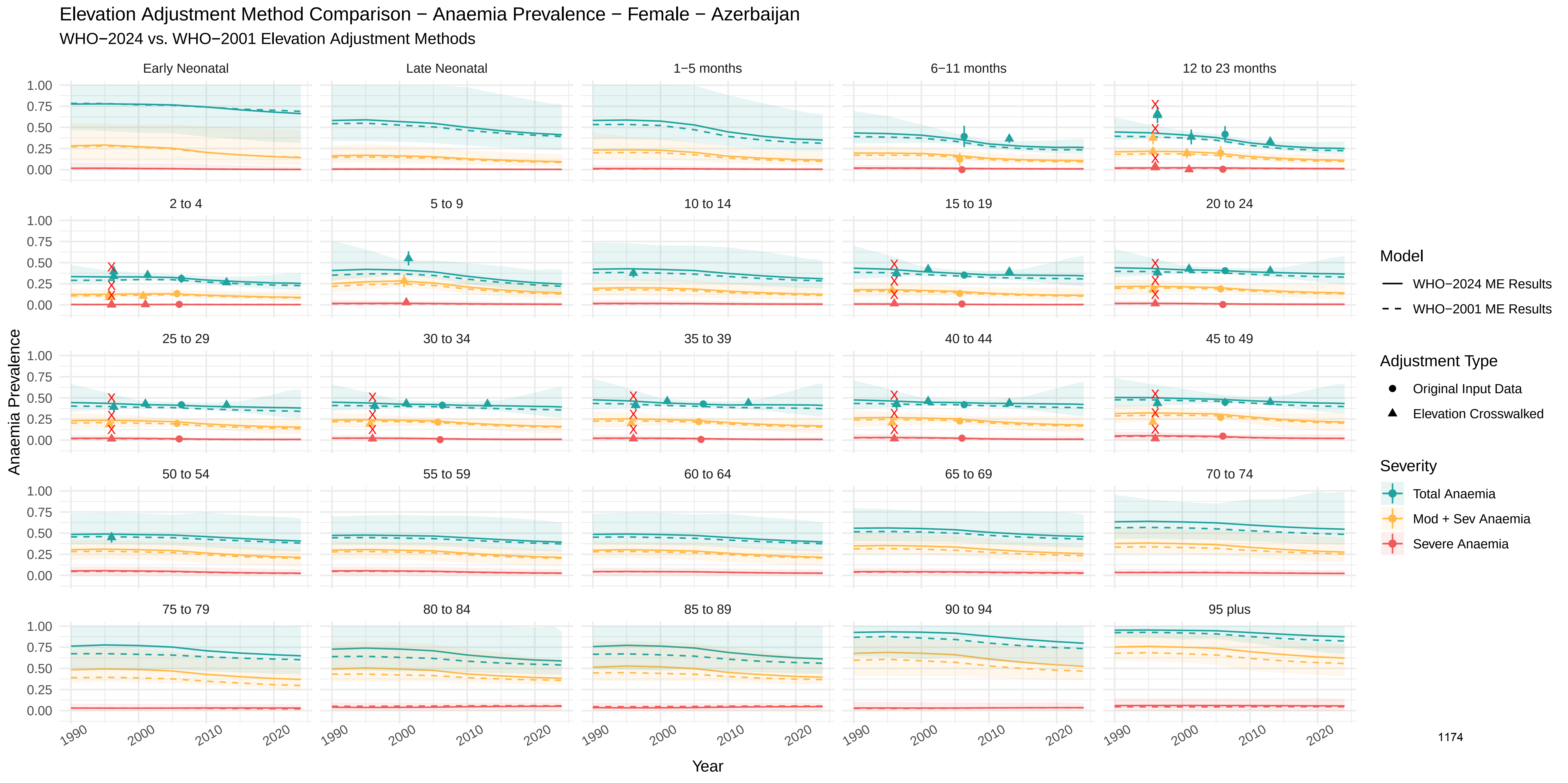
Elevation Adjustment Method Comparison − Anaemia Prevalence − Female − Azerbaijan
WHO−2024 vs. WHO−2001 Elevation Adjustment Methods
Early Neonatal
Late Neonatal
1−5 months
6−11 months
12 to 23 months
2 to 4
5 to 9
10 to 14
15 to 19
20 to 24
25 to 29
30 to 34
35 to 39
40 to 44
45 to 49
50 to 54
55 to 59
60 to 64
65 to 69
70 to 74
75 to 79
80 to 84
85 to 89
90 to 94
95 plus
Anaemia Prevalence
Year
Model
WHO−2024 ME Results
WHO−2001 ME Results
Adjustment Type
Original Input Data
Elevation Crosswalked
Severity
Total Anaemia
Mod + Sev Anaemia
Severe Anaemia

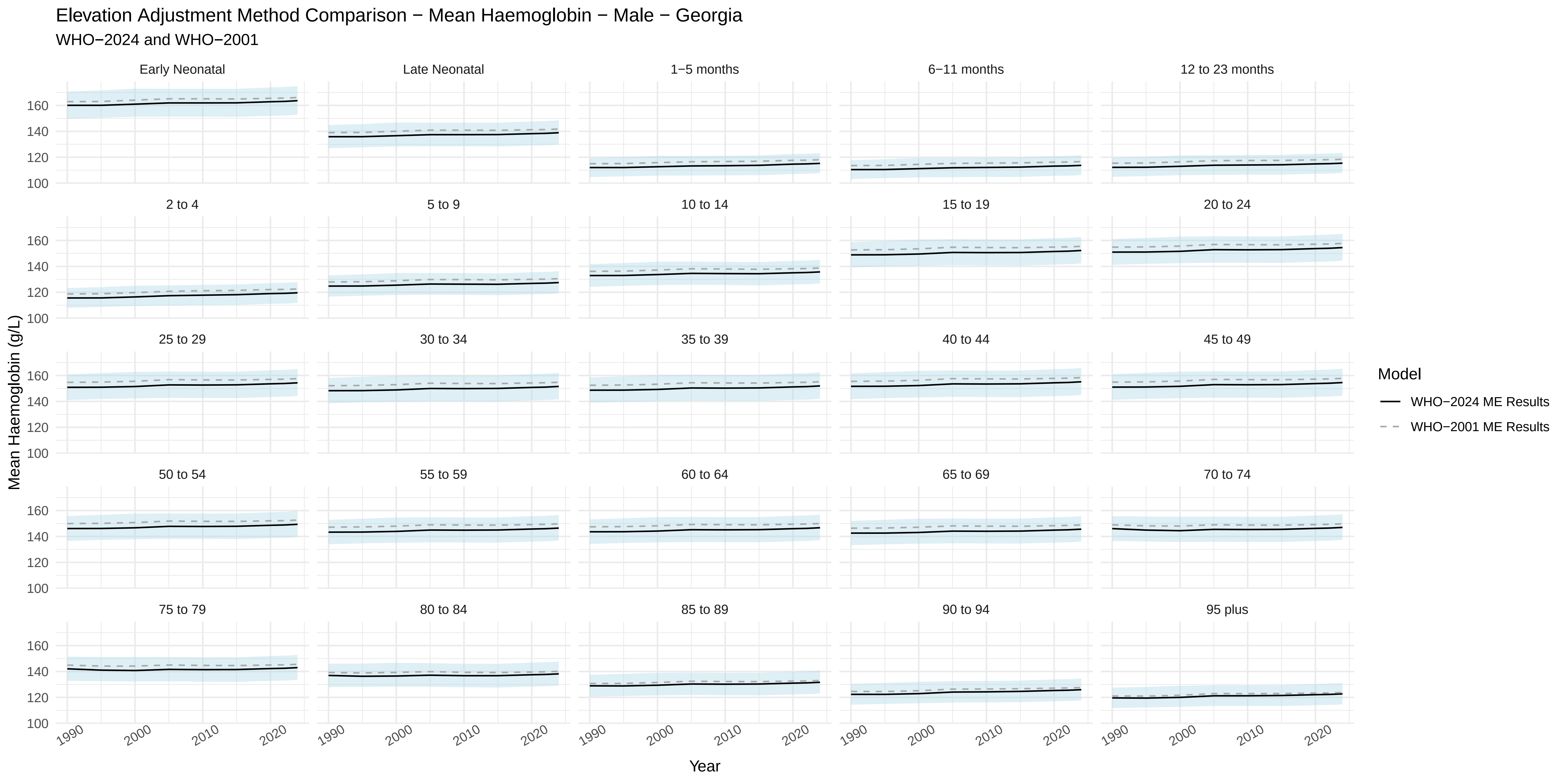
Elevation Adjustment Method Comparison − Mean Haemoglobin − Male − Georgia
WHO−2024 and WHO−2001
Early Neonatal
Late Neonatal
1−5 months
6−11 months
12 to 23 months
2 to 4
5 to 9
10 to 14
15 to 19
20 to 24
25 to 29
30 to 34
35 to 39
40 to 44
45 to 49
50 to 54
55 to 59
60 to 64
65 to 69
70 to 74
75 to 79
80 to 84
85 to 89
90 to 94
95 plus
Mean Haemoglobin (g/L)
Year
Model
WHO−2024 ME Results
WHO−2001 ME Results

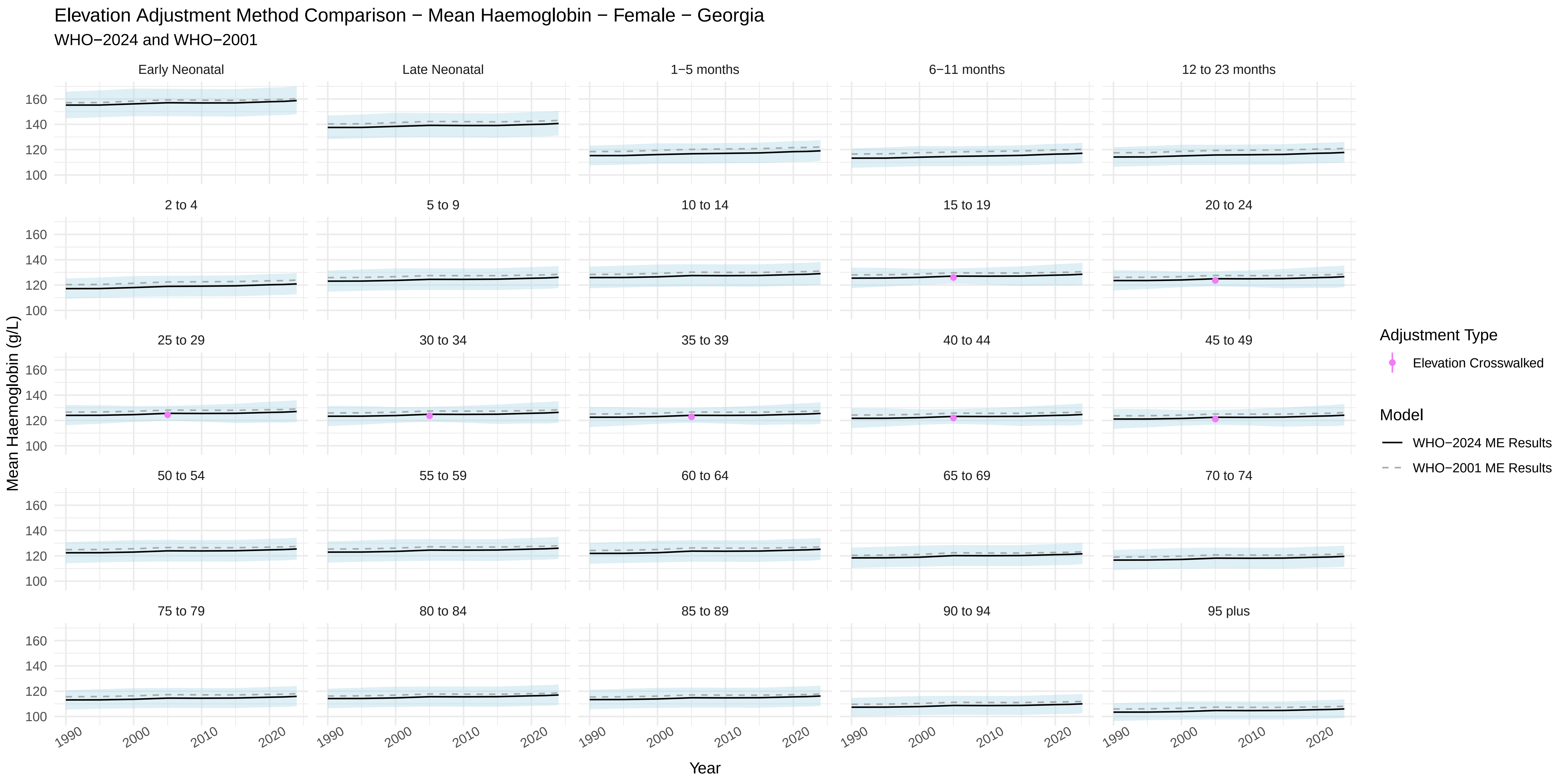
Elevation Adjustment Method Comparison − Mean Haemoglobin − Female − Georgia
WHO−2024 and WHO−2001
Early Neonatal
Late Neonatal
1−5 months
6−11 months
12 to 23 months
2 to 4
5 to 9
10 to 14
15 to 19
20 to 24
25 to 29
30 to 34
35 to 39
40 to 44
45 to 49
50 to 54
55 to 59
60 to 64
65 to 69
70 to 74
75 to 79
80 to 84
85 to 89
90 to 94
95 plus
Mean Haemoglobin (g/L)
Year
Adjustment Type
Elevation Crosswalked
Model
WHO−2024 ME Results
WHO−2001 ME Results

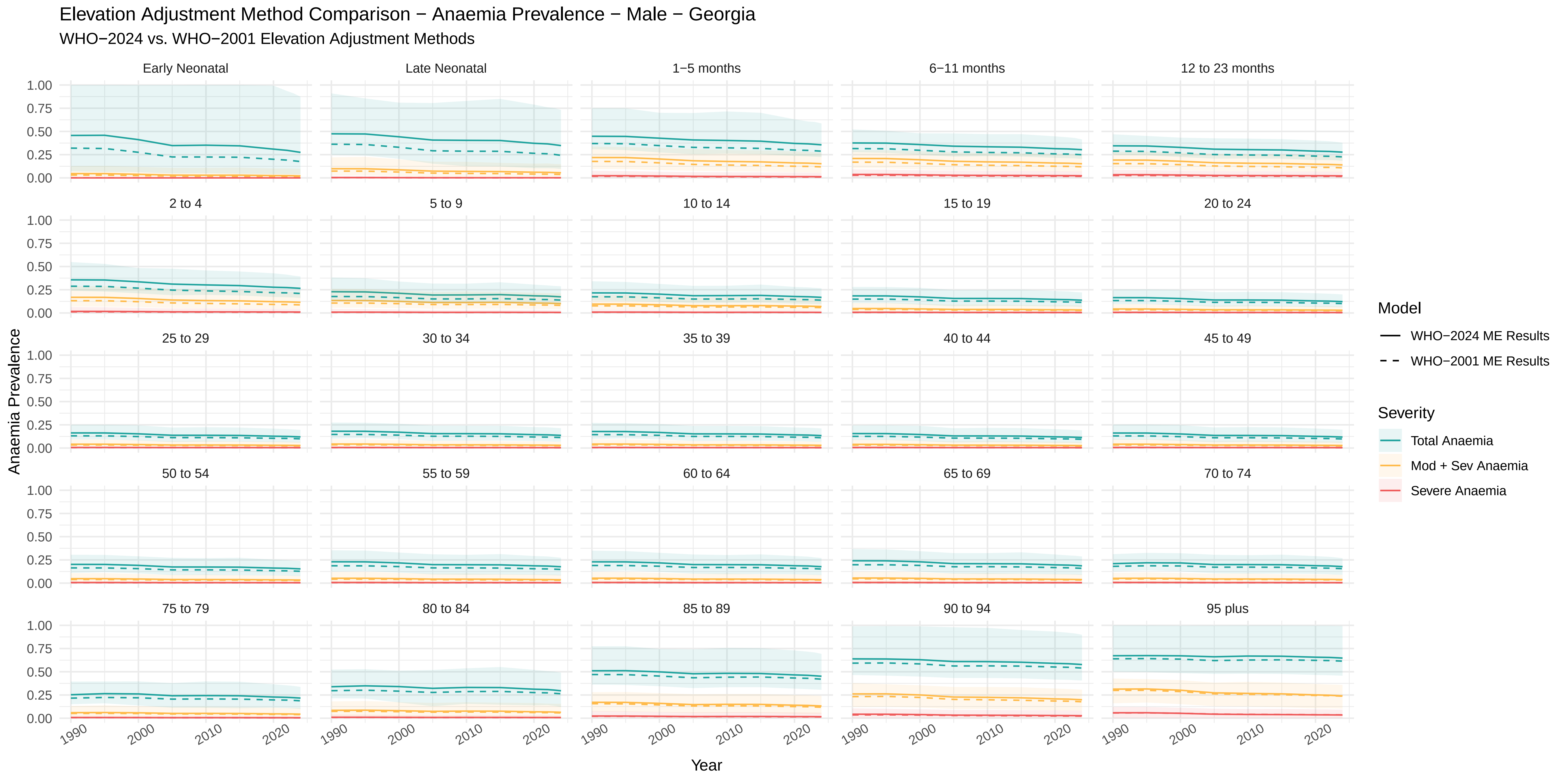
Elevation Adjustment Method Comparison − Anaemia Prevalence − Male − Georgia
WHO−2024 vs. WHO−2001 Elevation Adjustment Methods
Early Neonatal
Late Neonatal
1−5 months
6−11 months
12 to 23 months
2 to 4
5 to 9
10 to 14
15 to 19
20 to 24
25 to 29
30 to 34
35 to 39
40 to 44
45 to 49
50 to 54
55 to 59
60 to 64
65 to 69
70 to 74
75 to 79
80 to 84
85 to 89
90 to 94
95 plus
Anaemia Prevalence
Year
Model
WHO−2024 ME Results
WHO−2001 ME Results
Severity
Total Anaemia
Mod + Sev Anaemia
Severe Anaemia

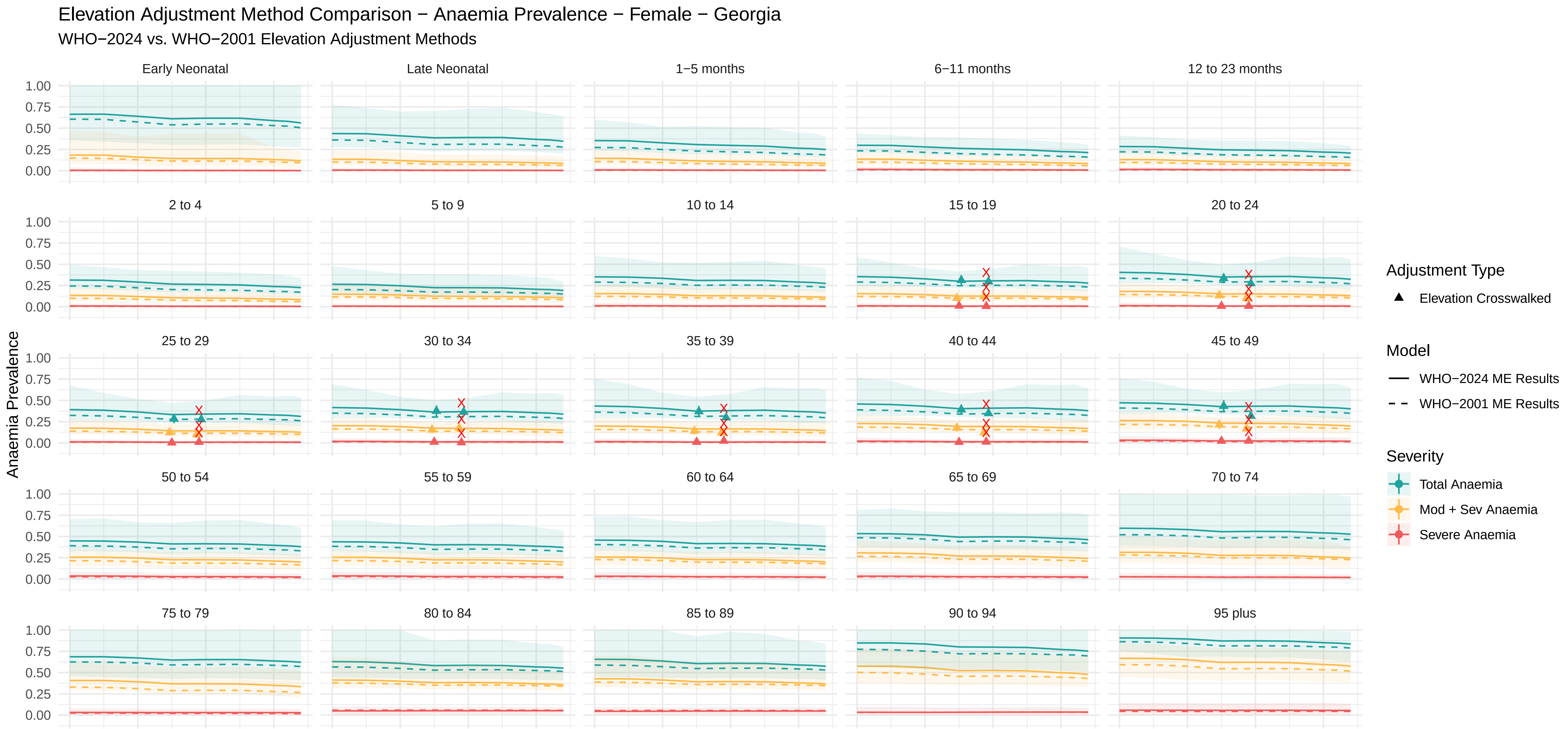
Elevation Adjustment Method Comparison − Anaemia Prevalence − Female − Georgia
WHO−2024 vs. WHO−2001 Elevation Adjustment Methods
Early Neonatal
Late Neonatal
1−5 months
6−11 months
12 to 23 months
2 to 4
5 to 9
10 to 14
15 to 19
20 to 24
25 to 29
30 to 34
35 to 39
40 to 44
45 to 49
50 to 54
55 to 59
60 to 64
65 to 69
70 to 74
75 to 79
80 to 84
85 to 89
90 to 94
95 plus
Anaemia Prevalence
Year
Adjustment Type
Elevation Crosswalked
Model
WHO−2024 ME Results
WHO−2001 ME Results
Severity
Total Anaemia
Mod + Sev Anaemia
Severe Anaemia

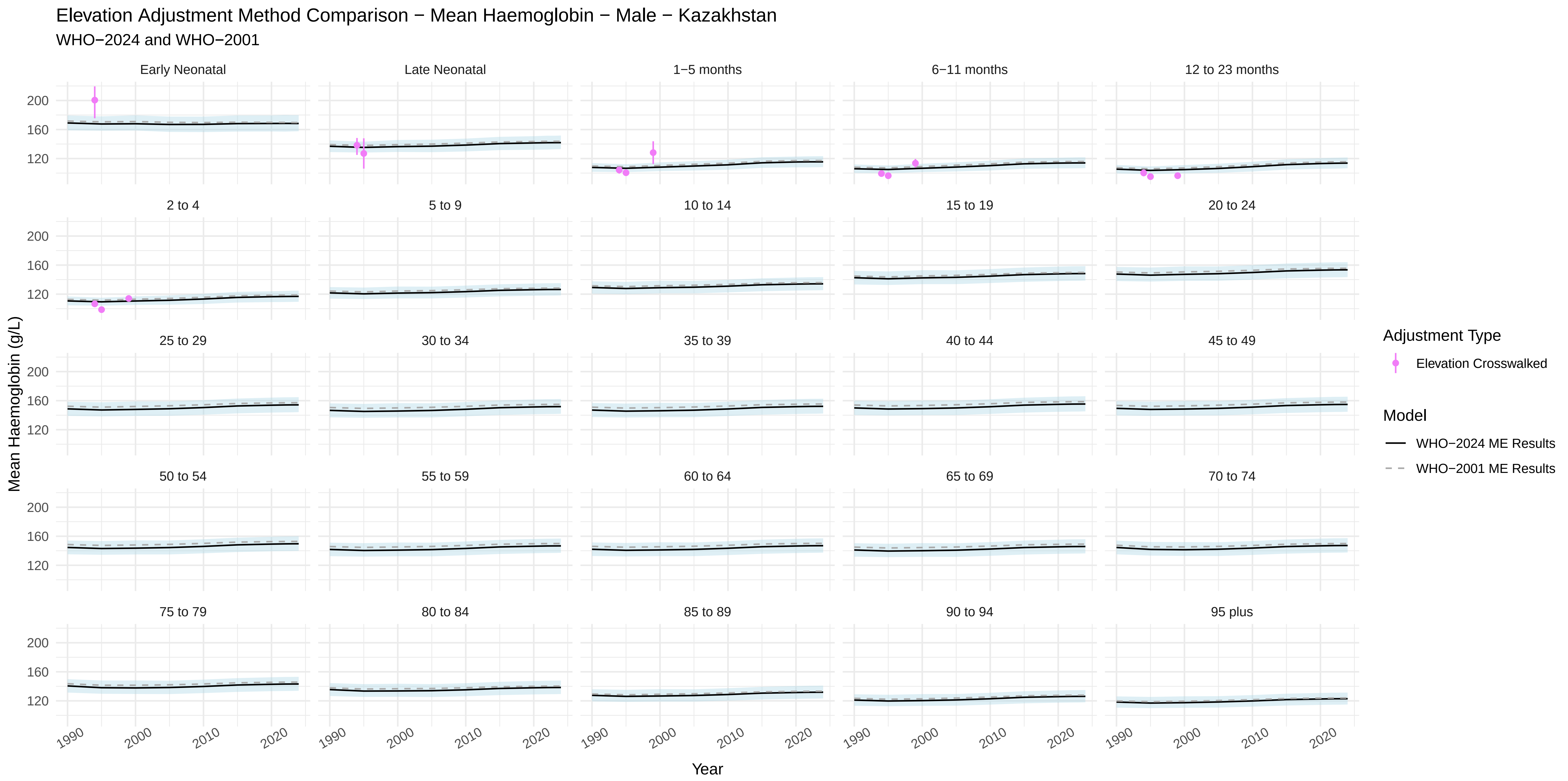

Elevation Adjustment Method Comparison − Mean Haemoglobin − Male − Kazakhstan
WHO−2024 and WHO−2001
Mean Haemoglobin (g/L)
Year
Adjustment Type
Elevation Crosswalked
Model
WHO−2024 ME Results
WHO−2001 ME Results

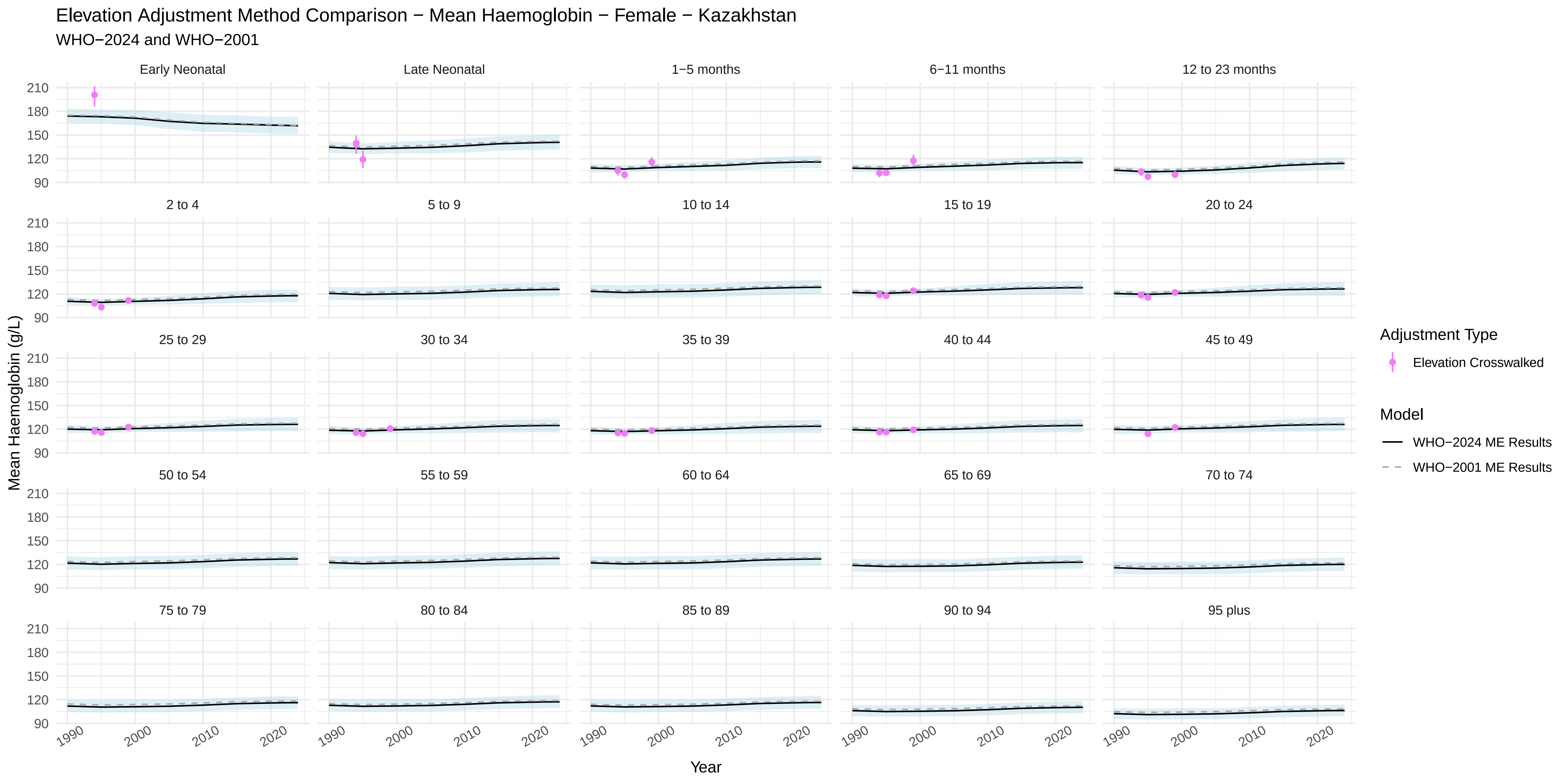

Elevation Adjustment Method Comparison − Mean Haemoglobin − Female − Kazakhstan
WHO−2024 and WHO−2001
Mean Haemoglobin (g/L)
Year
Adjustment Type
Elevation Crosswalked
Model
WHO−2024 ME Results
WHO−2001 ME Results

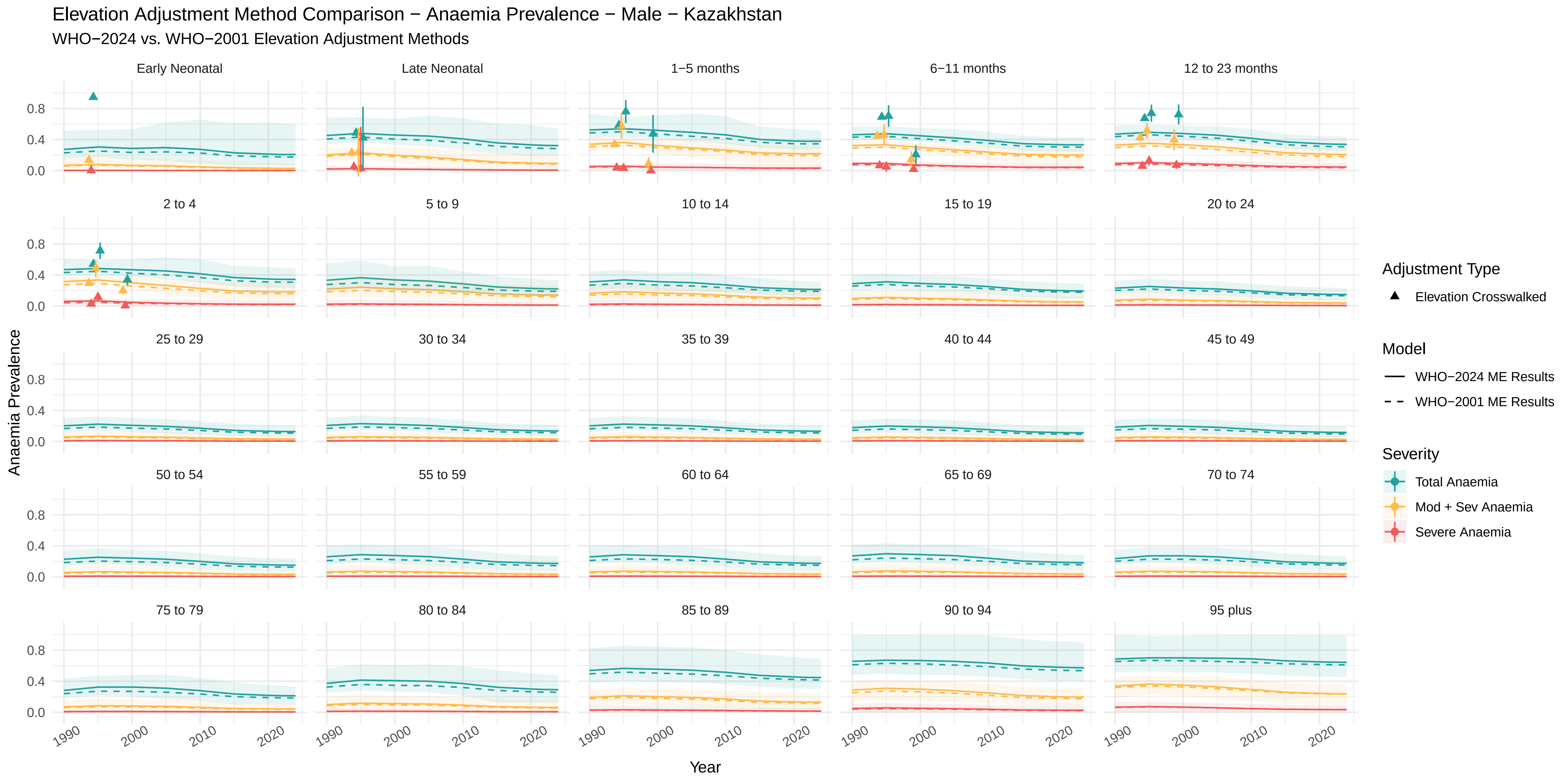

Elevation Adjustment Method Comparison − Anaemia Prevalence − Male − Kazakhstan
WHO−2024 vs. WHO−2001 Elevation Adjustment Methods
Anaemia Prevalence
Year
Adjustment Type
Elevation Crosswalked
Model
WHO−2024 ME Results
WHO−2001 ME Results
Severity
Total Anaemia
Mod + Sev Anaemia
Severe Anaemia

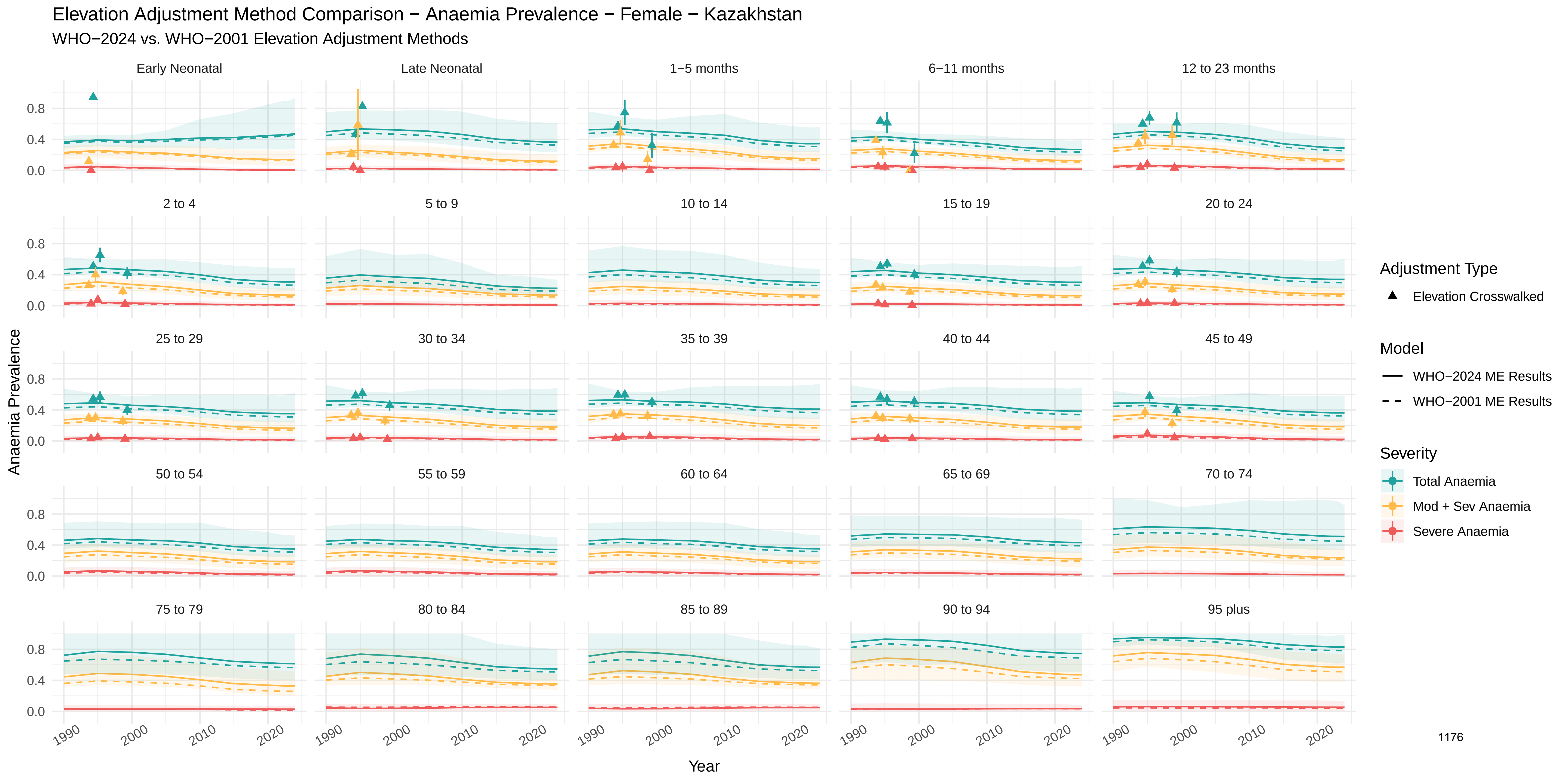

Elevation Adjustment Method Comparison − Anaemia Prevalence − Female − Kazakhstan
WHO−2024 vs. WHO−2001 Elevation Adjustment Methods
Anaemia Prevalence
Year
Adjustment Type
Elevation Crosswalked
Model
WHO−2024 ME Results
WHO−2001 ME Results
Severity
Total Anaemia
Mod + Sev Anaemia
Severe Anaemia

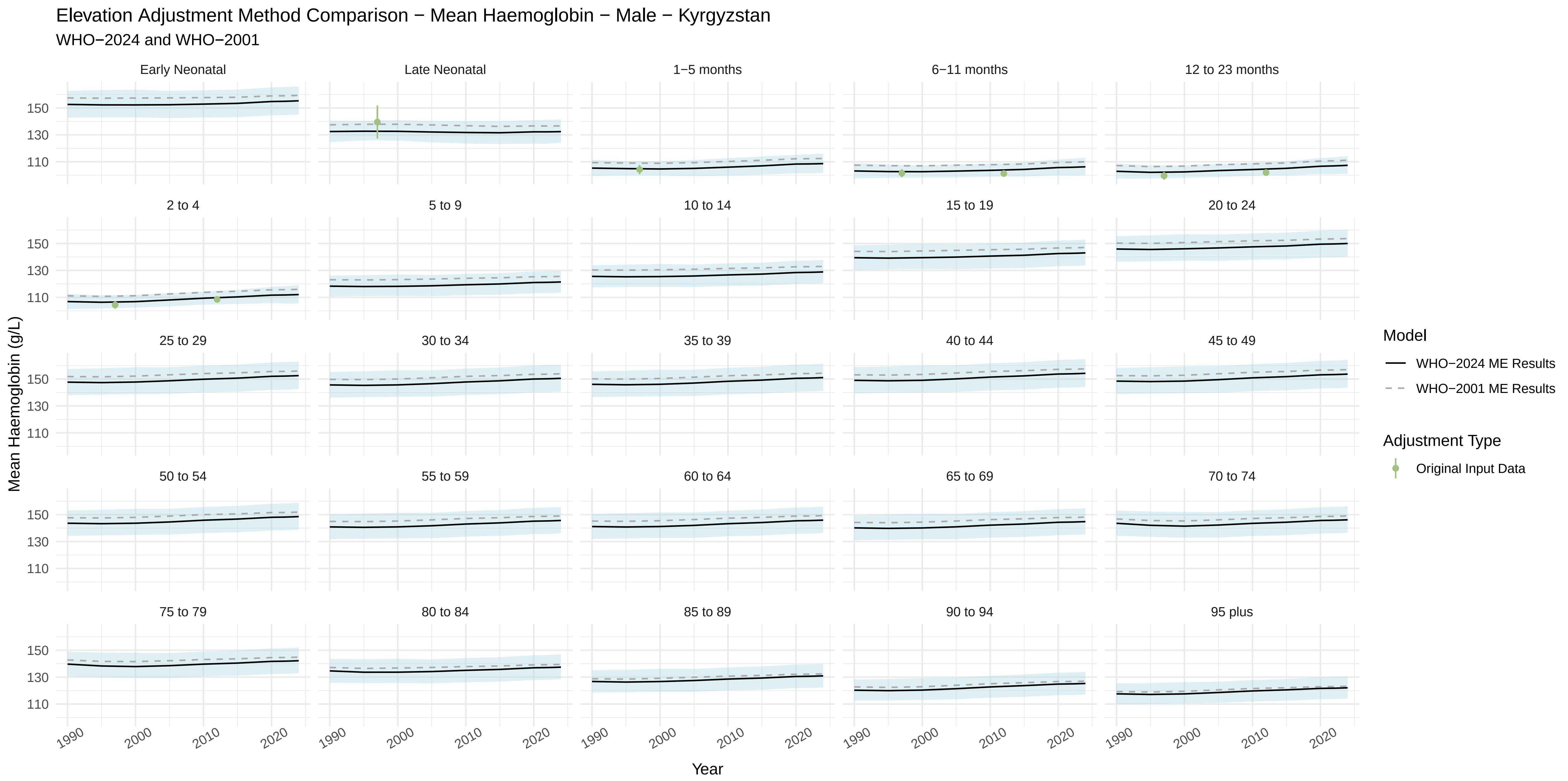
Elevation Adjustment Method Comparison − Mean Haemoglobin − Male − Kyrgyzstan
WHO−2024 and WHO−2001
Early Neonatal
Late Neonatal
1−5 months
6−11 months
12 to 23 months
2 to 4
5 to 9
10 to 14
15 to 19
20 to 24
25 to 29
30 to 34
35 to 39
40 to 44
45 to 49
50 to 54
55 to 59
60 to 64
65 to 69
70 to 74
75 to 79
80 to 84
85 to 89
90 to 94
95 plus
Mean Haemoglobin (g/L)
Year
Model
WHO−2024 ME Results
WHO−2001 ME Results
Adjustment Type
Original Input Data

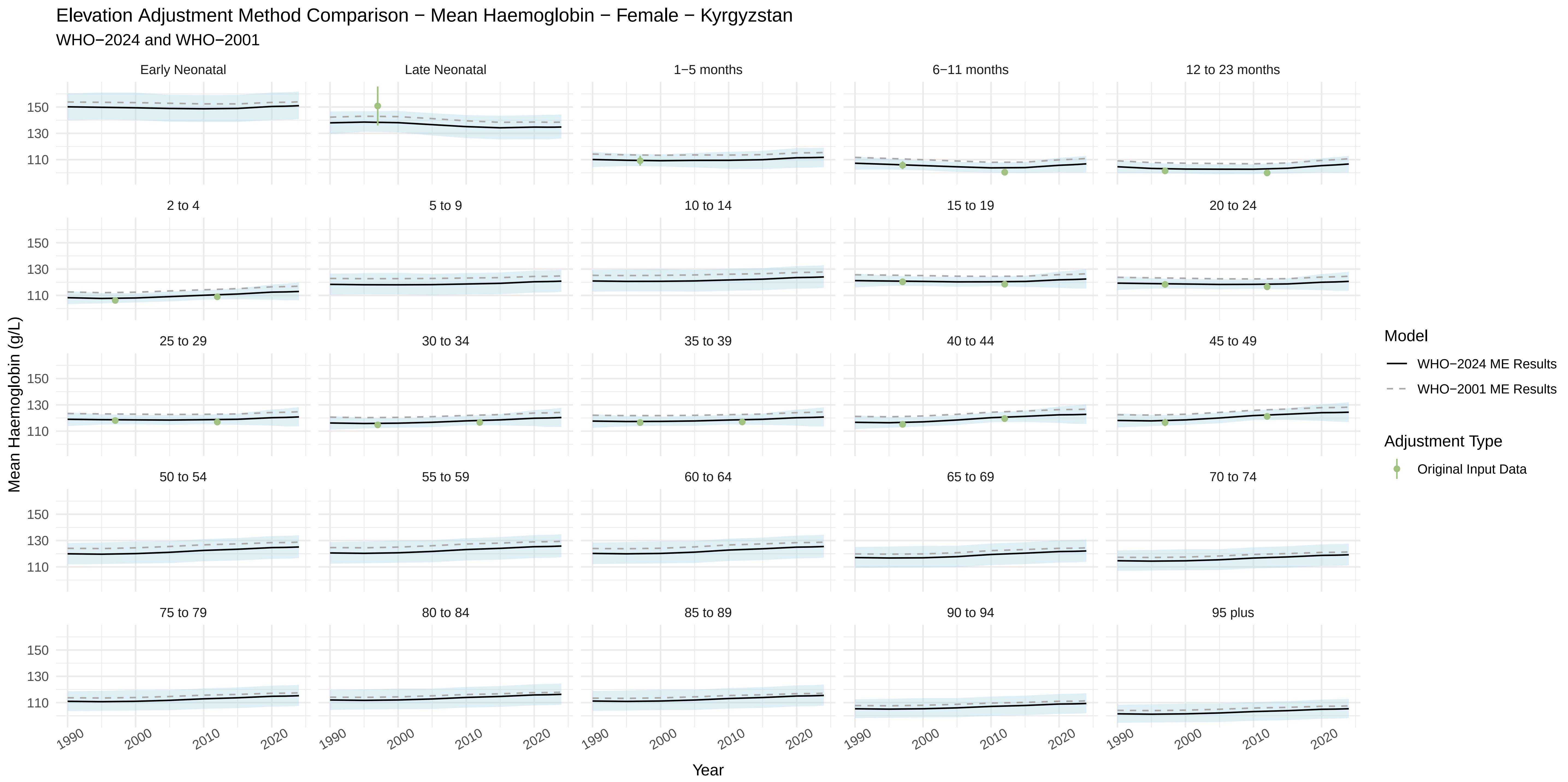
Elevation Adjustment Method Comparison − Mean Haemoglobin − Female − Kyrgyzstan
WHO−2024 and WHO−2001
Early Neonatal
Late Neonatal
1−5 months
6−11 months
12 to 23 months
2 to 4
5 to 9
10 to 14
15 to 19
20 to 24
25 to 29
30 to 34
35 to 39
40 to 44
45 to 49
50 to 54
55 to 59
60 to 64
65 to 69
70 to 74
75 to 79
80 to 84
85 to 89
90 to 94
95 plus
Mean Haemoglobin (g/L)
Year
Model
WHO−2024 ME Results
WHO−2001 ME Results
Adjustment Type
Original Input Data

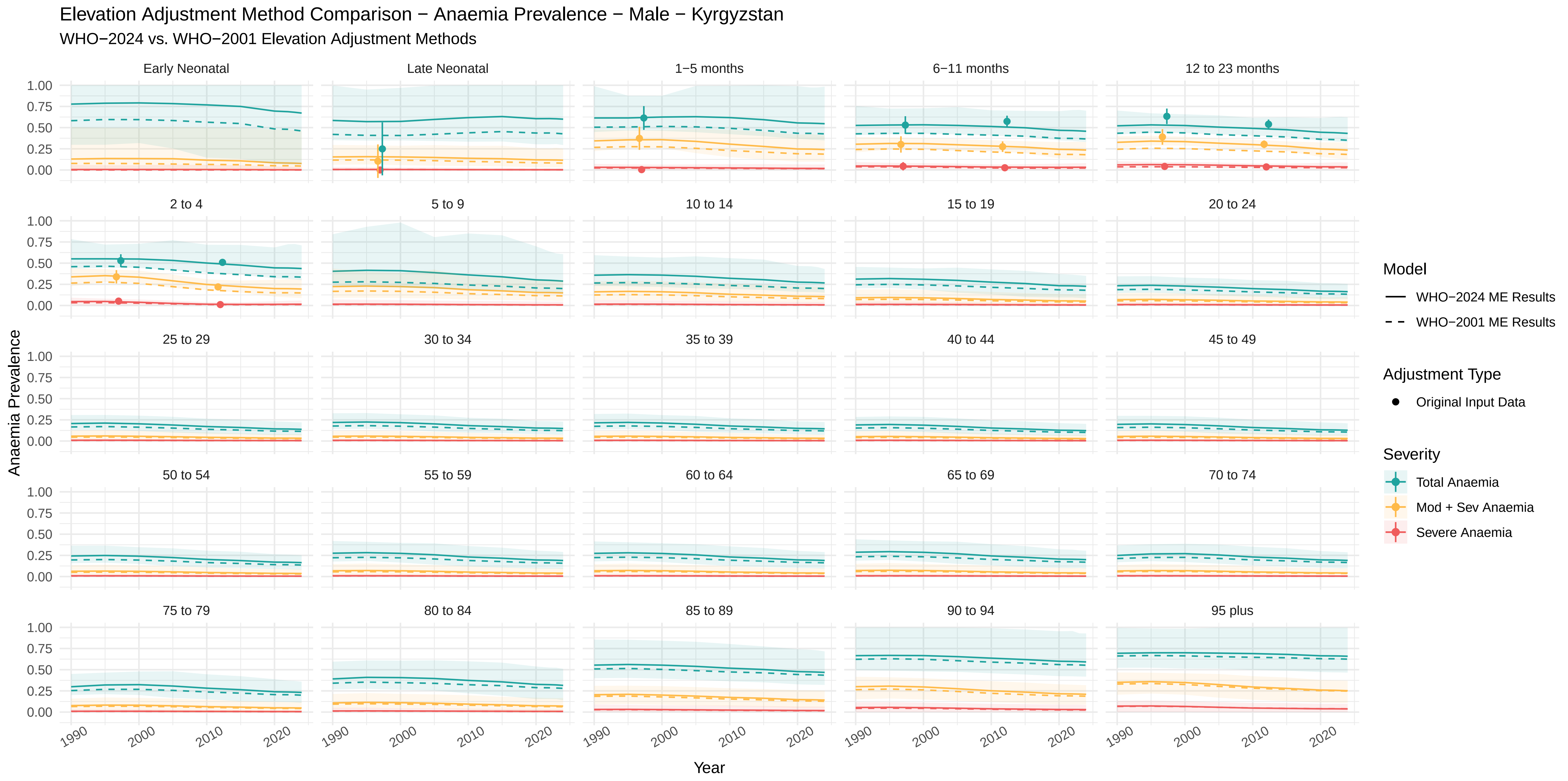
Elevation Adjustment Method Comparison − Anaemia Prevalence − Male − Kyrgyzstan
WHO−2024 vs. WHO−2001 Elevation Adjustment Methods
Early Neonatal
Late Neonatal
1−5 months
6−11 months
12 to 23 months
2 to 4
5 to 9
10 to 14
15 to 19
20 to 24
25 to 29
30 to 34
35 to 39
40 to 44
45 to 49
50 to 54
55 to 59
60 to 64
65 to 69
70 to 74
75 to 79
80 to 84
85 to 89
90 to 94
95 plus
Anaemia Prevalence
Year
Model
WHO−2024 ME Results
WHO−2001 ME Results
Adjustment Type
Original Input Data
Severity
Total Anaemia
Mod + Sev Anaemia
Severe Anaemia

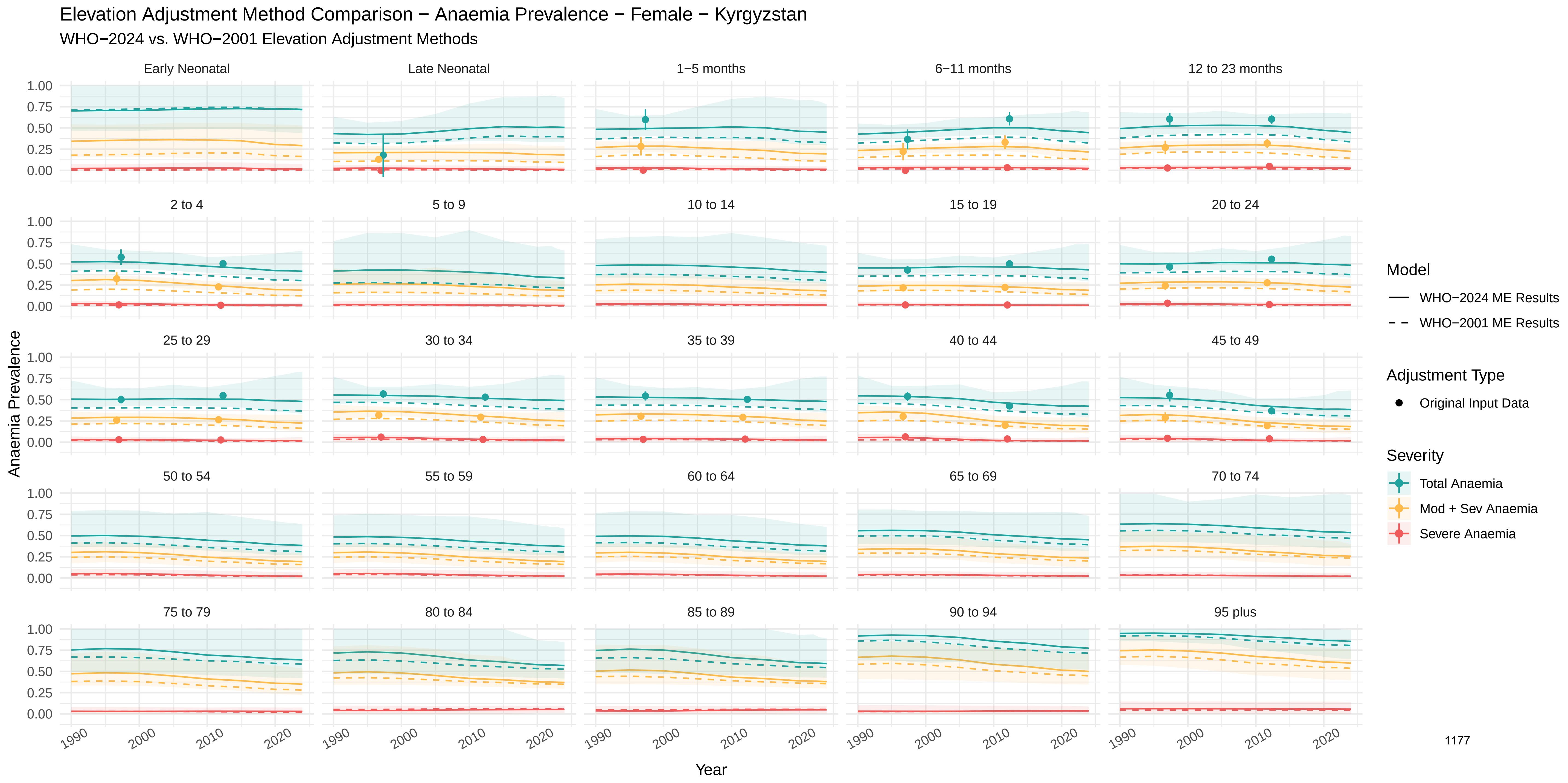
Elevation Adjustment Method Comparison − Anaemia Prevalence − Female − Kyrgyzstan
WHO−2024 vs. WHO−2001 Elevation Adjustment Methods
Early Neonatal
Late Neonatal
1−5 months
6−11 months
12 to 23 months
2 to 4
5 to 9
10 to 14
15 to 19
20 to 24
25 to 29
30 to 34
35 to 39
40 to 44
45 to 49
50 to 54
55 to 59
60 to 64
65 to 69
70 to 74
75 to 79
80 to 84
85 to 89
90 to 94
95 plus
Anaemia Prevalence
Year
Model
WHO−2024 ME Results
WHO−2001 ME Results
Adjustment Type
Original Input Data
Severity
Total Anaemia
Mod + Sev Anaemia
Severe Anaemia

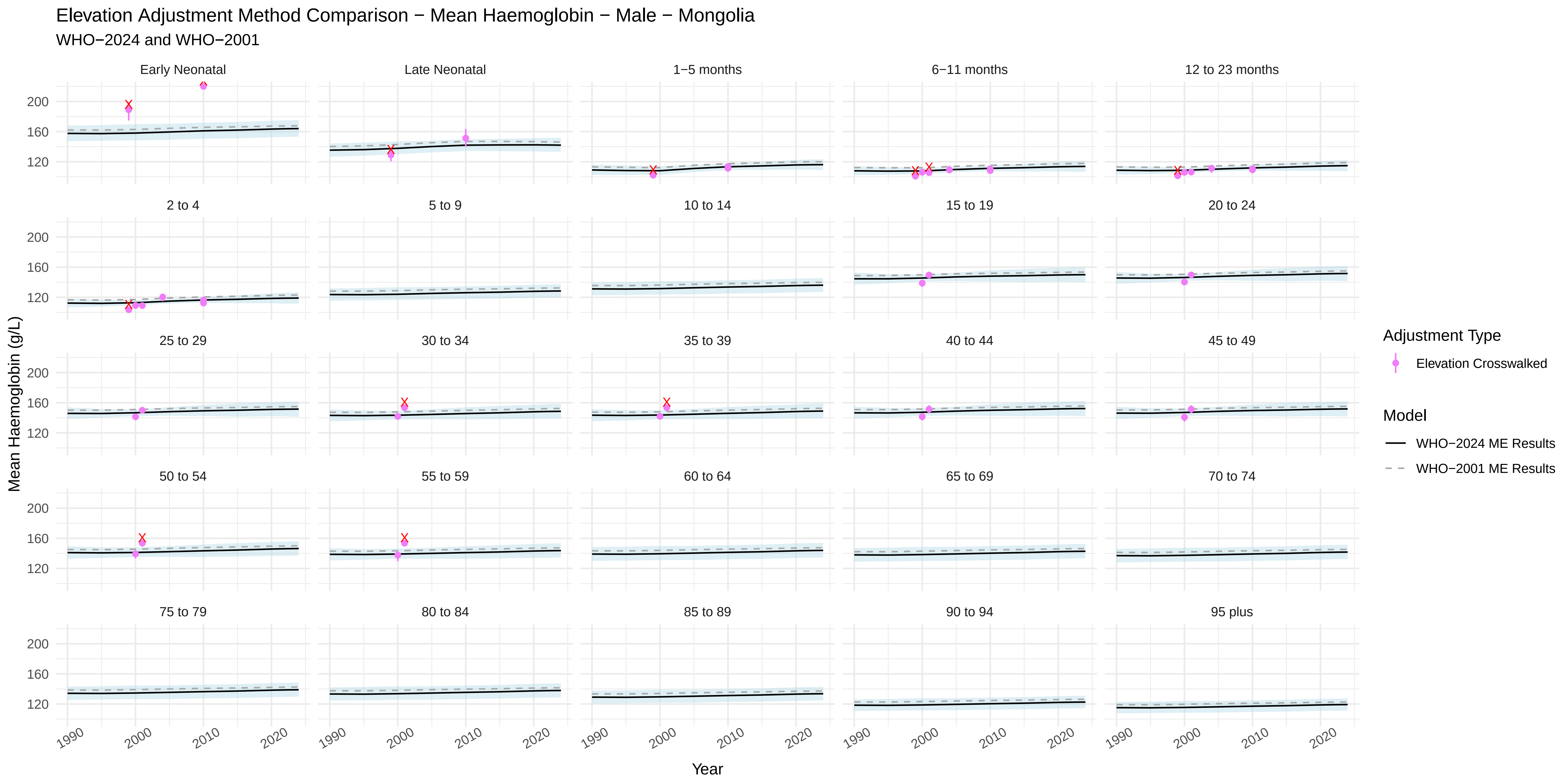
Elevation Adjustment Method Comparison − Mean Haemoglobin − Male − Mongolia
WHO−2024 and WHO−2001
Mean Haemoglobin (g/L)
Year
Adjustment Type
Elevation Crosswalked
Model
WHO−2024 ME Results
WHO−2001 ME Results

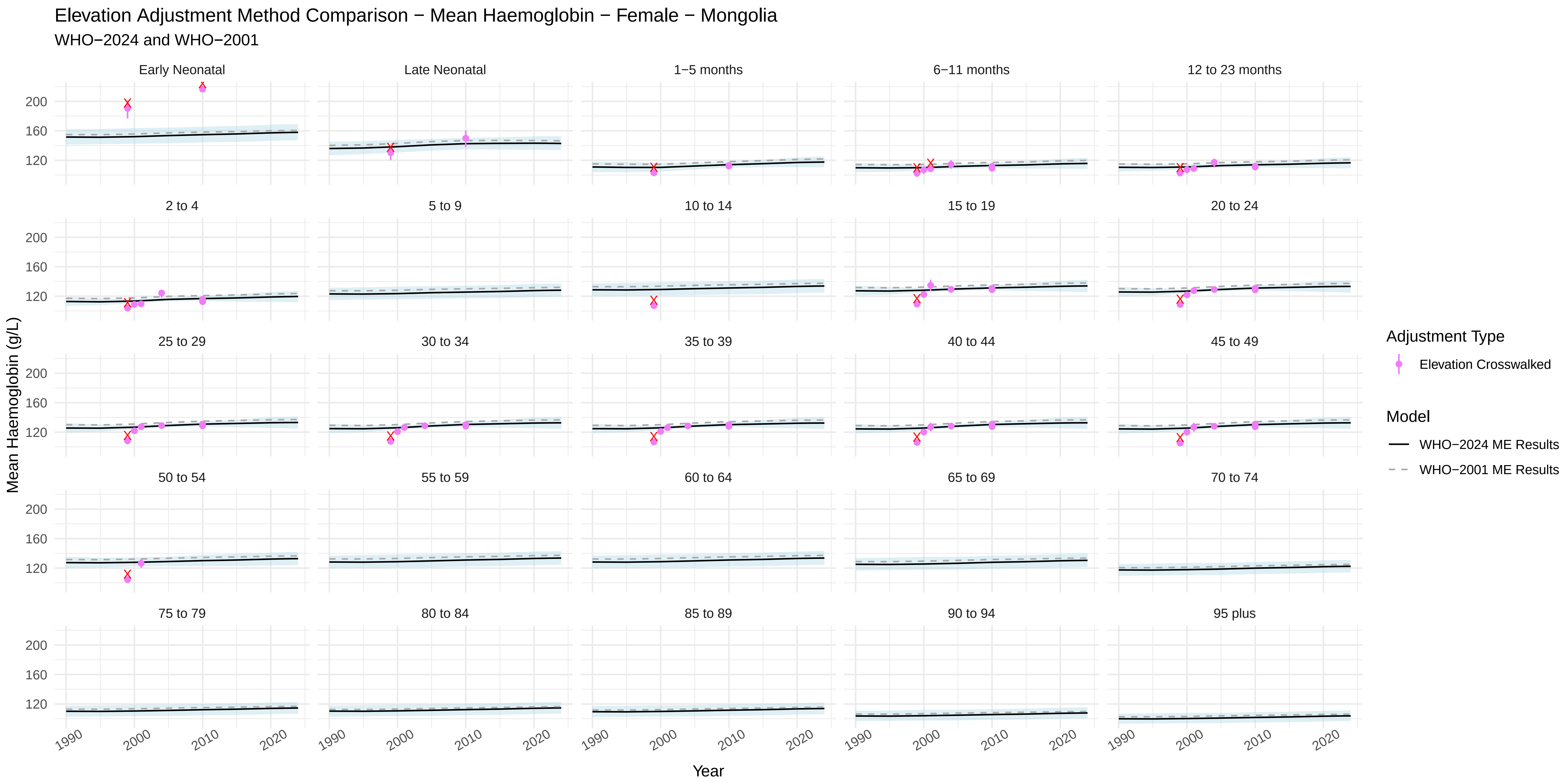
Elevation Adjustment Method Comparison − Mean Haemoglobin − Female − Mongolia
WHO−2024 and WHO−2001
Mean Haemoglobin (g/L)
Year
Adjustment Type
Elevation Crosswalked
Model
WHO−2024 ME Results
WHO−2001 ME Results

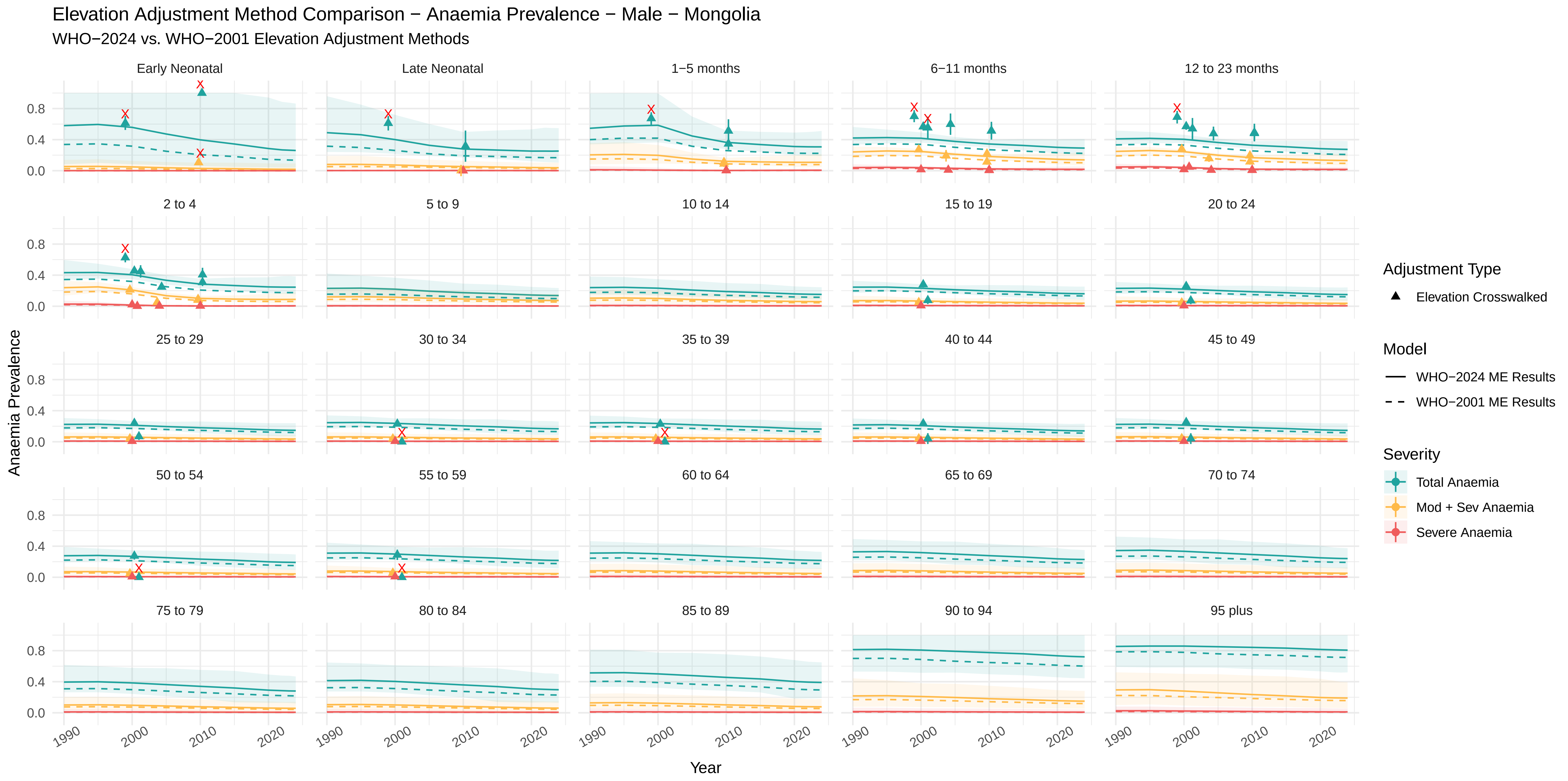
Elevation Adjustment Method Comparison − Anaemia Prevalence − Male − Mongolia
WHO−2024 vs. WHO−2001 Elevation Adjustment Methods
Anaemia Prevalence
Year
Adjustment Type
Elevation Crosswalked
Model
WHO−2024 ME Results
WHO−2001 ME Results
Severity
Total Anaemia
Mod + Sev Anaemia
Severe Anaemia

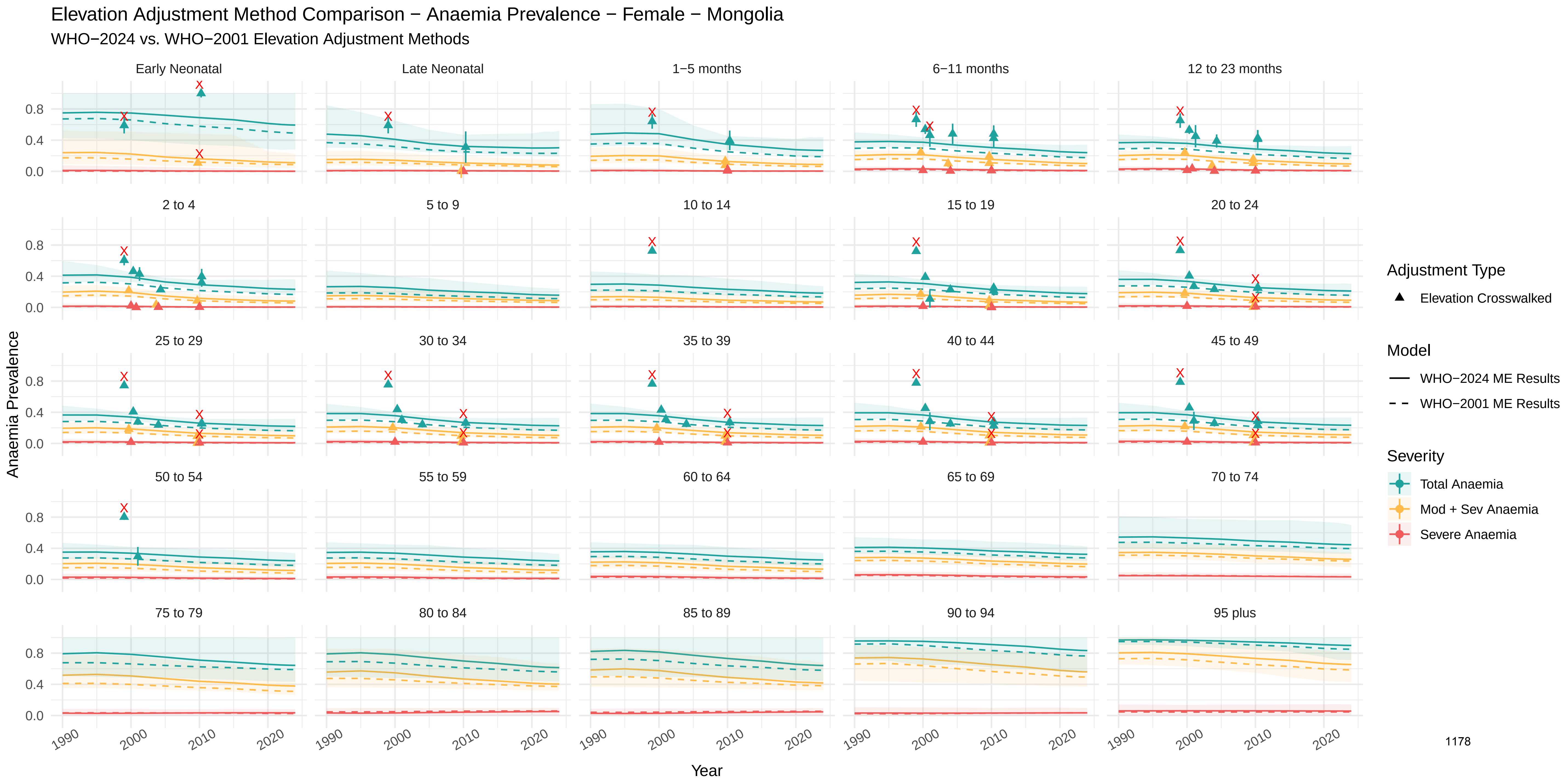
Elevation Adjustment Method Comparison − Anaemia Prevalence − Female − Mongolia
WHO−2024 vs. WHO−2001 Elevation Adjustment Methods
Anaemia Prevalence
Year
Adjustment Type
Elevation Crosswalked
Model
WHO−2024 ME Results
WHO−2001 ME Results
Severity
Total Anaemia
Mod + Sev Anaemia
Severe Anaemia

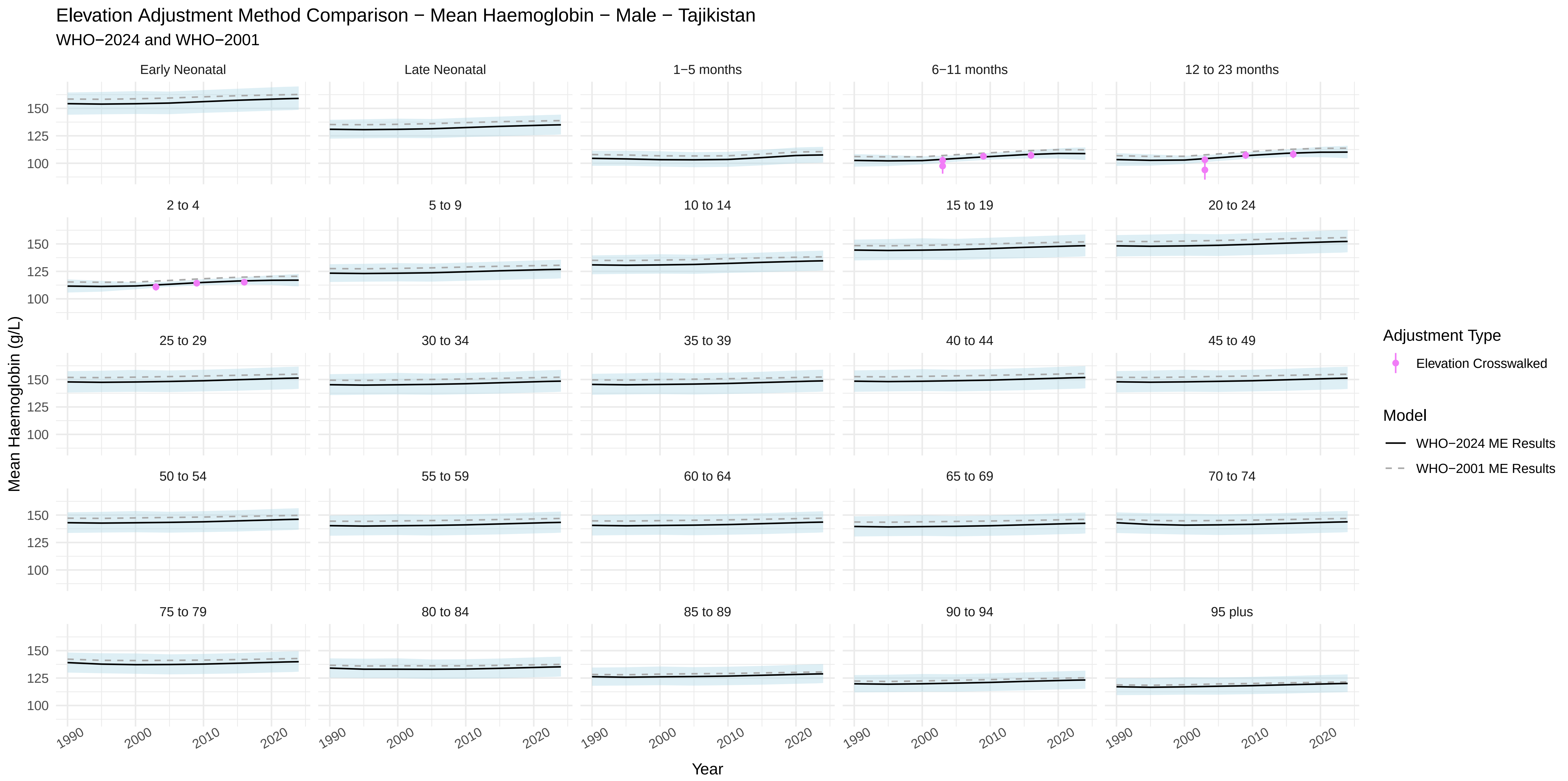

Elevation Adjustment Method Comparison − Mean Haemoglobin − Male − Tajikistan
WHO−2024 and WHO−2001
Mean Haemoglobin (g/L)
Year
Adjustment Type
Elevation Crosswalked
Model
WHO−2024 ME Results
WHO−2001 ME Results

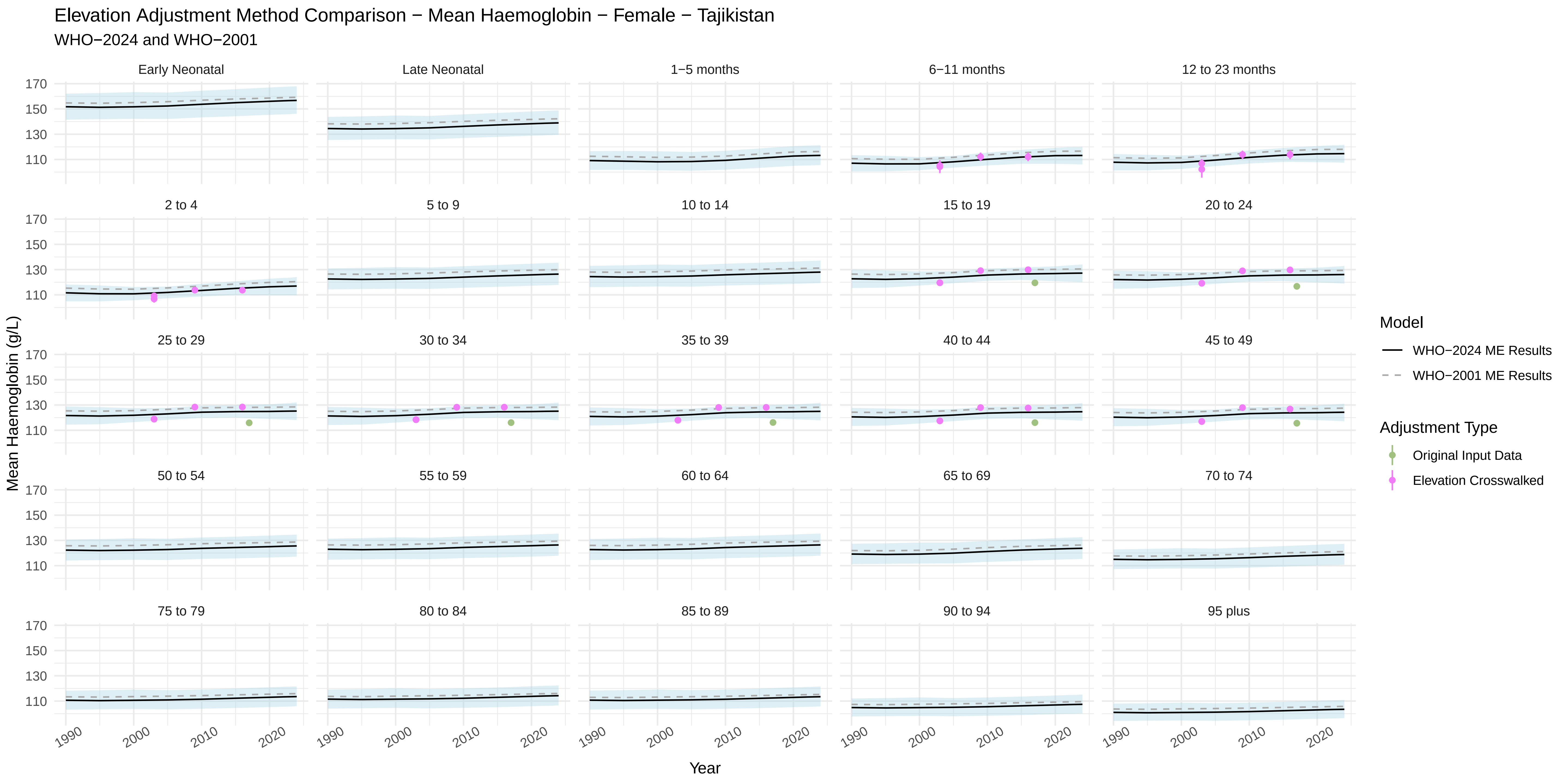

Elevation Adjustment Method Comparison − Mean Haemoglobin − Female − Tajikistan
WHO−2024 and WHO−2001
Mean Haemoglobin (g/L)
Year
Model
WHO−2024 ME Results
WHO−2001 ME Results
Adjustment Type
Original Input Data
Elevation Crosswalked

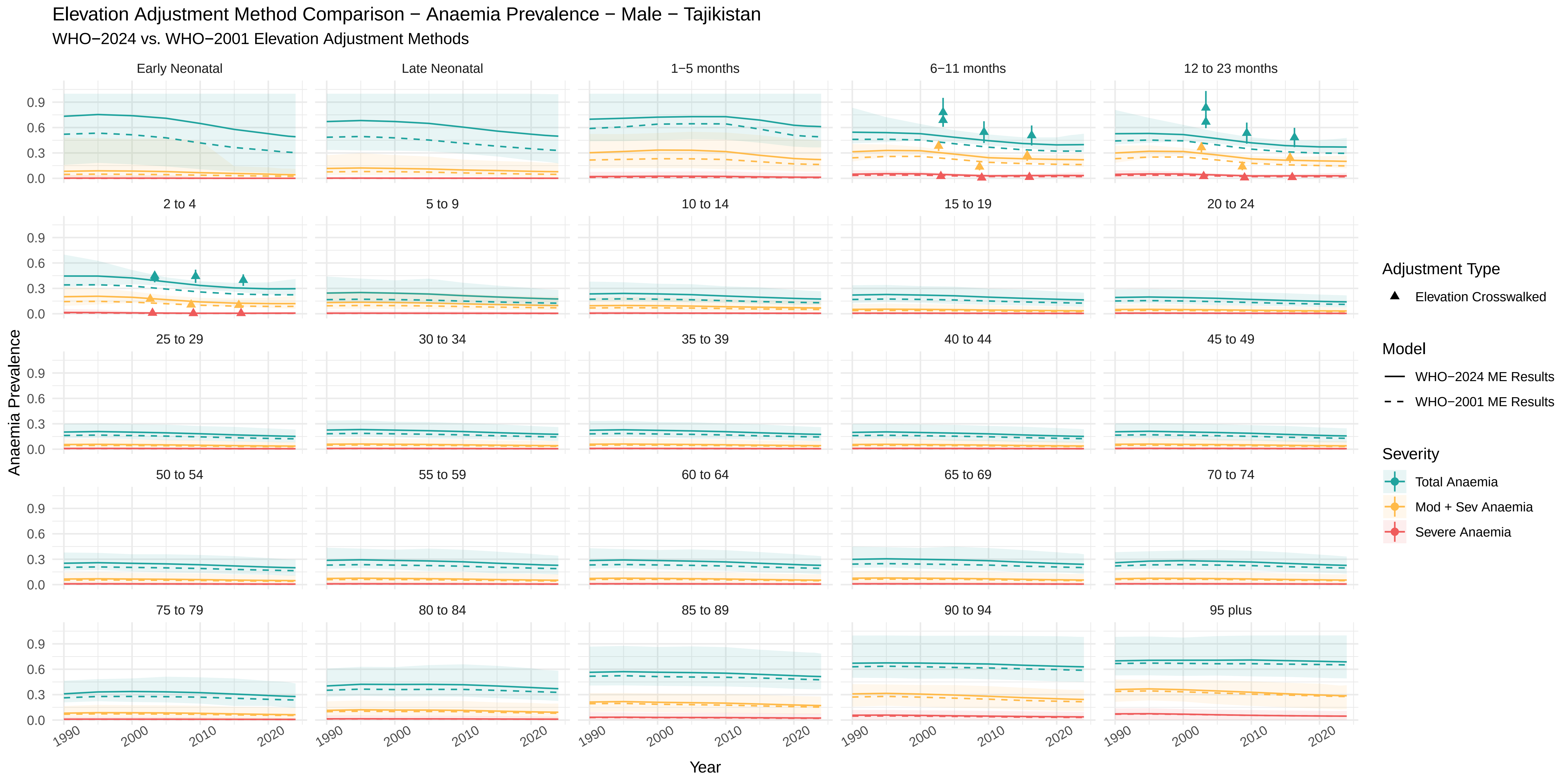

Elevation Adjustment Method Comparison − Anaemia Prevalence − Male − Tajikistan
WHO−2024 vs. WHO−2001 Elevation Adjustment Methods
Anaemia Prevalence
Year
Adjustment Type
Elevation Crosswalked
Model
WHO−2024 ME Results
WHO−2001 ME Results
Severity
Total Anaemia
Mod + Sev Anaemia
Severe Anaemia

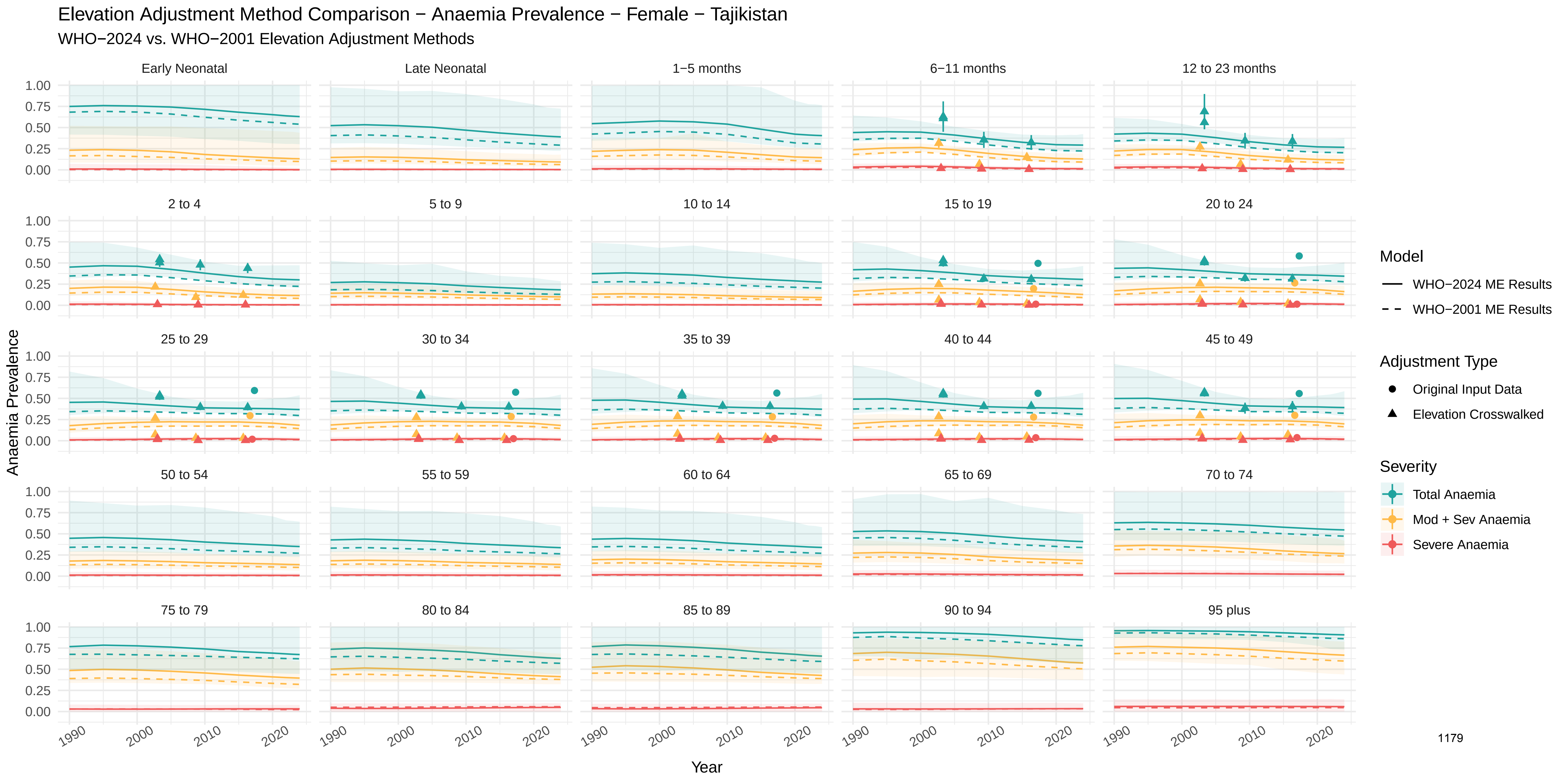

Elevation Adjustment Method Comparison − Anaemia Prevalence − Female − Tajikistan
WHO−2024 vs. WHO−2001 Elevation Adjustment Methods
Anaemia Prevalence
Year
Model
WHO−2024 ME Results
WHO−2001 ME Results
Adjustment Type
Original Input Data
Elevation Crosswalked
Severity
Total Anaemia
Mod + Sev Anaemia
Severe Anaemia

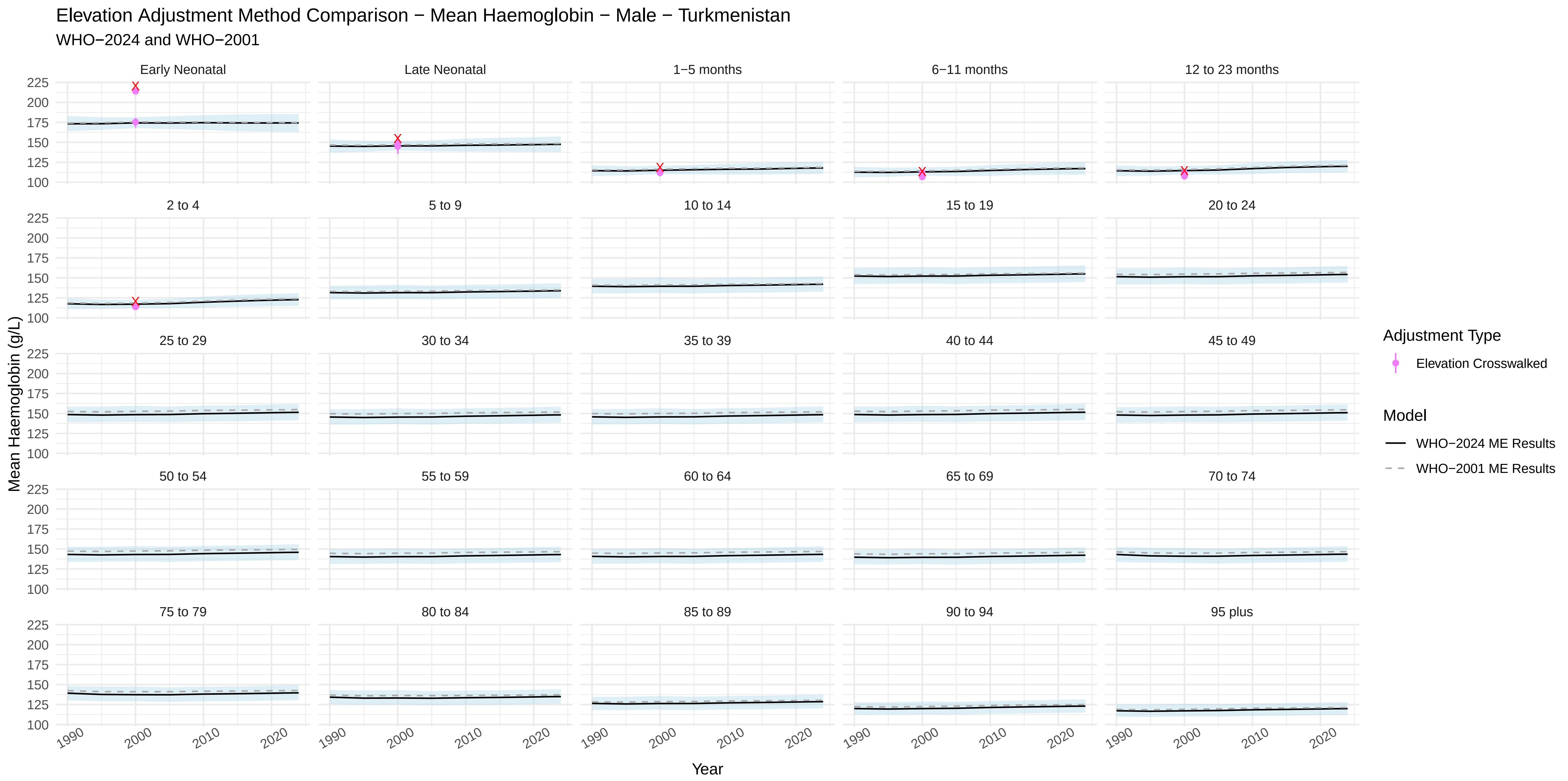
Elevation Adjustment Method Comparison − Mean Haemoglobin − Male − Turkmenistan
WHO−2024 and WHO−2001
Early Neonatal
Late Neonatal
1−5 months
6−11 months
12 to 23 months
2 to 4
5 to 9
10 to 14
15 to 19
20 to 24
25 to 29
30 to 34
35 to 39
40 to 44
45 to 49
50 to 54
55 to 59
60 to 64
65 to 69
70 to 74
75 to 79
80 to 84
85 to 89
90 to 94
95 plus
Mean Haemoglobin (g/L)
Year
Adjustment Type
Elevation Crosswalked
Model
WHO−2024 ME Results
WHO−2001 ME Results

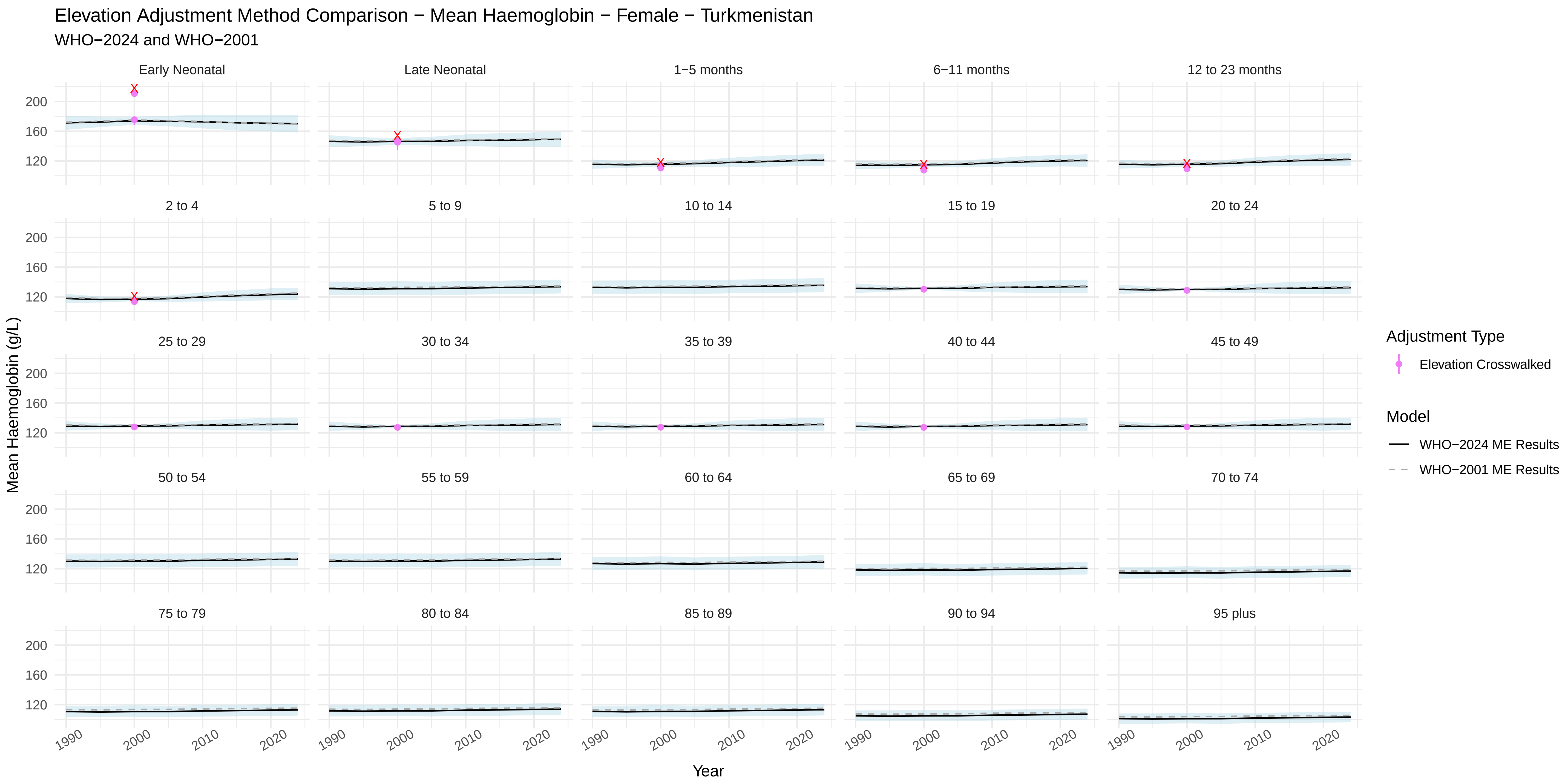
Elevation Adjustment Method Comparison − Mean Haemoglobin − Female − Turkmenistan
WHO−2024 and WHO−2001
Early Neonatal
Late Neonatal
1−5 months
6−11 months
12 to 23 months
2 to 4
5 to 9
10 to 14
15 to 19
20 to 24
25 to 29
30 to 34
35 to 39
40 to 44
45 to 49
50 to 54
55 to 59
60 to 64
65 to 69
70 to 74
75 to 79
80 to 84
85 to 89
90 to 94
95 plus
Mean Haemoglobin (g/L)
Year
Adjustment Type
Elevation Crosswalked
Model
WHO−2024 ME Results
WHO−2001 ME Results

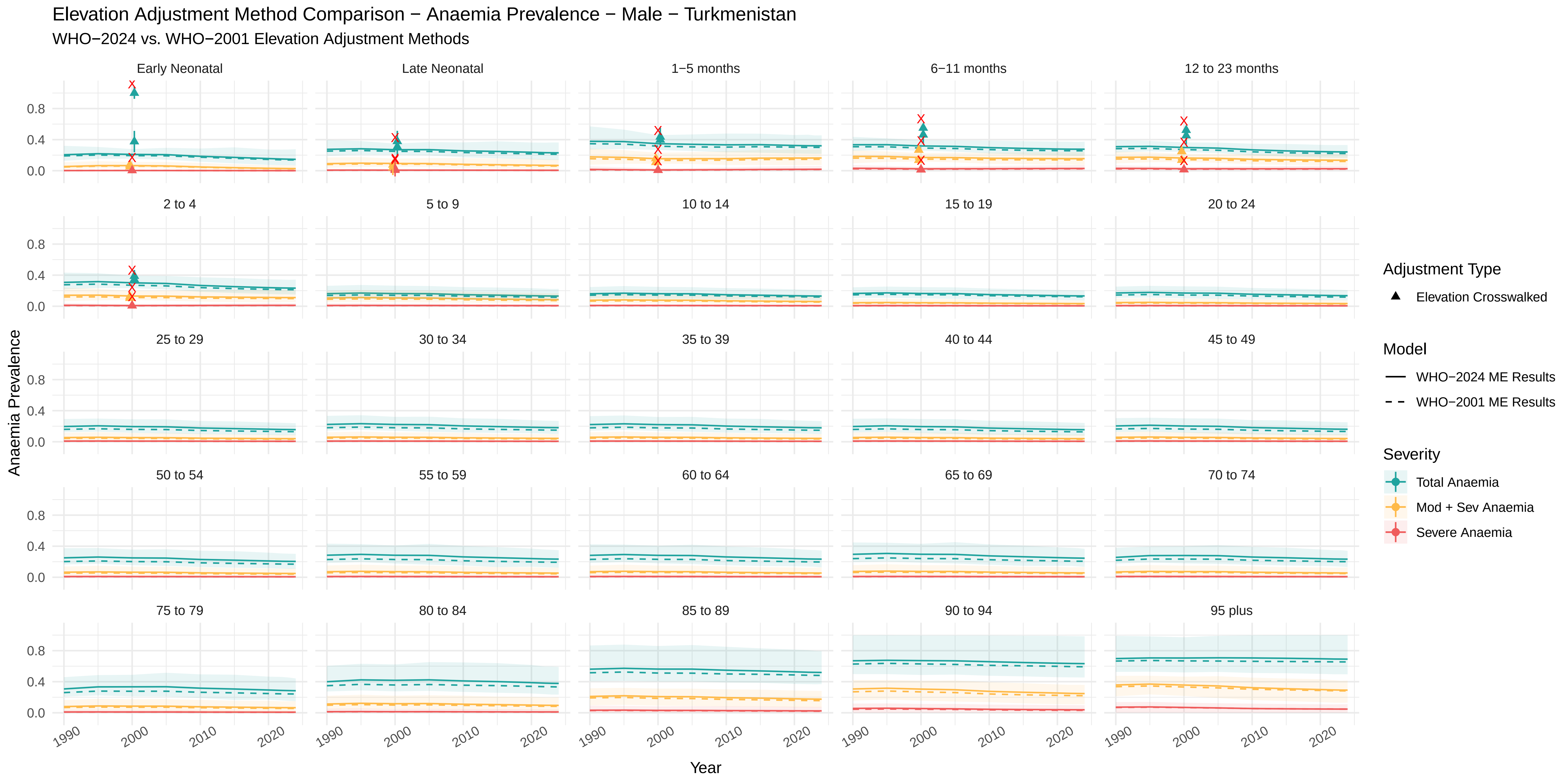
Elevation Adjustment Method Comparison − Anaemia Prevalence − Male − Turkmenistan
WHO−2024 vs. WHO−2001 Elevation Adjustment Methods
Early Neonatal
Late Neonatal
1−5 months
6−11 months
12 to 23 months
2 to 4
5 to 9
10 to 14
15 to 19
20 to 24
25 to 29
30 to 34
35 to 39
40 to 44
45 to 49
50 to 54
55 to 59
60 to 64
65 to 69
70 to 74
75 to 79
80 to 84
85 to 89
90 to 94
95 plus
Anaemia Prevalence
Year
Adjustment Type
Elevation Crosswalked
Model
WHO−2024 ME Results
WHO−2001 ME Results
Severity
Total Anaemia
Mod + Sev Anaemia
Severe Anaemia

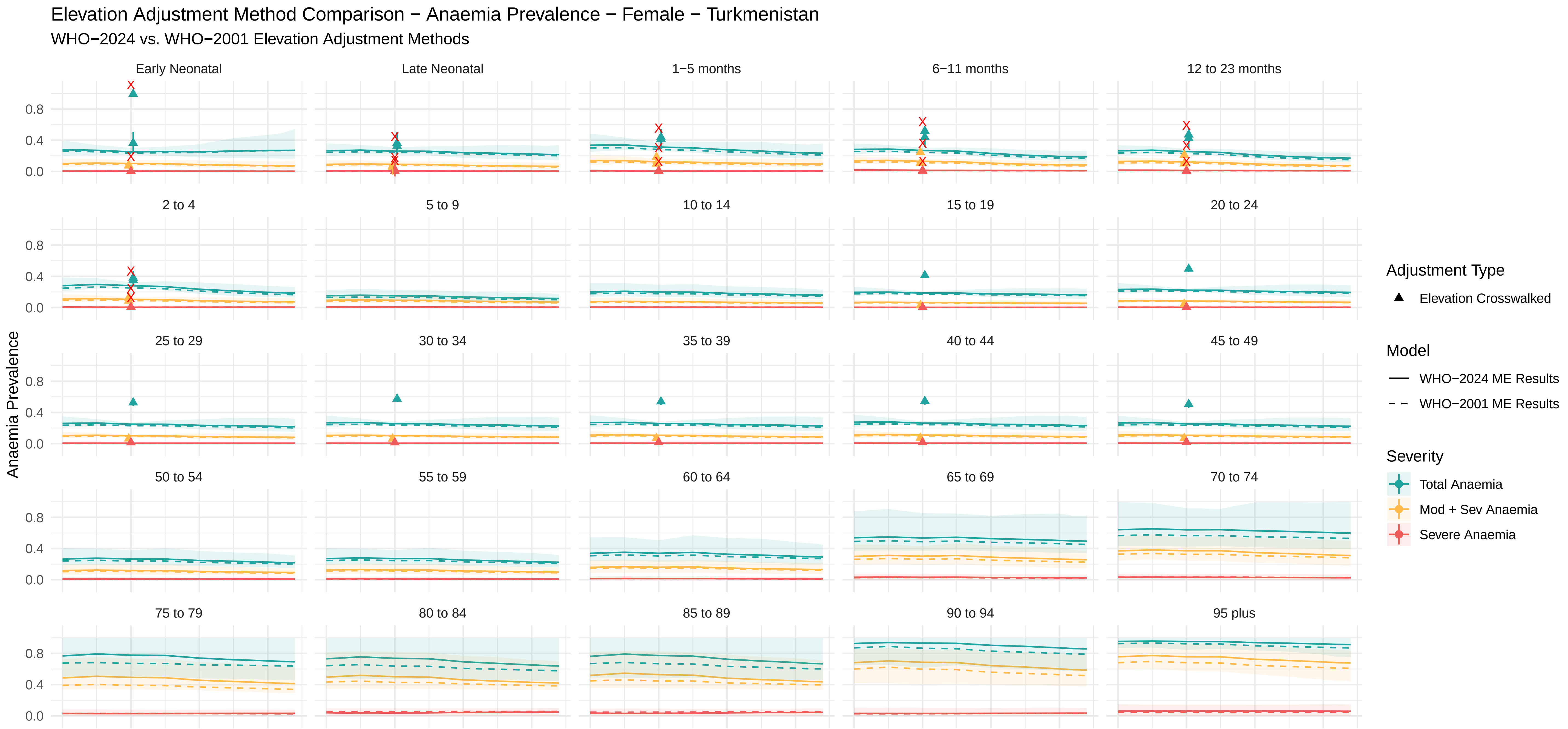
Elevation Adjustment Method Comparison − Anaemia Prevalence − Female − Turkmenistan
WHO−2024 vs. WHO−2001 Elevation Adjustment Methods
Early Neonatal
Late Neonatal
1−5 months
6−11 months
12 to 23 months
2 to 4
5 to 9
10 to 14
15 to 19
20 to 24
25 to 29
30 to 34
35 to 39
40 to 44
45 to 49
50 to 54
55 to 59
60 to 64
65 to 69
70 to 74
75 to 79
80 to 84
85 to 89
90 to 94
95 plus
Anaemia Prevalence
Year
Adjustment Type
Elevation Crosswalked
Model
WHO−2024 ME Results
WHO−2001 ME Results
Severity
Total Anaemia
Mod + Sev Anaemia
Severe Anaemia

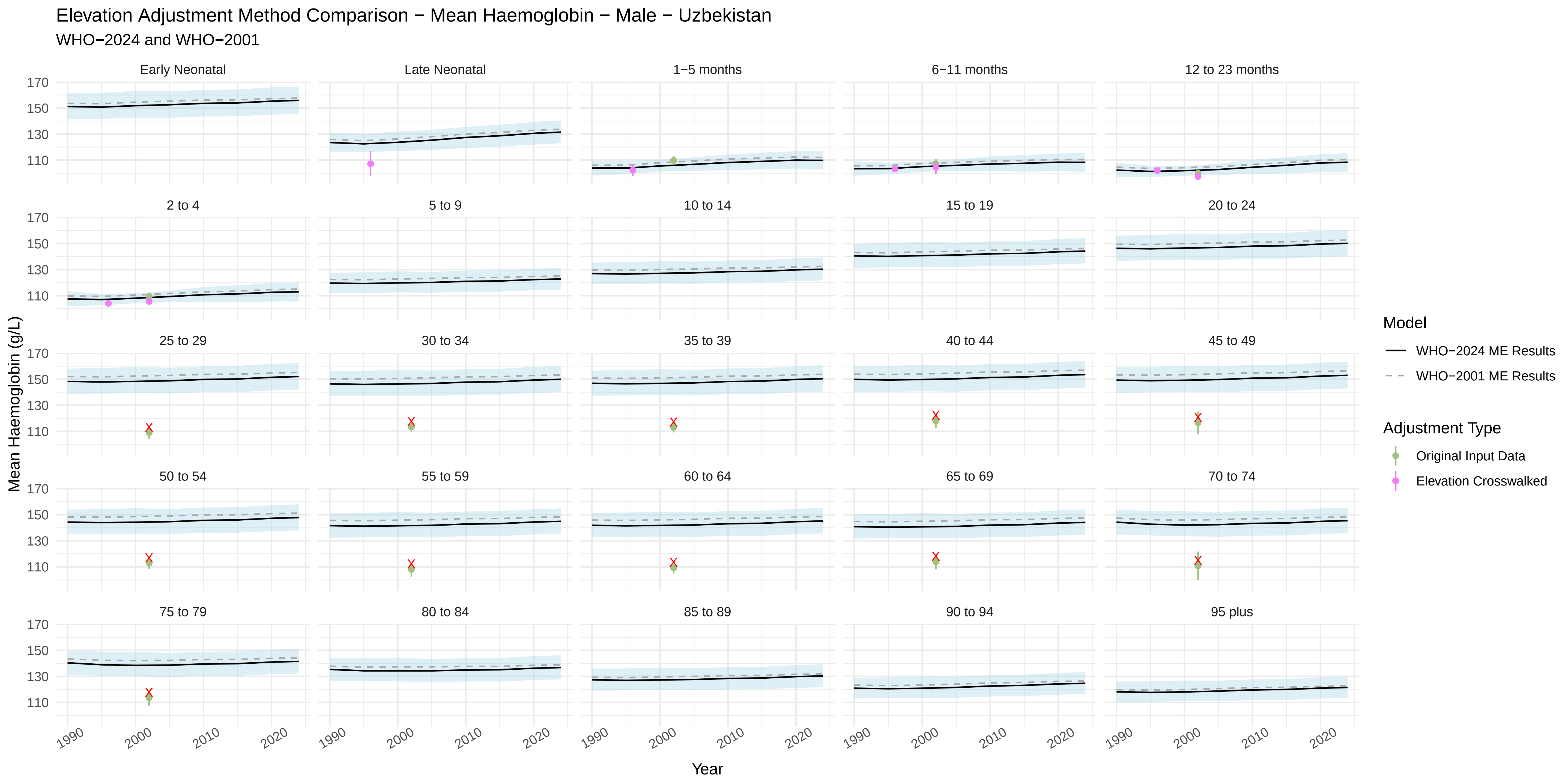
Elevation Adjustment Method Comparison − Mean Haemoglobin − Male − Uzbekistan
WHO−2024 and WHO−2001
Early Neonatal
Late Neonatal
1−5 months
6−11 months
12 to 23 months
2 to 4
5 to 9
10 to 14
15 to 19
20 to 24
25 to 29
30 to 34
35 to 39
40 to 44
45 to 49
50 to 54
55 to 59
60 to 64
65 to 69
70 to 74
75 to 79
80 to 84
85 to 89
90 to 94
95 plus
Mean Haemoglobin (g/L)
Year
Model
WHO−2024 ME Results
WHO−2001 ME Results
Adjustment Type
Original Input Data
Elevation Crosswalked

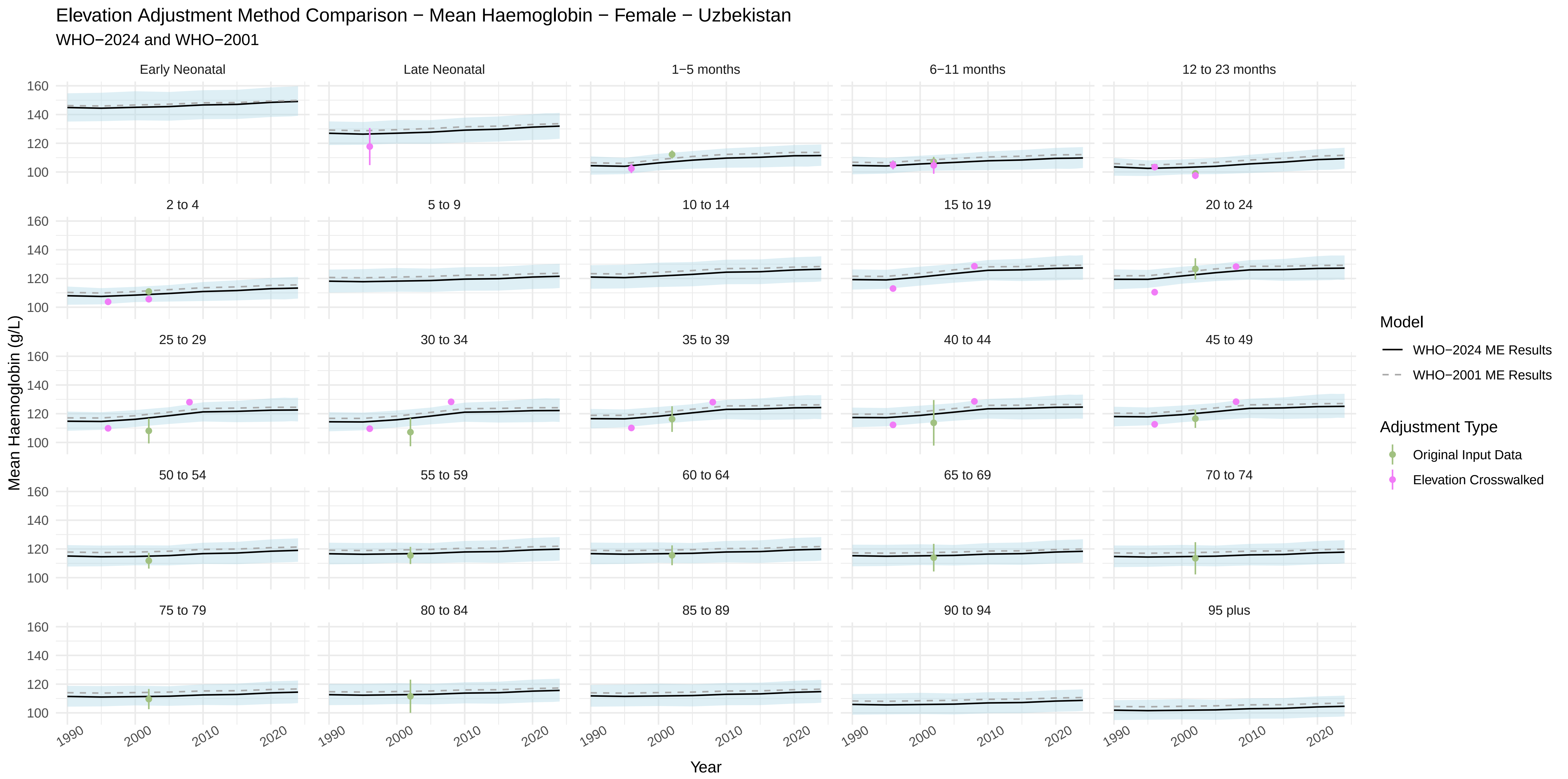
Elevation Adjustment Method Comparison − Mean Haemoglobin − Female − Uzbekistan
WHO−2024 and WHO−2001
Early Neonatal
Late Neonatal
1−5 months
6−11 months
12 to 23 months
2 to 4
5 to 9
10 to 14
15 to 19
20 to 24
25 to 29
30 to 34
35 to 39
40 to 44
45 to 49
50 to 54
55 to 59
60 to 64
65 to 69
70 to 74
75 to 79
80 to 84
85 to 89
90 to 94
95 plus
Mean Haemoglobin (g/L)
Year
Model
WHO−2024 ME Results
WHO−2001 ME Results
Adjustment Type
Original Input Data
Elevation Crosswalked

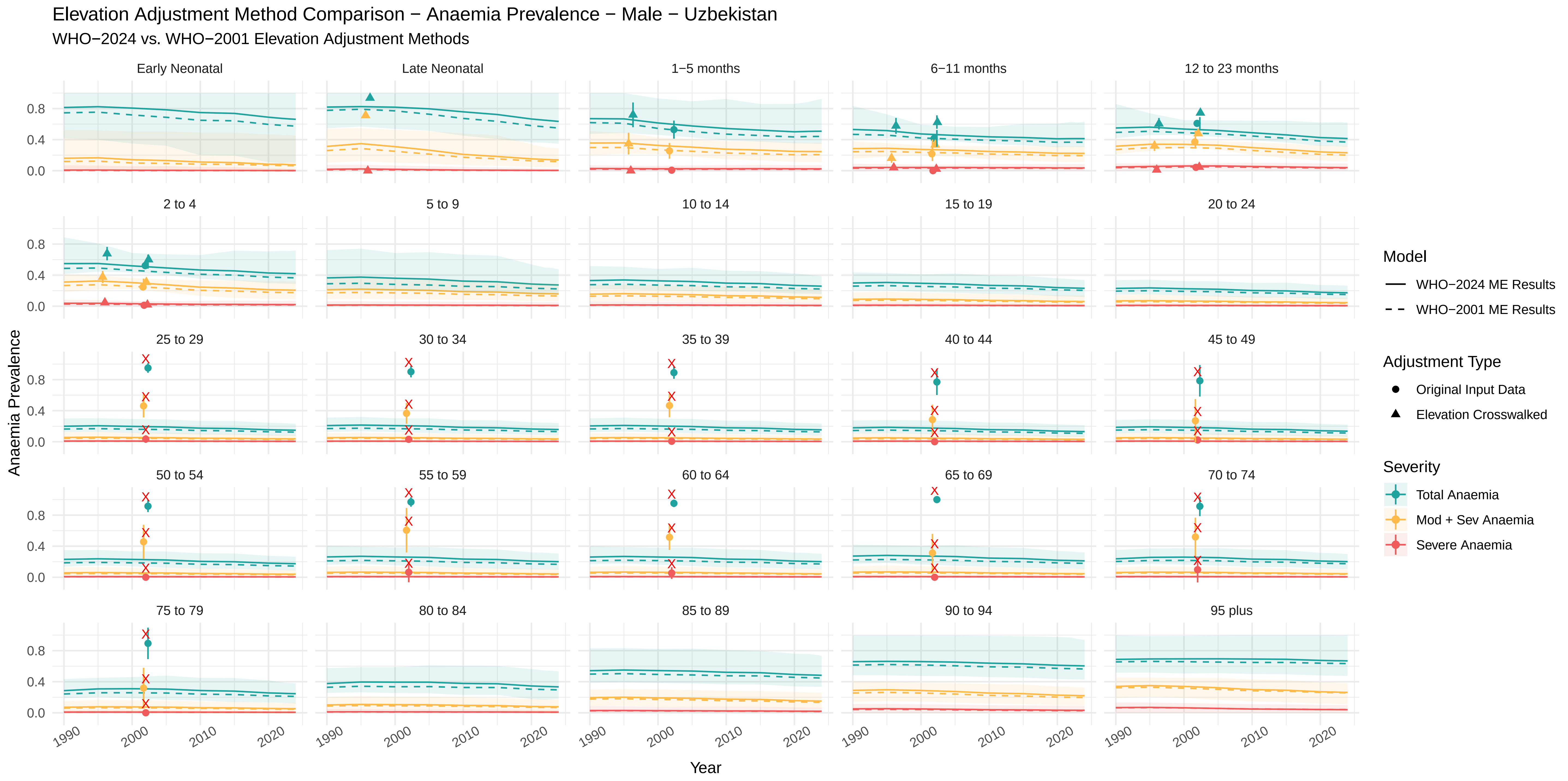
Elevation Adjustment Method Comparison − Anaemia Prevalence − Male − Uzbekistan
WHO−2024 vs. WHO−2001 Elevation Adjustment Methods
Early Neonatal
Late Neonatal
1−5 months
6−11 months
12 to 23 months
2 to 4
5 to 9
10 to 14
15 to 19
20 to 24
25 to 29
30 to 34
35 to 39
40 to 44
45 to 49
50 to 54
55 to 59
60 to 64
65 to 69
70 to 74
75 to 79
80 to 84
85 to 89
90 to 94
95 plus
Anaemia Prevalence
Year
Model
WHO−2024 ME Results
WHO−2001 ME Results
Adjustment Type
Original Input Data
Elevation Crosswalked
Severity
Total Anaemia
Mod + Sev Anaemia
Severe Anaemia

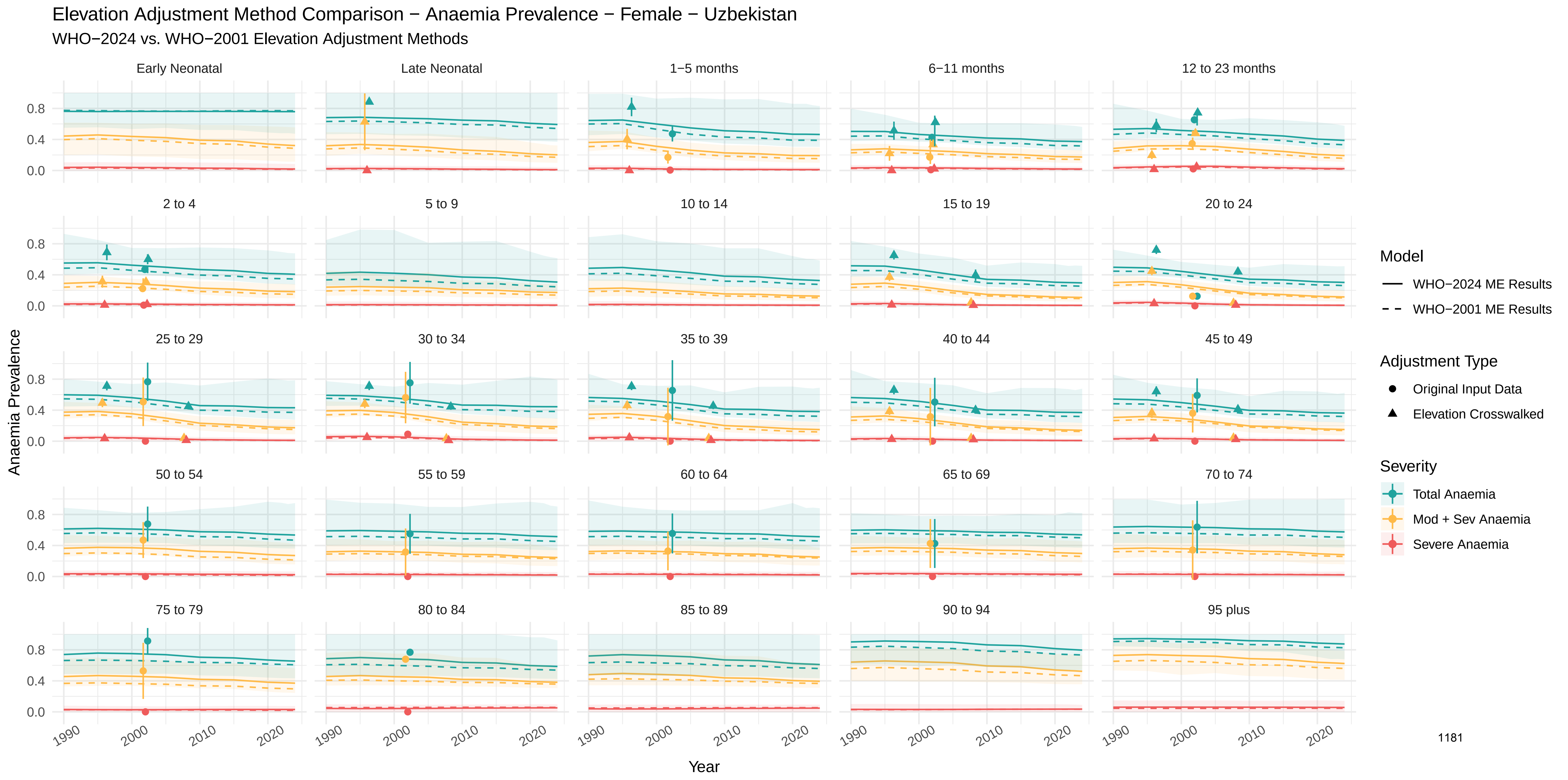
Elevation Adjustment Method Comparison − Anaemia Prevalence − Female − Uzbekistan
WHO−2024 vs. WHO−2001 Elevation Adjustment Methods
Early Neonatal
Late Neonatal
1−5 months
6−11 months
12 to 23 months
2 to 4
5 to 9
10 to 14
15 to 19
20 to 24
25 to 29
30 to 34
35 to 39
40 to 44
45 to 49
50 to 54
55 to 59
60 to 64
65 to 69
70 to 74
75 to 79
80 to 84
85 to 89
90 to 94
95 plus
Anaemia Prevalence
Year
Model
WHO−2024 ME Results
WHO−2001 ME Results
Adjustment Type
Original Input Data
Elevation Crosswalked
Severity
Total Anaemia
Mod + Sev Anaemia
Severe Anaemia

Elevation Adjustment Method Comparison − Mean Haemoglobin − Male − Central Europe

WHO−2024 and WHO−2001

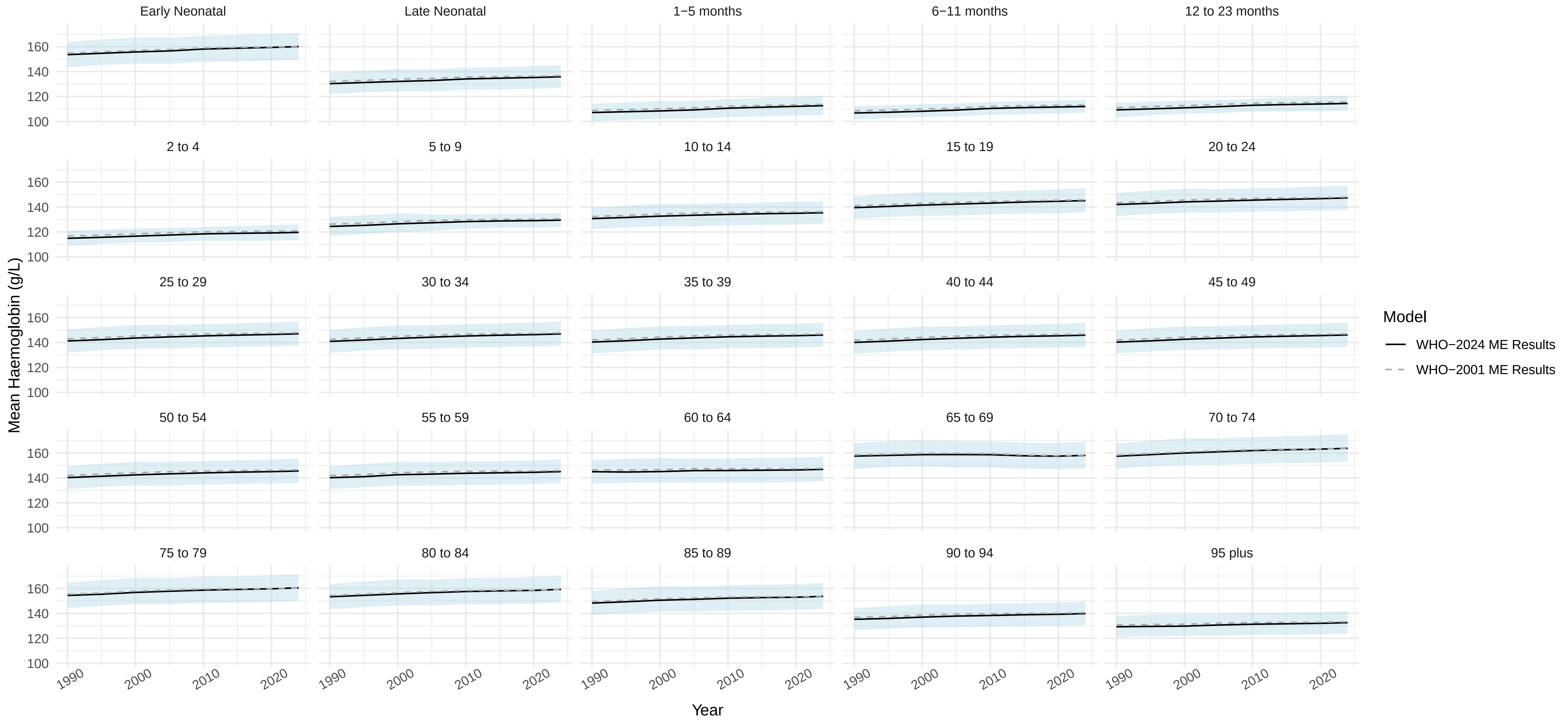

Elevation Adjustment Method Comparison − Mean Haemoglobin − Female − Central Europe

WHO−2024 and WHO−2001

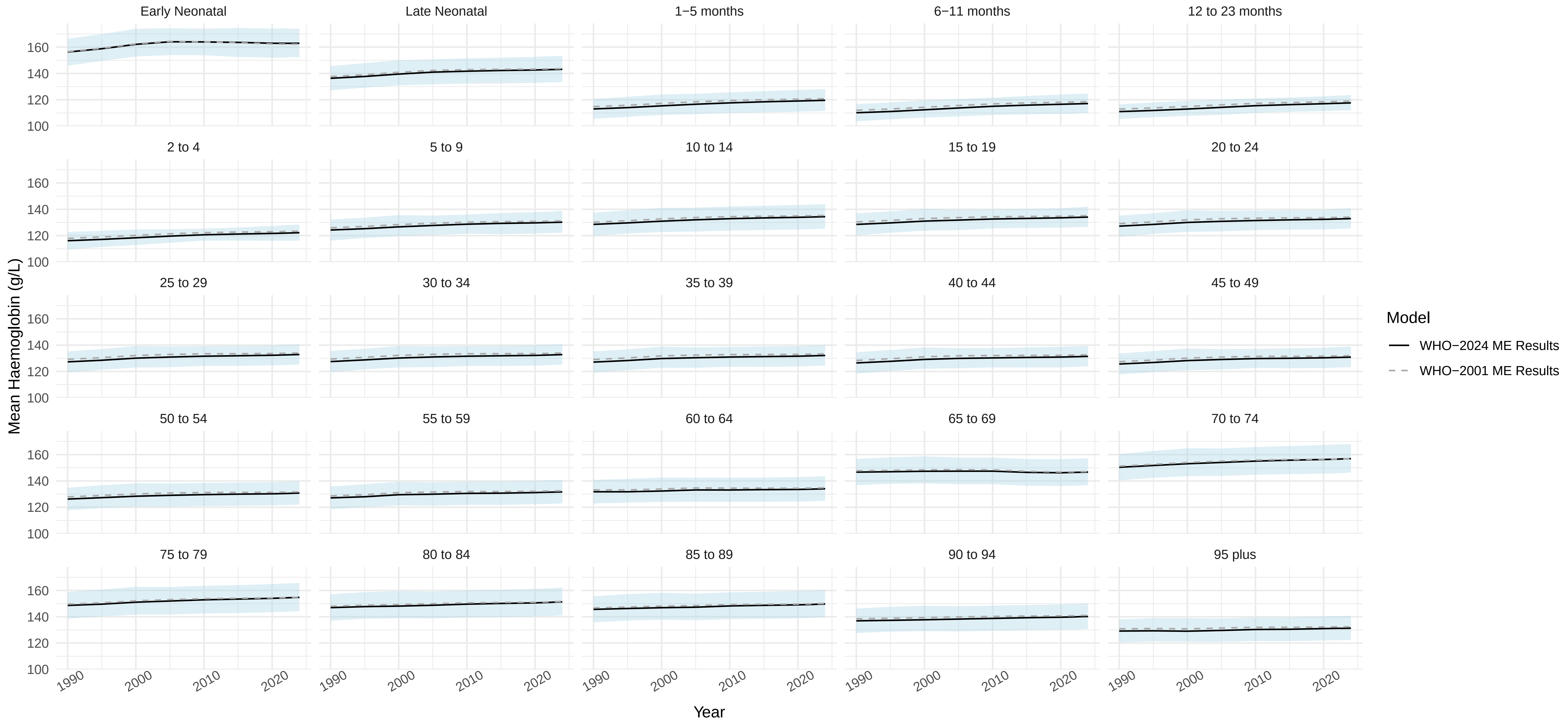

Elevation Adjustment Method Comparison − Anaemia Prevalence − Male − Central Europe

WHO−2024 vs. WHO−2001 Elevation Adjustment Methods

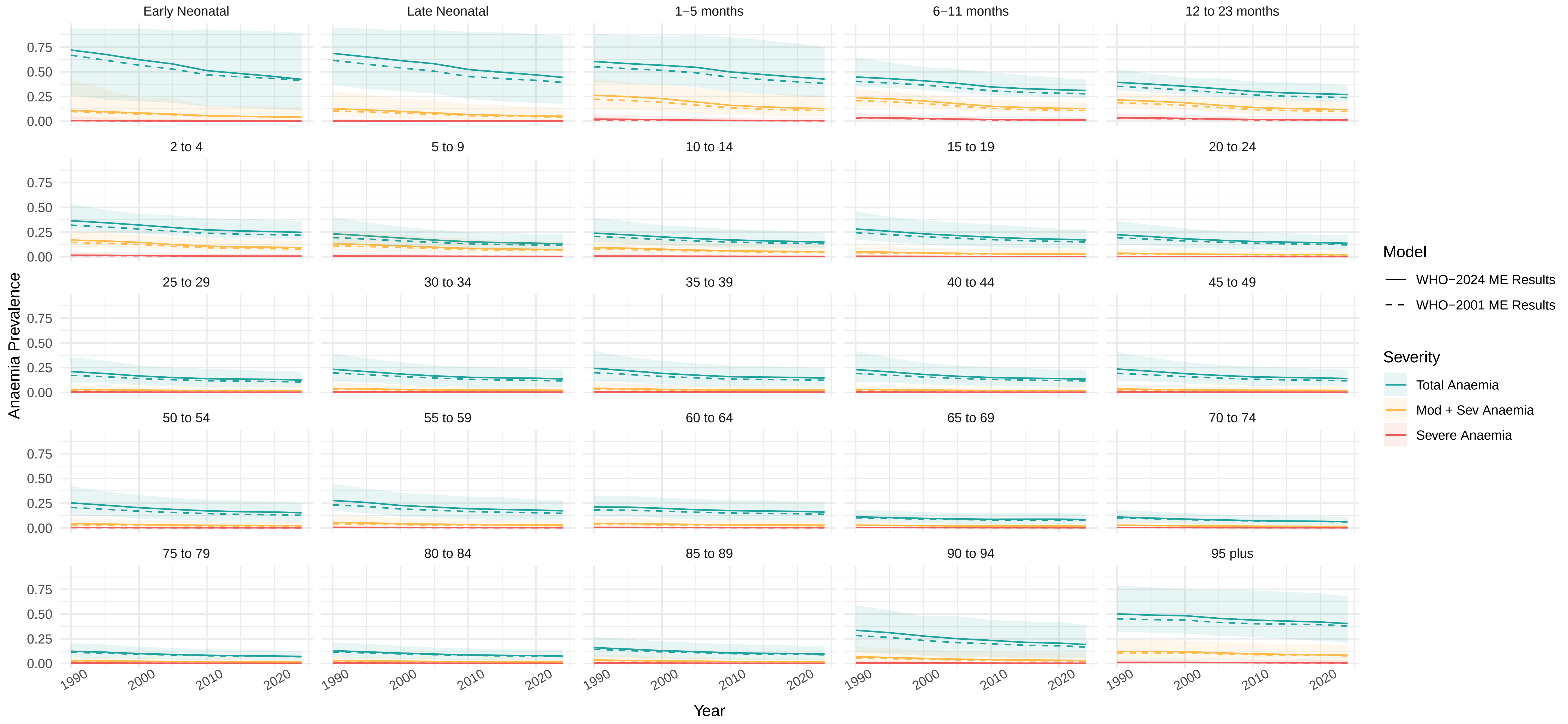

Elevation Adjustment Method Comparison − Anaemia Prevalence − Female − Central Europe

WHO−2024 vs. WHO−2001 Elevation Adjustment Methods

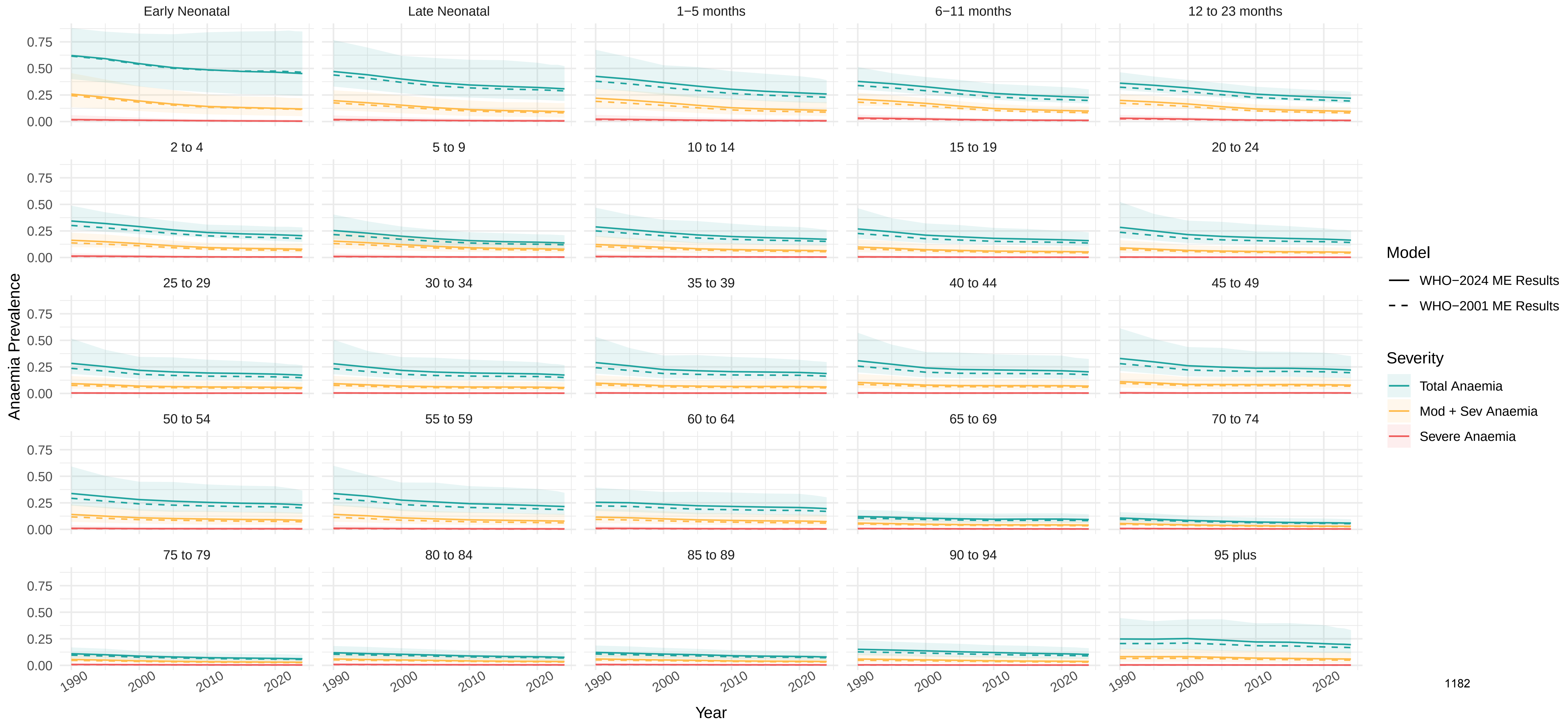

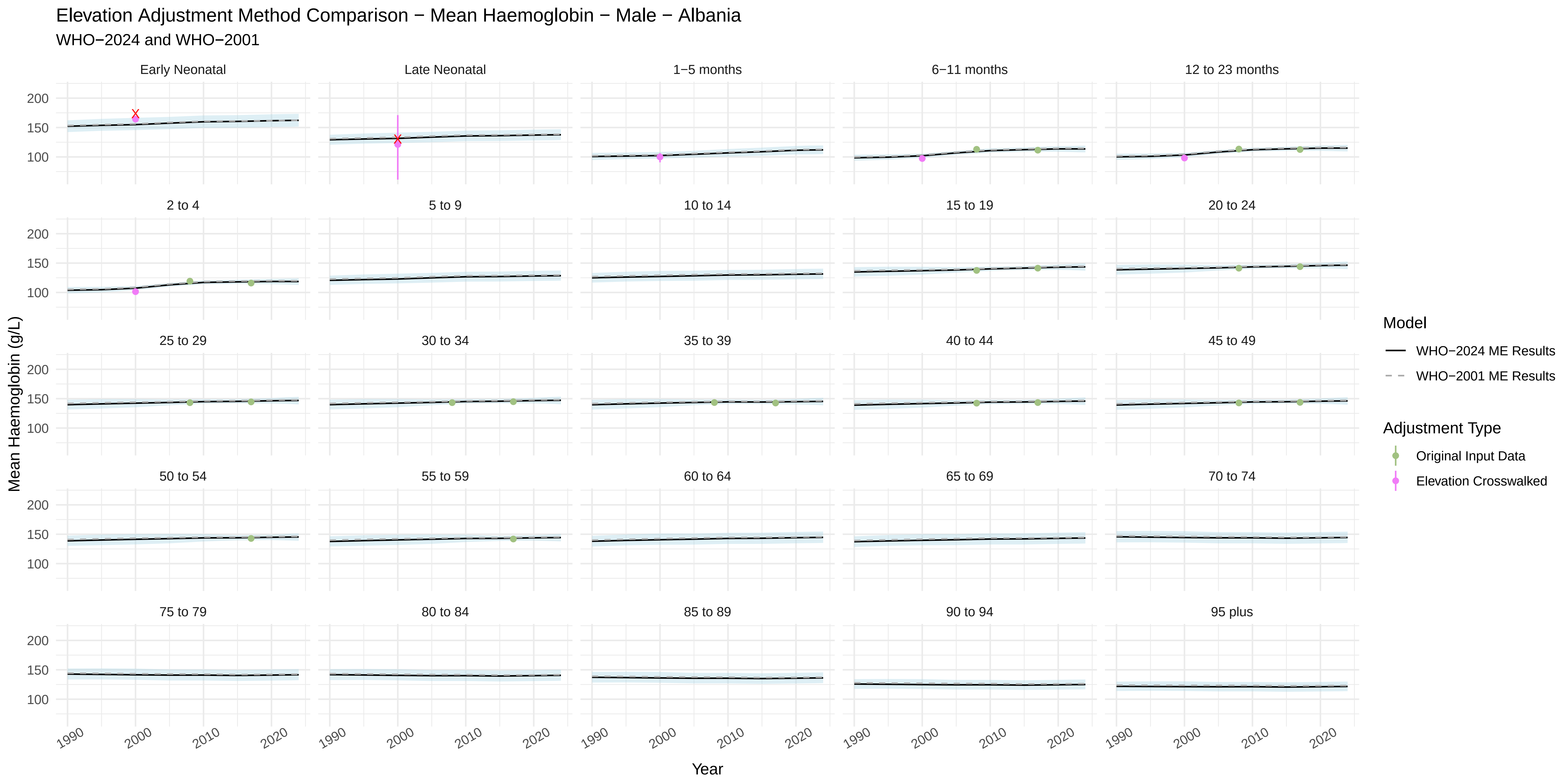
Elevation Adjustment Method Comparison − Mean Haemoglobin − Male − Albania
WHO−2024 and WHO−2001
Early Neonatal
Late Neonatal
1−5 months
6−11 months
12 to 23 months
2 to 4
5 to 9
10 to 14
15 to 19
20 to 24
25 to 29
30 to 34
35 to 39
40 to 44
45 to 49
50 to 54
55 to 59
60 to 64
65 to 69
70 to 74
75 to 79
80 to 84
85 to 89
90 to 94
95 plus
Mean Haemoglobin (g/L)
Year
Model
WHO−2024 ME Results
WHO−2001 ME Results
Adjustment Type
Original Input Data
Elevation Crosswalked

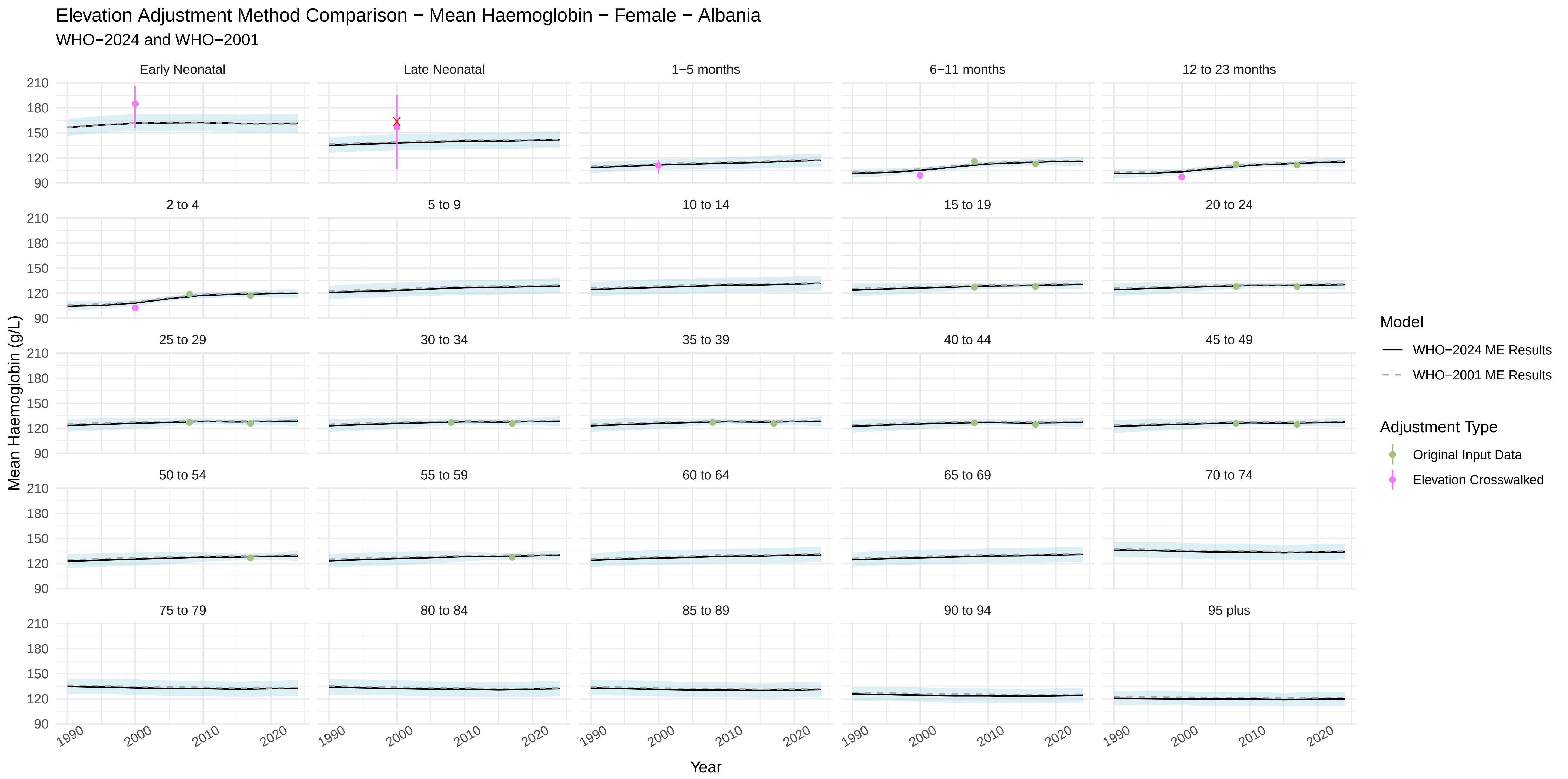
Elevation Adjustment Method Comparison − Mean Haemoglobin − Female − Albania
WHO−2024 and WHO−2001
Early Neonatal
Late Neonatal
1−5 months
6−11 months
12 to 23 months
2 to 4
5 to 9
10 to 14
15 to 19
20 to 24
25 to 29
30 to 34
35 to 39
40 to 44
45 to 49
50 to 54
55 to 59
60 to 64
65 to 69
70 to 74
75 to 79
80 to 84
85 to 89
90 to 94
95 plus
Mean Haemoglobin (g/L)
Year
Model
WHO−2024 ME Results
WHO−2001 ME Results
Adjustment Type
Original Input Data
Elevation Crosswalked

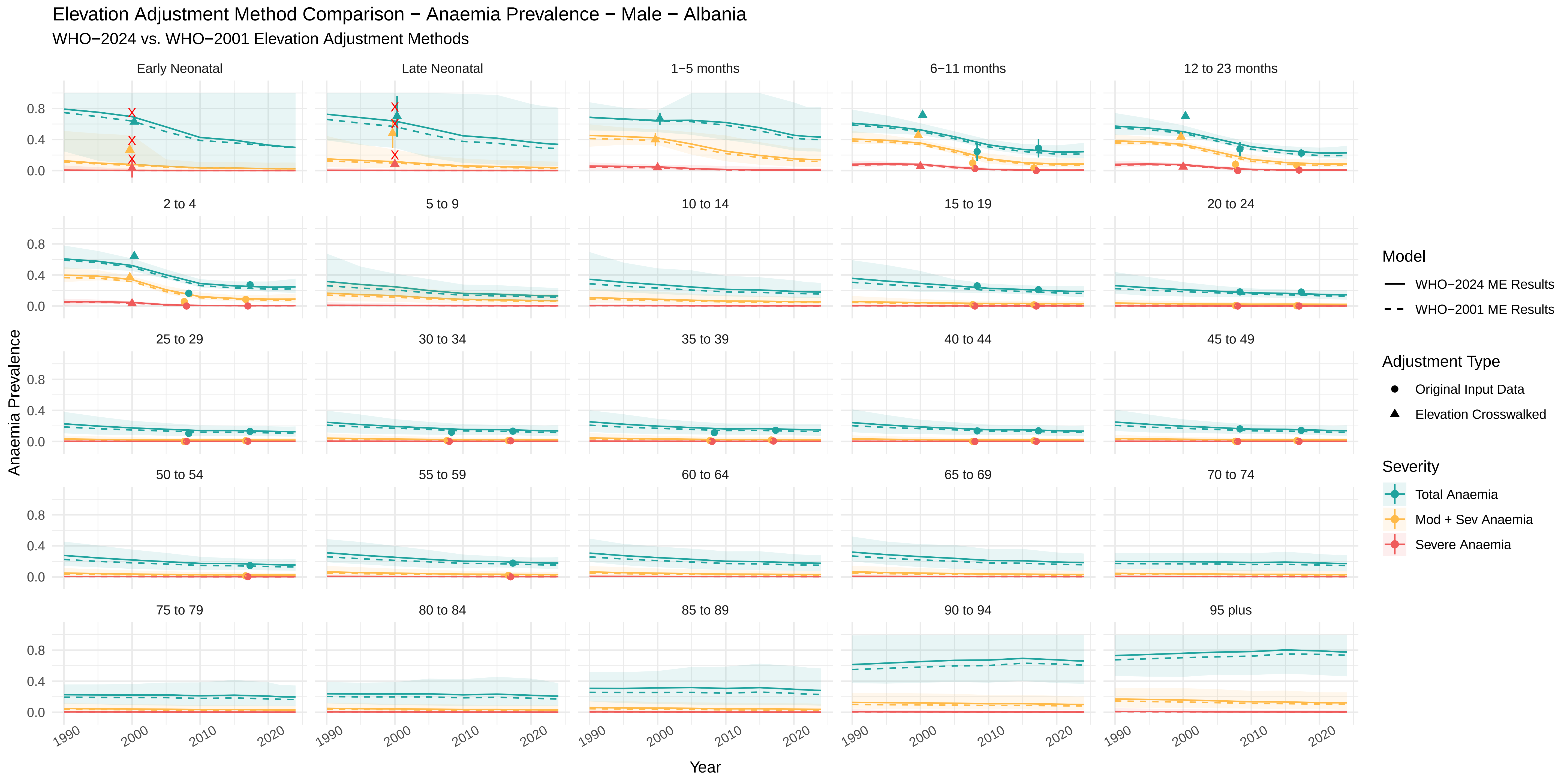
Elevation Adjustment Method Comparison − Anaemia Prevalence − Male − Albania
WHO−2024 vs. WHO−2001 Elevation Adjustment Methods
Early Neonatal
Late Neonatal
1−5 months
6−11 months
12 to 23 months
2 to 4
5 to 9
10 to 14
15 to 19
20 to 24
25 to 29
30 to 34
35 to 39
40 to 44
45 to 49
50 to 54
55 to 59
60 to 64
65 to 69
70 to 74
75 to 79
80 to 84
85 to 89
90 to 94
95 plus
Anaemia Prevalence
Year
Model
WHO−2024 ME Results
WHO−2001 ME Results
Adjustment Type
Original Input Data
Elevation Crosswalked
Severity
Total Anaemia
Mod + Sev Anaemia
Severe Anaemia

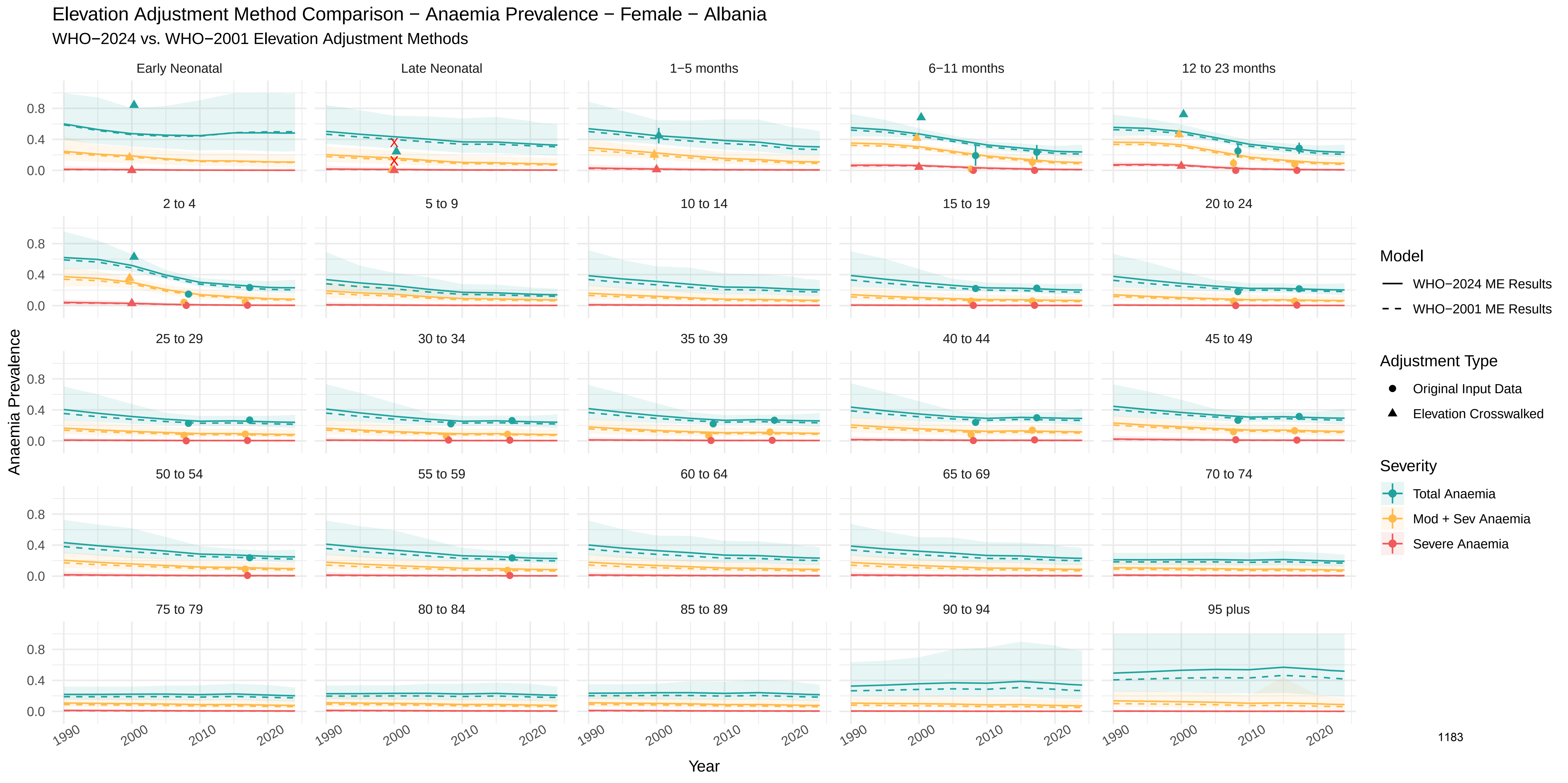
Elevation Adjustment Method Comparison − Anaemia Prevalence − Female − Albania
WHO−2024 vs. WHO−2001 Elevation Adjustment Methods
Early Neonatal
Late Neonatal
1−5 months
6−11 months
12 to 23 months
2 to 4
5 to 9
10 to 14
15 to 19
20 to 24
25 to 29
30 to 34
35 to 39
40 to 44
45 to 49
50 to 54
55 to 59
60 to 64
65 to 69
70 to 74
75 to 79
80 to 84
85 to 89
90 to 94
95 plus
Anaemia Prevalence
Year
Model
WHO−2024 ME Results
WHO−2001 ME Results
Adjustment Type
Original Input Data
Elevation Crosswalked
Severity
Total Anaemia
Mod + Sev Anaemia
Severe Anaemia

Elevation Adjustment Method Comparison − Mean Haemoglobin − Male − Bosnia and Herzegovina
WHO−2024 and WHO−2001

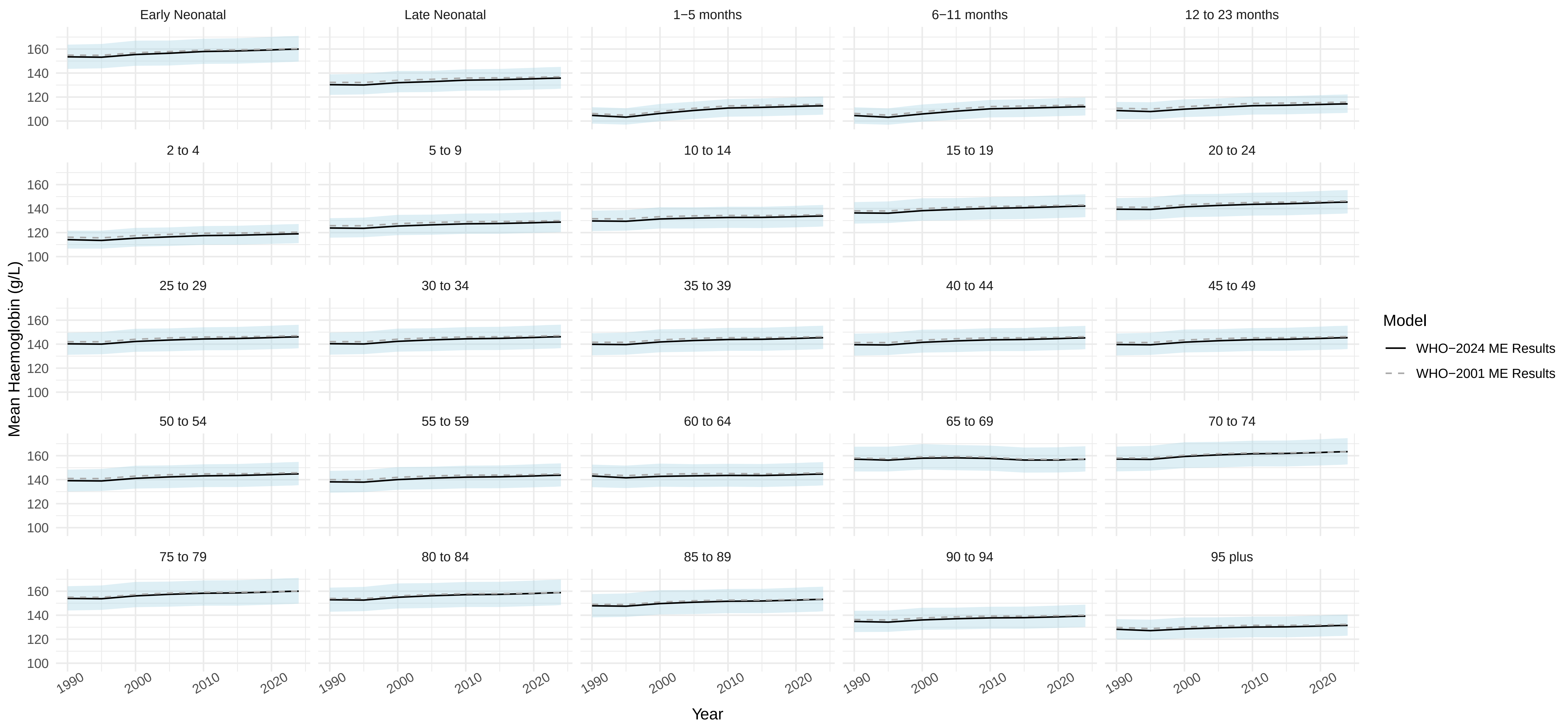

Elevation Adjustment Method Comparison − Mean Haemoglobin − Female − Bosnia and Herzegovina
WHO−2024 and WHO−2001

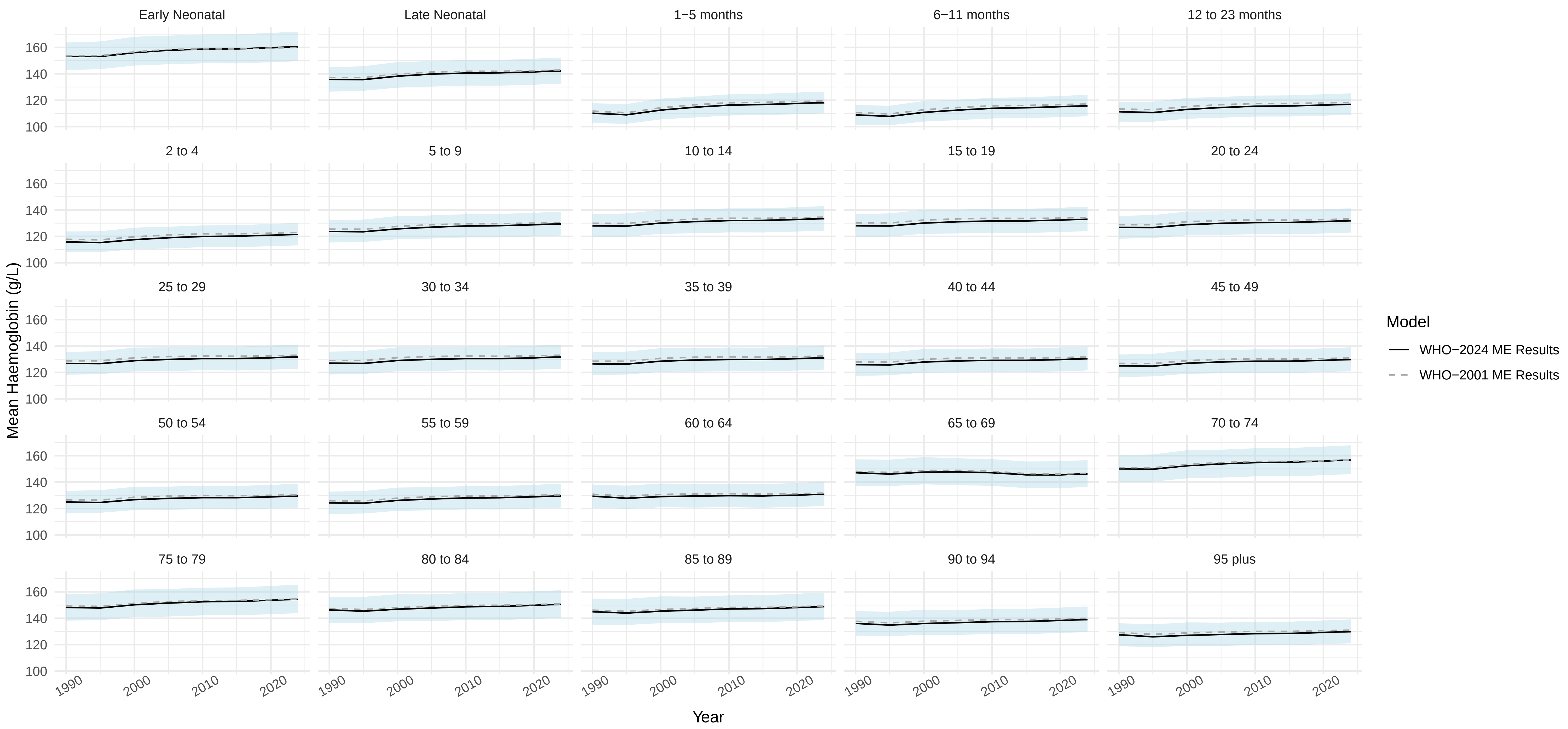

Elevation Adjustment Method Comparison − Anaemia Prevalence − Male − Bosnia and Herzegovina
WHO−2024 vs. WHO−2001 Elevation Adjustment Methods

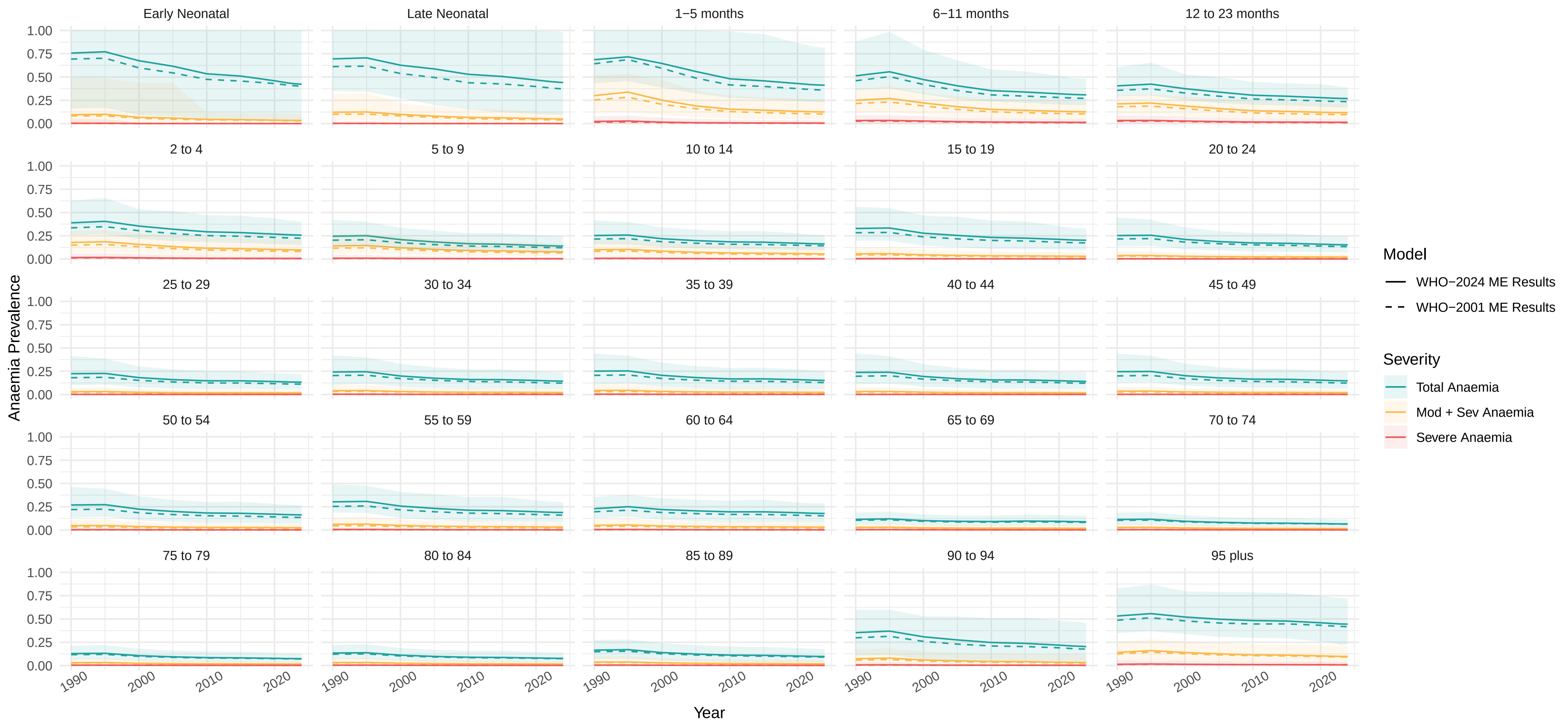

Elevation Adjustment Method Comparison − Anaemia Prevalence − Female − Bosnia and Herzegovina
WHO−2024 vs. WHO−2001 Elevation Adjustment Methods

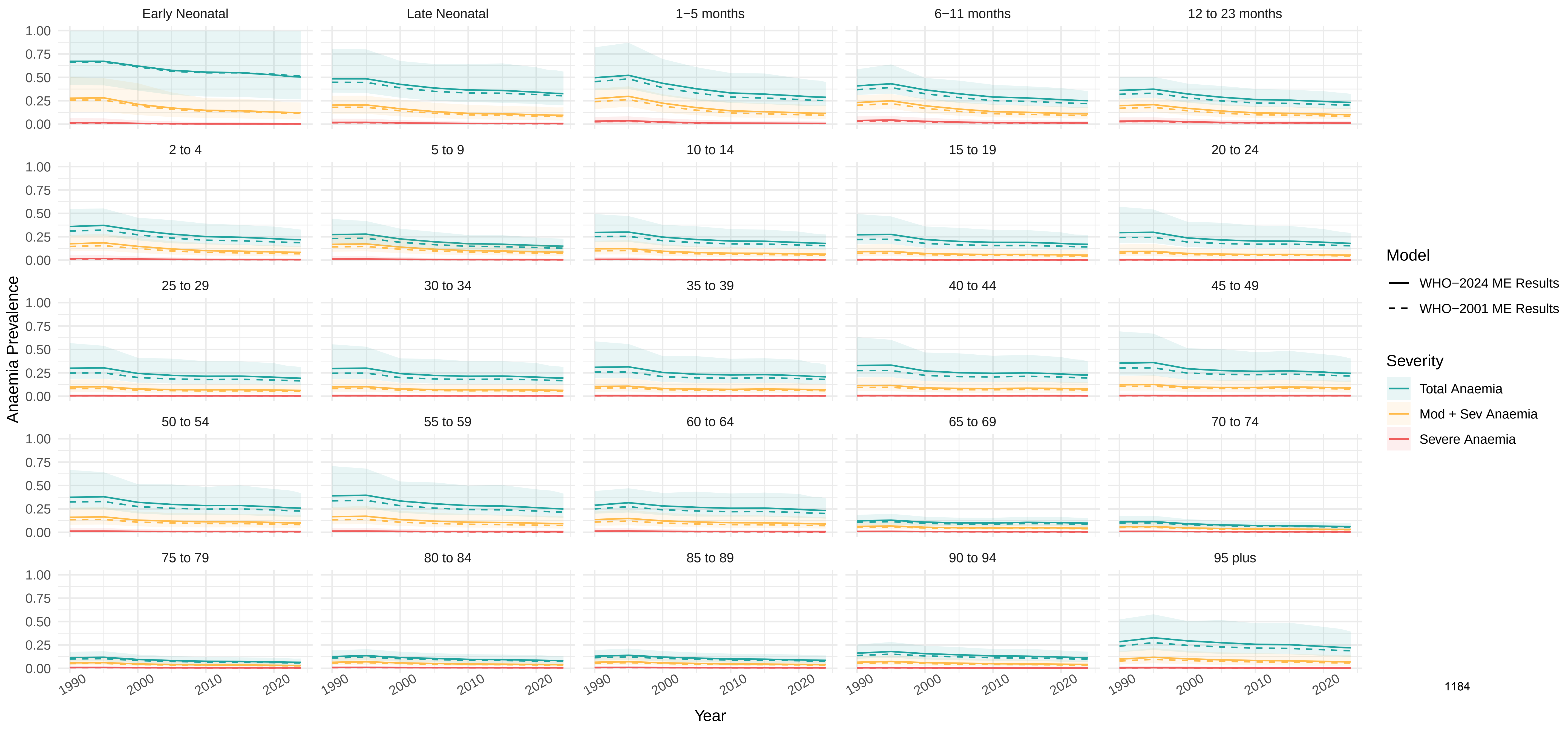

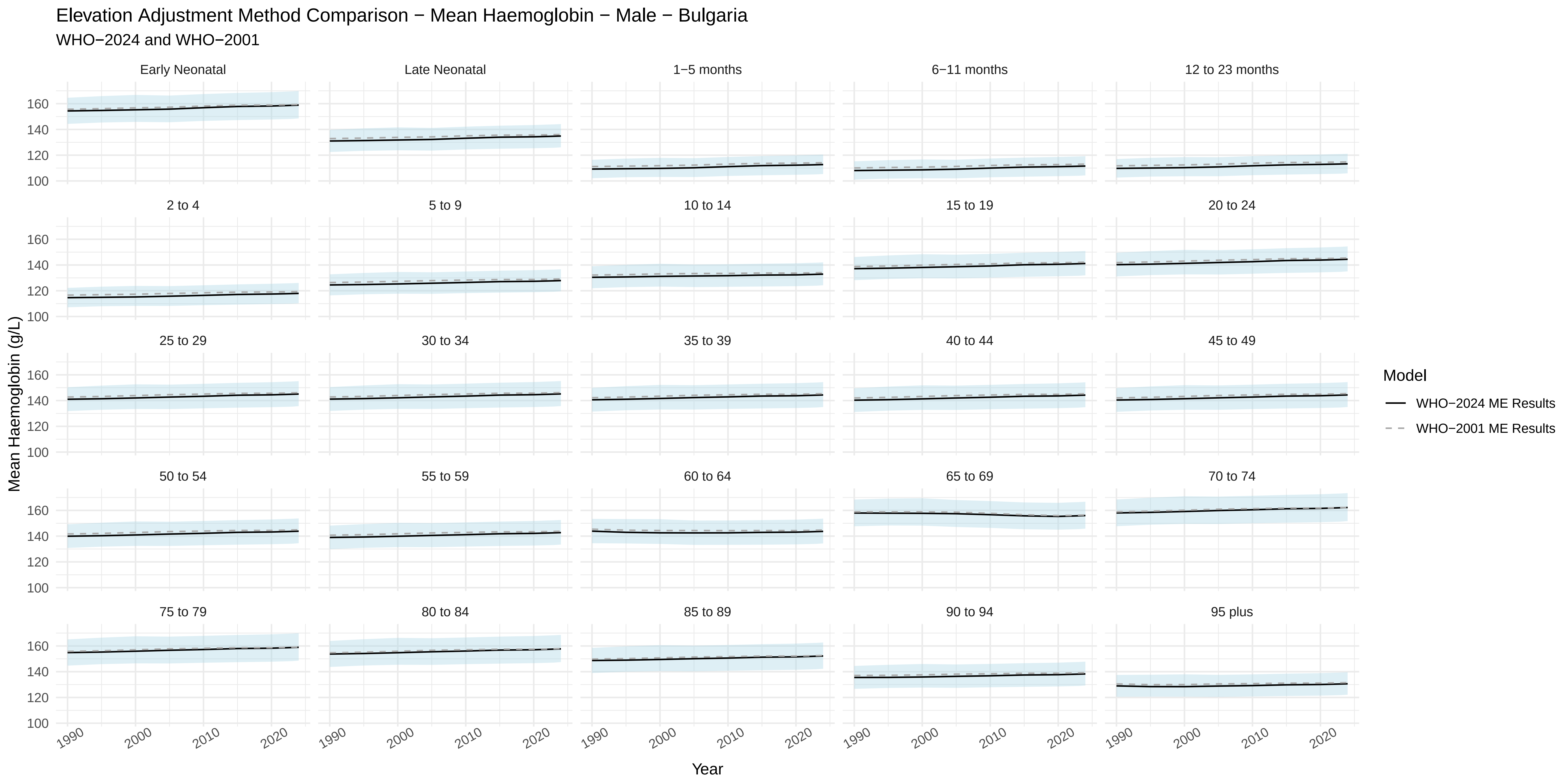
Elevation Adjustment Method Comparison − Mean Haemoglobin − Male − Bulgaria
WHO−2024 and WHO−2001
Mean Haemoglobin (g/L)
Year
Model
WHO−2024 ME Results
WHO−2001 ME Results

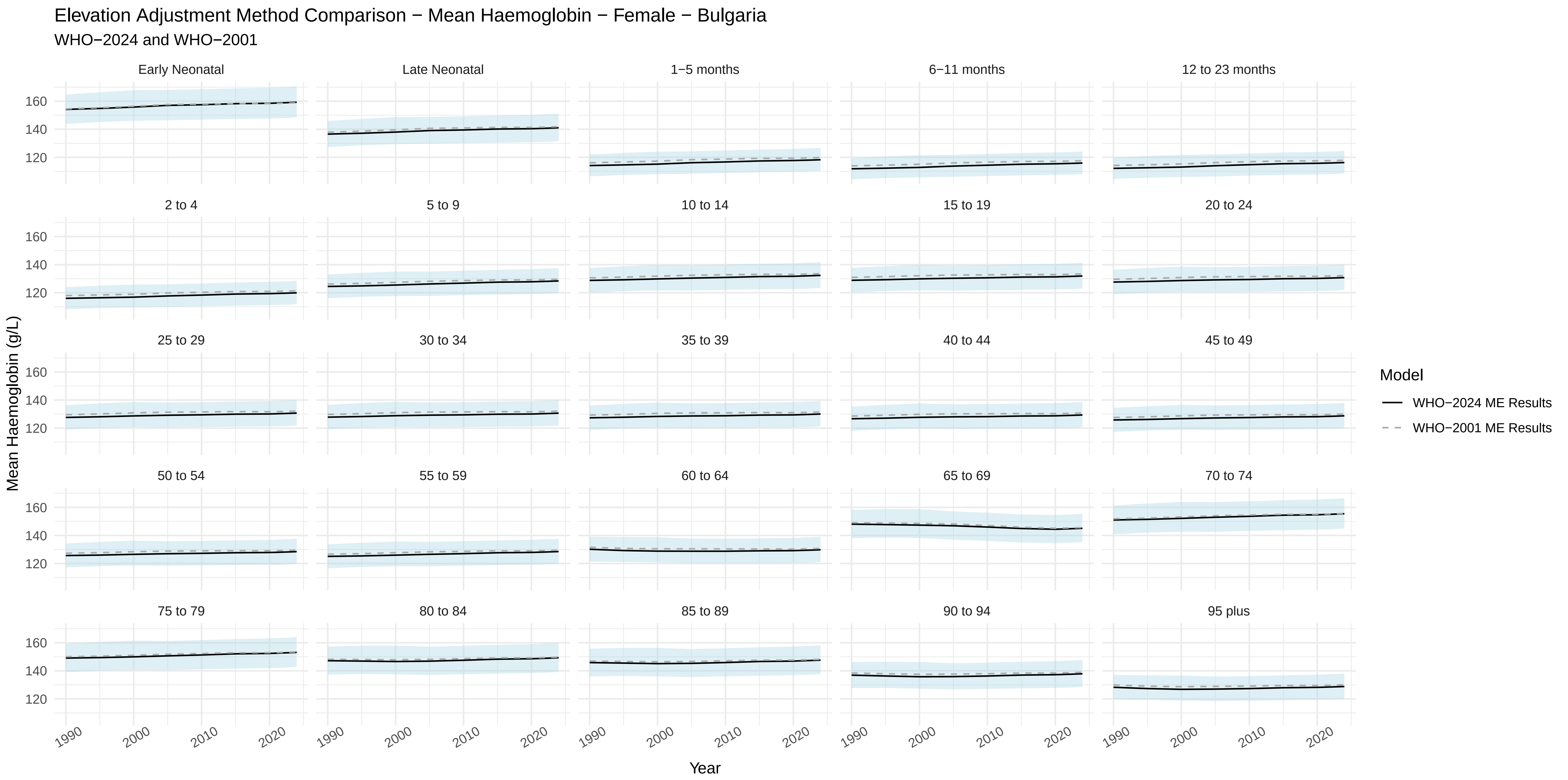
Elevation Adjustment Method Comparison − Mean Haemoglobin − Female − Bulgaria
WHO−2024 and WHO−2001
Mean Haemoglobin (g/L)
Year
Model
WHO−2024 ME Results
WHO−2001 ME Results

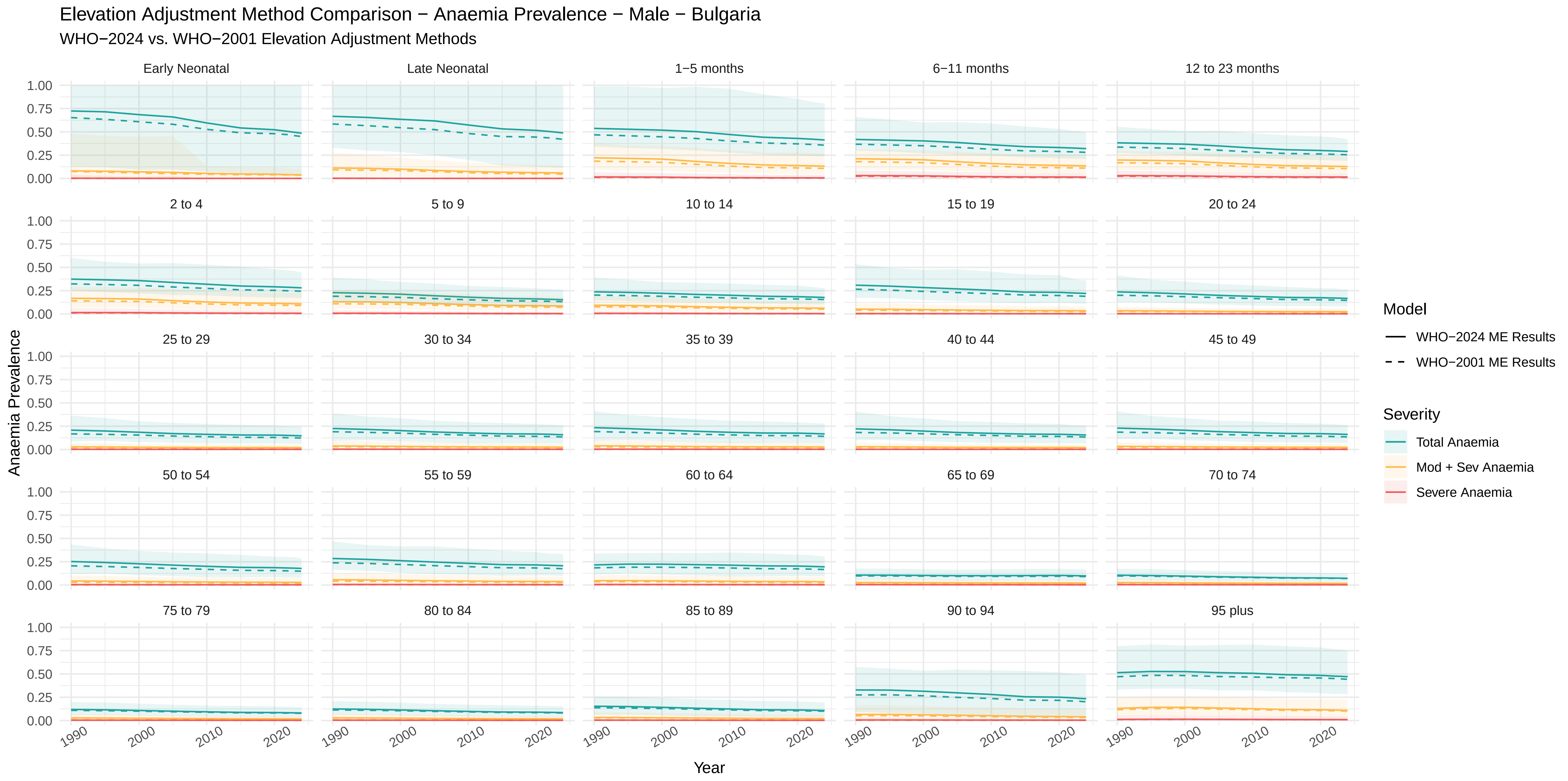
Elevation Adjustment Method Comparison − Anaemia Prevalence − Male − Bulgaria
WHO−2024 vs. WHO−2001 Elevation Adjustment Methods
Anaemia Prevalence
Year
Model
WHO−2024 ME Results
WHO−2001 ME Results
Severity
Total Anaemia
Mod + Sev Anaemia
Severe Anaemia

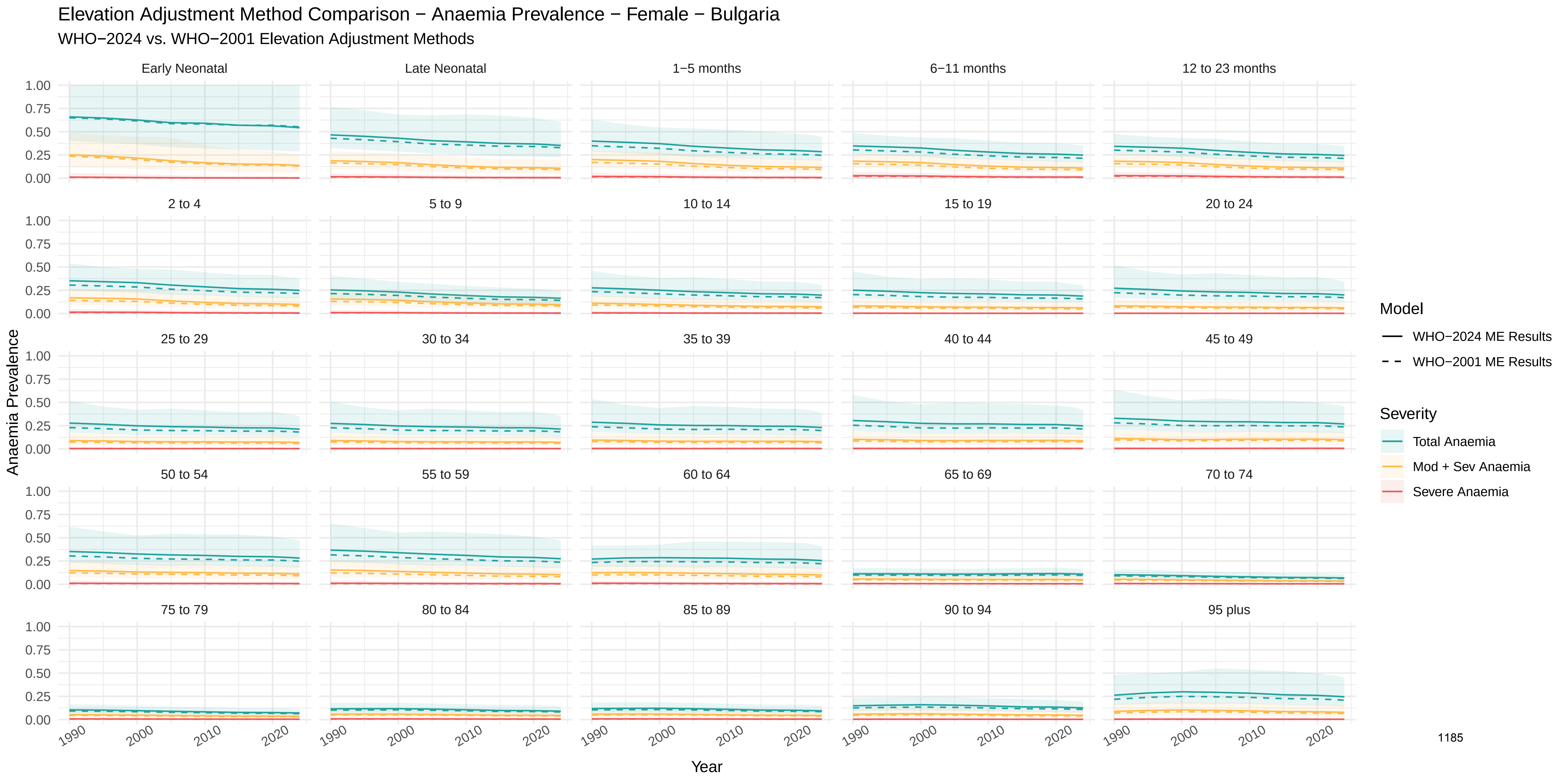
Elevation Adjustment Method Comparison − Anaemia Prevalence − Female − Bulgaria
WHO−2024 vs. WHO−2001 Elevation Adjustment Methods
Anaemia Prevalence
Year
Model
WHO−2024 ME Results
WHO−2001 ME Results
Severity
Total Anaemia
Mod + Sev Anaemia
Severe Anaemia

Elevation Adjustment Method Comparison − Mean Haemoglobin − Male − Croatia

WHO−2024 and WHO−2001

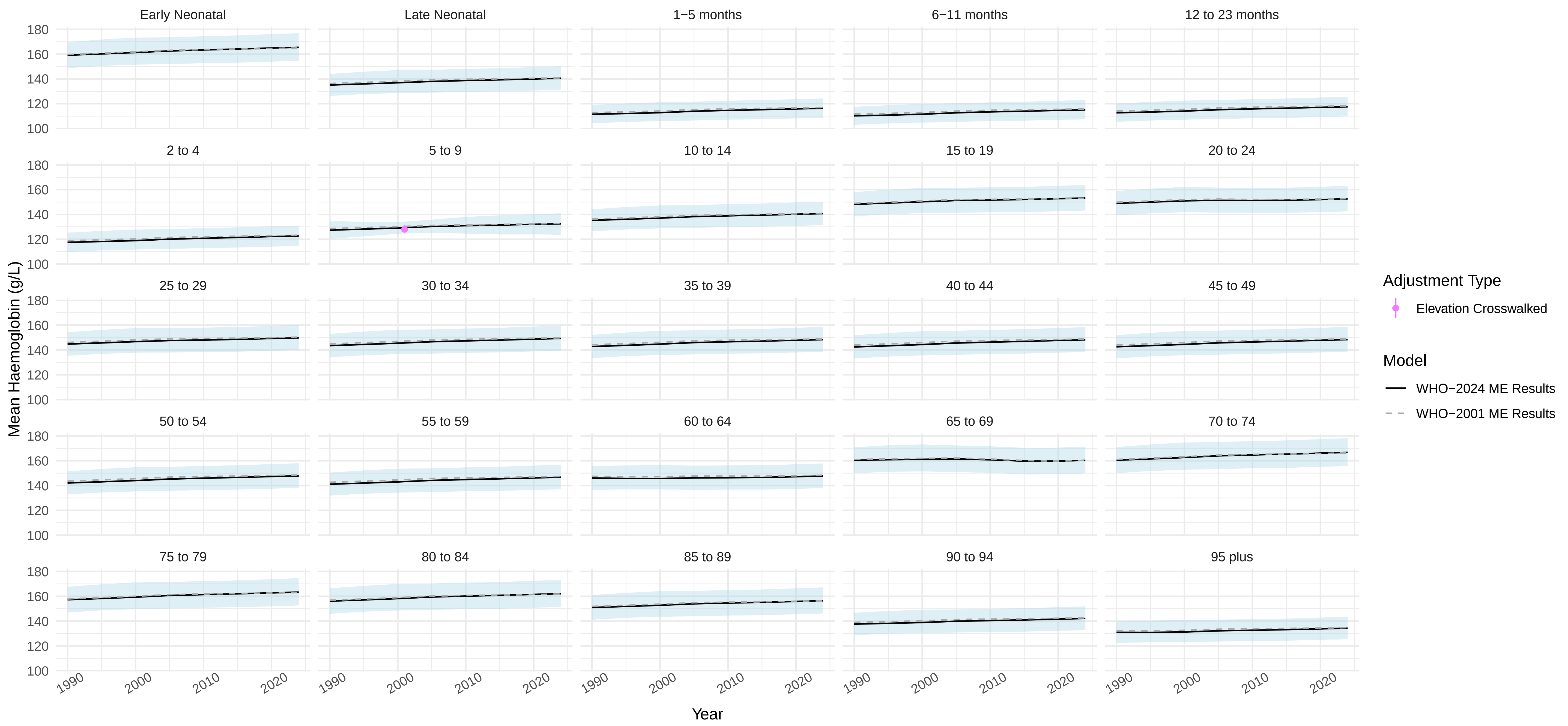

Elevation Adjustment Method Comparison − Mean Haemoglobin − Female − Croatia

WHO−2024 and WHO−2001

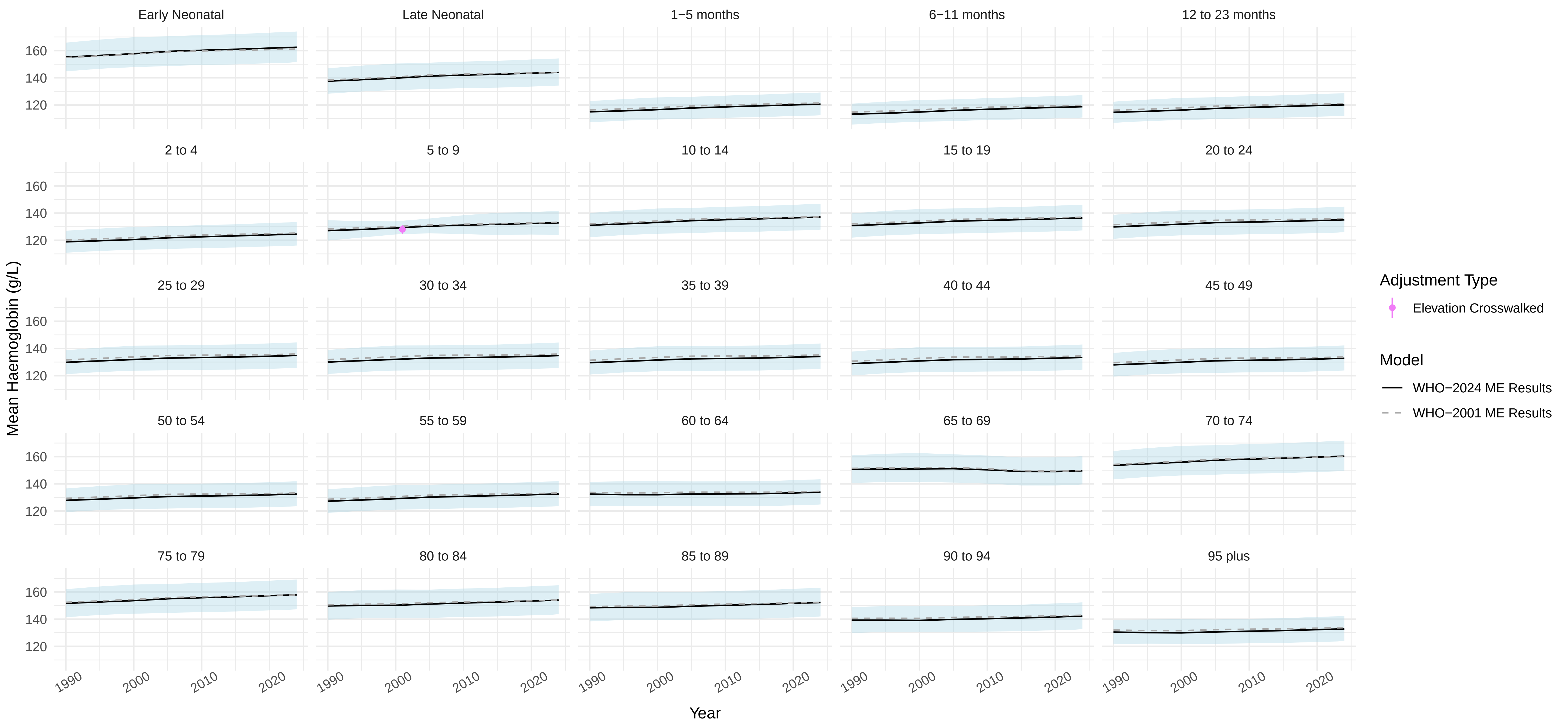

Elevation Adjustment Method Comparison − Anaemia Prevalence − Male − Croatia

WHO−2024 vs. WHO−2001 Elevation Adjustment Methods

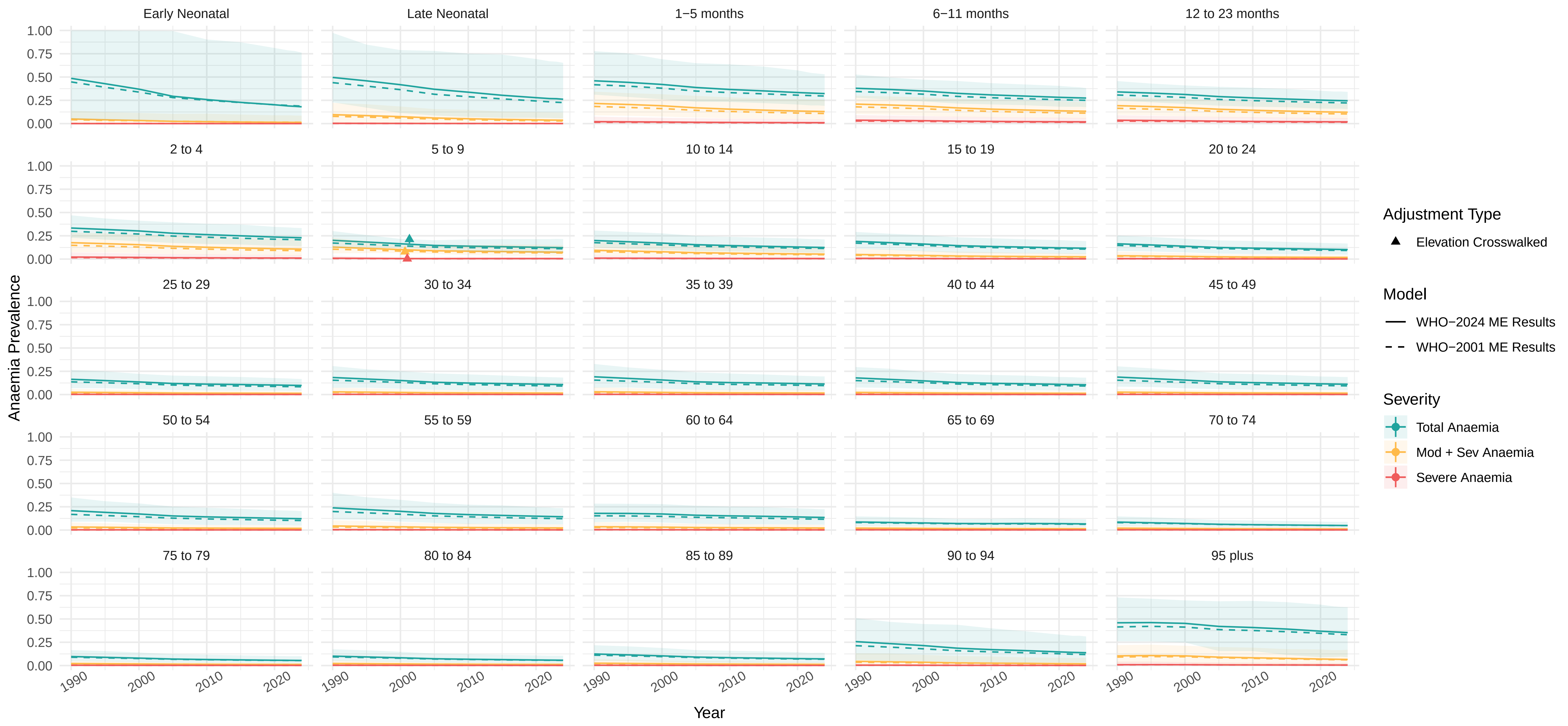

Elevation Adjustment Method Comparison − Anaemia Prevalence − Female − Croatia

WHO−2024 vs. WHO−2001 Elevation Adjustment Methods

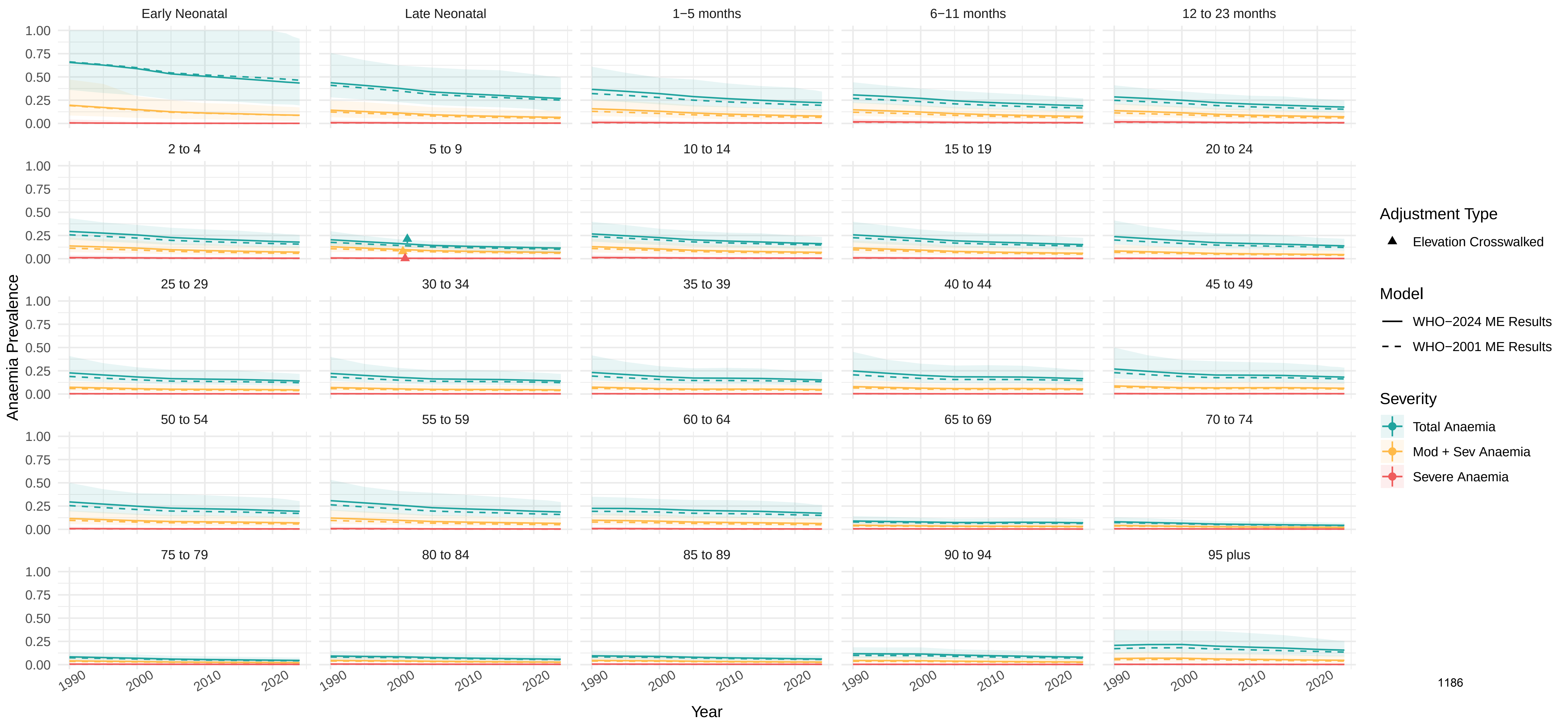

Elevation Adjustment Method Comparison − Mean Haemoglobin − Male − Czechia

WHO−2024 and WHO−2001

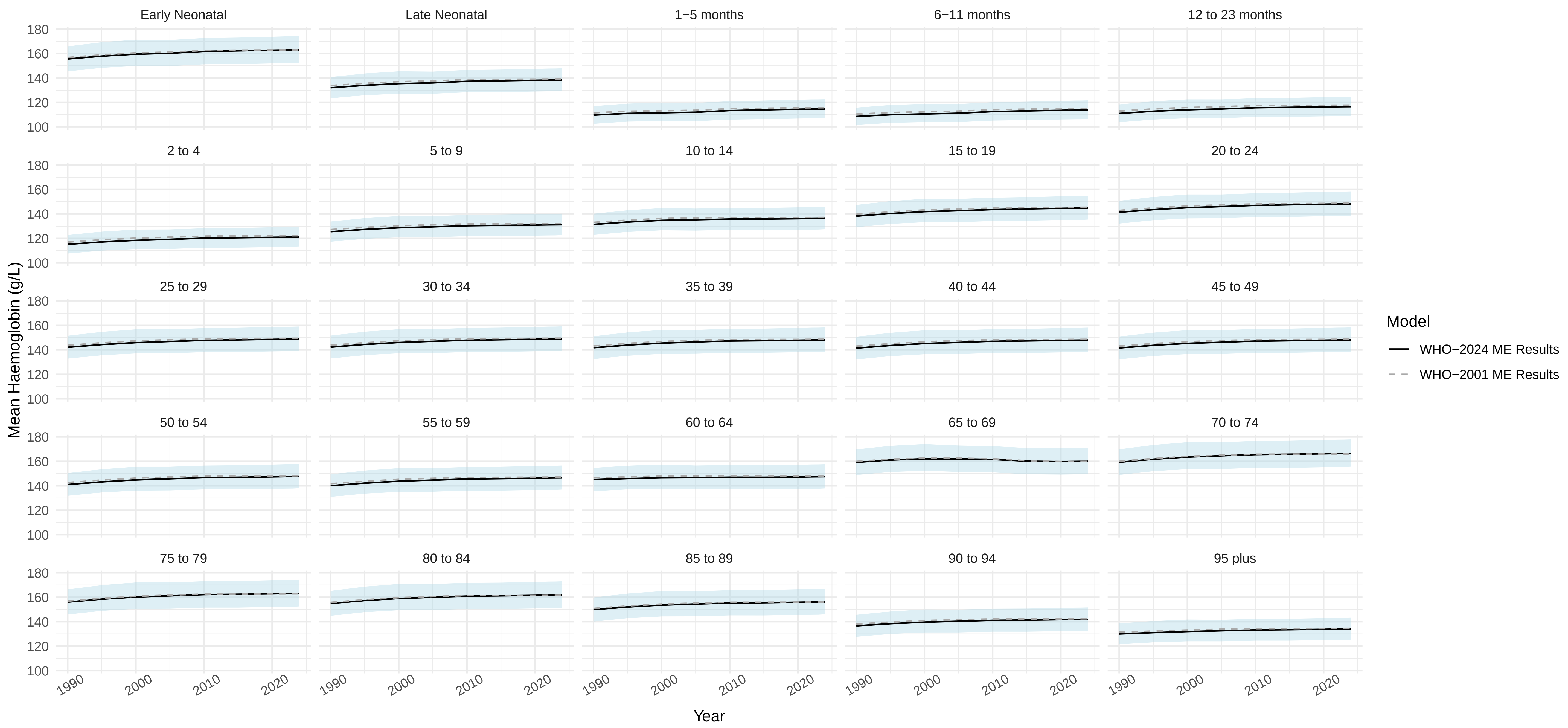

Elevation Adjustment Method Comparison − Mean Haemoglobin − Female − Czechia

WHO−2024 and WHO−2001

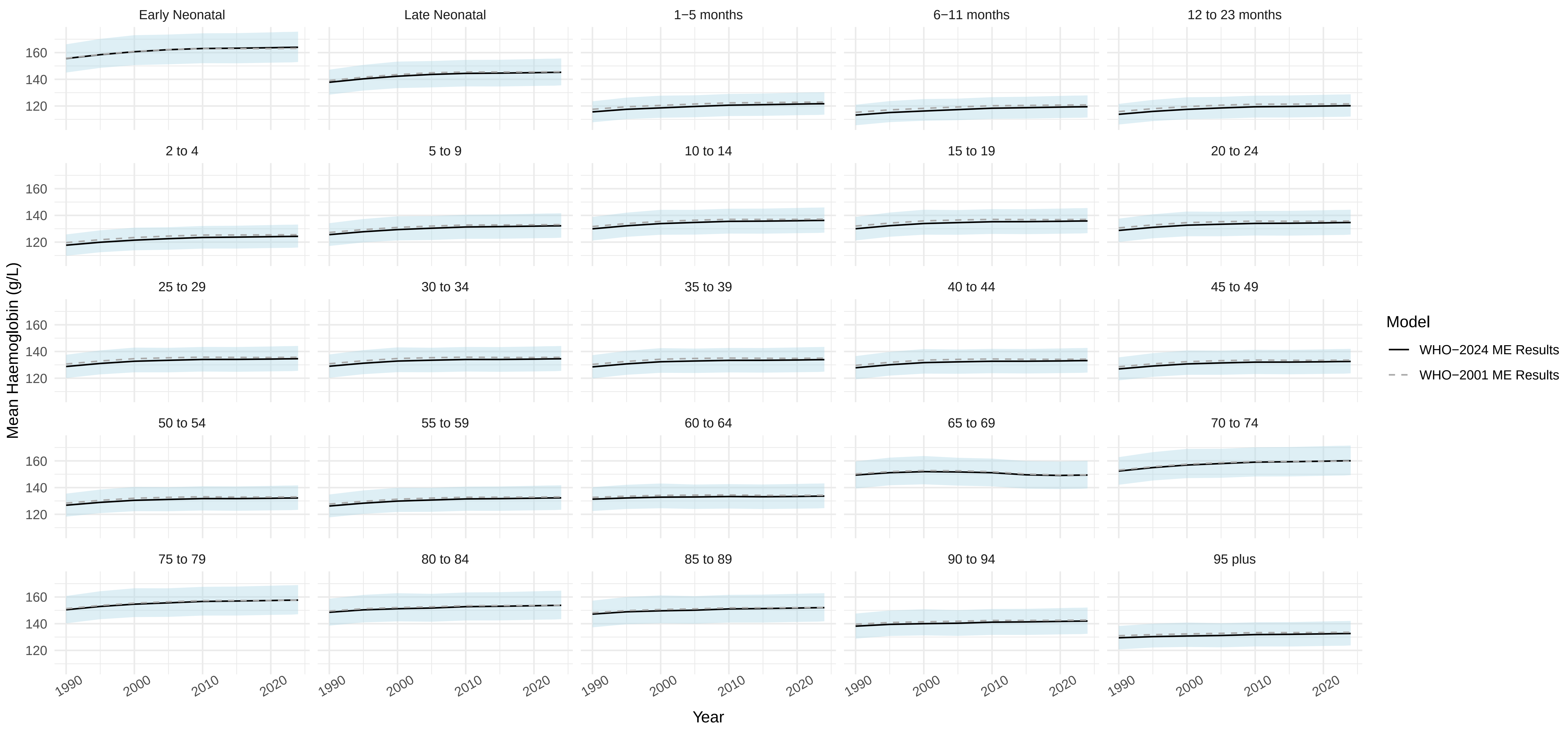

Elevation Adjustment Method Comparison − Anaemia Prevalence − Male − Czechia

WHO−2024 vs. WHO−2001 Elevation Adjustment Methods

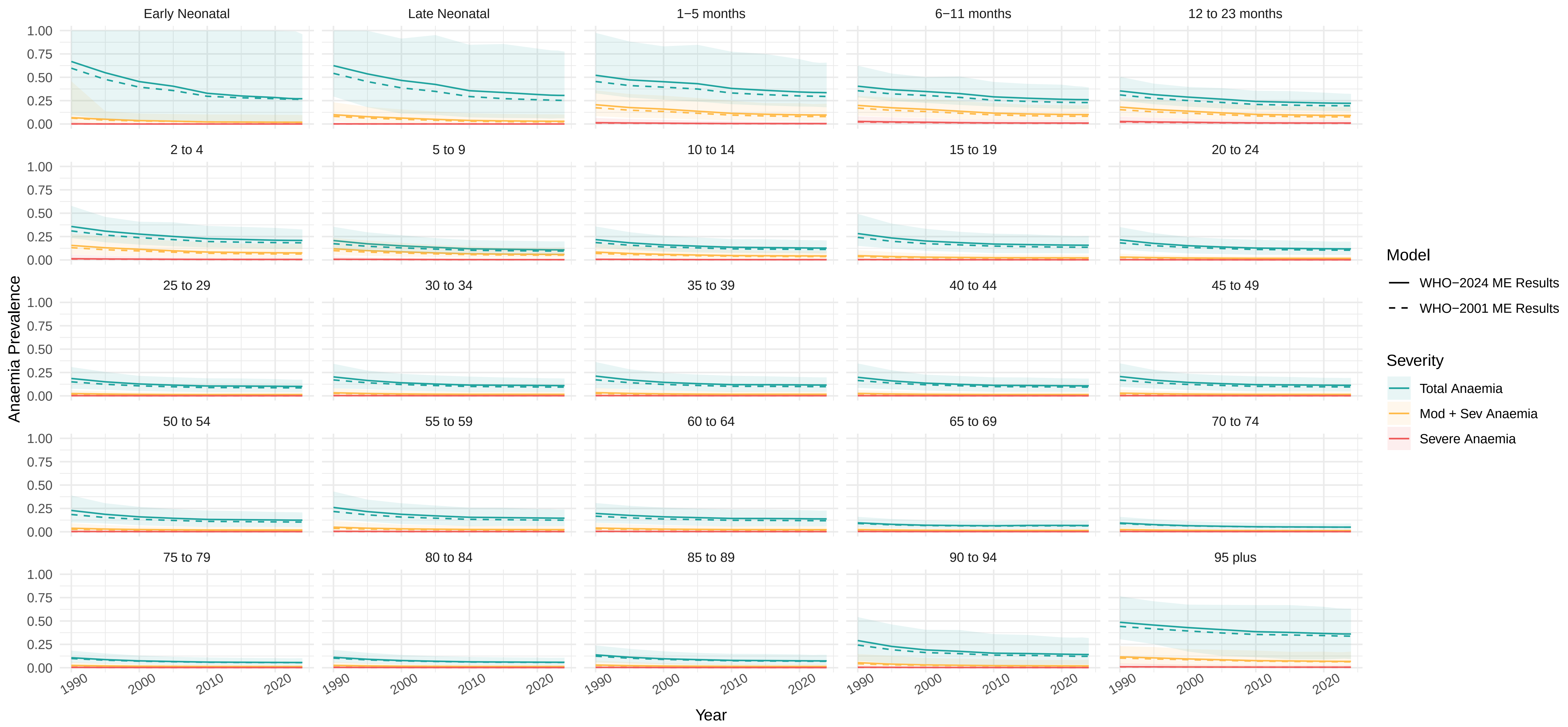

Elevation Adjustment Method Comparison − Anaemia Prevalence − Female − Czechia

WHO−2024 vs. WHO−2001 Elevation Adjustment Methods

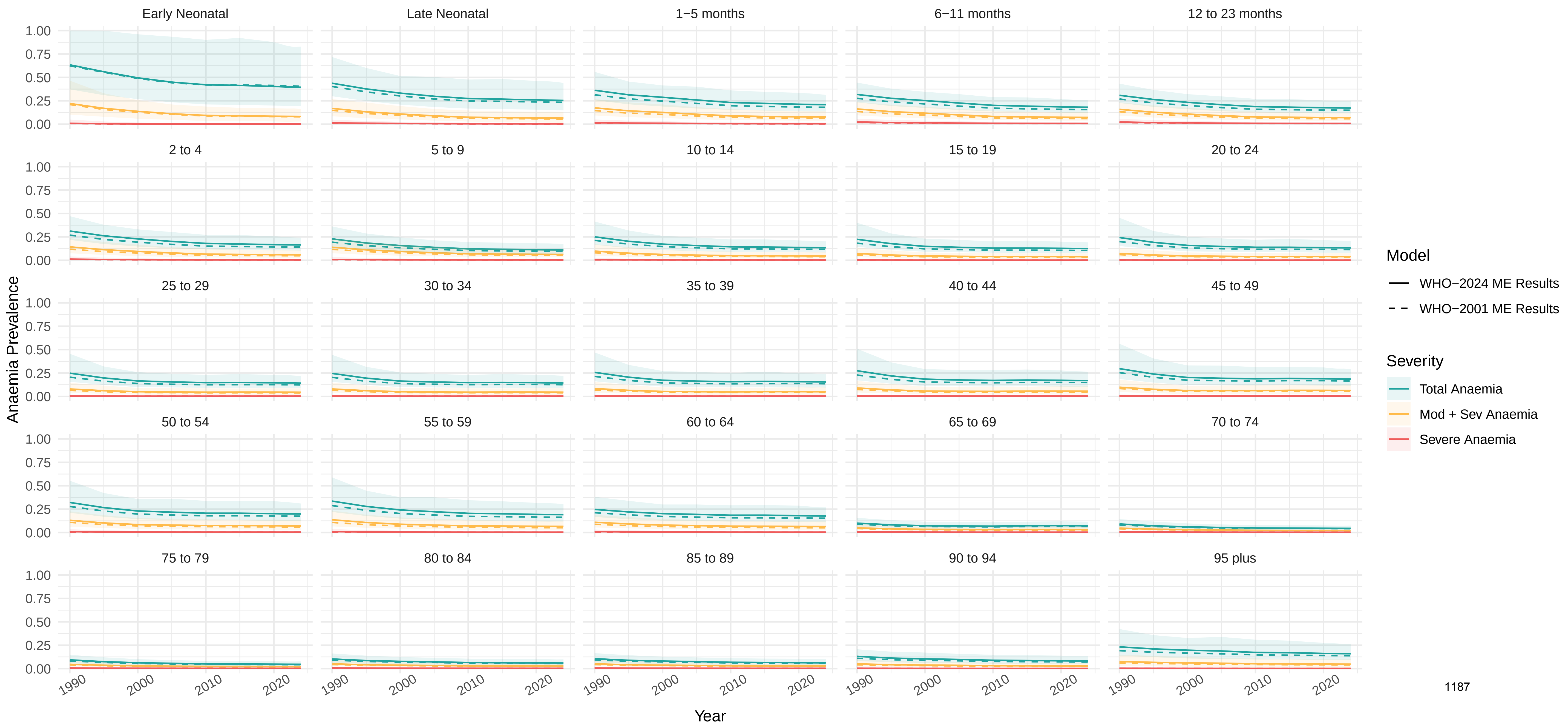

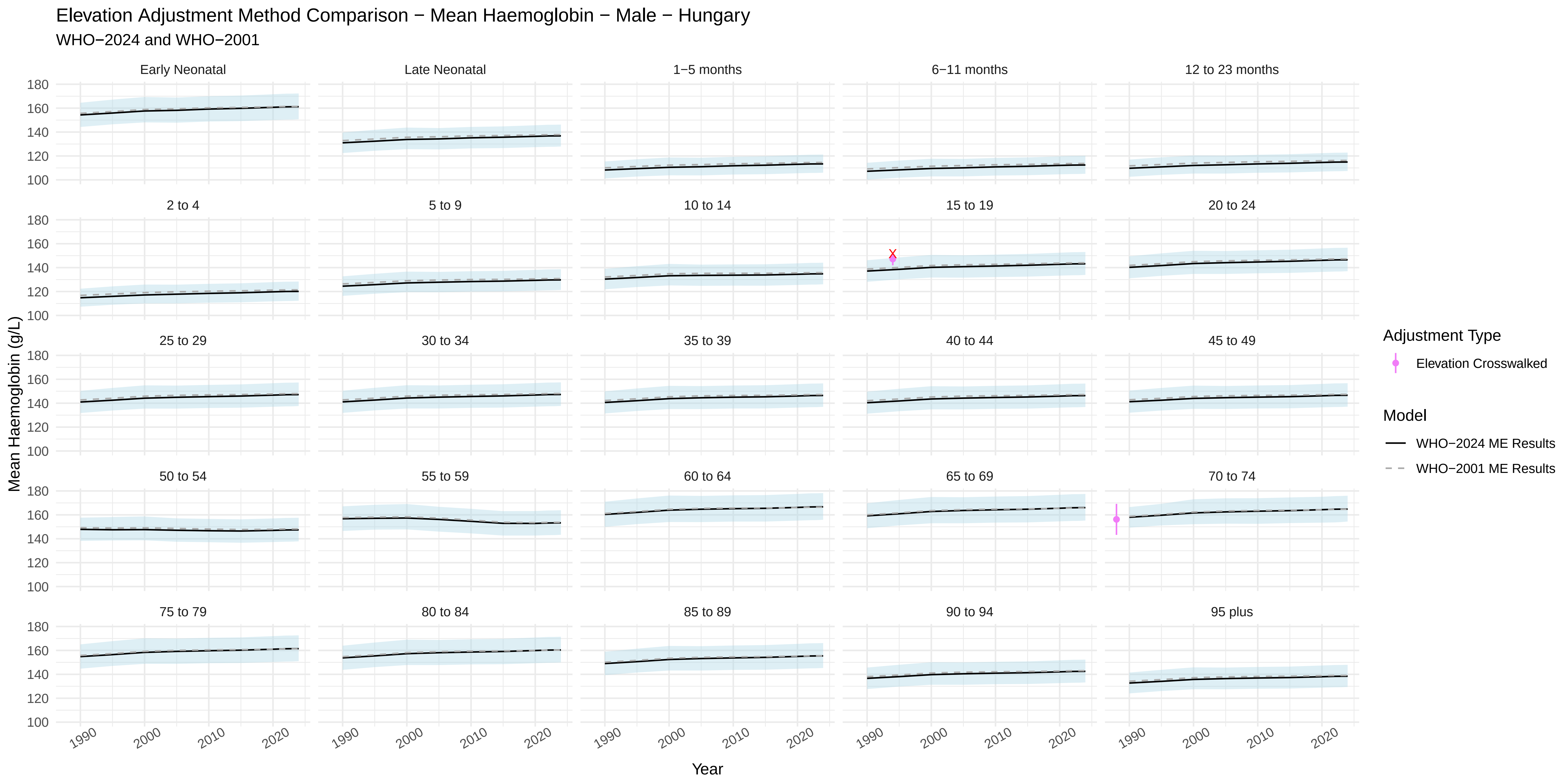
Elevation Adjustment Method Comparison − Mean Haemoglobin − Male − Hungary
WHO−2024 and WHO−2001
Early Neonatal
Late Neonatal
1−5 months
6−11 months
12 to 23 months
2 to 4
5 to 9
10 to 14
15 to 19
20 to 24
25 to 29
30 to 34
35 to 39
40 to 44
45 to 49
50 to 54
55 to 59
60 to 64
65 to 69
70 to 74
75 to 79
80 to 84
85 to 89
90 to 94
95 plus
Mean Haemoglobin (g/L)
Year
Adjustment Type
Elevation Crosswalked
Model
WHO−2024 ME Results
WHO−2001 ME Results

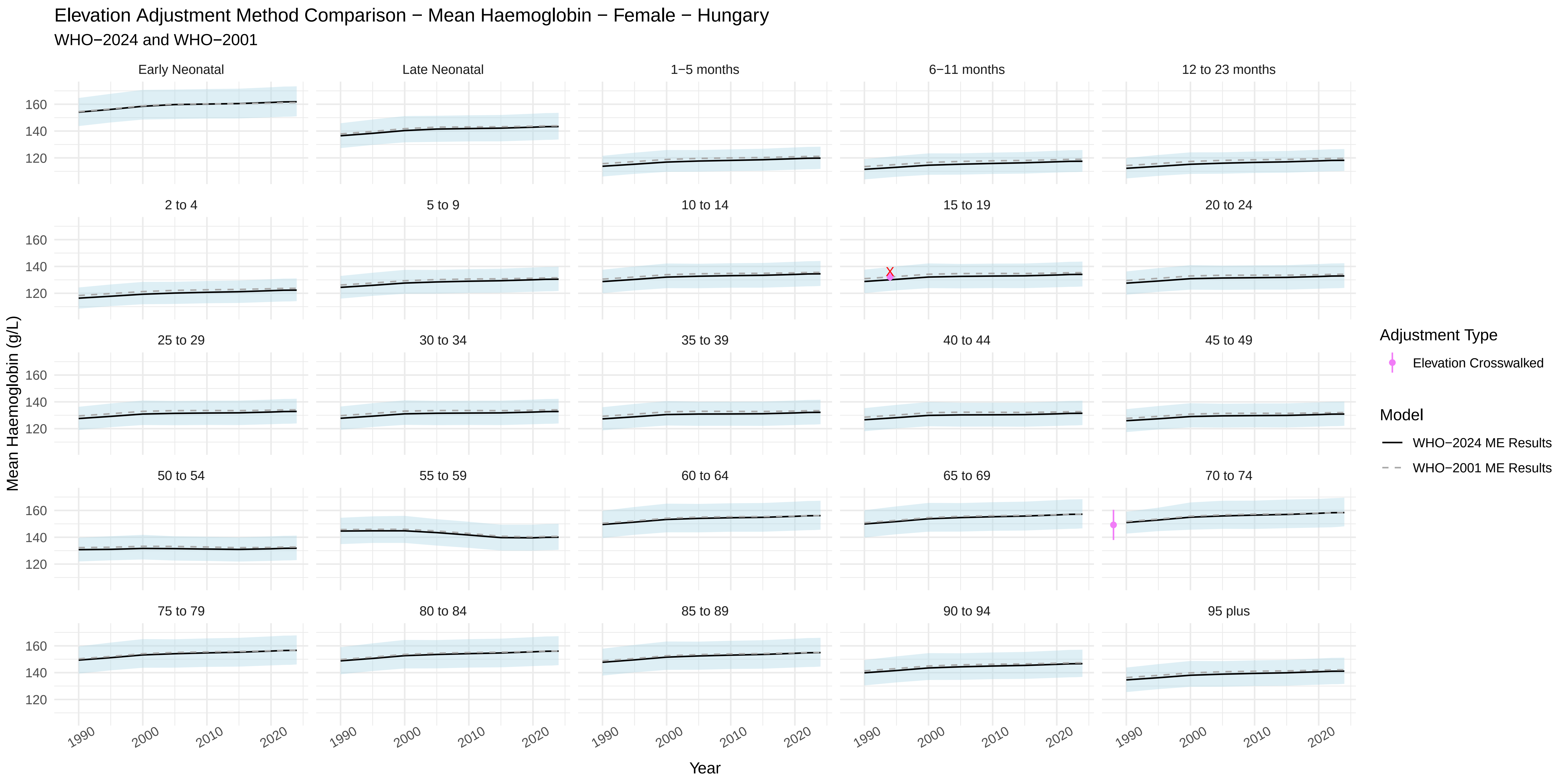
Elevation Adjustment Method Comparison − Mean Haemoglobin − Female − Hungary
WHO−2024 and WHO−2001
Early Neonatal
Late Neonatal
1−5 months
6−11 months
12 to 23 months
2 to 4
5 to 9
10 to 14
15 to 19
20 to 24
25 to 29
30 to 34
35 to 39
40 to 44
45 to 49
50 to 54
55 to 59
60 to 64
65 to 69
70 to 74
75 to 79
80 to 84
85 to 89
90 to 94
95 plus
Mean Haemoglobin (g/L)
Year
Adjustment Type
Elevation Crosswalked
Model
WHO−2024 ME Results
WHO−2001 ME Results

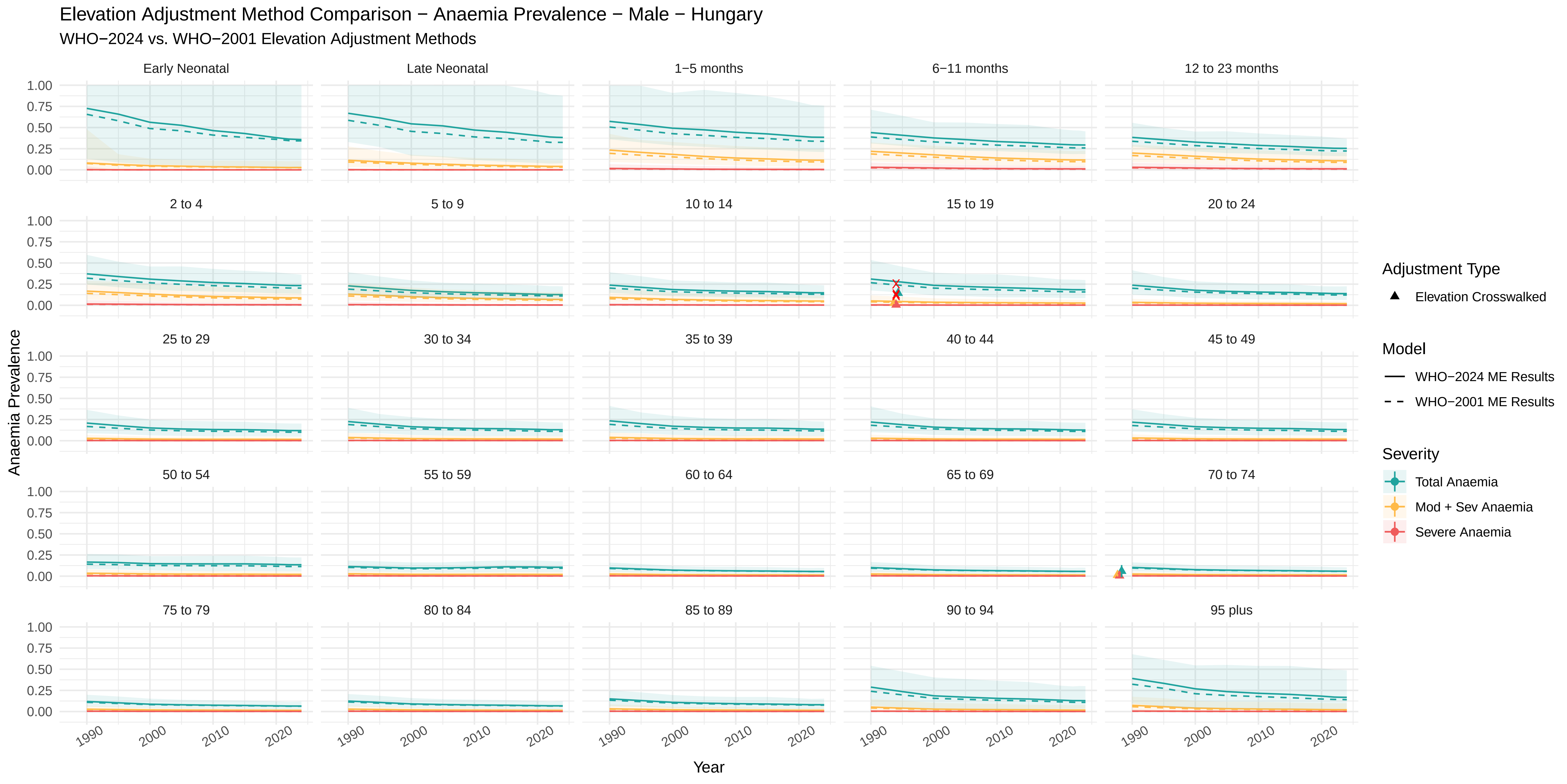
Elevation Adjustment Method Comparison − Anaemia Prevalence − Male − Hungary
WHO−2024 vs. WHO−2001 Elevation Adjustment Methods
Early Neonatal
Late Neonatal
1−5 months
6−11 months
12 to 23 months
2 to 4
5 to 9
10 to 14
15 to 19
20 to 24
25 to 29
30 to 34
35 to 39
40 to 44
45 to 49
50 to 54
55 to 59
60 to 64
65 to 69
70 to 74
75 to 79
80 to 84
85 to 89
90 to 94
95 plus
Anaemia Prevalence
Year
Adjustment Type
Elevation Crosswalked
Model
WHO−2024 ME Results
WHO−2001 ME Results
Severity
Total Anaemia
Mod + Sev Anaemia
Severe Anaemia

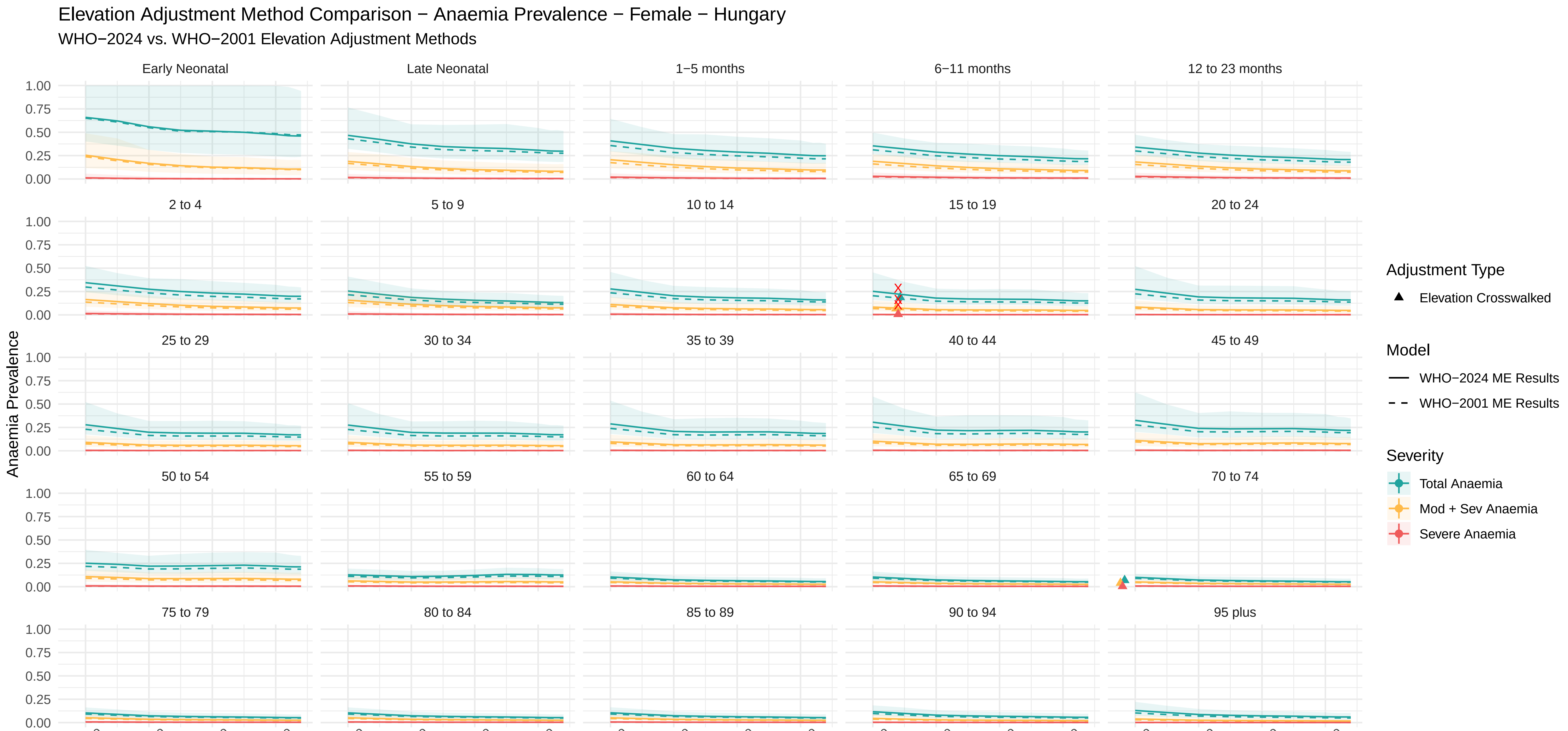
Elevation Adjustment Method Comparison − Anaemia Prevalence − Female − Hungary
WHO−2024 vs. WHO−2001 Elevation Adjustment Methods
Early Neonatal
Late Neonatal
1−5 months
6−11 months
12 to 23 months
2 to 4
5 to 9
10 to 14
15 to 19
20 to 24
25 to 29
30 to 34
35 to 39
40 to 44
45 to 49
50 to 54
55 to 59
60 to 64
65 to 69
70 to 74
75 to 79
80 to 84
85 to 89
90 to 94
95 plus
Anaemia Prevalence
Year
Adjustment Type
Elevation Crosswalked
Model
WHO−2024 ME Results
WHO−2001 ME Results
Severity
Total Anaemia
Mod + Sev Anaemia
Severe Anaemia

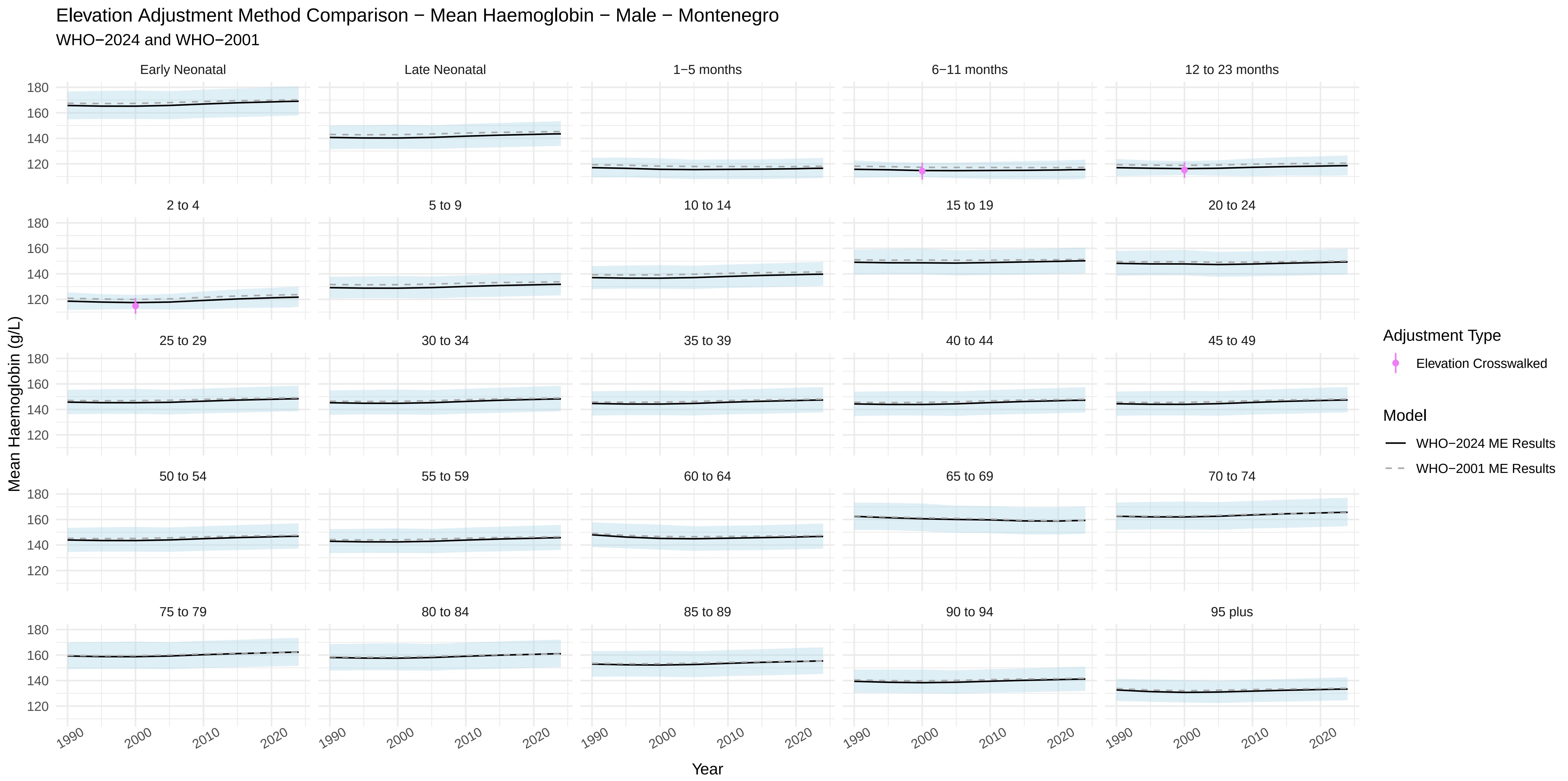
Elevation Adjustment Method Comparison − Mean Haemoglobin − Male − Montenegro
WHO−2024 and WHO−2001
Early Neonatal
Late Neonatal
1−5 months
6−11 months
12 to 23 months
2 to 4
5 to 9
10 to 14
15 to 19
20 to 24
25 to 29
30 to 34
35 to 39
40 to 44
45 to 49
50 to 54
55 to 59
60 to 64
65 to 69
70 to 74
75 to 79
80 to 84
85 to 89
90 to 94
95 plus
Mean Haemoglobin (g/L)
Year
Adjustment Type
Elevation Crosswalked
Model
WHO−2024 ME Results
WHO−2001 ME Results

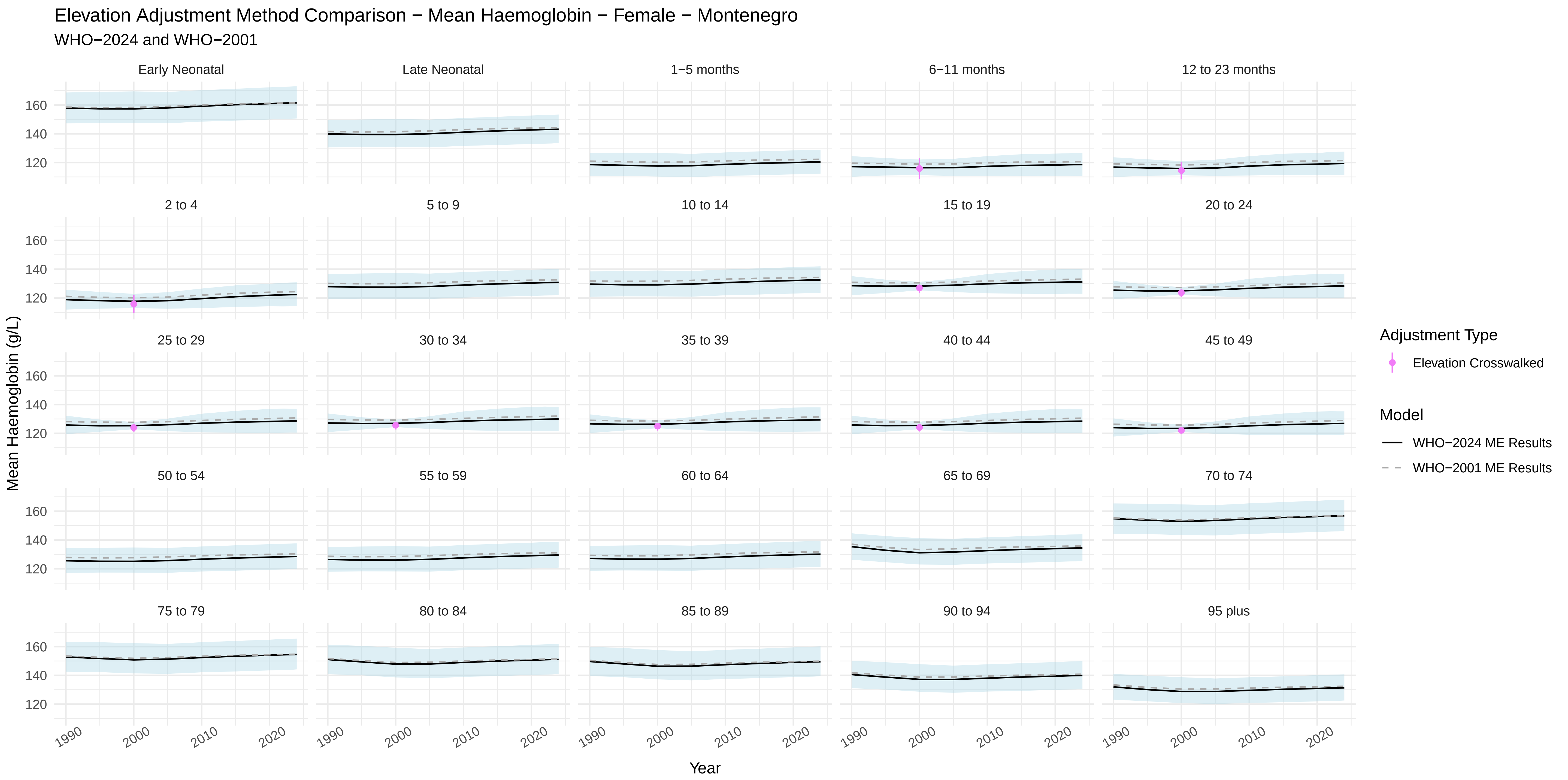
Elevation Adjustment Method Comparison − Mean Haemoglobin − Female − Montenegro
WHO−2024 and WHO−2001
Early Neonatal
Late Neonatal
1−5 months
6−11 months
12 to 23 months
2 to 4
5 to 9
10 to 14
15 to 19
20 to 24
25 to 29
30 to 34
35 to 39
40 to 44
45 to 49
50 to 54
55 to 59
60 to 64
65 to 69
70 to 74
75 to 79
80 to 84
85 to 89
90 to 94
95 plus
Mean Haemoglobin (g/L)
Year
Adjustment Type
Elevation Crosswalked
Model
WHO−2024 ME Results
WHO−2001 ME Results

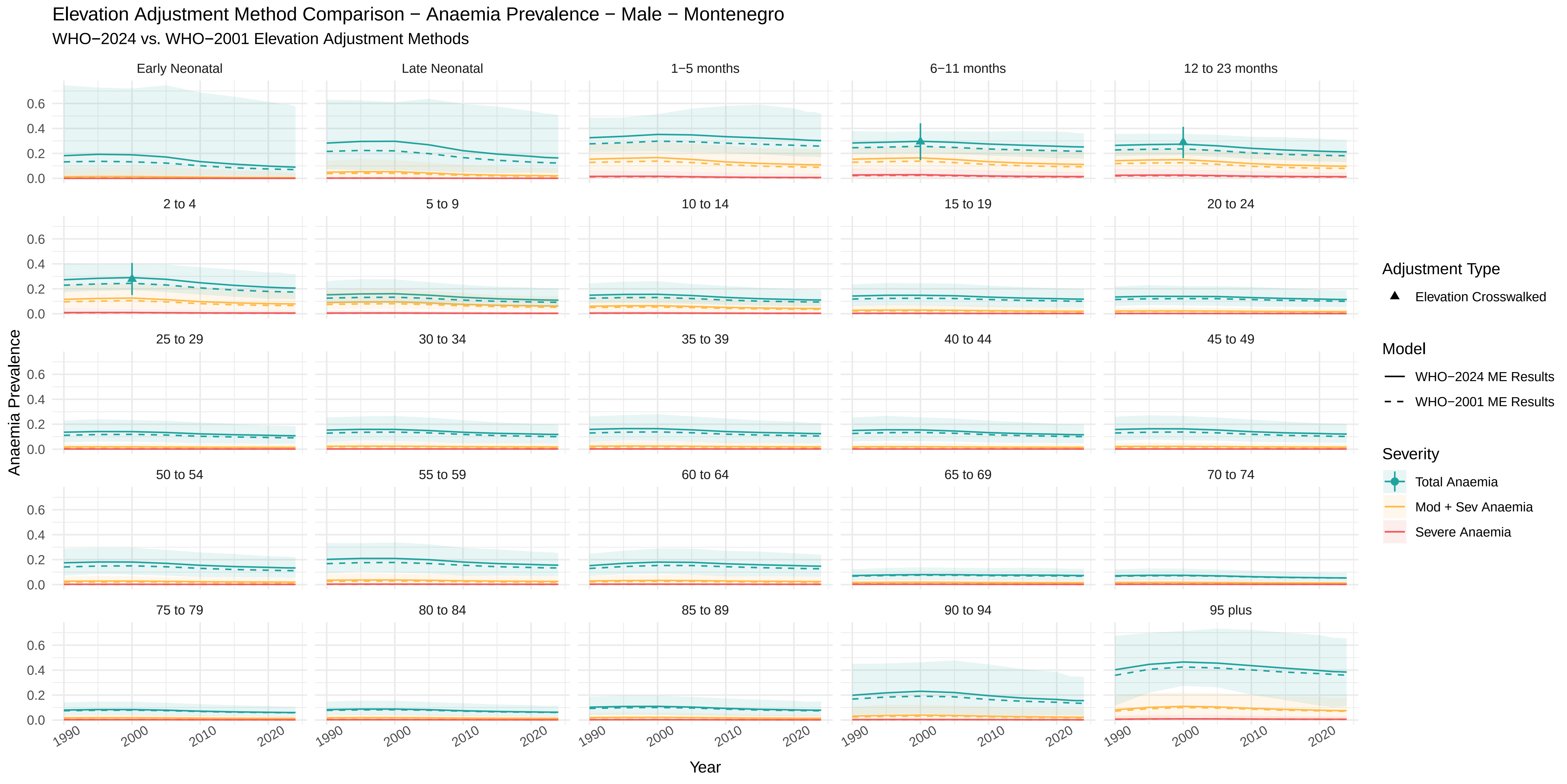
Elevation Adjustment Method Comparison − Anaemia Prevalence − Male − Montenegro
WHO−2024 vs. WHO−2001 Elevation Adjustment Methods
Early Neonatal
Late Neonatal
1−5 months
6−11 months
12 to 23 months
2 to 4
5 to 9
10 to 14
15 to 19
20 to 24
25 to 29
30 to 34
35 to 39
40 to 44
45 to 49
50 to 54
55 to 59
60 to 64
65 to 69
70 to 74
75 to 79
80 to 84
85 to 89
90 to 94
95 plus
Anaemia Prevalence
Year
Adjustment Type
Elevation Crosswalked
Model
WHO−2024 ME Results
WHO−2001 ME Results
Severity
Total Anaemia
Mod + Sev Anaemia
Severe Anaemia

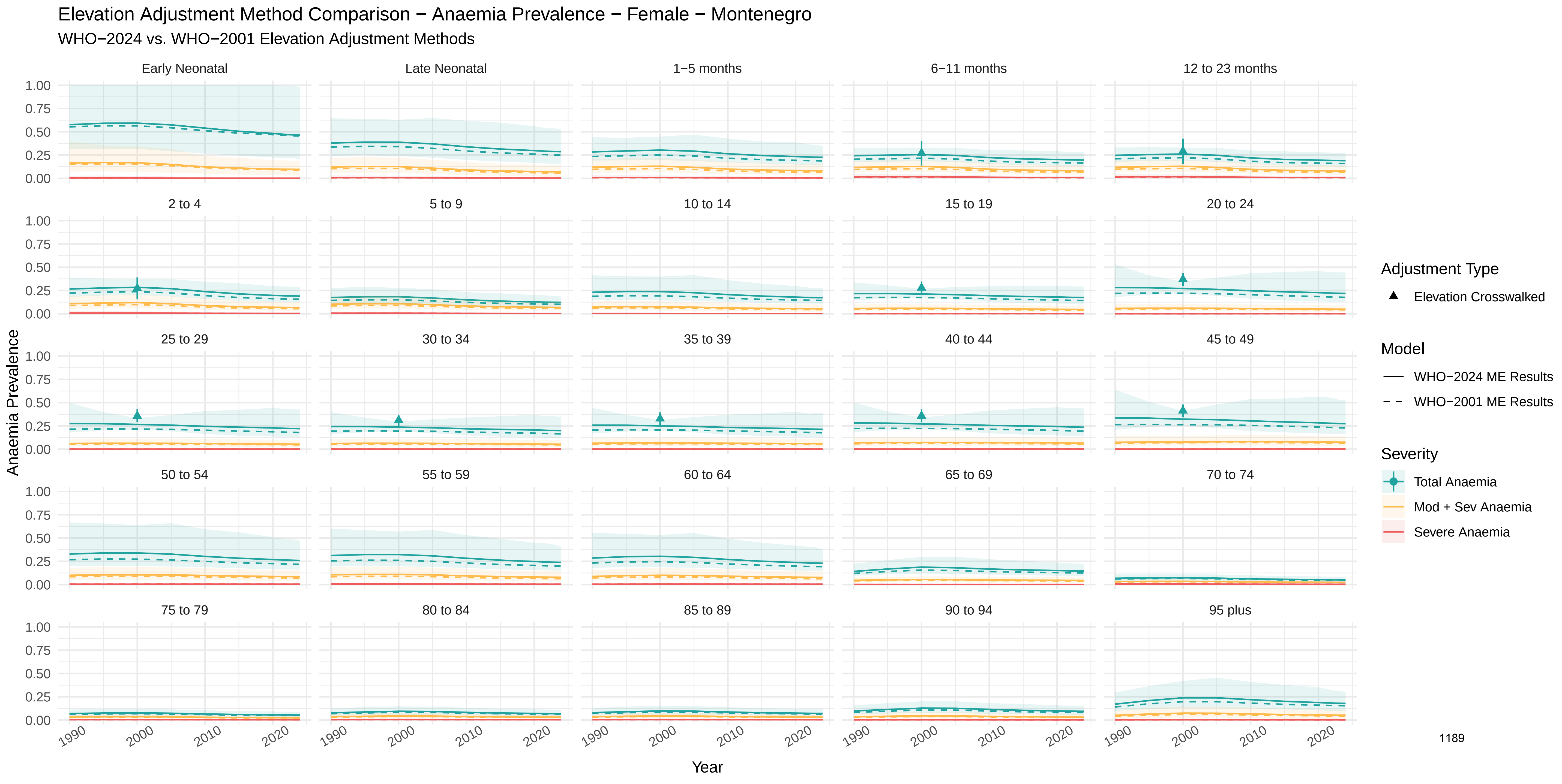
Elevation Adjustment Method Comparison − Anaemia Prevalence − Female − Montenegro
WHO−2024 vs. WHO−2001 Elevation Adjustment Methods
Early Neonatal
Late Neonatal
1−5 months
6−11 months
12 to 23 months
2 to 4
5 to 9
10 to 14
15 to 19
20 to 24
25 to 29
30 to 34
35 to 39
40 to 44
45 to 49
50 to 54
55 to 59
60 to 64
65 to 69
70 to 74
75 to 79
80 to 84
85 to 89
90 to 94
95 plus
Anaemia Prevalence
Year
Adjustment Type
Elevation Crosswalked
Model
WHO−2024 ME Results
WHO−2001 ME Results
Severity
Total Anaemia
Mod + Sev Anaemia
Severe Anaemia

Elevation Adjustment Method Comparison − Mean Haemoglobin − Male − North Macedonia

WHO−2024 and WHO−2001

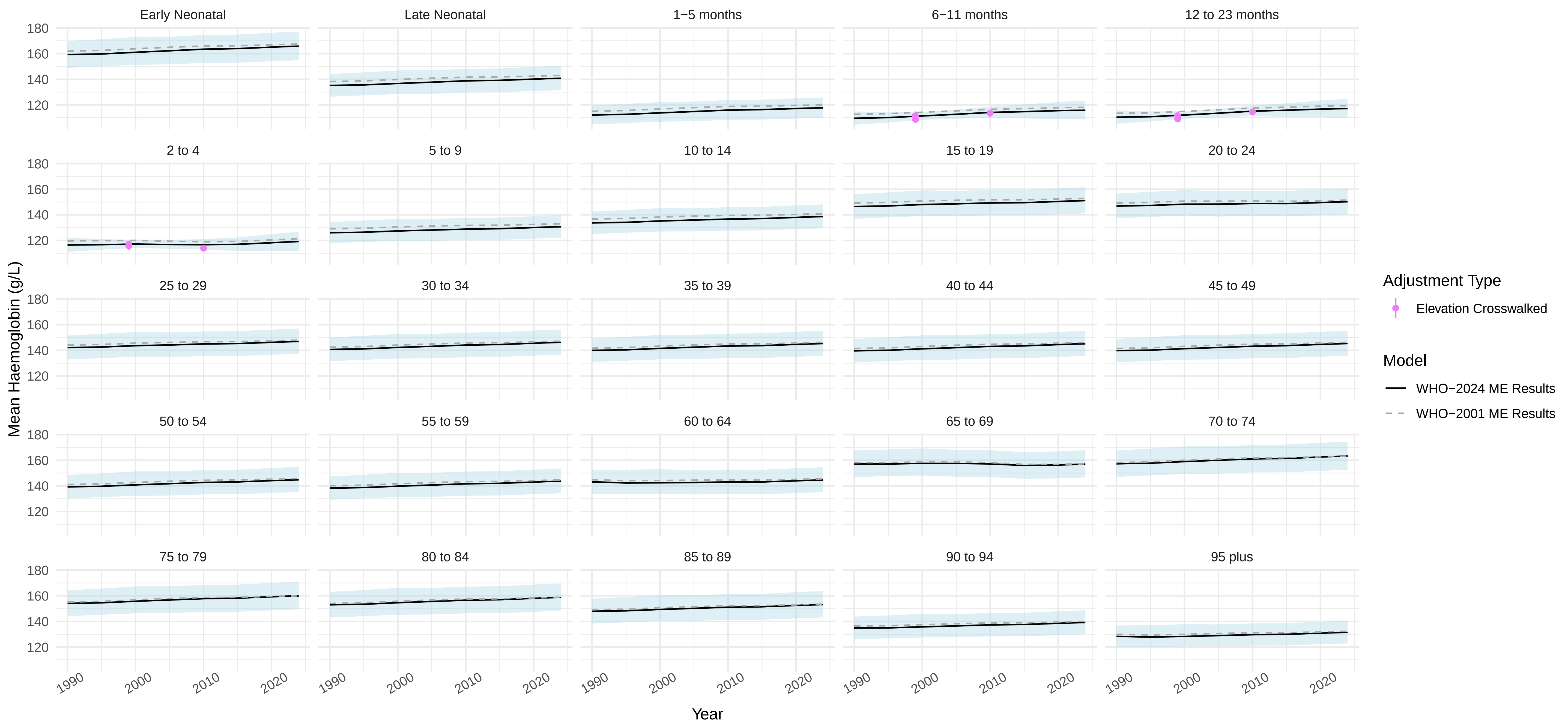

Elevation Adjustment Method Comparison − Mean Haemoglobin − Female − North Macedonia

WHO−2024 and WHO−2001

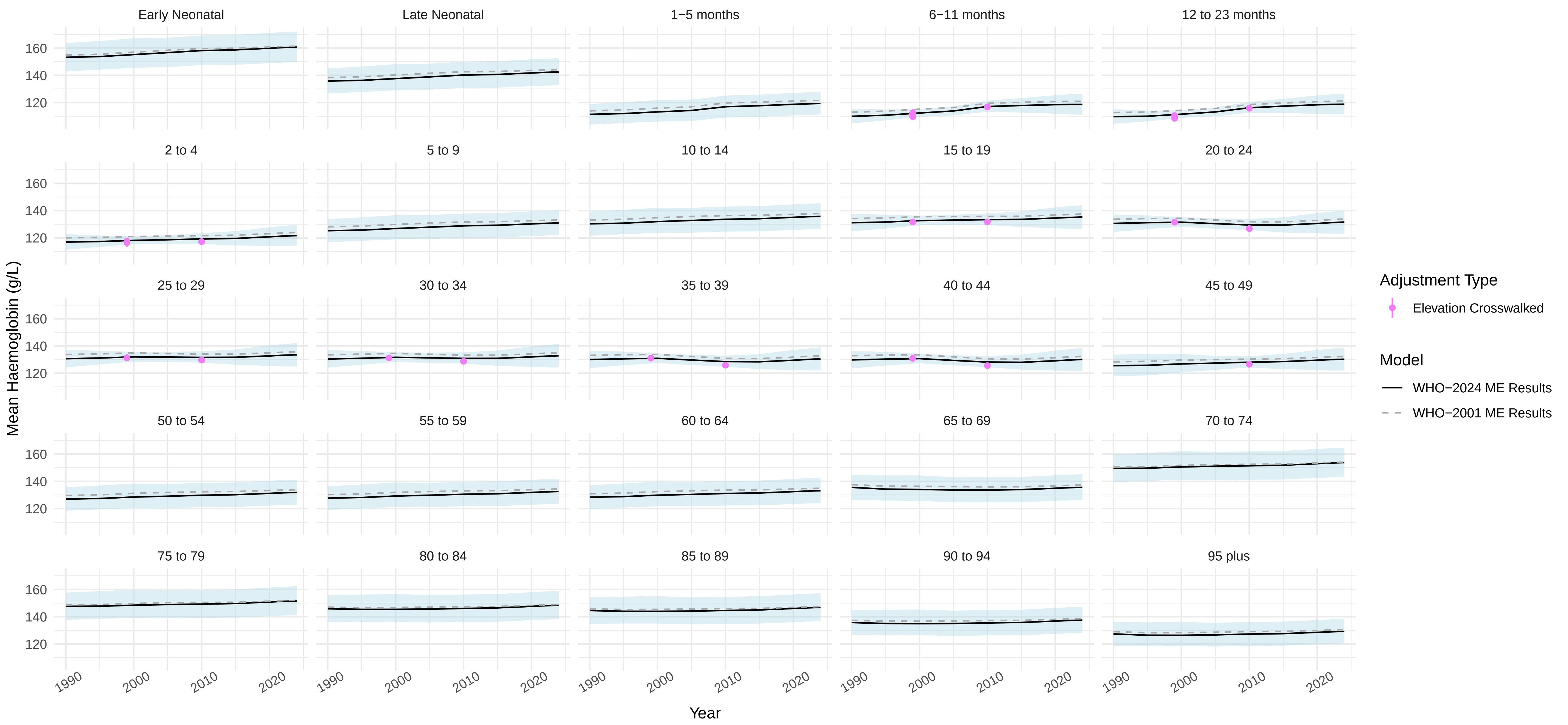

Elevation Adjustment Method Comparison − Anaemia Prevalence − Male − North Macedonia

WHO−2024 vs. WHO−2001 Elevation Adjustment Methods

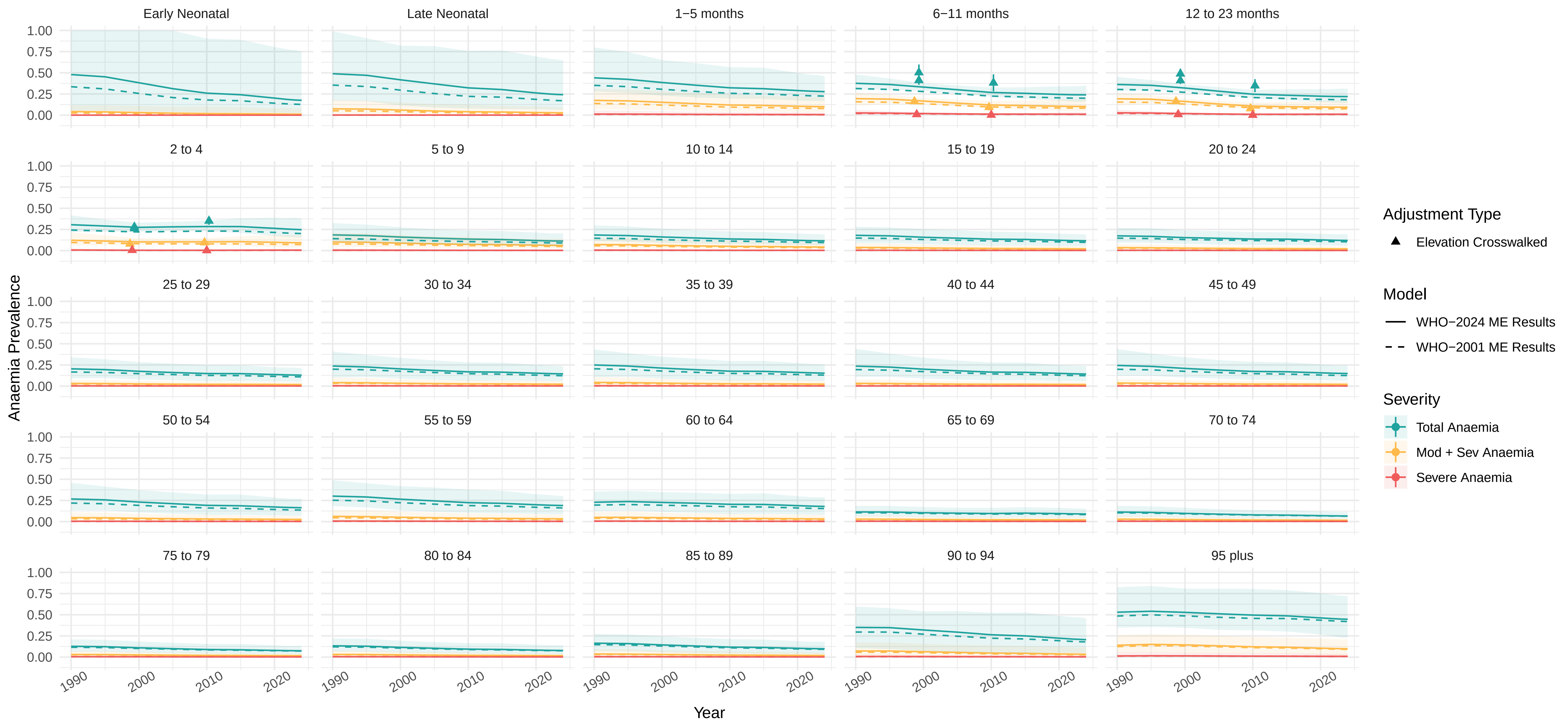

Elevation Adjustment Method Comparison − Anaemia Prevalence − Female − North Macedonia

WHO−2024 vs. WHO−2001 Elevation Adjustment Methods

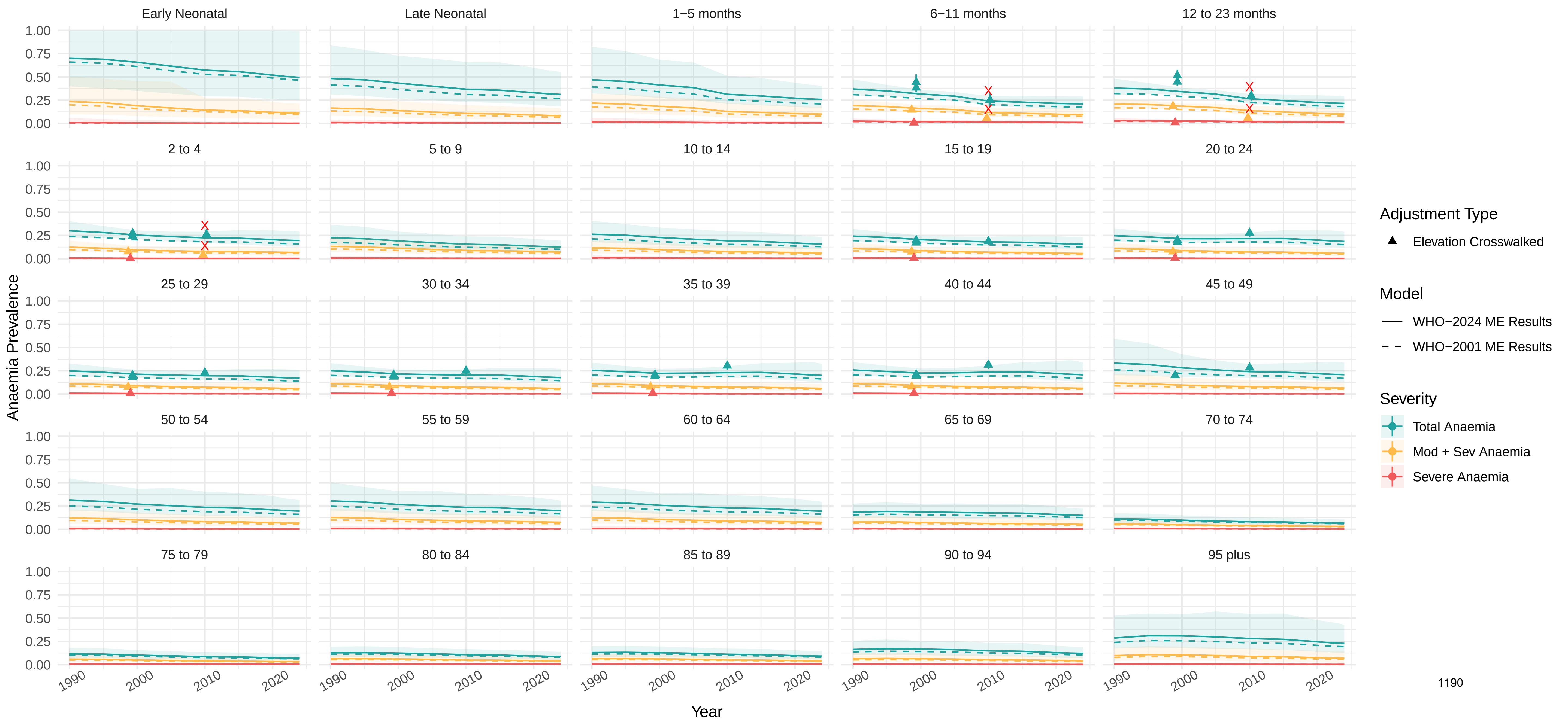

Elevation Adjustment Method Comparison − Mean Haemoglobin − Male − Poland
WHO−2024 and WHO−2001

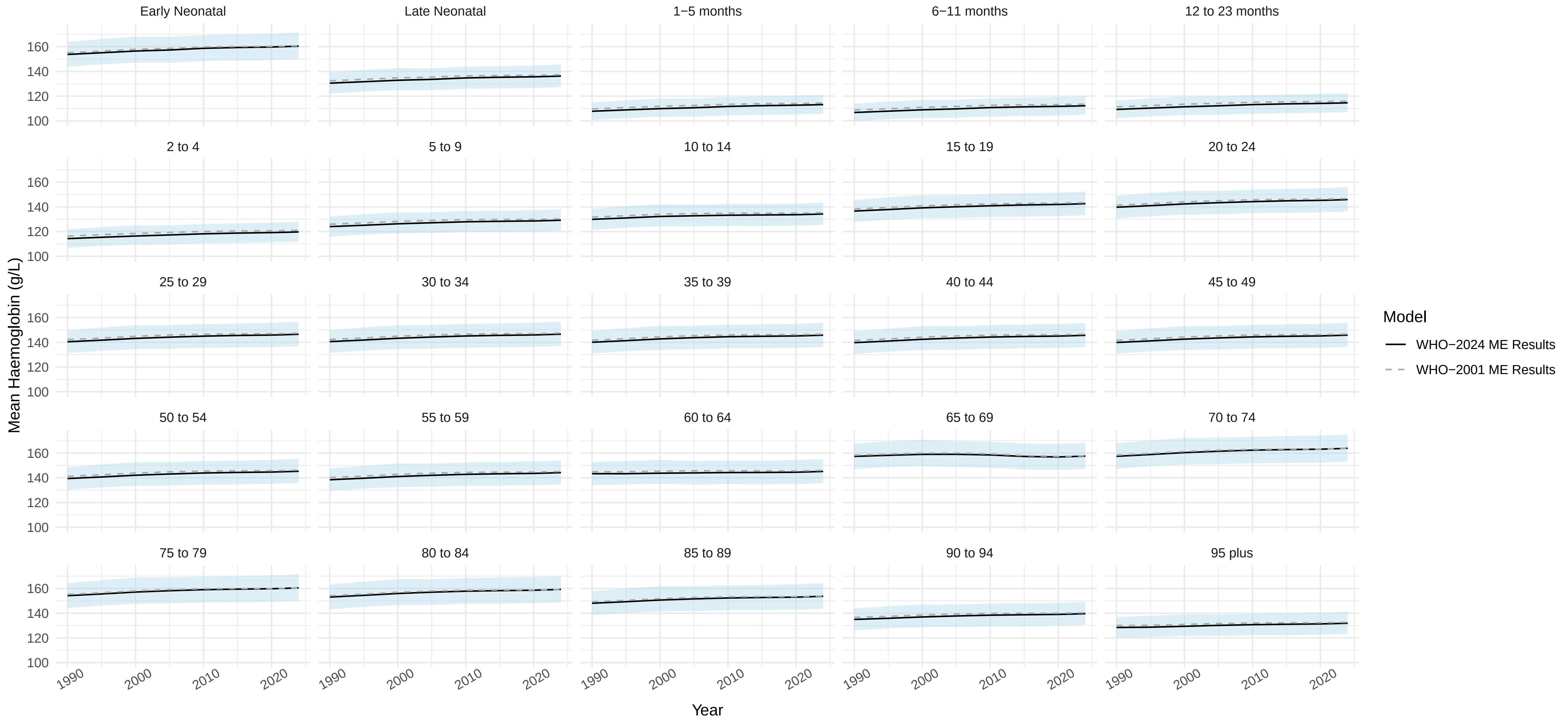

Elevation Adjustment Method Comparison − Mean Haemoglobin − Female − Poland
WHO−2024 and WHO−2001

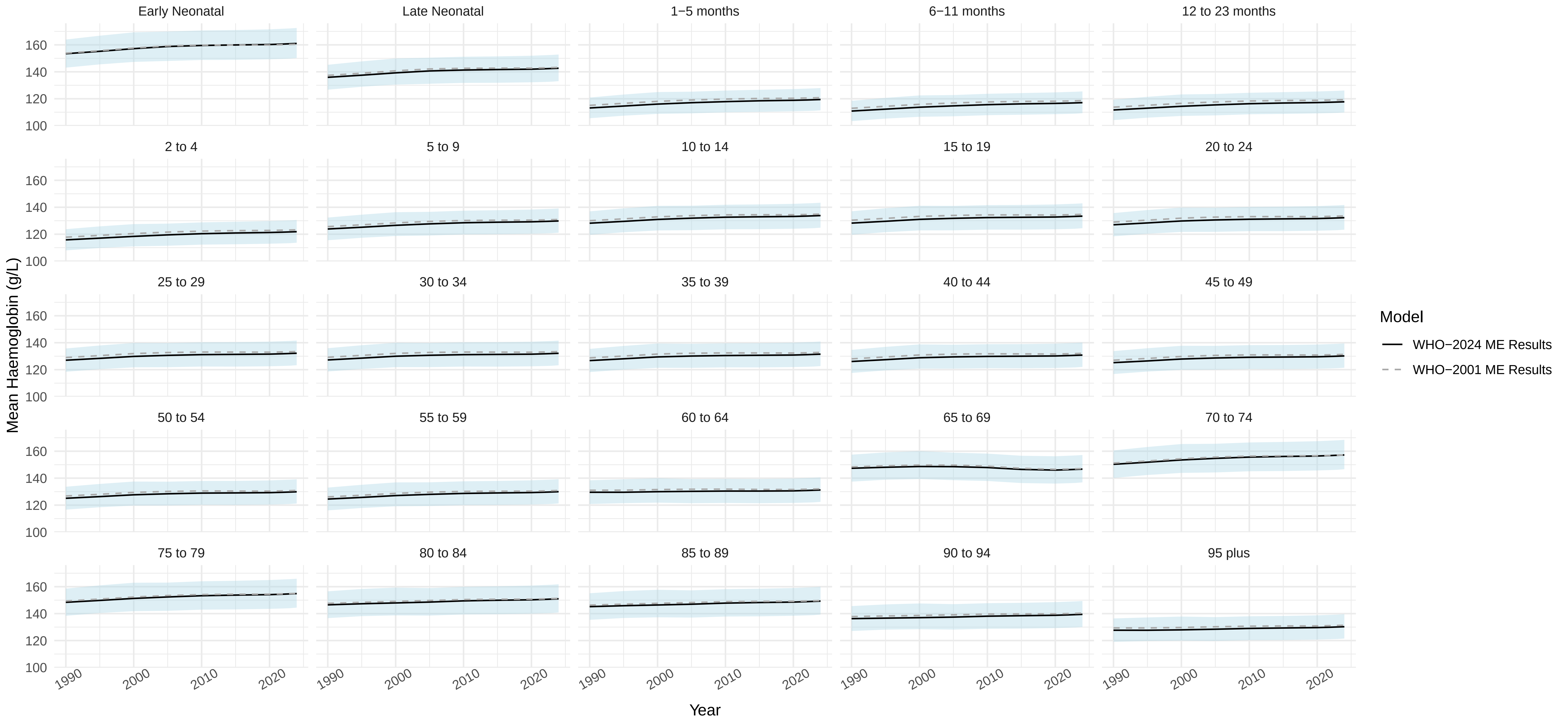

Elevation Adjustment Method Comparison − Anaemia Prevalence − Male − Poland
WHO−2024 vs. WHO−2001 Elevation Adjustment Methods

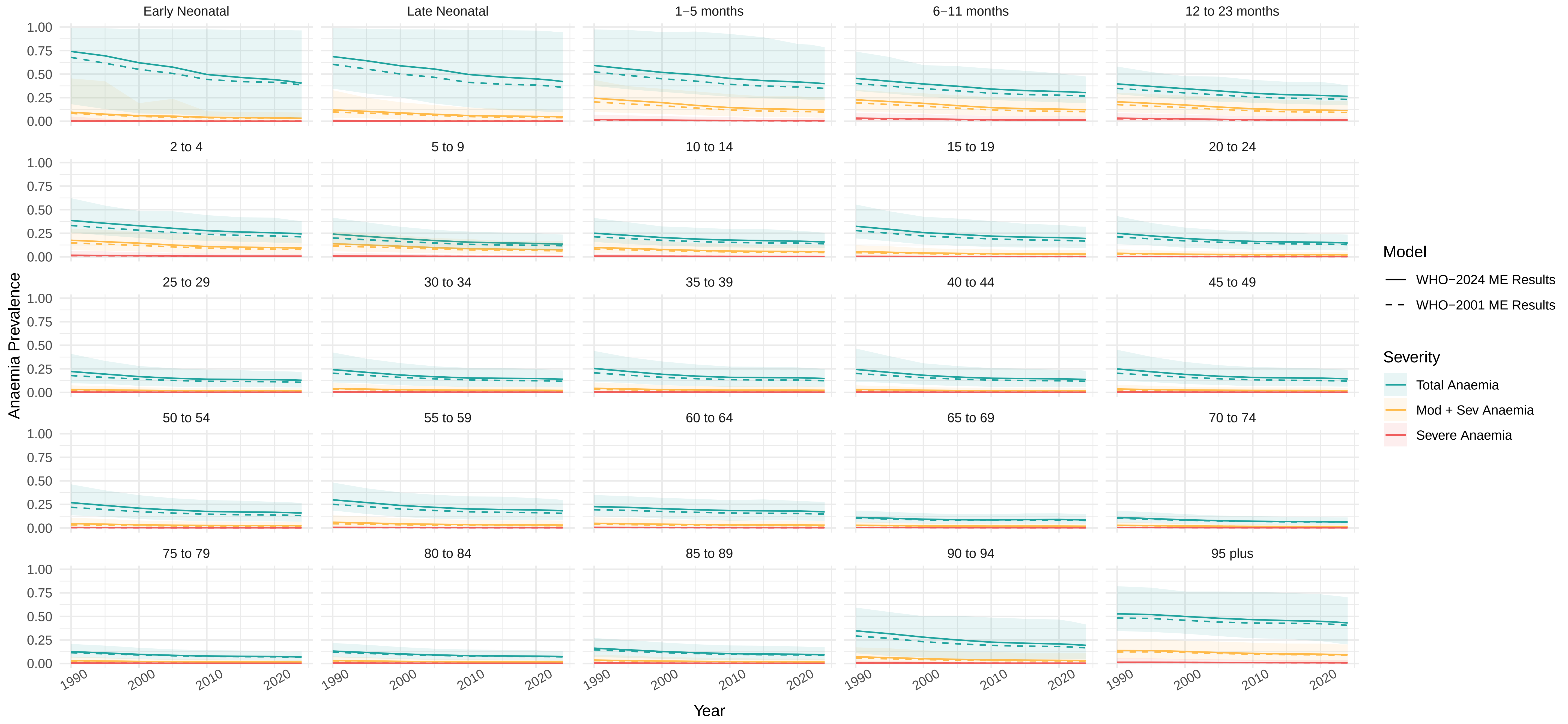

Elevation Adjustment Method Comparison − Anaemia Prevalence − Female − Poland
WHO−2024 vs. WHO−2001 Elevation Adjustment Methods

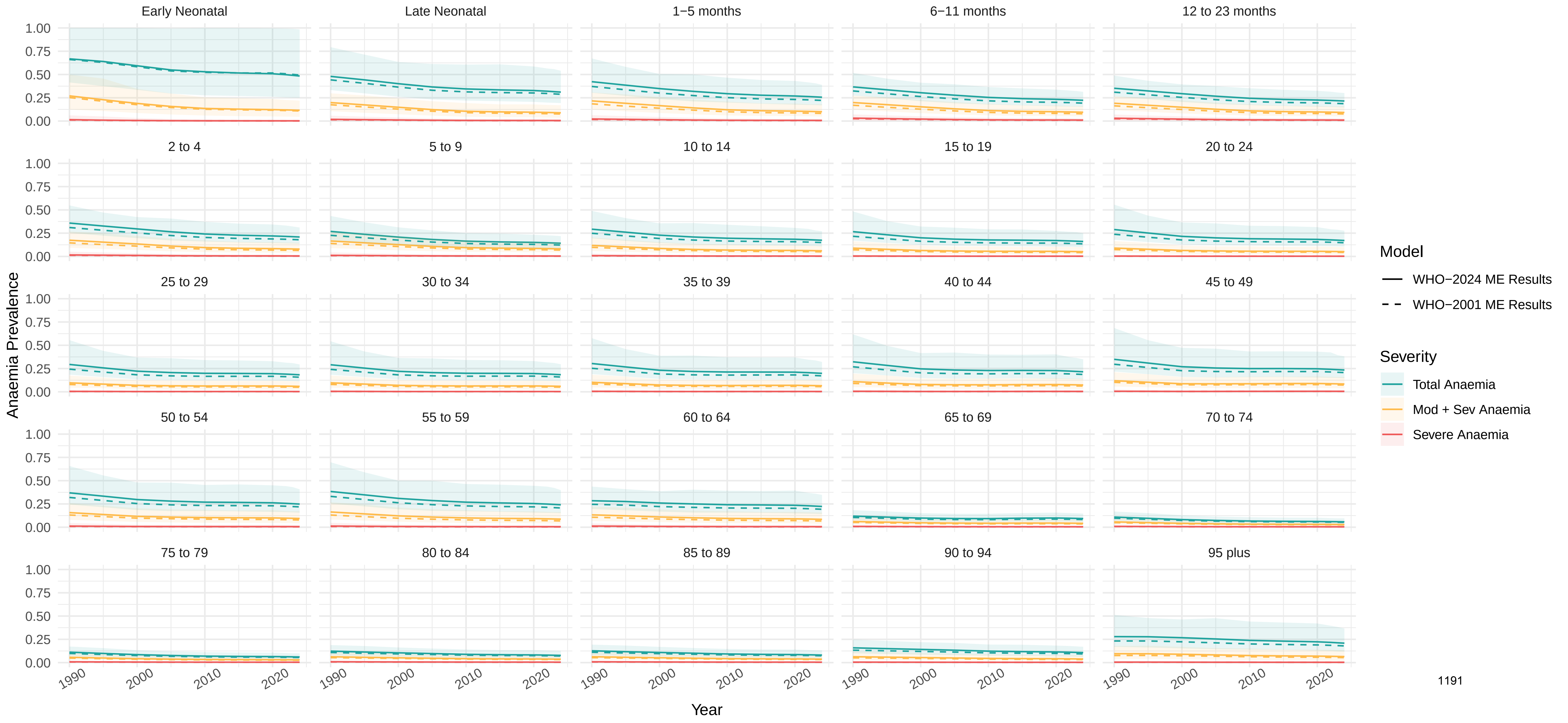

Elevation Adjustment Method Comparison − Mean Haemoglobin − Male − Romania

WHO−2024 and WHO−2001

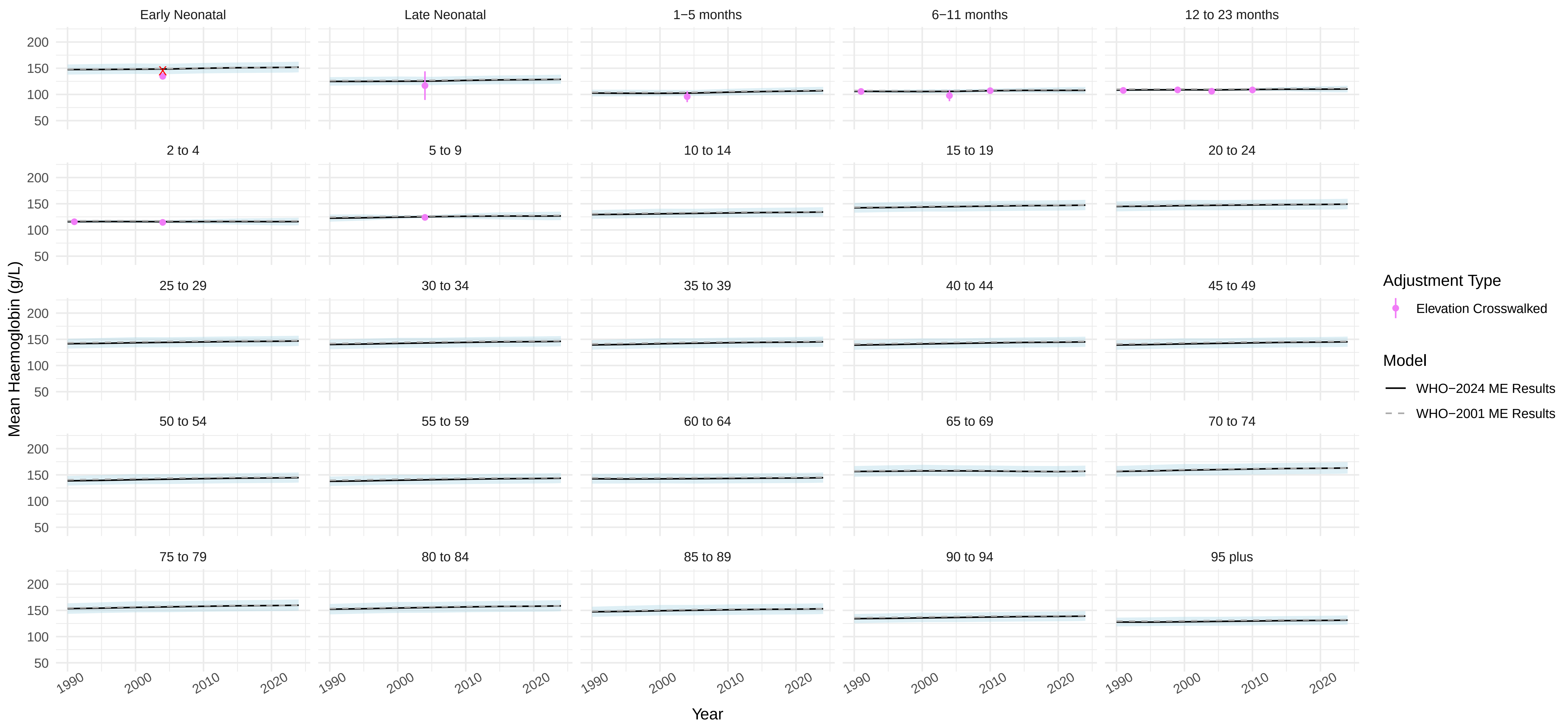

Elevation Adjustment Method Comparison − Mean Haemoglobin − Female − Romania

WHO−2024 and WHO−2001

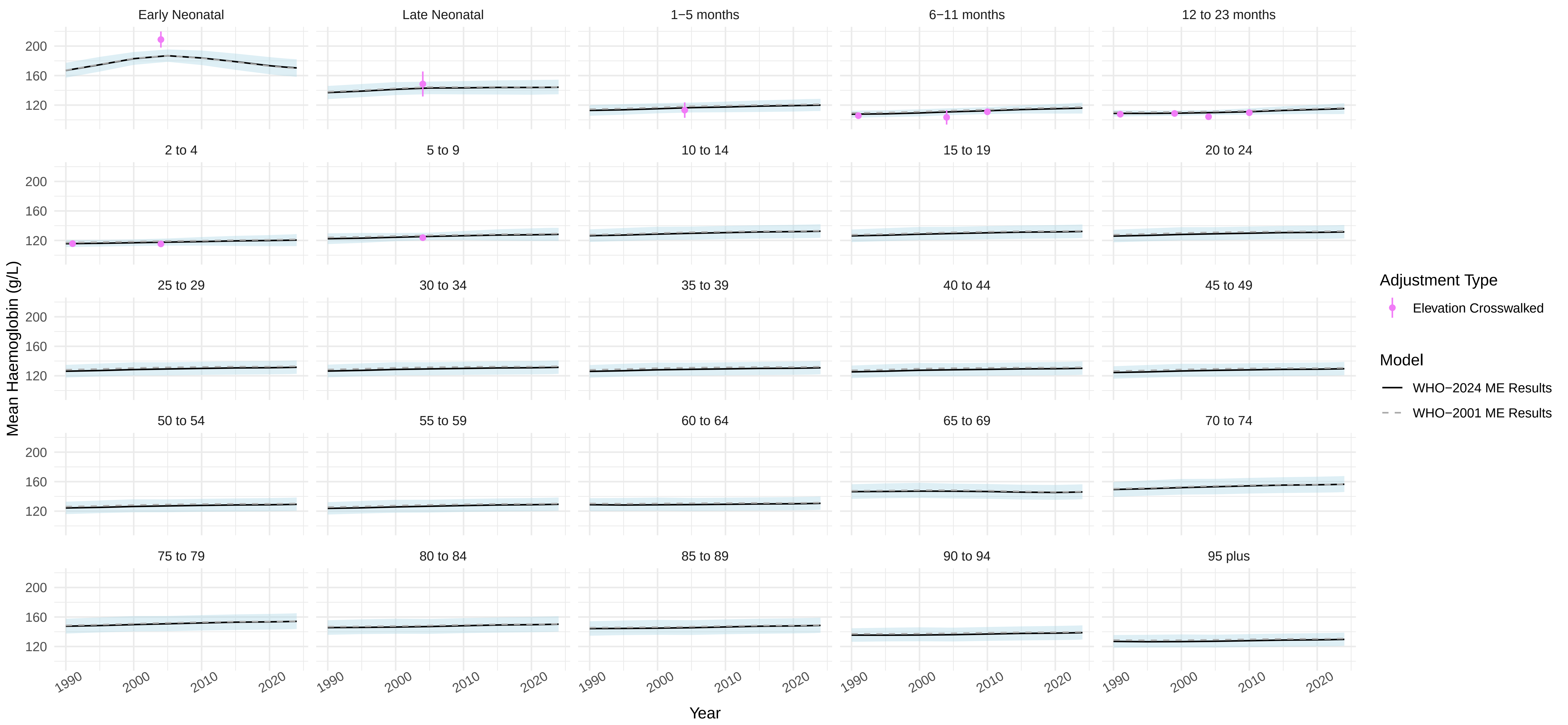

Elevation Adjustment Method Comparison − Anaemia Prevalence − Male − Romania

WHO−2024 vs. WHO−2001 Elevation Adjustment Methods

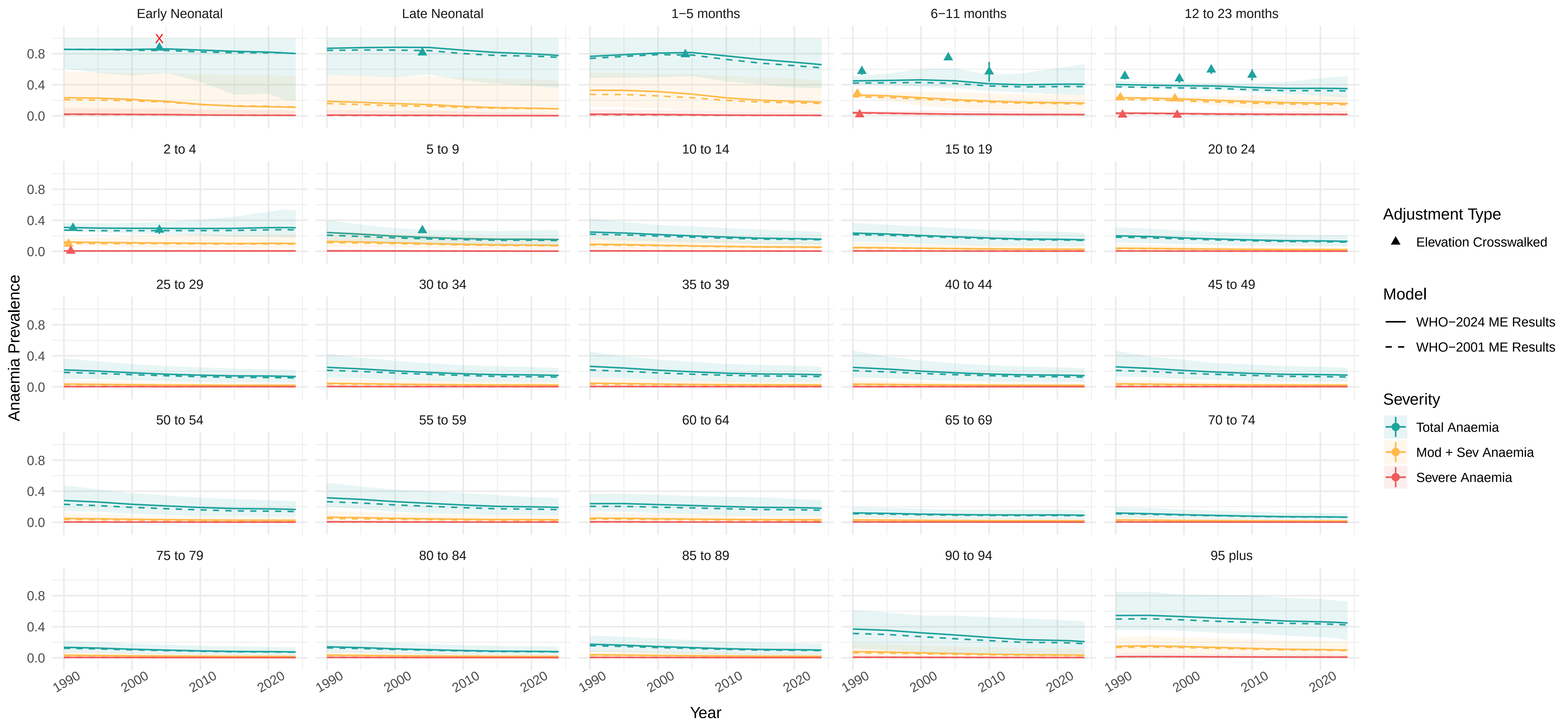

Elevation Adjustment Method Comparison − Anaemia Prevalence − Female − Romania

WHO−2024 vs. WHO−2001 Elevation Adjustment Methods

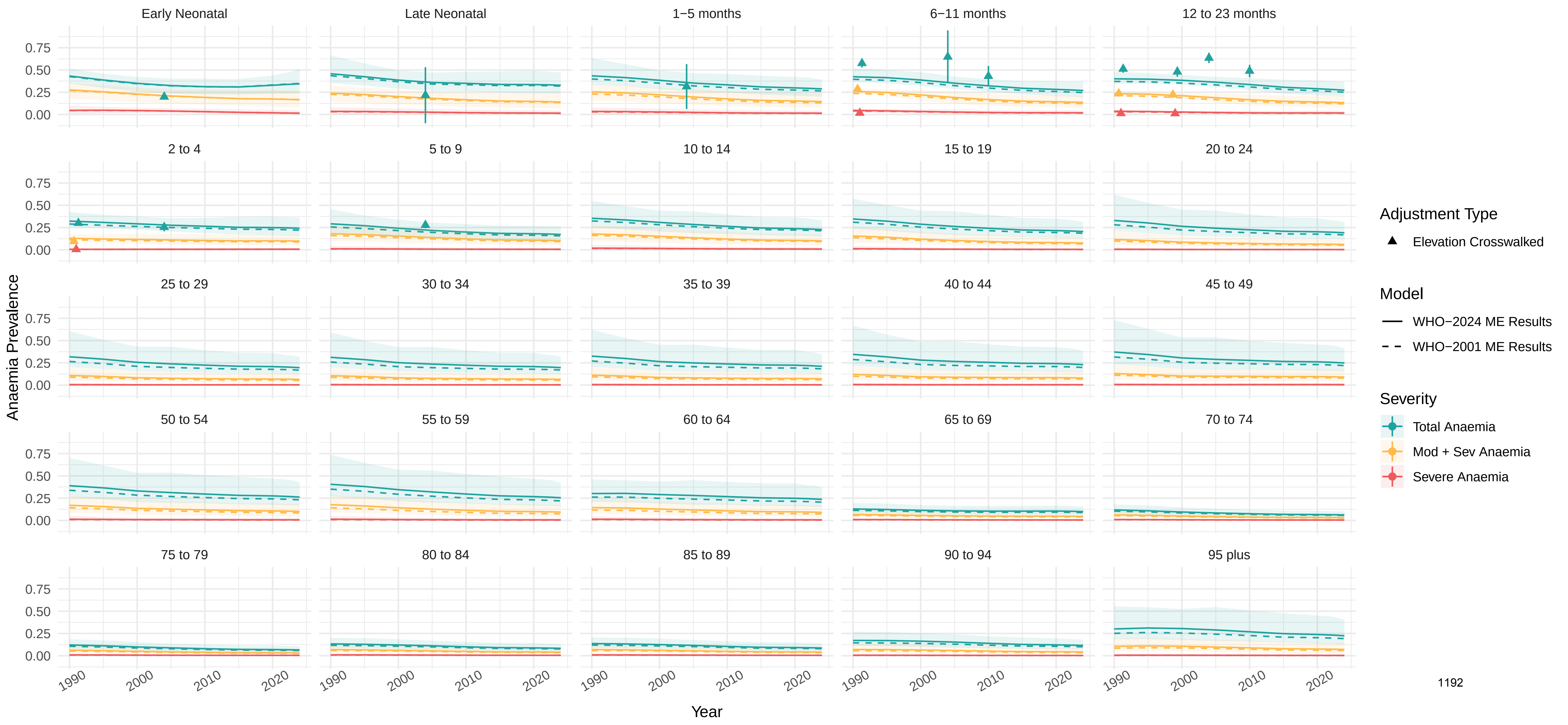

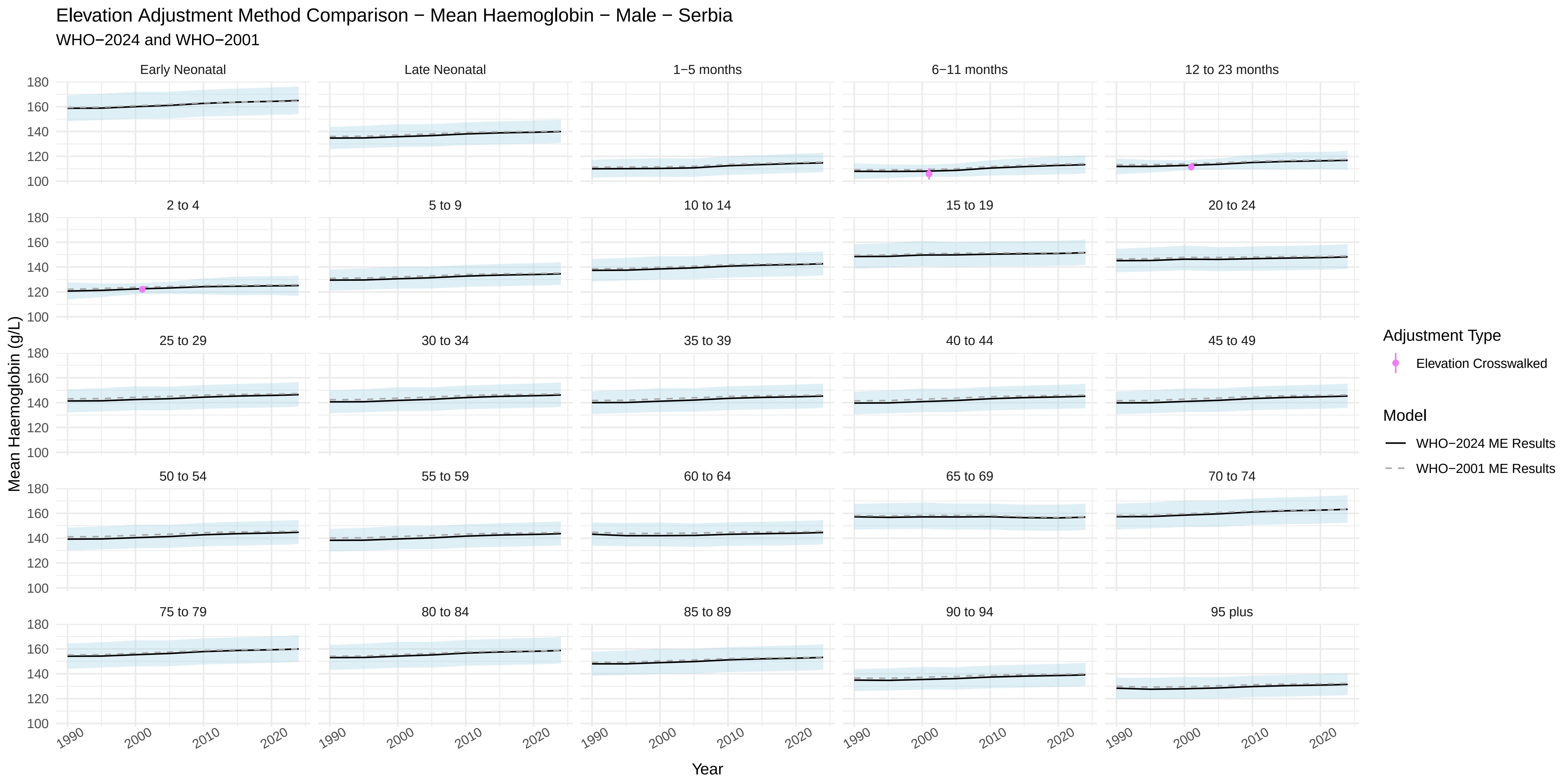
Elevation Adjustment Method Comparison − Mean Haemoglobin − Male − Serbia
WHO−2024 and WHO−2001
Early Neonatal
Late Neonatal
1−5 months
6−11 months
12 to 23 months
2 to 4
5 to 9
10 to 14
15 to 19
20 to 24
25 to 29
30 to 34
35 to 39
40 to 44
45 to 49
50 to 54
55 to 59
60 to 64
65 to 69
70 to 74
75 to 79
80 to 84
85 to 89
90 to 94
95 plus
Mean Haemoglobin (g/L)
Year
Adjustment Type
Elevation Crosswalked
Model
WHO−2024 ME Results
WHO−2001 ME Results

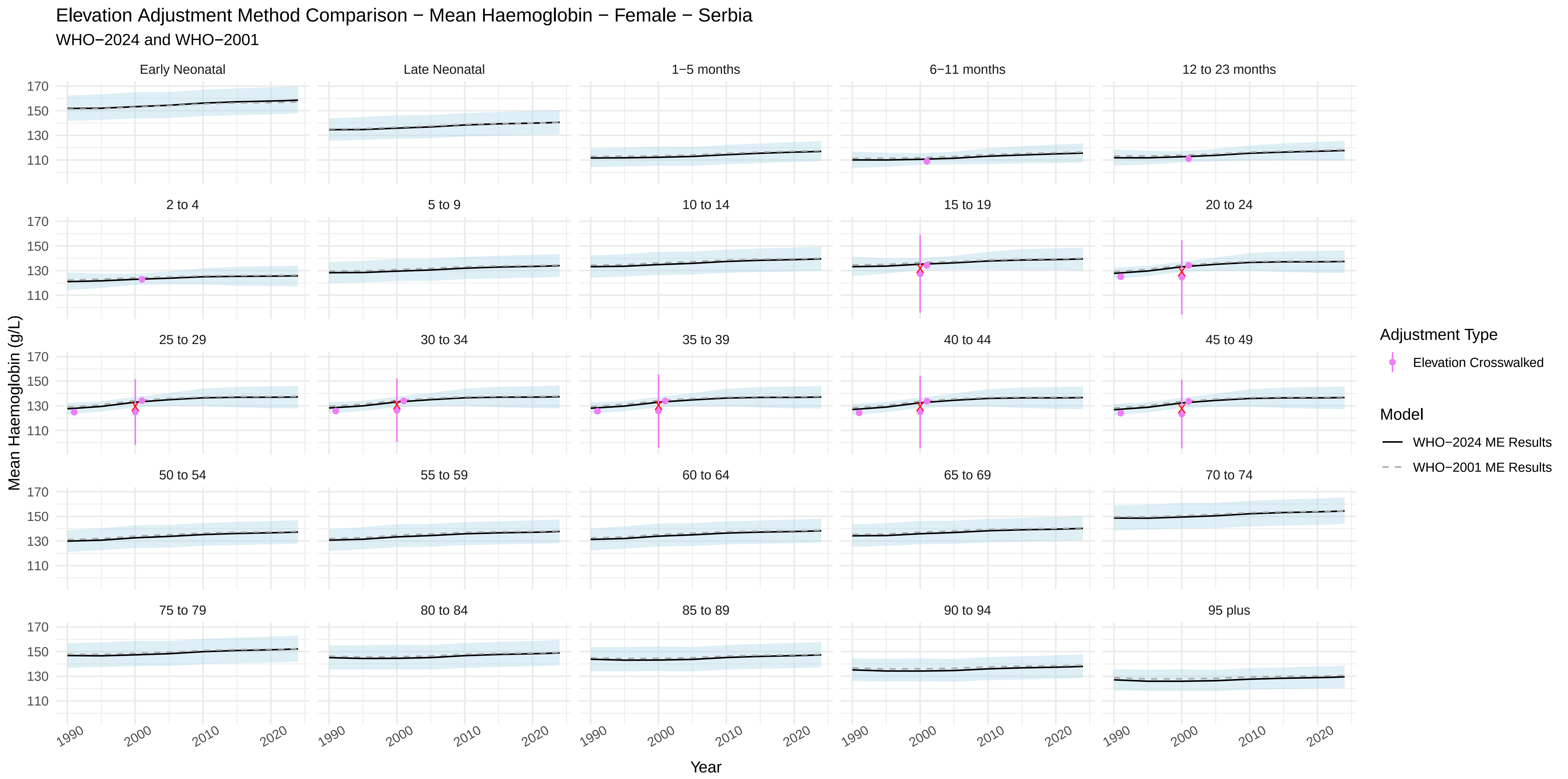
Elevation Adjustment Method Comparison − Mean Haemoglobin − Female − Serbia
WHO−2024 and WHO−2001
Early Neonatal
Late Neonatal
1−5 months
6−11 months
12 to 23 months
2 to 4
5 to 9
10 to 14
15 to 19
20 to 24
25 to 29
30 to 34
35 to 39
40 to 44
45 to 49
50 to 54
55 to 59
60 to 64
65 to 69
70 to 74
75 to 79
80 to 84
85 to 89
90 to 94
95 plus
Mean Haemoglobin (g/L)
Year
Adjustment Type
Elevation Crosswalked
Model
WHO−2024 ME Results
WHO−2001 ME Results

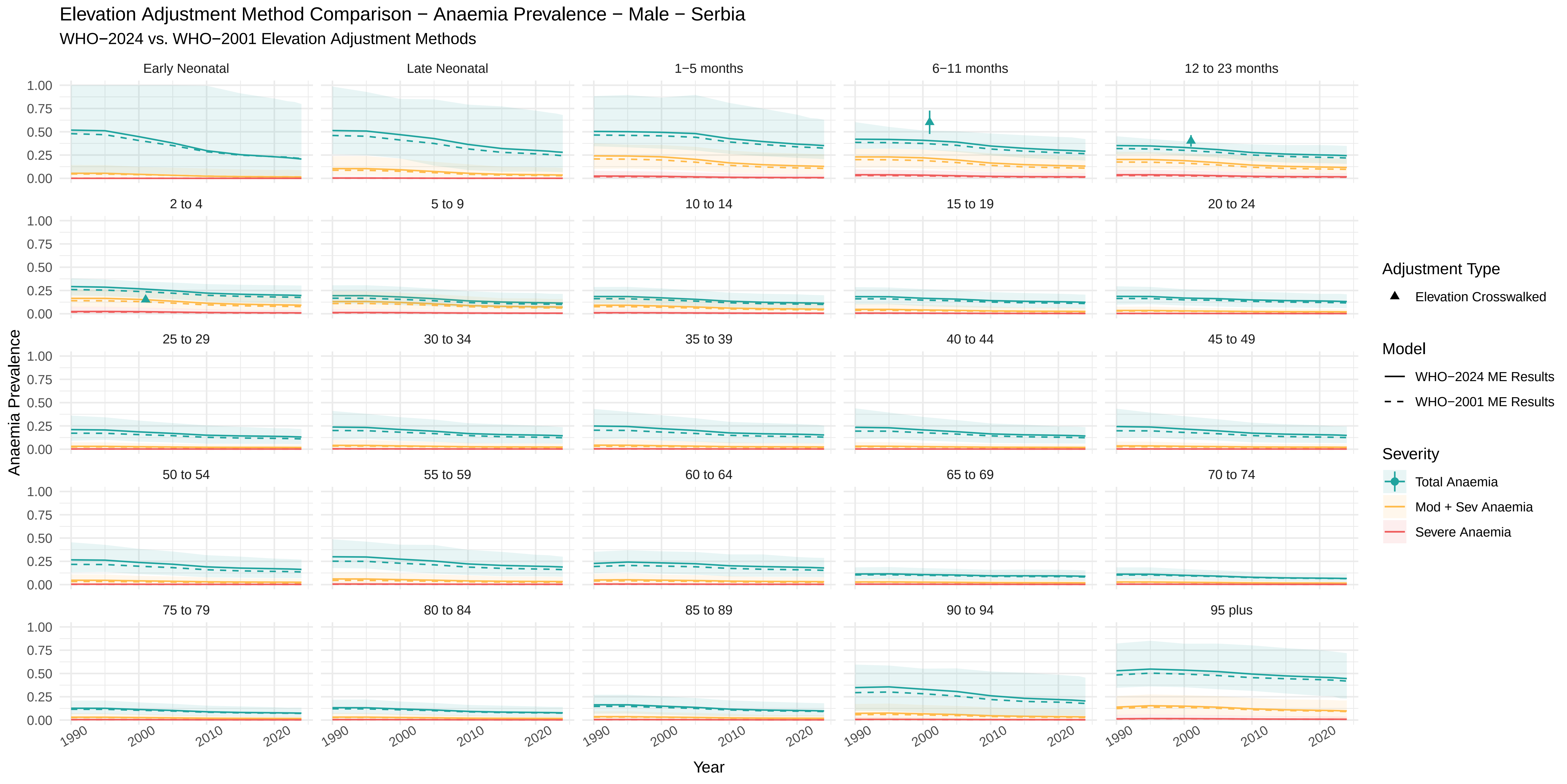
Elevation Adjustment Method Comparison − Anaemia Prevalence − Male − Serbia
WHO−2024 vs. WHO−2001 Elevation Adjustment Methods
Early Neonatal
Late Neonatal
1−5 months
6−11 months
12 to 23 months
2 to 4
5 to 9
10 to 14
15 to 19
20 to 24
25 to 29
30 to 34
35 to 39
40 to 44
45 to 49
50 to 54
55 to 59
60 to 64
65 to 69
70 to 74
75 to 79
80 to 84
85 to 89
90 to 94
95 plus
Anaemia Prevalence
Year
Adjustment Type
Elevation Crosswalked
Model
WHO−2024 ME Results
WHO−2001 ME Results
Severity
Total Anaemia
Mod + Sev Anaemia
Severe Anaemia

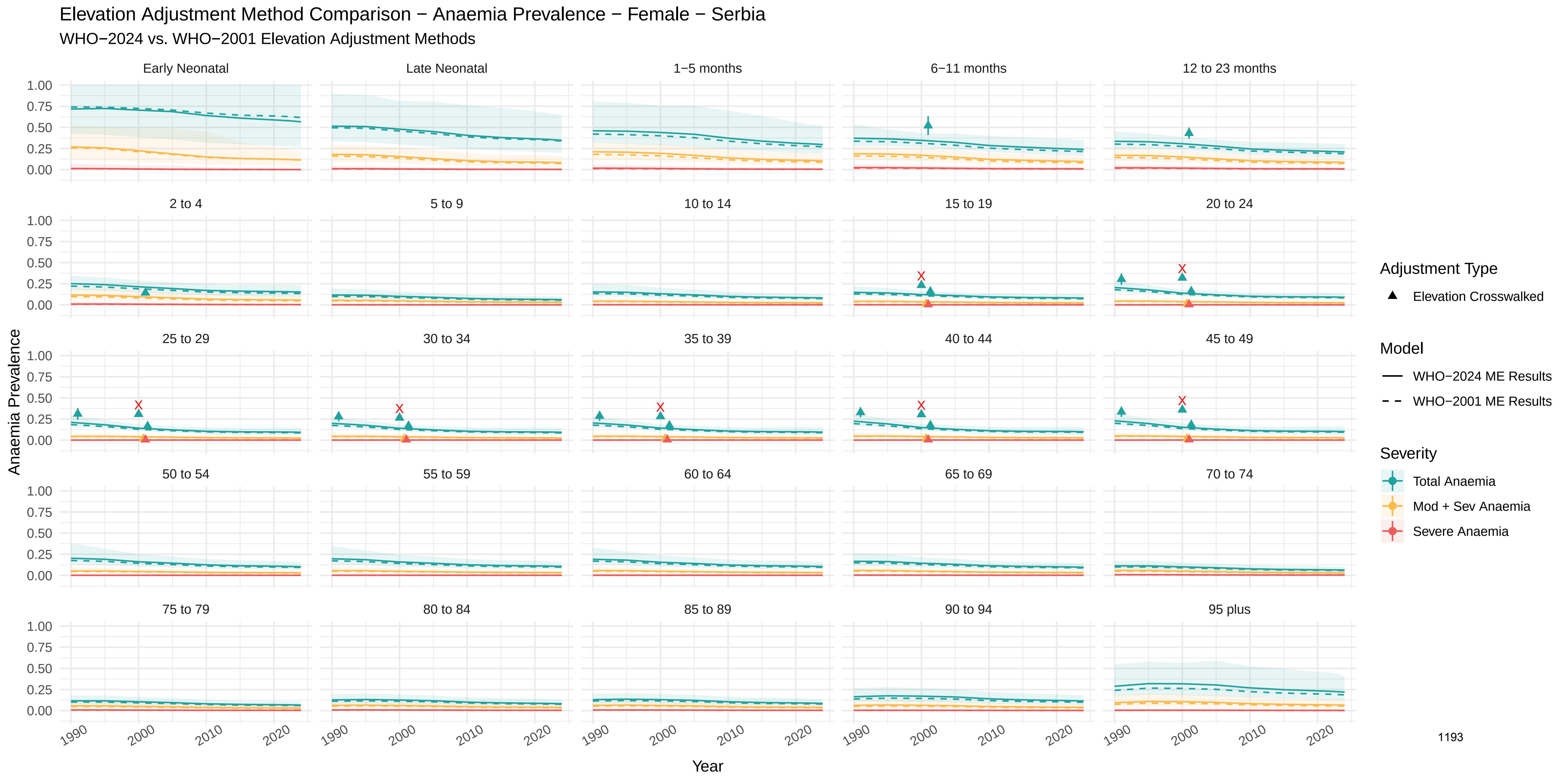
Elevation Adjustment Method Comparison − Anaemia Prevalence − Female − Serbia
WHO−2024 vs. WHO−2001 Elevation Adjustment Methods
Early Neonatal
Late Neonatal
1−5 months
6−11 months
12 to 23 months
2 to 4
5 to 9
10 to 14
15 to 19
20 to 24
25 to 29
30 to 34
35 to 39
40 to 44
45 to 49
50 to 54
55 to 59
60 to 64
65 to 69
70 to 74
75 to 79
80 to 84
85 to 89
90 to 94
95 plus
Anaemia Prevalence
Year
Adjustment Type
Elevation Crosswalked
Model
WHO−2024 ME Results
WHO−2001 ME Results
Severity
Total Anaemia
Mod + Sev Anaemia
Severe Anaemia

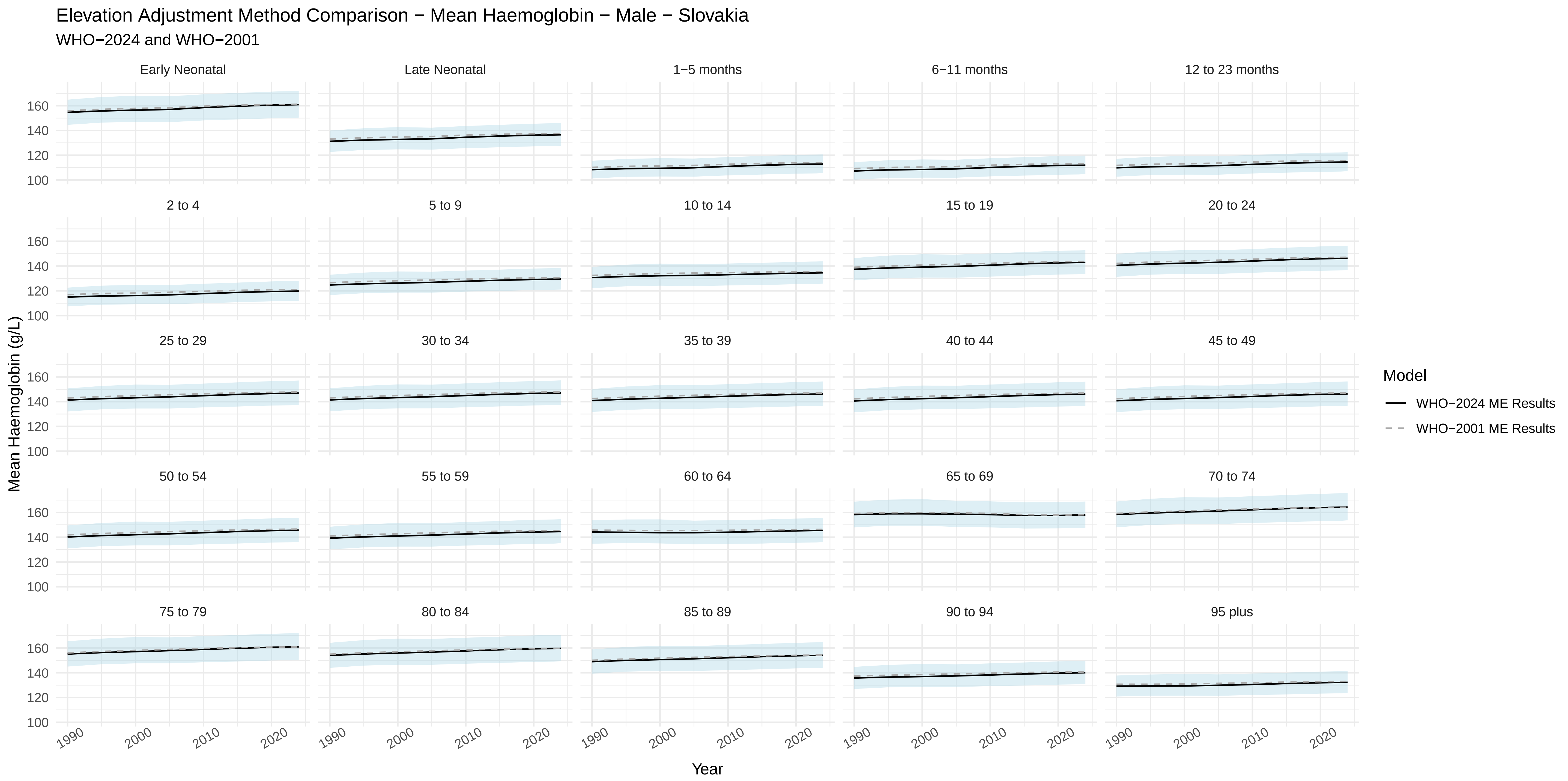
Elevation Adjustment Method Comparison − Mean Haemoglobin − Male − Slovakia
WHO−2024 and WHO−2001
Early Neonatal
Late Neonatal
1−5 months
6−11 months
12 to 23 months
2 to 4
5 to 9
10 to 14
15 to 19
20 to 24
25 to 29
30 to 34
35 to 39
40 to 44
45 to 49
50 to 54
55 to 59
60 to 64
65 to 69
70 to 74
75 to 79
80 to 84
85 to 89
90 to 94
95 plus
Mean Haemoglobin (g/L)
Year
Model
WHO−2024 ME Results
WHO−2001 ME Results

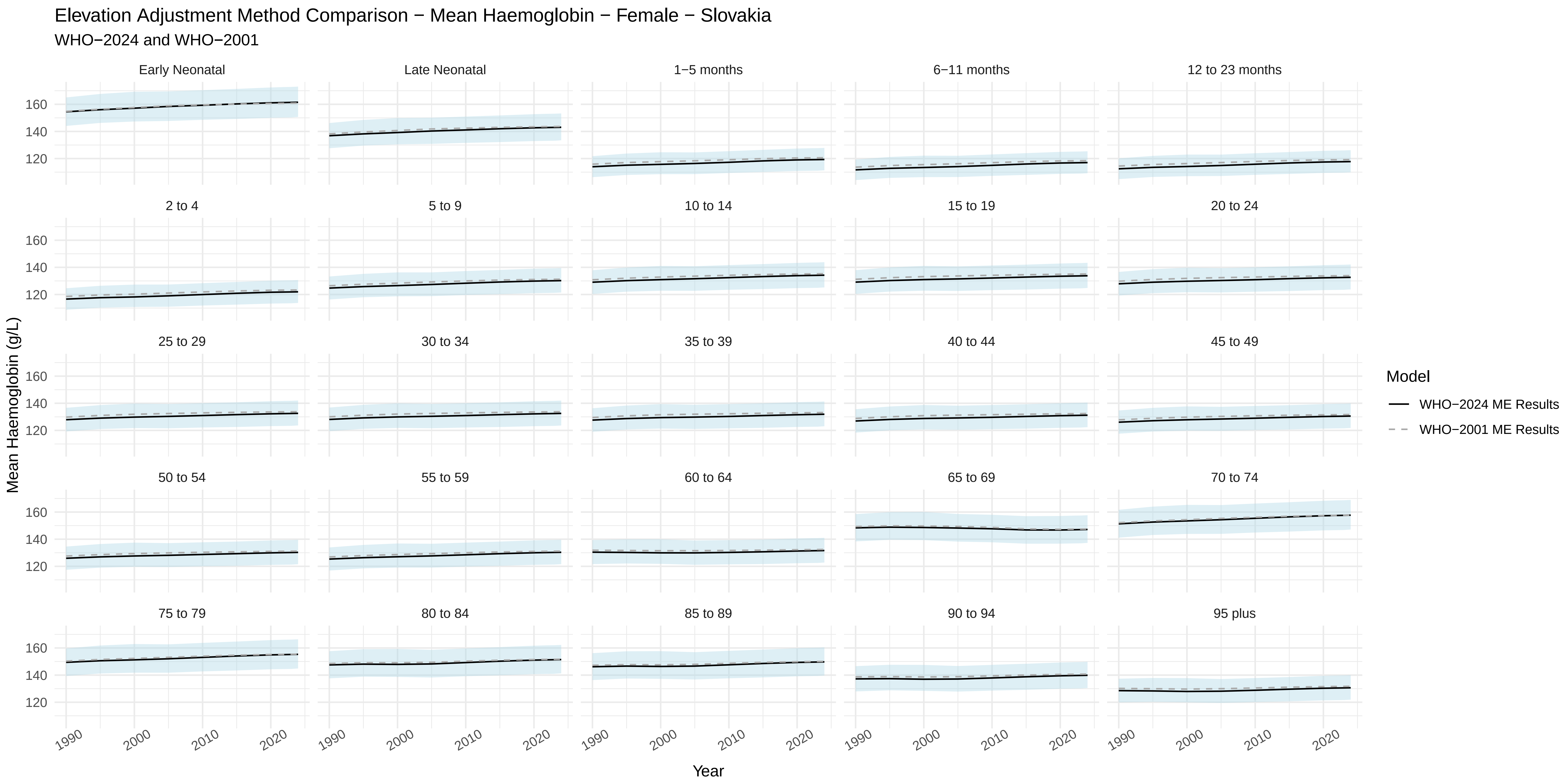
Elevation Adjustment Method Comparison − Mean Haemoglobin − Female − Slovakia
WHO−2024 and WHO−2001
Early Neonatal
Late Neonatal
1−5 months
6−11 months
12 to 23 months
2 to 4
5 to 9
10 to 14
15 to 19
20 to 24
25 to 29
30 to 34
35 to 39
40 to 44
45 to 49
50 to 54
55 to 59
60 to 64
65 to 69
70 to 74
75 to 79
80 to 84
85 to 89
90 to 94
95 plus
Mean Haemoglobin (g/L)
Year
Model
WHO−2024 ME Results
WHO−2001 ME Results

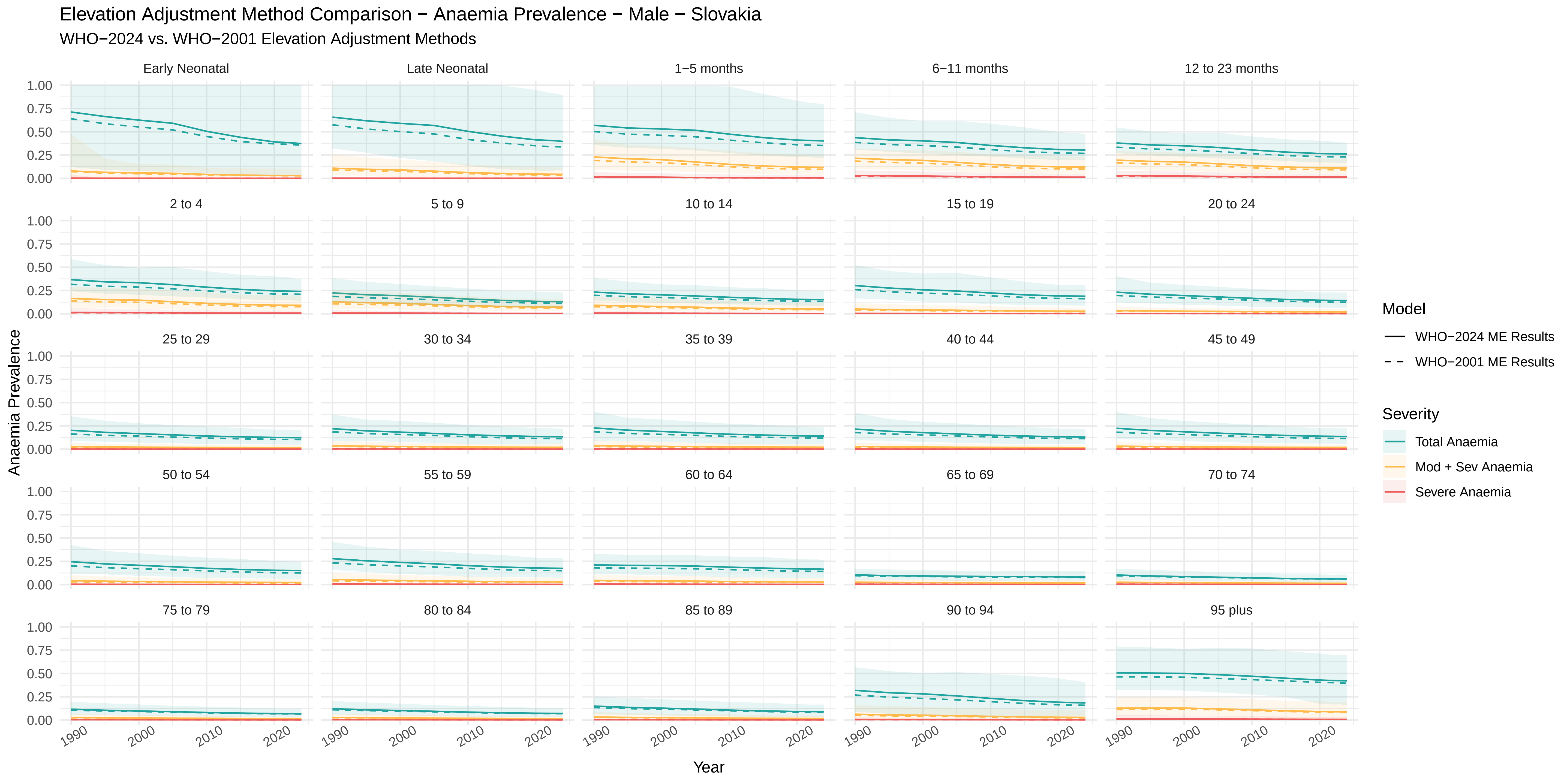
Elevation Adjustment Method Comparison − Anaemia Prevalence − Male − Slovakia
WHO−2024 vs. WHO−2001 Elevation Adjustment Methods
Early Neonatal
Late Neonatal
1−5 months
6−11 months
12 to 23 months
2 to 4
5 to 9
10 to 14
15 to 19
20 to 24
25 to 29
30 to 34
35 to 39
40 to 44
45 to 49
50 to 54
55 to 59
60 to 64
65 to 69
70 to 74
75 to 79
80 to 84
85 to 89
90 to 94
95 plus
Anaemia Prevalence
Year
Model
WHO−2024 ME Results
WHO−2001 ME Results
Severity
Total Anaemia
Mod + Sev Anaemia
Severe Anaemia

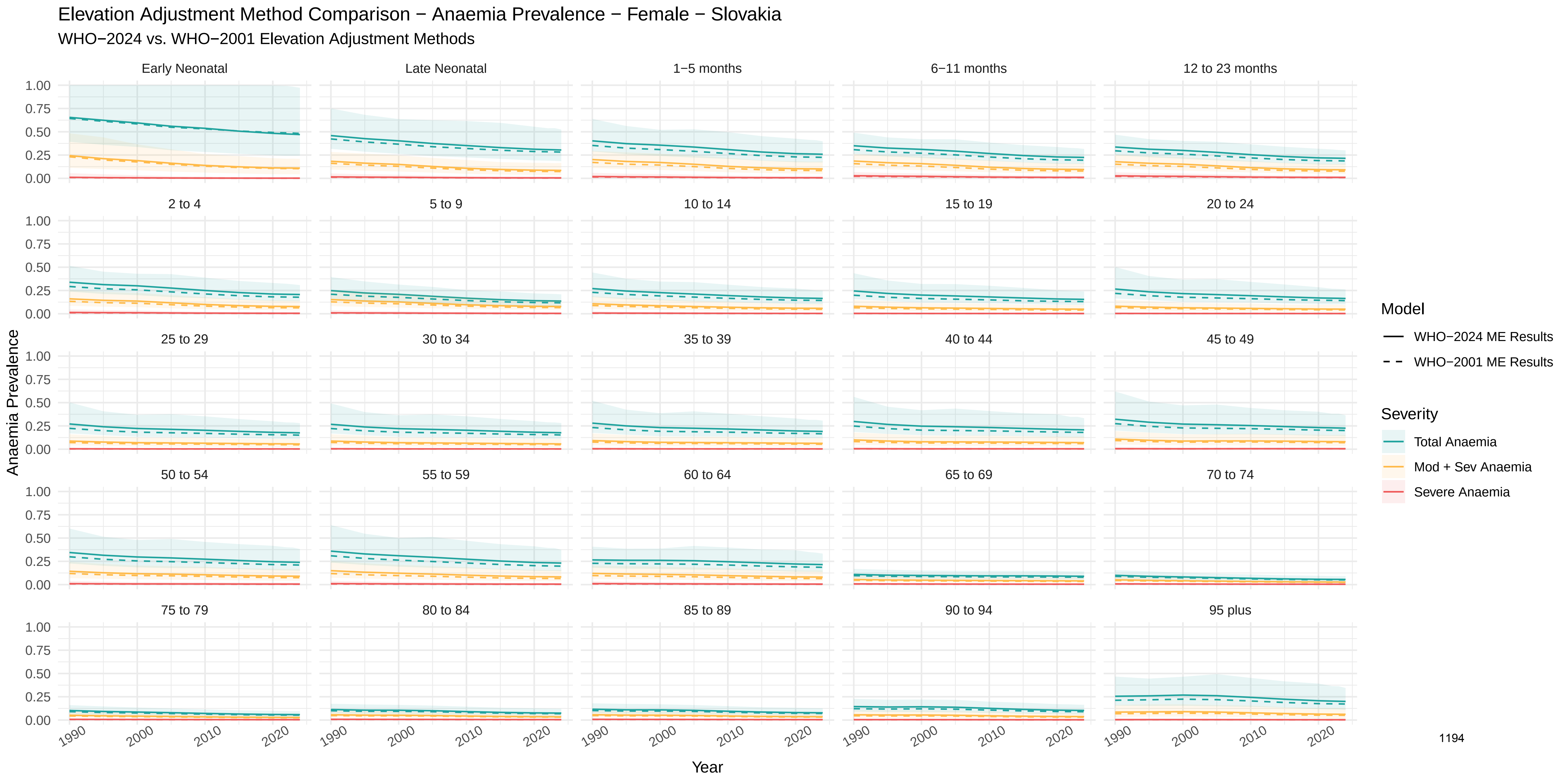
Elevation Adjustment Method Comparison − Anaemia Prevalence − Female − Slovakia
WHO−2024 vs. WHO−2001 Elevation Adjustment Methods
Early Neonatal
Late Neonatal
1−5 months
6−11 months
12 to 23 months
2 to 4
5 to 9
10 to 14
15 to 19
20 to 24
25 to 29
30 to 34
35 to 39
40 to 44
45 to 49
50 to 54
55 to 59
60 to 64
65 to 69
70 to 74
75 to 79
80 to 84
85 to 89
90 to 94
95 plus
Anaemia Prevalence
Year
Model
WHO−2024 ME Results
WHO−2001 ME Results
Severity
Total Anaemia
Mod + Sev Anaemia
Severe Anaemia

Elevation Adjustment Method Comparison − Mean Haemoglobin − Male − Slovenia

WHO−2024 and WHO−2001

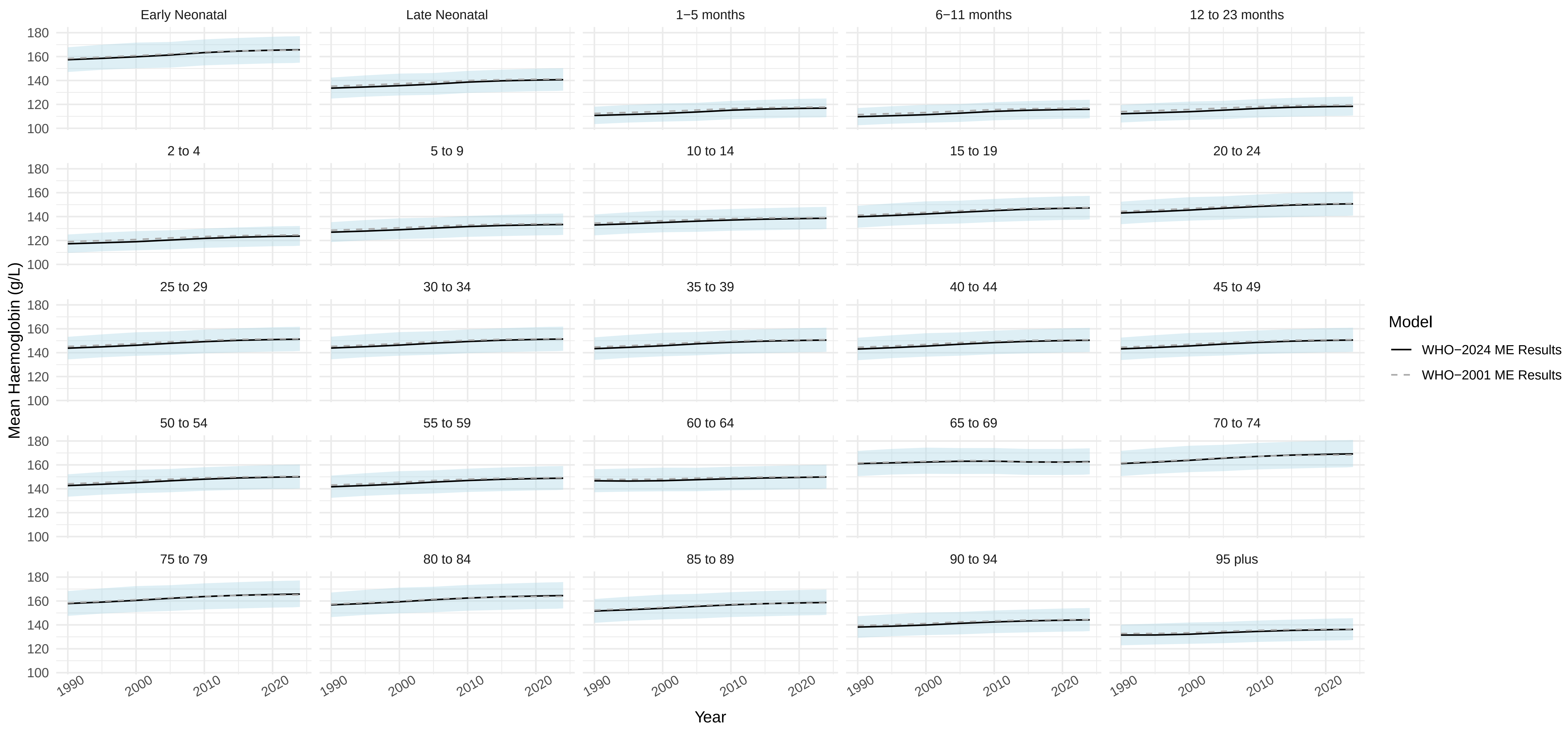

Elevation Adjustment Method Comparison − Mean Haemoglobin − Female − Slovenia

WHO−2024 and WHO−2001

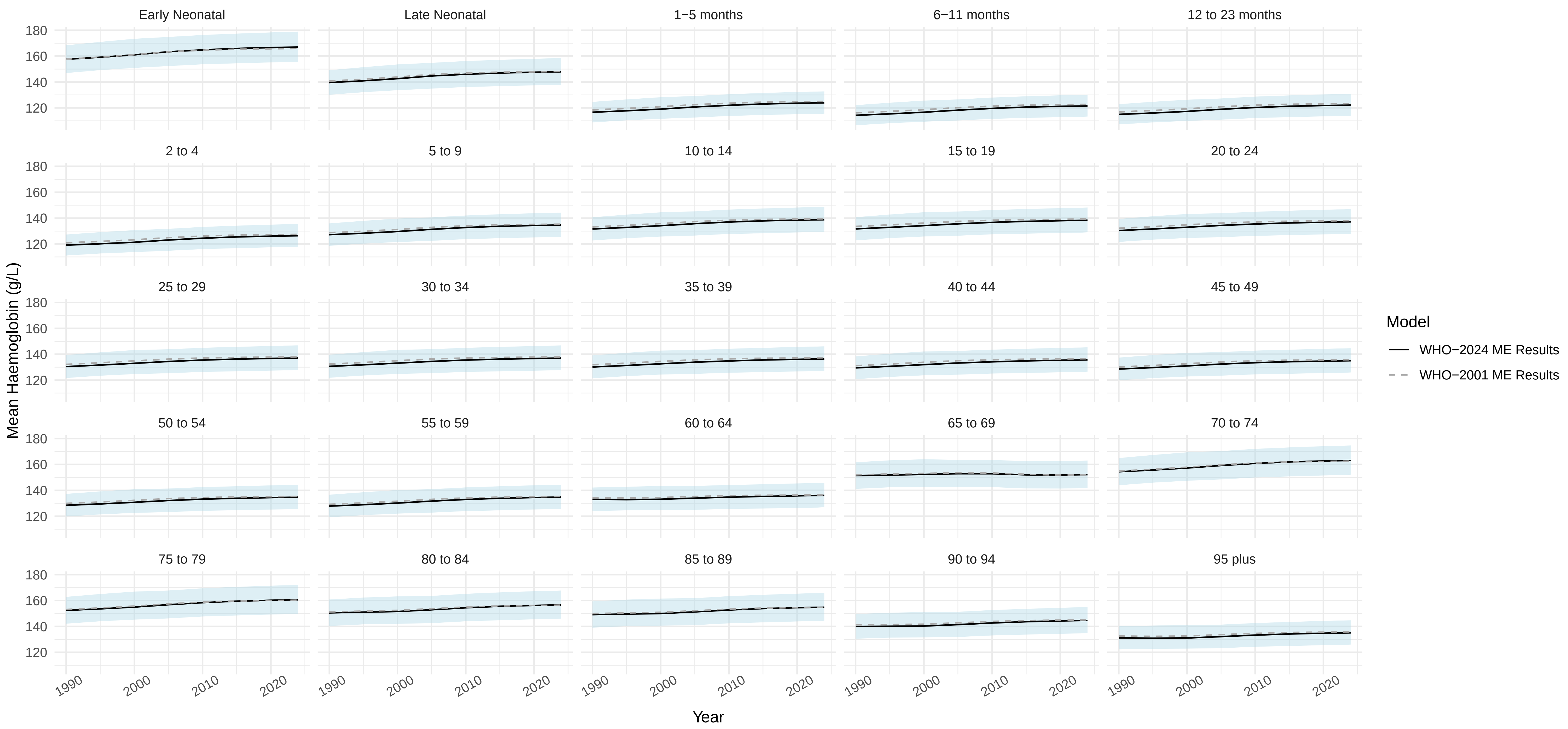

Elevation Adjustment Method Comparison − Anaemia Prevalence − Male − Slovenia

WHO−2024 vs. WHO−2001 Elevation Adjustment Methods

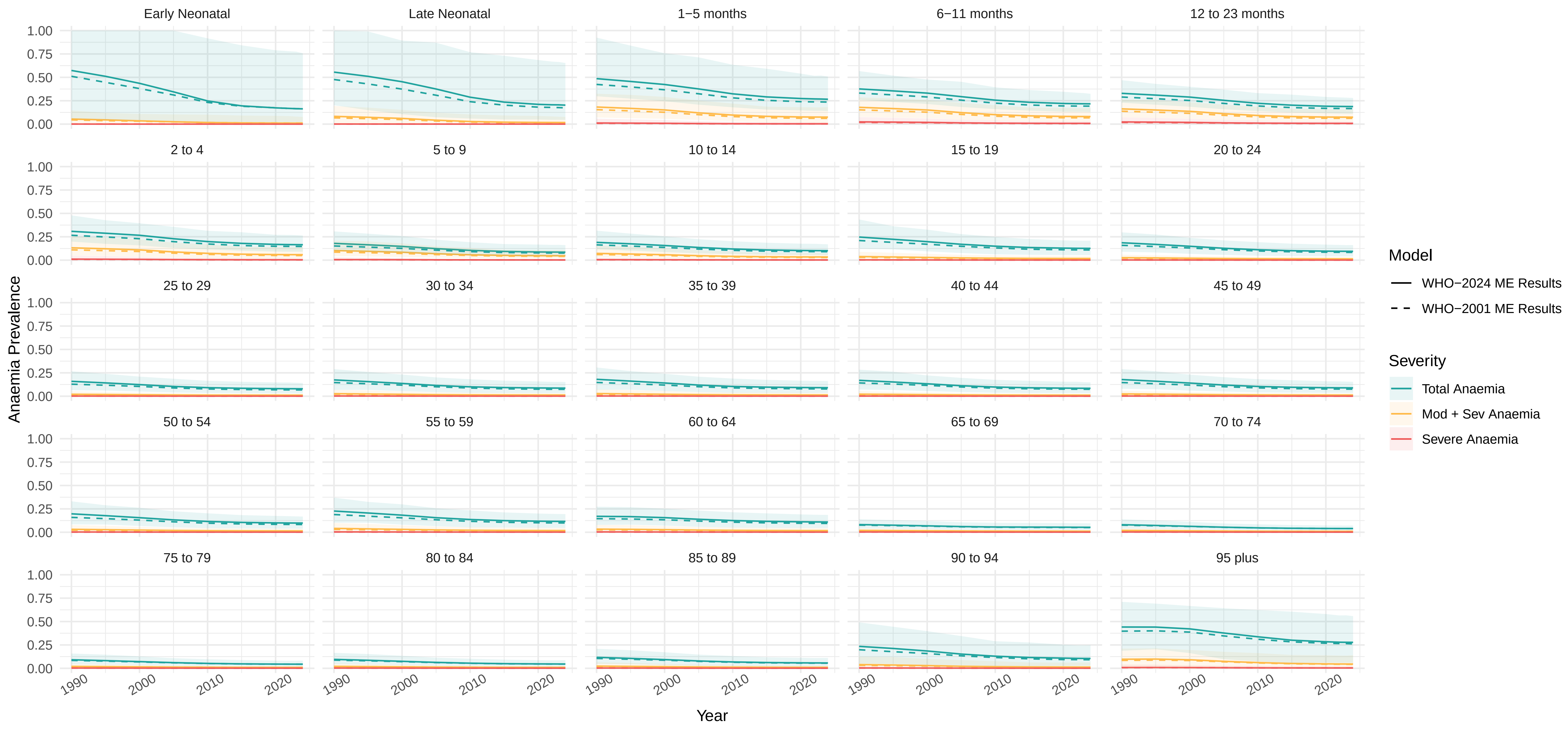

Elevation Adjustment Method Comparison − Anaemia Prevalence − Female − Slovenia

WHO−2024 vs. WHO−2001 Elevation Adjustment Methods

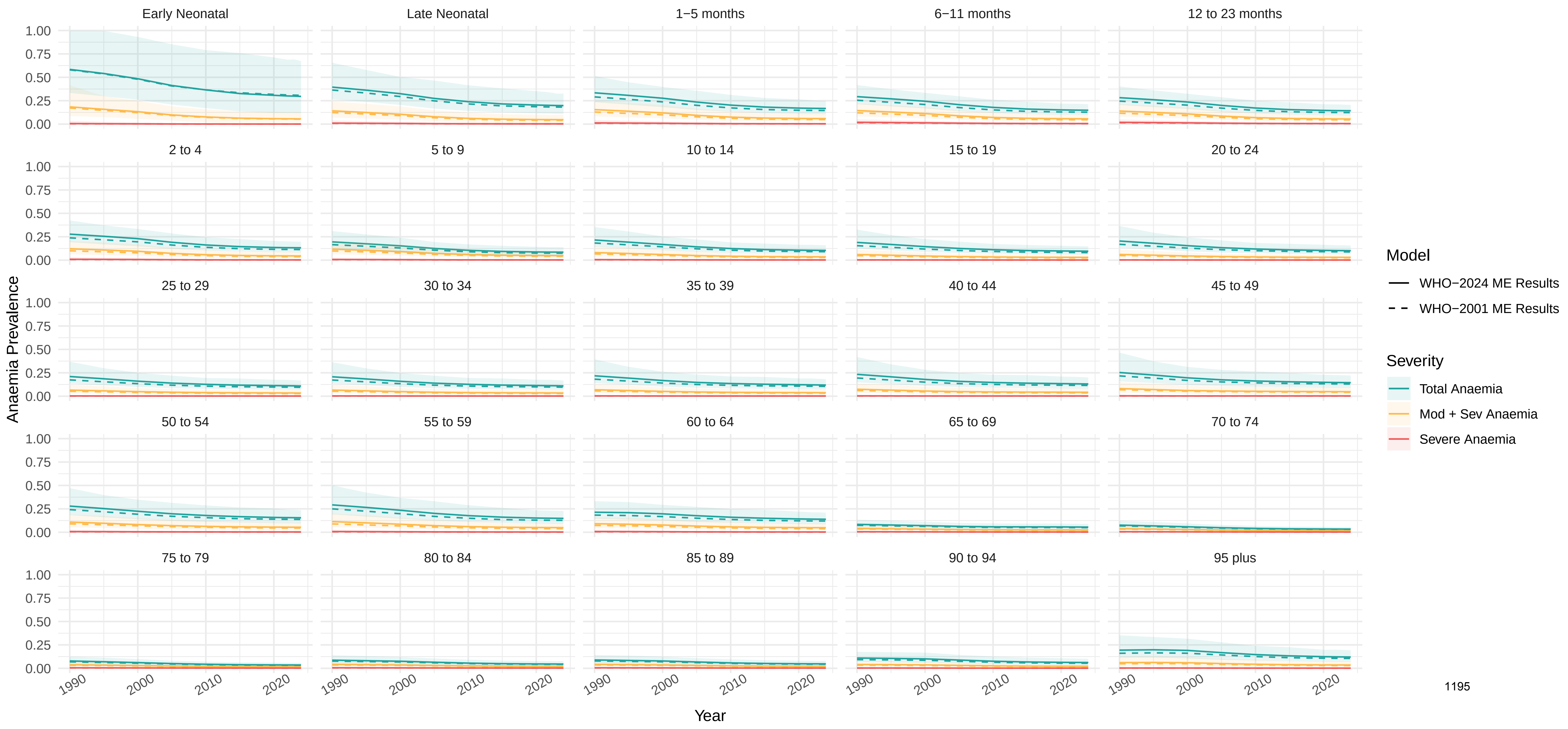

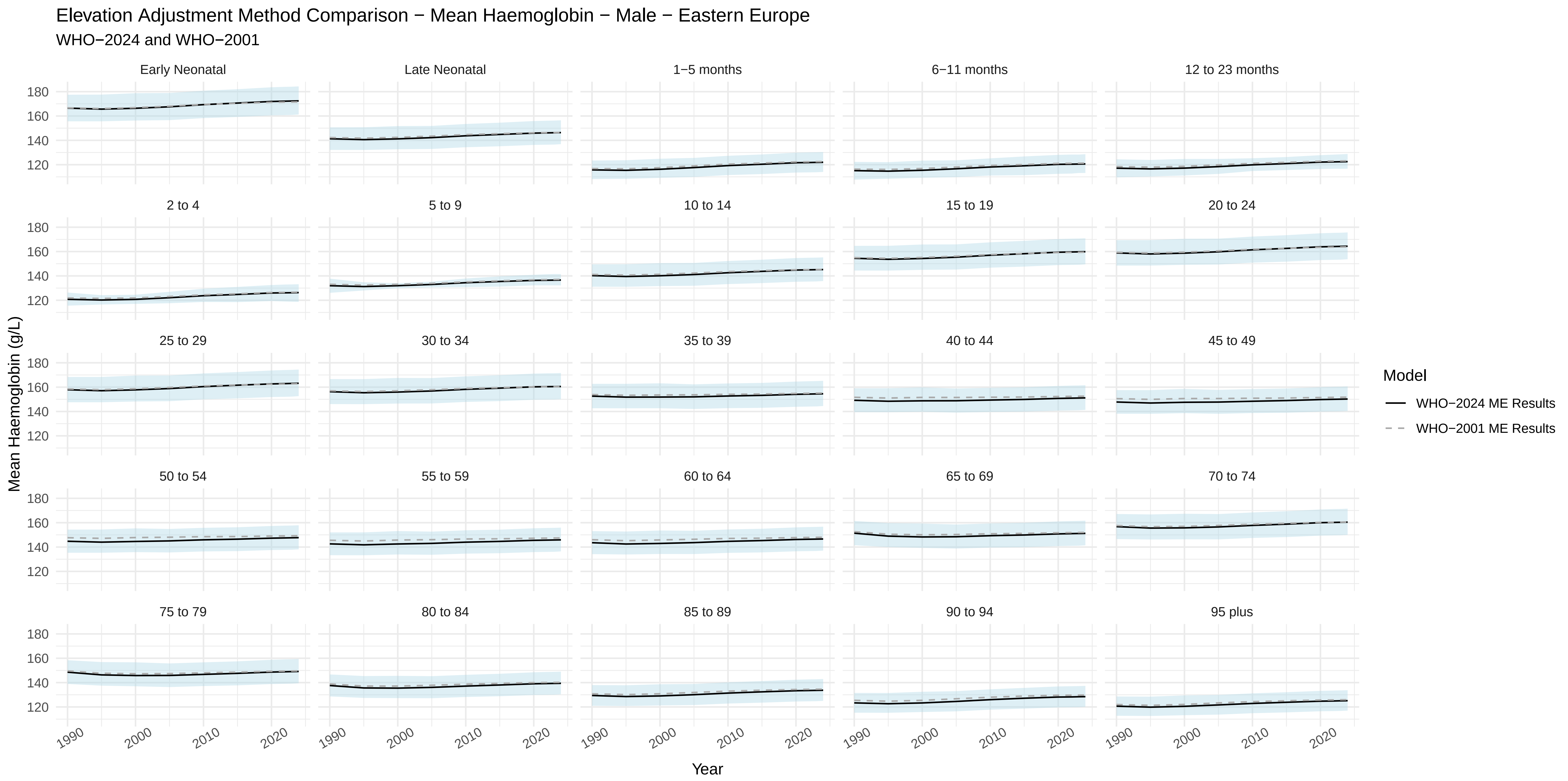
Elevation Adjustment Method Comparison − Mean Haemoglobin − Male − Eastern Europe
WHO−2024 and WHO−2001
Early Neonatal
Late Neonatal
1−5 months
6−11 months
12 to 23 months
2 to 4
5 to 9
10 to 14
15 to 19
20 to 24
25 to 29
30 to 34
35 to 39
40 to 44
45 to 49
50 to 54
55 to 59
60 to 64
65 to 69
70 to 74
75 to 79
80 to 84
85 to 89
90 to 94
95 plus
Mean Haemoglobin (g/L)
Year
Model
WHO−2024 ME Results
WHO−2001 ME Results

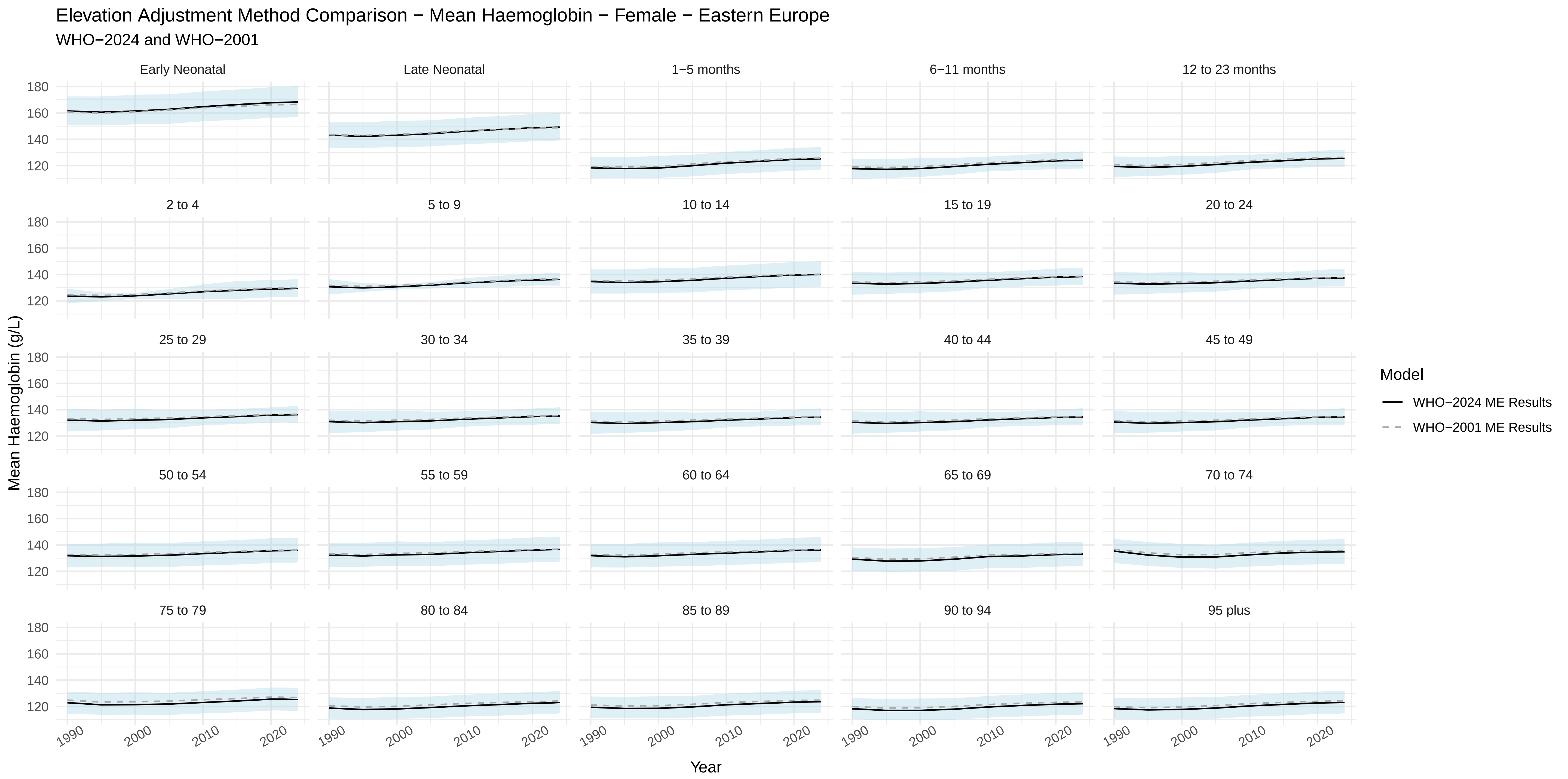
Elevation Adjustment Method Comparison − Mean Haemoglobin − Female − Eastern Europe
WHO−2024 and WHO−2001
Mean Haemoglobin (g/L)
Year
Model
WHO−2024 ME Results
WHO−2001 ME Results

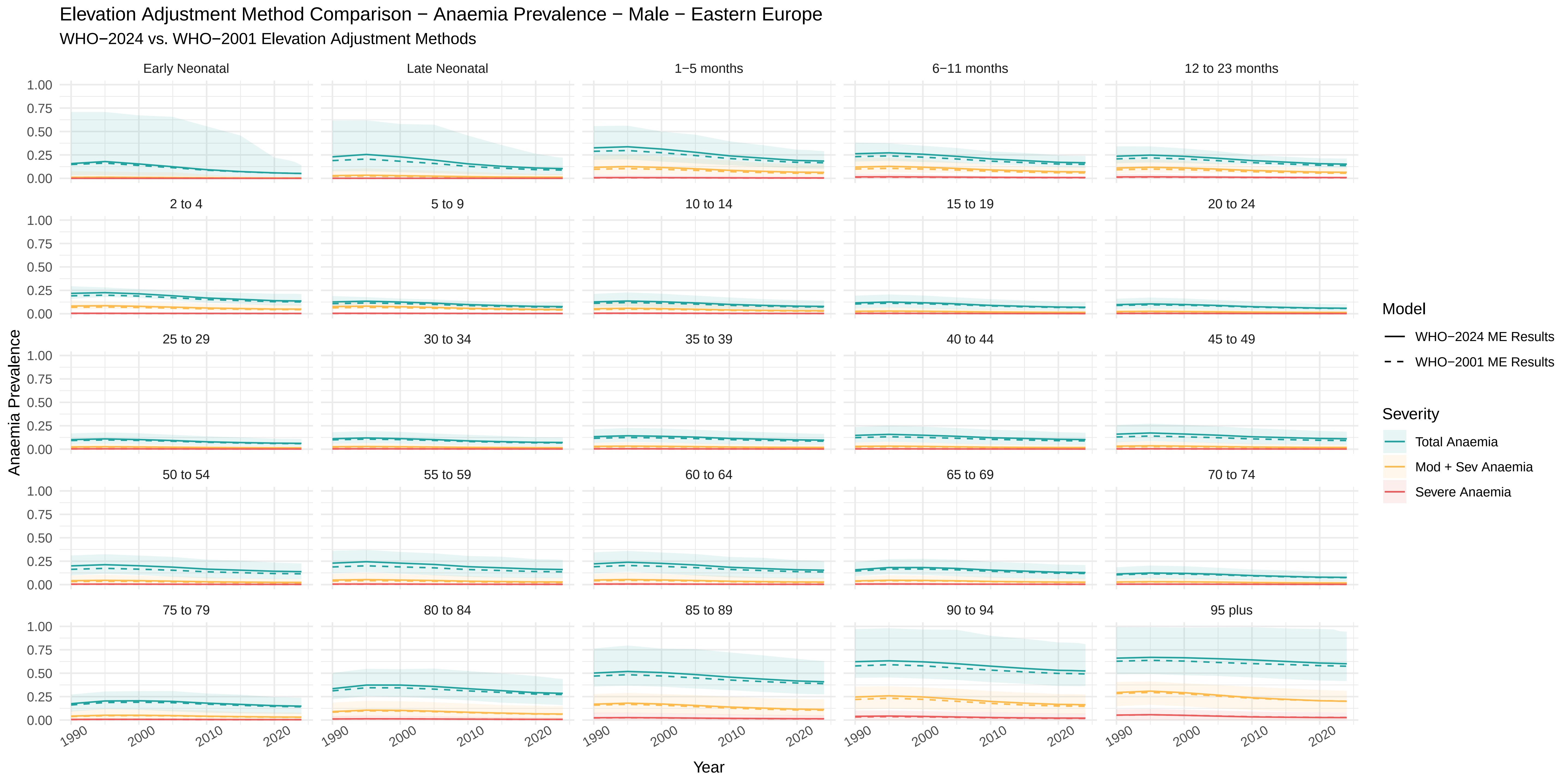
Elevation Adjustment Method Comparison − Anaemia Prevalence − Male − Eastern Europe
WHO−2024 vs. WHO−2001 Elevation Adjustment Methods
Anaemia Prevalence
Year
Model
WHO−2024 ME Results
WHO−2001 ME Results
Severity
Total Anaemia
Mod + Sev Anaemia
Severe Anaemia

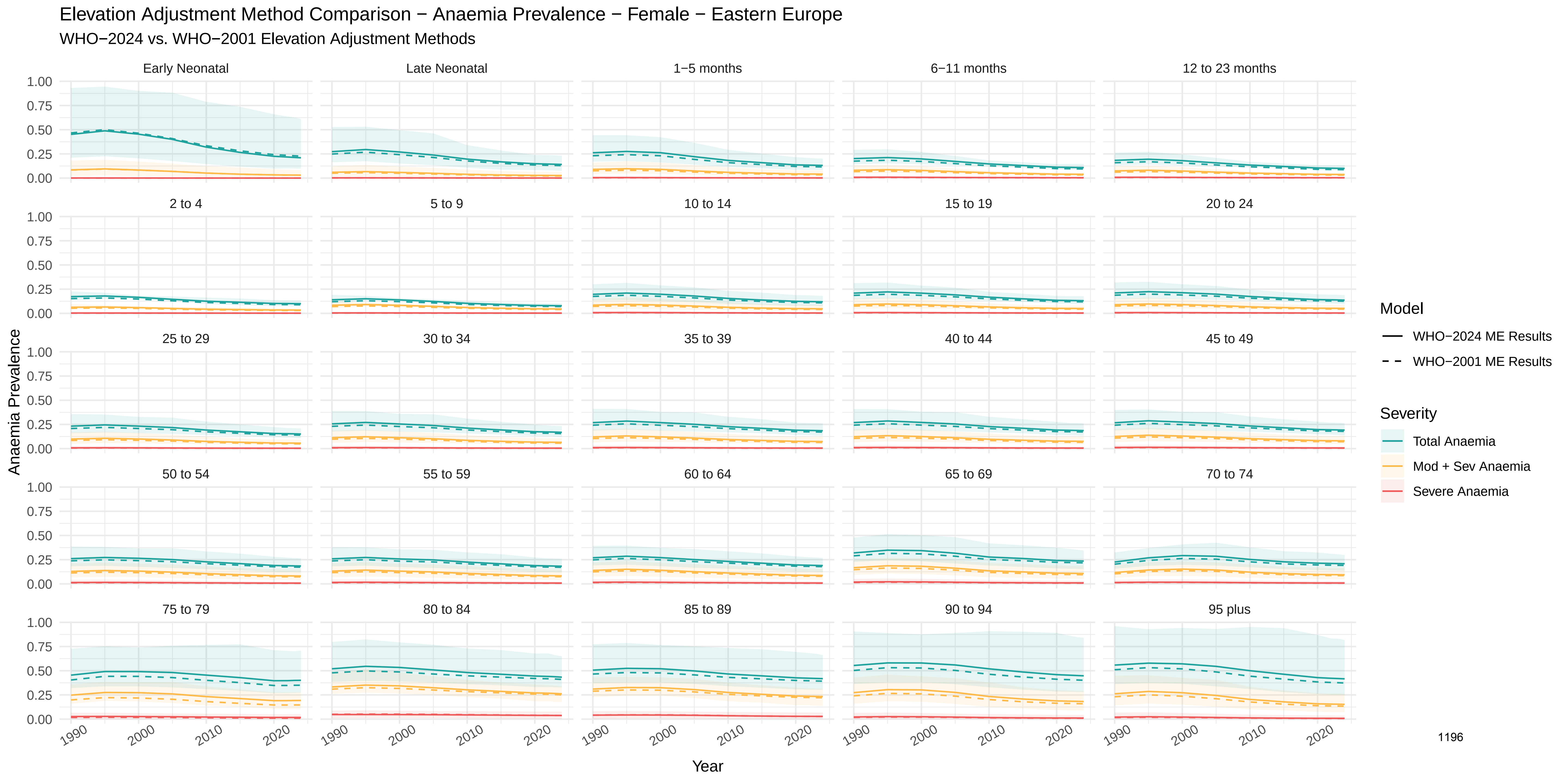
Elevation Adjustment Method Comparison − Anaemia Prevalence − Female − Eastern Europe
WHO−2024 vs. WHO−2001 Elevation Adjustment Methods
Anaemia Prevalence
Year
Model
WHO−2024 ME Results
WHO−2001 ME Results
Severity
Total Anaemia
Mod + Sev Anaemia
Severe Anaemia

Elevation Adjustment Method Comparison − Mean Haemoglobin − Male − Belarus

WHO−2024 and WHO−2001

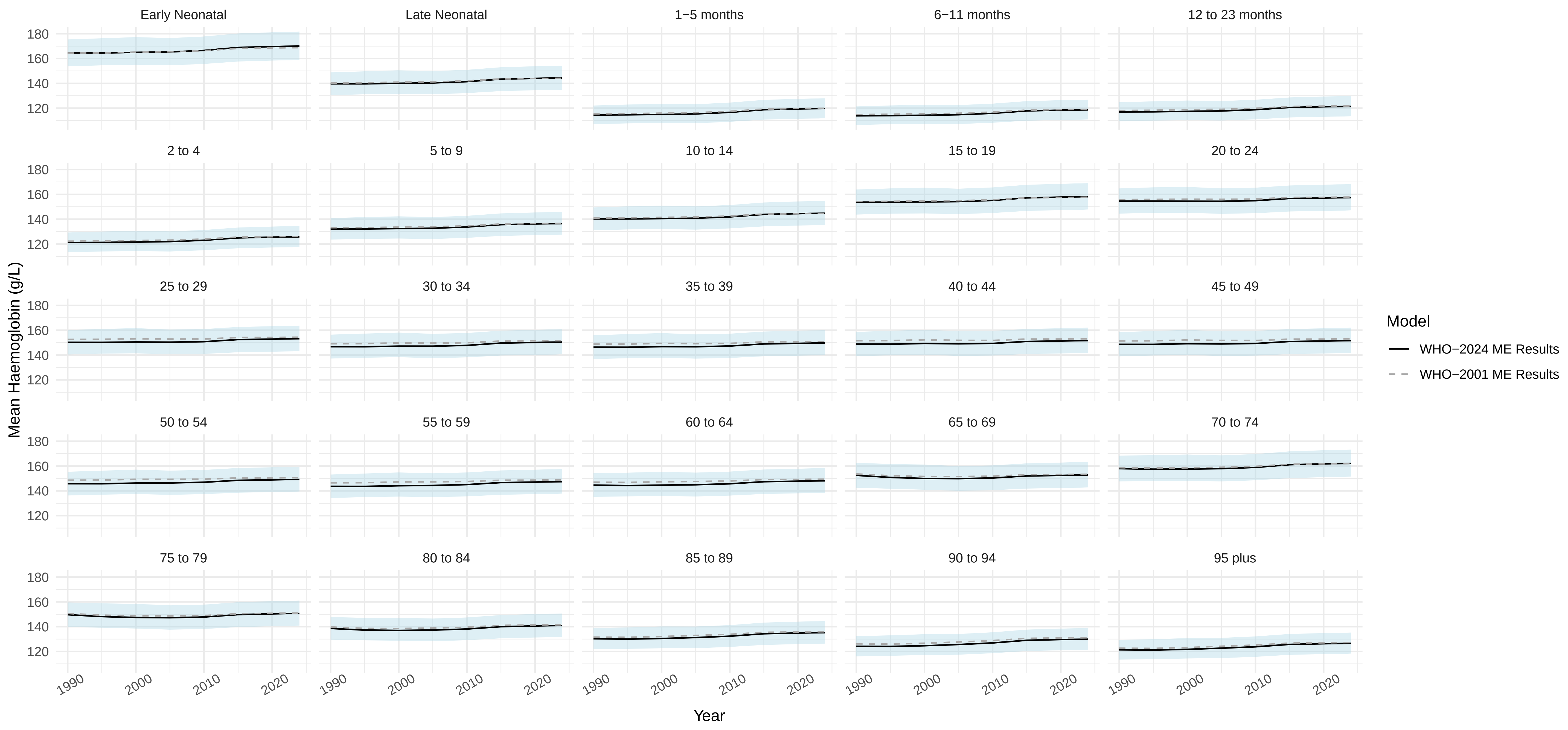

Elevation Adjustment Method Comparison − Mean Haemoglobin − Female − Belarus

WHO−2024 and WHO−2001

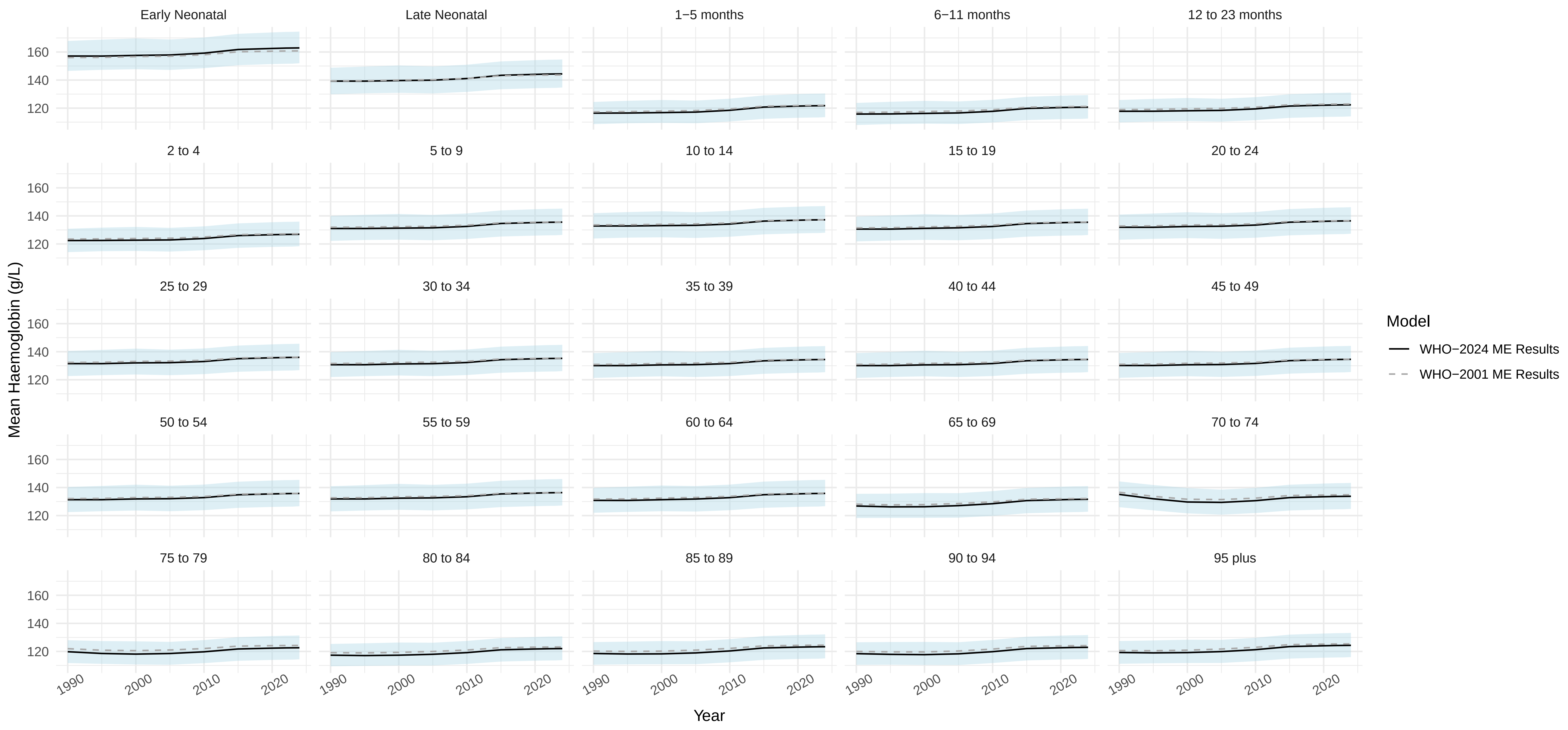

Elevation Adjustment Method Comparison − Anaemia Prevalence − Male − Belarus

WHO−2024 vs. WHO−2001 Elevation Adjustment Methods

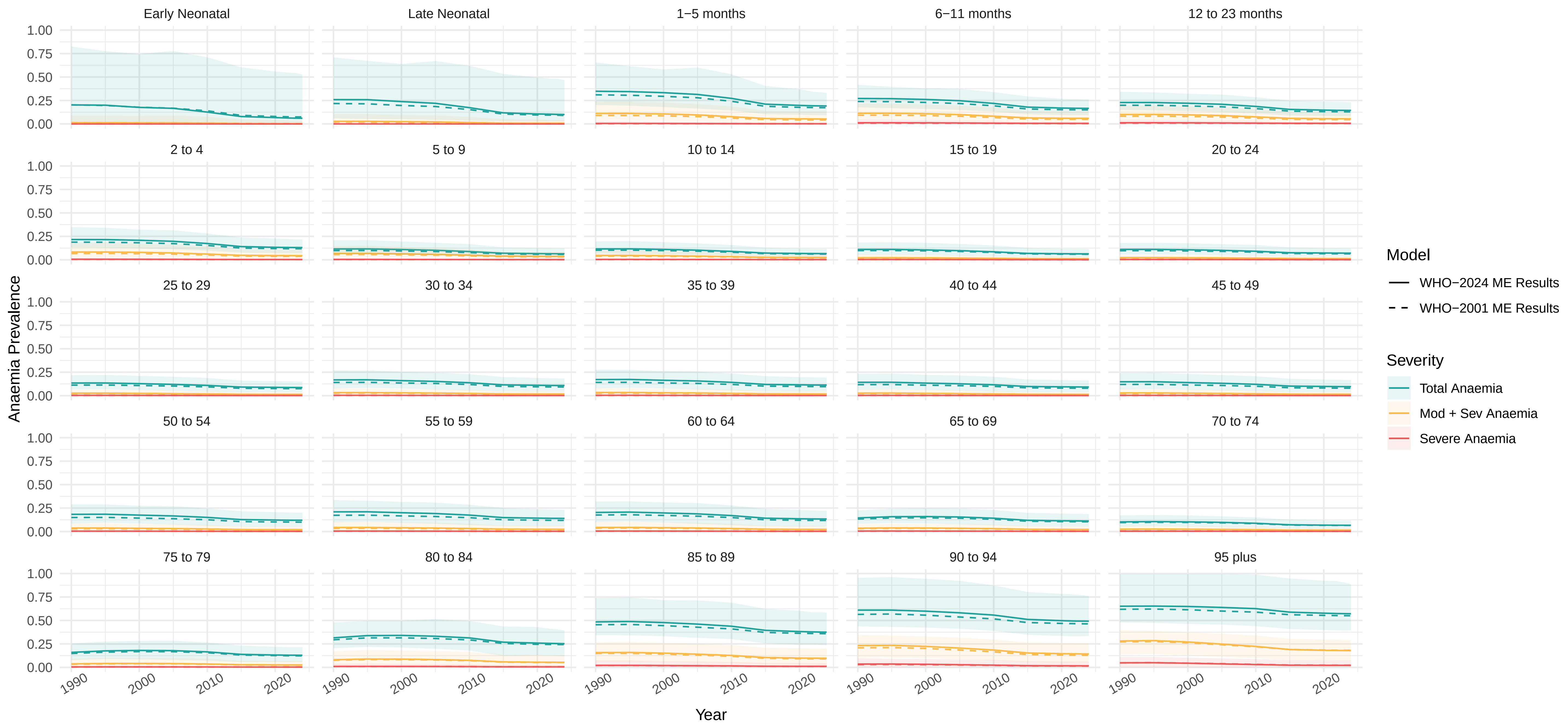

Elevation Adjustment Method Comparison − Anaemia Prevalence − Female − Belarus

WHO−2024 vs. WHO−2001 Elevation Adjustment Methods

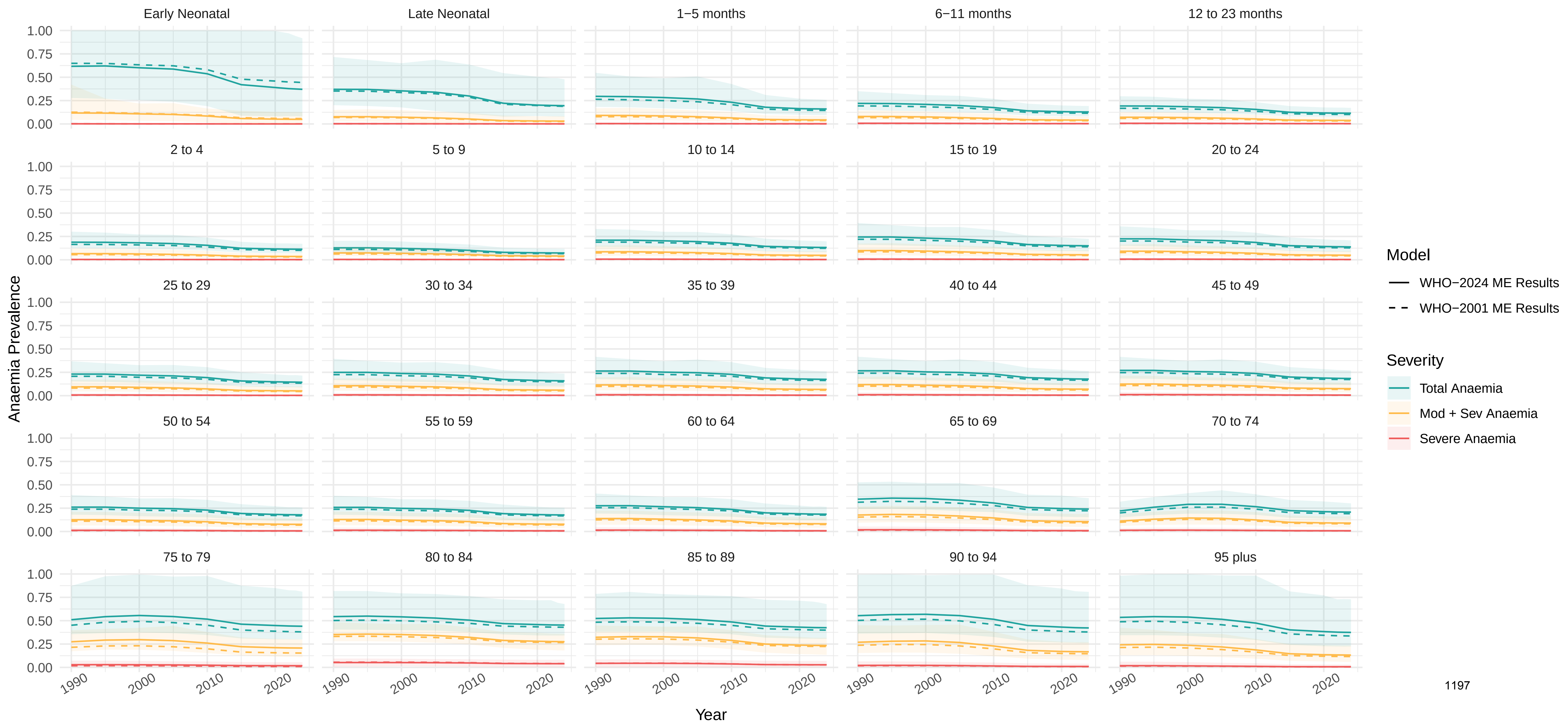

Elevation Adjustment Method Comparison − Mean Haemoglobin − Male − Estonia

WHO−2024 and WHO−2001

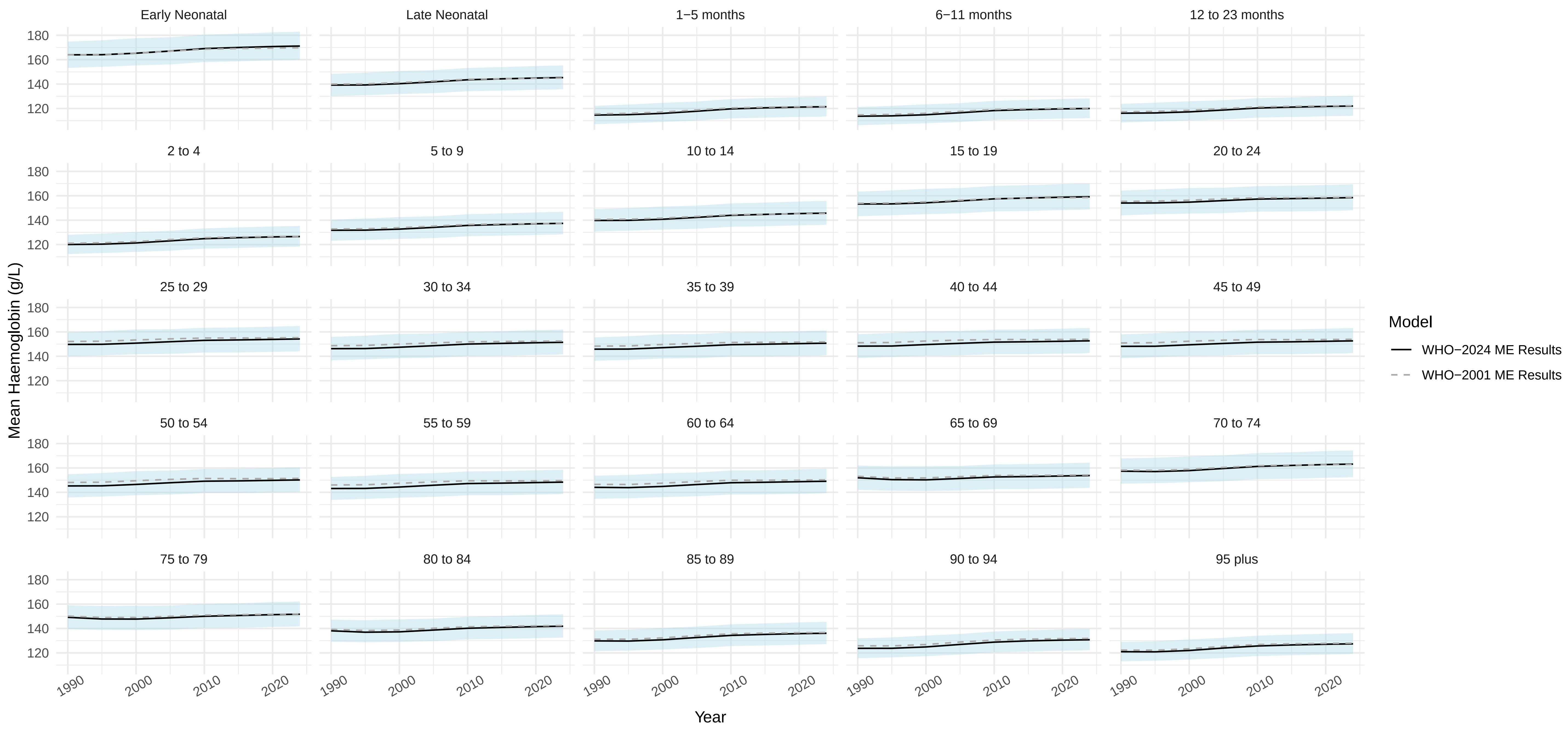

Elevation Adjustment Method Comparison − Mean Haemoglobin − Female − Estonia

WHO−2024 and WHO−2001

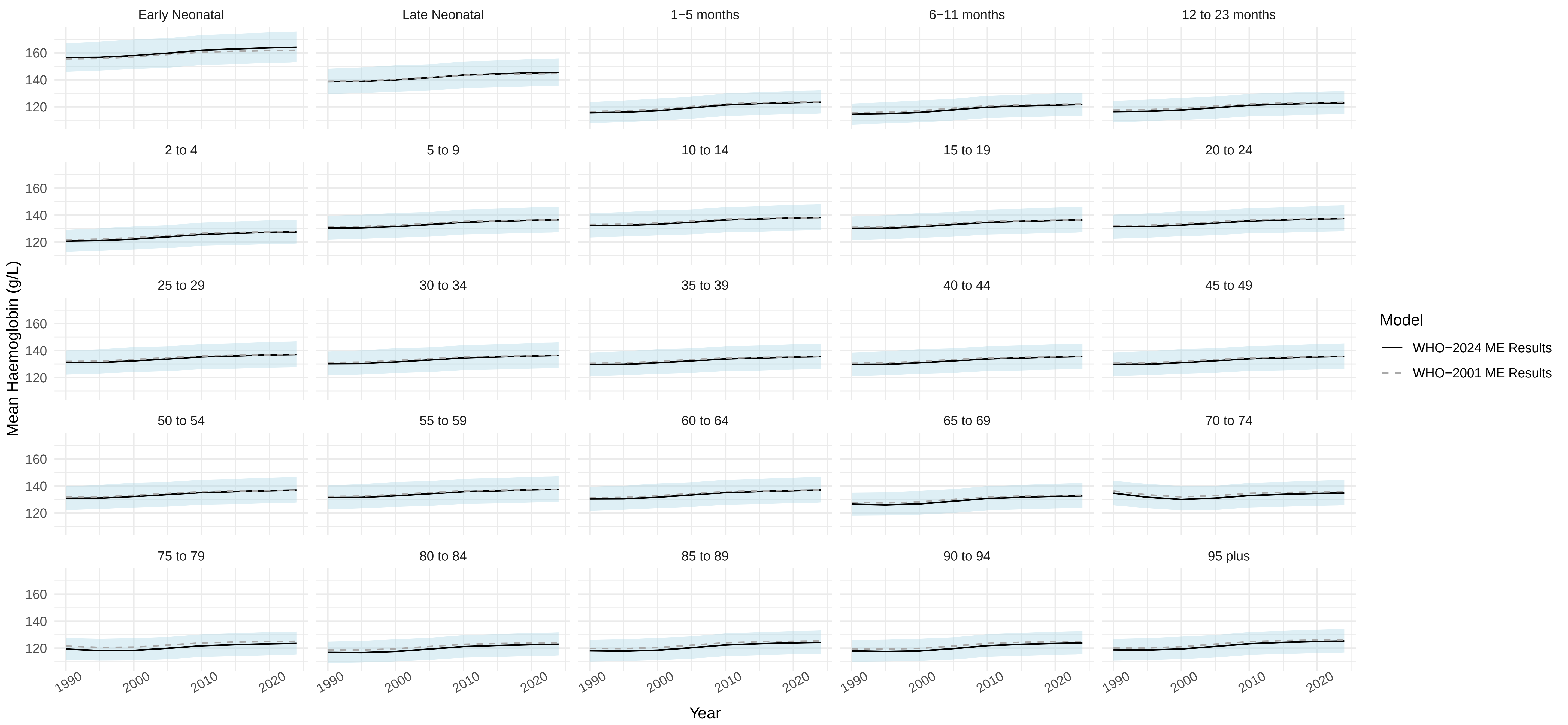

Elevation Adjustment Method Comparison − Anaemia Prevalence − Male − Estonia

WHO−2024 vs. WHO−2001 Elevation Adjustment Methods

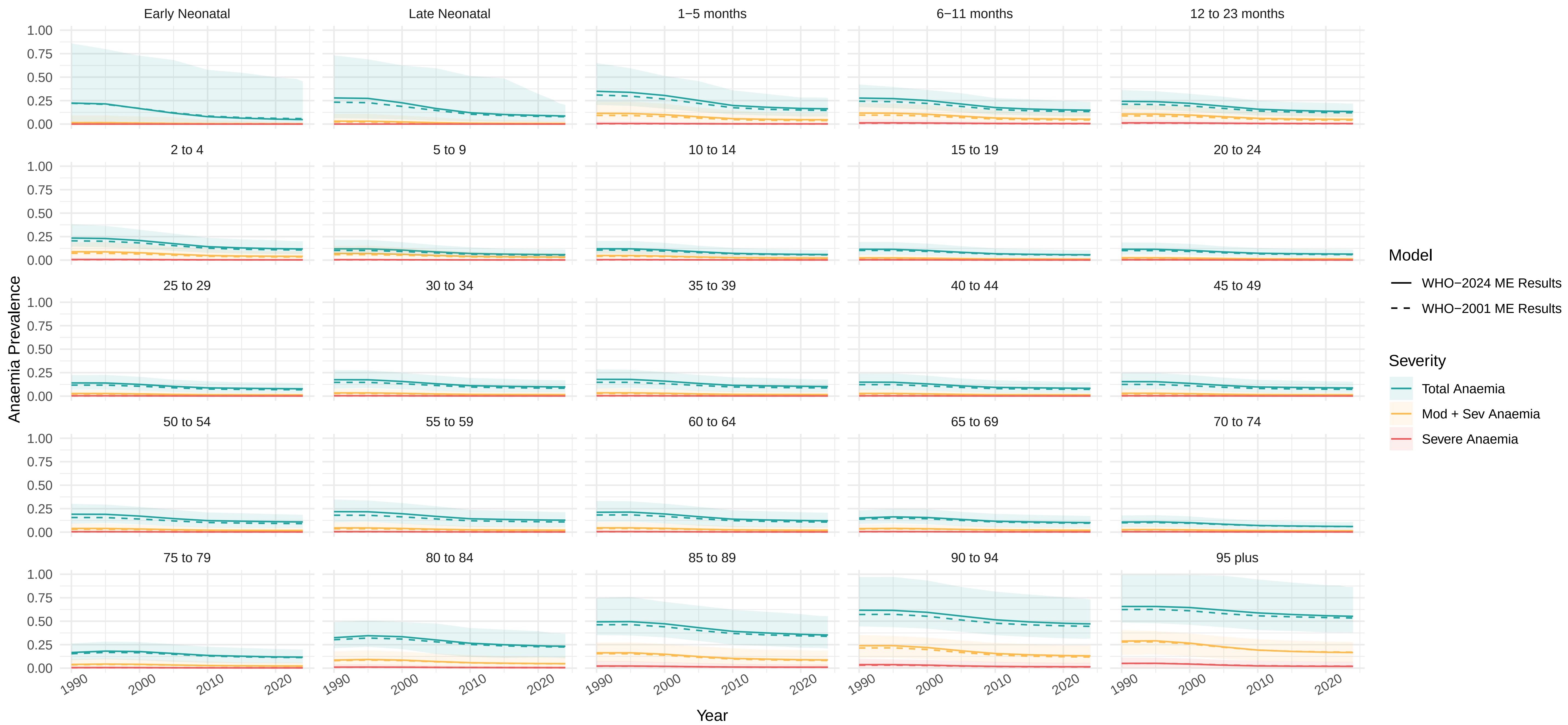

Elevation Adjustment Method Comparison − Anaemia Prevalence − Female − Estonia

WHO−2024 vs. WHO−2001 Elevation Adjustment Methods

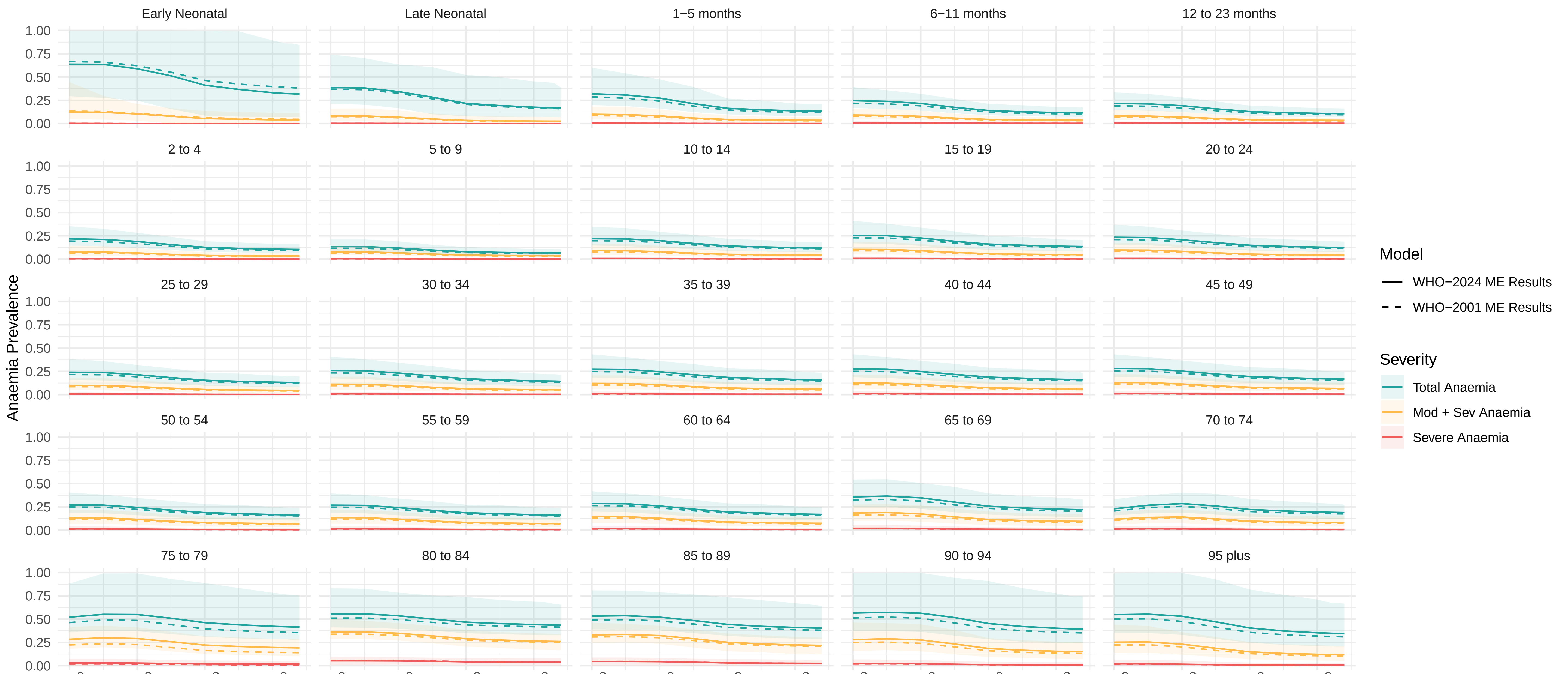

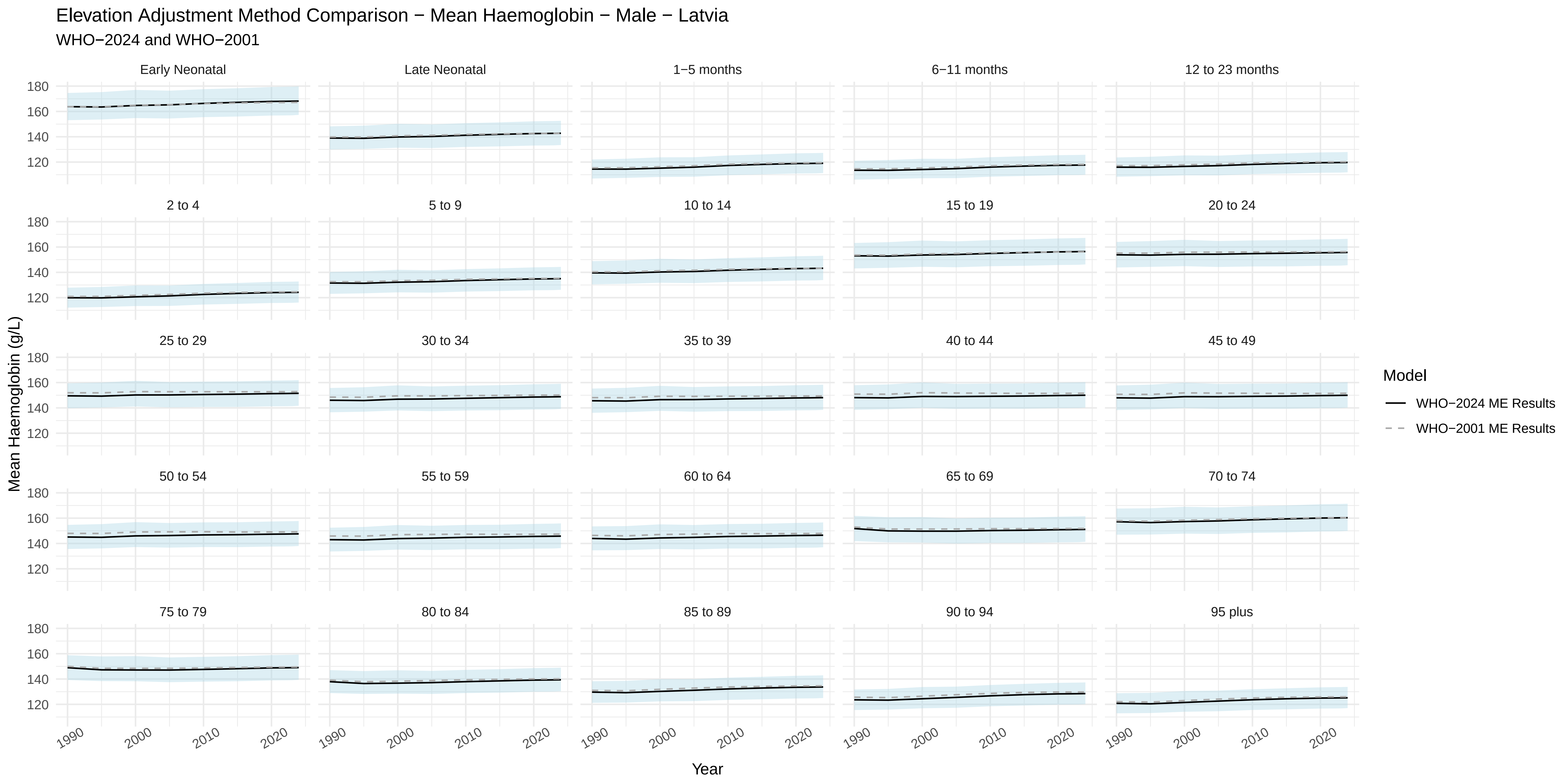
Elevation Adjustment Method Comparison − Mean Haemoglobin − Male − Latvia
WHO−2024 and WHO−2001
Early Neonatal
Late Neonatal
1−5 months
6−11 months
12 to 23 months
2 to 4
5 to 9
10 to 14
15 to 19
20 to 24
25 to 29
30 to 34
35 to 39
40 to 44
45 to 49
50 to 54
55 to 59
60 to 64
65 to 69
70 to 74
75 to 79
80 to 84
85 to 89
90 to 94
95 plus
Mean Haemoglobin (g/L)
Year
Model
WHO−2024 ME Results
WHO−2001 ME Results

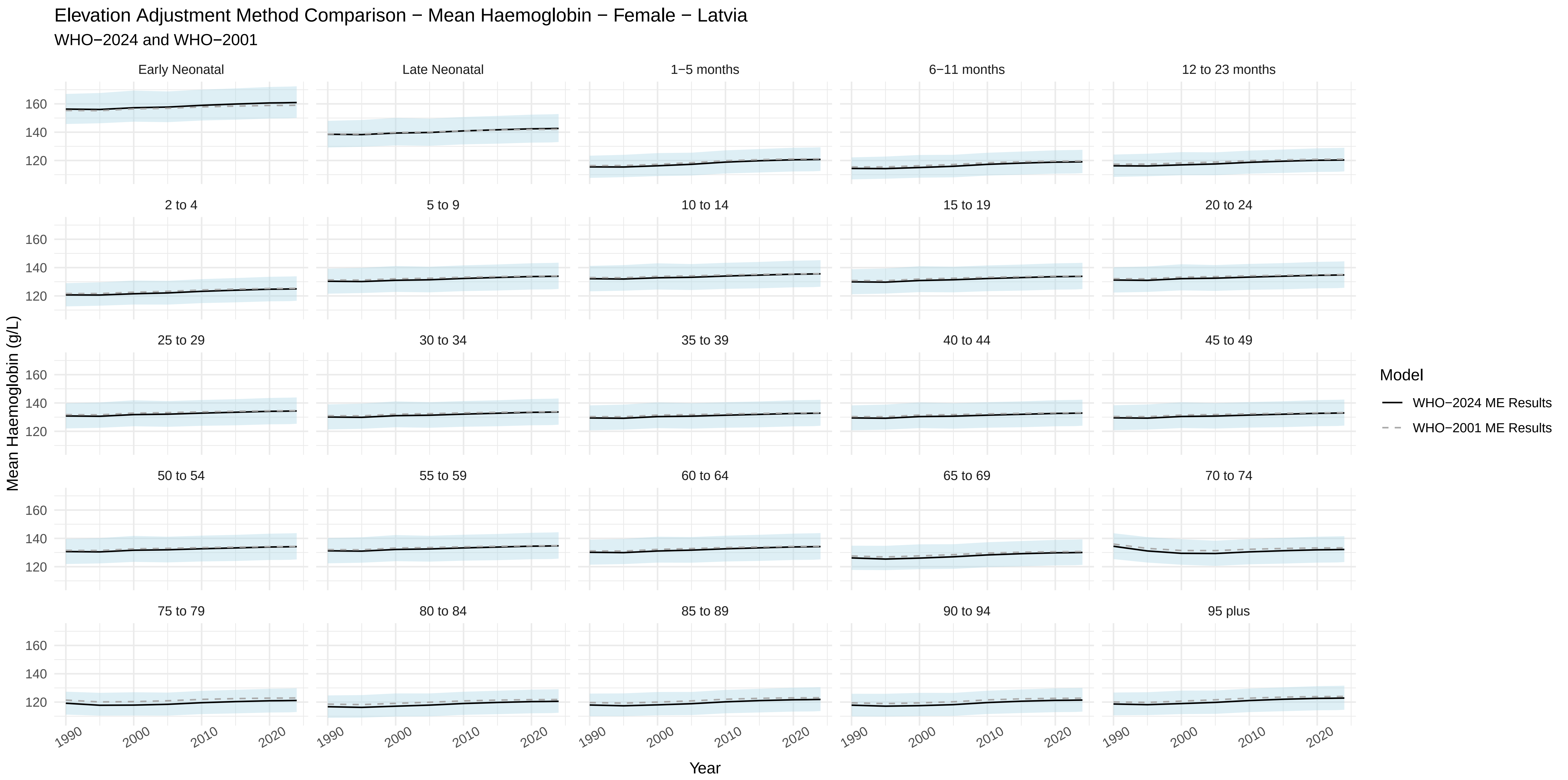
Elevation Adjustment Method Comparison − Mean Haemoglobin − Female − Latvia
WHO−2024 and WHO−2001
Early Neonatal
Late Neonatal
1−5 months
6−11 months
12 to 23 months
2 to 4
5 to 9
10 to 14
15 to 19
20 to 24
25 to 29
30 to 34
35 to 39
40 to 44
45 to 49
50 to 54
55 to 59
60 to 64
65 to 69
70 to 74
75 to 79
80 to 84
85 to 89
90 to 94
95 plus
Mean Haemoglobin (g/L)
Year
Model
WHO−2024 ME Results
WHO−2001 ME Results

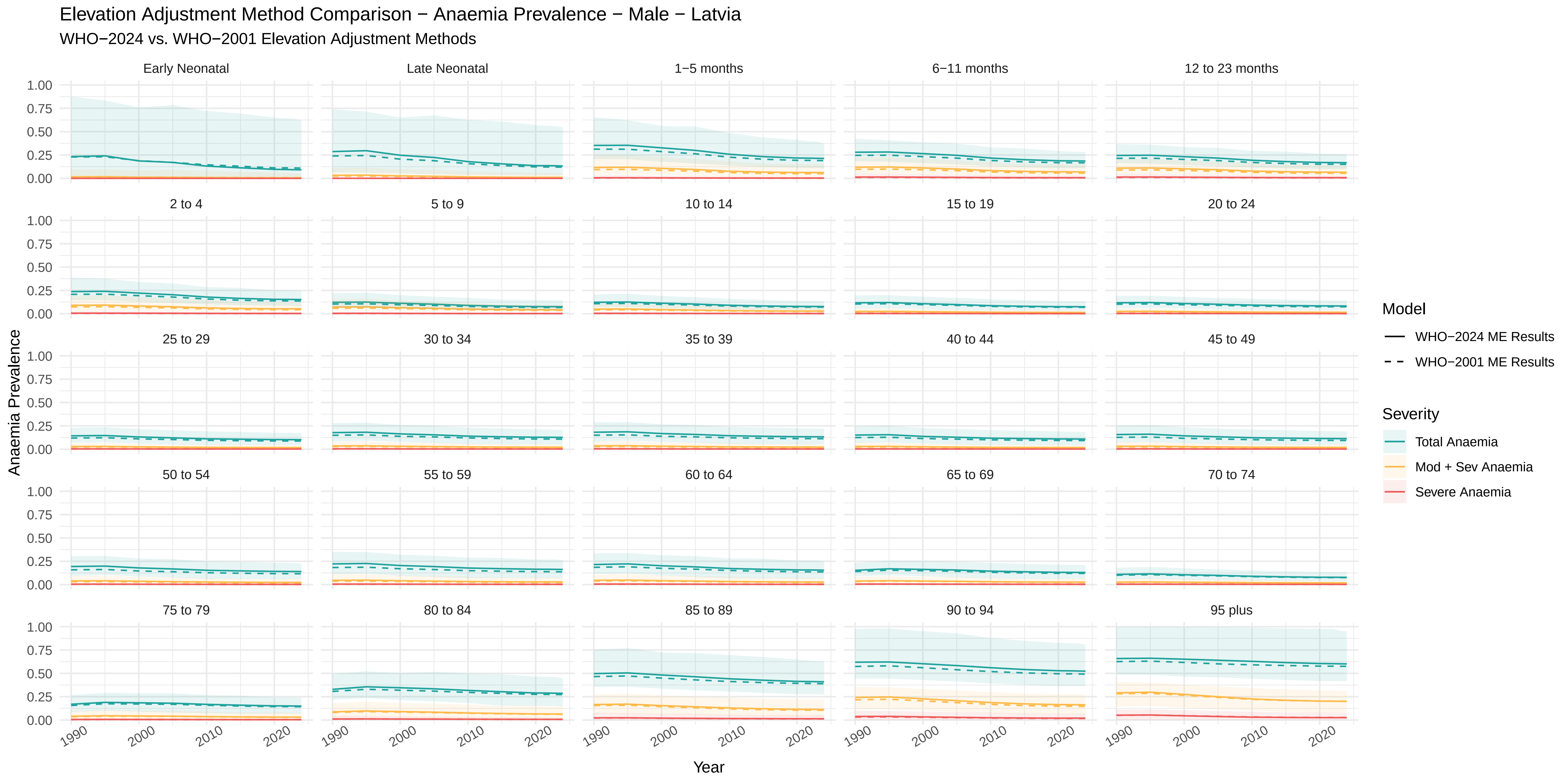
Elevation Adjustment Method Comparison − Anaemia Prevalence − Male − Latvia
WHO−2024 vs. WHO−2001 Elevation Adjustment Methods
Early Neonatal
Late Neonatal
1−5 months
6−11 months
12 to 23 months
2 to 4
5 to 9
10 to 14
15 to 19
20 to 24
25 to 29
30 to 34
35 to 39
40 to 44
45 to 49
50 to 54
55 to 59
60 to 64
65 to 69
70 to 74
75 to 79
80 to 84
85 to 89
90 to 94
95 plus
Anaemia Prevalence
Year
Model
WHO−2024 ME Results
WHO−2001 ME Results
Severity
Total Anaemia
Mod + Sev Anaemia
Severe Anaemia

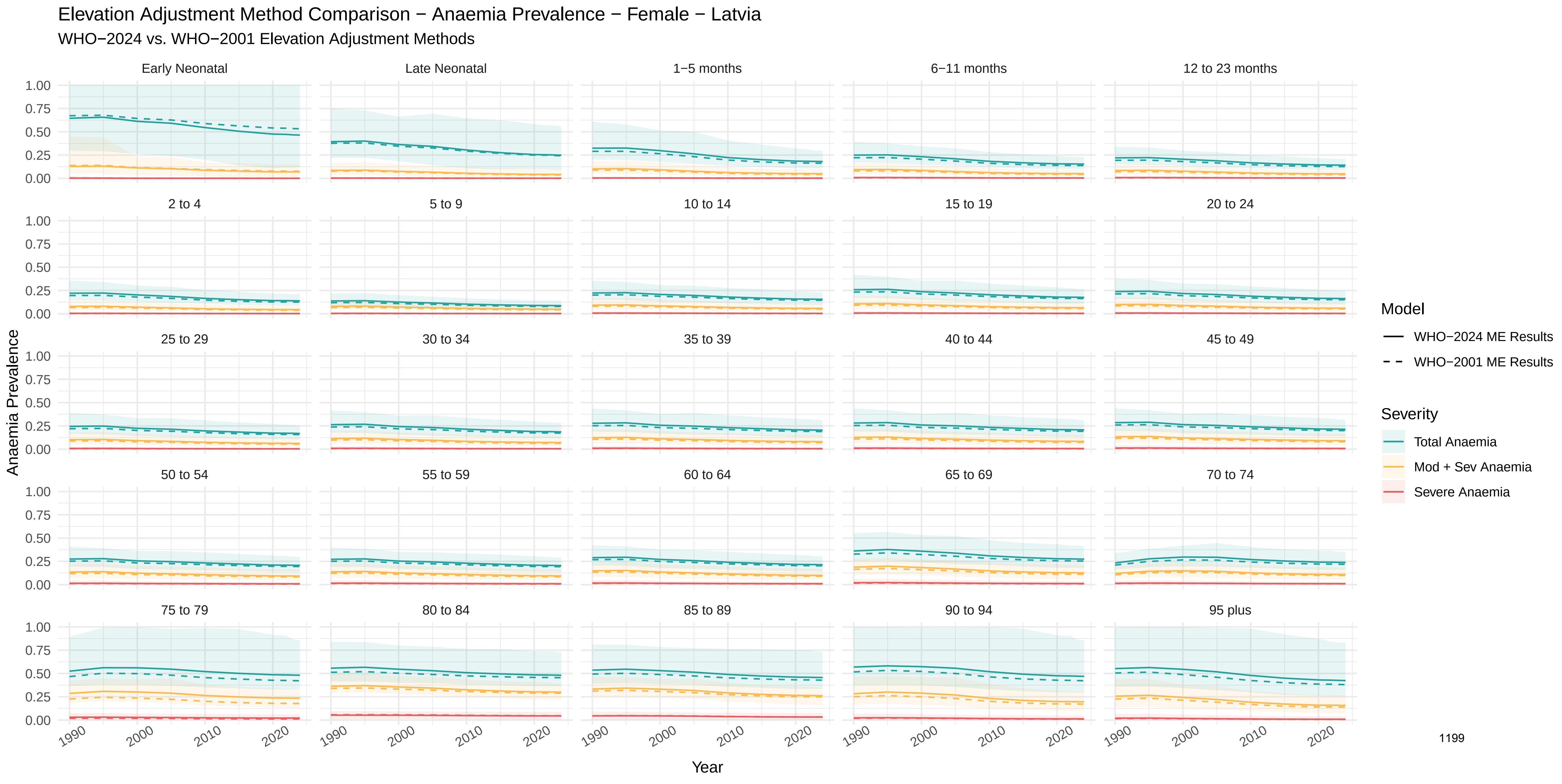
Elevation Adjustment Method Comparison − Anaemia Prevalence − Female − Latvia
WHO−2024 vs. WHO−2001 Elevation Adjustment Methods
Early Neonatal
Late Neonatal
1−5 months
6−11 months
12 to 23 months
2 to 4
5 to 9
10 to 14
15 to 19
20 to 24
25 to 29
30 to 34
35 to 39
40 to 44
45 to 49
50 to 54
55 to 59
60 to 64
65 to 69
70 to 74
75 to 79
80 to 84
85 to 89
90 to 94
95 plus
Anaemia Prevalence
Year
Model
WHO−2024 ME Results
WHO−2001 ME Results
Severity
Total Anaemia
Mod + Sev Anaemia
Severe Anaemia

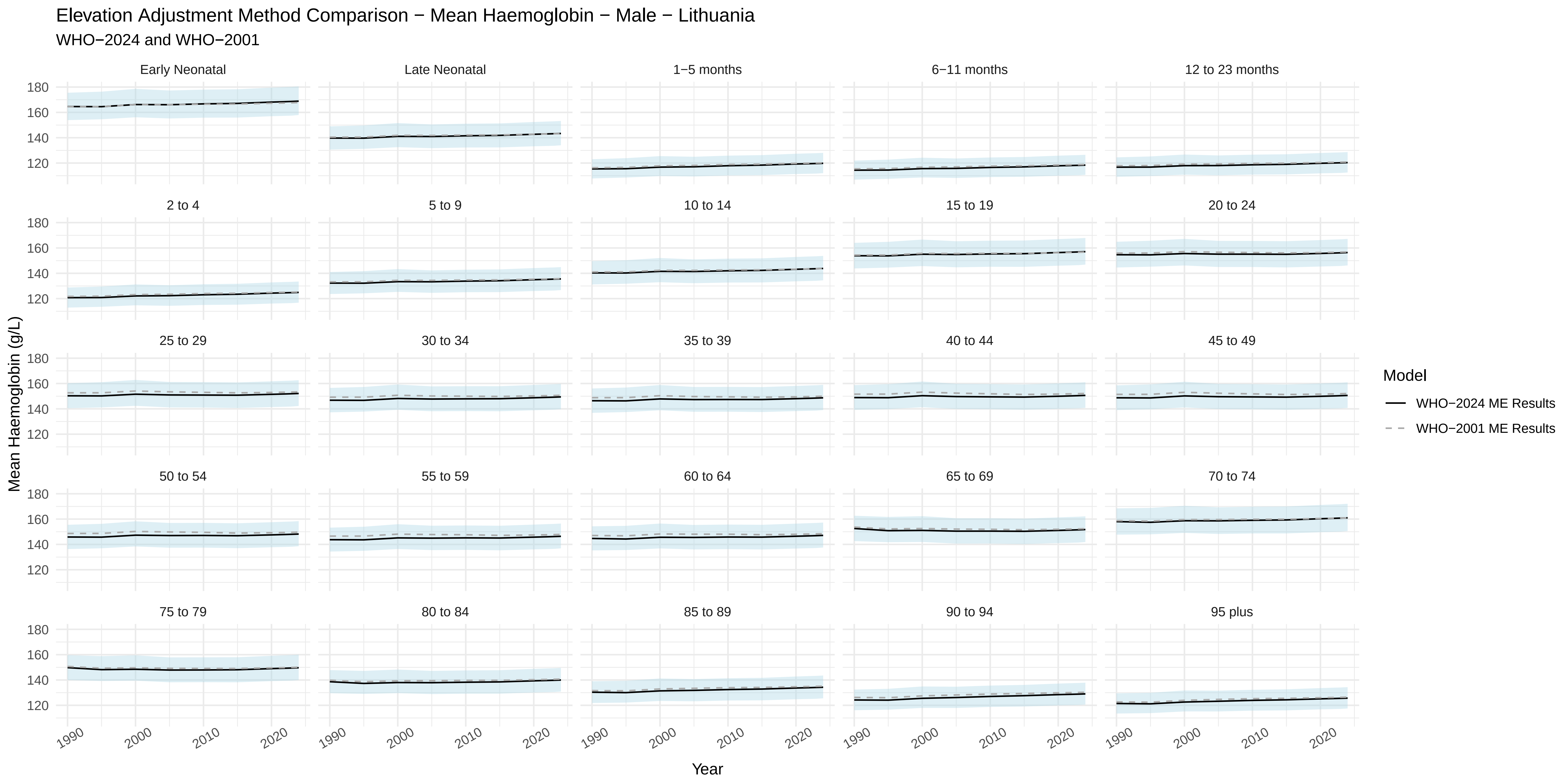
Elevation Adjustment Method Comparison − Mean Haemoglobin − Male − Lithuania
WHO−2024 and WHO−2001
Mean Haemoglobin (g/L)
Year
Model
WHO−2024 ME Results
WHO−2001 ME Results

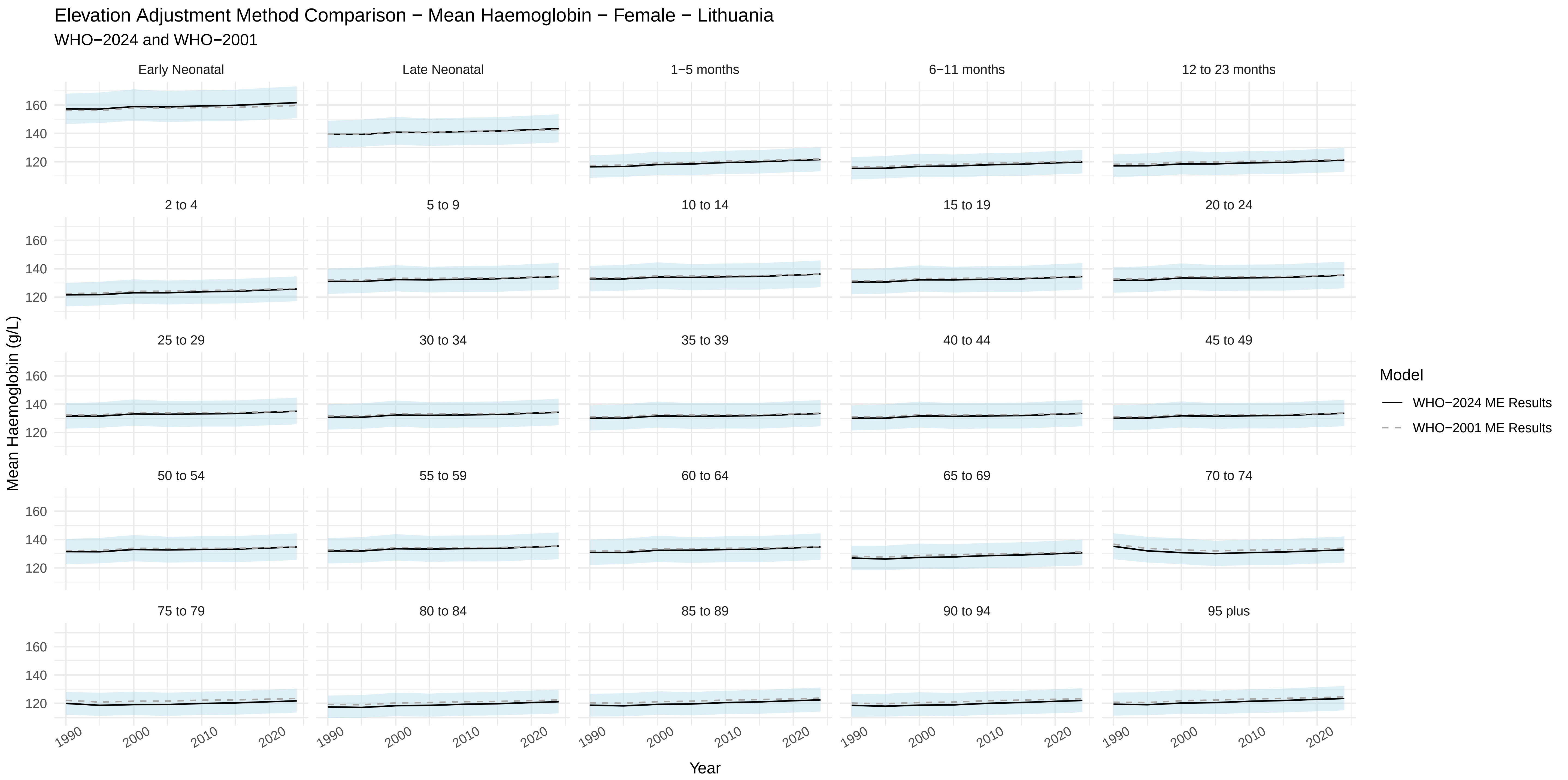
Elevation Adjustment Method Comparison − Mean Haemoglobin − Female − Lithuania
WHO−2024 and WHO−2001
Mean Haemoglobin (g/L)
Year
Model
WHO−2024 ME Results
WHO−2001 ME Results

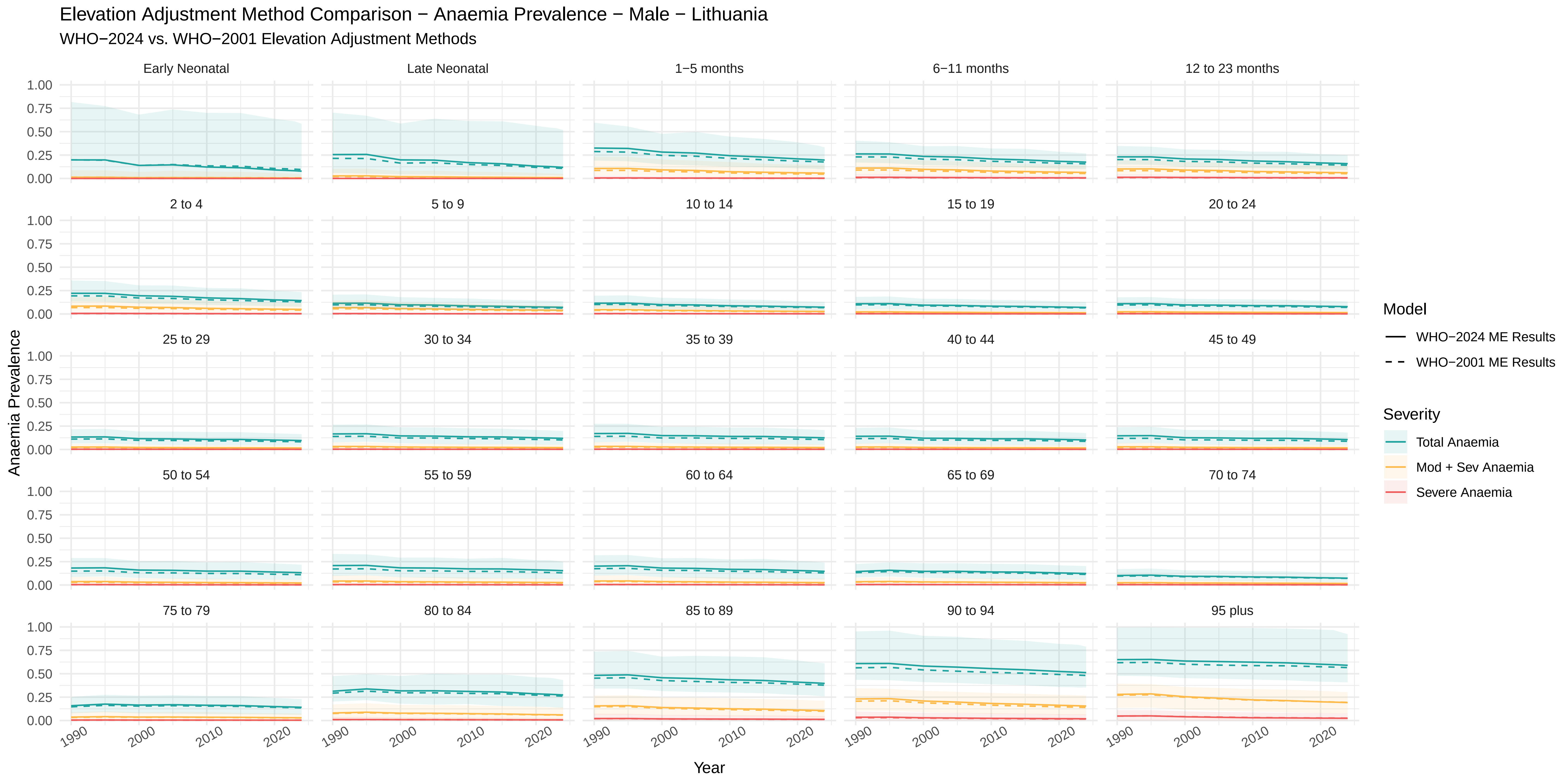
Elevation Adjustment Method Comparison − Anaemia Prevalence − Male − Lithuania
WHO−2024 vs. WHO−2001 Elevation Adjustment Methods
Anaemia Prevalence
Year
Model
WHO−2024 ME Results
WHO−2001 ME Results
Severity
Total Anaemia
Mod + Sev Anaemia
Severe Anaemia

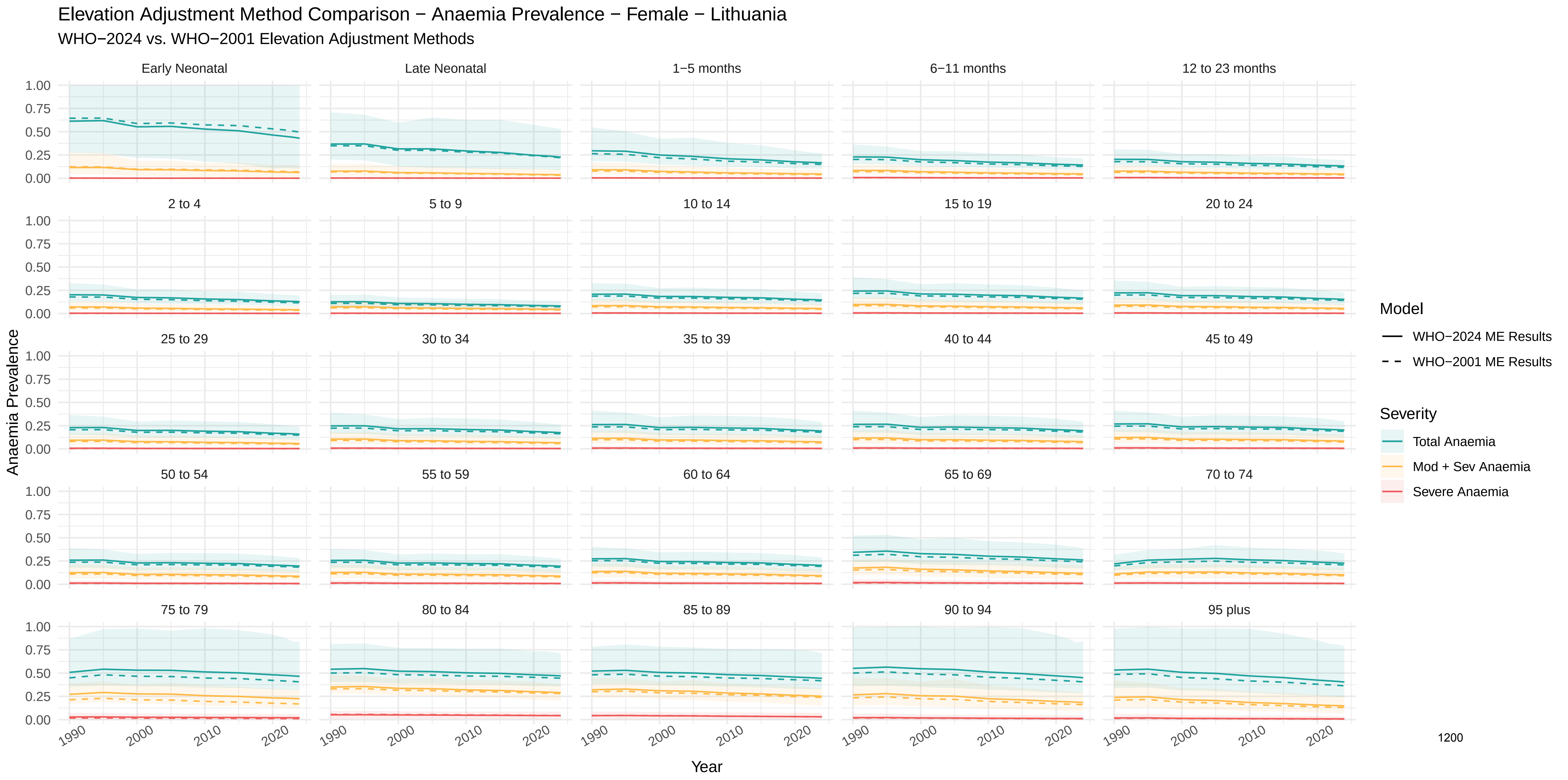
Elevation Adjustment Method Comparison − Anaemia Prevalence − Female − Lithuania
WHO−2024 vs. WHO−2001 Elevation Adjustment Methods
Anaemia Prevalence
Year
Model
WHO−2024 ME Results
WHO−2001 ME Results
Severity
Total Anaemia
Mod + Sev Anaemia
Severe Anaemia

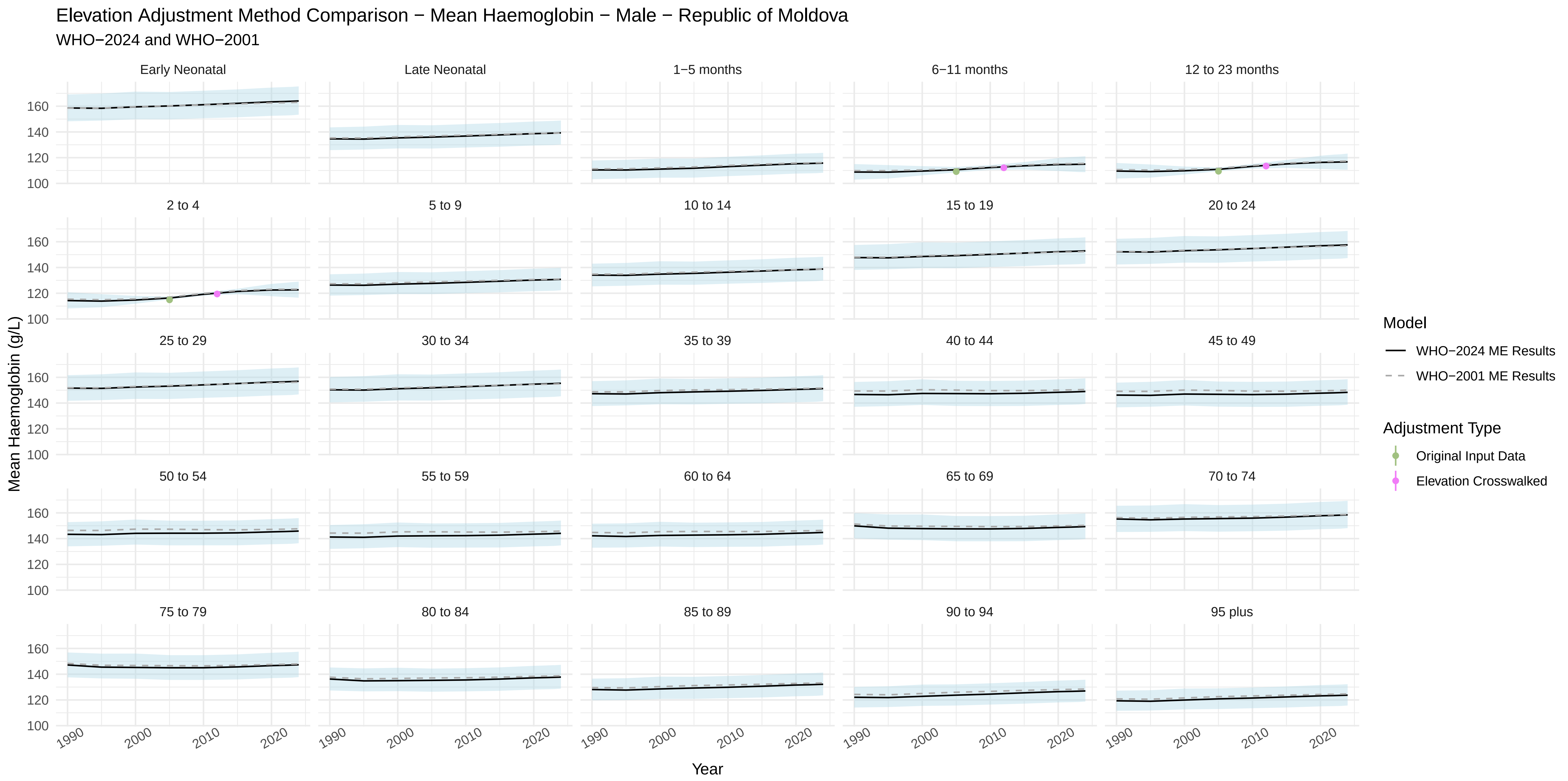
Elevation Adjustment Method Comparison − Mean Haemoglobin − Male − Republic of Moldova
WHO−2024 and WHO−2001
Early Neonatal
Late Neonatal
1−5 months
6−11 months
12 to 23 months
2 to 4
5 to 9
10 to 14
15 to 19
20 to 24
25 to 29
30 to 34
35 to 39
40 to 44
45 to 49
50 to 54
55 to 59
60 to 64
65 to 69
70 to 74
75 to 79
80 to 84
85 to 89
90 to 94
95 plus
Mean Haemoglobin (g/L)
Year
Model
WHO−2024 ME Results
WHO−2001 ME Results
Adjustment Type
Original Input Data
Elevation Crosswalked

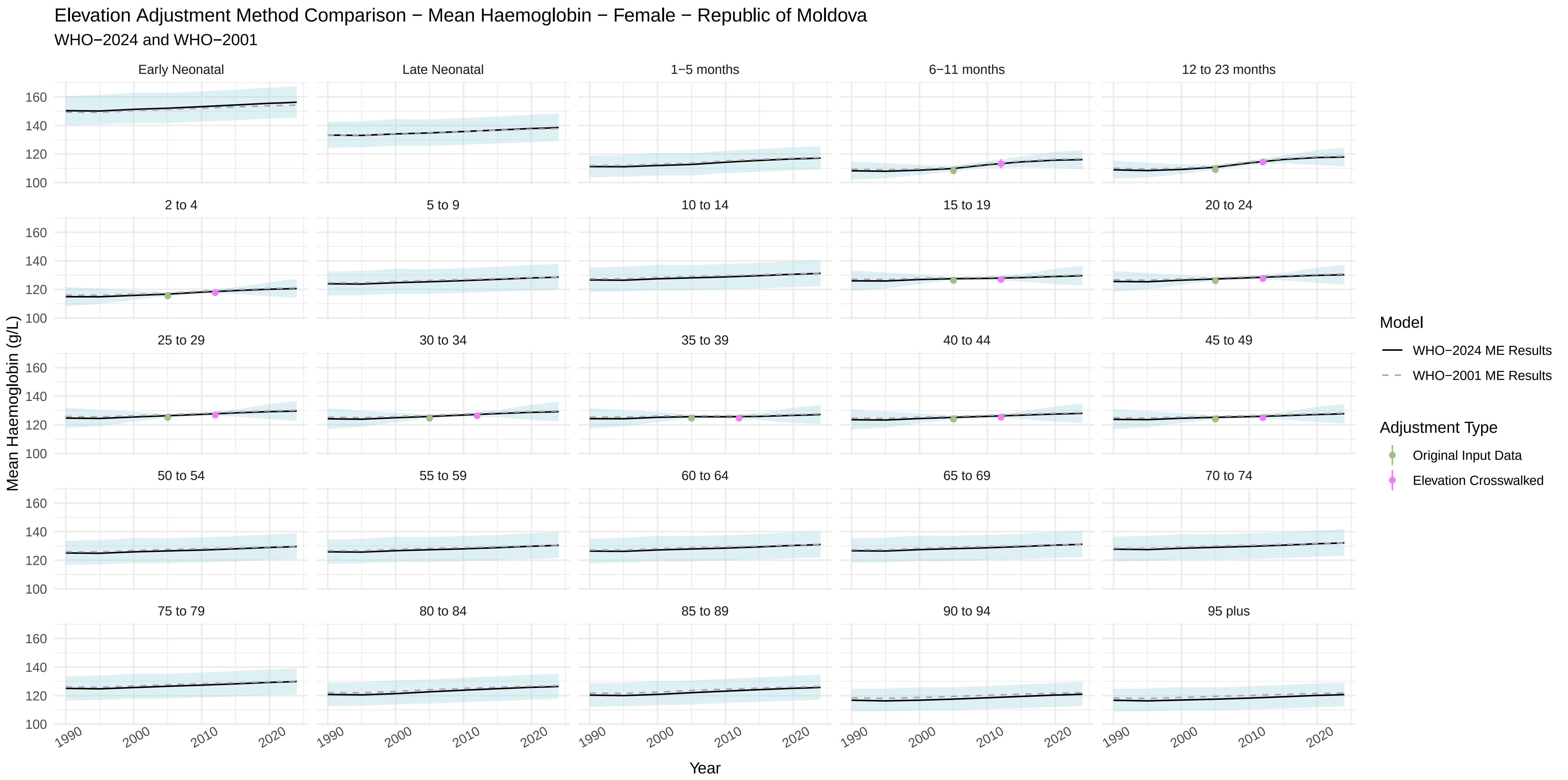
Elevation Adjustment Method Comparison − Mean Haemoglobin − Female − Republic of Moldova
WHO−2024 and WHO−2001
Early Neonatal
Late Neonatal
1−5 months
6−11 months
12 to 23 months
2 to 4
5 to 9
10 to 14
15 to 19
20 to 24
25 to 29
30 to 34
35 to 39
40 to 44
45 to 49
50 to 54
55 to 59
60 to 64
65 to 69
70 to 74
75 to 79
80 to 84
85 to 89
90 to 94
95 plus
Mean Haemoglobin (g/L)
Year
Model
WHO−2024 ME Results
WHO−2001 ME Results
Adjustment Type
Original Input Data
Elevation Crosswalked

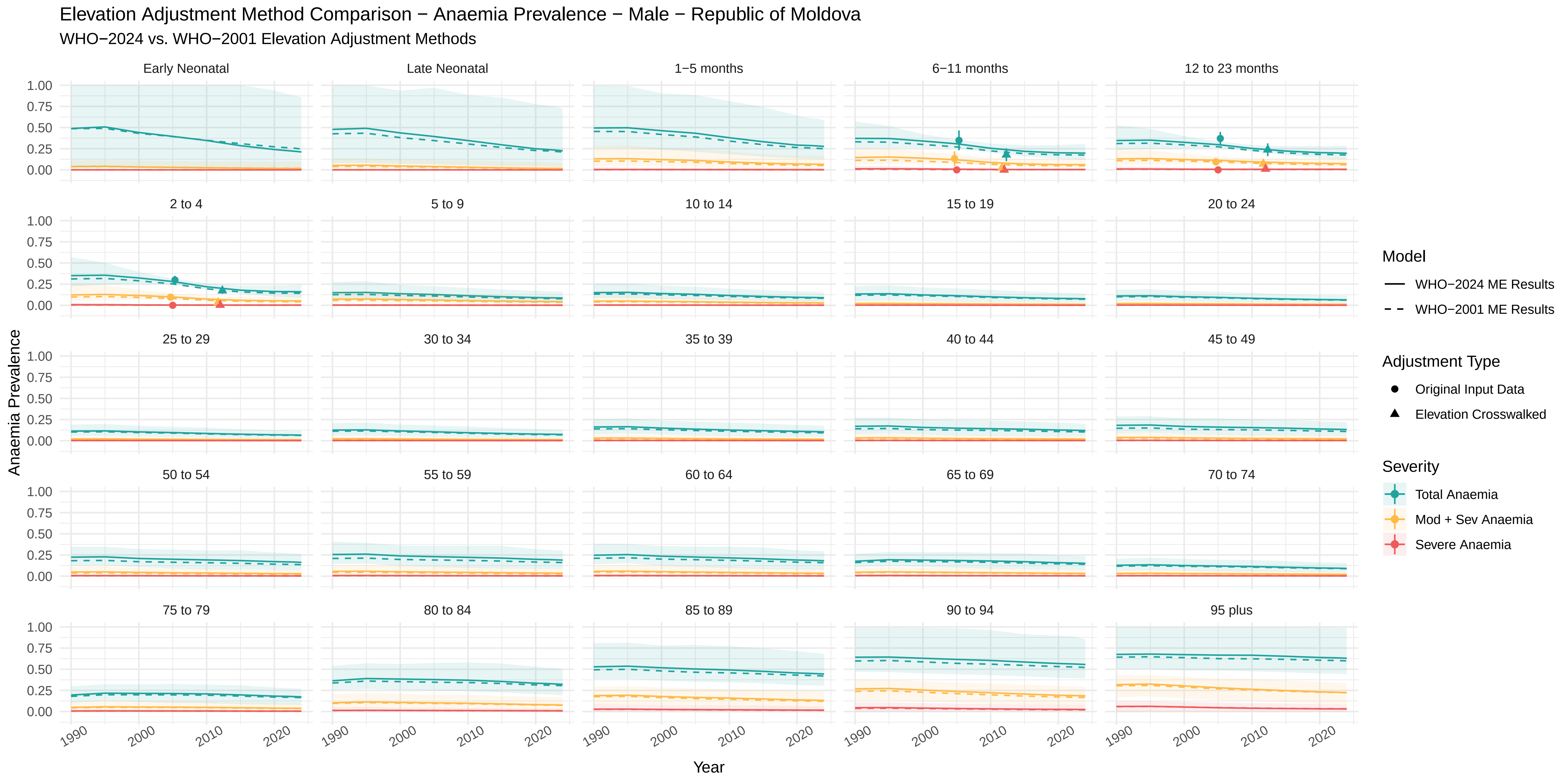
Elevation Adjustment Method Comparison − Anaemia Prevalence − Male − Republic of Moldova
WHO−2024 vs. WHO−2001 Elevation Adjustment Methods
Early Neonatal
Late Neonatal
1−5 months
6−11 months
12 to 23 months
2 to 4
5 to 9
10 to 14
15 to 19
20 to 24
25 to 29
30 to 34
35 to 39
40 to 44
45 to 49
50 to 54
55 to 59
60 to 64
65 to 69
70 to 74
75 to 79
80 to 84
85 to 89
90 to 94
95 plus
Anaemia Prevalence
Year
Model
WHO−2024 ME Results
WHO−2001 ME Results
Adjustment Type
Original Input Data
Elevation Crosswalked
Severity
Total Anaemia
Mod + Sev Anaemia
Severe Anaemia

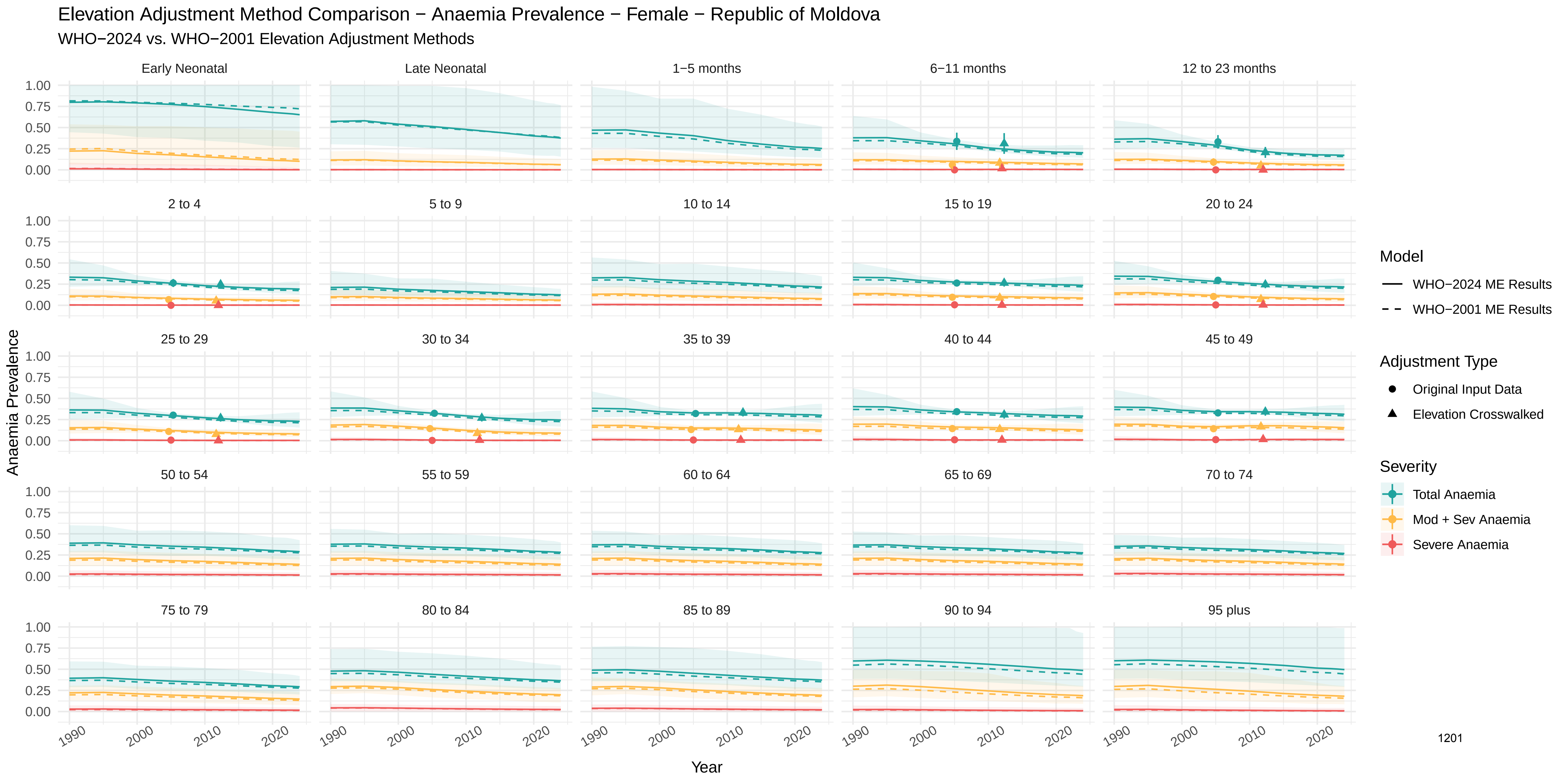
Elevation Adjustment Method Comparison − Anaemia Prevalence − Female − Republic of Moldova
WHO−2024 vs. WHO−2001 Elevation Adjustment Methods
Early Neonatal
Late Neonatal
1−5 months
6−11 months
12 to 23 months
2 to 4
5 to 9
10 to 14
15 to 19
20 to 24
25 to 29
30 to 34
35 to 39
40 to 44
45 to 49
50 to 54
55 to 59
60 to 64
65 to 69
70 to 74
75 to 79
80 to 84
85 to 89
90 to 94
95 plus
Anaemia Prevalence
Year
Model
WHO−2024 ME Results
WHO−2001 ME Results
Adjustment Type
Original Input Data
Elevation Crosswalked
Severity
Total Anaemia
Mod + Sev Anaemia
Severe Anaemia

Elevation Adjustment Method Comparison − Mean Haemoglobin − Male − Russian Federation

WHO−2024 and WHO−2001

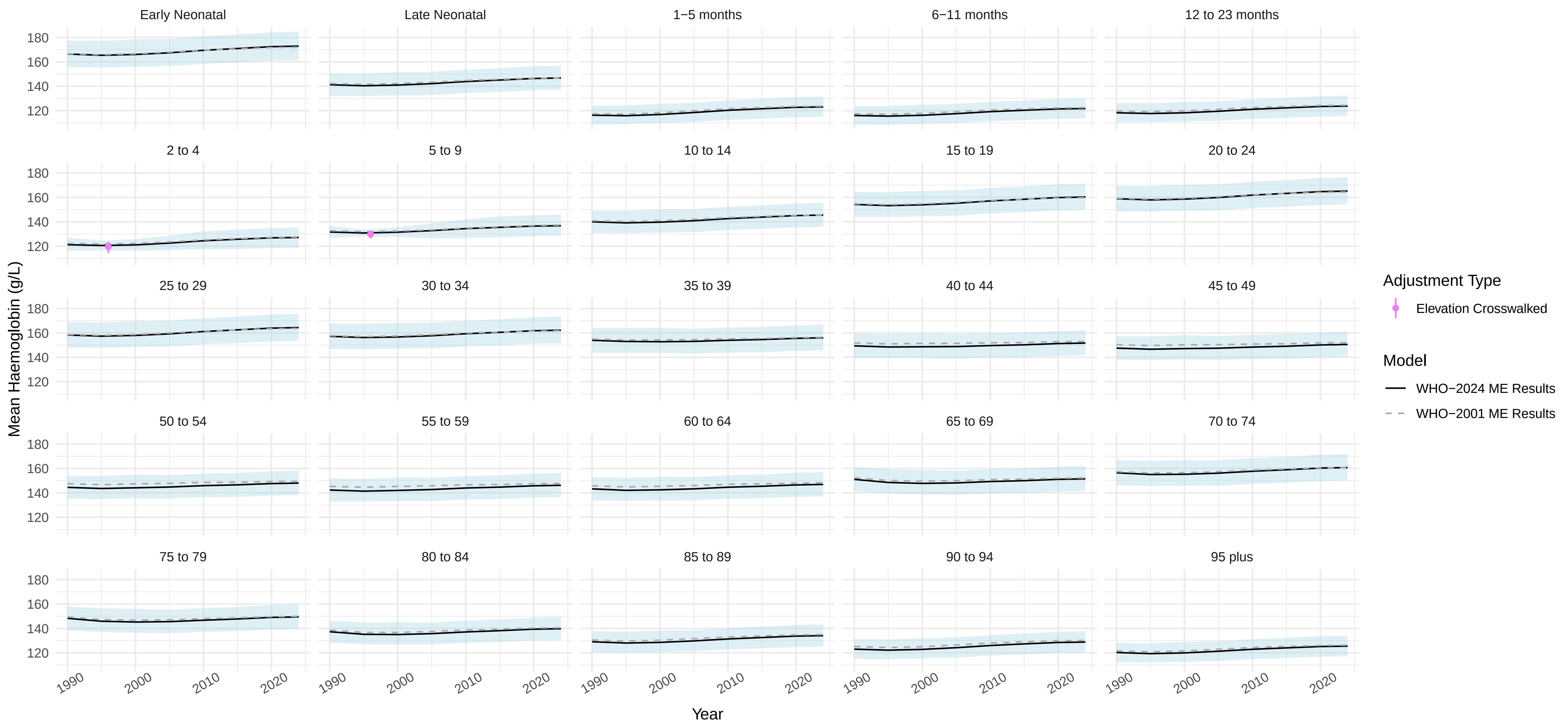

Elevation Adjustment Method Comparison − Mean Haemoglobin − Female − Russian Federation

WHO−2024 and WHO−2001

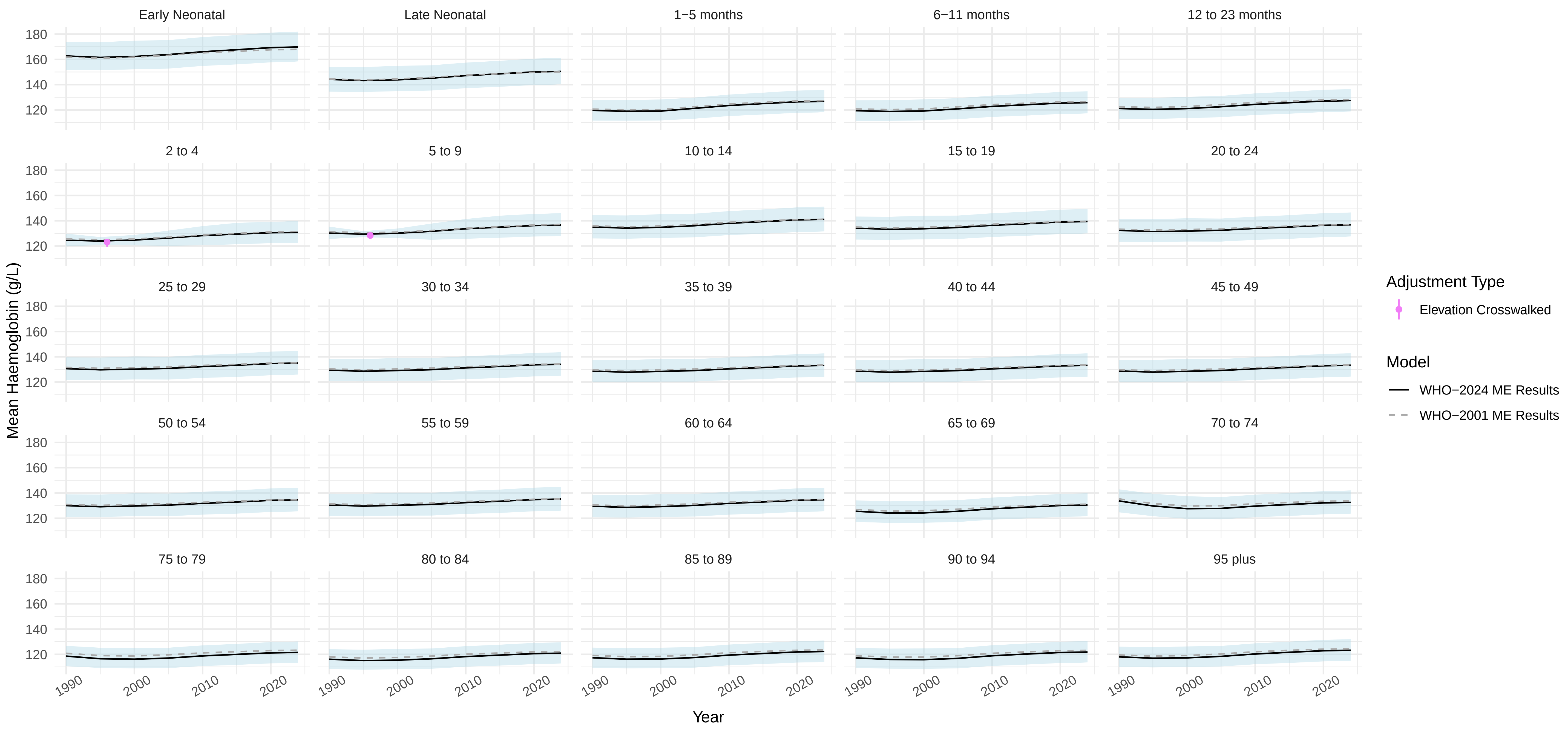

Elevation Adjustment Method Comparison − Anaemia Prevalence − Male − Russian Federation

WHO−2024 vs. WHO−2001 Elevation Adjustment Methods

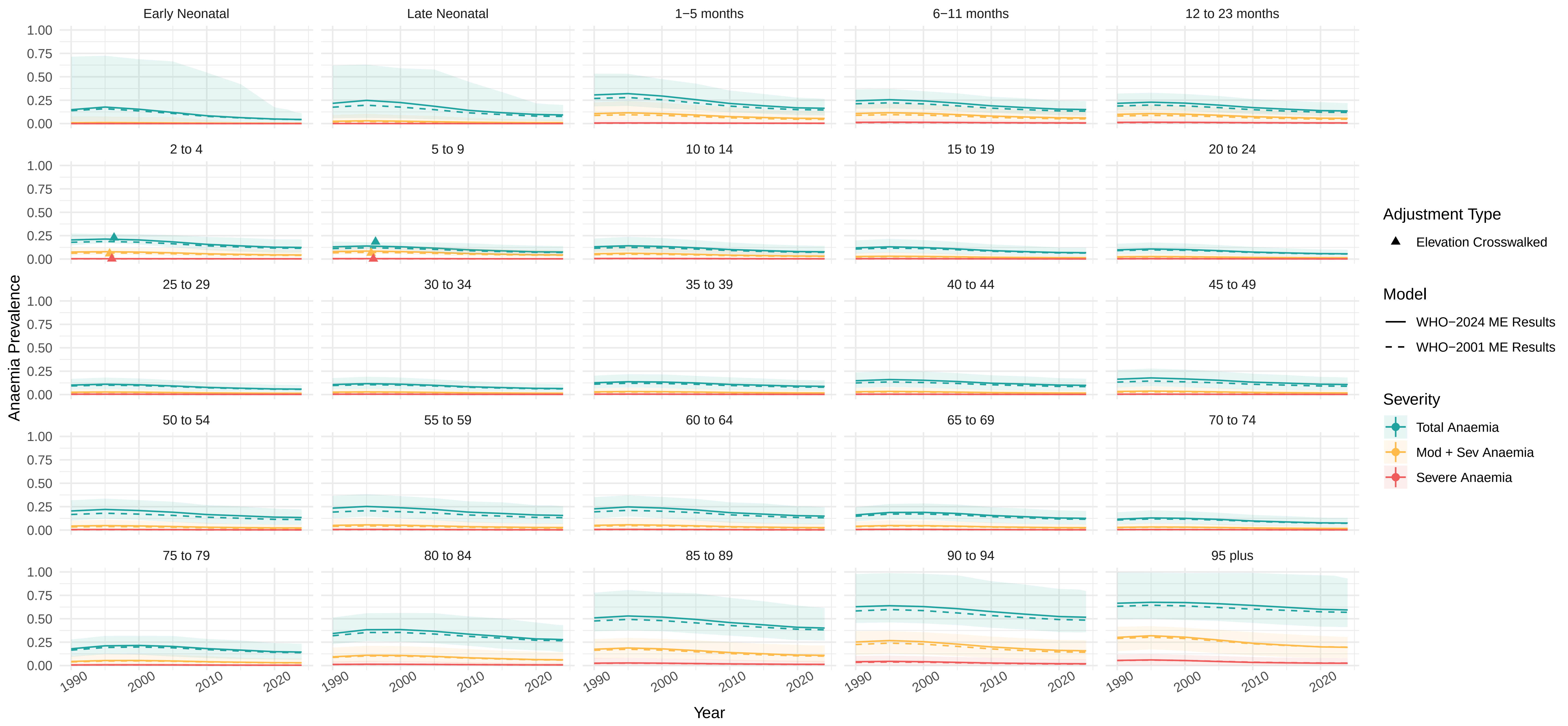

Elevation Adjustment Method Comparison − Anaemia Prevalence − Female − Russian Federation

WHO−2024 vs. WHO−2001 Elevation Adjustment Methods

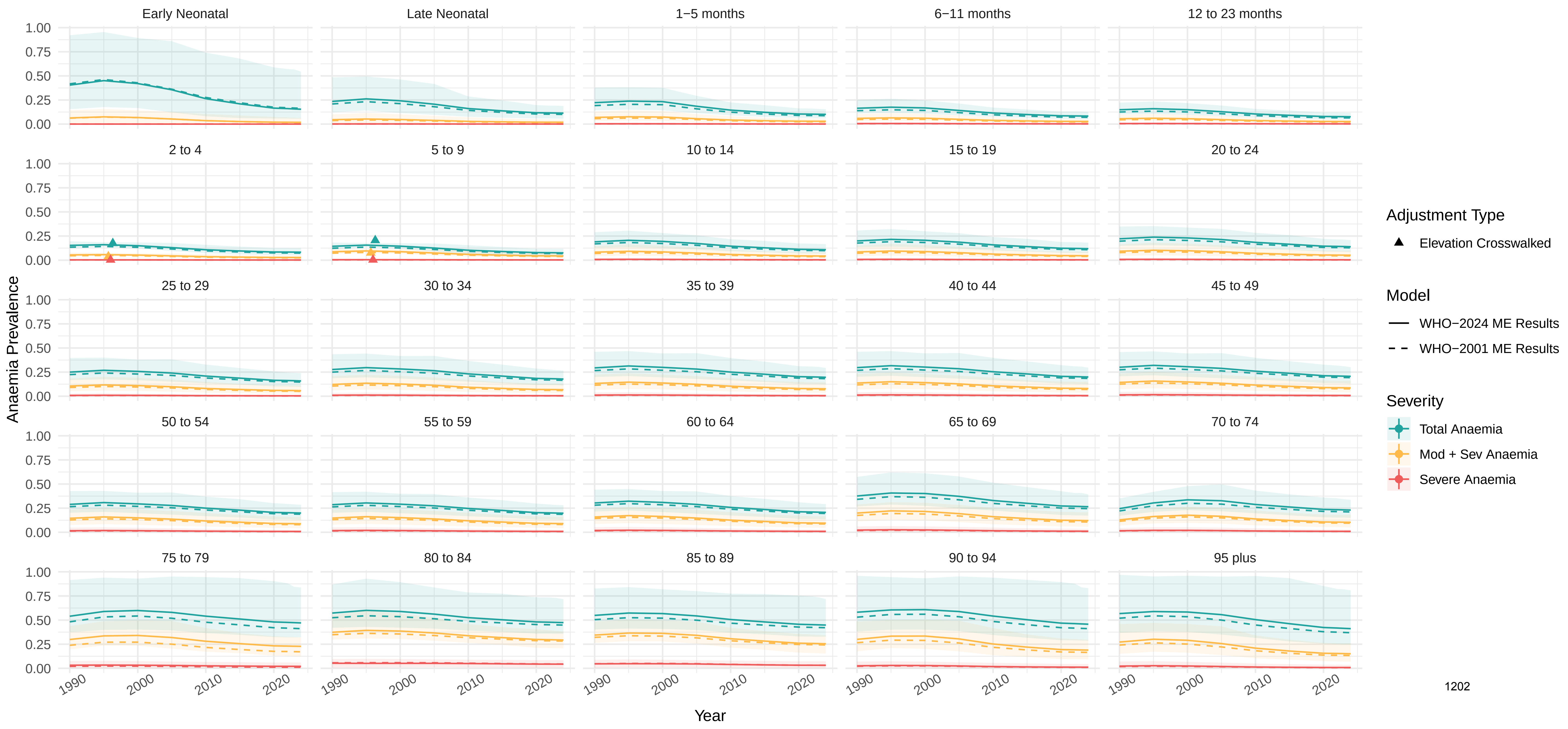

Elevation Adjustment Method Comparison − Mean Haemoglobin − Male − Altai kray

WHO−2024 and WHO−2001

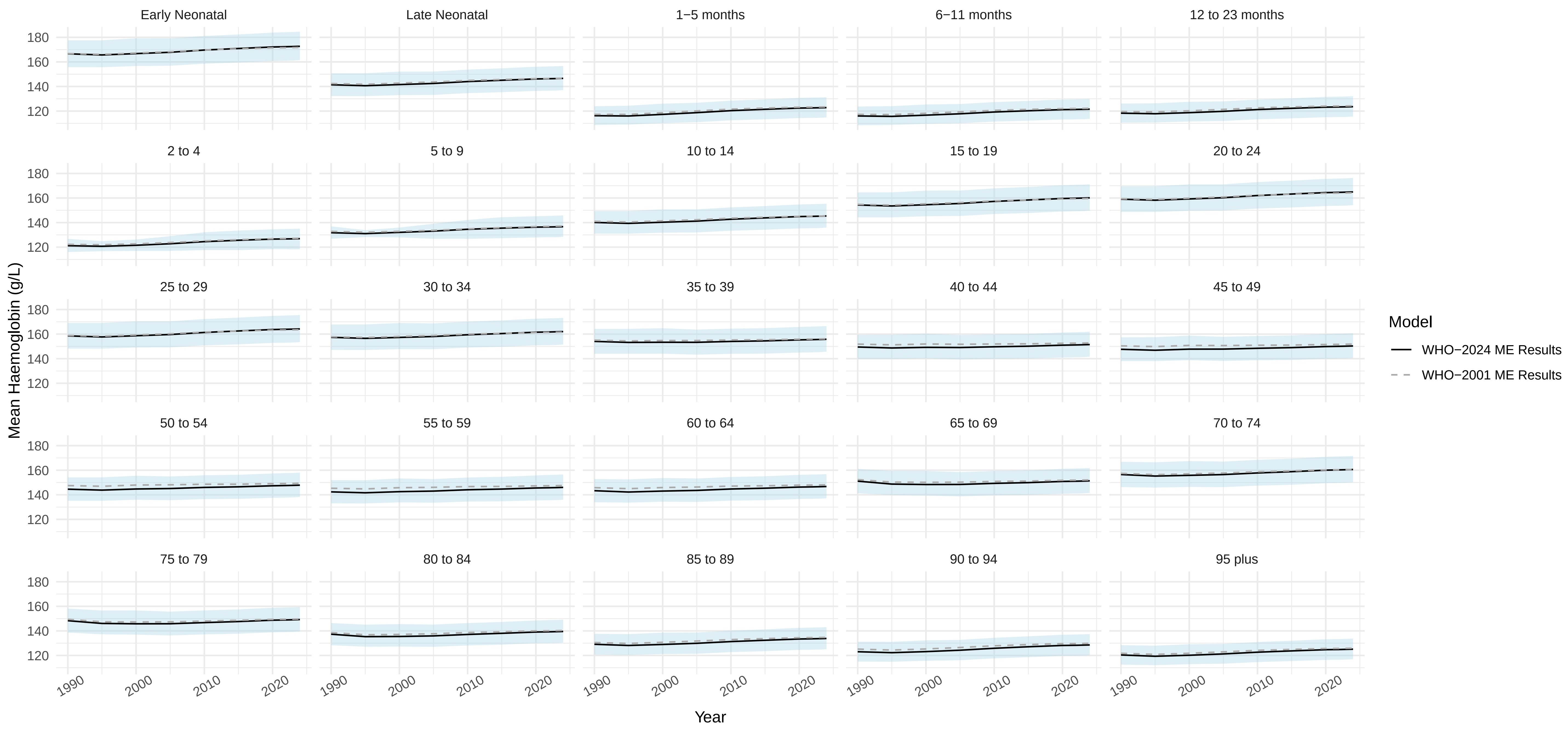

Elevation Adjustment Method Comparison − Mean Haemoglobin − Female − Altai kray

WHO−2024 and WHO−2001

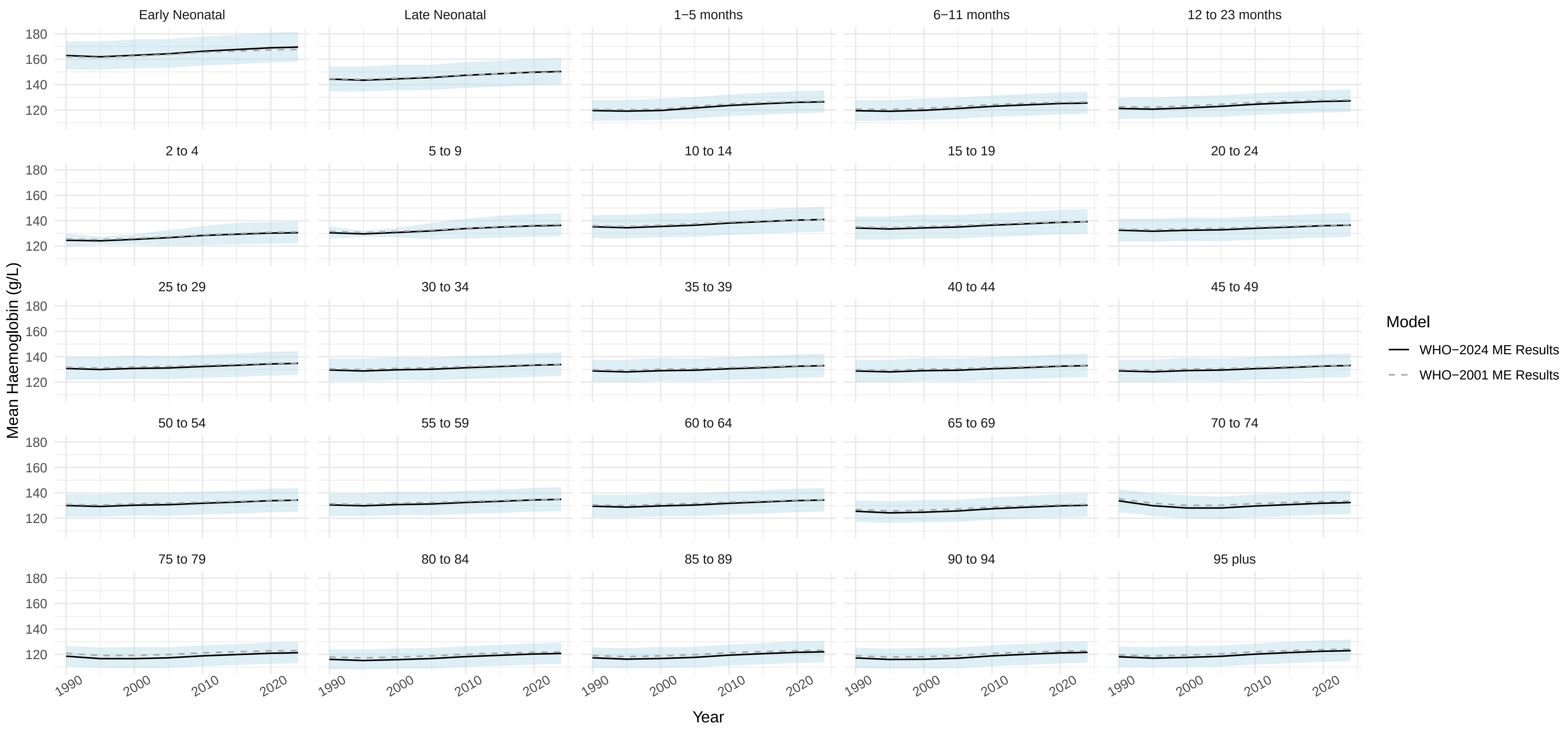

Elevation Adjustment Method Comparison − Anaemia Prevalence − Male − Altai kray

WHO−2024 vs. WHO−2001 Elevation Adjustment Methods

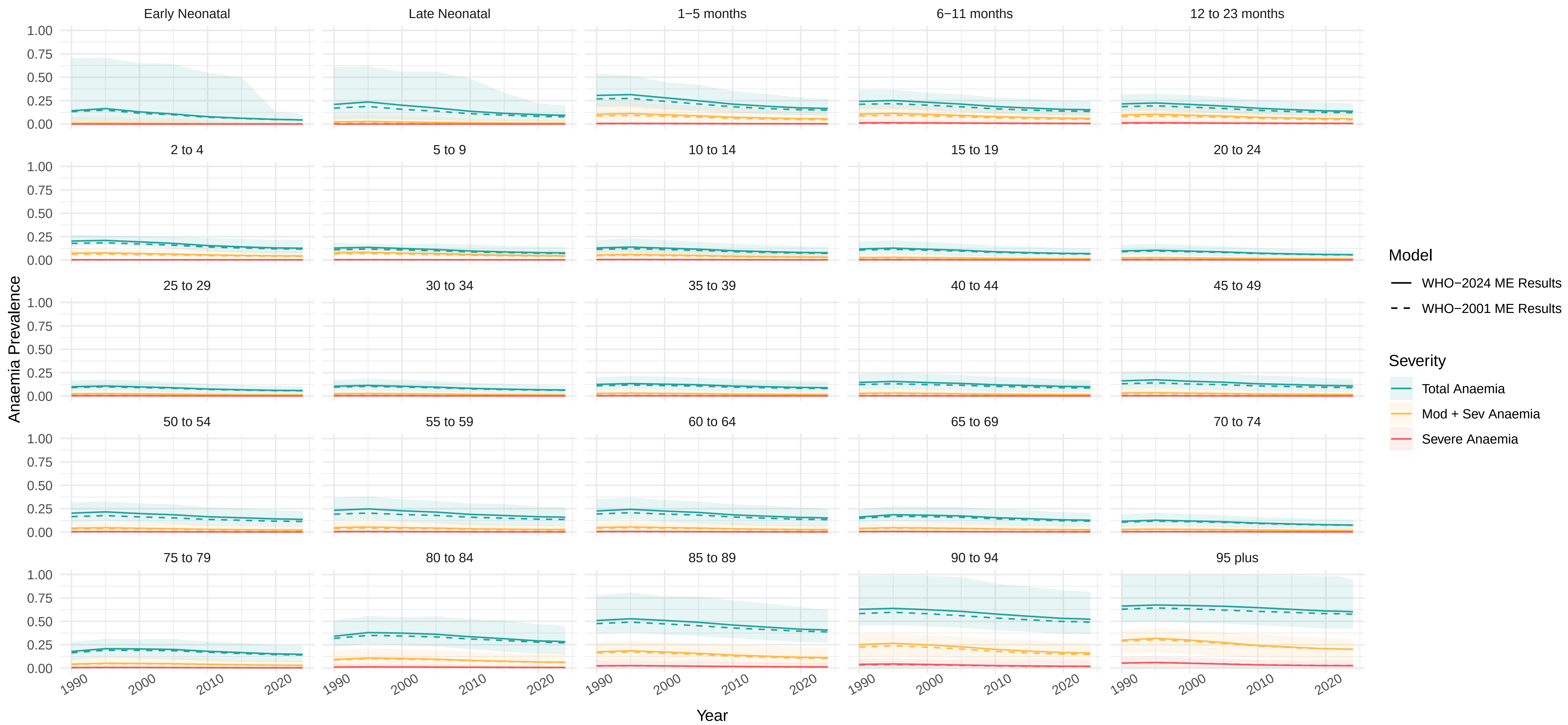

Elevation Adjustment Method Comparison − Anaemia Prevalence − Female − Altai kray

WHO−2024 vs. WHO−2001 Elevation Adjustment Methods

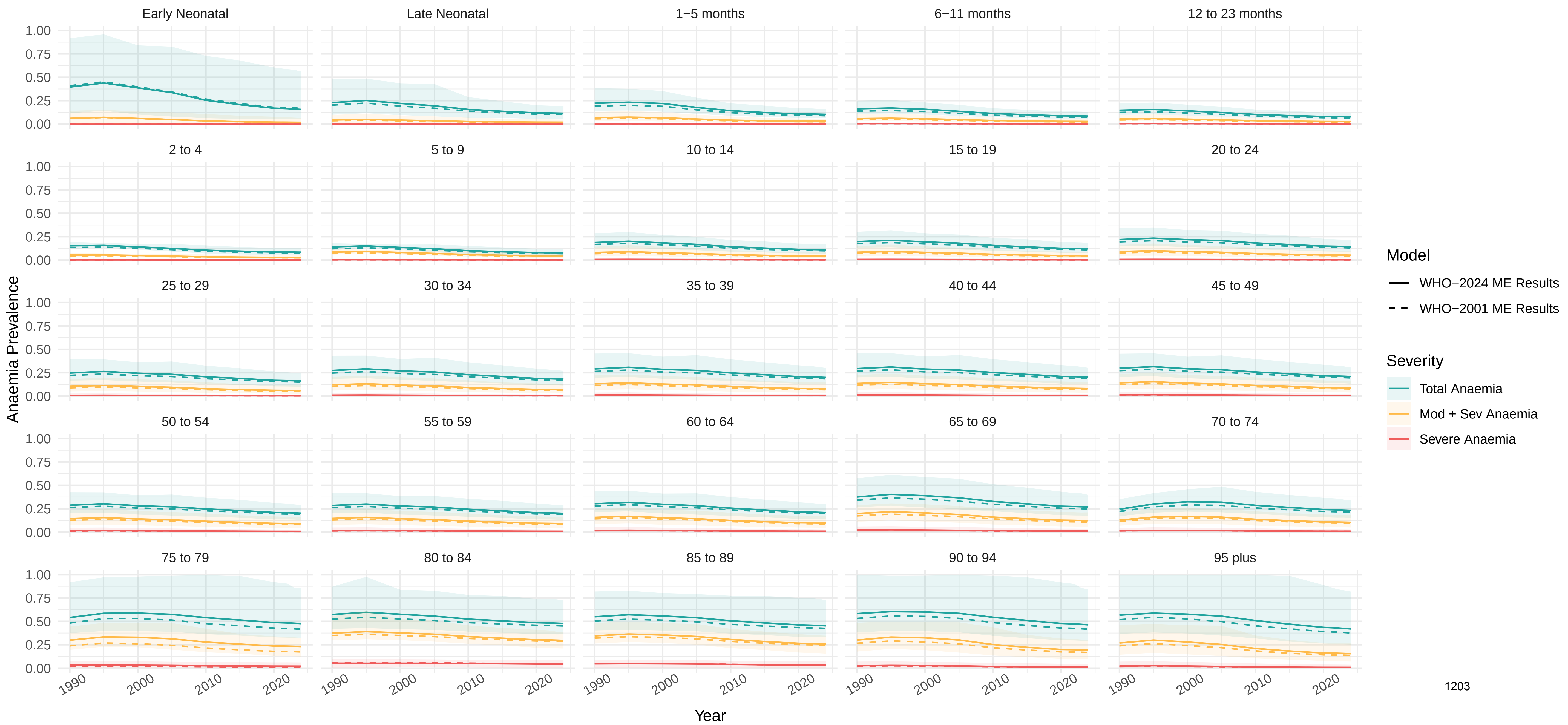

Elevation Adjustment Method Comparison − Mean Haemoglobin − Male − Amur oblast

WHO−2024 and WHO−2001

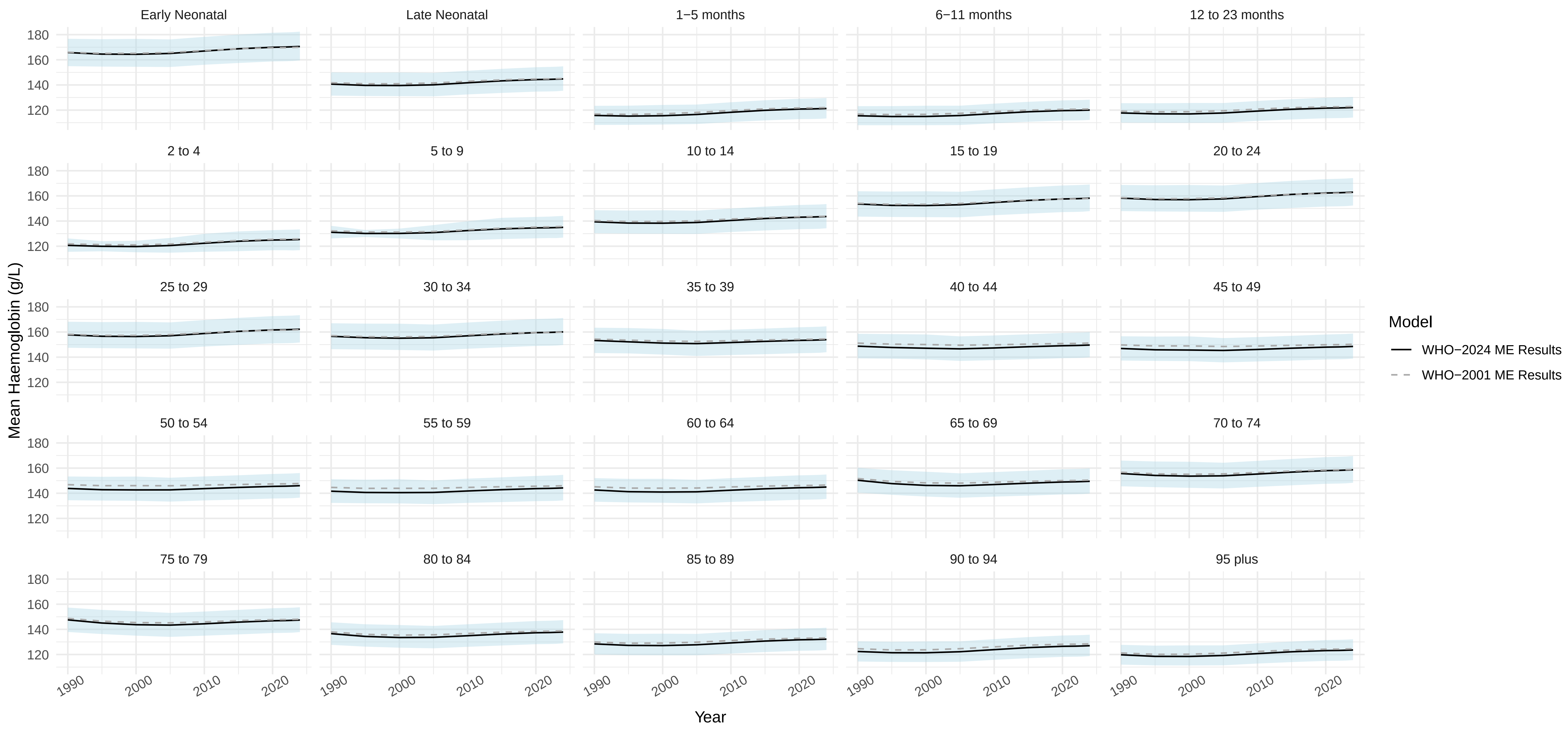

Elevation Adjustment Method Comparison − Mean Haemoglobin − Female − Amur oblast

WHO−2024 and WHO−2001

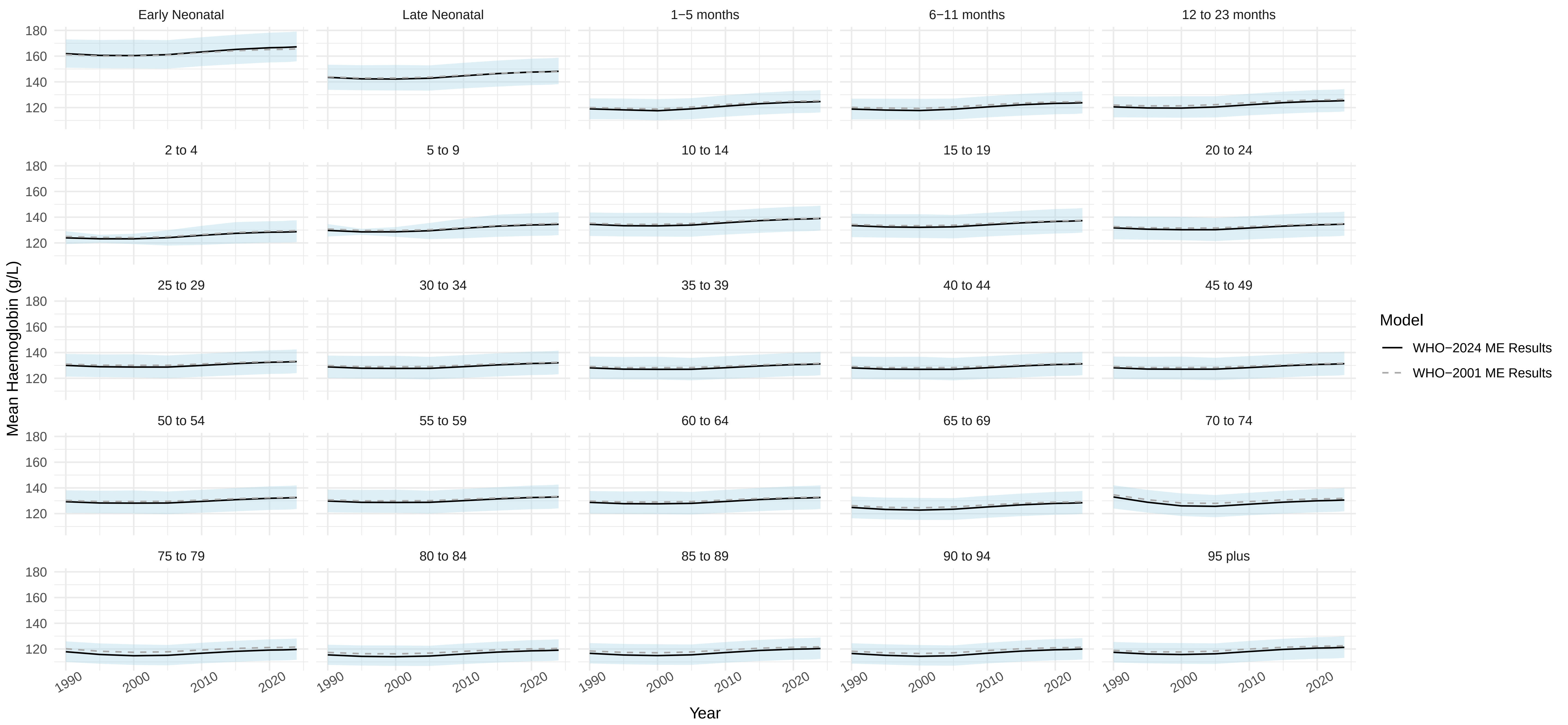

Elevation Adjustment Method Comparison − Anaemia Prevalence − Male − Amur oblast

WHO−2024 vs. WHO−2001 Elevation Adjustment Methods

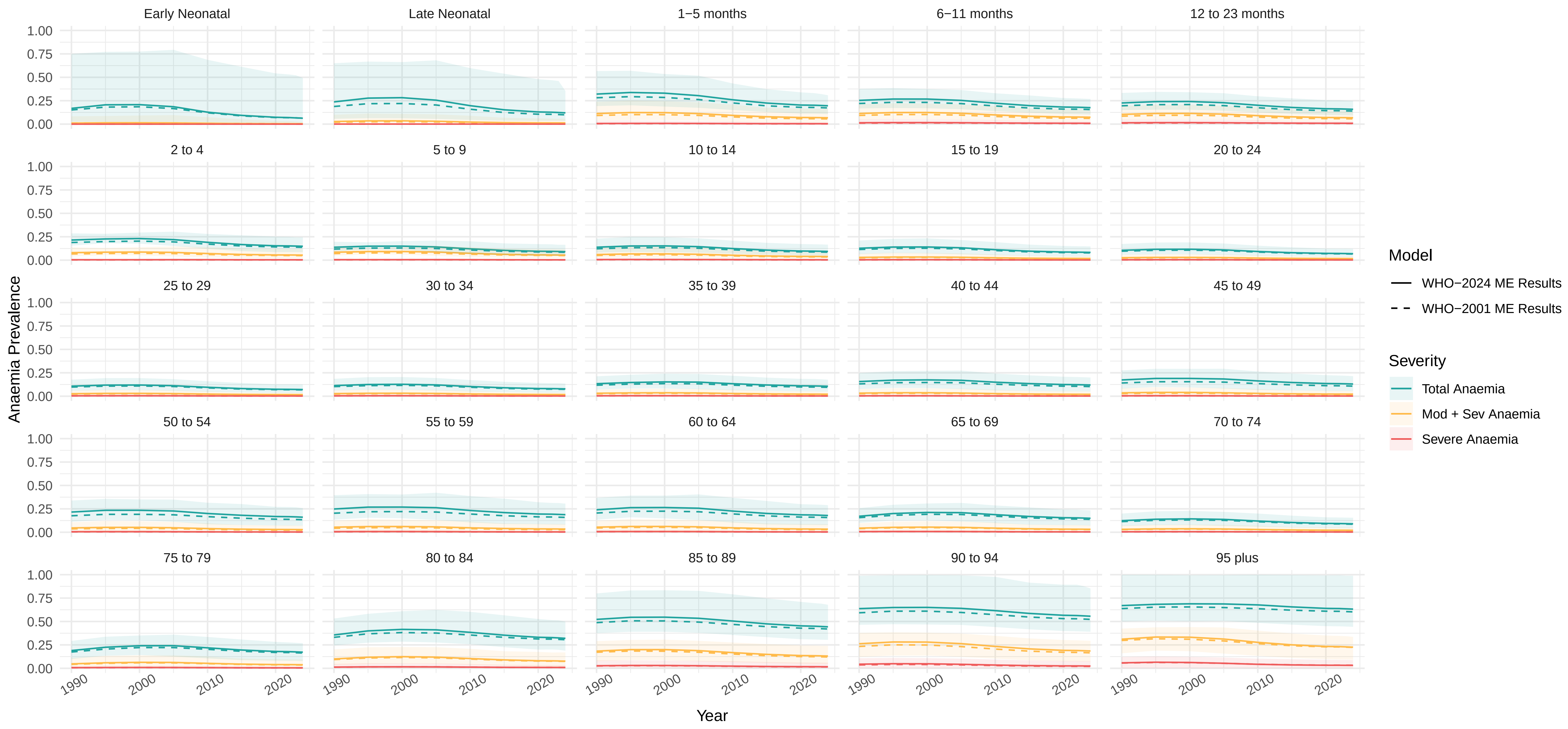

Elevation Adjustment Method Comparison − Anaemia Prevalence − Female − Amur oblast

WHO−2024 vs. WHO−2001 Elevation Adjustment Methods

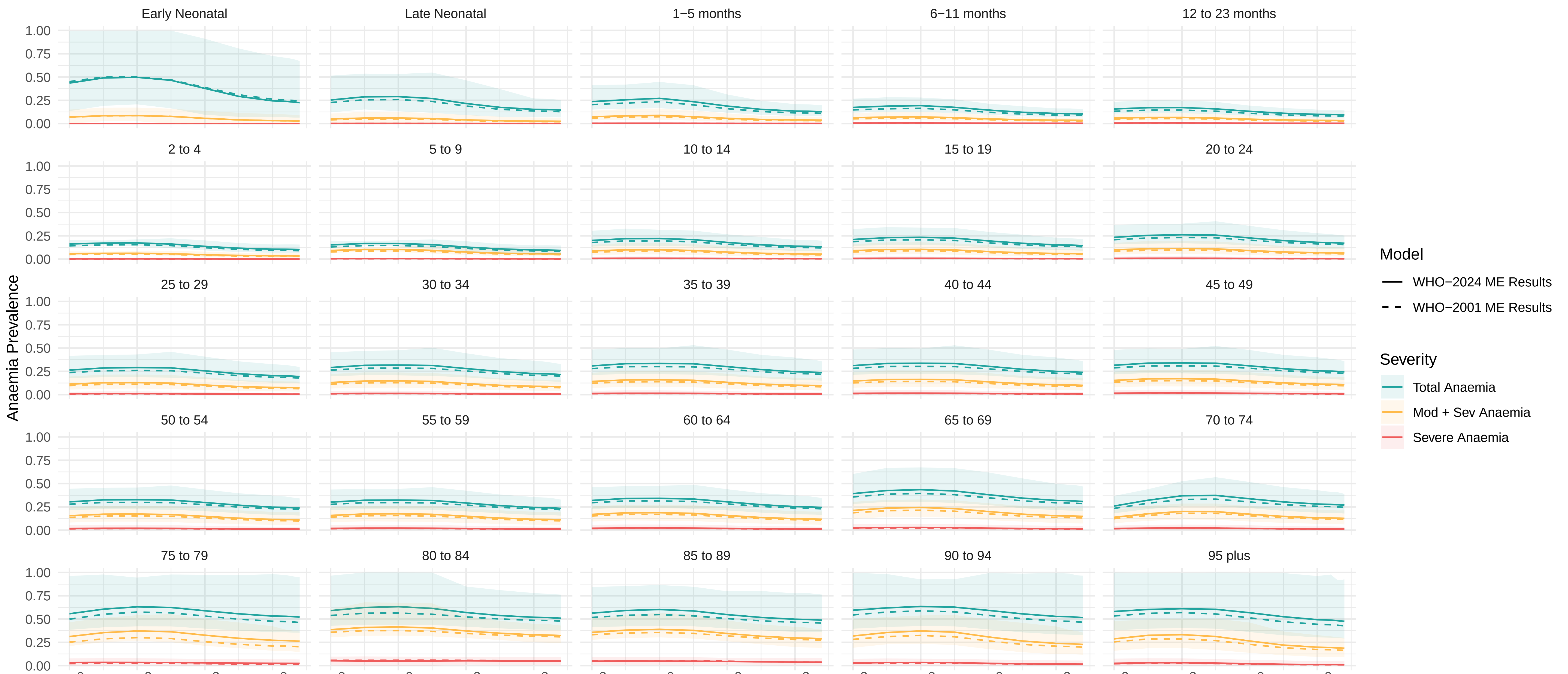

Elevation Adjustment Method Comparison − Mean Haemoglobin − Male − Arkhangelsk oblast without Nenets autonomous district

WHO−2024 and WHO−2001

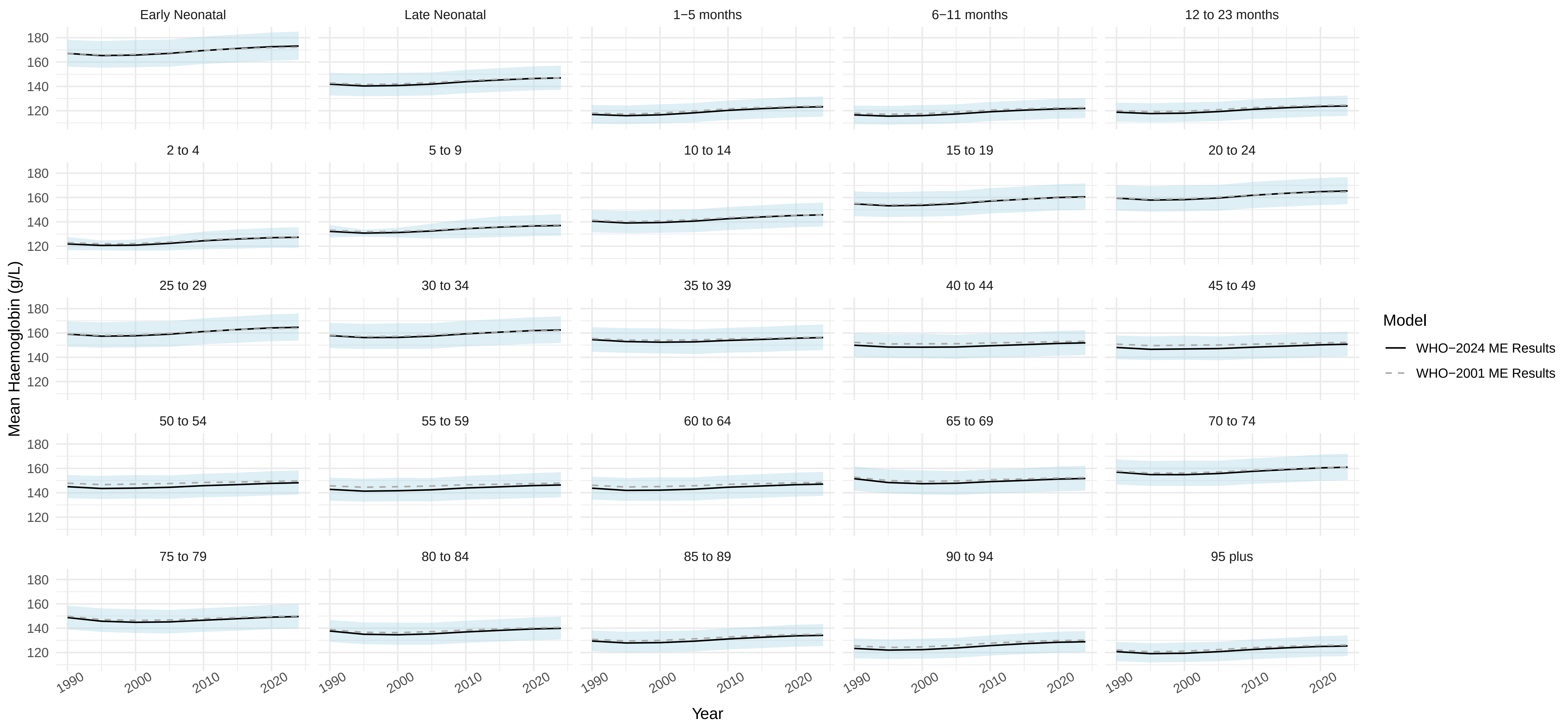

Elevation Adjustment Method Comparison − Mean Haemoglobin − Female − Arkhangelsk oblast without Nenets autonomous district

WHO−2024 and WHO−2001

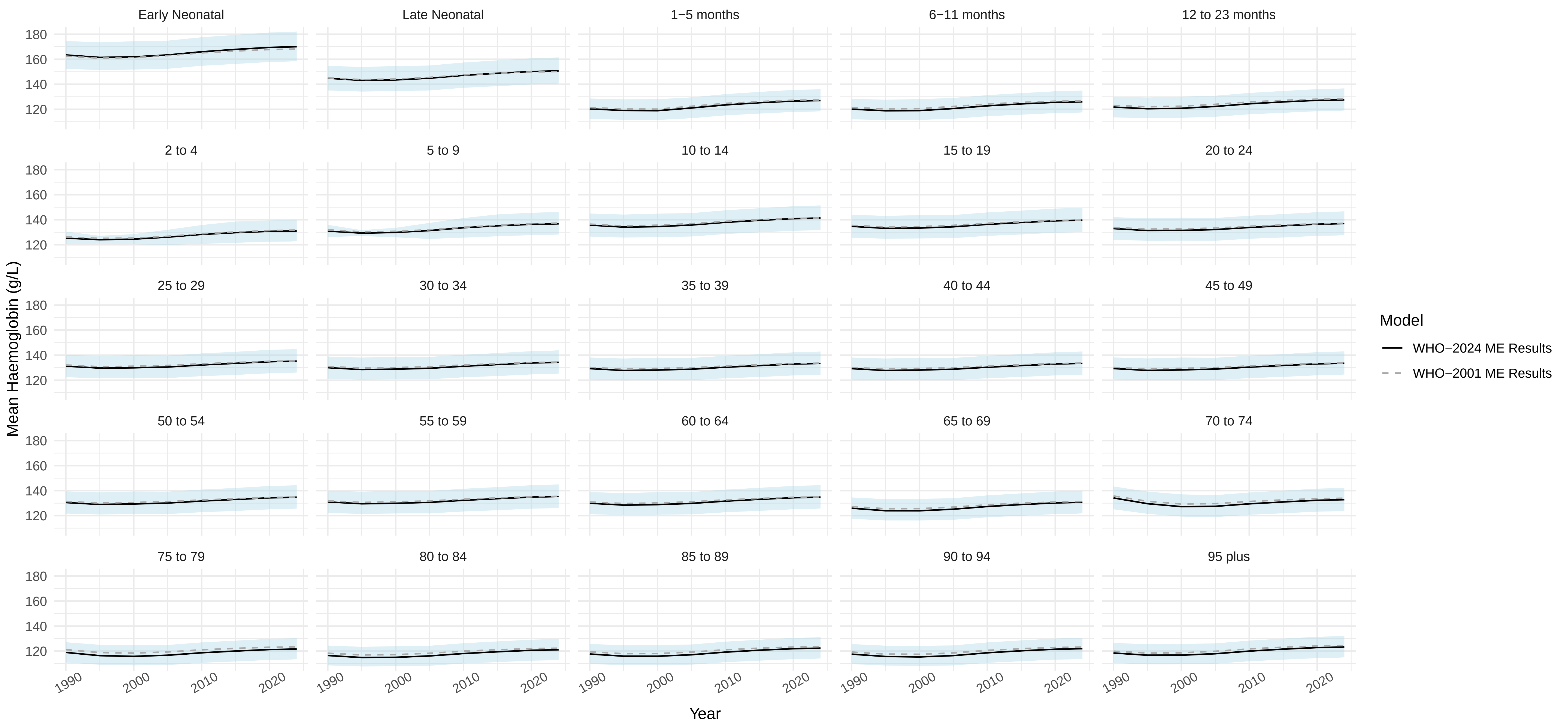

Elevation Adjustment Method Comparison − Anaemia Prevalence − Male − Arkhangelsk oblast without Nenets autonomous district

WHO−2024 vs. WHO−2001 Elevation Adjustment Methods

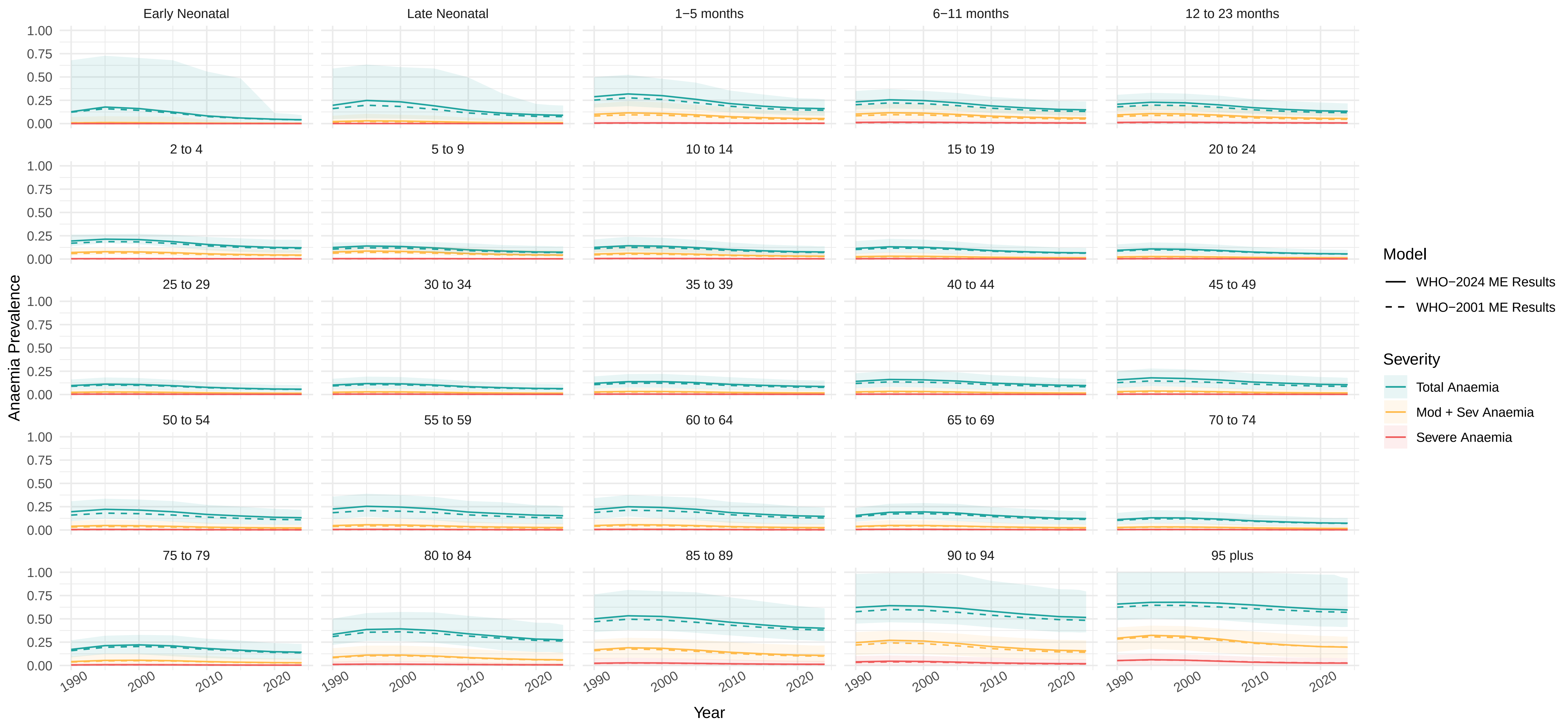
[truncated: 583,017 more chars]
